# Phenotypic and Complete Reference Whole Genome Sequence Analyses of Two *Paenibacillus* spp. Isolates from a Gray Wolf (*Canis lupus*) Gastrointestinal Tract

**DOI:** 10.3390/vetsci12010051

**Published:** 2025-01-13

**Authors:** Jessika L. Bryant, Jennifer McCabe, C. Cristoph Klews, MiCayla Johnson, Ariel N. Atchley, Thomas W. Cousins, Maya Barnard-Davidson, Kristina M. Smith, Mark R. Ackermann, Michael Netherland, Nur A. Hasan, Peter A. Jordan, Evan S. Forsythe, Patrick N. Ball, Bruce S. Seal

**Affiliations:** 1 Biology Program, Oregon State University-Cascades, 1500 SW Chandler Avenue, Bend, OR 97702, USA; bryantj2@oregonstate.edu (J.L.B.); klewsc@oregonstate.edu (C.C.K.); johnsonmicayla5221@gmail.com (M.J.); arielatchley@outlook.com (A.N.A.); thomascuzz@gmail.com (T.W.C.); kristina.smith@osucascades.edu (K.M.S.); peter.jordan@osucascades.edu (P.A.J.); evan.forsythe@osucascades.edu (E.S.F.); 2 Oregon Veterinary Diagnostic Laboratory, OSU Carlson College of Veterinary Medicine, 134 Magruder Hall, 700 SW 30th, Corvallis, OR 97331, USA; 3 EzBiome Inc., 704 Quince Orchard Rd Suite 250, Gaithersburg, MD 20878, USA; michael@ezbiome.com (M.N.J.); hasan@ezbiome.com (N.A.H.)

**Keywords:** genomics, probiotic, canine, antimicrobial, *Paenibacillaceae*

## Abstract

Inflammatory bowel disease (IBD) is increasing among mammals around the world, and domestic dogs are no exception. There is no approved cure for canine IBD with limited treatment options. Novel probiotic bacteria discovery from free-ranging animals for the treatment of IBD in domestic pets can likely yield promising probiotic candidates. Consequently, the overall aim was to isolate bacteria from free-ranging animals that could potentially be utilized as novel probiotics. Two bacteria identified as unique *Paenibacillus* spp. strains by small ribosomal RNA (16S) gene sequencing were isolated from the gastrointestinal tract of a North American Gray Wolf (*Canis lupus*). The bacteria were typed as Gram-variable, and both were catalase/oxidase positive as well as sensitive to commonly used antibiotics. The bacteria digested complex carbohydrates and lipids by standard assays. The isolated bacteria also inhibited the growth of *Staphylococcus aureus* and *Micrococcus luteus*. The whole genome sequence (WGS) length of bacterial isolate ClWae17B was 6,939,193 bp, while ClWae19 was 7,032,512 bp, both similar in size to other *Paenibacillus* spp. The genomes of both bacteria encoded enzymes involved with the metabolism of complex starches and lipids, such as lyases and pectinases, along with encoding antimicrobials such as lanthipeptides, lasso peptides, and cyclic-lactone-autoinducers. No pernicious virulence genes were identified in the WGS of either bacterial isolate. Phylogenetically, the most closely related bacteria based on 16S gene sequences and WGS were *P. taichungensis* for ClWae17B and *P. amylolyticus* for ClWae19. WGS analyses and phenotypic assays supported the hypothesis that the isolates described constitute two novel candidate probiotic bacteria for potential use in dogs.

## Simple Summary

The current market for animal probiotics is comprised of companies producing supplements that contain probiotic bacteria such as *Lactobacillus* spp. and *Bifidobacterium* spp. Consequently, bacteria utilized as probiotics in animals have already been discovered and are generally recognized as “safe” as determined by regulatory agencies. Many studies report that commonly utilized probiotic bacteria for animals and antibiotics have little to no effect in positively treating inflammatory bowel disease (IBD) in dogs. Therefore, it is important to explore new territory for probiotics to aid in treating IBD among dogs. All dogs are descended from the Gray Wolf (*Canis lupus*), and research was expanded to include the isolation of spore-forming bacteria from the gastrointestinal tract of a free-ranging wolf. Two bacteria from the genus *Paenibacillus* were isolated and characterized by biological properties and sequencing of their genomes. The bacteria were able to digest complex carbohydrates and lipids such that they would contribute to host dogs’ overall energy utilization. Both bacterial isolates inhibited the growth of *Staphylococcus aureus* and were found to encode a variety of antimicrobials important for probiotics. The bacteria lacked common antibiotic resistance and did not encode harmful virulence genes. These attributes support the idea that these *Paenibacillus* bacteria could potentially be used as probiotics for animals such as dogs.

## 1. Introduction

Chronic inflammatory enteropathies (CIEs), including canine inflammatory bowel disease (cIBD), are characterized as chronic dysbiosis of a dog’s gastrointestinal (GI) tract with no known cure and limited treatment options [1,2,3]. Host genetics, environmental factors, the immunological state, and an altered microbiome contribute to GI tract dysbiosis [4], with certain dog breeds being more susceptible to cIBD [5]. The canine GI tract microbiota has been characterized as consisting of a “core” group including the Firmicutes, Bacteroidetes, and Fusobacterium with taxa that include Clostridia and Bacilli. Many of the predominant bacteria produce short-chain fatty acids, such as *Faecalibacterium*. Bacteroidetes are reportedly abundant in feces, including *Prevotella* and *Bacteroides* [6].

The first domesticated animals were dogs (*Canis familiaris*), descended from the Gray wolf (*Canis lupus*) from eastern and southwest Eurasia with no known true progenitor [7]. This resulted in most breeds forming monophyletic clusters and 25 major clades [8]. It is hypothesized that dog domestication from wolves occurred during relationships with humans during the late Pleistocene in Siberia and accompanied humans into the Americas approximately 15,000 years ago [9,10]. Wolves are thought to have developed relationships with humans as undomesticated “synanthropes” that benefitted from humans and their altered environments [11]. Furthermore, changes in lifestyles that occurred during human evolution, such as diet, resulted in a switch from the ancestral state that included a depletion of the gut microflora and an increase in IBD [12]. Diversification and adaptation during mammalian evolution have altered the gut microbiota, especially during domestication [13], which can result even in the “humanization” of wildlife GI tracts [14].

The diet of modern dogs does not resemble that of its wolf ancestor [15], and starch in domestic dog diets [16] is resistant to digestion, which can potentially have negative impacts on dog gastric physiology [17]. Consequently, probiotics from free-ranging animals could be potentially useful for supplementing dogs’ diets to improve GI tract health [6,18]. The FAO/WHO defines probiotics as “live microorganisms that, when administered in adequate amounts, confer a health benefit on the host”, as put forth by the International Scientific Association for Probiotics and Prebiotics (ISAPP) consensus statement [19]. Ownership of companion animals has increased, and maintaining canine health, specifically by feeding probiotics to treat cIBD, will become more important [6,18,20,21]. Consistent with this practice, probiotics are now used during wildlife rehabilitation [22].

The genus *Paenibacillus* was first proposed in 1993 for the monophyletic lineage of endospore-forming bacteria previously assigned to the genus *Bacillus* based on morphological characteristics and comparative 16S rRNA sequence [23]. These bacteria were subsequently assigned as a new genus, the *Paenibacillus* spp. [24] based on the results of Ash et al. [23]. Species members include a variety of bacteria important to other organisms and the environment that can also be utilized for various practical uses, such as probiotics. This is because they produce a variety of antimicrobials and enzymes, such as lipases, amylases, cellulases, hemicellulases, pectinases, and lignin-modifying enzymes [25]. Many of these enzymes are included in monogastric animal feeds to improve digestion and promote growth [26,27]. Herein, we report two *Paenibacillus* spp. isolated from the GI tract of a Gray wolf (*Canis lupus*) that has potential use as a canine probiotic to help control cIBD.

## 2. Materials and Methods

### 2.1. Isolation of Bacteria, Phenotypic Characterization and Bacterial Growth Inhibition

From the ileum of a one-day deceased male gray wolf, *Canis lupus* (killed by an automobile accident) of indeterminant age, gastrointestinal (GI) tract material was collected following necropsy at the Oregon Veterinary Diagnostic Laboratory, College of Veterinary Medicine, Oregon State University in Corvallis, OR [https://vetmed.oregonstate.edu/ovdl] (accessed on 13 November 2024). No gross pathology was reported other than fractures of the radius and ulna (OVDL case 20V15449), with no apparent pathophysiology of the GI tract. As described previously by McCabe et al. [28], to eliminate vegetative bacterial cells, GI tract samples were suspended in phosphate-buffered saline (PBS) and treated with 3% chloroform for 30 min ([29], Honda K, personal communication). Chloroform-treated GI tract sample aliquots were cultured aerobically on brucella broth agar with hemin and vitamin K (BBHK) at 37 °C. This media is routinely utilized to propagate spore-forming bacteria [28,30,31]. Subsequently, several isolates were propagated on brain heart infusion (BHI) agar, and two designated ClWae17B and ClWae19 were chosen for further analyses.

Phenotypic characterizations, such as Gram stains and starch hydrolysis, with catalase, lipase, and oxidase assays were completed via standard microbial assays [30,31]. Isolates ClWae17B and ClWae19 were assayed for antibiotic sensitivities to vancomycin, penicillin, streptomycin, erythromycin, neomycin, novobiocin, chloramphenicol, and kanamycin via disc diffusion to assay for potential antibiotic resistances (BBL-Carolina Biologicals: 805081 [30,31]) as set forth by the Clinical and Laboratory Standards Institute (CLSI; [32]). Growth inhibition assays were completed to determine if the isolates had antibacterial activity against *Staphylococcus aureus*, *Escherichia coli*, and *Micrococcus luteus* [33]. Briefly, overnight cultures of both test and target bacteria (∼10^6^ cells) were propagated in 3 mL of BHI media. Target bacteria were inoculated into 15 mL of cooled 55 °C sterile BHI agar. Inoculated agars were poured onto sterile petri dishes and congealed under sterile conditions. The wolf test bacteria were pelleted and suspended in 200 µL of BHI media, into which sterile filter discs were saturated with the test bacterium and then placed on the inoculated target bacterial agar plates. Inoculated plates with discs were incubated for 24 h at 37 °C, and the formation of a zone of clearance (ZOC) was visually assessed after 24–36 h, as described by Grady et al. [33].

### 2.2. Bacterial Genomic DNA Isolation and Whole Genome Sequencing Analyses of Bacteria

Bacterial genomic DNA was purified from bacterial colonies propagated in 3 mL BHI cultures using the Illustra Nucleic Acid Purification^TM^ system to complete whole genome sequencing (WGS) and obtain 16S gene sequences [28]. Hybrid sequencing of the two bacterial isolates was completed at EzBiome (Gaithersburg, MD, USA). Briefly, the extracted genomic DNA was quantified by fluorescence-based Qubit dsDNA, DNA quantification System (ThermoFisher, Waltham, MA, USA) and sequenced on an Illumina NextSeq2000 (2 × 150 bp) and an R10.4.1 flow cell of a Nanopore PromethION (Eugene, OR, USA). The Illumina library was prepared using the NEBNext^®^ Ultra™ II FS DNA library kit for Illumina, while the Nanopore library was prepared using v14 library prep chemistry without fragmentation or size selection.

Resultant DNA sequencing reads were filtered using Filtlong v0.2.1 min_length 1000 keep_percent 95 [https://github.com/rrwick/Filtlong] (accessed on 13 November 2024) by removing the 5% worst FASTQ reads. Nanopore reads were then assembled with Flye v2.9.2 [34]. Illumina reads were aligned to the draft assemblies using BWA with the “-a” flag [35]. Alignment files and draft assemblies were used to produce polished assemblies using Polypolish v0.5.0 [36]. Polished assemblies were checked for contamination using CheckM [37], annotated with Prokka v1.14.6 [38], and circular genome maps were generated with GenoVi v0.4.3 [39].

Isolate species identities were determined by extracting a set of bacterial core genes [40] from the isolates and identifying their closest match using the EzBioCloud platform through whole genome average nucleotide identities (ANI) comparisons [41]. Based on the top hit identities, genomes and 16S sequences were downloaded from EzBioCloud. Core genes were extracted from genomes using UBCG2. The UBCG phylogeny used on EzBioCloud Pro uses 92 conserved loci for species determination [40,41]. Concatenated core genes and 16S sequences were aligned with MAFFT v7.508 [42] using the G-INS-i strategy, and phylogenies were constructed with 1000 bootstrap replicates using RAxML-NG v. 1.1.0 [43].

Antibiotic resistance gene profiles were produced by using a pre-built database [44] composed of NCBI’s National Database of Antibiotic-Resistant Organisms (NDARO, www.ncbi.nlm.nih.gov/pathogens/antimicrobial-resistance/) (accessed on 13 November 2024) reference genes. Each read of the metagenome sample was mapped against these genes using bowtie2 with the very sensitive option, and the output was then converted and sorted by Samtools [45]. Finally, depth and coverage were calculated for each gene using Samtool’s mpileup script. Virulence factor profiles were produced by using a pre-built bowtie2 [44] database composed of reference factors obtained from the Virulence Factors of Pathogenic Bacteria (VFDB) database [46]. Each read of the metagenome sample was mapped against these virulence factors using bowtie2 with the very-sensitive option, and the output was then converted and sorted by Samtools [45]. Finally, depth and coverage were calculated for each virulence factor using Samtool’s mpileup script.

### 2.3. Phylogenetic Analyses of the Wolf Bacterial Isolates

Reference genomes were downloaded from EzBioCloud databases [47]. Average Nucleotide (ANI) values, query, and reference coverage values were calculated with OrthoANIu [48]. Subsequently, a neighbor-joining tree was made from the ANI values using the “ape” R library [49]. The query and reference genomes were subject to SNP analysis using parsnp with default parameters [50]. SNPs were counted from the VCF file produced by parsnp, and a matrix of SNP counts was produced using custom scripts. To further support the phylogeny results, genomes were analyzed using the Type (Strain) Genome Server (TYGS) in tandem with the List of Prokaryotic names coupled to the Standing in Nomenclature (LPSN) [51], followed by preparing phylogenetic trees in MEGA12 [52]. Searches for genes potentially encoding bioactive compounds, such as antimicrobials synthesized by the isolates, were accomplished utilizing the antiSmash program [53]. Concurrently, putative prophage sequences were identified utilizing the PHAge Search Tool with Enhanced Sequence Translation or PHASTEST [54] searches of the isolates’ genomes.

## 3. Results

### 3.1. Isolation of Bacteria from a Gray Wolf Gastrointestinal Tract, Phenotypic Characteristics and Growth Inhibition of Target Bacteria

**Figure 1:**
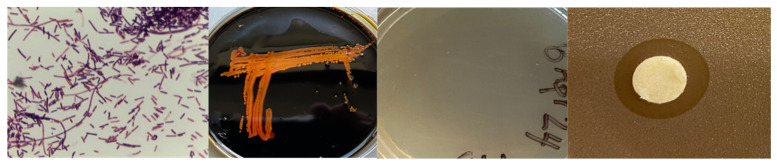
Phenotypic and Growth Inhibition Assays for Wolf Bacterial Isolates ClWae17B and ClWae19. Bacterial isolates were Gram stained, propagated on starch plates followed by staining with iodine, propagated on spirit blue plates, and assayed for growth inhibition of *Staphylococcus aureus* as depicted from left to right. Note: Duplicate results were obtained for both isolates.

Chloroform-treated gastrointestinal (GI) tract material from a North American Gray wolf (*Canis lupus*) was plated on BBHK agar media to isolate potential spore-forming bacteria. Two isolates designated ClWae17B and ClWae19 were then propagated on BHI agar media and further characterized phenotypically as Gram-variables that digested complex carbohydrates on starch plates and were lipase positive by spirit blue agar plate analysis (Figure 1). Both isolates were also catalase- and oxidase-positive but would not propagate on mannitol salt agar or Simmons citrate media, which is used for differentiating Gram-negative bacteria [28,30,31]. Both isolates were sensitive to antibiotics by determining at least a 4 mm zone of clearance (ZOC) as a cut-off for growth inhibition of each isolate [32]. These included vancomycin, penicillin, streptomycin, erythromycin, neomycin, novobiocin, chloramphenicol, and kanamycin. The only exception was that ClWae17B appeared resistant to streptomycin by the disc diffusion assay (Supplementary Figure S1).

Both the bacterial isolates ClWae17B and ClWae19 inhibited the growth of the target bacterium *Staphylococcus aureus* (Figure 1). Sterile discs were saturated with the Gray wolf test bacterial isolates and placed on bacterial agars inoculated with the target bacterium [33]. After a 24 h incubation, clear transparent growth inhibition zones were noted that were at least 2 mm with a defined edge of target bacterial growth. No growth inhibition was obtained when using BHI-media-soaked discs as controls.

### 3.2. Whole Genome Sequence of Gray Wolf Bacterial Isolates

**Figure 2:**
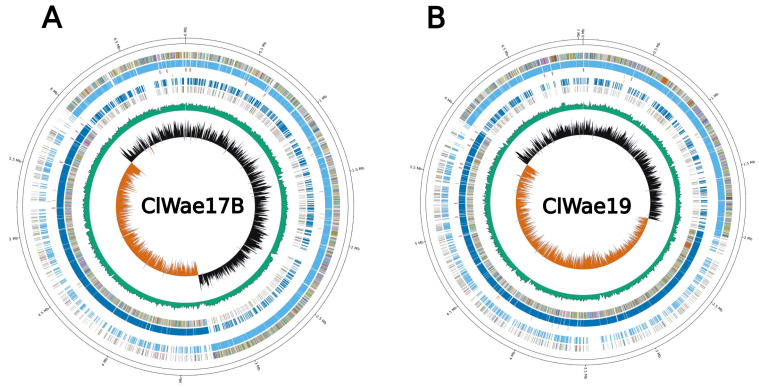
Genome maps and gene composition of bacterial isolates ClWae17B (**A**) and ClWae19 (**B**). The panels visualize gene content as circle plots from inner to outer circles: GC skew, GC content, negative sequence gene content, negative coding sequences, tRNA location, positive coding sequences, and positive sequence gene content with genomic coordinates.

Genome sequencing and assembly of wolf bacterial isolates ClWae17B and ClWae19 resulted in a complete whole genome sequence (WGS) for each isolate. The N50 for the genomes of both isolates was equal to the WGS with completeness of 99.85% for both genomes, with 33X sequence coverage for the ClWae17B genome and 41X sequence coverage for the ClWae19B genome (Supplementary Table S1). The genome of bacterial isolate ClWae17B was 6,939,193 bp, while the complete WGS for ClWae19 was 7,032,512 bp, both similar in size to other known *Paenibacillus* spp. [25]. The percentage GC content was 46.3% for the ClWae17B genome, while it was 46.1% for the ClWae19 genome. Circular full genome maps and composition are reported in Figure 2. A total of 4191 genes were annotated in the ClWae17B genome, while 4059 genes were annotated in the genome of isolate ClWae19.

In addition to the sensitivity to streptomycin of isolate ClWae17B by disc diffusion (Supplementary Figure S1), several other antimicrobial resistances were detected as being encoded by the genomes of both isolates (Supplementary Tables S2 and S3). The antimicrobial resistance profile for isolate ClWae17B revealed potential resistance to rifamycin, streptogramin, and macrolide antibiotics. Similarly, the genome of ClWae19 encodes genes that would provide resistance to rifamycin and chloramphenicol, although the isolate was sensitive to these antibiotics by disc diffusion. Both isolates had genes encoding the ATP-binding cassette F (ABC-F) proteins that are reported to confer resistance to several classes of clinically important antibiotics through ribosome protection of the positioning of tRNA substrates [55].

Several virulence factors were detected as being encoded in the genomes of both isolates (Supplementary Tables S4 and S5). These included genes *gnd* and *htpB* encoding immune modulation-antiphagocytosis and cell wall anchored adherence proteins.

The number of genes and their respective functions are presented in Table 1 for each bacterial isolate. The majority of genes identified are involved with metabolic functions such as carbohydrate transport and metabolism, transcription, signal transduction, amino acid transport and metabolism, cell membrane biogenesis, cell cycle control, and chromosomal portioning. Genes encoding exoenzymes involved in starch and lipase digestion, such as lipase, xylanases, cellulase, pectin lyase, and pectin esterase, were present in the genomes (Supplementary Tables S6 and S7).

The antiSMASH bacterial version [53] was utilized to identify secondary metabolite biosynthesis gene clusters, specifically potential antimicrobials encoded by the wolf bacterial isolates. These included genes encoding for the synthesis of bacillibactin, lanthipeptides, lassopeptides, chitinases, lysins, terpenes, cyclic-lactone-autoinducers, and NRPS-PKS domains involved with polyketide biosynthesis. Both isolates’ genomes also encoded genes that express lipases, catalases, hydrolases, pectin esterase, and pectin lyases. Forty-plus genes were identified as important for spore germination and 30-plus for sporulation, including genes encoding spore germination proteins, spore coat proteins, and acid-soluble spore proteins. Over 30 genes were detected associated with various cytochromes.

**Figure 3:**
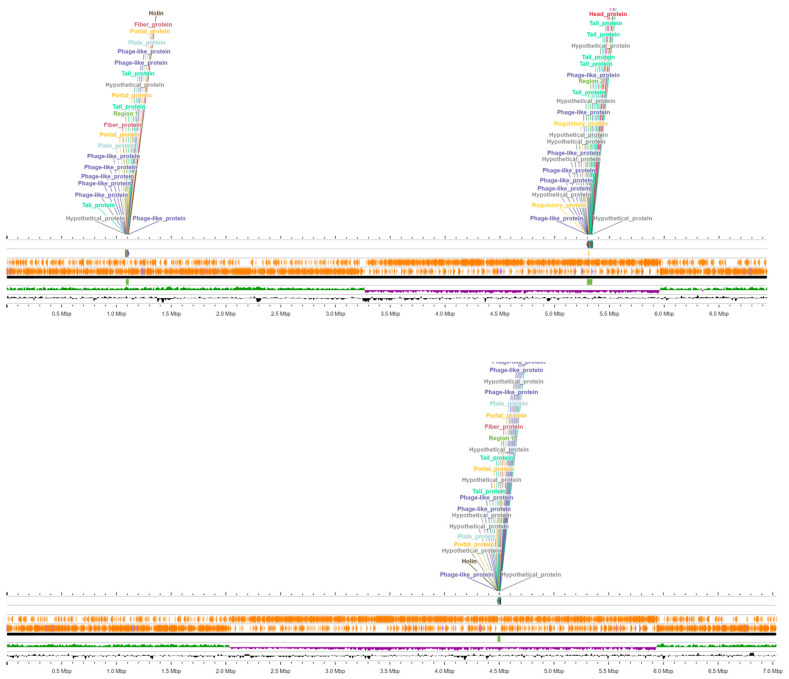
Linear maps of the bacteriophage regions detected in the genomes of bacterial isolates ClWae17B (**upper** panel) and ClWae19 (**lower** panel).

The bacterial genomes’ FASTA files were submitted to PHASTEST to search for potential prophage sequences [54]. ClWae17B contained two regions of prophage sequences, while ClWae19 had one prophage region (Figure 3). The prophage regions in ClWae17B were 26.2 kb from position 1085689–1111904, containing 32 CDS, and a 48.2 kb region from position 5295127–5343340 with 75 CDS. The most common phage sequences were similar to phage Bacill-BalMu-1 (NC_030945) and phage Paenib-Harrison (NC_028746), respectively. The prophage regions were 48.54% GC and 45.32% GC, respectively. The bacterial isolate ClWae19 contained one prophage region of 25.2 kb from position 4484195–4509475 with 28 CDS at 47.98% GC that was most similar to phage Bacill-BalMu-1 (NC_030945).

The total number of prophage genes detected in the ClWae17B was 68, and for ClWae19, it was 26 prophage genes. Common bacteriophage genes identified were those encoding fiber proteins, terminase, holin, portal, head, and tail proteins (Supplementary Tables S8 and S9).

### 3.3. Phylogenetic Analyses of Isolates of Gray Wolf Bacterial Isolates

**Figure 4:**
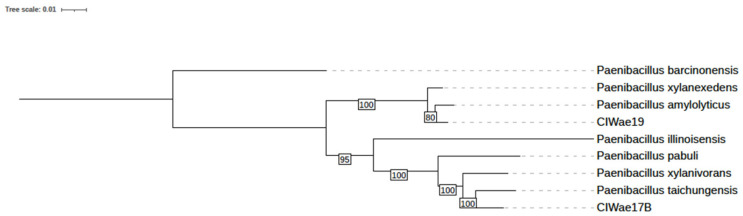
Phylogeny of the ClWae17b and ClWae19 based on bacterial core gene sequences. The ClWae17B and ClWae19 phylogenetic relationships were obtained using universal bacterial core genes. Bootstrap confidence levels are presented in the boxes. The scale bar indicates branch lengths in substitutions per site. Accession numbers and species designations for the phylogenetic comparisons are provided in Supplementary Table S11.

Initial phylogenetic relationships were determined by 16S rRNA sequences. The 16S sequence identity of ClWae17B was 99.7% to *Paenibacillus xylanivorans* and 99.4% to *P. taichungensis*. 16S sequence identity for CLWae19 was 99.9% to *P. amylolyticus* (Supplementary Table S10 and Figure S2). The phylogeny of both isolates was further assessed based on universal bacterial core genes, as depicted in Figure 4. The core gene phylogenetic analysis resulted in the ClWae17B bacterial isolate being most closely related to *P. taichungensis.* Also, the ClWae19 formed a monophyletic clade most closely related to *P. amylolyticus* with modest bootstrap support for this relationship.

**Figure 5:**
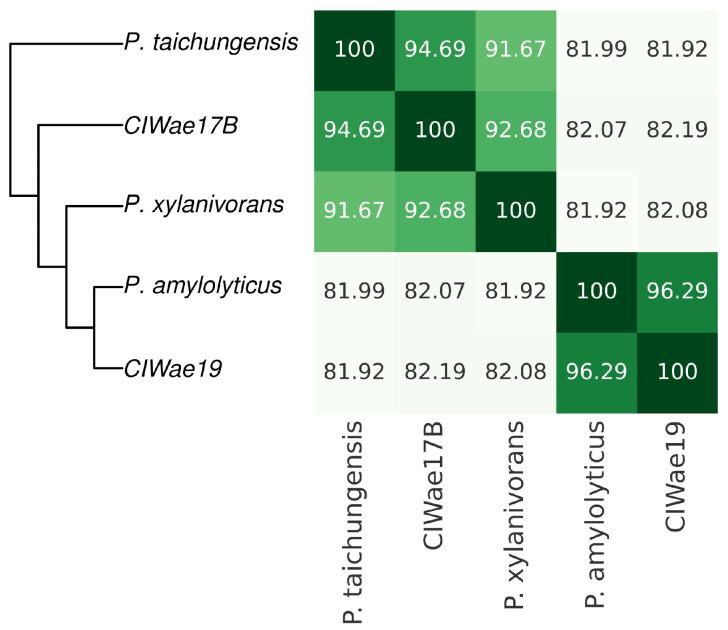
Phylogeny of the ClWae17b and ClWae19 based on genomic average nucleotide identities (ANI). ANI values, query, and reference coverage values were calculated with OrthoANIu, and a neighbor-joining tree was constructed from the ANI values using the ‘ape’ R library functions. Accession numbers and species designations for the phylogenetic comparisons are provided in Supplementary Table S11.

The average nucleotide identities were determined for the genomes of each isolate. The genome of isolate ClWae17B was 92.68% and 94.69%, similar to *P. xylanivorans* and *P. taichungensis*, respectively. The genome sequence of ClWae19 was 96.29%, similar to *P. amylolyticus* (Figure 5). Single nucleotide polymorphism (SNP) analyses also supported these results (Supplementary Figure S3), and this correlates to the phylogeny based on WGS. Furthermore, utilizing the TYGS analyses coupled with the LPSN supported these relationships (Supplementary Figure S4).

## 4. Discussion

Similar to other members of the genus, both wolf Paenibacillus spp. isolates typed as Gram-variable were catalase positive and digested both complex starch and lipid. The genus *Paenibacillus* includes a variety of endospore-producing bacteria that play important roles environmentally. They produce a variety of industrially important enzymes and synthesize a variety of antimicrobials [25]. Moreover, both isolates inhibited the growth of *Staphylococcus aureus*. Most antimicrobials that have been studied among the genus are polymyxins and nonribosomal lipopeptides produced by *P. polymyxa* that are active against Gram-negative bacteria [56,57]. No peptide synthase enzymes potentially involved with the synthesis of polymyxins were detected as encoded in the genomes of either isolate. However, genes associated with the synthesis of lanthipeptides, lassopeptides, chitinases, lysins, terpenes, cyclic-lactone-autoinducers, and NRPS-PKS domains involved with polyketide biosynthesis were present in the isolates’ genomes.

The biosynthetic pathways encoding antimicrobial lanthipeptides and nonribosomal peptides have been reported for 127 members of the *Paenibacillus*. These were mostly from *P. polymyxa* but did include a variety of other genus members, including *P. amylolyticus*, but not *P. taichungensis* [58]. Lasso peptides are a diverse set of ribosomally-synthesized and post-translationally modified peptides with antibacterial activity [59,60]. The ClWae17B and ClWae19 genomes encode biosynthetic pathways for these therapeutically important natural products that have only been previously reported in one other member of the genus, *P. dendritiformis* C454 [61,62]. These antimicrobial types could potentially inhibit the growth of *S. aureus*, and the genomes of both isolates encode potential antifungals, specifically chitinase. Chitinases are glycosyl hydrolases produced by bacteria that have been used to control various pests and have antifungal properties [63]. Many *Paenibacillus* spp. encode enzymes with the ability to digest chitin [25]; however, a literature search resulted in none related to the species described herein.

Cyclic lactone autoinducers are quorum-sensing molecules produced during Gram-negative bacterial replication [64]. *Bacillus* spores in the gastrointestinal tract (GIT) can prevent *Staphylococcus aureus* from colonizing the GIT by inhibiting quorum sensing of the bacterium [65]. The bacteria reported herein encode enzyme systems for synthesizing cyclic lactone autoinducers in addition to other antimicrobials and inhibit the growth of *S. aureus*. As recently reported, the *Paenibacillus* sp. *lzh-N1* gene also encodes cyclic lactone autoinducers that inhibit the growth of a plant fungal pathogen, and it was inferred that in addition to antifungal peptides, quorum sensing systems may influence the bacterium’s biocontrol activity [66].

In addition to the antimicrobials encoded by the two reported *Paenibacillus* spp., the bacteria encode enzymes that can digest complex carbohydrates and lipids. The first domesticated animals were dogs that have common ancestry with the gray wolf [7], which occurred because of relationships with humans [9]. Consequently, the modern dog diet does not reflect that of its ancestor, the wolf [15], and starch in domestic dog diets [16] is resistant to digestion, which can potentially impact gastric physiology [17,67]. GI tract microbial communities of dogs fed raw meat resembled somewhat that of wolves at baseline, but the gut microbial ecology of wolves fed dog food remained distinct from those of dogs [68]. The domestication of dogs includes adaptation to processed feed of carbohydrates, including seed grains. This has resulted in the GI tract of domestic dogs having a microbiota that supports the metabolism of polysaccharides [69]. Moreover, even when changing to a raw meat diet, a dog’s GI microbiota only resembles, to some extent, that of a wolf [70]. Since the gut microbiome of captive wolves may adapt to become more like domestic dogs [71], it makes sense to obtain new probiotics from free-ranging species to replace microbiota lost during domestication.

## 5. Conclusions

Herein, we report the isolation of two novel *Paenibacillus* spp. from the gastrointestinal tract of a North American Gray wolf (*Canis lupus*). ClWae17B is most closely related to *P. taichungensis*, and ClWae19 is most closely related to *P. amylolyticus*. Probiotics can potentially help reduce enteric pathogens, help maintain gastrointestinal health, facilitate the immune system, alleviate lactose intolerance, and improve digestion of complex carbohydrates and lipids. All these characteristics confer health benefits to the host, such as canines [6,18,20,21,72,73]. The reported isolates encode gene products that satisfy these criteria and could provide potential benefits for the health of dogs. This is a continuation of our research involving the identification of potential probiotic bacteria isolated from free-ranging species that could be utilized for domestic animals [28].

## Figures and Tables

**Table 1:** Gene composition of bacterial isolates ClWae17B and ClWae19. Clusters of orthologous groups (COG) and gene function are tabulated by the number of genes and percentage of each genome total in respective columns.

Gene Function	Number of Genes—ClWae17B	% of Total—ClWae17B	Number of Genes—ClWae19	% of Total—ClWae19
Cell cycle control, division, chromosome partitionin	265	3.8	277	4.1
Cell wall/membrane/envelope biogenesis	374	5.4	368	5.4
Cell motility	92	1.3	104	1.5
Post-translational modification, protein turnover, chaperones	286	4.1	272	4.0
Signal transduction mechanism	492	7.1	512	7.5
Intracellular trafficking, secretion, and vesicular transport	99	1.4	102	1.5
Defense mechanism	230	3.3	245	3.6
Extracellular structures	42	0.6	46	0.7
Nuclear structure	0	0.0	0	0.0
Cytoskeleton	11	0.2	8	0.1
RNA processing and modification	1	0.0	0	0.0
Chromatin structure and dynamics	1	0.0	0	0.0
Translation, ribosomal structure, and biogenesis	379	5.5	359	5.3
Transcription	768	11.1	745	10.9
Replication, recombination, and repair	191	2.7	185	2.7
Mobilome: prophages, transposons,	100	1.4	127	1.9
Energy production and conversion	244	3.5	245	3.6
Amino acid transport and metabolism	440	6.3	415	6.1
Nucleotide transport and metabolism	173	2.5	162	2.4
Carbohydrate transport and metabolism	861	12.4	800	11.8
Coenzyme transport and metabolism	314	4.5	278	4.1
Lipid transport and metabolism	220	3.2	230	3.4
Inorganic ion transport and metabolism	346	5.0	343	5.0
Secondary metabolites biosynthesis, transport, and metabolism	106	1.5	106	1.6
General function prediction only	607	8.7	603	8.9
Function unknown	306	4.4	273	4.0
Unclassified	0	0.0	0	0.0

## Data Availability

The data are retrievable at the National Center for Biotechnology Information (NCBI) as SUB14651639, BioProject PRJNA1145425, BioSample SAMN43065442 with a Localid of ClWae17b_contig_1 with Accession CP168020 as *Paenibacillus taichungensis* ClWae17b. The second bacterial isolate is reported as SUB14651639, BioProject PRJNA1145425, BioSample SAMN43065443 with a Localid as ClWae19_contig_1 with Accession CP168019 as *Paenibacillus amylolyticus* ClWae19.

## Supplementary Materials

The following supporting information can be downloaded at https://www.mdpi.com/article/10.3390/vetsci12010051/s1, Figure S1: Antibiotic resistance plates. Figure S2: Phylogenetic relationship based on 16S sequence. Figure S3: SNP matrix analyses. Figure S4: Phylogeny for ClWae17B and ClWae19 using TGYS coupled to the LPSN. Tables S1–S5: Genome statistics. Tables S6 and S7: GenBank files. Tables S8 and S9: Prophage statistics. Table S10: Percent 16S identities. Table S11: Accession numbers and species designations for the phylogenetic comparisons.

## References

[1] Vázquez-Baeza Y., Hyde E.R., Suchodolski J.S., Knight R. (2016). Dog and human inflammatory bowel disease rely on overlapping yet distinct dysbiosis networks. Nat. Microbiol..

[2] Jergens A.E., Heilmann R.M. (2022). Canine chronic enteropathy-Current state-of-the-art and emerging concepts. Front. Vet. Sci..

[3] Rhimi S., Kriaa A., Mariaule V., Saidi A., Drut A., Jablaoui A., Akermi N., Maguin E., Hernandez J., Rhimi M. (2022). The Nexus of Diet, Gut Microbiota and Inflammatory Bowel Diseases in Dogs. Metabolites.

[4] Doulidis P.G., Galler A.I., Hausmann B., Berry D., Rodríguez-Rojas A., Burgener I.A. (2023). Gut microbiome signatures of Yorkshire Terrier enteropathy during disease and remission. Sci. Rep..

[5] Kathrani A., Werling D., Allenspach K. (2011). Canine Breeds at High Risk of Developing Inflammatory Bowel Disease in the South-Eastern UK. Vet. Rec..

[6] Pilla R., Suchodolski J.S. (2021). The Gut Microbiome of Dogs and Cats, and the Influence of Diet. Vet. Clin. North Am. Small Anim. Pract..

[7] Bergström A., Stanton D.W.G., Taron U.H., Frantz L., Sinding M.S., Ersmark E., Pfrengle S., Cassatt-Johnstone M., Lebrasseur O., Girdland-Flink L. (2022). Grey wolf genomic history reveals a dual ancestry of dogs. Nature.

[8] Meadows J.R.S., Kidd J.M., Wang G.D., Parker H.G., Schall P.Z., Bianchi M., Christmas M.J., Bougiouri K., Buckley R.M., Hitte C. (2023). Genome sequencing of 2000 canids by the Dog10K consortium advances the understanding of demography, genome function and architecture. Genome Biol..

[9] Perri A.R., Feuerborn T.R., Frantz L.A.F., Larson G., Malhi R.S., Meltzer D.J., Witt K.E. (2021). Dog domestication and the dual dispersal of people and dogs into the Americas. Proc. Natl. Acad. Sci. USA.

[10] Feuerborn T.R., Carmagnini A., Losey R.J., Nomokonova T., Askeyev A., Askeyev I., Askeyev O., Antipina E.E., Appelt M., Bachura O.P. (2021). Modern Siberian dog ancestry was shaped by several thousand years of Eurasian-wide trade and human dispersal. Proc. Natl. Acad. Sci. USA.

[11] Tancredi D., Cardinali I. (2023). Being a Dog: A Review of the Domestication Process. Genes.

[12] Halfvarson J., Brislawn C.J., Lamendella R., Vázquez-Baeza Y., Walters W.A., Bramer L.M., D’Amato M., Bonfiglio F., McDonald D., Gonzalez A. (2017). Dynamics of the human gut microbiome in inflammatory bowel disease. Nat. Microbiol..

[13] Moeller A.H., Sanders J.G. (2020). Roles of the gut microbiota in the adaptive evolution of mammalian species. Philos. Trans. R. Soc. Lond. B Biol. Sci..

[14] Dillard B.A., Chung A.K., Gunderson A.R., Campbell-Staton S.C., Moeller A.H. (2022). Humanization of wildlife gut microbiota in urban environments. eLife.

[15] Arendt M., Cairns K.M., Ballard J.W., Savolainen P., Axelsson E. (2016). Diet adaptation in dog reflects spread of prehistoric agriculture. Heredity.

[16] Corsato Alvarenga I., Aldrich C.G. (2020). Starch characterization of commercial extruded dry pet foods. Transl. Anim. Sci..

[17] Richards T.L., Rankovic A., Cant J.P., Shoveller A.K., Adolphe J.L., Ramdath D., Verbrugghe A. (2021). Effect of Total Starch and Resistant Starch in Commercial Extruded Dog Foods on Gastric Emptying in Siberian Huskies. Animals.

[18] Yang Q., Wu Z. (2023). Gut Probiotics and Health of Dogs and Cats: Benefits, Applications, and Underlying Mechanisms. Microorganisms.

[19] Salminen S., Collado M.C., Endo A., Hill C., Lebeer S., Quigley E.M.M., Sanders M.E., Shamir R., Swann J.R., Szajewska H. (2021). The International Scientific Association of Probiotics and Prebiotics (ISAPP) consensus statement on the definition and scope of postbiotics. Nat. Rev. Gastroenterol. Hepatol..

[20] Schmitz S.S. (2021). Value of Probiotics in Canine and Feline Gastroenterology. Vet. Clin. N. Am. Small Anim. Pract..

[21] Lee D., Goh T.W., Kang M.G., Choi H.J., Yeo S.Y., Yang J., Huh C.S., Kim Y.Y., Kim Y. (2022). Perspectives and advances in probiotics and the gut microbiome in companion animals. J. Anim. Sci. Technol..

[22] Miller E.A., Amato R., Ponder J.B., Bueno I. (2024). Survey of antimicrobial and probiotic use practices in wildlife rehabilitation in the United States. PLoS ONE.

[23] Ash C., Priest F.G., Collins M.D. (1993). Molecular identification of rRNA group 3 bacilli (Ash, Farrow, Wallbanks and Collins) using a PCR probe test. Proposal for the creation of a new genus Paenibacillus. Antonie Van Leeuwenhoek.

[24] Information A. (1994). Validation of the Publication of New Names and New Combinations Previously Effectively Published Outside the IJSB: List No. 51. Int. J. Syst. Bacteriol..

[25] Grady E.N., MacDonald J., Liu L., Richman A., Yuan Z.C. (2016). Current knowledge and perspectives of *Paenibacillus*: A review. Microb. Cell Factories.

[26] Plouhinec L., Neugnot V., Lafond M., Berrin J.G. (2023). Carbohydrate-active enzymes in animal feed. Biotechnol. Adv..

[27] Sureshkumar S., Song J., Sampath V., Kim I. (2023). Exogenous Enzymes as Zootechnical Additives in Monogastric Animal Feed: A Review. Agriculture.

[28] McCabe J., Bryant J.L., Klews C.C., Johnson M., Atchley A.N., Cousins T.W., Dominguez A., Gabriel M., Middleton K., Bowles N.A. (2023). Phenotypic and Draft Genome Sequence Analyses of a *Paenibacillus* sp. isolated from the Gastrointestinal Tract of a North American Gray Wolf (*Canis lupus*). Appl. Microbiol..

[29] Atarashi K., Tanoue T., Shima T., Imaoka A., Kuwahara T., Momose Y., Cheng G., Yamasaki S., Saito T., Ohba Y. (2011). Induction of colonic regulatory T cells by indigenous *Clostridium* species. Science.

[30] Chapin K., Murray P., Murray P.R., Baron E.J., Pfaller M.A., Tenover F.C., Yolken R.H. (1999). Manual of Clinical Microbiology.

[31] Mahon C.R., Lehman D.C., Manuselis G. (2014). Textbook of Diagnostic Microbiology.

[32] CLSI (2022). Performance Standards for Antimicrobial Susceptibility Testing M100.

[33] Hardy B.L., Bansal G., Hewlett K.H., Arora A., Schaffer S.D., Kamau E., Bennett J.W., Merrell D.S. (2020). Antimicrobial Activity of Clinically Isolated Bacterial Species Against *Staphylococcus aureus*. Front. Microbiol..

[34] Kolmogorov M., Yuan J., Lin Y., Pevzner P.A. (2019). Assembly of long, error-prone reads using repeat graphs. Nat. Biotechnol..

[35] Li H. (2014). Toward a better understanding of artifacts in variant calling from high-coverage samples. Bioinformatics.

[36] Wick R.R., Holt K.E. (2022). Polypolish: Short-read polishing of long-read bacterial genome assemblies. PLoS Comput. Biol..

[37] Parks D.H., Imelfort M., Skennerton C.T., Hugenholtz P., Tyson G.W. (2015). CheckM: Assessing the quality of microbial genomes recovered from isolates, single cells, and metagenomes. Genome Res..

[38] Seemann T. (2014). Prokka: Rapid prokaryotic genome annotation. Bioinformatics.

[39] Cumsille A., Durán R.E., Rodríguez-Delherbe A., Saona-Urmeneta V., Cámara B., Seeger M., Araya M., Jara N., Buil-Aranda C. (2023). GenoVi, an open-source automated circular genome visualizer for bacteria and archaea. PLoS Comput. Biol..

[40] Kim J., Na S.I., Kim D., Chun J. (2021). UBCG2: Up-to-date bacterial core genes and pipeline for phylogenomic analysis. J. Microbiol..

[41] Chalita M., Kim Y.O., Park S., Oh H.S., Cho J.H., Moon J., Baek N., Moon C., Lee K., Yang J. (2024). EzBioCloud: A genome-driven database and platform for microbiome identification and discovery. Int. J. Syst. Evol. Microbiol..

[42] Katoh K., Standley D.M. (2013). MAFFT multiple sequence alignment software version 7: Improvements in performance and usability. Mol. Biol. Evol..

[43] Kozlov A.M., Darriba D., Flouri T., Morel B., Stamatakis A. (2019). RAxML-NG: A fast, scalable and user-friendly tool for maximum likelihood phylogenetic inference. Bioinformatics.

[44] Langmead B., Salzberg S.L. (2012). Fast gapped-read alignment with Bowtie 2. Nat. Methods.

[45] Li H., Handsaker B., Wysoker A., Fennell T., Ruan J., Homer N., Marth G., Abecasis G., Durbin R. (2009). 1000 Genome Project Data Processing Subgroup. The Sequence Alignment/Map format and SAMtools. Bioinformatics.

[46] Liu B., Zheng D., Jin Q., Chen L., Yang J. (2019). VFDB 2019: A comparative pathogenomic platform with an interactive web interface. Nucleic Acids Res..

[47] Yoon S.H., Ha S.M., Kwon S., Lim J., Kim Y., Seo H., Chun J. (2017). Introducing EzBioCloud: A taxonomically united database of 16S rRNA gene sequences and whole-genome assemblies. Int. J. Syst. Evol. Microbiol..

[48] Yoon S.H., Ha S.M., Lim J., Kwon S., Chun J. (2017). A large-scale evaluation of algorithms to calculate average nucleotide identity. Antonie Van Leeuwenhoek.

[49] Paradis E., Schliep K. (2019). ape 5.0: An environment for modern phylogenetics and evolutionary analyses in R. Bioinformatics.

[50] Treangen T.J., Ondov B.D., Koren S., Phillippy A.M. (2014). The Harvest suite for rapid core-genome alignment and visualization of thousands of intraspecific microbial genomes. Genome Biol..

[51] Meier-Kolthoff J.P., Carbasse J.S., Peinado-Olarte R.L., Göker M. (2022). TYGS and LPSN: A database tandem for fast and reliable genome-based classification and nomenclature of prokaryotes. Nucleic Acids Res..

[52] Kumar S., Stecher G., Suleski M., Sanderford M., Sharma S., Tamura K. (2024). MEGA12: Molecular Evolutionary Genetic Analysis version 12 for adaptive and green computing. Mol. Biol. Evol..

[53] Blin K., Shaw S., Medema M.H., Weber T. (2024). The antiSMASH database version 4: Additional genomes and BGCs, new sequence-based searches and more. Nucleic Acids Res..

[54] Wishart D.S., Han S., Saha S., Oler E., Peters H., Grant J.R., Stothard P., Gautam V. (2023). PHASTEST: Faster than PHASTER, better than PHAST. Nucleic Acids Res..

[55] Ero R., Yan X.F., Gao Y.G. (2021). Ribosome Protection Proteins-”New” Players in the Global Arms Race with Antibiotic-Resistant Pathogens. Int. J. Mol. Sci..

[56] Trimble M.J., Mlynárčik P., Kolář M., Hancock R.E. (2016). Polymyxin: Alternative Mechanisms of Action and Resistance. Cold Spring Harb. Perspect. Med..

[57] Slingerland C.J., Martin N.I. (2024). Recent Advances in the Development of Polymyxin Antibiotics: 2010–2023. ACS Infect. Dis..

[58] Baindara P., Nayudu N., Korpole S. (2020). Whole genome mining reveals a diverse repertoire of lanthionine synthetases and lanthipeptides among the genus *Paenibacillus*. J. Appl. Microbiol..

[59] Cheng C., Hua Z.C. (2020). Lasso Peptides: Heterologous Production and Potential Medical Application. Front. Bioeng. Biotechnol..

[60] Barrett S.E., Mitchell D.A. (2024). Advances in lasso peptide discovery, biosynthesis, and function. Trends Genet..

[61] Sirota-Madi A., Olender T., Helman Y., Brainis I., Finkelshtein A., Roth D., Hagai E., Leshkowitz D., Brodsky L., Galatenko V. (2012). Genome sequence of the pattern-forming social bacterium *Paenibacillus dendritiformis* C454 chiral morphotype. J. Bacteriol..

[62] Zhu S., Hegemann J.D., Fage C.D., Zimmermann M., Xie X., Linne U., Marahiel M.A. (2016). Insights into the Unique Phosphorylation of the Lasso Peptide Paeninodin. J. Biol. Chem..

[63] Kumar M., Chakdar H., Pandiyan K., Thapa S., Shahid M., Singh A., Srivastava A.K., Saxena A.K. (2022). Bacterial chitinases: Genetics, engineering and applications. World J. Microbiol. Biotechnol..

[64] Wu L., Luo Y. (2021). Bacterial quorum-sensing systems and their role in intestinal bacteria-host crosstalk. Front. Microbiol..

[65] Piewngam P., Zheng Y., Nguyen T.H., Dickey S.W., Joo H.S., Villaruz A.E., Glose K.A., Fisher E.L., Hunt R.L., Li B. (2018). Pathogen elimination by probiotic *Bacillus* via signalling interference. Nature.

[66] Li E., Liu K., Yang S., Li L., Ran K., Sun X., Qu J., Zhao L., Xin Y., Zhu F. (2024). Analysis of the complete genome sequence of *Paenibacillus* sp. lzh-N1 reveals its antagonistic ability. BMC Genom..

[67] Perry E.B., Valach A.A., Fenton J.M., Moore G.E. (2022). An Assessment of Starch Content and Gelatinization in Traditional and Non-Traditional Dog Food Formulations. Animals.

[68] Reese A.T., Chadaideh K.S., Diggins C.E., Schell L.D., Beckel M., Callahan P., Ryan R., Emery Thompson M., Carmody R.N. (2021). Effects of domestication on the gut microbiota parallel those of human industrialization. eLife.

[69] Lyu T., Liu G., Zhang H., Wang L., Zhou S., Dou H., Pang B., Sha W., Zhang H. (2018). Changes in feeding habits promoted the differentiation of the composition and function of gut microbiotas between domestic dogs (*Canis lupus familiaris*) and gray wolves (*Canis lupus*). AMB Express.

[70] Xu J., Becker A.A.M.J., Luo Y., Zhang W., Ge B., Leng C., Wang G., Ding L., Wang J., Fu X. (2021). The Fecal Microbiota of Dogs Switching to a Raw Diet Only Partially Converges to That of Wolves. Front. Microbiol..

[71] Chen L., Sun M., Xu D., Gao Z., Shi Y., Wang S., Zhou Y. (2022). Gut microbiome of captive wolves is more similar to domestic dogs than wild wolves indicated by metagenomics study. Front. Microbiol..

[72] Wang G., Chen Y., Xia Y., Song X., Ai L. (2022). Characteristics of Probiotic Preparations and Their Applications. Foods.

[73] Xia J., Cui Y., Guo Y., Liu Y., Deng B., Han S. (2024). The Function of Probiotics and Prebiotics on Canine Intestinal Health and Their Evaluation Criteria. Microorganisms.

