# Supplementary material for: Phenotypic and Complete Reference Whole Genome Sequence Analyses of Two Paenibacillus spp. Isolates from a Gray Wolf (Canis lupus) Gastrointestinal Tract
Source: Vet Sci. 2025 Jan 13;12(1):51. doi: 10.3390/vetsci12010051 (PMC11769508; doi:10.3390/vetsci12010051)
Supplement: Supplementary file 1 [file Supplementary Table S6 ClWae17B_GenBankFile.pdf › Supplementary Table S6 ClWae17B_GenBankFile.pdf]

LOCUS: contig_1 ClWae17B 6939193 bp DNA linear 20-MAR-2024

DEFINITION as Genus species strain: *Paenibacillus taichungensis* ClWae17B

ACCESSION: The data is retrievable at the National Center for Biotechnology (NCBI) as SUB14651639, BioProject PRJNA1145425, BioSample SAMN43065442 with a Localid of ClWae17b_contig_1 with Accession CP168020 as *Paenibacillus taichungensis* ClWae17b

KEYWORDS: genomics; probiotic; canine; antimicrobial; *Paenibacillaceae*

SOURCE: North American Gray wolf (*Canis lupus*) GI tract

ORGANISM: *Paenibacillus taichungensis*

COMMENT: Annotated using prokka 1.14.6 from https://github.com/tseemann/prokka

FEATURES: Location/Qualifiers

source 1..6939193

/organism="*Paenibacillus taichungensis*"

/mol_type="genomic DNA"

/strain="strain ClWae17B"

rRNA 304..1853

/locus_tag="PIKHDJKN_00001"

/product="16S ribosomal RNA"

rRNA 2161..5082

/locus_tag="PIKHDJKN_00002"

/product="23S ribosomal RNA"

rRNA 5169..5280

/locus_tag="PIKHDJKN_00003"

/product="5S ribosomal RNA"

tRNA 5322..5395

/locus_tag="PIKHDJKN_00004"

/product="tRNA-Ile"

/inference="COORDINATES:profile:Aragorn:001002"

/note="tRNA-Ile(gat)"

tRNA 5424..5499

/locus_tag="PIKHDJKN_00005"

/product="tRNA-Ala"

/inference="COORDINATES:profile:Aragorn:001002"

/note="tRNA-Ala(tgc)"

tRNA 5501..5576

/locus_tag="PIKHDJKN_00006"

/product="tRNA-Asn"

/inference="COORDINATES:profile:Aragorn:001002"

/note="tRNA-Asn(gtt)"

tRNA 5581..5652

/locus_tag="PIKHDJKN_00007"

/product="tRNA-Glu"

/inference="COORDINATES:profile:Aragorn:001002"

/note="tRNA-Glu(ttc)"

tRNA 5777..5852

/locus_tag="PIKHDJKN_00008"

/product="tRNA-Val"

/inference="COORDINATES:profile:Aragorn:001002"

/note="tRNA-Val(tac)"

tRNA 5873..5946

/locus_tag="PIKHDJKN_00009"

/product="tRNA-Met"

/inference="COORDINATES:profile:Aragorn:001002"

/note="tRNA-Met(cat)"

tRNA 5974..6051

/locus_tag="PIKHDJKN_00010"

/product="tRNA-Asp"

/inference="COORDINATES:profile:Aragorn:001002"

/note="tRNA-Asp(gtc)"

tRNA 6262..6337

/locus_tag="PIKHDJKN_00011"

/product="tRNA-Phe"

/inference="COORDINATES:profile:Aragorn:001002"

/note="tRNA-Phe(gaa)"

tRNA 6356..6431

/locus_tag="PIKHDJKN_00012"

/product="tRNA-Thr"

/inference="COORDINATES:profile:Aragorn:001002"

/note="tRNA-Thr(tgt)"

tRNA 6441..6526

/locus_tag="PIKHDJKN_00013"

/product="tRNA-Tyr"

/inference="COORDINATES:profile:Aragorn:001002"

/note="tRNA-Tyr(gta)"

tRNA 6534..6609

/locus_tag="PIKHDJKN_00014"

/product="tRNA-Lys"

/inference="COORDINATES:profile:Aragorn:001002"

/note="tRNA-Lys(ttt)"

tRNA 6615..6699

/locus_tag="PIKHDJKN_00015"

/product="tRNA-Leu"

/inference="COORDINATES:profile:Aragorn:001002"

/note="tRNA-Leu(tag)"

tRNA 6707..6781

/locus_tag="PIKHDJKN_00016"

/product="tRNA-Gly"

/inference="COORDINATES:profile:Aragorn:001002"

/note="tRNA-Gly(gcc)"

tRNA 6789..6865

/locus_tag="PIKHDJKN_00017"

/product="tRNA-Arg"

/inference="COORDINATES:profile:Aragorn:001002"

/note="tRNA-Arg(acg)"

tRNA 6879..6959

/locus_tag="PIKHDJKN_00018"

/product="tRNA-Leu"

/inference="COORDINATES:profile:Aragorn:001002"

/note="tRNA-Leu(caa)"

tRNA 6966..7039

/locus_tag="PIKHDJKN_00019"

/product="tRNA-Pro"

/inference="COORDINATES:profile:Aragorn:001002"

/note="tRNA-Pro(tgg)"

tRNA 7049..7119

/locus_tag="PIKHDJKN_00020"

/product="tRNA-Gly"

/inference="COORDINATES:profile:Aragorn:001002"

/note="tRNA-Gly(tcc)"

CDS 7438..9303

/gene="iolD"

/locus_tag="PIKHDJKN_00021"

/EC_number="3.7.1.22"

/inference="ab initio prediction:Prodigal:002006"

/inference="similar to AA sequence:UniProtKB:P42415"

/codon_start=1

/transl_table=11

/product="3D-(3,5/4)-trihydroxycyclohexane-1,2-dione

hydrolase"

/db_xref="COG:COG3962"

/translation="MTTIRLTMAQALLRYLDQQYISVDGVETKFVKGVIGIFGHGNVT

GIGEALERSSGSLTYMQGKNEQGMVHTAAAYAKQKNRRQIYACTTSIGPGALNMITAA

ATATVNRIPVLLLPGDNFATREPDPVLQQLEVSGDYTISATDPFKAVSKYWDRIVRPE

QLMIAATQAMRVLIDPAETGAVTLALPQDVQAEAYDYPESFFARKVHYLDRRPPVQAA

IERATQQIARGRKPLIVAGGGVLYAEASTQLAEFAEAFGIPVAETQAGKSALAWDHPL

NVGAIGVTGSLAANRLAREADVVIGVGTRFSDFTTASRSAFQHPEATFININLNGMDA

AKLGGEAILADAREGLQALQTALQGRQYHSGYGTSEIADLRDEWNAEVNRLYGLQHEA

GLAQTTAVGIINRSIDPSSVIVCAAGSLPGDLHRLWRASAPKTYHMEYGFSCMGYEVS

GAFGAALAEPDREVYAMVGDGSYLMLHSELVTSLQEQKKMTILLFNNNGFQCIHNLQR

EHGSDGFGNEFRYRESESGRLTGNYMPMDFAAHARSLGAKAYKAETTEQLEQAIRDAR

NETVTTLIEIPVVPGTNAGGYESWWNVGVPEVSNEEKVVTAHHAMQANRAKARLI"

CDS 9418..10320

/gene="iolE_1"

/locus_tag="PIKHDJKN_00022"

/EC_number="4.2.1.44"

/inference="ab initio prediction:Prodigal:002006"

/inference="similar to AA sequence:UniProtKB:P42416"

/codon_start=1

/transl_table=11

/product="Inosose dehydratase"

/db_xref="COG:COG1082"

/translation="MNKLPFQLGIHPINWVGEDVREHGDATTCEQILDDIQRLGLTGT

EMGRKYPTDPDTLREELGKRNIRLVSQWKSVLFSDPAYRQSELDSYCRHAEFLQSMGS

KVISTAEVGGSLHFDPRRTPHEKEVLRLSESEWHILAKGLNEAGAIAREHGLKLTYHH

HGGTVVEQPDEIDKLMELTDPSLVYLLYDTGHAYYGGADPLALLRKHYDRIAYIHLKD

IRPQVLEEARAEQSDFVGCIRKGVFTVPGDGCIDFAPILQELVTRGYDGWAMLEGEQD

PAIHNPYEYARRSLQYMESLYQHS"

CDS 10432..11442

/gene="iolC"

/locus_tag="PIKHDJKN_00023"

/EC_number="2.7.1.92"

/inference="ab initio prediction:Prodigal:002006"

/inference="similar to AA sequence:UniProtKB:Q9KAG8"

/codon_start=1

/transl_table=11

/product="5-dehydro-2-deoxygluconokinase"

/db_xref="COG:COG0524"

/translation="MTYVSFPDSRKMDFTAIGRLCIDLNANEINRPMEETMTFTKYVG

GSPANITIGMSRLGMETAFIGKIANDQMGRFINSYLERNGIDTSNVVTDDTGAVTGLA

FTEIKSPTDCSILMYRDHVADLLLQSREVKEQLIAESKVLLISGTALAQSPSREAVFQ

ALAYAKKHGTIIVFDLDYRPYTWTSAEETAVYYNLAAEKCDIILGTREEFDMMETFDH

NQDHNDQVTAQKWFDFSAKIVVIKHGKEGSIAYTGEGLSHQADSYPARVVKTFGAGDS

YAAGFLYGLMQGWTIKRSMAFGSAAASIVISSHSCSDAMPTVEQVNDYIERCNRGEIT

VS"

CDS 11516..12991

/gene="iolA"

/locus_tag="PIKHDJKN_00024"

/EC_number="1.2.1.-"

/inference="ab initio prediction:Prodigal:002006"

/inference="similar to AA sequence:UniProtKB:P42412"

/codon_start=1

/transl_table=11

/product="Malonate-semialdehyde dehydrogenase"

/db_xref="COG:COG1012"

/translation="MGKGISEASVTATMVQNWIGGAWVTPAATRTEQVVNPATEEVIA

HVPLSEQADVDLAVQTAREAFPSWSGTPVPRRARILFRYQQLLVEHWEELARLVTLEN

GKSYAEAYGEVQRGIECVEFAAGAPNLMMGKQLPDIATGLESGMYRYPIGVIGGITPF

NFPMMVPCWMFPLAIACGNTFVLKPSERTPLLAGRLAELFKEAGLPDGVLNIVHGAHD

VVNGLLEHKDVQAISFVGSQPVAEYVYTTASAHGKRVQALAGAKNHSIVMPDADLDLT

VKEITSAAFGSAGERCMSCSVVVAVGDVADTLVQKLVEAADRITIGNGMDEGVFLGPV

IRGPHKERTLSYIESGEQEGAALIRDGRKDEATGKSGYFVGPTVFDEVQSTMKIWKDE

IFAPVLSVARVATLEEAVELANRSDFANGACLFTRSGASMRQFRETIDAGMLGINLGV

PAPMAFFPFSGWKKSFYGDLHANGTDGVEFYTRKKMVTARW"

CDS 13061..14113

/gene="iolG_1"

/locus_tag="PIKHDJKN_00025"

/EC_number="1.1.1.18"

/inference="ab initio prediction:Prodigal:002006"

/inference="similar to AA sequence:UniProtKB:Q9WYP5"

/codon_start=1

/transl_table=11

/product="Myo-inositol 2-dehydrogenase"

/db_xref="COG:COG0673"

/translation="MGKDKVRIGIIGAGRIGKIHADNLLRNPHAEIVGISDLFAGPEL

EEWASSRGIPVVTTDSSQLIAMPNVDAVLICSSTDTHVPLIEQAAQAGKHIFCEKPVS

MDLHQTQAAVAAVQKAGVKLQIGFNRRFDHNFKRVRAHVQEGTIGDPHIIKITSRDPS

PPPAEYIRVSGGIFMDMMIHDFDMARYLSGSEVEEVYAQGNVLIHPVFAEHGDVDTAI

VTMSFENGAIGVIDNSRQAVYGYDQRVEVFGSLGSAAAANDHPNTAEISTANGLMRDK

PLHFFLERYNEAYVQETALFIDAIRNDTSVIVNGNDAVQAERIALAARMSMEQGRPVK

LREVPGISVESQTVAP"

CDS 14196..15005

/gene="iolB"

/locus_tag="PIKHDJKN_00026"

/EC_number="5.3.1.30"

/inference="ab initio prediction:Prodigal:002006"

/inference="similar to AA sequence:UniProtKB:P42413"

/codon_start=1

/transl_table=11

/product="5-deoxy-glucuronate isomerase"

/db_xref="COG:COG3718"

/translation="MSERVVKPVLNPKADGTLLTVTPESAGWEYVGFQVVQLAEGQTL

TRESGDQELCIVLLSGLANVSTREYTWENIGKRMSVFEKIPPYSIYVSSSDRVEFTAL

TALEIAICAAPGKGTYPARLIAPEDVGVETRGYGNLERQIHNILPEQKEADSLLVVEV

FTPDGHWSSYPPHKHDRDALPDESLLEETYYFRVQPEQGFAIQRIYTDDRAVDETLAV

NNGEVVLVPCGYHPVGAPPGYEVYYLNVMAGPTRTWKFHNDPDHDWLMNKK"

CDS 15109..16122

/gene="degA_1"

/locus_tag="PIKHDJKN_00027"

/inference="ab initio prediction:Prodigal:002006"

/inference="similar to AA sequence:UniProtKB:P37947"

/codon_start=1

/transl_table=11

/product="HTH-type transcriptional regulator DegA"

/db_xref="COG:COG1609"

/translation="MKATIYDIAREAGVSIATVSQVINGKGKISEKRRAEIMEIMERL

HYQPSAIAAALTGKQTYTLGLLVPDISNPYFAELARAVEDRSRQLGYSVVICSTDNKD

ERVERYLNLLQQKRVDGMMIGTGIDNAEILSPLLQQSIPVALIARHMPSLSVHTVAID

DRLGGALAAQHLLELGHTHVAVLSESFKVSSSQERVRGFREALEKEGFSLEADQVRES

SADLGSAKKEALVLLTKKNRATGIFCCNDMQAIGALQAAKELGLRVPEDVSIIGFDNT

ILASVTNPPLTTVAQPIEDLGRRAVDLLIDELKDEGNSPRKIVLKPELVIRDSTGNVS

NQS"

CDS 17424..17933

/locus_tag="PIKHDJKN_00028"

/inference="ab initio prediction:Prodigal:002006"

/codon_start=1

/transl_table=11

/product="hypothetical protein"

/translation="MKKRTKTILGVVVVIVIAAIAIPMYISRQHTTFRAEVTDHMSML

DTLDILVIRKITTTDPYDEEEVIIKGPQEIRNMLKLAKDIELRRLKHTDISERFEGNP

YYYDWQLRTKKEDRFTEGFGITFYNQHAVSIYSGYKTRDKLENYEITNDFNLNEMEQL

FTNLKEEKE"

CDS 17930..18424

/locus_tag="PIKHDJKN_00029"

/inference="ab initio prediction:Prodigal:002006"

/codon_start=1

/transl_table=11

/product="hypothetical protein"

/translation="MTEYQLRPIEERDIPFLWEMLYASLFKREGEEPFEPEIIHSPGM

SKYVEGWGREGDFGFIAVDSQGKRMGSLTLRFFNDQNAGYGYVNADTPEMGMAVVETA

RGKGIGTRLIQEALEEARKRGIDAVSLSVDPDNEAIRLYRRFGFVEQGMCGTSVTMVC

VINP"

CDS complement(18561..19361)

/locus_tag="PIKHDJKN_00030"

/inference="ab initio prediction:Prodigal:002006"

/codon_start=1

/transl_table=11

/product="hypothetical protein"

/translation="MGLFDKLRRRKKENATASGAVHTAPYSETIVGFVLLEHEDCNFD

HFINTMKNEWNIEIEERPEEGNLFFEVDGMQVVCAHINAPIPDHEVEENAKLNVLWQD

AVQVTSRHQSQIIVSVLNATDAIQAHILFTQTASALLQLDHALAIYMAPLVVEAGQYV

DISRSIKDDELPVSLWIFIGLFQSPEGASAYTYGLRNFGKEEMEITQSAKPLSEVFEM

MYMTATYVVENDVTLHDGETLGFSAEQKLPINRPEGIATEGNSLKIDF"

CDS complement(19481..19846)

/locus_tag="PIKHDJKN_00031"

/inference="ab initio prediction:Prodigal:002006"

/codon_start=1

/transl_table=11

/product="hypothetical protein"

/translation="MFLLISIIFVAIVAIEHFYIMVMEMFLWTRPRTMKTFGLTPELA

ESTKSLAANQGLYNGFLAAGLIWGLVYPDASVGQHIQIFFLACVIIAAIYGGVTATRS

IIIKQGLPAIIALLLVLFL"

CDS 20099..20431

/gene="yuaD"

/locus_tag="PIKHDJKN_00032"

/inference="ab initio prediction:Prodigal:002006"

/inference="similar to AA sequence:UniProtKB:O32079"

/codon_start=1

/transl_table=11

/product="Putative metal-sulfur cluster biosynthesis

proteins YuaD"

/translation="MWEPFNSSCWRMIHQRLEWLGANLLVRGVEKLTELPFGTRLLFP

DGTGLICEGENLPCVHPGKIIEQVYEQDGLRKKFVPAARKKRGIVCSVESEGVIRTGD

AIEIIHLS"

CDS 20454..20747

/locus_tag="PIKHDJKN_00033"

/inference="ab initio prediction:Prodigal:002006"

/codon_start=1

/transl_table=11

/product="hypothetical protein"

/translation="MQSSPFVSTVVNLSLSSGMDVIFRCASDNGLEVFDKMRLIVISK

VLGQPNQIAIHTPLHQLSSILQAVELDHHFGADAHILIEKALELSCVHIPGGR"

CDS complement(20661..21305)

/locus_tag="PIKHDJKN_00034"

/inference="ab initio prediction:Prodigal:002006"

/codon_start=1

/transl_table=11

/product="hypothetical protein"

/translation="MKHADHAVRPSMGLLNLDEGDNRYCLTRYAPSEALSSLVKHFWI

VSWDLTGHDPYPQHVVPNPCVNLVVERGNTFFFGPSGQKFSYLVRGKGSVFGVKFKPG

GFYPFIRTPVSTLYGNPLNVSGVLDVDAPLLEERLLGDGSDADKVSYMDQLLCKHLPP

PDDQAILVSQIVLYIEQHRDMLRVDDLSASWNMHTRKLQRLFNQYVGISPKMVI"

CDS complement(21302..21847)

/gene="cmk_1"

/locus_tag="PIKHDJKN_00035"

/EC_number="2.7.4.25"

/inference="ab initio prediction:Prodigal:002006"

/inference="protein motif:HAMAP:MF_00239"

/codon_start=1

/transl_table=11

/product="Cytidylate kinase"

/translation="MVGEESKIKFRPNKIHIIGSVGSGKSTLARHLSTRLDLPYYELD

NIVWHRVPQGQDIRNSPETRDTLLQKAVHSNQWIIEGVHYEWVARSFEQADLIVYLNT

PVWKRNVRILKRFTVQKLGLEQGNYRQTFTMLWRMYKWSYQHDRKEKALIMAFLQPHQ

HKLCIVRNVAELTRYLEESAK"

CDS complement(21872..22609)

/gene="tagA_1"

/locus_tag="PIKHDJKN_00036"

/EC_number="2.4.1.187"

/inference="ab initio prediction:Prodigal:002006"

/inference="similar to AA sequence:UniProtKB:P27620"

/codon_start=1

/transl_table=11

/product="N-acetylglucosaminyldiphosphoundecaprenol

N-acetyl-beta-D-mannosaminyltransferase"

/db_xref="COG:COG1922"

/translation="MNQTTKIMGIPFPNITMDQTVTILGDVIYQRQSELFHVITGNPE

IVMSCQKDQTLRSVVDQAGIVTADGAGIVMVSRFRGGQLTERVTGCDLLFRLLEEANR

KNWSFYMLGAEETVSKRAVEVIAQNYPGVIVRGRQHGFFTTDEEQSIVEEIHEAQPDF

LIVALGAPNAEHWINKYRHQLNARVAIGVGGSLDILAGKTKRAPAIWQKLNLEWLYRL

LSQPSRWRRQLILPRFAVRALLFREQR"

CDS 22860..23309

/locus_tag="PIKHDJKN_00037"

/inference="ab initio prediction:Prodigal:002006"

/codon_start=1

/transl_table=11

/product="hypothetical protein"

/translation="MELKYEFYINAGQEDVWNALISPDGTRSSFFGSELRSNFQPGQP

FAYVGPGNDGAETVHVYGDILEFEPLSLLSYLEHPGPSYHANHAELQSRVVFQLETVG

ECTKLTLINDQFTDNHPSFANAQSNWWMILSSIKTWVETGKTLNFGW"

CDS complement(23503..24918)

/gene="pyk_1"

/locus_tag="PIKHDJKN_00038"

/EC_number="2.7.1.40"

/inference="ab initio prediction:Prodigal:002006"

/inference="similar to AA sequence:UniProtKB:P80885"

/codon_start=1

/transl_table=11

/product="Pyruvate kinase"

/db_xref="COG:COG0469"

/translation="MLKTKIICTMGPACDSIELLKVMIQEGMTVARLNMAHGELEDHV

ARINNIRKAASELNTYVPIMMDIKGPEVRIGKLKEASCHLQAGKELILTTEEILGDAE

RISVNYPELNLVVKPGDRILIDDGLVDLTVLSVEGSDIHCKIISGGILKPRKGVNLPG

IKTTLPGVTERDVMHIGFGIENGIEIIAASFVRKGDDIREIRSILKERGVDHVQIISK

IENQEGMTNLDDIIEASDGIMVARGDLGVEVPIEDVPMMQKEMIDKCNRAGKPVIVAT

HMLESMQVNPRPTRSEVSDVANAVLQGADVVMLSGESAAGKYPVQSVRTMAAVARRAE

TMIDYKEQFNQKSAQQVADITEVISQGAVSSSLVLNAKAIITSTESGFTARMISKYRP

KAPIIAVTQHEEVLAKICLLSGVIPVMGDKVTTTDEMFESATRNAIKTGYIEKGDIIV

LSAGVPIGQSGNTNLIKVQQV"

CDS 25031..25810

/gene="rhaR_1"

/locus_tag="PIKHDJKN_00039"

/inference="ab initio prediction:Prodigal:002006"

/inference="protein motif:HAMAP:MF_01533"

/codon_start=1

/transl_table=11

/product="HTH-type transcriptional activator RhaR"

/translation="MVTPLEVRHINGVGWYEEAVQSKATWRLSLVTYGKCVYWVNGEK

QIMEKGELLLIPAGIPYYGKSIPTVTHTQIVVQLQGDHGQGLPALERSEALRHKPGCY

ELIHERMKAIQQQWQERPSYYVMMSQALLMEVLIYINRELDRGVIPPERHVHAERMKR

YIARHYREKVTKEELGDEIGKTPNYAAALFKSMTNQTISQYVHDQRMKRAVYLLTESQ

LSIQEIAEFLGYRDLSYFYRIFKRLMGTPPSDLLHERPPIV"

CDS complement(25957..26628)

/locus_tag="PIKHDJKN_00040"

/inference="ab initio prediction:Prodigal:002006"

/codon_start=1

/transl_table=11

/product="hypothetical protein"

/translation="MKALFLRWGHLLAILCIPLQGSIYVLLGSNTGSDVFYNYAWIDT

QIPFIKEFIYPYVSWMPILYLGFLYLGLTNKSLFWRMIITYNVGVIAANIVFAVFPTY

VPRPEAIGTGLSSALVQFIYTNDAPFNCFPSIHCLTSYLLFIIINRHLNFKPLARISW

SIWLWLIIASTVFVKQHSLLDVAGGILFAEAAYWAVHVFAVRAGLANKKTKQPITATN

TSSNA"

CDS 26917..27702

/locus_tag="PIKHDJKN_00041"

/inference="ab initio prediction:Prodigal:002006"

/codon_start=1

/transl_table=11

/product="hypothetical protein"

/translation="MKTPQSRQNQSFSTRRPVLTVVIIELLLLLAVFAAGAVATVKQL

DYTSPVLMSFTPIALVLIIYLTMRRKWGETGFRSLRTIPASHAKYYIPLLLVLGTIAL

KGFTELTLSKVTFFIFFTLLVAFVEETIYRGLIFKTLLRKSAVAAVVTSSVLFSVTHI

MNALSGQSMADTILQLIYALLLGAALALLMLKNGNIVPLILFHFIHNLIQFLGDDRQD

TGTLPYDIFILVVLVAYCVWLTFSVRTKLSSSESSTPGSSVVH"

CDS 27848..28084

/locus_tag="PIKHDJKN_00042"

/inference="ab initio prediction:Prodigal:002006"

/codon_start=1

/transl_table=11

/product="hypothetical protein"

/translation="MEKTAQTVAEWMVQEIKFTGTLHQEAAIEYVKSNFGEEFVFVNE

NGNTSLSKEVKKAFRKLHRGQIAWDRDAFMWAWT"

CDS 28473..28673

/gene="cspB_1"

/locus_tag="PIKHDJKN_00043"

/inference="ab initio prediction:Prodigal:002006"

/inference="similar to AA sequence:UniProtKB:P41016"

/codon_start=1

/transl_table=11

/product="Cold shock protein CspB"

/translation="MQNGTVKWFNADKGFGFIEVEGGEDVFVHFSAITGEGFKTLDEG

QRVQFKIVQGNRGPQAEEVVKL"

CDS complement(28674..29093)

/locus_tag="PIKHDJKN_00044"

/inference="ab initio prediction:Prodigal:002006"

/codon_start=1

/transl_table=11

/product="hypothetical protein"

/translation="MLNPISFRHRFQITIRIFKQWQHPGRANHLRSTYRTSRHAIKRK

IVPHPSFARIARTFPDCRIRLWNLFQRFFAIIHDFYPLCYGKTSNPTLFHRSHYLAEV

SSRPDFCLDNYWADFHYNCSKKRSCLYHYNMLKAAML"

CDS complement(29396..29755)

/locus_tag="PIKHDJKN_00045"

/inference="ab initio prediction:Prodigal:002006"

/codon_start=1

/transl_table=11

/product="hypothetical protein"

/translation="MYRINPVCEETVSPHVGRSVCLVMNDGKHMYGTLSQCRDGKVYL

NGCFEGPRLCSVKSKQQLVKSSKKNAVATKKVKSSAYGYGGYGGGYGRGYGGYGSGAG

IELALVATLFLLPFLFI"

CDS 29919..30659

/gene="COQ5_1"

/locus_tag="PIKHDJKN_00046"

/EC_number="2.1.1.163"

/inference="ab initio prediction:Prodigal:002006"

/inference="protein motif:HAMAP:MF_01813"

/codon_start=1

/transl_table=11

/product="2-methoxy-6-polyprenyl-1,4-benzoquinol

methylase, mitochondrial"

/translation="MDYHDLLARLGEGSAHPGGFTATMQLLDTLSLPAHSKVLEVGCG

TGRTACYLARNGYRVTAVDLNRQMLEKAVRRARRERLDIRFIQADVRSLPFPDHYFDV

VFVESVTIFTQWRSTLAEYKRVLKPGGLFIDREMVLSGRKTEVMSRRLKQFYGIRTLA

TMTTWKKRLRQSGFTEVLVKEQSRSMGKWGVDHDPHREMDMSLFEDEQVQRMGRTNDR

LLARYGKRLGYAVFEARVPMENGNTQKE"

CDS complement(30858..31502)

/gene="glaR_1"

/locus_tag="PIKHDJKN_00047"

/inference="ab initio prediction:Prodigal:002006"

/inference="similar to AA sequence:UniProtKB:P37338"

/codon_start=1

/transl_table=11

/product="HTH-type transcriptional repressor GlaR"

/db_xref="COG:COG1802"

/translation="MEAPKYRSLKDHVYDYIAQKIQDGTLLPNQKINEAEICKKLDIS

RTPTREALFQLTSDNLLQYIPRRGFIVAPFDAGKKLEFSQAIGALEALAATLAADHLK

PSELMEMEALVARMDEDITQLDLAAYSKNQYQFHNLYIQRCGNATVIEMLNTLKNSFI

RQSYVSDNKPKLSSVLNEVNEEHRQILSAFRAKDKPQLEALLKHHWRIIDNDML"

CDS 31818..32042

/gene="occQ"

/locus_tag="PIKHDJKN_00048"

/inference="ab initio prediction:Prodigal:002006"

/inference="similar to AA sequence:UniProtKB:P0A4N5"

/codon_start=1

/transl_table=11

/product="Octopine transport system permease protein OccQ"

/translation="MLPAIGNEFIIIIKDSSLVPMIGIAEIIYTARAVQGITFQPLAP

LLVAAALYFVITFTLANLLTWLEHRLSISR"

CDS 32109..33374

/gene="rocD"

/locus_tag="PIKHDJKN_00049"

/EC_number="2.6.1.13"

/inference="ab initio prediction:Prodigal:002006"

/inference="similar to AA sequence:UniProtKB:P38021"

/codon_start=1

/transl_table=11

/product="Ornithine aminotransferase"

/db_xref="COG:COG4992"

/translation="MVVAKHTLILYFVYNIIKEGCWEYMSDSKVLMDWSERYAAPNYH

PLPIVIEQAEGVWVEDPEGRRYMDMLSAYSALNHGHRHPAIIQALKDQADRVTLTSRA

FHSSAASLFYQKLSQFTGKPKILAMNTGAEAVETAVKAVRRWAYRCKDVSENQAEIIV

CSGNFHGRTLTVTSFSSSEEYKKDFGPFTPGFKIIPYGDIEALKLAITPNTAAFLVEP

IQGEAGIVIPPEGYLAEAFTLCKQHRVLTVADEIQTGFGRTGRRFASDWEGIEPDIWI

MGKALGGGVMPISAIAANAEILDLFEPGSHGSTFGGNPLACAVAVAALKVLEDEKLAE
RSERLGNYFMKQLQGIRSSAVREIRGRGLFIGVDLHVPARSYCEKLMSVGLLCKETHE
TTIRFAPPLTITEAEIDWALERIAQVLGD"

CDS 33429..34397

/gene="rocF"

/locus_tag="PIKHDJKN_00050"

/EC_number="3.5.3.1"

/inference="ab initio prediction:Prodigal:002006"

/inference="similar to AA sequence:UniProtKB:Q7M0Z3"

/codon_start=1

/transl_table=11

/product="Arginase"

/db_xref="COG:COG0010"

/translation="MGNNDIESAGTLNASNETSQLLLRHHDVAIIKVPFGLGGARGGA
ELGPDELITAGLKREIASLGLNVSKEIRVDCPSESVAPIECNRVKYLSEVRQVSERVC
NEVSGAISGGAFPLVLGGDHSVAIGSLAGVSAHYSNLGVIWFDAHADLNTEERSLSGN
MHGMSVAAALGHSAFNLSHIPGAGSFVNPSNLVYIGLRDLDVYEKEQIRALGIKSFTM
HDIDRIGIQQVVEQALAVVGEGTDGIHISFDMDCLDPREAPGVGTPVPGGLNYREAHF
ALELLASTNRVTSMDLVEVNPLFDQNRHTARLGVELIASLLGKRIL"

rRNA 35094..36643

/locus_tag="PIKHDJKN_00051"

/product="16S ribosomal RNA"

rRNA 36955..39875

/locus_tag="PIKHDJKN_00052"

/product="23S ribosomal RNA"

rRNA 39961..40072

/locus_tag="PIKHDJKN_00053"

/product="5S ribosomal RNA"

tRNA 40133..40209

/locus_tag="PIKHDJKN_00054"

/product="tRNA-Ile"

/inference="COORDINATES:profile:Aragorn:001002"

/note="tRNA-Ile(gat)"

tRNA 40236..40311

/locus_tag="PIKHDJKN_00055"

/product="tRNA-Ala"

/inference="COORDINATES:profile:Aragorn:001002"

/note="tRNA-Ala(tgc)"

tRNA 40313..40388

/locus_tag="PIKHDJKN_00056"

/product="tRNA-Asn"

/inference="COORDINATES:profile:Aragorn:001002"

/note="tRNA-Asn(gtt)"

tRNA 40393..40484

/locus_tag="PIKHDJKN_00057"

/product="tRNA-Ser"

/inference="COORDINATES:profile:Aragorn:001002"

/note="tRNA-Ser(gga)"

tRNA 40503..40576

/locus_tag="PIKHDJKN_00058"

/product="tRNA-Glu"

/inference="COORDINATES:profile:Aragorn:001002"

/note="tRNA-Glu(ttc)"

tRNA 40583..40658

/locus_tag="PIKHDJKN_00059"

/product="tRNA-Val"

/inference="COORDINATES:profile:Aragorn:001002"

/note="tRNA-Val(tac)"

tRNA 40678..40751

/locus_tag="PIKHDJKN_00060"

/product="tRNA-Met"

/inference="COORDINATES:profile:Aragorn:001002"

/note="tRNA-Met(cat)"

tRNA 40779..40856

/locus_tag="PIKHDJKN_00061"

/product="tRNA-Asp"

/inference="COORDINATES:profile:Aragorn:001002"

/note="tRNA-Asp(gtc)"

tRNA 40885..40960

/locus_tag="PIKHDJKN_00062"

/product="tRNA-Thr"

/inference="COORDINATES:profile:Aragorn:001002"

/note="tRNA-Thr(tgt)"

tRNA 41058..41133

/locus_tag="PIKHDJKN_00063"

/product="tRNA-His"

/inference="COORDINATES:profile:Aragorn:001002"

/note="tRNA-His(gtg)"

tRNA 41145..41219

/locus_tag="PIKHDJKN_00064"

/product="tRNA-Gln"

/inference="COORDINATES:profile:Aragorn:001002"

/note="tRNA-Gln(ttg)"

tRNA 41236..41311

/locus_tag="PIKHDJKN_00065"

/product="tRNA-Lys"

/inference="COORDINATES:profile:Aragorn:001002"

/note="tRNA-Lys(ttt)"

tRNA 41321..41407

/locus_tag="PIKHDJKN_00066"

/product="tRNA-Leu"

/inference="COORDINATES:profile:Aragorn:001002"

/note="tRNA-Leu(cag)"

tRNA 41416..41490

/locus_tag="PIKHDJKN_00067"

/product="tRNA-Gly"

/inference="COORDINATES:profile:Aragorn:001002"

/note="tRNA-Gly(gcc)"

tRNA 41497..41573

/locus_tag="PIKHDJKN_00068"

/product="tRNA-Arg"

/inference="COORDINATES:profile:Aragorn:001002"

/note="tRNA-Arg(acg)"

tRNA 41589..41664

/locus_tag="PIKHDJKN_00069"

/product="tRNA-Pro"

/inference="COORDINATES:profile:Aragorn:001002"

/note="tRNA-Pro(tgg)"

tRNA 41673..41743

/locus_tag="PIKHDJKN_00070"

/product="tRNA-Gly"

/inference="COORDINATES:profile:Aragorn:001002"

/note="tRNA-Gly(tcc)"

CDS 41831..42469

/locus_tag="PIKHDJKN_00071"

/inference="ab initio prediction:Prodigal:002006"

/codon_start=1

/transl_table=11

/product="hypothetical protein"

/translation="MKNKIEAPKIPLELEDLSLTLEAWESKEEIKHGRFTNISIENQS

ASRVSFDKVVFENVIFSQSSLPEVELTDVIFDKCDLSNMDFNGSFLHRTEFRNCKLLG

TNFANSRLQNVSFQGCVADLSLFRFSKFKQVKYTECSLVSGDFFQATAQKLEFSECNI

DQALLSGVKLKGVDLSDCSFERLHVDIEDLNGCIISDAQAASFVGLLGLIVK"

CDS 42557..43162

/locus_tag="PIKHDJKN_00072"

/inference="ab initio prediction:Prodigal:002006"

/codon_start=1

/transl_table=11

/product="hypothetical protein"

/translation="MKRALVIGGHGTLGKAVVAKLQEYSVEVITAGRQSGDYQVDMTS

TESITSLFETVTNIDYVIVAAGQTHYAKLEELTPENNMISVQGKLLGQVNIVLIGQHY

INDMGSFTLVSGIIQDHPILKGASSAMVNGAIDSFAKAAAYELPRGIRLNSVSPNLFV

ESAEKYKGVFIGFNPVPVERVANTFIQSALGIETAQTFKIY"

CDS complement(43324..44184)

/locus_tag="PIKHDJKN_00073"

/inference="ab initio prediction:Prodigal:002006"

/codon_start=1

/transl_table=11

/product="hypothetical protein"

/translation="MKKWKKVTASFMLSIVTLGSGLTFLEAPQASAAANAVPAYEVKF

LAKPELVLNTDGTPRSEVIQTLGLSSTARNISAEYFDTNALNLDQQGWNVRFRKKDDK

NNYELTYKKRYPVINGDVNAALTLANQEGFDASDDNYEAEIDWGYGKQTLSFSNTKKV

DTKATGVQLPSQQEALSMLLDKLPGKLKNWSSSNWGKQQLTASRAYGPVTFQRYEGTW

NGQELTLEVWPIRNAAGTGVENIVEISFKTADSTAVSGLRTQLMQVLENKNWLIPADG

LKTQLILERY"

CDS complement(44329..45237)

/gene="cmpR_1"

/locus_tag="PIKHDJKN_00074"

/inference="ab initio prediction:Prodigal:002006"

/inference="similar to AA sequence:UniProtKB:Q55459"

/codon_start=1

/transl_table=11

/product="HTH-type transcriptional activator CmpR"

/translation="MNLIKLQIVELIDKHHHMTSVAELLGIKQPTVTFHMKSLEEEMG

VRLFESRSGKTFLTEAGQALLHYSVKINALTQEARRVVQEYDSLYRGTLHIGASYVPA

TYLLPPILNTFSQEFPGIRIMLSVKPSPVIREMLIRHQIDLGVISSEPFVGPVLQAET

LCEDDLVLICSPQHALAQRDVLKPEHMAQVPFALHGDESSTRRLTNQWLEQHDIRLRS

TVEMDSLEAIKQLVLLGGHISFMSRMAVQWEEQHGLIKVLPIPGEQAPRHIYTVHNKD

RHPSVQVNRFKEVLRDMSHSLSLFNS"

CDS 45401..46423

/gene="btuD_1"

/locus_tag="PIKHDJKN_00075"

/EC_number="7.6.2.8"

/inference="ab initio prediction:Prodigal:002006"

/inference="protein motif:HAMAP:MF_01005"

/codon_start=1

/transl_table=11

/product="Vitamin B12 import ATP-binding protein BtuD"

/translation="MLMKMEIRGIQKSFNRTPALLPTDLTLQHGQFTTLLGPSGCGKT

TLLRMLAGLEQPDAGEIVADGKSFYSATKRIDVPTHKRNLGMVFQDFALWPHMTVYEN

VAFGLKAGKQKSDLRQKVTEALGMVRLQGMEDRYPHQLSGGQQQRVAFARAVAVRPGI

ILFDEPLSALDAVLREEMRIEMMSLVRDMGLTALYVTHDQVEAMSMSDEIVVMEKGRI

LQKGTPETIYASPSDPFVASFIGKSNWLTPNQSMVRPEHVSWSKTGHDDLCYQAVVLS

VSYVGERYEIRVQMEGLSVWTAYLDRRVRVGEKVQLYVAPERICRMNGQDTPNVKPRE

FIAAAN"

CDS 46684..47766

/locus_tag="PIKHDJKN_00076"

/inference="ab initio prediction:Prodigal:002006"

/codon_start=1

/transl_table=11

/product="hypothetical protein"

/translation="MKMLGMKTRKRSAMLLLSAVMSISLFGCSTGNSTTGNAGQAAGE

GSTATASAETTKPAGGKLVLYSAGPQKLADNIISGFTAKTGIEVEMFQGTTGKILARM

EAEKSNPVADVVILASLPSAQALKADGLTLPYPDAENADKLNKDWSDADGNYFSSSAS

ALGIVYNTKLVSTPPTSWAELATPAWKDVVNIPDPTLSGSALDFITGYLSVNGDKGWD

LLDSYKANGVAMAGANQEALDPVITGAKSIVAAGVDYMAYSSKAKGEPLDIVYPEEGT

VISPRPAAILKSSQNVENAKAFIDYLLSDEAQKLVADAYLIPGREDIEATNRANLKDI

PQLKVDWNWMSEHGDETAARFSETFK"

CDS 47855..49540

/locus_tag="PIKHDJKN_00077"

/inference="ab initio prediction:Prodigal:002006"

/codon_start=1

/transl_table=11

/product="hypothetical protein"

/translation="MKPVVVDNNRIFRRVGVILALFLLTISIGVPLLLIFWQSVYPDG

QWDWMAPIRTITGQHLSGVLLNSVWLGICVVAVTTLLALPLAWMMAKTRMGQHRWVDV

ILMIPFMTPPYIGSMGWILFMQKGGYLQQWVPSAASWSELFFSFWGMVLIMSLHLFPF

LYLLLRDAIIRIGGNLEEAGAVHGGRAGYRFRRIILPLLLSSYGMGIMLVFVKTIAEF

GTPATFGRRIGYYVMTSEIHKYISSWPIDFGKATSLASVLLSVCLVMWYMQSAMSRKF

TYRLVGGKGQRSKRYSLRGGAGWLCGLYLALLLILSVGIPYFSIIAASTMKLRGAGLS

FDNLTLDHYRELLSWGSVSMKAIGNSLGLSLAASTVAVIIGTGFALTIGKSSSFMQRV

IDLFSLLPNTVPGIVMVVGLILFWNSPWMPFTLYNSYGMVVLTYVVLFLPYTVQYVKS

SFTQIDGTLFQAGQVFGGRPLYILRRILLPLIIPGMLAGWMMTFTIATRELVGSLLIL

PPSMQTSATYIFAQFEQGQVSLGMAMAVVTVGMTVLMLLGIEVLNSKRKWNAS"

CDS 49537..51099

/locus_tag="PIKHDJKN_00078"

/inference="ab initio prediction:Prodigal:002006"

/codon_start=1

/transl_table=11

/product="hypothetical protein"

/translation="MIKLKVWGGAGEHGRSAYLLRGSRVQLLLDCGVKKEGAGEYPLI

EPEIVKELDVVLLSHAHEDHSVAIPLLYRMGYQGEVWTTRETREQLDTYFRAWRRNME

RAGHALPYEEGDEQLIRYRFLEDEAEGGTWFEMVPGVSVVWGRSGHLAGSVWFGLEME

DKRILYSGDYTSESMLLQEDCPADVFWQARVPRNRRGFDAPVSQRWKTDIRTVVNGRH

VLAGEPSASQTLPFAASEGMLRSHVSAGRTVEQGDAYAVSKGVEVNKASSSGLLDLVI

MDAAYGTDRDTQTNKLEQLEQAIHETIARGGKVLMPMPSVGRGQEIMLWSQQQFPDVP

IVVEQGLVDGLKQLLHAPYWLKEEEEHIPGSVKEAIGRFLEGHGWEMPVTKEERERLL

HQHAASLWFIPDGMMQSSLARWYYNQFADDENNLVLLTGHVSAGTYAYRLLQNPAKHG

ACEVRKIRYKVHQGWKDVEKMLHQVPARHTVLVHADRAETDKLREGLLREKWVSGKGI

LHSLSPGDELYL"

CDS complement(51271..51825)

/locus_tag="PIKHDJKN_00079"

/inference="ab initio prediction:Prodigal:002006"

/codon_start=1

/transl_table=11

/product="hypothetical protein"

/translation="MKTKLGCALVLCFLMIVGIGCAQKPVSESGSAEPAVQGGQSPNP

SETKVDLGTMNTQAVDVYFADAQVLELEKKEQEIEYKDNSEKYKKTFEALQNNTDSEL

VSLWEKVELLSQQYGEGKVTLDVHIPDEAKLGTSGELLALDALTQTMFQYDEVESIDI

LVDGEALDSLMGHSELEHPIQREP"

CDS complement(51867..53288)

/locus_tag="PIKHDJKN_00080"

/inference="ab initio prediction:Prodigal:002006"

/codon_start=1

/transl_table=11

/product="hypothetical protein"

/translation="MKKSILLMFLSLFLLVVLPHQGNAAASTSKIFLDGEELILPSDV

QVTIVNKNVMIPIRVVAENLKFKVDWNQNTRNVKIQQNDKTISLTVDQKQAMVADTQV

TLNTAPLILNNTMVVPIRFVSEQMGLNVKWNNKEKIVYLINTDTSKVPNVEPSNGSGN

GSPTSLTHVTDIQFANNQLVVSMDGESKPVMTTLKNPDRLVVDLPGAVFGDLSQPLNQ

NMNGKLDVDAYPNVTEVRYSLFKQDPGQVRIVVELNQVKDVQYSQNIIADKLIIDLNV

SGDNVTPVPVTPVGDSGRKVVVIDPGHGGKDPGSTSITNKPEKEYNLALALKVQALLL

NEPDIELVMTRDGDTYPTRPERVRLANQLNADVFVSIHGNNVESSPQATGTETFYYQR

SSSKELANIIHQRLVKAMGLKDRGVKNGNLEVIRDTTMAAVLLEVGFLSNVMDEELMS

SDVVQMKAAQAIADGIKEYLGIN"

CDS complement(53313..54182)

/locus_tag="PIKHDJKN_00081"

/inference="ab initio prediction:Prodigal:002006"

/codon_start=1

/transl_table=11

/product="hypothetical protein"

/translation="MLRVYKLSPAEGDTKVNNAVIWFSTVLLLGGLTTTSSSVVGAKP

AASELISHSSINAISVTPSIYSSVKPFKRNNSTNKIIWKGGNITLTATTVKLEGKPTF

EQNVIQSIVVTKGSQKHTIETRDLEDNWLDITSVVASNSNTWLAIHAERSSGSTLMLL

NLQTGEVTELNDRLKKAGKKNIETIADYNWAPNEDQIAFSYGDPSKSSIAIYNVQKES

FVYLPRATNYISTILILWQKNGKILDYISEYPSDQMILYRYSIESNKVKPLKKISRNE

FAEWFKLDKYSIQ"

CDS 54435..55664

/gene="anmK"

/locus_tag="PIKHDJKN_00082"

/EC_number="2.7.1.170"

/inference="ab initio prediction:Prodigal:002006"

/inference="similar to AA sequence:UniProtKB:Q8EHB5"

/codon_start=1

/transl_table=11

/product="Anhydro-N-acetylmuramic acid kinase"

/db_xref="COG:COG2377"

/translation="MDTGSYPMWEQYRLKREHYIIGLMSGTSLDGTDAALVRILTDDS

GALQKIDLVDFVCVPYSNELREVLIRLCSPETARVDELTAAHFGVSEWYAHSVRELIR

SSGISSGQVDAISMHGQTVWHAPVASAFPGPAGESLPVVSTLQIGECAVVRERTGLPV

IGNLRARDMAAGGEGAPLTPYADALMFRSPEEGRLVQNIGGIGNVTVLPSESSNEGIS

AFDTGPGNMVMDAIVRQATAGRQHYDPNGSIAAQGKFDQDLVDLCLDDNYFKRLPPKS

TGREVYGAAYAARLMEMATARSLSLEDTLATATCLTAETIVRAVKDFILPKMQISAML

ACGGGTSNATLMEMIRQRLPEGIRLERTADYGIPDDAREAIGFALLGHEALMGRTNTL

PAVTGAKHAVISGNLTL"

CDS 55780..56628

/gene="ybbH_1"

/locus_tag="PIKHDJKN_00083"

/inference="ab initio prediction:Prodigal:002006"

/inference="similar to AA sequence:UniProtKB:Q45581"

/codon_start=1

/transl_table=11

/product="putative HTH-type transcriptional regulator

YbbH"

/db_xref="COG:COG1737"

/translation="MEGMKGGLVRLRALMDDLTPSERKIGAYILDHPQETVQSSVAQL

SERSGGSPAAIIRLCKSLGVTGFQELKLKIAGDLQTGEQYQYTEIRPQDSMESIIQHV

SANNIQSVKDTVHILDPRLVEQAVDVLDRANRIFFYGVGASNLIALDAHYKFMRINRT

SFSFADPHMQVSSATTLREDDAVVCISYSGETSNVISCLKHAREGGAATISITKWGSN

TLSSLADVPLMITSTESDIRSGATSSRIAQLNVIDILYLAIASRDYNQSVEYLEKSRQ

AILEHK"

CDS complement(56771..57676)

/gene="murQ"

/locus_tag="PIKHDJKN_00084"

/EC_number="4.2.1.126"

/inference="ab initio prediction:Prodigal:002006"

/inference="similar to AA sequence:UniProtKB:P44862"

/codon_start=1

/transl_table=11

/product="N-acetylmuramic acid 6-phosphate etherase"

/db_xref="COG:COG2103"

/translation="MNHMLNSLLTEQPNPLTDHIDELPSAEIMELINKEDQRIAELIQ

PLIPVIAQAADLILEAFQSGGRLFYVGAGTSGRLGILDASECPPTYGTPPSMVQGIIA

GGFRAVKDPVEGAEDSEELGATDLDEHGLDENDVVVGIAASGRTPYVLGAMRHAKEIG

ATVISLSNNAGTPMTLLADVSIEAVVGPEVVMGSTRMKAGSAQKMILNMLTTTAMIRL

GKVYRNFMVDLNPSNEKLVHRAKRMIHLATGANEADIEQAFAGADGHVKTAIVILMAG

VDAVEAQRRLDLADGFVRSAIAGPS"

CDS complement(57699..58661)

/gene="murK_1"

/locus_tag="PIKHDJKN_00085"

/EC_number="2.7.1.-"

/inference="ab initio prediction:Prodigal:002006"

/inference="similar to AA sequence:UniProtKB:Q97ML3"

/codon_start=1

/transl_table=11

/product="N-acetylmuramic acid/N-acetylglucosamine kinase"

/db_xref="COG:COG2971"

/translation="MRVYVGVDGGGTNTDAAIISESGEILARFSGGPTNPHSVSTEQA

LSELQRVLEQLFNLKSDLSTNCEGICLGMSGVDTIQERQLIADAVNNYMKSSNRQRSE

GCPVWVVSEGEIALMASLGHTHGVLCISGTGSIVYGFTLEGERYRAGGWGHLLGDEGS

GYRIGQRALQVVMQSYDGALPPTRLTPLLLKQLNLRDISELKARVYQTDWGKTETASI

ARLAIEAAESGDEAARALIIDEAAQLADTAKALIARHPEFASTQVVLSGSLFRYAALF

RNTFIQKLSGYYGELDFVYREDAPASAIGAAQLARKRCSLNESF"

CDS 59023..60375

/locus_tag="PIKHDJKN_00086"

/inference="ab initio prediction:Prodigal:002006"

/codon_start=1

/transl_table=11

/product="hypothetical protein"

/translation="MKKKGLVWAMLLMMVWVTACGNSGGAASDDPNAEDTVTVWTYPV

HGTYEDELKDLIVDFNKENPNITVKTEMLSWAEGPKKFDVALNAGNPPDLYFHSVDGT

YVNTGLALELDSYLTPEIKDDYLPGTLELGQIQGKQYGLPLYQFQWAWGGNKRILEEA

GIDWKSIQQNGWTWSEFNAAAAKLTKTLDGGAKQYALVTDGTSLDFIEMLSRNNGMID

VLDKAGTFQWNDGRILDTLSFIKNLMDQGYMPKETAALAPAKRTDMFYAGETAIISKA

IPYYDVMIQNRNKDIDDGKVQGEKIDFVLLPVPHNDDQSAATTMGGEGYVAFKQKKDK

GEQHAKNTFLVMEALSGAKAGNSANELALPFVRQSQVDLFKGKELGQPDNLDAAKVMA

ENIAMPVVLELDIDKASQQKQFKEQVVKPNIQALFSGEKSPEQIAEDFKNKGQQMFGQ

"

CDS 60431..61354

/gene="araP_1"

/locus_tag="PIKHDJKN_00087"

/inference="ab initio prediction:Prodigal:002006"

/inference="similar to AA sequence:UniProtKB:P94529"

/codon_start=1

/transl_table=11

/product="L-arabinose transport system permease protein

AraP"

/db_xref="COG:COG1175"

/translation="MQTALAPKPSVPRTSRVARFWRDYGWAYLFILAPVLLFLIFTLY

PVLTALVMSFQKYNIMNSTWVGLDNYERLVKDETFWKSIKNTLIFTVGTVPVNILITF

VLSYFIFQMKSKWQTFFKATLYLPAVASGVTISIVWLAIFDPTDSGLLNRFLGLFGLD

PVIWLGQSGTALFSLILMNWLGSHGAGIILYLAAMGGIPKSLYEAADIDHASGWTQFS

KITWPLLKPTTLYLLVTGVITSFQVFISVYLMTQGGPNFATTTIAYLIYETAFKFYEF

GLASAQSFVLAIIIIVISVIQFKYFSSDIEY"

CDS 61378..62211

/gene="araQ_1"

/locus_tag="PIKHDJKN_00088"

/inference="ab initio prediction:Prodigal:002006"

/inference="similar to AA sequence:UniProtKB:P94530"

/codon_start=1

/transl_table=11

/product="L-arabinose transport system permease protein

AraQ"

/db_xref="COG:COG0395"

/translation="MKSTQKKTLLVVASILLVVWALVTVIPMYWMLVGSVQDSAMSAS

FKPQMIPEQLSLSPYERFFAKTDAWRWLYNSLLISVILTVTNVFFASLAGYAFAKLKF

PGSQAVFWTLLGTMMIPAQVTLIPLYILMVNVFDLGDTYTAIILPAAVSVGNIFLMKQ

FMSTLPTSLIHAARIDACSEFGIFWKVILPMAKPGIAVLAIFTFVASWNEFFWPFLIT

NSNEMRTVQVGLASFVFAESTDFGAMMAGATIGALPMIILFFSLQRYFLQGITIGAVK

G"

CDS 62484..63593

/gene="msmX_1"

/locus_tag="PIKHDJKN_00089"

/EC_number="7.5.2.-"

/inference="ab initio prediction:Prodigal:002006"

/inference="similar to AA sequence:UniProtKB:P94360"

/codon_start=1

/transl_table=11

/product="Oligosaccharides import ATP-binding protein

MsmX"

/db_xref="COG:COG3839"

/translation="MARVEFRQVRKEFKDDHKGTFTAVAGSDFVIEDKEFVVFVGPSG

CGKTTSLRMIAGLEKQTSGDIVIGERVVNDLHPKDRDIAMVFQDYALYPHMTIRENLS

FGLKNLKKPKSYIDEQVQNAASILGLEAMLERKPRELSGGQRQRVAVGRAIVRDPQVF

LFDEPLSNLDAKLRVQMRVELGELHKRLGATIVYVTHDQVEAMTLGERIVVMNHGDIQ
QVASPKELYASPRNMFVAGFIGSPAMNFIDARIEGTQVVVEGASFTLPEDVLARLQSY
QGKPVIMGLRPEHIFGDDVAPNIPTDHMLQARVQVVEHLGSENLVYFHSGARTVTSKV
HPETHAYVGMDKNFVLDLRKAHFFDPETELAIGRE"

CDS 63640..64350

/locus_tag="PIKHDJKN_00090"

/inference="ab initio prediction:Prodigal:002006"

/codon_start=1

/transl_table=11

/product="hypothetical protein"

/translation="MRKLSEVLVKSGAEIYRDIIPVALYSVVSSIVLVPILMFAPLPI
AVLLLAVIYMPILFGVSYAVHHRLERKERRNGLKDIWNGTMRGFIPGGAVGVLFAVLG
FILWSTWWYYGGQGGITGTAVSMFQTFFVLMALMSQFYTWQLVLQKNMGIIQAMGESV
KLFFRHPGYTMGACFQALCLTALLMLTVVGFGALFAGMFAIYQHKVALNVLEPEEEPV
TSGGNDHQHTRWISQGNV"

CDS 64350..65516

/locus_tag="PIKHDJKN_00091"

/inference="ab initio prediction:Prodigal:002006"

/codon_start=1

/transl_table=11

/product="hypothetical protein"

/translation="MGVRTGVDLLSGGFSHPWLTDARIGLMTNPTGITADFVSTVDVC
AGLADAQLTALYACEHGLDGELQAGVRFGDTVHPRLGIPVFSLYGEHKKPTPAMLAEV
DTVLFDIQDVGVRYYTYASTLFHMMEACAEAGKRLLVLDRPNPLGGDSVEGGLLSTGY
ESLVGAWRVPVRTGLTVGELALLVNSEMDVPCELDVIAMEGWQRSMEFTDCELPWMLP
SPNMPTMDSVRVYAGTCLFEGTNVSEGRGTTRPFEWIGAPWIEGERLAERFREHKMEG
VHVHPVYMSPTFSKHAGELCGGVRIFVIDSRAFRAVDTGLVLLHELASLYPEQFRWLE
PPQPGSRYFIDLLTGGRKVRDIIHDREELVRLMEDWNAEAEEWKERRKPFLLYP"

CDS 65586..67271

/gene="nagZ_1"

/locus_tag="PIKHDJKN_00092"

/EC_number="3.2.1.52"

/inference="ab initio prediction:Prodigal:002006"

/inference="similar to AA sequence:UniProtKB:P40406"

/codon_start=1

/transl_table=11

/product="Beta-hexosaminidase"

/db_xref="COG:COG1472"

/translation="MSGPKEIYPLKLKQMTLREKIGQMLLCGFHGTEAAGDVHAFLRK

YPIGGVIYFARNVESPEQVERLSSGLQRIAVDSGNMPLWISIDQEGGMVARITEGIAL

MPGQMAIAAAGSIEDAYQAAYISGVELRSMGINMNFAPVLDVNNNAANPVIGVRSFGE

SPQSVAAYGARSIVGVQDAGISATAKHFPGHGDTDTDSHLDLPVITHDRERVERVELI

PFRAAIAADVDAMMSAHIYFPALEPERLPVTLSRAVLSGLLRQELGYDGMIVTDCMEM

DAIAANYGTVDAAVMAVEAGADLVLISHTVHLQAGAFEALLAAVQSGRISEARIDESV

TRLLKYKAKRGLLEKGMGTGNDEVHGSVSTSLSAEASTDPASSKRQERNQPLHQEVAR

RISENSITLVRDQLNMLPLKPERTLVITVATSVTTIADEQLTQVVTLGSALSHCGLDV

VDITVTPEEVAIRSARLLQAAEEDDIRQIVVGTYNAGSASGDPQCRLIGWLQQLGKPI

AVVALRSPYDLLAIPEVQVYVAAYESRPLAMDSAARALMGDIPFAGKLPVSIK"

CDS complement(67390..68745)

/gene="dap_1"

/locus_tag="PIKHDJKN_00093"

/EC_number="3.4.11.19"

/inference="ab initio prediction:Prodigal:002006"

/inference="protein motif:HAMAP:MF_01960"

/codon_start=1

/transl_table=11

/product="D-aminopeptidase"

/translation="MGQASFKASAAQETQTVHIEVNGQEQSWKSAPLVFKGTTYVPLR

DVVTSVNGTLKWEARTKTATITVGRDKLVHQAGSDFIQVNHVRLSSGVQSRTINGTLM

LPVRALANALKADIKVQRTASGQMSVNIQTDKVSLLNSQVASVDSYLREIDFPGMALI

ARNGEVLLRQGYGLADEQTLNRPDQKTRIASLSKSFTAASILSLVEKGKLDVQDPISK

YISGIPKGDQITLHMLLSQTSGIPSAFGRGEGTSLEETVEEIRHKTLKFEPGSAYLYS

NSGYVLLAYVIEQVSGMSYADYVQQTILKPLGMKNSGEASRKVHTTSGFVSENKQWVP

APYYVSQSGSGTIYSTVDDLLKWDRALYTDKILSQGTIEQMYEPYSDKNYGYAWILKE

SDQNRTVFHNGSGGGFATAFSRNLTDDVTIILLGNHAGMDMTSLMDEVEAKTAQALNL
Q"

CDS 69055..71028

/gene="mcpB_1"

/locus_tag="PIKHDJKN_00094"

/inference="ab initio prediction:Prodigal:002006"

/inference="similar to AA sequence:UniProtKB:P39215"

/codon_start=1

/transl_table=11

/product="Methyl-accepting chemotaxis protein McpB"

/db_xref="COG:COG0840"

/translation="MKKRIRNWFTLTVKKRLIASLLLFLIVPSISVGWLSYLKAADQV
KEEIIHSAQAKTEMLSLQITQMLDMEKDNAAQFAAGITSTDIINKSPAVQRQMDRMSK
AHKELGVITAGAEDGSWMKSPDPGQQIYDPRERSWYTMGMSQDEPIISDTFQSVTTGE
WVVTAAAKLADGKGVIGANVSLNHLKESVDKIHIGEKGKLYMLDNGGKFLFHYNIESG
VQSNESYINEMYTKDQGTVKYTYDGQELEAVFYTNPETGWKIVGEMVPSEAADAVRPI
LIRAYTLVGTSLIIGIILLVFIIRSIHRPLLQLTQAASKVSAGDLTVRVGLQRRDEFG
QLGASFDTMTSSLRNVLGEVHDTSSQLAASSEELMASSEQTSRATEQVAELMQDAASG
TNKQNASLAATGQLVSEMSIGVKEISSSAEDTARIAVEASSKSEAGMVTVEEAVAHIQ
KVNNESEAMAAVIQDLRAKNEEIVEIVAEITNIAKQTNILALNASIEASRAGEQGRGF
AVVANEVKSLANNSGSAAERINELMREMQEKTNAVQSTFALTGEGMIKGTQMITEAGE
AFQNIRSAVQLVAAQAGEVSAASRQIDGGMSHVSKEVNDTIELSNQIASGTEDGSAAA
QEQLATMEEVAASSAALSRMAEDLQSMIEKFKL"

CDS 71217..71987

/locus_tag="PIKHDJKN_00095"

/inference="ab initio prediction:Prodigal:002006"

/codon_start=1

/transl_table=11

/product="hypothetical protein"

/translation="MTWKGDEPDLDKSIPLTSGPANTEEVLDDASFMQGVKDCIPTLL
GYLSIGFAAGVIEMTAGLSLAETALLSLIVYAGSAQFIAAGMLASSGSATAIIFTIFF
VNLRHLLLSAAVSPYFRHLTPLKNMWIGSLLTDESFGVAMTRAIGRKTLSERWMHGLN

ITAYLNWFVANMAGAYFGRWITNPERLGLDFALPAMFIGLVVLQLMQRKNKKLHINVA

IIAVVCVIFASMASLGSMSVIVAAVIAATMGVVMERWK"

CDS 71978..72307

/locus_tag="PIKHDJKN_00096"

/inference="ab initio prediction:Prodigal:002006"

/codon_start=1

/transl_table=11

/product="hypothetical protein"

/translation="MEVRWDVFWIIMGSALLTFIPRVLPLMLFSKIQIPMWLLRWLEY

VPVAVMAALIGQELFMSDNQLVPIIHNAALWAALPTIAVAIWTRSLLGTVLVGIVAMM

ILRYLMG"

CDS 72400..73203

/gene="udh_1"

/locus_tag="PIKHDJKN_00097"

/EC_number="1.1.1.203"

/inference="ab initio prediction:Prodigal:002006"

/inference="similar to AA sequence:UniProtKB:Q88NN6"

/codon_start=1

/transl_table=11

/product="Uronate dehydrogenase"

/db_xref="COG:COG0451"

/translation="MSNSKHRILITGAAGVIGRSLLEGLREKGKYDIVAADLHDDPQA

GIVAMDVTDGERLKELMQGVQTVLHIAWAKDEEDFLGKVLPINITGAYHVYEAARLNG

VQRVIFASSNHATGFYPTGEDIEVDDPYRPDSFYGLSKCYIELLGRYYVDKYDLSSFN

IRIGNFSGDAHPHSERSAHIWISPRDMVQLAECCIEADSSMKYVNLYGTSANTDNYYD

IGYLEQKIGYRPKDDAAELWDEAKRKGAPDKQDETEYQGGGYVAKSPKS"

CDS 73394..74050

/gene="axe2_1"

/locus_tag="PIKHDJKN_00098"

/EC_number="3.1.1.72"

/inference="ab initio prediction:Prodigal:002006"

/inference="similar to AA sequence:UniProtKB:Q09LX1"

/codon_start=1

/transl_table=11

/product="Acetylxylan esterase"

/translation="MSTHSSVILFQGDSITDGGRSRNDDPNHFLGHSYAYLISSKLGV

ELAAKQPTFYNRGISGDRASDLYARWNEDTFSLKPDLISILIGVNDAWRTMNGEPSGV

TDRLGRAYRHLLEETREVMPNTGLVLMEPFILKTGATVEQWDVWQERIGHYQNLVREL

ASEFGAIWVPLQETFNDALEQADAAYWLWDGVHPTAAGHELIARQWLSVVQGSPLKIQ

"

CDS 74227..74514

/gene="gsiB_1"

/locus_tag="PIKHDJKN_00099"

/inference="ab initio prediction:Prodigal:002006"

/inference="similar to AA sequence:UniProtKB:P26907"

/codon_start=1

/transl_table=11

/product="Glucose starvation-inducible protein B"

/db_xref="COG:COG3729"

/translation="MAQNNQNGKMSREEAGRKGGEATSKNHDQEFYQEIGSKGGEATS

KSHDREFYQEIGREGGSARGNNDGNDDQDGKMSREEAGRKGGQARSRNRKN"

CDS complement(74624..74902)

/locus_tag="PIKHDJKN_00100"

/inference="ab initio prediction:Prodigal:002006"

/codon_start=1

/transl_table=11

/product="hypothetical protein"

/translation="MLFGSLAPLQAYIYVILAHELGHAEDVELMDLSNLLDSPLTASE

QAGIRLRIEENAWRYAESLLFDMDPVFLRTIIDESLFSYRQAIEPHIA"

CDS 75279..77207

/locus_tag="PIKHDJKN_00101"

/inference="ab initio prediction:Prodigal:002006"

/codon_start=1

/transl_table=11

/product="hypothetical protein"

/translation="MTIHPGLKWTADHIIAAMRHGTSSGKEYICRWKDRLADMLNDPD

YASFWSDLRVYEQKANTENLPELSFSLYRQFRETGERQAYELAYFERRGRLAALGLLA

AASPSAERLQLLEDLIWSVCNEQTWCLSAHVPEGKEAGARQHIDLFAAETAQTLSEMI

VMLDDVLDPRVLRMMRAEIECRIFTPLYREQQTYGWENADHNWSAVCSGGCGIAALLL

LEDEHLRAKAVQQTIHSMNAFLRGYGMDGGCAEGIGYWVYGFGFYTYFAEMLRIFSAG

KLDMLSNPETRAIAAFPGNVHLSGGVFVNFSDSRERETIPPGLLSLLSERQQVQVNLE

LRIPLLTEDPCRRWAHALRNILWSNPAVYRRESEAELPAAPVYLENLSWGIARGVLAT

GEQKEMTAAFAVKGGHNAEPHNHNDLGHFILHGGGENILCDPGSGEYTQAYFAPGRES

IFQIGSQGHSVPVIEGAGQHSGRQVEAKVLHIKRLSPQGIEAAFDLTSAYPDAPTLAR

YTRHLSWNCLPEASEAELFLEDYYQWQQPSKTCREEHLIEKPPVVEYFISRINPVIQE

ALVQWEGNKAVVTMHYDADLWDAQVEELETVDHDHLPLKFYRAALSLRQTANEEEDPW

NHSVKETWCRLKFFIHAK"

CDS 77322..79214

/locus_tag="PIKHDJKN_00102"

/inference="ab initio prediction:Prodigal:002006"

/codon_start=1

/transl_table=11

/product="hypothetical protein"

/translation="MLAYTSDSAYPWKSELERFRTDAEQANLAEPSGWDRSRKIALIE

DIVRAYSVYQQENGAVVDPFSGRERYYSTPAYAFAAAVLVKEGRTDLLSSASAALTHS

ISLVVRGEVPDNHADFFPILMLRAYVLLQPLMPEQATQWAAGLKQIQPEETYVFTMSK

MNNPNRMINWNAIMISGEYLRYREQLASEGIEWMDQYLKRYHLPRFTGLGLYQDGPLD

RPNSPLAYDIATRYHLGVMLDAGYDESCASSLDESIRRGAFTSLLTLSPLGEIPPRGR

SSQHQWNEAAAAYVFSSHASAAKQIGDPIMAGVFARAANLCFEAVDRWKREDGRLNIV

RNRYAPEVRHGYEIYTNHTCYNLWTVAALANAYLCDPGDEVAELPIPSEIGSRVLQMD

GWFETITASVPGQQIVLHTALNDPYTVPGLVRIHQQGLPGLIGPAAAGHAESGFTEFS

EGETKAISYCPAWRTPDGQWYCLSAGIPSGFEYDRDAGINPSDGGGAVVFNVIKDQAQ

ETNTFTVDWVGPLVGLQALRTVYTQQPGLLTVTYEFEGEIEAAGAVIPLMFSDGEEQA

VITVSGDHVRSEFCGAYVESTVLDEGAEIHLQDHMVASRNGVLKEARMIVSGSQKITF

TVRLGK"

CDS 79304..80440

/gene="ugl_1"

/locus_tag="PIKHDJKN_00103"

/EC_number="3.2.1.179"

/inference="ab initio prediction:Prodigal:002006"

/inference="similar to AA sequence:UniProtKB:Q9RC92"

/codon_start=1

/transl_table=11

/product="Unsaturated glucuronyl hydrolase"

/translation="MNTSTSVTWVEEAWRQGAAKIIRNAHRIKDTFPHTAPKGMYDRN

DPEWWTAGFWPGLLWLVYGESPENEAVAPLSRIAESCERQLEGCLRDPESVDHDLGFI

WLLSGVANYRQTGSADGRRRGMLAANLLAARFNVRGEFIRAWNFSSSTMDTRGVAIID

SMMNLPLLYWASEQSGDPRFRWLAEAHADTVAREFVRTDGSICHVVEFDPLTGQKLQE

HGGQGHAPGSAWARGAAWALHGFALSFRYTGEARYLETAERAADFFLAMLGEEIVPVW

DFRAPAEHQVAWDSSASAIAASGLLELAKLSPRGEEYAAAAERILRGLHENYSSGETA

AEEGLIMQGTVHYPEGRGLNVPIIYGDYFYMEALAKLRGRPGLF"

CDS complement(80588..81208)

/locus_tag="PIKHDJKN_00104"

/inference="ab initio prediction:Prodigal:002006"

/codon_start=1

/transl_table=11

/product="hypothetical protein"

/translation="MSQPFDYKGYWEQTYSSGETSGRGSYGVLAEFKAEVVNGLIQRE

GISSVIEFGCGDGNQLQYMNYTTYLGVDVAASSVRLCASKFANDTSKSFMLYTPGLWI

NRGFLQADLTVCLDVLYHITDETDFRNTLYDILHSTSGWVVLYTRLKENGNPGVSTIQ

DRNIFDYLFDYPEFKVHEIIPQRYPDQSSADFVILRRTPGIENKTD"

CDS 81419..83791

/locus_tag="PIKHDJKN_00105"

/inference="ab initio prediction:Prodigal:002006"

/codon_start=1

/transl_table=11

/product="hypothetical protein"

/translation="MLKPRLTKCMVMMLVLVLSISNVGLAAAADKELSKIVVSKNELS

LEVGDSSSVTVTGVYTDNTSANVTISSSWSSSDTSIATVYNGAITAKKEGTATITTSY
LSQSQTVQVNVTKKVKALSKNVQSLDLRTGDSKDIILTATYSDNTTNADASKVAEWSS
DDEKVATVVNGKVTGQSAGTAVITGKVGSQSVTVDVNVEVVKRVDMDKKQVNLLLNKS
ETVKLTATYPDGTTKDVTDLAEWTSSNEKVADVLKGEITGYSAGSAKITAKYGTKSVS
VDVDVDLTSKLSVEKQSIFFRLSESNKTANVVLTASYPNSADVNVTDQATWTSSNEKV
ATVFKGQVTAIGAGSTTIKATYSGKTVEITVDVDTARYLDIAGVEDKLAMSVTGDNKT
KKLVANAEYIDASTENVTSKATWTSSNADVVYVSGGELIAYKSGTATITAAYGGKTVK
FTVNVDVPDKYEMDKKKASVAVGGTLSVKVLAVYGDTSKDVSEDADWSSSSEKLAEVD
SKGVVTGIATGKVTITAKIEGKTLTLPVEVGMASGLEADLNFVVLSSKETQQIVLTAT
DEDGLTKDVSSDATWKSSNSRVADVKKGVITANSSGKANITAEYGSNKVTIQVEVDVI
SRIEASEPALSLKTGDSADLTVTAYLSDGSERDVTDKAEWKTNSYKVAQVTKGKVKAT
GSGKAKITAKYGSKTVTIAVDVDTLKYLQTDKVTLTMKPGDKATLVATATYADGSEAN
VSKPALWKSSRIATASVKDGIIQANGKGKATITVTYAGVKTKVTIVVEAK"

CDS complement(83994..84539)

/gene="srpC"

/locus_tag="PIKHDJKN_00106"

/inference="ab initio prediction:Prodigal:002006"

/inference="similar to AA sequence:UniProtKB:Q55027"

/codon_start=1

/transl_table=11

/product="putative chromate transport protein"

/db_xref="COG:COG2059"

/translation="MLQTWWELFWGFFVANILGYGGGPASIPLMQEEVVNHYKWMTTE
QFGDVLAIGNALPGPIATKIAAFVGYQVAGWFGAFIASFATVVPSAVALILLLRLLNK
HRTSPKVKGMTLLVQPVIAVLMILLTWEFGQVSTNSIGIWQTLIIAGISLWVMTKTKL
HPAILIVIAFAYGALVLSHTM"

CDS complement(84543..85130)

/locus_tag="PIKHDJKN_00107"

/inference="ab initio prediction:Prodigal:002006"

/codon_start=1

/transl_table=11

/product="hypothetical protein"

/translation="MSWKDYNGLVIGMVRTGILGYGGGPSVIPLIRYEAVTRYKWVSD
DEFGEILAIANALPGPIATKMAAYLGYKTKGVLGAIVSVLAHILPTSIAIIALLGSMY
ALRESKIVAGMVAAVRPVIFVMLGMMAYEFAKKAWKGLGTTFAALFGVLAFVLLQLLD
IHPGIVIAVFLGYGVFHLDVVQRFKSKGNSDKGVF"
CDS complement(85143..85637)
/gene="decR_1"
/locus_tag="PIKHDJKN_00108"
/inference="ab initio prediction:Prodigal:002006"
/inference="similar to AA sequence:UniProtKB:P0ACJ5"
/codon_start=1
/transl_table=11
/product="DNA-binding transcriptional activator DecR"
/db_xref="COG:COG1522"
/translation="MSEKRSSKGGEIPQMHNLDAIDRKIIAALHQNSRISYTDLGAQI
GLSRVAVQARINALSEKGIIERFTVVINPGKVGLQVSAFFNVDVEPLFLDEVAAKLDE
EPAVTSLYHMTGPSTLHMHGIFADMEEMEQFLLEKLYKMPGIVKVESQLLLKRYKSRM
GMRL"
CDS complement(85914..87026)
/locus_tag="PIKHDJKN_00109"
/inference="ab initio prediction:Prodigal:002006"
/codon_start=1
/transl_table=11
/product="hypothetical protein"
/translation="MSSTRKNRLSGFADQPVRQLLEEIDSHITDTEFRSRLKGSLHQL
SDLKFALDESSIVALTDRKGKIQYVNDKFCEISQYNREELIGQDHRIINSGYHGKNFM
KNLWETISSGRVWNGEIRNRASDGSHYWVNTTIVPFLDNDGEPYQYLAVRSEVTKLKR
VEAELQTMMTQVMNIQEEERRRISRELHDGIGQSLFSLVIQMDRLLADQPQSGVEALR
KQVTGIMEDVRGMAWELRPSVLDDLGVVPAIRTYIENYTRHYGIEVELECNLRKRLEM
NREIAMYRIIQEALTNVAKYADVSEAHVTVVDSEEATVVKIEDRGAGFNGTTAGNGVG
LFSMEERARGAGGTLVVSSEPGEGTVVTLCLPKTVH"
CDS complement(87019..87669)
/gene="nreC"

/locus_tag="PIKHDJKN_00110"

/inference="ab initio prediction:Prodigal:002006"

/inference="similar to AA sequence:UniProtKB:Q7WZY4"

/codon_start=1

/transl_table=11

/product="Oxygen regulatory protein NreC"

/db_xref="COG:COG2197"

/translation="MIQLLVVDDHVVVRSGLIALLDGKNDIHIVGDAADGDEAIAKAQ

ELKPDVVLMDFSMPPGKDGLTATAELKKLMPDVSILILTMHDDEEYLFRAIHAGASGY

ILKSAPHEELLAAIRSVAEGSAYLYPSATKRLMSEYLDKAKQENAGPYDTLSEREKEI

LSWIAKGYANKEIAEHLVISVKTVESHKSNLMEKLGLRTRPELVKFAMKKGLLNFE"

CDS complement(87791..88789)

/gene="yxeB_1"

/locus_tag="PIKHDJKN_00111"

/inference="ab initio prediction:Prodigal:002006"

/inference="similar to AA sequence:UniProtKB:P54941"

/codon_start=1

/transl_table=11

/product="Iron(3+)-hydroxamate-binding protein YxeB"

/db_xref="COG:COG0614"

/translation="MTGVRSKWWAGLIILLLVIGIIGCSGQAEETTENNASAPAVTEE

QPATSEAETQVISTVNGDVEIPVNPQRIVTQGYLADFLALGIKPVGAPDYELESPYVL

DLVEGVADIGRIDGGSVEKILALQPDLIVTVGGDEKLNEQYRKIAPTLIIPYGTYDNV

HEEITAFGEIINKQKEANDWLSQFEVKVKAAKESIQGLVKEGTTFSNFSLFGKEWYIN

GDGVNRGGQAIYQQLGLKAPDIVQKELIDQDKDTVAISEEKLADYAGDYIFLDLSNGG

SLDEGSAVWSSLDAVKNNRVFKLDGERFWPYDPLAVEAQVQEIADMIKERFGKTEN"

CDS complement(88907..90517)

/gene="rhaS_1"

/locus_tag="PIKHDJKN_00112"

/inference="ab initio prediction:Prodigal:002006"

/inference="protein motif:HAMAP:MF_01534"

/codon_start=1

/transl_table=11

/product="HTH-type transcriptional activator RhaS"

/translation="MLVLTNASVIPWEAAQQTGFISIESPSLVTCTATVNFQYGQVHG

VLHKGQTLFLYPPCSLELIHTPASAFTVYHIGFLEYGLTKKSHEKRVYELTSENLPAH

GSKTGASSQGLRILAIIQELTDKRSGSIMTEEARSHFLLSELLELFRLAVKPATEMVD

SVIRDALYYINHHYGDHLTREDIARMTGFNASYFSRFFQKQVGRSFQAHLTRVRMDKA

KQYLLSTQATLNEIALLVGYADGLYLSRKFKQFTGISPSEYRLQPRARRIATVQYTGD

LLALGVQPVAASFLPWALSPLIQDELHDVIDLDEHGVEELLRAEPIDLIIAPDYLYYL

PHKLEQLERISQVIVLPWNQLDRLETVRLIGRIVGREQAAEDWIEHYTTRARTESDRL

KDFIKPGETVGLYELWEDGTICIWNATARAAYNVYYGLHLTPHPNILRDVLEPDNHQY

IEEDQLAEYAADHMFLVLPAHEGGLYHDSPDKLRERPGWRKVMNNGNRRIYPLQLEQF

WCNDALSLEKQLELMVDILIRENSVKAR"

CDS complement(90734..91165)

/locus_tag="PIKHDJKN_00113"

/inference="ab initio prediction:Prodigal:002006"

/codon_start=1

/transl_table=11

/product="hypothetical protein"

/translation="MHDELPSGIQEMIDGLRLSTSSDFCGLACLSGTMLRWKYTSGAS

NERVKRMEMRPGQDLVGTALRTGRTALSDAQSTGEHTPGRCPLMLAERLVCAIAVPVF

QQGSVPGAVLLLGSRLPCTFSPEMVRQTEQVAIQLQPKVYG"

CDS 91550..92326

/gene="fnr_1"

/locus_tag="PIKHDJKN_00114"

/inference="ab initio prediction:Prodigal:002006"

/inference="similar to AA sequence:UniProtKB:P46908"

/codon_start=1

/transl_table=11

/product="Anaerobic regulatory protein"

/db_xref="COG:COG0664"

/translation="MGTVFKERTTQATQQEQTEASKMTRETGGILSFVTAEQWGLIEA

KMQPKRVKSGYSLFLEGDEAGYLYYIRSGRVKLTKSTEDGKEIILSIQQTGDLIGEFG

GIGGLSHSYSAEMAEKGELGIISLSDLESVLSKHGDLALKFLQWMALAQRITQSRFRD

LLLYGKAGALASTLIRASNSYGRITPDGIVLDMKLNHTELSEMIGATRESVTRMLGAW

KEQGTLDTLDGKLMIRDLAALRCMCGCPTFPSCPVELCRL"

CDS 92434..92760

/locus_tag="PIKHDJKN_00115"

/inference="ab initio prediction:Prodigal:002006"

/codon_start=1

/transl_table=11

/product="hypothetical protein"

/translation="MSRLKQWYGLHSKLLGFVRREATVIQAYVHREHHSTNTISKWTE

NIAVYNVDKEVNLISMNGSLEFLMAMLSSSQREVIQRSYLDNEGEFDYISCVEMGISK

STKNYA"

CDS complement(92953..93132)

/locus_tag="PIKHDJKN_00116"

/inference="ab initio prediction:Prodigal:002006"

/codon_start=1

/transl_table=11

/product="hypothetical protein"

/translation="MYILALFILITTGLVSIIDILFGIKNSGEGIRLLVTISQALFFL

SLLFFVIKMPTKKKK"

CDS complement(93156..93722)

/locus_tag="PIKHDJKN_00117"

/inference="ab initio prediction:Prodigal:002006"

/codon_start=1

/transl_table=11

/product="hypothetical protein"

/translation="MKKVKQLGIASLSALLLSSFTLPAFASAQVANESSINSGTQVEL

PTGPIVKDARVELELRQVDYDALFRTVGAELGMTTAAGEVGTLGWKTKLAKEVAKDLI

SKLKNVGSRAWNEQIKVYVDKLPLLTDGAKTTLKGYLSYQVLMQALDIMVDFTGTAED

GLSNALQSVGVPGWLSDAAARAIVFFLL"

CDS 94179..94391

/locus_tag="PIKHDJKN_00118"

/inference="ab initio prediction:Prodigal:002006"

/codon_start=1

/transl_table=11

/product="hypothetical protein"

/translation="MMDLQSFVSDYTKDKKHLQLVIGIVREGDVEYYSFDNNKKKSMI

HPENMLFEIGSITKVFTSIILLEMEK"

CDS 94395..95249

/gene="ampC"

/locus_tag="PIKHDJKN_00119"

/EC_number="3.5.2.6"

/inference="ab initio prediction:Prodigal:002006"

/inference="similar to AA sequence:UniProtKB:P05193"

/codon_start=1

/transl_table=11

/product="Beta-lactamase"

/translation="MLSSDDIVGKFVQNVKNEYLNKITLRSLATHTSGLPILATNHNL

AKKRSNPYSMYTEQDLTAFLSNADYTDSIGSFGYSNIGVGLLGYILCKVSGSTYDELL

KKYITSPLKMNETAVMLDSEQNGRFLNGHASTGKRVPHWDLSIHEGAGGIKSSVSDLS

LFVQANLNDDHPIASTLQKSHLPIWIGNSNRYFGWGEDNLLDKGILWHNGGSAGFNSY

LAFNKELRVGVVLLSNYSFFSNSRMDYYFAQFIKMITKKEPSQGTMIDSTSKKILEEI

MRRETSLK"

CDS complement(95283..95825)

/locus_tag="PIKHDJKN_00120"

/inference="ab initio prediction:Prodigal:002006"

/codon_start=1

/transl_table=11

/product="hypothetical protein"

/translation="MQLETERLIIRDILETDWESIHSYTSMTEVTQHTAWGPNTEEDT

RAYVEFVLNSQQTQPREGFELAICLKKEGILIGGVGLHIEKTNAEIGYVLNPVYQGKG

YAAEAARAMLGFGFNTLGVHRIYAKCRPNNKASENVMQKIGMQREGLMREHWFYKGEY

HDSCLYSILAKEFINHKKTT"

CDS complement(96143..96298)

/locus_tag="PIKHDJKN_00121"

/inference="ab initio prediction:Prodigal:002006"

/codon_start=1

/transl_table=11

/product="hypothetical protein"

/translation="MNTTLQKADLQLDDYLDLLNLATNLGDTKWQKEIIRKLAYTYPA

VECKQPQ"

CDS complement(96451..96741)

/locus_tag="PIKHDJKN_00122"

/inference="ab initio prediction:Prodigal:002006"

/codon_start=1

/transl_table=11

/product="hypothetical protein"

/translation="MKFLFLLLLLIVPLIMVFVALRSRMVTRVFHVLALLCFYSAATV

IAGDVYATNAHMTTFTTEIHHFLLNGWFLYPSAYLGVYVPYVLWMSLFSKKS"

CDS 96911..98473

/locus_tag="PIKHDJKN_00123"

/inference="ab initio prediction:Prodigal:002006"

/codon_start=1

/transl_table=11

/product="hypothetical protein"

/translation="MKKAIWQHWIGGFLIFIMMATALLQPGSQGPVQAAAAPFTVSQA

IAAQSGGATATVEGYIVGHATGSLTAKFTSPYANDFNFLIADSATEKTNAKLLDVQIP

ASYRSQYGLASNPNLVGQKVIVTGTLGAYNSYAGVKNPTSIALSSGTTNPDPDPGTTL

PDGTGKKVLFDNTHAQTAGAADWVIDGAFSDFANGLRNAGFTVDQLNRSIPYTFDEQA

ITFNKLKDYDVFVIGEANVPFKATEQAALLQYVQNGGSVFFIADHYNADRNKNRWDSS

EVFNGYRRGAFLNPAKGMSSAEAESPAMQGVTSSDWLASNFGIRFRYNALGDVNATDI

VAPSQSFGITTGVSAVAMHAGSTLAIIDPNKAKGLVYVPSGVSKWGNAVDQGVYNGGG

RAEGAYAAIAKIGAGKAAFIGDSSPVEDATPKYLREETGAAKTTYDGFKEVNDGVFLV

NTVKWLAVKESYTSLSQVSGLTLDTPTSLLAIEAPASSNEPQTEPWATPAAGYKWYDP

TTFKAGSYGKAQ"

CDS 98618..99250

/gene="cdaS"

/locus_tag="PIKHDJKN_00124"

/EC_number="2.7.7.85"

/inference="ab initio prediction:Prodigal:002006"

/inference="similar to AA sequence:UniProtKB:D5TM67"

/codon_start=1

/transl_table=11

/product="Diadenylate cyclase CdaS"

/translation="MMMNRQADCDSSTMRQKLKGDLHRVADRMNQTLSTFDHDSVCLL

GQFAEIRAEIKQIEVLASSFYLDCYLSPFTEKFAELTASVQHLSDRRYGALIVIEREV

PLEPIIHSGVAVDARVTHALLESLFIPGAPLHDGAVLIRGNQIVSAGNVLPLSQAIVH

ERKIGTRHRAALGLSELTDAVVLVVSEETGQASFAVGGDLHPINVVETLP"

CDS 99448..100554

/gene="ldh_1"

/locus_tag="PIKHDJKN_00125"

/EC_number="1.4.1.9"

/inference="ab initio prediction:Prodigal:002006"

/inference="similar to AA sequence:UniProtKB:P13154"

/codon_start=1

/transl_table=11

/product="Leucine dehydrogenase"

/translation="MSWFEAMEHHDYEELVLCQDRASGLKAIIAIHDTTLGPALGGTR

MWTYASEEAAIEDALRLARGMTYKHAVSGLNLGGGKAVIIGDPRRDKNEAMFRAFGRY

IQGLNGRYVTAEDVGTTEEDMNLIYQETDYVTGISPSYGSSGNPSPATAWGVYRGMKA

AAKQAFGTDLLEGKTVAVQGVGNVAMRLCKYLYEEGAHLIVTDIHKDSVKQAVDQFGA

KAVDPADITGVDCDIYAPCALGGTINDDTLKQLKAKVVAGCANNQLLEPRHGDKLHEM

GIVYAPDYVINAGGVINIADELNGYNADRAWSKVGEIYNNLEKIFDTSRTEGIATYIA

ADRLAERRIELMKNTRSTFLQNGHHALSSRRLRG"

CDS complement(101118..101981)

/gene="gltR_1"

/locus_tag="PIKHDJKN_00126"

/inference="ab initio prediction:Prodigal:002006"

/inference="similar to AA sequence:UniProtKB:P94501"

/codon_start=1

/transl_table=11

/product="HTH-type transcriptional regulator GltR"

/db_xref="COG:COG0583"

/translation="MELSDIDIILAVARSGKISQAAKELNYAQSNVTTRIKKLEQEYQ

VQLFNRFPKGVVLTSKGEQFVQYATRIRDLLNDLEQDMTDPGEPSGTLKIGIVETAAS

SRFMEILNEYQVTYPEVSISLVNATSPKLLRKKIQEYEIDGAFISGACVKEGLKVEYE

MQDTVHIISKRMDVAPESLCQVSWAVFPKGCPYREITEEFLQEEGLSARNMIEVSTMD

NLLSCVESGIAFTIMPCSVIHKKPDEFSVHDLAERFTHTTTTFVRGEDRYVSSALDRF

VKLLNQKCITF"

CDS 102156..103208

/gene="nemA"

/locus_tag="PIKHDJKN_00127"

/EC_number="1.3.1.-"

/inference="ab initio prediction:Prodigal:002006"

/inference="similar to AA sequence:UniProtKB:P77258"

/codon_start=1

/transl_table=11

/product="N-ethylmaleimide reductase"

/db_xref="COG:COG1902"

/translation="MKNMSTLLSPVTINEWHLRNRLVMAPLTRGFANDQDGTATEEMV

AYYERRARDGVGLIITEGINPSLAGKGTYGIPGLYTVEQTDSWKKVTNAVHKHGGTII

AQLWHVGRLSHSDLIGNPPLAPSEIPAQGKVHKLHKPYQIPQAMSVLDIRDTIQHFQT

AARNAVLAGFDGIELHAAHGYLIDQFVNEKTNHRTDEYGGDIQGRLRFLKEIIIAVQK

EISVDRISVRFSEKKDDDPSYVWSDKAGVLNAYLNLFRETGITILHPSTDRYTTVWTR

EQNFHEAIRQQWEGIIIGVGDLEAKIAEQAVQNRSIDLAAFGRPFIANPDLVQKLRTG

QQLIEYDANAHLTVLV"

CDS complement(103279..104484)

/locus_tag="PIKHDJKN_00128"

/inference="ab initio prediction:Prodigal:002006"

/codon_start=1

/transl_table=11

/product="hypothetical protein"

/translation="MDMQPDVKSNSVPSWLILLLAAACGLIAANLYYAQTVIGPISVT

TGLSSAAAGLIVTLTQIGYVIGLLFIVPLSDILENKRLVTFFLIILVVALIGAAFSSH

AVLFLTASLVIGISSVVAQILVPYATYLTSEEQRGRVVGNVMSGLLLGIMLARPVASF

ITSLLGWQAVFVFSAIIIVLLTLLLSRALPARRPQPAMKYGQLIVSLGSLLKTFPLLR

RRALYQASLFGAFSLFWTTVPMQLANEYGMSQQGIAWFALAGVGGAIAAPIAGRWADK

GLTRVLTGLAMVIAAASFGLAYLFQGHSTATLILLVVVAITLDMAVSGNLVLGQRIIY

SLSEARGRVNGIFMSIFFVGGAIGSSLGSWSYAHGGWSLTTLIGLLMPLLALVYYLTE

KKVTVVSNP"

CDS 104744..105319

/locus_tag="PIKHDJKN_00129"

/inference="ab initio prediction:Prodigal:002006"

/inference="similar to AA sequence:UniProtKB:P9WMD1"

/codon_start=1

/transl_table=11

/product="putative HTH-type transcriptional regulator"

/translation="MTAKRGRPRNVETQNAILAASYELLLEHGFGAITIEKIAERAKV

SKATIYKWWPNKGAVIMDGYMSAAKARLPVPDTGSVFEDVRIHASNLVDFLSSREGKV

ITQIVGEGQSDPGLAEEYRTRYIQPRRREAWGIFEKGVERGELKKDCDIGLCIDLVYG

AMFYRMLVTGEPLDTEFVQRSLISLFEGIKS"

CDS complement(105423..106547)

/gene="limB"

/locus_tag="PIKHDJKN_00130"

/EC_number="1.14.13.107"

/inference="ab initio prediction:Prodigal:002006"

/inference="similar to AA sequence:UniProtKB:Q9EUT9"

/codon_start=1

/transl_table=11

/product="Limonene 1,2-monooxygenase"

/translation="MKFALFSLMMNLPNAVTGESLTTQQKFHNILEQAKLAERLGFDA

YGIGERHGAPFLSSSPPVVLTAVAAATSRIRLLTTVTVLSILDPVRVAEDYATLDQLS

GGRLEMIIGKGNDPRHYPLFGISEEEQWDSLGERYELLKRLWTEENVTWQGTYRPPLQ
EVTTQPRPLQQSIPIWHGSASSTRSTELAAKYGEPIFSSNSFHPQAKYKALIDHYRER
LDYYGHDANRAVVGSGAGSLYLANTREEAIRRYTPYYEAFHATAAAQHNQSPFKDLED
NIAHGPVLIGSPEQVIEKILNYHAAYGHQVLSISVDGLSHAEQLEQVERFAQDVAPVL
RREIPSFIWNEPPLLNQSLPSSTSHSPDSWPPAISPIFQM"

CDS 106817..108208

/locus_tag="PIKHDJKN_00131"

/inference="ab initio prediction:Prodigal:002006"

/codon_start=1

/transl_table=11

/product="hypothetical protein"

/translation="MEPTTTIRSFIEDYIRKQGYTLQYFADISGVNAGTLSAIIKGTR
PIAMAQLDLITKGMKLEEGHFYEIYGAECFVESAPHWRRLEPFLQRCAELDKLECIQR
VIQQVTDDRSYISELFEMAEGMLERGQKKAARMLYECVAECEKYQHSERLALCQYRIF
TLSLGQDQHENLRAAVHFEPYINRLDEERQLDAIKDLANTYLALRYWDKVSDLGGELE
QKTSFLNSYKKRKSENLRIASYPIFVYSAYSNLLRAAACDGREEYSEALRYTEAYSKL
IRIEQPNEEDLIFINKFSGWAEVNHYLYRLMMGDSEVIRPYIAFIEKNEAEILPALVK
IMEAANHHNIDVDAVLNRLESYISMYAKTYGDIGSYTEQITSGRYVQFLKEVAIYQLQ
RERYTTGFQYLLNCLALAKSMKSESIIIQCVALFERHRARASIELEIKYKTLIEEVYS
NYEKKSSFDRSFV"

CDS 108171..108299

/locus_tag="PIKHDJKN_00132"

/inference="ab initio prediction:Prodigal:002006"

/codon_start=1

/transl_table=11

/product="hypothetical protein"

/translation="MRKKAALIVVLFSAVLLFSLYPQPIPSNTMMYSPSTHGGAGL"

CDS 108468..109853

/locus_tag="PIKHDJKN_00133"

/inference="ab initio prediction:Prodigal:002006"

/codon_start=1

/transl_table=11

/product="hypothetical protein"

/translation="MKLAPTIRTYIENHIRERGYKLQQFSDVTGVNVGTLSAILKGSR

PLAMNQLDQITSGMGLEKGYFYEMYSVECFVEAAPHWRRLEPFLYRCAELNKLGCIKK

VVYQVTDDRSYISELFQMAENLYFKEMNEAALILYGCVAAGEKYQHSERLALCQYRIF

LLHKTMSKFDNLAAAAQLEPYIEKLDEEIQLDAIKDLANVYSTIHLWDKVYELAEELE

RKVDFQLELQSRRRKNRKRTAFYPIFTYKAYANLLKASVCEARKQYEKALEYTDVYVT

AIEISNPTEEEQELIERFKGWAEGNRYLYHLMNGNHEVIEPYLNYLDANPHEILIAFV

NIVQAANRHSLDIDSALDRFDPYIKQFNTDIHLKGTYNMQMLNYRYICFYYELAKYRL

NQQRYATGIDTLLTSLELSSSSNDDLMSIKSIDLYGKFRRHVTNQQEEQYIRLMEGLS

SQNFGSRIKSV"

CDS 109985..110146

/locus_tag="PIKHDJKN_00134"

/inference="ab initio prediction:Prodigal:002006"

/codon_start=1

/transl_table=11

/product="hypothetical protein"

/translation="MREIKQIKDQIEQNRQHLRRLVEKHGMHDDKVLKQSMVLDELIN

KYIRLIEKH"

CDS 110284..110793

/locus_tag="PIKHDJKN_00135"

/inference="ab initio prediction:Prodigal:002006"

/codon_start=1

/transl_table=11

/product="hypothetical protein"

/translation="MTTSMWIQMIIMFAVLVFTSIGRKPVRFYRLLLPFVIMGYYAAK

FLKDMIFTGNNVLFLLVAVIVGAVFGMIVLGITRMEFDATAGKWYTRVGIGSVVVWAV

AFLLRIVPVEWITHHPQQAYHYAIEDQLDLQNIGPAFILMTAAMVLVRVAGILIRSRA

SKVQHTIGT"

CDS 111041..111229

/locus_tag="PIKHDJKN_00136"

/inference="ab initio prediction:Prodigal:002006"

/codon_start=1

/transl_table=11

/product="hypothetical protein"

/translation="MREPKQIRDQIEQNRHELSRLAEYHGMQDYKVLQQSMVLDELIN

EYNRFKYKKHFMKRQPIA"

CDS 111459..113282

/locus_tag="PIKHDJKN_00137"

/inference="ab initio prediction:Prodigal:002006"

/codon_start=1

/transl_table=11

/product="hypothetical protein"

/translation="MMDKVQGYFGRNMNLRTKLLLLFLALTLLPLSLQGVVNYLHFSQ

TIDRKTEQFTIDIVRQINTNLNRLLKDFERLSLLPLYDQMVLGILGKYNAPMGSGTWA

RSDDYLKMKLYTSGQAYDRPEIRGIHLISNSGILFSNLDSLAVNPVWDSRQDDWFAEL

NGSEGEWRLLPPHEPSYYTGSHEEPYISVAREIRDPGTLQRLGYILIDIRLEAFGQLL

SNLNFEQNASLMIVDSKQHLLFERTSAGGLSAYDQLLKHGQLQGYAGNQKVVLDGKPY

LYVQHHSSYSGLSVISLTPIAVIQKESGEMLTFTFGFAVLCMAAVAVLAVLLSYRITR

PLIRLKHNMIRVEQGDFSQRVAHFSNDEFGQISRGFNRMMEEINRLFNEVFLLGIQER

EAELSALQSQINPHFIYNTLESINMMAVRQKHAEVSDMVTALGKLLRYTIDKVDRRVP

LQEELAFVQSYVRIQQVRYDGKLEVIYDIDEAITECLIPKLILQPLVENAVYHGIEGQ

ADGGVIWVSALKFDDELLIIVRDNGKGMAQAEIDELNESIARQPSSEALRCHAGDRLG

LNNISQRLRLMYGEGGSLSVDGSPGQGLAVTISIQLQQKGE"

CDS 113279..114055

/locus_tag="PIKHDJKN_00138"

/inference="ab initio prediction:Prodigal:002006"

/inference="similar to AA sequence:UniProtKB:Q5HJF7"

/codon_start=1

/transl_table=11

/product="putative response regulatory protein"

/db_xref="COG:COG4753"

/translation="MKRMYKVLLVEDETVIRQGLRELIVQAAPQFQVTGEASSGAEAL

DFLRCEVPDVLITDIRMREMDGLTLVSKARDMYPELLMIIISGYGEFEYARRAMEYGV

LNYLLKPIERHELALCIQKIQLLLDRRYGISTLPVTEPSGKAEHFGGDTRKIIRNVKE

HIKQHPDGDLRLQTIAALVNLNPTYLSQLFKNETGTNYSEYIAEARMERAKWLLINTG

LKIYDVARLSGHQSPKHFMLVFKQQVGWTAGEYRDRFSIS"

CDS 114159..115454

/locus_tag="PIKHDJKN_00139"

/inference="ab initio prediction:Prodigal:002006"

/codon_start=1

/transl_table=11

/product="hypothetical protein"

/translation="MKKAGLILILLLMMIASIGCTSSNSGSNPENAAGENGEVELKFM

MWGNQAHMDLYNKLIEGFTQENPGIKVTMESVPFAEYQQKISVLAAGGSLPDIAWVSE

RMVPQFKSNHILADVSEFKDDAQFKLDDYIPSTLDLFRDGDQLLGLPFSTPPVVMFYN

KTLFDKAGLTDPNTLASQGQWTWEQFEKSAKAITSKEATNRVYGANFFRDWKNWAVLS

SYSWSNGSGPFDEGMTKFTWNDAYGVQTFELLERMMFTDESHPKAGEQVSFDAGNVGM

FFDNYSYVSKAREITDFEWSIAPMPSGSQGSVPMLGQAGYAMFNDSKHPEETKKLLEY

FASEQGIQATATYFVPPRTSVLNSDAFINQPNNPSKEHIVQAVINEMPKARLIPGHIR

WQDIDNAVLQGFDRLFARSATPEDNLKQMQEEIQGVLQQ"

CDS 115482..116186

/locus_tag="PIKHDJKN_00140"

/inference="ab initio prediction:Prodigal:002006"

/codon_start=1

/transl_table=11

/product="hypothetical protein"

/translation="MMGKFKELLDRNSLSLVVSLPENDLSLARAAIEEGADALKVHYN

VGHRASGNHFGPLDNYAEVFQAIRSEFGGPLGVVPSGSIDGARREDVERLSGLGFDFY

SIYAHHLPSFMLDDLGLDPTFAINDEYEPSLATSAAHYGFTALEASIVPGNEYGTPLS

FADVLKYRRLVLQAQIPVLVPSQRKLVPEDVRVLHDTGVKAIMLGAVVTGRAEEQLRR

AVNGFRNAVDNLNRPD"

CDS 116220..117134

/gene="lacF_1"

/locus_tag="PIKHDJKN_00141"

/inference="ab initio prediction:Prodigal:002006"

/inference="similar to AA sequence:UniProtKB:P29823"

/codon_start=1

/transl_table=11

/product="Lactose transport system permease protein LacF"

/translation="MTHSRKRTGRGPLAREAQIAGWLFVSPMVLGFTLLLLFPMGLAL

YMSLTDWPLLGDHHFIGLENYRNVMTDAMFWKVLANTVYFTAGLVPLNIVLALLLALL

LSRSLRGIGIFRTAIFVPVMTSLIVWSIVWKLMYATESGLINQLLLMIGIKGPAWLYN

QDLAMPAVIVTSVLKNVGLNMVLFIAAIQQVPRSLYEAATLDGAGRRGTFFHVTLPMI

TPTVFLTMVMTVIGSLKVFGQIYVMTQGGPSNSTKVLVYYIWEKAFKLFQFGYASSLA

YVLFFIVLILTLLQWQLRKRWVFNEGDA"

CDS 117118..117972

/gene="araQ_2"

/locus_tag="PIKHDJKN_00142"

/inference="ab initio prediction:Prodigal:002006"

/inference="similar to AA sequence:UniProtKB:P94530"

/codon_start=1

/transl_table=11

/product="L-arabinose transport system permease protein

AraQ"

/db_xref="COG:COG0395"

/translation="MKAMPNPSIRKTGSALGTYLLLTLISLIMIVPFIWMISTSFKEP

QSIFTYPPQWIPDTFRFQNYIDVFRLIPFHRFYFNSVYIAAVVVLGTVFFASLAGYAF

AKIPFKGRNVVFLILLSAMMIPHEVTAIPMFLFMRQLGWIDTHLPLILLPIFGAGGVL

GIFVMRQFFITVPTELEEAAMIDGCNRFRIYARIMLPIAKPGMATLTIFTFVTIWNEF

FDPLIFINSRELMTLPLGLSLFTDEVGTAWQYLMSATVMATLPLLIVFFLAQRRFIEG

VAMTGLKE"

CDS 118003..119364

/gene="thi4"

/locus_tag="PIKHDJKN_00143"

/EC_number="2.4.2.59"

/inference="ab initio prediction:Prodigal:002006"

/inference="protein motif:HAMAP:MF_00304"

/codon_start=1

/transl_table=11

/product="Thiamine thiazole synthase"

/translation="MSERISMSMKKEEADVVVVGGGPAGIAAAIAAGRQGVRAVLVER

YGFVGGMSTAAMVYPWMTFHTESGEQVIRGIAQEIVDRLQARGGSPGHLRDTVGFVHT

VTPYHPAIYQVVAAEMLQEAGVRLLLHSFVDEVVVADDRVEAVRLTNKSGRMELQANV

FVDASGDADAAYLAGASVAKGRDGDHQSQPMTMKFRMRGVDLGRVKQYMQEHPEDFYA

KTPFSELDSIPLTGVSGFYSQWKKAGVPINRDQVLFFTGPAEDEVLINCTRVQGLDAT

DAEDLTSAEQEGRRQVLMIAEFLQRDVPGFERASISAVAPQIGIRESRRIIGHYALTK

ADVVAGRKFDDVIARSGYPIDIHDPSGQGVVAAFIEGDGAYDIPYRSLISRNIRNLLA

AGRCISTTHEAHATTRLTPSCMATGQAAGTAAALAVQMKLDPLELPVAVLQKELRRNG

AAI"

CDS complement(119478..120128)

/gene="glnP"

/locus_tag="PIKHDJKN_00144"

/inference="ab initio prediction:Prodigal:002006"

/inference="similar to AA sequence:UniProtKB:O34606"

/codon_start=1

/transl_table=11

/product="putative glutamine ABC transporter permease

protein GlnP"

/db_xref="COG:COG0765"

/translation="MDFSGAYAWPNLRFLLHGFLITLQVAGLSIVFSFVLGTVLGTVR

YTRIPVLSQIVAVIVDTIRNLPLLLIIFFIHIVLPQLGIKMSVFWSTVVGLSLFEGAM

IAEIVRSGLKSIERGQVEAARSSGLSYMQTLGGIIMPQALRRMSPPMVSQFISLLKDT

SLAIIISLPELMHNVQILGGQSFDYVIPALLLAAVLYFVINYTLSVVARRLEARMT"

CDS complement(120182..120904)

/gene="glnM"

/locus_tag="PIKHDJKN_00145"

/inference="ab initio prediction:Prodigal:002006"

/inference="similar to AA sequence:UniProtKB:O34671"

/codon_start=1

/transl_table=11

/product="putative glutamine ABC transporter permease
protein GlnM"
/db_xref="COG:COG0765"
/translation="MGKLDFSVLIKHSDRFLEGFLNTIQVSIMALIGSFILGAIMAIF
RISPIKPLNWIGTAFVEFIRNIPLLLVVFFFYLGLPALGISLDGFISGTLGLTIYTAA
FIAEAIRAGIQTVPRGQLEAARSSGLSYVQAMNLIILPQAIKIVLPSIGNQFINLVKN
SSILAVVAGMDLMYFADLINSDTFLPLSVYTIVALFYLVLTLPLSFLVHYMERRFGQS
DADASRVKGKPKKNKPTGQVTM"
CDS complement(121007..121840)
/gene="glnH"
/locus_tag="PIKHDJKN_00146"
/inference="ab initio prediction:Prodigal:002006"
/inference="similar to AA sequence:UniProtKB:O34563"
/codon_start=1
/transl_table=11
/product="ABC transporter glutamine-binding protein GlnH"
/db_xref="COG:COG0834"
/translation="MKNVLKWPSFMLVLILSLVLSGCSTGEESSSSGGSGGSTEAKGT
IEQIKERGKLIAGVKYDTKLFGLKDPASGEVEGFDIDIAKALAKQILGDETKVELKEV
TSKTRIPMLQNGDIDIIIATMTITDERKEQVDFSDVYFEAGQSLLVKNDSAITGLESL
SGVKVLAVKGSTSAQNIREKAPDAEVLEFDNYQDAFTALKAGKGEALTTDNIILIGMQ
QTDNNYKLVGGNFTSEPYGIAIRKGDTAFVEEVNSLLKNLKDSGEYDTLHEKWLGSKP
E"
CDS complement(121873..122601)
/gene="glnQ_1"
/locus_tag="PIKHDJKN_00147"
/inference="ab initio prediction:Prodigal:002006"
/inference="similar to AA sequence:UniProtKB:P27675"
/codon_start=1
/transl_table=11
/product="Glutamine transport ATP-binding protein GlnQ"
/translation="MIEFRGVQKHFGHFHVLKDIHLHIEEGEVVVIIGPSGSGKSTLL

RCINRLESITEGELVVSGVPLHQKKVDINLFRRDIGMVFQHFNLYPHKKVIDNITLAP

MKVRKQPKAQAASTAMKYLTRVGIADKADSYPSQLSGGQQQRVAIARGLAMEPKIMLF

DEPTSALDPEMIGEVLDVMRSLAHNGMTMVVVTHEMGFAREVADRVIFMDEGRIVEEA

SAAEFFDNPREERAKQFLSRLIHH"

CDS 123064..124677

/locus_tag="PIKHDJKN_00148"

/inference="ab initio prediction:Prodigal:002006"

/codon_start=1

/transl_table=11

/product="hypothetical protein"

/translation="MNTYYSPLGTGEGIESDIKAYEVSLPVIPARDFQFTDYGAVGDG

LTDNTEMFRLAIAACAEAGGGRIVIPAGVWLTGPIVLRSRIELHVQAGALVTFSRNFD

QYPLIASSFEGWQAVRCQSPIDGEQLEDIAITGEGIWDGGGEAWRPVKRSKMTASQWN

RLVSSGGVVEHAGGDEAIWWPSTSALEGGAIANRLHLEQVRDVAAYDEVRDFLRPNMV

SLRQCKRVLLDGPTFQNSPAWNLHPWASEHVTIRNVSVRNPWFSQNGDGLDIESCRHV

VVEQSVFDVGDDAICLKSGKDAEGRELGLSSEYITIRNCTVYHGHGGFVIGSEMSGGV

RHVRVSDCTFIGTDIGLRFKSARGRGGVVEDIQVEHIYMKDIIMEAISFSFFYANQEG

SARGSDLTQEVSEETPVFRDIRISDVVCAGAETALLVSGLPEMPLDGLVIQGYTVTAR

NGVQCAHAKHLRIAEMKAHITEGSLIHLHQCKGAELEAIEGVGADGRLLMVTGHESAG

IVCRESDADTEGRQISVGPEVRSGVIIRR"

CDS complement(124792..125643)

/gene="araC_1"

/locus_tag="PIKHDJKN_00149"

/inference="ab initio prediction:Prodigal:002006"

/inference="similar to AA sequence:UniProtKB:P0A9E0"

/codon_start=1

/transl_table=11

/product="Arabinose operon regulatory protein"

/translation="MSVIEDRIGPKYRIGESSFTIEHMSRHDGNAMPQPHAHPFYELY

YLLEGERVYSMNGQLLTARKGDLILINPHDVHTTSKGSIPGFERILIGFSPSFATGME

LGICGLLPFDRSRLLHFPDAEQPEIERMLWQMLQECRERRPHYDIVVRSLLAQILIQI

HRVEEDTRQSSPVPHHPMQDKISEIVTYVNNHYTDPLTLEEAAARFYISPSYLSRMFS

RFTGFRFSEYLRVTRVREAQRRLLSTQERVQMIAEKVGFEHTAHFNKTFKQVTGTTPL
RYRKEHR"
CDS 125902..126360
/locus_tag="PIKHDJKN_00150"
/inference="ab initio prediction:Prodigal:002006"
/codon_start=1
/transl_table=11
/product="hypothetical protein"
/translation="MTTHELSPLVAALDMQPHVEGGWYKEEWKASYQIPQSVLPDTYS
GPRFSASSTYFLLHSHEVSEWHTVLSDELWLWHSGSPVELKLGGNGENPENEEVLVLG
MDIAAGQSPQVLVPAGVWQTARPLGDEPVLVTCVVAPGFHFDDFKLVSKG"
CDS complement(126457..127284)
/gene="ytbE_1"
/locus_tag="PIKHDJKN_00151"
/EC_number="1.-.-.-"
/inference="ab initio prediction:Prodigal:002006"
/inference="similar to AA sequence:UniProtKB:O34678"
/codon_start=1
/transl_table=11
/product="putative oxidoreductase YtbE"
/db_xref="COG:COG0656"
/translation="MKHVQDTTTLYNGVKMPWLGFGVFKVKDGEEVVDAVKTAIQAGY
RSIDTAKAYNNETGVAQGIRESGIAREDLFITTKVWNGDQGYESTLAAFEASMERLEL
EYLDLYLIHWPVKGKYKDTWRALEKLHKEGRIRAIGVSNFQIHHLEDLMTDATIKPAV
NQVELHPLLIQTELREYCSKHQIQIEAWSPLGQGHLLEHPLLKDIAAKYSKSPAQVIL
RWDLQNGIVTIPKSVTPQRIRDNADLFDFELTTEEIERISQLNENKRFGSDPDNFNF"
CDS 127621..128877
/locus_tag="PIKHDJKN_00152"
/inference="ab initio prediction:Prodigal:002006"
/codon_start=1
/transl_table=11
/product="hypothetical protein"

/translation="MAKPKQTKKAAAWSLVVMGAGFAASLPFQGGVVGKLLVGSFEAG
LVGGLADWFAVTALFRHPLGIPIPHTALLPKNRDKLTEGLVSAVENNLLNKDSITEKI
ADFKAAEIVLDTMTRELHNDGVKTMIDTLCKRILAGLPLEQIAPLVAGEIKSQAGAFD
LGPILERASIQMSERGYDAKALDYGLKQAEEWLVKPETIMFLGESGLKAISGIQMNGL
MQFAMNAFMGYLNEERLGSILQGYLFDRVEDMKREGSALRYKVLDLLRTQAIRLARSE
SVQDGVNGWKNNMLDGWNAEETVLNKLIELRDKALTAMEDGQYVETFALPAIERVLSD
LRSDDALLAGLNAKIVEGVTTLLEKNHSKIGNLVRENVDKMDNASLISLIEDKVGQDL
QWIRINGAVTGFIIGIALTALRMVLE"

CDS complement(128965..129318)

/locus_tag="PIKHDJKN_00153"

/inference="ab initio prediction:Prodigal:002006"

/codon_start=1

/transl_table=11

/product="hypothetical protein"

/translation="MKKALTILALVIVILTCLWLFFMDPAKLTPDNPAGKTTYYTIVN
QSKVNKDDNGRYAYELVSYTDEGKSKTLSFSTSKLLKNGAYLKLYVSTLRGVTHWEEV
TADDLPGHAKKLTSK"

CDS complement(129347..130078)

/locus_tag="PIKHDJKN_00154"

/inference="ab initio prediction:Prodigal:002006"

/codon_start=1

/transl_table=11

/product="hypothetical protein"

/translation="MLRSISAEWLKLRHSRISLVLAILPIISLLIGCFNFYFNQAALQ
NGWYSLWTQVSLFYGEFFLPILIAICCAFVCRLEHSNRNWNMILASPLSVTSLFMAKL
VIVGILIFSAQAFFMLMYWAAGQLFTIPGSFPMETLVWSLRGWFASISIAALQLGLSI
RIRSFATPIGISLCAVFLGLGMYVLKIGMLFPYSLLTIGMGVLSQKGLTSSENIVFLL
MNCLFILLFATWSIRRLKYKDIASS"

CDS complement(130091..130840)

/locus_tag="PIKHDJKN_00155"

/inference="ab initio prediction:Prodigal:002006"

/codon_start=1

/transl_table=11

/product="hypothetical protein"

/translation="MKALSLEYFKIRRKKIWVMLTLFLAAEMIWASMSMSISISRSAA

NASWEALIFSIASMNGLFLPIISAIVVSRICDMEHKGNTWKMLMTTNTGRNHVYTAKY

ICANSLILYGIIAQAVFIVGFGLSKNIPGPLPLSLLVQFAAGTLLTTLAITALQQWLS

LTVRNQAFALCLGMLGGFIGTTSGLFPAGIRHLFIWSYYMDLNPVTYIYSASSGTYVS

GMLPVGLFIAALLMAAIFYFAGNIHASRQEI"

CDS complement(130840..131757)

/gene="bcrA_1"

/locus_tag="PIKHDJKN_00156"

/inference="ab initio prediction:Prodigal:002006"

/inference="similar to AA sequence:UniProtKB:Q5WNX0"

/codon_start=1

/transl_table=11

/product="Bacitracin transport ATP-binding protein BcrA"

/translation="MNTFPIVETHNLSKSYGGTNRVHQINLAVETGQIFGFLGPNGAG

KTTTLKMLLGLIKPSEGTVKVFGKDLNKHRPSILNQTGSLIESPSYYGHLTGLENMKV

MQRLRNVPNKNIDEALKIVRLENQKHKQTDQYSLGMKQRLGIAMALLAFPSLLILDEP

TNGLDPAGIGEIRELIKSLPGQYGITVLLSSHLLSEIEQIATSVGIISDGKLLFQGTM

QSLQANNKATIRFQTTDTARAEKILLTQGYMPKVQGKQLVFDYLRDEEVSRMNKVLVE

HDIPVIRIEEHKKSLEDIFLDLTGKERSL"

CDS 131916..132629

/gene="walR_1"

/locus_tag="PIKHDJKN_00157"

/inference="ab initio prediction:Prodigal:002006"

/inference="similar to AA sequence:UniProtKB:P37478"

/codon_start=1

/transl_table=11

/product="Transcriptional regulatory protein WalR"

/db_xref="COG:COG0745"

/translation="MELMKNKKILIVDDEPEIREMIERFLRKEGFFRVYTADNFVNAL

AVCRLEKPDAAILDVMLPDGDGFSLLSSIRSFSDMPVLFLSARGEDEDRLLGLGLGAD

DYMVKPFLPRELILRLMAILKRVYASNTVERLPVFRLGGQIVDLESAVVQGTGREFPL

TAKEHAILIKLYENQGRIVTSDALCQAVWGDESYGYENTLMVHVRRIREKIEDDPSKP

IFLLTVRGLGYKLMVPESR"

CDS 132629..134011

/gene="sasA_1"

/locus_tag="PIKHDJKN_00158"

/EC_number="2.7.-.-"

/inference="ab initio prediction:Prodigal:002006"

/inference="protein motif:HAMAP:MF_01837"

/codon_start=1

/transl_table=11

/product="Adaptive-response sensory-kinase SasA"

/translation="MDGVVRILRRFVGSTILVSVVLLLFNLILLGSLIFKEIHEGPSP

GAVVKQVASVLSKGEQGDYALKSDGMQLLDTYQAWAMLLDPEGKVQWSERLPAEIPRS

YNVTDVAKFSRYYLMEYPVYVWEREDGLLVVGYPKNTYGKYQFDFLAEWLKSLPVRIV

LLLVCNVALALLVSVLIGTRVIRSIRPLIHGIHALAKEEPVHVETKGIFNDLSQSINS

TSRMLQDKNDALKARDEARSNWIAGISHDIRTPLSMILGYASELEEQPDLPGEQKQQA

SIIRRQGERLRSLVSDLNLVSMLEYEMQPLQLKSIRLSVLARQIVSDFLNNDLDERYP

ISLHVQDESLHVMGDERLLYRAVTNLVQNSIRHNPNGCEIVLETIRAAEDLFCEFVVS

DDGKGIPKEYLNDVVVLPYTAGRIRPSRQGHGLGLPMVARIALAHRGRLILESESGEG

LRAVLKLPTT"

CDS 134288..134470

/locus_tag="PIKHDJKN_00159"

/inference="ab initio prediction:Prodigal:002006"

/codon_start=1

/transl_table=11

/product="hypothetical protein"

/translation="MKQFNFEALGAIEDISDDELMVITGAVSVVDAQASGGVFCTVSH

ECHYNSVSPSSWLTCC"

CDS 134576..137977

/locus_tag="PIKHDJKN_00160"

/inference="ab initio prediction:Prodigal:002006"

/codon_start=1

/transl_table=11

/product="hypothetical protein"

/translation="MSIQEQLNNSRWSDALYLTEKYKQFVQQYTPSVPDDIGEAVTDI

KEENIVASDQSVDKSVEETSDSGRMQLWLDKFFQDQEHLSDRFRAESMNVDTVQVLFN

DTGFRYEFAQTLWHQELRKTFLENEHLEIEYFIEGDTGEIPFFEFSRPFLQRSIQIMT

EGLIGGYDWLDKRRVTGLVLKTVSDKVFHMSAKTLIYELNKARLQGELRGEDSRQRYQ

HFVSQKIKSREQILAFLLEYPVLARGIVEHTLRISLNMMRVLERLTRDRHELESFFEF

KLDKLEDIVFLGDSHNGGASVLRLKFSEEVNVMYKPRDMAIDTAFAGLLQWFNDQKIG

LPFRIAKSLDRSQYGWQQFIPYEEVSNLEGVERFFERQGQYLAILYAVNATDMHMENI

VAQGEHPFFVDLESLLHNQPFKYTEEKDHYTALEKTLSILNDSVLKTSMLPTLDSNAL

YHSDLSGLAGDVDQILNTYEIHDKNTDTMRIKRSKVSVSRFSHLPSYEHTPIKPERYI

ESLKEGFYRIYSAIMEQKELFISIVRNHFHGCAVRTIARPTITYFTLIEASSHPKYLR

NGLDKSLLFDMMWIIVRQDRNREKLVKYECHDLLHGDVPMFQTLIGEEILFHHHDNKA

IDGVFSSDILTQTADKVRAMNQQDMDMQWEFTERTLQTKYVLERSFAMEKSTASVRPE

KLNEHRIPMTKEEYLHEAVRIGEYIKDIAIMGDEGHSVSWISMGMDADEKLIYKSSEL

GLYNGVTGIAYFYLYLSQETGDHKYVQMVDLCIQTAWEMIRKKIDKNISLFTGYGSLL

YLLVHKAKVCLEPGIHPDTLELLDILDEMIDNDQQLDVISGCAGTLMACVDMFTSFQS

QKALDVAVRCGDRLLKLAQPMDQGIAWVTSAINKTPLSGIAHGNAGICLSLARLYSAT

GNKAYLETCMEGLKYEDGLYSPEVNNWKDLRFTDGNMPEDHYVMYWCNGAPGLGIARI

GIAQHLSSLGAMVTVETDIRRAVAKTMEDGFTEVSYSLCHGDMGNLELMLLAADYLQD

EALEEYSYQMADYIVQQVRNDNHHWRCGIPGRQQIPGLMLGLAGIGYQLLRLYNRKLP

SVLMMEGPERKEMWEIVQTYVMETASAQQQINNAEQPSHLGQHYA"

CDS 137970..140189

/gene="hlyB"

/locus_tag="PIKHDJKN_00161"

/inference="ab initio prediction:Prodigal:002006"

/inference="similar to AA sequence:UniProtKB:P08716"

/codon_start=1

/transl_table=11

/product="Alpha-hemolysin translocation ATP-binding

protein HlyB"

/db_xref="COG:COG2274"

/translation="MPERPVFRLRGKRQRKVPVKLQMNQYDCGPSCLHMLLAYHGYEA

DFTDLKERFAMLGNGRDGSSMLKMKEVAQTYHLKGIAKKAPVESLNNNFLPVICFWEE

RHFVVLERIKANGHFIIVDPALGRLVISKYEFTEKYSGIFLLVYPDEGFVTLSEKKIS

KVAYFKDLIRSNKKYFVWTVLLALGLQMVSVAFPFLMQVSIDAATSGEGYSLFPILIT

SIVLLTVFQIGFSYLKDMCIVRLQELLDSQLTTSFVSHMLRLPYQYFEIRSRGDLMLR

INSNTTIREILSQKMISTLINLFLIVVTFTYIVIQSRLIAFTLLSIGLVQILIFVYTR

SRFKKLTQTQIVAQSLAGSFMTEVLEGISTIKSLGIEERTGEKWRALFKRQLATVKEK

AIFQTRVNTVTNTLNFMTPMLILLVGIYQIMQGNMTLGMLVSFQSLAALFLGPLNSLA

LMLNEFVMADALLDRIYDVIQAEESNKFKKKSTPALKAVKLNGDITIDQVNFRYTDYG

EDVLKNINISIKAGQRVALVGKSGSGKSTLAKLLVGLYTPTQGNIYFDGLNAEELEQQ

EIRSQISIVLQDNFVFNNTVYDNIRLHAEESTTEDVMIAAKLADIHADIEKMPMKYNT

LISEAGSNLSGGQKQRVALARALVSRPKILLLDEATSALDTVTEATIAHNLDMLKCTQ

IIIAHRLSTIRSADTIYVLDQGELVDAGTHDELIGRCERYNSLVCEQLAETGVRQYG"

CDS 140568..140849

/locus_tag="PIKHDJKN_00162"

/inference="ab initio prediction:Prodigal:002006"

/codon_start=1

/transl_table=11

/product="hypothetical protein"

/translation="MKESEKQAYFKEAETEAVKYLKNKYELDVVITNKELLPEMALDS

IAMEGHVVEHEAQEFTISYDYEDKKIKNFGMSPAIKEAIIAKGYDPFNK"

CDS 140891..142183

/locus_tag="PIKHDJKN_00163"

/inference="ab initio prediction:Prodigal:002006"

/codon_start=1

/transl_table=11

/product="hypothetical protein"

/translation="MSKSPQIDDETYNKMSELAYENLKSGIEFENLPGWQVLEDTHGN

KKSGFDAVTFYNPETKQAVIAYRGTEGSASLDRSAPDYYADAQIGLPELGRKIEQSTD

LTPDWLANGIQKVRDVTGVTKVENAVGELDKKLDKTFGLGPNQMYEAEDYAKDMQKKY

KDLDFSLTGHSLGGGNAQYAAAYTGLSAVTFSAPSVISSLTPEAKRKAEEGGFDSQII

NYGHPGDFVTSGFFGGYDRHVGSMYYINSNYKDMNDDMGMMDKISNTFGGEEYHGLKQ

YKFENGYISNDLYDPVTGEQISYSPRQPSNVLSDLAAFMRNLGGQAGNLARGMAAAAG

ASGLIQVTPEELKSVAARWKQNAQQCNAELNTVRSRMAHYLHSSRSRRLEPIVTHLDA

SINELSTWHMKHTSQFLNFIEEKAEAFRQADESQVSFK"

CDS 142237..142509

/locus_tag="PIKHDJKN_00164"

/inference="ab initio prediction:Prodigal:002006"

/codon_start=1

/transl_table=11

/product="hypothetical protein"

/translation="MSGQLLVELNDLRIAEKELTQLLARLQADEQEARSLYNRLDDWK

GQSADYTRQQIEAFFAGLSGRIQSIEQQKKSLLQYIEIMIQTDQGR"

CDS complement(142684..143454)

/locus_tag="PIKHDJKN_00165"

/inference="ab initio prediction:Prodigal:002006"

/codon_start=1

/transl_table=11

/product="hypothetical protein"

/translation="MKKTSWTSKLTSSLTAGAAALMLFAGFASAHVTVNPTVAQTGAW

QTYSIKIPSEKELPTTKVTLKVPQGVAFKQYQPLAGWKITTEKNDSNEVTSITWEVDG

DNEGILAGQFQQFNFVAQNPDAETEVAWDAFQYYSDGSIVEWTGGPSDSNPHSITAIS

QDPAATGNAAAGGGHDSAGTAGNAGHDDEAATGDDTKANDDTTLGDALNDTVTGEPNN

TDSDPGTLKLQQATLIVSILALILSFLGIALATRRKKR"

CDS complement(143731..145425)

/gene="ycnJ"

/locus_tag="PIKHDJKN_00166"

/inference="ab initio prediction:Prodigal:002006"

/inference="similar to AA sequence:UniProtKB:C0SP95"

/codon_start=1

/transl_table=11

/product="Copper transport protein YcnJ"

/db_xref="COG:COG1276"

/translation="MKLGKRHWALMGALMLLFCLVMPQWASAHAYIVQASPGENELVA

TAPERLTLEFNESLQTAFFDIKITGPDGTQADDGNVHVDEDRPHILETGLRTGLGNGT

YAVNWKAVSADGHPIQGAYVFHIGEPSGAPAGLADLTSGSGQVGGPLKWILSLTDWIQ

YLGLSVILGTLAFLLFRIAPASMAREPLEVPRSYRLLWISYGAASVAALISLPLNTLY

ESGVSIGEFSWALMGSALKLTSFGQIWMVQILIVMLLAVTILSGYDRDRSLRIRIWSS

YGSLVLVLGWLFTHAMTGHPAAAEQRALAIAMDFVHLIGAAFWIGALTAMAACLPPLA

DKLPSTVRGEVYWTAIRRFTAWGIGAVAALVATGIYSSLIILPAPILTSLFTTAYGLV

LIAKVVLLVMMLALAWRHARMARASSGNRLASSLKAELIAGAVVLALAAVLTHLSPGQ

PAVAGPYKEVQTTDDGTTITLQVSPNRTGENQFKVDVKKPDGSTVNDLEQITLSLTHL

DMDMGIYEITIPKNDTGVYEAEEYISMPGRWNIKVHLLTRSLDSLDTEFEIDTANP"

CDS complement(145606..146271)

/gene="degU_1"

/locus_tag="PIKHDJKN_00167"

/inference="ab initio prediction:Prodigal:002006"

/inference="similar to AA sequence:UniProtKB:P13800"

/codon_start=1

/transl_table=11

/product="Transcriptional regulatory protein DegU"

/db_xref="COG:COG2197"

/translation="MLNMELVRVLVVDDHAHAREAICSILSEDSLFEVIGTASTGLEA

LELTGQWMPDLILMDVQMPDMDGLEATRLIKLRFPYVIIVMVTVSDDVTYLFEALKQG

AQGYLLKNLAPSTWLEYLRAIVSDDAPLSKELAYRILQEFPAPRTEDVPDNPLTTREL

EILQWVSAGYTNREIADQLGISDQTVKNHLKNILQKLHLENRVQLTRYALESGLAGRK

LRK"

CDS complement(146264..147094)

/locus_tag="PIKHDJKN_00168"

/inference="ab initio prediction:Prodigal:002006"

/codon_start=1

/transl_table=11

/product="hypothetical protein"

/translation="MSYKQIKWMILLVPTFTVGLWEYIRHQFLMPYLSMDAGNWLTPL

ILYLISVTLLSRLFNMLESARAALEQERASKVALEARDQLARELHDGISQSLFLLSVK

TDKVERSLAGSGHEHEIQEIRKTVHEVNAYVRQAIAQLRYVPGQAAVPEGTSLLSQVE

LLIEETVPSARLEWDLDSTFFSSKEQVELHACIREALLNVRKHARATDIRVIAEGTPF

VWSIFVQDNGVGVSGDPLHLKDRYGLRITKERAAQMGWSFALDSRPGYTRITIGKGHA

"

CDS 147397..147564

/locus_tag="PIKHDJKN_00169"

/inference="ab initio prediction:Prodigal:002006"

/codon_start=1

/transl_table=11

/product="hypothetical protein"

/translation="MNQEVLERRSELLKKNIHQMLLQDNQHGISRQDNMFLQQMIKEL

HQTSYEMNTTR"

CDS complement(147694..149526)

/locus_tag="PIKHDJKN_00170"

/inference="ab initio prediction:Prodigal:002006"

/codon_start=1

/transl_table=11

/product="hypothetical protein"

/translation="MNFIRNLIHKADRSSMYVMLLSCTGIGIFLYMNKWSYLHLSSDE

WVMVYTMLGAALILDYFTFQIPPKGNQQSMDSSVYLACIFMFGGAFSLSVLLPVSLIL

LIKDRKLTWWKHIVNFSIYSLMIAGAAYVFTWTGGIPGALDGYNLLPYFAALAAYFII

NTITLGLFFHFSTKDALQQMKRVFVTESLLVYLCTLILALVLTILVVHNGVLGLLLYL

SLSILLSHAFKQLFVMYQSIEEKANTDQRTGLFNHSHFESMLESELGNARSQGTPLSL

GLIDIDDFKKYNDRFGHLQGDSLLALLGDFLSRKTEGTQVTAFRYGGEEFTLLMPGMD

LDEAYVFMNKLRKQLNDTPFEGVEVFPHGCLSFSAGVARYEVDMYNKSQLVDQADKAL

YYAKKQGKNNVHRHGSNDGMEQEIDLVQDVRDIEQQLNLFRYKDMDTFKHSKRVYKYA

LDLSELLELDNVEKRNFVLGALIHDIGKLEIPWSILNKKEKLTSEEWETIKGHVTWGK

KMAMTNERFADLIPYIELHHERYDGAGYPYGLKGCEIPRLCRMLTVIDSFDAMTTERP

YQETKNVDEAIKELQMCSGSQFDPELAELFIRYIQKRTVQQQLV"

CDS complement(149678..149782)

/locus_tag="PIKHDJKN_00171"

/inference="ab initio prediction:Prodigal:002006"

/codon_start=1

/transl_table=11

/product="hypothetical protein"

/translation="MKKFEGIKYSRLVINAIIVASVVVALTSGYKLGG"

CDS complement(149779..150411)

/locus_tag="PIKHDJKN_00172"

/inference="ab initio prediction:Prodigal:002006"

/codon_start=1

/transl_table=11

/product="hypothetical protein"

/translation="MVYDGILLGLIVGLFRGGFRYGLHQFAALKLRGGWIFPLLLLAQ

FFIFFLQERLDWVASINGYLFAAVYVTGLAFLWLNRHYKGFTLIWIGVFLNFIVMAVN

GGRMPVSVDASAVLGPYYVDMLREGGAVSKHYMMDASTHLSFLGDIIPLSSPYPRTQV

ISIGDVVMNIGIFLFIQYMMVNRTGEALQPVNSIRPEEIRTDSKEGRSFP"

CDS complement(150651..152093)

/locus_tag="PIKHDJKN_00173"

/inference="ab initio prediction:Prodigal:002006"

/codon_start=1

/transl_table=11

/product="hypothetical protein"

/translation="MKKITQITLAALLAAPVTFGSLSLPASVSAASATPAGQTTSQAV

KGPVAQAPSVYTESAADYAQFLQAKYNIQLPKQLTKGDFIKAIATILEADQAAPATDD

ASDSEVKAPAFTDLNSNDSAYKAAVSLYNNGILSGTEVRANDKLSTYAAVFIAVKAAG

FKELAYTYPEDKTAKALAKLGISPSLVQGQAAQELAAAIDTGLIPQSLYPALRKGGEA

SSDFANILLGRVLTSLGKYKNEIGRSGDADIYSKLYAAYRTADLIEAPELRTIVDQAL

RNDLVTGYNLKDSRFDSNFIDELTLTYGHDDIKHAVQLIGLLRSEGIDADVQFQPKTS

AFIYLKEWGEPKETPDYKVTQIENGNYIAYAKEYDIQFEFNNTADKARFNDIILKYAK

KNSDDTSALIHASWWQPLYYSPTALAEYPVIANNKVSLGNYYAQSFSLKENSKAIQEG

FRKLAPDADISSYTFWVDHPFFNYLNGESE"

CDS 152371..153402

/gene="tmpC_1"

/locus_tag="PIKHDJKN_00174"

/inference="ab initio prediction:Prodigal:002006"

/inference="similar to AA sequence:UniProtKB:P29724"

/codon_start=1

/transl_table=11

/product="Membrane lipoprotein TmpC"

/db_xref="COG:COG1744"

/translation="MKRGLSFVLSIIMLAILLAACGNSGSKDEQSAQGGDSEKSLRMA

LVLPEKIGVNPFFVQMDEGFKKAGEEFKVDTKTIESTDPAAFEQNLRAAVAENYDLII

TATFQAEDALKKVAAENPDKSFAIVDTTVDLPNVRSVGFHEYEGAYLLGAAAGLSTKT

DKVGMIAAMDVPLIKKYTEGFKAGLESVNPNAEFLVNYVGGFNDPAKAKELALVQFGK

GADFIAAASAVGDLGVFEAAKEKGFYTAGQDTDRTVEDPEHIVLSQLKSTDTVSYQTV

KDFVEGNFKAGAVNYGLKEDGVGLTYVTRDSETPLNAFVGQEVIDKVKAIKDDIVSGK

IVVKDPLQQ"

CDS 153514..155013

/gene="rbsA_1"

/locus_tag="PIKHDJKN_00175"

/EC_number="7.5.2.7"

/inference="ab initio prediction:Prodigal:002006"

/inference="similar to AA sequence:UniProtKB:P04983"

/codon_start=1

/transl_table=11

/product="Ribose import ATP-binding protein RbsA"

/db_xref="COG:COG1129"

/translation="MLLEMEQITKKYGGFTANRDIRFNLREGEIHALVGENGAGKTTL

MRMLYGMEQPTSGTIKVRGREVSFATPSQAMASGIGMVHQHFMLFPSFTVAENIVIGR

EPSAAGVFDRKQAAAQVNELGRTYGMPVDPWKKVFECPLGMQQRVEILKVLHQGADII

ILDEPSAVLTPLEVKELLANMKSLAKLGKTFVLITHKLQEVMDVADRITVLRDGQVTG

TLEAKDTNVEELSRLMVGRELVRMDKQPSVPGEAVLQVEAVNLTGAEGRSALKNIHLQ

VRKGEVVGIAGISGNGQSELIQIIAGLHKADSGRVLLSGQDTTNWPVRRIREHGLAHI

PEDRYMWGAAKDASVRENGLMGHHHRLQSRGIIKAKAARTMVENWIKQFSIKTGSAET

KAQFLSGGNLQKLIAAREFAQDTPFLIAAEPTRGVDIGAMETIHAELLRKRNEGAGIL

LVSSELSEILQLSDRIIVMYEGGIAGELKADEATEEQISLLMAGGKERI"

CDS 155010..156077

/locus_tag="PIKHDJKN_00176"

/inference="ab initio prediction:Prodigal:002006"

/codon_start=1

/transl_table=11

/product="hypothetical protein"

/translation="MNRVKETLRGLIQPLLAVFIGLLAGAVAILIVGGSVVDTYAEMW

KGAFGNFYFFTNTLARSTPIILAGLGVALAFRAGFFNMGAEGQMVLGGLSAALTALYL

PGPGWFVCIAAIVAGIIAGGVWSLFAGWLDARFGMNLLITTLLLNYIAIYFGGYMVSY

PFKDRTGSAAMAQTPMIDQSVWLPKLFQGMGLHAGFIIAIVAAILIYWFTHKTVTGYE

IRMLGSNPSFATYGGVRRIRMMMLSMIISGGLAGLAGAGEVLGTQYRFLDGSLSSASY

AWSGIMATLLARSHPLGTAVAAILLAALQTGAMGMERNTDVPLEVGSVIQAVLTLFVS

AQIGYSFLKRRKEKKSNATTV"

CDS 156061..156984

/locus_tag="PIKHDJKN_00177"

/inference="ab initio prediction:Prodigal:002006"

/codon_start=1

/transl_table=11

/product="hypothetical protein"

/translation="MQQLFDAAMFGSTLRIMTPILLAALGGALCSRVGLFNVGLEGLV

LIGAFSAIVGNYLFGNVLLAVIFSIVIVMLFSALFAFISINLKANAIVVGISLNFLAT

GLTTFALRAIFDVKGAYYDKDMVGLPKWDIPLIKDIPWVGDVISGHSPLVYLGIIIVI

ALQFYLFKSVSGFRLRSVGENPVAAQSIGIKVRGIQYGAVLMCGVLCALAGAQLSLGQ

VTMFTEGMTAGRGFIALVATMLGQANPLGVMGSSVLFGFMEALSIRLQGFALPTHFTM

MLPYIVTLVAMFFFKDRTYAQDALKAGGSSR"

CDS 157031..157702

/gene="msrP_1"

/locus_tag="PIKHDJKN_00178"

/EC_number="1.8.5.-"

/inference="ab initio prediction:Prodigal:002006"

/inference="protein motif:HAMAP:MF_01206"

/codon_start=1

/transl_table=11

/product="Protein-methionine-sulfoxide reductase catalytic

subunit MsrP"

/translation="MHNKAERLKKTKSPAPKAGAEHGDRLPPGQVLTEKFPILHEGEV

PEYDLSTWDLKVFGEVEEEKVFSFAELQAMPQVNTVSDIHCVTRWSKFDTPWEGIRFS

DFIKLLGVKPEAKYVMIHADHDYETNVPLEELMHDDVLLAFKFNGEPLTPKHGYPLRL

VVPQLYFWKSAKWIRGLEFMKEDRNGFWEVNGFHHFADPFKEQRFSGEDIPIPEDEWT

KKEFD"

CDS 157704..158462

/gene="deoD_1"

/locus_tag="PIKHDJKN_00179"

/EC_number="2.4.2.1"

/inference="ab initio prediction:Prodigal:002006"

/inference="protein motif:HAMAP:MF_01627"

/codon_start=1

/transl_table=11

/product="Purine nucleoside phosphorylase DeoD-type"

/translation="MLMPILQIHSEDMPAYAIVCGDPARAEKISRKLEQARELAFSRE

YRTFVGLYEGVQMAVVSHGVGSPGAAVCFEELIRAGVTTLIRVGTAGSYTADYPAGSV

IVSTAAVRTDGLTRQLVPDGFPAVADLGVTQALIEATDEHASGADASSAGKVGVGITV

TLDAFFTGVEEIPHRKYKQAGALAAEMEIAALYIVSTLRGARAGAIVAIDGFADSDLA

AEYDPHTNAVADAVEREIEAALRALAALARQDQA"

CDS complement(158643..159530)

/gene="gltC_1"

/locus_tag="PIKHDJKN_00180"

/inference="ab initio prediction:Prodigal:002006"

/inference="similar to AA sequence:UniProtKB:P20668"

/codon_start=1

/transl_table=11

/product="HTH-type transcriptional regulator GltC"

/translation="MNNSQLQLFVKIAETGSFTRAGQELNMTQPAVSRAISSLENELD

VTLIIRDRRNGIVLTDVGQRILVIFRRILQQFDKVQQVVAAEKGLEIGTIRIGSFPMS

SAHLLPKIIRSIRDRYPQIQFELHEGNILEIQEWLSSRAIDVALIIATDKAPIDMAYE

TLPLYSEEMLAVFRDDEPFANQEILPVQMLDKHPMIICKGGYEVPIVDLFNRAHAELH

FGFVVYNVNTCLSMIEQGLGTAMLPAISLSWLPPGVKALHTEPKAYRNIEIAVPSLVD

ASPASRLFIETAQQLFGQR"

CDS 159741..160631

/gene="eamA_1"

/locus_tag="PIKHDJKN_00181"

/inference="ab initio prediction:Prodigal:002006"

/inference="similar to AA sequence:UniProtKB:P31125"

/codon_start=1

/transl_table=11

/product="putative amino-acid metabolite efflux pump"

/db_xref="COG:COG0697"

/translation="MALSRAKAGWVLAFLVLMWGVNWPLTKYALNFTPPLIFAGMRLL

IGGLVLGLYAFPRYKTLRLKQTWHIYAISAVLNIIFFYGFQTVGLTEVPAGLFSSIVF

LQPVLLGIGAWLWLGESMYGLKIAGLVIGFAGVATISIGGMTGKVSPEGVMLGLFSAI

SWAIGTVYMKRTSMKVDGIWMTAIPILIGGVVLTLTGTVTESWADVQWVLPFILDTLF

ISVFVIAMGWLAFFKLVSSGEASKVGSFTFLIPLIALLCSVLFLGESVTVNLIAGLVM

IIASILLVNVKVKGSKVAKI"

CDS 160756..160938

/locus_tag="PIKHDJKN_00182"

/inference="ab initio prediction:Prodigal:002006"

/codon_start=1

/transl_table=11

/product="hypothetical protein"

/translation="MTQNNQPQDEAAIRNKLDEDGDSLMEKKKILSGVDIEPQADEWS

AKPSPVAFDEGKSSKE"

CDS 161087..161569

/locus_tag="PIKHDJKN_00183"

/inference="ab initio prediction:Prodigal:002006"

/codon_start=1

/transl_table=11

/product="hypothetical protein"

/translation="MSEINIRRAVIGDYTGVSALMDELHQLHVEARPDIYRELQTRMS

HKEYEQLLETDHRYLYVAELLDQGEIVGYTSAQLNVIQNVDLLKDRKMLYINEIIVGS

MHRGHGTGKQLMQELIELGREIQVDSVELTVSIFNNGAQAFYEQLGLAVRSSRMEYIL

"

CDS 161727..162128

/locus_tag="PIKHDJKN_00184"

/inference="ab initio prediction:Prodigal:002006"

/codon_start=1

/transl_table=11

/product="hypothetical protein"

/translation="MEHQSHPKEPLESSVTEVNEVSQTDDQQANSCHTAGSDGKHVRK

SHHSQQMKGNLISRLNRVEGQIRGIKGLIEKDTYCDDVLTQIAAAQSALNSVGKLLLE

GHMKSCIVERIQAGEHEVVDELLVTVRKLMK"

CDS 162177..162377

/gene="copZ_1"

/locus_tag="PIKHDJKN_00185"

/inference="ab initio prediction:Prodigal:002006"

/inference="similar to AA sequence:UniProtKB:Q7A3E5"

/codon_start=1

/transl_table=11

/product="Copper chaperone CopZ"

/translation="MSNITLNVTGMSCNHCVKSVEEAVKNAGANGQVDLAAGTVAVEY

DEQKVNVDQIKAAIEDQGYDVV"

CDS 162539..165067

/gene="copA_1"

/locus_tag="PIKHDJKN_00186"

/EC_number="7.2.2.8"

/inference="ab initio prediction:Prodigal:002006"

/inference="similar to AA sequence:UniProtKB:O32220"

/codon_start=1

/transl_table=11

/product="Copper-exporting P-type ATPase"

/db_xref="COG:COG2217"

/translation="MDKSSTTDNATTSEPVPSPEAAGLQTTLQITGMTCAACSTRIEK

GLSRMEGVNQANVNLAIEQATVSYDPKTTNINALRDKVEALGYGTVTESVDLDITGMT

CAACSARIEKGLSRLPGVSRANVNLALETGHVEFAAGVLKATDITAKIKQLGYGAELQ

QTQEDTQSVRERELQRKKWKWMISALLSLPLLWAMVGHFSFTSWIWVPDLFMNPWFQL

VLATPVQFVIGWQFYVGAYKALRNGSANMDVLVALGTSAAFFYSLYLTLTSGNAASTM

MDHGGTGMATAAMPTVELYYETSAILITLILLGKWFEAVAKGRSSQAIKSLIELAPRE

ARVIRDGQEVIVPSAYVAVGDLVLVKPGDSIPVDGVVEEGQSSVDESMLSGESLPVDK

KPGDAVTGATLNKNGVLRLRATRVGSDTALSQIIKVVEQAQGSKAPIQRIADVISGIF

VPIVVGIAAVTFLIWYFFASPGDFAGSLEKAIAVLVIACPCALGLATPTSIMAGSGRA

AEYGVLFKGGEHLESAQQIQTVVLDKTGTVTQGKPVLTDVVTAPEWTEAELLQRVGAA

EQSSEHPLAEAIVEGIRGKGLELTQSEQFENIPGYGVRARVQGQEILVGTRRLLTDAK

VNVSEETMQQMNNLEEQGRTAMLVAVDGQWAGVVAVADTIKETSREAIQRLHAMGIDV

IMITGDNERTARAVAEQAGINKVLAEVLPEGKAAEVKKLQESGLKVAMVGDGINDAPA

LATADIGMAIGTGTDVAMEAADITLMRGDLNSIADAIEMSRRTMGNIKQNLFWALGYN

VIGIPIAAVGFLAPWLAGAAMAFSSVSVVLNSLRLQRMKLKSND"

CDS 165403..166563

/locus_tag="PIKHDJKN_00187"

/inference="ab initio prediction:Prodigal:002006"

/codon_start=1

/transl_table=11

/product="hypothetical protein"

/translation="MLQALRILLKKPPVIVGIVTALMFQVIFSVIWMTAYSGVNDRMN

ELTVAVVNEDGEQSKGIADRMAETLPFHTVTNLSAAEALDQLNHRKIHMVLNIPAGFN

ELIQTAGSTAEIKYTINEANPVTIKSMMQGVSQSVTNTINKQATAQGVQAVLTASGAP

ADQAAEAAANLTTRVEGPTTSINPVNGMNNQMVPMMMVLASYVGAMIMGMNLQTAMGM

LSATHSRWTLFGARVVINLGSALVVSLLGSSLIVSLGGQIEQGFIVFWLFQALFLCTF

MFFSQFFLICFGPAGSLFNIISLSLQLVSSGAMVPRELLNSFYSGIGQYLPATYAVQG

VLSAQLGGPGVQSAAGSIAIVLLVAVALSLVVTSLKKQRMPAGAPSPAQMNS"

CDS 166791..167399

/gene="nicS"

/locus_tag="PIKHDJKN_00188"

/inference="ab initio prediction:Prodigal:002006"

/inference="similar to AA sequence:UniProtKB:Q88FX7"

/codon_start=1

/transl_table=11

/product="HTH-type transcriptional repressor NicS"

/translation="MTEPELDIKTRILLAAKKLFAQQGYDGTSVRQICDEAGANVSLV

SYHFGGKEKVFEALFEHFFPDHMMNELAEESMSSPVEGIRRIIGEVVKFTMTDREMSD

IVQLEITLRTHRTDTVFRFLDPVWTKVRELLQEGKEQGVFQIESVSYAMLQVMGVALA

HKRAKNSRFSFDYQDMNTDELAEQTIEFVLRGLGVNSNERNH"

CDS 167383..168126

/gene="nfrA1"

/locus_tag="PIKHDJKN_00189"

/EC_number="1.5.1.38"

/inference="ab initio prediction:Prodigal:002006"

/inference="similar to AA sequence:UniProtKB:P39605"

/codon_start=1

/transl_table=11

/product="FMN reductase (NADPH)"

/db_xref="COG:COG0778"

/translation="MNETIELMMKHRSVRKFKPDPVSEEQLAAIVAAGQMASSSSSVQ

AYTIIAVTEQEQKAKLAELAGNQSYVNECPVFLVWCADLYRLSDAAKRHLPEKESYED

STENFMVATIDVALASQNAALAAESLGFGIVYIGGLRNRIEEVSEVLGLPDGVYPVYG

MCIGVADQETGIRPRLPLDAVLHHGRYNAEQTLKCVESYDETTAAYMKERTQGERSTP

WSEMMAKRLTEPVRLQMKPFLEGKGFLKR"

CDS complement(168234..169040)

/gene="ycfH_1"

/locus_tag="PIKHDJKN_00190"

/EC_number="3.1.-.-"

/inference="ab initio prediction:Prodigal:002006"

/inference="similar to AA sequence:UniProtKB:P0AFQ7"

/codon_start=1

/transl_table=11

/product="putative metal-dependent hydrolase YcfH"

/db_xref="COG:COG0084"

/translation="MQSTAFAPLVDAHLHLDKYTPEEQREMLHSFPSQQVEAVIAVSM

NLASAQTNLELASQYPRTVYPAFGFHPEQPLPSLAEQDLFFKWIEKHMGQATAIGEVG

LPYYNRQEAEQAGQHFDQSGYIDLLERFIQLAKRHGKPIVLHAVYEDADIACDLLEQY

HHRRAHFHWFKGSKRTVQRMADNGYFVSFTPDIIYEEEIRELARQYPSEQVMAETDGP

WPFEGPFQGRMTHPAMTRQVIQAWSEVTGMGTERAARLFYQNTKRFYDLP"

CDS complement(169136..170035)

/gene="nrtD_1"

/locus_tag="PIKHDJKN_00191"

/EC_number="7.3.2.4"

/inference="ab initio prediction:Prodigal:002006"

/inference="similar to AA sequence:UniProtKB:P38046"

/codon_start=1

/transl_table=11

/product="Nitrate import ATP-binding protein NrtD"

/db_xref="COG:COG1116"

/translation="MTPEHNERVAAAPDEGATTPTSSGSPEYGAATLPAHHSSPALEV

LNVNASFRERRSTLPVLDGLSLTVEQGEFVAIVGPSGCGKSTLFHIIGGLLKPQEGQI

LMNGQDVTGQRGKISYMPQQPALFPWRTIEDNVLLAGEVSADAPPRAEALADARKWLS

SVGLAGFEQAYPHMLSGGMQQRVAFLRALLSPQELMLLDEPFSALDALTRSDMQRWLL

DIWEQNRRSVLFITHNIEEALLLADRIYVLSNRPAAVLHEVDVPFDRPRREAITEESA

FLERKRQISQWMREEQQKTRLSK"

CDS complement(170079..170837)

/gene="ribX_1"

/locus_tag="PIKHDJKN_00192"

/inference="ab initio prediction:Prodigal:002006"

/inference="similar to AA sequence:UniProtKB:A9WGD1"

/codon_start=1

/transl_table=11

/product="Riboflavin transport system permease protein

RibX"

/translation="MHAYWKSVWPPFVAVILFIAIWQGAVSLFHIEKWMLPAPSDIAH

EAASQAERLGMHAWATIQLTLIGFAAGTLVGLLIAMVLHLIPFLKSALYPLLILSQNI

PTIALAPLLLIWFGFGLLPKLITIILVCFFPVAVAAMDGLTRTDAAMMNYMRMAGAKR

GQIFWKLELPHALPSVFSGIKIAATYSVMGAIIAEWIGADKGIGYYMMLQKSAYRTDR

LFVAIMIIVALSLLLFLLIALLEKLLVRWRPQKR"

CDS complement(170837..171127)

/locus_tag="PIKHDJKN_00193"

/inference="ab initio prediction:Prodigal:002006"

/codon_start=1

/transl_table=11

/product="hypothetical protein"

/translation="MASTLLSIQVIPKTPNGENSYPYVDRAIEVIQQSGLKYQVNPLD

TTMEGELEELLEVVRKMHEVLVEAGSPSIISQIKIAHSPSGFSMDTLTEKYR"

CDS complement(171351..172391)

/gene="thiY_1"

/locus_tag="PIKHDJKN_00194"

/inference="ab initio prediction:Prodigal:002006"

/inference="similar to AA sequence:UniProtKB:Q9K9G5"

/codon_start=1

/transl_table=11

/product="Formylaminopyrimidine-binding protein"

/db_xref="COG:COG0715"

/translation="MRWRKMMGLMLLCVLLVAVAACGGKEAAPSGENGSSNTENSNEG

DNAALKDIKVVLDWTPNTNHTGLYAAVDQGFYKAEGLNVEIVQPGAGGADTMVASNEV

PFGVSYQESVTQARTQGVPLVSIAAVIQHNTSGFAAPADRNIKSPKDFEGKTYGGWGS

PVEEAVMQSIMEGEGADVSKVKNISMGDADFFTAVKRDIDFAWIFYAWTGIEAELRGE

PIDMLYVKDYSEALDYYTPVLVTNEKTIQNDPELVKAFLKATSEGYQYAIEHPEDAAN

ILIKAVPDLDKDLVLASQKWLSPKYQDDAPRWGEQKQEVWQNYTDWMFSKKLLDEQVD

VSKAYTNEFLPQ"

CDS complement(172871..173626)

/gene="udh_2"

/locus_tag="PIKHDJKN_00195"

/EC_number="1.1.1.203"

/inference="ab initio prediction:Prodigal:002006"

/inference="similar to AA sequence:UniProtKB:Q88NN6"

/codon_start=1

/transl_table=11

/product="Uronate dehydrogenase"

/db_xref="COG:COG0451"

/translation="MNILITGAAGKIGRDLSQHLSEAFHLRLTDLNIDRLHTYQGTSH

EIMTLDVTDPEACQHACEGMDVVVHLAGDPSPHAGFYESLLDINIKGTFNVFRAAKDQ

GVRRVILASSAQTIEGYPIDAQVYPDMPTRPRNLYGVSKCFGESLASYFAYTEGLQSV

AIRIGAYDDFQPDGPALEARDMSAYISPADLCDLMFKAITTTDLEPFTILHGISNNRF

KRLNLGTTQKQVGYDPKSDAFALSQISLYDSPR"

CDS complement(173643..174029)

/locus_tag="PIKHDJKN_00196"

/inference="ab initio prediction:Prodigal:002006"

/codon_start=1

/transl_table=11

/product="hypothetical protein"

/translation="MSFQKRIDHVGIAVRDLETTLRFYTEIVGLELKDRVTHTNGVIQ

LAFLGFNGSDETEIELIQGYSDKLPSEGTVHHFAIHVDDLEAEYNRIKATEAEFIDGE

IITLPNGYRYFFIYGPEKEWIEFFQR"

CDS 174216..174704

/locus_tag="PIKHDJKN_00197"

/inference="ab initio prediction:Prodigal:002006"

/codon_start=1

/transl_table=11

/product="hypothetical protein"

/translation="MPTTKKEQIVFGMMMCTGMVIVMMTFNLWHNGLLSKMSALEILL

QFILCFIIAFLVESFIVGPVARKIAFSLPFDKSNKILGVLAMSFFMVIGMVLIMSLYG

MISAYLANQLIGASVLSTYLHTIARNFSLALPYQIIILGPLVRYVFGKFIKDKGSVVP

ST"

CDS complement(174887..176122)

/gene="glcP_1"

/locus_tag="PIKHDJKN_00198"

/inference="ab initio prediction:Prodigal:002006"

/inference="similar to AA sequence:UniProtKB:O07563"

/codon_start=1

/transl_table=11

/product="Glucose/mannose transporter GlcP"

/db_xref="COG:COG0738"

/translation="MKKLLWIGCLSYFLIGLAHVVLGSILPVALEHYGKDYSQGGTLI

FAQFAGFLGGVLLSPWLNKRFGKRGGILIAAALLCIAELSYMLLPPWGWMFVIAPAAG

FGFGMIEAVIGTIIIAAIKDNTAVAMSRLEVLFGIGAMVMPLIASGLISAGYWRMSFL

VVAVCAAMTFIFWAKSSFGELDSELSRRNSDQTPGHTHAAESPIGTNPADIKPTRSTY

RGRNLALLSLFVLFFFLYVGTEMSLANFMPAILIEKMNMKEAGAALSVTCFWIAMSVG

RLFAGYIAEKFQYRVYVLYSCLAAVVLLMIFPFTNQIWSAFLIILLLGLALSGVFSIA

LVFASKMLPGTEESTPSILIASGGVGGAILPLVTGWSLDHLEVNQSAWMLAFFAVGLL

IISVIAYQWQLRHTVSSAS"

CDS 176504..177802

/gene="serS_1"

/locus_tag="PIKHDJKN_00199"

/EC_number="6.1.1.11"

/inference="ab initio prediction:Prodigal:002006"

/inference="similar to AA sequence:UniProtKB:P0A8L1"

/codon_start=1

/transl_table=11

/product="Serine--tRNA ligase"

/db_xref="COG:COG0172"

/translation="MLEMKWIREHAEEVQAAAVGKRMNISIRELLERDEERRALLLET

EEGRRVRNSLSADIGRLMQAGEREQAEGLREQVKQLNGQLEQVEAKLAVVQEEVTRLQ

WLVPNVVSPDTPMGASDEDNVELRRVGELPVFDYEAKDHVELGERHDLIDIPRGVKIG

GTRSYVLKGAGLLLHRAVQQLALDLLLKHGFTPMEVPLMVRENTLLNTGFFPTGRDQV

YEMQGENKWLVGTSEVPLVSYYADEIVDVQEPIKLAAVSTCFRSEVGSGGRDVRGLYR

VHQFAKVEQVIICAPDAKESERMLQEITGHAEELLQLLELPYRVVAVCTGDMSQKTYK

QYDIETWMPSRNAYGETHSSSNLHDFQARRSNIRCRDAQGKLAYCHTLNNTAVASPRI

LIPLLENHQQADGSIRIPAALQPYMGGAEYLVLPHQDEEN"

CDS 178020..178667

/gene="walR_2"

/locus_tag="PIKHDJKN_00200"

/inference="ab initio prediction:Prodigal:002006"

/inference="similar to AA sequence:UniProtKB:Q9RDT5"

/codon_start=1

/transl_table=11

/product="Transcriptional regulatory protein WalR"

/db_xref="COG:COG0745"

/translation="MNLLLADDEPLMLQILKAYFVKEGFQVFLAEDGESALNLFYNNQ

IDLAVLDWMMPGHSGVEVCRVIKRTSRAKVLLLTAKGESDDEFVALKAGADDYLRKPF

DSRILLLRVKKLLQLSSKIMVGELTVDLDGQKIYRRGVDVGATHKEFELMKVLGMNKG

QIVTRKILLDQVWGFDYFGEERTVDTHIRRLREKIGEDSIKTYRGMGYCLEDQDE"

CDS 178660..180045

/gene="sasA_2"

/locus_tag="PIKHDJKN_00201"

/EC_number="2.7.-.-"

/inference="ab initio prediction:Prodigal:002006"

/inference="protein motif:HAMAP:MF_01837"

/codon_start=1

/transl_table=11

/product="Adaptive-response sensory-kinase SasA"

/translation="MSKLTRKLLVRIISALCIVFFLSFIVNTFFLPTYFLYQKKQKLA

ELTTEISASEHSVPIPQIESLETQYEVAIAYASLKDSVNDINDQLLFQYNRKGIALSK

FWLTEESISALREGARVNKFYDQTKLKSSFLVNFVKVGDSVFAVGESISHSSETIRII

NQFNAYIWLGMLLLLILLSVLYTARIVKPLAKLSETAEAISNLSFAKVDIKTGDEIES

LGRSINVMSDKLQQAQHSLEIKNANLRTFISDISHELKTPLSLIRVYASGIQDGLDDR

TYAGVIQQQSEELAGLIDRLLELSRLQAEVYDFEPVDLRQLLSETLHTYETVFQQHGL

HVEVEDRWTAETWVRADRRKLESVLHNWMTNAVKYTSGDRITITLELKGEAAYFGIAN

ETDKEEHEQWDQVWEPFYVMDSSRSKKFSGTGLGLSIARTILENHHADYGLSVADGKV

RFYFSLPLIQR"

CDS 180215..181240

/gene="pseB_1"

/locus_tag="PIKHDJKN_00202"

/EC_number="4.2.1.115"

/inference="ab initio prediction:Prodigal:002006"

/inference="similar to AA sequence:UniProtKB:Q0P8W4"

/codon_start=1

/transl_table=11

/product="UDP-N-acetylglucosamine 4,6-dehydratase

(inverting)"

/db_xref="COG:COG1086"

/translation="MFNKTFLITGGTGSWGEELVKQLLVQEPKEVRIFSRNESLQAQM

RQAVSDPRVKFVLGDIRDRWELAGACKDVDYVFHLAALKHVPVCEDQPDCAVKTNIIG

TQNVIDAAIENGVRKVVYVSTDKAANPSSMYGMTKAIGEKLILLADSRSATSFACVRS

GNVLGSTGSVVPLFKRQLELKQPLSVTDSRMTRFFLPLGDAVEILLSAMEQCEGGEII

VPRMPSCKITDIARVLAEDAGQKDVPIHFIGARPGERLYETLISDWENVSDVEEKKSY

FVVIASPSAKLADKEGCGTFVLAGRGYHSEDVIMTKAEVRDMLWRGGFLCSSGGSYLQ

EAAPPVM"

CDS 181189..182286

/gene="murG_1"

/locus_tag="PIKHDJKN_00203"

/EC_number="2.4.1.227"

/inference="ab initio prediction:Prodigal:002006"

/inference="similar to AA sequence:UniProtKB:P65482"

/codon_start=1

/transl_table=11

/product="UDP-N-acetylglucosamine--N-acetylmuramyl-

(pentapeptide) pyrophosphoryl-undecaprenol

N-acetylglucosamine transferase"

/translation="MFKRRIIFTGGGSAGHVTANLILISRLLQEQWEIHYIGSKHGIE

RQLIGKIPAVHYHAVATGKLRRYWAWANISDVFKIILGVLQAYLLILRIKPHLCFSLG
GFVAVPAVVGARWNRVPVLILEPDLHPGLANRLSRRYAQMMCTTFMDTATFASSKASD
KTVYVGAVIREELKLGSRARGIHFCRFVSDRPILLIMGGSQGSEKLNHMVIETLPELL
KRYQIIHICGTGKLNSSFPRFAGYKVFEYVHEELADLMAMADMVVSRAGSNAIHEWLL
LRKPMLLIPHANGGSLVGQTLNAKYFQERGYARVLQEEELTPQTFLRDIDLVYKEREH
MIDHMKASGTDNAVDKVLQLIHTTVSSLEKN"

CDS 182626..183732

/locus_tag="PIKHDJKN_00204"

/inference="ab initio prediction:Prodigal:002006"

/inference="similar to AA sequence:UniProtKB:Q086E4"

/codon_start=1

/transl_table=11

/product="Ice-binding protein 1"

/translation="MNGMKLAIRGTLAFILVLAVLAPSLALAATGDVTSIEITNSSPQ
QMSVSQTTTLQVMAVIEGFDNKQDVTGGVTWSTSNEAVATIIKGKVKAVAAGEATIYA
QVEGAKAQLVVKVQDKIKSIKASPSSYNFVKGSESTLPKVSIVRANGKEEDVTSEIVW
TVSSASAVLESGKIKGITPGRVLLQGKYGSETVKVPVAITDKITKVEVTPAVMQLNIK
KSKALKVIGTYANGKTINLSKQVTWTSSNNNVAIVKNGTVKTLTEGKATLTGTYQNQT
IKAEVTVVPLLKKLITGQKTLVLSPQASTTLSVMAQYDTGKTTIVTSSAVWSSTKPSV
ATVTGGKIVAVGKGKTSITAKWGNKKVTIPVTVK"

CDS 184038..184589

/locus_tag="PIKHDJKN_00205"

/inference="ab initio prediction:Prodigal:002006"

/codon_start=1

/transl_table=11

/product="hypothetical protein"

/translation="MTDHLQPENWPAWATESVEVVEANPMWGLQAQDEILQLRDLLQQ
LNIHKFEHIGSTSIPGLPAKPIIDLMGEVQSWEDMDLIADQLNPVGWNYVPPELDDRE
YRRFWVRVKDGKRAVHLHLMRPGEERWDRQIQFRDVLRQRPDLVEVYAALKAKLADEN
KDDREAYTAAKTEFILQVLDDGI"

CDS 184593..184997

/locus_tag="PIKHDJKN_00206"

/inference="ab initio prediction:Prodigal:002006"

/codon_start=1

/transl_table=11

/product="hypothetical protein"

/translation="MMKAFFKERLIYFIAVIITMAAGLSSRHFGELLPDFVREHFGDA

LWAAMIYFGVRMVWINRSKELALIVSLIFSWAVECSQMIQTPWLNEVRSTVLGALILG

RGFLVMDLLRYAVGILCVYGIDRYFLRNKKAR"

CDS 185014..185571

/locus_tag="PIKHDJKN_00207"

/EC_number="2.3.1.-"

/inference="ab initio prediction:Prodigal:002006"

/inference="similar to AA sequence:UniProtKB:Q9HX72"

/codon_start=1

/transl_table=11

/product="Acetyltransferase"

/db_xref="COG:COG1670"

/translation="MNVDKLFAESPEFETERLVLRRLTLNDLDDYFVFASDPKVSQQS

LWNCHETLDDSVHYIQRVLDNYEKKTVYIWAFILKGTGKLIGRGGIFHLNESMQSAEL

GYAIASSCWGKGLAAEAMQPIVEYCFQELDCNRLEGKCNAGNIGSARVMEKLGMSYEG

LLRKQLKIKGVFTDQKLYSRIRDDL"

CDS 185623..185952

/locus_tag="PIKHDJKN_00208"

/inference="ab initio prediction:Prodigal:002006"

/codon_start=1

/transl_table=11

/product="hypothetical protein"

/translation="MIYSIISQAVWEQVSKQSVYAPDSLETDGFIHCSTKEQIPWVAA

QFYQGRTDLLLIGIDERALKPELVYEDLYELNELFPHIYGELNLDAVRKVISFEPNED

GTFSFPE"

CDS 185980..186576

/locus_tag="PIKHDJKN_00209"

/inference="ab initio prediction:Prodigal:002006"

/codon_start=1

/transl_table=11

/product="hypothetical protein"

/translation="MDMNQVFLDTAEKQFLYYKQLGEKAMEQLESEQLFQSWNEDANS

IAVIVKHLWGNMLSRWTDVLTTDGEKPWRERDAEFVNDISTREELLAKWEEGWNCLLG

AIRSFTPEQLSHIIYIRNEGHTVMEAIIRQLAHYPYHVGQIIYAAKMLKETSWNSLSI

PRNGSDQYNGGKFAKPKARKHFTDDELRIEKGKGEEQQ"

CDS 186573..187163

/gene="gpmB"

/locus_tag="PIKHDJKN_00210"

/EC_number="5.4.2.-"

/inference="ab initio prediction:Prodigal:002006"

/inference="protein motif:HAMAP:MF_01040"

/codon_start=1

/transl_table=11

/product="phosphoglycerate mutase GpmB"

/translation="MTQIALIRHGSTAWNKEKRSQGQTDNPLDQEGREQALLLAARLS

EETWDAIYASDLERASETARIIGDRLGIQEIHLDPRLREMGGGQVEGTTEAERVAQWG

ADWSDLDLGRELADAGTIRGSAAIEDIVRQHPNGRVIVVSHGAILRNTLRGLVPELDV

SAKLSNTSITRISWETEFWQCELYNCSVHLDSSKEL"

CDS 187167..187997

/locus_tag="PIKHDJKN_00211"

/inference="ab initio prediction:Prodigal:002006"

/codon_start=1

/transl_table=11

/product="hypothetical protein"

/translation="MDALALKQQLQHIQSANLSTHEVEHPYELALHMMQHIGSPDPVL

RDELIYVTFATWIGQGVFTEEQLSQLLQMALDDQHLFHGIGEQGTDSVFTRTFSVLLL

PPILSVDRQRPFLKKEDIEVIHHRLTTYLEREKDVRGYTDDKGWAHAPAHAADAVEDL

AQSPYMERAALRELLHALTVKITESSVVYIHDEDQRIAHAVVTILRRNLLEQNDISSW

IDSLNPNDKKEGKSHLEISQMRLNVRVFLQTLYLAIRTEDAEPFPAVRSLILQALEKK

"

CDS 188037..188747

/gene="pxpB"

/locus_tag="PIKHDJKN_00212"

/EC_number="3.5.2.9"

/inference="ab initio prediction:Prodigal:002006"

/inference="similar to AA sequence:UniProtKB:P60495"

/codon_start=1

/transl_table=11

/product="5-oxoprolinase subunit B"

/db_xref="COG:COG2049"

/translation="MTEMPYLWTEDVLSPLGETGVMIRCGDTISEAVHRRVMSVCALL

EKSQLPGIMEWLPSFASVTLFYDPLISPYHEVCGMLLHELNKMEEVTLDQPRTIIIPV

CYGGELGPDLDYVATEHGLTPDEVIAIHTSGDYLVHMIGFAPGFPYLGGLTEKIATPR

RSTPRLRVEAGTVGIGGKQTGVYPVTTPGGWQCIGRTPLALFRPMENPPILLAAGDRV

RFASISLQEYLKQKEGER"

CDS 188744..189772

/gene="pxpC"

/locus_tag="PIKHDJKN_00213"

/EC_number="3.5.2.9"

/inference="ab initio prediction:Prodigal:002006"

/inference="similar to AA sequence:UniProtKB:Q7WY77"

/codon_start=1

/transl_table=11

/product="5-oxoprolinase subunit C"

/db_xref="COG:COG1984"

/translation="MSIEVIRPGLLSTVQDEGRTGFRRYGIHPGGVMDTFAARAANAL

VGNSRDAAVLEMTMTGPELRFHETLLISLCGADLTATVDGLPVPLWRPVVLLAGKVLR

FGRCRSGLRSYMAIAGGIAVPEIMGSRSTDLKTGFGGFEGRALKVGDRLSAGEASREA

QAALRGLSVEAEKNNGRMHAPAWYLSSREWPAYHAEPVIRVMPGKDSAAFNENSLVRF

HKERYVISPQSDRMGYRLEGAKLELGQPMDRLSEAVIYGTVQVPADGQPIILMADHQT

IGGYPVIAQVARVDLPILAQARPGAPVAFERITYEEAQQLFLEQELEWRLTDQLIRRR

LAEMGDHQ"

CDS 189712..190536

/gene="pxpA"

/locus_tag="PIKHDJKN_00214"

/EC_number="3.5.2.9"

/inference="ab initio prediction:Prodigal:002006"

/inference="similar to AA sequence:UniProtKB:P42963"

/codon_start=1

/transl_table=11

/product="5-oxoprolinase subunit A"

/db_xref="COG:COG1540"

/translation="MAANRSADSQEIGRNGGPSMNTVDINCDLGESYGVYRMASDESI

LPLITSANIACGFHAGDPATMRQTVQQALEHQVAIGAHPGLPDLQGFGRRRMDITPRE

AYDMVVYQIGALDAFVRSYGGRMHHVKPHGALYNMAAEDNQLAEGIAEALYKVQPELY

LYGLAGSALIQAADRIGLRSVSEVFADRTYGPDGTLTPRSQPGAVIQQTEQALVQVLR

MVKEGIVVSTEGTPVSIKAETICIHGDGAHALAFAQEIRTLLESDGINLSAPGVTR"

CDS 190600..191058

/locus_tag="PIKHDJKN_00215"

/inference="ab initio prediction:Prodigal:002006"

/codon_start=1

/transl_table=11

/product="hypothetical protein"

/translation="MKTIRNSAKAVIVQDGRLLVIRLEDQYGDAYVFPGGGQEKGEEL

KDAVARECLEEIGQAVDVGELLHIREYIGKNHEFAVWDADVHQVEFYFECRLIDAKAT

IFEGSSPDDHQVAVEWIPLEELSKVRLYPKTIGELLLDQASSRIYLGDLN"

CDS 191526..191942

/gene="ydaG"

/locus_tag="PIKHDJKN_00216"

/inference="ab initio prediction:Prodigal:002006"

/inference="similar to AA sequence:UniProtKB:P80238"

/codon_start=1

/transl_table=11

/product="General stress protein 26"

/db_xref="COG:COG3871"

/translation="MNQTELEQNIVKALENNPFCSFSTVENGKPKSRYMALFNDGMNI

HLATNRRTHKVEELENNPNVSLLLGYEAGGSKEVVEIEGTCEVTKNEGLREQVWNDEL

KAWFDGPNDPNYVILDITPKRIEYIGKDHEHQVWEQ"

CDS 192421..194679

/gene="yfkN_1"

/locus_tag="PIKHDJKN_00217"

/inference="ab initio prediction:Prodigal:002006"

/inference="similar to AA sequence:UniProtKB:O34313"

/codon_start=1

/transl_table=11

/product="Trifunctional nucleotide phosphoesterase protein

YfkN"

/db_xref="COG:COG0737"

/translation="MRKSKKVLSSLTAALVALNVLAVFPVPVSAADATKVKLRIMETT

DIHDNLINYDYYSDKETDQYGLAKTATLIKKARDEAKNSLLFDNGDLIQGNPLGDYVA

KIDPLKKGETHPVYKAMNLLDYDAGNIGNHEFNYGLDFLDMTLEGANFPYINANVYVD

DGDDDETNDKNYFTPYKILDKKVTDESGKEHTIKVGVIGFVPPQVMQWDSANLEGKVI

AKDIIATAKKFIPKMKAEGADIIVAIPHSGFEDIPQTDLMENSVLYLSQVEGINAILF

GHAHKVFPSADFAGKKGVDLEKGTINGVPAVEPGFWGDHLGIIDLDLELVDGKWKVAD

SKVEARPIYDTANKKALVDADQEIVDAVHDEHEGTLEYVRGPVGETTAPINSFFALVQ

DDPSIQIVTNAQKWYVEKHMQGTEYEDIPVLSAGAPFKAGGRSGASYYTNIPKGTIAI

KNVADLYVYPNTVHAVLVNGAELKEWLEWSAGQFNQIDPAKGGQQQLVNMDFPTYNFD

VIDGVTYQIDVTQPAKYDGKATIVNASANRIKDLSFNGKPVDPAQKFIVATNNYRASS

SKLANPDGKRIVLAAPDENRQVIIDYIRENKTINPAADGNWSLAPIKPSAGVTAAALN

DLEVVFASSPDAKALVEANPAMSFIGTNDDGFAEYGLKLTGEATTTPETGTEPAPTPT

KPDPTPTKPTTKPQPEKPAGGKVVHVVKKGDNLYRIGLKYGVDWRKLVSVNKITNVHN

LKVGQKIVIPAS"

CDS 194893..195936

/locus_tag="PIKHDJKN_00218"

/inference="ab initio prediction:Prodigal:002006"

/codon_start=1

/transl_table=11

/product="hypothetical protein"

/translation="MNCSVCSPIHPIEDQGTLYMRPISPALLETLQTQGRHVEHSDEL

IWMHFLNLESVQRCIEDILKIESTLPDKLTVQVTPLYGQADADSWISLSMQEARLKHA

DLVSIILEHQFSSHMQPIVDASEQIIGFEFLLRPTEQGRPFSAYELFEVARDTGLHSF

LDRTARIAAIETSAVLLPHGVKRFVNFLPSSIYNPEYCLTHTFETIERLSLDPKDFVF

EVVETEQIQHMSILQHIFEVYRSHGMSVALDDVGAGFSTIEVMNWLEPDFVKIDRSLI

DHCDHDLNKQQQIIEIVEMSRRFGGRVLAEGIERIEEFEFCRSVGIDLAQGYYFGKPT

AYPPQGPYGKTSA"

CDS 196172..198268

/gene="mcpB_2"

/locus_tag="PIKHDJKN_00219"

/inference="ab initio prediction:Prodigal:002006"

/inference="similar to AA sequence:UniProtKB:P39215"

/codon_start=1

/transl_table=11

/product="Methyl-accepting chemotaxis protein McpB"

/db_xref="COG:COG0840"

/translation="MGHIRGNEQEKAATKPAKPKDNKPKKKNKKKKGFVWSIRNKLLV

SFLAVLLLPSLTIGIVTLIIADHTVEDQLMDSAKQSVDTTNSIIGTHVDAKIHDINYF

ASLIVTDMIKGQDESPELETKLKQYLGLHPDALNIFVGTKEGVMVRGKESAGSSQGSA

YDPREREWYKLAMEKPGTPVVSPVSKNTDGIAVVFVSKTLDDQSGVVGLSLDLTELRE

QASIKVGKEGYVVILDADKNYVVSPTVEAGTQEQTTTLDPMFESKEGEFSYIYNDDPK

NMIFSTNEETGWKIGGTMFKSEVLNASKDIRMATLLVILTATFLTLIFIIWFTRSMLR

PIKRLQESARAVSKGDLTVKMETGRKDEVGDLAKYFERMVDNLRMMILGVQETAEQVS

ASSQELSASADQTTKAIEHSTMAIQELAEGAEQQVKSVTDGSGQMSQMAEDVRMMSER

VQSITTNMRHTSGAAYSGNEAAGQAVEQMNSIQETVEQLATVVQSLNARSVEIGSMVD

VIATISKQTNLLALNASIEAARAGDAGRGFAVVAGEVRKLAEESGSSAAQIGELVHNI

RQDMDAALNTMNAAQTRVGDGIQAVNTSGQSFAQIREAVEDAVHTLDDLSETTKQLES

GASHVAKAMNDISIVTQESAANTESVSASSQEQLASVEEIASSSAHLNSMAEQLQGLL

GMFKMVEDTQKDGDKS"

CDS 198458..199750

/gene="arsB_1"

/locus_tag="PIKHDJKN_00220"

/inference="ab initio prediction:Prodigal:002006"

/inference="similar to AA sequence:UniProtKB:P0AB93"

/codon_start=1

/transl_table=11

/product="Arsenical pump membrane protein"

/db_xref="COG:COG1055"

/translation="MTYVAILIFVLTITLVIWQPRGLGIGWSAMGGALLALMCGVVSL

NDASDVASIVWNATLAFVGIIMISLILDETGFFEWAALHMARLAGGNGRRLFIYSILL

GAAVSALFANDGAALILTPIVLAMVRALKFDERMVLAFVMASGFIADTTSLPLVVSNL

VNIVSADYFGITFVEYAVRMIVPNLFSIVASTGMLFLFYRKSIPLHYDISAARDPREA

IRDPRMFRIAWFMLVLLLVAYASSEFLPIPVSVIVGAVALLFAILARKSEAVDLKRVI

KEAPWSIVVFSVGMYIVVYGLRNAGLTDHLSRWLDAIADHGLLVASLGMGVIAAVLSS

VMNNLPTVLINALAIQGAHTEGIVREALIYANVIGSDLGPKMTPIGSLATLLWLHVLS

RKGVKITWGYYFKAGIILTIPTLLITLAGLALWLWILG"

CDS 200528..201451

/gene="pstS1_1"

/locus_tag="PIKHDJKN_00221"

/inference="ab initio prediction:Prodigal:002006"

/inference="similar to AA sequence:UniProtKB:Q8DPB1"

/codon_start=1

/transl_table=11

/product="Phosphate-binding protein PstS 1"

/db_xref="COG:COG0226"

/translation="MFKKLPFILMTLTFVLVLAACGSKNDAGTEGSNGSTGETATELS

GNILAVGSTALQPLVEQAGQKFMAVDEYKNVMVQVQGGGSGTGLTQVSEGQATIGNSD

VFAEEKIDDAAKVSELKDHQVAVVAIAPVSNKDAGVQDLTKQQLIDIFSGKITNWKDV

GGKDQAIVIVNRPSSSGTRATFENFALGTKVEDIQGSIQEDSSGTVKKLVAETPGAIG

YLALSYLDDSLQVLKYEGVEATVENVEAGTYPVWAYEHMYTKGDPDAATKAFLDYILS

DEIQQNDVTELGYIPVSGMKVKRDAAGTVTQ"

CDS 201567..202502

/locus_tag="PIKHDJKN_00222"

/inference="ab initio prediction:Prodigal:002006"

/codon_start=1

/transl_table=11

/product="hypothetical protein"

/translation="MEQTEGGTVMNNKLKPRRPLREKHYWEEWTGRIYTSICVVFLIV

VMFSIVYFVASKGLSTFFQNGVSLREFLSGKTWNPTGEPAAYGALPFITGSFITTLLA

ALIASPLSLCAALFMTEIVPGKGKKILQPAIELLSGIPSVVYGFIGLSVIVPLLRSMF

GGAGVGIAAGCLVLSVMILPTVTSIMADALSALPKGLRESSYALGATRWQTIYRVIIP

TVLPALLTGIVLGMARAFGEALAVQMVIGNAPHVPTSLLESASTLTSVITLSMGNTTM

GSVHNNALWSMALVLLVMTFVFVILVRLLERRNRV"

CDS 202499..203392

/gene="pstA_1"

/locus_tag="PIKHDJKN_00223"

/inference="ab initio prediction:Prodigal:002006"

/inference="similar to AA sequence:UniProtKB:P07654"

/codon_start=1

/transl_table=11

/product="Phosphate transport system permease protein

PstA"

/db_xref="COG:COG0581"

/translation="MKMKAKTVDKVATSIIVVLALFIVVLLLGLLGFILVRGIGQINW

HFLTSAPQLLKGGGGIGPQLFNSIFLLVLTLIITIPLGWGGGIYMAEYAKPGRITSFI

RLVVEVLSSFPSIVIGLFGLLLIVNTFGLGFSLLSGAMALAIFNLPLMVRTTEQAFRA

VPKEQKEAGLALGLSKWKIITSILLPVALPSLITGTILASGRIFGEAAALMFTAGMSS

PPLDFTDWNPTSPRSPINPLRPAETLAVHIWKVNSEGIGPDSKEVAAGASAVLVLLVL

AFNLSARWIGRVVYRRMTASK"

CDS 203405..204160

/gene="pstB3_1"

/locus_tag="PIKHDJKN_00224"

/EC_number="7.3.2.1"

/inference="ab initio prediction:Prodigal:002006"

/inference="similar to AA sequence:UniProtKB:P0A2V9"

/codon_start=1

/transl_table=11

/product="Phosphate import ATP-binding protein PstB 3"

/db_xref="COG:COG1117"

/translation="MAIPFGTEQLSIYYGHFQAVKQISLTFPEASVTALIGPSGCGKS

TFLRSLNRMNDEIAGSRTEGHIWMDGNDLNEPGTDVIKLRQKIGMVWQKPNPFHKSIY

NNIAFGPRYRGIKNKKALDEIVEKSLRRAALWDEVKDRLNESALALSGGQQQRLCIAR

ALSVDPQILLLDEPASALDPVSTGKVEELISELKKELRIVIVTHNMQQAARISDYTAY

FYLGSMIEHGDTEHIFTNPDNRLTQEYIMGRFG"

CDS 204349..205485

/locus_tag="PIKHDJKN_00225"

/inference="ab initio prediction:Prodigal:002006"

/codon_start=1

/transl_table=11

/product="hypothetical protein"

/translation="MWIITTHKISHDTMATEIISHYSIEKPSVSFIRHNENLTYQVVD

KSTGQKYLLRIHKAAFASMTGIQHTRPALEAEMNLLHELRATSPLRVQTPVRNTSDEW

VTVWLNQTGEEICCTVLEWIEGRDIKQGERLTTAQIHDMGVQLQTLHQYGRRPKEAPK

PQVGSQKPESGQQVNDREQHAERAINSEMYTVSDRPALKQTGVRPAYGSIKENLVMLE

QLEEGVRRGIFTPEDFDLIRETFENINRQLETYPHNAETWGVIHGDITRGNLLVTDQG

ISMIDFCLYGYGYYLFDAGGAALIFNREERDVFLSGYTEQMGPLTKRDIRLMEGFMLI

FTFGYFAFQMENESRYEWMKERMPLICSKFCKPYLQNESIFYEL"

CDS 205515..206792

/locus_tag="PIKHDJKN_00226"

/inference="ab initio prediction:Prodigal:002006"

/codon_start=1

/transl_table=11

/product="hypothetical protein"

/translation="MKNIRAKRVASKGQSGGWKHAGLLLSGVGISNLGDFIYIVAINL

MVLNMTQSPAAVAGLWIISPIASVCTKFWSGSLIDRYDQRQLMIGADMLRAVLVATLP

LMSSLWMMYAVMFLISMSSSIFAPSSQVYITRLVPAERRKRFNSLQALISSGAFITGP

AVAGVMLIYLSPEAAIYANAASFAASALILVFLPKLGIGEQNIGTGSRLTPRMIAQDW

RAVLTFSRKSGYVVGIYTLFQIILVVGMSLDAQEVVFIRQVMNLPESQYGALMSITGI

GYLAGSMLVWVFAKRLPIRHMMGGGVLLVALGYFLFARSFSFELAAVAFILLGLCSAL

ANTGLVTFYQNNIPVDLMGRMSSVLGLVLSVLQVASILIAGVVAEQLSLRMVYTVVSG

VMLLAALGLWVTSMLPSRSRYYDEPAKPKAMVG"

CDS 207512..208201

/gene="sdhB"

/locus_tag="PIKHDJKN_00227"

/EC_number="4.3.1.17"

/inference="ab initio prediction:Prodigal:002006"

/inference="similar to AA sequence:UniProtKB:P33074"

/codon_start=1

/transl_table=11

/product="L-serine dehydratase, beta chain"

/translation="MRFKDVFSIIGPSMTGPSSSHTAGAARLGRIARQWLGCTPERAR

LTLYGSFADTYQGHGTDLALIGGLLDYVTDDPRIPDAEQYAEEAGMEVEFFTSGLPAP

HPNTVKIELWHGDRQCSLIGASIGGGSVSVHALNDFRVQISGEFPTLVLRHSDKAGVL

ASVTSTISSSGVNIGYMQVDRKARDGEALTAMEMDGVPNPEMLNRLRSLDHMLDIRVI

DLKRGVEPDAV"

CDS 208191..209135

/gene="sdhA"

/locus_tag="PIKHDJKN_00228"

/EC_number="4.3.1.17"

/inference="ab initio prediction:Prodigal:002006"

/inference="similar to AA sequence:UniProtKB:P33073"

/codon_start=1

/transl_table=11

/product="L-serine dehydratase, alpha chain"

/translation="MRFKHLHELNTICTAESKTIAELMIEEQVQETNIPEADVVRQMS

DYYQVMKEAVHKGLTEDTTSRSGLTGGDGKKMAEYIRKGETCSGDASALAMAYALCVS

EVNASMGRIVATPTAGSCGIIPGVFISSQERFGWSDEHLVNGLFCAGAIGYVIANNSF

ISGAEGGCQAEVGSAIGMAAGAMVELRGGTPEQVVHAVGLALKNTLGLICDPVAGLVE

IPCIVRNGLGAVTALAAADMALAGVRSAIPSDEVIDVMLEVGSAMPSRHRETAQGGLA

QTPTGRKMMEKLAKPKAKRAEPEAEVESPAENEPEAKA"

CDS 209395..210612

/locus_tag="PIKHDJKN_00229"

/inference="ab initio prediction:Prodigal:002006"

/codon_start=1

/transl_table=11

/product="hypothetical protein"

/translation="MKSVFQTSLLFLKNGATVVGLITAVLFQVFFNTIWLSGYDQVNA

RMNQLAVTIVNEDGAVAEPVAKSLATGLNFVIKPSESMEEARQMLTDRDVYMIIEIPS

GFMQQAGDLSSPVAVKYVMNESNVATVKSVMQTVSAQVTATLNREVQQNSIRGVLDQS

GMAADQANALAVGLSSRVEAEVERLNPVHNFAFSMVPMLIVTATFTGAMLLGMNLQKV

SGELSNKAGKWERFWARNIINVGAALIVSLVGAGMMHVMGVSSADGWWMLWLFQLLIT

LSFLFVAQLSLLLLGNAGAWLNSALLPLLMLSSGATIPRDVMPDFYQQIGHYLPATYA

VEGMMNLVLGGDGISHDVLLLVVIGAATLLLGAVCTWVKRSGPAQSGIPLESKSAATP

VALTGNLPSPDRG"

CDS 210731..211243

/locus_tag="PIKHDJKN_00230"

/inference="ab initio prediction:Prodigal:002006"

/codon_start=1

/transl_table=11

/product="hypothetical protein"

/translation="MNTQHVILGILHNQPCSGYEIKQYFEQYFSFFFDASFGTIYPTL

AKMEKSGLLTKESVRQEGKPDKNVYTLTPEGSAEFNTYLMSPLEAEVFRSDFLMRLYF

GELADEDTVAGWVQSELNRKELLYAELQRQMKQFGEQISPAQRLCMQVGLVQYEATIR

LLKEQLTAPQ"

CDS complement(211404..213158)

/locus_tag="PIKHDJKN_00231"

/EC_number="3.2.1.54"

/inference="ab initio prediction:Prodigal:002006"

/inference="similar to AA sequence:UniProtKB:Q08341"

/codon_start=1

/transl_table=11

/product="Cyclomaltodextrinase"

/translation="MLLEAIYHQPKRNWAYAYDKDTIRLRLRAKKNDLTEVHALTGDK

YAWDRTKELVPLTKYTSDSMFDYYEGQVKPPYHRLKYSFLLKSGTEQIWMTETDFQTH

EPDDPGHMFQFPYIHPGAVFTPPAWVKDAIFYQIFPERFANGNPDLDPEKVEPWGGEP

TPFNFFGGDLQGVIDHLDYFEELGINAIYFTPIFEATTNHKYDTEDYLRVDRHFGDAD

TVKRLVELCHARGIRVLLDAVFNHSGKTFAPFVDVQKNGEKSKYKDWFHVHEFPLDVK

DGIPTYETFGFEAHMPKLNTENPEVKAYLLEVAEYWIKEVGTDGWRLDVADEVDNAFW

RDFRRVVKAANPDAYILGEVWNESSSWLQGDQFDASMNYPFTEAVNRFFVKDVMHAAQ

FANSIGRQLSRYPLQASEVAFNLLDSHDTPRLLTLCEGDQRKMKLAALFQFTYMGTPC

IYYGDEIGLDGDHDPGCRKCMEWDETKQDRELFTFYQKLIALRHAHPALRAEGSIRFL

QAREGGSQLVMERQDEEERILVLFNRSEETAIVELEAGAGEWTELFGENYRTAKEEGI

LAVELPAYGYAVLSTDIA"

CDS 213438..214121

/gene="walR_3"

/locus_tag="PIKHDJKN_00232"

/inference="ab initio prediction:Prodigal:002006"

/inference="similar to AA sequence:UniProtKB:Q9RDT5"

/codon_start=1

/transl_table=11

/product="Transcriptional regulatory protein WalR"

/db_xref="COG:COG0745"

/translation="MKTKLLYIEDDTEIATWVRADLEERGYEVVWLGSGEGAAEAAGG

CSLVILDVMLPGLDGFTVGQRLKKEYPEVPIVMLSARTSIDDKLHGLDFADDYVTKPF

HPDELAARIEVQLRKAGTAISADTVVKLDHLSIYEKDHRIVNEETGEEIILSGKQFHI

FAYLLRHMGMIRTKEQIYEAVWNEPYLDGDKTLMVHIRHLREKLERDPANPTIIQTVR

GVGYRVKKP"

CDS 214118..215662

/gene="sasA_3"

/locus_tag="PIKHDJKN_00233"

/EC_number="2.7.-.-"

/inference="ab initio prediction:Prodigal:002006"

/inference="protein motif:HAMAP:MF_01837"

/codon_start=1

/transl_table=11

/product="Adaptive-response sensory-kinase SasA"

/translation="MSKKATMRDPSDRDRGVKRSLPGKRKMRFGRTLMSQYILLILAA

VLFVPVVLPITSIIYVVVVNNTNKDNTAPYGDSTKISNLWTRESEKLDGATSEKINQR

MAQLHRKYPKSSMYRVDQNGETAFILAGEDVDVLESTVDGTTMTTLRWTLDHPNTETR

IPSIWNANTTLEFMKEASYRDPLTVVSFVGGDSEDKGQGFMVIEVPRYLLQKPQNNWP

MELLYLGGLMMIIFLLFIIMSILFFARIRKRLIRLQTAMIATGKEGIPLPVEIRRSDE

IGHLEESFNEMVHQLTDSRHREREEEQLRKRLVAGLSHDLRTPLTVIRGHMHSLHKEP

LSTAADTSLRRMEAKMDDLSGLIDNMLSYNLLTSGKYTLKLEQKDVLRIVRETAAAWY

PVWEKEQFEIDIDLPEEPLIWQVDEQGLRRVLDNLFQNVIRHAASGKYIGISTEQIHG

EKALVIQDRGPGFQEDSDTKGTGLGLSIVDLLIREMGLRKRVDSSENGLRVCLYSGGG

AGKPRP"

CDS 215746..216660

/gene="bcrA_2"

/locus_tag="PIKHDJKN_00234"

/inference="ab initio prediction:Prodigal:002006"

/inference="similar to AA sequence:UniProtKB:Q5WNX0"

/codon_start=1

/transl_table=11

/product="Bacitracin transport ATP-binding protein BcrA"

/translation="MTDNIIQTANLWKTYRDRAAVRELDLHIKKGDIYGFLGPNGAGK

TTTIRMLLGLIKPTKGVIRIFDKDIRKDRMDILRRVGSLVEYPSYYGHLNAVENLETL

RRILGVPKSRIAEVLSIVDLTRDAKRSVKGYSLGMKQRLGIASALLGQPELLILDEPT

NGLDPAGIQEIRELIKRMPLEHGITVLVSSHLLSEVEQMASRVGIIREGKMVLQDTIA

NLHSQTGSSIRLTVSEPEEAMKLAREQGQFGQREGAALTFPYMDNSAIALLVRRLVEQ

DHAVYRVEEHRQSLEDLFMRVIGEGATV"

CDS 216657..217373

/locus_tag="PIKHDJKN_00235"

/inference="ab initio prediction:Prodigal:002006"

/codon_start=1

/transl_table=11

/product="hypothetical protein"

/translation="MTGRALSSDWLKIRGKGIWFLVFLAPIGLTAMQALNFGLRLGYL

KEQYGNHLWEGLLDNVVVFVPLALMLGATILSSMIANVEHEQGSWKQLLAMPITRPAV

YLSKFLLACLLLIISCLLLTAGTIGLGLLLGFDAGEIPWTHAIKLGLLPLAGALPVLS

LELWLTMVNKNQALPVTLGIVLAVMGMFALSISPYFPLAWAQMAWISPKPFVYAGLGV

GAGLLIMLLGMVHFSRKDVA"

CDS 217375..218109

/locus_tag="PIKHDJKN_00236"

/inference="ab initio prediction:Prodigal:002006"

/codon_start=1

/transl_table=11

/product="hypothetical protein"

/translation="MSFATTYFRILSSERLKMGKSPIWLLILLSPVIALLIGLLSTPS

GQWNVLMGSMVFLHGLLLLPILTGVFTSFVCRFEHAGGGWKQMLVLPLTRNGVYAGKL

TIVILLLAGTQLLLLGSILLAGMIQGMTDPIPWGFLAGKLLLGLFACVPLAALQMFVS

LVWSSFAAPLALNFALTVPNILIVNSATFGPYYPWAQPMILMTPFEGGGFGAYNVPLG

TMLAVVGGSAVLFILIGVLYFRKKEI"

CDS complement(218202..218762)

/gene="lacA_1"

/locus_tag="PIKHDJKN_00237"

/EC_number="2.3.1.18"

/inference="ab initio prediction:Prodigal:002006"

/inference="similar to AA sequence:UniProtKB:P07464"

/codon_start=1

/transl_table=11

/product="Galactoside O-acetyltransferase"

/db_xref="COG:COG0110"

/translation="MLPIRVKYAYHKVRGLTLFGKIQSSFGLLPRIRGKVYLNKFGDL

RVGKRLNIIGKPWGTQLTVVKGARLTIGDDVMINAGVGIAANIEITIGNNVMIGPRTS

IFDSAYHRIDSLDDGSNMTKKITIQDNVWIGTGVLVLPGVTIGKNSVVAAGSTVTKDV

PDNTLVAGTPAKVIRELTIHDGWVRK"

CDS 219177..221885

/gene="rcsC_1"

/locus_tag="PIKHDJKN_00238"

/EC_number="2.7.13.3"

/inference="ab initio prediction:Prodigal:002006"

/inference="protein motif:HAMAP:MF_00979"

/codon_start=1

/transl_table=11

/product="Sensor histidine kinase RcsC"

/translation="MDINKTETLKRIISEGSSYQSLFFNHPDAIYVMDIHGNYIDANP

SIERISGYTFEELRSLNRDRICPPESEESRKEYIREVLAGHSVSKSITFYHRDGSLKQ

AEITYVPITEQEEIVGIYGIARDVTEIVEVERKLHETQEKYQMLADHAQDLITTSSMD

GKLLYISPSVYSLLGYRPEEVTGKFLKDYCYPGDYPELLEMSSSGDGCKMRVLHKKGH

YIWMETLAKPVAGEEGKNTQIVSVSRDITQHKNAERRLRESRQRYKSLFEHNPAAVYS

LNLEGKYTAVNRNLVKMLDVPRSKLIGQSFLLHLDKSEVHGGQAYFELVKQGKPQHYE

TRIVNSGGRKVDVSIINVPIIVDQELVGVYGILSDITERKDYTEQIQELSKQHALILN

SVTDGIYGLDADGITRFMNPAAASMFGYEAEEFVGKNSHPIIHHSRADGSYFPKKECP

IHMTVMDGQRRTVKEDVFWRKDGTSFLVEYQVTPIIDEGQIRGAVIVFNDVTGDREIV

RAKETAELAAQAKSEFLSMVSHEIRTPMNGIVGMTELLIGTDLSEEQREYAEIIRESG

DALLNILNDILDFSKLESGKMALAYEPFSLRIMLEQVAELFSPKAEEKQLKIRYRLNP

DIPEFMVGDAMRIRQILVNLVSNALKFTDQGSIDVTVDIIRGNRPESSVLDFAVKDTG

IGIPEDKLDQLFQSFSQLHPVINRKYGGTGLGLVISKRLVEIMGGSISVESEDGKGST

FRFAVPATSVDATAEQSARQFSHDRTRQGDKVAMRILVAEDHPVNRKILSEYLEKLGY

QADVCTNGVEAIEAISQNAYDIVLMDLQMPVMDGLKATDLVHRLIPQERIPAIIAVTG

NAKREDKEACLEIGMRDFISKPIMLSELKRVLQQWGPADEPQLAPS"

CDS complement(222023..222976)

/gene="ldh_2"

/locus_tag="PIKHDJKN_00239"

/EC_number="1.1.1.27"

/inference="ab initio prediction:Prodigal:002006"

/inference="similar to AA sequence:UniProtKB:D8KFT1"

/codon_start=1

/transl_table=11

/product="L-lactate dehydrogenase"

/translation="MLGKSGKVAVIGAGLVGSSCAYSMINQSICREIMMVDRTYDRAV

AQALDFSHCMDFTHNRTKVYAGTYADCSSMDVIVLTAGANPKPGQTRLDILEEAESIA

KDIVVPIMNSGFRGIFVVAANPVDIVTYMVWKLSGLPRERVIGTGTSIDSSRLKTLLS

EVFSIDPRSVHGYALGEHGESQFVAWSHVTIGGKPIMHIMEQHKERFKHLDLEDIARK

TKDAGWEIFTRKGSTQFGIGNALAHITRSILNDEHKIIAVSAILDGEYGQSNVCAGVP

AIIGGDGIQEIIELNLDASEREKFERSCEVLSGNINRLTLV"

CDS 223478..224131

/locus_tag="PIKHDJKN_00240"

/inference="ab initio prediction:Prodigal:002006"

/codon_start=1

/transl_table=11

/product="hypothetical protein"

/translation="MIEEAGATTETAKSLGIGASTLRKYAAALEEQGYRFERSANKSR

LFKSDDIECIERLMTELREHNLPLADAVVTVLTPDVPEMVMEDASETECAATVMPAIF

PEGPEPTGALRSYEGLGQLVSEVTYGAVGQQDDQVQLLQHRVDELELTLQHLADTHLA

LQEQMEKQRLWMNEKLEEERDRELVTNLRSFQGRKRKPKGTSLRMLFGLLPKKHKEA"

CDS 224268..225089

/gene="ybdM"

/locus_tag="PIKHDJKN_00241"

/EC_number="2.7.11.1"

/inference="ab initio prediction:Prodigal:002006"

/inference="similar to AA sequence:UniProtKB:O31435"

/codon_start=1

/transl_table=11

/product="putative serine/threonine-protein kinase YbdM"

/db_xref="COG:COG0515"

/translation="MELRRSWQRLLGLWTDRPRRKGTKIAGRYTIYDLLGMGSYGLTY

LCNDEQTGKDVALKESKPSKGRLAVQLLDREADVMNRMDHPAIPKLLDVFTYRARSYI

VTEYILGETLEQSIFEQGRKYTEQDCLELVKQLLAPIVHVHEQGYIHGDVRIPNVILR

DGQVHLIDFGLARRMGEPLLPELKRRMRELPEPEDEPAAPDQDLQDIGHFLLFMLYSA

YEPEKGRKPASWQEELKLTPEVRHMLERLLGLRPGYEGNGLELQADIDRVLIKLR"

CDS complement(225214..225390)

/locus_tag="PIKHDJKN_00242"

/inference="ab initio prediction:Prodigal:002006"

/codon_start=1

/transl_table=11

/product="hypothetical protein"

/translation="MLRKMLGKLLDSKSSSHRKRYSSSSGKRNYRRYSSSDAKHRDSR

HGSGYYKSRKYSSS"

CDS 225600..226346

/gene="map_1"

/locus_tag="PIKHDJKN_00243"

/EC_number="3.4.11.18"

/inference="ab initio prediction:Prodigal:002006"

/inference="similar to AA sequence:UniProtKB:P19994"

/codon_start=1

/transl_table=11

/product="Methionine aminopeptidase 1"

/db_xref="COG:COG0024"

/translation="MITLKTKEQIELMKKAGEILAACHREIAGMIRPGITTQEIDQFA

EAFMKKNGATPEQKGYNGYQFATCASVNDVICHGFPGKYVLQDGDIVTIDMVVNLDGW

LADSAWSYAVGQVTPEAQHLLEVTKTSLYKGIELAVIGNRIGDISNAIQTYAEGEGLS

VVREFIGHGIGEKMHEEPQVPHYGPPHRGPRLKEGMVITIEPMLNIGTYRSKLDSDGW

TARTQDGSLSAQYEHTIAITADGPVILTAQ"

CDS 226521..227534

/gene="yfmJ"

/locus_tag="PIKHDJKN_00244"

/EC_number="1.-.-.-"

/inference="ab initio prediction:Prodigal:002006"

/inference="similar to AA sequence:UniProtKB:O34812"

/codon_start=1

/transl_table=11

/product="Putative NADP-dependent oxidoreductase YfmJ"

/db_xref="COG:COG2130"

/translation="MSENKQIVLASRPEGAPSREDFKFIDAPLPEPEAGQVLVRTLYL

SVDPYMRGRMKDTKSYAPPYALNEVIKGGTIGQVVDSSEPNLRKGDLVSGMWGWQQYA

AVNTGDISLIDTEEAPITAYLGALGMTGLTAYFGMEDIGKPKDGETVVVSGAAGAVGM

IAGQIAKIVGARVIGIAGSDEKCAYLKEKLGFDVVLNYKHEEDMSAAIERACPAGVDV

YFDNVGGEISDAVLRHINRNARIPLCGQISSYNLEKPDIGMRPQTLLLTNTALMKGFL

VGDYAKSFKEGRAKLAKWIKEGHIQFEENIVEGFDQTPEAFMGLFSGDNLGKQLVKVA

DPE"

CDS complement(227637..228122)

/locus_tag="PIKHDJKN_00245"

/inference="ab initio prediction:Prodigal:002006"

/codon_start=1

/transl_table=11

/product="hypothetical protein"

/translation="MAQISPSYLQIGATLGMLVMALLAIFIRMKASHRPVTIRKILIP

PLGMSTGFLMFVVPETHVPLLWALIALLVGWFIFSYPLIRSTRFERVNEEIFATRSRS

FAFILLGLLAIRLILHEVIQRYVSIPQTGGLFFLLAFGMIVRWRVYMYKHYKEVVAAE

S"

CDS 228376..229506

/locus_tag="PIKHDJKN_00246"

/inference="ab initio prediction:Prodigal:002006"

/codon_start=1

/transl_table=11

/product="hypothetical protein"

/translation="MNRNHRPLTVWTLACLTVVSVILVIPWILWQSQAPTPLNIMIVD

KSKPDLSYVGHKGLVWILNQQKIVQQTGEHYKYEEDYYGYDIQNGISRIERTLPDEVT

DTDLIYLTANHHISSTDPNERIQGKAYEGLTIYDAHKIREAATKGVTIVTEFSALSNA

VSNMTREQLYPILGMKSSGWQGRSVSDLQSLDEVPQRVRMNYERREKKEWPYHGAGIV

LVHEDGRVIVLKQGRDVKTGDIKLAFTQEGGKWSGITENIHYSEWFDIIVPEQQATVL

AWYKTDLTENGQQKLMNAGIPADFAALIRHEGYNRTYYMAGSFGELEHYSFWRRIKGW

DVVKTRLTPDQKGVPDRFYWKVYVPVMKKILQEVQTGQAPWQ"

CDS complement(229604..230173)

/locus_tag="PIKHDJKN_00247"

/inference="ab initio prediction:Prodigal:002006"

/codon_start=1

/transl_table=11

/product="hypothetical protein"

/translation="MSPRQGLDRGALLSAATQLADSDGFHSLTLAALAQRLDVRSPSL

YNHINGLPGLRQEMALMSAQQLRRALTEASVGRSGDEAIQEVAAAYIGFVRKHPGLYE

AFFHAPDRQEPQLVVESTAALDLLLRLLQPYPLSEAEALHAVRGLRSLCHGFASMGEK

GGFGMNFDPDESLQLSVTAFLNGLQHLHS"

CDS complement(230170..230895)

/gene="yflN_1"

/locus_tag="PIKHDJKN_00248"

/EC_number="3.-.-.-"

/inference="ab initio prediction:Prodigal:002006"

/inference="similar to AA sequence:UniProtKB:O34409"

/codon_start=1

/transl_table=11

/product="putative metallo-hydrolase YflN"

/db_xref="COG:COG0491"

/translation="MRITREHDVVQVSFLPRLFPVNVYLVEEEDGFTLIDAGMPFSLK

GILATAQSLGKPITKIILTHAHGDHVGALDGLKDALPKAEVCISRRDAPLLAGDASLL

PGEPQTPVRGSVPKAIRTRPDRLLDDGDRIGSLVAIATPGHTPGHMAFMDTRSRVLIA

GDAYQLHGGLAVSGRLRPLFPFPALATWNRELALASAKRLAELEPSVLAVGHGRMLRQ

PGAAMRAATADAEQRLRPAGGHL"

CDS 231331..231795

/gene="ywnA_1"

/locus_tag="PIKHDJKN_00249"

/inference="ab initio prediction:Prodigal:002006"

/inference="similar to AA sequence:UniProtKB:P71036"

/codon_start=1

/transl_table=11

/product="Putative HTH-type transcriptional regulator

YwnA"

/db_xref="COG:COG1959"

/translation="MNSEFTIAVHCLVFLSMKDECMANSEDLSQSVGTHPARVRKVLS

VLRKHGFLTTKEGAHGGYLLSRKSEEIKLGELYRLVAGGSLGPSWCSGESGSTCVVSS

NMQDVMGSIYDGGEEALSAYFDRISIEDVKHRIGNGEECSLSMEPLSMKDRS"

CDS 232047..232673

/gene="yodC_1"

/locus_tag="PIKHDJKN_00250"

/EC_number="1.-.-.-"

/inference="ab initio prediction:Prodigal:002006"

/inference="similar to AA sequence:UniProtKB:P81102"

/codon_start=1

/transl_table=11

/product="Putative NAD(P)H nitroreductase YodC"

/db_xref="COG:COG0778"

/translation="MSSIENNETLRVINERHAVKKYENGFVMPEADLNAILTAASEAP

SSWNLQHWKFLVIESEADKAKLLPIAYGQSQITDSSVTIAVLGDLEANRNAVIYDQAV

EAGAIPAEVRDALVGQINGAYKNPQIARDEAIRNASFASQNIMLAARSLGYDTCPMGG

YNPQQLIETFNIPARYLPTLLITVGKAAVPARPAGRFPLSEVVVKGSF"

CDS complement(232776..233459)

/locus_tag="PIKHDJKN_00251"

/inference="ab initio prediction:Prodigal:002006"

/codon_start=1

/transl_table=11

/product="hypothetical protein"

/translation="MKKNILATLTAGALLTLSLSAGPIHAAQAEFTDIQGIAGADKIE

SLHQDGLIKGVSDSLFKPEQELNTAQGIQLIADGLHLNLDTIRFIKQPLPSDYFSNVK

DGVWYSDAFIRAQFNGIKMSNDIDPSKALTREQYTLFLMQGIEAKGGLPMINIKPVDI

MDEKELTPEFQGAVQRSLVLKINTLDAEGNFNPKETITRAEAAVMMYNAIEYLEDFNA

PKIPETPEK"

CDS 233785..234840

/gene="pgl_1"

/locus_tag="PIKHDJKN_00252"

/EC_number="3.1.1.31"

/inference="ab initio prediction:Prodigal:002006"

/inference="similar to AA sequence:UniProtKB:O34499"

/codon_start=1

/transl_table=11

/product="6-phosphogluconolactonase"

/db_xref="COG:COG2706"

/translation="MEPTATNEAFFYTGTYASAEQPGIFLCALNKDTGELRIVNHMDG

VDNPSYLALSADGNGLYVVSETDEGEVLVYRRDAATGELHLMDRKLTKGASPCYVSVT

EDGKWVLTSNYSSGSVNVFLVGDQGTLEEMSALIEHTGRGQNDDRQEGPHAHSIQPDP

SGQYAVVCDLGLDQIIVYRQEEGRLVTHRETNQPPGSGPRHLVFHPSSKWAYVINELS

NTVTAFMYDQRRGEFTTLQHITTLPEGHSGEGTAADIRVSPCGRFLYASNRGDDSIVL

YHIDQESGELEAIEWTTTIGQTPRNFNLLPGGILLVANQDSNNLVAFQINGEDGRLTH

NGFKLEIPRPVCIAPVV"

CDS 235089..236963

/gene="yesW"

/locus_tag="PIKHDJKN_00253"

/EC_number="4.2.2.23"

/inference="ab initio prediction:Prodigal:002006"

/inference="similar to AA sequence:UniProtKB:O31526"

/codon_start=1

/transl_table=11

/product="Rhamnogalacturonan endolyase YesW"

/translation="MGKRSLWKRTLRTAGVSLLSSALLVTSLGLGYTPVTHAAGARQM

EFLDRGVVAVKTGTGVFVSWRLLGTEGSNVSFNVYRDGTKVNASPITNSTNLQDASGT

SSSKYTVRAVVSGTEQAASAAASVWGNNYLSVPLSVPAGGTTPDGVAYTYSANDASAG

DVDGDGEYELIVKWDPSNSKDNSQSGYTGEVFIDAYKLNGTRLWRISLGKNIRAGAHY

TQFMVYDLDGDGKAEVAMKTADGTKDGTGVVIGDASKDYRNSSGYVLSGPEFLTIFNG

QTGKALSTVNYEPARGNVSDWGDNYGNRVDRFLAAIAYLDGERPSLVMARGYYTRTVL

VAYNWRDGQLTKQWTFDSNTSGNSGYAGQGNHNLSVADVDGDGKDEIVYGAMAVDDNG

KGLYTTGLHHGDAMHLSDLDPDRAGLEVFQVHETASNAGVEFRDARTGQLIWGIPTTK

DIGRGMAADIDPRYKGAEVWADGGLYTAKGQKIGTTLPSSTNFGIWWDGDLLRELLDS

NRIDKWDYANSKTVNLLTASGVSSNNGTKSTPNLQADLFGDWREEVVWRTNDSSALRI

YTTTAVTDKRIYTLMHDPVYRLGVAWQNVAYNQPPHTGFYLGDGMSTPPVPNIRYAGK

"

CDS 237442..238521

/gene="sbp"

/locus_tag="PIKHDJKN_00254"

/inference="ab initio prediction:Prodigal:002006"

/inference="similar to AA sequence:UniProtKB:P0AG78"

/codon_start=1

/transl_table=11

/product="Sulfate-binding protein"

/db_xref="COG:COG1613"

/translation="MKKRIHKGILVGLALVLTGVLAACGSDSGGTNAAGTSGGTEGGK

EGAKAIELLNVSYDPTRELYEQYNKAFAAYWLKEKGQEVTVKQSHGGSGKQSRSVIDG

LDADVVTLALGYDIDAIEDKGLINAGWQDKYEHNSSPYTSTIVFLVRKGNPKGIKDWD

DLIKGDTQVITPNPKTSGGARWNYLAAWGYALKHNNNDEEKAKAFVAELFKHAPVLDS

GARGATTTFVERGIGDVLLAWENEAFLSVKELGPDKFDIVVPSVSILAEPPVAIVDKN

ADKKGSREVADAYLKYLYSEEGQTIAAENYYRPTLDSVKEKFKDQFPQLELFTLDDVF

GTWRDTQAKHFNDGGIFDQIYVPGS"

CDS 238604..239440

/gene="cysT_1"

/locus_tag="PIKHDJKN_00255"

/inference="ab initio prediction:Prodigal:002006"

/inference="similar to AA sequence:UniProtKB:P27367"

/codon_start=1

/transl_table=11

/product="Sulfate transport system permease protein CysT"

/db_xref="COG:COG0555"

/translation="MSKVMVARSRTLPGFGLTMGYSVLYLSLVVLIPLAALLFNSTGL

TWSTLIEVATNPRVLASFQVSFLTAGAAALIDLVLGLLLAWVLVRYEFPGKRLFDAVI

DLPFALPTAVAGVALTALYAGNGWIGQFVEPLGIKLAYSQAGITLALMFIGIPFVVRT
VQPVLQELETEVEEAAATLGAGRWRIFRKILLPDLIPPLLTGFALAFARGIGEYGSVV
FISGNMPMKTEIAPLLIMAKLEQFDYAGATAVALLLLLVSFVLLLIINSLQRWSRKAG
RA"

CDS 239481..240356
/gene="cysW_1"
/locus_tag="PIKHDJKN_00256"
/inference="ab initio prediction:Prodigal:002006"
/inference="similar to AA sequence:UniProtKB:P0AEB0"
/codon_start=1
/transl_table=11
/product="Sulfate transport system permease protein CysW"
/db_xref="COG:COG4208"
/translation="MAGTVPLTPASRVRTGPGPNRATTEAPWVKWLLIGLASLVLLWL
LILPLVIVLMEALKQGWGVYIAALTEPDAMSALKLTLLVAGITVPLNTIFGVAAAWVI
TKFQFRGKGLMITLIDLPFSISPVVGGLIFVLIFGSNGWFGPWLAEHDIKIIFALPGI
VIATLFITFPFVARELIPLMEDQGTREEEAAVTLGASGWRIFWNVTLPNIKWGLLYGI
ILCNARAMGEFGAVSVVSGHIRGETNTLPLHVEILYNEYQFSASFAVASLLLILALAT
LLLKSWLGHKTIPEK"

CDS 240582..240821
/locus_tag="PIKHDJKN_00257"
/inference="ab initio prediction:Prodigal:002006"
/codon_start=1
/transl_table=11
/product="hypothetical protein"
/translation="MAKPLKVDEVWMDRIAGQLNDMEFGSLHIVVHEGQIVQMERTER
KRFENTPSGSASKSGSTARSSSPRSLRSSNASAKG"

CDS complement(240969..241889)
/locus_tag="PIKHDJKN_00258"
/inference="ab initio prediction:Prodigal:002006"
/codon_start=1
/transl_table=11

/product="hypothetical protein"

/translation="MLQRWMLALFSYPGFVAVLLGMIAVLLGLYVWSATEVRRSRDRR

MHAIRDTLYISTTLLGQLTANEQRNVAITEHEHHELLTAMLSCRAAVHLSPQLQDQIR

SCIIDVDPARLALMHRALERESAALSDELNRSVHPDHWGRAIWNVIRPAAEPTAVAAV

LYLIADFVFRQQGLLYPFWHSWFNALPWIRFVSLIITVFYGYLLWSSPRQGSSGTVTK

ALVTLIAMCSLLHLIGLAAAPYALGLQLILFISGFSLTGTPSRSDRPYAGHTDSDKEL

NSSTEAHEDENLDMELNLYKPRTSGAISEK"

CDS 242214..247847

/locus_tag="PIKHDJKN_00259"

/inference="ab initio prediction:Prodigal:002006"

/codon_start=1

/transl_table=11

/product="hypothetical protein"

/translation="MRSLKRVFLLFLTLSILIGNFEVAIGFAASEGGKGNASIPVTKQ

NSQDKTSTDPEVGSVPIDQVDSDITNEVETPEKEAEEVVEETVPLPEEPVQVDSPKND

VSDVLNNISVIGDVHNNKPRSKFLVRLKKEFSVQLPSVTESVYKGSADQKSMKIQGLM

SEDPTSSISDIEYKLDLYNVKSLESVNSLSMELNTEEVNLLLSDPKIESIEEDKPIEI

AADNVTEVEDKSVKESAQTIPWGIHSTGAYMLQSKKGTGDRVVKVAVFDTGVVDHLDL

NIAGGISFDESSTSYTDDKGHGTHIAGTIAAIDNGIGVVGISPSTELYAVKVIDGTGN

GYTSSVIQGIEWAITNNINIINMSFVSSQYSEALHEEIVRAKEAGIIIVAAAGNSGLG

EDTIKYPAKYPEVVAVGAVDYSYHRADFSSTGSELDLVAPGFGIISTTMDGAYGVSSG

TSNAAAHVTGAASLLWSHNPSWTGEEVVQRIYETVTPLEDTHETGRGLVNVAKAANVI

EGSIAPLSEENLSGLNTVIPVEPEGEITTASYDLKNNGATIEPGDTVTVSLKLEGDQN

GNNPHSKIIIEVSAASNPNNIIASQTISNPSLNVDIPYTWRTSASTPTGTYYIKYRYP

GVTSGKFDDTFIINVAQQGSGQDTYEPNNTILTAKSVNPGNSYISYISSSSDIDYYKL

TADKDGQISITLDIPSSVDYEMDIYNESGIQVGESSNGTGITEHIDFEVTATKSYYIK

ITGFSGQFSTSPYTLTLSNITAQAFKAPTGLEAVPYATSIKLSWNTMPDATSYVVRLD

GKEVGTVKTSSYTFSSLEPLKAYKLEVAAVYAGGKSSFASIQSSTTIPELFVAKPEDI

DQPAGRDQVYSFTPATTGVYRIRTSAYQGSGPDVDTELSIYSSLQLTKQMASNDDAND

SVFSEITISLVGGQTYYVKVNGFDTTPLRARITADVVSSSIPYIYLNQPQDINEQAGN

SNVYVFIPASNGKFRIATSRYNGSASSKINDTELNVFSDADMLTPITNGYNDDKGESV

YSEVIVNLSAGTPYYVRVNEANKRKVLARLQITAAGQTGFDSLKLGEAVSLSKPSGEQ

AYLQFTPSTTGKYRFFTSYYPGSNEVNDTDIALYSDPEMERLLDSNDDVKGYRPYGDL

FSKLEVTLNAGTTYYMAVGSYGSAQGLRAQLTVESMAHSTRETAQLIPFGESVNQDRA

GNSLSISSLYDVDYYKFELSTPEELSIALSEGEGILEDDSGFTVSVSDSESTQVVELY

PGTYYLKVKSGQFKNTFNPYLYDLAVDINKVEYLNWVDGEEVVAMRMAKASAKDDLYL

DATPGSNSSATINYRVKNPTQKLYFEVRTIGKNQLVYNDFVEGNFKKGQDVAVVWKGQ

VSTFNKDKAEFSRHAVISGKDTYWAKSATYIVTISSYNERDRSKKDIQVVRVYVNNDS

KLKLNWIPVPPKKYKNSNKLIKAGETKCLECRDYFSRYVYIPNTKAYETQYDAWFSDI

YGKTGIQKFWAGAEKLATCQDLKGSKLVHCTIQNIGMIPILGEPFDGIDGVIYLIEGN

VAEALWSAGSMIPIAGNGLTGFKTVKRVYNNTCGCMPEGTLIKTKNGEIPIEKIKIGD

LVLAKDTDKGIQSYKPVEQLFNTTAEEIFTLQIGKTSLRSTGDHPFWIKNRGWVPVDE

LAVGDHVELQDGSLEKISSIEINYESVKVYNFTVSDFHTYYVSEAGILTHNLQKECDI

SNYINLKTKTTGEKGSSNGDKLAKEIEKSTGMKRPDGWDAHHIVPVGCTKAPCVKLQK

IFEDLDIDLNSSANGVYLPKDKGKATTVIDGTSMATHNGGHATSYYEFVLERIQPVKN

NKKKVEEQLEWIRGELMSRRLRIGNLGKNGESYAP"

CDS 247899..248708

/locus_tag="PIKHDJKN_00260"

/inference="ab initio prediction:Prodigal:002006"

/codon_start=1

/transl_table=11

/product="hypothetical protein"

/translation="MKVYELRNNDSSMDLTTIDWEHELVSDFNGDTKGGIWTPTKVKT

LYKKKYNDFPRLLIGITIMKETVKEVFEAFISREAEFLPLIHDELELYVVNVTNVLDC

VDWTRSDVRTFSDGEWADFNKLVFDFSKIPDDTYMFKIKETATVKVFVTDLFKKVVDE

HKFKGLNFSVVYDSEFTQEKEEEQQRLYDAALEEIERNKGQEYTYDAARELVDQGEAM

ASGPWKMQLNQHGQFLQGELLKDLSYQWMIPAYIPPVLLLKSWHKIKKSEI"

CDS 248728..252921

/locus_tag="PIKHDJKN_00261"

/inference="ab initio prediction:Prodigal:002006"

/codon_start=1

/transl_table=11

/product="hypothetical protein"

/translation="MRGLRHNIAATIALVMIISLLSPLFGGVGISYAADIPGAPITTH

NIKGNKSSNGIYYGAVELELIAQDTGSGVSITRFSLNDGDTWSTYTQPILFSDKKVYN
IIYQSVSNNSRVETPKELRFTIKKDTNPPETTIQVTGTQGQNSYYLSPVMISLQGTDT
QSSVDYTEYSLDGGLTWARYTTPITANEEQSAIYYRSRDLDGNLEKTKKSKISIDTTA
PTPPNFSYEPEEWSNSTYLVNIYNGSDEQSGVMKSQYRLDQGATWTDYTVPFNVSGEK
FRTVYVRTLDNAGNISEVEEFTLQFDKMPPTAPKIYLEYEEWRGHEIYVEIDEGTDED
SKVKGYEYRLGSSLEWKEYINSFPVQQEGITKVYARTLDRAGNVSATVEAEVKIDLTP
PSPPENLFKVSQLGSTAVIRWSPAKDELSGIYGYEIYNGDTLLGETTDTKFTITNLTL
NEMQSISIVAIDRAENYSENSRPLIFFTNNLAVSAYRDHTFAWNQQGQVWGWGLNSNY
QLGEATTANRTSATRITNLDGFSMISTGLKQNIGLRPDGTVWTWGANDYTNQRLPLTR
VPGLEKIVSISAGLQHYMALKEDGTVWTWGDNGLGQLGVGGKIPYNSLEPVQVVGLTD
VVAINGSYYNSMALKEDGTVWIWGDGSRAIGYKTSTSTIHYSPIQIAGLSNIVQIDMT
YLHGLALRNDGTVWSWGMNESGQLGLGDYIDHPTPTQIPNLNNVIKITAGYSHSMALT
NNGEVWTWGNNYYGEVGDGTTIQKRPTPVKAAHLKGATDIEAGEMYSFALKKNGSLWA
WGFNMYGQLGNGATTAQPTRVLVNGISYPVDKVAPTAPEQLRITGKTSSTAVLYWDES
EDNHAVKEYLVYSGSTLFSTLPVDGKSLESIINYTATGLASGQTYTFTVKAKDYQGNI
SNSSNSVTVTTEQSFTRQMSGGQSHSLALKSDGSVWAWGYNQMGQLGTKDAYLAKTPI
QIPTLTSITSITSGPEYNLALKSDGTVWAWGSNQSGQLANTNAIMQNVPMKIEGIDSV
IAISAGTSHALALKKDGTVWAWGSNYYGDLGLGHNNSQYAPTKIPTLSGVKSISAGAF
FSFAIKSDGTVWAWGANGFGQLGDGTKVQKNVPTRIAGLTGVESISLGFYHGLALKQD
GTVWAWGYNYNGQLGDGTNTDRLTPVRINSLSGISQIASGNFYSLARTSNAVYSWGQN
SAGELGTGGYYATNSPDKIASITSVQSIAAGITHGMALSNNNLLAWGLNRDGQLGNGL
TTNSYVPVNVTGMSTAKAAVASKAAAAPKTESMNKTSVEQELLTKVILTDFIAPTAPS
DVRVIRNGGKLVLDWTPSTDDVGVKEYWIYLNEELILKTQSYTNVQIQELAEGLVNII
IKAVDEAGNISAASAPVTP"

CDS 253094..253507

/locus_tag="PIKHDJKN_00262"

/inference="ab initio prediction:Prodigal:002006"

/codon_start=1

/transl_table=11

/product="hypothetical protein"

/translation="MDQINIERTPPSAAEYLSLRKSAGLSAMSKEGAEIGLPNSLFAV
CLREENEIVGMGRVIGDGGCFFQVVDIAVRPDQQGRGYGKLIMSEIMNYLREHVPARG

LVSLLADVPADRLYAQFGFTYTSPKSEGMWWRQGE"

CDS 253773..254405

/locus_tag="PIKHDJKN_00263"

/inference="ab initio prediction:Prodigal:002006"

/codon_start=1

/transl_table=11

/product="hypothetical protein"

/translation="MKVAIFGATGAIGKTILWELMDRGHEVTAIVRDPSKVEMEHERL

RVVQGDLLNPDQVADFTAGQEAVVSAYGPKFGAEEEMLEVTRSLIEGVRRAKAERLVV

VGGAGSLLTDSGEMLMDTPGFPEEVKPLAKAHVDAYNLIEASDINWTYMSPAATITTG

RRTGLFRVGMNRVITDDLGESSISVGDFAAALVDELDDPQFIQSRFTVGY"

CDS 254471..256138

/gene="APOA1BP"

/locus_tag="PIKHDJKN_00264"

/EC_number="5.1.99.6"

/inference="ab initio prediction:Prodigal:002006"

/inference="protein motif:HAMAP:MF_01966"

/codon_start=1

/transl_table=11

/product="NAD(P)H-hydrate epimerase"

/translation="MFIVTAEQMRAVDEYTIHKLGIPAASLMENAGRAIAEEVIRLCR

EDDTDRRSEQHGCWNKNGKRAHTGPGDLPAHGGDIIADPALVMEQPGDQQWYMLIGKG

NNGGDGLVAARHLVEAGLGVTLVYADAPDALRGEAAVQRDAAAQLGIPALVHGREAVD

FGRCTGIVDALLGTGSRGAPCGAYAALIEAANGSGKPVVSADVPSGLNADTGEVYEPC

IQARVTVCLALLKRGLVQYPGASAAGRIVVRSIGIPARLAPEHGPSVRLLTEEVLRSA

LRVDTGRLRAPDGHKGTYGHVLLAAGSLPMSGAGLLSAKAALRAGCGLATWALPASLL

PHVIGTVPELMLAAAADGDSGEWNAASADAVLRLAESRDVLATGPGLGRFKGDTDWLR

RLWQHSDRPLVIDADALNMLADAGPTGPRDWGKRSAATILTPHPGEMGRLLGMPTPEV

QRDRIGHAARYAREQGVTLVLKGARTVIATPSGEAYINTTGHAGMATGGVGDVLTGII

AGLLAQGLSAEQAATFGVFLHGQAAERAALLRGDPASLLAGDIIDAL"

CDS complement(256652..258304)

/locus_tag="PIKHDJKN_00265"

/inference="ab initio prediction:Prodigal:002006"

/codon_start=1

/transl_table=11

/product="hypothetical protein"

/translation="MKLQGKLIMNAIVSLLLCLLLVAYIIIQLLSMNAKNQNLVPAML

KVTELNANQIQTEQALDVYSFSMTAGNQDNVTRLLNEGKTMVKQLTEGLLETDEQLRL

IQSIQTKLTALDTGATEAITAMNSSDAKRFSSRVKGIQNDIYMLDEVTRDRYDQYTVD

LEQDIQRTWQIALAGAIILLVAVLLFNMYTSRKLARRIRTLKEAAGQIANGDLTQQLP

ETRGKDELDDLNRSFRLMTGNIRSIIQSIGAAGNRVDGMAQDIDRGNDTVQAIVQQVS

RTTEELSIGSQKIAEDLSGTVMVVDKMQHMFESNLQATSQSAIYGNEVLSSVQEGHIA

VQEQLRLAEVNRIAMSEVEKTVQELEESASRIATMTAYVSGIATQTTLLSLNASIEAA

RAGEAGRGFAVVAGEVKKLAEQSEQSVQHIFAAVGEITAAMGKVKNSVTQSIQLFAEQ

EQATGKTGESFSGIRHSVERISTSINQLTEDMQQSSELSAQVQQAIENISAITEQSAA

SSEEITASTAEQQRSFAEASTKVKTLRDISAEMHQELLRFRL"

CDS complement(258332..259339)

/gene="tmpC_2"

/locus_tag="PIKHDJKN_00266"

/inference="ab initio prediction:Prodigal:002006"

/inference="similar to AA sequence:UniProtKB:P29724"

/codon_start=1

/transl_table=11

/product="Membrane lipoprotein TmpC"

/db_xref="COG:COG1744"

/translation="MKTQWKIKFGFTMMMVLVVLVLGACSATDKVSPADQRTKVGIVL

TDVGLGDHSFSDASFEGLVQARNENSIVFDYKEPGKDLTSEAAFEQFAKDKVDLIIGL

SDTIKTDLEKIAQKYPDQHFLIIDGHSDLPNVTSMSFKAEEGSYLAGIIAGFATTEDH

VGFLGGMDIPVLHDFQQGFEQGVQAANPDATVHVVYAGDFGNPDLGGKLAAQMIQEQR

VDVIYVAAGLTGIGSLSEIQRLGKYAIGVDQDQFFLAEKAVLTSMLKNVDVSLHNAIN

TFNQNQHSFPEKEMFYGLAENGVGLTALHNIILTDEQQQTFEDLKAQIASGKTKITLD

Q"

CDS complement(259760..261370)

/gene="ywrD_1"

/locus_tag="PIKHDJKN_00267"

/EC_number="2.3.2.2"

/inference="ab initio prediction:Prodigal:002006"

/inference="similar to AA sequence:UniProtKB:O05218"

/codon_start=1

/transl_table=11

/product="Glutathione hydrolase-like YwrD proenzyme"

/db_xref="COG:COG0405"

/translation="MNYDPLYQPYPSYRVPVYAKQGMVATSQPLAAQAGLDVLKKGGN

AIDAAIATAAALTVLEPTSNGIGGDAFALVWTEGKLHGLNASGPAPQSISIEALKAAG

HSEMPKLGVIPVTVPGAPAGWAELSRRFGRLTLTEALEPAVRYAEEGYPLAPGLARHW

ARAADIYARQGDAEAGRAWFETFAPGGRVPAVGEMWRSPDHAATLRRIGESGARDFYE

GELAERIHSFMAEHGGYLTKEDLAAFQPEWVDPISVSYRGYDVWEIPPNGQGLIALAA

LNLLKGFDFEEKESVLAYHRQLEAMKLAFADGEKYITEERKMGVTVQELLSEAYADER

RKLIGDVALPPEAGDPRASGTVYLATADGEGNMVSFIQSNYMGFGSGLVVPGTGIALQ

NRGHNFSLDPDHANALEPGKRTFHTIIPGFLTHGDEAVGPFGVMGGFMQPQGHVQVVM

NTVDFHLNPQAALDSPRWQWTKGKTILVEPGFPQHIAQALARKGHDIQVALDPSMFGR

GQIIWRNPDNGVLCGGTESRADGSVAAW"

CDS complement(261575..262474)

/gene="ydaD_1"

/locus_tag="PIKHDJKN_00268"

/EC_number="1.-.-.-"

/inference="ab initio prediction:Prodigal:002006"

/inference="similar to AA sequence:UniProtKB:P80873"

/codon_start=1

/transl_table=11

/product="General stress protein 39"

/translation="MNPVYPFYGEKTVCKQQKLAFPPQHQDQQPGLETLMVPEPISED

PAYIGSCKLEGKVAIITGGDSGIGRAAAIAFAKEGADITIAYLYERTDAERTRERIEE

LGQRCLLIEIDLRLKKNCEAVIRTTMETFGKIDVLVNNHGVQYVQPSIVDITEEQLYH

TFQTNVFAYFFLIQAALPHLCKGASIINTASITAYKGEVQLIDYSSSKGAVVSLTRVL

AKSLAAQGIRVNCVAPGPIWTPLIPSSFSAEDVQVFGTETPMGRAGQPYELAAAYVYL

ASRDSSYVTGECIHVNGGDMVTT"

CDS 262642..263061

/gene="fosB2"

/locus_tag="PIKHDJKN_00269"

/EC_number="2.5.1.-"

/inference="ab initio prediction:Prodigal:002006"

/inference="similar to AA sequence:UniProtKB:Q81W73"

/codon_start=1

/transl_table=11

/product="Metallothiol transferase FosB 2"

/db_xref="COG:COG0346"

/translation="MNIQGINHLCFSVSNLERSITFYEQALGARIQVKGRKLAYFELA

GLWIALNQEDVIRNYTERTYTHIAFTVKEEDFDESVQQLRAAGADILPGRPRDPKDAL

SVYFTDPDGHLFELHTGNMKQRLDYYREDKPHMTFYT"

CDS 263192..265003

/locus_tag="PIKHDJKN_00270"

/inference="ab initio prediction:Prodigal:002006"

/codon_start=1

/transl_table=11

/product="hypothetical protein"

/translation="MIQFPKPDVEQYFQTYRISHFAVSADEKRLFFDSNLNGQPNIWA

MDLPGGYPYPLTYLNQSSQFIKPDPEGRHILTAFDRDGDENYHLYALKPEGGVPFPVV

PAEPNDRCYFSHLSDDGQRLYYMTSRDNPNYLNSRRINLETGEDELLHQGEEVTSNLV

AVSSDEQSYVILNVYSNTYQTAYVYRNGESESIIPVSERQSQVSYVLFADNNRLLMIT

NDNEPYTYVAEYRLDTQEFRLLCKVEGEDVDIIRWHKDSETLYFWTFTGPENRMYALD

KGSEQPRRVAMPLDTVEHVTVTKAGNVYISGRGAIQPHNIYRLMAGSESWEPLTDNRV

TGLDPSDLVYPDVIRYNSYDGLEIEALFFKAKPEQANGYTVFWPHGGPQASEAKFFRP

MFQMMLAQGYHIFAPNFRGSTGYGAEFGKMVERDWGEGPRLDCVAGINWLFDEGISSP

DRLFVVGGSYGGYMTLLLAGRHPELFRAAVDIFGPSNLFTFLESVPEDWKPMMDNWLG

DPVRDRERLTKDSPITYLDQMVNPMLVIQGANDPRVVKAESDQIVAALQGKGVDVEYI

VLDDEGHGFSRKTNEILVYRRMLEFLQKHQEVPVAQP"

CDS complement(265098..266135)

/locus_tag="PIKHDJKN_00271"

/inference="ab initio prediction:Prodigal:002006"

/codon_start=1

/transl_table=11

/product="hypothetical protein"

/translation="MTTRIYFGSNKLGDIEQASLQKALHDFNLGTLMDYKRTDEGVMG

QTLLIRSSEGEYILKGNPLYAGQLQEEQFFVEQLRTHTNIPVPDPYLLYEDTDILGWS

YAIMPRLPGMHLHDPSLQASLSQEDQEKIATMLAHSLAELHRWKVPDAGEYDPVAKQI

VPFAGKYLDWLYGTIHHWLQDAAKYSVITDEDIQWVDEQLKNAESAFHSMPIPGFVMG

DFKVENFVIQRNGDSLAADWQITGLFDFTTAYFGDGTADLTKMTAMYIREGQPELAKR

FLHDYRDVVCAADTERYQHFATRLRVHLLYQRILLWGECKATGRVTWAADMPFAHWAE

QYMDSVIALLD"

CDS complement(266153..267127)

/locus_tag="PIKHDJKN_00272"

/inference="ab initio prediction:Prodigal:002006"

/codon_start=1

/transl_table=11

/product="hypothetical protein"

/translation="MERELLEQLNKWHEQDQFDLIIERIQHIPESERDYELIGQLSRA

YNNEGHYREAVQQLLSVNGQGVSDPLWQYRLGYAYYHMAMYEQALKAFEMANELLPHD

ESTIEFLEWTRPKAEKMQQDRHRHQEILLELEHSGRLNHLRAASGSYDPVSFWEQSEY

ALESYVSTPFDEELIQTIEQELGYKLPASYIQLMNTQNGGIPAHTVFPTKEATSWAED

HIAITGIMGIARDKSNTLGGEFGSRFMIEDWGYPDLGVVICDCPSAGHDVVMLDYRFC

GPEGEPAVVHVDQENDYEITYLTPNFEAFIRGLVDADTFDLSDEEDED"

CDS complement(267319..268350)

/gene="nrdB"

/locus_tag="PIKHDJKN_00273"

/EC_number="1.17.4.1"

/inference="ab initio prediction:Prodigal:002006"

/inference="similar to AA sequence:UniProtKB:Q9KFH7"

/codon_start=1

/transl_table=11

/product="Ribonucleoside-diphosphate reductase subunit

beta"

/db_xref="COG:COG0208"

/translation="MQLQKIFNTEAPNQSTRIIEGECSGILNWNDIRMPHMYKLYKVL

LLNHWIADEIPMSKDASQFAQLDPEEQRTFKVNISLLAVLDSMQTMFVGDVKRYFTDS

SLEAISAIIGQQEVVHNQSYSYVLSSIVSDREQKEIFEYWKHDPVLLDRNRFIADIYQ

TFRDNPSPQTFFQAMVADLVLEGIFFYSTFAFFYNLARDQKMMATSQMISYIQRDENQ

HCYFFAEVYKQLLVDFPELNTPENMDYVYKTINRAVELETNWAHYTLSNVRGIDLNEL

EDYIKYIANKRLRLMGMEKAYEGVDVNCMPWIKPFSDEALNATKTDFFEAKSRNYGKV

GDDNGFDDL"

CDS complement(268756..271074)

/gene="nrdE_1"

/locus_tag="PIKHDJKN_00274"

/EC_number="1.17.4.1"

/inference="ab initio prediction:Prodigal:002006"

/inference="similar to AA sequence:UniProtKB:P39452"

/codon_start=1

/transl_table=11

/product="Ribonucleoside-diphosphate reductase 2 subunit

alpha"

/db_xref="COG:COG0209"

/translation="MPQVVTKPNNRQLAFDDMRISVYADRTLAGLDMLDKERLVRGVN

SKLRRDEVTGDEISSAFMMSALELVTKEEPNWKFAAARSLLTSLYKKAATNRRYKSYP

DEPYGAFHPLLVDLIKKGIYREELLECYTKEQIDELAECIDYRNDLLFDYIGLLTLAE

RYLAHDFDGKVMELPQERYMVIAMFLMHQEPAENRMNLVKEAYWAMSNMYMTAATPTM

SNAGKKVAGQLSSCFIDTVDDSLEGIFDSNTDVARLSKMGGGIGVYLGKVRARGSDIR

GHQNTSSGVIPWIRQLNNTAVSVDQLGTRKGAIAVYLDVFHKDILAFLDLKLNNGDER

MRAHDVFHGICLPDLFMERVSSRGEWSLFCPHETKKVMGWKDENGRALGLEDFYDESF

GEGAFRDKYEEAVNHPLLSRITVQAIDIMKRVLKSQLETGTPYMFYRDTVNRSNPNRA

HGMVYSSNLCTEIMQNQSATVIEKEELVTKDGQTRIVISKVPGDFVVCNLNSIHLARA

VPHNVLERLVPIQVRMLDNVIDINNIEVLQAQYTNSQYRAVGLGTFGLHHLLALEGIH

WESDEAVTYNDNLYEKINYLLVKASMELSKEKGHYPKFKGSDWESGHYFDQREYTSGE

RAGEFVTTEQWKELQAEVQQNGVRNAWLFAIAPNGSTSIIAGSTASIDPLYELLSYEE

KTTYKIANPAPDLSEKTSPYYQTAFMVDQHASINMAAARQRHVDQGQSFNFYVRPDIK

ATEFLELHLHAWRAGMKSTYYVRSRALTIEES"

CDS 271841..272173

/locus_tag="PIKHDJKN_00275"

/inference="ab initio prediction:Prodigal:002006"

/codon_start=1

/transl_table=11

/product="hypothetical protein"

/translation="MAIAEVTVIPIGTGTTSLSSYVADMQKVLENQRGITYQLTSMST

IIEGPLNEVFTAIAALHEAPFLSGAQRVSTSVKIDDRRDKPDASSVQKLQSVQDKLAS

RSRQARPN"

CDS 272384..272752

/locus_tag="PIKHDJKN_00276"

/inference="ab initio prediction:Prodigal:002006"

/codon_start=1

/transl_table=11

/product="hypothetical protein"

/translation="MPYTTGLLFNSPTGSDRATQLIVTCLNDSLNIPANIELQVFRWD

TSQTARIPIGHDLFQLPPQASKTLLYTLNTADYFEVQSDYFSATSAVIQAYSLDSVGN

VMQRVLQSEMVWVDRFTNIP"

CDS 272778..274010

/locus_tag="PIKHDJKN_00277"

/inference="ab initio prediction:Prodigal:002006"

/codon_start=1

/transl_table=11

/product="hypothetical protein"

/translation="MPVVNITGALGGNQFEPSIAVNELLPNIMCVVAVDTSTGPTLIG

FYRSIDGGQTWSTTTLLQPAGYAGAEAPTIDYTFPSTFIVTVHIFNGDNDGTIISYTS

MDDGLTWAPPVFVNKGYGLIVHNDEPYIACDRTPGSPYRGNAYVGYTPLATAASSIFF

QRSLDQAATWQTPSRISSPRGFHDRATIAIGFTGEVYAGYITTGPDSPYALLRISYDG

GATFQPPVSNQSTLIASVVPAPSPLPVIDYAFRVQTNLNLAADISNSIYSGTVYAVWN

DARNGYTDVFMCQSPDGLLWSEPVSITGAPAGSQNFFPSITVSPFTGAIRVIYYSNQI
NGFLLDVYVAESFDGGATFTNRRLTTTSFNPNGNSPVPTVLIGDYITAYTQSPDNLAA
VWMATTQPTGKLDVYFGS"

CDS 274113..274217
/locus_tag="PIKHDJKN_00278"
/inference="ab initio prediction:Prodigal:002006"
/codon_start=1
/transl_table=11
/product="hypothetical protein"
/translation="MSGVVGGYGGAWTSTGAILVLFILLVIISKAFLI"

CDS 274260..274367
/locus_tag="PIKHDJKN_00279"
/inference="ab initio prediction:Prodigal:002006"
/codon_start=1
/transl_table=11
/product="hypothetical protein"
/translation="MSEVLGGAGYGGWTSTGAILVLFILLVIITKSFWV"

CDS 274534..274851
/locus_tag="PIKHDJKN_00280"
/inference="ab initio prediction:Prodigal:002006"
/codon_start=1
/transl_table=11
/product="hypothetical protein"
/translation="MSEVKPNSPNGQGHVYGHMGGYEHMQGYQYTGHEMHGYGYGCTM
PTGYGYGYAHHANHANHAPMLHGYHQGCGTSYGHGGGSWGSMGTILVLFILLVIISKT
MFI"

CDS 275457..276023
/gene="sigK_1"
/locus_tag="PIKHDJKN_00281"
/inference="ab initio prediction:Prodigal:002006"
/inference="similar to AA sequence:UniProtKB:P9WGH7"
/codon_start=1

/transl_table=11

/product="ECF RNA polymerase sigma factor SigK"

/db_xref="COG:COG1595"

/translation="MRQIAERDASALELLYDRYERAVYSFAYRIVGDPMTAEETVQEL

FLRVWNNAERYEASQGKLTTWMFAITRNIAVDMLRRKSKSAATTSMENETLAAYADEH

TNTEEEIERKWEGVRIKEALSQLNGDQQQVIESIYYAGLTQQEVSSRFGIPLGTVKSR

VRLAMRQLQKLLADAEMHPDAGREGIHP"

CDS 276020..277000

/locus_tag="PIKHDJKN_00282"

/inference="ab initio prediction:Prodigal:002006"

/codon_start=1

/transl_table=11

/product="hypothetical protein"

/translation="MIERHEEWSDLAPVYVLGGLEAAEVAAFEAHLANCESCRQEVRE

LQEVTGFLPLAAEPVAPPPGMRARVLGNVLGHAQESAGTKPAAAPAEPEAPVVLQEDL

APQHQAAQPGQGLPPETAVPAARAEEAAQAQPWQPQGRARARSSRSWRIASAALAAAA

LVLGIYAGQLQGQVDSLKQQAEGSSTVQEQLAQTQAQNAQLQEQLASSLQPAQGMQTG

EAVKLSPATQDIVAQGLATIVIDSKGTHLVVQAENLPTLEGNEAFQVWLIKGDTPQNA

GTFLSHNGTGAVYYTLDSANDYDTVAITLEPDAMGDEPRGTMILAAKIKG"

CDS 277244..278767

/locus_tag="PIKHDJKN_00283"

/inference="ab initio prediction:Prodigal:002006"

/codon_start=1

/transl_table=11

/product="hypothetical protein"

/translation="MEVYRSFIFRLMRNYLIGSMGAVFVVGTVVMVSTLQIPNIQFVR

LIIIVLISLLFMLVAELITLWAQLGPIRQFFAAEHHEKAELTKMYESIHRFPGQTIYR

ILGPHMLGFSLPAAGLTIWMISTGWLEFPYGYVAVAAACALLIAVMHALIEYFLTVRT

VRPLLLEVRRRGKEQHGMEPSLGGRVLVSIQRKFQLSTALIGLFPLFLFFLATSIRLQ

YMDSEFAKEYILWAVLIVVLGGGFALVGSWLLIRDVRGPVAELTEEMNRIQEGDLGRR

TPDLYADEFSALISGFNMMINRLEMRQERNRQLLQSYFSTLAAALDARDKYTAGHSMR

VAEYSIMIGKLSGMNEEQADLLYKSALLHDIGKIGIPDEVLLKDGKLTDEEFAIIRTH

PVLGESILLQIEPIDAMADFLPGVRSHHERYDGKGYPDGIAGQEIPLFGRIIAVADAF

DAMTSDRPYRNGMSHEKALSILEEGKGTQWDPYFASLFIDEWRRQQHLQKSSERGVS"

CDS complement(278760..279413)

/locus_tag="PIKHDJKN_00284"

/inference="ab initio prediction:Prodigal:002006"

/codon_start=1

/transl_table=11

/product="hypothetical protein"

/translation="MHVLKSKSYWLSLSLMLSLAVMGMFYDILNSPERGFSVLDSPLD

RIIPYVPSMSIPYLGWYPFVFGVLAYLCAKDRLTYYRVLLSMNICVWICYLIYFNFQT

MVPRPELTGTGLGASLLGWLYGQDRPYNCFPSIHALHSYLVMRAVLSVQSIRKQVKVF

VAAGAALIILSTLMIKQHVIYDALGAVVLGECVFTIITGLSLHRRRRKESKWTEGIS"

CDS 279646..281061

/gene="rcsC_2"

/locus_tag="PIKHDJKN_00285"

/EC_number="2.7.13.3"

/inference="ab initio prediction:Prodigal:002006"

/inference="protein motif:HAMAP:MF_00979"

/codon_start=1

/transl_table=11

/product="Sensor histidine kinase RcsC"

/translation="MQFSKMFVLNMGMLITIAYLASVFYKYIVIRASSRVKQICSLFV

LIFAGWFSTVFGFQLNDEVVFDLRFVPLIVAVLTYRQPYSVIIVGVGIGMTRLTFGIH

EATVAALINMSILGVVCAGLNIWMRRSDYRLIVKGIIVILIVNVVNSLNIAIFGVIPA

TYFFSHIMPYTLPVGIVLSLIFAFILRDFQNEQNRILLIQSTNRLLSVQKEELQKAQI

VLEDRAKQLMIASQYKSEFLANMSHELRTPLNSVINFAQMISENGDTMEQEDIVHFAT

MIEHSGQELLTLINDILDLSKVEAGRLDIVLEDISVAQLTEDAMTHFQLVAEKKGVEL

KLDKKPGLPETLWSDPQRVQQILRNLMSNAIKFTHQGKVTLTVSTKQMKKSGILSQWI

VFSVQDTGIGIAEDKHQSIFEAFQQADGSISRKFGGTGLGLSISRDLARLLDGSIELK

SAEGKGSTFYLYLPLNREKMS"

CDS 281393..282427

/locus_tag="PIKHDJKN_00286"

/inference="ab initio prediction:Prodigal:002006"

/codon_start=1

/transl_table=11

/product="hypothetical protein"

/translation="MQRIRSRKRMLVLFVGIHRILWSSTGLIPFLFPKEWWVGIYIGV

YTVAFIANTIGGMIWTSLIGDIVPAKVRGRYFGIRNTILNALGSVCLFAGGIVLDRFP

GEMGFLILFIPVWICAIANTIIYCFYPDVPFERSTEKVFWGMFKKPFQDPSFLRAALF

LAAWLLIQTLIVPLFSYVMLDLLNINYQTVSIITVVQTLVMMAGFYIWGNLNARFSNK

TLLFWTLPIIALSCLSWGLMSFMPVLIALFLSHIFLGIGVGGFNQLAFNFTIGDTPKS

ERPMFVAVYSALTGVTSFLGPLIGGWLYERMETWPDAMNWVSAYGFQVGVGAAMLVLT

FTLGRRVLLK"

CDS 282448..283128

/locus_tag="PIKHDJKN_00287"

/inference="ab initio prediction:Prodigal:002006"

/codon_start=1

/transl_table=11

/product="hypothetical protein"

/translation="MENHERQALVIGATGLVGELLVRSLLQNPAYGRIRILVRRPLEL

EHAKLEQHVVDWEQLEEQTDLFRDVDDLYCCLGTTIKKAGSQENFRQVDLHYPVKAAI

LAKEHGVSQMLVISSMGADAGSRVFYSRTKGEMEEALSAIGFRSLHIFRPSLILGDRK

EKRLGEQLAAHVMTFLDRWMKGRVDKYRAIQASTIARAMMNIALVQTKGNHVYSNNVI

HVLGLDES"

CDS 283276..283650

/gene="tdcF"

/locus_tag="PIKHDJKN_00288"

/EC_number="3.5.4.-"

/inference="ab initio prediction:Prodigal:002006"

/inference="similar to AA sequence:UniProtKB:P0AGL2"

/codon_start=1

/transl_table=11

/product="Putative reactive intermediate deaminase TdcF"

/db_xref="COG:COG0251"

/translation="MKKPISTDQAPGAIGPYSQAVEAGDFIYTSGQLGLNPQTGEFGA
DVQEQTRLSLSNVKAILEAAGTSMDKIVKTTVFLKDMNDFVPVNEVYSTFFEQPYPAR
SAVEVARLPKDALVEIEVIALK"

CDS complement(283751..284509)

/locus_tag="PIKHDJKN_00289"

/inference="ab initio prediction:Prodigal:002006"

/codon_start=1

/transl_table=11

/product="hypothetical protein"

/translation="MSRNNPYKWLNAIGFIAVIVVNYLSNALPIGGRTNKEVSDMYPV
LLTPAGYAFSIWGLIYLLLAGFVIYQFVPSSWKRDSITRLGYWFLASCAFNVAWIFAF
QNLKIGLALVVIVLLLLSLIMLYVKTRAITLPTTAEIWLVKLPFSIYLGWVSVATIIN
AAVLLYKIGWDGFGLSEPTWTIIMLIVGLVLAILVSFPYRDSVYPLVFTWAYIAIALK
QKDVTSVYYTAIIIAIVLAIYAVWLFFARNQDRD"

CDS 285205..286170

/locus_tag="PIKHDJKN_00290"

/inference="ab initio prediction:Prodigal:002006"

/codon_start=1

/transl_table=11

/product="hypothetical protein"

/translation="MFTRETMEKQQTWFYVVALFVGAAIGLGTPEWGSALHYTVSPVL
AILLYSMFAQIPFLQLKESLSNLRFIGALLVANFIVVPVVVWLLTLAFPQPPAVLIGV
YLVLLTPCIDYVIVFTQLGRGNEKLMLAATPILFVIQMVLLPVYLWLFIGKEAAQVMQ
VGPFVEAFLFLIVIPLLLAILTQVVTKGKAGGERVMNATAWLPVPMMALALIVVVASQ
IGKVYNDFAVIVDVLPIYIIFLIVMPFISRILVALFRLDTGAGRAVIFSAGTRNSLVV
LPLALALPQEWATIAAAVIVTQTIVELAGELIYIRIVPSVILRDR"

CDS complement(286336..287967)

/gene="rhaR_2"

/locus_tag="PIKHDJKN_00291"

/inference="ab initio prediction:Prodigal:002006"

/inference="protein motif:HAMAP:MF_01533"

/codon_start=1

/transl_table=11

/product="HTH-type transcriptional activator RhaR"

/translation="MKALIVDDEKHVRDAIKLLGQWEAAGIDTLYEAADGHEAVAAIT

AHQPQIVLSDMRMPGKDGMALLEWVSVHAPHSKVLVISGYDDFELVRHAIRYGGTDYL

LKPVEADELNSSLFRAVDAWKEDDAVRVQSTRQSMVVNQMRPHYHDRLLTELVVGRGS

NNTQFQRLRDELNLPKQIDSCSVAVTSLSHLDAQCLAKYRSQPDLLVFSVLNICAEML

SPPAEGVVFRQLDQPDEVVLLYWGQCSTLQTILEKVNDGLEQTIQRRLHFGISSCEFY

PTGTSGAYQEASLRLWRRNALQTKQCIHDAMESSNGNKGLRLSTFEESLRLASMSGRA

SSVSNAVAEWIDPITRLDVVTAEQIQQWMDEMEWMIGRWLDDAAGAPPKEEDEAIEEQ

SIPFAELPLDCDGLLSLPLLRSLLEQRLLAAGKALTAHHHATPDPMSEIARYMDAHYQ

EDLSLQQIAARFYLSREYISRKFKQQFGLNWSEYLGKLRINNAKLLLQNPSLRIAKIS

EMVGYQDEKYFSKVFKKMEGITPGEYRKTLSDGNA"

CDS complement(287973..289799)

/locus_tag="PIKHDJKN_00292"

/inference="ab initio prediction:Prodigal:002006"

/codon_start=1

/transl_table=11

/product="hypothetical protein"

/translation="MSKYYSIRTKLIAFMLIATTIPLLASISMTFIQTKTALREQAVA

ENKRLIFQASTNLNNYVDNVARASLAVYNDPNFLRNLAKIPGDYRAVAEVYTTLQTIR

SAVPDVFQLYLHSFAANQSTLIANPFPKREERKQAYSDSLHGKSNAKSPDIWVESAHL

SHSYGFKAASADDPPRTVITLHRVIKDVPSTERLGVLAIDLNMDTIAEICSRLYDPAK

EQIYVIDNHNQIIFEGKSEVSGTDALRQEAATELNSARSGAGTVQTASGHFEQDHSMY

VYQHLGSQFADWTIVKQIPNETLYASATTLTWNNAMIAIAALLLVIVATLFISIRITG

PLKQLMRYMNQIQAGRLHVDIRLTSRDEIGVLARHFRDMMDTVNNLILREYRLEIANK

TNQLKALQAQIHPHFLYNTLQSIGTLALQQQGQRVYVLLSSLSKMLRYSMRDQTRVTL

HEEAEHARLYLELQKERFGDRLEVDLDFAEDTLRAEMPRMTLQPLIENYFKHGADVQP

GKGRIRISSHRTPDDWIQIRLENNGPCIPEDKLSEIQRWLSPASISSDSSQEPDETES

IGLRNVMRRLQLNSPPGYNATLTIGNLEPNGVQIIVKIYAGE"

CDS 289943..290824

/gene="melD_1"

/locus_tag="PIKHDJKN_00293"

/inference="ab initio prediction:Prodigal:002006"

/inference="similar to AA sequence:UniProtKB:O34706"

/codon_start=1

/transl_table=11

/product="Melibiose/raffinose/stachyose import permease

protein MelD"

/db_xref="COG:COG1175"

/translation="MKHRKSSQLLQQLVFVGPSIVFFILIIVVPFLLGMVYSFTDWNG

VSANIHWVGFDNFKQIFANDPKFLSAFWFTVRFTVVGVILTNVIGFFLAYFLTKPLKT

RNFLRTIFFMPNVIGGLLLGFIWQFIFVKGFSAVGDVTGWSFFNLPWLGDEPTAFWGI

VIVFVWQTAGYLMVIYISSLTNVSPDLLEAAEIDGASRWQVLRSIIFPLIMPGVTICL

FLAISWSFKMFDLNLSLTKGGPFGSTESVALNIYNEAFVNNRYGIGTAKALVFFVIVA

IITMIQVRVTKSKEVEA"

CDS 290825..291661

/gene="melC_1"

/locus_tag="PIKHDJKN_00294"

/inference="ab initio prediction:Prodigal:002006"

/inference="similar to AA sequence:UniProtKB:O34518"

/codon_start=1

/transl_table=11

/product="Melibiose/raffinose/stachyose import permease

protein MelC"

/db_xref="COG:COG0395"

/translation="METSKNYRFSTIVTEIVMVLIGLLFLVPFYFLFVNSVKTFGDLL

TNSAAWPEVFQWGNYANAWEKINFPSALMNSLIVTVVSNLLLVLISSMAAYRMVRSDT

RFNRILFGMFIAAMVIPFQSIMIPLVTVTSNLGLIDSLFGLIICYLGFGAPMSIFLFH

GFVKSVPLEIEEAARVDGSSAYGVFFRIVFPLMKPMYVTVIILNTLWIWNDYLLPSLI

LQSSNLRTIPIATFALFGQYTKQWDLALPALVLGIMPIIIFFLLMQKYIIQGITAGSV

KG"

CDS 291783..293081

/gene="msmE_1"

/locus_tag="PIKHDJKN_00295"

/inference="ab initio prediction:Prodigal:002006"

/inference="similar to AA sequence:UniProtKB:Q00749"

/codon_start=1

/transl_table=11

/product="Multiple sugar-binding protein"

/db_xref="COG:COG1653"

/translation="MKRMTKLTLLMLIAFSVMLAGCGNGDKSGSPVKTDAQEGGGAAA

GDKTIKIFQFKVEIAEALNRLKAEYESSHPGIKLDIQTVGGGSDYGAALKAKFAAGEQ

PDIFNVGGYRELDTWLEYLEDLSGEAWVKDVLDVAKEPMTKDGKLYGQPLALEGYGFI

YNKDLFQKAGITEIPTTLEQLDQAAQKLQAAGITPFSNGYQEWWVLGNHNVNVAFANQ

ADPVKFIQGLNEGTEKIPGNQVFTDWMNLLDLTLKYSNKNPLTTDYNTQVTLFATGEA

AMMQQGNWTQVQIDGIDPNLNLGILPMPITNEPNDKLFVGVPNYWVVNKNSQVKPEAK

EFLEWLVTSDIGKQYMTKEFKFIPAFSSIQASEEDLGDLATDIMKYSQENKTLSWNFN

RFPEGVPQEFGSTIQAYVAGKSDKKALLDALQQNWDSLKK"

CDS 293491..294111

/locus_tag="PIKHDJKN_00296"

/inference="ab initio prediction:Prodigal:002006"

/codon_start=1

/transl_table=11

/product="hypothetical protein"

/translation="MTNQKSISGSVKSKSSNPVNRTKAVEVAAQLFLRQGYSYVSMDE

VVRASGVSKSNIYYHFKNKEELLQAVVQYWIAQYESELYLLLSQREREVTERIYSFMA

LLSAGMEGRDYKGSCPFITLYMQTPASAPEVKESIARFFRELQPMMEKLLQQGVDRGE

FRKGIEPGPAAALFIAAFEGSLVLAETARDIGIIEQSARTFCQMLR"

CDS 294190..296196

/gene="menH_1"

/locus_tag="PIKHDJKN_00297"

/EC_number="4.2.99.20"

/inference="ab initio prediction:Prodigal:002006"

/inference="protein motif:HAMAP:MF_01660"

/codon_start=1

/transl_table=11

/product="2-succinyl-6-hydroxy-2,

4-cyclohexadiene-1-carboxylate synthase"

/translation="MSVHTSSLLNAELKIDPNPAKNNMATESTTILMTGSTGFIGKEA

VKQLGDSEAQLLLLVRSKSKVRMVLNAYGVTDFSRITCIQGDLSAPGLGLTKADRERA

LQATIIIHAGGTMDVTLQPKVAEQIFMNGARGIAELAEEIQRTQGLRHFIHVVGFMSP

YGREDSPSVSSTNEDVGDKSSRQKSLISKVFHSESAYEHMKFEADRYIRQHAEQHSYV

LSVVNPSTIVGPYPTGATEQTGGIGMMIQAMRRRRMPVIPGGRQHWLPLVSNDVVAKT

LVFLTQDSNPSGGTYPLLSRKADGPDMKELILLLAAELDVSAPRWSVPLPMIHAAMKA

GGTKISGIPPESIAFITKQEFEVQQTERLFQRMGQKLPEVNELLPYVTADLDYRFSNP

SQEDLPQGWTRTRKGTLAVLVHEGQGEPWVIAHGLMSSADDMIPLGDALWSMTGNPIW

LVDLAGFGRSPVHRNKARGEAFKGQVEAVRSVLDEVQGPVKLVGHSVGAAIAAVVMQE

SGRENIQLAMLQPVWRASTDGMQRLISRFPRRMLQGLLSWMTPQQVAGQLKRADASDI

NPTLLEGYTRRASSGLKSPRIAGANADLLHWIQSHSNDKVSQTSADAQRTLMVWGTND

KGYSRPENIHSSVQHVELPYGHHFPLFQAEETASLLVKWQMKRR"

CDS 296437..297387

/locus_tag="PIKHDJKN_00298"

/inference="ab initio prediction:Prodigal:002006"

/codon_start=1

/transl_table=11

/product="hypothetical protein"

/translation="MPLQKTLLALDALDSAYVNGESVKQLFVQYPQVSVEVLTVQGEK

GSTDFVKIIIPGTDGKLSGGQAPTMGIVGRLGGIGARPTRIGLVSDADGAVAAVAAGL

KLADMQTKGDSLKGDVIVTTHICPDAPTLPHEPVDFMDSPVDILQMNEHEVLPEMEAI

LSIDTTKGNRVINHKGIAISPTVKEGYILRVSDDLLRIMEMTTGQLPVTFPITTQDIT

PYGNDLYHINSILQPAVATHAPVVGVAVTAQSAVPGCGTGASHEVDIASAVRLVVETA

KEFTNGTCSFYNVEEYNLITRLYGSMKVLQTPGMQATPSA"

CDS 297485..298156

/locus_tag="PIKHDJKN_00299"

/inference="ab initio prediction:Prodigal:002006"

/codon_start=1

/transl_table=11

/product="hypothetical protein"

/translation="MIKQLGMITIGQAPRTDVAPVIERYLEGRAELIQVGVLDGLSAT

QIAALAPEPGEYVLTSRLQDGSAAIVSREKIRPMLNQKIRELEAMGVPNILLLCTGSF

PGLRSAKVHLIEPDRIIPPAVQAMADGRRLGLIGPLPEQAGQLDLKFSSLVQPAFAAA

SPYTSGEAEFRQATESLKGRVELILLDCMGYDERHRQWVANASAGIPVILSNALMGKL

VAEMV"

CDS 298176..299126

/locus_tag="PIKHDJKN_00300"

/inference="ab initio prediction:Prodigal:002006"

/codon_start=1

/transl_table=11

/product="hypothetical protein"

/translation="MNQQRIDVSKNVLVECLGLRSGETLAVIADDAKRELAESIYEAG

KALGADAVLMVMKERSKSGEEPPAPISEAMKRADVAVCVTRYSLTHTQARKQAAAAGT

RVATMPGMTDDMFMNGAITAEYPKVKALTDKVTAMLSAGRSVRIEKDGHQLSFSIQGR

NGVPSTGMYLNPGESGNLPSGEAYIAPVEGIGEGQILVDGSIAGIGAISEPVLLTVRE

GRLVTATGAEGDKLLSMLGEGDGRILGEFGIGTNDKARITGVVLEDEKVYGTIHVAFG

SNNTFGGTVAAGVHIDAVVQKPDVYIDDVLIMRQGELLDQ"

CDS 299355..299987

/locus_tag="PIKHDJKN_00301"

/inference="ab initio prediction:Prodigal:002006"

/codon_start=1

/transl_table=11

/product="hypothetical protein"

/translation="MLGLIRVITLHHEEDIHRHGALIEGRYGIQVRSRCIPDQPLGVY

DQATELESIPKIVDLARKLEQEGCTSIGISCAADPALAEMRAAVHIPVYGAGSCAAHL

ALTSAARVGVLTILEEVPLLIRGILGDAYIGMERPEGVVTTLDLNTAQGRIAAMKAAR

RLKAEGAESIVLACTGFATMGFALEVEKELHIRALDPIYSLGAAVSALNV"

CDS 300385..301917

/gene="pucR_1"

/locus_tag="PIKHDJKN_00302"

/inference="ab initio prediction:Prodigal:002006"

/inference="similar to AA sequence:UniProtKB:O32138"

/codon_start=1

/transl_table=11

/product="Purine catabolism regulatory protein"

/db_xref="COG:COG2508"

/translation="MKTTGITLKELLNIPILSKAKVISGHQGLDRVVRFVDIMEVPDL

QGWLREGMLMLTTAYSIRDNPGLLGELIYTLNEAGAAALAIKPARFLKEIPKEAVEAS

NDCGLPIIEIPPEIPYMDITQPVMELVLDRQAALLRRSEEVYRTLMTMVLENSGIQAV

GDNVAELLQAPVGVIDNAGQMIVSSPPDYDWKEAVDPLVWEINLDRRKVARLLVDKDS

LDEMEQVGIEQARLVLSLELMRNKVAEDTELRLRGNFIDELLTPPLLLPHEVESRGRK

LGMNPEHLWEVAVMEGETAPDEDLLTELLGQEARKRRVAPHIEFRTNRAVLFLPTPES

RDAVWMDDVQSWSDTIGLWLEDPANKLGQFRTGVGTQVPLWNMHQSYGEARNALLVSS

RLSGGRTTRFEDVEVYHLLSETANDPGFSALFERKLGKLRAYDDEHSGDLLRTFFYYL

ESRGSLIETANRLYIHRNSVKYRLERIRDITGFDLNHPREQFVCHLCLVYYYMKQDKQ

EV"

CDS 302263..303240

/gene="dppB_1"

/locus_tag="PIKHDJKN_00303"

/inference="ab initio prediction:Prodigal:002006"

/inference="similar to AA sequence:UniProtKB:P0AEF8"

/codon_start=1

/transl_table=11

/product="Dipeptide transport system permease protein

DppB"

/db_xref="COG:COG0601"

/translation="MIPALIGIVVITFILSRVLPGDPAIVMAGEQATDDVIAKIRMDM

GLDKPLFVQFFSYVGQLLQGNLGFAYHTGHTVLSDFATRFPATIELTLASVIIAICVA

IPVGIIAATRKESFIDHISRVFSLIGACVPIFWLGLLFIYIFYSILGWAPAPMGRISG

DLNPPTHITGLYVVDSLMTGDMVALKSSLAHLLLPAICLSTGTMAIVARMTRSSMLEV

IGQDYVRTARAKGLSETAVVGKHSLINALIPTLTVLGLQFGGLLGGAVITETIFSWPG

VGGYVTDSILAADYAPIQAFTLVSAILFSFINLAVDLVYGLIDPRIRYE"

CDS 303268..304167

/gene="ddpC_1"

/locus_tag="PIKHDJKN_00304"

/inference="ab initio prediction:Prodigal:002006"

/inference="similar to AA sequence:UniProtKB:P77463"

/codon_start=1

/transl_table=11

/product="putative D,D-dipeptide transport system permease

protein DdpC"

/db_xref="COG:COG1173"

/translation="MSTAAPTSMGTKPAPVVVPKPKTFLRLLLRNRLAAIGLAFIVMW

TVIAAIAPWIAPYDPYVTNTAVKLESPSGGHWFGTDNYGRDILSRVLYGARISIWTGL

IAVSISFVIGVTLGGIAAYYGGRTGNIIMRVMDVLLAFPSLVLSMAIAASIGNGLTSA

MIAVGIVGIPEFARLMYGQTVSLREKEYVEASRAIGVRDRVILFRHILPNALAPLLVQ

ATLGMGFAILTASSLSFLGLGVKPPTAEWGAMISEGREYIISGQWWLVTFPGLAIATS

ILGFNLLGDGFRDVLDPRLRSGK"

CDS 304431..306014

/gene="ddpA"

/locus_tag="PIKHDJKN_00305"

/inference="ab initio prediction:Prodigal:002006"

/inference="similar to AA sequence:UniProtKB:P76128"

/codon_start=1

/transl_table=11

/product="putative D,D-dipeptide-binding periplasmic

protein DdpA"

/db_xref="COG:COG0747"

/translation="MKKLKWFSAMIALTVVLGGCASNANQQPAASGSGEETKPAPTLT

VAYSEGGTTMDPAEANDLTSDTLVLATYDQLVTYGVKTVDGADVANTEDIQPMLAESW

EVSDDNTTYTFKIKSGLKFQSGNPVNADAVVYSFDRVAKSSSGSFLYGMADIKSVTAK

DDSTVEIVLNKANHMFTQIIAMYTFSIVDQKLVEEKGDDYLKTNAAGSGPFTLEKWDP

ASEAVFQANKDYWQGAPKLSKVTLKFTKEASNRVLLLNKSDVDMAIEIPPKDVAALQE

NSNLTIKSNASNRILFFAMNNNVKPFDNEKVRQAINYAIPYDQLINDVMYGQAKEMKS

AVASNTPGFTDAGYVYEYNLDKAKELLKEAGYAEGFSFDFTLGSGFDDWEYDAVLIQA

ELAKIGVKMNINKVARAQFLEQQKDGNLVSYISKWTSFVNDPGYHLGFLLYGEGSSNY

IHYNNAEVNKLWEEAGAEPDQAKRNELYMKAQEIITTEAPWAYLYEYNRVVGMNNKVS

GYVYYPDEVLRFYPLSKEQ"

CDS 306110..307447

/gene="dapE_1"

/locus_tag="PIKHDJKN_00306"

/EC_number="3.5.1.18"

/inference="ab initio prediction:Prodigal:002006"

/inference="similar to AA sequence:UniProtKB:Q8ZN75"

/codon_start=1

/transl_table=11

/product="Succinyl-diaminopimelate desuccinylase"

/db_xref="COG:COG0624"

/translation="MDWKKKVLDAIEEGQDELLELCSQLIRFPTENPPGDSREISAFI

IEYLHQAGIDTKVHAATETMFNLISTLNGEGADKSDKHLIFCGHTDVVPAGDLSRWDF

DPFCGEIKDGYMLGRGASDMKAGLAGLIFATVILAKLGVPLRGDLSLMIVPDEETGGD

LGVPWVLERGLATGTAAVIAEPSSPQHPTIGQKGSCWFEFTVEGTPGHGSLQPIVGDN

AIVKAAKGIEALQRLWDIKPDIPEEVKDIIRISQEYARDREKAGLAYQVFDHVTVNIG

SIQGGTKVNVVADRCTVQVDSRVPFGVDYRDVLDRARSLLLEAGIESELKPFGFQGNA

NWTPAHEPIVSQLVESISEVSGQEAYGVLQWASSDARHFRNHQIPVLQYGPAELSTIH

NFNEKAPVWQIIQCAKVYALSALKYLGVEGMEDEMSLKNPKDAASVEATTSKR"

CDS 307508..308485

/gene="oppD_1"

/locus_tag="PIKHDJKN_00307"

/inference="ab initio prediction:Prodigal:002006"

/inference="similar to AA sequence:UniProtKB:P24136"

/codon_start=1

/transl_table=11

/product="Oligopeptide transport ATP-binding protein OppD"

/db_xref="COG:COG0444"

/translation="MTELLEVSDLRTEFITDAGVIRAVDGISLSIRQGETLGIVGESG

CGKSITSLSIMQLLPKKIGRVASGEVRFQGKDMLKLSGREIRRIRGNRMAMIFQEPMT

SLNPVFKIGKQISEAARYHLKLGKKEAWARSVDMLRKVGIPRPDKIAEQYPHQLSGGM

RQRVMIAMAMVCSPQLLIADEPTTALDVTIQAQILDLMRDLQRTENMAIMMITHDLGV

VAEMCDRVMVMYAGQVVEETDVKTLFADPKHPYTRGLLASLPQLAGDQDRLQSIPGQV

PNPAHMPSGCRFAPRCPVKEARCETIQPELLETAPSHSCRCLLQQEGII"

CDS 308482..309444

/gene="oppF_1"

/locus_tag="PIKHDJKN_00308"

/inference="ab initio prediction:Prodigal:002006"

/inference="similar to AA sequence:UniProtKB:P24137"

/codon_start=1

/transl_table=11

/product="Oligopeptide transport ATP-binding protein OppF"

/db_xref="COG:COG4608"

/translation="MSAILEVRNLKKHYPIRKGLFSKQVGAVKAVDGVTLSVERGETL

AVVGESGCGKSTTGRAILRLIEPTDGEVFFEGKDVRKLPPEELRRFRTDMQMVFQDPY

ASLDPRWTVQRTLEEPLLTHHNLKKNELKSRIEELMEVVGLSPYQAHRFPHEFSGGQR

QRIGIARALALNPKFIVCDEPVSALDVSIQAQVLNLMQDLQEQFGLTYMFISHDLSVV

KFISDRVAVMYLGKVVELAPTSELFTEALHPYTKALMSAVPVPDPNVQKERIVLGGDV

PNPENPPSGCTFHTRCPYVKDECRSVIPELREISPGRQVACHLY"

CDS 309713..310153

/locus_tag="PIKHDJKN_00309"

/inference="ab initio prediction:Prodigal:002006"

/codon_start=1

/transl_table=11

/product="hypothetical protein"

/translation="MDEEVQIQYINFSVGNQICALRIDEVHEIIKMLPVTTVPFGSPE

IKGFTPLYGKVVSVVSVRVLLGMPDEETTPSTRIIVVPYHDDYVPLVVDSVDSVVMYD

RFEEPTEEYRRYTTGIFKEIAHNDDHEAGILNLDVLLGNLTKSQ"

CDS 310439..313642

/gene="rapA_1"

/locus_tag="PIKHDJKN_00310"

/EC_number="3.6.4.-"

/inference="ab initio prediction:Prodigal:002006"

/inference="protein motif:HAMAP:MF_01821"

/codon_start=1

/transl_table=11

/product="RNA polymerase-associated protein RapA"

/translation="MMQSLYGVWLGDVFFCFSGETSEPRVDAWSHVVRKLNFGDGGRL

FQPAALRLAELRWPNPMRNAAEAKGTKRRQLLGRTLEGLAISPKDTFKLLLHWDDSIL

NAAGIQAGEEIRYWVKAAQFTQELLLRGEFAPSAEFAAKTGARRRTGQETLTGVWRPR

LLQEADVERFRELAESMPPIGLSAPGAYASSEPESREEAGAAVLFSFMSGMVHAVVTG

EMESMDSELSRYRTPFRRGSSPVAELWWNSLISMFRPVTVQGPTEEMAELIGTLQEAG

GTARPVVGSEDTAPTEGELKLVLRLEPPLGDHESLWGITFWADSVQESGLRLPARTIW

AHPERDLDRGKVRYISAAEQLLMALGHASELAPELETALLHARPEGMKLEQQAFFEFL

TYAVPRLQKAGVTVLMPSRWSRAGKRRAGLRLQMLNRGIERPLGAPSALGMEQLVAFK

AEPMLDGKPVTAEELAALAEATVPYVMFRGEWIEVDTKEIRQVLRYMKKEEEQYMPLS

EWLHLAAEEGEDTAWKGLSVFGAESEGLLSFLLDGQVLRSIEPRQVPAELHGELRPYQ

ERGYQWLSAMRELGFGVCLADDMGLGKTIQVITCLLDLKHEEQQAAAEEAREHEAFMG

MEKESANVEQPHVHDAHLPSLIVCPTSLLGNWQRELKRFAPNLSLYIHHGGQRLHGNA

FQAEAQSHDIVLTTYHLAGRDGPDLASLHWSTVVLDEAQYIKNYRTKQAQSVMRLSTP

HRIAMTGTPVENRLSELWSIFQFLNPGYLGTASSFRQRYTGLGPSEENAASLRELHRL

VSPFMLRRLKSDPDIRKDLPEKLELKSYCSLTPEQTVLYQRVVDDLMGGLDGRNGIAR

KGIVLSSLTKLKQICDHPVLADSSRKEHGKAEASGKMERLLELVDAIRDNGESALIFT

QYVAMGELLVSRLTQRYEEEPYFLHGGVSKSQRDDMVETFQKGEGPSLFVLSLRAGGV

GLNLTRASHVVHYDRWWNPAVENQATDRVFRIGQNRNVQVHKLICQGTLEERIDELIE

SKKALSEQVVGSGENWLTEMSDDELRGLISLQGEAWL"

CDS 313695..314516

/locus_tag="PIKHDJKN_00311"

/inference="ab initio prediction:Prodigal:002006"

/codon_start=1

/transl_table=11

/product="hypothetical protein"

/translation="MSEKWNVELHMSPGGWTAEVSAAVDASKEALAAAGKTESDEGLF

TVTGTLHQLSEAEREAVLAQLRKRPLALYALLRGGPAPGELDGLLPRHAPSEAVPGTA

VAGGSLNAGGTAACTCGRSGCAHAAAAERAVAARFAAEPLLQLAHAGLPREALLAGVL

GSWGEELAHDPSGPGRAAETAGRPQARGGEGGAAVGEWIAEAAADGAMHQPGPGFGAV

KVRLAQPGKPPATPELAALLPGVPATAGLALIRERVAARMWEAVQKKKKDSPATK"

CDS 314836..315807

/locus_tag="PIKHDJKN_00312"

/inference="ab initio prediction:Prodigal:002006"

/codon_start=1

/transl_table=11

/product="hypothetical protein"

/translation="MKLRTGRQRQVAVMLSSLMICGALLSACQDGSGSEENQNLNAAG

SAQQESDGTTVHLTEDTGKDGNDTGGDDSSSSDNNNEGTDSGSGDASTGGEGSSDAGN

GATAEDPLMEKRSISALQTTIDAQAVVTNSEAMTVIVNKQRSLPEGYEPDDLVEPNVP

FSFDGPHEKRHMRKEAAEALEKLFAGAKADDIELRAVSGYRSYERQVSIYNNNVKTKG

KEYTDRVSSVPGHSEHQTGLAIDVSSPSVGNAIEEVFGTSKEGQWLAEHAAEYGFIIR

YPKGEEGITGYVYEPWHIRYVGTDLAPDVVKSGLTLEEYFDEANIKL"

CDS 316024..316440

/gene="rutC"

/locus_tag="PIKHDJKN_00313"

/EC_number="1.-.-.-"

/inference="ab initio prediction:Prodigal:002006"

/inference="similar to AA sequence:UniProtKB:P0AFQ5"

/codon_start=1

/transl_table=11

/product="Putative aminoacrylate peracid reductase RutC"

/db_xref="COG:COG0251"

/translation="MNRQQVFTGSPWEPLVGYCRAIRVGNRIEVAGTTAMQDGVVVGA

GDPYAQTRFILQTIENALKELGADMSHVVRTRMFVTDISRWEEVGKAHGEFFGQIQPV

ATMVEVSALIDPLLMVEIEAEAIVEVEAEVDAEITE"

CDS complement(316604..317689)

/locus_tag="PIKHDJKN_00314"

/inference="ab initio prediction:Prodigal:002006"

/inference="similar to AA sequence:UniProtKB:P43131"

/codon_start=1

/transl_table=11

/product="Protease inhibitor"

/translation="MKSTMNNNMKKVSALMVLSLALGGGAAYAANLDSTQPINSTSVS

ADSKGTEAVKVSVNGAPMADGYWNKDGQVPMIALRDLTEALGIELKWNKENKSAELTK

GTLWTIVTTGKDQYSVNKMLLKLGTAPEITNGTLFVPASFADKVLHAQVNTTGNQVTI

SSEEEVKTVTERGVITGINSEGKYQSIHIGGAGTDGIVLNVGEDTTFKSADGKDIKLT

DLTLGMNVEAEHSEIATLSLPPQTPTYKVTVLDTATSESEAQPKEVLGTAGTIENVKT

TEDNGTQIEISGSRLTETAPDHVILNISKDTELVNHQGETIKPEELTKGAKVIGFYSP

VLTRSLPPIGTAWKVVIETPADQPEAK"

CDS 317961..318437

/locus_tag="PIKHDJKN_00315"

/inference="ab initio prediction:Prodigal:002006"

/codon_start=1

/transl_table=11

/product="hypothetical protein"

/translation="MSDMLVALYHLPEQESGLKKLEESSIVIRRAIAPEKQVVLDWVR

SHFSQPWVDECDVAFARQPVTCYIALEHGKMIGFACYEATCRNFFGPTGVGQEARGKG

VGTALLLACMHAMKTNGYAYAIIGSAGPVDFYAQTLGAVKIENSTPGIYKGMLRVD"

CDS 318476..319546

/gene="iolG_2"

/locus_tag="PIKHDJKN_00316"

/EC_number="1.1.1.369"

/inference="ab initio prediction:Prodigal:002006"

/inference="protein motif:HAMAP:MF_01671"

/codon_start=1

/transl_table=11

/product="Inositol 2-dehydrogenase/D-chiro-inositol

3-dehydrogenase"

/translation="MSTSKMLHIGMIGTGSISDLHMRCYAKNEDAVIYAICDLNEQRA

AAAAEKYDAQSVYTDYREMLKDPHVDAVSICTWNNTHAEFAIAALEAGKHVLLEKPVA

TNVEDALRIEEAVKKSGCTFVVGFVRRYDNNMQMMRSFIDAGEFGELYYAKASILRRL

GNPGGWFADKNRSGGGPLIDLGVHIIDQCWYLMGKPKPVSVSGNTYRKLGNRAHIEHL

SFYKAADYSSAVNNVEDMANALIRFENGASLAVDVSFTLHARGEESSVRLYGERGGIE
LEPETLIVTEKHNTILNIEPQTDYKGLDIHGAFQNQIDHFVDCCLNGTEPISPIADGV
ASTRMLCGIYESAEKGQEIRLD"

CDS complement(319661..320701)
/locus_tag="PIKHDJKN_00317"
/inference="ab initio prediction:Prodigal:002006"
/inference="similar to AA sequence:UniProtKB:P99173"
/codon_start=1
/transl_table=11
/product="Zinc-type alcohol dehydrogenase-like protein"
/translation="MGNPIMMKAFGYYEPGLETDIPPFQEVEVEKPKPTGKDLLVEVK
AVSVNPTDWRSHLSKHPDNLSLTVPGRDVAGVVVDIGSDCNLFNVGDEVYYAGSNTRP
GGHSEFHLVDERIVGRKPKNLDFAQAAALPLTSLTAGEAFFERLGISRNASDNEGNSM
LIIGAAGGVGSIATQLAKLAGLRVIATASRPESVSWAKSNGADHVINHHQPFGPQLKD
LGLTGVQYIFVLNHIDEHMDEMAEVILPQGKICSILPFEKPAELQKLFSKSVTLVWEM
MFTRPMFQTDDMIEQHLLLNEIADLVDAGKIHTTMAERIEPINAANLLEAYRKSKSGT
TIGKIVLEGFEK"

CDS complement(320904..321272)
/locus_tag="PIKHDJKN_00318"
/inference="ab initio prediction:Prodigal:002006"
/codon_start=1
/transl_table=11
/product="hypothetical protein"
/translation="MQDGKKHYYVSVTHNHIEQTKNESTPFEIMVDDEQLNRLKDLMK
VLEDEDAYTLQRAPVPYKSADHDEATEQFSDGMTLLYTFLYDHGTSDTRQAIESMNVL
SRLQDTDYDDPGYENSPLNK"

CDS complement(321358..324042)
/gene="rep_1"
/locus_tag="PIKHDJKN_00319"
/EC_number="3.6.4.12"
/inference="ab initio prediction:Prodigal:002006"
/inference="protein motif:HAMAP:MF_01920"

/codon_start=1

/transl_table=11

/product="ATP-dependent DNA helicase Rep"

/translation="MLSPNSTFYPRPLGVTPAASLPQSPSAPLETSRQLVSSDERDAP

YFRSLEEVGIRLNGPQISAVRHGKGPLLTLAGAGCGKTTVLAARAGYLMEISGAHAGS

ILLVTFTNKAATEMKDRIASLPGIRPAAARAVQARTFHSFALTLLRHYGVQEEIFGES

RAQHTVLKMLLRQNGMSEAFQPESLLAMLSAWKMQGSETSDLPEKSQEERDAKRVLLG

YEAWKQDRNKMDFDDILLRAAALLRDPAVLGPLQQRFQYIMVDEFQDTNSLQYEIVQK

LADAHRNLMVVGDDDQTIYTFNGARQESILEFDKVYPGARIVTLDINYRSDARILGLG

SELVARNKRRRDKRLRAAGHRGDAPRFATPSNTEEEAAWVVNQLCQQVEEGLHTYRDI

AILHRTASSSRAIFEQLVLKDVPFVQHGASPVFYDQSLIRPLMDHMRLSLNPRAVDAL

PSALGPLYVSRDAGLEWIQRSEEQQAKKYPLIHLAKWDKLKPFQQEQVKERIKLIKSL

TKLKPVIAIQEMRRQFYDKYMESGDPSIFTHYKETMLETLDEFEAAAKKFETVEEFIQ

FADELTRRHREMESLRRSQDSDAVQLMTIHRAKGLEFPCVYWIGASEGIVPHSTALRQ

DIPEDQKAALTVQQTDAELDMALEEERRLAYVAITRAKKYLYVTSPASHHGKPADVSR

FLLEAFGMEVPDKRKKREDGPTSNHASYGNSSSNWKGNGNTNGKANGNESRTRNGNAA

YAHSGSRPQGRDAVSPVQRGSDLRSTDQRSAIRQSDRRDFEVLDERDEDRLSERRGSG

VIRNHTAFSTTGVNQAASSSSNSSGSHSSGNTERTETVAIWKCSSSTCKAWLRQKPDG

ASAKSSKKATSGPPPCPLCSGTMEASTRKVPVTGRFGR"

CDS 324387..325178

/locus_tag="PIKHDJKN_00320"

/inference="ab initio prediction:Prodigal:002006"

/codon_start=1

/transl_table=11

/product="hypothetical protein"

/translation="MKLLQRIKNGANKATERAQHAVEIGKINNQIVGLQQEQEVHFTD

MGRIFYEGYRAQDMTHAEKEMVDLSGLCDELQDEIDGLRNKIAQLKNERLCECGHVAS

LDANFCPKCGRKLGEFKPAAASVGATNPARQEAAVAQSPEGPIYDAPAEEELEEAEPY

HTVIPSIADLETESEYNSTEFTQEEKEAFDAEWERRRDEEMQRERERQQELDERIRYW

KENNPIVNTVDVQTEVPREMVNCQICAAELPKGSKWCPRCGAEQI"

CDS 325220..326053

/locus_tag="PIKHDJKN_00321"

/inference="ab initio prediction:Prodigal:002006"

/codon_start=1

/transl_table=11

/product="hypothetical protein"

/translation="MLTAWGDGNMDQLLHHLRHLGFTEMESKIMVELARQGSASGYEV

AKRLGVSRSNVYATLQRLEQRGFLRCSAGEPAKYSVLKPEEMTSMISGQMRASLEYVQ

SSMPQHEPEKPVFYNIEGDKNVFENLSRELAEAKHEIVVDVWREEAELLRADLKRAED

RGVRLLWSCDGGEGIMDQPAPWPGLPSYGTGNGRKFSLVVDRRWCMLGMRGESCATQA

MVTEHPVMTGLLLNHFAQELVLYELEQDMGEELESRYGPRYQNLSARYWSVPSEGINP

D"

CDS complement(326298..326981)

/gene="idi"

/locus_tag="PIKHDJKN_00322"

/EC_number="5.3.3.2"

/inference="ab initio prediction:Prodigal:002006"

/inference="protein motif:HAMAP:MF_00202"

/codon_start=1

/transl_table=11

/product="Isopentenyl-diphosphate Delta-isomerase"

/translation="MTPERFDIYDDQQNWIGTELRSVVHAKGFWHRSFHCWIVRDEGS

QRLVLFQRRRDIKDTFPGCYDITAAGHLTAGEQLHEASRELEEELGVQAPFETLTYLL

TARQQLEGQVRGVPFIDREFSSVFGLCLNQHLEAYTLQASEVDSLYEVPLDDLIALFR

GETHVIQAAGVQAVQSASDQTDQANSNSVPLHVQTRHMREIRAAEFVPHGTEYYIDVL

EALYHLPKN"

CDS 327061..327363

/locus_tag="PIKHDJKN_00323"

/inference="ab initio prediction:Prodigal:002006"

/codon_start=1

/transl_table=11

/product="hypothetical protein"

/translation="MECIVHFQVIYPQPQERKSLRGLIMVGQGQEPANSQLTTMFKDM

GFNVRLEDESQLLFKPVDASMNFDYIRVTELDTGEEVYKEDRDLKSILEHLLPRRF"

CDS complement(327653..328147)

/gene="mscL"

/locus_tag="PIKHDJKN_00324"

/inference="ab initio prediction:Prodigal:002006"

/inference="similar to AA sequence:UniProtKB:P0A742"

/codon_start=1

/transl_table=11

/product="Large-conductance mechanosensitive channel"

/db_xref="COG:COG1970"

/translation="MKGVLKEFKEFAVRGNVIDLAVGVIIGAAFGKIVTSLVNDIIMP

PVGKLLGGIDFSQKIINLDRDMKTASGQEITTLAQANEAGATVIAYGQFINVMIDFLI

VAFCIFMLVKGINYIKSKEHKKPEPQKTTKACKYCLSEIPAAATRCSHCTSELEAEGN

GATA"

CDS 328823..329410

/gene="xpt_1"

/locus_tag="PIKHDJKN_00325"

/EC_number="2.4.2.22"

/inference="ab initio prediction:Prodigal:002006"

/inference="similar to AA sequence:UniProtKB:P42085"

/codon_start=1

/transl_table=11

/product="Xanthine phosphoribosyltransferase"

/db_xref="COG:COG0503"

/translation="MQLLKDKVIQEGIVLSEQVLKVDSFLNHQMDPVLMKEVGKEFIR

RFEGENITRVLTIESSGIAPGIMTALELNVPLIFARKQKSLTLTEDILVEKVYSFTKQ

ETNEITVAKKFMQPGDRVLIIDDFLANGEAAFGLARIVEQVGAEVVGIGIVIEKAFQP

GGRLLKEAGYRVESLVRIGALSDGKVTFADEEVTI"

CDS 329412..330773

/locus_tag="PIKHDJKN_00326"

/inference="ab initio prediction:Prodigal:002006"

/inference="similar to AA sequence:UniProtKB:A0A2A5JY22"

/codon_start=1

/transl_table=11

/product="Nucleobase transporter PlUacP"

/translation="MARERIFQRHRHPIKTFSLGLQHVLAMYAGAVVVPIIVSKALGF

TTEQLTYLIAIDLLACGVATLLQVWGNRFFGVGLPVMLGCAFQAVSPMILIGMKSGVS

AIYGAIIASGIFVVLFSGIFGKLIRLFPPVVTGSVVTIIGLTLIPVAFNDLGGGQGAE

DFGSGVHLLLGFGVLVFIILMTRFTTGFVRSISVLIGLLVGTVAAGLMGEVNFAPIRE

ASWFHVVQPFYFGTPTFEIVPILTMILVAIVSVAESTGVFMALGKILDKDLSSKDLAR

GYRAEGLAIVLGGIFNSFPYTTYSQNVGLVQMTRVKTRDVIVVAGGLLVVIGFVPKIA

ALAQLVPGSVLGGAMVALFGMVVSSGIRILGSQVDLNRHENLFIIACSVGMGLGVTVV

PQLFAGAPDWAQIMLGNGIIAGSFTAIFMNLLFNGLGTQATAAKMAERQADVILGDNG

KSA"

CDS complement(331089..331427)

/locus_tag="PIKHDJKN_00327"

/inference="ab initio prediction:Prodigal:002006"

/codon_start=1

/transl_table=11

/product="hypothetical protein"

/translation="MSPMKSSSGLDENIAGMLCYLFTFVGGIVFLAVEKRSRFVLFHA

LQSVTVFGLIMVGHVLCAFLPLFGPLLASLLSLLGVVVWLLMVVTSLQGKWLKLPWVG

DFAEKQMRHL"

CDS 331710..333290

/locus_tag="PIKHDJKN_00328"

/inference="ab initio prediction:Prodigal:002006"

/codon_start=1

/transl_table=11

/product="hypothetical protein"

/translation="MNISATTYGQVRVTPYEIVRLQTLEVNKTMNGHARMRMTAVIPE

ARKDSYVQATTSQTDIKVKITDDEGHEKVFFSGVVLHVKVNVINDVHTLMVDAVSHTY

LLDVKLHKRSFQNPNLSYKNLVRKIISPYEGSDVIDFATDGRKIGDFVMQYEETDWQF

IKRMASHFYTGVVPDTASGKPKFYFGLPEGQYKGELQASHYTATKRIADYRSAKENKV

PGVKDPDYIVYEADSRQWFEVGNEVEFKSKKWIVGEAKTRFIDSLLNHTYSLFPAEGL

RYKKSYNTSIIGASIQASVLQVKGDKVRAKLDMDEQQDESTAYFFPYSTIYASENNTG

WYCMPEVNDSIRIYFPSKKEEDGIAISSVKRQDPEIVPATPKQASTASSAAGKGGASG

QSGGSPSGSNRNSSGAGGGGSKSSPASRVAPPPVPEDRMGNPDIKTFRTKYGKEIMLA

PDKIVISAGGMYITVSDENGIEIVSDQNVSITAGQDVVMSGQTIRIAGEKVELTGKGN

TITLEEELKMHGAEIKMN"

CDS 333316..334464

/locus_tag="PIKHDJKN_00329"

/inference="ab initio prediction:Prodigal:002006"

/codon_start=1

/transl_table=11

/product="hypothetical protein"

/translation="MNKAEAIAHLQQEILEPALQAHLSTLRVYIMQHQNSLIQSFVES

MRQLCVKAEWAQQELGKEPISILHCSMLRTSILEGSYTCLMEAYSDQWYWDEVECMHT

YDATWAFQPLSSLQQLLHIEMRRYGGVLNASDVERMMLQSAGRFNAYVITLARLAVPH

VVNLKEMEHINRADQFQIRVGEYKDWSEPVYIRDYGYRDMQNIRKKLSVAEGLECAYG

DYTSLSMAEGLYDHVDIRYSDFSHADLNKSNLRGAVMIGTKWEQSDLSGADLRGALLM

DAVFRECSLRGANLSDVSDGASMEFEKSVLGLQGICFAGANLEGADLRGAQLFHADFQ

GARLSQAQIWIHDRERYTHVLNEEQIESIRWVVSHEEEEELWSHDFMN"

CDS 334440..334937

/locus_tag="PIKHDJKN_00330"

/inference="ab initio prediction:Prodigal:002006"

/codon_start=1

/transl_table=11

/product="hypothetical protein"

/translation="MVPRFYELTEDHRILNPFRPPILQTSFDELPISTVMMIERRLNE

EPTDWISRPSYFVSDRMKRLMEMVDEALIFKSVTFIDHGSGEQLSYWACHIPAVQALS

DDSLFYRNGSIQQLVLQGEAVKDQRMFTLEGVRGSGVIVRLDVAESMLRRGLSGFVLR

KVDLE"

CDS 334934..335587

/locus_tag="PIKHDJKN_00331"

/inference="ab initio prediction:Prodigal:002006"

/codon_start=1

/transl_table=11

/product="hypothetical protein"

/translation="MNRYGDEMLLKMDRDFRLAKRIQEAEAASGELMTQEEVTYGVRQ

GLIETEAEIITLNNYRIWDGLLTLPLPDQLVQVPRGKLQEGRNSHERDEVMWIDEQKG

ISLSLSRSSHPLHVSQIEGMGSIITDQMRTLKKDAQWLHEETVQGDSLIVFYGESIQP

SNLGMAYDIVIVTSVKGRAVTGNVRFPVEQLHLWRLLTRAIFTCAQMSENGNGPIDY"

CDS 335604..335981

/locus_tag="PIKHDJKN_00332"

/inference="ab initio prediction:Prodigal:002006"

/codon_start=1

/transl_table=11

/product="hypothetical protein"

/translation="MLLPMLFKALMSGGFGGEFTYVVRGAEIFCSQGSDPGVLNLPLS

HGIYIKEQPVLNVADVIPLANVGRCGFCKMDGALCEPDTFFNKWSQGKEDVLVEGEAA

LLSRSELICRKGGIIKIEVDGQL"

CDS 336013..339063

/locus_tag="PIKHDJKN_00333"

/inference="ab initio prediction:Prodigal:002006"

/codon_start=1

/transl_table=11

/product="hypothetical protein"

/translation="MQTLEKSTLTYGDIHVHWPYGKIRLDKLELIQQAGAHGRLSFSG

WVDEEWEETIIQKAGSHDRVGFIRMDESGRELPLFKGQLHEIKVEATRGIVYVMAVVI

SHTYELDALQRTKSYQNENLRYLDIVNRVMKGYPEGDFIDFAFDETKTGAFIMQYQET

DWSFLKRLASHLGADLVPDVTAHKPRFWIGYPEGRGAFELKAIPFEKSRNIERFLHTK

AQGKHAVTEEKYTTYTFDLGQELQLGDEVSSDGHIYNITSRQAMLERSLLQWKYTCAL

PASHPLPRQLQTMITGAAIEGKIIDVKRNQVKVHLDMDEDQQAGEARWFPYAAEGSQV

WYMMPEPGGKVKLYFPSAEEDEAMVMQSVRQEPTEAGHVEKSGKVMQDPGVKSFGNPQ

GKSFTLGDSELVMNAQEGALYISMAAGNGVTLNSTTHVQIQCMGDLNISGASISLEGT

EGLYIQTMTDHVELVEEVNGKSENVVLEAEIHRSFDMIQSAFDQSLASMGESALKEAR

LKRNWEAREKGKLDGVIEEGKSLLSMVGDVADLAFTGIVGGDNAQGLYSWVKGEEVGS

LTERNATVQGTITGVNNVIDYTGDLVTGQKSGKEIWQDVSSAGEAYAKPFMDLKGEDN

RTMLTMTEQESHDAGLNDVGATMRVVDTAGAIFGGASLVKNGVKVLKGGGKKSGHGDG

AKGGVPHGHGDGPDGTSGGALKDGKLKTNAALMPSSLAELGERISQALGNMTAGAAAS

VPWRVQRMRLSNGQTVPVIVKNDSHMFSEPGGNRGKDSGPGNSKGSPNTSHVDSKENI

MSSKGTVSKESSTSSSVTSAKPKDASKPKEAPSSKDGTKVRDGDTEGAGKARTPKPID

PRGEPIKFVPNFKQEEIALQTYEKLRSTGLDAGELETFSRNTGLSFEEASQLKTHVIL

TEHMNLVDTVNGKYYYKGYFDPDIDMAYGWEKALKGELSAEEKAWFRQLADHELAESK

MMQEGLPYRDINYFKDGTMNESTKGAHELAPPQPGGFPGFKPKF"

CDS 339074..339586

/locus_tag="PIKHDJKN_00334"

/inference="ab initio prediction:Prodigal:002006"

/codon_start=1

/transl_table=11

/product="hypothetical protein"

/translation="MNSNFEQASERINNINWENNGMDKPSYAILVTEFIRRGNIFLDF

NSMDDNRRAVFNAAEIINFDTPAHIKEECNGLVNANVDWVTEYLCTFYLEWAYVVDKQ

MPIALKFHDLYDPIIKLFERGGRISYHHNELVSGKHGWPRNSGAITRELPPQDIRDEV

LDALDDENIE"

CDS 339851..340165

/locus_tag="PIKHDJKN_00335"

/inference="ab initio prediction:Prodigal:002006"

/codon_start=1

/transl_table=11

/product="hypothetical protein"

/translation="MPKLAQHTNSLWMKFVGTEEFRKRISAVPTQGILRIQRIQEMND

TQNRSYHPPMKNRIELIREHWAMIPAYRPDAAKERRINEEFRQLEQRSQKILLNDLRD

AS"

CDS complement(340269..340550)

/locus_tag="PIKHDJKN_00336"

/inference="ab initio prediction:Prodigal:002006"

/codon_start=1

/transl_table=11

/product="hypothetical protein"

/translation="MDQKDKEMLPLSHEKVEVDGIYTNEAGQEEHLHRGQQFPADPVL

GKTEWELTEFAFDNHHEGRTDERLVPKENDTDKIGKTTHPRRHFQRGDR"

CDS 340949..341137

/locus_tag="PIKHDJKN_00337"

/inference="ab initio prediction:Prodigal:002006"

/codon_start=1

/transl_table=11

/product="hypothetical protein"

/translation="MATKGHNEVKESLREMTRIFRPKDPKKFVKEYVRKYRITGGYEE

ELTSVVEHELVKMDSSVS"

CDS 341370..342131

/gene="map_2"

/locus_tag="PIKHDJKN_00338"

/EC_number="3.4.11.18"

/inference="ab initio prediction:Prodigal:002006"

/inference="similar to AA sequence:UniProtKB:P19994"

/codon_start=1

/transl_table=11

/product="Methionine aminopeptidase 1"

/db_xref="COG:COG0024"

/translation="MQVDPILKTKEEIGYMREAGRILRSCHEYIEKWLAPGVTTGDID

ERVEAFLAERGATPEQKGYKGYPYATCASVNEVVCHGFPNDRKLAEGDVVTIDMVVNK

DGWLADSAWTYGIGEQRRSIRKLMNRTERALHKAIAQAVPGNTLGDIGNAIERTARLY

RYGIVKPLIGHGIGQYIHEPPNVLPYGKRRTGMMLTEGMVITIEPIFTKGSSGAVVWD

EDGWTVRTVDGSWGVQYEHTVAITSDGPLILTDGT"

CDS 342711..343763

/gene="ctaC"

/locus_tag="PIKHDJKN_00339"

/EC_number="1.9.3.1"

/inference="ab initio prediction:Prodigal:002006"

/inference="similar to AA sequence:UniProtKB:Q04441"

/codon_start=1

/transl_table=11

/product="Cytochrome c oxidase subunit 2"

/db_xref="COG:COG1622"

/translation="MMKQWQVAKRILPLLAVFSLLLSACGREDLSVMKPQGPVAQGQY

DLMKLSITIMIVVLIIVFAIAAYVLIRFRRRAGQNEMPEQVEGNFKLEVIWTAIPLLL

VIVLAVPTVKTIFAQGEDLSNDKNAIQVKVTSHQYWWEFTYPQYDVTTAQDLIIPTGK

KIAFELKTADVLHSFWVPSLAGKMDTNPDGTLNKFSFSAPNEGVYRGKCAELCGRSHA

FMEFKVKAVSQESFDKWVNEMKAPAVLPEDTQLAEKFKTNCLSCHAVGDQGGPVAPNL

TGIGGKQSVAGILLNQGEGQEDGNPVLDNMKEWLHDPQSVKPGNTMPNPKDLGLTDEE

IDGIAEYLANYKLDYE"

CDS 343796..345685

/gene="ctaD"

/locus_tag="PIKHDJKN_00340"

/EC_number="1.9.3.1"

/inference="ab initio prediction:Prodigal:002006"

/inference="similar to AA sequence:UniProtKB:P16262"

/codon_start=1

/transl_table=11

/product="Cytochrome c oxidase subunit 1"

/translation="MAHAHSVKRYRGLMDWITTVDHKKIAVLYLIAGGFFFGIGGIEA

ILIRIQLMKPMNDFVSAQVFNELITMHGTTMIFLGVMPIIFAVMNAVVPLQIGARDVA

FPFVNALGFWTFLFGGLLLNLSWLMGGAPDAGWTSYTPLSGSEYSGTHGVDFYTIGLQ

IAGLGTLIGGINFLATIITMRAPGMSYMRMPMFTWTTFITSAIILFAFPAITVGLVLL

TFDRILGANFFDVAGGGNPVLWQHIFWIFGHPEVYILILPAFGIISEVIPTFARKRLF

GYSSMVFATILIAFLGFMVWAHHMFTTGLGTVANALFSISTMLIAVPTGIKIFNWLFT

MWGGQIRFTAANLFAVGFVPTFVMGGVTGVMLASAPADFQFHDTYFVVAHFHYVIVGG

LVLGLFSGLHYWWPKMFGRVLSETLGKWTFWTFMIGFQLTFFVQHFLGLMGMQRRIVT

YLPNQDFDLLNLVSSVGAFLMGVGVIMFLVNIVITMRKPADAPNDPWEDGRTLEWSIP

SPPPEYNFKQTPLVRGIDAYWKEKMAGHTEMTPAEPVGSIHMPSATPLPFVMSVGIFI

AGLGLMFSKDDFGNAFMNGLFNNYIVVIIGLLITFGAMALRSLYDDHGWHIEPEDQDE

KGVRT"

CDS 345682..346308

/gene="ctaE"

/locus_tag="PIKHDJKN_00341"
/EC_number="1.9.3.1"
/inference="ab initio prediction:Prodigal:002006"
/inference="similar to AA sequence:UniProtKB:Q03439"
/codon_start=1
/transl_table=11
/product="Cytochrome c oxidase subunit 3"
/translation="MTTSHAEPVNGKLPHEPEKATLEGRNKLVGFWLFLGGETVLFGT
LFATFLALRGQTNDGPTANELFHLPLVAAATFILLVSSLTSVFAIQAMHKGKRNALAL
WLGITVVLGLGFLGLEIYEFYEYVKHKEFGMTTSAFSSAFYTLVGFHGAHVAFGIMWI
GLIIGQLFRKGLTVVTAPKVYVSAMYWHFIDVVWVFIFTVVYLLGKVG"

CDS 346312..346632
/gene="caaD"
/locus_tag="PIKHDJKN_00342"
/EC_number="1.9.3.1"
/inference="ab initio prediction:Prodigal:002006"
/inference="similar to AA sequence:UniProtKB:Q03440"
/codon_start=1
/transl_table=11
/product="Cytochrome c oxidase subunit 4B"
/translation="MSVQDKSDQQPVKHRHRAEGPQKHVVVFIFSIVLTLIAFAAAAA
GGVNTTFTIIILVVMAILQVFVQLGYWMHLKDKGHLMPILFMCFGFFVAFTCIIMALY
WVWW"

CDS 346739..347662
/locus_tag="PIKHDJKN_00343"
/inference="ab initio prediction:Prodigal:002006"
/codon_start=1
/transl_table=11
/product="hypothetical protein"
/translation="MLGLQYFSFNDLWSPLIMALFLLIAAAYLVLVGPFSERIKGADQ
ATVAQKMMFITGLTVLYLAQAGPFNLLGHVMFSFHMVSMALSYLVAPPLLMKGLPLWV
WRGIIRWLPTRQLSFLAHPIVAAVLFNGLFSLYHLPVVHDYVMLNFTVHRLYYIVLFI

TSMLMWWTLLNPLPEGRQASGLSKIGFIFLNMVLLTPACGLIIFASEPLYATYSNPAV

WAEAMRYCVSGDSTALLRSFGGPAFFNFLSSAKEDQQVGGILMKFIQEGIFVSMLAYV

FFQWYRKEKREDDDDPYPVENTGGPLNPAAK"

CDS 347699..348163

/locus_tag="PIKHDJKN_00344"

/inference="ab initio prediction:Prodigal:002006"

/codon_start=1

/transl_table=11

/product="hypothetical protein"

/translation="MDMYFLLPTISTSFIVISAVLVGIGWVLIIRGKREAHQTAMIAG

AIAALLFFVIYTSRTIFVGNTAWGGDPEMEIFYRIFLIFHIILATVAAVFGISTLVLG

FKKKFKTHRRWGRFTSMIWFSSAITGVIVYVLLYLLYPGGHTRPVWEVILGV"

CDS 348275..348646

/locus_tag="PIKHDJKN_00345"

/inference="ab initio prediction:Prodigal:002006"

/codon_start=1

/transl_table=11

/product="hypothetical protein"

/translation="MKKTVHVSQTYPRLTVYSEEDYRGRSRVYRGNTGLRNLDNILGG

VESLRFFSTSSNATLVVFTRPNFQGGFRVFRGNTNLRDLDDLIRGNDVESLISTNQRL

TLAEIREIRSNRTLPSGYDLV"

CDS complement(348752..349072)

/locus_tag="PIKHDJKN_00346"

/inference="ab initio prediction:Prodigal:002006"

/codon_start=1

/transl_table=11

/product="hypothetical protein"

/translation="MDWLVEIGFVLLFLIVVIWLIVRDEPKRHSAKKGRHRVRRNNGY

TDSSPAYPVITTNDDSYTHHSKHTHDHKHHDGDSFSGSNHHHSSDSGTSNDSGGDSGG

GGGD"

CDS 349228..350025

/locus_tag="PIKHDJKN_00347"

/inference="ab initio prediction:Prodigal:002006"

/codon_start=1

/transl_table=11

/product="hypothetical protein"

/translation="MTGIGSGLYTVYIDIYSEHSRDGKAGNDMNEWKQAWWLTRSEMN

KDKLQWLWTAIFMIYTGGMSGVMLLGQQKTDFINPVVDTFFLIMVPFQGFVFSRRSFR

YIQEDSYTQMLAYYRRLPIPNETVMWSRLQQSLMAFTYNGIFFYGSLYVVGLHEEGFR

WDQYLAFGITCTGYGLLMTGLYIYGEFLYSGKKYLLLSSLFLPLTIIISLLIRTSGSY

GFSIVMDVSKSWGLLSPIMWVALVAGIAGLWLFSRLTLKKLSIRDLS"

CDS 350109..350507

/gene="ytrA_1"

/locus_tag="PIKHDJKN_00348"

/inference="ab initio prediction:Prodigal:002006"

/inference="similar to AA sequence:UniProtKB:O34712"

/codon_start=1

/transl_table=11

/product="HTH-type transcriptional repressor YtrA"

/db_xref="COG:COG1725"

/translation="MQDVKIPIQINENSAEPLYHQIENQLRSLIVTGQLGEGTHLPSI

REFAGSLNCSVITVRRVYQDLENEGLLRTRQGTGTFVAQVEAGDRENYRLKAAHEAMQ

AAVQSGKSVGCTEEELESLFREVLKAVYSK"

CDS 350523..351419

/gene="btuD_2"

/locus_tag="PIKHDJKN_00349"

/EC_number="7.6.2.8"

/inference="ab initio prediction:Prodigal:002006"

/inference="protein motif:HAMAP:MF_01005"

/codon_start=1

/transl_table=11

/product="Vitamin B12 import ATP-binding protein BtuD"

/translation="MEPIAVQLNGVSKMRKRRVIGPIDLTIPEGYVVAILGHNGSGKS

TLLNMLQQVVLPDAGQIIWFGKEHEGPLPIELRQQIGFVADNAGLEENRITAQEAADF

RAYWYPRWDMKLFDQLIHDMEVPVDVKLNKMSKGERRKFEIAAAIAARPRLLLLDEPS

SGLDPFAWKVMIEQFRTFMAEGDTTILIATHIADEVKRLADYIVLMHRGQSLGMAEKD

MVLDQWKEVWYEGDLRPESIPGVVESSSEERGLVRVITTRVSEAQERLELANNRVLKI

RNLELDEVLAFWIAGYAPVQWK"

CDS 351440..352342

/locus_tag="PIKHDJKN_00350"

/EC_number="7.6.2.-"

/inference="ab initio prediction:Prodigal:002006"

/inference="similar to AA sequence:UniProtKB:O86311"

/codon_start=1

/transl_table=11

/product="Multidrug efflux system ATP-binding protein"

/db_xref="COG:COG1131"

/translation="MNRLELKQVVKQYADKTAVNGVTLNVKEGEIYGLLGANGAGKTT

TMRMVLGLIHPDGGNIQYNGKPYSTELQQIMGYLPEERGLYPKVKVSEQINYLARLRG

MNGKDADQSLKYWLDRFEVPEYYDKKIEELSKGNQQKMGFIAAVVHRPQILILDEAFS

GLDPVNVELLKSTVKELRDEGTAILFSTHRMEHVEELCRQITILHRSNTVVQGEIKEI

KSRYPREHVFLSTTGNVGGLEQLPGVKKVEQNERGYLIHIGQMEAAQEILRAAMAQTT

VEHFEIKEPTLNQIFIREVGESNE"

CDS 352335..353630

/gene="yhaP"

/locus_tag="PIKHDJKN_00351"

/inference="ab initio prediction:Prodigal:002006"

/inference="similar to AA sequence:UniProtKB:O07523"

/codon_start=1

/transl_table=11

/product="putative protein YhaP"

/db_xref="COG:COG1668"

/translation="MNKMGTIIGFTFKNKVKTKSFMVTTIVLAVLITIGLNIPYFITL

FNGGPIGGGSSTNPVNIGLLSTGQTEVAEKLETYSSAQGNQTYRFVNDASKDEAALKA

DVESELLDGYLKFEPVAGQDFPQPVLYSSEDISPQVIAPIEAALQIVKLDVVVKDTLT

AEQKQLISTPVKLTEQSLDSDQSGAAAGSEGTMSGINYIVVYLLIILLFTSTMMTGNM

IASEITAEKSSRIMEILITSVSPLSQMFGKIIGIFMVGLLQIGIFGAVVAGNMLLPHN

RDVLSDFNMNLGDVNVAVIVYGLIFYILGYFLYAVMFAAIGSMVSRTEELGQAVLPIT

MLSLVSFYIAIFSIATPNILLLKVASFIPFTSPTAILVRIGAGVAPTWQIWTSLAILV

VSIMIFGWLAAKIYRTGVLMYGKRPTFKELFKAMKAYKI"

CDS 353836..355056

/locus_tag="PIKHDJKN_00352"

/inference="ab initio prediction:Prodigal:002006"

/codon_start=1

/transl_table=11

/product="hypothetical protein"

/translation="MKNWKRMLLSLTISVGLLASAVPAMASPQETSVKVNDETVEYTA

GAPINDKGTTLVPLRSTLDAMDVKLTTATDDTITGVVNGKTITLKSRLTRINGVTYAP

IRIVGDATGYEVRWDTATRTVLLVSKGGETETVQTGGRGFMWEVESNGNTVYLVGSMH

IADESFYPLRKEFEEAFAEADYLGVEIDISKAAEEAQQKLVLSLGSYQDGTTLKDHIS

SETYTQLGDILKQSGLEPNALDTFKPWVVESTLASLKSMKAGYEASAGVDLYFIQKAI

ERKLPVIELESYESQLGMFNDFSKELQEKTLKATLDNFDVLDDSVNQMAEMWKTGNDE

QLLELTNSISDDEEYNKAMLIDRNVGMADKIDGYLRNGKGEEYFIVVGAAHYLGDHGI

VKLLKDKGYTVVRK"

CDS 355496..356725

/locus_tag="PIKHDJKN_00353"

/EC_number="3.2.1.4"

/inference="ab initio prediction:Prodigal:002006"

/inference="similar to AA sequence:UniProtKB:P29019"

/codon_start=1

/transl_table=11

/product="Endoglucanase"

/translation="MRKNRGFSFSSKAVMMCCLAFLLIPASFAFAAPNKPFPQHTTYT

SGSIKPNHVTQSAMDSSVKAKWDSWKSAYLKTAGTGKYYVKYQSNGDTVSEAHGYGML

ATVIMAGYDGNAQTYFDGLYQYYKAHPSANNSKLMAWKQNSSFQNIEGADSATDGDMD

IAYSLLLADKQWGSSGSINYLQAGKDIINAIMQSDVNQSQWTLRLGDWATDNTFKNAT

RPSDFMLNHLKAFQAATGDARWANVIDKTYTIVNSLYNGYSSSTGLLPDFVVLSGSTY

KPASADFLEGANDGSYDYNSCRTPWRIATDYLMTRDSRALNQLNQMNSWISTKVSGNP

SNVKDGYKLNGTVTGSGGSGAFYAPFGVSAMTSSVNQNWLNSVWTKTAGSSNEGYYED

SIKLFSMIVMSGNWWTY"

CDS complement(356989..358314)

/gene="licH"

/locus_tag="PIKHDJKN_00354"

/EC_number="3.2.1.86"

/inference="ab initio prediction:Prodigal:002006"

/inference="similar to AA sequence:UniProtKB:P46320"

/codon_start=1

/transl_table=11

/product="putative 6-phospho-beta-glucosidase"

/db_xref="COG:COG1486"

/translation="MAYEQGLKIAVIGGGSSYTPELVEGFILHHAELPVRELWLVDIE

PGERKLNIVGNLAKRMVEKSGLPIEVHLTFDRREAIKGADFVSTQMRVGMLDARGRDE

SIPLKYGVIGQETTGPGGMMKALRTIPVLLDICRDIEELAPNAWLLNFTNPAGMVTEA

ILKYSNVKSIGLCNAPIGLIKQTSAKYGVEADRIYAEFVGLNHLHWITRIDVNGEDKL

DDMLADTAGYSAKNVPAREWNPEFLQSLHALPSYYLKYFYMTDAMLEEQLESLQKGGN

RAEVVKRVEEELFELYNDPDLKDKPKQLEQRGGAFYSEAAVNLMRSLYNGTNDIQTLN

VANQGIIDFLPDDASIEVNCVVTKTGPLPLPLTKVPPMAVGLIHAVKTYERLAIDAAV

TGDRGLAIQALAHHPLVPSVEVAIQMLDEMLEANKEYLPQFFTESAANA"

CDS complement(358369..359361)

/gene="murK_2"

/locus_tag="PIKHDJKN_00355"

/EC_number="2.7.1.-"

/inference="ab initio prediction:Prodigal:002006"

/inference="similar to AA sequence:UniProtKB:Q97ML3"

/codon_start=1

/transl_table=11

/product="N-acetylmuramic acid/N-acetylglucosamine kinase"

/db_xref="COG:COG2971"

/translation="MAYYLGIDGGGTKTYALLTDDFGNVLGKGVGGNGNHQIDRALAA

QSIREAAEGALNEAGLRIHEISHTYFGLAGADREVDYRILHPLVQGIGFTQNYSINCD

TMIGLRAGTNRPYGVALICGTGTNSAGRNPAGEHVQVGGFDYMYGDFGGGGSLNIEVF
RSVIRSWDGREQPTLLTPLLLNFLGYDSVSDMFDDFQDHGKHVPVHAAKLLFEAAAED
DAVALEILNRQGVEIGKSAAAVIHKLGMEKDTFDVILAGSLLTRGDRGWIRSQVEQTV
ATLAPNATIVTLATEPVVGALWSAMDASGHAVSQDVYNKMRVFRDFEHIKQTTR"

CDS complement(359396..360403)

/gene="ccpA_1"

/locus_tag="PIKHDJKN_00356"

/inference="ab initio prediction:Prodigal:002006"

/inference="similar to AA sequence:UniProtKB:P46828"

/codon_start=1

/transl_table=11

/product="Catabolite control protein A"

/db_xref="COG:COG1609"

/translation="MKVSIFDVAKKSGLSVVTVSRVLNGAESVREKNRQKVLEAIKEL
DYHPNAAARSLARGKTGIVGLIMTTLQDSFFDTVVKELNEVLALHGYFLAVSVSTGIG
SDETHYLIQEDRVDGLILLSPMEEDNYIVELKRRNIPYVLIDNQKPENDTFSVTIDNY
KGGYAATKHLLDLGHTSIAHLSGQNMFRSTRERRSGFLQALKEKDLAPFEMITGDFEI
DFGYDICRKWLREGRLPSAVFAGDDHIALGVVNALMEEGIKVPEQVAIVGYDDQYISS
KLHPHLTTVRQPADRIGVAAADMLLKRMDGTMKRGANVRIDPELIVRESTGSPTKGTT
G"

CDS complement(360535..361926)

/gene="msmE_2"

/locus_tag="PIKHDJKN_00357"

/inference="ab initio prediction:Prodigal:002006"

/inference="similar to AA sequence:UniProtKB:Q00749"

/codon_start=1

/transl_table=11

/product="Multiple sugar-binding protein"

/db_xref="COG:COG1653"

/translation="MKTIRIKKSFTLLLATSMLVVLALSGCSGGKSAGDVVPGNDGDN
AKSGSSGGQVTITHYTIDSEDRTFIEKLVPDFEAQHPNIKVKIEKAPYEQFDSKLQTL
IAGGKSPDVTSHYGYGGFAEYYNKDMLLDMNDIIKEDGFKASDYNIPDDLMKIYTVKD

QVYGIPVNMYVSLMLYNKDMFDAANVAYPPSDYEDKSWTFDKMVEDAKKMTHVSNDIA

KTQYGVDFTWSERDMRPLYFGAEPYSQDTWTNGGVPSETHFDSPEVIAAYNKLFNLIL

KDKVSPTTEWSKSVAGQNGDAFVTGKVGMTVSGSWSLAGSNDFPFKVGVAAVPAGGND

KVRSVLFVDPLLILKGSKHPKEAFEWIKYLVSDEIQEKSIDLSGGNPPVNTKAAETYY

KHFDGIDAADVKKVYEGAVEYGFESYNHLITHYSQINDMFINEMQPIDTGHNTVEEVM

PTIQKKVMEIIKR"

CDS complement(361980..362864)

/gene="araQ_3"

/locus_tag="PIKHDJKN_00358"

/inference="ab initio prediction:Prodigal:002006"

/inference="similar to AA sequence:UniProtKB:P94530"

/codon_start=1

/transl_table=11

/product="L-arabinose transport system permease protein

AraQ"

/db_xref="COG:COG0395"

/translation="MSTIPTAGPGASPSVNKRRRKIDAVSIASFIALVVTTFLMLLPL

FFMVSTSLKSKKELLTFPPTFLPESWQWSNYREIFDTLNFGQMYMNSLIIGILTVVGT

LFSSALAGYGFARYRGKGSNLWFMLMLSTMMLPYPAIMIPQFILFSKLNWIDTFLPLI

VPAFFGSAYNIFLLRQFFTTLPDELFDAGRIDGCSEFRMWRQIALPLSGPALATVAIF

AFIYSWNDLLTPVLYLSSSDKFTLPVGMSSFTSSRFRIPPWHLLMVASVLAMLPIVTL

FAIAQKRFVEGIVLTGIK"

CDS complement(362861..363718)

/gene="ngcF_1"

/locus_tag="PIKHDJKN_00359"

/inference="ab initio prediction:Prodigal:002006"

/inference="similar to AA sequence:UniProtKB:O50500"

/codon_start=1

/transl_table=11

/product="Diacetylchitobiose uptake system permease

protein NgcF"

/db_xref="COG:COG1175"

/translation="MYLFISPWLIGFFVFALYPILSSLYYSFTDYDIIHPPKYVGLAN

YKEMFQNDLFWKSVVVTVRYTFISVPIQLLLALGFALLLNAKIPFRGFFRTAMYFPSM

VSGVAMSLLWYWIFNPSIGLFNYVLSWFGISGPAWLMNPDYALYALMIMSFWTVGSGM

ILFLAGLQGVPASLVEAAKLDGAGRFRIFLNVTLPMISPVLLFQLIMGVIDSFQVFTQ

AYVMTQGGPNYSTWFYVYNLYTSAFKEYRAGYSSALAWVLLIVVMAFTALIIKLSNRY

VHYEGGGRK"

CDS complement(364695..365429)

/gene="kdgR"

/locus_tag="PIKHDJKN_00360"

/inference="ab initio prediction:Prodigal:002006"

/inference="similar to AA sequence:UniProtKB:P37728"

/codon_start=1

/transl_table=11

/product="Pectin degradation repressor protein KdgR"

/translation="MDQYEVATLKKGLLILDALQQGEPMRLTEIMHRFNLNKSTTFRL

LYTLEMTGFIQKDGHTYKASELKGHHSSSANTRLNWLVVPSLHQLAQELGETAYIGVL

DGMKVMTVQVVEGNHAIRSHTRVGDSAYAHLSAFGKVILAHLSDEQLKKMLTHLTQHK

ATKHTFDDNHLLKEHLNVIRQQGYAIDDEETEVGLRCVAAPIFYNGKVIAALAISGPA

ARLSRKRDRAVSKKLKECSAQISGLL"

CDS complement(365701..366159)

/locus_tag="PIKHDJKN_00361"

/inference="ab initio prediction:Prodigal:002006"

/codon_start=1

/transl_table=11

/product="hypothetical protein"

/translation="MDLKSLELLEEELEFASFSHQDALVIGNQIVQYAQENNVAVAVH

IERNRVPVFTHLMDGTSEENYVWLFRKKRIVDHYNRSSAYIDQRFIQSGASHAEHSLL

STAEYQAVGGSIPIRVKGVGVVGSVTVGGLTGELDHAYAVEGIRLFLKGE"

CDS complement(366160..367353)

/gene="ydhP_1"

/locus_tag="PIKHDJKN_00362"

/inference="ab initio prediction:Prodigal:002006"

/inference="similar to AA sequence:UniProtKB:P77389"

/codon_start=1

/transl_table=11

/product="Inner membrane transport protein YdhP"

/db_xref="COG:COG2814"

/translation="MNSKIFIALMSLAISAFAIGTTEFVIVGILQEVATDLGISVTQA

GTLVSGYAVALAIGTPIVVVITGKLPKKGYLVFLMVVFTLGNALAAIASTYFLLLLSR

IITAITHGVFFAVAATVAADMVPANKKGTAISIMFTGLTVATILGVPLGTYIGQEFGW

RMSFAVVAILGLIALLVSLVAVEKVPRSMHSPTLRDVGSLIVNPRIITALLMTILGFG

GTFALFTYISPILEEISGYSSSSISWLLLIYGVAVAIGNIIGGKLANGHPVKALRFVF

LFQGIALLLQIWLLPNKTLTILSLILMGFFAFMMSPGVQAYILMLAEKLVPSAKDVAS

ALNISAFNIGIAAGSTLGGLAVDHLHMMDTAWIGAIMAGFAFLLAAINYRMDKKQNLF

VEERG"

CDS complement(367523..367816)

/gene="ycnE_1"

/locus_tag="PIKHDJKN_00363"

/EC_number="1.-.-.-"

/inference="ab initio prediction:Prodigal:002006"

/inference="similar to AA sequence:UniProtKB:P94425"

/codon_start=1

/transl_table=11

/product="Putative monooxygenase YcnE"

/db_xref="COG:COG1359"

/translation="MNVITICAILKAKPGQEEQLRDELIKLIEPSRAEKGCIQYKLHK

SLEKIGTYVFYESWADENAIIAHVNTEHYQTYRKNIETIIDTMDVYHLENIET"

CDS 368485..369513

/gene="araR_1"

/locus_tag="PIKHDJKN_00364"

/inference="ab initio prediction:Prodigal:002006"

/inference="similar to AA sequence:UniProtKB:P96711"

/codon_start=1

/transl_table=11

/product="Arabinose metabolism transcriptional repressor"

/db_xref="COG:COG1609"

/translation="MDQFNVSKITVVNALTGLANEKLIARVPGKGSFVAEETDAVITV

FSESPTTMRRGLSTGLIGIIMPTIHDYFAIRLIEGIEQTLNQEGYRSMIMLTNGKIEK

EKDAIKELKALGAEGLLIFPVDEGNYNEEILGMKLSGFPFLLIDRYLPGVETHYIAAD

GRLGVRMAVEHLWDLGHRDIAICSDSPLQTVTVQERIDGYIEALKDKGALINPAHMIT

DFKPLSVLKDAESHPLYRYISNKMVTAYISLNGRLGVQIYQMAKQAGLRIPEDVSIVS

FDDPTSIVEEFSIFTHVKQFESEMGSRAAGQLVEVIRSSGDTIGFSKILIQPELVIRQ

TSGICPIS"

CDS 369675..371894

/locus_tag="PIKHDJKN_00365"

/inference="ab initio prediction:Prodigal:002006"

/codon_start=1

/transl_table=11

/product="hypothetical protein"

/translation="MLRDRKEERIGFQESAPYHPDYDLQTDFVMVYGIDETMQERIRE

WKNKGYVVHLMTGVAWGTYNSYLDGEVDGETHWDEAQTDRHGEMIVHGKEPIIPYMVP

TVSFGKYIAERIRVAVDSGVEAIHLEEPEFWVNGGYSESFKREWQLYYKESWRAPHSS

PDAQFRASKLKAYLYTRVLDRLCTEMKDYALKRYNRVLRFYVPTHSLINYTQWRIVSP

QSRLIELPSVDGYIAQIWTGTSRTPNVYEGVRKERTFETAYLEYGVMQELVRGTDRRM

WFLHDPIEDNPNYTWADYKQNYLKTVAASLLHPGVSHYEVAPWPRRIFKGNYPSDDGA

GKEQIPPDYATTLLHVMHTLGNMDQDNVEIEGESPQVGVLIADSAMFQRMKSEPEAPD

AGKYDGTDPEGFQEDGDTELLDFSPFYGLALPLLKHGIPVRPVQLDNVRRYPHYLAPY

RVLLLSYEFMKPEHSDIHYALAQWVQDGGALIYIGDDSDPYHNIRAWWNDDQRSESTN

TMYHSPREHLFEQLGLKVEQEGIHHVGKGLVQWLSVHPATCSSSKSGADQLRQSVKET

LEKLQDSNFRWEAKHYFKIKRGPYIIASVLEESVSEEPLVIPGPVVDLFDPALHVLQE

VRLAPGEQALLYDITAGRPQQDKVSLVSASSRIEDLTITNTGFQCVAKGPSNMQATAR

FYCPFEPISVSSIDQHKTVTTGWEWDTESKTVLLKYEHGSKNKVEIQVRWGRENTKA"

CDS 371922..374996

/locus_tag="PIKHDJKN_00366"

/inference="ab initio prediction:Prodigal:002006"

/codon_start=1

/transl_table=11

/product="hypothetical protein"

/translation="MPYEPIRLGHLNQMLSKLREAIYEPIAKLNVTAWVTPEPVSFEE

RMSGQKITLSQGERWGELWDCAWFCFTGSIPPSGSDAKVVLLLDVNGELCLVDHEGTP

VQGLTTINSEFEFTLGLPGKRVVELDHSSITVAEGAFELWADAGNNDLFGKYQGGTVK

EAVIAICHEGIRSLYYDTEVLLETAEHLPADTARREKILQSLYDVSLILTEFTPDRIH

RAKGLLNEQLSFRGGDSVMNISAVGHAHIDLAWLWPIRETIRKGARTFSTVLRMMERY

PDYVFGASQPQLYDWMKEHYPKLYDQIKERVQEGRWEPQGAMWVESDTNVPGGESLVR

QILYGKRYFQQEFGMEMKSLWMPDVFGYSASLPQLLKKSGVDYMMTQKLSWSEYNRHP

HHSFLWEGIDGSSVLTHMPPEDTYNSPAAPRSIAKAEREYLDKNVSNHALMLFGIGDG

GGGPGEEHLERLEREKNLLGLAPVVQEPSWRFFERLNEERERFQTWRGELYLEKHQGT

LTSQARSKRYNRKMEKALRELEFAAVLANRIGKSYPAETLETIWKEVLLYQFHDILPG

SSIKRVYDESLIRYEELLQQTEAMITETYGYLADHISSSEADSSGVVVFNSLPWEREE

WLHMDGRWCKVQVPSMGYTVVSDYGNQGVEREVASSIEIDSETLDSGFHVKAKERSME

NDILIVKFDQNGGITSILDKLEAREVILQGQRGNDLRVYHDEGDAWDFRHDYSAQAGM

PLILNDIRELRDGPRVGLVFDYSYGESTLTQTVTLTEGSRRIDFETGADWRENGKMLR

TSFPVNIRTDQVHCEIQFGSLSRPTHRNTMWDFAKDEICAHQWVDLSEPDYGVALLND

CKYGHRAINNVLDLHLLRSSSYPDPVADRAEHQFTYSLYPHQGNHVQAEIYRKGNELN

IPLRLSSLSVSSADVSEGKLPYMKAFLQPDHPHIMVESVKKAEDSNDIIVRLYETSGT

QVKTKLSIGWNAAEVWEVDLMENNISKLELQNQSDVVLSFTPFEIISLRLKG"

CDS 375045..375962

/gene="yteP_1"

/locus_tag="PIKHDJKN_00367"

/inference="ab initio prediction:Prodigal:002006"

/inference="similar to AA sequence:UniProtKB:C0SPB3"

/codon_start=1

/transl_table=11

/product="putative multiple-sugar transport system

permease YteP"

/db_xref="COG:COG4209"

/translation="MNKSNNLLMKRIWKHKIFYLFMLPGIIWFFLFSYVPLYGIQVAF

RDYTFVGGFSGSPWAGFKYFEQFFNYYQSGDIIRNTVIISLMKLLIGFPMPIILAILL

NEIRMIRFKKTVQTLSYLPYFVSWIVVVTLMQRLLTPYGGPVNDLLGSFGTDPIQFLY
NPTWFYQMILGSDIWKNIGWNSIIFMAAIAGIDQQQYEAAKIDGAGRFQQMWHVTLPG
IRNVAIILFILAVGNLMSAGYEQLLLLNGPGTASMGSVLDVHAINSGIREGRLSYAAA
VGLFQSVIGLILVLTVNRIARKVSDVSLF"

CDS 375975..376871

/gene="dasC_1"

/locus_tag="PIKHDJKN_00368"

/inference="ab initio prediction:Prodigal:002006"

/inference="similar to AA sequence:UniProtKB:Q9K489"

/codon_start=1

/transl_table=11

/product="Diacetylchitobiose uptake system permease
protein DasC"

/db_xref="COG:COG0395"

/translation="MTRKWSLFSVVNYTLLALVGFSMIYPFIYILAYSLNDGKDSMQG
AIYFLPRKFTLENYAQVFDNARIWKAYQITIMRTVLGTFLHVVLCTLMAYALSKKTLP
GRSFFTFYIFLPTIFSAGFIPFFITLQKLHLINSFWVYVLPLLFNFMHIIIIRTFLQG
IPEELEESAHIDGYGDFQIFVRIILPLSGPVLATISLFIGVAHWNDWFSGAYYVSNKD
LIPVQTLLQEMLTEAESLSNSMQRAAQQGGQTVGGAGGAGATPESLRMALLVITVFPI
LCIYPFMQRYFVKGVMIGSVKG"

CDS 376952..378607

/locus_tag="PIKHDJKN_00369"

/inference="ab initio prediction:Prodigal:002006"

/codon_start=1

/transl_table=11

/product="hypothetical protein"

/translation="MNKIWSKGVSVLLVSILVGGLLSGCTDSSGNGDGGEAADGSYKL
KILHNWNGSGGSDNGMTPVEEVIKEKTGVTLQWEYTKGSETEKVNQIFATQDLPDIYT
GPAWGGELDGIIKAAKEGQLLDISDKLGDYPNLAKSIAEENVPPALYEKAIDAYDGKK
YLLLQNQPAKNEDGMDWLYGFYVRKDIAEKVGVDPQSVVTKEDFYNFLKTIKDANLQE
NGQPVFPLGGFSNGWSVGIGNTMFYSGAGYVDKGDGTVEHHFFTKGYEEYTLFYRKLV
EEGLMDPEAFTQTDPIAKEKINQGRIAILAAHYPAILDASKEYVTSHPGSDYIPIGPL

EWADVDPNRPADLGIQGNNVTAITKDCKGACVDAALKFLDYMASDEGFMLARYGVQGV

HWDMKDGKPVANQEWFDKFTADQSGKVRKNEGISIGLESITGLDRINSAAGGDIWADQ

ERLDAMEQARKILRPNGIQVITAYNPGDVITKAPEWEMLKPSMDRINDAWKEAIFAKS

DEAAMGIINDLREQLRKTGYDQAMQFTNENLKGKDVVTVQMPN"

CDS 378786..380801

/locus_tag="PIKHDJKN_00370"

/inference="ab initio prediction:Prodigal:002006"

/codon_start=1

/transl_table=11

/product="hypothetical protein"

/translation="MTTNFRPPSVPLVTVDPYFSVWSAADHLYEDHTRHWTNKTNGMV

GLAIIDGKARRFMGKVALRDQSEFQEPEALVQTDLTVEPVTTRYTFQGGGIELKVQFN

TPLLLDDLDLLSRPVTYVTFQAKSIDGKSHQVKIYFDVTGEWCVHTTDQQVTWERHVI

EGQLNAMSMGTVEQPVLQLAGDDTRIDWGYMVLAVPQTPRIQTVIHSVTSREQYVRTS

QLPTEDDQHQPRAVSDNMTVMATEINLGVIQEEPVSQYLMLAYDDVHAIEYFHKPLNA

YWKRNGLTFHEMLLMAADQYEEIQQRCDSFNRELLAESQAAGGEKYRDILALTYRQAI

AAHKLVADEAGDVLFFSKENFSNGCIATVDVSYPSIPLFLRYNPELIKGMMRPIYKYA

MSRDWTFDFAPHDVGTYPKANGQVYGENKLEDQMPIEECGNMLLMAAAVSKYEGDIDF

AREHWGPLTKWAGYLLQNGLDPENQLCTDDFAGHLAHNTNLSIKAILGIAAYSYMCDK

LGLQEGAAYLEAARKMAHEWMTMADAGDHYKLTFDSPADSWSMKYNLVWDHLLDLHLF

PKEVAAKELEYYMHKQNRYGLPLDSRETYTKSDWLVWCASMSSTQAQFESLISPLWDF

MNETSARVPVTDWYDTITTKQMNFQNRSVVGGFFIQLLMQDSTGKFV"

CDS 380811..384029

/locus_tag="PIKHDJKN_00371"

/inference="ab initio prediction:Prodigal:002006"

/codon_start=1

/transl_table=11

/product="hypothetical protein"

/translation="MNVNNIIEEVCTDLIQLKQQFENPDATYRPQPFWFLNHELEKPE

LESQIQSMFEAGVGGVVLHARHGMQTPYLSPEFMEVLEFCTTECEKRNMVVWLYDEDN

WPSGTLGGKLTRDHPEYRMRYLRMEEKRYSRGPKQEGLKLDFASYDHNELIAILAYRS

VHHDGEWLLYDEPEDITHLWGQDWKSDTEDTYIVLACWSCEIAEGITFAKGYYLDTLN

PEAVQTFIQMSYEPFLPLQKYFGSTIQGVFTDEPGLMIHDGFSGVEAIRTVVDDVGAT

LPGMVFAWTHGMAKRYQHENGYDLIPRLGALLYNMADGSRSTKQQYYDTITRWYVEGY

HAAIRTWCERHGLLYIGHTLEEPVWGQARSQGNQTRVLQQFHYAGVDYLTPGIGTKEN

PHRIVSVKTAASVAQLDRKKRVICESFGASGHGYSMRQRRLDANFMAFLGVNLFIPHA

FYYSFAGYRKTDFPPTEFKHAPHWPHYRAFADYIGRLSLLGASGIRTPEVLLLSPIHT

VYQNMFASGESNMHPSSDVLFSLLSDRMLRGSVDYDYVDECQLRDATLVDGEGYRFGG

NENVYSILIMPEIEVMSKDIAAQLVSFVKQGGTLIAVGTTPQHSEALRQDPELLKYMS

ELFSSDRRHGKLHAVGKGYSMFYSMNDSAHEGDQAGDRLEAFCALLCDLLREPRAIPY

KLIKGQSEDLIRVERKIGDHVYVWFMNWSDQPVTIKLDPRENKDYLEEWDLESGGVRR

LDCEAVLSFVPGELRVLSVRPQERPSFPQEQMNNGERTSTDVVVLDEKWQFKTADPNV

LLLDQWHVTLNDRQSRMNATMPGQVNTYRTTFMMTQELIEIVQSQSDSGHSDESGKEQ

SAKIELILDDVEQKIPSHIGFLSRRRNLEIFVNGIRQEALKPSSWQDAYYDSVDITPH

LLVGENELEILTVSLLEPMPAISFPAFLIGPFAIEDHTTLTSDVEHVVGCWGSAGYPY

YSGTGFYSQHVDLTDVSLTANDELWLVAEDIRETACLYVNDIEAGIRLWPPYQWNITP

YVQEGNNKINIHAANTLENLYGKSALPSGMNGQVRLVRRALG"

CDS 384425..386461

/locus_tag="PIKHDJKN_00372"

/inference="ab initio prediction:Prodigal:002006"

/codon_start=1

/transl_table=11

/product="hypothetical protein"

/translation="MIRKLAVLCITLVCTTVFSVSAFAASNDSKHVDSLKWPKNQALP

NFGKVEHLDVANVYEAQGDIKLLLTTLSGVVNREKPRIYVLQNKVDPWLKDLKVPYKQ

YDNAMDVFKKYAKEIKGIIVYDPNLPDSINVATTLAGLKDAVVASPELAQQLTAAPYN

LSVLEDLQGKFKNRMDAYNWQYENLWSQTTQRMLVGLSPDTSIPIPPGNFDSFQTIAV

EKEQVRDGSNRKTFEYDLSSYLGKEAVYLRFEDAFPQDGWGPAVHQVTVKADGQVISD

FKTGTSEEETFLYDRHNSKFLPGSDHRFVDHGNYFVYEFKPAANTKQLTVSIEMWNQY

KVSAGNIKPLSSEEKEPYGFLRDYAVANKAMVFWLSTSVPEEKALFEKILSDVKPGTP

YMGWFDNDFEGEVAGLELTSRHSVYVIPSDWFHNMTVFSGTKVKQEPTPKRVDPKLEN

KIYVTYTFGEGDNLQYDLNHMRNLWDDPARGKVPINWTSSPLLYDAAPAVLNYYRSTA

TKNDLLVAGPSGASYFNPHVWPKETFPEYLKQSYSYLKKTDMSYPYVLNRELGKDIPL

SESTAAAYEKNYKLKGLFLAGEDKEDIKIMNGSLPVSMMRGTSTVQDGKNVLTDAKKD

WDGKSPKFISIGINAWSMTPSDVLAITESLGAEFQVVKADEYFSLVREAHGLSK"

CDS 386661..387017

/locus_tag="PIKHDJKN_00373"

/inference="ab initio prediction:Prodigal:002006"

/codon_start=1

/transl_table=11

/product="hypothetical protein"

/translation="MNISSAASSTTSYISTSSSSSNSTSELEKQKTKLEAELEKVQSS

KDDEKTKETKTKQLQQQIKQIEAQISQQSSQSSGTTGTKEAPPAKPPTNGMQAASPQE

IANATTESNGRFDIRV"

CDS complement(387155..388510)

/gene="mepA_1"

/locus_tag="PIKHDJKN_00374"

/inference="ab initio prediction:Prodigal:002006"

/inference="similar to AA sequence:UniProtKB:Q2G140"

/codon_start=1

/transl_table=11

/product="Multidrug export protein MepA"

/db_xref="COG:COG0534"

/translation="MNTNWTHPLERQSVGKAFVSYLVPSILGMLVVAFNFIIDGIMVG

HKLGSTALAGIGIASPVYTLFVAMSLWIGMGGATLYSTYMGKKDLTTAKQIFTKSITI

ILLITMVIGYTAYTFRDKLVLALGANTETFPFAADYMNIMLLFGFVFTIENALTIFVR

NDGNPNTSMYAQITFAVANIVLNYITLYVLEWGVRGVAIGTIVSASLALLVLLTHFFR

KNNNLKFTRFKWNTKLLASIVLIGFPSFLAELGMSVFSISHNISLDRIAGTDGVASFT

VLNYIHGVVLLAFLGLASAAQPLVSYYHGAKQMDREKQTIRLASRTALACGVLLLLVV

QLGAPYFVQIFGNFSASVTDNAVYGLRIFTFAYLFMGINFVMSTYFQSVGNAKMAIWI

TAAREMIIMVALIAILPSFFGVTGVWLAVPLSEMVVLVTIGMYYKKRSISERINGLTI

E"

CDS 388673..389407

/gene="cmoB"

/locus_tag="PIKHDJKN_00375"

/EC_number="2.5.1.-"

/inference="ab initio prediction:Prodigal:002006"

/inference="protein motif:HAMAP:MF_01590"

/codon_start=1

/transl_table=11

/product="tRNA U34 carboxymethyltransferase"

/translation="MKQNKYDEAEFFGNYSQMARSTQGLEAAGEWHELQTMLPELKGK

NVLDLGCGFGWHCRYAREQQASSVVGVDLSENMLQRAREMTDDPHIEYQRMAIEDIDF

APGQFDVVISSLALHYIERLDQVYAKINACLVQGGKFVLSAEHPIFTARAEQDWHYGP

QGEILHWPVDHYQEEGQRVANFLNQDVVKYHRTMATHLNELIQAGFVIQQVAESKPSP

EMMEQVPGMSDESRRPMFLMIAAVKA"

CDS 389571..390149

/gene="sigW_1"

/locus_tag="PIKHDJKN_00376"

/inference="ab initio prediction:Prodigal:002006"

/inference="similar to AA sequence:UniProtKB:Q45585"

/codon_start=1

/transl_table=11

/product="ECF RNA polymerase sigma factor SigW"

/db_xref="COG:COG1595"

/translation="MDYQYLAHVQTIDNYTLSSMMDDYGNDVWNYAYFLTKSTEQADE

LSQEVFIRAYSGIAHFRGECSLKTWLLTITRNTTFTYRKSRFFRSSLWGETLPIHTEQ

VNTSMGEMKPEKLVHPSAEAEAISREHVHEIWDIIMALPDKFRELLLLHLKYELTTGE

IAEMLGISAGTVKSRLSRVKAKVRKQWEERSE"

CDS 390146..391402

/locus_tag="PIKHDJKN_00377"

/inference="ab initio prediction:Prodigal:002006"

/codon_start=1

/transl_table=11

/product="hypothetical protein"

/translation="MKHMDDEWEKVVRQEPFAESPFTEEHKQNVLRQVAWLKSGSGQK

MSDVGDKSTEQNTLPNQHRRRLRSHKAVMACTTIAAVAAAVILWNGQIVEPVIEKVYP

TAALQYTDDAGMSLLTDKMKKNVAVTMRDYLGKQLLITKVKDLPVSGRVSVEAGKEGD

AEYALVWLDAATGNLREVQMRREMSANELEHRYLRQIPSLLQGIGGDPALTPISANRY
VGMKQGELEPESYTTLSLENEESYGEIIWLQDAAISVSGDVQPDQVSQTALTYAEDSI
EAFTGNADLDLTSIHRTKDDENGRDIIIFSFENNYFVQTTEGTLGINYAVIDANSYQK
DYTTWEEVEAYHEKLLQVKESTLREKVEPLIKKIFKVDLQAYKLHRNTEAPGIVTFEL
DSTRDVFQIRYNEDARITMITRDANL"

CDS 391463..391921

/locus_tag="PIKHDJKN_00378"

/inference="ab initio prediction:Prodigal:002006"

/codon_start=1

/transl_table=11

/product="hypothetical protein"

/translation="MKYLVSSCLAGVACRYNGTASLDAKIQELVEQEQAKMVCPELLG
GFSTPREPAEIIGGTGKDVLAGTAKVIEKSGKDVTDLYIKGAYQTLEWAQKMDVSCVV
LKEFSPSCGTQMIYDGNFANHKVVGEGVTSALLRQEGFTVISENKFMEQL"

CDS 392190..392867

/gene="yfkO"

/locus_tag="PIKHDJKN_00379"

/EC_number="1.-.-.-"

/inference="ab initio prediction:Prodigal:002006"

/inference="similar to AA sequence:UniProtKB:O34475"

/codon_start=1

/transl_table=11

/product="Putative NAD(P)H nitroreductase YfkO"

/db_xref="COG:COG0778"

/translation="MESSAKAITKENILNAYYFRHATKQFDASRIISAEDFEFILETG
RLSPSSIGLEPWKFLIVQNPDLRKRLSEVSSGAQKQLAKASHFIVILARSDASYNSPY
AEYMLKEIKGMPDDVFELTREAYGKFQKNQRMLENPRTLFDWASKQTYIALGNMMTAA
ALIGIDSCPIEGFNYEGVHRILEEEGLLEDGLWDVSVMAAFGYRAEDPPREKSRQPLD
KVTQWLD"

CDS 393137..393448

/locus_tag="PIKHDJKN_00380"

/inference="ab initio prediction:Prodigal:002006"

/codon_start=1

/transl_table=11

/product="hypothetical protein"

/translation="MEPILIFKALSNETRRQILLWLKNPEQHFPPLELSQHPDAGKSG

ICVGTIQVKAGLAQSVISSYLLTMLKAGLLISERRGQWTYYRRNEETIRQFAEYVQHE

L"

CDS 393613..394647

/locus_tag="PIKHDJKN_00381"

/inference="ab initio prediction:Prodigal:002006"

/codon_start=1

/transl_table=11

/product="hypothetical protein"

/translation="MSTENTASSHKKHINDVKLSILDLAPVVVGATPADALRNSLDLA

QHAERWGYHRYWVAEHHNMPGIASSATSVVIGYLAGGTKTIRLGSGGIMLPNHAPLVI

AEQFGTLESLYPGRIDLGLGRAPGSDRRTSLALRKDLNSGEDFPELLAELRAYFDASA

TSYHAPVRAVPGEGLNIPIYLLGSSDFSARLAGQLGLPFAFASHFSPDYTRIALETYR

SSFQPSDHLKEPHVIVGVNAVVADTDEEAAWLGTTMQQQFLNIIRGTTGLVQPPADME

GKWTDREKAGVEQTLKAAVNGSPETVRGQLESFITQTQADELIITSMIYDHQARLHSY

ELIAQLAGKA"

CDS 394963..396123

/locus_tag="PIKHDJKN_00382"

/inference="ab initio prediction:Prodigal:002006"

/codon_start=1

/transl_table=11

/product="hypothetical protein"

/translation="MEQPEQPRIWPDRFKRFFLNNKFVLFLLILLLVGLNVMVLTKVS

FVLHPLAVLIKTIVLPIILSGILYYLLNPIVDVMEKWKIKRGWSILILYLAIGGILTV

VVLAVIPVVRNQIMGLIENFPTYSETVKHQFEELTGSQLFSQFQETVNLNSQDWWGTI

SQKATEILNSTWTKLGGFLGAFTETVLSIVTVPFILFYLLKDGKKLPSKILSFLPIKS

RTGAMHVLEDINHQISSFIRGQIIVSFCIGILLYIGYMIIGLDYALILAIIASFTSVV

PYLGPAIAITPALIVALVTSPLMLLKMVVVWTVVQLIEGKFISPQIMGKTLKIHPITI

IFVILTSGNLFGVVGILLAVPGYAVLKVCVSHIFNWFKERSGLYDPKNNNLL"

CDS complement(396264..396635)

/gene="ziaR"

/locus_tag="PIKHDJKN_00383"

/inference="ab initio prediction:Prodigal:002006"

/inference="similar to AA sequence:UniProtKB:Q55940"

/codon_start=1

/transl_table=11

/product="Transcriptional repressor SmtB"

/translation="MEQPVKAPAECDAACSGTEADVQTIRASLMDRETSSEMADWFKA

FSDPTRLRIIDALLQKELCVHDLTVLLDMGQSAISHQLRSLRNMRIVKRRKEGKTVYY

SLDDAHIEQIFLQTLQHIKHS"

CDS 396901..399270

/gene="kdpB_1"

/locus_tag="PIKHDJKN_00384"

/EC_number="7.2.2.6"

/inference="ab initio prediction:Prodigal:002006"

/inference="protein motif:HAMAP:MF_00285"

/codon_start=1

/transl_table=11

/product="Potassium-transporting ATPase ATP-binding

subunit"

/translation="MGTGQEQVKRELLLDGLDCANCALKIENGVKKIKGITDCSVNFV

TKTLSMHTTSDMDEQVVEEAKRKVLRLEPHIRISEKGKSSQGSRGAADSINHANRNVH

AHTDSGAHTHEHTPSGLDHAGHSHDHDHGHHHGAQAHTHDHGSHAGHDHGHADGHDHD

AHAGHSHEHGAGQTKVLLARLAAGSVLVAAAIWSPLEGWAQLALYVVAYLIAGGDIVL

QAFKNIIRGQVFDEYFLMSVATIGAFAIGQYPEGVAVMLFYQLGELFQGLAVNRSRKS

IQSLMDIRPDYANMLIGSGEETKRVSPEEVRIGDRILVKAGERVPLDGIVKAGRSMVD

TSALTGESVPRELEPGSDVLSGFVNKNGMLTIEVTKTFGESTVSKILDLVQNASSRKA

KTEHFISKFARYYTPVVVILAALIALVPPLMLSGATFADWIYRALVFLVISCPCALVV

SIPLGFFGGIGAASRNGILIKGSNYLEALNDVKVVVFDKTGTLTKGVFKVTAIRPEGG

RNEDELMELAAIAEAHSNHPIAESIRAAWAKDIRTQGVDDYDEVAGHGIKVSVDGREV

LAGNAKLMEQAGISYTTPQTVGTVVHVAESGSYVGHLIIADEVKDDAAAAIQALKKLG

IRKTVMLTGDAKAVGEAVGRELGVDEVYAELLPQHKVEQLERLEAAKSPKEKMVFVGD

GINDTPVLARADVGVAMGGLGSDAAIEAADVVIMTDEPSRLASAIRIAKRTRTIVWQN

IGFALGVKAIFLLLGVFGIATMWEAVFSDVGVTVLAVLNAMRVLRVKDI"

CDS 399371..399730

/locus_tag="PIKHDJKN_00385"

/inference="ab initio prediction:Prodigal:002006"

/codon_start=1

/transl_table=11

/product="hypothetical protein"

/translation="MAGAIVGLICGVCYFVLGLMVYKKPPKKINGIYGYRTPRAMSNP

ELWEEAQRYSANLMMQFGVIITVFGILGFWFTDVQALVLSLFAVVFYTVRLFTKVEGR

LKEMQRVQQQGQKKQGA"

CDS 399977..400417

/gene="yfmP"

/locus_tag="PIKHDJKN_00386"

/inference="ab initio prediction:Prodigal:002006"

/inference="similar to AA sequence:UniProtKB:O06474"

/codon_start=1

/transl_table=11

/product="HTH-type transcriptional regulator YfmP"

/db_xref="COG:COG0789"

/translation="MSLYKIDDVAKECGLTKRTIRYYEEIGVMPSPQRTDGGTRLYTR

EDIDYLKKVVRAKEVLGFSLQELHTYVATADALNEQRFDYQQTTEVRERIEKLTKMET

TLDDQLQLIEQKLQSIHAVQEELEELRERVQNGIRRLEGHENDT"

CDS 400562..401782

/gene="yfmO"

/locus_tag="PIKHDJKN_00387"

/inference="ab initio prediction:Prodigal:002006"

/inference="similar to AA sequence:UniProtKB:O06473"

/codon_start=1

/transl_table=11

/product="Multidrug efflux protein YfmO"

/translation="MKREPSLPDELPSSRGGLLSQPRAVWAVAFACIISFMGLGLVDP

ILPAIADQLHASKSQVSLLFTSYNAVTGVAMLITGVVSSRIGVKWTLLSGILLIIVFS

FLGGTSDTVGALVGYRGGWGLGNALFIATALSAIVGLSTSGTAKAIILYEAALGLGIA

VGPLLGGELGSISWRGPFFGVAVLMAIAFISITFMLPKMAKPKTRSSLSDPFKALGYP

SLKTLAITAFLYNFGFFTLMAYSPYVMNLDEHGLGYVFFGWGLMLAITSVFVAPRLQR

RFGSVPSMSVMLTLFALDLLVMAVGTVMGSSTTVIVAVIVAGIFLGINNTLITTAVME

AAPVERSVASAAYSFVRFLGGALAPWLAGKLSEWFLPETPFYFGALMVLIGVVVLLVR

RRHLRDIDAAITHH"

CDS 401802..402224

/locus_tag="PIKHDJKN_00388"

/inference="ab initio prediction:Prodigal:002006"

/inference="similar to AA sequence:UniProtKB:Q7A551"

/codon_start=1

/transl_table=11

/product="Putative universal stress protein"

/translation="MLKRILVAVDGSDHAHKALEQAVVLAESLKQTASLLIVHVNPSI

SLNEPALGVDLEARIAEEGQHIIQPVNELLAARSVHYETLLIAGDPVNEIGRVAKDRD

CDLIVMGTAGKSMLAEIVVGSVSHGVLKHAGCPVMTVK"

CDS 402315..403739

/gene="safD"

/locus_tag="PIKHDJKN_00389"

/EC_number="1.2.1.73"

/inference="ab initio prediction:Prodigal:002006"

/inference="similar to AA sequence:UniProtKB:Q2BN77"

/codon_start=1

/transl_table=11

/product="Sulfoacetaldehyde dehydrogenase"

/db_xref="COG:COG1012"

/translation="MKKQHLFIGGKPTESVDYIALQAPYSKETLAEVSSASAEEVEAA

IVAAVQAGKEMRRMPAYKRADVLYKLSTLLEERKEEAARIIALEAAKPITAALAEVDR

TVETYRFAAEEAKRLTGETVPMDAAKGGEGRVGYTMRQPLGVIGAITPFNFPMNLVAH

KVGPALAAGNTIVLKPAEQTPLSSYYIANLLQEAGLPDGALNVVSGDGKTIGDVLVEH

PDVAHITFTGSPAVGTSIRSKAGLKRVTLELGSNAAVIIDADANLDNVVPRCVTGAFT
YQGQVCISLQRIYVHSAIADEFIRRFAEAAQKVVVGDPLNPDTVVSALITSKDVQRTL
DWIDEAKQAGAKVAAGGEAEGGVLRPTVLVNVPRDAKVSCQEVFAPIVVINTVDSVEE
GIEHVNDSIYGLQAGVFTNDIHTALHAADHIEAGGVMINDIPTFRVDHMPYGGVKQSG
MGREGVKYAVEEMTELKFVMFNKN"

CDS 404110..404925

/gene="mta_1"

/locus_tag="PIKHDJKN_00390"

/inference="ab initio prediction:Prodigal:002006"

/inference="similar to AA sequence:UniProtKB:P71039"

/codon_start=1

/transl_table=11

/product="HTH-type transcriptional activator mta"

/db_xref="COG:COG0789"

/translation="MAYSMVDVSGMSGVSLSELGQYAETGLLNPAFVGQDEGIYYEKP
ELLRLQQILFCKEVGMEEEEIGPMLRDTPRDMIRIMQQHRIEMLEKALHLHGMIQTLD
KTISYLRGEQELDEYKLYAGFSNKGRHQLLNETTSDHAAKNDYYEQLQTEASQKYSAN
TPESSTLPEGQEMKSKEDFLDSQAKIDRIHLDLQGAIEDGLEPASSEVQQIIGRHLDW
IKDYYTPTAEIYRDLGNLYVEHKNFRQMYDGYHPKLAEFLRDGMMIKAEQDLS"

CDS complement(405081..405569)

/gene="msrC"

/locus_tag="PIKHDJKN_00391"

/EC_number="1.8.4.14"

/inference="ab initio prediction:Prodigal:002006"

/inference="similar to AA sequence:UniProtKB:P76270"

/codon_start=1

/transl_table=11

/product="Free methionine-R-sulfoxide reductase"

/db_xref="COG:COG1956"

/translation="MFQAVSYEGTRSEQQTAVLGQLSALIRDEPSDIANLANAAALLN
VFMTDTNWVGFYLYDGKELVLGPFQGLPACIRIPLGRGVCGTSAAERRTMVVEDVHAF
PGHIACDAASNSEIVVPIIKNGELYGVLDIDSPLKNRFDDEDRVFLEKAVSLITEQLE

LS"

CDS 405756..406052

/locus_tag="PIKHDJKN_00392"

/inference="ab initio prediction:Prodigal:002006"

/codon_start=1

/transl_table=11

/product="hypothetical protein"

/translation="MHAEVQNLFVRIHLLYLAQKQDLTVSDALPLLEERGYRVGEREI

KQELEHLTQENFLTAHGDQWSLTGTGIEEFKEIIAVFGRVSEELLGTGKKTSTA"

CDS 406248..406820

/locus_tag="PIKHDJKN_00393"

/inference="ab initio prediction:Prodigal:002006"

/inference="similar to AA sequence:ISfinder:ISSpgl1"

/codon_start=1

/transl_table=11

/product="IS1595 family transposase ISSpgl1"

/translation="MYKCKGRIPELETERLRLRKMRRRDAAQMFACWSDREVTRYMNL

APMIGTSEAADMIGLLNQMAGEEEAIRWGIELKETGRLIGSCGYNTWQLEGAFRGEIG

YELGRDYWRHGYMTEVFSVMLPFGYETMGLNRIEALVDPRNLASGEFLTNRGFTREGL

LRQVQHTSTGYKDMVMYSLLYDEFLRKRDK"

CDS 407043..408569

/gene="bmr3_1"

/locus_tag="PIKHDJKN_00394"

/inference="ab initio prediction:Prodigal:002006"

/inference="similar to AA sequence:UniProtKB:P96712"

/codon_start=1

/transl_table=11

/product="Multidrug resistance protein 3"

/translation="MNTTGGLNRSFILAGLLLATFLSAIEGTVIGPAGPTIVSELGSV

QLLSWIFTAYLLTMAVSTPIFGKISDLYGRKPVFLIGCALFLLGSLLCCLSQNMEQLI

IFRAIQGIGAGAVVPVTFTIIGDIYRLEERGKIQGWISSVWGISSLAGPLLGGYFVDN

LGWQWIFGFNLPFGLLAMWFVFRYLKEEMSPRTAKIDYMGALTFTVGITALLFVLSAG

GQYYAWSSPIIVGLSIVAILFMILFFVVEKRAQAPMVPLHLFRIRDIRVANIAGLLTS

TLMIGLTSYLPLWVQGVRGGNATESGLLLAPMSVGWLIGSVLAGRLLMKIGSRLTAVI

GLTGIAIGSGGLFLVGGTSPQAVLFILTFIYGLGFGFAFTIFTIISQSSVGYKERGSS

TALHTFMRTLGQTIGAAAFGTWLNYRISTLSSEQHLAEAGISDGDLNQLLAPHTEAAL

SDDKWALLRNVLEGSLHSLFVIMFVIAIISWVTTLALRKRLIVPENADAPPQAQASGN

"

CDS 408689..409174

/locus_tag="PIKHDJKN_00395"

/inference="ab initio prediction:Prodigal:002006"

/codon_start=1

/transl_table=11

/product="hypothetical protein"

/translation="MSKQSLQSFWLIPFPMKLLAANLTCISLTLVVGLMKYVPLAERA

EHTYYYPFWYTLIISILINLALLLFLILPLSLIADSVLASLKMKGPVTKVICTILAYF

LLGVGSALLFSIFVYKMDAFQYAGPYQYVVITSFVFLLWQSGLGWILNRVRKQEQTNT

G"

CDS 409206..409391

/locus_tag="PIKHDJKN_00396"

/inference="ab initio prediction:Prodigal:002006"

/codon_start=1

/transl_table=11

/product="hypothetical protein"

/translation="MMFPNNLNSSKEDEAKSKYEETQREIEGHSRSGLSSYIVDLLIA

GGLVVAALTVLMWILYL"

CDS complement(409472..409933)

/gene="mhqR_1"

/locus_tag="PIKHDJKN_00397"

/inference="ab initio prediction:Prodigal:002006"

/inference="similar to AA sequence:UniProtKB:O31672"

/codon_start=1

/transl_table=11

/product="HTH-type transcriptional regulator MhqR"

/db_xref="COG:COG1846"

/translation="MLELQEIGTERSLHLYRTLAQTFKSVNEHAVSGSKVHGFNPTAY

GVLEVLYMKGAQPIQQVGAQLLLQSGNVTYVIDKLEQKGLLHRKHCPQDRRIIFVELT

EEGQRTMDDIYPGYAHKIDRAVSGLSEDDKTLLSELLERLALGADRLSANG"

CDS 410440..411771

/gene="mdtG_1"

/locus_tag="PIKHDJKN_00398"

/inference="ab initio prediction:Prodigal:002006"

/inference="protein motif:HAMAP:MF_01528"

/codon_start=1

/transl_table=11

/product="Multidrug resistance protein MdtG"

/translation="MTSGLTMQERRPLKHNRSFITLMAAQAISNLGDWLHLLAILTLV

GIRWNATPWEITFTTLCAALPVLLTGPFAGALADRANRKWLMIGADGARIVIVGGLIF

ADQIWHVYVLLILKSLFDVVFSPAKNGKLKEIVPQEQLGQAVSISSVIEQMSKIIGPA

LGGLLVAAFGITWCFVLDSASFLISGIILLWIPGARVIQSVNQNVEQVREETAGVAPA

GKKATFMKDTMEGIRMLASLPHVGTSLVLLASALLFLQFADSQTVVLFRQLPGISSDL

LGWCVAASGVGTLIAVMSVQKWKKAGHVIKMGLGTSLMGLVIGGVGMIVGVWPHAGLG

ANLLLISLFALAGVGVGFAIVPFQILLQEQTPESMTGRVFGTVGSIMTASNIMGPVVG

GFLVTSFGVIPAFISSGILLTLLGVTYVVIRRQKKKDMDGSLAANSMITGK"

CDS 411987..412778

/locus_tag="PIKHDJKN_00399"

/inference="ab initio prediction:Prodigal:002006"

/codon_start=1

/transl_table=11

/product="hypothetical protein"

/translation="MNVKLPKSIVRLGSIPLAILAWTLIIGLGSSHVYADDSQASSAN

KSSGLSLNLFSRDSDGGLNLTPSVSVSTPLLDVEVPSIKADKLTGKLSVSELKVDTPL

GSAGTSEIDIDANKGTVELPSVKADTPVLKADVSSSEVNLNEGTASLPGVTAEVPEVI

KAETSAVKTDLRQGKVELPSVKVDVPEVTWVNVSSSRVDLTKGKVQLPSVEANIPVVD

VSAGNPDKGTVEIPSLKPEKPTVTPEQRSVVNTSNVEKPTIGKTE"

CDS 412824..413351

/locus_tag="PIKHDJKN_00400"

/inference="ab initio prediction:Prodigal:002006"

/codon_start=1

/transl_table=11

/product="hypothetical protein"

/translation="MHVTGNVIDVTWQSEVRPQQPATPQAVPVEESNLEQDSSSNAVL

PDEEQFLVDQSLVKPAVIDPVQNNKGVNLEDDSNKASPLQPRTERSTDWSVTATSPGA

ANAAAGGSSGSSGVTGGGSTAPPAALPGATSGLATPDYGFAFRMERLDGFSQWSQAPP

GRPPQYISFSQTQWR"

CDS 413385..414428

/locus_tag="PIKHDJKN_00401"

/inference="ab initio prediction:Prodigal:002006"

/codon_start=1

/transl_table=11

/product="hypothetical protein"

/translation="MKKVNRWVKLSVLSGTLVAGLLGASQATYADSYSNNSSGNLDLN

AGLRLELGSLLSGHRDAGYGNNGSYGGSSSASGALNLDLGLNAGLSTESMNRYNDRSS

DHTAERSSSGNLGLGLNANVSGESTTMSDYSRSGEQRNVNSTSESESRGALDLGLNAK

AEGESATWAQTERNNSVNDRGRNDSSSYASESRGALDLGLNAKAEGESATVAQSERNS

SVKDRERNDSSSYASESRGALDLGLNAKAEGESATRTQTESNNNVNDRDRNDSSSYAS

ESHGALDLGLNAKAEGESATWAQTERNNSVNDRDRNESSSSESESRGALDLGLNVNAD

AESETSTVVESNR"

CDS complement(414503..414895)

/locus_tag="PIKHDJKN_00402"

/inference="ab initio prediction:Prodigal:002006"

/codon_start=1

/transl_table=11

/product="hypothetical protein"

/translation="MIIFLSVEEVVAAHYFMMKRMNDTAQAGVKNYSLLDSAVNRPQQ

TLFGEDAYPSIFDKASALLESLVKNHCFHNGNKRTAYLVTKSFLILNGYHLQMERQYA

VEFMVNIAEGKHSFESIANILKYHSVVR"

CDS complement(414898..415191)

/locus_tag="PIKHDJKN_00403"

/inference="ab initio prediction:Prodigal:002006"

/codon_start=1

/transl_table=11

/product="hypothetical protein"

/translation="MNGGAQMKEDNFKKKILSRKIGKMGNSLGVSLPKVLIDKLDVSQ

GDEIEFIENSQGEIILKKVRHLKIPENVRPEVLEAFFDVFEEDEGILQDLRDR"

CDS 415398..416129

/gene="ecsA_1"

/locus_tag="PIKHDJKN_00404"

/inference="ab initio prediction:Prodigal:002006"

/inference="similar to AA sequence:UniProtKB:P55339"

/codon_start=1

/transl_table=11

/product="ABC-type transporter ATP-binding protein EcsA"

/db_xref="COG:COG1131"

/translation="MEIENKDTAAQESILDVHITEAGYEVGRSTIRNIRLHVAPGELV

GIIGPNGAGKSTTIKTLLGLLEHAKYEVTIGGDGRYAYIPEQPVFYEYMTLWEHLDLA

AAAYEMEEEVFVARAEEMLVRFGMDHVRNDLPASFSKGMRQKMMLMIGFLSSPDVYIV

DEPFIGLDPRATKDFLKLLDDERRRGAGVLMSTHVLDTAERICNRFILIHSGRSAAEG

TLDEIRAAADLPDASLFDCFDTLTS"

CDS 416149..417399

/locus_tag="PIKHDJKN_00405"

/inference="ab initio prediction:Prodigal:002006"

/codon_start=1

/transl_table=11

/product="hypothetical protein"

/translation="METSRAYTPLSLYRRRRKEHFNEQLKNLKLVVDWTVWVYLLIPG

LLYLSGWYVSLWSKPLPSWATGLTLPTLTGLIDIIMLTGGILIFVEEADVLFLKSRSL

WMRTLMKQGIYRACLQHLGKMIAITALTSPLWSRVYDMSFIHIALIAIWFGAVASFQV

IVLHMTKVRYTGWRRWLRMFPLAIAMGIITIGLTSWMHGHIWTLLAGIGAAILLLIVV

GRMRLEMKGTFEGDVREDLRERLKFTALLLSRSVTKPKAPRTRTIIFRKQRKLLRHRT

ISNRTAETAFKAFFRNSSTMKLYLQLGGLSIAAVALPPFPVNIIVCGLLVIMLSVLFY

RSWEVFATSEYVQLVSYDSETLNRAGVTMVRMLFVPLGILMGFSLGAAWLGWLEGIVT

AAAVVAFGLFVLSITGWIRYIRSA"

CDS 417514..417897

/locus_tag="PIKHDJKN_00406"

/inference="ab initio prediction:Prodigal:002006"

/codon_start=1

/transl_table=11

/product="hypothetical protein"

/translation="MLDDSELQVHFADNAALFAAQATLEELGYKPYQSGPLELYIPTD

RQDTLSAVEIVQSHGGSAVFLSETDGLDQFQNISIPAHLVNEDWNEGYASGIQGGNPE

QEMSNRDEDPTFDDSVDGFSGSVKA"

CDS complement(418071..419339)

/locus_tag="PIKHDJKN_00407"

/inference="ab initio prediction:Prodigal:002006"

/inference="similar to AA sequence:UniProtKB:P44941"

/codon_start=1

/transl_table=11

/product="hypothetical protein"

/db_xref="COG:COG2081"

/translation="MYDVIVIGGGSAGLMACVAAAEHGASVLLLDKGDQLGRKLGISG

GGRCNVTNAKETDELIRHIPGNGRFLYSSFQNLDNQGIMRFFENLGIALKEEDNGRMF

PVTDKAKTVVDALVGKIVSLGVEIRTKQPVKELIQNGQHVQGVKLASGTTVLARSVII

ATGGKSVPQTGSTGDGYPWAEAAGHTITELYPTEVPIVSGESWIQSKELQGLSLRDVG

LSVLDAKGKSIISHRGDMIFTHFGVSGPIALRCSQFIRKVQMKSGNPQVTMSIDLFPD

LSAGALEAQVQQVLEQESRKAVKNILKTWVPERMIPLMMKRAEISEDLTFHHFPKGML

NTLVGLMKDFTFRVDGTRSLKEAFVTGGGIHLKEIYPKTMESKLLPGLFFCGEVLDIH

GYTGGYNITAAFSTGYTAGMHAAEYKNARS"

CDS 419536..421230

/gene="sasA_4"

/locus_tag="PIKHDJKN_00408"

/EC_number="2.7.-.-"

/inference="ab initio prediction:Prodigal:002006"

/inference="protein motif:HAMAP:MF_01837"

/codon_start=1

/transl_table=11

/product="Adaptive-response sensory-kinase SasA"

/translation="MVALKDILLQILLAGAAVFLIPLLHLGLSRQAVAKAGYHRLVQP

SFAATSVVSMLLCLLFAHYGSPVSVPISLGIVPLTLVILYCRPRIGLMLSLLQILFYF

LFAHPYNLYGFLLQTGILLYPIVWLSAKRFKHYTRSRKMGTVIALITAELVVSSVLYI

PSMESQPTFSIVQWVLLTLGYTIGAVLAGSLSMLWIEQMQHHRGLEQHLSEVHHRYIA

ETEKLNQILNAVPLSIATVDKEGTVQFVNDTMEQTARDQLPCTTTPDLIGQPASQFVE

QAQADKMESSIRKAIVHGEVSGLTVRYGSNVFQSRTVPIYAYSAELPREAIGAMLIIQ

DITELEMLRSELDNVDRLSLVGQMAASITHEVRNPMAVVRGFLQLMQEKSPESLDHYY

RIVLEELDRANSIINDFLSLAQNRIAEKEESQLHDIIHELSPLLWADANLRGQSIEFM

LAHNVPKLHLNSKEIKQVVLNLARNGMEAMNEKGVLTLETRLVDDTVELCVRDTGPGI

PRVKQEKLFEPFFTTKAKGTGLGLSMCLSIVERHNGTITIESEEGQGTTFKVAFQR"

CDS 421399..421833

/locus_tag="PIKHDJKN_00409"

/inference="ab initio prediction:Prodigal:002006"

/inference="similar to AA sequence:UniProtKB:P54170"

/note="UPF0403 protein YphP"

/codon_start=1

/transl_table=11

/product="hypothetical protein"

/translation="MSMSFDQYMKDMVQPMRDDLTRLGIQELRTPEEVEASLPDAKGT

ALVVINSVCGCAAGQCRPGVSQALQHDITPDHLYTVFAGQDKEATAKAREYFAPYPPS

SPSIALMKDGELVHFIERHQVEDRSAEEIAAELTSVFDRYCR"

CDS 421971..422780

/gene="nadE"

/locus_tag="PIKHDJKN_00410"

/EC_number="6.3.1.5"

/inference="ab initio prediction:Prodigal:002006"

/inference="similar to AA sequence:UniProtKB:Q5DZX4"

/codon_start=1

/transl_table=11

/product="NH(3)-dependent NAD(+) synthetase"

/db_xref="COG:COG0171"

/translation="MSLQQQIIAELKVKPSINEEEEVRKRVDFLKTYVKNANSKGLLI

AISGGIDSAVATALCKIATDELTAEENKEYKTLGVFQPYGEQSDIDHSYAVAKAYDLK

HVVETNIEDAVNEIALEVEQGFKSMGSPRHMTHQGKGNVKARTRMVMQYALSFEENLL

VVGTDHASEAITGFYTKWGDGAVDITPLSSLNKRQVRQLAAYLNVPQAILDKAPSAGL

WEGQTDEDELGISYEANSDYLEGKQIDPAAQERLEAFYTRTHHKRNAIPGI"

CDS complement(422880..423695)

/locus_tag="PIKHDJKN_00411"

/inference="ab initio prediction:Prodigal:002006"

/codon_start=1

/transl_table=11

/product="hypothetical protein"

/translation="MSTAFELPAGGDAVIRGNIFTARQPAQGIIVLAHGYKGFKDWGM

FPYAASQLSENHHVLTFNFSHNGVGEDLEQFSELEKFAVNTYSRELADLATLLDYVST

QPELNGLPLFLLGHSRGAGVSLVYALDHPEQIAGVLSWNGVTNLDLFTPEQKEEMRTQ

GRSHVTNGRTGQQMPLDVSILEDLDKHKDRYAIIDRLASSPLRVALIQGTEDGKRLRD

GSAALVQARPDIPWHQIPGGNHTFNTVHPFKESTPQLEQAIAETLRQIATWIN"

CDS 423924..424301

/gene="acpS"

/locus_tag="PIKHDJKN_00412"

/EC_number="2.7.8.7"

/inference="ab initio prediction:Prodigal:002006"

/inference="similar to AA sequence:UniProtKB:Q81JG3"

/codon_start=1

/transl_table=11

/product="Holo-[acyl-carrier-protein] synthase"

/db_xref="COG:COG0736"

/translation="MRKLLSGSHAEAFMKRILTHAEREIADHRGKRMVEFVSGRFAAK

EAVSKAFGCGIGSMMSFTDIEVLPDAKGRPVATLSDEAWERLQLPYGKQYDIHLSITH

QTELAAAFAIVEQMGKTLPGSEQ"

CDS 424357..425688

/gene="nth_1"

/locus_tag="PIKHDJKN_00413"

/EC_number="4.2.99.18"

/inference="ab initio prediction:Prodigal:002006"

/inference="protein motif:HAMAP:MF_00942"

/codon_start=1

/transl_table=11

/product="Endonuclease III"

/translation="MGLLEQKRHFSVNLLDWYMVNRRDLPWRRHNNPYFTWVSEIMLQ

QTRVDTVIPYFNRFIGNFPTVQALAEAPEEEVLKNWEGLGYYSRARNLQAAARQVMEL

HGGEMPQDKQAVFALKGVGPYTAGAILSIAFNQPQPAVDGNVMRVLSRYFLIDEDIMK

GSTRVLMEELAGELIPEGRARDFNQALMELGALVCTPKAPHCLTCPVMEQCSGRIAGR

ELTLPVKTKAKPPRPEQRLVAIVEGRGAHRGRVLVRQRPATGLLARMWELPHVLAAPA

AAGKAAGPLADEPAMALLAGSLWAEGFAARPEGLATHAEHVFSHIVWSLQVYKCTEQD

QSNELPLIAAEARAAYDAQAAAKEATALAQASEPGNIDAANISGSLDEQGEPLSAFAA

SSPAHDVMSGKGDGLTYRWIGPEDMDKMAFPNIFLKLISSYFAGAYDDVNE"

CDS complement(425871..426482)

/gene="sodA"

/locus_tag="PIKHDJKN_00414"

/EC_number="1.15.1.1"

/inference="ab initio prediction:Prodigal:002006"

/inference="similar to AA sequence:UniProtKB:P00449"

/codon_start=1

/transl_table=11

/product="Superoxide dismutase [Mn]"

/translation="MTFQLPALPYANDALEPHIDAQTMEIHHDRHHNTYVTNLNAALE

SAPELQGKSLEDLIANLDSVPEGIRTAVRNNGGGHANHSLFWEIIGPNGGGAPTGDIA

AAIDSELGGFDKFKEDFAKAATTRFGSGWAWLVVGKDGKLSITSTPNQDSPLFEGLTP

VLGLDVWEHAYYLKYQNKRPDYIGAFWNVINWDEVNKRYASAK"

CDS 427021..427446

/gene="ypeA"

/locus_tag="PIKHDJKN_00415"

/EC_number="2.3.1.-"

/inference="ab initio prediction:Prodigal:002006"

/inference="similar to AA sequence:UniProtKB:P76539"

/codon_start=1

/transl_table=11

/product="Acetyltransferase YpeA"

/db_xref="COG:COG0456"

/translation="MHVRSFQLSDASQMTELLQVALSEECYENTMGPFARQLSWDSDL

IMVAEEEGDLVGALIGTIDHNQGCIYRIAVHPDYRRRGVGKKLVEAMEQRFQQRKVSQ

IWVAGDEHNKVAMPLYEAMGYGANQIMSAFQKLSILSKA"

CDS 427867..428472

/gene="sipP"

/locus_tag="PIKHDJKN_00416"

/EC_number="3.4.21.89"

/inference="ab initio prediction:Prodigal:002006"

/inference="similar to AA sequence:UniProtKB:P37943"

/codon_start=1

/transl_table=11

/product="Signal peptidase I P"

/translation="MNSNNLSTEHVASKERNGPGVPDQPGKSWGAELWDWVKTIVIAF

VIMMLLNLFVFNLSMVKGQSMQPTLVERDRLFVNKIVYDFGTPSRSDVIVLRDPSEGV

EKKDFLVKRIVGLPGDTVEVRNHHLFVNGEQQAETYTDTEVQDPDFGPITLKADHYFV

MGDNRHEGKSKDSRVFGSIISSEIVGRAEFIFWPFSEIKKL"

CDS 428651..430540

/gene="queG"

/locus_tag="PIKHDJKN_00417"

/EC_number="1.17.99.6"

/inference="ab initio prediction:Prodigal:002006"

/inference="protein motif:HAMAP:MF_00916"

/codon_start=1

/transl_table=11

/product="Epoxyqueuosine reductase"

/translation="MTSVQTGAAKAAAVWEKLKQEIKAAAPEMGIDDIGFASAEPFVT

LKSILEQHRAKGFESGFEEPDIEKRVRPELKDGEPASLIAIAVAYPSKMVNPPKSEPG

AYRGILARSAWGQDYHQVLRAAMDKLVHFIRERVPEAMIESMVDTGALVDRAVSQRAG

IGFSAKNCAIISPKFGSWIFLGELVTNIPFQPDNPVTEDCGECTKCIDACPTGALVGP

GQLNAQRCISFVTQTKGFVDEEFMLKIGNRLYGCDTCQIVCPKNRGKNWDHHPEFHPD

PEIVKPLLIPLLDIGNREFKERFGQSSAAWRGKKPIQRNAVIALGNFKDKTAVPKLTE

VLKRDPRPELRGTAAWALSRIGGEDAMRAIGEAAANEQDGNVLSMLQKAKERLSSSKT

LPDKPQAEQTSNNKPEDQKEQNNPINQLDVQQPNQKTEEASEQESGQTTEPAKPEAAA

WRPSAVTGLHGTPVYYDEVLTPIGTLTLCATDKGLCHIDFGAFHVREAHLQQWARTWI

GEYRYEKNEEKLSEAAKQVHQYFAGERKAFDLKLDLFGTPFQLQVWQVLSDISYGEAS

THQQVAEIIGRPKAVRAVLDAISKNPIPIIIPCHRISGKDGTLVGYVGGLQTKEQLLT

LEQQS"

CDS 430556..431587

/locus_tag="PIKHDJKN_00418"

/inference="ab initio prediction:Prodigal:002006"

/codon_start=1

/transl_table=11

/product="hypothetical protein"

/translation="MKYVNVESVEAGEFLGKTIYSGNGTVMLSAGVQLTVFMVNTLKR

IGVTMLYIQDEAYKDVDTEDILDEATKKAVINEMSVTLESIRSGKDWSPKKVAISIDK

LLNDVLNGRELLVQLTDIRTKDNAQYVHAMNVCLLSSVIGLNLGLNYVQLKDLAVGAL

LHDIGKVGEPPGSGSAANSSLHHTWRGFELIKSKREFNLLVAHTALQHHEHVDGTGLP

RGVKGKDIHLFARIVSAANTYDNLINGLSGRSMLPHEACEEMMAMSGTKLDCDILIEF

NRSVSVYPNGTAVRLSTKETGVIVRQHRGLPGRPVIRVARGSTRYDLDVIEVDLAQHT

TVFIEAVLM"

CDS 431811..432074

/locus_tag="PIKHDJKN_00419"

/inference="ab initio prediction:Prodigal:002006"

/codon_start=1

/transl_table=11

/product="hypothetical protein"

/translation="MEIAIPIITLIVGLVGGFFIGVYYLRKQLEKMQSDPDMLQKMAK

QMGYNLNGKQMQRAQQMMKNQQPGAKMPQPQQHPARKNSGRRK"

CDS 432154..432747

/gene="folE"

/locus_tag="PIKHDJKN_00420"

/EC_number="3.5.4.16"

/inference="ab initio prediction:Prodigal:002006"

/inference="similar to AA sequence:UniProtKB:P19465"

/codon_start=1

/transl_table=11

/product="GTP cyclohydrolase 1"

/db_xref="COG:COG0302"

/translation="MAGVKDYLNSKVSDNREKIEYHIEQILQLIGEDSTREGLLETPA

RVTRMYEEIFGGYEVDPRDVLGVTFDENHEELVIVKDIVYYSQCEHHMAPFFGKVHIG

YVPSGKIVGLSKMARLVEAVTRRLQVQERITSQIADILTEAVEPNGVMVVVEGEHLCM

CSRGVKKPGSKTVTSAVRGSFRDNPAQRAEFLSLMKD"

CDS 433005..433859

/locus_tag="PIKHDJKN_00421"

/inference="ab initio prediction:Prodigal:002006"

/codon_start=1

/transl_table=11

/product="hypothetical protein"

/translation="MSVPSNSDQPGSPGAEQQMQLDHSLRLQIWETPLLRHGSSVLEA

FAWEEVMCRRVGEGQLPVAHIWRHPDAFVAGLRDRRLPQAVEAMERIRSQGTAVCVRP

SGGAAVPLNPGVVNVSLILPNPGRAINIHDDFREMASLIAESLIPWSSQARTGEIEGA

FCPGDYDVSVGGLKFCGIAQRRQAKAYIITAFIIVEGQGDQLAADVRKFYQDASDGAN

EGYPDVRPGTMASLQELAGVPSAAAYTASLVRTLRQRFPLAETSRVLTVDREAVKLTA

EQMKLRYD"

CDS 433930..434154

/locus_tag="PIKHDJKN_00422"

/inference="ab initio prediction:Prodigal:002006"

/codon_start=1

/transl_table=11

/product="hypothetical protein"

/translation="METLRYTYLYEVISTGEKSEFSQMATSKEEAAALIVARIADLEF

TGEEDIKLGDLIAISKQVGDNYVACEGCAS"

CDS complement(434294..435616)

/locus_tag="PIKHDJKN_00423"

/inference="ab initio prediction:Prodigal:002006"

/codon_start=1

/transl_table=11

/product="hypothetical protein"

/translation="MNWYGYGHLLPLRVLEEVRFWKEQEKEHTIVIRALVPELEPSYV

KWLAEWEAIFENSERVANQLLKQILPGTQPPAPYIIRCIEQLVAAACSQSREFIRQLY

VLLEQSTAVQNGPIAKVVILHFIRESEYFLGVLETLKQSGALRDLVSDPSFSLEHILQ

TSGQPANPNVLIASTPVSEQPYREAVEESSNTAKKINNPTPAVQAQSSAVVQSPNANA

EGTTNSSAAPVKEKPVPIGGHTLPPLPYAYNALEPHIDEMTMRIHHDKHHQSYVDGLN

TAEKKLAESRKKNNFELVKHWERELAFNGAGHYLHTIFWTIMNPKGGGKPSGMLAEQI

KRDFGSYEAFKNQFTEAANKVEGSGWAMLVWSPRAHRLEILQAEKHQNLSQSDIVPLL

PLDVWEHAYYLKHQNERKKYIEDWWKVVYWPAVAERYETARKLLWPPY"

CDS complement(435814..436842)

/gene="menH_2"

/locus_tag="PIKHDJKN_00424"

/EC_number="4.2.99.20"

/inference="ab initio prediction:Prodigal:002006"

/inference="protein motif:HAMAP:MF_01660"

/codon_start=1

/transl_table=11

/product="2-succinyl-6-hydroxy-2,

4-cyclohexadiene-1-carboxylate synthase"

/translation="MYPTSQSEAPLQTKVSDLPSSLSPRLIRFKHIVVALLLSVIFFL

LFCFIALHGYIAWVLSNPTVAPVFSNPMQAKNMKYQDITFPAADGSRTMQGWYIPADD

NANKTIIFSHGYGANREETWVPMYDLAHYAHQLGFNVVMFDYGFASQVNKAVATGGKA

ESQQLLGAIQFAKQRGAQELVVWGFSMGAGTALQTGLITKDVDAMILDSTFLLEPDTL
YHNIHNQIDLPRQPTLEIMNLLFPILNGTGLQQIPYQEVKKEDYPFPIFFIHGTEDEK
APYPIAELLAGNQTNPNSDEWIVDGAHHELIFREHPKEYLRRVSTFLSHVTKTSSDDV
ENTNTGES"

CDS 437055..437219

/locus_tag="PIKHDJKN_00425"

/inference="ab initio prediction:Prodigal:002006"

/codon_start=1

/transl_table=11

/product="hypothetical protein"

/translation="MQGILIYKKKIFTNIPIPNGAFWFTDETVSHIIYVTKFHVMKHE
EGNLPWKTES"

CDS 437201..437944

/gene="kipR"

/locus_tag="PIKHDJKN_00426"

/inference="ab initio prediction:Prodigal:002006"

/inference="similar to AA sequence:UniProtKB:P42968"

/codon_start=1

/transl_table=11

/product="HTH-type transcriptional regulator KipR"

/db_xref="COG:COG1414"

/translation="MEDRKLTVRAVERALDILLCFTTRSDLGLTEIASQIGLHKSTVH
RLMATLEDKGFVIRDAATEKYRLGIRIWELSAHMSRSDDPAILLLPAMERLRDRLGET
VSLYLRDGSERIRIQAVQSDQAIRRVAPVGVRLPLSVGASSKVLMAFATEEDREDLMN
GPEWPVFIDPKVYLAQMKDILEYGYATSYEEREPGAAAVSVPIMDRKGNIAAALSVSG
PVSRLSQETLHEYAPVLKEAATQMGLMLS"

CDS 438522..440327

/gene="thiC"

/locus_tag="PIKHDJKN_00427"

/EC_number="4.1.99.17"

/inference="ab initio prediction:Prodigal:002006"

/inference="similar to AA sequence:UniProtKB:P45740"

/codon_start=1

/transl_table=11

/product="Phosphomethylpyrimidine synthase"

/db_xref="COG:COG0422"

/translation="MSTGNRTMGQDKQDQQLAEEKGTTGRVQPFPGSRKVYIHGSRPD

IAVPEREIALHDTNTPQGVEHNEPLRVYDTSGPMTDPGFHADIRAGLPALRTRWITER

EDVEAYQGRTVKPEDNGLKAGGKRAGAEEYPGLRGKPLRGQAGRSVTQMHYARQGMIT

PEMEFAAIREGVEPEFVRQELASGRAILPSNINHPESEPMLIGRHFHVKINANIGNSA

VSSSIEEEVEKMTWAVRWGSDTVMDLSTGKDIHTTREWIIRNSPVPIGTVPLYQALEK

VNGEAEALTWELYRDTLIEQAEQGVDYFTIHAGVLLRYIPLTAKRMTGIVSRGGSIMA

AWCLAHHQENFLYTHFEEICEIMKRYDVAFSLGDGLRPGSIYDANDEAQMAELATLGE

LTQIAWKHDVQVMIEGPGHVPMHKIKENVDLQMEICKEAPFYTLGPLTTDIAPGYDHI

TSAIGAAMIGWFGTSMLCYVTPKEHLGLPNKDDVREGVIAYKIAAHAADLAKGHPRAQ

RRDDALSKARFEFRWRDQFNLSLDPERALSYHDETLPAEGAKEAHFCSMCGPKFCSMR

ITQDIRAFAADKGLSENEAVAAGMQEKAEEYRTRP"

CDS 440548..440850

/locus_tag="PIKHDJKN_00428"

/inference="ab initio prediction:Prodigal:002006"

/codon_start=1

/transl_table=11

/product="hypothetical protein"

/translation="MATPAQHLRWVFLQVQHSSDEVTRQACQPVAGHFFLRSMYVKSA

IINIANTITWLKEKFIGSPPTGGNLFPTTNWLYKMIIPFESLSFESLFILLHELNT"

CDS complement(440847..441542)

/locus_tag="PIKHDJKN_00429"

/inference="ab initio prediction:Prodigal:002006"

/codon_start=1

/transl_table=11

/product="hypothetical protein"

/translation="MMMTIGCFHAHYSNIVLIEEALAPYEVELVHYVDPGLDRLKHDA

DFTEAVTHEKVSQTLQWIAHCHADAILVTCTLFAAVLEQEAKHVPVPVVGIDDPLLQE

MQQNPGEYILAFTNPATIEGTMVRVNQASLQQGDEDGQRNGIEAIETEAVLIPGTFEL

IMRGDKEGYLAEVSTGLQQLVEKYPGSTVVAAQLSMAPAAARVTADTGMLIYSPLASL

AAYLEKKFNMAQR"

CDS complement(441744..441911)

/locus_tag="PIKHDJKN_00430"

/inference="ab initio prediction:Prodigal:002006"

/codon_start=1

/transl_table=11

/product="hypothetical protein"

/translation="MIRMPIQQMIPYHHQYPRNTATTAPKKENENTQGLSFHEILQQK

MAKKNAAPSLR"

CDS complement(442456..443277)

/locus_tag="PIKHDJKN_00431"

/inference="ab initio prediction:Prodigal:002006"

/codon_start=1

/transl_table=11

/product="hypothetical protein"

/translation="MMNVTICQSCGMPLTTPAQFGTETEGNTTREYCIYCYKEGKFEQ

PDITLDGMIEMCTAILNQEGMDEESARAMLRNQLPFLKRWNASGSERNVAFGAENSDE

SVVFNQTTRDNRLSTEPARYVTLPGKRLAGVSARTTNAIEISGKGCIQGLWNSYFASD

HQPAPEAARYGCYADYTDGINGEYTILVGHEVGPDEALPEGVSSVDLPPATYAVFTSR

KGPMAEVVGEAWGAVWAWNNQGERTFTGDFELYDERSLDPDNVQVDLYIAVRQEK"

CDS complement(443347..444297)

/locus_tag="PIKHDJKN_00432"

/inference="ab initio prediction:Prodigal:002006"

/codon_start=1

/transl_table=11

/product="hypothetical protein"

/translation="MKLDRLLAIVVLLINRGRVQAKDLADTFEVSIRTIYRDIDTLGQ

AGIPVVTYQGASGGIGLAEGYRLDRNVLTDKDLASIVTALRSVSTSHTNVARDLLMEK

LSSIVPEAKNEDFQANTSRFIVDYSMWTHPEALQIKLQLIEQGMDQLRPITFTYCSAE

GTQTHRTTDPHTLVLKKHSWYLYAFCHERNDFRMFKLVRMREVVLAQGSFERKPINLQ

DRPWQQEWATPDNKVALSLRFHARIRHIAEEWFGIENVVSDGEGYYISQVAFPEDNWL

YGFILGFGADVEVLEPLHIREEIRRIAEQIVQNYKAPPLT"

CDS 444381..445076

/gene="walR_4"

/locus_tag="PIKHDJKN_00433"

/inference="ab initio prediction:Prodigal:002006"

/inference="similar to AA sequence:UniProtKB:Q9RDT5"

/codon_start=1

/transl_table=11

/product="Transcriptional regulatory protein WalR"

/db_xref="COG:COG0745"

/translation="MKRITILIADDEVEIADLVALHLQKEGYHIIKAFDGKAAVQAVQ

TQAIDLAILDIMMPGMDGYEVTRKIREQYHLPIIFLSAKTSDMDKITGLVMGADDYMT

KPFNPMELVARVNSQLRRSLQFSQSAPVQRSILEKGGLIISPDQHSVTLYGKPVELTP

KEFDILYLLASHPKQVFSAESIFEQVWGEAYYESGNTVMVHIRTLRKKLGEDIDKNKF

IKTIWGVGYTFNE"

CDS 445099..446157

/gene="sasA_5"

/locus_tag="PIKHDJKN_00434"

/EC_number="2.7.-.-"

/inference="ab initio prediction:Prodigal:002006"

/inference="protein motif:HAMAP:MF_01837"

/codon_start=1

/transl_table=11

/product="Adaptive-response sensory-kinase SasA"

/translation="MILLLGLSMLASGAITFGVYKLMQYYYTGVRAEDQLAEYRHFMR

SIGDIYFFLILFIPLAILFFFWFTKPYVTYFKNISTGIRQLASGDFQHRVQISTKDEL

GAIAEDINLASLKLREAVERGDFAESSRDQLVVNLAHDLRTPLTSVLGYLDLLMKDDQ

LTEEQVRHFTAIAFTKSQRLEKLIDDLFEITRMNYGMLPIEKKRLDLSELLRQMNEEL

YPVFEKNRLVTRLHIDAELTISGDGEVLARVFENLLINAARHGTDGLYVDINGHRDAG

QVIIQIMNYGGHIHPDDLPHIFDMYYTGDRARTPQEGGTGLGLFIAKNIVEQHNGSIS

AQSDIVRTVFEVRLPALE"

CDS 446484..447347

/gene="vanY_1"

/locus_tag="PIKHDJKN_00435"

/EC_number="3.4.17.-"

/inference="ab initio prediction:Prodigal:002006"

/inference="similar to AA sequence:UniProtKB:P37711"

/codon_start=1

/transl_table=11

/product="D-alanyl-D-alanine carboxypeptidase"

/translation="MKKWGFLICIIVLGYIFSKSPALFEQKEEANLEVQDTGEIRAGY

TKTINVDMRNQVHKGNLLLVNSEYPVHAEGIRTDIVNLAEQHELVRGYGLLDNNIMLS

KYVAQAFQKMVEAAGKDDVRYFIISSGYRDFTKQEQLYEEKGSDYALPAGHSEHNLGL

SMDIGSTLAAMSEAPEGAWLEKNAWKFGFILRYPKDKVDITGIQFEPWHFRYVGLPHS

AIMYHNHLVLEQYLELLQQKKSISAEVGGEHYLVRYYDASKDETINIPEHGQYEISGD

NMKGVVVTVEE"

CDS 447376..447927

/locus_tag="PIKHDJKN_00436"

/inference="ab initio prediction:Prodigal:002006"

/codon_start=1

/transl_table=11

/product="hypothetical protein"

/translation="MKPKEFNMKMIWVAALIVYLYLLTKLILFKGHTVDWDIVRVQLI

AIAHQPDLIHTRTVNLTPFQEISRDWHSLSLHRPGTAIHLLGNVMAFIPLGIFIPVLM

GNKLLSGAKVLVLSLLLSFAFEATQLLTGMGIFDVDDLMLNTFGGMIGYVLYTMLISM

KRMIVGGTARTAKKEYNSNQSRV"

CDS 448196..448594

/locus_tag="PIKHDJKN_00437"

/inference="ab initio prediction:Prodigal:002006"

/codon_start=1

/transl_table=11

/product="hypothetical protein"

/translation="MYSLAKQLAGRMKGIMDIESEIAEAERNQQGEEFVCDLEQKRSD

LIKTCTRDELLVITTVMEVGQSERGYRHYFDSDNVELIYLPIELNEHELMKKYSHFLV

HKTKQEIADRIQFHTLVSSFLKEGMEILKL"

CDS 448630..448944

/locus_tag="PIKHDJKN_00438"

/inference="ab initio prediction:Prodigal:002006"

/codon_start=1

/transl_table=11

/product="hypothetical protein"

/translation="MIMKISYFTPDGFYYYVPDQYAEQMNKWRGEFSDFLQSIEGKHP

FTQYTEYIDHEGKKEYGVFVRCYGGDDFADWINVEKLNCRGVYRIPAPPDDSEVGLKI

DF"

CDS complement(449057..451771)

/gene="citB"

/locus_tag="PIKHDJKN_00439"

/EC_number="4.2.1.-"

/inference="ab initio prediction:Prodigal:002006"

/inference="similar to AA sequence:UniProtKB:P09339"

/codon_start=1

/transl_table=11

/product="Aconitate/2-methylaconitate hydratase"

/db_xref="COG:COG1048"

/translation="MSAKNHFSAARSLEVGGKSYRYYSLDALQEGGYGDLSKLPFSIK

VLLEAAIRQFDGRAITEEHVKQLTGWAEGRDNNKEIPFIPARIVLQDFTGVPVVVDLA

AMRDTVKKAGGDPKQINPLVPVDLVIDHSVMVDAFGTNDALDYNIKVEFERNEERYRF

LRWAQTAFNNFRAVPPSTGIVHQVNLEYLASVAATKTIDGETVVFPDSLVGTDSHTTM

INGLGVVGWGVGGIEAEAGMLGQPLYFVTPDVIGFKLTGSLSEGSTATDLALTVTQML

RKKGVVGKFVEFYGPGLSNISLADRATVANMAPEYGATIGFFPVDSETLNYMRNTGRT

DEQVDLVEAYYKAQNMFRTADTVDPEFTDVIELDLASVVPSLAGPKRPQDRIELTQMK

DSFNSIIRTPIDKGGYGLSDEKIAEKVPLKHPNGSTSELTAGAVVIAAITSCTNTSNP

SVMVGAGLLAKKAVERGLTKPGYVKSSLTPGSLVVTEYLEKAGLIEYLDKLGFNVAGY

GCATCIGNSGPLPDEVSEAISENDLTVAAVLSGNRNFEGRVHAQVKANYLASPPLVVA

YALAGTVNIDFATDPIGYDTNNEPVFLKDLWPTSEEIKDTIASSLNAQMFRNKYENVF

TANERWNTIPVPEGELYEWDPNSTYIQNPPFFQELGDKLTDIADIRSARVMALLADSV

TTDHISPAGNIAPSSPAGLYLKEHGVERKDFNSYGSRRGNHEVMMRGTFANIRIRNQV

APGTEGGITKYLPTDEEMSIYDASMKYQDEGQNLIVIAGKEYGTGSSRDWAAKGTFLL

GVKAVIAESFERIHRSNLVGMGVMPLQFQEGHGWKSLGLNGRETYDITGLSNDVKPGQ

ELTVTVTREDGTKFDFPVIARLDSMVDVDYYHNGGILQTVLRQMMKKS"

CDS 452152..453150

/locus_tag="PIKHDJKN_00440"

/inference="ab initio prediction:Prodigal:002006"

/codon_start=1

/transl_table=11

/product="hypothetical protein"

/translation="MEREWKSALYTYVNQYNRCEIDYRPQTSERIVTDPEFVLERGER

MARLDQWYRKRRAVPLRSETSAKLVRTLMDGVDESVVDVQLYSRLFYEKSGLTHREDR

IERERLTFLRQGGVWTIARVEREVPERRPVGEGRPFQQADFVQAMNRPLLNREVLGQG

RRSRQQLYRRDLAVAYADRWWNAGNPAFEEFEVDCTNYVSQCLFAGEAPIHYTGKREA

GWWYKGYVNGSEMWSYSWAVSNSLERYLSGSSWGLSATEVDRPEQLMLGDVILYDWDG

DGRFQHSTVVTAFDAGGMPLVNAHTVSSRHRFWDYRDSYAWNERTVYRLFHIADEF"

CDS 453284..454384

/gene="ddl_1"

/locus_tag="PIKHDJKN_00441"

/EC_number="6.3.2.4"

/inference="ab initio prediction:Prodigal:002006"

/inference="similar to AA sequence:UniProtKB:P63892"

/codon_start=1

/transl_table=11

/product="D-alanine--D-alanine ligase"

/translation="MSMDKLTVGVVYGGKSGEHEVSLQTAFAVTNAFDYEKYELVPFY

ISKQGTWKKGSVMHAPFAQIEDLKLEQAAGGTQEALNALFGRLYGGEEAMDVMFPLLH

GTFGEDGTIQGMFEMADMPYVGAGVLASAGGMDKVVMKKLFAQAGIDQCAFTYFNATQ

WKQTEHEMILQVEDQLGYPCFIKPANLGSSVGISKARNRDELKKAVEFALRYDTKVVI

EEFVEAREVEVSVLGNDEPMASVPGEIVSSGEYYDYAAKYIDGQSQMLIPAPLDPEAA

DRIREAALQAFRAIEGNGISRADFFIRKSDGALLINEVNTMPGFTPYSMYPLLWRETG

VSYAELLDRMIELALERYNRKQALNYENGVQA"

CDS 454416..454739

/locus_tag="PIKHDJKN_00442"

/inference="ab initio prediction:Prodigal:002006"

/codon_start=1

/transl_table=11

/product="hypothetical protein"

/translation="MGFQTEFNSVCKFKNEQELFELLEYGRGKMVKQGFRVFPTGQKV

IAYTPDNVAVAIVKIVVSIAEINFQGQEVTEVEMQLIRKLNEEESRIQTALADEMFFG

EQAQG"

CDS 454895..455896

/gene="uvsE"

/locus_tag="PIKHDJKN_00443"

/EC_number="3.-.-.-"

/inference="ab initio prediction:Prodigal:002006"

/inference="protein motif:HAMAP:MF_00606"

/codon_start=1

/transl_table=11

/product="UV DNA damage endonuclease"

/translation="MLVRFGYVAMSVLVENASPSRTMTMSSFKKIDDREAAIRKLERI

AAENLHNTLRLLRHNKGSHIHVYRFSSKLIPLATHEDLSDWDPFPALKQDFATIGDFV

KENEMRVSFHPDHFTVLSTPREEVLRNSMRDLRHHVRMLDAMGLNATAKNNIHIGGAY

GDKPSAALRFEENFLKLDRDIQERITLENDDKTFNAPETLAVCQRMGLPMVLDIHHQW

VNNEGEQPWDLWPDILKTWESPLAQAESPADKPLPPKIHVSSPKSEKDLRGHADGVEV

EPLLAFLRHIAADTLALDVMIEAKRKDEALVQLMQKLAFYHEEGVEWVDESTVIIHP"

CDS 456004..456867

/gene="suhB"

/locus_tag="PIKHDJKN_00444"

/EC_number="3.1.3.25"

/inference="ab initio prediction:Prodigal:002006"

/inference="similar to AA sequence:UniProtKB:P0ADG4"

/codon_start=1

/transl_table=11

/product="Inositol-1-monophosphatase"

/db_xref="COG:COG0483"

/translation="MNEKEKTPYVVTSKSYTAVAINAASKAGEWIKSRLGTVGEPGTK

YSPQDLVTEVDKGAEQMIRRLILTHFPHHAILGEEGVEPGPEASAKALKEAEEEEFLW

IVDPVDGTTNFVHGFPFYSVSIALAHNGEVIVGVIYDPSRDEMFVAEKGKGAYVHGNR

MLVSGEQELAQSLIAVGFPADTTYALPINMAAVQALAPQVRSLRAGGSAALHLAYVAA

GRLSAYTEVGLKPWDIAAGALLVEESGGKVTDTVGTPYQLSVSHVVASNGKIHDALTG

VLKEANATGLE"

CDS 456893..457177

/locus_tag="PIKHDJKN_00445"

/inference="ab initio prediction:Prodigal:002006"

/codon_start=1

/transl_table=11

/product="hypothetical protein"

/translation="MDESKELERRLSDMLTEEDMEQSGDADKRERQLIPPKYEVRIQT

ERDPIVEETLKYRDMAREVDDRYDRYLERSAGNKPEMDTDGSDSGDAKKS"

CDS 457390..458706

/locus_tag="PIKHDJKN_00446"

/inference="ab initio prediction:Prodigal:002006"

/codon_start=1

/transl_table=11

/product="hypothetical protein"

/translation="MKRKRIWRSTAAGLTASMLAGMLILASSAQSAHAADSNTLPAGS

VTSSLPVQKDAVAVTKEFRLVTLGDSITVGYEPNLKELPYGYVERLQEQGLLHGRTQV

DNYGIAGLKSSGLKNFADAIKEGKALTSEAIQPNLPDPRAAQVGANIAAIRESVAGAN

LVAITIGGNDVSELLGTADKLSDAELEAKVKELLSSYTANVSAVINDIHEINPNAKIV

IADQYQPMPEVAGKELYAKLMEASQGFTATIDGIATEFSAKGIDVKVAHVAKEFIGGE

GTMTHMIKDRDFHPNQFGYEAIAKVFAETIWGDYTKLTAPAAGQPMNIIVSGKTLDTP

YKPIIRNGKNFVAIQDIVNAVGATTVWDNKTSTATITYGDRKVAVKIGATAVQVNGAS

VAVDTPAFLNKVGTESKTYVPLAMVAEGLGFDVQYVAKLKTVFVNP"

CDS 458959..460746

/locus_tag="PIKHDJKN_00447"

/inference="ab initio prediction:Prodigal:002006"

/codon_start=1

/transl_table=11

/product="hypothetical protein"

/translation="MRKIKNAFTTLPIHHKTILLIGLLMLISFTFYVSVLGYVFSIYD

RQIYEKSSQVLNMSSVGIENQLREISNLSFKVMSDEPLQQYLLQLDKAESGYEKNGLR

KKITNRLVAYAGSEKYVYSMIFIDNDNNIMAAGNREGISEKKQRELVSLAQQYSGSNA

WYTGGDQQARLLSVRQVKSFTGGAFTLEKLGTLVIRVRLDRIVQDQMQHPAEDSQLLI

TDGREVIYPTESTVSKSEIESELKRTQPYGIAVFEQGRNFVARAHSSYTDWVYLYMTP

FDQMFKQIQFVKQLVTVIFIMIFLVALVFGAKFSRSITQPIAQLIKKMRNIEKGDLDK

LEEAALGNVPISSQNEVGLLHRTFKMMVQRIRELIDENYAKQLVIRETELKALQAQIN

PHFLYNTLESINWLAKVQKQHQISEMVEALGFLLRSSVSMSEKWITLERELEIVRSYV

TIQHTRFEERLDFDMDIAPEVGTARIPKLTLQPLVENAIHYALEPSIEPCRIRIRARA

DGDNVVIEVEDDGPGMTSEFLEQLQEGRIQTRGQGIGLSNIKERIRLTFGDEGEMIMS

SNPGSGTVVSIRIPWIREDDDDVQSDARR"

CDS 460721..462274

/gene="cheB_1"

/locus_tag="PIKHDJKN_00448"

/EC_number="3.5.1.44"

/inference="ab initio prediction:Prodigal:002006"

/inference="protein motif:HAMAP:MF_00099"

/codon_start=1

/transl_table=11

/product="Protein-glutamate

methylesterase/protein-glutamine glutaminase"

/translation="MYKVMLVDDERVILEGISQVVDWAAAGTELVDTARNGIEALDKI

GHSRPDIIITDISMPGLDGLGLIEKASEAYPGLRFIMLSGYKEFEYARRAMQYGVKHY

LLKPCNENQIRDALSELLQEHQDAQVKEHVAGEMKQRLQRVLPHVKEQFLLEFMTNQT

YGPVDLEYYQELFDLELEDNEVRLLLFRIADEHDYSHLFAIKNIASDLLPNVLLSTTI

EGKLLILLGDSADPVGLKESIEEVRAAFTRLYKLEVTAALSEADRMIQSRRLFREALQ

CLNHRFFIGEGKLITKNDLALAGECDGVHVEQDAEQLCQLIKSGNTEEAASEVDRLFE

LLSGQQLEIGITRSYVVQLYSAMIHVCPPEEATEFTQRMAELPHIDTLSGLKSFVASS

AARLTSGYYKNHISRQSSAVEKMMDIVDRHYGEADLSLNGVAHQMLYMNPDYLGKIFK

KVTGENFSNYVNRLRIERACDHIRRGGDVKVFELAELFGFGGNSQYFSQVFKKWTGMT

PTEFRRTGL"

CDS 462496..463779

/gene="yesO_1"

/locus_tag="PIKHDJKN_00449"

/inference="ab initio prediction:Prodigal:002006"

/inference="similar to AA sequence:UniProtKB:O31518"

/codon_start=1

/transl_table=11

/product="Putative ABC transporter substrate-binding

protein YesO"

/db_xref="COG:COG1653"

/translation="MVKKAIFLMMAALLVLTTACSSSGESGGTSGDGSVTLRIAWWGS

DARHEYTQKVIDLYKEKNPNVKIDVEYASFDDYWKKLAPQAAANQLPDIVQMDISYIS

QYAQNGQLEDLSPYLGNQIKVDDVSENVISTGIIKEKQYGVPAGVNVLGFQYDPALLK

KAGVDAIPENMTWESYEALGKQAAAKGLFLDGGVAPDIFFHYFLRTKGLSLYNAEGTG

LGYDDDKLFVEFFGLMRRMIEQGAAPSPDLANQTKGIIEESDLVKEKGIGIWQWSNQF

VAVQQVANRPLEFAPMPGPDMEKGLYMQPSMYWAVTSNSKVKEEAAKFIDFWVNDVEA

NKLIKGERGVPISGKIKEAIAPELSDATKQVFDFVASMEPKASPMSPPPPVGSPEVIA

SLADVVEELNFGKVTPEQAAATFRKNAEAVLANNK"

CDS 463923..464825

/gene="lacF_2"

/locus_tag="PIKHDJKN_00450"

/inference="ab initio prediction:Prodigal:002006"

/inference="similar to AA sequence:UniProtKB:P29823"

/codon_start=1

/transl_table=11

/product="Lactose transport system permease protein LacF"

/translation="MRQYSSLRRNLTGYAFISPFIIGFLGFTLIPMFVSLYMSFTSYN

LFTSPRWIGMDNYTKMFFDDPKYWNSVKVTFLYVFIGVPLRLVFALFVAMILNTGSRM

VGTYRTLYYLPSIIGGSVAVSIMWRNLFSNEGVINSALTAIGIGPISWFGDPNASLVM

LISLSVWQFGSSMLIFLAGLKNISPEMYEAAGVDGANPVRKFFGITLPLLSPIILFNL
IMQTIGAFMTFVPAYIISKGEGGPMDGTMLYSLYLFRQAFMFNNMGYASAMAWIMLIM
IGILTVAVFLTSKFWVFYESEGGK"

CDS 464828..465667

/gene="ngcG_1"

/locus_tag="PIKHDJKN_00451"

/inference="ab initio prediction:Prodigal:002006"

/inference="similar to AA sequence:UniProtKB:O50501"

/codon_start=1

/transl_table=11

/product="Diacetylchitobiose uptake system permease
protein NgcG"

/db_xref="COG:COG0395"

/translation="MVWRNVKWPIYHLFVAALALLMLYPVLWMLFSSFKESRTIFVTA
DSLFPTEWIWSNYADGWKGAGGRPFMDYITNSLVIVVISTIGAVLSSSLIAFGFARLN
FKGRNFWFSLMMLTLMLPHDVVLVPQYIIFTKLGWLNTILPIVVPTFFGMPFFIFLMV
QFIRTIPKELDEAATIDGCNKFRLYVQIIMPLIKSSLATAAIFSFYWRWEDLLGPVLY
LNSPDKYTVSMALKMFLDSESASNWGAMFAMSIVSLVPVVAVFFIFQKQIVQGMSTSG
LKG"

CDS 465725..466966

/locus_tag="PIKHDJKN_00452"

/inference="ab initio prediction:Prodigal:002006"

/codon_start=1

/transl_table=11

/product="hypothetical protein"

/translation="MNKAQGSQAVATEATAERGKNTVTHWKFNFGPDFGSAGEIEGYL
KVSASTAYVEQGEYGFESGSLVYEKQRISEDEVSDISRANNNNQGQANVSARLRSSFC
IPLKASFIVDVPDGTYQVLLIAGDELAETITRVKAGEGRLMLPTIRTLPGQCAEVRFS
VVVRGGKLRLSFSGPAPRINALEITLANQTMTVFLAGDSTVTDQPESGYPYCGWGQLL
PAQFKHDVAVDNHAQSGRSSRSFINEGRLDAILEKIKPEDFLFIQFGHNDEKPGPDRG
TDPFTTYKEYLKKYIDAVRGASAHPVLVTPVHRRYFADDGTLTDTHGDYIVAVRELAE
EEGVPLIDLAERSRILFEQAGVEGSKEDFMWVLPGEYVNFPAGVEDNTHFQERGARRL

AIQVAEAIRELRLQPLQMYLR"

CDS 467662..468072

/locus_tag="PIKHDJKN_00453"

/inference="ab initio prediction:Prodigal:002006"

/codon_start=1

/transl_table=11

/product="hypothetical protein"

/translation="MAQDQTNTSAHKTSSTAKGHTYWVIPDGYIPPDSSGSLESHESI

CVLNTGTVDAQLQMTIYFEDREPLEAIVAEVPARRTKHIRTASLRSGEQLIPPGVPYA

ITVSSDVPVIVQYSRLDTTQPELALMSVMAFPLG"

CDS complement(468146..469303)

/locus_tag="PIKHDJKN_00454"

/inference="ab initio prediction:Prodigal:002006"

/codon_start=1

/transl_table=11

/product="hypothetical protein"

/translation="MTRWMAKVSLFISCFILLTGCWDSKEIQSINFITAVGIDYVDSQ

YIAYAQLIDFSSIAKQEGPTSREASDIWIGRGEGSTLSMAIDDLYQTSQQQTLWTHVK

AIVLSKNVLNGRLEDVFNTLLHSGQLRYTPWIYGTDHNIPDVLSPSALLNQSAQTIEL

FEPMKLYKQHSGYEPIRLHQLLDGFREPASVILLPSITNQNETWHNGDKTPPLIRMDG

FYVISNGKSQGRITGKDADGTRFVNYDRVYQYPLYVVYRKGSKTPDLTLRLNNPKTTIK

ARKEGNGVTFDLVISVKSAIIEDHDSHRSPHVMEKEAEKQIESEIRNTFEKTKSRKID

SYGFVEYLYRHDLPLWRQEVYKRANPLERFKLGKVEVHVKILNASTYKYNE"

CDS complement(469300..470769)

/gene="gerXA_1"

/locus_tag="PIKHDJKN_00455"

/inference="ab initio prediction:Prodigal:002006"

/inference="similar to AA sequence:UniProtKB:Q9ZFB4"

/codon_start=1

/transl_table=11

/product="Spore germination protein XA"

/translation="MEHQTIDTTSHETLLASFEEQFNPCADVVIQTFPAKDETDMSVI

MLYCDGLVDGKQINQFIIPRLERHSLFIEEAHPKELGLTLNPVEDLTDAENLNLLIFS

GQLLLIFGNGDQSCSLDIASIPGRNPEESAIESSIKGPRDGFTEELSVNIGLVRKRMK

TSSMCYEKYVKGGRTQTSIGLLYVKDIINPDILEEARQNLDKIEIDGILGTSIMERAI

MGGIRSIFPLTDNTERPDYVVDSLLNGRFAVLIEGSPVAIIAPTTLFNQLKSPEDEST

PFFIVTFERILRISGLLIACFFPAFYIGLTSFNVEQIPLPFLATISGTRMGLPMPVTL

EAFLMIFMFELFNEAGRRLPRALGQTVSVVGGLIIGDAAIRAGITSPTIIVTVAISVI

SSYILVNTVLSGATAQVRIGMLFISTILGLFGFMLGLFALLVHLVSLECYGVSYLSPV

SPYIPGDFTQAVSMPPSMKRTKRPAVLHTQDSTRRRKRS"

CDS complement(470750..471856)

/locus_tag="PIKHDJKN_00456"

/inference="ab initio prediction:Prodigal:002006"

/codon_start=1

/transl_table=11

/product="hypothetical protein"

/translation="MKPTQPSFLQAMMLMMLSVGLISHVLIIPALLSSAKRDAWISVL

LSAGPYLLFTLMLAYVSRFLQHQTLHEWLTSRLGKPIGLLFRVGNSLFFLSMIYFTLH

DTTTWAKTNYLTETPILVTSIALMSLCAYAAYKGVRSLAFTAGLILPLVVLLGFYVAI

VNTQYKDYSRLLPVLEHGWHPVLHGTVYSLSGMFELLFIWYLQPHLSKRIRVWQYLML

ASIILGLTLGPLIGAIVEFDPFEAAKMRYPAFEEWRIASLGKYIAQTDFFSIYQWLAG

SFTRISLAMYIVVEIWNIKSASRRIICCVTLGVLFILSMLYPLDDMTFEKLLVQYIFP

FNLMYLSGLAVVATAVAYISSRHQRRKHHGAPND"

CDS complement(471926..472828)

/gene="trpS2"

/locus_tag="PIKHDJKN_00457"

/EC_number="6.1.1.2"

/inference="ab initio prediction:Prodigal:002006"

/inference="similar to AA sequence:UniProtKB:Q9RVD6"

/codon_start=1

/transl_table=11

/product="Tryptophan--tRNA ligase 2"

/db_xref="COG:COG0180"

/translation="MQLQDEYKTYILLADVQALTTHYDQPGLIADSVHQVTLDYLAVG

IDPNKATLFIQSMIPEIAELTVIFSMFITVNSLRHNPTIKNEARDRGYKDLYYGFLGY

PVSQAADIAFCKATLVPAGEDQIPHIETARKLVRRFNELYSPVLPEPRILTGERLGGL

DGVGKMSKSMGNAISLNAHPDDVYAKLARAKTYPARIHRSDPGHPDICPVFAYHQLFR

KDEAAEIHANCSNGTIGCKDCKKIAGTAINELLSPFRERRNYYEQRGHKVKEILVEGT

RQARQAARETMLEVREAMGIRYFD"

CDS complement(473413..474348)

/locus_tag="PIKHDJKN_00458"

/inference="ab initio prediction:Prodigal:002006"

/codon_start=1

/transl_table=11

/product="hypothetical protein"

/translation="MNNTQTRTYAYVAAVLYAAIIGLSFLFVKMTVSVAHPIDVLAHR

FALSLLVVSIPVIFGWIKIKLTLRDLWRIIPLGLLSPVLFFAFQAFGLVSSNSSEAGI

IQAMAPVFTLVLASIFLKERTSLLQKLFLLLSVAGVIFIFLMKGSGMSAGNFKGIALL

LLSTVCFAGYGVLARPLAQKYKPMELTWVTLMVGFIVFNAASLIRHASSGTMMDYVKP

LGDASYLGALAYLAVLSTMFSTLLASYALTHLEASKMSVFSNFSTLISIAGGAWILHE

PVHGYHFVGALLIIAGVLGTNMSGRKRKLVNPDKA"

CDS 474482..475876

/gene="norG_1"

/locus_tag="PIKHDJKN_00459"

/inference="ab initio prediction:Prodigal:002006"

/inference="similar to AA sequence:UniProtKB:Q2G1P1"

/codon_start=1

/transl_table=11

/product="HTH-type transcriptional regulator NorG"

/db_xref="COG:COG1167"

/translation="MRKYEIIAESLKQWIQDQLRQQNSGQWQDKGIKLPAVRVLAEQY

QCSVSTAIRAYEWLEERHLVYAVPQSGYYAVQNGAEVEDGEWQGPLDFASAAPDPRVF

PYSDFRHCVDQAMEKKQADLFLYGTERGLPSLIELLQKQFADYQVFAGKEQFFITSGV

QQALAVLALMPFPNGKKQVMLELPGYHNMPSLLQGLNVPIAGVRRSLEGLDWDALERQ

FREGDIKFFYVMPRFHNPIGTSLTAAEKKRLIRLAQRYDVYLVEDDFLADLEENTKQD

PLWSYDTEGRVIYLKSYSKILFPGLRIGVAILPASLTASFSKYKKMLDIDTAVLSQAA

LEVYIRNGMFAHHGKVIRSRYAARMNKLYQQLNAYPDFTPFANAPRTGGEHTVLPLPK
DMPLKVLLSRLDQRGVIADTTERYYPEDAQEIHQDKMLRLNISNVPKQRIKEGLRIIR
EEIAKLQKAPTQAE"

CDS complement(475948..477267)

/gene="hemL1"

/locus_tag="PIKHDJKN_00460"

/EC_number="5.4.3.8"

/inference="ab initio prediction:Prodigal:002006"

/inference="similar to AA sequence:UniProtKB:Q81YV0"

/codon_start=1

/transl_table=11

/product="Glutamate-1-semialdehyde 2,1-aminomutase 1"

/db_xref="COG:COG0001"

/translation="MNPSRSRSELLYEEALQHIVGGVNSPSRSFKAVGGGAPVFMKKA
QGAHFWDVDDNRYIDYLAAYGPIVTGHAHPHITQAITEAAANGVLYGTPTELEIKLAK
MLKEAIPSMDKVRFVNSGTEAVMTTIRVARAYTKRNKIVKFAGCYHGHSDLVLVAAGS
GPSTLGIPDSAGIPTSIAHEVITVPFNDLDSLKAALERWGDDVAAVMVEPIVGNFGMV
MPEPGFLEGLCAMTRDNGSLVIYDEVITAFRFHYGSTQTYAGLDNHAEIEPDLTALGK
IIGGGLPIGAYGGRKQVMEQVAPLGPAYQAGTMAGNPASISAGIACLEVLQGEGVYAE
MERLAIDLTTGLQASADRHGIALTINRIRGAFSTHFCDHPITNYDQAQDTDGELFASF
FRHMLNRGINLAPSKYEAWFLTTAHTDKDVQETLEAAEASFKAMAQA"

CDS complement(477354..478397)

/gene="tagU_1"

/locus_tag="PIKHDJKN_00461"

/EC_number="2.7.8.-"

/inference="ab initio prediction:Prodigal:002006"

/inference="protein motif:HAMAP:MF_01140"

/codon_start=1

/transl_table=11

/product="Polyisoprenyl-teichoic acid--peptidoglycan
teichoic acid transferase TagU"

/translation="MTRKTKRTIWISLAAFVLIVGGAAAFYFGSILNQLNGLQKEGDD

SPFAGIENVEKVSTPDPPKWEGTETVNILVMGVDARGLEKGEVPRSDSMMVVSLDPLT

KKINLFSILRDTYVNIDGYGKERINTAITHGPNVAMKAAGDLLGIPVQYYVYTDFQGF

IKLVDAVGGVDFDVEKDMHYTSKADNNEYDIDLKKGYQHLDGDTALMYVRFRHDAMSD

YARSERQREFLKAVAAKMQSTTTIAKLPSILEEVNPYVDTNLTLSDMWKLGGLGYQSS

MNGSEQIPPMKMLKEERTPGGAEVLSVTNEDKLKQYIQDIIHPPAQEEGSTTGAEDKT

ASGDEQESKQPAQ"

CDS 478602..479648

/gene="btuD_3"

/locus_tag="PIKHDJKN_00462"

/EC_number="7.6.2.8"

/inference="ab initio prediction:Prodigal:002006"

/inference="protein motif:HAMAP:MF_01005"

/codon_start=1

/transl_table=11

/product="Vitamin B12 import ATP-binding protein BtuD"

/translation="MLAIDVKDLRKSFSVQKSRGGLKGAFQDLFARQYQEVLAVNDIS

FQIPQGEICGYIGENGAGKSTTIKMLTGILVPTSGQISVGGYVPYQEREKFVQNIGVV

FGQRSQLWWDIGVIESFHLLRKVYRVGEVDFRKRLDELVVRLQLQDLLNRPVRKLSLG

QRMRCELVAALLHNPDIVFLDEPTIGLDIVVKSEIREFLKDMNKEHGTTILLTTHDLQ

DIEALCSRVIMLDAGNIIYDGGLDHLKSQWGKEREIRFKFGTAHPMEQMLEWTAALPV

RWTVENELSASVWIPLELNVSDVLGRVVGQADITDIQIIEINTDEIVRSIYQSGSAER

PEIVGAKNEAVGVS"

CDS 479650..480444

/locus_tag="PIKHDJKN_00463"

/inference="ab initio prediction:Prodigal:002006"

/codon_start=1

/transl_table=11

/product="hypothetical protein"

/translation="MNSAFFDFMRIRFLTMLAYRVNYYSGILIYTLNIGVYYFTWQAI

YGSSGELGGFTAAQMTTYVAVSWMARAFYFNNLDREIAADIRDGSIAIQFIRPINYVM

VKMMQGLGEGIFRFLLLMIPGMLIAILLFPVELPTAPSAWIGFLVMLFFSFLINSQIN

VITGLAAFFVENNEGMMRMKRVVVDLFSGLIIPISLYPGWMSAVMKVLPFQAITYLPG

SVFTGRVEGTGIWSVLGIQVFWFVILLIPMMLIWRQARKRLFVQGG"

CDS 480449..481234

/locus_tag="PIKHDJKN_00464"

/inference="ab initio prediction:Prodigal:002006"

/codon_start=1

/transl_table=11

/product="hypothetical protein"

/translation="MYYMGLIWEYLKNYMKTRLTYRADFWVEILSDLLFQATNLIFIF

VVFRHTDNLGGWSESEVLFVYGYFMVPYGIFSCFINLWGFSERYIVKGEMDRILTRPA

HNLFQILLENVDPPALVGSFIGLIIMVISGAEMGLVLEWWHIPALIILTLSSVLIYAG

IYITLTSLSFYSDAPTGILPLMYNIQGYGRYPVTIYNRAIQVLLTWVIPFAFVGIYPA

ALFLERSEMHRMAMLTPVMGLVFGSIGLLLWNYGVKKYRGAGS"

CDS 481279..481743

/gene="bcp"

/locus_tag="PIKHDJKN_00465"

/EC_number="1.11.1.15"

/inference="ab initio prediction:Prodigal:002006"

/inference="similar to AA sequence:UniProtKB:Q83CY8"

/codon_start=1

/transl_table=11

/product="Putative peroxiredoxin bcp"

/db_xref="COG:COG1225"

/translation="MTLTEGKLAPDFELPSSTGETVKLSDYRGQRVLIYFYPKDMTSS

CTQQACDFRDRHEEFKGLNTVILGISIDPMKQHDKFIAKYGLPFILLSDEEHQVAEQY

GVWQLKKMYGKEYMGMVRSTFLIDEEGLLLKSWLKVRVKGHIEAALEALQQL"

CDS complement(481835..482962)

/locus_tag="PIKHDJKN_00466"

/inference="ab initio prediction:Prodigal:002006"

/codon_start=1

/transl_table=11

/product="hypothetical protein"

/translation="MIIKDKITIRQFSLLTFLVMVGDMILIYPSLVTTLGKQDAWICS

FLSQPIGLFIIWILYKLHQTYPELSLIEICKKLLGPWIGSVLSAGYLFYFAIGAAICI

REVGDFMTTQIYLQTPIRVILIMLMCSLIWGLMKGLQTMGATSELLTPIVVVFMALLF

VGLLPNIKVSHLQPFLSIGWVHLIETVIRGSFTAFGELLVLTMILPYVMRGPHLKRDL

LLATFCGGLLLCVLLLFSLMVVGPFLTQHNIFISYTLSQKINIGNFFERIEAFMATAW

LIATYFKSLLYMFAFVIGLAQLFQLKTYKPLILPSSLLMFALAILISPNVIFYIETIM

PAWVDWDITCSFIIPLLLLLVHRIRFGKNKKNQTHPERLPT"

CDS complement(483007..484113)

/gene="yndE_1"

/locus_tag="PIKHDJKN_00467"

/inference="ab initio prediction:Prodigal:002006"

/inference="similar to AA sequence:UniProtKB:O31809"

/codon_start=1

/transl_table=11

/product="Spore germination protein YndE"

/translation="MLEQGRIGTRQLSILIFMMVVGDMMLIYPSVITSYAKQDAWICA

LVGVPLGMLLMLMFIKLCSLYPGKNLVQISRSILGFWPGSLVSVFYLFFFVIGASTHT

REVGDFLTSQIFQYTPIRVIILMFVITLGWGVYHGLETMGRSSELLMPILIVFILVLA

FCLLPQVDPSNVKPATDIGVVPILQGILISIIYPVGELLPIMMIVPYTLKQAHRTRDV

LVATGLGNLLLAILVTISLLVLGAFLTQHNIYASFVLSQKINIGGFFERIEAIMASSW

LISTYVKAMVYLYAFIVGCAELFKLKQYRMLILPASLLLFGLANLVSPNLTFIVITVV

PYWVDWDTTLSIILPGLLLLVHMFKKGWKNKPSG"

CDS complement(484133..484369)

/locus_tag="PIKHDJKN_00468"

/inference="ab initio prediction:Prodigal:002006"

/codon_start=1

/transl_table=11

/product="hypothetical protein"

/translation="MLLTLSVVVTAGLIGWYDLPGLIRRKEWKETAVYGVLLIIATFL

SIFAANLWEFPSPLYLIIWMYEPVNQFLAHLTGT"

CDS complement(484375..485577)

/gene="gerBC_1"

/locus_tag="PIKHDJKN_00469"

/inference="ab initio prediction:Prodigal:002006"

/inference="similar to AA sequence:UniProtKB:P39571"

/codon_start=1

/transl_table=11

/product="Spore germination protein B3"

/translation="MNKCLQLILSFAVLLPLLTGCWDRQELNELGIMLGLGVDKDGDM

IKVSAQVVVPNEVSSKSGGGKGTPVTQYQASAPTLFEAIQKLTESSPRRIFMAHIRIL

VYGEEYARKVGIYDVTEALMREPTVRPDYYVMVARNTTASKVLDVMTPLENLPAEKMF

NSLDVSTKTWSPTTTVTGDQLMEYILSPGIQPVITGVEVVGNPEKSGHQENIATIKAP

ARLNSTGLSIFKGDKLIGWLTEDESKGYNYIRNNVVSTISHLPCRKEGYVTFKALRTT

TKRSVKVVAGHPVINIKMKNVSSIGAVECGIKIGSMEVLKELERDSEERLIELMQKSV

KSVQRKFHVDIFGFGQEVYHADPKFFKKVEKDWDKYLEDLDVHYDVNVQIKRVGTLDD

SFKDQIRE"

CDS complement(485596..487107)

/gene="gerBA_1"

/locus_tag="PIKHDJKN_00470"

/inference="ab initio prediction:Prodigal:002006"

/inference="similar to AA sequence:UniProtKB:P39569"

/codon_start=1

/transl_table=11

/product="Spore germination protein B1"

/translation="MSTAAQSSQDQRISPNLTENTDYCKKVMGNSKDLMIRPLQCLHK

WPSVMLYIDGMVDVQIVNHSILEPLLQTRELPDFSAKNEHLSYLQNDVLVASNVILID

EIKDVLDALLAGFAVVLLEGSNQGLKIAAAGWEDRAVGEPISQTVVRGPMEGFNENLR

TNTGLIRKRIRDPHLWIEEREIGRVTNTRVAVVYLEHIVDQEVVKELRRRLDEIDIDA

ILESGYIEELVQDKTGTIFPTVYNSERPDTVCAALLEGRVAIIVDGTPFVLLVPALFV

HFFQSPEDYYQRADISSLIRMIRYLAFFIALLAPSFYIAITTFHQEMLPTNLLISLAA

QREGVPFPAFIEAILMELTYEILREAGIRIPKTVGQAVSIVGTLVIGQAAVDAGVVSA

AMVIIVSITAISSYVIPENGLSISVRILRFVLMILAAAFGFYGILMVLLITVTHLCSL

RSFGVSYMSPFAPYIGKDLKDTIFRVPWSRMKTRPLSTGTANETRQANKKTKQ"

CDS 487282..488592

/gene="entS_1"

/locus_tag="PIKHDJKN_00471"

/inference="ab initio prediction:Prodigal:002006"

/inference="protein motif:HAMAP:MF_01436"

/codon_start=1

/transl_table=11

/product="Enterobactin exporter EntS"

/translation="MLFRSSIVILLQKESEYRKFFVAGLVNGIGDRFCQVAMLSLILS

TTGSGVAVGLALGLRVLPFLLLAPAGGMLTLRFSRRKIMLLTNLIRVPLALSYLLVNS

ADDLWILYTASTLLACAEALYAPVRKSGIPLLVQPENLLKINGLEQVMNGTVLIVGAF

LGGITSSVMGPQAAFITNALCFLVAAVLIRKVSFPSGMGTESNGRSLENVERSTVSAS

DNPSVIARANGAFYVPSVIWRLVRSSVALQIIILFELWVPVINGIDNVLISVYAIEVF

ALGDWGVGLFYAALGTGLVLSNWCSRYFQHWLLPGVVMCLLVEGGLLMLLSMATLPVI

AVLIYILLALMSGVGNACLDTLLMRETPEKYRGLIYGLVTACSSSMLGLSMFGAGLLL

EVVEPRKLGFAGGLGFAAIAVLLTAYGWLRAREAFNIRRITKKT"

CDS complement(488740..489384)

/locus_tag="PIKHDJKN_00472"

/inference="ab initio prediction:Prodigal:002006"

/codon_start=1

/transl_table=11

/product="hypothetical protein"

/translation="MELLEKVQHLFGSYGYSVLFFGLLLEFIALPFPGETTMAYAGFL

SYKGHLDFGILAILAFLGTTIGMTITYFIGAKAGLPFITRYGKWFLMKQDKLDKTQKW

FAKYGNALIFIGYFIPGVRHFTGYFAGIAAVPFRKFALYAYSGALFWVMLFLGIGKIF

GPQWNAVFQLVHQYAPYIVAGIGLLLIAAVLYRYRKAWVARSLRKPAAIRQRQK"

CDS 489630..490898

/locus_tag="PIKHDJKN_00473"

/inference="ab initio prediction:Prodigal:002006"

/codon_start=1

/transl_table=11

/product="hypothetical protein"

/translation="MVTPIHTISSGKWSVLILRIVVLLCVFAASAGTTYASSVSGSDA

PASKEENPQDKVVYLSFDDGPGNHTREVLDILRKEQVLATFFVLGEQAERYPEMIRAV

VEDGHALGNHTFNHNYEQLYSDFKVFWKQIKQTEAVVERITGFRPNLVRAPGGTYGHF

DKSYFDLLKLGGYTVMDWNVDSGDSKRKGVPAKEILHNATKVPAGAHSVIVLMHDGGA

HAETVKALPGIIKYYRDQGFRFDTMKASDQPVQFRIDSNGKYKSRKAPGKEWVAENVQ

ENSNLWLAGKELKVEIGLAAATLKQGEFRVEDQRIMVPLRTFMKKFGGSARWNAETRT

ATAVWKDRTIHADSISGDLTTKRADQMNDRMTEGVVQSQGGTIWVPLRELMEHMGVTV

DSLKVNEKEWTVKASVPLAAANSMYLDKLI"

CDS 491002..491991

/locus_tag="PIKHDJKN_00474"

/inference="ab initio prediction:Prodigal:002006"

/codon_start=1

/transl_table=11

/product="hypothetical protein"

/translation="MSYIRMEHEHAVELLNRVMKRVESVIIGKKQEIRYVLTAMLSGG

HVLLEDVPGTGKTMLVRAVASALDCSMGRIQFTSDVMPADVTGTSMYHPHTGEFKFRP

GPVMSNIVLADEINRASPRTQSALLEAMEERRITVDGTTYALPRPFFLLATQNPLQFE

GTYRLPEAQLDRFIMKIGLGYPDPEQEVELLTRMQSRELLDELRPVMLAEEVVAMQRE

VKHVHVDSVVKQYLVAVAVASRSHPAVRLGISPRGTLAWMAAAQSFAYLQGRSYVIPD

DVKEMAVPVLSHRVQLKAQNRAEIWAQAQLIQEALSSVPVPVQMATQGRGRRQ"

CDS 491988..493355

/locus_tag="PIKHDJKN_00475"

/inference="ab initio prediction:Prodigal:002006"

/codon_start=1

/transl_table=11

/product="hypothetical protein"

/translation="MTALGQRMLGAVLVVAFASLYVWHGGKAALFLSIIAALMLASAL

IIHFFGPRNINIRRQMHANRVVAGERTRVAVELKFDCPIPLLWIILCENTPAGVHRKL

LFPGTRRQFTYQYELSGLRRGVYRWESGRLYWGDVFGWNTVSAETVNHTSLIVVPQAT

EWGESNSGEYSAVGEDALSERRSSQGNRSPEFREYQQGDPLGRVHWKSTAKTGRLQTF

LPETSDSVSLGILVYEGASGYKVRQSEKRDMPAFERAVRAAARWIHTAERDNIPYQVW

TEGGGSGSALQEKWKQELLHSPENHALDKLAQARIATAQVVPPSLRTEMLDRMAIGSR

IVVLTGRMDEALVEWVMCAIGLGYRVEVQLTEFMAGEDPSPISRAESLSSNMAQNRGT

QGDATSDTTVVSETAQSIATDRTIRNMGNESLEQLRQSGVQIHWITDSSLPSMRKAEV

TDVGA"

CDS 493342..495705

/locus_tag="PIKHDJKN_00476"

/inference="ab initio prediction:Prodigal:002006"

/codon_start=1

/transl_table=11

/product="hypothetical protein"

/translation="MWEHNGNPQVNSTVADRGHTASLGLESVHSIYKAETRTSGVDLK

SHGQNSEPLLYRIGLSAILLILSLEWIYPVTSTGQQGSERFLSVMAGLTGTLLLAGLL

RTGWITGILIRLLIALAALCCMYGGNDPAGWAVTYPATLSEDMGSFMSTWRVHSISVE

TRGLLMMCGWSMLMASVQSLVLLRRSVMLFGIATLVYLLLLESFAGLDVYASVIRSVL

WILLIQAMLQLLRLNGGVTTPIFKQSPYGRWCTVTLAVSAGMVLFSAIPGQLASIPPP

ERISLEQMGERLARWAGYTQHGSIPATTAVTGYSTADAPMGAPLVQGDTIFFTAKSPE

ATYWRGETRSYYNGSTWSDPVQSFETASPSGLVRTDGWEYPAYWQRIRQTVTMKREWR

GPNPLFTGGMPINLSFQDKNRDNQKNKRLLLSNEDSATLWLAATYDNQSVKSYTADIM

IPVATPEQLRLLGKKSNMKDPAAIRRTYLQLPTTLPGRVQALAKEIIQGSETRYDAVQ

AVKSYLADHAEYTLDTRMPPRGTDFVDDFLFVTRQGYCNHFSTAMIVLLRAEGIPARW

VKGFGPGEADADVSGQYIVTQGDAHSWVEVYFPGGGWMPFEATPGFTMTQGEGQDAAA

LSGQQPVAVTPPQTDGGVAHAGAWLLARARAIAAHPWLAAALAAAALLCAAGAIRMRR

LRPALRLRLLLAWPRSSFPDRERLLSAAAPVWTALARRYGQRPPGMTLREYAASPAVA

AGTDGADIARFAADWERLLYGPDRPLRADSLDFLRRALQLARRMQAL"

CDS 496022..497560

/gene="guaA"

/locus_tag="PIKHDJKN_00477"

/EC_number="6.3.5.2"

/inference="ab initio prediction:Prodigal:002006"

/inference="similar to AA sequence:UniProtKB:P99105"

/codon_start=1

/transl_table=11

/product="GMP synthase [glutamine-hydrolyzing]"

/translation="MNKQNEIVVVLDFGGQYNQLIARRIRDLGVYSELLPYNTPAEKI

AELSPKGIVFSGGPSSVYAENAPHVDPAIYDLGLPIFGICYGMQLMAQQLDGKVERSE

KREYGKADIEFAPGAALAQGIEGEHTVWMSHGDHVVALPAGFKLDAGTESAPIAAMSN
DERKLYAVQFHPEVRHSVRGNEMIKNFLYEICGCEGNWSMETFIEDTIKDIREKVGDG
KVLCALSGGVDSSVVAALLHKAIGDQLTCMFIDHGLLRKGEAESVMETFVGKFDMKVV
KIDAQERFLSKLVGVDDPEQKRKIIGNEFIYVFDEESKQFDDFAFLAQGTLYTDIVES
GTATAQTIKSHHNVGGLPEDMNFKLIEPLSALFKDEVRKVGEECGLPEAIVWRQPFPG
PGLAIRVLGEVTEDKLKIVRDSDYILREEIAKAGLDREIWQYFTALPNMKSVGVMGDA
RTYSYTVGIRAVTSIDGMTADWARIPWDVLEKISVRIVNEVDNVNRIVYDVTSKPPAT
IEWE"

CDS complement(497700..498977)
/gene="flaD"
/locus_tag="PIKHDJKN_00478"
/inference="ab initio prediction:Prodigal:002006"
/inference="similar to AA sequence:UniProtKB:P0C6C6"
/codon_start=1
/transl_table=11
/product="Flagellin D"
/db_xref="COG:COG1344"
/translation="MRINNNLPALNAKNRINNNSNSKKTSFERLSSGLRINRAADDAA
GLAISESMRAQIRALEQGARNIQDGISLIQTAEGALDSITNGLQRIRELLVQRENDTY
TYSDKETINLEITHNLRNIEDITNNTQFNGIYLLNGSLTNAAIHTGTETIPLSINDMH
LDLYYRENFNDQNRFSQWHPAQGSWTVQNGSLTQSTEGVGDKTILSEVIQGNDMTITV
NGKFMTTHPDGNIGIIIKGNDFNNQVFGWYSQYGGLYIGERVNGSYRLITSIVVDPAQ
LDQNYELTGTVVGNYYTFSVTGVGKIEGYSANTSMLEGYTGLFTAFSEASFDDYEVIP
HLTVSDIKTALDKVTGERAKLGAYQNRLEYAYNNTSNYSENLTASESRIRDTDMAKEM
IKISTLSILTEASQAILAQANSQPQRILELIRN"

CDS 499446..500486
/locus_tag="PIKHDJKN_00479"
/inference="ab initio prediction:Prodigal:002006"
/codon_start=1
/transl_table=11
/product="hypothetical protein"
/translation="MKERLELEPGWTREEVAALTAHPELKIVQYSEHNIPDVNLIQTL

NEELFANRPDVTFRIYGFHSQSGDLSVLKYMNHVEKLVIDCNRRIHGIEGLGELLKLR
EFAFDVFEAESLEVLTLVPTTLHQLRIGKTKTKKTDLSVLSRFIELNKLSVNGHRTHI
ETIGTLSSLRSLYISGMPLKELGYLKELANLRQLHLSFGGAENLDSLAGMKSLQLLKL
LRVKGLSDLSVLPELKGLHLFGIEDQPHLTSLPSLENLTELQRVFLNNVNVENIDWAK
TSKSLKELALLQMKHINAEDIESLIYHKSLERMIVHLKSAKQQKVAKKAIMAAGMWDE
SGWWYNDESLDI"

CDS 500583..500834

/locus_tag="PIKHDJKN_00480"

/inference="ab initio prediction:Prodigal:002006"

/codon_start=1

/transl_table=11

/product="hypothetical protein"

/translation="MRKTDNMSKWKMGIGSAFLMVMLAGCGSGMQDLALGAGDSSFVN
LEAADSSSDQAQNQELSEGSTDNTGQEQQQAQDVFQTEI"

CDS 501458..502834

/gene="adeQ"

/locus_tag="PIKHDJKN_00481"

/inference="ab initio prediction:Prodigal:002006"

/inference="similar to AA sequence:UniProtKB:P31440"

/codon_start=1

/transl_table=11

/product="Adenine permease AdeQ"

/db_xref="COG:COG2252"

/translation="MDRFFKLKENGTNVRTEIVAGLTTFMTMAYILFVNTLFLGSAGA
GMSDNAVFFATAVGAGLMTIIMGLFVNIPIALAPGMGLNAYFMTVVLSSNGAISWQAA
LGAVFISGIVFIILTVTKIRQMLLVAVPDSLKMAITVGIGLFITIVGFKLANLVAVTV
NVAPDADLSQPIPGSSFNLSLGNFITHHDALLALIGLLIIAVLMVMRVKGALLIGIIA
TTLIGIPMGVTNLSGLSGASWLPNFSDLAVGQLDLKGAISLGLFEIIFIFTFVELFDT
FGTMVGTATRMGIMKDKKKGEKTIGKAMLVDAVGVSAGAALGTSTITAFVESASGVEA
GGRTGLTSVTTGILFILALFIAPLALVVPSAATAPALIIVGVLMMSQVRSIEWDDFLQ
AFPAFLTIVLMPFTGGIANGISAGIVSYVILAVFSNLVTERKVKIHWLMWILAIIVIC
RYVFIGGE"

rRNA 504108..505657

/locus_tag="PIKHDJKN_00482"

/product="16S ribosomal RNA"

rRNA 505965..508885

/locus_tag="PIKHDJKN_00483"

/product="23S ribosomal RNA"

rRNA 508970..509081

/locus_tag="PIKHDJKN_00484"

/product="5S ribosomal RNA"

CDS complement(509271..509825)

/locus_tag="PIKHDJKN_00485"

/inference="ab initio prediction:Prodigal:002006"

/codon_start=1

/transl_table=11

/product="hypothetical protein"

/translation="MKNEFNILAISGSLRNQSSNTLLMHAMIKLAPPNLKFGVYGGLN

DLPHFNPDLDVEEGPESVQNLRAQLKHSDGVLICTPEYGNGVPGVLKNALDWIVSSGE

WLNKPTVAIAASPSPMGGDKAHASLLLTLNMINAQVLHEASLTIPHITLKMNKQGVIV

DSETQHALENSLLSLGDACNQMDK"

CDS 510129..511130

/gene="fabHB"

/locus_tag="PIKHDJKN_00486"

/EC_number="2.3.1.180"

/inference="ab initio prediction:Prodigal:002006"

/inference="similar to AA sequence:UniProtKB:O07600"

/codon_start=1

/transl_table=11

/product="3-oxoacyl-[acyl-carrier-protein] synthase 3

protein 2"

/db_xref="COG:COG0332"

/translation="MNQSMSTITAMGTYVPDRILSNADLEKLVDTSDEWIVQRTGMRE

RRIAADDQFVSDLATKAVEDMMRRYNVSVEDVDMILVATSTPEYSFPSTASRVQANLK

IPHTGVLDLNAACAGFTYGLQLADSLVTSGMYHKVLVIGAETLSKITDYTDRTTCVLF
GDGAGAFLVERAAGKGDFMAAISGTSGEGGIHLYKSGLSSEMNGVPLEGEGCLVQNGR
EIYKWAVRTIPEQLSGLIAKAEMKPEQIDWFVPHSANLRMIEAVCERGPIPLERTLTS
VEYRGNTSAASIPLALQLAVDAGKLKHGHQIALFGFGGGLTFAGLVLRWSVPDSPVK"

CDS 511339..512115

/gene="ssuB_1"

/locus_tag="PIKHDJKN_00487"

/EC_number="3.6.3.-"

/inference="ab initio prediction:Prodigal:002006"

/inference="similar to AA sequence:UniProtKB:P97027"

/codon_start=1

/transl_table=11

/product="Aliphatic sulfonates import ATP-binding protein
SsuB"

/db_xref="COG:COG1116"

/translation="MGETLLAISQLNKKFDTTGGSVQALYNVDLHVKEGEFITIIGPS
GCGKSTLLRIVAGLDRGYTGSVTLNGNEIKGPGIDKGFIFQEHRLFPWLTVEKNIASD
LPLGKPEVRRKVNELIELVKLQGFEQAYPRELSGGMAQRVAIARALLRSPQILLLDEP
FGALDAFTRAHMQSVLLDIWRTNKTTMIFVTHDIDEAVFLGERVVILEPRPGQIRKIV
PIDLPYPRKKASNSFQEVRLKVLSEFEKLEEFELKDGAGV"

CDS 512207..513013

/gene="ssuC_1"

/locus_tag="PIKHDJKN_00488"

/inference="ab initio prediction:Prodigal:002006"

/inference="similar to AA sequence:UniProtKB:P40401"

/codon_start=1

/transl_table=11

/product="Putative aliphatic sulfonates transport permease
protein SsuC"

/db_xref="COG:COG0600"

/translation="MSEQAATLKATDKKTGKAVKFALGLILPGIVLILWQTLGHYEII
SEMLFPTPYTIAVALVTLVVSGDLWANLQISAMRAFAGFFLGGGLGLFFGILVGLFRK

SEKLLDPTLQMIRMIPSLAVVPLFILWFGIGEESKVLLIAKAAFFPLYIQTFMGIRGV
DNKLFEVTRVLGYSRIKQVTRLVLPAAVPNVILGVRLSLGLSWLALVVAEMIASSSGI
GYMMSDARQFADTPVVFVGIFIFAAAGLLSDTLVRLLEQRLLQWKDSISG"

CDS 513067..513966

/gene="ssuC_2"

/locus_tag="PIKHDJKN_00489"

/inference="ab initio prediction:Prodigal:002006"

/inference="similar to AA sequence:UniProtKB:P40401"

/codon_start=1

/transl_table=11

/product="Putative aliphatic sulfonates transport permease
protein SsuC"

/db_xref="COG:COG0600"

/translation="MNEGTEAIMKTAKSALDSRDISGTAPIVAGRTKVISRLKLPSRS
WKTSLSDVVIGSLIPLGTLALWQIAGSIGWLSAEFLPTPLSILSALTELVATGELIHH
LGVSMGRAGLGFLIGGVLGLCFGILTGLFRQAEYILDPSVQVLRLVPHLAIAPLIILW
FGFGEVSKVVIIMSGSFFPLYINTFMAIRGVDKKLYEVAQVLGFSPYQRIRRLILPAA
LPGILLGLRLSLAVAWIGLVVAELIGSQSGIGFLINEGKQNANTAIIFVGILIFAIVG
KGIDSLFRMIERKFLFWRDSYQG"

CDS 513990..515597

/gene="frdA_1"

/locus_tag="PIKHDJKN_00490"

/EC_number="1.3.5.4"

/inference="ab initio prediction:Prodigal:002006"

/inference="similar to AA sequence:UniProtKB:P17412"

/codon_start=1

/transl_table=11

/product="Fumarate reductase flavoprotein subunit"

/db_xref="COG:COG1053"

/translation="MSEAVREDSLFLLADVLVIGGGPAGTWAALAAAAKGASVVLADK
GYCGSSGATAPSGTGVWYVKPDEKLREEAKASRYFMGGQLAEHRWMDRVLNRTYDNMN
RLGVSGYPFPINEEGQQHRRGLQGPEYMRLMRKLVKRAGVKILDHSPAMELLADEYGV

AGAAGVQTQSGKTWTVHAGAVVIATGGCAFLSKALGCNVLTGDGYLFAVEAGAALSGM

EFSNAYAICPTFSSVTKTAYYHYASFYHEDGTIVEGAGSKKGRSVIARHLMAGRQVYA

RLDQAPEDLQPLLRVAQTNFFLPFDRLGINPFKELFPITLRLEGTVRGTGGIRVTDEN

CATGVPGLYAAGDAATRELICGGFTGGGSHNAAWAMSSGSFAGEGAAGYVLELGHNAA

KRNPSGLSSAPLVQGQVKQGTAARTAEYVSAVQAEVKPYEVNLFRTEQGLSSALGRLD

ELWSIQRNIQVQRTPEGVKAREAAAMTATARWMYNSALARTESRGMHKREDYKESDTA

QHHRLISGGLDQVWVKTEEVAKESVPL"

CDS 515594..515926

/gene="cutD"

/locus_tag="PIKHDJKN_00491"

/EC_number="1.97.1.-"

/inference="ab initio prediction:Prodigal:002006"

/inference="protein motif:HAMAP:MF_02059"

/codon_start=1

/transl_table=11

/product="Choline trimethylamine-lyase activating enzyme"

/translation="MIEVISATKCIECNQCVAVCPTNVFDRVEGGIPVIARQDDCQTC

FMCELYCPVDALYVAPDSEALTGITEQELQEQGFLGGYREKVGWGKGRKPVASHNFMV

QLSARAGF"

CDS 515957..517039

/gene="ssuA_1"

/locus_tag="PIKHDJKN_00492"

/inference="ab initio prediction:Prodigal:002006"

/inference="similar to AA sequence:UniProtKB:P40400"

/codon_start=1

/transl_table=11

/product="Putative aliphatic sulfonates-binding protein"

/db_xref="COG:COG0715"

/translation="MKQAHRYKEKNTRGTTAMWLRLSVLVSIVMVVLTLQACGNNVGS

GNTNSATGSTDEGQSKATSGQRSEDIPTILNYGFIGSNKLNLPGGAEGWGLYKGIIQE

ELKKYGITEVKLTGFPNGPDQTESLISGRLDFGSLGDTPAIIAYASGAKTRLITQSMV

NNVGYLIGKKDGPKTVKELQGKTIAIQKGSFMHRYVVGLLQEEGVTDYKLVHMLTPDA

TAALARGDVDATTNLGVPALKLISEGYTHLDDASQHPDLLGSSVTVVGEDFLAKFSDF

PRVWNEARQKALADLKQHEDEYYQFLAEIGDTTPELAKQVNPISEIKDTAFTEEGLKL

LEGTKNFLVEEKLAKKDYLISDWQQQ"

CDS complement(517239..519173)

/gene="QRSL1"

/locus_tag="PIKHDJKN_00493"

/EC_number="6.3.5.7"

/inference="ab initio prediction:Prodigal:002006"

/inference="protein motif:HAMAP:MF_00120"

/codon_start=1

/transl_table=11

/product="Glutamyl-tRNA(Gln) amidotransferase subunit A,

chloroplastic/mitochondrial"

/translation="MTYHGSVLKKSGALLLAGAVVVTTWGGTVPSASAATATPSSKTT

VAAVTAGKVPVTAVAFVQAMEEAATLAGVPFSMDKLSGTTVQRKDAALALQTWLKLES

TPESFKDVPDQASFAGAVGALNTAGLMKGYTDALFLPNAVLTQNDVAILKDRIYNYIK

PFVLEEATITDLQAAMTQGKLTSKELVQKYLDRIEKYDDQGVSINAVLTLNPDALKIA

ETLDEERAARGARGPLHGIPVLVKDNFDTNDMPTTAGCICLKDSVPAHDAEQVKKLKA

AGAIILGKTNLHEFAFGITTSSSLGGQTLNPYALDHYPGGSSGGTGAAIASNFAAAGM

GTDTGGSIRIPSSFNSLVGIRPTIGLSSREGIIPLALTQDVGGPMARTVSDAAIMLDA

TAGYDKNDVATAYAVGKIPSSYTDFLDVNGLKGARIGVATELIPSTKAEEKAVADVIN

NAVEELKTLGATAVPISIPNLAEINKYPSLSGYEFKFQLNDYLESLGDEAPYHSLSEI

IASGQFDKSQEQSMKTRDARETLETAEYKDIVLKRTQITRESLLKVMADNNLDAIIYP

TSTQAAGVIGEGQNSGGNNRLSPFSGFPAITVPAGFTTEGLPVGMEFLGRAFDEGTLI

KLAYSYEQGTHHRQAPKLTP"

CDS 519557..521686

/gene="topB_1"

/locus_tag="PIKHDJKN_00494"

/EC_number="5.6.2.1"

/inference="ab initio prediction:Prodigal:002006"

/inference="similar to AA sequence:UniProtKB:P14294"

/codon_start=1

/transl_table=11

/product="DNA topoisomerase 3"

/db_xref="COG:COG0550"

/translation="MKTLVLAEKPSVAREIARVMGARDKHKSYMEGPKYIVTWALGHL

VGLAEPEDYDKKYATWNLEDLPILPDRTKLKVLKETNHQYKAVQQLMKRQDVGELVIA

TDAAREGELLARWIMQMAQWKKPFKRLWISSQTDKAIKDGFTSLKPGSQFDRLYESAR

CRAEADWMIGLNVTRALTVRFNAQLSAGRVQTPTLGMIMDRENEINGFRSQEYETLTA

DLGDFQAVWRAAGGDSRIFDPQETQELKKRIEGRKGSIAQVKKSEKVELHPLAYDLTE

LQRDANRKYGFSAKQTSNVLQRLYEQHKLVTYPRTDSRYLTSDMTGTLKERLDSVAIG

PYASLARPLLRKNLNITKRIVDDSRVTDHHAIIPTEQTVLLNQLNPEERKLYDLIVRR

FISLFYPAAKYDSVAITVQVGNDSFHVKGTTVKESGWREVYGGDYSDEDDDRADDSSD

LDRALLPDVQQGQSVTVQRCHIKSGRTMPPKRYTEAALLSQMEKHGLGTPATRADIIE

KLVSSDTIDRQGNSMHPTGKGKQLIELAAPQLRSPELTARWEAELERIARGQGKPEPF

LESIRSMAKELVSTVKGSKAEYKPHNVSNSHCPDCNARLLEKKGKRGKFLVCPTEDCG

YRRSAEKRLSNRRCAQCHKKMEIKEGKAGLYVQCLPCGITETLDKDKQHVNKRDQQKL

VKQYAKQESIGSNLGELLKAAMEKKGE"

CDS 521745..521882

/locus_tag="PIKHDJKN_00495"

/inference="ab initio prediction:Prodigal:002006"

/codon_start=1

/transl_table=11

/product="hypothetical protein"

/translation="MPHNKPEKIHNQQNKDSIQEESISVRPEKAAKRAPNLNGIPKQE

S"

CDS 522012..522275

/locus_tag="PIKHDJKN_00496"

/inference="ab initio prediction:Prodigal:002006"

/codon_start=1

/transl_table=11

/product="hypothetical protein"

/translation="MQTGLILWFLFINVVGYLVMSDDKKRAQRRRDRTPERTLFLLAF

IGGALGVWIAMYRKRHKTKHPSFTIGIPLLLFLNAVIYGYFLQ"

CDS 522462..522896

/gene="nhaX"

/locus_tag="PIKHDJKN_00497"

/inference="ab initio prediction:Prodigal:002006"

/inference="similar to AA sequence:UniProtKB:O07552"

/codon_start=1

/transl_table=11

/product="Stress response protein NhaX"

/db_xref="COG:COG0589"

/translation="MLFSKILVAYDGSKASNKALDRAIELAKVSPGAVLDVIHAFDFP

RVFIGEGLAPLPPSLNNDYYNLAVQTTDEAKERIQAAGVTANVDLIQGAAAEVLLDYA

KENGSDIIIIGSRGLGGIREFVLGSVSHNVVQHAQVPVLIVK"

CDS 523479..523940

/gene="purE"

/locus_tag="PIKHDJKN_00498"

/EC_number="5.4.99.18"

/inference="ab initio prediction:Prodigal:002006"

/inference="similar to AA sequence:UniProtKB:Q9WYS7"

/codon_start=1

/transl_table=11

/product="N5-carboxyaminoimidazole ribonucleotide mutase"

/db_xref="COG:COG0041"

/translation="MGSKSDWETMKYACEVLDELEIGYEKKVVSAHRTPDLMFEYAEQ

AIDRGFKVIIAGAGGAAHLPGMVASKTMLPVIGVPVQSKALNGLDSLLSIVQMPGGIP

VATVAIGKAGATNAGLLAAQIIGAFDQDVQRRSEARRERIKQEVLESSDEI"

CDS 523937..525139

/gene="purK"

/locus_tag="PIKHDJKN_00499"

/EC_number="6.3.4.18"

/inference="ab initio prediction:Prodigal:002006"

/inference="similar to AA sequence:UniProtKB:Q7A695"

/codon_start=1

/transl_table=11

/product="N5-carboxyaminoimidazole ribonucleotide

synthase"

/translation="MNSTRIQPGSAGKAERVLLPGKTTIGVLGGGQLGRMLTLAGTAM

GYRFVTLDPAADAPCGQIADQIKAGYDDEQAALELARQCDVITYEFENVDADVASLLE

RESYVPQGSALLYTTQHRLREKRAIEAAGVRVAPYREITSKDTMKAAVSELGVPCVLK

TVTGGYDGKGQRVIRETNQAISAYEELAATGAELVLEQFIKFDCEISVVVARSTNGEI

KTFPPAENIHVNNILHASIVPARVATEIQIEAQKLAAAVAQSIQAVGLLAVELFVAAD

GRLYANELAPRPHNSGHYTMEACATSQFEQHIRAICGLPLGDTSLLSPVVMVNVLGEH

LEGIIARTGQADAEALELGVIPKLHIYGKTEAKTGRKMGHVNLLCQDVEEGLQWIEQT

NLWRNTNL"

CDS 525136..526434

/gene="purB"

/locus_tag="PIKHDJKN_00500"

/EC_number="4.3.2.2"

/inference="ab initio prediction:Prodigal:002006"

/inference="similar to AA sequence:UniProtKB:P12047"

/codon_start=1

/transl_table=11

/product="Adenylosuccinate lyase"

/db_xref="COG:COG0015"

/translation="MIERYSRPEMRAIWTEENKFKSWLEVEICACEAWAELGVIPKEE

AALLRQNASFDIDRIYEIEQETRHDVIAFTRTVSESLGAERKWVHYGLTSTDVVDTAL

GYVLRQANEILERDIVNFIEILRDKAKTYQHTPMMGRTHGVHAEPTTFGLKMALWHEE

MKRNLERFRHAADNVQYGKISGAVGTYANIDPFVEEFVCEKLGTKPAPISTQTLQRDR

HAEYMATLALIATSLDKFAKEVRALQKSEFREVEEAFAKGQKGSSAMPHKRNPIGSEN

ISGLSRVIRGHMVSAYENVTLWHERDISHSSVERIILPDATMLLNYMLNRFGNIVKNL

TVFPENMKRNMERTFGVPFSGRVMTKLIDKGFSREQAYDTVQPRAMQAWEEQRQFQDI

VKSTPEITEVLSEEEIADAFNPSWHLKHVDTIFKKLGLND"

CDS 526673..527545

/gene="purC"

/locus_tag="PIKHDJKN_00501"

/EC_number="6.3.2.6"

/inference="ab initio prediction:Prodigal:002006"

/inference="similar to AA sequence:UniProtKB:P9WHN1"

/codon_start=1

/transl_table=11

/product="Phosphoribosylaminoimidazole-succinocarboxamide

synthase"

/db_xref="COG:COG0152"

/translation="MALSTAADLVKAPLLYKGKVRELYDLGENFLIVVTDRISAFDYV

LDPAVPEKGNVLNKLSSFWFELTGDMMDNHVVHTDVNQLGDIITDPELLKDRIMVTRK

AERIDIECVVRGYITGGGWRQYEKSGEVNGIKLPDGLRKNAKLDVPIFTPAAKNDVGH

DEDIPMDRMKELVGDALAIELQEKSLRLYEFARDYCDQRGIILADCKFEFGMVDGKVI

LIDEIFTPDASRFWAKENYELDIEIDSMDKEPVRTYLLGSDWDKNSKPDPLPQEVVEA

TTARYVDIYNRLTK"

CDS 528138..528383

/gene="purS"

/locus_tag="PIKHDJKN_00502"

/EC_number="6.3.5.3"

/inference="ab initio prediction:Prodigal:002006"

/inference="similar to AA sequence:UniProtKB:P12049"

/codon_start=1

/transl_table=11

/product="Phosphoribosylformylglycinamidine synthase

subunit PurS"

/db_xref="COG:COG1828"

/translation="MIKATVYVTIKQSVLDPQGVAVQGALHSMGFNEVESVRIGKVME

LTLDTTDRAEAEKRLTVMCEKLLANTVVEDYRYELEG"

CDS 528388..529077

/gene="purQ"

/locus_tag="PIKHDJKN_00503"

/EC_number="6.3.5.3"

/inference="ab initio prediction:Prodigal:002006"

/inference="similar to AA sequence:UniProtKB:P12041"
/codon_start=1
/transl_table=11
/product="Phosphoribosylformylglycinamidine synthase
subunit PurQ"
/db_xref="COG:COG0047"
/translation="MKFAVLVFPGSNCDIDCYKAVEDAIGQEVDYVWHTATDLSAYDC
ILVPGGFSYGDYLRCGAISRFAPVMNEVAKAAEQGKYILGICNGFQILTEAGLLPGAL
IRNMSLKFRCHDTVLKVANADTPFTRDYAPGEEIVIPIAHGEGNYYCDEETLASLQAN
NQIVFTYGSNPNGSLGDIAGICNEGGNVVGMMPHPERAVDSLLGSEDGKRMFTSILKA
WRDRHDAAAIR"

CDS 529055..531298

/gene="purL"
/locus_tag="PIKHDJKN_00504"
/EC_number="6.3.5.3"
/inference="ab initio prediction:Prodigal:002006"
/inference="similar to AA sequence:UniProtKB:P12042"
/codon_start=1
/transl_table=11
/product="Phosphoribosylformylglycinamidine synthase
subunit PurL"
/db_xref="COG:COG0046"
/translation="MTQQLSAKEPTAEQVAEHKLYAQMGVSDSEYELICEFMGRKPNY
TEIGVFSVMWSEHCAYKNSKPLLRRFPTSGPRVLMGPGEGAGIVDIGDNQAVVFKIES
HNHPSAVEPYQGAATGVGGIIRDIFSMGARPVALLNSLRFGKLESDRVKYLFEHVVAG
IAGYGNCIGIPTVAGEVMFDESYEGNPLVNAMCVGLIDHDKIQRGVAKGVGNPVYYVG
PPTGRDGIHGATFASVELTEESESQRTAVQVGDPFMEKLVMESCLELIDTGIVLGIQD
MGAAGLTCSSAEMASKAGNGLELYLDQVPQREEGMTPYEMMLSESQERMLFVVEPKDE
AQAMEIFERWGVICAKVGKVTDDGRLKLIHHGEVVGDMPVTALVDECPVYDKPSSVPA
YYEQSASIDTLRYDEVSDLGGALKQVLASPTVASKKWVYDQYDYMVRTSTAVRPGSDA
AVVTIRGTRKGLAMTTDCNGRYVYLDPEVGGRIAVSEAARNIVCSGAEPLAITDNLNF
GNPEKPDIFWQMEKAVDGMAEACRVLDTPVIGGNVSLYNENAKGSIYPTPVVGMVGLV

HDTDHITTQAFKSEGDVIILLGETKAELGGSELQYAVHGKTEGRPPELNLQTEKALLG

TVLEAIQSGLVRSAHDLSEGGLAVALAESCISGNVGAQVNVETALRADHALFSESQSR

ILLSASPEQAGKLEAFVRERGVPVAVIGRVEGSNLTIELNGTSAVNEPVGGLAQVWED

AIPCLMN"

CDS 531283..532761

/gene="purF"

/locus_tag="PIKHDJKN_00505"

/EC_number="2.4.2.14"

/inference="ab initio prediction:Prodigal:002006"

/inference="similar to AA sequence:UniProtKB:P00497"

/codon_start=1

/transl_table=11

/product="Amidophosphoribosyltransferase"

/db_xref="COG:COG0034"

/translation="MSHELTTGPLWTGDYYNEGSGKEGLDKLKEECGVFGVFKHPDAA

SLSYYGLHALQHRGEESAGMCVSDGNEFHYHRGMGLVKEVFTKDLMQTLTGDISIGHV

RYSTSGDSKLTNAQPLVFKYRDGDLAVATNGNIVNAPTIRRELEQGGSIFQTTSDTEV

IAHLIARSSKGLVEAAKDAFQRIVGGYAFLIMTNDKLLVASDPHGLRPLTMGKLGDAY

LFASETCALETIGAELIRDIEPGELLVLDADGLHEDRFDHHKHRKALCAMEYIYFARP

DSDMNGANQHAARKRMGSRMAIESFVDADLVTGVPDSSISAAIGYAEQTGIPYEMGMI

KNKYTGRTFIQPSQELREQGVKMKLSAVRRVVEGKRVVMIDDSIVRGTTSRRIVNMLR

DAGATEVHVRITSPPFKNPCFYGIDTPDSRELIASQLSVEEICREINADSLSFLSPDG

LITSIQGDNQDDYKGGLCLACFDNDYPTRLDFGGEEKFGCSC"

CDS 533310..534350

/gene="purM"

/locus_tag="PIKHDJKN_00506"

/EC_number="6.3.3.1"

/inference="ab initio prediction:Prodigal:002006"

/inference="similar to AA sequence:UniProtKB:Q5L3D0"

/codon_start=1

/transl_table=11

/product="Phosphoribosylformylglycinamidine cyclo-ligase"

/db_xref="COG:COG0150"

/translation="MSEAYKNAGVDIAAGNEAVERMKKHVKRTFRPEVMTDLGGFGAL

FGLNKDKYDEPVLVSGTDGVGTKLKIAFAMDRHDTIGIDAVAMCVNDIVVQGAEPLFF

LDYLACDKVIPEKIEAIVAGIAEGCHQSGCALIGGETAEMPGMYSEGEYDIAGFTVGI

VDKAKIINGTTIAPGDTVIGLASSGVHSNGFSLVRKLLLEQAGLGLQDEVAELGGKLG

DALLEPTKIYVKPLLSLLEKVKVKGMAHITGGGFIENIPRMLPSSVNVDINYGSWPIL

PIFNLLQEKGSVSNRDMFTTFNMGIGLVLVVNEADAVEALEQLKASGEEAYIIGRVTE

GDARVTFTGADV"

CDS 534350..534967

/gene="purN"

/locus_tag="PIKHDJKN_00507"

/EC_number="2.1.2.2"

/inference="ab initio prediction:Prodigal:002006"

/inference="similar to AA sequence:UniProtKB:P08179"

/codon_start=1

/transl_table=11

/product="Phosphoribosylglycinamide formyltransferase"

/db_xref="COG:COG0299"

/translation="MANYRIAVFASGEGSNFQALVDAARNGGLGASIDLLVCDKPAAR

VVQRAQEAGVECHLFTPKNYDSREAYEAEIVEVLESRNIDLVVLAGYMRLLTSVVVDR

YAGRLINIHPSLLPAFAGKDAIGQALEYGVKITGVTVHFVDGGMDTGPIIAQHPVPIL

PEDTVESVSRSIHAAEQKLYPEVVSWFAQGWVQLEGRQVTVMKPD"

CDS 535300..536847

/gene="purH"

/locus_tag="PIKHDJKN_00508"

/inference="ab initio prediction:Prodigal:002006"

/inference="similar to AA sequence:UniProtKB:P67544"

/codon_start=1

/transl_table=11

/product="Bifunctional purine biosynthesis protein PurH"

/translation="MSIKRALVSVWDKTGIVDFCRELSQMGVEIISTGGTSSLLSKEG

VPVIGISDVTGFPEIMDGRVKTLHPAVHGGLLAVRDSEEHKRQMEENGLDYIDLVVVN

LYPFQDTIAKPDVTYEDAIENIDIGGPTMLRSAAKNHAYVSVVVDTADYGKVLEEVRS
NGDTTLETRKQLAAKVFRHTAAYDAVISDYLSNLNGDPLPERLTVTYEKLQDLRYGEN
PHQQAAFYRKPLAAQDTLTTAEQLHGKELSYNNINDANAALQIVKEFEEPAVVAVKHM
NPCGVGIGESIYEAYGKAYAADPTSIFGGIVAANRIIDSDTAAKLSEIFLEIVLAPDF
TQEALDILTKKKNIRLLKTGELNAARNRESKFVVTSIDGGMIVQQSDVHSIEASELKV
VTDRAPSEEELKQLLFGWKVVKHVKSNAIVLAANDMTVGVGAGQMNRVGAAKIAIEQA
GELAKGAILASDAFFPMGDTLELAAKAGVTAVIQPGGSIKDEESIKVANEYGIAMVFT
GVRHFKH"

CDS 536977..538242

/gene="purD"

/locus_tag="PIKHDJKN_00509"

/EC_number="6.3.4.13"

/inference="ab initio prediction:Prodigal:002006"

/inference="similar to AA sequence:UniProtKB:Q8ZAR2"

/codon_start=1

/transl_table=11

/product="Phosphoribosylamine--glycine ligase"

/db_xref="COG:COG0151"

/translation="MDILVVGGGGREHAIIWALAKSPKAGKIHCAPGNAGIAQLAECH
PIAVNEFDKLTALATELNVGLVVIGPDDPLADGIVDAFDKTGIPVFGPRRNAAEIEGS
KTFMKDLLHKYNIPTAAYEKFDNYEQAQAYLNEQSIPVVIKADGLAAGKGVTVAYSRD
EADQALRSIMVDKVFGEAGAKVIIEEFLAGQEMSILAFVDGETVRPMAAAQDHKPVFD
NDQGPNTGGMGTYSPLPHIPASIIEEAVETIIIPTARAMVSEGRPFQGVLFAGLMISP
DGKPKTIEFNARFGDPETQVVLPRLKSDLIDIFWATVHGKLADIEIEWSDEAAVCVVL
ASGGYPGPYAKGVVIEGLDQVDEAVVFHAGTARNESGEWVTNGGRILGVVGLGSDIAE
ARNKAYAQAERIHFDGKHQRSDIAAKALV"

CDS 538969..540306

/locus_tag="PIKHDJKN_00510"

/inference="ab initio prediction:Prodigal:002006"

/inference="similar to AA sequence:UniProtKB:P9WFP3"

/note="UPF0053 protein Rv1842c"

/codon_start=1

/transl_table=11

/product="hypothetical protein"

/translation="MDIITILNIVLLIILIALTAFFVASEFAVVKIRTSRVDQLVAEG

NKKAILAKKVVSDLDYYLSACQLGITVTALGLGALGKPTVERLLYPVFNYLDVSASIS

SVASYAIAFILVTFLHVVVGEMAPKTMAIQFSEKLTLLLSPPLYWFGKIMYPFIWALN

GASRVLLRAFGVKPAGHEQAYSEDEIKIIMNQSYEGDENNKTKLSYLENVFVFDERDA

KDIMVPRTELVTLNQNMTYDDIIPILDEHNYSRYPVIEDGDKDRIVGIVNVKKILPDM

VALREHKLIDFVREIPFVSEVTSIQDAMIKMQQERVHMAVVVDEYGGTSGIITMEDIL

EELVGEIRDEFDADEVADIQVTGEHQYLINGRVLLDELERQFGLVFEGNEEMDTVAGW

IQFQKGVGVEQGDTVEHGDYVWTVVDAENFHIKQVLLERVNGAQVDESSSDLA"

CDS 540680..541762

/locus_tag="PIKHDJKN_00511"

/inference="ab initio prediction:Prodigal:002006"

/inference="similar to AA sequence:UniProtKB:P9WLQ7"

/codon_start=1

/transl_table=11

/product="hypothetical protein"

/db_xref="COG:COG1253"

/translation="MDGIIALNLFLVAVFIGLTAFFVGAEFAILKVRMSRIDQLILEG

NKKAVLAKKVAHNLDYYLSACQLGITITALVLGALGEPTVEKMLHPLFERLEVPAALS

TVLSYGIALAIITFLHVVIGELAPKTLAIQFAEKMTLLLAPPLYWFGKIMNPFIYALN

GAARLLLGIFGIKPAGHDTVHSEEELKLIMAQSYESGEINQTELDYLKNIFAFDERLL

QEIMIRKEKIVTLDKEMPLERMIEVLKQHEYTRFPVIEKGNQDQFIGFIHTKEMLTSV

AAGREFNMDTFVHDMLKFSELSPIKDVLVQMQQSRSHIATVHNAEGETVGLVTMEDIL

EEIVGDIKDEYDGKDLVQPSKRLKLT"

CDS 542061..543011

/gene="mhqO_1"

/locus_tag="PIKHDJKN_00512"

/EC_number="1.13.11.-"

/inference="ab initio prediction:Prodigal:002006"

/inference="similar to AA sequence:UniProtKB:P96693"

/codon_start=1

/transl_table=11

/product="Putative ring-cleaving dioxygenase MhqO"

/db_xref="COG:COG0346"

/translation="MFRTSGIHHVTAFVDDAQKNVDFYAGVLALRLVKKTINFDAPDV

YHLYYGNEDGAPGTIITFFPQQNARRGVIGAGQTGVTVYAIPVGSLPFWKERLTSFDI

PFENKTRFGEQYIRFFDKGGLLLELVERDGGQPSQWAFNGVQSEHAIKGFGGAVLFSH

VPDKTTDVLITMLGLEQVGEEDGIIRLQASGDIGQLIDIQSTGIQRGIGGAGTVHHIA

WRAKDYVEHEQMQQELQKAGYHPTPVIDRQYFNAVYFREPGGILFELATDPPGFARDE

PQESMGQKLMLPEWYEPQREQIEQLLPPIQVREWKGESKS"

CDS 543008..543631

/gene="mhqD"

/locus_tag="PIKHDJKN_00513"

/EC_number="3.1.-.-"

/inference="ab initio prediction:Prodigal:002006"

/inference="similar to AA sequence:UniProtKB:O34842"

/codon_start=1

/transl_table=11

/product="Putative hydrolase MhqD"

/db_xref="COG:COG0400"

/translation="MSTTNTMKHIYKAGSQPNAPTLLLLHGTGGTENDLVGLAEMIAP

GSGILGVRGNVSENGMPRFFRRLAEGVFDEEDLIARTAELGSFVDAAAVEYGFDRANV

FALGYSNGANIAASLIFHQADVFKGAILHHPMVPLHGLELPDLKGLPVFIGAGENDPI

VPKRETDELASLLSGAGAAVHMHLERQGHQLTRTEAEAAAAWFKTQA"

CDS 543902..544792

/gene="rutD_1"

/locus_tag="PIKHDJKN_00514"

/EC_number="3.5.1.-"

/inference="ab initio prediction:Prodigal:002006"

/inference="protein motif:HAMAP:MF_00832"

/codon_start=1

/transl_table=11

/product="Putative aminoacrylate hydrolase RutD"

/translation="MKPSHSSHSFGSSAFIQTRDGRKLHYMSRGTGDLIVIFESGMGF
SRSTWGLVAPAVAEHARAVVYDRSGTGRSEVDSSPRKLRRIAEDLDDLLTALGPGPFI
LVGHSWGGPIVRAAAASNLSRLRGIIMVDPSDEHCELYFSKAAKFNFALNSLLIPLMA
RTGLYRLVGSRPGSVQPDDVVRDHGAEDFTVQAARTMVAESKPFLNDLASLLVTPLDL
GDLEVSIISGTKTGMGERKIRPAIMEAHRRTVSTLTNAKWIGADQSSHMVMFTDPQVI
VDEILRMVNDASSGARTEQK"

CDS 544856..546148

/gene="ilvA"

/locus_tag="PIKHDJKN_00515"

/EC_number="4.3.1.19"

/inference="ab initio prediction:Prodigal:002006"

/inference="similar to AA sequence:UniProtKB:P9WG95"

/codon_start=1

/transl_table=11

/product="L-threonine dehydratase biosynthetic IlvA"

/db_xref="COG:COG1171"

/translation="MKPVEQEPTGTANPGRTGVGMEDIVRAHHVLREVIVRTPLQRDA
VLSAKYNCNVYLKREDLQVVRSFKIRGAYNMIRSLTPAELEKGIVCASAGNHAQGVAF
SCNALSIHGKIFMPSTTPNQKVKQVRRFGGDNVEVVLTGDTYDDAYEEAMRACNEQGM
TFIHPFDQPKIIAGNGTVAMEIMESLDEKADYVFVTIGGGGLAAGVGTYMKTVSPETR
IIGVEPLGAASMSEAMFRKQVVTLDDIDKFVDGAAVKRVGDLTFDICSGTLDDIVKVP
EGKACTTILELYNENAIVVEPAGSLAVAALEQYREQIAGKTVVCVISGGNNDIDRMQE
IKERSLIYEGLKYYFMVNFPQRAGALREFLVEVLGPNDDIARFEYTKKHDKENGPALV
GIELMYKEDYQPLIERMNRKGLAYTELNKNLNLFNMLI"

CDS 546148..546750

/locus_tag="PIKHDJKN_00516"

/inference="ab initio prediction:Prodigal:002006"

/codon_start=1

/transl_table=11

/product="hypothetical protein"

/translation="MNHSNMKSDRSEAQPLLRPARKEDVAQVIPLLYQAIGDIAYALA
GEGEHEKAMQILQQFYVQEDNRISYRNITVMEQNEQVAGILVAYDGGEADLLDQPILD

RPGRSTDEKYTLVKETRPGEYYLDTLSVSEVYQGQGIGKALMAAFEQQGRELGHSQVS

LIVERDNGRALMLYERQGYIKDEVIVIAGHEYNHMVKPIL"

CDS complement(546878..547843)

/locus_tag="PIKHDJKN_00517"

/inference="ab initio prediction:Prodigal:002006"

/codon_start=1

/transl_table=11

/product="hypothetical protein"

/translation="MTSSYLSDQRKGMRFFITVLTIATIFTGTAATSGAATTAPSSLD

TKVTTSSSTAIYKTFQSMLKKKNGLPAADTFLESNIKKVTTHHATLMVLQLESARLKA

LTSTTDRLLVPNVQDKMLKVYQPNDSFTKLMERSQDSDLRVLLKEARDSGYRLVMLEG

SLYPIMNYAALVKYTSDITEDISSYISIMTKETKNLPADDGALVIGYQEILLRALSQE

RFLELYPKSNRAKQVQDLLNSYTLYTFYGLNNTPLFDYDTNKMVANAQRGYNGVLQRL

ASVDSEFLTKLDAFMDVVKEAKYEKTAAVEKWLEQNVPTGADAGY"

CDS 548155..551319

/locus_tag="PIKHDJKN_00518"

/inference="ab initio prediction:Prodigal:002006"

/codon_start=1

/transl_table=11

/product="hypothetical protein"

/translation="MKKVMSALAVSAMLMSALPTSVMDAAARISIYINDAELSSAQAP

VMKGGRVLVPLRSIFEGLDATVKYTNKTKTITASRGDQEVSLTLGSKTAYINGEAIAL

DVPANTIKGNTMVPIRFVSEAFGEKVFWNSRNQRVDIKTTSTPPVDETKYAAWNIYGS

VSGSNGDGRDLTVSFTRPTSEKAVSQYRVMLVKTRDVNSFNESAASVVPSGNYTSISP

NGTDPRLTLNAQTRDVNGDLLNTSETYRLYVLTMGNSNNSYKNLLAWSSQSLKLNNVK

TTVQPVTGLRIADISDYGDGRDLEINFTQPSTTSNITYYRAFVVKAKDASSFNLTTAN

NVSSSNSTIIYKGNSTAVKTQLSSSTRDTSGELIKNGTSYVVYIVSVSSNAATADNKL

STVSSSLTLGVNTATAPVITSVRDNSDYGDGRDIQVSFNRSSDESKVANYRIFVVRNS

VSSSFNLTTASNLSSSLYYNVNKTGNNITTTLPSSMKDTSGYNVTNLQDYRIYVMAVG

NQQNGYTNALSSSSTLLRLTTNSNVGVASNIGVADVSDYNDGRDLKVSFNRASDETNV

SAYRVYVVRSAYAGSFTLSAANASNNYYQVSKTGGNQSFTLPSNTVDTSGSRITNGIS

YRVFVLSVSNSGNSSQNALSSYSSQITLVQNSTVAAPTYVNATDIGDNGDGSDLQVTF

NRSSNETNVYHYRVFVVKTANLGGFDLAEANRNPNYKVVNKANGSGNYTLTLNDVKAT
DGTKILNDENYRIFVLAVSNNSNSSNVLSTAYADIKLSVTSAVGAVKITDVEVKNEEQ
AAGDATDVTLRYTEPASKVGISRYVLMIAPTGSTFNLSMAKNIALANNSRSVGINGSP
VLLSNALLDINGQNIEAGKLYTAYILSESSDSKRDSSMTSSTFKINAKKQSQVPVATD
VTATASIETGINVSFNMPSGTDGISGYNLYIVKASKTSFDLTKPSGNINTADNFTANL
GLNAKDSDGADLEAGKDYIIYVVSVAGNQTTNKNSTSAASRSFTVPTSNTNQGGTGTP
NTEVSPNGKDGNSTNNQTSSTGSN"

CDS 551625..552542

/gene="rhaS_2"

/locus_tag="PIKHDJKN_00519"

/inference="ab initio prediction:Prodigal:002006"

/inference="protein motif:HAMAP:MF_01534"

/codon_start=1

/transl_table=11

/product="HTH-type transcriptional activator RhaS"

/translation="MLAFRLTGLPDSRLPLYLYCVGTQEEKVQFRPDGFPVYQLFLLR
SGKGEFKVPGEGAWTVSAGELFVMEPGVAHEYIPHSKTHGELGYIGIGGEAAGMVLRS
AGLLAMGPLRLSEFEQFWSRVTAIWHSLDDGMTEMWNSSTIIYQLILDMSRSTLTLHD
ETEEREARSRSLTRSERESESANEAFTRAVSLMHTHYQDDLLLKHVAEAVGYSVQHLN
RLFHQRHGVTGHQYMQRLRLQKAAEWLNKHPRASVREAAETVGMEVNYFIRMYKREFG
ETPGKEIKHRIQLKMEKDSTGPVDHLYEV"

CDS complement(552859..553788)

/locus_tag="PIKHDJKN_00520"

/inference="ab initio prediction:Prodigal:002006"

/codon_start=1

/transl_table=11

/product="hypothetical protein"

/translation="MRNESPSRILKYRQGLQITGDQNVCRQGEQSLGISYKKLKEIQN
RQMTEMSKMTPEHVKLFDQISTIARQAPADERTQEEWILSAGRAIVQAQRDGKPAREL
YGPDLEQHIYSQLGVADNNPAATASESNMDSSRASSTAVQGSGSKSNERSTSAKKTAA
VEAEPAETPDQVKRTPKWYFMISWAALSFVMLIQGAVGLFIGWTGGDTEPFSHISLFS
LIVAAVGGIALVEMLRRLAERPDDQGADKTARPQVNLRGIVIYIVIVVLVLFVGYPLR

DSLPVFTLAPWISLVIGIVGLATLRPLFGQKKA"

CDS 554110..554346

/locus_tag="PIKHDJKN_00521"

/inference="ab initio prediction:Prodigal:002006"

/codon_start=1

/transl_table=11

/product="hypothetical protein"

/translation="MDQQTDKLTRKLLLNSNSRGVTDEVVPMDETLEDAEYAEEYTDG

TFQSGSFWGSSNSAYEHEWQGRVETHEMFRRSYE"

CDS 554535..555416

/gene="menH_3"

/locus_tag="PIKHDJKN_00522"

/EC_number="4.2.99.20"

/inference="ab initio prediction:Prodigal:002006"

/inference="protein motif:HAMAP:MF_01660"

/codon_start=1

/transl_table=11

/product="2-succinyl-6-hydroxy-2,

4-cyclohexadiene-1-carboxylate synthase"

/translation="MINKMNMSREEWKGEIGMERQISIRHGQEELTATIHYPVVKDIK

EGNHQQRVPLAVICHGFVGSRIGVDRLFVKTARELAEDGYLVLRFDYIGCGESSGEYG

AEGLESMITQTRSVLDYAVNCSDVDPTRVTLIGHSLGGAVALLTAVRDKRVKNLVMWS

SVGYPFNDIVKITGRDVYDEGVKLGSADYLGYKFTPAFFESLAEHQPYQEAVKFNGDV

LVVHGTSDEIIPVDYAFLYQKVFWMRQEGRCDKEIIFQGDHTFSSGKEREQLITRTRE

WLGERQKIEQDWQHWMI"

CDS 555596..556381

/gene="ygiD_1"

/locus_tag="PIKHDJKN_00523"

/EC_number="1.13.11.29"

/inference="ab initio prediction:Prodigal:002006"

/inference="similar to AA sequence:UniProtKB:P24197"

/codon_start=1

/transl_table=11

/product="4,5-DOPA dioxygenase extradiol"

/db_xref="COG:COG3384"

/translation="MTLPALFIAHGSPALAVESNDYTHFLNQLGDRLPAPKAIVVFTA

HWDRPEPSVTMDDTHQTLHDFYGFPSNMYTMNYPAPGQPDLANEICALFTRSNLPHQP

VRGRGLDHGVWVPLLHMYPEANIPVIAVSVDSLRSPQEQYDIGRMLEQLRHDNVLIIG

SGGTVHNLRMLGTNDEPQDWAVEFDNWIGERLEQWNTRELFQYDKKAPHARTAVPSYG

TEHVAPLFYAMGTADMSRSAKRLFQSYPYGTLSLNCWQFGDGV"

CDS complement(556561..556773)

/locus_tag="PIKHDJKN_00524"

/inference="ab initio prediction:Prodigal:002006"

/codon_start=1

/transl_table=11

/product="hypothetical protein"

/translation="MPTQRRLETDADFQEAMEMKHKVRVFKDNHQIDSGGVIIRFDSN

TVVIQSSVSELAYHSRTACELFEIRK"

CDS complement(556935..557474)

/locus_tag="PIKHDJKN_00525"

/inference="ab initio prediction:Prodigal:002006"

/codon_start=1

/transl_table=11

/product="hypothetical protein"

/translation="MFSFNQWLRENKVAMWILTVLRVYIGYDWMTHGWGKLTGGFQAG

GFLAGAVGKATGENPTVQAWWGTFLENFAVPNAGLFDFIIPMGEFLVGLGLILGCFTT

LAALMAMVMNFAFLFSGTVSTNAQLVLMEVFLVVAGANAGKIGLDHWVMPYLRGLFTR

NKGSQPKDTPTINHTHKTA"

CDS 557814..558881

/gene="yerB"

/locus_tag="PIKHDJKN_00526"

/inference="ab initio prediction:Prodigal:002006"

/inference="similar to AA sequence:UniProtKB:O34968"

/codon_start=1

/transl_table=11

/product="Putative lipoprotein YerB"

/translation="MKQLKWNKTTSAASLLILALSLVACQNKEMVQIPKPESVPTPIQ

EEQETEVPATSLYTAPLTGLPVDEAITHRPLAVMINNAPAARPQSGLSAADIIIEVLA

EGGITRFIAIFQSEGGADTVGPVRSIRPYLIELGESYDGVLVHAGGSPDAYSILQKQQ

KQHMDEISNSGPYFWRSSDRKAPHNLYTSADKLREGADTKGYSHDFKSPVYTYNEESA

TSAGELVKQFDIHYLLESYRVTYDYDEVSGRYMRLVNGKADQDLDNGNPIGAANIIVA

GADHKVLDNVGRISVNLEQGGEAMLFQKGKMIRGQWVKKPGDIIRFVQNGSEVALVPG

KTFISIVPNQPDFAGHVEIEN"

CDS 559053..559715

/locus_tag="PIKHDJKN_00527"

/inference="ab initio prediction:Prodigal:002006"

/codon_start=1

/transl_table=11

/product="hypothetical protein"

/translation="MFHRLSAISRKGDRAMKLKKKKDIFFETLENMADTVVQAADYFS

QHVSNLQDVTLFANEMKKYESQCDDYVHTVITELNKTFITPIERDDIMELTTTLDDVL

DGLEATASRFYMYQLTDPDEYIVQFAEILRQSAYEIQKAIHLLSQKKLLAIREYTIRL

NDLENQGDEVLRNCIKNLFATVPDPIELIKRKEIYERLETTTDACEHVANVLESIIMR

NS"

CDS 559730..560719

/locus_tag="PIKHDJKN_00528"

/inference="ab initio prediction:Prodigal:002006"

/codon_start=1

/transl_table=11

/product="hypothetical protein"

/translation="METTIWVLGIVVFLALAFDFINGFHDTANAIATSVSTRALTPRR

AILMAAVMNFVGAMMFTGVAKTIGGSVTDPTTLDNGIEVVIVTLIAAIIWNLVTWWFG

IPSSSSHALIGALAGAVLVGAGADKVKWSGFIDIVGGLLLSPLIAFAIGYVVMTILKY

IFAKRSPHNVNKGFRTVQIFTAALQAFTHGTNDAQKAMGIITFALVAAGVQDHLDVPL

WVKISAATAMALGTSIGGWKIIKTMGTKIFKIEPINGFAADLSAASVIFSATLLHLPV

STTHAITSAILGVGSAKRFSAVKWGLAGRIIITWFITIPITGALAGLLYWIIF"

CDS 561072..562031

/gene="corA_1"

/locus_tag="PIKHDJKN_00529"

/inference="ab initio prediction:Prodigal:002006"

/inference="similar to AA sequence:UniProtKB:Q9WZ31"

/codon_start=1

/transl_table=11

/product="Cobalt/magnesium transport protein CorA"

/db_xref="COG:COG0598"

/translation="MIRTLAISRDHQVSVNVPLTQLDLQDYAWVWADFNQPSEEESQL

LDTYFHFHPLAIEDCMHVLQRPKLDYYDNLQFLVLHALNPTTLEAEEVDLFLGANFLV

SFHHGALEEVDEAWERLLHHAHERTIWARGPVAAAYTVMDKLVDHYFPSLFAIEDELA

ELENRGGKESVEDLMNQVFDLRSRLLKLRRTVVPMRDLLYRVVNSQHVQRTGEHTVYF

TDIYDHLLKLTDMIEADREMTADLRDSYISLNSNRMNQIMKTLTVITTVFMPLTLIAG

IYGMNFAYMPELQWKFGYGAVLLLMFILGGSMVAWFVKRGWFK"

CDS complement(562392..563483)

/gene="yhaZ"

/locus_tag="PIKHDJKN_00530"

/inference="ab initio prediction:Prodigal:002006"

/inference="similar to AA sequence:UniProtKB:O07541"

/codon_start=1

/transl_table=11

/product="putative protein YhaZ"

/db_xref="COG:COG4335"

/translation="MELFKDKYTPALIDRTGEWLHQFYPKLDKQQFRELVFAEGWEEL

EFKARIRRITSALTEVLPDNYEEALHVIEQAAPQMRGVEYLFVPDYIEVNGLAPENYE

LSMKYLAVFTPYSSSEFAVRPFIERYPIETMKRMMEWTGSPNEHIRRLASEGSRPRLP

WGSKLRGFITDPTPVLPILHELKQDESLYVRKSVANHLNDISKDHPELVLDLASSWYG

QHEHTDWIVRHASRSLLKKGHPKALSLFGYVEQDATHIEHLQLARDTVAIGEDLHFSF

DVVNESGQSQMLRIEYEIGYMKANGKQAPKRFKCSDKTFPTGRTKVATKQSFKVITTR

KHYAGLHTLTIVVNGQPTATATFVLEQTE"

CDS complement(563662..564294)

/locus_tag="PIKHDJKN_00531"

/inference="ab initio prediction:Prodigal:002006"

/codon_start=1

/transl_table=11

/product="hypothetical protein"

/translation="MPQHYYHRQPAPRQRPSSHTQRQGHTSAYMPPRALNEAQVQPMY

PGVEPYNPQFQEVEASGLVPYSPGGPGGPGGNFFGGGAPVAPPVSVEAPASSSGFSLA

NIGELKGMIDRFGGIDGIMSGIGKMQKVVGGFQQMAPMMKLVMGILPFGKGKKSSSAA

DKDYEEYSPPRSRKRRKKTTSAPRKRNTNSKTGRNKSNSTNRRPKRSKKL"

CDS 564742..565032

/locus_tag="PIKHDJKN_00532"

/inference="ab initio prediction:Prodigal:002006"

/codon_start=1

/transl_table=11

/product="hypothetical protein"

/translation="MQLKKLNDKSIEQLFEAILTLKDIEECYVFFDDLCTVNEIQSMS

QRLEVARMLGKGNTYNQIEAETGASTATISRVKRCLNYGNDGYKMTLERLGR"

CDS 565035..565814

/locus_tag="PIKHDJKN_00533"

/inference="ab initio prediction:Prodigal:002006"

/codon_start=1

/transl_table=11

/product="hypothetical protein"

/translation="MKKPGVLIISHGSQEKTWVESVDDTVSRLNLPVPLPVEAGFLEL

VEGRLIQDGINRLEAQDVTDILVVPLFVSSGSTHVDEIEYAIGAKDAPERETELEPFG

VKALVHFGYPVDNDPDIAVMVWDKVKALSQQPEQETILLVGHGSIHDGFRQRWEAGIS

SLAQRVQEVSGVAHTDYALLNPDSVYDKAKYWSEERGSRVIVAPLFLSAGYFTMKVIP

GRLKELDYAYSGETLLPHPLLGKWLERQIQLLLERCNEVKV"

CDS 566146..567504

/gene="bioK"

/locus_tag="PIKHDJKN_00534"

/EC_number="2.6.1.105"

/inference="ab initio prediction:Prodigal:002006"

/inference="similar to AA sequence:UniProtKB:P53555"

/codon_start=1

/transl_table=11

/product="L-Lysine--8-amino-7-oxononanoate transaminase"

/db_xref="COG:COG0161"

/translation="MTHYERLAAINKAHLWNPFTQMKDYNNSDPLIIERGEGVMLYDV

QGRAYYDGFSSVWLNVHGHNVPELNQAITDQLERVAHSTLLGMANVPAIELAEKLVEI

SPEGLNKVFYSDSGATGVEIAVKMAFQYWKNRGETGKTKFITMNQAYHGDTIGAVSVG

AIPLYHDVFRPMLFPTHVIPYPYVYRHEDGEREAMEATLTALRNVLETSADEIAAIIV

EPIVQGASGILIMPEGCLREMAALCRTYGVLFIADEVATGFGRTGAMFACDLEEVSPD

LMVIGKGLTGGYLPVAATLATDEVYSAFYADYEDQKTFFHGHSFTGNPLGCAVALANL

KLFEERNLVEGVRAKAAFVEQKLSVLKDSPHVGDIRQKGLMLGIELVRDKATREPYDW

AERIGVRVTKRARELGMLTRPLGNVVVFIPPLASTEAELEAMIDILMQSIFDVTEGGL

ES"

CDS 567501..568235

/gene="bioD"

/locus_tag="PIKHDJKN_00535"

/EC_number="6.3.3.3"

/inference="ab initio prediction:Prodigal:002006"

/inference="similar to AA sequence:UniProtKB:Q5NGB5"

/codon_start=1

/transl_table=11

/product="ATP-dependent dethiobiotin synthetase BioD"

/db_xref="COG:COG0132"

/translation="MNVIRGVFVTGTDTGVGKTIVTGALVATLRAEKLNTGVWKPVQS

GALLGSGETDAERLLQYTGIDERAEDVATFTFKAPLTPMLAAKQDGVDITLQEIINAG

QPLANRYESVLIEGAGGVAVPLTEDSLVVDLISELRTPALIVARTGLGTINHTLLTVS

YLQQHGIPIVGFILNDGESDGIYNDSSITTNAELIERYCGIPFLGRFPRLMDEMNTEM

LIQVIREKIQLTPIRQALTVQSMEEE"

CDS 568232..569236

/gene="bioB"

/locus_tag="PIKHDJKN_00536"

/EC_number="2.8.1.6"

/inference="ab initio prediction:Prodigal:002006"

/inference="similar to AA sequence:UniProtKB:P19206"

/codon_start=1

/transl_table=11

/product="Biotin synthase"

/translation="MKLKTTVTNLEWSSLANRSLNGECLTMEEGLAVLEASDDEVLPL

MQAAFQVRQYFYGKKVKLNMIINAKSGFCPEDCGYCSQSIVSTAPIQKYSLLDKETLL

AGAHEAMARKAGTYCIVASGKGPTNKELDQVVEAVKEIRETMPLKICACLGILKDGQA

ERLAEAGVHRYNHNLNTSRANYPSITTTHTYDQRIETVEKVKAYGMSPCSGVIIGMGE

SNQEIVEMAFALRKLDADSIPINFLNAIPGTPLEGAGRTPAMKALKVLALFRFICPSK

EIRVAGGREINLRTLQPLSLYAANSLFVGDYLTTAGQEISNDHQMIEDLGFEIELNAL

"

CDS 569250..570428

/gene="bioF"

/locus_tag="PIKHDJKN_00537"

/EC_number="2.3.1.47"

/inference="ab initio prediction:Prodigal:002006"

/inference="similar to AA sequence:UniProtKB:P53556"

/codon_start=1

/transl_table=11

/product="8-amino-7-oxononanoate synthase 2"

/db_xref="COG:COG0156"

/translation="MNWMEKELALLADASNERYLRDSAAVPDCPGYTWRGDRVLLNLA

SNDYLGLARHSAIIDTIREALLTEGGGSGASRLVTGNRLLYGRLEEELATWQHSEAAL

VFANGYMANSGVIRALVGRGDVVFSDQLNHASIVDGIVLSRAEHARYRHNDMEHLKLL

LNKHRDKRRKLIVTDAVFSMDGDQAHLEELVALKQEYGAMLMVDEAHSGGIYGKRGEG

LCFKLGLQNDVDVHMGTFSKSFGLYGAYVCGSRTLIHFLINKARPLIYSTALPPSLVA

GISTALTLVQADHWRRERLYLASSLFRSSLCDAGFNVGTGDSPIVPIIVGDNERALRL

SEALEADGIAGVAIRPPTVPDGTARIRFSLSADHTPKELNDASARICKIGLQLGVLSS

"

CDS 570425..571216

/locus_tag="PIKHDJKN_00538"

/inference="ab initio prediction:Prodigal:002006"

/codon_start=1

/transl_table=11

/product="hypothetical protein"

/translation="MNALDNSPLKIEQETSGTILWLTGWSMPDTVFDRLRLLLPDFRH

ISVDYSAADSPEEMLSLTETAVTDMLCIEGSACRERASHGPLLISGWSLGGLLALRLA

AKGYVDGLVLFGATARFTRSKEEFDRGLADAYVRQMIKGILKDRDEVEANFRKIMFTE

QEWEAGLAERLPHTGSWTTQALLAGLQLLRSEECLSQLPNIDCPVLLIHGTEDRICPY

GAGLELTAHLPQAKLITLTGCGHAPFLGKEVHIADEWRRWWHDHS"

CDS 571203..572066

/gene="tam_1"

/locus_tag="PIKHDJKN_00539"

/EC_number="2.1.1.144"

/inference="ab initio prediction:Prodigal:002006"

/inference="protein motif:HAMAP:MF_00560"

/codon_start=1

/transl_table=11

/product="Trans-aconitate 2-methyltransferase"

/translation="MTIRRSSAVQRQFNRSAISYDAHAHIQRSMADHLARSLAEWKNK

GFLSDPSILEIGCGTGALTQILVNEWPSANITALDIAPEMIEVASKRFQSAQHPGTGE

YTTDNLRFLQADIEMWAVNAPECSVDIIVSNACFQWLSSPQETLGHLRRMLRPGGLLI

YTTFGPDTFREMHEAFNEVYRANGMEPQRHGLSFHSPVQWEIMLQESGYSSFHCERSI

HMEKYASARDFLYSVKGMGASTSEAATIPGYSLRRLFTNMYKEYEDKFSIQGGVSATY

DLLFIQSVAPY"

CDS complement(572069..572629)

/locus_tag="PIKHDJKN_00540"

/inference="ab initio prediction:Prodigal:002006"

/codon_start=1

/transl_table=11

/product="hypothetical protein"

/translation="MDRRVVKTRKAITDAFIGLLEEQDFEQITINEIADRANVNRGTV
YLHYTDKFDLLDQSIETYLQQLVESCMVESPTTPLTIKDALLRTFRYLEQHAPVYTTL
LTQKGIPAFRNRLMTLLIQGVEEQIDACGIHKGINREITVQFLASASAGLLEWWIMNS
MPYPAEKIVEELMSLLELHLILPSQV"

CDS 572797..573720

/locus_tag="PIKHDJKN_00541"

/inference="ab initio prediction:Prodigal:002006"

/codon_start=1

/transl_table=11

/product="hypothetical protein"

/translation="MNILVYGAGVLGSQLAQVLVRGGNDVTILARGKRAEELERDGIV
IRHVFQFKTTVDPVRVTRTLEENDEYDLIFVVMKYNDFPSVLPILAANQSRNLVIVGN
NADARSMQIFLEENSKVEKKVAFGFQVSGGRRQEDRMLSIGGASGQMVIGGLDGEIAF
KSILDQAFEKTKYKLNYLDDIDAWLKSHIVPILMLNAVSFNEKREMIKLDGNKKQLQH
MIGAMDEGFSVLEAMGITIIPEIQAKLIRKHQRMFYLLLKMYSMLPIYKLVAGSFGEI
EALDNAFTDWKKATGVPTPHWEILKRGYSFL"

CDS 573810..574226

/locus_tag="PIKHDJKN_00542"

/inference="ab initio prediction:Prodigal:002006"

/codon_start=1

/transl_table=11

/product="hypothetical protein"

/translation="MREVPMRYVKANQIGIVLFVVLSFLFNQPLILGALWIIQVVGLA
SGGKLNLFVQIGKAVLTGKGTETQAVELQRFNNVLAVLFLTLALVSFGLGWQVAGYIF
SSMLLLAASAALLGYCVGCTIYFWYKQLRAGRKIGF"

CDS complement(574361..575077)

/locus_tag="PIKHDJKN_00543"

/inference="ab initio prediction:Prodigal:002006"

/codon_start=1

/transl_table=11

/product="hypothetical protein"

/translation="MKKLVLLLMVSLLACIPFVTDVEAAVINHDQVVGFQEVNPVTVT

QITAKRFHPYLKVYHGCVPFFPAVDQQGNTNAGLQTSGSPNGNCSSSTGQVYSRSAWHN

GVWAIMYAWYFPKDSPSSGLGHRHDWEGIVVWVDNPAAQNPQILSIAYSGHGQFTNVA

PSNTNLNGTNPLISYNSTWPLNHELGVTETVGGMQPLIGWDDLTSAARDALNTTDFGS

ANVPLNDSNFTNNLNKAWFR"

CDS 575689..576570

/gene="dagK_1"

/locus_tag="PIKHDJKN_00544"

/EC_number="2.7.1.107"

/inference="ab initio prediction:Prodigal:002006"

/inference="similar to AA sequence:UniProtKB:O31502"

/codon_start=1

/transl_table=11

/product="Diacylglycerol kinase"

/db_xref="COG:COG1597"

/translation="MKKARLIYNPTSGREEMKKRLADILQRLDQGGIEASCHATTGEG

DATREAELAIERGYDMIIAAGGDGTLYEVINGMAERENRPPLGVFPLGTTNDFARALG

IPRQWEDYVDLVINQQLRPLDLGKANDKYFINIAGGGSLTELTYEVPSRLKTMIGQLA

YYMKGIEKMASLSPQELVIRAAGQEEIHDEFMLFLIANTNSVGGFEKLAPGATIDDGL

FDVIGVRKCNLADMIRLVTLALRGEHLNDKKVVHFQTDYMEVTSPGYVQLNLDGELGG

TLPATFKNLRHHLMLYR"

CDS 576599..578206

/gene="rlmCD"

/locus_tag="PIKHDJKN_00545"

/EC_number="2.1.1.189"

/inference="ab initio prediction:Prodigal:002006"

/inference="similar to AA sequence:UniProtKB:O31503"

/codon_start=1

/transl_table=11

/product="23S rRNA (uracil-C(5))-methyltransferase RlmCD"

/db_xref="COG:COG2265"

/translation="MSNTNRSGRGKNRRNSAASQGQGNASASRQPSKSSRPFTRQQGK

EVRPQGASLSAVRPKGRERESAPIEGLPVSKNEETVIDIIGMNHDGEGVGRANGYTLF

VQGALPGETVRVRVMKTKKQYGYAKLLEIVKASPDRVSAPCPIYDQCGGCQIQHMSYA
GQLAWKRQLVVDNLQRIGKLNVLVEDAEGAEQQGIRVLPTMGMDEPWRYRNKAQVPIG
AAEGGLVGGFYAKGSHRIIDMESCLIQHEHNDEVVAKVKEIGSHFGISAYNEETGRGL
LRHVVVKKAFRTGEMMLVLVTNGRDIPHKDAWIGSIREAIPHVASICQNVNKKQTNVI
FGDETRVLWGRDVIYDYIGDVQFAISARSFYQVNPVQTEVLYGKTVEYAGLSGKETVI
DAYCGIGTISLFLAQHADQVYGVEIVPEAIEDARSNAMLNEMRNVKFEVGASEDVIPR
WKEQGIEADVIVVDPPRKGCDPRLLDTILEMKPERVVYVSCNPSTLARDLRVLEDGGY
RTVEVTPVDMFPHTVHVECCVLLVRKY"

CDS 578481..582803

/gene="malT_1"

/locus_tag="PIKHDJKN_00546"

/inference="ab initio prediction:Prodigal:002006"

/inference="protein motif:HAMAP:MF_01247"

/codon_start=1

/transl_table=11

/product="HTH-type transcriptional regulator MalT"

/translation="MEQKDFIGLSILLTDLVYRTHQQIAVIGCLNPSSISIQRDLKKA
HLSGNMEWNEVYQSPEQTGRVNRVADGRSDLYTLGLLFYEMLTGQLPFLPEDGEEWAA
AHVQMAPLPLSEIRREWDGPLQAILLKLLAKSPDERYQSAYGLLDDLKHCEEMLEKSG
ELTSFEIGRIDTIRSFHLTDSLYGCSAAIEQMQAGLQQAANGLPVFRWISGVEGSGKT
SLVQKLYPDIVARGGRFIEGRGKQFPSIKPFEPLLQALRQWIHQLWSEPDDEITRLKG
RLHAECGQETQTIVSVLPEAKSLFISQVDETLAPDADAWRRFGELLPLLIRCFANSKP
PLVMFMDDLQRADENTHAVIRALLLGEPIPGLFLIGAFQTELDCGVDDNDRNFTLKFP
WLADRIRTFAAEQVVIHPLHFADIRQYVTDTIHEDSARVRLLARLLYHETGGNPRAIR
LLLEGWVLEKKLLFDDKQRQWIWDAEIVRQLSDVKANPDLMEKGFTLLPVETKMLLAM
AAAIGSTFHLSLLSEVSNYATEVTLGFLHDAEVQGIVYWEEELRTKDVHERVYMFGHD
HLHQLAYSCDTDRNSQRHQKIGRLLQRRQSEGDDTLLEMVDHLNRGVASMSVQEMEQL
AEYNLQVAMNAMDDGHYWKGKPYIEAGLQLIVEEEEIQSGSQFFRLKQAYVWFEYMSG
NKESAKQLLLDMDKRGGKLNRVDRSQLWEELIHFHALDENEISIRYGKEALAAYGWKV
RGKVSKLSIVKEVIHTRLFLHQMQDQLHAITSSHDVEHAALCRYVELLFYPLLVHNPE
ALVDLYARFIRYGVRKGLNESLIGIIWSYELLLQRVSRRYVQAEFLFDRESIGASVFA
GAKLQHRLAYTRGLSKQLEHPVEASIYMGQALHYAVELGDKSFANIVMISCLITHNRD

LYVLSDLLDYFEAHMRPYASDKTLELVQIAGSYLAALQDESLQADFITIPVPHSEDRA

RDSEDNFSFICKLEVAYLSGKYREALYWAKRGRENELALDWGRIWKHRLYEALALAAV

YPEVDAVERKRIRQAIRRQLRLMKKRKGYLGADSSAYLLMKAEWTRIEGDEWGALRGY

TEAVKRARVEKYGLMEGISSERLASYYQNDMHSRSGAIIAAMDACTAYSIWGITSKVT

LIRHEHADLFRYITKTYGGQIHEELKGKADRELQLPHPHVAGHMRASANGDELLQQIL

DWQGWTNKADWPISFLKVAIRQAGADRGAVLRCRNNGFDIASSLGGHADEEVADGYAV

SVLRHTVMTNEPILLQDALHSHFLQDTYIQEQRPRSILCLPVRVPGERSAALLYLENR

HLSGVFTERDLNVLQLLATRIIYFNVLEDEAAAASAEDARSLASSLAPGTAQSSLVEP

LTGREVEILTAIAEGLSNRDIANRFGIAETTVKTHTSRIFGKLGAKRRGQAVVRAKEL

QLID"

CDS 582955..587229

/locus_tag="PIKHDJKN_00547"

/inference="ab initio prediction:Prodigal:002006"

/codon_start=1

/transl_table=11

/product="hypothetical protein"

/translation="MKLSKNWKRLVLQVLSSLLLLNVIFPIHPQKVEAAQRGIESRIV

KIAAGFFHSLALKADGTVVAWGDNNYGQTTVPAEVKEGVVAIAAGGYHSLALKADGTV

VAWGYNSNGQTTVPVEAKEGVVAIAAGLYHSLALKADGTVEAWGDNNYGQTTVPVEAK

EGVVAIAAGGRHSLALKADGTVEAWGDNNYGQTTVPVEAKEGVVAIAAGGYHSLALKA

DGTVEAWGYNYYGQTTVPLEAKEGVVAIAAGGYHSLALKADGTVEAWGSNGGGQRTVP

AEAKEGVVAIAAGWDHSLALKADGTVEAWGYNGSGQTTVPKDIASPVKGSKIAAGAYH

SLGLKADGMVEAWGDNYYGQTTVPVEAKEGVVAIAAGGYHSLVLKADGMVEAWGYNSN

GQTTVPAEAKEGVVAIAAGDSHSLALKADGTVEAWGYNVSGQTTVPAEAKEGVVAITA

GGYHSLALKTDGMVEAWGANNYGQTTVPVEAKEGVVAIAAGGYHSLALKADGTVEAWG

YNGQGQTTVPAEAKEGVVAIAAGWNHSLGLKADGRVEAWGYNYFGQTTVPVEAKEGVV

AIAAGLNHSLALKADGTVVAWGDNGSGQTTVPGDNGLKNLGLQEGVFDVPFSSEITSY

TSYIGPSVSQVHVETGLEDEEYAALYVNDQLQVSGSSAIVGISADTTTINVRVEPYFK

PSRTYTITVLRDLTPPAVTISSTASGTVNAPFPVKIDFSKEVTGFMVAGITVDNGTVT

DFVAEDAKTYTATIVPLISGQAVTVQVAANAATDTVGNQNVASNVLSYLYDTTKPVVT

FGGFTDHQVFIAPPASIHITVSEAVYGIANGDEVDVSDAATLLSMEKDGAPFSDYTVT

YDQSSLTFTLTFDGTLGDSAYHVKVAGDEVQNANHNSLDAASASFTVAVPVVTNITAQ

PASLAHTGGDVTVSIAGMHLIGQKLDVYVDGIYADTAVASNDTSAEAVVTLPRNHAAN
AKSHSLKVYLNGTEQAGPSATVTVMPAEQSNTGGGAPTPAPGPVPGPAPVPAPAKPYI
DVNGTRMNPEIIDLTKPSYTLEATPKEGAVHVSIPATVLSGFADKNASFVLEIKTPYG
SYLLPVQLGVLIPGLQDVLTTNTLKIDEISLRISLTDRSDDKNIQTTFTGDLPNGKVM
SAIVDFGMEIVNTKTGQSIGNAEGSNKALTRMIPLPKRLTVLPKQWGAFRYDEKVQKF
EFVAARSVQIEGVWYAMIQSNINSVYVVAENPLSFTDVKKDWSQPYVELAAAKGLVNG
VGGGKYAPERSVTRAEFTAMLVRALGRSSVTAAESSPYQDILPGEWYSSVVAQAKKLG
LLGFAGSDSFLPDQPLTREEIASMLMAAIVLEELPITREDSSPSGYKDLDSVDATNLE
SIRLMQKLNIMQGTGKNTFSPKSETTREQAAIVLIRIMRALGKIDL"

CDS complement(587804..589669)

/locus_tag="PIKHDJKN_00548"

/inference="ab initio prediction:Prodigal:002006"

/codon_start=1

/transl_table=11

/product="hypothetical protein"

/translation="MAFIPLNCPNCNGRIEYKEGEILKCPYCETELLLKQNHVYYVDQ
TINHYHGTPPKAPLKQTASVKVMLILMLVLTGAIGTYFYYSNSSTYPKTEANLPVRKM
PESEVLLSFLRDIFDKGSALPAEEELARLRYLTVEHSENDQWNFTYSLSDPFSDEQAE
KITYVTQDKKLNSQRIDQRDFEAFKGLTALDLTNTYEISQTDQTTLAHMSGLKSYGGA
FNESFSKFSGYFGDKSKITELSTQLRSNQELVLLLEFPNLSSLSITYVDESVTDFFLL
NKLPLKSLSLTFVDELEWLSSMTGLSSLSIQHSEATDLQPLYALTQLQELQLSYLTNV
KSIDFVQNMPALQTLDIENVNFSSLERLAGKNSITKLRLASLSQLSSVKAINSLPSLR
ELTLSGYYENAEALSLPNVGRVEIPSSFLPGLKAPAATSLTLLGGSGELNLAALGKFP
KLEQLSLWEISEITHLGALDGLSRLETLSIYDSSLFNESDALFRLKQVKSLMCSECRL
NFGQKSAAENGVLEHLTLEQPYVSINNTSVTEVDQMMSYFAKMSALRSFTLQDSNLAS
LEFMSNWQAIEELHLENNAISNIETLSQLPNLQKVYLSGNSVQNKSVLGTGVHVY"

CDS 590095..594546

/gene="xynA1"

/locus_tag="PIKHDJKN_00549"

/EC_number="3.2.1.8"

/inference="ab initio prediction:Prodigal:002006"

/inference="similar to AA sequence:UniProtKB:C6CRV0"

/codon_start=1

/transl_table=11

/product="Endo-1,4-beta-xylanase A"

/db_xref="COG:COG3693"

/translation="MRKSFKQVVSGVLAAALLLPGGLIAPADSAADLAASNSQIVYQE

TFQNGQGKVTQSGGAQLDAVTGKPFEGNDDGAALKVSNRSNNWDGVDITFGDLGLAVG

EEYSVTASVYVDTDSNPPAAGKALLEIVSNKGVTGSEGYTGISSADFKAGKGITLSAA

LSVTSVDKTALRIKSDDAGNGVQFYIGNITITQTAAPVEPAVVLNQSFEDGSTDGWGK

LGWGSNGDTAVVNDVASEGTKSLKFFNRADEKAVPALVLTGKMKSDRKYDISLKVRLG

SGEGQFHLASKIDSEILSDDQKYPWIIGNKLVTASDWTLFETKGYEVPSKTNEVIIYV

EPSNNTLTSDIYIDEVIITDVTPDQTAPEIVDKTGISADFENGLDGFKARNGRETVVL

SQNDNHTSGGKQSLLVTTTTQYDAALVDATGKMAKNHEYELSAWVKMAPGEAPTTLRI

SVQYADSGFANVSPNATVTDQEWVHLTGKYTLTTTPSVLNAYIETANNDGGNRTFFLD

DFALKYLGPVTAAPPIQENLPNLKHIYKDDFLIGNIVNPSTFDETRLKLLKKHHNVVT

FENDMKPDYAYDMDRQFDFTAEDALVQKVHDAGLDLHGHVLVWHAQSPTWLHTAVSGE

PLSREDALTNLRRHVKTVVEHFGDQVISWDVVNEAMNDNPANPEDWKNALRNSGWLQA

IGPEFIEEAFLAAKEVIEENGWDIKLYYNDYNDDNQNKATAIYSMVKELNEKYAVNHP

GEKLIDGIGMQAHYNLGTRLDNVKMSLERFISLGVEVSVTELDIMAGTNSTITEKEAK

QQAYLYAQLMDLYKKNSEHISRVTFWGLNDSTSWRAEQNPTLFDKNFQAKEAFYAVVD

PEKYMAENPPEALDYKRSSAYYGTPAIDGSVDSIWSKAPELKLDTKQMAWSGATGTAK

ALWDDDNLYVLVQVRDDQLNKTNPNVWEQDSVEVFVDENNGKTANFQDDDGQYRVNFE

NVASFNPAKIAAGFESKVSVSGTNYTVEMKIPFKKVKPANNAKIGFDAQINDAKDGNR

ISVNAWNDPSGQGYQDTSVFGELTLTGKPSSGGNNGGGNNSGGNNGGGSGGSNSGTTP

QPQPQPELPGSDSAIVPVIKVENGTATATISSDSLNKAIEKASPGANGKKQVTIEVPK

QANVQSYVVQLPTSILQSNNNVEFVLKTDNATLQIPSNMLSNISEQADQVSIQISKVS

ADRLSSDVRAIVGSRPVIDLSVKAGDRLLAWNNPAAPVTVSVPYTLAAQELANAHQIV

ILYIDNQGHATAVPSGRYDAKSGSMVFKTTHFSTYAVAYVTKSFADLQNVLWAKQAVE

AMAARDIIKGISDDSFAPAASITRADFITLLVRALELKGSGTNTAAFSDVQPTAHYAQ

VVAIAKELGITSGFEDHTFKPDSSISRQDMMFLTAKAMKAAGKQSASNANLAPYSDAA

SISAYAMDSVASLVGSGIVNGKDGKIAPAEPLTRAEAAMVLYRIWEM"

CDS complement(594642..594848)

/locus_tag="PIKHDJKN_00550"

/inference="ab initio prediction:Prodigal:002006"

/codon_start=1

/transl_table=11

/product="hypothetical protein"

/translation="MEISLDIIKDKVECLQAYDFNELERAIEDRIGMNKALLLRVKQV

QHQVTFDPLRNKMLYSAVVHFSAE"

CDS 595090..595227

/locus_tag="PIKHDJKN_00551"

/inference="ab initio prediction:Prodigal:002006"

/codon_start=1

/transl_table=11

/product="hypothetical protein"

/translation="MEKRKTRTHTVTSKGGIAPLFYASLLKSALLFGVGYIHLDVINR

Y"

CDS 595401..596387

/gene="dus"

/locus_tag="PIKHDJKN_00552"

/EC_number="1.3.1.-"

/inference="ab initio prediction:Prodigal:002006"

/inference="similar to AA sequence:UniProtKB:P67717"

/codon_start=1

/transl_table=11

/product="putative tRNA-dihydrouridine synthase"

/translation="MKENFWRDLPRPFFILAPMEDVTDVVFRHVVSEAGRPDVFFTEF

ANTESYCHPEGNKSVRGRLTFTEDEQPIVAHIWGDKPEFFREMSIGMAKEGFKGIDIN

MGCPVANVAENGKGSGLICRPALAAEIIQAAKAGGLPVSVKTRLGFTEVDEWRDWLTH

ILQQDIVNLSIHLRTREEMSKVDAHWELIPDIKKLRDEIAPNTLLTINGDIPDRATGL

KLAEQYGVDGIMIGRGIFQNPFAFETEPREHTTEEFLHLLRLHLDLHDQYSELEPRSF

SPLARFFKIYVRGFRGASELRNSLMNAKTTTRVRELLDEFGSKEHDGVEENE"

CDS 596403..596972

/locus_tag="PIKHDJKN_00553"

/inference="ab initio prediction:Prodigal:002006"

/codon_start=1

/transl_table=11

/product="hypothetical protein"

/translation="MSWSKLKQQLESFLSPTLVGTVEYRATSYRYSPDKSGNCYITVD

KKNVLNMSDITGSIRWYQTEQEIKNDPDVLIPVSHEEIEVVRKETKGTVPEDRLQVIA

RSRKSSGLAKELLSAQSSLSKSNFTVAATTFLSISIEESLESHDILLNILALIDRRVG

KKRILNMSEQIKLKHPAVQYFYELRRSTL"

CDS 597210..598370

/locus_tag="PIKHDJKN_00554"

/inference="ab initio prediction:Prodigal:002006"

/codon_start=1

/transl_table=11

/product="hypothetical protein"

/translation="MTDKFQIVGSLLRPDELLTYKTQIEHRDDIKYPFYESFEGYEKC

ETEAIKQVVKKEIEHDLTIITDGEFSKSMWHLDFVWGFGGVERYIADHGYFFRDVDGS

SKYETRKDIGLRITGELSGKNHHFIQLFKQLQDTAGDQQTKLCVPSPSHIFGELSWSD

NIGGTDAVYQNIQELKEGLVKAYKEFVEEFAAVGGKILQMDDCLWELFADDNPNSPFT

GEHINQEEVQGLATEFIDINNTVIDFGHSLGLKMWTHNCRGNYDSRNMGGGSYTKIAN

LFLKQLKYDRFFLEWDDDRAGSIEALEVFKDRPETEIVLGLLSSKTNTLDDEERVIRL

LDEASKIIDKDRLLLSHQCGFASCDGGNELSEAEQWAKIVQGQKIAKQYWEN"

CDS 598733..599452

/locus_tag="PIKHDJKN_00555"

/inference="ab initio prediction:Prodigal:002006"

/codon_start=1

/transl_table=11

/product="hypothetical protein"

/translation="MEKIELVLIKPGKTFTDYRHLIITEATEVCILNIIKGRLYPDKK

TMNPTYEPYPTKQETVDGLNELANELRTKGFVEAQREVLFQIPEKEIYVYDKAKWHYE

GDFPKELDITQAYVPTEMFITWIIKNDMIGKQSSRNDASEIELVKRNEMTGAQFFRKN

WDGVLSSKELSDEADVFAREYLDIHNDLYTAVDFTNVLATGLPTIYYVQDSIENYHII

EPIITERYREWKSRQRPVNQG"

CDS 599687..600394

/locus_tag="PIKHDJKN_00556"

/inference="ab initio prediction:Prodigal:002006"

/codon_start=1

/transl_table=11

/product="hypothetical protein"

/translation="MNLEEVMQELEALGKERTKKIYMSNGAHEPLFGVATGAMKPIAK

KIKKDQPLAELLYATGNYDAMYFAGVIADPQAMTEADFDRWIDAAYFYMISDFIVSVT

LAETDIAQAVSDKWIASGDELKMSAGWSCYCWLLGSRPDSEFSESKLNDMLKQVEKTI

HESPERTKYSMNNFLYTVAVSYLPLHDQAVETAKAVGPVEVNKDKPKSKFLLASENIQ

KAVEKERTGFKRKYVRC"

CDS 601200..602372

/locus_tag="PIKHDJKN_00557"

/inference="ab initio prediction:Prodigal:002006"

/codon_start=1

/transl_table=11

/product="hypothetical protein"

/translation="MNKKEVAHIRKQFKMDHDLLNIYDILNVYITKETNEIYHWERHP

FELVDREKQELYMGNFKKLLTGELDQKLFELKFQEQAEEPAQVMLHQALVTGDPEEWQ

DLMLLLVERMLADAKYERDMVITFVRGQYYLPTKARNDQAEESEKNEVFAHPFILCSV

NSTEKQRKTLLFDYVEREFKYNVIVDPVVKLSTPEQGFLYPSVTDNYSDVNRILYCTG

KSNFPDPHFVENVLNGERSVTALEERAIFEDIVKELTGEQLDASTLAHVYEEINRVIE

INEETHEEEPPKLDYKDVERVLTASGVEDLTSEKVERAFETIVDDKNYELKANSVMPK

FTSKSIKIETKVATISVSPQDLKYVKQVNYQGRRCIMIEVDEDAVIEGFTLTTEIL"

CDS complement(602483..603427)

/gene="rhaS_3"

/locus_tag="PIKHDJKN_00558"

/inference="ab initio prediction:Prodigal:002006"

/inference="protein motif:HAMAP:MF_01534"

/codon_start=1

/transl_table=11

/product="HTH-type transcriptional activator RhaS"

/translation="MPRKKQPIIEYRHYSLPIHFPILLLSGERWKISDIKSEHLHFHN

HLEIGICHSDSGIMEIKGESVPFKAGDVTFIPRYLPHTTYSSPNEASLWSYLFFSPED

LFQHSFKSSTYSNFEPNLWAIKGLNCILNREEHPRIYTLATSVVEELKQKNPFYQESA

YGLLLSLYIELLRIHSRNEPLADQETQHNLKGDLVISPVLEYITKNYMSPITIDFLAD

LCHLSTTHFRRKFHDIMGTAPLDFLNSTRIEEACKQLKSTDASILSISEQVGFQSISS

FNRCFSRLMGKSPKEWRKGAQSEVQSAKASILEFTGWM"

CDS 603600..604619

/gene="yesR"

/locus_tag="PIKHDJKN_00559"

/EC_number="3.2.1.172"

/inference="ab initio prediction:Prodigal:002006"

/inference="similar to AA sequence:UniProtKB:O31521"

/codon_start=1

/transl_table=11

/product="Unsaturated rhamnogalacturonyl hydrolase YesR"

/db_xref="COG:COG4225"

/translation="MLQVKYDREEILNVIDNVTKKTLAMDLTWDWPCGVAYYGVTRAY

QTTGNQEYLDRLVQWADEYIELGLPDWTVNTCAMGHMLITLYEETGNQKYWDIVMSKV

DYLQNHALRFGDNVLQHTVSISNDFPEQAWADTLFMAAFFLLRVGSKLKDQDMIQDAL

NQYYWHIKYLQDPSSGFWYHGYNNVNKDHMSGLYWGRANAWGAYTMSQVKPQLKDWYL

YPQCMDVECSLRDQLAALKLVQTENGLWRTVLDDEQSYEEVSASAGIAAAMINNGNPL

HTKYVQKALEGILNNISEDGRVLGVSGGTAVMKDRDGYRNIPKDWIQGWGQGLALAFL

SDMLK"

CDS 604633..606807

/locus_tag="PIKHDJKN_00560"

/EC_number="2.4.1.247"

/inference="ab initio prediction:Prodigal:002006"

/inference="similar to AA sequence:UniProtKB:A9KHK4"

/codon_start=1

/transl_table=11

/product="D-galactosyl-beta-1->4-L-rhamnose phosphorylase"

/translation="MSKPIKGSFTLPGESGYEALTLELAERWGADVIRDSDGTQLSDE

IINAGYGIYSTICIIRDHNEWASRNQDKLQQCFLITNPKVAVQDYVSFYLMEDFFAEQ

FKVNDSKEAFKYWQVFDRTTGEEVPKGQWNYERESGHVVITGIVPWHKYTVSFMAYRI

WEEISMYNHTTNHWDKEHLMQIDPIYSETQTYLLDWMEDWCQRHQETTVVRSTSLFYN

FAWIWGSDERNRHLFSDWGSYDFTVSSRALDLFAKKVGYSLSAEDFVNGGKYRVGHIP

AQQRKLDWMAFINDFVIEFGKKLIDIVHNHGKLAYVFYDDSWVGMEPYNDRFQEFGFD

GMIKCVFSGYEARMCSGVNVDTREIRLHPYLFPVGLGGLPTFKEGGDPTLDAKKYWIN

IRRALLREPIDRIGLGGYLHLVEPYPDFCDYIEKIANEFREIKELHNEGKPYQIKTKV

AVLHSWGKLRSWTLSGHFHETYMHDLIHVNEALSGLPVEVQFIDFEDIRQGVLQNCDV

VINAGSAGSAWSGGELWNDHQCVDLLTKWVYEGGTFIGINQPSAVEGYDSFFRMAHVL

GVDEDTGARVVHGKWTYETCDEHGLLPEGAKIAPKNNIYLTDGTAAVVDETDGMITLS

AHDFGKGKGIYLPSFEFSWENTRLLLNLIRFAGHEFHDSKYITDNLFTECTYYPESKI

LVVINNSDQVQTTTIDTEYGKQTVELDPYDTRITHIGLIKSV"

CDS complement(606967..608307)

/gene="gbpA"

/locus_tag="PIKHDJKN_00561"

/inference="ab initio prediction:Prodigal:002006"

/inference="protein motif:HAMAP:MF_01905"

/codon_start=1

/transl_table=11

/product="GlcNAc-binding protein A"

/translation="MFRFILPSSKLVLICCLLVLTTVSMLFNSPKALSHGYVEGPASR

AALCASGVNQDCGSIVYEPQSLEAPKGFPTAGPTDGHIASANGVFPKLDEQSATRWSK

VNMSSGSNTFTWKLTANHSTASWKYYITKTNWNPNAPLSRDSFDLTPFCSVPYNGSQP

PFSYTNSCNVPDRSGYHVILAVWEVADTPNAFYNVIDVNFSGTNPVDNVAPTPPASLV

ATATAATSTSLSWKASSDNVGVTGYKIYNGTTLIGNVSGTTTSYTVTGLTASTAYTFT

VKAVDAAGNESAASNSVSVTTTAPPANDTTAPTSPGGLHVMGTPTASSIQLMWTASTD

NVGVTGYKIYNGSTLVTTTSGTATSYTVTNLEANTTYNFSVYAVDAAGNQSAASTVSG

KTAAASTAPAWAANTQYTAGTIVSYNNLTYKCLLTHTSQVDWIPSATPTLWQLQ"

CDS 608704..608919

/locus_tag="PIKHDJKN_00562"

/inference="ab initio prediction:Prodigal:002006"

/codon_start=1

/transl_table=11

/product="hypothetical protein"

/translation="MENMCMNMMMNMCMEEMMCKMKSMKMMMECMEDKSMMEGMDMNK

MMSCMQECDEMMTTMMGMMSNMKKASM"

CDS complement(609063..609386)

/locus_tag="PIKHDJKN_00563"

/inference="ab initio prediction:Prodigal:002006"

/codon_start=1

/transl_table=11

/product="hypothetical protein"

/translation="MDSLTQTWVNDYLDLYNYARTIEDLEWAEDILRKLQDQKDALLE

EERKAILLRELLTSYDRINKQLIDIFSKLRVASEGYQTESLQEQWFKLKLMRIDVSRK

ILQYK"

CDS complement(609521..610444)

/locus_tag="PIKHDJKN_00564"

/inference="ab initio prediction:Prodigal:002006"

/codon_start=1

/transl_table=11

/product="hypothetical protein"

/translation="MSVNIYNKKRSIKSYLLEKYIGTLDSKKNLSTRENTLKHLEHMG

VENNKPYELGKVKLISSVNERSFEDIQVFTLNDQSSPEQRVILYIHGGAHTHQPLTFH

WKFMDRIAQSLNAKVIAPIYPKVPHFNYQHTYPRLLNLYREIIESIKSPAQLTIMGDS

AGGNISLSFAHYIKMNLLPQPKDIILLSPCVDMVLDNPVIFDYETRDPMLAVEGYDVI

RGIWAADKDLDDPLISPIYGDFKGLGKITIFIGTHEGLFPDNMKLDKLLTDQGIDHNT

FVYPKMNHVFVLYPIPEAKDAERKIINIIKQ"

CDS 610694..611680

/gene="thrB_1"

/locus_tag="PIKHDJKN_00565"

/EC_number="2.7.1.39"

/inference="ab initio prediction:Prodigal:002006"

/inference="protein motif:HAMAP:MF_00301"

/codon_start=1

/transl_table=11

/product="Homoserine kinase"

/translation="MFNQPTALRSVLNPKYLEFALSDQYDIGPWEECLFWLRGLNDTY

RVRTSSGMYILRVYHTEITENDVRYELSLLSQLKGILGSSVHTDIGEYIEKKDHTGYT

VLEAAEGKRVAVMFRYIEGTENNLEDEQSCYAFGQSAAELHKAMDQVVLEQPRYELDT

KLLIDEPLERIINYIGEKNEASSFLHTFARELKERIGVASKQGLDTGLCHGDMHGNNN

AFQQGNQFIHYDFEWAAKGWRAYDLAQVKARKRQSGEQKEVLWSALMAGYRSVRSFSA

EDEQAVDLFIIARRFWVMGLDVAFIESDMGALDYGSDWLDSFIEEFRDTGIV"

CDS complement(611778..612767)

/locus_tag="PIKHDJKN_00566"

/inference="ab initio prediction:Prodigal:002006"

/codon_start=1

/transl_table=11

/product="hypothetical protein"

/translation="MQEQHAARVELFVSNTQIIKKSFKWQNVMMHRLAALLYAAENKQ

ADGEAIRQSHELIKQNTNLFSTFRGNSAISIATLLSLTTDQEKKLEDTLLIYDLMKKI

KFRTSDFLVIAAYQIAAHATPDQFEHKVERAKSFYDHMKAQHRFLTGQDDYIFAAMLA

LSDLDVESGVTRMEQLYVELKPEFSPGNSVQALTQVLVLGDDNPEAGAHVIALNEAFR

RRGIRLDKMYTLPALGILSLLPADRDTLVEQVEETYEWLRTQKGFGAWSINKQELLLL

TSSLVAVQYVEDLRNGVLTTTVSTSITNIIIAQQAAMAAAATSAAVVASTSSN"

CDS complement(612978..613373)

/locus_tag="PIKHDJKN_00567"

/inference="ab initio prediction:Prodigal:002006"

/codon_start=1

/transl_table=11

/product="hypothetical protein"

/translation="MKKLSSTLFVFFMGISLMFGLTASANPAIVSYGGSYSNTLTAGQ

GYTYILPNAKTTGTSVKITASAANALQLHYYWSYNNTTIDLGTAIVNSTNYNGGSAYY

SPGTLVTVVERVPASTTLYPSVSYTISFN"

CDS complement(613542..613907)

/locus_tag="PIKHDJKN_00568"

/inference="ab initio prediction:Prodigal:002006"

/codon_start=1

/transl_table=11

/product="hypothetical protein"

/translation="MPDFLLVLFLFNLSLFLLHEMDAIRRSEWKLFMVLKVMEDEKAY

KCFTFVHLPLYTVILALLFSSYQTITFWVLDIFFIIHAVLHLFFEKHSRNEFKNSFSR

SFIYPMGIIGAIHLLALIL"

CDS 614078..616051

/gene="dap_2"

/locus_tag="PIKHDJKN_00569"

/EC_number="3.4.11.19"

/inference="ab initio prediction:Prodigal:002006"

/inference="protein motif:HAMAP:MF_01960"

/codon_start=1

/transl_table=11

/product="D-aminopeptidase"

/translation="MSSSIRKMACKMMLLVLLGTTLGATWLGVKAPVSAEAYSSGLSS

GNQEKGVAVNLDNKEKLAAFIDGIMDVQMDSLQIPGAVISIVKDGKILLSKGYGHSNI

EENKPVDPETSLFRIASTTKLFTWTAVMQLVEEGKLDLDTDVNTYLKTVKIPPTYAEP

ITLRHLMTHTAGFEEGGVGYQITTDPEKLPVSIAETMANHMPARVMPPGEMMAYSNYG

ASLAGLIVEEVGGIAYNDYIRKNIFDPLSMKYATVEEPVPASLKPYAVLGYARANGEY

VTKRPTFEGGFRPAGSGSVSANDMAHFMIAHLQDGRYEDKQILKPETARLMHSPAFQF

DERLPAMDLGFYELNMNGLRVISHGGADELFNTALYLVPDQQVGIFVSYSGGDGGTAA

GGLAQAFFDRYYPAHMTKQPAATSIELGEPLEKYAGSYQFTRRNHTKIDKFFSFLTQI

NIEVSDNRLSIGSGADQQVYTPIGVNLFQEVGGKHQMGFRTDTEGKVTYLFLDILNPM

PLEPTLLINQSKFWLPLLGISAVLFITVLLGAVYRRRKIKAMPRAQKWAVLLSVVSSV

WSLVTLGATLLVMNLDLIDRLSHIPLSLRLYLFMPLILVGWSVAIVTMNVMAWKNKYW

TLPKRVYYTFVTLAAVTLCLFFYHWNLLGWHFG"

CDS 616106..616780

/gene="hssR_1"

/locus_tag="PIKHDJKN_00570"

/inference="ab initio prediction:Prodigal:002006"

/inference="similar to AA sequence:UniProtKB:A6QJK3"

/codon_start=1

/transl_table=11

/product="Heme response regulator HssR"

/translation="MTQILVVDDDVHIRELITLFLRNEGFEIVVAKDGAEALDIVEKS

PIDLVILDIMMPKLDGWELCREIRRMDLNMPLLMVTVKGESAQKVKGFQLGIDDYLTK

PFDPLELVMRVKALLKRYLIVSSQTIQLGGITLNRRSFQVIRGEETLNLPLKEFELLF

MLANQPGQIFTREQLITKIWGSDYEGDDRTVDVHIKRLREKFAGDVHHFQIETARSLG

YRLVIT"

CDS 616777..618129

/gene="hssS_1"

/locus_tag="PIKHDJKN_00571"

/EC_number="2.7.13.3"

/inference="ab initio prediction:Prodigal:002006"

/inference="similar to AA sequence:UniProtKB:A6QJK4"

/codon_start=1

/transl_table=11

/product="Heme sensor protein HssS"

/translation="MIKTLYVRIIFTFLIVIIFSLLSSFLIGLFLFKKEVNHLGQNDM

IAAGEEMIRLYEQTHPEDREAFIKSMVKISTYPIHLYDVSGNVTYYDLDNTETVQIAP

EVVQMVLQGQVYRSPAEVDQTFIGLPFPFQGEPQAVFMQYSPQNENIINRMVFLVLLL

ALFIGSLCILIAARYLVKPIQVLTRATKRLAKGDFDVEIQTKRVDEMGALTHSFNEMA

SELKQLEQMRQDFVSNVSHEIQTPLTSISGFAMAMKNSSLVAEADRNYYLDIIITESG

RLSRLSDNLLELASLDSDHHPFEAATYNLDEQIRQIIVTCEPQWSTKNIQVHLELAEP

IQVTADRDQLNQVWMNLLGNSIKFTPAGGHIHIRTNQVANEIHFMITDTGIGIAPEQL

EYVFDRFYKTDLSRNRSISGNGLGLAIVKKIVMLHHGTVEMKSQEGDGTTVMVHLPLG

"

CDS 618306..618635

/locus_tag="PIKHDJKN_00572"

/inference="ab initio prediction:Prodigal:002006"

/inference="similar to AA sequence:UniProtKB:P9WMI7"

/codon_start=1

/transl_table=11

/product="putative HTH-type transcriptional regulator"

/db_xref="COG:COG0640"

/translation="MNQSIQQFKADFFKALAHPMRIQILELLSDGAKNVNELQSILGS

EGSAVSQQLAVLRSKNVVQGLKEGTTVTYSLRDPLIKDLLAVAKKIFDNHLVDAISML

EDIRKDT"

CDS 618803..620557

/gene="dauA"

/locus_tag="PIKHDJKN_00573"

/inference="ab initio prediction:Prodigal:002006"

/inference="similar to AA sequence:UniProtKB:P0AFR2"

/codon_start=1

/transl_table=11

/product="C4-dicarboxylic acid transporter DauA"

/db_xref="COG:COG0659"

/translation="MKWMGRFAGYNASAFRKDLLSGLIVGIIAIPLGMAFAIASGVKP

EYGLYTTIIAGVLISLLGGSKFQIGGPTGAFIPILFAIVMQYGYENLLIAGIMAGLML

VLMGVFKLGALIKFIPRPVTIGFTAGIAVIIFSGQIANFLGLSGIEKHEDFWSNMKEI

GVHISTINIYSLLTAGICLVVLLLVPKFAPKVPASLLGLFLSTVVAALFFKDQVATIG

STFGVIPGALPQFHIPEITWARVVSLLQPAFVIAMLGGIESLLSAVVADGMTGSRHNS

NRELIGQGIANMVTPLFGGIPATGAIARTATNIKSGAVSPFSGVIHAVVVLLVLVLFA

PYASHIPLASMAPVLMLVAWNMSERRSFIHVMKTKTSDSLVLLITFLLTVFTSLTTAV

EVGLILAVLLFVKRMSEMLKVAKVLPDPDHKHKKVMAHMVHEGHDCPQISMYTIEGPL

FFGAADVFEKSVMDSIHQRPGILLLRMGKVPFMDTTGEANLASLVKHFERCGGMILFS

GVQEQPLEMMRKTGLMERIHPDHMFEHTGEAINYALQHLNHQKCLGCKHFAFRECAVL

SREDAVAERQSLLEPTSI"

CDS 620643..620882

/locus_tag="PIKHDJKN_00574"

/inference="ab initio prediction:Prodigal:002006"

/codon_start=1

/transl_table=11

/product="hypothetical protein"

/translation="MLYGLIENTKANEYVRGQALNALVILVLHGLLPIYSMNPLPYIP

CCISAAHLVQFGEVILHCVNVNVKRGESFGIKATI"

CDS 620860..622239

/gene="yifK"

/locus_tag="PIKHDJKN_00575"

/inference="ab initio prediction:Prodigal:002006"

/inference="similar to AA sequence:UniProtKB:P27837"

/codon_start=1

/transl_table=11

/product="putative transport protein YifK"

/db_xref="COG:COG1113"

/translation="MESKQLSRGLKPRHVELIALGGTIGVGLFMGSASTIKWAGPSVL

LAYLLAGIIIFFVMRIMGEMLIQEPVTGSFATFAHKYISPLAGFLTAWSYWFLWVTVG

MAEVTAIGIYVGYWFPDIPQWLPALAGVLIIAAANLAAVKYYGEFEFWFALIKVTAIV

FMIVIGSGLIFFGWGNGGQPIGLSNLVSHGGFFPGGIKGFLFALCIVTAAYQGVEMVG

ITAGEAENPKITLRKAIKNIVWRILIFYVGAIFVIVTLYPWNEVGETGSPFVLTFAKV

GIVAAAGIINFVVLTAAMSGCNSGIYSAGRMLYTLAENGQAPAFFKKLSKGGVPRNSI

IITISLLLVGVVLNYLMPDSKLFLYIYSASVLPGMVPWFALAFSQFRFRKRWGNEMGD

HNFKSKWFPISNYIIIVYLTLVIIGMAFNPDTRLPLIVGATFMAIVVIGYFVFGIGKR

QRVDEGEHH"

CDS 622466..623926

/locus_tag="PIKHDJKN_00576"

/inference="ab initio prediction:Prodigal:002006"

/codon_start=1

/transl_table=11

/product="hypothetical protein"

/translation="MRTLVWIYLIFNVVMLVWLIGNRNKSRKMVWGGLVISVVLLLVH

GVIEGLRWPMIPAYLLTLVPLIVLIAKRSKSAVVRKFGRVRIIGTSLLVLVYAAVTMG

LPLLFPVFSFDNPTGPYGIGTVSYDWTDESREELLTSTAGDKRELMVQIWYPTDKDTK

GTTAPYVNDPKVYATAFNDVLKLPKLLFSSLSQVKTHAILEASLSDAEAKYPVILFSH

GLHGYENQNTFQVEQLVSQGYIVVGINHTYSSLVSVFPDGRVAQFESEGKEGFDQLQF

SYMDKLNETWVKDAQFVLDQVEKLAANDANGRFNGHMDLDNIGMFGHSFGGATTVQML

MDDPRVKAGMNMDGVLFGEKRIPAEGVGKPFLMMSADTTVAGTSVMSDDEIAAMGTTR

TEAEKYYEEVYARYEPVTAGGNYWMELSNTKHLSFSDLYLISPILEWSQGVKARGAHQ

IVNDTTIDFFNHYLKGQPLQLDSEVGEHTSYSLKKG"

CDS complement(623953..624936)

/locus_tag="PIKHDJKN_00577"

/inference="ab initio prediction:Prodigal:002006"

/codon_start=1

/transl_table=11

/product="hypothetical protein"

/translation="MNQSKDTYSIGEAAKMIGSTVKTIRYYDEIKLLRPSFHTEGGHR

LYTPQDVWRLELITTLRYLNFGISDIRKLMSGELGVSQALDLQIEALETQIGTMNSML

SILQQAKQQEVEDTLHSGQSLTYVSSLVDSLTVNAQKRKKFVSTKMEESHLLDGIPHE

WRVSFLHFFDKYMMKDTKLSAQQTLAWKEIQEIINDPAYIADLARLELPFFTMANHPQ

IEADAWVKKMETLRIRATEALEKRWPLDSPAVQSMVWDFVMMYASIEHAGDPEAFFRK

QARYMLDSVTERILRFNKLCKIVNPEWSQIVDGINLLQEGMRLRLKQMEED"

CDS 625270..626172

/gene="yghA"

/locus_tag="PIKHDJKN_00578"

/EC_number="1.-.-.-"

/inference="ab initio prediction:Prodigal:002006"

/inference="similar to AA sequence:UniProtKB:P0AG84"

/codon_start=1

/transl_table=11

/product="putative oxidoreductase YghA"

/translation="MANQDQHTMQDPTTQYPKATSDWKQQQEEPGLQREMTPVPDAGE

KSYKGSGRLTGRKAVVTGADSGIGRAAAIAFAREGADVVLAYLPEEEADAKEVVKLIE

EAGRKAVAIPGDLKDEKYCEELIESAVKELGGIDILANVAGKQQFVEQIADLTTEQFD

ATFKTNVYSMFWLCKAAVKHMKPGSSIINTSSIQAYKPSPILLDYATTKAAINTFSKA

LAQQVGSKGIRVNVVAPGPVWTPLQVVGGQPVEKLADFGSNTPLGRAGQPAEMAPAFV

FLASQESSYVSGETLNANGGTVSP"

CDS complement(626297..628834)

/locus_tag="PIKHDJKN_00579"

/inference="ab initio prediction:Prodigal:002006"

/codon_start=1

/transl_table=11

/product="hypothetical protein"

/translation="MKRLFLIMFTLTLLLAGCQSNDKESSPSENNPTEIVEGPSVLSL

KQKYGSESNKAIMPMYNVAQDEEFKFKFKVDLKNSRIQASDMISVHTDIKALESSKVY

AIPEINDNSVSIKPLGWGVLTSDKLRTQEQSTWGGAPIYYIRINYDLDAEKLTLLDQP

MIIPFTVKSELPVPNLKYEIDKDKRFKLTWDKVEGATEYKIYNRADRTVIFETNNVPL

QGAEKAFNGSNPLVLTTTTETSFNDFMEDGKGGLGTINEQMTSYQNHGMKGEYYVTAV

SGDKESNFSNGVATAPLSNQLPMALKEQLYMKKLKNMNELPKTTSIEYIDGSFSQGTI

VYDTTGVGISEFNETNIPFHIKGTALKSYVRVENITQEDLDKLNNQTVAESSNGLVEP

KNDTPYVPAPDVPTIINNHDTPASETATETETGPTVEEAPSTESTTDTGTSEDKTDPV

SNEASDDTSDKSSDKASEEITSESPDPASEIEEDNLVDEQKSNTQQNVEQGNQETVPE

PATGDAMINADSALEEVLAKNMVAVQDKISLKAFPEAQNFTTLSDVVLKVMYQNPLIL

GVEGFEYNYGTLTLSIHYNESADVIQKKQSEIIAKANEVVASIIKDGMKDDEKRKAIY

DYLNDNAKYDDAALENAEQNNFKNVDAQFNDSFTTYGILVKGVGVCASYASVYKLLSD

LSGLESIVVTGASSGVPHAWNKVKIGNEWFHVDATNNLTNSGIPYFLYNANDETAASQ

KTIADKDYWLDSELALFNGESDANDYYVQNDLEVASISEYKTKAESELKNGENRVILR

FASPVDSDELMTAAGEALATVDEALLNTAQLATLGSYAILEPNPDQK"

CDS 629152..629571

/locus_tag="PIKHDJKN_00580"

/inference="ab initio prediction:Prodigal:002006"

/codon_start=1

/transl_table=11

/product="hypothetical protein"

/translation="MTPEQIVRTFFEEVRSGRNLDYANTLMAEQVLAHQVISEEEVTV

TRTPSVYADHVREMIEAYGDFSLQIQELLAQGDKVYVRWRQVGTHVGEVDGYAPTNLP

VIEMASAVYRVESERIAEYWIQIDRLGIEKQLERNQS"

CDS 629653..630063

/locus_tag="PIKHDJKN_00581"

/inference="ab initio prediction:Prodigal:002006"

/codon_start=1

/transl_table=11

/product="hypothetical protein"

/translation="MAALAATAIITTAITTVSMNNETAVTTSSTLSVINNEDLAGTAT
TKQFNIPVGFEYVKVSFKNTGSKAFTFTINQDSTAGTAKMSYTVPADGKEHTYWSDQA
WSTGWFYVSVSSAQGMSGRLSVRLGTDLNKLKSL"

CDS complement(630104..630862)

/locus_tag="PIKHDJKN_00582"

/inference="ab initio prediction:Prodigal:002006"

/codon_start=1

/transl_table=11

/product="hypothetical protein"

/translation="MIPTSNSDYKTIEEISLNHWQPLSTLLFDGWVLRFAQGYTKRAN
SIQPLHYSTQDVYEKIEQCERIYASNQLSTIFKITPFIQPDHLDQLLHEKGYYVVDLS
RVQTRSLETIKEPEHSAVKMDEQLTGEWLDHFCRLNQVNDLQRGTMEQMLTNIRTRTG
FISLFIDGQVVACGFGVVERGYIGLYDIITDANFRNRGLAEQMILHLLHWGKENGATS
SYLQVVANNAPALKLYAKLGFSEIYTYWYRVKEA"

CDS 631033..631695

/locus_tag="PIKHDJKN_00583"

/inference="ab initio prediction:Prodigal:002006"

/codon_start=1

/transl_table=11

/product="hypothetical protein"

/translation="MLTIEINNHIINCHIQYGKRKKASITMDLPYMVTIKAPNGTSED
MIRQLVAQHGEVILTKSALMQQALDGPQAKEYEEEGKGKFLLFGKEHALHELINVEGC
SEEDLRANLKKFYVAECKRMIGERIGRYQQELKVKPKSVDIVESATKWGSCSWDKKLT
FNYRLAMAPLEVIDYVIIHELCHIHHMNHDRSFWRRVGSIMPDYKAKEDYLMRNGRAM
TL"

CDS 631739..632125

/locus_tag="PIKHDJKN_00584"

/inference="ab initio prediction:Prodigal:002006"

/codon_start=1

/transl_table=11

/product="hypothetical protein"

/translation="MSESTSFDVLVDEYIAQFPSDVQVKLQSLRQIIRDSAPNAVEKI

SYKMPTYAEHGNLVHFAAYSKHIGFYPGASGIEAFKEELSRYKGAKGSVQFPLDQPLP
EELIRRIVEFRVQVNVEKAREKKRKK"

CDS 632174..632707
/locus_tag="PIKHDJKN_00585"
/inference="ab initio prediction:Prodigal:002006"
/codon_start=1
/transl_table=11
/product="hypothetical protein"
/translation="MNKALIVLDVQYGITSLRDFTAQLGKIEAVIADFEQRNEPVIYL
KHVDYDHEGASLFYKNAAGLEIVVDTGRHTILEKSKPNAFSNPGLKKWLHEHQVEHVF
IVGFNTEYCCLFTAITAEHEGFKVTLIEDATGSVNTEETYEMPGLDIQDFVGSILNWS
NCIEVLYWHEYKELYED"

CDS complement(632965..633252)
/locus_tag="PIKHDJKN_00586"
/inference="ab initio prediction:Prodigal:002006"
/codon_start=1
/transl_table=11
/product="hypothetical protein"
/translation="MIESLESLAEPLIRTRLELLYNNLSHTQPDYTQLSEESDHYFHT
IRQALPEHLNQTIFLYEDTQLSMQTLLEREIYLQGFRDALQLMSELHIHRF"

CDS 633397..633606
/locus_tag="PIKHDJKN_00587"
/inference="ab initio prediction:Prodigal:002006"
/codon_start=1
/transl_table=11
/product="hypothetical protein"
/translation="MIKIHLSRIMGEKRINIADLSRLTGLHRNGIAKLYNEETDGVKF
DTLNRICEALDCEIQDIIEFIKDEK"

CDS complement(633755..634330)
/gene="yyaP"
/locus_tag="PIKHDJKN_00588"
/inference="ab initio prediction:Prodigal:002006"

/inference="similar to AA sequence:UniProtKB:P37508"

/codon_start=1

/transl_table=11

/product="putative protein YyaP"

/db_xref="COG:COG0262"

/translation="MRKLVLFLHASLDGFVEGPNGEMDIGWVAYDADLEKHAKEILST

ADTVIWGRGTYQMMHSYWPSVPSNPSASQHERDHAEWIEKTAKIVFSTTLETVEWNNA

RLVKEDIEEEINNLKQQPGKDMVILGSPRFAHHLMQLDLIDEYKITVSPVLIGKGLPL

FQGLKEKINLKLIGNKTFDSGAIGLVYQTVR"

CDS 634834..636417

/locus_tag="PIKHDJKN_00589"

/inference="ab initio prediction:Prodigal:002006"

/codon_start=1

/transl_table=11

/product="hypothetical protein"

/translation="MSKVIHKSLYLILLVFVGVFIVSSLFVRAQYNYTLYGDNPILGM

QQWSIFIPVIALLLVSGIGLYRLCLRLNKYSPKVVIPVVLLCSLAIQIIIIFVFPRVP

TDDSQTVLSLAMNMLYDQDYSSFETGGYLHMFPFNFSSVLYLKTLLYLFPDNYLVIKL

FNILFSTLTTLMIYLIYKQLNNKSAERDYGVLIFAATYLPSLFLNNLIYNDVIATAFL

TSCLYFLIRFIREKSWQTIVIAAILLAIGNYFRSIGVIVLIAAMLTILLNMRSIGFKK

VIVSIFVLAILFNVPGWTQNAVLKSSGAVSEPVGESSAPVYMWLNMGINLERFGFWDN

MESYQIYQREANYNKAESKELYKQEIERKLSEASLSDLAQMYYKKIIWTWTEGTYQMD

RYGIGNESSSGMGGGRGGGIAGSYSYTNAVTDLFKGDSVYRTGVLWVVYVMNFMMYCF

IFIRLVSGIRGKRYDEVSLILVILGFIGFYILWEIKSRYIYPVYPLLVVLSYMGFKDA

YNFMFHRKLGWERSSLRKR"

CDS 636423..636992

/locus_tag="PIKHDJKN_00590"

/inference="ab initio prediction:Prodigal:002006"

/codon_start=1

/transl_table=11

/product="hypothetical protein"

/translation="MRKNSYLTSAMLLLLACSVLLSACDAITAQNITNTGSAQGMNGG

GPGMNGGTQRGGGQGMNDRTGGQDDMRGMMNADLTGRVISATGSTVTISLLEVEDNSS

TDSNNGNGRRSGASRGGSMDMKDTGIEMKLNISEDVDITEGMGVGTPGNNQNANSSIQ

VSDLKEGDVVMVWYKENTETVERMVVTQS"

CDS 637381..638385

/gene="thrB_2"

/locus_tag="PIKHDJKN_00591"

/EC_number="2.7.1.39"

/inference="ab initio prediction:Prodigal:002006"

/inference="protein motif:HAMAP:MF_00301"

/codon_start=1

/transl_table=11

/product="Homoserine kinase"

/translation="MLKLKYLFHNNDLAEMILKNWSYDPESLDMFQYYRISSNAVYPF

RDQGKVHLLRFAPVEEKNQTNLAAELEFLSYLKLNQYGAMEAVPSHAGKELVEAHTPW

GMYYASVFKRVPGNQMGSIDLNDQIVYSFGEALGKLHGLSREYRPKQATRWTYTDVLD

WIKDVLDEFPGETAALNEVEVLRDCFADWPVTQQNFGLIHYDFELDNVFYDEESHSCY

AIDFDDAMYHWYAMDVEQTLDSLRDEIQPDEWEQKKRIFLQGYWKEAGDAYDMESMFP

ACRRFANLYGYVRVLRSGAEQWAHEPEWMSGLRARLDKAMAVKAKQFGQQQPLQLPTN

"

CDS 638596..639126

/gene="gph_1"

/locus_tag="PIKHDJKN_00592"

/EC_number="3.1.3.18"

/inference="ab initio prediction:Prodigal:002006"

/inference="protein motif:HAMAP:MF_00495"

/codon_start=1

/transl_table=11

/product="Phosphoglycolate phosphatase"

/translation="MDDKKLIIFFDSGDTIVDESTEIRDEEGIVLSADLIPGADITIQ

KLHEKGYTLALVADGDAQSFKNVFKQHGLHDYFSAMIISENIKASKPSPRMFKAAIGA

LDLSEGDFSRTVMIGNNLSRDMKGANALGITSIFQSWTPRYPHEPVDETERPTHTVGE

PLELIELIERLNAEVK"

CDS 639226..640401

/gene="araR_2"

/locus_tag="PIKHDJKN_00593"

/inference="ab initio prediction:Prodigal:002006"

/inference="similar to AA sequence:UniProtKB:P96711"

/codon_start=1

/transl_table=11

/product="Arabinose metabolism transcriptional repressor"

/db_xref="COG:COG1609"

/translation="MIVKTGTGVGVHMEEPHRKPLLYLQTADLVMEEIRNRKLQPHDP

VPSEGELAKLYSVSRMTAKLALQILEKQGIVYRLARRGTFVSGDYLGGPGKESLSGNV

TTDKKLMKRKIALLFPNMDDYVARIIASVEQEARKSGCQLLIRITTDKEDESFCLQEL

YEEGIDGIILFPRGRTRCSEKVLELNLLNYPLVIIDRIFREVNIDCVYHDHYQGAYNL

TEYLIERGHREIGYISMPFDGVTSREDRYKGYVQAMLDYELPVNGRNICLDCAEDYML

NLNGANLQLKDFIQSNPAMTAVVCADDYIATSCLYTSLFIHKRVPEELSIIGFSDIQL

ASLLPVPLTTARQTTEKLGQAAVNLLCKRMENSREGALSIKVNTTIIERSSVRNLNQ"

CDS 640790..642277

/locus_tag="PIKHDJKN_00594"

/inference="ab initio prediction:Prodigal:002006"

/codon_start=1

/transl_table=11

/product="hypothetical protein"

/translation="MTIKSLLAASFLAVSLFFPLAQPAQAAEDNAVTVYQDGDFGGSS

QQFGIGQFNVNQLNVVGNDKISSVKVAPGYRLTLYRDKDFSGDTKTLTVDAGWLDDFN

DATSSLKVEKIGGPNKPVIAYSNSPYGGFEQQFDIGSYNVDELKAGVGNDAISALRVA

PGYKVTLYKDYNFAGYSKVLTADAIYVGGDINDSVSSLKVEAISPLDATSVAVPGNDY

SDTSKREILQTFAPKIWFAQGEVYFPSSVEHTLPYVDRYVNPGSGLYEFKTKTELNPY

TLKLPYFTGDLANAPIYAFWVEKEYNNVDLVYFQFSPYDLGKTVFGTEVGDHVGDWER

VTVRLAKFTDQNSNYVKPVQVYYGAHSFGTIYSWDEVDKVNNTHPVAYSAFGSHGMWK

DPGNHVYQELVVTQLIDVTNQGTAWDTWNNIQAFQYYPNTRTGNGLGNAWPSWLDRDY

SNLNSLSVYKFGNPSQGSFFGQPLLAGGPTGPQEKGALTNDVILD"

CDS 642385..644499

/locus_tag="PIKHDJKN_00595"

/inference="ab initio prediction:Prodigal:002006"

/codon_start=1

/transl_table=11

/product="hypothetical protein"

/translation="MTNRKKVHVVFKTHLDIGFTHLAENVVKQYMEEYVPKAIALSAQ

LEAEGGREQFIWTTGSWLIRHYLNHASEEGKQAMEQAIHKGHIVWHGLPFTTHTELMD

PSLFRYGLSIGLELDKQFGKTTIAAKMTDVPGHTLSMVPLMNEAGLRYMHLGVNPASM

RPDVPKLFRWQAADGSEIIVNYADTYGEAVEIEGIDDILLFTHTGDNCGPPSAEEIRE

QFQHIQERYPDAEIMASSLDAFAVHLDQVRDQLPVVREELGDTWIHGGGTDPLKIARF

RQLQRLRDKWLTDGRIDPASAEYKGMSDAMLLVVEHTWGLDVKKWLPDFRNYAKPDFN

RAREMNDVDVEDIPAKFQYIGAFAMDEFDSHSSGLFSSETSRRTYSMIESSWAEQRGY

LDTAVAFLSDDKQKEAKQAFEQLNPVKSVSSGTVSAACHQVYSSGGYQLAFNNNGALI

QLTDEKEKNWVDAKHPFGAYAYETFGTQDYDRYFRTYMQNLPVTHPWADADFSKPGFE

FVRPLPSHQVYLPIVTDMHKSGNANGDTYGFRLSMPTESHELYGAPKELELVYTFSQA

GVVEVSLQWFGKEANRLPEASWFGCGLKVDNPNLWMMDKLGGRISPLNVVKDGNRNLH

AVDKGVFYEGTDGQASIEALDSALVAPGQRRLLQFDNSFVSLESGWQFNLHNNIWGTN

FPMWFGEDAKFRFRLNTAANRK"

CDS 644916..646862

/gene="agpA"

/locus_tag="PIKHDJKN_00596"

/inference="ab initio prediction:Prodigal:002006"

/inference="similar to AA sequence:UniProtKB:Q9X4Y1"

/codon_start=1

/transl_table=11

/product="Periplasmic alpha-galactoside-binding protein"

/translation="MKRKSNLLIVLVLITTILGGCSASSSNNENEESTVKEPVVTDNS

AKEAPMLTKLVDEGKLPALAERLPASKDIMVEEVVEEIGQYGGDWNMPWTGVPDKWGI

GQPTEEALFRFNKEGNKVEPNVAKSYEVNDDSTVFTIHLREGMKWSDGVPFTADDVLF

YWEHMLTKETFGKKIYDAYYSVDPVTGDRALAEVKKVDDYTFTVTHKYPSVLFLERVA

IDNKWFFAPAHFHKTILPEFVGDAKTLEITKQWGYEDPKVFLMETGYTDWLHYQIPTL

RAWVPTNDPNGDQFVMERNPYYWKTDKEGKQLPYINRIVATKIQDPSQKVLGTLAGDY

NLVIFDSKDFTVLKENEKKGDYRIIPWTQPAWSSAGVQLNQTTEDPNLRKLFQDIKFR

EALSIGVDRNQVSEIVTSGLGEPIQASVPEGVMGYQEGWAQQWAEYDPERANQILDEL

GLNKRDKDNFRLNPDGSELTLTVIENTTDTAPFLELLKKYYEDMGLRTNLKVVDGGTH

FDMKYANKIPMTTENISVVNVAFRPDTMVPLRVITPWFGHYGLYSQSGGKEGVKPEGD

VAKILEYWEKVKASKTLDEINQYSNEIVKLHQKNQWVIGYAGPTPVLITASNKIHNIP

TDLIWSDEYRSLGQGHPAQFFIKQ"

CDS 646958..647956

/gene="dppB_2"

/locus_tag="PIKHDJKN_00597"

/inference="ab initio prediction:Prodigal:002006"

/inference="similar to AA sequence:UniProtKB:P0AEF8"

/codon_start=1

/transl_table=11

/product="Dipeptide transport system permease protein

DppB"

/db_xref="COG:COG0601"

/translation="MFEYILKRILLAIPVVVVISFIVFYIIQLPPGDYVSSYSAKMSA

SGDIMTQAEMDDMRANLGLDRPIYEQYFVWVKKIILHGDFGFSFNYNKPVMAVIQQYM

GLTLVVSLVTMAFHYIIAIPVGIYTAVKQYSAGDYFFSGLSFIGMATPNFLLAIILMF

FSYKWFGNPLLGLFSNEYVNAPWSMDKVKDLMQHMVIPVIVIGLSGTADLIRIMRGQM

LDELNKPNVITARAKGLSEMQILFKYPTRAALNPIVSTFGWSLSSIFTGATITAIVLN

LPIQGPVMHNALLGQDMYLAGTFLLIMALLTVLGTLISDILLAWLDPKIRTEFRGS"

CDS 647959..649104

/locus_tag="PIKHDJKN_00598"

/inference="ab initio prediction:Prodigal:002006"

/codon_start=1

/transl_table=11

/product="hypothetical protein"

/translation="MRPLPLFKGQRPEVPVTTAPKDTVYSSQWRLIYQKFKKHKLARI

SMYILGAFYIIALFAQFIAPYGLQSYDSKYVNAPPMGLRFVDAEGKYHIKPFVYELKS

ERDPETLRKMFVPDTEKKHFFKFFVKGEEYKFLGLIPMSTHLFAVDEPGRIFLLGTDG

MGRDLFSRIVLGSQISLSIPLVGVGISFILGLIIGGISGYFGGWIDSLIQRIIEIIRS

FPTLPLWMALSAAIPPRIPVVTMYLYIVIIFAFIGWTDLARVVRGKFISLKNEDYVIA

AKIAGVGDAKIIVKHLLPGFMSYLVVATTLAIPSMILGETAMSFLGLGVRSPATSWGV

LLQEAQKIENVALYPWKLIPLAFVILTVLTYNFIGDGLRDAADPYKK"

CDS 649137..650177

/gene="oppD_2"

/locus_tag="PIKHDJKN_00599"

/inference="ab initio prediction:Prodigal:002006"

/inference="similar to AA sequence:UniProtKB:P24136"

/codon_start=1

/transl_table=11

/product="Oligopeptide transport ATP-binding protein OppD"

/db_xref="COG:COG0444"

/translation="MATQAVDKEQPLLSVQDLKIRIQMENGVMNAVDGVDFNIYKGKT

LGLVGESGCGKSLTSRSIISINPKECETSGTITYNSDSESTLHGLNLLSLKPTGKQIR

SIRGRKISMIFQEPMTAFSPMYTIGNQIMEAIRIHQTRNKKEAKNIALEMLSKVGISD

QAKRFDQYPHEFSGGMRQRAMISMALSCNPEILIADEPTTALDVTIQAQVLELMKSLQ

AEFGMAILLVTHDLGIIAEMCDEVAVMYLGRIVEQAPMREIFNNPKHPYTKGLLKSMP

RLGGNKSKRLDSIEGSVPMPINMPPMCGFYDRCKDRIEGVCNQQAVPKTAISDQHMVR

CFLYADQREGEV"

CDS 650177..651223

/gene="oppF_2"

/locus_tag="PIKHDJKN_00600"

/inference="ab initio prediction:Prodigal:002006"

/inference="similar to AA sequence:UniProtKB:P24137"

/codon_start=1

/transl_table=11

/product="Oligopeptide transport ATP-binding protein OppF"

/db_xref="COG:COG4608"

/translation="MTKPILEVLNLNKSFQVKSKKALFKKPKPVHILNEISFELLEGE

TLSIVGESGCGKTTLGRCIVRGVDASSGSVVYHAEDGKEVNYLNLRGKEIKAYRKDIQ

MIFQDPYSSLSPRMSVFDIIAEPLIANFKLSRAEVETKVMEIAEKTGLNVSYLKRYPH

AFSGGQRQRIAIARALITQPRLIVCDEAVSALDVSIQAQIINLLKDLQEQFKITYIFI

SHDLSIVQNISDRVAVMHLGRIVELATTESLFSNPQHPYTEALMSAVPQPNPDFKKER

IILEGEVPNPANPPSGCHFHPRCSYKTDKCIQHVPELREVGYNHFAACHYAEQLQLRG

VPIETEDEIQSRTS"

CDS 651311..652342

/gene="purR_1"

/locus_tag="PIKHDJKN_00601"

/inference="ab initio prediction:Prodigal:002006"

/inference="protein motif:HAMAP:MF_01277"

/codon_start=1

/transl_table=11

/product="HTH-type transcriptional repressor PurR"

/translation="MAKIDDVARLAGVSKGTVSNVFSQKRPTSKKVTEKVMQISKELN

YVPNHIARSLVTKKTMAIGLTIPHGKFFFSVFHNQFINGVILEASNYGYRVLLDMIAA

EEVKTPSLASYPIDGAIVLGPTEEDDRINLLYQHEIPFVTIGKIINKGLQGVSTVNND

NKQVIRDICDYLLGLGHRNIMFLNAGEQMTVSIERTEEFIRVLQEHGVMVQPGMIHYK

PGIHSDKYIKYGYDETLHNVKNHTERVTAIIADDDRTAFSALNAINDAGLRVPEDISL

FVICGDQSMMHQVRPSLTGMDLQPSELGVQSVRLLMNKLGILEGTYENNRIVPSKIIE

RNSCSSIKN"

CDS 652376..652879

/locus_tag="PIKHDJKN_00602"

/inference="ab initio prediction:Prodigal:002006"

/codon_start=1

/transl_table=11

/product="hypothetical protein"

/translation="MITFTSWMGNSVPFEYYVRILISAVLGLLIGFDRTHKNKPAGVK

TYTFVTVASALITIVSIESVEVYGQLHNRTMMDPMRLAAQIVSGLGFLGAGLIMKAGF

EVKGLTSAAMILFAGGVGIGTGAGFYGIVTFSMAVAFFFIRVGGWIEKWEHKKLVAKE

EKKDLSA"

CDS complement(652988..653200)

/locus_tag="PIKHDJKN_00603"

/inference="ab initio prediction:Prodigal:002006"

/codon_start=1

/transl_table=11

/product="hypothetical protein"

/translation="MDFREETPEEKRERLRQEELKGNPMGTFGDSINRAQFGSFADLV

GGLGWKGTGILIILLVIGYVIYKLVF"

CDS complement(653322..653927)

/locus_tag="PIKHDJKN_00604"

/inference="ab initio prediction:Prodigal:002006"

/codon_start=1

/transl_table=11

/product="hypothetical protein"

/translation="MRISCSPGFPRSMIGSIDLQPTKFNMGVSSNSEISHHVDPKLIA

IPYVDDPEFGSYFDAMKVMKGTYQEEFHVSYDVEFTIDVDKKGYITQFEHTFALDRYL

DLVRTQSYKVIKTNWKGHSFHVMTYSYMEEVCNPNNVIFKCNNAEDVFVVTELVPYSV

GGVVVQPHNRYLHLRALISARDDLYPIDYMCEPDFDLSIEP"

CDS 654087..655235

/locus_tag="PIKHDJKN_00605"

/inference="ab initio prediction:Prodigal:002006"

/codon_start=1

/transl_table=11

/product="hypothetical protein"

/translation="MEQSLQELLRNARAEENTFLYMLHEEARIDQLLFWKYVNSIVEL

TRLTAAQPLDREMASAVSFTYSKIMEHLQWHHSDRDVYEIQQFPYEYANLMVDRLGRV

VDGFFKGIVLEEERFDEEFPNPTSTGEMAEEQPAVLQLGYYKQNTNVYAIGFREEDGT

YRIVLNEEEDRELFDSKLSRREVEGTYLFMAPDASSAHQLFHEWVMGNHTPYRSALDL

KGAHSMNVKDNLIEQQSLTFVGIKRTFSCVDGENLREIPKMWQEALADGIENRLNEFN

NGAVSGLVGICIDQRELHSDQMEYWIATSHSGEVPEGLFSIEIPASHWMVFEANELAP

EAIQRLWKYIMSEWFGATPYKHAGIPELEVYRGQGLPPQVWIPVKPLS"

CDS 655345..655794

/locus_tag="PIKHDJKN_00606"

/inference="ab initio prediction:Prodigal:002006"

/codon_start=1

/transl_table=11

/product="hypothetical protein"

/translation="MLSNKVVDYCQNQGWWHEDVPAEYEEGLRKLGIDLESEFAHFYL

HADDGPTFYSRHQEIYQICWVMENTVYLEDMTVAQLTLGLPEAYIPLDSFEGEGGFFY

NRQTGDVALVELGESIERFLSGESKPQWASFNNFLEWYFELEKEVTE"

CDS 655791..656237

/locus_tag="PIKHDJKN_00607"

/inference="ab initio prediction:Prodigal:002006"

/codon_start=1

/transl_table=11

/product="hypothetical protein"

/translation="MKDLTPEHPELERQIIETVEAGNVLDEMQQHEQALIYYDQAWWM

LPEPKTDWEIASWIASCHVNAHMDLAQYALAKPWAEIALQTRGSDINTGPWIELGIVC

MRLEQHDEAFANLDLAYSYGQERAFQGSPRDVLQYYKEQKAKRERN"

CDS complement(656336..657547)

/locus_tag="PIKHDJKN_00608"

/inference="ab initio prediction:Prodigal:002006"

/codon_start=1

/transl_table=11

/product="hypothetical protein"

/translation="MHNFFAKKRITAFLFILVLVTFSVLNIIQSFGPIQKTLASADYH

YSEAKELIHELDDDINEHVFEKFGFVEAYGYMQSLMWKNEENNFEVVKDMEGKLHYTY

FATGPTDTKELSDRVAALGAQLGPKTKLTYVMTPDKYVRGYTQFPEGIPYNYNNETAD

GFLANLQQDGIDTVDLREGLLESGIPAKDLFFTTDHHWKIKTAFWAFGQLVNHLDGMY

DKSLDPEHYFTDLNNYNVVPYQDIFIGSQGRKAGKYFAGDDDFDLIYPKFKTDYSFYF

KTGETEATLNGRFEDALLTTYPLNVQGATYDLTSDKYFTYLYGNQGIVHVVNKDKPNG

LRVLFIKDSLAVPMISFLSTVVSEIYLIDPRYYTGDIVDFASKTDLDHVFVSISPQDL

VDEFFPYGSKD"

CDS complement(657562..658983)

/gene="patA"

/locus_tag="PIKHDJKN_00609"

/EC_number="2.3.1.-"

/inference="ab initio prediction:Prodigal:002006"

/inference="similar to AA sequence:UniProtKB:O25526"

/codon_start=1

/transl_table=11

/product="Peptidoglycan O-acetyltransferase"

/db_xref="COG:COG1696"

/translation="MLFSSVIFLFYFLPLVLGLYYILRFSVTAKNMLLLVASLFFYAW

GEPWFVLIMLASICFNYIFALLVDKFRERRNAAYIIITLMLIVNLGMLFVFKYMAFAL

RIMNENTGFGLNIPNIALPLGISFFTFHGISYVIDVYRGHGAVQKNLFYVALYISFFP

QLVAGPILRYNTIADQMVNRKESWDKFALGCCRFIVGLGKKVLLSNSMAIVADHIFSM

GGTSDIPASLAWLGAIAYTLQIYFDFSGYSDMAIGLALMFGFKLEENFRYPYISRSIT

EFWRRWHISLGTWFKEYVYFPLGGSRVTNKDKMIRNLLIVWGLTGVWHGAEWTFVVWG

LINFAFIALEKISNFDKGTRFNPLRHLYAMFIVVIGWVIFRSPDLLQAGNYLGNMFGL

YGNGFWSETTWMFLKEYALFFILGILFCMPIATRMNKLMVDGARFSKPLELVYPVTII

VLFLVCVSYLVKGTYNPFIYFNF"

CDS complement(659101..660252)

/locus_tag="PIKHDJKN_00610"

/inference="ab initio prediction:Prodigal:002006"

/codon_start=1

/transl_table=11

/product="hypothetical protein"

/translation="MIKKWKQLTLVASLVVLWNVYPIQAVQAEQLKVSGNTLHEWNNG

AKVDASVDNKGSIQIKTSTQGLKKGYYSAYVYELQNRDWSGYGALTFSIRNESDRKLP

LNIVLTRADKISLTVSDDRNVIVVEKSTGQAELVRPISGLIALEPGFVGQIRIPFSSL

MVQDKASTAGQVNLGKVIGWGITTTTPENAKLSFRIGNIKPLSKKQAVVENGLTTLQM

TGDERVVKPVAGESIVQYGVESFSEEKPNVKFQLDTPVQGVSITPDGLLTLHTDVDAE

RATIRALVNDKWNLTKTVMLDRSSAMNVQEKDGTPRSIPDPSDVTKVLSPADPLMKTS

VGWFIRLLLIAAGIHGLVLYWMWRRQRNTQGARKRRSRDDRQARRPFRM"

CDS complement(660270..662549)

/locus_tag="PIKHDJKN_00611"

/inference="ab initio prediction:Prodigal:002006"

/codon_start=1

/transl_table=11

/product="hypothetical protein"

/translation="MNYLSTRNKILVTATVIMSCIYVIWRTFFTIPFGHGPLAVTAGL

ALLIVELIGMFELAVHFYNMTRLEYPELPVVDEALYPDVDVFIATYNEPTSLLFKTIN

GCLNMDYPDRSKVHIHLCDDSNRPEMRELAAHLGINYLTRTEHVHAKAGNLNNAMKHT

SSPLIVTFDADMIPKHDFLTSCVPYFLTGEKIGFVQTPQSFYNPDQFQYNLYSENRIP

NEQDYFYRDVQVGRNKSNSVIYGGTNTVLSRQALEDVGGFYTGSITEDFATGIEMQSK

GYRCFATNKVLASGLAPGDLKSLIKQRQRWARGCIQTGKKMNLLFKKGLSFAQKLNYI

SSITYWYSGLKRLLYIMSPILFAVFGVLVVKCTILEILVFWLPMYLLTNTCLRMLSGN

IRTTKWTNVYETILFPSLLPAVILETLGITMNKFVVTRKDGAQNDDRNYQIKQMIPHL

ILAVLSLIGIVNCIHWTFSQGTIGFLVVLYWLLINFYNIMMSIFFLAGRKVFRNHERS

MATVDCTLTTEGETISCITHDLSEGGISVMLDTPKYIPSDMEIGIKLVNDRYSSEFTG

KVIHVASIGNRWKYAFTVKDIKEQEQRQLLHIIYDREPTLPQKLDKDISTFEDIRLNF

VRRGQSEFMSNRKLARIALNRELKTPDHGIVTLLDFNYEFVLVEMESHDLVNKIQLPL

GEDISLECVWVQSLRDKRGDLYRVTNIRDIARNHQMPTLEALLKQWNKEHSQLGKTGP

ASQGKNNISPDELNEMAYL"

CDS complement(662933..663337)

/locus_tag="PIKHDJKN_00612"

/inference="ab initio prediction:Prodigal:002006"

/codon_start=1

/transl_table=11

/product="hypothetical protein"

/translation="MEISYFLLPKAEVAYIKSSASMKDAIEQLELQHYTAIPVIDQDG

KYVATLSEGDLLWKMRSTPGLTFETMDQVQVHEIDNRVYNECVFIKAEMEDMLTLAAD

QNFVPVVDVDRVFLGIIRRKDIIEYYTRNISD"

CDS 663485..665575

/gene="pbp"

/locus_tag="PIKHDJKN_00613"

/inference="ab initio prediction:Prodigal:002006"

/inference="similar to AA sequence:UniProtKB:P07944"

/codon_start=1

/transl_table=11

/product="Beta-lactam-inducible penicillin-binding

protein"

/db_xref="COG:COG0768"

/translation="MKSKRKIIYGLLPILFAGGIGMYLYMQNSKTEEVKPEVTANQYI

EHLQKKEFDQLYTLMTPASLKESGMNREQFVEKYNAIYSGMEVSAIKAELEPKVNEET

TDGTGNENKNQNQNPDAYEVDYSLQLTTFLGEVDETHTLKLVREELEEGGKVWRINWK

PSMILTDMVKGSKVRVRTLFPERGDIVDREGMPLATKGNIYEWGIIPEKLGENPEAMI

ARIAEHYKVTEAMINTALAQKWVKPEYFVPIASTEDSRVPTALAGVQVQSKEIRYYPL

GEAAAHLIGYVRKATKEDLEKDTEGYYRAEDWIGKAGLEQSLEKQLRGERGGLIEITD

ESGNTRSELIRKDAVDGQNIQLTISSEQQRKLYKTLSSGGDAGAMVLMNPTDGNLLAL

VSAPAYNPNKMVTGLTQAEWDAYSADEKLPFINRFTNRYAPGSTFKAITAAAGLMEKV

TTADKSHDISGLQWRKDESWGGYYVKRVKSVSPVNMVDALVYSDNIYFAKEAVEMGSA

KFIDGIQKFGFGDNFGLDELYLKPSQYANEAHLDLSSEVLLADTSYGQGEMLMSPIHL

ASSFTPFINEGKMVKPVLIEEKETAEPEVVITPEVANTVKDALGEVVTRSGGTAHSLN

SLPEGLAAKTGTAELKAKKGEKGQENGFVVVFDTESPSFLIAAVIEQVNGRGGSHYVL

DKLKPFLEKKGVTQ"

CDS complement(665680..666435)

/gene="COQ5_2"

/locus_tag="PIKHDJKN_00614"

/EC_number="2.1.1.163"

/inference="ab initio prediction:Prodigal:002006"

/inference="protein motif:HAMAP:MF_01813"

/codon_start=1

/transl_table=11

/product="2-methoxy-6-polyprenyl-1,4-benzoquinol

methylase, mitochondrial"

/translation="MKKKMDYTTFYERIGRLNGWDFSSLKVTSEGIEWNFYEEVTRCT

KPSDLILDIGTGGGEAVLSIAHPALLLVGIDLSQGMIETAQRNLKASGSRSNVRFVNM

NAEQLAFPDQFFNVVSSRHCDFCATEVFRVLADGGVFLTQQVNENDKCNISKAFDRGQ

SLGVQPGTLMERYKRELREAGFRDIEAHEYNATEYYATPDDLLFLLTHAPIIPGFGEK

EYDLERFQQFVEQNRCDKGIRTNSARFMLTARK"

CDS 666707..667129

/locus_tag="PIKHDJKN_00615"

/inference="ab initio prediction:Prodigal:002006"

/codon_start=1

/transl_table=11

/product="hypothetical protein"

/translation="MVAVRAKQIFVNLPVKDLKRSVDFFTKVGFEFNVNFTDESTTCM

IIGDNIYAMLLVEDRFQSFISKKISNAADTAEVIVALSVDSREQVDLIVQAALDAGAK

PYNDPQDHGFMYGWSFQDPDDHLWEVSYMDLSSFPTQE"

CDS 667289..668533

/locus_tag="PIKHDJKN_00616"

/inference="ab initio prediction:Prodigal:002006"

/codon_start=1

/transl_table=11

/product="hypothetical protein"

/translation="MYCHVCGTKSNPGDSTCRKCRTKLKDSSSTDEQIWAETAVGLES

GTGKALDQLSRQHQSAPKQSNVVQGRAFSWIIPLLLAAVMGALLTYYYKQEMGINAEV

KSLHQQAEQAALDGKYQEALQLLDSALTKRPNVDGLVQDRQITAKAFNLMNQLNEAAT

SLKTGKLAAGDKTLQAVAKVLKEREEPVFAKVRTTLNNNKVTLAVLKVKKEIDSLTTV

QALAEKLDTVSKLKGKEAEAVQKQIIDKLAGLSYKQAEQQVKKKDFTAALQTVDLGLS

YAPEDNKLTAYRERVLSEKKAFEKAEADRIQLAEQQAAEEDLKNRTAAVSIVEVEATL

DMYGDLYISGSIVNNATRPISSVTVMLDIYSSDGAYLGQTYADVYPSWLDVGDQGFFE

TYYYGVYEEAEVSVVNATWYLE"

CDS 668548..669699

/locus_tag="PIKHDJKN_00617"

/inference="ab initio prediction:Prodigal:002006"

/codon_start=1

/transl_table=11

/product="hypothetical protein"

/translation="MAKRTWSTIISSAIIIGAGAGGVFWIHQYSADQLQDGPRLAVVT

TKETATTASKPSSQTAVKKTRKQIIEESQKKVVTIESSSGLGSGFLYNDQGDVVTNAH

VVEGSKEVTIRTLNHEEYKGTVIGIGEETDIAVVRVPDLKKVKPLPIAKSKAETGDEI

LALGSPLGLENTVTTGIISGVGRSFEIAPYIYSNLYQISAPITHGNSGGPLISADTGE

VLGINSAVVEQEGGIGFSIPITSVLKQVQAWSEKPSSSKTAVQTSGSQGAGANSASLE

AEGLVTEFYMNLNLNDYVTAYALLGSEWQSGISYAKFREGYINTSYVTIAEVSSSDKS

NDEIEVAAVITADERKDGELVTTKYKVTYQVGYENGQLKILHGQGKKVK"

CDS 669715..670419

/locus_tag="PIKHDJKN_00618"

/inference="ab initio prediction:Prodigal:002006"

/codon_start=1

/transl_table=11

/product="hypothetical protein"

/translation="MGAKPILLFDCLSLMVLLKYRTNVQYARGGVKRMSMNKKQIQTE

QTKKKLADASRALFVQKGYKATSIEDIVAATGSSKGNIYYHFKSKEGLFLYLIDEWDR

EWEENWAAKEHLYHTSTEKIYGLAEQLILDDMNHPLTKAADEFFTGEKKENDIEERIS

LMFERHIQFNKQLVEQGIESGEFKADNADNLALILESTIIGLSQLSRGMEPEQALALY

RQAASVFLYGIAKDKA"

CDS 670453..671673

/gene="tetA_1"

/locus_tag="PIKHDJKN_00619"

/inference="ab initio prediction:Prodigal:002006"

/inference="similar to AA sequence:UniProtKB:P02981"

/codon_start=1

/transl_table=11

/product="Tetracycline resistance protein, class C"

/translation="MALLTRNRGALLLLMFNIFLVFTGIGLVVPIMPAYMDLLHITGF

TVGLLVAAFSFTQFLFSPVAGRWSDILGRKKIIVGGMLIFAVSEFMFGAVNAPSLLFA

ARMLGGIGAAMIFPAVMAYTADITTDEERGRGMGLINAAITTGFIIGPGIGGYIADFG

IRIPFYAAGIAGLLASIITLIILPESTRITGEQTKPVAGAPKVKSPGMVSQLLHSYRE

PYFFSLIIVFVMAFGLANYETVFSLFVDHKFGFTTKDIAFIITFGSIAGAVVQVSMIG

WLLNRFGEKKVISVCLLFVAVFVLLTLFVNTYWMILVVTFIVFLGMDILRPAISTQMS

KLAEEQQGFVAGLNSAYTSLGNIAGPIVAGALFDVNINYPYVSAAVVLAICFLLSLRV

LRGVNSTGNAKAEM"

CDS complement(671771..673180)

/gene="bglC"

/locus_tag="PIKHDJKN_00620"

/EC_number="3.2.1.86"

/inference="ab initio prediction:Prodigal:002006"

/inference="similar to AA sequence:UniProtKB:P42403"

/codon_start=1

/transl_table=11

/product="Aryl-phospho-beta-D-glucosidase BglC"

/db_xref="COG:COG2723"

/translation="MKHTQLKPFPKDFFWGGSTSAYQFEGAWNEDGKGPSVIDMGNHV

EGVTDFKVTSDHYHMYKEDVALMAEMGFKAYRFSIAWTRIYPQGAGEVNPKGLAFYDS

LIDELIKYGIEPIVTMYHFDLPYALEENGGWSNRATIDAFEQYAKTLYENYGDRVKYW

LTINEQNMLILHPSSLGTLDTTLVDPQKTLYQQNHHMLVAQAKAMVLCHEMLPEAKIG

PAPNIGVIYPASSKPEDTLAADNYAAIRNWLYLDMAVYGRYNHIAWSYLEEKGYTPVI

EDGDMDILAQGNPDFIAFNYYTSQTVGESLNDGNDFSHTGDQHEIVGEPGAYRGSVNP

NLQKTEFGWEIDPVGFRSTLRQIYSRYHLPLIVTENGLGAFDKLEKGDVVNDPYRIDF

FNKHIEQIQLAITDGVDVFGFCPWSAIDLVSTHQGSSKRYGFIYVDREEFDIKELRRI

RKQSFHWYQKLIETNGKIR"

CDS complement(673234..675132)

/gene="bglF_1"

/locus_tag="PIKHDJKN_00621"

/inference="ab initio prediction:Prodigal:002006"

/inference="similar to AA sequence:UniProtKB:P08722"

/codon_start=1

/transl_table=11

/product="PTS system beta-glucoside-specific EIIBCA

component"

/db_xref="COG:COG1263"

/translation="MDHKKMGDDIVRLVGGEANINGLVHCATRLRFDLKDSKKAERET

LEKHEGIITVVESGGQFQVVIGSNVAHVYAEIVKNRDFGGDSSSSAESTGEKTSVLSK

VFEIISGSFSPLIPAMAGSGMLKALLTVLTMFGWMSDTSDTYLILSAAGNAVFYFLPI

FLGITLGMKLKANPYVAGVIGAALMEPNFTGLMDKGSDVSFLGIPVVMMNYSASVFPI

FISISIYAVLDKLLKKIILKDLQLFLVPMIALMIMVPLSAMAFGPFGTTVGDWISSGV

TWLIGVSGILSGIVLGGFMTFMVVFGLHWGFTPITIQNIGVGGDPIEAMAAAAVFAQI

GVAFGIFLKAKKNKALRTLAGSTSLTGLLAGVTEPIVYGLILRYKRVIPIVVIAGAIG

GAINGHFGVKMTAYVFHNIFAIPVYTPTVVYVIAIACSFAVAAVLTVMFGYESKTKDT

ASEKNESISNTSTESTPEELPVVPEAKTTEIKKEQIYSPLTGKAVALNTINDPAFSTG

AMGKGLAIVPEIGEVVAPVDGVVTSLFPTGHAIGLTTNAGTEILIHIGINTVALKGKH

FNPVVQEGDIVRQGDLLIQFEIDKIKEAGYETVTPVIITLTQQEVDVFETTQEHVQKN

DVLLTLVV"

CDS complement(675317..676177)

/gene="licT_1"

/locus_tag="PIKHDJKN_00622"

/inference="ab initio prediction:Prodigal:002006"

/inference="similar to AA sequence:UniProtKB:P39805"

/codon_start=1

/transl_table=11

/product="Transcription antiterminator LicT"

/db_xref="COG:COG3711"

/translation="MIIRQIFNNNVIRAENQVGQEFVVIGNGLGFKKKNGQPVDEDKI

EKTFVLKSDKIPQKLIDLIGETSVEYLKLADEIVGYAKQEMGDIFSDNIYISLIDHIQ

FAITRYRKSVGLKNSLLWQIKKFYKKEFWIGMNAVHLIQEQFGIQMDEHEASFIAMHF

VNARQDGQGMKQTVEITEVIDDIFNIVTNHYHIALDENSFNYSRFITHLQYFVQRMLS

NEQEQFASGDNFLYEQVKDKYAKAFQCTQLINQYLEEKFESAMSIDEKVYLTIHIHRV

TSRNEMLDAE"

CDS 676489..676656

/locus_tag="PIKHDJKN_00623"

/inference="ab initio prediction:Prodigal:002006"

/codon_start=1

/transl_table=11

/product="hypothetical protein"

/translation="MSKAKGKGGTGRGTGKKGWNRWQAAANRAKSAPKPYKSKGTKNK

TDAETPSDKSV"

CDS complement(676838..678604)

/locus_tag="PIKHDJKN_00624"

/inference="ab initio prediction:Prodigal:002006"

/codon_start=1

/transl_table=11

/product="hypothetical protein"

/translation="MLLDKIRWSTLPFFAKNLVISFGSIMLIGVILITAGYQLQKNIL

SQQLYEQAEVTTQKWFDDLDTHKVLEAVKEKSYSGPVQKELREYLDSIHELYPNIAQA

YIFGTELKEGNQTSIIAIPTNLLGPFEESNMAAGSMYPLPQKNVIALEGMKNDEEPAL

SSFYTDEYGTWTTIMYPIKNAEGKMYAALYFDVDANAVPKGLHKLITYSSIFLIGFLI

LFMVLQYILLKKTLKPIRDLMKGIETASAGNLDVTIQTGRDDLGIINEKFNTMIQRFN

DTMFKVQSTSYHLSDSSKKLLDISEKNNDNIQMISSNIRDISHGLRSQDQASVESARA

MTEMSTVVQTIASSSADVADEALNMEQRSNTGHEIMQQVVEQMRLISGAVQHTSESIQ

SLESRSNEISSIVNMITQIADQTNLLALNASIEAARVGEEGKGFAVVAGEVRKLAEQS

QDSAKQIRTLIDGIQRDILQSAEAMQLGSQEVEKGSQVTMETGQFFEDILTATNKVAN

QIQDISSSTEEISASTQEMSATADELSASVSKAAHSSKQIEQSIAEQESSMASIVAAS

DELSAVSGQLQELISFFKVRAN"

rRNA 679620..681169

/locus_tag="PIKHDJKN_00625"

/product="16S ribosomal RNA"

rRNA 681480..684401

/locus_tag="PIKHDJKN_00626"

/product="23S ribosomal RNA"

rRNA 684487..684598

/locus_tag="PIKHDJKN_00627"

/product="5S ribosomal RNA"

CDS 684826..685449

/gene="dgk"

/locus_tag="PIKHDJKN_00628"

/EC_number="2.7.1.113"

/inference="ab initio prediction:Prodigal:002006"

/inference="similar to AA sequence:UniProtKB:P37530"

/codon_start=1

/transl_table=11

/product="Deoxyguanosine kinase"

/db_xref="COG:COG1428"

/translation="MKSAPFIAVEGPIGAGKTTLATMLSQELNLPLVKEIVEENPFLA

SFYQDIDEWSFQLEMFFLCNRFKQLEDTGVHYIKKNTPVISDYHIYKNMIFADRTLKG

TKRDKYRQIYHLLTDDLPKPNLVLYIEAELDTLMNRINKRGRSFEQDMDPAYMEQLIA

DYKTGMDYLASSPNPPMIIKVNAEQLDFVEHPEHFKQIVNQVKEYIT"

CDS 685446..686102

/gene="dck"

/locus_tag="PIKHDJKN_00629"

/EC_number="2.7.1.74"

/inference="ab initio prediction:Prodigal:002006"

/inference="similar to AA sequence:UniProtKB:P37529"

/codon_start=1

/transl_table=11

/product="Deoxyadenosine/deoxycytidine kinase"

/db_xref="COG:COG1428"

/translation="MNNYGIPANALITVAGTVGVGKSTLTAALAERLSFKTSLEQVDH

NPYLEKFYHDFERWSFHLQIYFLAERFKEQKKIFELGGGFVQDRSIYEDTGIFAQMHA

DQGTMSATDFETYSSLFEAMVMTPYFPHPDVLIYLEGSLPSILNRITERGREMEIQTD

RSYWEHMHERYSVWINQFTACPVLRLNIDEYDVNDPASVDAILAQIAAVINPSNATQL

"

CDS complement(686384..687127)

/gene="mta_2"

/locus_tag="PIKHDJKN_00630"

/inference="ab initio prediction:Prodigal:002006"

/inference="similar to AA sequence:UniProtKB:P71039"

/codon_start=1

/transl_table=11

/product="HTH-type transcriptional activator mta"

/db_xref="COG:COG0789"

/translation="MAMKVKEVAELVGISVRTLHHYDEIGLLTPDEVTSAGYRLYSDA

NLEMLQQILFFKELDFSLKDIKEIINNPSFDREEALNMHRRILLEKRQRLDQMIATID

KSVQHVRGEIKMTAKEQFEGFNFSHNPYEQEARERWGDQAVDQANQKLHNHSPGEQKA

LSDQMNSIYTRLAAIRHTDPNSAEAQAGISEWYSYLNNMGNYSPEVFRGLGQMYVDDE

RFTRNIDQFGEGLAVFMRDAMAVFADQNS"

CDS complement(687291..687956)

/gene="thiE"

/locus_tag="PIKHDJKN_00631"

/EC_number="2.5.1.3"

/inference="ab initio prediction:Prodigal:002006"

/inference="similar to AA sequence:UniProtKB:P39594"

/codon_start=1

/transl_table=11

/product="Thiamine-phosphate synthase"

/db_xref="COG:COG0352"

/translation="MRHLDAEAVRRAMQVYLVMGSVNTTRDPVAVLRQAIAGGITLFQ

FREKGTGALVGEARITLAMRLREVCSQHGVPFIVNDDVELAVAVEADGIHVGQDDADA

ALVRARIGEARMLGVSAHSALEARRAVQAGADYLGVGPMYPTQSKADAHAVLGPPGVA

ELRAAGIAVPVVGIGGITPDTTAAVMAAGADGIAVISAIAGAADVRAAAAQFAAAVRG

MQA"

CDS complement(687956..688768)

/gene="thiD"

/locus_tag="PIKHDJKN_00632"

/EC_number="2.7.1.49"

/inference="ab initio prediction:Prodigal:002006"

/inference="similar to AA sequence:UniProtKB:O31620"

/codon_start=1

/transl_table=11

/product="Hydroxymethylpyrimidine/phosphomethylpyrimidine

kinase"

/db_xref="COG:COG0351"

/translation="MSIAQALTIAGSDNGGGAGIQADLKTFQELGVYGMTVITAIAAQ

NTTGVQGVFPISFEGIAQQLDSTGDDFQPTAVKTGMLYSAEIIRLVADKWQQYGWSNL

VIDPVMVAKGGASLLQQEAVQALIKELLPHALITTPNIPEAELLTGMSITNLSQREEA

ARRMVQMGSTYALVKGGHDEGSGIIVDVLYDGQSFHYLENVRVVTRHTHGTGCTYSAA

ITAELAKGSAVLAAVTTARAFIQAAIEDELGIGAGHGPTNHFAYQRRLRGGQ"

CDS complement(688765..689577)

/gene="thiM"

/locus_tag="PIKHDJKN_00633"

/EC_number="2.7.1.50"

/inference="ab initio prediction:Prodigal:002006"

/inference="similar to AA sequence:UniProtKB:P39593"

/codon_start=1

/transl_table=11

/product="Hydroxyethylthiazole kinase"

/db_xref="COG:COG2145"

/translation="MSYLERVRTQNPLVHNITNIVVAPFTANGLLALGASPFMAYAHE

EVADVAKMSGAVVLNIGTLDEKVVEAIRLAGQSANANNVPVVLDPVGAGATAYRTETV

QMLIRELRLTVLRGNVAEVANVIGERWSIKGVDAGQGEGDRIGIAERAAQKLGCVVVI

TGREDVITDGQMTFLTSNGHALLTQVTGAGCLLSSVIGAFAAIAKPGEDMLGSVVEAL

AFYGIAAEIAAERSADQGPGSFQIELLNQLSQVTPDVLAQRAQIREIDGGAQ"

CDS complement(689655..690722)

/gene="thiY_2"

/locus_tag="PIKHDJKN_00634"

/inference="ab initio prediction:Prodigal:002006"

/inference="similar to AA sequence:UniProtKB:Q9K9G5"

/codon_start=1

/transl_table=11

/product="Formylaminopyrimidine-binding protein"

/db_xref="COG:COG0715"

/translation="MNKKHIYSLIPILLITLLLIISGCGNTGTTTPATDSTSNAKDTE

SSSAERLSESKDKLSIMLDWYPNAVHSFIYVAQEKGYFADQGLDVDIQMPADTNDALK

LVAAGKIDLALSYQPQILLARGENIPVRSIAAVVRHPLVHLLTEAEGKVKSPKDLEGR

TVGYSSIPLYEAMVRTMISQDGGNPDKMNLVDVGFDLIPSLASGQADAIMGGFINHEQ

LILEKEGHAMKSINPVDYGVPDYYELVLTASDAGIEAKKDQLTRFIKAMQEGQKYVTE

HPEDALNILLAHENETSPLDPEIETKSLNILLPLMNEKGQPFGRQDASSWENVRAWLV

QSELIPDSVKAEDAFINLQGE"

CDS complement(690771..691583)

/gene="ribX_2"

/locus_tag="PIKHDJKN_00635"

/inference="ab initio prediction:Prodigal:002006"

/inference="similar to AA sequence:UniProtKB:A9WGD1"

/codon_start=1

/transl_table=11

/product="Riboflavin transport system permease protein

RibX"

/translation="MRDGNLRDSMKDSVRGWLARYGLFLLLMILLLVIWEWIVRMGWV

PSFIIPAPTAIASSLYEYRRLLLATHLPATLMEVIVGFGLSIGAGIVFATGMHMNRSI

EKALYPFIVISQTIPLIALSPVFILWFGYTLWSKVAVVFLIAFFPIVVSTYDGLRQGD

PEQRELLLTMGASKWDIFRKLQIPLALPSFFSGLKMSVVYCVVGATIGEWLGGSKGLG

YFSRRMSSNMNTDAMFAAIVLLSLLGIALFVLIAWMERRFGTPRHAGRRKTG"

CDS complement(691570..692337)

/gene="cmpD"

/locus_tag="PIKHDJKN_00636"

/EC_number="3.6.3.-"

/inference="ab initio prediction:Prodigal:002006"

/inference="similar to AA sequence:UniProtKB:Q55463"

/codon_start=1

/transl_table=11

/product="Bicarbonate transport ATP-binding protein CmpD"

/translation="MDNDITIHNMSYAFPGKRDSPVLSNVSMRVAKGEFVSLIGSSGS

GKSTLFKLLAGLLEPTHGTIEIPWVQEGKRLGQVAYMPQKDLLLPWRTVMENCMLPSE

LAAGRQDKVRIRADILAGLDRFGLSGHAHAYPDELSGGMRQRVALLRTLLTGGQLMLL

DEPFGALDALTKREMHRWLLELWEGLGKTVLFITHDIEEALLLSDRIILLTPGGQGQQ

LQDLTVPLPRPRHSDMIYEPALVQMRRLLEEQLHARR"

CDS complement(692722..693843)

/gene="hisC_1"

/locus_tag="PIKHDJKN_00637"

/EC_number="2.6.1.9"

/inference="ab initio prediction:Prodigal:002006"

/inference="similar to AA sequence:UniProtKB:P67725"

/codon_start=1

/transl_table=11

/product="Histidinol-phosphate aminotransferase"

/translation="MQSLSPVPSPPVKDLLNQIPAYTPAKSMEQIRRELGLESVDRLA

ANENPFGSSPLAADAVRSLGSDLLAQYPDGSSQLLRDKLAERLGVRTSQLVFGSGSFE

LLTLTAQAYLNPGEESLIPVPSFNWYKGATLLAGGVIHEVPLVNHSLDLEQFRQRINA

STRIIWLCNPNNPTGTIFTADALLGLLEHVPSHVVVVLDEAYYEFAESEDYPDTVKLL

EQYNNLIILRTFSKIYGLAGLRIGYGIGNESIIEGINRVKLPISLTATSQAAASASLD

DHEFVSHCLHYNRQGRETLIQSFDQWKLPYIPSETNFIMVDLLQDSGPITKELLNRGI

SVRGGAEFGMPTWLRITIGTPAQIVRLIGELDSLLHSND"

CDS complement(693868..694644)

/gene="ssuB_2"

/locus_tag="PIKHDJKN_00638"

/EC_number="3.6.3.-"

/inference="ab initio prediction:Prodigal:002006"

/inference="similar to AA sequence:UniProtKB:Q8KZQ6"

/codon_start=1

/transl_table=11

/product="Aliphatic sulfonates import ATP-binding protein

SsuB"

/db_xref="COG:COG1116"

/translation="MSTSQISPFLQVDGVQKSYDQRTILSDINVNVRQGEFVAIVGRS

GCGKSTLLRLIAGLEPPSSGSIMLGNRKLGSTAPETRFLFQEARLLPWKSVLDNVRLG

IPDKNKEDARQSLRNVGLGEREGDWPGLLSGGQKQRVALARALASHPRLLLLDEPLGA

LDALTRIEMQQLIERLWVEEGLTVILVTHDVSEAVALADRVLLIEDGHITMDTRITLE

RPRVRDSGFAHFENLILNRLLTPPQETAQQIAQKQISFSI"

CDS complement(694669..695454)

/gene="ssuC_3"

/locus_tag="PIKHDJKN_00639"

/inference="ab initio prediction:Prodigal:002006"

/inference="similar to AA sequence:UniProtKB:P75851"

/codon_start=1

/transl_table=11

/product="Putative aliphatic sulfonates transport permease

protein SsuC"

/db_xref="COG:COG0600"

/translation="MRSNTFNRAWFNQFIPFVVPLLILALWQWLTASGVVAANILPRP

AQVLEAFIRLLRNGELLNNISISARRALGGFLIGGGLGFLLGVLNGISSLAEKIADSS

VQMIRNIPHLSLIPLVIVWFGIGEEAKLFLVSLGVFFPVYLNTFHGIRSVDPGLIEMG

KVYGLSRWSLYKDIILPGALPSILVGIRYALGIMWLTLIVAETVAADAGIGYMAMNAR

EFMQMDVIVLSILLYALLGKLSDTIAKWLERRWLRWNPALSRK"

CDS complement(695482..696537)

/gene="ssuA_2"

/locus_tag="PIKHDJKN_00640"

/inference="ab initio prediction:Prodigal:002006"

/inference="similar to AA sequence:UniProtKB:P75853"

/codon_start=1

/transl_table=11

/product="Putative aliphatic sulfonates-binding protein"

/translation="MIRPNRHYTLPLTAFLSALLLLLISGCSAGADNQDAGTGSTPTA

ASAASNNDVTIRVGQTGWGNLELGLKAAGLDDTPYKVTYNVFQGGNLILEAMAANQLD

FGVTSEIPPIFASQAANGGSFKIIAVQQGSTLNQEVVVPKGSSIKSVADLKGKKVAFV

KNTTAHYFLLKILEEAGLTWSDISPVELSTADGLSALISGKVDALASYGNAIISAHLN

EATTLASAKDILSGSFLVAASDQAIANAEQKEVLADYLERINKFNDWARANKDAWAQI

TADNTHQPVEQALKTYTDGEAQRPSRIVAISDEAIKSEQDVADTFQQAGIFEQAVDVS

TFWSDELQDQLPAPGSQ"

CDS complement(696534..697676)

/gene="gldA"

/locus_tag="PIKHDJKN_00641"

/EC_number="1.1.1.6"

/inference="ab initio prediction:Prodigal:002006"

/inference="similar to AA sequence:UniProtKB:P0A9S5"

/codon_start=1

/transl_table=11

/product="Glycerol dehydrogenase"

/db_xref="COG:COG0371"

/translation="MITVKAPAVYVHEANILEQSGSRIAQLGQHVLIIAGESALKAVK

PYLLPSLEEHKIKFHVQLFHGEVTTGQIDTFTQQAFDLDVDLIIGVGGGRVLDLSKAV

AEQAQLPIVTVPTIAATCAAWSALTVLYDEQGQSAGYRPLRRSPNLVLADPHILASAP

RRYLAAGIGDTLVKWHEFAVNLSGNATSLTLRNSVSTAKLALDILEAHAIDVYESTDT

TGSTPQFRDVADAIIVLAGLVGSISDGSLHAAIAHGLHDSLTKLPETHASLHGEKVAF

GLFTQWILEGKAGDELHELTTLLHKLNLPITLAQLGIQQQSQEAAARIAAGLQLREGS

AAHLSFGVSTAQVSEAILQADRIGQTFRESSQSSESSSYTKEVSSL"

CDS 697970..698845

/gene="moaA_1"

/locus_tag="PIKHDJKN_00642"

/inference="ab initio prediction:Prodigal:002006"

/inference="protein motif:HAMAP:MF_01225_A"

/codon_start=1

/transl_table=11

/product="GTP 3',8-cyclase"

/translation="MKTFKKVYIEITSICNLACSFCPQTKRAKNFIDPEVFNNILDQI

KPHTKHIYLHVKGEPLLHPKIDLLLDSAHEKGFKVNITTNGTLLPKTQHKLLGKPALR

QMNFSLHSFDGHEGSTDREGYLGNVLSFVREAVKHNVIISFRLWNLTQDNFTNAQMNR

NRETLEVLEREFNLDFRIEEKVMPGSGVKIAPNVYLNQDHEFQWPSLDAPEDDGKGFC

HALRSQAAVLVDGTVVPCCLDGEGVINLGNVHEKSFSEIVDGERANNLFYGFSKREAV

EELCRKCGYRQRFGA"

CDS 699212..699880

/gene="pelA"

/locus_tag="PIKHDJKN_00643"

/EC_number="4.2.2.2"

/inference="ab initio prediction:Prodigal:002006"

/inference="similar to AA sequence:UniProtKB:D3JTC1"

/codon_start=1

/transl_table=11

/product="Pectate lyase A"

/translation="MKKMLTLLLSAGLVASLFGAIPAFAAPTVVNSTIIVAKGETFDG

KGQTYVANPSTLGDGSQAEDQKPVFRLEAGATLKNVIIGSPAADGVHCYGNCSISNVV

WQDVGEDALTLKSSGTVNITGGAAYKAYDKVFQMNAAGTINIKNFRADDIGKLVRQNG

GTTFAVNMTLDNSDISNVRDSIMRTDSSTSQGKITNTRYSKVPTLFKGFASGKTSQSG

NTQY"

CDS 700463..701056

/gene="ykoE"

/locus_tag="PIKHDJKN_00644"

/inference="ab initio prediction:Prodigal:002006"

/inference="similar to AA sequence:UniProtKB:O34738"

/codon_start=1

/transl_table=11

/product="Putative HMP/thiamine permease protein YkoE"

/db_xref="COG:COG4721"

/translation="MSTAVGSNRLKLTDILVTIVIAVVFGVIYKIWGPTYDLMKPFGV

HAEQMIYGMWFMAGTFAFVIIRKPGVAILAEVAASTVSAFLGSEWGMSTLVYGLLQGL

GAEIFFAAFLYRKSNLFVTCLAAIGAAAASLVLDYQYGYIDSLSAWNYTLFIGFRFVG

SILIAGIFAYYLAKALELTGVTRSLRPVSKQDYEALD"

CDS 701057..702565

/gene="ykoD_1"

/locus_tag="PIKHDJKN_00645"

/EC_number="3.6.3.-"

/inference="ab initio prediction:Prodigal:002006"

/inference="similar to AA sequence:UniProtKB:O34362"

/codon_start=1

/transl_table=11

/product="Putative HMP/thiamine import ATP-binding protein

YkoD"

/db_xref="COG:COG1122"

/translation="MNEVENVQAVGVTNLRCKFPGEKALVFQGLSLSVRQGEKVLLLG

PSGSGKSTLLQILSGLIPRSVEIPMKCDDIQVPEQSGIVFQDPDTQFCMSYTDEEIAF
VLENRNIPREQMPALIHQYLEQVGLHFEHNRTLIQSMSQGMKQRLAMASILAMEPDVL
FLDEPTALLDDEGTSQVWDTVKQIAQDKTLIIVEHKINEIVEWVDRIIVLSPEGKIVA
DGPANQVFTNERNMLREYGIWYPGVWEERGQTKAQTLVTGQFIRYGATETESMDNKEG
RASALPQPALEMQQFTGWRGKTPFIQVEQAKVWHGDWVGIVGANGAGKSSLLLSMMNI
LKTTGHYVVEGQSAGKTEQLADHIAFVFQNPEFQFVTNTVRDEVGFSLLSGKLTAEER
GIRTDHMLEQFGLTDLSGRHPYQLSMGQKRRLSVASALVREQRILLLDEPTFGQDARN
TFAMLSQLERLRREGTAIVMVTHDREIVKRYCTRIWTVDEGRLTDAALVSSS"

CDS 702540..703334

/gene="ykoC"

/locus_tag="PIKHDJKN_00646"

/inference="ab initio prediction:Prodigal:002006"

/inference="similar to AA sequence:UniProtKB:O34572"

/codon_start=1

/transl_table=11

/product="Putative HMP/thiamine permease protein YkoC"

/translation="MQLSFPHRETWLHAVNPGLKMIFLTMLFVFVILVHNLNVMANVA
VVMLLLLGWTGHPWLRLLLYASPFILVFISTSTGMIMFGKGETTWFQWGLIHITEESF
YRGLHLGFRSLSMAAAGLLFGLTTKPVRLFYSLMQQWRLPPKYAYSFLAAMRMIPILL
DEFQTLRYAIRIRGTRQRGSSWNVYGILKRYAIPLLAQSIRRAQRMAVAMEAKGFKEG
GSRTYYIQIGYSRADVWFMGYFIIMLTAAYILGTTFPYNSMLVDVR"

CDS 703444..705732

/gene="bamB_1"

/locus_tag="PIKHDJKN_00647"

/inference="ab initio prediction:Prodigal:002006"

/inference="protein motif:HAMAP:MF_00923"

/codon_start=1

/transl_table=11

/product="Outer membrane protein assembly factor BamB"

/translation="MKTESKWQKTGYLWITVTGISAVLLTGFMLLDTNTETRAAQGSG
STTQALSAQAKTTYKPGDILNLEAGMPLYTQVIGNKVPVDLMGIQYYTSSNEKIKVQA
ARGEWIKFQDYSRGELWIPGWYASKESRSLKTVTPQTFSLRPGSKLYLAPGSTTSWTP

SPTLTDQALIVAATKDWYGLSIAPRVWNKESFTYRPALLWVKAQAVEQQAIVADGWFQ

QDASQTALAVRHLTDIMLNKKTTSKQVGKWLGEPDWKENSANLNDTGYTMSIGQTWRY

ERADAQFLVTFNKNGKLARTRWNIPQDDRNNVVSDWSFGRSDEYEFTTKITGKSLPIT

LPWKPIWTNQGDINYTFLQAATDDVLLMKGDDGGFSGGYYEGSIYALNRHSGQKLWQI

NSGYGALQAYIDTNREAVTIYTSYDPDKKEYVDRIRHLRMKDGEVTWEYTPQKQIRLK

GITAAKNVLIVDKPAAEGSSNGRITVLNSVNGKTLWTRKLSTGYQLLNQSADDPYVLY

WEQDQLVAADPQSGRTIWSLKSKRSTIEQLVNDPYFDGVERLDPFAPAQPERWMLQGH

QWVLLNLNTGAQLANFPAREGQRFEVLNDGMLLIRENKKGDHYGEFSAFTTTLYDAKS

GTKRWTLNDKIERGFVEEDQLYVIKNGYPAALAYDTGETRWNTQNTILSLRYPTNQGS

YVVIKDQLLLPMGEDLLVMDKNDGTLIGRVHDVVMGNPEHRDRDAKNGTMNRLGNEVY

IGSSNGRFSIYDASDLDTDILR"

CDS 705864..706682

/gene="metN_1"

/locus_tag="PIKHDJKN_00648"

/EC_number="3.6.3.-"

/inference="ab initio prediction:Prodigal:002006"

/inference="similar to AA sequence:UniProtKB:O32169"

/codon_start=1

/transl_table=11

/product="Methionine import ATP-binding protein MetN"

/db_xref="COG:COG1135"

/translation="MISLYGVSKRYNERGSRADQGFEALSSVSLEVGQGEIHGIIGSS

GAGKSTLLRMLNGLEKPEDGEVIVNGQHLTRMSESSLREARRSIGMIFQHFNLVSNRT

VSGNVCMPLELAGVSRTERVARGLEVLRFVGLEDKANQYPAQLSGGQKQRVAIARALA

SRPDVLLCDEPTSSLDPQTTSGILDVLRHVNETLGVTIVLVTHEMEVARKLCDRISVM

KEGRIIKTLSEAEVRSIPAPKPDLLTSLLMGEDVSMSGRNTSLHGGQEDNNDDT"

CDS 706672..707340

/gene="metI_1"

/locus_tag="PIKHDJKN_00649"

/inference="ab initio prediction:Prodigal:002006"

/inference="similar to AA sequence:UniProtKB:P31547"

/codon_start=1

/transl_table=11

/product="D-methionine transport system permease protein

MetI"

/db_xref="COG:COG2011"

/translation="MIPESIVKYQHEIWQAIGETFVMVGISITAAVLIGLPLGTLLYL

FRKGQRYQNRPLFTILGSLVNIIRSFPFLLLVVFMIPFTRLIVGTSIGTLAATVPLSV

IAIAYYARLVEQALLDVPKGVVEAASSMGASTIQLVVKFLYVEARSSLVLGLTTATIS

FISYSTVMGIVGGGGVGDFAIRYGYQRFETEIMVFTIIIMIILVQMIQFTGSRLSRWL

DRRS"

CDS 707417..708256

/gene="metQ"

/locus_tag="PIKHDJKN_00650"

/inference="ab initio prediction:Prodigal:002006"

/inference="similar to AA sequence:UniProtKB:P28635"

/codon_start=1

/transl_table=11

/product="D-methionine-binding lipoprotein MetQ"

/db_xref="COG:COG1464"

/translation="MKAKLILMLLAVTLVAAGCGKKEETPAAEGNKEGTQAGQEVTLK

VATLIPPMTDVLDIVKPLLKEDGVNLEVVVLSDNVQPNTALANKEVDANFFQHVPYMN

QYNEANNANLVAVQPIYNAIYGAYSKKVKSIEELPEGATVAIANDPSNIGRSLVMMEQ

NGLIKLKEGVDFNATQADITENTKNFKFKEVDLLMLARMLDDADLVAMTPAYASPLGL

TPKKDALFTEKDDSHFAITLVAREDNKDSEAIQKLAKRMAGPEVKAFFEEKYADIAIP

AFK"

CDS complement(708382..709245)

/locus_tag="PIKHDJKN_00651"

/inference="ab initio prediction:Prodigal:002006"

/codon_start=1

/transl_table=11

/product="hypothetical protein"

/translation="MNHDVRLQALSVFLKNQRSKISPESVGLPAGSRRRTPGLRREEV

SQLAGVSTTWYTWLEQGRDIQVSHSVLDNIASALRLTADERKYLFSLGLDYHSELHIL

DEKPLQIHPSLQKILQELRNCPTIISDRRCHIVGWNDAARHVFMDFEQIPAEQRNMIS
LLFERKEFRRLAVNWEDFVSGFLAIFRAYYGQYVDDEWYNLFLDDMMDRYPDFHTLWK
QSNVSSAPDVLIEFRHSRAGKMLFDLTSLQVQGNADLRCSIYTPATDTATERKLIRLM
EPKVEHDSDKS"

CDS 709388..710626

/gene="fabF_1"

/locus_tag="PIKHDJKN_00652"

/EC_number="2.3.1.179"

/inference="ab initio prediction:Prodigal:002006"

/inference="similar to AA sequence:UniProtKB:P73283"

/codon_start=1

/transl_table=11

/product="3-oxoacyl-[acyl-carrier-protein] synthase 2"

/translation="MERVVITGMGIISPLGNDVHTFWNGLVEGKSGISPIEAFDTTSY
KTKIAGAVRDFDGEERFGRKEARRMDRFVQFAVAAADQALSDSGLVMDNVDRERVGVY
IGSGIGGIQTLMEQGKLLSERGPARVSPTLVPMMISNMAAAMVSMRFGCWGPTLSPVT
ACSIGNTAIGEAFRLIRHGGADVIFAGGTEAAVTEVSLASFGNATALSTRNEAYEGAS
RPFDAGRDGFVMAEGAGVLVVESLSHALARGANILAEIVGYGASSDAYHIVATHPEGR
GAYLAMKAALADAQLQPHEIDVINAHATSTEAGDLSETRAIKQLFGAEAYHIPVTANK
SMTGHMLGAAGGAEAISLIQSLRSGIIPPTINQEQSDPECDLDYVPNVAREADLKIGM
SNSFGFGGHNAVIILQKYSS"

CDS 710933..711352

/locus_tag="PIKHDJKN_00653"

/inference="ab initio prediction:Prodigal:002006"

/codon_start=1

/transl_table=11

/product="hypothetical protein"

/translation="MNVWITVKSLGKRKPALAKQAAELPETTDTLRQLIKNMVAQQLK
ALQDKNSEAEWLAYLMPEDIQEQGAAGKVGFGTIYNEGVPDVEGAMDTAVTAYEDGLF
KVFLNDEELQALDEPLIIQEDDNVVFIRFTMLAGRLW"

CDS 711375..716360

/locus_tag="PIKHDJKN_00654"

/inference="ab initio prediction:Prodigal:002006"

/codon_start=1

/transl_table=11

/product="hypothetical protein"

/translation="MNQEDKVQQYQQELQEKVKSLNGVEQELAGYVAEMAGFTYLRED

EKAYLDVEQQLKRMSAETKTPLFRPLLDVLEQLVSESFLARFRYIAERAANFPYSKHY

ERRPFRTTDPEQHIGQVLRKMIALFRMEMHGFSLNEYVSMREYKFDYLHEIRAVLPDC

IAYELDHETGDMKQALHDIIYGDNQNALLTTEMIKGIFMSDQVDAYRMVGELLVAARL

QEGLRQSIVERMDEGTLEAYIYILKIIIDNNLIRFSSVVRALAVWTGIGIEAANQRVA

AQLITQAHQALTDPAVREEWQQSANANQLFISLWATAVIEENEMERKVIQIMDHGQVY

QQIVAQYVLANSQNRGLRLNMARQYLEVQDLELLHWVIENYDAMYMFTWSFENGENQR

SVHVWPLPVFEDKAVRRRDFDLFKQMLAYIPQGGAGGPSGVLEFVQYRIDPDDVVKKM

LYLAAYDMDPEWIGEVIALKDKLSPDLRGELLSQFVHHPENEVQRQFIFDSLSDKSMN

NRENALLKAKLLTLTTEEMKLMEALLKLKTGSLRQKVIQVLMLQPADQLTMSLKSLLQ

AKSELQRLGALELLTEISADPQRAEQQEQIQPLIELIAKPTAKEQKLLDKLTEQGSRY

TASNGFGLYDPSWDNPLLNEQRDLGGYQPKDLFTLSLEKAKQFLEGFDALVHEHRDHE

YEVEYFPGHKETLLVGASLRSIAFITYEQRREIALMEQFPLHQVWQGYVQSSGLDNKE

LMQLLFTIRIEEMNKKLSDYYRYYSDVYEYSELEKHWLLEGWRKEFAEEVYPLKYIQD

LKKIVSELRYQDQVRTLIEAAHDDSEKVDTFEIAERTLYALMKMMPEDKVEKESGMLS

LLSGPWLYIVRDRVYDDASFKRFFQTFCQFRRLNKDSYRSSFLGLDDYLRAYELDFID

VQELYKQLLVEESRAQFIRDMTSSRYDWIAEKPLLADLRRTVVERILEIELTRGELST

EVSTLAMKLERIEGMEHWVHLVAAMDQDTFVRGYIYSYGDNTTRKETFSHLIQVCHPR

DGEDEVLLGQLLAKHPIQEKKLLEAAMYAPQWMEIVAKHLGWEGLRSAAWYFHAHINE

RFTAEKETIVAHYSPISPQDFNEGAFDIAWFEEAYEAVGEERFSLLYDCAKYISGGAN

HRRSQLFADAALGKLKLEDMRGSVEDKRNKDHLLTYSLIPFAEEREQDLRERYDFIQK

FLMQSKQFGAQRRASEGLVSQIALGNLARNAGYADVTRLMWDMEARKLDEMKSYFEPH

ELDDATTAQLVIDEEGQPELLIVSKGKTLKSVPARFKKDGYIAELKELRTDLVDQYRR

ARQELERSMTAGTSFTSEEVSSLLQNPVLRPLVRTLVFLADDKLGRFDVTSGGLDTPA

LDDSVSSIQTLTEQDRLLIAHPLHLFQSGSWSEFQRDLFNRQERQPFKQVFRELYLPN

EDELANGTVSRRYAGYQIQPKKAVALLKGRQWTVSYEEGLQKVSYEHNLIANLYAMAD

WFSPADTEAPTLETVQFYDRKTYKPVALKDVPPVFFSEVMRDIDLVVSVAHVGGVDPE

ASLTTIEMRHVIVNESLRLLKVDNVRLDGNYARIDGELGEYAVHLGSGNVFKQATGAL

HIVPVHSQHRGRIFLPFLDEDPRTAEILSKVILLAEDKKIKDPQILAQLQH"

CDS 716624..718813

/gene="yesS_1"

/locus_tag="PIKHDJKN_00655"

/inference="ab initio prediction:Prodigal:002006"

/inference="similar to AA sequence:UniProtKB:O31522"

/codon_start=1

/transl_table=11

/product="HTH-type transcriptional regulator YesS"

/db_xref="COG:COG2207"

/translation="MQTLEQTQLKTEQLLQTIDYSLIQYINTSRISESVRKPVRVKDY

PGINELREGLYKLQTYEKGIENVRLVSLEGRWVLDNSGYSSSFANLEMKTLSKFIRMS

ATSGWFTDLTSENVQLIKKIPVNSVREPAGLLEVTLPSHRLEQLVPARQNGAESLILD

SEHHRLTSPKDQAFSKTAMEQIVPVLLADHLASGYERLNIDGQPYGMSYRLSDYNGWT

YISLVSINEIAKESRAIGWYTFWICTGVFIGLAAFSLFASRRIYMPIRHAFEAAMGLE

PGDRNRRIHWREFSRKDGLKGVAGEAATVEWPQDELRAIGERVQSLKFSESRLMKEIE

NHTVQLRGLFVRKLLTGEMNPRDIQSGMEQVHYAMNAKAYCVIAVQADTFSDTRFRES

DRDLLLFAVSNMVADLVPACSRMDPVVIGEQQVTLLTTDETDEERRKLEVHRLAQSVQ

EAVKEVLGLGVSVGISRFLQDMAQATQAYKESVEALKYRIRFGENVVLHVEDVLPDQE

VQMMFPEWIEKRLIDALHVPDLDLARECLHELLEVTLRESHSHQEYQMILFRLLSDLI

REFQTTGEALLLPSGDVRDLFEQLVNLKTSADVERWFMKTILEPMVEMTTAKWGERNT

NISQQMMEIIHANVEQDLTLDTCAAKLNYHPNYLKTVFRKETGVNFSDYVSRHRLNQA

KQWLVETDMKIGDIAEQLGYRNAQNFIRYFRKVEEMTPGEYRKKYQK"

CDS 719178..720128

/gene="yteP_2"

/locus_tag="PIKHDJKN_00656"

/inference="ab initio prediction:Prodigal:002006"

/inference="similar to AA sequence:UniProtKB:C0SPB3"

/codon_start=1

/transl_table=11

/product="putative multiple-sugar transport system

permease YteP"

/db_xref="COG:COG4209"

/translation="MEAVSTELKTLTNSKRSVFMRRLRRQKWFFIMLTPGLIYFLLYK

YLPMWGILIAFQDYQPFEGIFRSEWVGFKHFQQFFSDETFWLLFRNTLILAVYNIVFF

FPLPIIIALMLNEVTKNKFKRIIQTLIYIPHFVSWVVVAGITFLFFSAQDGLVNNLLA

QLGSSPVQFLLSKEWFRTVITSQVMWKETGWGTIIFLAALAGVDPQLHEAARMDGAGR

WKQIWHITLPAIRSTIIIMFILRMGSFLDTGFEQIFLMINPMNREVGEVFDTYVYNMG

ISQGKFSYSAAVGVFKSVVALIMVIGANRLAKKFGEEGIY"

CDS 720147..721025

/locus_tag="PIKHDJKN_00657"

/inference="ab initio prediction:Prodigal:002006"

/codon_start=1

/transl_table=11

/product="hypothetical protein"

/translation="MADRSLLSRLADGLNYIILSMVALMCVLPFIYILAASFTAPEEL

LSKGFVLFPTQFSLEGYRYILSTETIMYSLGISIFITVTGTLLNLLFTALMAYPLAQR

DLMGGKLFMKLIVFSMLFSGGMIPTYLVVRWTGLLDSLGSLIIPGLISAFNLIIMRNF

FQQLPDGIEEAARIDGSSDLGTLFRIVIPLSAPVLASLALFYAVGHWNSYFNAVLYIN

DNTKWPIQVWLRQIVILSEGGIGDMSLMGDDYVAPPAETIKMAVIVLSTLPILAVYPF

LQKHFAKGALLGSVKG"

CDS 721054..722550

/gene="lipO_1"

/locus_tag="PIKHDJKN_00658"

/inference="ab initio prediction:Prodigal:002006"

/inference="similar to AA sequence:UniProtKB:P37966"

/codon_start=1

/transl_table=11

/product="Lipoprotein LipO"

/db_xref="COG:COG1653"

/translation="MNKTEKSSKFCHKRVALVLVASLFTSLLAACGSDKDSDGVTADG

KTVLNIMMIQQGAEPVKRDNPILKQIEEYTDTQLNITWVPASAYDDKVSATIASGKLP

DLMLARRNKESAMLNAQLSGYFWDVSSYLKETKNLSQMNETGVKNASVNGSLFGVPRE

RVLARYGMIFRKDWLDNLGLKVPKTPEDIYTVAKAFTQDDPDQDGQKNTFGIQEDNTM

ELLKQLTVYNGGPNGWGLKDGKVTPDFLYPEFKEALDLYRKMVSEGIISPDFSIAKKY
EYFNKGKAGMYFSVIDDAGTRHVDMMKQNDKINIDIAQNFEGTDGERVRGTNGYDSLL
VIPKSSVTSEEKLKKIIDFLDKLNDDDMRNLIAYGIEGTDYTIQDGKVKKIAGSTGAG
DLFNLRWGAQTGTKEVIQTDLEKKIEQTIKDNADIAVTDLSASLISDTNTEKGANLSK
DMTDANTKYVLGELDEQGWNDAVAKWRSGGGDQIMEEFTTYYNENMSK"

CDS 722621..723400

/locus_tag="PIKHDJKN_00659"

/inference="ab initio prediction:Prodigal:002006"

/codon_start=1

/transl_table=11

/product="hypothetical protein"

/translation="MTGTTGCPDWWAVTGDIQLWKSPDLKQWSPVITEPRRRSVVWNI
DRDGTWQKEIGLRDAAPFRPLWAPEIHYLKDTYWLTYCIPRLGSGILRSISGQAEGPY
VDDRSEQGPLSPYIDASLFQDDDGLVYYLCDNGKLARMNESMTALEEELVLLKPSNAA
HVGFEGTYLFKRQGRYYLSGADFVDGDYHGFMASSDHLYGPYEDRYIAVRHGGHNMYF
QDQQQQWWSTFFGNNDSAPLRERPAILRVELDSQGRWGPII"

CDS 723461..724393

/locus_tag="PIKHDJKN_00660"

/inference="ab initio prediction:Prodigal:002006"

/codon_start=1

/transl_table=11

/product="hypothetical protein"

/translation="MEHNILNQLQSPIVLQGNALIAYRDPAVIAHEGKVYLYYTLVET
EPTGEVFMYTAMSTSTDWIHWSRAVRLTPRDRRLNYSSPGNIVRVQDRWLICLQTYPR
PDGQQYGNEDARIWIMDSTDLLHWSTPELIRVKGPDIAVEQMGRMIDPYLVEDRDEQG
KWWCFYKQDGVSCSYSHDMTQWTYAGRESAGENVCIVHNSEEYIMFHSPENGIGVMRS
PNLHEWTADPVLLTLGQADWPWARGRITAGVVLDAREIRDVGCYVMFFHGTGPEDERT
IFDTHACIGLAWSDDLLNWDWPEQMKDGASYAHQ"

CDS 724390..724560

/locus_tag="PIKHDJKN_00661"

/inference="ab initio prediction:Prodigal:002006"

/codon_start=1

/transl_table=11

/product="hypothetical protein"

/translation="MIWDYEYIDLPRQLEHQIKVTLAAFYFLFFGKESLKSSEKLTQD

WQWWVQIENWAW"

CDS 724661..725119

/gene="yjaB"

/locus_tag="PIKHDJKN_00662"

/EC_number="2.3.1.-"

/inference="ab initio prediction:Prodigal:002006"

/inference="similar to AA sequence:UniProtKB:P09163"

/codon_start=1

/transl_table=11

/product="Peptidyl-lysine N-acetyltransferase YjaB"

/db_xref="COG:COG0454"

/translation="MLIASYREQDHDKLIEIWERAVRATHTFLEEHHIQFYKKVVSDV

LQQRQVEVWEALNAENEPVGFIGLDDNFIEMLFVDVSQHGQGLGRLLINHTLNIKGRH

LKVDVNEQNAGAARFYEKMGFIQIGRSELDGSGNPFPLLHLEIKADQAAR"

CDS 725171..726157

/locus_tag="PIKHDJKN_00663"

/inference="ab initio prediction:Prodigal:002006"

/codon_start=1

/transl_table=11

/product="hypothetical protein"

/translation="MTQIKVTPEQLDTVSGQFAQAHQQLSEFMSTLDSQIRVMRSNWD

GMERERFYNDYATAQGTMKSVLELVLSIQSELKKIAERFRTTDEDIVNKALMVALTAA

QTLTSLGKHKGDGPGKTSGPPENMDDWDKKDAEDYKNYEEMLKKAEEMGDEKLVQQLQ

ASMNIIRLQYEDEIYQTDPSTGKTVKITEDSIVGTYQVKSDKGETTSISLDKQGNVVD

YNKDTKKYEYWEQTHTTSHGEHLFGKGAQTATAYGIGLLLTSKSGSAFTEHATGLGSS

FVADKFLFSVPEEGETRTMIYRTNKDTGKIENMIVVTRGDNDIEYIPWREYN"

CDS complement(726362..727150)

/gene="nirC"

/locus_tag="PIKHDJKN_00664"

/inference="ab initio prediction:Prodigal:002006"

/inference="similar to AA sequence:UniProtKB:P0AC26"

/codon_start=1

/transl_table=11

/product="Nitrite transporter NirC"

/db_xref="COG:COG2116"

/translation="METEGLRNVEKLALKKHKIYKQSLIRYLARSMLASMFIGFGVIV

AFKTGNFFYMEDSPFTYPMAALTFGAAIILIAYGGGDLFTGNTFYYTYAALRKKLKWF

EVVKLWIASYSGNLMGAAVFALLIYLTGLFDSSQVNGFLLSVVEHKMEAPAMQLFFRG

ILCNWLVCLAFFVPMFMKENGAKMFAMMLFVFCFFISGYEHSVANMCTFAIALVLNHP

GTISFGGVIHNLIPVTLGNLVGGVLLMGFMYYAVNKPFLDEETH"

CDS complement(727253..727471)

/locus_tag="PIKHDJKN_00665"

/inference="ab initio prediction:Prodigal:002006"

/codon_start=1

/transl_table=11

/product="hypothetical protein"

/translation="MSNHSHLVEWVRKLQNAEGTEDELDEMLTELQQQLPHAEISNLI

FWDDRDLTPEQIVEEALAARPIILPPSP"

CDS complement(727865..728854)

/gene="ccr"

/locus_tag="PIKHDJKN_00666"

/EC_number="1.3.1.85"

/inference="ab initio prediction:Prodigal:002006"

/inference="similar to AA sequence:UniProtKB:Q3IZ91"

/codon_start=1

/transl_table=11

/product="Crotonyl-CoA carboxylase/reductase"

/db_xref="COG:COG0604"

/translation="MKAIIHSGQSGLEGLQYTESTSRAPEAGEVQIQLKSAGINHRDL

FIMAGRTFQDTPLILGSDGAGVIIAIGEDVENLSVGDEVIIHPTLGWDHALDVPIVPE

IVGGPTDGTLARYITLPARNALPKPSHLSWAEAGVLSLSALTAYRALFTRGGLKQGEH

TLIPGIGGGVATYALLMAVAAGAKVTVTSRSEAKRHEALRLGAFQALDSHADWSLQND
LDPVDFILDSIGQAMFPKYFDIIRPGGRIVMYGASSGDNLAVPIRSIFFPQVSLIGTS
MGSREEFVQMLQWVEEHNLHPVIDSIYPLQDTAKAFERMEKGEQFGNLAILVE"

CDS 728992..729858

/gene="yofA_1"

/locus_tag="PIKHDJKN_00667"

/inference="ab initio prediction:Prodigal:002006"

/inference="similar to AA sequence:UniProtKB:O34685"

/codon_start=1

/transl_table=11

/product="HTH-type transcriptional regulator YofA"

/db_xref="COG:COG0583"

/translation="MDAGDLKIFQTVAREGSISKAALALNYVQSNVTTRIKQLEDQLQ
VPLFHRSNRGMSLTPAGENLLGYADKILHLLYEAEQATQVGNPPAGILRLGAIETAAS
SFLTPLLAEYRLCYPKVQHSLITGGTHELNQKVIQHELHGALIYGPIDHPELNYMKVY
DEELVLIAEPGTREMHDLLCKPMLFFEVGCTHRMQAEAFLKDQGVHTLNVTEYGTLDT
IMNGVSAGLGVSLLPRSSVAKAERRGEVTVLSLPDPYRRLEVGFVHSRSEHRSGALDA
LVQMMTKQGPEQ"

CDS 729874..730974

/locus_tag="PIKHDJKN_00668"

/inference="ab initio prediction:Prodigal:002006"

/codon_start=1

/transl_table=11

/product="hypothetical protein"

/translation="MKDIELTLAEAFNQAEFIIGGHGSRKVKVLQEALEQVDGEQYSD
HYGNGKIIDQFQQQMADVLGKESAVFFPSGTMAQQIVLRIWCDRKGIKRVAYHPLSHL
EIHEEDGLKELHQIESILLADEDRLIRLEDVEGMDEEISCLLLELPQREIGGQLPAYE
ELEAISAYCRERGIKLHLDGARLFEITPYYQKTPAEICSLFDSVYVSFYKGIGGIAGA
ILAGDTDVMQESKIWKRRHGGDLIGLYPYILSSQYYFNERIGKMGLYYEQAKELATLL
NACHGISTLPEVPVSNMFHVHFPLAQAEVERILASMAQQFGIGITSYLQQTSAHSCAF
EFSTGDRYQNVPKDKLRSALEWLDEELRKHVN"

CDS 731209..732096

/gene="hcaR_1"

/locus_tag="PIKHDJKN_00669"

/inference="ab initio prediction:Prodigal:002006"

/inference="similar to AA sequence:UniProtKB:Q47141"

/codon_start=1

/transl_table=11

/product="Hca operon transcriptional activator HcaR"

/translation="MDIRKLRYFITVAEELHFHRAAEKLNMTQPPLSQQIQNLEEELG

VKLLERTKKMVRLTAAGAVFLEQARLIMAQLERSIQLTQKADQGIIGHLTVAFVDSAS

GSIMVNVLRKFRAAYSQIELTLREMTSSEQLQALADGQIHIGFLRYQDHNGNLSFRVC

QTETLIAVLPDYHPLASQIQVSITELADEDFILFPRRLGSPFHRLVLDYCREHGVDPR

ITQEAIQMYTIVNLVAAGMGISIVPSSVDVFERRGVVFLPLAESPPSVPLYTAWRTDM

NQEVISRFMNIVDECVDGE"

CDS complement(732134..733123)

/gene="gpr_1"

/locus_tag="PIKHDJKN_00670"

/EC_number="1.1.1.-"

/inference="ab initio prediction:Prodigal:002006"

/inference="similar to AA sequence:UniProtKB:Q46851"

/codon_start=1

/transl_table=11

/product="L-glyceraldehyde 3-phosphate reductase"

/db_xref="COG:COG0667"

/translation="MEYRKLGNSGLTVSEISLGNWITHGAQVDDGTAKACVRAALEAG

ITTFDTADVYSNTKAETVLGHALKEIRRESIELCTKVCHPTGSGRNDRGLSRKHIMEA

CNASLQRLQTDYIDVYYAHRYDPNTPLEETFLAFADLVRQGKVHYIGVSEWTAAQIEE

GSALAKELRVPFIASQPQYSMLWRVIEEKVVPASTQAGLGHITWSPLAQGILSGKYIP

HEALPAGSRAAANAGAPFFNNLAGRWLRDDVLTAVQQLVPLAKEIGLTLPQLAVAWVL

QHPYVSSVIIGASRPEQVLENVKASGVQLDREILSRIDEILSEWIERDPAKTG"

CDS complement(733264..734295)

/gene="rhaR_3"

/locus_tag="PIKHDJKN_00671"

/inference="ab initio prediction:Prodigal:002006"

/inference="protein motif:HAMAP:MF_01533"

/codon_start=1

/transl_table=11

/product="HTH-type transcriptional activator RhaR"

/translation="MIKEYLHINKILDLNKWKRLQDSLATVTKLAILTVDYKGIPVTS

HSSCQAFCQNVRKDPALLPYCQKCDSRGGLEAVRLNEPYVYLCHFNIVDIAIPITIDG

KYIGAVMAGQVKLADPEKGTELEQIVTSKNVPMHAAKLKELQDDYDQLPVMTYEEVVK

ISNMLSLLCNYIVEEALNKNLLVEMFEKASGHQESLNLSTILPGYSIRNIESIKKEMT

NAIADAYLKNSPSDTESSSPVLQPAFEYIHSHKSEQVSLKQMADLCHLSPSYFSRLFA

KETGENFTTYIARLKIKWAKQLLEVTDMPVSQISDELGFNESGYFIKIFKKFEEITPA

LYRKYMQEQ"

CDS 734666..735805

/gene="dhaD"

/locus_tag="PIKHDJKN_00672"

/EC_number="1.1.1.6"

/inference="ab initio prediction:Prodigal:002006"

/inference="similar to AA sequence:UniProtKB:P45511"

/codon_start=1

/transl_table=11

/product="Glycerol dehydrogenase"

/translation="MRKAFISPTKYVQGEDELLNLGYFVKSFGESALLIAHPDDVQRV

KAKLDATAQKFNITFVESGFRGECSREEVARLQEIAKEKGCTCTIGLGGGKAIDAAKC

VAEGEALIICPTIAATDAPTSHSAVLYTPEGAFDDYAYFKQSPSVVLVDTTVIANAPT

RFLVSGMGDALSTYFEARATAKSYSRVNASFPMGSREGYTPSAVGTNAALALAKLCYE

MLLSDGVKAKVASDSNMVTQALENIVETNILLSGLGFESGGLAGAHAIHNGFTILEGT

HHFFHGEKVAFGTIAQLVLENAPTEELHEVMDFCLAVGLPVCLADIGVDTISQEELLK

VAEIACIPEESIHAMPFPITVTEVAGAIATADRIGAEYKARREAK"

CDS 735805..737574

/locus_tag="PIKHDJKN_00673"

/inference="ab initio prediction:Prodigal:002006"

/codon_start=1

/transl_table=11

/product="hypothetical protein"

/translation="MKKIINQAENVVMEMCNGIALAHPELEFLKKYKVIKRRKINADK

VSLISGGGSGHEPAHAGYVGKGMLDAAVCGDVFASPSQIQVYQAIKATASKKGTLLII

KNYSGDMMNFKNAAHLAEEDGIDVQYVRVEDDIAVQDSLYTVGRRGVAGTVLVHKIAG

AAAEEGRSLAEVKTVAEKAAQNVRSIGFGFTSCTVPAKGTPTFEIAEDEMEFGVGIHG

EPGIRREKLVSADELAGRMVESLLSDMKLDNDASAEIAVLVNGFGATPLQELYLLNNS

VQRELAQRSGLGVATTFVGNYMTSIDMAGASVTILKLDDELKTLLFKESDTPAFKVSG

PPVAPVAYSEALEAVVTEDAPVSYEVETPATSAVINNNQFSLDNIVYLIDKMSEIIIK

NEVPFCELDSHAGDGDFGMSIAKGFRQLKREWNHIVNEEKKNIGSFLDACSLVIMEYC

GGASGPIWGSAFRAAGKAVGDKQSMNVAEFADMIQAAVQGIQSTGERSFGRGAVVGDK

TLVDALVPCADSWTQSATSGDDFKTAFAKGAEAAVEGAKKTEDIVARMGRAGAVGDRS

LGYPDAGAYALGVIFTELSESMK"

CDS 737948..739126

/gene="alaS_1"

/locus_tag="PIKHDJKN_00674"

/EC_number="6.1.1.7"

/inference="ab initio prediction:Prodigal:002006"

/inference="similar to AA sequence:UniProtKB:O67323"

/codon_start=1

/transl_table=11

/product="Alanine--tRNA ligase"

/db_xref="COG:COG0013"

/translation="MTQKIYYDSAYTQEWHTQITGRVDKEDGIYVTLAETAFYPHGGG

QPCDLGQIGGIDVLDVISEDGEVLHKLERAPEQNDVDCHIDWQRRFDHMQQHSGQHLL

SAITLKLADAMTLSFHLGTDYITIDVAAAELGANQLAAIEMEVNQQIYRNARISSSWV

TAEEAARLPLVKQPSVTEDIRIVEIEGVEYNACGGTHVSATGEIGIIKLLKTEKVKGG

TRIYFKCGYRALNEFNAAQSVLTGITAKLKTSREELADRIDKMEAEQKQLQAELNAVK

TSNDAYYAQELLSAREGLVIAQIFEDKSLKDMQSLATKLTSEHEGLVLFASISEAKVV

LAQNGQPPEWACGPFFKGNLGAYQGKGGGSDKMAQAGFANSEDAIAFYEFTKDQLGHH

"

CDS 739205..740509

/locus_tag="PIKHDJKN_00675"

/inference="ab initio prediction:Prodigal:002006"

/codon_start=1

/transl_table=11

/product="hypothetical protein"

/translation="MTERIPCKREGCPNTILPATAARTGGYCMPCKQEMEREAHQRYI

EANRRDVDLYEGITDPVEILKIMHTRQVHDPLIRYVAYEQSKEQVYLSLSTEQQALMI

DYAMKLIRTGDDDTGKDILVYLVCYHGTSLCAQIPELLEREIYYPVILYKSASAEVRN

QLLQRVNIDDENRNNLLLMLAYIGDEVVVRQFQQWRQSPPRWADQLHVAPEHYTTEAG

WELTNEGQRRDLFITPSYSLYKVKENEGAGDTSIGYPISILLTSADNCEWCGSHLTTL

IDLDVRHPALQDVAWNSERLQIQTCVICSCYGVVYMELDSAGEPCWSAHNVMPMGADD

LDPDDYVQLAPDAGRQFRIATAPRHPFHGSEWAMEPSTSQIGGHPGWVQDADYPNCPC

CASRMRVVGQMDWSEVEEYGDGMYYMFICEPCQMTAVSYQQS"

CDS complement(740964..741311)

/locus_tag="PIKHDJKN_00676"

/inference="ab initio prediction:Prodigal:002006"

/codon_start=1

/transl_table=11

/product="hypothetical protein"

/translation="MNMNVEVTEFIEQIPVPWQAQVASKLRELVHESIPEVEERVQYK

KPHFLKNGKYAAVISPSKPAVSFTIFNATGLELPDGIFEGPAERKTIKIKEKDTPDYE

WLAALLTQASVEL"

CDS complement(741308..741883)

/locus_tag="PIKHDJKN_00677"

/inference="ab initio prediction:Prodigal:002006"

/codon_start=1

/transl_table=11

/product="hypothetical protein"

/translation="MTLSTENLFYSKRLKMTPPRAEDVQTMLQWNEDPEYLRNVDTDI

AIPFSEKQLEDEGETKDKEVYFRLRTLEDEQLIGFVVIHSIEWNNRCGQLAIGIGLAE

HRNKGYGTEALNLILRYAFHELNLDRVGLDVIAYNAKGIRAYEKVGFQLEGRARSAVY

RDGKRYDRLLMGILRPEWEVHNQINVEGGQA"

CDS 742141..742596

/gene="ysnE"

/locus_tag="PIKHDJKN_00678"

/EC_number="2.3.1.-"

/inference="ab initio prediction:Prodigal:002006"

/inference="similar to AA sequence:UniProtKB:P94562"

/codon_start=1

/transl_table=11

/product="putative N-acetyltransferase YsnE"

/translation="MEIKVDDLSGHQVIGLIAEHLQGMAADSPPESIHALDLDGLKKP

EITFWCAWEGKELLGCGAMKELNSEHAELKSMRTASAHLRKGVARQILAHIIEVAKNR

GYKRISLETGSMDSFIPARKLYEDFGFVYCEPFADYSLDPNSAFMTKEL"

CDS 742956..744296

/gene="gabP_1"

/locus_tag="PIKHDJKN_00679"

/inference="ab initio prediction:Prodigal:002006"

/inference="similar to AA sequence:UniProtKB:P46349"

/codon_start=1

/transl_table=11

/product="GABA permease"

/db_xref="COG:COG1113"

/translation="MAQTEGTLQKKLKPRHISFMAMGGVIGTGIFKGSAETIGLAGPG

VIVTYIFAGLLLLVVMAAMAEMATVYKNKNMKDFVQEAFGSRVSFIMGWMYCFLWLSV

CVIEVIAAGSFLQYWFSEVPLWMLSLVCAVFIILINLLSVGVFGEFEFWLAGIKIAMI

IIFIILGAVLIFGIIPSDNTPYLQNYTQAGGFFPNGWSSIFSALLVVMFSYGGSELIG

LTLTETENADKVMPKIVGNFMLRIILFFTLPILIICGLIPWNELGPESSPFVQVLAST

GLPGAAHIMNFILVTAVLSAANSGIYGASRMMHSMAVGGEAPKALSQTNRNGSPVNTL

LVCAVILLGGSMLGLFAQDQLFRVLLAVPGFVVMLVWICIATSQMKLRKRYPVQPTFK

VWGFPYVTGAAVLCLGVIAVMFVFDQSNRFSISICLIVLAVLIIWSLIRFRKAD"

CDS 744476..746230

/locus_tag="PIKHDJKN_00680"

/inference="ab initio prediction:Prodigal:002006"

/codon_start=1

/transl_table=11

/product="hypothetical protein"

/translation="MVGFFKKRLVVRIVAIVTLVIMIISAGSMLLQLANMKLAAQEAI

SSYNIQIAESYVKQLDTGPYVEFAKDPKENDVFLKIRDELDDFRMSIGAMYVYFVKID

DTGTPLIMVDGMKDADKASPINEVTDIPGDAVQKLLQGKTASSPIINNEEYGEYISSY

APILDSNGALTGVIGIDTAVSVIGSIESDIMKSSIPLYALLLFITVAGIAVVLWFIVR

GLRPLKPLKASVEQMAQGELAEANRILTAYRLQSEDEIGSTFKAMIHMSGDLNKMIGD

MVAGVSVTTDILAESTEQFNRDAEDMLEMNKSVDQSVQQIRQGAHTQKQGASDSANAM

EEIAKGITDISESSTAVSDAATTALATAQSGKQSMTQMKKQMESISAVSAEVVAMVQV

LNNFSQQIGGALYTVRDFASQTKLLALNASIEAAHAGEHGKGFAVVADEVRKLAEASS

TSVQTISDLLLGIQKESQHIGSQMDVTSQEIGQGVNSTAEAELAFTHMVDTFRVVTHR

IQEVSAAAEEISAGSEEAAASVNTISQISSGVSDHSDEIYRLTGEQSSMFQKVAQTST

LLRQQTIELSEAVRKVKV"

CDS complement(746324..747229)

/gene="cysB"

/locus_tag="PIKHDJKN_00681"

/inference="ab initio prediction:Prodigal:002006"

/inference="similar to AA sequence:UniProtKB:P45600"

/codon_start=1

/transl_table=11

/product="HTH-type transcriptional regulator CysB"

/translation="MNLEQLEYVVEIAKTQSFSAASEHLHVTQSAISQSVHRLENELG

MILFERSRHGTFPTPEGKPFIAKALDILQRIDELKSLNAETFSLTGELHVATFPSVMP

YLVQTAADMKREHPQLNIAIEEKGSMEIIEDIRNNRTHLGFIAIYAKQLREFDGLHFS

PMYSGKLVVCTHHQSELAKLNRVTPEQLKEHKLALYRDGFIEDFLQEFTYDYGPLSIL

FKTNNSEAISTVLKNNIAATIGHDFSFHQHPLWKEGIAKMVEIASIDQPKMQIGFVQT

ESKEVAVAAERFAQRFRQAIELDQL"

CDS 747370..748413

/gene="ydjJ"

/locus_tag="PIKHDJKN_00682"

/EC_number="1.-.-.-"

/inference="ab initio prediction:Prodigal:002006"

/inference="similar to AA sequence:UniProtKB:P77280"

/codon_start=1

/transl_table=11

/product="putative zinc-type alcohol dehydrogenase-like

protein YdjJ"

/db_xref="COG:COG1063"

/translation="MKAAVWYAKKDVRVEEREIPVAQSGQVKIKVEYAGICGSDLHAY

HHGVGIQEGQNHPLSGQKAPLTLGHEFAGTVSELGSDVSGISVGDRVVVEPLYHCGQC

EYCIQGRYNQCTQFGFVGLNGDGGFAEYVVVEAYMVHKLPDNVSFEEGALVEPTAVAF

HAVRHSKLKVGQKVAVYGAGPIGLLTILSAKAAGASTIYAVDVFEERLNLAAKLGAIP

VNSAKVNATEVILQQSGGIDVAYEAAGVQPTMDSAVAVIKKGGEVVVIAAIPNPLQVN

FFDLLVKEANLTATLAYRHIFPEVISLIAEGALDVGQVITRKIKLDDIVQEGLELLMT

DKSHAKILVEIGG"

CDS complement(748561..749070)

/locus_tag="PIKHDJKN_00683"

/inference="ab initio prediction:Prodigal:002006"

/codon_start=1

/transl_table=11

/product="hypothetical protein"

/translation="MTTAVTIHPATEADHEEIVHILIESYSEYRDTFQTKEQWEHYLK

DIRESPSNPYIKQLWIAKIDNQIVGTVQLFETAHKAYPNFELPIDYPFIRLLGVDPKW

RGHGIARELLQQCVESAKDMGKNTVYLYTGGQMVNAIRLYERFGFVEDPEFSSESSGG

KAICYRYDS"

CDS complement(749237..750004)

/gene="araC_2"

/locus_tag="PIKHDJKN_00684"

/inference="ab initio prediction:Prodigal:002006"

/inference="similar to AA sequence:UniProtKB:P0A9E0"

/codon_start=1

/transl_table=11

/product="Arabinose operon regulatory protein"

/translation="MQKPLEHYLCGKFITEGNWSHMKRSMPVHEVILMLEGEMYIAEE
DMQFVVHANDLLFLRAGRTHYGYQVSDAPVSFYWVHYDSIEEEFDQYPTHATIQVPST
ANQLFKQLLHSSSFSSEEADAALLLLLKELERNTQAGYRPHNAIVDHICKWVDIHLHT
DITVGKIAETFNFNKDYISKMVKREKGIGLKTYILTERIRRAKQMLLNTNASVKEIAG
QCGFSDYKLFLRMFKQYEGNTPTEYRNNLYSTQLNRL"

CDS 750199..752529

/locus_tag="PIKHDJKN_00685"
/inference="ab initio prediction:Prodigal:002006"
/codon_start=1
/transl_table=11
/product="hypothetical protein"
/translation="MIETKKGFLGPEHVDLLNGVFQTSQEVGEKYLLSLDIDRFLAPC
FEAHGLPAKKERYAGWEARSISGHSLGHYLSALAVTYQATGNEMLKQMLDYAVSELAS
IQLTTGSGYVGGLSEEAFRIAFRADHIGGFNIGEYWVPWYSVHKIYRGLIDAYKLTGN
GQALEVVTRFADWAVDGLVPMTEEQMQIMLQCEHGGMNEVFAHLYGITGKSVYLEIAN
KFTHQLILEPLEHKQDDLQGKHANTQIPKVIGAAEIYNQDHNHESYRTAAEFFWDTTI
HHRSYVFGATSISEHYEAKGMESLGIKTGESCCTHNMMHLTKQLYTWNPDSAYMDYYE
NAIYNHILGTQDPDTGNKTYFASTLQGHYKIYGTRDTAWWCCTGSGMENPGKYAEAIY
FEDEQDLYVNLYIASQLDWRSQGISLKLETDFPYSEKVTLTITEGSASANLRLRVPSW
LQEPMTATVNGDTAHPHTRMEPGYLDIERTWTAGDVITITLPMSLHQYTSRDDAHKVA
FLYGPIVLAGALGSEGLPEDTIVDETALNPKTAPVPVIWTEQEDVREWIKVVDASTLT
FEISKDVTSDGNPVKLIPFYDVHHEFYTVYWPFNDEGDALEKELNDITIDRVQPDGQQ
DEIGHHLDSNCRADHHNGSYTDSRNKLHMWREAFGVSGAYFSYQLAVDGSAPNYVCVA
YWGGDHSPFEREGTRHERIFSIAVDGHVVGEQRIHMNKIGEVFYVTYDIPQTVTSGKD
SVRVAFQALDDNGCAGKVVDVRTTRSKPDPSFRKIK"

CDS 752880..753620

/locus_tag="PIKHDJKN_00686"
/inference="ab initio prediction:Prodigal:002006"
/codon_start=1
/transl_table=11
/product="hypothetical protein"
/translation="MIYMIKYIKLLGLIFGVVVMNVLLFSPGFIGLEFGGGAFTTALS

VTFLFGSVLALFYGSYTLLFRQPVVLPVKNIETHEDYVEALSFYRRIKVLEEDITLGL
SQLSRMKKKKETLLNVLNQRFDPGELSYKKFASVTLEVEKLLYLNIRSVLNRLNVFDE
AEYASLMKSKSATLPQKLFQEKTKVYNDYLSYVKNSLHTNEEILLKLDQLMLEISRLD
SFEAGDIEQMPCMQEIDQLIKHTKLYRQ"

CDS 753633..755321

/locus_tag="PIKHDJKN_00687"

/inference="ab initio prediction:Prodigal:002006"

/codon_start=1

/transl_table=11

/product="hypothetical protein"

/translation="MAKKGKFFFISILMLVLVFGLVYAGITLTSNVGKTSTQVTTENA
GKELGKLYADIAPATAEPVKGQIDLDPVDVAESLPDISKFPITVENTTNDYVEIFSSI
EKAGTGADGWLTEVSEEFNKANITVDGKQVSVKLRNIASGTAADYIKSGKYVPDAFTP
SNELWGEMVAASGVKTDLVSKRLVGNVPGIVISKAKYDALVDTYGSVNVKTVTEAIAN
NELAMGYTDPFASSTGLNFLVTALNTYDSSNPLGEKAIEGFEKFQANVPFTASTTIQM
REAAKSGRLDAFVLEYQTYVNTADLKSGYVFTPFGVRHDSPLYALGQLPQNKQEIIKK
YAEFVTQAKYQQSAEEFGFNGLQDYKSELNTVDGGTLLSAQKVWKEKKNGSKPIAAVF
VTDVSGSMDGEPLNRLKESLRKGQKFLGTDNSIGLVSYSSGVTVNLPIAKYDTNQQSM
FVGTVDSLQAGGGTATFDGIVVAMKMLEDYMATDPNVKPLIFVLSDGETNEGHTLKDI
RDLVETYKVPIYTIGYNADIKALESISSINEAASINADTDDVVYKIGNLFNVQM"

CDS 755402..756541

/locus_tag="PIKHDJKN_00688"

/inference="ab initio prediction:Prodigal:002006"

/codon_start=1

/transl_table=11

/product="hypothetical protein"

/translation="MSFSMEIPSQKEIQKVIEEEIKPVPAEVAELQQVANANVEMIMT
LDLESLEKRKEILQSIDGFGMNTMRSSSEKNALLQVSVGHLSKTGDEGGQVAKGLTEL
HMQLKDLDPSVVDFAKTGFLGKLFNPLRAYFLKYQKADAVIADIVTSLDKGRATLRND
NTTLEIEQQNLRELTKRLQKEIQLGVLMDESIDAQIEAAKVRNEDPEKVRFITEEVLF
PLRQRVMDLQQMLVVNQQGIMAIEVVIRNNKELIRGVDRAKNVTISALKIAVTVASAL
YNQKIVLQKIELLNRTTNDLIAGTSKMLKDQGIAIQKQAYEASISVDTMKQAFTDVLS

ALDSISLYKQEALPRMRETINQFRELADNGEQQIQRLEKGQKLGL"

CDS complement(756649..757356)

/locus_tag="PIKHDJKN_00689"

/inference="ab initio prediction:Prodigal:002006"

/codon_start=1

/transl_table=11

/product="hypothetical protein"

/translation="MASASDSTSDRKRVGHTVERLEPGLSRLLLSCVLVLISGFIDAV

GYLQLGKIYLSFMSGNSTKLGIALFEGDSAVLTNIGLVIFLFVVGAFIGTLITDRSGK

RRLAGILGFESLLFAGAIVFLLFLHTQSVLFPIAVAMGMQNTMHQMVAHADVGKGFVT

GTLFGIGQALAKAVRGKAPASEWAVLALSWVIFVIGAALGALLLTQGSLLLALIASLA

LLLLLIPYVLHVQRDLT"

CDS complement(757456..758496)

/gene="oxdC"

/locus_tag="PIKHDJKN_00690"

/EC_number="4.1.1.2"

/inference="ab initio prediction:Prodigal:002006"

/inference="similar to AA sequence:UniProtKB:O34714"

/codon_start=1

/transl_table=11

/product="Oxalate decarboxylase OxdC"

/db_xref="COG:COG2140"

/translation="MTSFNYRLEQKEPRMGPGGITRGASVRDFPASIGIAGVSMRLEP

GGMRELHWHANAAEWAYVISGSCRTTVIHPDGHAYTDTFEPGDVWYFPRGYGHSIQGL

GPDECHFILIFDNGDFSEDHTFSITDFLSHTPNNIVAQNLGITPERAAKLPQGEAYFV

KGPVPNDSSSSGTYRRNSELTTPHRYPLHAKQPRRAPGGGTQRTVTVEDFPISTTMAG

SLIELQPGALREMHWHPNADEWQYYISGTAEMTVFLAEGHAVTEQFEAGDVGYAPMGA

GHYIKNTGDEVCRVLIGFNSGHYEAIDLSEWLAGNPDDVLSTNLEMSKEEAEQLPNDT

LFIAPAPNKPKS"

CDS complement(758660..759268)

/locus_tag="PIKHDJKN_00691"

/inference="ab initio prediction:Prodigal:002006"

/codon_start=1

/transl_table=11

/product="hypothetical protein"

/translation="MAELPSSMDRRIKKSKAALKGALIQLMQKQSFKEISITDIVQLA

DLNRGTFYRHYQYKEDLFNEIIDDIIKDLVLSYREPYLDKEIFEVSHMPSSAIKIFEH

VHQHAQFYTLVVKSEASSNFQQMICDVLRDLALQDLNEIFPSHINRELLASYQSHAIF

GMIIEWIRQEFKHSPAYMADELFKIINYKPGNVVYHTSYKAP"

CDS 759459..760217

/locus_tag="PIKHDJKN_00692"

/EC_number="1.1.1.391"

/inference="ab initio prediction:Prodigal:002006"

/inference="similar to AA sequence:UniProtKB:C8WGQ3"

/codon_start=1

/transl_table=11

/product="3-beta-hydroxycholanate 3-dehydrogenase (NAD(+))

2"

/db_xref="COG:COG1028"

/translation="MRLQDKVAVVTGAGSGMGKAIATLYAQEGAKVVVSDINEASAQA

VAEDIKAKGGEATFVVANVAKEEDIQNLIDTTVNTYGTVDILVNNAGIMDSMEPAGDI

VDDKWERIFAINTTSVMRATRKVLPIFLEKQKGVIVNIASAGGLHGARAGAAYTASKH

AVVGFTKNTGFMYAQQGIRCNAIAPGGVETNIASTMTNINHFGASRQQLGMAINPRAG

KSEEIAQVALFLGSDESSFVNGTVVTADAGWSSY"

CDS complement(760342..761370)

/gene="malR"

/locus_tag="PIKHDJKN_00693"

/inference="ab initio prediction:Prodigal:002006"

/inference="similar to AA sequence:UniProtKB:P0A4T2"

/codon_start=1

/transl_table=11

/product="HTH-type transcriptional regulator MalR"

/db_xref="COG:COG1609"

/translation="MITIKDIAKLAGVSPSTVSRVISNHPRISSETSLKVKQIMKEMN

YHPNMMAKSLVSKTTQTLGIMLPRPAEELFQNYFFGELLRGIITYATRMNYELLLSTE

TSSSDELNAISRLVHGRRVDGILLLGSKREDPIISFLEAEKFPFVLIGRSETHPDAPM

VDNNNVQTAFDATTHLIAQGHKRIGFVSGPPDLTLSHDRMRGYKNALEEAGLDANNDW

IVEGEFLQESGFRAMSLFMSIPDRPTALVVIDDNVAFGVLRALAELGYNVPEDISVVS

FNNIALSELASPPLSSIDIGTYQLGYTAVQVLLKILNGEHLPQNPVIIPHRLIVRDSS

LFTLSGNA"

CDS 761828..763114

/gene="cycB"

/locus_tag="PIKHDJKN_00694"

/inference="ab initio prediction:Prodigal:002006"

/inference="similar to AA sequence:UniProtKB:O07009"

/codon_start=1

/transl_table=11

/product="Cyclodextrin-binding protein"

/db_xref="COG:COG2182"

/translation="MRKWQQAALAGFMVLTLTACSSGGGGAAPTTTDSGETGETTVTA

ENIQPEEGASLVIWEDKNQRSFIEERAKKFEEEYGVSVKIEELPPTDQVTKLTTDGPA

GLAADVVVFPHDKIGSASEAGLILPNDIFEQQTTQSTSDNAVKAVTFKDILYGYPYSV

ETYALFYNKKLYPEAPKSFDDIISFAKTFNDTKNNKYTLMWELQQFYYNFAYLASQGG

YIFGDEGMNSEDIGLNNEGAIKGGQFLQTLKSEVLPVKMGDVNYDIKKGLFSSGKLAM

DINGPWSIADYRNAGIDFGVAPLPAIEGKPMTSFSGVKAYYVNAFTTYPNAAKLFAAY

LSSADSQMVNFEMNGTLPANKDVAADPKIQGDEITKAFLEQFNNSTPMPSLPAMDSVW

GPISAAITDIWDGGKDVKTSLDNAVKQVKESLATVQ"

CDS 763209..764564

/locus_tag="PIKHDJKN_00695"

/inference="ab initio prediction:Prodigal:002006"

/codon_start=1

/transl_table=11

/product="hypothetical protein"

/translation="MKQQHVPVTVKPNRERQHRMTAAVLSLVLQGLGQLYNRQWIKGI

CFLILEGVGLAYLIPRLNQAVWGVWTLGEQTQRFVKVNGSTVLQQGDHSIFLLLNGII

VLLVFFVFLLLYIFNIRDAYITGKLREEGKQATGASASLRYLLDKQFPYLFLSIPALG

ILFFTIMPILFTITLAFTNYSAPDHIPPAKLVDWVGFKTFTDLIQLKSWSHTFFGVLT
WTVIWAILATVTTYFGGVLVALLIEQKGIRFKKVWRTIFILPYAIPQIISLLLMRNLF
NGQFGPINTYMKAFGLEGLPWLTDPFWAKVTVVIVNMWIGIPVSMVLILGVLTAIPRD
LYEAAEVDGASGFQKFRIITLPFIMFATTPVLIMQFAGNFNNFNVIFLLTNGNPLRGD
YQYAGSTDLLVTWLYKLTLDNNKFNMASAVGIIIFLIIASFSIWNFRRSKSFKEEDMI
Q"

CDS 764561..765397

/gene="malG"

/locus_tag="PIKHDJKN_00696"

/inference="ab initio prediction:Prodigal:002006"

/inference="similar to AA sequence:UniProtKB:P68183"

/codon_start=1

/transl_table=11

/product="Maltose/maltodextrin transport system permease
protein MalG"

/db_xref="COG:COG3833"

/translation="MKKRNNPMRLTISYVLLVIIAIVSVYPVLWIFLSSLRPGAALFS
ERLWPEAFTLAHYGELFNNPSFMYGTWYLNTLKIAFFTMIFSTLMVTLGMYALSRFRF
RGRKTILSTMLILGMFPSFMSMIAIYIILLQIKLLDTHAALILVYSSGAVLGGFIVKG
FFDTIPRSLDEAARIDGASHLRVFTSIILPLSRPMLTYVALTSFTGAWMDFIFARLVL
RTKENWTLAVGMWDLVSRYQDSNFTMFAAGAVLIAIPITLLFVFLQRFLVQGLTSGAS
KG"

CDS 765557..766843

/gene="entS_2"

/locus_tag="PIKHDJKN_00697"

/inference="ab initio prediction:Prodigal:002006"

/inference="protein motif:HAMAP:MF_01436"

/codon_start=1

/transl_table=11

/product="Enterobactin exporter EntS"

/translation="MGTLQSPEPELGPNGALAAAESQKMANRALWRNVAYRRILCGYG
ISVFGDCFNGIAISLWVLQTTGSAKSMAAVQVCNMVVSFLFGSFAGTFADRLDRRTLM

LSSDLFRGAIAFIIAVSLFVLHAPFAIVLLMLSLSMFSSLFQAPAFHASVTSLVGREH
LQQATGTIHLVDNIARISGLAAAGIAVSAFGGFTAIMITGVTFLLSAICVMLAGRFPD
VQRSSMHRGSFVKEWGGSFSYIFAHPLIRSIVILNPLLILFFMSAIMLVQVMAVKVWQ
ANPVQFGLIETCIPLGYMLGSGILIASGNRLKRRGRWVFIGLLVLGPLYMLLSNVSSP
LVALPLIIAGGAMFACCTMLTQIMMRAEVPDELQGRIYGVLGTITSTAPILGLTVVSV
LADQWGAQTVLESLGALLLVVGIIAAAGLKSIRTYR"

CDS complement(766943..769102)

/locus_tag="PIKHDJKN_00698"

/EC_number="2.4.1.19"

/inference="ab initio prediction:Prodigal:002006"

/inference="similar to AA sequence:UniProtKB:P30920"

/codon_start=1

/transl_table=11

/product="Cyclomaltodextrin glucanotransferase"

/translation="MFKWTKRIILSTTLSFSLLAGSALPIFPAASVFADADTAVTNKQ
NFSTDVIYQVFTDRFLDGNPSNNPTGAAYDASCSNLKLYCGGDWQGLINKINDNYFSD
LGITALWISQPVENIYSLINYSGVNNTAYHGYWARDFKKTNPAFGTMTDFQNLINTAH
AKGIKVIIDFAPNHTSPAMETDTSFAENGKLYNNGTLLGGYTNDTNKLFHHNGGSDFS
TLENGIYKNLYDLADLNHNNSTIDTYFKDAIKLWLDMGIDGIRVDAVKHMPMGWQKNW
MSSIYGYKPVFTFGEWFLGSSASDADNTNFANQSGMSLLDFRFNNEVRNVFRDNTSTM
VALDSMITSTAADYAQVNDQVTFIDNHDMDRFKTSAVNNRRLEQALAFTLTSRGVPAI
YYGTEQYLTGNGDPDNRAKMPSFSKTTTAFNVISKLAPLRKTNPAIAYGTTQQRWINN
DVYVYERKFGNNVAVVAVNRNLSTSTSISGLTTSLPSGTYNDVLAGALNGNNITSTGG
NVANFTLAAGATAVWQYTANTTTPTIGHVGPTMGKAGNTVTIDGRGFGTTKGTVYFGT
TAVTGSAITSWEDTQIKVTIPAVAAGNYAVKVAASGVNSNTYNNFTILSGNQVSVRFV
INNASTTLGQNLYVTGNVAELGNWSTGPLAIGPVFNQVIYSYPTWYYDVSVPAGTNLE
FKFFKKNGSTITWENGNNHTFTTPTSGTATVTVDWQP"

CDS 769813..771186

/locus_tag="PIKHDJKN_00699"

/inference="ab initio prediction:Prodigal:002006"

/codon_start=1

/transl_table=11

/product="hypothetical protein"

/translation="MNTHSVIECVEGFKASRLDNARKIFVYLPPCYKEHVDHRYPVLY

MHAGQRAFGSSTAGNETWHVDQAADQLIDQGLMDGLIIVGIAHVRPVTHNEFYHFIAP

AREAVSVGCSGLDYEHFIIHELKPFIDEHYRTLPDRDNTGLLGSSAAALSTFHMGTRH

PDIFGKLIMLSPFFVDVQLDETSESRLQEENMYRMPEGSAPIQMWMDIGDAEGLFLPS

QVRNVVHQMLALGYKPGKDIAFLEQPDAGHQEADWGERVHLPLLYMFGRIGNPVSLEL

MGRDVIGLKGGMSGHINALLHYDSGFTMSVLEGDYRSDHPDVLEVRPNGELVPVKIGN

AVVTLTVGELSASRTFTVVEEVSGHVTVCMSAEVPFQDMYDDSIYGGMGMKLLHTGGG

HYEGCFEVPRDSGFRFRFTRGFRQFETDAEGKALPNRVFRAHDNLSLHYKIQSWGSTA

AKAENRR"

CDS 771189..772604

/locus_tag="PIKHDJKN_00700"

/inference="ab initio prediction:Prodigal:002006"

/codon_start=1

/transl_table=11

/product="hypothetical protein"

/translation="MISQPFQPTMDIPYYYPCNFPLIHEILQRQGSISSLGLLASSRL

YSLTSCSDRGLIKPYFHKLDYEEPMWEVFGEREFDSFEQGKAYIRERLENEGPLVVTG

TSYCLPYGDDYRNPEYIHKLVKQDSRLHLVDHWLAVYGMDEGQFYVYDPVPSKYMGAV

SSPDFQEFWKGNKNISELEIARRKETLRTYGTMEIRAVETLDSAGYRNMLRSALATQA

HEFIAGRTIWEGNRSYYFGQAVTSQLLQRLHPDAEVDREQEKAISAFLFDMRWSRYFF

RDLLEEAAKWLDSPHDQYVAEFGAMIARWEQAHKLLQIARMKRSPGWREQLTDIIEQL

AADELRWYEALMTIHQHVDRFRQIPSTVENPAPSNREVIQRIVLDSCDELNRYHNAPI

PLEHGLQAPLYGSRGRLDSLELVTLLAVVEQSVEDAFGVGITLAEMAAASMPESPYRT

VESLVEYLEAQLKHCPKDDEG"

CDS 772601..774013

/gene="lcfB"

/locus_tag="PIKHDJKN_00701"

/EC_number="6.2.1.3"

/inference="ab initio prediction:Prodigal:002006"

/inference="similar to AA sequence:UniProtKB:O07610"

/codon_start=1

/transl_table=11

/product="Long-chain-fatty-acid--CoA ligase"

/db_xref="COG:COG0318"

/translation="MNITFLLERFAAYDHRPALIWKDEQYSYNWLLDQVHTFSEWISR

EGLEEAIVVLEEDYSPYAAAALIALLERNCIVVPLDRHLVEAKRNEYTALAQAEWRMS

VGEGEAIARHCSVQGSRSPILARLGQEKKAGLVIFSSGSTGRSKAAVHSADRLLDGFR

RQVRPLCTIPFMMFDHIGGLNTMLQSLSSGGCLCVIPDRSPLEVCKTIEKYRVQALPV

SPTFMNLLLLSGVYQEFDLSSLEVVSYGSEVMPEALLTAWNRQFPNVRTVQAYGMSEL

GILPTRSKDSGSLLFTVRDGEVRYRIVDGLLEIQTKTAMIGYLNAPSPFTEDGWLQTG

DEAEMEDGYIRILGRRSEIINVGGRKVYPAEVESILEQMEAIETAVVSGEPSGIMGQM

VKATIRLTGEQPLTELRRQIWNFCRDKLPQYKIPQKIVITQETLRSTRMKKIRNPGPI

SSVRDEVAASLEEDSGGTQY"

CDS complement(774243..776012)

/locus_tag="PIKHDJKN_00702"

/EC_number="3.2.1.54"

/inference="ab initio prediction:Prodigal:002006"

/inference="similar to AA sequence:UniProtKB:Q08341"

/codon_start=1

/transl_table=11

/product="Cyclomaltodextrinase"

/translation="MLLEAIYHRPKMNWSYVYDRSTMHLRLRSKRGDLDAVIAIIGDK

YAWDRTVLQLPMRIFARDALFDYWEVEAQPPYRRLRYGFQLIEGEESVWMTERGFEQA

LPDHPLEFFDFPFMNPADIFEPPAWVKDAVFYQIFPERFANGDPSISPKNAEPWGGEP

TPVNFFGGDLQGVLDHLDHLSALGINAIYFCPVFQATTNHKYNTADYMRVDPHFGTNE

KLKELVDACHERGIRVVLDAVFNHSGREFLPFADVLEKGAASRYADWFHVREWPARIE

DGIPTYDTFAFEPLMPKLNTEHPEVKEYLLEVARYWIQDMGIDGWRLDVANEVDHQFW

REFRQTVKSLNPDAYLLGEIWHDSIMWLQGDQFDAVMNYPFTNSVMDYAVLGKLDGLG

FANEIGKLLAAYSQPVTEAAFNLLGSHDTPRLLTLCEGDVRKMKLAVILLLTYPGAPC

IYYGDEVGLDGGYDPGCRKCMEWDEQKQNKDLLDFFTQAIALRKQHAALRSADITMIY

AEAGDPCIAIGRKDQDTGEQFVLVLNASEEPRSMELPLHQGTWRNVFSATTRETQQNK

LKLDLEAYGFSLLQLDVKSHVKA"

CDS complement(776268..776846)

/locus_tag="PIKHDJKN_00703"

/inference="ab initio prediction:Prodigal:002006"

/codon_start=1

/transl_table=11

/product="hypothetical protein"

/translation="MKKSWVLSIIMSFVLLGGVFAPAGSYAAAADDSTTTYSEDGYSE

VDPTEEEPELTADEADFLNYLDQLDSASVYEEKAFNALGGGFYITSSNRKSVFLKLTN

TAIPNYTKYVSKLKLIKPQNTELQKIHASLIKGSYTQLEGFQLAKKAVSKTTVNSAVL

KQANAKIDTGKKNIEKYQKELSAYANKLGYEF"

CDS 777051..778208

/locus_tag="PIKHDJKN_00704"

/inference="ab initio prediction:Prodigal:002006"

/codon_start=1

/transl_table=11

/product="hypothetical protein"

/translation="MTIERFHIHVSDEILDDLQYRLNHIRWPDQLEDSGWERGTDISY

LKSLVSYWRDHYDWRAQESRLNQFSQFHCQLDGVDVHFVHERGKGPNPLPIILTHGWP

DSYLRYQKIIPLLTDPASHGGNLEDSFDVIVPSLPGFGFSSRPDQPGVNNSRVSQMWA

KLMTKELGYSKFAAAGGDIGSGVTRYLASNNPELLVGIHLTDIGIIRDLLSSQDEAER

SEEEREYKKNASTWISQEGGYMSIQSTKPQTLAYGLSDSPVGLAAWIIEKFRAWSDCK

GDLNQIFSKDELLTHIMIYWVTNTIGSSTRMYYENSHSLPPLGRIEVPTGIAIFPADI

VLPPKSWTMQNLNVTRWTSMPSGGHFTALEEPELLADDIRAFYRPIRAMNN"

CDS 778432..781911

/gene="lacZ_1"

/locus_tag="PIKHDJKN_00705"

/EC_number="3.2.1.23"

/inference="ab initio prediction:Prodigal:002006"

/inference="protein motif:HAMAP:MF_01687"

/codon_start=1

/transl_table=11

/product="Beta-galactosidase"

/translation="MNQKKLFTDGWQFAKSKLEVTEPAGLTFEPVELPHDWLIYNTLE

LYEDSIGWYRKTFPYTKDEQQLLLCFDGVYMDSSVYVNGQLVGEWKYGYSAFEHEITS
ALVEGDNEIIVKVVHQSPNSRWYSGAGIYRNVWLKTRDRNHIVTDGTYVSIQQQPEGW
QVEVDTELSLNQNAQLVHTIWYQGNQIVSSKADVTATAGKDTGHGEIQSNSQQLTVDL
PKLWSPDEPNLYDLVTELQVATGEQGYETIETVTQRIGFRDIKLDASEGFHLNGVKMK
LNGVCEHHDLGALGAAFNLTALRRRFVLLKEMGVNAIRTAHNMPAKEFMELADEMGML
IVSEAFDMWERAKTPYDYARFFPEWAHTDVKSWVKRDRNHASLIMWSIGNEIYDTHAD
ERGQEVTRMLMEYVPEFDPKGNAGVTIGSNYMPWENAQKCADIVKLAGYNYAEKYYDK
HHEEHPDWIIYGSETSSVVQSRGIYHFPFEQSILADDDEQCSALGNSTTSWGAKSAEY
CILAERDTPYSLGQFLWTGFDYIGEPTPYHTKNSYFGQLDTATFPKDSYYIYQAAWTD
YKKSPMVHLFPYWDFSPGQLIDVRVCSNAPKIELQLNGRTIGTYDIDHATGTQLAGWW
KVPYEEGELKAIAYDEHGNVIATDVQRSYTDAKKIRLQADREQLQADGTDLIFVEIQV
EDEAGNPVQNANNRVQVKVTGAGRLLGLDNGDSTDYDPYKGLSRRLFSGKLMAIIGAT
NESGTVRIEVTSEGLESAAAEFESRAVAGAEDRTLGDFGAAASQKQTVPMSNTERPVL
TGRAQEIPLRKIEIISEGGQLFDTSKQQMTVSAKLYPENTSYRDIEWAVVNDAGIESN
IAKVEAKGLEVNVSALGDGEFRLRATSTNGTDQTKLISQLEFKATGLGTAYKDPYGFI
TGGLYDYTKGDVGNGNERGVATSRDGETHVGFRNIDFGPYGSDTITIPIFALSSEEYF
IQIWEGMPDEEGSTLLADVVYDKESRWNVYQEETYQLSKRLSGITSICFVLKQKIHIK
GFSFERQSRAFEQNTAASCDHLYGDTFKIEGDHVEGIGNNVSLEFENMDFTAEAPSKL
VIYGRSPIDKNTIHIRFAGEDGQSNQLVEFTQSDGYEERVFELEQVKGVQKVTFIFLP
GSQFDFGWFRFEK"

CDS complement(782026..783594)

/locus_tag="PIKHDJKN_00706"

/inference="ab initio prediction:Prodigal:002006"

/codon_start=1

/transl_table=11

/product="hypothetical protein"

/translation="MRSVNSSDDTAKEKRRPTSRLNHRPNSKSNSSRRFAIAMAAALV
ASSLLLELTAEPTTYAATNVQHSGNNTTVRSIATNTEKKLHYTAVEGAYYYTVALRSD
GSVWTWGRNLWGEIGVSEKVRYRHTFSPIRIPELSNVISISAAGGGINAAVQADGTVW
EWGAGKLPSKVESIHSAKDVVTGTFTVALLQNGTVWSWQHPAAQGSDSDHLRKPHAIS
GLKDIIQVGTAGLHGYALKKDGSVWTWNEPDESGKVLSKPTKIKGLTNISSITSSDTS
LLALDKNGKVWGLDETGKAFAFDYDFKVKKMDGNSQYMLLLTTSGEVYSYGRSVNGKE

GKVNHLSGITDISAGYYHSLALSSDGTVWGWGSDKYEEAGAPATSSGGMVYKPVQAKI

GTDVYLNGELFQSMYAAVETPKTVQLPIKAIAAALGAEFEIHKGEGSLNHYTLKYDHR

TITIRPNETQYVITSADASGERVMQLPEPINNYSGATTVPFEVLRGLGLNVSWDLEKA

MLTIDDADKKDSGS"

CDS 783793..784227

/locus_tag="PIKHDJKN_00707"

/inference="ab initio prediction:Prodigal:002006"

/codon_start=1

/transl_table=11

/product="hypothetical protein"

/translation="MKKVLLTLSLALMMLTACEANDPTVDSPVVLNSFPEIIEPHNPE

QAEQSGDVVVLLEGMRNKDKWKTFVKNVKNKQQDQVRVTMYTIEGGAIIYELIYDGKS

IQSTYDNSRDAYGSKQGITTNTCKGIGTMKSEQGRVFYVLTG"

CDS 784898..785071

/locus_tag="PIKHDJKN_00708"

/inference="ab initio prediction:Prodigal:002006"

/codon_start=1

/transl_table=11

/product="hypothetical protein"

/translation="MMKKFWMLCSLMLLIMGCGQNNREKAIEVLIKNQTHDVTLIVFT

NKPAESSFIKQCP"

CDS 785307..786230

/locus_tag="PIKHDJKN_00709"

/inference="ab initio prediction:Prodigal:002006"

/codon_start=1

/transl_table=11

/product="hypothetical protein"

/translation="MRYLNKRIATSSKLRSRRPTPKSRKKSPEKRKKCHIIRKPCRRK

RRSLRPQRPIRQLKIIWDKKVIALPRGIGVNPAFAEPGPRLAPVREHETKTVGPPGMQ

GLVGEQGAKGPAGPHGEHGLPGPPGAQGLVGPAGVPGLPGAQGPVGPPGVQGLPGLPG

APGLPGLPGAQGLVGPPGAQGPAGPPGAQGPAGPPGEQGPPGSIPGIEIIPTVNRYFY

FPDTDLNLSDSVTIPVEEFVNDEGGSVSEFAGIGLTSFYNLYINGIVQPGNSYDVSKV

MLFFPSQGGVIFAETPIILETIQLTAKITNG"

CDS 786313..786597

/locus_tag="PIKHDJKN_00710"

/inference="ab initio prediction:Prodigal:002006"

/codon_start=1

/transl_table=11

/product="hypothetical protein"

/translation="MPLVTPFQNSLRFAATIGDGTGTGATFAIAATTFTNDAGTAATA

FPGTFNYYNLYINGIMQTADTSTVTTTTITIPGGDALNAGIPVVVQFVVS"

CDS 786879..788045

/locus_tag="PIKHDJKN_00711"

/inference="ab initio prediction:Prodigal:002006"

/codon_start=1

/transl_table=11

/product="hypothetical protein"

/translation="MQRIEIHPDVLEEKARLVQQKKLELERMVRELEKSIYLLQSEWS

GVTGERFFWDFMQLKEVFPATLGLLDKIQNEFIFIAKNFRTADGSGEVALYIPEELKR

NFGVGLLDKSIGETVTGIGQTAEALFYDPFGTVTSVAYAMTLGRVVDVGRGIKFAWDA

TWGNGTARSDVGQFVEEQKKQVDESGAGYYSGSVMGQVLAYAFVGKALRSVENKHSDL

GGSSGGKPEPVKGNDIPRDLIKPSGSPNPEALPSGPRTKIPNNADKATKQSLELENGA

ADQLARSGYRIEQNPKVRDSDGIDSNRDPDFRIEGRIFDCYAPTENKGIRGIWSEVQE

KVVIKQQTKNVVINLTKWSGDDASLLKQFKEWEIQGLEEVIAIKKDGKIISVFP"

CDS 788064..788552

/locus_tag="PIKHDJKN_00712"

/inference="ab initio prediction:Prodigal:002006"

/codon_start=1

/transl_table=11

/product="hypothetical protein"

/translation="MEYSLKLNGKQDAFWLKKLIESKNFEVNYMNRKHNSLSIDEFDL

RGGLKIAAYDNNFPPDHPAASYETMFQEEDFIYQQTISYELENNQQYELWYKTMYEIS

FAIVESLKVNGVFYHTSGEEIFFFKNGKYTFNGTYLELLQMHYGELLENIEYSVFTKP

HN"

CDS 788641..789303

/locus_tag="PIKHDJKN_00713"

/inference="ab initio prediction:Prodigal:002006"

/codon_start=1

/transl_table=11

/product="hypothetical protein"

/translation="MILKKRFVMIGCCVGFVFLLVLTYSFYKEKRMYEQYLTMNVRQD

IIDMKSAIINNDIIFNRILKNEIITNEMVEDGEQASQLYYNFTDMTQIMNKYNLMNLS

FSEEANWLVKPAALFTNDFSQDDILPPTLHRMRMTFERLDISSYPVTAELKARIEEYQ

QLNHRWLNVVGDFESTEINENDWPQILNDLEQETQSYFQEKNVEFVDDDVWREALNPR

YK"

CDS 789527..792664

/locus_tag="PIKHDJKN_00714"

/inference="ab initio prediction:Prodigal:002006"

/codon_start=1

/transl_table=11

/product="hypothetical protein"

/translation="MKQGIYEQLINTITRQEISALDPDVYHIGTEKLDAEEARKLLST

YLAAVTRRALKIVREQTEDKTAVLAQIRTCNEIIATLKGTLGEEEYNELQLDEQGEVL

THVYSKLNSIRAVRDSKIVRPDTPISQSSLFTGAKSEPSMLLELQKEIVTADRIDWLV

SFIKFSGLRLLLEQLQQFTASGGKLRIITTTYMEATDLKAITELSKLPNTEIQISYDT

KVTRLHAKTYIFHRDTGFTTAYVGSSNLSNPALTSGMEWNLKLTEKDSLDVLRKIEAT

FESYWNDREFTRYVAEDEGHEAQLKAALNRKKDQSNHFHLDIQPYDYQKEVLEQLHAE

RTLYGRTRNLIVAATGVGKTVISAFDYKRFCASHAGAKLLFVAHREEILKQSRDTFRY

ILKDMNFGELHVGNHRAEALDHLFVSIQSLNSMKLTEVTSRGYYDYIIVDEFHHAAAP

SYQKLLSHYEPKILLGLTATLERMDGKDITAYFDHTIAAEIRLTDAIDRKLLSPFQYF

GVTDTVDLSQVKWSRKGYDLNELEKLYTHNKIRANQIIQSLNKYVTDLDDVKGLGFCV

GVDHALYMAKIFNEAGIPSMALHGGSIEQERHSAKGQLVNGELRMIFVVDLYNEGVDI

PEVNTILFLRPTESLTVFLQQLGRGLRMAEGKECLTVLDFIGQAHQEYRFQDKFRALI

GATKHSISYYVENGFSNLPRGTFIQLEKQAKEYIMRNLKQMSRNRKALIQKLQTFQQD

TGLPLTLAHFVEHHGMTLYELYGGRTGKRYFRGMLAEAGLTEPIEGEQEEYIRRLPSV

LTINARSWLTFLIDYIEKGKEPTTTDERRMLIMFYYTFHRAAPEKLGLSSIEEGVQRV

LSCEAFRAELVDIFKYNLAHLRFVDKSNSFPYTCPLDIHCMYSIDQVLAAFGYWNAEQ
SPAFREGVKYFADEQTDIFFITLNKSDKDFSPSTLYEDYAINERLFHWQTQSRVSEHT
ATSQRYIHHRETGNRIALFVREYKEEHNYTSPFVFLGEADYVSHEGNKPMSFVWRLRE
EMPAKMVAVANKCIV"

CDS 793035..794606

/gene="hsdM"

/locus_tag="PIKHDJKN_00715"

/EC_number="2.1.1.72"

/inference="ab initio prediction:Prodigal:002006"

/inference="similar to AA sequence:UniProtKB:P08957"

/codon_start=1

/transl_table=11

/product="Type I restriction enzyme EcoKI M protein"

/db_xref="COG:COG0286"

/translation="MAAKKKTKQKSIEETLWDSANKLRGSVESSEYKHVVLGLIFLKF
ASDKFEERREELISDGKETYIDMVEFYTMKNVFYLSEISRWSFLVENAKQEDIALKID
TALFTVEKNNPSLKGALPDNYYSRLGLDVSKLASLIDTINNIDTLKDKQQDIVGRVYE
YFLSKFALAEGKGKGEFYTPKSIVNLITELLEPYKGKIYDPACGSGGMFVQSVKFIES
HHGNKKDISIYGQEYTATTYKLAKMNLAIRGISANLGDMAEDTFFKDQHKDLKADFIM
ANPPFNQKQWRADDELTDDPRWAGYDLPPTGNANYAWILNIVSKLSENGVAGFLLANG
ALSGSGDEYKIRRKLIENKLIEAILVLPRNLFYATDISVTLWILNKNKKARSIDHNGK
LKKFRNRVNEILFMDLRQKGIPFEKKYTQFSDEDINEVTKTYHNWQQVDYEKTYQNTA
EYCYSATLEEVEKKDFSLVPSKYIEFVNRDENIDFDEKMELLQSEFIELLKAEENSKN
ELLTVFKELGYEIKL"

CDS 794590..795846

/locus_tag="PIKHDJKN_00716"

/inference="ab initio prediction:Prodigal:002006"

/codon_start=1

/transl_table=11

/product="hypothetical protein"

/translation="MKSNYKRLGDYIHKVDNRNTDLKVTKLLGLSMTKEFRETTSNTV
GTDMSIYKVMSKWQFACDFMSVIRVYKLPVVLKTDDEPNLVSPAYPVFEVNDTNELHP

EYLMMWFRRTEFDRYAFFKCDSAIRGGFDWVELCNTTLPIPSINKQKEIVKEYNVLVN

RINLNYRLIQKIEETAQVIYKHWFVDYEFPDENGKPYKSNGGEMEFNKVLNIEIPKGW

EVERLGDLIDYKKGYAFQSNDYIEKGVPIVRVSDFKDKSIDITSCYYIAENQAQEYSQ

YKLKTNDIIISTVGSWPNNPLSIVGKVVIVPELANNSLLNQNAVRLRSKKTGNLFLYY

RLIEKDFSDFVISGAQGSANQASVTLEHLFNFNITVPSSKVGSKLEFIFNILNENIMI

KRKVNYFLESLKDILLSKLATLEDNL"

CDS 795843..798992

/locus_tag="PIKHDJKN_00717"

/inference="ab initio prediction:Prodigal:002006"

/codon_start=1

/transl_table=11

/product="hypothetical protein"

/translation="MKFTEESLERAVIELFESEDYKHQNGMCIHKEISDVLLRDDIKT

YLLDRYSDDEITNNEIDSIIREMEVLPSSALYDSNKAIIKMISDGFIFKREDRSKKDL

FIQLIDFEGLENNLYKIVNQLEIQGYEKRIPDAIVYVNGLPLVVLEFKSAIKENTTIK

NAFTQLTVRYRRDIPELFKYNVLCVISDGINNKTGSLFAPYDFFYSWRKIEGNEPLDK

DGIDSLFTMVKGLFNQKRLMDVVRHFVYFPDTSTKEEKIVPRYPQYYAANKLFESIKR

NMRPQGTGKGGTYFGATGCGKSFTMLYLTRLLMRSVYFHSPTIVLITDRTDLDDQLSG

QFTNAKGFIGDDQVISVESRAELKTYLQKRKSGGVFLTTIHKFNEDIDLLTERTNVIC

ISDEAHRSQFNLDQKIKITKDGVEKKYGFAKHLHDSLPNATYVGFTGTPIDATMDVFG

DVEDSYTMKESVYDEITVRIVYEGRAAKVNLNEAKLKEIEEYYKKCEADGANEHQIEE

SKKAVTQMDVILGDPKRLKLVAEDFVKHYEKRIEEGATVKGKVMFVASSRAIAYDLYK

QIVALRPQWAEIRESDEGTVLTEKEKQGIKPIEKIKMVMTRDKDDEEELYKLLGTKDY

RKELDRQYKSEKSNFKIAIVVDMWLTGFDVPFLDTIYIDKPIQQHSLIQTISRVNRVY

EGKEMGLVVDYFGIKSSMNMALKRYAVVDGENFDGTEKAVVIVKDQLDLLARIFHRFD

STAYFKGTPLQQLQCLNQAAEFAQQTNELEKRFISMVKKLRSAYNLCCSSEDISQIER

DYIHYYFAIKSIIHKLTVGGAPDSSQMNEKVRQMIQEALISEGIEEIFKLDKNDPKDN

VDLFSDEYMAKIDKIKLPNTKIKLLQKLLARAIEEFKKTNRIQGIDFSKRLKKLIDLY

NERKDFQAMQSDVLDDVADQFADLFAELLNERNSFKDLGIDFEEKAFYDILKAIAEKY

KFEYAHDKLIKLSQEIKKIIDDKARYTDWAHRDDIKAELKVDLILTLAENGYPPVPRD

EIFKEIFEQAENFKKYRTD"

CDS complement(799224..799565)

/locus_tag="PIKHDJKN_00718"

/inference="ab initio prediction:Prodigal:002006"

/codon_start=1

/transl_table=11

/product="hypothetical protein"

/translation="MNEDREVLKLVGARIRALRKERGYSQESLGEKGGFHFSYIGQIE

RGEKNVSLVNLAKIAESLEVNLIQLFAYIDEDFHFTESELVIQEIVSMLRDASPESIH

LTKNVIREILK"

CDS 799707..800222

/locus_tag="PIKHDJKN_00719"

/inference="ab initio prediction:Prodigal:002006"

/codon_start=1

/transl_table=11

/product="hypothetical protein"

/translation="MARNWSITEAHCNHQVDFTPRLQDHDISTQPRSKHIPAYHMLRT

TIMAYHQHAKYHLKLAAIMCNHNQFKACLILCDWSLASMIKALYIHKYHSVHPPKELT

MNEILPLVHTDTELGLDIALFIGTMQHMSSLEDCPHDQPLKLNNIESLIQRTEEILDD

LSARLMKDSSE"

CDS 800368..800640

/locus_tag="PIKHDJKN_00720"

/inference="ab initio prediction:Prodigal:002006"

/codon_start=1

/transl_table=11

/product="hypothetical protein"

/translation="MKRVIAIAFFILSILELVTLILIGKSMMHGYMEPSTFKGIISFP

LGATYLSIWMFFSRQEFTGGRVAAQISFTAALLTTCPLVCLLIIFY"

CDS 800801..801835

/locus_tag="PIKHDJKN_00721"

/inference="ab initio prediction:Prodigal:002006"

/codon_start=1

/transl_table=11

/product="hypothetical protein"

/translation="MSTIKVTPEELLHISKQIEQGRQQLEGIRNDLTARIGFIQSQWA
GATQERFFYDFQQSRSVLDRALESMVKSSQDLFAIAERFDQADQEQVSLGAVAGQIAA
HTMMNHNTGNPEEQRRMVYNPLFGVNMPASEAEDGMPGQKEFVKYWEQGGTYEEMRAG
PKQPESGGGDIYDEQISAFKEGHHPVTGESIPAWQAAVSIGGLQTAKLVLAFTGTYNK
GYKVPTGGLKFPKSKLDNLNGVNPVIFNRDTVISSATAPKKGGETVVGHALQKHAGRN
PDIWGRVRGGPEQINQVAQKHLQDILDAPGEFKKITNNRGITFLEKNLPDGRGVRLNQ
DGSFKGFIDQ"

CDS 801832..802347

/locus_tag="PIKHDJKN_00722"

/inference="ab initio prediction:Prodigal:002006"

/codon_start=1

/transl_table=11

/product="hypothetical protein"

/translation="MMEVVNLESLEIILIDFNKNNLDQFIKNDLNIQVDRIKSSHFYD
NRSENDIEFQQIESLEEILSPKGTGNILLSQLNLGHTYNDVMIVFSFDEESGDIVMNF
PEEELFSGENSETTLNAQKLIEYILDIKKKYAIEKVRIGYEPAMDDDTCLVEIDKEMM
DTNVIVSKLLA"

CDS 802568..802894

/locus_tag="PIKHDJKN_00723"

/inference="ab initio prediction:Prodigal:002006"

/codon_start=1

/transl_table=11

/product="hypothetical protein"

/translation="MPTYNKLVRDKIPHIITSSGKECRTRILDPEEYKQELRTKLQEE
SEEYVSAANDQEALEELADMLEVIRALAEVHGANAAQLDKLRADKAEARGGFQERVYL
IDVDEA"

CDS 802878..805316

/gene="uvrB_1"

/locus_tag="PIKHDJKN_00724"

/inference="ab initio prediction:Prodigal:002006"

/inference="protein motif:HAMAP:MF_00204"

/codon_start=1

/transl_table=11

/product="UvrABC system protein B"

/translation="MSTKLNVKLITDNLADELITRMQHASGIYIMTSFIMQSGVKLLA

PHLKRAAERGAEVRMLAGDYLYITQPEGLRALCEVDPRIETRLWRSMGTSFHPKAYLF

DHDNGEGLLIVGSSNFSFTALKTGYEWNLAMNAEAEPYTFQSALDKFMQSFYHETPLP

VNPDSIALYEEEYRKYHQKNPEMIQRIMEMEEAEFGTGLPEETEEETAAELSLAPIQP

RFAQLDALEALHGTMEEQYDKAMVVMATGLGKTYLAGFFAQRFQRVLFVAHREEILFQ

AKKSFQRIMPERSHGIYNGQYKDGAADCVYASIFTLSMQKHRDGFAVDAFDLIVVDEF

HHAAAKTYMLVIEHFQPKFLLGITATPDRLDGKDVYALCDGNVAYQMHFIEAIRRGWL

APFQYYGVFDDTDYSQIRWIGTKYDEEQLMAVQLQEEHVETIYAAWLRHKQTRTIGFC

SSIRQADYLAAYFRSQGVKVLSLHSRTTEMSREEAIRQLDAGDLEVVLTVDLFNEGTD

IPRVDTLLFVRPTESLTVFTQQVGRGLRLAEGKSHCVIIDLIGNYRNADVKLSLLDVR

GELEGTAKDRDSAVPEVPDNCGIHLETRVVNLLQELSRKRLPRREKLHQDFLDVKREL

GRIPTYLELHLMGRTKSIGYRNEFGSYVGFLYWAELLSPTEGEVYYVRHEAWLREVEKT

LMNKSYKMIVLLYMLERGEAHWMDPITPGEMARFFHTYLTEKVYRKRKDFSDKGKLAL

WEWNEKTATRIEKLIIDMPMSMWSGAKGSMTRFEDGVFSLNVQVEDAEEGALLYRWTK

EICQYRLHAYFERG"

CDS complement(805446..805571)

/locus_tag="PIKHDJKN_00725"

/inference="ab initio prediction:Prodigal:002006"

/codon_start=1

/transl_table=11

/product="hypothetical protein"

/translation="MKKLITKLASVLGYGLLITLFIFFVIALIAIIILAIYEFKN"

CDS 805671..806585

/locus_tag="PIKHDJKN_00726"

/inference="ab initio prediction:Prodigal:002006"

/codon_start=1

/transl_table=11

/product="hypothetical protein"

/translation="MNNSSKLLKPVLAFSLLSAAFMPYAISSNTTYADSLSVEASTPI

TNGVNNAWTDGTVNNEQLKEEADLSTWNIRYDDEAKTVLKSIKSNNTSALKAQAQQEA

EAKFLEEVQQFQSSTNANDQLNFAAAAEKTKAQYRSDFNLVALSLGSFSCPFAGQALK

NSLQDSPSALDYAAGSSRSNAWSLTSAYTTVSIPIAKAIDNANAAGKTFVSGDSSVAT

SAGNAGLDFYLALGKVTVSWAAEKVSGSWKVYIHTYDKYDFQDKTVPSSFPNNIITIA

NNEGAKAQAAGAIVPYFIHIYTQQNYKP"

CDS 806830..808749

/gene="rep_2"

/locus_tag="PIKHDJKN_00727"

/EC_number="3.6.4.12"

/inference="ab initio prediction:Prodigal:002006"

/inference="protein motif:HAMAP:MF_01920"

/codon_start=1

/transl_table=11

/product="ATP-dependent DNA helicase Rep"

/translation="MAITVPEAIPKKAMAGERLLFETLKKHLPEDYIVYYEPEIRGRR

PDFVIIGPDLGLLVLEVKDYTKGTLHQVNRDEWTLHTTAGETTTEPSPLKQARGYAFL

IADQLKKDKNLIKEGTPGYLKFNYGYGVVFTRLKQKDFLHMDLYSVIDPVFVLTREEV

NPEDEDFSEEILIEKLLHMFTVPTRNRYSLTHDDIQAIRYHLFPEVRISAERKEIVPH

QDQLLLSLHNIKTMDLHQEKMAKQVGDKHRLIRGVAGSGKTLVLVSRARMLAKANPDW

DILVLCYGSTLSQYLRQAIVQKMAEPEDLFDFSDNDGVSTLGSNIQVSTFHSWLGKQF

RIMDKKLPALLEKMEQGIQELPKYDAILIDEGQDFEPDWLKLVSACLNEETQSLLLVE

DRAQTIFKRKNSLAQDTGLDFRGRSKILTINYRNTAQIVQFAWDFYKEHSRLQHKVIS

TAADGIEIIPPQFTKRKGPEPLIYQSQSTQEEMAFVAKSIDYLRREKSFALKDIAILY

RVQEHQGINIIGEMQQALNDNSLPYNWVTRDEQSKKSFDREEESIKMLTIDSAKGLDF

RAVFIIAIETMPRKVGKTDEMDEREVSRFYIGMTRALERLFLSYSGDSRFTQYLDEVQ

QQRTERPKDKAVSRG"

CDS complement(808899..809666)

/gene="mta_3"

/locus_tag="PIKHDJKN_00728"

/inference="ab initio prediction:Prodigal:002006"

/inference="similar to AA sequence:UniProtKB:P71039"

/codon_start=1

/transl_table=11

/product="HTH-type transcriptional activator mta"

/db_xref="COG:COG0789"

/translation="MKTPYFTVKDIIQITGITKRALHYYDKTDLLKPSKVEDNGYRYY

DQEALGNLQMILLFKEMNFSLKDIAAMMQLSKEEQKDILREHRSTLVQRKQKLETIID

QLDEYVDGTDISHLHLFDDSSILSIQEQYESEAKFVYGDTKKYQEFEANVNQLSAEEQ

EKAYQQFSVNMEQVFRELAKHQDLSPASGEVQALVLEWKSCLEQFMSCDAEILKCIAE

AYTTDRRYAGYFDQFGDKEFLSFFYQAIMVYVEEGEK"

CDS complement(809641..810567)

/locus_tag="PIKHDJKN_00729"

/inference="ab initio prediction:Prodigal:002006"

/codon_start=1

/transl_table=11

/product="hypothetical protein"

/translation="MKITELRSDKIYKEIIKAAPDDKLELYRERMMGPFMNKWNIQQI

PFRSSEPNGFDVIKMNNFMNIAPADITPEINESLAAISSKAFWQQCHEAVRTSLSTFT

DHGIELLVSEYLYTILLGDKNSTSLTMNEGISGDGGIPGYIIANLIPNGYTLPRMQSV

LAHECNHNVRYQYIQWNPQITLGEMVVSEGLAESFATSLYGEKLRGPWVGKTDMETLN

TLIKPKMKDQLHVTGFDQINPYLYGDELAIMQNFTPVGMPYAAGYACGYHLIQYYLNK

TGTPITEATITPASVILAETKDFWNEDTLFHR"

CDS complement(810769..811233)

/locus_tag="PIKHDJKN_00730"

/inference="ab initio prediction:Prodigal:002006"

/codon_start=1

/transl_table=11

/product="hypothetical protein"

/translation="MNVILNKEPDKRRINVAMLMYLILMILFYGIYVSLESDRIAQSQ

QWTSGGSISEQAIESMSQLGRWTSITESLFLVLFILVMIIMITRYRSKVRRLLPFALW

NVALFVGVGAVSLVGSQVTSMSIGNLAQPIIVPAFLLAALFIYLVSGLMKRG"

CDS 811369..812109

/locus_tag="PIKHDJKN_00731"

/inference="ab initio prediction:Prodigal:002006"

/codon_start=1

/transl_table=11

/product="hypothetical protein"

/translation="MKKKFLLLLLFVVLVTSAVIILWFDRLKSSAFQTEMGNDANSVG

LPNQASSLPNIIEIINEEATLQLSLNDVPEYKRHLMNEWDFQTEIDRTQLEVLKVPSS

ETYSMLKYGCGTKACSTLFVKTDDIGTHSLPLPEGILQDYKLSSDYQQVLIRYAYDEG

GLVKRQILVAVDLKKLEIIPYASTDLEEAFMLKPTWPIISYDWIDNNHFTIETAALET

SEYNVLKEWFDSNDRETRKVTIRLNSLD"

CDS 812238..812951

/locus_tag="PIKHDJKN_00732"

/inference="ab initio prediction:Prodigal:002006"

/codon_start=1

/transl_table=11

/product="hypothetical protein"

/translation="MSPSKQVTTKPEQETNSKLKLIGSSEDEQVKLYTVKETEDAIER

VTIDINGRTKDFDWSISHYSGTEPQLFYADLTGDGKEEAIVIIQIGRGTGLNIYEIHV

VNSDNLSEIKVQRYEDIVAEHIESRVSKNDDGTLAITVNSQGQVGNFNYDFDPAPDYH

QDELAFGGVVIYTLDNHKIKLSLPGSIGISPTYVCDFNITYTFDKADNEFIVDQIEVK

PFKKRLQTFNSTLPFPKRS"

CDS 813046..814344

/gene="galK_1"

/locus_tag="PIKHDJKN_00733"

/EC_number="2.7.1.6"

/inference="ab initio prediction:Prodigal:002006"

/inference="similar to AA sequence:UniProtKB:Q9R7D7"

/codon_start=1

/transl_table=11

/product="Galactokinase"

/db_xref="COG:COG0153"

/translation="MNTQPLELIQSTSGQELLAQMYGQQQLEEQTARYTKLNESFEQY

FGVQEGSKLFSAAGRSEIGGNHTDHNHGKVLAGSITLDTIAVAASTTDSVITFYSEGY

EQKYVIDLTDLAPKAEDDGTTALIRGMAAGFGQFGYKVGGFQAYISSNVFSASGLSSS

ASFEMLICTILNHFYNEGTLDVVVMSKIGQYAENQYWNKPSGLLDQMACAYGGLIAID

FENPAEPVIEPVKWDFQQNGYSLVIVNTGGNHADLTADYAAVPYEMRAVAKALGSEYV

REITGEAIYANLKQVREAAGDRAVLRALHFLEENNRVDGQVQALRDGRFADFLNLITA

SGNSSWKWLQNVYQSGSVKEQEIGIALALTENYLQKLGDGACRIHGGGFAGVILTILP

NEKVEEYMSWMHDMLDTPIIVVNVRAQGAVCLNTLIENVG"

CDS 814642..814923

/locus_tag="PIKHDJKN_00734"

/inference="ab initio prediction:Prodigal:002006"

/codon_start=1

/transl_table=11

/product="hypothetical protein"

/translation="MTTSTVAEQEEALLYDELRRALEEVEEGKQRYKMLKHMIRQLKF

AKWRKERFHRFDQYFQNVRPVTLWIMLATGGLMPVVLFSIFIWVSLWVE"

CDS 815169..815420

/locus_tag="PIKHDJKN_00735"

/inference="ab initio prediction:Prodigal:002006"

/codon_start=1

/transl_table=11

/product="hypothetical protein"

/translation="MPNQNKIIEQATEIGQQYFIDQYNTEVEFTDHKFMPKDLSHNVS

LSGHVKDDKETEISILIDYDTFEVQTAIVPKELLKQKKK"

CDS complement(815463..815795)

/locus_tag="PIKHDJKN_00736"

/inference="ab initio prediction:Prodigal:002006"

/codon_start=1

/transl_table=11

/product="hypothetical protein"

/translation="MNILNRKYLTLAFLFIWFIFAFIVLSGCISNEQKEIVQKAEEAS

LTYFKENYGIDVEFTNHKFIPADLSHTVSLTGYVKDNKDQEVFAMVDYDTYEVKTGSV

PDHIKPLK"

CDS complement(815833..816078)

/locus_tag="PIKHDJKN_00737"

/inference="ab initio prediction:Prodigal:002006"

/codon_start=1

/transl_table=11

/product="hypothetical protein"

/translation="MSTQEKVILADAEQFAHKYIFDNYGLDVDFTEHKFTPVDLDKSV

GIHGHVKGDDDQKVFVLVKYDPLKVETLSLPEGTDKK"

CDS 816249..817616

/locus_tag="PIKHDJKN_00738"

/inference="ab initio prediction:Prodigal:002006"

/codon_start=1

/transl_table=11

/product="hypothetical protein"

/translation="MQDYISDELYHKISTIAYQNDISVGTVISEDAKYKDWKAVEPEG

ATLHKKYSGFDSIIFRNEETNQVIIGYRGTEPEGIIGKTADIETDAFDVVGGRTRRLE

DAITDPERHNIFKNSPFKLMKDNMDWENNQFHQAEELYEAVKEAYPDSDISLTGHSLG

GGLAQYVAARQDVSGMTYSAPSVTNLLDDVSLAKVNEGHFDNKMVNVVHPNDSVGAGG

ISEYDRHVGSTVYKGQDFDTANAMYQNWKFQFQLKVPFQPLGAGSPMLVHDVRIGVDL

DKLHVGDIMRFFDSFSKDEGKGYHSMDQYEFDENGNLKGPLIDRNTGETIDISPRWTA

HQEALATFSNLMSGIIPPLIAAATGNVGGKGGGKIQLTTEELAQAAREMRLSLSGFSN

DTQASIQMFQTHISTSESQSFTPIAYNATATLQAINRWYQESISEIAEYIERKREDFI

IADQH"

CDS 817657..817953

/locus_tag="PIKHDJKN_00739"

/inference="ab initio prediction:Prodigal:002006"

/codon_start=1

/transl_table=11

/product="hypothetical protein"

/translation="MGRISVSLSDLRRAVQQCEQLQQRLMQQEQKMRSIHGRLQQDWI

GTSATELTHKMQSFMDGASAKLTELEAHKEELKRYIRKMEEADREDRRDRSRIQ"

CDS complement(818052..818582)

/locus_tag="PIKHDJKN_00740"

/inference="ab initio prediction:Prodigal:002006"

/codon_start=1

/transl_table=11

/product="hypothetical protein"

/translation="MRLKSIIVVGTVLTCSTLLWGCGNANMNHASQQKDTVQTESWSD

PKRLEASHLEALERMEKDHIHRMVSLNASMDEDKVMTTLERSTQKLEEMRPLAEMLQH

EGHPTLLQRIQEMAGDIQGSKQVVAEMKKLPQDGKIKMQSENSDSLHHIGSFRDYHQY

MQEKIQTLNKAKSATH"

CDS 818732..819103

/locus_tag="PIKHDJKN_00741"

/inference="ab initio prediction:Prodigal:002006"

/codon_start=1

/transl_table=11

/product="hypothetical protein"

/translation="MKLYTNNIWRWGTTLLYPILIFLVWSRMIPLQMWYVLAIVVVFC

FLWSGVKELFISTGLTWLVAIPCWWYFFELPKPSFGAENFAAHLVLIVPLFIFVVLLP

QTLILTTRMRIMEYYRKNASQ"

CDS complement(819192..820355)

/locus_tag="PIKHDJKN_00742"

/inference="ab initio prediction:Prodigal:002006"

/codon_start=1

/transl_table=11

/product="hypothetical protein"

/translation="MKRLMHRWLLGLLSLSLCFVTSGCWSAYEIQQVDYAKAIGIDYK

DGLYHMYVQTMDFASVAKSDSSAKSADTPPVWVGHAAGKTLNLAVNELFRSSQLHIAW

GHVTAIVMAESVLTSNHIKGVFDMLGRFPESRYTTWVYGTRDPLENILSATSIYNMSP

LDSILHNPLPSFQEESLFPPVLSFKLISTHNDASTTTYLPCIAMNKDHWSQNKKNHEL

FIIDGAFFERTGDDFEYLPHSKLPGYHWLLKDMRRAPLIIENGGTIYGVLSVGLPNIK

IVPVIRGNEVYFNIDAKYLTALYEYLTPISYDEMVQFSEKTLRDQILQTYREGLKRGV

DIYGLQEKVYRKNPSLWRKLSNNGTKMILTEDSIQKLDIHITIPYTGKFRRKV"

CDS complement(820352..821857)

/gene="gerXA_2"

/locus_tag="PIKHDJKN_00743"

/inference="ab initio prediction:Prodigal:002006"

/inference="similar to AA sequence:UniProtKB:Q9ZFB4"

/codon_start=1

/transl_table=11

/product="Spore germination protein XA"

/translation="MPLTTTPKKNPPLAEPFRINEHNLTTFFAGSDDVIISSHMIGES

PLQIVVVYCSGMVDSKSIYDIILPELTRTYERTHFVRASDIEKSITLQWTSMDLQNNP

LGTELMSLRVFEGHMLICIPSLQKMWSMDISNIPTRTPDESSTEVSIRGSRDGFIERL

SVNIALIRTRLRTAELACNIELIGSRSVTKVALMYIKDIANPELIDDVKNRLRKIDIE

RIMTANELEELLSPSKITLFPVTHYTGRPDFAAECLLNGRFIIIVDGNPSVIIGPVNL

FLLLKSPEDASFPFLPVNVGRMLRFIGLLVTVFLPGFYIALTSFHMDQLPFPLVATIS

VGRMGLPMESGVEMFLIMLLMELFREAGVRLPSAIGQTLTVVGGLIIGDSAIRAGMVS

PLMIVVIAVTVVAGATIVNQVMTSSVLILRFFCFVLGASLGIYGFILSIILFLIYLTD

LKSFGIPYLTPLSPLNFKQALASLFKLPKGWMKRRPVYLETQEPRKEGNDR"

CDS complement(821814..822950)

/locus_tag="PIKHDJKN_00744"

/inference="ab initio prediction:Prodigal:002006"

/codon_start=1

/transl_table=11

/product="hypothetical protein"

/translation="MSREKGEITIWLSFSIILLSAGLVCHVLSIPAILDYAGRDGWLS

VVAAAPFFLLFLCMMFVIIRRVRGQRLTDWITREFGAIPSWIFRITASLMLLSLGSHT

LYETTNWTVSTYLQFTPPYVLAGVGALVAAWAAAKGIRSIAMTSSLLLPFVILLGYFV

MSANMKYKDYGQLFPIMEYGAGPVLRGMIYSLAGLMEIWILMLFQHDIKSKIRWWHLL

LLGLFMLIMAIGPTLGAIVEFGPEEAAKQRNSPYEQWKLVNIGKLLQHVDFLSIYQWL

SGSFARVAISIYLIVDLLDFRRPMKRYVAVLIVTFIMCAIAVQWWRIDYVDYYVNHIQ

FPVMLAYVSTVTVLLTLAAFIHKKDKEAPAHAAHNHTKEEPPSG"

CDS 823147..824040

/gene="glxR"

/locus_tag="PIKHDJKN_00745"

/EC_number="1.1.1.60"

/inference="ab initio prediction:Prodigal:002006"

/inference="similar to AA sequence:UniProtKB:P77161"

/codon_start=1

/transl_table=11

/product="2-hydroxy-3-oxopropionate reductase"

/db_xref="COG:COG2084"

/translation="MTNTTKTPGETKVGFIGTGVMGKSMAGHIQQAGYPLHVYTRTAA

KAEALVKEGAVWHDTPGKLAAECDVIITMVGYPKDVEEIYLGEDGLVANAKPGSYLID

MTTSSPLLAARIYEAAEAKGLHALDAPVSGGDIGARDAKLSIMVGGRTEAFEAVRPLF

EQMGTNIVLQGKAGAGQHTKMCNQIAIASGMMGVCEALAYAKTSGLDAENVLKSIATG

AAGSWSLSNLGPRMIAGDYEPGFYVKHFIKDMGIALESAKAMGMKTPGLALAESLYQE

ISSNGLDEKGTQVLYTYYLQA"

CDS 824288..825019

/locus_tag="PIKHDJKN_00746"

/inference="ab initio prediction:Prodigal:002006"

/codon_start=1

/transl_table=11

/product="hypothetical protein"

/translation="MFKKILSLFKSQPELANQITASASSLPSTAPIPTRMVRSKRKTK

SDGDWTRQPEEPSTAQQLHGLPGEYKVLNDLLVANSKSRSGYSSIDHVVIGPRGIFVI

ETRNLTTGEIRGGRREANWTVSSSRIKMYNPLMQHRGHVEAIQAHLGDYKRVRLVSMV

TFTNRCRISVDPAVRYVNSDELIIYDHELVETILRKTERLETEVPETLYKEQDIQAIY

NLLSAVNSTDPQIRADHMEKAKGIK"

CDS 825356..826255

/locus_tag="PIKHDJKN_00747"

/inference="ab initio prediction:Prodigal:002006"

/codon_start=1

/transl_table=11

/product="hypothetical protein"

/translation="MRWNHRIKMFTAIFSLVAILTACGGGAKEATTKESAQDAGTVAT

TTTSSDTSTADSPAADTNAEDSADTSSTSDTTGSSSEEGKIGVDSTLEELKQKYADKL

GYIRVPLNDHPVRRVSEEGSKNINIANYSDAIVNMNTGIPIHSDSQRLIDDAFPPFFTT

VQAPEVSYVPWEEATNLERAMVKTLFISRQALLLTEDAMKNKDYTSQSFTDSMEFFKT

AAEFDSMAPVAQHTYDITLTKLYDKARDSWAKLAAIDPEQDEAAFAEKYKEARTEANN

VMGLLNILLSTNEEERLEQTYGK"

CDS 826429..828207

/locus_tag="PIKHDJKN_00748"

/inference="ab initio prediction:Prodigal:002006"

/codon_start=1

/transl_table=11

/product="hypothetical protein"

/translation="MRWIQLCKRFKFRTKLILFLSAATVLISGITGLITYRIHISLFN

EEVSRQYSLTAEQVLARLDSRVHDMYKVTDYITLNPSVKNAIKAQAAGISSYDQMKLE

DELDDQLYQVRLDAPEIMGIRIYDLKGNIFNLGTFAGSFQQMDPSYLAEMVHKLEGTG

GEYVWNRLDADAFLQEEKSNWILAGRLMRSVDLETYGVMLILFNTSLFESYLKDLRLN

EDIAAYLLDADGKLVYAFHNQDADPPPLTQLSMGATEIRDEQGTAHLYTKQTSDKAKF

TLISKVSLAQIQHKGKIIVKVAVFSAAATVLCSWIIITIISRRLLRPLASLVNAMKRV

RDGQFDTRVRIQTQDELGFIGERFNAMASRIDTLIHEVYERELSEKQAELKAIQAQLN

PHFLYNTLSMFFWKFYMLGDEKSARLVTALSEMLQYTLEPVQRLTTLQDEMTQIDHYL

QIQQARYQEALSIEISVPPELLRCQVIRLLLQPIVENVFVHAFADKRDNRRLEIRGFR

YAGNDAESDMLILEIADNGCGMAPSMMDRILAPLAHADEERQHIGMRSVLRRIELIHG

EPYGVQIESTAEVGTLVRLRLPYQVE"

CDS 828254..829330

/gene="rssB_1"

/locus_tag="PIKHDJKN_00749"

/inference="ab initio prediction:Prodigal:002006"

/inference="protein motif:HAMAP:MF_00958"

/codon_start=1

/transl_table=11

/product="Regulator of RpoS"

/translation="MNGRMLVVDDEAMFRQGLIHLVSNNPLGWEVVGEAADGEEALQA

VNSCTPDLIITDINMPVMDGLDLAEQIHDRGQDIMIIILTGYREFEYAQRAIRYGAME

FLLKPFSLDEACRVLRKAHERYCQRQSDNRIREQYSLVERADRLRDELAAFLLNQQMP

QMAARIDELFSNASRMPLPECKAEIQLLMKVMTDLLVQQFRVQQSASVDKFGPDPLLW

IHSVAEVMAWARCKSEEWLGMLVRLTQEQQDHVVTRVLQYIEMNYSSNCNLQAAAAHV

HVTPNYLSHLFKKETGHGFSQYVSKRRVDKAKLLLHSTRQSMADIAEQTGFDNSSYFT

TVFKQMTGLSPREFRKQPSHESKG"

CDS 829477..830448

/gene="yteP_3"

/locus_tag="PIKHDJKN_00750"

/inference="ab initio prediction:Prodigal:002006"

/inference="similar to AA sequence:UniProtKB:C0SPB3"

/codon_start=1

/transl_table=11

/product="putative multiple-sugar transport system

permease YteP"

/db_xref="COG:COG4209"

/translation="MKGELAAGPPRLEPPDSEVRQRIRQQRLRRFKTNIPLLLMFLPV

ILFYLIFRYAPIGGLVIAFKDYNFYDGLWNSPWVGFQHFQTLFSDPRTVEIIRNTLFL

SLLSIIVGFPIPIILAIMLNEVRNMAFKRTVQTVVYMPHFFSWVIIAMMIMTVFSLEN

GIVNRWVEAWTGEPYPFMYNKGSWIAVFVGSGIWKDMGFNAIIFLAALTTIDPSQYEA

AQMDGASKLRQIWHVTLPGIRSTIILLLILSMGRVMEVGFDQVYMLQNSNVNEVADVI

STYIYRTGLQGAQFSLTTAMGLFESLVAYILIYCANYIARRFNEGLW"

CDS 830464..831333

/locus_tag="PIKHDJKN_00751"

/inference="ab initio prediction:Prodigal:002006"

/codon_start=1

/transl_table=11

/product="hypothetical protein"

/translation="MRKTRGEQVFYMINYVLLTLVAISCILPLLNIVALSLSDARAVV

SGKVGLWPVDFTWFSYHSLLTGTPILNAFWNSVEITLIGTGLSMAVTIMAAYPLSRRH

FYHRRFFTMAMVFTMIFNGGLIPTYLVVQNLGLVNSYGALWLPGLVSTYNMLIMRSYF

ENLPGEVDEAARIDGCGELGLLFRIVLPLSKPLLATIALFYGVGYWNSFMSVMIYIND

TSKYNMTVLVQNMIMSNLNVQDFTDPTMISNLTPEGIRAAAVIVMVIPILAVYPFLQK

YFVKGVMLGSIKG"

CDS 831396..833051

/locus_tag="PIKHDJKN_00752"

/inference="ab initio prediction:Prodigal:002006"

/codon_start=1

/transl_table=11

/product="hypothetical protein"

/translation="MAISIKAWMKSGLVLGSIGGILAGCSGGSGSERAEGEGGRGNIT

STIYDRGAVPSGMGTIEDNMWSKWINENGPANVKYTAVPRWESQSKLNVLFASGSAPD

VIFEFGTPIRNTLFNQKQLMPLDELIENSSVEYKALMEKYPQLKKAGIKSDGKLYEVG

RMNEVFPLTSFFIREDWLEKLNLEVPTNEAEMLAVAKAFTENDPDGNGANDTYGIGGL

QFGDTAGLFRYMYNANWVNEEDGEIVVGPNHMKEVTAFKRALFEAGVVDKDFLTDKDG

AKSKQDFLNGKIGMYAAMTADYTGFAAKELDTLMQNVPDAKLKVIALPSTSVGQYTMV

WNNPVQMTAVVNARAKNPEAVIQYIDFLTKTETGRTVKNGFEGTHYTLNEQGCPRISD

QEKYKQEISWAGDYAMLYSRLEEGKCGYTEMLFSEEIPSQKEGLRLFKEAREVYMNDL

PVGEGVTHSEHMPQLPKELQVKLTNVTTAINDIFTRSIISGSKYTVEQAAAEAQQKWE

QGGGPEIETWYKEWWSKEKDNVLVWDDFYEIYEQQQADFKKSE"

CDS 833205..835097

/locus_tag="PIKHDJKN_00753"

/inference="ab initio prediction:Prodigal:002006"

/inference="similar to AA sequence:UniProtKB:P9WQJ1"

/codon_start=1

/transl_table=11

/product="putative ABC transporter ATP-binding protein"

/db_xref="COG:COG1132"

/translation="MRYNKRIGYVKFYIELWKETNGAGCVNIPNRHENKALRAGGMVM

IKLLYYLKKYRVAAIAALVMMLIELTVELAQPYLISKIIDNGIQQGDLSVVWLWGGVL

VGSAVVAFAAGIASSFFASHASLGFGYDLREKLYDKVQAFSYAVFNRFATSSLITRLT

GDVTQVQDTVFMSLRFMTRVPLVVIGSMIMAVVVNPKLGLLLVVMVPVLLVFVIWMIK

KAALLFRNVQRRLDAVNGVIQENLTGIRLIRVFVRMGHEIERFAGYSGKLMKGTISAL

RLTETTMPFMLLMMNGCIIAVLWFGRRDITTGNATVGEVVAVINYLLRTIGAMSALSW

ILVTFSRASASAQRLNEVFDTEEVSESAQTLNTGRSAQTGKGNPVIQGAVDFQGVGFS

YPGSEITVLEDITFSAKRGERIAIMGATGSGKSSLVQLIPRLYTEDQGEVRIDGNDAE

RLDISTLRGAIGYVPQEVVLFTGSVRDNIAWGREDATLEEIKEAARRAQIHETIEKLP

NGYDTQLGQRGVNLSGGQKQRLSIARALIRRPSILILDDSTSALDVATEARLLGALEE

LSCTTFIITQKISSTTSADLILLLDDGRLIGQGKHEDLMDSSELYRRIYESQYGEGTP

HVQSIH"

CDS 835078..836919

/locus_tag="PIKHDJKN_00754"

/inference="ab initio prediction:Prodigal:002006"

/inference="similar to AA sequence:UniProtKB:Q9WYC4"

/codon_start=1

/transl_table=11

/product="putative ABC transporter ATP-binding protein"

/db_xref="COG:COG1132"

/translation="MFKAFIEPFRQPPPPIDPETLKAGGGRKPKARAKNWSGTLGRIW

AYLARRKVKLTMVLLMVFASSALALLGPYMVGVAVDKFIDGEATSSSWTTFLSGLLAV

YVLFSLSSWLQNIWMIEIAQETVFRMRYDLFSHLHKLPIPFFGKRQQGEIMSRVTNDI

ENVSGTLNSSAIQIFSSVLTLIGTFGVMLWLSPLLTLLTFIVVPLMAIGMRWITRRTG

PLFKQRQRNLGELNGYIEETLSGQKIIKAFSQEERVIRGFEERNTKIRLSGFWAQTIS

GFIPKLMNGLNNLSFAIVAGIGGILAIQGSVTVGVIIIFVEYARQFTRPLNDLANQWN

TLLSAIAGAERVFEVLDEDEEAKDEGAAVSLEKVEGAVRFDKVSFGYDEGRNILHEIN

FEAKPGEMIALVGPTGAGKTTLIQLLSRFYDPTAGTLTVDGRDITTIRRENLRSHMAF

VLQDSFLFQGTIRENIRFGRLDATDEEVEAASRLANAHSFIIRMKDGYDKVLQADGSG

ISQGQKQLLAIARAILADPSILVLDEATSSIDTVTEIKIQEGLQRLMQGRTSFVIAHR

LNTIRQADRILVLKDGQLLEQGSHDALLEQGGFYSELYYSQLRNKAQ"

CDS complement(836980..837996)

/gene="tdh_1"

/locus_tag="PIKHDJKN_00755"

/EC_number="1.1.1.103"

/inference="ab initio prediction:Prodigal:002006"

/inference="protein motif:HAMAP:MF_00627"

/codon_start=1

/transl_table=11

/product="L-threonine 3-dehydrogenase"

/translation="MKAVVIENYGGSEVFAEQERDIPHISDTQVLVEMKATTVSSADQ

LIRTGTFRHFMPTQFPYVLGVDITGIVVATGKKVTRVQTGDRVIAVSRTGGGYAEHVA

VEESDLAVITQSLSDAEAASVSASGMTAWQALFHYGKLQPGQRILIHAGAGGVGHIAI

QLAKQHGAYVITTARAHNHSFVLQLGADEVIDYTTTDFAESLSPVDVVLDMVRDPVVD

KVTGIGATELKNYSILKNNGTFISLVNPAIAKQPPIRGIDAQFALISPNVNDWEAFTQ

WIQTNKLDIHVDNVFPFTRQGVAEAHKYYDTRNMRGKIVIQRDVITDNLSSIVAQETG

MSHL"

CDS 838173..838736

/locus_tag="PIKHDJKN_00756"

/inference="ab initio prediction:Prodigal:002006"

/codon_start=1

/transl_table=11

/product="hypothetical protein"

/translation="MDRRIQKSRESIIEAFIQLLSEKKFEQITINQIAERANVSRGTV

YLHFMDKFDLLDQCIKSHLVELIESSLFCGVTGIFPSKEALLHSFRYLKEHALFYGAV

LTDQSVPEFRTRLQAALLRGLDKQIDMSGINQGRNKEIMLQFLASALAGMIEWWITSA

EPYSAEELVDELWILMERNQMTPVPHV"

CDS complement(838861..840849)

/locus_tag="PIKHDJKN_00757"

/inference="ab initio prediction:Prodigal:002006"

/codon_start=1

/transl_table=11

/product="hypothetical protein"

/translation="MTRLFDQSKRSFGLFLAFVLIITLLLPAGALPKASAATITIDGN

VSDWSSVSALTTNSGTAQTLKVTNDGTNLYLLIQGSGLSTTMGNFWINTDNNTSTGYQ

AAGWGVTGVEWLLENTGLYRYGGSGTDWVWNFNSTLTSSQFYSSSTVIEVAIPLATLQ

ISAGSTVRVGYIDNSSDTSRLPASGQTLPAYLLTSAGSGGETGNTVVNPVELDSPLNN

PFKGWAPSAKSTTYAQPHRLVYAGVTWKELESTKGTYDFSAIEAANNFAYWKSKGVKV

VFRFILDSPTGQAHRDIPDWLYNDMVARGESPGTVYNDTTGGMGTGNNMGFSPNYNST

YLQERHKLAIEAIAARYNTNDRPVAFVQIGSLGHWGEFHTWPYVGPNGETNYTGAFPT

NDISNKYVQHYIDAFAGKEDKMQVLIRRSIALAKTNNKGMVMGMFNDVFGHKDSFDAD

WGWYTGTQNGYWDDIGQQQPAHANFWETRISGGEFYGGASGMLAALTTGSGFTETLRQ

TELSKPSWLGPNSPASLPLNHALQANIDALKKRMGYHFVVKEISHPATISGTTFTTTI

KVENKGVQHFPFAWPVEIQLRSGNTVVAKKTTTVNLTTWKTGTYTLTDSIPVSGLAAG

TYDIAIAIIDPETNKPGVDFANTNKLTDGSFKLSSVTK"

CDS 841492..842592

/gene="iolG_3"

/locus_tag="PIKHDJKN_00758"

/EC_number="1.1.1.18"

/inference="ab initio prediction:Prodigal:002006"

/inference="similar to AA sequence:UniProtKB:Q9WYP5"

/codon_start=1

/transl_table=11

/product="Myo-inositol 2-dehydrogenase"

/db_xref="COG:COG0673"

/translation="MGKIKMAFIGVGDMGSHHCIGFDLLEDSEVRYICDMNEANVART

LAELKNSNPTIVSDYRELLAKEDLDAVVISVPNYLHREVAVAFLEAGKHVFLEKPVAH

TIEDCDAIIEAAKASGRVLQIGLVYRYSNLYRRMEKELENGRLGDVKLMWCKEFRDPF

PPADWFYDKTKSGGAIVEKDCHHFDIFNWMIQAKPVRVFASGGQHVMKQGEPNRIQNS

YSHYPTKEISDTSIVDHAFITIDYDNGSKANLGLCMYLKPRNLMGEGLEIGLIGENGG

QMVARNDKTIDIVGGQDWTKDHLEIDVTSDSIMGGHTGGQTQRIDFLKCVQEGRQPFA

SAQVGRNALLIALAAEKSILEERYVYLEEISG"

CDS 842604..843494

/gene="ngcF_2"

/locus_tag="PIKHDJKN_00759"

/inference="ab initio prediction:Prodigal:002006"

/inference="similar to AA sequence:UniProtKB:O50500"

/codon_start=1

/transl_table=11

/product="Diacetylchitobiose uptake system permease

protein NgcF"

/db_xref="COG:COG1175"

/translation="MNRLRKYDTLLFFLFILPWIIGFITFFLFPFGTSVFYAFTDARL

PGATNFNFVGIDNFTHMMDDPIFLKSLMNTMFFVVFGVPIVTAGMLGLAMLLNFNVKG

IALFRTFFYLPTLVPIVATVIIWRLVFNSEFGILNAGLGALGIGKVDWIGGEHTIKPV

IIMLQVWISGSGVLIFLAALKNVPRHLYEAAAIDGASGIRRFFQITLPMISPSILFVI

IIQTMYNFQMFTEALLLSKGGPNYSAYTFVYNIYKSAFTDLKFSMAMTQSIFLFAVIS

LVTLILMKVSNRFVYYEGER"

CDS 843507..844340

/gene="araQ_4"

/locus_tag="PIKHDJKN_00760"

/inference="ab initio prediction:Prodigal:002006"

/inference="similar to AA sequence:UniProtKB:P94530"

/codon_start=1

/transl_table=11

/product="L-arabinose transport system permease protein

AraQ"

/db_xref="COG:COG0395"

/translation="MNTRKIAVKASQYTMLIVALMLFAGPLVWMLSTMLKTKAETYKV

PPTILPDTFSLEAFERLFAVQPMMWQWIQNSFIISFFVAAGAVVSSSLVAYGFSRFAT

KYRNILFPVVLVTLMIPPSIMMIPSYVLFSKLNWIDTWLPLIVPAWLGGAYYIFLFRQ

FFMTIPQELDEATYLDGGNRWTVYARVIMPLSKPIIMTTIIFAFVNSWLDFLGPFLYI

KNPEMFTLSVGLQLLIGQTSQDLPALAAGAFISIVPIGLLYLFAQRYIVEGVVLTGTK

G"

CDS 844411..845754

/gene="yesO_2"

/locus_tag="PIKHDJKN_00761"

/inference="ab initio prediction:Prodigal:002006"

/inference="similar to AA sequence:UniProtKB:O31518"

/codon_start=1

/transl_table=11

/product="Putative ABC transporter substrate-binding

protein YesO"

/db_xref="COG:COG1653"

/translation="MRAKSKRLFNVWALVLATMVVISGCGNGGSSENADSSGSGGSQS

EPVTITYSQWGTAEELHRTQELLDQFMKANTDIKVKLEGKDWGSYWDGLTANAAGGTL

PDVFKTSYAYVEKYAELGIFKELDGLLAENQFDLNNVDKSLLGLHQYQGKQVSLPIDA

NVIVWYYNKAIFENEATNPHKAPVPSLEPTWDEITDIATKLTLDKNGKSADEAGFDAG

NIVQWGLSISPGSTMDWFLEPELWSNGAKLVNDDGSLALETPEAMEVLNYFIDLTKNK

KINTTPAQIEGLGGQVNLAITTSKVAMNPGGSWNTTNYQEAGVDYGISYLPKFKTNQT

VVQPAGLAISSNTKHEEAAYKLLAWLAGPEGQTELAKQGYSIPANKAAADAYIATVGE

DNKIFLDAQQYGIVSPFTTKKTDLVWTYGEQSLKLPLAGDGDLNAALKDLASKMQ"

CDS 846007..847035

/gene="melR_1"

/locus_tag="PIKHDJKN_00762"

/inference="ab initio prediction:Prodigal:002006"

/inference="similar to AA sequence:UniProtKB:O34829"

/codon_start=1

/transl_table=11

/product="HTH-type transcriptional repressor MelR"

/db_xref="COG:COG1609"

/translation="MAKLKDIADRVGVSISTVSRVMKNDVNRSVSDETRKKIWDTAEE

LGYRSQRPAKKKKVQPKTAYAIGCVVAIPQNKYNSPYFSVIMEGIEKQLAEAGMRLEF

VYSIENDGDIVQLQSLVKEHQIDGMIVVERIDPEAYRWLKTNVRTVVGVDITDPDIPV

VGYDREEAAREATNHLIAQGHRIIAYIGGAEFKNDFLEEKRFLGYRRAMTDAGLPIDN

DWVIDVKWDVSLSYELTKQAFTNNANRPTAIFAASDMMAIAAMRAATEQGLRIPEDIA

FFGVDNIEISEFTSPPLSTIHVPKLEMGMFAVKQLLDYLDHQYEVAVKMIVPYRCIFR

QSSNGKIE"

CDS complement(847095..847511)

/locus_tag="PIKHDJKN_00763"

/inference="ab initio prediction:Prodigal:002006"

/codon_start=1

/transl_table=11

/product="hypothetical protein"

/translation="MDDQKNVLDNGPFFHGTKAELKIGNLLEPLYLSNYQDKKSNHIY

FTATLNAAKWGAELAKTNAKERIYIVEPLGDFENDPNLTDKKFPGNPTRSYRSKSPLK

IVAELASWERHSGEEINHMLTSLKKLSEEGKNVIYD"

CDS complement(847755..848660)

/gene="rhaR_4"

/locus_tag="PIKHDJKN_00764"

/inference="ab initio prediction:Prodigal:002006"

/inference="protein motif:HAMAP:MF_01533"

/codon_start=1

/transl_table=11

/product="HTH-type transcriptional activator RhaR"

/translation="MSFQQAVYPLQRTVTYKEWSPNVHYAQFQSLPPGKLAKRRLYDF

ELLYVSQGEAATHMNDKQHNLKAGQLILLPAGVYHQNEVVSSPEARFIGIHFDFFNEL

TILTEADMVVNEETVLHHKFAVEACVEGMTPLSINPVYTPSPATVQLMEQLVHEFTMR

PPGYELVCKALMLQISTHLLRSSWRSSSRHDSVHGEKLHELMKRIEAAPSDPWTNSSI

AKQLNLSVDHMAKLFKQIAGMPPNEYIQSMRLSEARKLLRETDHSIEAVGAQSGYPDI

HYFSRMFRKYEGISPREYRKLSRML"

CDS 848847..849488

/locus_tag="PIKHDJKN_00765"

/inference="ab initio prediction:Prodigal:002006"

/codon_start=1

/transl_table=11

/product="hypothetical protein"

/translation="MRSLPVERSPSYCCEPLRSVAQSGFPSTADWERCVPVELVDTVT

GRPPHQSTEVRFGWSREYLHIRFVCQDDHVVSDFTERDQPLYEQDVVELFIDEQGDGR

NYIELEVSPHNVIFDALIHNNSEGSELKADVTWQLEGLQTAVEVDRQGNRVYLIHIPA

SNFKSSLAKGVRWRVNVYRIDENVQGDREYQAWQPTGAVNFHLPARFGYFQLG"

CDS 849539..850387

/gene="iolO"

/locus_tag="PIKHDJKN_00766"

/EC_number="5.1.3.-"

/inference="ab initio prediction:Prodigal:002006"

/inference="similar to AA sequence:UniProtKB:Q9WYP7"

/codon_start=1

/transl_table=11

/product="5-keto-L-gluconate epimerase"

/db_xref="COG:COG1082"

/translation="MKKGINIWSFPGDASIADCIQTAKQAGFEGIELSLNGEGELSLS

ATDQEVRDIQGRLEEAGLEIAGLATGLYWDYPMTSARPEIRTKALDVCKKQLELAAAF

GVDTILVIPGAVGVDFIPDSEVTDYEVAYERAQEALAHLLPYAKSAGVSIGIENVWNK

FLVSPLELRTFIDSFSSDYIGSYFDVGNVVQNGYPEQWVRILGHRIKKVHFKDYRRQA

GGLHGFVDLLAGDVNYPAVMQALQAIGYDNYVTAEMIPPYTHHSKQIIFNTSKSMDAI

LGRSQE"

CDS 850446..851456

/gene="iolG_4"

/locus_tag="PIKHDJKN_00767"

/EC_number="1.1.1.369"

/inference="ab initio prediction:Prodigal:002006"

/inference="protein motif:HAMAP:MF_01671"

/codon_start=1

/transl_table=11

/product="Inositol 2-dehydrogenase/D-chiro-inositol

3-dehydrogenase"

/translation="MLKVGLIGFGFMGRMHFDNYVRLASEGEPVELVAICDLRIEELK

NGKAAGNMATEQEVYDLSPYRLYDNIDAMLEQEELDIIDITLPTPLHAELTCSMLEKG

YHVLCEKPVARHSAEGWKMAQTAKATGKTLMIGQCLRFWPAYAYLKSVVEDGRYGAVN

AGYFFRGSGLPQEWFLDGEKSGGCILDMHIHDADIIHWLFGKPDQVSTLARNVIPGSG

YDTVSTNYVYPDGKVLNAQADWTLGGDYGFSMTYRVNLEQGNLVFENGELKVNPNNAP

GFVAELSPDSGYYHQLKYYIQSVQAGMPVSVCTPESATGTLELIEAEMRSADERGALV

SL"

CDS complement(851552..853198)

/gene="yfkN_2"

/locus_tag="PIKHDJKN_00768"

/inference="ab initio prediction:Prodigal:002006"

/inference="similar to AA sequence:UniProtKB:O34313"

/codon_start=1

/transl_table=11

/product="Trifunctional nucleotide phosphoesterase protein

YfkN"

/db_xref="COG:COG0737"

/translation="MTSITTTSSFDIMVTSDLHGAIRPIHYNTNAYRPAGLALLASLI

RRERKRSPELLLVDNGDLLQGSPLASYAASFVSDHEVHPFINVLNELGYDAAVMGNHE

FNYGQDLLRKAVEDSHFPWLSANIVNGGKHDDEVPHTAPTGAAIPAFGPPYLIKTLSS

GVKIALLGATTHYIPNWEHPKNIEGLLFLDALETIRAWVGYIREHEQPDVLVVSYHGG

FESDLETGEPAERLTGENQAYAICRDIAGIDVLLTGHQHRQLTADIHGVTVIQPGFSG

NGAGHVSVQLERLSNGKWQIAGKQARLLLLDENSDVQPDAAVMKLTDELEAKAQAWLD

QPIGEVAGDLSITDSAALRLAAHPFIAFVHQVQMEATGAQISNTALLSEEARGFGSLI

TVRDVLSNFIYPNTLTVLELRGQDIRDALEQTARYFEVEASGEVVVNPAYMQPKPQHY

NYDMWAGVEYELDISKPVGSRVVKLEREGIPVAMDATYSVVMNSYRAAGGGDYAMYPG

KRVLHEGATDMAALVEDYIRRHQPLTVEQANNWRVVGSCE"

CDS 853451..854434

/gene="ptxB"

/locus_tag="PIKHDJKN_00769"

/inference="ab initio prediction:Prodigal:002006"

/inference="similar to AA sequence:UniProtKB:O69052"

/codon_start=1

/transl_table=11

/product="putative phosphite transport system-binding

protein PtxB"

/translation="MKKSALFLPLLILVLFLSACGSNSSTTGSANGANTSSNSSSADT

AKTDKAAEGYVPTELTVQFVPSQNADTLEAKAKPLEKLLGDKLGIPVKVSVSTDYNTI

IEAMASNKVDVGFLPPTAYVLAKEKGAAEVILQAQRFGVNDETGAPTEQLADSYKSMI

IVKKDSPIQSVEDLKGKKIAYQNVTSSAGYVWPAALLLDRGIDPLKDVQAITVKGHDQ

GVLAVLNGDVDAAAIFQDARNTVAKDYPTVFEDTRVLAFTEPIPNDTITVRTDMNADW

TAKIKQAFIDIGKDPQGHEIIKEIYTHEGYVESDDSKFDIVRQYNEKVKTE"

CDS 854608..855408

/gene="glnQ_2"

/locus_tag="PIKHDJKN_00770"

/inference="ab initio prediction:Prodigal:002006"

/inference="similar to AA sequence:UniProtKB:P27675"

/codon_start=1

/transl_table=11

/product="Glutamine transport ATP-binding protein GlnQ"

/translation="MIELHNVTKTYANGTKGLNNINLKFKQGEFIAVVGLSGAGKSTL
LRSINRLHDISDGEILINGKSITKAHGKSLRMIRRDIGMIFQSFNLVKRSSVLRNVLA
GRVGYHSTMRTILGRFPKEDTELAFGALERVNIAEKAYSRADQLSGGQQQRVAIARVL
AQEAKIILADEPVASLDPLTTKQVMDDLKRINQDLGITTIVNLHFIDLAREYATRIVG
LRAGEVVFDGPVSEATDERFAEIYGRPILADELLDKQAVQEQRGEVLV"

CDS 855405..856268

/gene="phnE_1"

/locus_tag="PIKHDJKN_00771"

/inference="ab initio prediction:Prodigal:002006"

/inference="similar to AA sequence:UniProtKB:A0QQ68"

/codon_start=1

/transl_table=11

/product="Phosphate-import permease protein PhnE"

/db_xref="COG:COG3639"

/translation="MKQPVNVSMDGSLQNESGKGKRPGTGSGSVVHRPKPPGRTKHLL
TLVIILLLLWASARQTDASFSELFEGLPNMLDLLREMFPPRWSYFDNIVQGMLETIRM
ALIGTTIGAIIAIPVSIICAGNLMPSRWIYYPARFVLNLIRTVPDLLLAALFVAVFGL
GPIPGILALAVFSVGLIAKLTYETLETIDQGPLEAMTAVGMNRIQLIIYGVVPQLAAQ
FTSYVLYAFEINVRAAAILGLVGAGGIGLYYEATLGFLEYDKTSVIILFTLVIVLIID
YVSTKLREKLL"

CDS 856265..857071

/gene="phnE_2"

/locus_tag="PIKHDJKN_00772"

/inference="ab initio prediction:Prodigal:002006"

/inference="similar to AA sequence:UniProtKB:A0QQ68"

/codon_start=1

/transl_table=11

/product="Phosphate-import permease protein PhnE"

/db_xref="COG:COG3639"

/translation="MMKNETSVIRRKPRKNPLRWVIVILLILVYAWALAGVPFTGLKE
TAGQIMKAIVAGIFSPDWDFVYLPEGEDLLRGLLDTLAISVLGTVISAVLCIPFAFWS
ARNMSSFRPVSGTGKMVLSFIRTFPEIIMALLFIKAVGPGSFAGVLALGLHSIGMLGK

LFADEVENIDYGPSEALLASGATRMQQLWFAVLPQVLPGFLNYTLYRFEINVRSATIL
GVIGAGGIGTPLIFALSTRNWPRVGIILLGIIVMITIIDLISGYIRKKLV"

CDS 857662..857949

/locus_tag="PIKHDJKN_00773"

/inference="ab initio prediction:Prodigal:002006"

/codon_start=1

/transl_table=11

/product="hypothetical protein"

/translation="MILRIVFLIVVGILSVCGGVIFLNYSIVLMLTAGEGFIIFDGPA
KGKTILNMSLTLLPFVFAVYFLNRKKQYVIMNTIIYPFILSFFRGFIWLVS"

CDS complement(858218..861028)

/locus_tag="PIKHDJKN_00774"

/inference="ab initio prediction:Prodigal:002006"

/codon_start=1

/transl_table=11

/product="hypothetical protein"

/translation="MGKLTRMRGVWVKSLLALSMALPLQIGLWNGDATVQAEWATDPA
PFIQAKVVNGNAGKKVLFDNTHAQTAGAADWVIDGGFSDFGNALANDGYDVKELRKTT
PFTYDDLKEYNVFVIAEPNVPFKTTEQAALKEYVEKGGSIFFIGDHYNADRNKNRWDG
SEAINGYRRGAFDDPAKGMSTEERNSEAMQNVTSTDWLSDNFGVRFRYNALGDINATN
IVPAGQAFGITEGVSAVAMHAGSTLAITDPTKAKGIVYLPKTNAAWPNAVDQGVYNGG
GVEEGPYVAVSKLGAGKAAFIGDSSPVEDASPKYLREETGARKTTYDGFKEVDDGKLL
VNTVNWLANQENYTSLTQVNGLQLDNATALLPFEQPAASTEPQAEPWAAPAAGYKWYD
RSTFKAGSYGGPASSANASYSFVKQETLPNAEDFQIRVVVENMPANTTVSGYSAGIYL
TGGTQVAMIQNEGGTWPTSYGYSSNFSVTSDSKGRAIKDLNVRIKSGTTGAASLRLRL
NGSNLITTAVTVGNVPAEQLPEEEGPIPATISIADARTKAAGSTVTVEGVVTTEPGAF
GGQSFYLQDTTGGLYVFQSTGGFHAGDVVKVTAATALYNSEFELTDIVAIEKTGTASI
PAAIEVPAVTAANQGQLVQLKEVTVQNIISATPIGSFEFDAVAADGTSTHVRVDTRTG
VTQTIFPYAAGDKISVTGVAAIFKDVFQLKPRSLNDFVKVEEGGEEGPSTPAVGAPGK
PALTSNNGYSNGLSEGSYTVTMNLWWGDNGTSYKLYEDGVLIDTQKLTDATPAAQSAQ
TTINGKANGTYTYVAELTNSKGTTRSEVLTVQVANAAPGKAVLSQDNWDGDGNYKVTM
NLWWGTNASEYRLYENGQLIDTKALNAVTPNAQSAVTDVSGHANGTYTYRAELINAAG

VTSTETITVQVTKSAVSLPAAS"

CDS complement(861234..861710)

/locus_tag="PIKHDJKN_00775"

/inference="ab initio prediction:Prodigal:002006"

/codon_start=1

/transl_table=11

/product="hypothetical protein"

/translation="MNNTTIHDAYVNYKSEVTRYLSQIVKNPQDAEDLTQECFIRMMN

VSADIPEERLLYYLKRIARNLAMDTFRKRTRTLKRDSKLEMQTYHCDTSELEISEGVN

DLVSIINNTEHRKILELRVIHGYSIKETAQLVNRSEGMIKSSVFHAVNRIRAKVIS"

CDS 861918..862631

/gene="regX3_1"

/locus_tag="PIKHDJKN_00776"

/inference="ab initio prediction:Prodigal:002006"

/inference="similar to AA sequence:UniProtKB:Q9F868"

/codon_start=1

/transl_table=11

/product="Sensory transduction protein regX3"

/db_xref="COG:COG0745"

/translation="MRPKQILIIEDEESIRDILSYSLRKEGFEMTEAATGREGLDLLR

ESRPDLILLDLMLPDMSGFDVCRQLSVNSKIPVIMITAKSDMLDKVLGMELGADDYIT

KPFDIREVVVRIRAIFRRIDLISETLENQSYEVVRLGRHIEIRKDEREVWKDGERAGL

TNKEYDLLLYLVNHHRKVHTRSELLDKVWGFDFPGDTRTVDIHIQRIRKKLDSGEQGI

SLIETVFGVGYKLNIQVHT"

CDS 862628..864091

/gene="sasA_6"

/locus_tag="PIKHDJKN_00777"

/EC_number="2.7.-.-"

/inference="ab initio prediction:Prodigal:002006"

/inference="protein motif:HAMAP:MF_01837"

/codon_start=1

/transl_table=11

/product="Adaptive-response sensory-kinase SasA"

/translation="MKWTIQFKMVVLFSVIVFIGFSALLIISNKVGEENMYREVQEDM

VQSKKNLDIALNQYFLIHNKRISEDSLGTGNRELAEQIGSAVGGKVMVYRSDGTPFST

VGAEGKLGAAQTSDLQAAMKSKIAYTTVVDKGRVTGNLSFPVLSEGSILGIIQLERDY

TDLFKRHLRFQNTIKGFAAVIFVFVFIGSVFISRQITKPIRVLTKRSAEVAQGSLNAD

IHISTKDEIGELASSFTVMIARVKEQIDVIERERDEVKHLQARNKVFFDNVTHELKTP

LTTILGYAQILRDNGFTDQAFFDKGLKYIINESQRLNVMVADILEVTVSSTPIQTYRF

ERVNVSNIIREACEDMSIKAGKYNIGIRYELEEMLYVQGDWNKLKEVFLNVLDNSVKY

GNVNSIIQVQSFRLGDSVAILIRDEGEGIPEEALKHVFEPFYRDNGMNRTEKGSAGLG

LSIVKNTVEQHGGTVEMKSILNKGTQVNISLPGESVV"

CDS 864088..865245

/locus_tag="PIKHDJKN_00778"

/inference="ab initio prediction:Prodigal:002006"

/codon_start=1

/transl_table=11

/product="hypothetical protein"

/translation="MKKRGFWSRHRDMLMLSGLLIVFVGGIFGIGSLFSIGHGKPRTV

ILPDKQFNSRLAPPPSSTIQIESATKLPNDFAIYDFEVADPNTVILNRPDRSYTGIKL

SQLHMDDNLLKDIATNTEYGITLSPDQSKLIYSQYRSTQAQKTTYEYDLKTGEHHKLK

NDNSYSRVFVGNESYIGYDDLVFNMVELNTGKKHELYSYDELVAKVAQTSNISSPDDT

LIMLDSYEISRDLSRVYVLVKLKENYAVYSFSLNDKNDITAYTPAQDIQQFKVLKNGD

MLIQGMINNEQGLYRYRADTKKFELLVKGPIWSFDLDEEESRIAYFLTLEGQKNEMHI

AYLNDRKLGSDTVIYRNIDYFIKLKWNDSNLFVVGSSMEKSELYRFSFRAW"

CDS 865297..866187

/gene="btuD_4"

/locus_tag="PIKHDJKN_00779"

/EC_number="7.6.2.8"

/inference="ab initio prediction:Prodigal:002006"

/inference="protein motif:HAMAP:MF_01005"

/codon_start=1

/transl_table=11

/product="Vitamin B12 import ATP-binding protein BtuD"

/translation="MLRTEPIGPFCDLNMLHKSKEGLSILKQLKSDFTNWIQVQIGPI
HLMAIITSITSMREEAGFMLRVEQVSHTFRNAQGTVPVLQDINISVGKGEMVALLGSS
GSGKSTLLHLMAGLMKPDTGKILIADQDIVRMSENRLAEFRRTHIGFIFQAYELLANL
TIRENVELPLVFQGIRPSRRKQKAMHLLEQVGLVGKSELFPSQLSGGQQQRVSIARSL
ITEPSVIFADEPTGNLDTRTEEEIISILQQLNREMHTTFVIVTHEAEVAEQMQVVITL
QDGCLVESGQSKAVEQGERL"

CDS 866184..867548

/gene="ytrF"

/locus_tag="PIKHDJKN_00780"

/inference="ab initio prediction:Prodigal:002006"

/inference="similar to AA sequence:UniProtKB:O35005"

/codon_start=1

/transl_table=11

/product="ABC transporter permease YtrF"

/db_xref="COG:COG0577"

/translation="MRVLDVLRMSWGQIVRRKVVTLLCMIGLSIGCAAMIIALSVGQS
VQVYGEKTLNENYKMDEITVSPNEGIGSGGSNGQTSTFERGALTARKIDIIASLPHVV
AVAPMLKLDMLEMATLDGKSSNVEVIGTELGSLSRFGYRFDKGGASGISGAAIVNYGA
TFGLVDAEVIRELNNKLMEDSFNDEFLQQYMALMSTQTELFQAQFNLKYQDMNDSSKV
KTSSPLRVTGVLQLPGGMSENNAMYDKKVYVSLETARMLAEQFQSSQAQAAAAGRLNS
ALVKVEDKKYVAQVQEQIQKLTLMTDSNLYQEQAMNEQLGMYQKAAMGIGIFVMLLAS
LSIIVAMIMSTHQRRKQIGVMKVLGANLWQIRQMFITEAAVLGVLGGIVGVFIAYGAI
QGVNGLLQEQISMEASTPLQVVIETSALPAGIAFAVLTGVLSGIYPAISASRTNALQV
IKSA"

CDS 867669..868718

/locus_tag="PIKHDJKN_00781"

/inference="ab initio prediction:Prodigal:002006"

/codon_start=1

/transl_table=11

/product="hypothetical protein"

/translation="MIGLLAGAGYFLYQQYKPVPEAPIEAPQPITFEVTQETITQEIQ
VKGKSAYAKQTDVYAPFASKIKQWNVENGESIHKGAVMFSLDSKALQTEVQQLETDIE

KSRLEAQLNEIVLTQGEGSEQLGVTEEERKKAFAERETKRLTNEMNKESIAVKEQEIQ

AKKAVIGLSTVYAPVSGVFLMNDTETKTRMVSEGQYVGTIVDTGKLQFVAAISEQELF

KIQTGMPVSVHMTGNKEVPLTGKVSKISKFAKKGTETDLKQPSQFDIVIDLKPNAKLI

GGLSLEGKIETLRKEKATVVPTLAVMRDQDMAYVMLDQGNGQYAQQTIQTGIETDDKT

EVLNGLKPGDVVVLP"

CDS 869267..871699

/locus_tag="PIKHDJKN_00782"

/inference="ab initio prediction:Prodigal:002006"

/codon_start=1

/transl_table=11

/product="hypothetical protein"

/translation="MTKSKTLSRGGKVLHRSVKQMLAATLLAAGIFPGMAPGVSQAAE

AHVDNPFVGATAYLNQDYSALVDTSIALTSDASLKAKMETVKSYPTAVWIDRIAAING

GADNAGRKSVEQHLDAALAQKKAGTPITASFVIYNLPGRDCHAFASNGELPLTQAALQ

TYENDYINVIADIFAEPKYQDIRIIAIIEPDSLPNLVTNLSTPACAQASSTGIYEAGV

KYALEKLHAIPNVYNYLDIGHSGWLGWDNNRTGAISLYTNVVQGTAAGLNSADGFITN

TANSTPLNEPNLPNPDLNIGGQPIKSSKFYEWNPYFDETDFTAALYSGFVQAGWPAST

GFLIDTSRNGWGGVNRPTSATGSDINTYVNSGRVDKRDHRGNWCNNSGAGIGEAPKAA

PGPAHLDAYVWVKPPGESDGSSSEIPNNEGKGFDRMCDPTFTTRDGVLTGALPNAPVS

GHWFHDQFVMLVQNSFPVLPASNGGGGETTAPAAPATLSATAGNAQVSLSWTASTGAT

SYNVKRALSASGPFTTVAAGVTSTSYANTGLINGTTYYYVVSAVNAAGESANSAVKSA

VPVAGVTAPAAPTALTATAGNAQISLSWAASAGATGYNVKRALSASGPFTTVAAGVSG

TSYTNTGLTNGTTYHYVVSAVNAAGESVNSAAASATPQSVVVPTSDLVLQYRAGDTNA

ADNQIKPYFNIKNVGNTAVNLSDLKIRYYFSKEGTAAMDSAIDWAQVGGANIQRTFTD

SYVELSFTSEAGAIQAGGQSGDIQLRMYKMDWSNFDETNDYSYDPTKTSYQDWNKVTL

YKGGTLVWGIEP"

CDS 872030..872164

/locus_tag="PIKHDJKN_00783"

/inference="ab initio prediction:Prodigal:002006"

/codon_start=1

/transl_table=11

/product="hypothetical protein"

/translation="MSIIIPDGLDEAALMEMEWKGDGVEEVCSQGLFFYNFVSVFSKI

"

CDS 872259..874676

/locus_tag="PIKHDJKN_00784"

/inference="ab initio prediction:Prodigal:002006"

/codon_start=1

/transl_table=11

/product="hypothetical protein"

/translation="MKSKITAPIHKGSVSGMHSNRRKVRMPLAAKMLSSALSVALLVA

GTAGASGAAASSENSAAEASVQSHKGGSSLKDIQLEYLDRGLVAASTSEGVFLSWRLL

GDEATGYSDKGLTGTDFNVYRDGRKIATVTDSTNYVDASGKSSSRYEVAAVSKKGKES

KRSASVKPWTNGYVDIPLQKPADGVTPVGEAYSYSANDMSVGDVDGDGQYEFFVKWDP

SNSKDVSQKGYTGKTYVDCYTLDGQLLYRIDLGVNIRSGAHYTQMLVYDFDGDGKAEM

MFKTAPGTKIIKYNKKGKVTSEKYITLPKQDRKAGYSNEDDYRLSADGYYDHVVDMFK

NWHKHEEVVKGNWPATLEEAFGIEKKYSYPLSQKDAESLADYFIDVYAPSRSDKNELR

KFEGFIVDGPEYVTVFEGESGKELETIPYEPGRQDDGLMWGDYAMARIEPANRVDRFL

AGVAYLDGKKPSAIFARGYYTRSTMVAYNWDGKKLKREWKVDSGWTPMKNPFNDGPHG

VDGTDPVYGSITTQGAHYFSVADVDGDGKQEIVYGSATIDHDGSVLYSSKDLMPAESA

APGTIARLGHGDALHVADIDPDRPGLEIFMVHEGGPWAPYGYSLRDAKTGEVIYGGYT

GKDTGRGMIGDVDPTRRGLETWAVGLWTASGEKISDKAPGTNMNIRWAADMTTQIVDG

AIDVTPTIKDWNRGRLLTANGTLTNNYTKGTPSLVADIFGDWREEMLVRTDDSSAIRI

YLSTEKTDRKLYTLMHDAMYRVGIAGQNSGYNQPSYPSFYMASDIDWSKVTLPKFHTP

KSGMGGK"

CDS complement(874934..875536)

/locus_tag="PIKHDJKN_00785"

/inference="ab initio prediction:Prodigal:002006"

/codon_start=1

/transl_table=11

/product="hypothetical protein"

/translation="MKTTFYLVRHALKEKRIGDVPITSEGVAQAKSTALHLVNVSVTK

IIASPLQRAIDTAAHIAHHKNVTVTEDPRLRERANWGDLPEQTFEEFIAMWDRSTSDP

DYLPPVGDSSKQAGERLASFLTELTNVELLDSHIVIVTHGGLITDFLVNTFPEHELNV

WHPDFIAMQSQLIPECSITTLIHENGKYTIQDFASVAHLK"

CDS 875720..876730

/gene="melR_2"

/locus_tag="PIKHDJKN_00786"

/inference="ab initio prediction:Prodigal:002006"

/inference="similar to AA sequence:UniProtKB:P0ACH8"

/codon_start=1

/transl_table=11

/product="Melibiose operon regulatory protein"

/translation="MEAKHSTGSLFQEKLRLEDYGPRFYAYYYKQWDNYQMSYHDHDS

TEIMYIISGMCRVDVQMQDGSSEQAVLKKGQFILLDAGVPHRLLVKDGVPCRMLNVEF

GVASSEPGHPSIRQLALEEEEVHAFLTQSSPYLVLPDPEEVYHIMKSLVLELDQRGLM

EQGRSSAPMRIIPPEERSHHRESRDLKSPEQGTLVRTLFIQLLVRVARLRGEINRSAL

DQTELYVKRTIEFMHHNMDRNIQMKDIAAAVNLHPGYLHRIFRQHTKRTPTDYLTMLR

MEKAKMLLQQTDIPISEISDYVGVGSRQYFHMLFKKYTGRTPVEFRSSMERHVSHYPA

EE"

CDS 876911..878416

/gene="melA_1"

/locus_tag="PIKHDJKN_00787"

/EC_number="3.2.1.22"

/inference="ab initio prediction:Prodigal:002006"

/inference="similar to AA sequence:UniProtKB:O34645"

/codon_start=1

/transl_table=11

/product="Alpha-galactosidase"

/db_xref="COG:COG1486"

/translation="MSFKVAFIGAGSIGFTRGLLRDLLAVPEFNNIEIAFCDINQHNL

DMVTELCQRDIRENGLNIQIQPTTDRREALKDAKYVLCTIRVGGLEAFATDVDIPLKY

GVDQCVGDTLCAGGIMYGQRGIAEMLDICKDIREQSAPDVLLLNYSNPMAMLTWACNK

YGGVRTIGLCHGVQHGHHQIAEAFGLKMNEVDIVCAGINHQTWYIKASHEGKDLTGDL

LEAFEKHPEYSRTEKVRIDMLRRFGYYSTESNGHLSEYVPWYRKRPEEIKEWIDLGNW

INGETGGYLRVCTEGRNWFETDFPNWMKDEPMQFAPEKRGGEHGSYIIEGLETGRVYR

GHFNTVNNGVISNLPDDAIIEAPGYVDRNGISMPHVGDLPLGPAAVCNVSISVQRLAV

EAAVHGDDKLLRQAFMMDPLVGAVCNPKEIWQMVDEMLVAQAEWLPQYGDAIATAEAR

LAAGNLIPTKEYEGAARLKVKTVEEMQQDRDAANKNAGESDKGKDREKVQQ"

CDS complement(878465..879103)

/locus_tag="PIKHDJKN_00788"

/inference="ab initio prediction:Prodigal:002006"

/codon_start=1

/transl_table=11

/product="hypothetical protein"

/translation="MHHNGQLSSLTLRLEDIREVDKIKFFITMRTLTSLVFLTTLLIL

SGCNPTPSNSTHGIASPPTTNAKEAEMGSTHAQEPELNLSQELPTPTIQSESGISIPA

IQGSYCWGNMCVDYAGDMELLEGKVPVSVLVGENISINLGTDVPPDEFTLVEYVNGEA

RPISLREGSFQFAQEKGTHYYGAFVRWSSPKNSQVSLGDTSFAFVIRGVEKK"

CDS 879193..879552

/locus_tag="PIKHDJKN_00789"

/inference="ab initio prediction:Prodigal:002006"

/codon_start=1

/transl_table=11

/product="hypothetical protein"

/translation="MDEQAINSDLIRGNIDPIILSVLIPKDNYGYSIIKEIYRKSGER

FELKEPTLYSSLKRLEKSGYVESYWGEETQGGRRKYYRITTQGQEVYTQHVQAWHAAK

TLIDCMIISGEKGDERI"

CDS 879549..880241

/locus_tag="PIKHDJKN_00790"

/inference="ab initio prediction:Prodigal:002006"

/codon_start=1

/transl_table=11

/product="hypothetical protein"

/translation="MTLEARITRHVNRLFDHAQDTLTNRELKEEIHSNLAARIEDYME

QGMDKEQAFQTAIQHVAGMDHIMSDHRRVQRVPYWTAVLQSALIYSLIAWIITIPMRV

MVQGATINNLLMLMSVIVGGAYVFYMLKNKNTSADPEDITVIRIPVFLQWTRRLWWLW

AGLIVVLWGTQAALRFGSNIWYSRPIQVEGPYQFTVIAIAFAIPLLSIIIPLIVQRAY

RIVGKYDVGDVI"

CDS 880238..880795

/locus_tag="PIKHDJKN_00791"

/inference="ab initio prediction:Prodigal:002006"

/codon_start=1

/transl_table=11

/product="hypothetical protein"

/translation="MTGKNKWIIALVMLGIFGVIVVEGVVNPRIEAKQAQYEAEQQDP

LTHDFAALAGYRSAYMGDFSNLSHLNQALPLSEQLNGYQLYPETLTAQINYRMNTSDM

KPEELERILVYNAAANFVWIDNLEHVLYTFENIQYTLSREAAQQWVGTELKTLQEPGE

WDSAVRKKLVEPMQVKEAFSQIVDN"

CDS 880927..881697

/gene="fliY_1"

/locus_tag="PIKHDJKN_00792"

/inference="ab initio prediction:Prodigal:002006"

/inference="similar to AA sequence:UniProtKB:P0AEM9"

/codon_start=1

/transl_table=11

/product="L-cystine-binding protein FliY"

/db_xref="COG:COG0834"

/translation="MRKQAIFLLLISICMIVVAGCSSSGSKNDNAIIVGIDDKFAPMG

FRDDKNEIVGFDIDYARAAAEKMGKEITFQPIDWSSKESELNSGRIDLIWNGYTITDE

RKDKVLFTKPYLENSQVAVTLADSPITKLNELDGKNVGLQALSSAADALAASPLKDKV

NASEFPDNVLALTDLKTKRLDAVIIDEVVARYYMSKEEGTFKLLDESLAPEQYGIGVK

KGNEELLNQLQKALDELNADGTSAEISTKWFGEDEVLK"

CDS 881807..882466

/gene="artQ"

/locus_tag="PIKHDJKN_00793"

/inference="ab initio prediction:Prodigal:002006"

/inference="similar to AA sequence:UniProtKB:P54536"

/codon_start=1

/transl_table=11

/product="Arginine transport system permease protein ArtQ"

/db_xref="COG:COG0765"

/translation="MSWEYLLGVIKPMLEGAQTTIFLFLVAIIVSVPLGFGVTLLMKS

RFKPLAWIAHTYVYVMRGTPLMLQLLFFCFGLPLLPGVGEYLVFDRFTAATLAFILNY

GAYFAEIFRGGLLSIDKGQHEAAQVLGLSKWQTMTKVIIPQMFRVALPATANESISLI

KDTALLYAVAVPELLHYAQAAVNRDFRLTPFLIAAIMYLLMTMVLTLFFKALEKRYTF

E"

CDS 882529..883284

/gene="artM_1"

/locus_tag="PIKHDJKN_00794"

/inference="ab initio prediction:Prodigal:002006"

/inference="similar to AA sequence:UniProtKB:P54537"

/codon_start=1

/transl_table=11

/product="Arginine transport ATP-binding protein ArtM"

/db_xref="COG:COG1126"

/translation="MTHIIEVNQLRKSFGTLDVLKQVSFNVEPGEVIAVIGPSGSGKS

TMLRSLIHLEDISGGTIRIQGQALAENGSYAGAADIRKITDRMGMVFQHFNLFPHLTV

QANLELAPKTLKKESSAIIRQRSLELLGKVGLSDKADAYPANLSGGQKQRVAIARALM

MQPDILLFDEPTSALDPELTGEVLRVIKQLAQENMTMMIVTHEMSFARDVADRVFFMD

NGEIAEAGPPEQIFGNAKLERTRTFLQRVEVEG"

CDS 883461..883799

/locus_tag="PIKHDJKN_00795"

/inference="ab initio prediction:Prodigal:002006"

/codon_start=1

/transl_table=11

/product="hypothetical protein"

/translation="MDQRHNDVDRYYSDPLPPPPYVVPKTNSKSIAALVLGILSLTIP

YLGFFIGIVAIIFASLALKELRIRMEQGKGLAIAGLVCGIIGTALYAILIILVLLFTV

VVSTNITSTY"

CDS complement(883927..886116)

/locus_tag="PIKHDJKN_00796"

/inference="ab initio prediction:Prodigal:002006"

/codon_start=1

/transl_table=11

/product="hypothetical protein"

/translation="MMKSFTVFGQDLKSAFKKPKVFIPILVVLFIPVLYSGLFLNAFW

DPYGKMNELPVAVVNTDQGATYNDKSLEVGQNLVNELKKSDDFNWKFVTREQAEQGME

DNTYYMTIVIPEDFSETATTLMDDHPQPAELIYEPNEGYNFLAGQIGGTAVKQIKSKV

SAKVTESYTETLLDQVEKISSGLADAGDGAGQINEGAVKLDEGASKLKENLSKLADGT

DKLETGITPLKEGTNTLAQGIGELHTGASSLSNGLSQLAAAGTKLGDGAAQAEAGGKK

LQAGIQSAQEGAAQLDVGLAATEEGSAKLTAGLQTSVGGSGKVSEGAKAVAQGLAQLA

EASPELAANPAVQQLLAASQAVAAGSEQVYQGGQQLLEGSKTLQAAQQQLHKGSSQLV

QGEQQLLEGATQLSAGQVQLATGLQQFNSKLSEAAAGGAKLSEGSSQLNAGAGKLVDG

LDQLSDGITTIADGSRKLDDGAGDLKEGTAKLTDGSGELATKLNDAADQTGSVKKTDE

LVTMYAEPVQVDEHKHNEVPNYGTGFSPYFLSLGLFVGALIATLVVPTRSSSVTDASG

WNRFVSRTLAFTIMSAVQSLLASWLVLSGLGLEVQSVPLFLLFSFVTSLTFMYIIQAL

VTWLENPGRFLAILMLIFQLTTSAGTFPLELIPNWLKGFNPWLPMTYSVTGYKAVISS

GQFSVAWDQIGILCIFAVIGLAATFAYFMMHRTNEVEEVNSEAVLHV"

CDS complement(886193..886771)

/locus_tag="PIKHDJKN_00797"

/inference="ab initio prediction:Prodigal:002006"

/inference="similar to AA sequence:UniProtKB:P44923"

/codon_start=1

/transl_table=11

/product="putative HTH-type transcriptional regulator"

/db_xref="COG:COG1309"

/translation="MKTIDRRQQVIDSAEKSFALFGYKATTMEQVARLANVGKGTIYT

FFENKEELFGEILHSIIADMKRITEQTVQDDKPFLENVHQSMDALLEYREEHELLIKL

FQEVNEFGTPQAKEGLQKVETAILEYLERQVQRAMELQQIREDDPRLVSFVLLKLYVT

LTSDWNKAHTSLHKDQIKDFVGLFLKSGLSPT"

CDS 887031..887240

/locus_tag="PIKHDJKN_00798"

/inference="ab initio prediction:Prodigal:002006"

/codon_start=1

/transl_table=11

/product="hypothetical protein"

/translation="MPEITIRLYEGRTDEQKQEIVEVFTRELSRIIDREPDYISVEFN

EIPWDENVPDNLKAMQSQKQGGEKT"

CDS 887237..888214

/gene="yteP_4"

/locus_tag="PIKHDJKN_00799"

/inference="ab initio prediction:Prodigal:002006"

/inference="similar to AA sequence:UniProtKB:C0SPB3"

/codon_start=1

/transl_table=11

/product="putative multiple-sugar transport system

permease YteP"

/db_xref="COG:COG4209"

/translation="MKPADIAPPSRGTARLNSSGLGSRLGDAGRYLWRYRILYLLSVP

GILYFFLFKYVPLFGSVIAFQNYNIFKGITGSDWVGLEHFQKMFSHYDFLRILNNTLL

LGLYDLVIAFPVPILLAILLNEVRMIVFKRLLQTIVYMPHFLSWVVISGIFMGIFSMD

AGVVNKALGFLGMQPIYFLGEDSYIRSILIGSGIWRDSGYGTIIFLAAIAGINPDLYE

AAEVDGAGRLKQIWSITLPSLLPTIMILLLLHIGKFLDLGFERVFVFLNPLNLESGEI

LDTYIYKAGLLSQQYSYTTAIGLFKSVVGLMLILIGNFFSKKTTGESLY"

CDS 888227..889105

/gene="araQ_5"

/locus_tag="PIKHDJKN_00800"

/inference="ab initio prediction:Prodigal:002006"

/inference="similar to AA sequence:UniProtKB:P94530"

/codon_start=1

/transl_table=11

/product="L-arabinose transport system permease protein

AraQ"

/db_xref="COG:COG0395"

/translation="MRTPSVRYRIFRIGNLVFLTLLSLTMILPFINVLAQSLSSSEAI

MGGKVSFWPVAFTWINYEYVFGDASFWRAFAVSVGVTLFGTLVNLAATASLAYPVSRP
EYKGRSLVVMFVLVTVVFSAPLIPNFILMKELHLVNNPLVLIVPGAINAFNFFVMRSF
FAQLPGELIDAARIDGCGEFGIIWRIVIPLSKPAMASLGIFYAVGHWNAYSTALYYLN
DPAWWPIQVTLKKLFESDDISVDPGSAVYSTLAHTSPEGIKMATIIIATLPIIIIYPF
LQKHFVKGIMVGSVKS"

CDS 889148..890185

/gene="rssB_2"
/locus_tag="PIKHDJKN_00801"
/inference="ab initio prediction:Prodigal:002006"
/inference="protein motif:HAMAP:MF_00958"
/codon_start=1
/transl_table=11
/product="Regulator of RpoS"
/translation="MFRILITDDEPMIRMGLAKMIKQAGLFDCEIRQAAHGEEALQVV
EAFRPHILFTDIRMPTMDGIELCRRLSEQGSTMRIIVVSGYSDFEYARACMDYGVKRY
LLKPVGRQELHELLLKLLATEEEKPTVSLVPVRELNDWAMRLEEAIWELRQSDVTELL
AAWSGRYPAYSLMPEQTAELFQELLELIVARMNARGNGTMSTSCEINESASSKECFEA
LGNEIHTLMKTIKEKRSGKRKHPVEEAKAYLEKHLRREVSLDEIAAKLGLNPSYFSQL
FKQTTGQTFIQYRIRSKMELAKRMLEQPGNRITDISYEVGYADHPHFTKTFKKITGLT
PSEYRSKLGIE"

CDS 890182..892020

/locus_tag="PIKHDJKN_00802"
/inference="ab initio prediction:Prodigal:002006"
/codon_start=1
/transl_table=11
/product="hypothetical protein"
/translation="MMKRSLSIRLFFHFAVVITLSLSAIGLFTYTYASTEMNDQLADN
IAQTMRNTAYQTDLYLQNYDRATYSILSNGSVKHFLDMNSEDSYAYYEYSRQIKTNVF
PPVFMLYPQIKFLYVIGDNGRVVIDDNQNSAGIPDIDAAQQYKELLAATPANGESTLL
TRSIRSGQSANVITIARRIRGVSSYTPNGVLAMEVNVLELDKIWGELDLGQGGYQYVM
DQNGTVIYTPGDEEAQTAMPSNTVNRLMHMEAGSLEQNTDGTKRLLISELSAYSGWRF
VASVPLSELQRPIATIRSATLWVGAGTLLAALVVAYRIGASQVEPIRVLMNGMRQTEK

GIWNKVEMKERRDEIGVLIRSYNLMVSRLSDMIESVYESELRRQKSEIELQQEALERH

RAEFQALQLQINPHFLYNTLETIKCYAVVQDSEEIAQMVESMAHMLRYSIQTNLEEIT

VANELKHVLAYLSIMKHRMDRELEVEVIIAPDLLLEKMVRLTLQPLVENVLQHAFPRG

MEPGHFIRIDARRLEDRFLVIVQDNGMGMSEERLEKLRDRLELNRLAGEDSDDVYHRG

GIGLMNVHRRIQLVFGEMYGLMIESERGLGTTITMALPADQHSKRI"

CDS 892050..892817

/gene="fabG_1"

/locus_tag="PIKHDJKN_00803"

/EC_number="1.1.1.100"

/inference="ab initio prediction:Prodigal:002006"

/inference="similar to AA sequence:UniProtKB:P99093"

/codon_start=1

/transl_table=11

/product="3-oxoacyl-[acyl-carrier-protein] reductase FabG"

/translation="MNINLQNKIALVTGSSGGIGAAIAGALARCGAKVAVNGLHNMDR

AEEVVTAIRYAGGEAAAFQADVTDTNAIESMVGEITIRFGGPIDLLVNNAGHLVERSP

IETMSEELYSRIMDVNLKSAVFVSKAVIPGMKAAGGGRIINLTSVAAHNGGGPGAAIY

AASKAACIALTKGLAKELAPGGITVNALSPGFIGQTAFHATFTSAEGRTSAVSSIPLG

REGTPDDVAGAALYLCSELGSFITGETIEINGGMYMR"

CDS 892852..895221

/locus_tag="PIKHDJKN_00804"

/inference="ab initio prediction:Prodigal:002006"

/codon_start=1

/transl_table=11

/product="hypothetical protein"

/translation="MEVKRKLAERPLYQPISGPFHVDYAPDEHTILAENPPRFTWMAA

QQEDENAYMLQVSVGSSFQEGETMTFAPLPYNFFTPDRVFEPGDYYWRYALLVNYPVQ

QVGEAEAHVSQGKQEEMSAWSEVRRFTVPAGLPETPIPSRAQRYVAADTSHPRLWLGE

SGLKALADAIASDATYCGWDAFMANSVEPWANREPICEPLPYPENKRVAALWRQMYID

CQETLYAIRHLSIAGRVLRDERLLGAAKTWLLHVAAWDTEGTTSRDYNDEAAFRVAAA

LAWGYDWMHDELNSEEQDVVRRSLLRRTEQVAQHVMVRSKIHHVPYDSHAVRSLSSVL

VPCCMALLHEEQQAAEWLDYAIDYYACLYSPWGGSDGGWAEGPMYWTTGMAYVTEAMN

LLRNYAGIDFFRRPFFQRTGDFPLYVYPPDARRASFGDQSTLGDPVNLKTGYLVRQLA
GVTGNHWYQWYFERVRQSDPGTEGAFYNYGWWDFDFDDLVYRHDYPQVEEESPVDIEP
LKWFRDVGWVAMHHRMDDPDEHVMLLLKSSRYGSISHSHADQNSFTLHAFGEPLAADT
GYYIAHGSSFHREWRRQTRSKNNLLIGGTGQYAENNKVLNMAATGQIEEAYWRDGDGY
VRAVATDAYASTVPHVKRVVREIHFLQSSYFVIVDHIDLEKPDSVQWLFHALHPLQLK
GQSFRLNGNKAGLEGTFVYASSGELVLSQTDQFAEVDPAEYEGLERHHHLSAETRPAS
SHRIVTLLVPYKIEEPKYVPYFIDDQDHGIHLYFTDNGVTKKIEVSKTY"

CDS 895386..896936

/gene="lipO_2"

/locus_tag="PIKHDJKN_00805"

/inference="ab initio prediction:Prodigal:002006"

/inference="similar to AA sequence:UniProtKB:P37966"

/codon_start=1

/transl_table=11

/product="Lipoprotein LipO"

/db_xref="COG:COG1653"

/translation="MKKWMVSGMALLLAAAVMTGCNKGSGAASGEGGGDGKTKFSISL
RTLAYTYVEKSPDINKDQWVKKLEDLTNTDLKIVLVPHKEYEQKMVQMFATNDIPDVV
QGDGGVNGKEMAGSVEAGVFQPLDELLQQYGQDLLKAVPKEAWDQVTHDGHIYAIPEY
LSNPSRRATWIRKDLLDQTGLPVPTTVEETMDVLRAFKKLGVENPYMGREDFKYADTF
FGAYDVQQFLSMMEQQGDQVVPKFMDNENMQQALTVYKTMYEEGLINKEFATINSTVF
KNTILSGKAGMWSMNANELIQWEKQIKASVPDAKIEIIPSPVGPDGKGGYYLYGPVTR
AYFINKDAADPASIIRFFNWMVSDEAEKFFTYGTEGETYTEDNGEISYTAPTDSAGVD
EERYRQSFLWFVQDTTYNKGSLSLTEEGRKLMNIYDTILAKEGRDGINFDPRLEAFVQ
NPDIAPNSDTPPQVLLTHMIKMVYGKEPISDWPKVVEEWKSKGGDQAIKEATEKFKKG
EGVSAPRR"

CDS 896994..901319

/gene="aes_1"

/locus_tag="PIKHDJKN_00806"

/EC_number="3.1.1.-"

/inference="ab initio prediction:Prodigal:002006"

/inference="protein motif:HAMAP:MF_01958"

/codon_start=1

/transl_table=11

/product="Acetyl esterase"

/translation="MNDVFKTWKKRVVLVAMSVILISGITVPVHEVRAMEDAVPHSIL

NTTQEENIPDPEGPELEIYAENFDDPDNFGSTGGIALRAPWLQEGGGGSKAKTSSSTT

APSLPNMIKIDGNDALALPLDLTGYGNIRLSYYTRASSYISGSIIIEWSKDGGISWAT

LETFELPPGTPEAKNKEGNMLKSWTLSSEANNNSTVKIRFRTGDAMQANMYIDNVAIY

GQAIPGITPAPSPVPPGEENTEFTPPQGVTLYEDVEIGTAGGRAIYSSIAVPETAGAE

PMPVMVYIHGGGWNHGDRKQALNSICNYVLKRGYIGVSLDYRLTHEAPFPAQIQDVKL

AIRYLRAHAAQYNLDPSRIGVWGSSAGGHLAALLGTTGDMVAGDTVMLDTGVTVDVPD

LEGSGGWAEYSDKVQAVADWYGPADFTTTFANNYSSVTALLGGHRAFDVPEQARLAMP

GTYASPDDPPFWIRHGDADATIPYTDSVTFAGQLQSAGVPIVDMKVVPGQGHGFTGTA

SEVANAEAWAFLDEHVKNRIVTEPIIFKSNPEDTSSGDEEEDEEKPLIEKVIASKLPS

DDAAIDSGKPDLNFNQATGSSTGLLSISSTPSTKKYVYFKFDMTGNEPEGDRYRLRIA

AKKGTSNIDTELSLYGLNAIDWSESSLTWSNAPVQSLSESSLVGKFQVTADRNGSPAV

YEVDVTDVVKSRPDTGQVAFLLADAGSTGVSVNVYTKEANGTSNPRPQLSVIALIEEG

SDTQSPEWEQGAELEIRNWGTDFAELRWPAASDDTAVSAYRIYRNGVLLAEQGKQAFR

DSGLTAGTSYTFQVRAIDEAGNISSALSTDLTTLAVPVSSLPVASVSASGSDGNLATN

TIDNNSYTRWSVAGEGQWITFDLGQAQQVGYVGIGFYKGDVRKTFFEVETSVDGNHWT

QVYGGESSGDTTEMQAFDIPDTSARYVRITGHGNSDSSIYTSLTDVHLYAPYAGGGTP

VALIPYIEPQPPEGTVPFIAPGLTETDGTPHTIHSPHAVTGRTIDVRDYGADLADNTS

DDRPAIQAAIDEANAGDEVFLPDGVYNLLSGPDGTTNLMLKSGVNLRGESSEGTVLKT

SLDQVTGSAVLKASAQHSILVSNMTITSSWSGSYTTEHKSNNPSAGGPDSMIHIANYG

EAPSYDITIDGVIVEKFKRMAIRIEHSRDVVVKHATFRNATDLGPGGSGYGISIQGTA

KTDRLGFDNDTIWNVVEDSTFEGPYLRHGALIQFVAHNNVLRGNTFNETKLDAIDLHG

ELEYLNEISGNIITDVLTGAGVGLGNTGGSAPSNHSKSGKGNYIHDNTIRNSQIGISV

TMGTPDTVIKDNLIENTTTVADAAGIKVLNGPGTVIRGNVIRNNTASGYWGVRLERDK

GDAGAGNIGEGNPENVLIENNRIEGNTNGIGLFAGVGILLKTNILNNVNEDYYKAAGV

TVTEL"

CDS 901663..903645

/locus_tag="PIKHDJKN_00807"

/inference="ab initio prediction:Prodigal:002006"

/codon_start=1

/transl_table=11

/product="hypothetical protein"

/translation="MNSLLRKVAVTALSVTMVTSSSFALVGSPNAAFAAEDTSTSTGI

TQAYESLFQTDNVIDVNVTIDDADWKSMLESPLDKDYKKVSVEVDGNKLDNVGFSTKG

NLTLKSVASMQDSDRYSFRLKFDKYDKTQTLLGLDKMVLNNNYADPSYMREVLHYEAL

RSIGMDVPMTNYVNLYINGELVGFYTGVEAVDDSYLERNYGEDYEDGVLYDTDEKSYL

QYSEGSDYSTITEDLGTDENKTKLKNFIKTLNDMPEGEKGDIESVLDVDSALKYIAGN

MVFGNYDSYSGDKGHNYMLYGDADGKFTVVPWDFNMSFNGYSGGGGRGTTGSTTTNTN

ATNVSVDEPVLGISMENVPMINNLLAVPEYKEKYLSYVNELTDYMEGIQDRITGLADI

IRPYVEADPTKFYTTEQFESNIAYSANADATGGMGGMGGTPPEGFEGMTPPEGMEGMT

PPEGFEGMTPPDGTTPQDGATGKGTTDSTGKTQTRPGGNFGGGGGMGSMAAGSLTTFA

LNRLANLQEQLGREVTPLPETSEDTGTDNGTGTTDKAITVSLDGKAITFPDQDPLEQS

GRVMVPVNAILEALGAEVTWDKTAKTVTAVLNDQTLVLQIGSSTATVNGETLEIDAPA

IIKNSRTLVPVRFISEGLGLTVDWDQTAAQVTLTTK"

CDS 903752..904396

/gene="vatD"

/locus_tag="PIKHDJKN_00808"

/EC_number="2.3.1.-"

/inference="ab initio prediction:Prodigal:002006"

/inference="similar to AA sequence:UniProtKB:P50870"

/codon_start=1

/transl_table=11

/product="Streptogramin A acetyltransferase"

/translation="MAPDKTKLFPNDNIRTVCYIQNLPPRSNVLIGDYTYYSDNSRPP

EQFYDRIQHHYDFLGDRLEIGKFCAIAEGVTFIMNGANHRMEGMTTYPFNIFGGGWER

VTPTLDQLPFKGDTLLGNDVWIGQNVTIMPGVTIGDGAIVASNATVTKDIEPYTIVGG

NPAKPIKKRFDDEIIALLLELKWWDQDEVWLDTHLERLVSTYDLQTLRELLKSK"

CDS 904625..906436

/gene="nikA"

/locus_tag="PIKHDJKN_00809"

/inference="ab initio prediction:Prodigal:002006"

/inference="similar to AA sequence:UniProtKB:P33590"

/codon_start=1

/transl_table=11

/product="Nickel-binding periplasmic protein"

/db_xref="COG:COG0747"

/translation="MRFKRAATPLVALFMSVTLFAGCQSVQPPSVSEPQGTVDTPTAT

TQNEPAPQTNKETPRNETLYINGLQWGPPTNFNLLSGNPAFPVNYGNSRELVYETLFM

VNQLDGGLEPLLGNSYEWTDETTLRIELNADAKWSDGTAFTADDVVYTYELGKKYDIN

WSSFWTYISEVKADGAQAVEIKLNPDNPNKLTVLDSIELIPMLPKHIWEEIEKKNNND

LTAIRKEINDNPVGTGAYKLHFYNDQKITIVRDDNYWGQKLFGKLPAPKYITHVIYKD

NAAGDLAFKSGQVDVSQQFIPQVWKMWEGGAAVKTYLKDAPYYLPGSMPSIFFNLSKA

GLDNADVRRAIAMSINYDKISELAMSGYSAPMQPSLTLNSDAESKYIDQDAIKSLQWT

MDIEGANALLDKIGATKGKDGTRVLNGTRLGPFEVECPYGWSDWNAALEIVAQSAKAI

GIEIRTKFPESPVWTNDLQTGKFDIIMNTPAGGVSPSQPWNRAMTIMYSKGVAPMGEM

AFWNWGRYQNDRADAIIEEIPSVSDEAKLKSLYTELNTIWLKDIPSIPLMYRPWVFDT

VNESVWKGFPTEGDGTNIPPQISMDGAGIKALYQIHN"

CDS 906526..907536

/gene="dppB_3"

/locus_tag="PIKHDJKN_00810"

/inference="ab initio prediction:Prodigal:002006"

/inference="similar to AA sequence:UniProtKB:P26903"

/codon_start=1

/transl_table=11

/product="Dipeptide transport system permease protein

DppB"

/db_xref="COG:COG0601"

/translation="MNAYSRYVAKKVFWYGLTLVIAIGLNFILPRLIEGNPVSMIASK

MTSGMTDSDSIKRVYETFTVEFGIDQPLWTQFGIYLKNLFTGNLGTSFGLYPKPVTDI

LASAVPWTVGLQLPAILVGWIIGNLLGAIAAYRKGVFDKVLFPVALFINSIPFFTLAI

IMLYVFALSLKWFPLSGGYDFQMVPQLSFDFIMSVVRHHTLPFLSIVLVTIGGQAIGM

REMSIYELNSDYVLYSKLLGIRDGRISRYVFRNAMLPQITGLALSIGTMVGGSLICEI

VFSYPGIGTWLFTAIRQLDYPLISGCTLLIAIAVLLANFTIDVIYGFIDPRIKAAQME

EQ"

CDS 907538..908374

/locus_tag="PIKHDJKN_00811"

/inference="ab initio prediction:Prodigal:002006"

/inference="similar to AA sequence:UniProtKB:P9WFZ9"

/codon_start=1

/transl_table=11

/product="Putative peptide transport permease protein"

/db_xref="COG:COG1173"

/translation="MKHSISILLKSPKFMIGAVTFVGMLLLVTIYPLINRSDPLEMIA

MAFQSPGDGLLLGSDNFGRDIFLELMYGIQTSIRVGLIAGVFATVIGLVMGLVSGYIG

GMLDNFLTAITNIFIVIPSFIILILISVSIDSRSSLVTAIIIGITSWPWTARAVRAQT

TSLRSRDHVHIAKISGHSTPRIIIHEILPYIASYVVMAFVLQTASGILSEASISMLGL

GPYNTISLGILMNWALVFEAPVAGAWWAFIPAALAIAVITFSLYLMNTGMDEIFNPKI

RS"

CDS 908379..909515

/gene="btuD_5"

/locus_tag="PIKHDJKN_00812"

/EC_number="7.6.2.8"

/inference="ab initio prediction:Prodigal:002006"

/inference="protein motif:HAMAP:MF_01005"

/codon_start=1

/transl_table=11

/product="Vitamin B12 import ATP-binding protein BtuD"

/translation="MNQPILQVSGLKTYYRTRLKENVFAVDGVEFGLKEGQTLGIAGE

SGCGKSTLALSLMGFYFPPLHYGSGSIRINGTDIMQLNKEQLRSRILGKEIAYIPQAA

MNALNPTLRIIRFIEDIMKEHRPELKRSDVRKMATERFQSLNLSPDVLDMYPNELSGG

MKQRTVIAISTILNPNVLIADEPTSALDVTSQKAVIRLLKDLLKRKVIKSLVFITHEL

PLLYHVTDEIMVMYAGEIVERGTAEQMIFNPLHPYTRKLMGSIIVPEEGMKEHKLAAI

PGAPPNLKNVPEGCRFAERCTLVTDECRQGKIENIHVDGRIYRCLLTPEMLEQIREQE

QDGHVQQESRDEEPQEREQNQEENASNKEEVRDIRTATDDIADQ"

CDS 909575..910375

/gene="oppF_3"

/locus_tag="PIKHDJKN_00813"

/inference="ab initio prediction:Prodigal:002006"

/inference="similar to AA sequence:UniProtKB:P24137"

/codon_start=1

/transl_table=11

/product="Oligopeptide transport ATP-binding protein OppF"

/db_xref="COG:COG4608"

/translation="MAMGKVLISGRHLTKVYGTGNRKKAAVDDVSFDFHEGEIISIVG

ESGSGKTTLAKMIMGLLKETGGAIEYDGKPRQLKRYRERKAYWKDIQAIFQDPFSSFN

VFHRVEKLLVDCIKLQGLKLNKDEEYAKMKEACSFVNLKFEELYNKYPFELSGGQMQR

LMIARIFMLHPKVLIADEPTSMVDACSRSTILDMLLKLRDENRMTIVFITHDVGLAYY

VSDTICIMEHGRMVEAGRAEEVIHHAEHPYTKQLINDVPKIHSPWVLD"

CDS complement(910647..910919)

/gene="hup_1"

/locus_tag="PIKHDJKN_00814"

/inference="ab initio prediction:Prodigal:002006"

/inference="similar to AA sequence:UniProtKB:P0A3H0"

/codon_start=1

/transl_table=11

/product="DNA-binding protein HU"

/translation="MDKEQLITEVAKNGGFSKKNAEKAVTVVLDSILEALKEGKEVQL

VGFGKFEVRNREARMGKSRRTGKEIQLPAGKVPVFTAGLTMKEALN"

CDS complement(911054..911254)

/gene="cspD_1"

/locus_tag="PIKHDJKN_00815"

/inference="ab initio prediction:Prodigal:002006"

/inference="similar to AA sequence:UniProtKB:Q45099"

/codon_start=1

/transl_table=11

/product="Cold shock-like protein CspD"

/db_xref="COG:COG1278"

/translation="MQTGTVKWFNADKGFGFIETEEGTDVFVHFSAIQGDGFKTLDEG
QRVQFEVTQGNRGPQAENVTKV"
CDS 911425..912375
/gene="xerD_1"
/locus_tag="PIKHDJKN_00816"
/inference="ab initio prediction:Prodigal:002006"
/inference="similar to AA sequence:UniProtKB:O31206"
/codon_start=1
/transl_table=11
/product="Tyrosine recombinase XerD"
/db_xref="COG:COG4974"
/translation="MDKGSGTSVQGEMVVQEFIHMLTIQGELTPKTLKEYASDLKHFT
EWYKGNDSRGEESSFHIEDVHTSTLVRYREDSQKVMQLKPSTINRRLITLKRFFKWAA
SESSFNSDPSKPLKFIPEDKVSPRRMTTEEERSFISAVEDGNSLRDQTILTLMFHTGL
RTMEVCNLAPSDIELGKRSGQLTVRADKRNGQRKISLNMTCIALLKMYMTGLAPDSPY
LFPSEKTSDRLTERALRHLIKKVMTSAGLEGLSSHDLRHRFGYAMAEHTPLHRLAEMM
GHINPDTTMIYVKGLNANLSSSAEEDGETEPQLKYMQHSL"
CDS complement(912419..912940)
/locus_tag="PIKHDJKN_00817"
/inference="ab initio prediction:Prodigal:002006"
/codon_start=1
/transl_table=11
/product="hypothetical protein"
/translation="MNYLKSGMVTTGIAAILICSGSITETIRTDRFEASTETSRWENQ
PQELQEKLTKSWSGGLDAIRNTLNENSEKENITEWKLFYSLEDSLNTLPHPEHSSFIA
AYYSNERRTMLENGDIMPALLLHPDHTKAILFWEKLDGTYAIITLESQINANNKPEWD
VSHSWITKQPSST"
CDS 913157..914776
/gene="rhaR_5"
/locus_tag="PIKHDJKN_00818"
/inference="ab initio prediction:Prodigal:002006"
/inference="protein motif:HAMAP:MF_01533"

/codon_start=1

/transl_table=11

/product="HTH-type transcriptional activator RhaR"

/translation="MYRVLLVDDEEDVREGLVVEVDWEALDLRIVGLAENGREALEMA

ERVEPDIVVTDISMPFMDGLELAKRLRERNPLVKVVILTGYDEFDYARQAVSLSVDEY

LLKPFSAGHLTELLTRLRAQMAAEVAEREDVQQLRDHYYTSLPLLQADLMATLLHRQK

SPQYIHGKAKQCGLDLHGERYGVSVLTLHMDGDQSEDAELKQFAALNIAAEVWTEHGA

GHAFMHQETIVLLYVDRWGGEDGEKRQQQALENVMRSINHYLRIPATVGSGSIVNTLA

GVKHAYEDALLALDYRLVPGTDPLIYIADVERQTAGKLRFDELKQQTLTRCLKAGTQA

ELEDALEIIFREITVEHGRSDIQLYLIEVLTNVWKAAQASGEAMEDIFGAGFQLYADL

FRLPGLSEAQGKVREVCLLVQHRIASGRQHVYKDIVEQALSFTKEHYAEPDLSIQKVC

GHLHISSGYFCGIFKKEVQLTFLQYLMQIRMEAAKELLRSTEMKSFEIAGQVGFAEPN

YFSFCFKKHIGVSPKEYRKQTALTASEGTSR"

CDS 914773..916587

/locus_tag="PIKHDJKN_00819"

/inference="ab initio prediction:Prodigal:002006"

/codon_start=1

/transl_table=11

/product="hypothetical protein"

/translation="MRTFAGWIRSLWLKLRSRLRSSRVSSIRFIITWSFSVFIVLVMT

IMALLLHDKFTQAAERSAELTTRQIVDQVSYNLEDYVRSMSHLYRAIEEHMLRDGTWE

GDQVDKQLDTLLSSREDIISITLLDSTGHLLKNRPSAELKPSAHVTQQGWFQSALRVP

DHLSFSLPHIQNMYTGPYKWVVSMSKGITVRQNGQDRQVILLVDINFKQMDELSRRVS

LGQRGYVYIIDESAGNIVYHPQQQLMYMGLKSENIEQALVATGSYEDEADGQKRLNTV

KSVANIGWKIVGVAYLDEIMTTRQEVNGYLIRVLLVVLVLVILVSLFLSSSLTRPIRR

MERKMKAVERGDFNVELPIEGPLEVERLSRRFNLMVNKIRTLMSEIIHEQEQKRRLEL

EALQAQINPHFLYNTLNSVVRMVGMSRNEEVITMITSLSRLFRISLSQGKTIITVREE

LEHAHHYLTIQQMRFKRKFNFSMNADEDTLDCLTLKLVLQPLIENAIVHGIEYHMDEG

CIEIRVYREDDKLVFRITDNGVGMTEEQMLKLLIDSPIVKSGAGSGVAVRNVHDRIQL

YYGEAYGLEFESELEEGTTVWIRIPIQSEREEDDAHES"

CDS 916577..917755

/locus_tag="PIKHDJKN_00820"

/inference="ab initio prediction:Prodigal:002006"

/codon_start=1

/transl_table=11

/product="hypothetical protein"

/translation="MKVNKINRTDWIAHMVMKITICTRVEAIRTRVLILRYMGMSAAI

CCLLLCMACGMSNADPAGSPPRIALITPAGTGELAEAIRLGAEAAAKEAGAELITVEA

FPSEGLTYAPSNPDFTAGQGRVKQEHLQNGTYPLSEREQAQVDAVSLALEQGASALLI

DPLSEKALSDIIQKAQTKDANGVIIPVILLNDEFPVKGITSVISIDNVEAGRQAGQAM

AVLLGGRGRIALLGPDPVNSVLINREQGVEESLAEYPDIHVETQSVCSTRDGCWQAAK

QLLDQQQVDGLIALHEPASLGAADELNRRTTDNNGIKVKIVGFGSEQQQLEQLQEGRI

QHLIVQNGFSAGYLGVNQAVARLNNQQVQARVTLETKLVSTDNMFWMDNQKLLFPFVQ

"

CDS 917877..918938

/gene="mglB"

/locus_tag="PIKHDJKN_00821"

/inference="ab initio prediction:Prodigal:002006"

/inference="similar to AA sequence:UniProtKB:P23905"

/codon_start=1

/transl_table=11

/product="D-galactose-binding periplasmic protein"

/db_xref="COG:COG1879"

/translation="MKKTWMTLLLTACMVAAAGCSSGGDSAGGSTGTDTGGQTAAETE

TPKIGVAIYKFDDTFMTGVRNAMTAAAEGKATLDIVDSQNAQPTQNEKVDLFVSKKYN

AMAVNPVDRTAAGVIIDKAKAASIPVVFLNREPVAEDMNKWDKVYYVGAKAEESGTIS

GQLIVDYWKAHPEADKNGDGKLQYVMLKGEPGHQDAELRTKYSVQAIQDAGIEVEALA

EDTAMWDRVKGQEKMQAFLASHGDKIEAVLANNDDMALGAIEALKAAGYFKDGKTMPV

VGVDATAPAIQALQDGTMLGTVLNDAKNQGAATVALASVLAKGETPTKENTGYDITDG

KYVWIAYKKITKDNIADAQ"

CDS 919013..920518

/gene="mglA_1"

/locus_tag="PIKHDJKN_00822"

/EC_number="7.5.2.11"

/inference="ab initio prediction:Prodigal:002006"

/inference="similar to AA sequence:UniProtKB:P0AAG8"

/codon_start=1

/transl_table=11

/product="Galactose/methyl galactoside import ATP-binding

protein MglA"

/db_xref="COG:COG1129"

/translation="MESPYLLEMDGISKAFPGVQALSQVTLKVKPGTVHALMGENGAG

KSTLMKCLFGMYRPDEGTIRMDGNEVEIPSSKAALQHGISMIHQELNPVPHRPVTENI

WLGRFPMRGLLVDEKRMYADTLALFKDLNLDIDPKTQAGTLSVSKVQSMEIAKAVSFQ

SKVIVMDEPTSSLTGKEVDQLFSIINQLRSRGVSIIYISHKMEEILTISDEVTIMRDG

FVVGTWDAADLTTDLIITRMVGRDLDERFPERTNVPGEVILKAEGLTSSQPNSFRDVT

FELRKGEVLGIGGLVGAQRTELIESLFGLRGLASGTISIHGRKVKIKSPAAAKRHNIA

LLTEERRVTGIFPVLSVYENTIIASLGRYRNRIGLLDEKKGRDEAREQTLKFRTKTPS

VNTLIRNLSGGNQQKVLLARWLLTDPEILLLDEPTRGIDVGAKFEIYTIITELARQGK

SIIMISSEMPELLGMSDRIMVMSEGRLTGIVDGAEATEQDIMRLAAQQRMA"

CDS 920533..921552

/gene="mglC"

/locus_tag="PIKHDJKN_00823"

/inference="ab initio prediction:Prodigal:002006"

/inference="similar to AA sequence:UniProtKB:P23200"

/codon_start=1

/transl_table=11

/product="Galactoside transport system permease protein

MglC"

/db_xref="COG:COG4211"

/translation="MNTQVINQVKQYVAQRAIFIVLILLVIGIAIADPHFLAFSTLRD

ILQQSSTRAIIALGAAFILVTGGVDLSAGRVVGLTAVVSASMLQISEYANRFFPDLPQ

YWVGLPIAIGIIAGLTVGLVNGIIVAKLHVPPFIATLGTMVAVYGLNSIYFDTEPNQS

QPIGGLRPDFTVIGSGYIDLGGGYSIPYIVLIAIGVALICWVVFNKTRLGKNMYAIGG

NIQAAHVSGIHVARNLIALYAIAGALYGLGGVLEAARTGGATNNYGNMYELDAIAACV

VGGVSTAGGIGTVPGVMAGVLIFGVINYGLTFIGVSPYWQLIIKGLIIVAAVAFDIRK

YMAKK"

CDS complement(921624..921959)

/locus_tag="PIKHDJKN_00824"

/inference="ab initio prediction:Prodigal:002006"

/codon_start=1

/transl_table=11

/product="hypothetical protein"

/translation="MIQQRKPLGIGTLSLLVLAMGLAFNFPWGTEKVKISHYLFNLLN

LPIYSNGHQGFNIPFIVAIIFWLSSVLIARKYQTHYGTKVAYKVGGFMLISSVMIFPL

YSLIWVIFH"

CDS 922222..923793

/gene="sasA_7"

/locus_tag="PIKHDJKN_00825"

/EC_number="2.7.-.-"

/inference="ab initio prediction:Prodigal:002006"

/inference="protein motif:HAMAP:MF_01837"

/codon_start=1

/transl_table=11

/product="Adaptive-response sensory-kinase SasA"

/translation="MLSYTMKMVIFPLGCLLVVLSSYFLGLTSHLALMICAGVLFVAS

IYKEKQYPVLRNIHWIFLGIFHYFSELNWCNMLYYLLIMSMIQDKQRVTQTLPISMLI

VFEYTVIRLSYVTIDTYNLLVSMFDMLTAIVIIFVYHTLINSEAEKRRLREKNHFLTL

HDPLTGLLNYEGYMNVLKETVDEQRSFLLIIMNIQNFNGFDKEVEDNWSHVIKATGES

VSKQFADAYGVSRYAGDRYAVVLPEIQNIEERMALLLSGELRGLQVNYSISLYPGMSE

TLQHFITVAEDRLFHQQRSKWLKNQEEVFRSERLRAVGELAAGMAHEIRNPLTAIRGF

LQLSRGQAFNIAPWYEVIMGEVARVTNLTAEFLQFSKPQANHMKPERVAQCLERVMSL

TESDAASRGHRITLEMTGDPVVVNMDRDKIVQVLINLIRNAFEAMEDPGEVHIDLLHD

GDKALISITDTGSGIPENSLATIFNPFYTTKEEGTGLGLALCQKIAQDHRGRITVRSE

IGIGSTFTLHLPMVP"

CDS 924077..924907

/gene="lpxC"

/locus_tag="PIKHDJKN_00826"

/EC_number="3.5.1.108"

/inference="ab initio prediction:Prodigal:002006"

/inference="similar to AA sequence:UniProtKB:P0A725"

/codon_start=1

/transl_table=11

/product="UDP-3-O-acyl-N-acetylglucosamine deacetylase"

/db_xref="COG:COG0774"

/translation="MYQKTIRNSFTLDGIAIHSGNLSEITLHSAQPNTGIIFRRVDVD

PYEIIEAKIGNVSNTERCTQLKNNHGYSVSMVEHTLSAVRGMGIDNVIIDVNGEELPI

FDGSSTGIAAKISEVGHLQQEIKKTYLHLQQKIRVEHGNSFLEAHPSSMISYCVCFKN

EHKISHLSDQTSNFNFIENNYLTDIAPARTFCFEEEIMYLKSKGLIQGASLEHGIVIG

EKKIYPNSRFHDELSRHKLLDLIGDLALNPPFHARIIGSGTSHYLNTLFAQELLNKLI

"

CDS 924928..925953

/gene="lpxD"

/locus_tag="PIKHDJKN_00827"

/EC_number="2.3.1.191"

/inference="ab initio prediction:Prodigal:002006"

/inference="similar to AA sequence:UniProtKB:P0A1X4"

/codon_start=1

/transl_table=11

/product="UDP-3-O-(3-hydroxymyristoyl)glucosamine

N-acyltransferase"

/db_xref="COG:COG1044"

/translation="MPCNLNEISEQLNGILIGDGNVVITGVGSINAAKEGEIAFVTDK

NLNKLDKCNASALIVPETTIIEFKVPVIKVKNPRLDFIRLIEMFSEKTKSSGVVSSEA

IHASVTLGYNVQIDEFAVIKEDVSIGDHTIISAHSFIGRNVTIGTNCLIHPNVTVLHD

TFIGDNVIIHSGVVIGADGFGFEWDGQKQVKIPHVGNVIIYDDVEIGANTTIDRGTID

STTIGKGTKMDNLIQIGHNVQIGEHCIIAGTSAIGGSSKIGNRVTMAGGSAVINNITI

GDSCVVLAKTMVTKNMPENSMVSGYTARSHKRQLRESAALSRLPQTIKKLNQKLDELS

KTKEGAK"

CDS 925950..926726

/gene="lpxA"

/locus_tag="PIKHDJKN_00828"

/EC_number="2.3.1.129"

/inference="ab initio prediction:Prodigal:002006"

/inference="similar to AA sequence:UniProtKB:Q2SWY6"

/codon_start=1

/transl_table=11

/product="Acyl-[acyl-carrier-protein]--UDP-N-

acetylglucosamine O-acyltransferase"

/translation="MIHQSAIIHPTAVIGDNVEVGPFSIIEENSKIGSGCRIGSHVII

GANTILGGNNNISHGAIIGSDPQDKSYQGEESYLIIGDNNTVREYVTICKGTSKGDCF

TRVGSNNFIMNYAHIAHDVLMGDHNVIVNNVQIGGHVNIEDYITFGCGSGVHQSCNIG

RFAMIGAGSKVSQDVVPYSLADGPRSYIHGINIVGLRRNGFSNEEISMIKKINTILFR

QKRTLDQSIEEINNLPPSEFKEHTLNFLYKSTRGIVRMKR"

CDS complement(926823..928646)

/locus_tag="PIKHDJKN_00829"

/inference="ab initio prediction:Prodigal:002006"

/inference="similar to AA sequence:UniProtKB:Q9WYC4"

/codon_start=1

/transl_table=11

/product="putative ABC transporter ATP-binding protein"

/db_xref="COG:COG1132"

/translation="MAKNTSSSIHQTVVPPIGRPGPPGRGPVPKVRAKNARHAIIRVW

SYMGRHRKGVITALVLTALGTLLNLAGPYLLGRTVNEHIVPKITDGLLKDCMLLLTVY

VLGSFMMWAQSYVMIGVSQLTVKDLRHALFSRLQQLPISFFDKNQSGDLMSRATNDID

NVSTTLNQSVTQLMSSAILLVGSLSIMFALDVRLTLLSLVTVPLITIATRLIASRTRK

HFTAQQKLLGELNGYAQETIAGQKVVAAYNRQEQAQKHFQNLNEKLRASSTQAQSVSG

LVGPTMNVMNNIGFAILAAVGGWMAYHDLTSIGLIVSFLAYSRQIERPINDLANQYNL

IQAAIAGAERVFHIIDTPSEYVEEQKKQLEQIQGKVVFEDVSFGYSPEREILKKVSFT

AKPGEMIALVGPTGAGKTTIINLLPRFYEITGGRITIDGCDISELEKDQLRRELGIVL

QDAYLFSGTIRDNIAYGKPDATDEQIQQAAKLANAHSFIRKLSQGYDTPIISGGSNLS

QGQRQLLTIARAILADPAILILDEATSSIDTRTEMQIQEAMRTLMKDRTSFVIAHRLS

TIREADQILVIDDGKIAERGSHDELMQQQGFYYQLHQGQLT"

CDS complement(928639..930360)

/locus_tag="PIKHDJKN_00830"

/inference="ab initio prediction:Prodigal:002006"

/inference="similar to AA sequence:UniProtKB:P9WQJ1"

/codon_start=1

/transl_table=11

/product="putative ABC transporter ATP-binding protein"

/db_xref="COG:COG1132"

/translation="MWTLKRFLTPYKSAAILAPLLMVLEVAMDLLQPKLMSSIVDDGV

LAGNLSHIMTTGLFMLLFALIGWVGGAGCTLFSSKAAVGYGTDLRQELFDHIQKFSFR

NLDTFQEGSLITRLTSDITQMQTFVQMLLRMFIRSPMLIIGSIIMAFTISVKLALILI

LSVPILFIILYILISASYPLFASVQNKLDKVNAVLQENLAGIRVVKAFARARQEKKRF

QQANEDYTHTAVKAWRIVSLNAPVLSLMLNATIVAVLWFGGFQVVGGDIAAGDLIAFI

NYVTVVLSSLTSIGMMMMSFSRAKVSATRINEVLHTQPDIQSGDDVSRKGAGQVEFRD

VYFRYDGEHTLSGITLTARPGEKVALLGSTGSGKTSLVQLIPRLYDASQGEVLVNGVN

VRNWDLQDLRSRVSIVLQESILFSGSIRDNICFGRPDATEAELRAAAQAAAADDFIMN

LKDGYDTELGQRGVNLSGGQKQRISIARALLMQPEVLILDDSTSAVDLRTEATIQKAL

HSLMKNSTTFLIAQRISSVKDADCIYVLDEGEIVASGTHEHLMAHSSHYQSIYYSQQR

KEDVQFG"

CDS complement(930431..930865)

/locus_tag="PIKHDJKN_00831"

/inference="ab initio prediction:Prodigal:002006"

/codon_start=1

/transl_table=11

/product="hypothetical protein"

/translation="MKSDRNERLMGQLSELVKLQRYKVHEKLINHPELYPGQPPLLFQ

LEREDGQTQKHLAEQLRRAPATVTVMLKRMESSGFIRREADPKDLRSLRVYLTDQGRA

ALKELREVFQELDRQAQKDFTPEETQLMSALAQRMVQNLREY"

CDS complement(931129..932076)

/gene="yfiY_1"

/locus_tag="PIKHDJKN_00832"

/inference="ab initio prediction:Prodigal:002006"

/inference="similar to AA sequence:UniProtKB:O31567"

/codon_start=1

/transl_table=11

/product="putative siderophore-binding lipoprotein YfiY"

/db_xref="COG:COG0614"

/translation="MLKKNLMLFMSICLMLVLAACGNANNSSSASEGSTTDTPNATQD

TNSASGDQRIASMSIHLTNDLLSLGITPVGSVIGGEAKAFLSHVADRLQNTTPLGPVK

DPDMEALLALKPDVIYLDEEFSGDDIAKFEKIAPVHVFNLDDGTWRDHLKDIGKLVNR

EKEAEQYIQDYATETEEVKSLIHDTIGDGTVMAIRVTAKELRVFSTRRPMGPILYEDL

GLTPAKGIEEFDSSKPYQVVSREVLPDFDADAIFVVVNSDDEAQNMFKELQNNPIWQG

LKAVKAGHVYPIGAQPWLDYSSIGNKMAMDEAKEMFSKK"

CDS complement(932167..933183)

/gene="feuC_1"

/locus_tag="PIKHDJKN_00833"

/inference="ab initio prediction:Prodigal:002006"

/inference="similar to AA sequence:UniProtKB:P40411"

/codon_start=1

/transl_table=11

/product="Iron-uptake system permease protein FeuC"

/db_xref="COG:COG0609"

/translation="MNTNAPSRGKRSIAVSITLLCIACAVIVISLNSGTIRLSPIAVL

HTLLGYGTPDDQIVLFDYRLPRILVTVLAGAGLGIAGAALQGITRNPLADPGILGLHA

GAAFGLMVFVSFSHSMNGSVALLIPLFAFAGSVAAALIIMLLSYDRHNGVSPIKLILV

GIAVAAGFHALTLYLSLRLDEDTYSFAARWLAGSVWGRDWVHVQALLPWIVLCISYIW

SRSKTLDAFNLGDAAATSIGTPVRSQRVILLLCTVALSAVSVSMAGGIGFIGLAAPHL

ARRLVGPMHRHLIPAAGLIGMVILVAADTVGRTIFQPNSIPAGVVVAAIGAPYFLYLL

VRTK"

CDS complement(933186..934232)

/gene="feuB_1"

/locus_tag="PIKHDJKN_00834"

/inference="ab initio prediction:Prodigal:002006"

/inference="similar to AA sequence:UniProtKB:P40410"

/codon_start=1

/transl_table=11

/product="Iron-uptake system permease protein FeuB"

/db_xref="COG:COG0609"

/translation="MTRIQHPGLSQRPSKSRKSAVTVGLMFLAAIGALLLSMFVAISL

GAKGLTLETVWTAIFQYNPALTPHQIIHELRLPRVIAAAVIGAAFAVAGALMQGITRN

PLADTGILGINAGATFVVALSFAFWPGLPYAWIMFLSFAGAVLGTLLIFLLGMAAPGG

LTSIRLTVAGAVIAAMLSSLSTGVAIYFDLSQDLAFWYAGGFGGIEWRHLKLILPVLL

VTLVLTLPMARRISLMSLGEEVAINLGINLRWTRLFALTAVVVLAGVSVSAVGSIGFV

GLVIPHISRKLVGVDYRLIIPMSSLLGAVLLVLADLGSRIVNPPQELAVGIMVAFVGV

PFFLYLARKERRAL"

CDS complement(934360..936078)

/gene="btr_1"

/locus_tag="PIKHDJKN_00835"

/inference="ab initio prediction:Prodigal:002006"

/inference="similar to AA sequence:UniProtKB:P40408"

/codon_start=1

/transl_table=11

/product="HTH-type transcriptional activator Btr"

/db_xref="COG:COG0614"

/translation="MLTHPSTNNNRNTTHMLTQPDQPKRTDESMTRTEMDAFLNDALL

ELRQAELVTLNTHWQWSQTELPHHTFVYMHKFTGKLLVNGIESLVARKSAFLCTPGTS

LELFSDADHEIELYIIHFDLFRVAEKTEERRIYERELGSPVTGWIQGPYYGIQRIAAQ

LTTEASQTSLKVQLLLTDLLHTLWPQEGQSDVDGQDEPEQWLRSTLQFMRENYMNEIK

LEKLAELAGMHPSYYSQLFKSRMQKNPIEYITHLRMNRAKELLMTSDLRIRDVARQVG

YRDEFYFSRRFRNHAGYAPTSYAKQVHRNIVSLSYPYTDHLMTLGITPCAAQIQGHLP

HVPKSLTLPFHAYEPWEQGRQAFLDVNPDLILTKDNAAAKAMEHIGDVAPIITIPWNQ

TDMFGHLQKIASIVDRTQAAKEWLDRHERKAERARKKIRETAGQVTVAICTLTAKGPR

MYSNRNFGHVFYRSLQLSPPDRIQKELTGKPAGVGFNWTSFTPGEWDGLEADVLLIAV

TSMNERTTLLQQITADPLWKNYPAVKAGRVHVIDWNSWVVYAPYSINIQLDEALSMLT

NKPAFL"

CDS complement(936112..936861)

/gene="fetB"

/locus_tag="PIKHDJKN_00836"

/inference="ab initio prediction:Prodigal:002006"

/inference="similar to AA sequence:UniProtKB:P77307"

/codon_start=1

/transl_table=11

/product="putative iron export permease protein FetB"

/db_xref="COG:COG0390"

/translation="MSLIALSFTIIFVMLTMLISVWQKLDLEKDIAIGTVRSAVQLLL

VGYVLQFIFNSDHPALIIAIVGFMIIVASTNAGKRGKGLRWISLYIAAAITVTELLMM

GLLLGLGIVEPTPQYIIPLSGMTIGNAMVVSGLFLNQMKREVESSKGEIESLLALGAT

PRRAFQDVLKRSVKSSMIPTIDGMKTVGLVQLPGMMTGMIIAGADPVEAVRYQILIVF

AFTSSAAITSILLSLMTYRQWFTSDMRLRSL"

CDS complement(936871..937611)

/gene="btuD_6"

/locus_tag="PIKHDJKN_00837"

/EC_number="7.6.2.8"

/inference="ab initio prediction:Prodigal:002006"

/inference="protein motif:HAMAP:MF_01005"

/codon_start=1

/transl_table=11

/product="Vitamin B12 import ATP-binding protein BtuD"

/translation="MSTLLTIEKLVKRIGLTEQHILFEHVNAVVNQGERIAILGASGQ

GKSTLLRILALLDIPDEGDLIWKGTSYRDSDPRIWRMRMTYVAQQAVMLSGTVEDNLK

TVHLLHRQPYDQDLARRLLAGMGLENLDIQKRAIDLSGGEKQRIALIRSMMLRPEVLL

LDEVTASLDRTNARLVEEQLTRWSQQEGTSLIWVTHDQEQAQSFSTTTWFMGNQTLLE

RQPTAHFFDNPATDLARQFILHPAPAAE"

CDS 937840..938841

/gene="iolU_1"

/locus_tag="PIKHDJKN_00838"

/EC_number="1.1.1.371"

/inference="ab initio prediction:Prodigal:002006"

/inference="similar to AA sequence:UniProtKB:O05265"

/codon_start=1

/transl_table=11

/product="scyllo-inositol 2-dehydrogenase (NADP(+)) IolU"

/db_xref="COG:COG0673"

/translation="MTKVVGRDKVNWGIMGTGWIASQFARDLEHAGNAVKAAVGSRTA

ESAEKFAAEYGFARAYGSYDEMLQDPEVDIIYVATPHPLHKENVLACLEAGKAVLCEK

PFTMNARQLEQLVDKARERKLFLMEGMWTRFLPPITQARAWIAEGRIGEVRLVKADFG

FRVGWEPEGRLLNPDLGGGALLDAGVYPISFASMIFGEQPQHVWSTANIGQTGVDEQF

SVLLSYSEGRSASLNGAIRLNLSNEAVIYGTEGYIRLPLFLAGKEAYLHVNGQDEPET

FTDDRTCIGYAFEAEEAGRCILEGRTESRTIQLDESLEIMKLMDTIRGQWGLRYKSE"

CDS 938863..939483

/gene="pcp"

/locus_tag="PIKHDJKN_00839"

/EC_number="3.4.19.3"

/inference="ab initio prediction:Prodigal:002006"

/inference="similar to AA sequence:UniProtKB:Q5XDD4"

/codon_start=1

/transl_table=11

/product="Pyrrolidone-carboxylate peptidase"

/translation="MVKILISGFEPFGGDAVNPTGELMEVLALEVIEGAELKTVLLPV

HFDECADLLIAEMESYQPDVVIACGLAKGRTAITPERIAVNVKDIPPGSYADNQGQHP

VDELIVEGSPDGLFSTLPIRAMVSNIGAAGIPAAVSNTAGTYICNNTMYRVLDHIRLK

QLPIRAGFVHFPASTEMAVLQPSVPSLPIPMMLDALRVMIRTVAAE"

CDS complement(939895..940737)

/gene="mqnD"

/locus_tag="PIKHDJKN_00840"

/EC_number="4.1.-.-"

/inference="ab initio prediction:Prodigal:002006"

/inference="similar to AA sequence:UniProtKB:Q5SI12"

/codon_start=1

/transl_table=11

/product="1,4-dihydroxy-6-naphtoate synthase"

/db_xref="COG:COG2107"

/translation="MKIAFSPCPNDTFIFHAWVHGLIPGAPELDVRYADIDITNGLAA

NPDGPDTPEVMKISYAALPYVLNDYALLPAGGALGRGCGPLVLTANGTTDPAYLSGRR

VAVPSERSTAYLLFRLWAAQNVPGGVGEIVVMPFDQIMPAVRDGKIDAGLVIHEARFT

YQNYDLKLMTDLGNWWESDTGLPIPLGAIIARRNLDAHALAGWARASVEYAWAHPDES

KTYVMEHAQEMDPDVTAQHIGLYVNEFSANLGDDGYGAITALLGRAMKEGLVTEFDLD

LLRI"

CDS complement(940734..941606)

/gene="mqnB"

/locus_tag="PIKHDJKN_00841"

/EC_number="3.2.2.26"

/inference="ab initio prediction:Prodigal:002006"

/inference="similar to AA sequence:UniProtKB:Q9KXN0"

/codon_start=1

/transl_table=11

/product="Futalosine hydrolase"

/db_xref="COG:COG0775"

/translation="MNEQKQNDSIERNRPETTSLSSSFETNSTSTASTQRVLIVTAVD

AEKEAVLRGLGDQAARFDVIAAGVGPASAAAGTAAALAFASAAAGTNALAQGSAAPSE

PDAAQASAASSRPGPLAGTGLTPAYTLVVGAGIGGGFPGRADVGSLVVADAMVAADLG

SQTPDGFLSVDELGFGSSRVAADAVLAARLRHELQRAGLAVSGGTAVTVSTATGTAET

AAELLRRVPDAAAEGMEGFGVATAAQQFGVPALELRAISNAVGPRNRDAWRIKDALDA

LQAASSILREVLTS"

CDS 942083..942472

/gene="ytrA_2"

/locus_tag="PIKHDJKN_00842"

/inference="ab initio prediction:Prodigal:002006"

/inference="similar to AA sequence:UniProtKB:O34712"

/codon_start=1

/transl_table=11

/product="HTH-type transcriptional repressor YtrA"

/db_xref="COG:COG1725"

/translation="MFELDVRSRKAIYEQLVDKIKEMIVYGVLQPDEQLPSVRTLSGQ

LTVNPNTIQKAYRELEREGYIYSLQGKGSFVSSSVEHPNAAMRDEIRAALVKLIAEAS

YSGLSKADMTLLFEEAMARIEKGEPHD"

CDS 942465..943367

/gene="ytrB_1"

/locus_tag="PIKHDJKN_00843"

/inference="ab initio prediction:Prodigal:002006"

/inference="similar to AA sequence:UniProtKB:O34641"

/codon_start=1

/transl_table=11

/product="ABC transporter ATP-binding protein YtrB"

/db_xref="COG:COG1131"

/translation="MIELNQVIKAFEQEKAVDGVTMQVHKGSIYGLLGSNGAGKTSLL

KILAGIYRQDSGTVRIEGQEIYENMDLKGRTIFMADAPYFFPQSSIHQMAAFYRSVYP

RWNEERFVQLGSVFKLDVKRKLHRMSKGMRRQAAVWLGLSCMPEVLLMDEPIDGLDPV

MRQQIKNLLFQEAAERQVTILISSHNLREIEDLCDHVAIMHKGRIIVEKDLDDLKADT

HKIQVAFRHPDHAKAMDEQVNILHEEQRGSVSLFIVKGNREAVTERFRAFDPYLLDVL

PLTLEEIFIYEMEDAGYDIQPIVL"

CDS 943342..945369

/locus_tag="PIKHDJKN_00844"

/inference="ab initio prediction:Prodigal:002006"

/codon_start=1

/transl_table=11

/product="hypothetical protein"

/translation="MIFNRLFCNRGILFQNFRQHGWIGILYLITLLFSLPLHIGTEYR

EVPQQITSLFQANGEVQILFAITFPVAAGIFLFRYVQSGAPSDLFHSLPLRRKHLLTV

NLLTGFIMILLPIWITTAITGWVWAALDQSAFIFGGSSIWMWGLSMSIYSLFMFMLTV

VVGMCIGQSVLQGLTVYALLLLPVVVWFIFSLHLQHYLLGYPYDEVGRNAEKISLVLR

MASISGAPVIGWELIIYSILTLLFIPLSYVFYAKRQVESATQAVTFTLVKPIFRFGLM

FTLVLIGGAYFTIIAPRGSSGWGIFGYILGGIVGYIGAEMIIRKTWLIWSSKLLPKFA

LYGIVAGLILYVPVANWNGYVIRVPELNKVNSIKLGGERYTYTNGVSSKMDDDPFYSS

DSEYMKAVIALHQKIVDSDLSLLDDPINPGMRNDEYVFINYKLDNGKVMKRKYYIPEP

EFRQELMALKQSQAYKKFAFDTYKLDEDALSIGIRSTVSSYRKVYISNPNQVREFKEL

LKQDIMDQTYEEQRSPWQSFAYAEMNQDSSSNEPSQPKEMWNFEIKPSYDRVLHWLKE

NKLYEQLEFPVDEVASAQFVKQEINSPGNPQMVYPQSEYVDGNLSGNKQIATKNKKII

SELLQRQRSGYQTERDTLYQIKLNVTRGENIYMILKEEDLTEEMKAILVGQ"

CDS 945676..946707

/locus_tag="PIKHDJKN_00845"

/inference="ab initio prediction:Prodigal:002006"

/codon_start=1

/transl_table=11

/product="hypothetical protein"

/translation="MQTGILEDKENHQVGHCSCSVQARQSRIIEGNEGKQQIMNQSSM

RSMTNHSFMSNMSNRYGPRPNLLANVWTRMSLGLIIIFIMSVMYYSSAAEAAGPELGV

TAQKAWETVLSKADSQTKLSLQRAYENVGNWTTQEQTWEQKTNTIHSANTKELEQLRV

SIRQIDDAKIAGLTEKVKQTEARYESLFALYSSVNRQLDAAKAIKNKEWSAAIRTQAD

TLKPVVQLARQDIRFKKKELAEARKRKSTEVKRLRTFLAGADPVKKQIQTGKKQASLS

KERYSNALQQFKQSAKLGQSSRVLSSLNSLSAAAEKWAGSKQHIYTLEQKVSATYAKV

RQELTKRPK"

CDS 946816..947337

/gene="nikR"

/locus_tag="PIKHDJKN_00846"

/inference="ab initio prediction:Prodigal:002006"

/inference="protein motif:HAMAP:MF_00476"

/codon_start=1

/transl_table=11

/product="Nickel-responsive regulator"

/translation="MADKEDLTRFGVAFPTPLIEQFDSYIEEQGYKNRSEAFRDLVRK

TLLEPSALHSEQDVAGTIVMVYDHHISDLPIRLMELQHDAHHDIISNMHVHLNHDQCL

EVIAVRGNLGRLRHLHQQIQVQKGVLYAELSVTYVDELNKMASHHSPHNHDHSQNQQS

HYHPHHSPDSSTD"

CDS complement(947234..947482)

/locus_tag="PIKHDJKN_00847"

/inference="ab initio prediction:Prodigal:002006"

/codon_start=1

/transl_table=11

/product="hypothetical protein"

/translation="MIQHFKLGIITKQKEQQIIGNGPIICCSKYVLWTKSTNANESVI

EILKVNPLKNQENGEDGNDFVDFGCGHDCADCGGLPFC"

CDS 947647..949290

/gene="appA_1"

/locus_tag="PIKHDJKN_00848"

/inference="ab initio prediction:Prodigal:002006"

/inference="similar to AA sequence:UniProtKB:P42061"

/codon_start=1

/transl_table=11

/product="Oligopeptide-binding protein AppA"

/translation="MRGFSWGKRSVNTGLGILLICTVGLSGCTQETSSTESVHRATVS

LPKDELVLAVGTEPEGGFDPTTGWGQYGSPLFQSTLLKRDAKLQLVNDLATEHSVSED

GLTWTVILRDDVKFSDGEPLTAEDVRFTFETAAKSGSVIDLTNMADVEAPDTTTVVFT

LKSPQSTFISLLTTLGIVPEHAYGPDYAEHPVGSGPYKLVQWDKGQQAIVEANEQYYG

KKSTFHKLTFLYLDEDAAFAAAQAGTVDIAAIPAAFSKQQVNGMKLESVKTVDNRGIM

FPMQTAGAKSGDGLPAGNDVTSDIFIRKAVNVAIDRTALVEGVLEGHGRPAYSVSDDL

PWSNPDSVFADANLDEAKQILADGGWVDTDGDGIVEKNGTKAEFNLLYFAGDLTRQSL

ALAAADMVAQAGIKVNVEGKSREETKKLAYTNAVLFGWGSHDPLETYNLYSSTHKGEG

YYNTGLYSNPTVDHWMQQALQATTEEEALPFWQKAEWDGSTGFSYQGDAPWAWLVNID

HLYLVKNGLNIGEQQIHPHGHGWPVTSNLEEWFWEEELK"

CDS 949333..950328

/gene="gsiC_1"

/locus_tag="PIKHDJKN_00849"

/inference="ab initio prediction:Prodigal:002006"

/inference="similar to AA sequence:UniProtKB:P75798"

/codon_start=1

/transl_table=11

/product="Glutathione transport system permease protein

GsiC"

/db_xref="COG:COG0601"

/translation="MEDRVGWARFVGFKMLRLLSLLAGVSVVSFILMQFSPVDPVEAY

IGGDMIRVSAEQRSLIEERWGLNDSPVERLLTWGQALIQGDLGTSMIYRQPVADIIKE

RFMNSVALMAVAWVLSGLIGFGLGVVAAMRRNSRLDRLICWYCYTLASTPVFWIALLL

LMVFGVWLGWLPIGLGVPAGMAADQVTWGDRAMHMILPALTLSLTGVAAIALHTRQKL

VDVLDSDYILFARARGERGVQLFRRHGFRHVVLPALTLQFASFSELFGGAVLAEQVFS

YPGLGQATVQAGLRGDVPLLLGLVLCSAIFVFTGNMIADILYRIIDPRMKEEAIG"

CDS 950325..951308

/locus_tag="PIKHDJKN_00850"

/inference="ab initio prediction:Prodigal:002006"

/codon_start=1

/transl_table=11

/product="hypothetical protein"

/translation="MKQTLERPSQPAAQTAQHPKVNSPKSEAGNKRNWRSVLPRTHGG

KGYSGGSNSRFRNPRKWAMIWGGLGVTWIATVWLTGRLLPAESTLTSLMDRNLAPAWA

HPFGTDWLGRDMFMRTLKGLATSIQVGLLAACGGGLIALLLGLAAASGKAADRVISWI

IDLFLSVPHLVSLVMLAFVFGGGLPGVALAIALTHWPNLARIVRAEMIQLRSAEYIHI

SRRLGHSRMRIAVQHMLPHLIPQLFVGVLLIFPHAILHEAAITFLGLGLSPQQPAIGI

ILSESMRYLSAGMWWLAFFPGLALLLVVRAFDVLGGSLKTLTGTGSSREVV"

CDS 951311..952267

/gene="dppD"

/locus_tag="PIKHDJKN_00851"

/inference="ab initio prediction:Prodigal:002006"

/inference="similar to AA sequence:UniProtKB:P0AAG0"

/codon_start=1

/transl_table=11

/product="Dipeptide transport ATP-binding protein DppD"

/db_xref="COG:COG0444"

/translation="MALLEVEGVSVSFRRARGWFGHEETHVIQDLNLSVNEGEIVAVV

GASGSGKSVLAQAIMGILPANATIEGRISYAGEPLTMDRQLHLRGDELVYIPQSVSYL

DPLLKVGRQVQPVSQKTGRKHGSTLRSTMQKAEQELTGRYGLPQGTSGKYPFELSGGM

ARRVLMATATSGAPKLIIADEPTPGIHPEVLAETMKHFRQLANEGVGILWITHDITTV

LTAADRISVFYAGSNVETAQACDFTGTGERLRHPYTKALWNALPQNEFQPLSGTQPVA

GRQRVEGCSFAPRCIRATTACTNQRLELRKVRGGEVRCIHAT"

CDS 952257..952871

/gene="nikE"

/locus_tag="PIKHDJKN_00852"

/EC_number="7.2.2.11"

/inference="ab initio prediction:Prodigal:002006"

/inference="similar to AA sequence:UniProtKB:Q2FYQ8"

/codon_start=1

/transl_table=11

/product="Nickel import system ATP-binding protein NikE"

/db_xref="COG:COG4608"

/translation="MPLEVRNAGYRYGKGAWVFQNISMEVQQGEVVGLWGPSGCGKTS

LGRILAGYVEPLAGQVLLDEKPLPKTGACPAQLVFQHPEKAVNPRWRMRRVLKEAMIE

DQELLDDLGIQMNWLDRRPGELSGGELQRFCVARALGTATRYVIADEMTTMLDAITQA

QIWHTVMNIARQRNLGMLIISHDRQLLDRLCDRIVQMPTIAYGV"

CDS 952912..953121

/locus_tag="PIKHDJKN_00853"

/inference="ab initio prediction:Prodigal:002006"

/codon_start=1

/transl_table=11

/product="hypothetical protein"

/translation="MNTNHGFEYRDSPFCNFKETNALTNRAHAIRRNYGVSVLERIVD

TLYARIACIFTEFCYANDNKVAEVR"

CDS complement(953202..954398)

/gene="patB_1"

/locus_tag="PIKHDJKN_00854"

/EC_number="4.4.1.13"

/inference="ab initio prediction:Prodigal:002006"

/inference="similar to AA sequence:UniProtKB:Q08432"

/codon_start=1

/transl_table=11

/product="Cystathionine beta-lyase PatB"

/db_xref="COG:COG1168"

/translation="MSKNNNHTFDEPINRLSTGSEKWDALEEIFGVADALPMWVADMD

FAAPPSVIQALKTRMEHGIFGYTVRTDAYHAALTGWMERRHNWRINDEWVVFTPGIVP

ALSIAVQRFTEPGDAVVIQTPVYAPFYDVVRGQGRELMTNPLVENDGHYSMDLKHLES

CLKTGRVKMLILCSPHNPVGRVWTREELQSLAALCVQYNVLIVSDEIHADLVHQRGAH

TPLALISEAVSDLSIICTAPSKTFNIPGLCTSNIIIPNAKLRESFMQGVKTMGLANIS

TLGAVAAEAAYNGAEEWLDDCLAYIRGNMEYVQEYVAEHMPQIGIQLPEATYLLWMDF

RKLSIPHAQLCTMLLKEAGLAFNDGSIFGAEGTGFMRMNVACPRSTVEEAMRRLSVLI

GRLSTK"

CDS 954729..955424

/locus_tag="PIKHDJKN_00855"

/inference="ab initio prediction:Prodigal:002006"

/codon_start=1

/transl_table=11

/product="hypothetical protein"

/translation="MKNKKWILTAGVFGMVLTGSAGVYAGTQLETIKAYLNHGLGIEV

NGQKFTPTGDKGKNLASITYQGSTYLPVRSIAEALNTEITYDSKTNKVSIGSTSSSSG

SSSTSNSGSTSTASTDKNNQAEIVKAKYLPADLPLPTDAKIISLIESSVDGGKNKIIF

TYTTKETLLTIGTSYKDYYQNKDLAQYAQDVKADGFSIVGREEGKFAVSITGSISTTY

KGVNEVTVVWGEE"

CDS complement(955669..957669)

/gene="dpp5_1"

/locus_tag="PIKHDJKN_00856"

/EC_number="3.4.14.-"

/inference="ab initio prediction:Prodigal:002006"

/inference="similar to AA sequence:UniProtKB:B2RIT0"

/codon_start=1

/transl_table=11

/product="Dipeptidyl-peptidase 5"

/db_xref="COG:COG1506"

/translation="MKSLRGITSEDLFQITWVNDPTPSPQGGQLVYVSRKTNEARDGY

SSHLRLLNLENQKDRAFTSGDKDHAPAWSPDGSQLAFLREHEGKTQVWLIASDGGEAQ

QVSHLKHGVSSLLWSPDGLTLLVKSSMDMSEKDEDETEDIDKKLLQELVVDRIRMKSD

SGGLWNGRRTHLFRVPTEGGEAIPVTTGHYDVGDYAWSPDGESIAWIAQFPEEGEQYN

DYTLTNHVYRAKADGSDVQQLTPEGYSFGRLSYAPDGQSLALLASDRSYGNATLLKLY

TLPISGGQPVCLSKDWDVQINHSLVGDMRSHLTNTGPVFSRDGSSILCLVTIEGSVRI

ARFARDGSHAEYILPDEKEFYQFAELENGQIVAGVADVLRPGDLFLYTQPDEVGAEPI

QLTHSNPQLDEEIQLSTPETFWFDSSDGLRLQGWMLKPAGMVEGVKVPTILEIHGGPH

MMYGFTFMHEFQILAAQGYAVVYINPRGGLGYGQQFVNACRGDYGGGDYRDLMEAMDY

AVSRYPFIDESRLGVTGGSYGGFMTNWIVGHTDRFKAAVTQRSISNWLSFYGVSDIGF

FFTEDQIGGNAWDDTEKLWKHSPLAYVGNVSTPLLILHGEQDLRCPIEQAEQLYVALK

RRKQTTRFVRFPGANHELSRGGHPHLRVRRLEHIAGWFNEHL"

CDS 958069..958581

/locus_tag="PIKHDJKN_00857"

/inference="ab initio prediction:Prodigal:002006"

/codon_start=1

/transl_table=11

/product="hypothetical protein"

/translation="MAYSGEALFVAVGFFLMFDDSSAKGSAISSVIGLFSILFFGLCL

CYSLVKMIKKEPSFVVDEHGFVDSSSYTSGGLIAWKDVENIFMYEFMGQKMIGVKLWD

EKAFLERQNGMKRKLMTVNSSMVDATVSIAQNSLTLPLDQLYIMMVERWKRSNEGASE

GSSFDLFNNE"

CDS 958617..959483

/gene="dkgA"

/locus_tag="PIKHDJKN_00858"

/EC_number="1.1.1.274"

/inference="ab initio prediction:Prodigal:002006"

/inference="similar to AA sequence:UniProtKB:Q46857"

/codon_start=1

/transl_table=11

/product="2,5-diketo-D-gluconic acid reductase A"

/db_xref="COG:COG0656"

/translation="MQQGNQTRTIQLPDGTTLPAIGQGTWYMGEKQSSRREEVQALRH

GIERGMTVIDTAEMYAEGGAEEVTGEAIKDCRDDVFLVSKVYPHHADRKQMITACERS

LSRLGTDRMDLYLLHWRGGVPLEETVEALEQLKQSGKILRWGVSNLDTEDMQELWNIP

AGKHCAVNQVLYHAASRGIEYELLPWLRERRIPVMAYCPLAQGGRLRRELLEHSVIRE

IAKDRGVSPSQLALSWVIRDGDILAIPKAVQLNHVAENAAAMDVILTQNEMVRLNEAF

PAPKGKVPLDIV"

CDS 959673..960083

/locus_tag="PIKHDJKN_00859"

/inference="ab initio prediction:Prodigal:002006"

/codon_start=1

/transl_table=11

/product="hypothetical protein"

/translation="MSIEFDNNLPIYLQIMQYIKRQIVIGTLKAGDKIPSVRELAAEL

QINPNTVQRTFQELEREEVVETKRGLGRYVTSEESKIMTIKKEMAGELLDRFLTGMQE

LGIEEQDIVNIVADAVAEGKQSLHRDGIGGTVNE"

CDS 960076..960774

/gene="ytrB_2"

/locus_tag="PIKHDJKN_00860"

/inference="ab initio prediction:Prodigal:002006"

/inference="similar to AA sequence:UniProtKB:O34641"

/codon_start=1

/transl_table=11

/product="ABC transporter ATP-binding protein YtrB"

/db_xref="COG:COG1131"

/translation="MSNILEMNHVSKTYGGKKALHDITLDIAPGRIVGLLGSNGSGKS

TLMKLVAGLLHPSSGDVRVTGKAVGLETKSLVSFMPDRPLTESWMKVRDAIAYYRDFY

ADFDEAKAREMLDFMKLGEGEKVRHLSKGMNERLQLTLALSRKARLYLLDEPIGGVDP

VARGKILDAIVKFYDEDSSLIISTHLVTDIERIFDEVIFIREGELVMREEVETLRLKY

GKSVDEMFKEVYAE"

CDS 960771..961511

/locus_tag="PIKHDJKN_00861"

/inference="ab initio prediction:Prodigal:002006"

/codon_start=1

/transl_table=11

/product="hypothetical protein"

/translation="MMTLLKYDFRRNWNTLLAGLAVLIIVQIGLSMFFSEITGSLIGV

IAYVGAGVAIYVKMIKTYRSNIRSYNRRLVPVTGLSHVLSPIIFGTLCGLGLLAVATI

HYFIYNATHTGINFTSYINDSGIHLSEIVSLLLFFWWVALFCIIIIFLSISIGGSFRF

KTGPWISIVAFFVLINVISWLENTIFSGRFNPDELFQVTEKSTGISIMSNRIMWSDGT

WASIIFEIIVAVILVGVAVYLNNKKVEV"

CDS 961647..962144

/locus_tag="PIKHDJKN_00862"

/inference="ab initio prediction:Prodigal:002006"

/codon_start=1

/transl_table=11

/product="hypothetical protein"

/translation="MELYFRDNFFNAGYTEIMNHEQEQAGHLDLKSAFGSSLDVFDNS

GLICSGKFRMLSNRWDVTSADGREWGVLRARISFFSKKYEYDAGPRGVYDVSAPAFSQ

EYDITGMGGKIVASFRRTSGWFGSGAFVLDNQSEHLDTYELIAVVMGVHAINKRRNSA

AGSGA"

CDS complement(962261..963154)

/locus_tag="PIKHDJKN_00863"

/inference="ab initio prediction:Prodigal:002006"

/codon_start=1

/transl_table=11

/product="hypothetical protein"

/translation="MNLFEKMFNYQMMTRLNETGLFTWTSQERAWLRMMLNHPAAPEA

LNPDTLHKMHDMLEAEQDLDLQDYLTEKAKSEENSVFHPQLKPLRQMILHHQGFRLTG

RVRNGRISQDQFGFPYKLEYSMVKKEWYVLWYAPLYNKLMSTKLHSIIAAEAQPLQSE

TASQYTAKIAILTEKRKTTVTIEVLPEYNQELSRILYAFSCFSKQVEYVEAQQTYRIE

LTIPRNEMDYVLSKMRFLGKRVRIVDNALLRERMSETAAKVLARYAESELEALPERTS

GGARSHHGEELIVKTEGSDTA"

CDS complement(963102..964457)

/locus_tag="PIKHDJKN_00864"

/inference="ab initio prediction:Prodigal:002006"

/codon_start=1

/transl_table=11

/product="hypothetical protein"

/translation="MAKESFDKEIQFLRMLSLTSGAYNRKQYAERLGISVHTFDKTQR

RLKEIMQTVTDQRSGSEQNKEMADLVRFQYGESAEPMLLFLFRAKSMKETEVQRLSVL

LHTLQDQALTAMELLDACCAELPEELALPDEKTIRSDLKYLEEVGVIRKEPGGRPYRY

LLQQDILTGLTVEEQLELYDFVDIMANTQVPSVQGYILRDSLKKAIGASYPQEEATEP

FIYKYHYYSRILDEAHLYTLLSAIRQRKHVQFLYYSPKKPSSYSSQNTNPRFEREAGG

RSNRIVPLEVVYDHQYGRWYVIGYQGRRGFVKFRMEGITQLVEQDSIEEQYMLELKQQ

WAEISRYSWLVDTGNTVTVQARFFHPQDGQRNFILDRVRLQGQWGTIMPETEHTFLYE

IQVNGITEIKPWLRSFGSSCEVIAPQRLRQEMIKEWKEIAQYYESVREDVQLPDDDEI

E"

CDS 964971..966464

/gene="rsr"

/locus_tag="PIKHDJKN_00865"

/inference="ab initio prediction:Prodigal:002006"

/inference="similar to AA sequence:UniProtKB:Q9RUW8"

/codon_start=1

/transl_table=11

/product="60 kDa SS-A/Ro ribonucleoprotein"

/translation="MSRAKQLFNQPQPTTRNHGGYGAYERLVEEQYIQMLITNTLNNT

FYADTQQLMDDAMVSHQEMAEIDAGFMARALVYARNEGLMRLQPLYGLALLSKVDPDQ

FAKVFSKVVQTPADLADFLTILRGTGRGQGGRAVKRQVSQFLNGITEYWAIKYNGRGR

GYSLGDMIATAHPKPTDMKQQALFRYLRGHEVDLTDLPQLQALEKLKATPNPASRMHL

IEKGKLPYSVVTSIMQPTRTVWEALMSQMPTFALLRHLNAMDRAGVFEKSKNIDYVTG

RLADAEALRKSRILPFRFASAYEMIAREELRYALREAVELSIGNLPSLPGRTAIFLDR

SGSMQGDYLRIGSVLALALYKQTRGNSLFWLFDHMVEDARPKMDESILSQAHRIRAQG

GTDTGRPVRELRDIGETVDQIIMITDEQQNEGSPLYVELERYRRMMNPDLKAFIVDIA

PYQQAMVPPRDGKTFYIYGWSETVLTYIAETLAGYDTLASRVREIEI"

CDS 966489..967295

/locus_tag="PIKHDJKN_00866"

/inference="ab initio prediction:Prodigal:002006"

/codon_start=1

/transl_table=11

/product="hypothetical protein"

/translation="MTHVHEEMRDTIRQQLKQIEQEEQVRIIYACESGSRAWEFPSQD

SDYDVRFLYVRPLEWYLSIEDKRDVIERPISDQLDINGWDLRKALKLFRKSNPPLLEW

LQSPIQYDEPYSVAEHIRALSPLTFSPKSCMYHYLNMAKGNFRDYLQGKQVKIKKYFY

VLRPLLACGWIERYDAMPPMAFEELVQELVPQSAPLYTEIHELLRRKKAGEELDLEPQ

LPAIQTFLAERIEHFEQLAAQLENEQIIQYEELDRIFRFALKEVWIPTCD"

CDS 967315..967533

/locus_tag="PIKHDJKN_00867"

/inference="ab initio prediction:Prodigal:002006"

/codon_start=1

/transl_table=11

/product="hypothetical protein"

/translation="MHIIYGMDYLAPDNLSRLSKRKTSKKERHALMESALGMEGIERF

VRMEPVERVHECVLATLALESEPVDPRL"

CDS 967781..968032

/locus_tag="PIKHDJKN_00868"

/inference="ab initio prediction:Prodigal:002006"

/codon_start=1

/transl_table=11

/product="hypothetical protein"

/translation="MASMILLVKQTEDDEKVIYRFGPNERKMGLIEMNKVKESVKELE

PVQVEGVSPSFYFNRAAQRLVRCLFREGGKFPERTTCES"

CDS complement(968228..969169)

/locus_tag="PIKHDJKN_00869"

/inference="ab initio prediction:Prodigal:002006"

/codon_start=1

/transl_table=11

/product="hypothetical protein"

/translation="MKKEAELMIADIMLEVVLPVFLLIAVGSWMQKVFKLDLYTLAKI
NFYCITPAAVFMSMYHSDMSGELLGTVTLFYALYVIILYIVGSVFARSLRMNKGMKAA
FNNSIMLDNAGNYGLPINSLVFRGDPLASSIQALVMSLQALLTFTYGVLSIQGAKLKG
NYRAVIIGFLKMPVPYALLLGILFHMGNIPLPTFLSMPLTYAQQSMVAVALLTLGAQI
VKYPIRLYRLDVYISTFLRLLIGPAIGISIVLLLGLQGIAAQALIIASGMPTGVNASI
LAEEYDNEPDFAAQTVLISTLLNIITITALISFAKTF"

CDS 969270..969785

/gene="mug"

/locus_tag="PIKHDJKN_00870"

/EC_number="3.2.2.28"

/inference="ab initio prediction:Prodigal:002006"

/inference="similar to AA sequence:UniProtKB:P0A9H1"

/codon_start=1

/transl_table=11

/product="G/U mismatch-specific DNA glycosylase"

/db_xref="COG:COG3663"

/translation="MIPDHLDYGLSILFIGFNPSVTSGETGHHYAYKGNRFWRILERS
GLTPRLYDAQEDGELLKLGYGFTNIVARPTRGMDDITKEEYAKGRQILRQKLEEYRPD
IACFVGKGVYTQYSQRAKAEWGFQDDPVVKEIHEFVAPSSSGLVRMSMDEIVAIYSQL
SDFVSEKSRED"

CDS 969985..970557

/gene="sigW_2"

/locus_tag="PIKHDJKN_00871"

/inference="ab initio prediction:Prodigal:002006"

/inference="similar to AA sequence:UniProtKB:Q45585"

/codon_start=1

/transl_table=11

/product="ECF RNA polymerase sigma factor SigW"

/db_xref="COG:COG1595"

/translation="MTQQIPEEELIQRIIAGDKQLFAVLVDRYKNKVFGILRGMGASH
QDAQDLAQDTFLRIYRYLPTRREGSSFSSWVYTIAVNRMRDFMRQQKPVMTAVHTGLE
QTNGETPEKHVLHKEMQREIYRQMDQLPESYRLVLLLKYTNELSYEEIADITDMSPAQ

VRNALYRGKKTLKKQMERKGGLSTYEAYSK"

CDS 970538..972169

/locus_tag="PIKHDJKN_00872"

/inference="ab initio prediction:Prodigal:002006"

/codon_start=1

/transl_table=11

/product="hypothetical protein"

/translation="MKPIPNDEDRLWEQHLFREEPDADFTLEVMRKLDGVSMENAEHG

NPFTKKASKSHWMRRTGIAAAAVVILAGGAWFAFDHTSAPEQPSVNAVNHPPLPNIPV

PEELKYSYFADDYKRLKPLGLVVNPNINIEDQGYTLKIENVLVDRSQIVMTLQQNAPD

GSMINPVYPELGRVHITDEKGNSIASLSSPGDQTLYSSINQRFVYQLQDGIPDKVVVR

GELSHLKIPGYYNSEKGEFVEPSATVDWSFQFNIDMTKAKSLAVEAPMNNTYTTPEGL

KLDMTQLVRTPNGTKLDMNVSLDDELRAKVEEGWANYMDLIYHIEISETNEYRIFNGS

RPSARQAKFRLRDQSGIRDGGPLKLSETWDPAFVTVDAKNIRFVLDGYTLPVKEEKSV

EVDLDNMRKDMKDTSKPYTFVQFEQFGDQIVLYEFNYQSVQGSYNLPKGNVKGNSSLV

LVGNGRFRNAFNGDQWVAIDSQGTEYPVEVMGIPDKPNNNEENVVNDLKLVIRGFYKE

DGTKLTLKRTVVNREYRDVKWEVDLPSYTNLPWQK"

CDS complement(972317..973375)

/gene="melR_3"

/locus_tag="PIKHDJKN_00873"

/inference="ab initio prediction:Prodigal:002006"

/inference="similar to AA sequence:UniProtKB:O34829"

/codon_start=1

/transl_table=11

/product="HTH-type transcriptional repressor MelR"

/db_xref="COG:COG1609"

/translation="MSRKVSIQSLADQLGLSKYAVSRALSGKTGVSEATRARVLELAR

ALGYRQSTPGSSTVPTAIHTEQSEPPFALICMNQLNRGEPHYWQRVLSGMISACNERG

WHHAIVSPSLGIADEHTPPERAIAPHLDWERCAGVIAMGAFPHAVLQRLTQTGRPIVL

VDHQEPLLNCDTISHDNLEAGITVARYLMSMQCRRIGLITDDGRAASFAQRKIGIELA

LNHFHADSSRLTTFREWNIPYENGEWIDQLAATIKNMPADERPDAWIGANDDIALQWM

NKLQEMGFSTPGDCLVIGIDNVHAAATSSPPLTTVNLCKEELGQRAIEALQRRIERPG

TPKETVMLSTSLIPRESA"

CDS 973624..974943

/locus_tag="PIKHDJKN_00874"

/inference="ab initio prediction:Prodigal:002006"

/codon_start=1

/transl_table=11

/product="hypothetical protein"

/translation="MEQFRLPKIPMPHLGLPQAVQNILTEAEERLQHRPRLLAQFRNC

FPNTLETTTKLLDDGTTFVITGDIPAMWLRDSVEQVMHYVPLAKEDEQLQRIIGGLIK

RHTFYADIDIYANAFNETANGWHWDANDETQMSPWVWERKFELDSLCFSMRLAYAYWR

ETELTDIFDDRFKRVMESIVRLWKTEQRHGEQSPYRFMRRNCPAHDTLRNEGLGMPVN

YTGMIWSGFRPSDDACDFHYNIPANMFAAVTLRQMGEISRWVFRDEKLVSEMALLEEE

IRHGISLYGTYRHPEYGQIYAYETDGYGNFCLMDDAGTPGLMSIPYLEYASTEDIVYQ

NTRRFILSKENPFYYEGQAAKGIGSPHTPPGYIWHMALSMQGLTADNDKEMLEMIDLL

ENTDAGTGYMHEGFHSDDPNTFTRPWFAWSNSLFSQLVYKAMKKGIL"

CDS complement(975247..976302)

/gene="afr_1"

/locus_tag="PIKHDJKN_00875"

/EC_number="1.1.1.292"

/inference="ab initio prediction:Prodigal:002006"

/inference="similar to AA sequence:UniProtKB:Q2I8V6"

/codon_start=1

/transl_table=11

/product="1,5-anhydro-D-fructose reductase"

/translation="MSHPYRVVVAGCGAMSNTWVDYAMQRPDTEIVGLVDLYEQTAVA

LAARHGLSCPTFTDISEAIRATDANIVFDVTIPASHYGIAMAALQQGCHVFGEKPLAE

SFSDCEDIVQTARRTGNIQAVMQNRRFDPRIRAYRQLISGGTIGQVGFAGADFFLGPH

FGGFRDLMDSPLLLDMAIHTFDQARYILGANPVSVYCHEFNPPGSWYQGNAMALCIFE

MSDGSVFNYRGSWCTEGVPTSWEASWRVTGEKGTAIWDGNDDIYAEVVSAQHLDAEGK

PSFFQPCERIEGQLPVMEKTGHHGCLEDMFAALESGQLPETDCSDNQFSMAMVLASLE

SARTGQKVLIADLLKTV"

CDS complement(976378..977265)

/locus_tag="PIKHDJKN_00876"

/inference="ab initio prediction:Prodigal:002006"

/codon_start=1

/transl_table=11

/product="hypothetical protein"

/translation="MNNPLRIGTLVGGHDAIRVIPQIMKHGFESFNLTFWQTTGDLDL

AETAKRVREIVDEQGIVISAVSVFGNPLTGAGDNADTLASWERVIDHAQLFGADIVSG

FTGRLTDQPIDQSIPRFKEVFGELARRAADRGVRIAFENCDMGGTWQTGDWNIAHNPT

AWEMMFDAVPDENIGLEWEPCHQMSSLIDPIPQLRKWAPKVFHVHGKDATIAWDIVKE

YGVHGPREFVWHRTPGFGDTNWSDIITILRQNGYQGTIDIEGWHDPVYKDELEMTGQV

HALKYLKQCRGGDYVPNPV"

CDS 977396..978283

/gene="rhaR_6"

/locus_tag="PIKHDJKN_00877"

/inference="ab initio prediction:Prodigal:002006"

/inference="protein motif:HAMAP:MF_01533"

/codon_start=1

/transl_table=11

/product="HTH-type transcriptional activator RhaR"

/translation="MLDLKALHENTRIDHKSHPFQLFQNRCWRMKTEECILYLHWHEH

FELIFMRKGSALFHIDSRPYVAQAGEVIIIPGGALHVGYALEEGDIHYDSVVLNSALF

HDFTHDPVHLKYVAPYLEGKVRFPVKPAEHDIACQGYYSLLDEAIEEMASQPLAYQLV

VKSKLHALFTLLARTFMPHQLPDKTTGSYFPNRERFKHLIEQIEAEPTGKMSVTEAAG

QVGLNTYHFCKMFKKLTGRTFVEYVNGCRMTKAEQLLQESSLTITEIAAQVGCDNANY

FTKLYKQYKGMTPSQGRLK"

CDS complement(978403..979086)

/locus_tag="PIKHDJKN_00878"

/inference="ab initio prediction:Prodigal:002006"

/codon_start=1

/transl_table=11

/product="hypothetical protein"

/translation="MNRPDWFQAEEALQQIQVIGSDRNELVNIIGHVEGLNCIGTGTD

AAVFTYDGLPQYAFKMYSDHALDKLENEKQVYEQLKGLPYFPTYYGSGRNILVISFEA
GDTLLECLEKGIPVPEQVMLDVDEAREAVRSRGLNPRDIHLKNVILQNGRGKVIDVSE
YIQDGNDNRWEHLVWAYHNIYPRIKGTPISPRMLQTIKWGYNQLDQANVKMDDLSKKA
NRLFSKFMK"

CDS 979458..980459

/locus_tag="PIKHDJKN_00879"

/inference="ab initio prediction:Prodigal:002006"

/codon_start=1

/transl_table=11

/product="hypothetical protein"

/translation="MRRFCPFLYTVSIKGSHADPAYRKAEYLMAQYPQWSYSQSRASM
FDECLRKYYYHYYGAHNGWKAESADEMQVRLYRLKQLSNLYLVFGDLAHRMCESAVRS
REEGKDKPREHFLELTMRKLLNQAYVESMDQDQWRLDPKNRVMLSEMYYGDDTLNDRI
ATIKERASACVGNLYQTLTWEDLSRASTDILEIEKWDTMMLHDTRVYVKMDLLYRRSN
GNIVIVDWKTGKEDDFSDQLMLYASYVREHYRVPLEQIELRVEYLLTGTHREFTATEE
DILKVEENVGRYIVEMRSCVDDEHYNRPKDVSYFTPMPSHRACRDCNFREVCSERVV"

CDS complement(980609..981742)

/gene="ald"

/locus_tag="PIKHDJKN_00880"

/EC_number="1.4.1.1"

/inference="ab initio prediction:Prodigal:002006"

/inference="similar to AA sequence:UniProtKB:Q08352"

/codon_start=1

/transl_table=11

/product="Alanine dehydrogenase"

/db_xref="COG:COG0686"

/translation="MRIGIPKEIKNNENRVAMTPAGAADFVRAGHQVMIERGAGLGSG
FTDNEYQSAGAELRDTAAPVWAEADMVIKVKEPLASEYAYFRPGLILFTYLHLAAEPA
LAKALIESRVTAIAYETLEVNGTLPLLTPMSEVAGRMSAQIGAQLLEKTEGGKGILLS
GVPGVSRGKVVVIGGGTVGTNAAKIAIGLGADVTILDLNLNRLRQLDDIFGNQIHTLV
SSPSNIASAVAAADLLICAVLIPGAKAPTLVSEQMVTTMAPGSVIVDVAIDQGGIVET
IDHITTHDEPTYVKHGVVHYAVANMPGAVPRTSTVALTNATMPYALQLANHGAAAAIR

GSSSIRSAVNVLNGHITYEAVARDLGHAYVPAGQALENTAAVQ"

CDS 981933..983603

/locus_tag="PIKHDJKN_00881"

/inference="ab initio prediction:Prodigal:002006"

/codon_start=1

/transl_table=11

/product="hypothetical protein"

/translation="MRNDRVFTIKDILARPVFAKAQLAAGKDGTSRQVGWVHVLEITN

VSPFVSPHDLILSTGLWLQSEEGREEYLLQLIRSEAAGLCVEFGTSIHGIPEELIDLA

DRHQFPLIVFEQPVRFVEITQDIHALLINHQHQLLKSLETYSRQLQQRTLQSTDMSAV

LHLLHEYAAKPVVYISSMEPGSFVPELPSETQQAIYTWYEQEVEHLDLNDSDTELWFH

LDQEQVLLCHPVVCFGQVFSAVGMVVHPSAPVEYLKLLLDYTAKAAAALTLRSQFLEE

KMVRNQNELIQDLMNGNILHEEQAQTRMGLRLLVKGQYWFAGGVIEIEHRLKGIGRER

METNHQDILVLLRSLLKKNNLASLIMLKNNQVYVCCAKEESKTSTRNQLVKSLEGIIG

DVKRFASRNLKQVTIHAGFGKVRNRLTGLPDSLREAYQVIEVSRSVDRLEHIHFYERM

GIYQMLKALPRSFLQPFVNDHLGVLIDYDRSHHLRLVETLDAFLQNFGSKRDAAAQLF

IHRQTLYNRLEKLEELMGSDFMDQERRICLEMALLAHAMIEEEQDFPS"

CDS complement(983658..983942)

/locus_tag="PIKHDJKN_00882"

/inference="ab initio prediction:Prodigal:002006"

/codon_start=1

/transl_table=11

/product="hypothetical protein"

/translation="MRKNLDICNLSGQLGINRERLDEWLRQAGVSSKKMETETLPERS

SSPYRLAAPTSLSLAPSSGQPKLFRGRRPASGNSVLPRALRHARRISKNC"

CDS 984250..986319

/locus_tag="PIKHDJKN_00883"

/inference="ab initio prediction:Prodigal:002006"

/codon_start=1

/transl_table=11

/product="hypothetical protein"

/translation="MENSSEIQYDSHYGHGKRFVGRAKELMMLDRWLNNPEAPLTIFS

VTGMGGIGKSSLLSEMLSFARNQGATAIWMDGRSCGSTPSVFLEYLSSTLGLETMGSD
GNTRPLSLLSETSVKRRLVLAVDNYEELALLESWFMEVFVSKLPLHGVLIILASRPEP
ASAWRTHPRLRQRLVQMPLQHFTGEEVSEYIAVAGSLNKSVAGTIARMTDGHPLGLAL
AVEAADQRRDIPISDWTELSQMISARLLLELTLPRLHPMVEVLTLLETANQELLASVL
QTEVTLEEYNVLRRMSFIRSGPNGLALHDMARLHLLRDFRQREPQRLQAMRVRIAGLL
KPLHEQAGPHERRQITRKMLLLCQESMLQHRKYADVSADSLFSPMEVMRAEDLPALHK
LLQQWYEYSVEPWQAPSYPAFLDELAQRYPEGIVLMRDKEGDPIAMFITVLVHRESSG
MLTNYFPAEMQECFTPEELDCDPDQTDTHFAVLAAARDDVPGYKREELVGYMSLDRLS
LLGEGARVILIATNPHLKLFLQSIGFQSRRTRTRLCDVYADEADVLDLDLRSGQFGDW
VMSLLCPDTKSSGLQQGSSDVSQVPWSEQDVRKMLTHLHSPGALQESAERVNGMTDGI
HLQSHILDLLEGRVHGISQQDQMLLYAAYWTHAGNPTAAAQVCSMSRATFYRHLRAAL
IRLARVL"

CDS complement(986499..988448)

/gene="rhaS_4"

/locus_tag="PIKHDJKN_00884"

/inference="ab initio prediction:Prodigal:002006"

/inference="protein motif:HAMAP:MF_01534"

/codon_start=1

/transl_table=11

/product="HTH-type transcriptional activator RhaS"

/translation="MMLWNHASFNIFDIRRVVVQAGQTLRSYVLPASVFIMSVRGSAQ
IILDETSYNTRRFQIFHAGKGSILNVEAGQEGFEYYCLYYKAKLSLPAHNELAALAER
LRPFHVTYSLVPAEPVILYRCLTDMLKEWGKAEALGHLRAKTLLFQFITEVLNQMQTQ
GARSQKRDLAAQIITLIQSRYAETITLELLSENLNYSVPHLSSYFKSRTGLSPIDYLI
KVRIDKTAALLLETDATLKEIASSVGYQDPYYLGRLFKKYKGVSPSHFRAEHRAPQLE
DSPSTTMRLSIGPPKTLSYTVVDDNDYQRVGDGEDELNMYRGSRPPITAVLLLSFMLL
ISACGTEATGNSSSGASENATTPTNTKQGQAAGTTTAEQPQTKTVSTITGDIEVPMQP
KRIVAGEYLGSLIALGVTPVGTSSHHIANPYLHDALKDVEDLGDGNGNLEKIAAMEPD
LIIMDDMYAEMNEQLTKIAPTVIVPYASFKTVHEEVSYFGELLGKQEEAKAWLADYDR
RTAAAKERVLQVIPADSTFSILEYMGKDISTVGANYGKGGPVIYNVLGFKPPAEVIAE
LTDPGWASLSTEVLPKYAGDYIVLTTDNQTMEDLKADPIWGGLDAVKNDHVYIWGADR
SWYWDPIAILTQTEELADWLVETKH"

CDS complement(988542..990236)

/gene="norR"

/locus_tag="PIKHDJKN_00885"

/inference="ab initio prediction:Prodigal:002006"

/inference="protein motif:HAMAP:MF_01314"

/codon_start=1

/transl_table=11

/product="Anaerobic nitric oxide reductase transcription

regulator NorR"

/translation="MKHLLPELMTLLRTNMTILDSDLSMPVIPSTTGLADAIPLFDTS

PYLLVQDDGPLLGYIAVSDVLQEMMHAHRLMEAYFETTLETAGSALTLINEEAKVTYW

TSGAEHVFSINKKDIIGKPAADFFPPDRLQSLKTLYTGETVYRKQHQPRPDLFALINA

RPVQLDGRIVGAVAAEVDITTEIRLHQELLHMTSKVQHLEKAVARLRPDSDPFARIKG

SSPVINQCLETIRKISTTSATVLILGESGTGKELFAKAIHDLREPQTAPFIAINCGAI

PGSLFESELFGYEKGAFSGADPKGKKGKIELAEGGTLFLDEIGEMPLELQVKLLRVLQ

EKSYFPVGGTRMKQAGCRIIAATNQNLLGMIARNQFREDLYYRLNVINLVIPPLRMRK

EDIYELTQTFLQEFSLLYNRHIELVPPEVFKLLFHYDWPGNVRELRNVIERITILTTD

GEVKPEYLPDTFISSIDQEQSILHGTVQLHSQPDLESGSSPLPGSTDTSVSTHVGEQD

EPADTSTLSYQEQLDAHESELLLQYLKAAGGNKRTLARQLGISRATLYNRMKRLGL"

CDS 990734..991720

/locus_tag="PIKHDJKN_00886"

/inference="ab initio prediction:Prodigal:002006"

/codon_start=1

/transl_table=11

/product="hypothetical protein"

/translation="MAILSTGPIENNISGMTGARPTQFVTIKIDNRNLTDVFNIHLLG

YYLDGVRTLYVEELINVLPNQVITKDYSGNFDAFEFVFSMTDTAASEAQVSVWGKDAE

GELVTAHRLVSQELLGETMGIVGVTGATGATGVTGATGIAGVTGDTGVTGVTGNTGVT

GATGDTGVTGDTGVTGATGDTGATGATGATGQGLSSYGYIFNTSAQSVATETDITFDS

NENLTNITHTPGTAEINIGNAGDYAVFFIVTGVQANQFTLYQNGAPVGGSVYGTGAGT

QPNPGMIIITAAPGDVLTLRNHSSASAVDLQTLAGGTQINANASILIQQLSS"

CDS complement(991918..992841)

/gene="fadM"

/locus_tag="PIKHDJKN_00887"

/EC_number="1.5.5.2"

/inference="ab initio prediction:Prodigal:002006"

/inference="similar to AA sequence:UniProtKB:O32179"

/codon_start=1

/transl_table=11

/product="Proline dehydrogenase 1"

/db_xref="COG:COG0506"

/translation="MSIGTEMYRKTLLTVAGNKAVENLSIKYGKKLAGKFIAGNTLEE

ALEEIRVLNNKGIMATLDHLGEGITHLREAALYRDEYIRLVEGIARQGADSNVSLKPT

QMGLALDPEEGYRNIRAVATQAKLHNLFVRIDMEDSPFTQATLDIVRRLHSEGLDNTG

TVLQAYLHRTVEDTRDMIREGIRLRLVKGAYKEPASVAYQNTSEVIDQFKTMIRNHLD

QGVYTAVASHDDHIISWTKQYAKDRGISPDAFEFQMLYGLRMGEQERLAREGYRIRCY

VPYGTMWYPYYTRRLAEKPANLWMVVKNMFR"

CDS 993088..994635

/gene="rocA1"

/locus_tag="PIKHDJKN_00888"

/EC_number="1.2.1.88"

/inference="ab initio prediction:Prodigal:002006"

/inference="similar to AA sequence:UniProtKB:Q9K9B2"

/codon_start=1

/transl_table=11

/product="1-pyrroline-5-carboxylate dehydrogenase 1"

/db_xref="COG:COG1012"

/translation="MNIPFVNEPFTSFAVPANREAFENALRKVEAELGQEYSIIIGGQ

KITSARTLSSVNPAYKNQIVGKIYQADQELAEQAIQTAAETFRTWKHTDPNERARYLY

KAAALMRRRKHEFSAWMVYEAGKTWPEADADTAEAIDFMEFYAREMQRLSEPQPLTRI

AGEDNELSYIPLGVGIVIPPWNFPLAIMAGMTSAALVSGNTVVLKPASTTPVIAAKFM

ELLAEVGVPDGVVNYLPGPGSEVGDYLVDHALTRFISFTGSRDVGLRINERAARTAPG

QKWIKRVIAEMGGKDSIVVDSDSDLELAAESITASAFGFSGQKCSACSRAIIHKDVYD

EVLQKVIERTKQLSMGSPLEVGNQVGPVIDDKAYAKIAEYIEIGKAEGRLVLGGSTGN

EAGYFIEPTIIADVDPQARISQEEIFGPVLAFIKAESFEDALHIANNTDYGLTGGVIS

RNREHLEQARREFFAGNLYFNRKCTGALVGTHPFGGFNMSGTDSKAGGRDYLLLFTQA

KLVSEKY"

CDS 994757..996061

/gene="rpoN"

/locus_tag="PIKHDJKN_00889"

/inference="ab initio prediction:Prodigal:002006"

/inference="similar to AA sequence:UniProtKB:P06223"

/codon_start=1

/transl_table=11

/product="RNA polymerase sigma-54 factor"

/db_xref="COG:COG1508"

/translation="MLGIQLVQEGRIRLSITPEMKQSIHLLTLSGQELARYLQEAADE

NPVLELEEPSTLRTRLPRRLNQERFSSYDPLLQMKGAEPTLEHLLISQLRLQNLPPAM

ETTAVYLAGCVNDDGYLPMELTEVASVRGISMPEAEAALDILQSLDPAGVGARNLREC

LLLQIRRDPLANQHAEHMVAKGLEELVRFHPGRAGTRLGISSQQAQAAYDYIACLDPK

PCRSIGCTERAHYIIPDAVVQIRNDELSLRLNSAGVPRVSLNEACCRWIKEIDADEVW

SGRAAEARAIIRSVRMRRRTLMRVLAAIMSEQESFLREGPAGLKPLNLAIIAEAIGMH

ESTVSRAVNGKYVQTPHGVFELKAFFATGLETTNGEMASAPAVKLRLKELIRSERAER

PYSDSQLAALLTNEGIVISRRTVAKYREELQILPSLERKRWA"

CDS complement(996220..997650)

/gene="aspA"

/locus_tag="PIKHDJKN_00890"

/EC_number="4.3.1.1"

/inference="ab initio prediction:Prodigal:002006"

/inference="similar to AA sequence:UniProtKB:P0AC38"

/codon_start=1

/transl_table=11

/product="Aspartate ammonia-lyase"

/db_xref="COG:COG1027"

/translation="MSTMSTRTEKDFIGEKEIPAYAYYGIQTVRAVENFPITGVPVHR

ELITALAAVKKAAAITNMELKMLPRKIGDVIVMAAEEMMKGHHLDHFIVDSIQGGAGT

SMNMNMNEILANRGLELLLQNKGDYFHCNPNNHVNMSQSTNDVIPTALRIAAYRLSET

LLDTMKRLQDAFRKKEQEFNDVVKVGRTHLQDAVPIRLGQEFGAYARVIGRDIERLEF

ANRRLLTINLGATAVGTGLNAKPEYIVKVTEHLSDVTGLNLQTAEDLVDATQNTDAYL

ELSAALKVCAVSLSKICNDIRMMASGPRAGFNELRLPPRQPGSSIMPGKVNPVMAEVI

NQVSFQVMGNDHTICMACEAGQFELNVMGPVIAFNLLQSLKIMNNGIDVFTRYAVEEM

EANRERCALIMKQSFSVITALNPHLGYDVAASIVKEALKTGNTLQEIILERGLLTPEE

LEEILHPEQMTTPGIAGEHFLHNMER"

CDS 997971..999248

/gene="mcpB_3"

/locus_tag="PIKHDJKN_00891"

/inference="ab initio prediction:Prodigal:002006"

/inference="similar to AA sequence:UniProtKB:P39215"

/codon_start=1

/transl_table=11

/product="Methyl-accepting chemotaxis protein McpB"

/db_xref="COG:COG0840"

/translation="MKWTLGAKTVAGLVLISLITYGTSGFFIFFLQDWLSFSMPSWLY

ISIVLIMGVCWNAILGWFASRWLTRPIVHLSRAAQQVSSGDLTAEIPERRTQDEITVL

YDAFRVMVFNLRSIVNDITDSTRTTSQNAQSLSEAIAQAAEQIEMMSDAVDHIAVGVE

EQKTTSHQSLITADEMLNDFQRMHSQSIGMTEMSGQMERSVDHTKQTFSSLMKGMDEL

TESHNRSRDIMVLLEKEASDIEAITQSVKNIAEETGLLALNASIEAARAGEEGSGFAV

VAQQIRKLADESKQSVHRINELISRVQQRIKDTAQLIHEQHGLVVNESERTISVDQTL

QELTGTVEVFMKGAHEIGSKIGEQTSRVEQTHGHVKKIQGKAGSFSDEARRIMDAAHE

ETAIMQEISSSAEELRQLTDRLMDKTKAFRMQP"

CDS 999418..1000263

/locus_tag="PIKHDJKN_00892"

/inference="ab initio prediction:Prodigal:002006"

/codon_start=1

/transl_table=11

/product="hypothetical protein"

/translation="MKTWTGILLMIILAVATISTTSSARTMNESQKQLNIDDQSSQTS

AVVASNIETMNEAAFTTKESIPVKGEYVSLAPENENVRLYPMKVEGSGYIYNGMVLEV

NGKKREFSKWQGEGGSYKPEVHELDLNGDGKKEIVVLFTAGHGTGIYLGEAHIIDPDT

LEEMKMQSLNEIVKQHVNSKVSVTPGQVTINVTVDGVHAEPSLMEEDTSEGVYNEQLK

FGGVVYYSVENGKLVVSASGAYGMGLYDGDLNFAYDYKDGEWQAVNLEYSTEEYEDEY

YESFD"

CDS 1000512..1001225

/locus_tag="PIKHDJKN_00893"

/inference="ab initio prediction:Prodigal:002006"

/codon_start=1

/transl_table=11

/product="hypothetical protein"

/translation="MKKKVVLGLMVGTLTLGIGTGALAATGLEPIKAYLNSKISLKLN

GTTVTAKDANGNTVLPITYNGTTYLPVRAVGTLLGTEITYDSATSSVNIGSNNVSAPP

SETGKLTLSSLGTAALGTSDWHTKDPAETAYKGKDYKDVYLHTDTAKQGKSFQVMTQK

KYAKLHLDLAVLGGSQKLEIMDENNTTLKTVSLSPEDGLIGVDVDVADTDFIFVEIVD

EAPGSSLFVPLTTSYLTKK"

CDS complement(1001328..1002302)

/gene="mhqA"

/locus_tag="PIKHDJKN_00894"

/EC_number="1.13.11.-"

/inference="ab initio prediction:Prodigal:002006"

/inference="similar to AA sequence:UniProtKB:O34689"

/codon_start=1

/transl_table=11

/product="Putative ring-cleaving dioxygenase MhqA"

/db_xref="COG:COG0346"

/translation="MKIKGLHHVSALTAHAAENYRFYTNVMGLRLIKKTVNQDDVSVY

HLFYGDEKGNPGTELTFFEIPMAGQTREGVNSISATSLRVPSDAALTYWLQRFDEFDV

PHGEITERGGRATLSFTDFEGQRLILVSDEHNTGVAGGKPWDQSPVPVEYGIVGLGPV

HLTVKDASLTTPVLTELLGFRQKGTYPAFVAGQPDVLVFESGEGGSGSEVHVEERNDL

TQERPGRGSVHHVAFRVDNEEELKQWVERVHNFRFPNSGFVDRFYFRSLYFREANGIL

FELATDGPGFDTDEELAYLGESLALPPFLEGRRAEIEANLKPLDTVIR"

CDS 1002531..1003292

/locus_tag="PIKHDJKN_00895"

/inference="ab initio prediction:Prodigal:002006"

/codon_start=1

/transl_table=11

/product="hypothetical protein"

/translation="MAYPQWLDPYDAEPFMLFSTSHLWAIGVIAALVILMFLFRNWLR

SFSSNTRRTFRIALACVMFGCEIVLQLWYVYGDVWSLQTSLPLELCSLSLLLSALLLM

TRSRLLHSALLFAGIAGALMAIVTPNLGYAYEHFRFIQFFTAHACIILALLYMTWVEQ

LRPSWRSVLGSMIFVNVAALVVYGVDVLLDANYMFLRHKPNTPSVLDMLGPYPLYILG

EEVLALVMFSLMYVLLFAIPERLNNRVKRGKSSAL"

CDS complement(1003448..1004242)

/gene="lvr_1"

/locus_tag="PIKHDJKN_00896"

/EC_number="1.1.1.-"

/inference="ab initio prediction:Prodigal:002006"

/inference="similar to AA sequence:UniProtKB:Q9LBG2"

/codon_start=1

/transl_table=11

/product="Levodione reductase"

/translation="MTEQRLQGKVAIVTGGGSGIGQATAIRFAEHGAKVFMLDRTPEN

AEKTKQTIENAGGEAHVIECDISKPDNVQKAINQVAEQAGQLDIVFANAGINGTMAPI

ETMEPEDWDQTMGINMRGTFATVKYAIPHLKDRGGSIIITSSINGNRVFSGIGFSAYA

SSKAGQTAFTKMAALELARYKIRVNAVCPGAIDTNIDDNTYPSDDLKEVQIPVEFPEG

HEHPLKGEPGTSKQVANVVLFLASDESSHVTGTRIYVDGAESLLRG"

CDS complement(1004401..1004658)

/locus_tag="PIKHDJKN_00897"

/inference="ab initio prediction:Prodigal:002006"

/codon_start=1

/transl_table=11

/product="hypothetical protein"

/translation="MAKSKKHTPAPKAAQDKPTTLKDLLSSDVLEKLKAQADEAKAAE

ADRKEQERLQAEEVRKAEQKRKDNDFEYLLNNSSLDWKKHK"

CDS 1004787..1004969

/locus_tag="PIKHDJKN_00898"

/inference="ab initio prediction:Prodigal:002006"

/codon_start=1

/transl_table=11

/product="hypothetical protein"

/translation="MKTGINSYIITDTATIPWRKESLFVEVNRSLLKGLAWGVAFSCP

LWIAIIGWLRLMGWMY"

CDS complement(1005152..1005799)

/locus_tag="PIKHDJKN_00899"

/inference="ab initio prediction:Prodigal:002006"

/codon_start=1

/transl_table=11

/product="hypothetical protein"

/translation="MSFRWKKTTAVSLVLAMLLIVISGCGRPSSSATETPAPVEPAGP

NPVATIEMSDGQKIVIELYPEIAPNTVNNFISLANKGFYDGLIFHRVIPGFMIQGGDP

DGNGSGGPGYAIKGEFTSNGHKNHLNHTRGVISMARTNDLDSAGSQFFIMLADADYLD

NAYATFGKVTEGMDVVDGIAAQQIGEQDKPVTDQVMKKVTVDTHGVEYPEPVKMP"

CDS 1006057..1007268

/gene="sbnD_1"

/locus_tag="PIKHDJKN_00900"

/inference="ab initio prediction:Prodigal:002006"

/inference="similar to AA sequence:UniProtKB:Q2G1N0"

/codon_start=1

/transl_table=11

/product="Staphyloferrin B transporter"

/translation="MDFSWKRNLVILWIGVFFCSTAYSISIPFLPLFLSGDLGVRDHL

EFWSGLAFGITFLASALVSPFWGSLADKYGRKPMLIRSGYSLAVLYLINYFVQDPYSL

IVVRLFQGLLAGFVPAAIALVGTNTPEEKTGYALGIMSTAGATGGIIGPLIGGVVSHY

YGNRNAFLFSSIVVLVSALIATFWVKEENFNRNKARSHVMDDIREARANRLFITVLGM

MGICTFSVMILEPLLTVYVMEMGIQPDRASLSSGIIFSAVGVATVIMAPRWGKIGSRI

GYGKVLIIGLVGGAIGNLLQFFTTGYIGFGILRFVYGLFFAAVFPAINAMIVQVTEPG

FRGRAFSLNQSASQIGTMAGPIIGGVLGGWLPIRWIFIINGVALLITAIVAKWSRLEE

KLPGSGKPLAK"

CDS complement(1007448..1007645)

/locus_tag="PIKHDJKN_00901"

/inference="ab initio prediction:Prodigal:002006"

/codon_start=1

/transl_table=11

/product="hypothetical protein"

/translation="MKDDDHKRQVHEPDNLVTERDIDPNFGLFTEDSFPEALADEDQL

DAVEHAIPKEKVTRNDSSEDE"

CDS complement(1007684..1008052)

/locus_tag="PIKHDJKN_00902"

/inference="ab initio prediction:Prodigal:002006"

/codon_start=1

/transl_table=11

/product="hypothetical protein"

/translation="MSDRPISNEESERYYDRYQRSRDIPLETEVPEGDIDTFDEVTGK

RIDTDNTLNPPLLAATEEPELESRIAEPALESVPDADLIQPNSPVDPAAPDPNALHGT

DLLNGAGADAKPENDIPPRR"

CDS complement(1008253..1008414)

/locus_tag="PIKHDJKN_00903"

/inference="ab initio prediction:Prodigal:002006"

/codon_start=1

/transl_table=11

/product="hypothetical protein"

/translation="MNEPHRTVEVEHKDVQHKSDSSMVASTFIKYAAYIIIFFGFLYF

LVKYVFPKF"

CDS 1008559..1008840

/locus_tag="PIKHDJKN_00904"

/inference="ab initio prediction:Prodigal:002006"

/codon_start=1

/transl_table=11

/product="hypothetical protein"

/translation="MDWFTLGNMITQIRIGQKASTPGFSRTVIRRPDGLFWVGGIWSG

QIVQLRDYLFSDIWTIYDDEETEQWLEYRTKIEQKEREMIENQFEDLRG"

CDS 1009004..1010905

/gene="rcsC_3"

/locus_tag="PIKHDJKN_00905"

/EC_number="2.7.13.3"

/inference="ab initio prediction:Prodigal:002006"

/inference="protein motif:HAMAP:MF_00979"

/codon_start=1

/transl_table=11

/product="Sensor histidine kinase RcsC"

/translation="MKNVPKAIIILWISMLNILCLPHDFVSATAGEVDRPVSITGWEV

KWGNVHDQGFISEVQGSEEIWEKQGAEKLEYSSVEAPSGSMWTRVTIPKINEDSSAIR

FENIKGNHIVIYLDDRKVYENYHYNYDNNAVLLPLSSENSGGKLYVWSENEKGRLGIY

GTVQVGPYATLQEKYIHNGLLDVILGATFVFTALTMLSCTFFLGKFHKGMWISLCIVM

GSIGTMIITYSQFLYTFYQVYGNLYSVLFDLAMLMGMPALCYFFEQIIGPGRYGIFTK

LRKFQFIYSVVAVAALMIDFISGGQWDMLYSLLVQNVIGFVLVILLSILMIGTISKAL

QRSREALLLATGFGTFALISVAELLWYYQRNGTYHLIWWKWSMVAFIISLIAILGSRF

AEKHTKVLEYSKELELFNNELQRSEKMEIISELAASVAHEVRNPLQVTRGFLQLMTEQ

EDNKNKGYVRIALEELDRASGIITDFLTFAKPEFDHIVSLNIADEFNHIEGILVPMAN

LEGGKITTDIPPDLWIRGNSSKFKQAFINIIKNSIEALQGQGQIDIWAYAQDGIIKVH

VRDNGEGMDEEALVRLGEPYFSNKIKGTGLGMMVTFRIVEAMHGQISFTSTKGVGTEA

VVSFAAFVE"

CDS complement(1010982..1011224)

/locus_tag="PIKHDJKN_00906"

/inference="ab initio prediction:Prodigal:002006"

/codon_start=1

/transl_table=11

/product="hypothetical protein"

/translation="MMVSEKTLHEDIIEKAWTDEHFRQQLHSNPKQALREAFGIDIPE

HIQVRTVEEQQNDYVLVIPPNPSKVDYDVNCGPWRS"

CDS complement(1011279..1012226)

/locus_tag="PIKHDJKN_00907"

/inference="ab initio prediction:Prodigal:002006"

/codon_start=1

/transl_table=11

/product="hypothetical protein"

/translation="MRREWFDPFRSDVEVVFAAAEQLVQKYPEPMSEHALEQLHLVNP

LLRDSGHSYIGYIIPLWMQLSDGLPPEKAHKLSTACLLHMLYFLNQDEVMDEQPEDVT

LKLSLGNLYYVDAICAYSELFSLSSPFWTYFRQYVEDWAMSVTGENSIDYFKRSPLLI

AQKAAPLQLGAAGSLLLLEQEHRITPVCSAVNIALMTLQMTDDFTDTKQDAVHGNYNS

FLSHISAALEHNYPAHPVSERVHDNVYNTQLMNSYVDIAQQYNIRLTSSNSGISHLEA

FNSYLCGILGQTVHDIEQHKKMLFQGGFHYWISEKQHLS"

CDS 1012447..1013181

/locus_tag="PIKHDJKN_00908"

/inference="ab initio prediction:Prodigal:002006"

/codon_start=1

/transl_table=11

/product="hypothetical protein"

/translation="MKYRWKKWMAVGLAAVFITASTGYTASADPNGSVEDQVATPSSS

TNQTKPCMQAGHGLFMIGETADLLGIGLRDLKEQMELGKTLTQIAKEHKGLSEDQLLE

KLKPSLSQRLDKAAEEGCLTKEQAAAAKAGMDTKLKKVINTPLRELRREFAHHGNRHP

MMDKGAIARFIGVTPEQLHQQLQSGKSLAEIAQAKGISETQLVDKLKEQLTGDLKRFV

NQKGNAHHAPGPHEPVPGRSASTEVK"

CDS complement(1013232..1014482)

/locus_tag="PIKHDJKN_00909"

/inference="ab initio prediction:Prodigal:002006"

/codon_start=1

/transl_table=11

/product="hypothetical protein"

/translation="MNKPRIVEWTQLRGLAFLAIVMQHNIAEYIYRADIEQPDSIMLT

MIYHLTRFGTPTFVFLSGVMLFYHHSHTKPDYPKFIRKRFGDIYVPFVVWTFIYWLFV

RIFTPEFWLSGMPDFRSLVRELFLPQTGYHLWFVIMVFQFYILFPLFLAGAKFIQRRI

EKVTRFTPMQSVITLILIGMALYTLLMKWSYYDMGGWTASLPQPWSGLLQYRSYSWVM

YWFYFLLGAVCAWSVDSWRSWTAKVLPWMVCLFIGMYIWLGYDVLRGSGDVVNLNIST

YLKPTTFIIIMAQMFMFYGFLVLLRGKDTPFQRLLTWIGRYSFGGYLVHALVLYCIAY

ITRPLTLTGWHLPITFLSFLVTVATSLAISWGLSQLPGSRFTIGLQRKKPLSPRLHDT

VSASRTSSPERNPSASANRSPETS"

CDS complement(1014645..1015634)

/gene="msrR"

/locus_tag="PIKHDJKN_00910"

/inference="ab initio prediction:Prodigal:002006"

/inference="similar to AA sequence:UniProtKB:Q99Q02"

/codon_start=1

/transl_table=11

/product="Regulatory protein MsrR"

/translation="MLKKWLWGTSLTLALAIAGVIVYYGYSIVHFANSISTASETSSS

DTNQNTDTPTTPVPKWEGKERVNILLLGGDTRGDDAGRSDSVMVASIDPVSKKAHLFS

VLRDTYVDIPGHGKSRLNAAFSYGGAELTKQTVGDLLGIPIQHYVYTDFIGFMALVDA

VDGIDIDVEKDMYYTSKADKHMYDIDLKKGLQHMDGKTALQYVRFRHDATSDFTRTER

QRIFMTELAKKMQSTTSLFKIPEILEAVAPYIETDLSPTQMLKLASLGFDINVNEIDK

QQIPPNKLLTNELAGSAQVLGVNKPKLQSYIQNLFEEDAKSSEDESTKVQDLN"

CDS 1015819..1016370

/gene="bcrC_1"

/locus_tag="PIKHDJKN_00911"

/EC_number="3.6.1.27"

/inference="ab initio prediction:Prodigal:002006"

/inference="similar to AA sequence:UniProtKB:P94571"

/codon_start=1

/transl_table=11

/product="Undecaprenyl-diphosphatase BcrC"

/db_xref="COG:COG0671"

/translation="MNQSVFNWINQFADRIPFLDWFMITSAEYAVWVMIGLLVIVWFL

GNPSKQRIVFYACVASIVALGLAKWGISPVVGHPRPFMEGTVNQLVPHVPDPSFPSKH

ASFVFALAAASFFIGRRFGGWMLLLAVLTGVSRVYVGVHYPGDILGGFILGSLVSVIL

IATRNYTKTIPDFFINIHRRIFR"

CDS 1016573..1018309

/locus_tag="PIKHDJKN_00912"

/inference="ab initio prediction:Prodigal:002006"

/codon_start=1

/transl_table=11

/product="hypothetical protein"

/translation="MKDARGNVTGKLRLTIRMKLLGGFLIVVALLAFVSIYALTQIHD

MSKKADEIDKMWMPSVSLLGMMNGDISDVERLALAVIVETNPTEITKMNEALEQLQAK

IENERKELVTLIAGNTEAVGMYNTFSTNYEAYLAKMPEFMKLGMANNYEEASRLHSEA

YPLWFTANDTIAQLITMGNSLSDAATNTSVESAEQAFNVILGVTIVAFLVAMFIAFFI

ASIISRPIQKMNAAAMLIANGDLTSETIVLKNKDELGTLAASFNTMTGNLREMIQSVS

MTSEQVAASSEELLASAEQNTRASEQISETVEELAVGTSDQVDMVKRSSQAMSEMALG

SEQIAELAQSVSVSAVDAANQSAEGNMIIQQAVEQMGSVRNSIASLTELVTGLGERSA

EIGTITEVINNIARQTNLLALNAAIEAARAGEHGRGFAVVAGEVRKLAEESSTSAQRI

TDLVQLIQKDTDHAVQAVKVNSNETEAGIEIVTAAGQAFEQISNVVNKVAGEIQEVSA

GSEEMSASTNEVVGYVDQISNIAGEAAGGVHNVSAATQQQLASMEEIASSAGSLSKMA

EELQEQINKFRV"

CDS 1018633..1020702

/gene="eglA"

/locus_tag="PIKHDJKN_00913"

/EC_number="3.2.1.4"

/inference="ab initio prediction:Prodigal:002006"

/inference="similar to AA sequence:UniProtKB:Q5YLG1"

/codon_start=1

/transl_table=11

/product="Endoglucanase A"

/translation="MKMKGSWWRRIAILALSAGLLAGSTSIQAWNGKADAAAGNPNYA

EALQKAVYFYETQRSGNLPEDNRVEWRGDSGLNDGADVGVDLTGGWYDAGDHVKFGLP

MAYSATMLAWSVVEFREGYEQAGQLEEIKDNLRWATDYFVRAHTKTNELWGQVGAGNI

DHAWWGPAEVMQMNRPAYKIDASCPGSEIAGETAAALASSSIVFRDSDPAYANKLLQH

AKELYSFADTYRGKYSDCITDAQSFYNSWTGYYDELAWAATWLYMATNDSAYLSKAIA

TANLWQADGQNGNWAYTWTQGWDDKHYGAQILLARITSDLNMPEATRFIQSTERNLDY

WSVGTNGQRIKYTPGGLAWLDTWGSLRYAANASFIAFVYSDWVSDPVKKARYQDFAVS

QMNYILGDNPRQSSYVVGYGQNPPKHPHHRTSHSSWTNNENVPSEHRHTLYGAMVGGP

DASDAYTDSIGDYVSNEVATDYNAGFTGALAKMNLLFGQNDQPIANFPAPEVKTDEFF

VEAAVKASGSNYTEIKAELNNRSGWPARMGDKLSFRYFLDLSEVYAAGFTVSDVNVTT

SYAEGATVSQPVVVDTAKRIYAVTADFTGTKIYPGGEGHYRKEVQFRITGPQGAWNAN

NDHSFQGLGTGNVAKSVYLPVYDAGIRIYGQEPGVTPVVMPIAPSGVARLRCSIVTEG

QVLPVMR"

CDS 1020657..1021100

/gene="celI"

/locus_tag="PIKHDJKN_00914"

/EC_number="3.2.1.4"

/inference="ab initio prediction:Prodigal:002006"

/inference="similar to AA sequence:UniProtKB:Q02934"

/codon_start=1

/transl_table=11

/product="Endoglucanase 1"

/translation="MQYRHGGSGASGNAVTPQFNLKNTGTQAIDLSTVKLRYYFTKDG

AGDLNFWCDYAQIGTANIEGEFVTLTPAKGTADTVLEISFKSGTGSLAAGAETGVIQG

RFSKNNWGNFDQSNDYSYDATKTAFTAWNQVTGFQGGTKVWGIEP"

CDS 1021178..1024453

/gene="cbhB"

/locus_tag="PIKHDJKN_00915"

/EC_number="3.2.1.91"

/inference="ab initio prediction:Prodigal:002006"

/inference="similar to AA sequence:UniProtKB:P50899"

/codon_start=1

/transl_table=11

/product="Exoglucanase B"

/translation="MLKSAAKKSLSAMLAGTVMLTGYTGLWAGPQTVYAEEQNIDAQA

DGINEARFLQLYDQLKDPANGYFSPEGIPYHSIETLMSEAPDYGHMTTSEAYSYWLWL

ETMYGHYTGDWSKLEAAWDSMEKYIIPVNEGDGKEEQPTMSYYNPNSPATYAAEKPFP

DQYPSAINGQYAAGKDPLDAELKSSYGNNQTYLMHWLLDVDNWYGYGNLLNPNHTAAY

VNTFQRGEQESVWEAIPHPSQDNKTFGKTGEGFMSLFTKETQTPAAQWRYTNATDADA

RAVQVLYWAKEMGYTNTAYLDKAKKMGDYLRYGMHDKYFQKIGSAKNGTPTAGTGKDS

NMYLMAWYTSWGGGLGEGGNWAWRIGASHTHQAYQNPVAAYALSDPAGGLIPKSTTAK

ADWNASLKRQLEFYTWLQSHEGAIGGGATNSFDGSYKAYPAGTSTFYDMAYQEAPVYR

DPDSNTWFGFQAWSLERVAEMYYILAESGDLSSENFQMAKKVISKWIDWSKDYVFVDE

RPVTDAQGYYLNAAGQRILGGTNALVATTPAPGEFWIPGSQEWEGQPDKWNGFSSFTN

NPNFHVVTKDPAQDTGVLGSYIKALTFFAAGTQAEGGVLSAKGQEAKDMAEKLLNTAW

DYNDGVGIVTEEVRKDYFRFFAKEIYIPANWTGTFGQSNTIPGTAGVPSDPAKGGNGV

YIGYSDLRPAIKQDPAWAYLDNLYKTSYNPTTKKWENGAPTFTYHRFWSQVDMATAYG

EYDRLLGDAGSPGVQVPGAPAGLTAAAGSEQVVMNWTASTGAASYTVKRAEVNGGPYT

SVATGVTGSTFTDTGLTNGKTYYYVVTAVNTAGESAPSAQASATPQAGISIPGLLTVT

GTAGNNQAVLTWTASTGAASYKVQRSVAGGAYADLATGLTALTYTDATAVNGTAYNYR

VVAVNASGQTLSNVVTVTPTAPPVTTGALEVQYRNGGSGTSGNAVTPQFNIKNTGTTA

VDLSQVKLRYYFTKDSASDLSFWCDYAQIGSANVEAHFVTVDPAKGTADTYLEIGFKS

GAGSLAAEAETGIIQGRFSKNNWTNFDQTNDYSFDATKTAFSAWNKVTAYIGGVNAWG

IEP"

CDS 1024788..1025696

/locus_tag="PIKHDJKN_00916"

/inference="ab initio prediction:Prodigal:002006"

/codon_start=1

/transl_table=11

/product="hypothetical protein"

/translation="MIVMDGPFYNGASPANEARLKFCKHQGEHDIMNWKSKIITASVT

ATMLMGSLTTGALTTPASAAAVENSKVKVIWGVNLRTSPSSSASIVRLVPKGETVTVV

QKSGSGWYKVTDSGNRTGYISSSSKYTQAVKGNTTTGSGSTSGSGSGSSTSVTGSASV

EKVIAAGMKYWGTPYEFGASRNSTATFDCSSFVRQAFIDALGIKLPADSRKQGAYVKA

NGTVKTNWKNLKRGDLMYFMSYKGSSASSYSSVNKSTAKITHTGIYLGDGKILHTYSN

AGGGVTISDISGKHWEYRFLFGGSAL"

CDS complement(1025781..1026689)

/locus_tag="PIKHDJKN_00917"

/inference="ab initio prediction:Prodigal:002006"

/codon_start=1

/transl_table=11

/product="hypothetical protein"

/translation="MIRANTDAEWLPLYEALASEVRLQIIRLVAETPMNVKDIAASLG

LSSAIVTMHVRKLQDVGIIQSKMIRKDGGTHKMNSLAVDWIGISMPQETGASRKLHEV

AIPVGHYTHFDVYPTCGLATSNQVIGQYDDPRYFLDPERMHAHILWFGRGFVEYKIPN

YILSGQQISAIELSLEIGSEAPSVNPNWPSDITFMLNGIRLGEWTSPGDSGNGRGMFT

PEWWSDSVNQYGMLKVLRITHEGTFIDGQHMSEITLADIPVERNQWTLRLSVEEDAQH

VGGLTLYGQGFGNYDQDILFRLYYQD"

CDS 1026868..1028373

/gene="abfA"

/locus_tag="PIKHDJKN_00918"

/EC_number="3.2.1.55"

/inference="ab initio prediction:Prodigal:002006"

/inference="similar to AA sequence:UniProtKB:Q9XBQ3"

/codon_start=1

/transl_table=11

/product="Intracellular

exo-alpha-(1->5)-L-arabinofuranosidase"

/translation="MEKAKMTVDKDFTIGVVDKRLYGSFIEHLGRAVYGGIYEPGHPT

ANEAGFRGDVLEMVKELQVPIVRYPGGNFVSGYNWEDSVGPVSERKRRLELAWRTIET

NEFGFNEFVDWSKQANSEVMMAVNLGTRGVDAARNIVEYSNHPEGSYYSDLRIKHGYK

EPHAIKTWCLGNEMDGPWQIGHKTAEEYGRIALEAAKVMKWTDPTIELVACGSSSLGM

PSFPEWEATVLDHTYDHVEYLSLHQYYGNAEQDTPTFLARSLEMDRFIETVKATCDYI

KAKKRSKKTMFLSFDEWNVWYHSNESDKKLDPWQIAPPQLEDVYNHEDALLVGCMLIS

MLKHADRVKMACLAQLVNVIAPIMTDNGGAAWRQTIFYPFMHASVFGRGTALVPLIQS

PKYDTKQITDVPYLEGIAVHNEEQGEVTVFAVNRHLEESLPLEVDLRSFGKCRLIEHI

VLESDDLKAANTAAQPNRVAPHNRGDAEVSDTLITASLAKASWNVIRLKVQ"

CDS complement(1028579..1029352)

/locus_tag="PIKHDJKN_00919"

/inference="ab initio prediction:Prodigal:002006"

/codon_start=1

/transl_table=11

/product="hypothetical protein"

/translation="MKIQLIRNATLWLEYGGLNILVDPMLMDKEVMPAFPNTPNELRN

PRVSLPETETDYVNPDLVIVTHTHPDHWDEAAAKQLRKDVPLLCQPGDEAVFTGAGFT

NVTAVDDKHEYHSVRLARTSGHHGTGEIGERMGKVSGFVLEADEEPVSYIAGDTIWCE

EPAEAIRQYQPEVIVVNAGGARFLQGDPITMDGPDVATVKRCAPDAHVIAVHMDAINH

CMMSRVDLASYLASEQLDGQVLIPRDGESFAFGPGAGLK"

CDS 1029480..1029911

/gene="lrpC_1"

/locus_tag="PIKHDJKN_00920"

/inference="ab initio prediction:Prodigal:002006"

/inference="similar to AA sequence:UniProtKB:P96582"

/codon_start=1

/transl_table=11

/product="HTH-type transcriptional regulator LrpC"

/db_xref="COG:COG1522"

/translation="MNETIDDTDIRILKSLIRDSKRSHKEIGEEVHLTGQAVGARVRK

LHDLGVLEGYTVKWNPERLGLGLQAFVTIFLNSGDRHAAFREFMAAREDIVEVHRVSG

EGCYLMRVRTGTMEQLGSLLEAILPYGNYRMNLSVGIEKSE"

CDS complement(1029925..1030440)

/locus_tag="PIKHDJKN_00921"

/inference="ab initio prediction:Prodigal:002006"

/codon_start=1

/transl_table=11

/product="hypothetical protein"

/translation="MKPELYKSSESKLEHRQIGYRRFTAVWKGVLCLLFLLLCVTLLW

IIYDDHAGQLETLYYSIAAILGMWFIGPLFFMFLSRAIASSPVLLSWNDEGLHTLKRP

IPWNEIRKIELGSPAFSKWILSASPMIALYLKDGTRRHIQTDHLLTKKELSRAVVILQ

ETLREKQQQDI"

CDS complement(1030546..1032405)

/gene="yheH_1"

/locus_tag="PIKHDJKN_00922"

/EC_number="3.6.3.-"

/inference="ab initio prediction:Prodigal:002006"

/inference="similar to AA sequence:UniProtKB:O07549"

/codon_start=1

/transl_table=11

/product="putative multidrug resistance ABC transporter

ATP-binding/permease protein YheH"

/db_xref="COG:COG1132"

/translation="MTDRNAEVRSDAGAQAPSGHGSGSDAGADQSKRTSFKAMMSYAQ

PHKWAFAGIFLCSLLGISADLLQPYLVKIAIDDHLAIGQTSVGFLIQLAAIFLGLAVI

SFIFTYVQNNLLQHVGQNIVSRIRKDLFKHISKMSMSFFDRFHIGSLVTNVSSDTETI

SSFFTQVLLSLIRDGMMLVLIIVFMFQLDPVLAGYSLIVLPVIAIVAVLFRSQLRRAY

QNARTRLSRLIAFTAENLSGMFLIQAFHQEEEQKKRFTEQNQLHLKANITQARSNVIF

NRTFDILGNAALVMMVWLGGRAVLGESLQVGVLYAFISYIRQFFQPINQITMQWNTFQ

STTVSMDRIWNILSTRPEVADPKPGISSTLEPRNVMGQIDFNDVSFGYRTDRPLIQHM

NLHLYPGEMVGIVGTTGAGKSTLISLLNRFYDVNQGSIEIDGIDIRHFPQANLHRIVG

LIQQEPFLFSGSIIDNVRMFREDITREQAIQACRFVGAHAMISRLPQGYDTRLSERGS

GLSAGERQLISFARIVVFQPRVLILDEATANLDSHTEQLVQQALESVSQGRTTIVIAH

RLSTVMHADRILVMENGEIVEEGSHQKLIDAEGVYADLYTHAREAGNDSAISG"

CDS complement(1032398..1034140)

/gene="yheI_1"

/locus_tag="PIKHDJKN_00923"

/EC_number="3.6.3.-"

/inference="ab initio prediction:Prodigal:002006"

/inference="similar to AA sequence:UniProtKB:O07550"

/codon_start=1

/transl_table=11

/product="putative multidrug resistance ABC transporter

ATP-binding/permease protein YheI"

/db_xref="COG:COG1132"

/translation="MSKKGLLRGYVIANWPVYLFAVLLIIASNVGQASLPRILGSFTD

QLMQNTLQMNTVIRYSLSLLAIAIAYNLLFGTGQFMIMKLGRRFEFMTRERIFGKFSE

LSEYYFSKQGNGKLLSYVMNDVTSVREAISNGVTMMTNATFLLLSCIVMMLLSGIPMR

LILISIVPLLAIPFLVVFFGPRIRKRSRDVQDALATMTESAEEQLGGIRVTKTFAIED

SASERFGITVDAIKSKQLRLVRLSSLFQALLPLLGAISLVVSLLVGGILTMQNSITLG

SFVALTLYLRIIMGPLQQIGNVINTMQRSGASLERVNDLLTEVPDVRELPNATSLKAV

NDITIENLSFSYPGSSSPALKNISLNIQAGRTVGIVGKTGSGKSTFVKLLLRTYEPPE

GTIRINGTDIRQLSLESLRSRIAYVPQDGFLFSTTIRDNIAFSDREVPLDTVEHSARQ

AMIYENIIRFPDRFDTLLGERGLTLSGGQRQRTSLARGLIKKAQVLILDDSMSAVDAV

TESGILRSLREIGKGKTTLIISHRISAVRHADEIIVLDEGRIAEQGTHAGLMAAKGLY

AATYRLQEEGLHHD"

CDS 1034500..1035423

/locus_tag="PIKHDJKN_00924"

/inference="ab initio prediction:Prodigal:002006"

/codon_start=1

/transl_table=11

/product="hypothetical protein"

/translation="MKHKKGLAATLALCVSLTAGGASVLAFSDVKDEGQKTIVDSLHS

KGVVNGVTADLFRPDLALSEPQGVQLIVNAFGLKNEYAAASAQNKISPTTWYADAVQA

ATQNGLSIPVELNPQAKMTREQFAILLLEGINTTGEYPVIMLYNDIKDENKIGKDAKS

AVQNLLNMDIIELDKDGNFRPDQSLTRMEAASMIFNALEFVDKHGNGGSTEPTPTNPG

EGQQGIVPTVTSTKVDDKTVKVKLTAQMPHPGYGLKIEDVKLEKDGRAIVLYSIIQPD

PDMMYPMVITEVTAETDIPTGYTAEAQPSGK"

CDS 1036611..1037582

/gene="yteP_5"

/locus_tag="PIKHDJKN_00925"

/inference="ab initio prediction:Prodigal:002006"

/inference="similar to AA sequence:UniProtKB:C0SPB3"

/codon_start=1

/transl_table=11

/product="putative multiple-sugar transport system

permease YteP"

/db_xref="COG:COG4209"

/translation="MQPEGKSALSQNLSVHTIPNRKKNKLGRRMIRNWELYLFIAPAF

LYFLIFHYGPMYGIQIAFKNFIPTLGVTGSPWVGFDHFIRFFNSYYFWDLLWNTLSIS

LYELAIGFPLPIILALAFNEVKDSFFKRTVQTVTYAPHFISVVVMSGMIITFLSPSSG

MIVNLVEALGFQAPQFLTDPAWFKTVYVLSGVWQSAGWGTIIYLAALSGVDPQLHEAA

VVDGASRFKRILHINIPAIIPTITILLILNMGSILGVGFEKILLLQNPLNMGSSDVIS

TFVYRSGLVDAQYSFSTAVGLFNSVVNAILLITVNQIARRTSENSLW"

CDS 1037599..1038507

/gene="dasC_2"

/locus_tag="PIKHDJKN_00926"

/inference="ab initio prediction:Prodigal:002006"

/inference="similar to AA sequence:UniProtKB:Q9K489"

/codon_start=1

/transl_table=11

/product="Diacetylchitobiose uptake system permease

protein DasC"

/db_xref="COG:COG0395"

/translation="MSTAVKESRSDKVFLWCNYIYLTIALVIVLYPLLYIISASISDP

KFVSSGEMWLLPKGITFEGYARVFENTNIWIGYKNTIIYTVVGTLVNLMVTLPAAYAL

SRSDFVGRGFFMAMFMVTMFFSGGLVPSYLLIKDLGMVNSMWALILPGAASIWNIIVC

RTFFQSTIPKELQEAAHIDGCTNTRLFIRIVLPLSMPIIAVMALFYGVGHWNSYFSAM

IYLNDSSKYPLQLFLRQILVLQEMAAQGGGAIDTSSATAMNSKAEIAALVKYAVIIVS

TLPVIAIYPFLQRYFVQGVMIGSVKG"

CDS 1038549..1040144

/locus_tag="PIKHDJKN_00927"

/inference="ab initio prediction:Prodigal:002006"

/codon_start=1

/transl_table=11

/product="hypothetical protein"

/translation="MKKLRKASSIVLCLTLSAALLAACGSNEDGGSVTSNVEGVKKEG

FPIVDKPLTLKVMSQDAGVADWNTMPVLQEMEKLSGIKLEYQLSPIDSFETKKNLVFA

SGDLPDMFYAADLKPAEQVTYGTQGILIPLEKYIDEGYAPNIKKILDENPDVRKSFTT

PDGHMYALPFIDTAAVWYRGPMWYNGEFLKALNVEEPKTTEELYTYLKRVKEEDPNGN

GQQDEIPLTSVKLDDLRMYFFGFWGMYNEGIYADKDGKVHYPYQEEGYKGYLTFMNRL

WKEDLLDHETFSQTGDQKKAKGESNKLALFNDYHPYFTLGGEPSTKHPLMTPVKSEIA

DSPVYGKHPGISARGTFAITSSNPSPEATMRWIDYMYSYDGATLFNQGPEGLLWKFKD

KENLIKEWLPVPGGGDREEYRGKITPNYGILTPGINDPDIAKGLRTEFDEWLDQQNQE

KLVPIGKSPFPNVYLTNEEQSEATALLSDLDTYVKQMEAKFVTGQEPLENWDKYIAQI

KKMGSDRIVELYQGAYDRWNTGQ"

CDS 1040178..1041464

/locus_tag="PIKHDJKN_00928"

/inference="ab initio prediction:Prodigal:002006"

/codon_start=1

/transl_table=11

/product="hypothetical protein"

/translation="MQKWFEEAKLGIFIHYGIYAVDGVSESWSFYNGRISYEEYMKQL

DGFTASKFNAEKWADLIEKSGARYAVLTTKHHDGVALWDTQYSDLNVVKQTPANRDLV

REYAEAIREKGIHLGMYFSLIDWSHPDYPSVFEGGKVPVDLGGVNRHSSPVDGVQDQE

KWKKFLEFNNHQLQEIMTNYGKVDLLWFDGDWERSAEQWNLPEFKHYLQSFNPDVIIN

SRLQGYGDYKTPEQGIPITRPEGPWEFCTTINTSWGYVPTDHKYKSLNQIIRMFCDCI

SMGGNMLLDIGPREDGTIDKRQEDILLGLGAWIRTHEEAVFGTGEGIMPRYYLGGSTV

SEDRKTLYLFVYDDPKENVCIKGLCNKIKKITVLHSGKELNHEIHGGVPWFNIPGTTW

IKMTPEDTHEQVTVLKLEFDEELEMYGGSGAVVTHN"

CDS 1041528..1042550

/locus_tag="PIKHDJKN_00929"

/inference="ab initio prediction:Prodigal:002006"

/codon_start=1

/transl_table=11

/product="hypothetical protein"

/translation="MNQSGVPEPKIEYAPKHYICKRAKEPLVLDGCVDKAFWDAADWT

DDFVDIEGDLRPKPGKQTRVKMLWDDDYFYFAAELIEDQIWATLTERDSVIFYDNDFE

IFIDPDGDTHQYYEFEINALNTVWDLLLVKPYRDGGPPVNGWDISGLKTAVHIDGELN

TPGSDNMKWSVEVAIPWTSLKECAEGNRPPAPGEFWRVNFSRVEWQTEVQDGEYRKVL

NPDTGKPYPEDNWVWSPMGIINMHYPELWGYVVFSDDGMDQSFELPEDERIKWELRRL

YYRERNYFEAHGEFTQDVKQLMGEESLICQPVIETTRSLFQISTPSSDGTTLICIRED

GKLWKE"

CDS 1042558..1045191

/locus_tag="PIKHDJKN_00930"

/inference="ab initio prediction:Prodigal:002006"

/codon_start=1

/transl_table=11

/product="hypothetical protein"

/translation="MTSQTLVFSMDAQSLERIEQKFQEKLNFADARKDKLFHVFQQPL

TEEEAWALKYVYAYMPVNDLADYDGELFLSHVRRALDIRKRVPWGERVPDHLFLHFVL

PYRINTENIEDFRGIIFDELADRTAKLSMADAILETNYWCHEKATYIGSDLRTISPLT

MIRNARGRCGEESTLAVAALRSIGIPARQVYTPRWAHCDSNHAWVEAWADGQWYYIGA

CEPEAGLNQGWFSPPARRAMLINTRIFADYPGPEDITLSEKGYTEINLLENYAPAHTI

CVKVINEQGAPVPEASVRFEVYNSAEFYPIAEIKTDAQGEASFKTGHGDLVIRAVSGG

KWGEILFKAQDGAHVELVLNSLEQPSGIVDFDMVPPPEGEGEVVTALPEEKILDHNRR

LEEGTKIRSGYEDTFLSEEEAFALARSAGLPPERVWDILQKARGNSREIAAFLEECSG

EFGEWPLQLLESMNEKDLIDTFRLTLDDHLIGALSQRGQWAEETFVKYILCPRVSYEM

VVPYRSVFQNAYSEEEAAAFREDPSMLACNLEQSYSYYEDLPNLKGKGNPVGTYSLMK

GDPQSLDILFVAVCRSLGIPARLHPSEQKPQYLSDKGWEDAVFSLISSRNQESSSWGM

LQLLKDSEAREDAPSASYAENFSLSRLEDGVYKTLIYPYGHTDIYNDPFEVEPGAYRL

TTGIRLKEGTVRVRFIYCTVRAGDTTEVVLTYRETEVDIPVLGKLATGNELTRLDGSE

VVLKDLLGSEGTIAAWIEPEREPTKHLIRELCELAEPLDKLGVPIVLVIGDTEWNAFF

DSGAYPKLPVRTIFVRDSSYASLSGFISKPAAHEAGYPHLFVLDNQDQIRYSASGYKI

GTGKEALQIAAAISQSLNGLE"

CDS 1045197..1046243

/gene="chiA1_1"

/locus_tag="PIKHDJKN_00931"

/EC_number="3.2.1.14"

/inference="ab initio prediction:Prodigal:002006"

/inference="similar to AA sequence:UniProtKB:P20533"

/codon_start=1

/transl_table=11

/product="Chitinase A1"

/translation="MTKYIAAGYVVDSVLPDITDEDLLKLTHLNVAFGHVKNDEITTE

HLKNGEVVRNIKQKYPNLTVLLSVGGWSAGGFSEAASTQAGRDSMAASAVRVLEEFPF

DGIDLDWEYPSYGEAGIASSPDDKRNFTLLLKTIRERLDAKGSEDGRHYLLTIAAGAD

QYYVDGTEMAEVQQYLDFVQLMTYDMRGGFQVLTGHHTNLYTPTGDLFRISTDASVNL

FIRAGVPKEKIVIGAAFYSRIWTEVPDRNHGLHQMAGSTGGYGPAFAELEAKYINKNG

YIRYWDEEACAPFLFNGSSLISYDDEESIQCKCDYVKDQGLAGIMFWEYGCDPTHRLL

GAIHQGLQDVPVGK"

CDS complement(1046357..1048657)

/gene="rhaR_7"

/locus_tag="PIKHDJKN_00932"

/inference="ab initio prediction:Prodigal:002006"

/inference="protein motif:HAMAP:MF_01533"

/codon_start=1

/transl_table=11

/product="HTH-type transcriptional activator RhaR"

/translation="MKWNHFKSKLLLKYTLSYISIFLIPLVILTIIIYHNAVDTLRSE

IEQTNVNQLTQAKTVIDDRMKELQDIAFRIAYDEQLTRYWTHHPYYSRESIAALVKYK

ATSSIIDELFLFFRGDDSIYSSQGSENLDVFTGRYKFNTWNKTDMIRDLNKVQFPTIR

PAEQVVQGTKVPNSMLAYLVPIAPNNTPAHGTVMYLIHESNLTGLIDSILSDYHGMTY

IFDNYGQVLAANYKGETITEQEVKALFELEPGTHSLSLNQEPHSVVSVKSDAGWTYVT

AMPSNQFFSRIVHIRTFVVLVFSFVVVMGTFLAILLARRQYHPISDLMEFIRLKNNPE

PSTTSNELEWIKKTLHDYSQRVDLQEPYARNHILLMLLKHGHTGELPSEFTDNLGIRF

NRSHYFVMTLGLEMHDLPSGDHPEQRTVMQLINDMELPELSAYAYGVELPQPDQVALI

VGFDAEVGHESSLNARMESIVESLQSMVADHVGVAPAIGIGNSYSSPLQLNQSYIEAS

TALEASMLHGQGSSTFFNALSGSSEQDSSFWVPKDVLLKLIQSLKQGSYDVAVQMVST

ALNTLKSEMPSVPLLRCICFDILNTMLKTASELGIHHVVNQLPRITSYDSLEDLEKKL

TGLAAEICAHVEAKSETEESSLMDEIVAYIDANFSDYDLSLGTISSKFTISSSYFSRS

FKEKIGMNFTQYIWQKRMDEVIRLLLHTTDPLKDIITRVGYLDTPNFIRKFKKETGYT

PGQYRKMHRPNDSADSPEDDDEECLG"

CDS 1049195..1049554

/locus_tag="PIKHDJKN_00933"

/inference="ab initio prediction:Prodigal:002006"

/codon_start=1

/transl_table=11

/product="hypothetical protein"

/translation="MRETCVPTGVALKETGFGYTLSLVNGKYKMVILYWLSENKVMRH

NELKRSIGTISHKTLSVMLKELEEHDLILRREYPQIPPKVEYSLSERGQSLMPLLDMM

CDWGEANMVSTPGLMQR"

CDS complement(1049642..1050169)

/gene="ywrO_1"

/locus_tag="PIKHDJKN_00934"

/EC_number="1.6.99.-"

/inference="ab initio prediction:Prodigal:002006"

/inference="similar to AA sequence:UniProtKB:P80871"

/codon_start=1

/transl_table=11

/product="General stress protein 14"

/db_xref="COG:COG2249"

/translation="MKTLVIVAHPNLETSVINKRWIEELQQYPEKYTIHELYKVYPDG

RIDVQQEQQLIEGHDHLVLQFPFYWFSSPPLLKQWLDEVFTYGWAYGSKGDKLQQRKV

ALAVSAGIKEADYTEEGKYRYTLGQLLSPFETTFLYCDADYRPFFAFYGAEHEPAANE

LEQSAQNYLRFMDNL"

CDS complement(1050392..1051309)

/locus_tag="PIKHDJKN_00935"

/inference="ab initio prediction:Prodigal:002006"

/codon_start=1

/transl_table=11

/product="hypothetical protein"

/translation="MIYIKDLMSGVNIFKALSSEIRIQIIELLAKNQSLNLNDLATKL

GLSNGAITMHIKKLEESGLIEINTAVGKHGIQKICYLNEEKLMVDLRSQEIFNRYEVE

IQVGHYSDYQAAPTCGLATRDSIVGEFDDPRYFADPLRIDAEMIWLAEGYLEYRIPNY

LKPNQTFSEIQLSMELGSEAPGFCDNYPSDIYFYVNGIEIGCWTSPGDFGNTRGTFNP

DWWPPHLNQYGMLKLIRITQEGSYIDGCRISDVTLDQIGLDYKSDIHFRIAVTNGPVN

KRGLTIFGKNFGNYGQNLLARVLYNVQEE"

CDS 1051643..1052620

/gene="abnA"

/locus_tag="PIKHDJKN_00936"

/EC_number="3.2.1.99"

/inference="ab initio prediction:Prodigal:002006"

/inference="similar to AA sequence:UniProtKB:P94522"

/codon_start=1

/transl_table=11

/product="Extracellular endo-alpha-(1->5)-L-arabinanase 1"

/db_xref="COG:COG3507"

/translation="MEKFKGMTGFRKKRWALPALLVLVLLLAGQSRAFAAFWNLSGDI

AVHDPSIIKEGNSWYTFSTGPGIQVLKSDNGSSWYRVPQIFLSKPSWWASAVPGQSGL

DVWAPDVEQYNGKVWLYYSISTFGSNRSAIGLASATSIGAGQWKDEGLVLQTTTANNY

NAIDPNLVIDASGNPWLAFGSFWSGLKIVKLDKNTMKPTGSISSIAARPNNGGAIEAP

SIVYRNGYYYLFASIDSCCQGVNSTYKMVYGRSTSITGSYVDKNGVSMLNGGGTILDS

GNVKWKGPGGQDVYNGNVIARHAYDADDNGNPKLLINDLLWDSSGWPTY"

CDS 1052753..1054093

/gene="yesO_3"

/locus_tag="PIKHDJKN_00937"

/inference="ab initio prediction:Prodigal:002006"

/inference="similar to AA sequence:UniProtKB:O31518"

/codon_start=1

/transl_table=11

/product="Putative ABC transporter substrate-binding

protein YesO"

/db_xref="COG:COG1653"

/translation="MVKKKSLVTLMLLMLVSALVFSGCGGSSGSSGDKELTFMFRGGT

DEQKAYQAVVKKFEEEHPGVKVKIIVTAADQYATKLRAAITGNSLPDVFYFNPGDIKA

YVNSNVLMNLTPYVENNADVDLNNIWKYGLDLYRYDGKMAGQGDIYGMPKDLGPFALG

YNKTLFEKEGIPFPDKDRPYTWEEFIKVNQQATKDTNGDGKPDVFGTGFNVQWALQSF

VWSNGADWLDDSKTKVTIDDPKFAEALQFFADMQNKYKITPSIEEAQTLDTYQRWMKG

EMAFFPVGPWDMSTFEKLPFEYDLLPFPAGSTGKSATWIGSLGIGVSAKTKHPEEAAA

LVNYLTASKEGMQHLVDAKVQIPNLLDMADEWAKDTSTKPANKQEFIDIVEDYGRSLP

GNYTYNAEWYDLFFTDIQPVLDGKITAADYVKQQQPKMQKLLDKAVEQEKKSQK"

CDS 1054387..1055229

/gene="lacF_3"

/locus_tag="PIKHDJKN_00938"

/inference="ab initio prediction:Prodigal:002006"

/inference="similar to AA sequence:UniProtKB:P29823"

/codon_start=1

/transl_table=11

/product="Lactose transport system permease protein LacF"

/translation="MFILPPVLGLLIFVLFPFLYSLYGSFTDWDGLGQMNFIGLANFK

DLLTDDLFYKAMFNTFYLMLGIPIGLLLALLLAMGLNRKIPGTTTFRVIYYIPVISSL

AAVSIMWNWAYNGDYGLVNQFLDLFGIEGPNWLANKDTVKPALIIMTVWKGLGYTMLL

YLAALQSVSRTYYEAAELDGANGFQIFRNITWPMVKPVTFFLVVTNIIGGSQIFTEMN

IMTPTGGPEYSSASIVFYIWQKAFSNLQMGYASAMAMILGIFIFVITLVQFKMNEKSA

YDGD"

CDS 1055256..1056098

/gene="araQ_6"

/locus_tag="PIKHDJKN_00939"

/inference="ab initio prediction:Prodigal:002006"

/inference="similar to AA sequence:UniProtKB:P94530"

/codon_start=1

/transl_table=11

/product="L-arabinose transport system permease protein

AraQ"

/db_xref="COG:COG0395"

/translation="MSYSQKRKLTNSIIFIVLALGAIAMIAPLIWMLSTSVKEKQDVF

ALPPVWIPEVFQFGKYKEIWEAGPLLSGIKNSLIVAISVTVVGTFTSSIAAFAFAKLR

FPHKNKLFLALLASMMIPYPTVMIPQFIMFSKLGWVDTLLPLIVPGLFGNVVMIFFLR

QYLLSVPDAIIEAAKIDGSSYFRLYSSITFPLIKPAIAAQLILWFMGIWNDYLAPIIY

LNSPEKQTLQLVIANFNATYAIQTDYPLIMAASIVALLPVLIIFLIFQKQIIESVAIS

GVKG"

CDS 1056155..1057576

/locus_tag="PIKHDJKN_00940"

/EC_number="3.2.1.99"

/inference="ab initio prediction:Prodigal:002006"

/inference="similar to AA sequence:UniProtKB:A5IKD4"

/codon_start=1

/transl_table=11

/product="Extracellular endo-alpha-(1->5)-L-arabinanase"

/db_xref="COG:COG3507"

/translation="MLLMLLVGATGCSGEGAGEVVSPPVFPEAPQDTQLYDTSILDDE

SRWTVNNAHDPAIIKTDQGYYVYSTDVRVAGEAKPGVMVRKSDDLINWSWVGQALPGI

PKEALDWTGAVNLWAPDVIKVGDTYRMYYSASTFGSTRSAIGLQTSSSPEGPWTDEGL

VVKTSENEKDKLNAIDANPVMDAEGNSWMVYGSFFDGIYIAPLDPETGKFKEEGYGTR

IAARDRATEEGAVEGPYIVYNPEFKKYYLFVSYDSLFEDYNVRVARADSITGPYTDVN

GMNMLDTEHLPQYEVGTKILGGYRFTEGEGWVAPGHNSVLKDGDDYYIVNHARGETDK

NWPYLHVRKMLWTKDGWPVVSPERYAGEYAQDIPKSMLAGEWEGLAVDPSVDGQVQAV

PYTLQANGKLKSENGSGKWTFDGKQTLTLEWKESPWGGASTDELKLLPSWDWERSQPA

LVVTGLSDRGIAVWGKQISAAEK"

CDS 1057880..1058860

/locus_tag="PIKHDJKN_00941"

/EC_number="3.2.1.55"

/inference="ab initio prediction:Prodigal:002006"

/inference="similar to AA sequence:UniProtKB:Q82P90"

/codon_start=1

/transl_table=11

/product="Extracellular

exo-alpha-(1->5)-L-arabinofuranosidase"

/db_xref="COG:COG3940"

/translation="MTDSITFTNPILEQRADPWVYRHTDGYYYFSASVPAFDRIEIRR

AQTLEELRDAEPVTAWHKHDTGPMSANIWAPEIHFIDGKWYIYYAAAHTSETNEGLFD

HRMYVLENDSANPLEGDWVEKGQIRTRWETFALDETTFEHKGIRYLVWAQKDPDIVGN

SNLYISEMENPWTLRGEQVMIATPEYDWEIIGFKVNEGAAVLHRNGRIFIGYSASATD

YNYCMGLLTADEDADLLDPASWVKSPKPVFQTCEANGQYGPGHNSFTVSPDGKTDILI

YHARNYKDIEGDPLYDPNRHARAQVIRWNEDGTPDFGVPIPDGKATAEAK"

CDS 1059054..1059902

/locus_tag="PIKHDJKN_00942"

/inference="ab initio prediction:Prodigal:002006"

/codon_start=1

/transl_table=11

/product="hypothetical protein"

/translation="MDSRETLDRELEQILKWEKGQKDLFIWDKIGRLPFAMLDKVMPK

ALKQKIGDSLNEVGQYVQNGGKFLVQKKKVAELLQEEAIKSGYSMKADTSYDEQNGEA

KDTTKIHSVEGLPLEVLDRTADSITDSRTRFAAAQGAATGIGGIVTIAADIPMVMGLS

LKVLQEMALCYGYDPDEPQERIFIVKCLQFSSADIVGKKAIIDELADYDNPDKQVEVI

SQMQGWREVFNSYSESFGWKKLFQLIPIAGMVFGSVSNKNTIRDVAEAGKMLYKKRLI

LQRLSQ"

CDS complement(1060434..1061708)

/gene="fsr_1"

/locus_tag="PIKHDJKN_00943"

/inference="ab initio prediction:Prodigal:002006"

/inference="similar to AA sequence:UniProtKB:P52067"

/codon_start=1

/transl_table=11

/product="Fosmidomycin resistance protein"

/translation="MSMQNSLSKDAAARSKAPPGLDAQGTVYRILIAISLVHLFNDSI

QSVIPAIFPILKDSMHLTYTQIGWISFAINFTASIMQPVVGYFADKKPTPSILPIGMG

FTFTGMLLLAFADSYMAVLISVIFVGLGSAAFHPEGSRVSHMASGPRRGLAQSIFQVG

GNAGQSLAPLLTRWIFIPFGLFGAIGFTGIAAAGIAVQIYIARWYGRMLQSGGYLRRQ

AAARRAPNPALRKKIAAAITILILLVFVRSWYVASIGSFYAFNLKDTFNLSTEDAQIY

IFLFLAAGALGTFFGGPLADRFGKRNMIFLSMAGAAPLALLLPYANLFWTAVLLSIIG

FIMLSSFSVTVVYAQMLIPGKIGTVSGLITGLAFGMGGLGALVLGNWIDVFGVSPVMQ

MCSFLPLIGIFTFLLPSDKLLNRWAEENGSEE"

CDS 1061932..1062144

/locus_tag="PIKHDJKN_00944"

/inference="ab initio prediction:Prodigal:002006"

/codon_start=1

/transl_table=11

/product="hypothetical protein"

/translation="MRSLSPYVLAKRNLAMTILFMAVMTFLTIYVASSSLYFAIGFFV

VVQSIGVLMLRHYIKKVKELKEDSNP"

CDS complement(1062296..1062850)

/gene="queF"

/locus_tag="PIKHDJKN_00945"

/EC_number="1.7.1.13"

/inference="ab initio prediction:Prodigal:002006"

/inference="similar to AA sequence:UniProtKB:O31678"

/codon_start=1

/transl_table=11

/product="NADPH-dependent 7-cyano-7-deazaguanine

reductase"

/db_xref="COG:COG0780"

/translation="MPIIYTGKLLAPPEIRRVYEPMRQPDEMQDLTLLGNQGVKYTFE

YDPGILENFDNKHPYRDYFVKFNCPEFTSLCPITGQPDFATIYISYIPDVKMVESKSL

KLYLFSFRNHGDFHEDCVNIIMNDLIKLMDPRFIEVWGKFTPRGGISIDPYTNYGKPG

TKYEQMAEHRMMNHDMYPETIDNR"

CDS complement(1062911..1063756)

/gene="queE"

/locus_tag="PIKHDJKN_00946"

/EC_number="4.3.99.3"

/inference="ab initio prediction:Prodigal:002006"

/inference="similar to AA sequence:UniProtKB:O31677"

/codon_start=1

/transl_table=11

/product="7-carboxy-7-deazaguanine synthase"

/db_xref="COG:COG0602"

/translation="MSSLELSNLTTSTRTDGTNTAPAEGLAANKRPHRQERNKEARIP

VMEIFGPTVQGEGMVIGQKTMFVRTAGCDYRCSWCDSSFTWDGSGKDLIRMVTPEDVW

EELRRVGGSRFSHVTISGGNPALLASLGGLVKLLRENGIRTAVETQGSRWQPWLADID

EVTVSPKPPSSGMTTDWAVLDDLIHRLAAGSVKRSHSLKIVIFDETDLDYARRVHARY

PGTDLFLQTGNPDVTSADTPDLASSLLARYEWLIDQISASDELNDVRVLPQLHTLVWG

NKRGV"

CDS complement(1063749..1064240)

/locus_tag="PIKHDJKN_00947"

/inference="ab initio prediction:Prodigal:002006"

/codon_start=1

/transl_table=11

/product="hypothetical protein"

/translation="MREPGTFRIVERLQRIGEDILPAQLRYHRKRVLVSKEFTFDAAH

HLHCYEGKCKNLHGHTYKVVFGISGYPGETGLTVDFGHIKEIWKTQIEGYLDHQYLNE

TLPLMNTTAENMVVWLFEQMEHALQTEPYAALTEGGRTEFVRLYETPTSYAEARREWM

IDE"

CDS complement(1064237..1064929)

/gene="queC"

/locus_tag="PIKHDJKN_00948"

/EC_number="6.3.4.20"

/inference="ab initio prediction:Prodigal:002006"

/inference="similar to AA sequence:UniProtKB:O31675"

/codon_start=1

/transl_table=11

/product="7-cyano-7-deazaguanine synthase"

/db_xref="COG:COG0603"

/translation="MLNEEKAVVVFSGGQDSTTCLFWAKQQFAEVEVVTFDYGQRHKL

EIECAGEIARDLGVQQTVLDMSLLNQLAPNALTRTDVEITHEEGELPSTFVDGRNLLF

LSFAAIMAKQKGARHLVTGVCETDFSGYPDCRDSFVKSMNVTLNLSMDYPFVIHTPLM

WLDKAQTWKMADELGAFDYVRERTLTCYNGIIGDGCGECPACKLRKAGLDRYLEQRTN

SATVGTPGADVR"

CDS complement(1065289..1065993)

/locus_tag="PIKHDJKN_00949"

/inference="ab initio prediction:Prodigal:002006"

/codon_start=1

/transl_table=11

/product="hypothetical protein"

/translation="MHIHLNNAEAGGAGQVVTFSLIQDDRLHILHPQQIVAFRGPSSS

RNDKFMNISGIYRKKKLIKSEITGPCQFVAALPPGFTMKEVELSENSDLLYDFRHLFF

YSDGVTMHTKIQKIKNMLITRDAVKMKFSGKGKIGLLTQGQVCQQELHPTAPLYVDAG

SIIAYPENAQLELTVYGNNLASQHMNYHWKMTGHGSVLFQAGRENHRLEQDMNDEGLF

KRILKEVIPFGNVLIK"

CDS complement(1066012..1066467)

/locus_tag="PIKHDJKN_00950"

/inference="ab initio prediction:Prodigal:002006"

/codon_start=1

/transl_table=11

/product="hypothetical protein"

/translation="MPKSIQGYFIRVLFTTCVLFTVSLTSTVSAANANIFSDIYSGWE

RVSELPGEVNALQESYKQTLEQLNQTSAQLGQAQANVEAFKAQNELLLAQNEKLTDMV

SKLQNDQDAKTATAKRIQTMIITAVSLILGYFILIRVMRWGMRRRSGRV"

CDS 1066808..1066975

/locus_tag="PIKHDJKN_00951"

/inference="ab initio prediction:Prodigal:002006"

/codon_start=1

/transl_table=11

/product="hypothetical protein"

/translation="MYTTKQAATTKTIMITVNGEPIGRELIIDSITYAPVTEPDSVKN

LHKMGINPSTI"

CDS 1067183..1067887

/locus_tag="PIKHDJKN_00952"

/inference="ab initio prediction:Prodigal:002006"

/codon_start=1

/transl_table=11

/product="hypothetical protein"

/translation="MNSVFEVSYSWNREAIPTGGADPVYMMVEWRNGAPAKRLRKIAP

KIVSRDLELLLKPEFGIQLKGIYGCRSKETDIGWVLRLGDVYKGESKQILLEFAMGPR

VSGKSAVCSAYWSTRKLKQSQRVLLRREQLYIQYTSHLGMLRQPEDPKVEKTIKLNET

VPLIKQALRAYERGRTQEGSDMLRRHADALLIEAARKQDLDYYVEAEIVEKLRHHYGI

TFNSMAHNRGKTMLGE"

CDS 1068050..1069015

/locus_tag="PIKHDJKN_00953"

/inference="ab initio prediction:Prodigal:002006"

/codon_start=1

/transl_table=11

/product="hypothetical protein"

/translation="MTDRLIRLMRIITLVQAKPGILARELAERCETTERTIYRDMEAL

SAMHIPIANMGHGRGYMFISHFAMYPLNWSDEEAQAFMHLAEVMENIRPLLKPAFESA

YEKVVASSQKNKTERVEWSEQISGLFKVGTSAWQNESTELLNHSLVILLQAGISQNTI

EGEYLFQGKKGIARMDPYCLIPREYRFDLLAYCHLSEKLRIFQVNRLYRVRILPRTFR

KDDYLLQSHFRTPQELSGSTEWTAFKIRFSPHAVERVMQEQFFARPILTIEPEGTLLF

EAILSDEEGFLTWLSQYGPDAEILEPEKCRLMMKERLERWREIYN"

CDS complement(1069070..1069741)

/gene="polC_1"

/locus_tag="PIKHDJKN_00954"

/EC_number="2.7.7.7"

/inference="ab initio prediction:Prodigal:002006"

/inference="protein motif:HAMAP:MF_00356"

/codon_start=1

/transl_table=11

/product="DNA polymerase III PolC-type"

/translation="MPYIIYDLEFTVSRNTRYSSEIIDIGAVKVTEGENGLYVSDTFH

TYVRPSNKSVLSADTIQFTGITQKDIDAAPLFPEALNQFIAWMGNETYYMCSWGPDDR

AKLISHCRTHQLDVAWITNHNDLQQQWSRTVRKEGKFRQLGLAQALELCGIEFDGTQH

RALDDAINTAKVFMHQFDQFKLETNCAADDEGITSKVVYSSSTEDDDKDSPFGNLTHL

FNTNE"

CDS 1070272..1072041

/gene="proS_1"

/locus_tag="PIKHDJKN_00955"

/EC_number="6.1.1.15"

/inference="ab initio prediction:Prodigal:002006"

/inference="similar to AA sequence:UniProtKB:Q831W7"

/codon_start=1

/transl_table=11

/product="Proline--tRNA ligase"

/db_xref="COG:COG0442"

/translation="MRQSEMLVPTLREAPAEADAAGHRWLLRSGMIRQLAAGIYSYLP

LGRRILLKVERIVREEMDRAGCQEVLLPIMQPAELWEESGRYTQYGPELMRLKDRHAR

EFALGPTHEEVVTALARDEVNSYRKLPFTLYQIGTKFRDERRPRFGLLRGREFIMKDA

YSFASDWEELDRTYQAMNTAYSRILERCGLEYIRVEADAGTIGGQGETHEFMALADVG

EDTIVTCKHCGYAANLEKAGYQTSESSVKNPGVAEASAVPTTVDKAEGNAEGDTLVRI

QTPGVRTINELSAFVGEDAQHMIKTLLYQADGQLIAALVRGDHEVNDIALKQVLGAEE

LILADETALAGHPKLTVGFLGPIGLDLPIVVDADVAAMERAITGANEVDVHYSNVVPG

KDFALDKVDRIRFAAEGDGCPTCGESLVFTKGIEVGHIFKLGTKYSDAMGASFLDRNG

RQCAPVMGCYGIGVSRLMAAIAEQYAGEDGIRWPATIAPYHVHLIPMSWKDEQQRELT

LELEQQLLDAGYSVLIDDRDERPGVKFKDSELIGLPVQIVIGRGAAEGQVEFGSHVLA

AQGESKRISMTAQEAVSQVKEQL"

CDS complement(1072625..1074424)

/locus_tag="PIKHDJKN_00956"

/inference="ab initio prediction:Prodigal:002006"

/codon_start=1

/transl_table=11

/product="hypothetical protein"

/translation="MKIWKTYVSLVLTLCLLFGSVGIVAAATDTTPGTGKHITILHTN

DTHARAVESSPAMGFAKIAGIADKYRSENPNTLLLDAGDAVHGTTFATLVNGESIVKV

MNEIGYQAIVPGNHEFNYGSERLIELADMMNFPMLSANVKKKDGTRLFDPYLIKEVDG

VKIGIIALTTPETMYKTNPKNVEGLDITDPAAEAKVLVNEIRSKVDVVVVLGHLGQDA

SSTDTSFKVVKEVPGIDVFIDGHSHTVLQDGLVSDNGTLIASAGEYTNFVGVIDLWVD

GGKVTNKQATLIDGTEAKDIKPNEKVAALVNSIQKEQEPILKEEVANTAILLDGKREQ

VRAGETNLGDLLADAIRDISKADIALTNGGGIRSSIEKGIVTKGDIITVLPFGNQVVT

LEVKGSDVLAALENGVGSYPEPSGGFPQVSGMTFSIDSSAAKGSRVHSVMIGDKALDP

KATYTMATNDFTAVGGDEYTMFAKYTTSGMYGAMDEALIEYMQKLGAVDIKTDGRIKE

AKAPAVEPEAPVTETPAPTTPKPETPKPETPSKPTPSKPAPAKVHVVKSGDSLYSISK

QYGTTWQALQKLNKIKNPHWIYPGQQLKLPAVS"

CDS complement(1074616..1075023)

/locus_tag="PIKHDJKN_00957"

/inference="ab initio prediction:Prodigal:002006"

/codon_start=1

/transl_table=11

/product="hypothetical protein"

/translation="MDDIVTKLIRKHRTNCPFSIARAIGIQIRFTNLGKSTKGLYFRK

LRRRFIVIHNDLPPEWQRFVCAHELGHDRLHKGINRFFLEEHSYFAPGKLERQANRFA

IQLLTSGVMPEPDESLEKFCLRTGLPREVQHFF"

CDS complement(1075066..1075509)

/gene="rghR"

/locus_tag="PIKHDJKN_00958"

/inference="ab initio prediction:Prodigal:002006"

/inference="similar to AA sequence:UniProtKB:O32236"

/codon_start=1

/transl_table=11

/product="HTH-type transcriptional repressor RghR"

/translation="MEHAFGPYLKQLREQQGYSINQLAEAAGISNSQISRIENGVRGV

PKPATIRKISDALSVPYADMMKQAGYLETGKPASELHDAPEWATYKDRRDFKKMLEDE

DDLMFDGIPLDDEDKKRIKDVLTGLFWEAKQMNKRKKTNDPDERP"

CDS 1076495..1076977

/locus_tag="PIKHDJKN_00959"

/inference="ab initio prediction:Prodigal:002006"

/codon_start=1

/transl_table=11

/product="hypothetical protein"

/translation="MELMLPELDRRKTQTAVETALEKYRIYKTIAFEEREVMVTASYA

ERFHGATNVTGDSTARTAIYNVDVQRARQAYCDTIDFVVSRLSEKERVLVSERYLKDD

DVFDYKVYNHVFDPPVSKDTYTKIRTRAFYKMALALSDRGLINMEPLSASRKERKKLG

"

CDS 1077073..1078743

/locus_tag="PIKHDJKN_00960"

/inference="ab initio prediction:Prodigal:002006"

/codon_start=1

/transl_table=11

/product="hypothetical protein"

/translation="MTEQAGMQEMYTLKTIAETLNTSNDLNLMLDTVLGKLMELTGLT

TGWVFLIDAQGEYSCIADYSLPPALLHHDKQPMRCGSCWCVNRFRDGRLDNAVNIINC

KRLEDAVDHQWGDTRDITHHATVPLRSGEKMLGLLNVAAPGKEHFSDSELALLQAVAY

QIGSAMERMRLYSAEQRRADLYVRLGEFSRSLGVMVNDCNSGDDMAKTVVKLLGHHYD

WPFAALLSQKNGSFMVQAAHAHGKSEMMTISELSPEVETRLHRVIDSHRAMVLSATET

QDVSANCNNWLPMSVLASGLAAPIPLSSPGETAVLVVGLSSVNDFLQADREVFDALAE

HITASWESLRLVYKRRELTRLEERNRLARDLHDSVNQILFSLSLTAKGAESMLAGSLQ

LHPAAEAMKDIRSLSQEALKEMRALIMQLRPAGLESGLLSALQEYGTGQGLQVVVHRT

GMRSLPQSIEEGLWRIGQEALNNVRKHAGVPSAEISLQLSDQEAVLIVTDRGKGGAKR

REALPASSLGLSIMRERAQSLGGRLELVSSSRKGTSVTVVIPLPSESV"

CDS 1078762..1079484

/gene="liaR_1"

/locus_tag="PIKHDJKN_00961"

/inference="ab initio prediction:Prodigal:002006"

/inference="similar to AA sequence:UniProtKB:O32197"

/codon_start=1

/transl_table=11

/product="Transcriptional regulatory protein LiaR"

/db_xref="COG:COG2197"

/translation="MPITILLADDHAMVRRGLHVFLSTQPDMEVVGEASNGQEALEQA

EQLHPDVVLMDLHMPVLDGIETARRLRAILPGIRIIVLTSFSDQDHVVPAVRAGVKGY

LLKDIEPEDLAVAIRNVHAGQVELHPAAAGQLMHVMASSELSMNEAHGRANPHTSNRL

PEQERVGLGKETSHGPDMLTRREREVLGLIAQGLSNKEIAIKLIITEKTVKTHVSHLL

DKLGLADRTQAALHAVRNGWVV"

CDS 1079667..1080242

/locus_tag="PIKHDJKN_00962"

/inference="ab initio prediction:Prodigal:002006"

/codon_start=1

/transl_table=11

/product="hypothetical protein"

/translation="MNIIILAGSNRKNATSTRLGEYAVEVISGQGHEASLFDLYQTPL

PFYAPDEKQNEDQNLSDLNTRMLAADAIILSTPEYHGSISGVLKNALDHLSQAHFSGK

PVLSISSAGGAVGVSSLLQLQAIIRNLHGINAQEWISIGGAQRRRFEATFDGYEEFEG

SQDIEDRIKRVIGSFLHLAETLTAVRKPSGS"

CDS 1080262..1080732

/gene="fosB"

/locus_tag="PIKHDJKN_00963"

/EC_number="2.5.1.-"

/inference="ab initio prediction:Prodigal:002006"

/inference="protein motif:HAMAP:MF_01512"

/codon_start=1

/transl_table=11

/product="Metallothiol transferase FosB"

/translation="MIKGIFETHLNVTDLERSHHFYEQIVGLQHAYGQKERGNSFYWI

GGEGNAMLGLWQKDPSEVKRQHFAFQVSLEDMKQAVTYLEDKGIKTHNFLDDDIGELY

VFGWMPAVSVYFTDPDGHSLEFISMLPDEAKQELGMVPWSEGEKMHGRVKEDTA"

CDS 1080729..1081364

/locus_tag="PIKHDJKN_00964"

/inference="ab initio prediction:Prodigal:002006"

/codon_start=1

/transl_table=11

/product="hypothetical protein"

/translation="MIFEEVKLYTALLEDIKTFYVDTLGLELLEESRHSFAIRVGLTK

MIFAESGTDQNPFYHFAWMIPANRFQEAKAWAAARVRLSRHEGEDETYSTSWNSNSLY

FEDPVGNILELIAHHTVHNESDRPFSEKDLLQVCEVGLVTEDVLSAVDELEQMGLKRW

RAISDTFAPVGDVNGLFIVVKKERTWFFSEQKANIYPLEVSIRDVGRLRIG"

CDS 1081509..1082462

/locus_tag="PIKHDJKN_00965"

/inference="ab initio prediction:Prodigal:002006"

/codon_start=1

/transl_table=11

/product="hypothetical protein"

/translation="MLDRRLLEEGLAVIVSSRERTGDLWHAHYGAGAIAAYFWVRENR

LTALAAGSVTAEAKAMLRQHGLNDGLTSRNGEVLPREEAEARITEALDRTIGELHWVG

HQAIYGALSLKAIRELDGWGTEHDMNQMVELIDSFERTIPGRSWVNTTVKEIRSLKLS

EADGFPDIERPEQLSRLILDELGAFRTIYQAEAHHDLIGHMLTYGHALNILFELDYPA

LFHKGIPPFLRMVKALRLSRDVDVEQEMPTLQSPVDRLPLIRAERSVALPHELEYWLT

DRRSRDWHGGHVFKFPFSFYHHARNGNSVPESWENFRYIIA"

CDS complement(1082519..1083001)

/locus_tag="PIKHDJKN_00966"

/inference="ab initio prediction:Prodigal:002006"

/codon_start=1

/transl_table=11

/product="hypothetical protein"

/translation="MKKKLWFYLWIAVTSYMAGPVMYALTMYTFYQETDVVTSSLIGW

TAATFSSVGILFILVTVILLRVLHIYYFWLQTLLFELLFLVLVYMTTVLLGARNTGLP

SLSFPFTPEGIPLWVFWGSIALMSSWGIWSARQPMRKSPYMLVSRVILILFILEIWSR

"

CDS 1083545..1085398

/locus_tag="PIKHDJKN_00967"

/inference="ab initio prediction:Prodigal:002006"

/codon_start=1

/transl_table=11

/product="hypothetical protein"

/translation="MKTVLYVPLDDRPANLDDVVVQGKAAGIHFITPHLGDIQNRLDS

EKMVEGTTLLGTSTPTYGKPSNIHDFILKNAAKVDGFIISSDMLAYGGLIGSRQLREG

GGGTYPDYDQDTTRLLDVIKAVKGKYPRKPVFVMDTIMRLATTSFADGLALDAYNESR

ALMQQPRQSFTAFEDIVNGYNLSPESTEYGETTYFNKEQYYNTRQHKFKTNLYILDKL

ARKGYIDFLGVGVDDANTQGVQINEINYVEARINEWLGGTDGQNPDRAIILPDADGLG

HALVARMANQLLRGGKKNRYAVKYFGPHGSTIINPYEYMNVHENVVRHVDIVGGVVVA

DSTYPEPDVATDTTSDVENENELTSATSVDEASSFDMASELDRMTNRHPGKPGKKPVD

IEIIAITALDQVQAAVAQLTSNSEKGLPSVLIDFVGKGPANVDVAEALLNSPYTGRVL

GYSAWNTPGNKIGMAVGMGQSRYALITTETHAHVLRDAMNAQGSLLFKRFLKDYYYKA

VAIADIRTYSRAHALYTNVATLADQNMSLFNSEEDYAHLQTLLRDLMQTYTTALANKT

AFAQGNVAIKQICNGEAAYAEYCSALLEYANPDFIWGRAFEITLNPKVTLK"

CDS 1085734..1086639

/locus_tag="PIKHDJKN_00968"

/inference="ab initio prediction:Prodigal:002006"

/codon_start=1

/transl_table=11

/product="hypothetical protein"

/translation="MSMSSGVNNTVSTGQVSNVIRRTLKQKLEALVPAWNGRVKDIPA

PGEVLAGPCAVIAFAEEVPKSAWAGYRRIIKISPYARPEDGGAEQVEVWSAELMEGLH

QIRLEDEEGGAFTCIYLGSSDCDRVDASSGLVTRSLRFGVYVPETVEYAQAETTDSWM

AALQGWTRTQLGQEWSVYGDVWPEGYKAKSILWRLTGCSTTNAGTSALEIRKQWIGHV

LAANVGDVRHTVTHLVEQLAVQTRIALTDTDGTRYVTVDEVSADLQSDAFLNGQIRLT

LQQRIRRPGTDVPLIREIHHSKGIE"

CDS 1086821..1087021

/locus_tag="PIKHDJKN_00969"

/inference="ab initio prediction:Prodigal:002006"

/codon_start=1

/transl_table=11

/product="hypothetical protein"

/translation="MASSAKKNKQAAPQYTRAELMNHAEALFAVKAEVLYGALYEAAQ

ETFSIEEAKERINQFMKAKVKG"

CDS 1087021..1088484

/locus_tag="PIKHDJKN_00970"

/inference="ab initio prediction:Prodigal:002006"

/codon_start=1

/transl_table=11

/product="hypothetical protein"

/translation="MAGGTWEQTNRPVLPGLYMNFQAAASSAIQAGNRGTVVVPIKAN

WGPVGTFVEVGSEAAIERIFSAHALDNGTAYTSLKLALLGGPKKLLAYRVAGETAKAA

TLTLKDSSDAAVLQLDAKYPGDRGNGFYVTIQPGVIDNTKHEVRLFEGNRMLYALLTA

DISAASLAKEINADESNIWIKAQAIGDGTGVVATVAGAAFKGGASGNDGLTNAEYIAV

QGALEGEQFDVLALDHAADAPLLASFAAWVKRVRSEGKPVMAVFGGTTAEDTSATAAQ

KAAARSLTLNHEGVINVGTGVRLGDAFYSSAETSAYVAGLIAGQRLNESTTYAPSPFD

DVTRRWTRAEQEQAVQNGVFIFFHDGRQVKALRGVNTLVTPSAGQNNAWKKIRSIRVM

DAINTDLQRSAEDTYIGKVNNTEEGRQALIGAMKAYLALLAQSNVIEAEGYDVVLDPA

YYGAAPILKPEADQVFLQWNVKLTDVMEQLFGTFYVQ"

CDS 1088579..1088986

/gene="xkdM_1"

/locus_tag="PIKHDJKN_00971"

/inference="ab initio prediction:Prodigal:002006"

/inference="similar to AA sequence:UniProtKB:P54332"

/codon_start=1

/transl_table=11

/product="Phage-like element PBSX protein XkdM"

/translation="MLDASRVILGTHGQLHIDGVWQTNINKLEASVEIEKRELNLVGN

DWKVHKNGAKKGTGTMTGYKVTSDMIQRGFTKFQIISKVDDPESYGHESVLLKGCMVD

KIQLANWTAGEEVPEETSFTFEGFELLNPIVAN"

CDS 1089169..1089603

/locus_tag="PIKHDJKN_00972"

/inference="ab initio prediction:Prodigal:002006"

/codon_start=1

/transl_table=11

/product="hypothetical protein"

/translation="MSMNENMSEEQILDQLFEAAERLPEENVRIQRLDLLLTLRGLTS

SKVDQIRERCTIRKTVKGRTEEKVDTETFNALLISEATVKMNVRGLELSGWGDNRITG

RMKLSGGEQAVRRMLLAGELDAVGDKVLELSGFGVEIEDLKN"

CDS 1089787..1091592

/locus_tag="PIKHDJKN_00973"

/inference="ab initio prediction:Prodigal:002006"

/codon_start=1

/transl_table=11

/product="hypothetical protein"

/translation="MAEMIVGLSKSNAEMRTTIRYLDQIQRSTERLGRVRYQSLIKVN

NELRTTGRRLESIYSTAVRLSRLRITPKIGLDDRLSPALDRALAKLNSFRNQMVKASG

TVSVEVKQKVEVAMGKMSPASGPSLSLNIGSNNNTVKNAPKEEEISWIREMAQKSNDF

LDPLNNATDLLGKLGSGVKFFYEKIKGKSSSSNTSQCCCCSGGSLVGRISKGGAKGGK

TKNAGGGNAKTVAATEGGKKGPYKSGSRSYQERAPGTVKTSASDRAKKIITNMPHAEE

KKLKMDFDQKLNPNRHGFSANLPGSPMSNMFSGNGMFGKLSGGLAKGAGKLLGPISML

ADVANVATAPPEQRGRAVGSMIGGTAGTAIGSAIGSFILPGIGTYIGGALGGWAGSSA

GGWIGDKAKDIGNFMSNATEGAGKALSTAADFVSEKTKNIASGISNFFGFGSKKEEKT

VSATTAVANPTPVPTGPSMPPAYIPSVLTTTGPMAYANSNGGQSTTAALLGTSVMQSQ

AMALGNGSQTNANGKSSTMTVQISEDQMSSLSGYLKDFKTETTNQISVNVPQGAVQVT

VRENAIDYDAISHQVGMRFAGEVRRAMENRKTIMA"

CDS 1091611..1092276

/locus_tag="PIKHDJKN_00974"

/inference="ab initio prediction:Prodigal:002006"

/codon_start=1

/transl_table=11

/product="hypothetical protein"

/translation="MTVFEDKVEGIKMEFTLIDGKTKFQFPVKPEELTISRSKGYETI

NMLEHGEFDFAQGEKVKEITFSSFFPKEYDASYCMYEPLPDPRVAMNMLNTFLVSKKP

LRFIITNTGVNVPVYLISHNTTFRGGESGDIYFDITLRTWRDSKVEKVGGATSASKSG

SRTDLKTSSKTYTVKSGDSLSKIAKLELGSSSKWNEIYKLNVKAIGSDPNRIKPGQKL

VMP"

CDS 1092273..1093259

/locus_tag="PIKHDJKN_00975"

/inference="ab initio prediction:Prodigal:002006"

/codon_start=1

/transl_table=11

/product="hypothetical protein"

/translation="MTYKVIVDDKYDITKLVETITLKDSLDQIAYQANIRLAVSASVA
LPAISPGMAVRISGVPFGEKSMVHLLHPAVIWEVESSNSGTKRLSLTVYDRMIYLEKS
EDEFLLPKDQTATQRLKTYAKEWKIPYAALPDTKTKLSKAVYRSQTIFSMMFADLKET
VKSGGDMYHPRMTPGGLQLFKVGSNAKVHELDRLIDLTQMRTLEGAVTKVKVMAASES
SSGKEVPSKVLAIEQDGVAELGTLQKLIEDDQVKTATAAKKLAKSRLTGIQETFTVSA
PDVNTIRAGDAVLLKGLKLIVMSVSRDLSAGPGTMTLELGTVEMVKRRVYLE"

CDS 1093252..1093662

/locus_tag="PIKHDJKN_00976"

/inference="ab initio prediction:Prodigal:002006"

/codon_start=1

/transl_table=11

/product="hypothetical protein"

/translation="MNKDDPYGHFAEVMRGAMSTHTRQAVSGMGAVLGTMTSSGVKLD
DFKHEVQDYLVAELPGTLGLPEREAAGAISGIPDVANGGTTGTGRFLLQKGEVEEAVW
SLGKGLKAGDRVLAMRVNGGNDIVVLCKVVSANA"

CDS 1093655..1094110

/locus_tag="PIKHDJKN_00977"

/inference="ab initio prediction:Prodigal:002006"

/codon_start=1

/transl_table=11

/product="hypothetical protein"

/translation="MPSLFPETGLVWGDEEDLSGVASEEVRFGRSWRFDYDAGDFVLT
PSGKVAAAGAHEAWVQWCIKAVKTPRYRHVIYSRNYGSELEDLLGQGDSWGVMESEIT
RMVTETLLADPRTDSVDQFTFDWNREQCMFSCRVASVQEEMFILESEVI"

CDS 1094115..1095254

/locus_tag="PIKHDJKN_00978"

/inference="ab initio prediction:Prodigal:002006"

/codon_start=1

/transl_table=11

/product="hypothetical protein"

/translation="MAEIPRYLEDQTEEQIMQRMLDRLPADLDKSEGSFLWDAEAPVA
FMLSEAALWAQELLRRGFASTAASSDPNFRSEELDLRAGEHGLTRRAAVAAQGTVRFV

GAPGKVIPAGTVVATLADEISGEASLEYETVGRVELGEDGLGSVGVRALVAGKESNVP

AGTVTVLSTPVSGVTSVTNVEVIKGGADIEADTALLERFCAKVRNQGTSGNKSQYVQW

ASEVPGVGATRVIPLWKGPGTVGLYLLDTDKRAAGSDLVAAVQKYVDPTQDGQGEGVA

PAGPVVSVMPAKEVPMNIQVKLTLASDATLADVWALIERGVTAYLKQLAFADPLVRFT

RIAAILLDIPPIIDYSELTVNGVSDQNIEMAANQVAVLGTVDVHE"

CDS 1095247..1095504

/locus_tag="PIKHDJKN_00979"

/inference="ab initio prediction:Prodigal:002006"

/codon_start=1

/transl_table=11

/product="hypothetical protein"

/translation="MSRGVMEEAKRRCGGSGSVGHRECSDYAASGSAGNEESGGQEEC

LGRAVRRSGTQHLSEGVYVDVKGREGTEHECSFCCKCWTDE"

CDS 1095464..1096054

/locus_tag="PIKHDJKN_00980"

/inference="ab initio prediction:Prodigal:002006"

/codon_start=1

/transl_table=11

/product="hypothetical protein"

/translation="MSAPSVVNVGLTSEKGRELFSYLPSYYETSRVMQADMQSKGTEM

DLLYQALDETLDQFFVRTATWGLNFWEQELGIETDRLKPVDQRRAVVESKLRGAGKFS

GRLVANVAEAYAGGKVDVSFQPEVWSFTVSFVDTMGIPPNIDDLKRAIDELKPAHMAV

EYEYRYLIWDDLDNKQMTWDELDAASLTWNELEVWA"

CDS 1096054..1097337

/locus_tag="PIKHDJKN_00981"

/inference="ab initio prediction:Prodigal:002006"

/codon_start=1

/transl_table=11

/product="hypothetical protein"

/translation="MPKETDRLKLPLPLGNENVNRESINGIFEKIDAGVATQADLDTL

REAVSKMDIPDASLTQKGKVQLSSKTDGTSETVAATEKAVRDARVAAETNAKNASLPR

TGGELTGKVKFSSWGGFSSNSTGLALYGSNCYLDGSTYRFENTHANFGARGLVMRFAG

GSNPEIYMFDTGLIATTNGVAFTPTLKRVLNQTDYDALFQSVSNGKASIAAAITGKGV

PTAADAQFVTIASNVEKIQQGRYQNLNITNNQSNVTISTSYDGIKWASFAKFPAGTKL

ITMNTGSINFGTSYTPTRINQYAASMDLCLVDTNANRLSLLSIAGGNINNDNYTWYNF

DVSANLTSLFLDSTRKSFGCSGSVNYVSYNNQNSYSTRNKPYASFNIPAGFNVDGELE

LALYITKSNRTDGAWAGGEGTGIHYYSSGTTIVSV"

CDS 1097497..1097928

/locus_tag="PIKHDJKN_00982"

/inference="ab initio prediction:Prodigal:002006"

/codon_start=1

/transl_table=11

/product="hypothetical protein"

/translation="MITNQLTTAIASKLTQHFPNIPIHLTTGTISPNPDAKGIIYRLL

SAQLIRERSDRFVQSHAFEIRWLEANDIPATLPDELFEALETIDVEGTPYRATELRWE

TESDTARMLVYYTIRTTKVSESAATIQQLEQRPTALKATRE"

CDS 1097951..1098187

/locus_tag="PIKHDJKN_00983"

/inference="ab initio prediction:Prodigal:002006"

/codon_start=1

/transl_table=11

/product="hypothetical protein"

/translation="MKGIGGALAMFTKKEPDPKKTEAQQKNNQKYSKSKFAESRQFSR

MEKDILAAVLLQQQTYTLQEAQQHIQQFMNGEAQ"

CDS 1098187..1099503

/locus_tag="PIKHDJKN_00984"

/inference="ab initio prediction:Prodigal:002006"

/codon_start=1

/transl_table=11

/product="hypothetical protein"

/translation="MAGGTWTTQNKVRPGVYMNFASEGSLPGTVGERGTVALALPLSW

GQAGTILTVQAGQDVQAKLGYDWTAPQLLLIREALKRAQTLLLYGLNTGTKAKATLDK

LTVTAQHGGVRGNDLAVVISANINDPDQFDVSTLLAGKEVDKQTVPTIEELESNAYVT

FTGEGALTTTAALPLTGGQDGNATNQEHADFLTKLEVLDFNTVGLISDDVTLKSVYTA

YIKRLRDTEGKKVQLVLSDYPAADHEGVISVKNGVVLADGTILTPKQTVAWTAGATAG

ANLNESLTFRAYDDAVDVNGRLTHSETEAALRNGEFVFTASSNRAVVEQDVNTFRSVS

PDKARHFAKNRVVRVLDGIANDMKRIFESYFIGKVNNNEDGRSLFRSQCVTYLKQLQD

IGAIQNFDSKTDITVTPGNETDSILIEIQVQPVDSVEKVYMKVKVV"

CDS 1099505..1099963

/gene="xkdM_2"

/locus_tag="PIKHDJKN_00985"

/inference="ab initio prediction:Prodigal:002006"

/inference="similar to AA sequence:UniProtKB:P54332"

/codon_start=1

/transl_table=11

/product="Phage-like element PBSX protein XkdM"

/translation="MAFLKASDTISGQEGRAYATINGQTEEMFYVKTLEATVEKQKAE

VKTLGRRGVQHKATGWSGSGSMTIFYTTSRFRELMLQYMQNGVDTYFDIEVTNEDPSS

TIGKQTVTLKGVNLDSVIMASLDTEAEALEEEVSFTFEDVDMPVSFNLPK"

CDS 1100215..1100637

/gene="yqbN"

/locus_tag="PIKHDJKN_00986"

/inference="ab initio prediction:Prodigal:002006"

/inference="similar to AA sequence:UniProtKB:P45930"

/codon_start=1

/transl_table=11

/product="putative protein YqbN"

/translation="MSGLSMFFAQNAVADTTEEFIVSSRFKDEKGEPVAWKLRSMTED

ENQECRKAATRKIKGKNGVYTPDIDANDYMARLMSASVVYPDLKNAELQRSYGVMGAE

SLLRKMLLPGEFASLGEQVQKLNGFNQDMNELVDDVKN"

CDS 1100839..1102812

/locus_tag="PIKHDJKN_00987"

/inference="ab initio prediction:Prodigal:002006"

/codon_start=1

/transl_table=11

/product="hypothetical protein"

/translation="MYAMFARLYLTSTRIVTQFQQLPAAISAMFNSARLNSIAMASRL
LQGVSEEQAKVNAAFSEGAGRVRAWISMIKSAGQAVLVPAAQEEDLKHRYMATTGDDA
QGETIFNRYRAEAFKSGQNVTDALKGALSLIPYAQNTDQVDQLRDMTKRLSMLSPDGK
SISDASGALVAAMNGDNGELASSFNIPEEILNGAGLQQSIDSSNLDAFIQKLDVILQK
QGYTQQAFDTMLDSPLQKWNILVNQFNGVLGQIGQAALVALTPLLDRLNEAFANGEFT
GIIEWISNAFTVAANALTMLVDGILYIASVVQQNWDIIQPILMALALVVLALVIIQVY
SLVAAWLLLNWPILLVIAAIAAVIGILSMMGITGTEILGAIIGTFMLLGEMVRVVIAT
MWNYFAIFADFIANVFKDPIYAFQKLLYDLGMFAMQMFYNIIVGIEDFLGRAVGAIGK
VASFINDTFGTNLSVMSESDINIGSKNIKEWKDTLESWEPQSDKDVFHMNRMDGEFNP
KVADKGQDVAKKLLSKIPSLPDPNSKETGLPGNFGGSTPKTPSMPSMPTAPAPTVVPN
NNMSNINKINNIGQVDKIGDVDGTVDVTSEDLKLMRELAEMQAIQRFVSLTPTVQVTT
GDINSGHDVDSIISKITDGLNSQIVSSAQGVYG"

CDS 1102813..1103508

/locus_tag="PIKHDJKN_00988"
/inference="ab initio prediction:Prodigal:002006"
/codon_start=1
/transl_table=11
/product="hypothetical protein"
/translation="MEYYIQLSFNNRSEYMFFPVTPESIEFSDSGDGSTFNVSALGEI
NVIKSPKLREVSFSGVFPADYSPYHLNFDASHPAVQKQFYRDPYEYVKKFIRWMQTGR
PVRLFFSSARYTINMAVSIESFDWKETAGTVGDIQYDIKMKQFIFYAAKKVVPLKDSK
DTAASKTKTKASRPNEKIQAKTVTLKAGDSLWSVAKANLGDGSRWKELQKLNGIKDAQ
LKKLPIGLVIKLP"

CDS 1103522..1104532

/locus_tag="PIKHDJKN_00989"
/inference="ab initio prediction:Prodigal:002006"
/codon_start=1
/transl_table=11
/product="hypothetical protein"
/translation="MQQQISLDDKQGNMKERLWLDDKQGNIWDISEIAGDITYKTSRI
GKPSSLEFTLIKGSLYQNTKFTYENGYVVKYISNEVGIFYGYIFSVDSGKDESVKIKA
YDQTRYLTANQTYKFVNATATDVIKRIATDFQLKVGELIQPKYVIPRMLFDNKKLIDM

ICEALDRTLIYGGKNYIFYDDFGKLVLRDVEEMPYGFVIGDNSLLTDYSYTRSIDDQT
YNKIKLYRDNKDTGKRETFVHQDSGSIRQWGLLFLYQKADDGLNEGQIDAMLKTLMTL
RNRETQTLKVDALGDFKVRAGSFVNIQINELKINQYFLVDECTHKVQGGVHTMSLDLK
VV"

CDS 1104539..1104796
/locus_tag="PIKHDJKN_00990"
/inference="ab initio prediction:Prodigal:002006"
/codon_start=1
/transl_table=11
/product="hypothetical protein"
/translation="MMLDVIKKAAVAAVDAKAPVQVMYGSVTNTQPLEITVEQRLALA
EPFLVLPESVVNKAWTVGDHVLLLRVQGGDSFVVLDRLVNP"

CDS 1104793..1105206
/locus_tag="PIKHDJKN_00991"
/inference="ab initio prediction:Prodigal:002006"
/codon_start=1
/transl_table=11
/product="hypothetical protein"
/translation="MIPQGAQISTEDQEETAVLPSLTYVLQPSGQRIGRLQLDGKDAV
KQAVYKALSTHRYEHLIYSSDYGMEWSWEGIAGRSMVESELERWIREALLPDDRISDV
TEFDFVHEADGVRVSFTVETDFGSFRGETEVNLNV"

CDS 1105199..1106257
/locus_tag="PIKHDJKN_00992"
/inference="ab initio prediction:Prodigal:002006"
/codon_start=1
/transl_table=11
/product="hypothetical protein"
/translation="MYEGQTFEVILDRMLDKVPDGVDKREGSIIYDALAPAAVEMAQM
YIELDVNANLKFADTASGEYLDRAVAWSGISRKAATKARWIGSFRDNEGKPVEVPLES
RFSTGDRVYYVVVERISAGQYVLECEVAGAEGNEYTGALLPIDYIAGLTTAELTQLLVP
GEDEETDQALYDRYQDKVSRPVTSANKYQYELWARENSGVGKAKAFPLWDGPGTVKVA
LLNNEMHAPAEAVIEAVQKYIDPTQDGMGEGAAPIGPVVTVVGAEEVPIHVEVQVTLA

SGSTYDGVKTLIEAGVTAYLKELAFADPLVRWTRIANVILDIPPVIDYSDLLVNGGMS
NLEIAPGAVAVLGTVKVT"

CDS 1106254..1106799

/locus_tag="PIKHDJKN_00993"

/inference="ab initio prediction:Prodigal:002006"

/codon_start=1

/transl_table=11

/product="hypothetical protein"

/translation="MSKAEVLMTLLPPLYENVLEMQLLTQTEGQELDRLVKGMDGVLD
QFYPESATWALDRYERDLQIPTNQAKPDDQRRSVIISKMRGSGKVSGSMLKNVAQAYE
SGGIDVSVSPEDYSIRIRFIDTWGLPPNLDDLKAAIEDIKPAHMTVEYRLRYLTIAEV
ESMTLDEIEQTRQDKFAGGGA"

CDS 1106800..1109847

/locus_tag="PIKHDJKN_00994"

/inference="ab initio prediction:Prodigal:002006"

/codon_start=1

/transl_table=11

/product="hypothetical protein"

/translation="MNEPKTPNLGLNKIDRSSPSTTYFDLDKYLDQNWEKVDDFAEQL
EGKSGETAEQVKSIQDRLNTEKRRSVKLEPGLQIINAERASAIKLEGIKGRTLVNLLG
RDGNFGDVTRWNVGLGTRTVANNVIMVTGNGTGVNPQLTNYNHISALNPKVGDKLFLR
VRATHIVGTAKQLQLYLYSNTLNRFTAMNIDNPVSGSTYDMYGVITVNQAIVDGWDTT
FGFKLTAEYATYEASNGSQVQFSNAAIYKVSDEDKSLTSSQMALKYPYVDSLQPVRNP
YAIRYGENLLPSLMNSSFVLSSNYVNGPYEWSNLLGADATGYTIVKFNVMPNTNYTFS
AETFLNLSTSVYPLDLQTPAPLRPWTNDSTFTFNSGQNSEINFLWRASSGGNPASLKN
PMIALGTTTKPFKPREDAMLALQTDLYADPITGANADEVFEKEGQYYKLTKWKKISFD
STYTYSFFNSYAGGKCVRLMDGITDRNKKYLPMATKFNGVRLSDGSPSLNVDQYSRGD
WDTGANGVFLGIGVSSADSGWGDSYTPTPDEIKAYFMGWKMSVNGSPRATAYDGTGTK
VWIRITRMLDSIALANGVDYTTALPTTLAGTDSLGNLYTPYQLAYQLATPTVESITSE
GQLTFTEGSNQVEVGTGIVLREPAKPIKFDNYDINTGKTMGNATLYAVDKWIALYENG
HLKPTGWGVFEGQDNGKAIAIYGHQYNPSAAYSVTYLMKDVSPVTSFLGSISVNEKAL
LTDLTKSVHQNTASLSMLEISAADGGVLKNYVDNKPWQKYKITQDNGSIIQLPHNSNL

NNIKNGGQYDCFNAVNVPGGVVNAWFYVEVLVHSNAPTHVIQRASRLDTHNTPTLYMR

TCMDNIWSEWSPDVFQSGVDAKNGIVDAINAMGSSASMNDTWLTLASKIHLIKNGKRF

ATGQFVIGSNGAGSATGLGFKPRNITFANATNTPSNIYFGVYSEDSPRNNYSLGGTRF

LNRVVGLDNGLSTDDKIVPTVNGFNVSGTGITDQRTYNYFATE"

CDS 1109991..1110488

/locus_tag="PIKHDJKN_00995"

/inference="ab initio prediction:Prodigal:002006"

/codon_start=1

/transl_table=11

/product="hypothetical protein"

/translation="MERWDTLWKWLLALLTSSATYFFGGWSGVLGVLLVFVILDYLTG

IAAAGMSGKLESNVGMFGIARKVFIFAMVSVAHLVDGVLGDGHLFRDAVAFFYIANEL

LSIIENGGKLGAPIPPVIRQAIEVLKGKGGDGGIPGNYTPDSKESFVQSDHEDTDKQT

RDETK"

CDS 1110966..1111904

/locus_tag="PIKHDJKN_00996"

/inference="ab initio prediction:Prodigal:002006"

/codon_start=1

/transl_table=11

/product="hypothetical protein"

/translation="MQTRSSGNTQGIDVSRYQGTIDWAKVKANGMTFVFIKATEGQTY

IDPNFQKNVTGALGAGLLIGTYHFFRATTTDGAKAEAAHYANTLQKVGGAKALQLPPV

MDYENNPGNLSKAQINTVAKAFLTELQRLTGAKPIIYTGNSFAGNFEPSMSSYDLWIA

RYSNTRVPNDQPAWKRWTFWQYTDSGKVNGISGNVDMNEFEGSTAELRARYATATPKP

PEPSEPTNPTNPTEPPKGGEPMTAEEKAAFDALKFQVDKLQARQQMEVPVWAKAAVDA

ALAYDTKNPLFSIDNGASYDFYRFITVMYRRGLFKK"

CDS 1112706..1114364

/gene="hbpA"

/locus_tag="PIKHDJKN_00997"

/inference="ab initio prediction:Prodigal:002006"

/inference="similar to AA sequence:UniProtKB:P33950"

/codon_start=1

/transl_table=11

/product="Heme-binding protein A"

/db_xref="COG:COG0747"

/translation="MRMRKKWLSGVMAMAMSTVLILSGCSSASNEGTGNSPAPAEGSE

PPAEVATQDTMIMGRGGDSVALDPAIVTDGESLKIGHQVFDSLLDYKEGGTEVVPGLA

ESWEISADGLKYDFKLKSGVKFHDGTDFNAEAVVFNFNRWSDPASEYKFEGDSFDYYD

SMFGPEDGRVIKEVKAIDETTVEFTLNQPQAPFLQNIAMTPFGIASPTAIQEKKENFK

SEPVGTGPFVFKEWKRNDSITLEKNPNYWKEGLPKLNKVIVRSIPDNTARFNALQNGE

IDIMEDLNPDDLSILEGNSELQKIERPPFNVSYIGFNFKKKPFDNVKVRQALNHAVNK

QGIIDAFFAGQAQPAVNPMPPTLWGYNDSIEDYAYDLEKAKALLAEAGYPDGLPDPVT

FYAMPVSRPYMPDGKKVAEAIQADFEKIGVTVNIESPEWATYLDDAKAGEKDDIYMLG

WTGDNGDPDNFLYTLLDKDAIPGNNRSFYVNEELHTLLTSAQKETDQDKRAELYKQAQ

VIIKEDAPWIPLVHTTPILAGKANLKNFVPSPLGTESYANAYFE"

CDS 1114520..1115524

/gene="dppB_4"

/locus_tag="PIKHDJKN_00998"

/inference="ab initio prediction:Prodigal:002006"

/inference="similar to AA sequence:UniProtKB:P0AEF8"

/codon_start=1

/transl_table=11

/product="Dipeptide transport system permease protein

DppB"

/db_xref="COG:COG0601"

/translation="MNSYIVKRVLVLLPVLLGMTLIVFSIIHAIPGDPAETILGQKAT

EQSKQALRDQLGLDKPWTQQYFAYLGDLFKGDLGTSIRTKVPIAQEIVPYLTATLELT

MASMLFAIIVGVNAGIVSAWKHNSWFDYCCMVIALVGVSMPIFWLGLMEQWLFANKLH

WLPSIGRMNARDPIEAITGLYVLDTMIAGRWDQLWTVTKHLLLPSIALGTIPMAVIAR

MTRSSMLEVMSSDYIRTAKAKGLGPFFVVYGHALKNAFIPVLTVIGIQTGSLLGGAVL

TETIFAWPGVGRYIYEAISSRDYPVIQSGILIVAFFFVVINLIVDLLYAVFDPRISYK

"

CDS 1115778..1116677

/gene="gsiD_1"

/locus_tag="PIKHDJKN_00999"

/inference="ab initio prediction:Prodigal:002006"

/inference="similar to AA sequence:UniProtKB:P75799"

/codon_start=1

/transl_table=11

/product="Glutathione transport system permease protein

GsiD"

/db_xref="COG:COG1173"

/translation="MAKLSTNTGASIDAASAPASGPWREAWRTFRKNRLALAGLIIIV

FFILMAFAAPYIAPYDYKEQVLTDRLQAPSAEHWFGTDDLGRDVFSRVLYGARISLWV

GFFSVIGSIIAGTLLGLIAGYYGKWADMLISRLFDILLAFPAILLAIAIVAILGPSLQ

NALLAIAIVNIPTYGRLVRSRVLSLRQEEFITSARTLGASNMRILFRHILPNSLTPLI

VQGTLGIGTAIIEAAALGFLGMGAQPPDPEWGKMLSDSRQFLQKAPWTLIFPGLSIMF

TVLGFNLLGDGLRDTLDPKMAKK"

CDS 1117674..1119554

/gene="htpG_1"

/locus_tag="PIKHDJKN_01000"

/inference="ab initio prediction:Prodigal:002006"

/inference="similar to AA sequence:UniProtKB:P9WMJ7"

/codon_start=1

/transl_table=11

/product="Chaperone protein HtpG"

/db_xref="COG:COG0326"

/translation="MAKKEFQAESKRLLDMMINSIYTQREIFLRELISNSSDAIDKIY

YKALTDDTLVFNKEDYYIKLTIDKENRTLTLTDTGIGMTQEELENNLGVIAKSGSLAF

KKENEAKDGHNIIGQFGVGFYSAFMVADKLTVTSKTLGSDEAWKWESEGADGYTISPA

EKDSVGTEIVLTIKQNTEEDSYDEFLEEYRLRSIVKKYSDFIRYPIKMDVTGQRPKEG

TENEFEEYKEEQTVNSMVPIWRKNKSELTEEDYTNFYMEKRYGFDKPLKHLHISADGA

VVYNAILFIPENTPFDYYTKEYEKGLELYSNGVLIMDKCGDLLPDYFGFVKGMVDSED

LSLNISREMLQHDRQLSLIAKNIKNKIKSQLQSLLKDERENYEKFYQAFGRQLKYGVY

SDYGVNKDTLQDLLLFSSSKEKKLVSLDEYVSRMPEDQKYIYYASGESIERIEKLPQI

EGVLDKGYEVLYFTDDIDEFAIKMITNYKEKEFKSISSGDLGIEDSADKEETDAQDND

NKELFEAMQAQLDGKVKAVKASKRLRSHPVCLSTEGELTIEMEKILKAMPNSENVQAD

KVLEINVNHDVFKSLKDAFAQDKEKLSLYTSLLYHQALLIEGLPIQDPVEFTNDICKV

MV"

CDS 1120143..1121558

/locus_tag="PIKHDJKN_01001"

/inference="ab initio prediction:Prodigal:002006"

/inference="protein motif:HAMAP:MF_01212"

/codon_start=1

/transl_table=11

/product="Deoxyguanosinetriphosphate

triphosphohydrolase-like protein"

/translation="MQWNDLREHRQYPELTKLDGARAAYERDYSRLIHSPTFRRLQGK

SQVFGAGTGDYYRTRLTHSLEVAQIAREAARSLLRRFPEVDWSQADNPGLIIDSEVVE

CAAIAHDFGHPPFGHKGEEVLDGILDDLINTEVKKIMKKSRGAKSPQQEPEIRAELKR

KYEHFEGNAHNFRLIMYLEKREDIDGLNLSDAVLLGINKYPYPGTESKKGMYHHEWQY

IHEIRNRWAIPAGKKTLEAQLMDLCDDIAYSAHDLEDGIKAGKIEVHEHFLQDPHINR

LIVDKITTLEDLFWNGWTREAIGKKVEEVLASFLRIWNEKMPFCEHDYSRTRREVKAY

WVSLFVASLGVIDNGDWKKVTFVREGAEDLDMLRTVSVLKSFAWVTMIRDLRVQRLQK

RSEWMIKRLWDAFLDPETSKSIIPSDWLQRYEKDQAKANPIWTWEHMVIDYIAGMTDA

FAEKIYNELYGLKVGSIYDLD"

CDS 1121589..1122584

/gene="fgd_1"

/locus_tag="PIKHDJKN_01002"

/EC_number="1.1.98.2"

/inference="ab initio prediction:Prodigal:002006"

/inference="protein motif:HAMAP:MF_02123"

/codon_start=1

/transl_table=11

/product="F420-dependent glucose-6-phosphate

dehydrogenase"

/translation="MGVNISEKYNLGVLDLVPRLNGATAEQALQQSVTLAQHAEDWGY

SRYWTSEHHDMDELASASPEVLLAHIGARTNTIQLGSGAVLLPHYSPLKVAESFRLLA

ALYPGRIELGLGRAPGGGPHATMALSGNYLQHVSKLPESLAALTELLEDRYTYEEHPV
TARPIPELPLSLWMLGTNVKSAEFAARFGMGYVFGQFMSDADGTEAVRRYREGFIPSA
NRKEPEVMVAVSVLCAETQEEALSWSLEMVERRRAVGKEITTQSDNISTSTSTNERDD
ANENEVQRHYAGTPAQVWNQLQQVCQRLDTDRLLVVTTGPDYERRLASYKLLANA"

CDS 1122946..1124658

/locus_tag="PIKHDJKN_01003"
/inference="ab initio prediction:Prodigal:002006"
/codon_start=1
/transl_table=11
/product="hypothetical protein"
/translation="MFSRFRIKSIGLRISIAFYLLILCLILLSVTIVTRMNSMERNTN
EITNNWMPSLQQINRLNYTTEHILSLSYRHYDAQAGDKASLAEERTKYIRETAQAIKI
YDQQEKTTEEKEQWDAFKTKWAAYLKINTQSIKLSDEGQEQLAKEVSEKGADSFDAMQ
VDLDYLVEYNQEQSSISAAQTIRSVQDGRMIIVVGVLVMIIITAIAIPIIRSQVVKPL
LRVISAVKLIAEGQLNVQDIHTKHEDEVGVLAKAVNDMKGNLTSMVLNVRRIAEAVNR
QSSELAISSEEVKIGSRQIAITMEESAKAAESQAETAVESARTVEGLNANIQQHSEQG
NQLRLMSDLILKQGRTGRESMEQSVNQMQQIAGAVSSSMNKMEQLDRKNEDISQLVQV
IHDIARQTNLLALNASIEAARAGESGRGFAVVAAEVRKLSEAVRTSVEEITAITGDIQ
KDSQGVVEELRTGVLETERGQQQVRNSGNLFRTISESVEGMVQVIGTMTDGLEGMQEA
SGRMHNFSQQISAVSEESAASVEEVSASAEEQVSSMETISGNIQTLKDLSEDLLASIE
KLKI"

CDS 1124700..1125677

/locus_tag="PIKHDJKN_01004"
/inference="ab initio prediction:Prodigal:002006"
/codon_start=1
/transl_table=11
/product="hypothetical protein"
/translation="MLSSTNSKDSVVGSYVYTFACHENERALCEMELGTMLVPGTDIG
SGDHAYVRSNICIPPGRSPFVRGRLDVMAEADTVAGLKPAASQIDLSSGETFKVVCLK
EGDDMPDYAHCRLLEKELGMCIKGKAMMKQPDVTFGLMHVNGKWLFGQWTEADRSWQT
HHQKPQNYSTGLGVTLARALVNIAIPQVEGHQLLDPCCGMGTVVIEALSMGIEAKGND
LNPLAVQGARINLPHYGYDPARISLGDMNDLEGFYDAVILDMPYNLCSVLPDEEQLAM

LTSLKRLAGRAVIVSTEWVEGHILDAGWNIDQYRTVSKGTFIRHIWLCT"

CDS complement(1125814..1126770)

/gene="ghrB"

/locus_tag="PIKHDJKN_01005"

/EC_number="1.1.1.79"

/inference="ab initio prediction:Prodigal:002006"

/inference="similar to AA sequence:UniProtKB:P37666"

/codon_start=1

/transl_table=11

/product="Glyoxylate/hydroxypyruvate reductase B"

/db_xref="COG:COG1052"

/translation="MGKIVCFPSLSTKQQQRILDAAPGYTLTVSKAKEIDSSELKEVE

IIVGWSPLVTEHALKENSPLKWVQVWSAGVDNLPFSDLEQKSILVTSANGVHAIPITE

IILGMMLSHSRWLRQAMLHQQQAEWKSPGKPLPELHGKTAVIVGVGEIGSETARILKA

LGMNVIGVRRSGKDVPNVDQMYNMSGLHEALSQGDYVINILPLTDETHHIYDQAAFEH

FKSGSCFVNVGRGPSVNTEALLGALDSGQVAFAALDVFEEEPLPADHPLWGKDNVLIT

PHIAGSTEQYTERALDIFVQNLEAYIAGKTLPLNLVNYSHKY"

CDS 1126961..1127563

/gene="ytlP"

/locus_tag="PIKHDJKN_01006"

/EC_number="3.1.4.58"

/inference="ab initio prediction:Prodigal:002006"

/inference="similar to AA sequence:UniProtKB:O34570"

/codon_start=1

/transl_table=11

/product="RNA 2',3'-cyclic phosphodiesterase"

/translation="MNKYSYTDLPSRRERLFTALRVPAHIQQSLRMSAELVQGKLDFR

KWTDHRDYHITLQFLGDTLVSDIGQLRKTLRSASVGFQPFELQLSGWGTFGLEETPKV

LWRGVEGEKDQLNLLQKRIVEATSSLGYEAELRPYSPHITIARKFLGQVPGNEDKGIF

GVLPEHSLGVNSWMIQDFVLYVTQPGQSPMYEVVDTFSFS"

CDS 1127694..1128551

/locus_tag="PIKHDJKN_01007"

/inference="ab initio prediction:Prodigal:002006"

/codon_start=1

/transl_table=11

/product="hypothetical protein"

/translation="MDIISKNRWVAPMLSVLLVCIVGANVVQATGKWNEASDSKQMTE

LAASAENSENSFSGDRRMMEDGTSLSTDGSIQTSNWTNSLPAKNWLTAAQEEQQQKEA

KAKHAARAAAAAKAKKEAALKLAKIEHAKAVAAITTPPQKLYFTRTKLLNQEDSKLAT

WSYSVSDKELLLLQKIVMAEAEGEPYEGKVAVANVVLNRLRSANFPDTIYKVIYQKSQ

FSPVANGRLKRVTPNEDSIKAVNAALNGKKEVADDTYYFLSLTLADDLTVARSQKKVK

TIGHHTFYK"

CDS complement(1128829..1130049)

/gene="ugtP_1"

/locus_tag="PIKHDJKN_01008"

/EC_number="2.4.1.315"

/inference="ab initio prediction:Prodigal:002006"

/inference="similar to AA sequence:UniProtKB:P54166"

/codon_start=1

/transl_table=11

/product="Processive diacylglycerol

beta-glucosyltransferase"

/db_xref="COG:COG0707"

/translation="MRKPRVLLLSEGFGTGHTQAAHALASGIKKVSPHVHSRVIELGK

FLNPTMAPLILSAYRKTLTVQPKLVSLLYRTQYNKSLNGFTKLALHRIFYTQTAQVVS

QLKPDAVICTHPFPNAVISRLKRQGLNIPLYTLVTDYDVHATWINPEVNKYLVSTPQV

KALLEIRGVEPSRIQITGIPVHPDFWEAGDKVSLRQEMGLQNMPTALLMGGGWGLSFD

EEHMKALASWADRVQLLFCLGSNEKMIAKLKEMSCFQHPNIRILGYTREVSKLMDVSD

VLITKPGGMTCTEALAKGLPMLFVPPLPGQEEENCEYFVQAGYGQVIHSADVIANRFR

HLCEQIYAPREAESQLKSVHKPESRNTYDPTCCAQAVHDLLFPATAEVTSAPKERFTA

GIGATSLSGTRIPF"

CDS 1130407..1130988

/locus_tag="PIKHDJKN_01009"

/inference="ab initio prediction:Prodigal:002006"

/codon_start=1

/transl_table=11

/product="hypothetical protein"

/translation="MAPIDRRQQVIHAAAQSFAMFGYKATTMDQVAKIANVGKGTIYT

FFTNKEQLFDQILVEVIQEMKNIAHREVHQESAFFDNLFRVLDSLLEFRRDHDLLVKL

SQELKDFGTLQAKEGLEKVEKVISDFLARELEKARDNGEIRECDPQVVAFMMIRLYIA

LTSDWSKQHEPLSKEEIKNYFRLFLMEGIAAAT"

CDS 1131196..1133478

/locus_tag="PIKHDJKN_01010"

/inference="ab initio prediction:Prodigal:002006"

/codon_start=1

/transl_table=11

/product="hypothetical protein"

/translation="MKSLSVFFKDVGSAVRNPKVLIPVIAILFIPILYSGIYLAAYWD

PYGHVDEMPVAVVNLDKGAELEGKSLHVGSDLVDELKKNADFKWDFVSASQAKEGMEN

DKYYMQITIPENFSSQATTLLDDKPEPADLIYEPNGNYSFVGAQIGKTAIKDLKAKVS

AKVTESYAETLLDKFSEVSDGLAEAGDGAGDLNTGAGKLDDGAVKLKDNLAKLASGTL

ELQDGLSPLSDGVNALHTGATKLESGTSNLVSGLQQLQAAATSQLQSGADQLKDGSAK

LETGLKSSVDGAGKLQAGLKSSEQGSAKLSEGLQSAVQGSGTLATGLQSAVDGSIKVA

DGAQGVADGLKQLAASNPELAASEDVQKLLAASTAVAEGSKQLRESEQKLAQGADQLH

QGNQQLAAGASELHGGQEQLLTGANQLVDGQQQLLAGAGQLSQGGAKLSDGLKQFSGK

LGEAASGGTLLADGAKQLGSGTTALQTGVGKLSGGVSSLTDGSKQLGDGAGKLADGLT

ELKDGSSELATKLNDAAQKTSEVKKTDDVVNMFAEPVNTSENTTENVSNYGTGLTPFF

LSIGLFVGSLISTIVLKMRETSVPGATGWNRFVSRTLVFGFVSIIQSVIVASFMLYGL

GLETHSVPLFYLFTIFTGLTFMFIVQALVTWLDLPGRYVVILLLVFQLAASAGTFPVE

LIPSWLQAFSPWLPMTHSIMGFKAVVSSGNFDVMWHQVGILAIYAGISVLLTLAFFLW

NGRRRKKEVEVAESGQVVTA"

CDS 1134154..1138752

/gene="gltB_1"

/locus_tag="PIKHDJKN_01011"

/EC_number="1.4.7.1"

/inference="ab initio prediction:Prodigal:002006"

/inference="similar to AA sequence:UniProtKB:P55037"

/codon_start=1

/transl_table=11

/product="Ferredoxin-dependent glutamate synthase 1"

/translation="MRHIGLPPKQGLYDPQFEKDACGMGFVANIKGVPSHDIVSQALT

MLSNMEHRGGQGSEPNSGDGAGILIQIPHRYFAQEADRLGFALPEQGFYGVGMLFLSQ

DPVIRSAHEESLKKIIEEEGQTFLGFRDVPTFDEMLGRSALAAKPYVRQVFIGRSTEV

KDELGFERKLYVIRRRAELAIRYSADEAEGGSFYLPSLSCRKIVYKGMLTTEQVGQFY

LDLQEDLVESAMALVHSRFSTNTFPSWERAHPYRFMIHNGEINTMRGNVNWMHARQSL

FESELFGNDISKVKPVINPDGSDTAMFDNTLEFLYLSGRSLPHVAMMMVPEPWSTDEG

MDPAKKAFYEYHSTMMEPWDGPAAMAFTDGLQIGATLDRNGLRPARYYVTKDDRIILS

SEVGVLDIAPEEILYKDRLRPGRMLLVDTQQGRIIADEEVKALIAAENPYQDWLDEHL

MDLNELPDAPELPDPKHDNVTQLQLAYGYTFEELRKVLEPMASTGMEATGSMGYDAPL

AVLSDRPQRLYNYFKQMFAQVTNPPIDAIREEIVTSTATTIGPERNLLNPEPESCRQI

RLDTPVLSNEDFAKIRHVRRPGFRSMTIPIFFTAKEGAEGLRKAMELLFEAADRVIDK

GHNILILSDRGVDAENAAIPALLAVAGLHHHLIRKGTRTKVSILLESAEPRDIHHYAL

LLGYGVSAVNPYLAFETLDDMIQQGLLRGISHEKAVKNYIKAATKGVIKILSKMGIST

IQSYRGAQIFEAVGLKSDFVDRYFTWTPSRIGGIGLEEVAAEALTHHNRAFTDKDGND

KVLDSGGEYQWRNDGEEHLFNPQTIHTLQHAVRTGDYKLYKKYSKLVQGENDQLLTIR

SMLRLKPVGPAIPLEEVESVEDIMRRFKTGAMSFGSISKEAHEDLAIAMNRVGGKSNT

GEGGEDPARFIKDSNGDSRRSAIKQVASGRFGVTSNYLVNADEIQIKMAQGAKPGEGG

QLPGRKVYPWVAEVRGSTPGVGLISPPPHHDIYSIEDLAELIYDLKNANPRAEINVKL

VSEVGVGTIAAGVAKGRADIILVSGYDGGTGASPQGSIRHAGMPWELGLAETHQTLML

NNLRDRVVLETDGKMLNGRDLAIAALLGAEEYGFSTAPLVALGCIMMRVCQMDTCPVG

VATQNPELRKNYTGDPAHVVNFMRFVAEDVREIMADLGFRTIQEMVGRTDCLETVEAV

DHWKKKGVDLSVLLHVPEMPEGSARYRTQHQNHQLEETLDMQQLLPLAQPALESGQPV

EAVLPITNVNRAVGTILGSEITRKYGIAGLPEDTVQFKFVGSAGQSFGAFVPKGMTLT

VEGDSNDYVGKGLSGGKLIVMPSPKATFDAEDNIIIGNTALYGATSGEAYIRGIAGER

FAVRNSGAKVVVEGVGDHGCEYMTGGRVVVLGDTGRNFAAGMSGGIAYVYDPKGTFLK

RCNLEMVLLERIEDVSESADLRGMIQRHVANTGSAAGQRILDNWQQSVDQFVRVIPKD

FKRMTEQIERIQATGLTGEAALLAAFEANMRELARAGG"

CDS 1138974..1139129

/locus_tag="PIKHDJKN_01012"

/inference="ab initio prediction:Prodigal:002006"

/codon_start=1

/transl_table=11

/product="hypothetical protein"

/translation="MNMKQPNRGDRKPKGKPVEATQMDIMKVLLQCGIDPERWNHFAS

SDNNKRA"

CDS complement(1139232..1139600)

/gene="xre"

/locus_tag="PIKHDJKN_01013"

/inference="ab initio prediction:Prodigal:002006"

/inference="similar to AA sequence:UniProtKB:P23789"

/codon_start=1

/transl_table=11

/product="HTH-type transcriptional regulator Xre"

/db_xref="COG:COG1396"

/translation="MSYGNRIAELREQRGLTQEELASSIHITRAALSHYEKNRRKPDF

EVLTRLADIFGVSIDYLIGRTKQSDVVMDEDVRQFVDALELSDKEVLERFGLMIDGKP

LTEEEARRFIAFVRMERSMD"

CDS 1139934..1140368

/locus_tag="PIKHDJKN_01014"

/inference="ab initio prediction:Prodigal:002006"

/codon_start=1

/transl_table=11

/product="hypothetical protein"

/translation="MNSMIQEWADLKLNVHMYIQEFHGKLVDRKRRPVLLLGMDTRAI

TFLSDLDLPISSEMLIGLDVEDSHVCVRLHAKLQWKQPFGELTLYHSSLHIQQEQTLY

ITGQLNTMIREVQFPTVSRFQYEKASEQPNLQETYHYIDFTI"

CDS 1140520..1141278

/gene="ywqE"

/locus_tag="PIKHDJKN_01015"

/EC_number="3.1.3.48"

/inference="ab initio prediction:Prodigal:002006"

/inference="similar to AA sequence:UniProtKB:P96717"

/codon_start=1

/transl_table=11

/product="Tyrosine-protein phosphatase YwqE"

/db_xref="COG:COG4464"

/translation="MVEMHCHILSGLDDGPVHMEQSIAMAEKAAASGITSIIATPHHL

NGQYNNKPNVVNQAVDLLHAELRKRNICLEIRPGQEIRVHDNLIADLYAEKCCTLAGS

RYMLLELPFGHIPSQFPGILQELKLAGIIPIIAHPERNRPIQNNPDLLVEYLGQGALC

QITAQSVMGLFGRKVQKWCFHFCKENGFHFISSDAHNMEERTFSMKAAYHLLERRFGS

RLVQTLQNNACSVWNNQIVEPEEPTIMRRRLLFW"

CDS 1141338..1142090

/gene="ywqC"

/locus_tag="PIKHDJKN_01016"

/inference="ab initio prediction:Prodigal:002006"

/inference="similar to AA sequence:UniProtKB:P96715"

/codon_start=1

/transl_table=11

/product="putative capsular polysaccharide biosynthesis

protein YwqC"

/db_xref="COG:COG3944"

/translation="MELKEYMQILRKRIWIIIGFVAVACIGAGVKNYFLTVPIYEANA

KLIVNQAYNAQGIPNLDISSIQTNIKVINSYIEIIKSSAILDKVAATYPDLDMSGNQL

AQSIRVTTANESQVMSLTATGLSSEKAAKTVNAVAKVFKSQIPVIMKVDNVTILSEAK

PTDSSRPINVNPVINILISFLVGLLLSVGFVFLLEYLDDTLKTEDELEKELGIPALAV

ISKIRKEEARFSKQSTTSQKQVGDGQYATVNQ"

CDS 1142110..1142754

/gene="ywqD"

/locus_tag="PIKHDJKN_01017"

/EC_number="2.7.10.2"

/inference="ab initio prediction:Prodigal:002006"

/inference="similar to AA sequence:UniProtKB:P96716"

/codon_start=1

/transl_table=11

/product="Tyrosine-protein kinase YwqD"

/db_xref="COG:COG0489"

/translation="MANPKSTSSEAYRKLRTNIQFSSIDSQIQTIMIASALSGEGKTT

TIGNLAVTYAQEGKKVLLMDTDLRKPAVHRMFNVPNHVGLTSVLSSQYTVTEVLRETG

VEGLHVLSSGPIPPNPSEMIGSRKMTALLEDLKEEYDVILFDTPPVLAVTDALIISSL

CDGVILVVSAGKVKKDLVKKAKAHLEHVNARILGAVLNNVQVPKSRNAYYGENA"

CDS 1143013..1143921

/gene="gtaB_1"

/locus_tag="PIKHDJKN_01018"

/EC_number="2.7.7.9"

/inference="ab initio prediction:Prodigal:002006"

/inference="similar to AA sequence:UniProtKB:Q05852"

/codon_start=1

/transl_table=11

/product="UTP--glucose-1-phosphate uridylyltransferase"

/db_xref="COG:COG1210"

/translation="MTKVRKAIIPAAGLGTRFLPATKAMPKEMLPIVDKPTIQYIVEE

AVASGIEDIIIVTGKGKRAIEDHFDSAFELEQNLLEKGKLSLLEDVRKSSNVDIHYIR

QKEALGLGHAVWCARNFIGNEPFAVLLGDDIVVSEVPCTKQLIQQYEKVNQSVLGVKT

VSPSETYRYGIIDPLHTEGRLSSVNSMVEKPPLGTAPSNLAIMGRYILTPKVFEYLEE

QNVGAGGEIQLTDALQMLNRDEGISAYDFEGSRFDVGEKLGYILTTLDFALKNKELRA

PILMAIEEILMKEQLNQMLVPGGDSL"

CDS 1143918..1144586

/locus_tag="PIKHDJKN_01019"

/inference="ab initio prediction:Prodigal:002006"

/codon_start=1

/transl_table=11

/product="hypothetical protein"

/translation="MKLPRPEYFGSGEALGKDGTSAVLEQPYSQRVGGYADVIKPFID

FIIAAVMLLITSPVMLVAAIMIKLDSKGPIIFRQERYGKNGVKFSIYKFRTMRTDAPK

YGISPTTSDDPRITRLGRLLRKTSLDELPQLLNIIKGDMSFIGPRPEMKRIVEESYTD

LERRRFLVKPGITGLWQVSDVRKEPIHHNLQYDFHYISNVSLKIDIIIIFKTVGVMIK

SNTF"

CDS 1144620..1145582

/locus_tag="PIKHDJKN_01020"

/EC_number="5.1.3.2"

/inference="ab initio prediction:Prodigal:002006"

/inference="similar to AA sequence:UniProtKB:A0R5C5"

/codon_start=1

/transl_table=11

/product="UDP-glucose 4-epimerase"

/db_xref="COG:COG1088"

/translation="MKHVVVTGAAGFLGSHLVKSLIDQGHQVTGIDNLSTGVSKNLQE

VANSPLFFFREADVTLSDSLDFLIDQPVDEVYHLASPASPKFYQADSIGTIWVNTLGT

YHLLELARTKGAKFLYSSTSEAYGDPEVHPQPESYRGNVNTWGPRACYDEAKRLGEVF

CYEYYTKHSVPVKVARIFNTYSSGLRNDDGRVISNFVTQALTGQDITVYGDGSQTRSF

CYVDDNIRGLQKLMETEEANGEIINIGNPTEYTVLEVAYLVKKLAQSDSKLVFLPLPQ

DDPKVRRPMIGKARTVLGWEPEISLETGLKLTIENVRATLQETP"

CDS 1145608..1146792

/gene="mshA_1"

/locus_tag="PIKHDJKN_01021"

/EC_number="2.4.1.250"

/inference="ab initio prediction:Prodigal:002006"

/inference="protein motif:HAMAP:MF_01695"

/codon_start=1

/transl_table=11

/product="D-inositol-3-phosphate glycosyltransferase"

/translation="MHKIMIAHSLYPPHVIGGAEISTQILAQTMSSHYEVEVLTVGEH

RHREIRLDHVNGINVVRLPYNNRYWIGDADRSVSIPSKIMWRLQDVFNVRQYHHIKQY

LARTRPELVHTQNLPGLSLAIWRAAYEMRIPVVHTLRDFSLIDPINLSLYSKMYRTIS

KRFSRTISSVIGISNHILGSHTSLGFFEDSNKHVVHNIVENAPSVAELYKKKVANVNR

PLKIGYFGQLTEVKGVHYLIDAIKLLDRNIAGQLCIFGEGPLLSPLQNSAASDRRIEF

KGKVANSEIAKQMAAMDVIIVPSTWDEPFGRVVIEAYQVGTPVYASRVGGMSEILLDT
EEFAFPAHSAEAIARSITHYYRLREPEKIQLKERCHLHSQTFNEAYLLQEHMDIYEKL
IR"

CDS 1146804..1148117

/locus_tag="PIKHDJKN_01022"
/inference="ab initio prediction:Prodigal:002006"
/codon_start=1
/transl_table=11
/product="hypothetical protein"
/translation="MHIVSMNRGTPVYVKAQDESNHALFKTMMHILVVYLSCYGLISI
YVDNILIRSLKDAGMALIAVFALSKVYRSQQEFIIPFLFLLGSIVVIGSLSLIMGKSI
VTLIYGFKITLLPILMIFAGMFIAKSKGIYLFARTNLIIFFLLIAGWLIQYSLGIEKL
ISLGFVYGVNIKHYLEGVPRLSSITYSPDGYAYALLISGLIAERIRLAEKHRLIRLSI
QLLSISFLLLSTIRSALVLWIVYQVVMFLLRVRKYNKHNVLLLGGVFLLLPAAVMLGI
QMLNSYNLLSVSSLTDRFQTWGANLTSPFSTAGMIGNGIGSVGAASRRTHVIGIQSNN
FAVDNQYFSFYEQTGWLGMLYWLALFMWILINLVKRMNRIPSVNVMGLPQMAIALGVG
VAASSLTTNVLEMFPGNISIWIVVGMALYQQKEIGDIPYEAGELK"

CDS 1148114..1149280

/gene="mshA_2"
/locus_tag="PIKHDJKN_01023"
/EC_number="2.4.1.250"
/inference="ab initio prediction:Prodigal:002006"
/inference="protein motif:HAMAP:MF_01695"
/codon_start=1
/transl_table=11
/product="D-inositol-3-phosphate glycosyltransferase"
/translation="MKVLHIGEYVVGGVATYLNEVVAYQRRFFDVYLLMSDYNSASDF
DLEPDRIVRYKYKRHPKHFFSVMRQIHQAIKRIQPDIIHVHSSFAGMLVRGLFFVSRR
QAGVIYCSHGWSFLMDTKHWLKKVYFMIEKILEFKTDCIINISKREQEQSIRFGMSPF
KSKLVYSGISEKRSSKSPHLQRPEYQSLEESSRIQLLFVGRYDWAKGLDIILDVFQRH
QHLQQRITLNVIGEPVLDDIKLNFSDNIVRIGWVNHEEIDEHYQKCDAVIVPSRWEGF
GLTAVEAMKNKKPVIASNRGALPELVSHGINGYVFDVDQSDELSELLMSLNKATLSDM

GNKGYDIYKEKFSSTRMNEEIVNLYYEVSNLRGKIQVPTTKQPKFSERMGDAHD"

CDS 1149273..1150355

/gene="mshA_3"

/locus_tag="PIKHDJKN_01024"

/EC_number="2.4.1.250"

/inference="ab initio prediction:Prodigal:002006"

/inference="protein motif:HAMAP:MF_01695"

/codon_start=1

/transl_table=11

/product="D-inositol-3-phosphate glycosyltransferase"

/translation="MIKIYFNARFLTQTVTGVQRYAIELVKEIDRLIEQGDIDLNQYQ

FFLLSPNNVITKLELKHICHRVVGKLKGHLWEQFELPLYSREGMLINMCNTGPAFKKN

QIVTIHDAAVFSSPEGFSFAFRNWYKFLLARLGKSSKKIITVSHFSKKELIHNCKIDE

EKIEVTYLGIDHIHRIQAAQGILEKYQLKGPYILAVCAMNPNKNFRMIFDVLPELEGH

NLKVVIVGSKNSRVFGEQDTPHSDAVHWLGYVSDEELLALYEHAAGFIFPSLYEGFGL

PPLEAMALGCPVVVSSRASIPEICGDAGLYFNPLVPQEAAAHLIEITTNLELGQKMSV

RGKEHSAFFTWNKCAKDTVNIIMNTV"

CDS 1150392..1151537

/gene="pimB"

/locus_tag="PIKHDJKN_01025"

/EC_number="2.4.1.346"

/inference="ab initio prediction:Prodigal:002006"

/inference="similar to AA sequence:UniProtKB:A0R043"

/codon_start=1

/transl_table=11

/product="GDP-mannose-dependent

alpha-(1-6)-phosphatidylinositol monomannoside

mannosyltransferase"

/db_xref="COG:COG0438"

/translation="MNKVFVAIDFPPEKGGIHDYAYGLISQMSAEETTVLTNKIKNTE

QSKQFDQKTDFEIIRKRIFWNSNKVLLLISQIILIIQLVVLKWRRKADEIHFINVFPV

GIAGPVMKACFRTRYFTYVHGLDVMGMLNSKLFPVLMHILRRSDKVIANSQYTRSRLI

ELGIREEQIVIIPPGLNIGKLKSVSSEEEDVRNKYDLHGKKVMITVSRLVERKGHDIT

LRAIRHVAPRIPNLKYVICGEGPYRSELERLVGQYALEDIVVFTGGIPDHELHQLYQC

SDLFIMPSRNIKEKGDVEGFGIVFLEANYYRLPVIGGNSGGIPDAIKDHVTGYLVDPL

DENEIADRIEQLVVDEELAQKLGDNGYEWVTNHCLWSHRGQLLRQLA"

CDS 1151661..1152467

/locus_tag="PIKHDJKN_01026"

/inference="ab initio prediction:Prodigal:002006"

/codon_start=1

/transl_table=11

/product="hypothetical protein"

/translation="MTYEILIIDNRSTDNTKELFDSLNFPEHVRYVFEPQLGLSYARN

RAIEEANGEFVLFLDDDALAAPTWVAEVIKIFDMDPAIGCVGGKIVPIWEGGKPEWIP

DDIVSLYTVMDFSESIVEMKAPHIPFGANVSFRKSIFREIQPFRVDLGRVGNNLMSSE

ESELIGRVRLVYKIYYSPYAVVEHKIAKSRLNKRWLLRRVYWQGISDATRYQKSVWGI

YKHLIRIAQSALLIAISFNNVKKVTSQMAKISYRNGTIVGSLRNGKGLNT"

CDS 1152467..1153852

/locus_tag="PIKHDJKN_01027"

/inference="ab initio prediction:Prodigal:002006"

/codon_start=1

/transl_table=11

/product="hypothetical protein"

/translation="MSIATAKPSRQRSMLQTIFLTSFANIAIMALTTITSILTARMFG

ATGKGELATVLFWPALLSGLAAFGLPTSLIYNVKKHQQQLSTYIGMSFVVQLPISLIV

GLISWVGMSSWLAGYSETLVALARWYTVLMVPVLLLTGVLAAAAQGTERFHIYNLSRL

LVPLLNLVGLIGFWLTGSLTVERAIAVSLASTLLVLVVYMFMMRRQIFASISYLIHQF

KACVHLFSYGIRVYGVELLGTLYTQFDKIIIIALLTPRDFGLYSVVYALSRMFNVVQN

AIINVIFPKVAGMEKERVLLLVGRAFRISMVLMTIVIIPSMLIGRFFIGLLFGPEFLQ

ASGVFYLLSLECIVGGGSWILASAFNALGRPGVIVLRQIIALAVTVTLFFVLTPMFGL

MGIGIALLTGSFVRILMSLISISVLFKLPIRSMLYDKEDIKFLKQQFREKNLFKGARK

DAKLAKNSSHG"

CDS 1153818..1154816

/gene="epsO"

/locus_tag="PIKHDJKN_01028"

/EC_number="2.-.-.-"

/inference="ab initio prediction:Prodigal:002006"

/inference="similar to AA sequence:UniProtKB:P71065"

/codon_start=1

/transl_table=11

/product="Putative pyruvyl transferase EpsO"

/db_xref="COG:COG5039"

/translation="MPNSQRTHPMDELKKQLETILEVIPSNSKIYYIDYPVHSNCGDL

LIMKGTETFFKDHRIHVTHRYSVYDFDYPVEIPRDQIIVLHGGGNFGDLYEAHQKLRE

NIVQHYPNHKIVILPQTIFYKDQSALQRTAAIFNQHPDLHIYVRDEISFQIASENFPN

CEVALIPDMAHQLWPIELKRSPRKEKLNFFRTDIEQISQQSSISTEQQGDYLDWPTLY

NRYEKKLIYYISKGLKKRAPRAISQFLWMKYSDYLVNKAIRCFADYRMIHSSRLHGHI

LSCLMAKPNVLIDNSYGKNSGYYRIWTHEVNTANMDTSSKTISPPTPKYMETTATV"

CDS 1154864..1156252

/gene="algA"

/locus_tag="PIKHDJKN_01029"

/inference="ab initio prediction:Prodigal:002006"

/inference="similar to AA sequence:UniProtKB:P07874"

/codon_start=1

/transl_table=11

/product="Alginate biosynthesis protein AlgA"

/db_xref="COG:COG0662"

/translation="MRIVLLSGGSGKRLWPLSNDTRSKQFLKVLEGPEGQTESMVQRV

WRQLKHTGLSEQATIATSKPQVEILRSQLGDEVDLVVEPERRDTFPAIALAAVYLYSV

QGVSLNETVAVLPVDPYVEESFFYKVTELEGVLDESGAELALIGVKPTYPSEKYGYII

PDPHETEKHNHYAKVSRFQEKPCETEAAEMIEQSALWNCGVFAFKLNYLINLLISLEL

PIQYEEMLKQYGRLQKISFDYQVVEKASHIVVTPYEGYWKDLGTWNTLTDEMSTHIVG

RGVMTNSSVNTHLINELNIPVCVLGVSNLIVATSPDGILVSEKEASPQIKEILKDDTQ

RPMYEERRWGCYKVLDYTRNARGEEVLTKRIIVGADKNFSYQYHLKRNEVWTVVSGQG

ELILDGELRLLNQGDVISILAGSKHSVKAVTELEIIEVQMGSELVEEDIVRIEMEWTD

IIQNCVNWGKTR"

CDS 1156249..1157580

/gene="tuaD"

/locus_tag="PIKHDJKN_01030"

/EC_number="1.1.1.22"

/inference="ab initio prediction:Prodigal:002006"

/inference="similar to AA sequence:UniProtKB:O32271"

/codon_start=1

/transl_table=11

/product="UDP-glucose 6-dehydrogenase TuaD"

/db_xref="COG:COG1004"

/translation="MNIAVIGTGYVGLVSGVCFSEIGNQVICVDNNEAKVEMLNEGNV

PIYEPGLKELMASNMKAGKLSFTSNIAEAISQSDIIFIAVGTPSLPSGEANLSYVEQV

AIEIGSHIESYKVIVTKSTVPVGTNDRVRELISSISSQPFDVASVPEFLREGSAVNDT

LNPDRIVIGTNSDRAIKTLKKLHRPLTENLIITDIRSAEMIKYASNAFLATKISFINE

ISNICEKVGADVTRVAEGMGYDKRIGASFLKAGIGYGGSCFPKDTQALIQIAGMVDYD

FRLLKSVVQVNQDQRFHVIHKLEEALGSLKGKTIAVWGLAFKPETDDVRDAPAIDIIQ

HLSDAGAVIKAYDPIATANFRKEVDVASITWEENPLHAAEGADALCVLTEWKEFAHID

LKELAEIMNDPIMIDGRNLYSAEQVQASNLQYYSVGRPGLTKLAGKAAAII"

CDS 1157620..1158537

/gene="tagU_2"

/locus_tag="PIKHDJKN_01031"

/EC_number="2.7.8.-"

/inference="ab initio prediction:Prodigal:002006"

/inference="similar to AA sequence:UniProtKB:Q02115"

/codon_start=1

/transl_table=11

/product="Polyisoprenyl-teichoic acid--peptidoglycan

teichoic acid transferase TagU"

/db_xref="COG:COG1316"

/translation="MKKWMKVTIWIVSFFVVVVFGYAIYLYQSVKSTADQIYEPRNPV

KPVSVTDSRGGLPVDINSKEPFNALILGVDERPNDRGRSDTIIVLSVNPGKKQVLMFN

IPRDTRTDIVGHNTVDKINHAYAFGGVDMSVNTVEQFLGVPIHYYMKVDMEGFAKIID

LVGGVDVNNPFAFDYEGQRYDQGNIHLDGVAALGFSRMRYDDPKGDLGRNDRQREVLK

QMLKNTMQISSVLNIQNMLDELGTHVRTDVTFDEMKELLLDYLNDLENVDTVEIKGKG

EKINGIYYYIVDQQEKDRIHGIIEEHLEK"

CDS 1158582..1159148

/locus_tag="PIKHDJKN_01032"

/inference="ab initio prediction:Prodigal:002006"

/codon_start=1

/transl_table=11

/product="hypothetical protein"

/translation="MEEDYGKRRHKHRRKQRPYVYILSLLLITVWVLVIWHFSTQSYQ

QQTIQPWLHKWSEKVKIGFALPDVRFTYGELDYSLRQRPYDFVEFIFRKSAHLFVYAV

LAVLIYGGLRYRKMRMLACIAAALAVVAVIASIDEYIQQFSPNRTSSIRDVGVDLLGG

CGGIALYMGVNVVFRRFRSPRRSSVKRK"

CDS 1159236..1159754

/locus_tag="PIKHDJKN_01033"

/inference="ab initio prediction:Prodigal:002006"

/codon_start=1

/transl_table=11

/product="hypothetical protein"

/translation="MLRRRNRNALLKKIGFIALVLLLLWLIGRTIPYLFRADAPDEAA

AIAQEFYKYEQTGDFGSSWELFHPLMKERFPKSAYLQSRAHIFMQHFGVETFDLEMEK

PEREFDVTVIDGVKPFAEAYRIGVTQKFTGTFGQFKIVQNCYIVKDGDDWTLLWYYPK

KGDTETEHDKTE"

CDS 1159998..1160249

/locus_tag="PIKHDJKN_01034"

/inference="ab initio prediction:Prodigal:002006"

/codon_start=1

/transl_table=11

/product="hypothetical protein"

/translation="MIETDRFYCVSCGMIVSVARGSVELKEEDCGGNHVFRTGFYRSE

MPLGCCEACVVHTKRQGKSQFAGQYTKDYDTLSSYEKRA"

CDS 1160282..1161190

/gene="bcrA_3"

/locus_tag="PIKHDJKN_01035"

/inference="ab initio prediction:Prodigal:002006"

/inference="similar to AA sequence:UniProtKB:Q5WNX0"

/codon_start=1

/transl_table=11

/product="Bacitracin transport ATP-binding protein BcrA"

/translation="MQQEPVVRLQGVSKIISSRSLVSDLTLDISPGQVFGFLGPNGAG

KTTTIRMMVGLMSISKGDIIISGHSVKNEFEQAIAQVGAIVENPEMYKFLTGYQNLVH

FARMSPGITKERIAETIERVGLTARIHDKVKTYSLGMRQRLGVAQAILHKPKLLVLDE

PTNGLDPQGIRELRDYLRQLCQEEGITVFVSSHLLSEMELMCDTVAIIQNGKLIDVRN

LRAEAGVDVLTEVAFEVNDAPRAAELISEATVQGNAIVIRLSREQIPDLNARLVSEGF

QVYGIRNVTHTLEDQFLQVTGGGSIE"

CDS 1161183..1161977

/locus_tag="PIKHDJKN_01036"

/inference="ab initio prediction:Prodigal:002006"

/codon_start=1

/transl_table=11

/product="hypothetical protein"

/translation="MSNFGNLIMNENMKIFRRPRTWIMLSILALISLLMPVLLNEGMG

SSEVPYWQAAITTVQVVFFLNTIFCVVIAAEAVAGEFTWGTIKLLLIRPWSRSKILAS

KYLSVIGFSILSTLLVVILATVMSYMLFSHSVTQDGSGASASHAFALWGYLYVDLFIT

LAIAFMVSSVFRSGALAIGLSLFIMFTQNLFSMIFNPVRYEWAKYVLFNNMDLSRYMN

SAADYGLLGDGNAGMTLGFSIAVLAVYYVIFMLISWVVFNKRDVAG"

CDS 1162102..1163277

/locus_tag="PIKHDJKN_01037"

/inference="ab initio prediction:Prodigal:002006"

/codon_start=1

/transl_table=11

/product="hypothetical protein"

/translation="MEHAAQAVLSCITYISPPQSYLAEQRMEGSRFLINNKFYRICTA

IILLLLIVYLGDKVNFIFSPLTSLIHIIIIPLLIAGFFYYLLRPLVDYMERHKMKRAL

SVLIIYVVIALIMAGFSVLVWPSLREQLLNFVENAPVLVTSLSDQLAEFEKSGFLSKY

IPEDSDLFSRFSDFLSQGIAQVTDYVSGLFSVVSNLIIILATFPIMLYYMLKEGSKFG

TSLTLLLPRRYRKDGEETVHEIDEALSSYIVGRVIVNVALGILMYIGFLFLGLPYALL

LTAVSIILNFIPYVGALLAAIPVVIVGFIESPSMAIWSLVIIVIAQQIQDNLISPYVY

GKQLDIHPLTTVILLLVGADLGNILGMIIVIPLYMIIKIIFRKIQNRIVEDKADPAN"

CDS complement(1163443..1164786)

/locus_tag="PIKHDJKN_01038"

/inference="ab initio prediction:Prodigal:002006"

/inference="similar to AA sequence:UniProtKB:P9WFP3"

/note="UPF0053 protein Rv1842c"

/codon_start=1

/transl_table=11

/product="hypothetical protein"

/translation="MDSERYALNLVLVAFLIGLSAFFVAVEFALVRIRPSRIDQMIAE

GNKRALAVKQAVANLDGYLSACQLGITITSLGLGWLGEPTVENILHPVFESMHMPETV

SSFLSFVIAFVAITYLHVVVGELAPKTIAIRKAEAVALLTSTPIIWFNRIMYPFIWLL

NSSANQLVKLFGIKPASEHEDAHSEEELQFIINESFESGKINQAEFGYVSRIFAFDEM

LAKEIMVPRTDMVCLYVNRSNEENLDIIRQEQYTRFPVVNESKDDIIGIINTKQFFLE

LYGNDEPVDLSSLIQPVSAVHETTPVKDLLKKMQKDGVHIAVLVDEYGGTSGIVTIED

VLEQIVGEIRDEFDADEVEDIQVINENYVIMDGKVSLSKVNDMFTSNLDADEWDTIGG

WLYSHRPEMNEQEEYEFENLTFVLLEKDKNRFYKVAVVPKEPLTMSDYTDEDETN"

CDS 1165163..1166602

/gene="clsA_1"

/locus_tag="PIKHDJKN_01039"

/EC_number="2.7.8.-"

/inference="ab initio prediction:Prodigal:002006"

/inference="similar to AA sequence:UniProtKB:P71040"

/codon_start=1

/transl_table=11

/product="Major cardiolipin synthase ClsA"

/db_xref="COG:COG1502"

/translation="MFWLVIILLVFTFQAATILLLEFRNPAKAVAWLFILFCVPLIGF

VVYYFVAQDYNKRKKVRKGGSRIFREIRETIWEQAHIVDHADQMSGSRYNHQHRLFNL

LSHLSESPITGCNRSQVLTNGEETFEAMLRAMEQAEHHLHVEFYIFRDDVISTKFQDV

MIRKAKEGVKVRLICDGLGSHKMSWRFIRKFQDAGVEFHYFLPPLIATIDRRVNYRNH

RKIVVVDGRIGFVGGINIGDDYLGQYPEVGFWRDTHVQIEGDAVYFLQNTFLNDWKLA

SGEQITEPQLTELFPPHICSGEERIQILASGPDQDWDAIQEMCFGAISVACERIYITT

PYFIPDPALYEAIKTAAVSGVDVKIIIPYQSDSRLVHLASMSYVEELLRAGVKFYQYR

KGFVHAKVLIVDDLLATVGTANMDMRSFFCNFELTAVLFEISSMERLTEDFERDLDEC

SQIDVKVFQQRSRWQKGAEMLSRMLSPLL"

CDS 1166950..1167858

/locus_tag="PIKHDJKN_01040"

/inference="ab initio prediction:Prodigal:002006"

/codon_start=1

/transl_table=11

/product="hypothetical protein"

/translation="MSNVSIKELTSKPDRNVKSSVFSLKLLQRIVMILVGAALMAVSL

EVFLVPNGVIDGGITGISIMVSELTHLPLGIFLTLLNLPFLILGYKQIGKTFALSTLL

GIVVMSIGTSLLHHVPALTPGEPLLGAVFGGLILGVGVGLVIRSGGSLDGTEIVAILL

SEKSPLSVGQIVLFINVFIFAGAGFVFGWPNALYSMIAYYIAMKMIDIVNEGLDQSKS

VWIISEKYRDIGSALTDRLGRGVTFLDGEGGFSGDEKKIIFVVITRLEEAKLKSIVED

WDPHAFVAIGNIHDVKGGRFKKKGIH"

CDS complement(1167918..1168808)

/gene="ligd"

/locus_tag="PIKHDJKN_01041"

/inference="ab initio prediction:Prodigal:002006"

/inference="similar to AA sequence:UniProtKB:O34398"

/codon_start=1

/transl_table=11

/product="Bifunctional non-homologous end joining protein

LigD"

/db_xref="COG:COG1793"

/translation="MAHTIKGTITINGMELTVTNPDKLLWPEAGVTKAIYLQKLAALA

PYLLTYTRNRLLTTIRYPHGAGGEFFYQKNAPEPVPEFVRTEMHDGIRYVVMNDLPDL

LWLGNLAALEFHPSLHAVSSHLPCEWMIDLDPSQEEEPRIMQAALIVGETLTSLGLRS

VPKTSGATGVQIIVPIRQGVTFDELRDIGYFVGKYVTQKHPDLFTLERLKKDRGDRIY

FDYLQHYGGKTLAAPYTPRAKPGGTVSTPLTWDEVRNNVSVKDYHLMNIVERLTKTGD

LIAAVEPQPVELIIQHLKQK"

CDS complement(1168889..1169854)

/gene="ligD_1"

/locus_tag="PIKHDJKN_01042"

/inference="ab initio prediction:Prodigal:002006"

/inference="similar to AA sequence:UniProtKB:A0R3R7"

/codon_start=1

/transl_table=11

/product="Multifunctional non-homologous end joining

protein LigD"

/db_xref="COG:COG1793"

/translation="MFNPLVPFEPISRDVIPTGQNWIAQIKWDGVRMLAYEDGQGIQL

INRRLHNRTAQYPELVQPRNLCSVPSYILDGEIIALDPDTRKPSFYHVLRRDRMSRPD

GIAQAVNQIPVTYMVFDILFCDGVWVTDQPLADRQRLLHQVLNPASHVQEVTNTSDPD

ALLSVMRQHQMEGIVCKDLSSTYGINGKDQRWQKVKIFHDLYAMIGGVTYRSGIVNAI

AVGVYDGPNFVYIGHVGTGKLNSDSWRKITEEVQPLIRKDRPFHNIPERSAETTWIEP

QIGVKVQFMELTHHRTLRHPSIQTFASVTREECQMSQISHLIPER"

CDS complement(1169923..1171020)

/gene="yndE_2"

/locus_tag="PIKHDJKN_01043"

/inference="ab initio prediction:Prodigal:002006"

/inference="similar to AA sequence:UniProtKB:O31809"

/codon_start=1

/transl_table=11

/product="Spore germination protein YndE"

/translation="MIEKGRLTVRQLGSLMFLCTIGEQIIVFPSMVTSYAHQDAWISA

LMGVAGGAGVLSIMLVVYKLQPRMNLIQHALYTLGPWIGAFISCFYLFYFLMGTSAFI

REIGDFMTTQILPESPLFIVSLLFVCTLAWGLFSGLEPIGRSAEVFLPLIVLFLLILT

VCLFPHSHLDNIKPVLAEGFLNPFKGFVAVLTYPYCELCIFLMLFPYAKKEAHWEKDI

LLAAIIGGLMLTLTLTICLLVMGPFMTQHHWFAAFNLSLKINIGNFLQRIEAFMASVW

IIAVFFKSILFFYSFVLGITHLFKLSSYRPLILPGAMLILSMSIVLAPNENFYLKVVI

PYWIDWDLTCGIGIPLLLIMVHKLKARLRKS"

CDS complement(1171241..1172110)

/gene="catE"

/locus_tag="PIKHDJKN_01044"

/EC_number="1.13.11.2"

/inference="ab initio prediction:Prodigal:002006"

/inference="similar to AA sequence:UniProtKB:P54721"

/codon_start=1

/transl_table=11

/product="Catechol-2,3-dioxygenase"

/db_xref="COG:COG2514"

/translation="MTSTYQIPATTHLGEVSLRISNLDRSIQFYTEVVGLKLLERSEK

VATMTADGKQSLLRLEQLTDAVTMPVRSTSGLYHFAILLPDRKSLGLALRNLAESGIE

IGQGDHLVSEAFYISDPDQNGIEIYADRARDTWKRDADNNYIMSSDPVDVDSLFALAA

NEPWQGLPARTVIGHVHFHVRSLEESRRFYTGVLGFDIVGNFANMSALFVSAGGYHHH

IGLNIWAGTGAPVNPANATGIDYFTIIYDGKEQLEQAVEQLRQSNASIEQQGKDWLTV

DPQNIRIRLTSTN"

CDS complement(1172418..1173329)

/gene="ku"

/locus_tag="PIKHDJKN_01045"

/inference="ab initio prediction:Prodigal:002006"

/inference="similar to AA sequence:UniProtKB:O34859"

/codon_start=1

/transl_table=11

/product="Non-homologous end joining protein Ku"

/db_xref="COG:COG1273"

/translation="MHTVWKGAISFGLVHVPVKMFSATEDKDISMRYIHKVCGSPLAY

VRKCPSCEVEVQWEEITKGYEYEKGKFVLFEKDELEALNDSSSKTITILDFVDLTEID

PIYFQKTYYLSPDQAGGNAYQLLMTAMRDTGKIGIAKISIRSKSSLAAIRVLEDCLSM

ETIFYPDEIRPVSQVPNLPEVLNVNEKELTMAKLLIDQLSTPFEPGKYTDDYRAKLLD

LINHKVAGEEIKIAPAKPEANVMDLMAALQASIEAVKPIPTDPGTTTAKPKKRAPKKT

SVQAAAGGETETPAPAKRKKAAPKPKA"

CDS 1173527..1173823

/locus_tag="PIKHDJKN_01046"

/inference="ab initio prediction:Prodigal:002006"

/codon_start=1

/transl_table=11

/product="hypothetical protein"

/translation="MKNKRDEDEAHAHTFSPYPRVKSEGSEGFHTPATAVNWEAVTSE

ELPLDRESFMLDIDRMVNEGLGGGQVTEDNGYIGESTTDSMVRESHDDPEGEYE"

CDS 1173823..1174002

/gene="sspH"

/locus_tag="PIKHDJKN_01047"

/inference="ab initio prediction:Prodigal:002006"

/inference="protein motif:HAMAP:MF_00667"

/codon_start=1

/transl_table=11

/product="Small, acid-soluble spore protein H"

/translation="MLDVKRAKAIYDSKDTIAVTLEGDPVWIENVDEANGMATVQVGS

RPGNTQTVRVDRLEE"

CDS 1174196..1174678

/gene="tsaE"

/locus_tag="PIKHDJKN_01048"

/inference="ab initio prediction:Prodigal:002006"

/inference="similar to AA sequence:UniProtKB:O05515"

/codon_start=1

/transl_table=11

/product="tRNA threonylcarbamoyladenosine biosynthesis

protein TsaE"

/db_xref="COG:COG0802"

/translation="MNQTHEQWVYHSHGIADTEVLASALAEQATAGMVIALDGDLGAG

KTAFSQKFAWHLGVRDVVNSPTFTLIKEYEGRLPLYHMDVYRISLEEADELGLDEYFY

GTGVSLVEWSSIIPELLPQEHLHVQIETTGPEDRTITLDGYGETYAAMCRQFRQNGVK

"

CDS 1174678..1175529

/gene="tsaB"

/locus_tag="PIKHDJKN_01049"

/inference="ab initio prediction:Prodigal:002006"

/inference="similar to AA sequence:UniProtKB:O05516"

/codon_start=1

/transl_table=11

/product="tRNA threonylcarbamoyladenosine biosynthesis

protein TsaB"

/db_xref="COG:COG1214"

/translation="MMEDLQKQPRQRFLALDTSTAVMAASVMEGHALLEERNERAERN

HSVHVVPVMEQLLEASSTQPGQLDGIAVGIGPGSYTGIRIAVTAAKTLAWAWDIPVVG

VSSLQALAWGGWHSGLAAKAEAAAEVAAGGAGGTPSADQGGTGAAPVHWIVPLVDARR

GQAYTALFASAGSAAPRRLAPDAIRLVDGWLEALAARVAEAAPEERPAAVWIVGETGP

HAAAAAELRCPAGTALQLVPYELEGRWVGRLGAEALLAGQRDDVHALVPNYTQLAEAE

ANLLRKG"

CDS 1175547..1176068

/locus_tag="PIKHDJKN_01050"

/inference="ab initio prediction:Prodigal:002006"

/codon_start=1

/transl_table=11

/product="hypothetical protein"

/translation="MDNVENDEQEAALQFRFMTLDDIPDVMIIEHEAFTLPWTEEAFQ

NELTHNHFAKYMVMELEGTAIGYAGMWTIMDEAHITNIAVREAYRGRKLGEKLLDELM

RTAAYLGMERMTLEVRVSNRIAQSLYQKKGFESAGLRKGYYSDNGEDAMIMWANLPPA

SRSGEEEGSVTDS"

CDS 1176065..1177120

/gene="tsaD"

/locus_tag="PIKHDJKN_01051"

/EC_number="2.3.1.234"

/inference="ab initio prediction:Prodigal:002006"

/inference="similar to AA sequence:UniProtKB:O05518"

/codon_start=1

/transl_table=11

/product="tRNA N6-adenosine threonylcarbamoyltransferase"

/db_xref="COG:COG0533"

/translation="MKDFNEKISSAPSYILAVETSCDETSVAVVKDGREVLSNLISSQ

IETHKAFGGVVPEVASRKHVEVITLMLEQAIEQSGIRPRDLSAIAVTQGPGLVGALLV

GIVAAKTMAMALGKPLIGTHHIAGHIYANRLTHELQYPAMALVVSGGHTELVHMESEG

KFKLIGRTRDDAVGEAYDKVARALGCPYPGGPHVDRMAAEAKQAVPLPRVWLEADSYD

FSLSGLKSAVLNVLNQAKMRGETLEPSAVARGFQEAVVEVLVEKAVRAVREYGSRQLL

LCGGVAANRGLRSALQERCAKEGLELLIPPMEYCTDNAAMIGAAAYLKWQRGEISEFD

AKADPGLSLEAWSVQSL"

CDS 1178198..1179754

/gene="leuA_1"

/locus_tag="PIKHDJKN_01052"

/EC_number="2.3.3.13"

/inference="ab initio prediction:Prodigal:002006"

/inference="similar to AA sequence:UniProtKB:Q71Y35"

/codon_start=1

/transl_table=11

/product="2-isopropylmalate synthase"

/translation="MTTNNNKRMIEIFDTTLRDGEQAPGASLQPEQKIELAHQLASLG

IDVIEPGFPISSPGEFAAVQAISRQLQNVEICGFARAVKGDIDAAVRATADAAHRRIH

LFISSSDIHIEHQLRRPRSEVVATAREMVSYARQFTDIVEFTAMDAARTKMDDLIEMV

EVAIEAGASIINLPDTVGYALPHEYGEMFRQVREGARGGDKVRYSAHCHNDLGLAVAN

SLAAIANGASQIEVTINGVGERTGNCALEELIMALETRGDVIGATTNIKLNQMYETSR

QISRAMHFPIAYNKPVVGRNAFQHESGIHQDGLLKNRSTYEIMDPEALGIPRSMIILG

KHSGRHALKDRVRKYGFEPDEQQMEQLYEIFKETADQQKVVSDDQLLQMVSQTMNIPA

QDYELVELQVTAGSMTDRMAAVRIRTSAGEQSYSAVGGGPVDATIRAIGQSISDDITF

VDMEMHALSGGEAASAEAAVTVERAGREFRGTATHNDIVMAAGLAYVAACNAAGLKAE

SSESDESVHA"

CDS complement(1180277..1182235)

/gene="yheS"

/locus_tag="PIKHDJKN_01053"

/inference="ab initio prediction:Prodigal:002006"

/inference="similar to AA sequence:UniProtKB:P63389"

/codon_start=1

/transl_table=11

/product="putative ABC transporter ATP-binding protein

YheS"

/db_xref="COG:COG0488"

/translation="MLLQVSGIIKRFGVDPILDGVNLQILERERIGLVGVNGAGKSTL

LKIVAGEMSYDGGQIFKSKETTLGYLAQNSGLQSDRSIWEEMMNVFGHLTQAEADLRQ

MEQDIADPAQMEDEKKYADLLERYAKRSDWFKDHGGYEMETRIRSVLHGMGFGEFSPD

TQIATLSGGQKTRLALARILLQAPDLLMLDEPTNYLDIATLTWLEDYLRGYSGALLVV

SHDRYFLDRLVTNIVEIERHRSKKYTGNYSRYMELKAAEYESQMKQYEKQQDEISKME

EFVQKNIVRASTTKRAQSRRKALDKMDRLDKPMGDLKKAHFSFEPAVMSGKEVLRVDQ

LSVAYDEASPLFRNVSFDLRRGETVALIGPNGIGKSTLLKCLTGSLRPVSGEIQWGTK

VQIGYYDQEQTGLNPSNTVLEELWSAYPGMEESRIRTVLGNFLFSGDDVLKKISSLSG

GEKARVSLSKLMLKEANMLILDEPTNHLDLFAKEVLEAALMDYEGTLLFISHDRYFLN

KMAERIVELHPGGTEHYLGNYDDYVEKKQELEDIAREAAEARQASNKNSAKSDPGLSA

TEKSGAASFEADKQAKREERNRQRKQEALEQQIAKLETEITELEAQMALPEIYQDYMK

LQELQEKAEDHKLQLAKAYEEWEELALE"

CDS 1182514..1183152

/locus_tag="PIKHDJKN_01054"

/inference="ab initio prediction:Prodigal:002006"

/codon_start=1

/transl_table=11

/product="hypothetical protein"

/translation="MQDHTEQKIRLRSKLRQSRDLMDESMRQQAMTEINDVTKRELER

LRGDKWKLGNKRLMIFSYLSYGSEASTAFLFEEGWKYGDRMLAPKVLANPARMELRRV

RGEQDIQPGIWGIPEPKDSCEVLQPESWPDIDLVIVPGLGYDPHGGRIGYGGGYYDRF

AETLAAKCAMTDKRPLMAALVLPGQLQDEIPMDPLDLRIDLLITTEGILHIE"

CDS 1183168..1183671

/gene="moaC"

/locus_tag="PIKHDJKN_01055"

/EC_number="4.6.1.17"

/inference="ab initio prediction:Prodigal:002006"

/inference="similar to AA sequence:UniProtKB:Q5L3F4"

/codon_start=1

/transl_table=11

/product="Cyclic pyranopterin monophosphate synthase"

/db_xref="COG:COG0315"

/translation="MNSETNNDQASGSKLTHFNEQGRARMVDISGKESTVRTAVAVSK

VTMNPATLEAIREGRIGKGDVLAVAQIAGIQGAKKTSDWIPMCHPLALTGVDIRFHDN

GVDELQIEVTVKTEGKTGVEMEALTAASAAALTIYDMCKAMQKDMIIGPTMLNSKSGG

KNGDFSR"

CDS 1183709..1184194

/gene="mog"

/locus_tag="PIKHDJKN_01056"

/EC_number="2.7.7.75"

/inference="ab initio prediction:Prodigal:002006"

/inference="similar to AA sequence:UniProtKB:Q9ZL45"

/codon_start=1

/transl_table=11

/product="Molybdopterin adenylyltransferase"

/db_xref="COG:COG0521"

/translation="MVWRTAILTASDKGARGEREDTSAQVIRELVEEELGGEIVEYRI

VPDEPDEIIAALIEMTDYFHADLVLTTGGTELAIRDITPEATRRVIEREVPGMAEAMR

YSVMGKNRSAMLFRGVCGIRGRTLIVNLPGTPKGVHEHLAAIMDQLPEALLMVTGQFK

Q"

CDS 1184397..1184645

/gene="tatA"

/locus_tag="PIKHDJKN_01057"

/inference="ab initio prediction:Prodigal:002006"

/inference="protein motif:HAMAP:MF_00236"

/codon_start=1

/transl_table=11

/product="Sec-independent protein translocase protein

TatA"

/translation="MLSSIGPTGFILLAVIALLLFGPNKLPELGRAVGRTFREFKEGA

REIISDDDSSSRKEQEKAKPLASETASADKPEDKRLPE"

CDS 1184724..1185506

/gene="tatC2"

/locus_tag="PIKHDJKN_01058"

/inference="ab initio prediction:Prodigal:002006"

/inference="similar to AA sequence:UniProtKB:O05523"

/codon_start=1

/transl_table=11

/product="Sec-independent protein translocase protein

TatCy"

/db_xref="COG:COG0805"

/translation="MTQQKEEMTLTEHLSELRKRLIYVLSIFILGLVAGFLVADPVYQ

YLTKAETAQGFVLHAFSFWDGIGIYMKIAGLFSLVITLPFTVFQLWKFVSPGLRPRER

KATLKYVPYVFLLFLIGLAFSYYVVFPMALSFTSSITEKMGLVETYGMKQYFSFLFGI

VLPVSLLFELPLIIMFLTGLRILNPIRLRKMRRVAYFVLIFIAVVVTPPDFISDFLVM

IPLLLLYEISVFLSGFVYRKQLAADAEAESRYIRAEDKQSAV"

CDS 1185496..1185729

/locus_tag="PIKHDJKN_01059"

/inference="ab initio prediction:Prodigal:002006"

/codon_start=1

/transl_table=11

/product="hypothetical protein"

/translation="MQYNKREIPCDFCNCDNLVWCPSRDKATNSYPGYKGRQAFAAYI

VTNGTSRHIMFNNGYNGKGLPENDAGGLTDSVE"

CDS 1185958..1186239

/gene="groS"

/locus_tag="PIKHDJKN_01060"

/inference="ab initio prediction:Prodigal:002006"

/inference="similar to AA sequence:UniProtKB:P28599"

/codon_start=1

/transl_table=11

/product="10 kDa chaperonin"

/db_xref="COG:COG0234"

/translation="MIRPLGERVLVEPLEQEQTTSFGIVLPDSAKEKPQEGRIIAVGA

GVLKDGVRVALEVKEGDRVIFSKYAGTEIKFEGKEYLIMKESDIHAILD"

CDS 1186313..1187947

/gene="groL"

/locus_tag="PIKHDJKN_01061"

/inference="ab initio prediction:Prodigal:002006"

/inference="similar to AA sequence:UniProtKB:Q4MPR6"

/codon_start=1

/transl_table=11

/product="60 kDa chaperonin"

/db_xref="COG:COG0459"

/translation="MAKDIKFSEDARRSMLRGVDALANAVKVTLGPKGRNVVLEKKFG

SPLITNDGVTIAKEIELEDAFENMGAQLVKEVATKTNDVAGDGTTTATVLAQALITEG

LKNVTAGASPIGIRKGIDKAVKAAVAELQSISKPIDSKQSIAQVAAISAADEEVGELI

AEAMEKVGKDGVITVEESKGFATELEVVEGMQFDRGYISPYMITDTDKMEAVLDNPYI

LITDKKISSTQDILPLLEKIVQQGKPLVLIAEDIEGEALAMLVVNKLRGTFNAVAVKA

PGFGDRRKAMLQDIAALTGGQLITEELGLDLKSAVVEQLGTARQIRVTKENTIIVDGA

GNKSDIDARVSQIRTQLEETTSEFDKEKLQERLAKLSGGVAVIKVGAATETELKERKL

RIEDALNATRAAVEEGIVSGGGTALMNVYSAVAAVALSGDEQTGVNIVLRALEAPIRT

IAANAGEEGSVIVERLKKEQTGIGFNAATGEWVNMIEAGIVDPAKVTRYALQNAASVA

AMFLTTEAVIADKPEPAGAGGGMPDMGGMGGMGGMM"

CDS 1188063..1188299

/locus_tag="PIKHDJKN_01062"

/inference="ab initio prediction:Prodigal:002006"

/codon_start=1

/transl_table=11

/product="hypothetical protein"

/translation="MRKSTKWTLWILGLMLGAIFINFYMNLYSFAYALITVLLLVFMG

WGFLAKLKNDVRQHEDHHRREHQSAIDDIDRYTR"

CDS complement(1188504..1189235)

/locus_tag="PIKHDJKN_01063"

/inference="ab initio prediction:Prodigal:002006"

/codon_start=1

/transl_table=11

/product="hypothetical protein"

/translation="MQYLFTTLFILSFIAFFVFLVLMVLSIRKHNRIPIRHVMLTAAC

FAIASIGMYGMLTLPSNPPQSSGTDQTEKHESGTATTEEDHAPLAQKNASSFHMTTEE

FKNKFNGAVGKYRLNGLSITRLNMKDSSEQDTGAFEYVFNDKLRLVGKLNSDQKVQEV

MLYGNGDASEPTGGTLLTAIATLILTTNNEYTYNDAQNVIQDIGLLDRDVSQSEFDGA

TVRNGFEYRFSIQEDISTFGITAVH"

CDS complement(1189378..1189962)

/gene="comR_1"

/locus_tag="PIKHDJKN_01064"

/inference="ab initio prediction:Prodigal:002006"

/inference="similar to AA sequence:UniProtKB:P75952"

/codon_start=1

/transl_table=11

/product="HTH-type transcriptional repressor ComR"

/translation="MARTREFDEEKVLDAAMQLFWEKGYEATSLSDLTSRMGIQRPSI

YSTFGDKRELFETSLRKYTMARAAEMRTRLAGIASVKEAFRTFFAEIVDEEYNAERLS

RGCFCINTMVELAPHDEKFEILTREHQMYLSVIFQENIERGMRSGELNPSMDAKAVAQ

ALIVSLIGLTVFMKSRPNRSFVDNSIRVTLSLLD"

CDS 1190265..1191329

/gene="fgd_2"

/locus_tag="PIKHDJKN_01065"

/EC_number="1.1.98.2"

/inference="ab initio prediction:Prodigal:002006"

/inference="protein motif:HAMAP:MF_02123"

/codon_start=1

/transl_table=11

/product="F420-dependent glucose-6-phosphate

dehydrogenase"

/translation="MEIGITSFVETTPDLRTGEVISHAQRLREVVEEIVLADQVGLDV

FGVGEHHRKDYAASSPAMVLSAAASLTKRIRLTSAVTVLSSADPVRVFQDFATLDGIS

NGRAEIMAGRGSFTESFPLFGYELGEYDELFDEHLELLLNLQKSEIVNWRGGHRPAIH

NLGVYPRPVQSPLPIWIGSGGNQESVVRAGLLGLPLTLAIIGGSPERNFGPLVQLYKQ

AAAHAGHDASKLTVGSHSLGFVAEDTELAADLFFPSTQAGMNKIGKERGWGHYSRASF

DAARSHDGALYVGDPETVARKIVHLRKNVGITRFMMYVPIATMPHDQVMRAIELLGTE

VAPRVREELAEWEAANGQKA"

CDS 1191358..1192326

/gene="nlhH_1"

/locus_tag="PIKHDJKN_01066"

/EC_number="3.1.1.1"

/inference="ab initio prediction:Prodigal:002006"

/inference="similar to AA sequence:UniProtKB:P9WK87"

/codon_start=1

/transl_table=11

/product="Carboxylesterase NlhH"

/db_xref="COG:COG0657"

/translation="MSFVDRVVPELRDTLAQFPGFNLKGGLQVSRAMLMNPPIEKSDD

VRTTKRIIAGAAGDMLVKIYEPSERTGDKLPAMLWIHGGGYVLGHPDMDDALCERFVQ

AAHCVVVSVDYRLAPEHPYPAGLHDCYAGLVWMTDETESLGIDLTRVAIAGASGGGGL

TASLAQMVRDKGGPSLIFQMPLYPMLDDRNISASSHEITDQNATWNRTNNLEAWSMYL

GGGMEGLGEKEEHNIPSYAVPARTENLAGLPPIYTCVGQLDLFRDETIEYVTRLATAG

VDVEMHLYPGAYHCFEVFVPDAEVSQRASQDYIRAMARALNPSWEQ"

CDS 1192549..1193460

/locus_tag="PIKHDJKN_01067"

/inference="ab initio prediction:Prodigal:002006"

/codon_start=1

/transl_table=11

/product="hypothetical protein"

/translation="MDPSLHPLFRPIQMNGTDKNTYYIERIPSVSLMAHVACFWESGS

IPHTGEQDRETISVPARVLPDGCTDMLITYDSVRSVHSYAYCGNYSHPFALPEPSDVS

PSADYTFGVRFFPGGAYVFHGLPLEGFTDMRIPLEECWPVRLNELQERIAGTRTFEER

VQVMNAYLIPLSVQTSTYENNLMKNALHRIFIDGGRMSVSELAMREVVSERQLHRKFA

EWIGISPKRFSEVVRFHRVLGSIHQGHAADGIALALNHGFFDQAHLIHQFRKFYGETP

LTAAKEHQRRLSDLYNNSVVPSVILKS"

CDS 1193487..1193975

/locus_tag="PIKHDJKN_01068"

/inference="ab initio prediction:Prodigal:002006"

/codon_start=1

/transl_table=11

/product="hypothetical protein"

/translation="MNGTLQIRDHLLNELETGVRTGASLIRLIRPEDWAYRPQDNMRS

LVELVHHFIQITASDLAIMQEKSEAEVGVVENSLSGIEDIEKLEATLWGNLEAYKAYI

VSLSEEDLLNRSTKAFYMEHGHLQAQWQIETLTHVFHHRSQLYNYLKQQGHELNFFML

YA"

CDS 1194152..1194439

/locus_tag="PIKHDJKN_01069"

/inference="ab initio prediction:Prodigal:002006"

/codon_start=1

/transl_table=11

/product="hypothetical protein"

/translation="MAILLPQQFYALAASVGKSYYENLAGGTNASITVNNFGPFPVDL

VVTRVNAPVITYLIPAGNSLTLSVDLLLVAALLTTPAGASTGTIQIATSDF"

CDS 1194676..1195197

/locus_tag="PIKHDJKN_01070"

/inference="ab initio prediction:Prodigal:002006"

/codon_start=1

/transl_table=11

/product="hypothetical protein"

/translation="MNRTTFLETERLVFSTWSEEDRALASALWGDHEVSKWISSKGFL

SEDEVEARLTQEIKRQEEVGVQYWPLFEKDSEVFVGCCGLRPYSPEEEIYELGFHLTR

DHWGKGYAMEAARAVIDYAFGELKAKALFAGHHPDNEVSRDMLSKLGFEYVRDERYEP

TGLMHPSYLLKKE"

CDS 1195590..1196441

/gene="rhaD"

/locus_tag="PIKHDJKN_01071"

/EC_number="4.1.2.19"

/inference="ab initio prediction:Prodigal:002006"

/inference="similar to AA sequence:UniProtKB:P32169"

/codon_start=1

/transl_table=11

/product="Rhamnulose-1-phosphate aldolase"

/db_xref="COG:COG0235"

/translation="MNVTLTNETTQAAGFHIPFVREMAEITQHMWKNGWDERNGGNVS

YLLEEEEVAQYIDINHVIRKIKPAFSMHELAGKYFIVTASGKYFKNVLADPESNLGLL

RVSNDGQELEVLWGLKSGANPTSELPTHFMSHIERLKVDPKHRVVMHNHATNVLAMTF

IHELDEMKFTKTLWQMCTECVVVFPDGVGIIPWMVPGSNKIGRETAEKMKEYHAVIWP

QHGIFGTGTTIDEAFGLIETIEKAAQVYMLVAGHEIRQRITDQQLATLAQAFGVTPRP

GILGMEV"

CDS 1196726..1198543

/gene="kimA"

/locus_tag="PIKHDJKN_01072"

/inference="ab initio prediction:Prodigal:002006"

/inference="similar to AA sequence:UniProtKB:P96589"

/codon_start=1

/transl_table=11

/product="Potassium transporter KimA"

/db_xref="COG:COG0531"

/translation="MLGKMKRILIGKPMKSAELDAEKLGKWKALAILSSDALSSVAYG

TEQILLVLVAAGFAALWYSVPISIAVLGLLVILIFSYRQTIFAYPTGGGAYIVAKDNL

GTAPSLIAGGSLLVDYILTVAVSSSAGTDAITSAFPMLHDYSIVIALIMIVFLTIMNL

RGVTESASVLAIPIYLFIFSIAILIISGGIKFLAGGMEAAAPEFGTSMSHVSLFLLLK

AFSSGCSALTGVEAVSNAIPNFKQPAEKNAAGTLMLMGCILGAMFIGITLLAYGYGVK

PDPKATVISQIAEATFGRGTMYFIIQGVTALILFLAANTAYSAFPLLSFMMAKDKYMP

HMFMVRGDRLGFSNGIIFLSVMSALLVVGFKGNTESLIPLYAVGVFIPFTLSQLGMMI

RWIKVKPTGWGVKLLVNTIGMLTTLSITLIFIFTKFTQTWVIFIFLPLVVYVFMRIHR

HYCNIADELRIDIKLEKPVRKGNTIVIPVAGITRVVMNTISYAQTMSDHVVALYIGFD

DEAIRKMEQKWEEWNPGVRLVVIKSRYRSIMGPLKKFIDTVEWKTAETDHITILIPQF

ITKHWWQNVLHNQTSFMIRAYLINYKDVIVTTVPYHLNR"

CDS 1198946..1199908

/gene="ugpA_1"

/locus_tag="PIKHDJKN_01073"

/inference="ab initio prediction:Prodigal:002006"

/inference="similar to AA sequence:UniProtKB:P10905"

/codon_start=1

/transl_table=11

/product="sn-glycerol-3-phosphate transport system

permease protein UgpA"

/db_xref="COG:COG1175"

/translation="MSELDNRIGLPAARRRVTNTSQRRTASLRVQRMRENMLAYGFLA

PSLLLFAVFLFYPMFKSVYLSLHSTDPTGQIAAYVGLDNFKAVFQSGLFLQGMKVTLL

FVLFTVPTGMLAALILAALTHNRFKGMRIFQFVFSLPVVLSVGSSAVIWKFLFHPTLG

MLNYLLGKVGIDPIPWLTSPDWALISISIMTIWMNLGFNYIILSSGLQGIPDEIYESA

KIDGAGPLLTFRKISMPLLSPTLFFVTVVSIIGAFQSFGQINILTRGGPMDSTNVFVY

SIYQEAFVNFRFGTGSAQALILFAVIMLLTLIQFKWVERKVHYQ"

CDS 1199905..1200726

/gene="araQ_7"

/locus_tag="PIKHDJKN_01074"

/inference="ab initio prediction:Prodigal:002006"

/inference="similar to AA sequence:UniProtKB:P94530"

/codon_start=1

/transl_table=11

/product="L-arabinose transport system permease protein

AraQ"

/db_xref="COG:COG0395"

/translation="MRTPWTKTLLYVLLTICAALVLYPVLYTFFMAVMTPEDASAYPP

HIIPQSIDLSNFTEVFDIVPIGSFIGNTFLVAGLTMLGQLVTASMAAYAFAKMEFKGK

NVIFSMFVATMMIPWEVTMIPNYLTVRSWGWLDTYQGLTIPFLATAFGTFLLRQFFMQ

LPKELFEAAKIDGCGHIRYFVSHVLPLSRPALGTLAIYSFLSMYNSYLWPLLVTNTPE

MRTVQIGISMLEFQESTAWNLVFAGTALVILPSLLLLVFGLKQLVRGMAAGALKG"

CDS 1201025..1202398

/gene="ugpB_1"

/locus_tag="PIKHDJKN_01075"

/inference="ab initio prediction:Prodigal:002006"

/inference="similar to AA sequence:UniProtKB:P0AG80"

/codon_start=1

/transl_table=11

/product="sn-glycerol-3-phosphate-binding periplasmic

protein UgpB"

/db_xref="COG:COG1653"

/translation="MIGFRLKKRGTFALMLAALMLVLSACGGKTETTSTTGGAQADAA

AAEPVQLTWWHSMSGAGEKAINQLASDFNASHPDIQVKAIYQGKYDESLNKLKASMGS

DSGPDIIQVYEIGSKFMIDSGMITPVQQFIDKDKFDLSQLEPNIIRYYTIDGKLNAMP

FNTSNPILYYNKDMFKAAGLDPENPPKTYEEFEQAAKALAKDGKPGASMAIYGWFMEQ

FFANQNADYVNNGNGRNGAATESMLNSEAGVKTLTWWKKMMDEKTISNLGRSTDDTTA

AFTAQQIGMTLDSTAGLRKIVEGAGGKFELGTGFLPRPADAKEGGVVVGGASLYIMNN

KSEAQQQAAWEFIKYLATPEVQAHWSVATGYFPITTAAYDEQVLKDNMAKYPQFQTAV

DQLHASADSTATSGAVMGVFPEARQIVEGAIETVLNGQGTPQDVLDAAAKQITDKIGQ

YNSTVKK"

CDS 1202885..1208482

/locus_tag="PIKHDJKN_01076"

/inference="ab initio prediction:Prodigal:002006"

/codon_start=1

/transl_table=11

/product="hypothetical protein"

/translation="MHSKQVKSWLRLFLAMVVLITGAIPAGLFSNKVAAADDPLTVAQ
AIARNNDGLPGTVEGYVIGYFNNNSKNQLQTEAPFSGGSNFGLADTPGETDLSKIMSV
QIPSDTSVRDIFGLNKNPELFGKKVRITNGTLASYLSLPGLKYIDSTSTTFQLIDETP
VTKVANVTANPTSGAVPTGAKVALETTTVGATVYYKQNADVDYLPYTVPITVNDATHI
ETYAAKEGIDNSDVTRFDYTIVNNTPISISEARNKSENTPVTVQGIVTYREESGGLAN
LYIQDEHAGIVIRGTDATVERGDRIEAYGPLTIYNGLLQVEKDKTGFPGGYIKVLDKT
LDIPEPVTLTSKDFTPAEGGGKGAGGIYEGMLVEVNAVTVTRSSSSTFYATDADGGEI
TIYAKNSPTALATGKTYEKVTGVMTYHTSYGLELIPRKASDVVENLLSVTASVPSGGI
VKGSTVTLSSPTAEAEIYYTVDGTEPTQTSTKYSAPITVDEDMVVKAIAVSDGNTSDV
YSFTYKVLHELDNLRIHDIQGASHNSLYDGLAVQNVEGIVTYVVNASSFYIQEIPGME
DDDVKTSEGILIYKSSHGMTVGNKVKVSGQVKEYATATTELSTTEIVATAITVEDNNQ
ELPEPVILGTNGRIIPTVIDSDQFGEFNPEMDAIDFYESLEGMRVQLDNATITGPYSS
EPGLAVVVDNGLNNPVRTPAGGVILTADGTEPYESTLNPQRLFINKKPSQAVKTGDKL
TETVKGVMSYSNGNFKVNPEGNLPVITSGGLEQATTTIQQADGKLTIATFNVENFSKK
DAARAVKIGGIVVNNLKNPDIIGIMEVQDNDGATDSGTTVADASFQTLIDAIASKGGP
TYKYTDIAPENNKDGGAPGGNIRVGFLYNESRVTLKQGNKGDAVTGIQVETDGSLSLN
PGRIDPTNDAFASSRKPLAAEFEFNEERVVVIANHLNSKGGDLKPYGSIQPAIRSSEI
QRARQATAVNSFVKELLSKDPEVNVAVLGDFNDFQFSKTLNIIEGNELDNLVNKLPEN
KRYSYIYDGNSQTLDHILVSKNLSDTAAIEAVHVNADFEDSHGRVSDHDPLLAQLSIG
STSEEGDFNLRVLHTNDTHAHLDNIPRRVTAIKETRNDNTLVLDAGDVFSGTLYFNLF
NGLADLEFMNMIGYDVMTFGNHEFDKGPSVLKAFIEQAEFPFVSANIDYTKDANLGGL
YNGNIGNPGENSQIYPAIITEVNGEKVGIFGLTTPDTVSLSSPGDDLKFEDYKASAQA
TVNMLQDEGINKIIALTHLGYSEDLKLAEAVEGIDIVVGGHSHTILNKPVVVDTHTDP
TLVVQTGEYDVSLGQLDVTFNEAGVLKTWNGKLLNLDAKDATGNYVYADDKEAAKKLE
GYAEPLAEFKKEVIGKTNVLLDGERGSVRKQETNLGNLMTDGMLEKVKSIVKENDVKG
YVAIQNGGGIRASFQKGNITLGDLLTVMPFGNNLSALKMTGKEITAALENGVSGVETG
EGRFPQVSGMRFYYDSTKPGEKIDAITNTVTQVGQRVLKVQIKNANGTYTDIDPNGYY
IVATNSFMANGGDFYRAMRAAKDDNRYYELNLVDYEIFHEHLDRVGTVNQTTEGRSTD
LKGAPLPGDGNGSNPGNGGGDNGSNPGSGNSGGGTTTPTTPVTPTDPTTPTTPTMPTT
PSVPGNGNGGGTTPTNPGVILKDIGNHWAAAAIEQGISRGIVNGYQDGNFRPNAPATR
AEFIVMLARAFELPASSKALTFKDASKIPAWAQSFIAQAVEQGIISGYTDETFRASGK
VSRVEMTVMLVRALGLPVKSNATLIFADANKVPAWAVPYIAAAYDAGLVKGAGKNMFN

PLAEATRAEVVTLLMSASEFQVK"

CDS 1208640..1208852

/locus_tag="PIKHDJKN_01077"

/inference="ab initio prediction:Prodigal:002006"

/codon_start=1

/transl_table=11

/product="hypothetical protein"

/translation="MQDQPVLIALLAIAAGIFSLLGGINNWDWFMKSFRAGLFVKTIG

RQGARVVYSILGIVLITIGVLLLLIG"

CDS complement(1209034..1211625)

/locus_tag="PIKHDJKN_01078"

/inference="ab initio prediction:Prodigal:002006"

/codon_start=1

/transl_table=11

/product="hypothetical protein"

/translation="MIPKIPLASWIEAIVDWMSTSLSGLFKVISVVIQEVVGFFSGLF

MLPHPLLFIVILGVLAYLVGRIPLTLFTVIGFLLVDNLGYWSQSMDTLGLVITSGLVS

ILLGVPIGIWLAYSKTAARIITPLLDFMQTMPAFVYLLPAVTFFSLGVVPGVIASVIF

AIPPTIRLTHLGIKQVSGELVEAADAFGSTSMQKLFKVQLPLALPTVMSGINQTIMLS

LSMVVIASMIGAQGIGAEVYRAVTQLQIGKGFEAGLAVVVLAIVLDRFTQNLFMPGRK

KSSRFSAKQKAWITAAATFVVLVAGFSQYFVGGNSTSAGGNNTPANAVGEEVKYQIIG

IDPGAGIMKSAAKAIEDYHLTDWTLIEGSGAAMTATLDKAIKAEQPIIITGWTPHWMF

NKYDLKYLDDPEKSFGDAEEIHTIARKGLKGDHPVAYEFLSRFQWTSDEMGEMMSAIQ

NGTSPEEAAKDYAEKHADQIDEWTKGLTPVNGDAFKLSYVAWDSEIASTNLLKYVMES

KLGYKVNALQVEAGPMWTGVASGDVDASPAAWLPLTHADYWERYKDQVDDLGANMTGV

RTGLVVPAYMTDVNSIADLETGASSSTPSANANVGNEVNHQIIGIDPGAGIMKSTASA

IEKYGLSDWKLVEGSGAAMTATLDKAVKNKEPVIVTGWTPHWMFNAYDLKYLDDPEGV

YGEAEQIHTIARKGLKEDKPVAYEFLDRFSWTPEDMGEIMVAIQNGDDPQKAAAAFAE

KHSDKVAEWTKGLTPVNGDPIKLSYVAWDSEIASTNLLEYILKEKLGYKVTSLQVEIG

PMWTGIANGDVDATPAAWLPLTSADYFNKYKDQIDDLGPNMDGAKTGLVVPTYMDINS

IEDLKDN"

CDS complement(1211591..1212820)

/gene="opuAA"

/locus_tag="PIKHDJKN_01079"

/EC_number="7.6.2.9"

/inference="ab initio prediction:Prodigal:002006"

/inference="similar to AA sequence:UniProtKB:P46920"

/codon_start=1

/transl_table=11

/product="Glycine betaine transport ATP-binding protein

OpuAA"

/db_xref="COG:COG4175"

/translation="MTILEVKNVSKLFGPQTEQGLQLLEQGWGKEKLAKEKQITVGVN

RVNMEIQEGEIFVIMGLSGSGKSTLVRMFNRLIEPTSGEILVHGKDLRKMNKEQLREV

RRKTISMVFQKFALFPHRTVLDNVEYGLEVQKVDKDERREKAKTSLELVGLKGWEDKM

PDELSGGMQQRVGLARALANDPEVLLMDEAFSALDPLIRRDMQDELIELQDKMKKTII

FITHDLDEALRIGDRIALMKDGAVVQIGTPEEIMIQPANSYVARFVEDVDLSKVLTAS

HVMRRPETITLDRGPRVALELMRERGISNLFVIDRSKKLLGVITAEDATRAMRENKVL

NDILITDGPTVAPETLIHELFEIVSSAHVPLAVVGETGRLQGVIVRGALLGALSGEVA

VKEELVNDSQNTTSIVD"

CDS 1213318..1213890

/gene="opcR_1"

/locus_tag="PIKHDJKN_01080"

/inference="ab initio prediction:Prodigal:002006"

/inference="similar to AA sequence:UniProtKB:O34709"

/codon_start=1

/transl_table=11

/product="HTH-type transcriptional repressor OpcR"

/db_xref="COG:COG1510"

/translation="MSLDHLQEEQQATVLRIRKRVIEAIGRNMDLYGVTLSTGHLYGL

LFFADKPMTLDDMGREMEMSKTSMSTGVRTLLDLKMVNKVWSKGSRKDLYEVEYDWHQ

TFTDYFVIKWRKAVESNLQVLRKSVDELNRLIADLDEQADAELLHILKEDKHKVLQAE

AYYKWLDRLIDTMEDEEIYKLVPKEEIKEH"

CDS complement(1213989..1215617)

/gene="rhaS_5"

/locus_tag="PIKHDJKN_01081"

/inference="ab initio prediction:Prodigal:002006"

/inference="protein motif:HAMAP:MF_01534"

/codon_start=1

/transl_table=11

/product="HTH-type transcriptional activator RhaS"

/translation="MTEQLPFISETSSLPLLSSMCRVRRGEHFRVQGKTVSRPMLCLI

LQGDGVLVLNDTVYTAEANQLYVLKPGTTIEAAARSTVTEFIILSMDTVCLQQVRGQW

KMSSSSGFPLDWETGRLMVRYDQQARVRFEQLYEAYRGVHPDHFISIHSQLHELLQHL

SENRLEENYEKVDPALERSIMYMRRFMSEGISMDQLAKIAGLTPSSYSRSFKKAKGMS

PTDYLNRLRIDEAKKQLKQESCVLKDVAVSVGYGNEYYFSRKFKQTLGIAPSLYMKKD

QIRVATASMMGLHENLSSLGLHPVASMDGFHDRELDEVEQQQRLATQLEKLRQAKPDL

IIGDFYHKSFYDKLKSIAPTVILDQAHDWKENHLRLAELVGREQQAMQNFKQLELRNL

DIGLRLKHHFGQERIMLMQVTNQSIRLQEDTEHPLNRLIYGELGLNPAQIISTDLMSD

EYAADSIPMLDTDHLFMNRVSNSPVSEKLLRRMKQTTAWNRSPAVLKGNVHDISDWSV

LSWTSNGRHQIMDELEQIVQQIVTLSRVGLIGQL"

CDS complement(1215805..1217091)

/locus_tag="PIKHDJKN_01082"

/inference="ab initio prediction:Prodigal:002006"

/codon_start=1

/transl_table=11

/product="hypothetical protein"

/translation="MNKWTTRMQRTVCTLLLFTLIAAAVLGGGANAQAASLSQTTVYY

ESDGVLYKVSGDGSGTTEVLLDYEGDGLSAAGSYLYYTRSESSTTLLRVPNDGSSNSA

ETYATDVLSYFTDNGFIYYMDSKGTIYRGNGNSEASAATKIADKADTDFPLLAVTKGR

VYYNGLVNGNPWIVSKASNGSGAVQRIAAGAVESRYFMNVAKNELQLMVNTDPYEQFY

STNAILMYKVNYNTGKGTAVNPKAKLDVNAVYAGGWGNNLYVYNKGIKLDSNKDYNYS

TGKAFALTTSGKTLQIHSKSIREVSPLGTDKVVVVDADKKAYAKTIAGNKVSKSANLN

LNSVTFVTAQLTNGTSTLAYLSGTNGLYSVSTALKVTKLAGEEWATFQLHDDVPGMFY

INANDLYRLYHVQANGTGKKVLSDVFLDDILLVVPN"

CDS complement(1217246..1218079)

/locus_tag="PIKHDJKN_01083"

/inference="ab initio prediction:Prodigal:002006"

/codon_start=1

/transl_table=11

/product="hypothetical protein"

/translation="MNNRQAIRALVRKDIRAITASVQLWLPMLIVPLVIGIIMPGVLI

WIASRMELASLGNMSFLLDTLDNLTHAGQIPQLASMPTDNHRIVYYLALYMFAPLFLI

IPVMASSILTANSFAGEKERKTLEGLLFTPMSMDTLFKGKVLAAFIPSLLLSWITFII

YGIIANILMYPMFGAWMFPNLNWIILVLWVVPACSLVVILLNVLISAKVRGFQEAYQL

GGLVVIPLLALIAGQASGMLLINPLMLLLVGAALLLISFILLRLVTLWNSRQQLAESQ

I"

CDS complement(1218072..1218983)

/gene="btuD_7"

/locus_tag="PIKHDJKN_01084"

/EC_number="7.6.2.8"

/inference="ab initio prediction:Prodigal:002006"

/inference="protein motif:HAMAP:MF_01005"

/codon_start=1

/transl_table=11

/product="Vitamin B12 import ATP-binding protein BtuD"

/translation="MYMIEVRNISKQFKVHQALNDVSFTVEQGSVTGLIGPNGSGKTT

LIRIMNGVLGASGGQVSINGLDPFRETENVLAICGTLTEQSGLYENMSGRDNLIFFAE

VFGVQHAKARIDELVDLFELQEYQHRKVGTYSTGMKKRVGLARVLLHRPSILFLDEPT

NGLDPDGIQMVLRIIRRLNQEEHMTILVSSHVLSQLSAVCDRYIFMEKGRKVEEGTEQ

EIVNRYLTSPRIEVEANMPNGWQTVTGYTPEVVSANKAIFQLQTREDIPLLLRQLTER

GQVYQARITGSDLESIYFAIREAHHHE"

CDS 1219396..1221261

/gene="htpG_2"

/locus_tag="PIKHDJKN_01085"

/inference="ab initio prediction:Prodigal:002006"

/inference="protein motif:HAMAP:MF_00505"

/codon_start=1

/transl_table=11

/product="Chaperone protein HtpG"

/translation="MCLDQRKVSEMTASNEYRFQVNLSGMIQILSNHLYSSPKVFLRE

LMQNATDAITARTEAEPGFQGEVRVELTGTGEQLTMMVEDNGIGLTEADIHEFLAMIG

QSSKRGQQALLDGETSFIGRFGIGLLSCFMVSHEIVMLTQSAKGGPSMEWRGKPDGTY

TIRQLETQLSPGTKVYLRCTPDAAHYFETEYVKEALFYYGALLPYPVILHSDGVQHIV

NSESPIWLMEPELSRGRRAEVLAFGERLLGEKFQDFIPLTTASGRTGGIAFVLPHAVN

LNAKRSHRVYLKRMLISEKAENILPEWAFFVKCLIWTDELQPTASREHFYENEKLEEV

RSELGDALRKGLADMAESQTERLQKLIRLHALSMKALAVQDQEFYAMIHRWLPFESTR

GHRELGELMSEGETLYFTTTVDEYRQIHHVASAQSMLVINGGYIYDSDLMAMLPLAVQ

HVQTQRLQPDEVSMSFTDVPPAERNQYYDALRLADSALQRFRCRAEVKGFKPADLPVL

FTLSQESSTLRALEKASEESTELFSSVLGSLSSGISSAGYSTLYLNISNPIIQRVLTS

PDAQMTPIAIEMLYVNALMMGHYAMNRQELEVLNQGIVRFIDWGLRANTDRKGDA"

CDS 1221261..1222325

/locus_tag="PIKHDJKN_01086"

/inference="ab initio prediction:Prodigal:002006"

/codon_start=1

/transl_table=11

/product="hypothetical protein"

/translation="MKTEMDFEDLMDEAYGLPNGTAKLGMLEEAAKVADVNGMEEEAY

EARSEIVETATFCGYPMKALIAFSWQLGKFDQQPERYDEETLMWSYKWILGELSSFPE

VSRDKMMELLEDFGRRFKSFGYSERSYWYYRFRISMDLGDLEEAGTSYTKFRNMDRDF

MSDCEACEQDEIMRYWILAGDDEKVLEAAKPILRGRMSCAEIPHLTLSEILMPLYRLG

KIDEADKHQAKGYRMIKGHNDFVQSFAEQMDYLARTNPAKGIDVLEESLVLAMDHEDP

FAKMMFYARAAQLLRRWADESPGYRLRLPASFPYEGDPGDLRKLADYFTVYAKAAADK

YDQRNGNQHVSSMLSEVFNP"

CDS complement(1222482..1224656)

/gene="yfeW_1"

/locus_tag="PIKHDJKN_01087"

/EC_number="3.4.16.4"

/inference="ab initio prediction:Prodigal:002006"

/inference="protein motif:HAMAP:MF_01034"

/codon_start=1

/transl_table=11

/product="Putative D-alanyl-D-alanine carboxypeptidase"

/translation="MHLSNRSTRFRFTSLTGAFMAFILSLSLWAPAVQAETAPAAVNE

KGKVTALTTESATAFLDTFFDSAEAKAQYVGASVVVVKDGRVVAEKGYGYSNLESKTP

VDPKNTTFRIASVSKTFTSAAVMQLVEQGKVDLKADFQTYVKGLEFDNPFDKHVTVEN

LLTHTTGFEIRDPQQEDIHADFDKSVSMEDYAKLHMPPVVREPGSAYMYDNFSFLLLG

MIVENVSGEPFETYMQQHIFKPLGMNNSSFMLNGKFKNQLATAYDAGHNPLDLYTLSP

TPMPQGGMLSTAEDIGNFMIAFLNDGVKDNERIWKESTVKSMEQYRSSIHPLLPNTTY

GFEAAFQLPGAGSSSKIITKAGDLIGFSSYLFLIPEQNTGVFLTYNQTGALRNFFYPA

FIQTFFPQYAEPVKFKEYTPQSADELQRFAGLYADLRLSTIVSSLKDGGKEPGQLSIS

DVFLGPRTLIQVEDNLFKDELTGQFTAFKENADGTIYMKEPYLNPMGYEKKGQKPQGF

RDVQANSPYAEAIYGLQSLGYYTNDASEAFQPKNAVTRAEFIEDVLKMSGLKASKTAP

PADTDWATHPAAGYIQLGYESGMITGADEKQFKPDQAITRQEAMVMIWRTLQLQYPNE

LFEDVKLAGQTDAWAVPAIQMMVKLGIHGPEVKVLGDGSVDFLSRKPLIRQEEAAIMY

QLFTQPTDKIVADLMTNQPHSETSSDEETAEDTTAPVTVTPE"

CDS complement(1224813..1226063)

/gene="mdrP_1"

/locus_tag="PIKHDJKN_01088"

/inference="ab initio prediction:Prodigal:002006"

/inference="similar to AA sequence:UniProtKB:A0A1C7E424"

/codon_start=1

/transl_table=11

/product="Na(+), Li(+), K(+)/H(+) antiporter"

/translation="MKYTDLHRNIKIRIITDFFTDLTQKSIIPFMAIYLSVQLGAGWA

GMLLTVNIVASMIVGLGAGYWSDRVGRKKLMVTAQMLQVFALFWLAAANSPWMDSVPL

TCAMFLLSSISSGITGPIANAMIVDVSSEHERQYVYGLQYWTTNAAVTIGALMGGLLF

ESFRFILFSLVCMESLVTLFILLFYIHETMDKKHFEQLDAPINRNILKTYDGVFRDKR

FMVFFTATVLAVSLEFQMDKYIAVRLKTEFNAQLFSWDISGLQMFSLIMAINTVLVVI

MAIPFSKWMRRFQSRFIMTVGMCLYTTGFAVLAFSNWAWLLVTSAVLLTIGELMYAPV

RQVMLAGMIPDSDRAAYMAADGMSYNAAALLGSLGLTIGAFLPSYAMAGLYVLMGIGA

LVFFRQSLRPSTVQSEPQPCADAS"

CDS 1226190..1228046

/gene="sgrR_1"

/locus_tag="PIKHDJKN_01089"

/inference="ab initio prediction:Prodigal:002006"

/inference="protein motif:HAMAP:MF_01449"

/codon_start=1

/transl_table=11

/product="HTH-type transcriptional regulator SgrR"

/translation="MVVEVMDHYIQLRMAFSNVDEEEEIHTTIGELANHLCCTMRNMN

LIMNKFMDHGWIRWSPQRGRGKTSVLVFCLPLLQAARERFDRFLQGYKLEEAYVLAST

LPFSMREILLQGLQGQFGLRSDQCARGRVDTLRIPQETPFETLDPTRAAMWGEVGIVA

EVFDRLVQYNAERQVCEPSLAVAWESNPDGTEWTFYLHKGILFHHGRVLDAEDVRYTF

ERIISDQENPCRSLFGSIHRIETFDELTVRFVLRFPNFMFPDLMSSMNASILPRDMEM

DPMHPIGTGPYRLTRNHPKLLVLDVFTSYFRGRAFIDRVEIWQLPQSGLVESVIKQDL

FPDGQARSVQHEVQGGVYMTFNMRKEGPQQDLYFRQAVQQLMDPQEMMGALARSSVQA

AYSLVRDKSEEKQMLEQIEKNEQSEQLSPPADSSLVRASELLQCSCYAGQIMNVWVEE

GEKMETDMAWFAQRCELIGLKVNVMPGNPVDAVYHDEFASYDVIYTGEVFDEHVTLSL

LTMYTFHNTLFLMALDDQRKSELERECRHVVMIQDSAERLRTLMTLEGRLIQEALLLP

TYSFREEHAHHVSLQDYRVVAFGMPDLRRLWVKRSPNIGEDTASYPAYIPLW"

CDS complement(1228090..1229583)

/gene="dap_3"

/locus_tag="PIKHDJKN_01090"

/EC_number="3.4.11.19"

/inference="ab initio prediction:Prodigal:002006"

/inference="protein motif:HAMAP:MF_01960"

/codon_start=1

/transl_table=11

/product="D-aminopeptidase"

/translation="MNTTYLHSLLNGLDDFAEEQLQRWKGVGVAVAVIHKDEVILQKG

YGYRDLESKLEVTPHTLFAIGSSTKAFTASTAALLVDQDILKWDTPVRTYLKDFKMFD

PVATERLTIRDMLCHRSGLPRHEMAWYNSSRSREELVNALQYLEPNEDFRNKWQYQNL

MYMAAGYLVGQLKGTSWENVVQESLFNPLGMNSSVFSVDEMQNQPDFAFPYMDQNGES

TRIPFRKIDAVGPAGSINSNLVDMIAWVQFQLNQGQQGENQLISKEQLAVIHSPHMAC

DTPFHSKELPVSTYGLGWMIDSYRGHAMIHHGGAIDGFASQVAFLPEEQIGVVVLCNT

NGSILPYTISFNVIDRLLEVEPVDWSGRLAKLMTGDSTDSEAATDTIETPTTSETSVE

EKPVETQVHPHDRPIHAYAGSYTHPGYDEIVIQQTNDGLQATLNAMEMPMEYTGKDTF

SVELVLFGLKITFTFKTDEEDTISSLSIPLLLEPGTNPIEFIKTPSE"

CDS 1229907..1231082

/locus_tag="PIKHDJKN_01091"

/inference="ab initio prediction:Prodigal:002006"

/codon_start=1

/transl_table=11

/product="hypothetical protein"

/translation="MVLVLSTLLAACGKKDAAAVVKDLNEVVGEMESYQGAGVMTLHT

GDTPQQYKVEVWHQKPSYYRIALTNAKKDVTQIVLRNDEGVFVLTPSQNKSFRFQSNW

PDNQGQVYLYETLIRSITGDSTRQFADEKESYVFDVAANYNTHALVRQKIWLNKSDYA

PKQVEVSDSNANVVVDVKFDSFKFGTEFEKDAFDMQRNMTAATENGGKVGTDSGTAPA

GEGKETTNPQATTEEGSASNPSGTVSDGQTGVSDNAEQQGSEDPASGQGTEEPTSAEP

EGTGSFGVIQPTYAPEGVKLKDDQIVEGSGDYSVMLRYEGTYNYTIFEARPQDRAVSL

APSSVVDLGFTLGMLSGDALQTLTWMTDGVEYRITSADLPQNEMVQIATSMQEESGK"

CDS 1231542..1232729

/gene="alr2"

/locus_tag="PIKHDJKN_01092"

/EC_number="5.1.1.1"

/inference="ab initio prediction:Prodigal:002006"

/inference="similar to AA sequence:UniProtKB:P94494"

/codon_start=1

/transl_table=11

/product="Alanine racemase 2"

/db_xref="COG:COG0787"

/translation="MQEQYRPTQAEINLDHLCNNVDAFRETLPQGKKLLACVKANAYG

HGAVEMARELERVGVDYLSVAFLDEALELRQHGITLPILVLGYTPPEGIAEAWKHDVT

ITLFSKEVLEAIQHLETGTSSRKLKVHIKIDSGMGRLGLLPGDEALAFIQEVANLKQV

MLEGMFTHFAKADEKDKTYTLEQYRRFRGVVDALRDQGCVIPIIHTANSAATIDTPEL

SYDMVRVGISLYGLYPSGEVNHQVVKLSPVLTLKTKAVLVKTLPPHWGVSYGTRYVTQ
GNERIATLPIGYADGFSRMLSGKAHVLVRGRRVPVVGTICMDQCMVSLQSFAAEAEEI
QAGEEVVLIGHQSGAVITADEVASQLGTIPYEVICMMAHRIPRVYTRNGAVVARINPL
LTS"

CDS 1233000..1233272
/gene="ndoAI"
/locus_tag="PIKHDJKN_01093"
/inference="ab initio prediction:Prodigal:002006"
/inference="similar to AA sequence:UniProtKB:P96621"
/codon_start=1
/transl_table=11
/product="Antitoxin EndoAI"
/db_xref="COG:COG0864"
/translation="MQNTKRIMISLPDYLLQEVDGIVALENSNRSELIRQAMKLYLTE
RKKRYIRESMQRGYMEMAKINLTMASEAFHAEEDADSTLDRLVSGV"

CDS 1233276..1233626
/gene="ndoA"
/locus_tag="PIKHDJKN_01094"
/EC_number="3.1.27.-"
/inference="ab initio prediction:Prodigal:002006"
/inference="similar to AA sequence:UniProtKB:P96622"
/codon_start=1
/transl_table=11
/product="Endoribonuclease EndoA"
/db_xref="COG:COG2337"
/translation="MIVKRGDVFFADLSPVVGSEQGGVRPVLVIQNDIGNRFSPTCIV
AAITAQIQKAKLPTHVEIDAAAHGFDRDSVILLEQIRTIDKQRLTDKITHLDEETMKL
VNEALQISLGLIDF"

CDS 1233861..1236050
/gene="agaA_1"
/locus_tag="PIKHDJKN_01095"
/EC_number="3.2.1.22"

/inference="ab initio prediction:Prodigal:002006"

/inference="similar to AA sequence:UniProtKB:Q9ALJ4"

/codon_start=1

/transl_table=11

/product="Alpha-galactosidase AgaA"

/translation="MSIYIDQEKLQFHLQTQKASYVFQVLPSGYLVHLYYGKRLRDTD

LSWLHVRTERASFSPNPVPEDRTISFDTLAVELPVYGTSDFRNPAIQLELENGSTVSE

FTYTGYQLIKGKAALTGLPATYVESDDEAETLIVELEDRVAGIKIELSYTAFTAFNAI

TRSMRIINESATSVNIARALSSSVDFPHADYELLQLSGAWTRERDIVRRPLASGLQGI

ESRRGSSSHQQNPFIALMTPGTDEDEGEVYGFSLVYSGSFAAQAEVDQFHTTRVSLGI

NPFEFSWKLDSQESFQTPETVMVYSDAGLDGMSQSYHKLYRERLARGKFRNAERPVLV

NNWEATYFGFNADKIEQIAQAGKKLGIELFVLDDGWFGHRDSDNSSLGDWIVDKNKLP

QGLDDLANRVTDLDMQFGLWFEPEMISPDSELYRAHPDWCLHVPDRRRTEGRQQLVLD

FSRQDVRDEIVRLLSDVLGSAPITYVKWDMNRNMTEVGSALLPADRQRETAHRYMLGL

YEVMERITSAFPNILFESCSGGGGRFDPGMLYYMPQTWTSDNTDAISRLRIQYGTSLV

YPVSSMGSHISAVPNHQVNRITSLEIRGHVAMSGNFGYELDLTRFTEEENDIVKAQVE

LYKEIRGTIQYGTFRRLLSPFEGNETAWMFIAPDGSEAVVFYFRVLSEPNAPLQRLKL

KGLDPNADYRLKGGSETFTGDALMYGGISVGRASGDYWSEMFRFERV"

CDS 1236325..1238547

/gene="yhgF"

/locus_tag="PIKHDJKN_01096"

/inference="ab initio prediction:Prodigal:002006"

/inference="similar to AA sequence:UniProtKB:P46837"

/codon_start=1

/transl_table=11

/product="Protein YhgF"

/db_xref="COG:COG2183"

/translation="MSEQETVLEPNEETIKAERHERIIKQVAKELSLSLKQVRTTSEL

LDEGNTIPFIARYRKEMTGELDENQLRLIEERIVYLRNLEDRKVEVIRIIEEQGKLTG

ELKQSIKQAVKLQEVEDLYRPFRQKRKTRASVAKEKGLEPLAIWIWGQPKQGNVLEEA

ARYINAELGVEDAEVALQGAKDILAENIADDAAIRAWIRRYTLDHGMLTSEAKDAEQE

SVYENYYDYRELAKKMPPHRILAINRGERENILKVGLDVQPEPAHRHMEGQIIKGASA

VQDILRAVVEDAYKRLIAPSIEREVRAELTEKGENQAISVFSANLRNLLLQPPIHGKR

VLGVDPAYRTGCKLAVVDDTGKLLEVAVTYPTPPHNKKREAAEVFHRMIKQYDIGLIV

IGNGTGSRETEQFVAEIIQENGDESLVYLIVNEAGASVYSASKLAQEEFPDLDVAERS

AASIARRVQDPLAELVKIDPKAIGVGQYQHDVSQKILEESLKAVVESAVNHVGVDVNT

ASPSLLSYVAGVNATIAKNIVKYREENGRFTNRRQLQKVPRLGAKTYEQCVGFMRIGE

GENPLDRTPIHPESYKVVDQLFKELQVALDKLGSKELSVLLSEQQPEQLAVKLDVGVP

TLRDILDSLQRPGRDPREEMPLPIFRTDVLKIEDLVEGMELQGTVRNVIDFGAFVDIG

IKSDGLVHISQLSNGYVKHPMDVVSVGDNVTVWVMNVDTKKGRVGLTMKKPASSKQSS

"

CDS complement(1239076..1239432)

/locus_tag="PIKHDJKN_01097"

/inference="ab initio prediction:Prodigal:002006"

/codon_start=1

/transl_table=11

/product="hypothetical protein"

/translation="MPTMRYVILQQEQQLQFVEMPADYAYQLSALNLRLHKEIDKLTA

ADVPVLPWAIAECDNLDLLDEQLSIIGGLDYINALEQSFAALRESEYPLISLLTEIRA

LQAQLEQWYEEEMESL"

CDS 1239549..1240010

/locus_tag="PIKHDJKN_01098"

/inference="ab initio prediction:Prodigal:002006"

/inference="protein motif:HAMAP:MF_00745"

/codon_start=1

/transl_table=11

/product="Protein SprT-like protein"

/translation="MENEELQHWIEQVSLDHFGVPFTHEALFNRRLTTTGGRYMLKSH

RIEINPHQLEAYGREEVEKIIKHELCHYHLHIRGRGYQHRDPEFKALLQKVGGSRFCQ

SLPYGKGRKPLPYRYKLVCKSCGTEYLRKRKVDPKRYRCGRCAGKLGIHTI"

tRNA 1240182..1240257

/locus_tag="PIKHDJKN_01099"

/product="tRNA-Asn"

/inference="COORDINATES:profile:Aragorn:001002"

/note="tRNA-Asn(gtt)"

tRNA 1240262..1240352

/locus_tag="PIKHDJKN_01100"

/product="tRNA-Ser"

/inference="COORDINATES:profile:Aragorn:001002"

/note="tRNA-Ser(gct)"

CDS complement(1240585..1241283)

/locus_tag="PIKHDJKN_01101"

/inference="ab initio prediction:Prodigal:002006"

/codon_start=1

/transl_table=11

/product="hypothetical protein"

/translation="MSTYPNISVIASLIADPSRAIFLEALLDGRALPAGELAYMAGVT

PQTASSHLAKLVEGGLLVVEQQGRHRYYRLAGKEVAFLIETMGSIAAPVQVRSLKQSN

QLQHLSFARTCYGHLAGKLGISLCEALLREGYLEEPEDEQAKDYHITEKGIQWFTSFG

IILDGKAGSRRAVARKCLDWSERRHHISGVLGDELGRRLSELDWTRQKAGSRSVEVTE

AGKKGLYEVLGISL"

CDS 1241386..1242054

/locus_tag="PIKHDJKN_01102"

/inference="ab initio prediction:Prodigal:002006"

/codon_start=1

/transl_table=11

/product="hypothetical protein"

/translation="MHTNDRAQEEFEPVNHRDLNKEVMQHEVITPSILYYGTPVLLLS

TLNGDGTTNLSPLSSSWALGDCLVLGVGIQGKAFENLSRHPECVINLPDASMWEQVEG

LGRYTGIYPVPEEKRRMGFEFCNDKFAVSGLTPQGSIQVEPDKIAECPLQIEAAVQHI

RIPEHTPFMAIVEVKALKVHAHARLISGLNKINPEKWNPLIYNFRHYYGLGERRGESF

RAEK"

CDS 1242265..1242936

/locus_tag="PIKHDJKN_01103"

/inference="ab initio prediction:Prodigal:002006"

/codon_start=1

/transl_table=11

/product="hypothetical protein"

/translation="MEMEYLMRVLIAGICGVLIGYERKNRMKEAGIRTHFVVAVGAAL

MMIVSKYGFQDQAGWANLSLDPSRIAAQVVSGVGFIGAGMIFTQRHTVRGLTTAAGIW

ATAGMGLAVGSGLYWTGAGVTLLIVVAQMLLHRPTRWLVSARTETLTIRLLKEGDTLK

TVLALLGQEKISVVGFKTEQQISTDSGEETVLEFTLQLPGSYRAEQLIVLLQDVPHVR

SVDMR"

CDS complement(1242994..1243890)

/gene="ywpJ_1"

/locus_tag="PIKHDJKN_01104"

/EC_number="3.1.3.-"

/inference="ab initio prediction:Prodigal:002006"

/inference="similar to AA sequence:UniProtKB:P94592"

/codon_start=1

/transl_table=11

/product="Phosphatase YwpJ"

/db_xref="COG:COG0561"

/translation="MKLFATDLDGTLLNRDSQISPENAAAIHKAQQEGLKITIATGRV

YSDVLTISSEGGINTPVIGSNGATIHDVNGERLFHLPLERETAASVMQWLEDHHCYYE

ASTQQGIYAPISSHEAMLAEMDRILGSSPGEDIERLIRAVKKHYDKKDYHRVNSHQEI

PSEAYIYNIMAFSMDPAQLQQGREYFASRSDVAMVVSSEHNFEMQHPDVSKGNALSKL

AAHLNISMEDTAAIGDNFNDVSMLKMAGLGIAMGNAEPEIKALAKAISLTNVEHGVAH

AIQCVLEGKPVSRPETVVEGGQ"

CDS complement(1243894..1244670)

/gene="glcR_1"

/locus_tag="PIKHDJKN_01105"

/inference="ab initio prediction:Prodigal:002006"

/inference="similar to AA sequence:UniProtKB:P94591"

/codon_start=1

/transl_table=11

/product="HTH-type transcriptional repressor GlcR"

/db_xref="COG:COG1349"

/translation="MFQEERMQLIIEHLRKHNRISADDIVSLFDVSRDTARRDLIKLE
EQDAIIRTRGGAILPVPPDEHRSYKDRLLHISDEKRAIGKLAAAIVRQGEHIILDSST
TVQTCAENLNGKSCTVITNSIYSAELLSNHTAVEIRLLGGKVDKEQRYVYGTSVIETL
SHYYVDKAFIGIGGITMDGFSASEEEGKIKHKMMQAAKQVIVLADHSKFDRRYGYRFA
DWSLVDMLITDQWPSQDWRTFLAEQQVEILIPEPTIEKEL"

tRNA 1244978..1245049

/locus_tag="PIKHDJKN_01106"

/product="tRNA-Glu"

/inference="COORDINATES:profile:Aragorn:001002"

/note="tRNA-Glu(ttc)"

tRNA 1245157..1245232

/locus_tag="PIKHDJKN_01107"

/product="tRNA-Lys"

/inference="COORDINATES:profile:Aragorn:001002"

/note="tRNA-Lys(ttt)"

tRNA 1245249..1245334

/locus_tag="PIKHDJKN_01108"

/product="tRNA-Leu"

/inference="COORDINATES:profile:Aragorn:001002"

/note="tRNA-Leu(gag)"

CDS 1245505..1245921

/locus_tag="PIKHDJKN_01109"

/inference="ab initio prediction:Prodigal:002006"

/codon_start=1

/transl_table=11

/product="hypothetical protein"

/translation="MQSIYERIEHLIAERGMTKKAFCQQLKISTGNLGDWKRGKSIPS
TNKLIEIASFFDVSLDWLMIGRPSKEAMVREKREDYFFDVLRQLNCQESELSTVEQSF
ISEYIEFTRYRKSKESRDSGGFRYKAEDKPKGDELL"

CDS complement(1246091..1246852)

/gene="ureH"

/locus_tag="PIKHDJKN_01110"

/inference="ab initio prediction:Prodigal:002006"

/inference="similar to AA sequence:UniProtKB:Q09067"

/codon_start=1

/transl_table=11

/product="Urease accessory protein UreH"

/db_xref="COG:COG0829"

/translation="MTDRYYSAPLRFSRSFRPPGGGDELCVYTSDVSPGVLNGDHYRS

EWQLGQNTHLMLSSTSATRLHPTPTIPSSVNHHFRLEQGAVLEYFPECVIPFKGSSSS

LTTTFELGEQAILAYADIWSAGRIHRGEVFQFKRYRSLTEIWQGKQLAVWDRFGLEPE

SDDPKSPAALLDYTHTAALWMIAPGLGAAQLESVRSILPPDGRMLAGASLLATGGIGV

RLLGMAAWELQEQCLQIWNTLRSDLLGKPSLTFRK"

CDS complement(1246968..1247690)

/gene="ureG"

/locus_tag="PIKHDJKN_01111"

/inference="ab initio prediction:Prodigal:002006"

/inference="similar to AA sequence:UniProtKB:Q9RP19"

/codon_start=1

/transl_table=11

/product="Urease accessory protein UreG"

/translation="MCGGANHTHHAEWERKVFDRSRPMRIGIGGPVGSGKTALVEKLS

KALRTRYSLAVITNDIYTKEDAEILLRQNALAPERIIGVETGGCPHTAIREDASMNFE

AVDELIERFPDLQLIFIESGGDNLSAAFSPELADVFIYIIDVAQGEKLPRKGGPGISR

SDLLLINKTDLAPYVGASLQVMKDDTERVREGRPYVMSNLMSGEGVSEIVHWLEHQYM

DDDASAAHLHTHTHEHGTTHSH"

CDS complement(1247724..1248419)

/gene="ureF_1"

/locus_tag="PIKHDJKN_01112"

/inference="ab initio prediction:Prodigal:002006"

/inference="similar to AA sequence:UniProtKB:Q79VJ2"

/codon_start=1

/transl_table=11

/product="Urease accessory protein UreF"

/db_xref="COG:COG0830"

/translation="MMHSGTKLLRYVQLLDSALPIGGFSHSFGLEAYTHDGRIRTTAQ

LEQFIRGQLHSSLVRFDGLAIKGVYQAIEQQDAALLALYDKRVHAQRTPRELRESGHK

MGKRLLKLARSLYPWMDFSLIDEAVQEHGAFCGITTIHGYINYQLEIGLDEAVTGHLY

TSVNAYVNSALRLLPIGQTEAQMLIQKLLNDIDSEWSQFRNNDPEDMHSFGIAQEIYA

MRHESLPARLFMS"

CDS complement(1248438..1250156)

/gene="ureC"

/locus_tag="PIKHDJKN_01113"

/EC_number="3.5.1.5"

/inference="ab initio prediction:Prodigal:002006"

/inference="similar to AA sequence:UniProtKB:Q07397"

/codon_start=1

/transl_table=11

/product="Urease subunit alpha"

/translation="MKRMSREQYASMFGPTTGDAVRLADTELWAEIEHDYAVYGDESK

FGGGKVIRDGMGQSTSALRSDGTPDTVITNAVIIDHWGIVKADIGIRDGLICAIGKSG

NPDTMDGVDPALVIGASTEIIAGEGMIVTAGGIDTHIHFICPQQIQTALSSGVTTMIG

GGTGPATGTKATTCTPGAWHIHRMLESAEAFPMNIGYLGKGNSSSTAPLIEQIEAGVI

GLKLHEDWGTTPSAIDACLTAAGEHDVQVAIHTDTLNETGFLENTLAAINGRTIHTYH

TEGAGGGHAPDIIRAAGESYVIPSSTNPTRPYTRNTVEEHLDMLMVCHHLDPSIPEDV

AFADSRIRPETIAAEDILHDLGVFSIISSDSQAMGRVGEVIIRTWQTADKMKKQRGKL

ELTSGSPSDNDRIKRYVAKYTINPAIAHGIGHLVGSVEVGKLADLIVWNPAYFGVKPE

MVIKGGMITFAQMGDPNASIPTPQPVFGRPMFGAYGGALAKGSITFVSQAAADAGIKE

ELGLNKRVEPVKGCRSVSKKDMIHNDVTPIIEVDPETYEVRADGELLTCEPADELPMA

QRYFMF"

CDS complement(1250153..1250542)

/gene="ureB"

/locus_tag="PIKHDJKN_01114"

/EC_number="3.5.1.5"

/inference="ab initio prediction:Prodigal:002006"

/inference="similar to AA sequence:UniProtKB:Q07398"

/codon_start=1

/transl_table=11

/product="Urease subunit beta"

/translation="MIPGEYRLKKDDEIVCHPDRSTVRLIVLNRGDRPVQVGSHVHFY

EVNAALDFDRPSAFGHRLHIPAGTAVRFEPGEEKPVELTTFGGKRQIYGFNGLTEGSA

DQPPDPQKLETFLKAFAPPMQDGGNPL"

CDS complement(1250561..1250863)

/gene="ureA"

/locus_tag="PIKHDJKN_01115"

/EC_number="3.5.1.5"

/inference="ab initio prediction:Prodigal:002006"

/inference="similar to AA sequence:UniProtKB:Q07399"

/codon_start=1

/transl_table=11

/product="Urease subunit gamma"

/translation="MHWTEQEKEKLLITVAANLARERRGRGLKLNVPEAIALLTSELM

ERARDGMSVAELMRYGGTILTREDCMEGVPDMIPEVQVEATFPDGTKLVTVHEPIR"

CDS 1251124..1252374

/gene="amiC"

/locus_tag="PIKHDJKN_01116"

/inference="ab initio prediction:Prodigal:002006"

/inference="similar to AA sequence:UniProtKB:P27017"

/codon_start=1

/transl_table=11

/product="Aliphatic amidase expression-regulating protein"

/translation="MKKRSVKLWSVLLGAVIALTGCVEGGTPPEASSSGGAGEPASSG

DTIKVGVLHSLSGTMAISEVSVKDAEMLAIEEINAAGGVLGKQIEPVIEDGASDWPTF

AEKAGKLLQQDKVAAVFGGWTSASRKAMLPVFEQNKGLLFYPVQYEGLESSPNIFYTG

ATTNQQIVPSVTWLLENRGKKFYLLGSDYVFPKTANQVIKAQLAAEGGEVVGEEYTPL

GHTDYSTIISKIKAAKPDIVYNTLNGDSNVAFFKQLNDAGISSEQLTTLSVSVAEEEI

RGIGADVLKGHLASWNYYQTTDTPENKTFVEKYKEKYGADRVTADPIEAGYVAVYLWK

AAVEKAGSTDVEKVKEAAKGLEFDAPEGKVTVDGENQHIYKTVRIGEVQEDGQFKELW

NSGAPVKPDPYLKTYEWGAFLSTK"

CDS 1252529..1253434

/gene="livH"

/locus_tag="PIKHDJKN_01117"

/inference="ab initio prediction:Prodigal:002006"

/inference="similar to AA sequence:UniProtKB:P0AEX7"

/codon_start=1

/transl_table=11

/product="High-affinity branched-chain amino acid

transport system permease protein LivH"

/db_xref="COG:COG0559"

/translation="MDMFVLQMFNGLSISSILLLIALGLAVTFGLMNVINMAHGELIM

IGAYATYVTQNLFISYAPQAWFDVYFIVALPIAFGVAALIGWLLEVVLIRHLYGRPLD

SLLATWGVGMMLQQLARTIFGAPNVGVSSPAWLNGGLTIADDIVFPYKRLFIIALVAV

VLLCMYLYIYRTSSGRRMRAVMQNRSMAGCLGISTRRVDGMTFAIGSGIAGIAGCALT

LIGPIGPSLGTYYIVDAFMVVVLGGVGKLVGTVCGALGIGMFNTLFETYTSASIGKVL

VFVCIVAFLQWKPRGLVAMRTRSLD"

CDS 1253456..1254547

/locus_tag="PIKHDJKN_01118"

/inference="ab initio prediction:Prodigal:002006"

/codon_start=1

/transl_table=11

/product="hypothetical protein"

/translation="MSALLKTGSLKMKIIWAVVLIMMCLAPLISTEFRLSLLAKFLAL

AILAIGLDLIWGYGGVLSLGHGVFFGLGGYAMAMYLKLQASGTTLPDFMGWSGLSGLP

WFWEPFRSFPVALLLGIALPALLAFALGWFTFRNRITGVYFTILTQALVLIIVTLFVG

KQEWTGGTNGITGYNAIFGFTLHSAGTTIALYYITLAVLVLAYMLCRRVVNSRFGQVL

EAARDGENRVRFLGYDPAGFKTLAFALSGALAGIAGMLFVLQVGIISPSMMGIVPSIE

MVLWVALGGRGTLIGAVIGAVVLNAAKTGISEAYPEGWLFVIGGLFVTVVLFMPNGLV

GVYRHVVRLLKRRGEVVHVPVSQEKPKVY"

CDS 1254510..1255319

/gene="lsrA"

/locus_tag="PIKHDJKN_01119"

/EC_number="3.6.3.-"

/inference="ab initio prediction:Prodigal:002006"

/inference="similar to AA sequence:UniProtKB:P77257"

/codon_start=1

/transl_table=11

/product="Autoinducer 2 import ATP-binding protein LsrA"

/db_xref="COG:COG1129"

/translation="MFQSVRKNQKSTDIPVVLVAEEITVAFGGFVAVKGMNLKLHEHD

LHFLIGPNGAGKTTMLDVICGKTKPLSGTVKMADGTDLTRLKEHQIVRKGVGRKFQAP

SIFAGLTVQENLTLAAETRRSPLHALGIQRYAKISPAMERITLQIGLQDRVDARAGAL

SHGEKQWLEIGMLLLQEPRVLLLDEPAAGMTDEETHKTGRLLQEIARERSVVVVEHDM

EFVREYAAKVTVMHEGKLLKEGTMAEVQEDPRVAEVYLGKRRDDHAVAATN"

CDS 1255297..1255992

/gene="livF"

/locus_tag="PIKHDJKN_01120"

/inference="ab initio prediction:Prodigal:002006"

/inference="similar to AA sequence:UniProtKB:P22731"

/codon_start=1

/transl_table=11

/product="High-affinity branched-chain amino acid

transport ATP-binding protein LivF"

/db_xref="COG:COG0410"

/translation="MLSLQRIESGYGESNVLRGVNLDVQPGQVVCLMGRNGVGKTTLM

KTLMGLLKTRKGSIQWKGQELSTHDPAKRARSGIGYVPQGREIFPQLTVKENLLLGLE

TSGAGVKTFPEDVLAMFPVLATMYGRQGGDLSGGQQQQLAFARALASRPGLLLLDEPT

EGIQPSIVEDIRQVILQIKEKGEISILLVEQSIDFVRGAADYIYVMDKGAIALQGTPR

ELDMSQFEHHLSV"

CDS complement(1256018..1256671)

/locus_tag="PIKHDJKN_01121"

/inference="ab initio prediction:Prodigal:002006"

/codon_start=1

/transl_table=11

/product="hypothetical protein"

/translation="MRYLISDLERLSFQSMQHQLIIVDHNWIIRSCNTAWQHGLGQSL

PKPSVSGPSRTAPVPPHYLHLVEGWTLREEKAKNAKIVQELKSITAPFKDSYTYDISV

QTAHNEQRWFRLELTPLIQADPSLLSDLALIAHTDVTEQKQTERQLKKALSEVRTLRG

LLPICAVCKQIKDEQDSWNSVESYLEKHTHAEFTHDICPDCIRRLYPKYSNILDKRS"

CDS complement(1256699..1256902)

/locus_tag="PIKHDJKN_01122"

/inference="ab initio prediction:Prodigal:002006"

/codon_start=1

/transl_table=11

/product="hypothetical protein"

/translation="MNKKKPVTPFGWAIKRRLTELQVDQKTFCEQYGIPPYRLSNLIH

GTRKATRFRNQVADILEIPDDLR"

CDS complement(1257178..1257924)

/gene="ureF_2"

/locus_tag="PIKHDJKN_01123"

/inference="ab initio prediction:Prodigal:002006"

/inference="similar to AA sequence:UniProtKB:Q09065"

/codon_start=1

/transl_table=11

/product="Urease accessory protein UreF"

/db_xref="COG:COG0830"

/translation="MLFNITFNERRRRKIVNRGNKLLDYVKLLDSSIQVGGFTHSFGM

DAHIAEGTIRSAEDLESFMRCQLHPSIVRLEGMAIKGIYTAADHNDAWRTALIDKLVH

VQRTPVNLREHASTMGKRLIKLARALHPWIDFSLLEQTLAKYESVGCLSTVHAWINHH

LEIPVEEAVLGYLHSAMSACITEASKAIPLNTDTTQDLLVRLAADLEHEWHTVSASAA

DGLVQPTSMSTKTLFPSFHMLGAGLHAYRA"

tRNA 1258300..1258385

/locus_tag="PIKHDJKN_01124"

/product="tRNA-Leu"

/inference="COORDINATES:profile:Aragorn:001002"

/note="tRNA-Leu(gag)"

CDS complement(1258533..1259009)

/gene="gpx1_1"

/locus_tag="PIKHDJKN_01125"

/EC_number="1.11.1.22"

/inference="ab initio prediction:Prodigal:002006"

/inference="similar to AA sequence:UniProtKB:P74250"

/codon_start=1

/transl_table=11

/product="Hydroperoxy fatty acid reductase gpx1"

/translation="MSVYDYKVNTLRGQEVEMSNYRDKVLLIVNTASQCGLTPQFKGL

QELQDKFKDSPFEVLGFPSNQFAQEKGSSDDIAEFCQMNYGVSFPMFEKIDVNGSSAH

PLFQHLTKEAPGVLGSKAIKWNFTKFLVDQNGRVLKRYAPKTTPDKIEADIKELLK"

CDS 1259673..1260068

/locus_tag="PIKHDJKN_01126"

/inference="ab initio prediction:Prodigal:002006"

/codon_start=1

/transl_table=11

/product="hypothetical protein"

/translation="MDKEMILVTAPNEAGRKFIKLLMYKKMPFAVLTNSAGEERRLRR

IGVEHVIRMNTAAAQKWFLPQDSVGNVFIFENSLNLTCRYLQICRSWTSKSLCVITEQ

SHPKGIYRGMGADRIVYSLNGEVGFLLNG"

CDS 1260234..1260926

/gene="yjjG_1"

/locus_tag="PIKHDJKN_01127"

/EC_number="3.1.3.5"

/inference="ab initio prediction:Prodigal:002006"

/inference="similar to AA sequence:UniProtKB:P0A8Y1"

/codon_start=1

/transl_table=11

/product="Pyrimidine 5'-nucleotidase YjjG"

/db_xref="COG:COG1011"

/translation="MPDVRGIQWLFFDVGDTLVDEWEPVDDIIGQFVREACALGYQVE

MQTVRDIFAASYRNYEQWPMKLAIRTLISDEGHREQIQEKLKFRKELERPFASADAVL

KTLSQHYKIGIIANQSPGTENRLDSYGLRKYVDVLACSAEEGVSKPDPELYAVALKQA

GCKPEEAVMIGDRIDNDIIPAKKLGMHTIRILQGYGRFQPELADAERPDWTVESLEEL

LPLLMVTQNSDN"

CDS complement(1261072..1262421)

/gene="mntH"

/locus_tag="PIKHDJKN_01128"

/inference="ab initio prediction:Prodigal:002006"

/inference="similar to AA sequence:UniProtKB:Q99UZ7"

/codon_start=1

/transl_table=11

/product="Divalent metal cation transporter MntH"

/translation="MSKKNPFNAPSNEPLAAGMAPSLGEAHSSMKVPQNAAWWKKFLA

FVGPGYLVAVGYMDPGNWATDIAGGSQFGYTLLSVILISNLMAVVLQSLAGKLGIVTG

RDLAQACRERFSMPVVIVLWILCELAIAATDLAEVIGSAIALKLLFNIPMLYGVIITA

VDVLVILLLQNKGFRALESLVIVLMATIALCFGIDLFLAKPDMGGVMHGFVPNVEILQ

NPAMLYIAIGIIGATVMPHNLYLHSSIVQTRQIEQTPKGKKEAILYSTMDSTIALTLA

LFINAAILIVSAAVFHSAGMTQVAEITDAYHLLTPLLGTTIASILFGVALLASGQNST

LTGTLSGQIVMEGFLNIRIPAWLRRLVTRLIAIIPAVIVTAIAGEHGTEELLILSQVV

LSLQLPFAVIPLVMFTSDKKSMGAFANKLWLKVISWIIAGVIVVLNVYLIIQTILLF"

CDS 1262812..1263231

/gene="ohrA_1"

/locus_tag="PIKHDJKN_01129"

/inference="ab initio prediction:Prodigal:002006"

/inference="similar to AA sequence:UniProtKB:O34762"

/codon_start=1

/transl_table=11

/product="Organic hydroperoxide resistance protein OhrA"

/db_xref="COG:COG1764"

/translation="MEALYTATATVKGGRTGSVASSDGVLKHDLKMPKELGGSGGEGT

NPEQLFAAGYGACYESALANVARKAGVKLENVVVTSNVSIGKDPADDGFQLSVRLDVS

MPGVDHTQAEDLARKAHDFCPYSKATRGNIDVVLNVV"

CDS 1263261..1263440

/locus_tag="PIKHDJKN_01130"

/inference="ab initio prediction:Prodigal:002006"

/codon_start=1

/transl_table=11

/product="hypothetical protein"

/translation="MEQGIFGYLMFEYKTLLAFGRVLYFIIELRCRRLRVEQRTILHN

LLLLEVIHSNKGERQ"

CDS 1263437..1263682

/locus_tag="PIKHDJKN_01131"

/inference="ab initio prediction:Prodigal:002006"

/codon_start=1

/transl_table=11

/product="hypothetical protein"

/translation="MSIEEMIERRFVCTKCRGTDCNIKEVSMSGAGLSKMFDIQHNHY

LFVACSSCGYVEVFDPDVLKGKKQGQVGTILDILFGG"

CDS 1263782..1264060

/locus_tag="PIKHDJKN_01132"

/inference="ab initio prediction:Prodigal:002006"

/codon_start=1

/transl_table=11

/product="hypothetical protein"

/translation="MSYRPRIANLELASGNKEDGLYEFKMKLVDGTQCRVFYSHSPEW

KLTNISRLQKTPCPVCRKDFICKCMDQWAGDLHQQMIDDQWMEKAIAE"

CDS 1264205..1264627

/locus_tag="PIKHDJKN_01133"

/inference="ab initio prediction:Prodigal:002006"

/codon_start=1

/transl_table=11

/product="hypothetical protein"

/translation="MTANQTDQHQGHPIVLVDGVCHFCQGLTKWIIKRDPEGKYHFAS

LQSDVAKKLLENGNLSTDSMDTFVLIEDGKYYTRSTAALRLAKGLKFPYPLLYVLIIV

PKFIRNAIYNMVARNRYRWFGKDEACMLPTPEIKDRFL"

CDS 1264726..1265589

/locus_tag="PIKHDJKN_01134"

/inference="ab initio prediction:Prodigal:002006"

/codon_start=1

/transl_table=11

/product="hypothetical protein"

/translation="MKKWTTLIIGALLAVSMTACGSDADNSTATPPAGNETSNEGNTT

AEQETKIPTLDELIEKTNAATKDMKSFTTEANIDQNLKLVAGEQSQDQQVKTSLKMDI

IKDPMMIYQEMKMEMSGQEAQNVKQYITSDKIYSQVGDQWVKIPDEQTKPLIEQMQAS

MNPEGELEQFKKIAEDTKITEEGDNYVINADVSGDNVKELAKAVMEQNGSDAQTQAML

DQMNITSMKMKYMINKETYLPASTDVNMVMDMEQEGQKISMEMKMTSTFSNHNGVKEI

KIPQEALDSAK"

CDS 1265858..1266241

/locus_tag="PIKHDJKN_01135"

/inference="ab initio prediction:Prodigal:002006"

/codon_start=1

/transl_table=11

/product="hypothetical protein"

/translation="MIEYAHIHHVSLAVRDLEVAKEFYSGLLRMQEIERPSFNSTGTW

YALGSQQLHLLQHPQGHTLREAGIDTTDGHFAIWVKSYKETLAWLEQQGIEYEARPDS

VAGFAQIFVLDPDRNIIEFDAPYHS"

CDS 1266402..1267625

/locus_tag="PIKHDJKN_01136"

/inference="ab initio prediction:Prodigal:002006"

/codon_start=1

/transl_table=11

/product="hypothetical protein"

/translation="MIVLKAVLASKDKEYISAWLDFVQGSPSGSGIRFTAFSQWEPFR

DHMNEQEGRELPDLVIAEPEFLNNWLGNGGETSGIPWLMLSEGLEEVDEEKRLMKYQP

LPTLMDTVLNACRQPRRKKAHRPGQETLSIGVVSASGGSGKTAVAMHMAKQLGLAGYA

VLYLNLETLDSSMPFLEKGLSRSGQRIPDAETGLSRLLYDLKVGRKESGKQGQVQSSS
KGVDGYVLRHEALKSDVFWPLSNRKELLQMSREDTKSLIRYLADCGQYDVLILDGDSG
WDGRSDGIFDAADAFVWLVEDDISSMHRWGQWMQHMERTWPDLHESVLDRARFIVNKY
RDNVINALPRPDLHLDAVLPYIPSWKQLSQEEVMLSSPIFQREVKRLCAMIVQDGEEE
LKQTGRIQKGDGWSL"

CDS 1267622..1268926

/locus_tag="PIKHDJKN_01137"

/inference="ab initio prediction:Prodigal:002006"

/codon_start=1

/transl_table=11

/product="hypothetical protein"

/translation="MMDSSKMILDREEQFQIMRREVRAGLDLTSSAGDEELWQGIERK
VLSDPKLDDLTSGERHTLVQRLFDSFRGLDILQPLVDHPEITEIMINSHREIFVEQEG
EVRQITLEFESRERLEDIIQMIVSGVNRIVNESSPIVDARLKDGSRVNIVLPPIALKG
PTMTIRKFPSEPMKMSDLIGKGALHEEAAELLQQLVRSKYNIFIGGGTGSGKTTFLNA
LSQFIPADERIITIEDSAELQIVTVPNLVSLETRNANTEGKGQISIRDLIKSSLRMRP
NRIVIGEVRGAEALDMLQAMNTGHDGSLSTGHANTISDMISRLETMVLSGADLPIAVV
RQQISSAIDIFVHLSRLRDRSRRVTEISEVIGMHDGEVLLNPLFRFQEIEEKEGKIIG
GLVRVGTLRQVDKIQMAGLGEWLNEYIERNSDELDKSDNNVN"

CDS 1268952..1269713

/locus_tag="PIKHDJKN_01138"

/inference="ab initio prediction:Prodigal:002006"

/codon_start=1

/transl_table=11

/product="hypothetical protein"

/translation="MGEARQALTDYTVYVLSRRQRIVCMLISGLLFFGVGILFYHHWL
AGLILAAGCIWVPKHWTKVLLERRRMTLSLHFKQALYALSSALAAGKSVENGFKESVE
DLRMLNPEADTDLIREFTILRTRMEYGQPIEEALQDFSDRAKIEDITNFADVFITCKR
TGGDLVEVVRRTSAVIGEKLDIQQDIMVAVAQKKFESKVMFAAPFIFLIFLNFTAKDF
MEPLYSGMGYMISSGALALLACCYLWITRIMDIKV"

CDS 1269726..1270595

/locus_tag="PIKHDJKN_01139"

/inference="ab initio prediction:Prodigal:002006"

/codon_start=1

/transl_table=11

/product="hypothetical protein"

/translation="MLLPVIFGGVLGAGWLVLDRTRGQTYRHLRKLDMEGLRLKKLHG

PFLYLLDKFEVGRRLPVLMFRMQHAIQKMYGIQHSGEKTMLYCAEMLTYSWLMLLVGC

LLSLVGDMGLGGMVGGLALGVALPFALYKDLNTKVQRRDQDILMELPELLNRIVLLVG

AGETVQRAIVHCVASQRERDHPLYNELRKTVDDWNNGYSFQQSFEQFSRRCGVQEVTI

FTTTMLLNFRRGGGDFVLALRDLSHVLWEKRKAVSRAKGEQASSKLVFPMVLIFFSIV

VMIGAPAFMMMNM"

CDS 1270607..1270795

/locus_tag="PIKHDJKN_01140"

/inference="ab initio prediction:Prodigal:002006"

/codon_start=1

/transl_table=11

/product="hypothetical protein"

/translation="MLELMKNKVTGFWKEEDGLGTLELILIIGVIIIIALIFKDQIKT

LVTRLLGQVSDKSNEFFE"

CDS 1270755..1271453

/locus_tag="PIKHDJKN_01141"

/inference="ab initio prediction:Prodigal:002006"

/codon_start=1

/transl_table=11

/product="hypothetical protein"

/translation="MVKLATKVMSSSNNKFKKEEGSFTIEASLVFPIVLFILVLILFF

SMYMYQKTFLNQHAYAASERAAYSWDNSHKQAMTGEVVAGQHDNLYWRLTDDRLLGAL

FGWAGADNEVIVSVPAGEGGSLSEKKLSQAAQGMPSGMKGTIEYQNSLIQRKVTTKLE

QVISLPLPSFLFDSGNSVLTQGPSAVVEPTEFIRTVDLVRYYAAKFKGKGGAAASTAA

EAGQVVQHFGKTKK"

CDS 1271511..1273661

/locus_tag="PIKHDJKN_01142"

/inference="ab initio prediction:Prodigal:002006"

/codon_start=1

/transl_table=11

/product="hypothetical protein"

/translation="MFLILILAGIFMFVSIFIDYARIAAFKVQAERMTHAAMRSVMSA

YDTSLREYGLFGYGDSSGEAIMAKVLNDSVKPSAVKDGFPILDIQWDTTSLSMERELG

RYDIFNRQIQEDMKYRAPVDFTLEVVNRFKPMSEEMKEAAHTVDLLKKLQKLYDKREE

LLDEAIANQTESADKLKGLPALIMNPPSSAIYDEMLRETPETAAEVAARYADYLNKKQ

MDEGLPFNQQQYILELARYRTGVGNVATGISMIIQPALQAHQTKLEEAQAKIEEARKI

NEEMKRVIAEGENRSQNASYEKVGNSTLPGTSPPSTGSGSEAKSTRSLASELVRADTL

FDQLNARVRSQNSDFTNVSNESMELNTQLRTVTVMSGVSIKPVVRQASQVTERYMDTY

VRSGPSNVILQSKQLLESGRGSDAQRKANDKESKGKLKDVKRILSQIEKGSSGSMEAF

KQLEEYYNTNIAFNQASREEAKKAEIAGDPYDSGGDAMNGMDSLFGGMSNLLDSLGDE

LFQNEYALAYFNSMDFGQLLGWTEGSGDAGEVFKLENQELEYIVYGFHNPSGNIAAAY

AEIFGMRLAIRTAEGLIENSKLGNPLLVLSAAILYGITNAIADMVKLAKDGSVELSKY

IKVKLTYRDHLRLFLLMHSNNERKMSRMLALIRLNTGVNPDEKQTYASGNMRSSIKLW

FLPGVTRSIGAVMGSADQVEDGRFFIDRKADYSY"

CDS 1273691..1274740

/locus_tag="PIKHDJKN_01143"

/inference="ab initio prediction:Prodigal:002006"

/codon_start=1

/transl_table=11

/product="hypothetical protein"

/translation="MDKQHQQEKVLASLHNRLTRIHRLERLKSLTKQIRSIQKVHSPK

REQGSIVLEASLVLPVFLFFVMFLIYIVQMTLVSTALQSTAGEAVKQLSTKIYPVSLV

FTPSDSAGSGGSEGGWKIPELSLTEWAEEYASSLPEPLSDWVRAAAARGEQPLQEIKT

SVLETVLDPTVKPLLQPFINTSVLDVERVHVNGISIPDLKNKTNPYFRLELSYELPVK

VPFLGKSLRIQAAAAERVWIGDTGEGSNSSGGDADSAGTVTVLSKPEPAYIGNNATIK

VKVEPGATANLTIFYKSGESSAKHIGWATADENGVIEWNWFVGTRTTEGTWTFVVETA

DGAKTVTEFNVASRK"

CDS 1274758..1275261

/locus_tag="PIKHDJKN_01144"

/inference="ab initio prediction:Prodigal:002006"

/codon_start=1

/transl_table=11

/product="hypothetical protein"

/translation="MEWFYLACGLYVIVAFITDVRSMKIPNRLTLPVTIAGVLAHIIW

GGWEGFLFSAAGFAIGFGVLFLMYLIGAVGAGDVKLFGGIGAWTGLAFGVHVIIYSVL

YAGAIGIVILLFRRDSAKRIRGMAGNLAGFFMLGSLKLVNQEKTLKFPFMLAVLPGFI

SAYIYMT"

CDS 1275288..1277198

/locus_tag="PIKHDJKN_01145"

/inference="ab initio prediction:Prodigal:002006"

/codon_start=1

/transl_table=11

/product="hypothetical protein"

/translation="MYGLTRDFIRNGGSFMVLEKVEGLRMDELSRVQMGMLSSNQIPR

LLPVHIREVDRNVTLQYDISGYKMLTQMLKSGKIKLRVLYGLLFQLADTFTECRQYML

DSRKLQIQEEYIFINGSLELGELGLVYIPIVDAVEVDPTPQQFRELVIRLMAHVQELQ

GEGIQRVLQLCDSEQWDIRQLRELLLELYADEQSGSGQASFMSSRGSSGTTDSQFGMP

LSAVPEQGNDPFSNRSGQDQYRQLDRNGEAEQQLKSNHQPRVSTSEEDKPFNKPFSGF

SGRRPPIESSLQSKGVKTNPSERQTFDSIQDDDMEKDEKQESSKTTYIILGCMIAMAL

VWRFIYMEQPGQTQMILSAALSAGLLGIAGWAWVTKGKFEHSKSNLLRKKMPNEEYTD

GELQEGSTNKRSAFFGLGRGKDKQKESEEEMFQESWRWNASEMSPEGTAYAQGYTSQH

SSGDMQSAQSERGNSFESRSSVAGNSRFQNLHIPSEVSDEPFVQHNVEAVAATSELVR

HHAAADATVNLQSVTGGSITGAGPVVPSFYLERRSATGDKNERMDIRGASFVIGRSAD

MVQWVDSATGVSRAHVELSRNKSGYVIKDLGSVNGTILKGDVLAPYKEYPLEDGDTFA

LAESVYTYRSVG"

CDS complement(1277187..1278098)

/locus_tag="PIKHDJKN_01146"

/inference="ab initio prediction:Prodigal:002006"

/inference="similar to AA sequence:UniProtKB:Q7A6Q5"

/codon_start=1

/transl_table=11

/product="Epimerase family protein"

/translation="MKIAICGGTGFVGGAIVDYWLQAGHHVKVITRKLPNLHNPSQNL
TYISWEQVEEQPHLLEGMDALVNLAGETLNQRWTTKAKLEIVESRVTTVARVAKLIDS
LEHKPEVVVQASAMAIYGTSPTETFDENSPQKSVNFPSRVSQQWEVAADSIKHVRLVK
IRVSLVLGHKKGAFPLMKLPYMLGVGGRVGSGKQWTSWIHIMDIVRLIDFTIQNKEIS
GPVNASSPTPVTNDEFGRTVGKVYHRPHWFPVPGILIKTLVGELSVVLLQGQRVIPQK
ALDHGFQFTFPTLIDALEDLKHRGLSD"

CDS complement(1278255..1278725)

/locus_tag="PIKHDJKN_01147"

/inference="ab initio prediction:Prodigal:002006"

/codon_start=1

/transl_table=11

/product="hypothetical protein"

/translation="MIFGSYALTLTSSSPSNWFMNTIAFWTVLLLGSMCVGGFFMMRK
FLKVLPKADGKSKLDWQNHWVETSRHLWTDEAKAFLDQLVEPVPGPFRDIAKHSIAAE
IGKIAFENNAPEVSRDHCIKGYIIATPKRDNKFLVKFLEKNQIDYSPYKHLMNK"

CDS 1278895..1280022

/gene="nfo_1"

/locus_tag="PIKHDJKN_01148"

/EC_number="3.1.21.2"

/inference="ab initio prediction:Prodigal:002006"

/inference="protein motif:HAMAP:MF_00152"

/codon_start=1

/transl_table=11

/product="endonuclease 4"

/translation="MLKIGSHVSFSDKGLLSATKEASSYGSSSFMIYTGAPQNTRRKP
IESMFIEEGKLAMQEGGMEDIVVHAPYIINLGSYKENTYELAVSFLQEEIRRTHAIGV
KNIVLHPGAFTDKDAHYGIGRIAEGLNEVLDGVKETDVNIALETMAGKGTEMGRSFEE
IAQIMEKVTHNERLTVCMDTCHIHDAGYDIVNDLDGVLEQFDRMVGLDRIAVMHINDS
KNPVGAHKDRHTPIGSGWIGFEAINRIVHHEALKGRPFILETPWIGKEAKTQRPMYEA
EIALLRGDVAGRFGPEFLTEVEQLHHFFKGKEIESRSYVLDIWTLLKNDAKAKKADPR
EPLERLYDLVAEAALFPHLNEEQLNHRLIAWLAGKEIFVTA"

CDS 1280045..1280938

/gene="purU"

/locus_tag="PIKHDJKN_01149"

/EC_number="3.5.1.10"

/inference="ab initio prediction:Prodigal:002006"

/inference="similar to AA sequence:UniProtKB:P9WHM3"

/codon_start=1

/transl_table=11

/product="Formyltetrahydrofolate deformylase"

/db_xref="COG:COG0788"

/translation="MEIHAKQVRPDVKNRADRARMLISCPDGPGIVAAVSHFLHQHGA

NIVQSDQYTMDPAGGMFFMRIEFDLPQLSVNLPKLEADFEEVANRFKMDWSLSAVSRK

KKLAIFVSKEDHCLVELLWQWQAGDLDADIALVVSNHLDMKDYVESFGIPYHHIPVTA

DTKKEAEQRQLDVIGNDIDVIILARYMQIISPMFIEHYRNRIINIHHSFLPAFVGGKP

YAQAYNRGVKIIGATAHYVTEELDGGPIIEQDVQRVSHGDDVTELKRIGRTIERVVLA

RAVKWHVEDRVLVHENKTVVF"

CDS 1281940..1283982

/gene="tkt"

/locus_tag="PIKHDJKN_01150"

/EC_number="2.2.1.1"

/inference="ab initio prediction:Prodigal:002006"

/inference="similar to AA sequence:UniProtKB:A0A0I9QGZ2"

/codon_start=1

/transl_table=11

/product="Transketolase"

/translation="MTDKNEAIQKEENNTIDNLSITTVRTLAIDAIEKANSGHPGMPM

GSAPMGYQLFAKTMTHNPDHPTWVNRDRFVLSAGHGSMLLYSLLHLSGYDLPMEQLKQ

FRQWGSKTPGHPEFGHTAGVDATTGPLGQGIAMAVGMAMAEAQLGATYNKDKFNVIDH

HTYAICGDGDLMEGVSHESASLAGRLHLGKLIMLFDSNDITLDGKLNLSSSESIAKRF

EAYGWQVLRVEDGNDLPAIQKAIEEGQADTLRPTLIEVKTVIGYGSPNKQGKGGHGGT

HGSPLGADEAKLTKDFYKWVYEEDFHVPTEVREHFAQVKDRGISANKAWDEKFAEYKK

AFPELAAQFETAINGDLPEGWDRDLPKYAATDKAVSTRVASGNALNGLAHNVPQLTGG

SADLESSTMTHLNNLENFSPEDYSGRNIYFGIREFGMAGAMNGMALHSGVKVFGGTFF

VFTDYLRPAVRLASLMGLPVTYVLTHDSIAVGEDGPTHEPIEQLASLRIIPNLTVIRP

ADGNETSAAWAYALENKSNPVALVLTRQNLPILEGTVEGSRENVKRGAYVVSDAKEGK

AVAQIIATGSEVQLAVKAQAALAEQGIQVRVISMPSWDLFEKQDKAYKESVLLPNVKA

RLAIEMAHPMGWEKYVGDHGDILGISTFGASAPGDRVIQEYGFTVENVVSRVKALL"

CDS 1284124..1284441

/gene="ppnP"

/locus_tag="PIKHDJKN_01151"

/EC_number="2.4.2.2"

/inference="ab initio prediction:Prodigal:002006"

/inference="similar to AA sequence:UniProtKB:Q6FF51"

/codon_start=1

/transl_table=11

/product="Pyrimidine/purine nucleoside phosphorylase"

/db_xref="COG:COG3123"

/translation="MSQFDQVSVVKEANIYYEGQVTSRTVILGDGSKVTLGIMLPGSY

EFGTDSREIMEILSGDLRVLLPGEEDWQEIQGQATFHVPAESKFKLEIRSVTDYCCSY

PAE"

CDS 1284629..1285984

/gene="pgi"

/locus_tag="PIKHDJKN_01152"

/EC_number="5.3.1.9"

/inference="ab initio prediction:Prodigal:002006"

/inference="similar to AA sequence:UniProtKB:Q81K75"

/codon_start=1

/transl_table=11

/product="Glucose-6-phosphate isomerase"

/db_xref="COG:COG0166"

/translation="MAKKVKFDYSTALQFVNQHEVDYFAEPIRLAHEQLHNGTGTGSD

YLGWIDLPTAYDKEEFSRIQKAAAKIQSDSEVLIVIGIGGSYLGARAAIEMLTHSFYN

NLPKDKRKTPEIYFAGNNISSTYVTHLLDLVEGKDFSVNVISKSGTTTEPAIAFRIFR

AALEKKYGKEEARKRIYATTDKERGALKKLANEEGYESFIIPDDVGGRYSVLTAVGLL

PIAAAGISIEEMMQGAADASKEYSNPNVAENEAYQYAAVRNALYRKGKGTEILVNYEP

SLHFVSEWWKQLYGESEGKDYKGIYPASVDFSTDLHSMGQFIQEGSRNIFETVIQVAE

VSEHISIQADPDDLDGLNFLEGKTMDFVNKKAFQGTLLAHTDGQVPNLIVNIPDMSPY

SFGYLVYFFEKACGISGYLLGVNPFDQPGVEAYKKNMFALLGKPGFEEEKAALEARLS

E"

CDS 1286149..1286781

/gene="yigZ"

/locus_tag="PIKHDJKN_01153"

/inference="ab initio prediction:Prodigal:002006"

/inference="similar to AA sequence:UniProtKB:P27862"

/codon_start=1

/transl_table=11

/product="IMPACT family member YigZ"

/db_xref="COG:COG1739"

/translation="MIERYRTVRGPGNLEIVIKKSRFIGHIMQVTTEEEANAFIDEIK

KKHWNATHNCSAYMIGERDEIQKQSDDGEPSGTAGKPILEVIKNQQLKNVAIVVTRYF

GGIMLGAGGLIRAYTDGAVAAIEAGEAITKVLHREVFVELDYTWLGKVENELRSREVR

TGETGFTDKVTLTCLPPDSETEAFVAWITDLTQGQSRITEGQRLYFIEGE"

CDS 1286787..1287389

/locus_tag="PIKHDJKN_01154"

/inference="ab initio prediction:Prodigal:002006"

/codon_start=1

/transl_table=11

/product="hypothetical protein"

/translation="MARRAVEQELSRERILEAARHLFITKGYRAISMRSIGQHLGYSH

GSLYYHFKEKAELFYAIVVEDFNHLGHLLLQAMVRPVMGGVSKVEHIMMEFIRFGLEN

PYQYEIMFMIRDEELLSYCRTEQGRCFELFASIIRQYMNEEGCTEEDIQCVPRTLFLS

MHGFISYYIQDRLTFAEIESAALSHVKVLCRNLQHQPGVQ"

CDS complement(1287583..1287984)

/locus_tag="PIKHDJKN_01155"

/inference="ab initio prediction:Prodigal:002006"

/inference="similar to AA sequence:UniProtKB:P9WFP9"

/note="UPF0047 protein Rv2556c"

/codon_start=1

/transl_table=11

/product="hypothetical protein"

/translation="MLYTKNLSTSRRDEMKDITREVEQVVRDSGVQNGTVLIYCPHTT

AGIAINENADPDVKHDVLLRLNEVYPWEHPEYRHAEGNTASHLKSITTGPSQTVIIHE

GRLLLGRWQGIYFCEFDGPRQREYFLKIMED"

CDS 1288349..1289164

/gene="cysT_2"

/locus_tag="PIKHDJKN_01156"

/inference="ab initio prediction:Prodigal:002006"

/inference="similar to AA sequence:UniProtKB:P27367"

/codon_start=1

/transl_table=11

/product="Sulfate transport system permease protein CysT"

/db_xref="COG:COG0555"

/translation="MNTVLRHKGWTWGFRSTVLLYFIVLIVLPIIGVYVNSFSEGWSN

FIQSIMDPIAWKAVLLTIRLAVIATVINVILGTMIAWVLTRYRFFGRSFLNSLVDLPF

ALPTAVGGLMIMLLLGPISAIGKLAESMGVEIVFHQPAIVIAMTFVTFPFVIRAVQPL

LEEIDSSEEEASYTMGASKARTFRQVILPSMAPGMISGGMLAFSRALAEFGAVVLVAG

NIPGRTLTASVFIYGEIESDNPTGAAAVSVLLLTLSFLILWLINLVQMRGRRR"

CDS 1289161..1289958

/gene="cysW_2"

/locus_tag="PIKHDJKN_01157"

/inference="ab initio prediction:Prodigal:002006"

/inference="similar to AA sequence:UniProtKB:P27370"

/codon_start=1

/transl_table=11

/product="Sulfate transport system permease protein CysW"

/db_xref="COG:COG4208"

/translation="MRRLLIGLTFAVFIVLLIIPLGRIFIGAFEDGAGGFLEGLLRPE

ALHALMMTGLVVLVVTLLNTLFGVMMAIYLVRAGWLSDRVKGLLNSIVDLPYAVSPVI

GGLMIVLLLGPNSIMGAFFEGIGFNVVYAFPGMVIATLFVTFPLMVREVMPVLQELGS

QQEEAASTLGAYGWRTFWSVTWPSIRWAVVYGLVLTVARSLGEFGAVLVVSGNIMNKT

QTATTLVYQDVENFNVAAANGVALVLVTFSVGLLLMMEWAKKRKEVH"

CDS 1289960..1291024

/gene="cysA"

/locus_tag="PIKHDJKN_01158"

/EC_number="7.3.2.3"

/inference="ab initio prediction:Prodigal:002006"

/inference="similar to AA sequence:UniProtKB:P14788"

/codon_start=1

/transl_table=11

/product="Sulfate/thiosulfate import ATP-binding protein

CysA"

/db_xref="COG:COG1118"

/translation="MHVEVRDLNKHFGDFHAVKDVSFDIAKGHLIGLLGPSGGGKTSI

LRMLAGLENPGSGEIRFHGKVVNHLPPQERGIGFVFQNYALFKHMTVFDNIAFGLKVK

KTPKAQIRDRVMELVELTGLKGFEQRYPHQLSGGQRQRVAFARALAPEPQLLLLDEPF

AAIDAKIRQELRSWLRELIERVGITSIFVTHDQDEAIEVADEIMIISQGRLEQKGTPW

DIYKNPETPFVATFIGESTVVEDASQLKGFEHAVEGERTRALIRPEYIEIGLKNEFSM

LSATEKGIVKHLHFRGSEWMVEVEVEGHKLITYRSLEKTTLQVGQQIRVLVHRAYLFN

DNDSWVVENRLKEDPMPVII"

CDS 1291080..1292144

/gene="sbpA"

/locus_tag="PIKHDJKN_01159"

/inference="ab initio prediction:Prodigal:002006"

/inference="similar to AA sequence:UniProtKB:P27366"

/codon_start=1

/transl_table=11

/product="Sulfate-binding protein"

/db_xref="COG:COG1613"

/translation="MQTRKKKITPLYVVLMLLLVCMTVGCSRQEDASGQNGTSTDDQS

NTLVIGAYSVAKDAVGELLPKFQEEWKAKTGQTVHFQESYEASGTQARAIVGGFEADV

ALLAMESDIDKLVKADLVSSNWKQAPNDGMITRSIVVLGTRAGNPLGIRDFEDLTKPG

VKVLYPNPKTSGGAQWDINAIYGAGLKLSEKEQGKKDPAVAKAFLEKVHENVESLDKS
GRSSMAAFEYGVGDVIVTYENELLARIAKGVNYDIVVPKNTILIENPAVVVDKYADKH
GNRELAEAFVAYLRTPEAQRIFAKHGFRSVDPDVFAETKSSFPTPEGLFDINYLGGWD
KVRSTLYSKRGVWYQVLAGL"

CDS 1292367..1293974

/gene="appA_2"

/locus_tag="PIKHDJKN_01160"

/inference="ab initio prediction:Prodigal:002006"

/inference="similar to AA sequence:UniProtKB:P42061"

/codon_start=1

/transl_table=11

/product="Oligopeptide-binding protein AppA"

/translation="MKSKKMWLSLMLVLVVAIMSACGSSGDGTSSSSNTAENTATTDD
GQPKDGGSIIIAVQDDPKVMNPIYAGDRVTLTIDQSLYAPLFNVNDGKKTYVLADSDS
FSDDHLTYTLKLKDGLKWHDGQPLTADDIVFTMDSILDEKQHSSSRSQYVFDGKPVKV
TKVDDLTVEFKLPEVSAAFEGALVSFSPIPKHIFEGEADIEKSDKNNNPIGSGPFKFK
EYRPGEYVTLERFDDYFAGKAHLDSVTYRVAKDPNAANLALQNGEIQMRMVDTQDYNK
LNDTGKFNMLTYPEGRLQYMVFNLNVPSMQKKEVRQAIAYAVDKNELITASYSSADFA
EPAPSILTPDTLYQTDDVEKYDYNLEKAKQLLADAGVSNLKLRLAYTNTNKPQTSQAL
YIQQKLKDVGIEVELLPMDGTAYGNRTLDMNNTDFELSFGGYIMGYEPDAYKSLFLSD
AAYNYSHYKNTDFDKLWNEAAVQIDETKRGDLYKQIQQTVANEMTVYPIAYTKAVVAV
DNTYGGVEDANPKPVVMLEDLSKIFKK"

CDS 1294285..1295235

/gene="nikB"

/locus_tag="PIKHDJKN_01161"

/inference="ab initio prediction:Prodigal:002006"

/inference="similar to AA sequence:UniProtKB:P33591"

/codon_start=1

/transl_table=11

/product="Nickel transport system permease protein NikB"

/db_xref="COG:COG0601"

/translation="MNNYIVRRIAQAIPLLFVISIVSFTLIKLAPGDPVLSFVTPNMG

AEDVERIRQNLGLDQPMYVQYVVWLKNVLSGDLGYSLVNHRPVAEMIIERLPATFGLM

GASLLLSLLISIPLSMIAGSRQNSLFDRGLSLISYIGISIPSFWFGMMLIYFFAVELH

WLPSMGMRTVGMDSAWDIIKHGILPCTVLTFMNVSVYMRYIRSNTISQLEEDYVQIQY

AYGATKGTVLLRHVLKNVLLPVITILGMSLPELIAGAFITESVFSWPGMGSLGISAVH

GLDYPIIMGITMMSSVLLVVGNLVADILYGVVDPRIKTTG"

CDS 1295243..1296088

/gene="dppC_1"

/locus_tag="PIKHDJKN_01162"

/inference="ab initio prediction:Prodigal:002006"

/inference="similar to AA sequence:UniProtKB:P26904"

/codon_start=1

/transl_table=11

/product="Dipeptide transport system permease protein

DppC"

/db_xref="COG:COG1173"

/translation="MNSMRWRNLRTQFRQQKLGLAAVIILGIFVLASLFAFLSPHDPN

EIVVMERLQQPSADHWFGTDDYGRDYLARALYGGRVSLAVGFLSMAIAVIVGTLVGTV

SGYFGGWVDNTLMRIVDVLMSIPSFFLMLILNAYLKPGIGTIIVIIGVLSWMNIARIV

RAEAMSVKQREYVLYARVSGQKPGRIIMKHIIPNIIPTIIVAATINIASAILMESSLS

FLGLGVKQPSSSWGSMLNDAQGFISEAPYMAIFPGLFILLTVLSFNFLGDVFRVAFEP

KANKR"

CDS 1296220..1297245

/gene="oppD_3"

/locus_tag="PIKHDJKN_01163"

/inference="ab initio prediction:Prodigal:002006"

/inference="similar to AA sequence:UniProtKB:P24136"

/codon_start=1

/transl_table=11

/product="Oligopeptide transport ATP-binding protein OppD"

/db_xref="COG:COG0444"

/translation="MTDLLSVKQLKTSFATREGEVQSVRGVSFTLKEGEVVGLVGESG

SGKSVTARSLIRLIQSPGRITDGQILFRGEDLLTKSDKELRKIRGNRISMIFQDPMTS

LNPVVRVGKQMTEVIRRHRKMDKSAARKEAIELLRQVGIPSPEERIDQYPHEFSGGMR

QRVMIAMALSCKPELLIADEPTTALDVTIQAQILQLMQELKESTQTSILMITHDLGVV

AQVCTRVIVMYGGLIMEEGPVERIFAHPQHPYTQGLLRSIPKPGEEGGHQRLATIEGT

PPNLLHPPTGCPFMERCPHAMEVCKQMPAYTETAPGHRALCWLLDDQGREQHQVQTGA

SSEEVEQ"

CDS 1297242..1298177

/gene="oppF_4"

/locus_tag="PIKHDJKN_01164"

/inference="ab initio prediction:Prodigal:002006"

/inference="similar to AA sequence:UniProtKB:P24137"

/codon_start=1

/transl_table=11

/product="Oligopeptide transport ATP-binding protein OppF"

/db_xref="COG:COG4608"

/translation="MSTGTDQREVLLDVRGLKKHFPVRGAGKGKDVVKAVDNVSFHIH

KGETFGLVGESGCGKSTTGRSIVRLYDVTDGSIMFGGQDVAKMNERQLKPFRKRMQTI

FQDPYSSLNPGMNVLQIISEPMEIHNYASKAEMKGRALDLMNRVGLKPEHAERYPHEF

SGGQRQRISIARALSVNPEFILCDEPLSALDVSVQAQVVNMLEDLQAELGLTYLFIAH

DLSMVRHISNRIGVMYRGKLVEVAPTEELYRNPVHPYTQALLAAIPVPDPTVRNVMPP

IYSGGTDPSARGRDEEPEWMEVSPGHFVSGYRDRI"

CDS 1298234..1298680

/locus_tag="PIKHDJKN_01165"

/inference="ab initio prediction:Prodigal:002006"

/codon_start=1

/transl_table=11

/product="hypothetical protein"

/translation="MSAEFKLSQNTAIRLEQARSKGMSDAALLEAIQAKDVAAFDVVS

EEYYRYDDLFSYADEHGENLEGAVKDGYRITFNTRGGLGIYLQKSFGLQPEKDFTVGE

GIVTGLKLKQEQADVLAKRLASNWVITESKDVPEGQELTLKLRALV"

CDS 1298837..1299616

/gene="rbn_1"

/locus_tag="PIKHDJKN_01166"

/EC_number="3.1.26.11"

/inference="ab initio prediction:Prodigal:002006"

/inference="protein motif:HAMAP:MF_01818"

/codon_start=1

/transl_table=11

/product="Ribonuclease BN"

/translation="MNQLTFLGTGDAMGVPRVYCDCDVCKEARITGINRRKRSAVLIH

SDGEHEPFMIDCGPDWRSQMEDQGMRMVQTLLITHAHFDHIGGLPEWADACRWLGVEG

QLYAPREVIATIQSQFPWLGRHMNFLETDADIELGGWKVHSWKVCHGHNGYSYAYRLE

REGFTWAYCSDAIDLKASEKQPLYGLDLLVLGTSFVHELAKFSTRSVYDMVEAQALLQ

DLKPGQTYFTHMSHDVDVRKNYDLDQGITLALTGLKVKLGE"

CDS 1299750..1300616

/locus_tag="PIKHDJKN_01167"

/inference="ab initio prediction:Prodigal:002006"

/codon_start=1

/transl_table=11

/product="hypothetical protein"

/translation="MAFVKSVDCRINRPHDDKQVELYSHSDVLIGYTLEQTIEAALPN

LMGSLYAYCMSLTGSVWETEDLVQDTCVKALSSSAARRTGMTEDMNWEAYLIRVARNT

WIDAIRKRENLASKLESMKPFMQEFEEEERFEEVESAVQILVDELPPWQRAIYMLRDL

MGYTTVETAELLDTTEGAVKAALSRARSALAKVRHSLNEADSDQTNHLEEHQQNREEL

RSYLLAFRSGDTARLIDLCLNQTDDPMAVAGTILQQTLPSQSMQPVMYSYSSSGTNSM

SYGGGYTVSMVA"

CDS 1300635..1301216

/gene="clpP_1"

/locus_tag="PIKHDJKN_01168"

/EC_number="3.4.21.92"

/inference="ab initio prediction:Prodigal:002006"

/inference="similar to AA sequence:UniProtKB:P80244"

/codon_start=1

/transl_table=11

/product="ATP-dependent Clp protease proteolytic subunit"

/db_xref="COG:COG0740"

/translation="MNVVPYVVEQTARGERSYDIYSRLLKDRIIMVSGEIEDQMANAI

VAQLLFLTAEDPEKDIQMYINSPGGSVTAGFSIYDTMQFVKPDISTICTGMAASFGTI

LLVGGTKGKRLALPNSEIMIHQPLGGTRGQASDMLIHANRIIQHRQRLNKLLADHTGQ

PMERIEKDTDRDYFLTAAEAVEYGLVDKVISST"

CDS 1301476..1301814

/locus_tag="PIKHDJKN_01169"

/inference="ab initio prediction:Prodigal:002006"

/codon_start=1

/transl_table=11

/product="hypothetical protein"

/translation="MQILTQIRQSILSGELVAGESLPSIRQLAKDLQVSVITTKRVYE

ELEKEKLIDSVVGKGCFVSGANQEFIREQRLKRIEDKMLDILRESKELGMSVQDVVKH

FTLLVEEEHT"

CDS 1301811..1302680

/gene="btuD_8"

/locus_tag="PIKHDJKN_01170"

/EC_number="7.6.2.8"

/inference="ab initio prediction:Prodigal:002006"

/inference="protein motif:HAMAP:MF_01005"

/codon_start=1

/transl_table=11

/product="Vitamin B12 import ATP-binding protein BtuD"

/translation="MMNAIELCNLSKTYPLFKLDQITLDVKQGYITGLIGPNGAGKST

LISLISGLISPDSGSIKTLGKEMPAQEVNVKERIGIVSDECFYYEHLTIREMKSMIAP

FYKKWDEKQFNNFLEQFELSPKKKIEELSKGMKIKFSLAIALSHDAELLIMDEPTSGL

DPVFRRELLDLLADMIQDETRSIIFSTHITTDLERIADYITFINKGKIVFNEAKDDVL

ERYTIVKGDMKLLDADTRRQFVGIRETAVGFEGLADNRAEAERLFGNFALLHRPSLED

LIYFTAKGEQHHA"

CDS 1302783..1303298

/locus_tag="PIKHDJKN_01171"

/inference="ab initio prediction:Prodigal:002006"

/codon_start=1

/transl_table=11

/product="hypothetical protein"

/translation="MYFVGIYTAFGSIMLATMIDIKNHNHKFLVTLPLSRRNIVQAKY

LSAILYTLFGVFASSGAHWLVNFLFPELNKPDFSVLDILISVGMVLVLISIYMPLFYA

LSKKGAGIINTVFMFVLIILAQPVASLMNMAGENGMARASVYVFVSVCILLLFIASYF

VTVRLFARKDL"

CDS complement(1303478..1304899)

/gene="hisC_2"

/locus_tag="PIKHDJKN_01172"

/EC_number="2.6.1.9"

/inference="ab initio prediction:Prodigal:002006"

/inference="protein motif:HAMAP:MF_01023"

/codon_start=1

/transl_table=11

/product="Histidinol-phosphate aminotransferase"

/translation="MGKAIMMTDNSLKLYEQVVHYLVVRIEAGEWKEHEKLPSVRSLS

ELLGVHRLTVFKAYQELKERGNVYVKDKSGYYVSPADPSPVTDQADDPAVSAWLHWDS

LARVQSLEAEYQFSKSLIDPALLPNRYWGELMRDLLDLYPRLLGTYSTIQGDLELRSA

LASHLTTRERFYISADEVLITSGAQQAIDVISRSLVRPGDRVLMERPTYGPAMEIFRK

QGAKLIFTDIHPDGYDLEQIECLMKQEKPRVFYMTPTFQNPTGINIPTEQRKQLPELA

EQYGCFLVEDDSTYDIYFKEKPPAPIFTYDTTGHTLYIRSYSKYVVPGLRIAAIMCRP

RFMPGLQAVKALTDNGSPLLNQKLFLRYFQSPRMHQHLSKLRTAIQLRMEVMEQCLQQ

TDWTWTRPEGGLNIWAELPEGVDTGRLLHRCMEHSVAFVPGTVFDPSDANASRKLRLS

FSYAHEQQIREGMSRLIALAQST"

CDS 1305020..1305958

/gene="yedA"

/locus_tag="PIKHDJKN_01173"

/inference="ab initio prediction:Prodigal:002006"

/inference="similar to AA sequence:UniProtKB:P0AA70"

/codon_start=1

/transl_table=11

/product="putative inner membrane transporter YedA"

/translation="MVALAFTVMCLIFGTTFLAIKIGVEAGMPPFLSAGLRFIIAGAL

MFAWMWMKGKVNLSLLWRKEMLLTGAGLTFGTFATLYWAEQYVSSGVGAILSATGPLM

IMVMQTALLRQKTSVRMITGCLIGFAGVILVVLPGVSIGGNSLWLWGCIAVLVGEVCY

SAGALYSKKVINQFREANPVAINAVQMMYGGVLLSALSAVTESWNTTGLDWMPAVSSL

IYLTVIGSMVGHSLFYWIMSRTNPLFPATWLYISPPIAVGLGAVLYNEHVSWITWLGV

ALIVSGLLTMNEKVMGWIKRGKRTTSLSQTSKSMVK"

CDS 1305999..1306307

/locus_tag="PIKHDJKN_01174"

/inference="ab initio prediction:Prodigal:002006"

/codon_start=1

/transl_table=11

/product="hypothetical protein"

/translation="MNRQGIGAVLLFAGIVLYGIIHIGTIIHLPDVTIWSNQWGRYLQ

AAFETGGIIGLVLAILLFITGVTLLLPRPLADLSKGRLMKEISERNREFDEKYGKQNN

"

CDS complement(1306417..1307220)

/gene="yidA_1"

/locus_tag="PIKHDJKN_01175"

/EC_number="3.1.3.23"

/inference="ab initio prediction:Prodigal:002006"

/inference="similar to AA sequence:UniProtKB:P0A8Y5"

/codon_start=1

/transl_table=11

/product="Sugar phosphatase YidA"

/db_xref="COG:COG0561"

/translation="MTYKLIAIDIDDTLINDNKEVTPATQTALEQAVAHGVVVTLATG

RAYASAQALARQTKLNVPIITYQGALVKNLLDEKVLYERYVPQDASRKLYDYCLEHNL

HLQTYIDDKLYAREENDKLRDYAKLNGTQYHIEPDFIKVIEQKTPKLLIIDEPDYLDE

VAVALRELLGSEVHITKSKPYFLEIMHREGTKGHALTFLADHFGHQLSECIAIGDSWN

DHEMLEVAGLGVAMGNAIPALKELADYITASNNEDGVKQVIEKFVLNAE"

tRNA 1307360..1307435

/locus_tag="PIKHDJKN_01176"

/product="tRNA-Gly"

/inference="COORDINATES:profile:Aragorn:001002"

/note="tRNA-Gly(tcc)"

CDS complement(1307502..1308503)

/gene="lplJ"

/locus_tag="PIKHDJKN_01177"

/EC_number="6.3.1.20"

/inference="ab initio prediction:Prodigal:002006"

/inference="similar to AA sequence:UniProtKB:O07608"

/codon_start=1

/transl_table=11

/product="Lipoate-protein ligase LplJ"

/db_xref="COG:COG0095"

/translation="MLFVDNQGITDPSVNLAIEEYILKHLPMDDSYLLFYINRPSIII

GKHQNTIEEINIEYVQDNGVQVVRRLSGGGAVYHDLGNLNFSFITKDDGQSFHNFRKF

TQPVVEALQELGVNAELTGRNDLQVGEKKISGNAQFSTRGRMFSHGTLMFNLNLDHVQ

ASLNVNPEKFKSKSTKSVRSRVANIRDLIDSNLTIEQFRDELLRHIFRMEPQDVPQYE

LTEKDWDKINEISAERYNNWDWNYGLSPESNVKHTRKFPVGIIDLRMNIKDGRIEDIK

IFGDFFGVGDVADIEDMLRGKRYEESEVRTALEGLDVKHYFGNLELEDFIGLIFLEE"

CDS 1308713..1309861

/gene="cobD_1"

/locus_tag="PIKHDJKN_01178"

/EC_number="4.1.1.81"

/inference="ab initio prediction:Prodigal:002006"

/inference="similar to AA sequence:UniProtKB:P97084"

/codon_start=1

/transl_table=11

/product="Threonine-phosphate decarboxylase"

/db_xref="COG:COG0079"

/translation="MTGLIEVFGHGGDVETAAARFGGNPADFLDFSANINPLGPPREV

LEALGQGLHTVLRYPDPGHRAFKMLLSDRLGLTPDHISVGNGAAESMALILLGIAPRR

VGTVEPGFSEYSALSRQFGAEVHHVQGREELDWRAESEDIEKLMERVDLLFLGQPNNP
NGVQYPVDVLQRLARKAETTGTILVVDEAFLDFIPESQRQSLAPSLKEYPQVIIIRSM
TKFYAIPGLRLGYAMGRPELIHAMTEKQVTWSVNGLALIAGEACLRSGETFEQETMAL
VSHERESLTEGLEALGCKVTRGEANFILAALPEPWTATSMQAALGERGILIRSCAMYP
GLGERHVRFAVKDAEANGRLLEIIGNVIETNPTTGLEAGGHDDGTAIL"

CDS 1309842..1310660

/locus_tag="PIKHDJKN_01179"

/inference="ab initio prediction:Prodigal:002006"

/codon_start=1

/transl_table=11

/product="hypothetical protein"

/translation="MALPFYNYYSHVQKDQNEYRSSVWPGLFVSAHERHIKASSPSVV
TALSSAVYGGGMIELDRIFNIYVDRHYRCDDPPRDIADLLSEWQEQQDQCAGLLTAVR
LEHTAIQEYESDEFGLLCCTTAGVSNAARAGSARTVFDAAGNEMSNGTVSQKIGKVSA
STYTPGTINIMLWINGRMTAGAMVNAIQTAVEAKAAALADFGVVDSENGLTATGTTTD
AIVLAVSQAEVNKPLISYAGTATVMGAAIGRLVYDTITESLQAAQEWKERNNKQ"

CDS 1310657..1311685

/gene="cobD_2"

/locus_tag="PIKHDJKN_01180"

/inference="ab initio prediction:Prodigal:002006"

/inference="similar to AA sequence:UniProtKB:P9WP93"

/codon_start=1

/transl_table=11

/product="Cobalamin biosynthesis protein CobD"

/db_xref="COG:COG1270"

/translation="MTSLLHQWATWPIWTGIAGAWIIVGAYILDRCIGDPRWIPHPVI
GMGKAISALERAIRSRVDSNQALKRAGVLFPFLIAGGAFALSWGLIYMLGLIHPAVAV
VAEVALIATTIASKGLKDAGNEVYRHLSQQDWPAARRSLGMIVGRDTAHLDEPEIVRG
TVETVAENIVDAIVSPLFYALIGGAPLAMAYRAVNTLDSMVGYKNGKYLHLGWASARL
DDWANWIPARLTALLLIIGAWFMKLDARGAARMVSRDARLHPSPNSGFPESAVAGALG
IRLGGHNVYHGVASFRAYMGEPSRRMEAEDIVRTTRLMFWSAGSFVLVCWLVTLGIWS
AGGALLWK"

CDS 1311676..1312323

/gene="cobC"

/locus_tag="PIKHDJKN_01181"

/EC_number="3.1.3.73"

/inference="ab initio prediction:Prodigal:002006"

/inference="similar to AA sequence:UniProtKB:P52086"

/codon_start=1

/transl_table=11

/product="Adenosylcobalamin/alpha-ribazole phosphatase"

/db_xref="COG:COG0406"

/translation="MEIEAGQGGMQSREIILIRHGTTAWNLEKRYLGHTDIGLLPEAK

QELSSLREQFSNIACEALYCSDLLRCQQTLTHIASSQMGQAKLDARLREIDFGQWEGL

TYDQLKDNPQYRDWIDAPQEITPPNGEPWTAFAARIDSFMEECLMSFSPILHSGSINV

PKVAVITHGGVIRYMVSRLVPDQGFWDTQVIPGQAIKIRLDGDGDSWVGTRLTFP"

CDS 1312819..1313820

/gene="btuF"

/locus_tag="PIKHDJKN_01182"

/inference="ab initio prediction:Prodigal:002006"

/inference="protein motif:HAMAP:MF_01000"

/codon_start=1

/transl_table=11

/product="Vitamin B12-binding protein"

/translation="MNTMNFKTWKGVASLLSAAMLALTLAGCGTTTTNEGTGTSQPAQ

EQSQEQAKTDLKTQYPLTVTDATGESFTFDKAPAKIVSVSPAETESLFALGLDEQIVG

VSDYDDYPEAATKKPKMGGITKPNEESIIAAEADIVFTGISMSEESVKKLRDLGITIF

KTDSKSVDDVMNNIETFGEITDHQEKAQEITTQMKQDVTDVTEAVKAVKPEEKKKVYV

EFSPGWTVGKGEFMDELITLAGGNNVASDLTGWNQINEENIIASNPDVILYANDVVED

NKTLDQIIKARSGWDQITAVKNDAVIGLDANLLSRPGPRVTQGLKEVAKAIYPDLFK"

CDS 1313817..1314863

/gene="hmuU"

/locus_tag="PIKHDJKN_01183"

/inference="ab initio prediction:Prodigal:002006"

/inference="similar to AA sequence:UniProtKB:Q56992"

/codon_start=1

/transl_table=11

/product="Hemin transport system permease protein HmuU"

/db_xref="COG:COG0609"

/translation="MSKKLAVYGTAGIVLLVLTVLICTGIGSVALPVRDIAGILLHRI

PWLGDWIVPDWNTAAEQIIWKVRFPRVLLAVLVGASLAIAGTGFQGVLRNPLADPFTL

GVSSGASVGAAFLIFFGLQYALIGIWTLPLVAFLTGVITLWFVLALAREGRKIPTHSL

ILAGVVMQSFLGAVVSFLSTMSKQTINEIIYWTMGSLALRGWSYTAILFPYFVLGLIF

LWSRARSLNVLALGERQAAHIGVKVDGLKLSVLAVGTLLTAGAVSVSGVIGFVGLVIP

HILRLIVGPDYRLLVPLSAIGGAIFMVWADTIARSLLAPTEIPLGVVTAFVGAPFFAY

LLHRNKKLRKGMMP"

CDS 1314860..1315681

/gene="fepC_1"

/locus_tag="PIKHDJKN_01184"

/inference="ab initio prediction:Prodigal:002006"

/inference="similar to AA sequence:UniProtKB:P23878"

/codon_start=1

/transl_table=11

/product="Ferric enterobactin transport ATP-binding

protein FepC"

/db_xref="COG:COG1120"

/translation="MSTFMKTDLTESRPMIKVTGAGKMYGNHRALHAVDWQVTEGEWW

GVVGPNGSGKSTLLQLLAGTEQPNAGSIQIDGRDISSYTRKDLSRMIAVLQQDGLPAI

SYPVRDVVEMGRYPYQNWLGKEPSDGAAVVDKVLEELGLSDMADRPLDALSGGQRQRV

ALAKVMAQEPRLLLLDEPTTFLDIKYQLQFMELLSAWRQKNHITIVAVLHDLNLAAQF

CDHILALRDGTCVGSGTPHSLMTEENIREIFRVSPAMVNHPDNGLPQLLLRREKK"

CDS 1315678..1316859

/gene="cobT"

/locus_tag="PIKHDJKN_01185"

/EC_number="2.4.2.21"

/inference="ab initio prediction:Prodigal:002006"

/inference="similar to AA sequence:UniProtKB:Q7SIC7"

/codon_start=1

/transl_table=11

/product="Nicotinate-nucleotide--dimethylbenzimidazole

phosphoribosyltransferase"

/translation="MNERGGTPYFDKGSAGCMNNEQVLEQLIGRIVGPHEAVAAEASA

HVDALTKPPGSLGKLEQLVIRLAGITGEARPCFDRRAVIVMAADHGVVEEGISAFPAE

VTPQMVLNFLAGGAAVNVLARHAGAHVICVDIGVNADLDHPDLVSRKVRKGTANMAKG

AAMTRGEAIQAILAGAEVVSTEVAKGTQLFVTGEMGIGNTTASAAVMCALTGTAPASA

VGRGTGVDDVGLQRKAAVVSQALSVNAPDADDALDVLCKVGGLEIAGLTGVILAAAAN

GCPVVVDGFISTAAALIASQLAPVSTEYMIASHTSHESGHAALLRELDLKPMLDLDMR

LGEGTGGVLSLHLIDASCRILNEMATFASAGVSGGTNDTQPTESSSASVDSSVQGEVS

S"

CDS 1316856..1317416

/gene="cobP"

/locus_tag="PIKHDJKN_01186"

/EC_number="2.7.1.156"

/inference="ab initio prediction:Prodigal:002006"

/inference="similar to AA sequence:UniProtKB:P29931"

/codon_start=1

/transl_table=11

/product="Bifunctional adenosylcobalamin biosynthesis

protein CobP"

/translation="MSVLVTGGARSGKSSFAERLCMQRSSEAWYVATAQAYDDEMRER

IRLHQKQRDEAGYLWHTVEEPLALPELITRMGESHTGTSAPTILVDCLTLWLSNVLLA

HEHEPVQVLYDHMDALVAAIHSYPGLLVFVTNEVGDGIVPEYALGRRYRDLAGILNQR

IAAICREVFLVTVGIAIELKSREYRL"

CDS 1317413..1318303

/gene="cobS"

/locus_tag="PIKHDJKN_01187"

/EC_number="2.7.8.26"

/inference="ab initio prediction:Prodigal:002006"

/inference="similar to AA sequence:UniProtKB:P36561"

/codon_start=1

/transl_table=11

/product="Adenosylcobinamide-GDP ribazoletransferase"

/db_xref="COG:COG0368"

/translation="MSKGDLEQKHAAAAAFQFLSRFPVRMQLDFVPPLLRESVVYYPL

VGAVIGLCVWLGGALTGALLPSFPAAVLTLTLWVWLTGGLHLDGWMDTADGLLSYRTR

ERMLEIMKDSRVGAMGVIACVLLLMMKAALIADFIARGNWAYGALLILPMIWSRWFMV

YAMAAWPNARKDDGLAVLFKGLGERREVQRARSSAVGLTILAGVITWAVVWIFQPDTA

MFQVLATENGLGTLPWWLYPVAAIIVVPLAGYVIGKFVAGRISERLGGLTGDTYGAMN

ELLEAALLTVLSLLQGFFLI"

CDS 1318328..1319917

/gene="cobQ"

/locus_tag="PIKHDJKN_01188"

/inference="ab initio prediction:Prodigal:002006"

/inference="similar to AA sequence:UniProtKB:P9WP95"

/codon_start=1

/transl_table=11

/product="Cobyric acid synthase"

/db_xref="COG:COG1492"

/translation="MEDTSAQTSEQTAAVLMLQGTASDVGKSVITTALCRIFKQDGFR

PAPFKSQNMALNSYVTEDGKEIGRAQGAQAEACGIEATTDMNPILIKPVRDMHSQIVV

HGVPLTQMSAWDYRQHFLPKAKQTVMDALNRLRATYDIVLMEGAGSPAEINLKDRDIV

NMNLAGWADAPVILISDIDRGGVFASIVGTLELLEPHEVQRVKGFIINKFRGDLSLLQ

PGLDWLEERTGIPVLGVLPYISDIQIEAEDSVVLDSMRHGKSGQQELDLAVIRYPRIS

NFTDFDALSREPDVNVRYVTSPDELGHPDAILLPGTKDTIGDLAFLRASGLEQAIASQ

TEREHVQLVGICGGYQMLGQHLKDPFAVEANQIQEANGLGWLPLSTRFLQNKQTVRAS

GNVHSHHPFRLSKESDAVNSLPINGYEIHMGVTECLEPEHVTALFQIAHPGGQSFNEG

WGTIDGKVWGTYLHGLFESDQFRRSWLNGLREGKGLAPLLETYSARERKEMEFDRVAE

SLRSALDMQRVYEIMGVQAPE"

CDS complement(1320068..1321768)

/gene="mcpA"

/locus_tag="PIKHDJKN_01189"

/inference="ab initio prediction:Prodigal:002006"

/inference="similar to AA sequence:UniProtKB:P39214"

/codon_start=1

/transl_table=11

/product="Methyl-accepting chemotaxis protein McpA"

/db_xref="COG:COG0840"

/translation="MFRNRTVAGKIRGTLFLVLLVASLLFSISFYAVSMNIMQSYVLP

QFDKVLGTSIQDIYKNTSASKILQVQSGGAGSEGAALTVESYLAGKAKEHNLDAAYVV

AIQDGKATVVIANTSSGMKAGDEIEVEAAMNEATEKKEMEISQVYADSFGVHKAAFIP

IAGSNMIMAVSMDAQFIQDKIAQIFWLCLGITALVFLLGWLISTSMIKRVTKPIIQLV

QHSKQISQGDLTAELQIKGKDEIAQLASSFQTMTHNLKEMISRALSTSNEVVEGSNDL

LKRVESMSGMVKDSSRSAESAEKGSVSIASSAAENARAMEEITQGIMHIASSSAEVSE

QIGDAANEAVNGNRLAQDAIQQMERVGQTASESLRYVETMNERSIAIGTIVQSIFEIT

KQINMLSLNASIEAARAGEHGRGFAVVAGEVRKLAEQSKTATEEISDYLGTIREDAER

SVDAMNRVTQEIGSGTHVVQQAGSAFQQLNELIQNVNLTIQTVSASTQQVSAGAEEVS

ASVEETAQITSKSRQSMQEIASTADLQLNEMDSHSSTVRHLHEQAVELQSAMKNFKIN

"

CDS 1322104..1323501

/gene="tcyP"

/locus_tag="PIKHDJKN_01190"

/inference="ab initio prediction:Prodigal:002006"

/inference="similar to AA sequence:UniProtKB:P54596"

/codon_start=1

/transl_table=11

/product="L-cystine uptake protein TcyP"

/db_xref="COG:COG1823"

/translation="MNTFLIILNVVVMLALLGVLFWMQKKHISFTKRVFAGLGIGVVY

GVILQLIYTSDSEIITKSVDWFNLAGSGYVRLLQMVVIPLIMVSIISAIMNLKGKQNL

GKMSLSIIAILLITTAIAASVSIVTSLSFNLTSIEIEGGDRELAQGQKMEERLVDVKD

QTIPQQVLEFIPSNPFADMTGERRTSTLAVVIFSAFIGVAVLGLDRKKPQQAETFRGM

VNAVYAVVMRIVTLVLRLTPYGILALITKVTATTNPDEILKLIKFVIASYVALIVMFI

IHLIIISLFGFNPITYVKKVLPTLIFAFTSRSSAAAIPLNVETQTKKLGVSDGIANLS

ASFGATIGQNGCAGIYPAMLAVMIAPTVGIDPMSWDFIITLILVVVISSFGVAGVGGG

ATFASLIVLSTMNLPVALAGLLISVEPLIDMGRTALNVNDSMTAGLVSSKILKENDQD

TFNDQSRDLDSAVQV"

CDS 1323761..1324444

/locus_tag="PIKHDJKN_01191"

/inference="ab initio prediction:Prodigal:002006"

/inference="protein motif:HAMAP:MF_02088"

/codon_start=1

/transl_table=11

/product="queuosine precursor transporter"

/translation="MFNLGWGALFVLVTYGFFLLCYRLFGKKGLYAWIGVATVIANIQ

VTKTIDIMGIVLTLGNTMYVSMYLTSDLLNEKYGPGEARKAVWFGFFTLIMTTVLMQM

VLFFEPASTDFAQESMEKLFGMLPRLALGSLTAYFISQFLDVRLYTWLRKVAPGRNQL

WIRTNGSSIISSFADTLVFCTIAFAFIYPWDVWLEIFLTTYVIKFVLTAVGTPFLYAA

RSFKFKDEA"

CDS complement(1324547..1325638)

/locus_tag="PIKHDJKN_01192"

/EC_number="3.4.-.-"

/inference="ab initio prediction:Prodigal:002006"

/inference="similar to AA sequence:UniProtKB:Q7A552"

/codon_start=1

/transl_table=11

/product="putative peptidase"

/translation="MNQTPLSRLEAGLSTQGLDAMLITDPKHIYYLTGFASNPHERFL

GLVLARGEDPLLIVPALDEEAATAASSVSNIATHSDTDNPYALFERYQGRLGRVGLEK

EYVTVARYEQLTAALGAANFEDVGPLLRTLRVKKTPDEVARIRHAIHLIEETLRQGLS

HVRTGVTEIELVAEMEYQMKKLGADGPSFDTMVLTGPKTGLPHGTPGERKLQHGDLLM

FDMGVYAGGYASDITRTFAFGDISPELKTIYNTVLAANEAAIRAVKPGVTCADIDRAA

RQVTEEAGYGERFLHRVGHGMGIDVHEYPSLHGQNMDFLEEGTVFTIEPGIYVTGAGG

VRIEDDVLVTETGVEVLTSYPKELQILTD"

CDS 1325888..1327096

/locus_tag="PIKHDJKN_01193"

/inference="ab initio prediction:Prodigal:002006"

/codon_start=1

/transl_table=11

/product="hypothetical protein"

/translation="MNNRLSVPLYASLLSMALLTACASGDPATESQQTSQGQGAGQGQ

TGEGGKDGASGGEEETDENAVIPYEASVLADGLNVPWELVSVPDGRMFVTERPGTIRV

IENGELASEPLIEFAAPFNEEGEGGLLGLAADPDFDDNGYLYAYHSYLEGGGDIANRV

LRLKVSDGKATMDKELLSDIPGGVNHNGGRIKIGPDNLLYITTGERYEPELAQNEDSL

GGKILRIGLDGSIPEDNPWPNSPVYSMGHRNAQGLAWNSDNGYLYATEHGQRNFDEIN

RIEAGENYGWPEVEGDDDDNGTYQAPLAHSGNETWAPSGVAFIEEGPWAGSLLAANLR

GEQLLKVTLSEDGTQVTQVEPIFKNEWGRIRNVTAGEGGKLYVLTNNRDGRGSPSDGD

DQLIVLTPYP"

CDS complement(1327198..1327929)

/gene="yflN_2"

/locus_tag="PIKHDJKN_01194"

/EC_number="3.-.-.-"

/inference="ab initio prediction:Prodigal:002006"

/inference="similar to AA sequence:UniProtKB:O34409"

/codon_start=1

/transl_table=11

/product="putative metallo-hydrolase YflN"

/db_xref="COG:COG0491"

/translation="MNNQSMDITVLNLHIVTPAGKNPIYPVMLRDEDGITLVDTGMIG

QFAELQSALEHEGVQLSDIKRIIVTHSDIDHIGNLGALVNAVPDVEIWAHSDEIPYIT

GKEPMIKFTPERKALLPEPIAELAEQLISQRTEFKISKVLEDGDMLSLQGGIQVIHTP

GHTPGHICLYFREQQCLLAADALRVVDDELVGPSPPATPDMPEALRSLKRLLGLKLEK

VLCYHGGEYTNEPSQRIAELAESAD"

CDS complement(1328001..1328546)

/locus_tag="PIKHDJKN_01195"

/inference="ab initio prediction:Prodigal:002006"

/codon_start=1

/transl_table=11

/product="hypothetical protein"

/translation="MLTKEDFKKLKKEAKLEIALIEQEVQNLQQKIDSSLYEKDKLWN

DEEIGELTQKRKERKYSSWTIELCSIIEDLLNQLYQQTYQKKFNSIQLMKTPAYRSLS

NIEILQAELKNQHLSLKSGEEKFEEEIAKVFQQRNKLIHSNFSYASIIRENHDAKQEF

ESILDTVKKYRKHLKYNQPEN"

CDS 1328676..1329539

/locus_tag="PIKHDJKN_01196"

/inference="ab initio prediction:Prodigal:002006"

/codon_start=1

/transl_table=11

/product="hypothetical protein"

/translation="MSIPTAIVTGANSGMGLATTIELARQGYHVIMACRSEKRGQQAL

EEALRQSGSTAIELMLCDLGSLESIRHFAQSFRERYDILDVLVNNAGVVMLKRQETSD

GFEQAIGINHLGHFLLTLLLIEPLKSAKQGRIVNVSSGAYKAGKIHFEDPHLHKGYNP

IKSYAQSKLANVLFTRVLARKLADTRITVNCLHPGAVGTSIGVDRNTGFGTRIMALVG

KLPFFLSPEEGAQTTIHLATSPEVAGVTGRYFYQQKDQELKAHALDAAAAERFWTWSE

EQVGLRPEEKL"

CDS complement(1329654..1331630)

/gene="thrZ"

/locus_tag="PIKHDJKN_01197"

/EC_number="6.1.1.3"

/inference="ab initio prediction:Prodigal:002006"

/inference="similar to AA sequence:UniProtKB:P18256"

/codon_start=1

/transl_table=11

/product="Threonine--tRNA ligase 2"

/db_xref="COG:COG0441"

/translation="MSNPNNNSQSSDQSNDQRQPGHPIEIRLQGGAVRSYDAGITVGA

VASSISTSLGKQAIGGIVNGKNVDLDWVLEQDCELVIVTLDSAEGLYRYRHSTAHVLA

QALKRIYGAEQVKLGIGPVIEDGFYYDVDLEHALSISDLAAIEQEMNKIIKENEKISR

RVVSREEALRIFGEIQDPYKLELIQDLPEDAELSIYDQGEFFDLCRGPHLPSTGRIKA

FKLLNVAGAYWRGNSDNKMLQRIYGTAFPSKAQLDEHLHMLEEAKKRDHRKLGKELEL

FMFSEEAPGMPFYLPKGMTVRTELEQFSRELQLQEGYQEVRTPLMMNNRLWEQSGHWE

HYKDNMYFSEVDDATFALKPMNCPGHMLIFKNTLHSYRELPIRMMEFGQVHRHEFSGA

LNGMMRVRTFCQDDAHLYVMPEQIEEEINQAISLIGRMYDIFGFEYNIELSTRPDDSM

GSEELWDQAERALQNVLDRRGVKYRINEGDGAFYGPKIDFHILDALKRSWQCGTIQLD

FQMPEKFDLTYIGEDSLKHRPVVIHRAIYGSIDRFIGILTEHYAGAFPLWLAPVQVKL

LPVSDHYADYALQVQSQLRAAGIRVETDLRSEKLGYKIREAQMEKVPYSLVLGENEKN

ASSASVRAYGQGDQGIQRIEAFIERIQQAVKAKA"

CDS complement(1332108..1333868)

/gene="sasA_8"

/locus_tag="PIKHDJKN_01198"

/EC_number="2.7.-.-"

/inference="ab initio prediction:Prodigal:002006"

/inference="protein motif:HAMAP:MF_01837"

/codon_start=1

/transl_table=11

/product="Adaptive-response sensory-kinase SasA"

/translation="MSIRRRLMTRFIGMLAGAVVLIILLGSAATYWVVQKVNEVSLVD

DFATSGLDQLINTAEIMPDGTVRYDPRLLKQVEKNQGWLQVVDEKGYVIDDFDTPDDV

PKQYKPGELIAYWEAKKPFPYQIVMLIREKNGEMFTLLYGERNQVKSLLDQAREDSHY

TDGELMLRPKQEDAIRSKGAYLQILDAYGQELASYNKPAMGVPSSYNIQELALRIRYP

NRYGISIATFYDEQNGTTWMLSIPVDPTATGEENPYNFILTPVLIVLILSVVILLILL

ALWYANRFGYPMLHMLQWLQRLEQAHYEEPKGIGGVPRSQRRNGKWKRKYHVYAEVLR

SMQALSATLKQDEELRKQTESLREEWIAGITHDLKTPLSSIQGYAHMLEAQKYSWSAE

EVREFAGIMLDKSMYMDRLVNDLAMTYRLRSGGYQPPVEETDVNTLLHDLVQRAERNP

VYGEERIVFRPAKVPVYGHVHIPSFERIVDNLTANALLHNPAESTLTVSVHPGKQAGE

FSVHFADDGQGMDPELVWRLFERYYRGTDTGTSDVGSGLGMAVTKGLIEAMKGRIEVQ

STSGEGTVIRLIWDGQSEHG"

CDS complement(1333865..1334632)

/gene="regX3_2"

/locus_tag="PIKHDJKN_01199"

/inference="ab initio prediction:Prodigal:002006"

/inference="similar to AA sequence:UniProtKB:P9WGL9"

/codon_start=1

/transl_table=11

/product="Sensory transduction protein regX3"

/db_xref="COG:COG0745"

/translation="MDHVSILLVDDEQAILHMLKTVLLKEQFVDIDTVTTGEEAITAC

ENKTYHCIVLDIMLPGKSGLEICPFLRQVTDAPILFLTAKTTDYDKLTGFAVGGDDYV

VKPFNPLEVVARIKSLLKRYLPTKIAKDTTATPPSYDANTSSPAVQEGVYDFGRFQVF

EQAGELQVEGQPVTCPALVYQLLLFFCKHPNRIFTKSELYERVWGSEAISDDNTVMVH

IHRIRERIEADPSNPAFLVNVRGLGYKLIQPNQVSRA"

CDS 1334851..1335999

/locus_tag="PIKHDJKN_01200"

/inference="ab initio prediction:Prodigal:002006"

/codon_start=1

/transl_table=11

/product="hypothetical protein"

/translation="MNRLKEGSKIRANHLNGGESLPKSGYAAHEKLQSKKSWKLLAIA

LGACVMLSACSNSNSITSEQAEQQSKEQTTNTNEASESTHQEENPGTEVSAIITPDNF

IDTLMNGSKDAIYSQMSPELKEALTLEQFKTSADQFLEGVTSFDLVVDAKANHLTEFA

WKDQTGTKGIQAYFTEANQIEGLLIQPLEPHEDTDQTFTKTEFQFPMKGEWYVFWGGN

DVMSNYHYEHETQRYALDIIRTKEAFSYNGGAKVNENYYAFGEPLYAAADGTVVEIKN

DIPDNTPGVMNPEEPAGNNVVIDHGNGEYSITGHIKEGSVAVKKGDKIKQGDPIGELG

NSGNSSEAHLHFQVSDGPDLFTSRSVNIRWVDQSQQLTRGNTIQGLPE"

CDS 1336100..1336603

/gene="ywnH"

/locus_tag="PIKHDJKN_01201"

/EC_number="2.3.1.183"

/inference="ab initio prediction:Prodigal:002006"

/inference="similar to AA sequence:UniProtKB:P71043"

/codon_start=1

/transl_table=11

/product="Putative phosphinothricin acetyltransferase

YwnH"

/db_xref="COG:COG1247"

/translation="MKLDSVQIEYARREDLPRIVEIYNSTIESRMVTADLEPVTVEQR

IPWFEEHSSDHRPLWVMRHEGQIVAWASLSSFYGRPAYNGTVEVSVYVDQQCRGIGAG

GRLLNTIFDACPALGIKTILGFVFGHNEPSLGLLRKHGFEQWGYYPEVAVLDGVNRDL

AILGKKL"

CDS complement(1336693..1337193)

/locus_tag="PIKHDJKN_01202"

/inference="ab initio prediction:Prodigal:002006"

/codon_start=1

/transl_table=11

/product="hypothetical protein"

/translation="MFTRNDDIRSQIWEAVSGLNEEQLNLQPTPDHWSIMQVLRHLNL

MESIIVKQARAALEKEQTVSVDKKPYELTLDRSHSVNAPSHLQPPAEPETLANVRHDL

EDSHQALMHVAQDHEAELLRSKSFLHPIFGEMDLVQWIDFASYHEERHLAQIQDIKQR

LGVSHT"

CDS 1337473..1337886

/locus_tag="PIKHDJKN_01203"

/inference="ab initio prediction:Prodigal:002006"

/codon_start=1

/transl_table=11

/product="hypothetical protein"

/translation="MLVIDEVYHHTALQISSSDLLYLIQQLKVKKENEIETLKHKIEQ

FEQKKRAEEVAYQSLSTVRKWFAGRPASHHQAVEYMVQVKERFRKMEQIRRRIRELDR

IAERIKHPDSIERDEIELTPETIREIRQLRETEDV"

CDS 1337889..1339178

/gene="nrgA_1"

/locus_tag="PIKHDJKN_01204"

/inference="ab initio prediction:Prodigal:002006"

/inference="similar to AA sequence:UniProtKB:Q07429"

/codon_start=1

/transl_table=11

/product="Ammonium transporter"

/translation="MTLETLSSGIDTVWVVLSAAMILLMEGGFALLEAGFVRYKNSVN

IIMKVFADITIGTLLFYAIGFGLMYGSDAGGFVGITGFFLNGDLSHLDVPVSLETFWL

FQAAFTIAVISIVSGAVAERINFRAYLLYIILMTAIIYPIGGHWAWGGGWLSKLGMQD

FAGSAVIHALGGFSALAAAIIIGPRKGKYTPLGVSAIALPSNLPLASVGAFLLWFGWF

GFNAGSTLSATDVRIGHIAIVTMLSAASGGAVTLLYTLFRFNRSDAPSVINGSLAGLV

GITAGCAFVGDVAAIFIGAVSGLLMMAATNWLDRRQIDDPVGAFPVHAVSGMWGTIAV

GLFATDGGLFMGGGWRLLGVQALGLTALVVWGFVMTWIGLKLIGKIVPVRSTEEEEDL

GLDISYHGVMAAHQSHEFLDGEEHIRAFQDDSPNRDR"

CDS 1339334..1340278

/locus_tag="PIKHDJKN_01205"

/inference="ab initio prediction:Prodigal:002006"

/codon_start=1

/transl_table=11

/product="hypothetical protein"

/translation="MIKPEQCERLTRKARKTLEEYGLGNAADLLLSSSRWGIRLDVST

IDEYRRTGNSRVGGNPDLPGNMKWPLTQDGVPMTFLAQLNLVDLTPYTPNDGRGSLPE

RGMLYFFIGVDEPAYHIDHRVIYEPDAHNLIRREPDGETALNEDDFVAHSVTVLPNLE

FPTYAYIDSLALNAIAAPLSGGTVAVEGEEGLYDRYLEFESSWNHPSTQNWGGMFGYP

DGQHPDAEHQALLQIVLGEEYAYDKQACEKKLTAHYGGDEERTQQELADTLLLLKIDT

HDAIGFQWWDCGELQFFIRKADLLAGRFDQTYCSLYSS"

CDS 1340462..1340920

/locus_tag="PIKHDJKN_01206"

/inference="ab initio prediction:Prodigal:002006"

/codon_start=1

/transl_table=11

/product="hypothetical protein"

/translation="MIQSAFKHIDVAVTSLINLCDQLSEQDLALIPIEGKRPVGELLS

HLSVICRADVHISEGASEEEMALFYEENQPNTLSEIREALIENQMYLYHRYRQFNTEE

LLQVTDSYWGASYSRLEWLLEIMGHVYHHRGQLHTMLTLTGIEPKVALFE"

CDS 1341284..1341988

/gene="phoP_1"

/locus_tag="PIKHDJKN_01207"

/inference="ab initio prediction:Prodigal:002006"

/inference="similar to AA sequence:UniProtKB:P13792"

/codon_start=1

/transl_table=11

/product="Alkaline phosphatase synthesis transcriptional

regulatory protein PhoP"

/db_xref="COG:COG0745"

/translation="MAQPATILLVDDEQDIIKLMEIYFGNEGYRVLTASDGIEALEQL

KKEPIDLIILDVMMPNMDGIEACMKIREEQKMPIIMLSAKSMDMDKITGLSIGADDYV

TKPFNPLELVARAKSQLRRYHTFNEGRESKEHEWVIDDLVINTDTHEVVWVDEQPVRLT

PREFAVLELLARHQGSVLSMEQIYRQVWKEEFMESNNTVMVHIRKIREKIELDSKHPK

FIQTVWGVGYKMIKPQ"

CDS 1342044..1343180

/gene="rcsC_4"

/locus_tag="PIKHDJKN_01208"

/EC_number="2.7.13.3"

/inference="ab initio prediction:Prodigal:002006"

/inference="protein motif:HAMAP:MF_00979"

/codon_start=1

/transl_table=11

/product="Sensor histidine kinase RcsC"

/translation="MNSKLINTVRWKFIYAFLLSGILTAAILYGGSKVVHSILAAQTY

PDYSIPARGIRWLVNNIGSVPLMIMVGILGFVLFFFLFTRRVMLVLDEITAGIQEVAK

GELSHRIEVKTSDEFGVVAASINQMAEQLQLSLQEERSAVAAKNDLITGISHDLRTPL

TSILGFLEYIEKDRYQDEIEMRYYVSIAYEKSLTLRKLIDDLFEYTRVSGGSLPLSLT

SLNLNPFLMQLAEEFAPMLEEAGMTYKIIGGQEPLWINAAPGELVRAYENLFSNAIRY

GAQGKVMEIGLSLEGEEAVVRISNYGDPIPAQDLLHLFDRFYRVDKSRSRDTGGTGLG

LAIAKSMIELHHGSIAAYSEQGRTDFVTRFPVTAPPMNSDPEER"

CDS 1343349..1344137

/locus_tag="PIKHDJKN_01209"

/inference="ab initio prediction:Prodigal:002006"

/codon_start=1

/transl_table=11

/product="hypothetical protein"

/translation="MRLLLRTVRLSYIYICGASALLSAALLFVVYHMLRFAHHHILSE

TTGITRIMYWMINHIGTTPLVIFIGGGMFALFFWIRSQKIADDLKVIGRGTHELANGR

IPAQIEVLNGGELRQIAADLNDIAIRIQAERLNSSDQEWTDRDRRGEEWFSSGMYDDE

SSRQQMMLESERIHKQRIQADLVGSQSGRISADLPIKLTIYGVRSSLDTIIEGRCRDE

AEIQHWVRLAYEQTLRLELALDSVEMKPQEHSSSNPHVFREPEC"

CDS 1344357..1345988

/gene="pbpF"

/locus_tag="PIKHDJKN_01210"

/inference="ab initio prediction:Prodigal:002006"

/inference="similar to AA sequence:UniProtKB:P38050"

/codon_start=1

/transl_table=11

/product="Penicillin-binding protein 1F"

/db_xref="COG:COG0744"

/translation="MKDMPDLLKQAFLATEDRRFYNHDGLDFIGIGRAVVQDILHMNL

SQGGSSITQQLARNLYLNGDKTLVRKVNEMSIAMAMEKRFSKDELLELYLNHIYMGRQ

QYGVKAAAWRYFGIKDLHQLEIWQMATLAGIPKGPSIYNPVDREDLSKERRSVVLGLM

HEQGIITKKQMEEARSVDYPPSNSTSASTPGSTVPAYVSALDAVIEEASRLTGKSGAE

IKSAGWTIRTGLDDQAQLAMEQAFRESSRFIDDREDEQVQASMVILDQHNGEVKAMMG

GRDPVKGGINRATTDARQPGSAFKPIIAYGPALESGRFKPESMLPDQRMKYGSYQPSN

LGGRYRGTVTMSQAIENSINAPAVWLLHKTGLGRAHQFAGRLGIELGKEDLNLSIALG

GLHKGVSPLKMAQAYSVFANNGKSNTAHLIREITDSRGRTIYVFKPENKQVISTSTAT

AMTGMLQNVVSQGTGSRAQLSQHKVAGKTGTTQAALPGVSKEANRDLWFVGYTSKWTA

AVWMGFDRTDAEHYMRSGSGVAADLFASVMKRTMP"

CDS 1346054..1346158

/locus_tag="PIKHDJKN_01211"

/inference="ab initio prediction:Prodigal:002006"

/codon_start=1

/transl_table=11

/product="hypothetical protein"

/translation="MDRTAIKQMDTTMKTKHCILDVHGSTLAGNIEDP"

CDS complement(1346231..1346680)

/locus_tag="PIKHDJKN_01212"

/inference="ab initio prediction:Prodigal:002006"

/codon_start=1

/transl_table=11

/product="hypothetical protein"

/translation="MLHFIEQALTSFVASAAFGIIFNAPRRMLLHGGFVGMIGWIIYI

VLEYAADAVPATLAATIAVGVISQLFSRMFRAPVIIFSVAGIIPLVPGGLAYNAMRNF

VQNDYGAAMEMAAKALMLSGAIAIGLVLSEVLNQMIRRIPESLRAKS"

CDS complement(1346700..1347512)

/gene="yjjP"

/locus_tag="PIKHDJKN_01213"

/inference="ab initio prediction:Prodigal:002006"

/inference="similar to AA sequence:UniProtKB:P0ADD5"

/codon_start=1

/transl_table=11

/product="Inner membrane protein YjjP"

/db_xref="COG:COG2966"

/translation="MEYIPHDPPPVPQSETELVGTNHTEQSRVIAICLLAGKIMLQSG

GETYRVEDTMKRMAAALGLPHSHSYVVPTGIFFSVDATEPAKLIRISERTTDLHKVSE

VNAVSRRIGQGELSVEDAYALLQQIEGQPSSYSAVVRLTAAALSSGCFTIMFGGGWVD

FLPAVLCGGIGYAAVIAFHRLVLVKFFAELTASFVIGLLAFLLVYMGAGHERDKIIIG

SVMPLVPGLLITNAVRDLMAGHLVSGLSKGAEAFLTAFAIGTGIAVVFSLFT"

CDS complement(1347763..1348077)

/locus_tag="PIKHDJKN_01214"

/inference="ab initio prediction:Prodigal:002006"

/codon_start=1

/transl_table=11

/product="hypothetical protein"

/translation="MCIMSESTSTPSARVVYQANQPMLQSVQSVRDMLHHTARQHVGK

KVQVQNIDGQVWEGVIISADRGILYLQVTPMHGYPEPRALFGPTILPLVLYELLVITL

LM"

CDS complement(1348133..1349176)

/gene="mccF"

/locus_tag="PIKHDJKN_01215"

/inference="ab initio prediction:Prodigal:002006"

/inference="similar to AA sequence:UniProtKB:Q47511"

/codon_start=1

/transl_table=11

/product="Microcin C7 self-immunity protein MccF"

/translation="MNNRSIRYPQPLAPGNTIGVAAPSSGVSESLHHYLEESRHNMER

LGFGVQESPFLRHNAKCVSSSKEDRAAEINAFFRDPEIQAIIPPWGGEFLMDILPLLD

WKALKTLPPKWVMGYSDISTFLFAYTLITGTASAHGTNYVDLRSSELDPVTAQWIDVL

QTGQGGQITQSSSTHYQSEWKPELAGFNLDTPSRWKQLGQPEGANSQVTFSGRLIGGC

MDTITSLIATPYAPVEVYLDQYCAEEGTIWYLESCEMNAGDIYRHLWQMRQAGWFAGV

KGFMLGRPAGYSDTGDFNFTDALSSALGDMNVPVLYDVDLGHIPPQLTFVNGALGNVA

YENGHGRLEMAFV"

CDS complement(1349271..1350608)

/locus_tag="PIKHDJKN_01216"

/inference="ab initio prediction:Prodigal:002006"

/codon_start=1

/transl_table=11

/product="hypothetical protein"

/translation="MSDEMKPFEAIEQRLNDRKTEYDAMSVPDAAAYQAVQAGIRQAA

RKRKSRLRWYMSTISAAAIILVFTGCIRVSPAFASFVEQLPGMEGIVSMIRQDKGLMM

AIDQSLLQKVGVTDEHNGASMTVDSIITDESRMVIFYTMKGMKDPDKGMYDIDLLDEH

GKNLPVGFSYSSPKPTSDENVYENYIDVIFTGDASPPEELSVVFKSRYKGSSKEWKVT

FPVDTDLTKGMKKIIPVNQTLMVDGQRIDVKQATLYPTRFVLDVKFDPKNTKKIFGLD

DLQLVDEQGRAWRTNTTTGEDERSIYFESMYFSIPKKLTLQGSGFSGLNKNELDIVVD

LKSHKITGGPSGLKMLGSKVVGKDLVIHFSVDGSMEGKFILAFGDVMDKAGKSYQIPG

SNWSSGDGTDERHTIISSTIQNGAKIKGEIKMHISTYPMKFKSPFSMEIPVNP"

CDS complement(1350598..1351119)

/gene="sigV_1"

/locus_tag="PIKHDJKN_01217"

/inference="ab initio prediction:Prodigal:002006"

/inference="similar to AA sequence:UniProtKB:O05404"

/codon_start=1

/transl_table=11

/product="RNA polymerase sigma factor SigV"

/db_xref="COG:COG1595"

/translation="MTPAQLTIAAQKGDAEAFAALMEMHQSRLFRIAYAYLHNEGDAL

EAIQESTYRAYRKLKKLKEPSYFATWLIRILLNYCADERKRKSRFSHVTEIHEPSSWD

RPADPDLAAAVSALDRDCKQIIILSYFEGFSLTEVADILEIPTGTVKSRLHRALGQLR

NQLETKGDVTHER"

CDS complement(1351360..1352460)

/gene="rppH_1"

/locus_tag="PIKHDJKN_01218"

/EC_number="3.6.1.-"

/inference="ab initio prediction:Prodigal:002006"

/inference="protein motif:HAMAP:MF_00298"

/codon_start=1

/transl_table=11

/product="RNA pyrophosphohydrolase"

/translation="MAPRHLNAFQGSKVRLAAPCPEDCMRLSRFTDSYDYLRNLDTDI

AVPLTPDETGSSAVRRDNGFEFALRTLEDNRFIGFTALYRIEWNNRSARLAIGIGDDK

DRGKGYGSDALALIIRYGFHELNLNRIALEVIEYNETARRAYLKAGFREEGRQRSAVL

RDGTHYDLILMSILAEEWSTQTKLPLAFQSTHAQGTKQNEDHSDSRLVAAEGSALSLL

NAKGTENSPVQPRIGVGAVILNERNEVLLVWRNRQPEQYTWSIPGGKVDPYESIETTV

IREIKEEVDLDIIIDKLLCTAETIRPEQQEHWISVLYSTRVIGGVARNLEEGGAIGEI

GWFPLDDLPSPLASFAVPGLEAAKKLHLYSED"

CDS complement(1352690..1353100)

/gene="flgB_1"

/locus_tag="PIKHDJKN_01219"

/inference="ab initio prediction:Prodigal:002006"

/inference="similar to AA sequence:UniProtKB:P24500"

/codon_start=1

/transl_table=11

/product="Flagellar basal body rod protein FlgB"

/db_xref="COG:COG1815"

/translation="MIETNTSRRNESLLQALNVQHKVTTENIANADTPNYKKKSVEFE

EELRRIIENDKTDQLDMKRTHEKHFPVSNPNDSIVNYRIVENNETSMNNNNNNVDIDK

EMANLSENQLMYNYMVDRVSGHYKKMKNLLQDMK"

CDS complement(1353282..1354097)

/gene="speD"

/locus_tag="PIKHDJKN_01220"

/EC_number="4.1.1.50"

/inference="ab initio prediction:Prodigal:002006"

/inference="similar to AA sequence:UniProtKB:P0A7F6"

/codon_start=1

/transl_table=11

/product="S-adenosylmethionine decarboxylase proenzyme"

/db_xref="COG:COG1586"

/translation="MTLTPEQRIQLHGFNNLTKSLSFNMYDICYTKTKDEREAYIEYI

DEQYNAARLSKILNNVSDIIGAHVLNVAQQDYVPQGASVTMLVSEGPIVEIPEESFDE

SPGPLPDNVVMQLDKSHITVHTYPEFHPSEGISTFRADIDVSTCGEISPLKALNYLIH

SFDTDIMTMDYRVRGFTRDTSGRKLFIDHEIGSIQNYIPDEIKSSFDMIDVNVYQENI

FHTKCKLKEFDLDNYLFGYTKDKLSKAEQQEITEWLKLEMDEIYYGKNINRPS"

CDS complement(1354221..1354802)

/locus_tag="PIKHDJKN_01221"

/inference="ab initio prediction:Prodigal:002006"

/inference="similar to AA sequence:UniProtKB:P50838"

/note="UPF0398 protein YpsA"

/codon_start=1

/transl_table=11

/product="hypothetical protein"

/translation="MLKNVLITGYRAHELQIFGQKHEGIPFIKKAISSRLTPLVEDGL

EWVLTPGQYGVDLWACEVVLELKKTFPDLKLSIITAFQNPEEQWKEDKQEYYRSILQG

VDYYGAISRQPYIGPWQFTARDDLLLRKSDGLLLVYDEDAGDGSPRFIKEKAVKKQQN

EDYTIISITSEDIQSVAEDERMNDVGDYEDPSF"

CDS complement(1354914..1356047)

/locus_tag="PIKHDJKN_01222"

/inference="ab initio prediction:Prodigal:002006"

/codon_start=1

/transl_table=11

/product="hypothetical protein"

/translation="MNKRKRPTGNTSAKGKTSAGSSTARSGRSTFTSAKSSGPSASRN

TGATKKPVASKSSAAPKKAGAPKPKAAKRSGNSYYRKQEPPRPYKVTEPDELLNFLLK

HLKSGRNAVKSILGRGQVSVDQKVVTKFNLELTPGQIVTISKEGAVAAPSLTGINILH

EDDDIIVIRKEAGLLSIAADKSDDLTAYRQLTEHVRRTDALNRIFIVHRLDRDTSGVM

MFAKSEEVQQKLQDNWKENVQERLYVALVEGAVAKEEGTISSWLKETKTLKMYSSSRP

NDGQHAITHYKRLQSNREFSLLEVRLETGRKNQIRVHMEDLGHPIAGDKKYGAHTKTL

GRLGLHARVLSFIHPTSGELMRFETDIPKQFLYPFRTDAPPAN"

CDS 1356237..1356653

/locus_tag="PIKHDJKN_01223"

/inference="ab initio prediction:Prodigal:002006"

/codon_start=1

/transl_table=11

/product="hypothetical protein"

/translation="MGVFLDMRTSMNSGTVGQPGLSLGPSPEAFGSIGLQTLGVPNPI

ITLNGTVGVTGEVGDTFVVELVRGSVYDPFFVIYRAEGTIGQNGGAEFHSFTAQDLSA

PPALESVYTSFISGVSTSVRTGPEVFYGIAATDWFL"

CDS 1356622..1356747

/locus_tag="PIKHDJKN_01224"

/inference="ab initio prediction:Prodigal:002006"

/codon_start=1

/transl_table=11

/product="hypothetical protein"

/translation="MVLQPPIGSYEQKFQQEECYHMASYDEQTKIPALRSPGFLL"

CDS complement(1356764..1357474)

/locus_tag="PIKHDJKN_01225"

/inference="ab initio prediction:Prodigal:002006"

/codon_start=1

/transl_table=11

/product="hypothetical protein"

/translation="MSPIYLEMDGVLDQLEAAVRDKRPFSLVRVGDGENIVMSQETVW

STEQVLQERWAIKANLGQKGLRLPNLQLRDEVAASLQRADIVGVLPHGDSTIKAPEHL

KRQLTDTVFAHFGIAPALTCHACVNRELAQVPRFWNMLAGKRILLVTREIEQLRAMLV

QEPYHLNITAALPFDGWDQMQETLQWIQTNQDTFDVALFSCGVNAVVLAERTAALAGK

VAIDFGKANNIILKGRAN"

CDS 1357828..1358517

/locus_tag="PIKHDJKN_01226"

/inference="ab initio prediction:Prodigal:002006"

/codon_start=1

/transl_table=11

/product="hypothetical protein"

/translation="MSRKVVIEINFNNYGMDPQRLTREWLERRMSIFRTFTLHCLKAQ

TNQDFLTVVKLSKESGELMQDILAEQEPLPSNIRFGTTIESVRAILAFAEGAEEIYMA

RLDSDDLYHRTFVQQLYDVQPQPGTIALINQNGYLWDSVNHEMAPAFHRSPQFYVYLY

KTAEYAAGYRVKLPGRGTHGNVIELPHELLAPRNYVNIVHSNNTSIKKVPPKDRLSRE

EMAAVLREFMI"

CDS 1358535..1359524

/gene="pseB_2"

/locus_tag="PIKHDJKN_01227"

/EC_number="4.2.1.115"

/inference="ab initio prediction:Prodigal:002006"

/inference="similar to AA sequence:UniProtKB:O25511"

/codon_start=1

/transl_table=11

/product="UDP-N-acetylglucosamine 4,6-dehydratase

(inverting)"

/db_xref="COG:COG1086"

/translation="MIKGQTILITGGTGSWGQKLTEVLLEQNPAEIRILSRNEYAQIT

MQRDFGHDARLRFVIGDIRDYRAVEEACRGVDVLFHLAALKHVPVCEDQPDEAFKTNV

IGTQNIIRAAIRMQVAKVIDVSTDKAVDPINVYGMTKALGEKMMIRANGLSESTRFVC

IRGGNVLGTSGSVVPLFRRQIDEGKSLTITDKGMTRFFLTRTEAIHLLLKAAEAAVGG

ETFVMKMKACKMTDLASVMLEQAGRPAHDYKVTGIRPGEKLHEVLISPFEAPRTYKYD

EQYYVILPQEPDKSLTDPYSGLPRVIFSEYRSDNAMMNRSAIAQFLRAGGFID"

CDS 1359559..1361112

/gene="wbpA_1"

/locus_tag="PIKHDJKN_01228"

/EC_number="1.1.1.136"

/inference="ab initio prediction:Prodigal:002006"

/inference="similar to AA sequence:UniProtKB:G3XD94"

/codon_start=1

/transl_table=11

/product="UDP-N-acetyl-D-glucosamine 6-dehydrogenase"

/db_xref="COG:COG0677"

/translation="MVESSKHEGNKETQGDTTLQNINNRSLKAAVIGLGYVGLPMAVE

MAQAGYQVQGIDIDGAKVAKLRAGKSYVIGIEDKVVQSLAQAGLFMAGTDFAAVGQAD

VIVICVPTPLTAEHQPDISFIAAAVDGITPYLQEGGLVILESTTYPGTTEERVKQPIE

AAKGWKVGQQFHVCYSPERVDPGSVHYGVKNTPKIIGGSTPACLSYGKAFYGSFLNEI

VPVSSTTVAETAKLFENTFRSVNIALVNELTPACEQMGVNIWEVLDAAATKPFGYMPF

YPGPGIGGHCIPIDPIYLSWAAGRQGSELQFISLADATNRQMPERVVKRAAELLKQNG

SSMKGARIVLAGMAYKKDIDDLRESPALDVYRLLREAGADVVFTDPMVPLFRKDDGTV

AQAQPASPALWAGADLVMITTDHTGFDYQEMADHAKLIFDTRNATAGCVGGNIVVLGQ

PIRPEVNVVQDVNEVHEMQIHENMRVSQTVDGDKRGHTETIKSDHEAHSEPHLKQSHE

NSGGSHGES"

CDS 1361099..1361845

/gene="COQ3_1"

/locus_tag="PIKHDJKN_01229"

/EC_number="2.1.1.222"

/inference="ab initio prediction:Prodigal:002006"

/inference="protein motif:HAMAP:MF_00472"

/codon_start=1

/transl_table=11

/product="Ubiquinone biosynthesis O-methyltransferase,

mitochondrial"

/translation="MANHDRVSERYYGEINSEESHEATRTRVHWMCREATGKRILDVG

CSQGITSILLAREGFRVTGIDLEEESIQYAREELAKESRPVRNNVDFRLLDITQWKAR

TTFDTVLLGEVLEHFAHPETLLLQIHRLLQEEGTLVVTVPYGYHPFYDHKQTFYAGNL

AMCLLPYFEVLKLEVHHKYLCCVARKRRKTQLYMSPTLNQLTEWMELDHVHFAAVEKN

HLRLMNQRKKAMDNAVDQVKRLRRQETGEG"

CDS 1361906..1363042

/locus_tag="PIKHDJKN_01230"

/inference="ab initio prediction:Prodigal:002006"

/codon_start=1

/transl_table=11

/product="hypothetical protein"

/translation="MITISLCMIVKNEQRTLARCLDSVAGIADEIVIVDTGSSDRTME

IAAQYTDRVYTYEWNDDFAVARNYSFDQATQEYILWLDADDVLLPAERVKLERLKQQL

PSDGETAAVILGYTLAEGPEGSPLVTDRRLRLVKREAGCRWHGRLHEQLGFPQSGVIT

ADIAVTHRREAGHHSARNVRILRKWIAEEGVAQGRLLFYYAGECYDRRCYAAAVRAYA

KLLLEPSGYREDRLIACARLAECYERLGEPGRKLGALLQSFQFDLPHADFCCAIAACF

QERQEIVSAIYWYMQALDVSSRDPGLRPVPAACRTWLPHARLSLCYAHIGNWEQALLH

NSKAREYLPNDPGLLANRQKLEAVVSKGNRGPEEKEKEKEKEKE"

CDS complement(1363123..1363647)

/locus_tag="PIKHDJKN_01231"

/inference="ab initio prediction:Prodigal:002006"

/codon_start=1

/transl_table=11

/product="hypothetical protein"

/translation="MSKQHAHASDCSNISPPKSWIRRHPIITTFSILIFVLFAAVFLY

FWLRPYFSTFDRSEQGDLSYSMPSRNGEYTAEIYGVPYGGAAGGVTIWVDVKKTNDPN

DSTVKTIYRAKHHGHNHLEWEDEHTLRIENWNEYVDETITLNIDEEIYDGRGWACRSL

RTKDQYVRCFAREK"

CDS 1363979..1364833

/locus_tag="PIKHDJKN_01232"

/inference="ab initio prediction:Prodigal:002006"

/codon_start=1

/transl_table=11

/product="hypothetical protein"

/translation="MSDLLDPRNDFVFKRIFGSEENKDVLLVFLNRTFAESGEQPLTE

IVLLNPYTDKDAPLDKQSIFDIWAKTAEGKLINIEMQLFNKYDIEKRTLYYWSKRYSS

QLQEGQTYKELKKCVTINILNYSFIENERYHNVFHLREDHTGLELSDDLEVHFMELSK

LDDQSVPMEGGLINWLLFLKGADKSNWEVLQMNEPTLKKAMDTLEFLSQDREARRLYE

ERQKYLHDEASMIEWATEKGIAKGIVEGEQKKAIEIAKNMLSFGIEVSVIAKTSGLTE

SEVEALKD"

CDS 1364887..1365144

/locus_tag="PIKHDJKN_01233"

/inference="ab initio prediction:Prodigal:002006"

/codon_start=1

/transl_table=11

/product="hypothetical protein"

/translation="MNEPTLKKAMDTLEFLSQDREARRLYEERQKYLHDEASMIEWAT

EKGERKKALEIAKNMLSFGIEVSVIAKTSGLTESEVEALKD"

CDS 1365489..1367369

/gene="recQ"

/locus_tag="PIKHDJKN_01234"

/EC_number="3.6.4.12"

/inference="ab initio prediction:Prodigal:002006"

/inference="similar to AA sequence:UniProtKB:P15043"

/codon_start=1

/transl_table=11

/product="ATP-dependent DNA helicase RecQ"

/db_xref="COG:COG0514"

/translation="MSVQTPTLEEAQGLLQKYYGYPDFREGQKKIVASLLEREDTLGI

MPTGGGKSICYQIPALLYSGLTLVVSPLISLMKDQVDALTTAGIAAAYINSTLSGKEV

NDRIRAAQRGELKLLYVAPERLELDWFREEMGYLPISCVAVDEAHCVSQWGHDFRTSY

LAVAPFVDSLPERPVVAAFTATATPEVMGDILRLLRLQDPQTYVTGLGRDNLAFSVLR

GESKKDFVLNYAREHASEPGIVYAATRKDVDDLHQRLLQAGLPAGRYHAGMTDDERSQ

SQEAFLYDDIRVMVATNAFGMGIDKSNVRYVIHNSMPKNMEAYVQEAGRAGRDGEPSQ

CILLFGAQDIITQKFLIEQNPMEGDRKQNDYRKLQQMVDYCYTTRCLRSAQLDYFGEV

HEDKPCGICSSCTDDRELVDMTIDAQKIFSCIHRMRERYGVSLVASVLKGSRAKKVLD

YGFNSLPTYGVMGNRTEREIAEIINVMVSEGYLMLSEGQYPVVRLQPLAVEVLKGQRE

VMQRVVRKTAATASGTNYGGRRGRDAMPSAVNETVFEQLRLIRRDLAAKEHVPSYIIF

NDATLREMSVVCPQTERDMLDIKGVGEVKYRKYGLPFLEFFQKQTGEEVDTDDDAFYE

YE"

CDS 1367442..1369451

/locus_tag="PIKHDJKN_01235"

/inference="ab initio prediction:Prodigal:002006"

/codon_start=1

/transl_table=11

/product="hypothetical protein"

/translation="MKVILSTLNAKYIHTSLALRCLKAYSEKDFDIELAEYTIKDPVM

NIVSDLYQRGADMIGFSCYIWNIEETIKVIDILKKVMPEVKILLGGPEVSYDTEYWMN

RIPNVDFIVMGEGEETLHQLLTELEGDKKFHFVYGLAYRKGEEVILMPGRPKADLNDL

PSPHRFEEDIPELGKRVVYFETSRGCPFSCQFCLSSIEVGVRYYDIERTKSDILYLIE

KGAKLIKFVDRTFNIKRDYALEMFKFLIENHQGTVFQFEITADIMRPEVLDYLAENAP

PGTFRFEIGVQSTNDPTNELVKRRQNFSKLSRTVNKVKASGKIDQHLDLIAGLPEEDY

NTFRKTFNDVFALGPEELQLGFLKMLRGTGLRLDAEKYNYTYMDHAPYEILGSDVLPF

SDIVRLKRLEDVLEKYWNAHRMDHTLKYLMEQEFDSPFDFFQAFGDYWEGQGWQKIGH

QLEDLFTRLHGFLESRNTPHMDIVLGLMKLDYFLGHKYKPRKIWWEDPLQKDQWAGYM

KTLAERPEDVRLPRIAGATGTAWLESSGTSAIAAGENPAAAGEDSAADAAGVIGSDAI

GSAGDVAADGVDGNASRALPMTSAMTAQTVMGARAFADLGLGEKELQKHTVLDVLPFR

LERVLAGASPLAAKGRTLLVVVYQQHEGQQAQYYTLPLGEEAAAM"

CDS 1369770..1370450

/gene="xerC_1"

/locus_tag="PIKHDJKN_01236"

/inference="ab initio prediction:Prodigal:002006"

/inference="protein motif:HAMAP:MF_01808"

/codon_start=1

/transl_table=11

/product="Tyrosine recombinase XerC"

/translation="MTNKATTKETITITVGSTDSLHIRIPYNLAYVQCIRQITGRRWE

PQLKVWIIPYTIDAVQEFISQFDPNDVHITPELWIESEDLQRWKASRGNNNSWSKEPL

QKALKLRGYSRKTIKAYCNQVERFLKHIPFEKTDVKTSNVQTYCLDLLERGISHSSVN

QTISALRFYCKHVLHHPTEIQYIRPKKQTKLPQVLSEKEVAQLLKSVTNPKHKAILFH

LFFWTSCK"

CDS 1370649..1370885

/gene="xerD_2"

/locus_tag="PIKHDJKN_01237"

/inference="ab initio prediction:Prodigal:002006"

/inference="similar to AA sequence:UniProtKB:P9WF33"

/codon_start=1

/transl_table=11

/product="Tyrosine recombinase XerD"

/db_xref="COG:COG4974"

/translation="MQKVFEEARQRAGIVRKVSIHVLRHSFATHLLENGTDLRYIQEL

LSHTSARTTQRYTHVSTKNIQRIQSPLDRMDLRD"

CDS 1371101..1371553

/locus_tag="PIKHDJKN_01238"

/inference="ab initio prediction:Prodigal:002006"

/codon_start=1

/transl_table=11

/product="hypothetical protein"

/translation="MTKNKLLRMDNVGIVVESLDDAISFFEEIGLNLEGRATVEGEWA

GRVTGLGSQCVENAMMVTPDGHSRLELSRFLNPPTISDHRTAPVNSLGYLRVMFTVQD

IDEMVSRLTKYGAQLVGEVVQYEDSYRLCYIRGIEGLLIGLAEQLGNK"

CDS complement(1371888..1372175)

/locus_tag="PIKHDJKN_01239"

/inference="ab initio prediction:Prodigal:002006"

/codon_start=1

/transl_table=11

/product="hypothetical protein"

/translation="MHNSLEILLDSLIRNRIEALYSDLLKNNTSYNQFSSDRNLYFKQ

LHELLPQDMHKTLFLYDDADLSVQTILEPEIYLQGFKDALQLHNELNITST"

CDS 1372346..1372546

/locus_tag="PIKHDJKN_01240"

/inference="ab initio prediction:Prodigal:002006"

/codon_start=1

/transl_table=11

/product="hypothetical protein"

/translation="MISYKPFQKLLIDREIKKQDLLKMTGISSATMAKLNTNEYVSLE

VIDKLCTALECQPGDLLEHLTD"

CDS 1372760..1373320

/locus_tag="PIKHDJKN_01241"

/inference="ab initio prediction:Prodigal:002006"

/codon_start=1

/transl_table=11

/product="hypothetical protein"

/translation="MPYPFDCISEFMFFETAIETSDVILIPGGSHPQLMERAAELYHQ

GLAPYILPSGGSTPNVSSTEWEFLRDVGLNLGVPESSILREDKATNTFENARYSWESL

QQLGINPRKAILVCKSYFARRALLTYQVEFPSEVEFYVCSVTDKTGTTRDNWFLDEQK

INYVMNELTKIGQYFKHHIPNWINNK"

CDS 1373534..1374181

/locus_tag="PIKHDJKN_01242"

/inference="ab initio prediction:Prodigal:002006"

/codon_start=1

/transl_table=11

/product="hypothetical protein"

/translation="MQLIHRRGWGIFEDEEEVYFHLMGEKGEDLGYAKWSNSQVNTIS

KQEYFYHKYGPYYVEIMNYFQDNITDSCRSLDGSVYVSHYQQSDIHKFDSNGNLEFIH

QDMFDTVYGFAVSEDLIWVAYPTSNTIMCYNLKTYKELYSIGDIEGTLFNLPESIYFY

DELLYVCDMENQRICTIDPKSKVLSSYKVFREPVWEYIRSNNYEIVRLKSGIFRV"

CDS 1374384..1375175

/locus_tag="PIKHDJKN_01243"

/inference="ab initio prediction:Prodigal:002006"

/codon_start=1

/transl_table=11

/product="hypothetical protein"

/translation="MKTILEIKDSFFNKLTILRNYYMSSLLENELANTQIEKNKYDSV

SLESKYKNEENKFIFGRKDDGFLATEVEVNECIHMITSNKEKTKMLINLLIINLCSAF

ESFLIELFEYYLKSYPEHINNSQKQMKLQDVFKFNDFNELKEDLIVQEIKSYVFRSLE

KQITDLEEHYKVKFGFSSESRAAKVIFIKRNCIVHNQSLVNKELFEMLTHEQKSRNSI

GKPINTSEFEFSEEIHLLNIMGRRIVTTIQTEISEQSEKHKNLIT"

CDS 1375304..1375750

/locus_tag="PIKHDJKN_01244"

/inference="ab initio prediction:Prodigal:002006"

/codon_start=1

/transl_table=11

/product="hypothetical protein"

/translation="MKKNKTPILELTTQERKELRKNKILISEIYKLTNNEICLIMNVS

ENRAKEIKALSEFQSLPSIGPKFAKDLLMLGYYSLNEIRDKDGARLFDDLEHLYGERI

DPCVEDQFRFVIYCANNSYCGNQWWDFTEERKHYRDTYGYSKERPE"

CDS 1375789..1375947

/locus_tag="PIKHDJKN_01245"

/inference="ab initio prediction:Prodigal:002006"

/codon_start=1

/transl_table=11

/product="hypothetical protein"

/translation="MTSDNTIFKLRGIRPSVRPKLDVDSEGHFSSEVANSAGHIPPPN

RIAAALRA"

CDS 1376039..1376326

/locus_tag="PIKHDJKN_01246"

/inference="ab initio prediction:Prodigal:002006"

/codon_start=1

/transl_table=11

/product="hypothetical protein"

/translation="MAICIKFKLYRMDDNKAVYAYGDCSENFEGLFELDLEKLISGEI

PSDTDMREVVKVIKPCISDIDYQQKANRAFSKIYKHYKETRTYLLEGGYYA"

CDS 1376571..1377002

/locus_tag="PIKHDJKN_01247"

/inference="ab initio prediction:Prodigal:002006"

/codon_start=1

/transl_table=11

/product="hypothetical protein"

/translation="MNYNQLKDLVNVDLVDYKDTPDLKLILRFRIHKETNEWPYLDLI

NTQGSSMIIQDDDRFPIIEIQFDSYIGFSIRNESYTSWDTYEEFEGKIFRIFERSRYL

DFIRLSTFATEDYPGPYKHYGVAGLNHIVDIVSIKDPIIRV"

CDS 1377157..1377600

/locus_tag="PIKHDJKN_01248"

/inference="ab initio prediction:Prodigal:002006"

/codon_start=1

/transl_table=11

/product="hypothetical protein"

/translation="MKTNKAMRNLLNELAQSNESISSNLFNPTFHEWDNCIVVDASSL

RETDSIEETIYQDRTDLEASINHYHIEDLRTGIKLISNWKENIKLNYSEKDFILILSS

TIEGEDVVIRFYQFRHDEPEWIKLNDLEGYKEEAILVIEILNAVQ"

CDS 1377773..1378237

/locus_tag="PIKHDJKN_01249"

/inference="ab initio prediction:Prodigal:002006"

/codon_start=1

/transl_table=11

/product="hypothetical protein"

/translation="MIRGISFEIPNEYGRFLEDILKPFKVEDHNWWIGGEESYIILDG

KLDALFSGLIECMEGETLRNVIKNNDYYLIFQDLKAFPKDSKPIEIKTYDDFLRSNCE

LALLVVDSSYTTIYCKNVELVEGLYSNAKKQDYTNIKYITDENDFRTGLSVW"

CDS 1378478..1379245

/locus_tag="PIKHDJKN_01250"

/inference="ab initio prediction:Prodigal:002006"

/codon_start=1

/transl_table=11

/product="hypothetical protein"

/translation="MSKYIEYLCTSTTAFVDKEIVVNFDQGEITAVKIQLSTEGDQPK

SGLVRITVYFKETIEFDQAEELGNHKVKQIINSLVFVKGSQINDPYIFKENIKPNSTI

VARMQGRFSILSDFGQEQIDEFVELTKNEKANHIYYTLYKQSMNHNDNLGKFMFLYSL

LIVMIDAKKQKDIDSYIISNYPDEEFNQRQSTRMVKRDGIETRSDELETRFTWLRNQL

GHTQHDSEITVVINEINECWRELDRIVKNVIKDKVSK"

CDS 1379399..1379794

/locus_tag="PIKHDJKN_01251"

/inference="ab initio prediction:Prodigal:002006"

/codon_start=1

/transl_table=11

/product="hypothetical protein"

/translation="MKVKLIFVSTAMIFFLLLIGCSEKQIAIDQIIGEWKVTSEQVNL

EKFTSTHGMIRIYEGLRIAEHFEEDNVITILGDGSIEFPTITGGYELDYQDGYNYISI

IGDSSESVHPVYVNDDTLQINTYILKKIK"

CDS 1379999..1380697

/gene="hdl IVa"

/locus_tag="PIKHDJKN_01252"

/EC_number="3.8.1.2"

/inference="ab initio prediction:Prodigal:002006"

/inference="similar to AA sequence:UniProtKB:Q51645"

/codon_start=1

/transl_table=11

/product="(S)-2-haloacid dehalogenase 4A"

/translation="MAKIRAVIFDLYETLITEYSNESRKVSRISRDYQELIGLSNKEF

SKEWNNRQEIRMTGAFPDYFSVIKDIIEKQSLIYEDKVIRALYRERVHEKSVPFLDIL

QEIIELLEVLRKKDIKLGLISNCTEEEVQSWKYSELAQYLDEVIFSYQVGFAKPDNRI

YELVCSRLGITANECIFVGDGGSNELDGASRNVMNAYHATWFIPEFISSRITDYKKLK

KPIDLFKELETYEI"

CDS 1380921..1381367

/locus_tag="PIKHDJKN_01253"

/inference="ab initio prediction:Prodigal:002006"

/codon_start=1

/transl_table=11

/product="hypothetical protein"

/translation="MKKLTVLVLFILFILTGCSGDQDNKDTKLRSVNIDFSKQNNLND

WKVIMLPKVDSVDQQVYDLEFQYTGESPIKDVTVYYRIDTKEKLSKSIVKADSKTIVS

DLDKNEKITLSELILPPNTPISVEVDWLKGNFIDKGVGTFNIKEIK"

CDS 1381512..1382120

/gene="nodA"

/locus_tag="PIKHDJKN_01254"

/EC_number="2.3.1.-"

/inference="ab initio prediction:Prodigal:002006"

/inference="similar to AA sequence:UniProtKB:P02962"

/codon_start=1

/transl_table=11

/product="Nodulation protein A"

/translation="MTGSLGSDLPIIAANTYEHELRWVLHWESDLSESDHRLLTRLLA

DIYPAHTARFKEACSWSGAQPEGRIVGYQGEHAVAHLGFVRRILRTEANEIGILTADV

GLVGIRSDLQGKGVGRKLLHQIANVFKSLNIPYAFLTCREAVVPFYESGGWVRLLDSK

VKMIDNHYQPELYTGPAMILPSAVPFEQWPHGETIIRDGLEV"

CDS 1382523..1382936

/gene="hit"

/locus_tag="PIKHDJKN_01255"

/inference="ab initio prediction:Prodigal:002006"

/inference="similar to AA sequence:UniProtKB:O07513"

/codon_start=1

/transl_table=11

/product="Protein hit"

/db_xref="COG:COG0537"

/translation="MDCLGCRIANGIEPNLNIVYENELITCVLDIAPFNEGHTLILPK

KHYLDIDEMESETVYAIMDASKKLSIALKNLFKPDGIRICQDGGKFNDLTHYHMHLIP

RYEGDGFIWGEPLHPHGAEKRLSQTKEKILKALHE"

CDS 1383099..1383389

/locus_tag="PIKHDJKN_01256"

/inference="ab initio prediction:Prodigal:002006"

/codon_start=1

/transl_table=11

/product="hypothetical protein"

/translation="MYVIRATQKLLKEMDCKVQKTDISVPLNTWHANMFTLNRRKTIV

FMNDLSRLSLTIEGVKKAELKNVEELFVNELRNYLMSEEIEGKVIQQLYQRK"

CDS 1383774..1384541

/locus_tag="PIKHDJKN_01257"

/inference="ab initio prediction:Prodigal:002006"

/codon_start=1

/transl_table=11

/product="hypothetical protein"

/translation="MILEKVINRIVIQSEYKDFVDKYIDHILTEFKGKIHSIYMCGSI

PKGTAKPFKSDADFTIVCVNPKDIDYERLSTIKDKLLKEYPIVTKIDTIICSIDDVLS

KPNEWGFWVKIICVCLYGHDIGEKVPPIIISPEFILDLNTETKEEVDRIHRLLSNASD

HTMKTRYIKGYSKRLIRALYSLVLEDTGVWQDDINKMKNAILNYCEIDSTLVDYLYAC

YLDSNVLVEEFLGIADEVYSYFENALNAMAASRTSFD"

CDS 1384730..1385449

/locus_tag="PIKHDJKN_01258"

/inference="ab initio prediction:Prodigal:002006"

/codon_start=1

/transl_table=11

/product="hypothetical protein"

/translation="MNITFSKWVQYYRRNNQNLKYIHWDDNYKLTTNEREIIIKSIQQ

FQLGENSEGKYLIKRAQEYVHQTQDQDYYEALIEFIKEEQRHARDLGRFMKVQGIPLL

RRHWVDNVFRRLRRYASLEQSVIVLLTAEIIAKLYYKALQKSTKSEVLIDLCNQILSD
EEKHVQFQSETLHKFAQNRNILFNRIVYILRRILFEGTLIIVWYQHKPVFKAGEYKLK
SYYYECRHEFNLTEKIIANSQ"

CDS 1385723..1386232

/locus_tag="PIKHDJKN_01259"

/inference="ab initio prediction:Prodigal:002006"

/codon_start=1

/transl_table=11

/product="hypothetical protein"

/translation="MKRELLDKAIQLREEGRAKQDQAILAEARTMLLDLLFTYPDDAE
INFQTAVAHDNSGLGKEAIPFYVRAIEQGLSGPDLERALLGLGSTYRYLGFYQEAEET
LRRGVKEFPDHRGLQVFLAISLYNTGKYKESTELLLSNLLETTSDEKLQYFSRPIQYY
AQHLDETWG"

CDS 1386495..1386842

/locus_tag="PIKHDJKN_01260"

/inference="ab initio prediction:Prodigal:002006"

/codon_start=1

/transl_table=11

/product="hypothetical protein"

/translation="MIKGFGGIFWRTKNLEVIKKWYSDVLKIEIENWNGTVIKPEPGN
ETVFSFFPENDSYFPTEQQVMLNFQVHDLNEIIQHLEHIGVPLVKKKEISEFGKFVWI
EDPEGRLIELWEK"

CDS 1387068..1387748

/gene="dauR"

/locus_tag="PIKHDJKN_01261"

/inference="ab initio prediction:Prodigal:002006"

/inference="similar to AA sequence:UniProtKB:Q9HXE2"

/codon_start=1

/transl_table=11

/product="Transcriptional regulator DauR"

/db_xref="COG:COG2964"

/translation="MGSENRQYLERFFPIASFIAAIIGPKCEVVVHDISDPEHSIVFI

ENGYISGREVGDASTDLVLKILKSEAYHEEQYIANYKASGKFGQSFRSSTYYIKNDQN

KLVGLLCLNIDVTHMEVAAEWVQHILQGGPNPYVMPAAAEKSKEEKQTTEYLQGNADD

LLQHMISSVIGKLKIPPERLSSQEKIEVVRELNELGVFLLKGGVSQVASSLSISEPTV

YRYLQKLK"

CDS 1387790..1388959

/gene="patB_2"

/locus_tag="PIKHDJKN_01262"

/EC_number="4.4.1.13"

/inference="ab initio prediction:Prodigal:002006"

/inference="similar to AA sequence:UniProtKB:Q08432"

/codon_start=1

/transl_table=11

/product="Cystathionine beta-lyase PatB"

/db_xref="COG:COG1168"

/translation="MSYNFDQVISRIGTNSAKWDGVVNSLGDDMIVLSVADMDIQAPP

QVVNKVSEMARHGIYGYTDVFPPYYKVVQHWLDKAYRWHVPQEWIVFCPRIVQAVSII

IERFTEPGDRILMHTPAYQPIANAVEINHRKLVESRLLLRDGRYEIDFDDMERQMSAG

VKIVLLISPHNPTGRVWTKAELERIAELCIRYDALLVSDDIHADFIHEGHEHTIIAKL

SEQIAERSIICTSPGKTFNLASLEIANIVIPNMQLRERFQLGLRQVGIHNPTFFAIPA

LDVAYTECDDWLNELRSYITDNISFTEDFFTEHMPELKIVRAEGTYLLWVDCTALSRN

ESDLIEWIQQKSRVSVSFGSSFGVDGEGFIRLNLGAPRALLQEAFERMVSNYPLK"

CDS 1388999..1390486

/gene="mleN"

/locus_tag="PIKHDJKN_01263"

/inference="ab initio prediction:Prodigal:002006"

/inference="similar to AA sequence:UniProtKB:P54571"

/codon_start=1

/transl_table=11

/product="Malate-2H(+)/Na(+)-lactate antiporter"

/db_xref="COG:COG1757"

/translation="MAMPNKEKTVKQPTLFLALLPIVTMILLLCIGYVLFELPPEPLI

IISTVVAGIIAIKLGYNYDDIMGSIAQKIAKTMPAILILITVGFMIGAWMIGGTIPMM

IYYGLNIINPQFLLITAFIVTSIVSLCTGTSWGSAGTIGVAFMGVGAGLDANLAAVAG

AVVAGAYFGDKLSPLSDTTNLAALSTGVNLYEHIGHLLYTTVPSFIVAGAVFLITGFN

TDASNIAIPDKVITIMDTLSTVFHWNLLLLLPVLIVLYGSIRKKPTIPVMLISSFVAM

ACGMIFQGFTLHDVTSSVINGFDVAMINVQGFDAATVISDIPTLLNRGGMNSMMGTLL

ICFCAITFAGTISLTKSLELIVGKILKFVHSTGSLILATIATGLTMIGVTSNGQVSIL

MPGEMLREAYIRRGLHPKNLGRTIEDSATIIEPILPWTAAGAYMAGTLGVATLSYLPW

AVLCWTGIIFAIIWGFTGFGITKLTPEQQQEMLKEYDDAKAAKSL"

CDS 1390566..1390949

/gene="ridA"

/locus_tag="PIKHDJKN_01264"

/EC_number="3.5.99.10"

/inference="ab initio prediction:Prodigal:002006"

/inference="similar to AA sequence:UniProtKB:P0AF93"

/codon_start=1

/transl_table=11

/product="2-iminobutanoate/2-iminopropanoate deaminase"

/db_xref="COG:COG0251"

/translation="MTKQERKTIATSNAPSAIGPYSQAVQFGNMIITSGQLGMNAEGE

FPDSVSEQAKQSLTNVKAILEAADYAMEHIVKTMVFLKDMNDFQTVNEVYATFFGEPY

PARSAVEVAKLPKGGLVEIEVIAVK"

CDS 1391173..1392225

/gene="cytR_1"

/locus_tag="PIKHDJKN_01265"

/inference="ab initio prediction:Prodigal:002006"

/inference="similar to AA sequence:UniProtKB:P0ACN7"

/codon_start=1

/transl_table=11

/product="HTH-type transcriptional repressor CytR"

/db_xref="COG:COG1609"

/translation="MPKLKDIAERVGVSISTVSRAISNDTNRPVNEGTKRKIREAALE

LGYILDESSKSIGKEITSAKRIACIVPQILMDDHPYFSQVLTGFHVKMTELGLPPAIV

RTCEELSDTERIKMMLQETGTQGIVAISWYDEKLFELLEQENITVLGVSLNDERLTVP

VVDCDRIFSARTAVRHLIEQGHTRIGFIGGPAYSEQIESEERYVGYKFAMLEANLNLN

EHWIINTNWNVNLSYSRLTDLLTETPKSDWPTAIFCASDMLAIPAMRAVVEQNGRIPQ

DIAFVGMDNISMAQYTTPPLTSVQVPKYEIGKVAAQLINDYLEGRYSSLHKVLLPSTL

IVRESSVYDRTNRTID"

CDS 1392834..1393796

/gene="yteP_6"

/locus_tag="PIKHDJKN_01266"

/inference="ab initio prediction:Prodigal:002006"

/inference="similar to AA sequence:UniProtKB:C0SPB3"

/codon_start=1

/transl_table=11

/product="putative multiple-sugar transport system

permease YteP"

/db_xref="COG:COG4209"

/translation="MANTTVQPAMNAVHEKKRRRRQKWNPILRNWQLYLLISPVVAYY

IVFQYLPMYGIQIAFKDFIATKGIWGSPWVGFEHFERFFHSYFFWRLIKNTLGLGLYS

LAVGFPVPIILALLMNEVRAEKFKKFVQTITYAPHFLSTVVVVGMMMMFLSPRYGIIN

HFIGMFGGNPINFLSEPSWFKTLFVMSDVWQTMGWSSIIYLAALAGVDSQLHEAARVD

GATRLQRIWHINIPSIMPTIIILFILNIGSIMGIGFEKVLLMQNTLNLETSDIISTYV

YRSGIQDAEYSFSAAIGLFNSIINFILLVTVNQISKRMSETSLW"

CDS 1393812..1394696

/gene="melC_2"

/locus_tag="PIKHDJKN_01267"

/inference="ab initio prediction:Prodigal:002006"

/inference="similar to AA sequence:UniProtKB:O34518"

/codon_start=1

/transl_table=11

/product="Melibiose/raffinose/stachyose import permease

protein MelC"

/db_xref="COG:COG0395"

/translation="MLVESRGDRIFNIINHFLLITITLIVIYPLVFVLSASFSDPQAV

LRGEMFLWPKGINLHSYEKIFQNKDILRGYTNTLIYTSVGTLINLVMTILAAYPLSRK

DFIGRNAIMALFVFTMFFSGGLIPTYMLIKNLGMLNTFWVMIIPNAVSIWNIIIMRTF
FQQSIPNELHEAATIDGCSNIQTLTRIILPLSMPIIAVTILFYAVGHWNAFFNAMLYL
SDKNKFPLQLILREILIQGQTNDMVKMSTESAIKQQREVEGIKYAVLVVANIPVLLLY
PFLQRYFVKGVMIGAIKG"

CDS 1394738..1396363

/locus_tag="PIKHDJKN_01268"

/inference="ab initio prediction:Prodigal:002006"

/codon_start=1

/transl_table=11

/product="hypothetical protein"

/translation="MTLKKWGSIALVCMMSVSMLAGCNSKDADPGSTKDKGAANGIVN
ATGFPIVNDTIKVSGFAGKFFANADWNDLRLWKEYEKMSNVHIQWDTVQKDNLKEKRN
LLLAGGAYPELFYASAFSRSDLLKYGKQGAFIPLNDLIDQYAPNFKALMKKYPVIGKG
ITMPDGNIYGLPTLYDPEFKSVLYGTPWVKKEWLDKLGLQEPQTLDDFYNMLKAFKEK
DPNGNGQKDEIAWGGVGTAGVVNYLRGSFGLNNHGTSNINIDTDPQTDKVRFIPTDPQ
YKELLQFVNKLYKDGLLEQDIVSVKSTEVDAKGMEGLIGVVDNVDPVAIYNQQGYVGM
PVLKGPHGDQTYNAYGSPLGNIGMFVLTDKAQNPEAMIRWMDYFYSDEGIKTFFMGWK
DETYKEDAEGNVDYVDNIKNNPDGLNLDQAVGQYLIWPGGYYPGFVKQKYFKGAEGLP
TSVDNAKKAEPHILPQDQIWPPFNFTQEEQRDLTTFQTDIETYVDEMRDKFITGNESF
DNWDQYVAALQKMGVERYVAIYQSAADRYTQAD"

CDS 1396407..1397678

/gene="yteT_1"

/locus_tag="PIKHDJKN_01269"

/EC_number="1.-.-.-"

/inference="ab initio prediction:Prodigal:002006"

/inference="similar to AA sequence:UniProtKB:O34371"

/codon_start=1

/transl_table=11

/product="Putative oxidoreductase YteT"

/db_xref="COG:COG0673"

/translation="MSQLKAVLIGAGARGAGGYAPYALDYPHELTFIAVAEADPVRRA
RFAEKHGIPPERCYESWEPLLAEPKLADIAVICTQDRMHYGPTMQALDKKYHVMLEKP

MSPDPKECLEMEQAARDNDRLLTICHVLRYTPFWNTIKKVIQEGRIGEIVSIQLNENV

GYWHIAHSFVRGNWNNSNKSSPMILSKSCHDMDVLSWLVDQPCLKVSSFGSLKHFHEG

NAPEGSADRCLDCAVEPTCAYSAPRFYLSDQFRGWAGHFTQELTKPNIIQGLRDTDYG

RCVYRSDNNVVDHQVVNMEFEGGATAMFSMCGFTFEQERRIQIMGTKGELRGEEDQIT

VYDFLTHQKTEITIPSQTSGHGGGDSGIVRNFLDEVRHYNGQESLTSAAASVRSHLIA

FAAEKSRLNSGESIDLNEYARQLMVKEELSK"

CDS complement(1398064..1398765)

/gene="opuCD"

/locus_tag="PIKHDJKN_01270"

/inference="ab initio prediction:Prodigal:002006"

/inference="similar to AA sequence:UniProtKB:O34742"

/codon_start=1

/transl_table=11

/product="Glycine betaine/carnitine/choline transport

system permease protein OpuCD"

/db_xref="COG:COG1174"

/translation="MESEHLTFADFLSYISRNKGLLWEYFIQHVTMVVIGLGLAVIIG

VPLGILCSKSKWAAKIILFITNILQVVPSLAMLVVLMLWLGLGTKTVMVGLFLYSLNP

IARNTYVGLRQVDPSYLESGKGIGMSAFQMLVKVRFPLALTYIISGLRIAAVIAIGVV

TIAPLVGGGGLGREIYAGLNSNNSLRIFAGAIPAAMLAIVADLVLGILQRKMDLSKRK

SPNAAPSPAKIQNIK"

CDS complement(1398778..1399731)

/gene="opuCC"

/locus_tag="PIKHDJKN_01271"

/inference="ab initio prediction:Prodigal:002006"

/inference="similar to AA sequence:UniProtKB:O32243"

/codon_start=1

/transl_table=11

/product="Glycine betaine/carnitine/choline-binding

protein OpuCC"

/db_xref="COG:COG1732"

/translation="MSNNNRRRRARRNKEGVMKLIKLKWIPMLVCMIALAAFNSACGI

KSEITIGTQTYTETKIIAEMYKALIEDQTNLKVNIIPDLASSSLVINAMKRNDVQMAT

LYTGEIFNNHFPISGTKERAKVLKEAQDGFLEHFGFIWMNPLGFENTYAFTIRKDLSE

SNGYTKISDVKQDMTNMKLGVDTTWLERNTDGYPAFSKVYDIKFGQVFPMEISLVYSA

VANEQVDIVLAYSTDSRLKAYQLQTLQDDKQFFPPYDASPVLRSDLLKEHPEIQDTVA

PLIGHLDADTMISLNYQVDIEKKSEREVAITYLKEQGLLKS"

CDS complement(1399728..1400393)

/gene="opuCB_1"

/locus_tag="PIKHDJKN_01272"

/inference="ab initio prediction:Prodigal:002006"

/inference="similar to AA sequence:UniProtKB:Q9KHT8"

/codon_start=1

/transl_table=11

/product="Carnitine transport permease protein OpuCB"

/db_xref="COG:COG1174"

/translation="MNAFWSFITDRYPDILKALQEHLILSSSAVLLGTIIAVPIGILL

VYNRIGWINSIVFFITNLFQTVPSLALLAILIPLLGIGMKPAILALFLYSLMPILRNT

YDGFHSVDKGVLNSARGMGYSTAQTILRIQLPLTLPYIMSGIRITTVYIISWATLASL

IGAGGLGQLIVSGLGVNKPEMIFIGGIGAILLALVADGLLGLLEGWLSKHYHQNQTRD

AAI"

CDS complement(1400395..1401558)

/gene="opuBA"

/locus_tag="PIKHDJKN_01273"

/inference="ab initio prediction:Prodigal:002006"

/inference="similar to AA sequence:UniProtKB:Q45460"

/codon_start=1

/transl_table=11

/product="Choline transport ATP-binding protein OpuBA"

/db_xref="COG:COG1125"

/translation="MIVLEHVNKVYDNGFHALKDLNLEFKKGEITVLIGPSGCGKSTT

MKLINALNTPSSGKVMIDGKDISRLNPVELRRNIGYVIQSVGLFPHMNISKNAGVVPR

LKKWEKDKTDQKVNELLDMVGLDHTTYATRYPSELSGGQQQRVGVVRALAAEPDIILM

DEPFSALDPISREQLQDELIRLQQDLHKTIIFVTHDMDEAIKIADTIVLMKDGEVMQT

GKPDTILRHPANDYVRDFIGSKRLQDENESAYEIPLVDEVMITNPVTAFPNRGLAEAI

KMMEMKRVDSLLIVDRNRQLQGVVSIYRVLDQYGEEGKTVADVMHPVRFSVASGSTLP

QAIETMDSHQLSNLPVIDSNNRFLGLITRGSVVKHLAEVYPMMVPPVLNGEDE"

CDS 1402317..1403435

/gene="purR_2"

/locus_tag="PIKHDJKN_01274"

/inference="ab initio prediction:Prodigal:002006"

/inference="protein motif:HAMAP:MF_01277"

/codon_start=1

/transl_table=11

/product="HTH-type transcriptional repressor PurR"

/translation="MHLYEQIYVYIVQEIKEGRLKEGDRVSSEKELAEKFGVSRITSK

KALEKLADSGVIKRIQGKGSYVADGIIGGQEPKHYSEVKTTLVPEDDKRLIGFIIPNF

SDEFGLQLLRSMEKRSAEHNYQLIIKRTCGDRDEEKRAIDLFIGLGIKGLIVTPIHGQ

HYNSELLRLVLDRFPIVLVDRYLKGIPVCSVYTDNKKAAMDLTRHLISKGHKKIAFLS

PPEENTSTLEERLLGYTFAFTQEGMNLNKDFLLTNLKGTLPMNINGTAIESDKETVKH

FLEQHPDVKAFVASEFLIATMLAQVIHSMGKSLEEFEIVCFDSLNDHLGQPIFTHIKQ

DENQMGELAVDMLISQLHGTAVPELNIIKHRMVVKNNR"

CDS 1403855..1405156

/gene="malX"

/locus_tag="PIKHDJKN_01275"

/inference="ab initio prediction:Prodigal:002006"

/inference="similar to AA sequence:UniProtKB:P59213"

/codon_start=1

/transl_table=11

/product="Maltose/maltodextrin-binding protein"

/db_xref="COG:COG2182"

/translation="MRRSKKIAFPILVYFLAMSLLVTACSTKGDSSDQAGEGNTTVTM

WHGLTSIDLDNMNKVVKAFEEKNPTIKMKLVYTESSEGSDQKLLTAVAGGNPPDVALF

DRFKVGTWAAQGSLTDLSSMAAESGIRKEMYYPFAWEESSYQGKLYAMPMDTDSRLLF

YNKDHFKEVGLDPNKPPQTIAELEAAAEKLTIKEGKRFKRIGFIPWYSQGWLYTWGWA

FGGEFKDAATGKITANDPKVVEALQWMTDFGKKYNVEDIAGFTSAAGTEEMDPFISGQ

VSMKISGNFTVKGIEKFKPDLNYGVAPIPTPTGTNFTTWSGGGSAIIPKGAKNVAAAW

KFLEFLGKEEGQTLLNADSQISVIDSVNDKYGYKDDPIMKEFINILPNSHNRPVIPEG

QLLWNELASATEKATRGNGTPKENLDRVTEIVNKALEKYDK"

CDS 1405227..1406162

/gene="lacF_4"

/locus_tag="PIKHDJKN_01276"

/inference="ab initio prediction:Prodigal:002006"

/inference="similar to AA sequence:UniProtKB:P29823"

/codon_start=1

/transl_table=11

/product="Lactose transport system permease protein LacF"

/translation="MAKLADSVQVNTIPSARKRALHRTNVIGWLFASPWAIGLLCFYA

IPMLMSIYFSFTTYSILQPGEFVGMSNYIDLFHDPLFWKSIYNTLYFTVFFVPLSIFF

GVALAMMLNMKVKGMAIFRTIFFLPTLVPQVALAVLWMWLLHPNFGLVNGMLANIGID

GPSWLGGESWSKPSLILMSLWGIGQAVVIYLAGLGDIPEEYYEAAQIDGANWFQKTRK

VTLPLLTPVIFYNLVMGMIGAFQQFTLPYTLTKGQGTPANSMTFYVMYLYENGFRYFK

MGYASAMAWILFIIVMALTGIVFITSKRWVHYQGK"

CDS 1406180..1407016

/gene="araQ_8"

/locus_tag="PIKHDJKN_01277"

/inference="ab initio prediction:Prodigal:002006"

/inference="similar to AA sequence:UniProtKB:P94530"

/codon_start=1

/transl_table=11

/product="L-arabinose transport system permease protein

AraQ"

/db_xref="COG:COG0395"

/translation="MNPIAIKRLNRALAYLCLIVASAFFIIPFIWLLSTSLKPLTQIF

TFPPEWIPHPILWSNYSRAVEYIPFWTYLKNTAIITIVSTLGVIISCPLVAYSFAKLE

YRGRGILFFVTLAVMMIPGQVTMIPLFLLFTKLGWVGTSLPLIVPQFFGVPIYIFLLR

QFFMGLPDALREAARLDGASEFRIYLQIMFPLAKSAVLAVALFQFMGSWTDFMGPLLY

LTNEPSYTVSLGLQQFQSQKGSEWGLLMAVSTLMTLPIIVLFFFLQKTFISGITFSGI

KG"

CDS 1407357..1407941

/locus_tag="PIKHDJKN_01278"

/EC_number="2.7.1.-"

/inference="ab initio prediction:Prodigal:002006"

/inference="similar to AA sequence:UniProtKB:P9WL55"

/codon_start=1

/transl_table=11

/product="Putative O-phosphotransferase"

/db_xref="COG:COG3896"

/translation="MERGLIIFLNGTSSAGKTSIAMELKNQGEVPFHHLSVDQFAQNY

DQFIDNTYPDMKPTREVEHHVMTDILFDPINSLYCATIKLFSEMGLNVIVDTVISNDK

WFNGFYDLLSDYPILFVGVHCSKEELTRREQSRGDRAIGLAHSQFDYIYSYDEYDLEV

NTEELSSAECAEKILSYMNSEQEYLAFKKLSRRD"

CDS 1408073..1408351

/locus_tag="PIKHDJKN_01279"

/inference="ab initio prediction:Prodigal:002006"

/codon_start=1

/transl_table=11

/product="hypothetical protein"

/translation="MNPEDHIQHMLQAIIEKTQSIINDSHKQPFGSLKYFLEHIIEYR

DKQQYMSNEWHIRTPRWLGEYGNTPEEEELLSDIYRLQAYIAEKLKGG"

CDS 1408488..1409528

/gene="yfiY_2"

/locus_tag="PIKHDJKN_01280"

/inference="ab initio prediction:Prodigal:002006"

/inference="similar to AA sequence:UniProtKB:O31567"

/codon_start=1

/transl_table=11

/product="putative siderophore-binding lipoprotein YfiY"

/db_xref="COG:COG0614"

/translation="MNQGTKGQAARSKAYAAHKSRLFMGLMLALILVLTACGAGTGTD

SGKPSAATPAETPANTENQTDGAFPVTIKHMKGELTLNEKPKKIAVLDVKFLDQMLAV
GEKPAGSVIAGGNTDFPEYLGDQPSDVQVLGTRDEPNLEAIVALGPDLIIMTDFQEKQ
YENVSKIAPTLVLDFYEDWHDTLATVAKITDKQDEAEKVRKAYEDKVAGLKAKLSEKM
GDETVALIRPRKEGIRVHGIEHRTGGILYQDLGLNMPALVQEIKDDASVEISMEKVPE
IGADHYFVLSDELFAAEAEAMASNQVWKSLDAVKDNRAYDVNSTLWIAYYGPLAINLI
VDQASEALLGSN"

CDS 1409530..1410306

/locus_tag="PIKHDJKN_01281"

/inference="ab initio prediction:Prodigal:002006"

/codon_start=1

/transl_table=11

/product="hypothetical protein"

/translation="MDRYLSTDLWKQTAEKHSIWLGEPPEHSIRTIALSELHDEAACR
KYIRWFQEYIDAPDMKVAASMLAKRIGYLWTAPLVIAMTFHHQHVTFQLENSFLYHPT
LSANEGGTRFPFLAVNGLRAEPLSEDREFSREKVVEEMFAVQLTPLLKMLAAIAPLSM
SILWENIMVRIGPLYTLEAEEAERESEHIQADFSYLTQGAPGHVFGVKRNPLTRFTDH
KDNIPVAKSERITCCFYYQMSGEYCRKCPKIDNENKSQLK"

CDS 1410418..1412139

/gene="rhaS_6"

/locus_tag="PIKHDJKN_01282"

/inference="ab initio prediction:Prodigal:002006"

/inference="protein motif:HAMAP:MF_01534"

/codon_start=1

/transl_table=11

/product="HTH-type transcriptional activator RhaS"

/translation="MPLQEQTSLWSDTTIKMLDGYSGTLQTGSVLSETELNSNVLLLA
YGGEGELAMNGELCHIGASFAGHVVKGTSFTLTARSDELYYIVIMYKASSMEGASHAI
PSYRKHPLQTSFIQNPVTQAEWIQNAEKIVAKWRRGEGLERFHANALLQGMIYELIME
YECGQGGTESDMVDVVVSYISSHYRQNLELKELAALAGCSVRQLQRRFKQEKQLGPME
YVIQLRMENASRLLHHTDAPIGEIADRIGYRDMYYFSRAFKKYYGVPPLRYRLAAALE
TDANYAHSLLQNRLVSSYESDQGPVICHMRGEYQVTSAPQRIAVLDVQYADHLLALGL
SPAGSVGSGSTVVNFPQYIRAGLQGTKLLGTYEYPDLLGVERLLPDLIICTEVHDQHY

ERLSRIAPVLMFKRNESWQTILSLFGELTGKRAEAKRILADYHRRTALLSEELATVLA

GKSVALIRPLDSLVRVHSASHRTGAVLYRDLGLPAPSFVADASDTAYHISVERLPAVH

ASHYFLLSNEMMQEGISATEQRVWGMLDASERQHIHSVDAATWIGCYGPTGINGIVDQ

IEQSLLA"

CDS 1412513..1413250

/gene="pyrH_1"

/locus_tag="PIKHDJKN_01283"

/EC_number="2.7.4.22"

/inference="ab initio prediction:Prodigal:002006"

/inference="similar to AA sequence:UniProtKB:Q97R83"

/codon_start=1

/transl_table=11

/product="Uridylate kinase"

/db_xref="COG:COG0528"

/translation="MSRYKRVLVKLSGGAVAGNSEFGFEPERLDHIADEIMSVVNLGV

EVSLVIGGGNIFRGNMAESWGIERAEADNIGTLATVVNSLMLRGVLKAKTKKEVRVMT

AIPITSVAEPYIRLRAIHHLEKGYIVIFAGGNGQPYVTTDYPSVQRAIEVNCDALLVA

KQGVDGVLNADPKFDKNAKKFRSLHYNDVLEHNLKVMDQSAFILARDYNLPMHVFNFD

KPGSMKEICEGMNNGTIISGESVSELE"

CDS 1413826..1414425

/locus_tag="PIKHDJKN_01284"

/inference="ab initio prediction:Prodigal:002006"

/codon_start=1

/transl_table=11

/product="hypothetical protein"

/translation="MKKTILACMMIMLMLASACGNVEKTSNPNTSSEPKPETQEAIIQ

KNTSDYCTVDRISLDSETEKYFYGTWKVEKLLGFANSYNDASEYPTGQKFIGDKVIIE

KDFFSSKGLKKYKKYQYELKNPLYQITATCYNSDSFYRYYKIDIPDLHINDEIKAIDI

SDSSTKMSIPVSLSFLAVNNDRLILLSEATIFELKKIID"

CDS 1414866..1415471

/locus_tag="PIKHDJKN_01285"

/inference="ab initio prediction:Prodigal:002006"

/codon_start=1

/transl_table=11

/product="hypothetical protein"

/translation="MRKNIAIIILMVGLIISMYFNYQFKAYKDNQKVDYTIKLNHGTQ

IGIKESIYNLDNVIKSLEDNSSKETVIHALGNVAVSLKVGEESFVFLKSDFREDQLSS

TYLIYNVFRDMYTHIRVEIIGEILSNKISLEKESRNQLIKDIGVLKHDLEYIDSQFNE

DILKEQSPKWIEDKWKELIGEIVNRNTDYKLYERIKIKYNL"

CDS 1416329..1416730

/locus_tag="PIKHDJKN_01286"

/inference="ab initio prediction:Prodigal:002006"

/codon_start=1

/transl_table=11

/product="hypothetical protein"

/translation="MAYFSLFGDEFDPNYVTSVLKIEPTNTAYKGDIINKKHRIKETS

WTLGTDYEESLDVNHQLTKVVDLLRNKVKEINSIRIEHRLSTKFFIVIRIEEGKTPAL

YFDSDFIEFVNMIHAEIDVDLYANPYNEELE"

CDS 1416821..1417117

/locus_tag="PIKHDJKN_01287"

/inference="ab initio prediction:Prodigal:002006"

/codon_start=1

/transl_table=11

/product="hypothetical protein"

/translation="MINSQEVIKRFLEDEIPSQELYEAIFDFIDSAHIRSGEFEGNYF

IIKKMDLNNFIIYAENIHPDENHREIPYSTSIYKESLLKAINNQARENNYILKP"

CDS 1417143..1417316

/locus_tag="PIKHDJKN_01288"

/inference="ab initio prediction:Prodigal:002006"

/codon_start=1

/transl_table=11

/product="hypothetical protein"

/translation="MNKWIDCNIFYSCCVHTLPKGGLTQRKQAVLFLTYFSGLRVGEI

VRLHETEEGAKGS"

CDS 1417480..1417575

/locus_tag="PIKHDJKN_01289"

/inference="ab initio prediction:Prodigal:002006"

/codon_start=1

/transl_table=11

/product="hypothetical protein"

/translation="MSARTTQRCTDVSKKNIQRIQSTLDRMDMGD"

CDS 1417746..1418150

/locus_tag="PIKHDJKN_01290"

/inference="ab initio prediction:Prodigal:002006"

/codon_start=1

/transl_table=11

/product="hypothetical protein"

/translation="MEETSPKYADKESIESLIKKLHLSPLTPFSQDWEYTSADSSRVN

EFITYYEKNKLNNKEKFTLTIVIISSFDDYLSKGIGTEDIKIWNRIKQNILKDYKLHI

NTIIYWALEENDLEDCFAVTPYIREILEQKSM"

CDS 1418426..1419205

/locus_tag="PIKHDJKN_01291"

/inference="ab initio prediction:Prodigal:002006"

/codon_start=1

/transl_table=11

/product="hypothetical protein"

/translation="MFSIYEISQEEHEMIVRANDQFGLIFEVCLEAIELSWTFTKNYQ

ISAWIFAGFLSQTNNSLYLAFLSLIRRHTVQTSFNLRLSIESAVLACYALYNPKIDSF

AIKNDQGSMVEKSGVKSKANNWIENNYKDMSDTIHVYKKQINSKFAHSSILSAFGNFE

LSEENYQGVSWIFDRTEDRDIHFQLLSLVNLVLIQVEMFYRVIKDYPIVGIVDDFTSR

YEKIHGTVTLLNDFYKLIAEELAKGEEVEIPILRTLNEEET"

CDS 1419370..1420956

/locus_tag="PIKHDJKN_01292"

/inference="ab initio prediction:Prodigal:002006"

/codon_start=1

/transl_table=11

/product="hypothetical protein"

/translation="MLKLKEESLNWSLKHINKYGDSYVSPKLFEFEAINENWDDVKNH

LLTTDVYSNGVRPYRTTITPKSNVGFRICTQLDPLDSIVYNALIYEIYEEIENKRIPA

VHDVVYSFRLNPESDGTLFDPKYNWKEYEDKTRDLLESEEYSYVVMTDITDFYPSIYL

HNIETHLRECVRLSGKNAHAETLINIIKAMHISQTHKGLPIGPQFSRPIAELILNEID

MILIENDIMFIRYVDDYRLFCKSENDAYKSLAFLAQKLYDILNLKLNEQKTKILTTES

FISKHLYLYKDREKDRVIEEFYELLDKLGFSRDFYGEIDLSLLGEEEIKKLHELNIFE

LLEEELDKDDFDIGFAKFLIGNLARFDNTEVAELILTEENMRKLFPIIKTIINHLELV

RSYSEEQKHEIGNKVLSLLQSSFMSELEFNRSWLLHLFSNNDEWNNKHFFSELIKKYH

DNFTVRELILDLGRSKNHRYFRENKFSIISDPWVRRAFLVAISCLPKDERNPYYKSRG

LTQRDLLEQVIEKWAQKNSF"

CDS 1421148..1421405

/locus_tag="PIKHDJKN_01293"

/inference="ab initio prediction:Prodigal:002006"

/codon_start=1

/transl_table=11

/product="hypothetical protein"

/translation="MDNLEKNIKMFCRTCGNDQFSILDECIYDLQSAPNETKIKCSDC

GLVTTKEELIEDNSHIIAANFEDLKKEAIKKLEKELKNLFK"

CDS 1421414..1421911

/locus_tag="PIKHDJKN_01294"

/inference="ab initio prediction:Prodigal:002006"

/codon_start=1

/transl_table=11

/product="hypothetical protein"

/translation="MTITDLWNQFKDVFADLVKYGFPTVAIIVSLLSFKDSRKANKVR

DRLNELEEKLKQFALEEKEKARLDETKASIEARIINISKGKYKLKIWNSGKATAYNVN

LEIPDEFKGIIYTDKVPYEFLEAGKNFEEHILVHSGTPRKFTIKTTWEDSKGINHLKE

LILTI"

CDS complement(1422205..1422519)

/locus_tag="PIKHDJKN_01295"

/inference="ab initio prediction:Prodigal:002006"

/codon_start=1

/transl_table=11

/product="hypothetical protein"

/translation="MHELFLQAINEKRKIMVTFFSNEDGHSLKRLCAPFDYGPSNKYK

DKSPRYHMYDYTSDSGSHVLSILPGQLKEILLTDEYFNPLDLVTWNNPKWHVQRNWGI

VS"

CDS 1422760..1423257

/locus_tag="PIKHDJKN_01296"

/inference="ab initio prediction:Prodigal:002006"

/codon_start=1

/transl_table=11

/product="hypothetical protein"

/translation="MIQSRYLFVSRAGKVIKIARPKDHKHKSVPQLAGEEVLEVILYY

RNKDRKPHKLLMVEFDRFHLDSDGNYKQTEEDRKRAIHNFLSFGMADLHEGTEVEDKP

LPIPVAPIIPTTAEKKSLYDYINEKLPSFSDDAPYVVESKIKAAKEKYEEFKSMAKKS

NKLSK"

CDS 1423423..1423974

/locus_tag="PIKHDJKN_01297"

/inference="ab initio prediction:Prodigal:002006"

/codon_start=1

/transl_table=11

/product="hypothetical protein"

/translation="MIKRLIEIFENANRDFLESDLDLLISKVSERTLCGALMLHINNI

IKKSEFAEYKVDVEYNRNKNGKIKTIVKTIYGPKDVGVKINCDLIVHSRGNNINQDNL

IAIEMKKSDRTKEEKESDRIRLIALTKDSYDDVWSFDGNTFPEHVCRYALGVYYEVNY

KKRSINIEYYKEGHIFDEKEVKF"

CDS 1424072..1424680

/locus_tag="PIKHDJKN_01298"

/inference="ab initio prediction:Prodigal:002006"

/codon_start=1

/transl_table=11

/product="hypothetical protein"

/translation="MDFEEVFQQTTPTRFMNTETLYEISAKLKKGPIIMNYQYNLQFE
TLCNSLELGELIKIPEAISGGLLHKMFVIQTSTSKYAVKALNPQIMARPTALNNYIKS
ERIVSIVSNYIPAQPAKIYNLGFLQNIDNQYYIIFDWIEGNSLKSHEITKANCKKIGS
ILAEIHKTDSSQIGNSSINELSNGIQQIDLELLFKYGERNSF"

CDS 1424754..1425197

/locus_tag="PIKHDJKN_01299"

/inference="ab initio prediction:Prodigal:002006"

/codon_start=1

/transl_table=11

/product="hypothetical protein"

/translation="MLAADSVFSHRDLEPKNVMWKQGSPILIDWESAGEINPKHDLIE
TAVYWSMNEPSGIDKDKFHAFVCGYQECYGSIDADWRTVLELGYLSKLDWLEYSLKRS
LQIDCADESEQEMGTKHVTGTINALKEYEEMVSVLETWLHNLNEA"

CDS 1425424..1426134

/locus_tag="PIKHDJKN_01300"

/inference="ab initio prediction:Prodigal:002006"

/codon_start=1

/transl_table=11

/product="hypothetical protein"

/translation="MTDNYEITLKTNFSFNHIQSAVFLARNALQVEKNKNNSSDVVAR
IQYESYVTGALLCTVGYLEATINEFFSNIKDENHNNNKRLKPKKRELINSMWELGIPK
TAKYSILDKYQIALTLADKNLFKKGVSPYQDVYLLIQVRNAITHYEPEWVTTESEFPE
KITEQALEKKLKHKFGLNPFANSPDPFFPDKCLSYEFIKWGIHSSMKLTEVFFEELDL
NYPISWLLNNEIYNLNLE"

CDS 1426299..1426859

/locus_tag="PIKHDJKN_01301"

/inference="ab initio prediction:Prodigal:002006"

/codon_start=1

/transl_table=11

/product="hypothetical protein"

/translation="METIIYMVRHAESPYDEGDERTRGLTAKGKVDGEKVTKLLVGEG
IDIIISSPYSRAVLSVEGLAQHLNVEIETFEDLRERHFASDYIIDLMSEIRNNFHKPE

YALPSGESNLDCQKRSIAVLKTILKEHKGKKIVIGTHGLVMTLMMNHFDSNYGLDFLD

QLKKPDIYKMQFEDLELKEVIRIWNE"

CDS 1427101..1427997

/locus_tag="PIKHDJKN_01302"

/inference="ab initio prediction:Prodigal:002006"

/codon_start=1

/transl_table=11

/product="hypothetical protein"

/translation="MKSFIQELSERKYSEFDSFPFYFIPGDYHDLDFHEFEDGSMYEL

NYEDDFFVSLQINRFDDILLFEFIKLGDDLGYYKDWSFRKFVSRMKAVVDQSDNMFVN

NYDDSDNEIARAFFLTMTVSVEEFPNFFEAYLHLTKRCKDIIRITENSLNSIIWDKEF

EKDEKLFCDVFLTRYFKSLGFDKVKYNHGNKEFGKDYILEIVNIFGDREYFAVQVKAG

DLSGNAKGSIFEITNQIKMAFKVPYNDFYGKEIYISKMLVIFSGVISENAIQIIKSDI

DNYMKANILFFDKKHLLSLREL"

CDS 1428188..1428721

/gene="yfiT_1"

/locus_tag="PIKHDJKN_01303"

/EC_number="3.-.-.-"

/inference="ab initio prediction:Prodigal:002006"

/inference="similar to AA sequence:UniProtKB:O31562"

/codon_start=1

/transl_table=11

/product="Putative metal-dependent hydrolase YfiT"

/translation="MDQIKYPIGQFQPKNNLSNDEIINLIKQIPELIKRLNALLVGLE

QYQLETPYRPNGWTVKQVIHHLADNDMNAYLRFKRGLTENNPLANTYREDSWGELIDY

KKLPVENSLSLIEILHHRFLVLLKELSIEEYKRTIQTEVLGIITLETAIQRFIWHNKH

HASQIENLIRREKWKDI"

CDS 1428928..1429188

/locus_tag="PIKHDJKN_01304"

/inference="ab initio prediction:Prodigal:002006"

/codon_start=1

/transl_table=11

/product="hypothetical protein"

/translation="MKRYRVLNFSFDSRSTLLNTEINEDWEEEAKLRWKKNKQFIYEG

IIHTYGVENYDSKLNNFSSFGLLPFSIIAFHNKFFKQIRDLS"

CDS 1429251..1429757

/locus_tag="PIKHDJKN_01305"

/inference="ab initio prediction:Prodigal:002006"

/codon_start=1

/transl_table=11

/product="hypothetical protein"

/translation="MVLLLRDEYKNTEHYKNVYSKKAFDNWDKMITPLNDWGVLLPEV

VEKFNELKKLRNYSIHFNPDTDVQDKEYAQSAIDILKAIIELQFGVGGPVLRPWFINS

IPGAFYISKEAEELPFVKKIILPNCALVGHKHRLENEANWFKVIDDYEYEDKEITDEE

FKDLLVTQ"

CDS 1429957..1431354

/locus_tag="PIKHDJKN_01306"

/inference="ab initio prediction:Prodigal:002006"

/codon_start=1

/transl_table=11

/product="hypothetical protein"

/translation="MNEMNKDTKMVELAVGLDFDQNIDVLIAELSEILKNKNAQIITK

EVNDETVVTCIYKDQIRTFEDLNLVLEFNFTKDIFKSMKVSVAHHQINENDLELYIYK

NIQIKLLNSVYKENSSKYTVRVYKCIFNKTTIKGVYTINWGNKIQMFPLMDFEKDESF

SEHIIVFDVEVRAVNLDQARSMAYNIVSDFTAFLSLLIDVGFQDLRSKYMNFLMKDNF

GRLQASRYRTGAYDKELGLYIKDNFYGITPEDKPGYDYGPIYLLAEETKAKAKVTYQL

DSDAFLEDKFKNREIKKKSTMKNEYLEGSFAEIHNPYNSIKVPRKIREYFREIKQLEE

KNVNKYEFFRNASRIYALAKNIEQFNETSAISYMVASVEALGKSEKMSFSEICRKYLG

EDVDLKFLDYLYGNVRSGHFHSGEFAFLEYEVNFNRSMDSLYTNLIRKNRQAKKSLRK

VLVSWIEDNLNMRYH"

CDS 1431598..1431720

/locus_tag="PIKHDJKN_01307"

/inference="ab initio prediction:Prodigal:002006"

/codon_start=1

/transl_table=11

/product="hypothetical protein"

/translation="MDIEDLFMNDGMLGPKRFYEFETFILKMLKHHIEDQNKCF"

CDS 1432165..1432824

/locus_tag="PIKHDJKN_01308"

/inference="ab initio prediction:Prodigal:002006"

/codon_start=1

/transl_table=11

/product="hypothetical protein"

/translation="MSELQIMRDERQKNELLLNQEFFQKDFNINFHKSVRNVILLTIL

LGGFLAWCSAGTESNIYLFLSNYNLDVSESNALFLVIGFLILMISLFSFPITVKNTYK

KIEVEALLFSSTQSEIFKNFICTKLSYLDSTPITNIQDVFKYIKTEKAEDIAFTEIEF

EKYLSSYLQKKRIIMSALFYGRYAKLILLNQCIEKTLVQIKSNPYGEYILITKDNYSI

L"

CDS 1433560..1433745

/locus_tag="PIKHDJKN_01309"

/inference="ab initio prediction:Prodigal:002006"

/codon_start=1

/transl_table=11

/product="hypothetical protein"

/translation="MSRLLDLLEEERRKLNQLGEASLKQAIPLWDNPEVQEQSRRVDE

LVAQVGEMKARHNRVVR"

CDS 1433782..1434084

/locus_tag="PIKHDJKN_01310"

/inference="ab initio prediction:Prodigal:002006"

/codon_start=1

/transl_table=11

/product="hypothetical protein"

/translation="MNFPPWLEQAIQARLDEVSAQIEHDSKLSRVREETDEAFEALFA

GKDTEQTAEYAEWENRYIVSKGIENERLYMQGLRDGIELTVSLLGQSMPEEGNTKA"

CDS complement(1434209..1435891)

/gene="lipO_3"

/locus_tag="PIKHDJKN_01311"
/inference="ab initio prediction:Prodigal:002006"
/inference="similar to AA sequence:UniProtKB:P37966"
/codon_start=1
/transl_table=11
/product="Lipoprotein LipO"
/db_xref="COG:COG1653"
/translation="MIKFKTWWSLLMVIALITITACGSGETAKENGTKDDTPATVGSA
EAEAAFAKGKYDPPIEFSSVLMPKKYVQGDTKENNVHDRWMLETLGMKHKDTWYPAND
DQYRQKLQLAIASGEKLPDFVSVPTNAVLTNQLIDSGQFIAIDELFDKYASQTLKDHA
AAHPELWYPFTKDGKKYNMPIMEYTDNDDTLLWLREDWMEKLNLEAPKTIADLENIMD
KFKNENPDGLSPDKVFPLAISLKNNTNTWMGQLDWLFGAYGTIEEQWNKDANGNLEYG
SVNPGAKQALAKLAEWMNKGYIHADSALWDEGKSAESWTAGKAGILPGANWVPDWPAP
DLLKNVPGSKYKAYPVPAGPDGKIGTKWQNSGVNASIMINKDAKHPEAIFLYYNYLLD
NLANPAAGSEYEYGFAKGYDWDIIDGQPSSDKEKIKDFSNEFPFLTGPARIPDLYMKT
LVKLANGEKPETPYEKQMAEFRKPENWYAGKVVMSQIDIRKQNYFTGAATPTMVSKWN
LLRQSEMETFNKIIYGQLPVDAFDQFVTNWKANGGDQITQEVNDWFKSVSGK"
CDS complement(1435996..1437606)
/gene="rhaR_8"
/locus_tag="PIKHDJKN_01312"
/inference="ab initio prediction:Prodigal:002006"
/inference="protein motif:HAMAP:MF_01533"
/codon_start=1
/transl_table=11
/product="HTH-type transcriptional activator RhaR"
/translation="MQMIIVDDEAHWVDNLSMTKPWHTLGIEQVHKAYSAHEALQLID
THPIDIVISDIQMPEMTGIELIERIRVRDKKIKCILLSGHSEFDYAKKAIQFEAVDYL
LKPPTDDELMGAVQKAIDQLNTEWELVSSLKRTQFTLRENLPHLRGRLLLEALQGHRI
ASSEWARKLANYDLPFHAGDCALMLVRMEEEFGKYDNNDQTLLEYAVINMAEEIMGEF
MEVWGVKEEHGYLVFLLQLKVNNTDIGKETILEKLSVQLQSKVKQFLKGSLSIVITEW
FAFPDQLYDRFRQASAYFRQIVGDEREFVMRVSDVETPSAQGPLDVLYTPPTFIHLLE
SGQWDAAEEKILAVCAELDEKWSESWEHCMEAGFLITASFTNLAHRNKLTLANLMGDD

IEWLQSGEAFTTISKLRKWSLSVLSKLKEGTSNEIKDIRSEYVKKIQDFTDKNLHLDV

SLRVLADHVNLHPTHLSKIYKIETGEGISDYISRLRMDRACHKLVTTTKKVYEISMEI

GYMDPAYFIKVFKRQFGVTPQEYRDQNK"

CDS complement(1438033..1439835)

/locus_tag="PIKHDJKN_01313"

/inference="ab initio prediction:Prodigal:002006"

/codon_start=1

/transl_table=11

/product="hypothetical protein"

/translation="MNTWKRFISFGRMSRSRFSLFAKINCLIVVLFIPIIIMYTYSNN

VTYDVVSKELQVSNTKQLTFLSSQIDSRINQMMDFSLILSRDPNVRAFNGLNIWNDRY

DKMQTRYVIQEKLMLQSGVTDIWPTRYAVHSQQNQDVIANYNRTTGYDEDYLKRNMSG

QWTYGDGAESKEDMKSFYWFFTDSLAQPGMLTGSNLVIEASFSYENIQNMLDTYKEGG

QGDPFFYHKGNSPILNRSADKQLSEELIRYLDEHSTEDTTQDVVKLNGKKYLVSSVKS

SYLDWHLVDVVPLYQILQPISLSRNLFYLFMILLFVVGISASILLYRNIQYPIKKLIQ

GLRRVQRGDYSVRLHSKDQNEFSFLFHRFNDMSHQIQDLIENVFQEKIRAKEATLKQL

QAQINPHFLYNCLGYIINMAQMKDEQAVVSMAHNLSAYYRYTTRVERETSSLKEEINL

LVNYLDIQKLRNGRIEYHIDIPEDMLAQSVPRLMLQPIVENSVIHGVAKSYSSGEIRI

TGESSNGFCKIYIDDDGPGLSPDQLEALNLKMQQPLQEEMGCGLWNTNQRIMHLFGNQ

SYLLFGPSPLGGFRTEMIWEIPKEDTERGDTCKS"

CDS 1440007..1440969

/gene="yteP_7"

/locus_tag="PIKHDJKN_01314"

/inference="ab initio prediction:Prodigal:002006"

/inference="similar to AA sequence:UniProtKB:C0SPB3"

/codon_start=1

/transl_table=11

/product="putative multiple-sugar transport system

permease YteP"

/db_xref="COG:COG4209"

/translation="MEANMEANRVPQADIGSKPNGKTKKRYKQPWILHLMVLPAAIMV

FIFSYIPMSGILMAFQDYKPALGFFDSEWVGLKHFRYMWENDYFLQITWNTLFFACSK

IIMNLIIPFVFALLLNEVRKMALKRTIQTLVYLPHFLSWVTLSGILIDILAQTGIVNQ
FLVSVFGIKPIFFLGDGSWFRFTIIASDVWKEFGFNTIIFLAALSGINPSLYEAAEVD
GAGRWKQTMYITIPALIPIGIVIATLALGNVLNANFDQIFNLYSPLIYQQGDIIDTFV
YREGLLSGQFSFATAVNLFKSVISLILIVISYRLAYRFAGYRIF"

CDS 1440986..1441858

/gene="lacG_1"

/locus_tag="PIKHDJKN_01315"

/inference="ab initio prediction:Prodigal:002006"

/inference="similar to AA sequence:UniProtKB:P29824"

/codon_start=1

/transl_table=11

/product="Lactose transport system permease protein LacG"

/translation="MYHKTMPYRIFSIINNVLLTILSLLCLLPLYHLLMVSLSASAPA
NAGLVTFWPIGFTLEAYAKTFANSNFLSSLWVSVERTVLGTGLALIVNTIAAYALSKE
TRVFRARNIYLWYFVITMLFSGGLIPGYILILKLGLMNTLLALILPGLVAVFNIILLL
NFFRTVPKDLEEAAFIDGAGHLQTFIKIYLPVSVPVIATVSLFMMVGHWNSYFDGIIY
IRDAEKLPLATFMQTIIVQADMSKLDPEAVANLSQRTIRASQIFISALPILLVYPFLQ
RYFVTGIVVGAVKE"

CDS 1442051..1442707

/locus_tag="PIKHDJKN_01316"

/inference="ab initio prediction:Prodigal:002006"

/codon_start=1

/transl_table=11

/product="hypothetical protein"

/translation="MKKMMIVTLVLSLLSASLAFAAASAPQKISIVLNGKELKTRAGT
EVKIIDNHAYIPASVLRDARFAVEYKNSKLNLVNSYSHYTKNLIEMHLSNHEFTSQFN
KIEQEVIDILSKVILEEQVDISRLQQLIKEAESNVVIRPEVFTPVQDYSSSDLSDAFY
SLEAYKKASIELIAYVHTGDKERLKSFHEERKKALEYYDSYTYMYDRIFKLSYISATK
"

CDS 1442779..1443360

/locus_tag="PIKHDJKN_01317"

/inference="ab initio prediction:Prodigal:002006"

/codon_start=1

/transl_table=11

/product="hypothetical protein"

/translation="MIQNHNSWSVCISSDFELNFLIYAASTYGLLKEDINENNLWPSR

QRVESDQLHQKLEIQWKWFWDRSIQQKAHKNNNHVSSVLGRPDFELVEQHELREVLVH

LWPSFRQWWYMPAGGQAAMNYWEGKPDLIRYVQEFEDEVDRKIKPFHLNMDLIYNGPS

EPIEVNEEYIIMPIRTEYLMKKDWWISRFKERY"

CDS complement(1443658..1444245)

/gene="kefF"

/locus_tag="PIKHDJKN_01318"

/EC_number="1.6.5.2"

/inference="ab initio prediction:Prodigal:002006"

/inference="protein motif:HAMAP:MF_01414"

/codon_start=1

/transl_table=11

/product="Glutathione-regulated potassium-efflux system

ancillary protein KefF"

/translation="MNVLVVVSHSRKDSLTFQVTDRFVQGLAEAGHGYEILDLHEIGF

DPVLQGIDEPDFTKEHQVFSPEIEAEIERLKKHDAVAFVFPLWWWHLPAMLKGYVDRV

MNNGFAYGSNKLHHQQMLWIALAGVTEEQMQKRNYDESITNLLNVGIANYCGVPQSSV

EFLYETLDAQPEHYEALLKQAYQLGLNYANDNPTL"

CDS 1444492..1444878

/gene="ytcD_1"

/locus_tag="PIKHDJKN_01319"

/inference="ab initio prediction:Prodigal:002006"

/inference="similar to AA sequence:UniProtKB:O34533"

/codon_start=1

/transl_table=11

/product="putative HTH-type transcriptional regulator

YtcD"

/db_xref="COG:COG1733"

/translation="MTEHGKTSTPKKYKVGVEAALEVMGGKWKPLIIYHLMTGRKRTS

ELRRLIPDITQKVLTTQLRGLEKDEIVQRKVYSEVPPKVEYELTSYGWGLKPALDFLC

YWGEEHLEKVHGDKSKVLEEFDSPEC"

CDS 1445143..1445532

/locus_tag="PIKHDJKN_01320"

/inference="ab initio prediction:Prodigal:002006"

/codon_start=1

/transl_table=11

/product="hypothetical protein"

/translation="MLAIEVILLSNVIFDENGILKYFGIVILTFILGYIILLIRRLFF

TKPSITLESDHVISTKHRKYDASQIECIYVNYKRIGIKLYGKRLVPMDLCFYFQRNQE

TEGLEELYAWAARNHKDMKHKFFQTLT"

CDS 1445681..1445911

/locus_tag="PIKHDJKN_01321"

/inference="ab initio prediction:Prodigal:002006"

/codon_start=1

/transl_table=11

/product="hypothetical protein"

/translation="MADNPDGEFPEGEEPDEGDRSEVVEVLGFTRSVIANNIIEYDMV

KNHPKQLTAYFKRTRIPGAAKFSSQNKRYMLQ"

CDS 1445993..1446856

/locus_tag="PIKHDJKN_01322"

/inference="ab initio prediction:Prodigal:002006"

/codon_start=1

/transl_table=11

/product="hypothetical protein"

/translation="MKYLTKEWYETCQRTGLHFSMRVHSEAYELNEALFQRLYKRKEK

AHVKQERELYNLDPRFMLEHDGQVLTRVDKAFSGGAVKEEDQMVYHMPLEERTHIENL

IAEYDARPPFDEKKCKEKYKEAMGWNFEHKAERLPQNIVEQIADIRLFTLGYCTREIL

LQLKKQSAENRREMVRVSKEYREAIVAQDIPGEIHSQVQFHDCTVTELLTGDEVVIRF

DTRGGFTKINKLTLVTPEIIKQEEEIAGSYWLYQELYRIDQGYELHVLFGGEHMPELI

VRCEDILAEEE"

CDS complement(1447002..1447799)

/locus_tag="PIKHDJKN_01323"

/inference="ab initio prediction:Prodigal:002006"

/inference="similar to AA sequence:ISfinder:ISBth8"

/codon_start=1

/transl_table=11

/product="IS3 family transposase ISBth8"

/translation="MVWLCKIAEISRAGYYKWKKNIVLREKRADRDADLKAHILGIHR

LRPYFGYKRMRTALGKEGLVVNHKKVRRLMRELQIRSVIRKKRPFAGRKPSVLFRNVL

NREFSAAAPLQKLVTDITYVRIGHDFAYLSVVMDLYNNEIVAWELSERNDLKLVMDTV

KKLKCGPTLLHSDQGFQYTTQSYAKLLEEKKLTGSHSRRGNCYDNACIESFFSHLKTE

KLYLEKPQNLEQARNRITEYILFYNKERFQNKLGDLSPIEFREKVAA"

CDS complement(1447814..1448152)

/locus_tag="PIKHDJKN_01324"

/inference="ab initio prediction:Prodigal:002006"

/codon_start=1

/transl_table=11

/product="hypothetical protein"

/translation="MPAKKGQTFNQYSEETKKEAVRLRLEEGWTYSRIMEKFGIKSES

QIITWVRKSHNGESFEDYRGRWTKKHFSTAEEENAYLKAQVEYLKKLNPNLHGEGSWI

SKPGASPFEK"

CDS complement(1448304..1449359)

/locus_tag="PIKHDJKN_01325"

/inference="ab initio prediction:Prodigal:002006"

/codon_start=1

/transl_table=11

/product="hypothetical protein"

/translation="MSKTPHDEEHVFQEEDFEYQRPEWGHKQFKRMVWKTRWKFFLNA

GGALFLVFMVYHIYVSSLHIYFDQSKVRNDFIRSIVSVVEMHGDGLRVEKTNYELFEV

TPLLTQKANLKIYRHVGSWEVITGEIQAELSITGKFTYTITNTGAYMNGNNPGPFYLP

YSLVDGIPTKDKDASDESSSLNRLRKIDDGHVAEMSLSLKNLASPDQLMQLLTDYDVA

VTAIPIYSGELKDLDISYSRSGMFNYNIPHLSLKPLSEFKEDGAKWYNYFVPEDSGQM

QEQVQAMMSDLEWMSNHLEYSGADLDKQRLAYLKKNGVQVYGAVVTGPVRELEKLTKV

PEFQHFHLNRVEIWNWD"

CDS complement(1449362..1449871)

/gene="ylaC"

/locus_tag="PIKHDJKN_01326"

/inference="ab initio prediction:Prodigal:002006"

/inference="similar to AA sequence:UniProtKB:O07627"

/codon_start=1

/transl_table=11

/product="RNA polymerase sigma factor YlaC"

/translation="MRSDLYAELFKQYQSSLHLYLYRMCGSQETAEELVQETFYRAMV

SMKAEHANYARAWLYKVARHLFIDWYRKYRGEIQMNREMEQQATENTYRSPEEVLTAR

ERTLRIQQVMKKLPEQYRTILVLREMNELSYKELCEILDMNLNKVKVTLFRARERFKA

EMLQMKGDV"

CDS 1450107..1450532

/gene="gph_2"

/locus_tag="PIKHDJKN_01327"

/EC_number="3.1.3.18"

/inference="ab initio prediction:Prodigal:002006"

/inference="protein motif:HAMAP:MF_00495"

/codon_start=1

/transl_table=11

/product="Phosphoglycolate phosphatase"

/translation="MTKSNLDVQAVFMDRDGTIGGTGHFIHPRDFELYPNAKEALMLL

KTCPHEQECSCRKPKPGMLLHASEQHDLDLSKCVVIGDVGDTDMLAAHAVGATKIMVK

TGWGESSLTKFRDRWSETEPDYVAEHILDAVQWIIRGPE"

CDS 1450588..1451061

/gene="rppH_2"

/locus_tag="PIKHDJKN_01328"

/EC_number="3.6.1.-"

/inference="ab initio prediction:Prodigal:002006"

/inference="protein motif:HAMAP:MF_00298"

/codon_start=1

/transl_table=11

/product="RNA pyrophosphohydrolase"

/translation="MGMSDYYKNLREKIGNELIFMPGVAGVIRNEQGEILFGRKHNET

NWGLIAGAIELGETLAQAMIREAWEETGLIIDPEKVIGIYGGEEQRFTYSNGHQVEYL

TIVFECSIKSGQLASDNEEIKDLQFFPENELPPIANKYPDYIFSSNQEERTHFIK"

CDS 1451413..1452276

/locus_tag="PIKHDJKN_01329"

/inference="ab initio prediction:Prodigal:002006"

/codon_start=1

/transl_table=11

/product="hypothetical protein"

/translation="MKAWLLDYYPPNNTIEFSDSEELDQVYEALGSDKPIIQEWSAPI

VEFNNVGKPADILNRYTGALVISRKVKELLEQNHIEHIEFLPLSTATEEEYYILHVLS

VIDCIDPENSKVRTYYDDVPQFDEYSLVLDQKALQGQHIFRIKLPSEDRELSYIYVSD

FLQEILSQSIVGYQLVEIWDSEFSWKAKEEKHEQMSKEIDSSLLEVFDFDKAKKFARK

NKGHVVYSGKWAIKADKEKRIWLGNLQLDGSYSWINPIFYPPILLELQWGIPKRSKTM

FSRLKESVGFK"

CDS 1452318..1452695

/locus_tag="PIKHDJKN_01330"

/inference="ab initio prediction:Prodigal:002006"

/codon_start=1

/transl_table=11

/product="hypothetical protein"

/translation="MRSSRFTPYLSFIGLGLIIMTLAINLIFHYGRGLDEGSLMLLSV

ANAVSLFFTLVWGLFGIIELYLLLISNKKLKSRLDTGRISKEEYMKLAKNHKFSFVVN

ISYLVMLLIQLGYVIMNWDEVNI"

CDS 1452708..1452995

/locus_tag="PIKHDJKN_01331"

/inference="ab initio prediction:Prodigal:002006"

/codon_start=1

/transl_table=11

/product="hypothetical protein"

/translation="MKVLSIIGIVLFTLTFLLIFGLGPEDPDSAAILGLLSMLYAIPF

SIIALVVSSRRKKQPVNVHEQLLQLGELKEKNIISEYEFDQKKEKLLSKIK"

CDS complement(1453106..1453522)

/locus_tag="PIKHDJKN_01332"

/inference="ab initio prediction:Prodigal:002006"

/codon_start=1

/transl_table=11

/product="hypothetical protein"

/translation="MTNKAFFPILLLILLLTACVTENHDTEVILRKEAIPIITNVETN

YPEKEGWIVIPTDSTELNVTVEATNAETLLFWAVPTGTETWGERELIGYDTNGNDGWK

ITWDIAGKSLHQHVIVQALGSDGMTITDETLNITNE"

CDS 1453666..1453878

/locus_tag="PIKHDJKN_01333"

/inference="ab initio prediction:Prodigal:002006"

/codon_start=1

/transl_table=11

/product="hypothetical protein"

/translation="MELFGAFIALILCFIVFLIVAAVIRYAIDSSKTSKRLEDLIKEV

HYLRTEIKKQNNNKQGDNKYIIDEKV"

CDS 1453945..1454361

/locus_tag="PIKHDJKN_01334"

/inference="ab initio prediction:Prodigal:002006"

/codon_start=1

/transl_table=11

/product="hypothetical protein"

/translation="MRSDFIELVEESDERYKCYVLKNTVQIFKQSIKDEDLKDVRIYI

STTIQLDAIADVIDSYLHWFTECEAVFRNYYENELHEQVHKDWFNEIEVYQVDITFNS

KEDYGATIACGDNVLQGHIMIIDFDREHIQAIHLNG"

CDS complement(1454363..1454491)

/locus_tag="PIKHDJKN_01335"

/inference="ab initio prediction:Prodigal:002006"

/codon_start=1

/transl_table=11

/product="hypothetical protein"

/translation="MGKFQKFMSVILVLITLLLFLNYLELRENNRLQNIIIDGIYN"

CDS complement(1454665..1455633)

/gene="czcD"

/locus_tag="PIKHDJKN_01336"

/inference="ab initio prediction:Prodigal:002006"

/inference="similar to AA sequence:UniProtKB:O07084"

/codon_start=1

/transl_table=11

/product="Cadmium, cobalt and zinc/H(+)-K(+) antiporter"

/db_xref="COG:COG1230"

/translation="MSGHHHNHDHGHNHGSTNNKKVLLFSFIIITVYMIVEAVGGFIT

NSLALISDAGHMLSDSIALGIALLAFTFGEKAVNTGKTYGYRRFEILAATLNGVTLIA

ISLYIFYEAIGRFINPPEVATVGMLIISIIGLLINILVAWIMMRGSDTEKNLNMRGAY

LHVISDMLGSVGAIAAALLMMFFGWGWADPLASVIVAALVLRSGFFVTKSSLHILMEG

TPANVDVNDLVQTIKQVDGVKGVHDVHVWSITSNLNALTAHIVVDGTMDVYASEVLVQ

KIEHLLEHKEIKHVTLQVESENHLHDTSVLCTVKGDEPDAHAHHHH"

CDS 1455939..1456616

/gene="hssR_2"

/locus_tag="PIKHDJKN_01337"

/inference="ab initio prediction:Prodigal:002006"

/inference="similar to AA sequence:UniProtKB:A6QJK3"

/codon_start=1

/transl_table=11

/product="Heme response regulator HssR"

/translation="MKNLLLADDDANIRALLRHVMTKEGYRVHEAQDGVEAVKVMQAT

PIDLAILDVMMPGMDGLELCDFIRQHYDIPIMLLTARDQLSDKRDGYLRGTDEYVTKP

FEPEELVYRVKALFRRYHRTSSDIIRMNRIVIDRNNVEVSDGQSILFLPMKEFELLSQ

LAQFPGRLFSRDELIRLVWGADYEGDDRTVDVHIKRLRDRFADYGDDFVIQTVRGIGY

KIEVKAH"

CDS 1456613..1457989

/gene="hssS_2"

/locus_tag="PIKHDJKN_01338"

/EC_number="2.7.13.3"

/inference="ab initio prediction:Prodigal:002006"

/inference="similar to AA sequence:UniProtKB:A6QJK4"

/codon_start=1

/transl_table=11

/product="Heme sensor protein HssS"

/translation="MRSLYFRVFMITIAVILVSAMLGFLLSNIYYHAKLKSFNDEKLV

GIATQMKQFVEQEPATVEHYLNNAAALGYEIYVTDGKGNDQFYGREFRQKDLDKQAVD

QVLSGEIYHGVAEFPSKPFITGFFDNQLSNTVGVHLEMGNASYALFMRPDVILQFGEL

RIFFALIGAFTVGISILLFIISTRYLVNPIERLSEATKRIAQGNYNLKLSTTRRDEIG

QLAQHFMTMSRELERVDQARQQFVSNVSHEIQSPLTSIQGFAQLVADRDLPEQEREHY

ASIIEEESRHLSLLSKQLLLLSSLEQGHEDLTKVRFSLRDQFRQAVQVLQWQLEGKEL

LLRISVPESIQLYGNEVLLMQVWMNLLGNAVNHLPQGRSIEIRAEETNAQCVIYIWDT

GDGIAAEHLPFLFDRFYRVDRARERSSGRTGLGLAIVQKIIRIHDGTIEVSSSTEGTV

FTVTLPQM"

CDS 1458056..1459180

/locus_tag="PIKHDJKN_01339"

/inference="ab initio prediction:Prodigal:002006"

/inference="similar to AA sequence:UniProtKB:P9WG17"

/codon_start=1

/transl_table=11

/product="putative ABC transporter permease"

/db_xref="COG:COG0577"

/translation="MYLAIREMRFAKGRYALIATIMVLVSFLVLFVTGLAQGLAYDNA

ASIKNMAATHFVLEQDSNHRFTRSQVNQDQLEQTRSVVGQENAEPLGVKMTTVSPAGD

TKKIDVTLFMVNPEGWLAPTVTEGSTITDLKDGQVVVDQKLSESGVRIGTELVDQASG

TKWTVGGFVENESFSHSPVVFLNEQEWLAIQEGSRTSQGTANSNAPVYNAIAVKDAGE

KVDSLSTALPNTEVITKSDAVSAIPGYKEEQGSLLMMIAFLFVISAFVLAVFFYVITI

QKTSQFGILKAIGTRNSYLAGSVSLQVLLLSIGSLVISVLLVQLFESILPASMPFQLG

LSTLALTCVLFVVMSMAGSLFSVWKVTKIDALDAIGRTAA"

CDS 1459177..1459854

/gene="hrtA"

/locus_tag="PIKHDJKN_01340"

/EC_number="3.6.3.-"

/inference="ab initio prediction:Prodigal:002006"

/inference="similar to AA sequence:UniProtKB:A6QJK1"

/codon_start=1

/transl_table=11

/product="Putative hemin import ATP-binding protein HrtA"

/translation="MRNRLVLQGITQSFEDGGVKRTILDKLDLKVAEGELVAVMGPSG

SGKSTFLSIAGALLEPTEGQVMLDGASIMGKNKQDISDVRLQQLGFIFQSANLIPYLK

VEEQLMVVAKLAGTDKNKAEKRVDELLDTVGLSHRRKAYAEKLSGGERQRVAIARALM

NDPAVLLADEPTASLDAERGLDIVSMIARLVKEQGKSAVMVTHDERILPLCDRVLFLE

NGKLVHH"

CDS 1460022..1460564

/locus_tag="PIKHDJKN_01341"

/inference="ab initio prediction:Prodigal:002006"

/codon_start=1

/transl_table=11

/product="hypothetical protein"

/translation="MKTLDREQFPALSTSIHWGIVKAEFRLNDIVDETLVSNVSIIPF

VGKQVVVFQIDNGKWELPGGTLEVGEKYMDGLEREVMEELGAKLVTYRVFGQFHCMSS

APESYRPHIPHPHFVRLIGYGEVELVGQPLNPVDGEQVIAVEVVEIGEAIKRFEEQNR

YDIAEMYRLAYLLREEANFE"

CDS 1460626..1461129

/locus_tag="PIKHDJKN_01342"

/inference="ab initio prediction:Prodigal:002006"

/codon_start=1

/transl_table=11

/product="hypothetical protein"

/translation="MIRDEINELLDALPDHELNVVYSRIELVHRKYMYNKNLEDKGVL

VTELCEESEEIIQKWDNTFAKNISEEVKEAIYYSQYKWHMFSYEKQDCLTDNAARDAF

NAENKNELYVMYQHTPFIQIFQNANNVIAEDFYSEQDIYIFDQEFTWTYVHTHESMCG

PYFYKVK"

CDS 1461215..1462249

/gene="rutD_2"

/locus_tag="PIKHDJKN_01343"

/EC_number="3.5.1.-"

/inference="ab initio prediction:Prodigal:002006"

/inference="protein motif:HAMAP:MF_00832"

/codon_start=1

/transl_table=11

/product="Putative aminoacrylate hydrolase RutD"

/translation="MRKRSIKIFRRTMLSILLIVILALIFPTWTPGIKGENSISTLGQ

VEINGVGHEVMIRGVDRNNPILLFVHGGPGCSEIPYVRKYQQELEQHFTIVHYDQRGS

GKSYHLLEDYSDVTSDVLVDDLLALTDYVTAELGQRKVTLVGHSFGTYIGTKAAAKAP

EKFIAYIGIGQMADTVQSELDSLEYTIAQAKQANNTADAESLERLRGPIERGEQYTPR

KLVRKYGGAARLIDDNRDYMLGFLLGTEYNALDVIRYSMGVQATQEILLKEGSSNHMP

DIVDQLEIPTYFVMGKYDYMTSVDAARDYFDELGAPEKDFVVFEESAHYPQFEEKERF

AAWLIGVLNF"

CDS 1462267..1462770

/locus_tag="PIKHDJKN_01344"

/inference="ab initio prediction:Prodigal:002006"

/codon_start=1

/transl_table=11

/product="hypothetical protein"

/translation="MEQINTKLITRLALPEEASIVLNLWQESARWLNSKEIFQWRPEY

FNLEQVIEFMTEGSDVYLAEINHEIVGTYTLTWSDPLIWEELDNTNSGYIHRFAVNRD

FKGQNIGLLLLKSAEEDIRQKGKTLIRLDCMADNLRLNQYYKDYGFQYIRRMKQGNWS

ASLYEKE"

CDS 1462826..1463299

/locus_tag="PIKHDJKN_01345"

/inference="ab initio prediction:Prodigal:002006"

/codon_start=1

/transl_table=11

/product="hypothetical protein"

/translation="MLLSYHPMKIVDAEKIATWKYEHEYAMYSFSESNEDIQELMNGE

YFSVLDELERVVGFICCGESARVPGGTSIGLYQEEGYLDIGLGLDPDLTGRKIGSVFL

KQSLVFLQNHFKSSQFRLVVATFNERAIKVYERTGLKEIRSFIAKCMTRTCYLSV"

CDS 1463266..1463802

/gene="bar"

/locus_tag="PIKHDJKN_01346"

/EC_number="2.3.1.183"

/inference="ab initio prediction:Prodigal:002006"

/inference="similar to AA sequence:UniProtKB:P21861"

/codon_start=1

/transl_table=11

/product="Phosphinothricin N-acetyltransferase"

/db_xref="COG:COG1247"

/translation="MHDEDMLFISMKMEMPIYSFEEITEQDLTEVREIYNYYVSNTTI

SFHTEELTLEEIKASVMNKNPRFKSFVIKEEDELKGYVLITQYKSKQAYDFSGEVTIY

LKPDIVGKGIGGTALRYIEDVAREQGFYTLIATVCMENTRSKSLFEKYGYEQCAMFKG

VGYKFDRRLDIGSFQKVL"

CDS 1463806..1464846

/gene="thrB_3"

/locus_tag="PIKHDJKN_01347"

/EC_number="2.7.1.39"

/inference="ab initio prediction:Prodigal:002006"

/inference="protein motif:HAMAP:MF_00301"

/codon_start=1

/transl_table=11

/product="Homoserine kinase"

/translation="MNNVQYNLKFEKLVAELELGELISKPKAISGGLLHRMYAIETTH

AKYAVKALNPQIMSRPTAMQNYIQSEKIANVAANFIHAQPAKVINGSSMQILDHQCYL

IFDWIDGKSLELHEVIETHCEHMGMILADIHKTDFSQLHIPDLQLDHSKETDWNDYLN

KGQTENSDWTILLADNIQKLYEWSAQAKNASVILASNTVISHRDLEPKNVMWQQDTPI

IIDWESAGYINPMHDLVETAMYWSVDHTGIVEKERFLAFINGYRSRVGNLNANWRRVL

ENGFSGKLDWLEYSLKRSLWIECTDNEEQQMGTSQVTWTIEALKQYEDMISDVEHWLS

KSEIIVIRYRKG"

CDS 1464852..1465145

/locus_tag="PIKHDJKN_01348"

/inference="ab initio prediction:Prodigal:002006"

/codon_start=1

/transl_table=11

/product="hypothetical protein"

/translation="MANTKGTLRTCEQGHSYYKSSDCPTCPVCEKERKPKEGFLSLLS

APARRALENQEITTLPLLSKYTEKEILKLHGIGPSAMPKLRQALEEEGLSFNV"

CDS 1465292..1465798

/locus_tag="PIKHDJKN_01349"

/inference="ab initio prediction:Prodigal:002006"

/codon_start=1

/transl_table=11

/product="hypothetical protein"

/translation="MEPQFEEQKVDYRPLGISGLGGWLILVHIGLYLTIIMLVVQLFQ

YLVPSLTTETWELLTSKQSDYYHPWWGPVLVFETVYNVLFLVFSVYTLITFYRKKSIF

PRLMIIFYSVSLAVGIIDYLLLLQIPMAVELEDGSSLKGIGRSVLTCAIWIPYFIKSV

RVHNTFVR"

CDS 1465877..1466491

/gene="gpmA_1"

/locus_tag="PIKHDJKN_01350"

/EC_number="5.4.2.11"

/inference="ab initio prediction:Prodigal:002006"

/inference="protein motif:HAMAP:MF_01039"

/codon_start=1

/transl_table=11

/product="2,3-bisphosphoglycerate-dependent

phosphoglycerate mutase"

/translation="MKIFLVRHGMDEEGYRGGWSQRGLIEEGVTQSERLGDYLYLHSE

QYKINTVISSDLPRAVQTAKELEKRLNIKGLYLKDWREMNNGDLAGMPNKEAEVKYAG

VYFNTLGMDTPFPGGESPRNFYNRICTSFRNLCRSIEEQQIKSNVLLVTHGGVINILY

YLLGNQNWTNTSVFHSIGNTSVHTVEKGVDGWEFSSMNMMSHLG"

CDS 1466625..1467281

/gene="pdxH"

/locus_tag="PIKHDJKN_01351"

/EC_number="1.4.3.5"

/inference="ab initio prediction:Prodigal:002006"

/inference="similar to AA sequence:UniProtKB:P21159"

/codon_start=1

/transl_table=11

/product="Pyridoxine/pyridoxamine 5'-phosphate oxidase"

/db_xref="COG:COG0259"

/translation="MSDKPSDLLRGLKSLSGPFPTWDTEQLPDQPGELFLEWLRRAID

YEVKEPHAMTISTVDSNGFPDARVLILKDVVDEAFYFASGSESRKGQQLESNPHVALT

FYWPTLGRQIRIRGDAVDMGEEAGAADFRKRSAGARAVAMTGQQSQELESEEELEHVI

AVQKERISRDPDVITPNWRLYAVNAKEVEFWQGDPERKHVRIQYILQHGQWKHQRLWP

"

CDS 1467303..1467611

/locus_tag="PIKHDJKN_01352"

/inference="ab initio prediction:Prodigal:002006"

/codon_start=1

/transl_table=11

/product="hypothetical protein"

/translation="MSRRTTGTLLLVIAAFLYGVRYLSAAIFGSGLASWNSGLFQDML

DYVGTAPRNLSIAALILGLFYLIWAEYEATSSKTRESLKRFVSYETVDQEAESQNKTE

"

CDS 1467678..1468247

/locus_tag="PIKHDJKN_01353"

/inference="ab initio prediction:Prodigal:002006"

/inference="similar to AA sequence:ISfinder:ISSpgl1"

/codon_start=1

/transl_table=11

/product="IS1595 family transposase ISSpgl1"

/translation="MKSIESYFDPFPVLETERILLRPITYADLDEMYRYCAVPAVSEY

TTWDTHQSKEDTKAFIDFVMSRYEMDKLGPWGIEDKQTKKLIGSCNYLGCDSNSMRVE

LGYVLSNEYWGRGIMTEVISRIIQFGFEDIGLERIQAKCLVGNIGSAKVMEKTGMKFE

GLLRKYMKIKNEMQDLKMYAMTREDFQLL"

CDS 1468280..1468906

/locus_tag="PIKHDJKN_01354"

/inference="ab initio prediction:Prodigal:002006"

/codon_start=1

/transl_table=11

/product="hypothetical protein"

/translation="MTKGILFVVLFMTLLSNSEVSDSLSEVSNLPPAPMIFTEEEFKV

QNKAKNITIQIGDTRAEVEQQLGKHVDYYYHTNVYQYKGMWIHYKEGIVDGIVIDDAA

ANSKKYKTPSGIGYGSTIQEVIQQYGKKAFIDSNQRKVRSITYLMERDKQGNYSVITS

FDKFVLEGGYENLMILSMVLDQQSRVSFLMIADYEFAYHPEHRWTETQ"

CDS 1469032..1469988

/gene="moaA_2"

/locus_tag="PIKHDJKN_01355"

/inference="ab initio prediction:Prodigal:002006"

/inference="protein motif:HAMAP:MF_01225_A"

/codon_start=1

/transl_table=11

/product="GTP 3',8-cyclase"

/translation="MTTKNRYKSLELETPGVYELEGLEVGVTSNCNYKCDYCCAYNRD

DGQCISGQEVIQIIRELPQLKRVRLSGGEVALKYQDCLEIVTYCGAHGIDTQLNTNAS

LLTEERIHALRDAGLSNIHISFNYTDRDAYAAYYRVHPRMYERLEHNIRLCTEAGLET

VLETLLFEGTQTNMQAISDKVYELGVRIHEIQISIVMSHSQWSQIASKESLIHSVNEL

IKHKKEDTTLYFTCMDRFAEQLGLREQPGVYFSNCVDGTKQLHLHGNGDILICELCHP

VVIGNIYEGTSLKDIYTKQPQALTDYLEKKPCPAYDALFAGE"

CDS complement(1469934..1470146)

/locus_tag="PIKHDJKN_01356"

/inference="ab initio prediction:Prodigal:002006"

/codon_start=1

/transl_table=11

/product="hypothetical protein"

/translation="MNLKQAERKKRRSSFPPRGNQTTFHISDNDGSQSIYVPQIGTST

MLNSFYKRDYSPAKRASYAGQGFFSR"

CDS 1470204..1471190

/locus_tag="PIKHDJKN_01357"

/inference="ab initio prediction:Prodigal:002006"

/codon_start=1

/transl_table=11

/product="hypothetical protein"

/translation="MMKIYPKTNENDPYKNAKPRRKWLIITLVIVLGLGTVIYSLADR

YLIRHVQVVVADDNTSATSASSDTSTTATEVSATSDDWNYSSDDMQVKIDKVQTGSGS

DQITYYVADVQLTDASSLRSALADNSFGTNITENTSEIAAANNAIFAINGDYYGFRDD

GVIIRNGTVYRDDPVRDAMALFSDGTMKTYNETEESSSELLEEGATNTLSFGPILIQD

GEIVSDFSSVKIDNNFGNRSIQDANPRTAIGMIAPNHYVFVVVDGRQDDSRGMTLAEL

ADVMKGLGATEAYNLDGGGSSTMYFMGRVVNNPLGRNQERGVSDILYLKEGQ"

CDS 1471187..1472206

/locus_tag="PIKHDJKN_01358"

/inference="ab initio prediction:Prodigal:002006"

/codon_start=1

/transl_table=11

/product="hypothetical protein"

/translation="MILTILIPSYEPDVRLLNLVLQLQTFQLGPIVIVDDGSGPGYRG

IFETAEAYGCTVLTHTVNLGKGRALKTGFQHIKEHGPQGGIVCADSDGQHLPHDIKRI

FDVLLEQTTPGIVLGSRRFSGTIPARSRFGNTVTRGVFSLTTGTKVYDTQTGLRGFPY

SMLDWLCQIPGERFEYEMNMLLTAHKEGYEITEEFIDTVYLDHNKSSHFRPLVDSFRI

YMPILMFSTSSVLSALIDFGLLFVIQYFTHNLFLSVVAARLCSSIFNYTINRKYVFSA

GKTSRVRQSLPKYFSLVILVLLLNYGLLYFYNEKLIIPLIAAKLLTEVSIFVFSYWAQ

RRFVY"

CDS 1472371..1472799

/locus_tag="PIKHDJKN_01359"

/inference="ab initio prediction:Prodigal:002006"

/codon_start=1

/transl_table=11

/product="hypothetical protein"

/translation="MVPQRVSLVTIGAMHLPELRSFYQKLGWSETAISSDQYSVFETT

GVLLSLYPIQELEKDTGMTFSASDGLKGTLLAINVDKAEDVDDTIRKIREAGGNVIKE

PYDAAWGGRIAYFLDPENNCWEVTWNPTAVFDEKGAMITF"

CDS 1473194..1473901

/locus_tag="PIKHDJKN_01360"

/inference="ab initio prediction:Prodigal:002006"

/codon_start=1

/transl_table=11

/product="hypothetical protein"

/translation="MYLRSVELLSAKVENPDQYPFHIPSIQSLERLEFNNNITFFVGE

NGSGKSTLLEGIAHQCGFNTAGGGRNNYFEIDAAESSLGDYLRLSWLPKISKGFFMRS

ESFYQFASHVDATDSIDYYGGRSLHEQSHGESFLNLFVNRFNSKGIYLLDEPEAALSP

ARQLSLLRILHDLSDNSQFIIATHSPILLGYPGARILSFDNGGIQEVEYEDTEHYQIT

RSFLENRDRMLNELFKD"

CDS 1473938..1474423

/locus_tag="PIKHDJKN_01361"

/inference="ab initio prediction:Prodigal:002006"

/codon_start=1

/transl_table=11

/product="hypothetical protein"

/translation="MSTKLVLLDQLAACHHDKSWFMPLDEMLRDVTAAQALWLNDEGQ

SIWRLVNHLTFWNETWLERFIKGEVGSDNSINNDETFIVDPSHVSEEDWQKSIAQIGA

TFSRWIQAVEDSDDAKLGMTIPSYFNAPWWNVISNLCIHNAYHIGQIMMLRKKVRGEM

I"

CDS 1474420..1474908

/locus_tag="PIKHDJKN_01362"

/inference="ab initio prediction:Prodigal:002006"

/codon_start=1

/transl_table=11

/product="hypothetical protein"

/translation="MKIGFLIVDMQESIVRDKLDQKEIDRTCEYINHVADVLRSSGHV

VVHVQDVEGMEDSNSEAYRIISEVDVNEKDLTVTKESSNAFWQTDLEQILKSHGVELL

IISGFAAEECVLFTYNGAMERGFRPVMLQNGILSTHSEAVISTYRDRNVISYPVVNYL

IK"

CDS 1475114..1475629

/locus_tag="PIKHDJKN_01363"

/inference="ab initio prediction:Prodigal:002006"

/codon_start=1

/transl_table=11

/product="hypothetical protein"

/translation="MNVQITRIDKSQKELFLNLYNLYLYDLSEFSGEDLLKEGKFDPT

NTYLYLERAELHPFFIQYDGKVIGFVLVCSPPYVDDDVDFTVQELFLVKKYRGQGLAA

QAVELVFAQFQGRFKVEQLANNQSAVSFWKKYYEQHHIEYVESEYSIEIENIAGSHRI

LSQTFVCARMD"

CDS 1475665..1476249

/locus_tag="PIKHDJKN_01364"

/inference="ab initio prediction:Prodigal:002006"

/codon_start=1

/transl_table=11

/product="hypothetical protein"

/translation="MEFKLRVQNSFAFTNTWVMMNEKGTARENGGSDSMPIHNFVIEN

KSEINESIYTSYSWDTHGVIHKSGLMAEEHAYLEDEFISYIQDSLNWLNTWNPSICKR

CSGLNNYGITVIEEKDALLKFHQLIRAWIDVFNLAPDQIKLTGDYCFDGNEQRGYYEK

LIYNRVELINELNKLANMAKDAELNGKCILHFGI"

CDS 1476329..1476790

/gene="rppH_3"

/locus_tag="PIKHDJKN_01365"

/EC_number="3.6.1.-"

/inference="ab initio prediction:Prodigal:002006"

/inference="protein motif:HAMAP:MF_00298"

/codon_start=1

/transl_table=11

/product="RNA pyrophosphohydrolase"

/translation="MTSRIVVTGGAIIRDHMGRILLQKRSDYGDWGLPGGGMEPGESI

EETMIREVKEETGLEVSSYELASIYTGEKMKYTYPDGNEVVFVMFLFNVTAELGGKLD

HDQTTLLFEDEQNESLELVFKSLDEIDISTINKVQKPVLVDLMQGELTILR"

CDS 1476882..1477517

/locus_tag="PIKHDJKN_01366"

/inference="ab initio prediction:Prodigal:002006"

/codon_start=1

/transl_table=11

/product="hypothetical protein"

/translation="MPWPMVHFAIASELLSDPTPEFIVGSLAPDSIHVRTNERAEKAK

THLMPETARFATDEELRVFFNSNKERAHNDPKFMQYLCGYIAHVYTDRVWTFEIYPVF

EEHPNGRSIYTEDVTKLEFMILRKGSDGHDLLNKLEAGKAFDLGGLLEQEVYQYRKEK

VDFLKNLENEPLDDLNILSMEAIEEFIQTTAQRLRQLFTKWDVFEDLCCIA"

CDS 1477534..1477842

/locus_tag="PIKHDJKN_01367"

/inference="ab initio prediction:Prodigal:002006"

/codon_start=1

/transl_table=11

/product="hypothetical protein"

/translation="MKVYKKQIRKMVIKDILFLYVVDEQAQDIIIRIFSNTYKSTFVE

FVVPWEDTWDIFVYEPKLISNLIQYALEQGWDCRQKNNRMKFDNATSIIRVLMAKKEA

"

CDS 1477906..1478388

/locus_tag="PIKHDJKN_01368"

/inference="ab initio prediction:Prodigal:002006"

/codon_start=1

/transl_table=11

/product="hypothetical protein"

/translation="MKKLISLAPYNEKYDQDLANFSLSDEQVQFTAMPAEVIEEALQN
PNKYPVLILNDETPVGFFILHKNSEYVEERDTSRTILVRALSITLEHQGNGFALAAMR
ELPTLVKQLDPKVNEIILAVNEGNIAAQKLYLKAGFLNMGERKMGINGLQLILQYRMI
"

CDS 1478445..1478846

/locus_tag="PIKHDJKN_01369"

/inference="ab initio prediction:Prodigal:002006"

/codon_start=1

/transl_table=11

/product="hypothetical protein"

/translation="MYAVIANVIKDNQLRTGAKVYILYCHGMAESPLVYGVAKGGKRI
EKYISYKKLKKFRSQWVPQHIRHRVQWSYEDRREADKTAKSLELMWTNVRLFDSAGHI
IQEGVTASQAFIIGSNIKFIERRYIFDERED"

CDS 1478848..1479351

/locus_tag="PIKHDJKN_01370"

/inference="ab initio prediction:Prodigal:002006"

/codon_start=1

/transl_table=11

/product="hypothetical protein"

/translation="MANIHKGRYSAEIDGEFVVFIIGMRVNRLWAIHKWLPVFKSMGP
MIKELYMNPDTGFLGTEFFMSWRGVTLLQYWRSYDELEKYARGGLHLEAWKRFNRSVG
SDGSVGIYHETYKAQSCSYETIYANMPKFGLAKVSNHVPATGRKETSRRRIGGESDPA
VETPKNP"

CDS 1479408..1479920

/locus_tag="PIKHDJKN_01371"

/inference="ab initio prediction:Prodigal:002006"

/codon_start=1

/transl_table=11

/product="hypothetical protein"

/translation="MKVKVKPEDLGSEQLSWLCVQPMLISVRGKDVAAKVEVYRQLHE
GQQAIFLFYSYHNHTKSLAEFYWFSAYNIIDMKSWNGIKTGMRYFNQNEMVTLLDDIE
LLITDKNKDGDTWREVLPTDLEKDPILLQRTEELYVKYRAAAQEAILHMNEIIRQNQE

KYLEITEINA"

CDS 1479953..1480336

/locus_tag="PIKHDJKN_01372"

/inference="ab initio prediction:Prodigal:002006"

/codon_start=1

/transl_table=11

/product="hypothetical protein"

/translation="MKSPGGSIFPYYYKGGEIHCLKYGSKHKDQNKLFDVMRQEEAFI

LGINKKLKVWVDMYETKITKEVLDQLICNMNNLKNHVDKLSFVGLSGKDRWKLKRRLK

SLGMGQFVSFYADPEEAKTWLITDR"

CDS 1480564..1480842

/locus_tag="PIKHDJKN_01373"

/inference="ab initio prediction:Prodigal:002006"

/codon_start=1

/transl_table=11

/product="hypothetical protein"

/translation="MNEEEILELYGLDPDTRHRQQIREFLQQEIENQEAVHHEYLKTL

CIMLFCIGNVEDTGLIWQAKRKNQDTGSYIDVQLLCGASYEKTEQPMS"

CDS 1480868..1481278

/locus_tag="PIKHDJKN_01374"

/inference="ab initio prediction:Prodigal:002006"

/codon_start=1

/transl_table=11

/product="hypothetical protein"

/translation="MASVHDNIILSYEVDLEYELIKMRTRSYHSDLSEDTDILFSDVL

AHSFDTPLQGSIIFDIDDVGLDCFISYNRELLDKGKGQGWPILYNTDEELQTKLIEGE

YLYFVITSSYGLSGWVLAKKVEMITKMRNEKDEE"

CDS 1481268..1481609

/locus_tag="PIKHDJKN_01375"

/inference="ab initio prediction:Prodigal:002006"

/codon_start=1

/transl_table=11

/product="hypothetical protein"

/translation="MRNRFGYEPNLIRKLPVVMSLVTLVVIMIITNPTRTDFYRWLES

EYGIHVSYDINAGKTYTQITNGQEKSLIFRSGHIQHVGIYTTYDELFTDAEGNDINIK

ALGIMNMFFKR"

CDS 1481840..1482517

/gene="gph_3"

/locus_tag="PIKHDJKN_01376"

/EC_number="3.1.3.18"

/inference="ab initio prediction:Prodigal:002006"

/inference="protein motif:HAMAP:MF_00495"

/codon_start=1

/transl_table=11

/product="Phosphoglycolate phosphatase"

/translation="MKAIFLDFYGTVVHEDDDILPVIYKQVQATAQVECTTDEIGAYW

WKAFSTLFYASHGSQFTTQRMLNVQSLAETLLHFKSDLQAESLNQEQLAHWRNPGIFA

DSIHFLHSLEVPVYILSNIDTDDVKAAMKAHNIEVAGVITSEDVRSYKPRSEMFDEAI

KRYGLQRDEVIHIGDSLVSDVQGAQNAGIKAIWLNRKGKLKPQQITPDYECKDLGEVV

HMLEAWI"

CDS 1482514..1483050

/locus_tag="PIKHDJKN_01377"

/inference="ab initio prediction:Prodigal:002006"

/codon_start=1

/transl_table=11

/product="hypothetical protein"

/translation="MKKLTVLSLLFLFLIACQDKTTHEAVQKPTIPVTESVTDDLIEQ

KDDESEAPKAPKEDKGIPPVYLDETKYTGDELEIVKLMNARMRYMWEEDEKSYMSLFD

PQSPVSGMPVSGFSGFKVRKIISMSDINIREQKKLYEALVMVTELRENEEEASSSMVF

WKKKADGDAAKWIFADID"

CDS 1483089..1483937

/locus_tag="PIKHDJKN_01378"

/inference="ab initio prediction:Prodigal:002006"

/codon_start=1

/transl_table=11

/product="hypothetical protein"

/translation="MTNQLILIEGLPGSGKSTIAKMVSEILIEQGKKVRLIQEGNLDH

PADYDGVAFYSAEEFRSMVDAHGACKHILESKATAYQDGFLIPYRKMKEGFGVDFPDY

VVQEIFSKDIYELPFEQNVKLITEKWRSFTESVISTADDSITIFECCFIQNPLTIGLV

KYNQSREENVQYVLELERIVQPLNPLLIYIHQEDLAHTFDKAIQERPKEWSDGFIQYY

TNQGWGLAKGYHGVEGTVKVLQARKELETEIYQLLKMDKHWLDNSEYNRAVCQEELDK

ILKISL"

CDS 1484003..1484188

/locus_tag="PIKHDJKN_01379"

/inference="ab initio prediction:Prodigal:002006"

/codon_start=1

/transl_table=11

/product="hypothetical protein"

/translation="MLFLEATLIFITAILFIVGVRSKRKTWIRWGIGSLTLLIVLFIP

SFVNGFVEGLSSGWSAK"

CDS 1484303..1484869

/locus_tag="PIKHDJKN_01380"

/inference="ab initio prediction:Prodigal:002006"

/codon_start=1

/transl_table=11

/product="hypothetical protein"

/translation="MDITFIRHGHAEHLLQYPHQLNQLHPGLTEQGKRQATALRKQIK

ILPEDIVLVSPTKRTIETAFLLTSSDKLIVCPLVGPRMFPQNPEFSVLRCDHIYSKDE

LNAQYTGLRILDFELDCWLDGINRMEEHLFEAYAGHLIQWCREQSGRTFIISHDGTIT

SYRAFLGEQGLTRSDFLGEAGHYTIRDL"

CDS 1484895..1485272

/locus_tag="PIKHDJKN_01381"

/inference="ab initio prediction:Prodigal:002006"

/codon_start=1

/transl_table=11

/product="hypothetical protein"

/translation="MKSIPFSILLASVILGISFIVGCLLLSNQGRASESTQAVEQDVP
NNNKTLMTIQETAEYLSMTKEQVMDIIKAEQGSFAASGFFEGKMFPFIKVQDQFLVSR
IELELWAQDASSSHRRYINGQMQ"

CDS 1485318..1485899

/gene="speG"

/locus_tag="PIKHDJKN_01382"

/EC_number="2.3.1.57"

/inference="ab initio prediction:Prodigal:002006"

/inference="similar to AA sequence:UniProtKB:P0A951"

/codon_start=1

/transl_table=11

/product="Spermidine N(1)-acetyltransferase"

/db_xref="COG:COG1670"

/translation="MEFAGQKIRIIPVTEQDLDFISKLECDASIWSFEENVETDTEKV
RDKYRSHFFTTDEKPYAYDFIIRRLEDPEGEPIGLIQMWSYVDHRKSWELGFALLPNY
VGEGYGSEATNLLLEFAFNGLHVHKLIGMCNAQNVRSAALMQHVGMTREAVFKEELWW
NNQWTDQYFFSILEKEFSERRGRGSDDQVGLVK"

CDS 1485874..1486512

/locus_tag="PIKHDJKN_01383"

/EC_number="3.4.21.-"

/inference="ab initio prediction:Prodigal:002006"

/inference="similar to AA sequence:UniProtKB:P58495"

/codon_start=1

/transl_table=11

/product="putative peptidase"

/db_xref="COG:COG3340"

/translation="MTKLVLLSDLYFESNHELNQRILSLFNSDQPSIGYVPSCSDPDR
RYFEHTRRYYNQLGIQTIDYYDLDLEYEENRFNTIFETDAIHLSGGNTFYFLSLLQKR
NAMELLRSYVHKGGILIGVSAGSILTTPTIEMAGYGEDADENHVGLMDKQALALVDFE
FSPHWDGTEEVLDSLRDYARDNSTTIYACKDGGGIVIDGEVVESYGEARCIN"

CDS 1486531..1487424

/gene="yddE"

/locus_tag="PIKHDJKN_01384"

/EC_number="5.1.-.-"

/inference="ab initio prediction:Prodigal:002006"

/inference="similar to AA sequence:UniProtKB:P37757"

/codon_start=1

/transl_table=11

/product="putative isomerase YddE"

/db_xref="COG:COG0384"

/translation="MSVITVYHYDAFSTVPGKGNPAGAVFHADSLSEEVMQQIAHKVG

FNETVFVISSEVADVRLRYFTPGHEINLCGHATMASMYGLKTRGLLEDKQSITIETNV

GILPIQFQQDGDTINIEMKQDQPRFMPFQGDIEKLANSINLTTDDIDLSIPIVYGSTG

TWTLLIPIRKLTSFQQMKPDSSLFPEILVENPKASLHPFTLETRDSDALMHARHFSSP

FSGTTEDPVTGTASGVMGAYYLEYMNSEIDQAHFVVEQGHEVGRDGKVHVSVVRDGQD

MDVRIRGTAVFVEEMKVKLDT"

CDS 1487448..1488239

/locus_tag="PIKHDJKN_01385"

/inference="ab initio prediction:Prodigal:002006"

/codon_start=1

/transl_table=11

/product="hypothetical protein"

/translation="MDEQAVLHTVTLLLKEECSDSLVGIYLHGSMAMGCFLPNQSDID

ILVVCREQLSADTYRRIAKRLMLIEDEMHIVTGFELSIVLESTVAKLTFPTPFEFHYS

AYHREKYRNDDQYLCGGYEDPDLVAHLAVIVDRGIVLVGKPIKELFQPVNREYMLASI

TSDVGSAMEEITENPVYYVLNLARVLLYVEDSVIYSKREAGDWALIHILPKYKEVISQ

CLAKYNGELESVNLSDDLLLDYAKYMLNEIKGLAESGLEHGRGKR"

CDS 1488324..1488755

/gene="bsr"

/locus_tag="PIKHDJKN_01386"

/EC_number="3.5.4.23"

/inference="ab initio prediction:Prodigal:002006"

/inference="similar to AA sequence:UniProtKB:P33967"

/codon_start=1

/transl_table=11

/product="Blasticidin-S deaminase"

/translation="MTITTEDLELIEAAKNVISRLYQEGKHHVGAAVRTRSGKIFAAV

HLEAYIGRVSVCAEAIAIGKAISEGEHEFETIVAVRHPEVDPNDATNGAEIQLDVVSP

CGICRELISDYGKDTKVILQGEEGYSKTTIGKLLPQKYTRT"

CDS 1488774..1489232

/locus_tag="PIKHDJKN_01387"

/inference="ab initio prediction:Prodigal:002006"

/codon_start=1

/transl_table=11

/product="hypothetical protein"

/translation="MFEIVDIRQKPDMLQAAVQYFWKQWGSESSYHFYRDCIERSVET

ESDVPRFYVLLDGDRIVGGYALLRSDLNSRQDLFPWFACLHVDPEYWGQNLGGQLQNH

AINEVKTKGYDKLYLCTDLTDYYEKNNWAYIGKGYLLDDEETRIYEICIK"

CDS 1489716..1489967

/locus_tag="PIKHDJKN_01388"

/inference="ab initio prediction:Prodigal:002006"

/codon_start=1

/transl_table=11

/product="hypothetical protein"

/translation="MMRLWAPTLQWCMLAKGDIDGIVLYNSEGDDLYSGILMAKEAGA

IVMDFEGNPFEGMNSEPYLIACHPDHRDYFLSVVQEGMN"

CDS 1490018..1490614

/locus_tag="PIKHDJKN_01389"

/inference="ab initio prediction:Prodigal:002006"

/codon_start=1

/transl_table=11

/product="hypothetical protein"

/translation="MNESLQEQIQFLIEIDKLKTIERKTRIIHGDRLENDAEHSWHLA

MMALILQSHSNKEVDILKVIKMLLVHDLVEIDAGDTFAYDTVGHADKYDREIKAAHRL

FGILPEEQAQELLNLWLEFEAKETNEAQFASSMDRLQPLIHNHQNEGDTWQKYNITSE

QVLNRNREIENGSETLWTYAQQIIQNSVDKGILTKSKS"

CDS 1490661..1491110

/gene="rppH_4"

/locus_tag="PIKHDJKN_01390"

/EC_number="3.6.1.-"

/inference="ab initio prediction:Prodigal:002006"

/inference="protein motif:HAMAP:MF_00298"

/codon_start=1

/transl_table=11

/product="RNA pyrophosphohydrolase"

/translation="MGYIMELRKLVGSRPLIMAGSCVLVFNEQGHLLLQRRTDSLDWG

TLGGSLEPGESLEEAAARELYEEAGLRAGAYKLITVFSGQDMYYKYPHGDEVYNVMAV

YEATEIQGEPTVMDDEGLELRYFDLSMPIPEINPFTEYVLKKAGYID"

CDS 1491131..1491463

/locus_tag="PIKHDJKN_01391"

/inference="ab initio prediction:Prodigal:002006"

/codon_start=1

/transl_table=11

/product="hypothetical protein"

/translation="MTQDFYCDEVLSGNTKVNIVMETDKVLAYHHTRPYYKHHIVVIP

KIHIQSFISQEEDNNDELLLEIMRVIKKVAADMVVQTGSSKIVTNLGTYQDSKHQHWH

VVSGERIQ"

CDS 1491688..1491843

/locus_tag="PIKHDJKN_01392"

/inference="ab initio prediction:Prodigal:002006"

/codon_start=1

/transl_table=11

/product="hypothetical protein"

/translation="MSFSFISTFAVLFNLVLFGLGVYALILFIKLAKRGIQALDIYLY

EKRGERF"

CDS 1491953..1492729

/locus_tag="PIKHDJKN_01393"

/inference="ab initio prediction:Prodigal:002006"

/codon_start=1

/transl_table=11

/product="hypothetical protein"

/translation="MDTFLEQINELQELQKDLVSIHPLFAEHYPVVVAFETLLYIYDY

SSKTKQYEWVKTVPDDLNIPDECLAAFPVHHMDGRMCAVVTDAAFQNFEQKVYLFHEF

VHCYVNEQYTDMGDRLEIKRKMEQIKRVTWELDYEFPYDNEMVVQRITSLLSALKSKD

LLQVKEARVALFSSLSEEQGEYFNWLEWNEGYARYLENRIREKFGLEINHMGDSSPFN

RLVFYECGSEYISLLVNEQPDLHTDLEQLFVRIQTERVQA"

CDS 1492928..1493356

/locus_tag="PIKHDJKN_01394"

/inference="ab initio prediction:Prodigal:002006"

/codon_start=1

/transl_table=11

/product="hypothetical protein"

/translation="MLMKKAGILLGLFVVLLAGCNPSNGTNSNPKEVLPSLITLTCNP

NFTIEACEDVEFKDPVEIGIVIEAINKAERIAGSLDYSVEYKMNLTDADGSLTKYDFS

IGKDPKQSALLVNHEDTHVGYSIPIEDANRVRKLIQSHTD"

CDS 1493614..1494180

/locus_tag="PIKHDJKN_01395"

/inference="ab initio prediction:Prodigal:002006"

/codon_start=1

/transl_table=11

/product="hypothetical protein"

/translation="MSHLITIQPLTASDMEGAWRVFETSITNAFTQEGLGSLHEDIRD

EIEHKKAMLYLALHPDNKQESNVFFLLAKREDTVVGTISYGPCGEEIKECTDHQLNYV

GELGSLYVLPEVQGQGIGSALIHALATELQRSEITQFCLDSGYRIAQKKWQRKFGEPY

AVAKNYWGEGTDHMVWLCNVKDFAAIND"

CDS 1494285..1494782

/locus_tag="PIKHDJKN_01396"

/inference="ab initio prediction:Prodigal:002006"

/codon_start=1

/transl_table=11

/product="hypothetical protein"

/translation="MQYIQSTPEQRLSPDAITIWRISAIISNGIGLIVLAALLILDSV

FGWRAWIGWLLWGVTAISVLYAVWEVFIRPSLLYKHWYYDANEEFLQLKHGALKKVHQ

IIPMAKVQSVTTNQGPLLRKYGLYSVSVGTMGSSHTIPALPEEVALELRNQIASYARI

NEVDE"

CDS 1494779..1496311

/locus_tag="PIKHDJKN_01397"

/inference="ab initio prediction:Prodigal:002006"

/codon_start=1

/transl_table=11

/product="hypothetical protein"

/translation="MNEMKRQHPLAMLWSLWKLVKNSFAFIVFLFILRHGTASTWIMY

GRIAFYVAIAFSVISIIWSWFTLRYTADDKAFQIYSGVFNRTRRTIPYTKIQNVNRHT

SLFHRLFKVTSIRFETGIKGEDATFQLQVVSLSESDRLEKVVAGHMSEANVASDAVEP

NEEPVTRPEHVTLEDIQLPVKEELERIVHYKSTRKDIFKASFTSLSFLVLIPILGSLY

SSVKDFFPDEKVTESILLTWLDTWWITALIITGLIAVSIMLGIVRTFVKYGDFQISSD

RKRIYITKGMLEQSAFSILKERVQAVKITQSPMKRLLGLAEVELTTAGGVGESDQEVN

SLYPFLPVKQAYDMISEILPSYQVTQEMQKLPRKSLWLRMLTPGWVWIIATGLLFYFK

PVILGQNQAWWMISAILLLWIVICRITDFLHTRYVLNQNFIQLRTGALTSTLYISKRE

KVIEVQITRNLLQRWFGVASIQTVNRAKPVLHHTLNDIPLEAVEAFQLWYMERSQNIQ

TR"

CDS 1496409..1497188

/gene="yidA_2"

/locus_tag="PIKHDJKN_01398"

/EC_number="3.1.3.23"

/inference="ab initio prediction:Prodigal:002006"

/inference="similar to AA sequence:UniProtKB:P0A8Y5"

/codon_start=1

/transl_table=11

/product="Sugar phosphatase YidA"

/db_xref="COG:COG0561"

/translation="MIKIVFMDVDGTLLSEKDRSLSPSTEDSIRKLVHRGIEVVLVTG

RPYNLCRDFRKLGIDTMISANGALIKSGEEVIHKSVLSTRMVREISEFAELNGSSISY

FTESFEMNGLCTADVRVTDALRDTLSLMGYPLSISSLEQEVYCICLYADQAEAEKFHS

RFPALKFVRFHPYVSNVLEENEVSKSIAAGKVLAHLNISKEEAMAFGDGENDIDLLEY

VGLGIAMGNGGERVKKSADYVTLRASEDGITHALRKFKIIE"

CDS 1497586..1497747

/locus_tag="PIKHDJKN_01399"

/inference="ab initio prediction:Prodigal:002006"

/codon_start=1

/transl_table=11

/product="hypothetical protein"

/translation="MGFIGIICVIIMTVTLISIESNTKRLIQEQAETNRKLEQLLRKE

RFTDQTGGA"

CDS 1497750..1498412

/gene="yjjG_2"

/locus_tag="PIKHDJKN_01400"

/EC_number="3.1.3.5"

/inference="ab initio prediction:Prodigal:002006"

/inference="similar to AA sequence:UniProtKB:P0A8Y1"

/codon_start=1

/transl_table=11

/product="Pyrimidine 5'-nucleotidase YjjG"

/db_xref="COG:COG1011"

/translation="MDHIKAIIFDLDNTILNRTSTFEGFSQSLINTYFAHLQTTDDIL

KRIIELDEDGYKDKGVLFNELLHELPWAENPPHVELMEFYGREYVKSAVLMEQAREVV

QHLRGKYKTGLITNGKTEIQYGKIDQLGIRDDFDHIIVSEEAGVKKPDPRIFKLALDH

FNLSPEQCIYIGDHPVNDVEGAAKVGMSTIWMKVNQPWQDSITTKPLHAIEHLGELKG

LL"

CDS 1498484..1499002

/locus_tag="PIKHDJKN_01401"

/inference="ab initio prediction:Prodigal:002006"

/codon_start=1

/transl_table=11

/product="hypothetical protein"

/translation="MEIRQMATAFLSNGNDMLMMKKAGSRLFDFEFWGGIGGHLEHGE

LNSPMTASYREIEEETGFKQAEIDNFRLRYVLLQYSDGEVRQQFVYFGETTHRAFIPS

DEGDLFWVPLDELLDLHTSILIKAAIRHFLQNQETDEIWIGNVLNGEGATAKPRVEWS

IMQDTISFEPVV"

CDS complement(1499244..1500401)

/gene="mgtA"

/locus_tag="PIKHDJKN_01402"

/EC_number="2.4.1.-"

/inference="ab initio prediction:Prodigal:002006"

/inference="similar to AA sequence:UniProtKB:P9WMY5"

/codon_start=1

/transl_table=11

/product="GDP-mannose-dependent alpha-mannosyltransferase"

/db_xref="COG:COG0438"

/translation="MRLALFTDTYVPETNGVAGTLHRLSNHLNRKGIEHLLFTPQSVI

EGNDAAPLRSVTNIPFFLYPECRIALPNRASIHRELKTFQPDLLHIATPFNMGLFGLR

YALKYQLPHVISYHTHFDQYLEYYKLKSVIPLYWKYIQWFHRACEATLTPSQDTLNSL

QSKGIQRLKLWSRGIDCSLYSPDKRSSDMRTRYNITAPLILLYVGRIAPEKDIATLTL

AMQQLPEHMQARVHWIIVGDGPLLPKMRMQSPPNVTFTGYLHGEELAAMYASADLFVF

PSSTETFGNVVLEAMASGLPVLAANGGGVKDLVTHHRSGVLFEPGNADALIREICLWE

DHMDQLRNMGFEGRKLAEQRSWDHIFDRLVGDYEEAIENRRRRTKDRIITA"

CDS 1500645..1501214

/locus_tag="PIKHDJKN_01403"

/inference="ab initio prediction:Prodigal:002006"

/codon_start=1

/transl_table=11

/product="hypothetical protein"

/translation="MNKLQEELQQLLPLDQLESMSGEEVVGSVAMDLYRAEFATIREC

GRELPQVLRDTILIVDLDTELSMSGITGFLENASGQFLGETTEAMQRIGNDADAEILK

NIQHMLSENGVTPVQLRENVNALSEQDVTTTLNTHGQQIHEVLQRVELEAGNLSMQSD

NEEVFELLYQYVDTNKDRLKQELEHLLSN"

CDS 1501240..1501776

/gene="tmrB"

/locus_tag="PIKHDJKN_01404"

/inference="ab initio prediction:Prodigal:002006"

/inference="similar to AA sequence:UniProtKB:P12921"

/codon_start=1

/transl_table=11

/product="Tunicamycin resistance protein"

/translation="MIVMINGAFGSGKTSAATKLQPLIPNSMIYDPEEIGYMLRNVIA

DDIRMEVEKTDDFQDMELWKVLTVKVAREIRQKYNKHLIIPMTIYKSEQFNYIHRGLK

ELDAELFHFTLMTSMETLHERLLKRGDKSDGWTFKQAVKCVEAFHGARFEEYIQTDGQ

TTDDVVNVIISKVLSNRK"

CDS 1501789..1502079

/locus_tag="PIKHDJKN_01405"

/inference="ab initio prediction:Prodigal:002006"

/codon_start=1

/transl_table=11

/product="hypothetical protein"

/translation="MSINKRLGVESLLVTLLFTLGFMAWNVVQGMLMTLNYTPEMMNT

DSSTSTLQSKVAFGSIARWDTTSILIAAVGFLLFAAAYYGLRSGIQRWTQRS"

CDS complement(1502180..1502953)

/gene="spo0M"

/locus_tag="PIKHDJKN_01406"

/inference="ab initio prediction:Prodigal:002006"

/inference="similar to AA sequence:UniProtKB:P71088"

/codon_start=1

/transl_table=11

/product="Sporulation-control protein spo0M"

/db_xref="COG:COG4326"

/translation="MSFFKKMLASVGVGAAKVNTELHTPEVTPGGIISGIVYIEGGDV

EQNVDRIYLTIKTHYIREHNDRKVNETAVVAKYLLTEGFILQPGAKLEKSFSFDLPEN

LPITLHRAEVWVETGLDISSAMDPSDRDRLHVVPTKNMNTVLDAIDLLGFKLREVTND

YAPKLGGNLPFVQEFEFVPTSKFRGYLDELEVLFYPMGDSLELLLQIDRRARGLGGIF

SEAMGTDESFVRLHLYERHLERGAHSVAQGLEEIISKHL"

CDS 1503278..1503718

/locus_tag="PIKHDJKN_01407"

/inference="ab initio prediction:Prodigal:002006"

/codon_start=1

/transl_table=11

/product="hypothetical protein"

/translation="MDRETLERNLGPDNVNTSFEQMANGERKYKMVSEDGSYYCRTVA

SSQGAWQNSHFHNHITEFYVVQSGWIAYANFANDQTLEIRVMGEGEHIIVQPGVHHNL

YMSANSVIHTIKYGLNTDSDWSASPELDSVTQKLMESELLQTYG"

CDS 1503739..1504281

/locus_tag="PIKHDJKN_01408"

/inference="ab initio prediction:Prodigal:002006"

/codon_start=1

/transl_table=11

/product="hypothetical protein"

/translation="MRKSTIFWLTGIAIIIVAAFIVYNNLAPSITAQDVNMKGTIRQT

ATDEYEVQLAITRTKADKGQHTIYPLVDGWGSISFYDKQDNRFIRPTSVGSTGAMEEI

LRQVLQRQSSTAITEFAGFSIPDAVGTYKVRFTIHPLGEEAELIRQPMIYYVHQEKLL

GKDLSWVSGEPLEIIKEYAE"

CDS 1504281..1504820

/gene="aacA-aphD"

/locus_tag="PIKHDJKN_01409"

/inference="ab initio prediction:Prodigal:002006"

/inference="similar to AA sequence:UniProtKB:Q7ATH7"

/codon_start=1

/transl_table=11

/product="Bifunctional AAC/APH"

/translation="MLIYQSNEITVRLLDEKDEQLLVQWLSNPVVLQYYEGRDQPHDL

NLVREHFYRQEDTAHRCMVEHEGKPIGYIQFYELEEEERNEYGYGDTDEIIYGTDQFI

GEADYWNRGIGTQLVKSMLGYLVNEKQARKVVMDPQSWNERAISCYEKCGLRKVRLLP

EHELHEGQMRDCWLMEYAP"

CDS 1504868..1505392

/gene="yfiT_2"

/locus_tag="PIKHDJKN_01410"

/EC_number="3.-.-.-"

/inference="ab initio prediction:Prodigal:002006"

/inference="similar to AA sequence:UniProtKB:O31562"

/codon_start=1

/transl_table=11

/product="Putative metal-dependent hydrolase YfiT"

/translation="MDLRYPIGTFEHTGEVTSEQRKQWIQDIAELPERAREAVKGLSE

EQLRLPYREGGWMLKQVIHHMADSHMNSMVRFKLALTEDNPTIRPYYEERWAELSDSR

ELDVEFSLQILDALHRRWVALLNTLTDVDYAKTFYHPASKETTRLDCNLGVYAWHGRH

HVAHITSLRSRLGI"

CDS 1505429..1505935

/locus_tag="PIKHDJKN_01411"

/inference="ab initio prediction:Prodigal:002006"

/codon_start=1

/transl_table=11

/product="hypothetical protein"

/translation="MNIHIRLLDESDPVLISQAFQEQGWAKSAEQYIQYLAEQQNGER

VTLIAELDGAFAGYANVLWNSYYPSFREQGIPEINDFNVLIKYQRQGIGSRLMDRAEE

VIHERTETAGIGVGVFSDYGKAQILYARRGYIPDGQGIHKHGRYLKWGDETIIDDDVV

LYLTKKLS"

CDS 1506203..1506925

/gene="COQ3_2"

/locus_tag="PIKHDJKN_01412"

/EC_number="2.1.1.222"

/inference="ab initio prediction:Prodigal:002006"

/inference="protein motif:HAMAP:MF_00472"

/codon_start=1

/transl_table=11

/product="Ubiquinone biosynthesis O-methyltransferase,

mitochondrial"

/translation="MEKTIKNVQDLYDMLDAEFRSAKQFWEPFYEDRERPIPFFPNKP

DENLVAHVNSGILSGGKALELGCGPGRNALYLTRQGYKVDAYDLSEQAIAWAKERAAE

EKLEVNFECKSVFELAPQEEYDLVYDSGCLHHLLPHQRIPYIQMIHNALKPGGYFGMT

CFAPGFGGQGGPESIMDDWEVYREKSMKGGLAFTEEKLRYLLEDPFECVELRPMKMKG

QDDMCFGLPILWVTLWRKPIHP"

CDS 1506973..1507713

/gene="yueD"

/locus_tag="PIKHDJKN_01413"

/EC_number="1.1.1.320"

/inference="ab initio prediction:Prodigal:002006"

/inference="similar to AA sequence:UniProtKB:Q8RJB2"

/codon_start=1

/transl_table=11

/product="Benzil reductase ((S)-benzoin forming)"

/translation="MGNRHFIITGTSKGIGKQLAKLLVEKGDYVYGIARGTADMKEAT

DRYKHFQYDLGDLSGIDELVSHILKQIPLQEAEFIGLINNAAMLEPLKSIDRCSAEEI

SLNLNISLAAPMILTSSFIQHTNHLTTRRKIVNVSSGSGSYPAPSMASYCASKAGINM

FTQCVAMEQSGQPNPVEVIAFDPGMVDTELQAVARGKSAEEFALSEVFGQVYEAGQLR

SPQDVAKQLIERMEEISDPSKVLHTMEG"

CDS 1508031..1508708

/gene="graR_1"

/locus_tag="PIKHDJKN_01414"

/inference="ab initio prediction:Prodigal:002006"

/inference="similar to AA sequence:UniProtKB:Q5HI09"

/codon_start=1

/transl_table=11

/product="Response regulator protein GraR"

/db_xref="COG:COG0745"

/translation="MLIEDDESLFSEIKERLSQWSYDVYGVQDFGKVMQEYSAIQPQL

VIIDIQLPQYDGFHWCRMIRAHSNVPIIFLSSRDHPTDMVMSMHLGADDFIQKPFHFD

VLIAKIQAVLRRVYNYNTERIELRTWRGATIEYVKNTLTHDNESVLLTKNEMFILKVL

VEHKNQIVTREDLIRSLWDHEHFVSDNTLTVNVNRLRKKLEPLELDGYIETKVGQGYM

ATEEAEV"

CDS 1508705..1509709

/gene="graS_1"

/locus_tag="PIKHDJKN_01415"

/EC_number="2.7.13.3"

/inference="ab initio prediction:Prodigal:002006"

/inference="similar to AA sequence:UniProtKB:Q7A6Z3"

/codon_start=1

/transl_table=11

/product="Sensor histidine kinase GraS"

/translation="MIGKYIREKLSWLVLLISIQLIFLFVAYIDTSIPFRSVSYIVVL

NVVVCIVFIWARYPRETRFYKRLTAWDHTYDPGDLDIAESPYERIVHDAVTSQTERYR

KESTAHFILLEQEKDEMLSWIHEVKTPLTALQLMIERMPDDKLQGQMTYEWLRIHQLL

DQQLHQKRIPFMHNDLFVEETELEPILNGEIRALKSWCISKRIGFDVSLDVTKVLTDS

KWLGFIIRQLLSNAVKYSHSSDIVIESKEQDGHIILTIQDYGRGIEAQDLPRIFDKGF

TSTQGRLEGTATGMGLYLTRQVAQALHIDIKVQSAPGEGTSVKLTFPRKNDMIHLAGV

"

CDS 1509810..1510565

/gene="bceA"

/locus_tag="PIKHDJKN_01416"

/inference="ab initio prediction:Prodigal:002006"

/inference="similar to AA sequence:UniProtKB:O34697"

/codon_start=1

/transl_table=11

/product="Bacitracin export ATP-binding protein BceA"

/db_xref="COG:COG1136"

/translation="MWILEAKKIHKVYGNRLNKQEVLKGIDLGVSKGEFVGIMGPSGS

GKTTLLNVLSSIDRVSQGTIDIEGQEFTGMKEKQLAEFRKFHLGFIFQDYNLLDTLTV

KENVMLPLSITSMPKHEAHLKFEQIARELGIYELKDKYPSEISGGQKQRTSAARAFVH

DPSIIFADEPTGALDSKSASDLLGKLSDMNSKHQATIIMVTHDPFAASYCSRVIFIRD

GQIYTQLNKGDESRQSFFNDIIKTQGVLGGVQP"

CDS 1510562..1512487

/gene="bceB"

/locus_tag="PIKHDJKN_01417"

/inference="ab initio prediction:Prodigal:002006"

/inference="similar to AA sequence:UniProtKB:O34741"

/codon_start=1

/transl_table=11

/product="Bacitracin export permease protein BceB"

/db_xref="COG:COG0577"

/translation="MSLNYIIFRNLRKNLKNYYLYVFALIFSVALYFSFVTLQYDPSM

DEVAESTKGAAAIGASSVLLIVIVGIFLLYANTIFIKRRSKEIGLFQLIGLTKGRIFG

ILSAENCILYFGSMVIGILIGFLGSKLILMILFKIIGVDVITKLYFSPMALVQTLIVF

AVMYLLIMLMNYTFIRAQSILSLFRVSSTTEQQVKKLSVGQILMGILGIALIVFGYIL

SARLFSVEKLDMQQLMYTMILILFSVILGTYLFYKGSVSFLFNLIRKSKKGYLSINEV

LSLSSIMFRMKSNALLLTIITTVSALAIGMLSLSYISYYSAEAQAKESLPEDFSFAQA

NVKNRFVAELDNKQIQYEEKHRQPLYIEIDANKVMNDSTMEELLFSSVVSDSMVDNID

LKPGEVIFMGYGNVIQQFFSIKDEGPIVLHGLKKTIDQQLIGTLKQGVLPVYYTKGTP

VAVVDESVYAELAQDLDPKLQEGEPTDYYGVHITDPGQDENAYAIFAELQLEAPSFSQ

IEFRNNQRTSMGLIMFIVGFLGLTFLITSGCILYFKQMNESEEEKSNYTILRKLGFTQ

GNLLRGIQFKQLFNFGIPLVVGLSHSYFAVKSGWFFFGTELATPTVIVMIVYTLLYSI

FGLLSIWYYKRVIKESL"

CDS 1512645..1513223

/gene="pgsA_1"

/locus_tag="PIKHDJKN_01418"

/EC_number="2.7.8.5"

/inference="ab initio prediction:Prodigal:002006"

/inference="similar to AA sequence:UniProtKB:P63756"

/codon_start=1

/transl_table=11

/product="CDP-diacylglycerol--glycerol-3-phosphate

3-phosphatidyltransferase"

/translation="MNLANKITLARMALIPLFMLCFIPLPSWIQESSELIRFLDQHGL
VIGVIIFALAAGTDKLDGYVARKYNQITNLGKLLDPLADKLLISVALIMMVQEDLIGS
WIAVIIIGREIVITALRMIATEQGIALAADRYGKIKMVLQVAAIIAVLLNNIPFSLLT
DVRVDVILLWLAAGVTLLSGLNYILVNYRLLK"

CDS 1513266..1513691

/locus_tag="PIKHDJKN_01419"

/EC_number="2.3.1.-"

/inference="ab initio prediction:Prodigal:002006"

/inference="similar to AA sequence:UniProtKB:Q7CXI0"

/codon_start=1

/transl_table=11

/product="Acetyltransferase"

/translation="MSQININPSTSEDSEYVRQQLIAFNAAHVSEELRHRYEELNFNI
KNEVGEIVAGVLSTLCWNWLEVDILWVDAEQRQQGYGSQLLREVERLAREKSCDFIQL
NTFSYQAPEFYKKHGYQLITTIENAPTGHSHYYFKKDLT"

CDS 1513729..1514163

/locus_tag="PIKHDJKN_01420"

/inference="ab initio prediction:Prodigal:002006"

/codon_start=1

/transl_table=11

/product="hypothetical protein"

/translation="MVTIKAIELEDLPALSQLYNELMGTPTNELQMQKMFHYIQQNGH
YHVLGAFYNGELAGSVMGIECMDLVGQCKPFMVVENVIVSDQVRRQGIGQKLMLQIEH
IARDLGCEYMILVSGDQRKEAHIFYEKLGFKDEKVQGYRKHL"

CDS 1514272..1514364

/locus_tag="PIKHDJKN_01421"

/inference="ab initio prediction:Prodigal:002006"

/codon_start=1

/transl_table=11

/product="hypothetical protein"

/translation="MFKKWVIKSKKRAEGSLFLLFRLFFGAGRG"

CDS complement(1514326..1515783)

/locus_tag="PIKHDJKN_01422"

/inference="ab initio prediction:Prodigal:002006"

/codon_start=1

/transl_table=11

/product="hypothetical protein"

/translation="MIYFRKRKTGAVLALTTAILASSLYPTGHAGAAAASNMETIQGQ

VTQVQSAKTSDEKIAEWNEWAKDHAYRLDSIQPVKTGHERDSFSDLNMLKPLLYDKRI

VFLGESSHGVAEFNLAKTRLIQFLHQEMGYNVLAFESGLSNTSLANATIAQQSPEQTM

KNSIFGVWWSKEITPLFDYLKTNAQTEQPLILTGFDMQLQFPLLDGNWLKDKKLAKRL

AQTEKKLSEFSAGTDLKAYHAEKNEIIQVYKDVLQILKSEANQTYLQKKYPNQPKLSM

LLERSLNDRIRVAREYVDLSIRSMAEINEGKMESFIDSMEWRDQAMHDNLMWLTTEVY

PNEKFIVWGHNDHIRKAQSEVMGSPYPIALMGEKLSKEMKQSSYVLGLYASSGQTADN

AGGVHAVEPAEPGSIEGIMSATNAKYSFLDLRYQSRETGNSWMFEPRFAYSWGIYPES

FVPRDQYDGILMIDDVHPPQYIRSSSSGSKEQTKE"

CDS complement(1515840..1516043)

/locus_tag="PIKHDJKN_01423"

/inference="ab initio prediction:Prodigal:002006"

/codon_start=1

/transl_table=11

/product="hypothetical protein"

/translation="MKNIRLKVARVEKDLSQDDLARIVGVSRQTIGLIELGKYNPSLS

LCIAICKALSRTLNDLFWDEEQA"

CDS complement(1516067..1516426)

/locus_tag="PIKHDJKN_01424"

/inference="ab initio prediction:Prodigal:002006"

/codon_start=1

/transl_table=11

/product="hypothetical protein"

/translation="MLTEVIILLAGSIYFGVRSVMLGIYSDEVEVHDQTSKISYSLRN

IITGLIIGISMALFLGIRSSVLFAETAHEKWWFFFLVFITSLLLYIPFFAGFNVIVHH

FANKASQKAARKDQWDS"

CDS complement(1517183..1518319)

/locus_tag="PIKHDJKN_01425"

/inference="ab initio prediction:Prodigal:002006"

/codon_start=1

/transl_table=11

/product="hypothetical protein"

/translation="MKTYDAFPEPIGGYTVGRTQMDLEYTASDHSKRELTAFVYYPSD

SSEGKTTSTYMFPEVYEMFNEQPLITALKDVFSIDIKTQCYDDLALSGKEKRYPVLFY

VCGGGGSPEWGTVICTDLASMGYVVVSIGHQNSTMYKRKDGRLFNVSKDFSDVIMALS

EDPEMLALAGKMEMRPDDETAIEMCRNVLTLPILAKVTEYSELQAEDVRYVADYLYKL

DSGELNSIFKGRLLLDIGMGIVGHSYGGPTTAIVCRDDDRFACGIGLDSGAFGLQGSD

LKKPFLLLFCEPNYNMNAIIGANNSMETYYFSVDRVAHLDYCDIVFTSVNEELRGERD

AMEMRNLVTDYTKDFFDHYLLQKAASVESLAYDGVDLIKKTSNK"

CDS complement(1518432..1519091)

/locus_tag="PIKHDJKN_01426"

/inference="ab initio prediction:Prodigal:002006"

/codon_start=1

/transl_table=11

/product="hypothetical protein"

/translation="MPKNSFFRLDEARREEISNSAMHLFVDNLYEDITMKMVLDSLSM

HPGTFYRYFEDKDDLYCLLVRNVTQKRAAYFNNSNEDSLYRFFLTGLFGNVNGTVTEP

LNELEIKLTKTFLYIPENVLLKVYLNVLKGESFPLIKDILRRMRVDGYLRPDIDDDLI

SFMFESMQFNLVMFFREFDIKDSTLQHKLSKYFADFMSHGLLEDHKYSEMVSDIKKAR

E"

CDS 1519450..1520670

/gene="rtcB_1"

/locus_tag="PIKHDJKN_01427"

/EC_number="6.5.1.-"

/inference="ab initio prediction:Prodigal:002006"

/inference="similar to AA sequence:UniProtKB:P46850"

/codon_start=1

/transl_table=11

/product="RNA-splicing ligase RtcB"

/db_xref="COG:COG1690"

/translation="MRIVDNIKVWGEPLENAVSQAVTCSQYGDVLGVALMADHHKGYS

QPIGGVVAYRNMISPSGVGYDIACGNKAVRTNLMWDDIRDQIAGIMDDINATISFGVG

RNNPNPVEHELFDDASWKLFDSIEPGLQHKLKTLARNQLGTVGSGNHFVDIFVEEATG

KVWIANHFGSRGFGHKVASGFLNLAAGRAFSGKAPGESMDQPPTLFDLKSELGDMYWD

GMTLAGRYAYAGRDYVIEQVLGILGASAEYSVHNHHNFAWKEQHMSEEVVVVRKGATP

LAPGQLGFVGGSMGDISVIVEGVHSEENTESFRSTVHGAGRTMSRTQAAGKMNYKLRT

RMGGEISEEQMQAAIRAYGVELRGAGTDESPFVYKKLQDVLNAHAHTLKINHTLRPVG

VAMAGGNEFDPYKD"

CDS 1520885..1522063

/locus_tag="PIKHDJKN_01428"

/inference="ab initio prediction:Prodigal:002006"

/codon_start=1

/transl_table=11

/product="hypothetical protein"

/translation="MRNCAIYSSQFDLDQLFEMIQSIYPQDTIERKEDKTHIQVTRKK

LFSKKTKGFNIMTSQTHPEEFTAMIQGMLGFLSQIEGKNASLQEKVLIKCSTLNMVVG

IETEEDISEEFFNELLHLADALDAVIFWGGGSLLNAQGQLLLDVNGESEVEDYKVTAH

TSFLDDARPPSESAMQRKERSEQLLTEQGIPFNVHLPARAGDEHTTIRSVEQVAERAV

ALCIAALKGECLGAGESAEDTAALVQEVIDKYDATSFFSPVERRFVEQLGAEHQEVIS

FSWGYEAYHVMLWALGYVEELGAPVNLCNVGQDVGYLQQCDDLEAFVAGSHLRSKSEI

LDAADLIYRYNWVCVDSRMKDQTPPGGLNGGVAYERHRALNWLICYLDQDWDDVRTDT

"

CDS complement(1522175..1523212)

/gene="exuR_1"

/locus_tag="PIKHDJKN_01429"

/inference="ab initio prediction:Prodigal:002006"

/inference="similar to AA sequence:UniProtKB:Q9JMQ1"

/codon_start=1

/transl_table=11

/product="putative HTH-type transcriptional repressor

ExuR"

/db_xref="COG:COG1609"

/translation="MKTKVTIQEIAHFTGLSKFAVSRALSGKSGVSEQTRDVILKAAG

KLGYFKDNSMLSGDMYSSNELQDPQNTRWSGTILILFPNVRYQNQESVYWGPVFNGIS

SRLNQKGINILTLTEPSADQLFTLLNPDAIRGIITVGSISTPILLEIKRLGIPVVMVD

HLDPVFHSDSIFTDNFASMRDIMLYLLRKGFKRFQFVGNTSDAHSFYERWVAYTSVLM

GHGLEMNQIPELSSPDVDDFRKTFTSVIHEGNLPEVFVCANDFYALYTIEALESIGIS

VPDQCVVTGFDNVYDNIPVLATVNVNKELLGARAVDQMLWRIVNPESNVEKKLILADV

MIREQYGRHSG"

CDS 1523962..1525338

/locus_tag="PIKHDJKN_01430"

/inference="ab initio prediction:Prodigal:002006"

/codon_start=1

/transl_table=11

/product="hypothetical protein"

/translation="MTRKWNVLPLMLLAVMLFVSACSGGGTAQPANDAPANSGNSTGT

VETDKEGGTEEVAVDVPDLGGRVIKVAAWWDLKPAGETASDKARLDKIAEVEKKYNVK

IEFVNVPFEEYMNKFTTTALAGEPFADIVQMEYKSALPAILKGQLLPISEFTTAENNI

NQEANLITKYPAIAGNYYAFESPTSIGLGLHYNRDLFKKLSLPDPQELYNKGEWNWDK

FMELAKQATKDTDNDGKIDVYGFSGWAIDVLRHFTAANGGTIVDDEHSKEGLSDPKTI

EAAEFVKRLYNVENVVKVKTGDKTNWEESNTFKDGDVALFTAAEWQLGDITFAAGVVP

IPNGPQGNKDVTYANNAASAKFIPKGVEDPKIVYQIYEETFDIPQIEEYPGQDYLESR

YTDEKDIAMIREHIAGTGRILLDDAYAGYPIGDYVNDIIKNNASVTATAEKYKAQAQA

AVDKLGKP"

CDS 1525455..1528382

/locus_tag="PIKHDJKN_01431"

/inference="ab initio prediction:Prodigal:002006"

/codon_start=1

/transl_table=11

/product="hypothetical protein"

/translation="MAGILQRKKWILPIVIAAAAACVILLLTSGAGVKSAVVPSGSLP

AIEVDAILQQTRQKNGYEQYLSQAGEASRPNVDMTIEAGDYIRAEGEEVRKLNNYEGK

PGVSLHTGETGEIDWVVEVPETGLYNLSAVYFPVEGKSSAIERALYIDGELPFAEAAY

LQFDRVWGNEKQAVEQDNQGNDLRPKQVEKPRWMEETFQDSDGYEQAPFKFYLEKGKH

TLTLKSSREPMVIRSIRLFNEKPAVAYAETAGAKQTDSSGQPKDVLIRIEGEAAVAKS

SPTLYPTSERSSSAVSPYSASQVRINTIGGFNWRLPGQWIEWEVEVPESGLYHIGFTA

QQNFVKGIYSTRKLTIDGEIPFAEMTKAPFRYQSDYRIDVLGGEEAYKFQLDKGKHVI

RLENSLGDFAPLIRNVEDSLYNLNSMYRRILMITGTKPDEFRDYRVEKQIPNLLEVFS

GESKRLKEVAGQLRLLSGQSSDQEALIKTMAQQLDEMIDKPDTIPRRLAAYKTNTGGL

GTWVQQAREQPLEIDALYVASLDSSIPGTGMGPLAKLGHEAKTFYHSFFIDYNQIGNV

ATSEDQRTVTVWIGSGRDQANTMKAMIDETFTPESGINVNLKLVNMGTLLPATLSGEG

PDVAMQIGNDLPVNFAMRNSAVDLTQFSDYTTVEQEFRDSAIVPYAYDSGVYALPETQ

TFNMLFYRKDVLAELGLEVPQTWDDVESLLAILSKNHMEFGMPVVAQANMQGVNIPPN

SQYATMLLQNGGSFYRNDDKESDLDSRIGIETFKKWTEFYTDYKLEREYDFANRFRTG

QMPIGLTDYTMYNQLSVFAPEIRGLWGFVPVPGTVQPDGTLNRDVPGGGSAVMMLESA

KDQEAAWEFMKWWTSTPVQAEFGREMEGLMGAAARYPTANIKALDSLPWPAEDYANLK

AQFETVKGIPEVPGGYFTGRHLFNAFYKTVVGQVEARESIMDYTQYIQDEIRVKRNEF

GLP"

CDS 1528401..1529369

/gene="araP_2"

/locus_tag="PIKHDJKN_01432"

/inference="ab initio prediction:Prodigal:002006"

/inference="similar to AA sequence:UniProtKB:P94529"

/codon_start=1

/transl_table=11

/product="L-arabinose transport system permease protein

AraP"

/db_xref="COG:COG1175"

/translation="MSQISLRDGQNSPPEKTPGMGMKSWWSWKLKEMKASKHSYALLA

PYMILFIMFTVIPVVISIILSFTYFNMLEFPRFIGWQNYTRLFLEDDVFLIAIKNTLL

FAIITGPISYIACFVFAWIINELTPKWRAFMTLIFYAPSISGNVYFIWLMIFSGDRYG

IANGLLIKWGFLLEPIQWLKTEAYIMPILILVQLWLSLGTGFLAFIAGLQTVDRTLYE

AGAVDGIKNRWQELWYITLPSMRPQLMFGAVIQLTTSFAVADVSIALAGFPSVNYAAE

TVVTHLIDFGTTRFEMGYASAIATVLFMIMVGTNLLVQKLLRRVGE"

CDS 1529372..1530235

/gene="ngcG_2"

/locus_tag="PIKHDJKN_01433"

/inference="ab initio prediction:Prodigal:002006"

/inference="similar to AA sequence:UniProtKB:O50501"

/codon_start=1

/transl_table=11

/product="Diacetylchitobiose uptake system permease

protein NgcG"

/db_xref="COG:COG0395"

/translation="MAAIATGKKRVNRSLSGSISLFALLLVFGAFMVLPLIYAINNAF

KPLDEIFTFPPTLLVKNPTFSNFTDLLNLLSDSWVPFSRYIFNTVFITGIGIVGHVLL

ASAAAYPLAKHKFPGKVFMFQVVVLSLMFTPAVTAIPNYMIMSWLGWIDTYWAVIIPA

FAYSLGLYLMKQFMEQIPDALLEAAKIDGASEYRIFWSIVMPNVKPAWLTLIILLFQM

LWGSDGNGYIYSEQLKTLHYAAGQIIQGGISRAGAGAAVALILMSVPITLFIFSQSRI

IETMASSGMKE"

CDS 1530247..1531713

/locus_tag="PIKHDJKN_01434"

/inference="ab initio prediction:Prodigal:002006"

/codon_start=1

/transl_table=11

/product="hypothetical protein"

/translation="MARTVKRWLVLLAAVSLLLVNAVPAAASPAPYESYNYNYWKEAV

PSPDAYLPKRTISGSDLGIGEFKDPGDVNVSPSGQIYILDSGNSRIVVLDADYKLLRV

IEGFMMDGSKESFNTPGGLFVDEEDRVYIADTGNSRVVVLDAEGQLVHTITKPESDII

ATNFQFQPLKLTVDRVGRVYVVAQGVYEGIMQFDEQGQFIGYVGTNKVERDYGEYIWR

MLSTKAQRAQMVLFVPTEFSNVDIDSKGFVYATNIDPGSSEPIKRLNPSGEDVLKRFG

YFEVKGDIRFRNNPGPSKLIDVKVLGNGMYSVLDATQNRVFTYDDEGHLLYVYGGKGN

QVGTLKTPVAMEQSGDHQLVLDRGKSNLVIYEPTRFGASVNEAVALHYQGEDTEAVHI

WKEVLKLNANYDIAYIGIGKSLLMEKKNEEALGYFELGMDRKSYSVAFKRYRREMMKE

HFGTFLTIAIALIFILILVRVAVKWRRRRQIDREAGLH"

CDS 1531694..1532314

/locus_tag="PIKHDJKN_01435"

/inference="ab initio prediction:Prodigal:002006"

/codon_start=1

/transl_table=11

/product="hypothetical protein"

/translation="MKQDYIKFPLHLIFHPIDAFWDLKSDQRGRMTVAFGALALTIIM

MILQKQYAGFLVNYIDPRTINSIGEIATVAVPFFLWCIANWAVTTLMEGEGKFREIFL

ATGYSLIPIILIYVPMIIISRFMVQEETAFYYLFNSIAFFWFLLLLFIGTMTVHQYTV

IKTILTMLLTLIVMGIIVFLGALVFSMLQQLYEFGYNIYRELIFRT"

CDS 1532329..1534914

/locus_tag="PIKHDJKN_01436"

/inference="ab initio prediction:Prodigal:002006"

/codon_start=1

/transl_table=11

/product="hypothetical protein"

/translation="MNKRQRIYTVLASGAAVILIAAGLLYINNRGVPAVEATAYLEAT

TEAMPNETESLQFLTDSSQEVPGMQLVAEDQGLALYYNEETTEIAVRDGASGQIWYSN

PNERNQDGLASAYEKEVLSSQLNVSFRDAIGTLENFPNFSSSISNKQFTAANVDQGIR

VTYTLGDTSLGIDALPKLISKQRLEEKVLSKLDATVARYTSARYYPTKNNPDVLERLD

GQISKQLVLNKMLDAFETAGYTADDLAFDNEENGVEGGGTSDKPSFVIPVEYRLDQGS

LVVTVPLSQVKESGQYRIRNIDLLAYFGAADTKGEGYMFVPDGSGSLIHLNNGKVKEE

QYVQRVYGADPNDNSLSRPQVSESVHMPVFGLKNGEHAWFAVIEKGDGMASISADIGG

RQNSYNHVYGTFSLRGEDELEMYTSQKMQEIQLLSEEPFRGDIQVRYHFLNGKDASYS

GMARLYQQQLVEQNVLKSLEDESALPFYVDVLGAVDKKASFLGVPYRTTLAMTTYEQA

AEMAAKLQQEGVNRVQMRYQGWFGGGFSHHTPTQVKLDSEVGSRSELQDLSEQLKKSG

GALFPDVAFQRIYHDDWNFAPSSDAARFVTKETAELYPYSPALNRMDQSKDSYYLLSA

AKLPYVVSEFAGKINKMELSALSLRDLGQVLSSDYRDSRVIHRETAKNIVKEQLGKLQ

QEYPNMMLSSANAYAWGYTQHIVNAPSGSSRFNITDEEVPFYEMVIHGYMDYAASAMN

TSGDQDLRKQLLRSLELGSAPQFQWTYEPSSKLKLTSYDSAYATDFTYWVDDAAALYK

EANEVLGHLRNQPITEHERVQDGVVRITYSGGATILVNYTDDPVTIDGITVGGADYAV

EGVNR"

CDS 1534911..1535810

/locus_tag="PIKHDJKN_01437"

/inference="ab initio prediction:Prodigal:002006"

/codon_start=1

/transl_table=11

/product="hypothetical protein"

/translation="MRTLRLSLKSRRALLGLAFISPWLIGFIFLFATPLLQSIRFSLS

NLSVAPGGYVLDFVGLKNFKDAILVDATFNRILVDSVGAMLLNVPMILFFSLFTATLL

NQKFKGRTMARAIFFLPVILASSAVAAAESAGLINLMGDASAIESSTEGGASFNVISI

VRMLNDVGLPMAYVDYIVEAIMRIYDIVSSSGVQILIFLAALQSVPGSMYEVAKIEGA

TAYESFWKITFPMVSPLILTNVIYTIIDSFAGSPVTEAIYQTAFKTQNFGLSSAMSWL

YTLVIGLVLVVVGWVLSRRVHYN"

CDS 1535843..1536856

/locus_tag="PIKHDJKN_01438"

/inference="ab initio prediction:Prodigal:002006"

/codon_start=1

/transl_table=11

/product="hypothetical protein"

/translation="MAVSGTDRMVVVPKENYYRMQRVRNKTSDLLYSIFRYALVIGIS

FIILYPLFLKISVAFKDKQDIYNPTIYMIPQHFTLDNIKLAAQVMDYLPLLANTLLFV

TITTLLTAISCALAGYGFARFSFPGSNVLFVLVILTILVPTSTLMVPMYLHFRNFDIL

GMVHLFTGREGINLLNTYWPSMITAATAGGLKAGLFIYIFRQFFKGMPKEIEEAALID

GAGGFRTFARIMLPNAISPLITVILFSFVWQYNDTFYSALFMSESPLISLKVASLPAQ

ANQLIPQLMGFGSNSGMKADPNYVAMIVDTGILLAIAPLIILYLFVQRYFVESIERSG

VVG"

CDS 1536948..1537910

/gene="manA_1"

/locus_tag="PIKHDJKN_01439"

/EC_number="3.2.1.78"

/inference="ab initio prediction:Prodigal:002006"

/inference="similar to AA sequence:UniProtKB:P49425"

/codon_start=1

/transl_table=11

/product="Mannan endo-1,4-beta-mannosidase"

/db_xref="COG:COG4124"

/translation="MTEQKISSNLVNKDATSEARELMKYLAERYGKGVLSGQQDYGNL

NWINENTGRKPAVIGFDLMDYSPSRTERGAISQEIRDAIAWHQQGGIVTLCWHWNAPT

HLVDEPGKEWWRGFYTDATTYDLASALAHPESQEYALLIRDIDAIAVHLQELKNAGVP

VLFRPLHEAEGGWFWWGAKGPEPAKHLYRLLYDQLVNKYELGNLIWVWNSEKPEWYPG

DDVVDIVSVDVYPEAGDHSPLAKRHENLRELVNDSKIIALAENGSIPDPKQMKEQGVH

WSWFCTWTGEFLKGGNHNAVAYLKEVYNSEDVITLDQLPKWSWR"

CDS complement(1538032..1538301)

/locus_tag="PIKHDJKN_01440"

/inference="ab initio prediction:Prodigal:002006"

/codon_start=1

/transl_table=11

/product="hypothetical protein"

/translation="MLGIKVQQVENKLIIRWQLSKIEIPISDIKAVTLDDTYGGSEPS

AVRIGAAYGASETILIRTNHQSYILFTSNETLFPKISTMLSNNAG"

CDS 1538496..1539530

/locus_tag="PIKHDJKN_01441"

/EC_number="1.8.3.7"

/inference="ab initio prediction:Prodigal:002006"

/inference="similar to AA sequence:UniProtKB:D1A7C3"

/codon_start=1

/transl_table=11

/product="Formylglycine-generating enzyme"

/db_xref="COG:COG1262"

/translation="MHDQLPKKPSSCCCAVSRDGKLEEKSLPEETAASVEAVEDKPSS

HTSYKADFKIALTNRAEKKESIRIEGGRFLMGTNDPEAFAADGEGPVREVQVNSFYLD

ACTVTNAEFARFVRETGDRTEAERFGWSFVFHLFVSPQTAAQVRTVVQQTPWWWVVEG

ADWAHPEGPDSDVKDRSDHPVLHISWNDANAYCRWAGKRLPTEAEWEYAARGGLIQKK

YPWGDTLRPEGQHYCNIWQGVFPTKNNAADGYAGTAPSQSFPPNAYGLYNMSGNVWEW

CADNYTTDHEKVTAADMELSPYEERKVLKGGSYLCHRSYCNRYRVAARIPNTPDTSTG

HIGFRCAADV"

CDS complement(1539566..1540672)

/locus_tag="PIKHDJKN_01442"

/inference="ab initio prediction:Prodigal:002006"

/codon_start=1

/transl_table=11

/product="hypothetical protein"

/translation="MKKIIFPGIALIAVCYAFGRFSYGLFMPEISEALQLDDAQSGAI

NSATYIAYCLSLLSAPLLINRIGHYHVIQMAGVIAVLGLLGIALSPNVFVLTLGVFLA

GLSTGLASPALGNTVYAELAPDQQARGNSWINTGTSFGIIVSGPIYWLFTEYWRLTYI

FFAVLGVVVVLWNSRVLSPRKTEPCTKSLWTCMKPTRPGVALLMATLLTGISSAIYWT

FARNFLTDEKGASDSEAVLFWIVMGIMGILGGCAGRIIERIGFGWSYRIGLLLLSVSL

GVILLPSMAASLVSAVMFGSTYIFLTSVFIVWATRLFRPNTSIGISLAFLALGAGQFL

GSSIAGYTIEIFSNTTAFLAFAVLGLFGLFIRVK"

CDS complement(1540761..1541291)

/locus_tag="PIKHDJKN_01443"

/inference="ab initio prediction:Prodigal:002006"

/codon_start=1

/transl_table=11

/product="hypothetical protein"

/translation="MSSKKEMLLNVAEELFYMHGFHSIGLKRIIKDAGIAIMTLYNHF

DSKDDLIVEVLRRREQRYLEQLRQYADNKAQPVFLNLAEGHAGWLMEHESRGCLFLRA

KEEFGGDTDNVIVQTVNAHKKHVITLIKNLDPAASDRAVLQFNLLLEGSTALAETENV

NNVCRELIHMTQNSFK"

CDS complement(1541368..1542138)

/gene="ydjF_1"

/locus_tag="PIKHDJKN_01444"

/inference="ab initio prediction:Prodigal:002006"

/inference="similar to AA sequence:UniProtKB:P77721"

/codon_start=1

/transl_table=11

/product="putative HTH-type transcriptional regulator

YdjF"

/db_xref="COG:COG1349"

/translation="MSLTYEERRHTILIQLATQGKVQVQVLADLFQVSTETIRRDLDR
LEKEGELRKVYGGAVRVRSGMIEAPFQKRAQLQLNEKQAIGVAAASLIEDGETVMLDN
GTTTLEIMRQLRHRSQVTVITNSVPILTCALEEFSGKIIFAGGEVTPVVQASTGPIAH
ELLSQFKVNKAFISAGGVSLTDGITDYVLEEALISRKMMERAEEAILVADHTKFGRST
FAQIASINQISMVITDSGCPTEWVKALHQMEIEMVHSI"

CDS 1542399..1543364

/gene="cpdA_1"

/locus_tag="PIKHDJKN_01445"

/EC_number="3.1.4.53"

/inference="ab initio prediction:Prodigal:002006"

/inference="protein motif:HAMAP:MF_00905"

/codon_start=1

/transl_table=11

/product="3',5'-cyclic adenosine monophosphate
phosphodiesterase CpdA"

/translation="MKRQLAFKENGTFKIVQFTDLHWKDGRPEDFRTRQLMKTVIEAE
QPDLVVFTGDVIYTGPVDPGNKECEHPEQAFRDAVSVVEERGIPWAFVYGNHDTENRI
TPNGLMDVVQEHAHTVTEPGPREIAGLGNYTLEIDGADGRPAAVLYLLDSGNYSQFES
IPGYGWIQPNQIQWLMSESTRVNPGRSRGDKLPALAFFHIPIPEYQSMWDTQICYGSK
YEPVCSAQVNSGLFSALLEMGDVMGTFCGHDHVNDFQGTYHGIRLCYGRSSGHSTYGR
EGMLRGGRVIQLQAGQRSFDTWIRLEDGSVVKEQPEHQPESTSAH"

CDS 1543407..1544264

/locus_tag="PIKHDJKN_01446"

/inference="ab initio prediction:Prodigal:002006"

/codon_start=1

/transl_table=11

/product="hypothetical protein"

/translation="MILPIVLVLASGMAHAVWSMFTKRSLNKSVFLWSIMMIPTVILL
PVLIVELVREPLTMPAYALLVLSMALQALYSWLLSQTYELGDLSQIYPIMRGTSTLLV
PLIGVAFLGETLSMFGWLGIACMIVGFTVLSGVGSRDGKSGSRVTGSKPVLMALCVGL
CTTSYVFVDKLNLEHISPLSLLEVTNIGFVAGLTPALLASRQLRQEWKKNRSTIMLGA
LLNPGSYLLFLFALQQAPMARLGPLREVGTVFAAFLGILLLKEQQGKKRILCSIVIFG

GILLIGMWG"

CDS 1544337..1545224

/locus_tag="PIKHDJKN_01447"

/inference="ab initio prediction:Prodigal:002006"

/codon_start=1

/transl_table=11

/product="hypothetical protein"

/translation="MNVHRLEDIHFRDPYILAILSRKKYYLYGSIGQNVWNGPAIGFD

VYESEDLIHWSGPSPCFRAPAAFWSDHHYWAPEVYEWEGRFYMFASFKAEGIPRVTGV

LVADQPEGPFELWCRSLTPPDWECLDGTLYVDDQGDPWMIFCKEWVQVEDGEMYAVRL

KPDLKGTIGKPDLLFKASEAPWSVGGGEQQNRYVTDGPFLYRMQNDELVMMWSTSGQD

GYTLGLARSVSGKPQGPWKQDDKPLFANNGGHGMLFRTFEGQLCLTIHAPNNHPNERP

AIFRMQEKNGQLTLMDNHT"

CDS 1545486..1546037

/locus_tag="PIKHDJKN_01448"

/inference="ab initio prediction:Prodigal:002006"

/codon_start=1

/transl_table=11

/product="hypothetical protein"

/translation="MLHEEKLIQAITEHEQLMHDLRRVRSLDLPQCYIGAGYIRNYIW

DVLHGYPLRELHSDIDVVYYDREDKREERDLLLEQRLREQTGNSKWSVKNQARMHRRN

GNEPYQSTEDALRYWPEIVTAIGVQLDEQNLVGICAPHGLEDLYALIVRQSPFFSGAD

YYNQRVEKKCWQQQWPKLTIIKS"

CDS 1546090..1546821

/locus_tag="PIKHDJKN_01449"

/inference="ab initio prediction:Prodigal:002006"

/codon_start=1

/transl_table=11

/product="hypothetical protein"

/translation="MKKFIREKGLILDLIFIACFVFFLVFWGWNFAVKFFGMITVAFI

VLRRIDYQKHILLKRILLTGFGIAAVSFVIIEALVFTQLSANDPEEADYVIILGSGIK

GTELSLTLKQRLDASLDYIRSHPQTPVIVSGGQGPGESIPEALAMKNYLVDQGISPTQ

VIMEDRSTSTQENMAFSKKIIDASGLEHPKIMIITSDYHMFRSKYLAAKNGYAAEYGI

SAPSPGYLKPINMIREYFAAVKAFI"

CDS 1546976..1547503

/locus_tag="PIKHDJKN_01450"

/inference="ab initio prediction:Prodigal:002006"

/codon_start=1

/transl_table=11

/product="hypothetical protein"

/translation="MIITAYMLPALYEQKKVSAHDMEEIVRLLAHAPLLYDDGLTIQV

QDFMEGLEIELEHEHEVRRAVIELYELAVQACRPFSEPSAYEQLQDALGLQAELWQEE

VLTLAEWMEWLQQIGKGQRKLPEYNFTAMLGNLPEGFMIHDFHDELMYQLEQNSANAW

AIEERNRLYAALGAN"

CDS 1547585..1548466

/locus_tag="PIKHDJKN_01451"

/inference="ab initio prediction:Prodigal:002006"

/inference="similar to AA sequence:UniProtKB:Q7A6Q5"

/codon_start=1

/transl_table=11

/product="Epimerase family protein"

/translation="MKKVVLAGGTGFVGQDFAQRFKELGYEVLIISRQPGHIAWENQA

GIIQALECAELLINLAGKSVNCRYTDENRKIILESRTRTTRILGESILACSNPPELWI

NSSTATIYRHAEDRPMTEKEGEIGSGFSVDVATAWEKAFFEFSLPSTRQIALRIAIVL

GEGGVMEPLTNLVRFGLGGSQGPGTQQFSWIHIEDLFRMVIYVQQHPQLEGVINASSP

HPVTNRQLMESLRRQMGVRIGLPSPRWMLELGARFIRTETELVLKSRWVIPERLEREG

FTFRYDTLDLALAEILK"

CDS 1548676..1548810

/locus_tag="PIKHDJKN_01452"

/inference="ab initio prediction:Prodigal:002006"

/codon_start=1

/transl_table=11

/product="hypothetical protein"

/translation="MQLRRVRIVGTGKYLPEQEVTDEELDRRLPCKGRVSVRAAARGL

"

CDS 1548851..1549201

/locus_tag="PIKHDJKN_01453"

/inference="ab initio prediction:Prodigal:002006"

/codon_start=1

/transl_table=11

/product="hypothetical protein"

/translation="MRAGEEFPFPEVYDIFGDGSLLGVEVSGHAEGMMGILLSTESHD

YFLCADAVRSSRAFQEQRRPHALAGIIMSDRQAYRRNFDRLVQLHQQFPAIRIVPSYF

REALDAWGSKGEDI"

CDS 1549511..1550431

/gene="hcaB_1"

/locus_tag="PIKHDJKN_01454"

/EC_number="1.3.1.87"

/inference="ab initio prediction:Prodigal:002006"

/inference="protein motif:HAMAP:MF_01647"

/codon_start=1

/transl_table=11

/product="3-phenylpropionate-dihydrodiol/cinnamic

acid-dihydrodiol dehydrogenase"

/translation="MTEQKIQTEDDHIQGETGSTVEKSKEFDSNAPIALITGTSSGFG

MLTAVTLAKQGYRVVATMRDLSRNVELARLAEQAGISDRLQYIRLDVTDADSVQEAVQ

TVLQNNGRIDMLVNNAGFALGGFIEEVSMDDWRRQMETNLFGLIAVTRAVLPVMREQK

QGLIINLSSVSGLSGFPGYAPYAASKFAVEGFTESLRHEMSSLGVRVVLVEPGAYRTP

IWSKGLGEIHRSDQSPYKDKLDAVLRYSQHAGETAPDPQEVADLIARIARMRAPRLRY

ALGKGSRLLIIGKLLLPWKWLEWIIAHGLK"

CDS 1550483..1551064

/gene="inhA"

/locus_tag="PIKHDJKN_01455"

/EC_number="4.2.1.103"

/inference="ab initio prediction:Prodigal:002006"

/inference="similar to AA sequence:UniProtKB:Q8G9F9"

/codon_start=1

/transl_table=11

/product="Isonitrile hydratase"

/translation="MKIAFVLFDGLTFLDFAGFYDVINRLNFFEQTKGTAWETCAMTD

QVTDESGLTLKVDRVKPDLAEYDLVFVPGGMGTRKLRFDEAFVGWLRQAENVPLKVSV

CTGSLLMGAAGFLTGKKATTHPNVYELLEPYVAEVIQTRIVKDGNVITAGGVATSIDL

GVYVVGLLAGQEAADNVKLQIDYPYDMQGVVEA"

CDS 1551061..1551642

/locus_tag="PIKHDJKN_01456"

/inference="ab initio prediction:Prodigal:002006"

/codon_start=1

/transl_table=11

/product="hypothetical protein"

/translation="MNEGQKENVFILRNIRRDEAEKYWPLRLEALKNHPEAFGASFEM

SIQLPMSEVQERIHTEPDDYILGAYTEEGILAGTMGFKREQGLKLRHKGYIWGVYVSP

SYRGCGLASRLLREVLDRGRQLEGIEQINLSVVTTNESARRLYERYGFETYGIERNAL

VVQGKAYDEAHMTYFYAERLNVSDEHVEAGDDI"

CDS 1551639..1552520

/locus_tag="PIKHDJKN_01457"

/inference="ab initio prediction:Prodigal:002006"

/codon_start=1

/transl_table=11

/product="hypothetical protein"

/translation="MTDTVQLELDGISFELKEPHSFEWIARLGTVFRVFDQQDSGNLS

FGVLGRDGERKFVKYAGARTIHASSIGAPAEAINYLKSSVSIYEDLAHDTLIHLKDHF

ATEHGYACVFDWVEGECLHSHWDFPPPAKYDDPRSPYYRFRQLPVKTRIHAMEQILDF

HIEVQRRGYVAVDFYDGSLIYDFDKQTMKICDIDLYRKDSFTNTMGRMWGSSRFMSPE

ELELGAPMDGVTNVFNMGAMAFSLLGGELDRSYARWDAGEALYQVVIRAVDPERANRY

ATVAELGVAWKKAVLQF"

CDS 1552758..1553453

/locus_tag="PIKHDJKN_01458"

/inference="ab initio prediction:Prodigal:002006"

/codon_start=1

/transl_table=11

/product="hypothetical protein"

/translation="MNPISNLSPMDMMKIQADTLYTMDNQKRLLNINEPDGGQAPALF

VGITSVGQCSYYHEQLPFTLIEELGCDTELPLDITKLIRRVETFKPVKSVWMGPAYAF

PEKCGGWHPNVQLIESHRTFLLEEHFPDLIERLHEKMPVAAYVIENSAVAVCCSARVS

QQGAEASLNTAPNFRGQGYAAEAVNCWQYHVKESGRIPIYSTSWDNIASQTVARKLGL

IQFGVDFSITTSS"

CDS 1553683..1554069

/locus_tag="PIKHDJKN_01459"

/inference="ab initio prediction:Prodigal:002006"

/codon_start=1

/transl_table=11

/product="hypothetical protein"

/translation="MIRKRIASATVMLCTAWMLVACSNQAESQDVSWKIESNLQQIVS

EPEVLTSSNPGDYIAANTEAYVQIRDTGEEGLNVLIQQLESSADNGLKEWLMAQASTE

LLGERNPVERWQSGKDWLRQYKMKVE"

CDS 1554325..1555149

/gene="yrpC"

/locus_tag="PIKHDJKN_01460"

/EC_number="5.1.1.3"

/inference="ab initio prediction:Prodigal:002006"

/inference="similar to AA sequence:UniProtKB:O05412"

/codon_start=1

/transl_table=11

/product="Glutamate racemase 2"

/db_xref="COG:COG0796"

/translation="MRGSRNLKEYVIEVKPLKKKIAFFDSGIGGLTVLHKALHQFPEE

AFLYYADTLHVPYGTKSADEVRGHIFNCVEAIVQENVQAIVIACNTATSLTVKDLRAK

YDIPVIGMEPAVKPAVEMNRKTGKRVLVFATALTLSQTKYHELVSRVDDHHSVDSIPL

PELVEWCEQLDFDSQKIAAYFRFKLADLDLDLYGTVVLGCTHYPFYTSILRTVLPDHI

QIIDGSTGTVNRLKQLLGLADQKGVWTEDKVTFLSSSGRSEDRENMRIALQYLEKH"

CDS 1555181..1555732

/locus_tag="PIKHDJKN_01461"

/inference="ab initio prediction:Prodigal:002006"

/codon_start=1

/transl_table=11

/product="hypothetical protein"

/translation="MQSIYLIRHAKATGQEPDAELTDEGLRQAEELAEMLAEHSIQYL

VSSPWKRALQTAAPLGKTVLQHIHTDERLQERVLSTLHLDNWMDVLERTYTDEDWTEE

GGESSRFATARGLEVLGELWSRPEHSGAVITHGNLLSLLIRSHDESFGFEEWKNLSNP

DVYVLEKQQGDKVTHSIRRIWRS"

CDS complement(1555802..1556164)

/locus_tag="PIKHDJKN_01462"

/inference="ab initio prediction:Prodigal:002006"

/inference="similar to AA sequence:UniProtKB:P39619"

/note="UPF0382 membrane protein YwdK"

/codon_start=1

/transl_table=11

/product="hypothetical protein"

/translation="MQTLIILGSIMMFLAVALGAFGAHALKRRLSADMIKIYETGVQY

HLVHGIGIILLGLLADRLEQPSLVLLAGWLLFAGILLFSGSLYVLSVTGVRKLGAITP

LGGVAFLVGWVMLLVAAL"

CDS complement(1556222..1557151)

/gene="coaA"

/locus_tag="PIKHDJKN_01463"

/EC_number="2.7.1.33"

/inference="ab initio prediction:Prodigal:002006"

/inference="similar to AA sequence:UniProtKB:P9WPA7"

/codon_start=1

/transl_table=11

/product="Pantothenate kinase"

/db_xref="COG:COG1072"

/translation="MNLYSPYTEFNRKEWADLKKYQTTLPLTKAELEQLKGLNEEVSI

QEVEDIYLPLTHFIDLYAGASRELNRLTASFLKKEAHHAPYIIGIGGSVAVGKSTTAR
LLQAMLARGKGNPKVDLVTTDGFLYPNAVLQEKGIMNRKGFPESYDIKSLIQFMGDVK
SGKPEVMAPVYSHLAYDVIAGEENAICQPDILIIEGINVLQVKKETPLLVSDFFDFSI
YIDAEEEHIRHWYIERFKLLRNTAFQNENSFFHSRFANLEEDEAVLTASQIWKDINAK
NLHENILPTKGRARLILKKEADHSIQRIQLRKL"

CDS 1557344..1558129

/locus_tag="PIKHDJKN_01464"

/inference="ab initio prediction:Prodigal:002006"

/codon_start=1

/transl_table=11

/product="hypothetical protein"

/translation="MIVYEREHDFVLTAQHEHGLVAGEMASHWKRELLTDEAHREDLI
LAAKEHDRGWIELDSAPFWNDYSQIPYSFRDFPLRPRFVFYRKGIEEVREKNLYAGLL
CSMMYTDLFQNNLGANANDDEDIREYLNDEREHQLRMEQQIGGGEALKLRLKHDVEIM
LFCDQLSLFLCMEEPGTPAARYDFFADGLGSTFDACSRQPIQAEWLSNEKVGLSFVPF
DEDFTVVMPYKSVPKASIRKFGLLQAYRRAEWKERRVLITGMS"

CDS 1558484..1558993

/locus_tag="PIKHDJKN_01465"

/inference="ab initio prediction:Prodigal:002006"

/codon_start=1

/transl_table=11

/product="hypothetical protein"

/translation="MDTRTEILTMCMVWDKRNDQVLLMNRPNQKGFPGYIAPGGKVDF
PESIVDGAVREVKEETGLVVKEITYKGLDEFCDPEQGLRYMVFNYVATSYEGELLNEP
PEGELLWVSLGQALELPMQDWFAERFPRFFQAGTFERSVIWEKSTGRTLKETFMVYSD
SVLEQSEVK"

CDS 1559020..1559388

/locus_tag="PIKHDJKN_01466"

/inference="ab initio prediction:Prodigal:002006"

/codon_start=1

/transl_table=11

/product="hypothetical protein"

/translation="MGYQHALEGYIAATNTHEFDEVSKWLSPDAIYWFTGTSCTTLNE

IRAYFENAWETVKDEVYRAEDVTWITTGEEQAVCIYTYHWKGMYQGEPAAGKGRATNV

FVAGINGEWKLIHEHLSTGE"

CDS 1559556..1559978

/locus_tag="PIKHDJKN_01467"

/inference="ab initio prediction:Prodigal:002006"

/codon_start=1

/transl_table=11

/product="hypothetical protein"

/translation="MDITFRESLEGIGIDQLSGDFFEGWPNPPSKPTFLKLLEQSYAV

ELAVDEENGNVVGFIQAISDGVLSAYIPLLEVLPEYQGKGIGTDLIKRMFNRLGDLYM

IDLLCDNELQEFYEKQGMQKASGMVIRHYQNQAGAVNS"

CDS 1560146..1560664

/gene="yvbK"

/locus_tag="PIKHDJKN_01468"

/EC_number="2.3.1.-"

/inference="ab initio prediction:Prodigal:002006"

/inference="similar to AA sequence:UniProtKB:O32248"

/codon_start=1

/transl_table=11

/product="putative N-acetyltransferase YvbK"

/translation="MNMYEEKVEIGVKLSFRILDWEEEKPYELLLMADPSKAIVDEYL

SRGVCFIAEYEGEMVGEFVLLKTRPETVEIVNIAVQEELQGQGVGKHMIKEAIEAARR

LGGRTIEIGTGNSSFHQLKLYQRCGFRIIGVDRDFFVKHYEEEIIEDGIRCVDMIRLA

LDLDTVAEENKK"

CDS 1560661..1561023

/locus_tag="PIKHDJKN_01469"

/inference="ab initio prediction:Prodigal:002006"

/codon_start=1

/transl_table=11

/product="hypothetical protein"

/translation="MKIYRLKSEDRRSITAYGSEGAEITPILRSTTACHVAQLKLSAG

GSVGLHPAVGEQVFLVLEGEGWVEGETGERVAVRSGEAAYWTNGENHQSGSNKGLTAL

LIEGEELIVRLSLESGDS"

CDS 1561166..1561597

/locus_tag="PIKHDJKN_01470"

/inference="ab initio prediction:Prodigal:002006"

/codon_start=1

/transl_table=11

/product="hypothetical protein"

/translation="MDRYTHMGVYGLVSWERKFLLVHKARGAYIGEWDLPGGRLEFGE

QPGAALHREIEEETGLSNLQLVIRSAESNVLEWVYEGEPEELHHIGILYDVYLTSESR

PYEIKREPDGEDSLGAQWFTMEEVGALSLTPFAEYMINKPI"

CDS 1561941..1563227

/locus_tag="PIKHDJKN_01471"

/inference="ab initio prediction:Prodigal:002006"

/codon_start=1

/transl_table=11

/product="hypothetical protein"

/translation="MKKQMRSNVRKSKRNVLIVSLLTTALMAGTLLTTGTVGLVSTVD

AATSTNGAGTMASALTKFKDIKGHWAQATIVKAYDKNLISGYQDGTFRPNAKITRGEY

ATLLSRATSLEKVQGENPFADLKGHWSESAVTQLVGQGFIHASDYPKGFNPNAELTRY

EMMKWIANGLIKSGTSFQDAFDDSKNTLLPTPEVNRGTIRAEQIPYLALVRGTGIVGG

FEDGSLKPADPTTRAEVSAILLRYMDVEGTDAGKYSDLNEMREVGTTGTNLTTISNYF

YTKNSVTVSDLSKGDIKLSNNSGVLTFHRFIVVKVQNGKPQGIYTKLFVEPKISSGDY

YLTFTDLSFTSNTDNTDLITYSSGLNNGLLKFRRLDLDYAKKNGFTTIPNSPKELITK

GKKVRFWTRSSLDFKNGTYHITSENIGEMKIQNMYY"

CDS 1563246..1566926

/locus_tag="PIKHDJKN_01472"

/inference="ab initio prediction:Prodigal:002006"

/codon_start=1

/transl_table=11

/product="hypothetical protein"

/translation="MKKRMRLPVFNWSKTLVVFFVISFGLAGLSMNNSIYAVSDPYGP

KSMKSPILKSIQAEKTIGTPGVYWYQIDDGRFRADHSGGPTYSNNDGGCATPYPALKE

VPDSVDFSRWPAVSWPQYNGNNLSASSFKEVKIKSVEYQTKPEQESYTGVGRTVITPP

STIVGIRTLTGGNHEWTQNDPFENGGKTTAGCIKHKVAYFTPMDIIWEGDLEEEKEID

VTPDSTLTVGETKQMVAKVKTKNYGATQFSEGIDVSRREAETTWWSSDPSIVSIEPKT

GMVKAEKPGTAFVRAIWNNGTYLISDTADITVTSEPGLIVNLPNGCKADTATPLQAKA

ILTKSDLSVHELTAHPKLTWQSSNPAVATIGADGKMTIKGIVGSTTITARFLDTAQQL

DEEGTQVLDVKDCTGNGGDGGTDPGNGGGVVGCPVTISPPNKGALIDSAAMDPSVRGV

LKADDRGSEKFDVTRGIPTSEDLYANVMAKGYLFQHRWVNMTGTVTYTVNVKKKYHKT

WTIPGRASTGPNDPGTPPQPKELDVPVEKPMQVIRQYSYWQIDNLEVYQLNQATISNY

ALGGYGGTVTLTPNGYTPPTLQSANDDAVTAHVKPAPCKEIDLGTETKSGGDSEPPTP

DETSLFQSKAETEVKENTVNNDKVVFNGTTVMDPASMDKTAPRPGTIPQPDMIGDNVL

YQNRLTIQNTLVNKADQPTTGEIAYGLIPGNIKGGQNQKFSIQGINSVTVHTPVVNYA

WVSDDQPHNQKTVPDSTSSALILERPFIVRLPTSGQHLDVTSYPGYGNRDYAKYFRIK

QIRFPFDVYNADRSQFIPAKTWVDIPVNQLDTVFYLPVWVDEGHYRIEFRNIAENAPS

TFTEQQDANTNLTHHVAADTVPVEVIGRLYDFHVTDIADYNWENVFRKQLGSSEPTGV

SYWTGLNSIDGDPRGNLAPFVLPVRPGSHPVQGFSNIAVKTGYHIKFDLKTKGNMFGK

QDGVRITPTFYFVSKDGSSRQEVDLYYHRGQERLIRIGSAQDLEKRFVVLNSRLRNVP

GTELGDTARYQYTYELTADERNQRSLADYMVTLVDQTSHQKTWVGHYDWMILPASIRT

LIGPKTDIPSGVSVDRANAAIQRWYGEYSLPADVYAVPKGTNLESLARQNQLDEKASV

FLKDGYIVVNFNIETLRDGNTEAPHLQYIYAPLMNQWKMEGFNNSPVDSQGRTWPLKD

GDVVFYHADQSSRSDFQSQVPH"

CDS 1567606..1570479

/gene="odhA"

/locus_tag="PIKHDJKN_01473"

/EC_number="1.2.4.2"

/inference="ab initio prediction:Prodigal:002006"

/inference="similar to AA sequence:UniProtKB:Q99U74"

/codon_start=1

/transl_table=11

/product="2-oxoglutarate dehydrogenase E1 component"

/translation="MTIEEGNKKPWESYYGPNMGYVQEQYELYSQDPGAVTPAYRELF

EQWGAPPMSGRDAHKASYSGNAQKASGSVDIQLLQKAVTAGKLVMNIRTYGHLAADID

PLGISEDSDTSLLEPKHFELNEEDLKALPASLIWEGADGQTVTGLDAIQRLRQIYTGP

IAYEFSHVHEVHEREWLNRRAESRTSPAPLNPEERKALLERLVEVEQFEDFLHKTFVG

QKRFSIEGNDVLVPMLDEAVRIMAHAGSSHILMGMAHRGRLNVLAHVLGKPYSKIFSE

FHHSPNKDLVPSEGSTGINYGWTGDVKYHLGANRYVKDGETVQARLTLANNPSHLEYV

NPVVQGFARAAQDDRRDPGYPKQDVTKAATILMHGDAAFPGEGIVAETLNFKALPGYQ

NGGTIHIIVNNRLGFTTDSSDSRSTYYASDLAKGYEIPIVHVNADNPDACIAAIRMAA

EYRNLFKKDFLIDLIGYRRYGHNETDDPETTQPTVYDKVKAHPTVSHLYQDHLKEESV

IDDASINSIREAVTSRLKEAYEQMKNNEVHEYYQRQISEPEAVSIIPTAVPLENLRTI

NADLLKWPENFNVYPKLQRILQRRSTSLNEGEKVDWSLAETLAFATILADGKPIRISG

QDAERATFAHRNLVLHDSENGAKFCPLHHLPQARASFAIYNSPLSEESVVGFEYGYNV

YSPDTLVIWEAQFGDFANCAQVIFDQFVSAGRAKWSQKSSLVMLLPHANEGQGPEHTS

ARLERFLQLCAEDNMTVANLTSASQYFHLLRRQASLTETEDARPLVMMSPKSLIRNPR

VASPATEFSEGKFEPVLEQAGLGTKPDRVERIVLCSGKIAIDLEDAFEKDKADWSWLH

IIRVEQLYPFPAEEIKRVLARFSKLKELVWVQEENKNMGAWTYMEPRLREIAPEGTTV

KYEGRPEHASPSSGYQLVHGMEQQQIITSALKQTTKNNIPLGR"

CDS 1570485..1571762

/gene="odhB"

/locus_tag="PIKHDJKN_01474"

/EC_number="2.3.1.61"

/inference="ab initio prediction:Prodigal:002006"

/inference="similar to AA sequence:UniProtKB:Q7A5N4"

/codon_start=1

/transl_table=11

/product="Dihydrolipoyllysine-residue succinyltransferase

component of 2-oxoglutarate dehydrogenase complex"

/translation="MSEIKVPAMGESITEGTVSRWLVKEGETVNQGDVLLELETDKVN

IEISAEESGVLEKIVRQEGETVEIGETIGTLSAGTGGGSGAPASQPAATEQKQAAAPA

PEAPTPPPAPVAAAPESSDSSSKTASPSARKLARERGIELDQVQSKDPIGRVYQDDVK

THSTQGAAPSASPAKAPAAAPSAPAAGGSTYTKPVERQRMSRRRATIAKRLVEAQQTA

AMLTTFNEVDMTAIMDVRKRRKDKFKEKHEINLGFMSFFTKAVVGALKKFPTINAEID

GEDVVLKKYYDIGIAVSAKEGLVVPVVRDADRLGFAEIEKSIADLASKARSNTLALSD

LQGGTFTITNGGTFGSLLSTPILNTPQVGILGMHKIQLRPVAIDAERMENRPMMYIAL

SYDHRIIDGSEAVRFLVTVKELLEDPESLLIEG"

CDS complement(1571890..1572867)

/locus_tag="PIKHDJKN_01475"

/EC_number="3.1.1.23"

/inference="ab initio prediction:Prodigal:002006"

/inference="similar to AA sequence:UniProtKB:A0QNZ7"

/codon_start=1

/transl_table=11

/product="Monoacylglycerol lipase"

/db_xref="COG:COG2267"

/translation="MQESTFSLVGDEGTRIHVYRWLPDPECNIKGVVQIAHGMSETAA

RYAEFAQHLTTHGYAVYANDHRGHGKTVENSNLLGNAGDDAFRWMASDMMNLGESAAK

ENPDVPLFLMGHSMGSFLVQHLMYAGHERYHAFILSGTNGKRGLLRIGEKLAFLQCGI

QGTGHPSMLLNALVFGGFNRSFRPATTPFDWLSRDPEEVKRFMDDPLCGAVCSAGFFR

DFFKLLLDIHLPRNMKRIPKDKSVYLFSGEQDPVGLHGKGVLNLVSQYRELQLEDIEY

RLYPGGRHEMLHETNRTEVAGHVMEWLERHTPDCSAHHSTVHVETSDSV"

CDS complement(1572951..1573928)

/gene="manA_2"

/locus_tag="PIKHDJKN_01476"

/EC_number="5.3.1.8"

/inference="ab initio prediction:Prodigal:002006"

/inference="similar to AA sequence:UniProtKB:O31646"

/codon_start=1

/transl_table=11

/product="Mannose-6-phosphate isomerase ManA"

/db_xref="COG:COG1482"

/translation="MPTPYPLQFQPEFKERVWGGRALEQFGLTPPEGHIGEGWMIADH

PNGTTKVLNGVLAGKGLDEVREQLGTAWLGTKGVSEKGGRFPLLIKLLDCNDDLSVQV

HPTDDYEALPPGELGKTEMWYVLDAKPGAHIIYGLNEGVDREVLKEALENGTVMDTLR

QVPVEAGDTFFIPAGTVHALCAGVVVAEIQQNSDTTYRIYDYNRPGLDGKPRELHVED

SLNVTAYEGAGATTMKTNNATPGEWLKLAECPYFVVEKGVVTERWELSTSTESFTILV

VCEGSGTLEWDNAESDKIELKAGQCYLLPANLGSYTLDGNTTVLRSYLP"

CDS complement(1574116..1574700)

/locus_tag="PIKHDJKN_01477"

/inference="ab initio prediction:Prodigal:002006"

/codon_start=1

/transl_table=11

/product="hypothetical protein"

/translation="MGFLSVLSCAHQWISSRLQPGDLAIDATVGTGADTLFLAQKVGK

RGQVIGFDIQSEALTLAQARIRKQEDSAKLGSISMLQLSHDRMAEAVPETWQGNVGAV

MFNLGYLPSEGADSTIITETDSTIAALQAALALLKPRGIITVVLYPGHDGGSQEADAV

LEWSSALPVEQAQVVIYRQLQRDASPFLIGIEKK"

CDS complement(1574702..1575655)

/locus_tag="PIKHDJKN_01478"

/inference="ab initio prediction:Prodigal:002006"

/codon_start=1

/transl_table=11

/product="hypothetical protein"

/translation="MNAPSIQPPLLWGDKRFHTWNYEMRDEFNNKVFKVMLDAGFTCP

NRDGSIAKGGCTFCSARGSGDFAGSRRDDLVTQFNTIRDKQHLKWPTAHYIGYFQAYT

NTYAPVEELREYFEEILKQPGVVGLSIATRPDCLPDDVVDYLAELNERTYLWVEMGLQ

TIHESTSTLINRAHDTHCYEEAVAKLRKRNIRVCTHIIYGLPQETHEMMLDTGRAVAN

MDVQGIKIHLLHLMRKTPMVKQYEAGLLRFLEQGEYIKLIVDTLEMLPPEMIVHRLTG

DAPRDLLIGPMWSMNKWEVLNSIDRELRERDSWQGKYWRGA"

CDS 1575928..1576128

/locus_tag="PIKHDJKN_01479"

/inference="ab initio prediction:Prodigal:002006"

/codon_start=1

/transl_table=11

/product="hypothetical protein"

/translation="MNSQVKNNDLHHVSVELTAEEALALTGVRFNGNPKVKAAARQKV

RDAFEKTFDFSHQDKVDYELLK"

CDS 1576281..1577030

/gene="trmB"

/locus_tag="PIKHDJKN_01480"

/EC_number="2.1.1.33"

/inference="ab initio prediction:Prodigal:002006"

/inference="similar to AA sequence:UniProtKB:O34522"

/codon_start=1

/transl_table=11

/product="tRNA (guanine-N(7)-)-methyltransferase"

/db_xref="COG:COG0220"

/translation="MRLRGRKGIRENLEQQVDLVVLDPKQYKGKWSELFGNDHPIFVE

FGMGKGQFISQMSYKYPEFNFIGIDMYDELVRRASEKARNAWSQAEVETPPNLKLALA

NIEQIEDVFEPEELERIYLNFSDPWPKAKHARRRLTHPRFLKKYTELLNTKGQIHFKT

DSETLFDFSLNAIADFGLQMTNLSLNLHRDGLNEEHVMTEYEQKFMGKGMNIHRVEVI

VGEEALREYQQIRLDKYKVREATDESGEDQE"

CDS complement(1577116..1579362)

/gene="rhaR_9"

/locus_tag="PIKHDJKN_01481"

/inference="ab initio prediction:Prodigal:002006"

/inference="protein motif:HAMAP:MF_01533"

/codon_start=1

/transl_table=11

/product="HTH-type transcriptional activator RhaR"

/translation="MRKRSEDSQKVFARILIGIIVSTVATLLVASTILYVNYNRIALR

QVYRTDMNSLTQTSREVSKMTETAKSLSYQIYQDYTISALLLYSNPSIYEITSAMEQL

DNYRMSLPFIESIYVYNSKNDEFFISSNEVRNGQQSISEIDDQGITSILDRFHDYKPF

VPIPRTYQVGSTEETQVSSYTYLCYDTINDNATLNYAVVVNIKDDWLSPNMNSVDQPG

KTFIINEQGQLLSDFSNRALMKDLSNETFMQPILENKEQSAYFTAEVDGKKSLITYTA

PDNLGWRYVRITPYDLITADIRNMRTHTVLFCLGLLIAGVLLSYLVSRKLYHPIDKVL

VRMRVMEAERRGSLHLLRQDFLRGALQGRETVTGGMLEERMKFYGSSVDVHRLTRLVL

LRIDHFTDFSDTYRDEVQLIKYAMMNICTETADLYYYTEAVDMGGDLITLIFNDKIQE

EADYEYSSIEELLCMMQAAVMTHLRCSISFTIGPAEDSLENSIASYTRSAEASLHRLF

MGPGCLIYTSDIMAYHAKEYAFPVGKERQLIDCLMTGKTGEAKQVYADIVSETATYPF

TVFQLALSHLTMTLNHVRNTLKKNNQLTLESISDRSMLPIHDAEDLSEVHAHFYRMFD

ELGSKVEEKRTLKHEELIRKINCIIERDYANTNLCLTSIADELAMSPIYVSRLYKQLT

LKGLTDVINETRIAKAQYLLIETENSVADIAEQTGFTNSSYFYRMFKKFNGVTPNDYR

RKELHSEL"

CDS 1579762..1580667

/gene="yteP_8"

/locus_tag="PIKHDJKN_01482"

/inference="ab initio prediction:Prodigal:002006"

/inference="similar to AA sequence:UniProtKB:C0SPB3"

/codon_start=1

/transl_table=11

/product="putative multiple-sugar transport system

permease YteP"

/db_xref="COG:COG4209"

/translation="MLKELNKNKIMFLMLLPTLIFFLINSYFPMVGIYYAFTRYDFEG

GLFGSPFVGLENFKFLWQSGMLLKLTTNTVGYNLAFIILGNGLAIFCAILLSEIRGKV

FKKITQSVMFLPYFISFVLLSVIAYNMFNYESGFVNTVLKRFEAGPIDIYNTPWIWVF

LIIIFYLWKNLGYSMVIYLAAITGISDEYYEAARIDGANIFQRIWYITVPMLKPTFVI

LLLFSLGSIMKGQFDLFYQLIGNNGVLYNATDIIDTYVYRSLKVTFDIGMATAAGLYQ

SLFGFILIMTVNYIIRKVNEDYALF"

CDS 1580697..1581596

/gene="araQ_9"

/locus_tag="PIKHDJKN_01483"

/inference="ab initio prediction:Prodigal:002006"

/inference="similar to AA sequence:UniProtKB:P94530"

/codon_start=1

/transl_table=11

/product="L-arabinose transport system permease protein

AraQ"

/db_xref="COG:COG0395"

/translation="MHTRTRDTEFTLLFQIIAYSMILILSLICIIPFLLILSGSFSSS

ESIVRDGYHLFPTNFSLEGYKMVFKFPTQVLKAYGVTVFTTVVGTALGLFLITMAGFV

LQRKDFKYRNAFSFFIYFTTLFGGGLVPWYIMLANYFNLTDTYTVLIFPGLMTPFLII

LMKNFIRSAVPDELIESAKIDGANDFRIYFSVVLKLAMPGIATVGLFLALGYWNDWFT

SSLFINNPDMYQLQFYLYNTMNTITFIDQMAIGTGITLSQDVPTESTKMAMAIVVTGP

ILFLYPFVQRYFVKGLTIGAVKG"

CDS 1581710..1583257

/locus_tag="PIKHDJKN_01484"

/inference="ab initio prediction:Prodigal:002006"

/codon_start=1

/transl_table=11

/product="hypothetical protein"

/translation="MRNKTIRHLSMVLALMLFAGVLAACNNGSGGSAQGSEQGGSSEN

KGEKVTLQFYMLGDAPKDLPAIESEINKLAEADLNVNVKFNYTSWTDWDQKYKLLLSS

GQPIDLIFTADWTFYQSYAKKGAFLPLDELLPKAAPTLKAYVPDDMWNAVKVNDKIYT

VPSTWTEYVTEGIAYREDLREKYNLPKPESLETLEAYLEGIKANEPNMIPIADSNANH

THGIRQLTSKLVNTAGQLPYGLDIMYDTPSNINSYWGSAQHLEDLKTYKRWMDKGFFP

KNVLNVKDTSNSLLQNGKSAVVLSGENPNKFNADVIKVQSTHPDWKLGYFPYPNAKGV

AQPVHPIHNGFAIPRSSKNPEKALAFYEKLVTDKRYNWLTEYGVEGKNFEVQDGYYKM

VGDAQTNGFPREGMNGWAWRNPEFMLYDKSFDDVLAIFKELDKMKKPDIFTGFAEDWT

PYQAEKAALEQVEKQYLYPLNVGLVEDVEAGLNTFMEKAKQAGLEKIQSEYTKQWQDY

LKSAGIQ"

CDS complement(1583341..1584489)

/gene="ugtP_2"

/locus_tag="PIKHDJKN_01485"

/EC_number="2.4.1.315"

/inference="ab initio prediction:Prodigal:002006"

/inference="similar to AA sequence:UniProtKB:Q5HH69"

/codon_start=1

/transl_table=11

/product="Processive diacylglycerol

beta-glucosyltransferase"

/db_xref="COG:COG0707"

/translation="MEKKRVLLLSEGFGAGHTQAAYALSSSLRKLSPNVQTKVLELGS

FLNPRVAPLIITAYKKTVINQPKLIGYVYRHQYKKSLNRLTTLALHKLFYTHTRSIVR

QLRPNVIVCTHPIPSAVISRLKRLGVQVPLCTVITDYDAHGTWISPEVDLYLVSTDEV

KSKLMLRGVSLDKIRVTGIPIHPNFWEHPGREEIRGKFNLKNMPTVLVMGGGWGMLSD

EVVNQLLTRWHEDVQIIFCLGRNDKSRISMEQNPLYQKDNIHIIGYTNEVDKLMEVSD

LLITKPGGMTCSEGLAKGIPMLFHNPIPGQEEENVQYFTARGLGEPITSLDVVVKWMN

KLLHNYPDIVRKRKRHMAQIAKYHPMQSAQSIIDLLDLRPYSADQARL"

CDS complement(1584540..1585067)

/locus_tag="PIKHDJKN_01486"

/inference="ab initio prediction:Prodigal:002006"

/codon_start=1

/transl_table=11

/product="hypothetical protein"

/translation="MSRLFVKFQEYDRNVFMWINGRLHNRFMNFWLYYFTHLGGATSS

IAVSLLIWLLAPAPWSTTGLQACIALAVSHIPVAIAKKLYPRIRPYLALPDTITFRNP

LTDHSFPSGHTTAVFSVTVPFMAMEPFLLLLLLPVALIVGFSRIYLGLHYPSDVLAGA

TIGTLVALATVALWT"

CDS 1585780..1587018

/locus_tag="PIKHDJKN_01487"

/inference="ab initio prediction:Prodigal:002006"

/codon_start=1

/transl_table=11

/product="hypothetical protein"

/translation="MLDAIFVTMQVILALLAVYQFTFSLFGLIKKKKKKHYPATKSFA

VLVAAHNEEQVIGALMENLKQLDYPEDLYDVFVICDNCTDGTAQIVRQHGLNACVRTN

ADLRGKGYAIEWMLKYLWKLPRQYDGVVMFDADNLVDRNFLLEMNDDLCNGSRVIQGY

IDTKNPEDSWITAAYGVSYWYINRLWQLSRHNLNMANFLGGTGMCFETNLLKEIGWGA

TSLVEDLEFTMRSVQRNVYPVFNYDAKVFDEKPLTFKASARQRLRWMQGHFTVARRYF

FPLLWQSIKERSLVKFDLAVYGANVYVVLLTFLMTAVLWVDTAIFSGPHIANIYGYFP

LWVGFLAIGLNVLTFLLSMALEKVTFAKVYLYLILFPIYLLSWYPITFYAFFTQNNKQ

WSHTQHTRVVRLDEVQSKQG"

CDS 1587293..1587823

/gene="infC_1"

/locus_tag="PIKHDJKN_01488"

/inference="ab initio prediction:Prodigal:002006"

/inference="similar to AA sequence:UniProtKB:P03000"

/codon_start=1

/transl_table=11

/product="Translation initiation factor IF-3"

/translation="MTWRWTVISKDHMINDEIRAKEVRLVGAEGEQIGITPIREALQM

AIDLNLDLVNVAPQAKPPVCRIMDYGKFRYEQQKKDKEARKNQKIVDIKEVWFRSNIE

EHDYQTKLRNVVKFLKEGDKVKCSVRYRGREIAHAAIGQRILERVKVEVAELCTIERQ

PKLEGRSMIMILAPKA"

CDS 1587848..1588048

/gene="rpmI"

/locus_tag="PIKHDJKN_01489"

/inference="ab initio prediction:Prodigal:002006"

/inference="similar to AA sequence:UniProtKB:P55874"

/codon_start=1

/transl_table=11

/product="50S ribosomal protein L35"

/db_xref="COG:COG0291"

/translation="MPKMKTHSSLKGRFKITGSGKVLRYKAHKNHLLSHKSKRAKRVL

NGNPVMAAGDVRRLKQGLANLK"

CDS 1588126..1588485

/gene="rplT"

/locus_tag="PIKHDJKN_01490"

/inference="ab initio prediction:Prodigal:002006"

/inference="similar to AA sequence:UniProtKB:P55873"

/codon_start=1

/transl_table=11

/product="50S ribosomal protein L20"

/db_xref="COG:COG0292"

/translation="MARVKGGFVVRRRHKKVLKLARGYFGSKHRIFKTANEQVMKSLV

YAYRDRRNTKRNFRRLWIVRINAAARMNGLSYNKLIHGLKLAGVDMNRKMLADLAVND

INAFNSLATVAKGKINA"

CDS 1589065..1590105

/gene="tmpC_3"

/locus_tag="PIKHDJKN_01491"

/inference="ab initio prediction:Prodigal:002006"

/inference="similar to AA sequence:UniProtKB:P29724"

/codon_start=1

/transl_table=11

/product="Membrane lipoprotein TmpC"

/db_xref="COG:COG1744"

/translation="MKKMLSLSLVMLLAVSVMLAGCGSKPKEETNAGGTTGGENTEAK

SDLKIGMVTDVGGVNDKSFNQSAWEALQATETETGVAVKYLQSKSDEEYIPNLNEFVK

GGYDLTWGIGFQLADAIKTVAEQNPDSKLAIIDSVVDVPNVKSVTFAEEEGSYLVGVV

AGLTTKSNKIGFVGGMESPLIKKFEVGFREGVKAVNPNAEFISNYTGAFDKPDLGKAA

ATTLYNKGVDIIFHASGATGNGVFKEAIARKEQGQDVWVIGVDKDQSLEFGDDVTLTS

MIKKVDEAVKRVNQEIIDGTFKGGAENLTLKENGVGIADTSTKNVSADTLAKVDEYKE

KIINGEIKVPTE"

CDS 1590448..1591986

/gene="mglA_2"

/locus_tag="PIKHDJKN_01492"

/EC_number="7.5.2.11"

/inference="ab initio prediction:Prodigal:002006"

/inference="similar to AA sequence:UniProtKB:P23924"

/codon_start=1

/transl_table=11

/product="Galactose/methyl galactoside import ATP-binding

protein MglA"

/db_xref="COG:COG1129"

/translation="MGAATPVVELKQITKRFPGIVANDAISLQLRKGEIHALLGENGA

GKSTLMNIVFGLYQPDEGSIEVNGKPVIIDSPNKAIDLGIGMVHQHFKLVQPFTVTEN

IILGSEPRKGLNINYKKAAAEVQRLSEQYGLKVNPHAKIHDISVGMQQRVEIIKTLYR

GADILIFDEPTAVLTPQEIKELMVIMKKLVAEGKSIILITHKLKEIMEISDTVTIIRR

GKVIDSVITSETNPNELAEKMVGRNVTFKVDKKPATPGNNVLEVSKLTAKNKEGISVL

NELNLNVRAGEIVGIAGVDGNGQSELIEALTGLRKVDSGSIRLEGKELSNHSPRHISE
SGVAHIPEDRHKHGLVLDFSVSENIVLESYYKAPYTRKGFLNFEAIKKQAKRLVEAFD
VRTPSIETKARSLSGGNQQKAIIAREIDKNPELLIAAQPTRGLDVGAIEFVQKQLIAQ
RDQGKAVLLISFELDEIINVSDRIAVIYEGQIVGEVLPEETNDRELGLMMAGSTQKRG
TVHE"

CDS 1591979..1593058

/locus_tag="PIKHDJKN_01493"
/inference="ab initio prediction:Prodigal:002006"
/codon_start=1
/transl_table=11
/product="hypothetical protein"
/translation="MNNVLKWFTRDSFILPIVAIIMGLILGGIVMLIGGYNPIEAYSA
LFTKVFGDMYNFGEAVREMTPLIMTGLAFAFAARAGLFNIGGEGQFLVGMTAATFVGV
KFAGLPIYIHAPLALIAGAVFGGLWAAIAGYLKAARGVNEVISSIMLNWIGLYLANLI
VRQFLLLKGENRSVDISDTASISLTWLSELMGNSRVHMGTLIALVMAVLFYIYMWKTK
QGYEIRAVGYNPNAAEYAGMHVNRNIVKAMFISGMLAGLGGAFQVLGVFQYQTVMSGS
PGTGFDGIAVALIGLNHPFGVLLGAVLFGTLTYGSAGMSFAADVPPEIIRIVIGSIIF
FIAAQGIVRWVLKPFYSKRKKEKVL"

CDS 1593059..1594021

/gene="rfuD"
/locus_tag="PIKHDJKN_01494"
/inference="ab initio prediction:Prodigal:002006"
/inference="similar to AA sequence:UniProtKB:O83324"
/codon_start=1
/transl_table=11
/product="putative riboflavin import permease protein
RfuD"
/db_xref="COG:COG1079"
/translation="MDLLTIGQIINTTLVFATALIFASLGGIFSEKSGVTNLGLEGFM
VFGAFAAGIAAHYAQEAGMGGTTSAWMGILFAVVLGVVVSLIHAVASITFKADQIISG
IVINFLAAGSTLYLVKLLFEGSGDSPLVQGFSKFNVPLLKDIPLLGEAFFKNVYPTTY
LAILFVFLTYYIMYKTPFGLRLRSVGEHPSAADTVGVKVRRYRYVAVMISGALAAIGG

AAITLTTTGTFSHNTVSGQGYIAIAAMIFGKWNPIGAFGAAVFFGFSQAIRNYVQLFE

WSQSIPQEIIYMLPYLLTIIVLVAAVGRSSAPSALGEPYDPGKR"

CDS 1594237..1594626

/locus_tag="PIKHDJKN_01495"

/inference="ab initio prediction:Prodigal:002006"

/codon_start=1

/transl_table=11

/product="hypothetical protein"

/translation="MKQHVTRDEAMKLIGKNIVAIKKDGTRVTGRLLKVSGNKLVLKR

AVGKKVHTKAILPLVLFDLLAVATLPYAYGPGGPGFGFGPGGGPGFGPGPKPGPGYGP

GFGGGYGPGPGPGPGYGPYGPRPPGFF"

CDS complement(1594633..1595079)

/locus_tag="PIKHDJKN_01496"

/inference="ab initio prediction:Prodigal:002006"

/codon_start=1

/transl_table=11

/product="hypothetical protein"

/translation="MVIRQRKSKLDDAAIMRLIDSQLVPLSHMSESEINKIRKEIPLR

MNRGMTFVVSQNPDNEAVAFIHFLMHGELLYVDMMAVATKEQRKKYGQTLLLKAENFA

ASRGCKRSKVMVDEGNTKGLRFYQKNGYSTIRYIMLSRCYELEKTL"

CDS 1595677..1597428

/gene="ilvB"

/locus_tag="PIKHDJKN_01497"

/EC_number="2.2.1.6"

/inference="ab initio prediction:Prodigal:002006"

/inference="similar to AA sequence:UniProtKB:P37251"

/codon_start=1

/transl_table=11

/product="Acetolactate synthase large subunit"

/db_xref="COG:COG0028"

/translation="MGAQIPEVRSTDELREKWMKPEVISGSEILLRSLLLEGVECVFG

YPGGAVLYIYDAMYGFEDFKHVLTRHEQGAIHAADGYARASGKVGVCIATSGPGATNL

VTGIATAYMDSVPLVVITGNVISSLIGSDAFQEADITGITMPITKHSYLVKDVEDLPR
VIHEAFHIANTGRKGPVLIDIPKDVSANKTLFEPVTEPVTLRGYNPRTVPNKLQVDRL
AQAIQEAERPMILAGGGVVYSGGHEALFEFVEKTGIPITTTLLGLGAFPSGHELWTGM
PGMHGTYTSNQAIQKSDLLINIGARFDDRVTGKLDGFAPHAKIVHIDIDPAEIGKNIA
TDIPIVGDVKTVLEIANKEVQRAERADAWRDQIKQWKQEKPYSYTDSEEVLKPQWVVE
MLNDTTKGEAIVTTDVGQHQMWAAQYYKFNKPRSWVTSGGLGTMGFGFPSAIGAQMAN
PDRLVISINGDGGMQMCSQELAICAINNIPVKIVIINNQVLGMVRQWQEIIYENRYSH
IDLAGSPDFVKLAEAYGVKGLRATNKEEAERAWQEALDTPGPVVVEFVVRKEENVYPM
VPQGATIDQMLMGDAEE"

CDS 1597425..1597907

/gene="ilvH"

/locus_tag="PIKHDJKN_01498"

/EC_number="2.2.1.6"

/inference="ab initio prediction:Prodigal:002006"

/inference="similar to AA sequence:UniProtKB:P9WKJ3"

/codon_start=1

/transl_table=11

/product="Putative acetolactate synthase small subunit"

/db_xref="COG:COG0440"

/translation="MIRHTISILVNDQPGVLQRVSGLFGRRGFNIESITVGQSEEPGL
SRMVIVTIGDDKTLEQIEKQLYKIIDVIKVVDFSLKPMVARELALIKVKAEPSERPEI
LGVVETFRASVVDVGPGSLIVQVVGDTDKIDAMIELLKPYGIRELSRTGITALVRGNV
"

CDS 1598211..1599203

/gene="ilvC"

/locus_tag="PIKHDJKN_01499"

/EC_number="1.1.1.382"

/inference="ab initio prediction:Prodigal:002006"

/inference="similar to AA sequence:UniProtKB:K4LVZ1"

/codon_start=1

/transl_table=11

/product="Ketol-acid reductoisomerase (NAD(+))"

/translation="MPVTTYYEQDAELNVLKGKTIAVIGYGSQGHAQAQNLRDSGLNV
VIGLREGKSFDTAKNDGFEVLSPAEATSRADVVQILLPDETQASVYKNEIEPNLKKGA
ALLFSHGFNVHFGQIVAPKDSDVLLVAPKSPGHMVRRTYVEGFGVPGLIAIEQDATGK
AKDIGLAYAKGIGCTRAGVIETSFREETETDLFGEQAVLCGGVSALVKAGFETLTEAG
YAPEMAYFECLHELKLIVDLMYEGGLASMRDSISNTAEYGDYVTGPRVVTEDTKKAMK
EVLTDIQQGKFARDFILENQSGRAFLTATRRNEAEHPIEVVGGQLREMMHWIKK"

CDS 1599327..1600868

/gene="leuA_2"

/locus_tag="PIKHDJKN_01500"

/EC_number="2.3.3.13"

/inference="ab initio prediction:Prodigal:002006"

/inference="similar to AA sequence:UniProtKB:Q71Y35"

/codon_start=1

/transl_table=11

/product="2-isopropylmalate synthase"

/translation="MRKIYVFDTTLRDGEQSPGVNLNTREKVEIAHQLERLGIDRMEA
GFPAASPGDLAAVNAVAKAVKNVTVIGLSRSREQDIDAVKEALKGAQDPCIHIFLATS
PIHRQHKLRMDRAQVLDTARSAIRYAKKTFSKLEFSLEDAGRTEYDFLVEMVNMAVEE
GAAVVNIPDTVGYLSPYEYGNIFKHLKENVHNIDKVQLSAHCHNDLGMATANTLAAIQ
NGADQIEGTINGIGERAGNTAIEEIAMALETRQEFFQAKTSLQLSEIARTSRLVSRLT
GMVVPGNKAIVGANAFAHESGIHQDGMLKEKTTYEIMTPETIGLKESKLVLGKHSGRH
AFRERLIELGYELEDEALNRAFAQFKDLADKKKEVTDEDLLAVIEEKLQDAPEVFKLE
SIFVTYGDESTPTAKVRIATLDGDTVEQQAEGNGSVDAIYNAIDQVSGEDVTLSDYSI
KSVTHGKDALGEVHVVLTQNQISVQGRGVSTDILGASARAYVDGLNKLIEKRKTYTNR
VNVNL"

CDS complement(1601095..1602075)

/gene="yajO_1"

/locus_tag="PIKHDJKN_01501"

/EC_number="1.1.-.-"

/inference="ab initio prediction:Prodigal:002006"

/inference="similar to AA sequence:UniProtKB:P77735"

/codon_start=1

/transl_table=11

/product="1-deoxyxylulose-5-phosphate synthase YajO"

/db_xref="COG:COG0667"

/translation="MQYTYLGKSGLKVSRICLGTMNFGPATDEKEAFRIMDAALDAGV

NFFDTANIYGWGENSGLTEEIIGRWFKQGGGRREKVVLATKVYGSMHDDTDGPNNDAG

LSAYKIRRHLEGSLRRLQTDHVELYQMHHVDPAISWDELWGAFENAVHQGKIGYVGSS

NFAGWQIAVAQSEAKNRHFLGLVSEQHKYSLNCRLPELEVLPAAKELGLGVIPWSPLD

GGLLGRNALQKLEGTRSGGIAERIESHQTQLEQFAALCRELGEPQDTIALAWVAANPA

VTAPIIGPRTLEQFETALKCLDVPLDETVLKRLDEIFPGPGGQAPNAYAW"

CDS 1602307..1603392

/gene="leuB"

/locus_tag="PIKHDJKN_01502"

/EC_number="1.1.1.85"

/inference="ab initio prediction:Prodigal:002006"

/inference="similar to AA sequence:UniProtKB:P12010"

/codon_start=1

/transl_table=11

/product="3-isopropylmalate dehydrogenase"

/db_xref="COG:COG0473"

/translation="MADVKKIAVIAGDGIGPEVVAEAEKVLKRTEEVFGYRFETEHAL

FGGIAIDEKGTPLPEETLTVCKSADAVLLGAVGGPKWDNNSKELRPETGLLGIRKALG

LFSNLRPAVVFDCLKDASTLKPEVLEGTDLMVVRELTGGIYFGEKFRREGAQGEEAVD

TCVYNVTEVERIVRQAFEIAQGRSKRLASVDKANVLETSRLWREVVNRVAPEYPDVEV

EHVLVDNCAMQLLRRPSSFDVIVTENMFGDILSDEAAMLTGSIGMLASASLGEGSFGL

YEPVHGSAPDIAGQGLANPIATILSLALMFRTTFGYAEGADAIEAAVSDVLNAGHRTS

DIAVDKSKAISTTEMGDLIVAAIQKQA"

CDS 1603666..1604205

/gene="ahpC"

/locus_tag="PIKHDJKN_01503"

/EC_number="1.11.1.15"

/inference="ab initio prediction:Prodigal:002006"

/inference="similar to AA sequence:UniProtKB:P56876"

/codon_start=1

/transl_table=11

/product="Alkyl hydroperoxide reductase C"

/db_xref="COG:COG0450"

/translation="MAERLVGRPAPDFAMETVSGDGQDFGSVKLSDYRGKWLVFFFYP

LDFTFVCPTEITALSDAAEQFKALDTEVLGVSVDSVHSHKAWINTPKDSNGLGQLNFP

LASDITKQVAKDYGILIEEEGVALRGLFIIDPEGELKYQVVNHNDVGRSVEETLRVLQ

ALQSGGLCAMNWKPGDNNL"

CDS 1604563..1604979

/locus_tag="PIKHDJKN_01504"

/inference="ab initio prediction:Prodigal:002006"

/codon_start=1

/transl_table=11

/product="hypothetical protein"

/translation="MSYCCGASMVGTKGTLKHYRTQVHNVPLLFCPVCHRVEVHYKVE

NEYEILAEYAHGDGASEIDFQDYVTEDEDAIFENCVNRESEDAMVIVQRQIDMSLDLL

RLAKEMKDEKWESELKRRLAVMSQRRLKIQHNKSGL"

CDS 1605422..1606525

/gene="sasA_9"

/locus_tag="PIKHDJKN_01505"

/EC_number="2.7.-.-"

/inference="ab initio prediction:Prodigal:002006"

/inference="protein motif:HAMAP:MF_01837"

/codon_start=1

/transl_table=11

/product="Adaptive-response sensory-kinase SasA"

/translation="MISEFQDTVPRPTNGFPPVHLDNNKYENVLEHLDSGIMLFDSNG

VLTFINVQMAKLLELPRSLLSGCTLMQMLHHPQMSRFKKKKILRIYRETIFHRKRYHE

LIDEYGRHWLVTVTYGDQMDGDFLFSVKDVSDYKQIEQTAYQNDKLAMLGRISASIAH

EIRNPLTAIRGFIQLLRPHLLQLGKDEYARIILTEIDRANDIIYEFLNSSKPSAPQKT

IMSVDSLLKEVVLLTESEGLMKGCEITLDEAEVPLNVSIDVKQIKQVILNMVKNAMDA

IEDVGEEHTGLIQISTGTENKYVQISIADNGQGMDHNTLVRLFDPFFTTKESGTGLGL

SVSYRIIKNHGGTISVDSKKGEGTRFIIMLPLV"

CDS 1606652..1607620

/locus_tag="PIKHDJKN_01506"

/inference="ab initio prediction:Prodigal:002006"

/codon_start=1

/transl_table=11

/product="hypothetical protein"

/translation="MNIQGMEQMNKQLMDHVGKIIVGKDHTIELVMTAIIASGHVLLE

DVPGTGKTLLAKSVASSLDCIFQRIQFTPDLLPSDLTGIHFFNQKEGDFEFRPGPLFA

NLVLADEINRATPRTQSSLLECMEERQISIDGSTRQLERPFIVIATQNPVDNQGTFPL

PEAQMDRFMMKIRMGYPSSEESVEILRRTVASRSVADLSAIISREQLLEAQSTYTSVQ

IDEDLLRYIIRLTEATRQHPELSLGVSPRGAQALLKASQAWAALHGRDFVLPDDIKIL

AEPVLAHRLVFRNRVRQQDGLAERIIQELLSQTEVPTENLATGSGR"

CDS 1607624..1608739

/locus_tag="PIKHDJKN_01507"

/inference="ab initio prediction:Prodigal:002006"

/codon_start=1

/transl_table=11

/product="hypothetical protein"

/translation="MALLWLVIVGGIVIGVHGIWFGRPALRKLKYTRQFSKMRCYAGD

ELEMVETIANEKRISVPWLRLEAMMPVSFVFRSGSGMDISQGDIYQNHKSIFTLKPFT

RITRKHPFVCSRRGIYTLNTVTMTGGDLFGLWRTSKPIPVHLSMVVYPSLVHAEDLPA

IYQVWQGEVEVSRWIVEDPFLILGVRPYGAGDPMNRIHWKASARTGELQVYKQGWTAD

PQSWIVVNIQESADMWSVVTRPEMIERALRYAATAAVDAIGRGLPAGFAHNGYRVSGG

RDPLRIEPDYGSPHLEMLLEAMAETELKCMVPMEQFLSDEVIRNEEAQQVRSYLLITS

YVSPAMEHEIARLHEQGHRVTILPVEGGKSDSKAVSA"

CDS 1608736..1610052

/locus_tag="PIKHDJKN_01508"

/inference="ab initio prediction:Prodigal:002006"

/codon_start=1

/transl_table=11

/product="hypothetical protein"

/translation="MNESNSNSIKEKLLLSISAVLLIGVYFYPLLTLTSLYATGHLPY
TLMAIFVIAGWLGQWVQHRLPTISEKNTFIRWGAALLMGLLVSLVIALVRAFPLPELL
TAVVWGVISAYIGLNFQPVFRSILIWRLQIWGVVSAIVLSGAANGIEFMQPLLAYTGT
IYAAGVIGFVGWLVGQYSVQLDRAILNDGSKRIVLREFARANHQRLLWMLIVIAGIGA
FPSLVAWLGPLRDRLLAWIRGLIGSSSNQEPPITPTAPNEPPMIPEGMEPAGEPSIFW
DILGWVVMGAVAVVILWLILRLGQKAINKLMERFKGLMQPGQKKVEPRTEYVDISETL
EAPAKTRKSWFSKKESLPSQEAERVRFYYRKWIDRAAHRGVHIQGAHTPLEAAETIVQ
HGAREDDQKLSAMLPDTYNAVRYGKKSPDDSEMSEIDRIWKSYRSK"

CDS 1610136..1611092

/locus_tag="PIKHDJKN_01509"
/EC_number="4.1.3.39"
/inference="ab initio prediction:Prodigal:002006"
/inference="similar to AA sequence:UniProtKB:Q53WI0"
/codon_start=1
/transl_table=11
/product="4-hydroxy-2-oxovalerate aldolase"
/translation="MKTNHCKIVDCTIRDGGLVNNWDFSVDFVQQLYAGLNEAGVDYM
EIGYKNSPKLLKGAEEAGPWRFLNDDFLRKVIPQKGNTKLSALVDVGRVDENDILPRS
ESMLDLIRVACYSKDVDKALALVQTFHDRGYETTLNIMALSNVMENELLEAFELIKES
AVDVVYIVDSYGSLDHNDVKYLVEKFKTHLPNKRLGVHTHNNMQLAFSNSLVAAELGV
ELLDASVYGMGRAAGNCPTELLVAHLKGTKYNLRPVLGVLEQLMVPLREKEEWGYILP
YMITGTLDEHPRSAMALRSSEDKDKVVDFYDKLTTPEVNFDK"

CDS 1611497..1613623

/locus_tag="PIKHDJKN_01510"
/inference="ab initio prediction:Prodigal:002006"
/codon_start=1
/transl_table=11
/product="hypothetical protein"
/translation="MKANQQDQRKIGWVAIGGLLCFLASQWFRSSASSDLIISGYPVL
TLLGGFAAAAACIGIYNQSWLFQTQRLTLRRVLMTTLFLLIGLFELVHIVSFAEEMPN
GAMVESEFSLKMMSLGSLVCAAGLLFIYAVNEREIALPRKFLIFSGTLGVFVVLYTVA
IQEWAVLPTLLEGNVLGGMFTRIHFLIGILYGVTAVVLFVQWKKGKDGDLPTILCAIL

CFFFGECYFVSATKINDLNLLLGLLSDCLGYFFIQKGLYSSVVDTPFLKQQVAEAKMN

FIAHHDDVTGLPNRRRLSQRLKVMMDEAMVEEQLVGVLVLNINRFKTINDSLGQQAAN

RVLRQVGQRLKHSSLPGEEVFGLGRDEFVLTMTDFCTTDTALRRTRSILQLFEKPVTV

DGNEYHLTLGIGMAIFPHDGDSPEEIIQNADTALHNAKEQGVELNRYAHAMQMKAQER

LQLENDLRKALDRGQFYLVYQPQVNLASGLIVGMEALVRWQHPLRGSVSPGEFIPLAE

ESGLIVPLGEWVLREACTQNKRWQEAGYRKLCVSVNLSMRQFRHSHLLDSISGILKET

GLEPVWLELEITESMTFDKDRAFEQLRKIKEIGVHISIDDFGTGYSSLHYLKDLPIDR

LKIDRSFVNEVMEDSNNAAIVSTITSMAHHLQLKVTAEGVENEDQLSFLRDQHCHEAQ

GYFFSKPIVAADFEKKFLRDVDKPTG"

tRNA 1613766..1613839

/locus_tag="PIKHDJKN_01511"

/product="tRNA-Pro"

/inference="COORDINATES:profile:Aragorn:001002"

/note="tRNA-Pro(tgg)"

tRNA 1613851..1613921

/locus_tag="PIKHDJKN_01512"

/product="tRNA-Gly"

/inference="COORDINATES:profile:Aragorn:001002"

/note="tRNA-Gly(tcc)"

CDS 1614408..1616297

/gene="uup"

/locus_tag="PIKHDJKN_01513"

/inference="ab initio prediction:Prodigal:002006"

/inference="similar to AA sequence:UniProtKB:P43672"

/codon_start=1

/transl_table=11

/product="ABC transporter ATP-binding protein uup"

/db_xref="COG:COG0488"

/translation="MTMVRLHEVSKEWNGNELFAGLNLEINEGERLAILGRNGCGKTT

LLRIILGEEDGGGRIERHIPQQEWGFMRQRSLIKPEMNVLDAVRQESGRIYEVKRDLE

ELEQRMSNSGAADEQRLLEAYTIAMEQYEQLNGYMWETEVEKVLTRLGLSSEHWIRPY

HSLSGGQKTKARLAGLLVSKPKFLILDEPTNHLDEESMRWLEEWLSAYAGTLLFVSHD

RTFIDEVATGVIEFSPDALTKYKGGYTDYKGHKERELREQEATYRKQELERKALEETI

RNYQQWFHQAHNSASDVEVKITQSFYKAKANKNISRYHAKQKQLERLEKERVDKPREA

AKLNMELQVNTLAARQLLALEDVSFGYPGSPTLLHNLRIAVERGDRLAVRGPNGTGKT

TLLKLMIGELEPSQGKVTRHPQLKIGYFSQELEGLPESETLLDSLLSLPSMTQSAART

ILGCFLFSRDDVFKRIGDLSMGEKCRVAFLRLYFGGSNVLVLDEPTNYLDIDTQEVME

NILKQASGALVLVSHDRMLTKVLASRLLDLEPGGKATLYEGGVEDWEQSIKLRESALE

VRESDDERLRLEMRLSELLSPMNSAGNDMLTKPDDVIERAAEVAEVREIQRRLKQLHE

LRKQK"

CDS 1616756..1617790

/gene="pheS"

/locus_tag="PIKHDJKN_01514"

/EC_number="6.1.1.20"

/inference="ab initio prediction:Prodigal:002006"

/inference="similar to AA sequence:UniProtKB:Q4L5E3"

/codon_start=1

/transl_table=11

/product="Phenylalanine--tRNA ligase alpha subunit"

/db_xref="COG:COG0016"

/translation="MKERLEALKIEALEQLSGVNDPQTLSDLRVKYLGKKGALTEILR

GMGALSAEERPVIGQVANDVRGAIEEVIDSKQEQFQKEETAKRLQSEKIDVTLPGRRG

RQGGLHPLTKVVQEIEDIFIGMGYRVAEGPEVEMDYYNFEALNLPKNHPARDMQDSFY

VTEDLLMRTHTSPVQVRTMQSMKGEVPVKVICPGKVYRRDDDDATHSFQFNQVEGLVI

SENIRMSDLKGTLLQFVREMFGSHTEIRLRPSFFPFTEPSAEVDVTCVQCGGSGCRVC

KQTGWLEILGGGMVHPKVLEMGGYDPEKYSGFAFGMGVERIAMLKYGIDDIRHFYNSD

MAFLKQFGRL"

CDS 1617863..1620316

/gene="pheT"

/locus_tag="PIKHDJKN_01515"

/EC_number="6.1.1.20"

/inference="ab initio prediction:Prodigal:002006"

/inference="similar to AA sequence:UniProtKB:Q4L5E4"

/codon_start=1

/transl_table=11

/product="Phenylalanine--tRNA ligase beta subunit"

/db_xref="COG:COG0072"

/translation="MRVSTDWLSDYIALEGVVPQELAEKFTRAGIEIDVVENRNQGVN

KVVVGYVKSKEKHPDADKLNVCVIDAGQEEDLQIVCGAKNVDAGQKVVVALVGAKLPG

GLDIKKAKLRGVVSLGMICSAKELGMNDKLLPKDQQEGILVLPAQTEIGTPISQVLGL

DDHVLELDLTPNRSDCLSMLGAAYEVGAILGREVKLPDPTQKLVEIGDVAANHISVEI

TAGEQCSHYAARYVTGIKLGASPQWMQNRLMAAGIRPINNIVDITNYVMLEYGQPLHA

FDADKLEKGHIEVRMAKEGETIVTLDSQERKLEPHMLLITDGVKPVAIAGVMGGENSE

VTETTVNLLLESAKFDGGTVRKTSRQLGLRSEASMRFEKEVDPAAVITALNRAVELIQ

RYAEGEVHQGIVEAGTAAADKHVVQLSLDKLNRYLGTELSLLEVKTIFARLHFACGDA

QQGLLDVEVPSRRGDITLDVDLFEEIARLYGYDNIPTTLIEGPTTPGTYTRSQAMRRT

IRGLFAGSGWQEMISYSFIHPDKVSLFPALTEGSKAVKLAMPMSEDRSVLRKSILPQM

LDATVYNMNRKHDSLAVFEVGSVFFTEEEKLTKQPHEIPVLSLLLTGNRASQQWNVGV

EKVDFFDLKGALENLFAYVGLEQRIRLVANSPEGFHPGRSASVYLEAKDGGEGKLIGT

MGQLHPELQQQYDLNDVYLAEIGLEAIYSEADADIRYRELPRFPAVERDIAVVVSEDI

EAGDMLRSIRESAGELLQTVQVFDVFTGSKLGEDKKSVAMALVYRNRERTLTDEEITE

VHARVVARLEERFGAELRK"

CDS 1620455..1623175

/gene="zapA"

/locus_tag="PIKHDJKN_01516"

/inference="ab initio prediction:Prodigal:002006"

/inference="protein motif:HAMAP:MF_02013"

/codon_start=1

/transl_table=11

/product="Cell division protein ZapA"

/translation="MTTPDRTRVTVEIYGTSYKLVGSSADYMKQVANLVDERMSAISK

HNSRLDTPRIAVLAAVHMAEQSLQTQEIRNELKMLTGERTEMRNELNRLNAIQNEHQQ

ELERRDQELAEAMKLKAEAEQAIAKLQKDQQAEVAKLNTLLEQERAQAAGREQKLQAQ

TAAQLKAAEEKAQARLKEAEEKAANQIKNAQTQAAAQLKEAQTRSANELQQAQAKAAA

QYKELQDKTAAQLKEAEARAVAERQKIEVQAAQQVQTAKEQAEAAILSELEQAEAQLQ

KVQEEAQLQYSELQSEMEQRIQQAEQAAVNQAQELTEKAKEEQAALQQQAEQKRLAEI

EQLNATFKESSEQAELEWMEKEAAYEEQLQQIHEAAQTQAKEAAATAEALLQEEQGKL

KQQLEEERKQAKSRLTDVEGQLAEVRKQLKNAQEVVVDQELQLQHERSQTETLRNEQA

VYKQEQDVQLRRITELEQQLDLLKADSAQLTEQLRNTEAEAEHFREAEQQWKQQYNET

VERESSLTAQLRKVQDQHGQLEQEIKKLREAEVKSEDEQRRLQKVLEQAHAAVRTLQH

ELGALTQSEQSWKEQAEQRLIEIGELETQVLEAAEQNETLESGVQSLHDELSVVKEEA

RHASEEAERYLAQAGRLELQHKELDGELNEAREELNKLQENYKQIRKDYGDALQREED

YTRKLNEMSEEKKEIVRALEESRQNANQLSGRIDEQGSLLTQTEEEALEWQIKHEELS

AKQAELSSRLKELTDREVELSSRVEQAEQQAEQQDQVWQRRADEWAAQEQSWQDRWTT

LENELNLWQKESAASVEKVEELEHERQQLAQQRAEAIEEKQTMENELLDIGERYELAA

NQLRLLQVEREMEQEKAEQLNTDYRQLRDEYTKLQTEYNEWIELIEQDQT"

CDS complement(1623280..1623663)

/locus_tag="PIKHDJKN_01517"

/inference="ab initio prediction:Prodigal:002006"

/codon_start=1

/transl_table=11

/product="hypothetical protein"

/translation="MKKGMAWLTACMLILVLAACGGTGQSAESNGESDSGATASEELV

IKASNYEFDQPEYHLKKGVPVKIVYENANGNHGVLVPELNLQLDTKNSSKVITPDKAG

EFEMSCSVFCGSGHSSMISKIIVEE"

CDS complement(1623880..1624245)

/locus_tag="PIKHDJKN_01518"

/inference="ab initio prediction:Prodigal:002006"

/codon_start=1

/transl_table=11

/product="hypothetical protein"

/translation="MNFLGHVVRFIIAAIVLMVVSWIVPGFAVGGFWSALMLALVIAL

LGWVVEGIFGKRVNPFGRGIVGFIVSALVIWIGQYVVDHVHVSLLGAVLAALVIGIID

LFIPVSTPFDAGRRSKDHA"

CDS 1624433..1626802

/gene="mutS2_1"

/locus_tag="PIKHDJKN_01519"

/EC_number="3.1.-.-"

/inference="ab initio prediction:Prodigal:002006"

/inference="similar to AA sequence:UniProtKB:P65496"

/codon_start=1

/transl_table=11

/product="Endonuclease MutS2"

/translation="MDTKILHTLEYRKILNTLQSFAQTTMGKRKADQLEPISELEEVK

RLLQQTDEAFTFDRLKGSPSFGGIVDITASIKRAEIGGTLNPHELLGISNTTFAARRL

KRQIAALHDDEKVESIFYISDQLSEQKTLEDAIRICIDDNAEVSDSASVTLAQIRREL

RGGEARIREKLDSMIRSSTVSKMLQDQLITIRGDRFVIPVKSEYRSYFGGIVHDQSGS

GATLFIEPESIVAMNNKLRETRIREEREIEVILQKLTALVSEQAEWLLYDVDLLGSLD

FIFAKARLARELKATLPRMNDRGFIKLKKGRHPLISIENVVPTDIELGNDYTSIIVTG

PNTGGKTVTLKTIGLLSLMAMSGLFVPAEDGSQLCVFDAIYADIGDEQSIEQNLSTFS

SHMTNIIRILKNMTTKSLVLLDELGAGTDPAEGSALAISILEHMHQTGCRMVATTHYS

ELKAYAYERKGVINASMEFDVNTLSPTYRLLVGVPGRSNAFAIAERLGLPGYILDYAR

GEVKEEDQRIEHMIASLEENRLTAEQERESAERLRQDMEKLRSRHQAELEKLEQQRDR

RIEKAEEDARNIVDKARAEAEKIIADLRQLAMEEGASVKEHKLIAARKQLDEAEPEKR

KKTVKRTATATKSRAIGPGDEVLVYSLNQKGHVVEMSGSKEAIVQLGIMKMKVSLNDL

ELQQSAPSEAKPKQKPVTGMKRTRDDNVKSELDLRGTNLEEALMETDRFIDEAFLANL

GQVYIIHGKGTGILRSGIQDYLRKHRHIKSYRLGNYGEGGNGVTVAELE"

CDS 1626875..1627294

/locus_tag="PIKHDJKN_01520"

/inference="ab initio prediction:Prodigal:002006"

/codon_start=1

/transl_table=11

/product="hypothetical protein"

/translation="MKGTIDQLLSHPLGMVLGFFSVAIMELLVFLACFELVTKYKCWH

EIKKGNVAVAMATGGKIFGICNVLHFCIQAKSSVYEAMTWSFVGFVLLLIAYFLFEFL

TPVFSIDKEIEADNRAVGLISMIISVSLSFVIGASIM"

CDS 1627415..1627981

/locus_tag="PIKHDJKN_01521"

/inference="ab initio prediction:Prodigal:002006"

/codon_start=1

/transl_table=11

/product="hypothetical protein"

/translation="MEPTVCPWCHTEIVWDEELGPEEECPYCHNELKGYRTLNINIGD

EENNEAEEDIYDMDDEEDGEQTTDLANLWGDEVELKLPELRTLTKYADEGNDLIQYEE

GVEKQLDLQEEVPECPHCREFMILTGTQKATKDSFNPVANAVSGTDSLLDAPFSVQMY

VCSGCFQVQYNLSEDDRLRLIQNLSARK"

CDS 1628108..1629322

/locus_tag="PIKHDJKN_01522"

/inference="ab initio prediction:Prodigal:002006"

/codon_start=1

/transl_table=11

/product="hypothetical protein"

/translation="MKISLGRQSILLLGVNGLFVLAGALSGTFLNVYLWKSRPDYAML

GWFTISQQIALGLTFWLAGKWVKEHNKMSALRVGTGLSGVFYMLVLWAGPKAVDWIWP

LGILFGCALGLFWIAFNVVYFEVTDRENRDLFNGWVGLLGSMTGIIGPWFSGLIISRM

VDNSGYRLIFTVSLVIYVIAVVFSFFLKKREVSGTYRWSEPWRQLSAKDSAWRPLGMG

LFAQGVREGVFAFLIALLVYVATKQEYKLGQFSLITSAVALVSYWAAGKWFKPQYRSR

GMLAGAVLLFIVLIPLLWQVSYGTLLIMGIGSALSMPLYILPMISAGFDLMGTSGENV

EKRVELVVLRELCLMVGRLFGLAVFILTVMNGPSLRTLTWLIVALGAFPLIGWIFMRK

MLVHPDQQEPSA"

CDS complement(1629385..1630605)

/gene="puuB"

/locus_tag="PIKHDJKN_01523"

/EC_number="1.4.3.-"

/inference="ab initio prediction:Prodigal:002006"

/inference="similar to AA sequence:UniProtKB:P37906"

/codon_start=1

/transl_table=11

/product="Gamma-glutamylputrescine oxidoreductase"

/translation="MDLVYGTTFWPSTFNTNPNYPSLQTDISCDCLIIGGGMGGALTS

ELLTERGVNTVVIDKRKIAHGSSSANTGLLQYTNDKTLTSCIHTFGEKAGVRFYELCL

EAMQKLTQIADRLDTDPWFIPRTSLCFASSIDDVAMLEEEYQTLKRYGFEAELWNSDK

ISNHFPFSKPAALYTLGDAEVNPYRFVHALFKSASKRGARIYGQTEMIHCEFDDYGVL

CHTLGGKIRARKVVFAAGYETQAIKKDQGAYLQTTYAIATKPLPDLKEWYEHSLIWET

ARPYLYMRTTPDGRIIAGGLDEETPREDQREIRAKHRGDTLLEEVRSYFPLDGLEVDY

AWGAVFGNTNDGLPFIGPHPQYPHSYFLEGYGGNGTVYSMFAASILADAVTGVKNGDM

ELFSLTRTSKPSPV"

CDS 1630702..1630935

/locus_tag="PIKHDJKN_01524"

/inference="ab initio prediction:Prodigal:002006"

/codon_start=1

/transl_table=11

/product="hypothetical protein"

/translation="MQNQSMQALSPKELNYIADSITNEELLIKQCAATASISHNPQIQ

QALNHYVRSHEQHMNTLVNALQQHQSLAPTQVQ"

CDS 1630966..1631361

/locus_tag="PIKHDJKN_01525"

/inference="ab initio prediction:Prodigal:002006"

/codon_start=1

/transl_table=11

/product="hypothetical protein"

/translation="MYSQNLSSNGSFMQEQDLLKSILADLRRTAREYTTATSEASCPI

TRRMFTDLTNDTLRLQGELFNVMQQNNMYSVGSKALRQDVDKQIQSAHQTQQKCQQFI

QEKNTQASTYNQAPNVPQHQPNYGNPYYM"

CDS 1631520..1632020

/locus_tag="PIKHDJKN_01526"

/inference="ab initio prediction:Prodigal:002006"

/codon_start=1

/transl_table=11

/product="hypothetical protein"

/translation="MKDLNDLQLKVLDLLKEDARRTPALLSTLLGESEDKIKNAVAQL

EQDHVIVKYATVVNWSKIDDEKVTALIEVQITPERGRGFEGIAERIYLYPQVKSVYLM

SGAYDLLVEVEGGNLREVANFVSEKLSPIDSVLSTKTNFILKKYKQDGIIFEDHQEDN

RLMISP"

CDS 1632034..1633242

/gene="dapX"

/locus_tag="PIKHDJKN_01527"

/EC_number="2.6.1.-"

/inference="ab initio prediction:Prodigal:002006"

/inference="similar to AA sequence:UniProtKB:P16524"

/codon_start=1

/transl_table=11

/product="putative N-acetyl-LL-diaminopimelate

aminotransferase"

/db_xref="COG:COG0436"

/translation="MIVNEQTTGNNKKMTSYLAPLVQQIPPSGIRKFFDLVGDNKDII

TLGVGEPDFVTPWHMREACVYSLERGMTSYTSNAGMPKLREAISEYLDTQFDTKYDPK

DEIIVTVGGSEAIDLALRALIVPGDEILIPEPSYVAYSPIASIGGGIPVGVETYAKDQ

FKLTAEALEAGITPKSKVVILCYPSNPTGAIMTYEEWLPIAEVIKKHDLIVIADEIYA

ELTYTQKHVSFASIPDMKERTILVSGFSKAFAMTGWRIGYMCGHPELIAAMLKIHQYT

VMCAPAMGQVAALEALTNGLGEKDRMVESYNQRRRLIVQGFRDIGLDCHEPQGAFYAF

PSIQKTGLSSDLFAERLLTENKVAAVPGNVFGPQGEGFLRCSYATSVTQLNEALERIG

NFVYKLQKEG"

CDS 1633388..1633654

/locus_tag="PIKHDJKN_01528"

/inference="ab initio prediction:Prodigal:002006"

/codon_start=1

/transl_table=11

/product="hypothetical protein"

/translation="MFCVEYDLPTNRGHYLAEGDHGDRWSVKPDHASLHNVSLEDEIH

MLRRRMEQIFLEEKSFTSDIVIEISSLLDLKINEYMKANPIKGK"

CDS 1633816..1634367

/locus_tag="PIKHDJKN_01529"

/inference="ab initio prediction:Prodigal:002006"

/codon_start=1

/transl_table=11

/product="hypothetical protein"

/translation="MKRFWGLTAIISMIFLLAGCGGKDGFTIFIIDNQGNPSVISEQL

QANLQQKLGEEPKVEVITSAMYDVQKIMVEYAAGGHDIFILPEADMKQYGSNGSNIPL

DDTFDPEKYKRGVFEGGVLVEKGEGDDGSDIKTESHLYGIPLEDMQMFKDVDYAAQNL

FATIPVSASNVEESKKVLKALTE"

CDS 1634405..1634941

/locus_tag="PIKHDJKN_01530"

/inference="ab initio prediction:Prodigal:002006"

/codon_start=1

/transl_table=11

/product="hypothetical protein"

/translation="MLITVTGGIGSGKTRFALGYAARISREGIYLSTGDHDPVIPELP

SAHYRAIQAGNGQHLTEVLHQINRESNLFLADQRIVIVDSLTAWMAAGFRASDDLDQQ

RSETQLLLDALLSYQGKLLVITNEMHGSLYPSEEERIFAARMASVNRALQAHSEQMYL

LISGLPVDLKDRGLRYED"

CDS complement(1635050..1635661)

/locus_tag="PIKHDJKN_01531"

/inference="ab initio prediction:Prodigal:002006"

/inference="similar to AA sequence:UniProtKB:P9WP09"

/codon_start=1

/transl_table=11

/product="putative membrane protein"

/db_xref="COG:COG0586"

/translation="MQNWITDFMEQYGYIGIALIIALENVFPPIPSEIILPFGGFMTT

YTSLTLPGVIIAATIGSVLGAVILYGIGLLIDVNRLEKIVERWGHILRIKKEDIHRVD

AWFDKYGMWTVLFCRMVPLVRSLISIPAGMSNMKFGLFLLFTTIGTLAWNIILVSVGA

ALGASWESILNFMDVYSLVVYVLLAIIVVGCIIWWIRRNKTRK"

CDS 1635888..1636469

/gene="yvqK"

/locus_tag="PIKHDJKN_01532"

/EC_number="2.5.1.17"

/inference="ab initio prediction:Prodigal:002006"

/inference="similar to AA sequence:UniProtKB:O34899"

/codon_start=1

/transl_table=11

/product="Corrinoid adenosyltransferase"

/db_xref="COG:COG2096"

/translation="MGIYTRTGDEGQTSVIGGRVIKDDDRVEAYGTIDELNCFVGQAI

SFIDQANGEFEDLREHLLEVQQELFDCGSDLAFVKISETKYKVRDEMVTRLEQWIDQY

DAENPKVERFILPGGSLLSSTLHVCRTVCRRAERRAVTLGQHTDINPSVRRYLNRLSD

YFFVVARTANARQQVADIEYVRSKKVFRRKDKE"

CDS 1636466..1637449

/gene="rluD_1"

/locus_tag="PIKHDJKN_01533"

/EC_number="5.4.99.23"

/inference="ab initio prediction:Prodigal:002006"

/inference="similar to AA sequence:UniProtKB:P33643"

/codon_start=1

/transl_table=11

/product="Ribosomal large subunit pseudouridine synthase

D"

/db_xref="COG:COG0564"

/translation="MSGYYSPLTYTVPEHEDGWLLKTVLQRRLQVSRKLLSRLKLTEQ

GIMLNGERVYISVKVSAGDVLEVRMEQEESDDILPEPIPFTILYEDEHLLIVNKDSGV

IVHPTHGHYTGTLANGVVHYWKSKGERFRFRPIHRLDQETSGVLAIAKNPYVHQHVSE

QMIAGTVDKQYIAFVHGSPAQEEGAIDGPIDRDPEEPHRRIVTPDGYAARTLYTTVAR

WAGGSASAVNLKLESGRTHQIRVHMTSIGCPLIGDRMYKTLPVHEIDEQIMAVREERD

TWISRQALHACELSFDHPILQSRMTFQAPLPADMAALEKRLDDEASPRETL"

CDS 1637809..1638174

/gene="spxA"

/locus_tag="PIKHDJKN_01534"

/inference="ab initio prediction:Prodigal:002006"

/inference="similar to AA sequence:UniProtKB:Q8DU17"

/codon_start=1

/transl_table=11

/product="Regulatory protein Spx"

/db_xref="COG:COG1393"

/translation="MSDLKVYQYAKCGTCRKAVKWLEAQGHELELIPIFDTPPSEAEL

TGLIQKSGLEVKKFFNTSGEVYKEQQLKDKLPGLSTEEQIRLLASNGRLIKRPIVTDG

HKVTVGFKEDTYEQEWNNA"

CDS 1638580..1639473

/gene="ypcP"

/locus_tag="PIKHDJKN_01535"

/EC_number="3.1.11.-"

/inference="ab initio prediction:Prodigal:002006"

/inference="similar to AA sequence:UniProtKB:P54161"

/codon_start=1

/transl_table=11

/product="5'-3' exonuclease"

/db_xref="COG:COG0258"

/translation="MNQRNEPTLLLVDGMAVLFRAFYATSASGYIRRTKAGMPTNAVY

GFIRYFWDAVQTFGPSHVVCCWDMGGKTFRGEEYAAYKGNRPEAPDDLIPQFALIREV

MDSLNIPNIGAQGYEADDCIGTLAKYYTEQTDMNVMVLTGDHDMLQLIDDRTSIIIMK

KGHGNYMVYTPESLMTEKQLTPRQVIDMKGLMGDASDNYPGVRGIGEKTALKLVQEFG

SIEGVLDNMDKLTPSVRNKIENDLEMLHLSRKLAEIHCAVPVACALDVCELRLDPDMV

MDKFEQLEMKSLGSWMGVAIG"

CDS 1639521..1640585

/locus_tag="PIKHDJKN_01536"

/inference="ab initio prediction:Prodigal:002006"

/codon_start=1

/transl_table=11

/product="hypothetical protein"

/translation="MALVLALPVILSGAVSAPQTADAKAAISTKVQKVKAAGRSFTVQ

TVSIPKGTPVTVGLAKKQVGQTATLPSIIKAYGAQAAINGAFFEAYNGAPDPYGMLIA

NGKVIHIGRYGTTIGFKEDGSAIMDSLQVSLTGKVTDTEGKSRSWYATFINRTPSANA

SITMLYTTERGSTVGFKGGTAVVMEKGIVTKKVPNTNVAIPKNGSVLVFTGNQKSSSD

RFTVGSTVEMNYKYTNAAGKEIPWQDVVTAVGAGPRLVKDGQVAVNPVSEGFKDTKIL

NASGARSGIAIMADGSVMLATVSGATIKEWAAVMQKLGAKQAMNLDGGASSGMYAGGK

MLTSPGRLLSNTLVFGGSVR"

CDS 1640615..1641310

/gene="yiiM"

/locus_tag="PIKHDJKN_01537"

/inference="ab initio prediction:Prodigal:002006"

/inference="similar to AA sequence:UniProtKB:P32157"

/codon_start=1

/transl_table=11

/product="Protein YiiM"

/db_xref="COG:COG2258"

/translation="MTLTHNRKPDESKTAVLAVNVGQPQALPGQKREVMSGIVKHPIS

SPVFLSFTGMTGDGQADLEHHGGPDKAVCVYDYSRYPALEQLMDRKLDWGACGENLTV

EGCMEDEVRIGDVYELGEAKVQVSQPRKPCFKLGVRYDYKELPVYFQDSGHTGFYFRV

LQEGEVTSAAIFRRISTDPTSMTILEANRIMHQGKHDAVGMRALLAIPTLADSWKQTL

LKRLAKLEPAELD"

CDS 1641382..1642260

/gene="gmuE_1"

/locus_tag="PIKHDJKN_01538"

/EC_number="2.7.1.4"

/inference="ab initio prediction:Prodigal:002006"

/inference="similar to AA sequence:UniProtKB:O05510"

/codon_start=1

/transl_table=11

/product="Putative fructokinase"

/db_xref="COG:COG1940"

/translation="MTILGAIEAGGTKFVCGIGTEEGEVLERVSFPTTTPEETMAQVI

AFFEGKGIEALGVGSFGPIDPIEGSPTYGYITTTPKPHWGQYNLIGKLKEHFDVPMTF

DTDVNGAALGEATWGAAQGLDSCLYITVGTGIGAGAVVGGKMVHGLSHPEMGHIIVRR

HPEDTYEGFCPYHGDCLEGLAAGPAINKRWEQPAYELPSDHKAWEIEAHYLAHALMNY

VLILTPQKIVMGGGVMKQEHLFPMVRSKLQELLAGYVQHPALQSEIDQYVVSPGLGDN

AGLCGSLALAKLALNK"

CDS 1642445..1643446

/locus_tag="PIKHDJKN_01539"

/inference="ab initio prediction:Prodigal:002006"

/codon_start=1

/transl_table=11

/product="hypothetical protein"

/translation="MEVIFMNRLSRMSGQNEEHAQLWIGEEEGAWHLGWSRYEEGDRT

DAIWYTGSSWDELLHVYRHQLAIQMGEGYRPLLQGLFHENADLKSRSYGGQRLHCYSE

LYGNEGLYMDLCIWRRKRAASDRKAPYFIATNRLLRMISAFVPQTIDELMQLPGVGES

KASEYGAEWLELTNGLERSTSFPLEWVYSVLKEQEYESWLYKQREQKYKQELDRFTTR

KQVLEGMKEGYTLEEIVNRSGLSRRELIELLETLDLEGYDTDCLLDAELAVMPEEEQE

AVWSAYEELGDTFLKPVLHKVYGEEKPGGSNLEQVYERLRMIRIRFRRHTETEQNAG"

CDS complement(1643540..1644475)

/gene="corA_2"

/locus_tag="PIKHDJKN_01540"

/inference="ab initio prediction:Prodigal:002006"

/inference="similar to AA sequence:UniProtKB:Q9WZ31"

/codon_start=1

/transl_table=11

/product="Cobalt/magnesium transport protein CorA"

/db_xref="COG:COG0598"

/translation="MKIRLVNNGVFIPVDDIQQALTPPAEGFYWIDADVDDLAVLQPL

FMMHDLAVEDCLSDEEQRPKIEIYESHYFIVINSIRFDDEEIFLRAVNLFLGRHFIIS

VTKQKVSELRTLKPILWEQEISTPDKLLYLLVDLIVDNYFTVGDRIEARIEKLEEDIL

MHTKKSHLNEIIGLRSEILWLKKVLGPQKEVINTLNKKDLRLIDDQLQKYFSDIYENA

VKISETFETYRDLMGNLREAYQSSIANRANEIMRVFTAITTVFMPLTVITGIYGMNFT

NMPELDWKYGYFVVIGLMVTLGLSMFFIFRKKDWV"

CDS 1645288..1646205

/gene="metAA"

/locus_tag="PIKHDJKN_01541"

/EC_number="2.3.1.31"

/inference="ab initio prediction:Prodigal:002006"

/inference="similar to AA sequence:UniProtKB:A0A0D8BWP6"

/codon_start=1

/transl_table=11

/product="Homoserine O-acetyltransferase"

/translation="MPIKIPDTLPAKEVLAGENIFVMDETSAYQQDIRPLRIAILNLM

PTKETTETQLLRLVGNTPIQVDIVLVHPKSHTSKNTSQEYLDLFYKTFDEIEHRRFDG

MIITGAPVEQMDFEDVNYWKEIQEIFEWTKTNVTSTMHICWASQAGLYHHFGVPKVPL

DEKCFGVFSHTINRSNVQLLRGFDEVFNVPHSRHTEVRREDIEKNDNLEILAESEEAG

IFLVATKDGKQIFATGHAEYDPLSLKWEYDRDAAKGMDIALPRHYFPNDDPSRVPPAT

WRAHANLLFSNWLNYYVYQETPYDIGPQI"

CDS 1646230..1647417

/gene="metI_2"

/locus_tag="PIKHDJKN_01542"

/EC_number="2.5.1.-"

/inference="ab initio prediction:Prodigal:002006"

/inference="similar to AA sequence:UniProtKB:O31631"

/codon_start=1

/transl_table=11

/product="Cystathionine gamma-synthase/O-acetylhomoserine

(thiol)-lyase"

/db_xref="COG:COG0626"

/translation="MEDNKLKIESRLAQIGSINEPVTGAINFPIYQSTAFRHPKLGQS

TGFDYIRTTNPTRKVLEEAAAALESGDAGFACSSGMAALQTIFALFGQGDHLIVSLDL

YGGTYRLLERILSRFGVTASYVDTNDLAALESIRQPNTKAVFIETPTNPLMMITDVEA

VCSWAKSHNLLTIVDNTLLTPFFQRPIELGADIIIHSATKYLGGHNDVLAGLIITKGE

ELSAEMAFLHNSIGAVLSPSDSYQLMKGMKTLALRMERHEYNALTIAKYLLEHPAVNE

VYHPGLSDHPGFEIQNKQSSGNTGIFSFKVKDARYVEPLLRNIKLIAFAESLGGVESL

MTYPAVQTHADIPIEIRDAVGVDDRLLRFSVGIEHAEDLIADLGNALTAAQQEIEGGT

HHE"

CDS 1647410..1648603

/gene="metC"

/locus_tag="PIKHDJKN_01543"

/EC_number="4.4.1.13"

/inference="ab initio prediction:Prodigal:002006"

/inference="similar to AA sequence:UniProtKB:O31632"

/codon_start=1

/transl_table=11

/product="Cystathionine beta-lyase MetC"

/db_xref="COG:COG0626"

/translation="MSEQNNNANNHERNVHSDLKFDTKLLHFGDEIDKTTGASSVPIY

QASTFHHFDIFNPPQHDYSRSGNPTRQALEDYITLLEGGARGFAYSSGMAAISSVFMM

FSAGDHMIVTEDVYGGTYRLLTSILSRMQIETTFVDMTNIEEVKAALKPNTKAVYMET

PSNPTLRITDIAAVTSWAQEHELISILDNTFMTPYYQRPIELGVDIVVHSATKFLGGH

SDVLAGLAVARTDSLGTQLKQLQNGLGTVLGAQESWLLMRGMKTLGARMAHSEQSTAK

LAAWLSGRSDITAVFYPGLLDHPGREAHERQSSGYGAVVSFDVGSGDRAKAILNRVKL

PIVAVSLGAVESILSYPAMMSHAAMPAEVRRDRGITDGLLRFSVGLEDIDDLIADLDQ

ALQDS"

CDS 1648847..1649983

/gene="mqnC"

/locus_tag="PIKHDJKN_01544"

/EC_number="1.21.98.1"

/inference="ab initio prediction:Prodigal:002006"

/inference="similar to AA sequence:UniProtKB:Q9K864"

/codon_start=1

/transl_table=11

/product="Cyclic dehypoxanthine futalosine synthase"

/db_xref="COG:COG1060"

/translation="MSTVDRILDKALRGERLDLEDTIQLFESNEVDKIGAAANVIVKR

MHPEPYRTFVIGRNVNYTNVCDVYCRFCAFYRRPGSDEGYVLPDEVIFQKIQETEDVN

GTEILMQGGTNPNLPFSYYTDLLKAIKERFPNITMHSFSPAEIMKMVEVSDGLTLEEV

VRAIHEAGLDSLPGGGAEILDDRTRRKISRLKGSWRDWMDVMQTAHRIGMNTTATMVI

GLGETMEERALHLLRVREAQDECIANKYDSEGFLAFIPWTFQPDNTNLKLERQTPEEY

LKTVAISRLVLDNIKNIQSSWVTMGPEVGKKSLEFGCNDFGSTMIEENVVSAAGATYK

VNIGSITQLIRESGYIPAQRNTRYDILRTFDEDNAIQQDFVMQN"

CDS 1650213..1651211

/locus_tag="PIKHDJKN_01545"

/inference="ab initio prediction:Prodigal:002006"

/codon_start=1

/transl_table=11

/product="hypothetical protein"

/translation="MLGRGRLMKKRRLGKTGLEVPVLSFGASSLGSVFRDIDQDEGVR

TVHAAVDAGMNYIDVSPYYGLTKAEIVLGEAIRSLPRSSFTLSTKAGRYGENAFDFSR

KRIHDSVQESLDRLHTDYIDIMFLHDIEFVPAEIIIEEAFPTLLRLKEQGVIRHAGIC

GLPLPLFEKVLPQIDADAIISYCHYALNDTSLLSLLPLLENKEVGLVNASPLSMGLLS

TRGAPAWHPADDRVKQICLQAARFCAEQGTDIAKLAVQFATANERIPTTLVSSASEHN

IRNNAAWIDEPLDHKLLTEVLRILAPVHNESWASGRPEYNIEHNMKSQASSKGEQE"

CDS 1651208..1652221

/gene="lgoD"

/locus_tag="PIKHDJKN_01546"

/EC_number="1.1.1.414"

/inference="ab initio prediction:Prodigal:002006"

/inference="similar to AA sequence:UniProtKB:P39400"

/codon_start=1

/transl_table=11

/product="L-galactonate-5-dehydrogenase"

/db_xref="COG:COG1063"

/translation="MRAVICEEINRLALRDIAEPERTENQALVTIRRIGICGTDLHAY

KGNQPFFTYPRVLGHELAGIVEEIGENEAGLKAGDQVSIIPYLHCGHCIACRSGKTNC

CVSMQVMGVHVDGGMRERIVVPVSHLIQADGLTLDETAMVEPLSIGAHAVRRAALRPG

EQVVVIGAGPIGLGVMAFAKQAGARVIAIDRNLDRLELSRTWAGVDNMVQANEQAAQQ

VAEITNGDYATAVFDATGNMQSMNDAIQYVAHGGQLIFVGLVKADISFHDPDFHKREM

SILGSRNATREDFEEVMSILREKKLNMDGYITHRASFDQLPVSIDTWLRPDSHVVKAI

VEL"

CDS complement(1652321..1653226)

/locus_tag="PIKHDJKN_01547"

/inference="ab initio prediction:Prodigal:002006"

/codon_start=1

/transl_table=11

/product="hypothetical protein"

/translation="MKAPLISANPVPQTAKTRLSSARRMLNLLFVIAGGILASVGLEL

FLHPNKIIIGGITGISSLFAHWTEMRIGLFLFLFNVPFIFLSYRLVQKKFVLVTVLGL

VVFSIGAIVLHPMPPLVEHPLAAAMFGGLCLGLGIGLVVRYGGTLDTLEIGDPSSRPP

ERVFSGKRMLIEKIIMLLNLLILTAAGVIFGWDQAMYSVIAYLIAYEMVYIAFRGFTA

KRKICILTTQSAQVEKAIRKRLRREPGTLDSSTDSGHPAVAEGWIQHVPGALYYEIHF

LEMIWLKSIVRHIDPHAGIVTNPEK"

CDS complement(1653339..1654577)

/gene="khtU_1"

/locus_tag="PIKHDJKN_01548"

/inference="ab initio prediction:Prodigal:002006"

/inference="similar to AA sequence:UniProtKB:O07536"

/codon_start=1

/transl_table=11

/product="K(+)/H(+) antiporter subunit KhtU"

/db_xref="COG:COG0475"

/translation="MDHLIFEVGLALVLIAAAGLLSAKLRFSVVPFYILIGMAVGPHA

MKIWHLDFRFIESQPFIEFMGRIGILFLLFYLGLEFSVGRLIKSGKSIAVGGSIYIGI

NFTLGLIFGFMSGFPVAEALIIAGITTISSSAIVAKVLVDLKRTANPETEMILGIIMF

EDVFLAVYISILSGLVLSDSSSIGGVIMSALIALGFMLIVIILGRKATPLLNKLLKIR

SNELFSLVVFGALFLVAGFSETIHVAEAIGALLVGLVLAETEHAKRIEHLILPFRDFF

GAIFFFSFGLSIDPLSLGGEAVWLALIAVVITLFGNFLAGMMAGRSAGLSPKASANIG

LTIVSRGEFSIILANMGKAGGLLPMIQPFAALYVLILAILGPLLTKESKHVFRLLDKI

FKFKDPRKKKEEPKVLQKEG"

CDS complement(1654582..1655076)

/gene="khtT_1"

/locus_tag="PIKHDJKN_01549"

/inference="ab initio prediction:Prodigal:002006"

/inference="similar to AA sequence:UniProtKB:O07535"

/codon_start=1

/transl_table=11

/product="K(+)/H(+) antiporter subunit KhtT"

/db_xref="COG:COG0490"

/translation="MDVRETDLPGIGKKFQIETSSGDKIIVIIHDDGRREMYHFEYDD

PDQSISMITLDDYEARQIAAIVGGLTYKPKQLENIEVTFDDLIIEWYKIEPSYGCIGK

SIGELDVRQNSGATVIAVVEKNHKKHISPGPEVVISAESTVVAVGERNQQKLFKSLLL

TGRG"

CDS complement(1655388..1655792)

/locus_tag="PIKHDJKN_01550"

/inference="ab initio prediction:Prodigal:002006"

/codon_start=1

/transl_table=11

/product="hypothetical protein"

/translation="MRQQPRILRYAFIIILCVFQALSFPIPMQSESAVEVTVETTSNK

EVLVHTLESQQKPAIRKYPQVMSKLIAVTHNKPVFLVLALLLMLRIPTPRLPFKPLIC

LFKRRLFLLPIKFTSMYVSPTPIAPIYVNALS"

CDS 1655973..1656917

/locus_tag="PIKHDJKN_01551"

/inference="ab initio prediction:Prodigal:002006"

/codon_start=1

/transl_table=11

/product="hypothetical protein"

/translation="MELFAVWTDVSGQEEADQFYRCVKEKTKGLHISKRGFRLTFKHN

DGRAAWVCRGNENHADFQQWLPQISDLLATGLADFIMDTRERIMVEEMISKACSVMDE

HEVEKVNRICMPLLFNGDLDQPGLRERRRTTLANGLKQDLEEVHFFHLEGILNFRIRP

YKQELHELVEYALDEFWMDRQYEEFMGLLKYFVFFQEAKVPLVHLIHKGLHEFQVLDA

ALNVLPVQKDEQVVVEMPGLELEMDIEDMIVSTLISISPEKVMLHTRTPDLPIISTIR

QIFEDKVQLCHHCPECEVLRSGLLTGLDASDIGNYNNC"

CDS 1657346..1659283

/gene="thrS"

/locus_tag="PIKHDJKN_01552"

/EC_number="6.1.1.3"

/inference="ab initio prediction:Prodigal:002006"

/inference="similar to AA sequence:UniProtKB:P18255"

/codon_start=1

/transl_table=11

/product="Threonine--tRNA ligase 1"

/db_xref="COG:COG0441"

/translation="MAVNIKLPDGSVREYADGSSIEDVAASISSGLRKNAVAGKLDGI

VVDLSTTLHEGALVEIVTLDSPEGLEVMRHSTAHLLAQAVKRLYGNKEVHLGVGPVIE

DGFYYDMDLEQPLNPEDLQKIEKEMERIVNENLPIVRKEVSREEAIKIFEEVGDPYKL

ELIRDLPEDSVITIYEQGEFFDLCRGPHVPSTSKIKVFKLMNVAGAYWRGDSKNKMLQ

RIYGTAFVKKAQLDEHLHFLEEARKRDHRKLGKELEIFTFSQLVGQGLPIWLPNGAKL

RRTLERYIVDLEESLGYQHVYTPVLGNVELYKTSGHWEHYQEDMFPKMVMDNEELVLR

PMNCPHHMMVYKSSMHSYRDLPIRIAELGMMHRYEMSGALTGLHRVRAMTLNDSHIFA

RPDQIKEEFARVIELIQTVYKDFGIHDYRFRLSYRDPQDTEKYFQNDEMWEMSQRMLR

EVVEELNLPFYEAEGEAAFYGPKLDVQIKTALGKEETLSTVQLDFLLPERFELEYVGD

DGQKHRPVVIHRGVISTMERFTAFLLENFAGALPLWLSPVQAKVIPVSGNFDDYAREV

EAKLKRAGISAEADLRNEKLGYKIREAQLEKMPYMFVVGENERNAEGVSVRKRGEGDL

GMKPLDEIVAQLKEEITTRQV"

CDS 1659465..1660238

/locus_tag="PIKHDJKN_01553"

/inference="ab initio prediction:Prodigal:002006"

/codon_start=1

/transl_table=11

/product="hypothetical protein"

/translation="MKKQFLIQKKNIRFIISCCVVLTGVVLNWNQAEAYTFEEENEVM

YVENKHSEIPNEFEGVYPETTVYEYSDQAQLTTIVYMGFLWSPQPIVIPRPEIPEKHI

AVDPSMPVLAPRTEQVLDTHKVTATGYTAGVESTGKGPKHPQYGITYSGVKVRRDKET

VSTIAADPKLFPLGSILYIPGYGYGIVADTGSAIKGNKIDLYFPTTKQVYKEWGKKDV

EVQVIKQGAGKCTEKMLEELSEAIDVYKSVPDAWLDKAV"

CDS 1660351..1660536

/locus_tag="PIKHDJKN_01554"

/inference="ab initio prediction:Prodigal:002006"

/codon_start=1

/transl_table=11

/product="hypothetical protein"

/translation="MAKKSQGSQTPNEKYNAEFGEENAASKVKQSQQNKVKDNFQTPD

EKYNAEFGTENTGKTQK"

CDS 1660706..1661761

/locus_tag="PIKHDJKN_01555"

/inference="ab initio prediction:Prodigal:002006"

/codon_start=1

/transl_table=11

/product="hypothetical protein"

/translation="MKRSTRDRWWVGIPLIAIGAIILFRQLGYDIDIGYMFRTYWPLF

LIWWGVKGITEIRRNGSHALIGPAIVLAIGGYFLARNLGWIDYSMGEFIRYLIPVMLI

GGGLFVLIGPRRRDRKHHDQVQPPPPPEPPYKPLSPEDLEMPSSFDEQFEKTFGKPKQ

EQKNDSHGSFYTDKEDHKSHGHQHHHKHQHHQHKHKGFGSNKKAYGTFDDDDHYDDDP

NYYGGHGNTINKSAFIGDMYMGQEVFSLKPMNISAFIGDTVIDLTKAQIPYGETKINI

SSFIGDVKVFVPEDMDLGVTVTTNSFIGDMSLLNQKRGGFLSSAQAETAHYREAGKKV

RIVVSVFIGDVKVNKVG"

CDS 1661765..1662802

/gene="liaS_1"

/locus_tag="PIKHDJKN_01556"

/EC_number="2.7.13.3"

/inference="ab initio prediction:Prodigal:002006"

/inference="similar to AA sequence:UniProtKB:O32198"

/codon_start=1

/transl_table=11

/product="Sensor histidine kinase LiaS"

/db_xref="COG:COG4585"

/translation="MIRTILKANKWELMMYFALTGLITLGGFYLLYGEVLMGTGRQRA

WTYVAVVLLATVITGYIAALRLQRKIDLLDLNMLKVSKGNLSVRMPEADDASFGRVYQ

EFNVMMDSIEKKMRLLQRLGEQEVIEKEQASERAVIEERKRMARDLHDTVSQQLFAMH

MSASSLPRLLEMNPEHGSKVLDQLIQMSHIAQRQMRGLIAQLRPVELEGRNLGEALDS
WFPDYCRQNGLKGVKELELDGGISDAIEHQLFLVIQEAVANVVKHAEAGLVSLSIRES
EHQISMSISDDGQGFLQQAERPGSYGLSTMRERAEKLGGQVQIISKPGAGTTVRVFIP
KFPKEPEGETE"

CDS 1662799..1663461

/gene="liaR_2"

/locus_tag="PIKHDJKN_01557"

/inference="ab initio prediction:Prodigal:002006"

/inference="similar to AA sequence:UniProtKB:O32197"

/codon_start=1

/transl_table=11

/product="Transcriptional regulatory protein LiaR"

/db_xref="COG:COG2197"

/translation="MSGKVNVMIVDDHDMVRMGLKTYLMLEPTFHVMGEAGNGQDALN
QLRKLGENDLPDLILMDLMMPVMNGAEATQAIMTEFPGMKIVMLTSFLEDDLVVQAIE
AGAVSYVLKTVSAEELIYALQGAYRGMPVMTGDVSQALTRGIRQRTARENESGLTERE
KEVLLLIAEGKTNKDIGEELHISIKTVKTHVSNLLMKCEMDDRTQLAIYAHRQGWVKN
KG"

CDS 1663610..1665295

/locus_tag="PIKHDJKN_01558"

/inference="ab initio prediction:Prodigal:002006"

/codon_start=1

/transl_table=11

/product="hypothetical protein"

/translation="MDERNYRANRQDEENETKQAENRNSSGTDDSSYYYSYGPFQSVN
QEDTASYNGEQNQREEGNVEITRPDPVKPVPTYYSNYSNESSDQANKSSRGGGNGSGN
GGDQGNGGKNNGNWNYNNRRPRSSVRSLLFSFIAGMLVITVLMYTADRTNMFTPQEAL
TSADNESSSQSSPPTNSGSSNNVTASLLPTGKEDVSSVVTSTSPAVVKIETLAKQSTR
SSSQGGSTMSDPLYQYFFGNGSNGGTDSYQGQNQQQSSNQLVPLGIGSGFIFDKEGY
ILTNQHVVQGADVIQVTLENNSKPYEAKLLGSSFDLDLAVLKIEKNSGDDAFPVAPLG
DSNSTQVGEWLVAIGNPEGFEHTVTAGVLSAKERTISIPDEETGKTREYSHLLQTDAS
INPGNSGGPLLNLNGEVIGMNVAVSADAQGIGFAIPSSVISDAVKYLKENKEVPKEPV

PFIGASLMALTPEVAKQMGTDITEGSVVASTIYQSPAYQADLRAYDIITGANGTAYNT

SQDLIDFIKKQEIGSEVTLNVVRDGKKMDVKLKIGNKNDYDTTQTTDQQQQQP"

CDS 1665494..1666183

/gene="mprA"

/locus_tag="PIKHDJKN_01559"

/inference="ab initio prediction:Prodigal:002006"

/inference="similar to AA sequence:UniProtKB:A0R3I8"

/codon_start=1

/transl_table=11

/product="Response regulator MprA"

/db_xref="COG:COG0745"

/translation="MRSTILIVDDDEKIVSMLRRGLAFEGYDVQTASNGAEGLSKLMD

KEPDIVVLDVMMPQIDGFEVCRRLREAGSKVPVLMLTAKDEVQSRVIGLDTGADDYLV

KPFALEELLARVRALLRRKADIADTPDNRLMYEDIILDNDSREVLRDGQRLELTAKEF

ELLNLFMQNPKRVLSRDLIMDKIWGYDYSGESNVLEVYIAMLRQKTEEYGGKRLIQTI

RGAGYILRGDS"

CDS 1666185..1667645

/gene="sasA_10"

/locus_tag="PIKHDJKN_01560"

/EC_number="2.7.-.-"

/inference="ab initio prediction:Prodigal:002006"

/inference="protein motif:HAMAP:MF_01837"

/codon_start=1

/transl_table=11

/product="Adaptive-response sensory-kinase SasA"

/translation="MSIRLRLTAWYSGILAAVLIFWGVVIYAFVYFNTYQEVEQQLKD

KSERITNQIGVNPLSQSLDLDPFTESQLQEAQIYIQLWDYQSRSGKISGNMEKLQIQF

PVVKSNEIQKKRGISKIYVNGTPFLVNQQPLSLQGTNEIRGLLQVGANVSSQERLLEA

LRNILVFGWLVAMALAITSGLILARKSMRPLVNVIDAANQIQSGDDLSVRIQYTGPKD

EIGRLIETVNNMLERTELSFRGLEETNKAQRRFVSDASHELRTPLTTIRGNVDFLKKL

WDQESTDRPNLDEETVKQMSLEAIEDMADEGKRMSRLISDMLSLARADTGQKIELNPI

PLQILVQEVARRSQFLDHQADWRPGDLSILNGIYVNGSKDYLQQMLFIFIENAFKYTP

DGSVTLDAILYKGQVGLRISDTGIGMDRDEVPFIFDRFYRADESRGATPGIGLGLSIA

KWIIEEHQGSVEVVTRRGEGTTFIIWLPVVFAPPIE"

CDS 1667714..1668667

/gene="ispH"

/locus_tag="PIKHDJKN_01561"

/EC_number="1.17.7.4"

/inference="ab initio prediction:Prodigal:002006"

/inference="similar to AA sequence:UniProtKB:P62623"

/codon_start=1

/transl_table=11

/product="4-hydroxy-3-methylbut-2-enyl diphosphate

reductase"

/db_xref="COG:COG0761"

/translation="MEVLRISPRGYCYGVVDAMVLARQAARNLDLPRPIYILGMIVHN

QHVTDSFEDEGIITLDGPNRIDILSQVETGTVIFTAHGVSPEVRKMARDKGLTTVDAT

CPDVTKTHDLIREKTAEGYDIIYIGKRNHPEPEGAVGVAPNHVHLIEQEEEIDGLNVP

PGKILITNQTTMSQWDIKHIMSRLLEKYPGAEIHNEICLATQVRQEAVAEQAGQADLV

IVVGDPRSNNSNRLAQVSEEIAGVTAYRVSDVAEIQQEWLKGVNKVAVTSGASTPTPI

TKEVILYLEQYEHDKPETWEIKRTVNMSKLLPPVREKTRTT"

CDS 1668900..1669964

/gene="aroF"

/locus_tag="PIKHDJKN_01562"

/EC_number="2.5.1.54"

/inference="ab initio prediction:Prodigal:002006"

/inference="similar to AA sequence:UniProtKB:Q9WYH8"

/codon_start=1

/transl_table=11

/product="Phospho-2-dehydro-3-deoxyheptonate aldolase"

/db_xref="COG:COG2876"

/translation="MIVIAGKATPEEHIQDIVATIEKEGLQVHISRGEDRTIIGLIGK

VEPKMAEHLRQMKGVENVVKISKSYKLASRDFHPQDTVISIKGVDIGGKELVVMGGPC

AVESAAQIDEIAGLVKAAGGQVLRGGAFKPRTGPYSFQGTGVEGLIMMAEAGKKHDLL

TITEVMTPEYVDICAEYADILQVGTRNMQNFDLLRKLGECGKPVLLKRGFSATYDELL

NAAEYILAGGNPNVMLCERGIRTFESYTRNTLDLSAIPVLQSLSHLPVISDPSHGTGR

RELVEPMTKASVAAGANGLIIEMHTDPDNSMTGDGVQSLFPDQFANLLQDLEKLAPIV

GKTFNTAKQPAEFFPARVGV"

CDS 1670379..1671803

/gene="glnA_1"

/locus_tag="PIKHDJKN_01563"

/EC_number="6.3.1.2"

/inference="ab initio prediction:Prodigal:002006"

/inference="similar to AA sequence:UniProtKB:P77961"

/codon_start=1

/transl_table=11

/product="Glutamine synthetase"

/translation="MSVENVLKSIQENNIEWVDFRFVDLSGRAHHISLPASAVDADTF

VNGVAFDGSSIQGFRGIEESDMVMIPDPEATYVDPFTAHPTLNVMCDIFTPDGERYER

DPRGIAVKAEEFLKESGVGTAAFFAPESEFFIFDDVRYESGTNSSSYFVDSEEASWNT

NRKEDGGNLGFKVRTKGGYVPVAPVDTQQDIRSEMCRILEEAGLSIERHHHEVATAGQ

AEINFRFDTLKKTADNLLVYKYIVHNTARQYGKVATFMPKPLFGDNGSGMHVHQSIFD

GDSPLFYDKAGYANLSEMALHYIGGILYHAPALIALTNPSTNSFKRLVPGYEAPVNLV

YSKGNRSAAVRIPVAAVTPKGCRIEFRTPDSTANPYLAFSAMLMAGLDGIKRKINPTE

LGYGPLDKNIYDLSDADKENIRSVPASLEEALDALAADNEFLTEGGVFTKEFIENYIN

LKRDEAKAVAIRIHPHEYSLYFDC"

CDS 1672037..1673125

/gene="serC"

/locus_tag="PIKHDJKN_01564"

/EC_number="2.6.1.52"

/inference="ab initio prediction:Prodigal:002006"

/inference="similar to AA sequence:UniProtKB:Q59196"

/codon_start=1

/transl_table=11

/product="Phosphoserine aminotransferase"

/translation="MTKRAYNFNAGPAALPLEVLERAQAEFVDFRNTGMSIMEMSHRG

AVYESVHNEAQERLLSLLGNPKGYKVLFLQGGASTQFAMLPMNLLGEGQTASYVMTGS

WAKKALSEAKLIGETQVAASSEADKYMKLPDVSNLSFPDRTAYVHLTSNETIEGTQFK

SFPDTGSVPLIADMSSDIFCKPFDLNQFGMIYAGAQKNLGPSGITVVIAREKLVSESP

KTIPTMLRYSTHVDNNSLYNTPPSFSVYMVNEVLKWIEEQGGLAGIEQKNVKKADLLY

NAIDSSGDFYRGCVDPADRSLMNVTFRLANEELEKQFIKASEQEGFVGLKGHRSVGGL

RASIYNAAPYESVKALTDFMSHFQKTNG"

CDS 1673263..1673727

/gene="trmL"

/locus_tag="PIKHDJKN_01565"

/EC_number="2.1.1.207"

/inference="ab initio prediction:Prodigal:002006"

/inference="similar to AA sequence:UniProtKB:P0AGJ7"

/codon_start=1

/transl_table=11

/product="tRNA (cytidine(34)-2'-O)-methyltransferase"

/db_xref="COG:COG0219"

/translation="MALHIVLVEPEIPANTGNISRTCAATGTHLHLVRPLGFRTDDAT

LKRAGLDYWHAVHIEYHDSFAEVQEQYPEGRFFYATTKAKNRYSDFKFQDGDFLVFGK

ETKGLPPELIAANPDTCMKMPMTGDVRSLNLSNSAAIIVYEALRQLNFPGLD"

CDS 1673898..1674152

/locus_tag="PIKHDJKN_01566"

/inference="ab initio prediction:Prodigal:002006"

/codon_start=1

/transl_table=11

/product="hypothetical protein"

/translation="MKPAGVVRKVDQLGRIVLPKSLRKRYQMNEGDPVEILVQGDHII

LERYRPKCIFCGSMEEVNEFKERYICAQCLDEMTQLPQHG"

CDS complement(1674248..1674679)

/locus_tag="PIKHDJKN_01567"

/inference="ab initio prediction:Prodigal:002006"

/codon_start=1

/transl_table=11

/product="hypothetical protein"

/translation="MNIRAATQADYAELRNIYLESRRARFHWANPEELSLQDFDTDTV

EEYIIVAENEEGQLLGFASLYLPDNFIHHLFVHPGFIGIGVGARLLEASIANMEQPIR

LKCVSENHNAMGFYERQGWKKVIEEGTPGKKYWVMEYDTNS"

CDS complement(1674676..1675593)

/locus_tag="PIKHDJKN_01568"

/inference="ab initio prediction:Prodigal:002006"

/codon_start=1

/transl_table=11

/product="hypothetical protein"

/translation="MSSIINSFVHHIDEQQLNVHSVRVLQKGVTIGAWDRTSDMRRLQ

HSVSKSFTCMAIGLALDEGTLTLESRLGDYFTSPTPASHLTAFPPADLTLYDLLRMSS

GHDVPVLWAEERATLPEKDWAKYYMSLPLNRVPGETFTYSSADTFMISAMFQAATDQT

VKDYLIPRLFEPLGITNVEWETSPLGITLGCAGLQLTNEELGRFGQLLLQEGHWNGKQ

LIPTEWIRLATTQQIPTPGDGDWGQGYGYQFWRCSHNAYRADGANGQLCVVLPTLESV

VAINSDEQNMQAILDNVWSDILPILEGNI"

CDS 1676058..1676822

/locus_tag="PIKHDJKN_01569"

/inference="ab initio prediction:Prodigal:002006"

/codon_start=1

/transl_table=11

/product="hypothetical protein"

/translation="MAHITIETGSPTLCMNTSIHVVSSDSGDTSGGTLYLLHGAGDNA

STWQRLTTIEMYAEQYGCTIIMPEANRSYYTDMEYGLNYFHYITQELPEFCKRQLNLN

TDPAKTYVAGLSMGGYGALKCALTYPERYRKAVSLSGVTDIQTRLHDPEMPSGMIKEM

KAVFGERLQVKPDQDLYALSAKLLKQGGNLPDILSCCGDIDPFVDMNRKFAEYMQETD

FKFRYVETPGTHEWRFWEQHLRTMFDFLYNDKTKVE"

CDS 1676834..1677793

/gene="yteP_9"

/locus_tag="PIKHDJKN_01570"

/inference="ab initio prediction:Prodigal:002006"

/inference="similar to AA sequence:UniProtKB:C0SPB3"

/codon_start=1

/transl_table=11

/product="putative multiple-sugar transport system

permease YteP"

/db_xref="COG:COG4209"

/translation="MKPAAEGPSKKLKENLKGNSLWSEIWKHRMTYTLLIPGLVWLIL

FAYMPMGGLSLAFKDYKANLGIWGSPWSGFENFKYVFRDPTFIDAVWRTLYINILKLI

IQFPFPIILALLLNELRMRRGKKWFQTVLTFPHFLSWIIVSGVVINVLAYDGLVNSAL

GLLGLPTINFLGSESNFVPMLLLTDIWKSSGWGAIIFLAAISGIDQDQYESAQIDGAS

RMQQMFKITLPNILPTITVMFILSVGGLMSSGFDQIFNLANAATKNVSEVLDVYIYRI

TFQSSTDFSFSTAVSLFRSLVNMVLLLLADRFAKWLGGDGLFR"

CDS 1677812..1678714

/gene="ngcG_3"

/locus_tag="PIKHDJKN_01571"

/inference="ab initio prediction:Prodigal:002006"

/inference="similar to AA sequence:UniProtKB:O50501"

/codon_start=1

/transl_table=11

/product="Diacetylchitobiose uptake system permease

protein NgcG"

/db_xref="COG:COG0395"

/translation="MSKKSNKKPRIGTEKMTFLDYIIFAILLVLALMILIPFWNVIMI

SFATQKEYADNPFLMFPKEWTLDSYKALFADGTILSGYKNTIILLVIGLPLSLFLTTS

MAYGLSRNKFPFKKFLFFLVLFTMIFNGGIVPLYLIMKSLHLTGTLWSVILAGSFSAF

NMILMMNYFYTLPESLMESARLDGAGEWRILFSVVLPLATPIMATITLFYGVAYWNSW

YDAMIFLRKADQLPLQNVLRNIIVTSQTNASNASSVDAAQASNFSMGMKMAAVFVTMV

PIMCFFPMLQKHFAKGVLTGAIKT"

CDS 1678769..1680367

/locus_tag="PIKHDJKN_01572"

/inference="ab initio prediction:Prodigal:002006"

/codon_start=1

/transl_table=11

/product="hypothetical protein"

/translation="MKTNKKLSVRALFAITLSVVMLMVTACSSQGASGGNEKDAEGNY

KDNLTISVANLTEIKNGNLDNDFHKFWTDKFNVTWDYNYIDWDSWGEKLRLWINSGDL

PDVAVWNYVHGDAMNSIDQGLFYKFPDDWKERWPNVAAAYKLTGLGDKLEELTGGTYV

LPRPIYFENKPADPLTNQIGVIALRKDWAEAVGFELKDAYTTTELNEYAELVKEKDPG

KVGNKLVPLSYNAADAMTNIIMPNSVHAMTDSPFYKGEDGQFHWGPADPETLTGLKLM

QKAYKDGLLNPEFYTWKNNEGQNNFKVTGTAAVTSLGGLASYRQGLDSDMRKNLGVDS

DELVHTAIVLGEDGKYRNMEQANFWSALIFNPDISDEKFERIMDLIDYSTTKEGQLLI

NMGFEGTDWKYDENNELVSLLPEGTVLEDKYPARFEGLYLLGDDFSVVNPAIKKDYRD

RAVKLFEEKAKLGTEGQSLATYDWDVNLYDSKAKSQASFDYQNEYANLILKNGDLEAN

WKEWVNSKMSLVQPYLDELNKEFK"

CDS 1680502..1682667

/locus_tag="PIKHDJKN_01573"

/EC_number="3.2.1.-"

/inference="ab initio prediction:Prodigal:002006"

/inference="similar to AA sequence:UniProtKB:T2KMH0"

/codon_start=1

/transl_table=11

/product="Beta-xylosidase"

/translation="MNNEQLLDLVKQMTLEEKTAQLLQLTANFYEGTNVEGQITGPME

EMGITEQSVDASGSILGLSGAQAIIDVQQAYLKKSRLGIPLLFMADVVHGFKTIFPIP

LAIGCSWDTELAEKSAEISARESAVSGLHVTFAPMVDLVRDPRWGRVMETTGEDPYLN

SLFSAAFVRGYQGDSLKNEPDRLAACVKHFAAYGAAEGGRDYNTVDMSERNLREYYLP

AYKAALDAGVEMVMASFNTVEGIPASGNEKLMRDILRDEWGFDGVLISDWASIREMIA

HGAAEDDREAAYRAIRAGVDIEMMTPCYVHHLPELIKNGEVEEKLIDEAVLRILQLKE

KLGLFDNPLRAADPEREREIVFSKEHRQVSYELATKSAVLLKNDNDVLPLKPEANVAL

IGPFAQSEDILGWWSCEGVKEDAVKLGTALQERLTAGKVHMAQGSNVHTITAEQIAEA

LEVASKADLIVLALGEDSEMSGEGGSRTDIRLPAAQLELVKQLKAAGKPIASVIFNGR

PLDLHGVFEESDAVLEAWFPGSEGGAAIADVLTGVVNPSARLTMSFPQSVGQIPVYYN

HFNTGRPLNPQKTEERYVSKYIDSPNDPLLPFGYGLSYTSFEYGDLEVSSNEMTADQP

LTIQVRVTNAGERAGAETVQLYVRDVTGEVVRPMRELKGYVKLSLASGESGTATFTLN

EEQLRYHHSDLSHRSDKGTFHIFVGPNSRDTLESKFKLV"

CDS 1682715..1686086

/locus_tag="PIKHDJKN_01574"

/EC_number="2.4.1.333"

/inference="ab initio prediction:Prodigal:002006"

/inference="similar to AA sequence:UniProtKB:Q92AT0"

/codon_start=1

/transl_table=11

/product="1,2-beta-oligoglucan phosphorylase"

/db_xref="COG:COG3459"

/translation="MKTMNKSQINVQSGDLCFTFWESGDLFKAVSGTTMINQWMASPV

DGSMNNLYLRFHSDSGIRFVPMLGIHSDSQVSTDGSRMVWTGTAEGVGYQVVFAPTTQ

GVWFWDVTVNGNGEEVDVVYGQDVGNADPGAVRSNEAYLSQYIDHAVFEDAKLGYIVA

SRQNQPQGGTFPYLQQGSLTCAAGYSTDGFQFFGLSYKETNVPESLSKPTLANEVYQY

EFAYTALQSEKVVLSGEARFVFYGLFRANHAEAVKELEYQDVVQQAWDEYEAKRQENT

SMVQAVSNVFPKVSLHPVMGEPIPTLSMTEEEVNSLFPGSKRHQEERADGELLAFFTD

TYEHIVLKEKEMRVERPHGHILMSGNNVKLGQNVITTSSYMYGIFNSHVVVGNTNFNK

MMSNARSALNIPKSSGQRIYVEVEGIFRLLTMPSLFEMGFNYVRWYYKTAEDTLIITN

YTTLDTPEVHLHVKSEKGIAYRYLITNQITMNVNEYELPVHVAEQNGELSFKADHSSL

SAEVYPNLEYRMRVSGAGVKVGDETELASGVNAGDDSLTTLRLDSSAEWTLTIQGLLE

GGQTASSTRSFEEEVAAYRTFYAEVMNGFHLSQPQKQGADELFKVNALAWWYTHNMLV

HYSVPHGLEQYGGAAWGTRDVCQGPVEYFMATQKYEQVRDILKTVYSHQYEDDGNWPQ

WFMFDRYFKVQQEESHGDIIVWPLKVLSDYLIATKDYSILNEEVPYTVKHGFDFTDQT

ASVLEHAQKELEYIRSHFLHDTFLSSYGDGDWDDTLQPANAQLKQYMVSSWTVALTYQ

TLKQLSRALAPVKPELSSELSDMAAGVKKDFSKYMLDTDVIPGFVYMEDPAQAKRMLH

PDDSETGIQYRLLPMTRSMIAELLEPEQAQSHYELIKDKLYCPDGVRLMNRPAQYDGG

VSTHFKRAEQASNFGREVGLQYVHAHIRYIEAMAKLGNPDEVWNGLGIINPVGIQEAV

PNADLRQSNAYFSSSDGKFNNRYAAQEQFDKLRTGDVQVKGGWRIYSSGPGIYMNQLI

SNALGIRQEEEDLVLDPVLPVSLDGLHFDFQFAGKKVTFVYHFSGEAVQRIVLNGKEL

DATRLQQPYRLGGLRISRKELEQSLSENNVIEIYS"

CDS 1686186..1687202

/gene="rbsR_1"

/locus_tag="PIKHDJKN_01575"

/inference="ab initio prediction:Prodigal:002006"

/inference="similar to AA sequence:UniProtKB:P0ACQ0"

/codon_start=1

/transl_table=11

/product="Ribose operon repressor"

/db_xref="COG:COG1609"

/translation="MVSIKDIAKQAGVSISTVSYALNGSNKVTDETRSKILAIAKELN

YVPNAAARTLKKRESKILGVFLTDFSGDVYGDLLSGMKSVCNAQGYDLIVCSGKQSHR

MLPERMIDGAVILDHTFASKELLQYADRGHKIVVLDRELDHPNINQVLLDNKAGATLA

MEHLIEQGHTKIYVVTGPEGSFDSAQRLKAVRQVSEREANVEWIEITGDFEKSGGERA

AERIIQEYTEPVGVFCLNDEMAIGMCDRLADSELNIGQEIDIIGFDNIELSKYVQPRL

VTIDYSKRKWGALAAEQLIKIIAGEPVDHERIYVTLVEGGSVNSQVQSEYLAQAQGEK

AVSY"

CDS complement(1687376..1687480)

/locus_tag="PIKHDJKN_01576"

/inference="ab initio prediction:Prodigal:002006"

/codon_start=1

/transl_table=11

/product="hypothetical protein"

/translation="MSFSLTFWIIAIVLALFLTGILTSSYNNDKTKGL"

CDS complement(1687556..1688620)

/locus_tag="PIKHDJKN_01577"

/inference="ab initio prediction:Prodigal:002006"

/codon_start=1

/transl_table=11

/product="hypothetical protein"

/translation="MITPVKKVNRLFMLALAPFVGLMLCMLLIRPPLEPGDFINSGLA

EENVTPRTQAISQELAGAKEAAVQTSSSIKRTTQLYNQTTSTMSTLVQKATVQAARPE

TIYNQRISSKLGVPFERIDSDRLTIELYRVNPGPYKGYAMKIKLKDPTAMKMALDSEP

GRSETTMQAVKRSGAIAGINAGGFADSGGKRYPLSTTVMDGKYVNGFQASFKDLFFVG

LDDAGKLVGGKFFDKSSLDRLQPQFGATFVPVLLQNGQKTVIPAKWKVSPKRAPRTVI

GNYKDDQLLIIVVDGYNEGGSSGATLEELQGRLYKLGVVDAYNLDGGGSSSLIVNNRV

VNNPSDGSLRPIPTHFLFYK"

CDS complement(1688617..1689027)

/locus_tag="PIKHDJKN_01578"

/inference="ab initio prediction:Prodigal:002006"

/codon_start=1

/transl_table=11

/product="hypothetical protein"

/translation="MKPSVQPSDIHHTRSAKGKAGSSVKLFLVMWIVLIALGVLGTYY

YSNHLQQQMINQLQTHNQQQIAALKADYEKQLTTISKEVADLQGQVQSFNELLTFTKD

NANDKTDNSNKLYTQLSEVKKQLETLQKKMDLLK"

CDS 1689469..1690125

/gene="comA"

/locus_tag="PIKHDJKN_01579"

/inference="ab initio prediction:Prodigal:002006"

/inference="similar to AA sequence:UniProtKB:P14204"

/codon_start=1

/transl_table=11

/product="Transcriptional regulatory protein ComA"

/db_xref="COG:COG2197"

/translation="MKKVWQVVIVDIHPTSMLGTKLILEEQQDLLVRGMTSTGSEGLD

LVNIHQPDLILMDYRLPEGQADQYISQMKNLSAHSHIIILTDEDNVKLFRHLISLGAS

GMLSKQASPSQLIHLISGLREGCVSIPLSWLASSEWAQPVESPAEELIVELTETETFI

MERIVQGVTYDKIASEINVSRRSIDNYLRKIYVKLDVSSRAQAIERYALYARQTKTVS

"

CDS 1690267..1691490

/gene="lysN_1"

/locus_tag="PIKHDJKN_01580"

/EC_number="2.6.1.39"

/inference="ab initio prediction:Prodigal:002006"

/inference="similar to AA sequence:UniProtKB:Q72LL6"

/codon_start=1

/transl_table=11

/product="2-aminoadipate transaminase"

/db_xref="COG:COG1167"

/translation="MKYFFASRTNRLLTSPLRDIREMSGRDYFISLAEELPAEELFPF

KLLEEAAVSVFSSGPSALQYGEPAGYRPLREWLDNDWNARKGIRTVPEQILLTTGTQQ

AIDLVMRLLLEPGDSVLVEHPTSPGCLEVLEMQGAKIVPVTGDGDGILPDLLEHHMQQ

VRPKLLFAAPSFSNPTGVLWSMERREAVLDLCSRYGVLLVEDDSYGELHFDGLEPGDF

YRKYPSLFALDTADQGGHVLYIGSFSKTVAPALRTGWAAGHPALIQAMASVKRIADGQ

SSPMNQRLLYQLLAHSPFKWSDHLSMLNREYKTRLKLMLELLKRPGWKGSQYSIPEGG

MYLWVQLPDGLDSGALLKAALLKGVSFLPGSLCSTGEQDHRYIRLNFSHPGRDELLLG

MNLISESITEFTARS"

CDS 1691730..1692356

/gene="azoR4"

/locus_tag="PIKHDJKN_01581"

/EC_number="1.7.1.17"

/inference="ab initio prediction:Prodigal:002006"

/inference="similar to AA sequence:UniProtKB:Q81JP2"

/codon_start=1

/transl_table=11

/product="FMN-dependent NADH-azoreductase 4"

/db_xref="COG:COG1182"

/translation="MSNILFVKANDRPADQAVSVQLYDAFLSAYKESHPGDTVTELDL

YNTDIPYYGNTVITGGYKAANGIEATAEEQKAAALAAQLQDQFLAADKVVFAFPLWNF

TVPAPLVNYISYLSQAGKMFKYTAEGPVGLVGDKKVALLNARGGVYSVEPMASAEMSV

KYVTNVLNFWGIQNPELVIVEGHNASPDRSQEIVAAGLKLASEVAASF"

CDS complement(1692462..1693310)

/locus_tag="PIKHDJKN_01582"

/inference="ab initio prediction:Prodigal:002006"

/codon_start=1

/transl_table=11

/product="hypothetical protein"

/translation="MAVKYETELYSPVKAFFERRGYDVKAEVKHCDLVGVKSDQNEPL

IVEMKKTFNLSLLLQGMQRLKLSPFVYLAVERNRSKRGAVNQRWGELTALCRQLGLGL

ITVTFYKTKSPLIDVLCEPAAQAPMSGSGSTSVRKGGIRRQRLLKEFDERSGDYNTGG

STRRQLVTAYREKALRVASALRAQGEASPASISKQTSVGSAAAILQKNYYGWFERLSR

GRYVLTVKGVQALTEHAHMLEDNDMIERSINELEMDFSSNQHDDSELELIAEVAEPYL

IHGRDD"

CDS 1693671..1695566

/locus_tag="PIKHDJKN_01583"

/inference="ab initio prediction:Prodigal:002006"

/codon_start=1

/transl_table=11

/product="hypothetical protein"

/translation="MNIFERVAEHRAESDRLSWNGTFEDYIALLREDPTPAMTAHARV

YQMIESFGVEEVGGHKRYKFFEQEIFGLDRSIEKLVEEYFHSAARRLDVRKRILLLMG

PVSGGKSTLVTLLKRGLEQFSRTEKGAIYAIDGCPMHEEPLHLIPLELRPEVEKEIGV

RIEGNLCPSCQMRLRTEYGGDISKVRVERVTISEDNRVGIGTFSPSDPKSQDIADLTG

SIDFSTITEFGSESDPRAYRFDGELNKANRGLMEFQEMLKCDEKFLWNLLSLTQEGNF

KAGRFALISADEMIVAHTNESEYKSFISNKKNEALQSRMIVMPIPYNLKVSEEEKIYG

KLIQQSDMKHVHIAPHALRTAAIFSILTRLKETKKQGMDLVKKMRMYDGEEVEGYKEA

DLREMQNEYLDEGMSGIDPRYVINRISSALIKQNLQCINALDILRAIKDGLDQHASIT

KDERERYLNFIALARKEYDELAKKEVQKAFVYSFEESARTLFENYLDNIEAFCNWSKI

RDPLTDEEMDPDERLMRSIEEQIGISENAKKAFREEILIRISAYSRKERKFEYSSHDR

LREAIEKKLFTDLKDIVKITTSTKTPDATQLKRMNEVIKRLIEEHGYTAASANELLRY

VGSLLNR"

CDS 1695811..1696815

/gene="hemAT"

/locus_tag="PIKHDJKN_01584"

/inference="ab initio prediction:Prodigal:002006"

/inference="similar to AA sequence:UniProtKB:O07621"

/codon_start=1

/transl_table=11

/product="Heme-based aerotactic transducer HemAT"

/db_xref="COG:COG0840"

/translation="MSIAIARQQQLDYIGLTAGDLQLLADHRPAFEKVVDEVVDHFYN

HVGNYPNLVDLIARFSSIDRLKETQKLYWLSMTDGVVDDAYIEQRIAIGLVHSRIGLS

EDYYLGTYMVYLDIATSIFQQVIPERWHLVIQALSKMFNLDSQLVLEAYEKKEKEKLN

QLAEDQQHTLLAITQITQQLTGMISELNENAQAISDVARETAASQDQANGLLEELTKE

IHQIGKMGEIIREISDQSHLVGLNAAIEAAHAGEFGRGFEVVASEVRKLAASSREAQG

KIQSNLAQIMKKLGSVQQESEHTASGARRQASRSEELAVFATTMEKLALDLRKLDHQE

"

CDS 1696945..1697949

/gene="ccpA_2"

/locus_tag="PIKHDJKN_01585"

/inference="ab initio prediction:Prodigal:002006"

/inference="similar to AA sequence:UniProtKB:P25144"

/codon_start=1

/transl_table=11

/product="Catabolite control protein A"

/db_xref="COG:COG1609"

/translation="MASIHDVAKEAGVSVATVSKVLNDYPDVSDKTRKKVNIAIELLK

YQPNVVARGLVKRRSWTVGVLLTVPFTNPFVSELLEGIKTALENSGYDLVRLSTRFDD

PTYSFIKHCRSRNVDGVVVFGEGRDNKSIQELVTAEIPTMFVDTDLFGKRAGYITTDN

ANAIAMSVKHLHELGHQKIAYISGTLGPAVANLRLDGYREGLRVCGIPYSTVYLEVCD

YSFDGGSKAARRLLALKDQPTGIVCASDMSAFGAIHEIEKHGLRVPEDVSVVGFDNTY

YAEVFKPGLTTINQNIHSIGIKSIEYLIAMIENPAYTPPVVTEPSNLVIRQTTAPVQA

"

CDS 1698190..1699806

/gene="cheB_2"

/locus_tag="PIKHDJKN_01586"

/EC_number="3.5.1.44"

/inference="ab initio prediction:Prodigal:002006"

/inference="protein motif:HAMAP:MF_00099"

/codon_start=1

/transl_table=11

/product="Protein-glutamate

methylesterase/protein-glutamine glutaminase"

/translation="MHRVLLVDDEVYARKGLRKLIRWEACGFEVAGEANDGDEACTRI
EELQPDVVITDIRMPETDGLELIRRTVERDEQVLTKCKQPYFIIVSGYNDFAYAQQAV
RYGVQDYILKPIDEIELEGTLRKLAEELNRHKHAEQTRRKQEYVRLLDQFIYGESESG
TLEQWTELTGTRAGDPLAYLLVENNTLHFWVSDKAQEEGPGARSGENFGRIVQSVLGK
MYANRSYLHVKEHGERIGVLLTAEHLRSYSMNLHAVAGQMQSCFAEMGHDSVSIYIGK
VCYEPLLIGHAYQTALKAISYKFISEKSGIVLYDDMQHEPVQWVHTDHEKEQELLTLM
EEQRIQALEHAVGEWFTGMRTANYAPVAVTSAIHQLISAVLSILKSAEANVKEIPSFK
PILAWQADRGTLAEARQLLTVFMLDSARKLAEQRQYNHRGAMTKIKQYIEANYSSNIS
LRSIASKFYMNPVYLGQRFRKVYGVYFNDFLLQLRMEEAKKLLRQTDMRVYEIAERVG
FGSSDYFATQFEKTESCAPTEYRNRIMDEG"

CDS 1699819..1701588

/locus_tag="PIKHDJKN_01587"

/inference="ab initio prediction:Prodigal:002006"

/codon_start=1

/transl_table=11

/product="hypothetical protein"

/translation="MKLNFFNNIRLRDKMLILYLFFVLVPVILTNVIFYQVTIANVKS
QRMADLSKGMEQIRSQFMGEIRNAADFSSSFYTDSILNDMLDVNYTNPVQYIEAYDSY
LRKILNTYYSGYRSLQGLTIYVDNESVLPSGGVEFLTEEVRQSEPYRLLANSEHSAPL
LMRSGPEGNRQTFSILRKLNYYEALPGWKEKFVRLDLKPSTIRQIFTNLNVQGNLYLV
NPAGQIDYSTDSGIPWTSQLVNYGDVMDQQSKKEFEMAYPLSGEMEGWKINGVFDESE
IYKELGKSSHLVVVLACINLILPTWIIIWMTRTIHVRLARILKHMEKVKHQRFEPIRQ
PESSDEIGQLTAEFNRMTMTIHRLINDVYVADIQHKNLELQRRHAQLNALQSQINPHF
LFNALEIIRMRSLMKDEDETARIIYHMAKIFRNALTWNRDKVSIQEELEYVTCFLEIQ
KYRFGHKLNYTIVLDPEAADCSIPKMTLLPFVENASIHGIEPLKEGGEIRLCIRRTGT
ELICVVEDSGAGMTEDKRKQLLSYVETEDSMGDRVGVQNVIYRLKLIYDSRFHIQIDS
AARAGTRVEIGLPLEDSSNPPTQ"

CDS 1701622..1703304

/locus_tag="PIKHDJKN_01588"

/inference="ab initio prediction:Prodigal:002006"

/codon_start=1

/transl_table=11

/product="hypothetical protein"

/translation="MFILVNHMLKRWLPAVLALTMVTACSWGRSEIKPEAGAVGDDKV

STEKVTFSYFKAGSGKDINTNETTIGKKLEEQTGVNFKIEYLVGDINTKIGTMIASNK

YPDVMVPDGAIDKIVDAGAFIPLNDLIDQYGPNIKRMYDPYYNLMKEEDGNIYFLPMS

AVVGDYLPNPNVDQGAFWIQRRVLKETGYPKITTLDEYMALIKQYADKHKDEGLTGFL

SLTYDDKFFTITNPAMHLAGYPNDGAIMVDMQTQEARDYGLTEETKRWMQKLNEMNSL

GLFDKSSFVDNYDQYLAKVTNGKVLGFFDYAWQVGQEMNHLKEAGNDDLRYMALPIVY

DANNKDQYTDPPSFVNNRGIGITVSAEDPVRIIKFFDNLLTDENQILSNWGIEGETYE

VDDKGRFTRSPEMIQKTDSDEFRQSFGFKYFEYSWPRYGNGSTLPDGNSVGVERQPEV

AQFSYTDGDKLILKKYGVETFSQLFAAPDERPWYPAWSIAFDQGSPAHIYGQKRTDLQ

KKYAPKLVLSSPDDFNKVWDEYVKEFNKLDVKGYEQTMTEEVRKKAKLVQGE"

CDS 1703608..1706079

/locus_tag="PIKHDJKN_01589"

/inference="ab initio prediction:Prodigal:002006"

/inference="similar to AA sequence:UniProtKB:Q9ABX9"

/codon_start=1

/transl_table=11

/product="putative signaling protein"

/db_xref="COG:COG2199"

/translation="MEHLHGTYNLELVILSYIIAALASYAALDLAGRVSLAKGMSRNV

WLTCGAVSMGLGIWSMHFVGMLAFVLPMHVSYSTGEVFLSVLLAIIASGVALNIAGQQ

SGKISKLLISGVLMTAGISSMHYIGMAAMSVPITYEPDRVVLSILIAALASFAALWLM

FFFRQHQSRYTWVYKMGSGLIMGLGILGMHYTGMSAARFHHAHDAMAGSRMQIEPSLL

AYLIAAGTFIALGLTLFGIFINQRLSQKDRRIHENEQWYQALYENHSDAIISVDKQGA

IKGINAAVTRITGYQEREIIHRSIDEIAQHVDIHWASEMHHVVWDDDSREQQHFMGKM

KNVQGNILDLSIAVVPVLIDGKHQGSHILIKDITEEKQAQESIRHQALHDPLTGLPNR

RKLDDVLESTIATSGAAGSSFAVMVMDIDRFKMINDSLGHSIGDVFLREVSSRIMSAI

QDSDPNATDNVMLARMGGDEFTLVVTNEQGNEDRVAELAERIVRAIQLPYRLKENDFY

VTASIGIAMYPYHGTGADALLKHADSAMYEVKKNGKNGFQFYTAQLDSELYERIELEG

YLRKALERDELVLYYQPQIRTEDSRMIGVEALIRWNHPDKGVLSPNVFIPLAEETGLI

YEIGNWTLREACRQMKLWHASGGPLIPVSVNLSSQQFHQSNLVEQVKNALTETGLDAR

YLELEITESMMMDASVSTGILNELTALGVKISLDDFGTGYSSLSYLKHFPIHKLKIDR

SFVTDITESRSDQAIVATIISMAQNLKMEVIAEGIETKGQLDILMQNDCREIQGYYFS

RPLPASEVEHDFFVPLRLHGAPSIL"

CDS complement(1706144..1707583)

/locus_tag="PIKHDJKN_01590"

/inference="ab initio prediction:Prodigal:002006"

/codon_start=1

/transl_table=11

/product="hypothetical protein"

/translation="MTDEIRDLEYAIAEIMEIADGFGLDYYPMRYEICPADIIYTFGA

YGMPTRFSHWSFGKTFHKMKMQYDFGLSKIYELVINSNPCYAFLLDGNSLIQNKLIVA

HVLAHCDFFKNNARFSKSNRNMVESMAATADRISNYEMEYGTEAVEAFIDAVIAIQEH

VDPQLIKPRHLDKQRYMEMKMREQRGEKTPRPEGRYDDLWSLDDTQPEETPTDGIQVH

HFPPEPEKDVMWFIQEFSEVLTDWQRDIMSMMREEMLYFWPQMETKIMNEGWASYWHQ

RIIRELDLNSEDTIEFAKLNSSVVQPSKQSLNPYYLGLKIFEDIERRWDNPSREERER

FGRQPGQGRAKMFEVREFDSDTSFIRNYMTKQLTEDLDLYVFEKKGPDWKITDKSWQN

IRDQLVFARINGGSPYLVVQDADYLRTGELVLKHQYEGIELDLKYMERTLPYLYRLWG

RTVHVETRVEDKPIWFTYDGKKHHRKFLS"

CDS complement(1707580..1708746)

/locus_tag="PIKHDJKN_01591"

/inference="ab initio prediction:Prodigal:002006"

/inference="similar to AA sequence:UniProtKB:P45742"

/note="Stress response UPF0229 protein YhbH"

/codon_start=1

/transl_table=11

/product="hypothetical protein"

/translation="MSNSHQPYSFVVSREDWSLHRKGYQDQQRHQQKVKDVIKQNLPD

LITEENIIMSDGQQIIKVPIRSLDEYRFVYNYQKQKHVGQGDGDSQVGDVIGRDPASK

KPGKGEKAGDQPGHDIVEAEVSIEELEDMLFAELELPDLKQKDKDQIETHTVVFNDIR

KKGMQSNIDKKRTILENLRRNATTGNPGIHHISPDDLRYKTWEDKIIPQSNAVIIAMM

DTSGSMGSFEKYCARSFFFWMTRFLRRQYEKVEIVFLAHHTEAKEVTEEEFFTRGESG

GTICSSVYMKALEIIDSRYPPSSYNIYPFHFSDGDNLTSDNERCVKLIGELMKRSNMF

GYGEVNQYNRSSTLMSAYRHIKIDQFMYYVIKEKGEVYKALRSFFQKREGGSVR"

CDS 1708968..1710350

/locus_tag="PIKHDJKN_01592"

/inference="ab initio prediction:Prodigal:002006"

/codon_start=1

/transl_table=11

/product="hypothetical protein"

/translation="MASLKGMYAKEGYDLDKRAVKAWIMYDWANSAYATTVLAAVLPV

FYASVAAATLDTDTAASYLAYTHSIGMLCVALLTPLLGTLADLSGRKGDFLRVFSIIG

IVATLGFSIIGEGDWLLASALLVISTIGFAGGNTFYDAMLPDLVSADRRDMVSSKGYA

YGYIGGGLLLAVNMLMIQQPGWFGMASTLAGTRLAFISVALWWLVFSIPLFRHAPRRP

AAPDMPKSWKGYAGVGVRRLGQTFRQIRRFPQLMRMLVAFWFFNDGINTIILMATIYG

TSIGIGTADLMLALLLTQFIGFPCTLLLGAWAQRWGAKQVLMVSLSIYICIVILGYFM

TSAIHFYLLAGLVGVVQGVSQSTARSLFSNLMPAGRTGEYFGFVNITGKFSSIFGPFV

FGWVGQLTGSTRWGILSLIFFFAVGIAVLVTVKVQQGMSDAIQADPEEGQGWMHHLIS

GKSSEARSTG"

CDS 1710733..1712001

/locus_tag="PIKHDJKN_01593"

/inference="ab initio prediction:Prodigal:002006"

/codon_start=1

/transl_table=11

/product="hypothetical protein"

/translation="MVDVHGNGLVLLLCVPRSGSSLSTVMLQNHSRVFATQEMWFLMN

LVDLQKADSRPYGGSAIIRQFYSAMVSDEGFEKACRSFALEIYNGFLEGSGADFIVDK

SPRYYYMLEWLDRLFPQSKRIHLQRNPLAIAASYKKVNRHTGEGFELIGSLQSPNLNM

KAVDLTLGMLRLNDYFADKHTNAYEVQYEQLVSNPRDEMEKLCGFLGIGYEEGIEQYG

RFVDSTKSNMFYSMGVGDPFLSSHQEAHKDSVNNWKKILDPQEVEFYCRVIGADLFHR

MGYSEQLEEAEKWTGVRYEAGPDEEVISKLTRQFSEATGSSWQRGYRMQPVDLVSNTP

DENRDNQAANEPDAIDPTLAALATIRQLEAALRAADNRLERGYNERERLKVQLASAHS

KIQRIKSLIPFGNQISAWASQRKILRGGKS"

CDS 1711998..1713929

/gene="asnO_1"

/locus_tag="PIKHDJKN_01594"

/EC_number="6.3.5.4"

/inference="ab initio prediction:Prodigal:002006"

/inference="similar to AA sequence:UniProtKB:O05272"

/codon_start=1

/transl_table=11

/product="Asparagine synthetase [glutamine-hydrolyzing] 3"

/db_xref="COG:COG0367"

/translation="MSAIAGIVHTDGQQALWEDSWRLYASLGHVPADTTGVWKGNEAF

LSCHAQWITPESVSEQLPLYDEVSGLTITADAILDNREQLADQLSISRAELAGLSDSE

LILRAYQRWDEHMAEKLLGDFAFAIWDDRKRRLFAARDITGMRSFYFRHDGERFAFCT

LMKPLLQLEGVHKELDEIWLSEFLAIPDMHDSADIHSTVYRGVSQLPPAHTLSFRDGR

MEQKQYHRWDEVEPLRLKSDGEYVEAFREVFGQAVGSRLRTHRQVAAALSGGLDSGAV

VGFASGTLRSQGKTLNAYSYVPVSDFTDYTSKTLLADERPFIQSTVNHVGNITENYLD

FEGRSPLSEVDTWLDIMEMPYKYFENSFWIRGFYEKASQQDAGILLTGARGNFTVSWG

PALDYYASLLKSGRWFRWLREMQQYSERTGMKFSRIAKITGRKAYPELFKSMSKGAAQ

AASVQLIHPEFARRTGVLDRLKSIIVLQGGAQADALKVRAEKFNNLAIANKNGAVATK

CSLRYRAWERDPTSDARVIRFCLSVPIEQYVNQGTDRALIRRATSPELPDKVRLNQRV

RGVQPADWLHRMIPNWGDFTAELLALCSDSRVAGILNTERIKSALANFPNPRPELASH

PDLRLMMHSLIVYRFIRKF"

CDS 1714006..1714143

/locus_tag="PIKHDJKN_01595"

/inference="ab initio prediction:Prodigal:002006"

/codon_start=1

/transl_table=11

/product="hypothetical protein"

/translation="MQMEKKEWQAPALEVLEVNQTMAGVGYRQIDWITVHDADLYDPT

S"

CDS 1714287..1715234

/locus_tag="PIKHDJKN_01596"

/inference="ab initio prediction:Prodigal:002006"

/codon_start=1

/transl_table=11

/product="hypothetical protein"

/translation="MLPVQYEAYGLRWLSQIPMPELQIVQSGTDSLTAHTDVHIEIAD

LAAQWEEWDVGSDNFVSRNGSLFFRIEDTGLFLVEQGRRIVVSPQAGADDKKVRLFVL

GTCMAVIMMQRGILPLHGSAIVIDGWAYAFVGHSGAGKSTLSAALASRGFPLLTDDVV

ALTWDAGGRAIVSPGYPQQKLWQPSLDGFGMKEQDYATVHAEITKYAIPVQHYFHDRP

VPIAAVFELDPKPEEMLKPVQLKEVAGLERLHMLCSHTFRSGLVSRQGLAQWLFETVS

RLSAGIEVFRITRSGSDFTAFEMADRITEYARKGVYTGQ"

CDS 1715231..1715530

/locus_tag="PIKHDJKN_01597"

/inference="ab initio prediction:Prodigal:002006"

/codon_start=1

/transl_table=11

/product="hypothetical protein"

/translation="MTATKPMNGEDRVLRKEGNLVSDMGGEKVMMSIHNGKYYNLGST

GGRIWDLISEERTLTELVEVLASEYEIEQDICREQVVQFLEHLTHEGLIDVTRGE"

CDS 1715534..1716016

/locus_tag="PIKHDJKN_01598"

/inference="ab initio prediction:Prodigal:002006"

/codon_start=1

/transl_table=11

/product="hypothetical protein"

/translation="MLRKIKAYRSLPREIRGLVWEAYVHLGWARILKAMPFAKIAPGL

GTPMYETPMTGLNRSDVTTIRNISKAILIASRYTLWESRCLVMAIAAMKMLERRKVES

TLYMGTARNKQGQMMAHAWLRSGKLIVTGADTMDQYTVVGVFGKRCPEKGAGEIVYDT

"

CDS 1716000..1717196

/locus_tag="PIKHDJKN_01599"

/inference="ab initio prediction:Prodigal:002006"

/codon_start=1

/transl_table=11

/product="hypothetical protein"

/translation="MSTIHEWEQYTAEFPQELKLILSMMKGDLTAITPEDAKIRMKDM

DWQLFLQLAHHHRLYSVLYLKIKELDSAVIPAFVVENLRQQYTANTFRMLHLTAEMEQ
VCGAFRERGIRNITLKGPALAHDLYGDISLRTSKDLDILIPFDDVESAEEILESLGYE
SKEGKRTPTVASWKWREHHICYKHPVKRTQVEIHWRLNPDSGRETDFELLWKRSRFST
YTQTPVRMLEREDLWAYLVTHGARHGWFRLRWLLDIDQMIRNMPLDVKKVERRLKAED
RLSIGSQALHLASVLMDTPLDANYLALLSSHERAGEKLARRGVVFMNVMIESPADVKS
YRSYLFALRSTRQKLFFFIERLYPSTWDVDQLPLPRSLHFLYFPLRPFLWFWRRIKRN
TMTERG"

CDS 1717200..1719101

/gene="btuD_9"

/locus_tag="PIKHDJKN_01600"

/EC_number="7.6.2.8"

/inference="ab initio prediction:Prodigal:002006"

/inference="protein motif:HAMAP:MF_01005"

/codon_start=1

/transl_table=11

/product="Vitamin B12 import ATP-binding protein BtuD"

/translation="MISEMQFFMRKLHRVTGPVLYWNLLGMICISLMEGIGIYMLVPM
LSLIGIFEMGPTGLHLPWISEFLNRFPENGRLLLVLLAFVLIVSGQAWLQRLQTIRNT
RIQQQFVRTLRMETYRAIIMAQWSFFLQKRKSDFNHILTTELARVSQGTNIMLQMAAS
LIFTGIQIGLAFWLSAKLTTLVLVCGLVLFIVLRKFVKRAKQIGDQTSEYSQSYYNGI
TEHFNGIKDIKSNMLEKSHISWFERMCGRIERNVIQFSQLNSGTQLIHRISAAVIIAA
FIYLSLHVLAVPPASLLLIILIFSRLWPRFTSIQSNLEYMSSMLPAFQIVRELQTETE
KSREISEKMVNAGEPYDEELTVPGPRSVKMKAQAKLNEQEDGSVRPIEPLLLKESIEC
QQVNYRYEGSDIYSLFEAYASIPAKGMTAIVGKSGAGKSTMIDLIMGLIRPESGQILV
DGVPLTENRLLAWRSSIGYVSQDPFLFHTSIRENLLLVDPNASEEQMWHALQFSSSAT
FVRKLPQGLDTIIGDRGIRLSGGERQRLVLARAMLRNPSVLVLDEATSALDSENEQHI
HEALERLKGHVTVIVIAHRLSTIRTADRVIVLDEGRVIQQGGYQQLSTDPVGTFSKLL
NMQAGVVGQ"

CDS complement(1719211..1719849)

/gene="cysC"

/locus_tag="PIKHDJKN_01601"

/EC_number="2.7.1.25"

/inference="ab initio prediction:Prodigal:002006"

/inference="similar to AA sequence:UniProtKB:O34577"

/codon_start=1

/transl_table=11

/product="putative adenylyl-sulfate kinase"

/db_xref="COG:COG0529"

/translation="MSKEERNITWQQSSLNRQDREKHNGHRSRALWFTGLSGAGKSSL

AFALEQYLYEKGVHCYVLDGDNVRHGLNRDLGFTAEDRQENLRRIGEVSKLMVDAGLF

VLSAFISPDAQDRYMVRQLFEADDFIEIYVRCSIEECERRDPKGLYKKARSGAIPHFT

GISAPYDIPEDPTLIIDTEVLSIEEAVHEIVQHLERVGAFHLPKPVSNAIVH"

CDS 1719997..1720395

/locus_tag="PIKHDJKN_01602"

/inference="ab initio prediction:Prodigal:002006"

/codon_start=1

/transl_table=11

/product="hypothetical protein"

/translation="MNNNIHNIQVYFQCADCDQDIRAIFAQEQVSAVRCSGCGRAYTL

MKPTIGDEILLDWEQEALYHKLRADRSEHDNHNMSALIIKVIELLTWRDSGDGQTRAI

EMVREWLASSGKPQTLLELFHHPERDNKRF"

CDS 1720552..1721331

/gene="pdaA"

/locus_tag="PIKHDJKN_01603"

/EC_number="3.5.1.-"

/inference="ab initio prediction:Prodigal:002006"

/inference="similar to AA sequence:UniProtKB:O34928"

/codon_start=1

/transl_table=11

/product="Peptidoglycan-N-acetylmuramic acid deacetylase

PdaA"

/db_xref="COG:COG0726"

/translation="MKRMVLYMLLAVLTFGILPAHTEASPINGAYHFGFKKSQNGQLP

SIDQEGFKEILQHNDAIFLGDTKQKELYLTFDNGYENGFTPAILDVLREKKVPAAFFV

TGHYLKDQPELVKRMSAEGHIVGNHSWSHPDMTRVSNEKIRTELDQVRSEVNRLTGQQ

ATFLRPPRGIFNNRTLAESHAQGYVNVFWSVAYKDWDTKVQRGTEYARQQVLKQLHPG

AVILLHSVSKDNTEALASIIDEARKQGYAFKSLNDLKTKSY"

CDS complement(1721467..1722315)

/locus_tag="PIKHDJKN_01604"

/inference="ab initio prediction:Prodigal:002006"

/inference="similar to AA sequence:UniProtKB:Q9X1H9"

/codon_start=1

/transl_table=11

/product="Fatty acid-binding protein"

/db_xref="COG:COG1307"

/translation="MSRIKIFTDSTSDLAPAWIQQHDIGIIPLYVVFGEESLKDGAEI

KPEQLYERVSRDGRLPKTAAPSPADFMTAFQPYIEQGYDILYISLSSELSSTYQNARL

ASSEFPEGRISVVDSLNLSSGIALMVMKAVHAVEKGQSLTEITHLVEAIKPNVRTEFV

IDTLEYLYKGGRCSGMQNLIGSLLKIRPVIRVADGKMSPAYKVRGKREKALEQMLQNT

LNHKEQIDRDLVIVVHTMAEEDALDLQKSLQEQTGARVELTTAGCVICSHCGPKTIGI

IYNTVL"

CDS complement(1722437..1722913)

/locus_tag="PIKHDJKN_01605"

/inference="ab initio prediction:Prodigal:002006"

/codon_start=1

/transl_table=11

/product="hypothetical protein"

/translation="MDNQNNQNNHHPYQSGSATDYSDPNHDNLYTPYPSALEDPSMGR

LKHSGPGIASFVIGLVSIIGYMLTFFIATMAINSSIGVVTTPIQVEEIALHPAVVLAS

LAVLVCLILNLAGIILGVIGLILKNRKKVFAIIGTILSGAMILAFAGLIIAGIYMM"

CDS 1723193..1723579

/locus_tag="PIKHDJKN_01606"

/inference="ab initio prediction:Prodigal:002006"

/inference="similar to AA sequence:UniProtKB:P39619"

/note="UPF0382 membrane protein YwdK"

/codon_start=1

/transl_table=11

/product="hypothetical protein"

/translation="MQRKWMMLGAVLTMLSVAIGAFGAHMLKEKIGADAIAVYETGVQ

YHMIHAIGLLIIGLTAGQLGPSTKLKWAARLLFIGIIIFSGSLYVLSISGIKILGAIT

PIGGVAFIVGWLLLAIDVWQRGRDRS"

CDS complement(1723726..1724304)

/locus_tag="PIKHDJKN_01607"

/inference="ab initio prediction:Prodigal:002006"

/codon_start=1

/transl_table=11

/product="hypothetical protein"

/translation="MKETLSNEEEIGFLFNVDILIKSRSNALALQSLLEFINKEEQIA

DFRIQSGIELGKIIEVTLQSKKDSFAFLHKERKKSQSNARQKNSSLNQATASTVPVKP

QSEIETPASQKAPSTEASNTTNGNSFDVWIDTLIKENRLTRIVVNNKNGRHQSIPCRI

LNFDRDTSIVSVYHVDEKQVYTFRTNEIDEFL"

CDS 1724534..1724836

/locus_tag="PIKHDJKN_01608"

/inference="ab initio prediction:Prodigal:002006"

/codon_start=1

/transl_table=11

/product="hypothetical protein"

/translation="MVTMEPIVVGEHVLVGVEVKLPKTTLLTINTSKGYIMCGALDVG

LLNEKLGDRKIIAARAVGVRTLEQLLHAPMESVTTEAEAMGITVGMTGVEALLKMI"

CDS complement(1724943..1726016)

/gene="drdI"

/locus_tag="PIKHDJKN_01609"

/EC_number="5.3.1.-"

/inference="ab initio prediction:Prodigal:002006"

/inference="similar to AA sequence:UniProtKB:P0DTQ1"

/codon_start=1

/transl_table=11

/product="5-deoxyribose 1-phosphate isomerase"

/translation="MTNPEHQPLSSLIWKQDKLEMLDQRLLPETILMLKLYTPEEVWE
SIHSMKVRGAPAIGIAAAFGVVLGAKAYDGLAVQGWLDHVKSICAHLATSRPTAVNLF
WALDRMMQKASDLAESGASIEDSNASLEAEALLIQKEDEEVCRLIGENALPLFADGMG
VLTHCNAGGLATAKYGTATAPMYLAHERGINLKVFADETRPVLQGARLTAFELQQAGI
DVTLICDNMAGMVMSKGWIQAVIVGTDRVAANGDVANKIGTYSVAVLAKAHNIPFYVA
SPLSTIDLSTPSGDLIPIEERAAEEVTEGFGKRTAPQGVKVFNPAFDVTPNEYVTAII
TEKGVVRAPFKENLAALFAKDEA"

CDS complement(1726039..1727256)

/gene="mtnK"

/locus_tag="PIKHDJKN_01610"

/EC_number="2.7.1.100"

/inference="ab initio prediction:Prodigal:002006"

/inference="similar to AA sequence:UniProtKB:Q9F0P1"

/codon_start=1

/transl_table=11

/product="Methylthioribose kinase"

/db_xref="COG:COG4857"

/translation="MSQYHPFTPQDAIELAKTLPGPFAADANLECREIGDGNLNLVFH
ITDQNSDKSIIIKQALPYAKVVGESWPLSLVRARIEREILQEEYRLCPGMVPEVYHYD
DDLALTVMEDLSDHVIMRKGLIDGRTYPLFAQHIGEFMARTLFFTSDLGMNQQLKKEK
QGRFVNPDQCKITEDLIFDEPYRIAEKNNYEAAIEDEAEALRTDGELHLEVALLREKF
LTHGQALLHGDLHTGSIFVTPESTKVIDPEFAYFGPMGFDVGAVLANLLLHYASLPGR
FQDQTALHERETILLNMVRDVWTEFESRFRALWTSDLVDPMAKAPGYEDLYVQQLFRD
SAGFAGAKMVRRIVGLAHVADIDTIEDATQRERAQRKALAIGKALIKRNRNLNTIGEL
LDTVATAVTFIKA"

CDS 1727556..1728017

/gene="dps2_1"

/locus_tag="PIKHDJKN_01611"

/EC_number="1.16.-.-"

/inference="ab initio prediction:Prodigal:002006"

/inference="similar to AA sequence:UniProtKB:Q8RPQ2"

/codon_start=1

/transl_table=11

/product="DNA protection during starvation protein 2"

/db_xref="COG:COG0783"

/translation="MATKNKTDQAKSVEQVLNRQVANLNVLYVKIHNYHWYVKGPNFF

TLHVKFEEFYNEVTVQMDEIAERILTLKGSPAATMKEYLEISSIQEAAGGEDANTMVQ

NLIEDFATLSNEYQEGIEVADAAEDQPTSDMLTGFKADLEKHMWMLRSFLG"

CDS 1728145..1728720

/locus_tag="PIKHDJKN_01612"

/inference="ab initio prediction:Prodigal:002006"

/codon_start=1

/transl_table=11

/product="hypothetical protein"

/translation="MIGTTIPALKEWASAIKALETGRQVMVMRKGGIVEETRRFELKS

PAFYLYPTYEHQRKELIKSSDHFYVEESLAEWVPEASTIHLTAYAEVTQDLEIRDQEM

LDRLLDFHMWTADFAEDRLKWKRKDPLHVLILRVYRLKEPMEIPVLPEYNGCRSWISI

PNGPVPREMTPVVDVADFDEQVQKINEMLKM"

CDS 1729092..1729871

/gene="sufC"

/locus_tag="PIKHDJKN_01613"

/inference="ab initio prediction:Prodigal:002006"

/inference="similar to AA sequence:UniProtKB:P80866"

/codon_start=1

/transl_table=11

/product="Vegetative protein 296"

/db_xref="COG:COG0396"

/translation="MATNFVIEGLKATIEGKEILKGINLEMKGGEIHAIMGPNGTGKS

TLASALMGHPKYEVTDGTITLDGEDVLDMAVDERARAGLFLAMQYPSEIAGVTNSDFL

RSAINARRGEGNEISLIKFIRQMEGKMKELDMNPEFAHRYLNEGFSGGEKKRNEILQM

MLLDPKIVVLDEIDSGLDIDALKIVANGVNAMKSEDRGFLIITHYQRLLNYITPDYVH

VMMQGRIVKSGGPELAHRLEAEGYDWVKEELGITDETVGQA"

CDS 1729896..1731200

/locus_tag="PIKHDJKN_01614"

/inference="ab initio prediction:Prodigal:002006"

/inference="similar to AA sequence:UniProtKB:Q7A6L4"

/note="UPF0051 protein SA0778"

/codon_start=1

/transl_table=11

/product="hypothetical protein"

/translation="MTTQTILPVESEALRALSESNNEPGWLTEQRLEALKLASGLALP

KLEKQKIERWNVSEYGTYKASEAIASLEEVPASIKDLVKDQAEGSLVIQRNSGTVYSK

LSADLAAKGVIFTDLATAVREHSDLVKPYLNTAVKADEHSLAALHAALWNGGVFLYVP

KNVEIEVPLQAVLLTDDASATFAPHVLVVAESNSSVTYVDNYVSGELSSPVFHNGVVE

VFVKAGAKVRFASVHQLSTSVTDVSFRRAVVENDGSIEWIVGEMNNGDTASNTMTVLK

GNGSSSDSKVIAVGSGSQKLNYTTEARHFGKNTPSQMITRAVMREEASAIINGITKIE

KGATKADGQQTEKVLMLSPKARGDANPILLIDEDDVTAGHAASVGQVNVEQIHYLMSR

GINRTDAERLIIYGFLAPVVADIPLEALRTQLQSLIERKLGQ"

CDS 1731197..1732417

/gene="sufS"

/locus_tag="PIKHDJKN_01615"

/EC_number="2.8.1.7"

/inference="ab initio prediction:Prodigal:002006"

/inference="similar to AA sequence:UniProtKB:O32164"

/codon_start=1

/transl_table=11

/product="Cysteine desulfurase SufS"

/db_xref="COG:COG0520"

/translation="MNPAIREQFPILHQEINGHPLVYLDNAATSQKPLAVIEAIKHYY

EYENSNVHRGVHTLGSRATDAYEGAREKVAKFINARRTQEIIFTRGTTTALNLVASSY

GRAVCKEGDEIVITQMEHHSNLIPWQQVAKETGATLKYIPLQPDGNIDLADVEKTITN

NTKIVAIAYVSNVMGVVHPVKQIAEIAHRKGAVIVVDGAQSTPHMKVDVQDLDCDFYA

LSGHKMCGPTGIGALYGKKALLESMEPVEFGGEMIDDVGLYESNWKELPWKFEGGTPI

IAGAVGLGAAIDFLEQIGMDEIAHHESVLAAYATERMAEIDGLTIYGPAKRHVGVVTF

NLGDVHPHDVATVLDASGVAIRAGHHCCQPLMRWLEVSSTARASFYLYNNEQDVDRLV

SALIQTKEYFGDAT"

CDS 1732407..1732838

/gene="sufU"

/locus_tag="PIKHDJKN_01616"

/EC_number="2.-.-.-"

/inference="ab initio prediction:Prodigal:002006"

/inference="similar to AA sequence:UniProtKB:O32163"

/codon_start=1

/transl_table=11

/product="Zinc-dependent sulfurtransferase SufU"

/db_xref="COG:COG0822"

/translation="MQLDDLYRRVIMDHYKNPRNRGTFDNEAVTVNLNNPTCGDRISL

QLLLKDGIVQEAKYTGEGCSISMSSASMMTEAVKGKTMDQALDMANRFSSLMKGEEVH

FDDYEDLEALSGVNKFPARIKCATLAWNALRKGIDEEDNVQ"

CDS 1732859..1734256

/locus_tag="PIKHDJKN_01617"

/inference="ab initio prediction:Prodigal:002006"

/inference="similar to AA sequence:UniProtKB:Q7A6L4"

/note="UPF0051 protein SA0778"

/codon_start=1

/transl_table=11

/product="hypothetical protein"

/translation="MAKKAPEMEEYKYGFRDEHKSIFQTGKGLTAEVVTEISKIKNEP

EWMLEFRLKSLKQFEKMPMPKWGGDLDGLDFNEIQYYVRASDKQGKTWEEVPSEIKET

FDKLGIPEAEQKFLAGVSAQYESEVVYHNMQKELEDQGVIFMDTDTALREHPEILREY

FATVIPPADNKFAALNSAVWSGGSFIYVPKGVKCEVPLQAYFRINSENMGQFERTLII

ADEDSFVHYVEGCTAPIYSTNSLHSAVVEIICKKNARVRYTTIQNWAPNIYNLVTKRA

VAEENATMEWVDGNIGSKLTMKYPAVVLKGRGAKGSVLSIAVAGKNQHQDAGAKMIHL

APDTTSTIVSKSISKHGGKVTYRGLASFGRQAEGAKSNIKCDTLILDNESTSDTIPYN

EIMNDNIMLEHEATVSKVSEDQLFYLMSRGLTDAEATQMIIMGFIEPFTKELPMEYAV

EMNRLIKFEMEGSIG"

CDS 1734501..1735220

/locus_tag="PIKHDJKN_01618"

/inference="ab initio prediction:Prodigal:002006"

/codon_start=1

/transl_table=11

/product="hypothetical protein"

/translation="MSINPFKRYRITSSFGYRIHPISGGQTFHRGIDLVTEPWNGPIH

AFMDGAVCFSAEGATGSGFGGYGLTVAVQDHRGYLHCYAHLSRIAVKVGQQVKQGQLL

GNQGSTGESTGPHVHYEVRKTSSPSYGYTASENGVVDPGIYLQSEYNSTVEGQEGTPM

TTEERARLTAMEKMLESQSAWIQQQKSISSMACPSWAEEAYHYYQPYIMNETGSYDFW

RLLVIMYRKEKGIKVSSSSSS"

CDS 1735363..1735494

/locus_tag="PIKHDJKN_01619"

/inference="ab initio prediction:Prodigal:002006"

/codon_start=1

/transl_table=11

/product="hypothetical protein"

/translation="MSEVGYGCGGNVGGVGGYNMFTNTGAILVLFILLVIITKAFFI"

CDS complement(1735637..1736686)

/locus_tag="PIKHDJKN_01620"

/inference="ab initio prediction:Prodigal:002006"

/codon_start=1

/transl_table=11

/product="hypothetical protein"

/translation="MRVHVTDLKTGDLLKNDVYNSSGLHMLMKGSTLSDDDIAKLLQH

GIDYADVEHTTSDLHSDAQTLPSDQTRLLTNLFNDTIKGTESLFEQAMEKGFIDETQV

DEILITLTGQLEKQKDVVSLLLMLDGDNDYTYNHSLQVGMLAFYIAGWMGYSPEECLT

VAKAGYLHDIGKSKIPSEMLHKPGKLTPEEFDEMKKHTLYGYEIVKESTQDEILATVA

LQHHEREDGSGYPYGLKGDEIHPFARITAVADVYSAMTTTRVYQTKQELISVLCELYS

LSFGQLNAEVTQELIKHMLPNFIGKRVLLTTGETGLIVLNNPADYFRPLVQTSTAFID

LVKERDVAIVEIYIQ"

CDS complement(1736811..1737557)

/locus_tag="PIKHDJKN_01621"

/inference="ab initio prediction:Prodigal:002006"

/codon_start=1

/transl_table=11

/product="hypothetical protein"

/translation="MKLTIQLFAGIAERLGTSLLEYEYPGTDPTPVQLKEKLGLAYPE

AAAQIRVSFLAVNQQYASADIIIKAQDELALIPPVSGGDGTHLNGDGHRYSHPEHRTD

DGLFLITQSPLSVEATTELVITANHGAALTFVGTTREMTGQQRTVHLEYEAYVPMALS

QMAAIGIEINERWPGVKCAISHRIGKVDVAEISVVIAVSSPHRSDCYDASRYAIERLK

QNVPIWKKEIWEDGSEWKGHQLGPWNPTDN"

CDS 1737677..1739107

/locus_tag="PIKHDJKN_01622"

/inference="ab initio prediction:Prodigal:002006"

/codon_start=1

/transl_table=11

/product="hypothetical protein"

/translation="MSTKKAQTLTILHTNDIHSHFGSMSSIAAMIEHEREQTGELLVV

DIGDHMDRMAVETEGTLGAANVDVINLTGYDAITIGNNEGLTFTPEQLGQSYSGLLCP

VVCGNVVDQATGRPPVWMKSSLVLEKGPFRIGLLGATAPFTAFYELLGWDVLDPVETL

REQVEALRAQVDVVVILSHLGLSTDRRLAEQIQGIDVILGGHTHHVLEEPLVIGQTVL

GAAGKFGQWLGKVVLEREYEANELTLVSSGCFPVQNMMLKESVSLAIDTHRTEAERTL

GQTAVITDRTLTIDYDHESPFASLLAQAVRQFTGAQLSLVNAGQLLADLPQGNITKGM

LHSLCPSPINACTICLNGIQIREALEQSMLSEFSRKPIIGFGFRGDILGTLCMDGMVV

EYDPHSPPYEKIRAIYIDGEPIRDEHEYVVGTLDMFTFNVGYPSLALGTDISYRLPEF

IRDLLETELKRPGALDDSLRSRWHEI"

CDS 1739177..1740295

/locus_tag="PIKHDJKN_01623"

/inference="ab initio prediction:Prodigal:002006"

/codon_start=1

/transl_table=11

/product="hypothetical protein"

/translation="MRLVHINLLQPGMKLGKRIYSEEGLVLLSEGVELTSRLIGRLKD

LGIGYVYISDAATEDIIIPDMLQEETRRRALVEIKQQFQSMSGLKTKSRIPHFGKALS

GLMNTILEDIGSQKEAMIMLMDMNASDFDLYNHSLNVCVYTLVLGVASGYTRQQLMEI

GLGALLHDIGKTQIAPEILHKPAKLSDEEFKIIQQHTTYGHRILKDEPGIPLLAAHCA
LQHHERIDGSGYPFGLKDKEIHEYAKWLALADSYDAMTTNRVYKQALLPHQAVEVLYT
GSGTLYEQRMLEKFRDCVAIYPLGISVTLSSGEVGVVADIHTRVPQRPRIRVLKDADG
QTLSAPYEIDLSTALSVMITGVEGVEDATPAAARGELF"

CDS 1740524..1741654

/locus_tag="PIKHDJKN_01624"
/inference="ab initio prediction:Prodigal:002006"
/codon_start=1
/transl_table=11
/product="hypothetical protein"
/translation="MLNSGSVEPMPLSPTNDPWDPIGSLRTYGRHVLTSVEMTVTHLC
NMRCEHCAVGDMLTMREAPALPLPLMLKRLDEVEHLQTISLTGGEPSFSQKTVDEMII
PLLKYAKERGIRSQINSNLTLDYRRYEQLLPYLDVMHISFNYLNADDFHQVGFANSGR
PVKREVAVKMYEKMIENSQKLSEAGMFISAESMINFRTHDKLEGIHQLIREMGCVRHE
VHPMYNSNFASALPVLSLDHMRAAIHRLLDVRDKDMWMLFGTLPFFACSAADQDRELI
NRLYSEPKVTVRNDPDGRNRVNVNMFTGNVYVTDFADIPAFGNIRDRKLDDVFHEWSA
EHPLNQTVNCHCDAASCCGPNLLVADMYYKGVDFKSRKAITR"

CDS 1741701..1743095

/locus_tag="PIKHDJKN_01625"
/inference="ab initio prediction:Prodigal:002006"
/inference="similar to AA sequence:UniProtKB:P9WFP3"
/note="UPF0053 protein Rv1842c"
/codon_start=1
/transl_table=11
/product="hypothetical protein"
/translation="MGIHTEFHLGQLVFNLVCVFLLVFLNGVFVAAEFSLVKVRQTRL
TQLQSEGNKLAGYALKVNGKLDAYLSATQFGITLTSLGLGWLGEPAISELLVEPLMFK
LGVADTGLISTVSVIIGFCIITFLHIVLGELAPKSLAIQKTDRVALLLSAPLLLFYKI
FFPFIWVLNASANALLRLAGIEPASEGEAHSEDELRILMKQSAKSGVIDKDEIKLMDN
IFDFSDMLAREIMLPRTDMDCLYTHMSLAENLKIINATKHSRYPVAVEDKDEIIGFIH
ITDLLLAEPEQQHDLAALVRPILNVPESMEISHVLRLMQKKHSQMTLVVDEYGGTAGL
LTAEEILEEIVGDLYDEFEDERPHMERSGEAFSIDGRSLIEEVHEWTGAVIEDEEVDT

IGGWLFKELEGSVVKGKTREQNGYVFEVEESTRLRITRVKVYKRPDSEEDKVEIGEQE

QGSMQADDRERNRD"

CDS 1743321..1743560

/locus_tag="PIKHDJKN_01626"

/inference="ab initio prediction:Prodigal:002006"

/codon_start=1

/transl_table=11

/product="hypothetical protein"

/translation="MSDPQLRAYAPFVGPFDPCPPKAIRTYLVPPQLFIPFQPMGWPQ

FSPAEALRIGTLWPALYSPYTPASSKGRKVEADGT"

CDS 1743550..1743825

/locus_tag="PIKHDJKN_01627"

/inference="ab initio prediction:Prodigal:002006"

/codon_start=1

/transl_table=11

/product="hypothetical protein"

/translation="MEPEAPKACDAKYYELLEELQALDFVLVELNLYLDTHPGDYQSI

EQYNKFSQERMRVAHEFQQLYGPLMNFGHAFSKYPWEWSQTPWPWQV"

CDS 1743919..1744488

/gene="ydbD_1"

/locus_tag="PIKHDJKN_01628"

/EC_number="1.11.1.6"

/inference="ab initio prediction:Prodigal:002006"

/inference="similar to AA sequence:UniProtKB:P80878"

/codon_start=1

/transl_table=11

/product="putative manganese catalase"

/db_xref="COG:COG3546"

/translation="MWVYEKKLQYPVRVSKCDPHMAKLLAEQYGGADGELAAALRYLN

QRYTIPDKIIGLLNDIGTEEFAHLEMIATMIYKLTKDATVEQLEAAGLGPNYAQRDSA

LFYTNSSGVPFTAAYIASKGDPIADLYEDIAAEEKARATYQWLIDLTDDVDLQDSLKF

LREREIVHSLRFREAVEILKDDRETKKIF"

CDS complement(1744707..1745699)

/gene="fabH_1"

/locus_tag="PIKHDJKN_01629"

/EC_number="2.3.1.180"

/inference="ab initio prediction:Prodigal:002006"

/inference="similar to AA sequence:UniProtKB:O67185"

/codon_start=1

/transl_table=11

/product="3-oxoacyl-[acyl-carrier-protein] synthase 3"

/db_xref="COG:COG0332"

/translation="MAGIRIVDIDIYHPTNKVGNDFYIEHFDARGVDIRGLLKALGRD

TRYKIDNDDENSLSMAFEAASNLLEKTGLTGADIDLIAYASQTPEYIFPTNSLMIHRL

INGASHTICIDSNANCAGMTAAFEQVSRQMLGNPRIRRALIIGSDNVAPHASPDDPVY

FANFGDAAAAVIVERDEQAVGFIDSIYQTDTCVYGNSLFPAQGLAKLGQTGVDAGAFH

VKFIPFDDSICVGAASESIRTLLSRNEIGADEIKAACFSQLSIGNIRAVSENIGIGED

IAVYIGDEFGYTSTSSPFIALHRAITSGQIQRGDKVLFWTVGAGWQNVAMVVEF"

CDS complement(1745755..1746105)

/locus_tag="PIKHDJKN_01630"

/inference="ab initio prediction:Prodigal:002006"

/codon_start=1

/transl_table=11

/product="hypothetical protein"

/translation="MGSFILKTDVPNKVLNIELEGSFSQEDGLRSISAYQDTIAPINT

KEYQLNIDCKKLNVTAPEIVPLLEGCLQMFKKDEYKKVVLTLENNAILKMQLSRLGRS

VGLENLEIISTVTT"

CDS complement(1746291..1746659)

/locus_tag="PIKHDJKN_01631"

/inference="ab initio prediction:Prodigal:002006"

/codon_start=1

/transl_table=11

/product="hypothetical protein"

/translation="MSEQVTQGLERFAKLSGEYGAKALAPIKDQFPELAEFIMGTAYG

DIFQRTSITDQWKEVAIISSLISQGQYEQLGVHYTMALSVGVTVDQLKGILLHLAPCV
GAPRIISAFNILLATLDEIQ"

CDS complement(1746672..1748303)

/gene="ilvB1"

/locus_tag="PIKHDJKN_01632"

/EC_number="2.2.1.6"

/inference="ab initio prediction:Prodigal:002006"

/inference="similar to AA sequence:UniProtKB:P9WG41"

/codon_start=1

/transl_table=11

/product="Acetolactate synthase large subunit IlvB1"

/db_xref="COG:COG0028"

/translation="MRTVADYLAESLRNLGVTHVFGIIGKSICPAVLKMVDYGLEFIP
GRHESSSGFAASGYALQTGKLGVAFATSGPGGTNLLTAAAHAKANNLPVLFITGHQSI
QELGLPQCQDSSSYLADLAEMFRPATLFSKLVERGDHFSTILNHALSIALGPNKGPVH
LCLPFDVQTEQLADFRVVIPEPEPLISVSNLNHILPLLQQSSHPLIIAGKGVSRARAH
DELLHLAEHFNIPVITSPGGKGAITWDHPLYHGPCGVGGFPHADELLNQSDLYIVFGS
RLSDMTICNLKPVNHPAHLIQFDADPTFVGKILNAQTLHITGDLKDNLQFLLGTLTEQ
PIVPRETTTVDYTVPLPDLPNLSLASVLEEMSELLPYDHKLFVDDGSHGFHAVQRYKV
KKPGSFVFDAYFACMGNAIGMAIGAKTAAPEETIVCITGDGCFMMLGTEINTAVCNNL
PVIFIVVNNKQLDMALKGMEKTTGRIDGTLFEVPVDAAKFAESLGAVAFRAETRAEFT
SALQAAQQLNQVTVIELLTDRDEIPPTAHRTVTLN"

CDS 1748709..1749680

/gene="iolG_5"

/locus_tag="PIKHDJKN_01633"

/EC_number="1.1.1.369"

/inference="ab initio prediction:Prodigal:002006"

/inference="protein motif:HAMAP:MF_01671"

/codon_start=1

/transl_table=11

/product="Inositol 2-dehydrogenase/D-chiro-inositol
3-dehydrogenase"

/translation="MTTIKMAVIGLGKMGIHMIHRATEAELSEKVEIVAVCDSSEDNL

TLFASSHPETKTYTDFKQLLDTHPVDLLYIAVPPKYHYPVVMDALKRKIHVFCEKPLA

NSVEEARAMLEAAEKADVVHAIHFSMPHEPSVVKLQELISQETIGEIRKIDLILQFPL

WPRAWQQNAWITSREQGGFILEVGIHWIHMIQKVFGAIRVISSQVQFPQNTGECEHEV

QAAMELEDGTRVQLNGIAHFAGEERVSMVVYGTEGTIALENWDQLWSGRVGEPLVPVV

VEESLSMLPVLKHVIARIQGQPAKIYDFHDGYHAQLVLEALRNPDQS"

CDS 1749715..1750524

/gene="gph_4"

/locus_tag="PIKHDJKN_01634"

/EC_number="3.1.3.18"

/inference="ab initio prediction:Prodigal:002006"

/inference="protein motif:HAMAP:MF_00495"

/codon_start=1

/transl_table=11

/product="Phosphoglycolate phosphatase"

/translation="MCITLMTQMFYIARFTLPNVYVYHGRGTIPNTYPNPKGTLDMTK

ETVLRKPEAMIFDMDGTLFQTETLLLPAYHQLFDTLRAEGHFEGETPPEERMLGSLGM

LLEDIWKVVMPEASVAAHRRADELLLQLEIEGLDGGSSQLYPQVKETLEALKEQGVRL

FVASNGLEDYVKGVAFAHEIMPLFEGVYSAGQYKTPSKVNLVQILLEKHRIQDAWMVG

DRSSDVEAGKMNNQTVIGCAYAGFGRDEELAGSDVLISDFTELLRLYREAE"

CDS complement(1750620..1751816)

/gene="yheD_1"

/locus_tag="PIKHDJKN_01635"

/inference="ab initio prediction:Prodigal:002006"

/inference="similar to AA sequence:UniProtKB:O07545"

/codon_start=1

/transl_table=11

/product="Endospore coat-associated protein YheD"

/translation="MSSPVLGIMTLYLNEHRALEERSIYRRMILEGRKRGLDIYVFTP

ADVHSGGKQIEAMVFHSEKGWSREWRSFPDMIFDRCRIQRNRRFQQLLAFREKYGHLL

FLNRPLRNKWTIHQTLSAKSAFREHLPDTVLFQDMSDVNRMLKASSLIYLKPINGTGG

RGILRIERSGSEANTVLVQGRDQKRRIITPRKVHLSRLGSLLQHWNMKDKYLVQQGIQ

LQLPNGRVHDYRMLVQKNGEGQWELTGCAGRMGAEKSVTSNLHGGGQAIAMHKLMKQW

ITDEKERAEINTTAENFGIDVASFLESTYGTLCELALDLAIDKSGRIYLLEVNPKPAR

EVFARIGEQDTYSKAITQPLEYALWVYRNHPNANRPPRGVKVTKPVKRVKGKSATLKR

TRRSRL"

CDS complement(1751852..1752958)

/gene="yheD_2"

/locus_tag="PIKHDJKN_01636"

/inference="ab initio prediction:Prodigal:002006"

/inference="similar to AA sequence:UniProtKB:O07545"

/codon_start=1

/transl_table=11

/product="Endospore coat-associated protein YheD"

/translation="MSPHDDFKPVIAVLTMHDDQRMFRGNHQNFQDIVQTGENMGYVV

YIVTVRDLNVNGPTVKGYTYNKGSGKWTSQSFPLPHVLYNRIPNREDERKPSVQRKID

ECMQSGIELYNPFFFNKWNLFEWLKKSKSTQHLVPYTRRMRNGSALGTVLRSYPYLYL

KPESGKAGKGIMMLKFQEKERLPYRLKIQSTRKSTTYKAATLSKLWARIRKETGHTPY

IMQQGIELASSRKRPFDLRVLVQKNGKGHWSVTGIGARLAGSRSITTHVPRGGSVEDP

EKLLTDLFGEETSITLMKRVKSTSLLIARQVERGSGHTLGEMSMDLGVDDLGEIWFFE

ANAKPMKFDEPQIRRQSLERIFEYSAYLARQSKR"

CDS complement(1752958..1754331)

/gene="yheD_3"

/locus_tag="PIKHDJKN_01637"

/inference="ab initio prediction:Prodigal:002006"

/inference="similar to AA sequence:UniProtKB:O07545"

/codon_start=1

/transl_table=11

/product="Endospore coat-associated protein YheD"

/translation="MSLTFCNLHFTQQPDKVVYVSNALMKSLNLSGKKTIHLRFGRDR

VPATIKPIKKAGKHLYLASGIRNLMNVPKRGSIYLRNLQNDEVQLGPLIGVLSDGPSS

GSNPFGSRTGFIKQLLREGSRKSYIYAFTPRDINWQNETVSGFFLNDNGSFSRKTVPL

PDVVYNRLPSRRSDFSPAINQLRERFIRRKIPFFNWSFFNKSDIYKLLENEPAAGRHI

PESITNPTVEQMREMLERHQFVYYKPTAGSLGNGIYRLTYSPKRGYFARYRKKGGNAL

LRFGSFNSLMRMLQGRHGKQLRGYVVQQGIRLIEIDECPIDFRFHMHKNGNNHWVVVG
IGAKKAGRGSVTTHIKNGGSLMTPEQALSRNFGDRAGEVLQHAKSVAITLAQAIETQH
QHLIGEIGFDLGIDQEEHVWMFEANAKPGRSIFRHPSLRVEGKSSVEHILEHCLYLSK
FRKQDGN"

CDS complement(1754393..1755583)
/locus_tag="PIKHDJKN_01638"
/inference="ab initio prediction:Prodigal:002006"
/codon_start=1
/transl_table=11
/product="hypothetical protein"
/translation="MFLQHQTNTVAILVRATEGSPPFVDELFCRRLSLESHRYDLNII
VLAVANKPSASLLTQGYAFDKGKWNIVPLPAVDLIMDRCLLPLPRRFKQQLQQLVSSG
NGHHGRYWSASLPGKWKVYCALSADTRLRALLAPTTLLQSDTLWEQSLERWPKGIFFK
PVSGTHGKNTFRLYREDHHASWIIEGRNTRNELIHRTFDRRQDVSAWLDSHKAGKKMI
LQPYLELCHHGRPFDIRALVQKNGQGRWSLTGCMVREGPAGSLTSNLHGGGKAYPVHP
YLLERYGSNRTNDLLETIRQAAAHIPALLESRFGRLAELGLDFGADVQGHLWLLEVNS
RPGRSSFAEAGDPRMYSLTYTRPLAYARYLLQQHVLTDVKRPMKMPNTSSTAGLKPIP
IHGG"

CDS complement(1755614..1756981)
/gene="yheD_4"
/locus_tag="PIKHDJKN_01639"
/inference="ab initio prediction:Prodigal:002006"
/inference="similar to AA sequence:UniProtKB:O07545"
/codon_start=1
/transl_table=11
/product="Endospore coat-associated protein YheD"
/translation="MSTKKVTIQVTGSGILQDDVIMLGEGLLKALKIPSGRPLQLQFG
SFSREVTVIPVPRYVGLRINQTVASKTGLVPRSVLSISYRSASRTLRLGPYISVLVSQ
DYPDQPDRPFGSITMFCQELVNACRKKGAYVSFFTPEDIGAVTGYMKGWVYDDGWKKT
VLPIADVVNNRLTSRKLENKPSVQHFMKEVKSLYGTQTFNEKFLDKNEVFDALKSIST
LKRVLPESHLLKASATLKTMCNRYPVVFLKPVRGSLGKGIIRVSRQSDGSFLTLATSV
GGTRKQTYASLDKLYSSLSGKMKTTRYQIQQGLSLIDNSGRPVDFRALVQKNRTGKWS

VTSIVARIAGGSHYVSNLARGGSLSTVKEAVAKTQLSGSAKASAYAGLHTAALDIAKG

IEGAIPAHFGELGIDLALDTSGRVWLLEVNSKPSKNDNTPLSESKIRPSVKAMLEYST

YLAGF"

CDS 1757263..1757610

/locus_tag="PIKHDJKN_01640"

/inference="ab initio prediction:Prodigal:002006"

/inference="similar to AA sequence:UniProtKB:O07542"

/note="UPF0342 protein YheA"

/codon_start=1

/transl_table=11

/product="hypothetical protein"

/translation="MNIYDKANDLAKALRESSEVEEITSAMKLIETDPEAKAMLDNFR

DQQMELQQRMMSGDMPAPDEMEKMEKLFEVLSLNLNIRRLFDAERRLSVIIEDVNKII

ADSLGHLYGGAEA"

CDS 1757713..1758000

/locus_tag="PIKHDJKN_01641"

/inference="ab initio prediction:Prodigal:002006"

/codon_start=1

/transl_table=11

/product="hypothetical protein"

/translation="MVKRLKKAKHVIYLLIALSMLVIALPRISFAGGMDWVNIFGIVW

VLFSLLIIGAHLHFILGVDEEKKRALEAVRRAKLRQWEMKLVDKQEQSKSV"

CDS 1758237..1759571

/gene="IMPDH"

/locus_tag="PIKHDJKN_01642"

/EC_number="1.1.1.205"

/inference="ab initio prediction:Prodigal:002006"

/inference="protein motif:HAMAP:MF_01964"

/codon_start=1

/transl_table=11

/product="Inosine-5'-monophosphate dehydrogenase"

/translation="MDGQEENVTKHEQLLQHIEQLKVGSKISVRGLARELGVSEGTAY

RAVKEAENFGLVVTKERIGTVRIEKRPRGMTEQLTFADVVTIVEGHVLGGSDGLAKPL

HKYVIGAMKEQAMARYIDAGSLLIVGNRDNAHSLALEQGAGVLITGGFGTSREVRVMA

DELGLPIISSRHDTFTVASMINRAIFDRLIKKKIMLVEDIIGQKPRLQVLKVTSSAAD

FHLLVSETGEHRFPVVDEWNRVIGIVSLKDVSELKEDQSIEKCVVRRPITASLQTSLA

SAAQIMTWEGIDFLPIVDRNRKLIASVTRKEVLQAMRDAQKQPQLGETFDHLIWNGFA

EERGEQNELFFHGFIIPQMATDLGTISEGVLLNVMTQAGRRAAWDVTGNDHVVDNVTT

YFVRPVQIEDQILVRPIILETSRRTCKMDIVISRDGNVVCKAVMTLQSIDHA"

CDS complement(1759738..1760079)

/locus_tag="PIKHDJKN_01643"

/inference="ab initio prediction:Prodigal:002006"

/codon_start=1

/transl_table=11

/product="hypothetical protein"

/translation="MFIDVLKYALIAIFALAMVCSALNSIRSRRTTDPIATGLYRSWT

NIWMGSMLIILALILMFVFTGSTLSVVVEALFLIMGAYNLFAGLRNRSYYARLQQHAD

GGTGKTSGQSA"

CDS complement(1760178..1760666)

/locus_tag="PIKHDJKN_01644"

/inference="ab initio prediction:Prodigal:002006"

/codon_start=1

/transl_table=11

/product="hypothetical protein"

/translation="MRIPPFARYRPLMRMTAVFVLGMIAGCVVYNGVFHLSYNELWLN

NQELQLQLHQNEEDIKTLKKYSKRQTVIKEIKVRAEDSDKGPDEASLKVMLQKLGDEL

EVLRGRDVFNIDEYSKMTRIMLNQKVYTVREKEYTVQVKTMLVMEGVLQVWVQIRMHV

PA"

CDS complement(1760688..1761017)

/gene="ytrH"

/locus_tag="PIKHDJKN_01645"

/inference="ab initio prediction:Prodigal:002006"

/inference="similar to AA sequence:UniProtKB:C0H3P8"

/codon_start=1

/transl_table=11

/product="Sporulation membrane protein YtrH"

/translation="MSTFLSKAILDFFIAFGIVLGGAMIGGIGAVVSLQPPTQTMLDV

AGKIKIWALAAAVGGTIDPMRVIESNVLDGNLSPAVKQILYLISAFMGAHMGTELVKW

VCGGGRG"

CDS 1761325..1765419

/gene="dnaE2"

/locus_tag="PIKHDJKN_01646"

/EC_number="2.7.7.7"

/inference="ab initio prediction:Prodigal:002006"

/inference="protein motif:HAMAP:MF_01902"

/codon_start=1

/transl_table=11

/product="Error-prone DNA polymerase"

/translation="MSSFVHLHVHSEYSLLDGAARIPDLVNKAADLGMTTLALTDHGV

MYGAIPFYKACVERGIKPIIGCEAYMTAGSRKERGSRKDQPIHHLILLAKNMTGYRNL

MKLCSIGHLEGFHYKPRIDMESLAAHHEGIICLSACLGGEVPQHLLHGREEEAKRAAL

RYKNIFGADFYLELQDHGLSEQKRVNPQLIRLAAELDIPLVATNDAHYLSEEDAELQD

VLICIGTGKTVDDENRLRIGTNQLYLKSEQEMARLFPHVPEALENTVRIAESCELQLE

FGKSILPEYRPLPEGESPSAYLRQLCEEGMEDRYIESSRWTDTELRAELEQRLDYELR

VIDSMGFSDYFLIVWDFIAYSHRQGIVTGPGRGSSAGSLVAYTLRITDVDPMKYNLLF

ERFLNPERISMPDIDIDFSDERRDEVIEYVAKKYGKAHVAQIITFGTMAARAAVRDVG

RALNVPYGEVDKAAKLIPAQLGINIQRAMEATPELKALYETKPKTRELLDMAMKVEGM

PRHASTHAAGVVISRDPLTDAVPLQEGSEGTALTQYSMENLESIGLLKMDFLGLRTLS

IIERCLRWIGENGEIPDFRHIPDDDALTYEMLGRGDTMGIFQLESAGVRRVLKELKPS

VFEDIISVLALYRPGPMEFISKYIQGKHGQIEVDYPHTDLESILKDTYGIIVYQEQIM

QIASRMAGFSLGEADLLRRAVSKKKREVLDLERGHFVQGSLKQGYSEAEADLVYDMIV

KFANYGFPRAHAAAYGVLAFQTAYLKAHYPIHFMSSMLTAVMGSHRKVAEYVVECRRM

DIEVLPPDVNESGILFTPVLASSTSTRPGQAAGTQRLGGEHGEDGTASPTMEHAVHDD

HYSGQAEYGEAPLPDDPGPVPDEDGGAYDWQAATAVPEQSEDRINSGVVTVSTAERLH

GNGEEESSHAEQRGMSDVREQDRLPDHNSNADKATGAIRFGLAAVKNVGTQAMESIMI

VRQERPFDSLLDFCRRVDLRVCNKRVIESLIQAGAFDTLPGHRAQLLAMLDETVEAAL

KWRKEREDLQIQLFDFVETPNWEIEYPEIPPYSGSQQLELERELLGLYLSGHPLDDYE

DVLESSGADRIMELTEAADDTMAVAAGMVVSVKSITTKQGKAMAFMELEDQIERCEVV

LFPEVWRRSQQHVGKGELLVVRAKVQQQDEGFKLLAEEVAPLSPAALEQQLRSRERRG

KPGSGSVSRPAAPPRQAGGAGAGAQRSSAGVDAGAARSSGGQGPAASHSAGGGSGAAN

ANGAASVARRAPEAAGGTEQSRGPRVVEQRVFVKITPQAEKPELLARLKQLLQQHSGP

VATVLFYEQQQKLLALSDAYRIKPSPSLFSEMEKMLGEGTVKIK"

CDS 1765974..1766465

/gene="ypjQ_1"

/locus_tag="PIKHDJKN_01647"

/inference="ab initio prediction:Prodigal:002006"

/inference="similar to AA sequence:UniProtKB:P54173"

/codon_start=1

/transl_table=11

/product="putative protein YpjQ"

/db_xref="COG:COG1267"

/translation="MSYEIVEAMLARRGVQIDAIAEIVYRLQKGYHPELTMDDCVTSV

KSVLQKREVQYTLYTGIALDELAEKRLLPQPLQAIMEADESLYGVDETLALGITHVYG

MIGLTSFGYLDKEKIGVIHDLNEAGPAIHVFLDDLVAGLAAAASARIAHKNIKAKKYP

SDI"

CDS 1766751..1766939

/locus_tag="PIKHDJKN_01648"

/inference="ab initio prediction:Prodigal:002006"

/codon_start=1

/transl_table=11

/product="hypothetical protein"

/translation="MWTVIYIAPTAKVADKIKTKLSEEGFLVQTRPISLSKQQFEIRV

PSGELEEVQEVLNSILHS"

CDS 1767346..1768239

/gene="accD"

/locus_tag="PIKHDJKN_01649"

/EC_number="2.1.3.15"

/inference="ab initio prediction:Prodigal:002006"

/inference="similar to AA sequence:UniProtKB:C0SP93"

/codon_start=1

/transl_table=11

/product="Acetyl-coenzyme A carboxylase carboxyl

transferase subunit beta"

/db_xref="COG:COG0777"

/translation="MFKDIFQKKRKYATIPSERALRGEGQEAGERPKREIPEGLMNKC

SKCGTIQYSKELEKNLKVCPACGYHMRLNAMERIAMVLDEQGFVEYDADMVSVDPLEF

PGYSNKLEQQRLKSGLKEAVITGEGTLEGLPVVVAVMSFDFFTGSMGSVVGEKITRAI

EHATEKRLPLIIFSTSGGARMQESILSLMQMAKTSAALSRLDEQGGLYISVITDPTTG

GVSASFASLGDIIIAEPGAVFGFAGRIVIEQTIRQKLPDDFQTAEFNLQHGQLDMVVH

RKELRATLGKLLDMHSEKGGV"

CDS 1768240..1769271

/gene="accA"

/locus_tag="PIKHDJKN_01650"

/EC_number="2.1.3.15"

/inference="ab initio prediction:Prodigal:002006"

/inference="similar to AA sequence:UniProtKB:Q6GG07"

/codon_start=1

/transl_table=11

/product="Acetyl-coenzyme A carboxylase carboxyl

transferase subunit alpha"

/translation="MAGELPYEAPLVEMRKKIEELVQFGQEKGIDFTDEIARLEERYH

RLEEEIYTGITAAQKMHLARHQQRPTALDLIQLIFTDFIELHGDRMFGDDLAVVGGLA

KLNGKTVTVIGQQRGKDTKDNIARFFGSAHPEGFRKGLRLMKQANKFGRPIITFVDTK

GAYPGNTAEERGQSEAIARNLLEMAKLSVPVIIVVIGEGGSGGALAMAVGNRVLMLEH

AIYSAISPNGAASILWKDASKADQAAEAMKITAKDLLEMEVIEEIIPEPRGGAHRDYE

ESAAAISEALVRHLDEMRGWTGDQLKQDRYEKFRKIGSVTFEPEVQIEAAQVPVEVDT

AGNLSGNAE"

CDS 1769377..1770804

/gene="pyk_2"

/locus_tag="PIKHDJKN_01651"

/EC_number="2.7.1.40"

/inference="ab initio prediction:Prodigal:002006"

/inference="similar to AA sequence:UniProtKB:P80885"

/codon_start=1

/transl_table=11

/product="Pyruvate kinase"

/db_xref="COG:COG0469"

/translation="MRKTKIVCTIGPSSESLENTKKLIMAGMNVARLNFSHGDFDEHG

GRITAIRQACEELNKTVAILLDTKGPEIRTGKLEVEPIELVQDEYITLTTEEILGTKE

RLSITYADLPSDVEPGSTILIDDGLIGLTVVEVQGTEIKCRIVNGGTIKSKKGVNVPG

VAISLPGITEKDANDIVFGIEQGVDFIAASFVRKASDVLEIRELLEKHNAGHIQIISK

IENQQGVDNLDEILEVSDGLMVARGDLGVEIPAEEVPLVQKRMIEKCNVAGKPVITAT

QMLDSMQRNPRPTRAEASDVANAIFDGTDAIMLSGETAAGKYPVESVLTMSRIAEKAE

SALPYQELYLKQRVAQQTTVTEAISQSVALSAQDLNAKAIITSTESGHTARMISKYRP

ESPIIAVTTEDRTSRRLALAWGVTPVKGRLVDSTDALFENAIEGGVKSGLVKEGDLVV

ITAGVPLGRSGSTNLIKVSQIPNNA"

CDS 1770935..1771441

/locus_tag="PIKHDJKN_01652"

/inference="ab initio prediction:Prodigal:002006"

/codon_start=1

/transl_table=11

/product="hypothetical protein"

/translation="MQQQSNGSWYTARLRVRYQESDQMGVVYHANYLNWFEIGRTEMI

RQMGYTYRKMEEQGLLLPVTGLDVKYHKPARYDDDIIIFTRIAAFSGLRLNYEYDIRR

MTPDLNEQIGIGQRVWSSEESLPGERLVTGSTQHVWVNGDWKPVRLDKAASELYSALE

KVWLSGKG"

CDS 1771448..1771849

/locus_tag="PIKHDJKN_01653"

/inference="ab initio prediction:Prodigal:002006"

/codon_start=1

/transl_table=11

/product="hypothetical protein"

/translation="MRRWMWALLLIIPVIELFGFILMSDWIGAGKTLLLMILTSLIGI
AMLQFEGRKVLIDAKSEMERGKVPGRKMVDGLFIFVGGFLLLIPGFITDLIGFTLLFP
VTRSVYRLFFLGWLEKKMRNGSIKFYRGPKK"

CDS complement(1771961..1773079)

/locus_tag="PIKHDJKN_01654"

/inference="ab initio prediction:Prodigal:002006"

/inference="similar to AA sequence:UniProtKB:A0A1W5X0D5"

/codon_start=1

/transl_table=11

/product="Sodium-lithium/proton antiporter"

/translation="MDRIIMKRLLRGLWVIIATILIAVAIYLLFPLLYPFAIAWIIAY
AMNPLVKLLQYKARFPRWLAVTLSLIIYFGAIVVVLSAAVTRMVKEVISLTTSFDLHV
DEIKATFIRWTQNDTIQSLIGQINEFYKENPNYQETINSNISKTTETVGTAVTDLVTG
FFNMILNLLTSLPNMGAVLIVVLLSTFFISKSWTRHSITVSGWVPSSIRKPITDIWND
LKKALFGYARAQLIMISITALFVMIGLLVLQVKSAFTIALLIGLVDLLPYLGVGLVMV
PWAAYLLMNGDLYLGIGISIVYLIVLIARQIIEPKVLASSVGLDPLATLVGMFVGLKL
FGVLGLIIGPVSLVILDAFNRANVFRDLRTYIINGRVR"

CDS 1773558..1774670

/gene="citZ"

/locus_tag="PIKHDJKN_01655"

/EC_number="2.3.3.16"

/inference="ab initio prediction:Prodigal:002006"

/inference="similar to AA sequence:UniProtKB:P39120"

/codon_start=1

/transl_table=11

/product="Citrate synthase 2"

/db_xref="COG:COG0372"

/translation="MTATKGLEGIVATTSSISSIVDGVLTYRGYDIDDLAEHASFEEV
AYLLWFGKLPTTDELKSLRKSLSDYAPIPSELIAQIQLYPKNMSTMAALRSAVSALAL
YDEQADEMTAEANENKAVKLQAQLPTIVAAIARIRQGKEPVAPKEGASIAENFLYMMT
GEQPAETAVKALDQALVLHADHELNASTFAARVTVATLSDIYSGVTSAIGALKGPLHG
GANEAVMKMLNEIGTPDRLEAAIQEKLNNREKIMGFGHRVYKNGDPRAKHLQKMSKEL

GEMNSDTRLYEMSVKIEELVTGQKGLKPNVDFYSASVYTQLGIEEVLFTPIFAISRVS

GWTAHILEQLADNRIIRPRAEYTGPTDQKYVSIELR"

CDS 1774821..1776113

/gene="icd"

/locus_tag="PIKHDJKN_01656"

/EC_number="1.1.1.42"

/inference="ab initio prediction:Prodigal:002006"

/inference="similar to AA sequence:UniProtKB:P39126"

/codon_start=1

/transl_table=11

/product="Isocitrate dehydrogenase [NADP]"

/db_xref="COG:COG0538"

/translation="MKLEKFAHPTEGEKIQIDNGTLQVPNNPIIPFIEGDGTGRDIWK

ASKRVLDAAVEKAYDGNKKIAWYEVFAGQKAFDTYGEWLPNDTLEAIREYIVAIKGPL

TTPIGGGIRSLNVALRQELDLYTCLRPVRYFDGVPSPVKRPELVDMVIFRENTEDIYA

GIEYAEGSEEVKKVIQFLQQEMGANKIRFPETSGIGIKPVSSEGSKRLVRAAVQYAID

HNRKSVTLVHKGNIMKFTEGAFKNWGYEVAEDEFADKVFTWAQYDIIKEKDGTDAANA

AQKAAEDAGKIIVKDAIADIALQQVLTRPGEFDVIATLNLNGDYLSDALAAQVGGIGI

APGANINYVTGHAIFEATHGTAPKYADKDVVNPGSVILSGVMLLEHLGWQEAANLIYK

GMETSINNKTVTYDFARLMDGATEVKCSEFADQIIKNL"

CDS 1776129..1777070

/gene="mdh"

/locus_tag="PIKHDJKN_01657"

/EC_number="1.1.1.37"

/inference="ab initio prediction:Prodigal:002006"

/inference="similar to AA sequence:UniProtKB:Q6HSF4"

/codon_start=1

/transl_table=11

/product="Malate dehydrogenase"

/db_xref="COG:COG0039"

/translation="MTIQRKKITVVGAGFTGATTALMLAQKELGDVILVDIPQLENPT

KGKALDMWEASPVQGFDSNIVGTSNYEDTAGSEIVIITAGIARKPGMSRDDLVNTNAG

IVKSVCENVKKYCPDSIVIILSNPVDAMTYAAYQTLGFPKNRVIGQSGVLDTARYCTF

IAQELNVSVEDVRGFVLGGHGDDMVPLVRYSSVGGIPIDTLIPAERIEAIVQRTRVGG

GEIVNLLGNGSAYYAPAASLVQMTEAILKDKKRIIPVIAYLEGEYGYNDLFLGVPTIL

GGNGIEKIFELELTAEEKAGLDKSADSVRNVISVVNL"

CDS 1777209..1777634

/locus_tag="PIKHDJKN_01658"

/inference="ab initio prediction:Prodigal:002006"

/codon_start=1

/transl_table=11

/product="hypothetical protein"

/translation="MTMIMYLLHIIGALAMGFYLILPFVVGKIRTLSVAAQEGAFASL

RSLNRIAQYGLVIQLLTGGYLMTKGEYSYLWMGVVVVLLLAIAAIGGIMGKPLRLAAE

AVKSNRDVSAEQSKIRTFSTLLAVFLLIMVYLMVNNQVI"

CDS complement(1777861..1779420)

/locus_tag="PIKHDJKN_01659"

/inference="ab initio prediction:Prodigal:002006"

/codon_start=1

/transl_table=11

/product="hypothetical protein"

/translation="MESHINSYITFVATSAVLNVFLCLYTYVRRAEIPSSKIFILYTA

ALSVYTFGYAIELASNTLEQMKFWTVVEYIGMPFSASLGLMLMMQYTGKKLSNKASAA

LFVIPSITLFMVATNDFHHLFYKRVWLREDSSVPLMDIAVGQWYVVHGAFTFSCLLCA

CLILVGQWKHTKKMYRRQLLTLITSQLIPMVAAFVYLLGLTPGGMDPVPVLMCITSAM

YIWAILSSRLLTIVPIAKDSIFESMREGVIVLDSSNRLVDYNKSLRDMLPELDFSMVG

QHLNDVWLSLAGETFPVEYGREGLQTDLYWQLNGETVCYQVRTSYVYNKEGQAVGTLI

MLIDITEQRFLQEQLKQLAYFDGLTKIYNRTQFLLRGREILSEAQLNLQPAAFILFDI

DYFKRINDTYGHDVGDQAIIHVVSVCNRYLNSEMLFARYGGEEFVIAIPNASLQDGEQ

LAEKLRVALLNDPLEVQGTRIPLTSSFGVAQYNGGSDSLESLLRDADTALYESKRNGR

NTVFAHAVSLG"

CDS 1779830..1780699

/gene="ydaD_2"

/locus_tag="PIKHDJKN_01660"

/EC_number="1.-.-.-"

/inference="ab initio prediction:Prodigal:002006"

/inference="similar to AA sequence:UniProtKB:P80873"

/codon_start=1

/transl_table=11

/product="General stress protein 39"

/translation="MSTDTQNKKTMPAQHQDQRPGIESEMHPKPEFEKPEYKAAGKLL

DKVALITGGDSGIGRAVAVTYAKEGADVAIVYLSEHEDAEETKRQVEQEGRKCILIAG

DIGDNAFAKKSVQQTVDQLGKLDIVVNNAAEQHPQQQLEDITPEQLERTFRTNIFGMF

YITQAALPHLKKGSTIINTTSITAYRGNPTLIDYSSTKGAITSFTRSLSMNVVEKGIR

VNAVAPGPIWTPLIPSTFDEKKVSEFGATQPMKRPGQPDELAPAYVYLASDDSSYVSG

QVMHVNGGEVVNG"

CDS 1781170..1782999

/gene="phoR"

/locus_tag="PIKHDJKN_01661"

/EC_number="2.7.13.3"

/inference="ab initio prediction:Prodigal:002006"

/inference="similar to AA sequence:UniProtKB:P23545"

/codon_start=1

/transl_table=11

/product="Alkaline phosphatase synthesis sensor protein

PhoR"

/db_xref="COG:COG0642"

/translation="MRPFRIRLAIIMMVLIGISVIVAGYTMGRVFKTTHINALEQTMV

REINLLKATFLFHDATNPKNAETQKYYSERAVELDRLTDSRVTFINKDGTVIGDSESD

PNSMDNHLNRQEIKEAVGQNYGQSIRYSATLGQDMLYVALPVNSDQSDMIELPSGKFD

GYIRLSMSLEAVDQGLQQGWILMFFTLGLLFLIVALVSYRVARGLTSPIEHITKVAHR

ITKLDYDARVDVTRNDEIGQLGLAINGMADSLQMQLKTIRDNEALLQSVLANMTGGII

MIDAGQSIALVNREAERMLGIQAERVTGKPYIELKRHYELTRTIEESVALREPMHEEV

SVFNPVEKLIRIDGVPMSEDDGAYRGMLFLLQDVTAIRRLESMRSEFVANVSHELKTP

VAAVKGFAETLLSGGVQDKETERSFLQIIYDEGDRLNRLIGDILELSKIESKRAPLQC

SPVHVYSFFEMVLGTLSKVAEKKEIRLEMNVPEELYIEADEDKMKQIFINLLSNGINY

TPEGGRVKLQVSVEHNEDEEEVVFAVSDTGIGIPKKDLPRIFERFYRVDKGRSRNSGG
TGLGLSIVKHLVELHHGKLSVESELGMGTTFRVILPLIQDEEI"

CDS 1783115..1783843

/gene="phoP_2"

/locus_tag="PIKHDJKN_01662"

/inference="ab initio prediction:Prodigal:002006"

/inference="similar to AA sequence:UniProtKB:P13792"

/codon_start=1

/transl_table=11

/product="Alkaline phosphatase synthesis transcriptional
regulatory protein PhoP"

/db_xref="COG:COG0745"

/translation="MAQRLLVIEDEPTLSRLLTYNLTQEGYDVIAEDHGSSGYDRALS
QEFDLILLDLMLPGMNGLDILNKLRIQGVSTPVIILTAKNGEAEVVQGLKSGADDYIT
KPFGVSELLARVDAVLRRYSNGEDLPQPEDKDGSRIILGELEIYPLKYEVTLGGQSIS
LRPKEFEVLLYLAKKPGVVLTRDDLMNAVWGFDYIGGQRTVDVHVSSLRKKLELDPES
VHIDSIRGVGYKLVVKRKTPHHSS"

CDS 1783995..1785188

/locus_tag="PIKHDJKN_01663"

/inference="ab initio prediction:Prodigal:002006"

/codon_start=1

/transl_table=11

/product="hypothetical protein"

/translation="MPMLLEVKPDQPEELINTQQEQVVAPMGEQKDEDEVGVTKENVI
SLWDYCRQIPVVDVHTTCGEAASLLNQDQEYPCIVLCDDHMQPLGLLMRETLYRMLNG
RFAADLFYRKPVVHAADSSPVIVDVHAEAIAIIDIALARSEQYFYDCLIVTEEGRLLG
VLTMRDVMSLSRKLQHVASEERVHIITQSRQEITRINDSVTKLVQAARKAGEEASQIM
QLSERGASSLSQVDASYNRVNQHMEGQQKHADDMLSSIDMGSGMARSIRSLADQSGLL
AMNASIEAAHAGEYGRGFQVVAGEIRALAKQTREVAGNMSSLLDDIGGLTRQTVELVR
ASAAEIDNSSVHVNEGGAAFKQLNTAVAGMSHIAEDIAMEGKTAGELSEHIRERLEDM
VQVDD"

CDS 1785407..1786162

/gene="pstB3_2"

/locus_tag="PIKHDJKN_01664"

/EC_number="7.3.2.1"

/inference="ab initio prediction:Prodigal:002006"

/inference="similar to AA sequence:UniProtKB:P0A2V9"

/codon_start=1

/transl_table=11

/product="Phosphate import ATP-binding protein PstB 3"

/db_xref="COG:COG1117"

/translation="MKDLIHIEGLNLYYDTHHALKNISMDLPEKTVTAFIGPSGCGKS

TLLRTLNRMNDMIPGVRVEGQVMLDGSDIYSNEIEVETLRKRVGMVFQQPNPFPKSIY

DNVAYGPRLHGVTKKAELDQLVEQSLVHAALWDEVKDVLKKSALSLSGGQQQRLCIAR

ALAVQPDVLLMDEATSALDPISTLKIEELVKELHHKYTIVMVTHNMHQAARVSGRTVF

FLNGEVVEAADTETLFSTPQDSRTEDYISGRFG"

CDS 1786226..1786885

/gene="phoU"

/locus_tag="PIKHDJKN_01665"

/inference="ab initio prediction:Prodigal:002006"

/inference="similar to AA sequence:UniProtKB:Q97ID9"

/codon_start=1

/transl_table=11

/product="Phosphate-specific transport system accessory

protein PhoU"

/db_xref="COG:COG0704"

/translation="MIRRKEFDQELEELRILLRQMGEHVGTALDGAIESLQTMDAEKA

QVIIKNDANLNALEDKIMELGSKLIITQQPVAKDLRRIIVAFKISSDLERMGDLALDV

AKVTLRMEGQELIKPLVDIPRMAEIVKAMVDESIESFLKENTDLAYKMAQTDDQVDQL

YSHMISDLYVFMTDHPNKASQAMLLMMVGRYIERIGDHATNIGESTVYLVTGKRPDLN

Q"

CDS 1787104..1789758

/gene="polA"

/locus_tag="PIKHDJKN_01666"

/EC_number="2.7.7.7"

/inference="ab initio prediction:Prodigal:002006"

/inference="similar to AA sequence:UniProtKB:P52026"

/codon_start=1

/transl_table=11

/product="DNA polymerase I"

/translation="MDKFILIDGNSIIYRAFFAMPPLTNSKGLHTNAVYGFTTMLLRL

LEEHKPTHVMVAFDAGKVTFRHEGYQEYKGGREKTPPELSEQFPLLKELLQGFGIAQF

ELAGFEADDIIGTLTKRADEAGRQVLVVSGDKDMLQLASEHVHIGLTRKGVTEIELYD

PAQIKERYGLTPLQIIDLKGLMGDTSDNIPGIPGVGEKTALKLLHQFGSVEEVLNGTG

ELKGKMKEKIEAHAEDARMSKQLATIHREVPLEQTWEDMQFAGLKEELAGPALAKLEF

KSLLERLSFRGSIVSEQEAVPAAAVESSIATDETISELFGSLESIDVLHVETHGDNPH

QAKLIGLAVGSAGTYTFLSPELLQSDVAAPVREWLGNAEQPKRGYDLHRVDLALHAHG

IEFAGASFDVQLAAYLLDPTESNQTISGLTTKYGLPSLVEDDTVMGKGAKYRVPEADI

LGDFLCRKAAAVAAIIPLQEQALESNEMNALFHELEMPLSRILADMEKQGIKANTADL

KALGSEFEENISRLMAEIYKLSGTEFNLNSPKQLGEILFDKLGLPVVKKTKTGYSTDA

EVLEKLAPYNDVVKHILQYRQLAKLQSTYVEGLLKEISDKDGKVHTYYRQTIAATGRL

SSQFPNLQNIPIRMEEGRKIRKVFVPSESGWSILAADYSQIELRVLAHISDDERLKEA

FVNDMDIHTKTASDVFGVKAEDVDGDMRRSAKAVNFGIVYGISDYGLSQNLNITRKEA

AQFIDQYFEVFQGVRRYMDDIVKEARQDGYVKTLLERRRYLPEINASNFNLRSFAERT

AMNTPIQGTAADIIKLAMVQMDEALRERKLKSRMLLQVHDELVFEVPADELELMKELV

PSVMEKALELSVPLKAEVSYGANWYEAK"

CDS 1789875..1790705

/gene="mutM_1"

/locus_tag="PIKHDJKN_01667"

/EC_number="3.2.2.23"

/inference="ab initio prediction:Prodigal:002006"

/inference="similar to AA sequence:UniProtKB:P42371"

/codon_start=1

/transl_table=11

/product="Formamidopyrimidine-DNA glycosylase"

/db_xref="COG:COG0266"

/translation="MPELPEVETVRRTLNQLIVGKTIDSVTVSLPRIIQRPDDIDAFA
MELAGHTVIGVERRGKFLRILLDGLVLVSHLRMEGRYGVYQQHEEVEKHTHVIFHFND
GTELRYKDVRQFGTMHLFNAGEELVSKPLLKLGLEPLDPAFTVAAFRDAVGKRMTKIK
AVLLNQAYVVGIGNIYVDEALFRAGIHPETIAKTLTAAQLTVLHEAIVVTLQDAVDAG
GSSIKSYVNGQGEMGMFQHQLKIYGRKSEPCATCGTLIEKSVVGGRGTHYCPKCQPLL
"

CDS 1790860..1791633

/gene="ytaF_1"

/locus_tag="PIKHDJKN_01668"

/inference="ab initio prediction:Prodigal:002006"

/inference="similar to AA sequence:UniProtKB:C0SP79"

/codon_start=1

/transl_table=11

/product="putative sporulation protein YtaF"

/translation="MLHHFISLLALALALSLDGFGVGITYGLRRTKIPLLSIVVISIC
SGLVIALSMQVGVLLSHVVSPDVASIVGAVILIGIGAWSLLQLIRKRGKEQLETDNGV
GEETGAGTIRSGTQASQEAQSKGRNQVLALELEPSASGGSLERMVFTLELRKLGVVIQ
ILRSPSKADMDNSGSISVQEAMWLGIALSLDAFGAGLGAALLGFPTLWTALVIALFSG
VFLSLGMKVGLRFAALRWMRRLSVLPALLLMFMGIMKLL"

CDS 1791643..1792239

/gene="coaE_1"

/locus_tag="PIKHDJKN_01669"

/EC_number="2.7.1.24"

/inference="ab initio prediction:Prodigal:002006"

/inference="similar to AA sequence:UniProtKB:P63831"

/codon_start=1

/transl_table=11

/product="Dephospho-CoA kinase"

/translation="MNIGLTGGIATGKSSVSAFLASKGALLIDADVIAREVMLPGHPV
LAAAVQRFGQAILNEDGTLDRKKLGSIVFQNPEERKVLEAITHPAIRKEMRKRAALYE
LEHPDKLVVSDIPLLYESGLEDGFEEVMVVYVPRSVQRDRLMSRDGMTAEQADARIDV
QMDIEDKKQRADVVIDNSGLWSETEQQIISFLHRKGLL"

CDS 1792236..1792799

/gene="mltC"

/locus_tag="PIKHDJKN_01670"

/EC_number="4.2.2.-"

/inference="ab initio prediction:Prodigal:002006"

/inference="protein motif:HAMAP:MF_01616"

/codon_start=1

/transl_table=11

/product="Membrane-bound lytic murein transglycosylase C"

/translation="MRLLRKKRVLLLMFVSFVLVLFLNTNWMSWFYPIHYKEEIRAQS

QSYEVDPFLIASIIKVETNFKTSKESKRGAIGLMQLMPDTANWIMEQAKIPGTSLEEL

KHEPERNIQLGTWYLRNLSDQFDGNEAAMIAAYNAGPGKVNSWLRDGVWDGSFETVKD

IPFGETRHYVQRVIYYYNQYVKIYNTF"

CDS complement(1792907..1793128)

/locus_tag="PIKHDJKN_01671"

/inference="ab initio prediction:Prodigal:002006"

/inference="similar to AA sequence:UniProtKB:P22065"

/codon_start=1

/transl_table=11

/product="Small, acid-soluble spore protein alpha"

/translation="MAQSNGNSNNLVVTKASAALEQLKYEVAQELGISIPQDGYQGNM

TSYENGSIGGYITKRLVTIAEQQLAGQYQ"

CDS 1793244..1793711

/gene="nrdR"

/locus_tag="PIKHDJKN_01672"

/inference="ab initio prediction:Prodigal:002006"

/inference="similar to AA sequence:UniProtKB:P67318"

/codon_start=1

/transl_table=11

/product="Transcriptional repressor NrdR"

/db_xref="COG:COG1327"

/translation="MKCPYCAYLGTKVLDSRPANESKSIRRRRECEQCSRRFTTFEMV

EETPLIVIKKDGSREEFSRDKILRGLIRACEKRPVSVETLEMMVSEVEKSLRNTADAE

VESRQIGELLMEQLFPVDEVAYVRFASVYRQFKDINMFMKELKSLLPKDNNED"

tRNA 1793897..1793971

/locus_tag="PIKHDJKN_01673"

/product="tRNA-Ala"

/inference="COORDINATES:profile:Aragorn:001002"

/note="tRNA-Ala(ggc)"

CDS 1794250..1795896

/gene="rhaS_7"

/locus_tag="PIKHDJKN_01674"

/inference="ab initio prediction:Prodigal:002006"

/inference="protein motif:HAMAP:MF_01534"

/codon_start=1

/transl_table=11

/product="HTH-type transcriptional activator RhaS"

/translation="MNNSEWAESYFPIYTADSIQSLYSTSTMPIHRIHENLTVLLAVS

SGQGSLHLDDHIYELTDGMVILIPAKSDVVIQGNQMHPLHIYTLSIRTQEQKRSLPMI

AMGRSSVLAAETLIKFYEPAIAAQLEELYMYRLPGNEVKHMSNQILFHQVLLSLLERM

EARYIASEQPSMERSIAFMENHFSEKITTEGLSEIAGVSRSHYSTLFKQITGFAPNEY

LSRLRVHRAKELLIGGSASLREIALKVGYKDEFYLSRRFKQQTGESPSGYTRRRPFQR

VAVWCAPYASHLMLLGLEPAIVISESSEYVSTEGVSPPQTIRFIHSDSSPEQIKPALL

DANIELIIAANQHLHMNGLSSERLRSIAPIVEIAWMELGWKEHLRFIAQAIQRVEKAE

QWLADFEREEQQAREAIQTSQIVNETLTILVIKPDTLQIYGIRNVGYVMYQSLALRPP

AKIAQEIQRFGDQFHSVSIQLSELQDYEGTRMLVIVFPDEKGSKAHSEGIFNSEYWKG

LEAVKQNRIYHLEQEEWIPYNPVSIRLQLGRAVSLWTGIQ"

CDS 1795943..1796986

/gene="fhuD_1"

/locus_tag="PIKHDJKN_01675"

/inference="ab initio prediction:Prodigal:002006"

/inference="similar to AA sequence:UniProtKB:P37580"

/codon_start=1

/transl_table=11

/product="Iron(3+)-hydroxamate-binding protein FhuD"

/db_xref="COG:COG0614"

/translation="MPIISYCNENDFHIQREKGESHLMRKLFVPFILVLVLLLSACGS

NSAKDTTGNATTSNESTSSNTDSAGSSSESDSGTFTYQSETGPVEVPSHPQRVVVLTR

FLTGNVMALDVPLVGVDEMSKENPNFKDKLANTEAVTDESLEKIIELNPDLIIGLSDI

KNVDKLKQIAPTVTYTYGKVDFLKQQLEIGKLVNKEKEAQAWVDDFDARSKKVGEEIK

AKIGANASVSVIETFNKQLYVYGEKFGRGTEILYDQFGLGMPDKVKEATKKDGFFAVS

NEVLKDYMGDFVIFSKNADEDNSFQNTETYNNIDAVKNNRVFEADAKAFYFNDPLSME

YQLEFFIQHFLGK"

CDS 1796999..1798006

/gene="feuB_2"

/locus_tag="PIKHDJKN_01676"

/inference="ab initio prediction:Prodigal:002006"

/inference="similar to AA sequence:UniProtKB:P40410"

/codon_start=1

/transl_table=11

/product="Iron-uptake system permease protein FeuB"

/db_xref="COG:COG0609"

/translation="MKNKRLPFRFATKLLGSVLILIVCFIIAMMFGAEETTFRNLWLT

LTSATKNDNILILREIRLPRELAAMLIGAALAVSGGIMQGITRNPLADPGLLGLTSGA

NMALALAFVFMPSLGYFGIMVACFVGAALGAAWVIVLGSMRRGSLSPIRVVLAGAAVS

AFMYAISDGISIFFKISKDVSMWTSGGLIGTTWGQIQAIAPVILIGLAVVLFLSNQIN

ILSLSDEVATGLGQKLVLVKTVLFLLVIVLTGASVALIGNMAFVGLMIPHIVRKLVGT

DYRYIIPVSIFAGASFMLLAETLGRTINAPYETPVVAIASMLGLPFFLFVVRRGGKAF

S"

CDS 1798003..1799010

/gene="feuC_2"

/locus_tag="PIKHDJKN_01677"

/inference="ab initio prediction:Prodigal:002006"

/inference="similar to AA sequence:UniProtKB:P40411"

/codon_start=1

/transl_table=11

/product="Iron-uptake system permease protein FeuC"

/db_xref="COG:COG0609"

/translation="MTMSARIRKQRLILLVCLLLILITAVVGMSWGYSSLSFKRVIPV

LFGQGTFKEDFVLFSVRLPRIFITLLAGMALALSGSILQSITRNDLADPGIIGINSGA

GVAVAIFFLYFPIEAGTFVYMLPLAAFIGALLTAALIYLFSYSRTKGLDPIKMVLVGV

GFSMALSGIMIVIISSAERSKVDFIAKWISGNIWGADWPFIWALLPWLIVLIPLTLLR

TRRLDLMGLNEETAIGVGLKLERERLVLMVIAVAAAASAVSVTGGIAFIGLMAPHIAK

ALVGPRNSLFIPVAVLLGGWLLLIADTVGRNLVDPDGIAAGIMVSLIGVPYFVYLLLK

K"

CDS 1799203..1800018

/gene="rhaS_8"

/locus_tag="PIKHDJKN_01678"

/inference="ab initio prediction:Prodigal:002006"

/inference="protein motif:HAMAP:MF_01534"

/codon_start=1

/transl_table=11

/product="HTH-type transcriptional activator RhaS"

/translation="MTDVDQLASIFADNSFKIDGINRLVLQPKSILRDFRTLNHGFLF

VVRGEASMSVNGTIYELQPGSVFHAAPGMQLDSQVYGHSEYEYYLVFYSFDQLEKGDS

IQECDSHFKLDSGANPRVVELLVMLQEQLHAQGGIGKLRVKELFLSIMVQVFTGCSQR

EGGTSPSEKVIEQAISYIHGHYMNLLTLDELAELHGMSSKRFSYFFHKYTGFRPIDYV

IHYRMERAGELLKSGSYLIRDVAISVGYTNPLYFSRIFKSKFGVSPSEYTHLD"

CDS 1800213..1801262

/locus_tag="PIKHDJKN_01679"

/EC_number="1.18.1.2"

/inference="ab initio prediction:Prodigal:002006"

/inference="similar to AA sequence:UniProtKB:Q2FVP8"

/codon_start=1

/transl_table=11

/product="Ferredoxin--NADP reductase"

/db_xref="COG:COG0492"

/translation="MKDHEVFDITIIGGGPAGLFSAFYSGLREMKTKIIEFQPYLGGK

VHVYPEKMIWDIGGLQPVPGAQLIEQMVNQGLTFQPEVVLGEKVTSISKDEQGTFVLH

TASEEKHYSKTVILAIGGGILKPIKLEIEGAERFEVTNLHYTVKSLSRFKDKTVLISG

GGNAAIDWANELALIAKQVYLTYRKDTLKGHEAEVSRLENSPVECLLNTMIEKLVATD

DHQQIETVVLKRNEDGASLELAVDEVIINYGYERDKELLVNSHIKIDMKDHWIAGTPV

SETSVPGIFAAGDILHHEGKLHLIAGAFQDAANAVNKAKLYVAPEANTYGMVSSHNDL

FTQKNRELMKHLYTK"

CDS complement(1801409..1801705)

/gene="ycnE_2"

/locus_tag="PIKHDJKN_01680"

/EC_number="1.-.-.-"

/inference="ab initio prediction:Prodigal:002006"

/inference="similar to AA sequence:UniProtKB:P94425"

/codon_start=1

/transl_table=11

/product="Putative monooxygenase YcnE"

/db_xref="COG:COG1359"

/translation="MIIIHAHLQVKPDQEQAFLEATKVLIPATRNEEGNISYDLAKST

EREHHYTMIELWKDEAATASHNASAHFQAFVQQAAAFMAAPMNVEVFAGEAVKR"

CDS complement(1801792..1802442)

/gene="yodC_2"

/locus_tag="PIKHDJKN_01681"

/EC_number="1.-.-.-"

/inference="ab initio prediction:Prodigal:002006"

/inference="similar to AA sequence:UniProtKB:P81102"

/codon_start=1

/transl_table=11

/product="Putative NAD(P)H nitroreductase YodC"

/db_xref="COG:COG0778"

/translation="MSTFQSTFQKTNDFNEITYGRRSVKLYDPEVKISREEMTEILAE

ASRAPSSINLQPWRFLVVDTAEGKEKLAPLAKFNQNQVLTSAAVVGVFIDMNNVEYMD

EIFGKAVELGYMPQEVKEMQLKTVMPYYENMPASALRDVNLIDAGLASMQLMLAARAH

GYDTNPIGGYEKDQIAEAFGMDKDRYQPVMLISIGKSAKEGHPSYRLPVEKITTWA"

CDS complement(1802470..1802889)

/gene="slyA_1"

/locus_tag="PIKHDJKN_01682"

/inference="ab initio prediction:Prodigal:002006"

/inference="protein motif:HAMAP:MF_01819"

/codon_start=1

/transl_table=11

/product="Transcriptional regulator SlyA"

/translation="MTRVFSKEEQIINLLNGLGNKISPKFERCTGISSSRFEILHELD

QVDEINQSMLQKIINIDSAAITRHLKQLEADSMVTRRKNPEDNRVTFVRLTDEGRKQI

EGYKVEKTNFINQILQDFSEEEVHLLADFLERMQNNF"

CDS 1803454..1803975

/locus_tag="PIKHDJKN_01683"

/inference="ab initio prediction:Prodigal:002006"

/codon_start=1

/transl_table=11

/product="hypothetical protein"

/translation="MGKNAAVSVNRRSDIISAAIEVFAEIGYFRATTAQVAERAKISQ

PYIFRFFKTKEALLLTALEVSWTRVIDSFRAVVETATPDQLENELTEAYEEILKSHKN

EILLQMQAQTVQEEVIRQAMQKGFAEVRDMVLEAFTAAGSANPQQRTLIFLAIGMLCN

VSTALDMPELKER"

CDS 1804092..1805045

/locus_tag="PIKHDJKN_01684"

/inference="ab initio prediction:Prodigal:002006"

/codon_start=1

/transl_table=11

/product="hypothetical protein"

/translation="MKKAIVVGATGGTGAAITEELIKRGISTIIFGRSRQKLEQLAVK

LGSPDYLQIAVGDAFRPDDIVAASLGADVMFHCANVPYNEMEGKLIPLGESVMEAAEQ

LGLKVVVIDGIYPYGRRQMKEVTEEHPKQPHTRKGKTRLAFEQMLFSSKWRKAQVMIV

RLPDYYGPTANQASYLGSTLEAIAEGKMTFFIGTMKMPREYIYLPDAAVMVVELASRD

DAYRQNWNIPGAGVISGKEIVRIAQQAAGRAKPVIALGKTGLSLLGLAVPVMKEVVEM

LYLTEEPLVLSKKKYEDSIGPVKMTSFEEGIAQTISELKKK"

CDS 1805643..1807070

/gene="hpyO"

/locus_tag="PIKHDJKN_01685"

/EC_number="1.14.13.-"

/inference="ab initio prediction:Prodigal:002006"

/inference="similar to AA sequence:UniProtKB:Q8PDQ6"

/codon_start=1

/transl_table=11

/product="FAD-dependent urate hydroxylase"

/db_xref="COG:COG2072"

/translation="MSLETLNQRVQDDLAYLAYGGADWIRPRELDEGHIYDVVIVGGG

QSGLGAAFGFLRERISNILVIDENAAGLEGPWETYARMVTLRTPKHLTSIDLGVPSLT

FRAWWEAQAGPSGWDEVDKIPRNEWMNYLRWYRQVLNLPVINEVKLRLIEPADEGIHR

LHITGAGAPSNILLARKVVLATGIQGGGEWHVPPLIAEKVPRHLYAHTSELIDFERLQ

GKRIAILGGGASAFDNANFALATGVAEAHVFVRREQLPSINPIRQMEQSGMIERFHVL

SDEDKYATISHFFKYNQPPTNDTFNRAAAWPGFQLHLHAPWLDIQMKDGKAVVTTPQG

QHSFDFLVISTGMVSDPSLRPELRMVEAYIARWADRFQAPTEQRNALLDAHPYLSPGF

AMTSRDGDGEDKLRGLFVFNYSALASCGLSASAISGTRNAVPKLVSAVADQLFLDDRE

QILEQFFKYNEQEFTASWVKSEVGI"

CDS 1807202..1807687

/locus_tag="PIKHDJKN_01686"

/inference="ab initio prediction:Prodigal:002006"

/codon_start=1

/transl_table=11

/product="hypothetical protein"

/translation="MRIMDYEGFRTHLKKASRKRNEPLIKIVAFQEKYMKIDEIQYYD

VEQNYMSVQACNTLWMNLKDKSFRNLVSHDLKFFQTMDNLGRHSLENLIKELYEMAVP

ILLDYDPSDYYSVQQLSEILVLDEMKLIEKLEMGRFKGAFINEEGNWVKPKPDRVELF

L"

CDS complement(1807803..1808486)

/locus_tag="PIKHDJKN_01687"

/inference="ab initio prediction:Prodigal:002006"

/codon_start=1

/transl_table=11

/product="hypothetical protein"

/translation="MANNRNKRRKPCIPHTGKTNSSLRFGWISSNWSGYARAGDKAEF

RRISAEWNVPFVLPSSKSSYSSAWIGIDGYENSNLIQTGTGHDWVNGQASYYAWWEIL

PDVETVIPFPVSPGDRMRAAIYRINRTRWCITLHNLTQNWTFRTVQQYTGQQSSAEWI

VEAPSVGSAISRMAKITPVTFTCCRLNGRSPGFVAKEKGIMIQQKQVVSIPGAPAPRG

DSFVVKRND"

CDS 1808748..1809494

/gene="glcR_2"

/locus_tag="PIKHDJKN_01688"

/inference="ab initio prediction:Prodigal:002006"

/inference="similar to AA sequence:UniProtKB:P94591"

/codon_start=1

/transl_table=11

/product="HTH-type transcriptional repressor GlcR"

/db_xref="COG:COG1349"

/translation="MLTEERYAAIIERLHERGIVKLQELVDVLGASESTIRRDLIDLE

SRQMLKRIHGGAALVNEKTPEPGMEEKTFKNIQQKTAIARLAAQEINNGECIYLDAGT

TTLAMIPYIEAKDVTVVTNGLSHVEALVSKRIRSYLLGGMMKIHTKAVIGSIALQNMD

NFRFDKCFIGTNGVDAEMGYTTPDPEEALIKRRAHQLSGRSYVLADSSKIGEITFAKL

FDLEEADLITERMPEHWRSVIAQKTKIIEG"

CDS 1809497..1810408

/gene="lacC_1"

/locus_tag="PIKHDJKN_01689"

/EC_number="2.7.1.144"

/inference="ab initio prediction:Prodigal:002006"

/inference="similar to AA sequence:UniProtKB:Q833W9"

/codon_start=1

/transl_table=11

/product="Tagatose-6-phosphate kinase"

/db_xref="COG:COG1105"

/translation="MIYTVTLNPSIDYIVEVDDLKLGDLNRMNRDLKLPGGKGINVSR

VLNQLGAENTALGFLGGFTGRFINDKLQEDEILTDFVTIADDTRINIKLKHGEETEIN

GLGPAIRPEEAAGLLNKLSALQKGDIVILSGSVPPSLGADFYDRLINVCKQTSAEFVI

DTTGPALMDALVYEPLLVKPNHHELAELFGVTINTQEELVTYGRKLLEAGAKHVLISM

AGEGALLITKTDVYHASVPKGIVKNSVGAGDSMIGGFVGTFGLNEDLLEAFRTGVASG

SATAFSDDLATRELIEELRTQVTITQI"

CDS 1810546..1812540

/gene="fruA"

/locus_tag="PIKHDJKN_01690"

/inference="ab initio prediction:Prodigal:002006"

/inference="similar to AA sequence:UniProtKB:P71012"

/codon_start=1

/transl_table=11

/product="PTS system fructose-specific EIIABC component"

/db_xref="COG:COG1299"

/translation="MRITDLMIQETMIMDLQATTKEEAIDELIASLNRSGRINDPVLF

KEMIYKREAESSTGIGGGIAMPHAKTTAVNEPTVVFAKSRKGLDFEALDDELAHIFFM

IAAPEGAANTHLRTLAALSRLLIDSDFISQLMSTDTPEEVTALFDAKQAEEAEKKAAK

EKAEAEKAANAANGTNAVSGSGNANGQKQNTSGLIVGDAASEDFVVAVTACPTGIAHT

FMAEDALKKKAQEMGINIRVETNGSEGAQNVLTADEIARAKGVIIAADKNVEMARFDG

KPVLQRPVSDGIRKSEELIRKAVNGDAPIYRSQGGNGGKSEGESQGKVSMGSKIYKDL

MNGISHMLPFVVGGGILLAISFLFEQIASPENPIVQLLQTIGGGTGAFHFLIPVLAGF

IAMSIGDRPALMPGMVGGLMAVNSNAGFLGGLAAGFLAGYVVIGLRKLFKGLPKAIDG

LKPILLYPVFGLLIVGAISFYVFDPIFGSLNTWLVDALGNLGTGNAVLLGLLLGGMMS

IDMGGPFNKAAYTFAIGVFTSSGNTDGAWMAAVMAGGMVPPLAIALATTFFKSKFTEQ

ERKSGLTNYVLGFSFITEGAIPFAAADPLRVLTSCILGSAVAGGLTQLWSINVPAPHG

GIFVAALANHALLFLLAVAIGAVISGLILGLWKKSPTVVK"

CDS 1812737..1813522

/gene="ycgJ"

/locus_tag="PIKHDJKN_01691"

/EC_number="2.1.1.-"

/inference="ab initio prediction:Prodigal:002006"

/inference="similar to AA sequence:UniProtKB:O31474"

/codon_start=1

/transl_table=11

/product="putative methyltransferase YcgJ"

/translation="MSNDQIKGQVRKQFAKNAEKYVTSVGHAKGDDLALLVASSQASS

DMKVLDIATGGGHVANALAPLVEQVIALDLTDEMLQVAERFIRGNGHQNVDFVAGDAE

HIPFEDDFFDLVTCRIAAHHFPDVPSFVSEALRVLKPGGRLLLIDNVAPERDENDQFY

NEIEKWRDGSHVRAWRKTEWIRMLELAGFRMETMVSYEKHFIFEDWCNRAGLPEPESR

ELEASMLSAPSVIKKYFNFEVTQPGRLVSFKGESVYIQAVKTK"

CDS complement(1813623..1814762)

/locus_tag="PIKHDJKN_01692"

/EC_number="1.1.1.1"

/inference="ab initio prediction:Prodigal:002006"

/inference="similar to AA sequence:UniProtKB:O07737"

/codon_start=1

/transl_table=11

/product="putative zinc-binding alcohol dehydrogenase"

/db_xref="COG:COG1063"

/translation="MKAVTFQGTKDIQVKEVEDPKLQQKDDIIVRITSTAICGSDLHI

YQGALPAQKDYVIGHEPMGIVEEVGPEVTRVKKGDRVVLPFNISCGECFYCNHDMESQ

CDNSNRNPEIHTGGYFGFTERYGNYPGGQAEFLRVPYGNFMPFVIPESCELEDEALLF

LSDVLPTAYWSVENAGVKPGDTVTVLGSGPIGLMAQKFAWMKGAKRVIAVDRLPYRLD

KAKRMNDSEVFNFEDYDDMGEHIREITRGGADVVIDCVGMDGKKSTLEEIGQKLKIQG

GTLSAIEIGIKAVRKFGTMQLTGVYGSTYNMFPLGNLFERNINLRMGQAPVIHYMPEL

FRKITAGEFDPTEIISHRIALENAKEAYRIFYEHEDECTKVILKP"

CDS complement(1814838..1816001)

/gene="oxdD"

/locus_tag="PIKHDJKN_01693"

/EC_number="4.1.1.2"

/inference="ab initio prediction:Prodigal:002006"

/inference="similar to AA sequence:UniProtKB:O34767"

/codon_start=1

/transl_table=11

/product="Oxalate decarboxylase OxdD"

/db_xref="COG:COG2140"

/translation="MKNENNNNGKPLIIPQPIRKDGAGGPDLGPRDVMRDLQNPDMLV

PPVTDNGLLPNLRMSFSDTHMQLNHGGWSREITVRDLPIATTLAGVNMSLTPGGVREL

HWHQQSEWAYMIWGTARITSVDQQGRNFIADVGPGDLWFFPKGLPHSIQGLADGCEFL

LVFDDGSFSDLNTLSISDWFAHTPPEVLSVNFGIPESAFQSMPTEQVYIFQDQVPGSL

ASQEVQSPYGTVPLSFKHRLLAQKPIITPGGSVRIVDSTNFPISTTVAAALVEIKPGG

MRELHWHPNADEWQYYLTGQGRMTVFGGNGIARTFDYRAGDVGYVPVAMGHYIQNTGT

DTLWFLEMFRSDRFEDISLNQWMALTPEELVHDNLNASPELLHALRKEKWPVV"

CDS complement(1816217..1817101)

/locus_tag="PIKHDJKN_01694"

/inference="ab initio prediction:Prodigal:002006"

/codon_start=1

/transl_table=11

/product="hypothetical protein"

/translation="MNSNHTLRTALITGSTSGIGLELTRTLLAEGWQVIGLNRSAFPK

EDSDIQDGLRSGQLRWVQANLTQYDSLRKALNQIKSETDTIDVLFNNAGGSGYDLRLS

DQGHEMHFELQTVVPYIIYVELQDLLHKSTLKTVINTSTSAFNMVKQFNMDILEHPAE

FKKLFGPYATSKLALSLWTREAASTPAAKGIRLLSVDPGGNNTLRGNKTSGLPFYIKP

IMKLFFPHPSHGASLLYNAAIAQTSLTSGTFLMKNKPTNLRFTEQGSAILNRVHEIYE

LEFLQFDSKTDPVSKKSV"

CDS 1817310..1817744

/locus_tag="PIKHDJKN_01695"

/inference="ab initio prediction:Prodigal:002006"

/codon_start=1

/transl_table=11

/product="hypothetical protein"

/translation="MNDAEVFSIKETSEQAGLSEDTIRYYEKIGLLPRAERKANRHRV

YRPEDIQTMKLITCLKKTGMSLEEMKPYLQMSMDSDLEDFPDEREMLLNHRKKIEAQI

ASLQQIVDFIDEKMEKRSMFPDECPITGENQMSVFEKKNIFS"

CDS complement(1817807..1818742)

/gene="rhaS_9"

/locus_tag="PIKHDJKN_01696"

/inference="ab initio prediction:Prodigal:002006"

/inference="protein motif:HAMAP:MF_01534"

/codon_start=1

/transl_table=11

/product="HTH-type transcriptional activator RhaS"

/translation="MLSKMEPDTAIIQQQQQELARLIERFTHEDGVHLTAIPSLALIR

ASQVSEPIHAVHEPALCIVAQGSKLVILGQESYTYDSSQYLVASINLPISGQVVQATT

EHPYLCLRLDFDSGQIFDLIQDSPTVQTKPDKSTRRGLFVSSTKPPLLEAVIRLVRLL

DTPEDIPVLAPLMTREILYRLIQDEHGHSIRQFAVQNSHAQHIAQVIEVIQSEFAHPL

RIEQLASMINMSTSSLHYHFKAITAMSPLQYQKQIRLQEARRMLLAGSNDAADAAFQV

GYESPSQFSREYARMYGLPPKSDINRLRHTLHIDC"

CDS 1818908..1819183

/locus_tag="PIKHDJKN_01697"

/inference="ab initio prediction:Prodigal:002006"

/codon_start=1

/transl_table=11

/product="hypothetical protein"

/translation="MVGNRSGERQEVEHPYVGMWVTKDGYIRHELLPDGRYDEARGNR

QSAYQGRYVVDGDHIEYVDDTGFTADGDFKDGVLYHAGMVLYRENAQ"

CDS 1819209..1819946

/locus_tag="PIKHDJKN_01698"

/EC_number="1.-.-.-"

/inference="ab initio prediction:Prodigal:002006"

/inference="similar to AA sequence:UniProtKB:Q7A3L9"

/codon_start=1

/transl_table=11

/product="putative oxidoreductase"

/translation="MFNVKGKVVVITGASSGIGEATARLLTEHGAHVVIGARRLDRLE

ALASSIRSEGGSVEYHTLDVTQLEEMQTIVGVALNRYGRLDVIVNNAGVMPLSPLEAL

KVDEWNRMIDVNIRGVLHGIAVGLPIMKEQGFGQFINIASIGAYAVTPTAAVYCATKY

AVRAISEGLRQEVGADIRVTLVSPGVTESELAESISDDEARELMKDYRRISIPATAIA

QSILYAINQPAEVDVNEIVVRPTASMA"

CDS 1820089..1820991

/gene="gltC_2"

/locus_tag="PIKHDJKN_01699"

/inference="ab initio prediction:Prodigal:002006"

/inference="similar to AA sequence:UniProtKB:P20668"

/codon_start=1

/transl_table=11

/product="HTH-type transcriptional regulator GltC"

/translation="MTLQQLKYVIEVATRGSMNEAAKRLFISQPSLSNAIRDLEQELR

ITIFERTNKGITLSKEGVEFLSYARQVVEQAELLENRYLNAKPSPQHFSVSTQHYAFA

VNAFVKLVQQYGQDEYELALRETKTYEIIQDVKSLRSEIGILYLNEFNSKVINKLLKD

AGLVFNSLFVAKPHIFISVQNPLAKQESVAIEQLQDYPYLSFDQGEYNSFHFSEEILS

TLSHPKSIQVNDRATLFNLLIGLNGYTISTGVLSADLNGNEIIPVPLECEETINVGWI

SHKNASLSNLGVEYVQALHEAIQS"

CDS 1821089..1821838

/gene="speE_1"

/locus_tag="PIKHDJKN_01700"

/EC_number="2.5.1.16"

/inference="ab initio prediction:Prodigal:002006"

/inference="protein motif:HAMAP:MF_00198"

/codon_start=1

/transl_table=11

/product="Polyamine aminopropyltransferase"

/translation="MRVLYRNKSEQNELTIYDTSKLYGEKGRFRVLEFSNAAVQGAMD

LDEPSRMVLEYPRAMVHLMERNNPEYESVFVVGHGIGTLSTYLSNRQVKVAELDAEVV

ELSRTFFGYIGDHVLVGDGRELLEQETSGSYNYIIVDAFTATGTPVQFTSSSFFAMLK

DKLHSGGIVLLNVFGRAGNDRLVNAIHTTLQEHFAYTRSFALPAETTDEIQNRILVGS

DEPISFQSRHMAGFVEQQPGEGYVIVDSNES"

CDS 1822019..1823635

/locus_tag="PIKHDJKN_01701"

/inference="ab initio prediction:Prodigal:002006"

/codon_start=1

/transl_table=11

/product="hypothetical protein"

/translation="MIAKNKTSRLRRKVREIKKISLTALLSGLVTIAVLMTLTIMFIS

SYTSQKQSLIDNTLSLNYSSAVQMSQTLDSLFQSMQGSLKYSANYFPDIDYADAKKLD

STLDLIRNSSNYFNSVSLIDESGIIRGSSPYLEKSLGQPITSDAAKYAFKSRDSFISE

AYRAPYSERRVVFVSEPIFSPSGEFKGLISGSIYLQENNILNLSFGSQLKTSNGSYFY

IVDAKGTLLFHPDTERIGENLSKNQVVQKLLNDQTGKEQYKNLAGVDSLAGYYKVPTT

DWGVVVVSPTQMVYDQLNQHIRMLLLYTSIPFIILTLIVIRVARKLASPFVYLADLVN

QVDQGKVDLPVMKPHWNREADLLTRTVLTALANFRKQKDQLTYDARTDVLTGMTNRRT

FEEVIQQWIDEEVPFSIVVLDIDRFKSINDTYGHHAGDQVLKHIANIIKLSVRTQDVC

SRFGGEEFVVLLRHTNSKTAYQIAEHIRVSVEQSILPIDRSVTISAGIAEYPLHSTTA

TELFHLADNALYQAKEEGRNRTITIQTVIK"

CDS complement(1823713..1824072)

/gene="yybR_1"

/locus_tag="PIKHDJKN_01702"

/inference="ab initio prediction:Prodigal:002006"

/inference="similar to AA sequence:UniProtKB:P37486"

/codon_start=1

/transl_table=11

/product="putative HTH-type transcriptional regulator

YybR"

/db_xref="COG:COG1733"

/translation="MRKNPNDACPSPYGCSVEVTLSVIGGKWKGAILYHLFSGSLRFN

EIRKLFPDITQRMLTLQLRELESSGIVHREIYPQVPPKVEYSLTPFGETLQPIIYSMR

DWGEQYTNEVLARSQEV"

CDS complement(1824275..1824670)

/gene="mhqP_1"

/locus_tag="PIKHDJKN_01703"

/EC_number="1.-.-.-"

/inference="ab initio prediction:Prodigal:002006"

/inference="similar to AA sequence:UniProtKB:P96694"

/codon_start=1

/transl_table=11

/product="Putative oxidoreductase MhqP"

/db_xref="COG:COG2259"

/translation="MLDAGLLIIRLVIGFIMMGHACKKLFGWFGGEGISGTAEFFGAI

GLKPARVMAVCGGLIELIGGFLFGAGLWTGFAAILLIIPMVVAIAKVHAGKGLWNLNG

GFEYNLVMLGSLIGVALAGAGAYSLDTLI"

CDS complement(1824728..1825720)

/gene="iolG_6"

/locus_tag="PIKHDJKN_01704"

/EC_number="1.1.1.369"

/inference="ab initio prediction:Prodigal:002006"

/inference="protein motif:HAMAP:MF_01671"

/codon_start=1

/transl_table=11

/product="Inositol 2-dehydrogenase/D-chiro-inositol

3-dehydrogenase"

/translation="MKKAKLCFIGAGFHASTNIYPSAVEAGADIQAIATRSIERSGAA

LLRFGSSGKAYDNTQLMLQNEDCDGVVVVAQPEDQTALALECIRAGKNVYVDKPLGWN

AAEAAAVAEAAQKAGVVVMVGFMKRYAPVYMKLKELIDGGSLGRARSFQMKFAVDSTP

FCKDEEQFIKLAAIHMVDLMRFLFGEAIQVTGTTVTDGEHINQSISLKFDHGVVGSAY

FVGMSAWSRESESVLVTFDNGFALADEINTLTVHQSRTGDNLPWKSLEEQDTVFTPSG

SPMSGAYRDLYLRGFVGEMAHFMECCQNKSDPHSSGKDNIETMALCDTILESLI"

CDS complement(1825761..1826948)

/gene="ahcY_1"

/locus_tag="PIKHDJKN_01705"

/EC_number="3.3.1.1"

/inference="ab initio prediction:Prodigal:002006"

/inference="similar to AA sequence:UniProtKB:O51933"

/codon_start=1

/transl_table=11

/product="Adenosylhomocysteinase"

/db_xref="COG:COG0499"

/translation="MNIKNTTHERIEEGKRSIHWTEMHMPLLGELKSQFAKELPFAGL

TIGICIHVEPKTAVLCRTLQAGGAKVVLTGSPGTTKDAVAAALQEEGITVYGQRADRR

NEHLQNIHSVLRHQPHLLMDNGADLARTFIQQYDTNSLIGGTEETTTGANLLREETGE

NIPFPIIVINDSPLKRIMENEHGVGQTIIEGFMRTTNLILPTRRFVIVGYGTCGRGIA

RYLRNLGSQVVVVETDPIAGLEAALDGFRVARLEDTFAFAQVYITVTGRPNAILKEHF

NQMNDGTILANAGHFSWEMDLESLRQDAKHTEQLTADIEQFTLTNGRRLMLLTQGEML

NLAGGSGNPAETMDLGLSLQAASLLYLVQQRQHLTLGPQPVPHSVNNTIAGQMLKHLS

YKI"

tRNA 1827181..1827267

/locus_tag="PIKHDJKN_01706"

/product="tRNA-Leu"

/inference="COORDINATES:profile:Aragorn:001002"

/note="tRNA-Leu(cag)"

CDS complement(1827327..1827506)

/locus_tag="PIKHDJKN_01707"

/inference="ab initio prediction:Prodigal:002006"

/codon_start=1

/transl_table=11

/product="hypothetical protein"

/translation="MKLTKEIGISLGFLAGTTFGSGIAFLFRFQAYEVMASVALFGIA

GAIAGLCVQQFIFNK"

CDS 1827666..1828820

/gene="egsA"

/locus_tag="PIKHDJKN_01708"

/EC_number="1.1.1.261"

/inference="ab initio prediction:Prodigal:002006"

/inference="similar to AA sequence:UniProtKB:P94527"

/codon_start=1

/transl_table=11

/product="Glycerol-1-phosphate dehydrogenase [NAD(P)+]"

/db_xref="COG:COG0371"

/translation="MNMNERIAAWNEEAQNCTCGHDHRLVQMQVYLEHGAIQRLPGYL

SEQGYRRVTVVYDRFTGPAAGYEVVTNIREAGVHVDEICMPENKAGDVIADEAGIVQV

LLGVKQGSQAIIAAGSGTIHDLVRFVCSKMNKPFLSVPTAASVDGFTSAGAPLIVSGV

KQTFQAVPPEAIFADIDILEQAPQEMNAAGFGDMLGKYTSLADWVVSRDLGGEPFCPV

AYRMTEEALTTCVEHVQGIAEGRADGVTVLMDALIASGISMLIIDHSRPASGGEHHLS

HRWEMDLMEAGHKPVLHGAKVGVACALLTVKYKDLAQESDEPVFRIYDNLPDASQLTA

WLEQVGGPITTEQLGVSMEMVEHAFTTAHLLRDRYTGLKYINEILNVGSK"

CDS 1829211..1829399

/locus_tag="PIKHDJKN_01709"

/inference="ab initio prediction:Prodigal:002006"

/codon_start=1

/transl_table=11

/product="hypothetical protein"

/translation="MGISRRPFIQSDFRLVQRASCIGAGKGVTGEEGMMHPKLVGGQE

GRLQSLRDDMKCIRLEKW"

CDS complement(1829488..1830084)

/locus_tag="PIKHDJKN_01710"

/inference="ab initio prediction:Prodigal:002006"

/codon_start=1

/transl_table=11

/product="hypothetical protein"

/translation="MPNQPEQHSIQAWSLINRKYLGKGVRVKRFRKPTRCQIRNRVLL

AVLMANDIKLSQLAEDLSISSRSVSAWVYEGRIPGSTNLDKACQLLGYPRHILFNEEV

VRKSPVICQPEPSRFMKRTVTRSPVSNRILTGLCMVHDLSVTDVSHWIGVHPGTFRKW

LHQGTLPSAAFQEQAEQFFRIPKTILFADVILKDRHNN"

CDS 1830532..1831080

/locus_tag="PIKHDJKN_01711"

/inference="ab initio prediction:Prodigal:002006"

/codon_start=1

/transl_table=11

/product="hypothetical protein"

/translation="MSPSVSTFAAISGAFLTFAFGGWDQLLSLLAVAMAVDYITGLAA

AVRTGKGLNSNVGFWGLARKGLMLTVILLAHRIDLLMGTDIIKGGAIYFYLVNELISI

TENYARIGLPLPDKLRQAIAVLKKQEGLSDREWPQPQPNVENKETKEQAAEVDPENQT

NQSEQTDNMSQEKSKANESSPD"

CDS 1831200..1831487

/locus_tag="PIKHDJKN_01712"

/inference="ab initio prediction:Prodigal:002006"

/codon_start=1

/transl_table=11

/product="hypothetical protein"

/translation="MIKHIVLFKMKDRSPENIEAAANVLRNLQGKIDVLISLEVGIDV

LRSERSFDISLTAEFASLEDLQAYQVHPLHQEVIKYMNEVREQSIAVDYEI"

CDS 1831661..1832122

/locus_tag="PIKHDJKN_01713"

/inference="ab initio prediction:Prodigal:002006"

/inference="similar to AA sequence:UniProtKB:O32126"

/note="UPF0331 protein YutE"

/codon_start=1

/transl_table=11

/product="hypothetical protein"

/translation="MYYVNREQIARRLAAVPEVAEGLRRASQAWDGSLMLGLVQERCL

HLAIEIVTDVGSYLIDGFIMRDASSYDDIIQINYEEKVFDHATYDVLSRLVSLRKPLV

QDYYSWERSELHPLSSDLPGVLEHFSHQVNTYVEKELGPFQIAQAEGQSKE"

CDS 1832134..1833039

/locus_tag="PIKHDJKN_01714"

/inference="ab initio prediction:Prodigal:002006"

/codon_start=1

/transl_table=11

/product="hypothetical protein"

/translation="MTNEWKEAREGNNETNDIPSGFHPVYMIELLFQERPVIDQGRLQ

DALSRHTGQVRLDVKQGNEEHNPEMLVFYHLDHKVSFQEGNIPAQTCMLPITEIVDRA

RFGGALQQAWHWPEAGQAMETIQYSIRLHDMFTAAMPRKQRLELFQMTIQAVMEVLPC

EALYWYGSDKLVEPEAYVLSQEREEHLYAAMNVRMYQAGGTEEQRQLVMDTVGLSALG

VPDVQCHFIGLDPDTVAQTLLGAAYYIFDQGDVLQDGQTLGSSGGRRWRCEHQAAMIA

PGRYVIDLDPGDEHAAAPLEPARQT"

CDS 1833226..1833612

/locus_tag="PIKHDJKN_01715"

/inference="ab initio prediction:Prodigal:002006"

/codon_start=1

/transl_table=11

/product="hypothetical protein"

/translation="MKDSNKSLLWGALIGSVVGSVTALLLAPKSGRELRQDISEGARQ

VSEKGQELASKVGEQSAQIVSKVKETADVVIQDIQSWRNCAEGKEVHVSAVITDSDSS

IDADAANESDIEIIAKLPADDSKDNI"

CDS 1833729..1834625

/gene="murI"

/locus_tag="PIKHDJKN_01716"

/EC_number="5.1.1.3"

/inference="ab initio prediction:Prodigal:002006"

/inference="similar to AA sequence:UniProtKB:O31332"

/codon_start=1

/transl_table=11

/product="Glutamate racemase"

/translation="MPENSYGREKALGTNVLQNQFNEEGSGVFVQQAIAILDSGVGGL

TVAKEVMRQLPREKIIYFGDTARTPYGPRSSEQVKQFTEQIVDFLIQFDPKVIVIACN

TATAAALDYIRAKVNVPVIGVIHPGARAAITATRTGRIGVIGTVGTIGSGAYTSALKQ

LSPYIDVVSQACPALVPLVEQGEFRSEQTTSTVEQSLGQIKQQPIDCLILGCTHYPFL

MDTIQEVMGQEVKLISSADETAREISTILYDKRKLASGDETPVHQFFCTGDPRMFQNI

TRQWLGEQISKTPVVWQVTQLS"

CDS 1834865..1836079

/locus_tag="PIKHDJKN_01717"

/EC_number="3.4.19.11"

/inference="ab initio prediction:Prodigal:002006"

/inference="similar to AA sequence:UniProtKB:Q03415"

/codon_start=1

/transl_table=11

/product="Gamma-D-glutamyl-L-diamino acid endopeptidase 1"

/translation="MELQWIIVHQGDTLSRIAAANHMTKELLAALNPEAASQPYLLTG

QMLRITPGTGRRYAVEPGEKVAVIALRFGLNEEELREANPEIANIPDWCGRCIHIPDS

NGKTIVKLQGEYGYREMNRDIDLLAKKYPFIEIGSIGSSVMGKDLPYLRLGQGPRHIH

VNASVHANEWLTTAVLMRFIEEYAKAYSTHTPWHQFQTERWMQETTLWAVPMVNPDGV

ELVQEGVVNDHPHAEELLEWNAGRSHFTHWKSNIRGVDLNDQFPAYWDEEAARRGITS

PGPRDYAGTAPLSEPEAWALAQWTEQHHFDAVVSLHSQGQEIYWNYRDLEPKESAPLS

RRLARASGYKAVKLGGSDAGYKDWFIQKFRKPGFTVEVGLGVNPLPMDQFDDICVEVG

MLLAELLSDREH"

CDS 1836309..1836566

/locus_tag="PIKHDJKN_01718"

/inference="ab initio prediction:Prodigal:002006"

/codon_start=1

/transl_table=11

/product="hypothetical protein"

/translation="MKWRRLLSFKRWTQVFKRLPRLLRAPQIPLGEKLLFIIPALLYW

VLPDVMPFMPIDDIGVTLLLMNWFVSRAERKYPVLTAGASS"

CDS 1836711..1837013

/locus_tag="PIKHDJKN_01719"

/inference="ab initio prediction:Prodigal:002006"

/codon_start=1

/transl_table=11

/product="hypothetical protein"

/translation="MNCKITRNAAKVLKLELDKPENEGKLLRVVITHAHGDHAHYGLD

IDTPKENDTVVSTDKEIDVILENDQPLLDGVKIDYLYFPEEGFVITNPSKGNHGDH"

CDS 1837035..1837256

/locus_tag="PIKHDJKN_01720"

/inference="ab initio prediction:Prodigal:002006"

/codon_start=1

/transl_table=11

/product="hypothetical protein"

/translation="MANDIRVCEKCNHIRLKSIVAKLEKMAPDTEIKIGCKSYCGPCA

KRAFVFINGRYISAPSEEEVLAKVAKFVK"

CDS complement(1837351..1837749)

/locus_tag="PIKHDJKN_01721"

/inference="ab initio prediction:Prodigal:002006"

/codon_start=1

/transl_table=11

/product="hypothetical protein"

/translation="MKNFWILFILVPLLLLTSGCTPSTYEITGYTGSSINDEIPVPEN

AKQLSVSTYSDNPNIKTGIKYELKHIGGEQGLYVPSDYFEKLSEAGWVEVKEERMGNV

HYFKKSDTVIAIEIQEDTFKIFEMRQDFTF"

CDS 1837953..1838234

/locus_tag="PIKHDJKN_01722"

/inference="ab initio prediction:Prodigal:002006"

/codon_start=1

/transl_table=11

/product="hypothetical protein"

/translation="MPKRYDSSLQADTTVSQAQNAVNKLHFAVSQAMSHPTEQTIEQA

ERRLAHTEQAMLQAERSLGGQGVELAEEMFIEEKRRLNSIQSQNGQGDL"

CDS 1838410..1839240

/gene="yvgN_1"

/locus_tag="PIKHDJKN_01723"

/EC_number="1.1.1.-"

/inference="ab initio prediction:Prodigal:002006"

/inference="similar to AA sequence:UniProtKB:O32210"

/codon_start=1

/transl_table=11

/product="Glyoxal reductase"

/db_xref="COG:COG0656"

/translation="MTKHITDCTILNNGVTMPWLGFGTYRAKGKEVQQAVETALEVGY

RSIDTASVYGNEEEVGQAIASSGIARNELFVTTKLWNEDQGFDSTLRAFEASQKALGL

NVIDLYLIHWPGRDQYKETWRAFERLYSEGSVRAIGVSNFEVHHLRDIIDEGGTVPAV

NQVELHPGLIQQELQDFCGEQGIQLEAWSPIMRGKLNKESALKSLAQKYGKTPAQVIL

RWDIQNQIVTIPKSVTPGRIRENADIFDFELTPDELKLIDTLDSDKRTGPHPDQLFWD

"

CDS complement(1839582..1840316)

/locus_tag="PIKHDJKN_01724"

/inference="ab initio prediction:Prodigal:002006"

/codon_start=1

/transl_table=11

/product="hypothetical protein"

/translation="MNIEIWSDFMCPFCYIGKRRLENVLAQFPHRDEVKLQFKSFELD

PNAELNSGKTNAEYLAAKYNMSVEQARGMNAQMNANARTAGLEYNIDEMIPTNSFSAH

RLTHWAETQGKMLELSERLFQAIFIEGKHVGNTDALIELAEEVGLDRNAAAEVLSSNQ

FADQVRADQAEGEQLGIRGVPFFVFDRKFAISGAQPDEVFLDAIQKAWDKRSPFTMVE

SNTTDADGSGVCTEDGCEVPQPDQSK"

CDS complement(1840473..1840898)

/locus_tag="PIKHDJKN_01725"

/inference="ab initio prediction:Prodigal:002006"

/codon_start=1

/transl_table=11

/product="hypothetical protein"

/translation="MTSHTDQFNIEQKLFEEAANFVKHRYPQGWGGAAAVYTEAGSLL

ISVAPEVINDATHLCMETGAYLEAHKLNERVTHSICVARDDEHSEFKVLTPCGVCQER

LFFWGEDVKAAVYDPDGALIYKRLDEIQPYHWTKAYRDK"

CDS complement(1840995..1842992)

/gene="ltaS2_1"

/locus_tag="PIKHDJKN_01726"

/inference="ab initio prediction:Prodigal:002006"

/inference="similar to AA sequence:UniProtKB:O34952"

/codon_start=1

/transl_table=11

/product="Lipoteichoic acid synthase 2"

/db_xref="COG:COG1368"

/translation="MFVTKPTRSLTPRGLLNHPLTLYLIFLVLMLLKLMWLHHNLHAY

NITMGLLDKVIAIGSLLILSFWTWWLPRRGMIISLVILNLLLTALIYADMVYYRYFQD

FLTIPVLMQARQVDALGDSIATLIYASDLWFFVDWLVIIPYAAILLFSKRNRRRDYSA

YSNGLNSYSSNSGKSSVRRRLTAGSIALVLGLGLAVGPIYFYSKTWAKGLFDNNWWNV

SMYNVTGLLAFHGYDLYNYAKDQLGSGPEVDPADLEQAEAYFASKQQADPLKDAMFGK

YKDSNVIIVQGEAFMNFMIGQSIGGQEITPHFNELMKESQYYSHFYHQTGQGRTSDAD

FGANISLHPLSVGSAFVRYADHSYDSLPSILKDHGYSTNVFHAYESGFWNRYTMYQNM

KYDKFYSKNDFEQDDPLGWSLSDESFFRQSVEKMSSEVTEPFYSFLITLSSHHPYALP

QDKQQLDVGEFKGTMFGNYLQSVHYVDSALGNMVEDLKNRGLWDKTIFMFYGDHDNSI

KEQPQYEKFLGRSLNDLDMEQIMNEVPLLVHLPDGAAAGTIDEPAGQLDITPSVLHLL

GISDQSYYHMGNDVYDSSSRMVILRSGAFTDGSLFYIPSDDYLYDSGSCYDLSTGTKT

DINACRSGHDEATKRLHVSDTVNTFDLISRFREEQASESSS"

CDS complement(1843206..1844366)

/gene="rlmL_1"

/locus_tag="PIKHDJKN_01727"

/EC_number="2.1.1.173"

/inference="ab initio prediction:Prodigal:002006"

/inference="similar to AA sequence:UniProtKB:Q9K0V4"

/codon_start=1

/transl_table=11

/product="Ribosomal RNA large subunit methyltransferase L"

/db_xref="COG:COG0116"

/translation="MNHMAQLQLIATSAMGLEAVVARELKQLGYEDVTIDNGRVFFTG

DYIDICRCNLWLRSSDRVLVKMGEFPATTFDELFEGTKALPWEEWIPADGEFPVEGRS

QKSQLSSVPASQGIVKKAIVEKLKLTYDTEWFPEDGSRYVIEVILLNDRALLTLDTTG

PGLHKRGYRKLVTEAPLKETLAAALIQLSRWNVSRPFYDPCCGSGTMLIEAAMIGWNI

APGLRRTFNSENWAVIPEELWEQAREEAFDAVRDDIPLQISGSDIDPEAIEVAQAAIK

SAGLAKDIEVSVLPAHRARPQGEYGVIITNPPYGERLSEEKEVQKLLRSLGRSYLDLP

TWSFFAITSTKSFEENFGHKADKRRKLFNGRIETQYYQYLGPLPPRNKTPQQ"

CDS 1844598..1845230

/locus_tag="PIKHDJKN_01728"

/EC_number="2.1.1.6"

/inference="ab initio prediction:Prodigal:002006"

/inference="similar to AA sequence:UniProtKB:O07431"

/codon_start=1

/transl_table=11

/product="Catechol O-methyltransferase"

/db_xref="COG:COG4122"

/translation="MNLTPDEYVNQLFQEDDLLLKVKEAIRSNGMPEVSVAAAYGRLL

TFLAKTSKAEAVLEIGVLGGYSGICLARGLSDGGTLTSLELKEEYAAMARGHLEEGGF

GDKVEYRIGPAAASLEQLAQEGRTFDFFFIDADKENYPVYLDYAIKLARPGAVIVGDN

CFLRGRTLNSDKQGPAVLAVRRFNEQMASDPRLVTTMLPDYDGLALAWVK"

CDS complement(1845362..1846573)

/gene="ansZ"

/locus_tag="PIKHDJKN_01729"

/EC_number="3.5.1.1"

/inference="ab initio prediction:Prodigal:002006"

/inference="similar to AA sequence:UniProtKB:O34482"

/codon_start=1

/transl_table=11

/product="L-asparaginase 2"

/db_xref="COG:COG0252"

/translation="MKKTSNLRPWAVWSTAALTALTISLSPIGTYAAQAATVEVSGTT

SAVPAASSTPARNTTIPALSDSFKQSALPNVLVIGTGGTIAGQSEDATSFQNYKAGTL

PIGEMVDALPDKQKIADVSTLQFGNSGSGSYTMADLYDLSQTVDKALAQYDSVVVTTG

TDTMEEIAYFLDMTVQSDKPVVITGSMRPWTVIGSDAQANLYNAIKLAGSGRTTSFGT

VLMLNDTIQLARGVTKTNDYRTDTFETPMLGAVGYIDEENIRIYRAPARALKPEGTLK

PAFDLSKIKKADLAKVEIAISYQEAGGEAIEGFVKGGAKGIVTSGTGAGGISRAMGQA

RTKAVEDGVIFVTTTRTGSGSVYGGGKGVIAGDNLSPQQARILLMLGLSFSDDFDTIQ

KWFETYGTPEV"

CDS 1847081..1848283

/gene="yjiB"

/locus_tag="PIKHDJKN_01730"

/EC_number="1.14.-.-"

/inference="ab initio prediction:Prodigal:002006"

/inference="similar to AA sequence:UniProtKB:O34374"

/codon_start=1

/transl_table=11

/product="Putative cytochrome P450 YjiB"

/db_xref="COG:COG2124"

/translation="MNRAPRKYANYIPIRELEAVEQQLFPFQVYAELRDNTPVRYDNH

RECWDVFRYEDVQYVLKNPKLFSSERDRANASMLTTDPPKHKQLRDLVNQAFTPKAIE

ALAPRIQEIADELLTPHFSEGRMRLIDDLATPLPVIVIAELIGVPATDRQKFKDWSDA

LVKGARDDSDEAFQELMEEKKRNQQELASYFTAIMEERRQRPKDDLISLLLAAEIEGE

KLTAEEVVGFCILLLAAGNETTTNLITNAVRILSEQPELQKQLREHPELISTAVEETL

RYYPPIVAIGRIAKENVELNGQTIQAGEQVISWVGSANRDSAQFDQAEKFVPDRKPNR

HMGFGFGIHFCLGAPLARLEARVVLQTMLRQMEDIQLVPETVLQPIQSAFVFGVKEYP

IQFRSSIH"

CDS complement(1848405..1849616)

/gene="sbnD_2"

/locus_tag="PIKHDJKN_01731"

/inference="ab initio prediction:Prodigal:002006"

/inference="similar to AA sequence:UniProtKB:Q2G1N0"

/codon_start=1

/transl_table=11

/product="Staphyloferrin B transporter"

/translation="MKTWKVNLIVLWFGQFLVNSGMTMITPFLSLYLARDLGVVGEHE

IGIWAGFIFAANFLTSFLFQPLWGKLSDKYGRKIMLLRSGFGMAIVIALMGFAQNPWQ

LLLLRLLNGTISGFNPAAVALISGTTPKDRMGFAMGISQSGQVAGTILGPLIGGLLAD

AVGFRPIFYITGGLIFAASMLAMFLVRETFDRKEAAKLPEQSVLSGLKELNKSPQLPA

LFAVTFLLQFAMISPMSLLPLYVQKLHASDVNVAFWAGLVGAVTGLSNMAMSPILGKL

SDKIGSHKVLTFSLIGTGLMLIPQAFVQTVWQLIIVRFMMGVFMGGLLPSVNSLIRSY

TPDSMISRAFSFNTSTLALGNMLGAVIGGFMAGFIGIEGLFIVSGGLLLLNMVWVRFK

LYKKPPLIRES"

CDS 1849821..1850885

/gene="hemE"

/locus_tag="PIKHDJKN_01732"

/EC_number="4.1.1.37"

/inference="ab initio prediction:Prodigal:002006"

/inference="similar to AA sequence:UniProtKB:P32395"

/codon_start=1

/transl_table=11

/product="Uroporphyrinogen decarboxylase"

/db_xref="COG:COG0407"

/translation="MSYNDRLIQASFKQQVDRVPVWYMRQAGRYDPEYRKIKEKYSLL

EICRQPELAAEVTLMPVRKLGVDAAILYSDIMNPVASLGIDFDIVKNIGPVIDNPIRT

AADVDRLRPIDVEGDLSHILETIRILDKELDVPLITFAGAPFTIASYLIEGRPSKGYI

RTKTMMYSEPEVWHKLMQKLGDMVITYVRAHIANGGKAFQLFDSWVGALSPNDFRTYV

LPTITRIFTELSDLNVPKIYFPGVASGELLPTLHDLQADVIGLDWRVSISEGRKRLGG

KFAVQGNLDPYVLTAPMDLIKQHAKLIIDEGIKEPGYIFNLGHGLFPEASLDKLRELT

AYIHEYSAKALKTGVTVTND"

CDS 1850905..1851828

/gene="hemH1"

/locus_tag="PIKHDJKN_01733"

/EC_number="4.99.1.1"

/inference="ab initio prediction:Prodigal:002006"

/inference="similar to AA sequence:UniProtKB:Q81U22"

/codon_start=1

/transl_table=11

/product="Ferrochelatase 1"

/db_xref="COG:COG0276"

/translation="MSYGTPENMESIEAYYTHIRRGRPPEPEQLKELTDRYEAIVGGV

FPLRENTDNQVKALQETLNKDDRSKEVEFRCYQGLKHAYPFIEDGVEQMAKDGIQTAI

GIVLAPHFSTMSVGSYIKRAREKAEELGIQMSFIESYHLHPKLIQALSTRVSAKLDAF

EEAGAKRGDVRVLFSAHSLPARIVDIGDPYPQQLLETSEVIASRLGITNWQFTWQSAG

RTAEPWLGPDILDTLQGLAREQVEDVLVAPIGFVSDHLEVLYDLDIEAKAIAKEIDMR

LMRIDSLNSDSLYMETLSDIIINQWQQGTDE"

CDS 1851828..1853252

/gene="hemY"

/locus_tag="PIKHDJKN_01734"

/EC_number="1.3.3.4"

/inference="ab initio prediction:Prodigal:002006"

/inference="similar to AA sequence:UniProtKB:P32397"

/codon_start=1

/transl_table=11

/product="Protoporphyrinogen oxidase"

/db_xref="COG:COG1232"

/translation="MEDHKRRVVVVGGGLTGLSAAFYIRKHYREAGIEPVITLVEKSS

SMGGMIETLHRDGFVIEKGPDSFLARKTAMIDLAKELEIDHELVSQNPESKKTYIMQR

GKLHPMPAGLVLGIPTELRPFLRSGLVSPAGKLRALMDFVIPPRRTTEDESLGYMIER

RLGTEVLENLTEPLLAGIYAGDMRRLSLQATFPQFGEVERDYGSLIRGMMTGRKPAET

HTGTKRSAFLNFRQGLQSLVHALVHELQDVDQRLNTSVQSLEVLNGDAARYNIQLDNG

ERLEADDVVITVPTYVASELLQPHVDTAALDAINYVSVANVVLAFEKKDIEHVFDGSG

FLVPRKEGRNITACTWTSTKWLHTSPDDKVLLRCYVGRSGDEQNVELPDDALTDLVLK

DLRETMGVEAVPIFSEITRLRKSMPQYPVGHLQQIAALREQLGSKLPGVYIAGAGYEG

VGLPDCIKQAKEMSVQATQLIAAK"

CDS 1853436..1854845

/locus_tag="PIKHDJKN_01735"

/inference="ab initio prediction:Prodigal:002006"

/codon_start=1

/transl_table=11

/product="hypothetical protein"

/translation="MYPPRSKRSVQKKKVKRARKRRIWIINLVLIAAIGLVGIYYAVG

MQKQQIDPAVTETAVNQEPVNEEGPQGGDQVSSPNTNSEAGNTSDEESEDSAEQVTEN

PGATSGEKKPDATGSEANGKTDTGTKKPTDTPASSGSKGSEGAGTGATQPSHSAKDVT

INFVGDIQFSGKVAELLEKNGYDYPFAKLGNLFKEDDLTIGNLETPVTLGGTGAADKT

YVYKSSPKALEAMASAGFDAVNLANNHILDQGVEGLVDTLTYLEQYGIAHTGAGMNRD

EAYAPAYLERKGMKIALLGFSRVVPETSWKAEGNRAGVAETYDSTGAVKAIQEARQKA

DLVIVVAHWGEERVSTPNNDQTRLAHEFVDAGADLVIGGHPHVLQGVEYYKGKWIAYS
TGNFIFSKSTTEETWKTAVFQASCSKDAKCSMKVIPYEAGLGQAIPMVDQANKLLLEQ
MTQLSPGIRFDANGFASTS"

CDS 1854877..1855629

/gene="glpQ_1"

/locus_tag="PIKHDJKN_01736"

/EC_number="3.1.4.46"

/inference="ab initio prediction:Prodigal:002006"

/inference="similar to AA sequence:UniProtKB:P37965"

/codon_start=1

/transl_table=11

/product="Glycerophosphodiester phosphodiesterase"

/db_xref="COG:COG0584"

/translation="MNNLCVAHRGFSSIAPENTMAAFLLAMEQPEVQWMELDVQLSRD
GVPVVIHDFTLNRTTNGEGFVRETDWADIKQLDAGSWKGKAYKGERVPALSEVLDRTC
GKVRLNIELKTQGNMYPGLAAAVIYEVRKRHMQHDVVITSFEPAALVEVKKLAPEFKT
GLIIDARPGDLAAVLRQMNCSFLSIGYTNVDKSLMREMRIEGIQVMAWTVDDKIIMKR
LAAIDPELMLCTNRPDVWEQAFQETSSRFFRP"

CDS 1855734..1856369

/gene="ycgM"

/locus_tag="PIKHDJKN_01737"

/inference="ab initio prediction:Prodigal:002006"

/inference="similar to AA sequence:UniProtKB:P76004"

/codon_start=1

/transl_table=11

/product="putative protein YcgM"

/db_xref="COG:COG0179"

/translation="MLTNIRNVYCVGRNYKLHAEELGNAVPDEPMIFMKPSHAVVALN
SETLQLPATKGEVHYEAELVVQIGRDYEPGMAVDELVDAYAFGIDFTLRDVQTVIKKK
GHPWTAAKGFKNSAPVTAFQPFPGVQALLEKDFTLTKNGTEVQRGNIRNMIFSLQDIV
DYVGHHYGLGPGDVIFTGTPEGVGPTAAGDVLELAWDGESLGKCTIAAAVQ"

CDS complement(1856488..1856895)

/locus_tag="PIKHDJKN_01738"

/inference="ab initio prediction:Prodigal:002006"

/codon_start=1

/transl_table=11

/product="hypothetical protein"

/translation="MNMKRVIIVGTMIVTMSFAGTAWGRSAISPIPLTKWSIVNIDTR

DESGDELLSALNQQSEDDLAQDLYAGKSLRMIADENDGDFNEVVALQIRQLKEQLDQR

LASGSISPDQHAVQSSELEELVTESANTVYSFA"

CDS 1857253..1857810

/locus_tag="PIKHDJKN_01739"

/inference="ab initio prediction:Prodigal:002006"

/codon_start=1

/transl_table=11

/product="hypothetical protein"

/translation="MSNKDWEALEKTDWLFRKMVRRFVKERDRISVEGVSLPGMLILH

KIIREGEQRLGDLAEQLDFTSGAITALTDKLEKKGLTIRRRKEDDRRTVLLDITASGR

EMFARNSNIGARCITLLFEGFTTEELEQQSQFYERVIANLEGFSDTLLELAENNGKKE

HDPSTESDPEQKQRENTGKSNYLSY"

CDS 1857857..1859296

/locus_tag="PIKHDJKN_01740"

/inference="ab initio prediction:Prodigal:002006"

/codon_start=1

/transl_table=11

/product="hypothetical protein"

/translation="MGHSTIYPTGATVYNPDKAWGGYTVYQAGEEGVVLIDMNGKEVH

LWKGLLGFPAKVFPGGFVLGSTGRRDPKFGIQDNVDLVQVDWDGNIVWKYNSYEHIED

PGYEPLWYARQHHDFQREGNPVGYYAPGLAPKTQSGKTLILAHKNVNNPSISDKPLLD

DAIIEIDWEGNVLWEWLPNEHFEELGFDEAARNVLFRDPNTRSFGHLGGGVGDWLHIN

SASYVGPNHFYENGDERFHPDNIIWDAREANIIAITDRQSGKIVWRLGPDYSLPEVKH

IGTIIGQHHAHIIPKGLPGEGNLLVFDNGGWAGYGLPNPASPYGLKHAVRDHSRVLEI

NPVTLEIVWQYTSAEAGFSVPTDSYKFYSPYISSAQRLPNGNTLITEGSNGRLFEVTS

EYELVWEYISPYKDGRNTNMVYRSYRVPYHWVPQLEKPKEVAIEAIDVSAYRVPGAAP

KGSASVVSVEITLPFVEGAACVATSDEGK"

CDS 1859318..1860373

/locus_tag="PIKHDJKN_01741"

/inference="ab initio prediction:Prodigal:002006"

/codon_start=1

/transl_table=11

/product="hypothetical protein"

/translation="MIRTNGVHIVRKPSTLTGALLMLLSMLLILSGCSANESVSEAKG

ASGTEAKKVKIRIADTSTNPTFRVAIAKGFFASHGIDAESITFGSPAEGVNALFIKQV

DVAYGADFPVLNALAKGDYSVIASAGQATNEAAAAWKLYAKNDIQSGADLKGKKVSFI

RGTFIPYLWDEYLKDQGLELTDVTQVGQGAFDEAYIALKQGDLDATWVIGSALTDKFD

ALEGVHQLTDMSQTPVRLGMGLVASNEFIQANPETVSGFLAALNEASVYAQAHPEEVA

DLMYKETKQPKEATLKDLPINPWEVGFTQAAYDSLAGQKQYMVDNGIIEHDFDLDNKL

NLELLKQTLPDKVTYSK"

CDS 1860401..1861177

/gene="nrtD_2"

/locus_tag="PIKHDJKN_01742"

/EC_number="7.3.2.4"

/inference="ab initio prediction:Prodigal:002006"

/inference="similar to AA sequence:UniProtKB:P38046"

/codon_start=1

/transl_table=11

/product="Nitrate import ATP-binding protein NrtD"

/db_xref="COG:COG1116"

/translation="MSLPADQHTIRIEQLRKTYNAPTHGDVHYIIKDVDLVIKGGEFF

VLLGPSGCGKSTLLNMIAGFISKSGGQLKVDNKEIDRPGKDRAMVFQQADSSLFPWLT

VRENVEFGLRMSKVPKSQRRTISDRYIELVGLGSHEAKFPKELSGGMKQRVQLARVLA

NDSAILLMDEPFGALDAMTRRTMQKELVNIWQETHKTVIFVTHDIQEALLLGQRIGIM

SVGPSSKITDIYDNALSYPRNIASSEFNTLYDQIQSHFEE"

CDS 1861194..1861967

/gene="ssuC_4"

/locus_tag="PIKHDJKN_01743"

/inference="ab initio prediction:Prodigal:002006"

/inference="similar to AA sequence:UniProtKB:P40401"

/codon_start=1

/transl_table=11

/product="Putative aliphatic sulfonates transport permease

protein SsuC"

/db_xref="COG:COG0600"

/translation="MRWLEKKWVSIPLLWVTVILIWQLGAMIYGPDVIPGPWDTILGA

RELLADGTLMQYIGISFTRVLAGWVLGSVIAIPVGLIIGKVHMIRLFAEPFLNFIRFI

PPIAFITLFLVWFGIGEQSKIALIMYATFFIVVLNTLTGVLSIEEDKIRSARSMGANE

WQILLHVVVPATTPYIFTGVRLAMGTSYMAIIGAEMIAANEGVGYLIWNSRLFFRTDW

IFVGLISLGFMGFLTDRLFNWFGRSVLYRYGVIGGTKRV"

CDS 1862205..1863014

/locus_tag="PIKHDJKN_01744"

/inference="ab initio prediction:Prodigal:002006"

/codon_start=1

/transl_table=11

/product="hypothetical protein"

/translation="MDWIIGAVCAFIVAGAAYAKKSLTLSGCLAAVIMGTIYYGAGNL

FWFGTLLLFFITSTLFSKFRKDRKQELEKSYAKTGNRDAGQVMANGGIGMFLVLGNWV

FPHPAWMYAFIGVMATVTSDTWATEWGSLSRKPPRSVVTWRVLTPGTSGGVSLLGTLA

AAVGGALIGVGAFFFSWIAGIGSLSLLSWMCVGLVGGLAGAFADSYLGATVQYMYRCT

VCGREVEVSEHCGHKTVRSRGWSWMSNDLVNVLSSLIGGCLAIGLGIILAS"

CDS 1863028..1863606

/gene="fadR"

/locus_tag="PIKHDJKN_01745"

/inference="ab initio prediction:Prodigal:002006"

/inference="similar to AA sequence:UniProtKB:P94548"

/codon_start=1

/transl_table=11

/product="Fatty acid metabolism regulator protein"

/translation="MSTVQGDKSEAILDAAYGIFGSKGFYETKMSDIADEAGIAKGTI

YLYFKNKEQLFIAVSKRDCDSFISRLDHALNSHQSTGDKLGAIAKTHLTYYYERRNHT
KLFFMAPNNDPDLMKFMKAFMNEYMSMVREVLENAGVPEPVLLAEAYIGILDRLKMDI
MLNPEFNEEHLNKRIAFAAALFLDGCRSFLQV"

CDS 1863681..1865615

/gene="taeA"

/locus_tag="PIKHDJKN_01746"

/inference="ab initio prediction:Prodigal:002006"

/inference="similar to AA sequence:BARRGD:NG_063829.1"

/codon_start=1

/transl_table=11

/product="ABC-F type ribosomal protection protein TaeA"

/translation="MNIMTVEQVAKSYGEKILFKDASFGMGDQDKIGVVGVNGTGKST
FLRIIAGMEPADEGQISIGNDVRIQFLAQNPQFNPDNTVLQQVFEGDSPEMKTVREYT
ETMELLELNPSDPALQERLLRLNQEMEQLQVWQMESEAKSILSKLGIRQFDALMGTLS
GGQRKRVALASALIHPCELLILDEPTNHIDNDSVVWLEQYLQKRRGALLMITHDRYFL
DRVANVMLELDHGRLFRYEANYTRFLELKAEREERESASEQKRQNLLRTELAWIRRGA
KARTTKQKARIDRFEELKDQQGPNRSGSLEVSVGSTRLGKKILEIEHLCKSADGRKLI
DDLSYIAVPGDRVGIVGPNGSGKSTLLQMISGKLEPDAGEVVVGPTVNLGYFTQEHQE
MNESLRVIEYIKEVAENVKTADGSLITAAQMLERFLFTPASQWTPISRLSGGEKRRLY
LLRVLMAAPNVLLLDEPTNDLDIQTLAVLEDYLDDFPGVVFVVSHDRYFLDRTVDKVL
SFEGGGAVRVHVGDYSEYAEWMLKNAPGSSQESTTGTAAKVKPATEENKPATSAAKPK
LKFSFKEQREYDQIDENIEKAEANLARINKEMEESFSDSARLQELMAEQAEAERHLDE
LMERWTVLNELAEQIENSKS"

CDS 1865782..1866720

/locus_tag="PIKHDJKN_01747"

/inference="ab initio prediction:Prodigal:002006"

/codon_start=1

/transl_table=11

/product="hypothetical protein"

/translation="MYRELNEQLAVIKEKGRMYEKWNDRLEKLNQEEAEWKRKVQQRL
EHLQKEQNDVDRLNSMTLSAFFYHLIGKKEDRLEKEELELMESKAEYDTACQMLTDIQ
EQRSHVQQELDGQRQYQFWQSDYKVLWGKKENDLLEKDAELQQMAEDREHLSGELQEL

NEADREGQYLLDALERAEKALSSAGNWGVYDMMGGGMISTHIKRSRMDDAQVAIMDAG

RRLRRFQKELEDVKMAVHTELHLGGLLSFADYFFDNLFVDWMVQDKIRKAETQVKDGL

SAVRSTLRVLKNEIREHKVMLELLERKYCEHVERAD"

CDS complement(1866903..1867943)

/gene="ysdC_1"

/locus_tag="PIKHDJKN_01748"

/EC_number="3.4.11.-"

/inference="ab initio prediction:Prodigal:002006"

/inference="similar to AA sequence:UniProtKB:P94521"

/codon_start=1

/transl_table=11

/product="Putative aminopeptidase YsdC"

/db_xref="COG:COG1363"

/translation="MSFTIDESYVLSFLKKLLDTPSPSGYTHDIIEMIRKEAESLNVS

FELNNKGGAVLTLPGQDSSRTIGLSAHVDTLGAMVRSITSYGTLKLTSVGGFTMNSIE

NEYCTIHTRDGKTYTGTILSLHPSVHVYPDARTFERSESHMEVRIDEVVSSKEDVLAL

GISVGDFISFDARAVITPSGYIKSRHLDDKASVAALFGILESAHRENWKPLHNVSLLI

SNYEEVGHGASYIPAEISELIAVDMGAMGDDLSCKETDVSICAKDSSGPYDYDMTTRL

IELAKQASLDYVVDIYPQYGSDGSAALRGGNNIRAALIGPGVHASHSMERTHKDAVLN

TARLLAAYITTK"

CDS complement(1868061..1868450)

/locus_tag="PIKHDJKN_01749"

/inference="ab initio prediction:Prodigal:002006"

/codon_start=1

/transl_table=11

/product="hypothetical protein"

/translation="MKMQWALIAGLVFALLTGIFAVINVDSVQVNLLFNTVQIPLILL

ILGCALIGGIIVGSYGIFRQYRLQRENKQLKLRVSELESSAASSSSSSLDSFKSMDTF

GTIDSDNASVESDSIQSQRKGNPSDTL"

CDS complement(1868586..1870376)

/gene="pepF1_1"

/locus_tag="PIKHDJKN_01750"

/EC_number="3.4.24.-"

/inference="ab initio prediction:Prodigal:002006"

/inference="similar to AA sequence:UniProtKB:P54124"

/codon_start=1

/transl_table=11

/product="Oligoendopeptidase F, plasmid"

/translation="MSQLLKRSEVPTEQTWKLEDLFADQKAWDQEYEEVSSLTKKASE

FQGKLNKPETLKSCFELEDEISLKTERLFVYARMHQDEDTASPTYQNLSQKAQKLSVQ

VGEALSYVTPEILSLPDDQLDALISNEKLSAYKFTLEEMKREKAHVLSQAEEALLAQV

GNLSQAPQTIFSMLNNADLKFPKIKDEHGNEVELTHGSYIQFLENPNREVRERAFKAV

YETYGKQKNTIAAALNANVTKNMFYADVRKYPSVMEMSLYGDNIPKDVYTNLVDTIHE

SLPLLHRYMDLRKKLLGLDKLHMYDLFAPLLDEYKMDITYEEAKQTVKDGLKPLGKDY

ADALQAGYDDRWIDVYENENKRSGAYSWGAYGTHPYVLLNHKDNLNSMFTLAHEMGHA

LHSHYSDTTLPYRDAQYTIFLAEVASTTNEALLMDYLLNKSTDPKEKLYLLTYYADQF

RTTVFRQTMFAEFEKIIHERAEQGEALTPQLLSEIYYDLNVKYHGDSMVVDKDIEMEW

ARIPHFYNSFYVYKYATGFSAATSFSKQILEEGQPAVDRYLGFLKSGGSDYSINILKK

AGVDMSTPEPIREAMSVFKELIEQMEQLTK"

CDS complement(1870577..1870762)

/locus_tag="PIKHDJKN_01751"

/inference="ab initio prediction:Prodigal:002006"

/codon_start=1

/transl_table=11

/product="hypothetical protein"

/translation="MQLDSSNVVIFIILAFSLGVVLILGRNSIPDRLRRGMALIATLL

ICFAFFLIVYFLVNMGA"

CDS complement(1870766..1870999)

/locus_tag="PIKHDJKN_01752"

/inference="ab initio prediction:Prodigal:002006"

/codon_start=1

/transl_table=11

/product="hypothetical protein"

/translation="MIKKHEIYKTDKWNMMTVEVQGKYLVLREISEEWGEECHTFLSR

PALMHWAERRFPKEDFENREDEWRQLMAAFKGV"

CDS 1871163..1871360

/gene="cspD_2"

/locus_tag="PIKHDJKN_01753"

/inference="ab initio prediction:Prodigal:002006"

/inference="similar to AA sequence:UniProtKB:Q45099"

/codon_start=1

/transl_table=11

/product="Cold shock-like protein CspD"

/db_xref="COG:COG1278"

/translation="MKGTVKWFNAEKGYGFISVEGGEDVFVHFSAIQGDGFKTLEEGQ

AVEFEITDGNRGPQAANVNKL"

CDS complement(1871498..1872667)

/gene="yegT"

/locus_tag="PIKHDJKN_01754"

/inference="ab initio prediction:Prodigal:002006"

/inference="similar to AA sequence:UniProtKB:P76417"

/codon_start=1

/transl_table=11

/product="Putative nucleoside transporter YegT"

/translation="MKSSLTARPDQTWLRALMFTIFGSTVLVVSYFQLYFSHLGFSRA

EIGYLYGIGPLVSVFSNMFWSMASDRYKTVRKVMIILLAGQLITGVMLANATTFGQVF

VLVTLFYFFYYPVYPLSDTMAITTASKYGRNFTSIRVFGSIGYAFFALSIGYFLGSFG

PGWTMWVCIALAATTLLIGFRLKDQPSGSSTKMDLSGLWSILKRRDVLTFFGCVFLLA

LGHRMNEAFLTITLKDLGASEGLIGWSLLISSVSEIPVFLLLSRYGNRYKELPLLTIA

ALMYTIRLFLMSISDTPAAVVAIQTMHSVTFGIFYVTAVRYITRLVPDGYRATGMALF

TIVWSSASGLLSGTLGGLLLEYTNRNTFYLTAMAFSLAALVGFSMKLWSSMTSRA"

CDS complement(1872704..1874044)

/locus_tag="PIKHDJKN_01755"

/inference="ab initio prediction:Prodigal:002006"

/codon_start=1

/transl_table=11

/product="hypothetical protein"

/translation="MTSNEPLSWRRLFAFFVPLGISASLVTISHVIINSTLARSAHPE

TVIASYAIAGSLLTLTERPSTLLRQTCSALVRDRLSFQALTFVTKIFLACVLLIGFLI

VYSPIGTGVFKYLFGVSPDLLSKVIDVYEILMYVSIFSVIRNIYQGIIITNNRTKWLT

IGMLFRLAGMYGLSLYFIYTDSIDSGRVGAIIFAVGMMIEALVSFLEGNSIKRKMPAK

LEDHPVENKADVFRFYKPLLLSSFVALFIGPVINIVLGKTTGIALAISSFAVASSLMQ

LMLSFFTYIHQIVLNFYLVDAKLVKKFALVTGFVPFAMTVSIAYTPLGPWVLENVMSV

QGNLLQQSLWTLRAFVLFPLISPFLDFSNGLILLRGQTKTMFRSQTANAICTVIVLLI

LVSIFPAWNGMIGAVAQSLGLLAELVIVWLVIRRTQKEPTMSVPSSGKSSSLKG"

CDS 1874224..1875303

/gene="ytpB"

/locus_tag="PIKHDJKN_01756"

/EC_number="4.2.3.130"

/inference="ab initio prediction:Prodigal:002006"

/inference="similar to AA sequence:UniProtKB:O34707"

/codon_start=1

/transl_table=11

/product="Tetraprenyl-beta-curcumene synthase"

/translation="MSQSNRNRHQYPRGPLALMRGVYKYTIPETRKELDGWRAQAEKI

PNEELRNQALASLRDKQFHCEGGTVYALPDLPNRHILIPLIVSYQTISDYLDNLCDRS

TSMDPNDFRLLHQSMLDAVDPEASPVNYYALREEQDDGGYLRNLVTSCQELTRQLPGY

ASAKPQIQDLAGLYTDLQVYKHIKPELRETALLEWWSEHRHRTPQFRWNEFAAATGST

LGVFMLFLAASDDQLTEEQAASIHTAYFPHVCALHIMLDYLIDQDEDRIGGDLNFCNY

YENVETMLDRIAFIVEMARSDVQKIPGSAFHRMIIEGLLAIYLSDPKVSEQQEVRVVS

KRLMKNSPMTRVFFFIFSRWIRKHM"

CDS 1875358..1876329

/gene="pfkA"

/locus_tag="PIKHDJKN_01757"

/EC_number="2.7.1.11"

/inference="ab initio prediction:Prodigal:002006"

/inference="similar to AA sequence:UniProtKB:P00512"

/codon_start=1

/transl_table=11

/product="ATP-dependent 6-phosphofructokinase"

/translation="MTAVKKIAVLTSGGDSQGMNAAVRAVVRSGLFHGLEVYGVQRGY

QGLLNNDIFPMDLRSVGDIIQRGGTVLQSARCKEFYTAEGQQKGADILRARGIDGLVV

IGGDGSYNGANKLSKLGINTMGLPGTIDNDVSFTDYTIGFDTAVSVVVDAVNKLRDTM

SSHERSSIVEVMGRHCGDIALHAGLASGAETILVPEVPFDMDEVADRMKANFAHGKRH

SIIIVAEGVGKGEDVAKELMERCPTYEPRVTVLGHIQRGGTPTPFDRNLASRLGDFAV

RSLIAGETDKGCGIIKGELTLTDIDKVVNTKKDFDMVTYELAQRLSQ"

CDS complement(1876392..1877759)

/gene="mdtK"

/locus_tag="PIKHDJKN_01758"

/inference="ab initio prediction:Prodigal:002006"

/inference="similar to AA sequence:UniProtKB:P37340"

/codon_start=1

/transl_table=11

/product="Multidrug resistance protein MdtK"

/db_xref="COG:COG0534"

/translation="MNMTTTSFSQKVKQFLIIFLPIFTTQIALSAMSFFDTNMSGKFS

PADLAGVAIGTSLWLPVQTGLSGILIGITPVVSHLLGSKRNDKIGHSVIQALYLGVAV

GIVVLAAGTLLLSPILNGMPLEPRVGQVAFYFLCALAFGVIPLFGYTVLRSFMDALGQ

TRITMMITLVSLPVNIFLNYLLIFGRWGFPQLGGVGAGVATASTYWLIFLISLFFVHR

IEPFAQYGIFRQMPKVSLTKWKELLKIGVPIGFATFFETSIFAAVTLMMSRFDTITIA

AHQAALNFASTLYMLPVSICMALTILVGYETGAGRVRDAKQYSLLGIGGAIGLSLLTA

VVLLLFGEQIASVYSNDHEVIALTQHFLIYAIFFQISDAIATPTQGALRGYKDVNPAL

IITFVAYWIIGLPVGYITATYTSLGAFGYWVGLIAGLAVGATALLWRLFLVQKQASVR

MSEKK"

CDS complement(1877953..1879134)

/locus_tag="PIKHDJKN_01759"

/inference="ab initio prediction:Prodigal:002006"

/codon_start=1

/transl_table=11

/product="hypothetical protein"

/translation="MNMFWALLAMAWGTFSGTPATADQGNQPFQSLTNSFNTAIIQSQ

KDHVIIDADNPPAKIDPQPDAPVVKGIYVTAYSAGGARMTLLLDLMDNTELNSMVIDI

KDDLGYITYPTENKTLQEMGKSQPFIRDIDALMKRLQKHDVYPIARVVVFKDTILAKK

NPELSFRNKDGSVWANGKGDSFVNPYSKEVWDYNIEIAKEAAKLGFKEIQFDYVRFPE

GFETRADALKYTKSDKSRVDIVAEFVQYARKELAPLGVRVSVDIFGYAASVPAAEGIG

QDFVKISQNVDVISPMVYPSHYSTGWYGVKDPDKDPYTTIKGSMKDTHKKLDPTKELK

PVIRPWIQDFTASWLGSGHYIKYGKKQVEDQIRAMKDMDVDEYLLWNASNRYTPDVNY

K"

CDS complement(1879296..1879499)

/locus_tag="PIKHDJKN_01760"

/inference="ab initio prediction:Prodigal:002006"

/codon_start=1

/transl_table=11

/product="hypothetical protein"

/translation="MIIVDMETVKLFEIVNKWCPEMLPFLKPNELDSLIVLRDGLGIL

EQGDAMEIIQYSICEHQNEIYIQ"

CDS complement(1879660..1880481)

/locus_tag="PIKHDJKN_01761"

/inference="ab initio prediction:Prodigal:002006"

/codon_start=1

/transl_table=11

/product="hypothetical protein"

/translation="MPKMIWHFLVIVLGAAAIACGFNWFLVPHQLLSGGVSGISMLLG

YFTNLNLSIMYFVLNIPLLIAGWFILGRRFITLSIVSVAATSWFIGLLPVFAVATDPL

LSCVFGGVLVGLGTGVSFRVGGSTGGLDIVGSIFTRNRDFPIGTVMAGMNGAIIMLAG

YLNDNWNIALASMVSIYITGKVLDLIHISHVKVTLYIITNETEAMLKKLLVRPRGVTK

IKTQGAYTDIEKDMLMTVTTRYELVEIKRIIKETDPNAFVNIVETVGVMGSFRRS"

CDS 1880787..1882373

/gene="cshA_1"

/locus_tag="PIKHDJKN_01762"

/EC_number="3.6.4.13"

/inference="ab initio prediction:Prodigal:002006"

/inference="similar to AA sequence:UniProtKB:P96614"

/codon_start=1

/transl_table=11

/product="DEAD-box ATP-dependent RNA helicase CshA"

/db_xref="COG:COG0513"

/translation="MTNLNFTDFNLEPLVLQAITELGFEEATPIQSQAIPLALEGRDL

IGQAQTGTGKTAAFGIPLISKITKSDEKIRALIMAPTRELAIQVAEEIEKLTRFKGLR

SLPIYGGQDIVRQIRALKRKPQIIIGTPGRLLDHINRKTIKLDDVQTVVLDEADEMLD

MGFMEDIQSILKQVPDERQTMLFSATMPPNIQKLAQQFLNNPEHISVIPKHVSAPLID

QSYIEVPERQKFEALSRLLDMESPELAIVFGRTKRRVDELAEALQKRGYSADGLHGDL

SQNQRDAVMRKFRDGSIDVLVATDVAARGLDVSGVTHVVNFDLPQDPESYVHRIGRTG

RAGKEGAAWSFVTPREIDHLHFIERVTRHRIPRKPLPTMAEAVEGKQRLTAERLLEIV

QSGELNEYKGIAIQMLEQYDSVQLLSAALKLLTGDKKDAQVDLTPEDPIRAKRRKPDV

RSGGRKPSGYSGNRSSSGGGGSYNRDRNSSGGGRGGYNRDRNSSGSGSGSREGGYNRD

RKPRPSNNEGRRPAKDSSFE"

CDS 1882557..1883393

/locus_tag="PIKHDJKN_01763"

/inference="ab initio prediction:Prodigal:002006"

/codon_start=1

/transl_table=11

/product="hypothetical protein"

/translation="MEFKGAMGGIYRLTEWITRLAATNLLWAICSSPFLFFLFMKLLV

MQQNLANESLQMNWAIAIVAPLTLFPATSALFTVVRKWNMGDTDVPIFRTFFVGYKEN

YKQSLIGGIFYTLLFVIMYLDYTVYMTQFKNMQLVGIIMLVLLLLLFVSLFNFFSMVV

HYHMSIGLIIKNAVLLTLIRPFRVFSTLLGSGLLFYIGFRYPVLFIFFIISIVAWFAF

FNFYATFNKMQEQMEKMQLKKEEEEAAQAAEQAGESVDTIETAEPSKLKPSDEKHDNL

QK"

CDS 1883823..1884215

/locus_tag="PIKHDJKN_01764"

/inference="ab initio prediction:Prodigal:002006"

/codon_start=1

/transl_table=11

/product="hypothetical protein"

/translation="MTLKRTLVGIIRSMEGTSDRAKDPKLKTRYYNLSKDRAWEEVSS

TLKKIPGYKVLHEVPSVGEVILEKRTTFGRTMDITVSIISVSPVRSAVDMYSASRGSL

GDLGSNYRTIMNLFSVLDKKLSKYKSND"

CDS complement(1884289..1884807)

/gene="tpx"

/locus_tag="PIKHDJKN_01765"

/EC_number="1.11.1.15"

/inference="ab initio prediction:Prodigal:002006"

/inference="similar to AA sequence:UniProtKB:P80864"

/codon_start=1

/transl_table=11

/product="Thiol peroxidase"

/db_xref="COG:COG2077"

/translation="MAQERTGAATFKGNPITLIGPELKVGDQAPDFTLSKNLVEDASL

KDFAGKVKLISVVPSLDTGVCDAQTRRFNVEAGDLGDNVVVLTVSVDLPFAQARWCGA

AGVDRVVTLSDYKTRSFGEDYGVLIKEFQLDMRSIFVVDAEDRITYVEYLPEMTDSPN

FEQAISAVKALL"

CDS 1884956..1885582

/gene="gluP"

/locus_tag="PIKHDJKN_01766"

/EC_number="3.4.21.105"

/inference="ab initio prediction:Prodigal:002006"

/inference="similar to AA sequence:UniProtKB:P54493"

/codon_start=1

/transl_table=11

/product="Rhomboid protease GluP"

/db_xref="COG:COG0705"

/translation="MIFIRYENWKSYLKFFPLTSILLVANVVMFIVLSLNGGSTNSMT

LLKFGALVNHELFAADWWRYITSMFLHAGFSHLLFNSFALIVFAPPMERLLGSVRYGV

LYLGGGILGNILAVAWYNSVGATTISVGASGAIYAIYGAFLYVALFQRTMMDETSRKT

LYTLLVFGIIFSFAMTGINWMAHLGGLLGGFFIYGLLIRLWKPRSFKQ"

CDS 1885635..1886564

/gene="gltC_3"

/locus_tag="PIKHDJKN_01767"

/inference="ab initio prediction:Prodigal:002006"

/inference="similar to AA sequence:UniProtKB:P20668"

/codon_start=1

/transl_table=11

/product="HTH-type transcriptional regulator GltC"

/translation="MERSGAVELRQLHYFLKVAQKEHVTQAAEELHVAQSAVSRQIHQ

LEEELGVDLFMQKGRNLQLTAVGQLFCKRVEGILKDLDKAVGEVHEFLDPEHGEIRIG

FPHSLGIHLIPSVVAAFRQRYPNVKFRFKQGMFPTLIRDVLSAEVDLAFISPFPEKHD

QVDGDIVLTEELHAILPPNHPLAGEDSIALEQLKDDKFVLFSKGYSLRPIVWHACLEA

GFTPKIAFEGEETDTIRGLVAAGMGVSLLPEMALFQTNPLQPAHVAISHPKVTRTIGL

IHRADDKLPLVAQSFRSFLLNYFGLQQNNTPSD"

CDS complement(1886598..1887278)

/locus_tag="PIKHDJKN_01768"

/inference="ab initio prediction:Prodigal:002006"

/codon_start=1

/transl_table=11

/product="hypothetical protein"

/translation="MSFNNGMFVLIIIAFLLSLWAQFRVKSTFNRWSDVKNLNGMTGY

DAARHMLDSNGLHDVPIEPVRGALSDHYDPINRVVRLSEPVYYENSISAVSVACHEVG

HAIQHKESYPMLALRHRIFPIVNFASGLAPFLLIAGFIFNAMNLVGIGIIFFSVTVAF

QLITLPVEFNASNRAREIMVSEGYIRNEEERGVAKVLNAAALTYVAAALISLLELVRY

IGIFNSRD"

CDS complement(1887308..1887808)

/locus_tag="PIKHDJKN_01769"

/inference="ab initio prediction:Prodigal:002006"

/codon_start=1

/transl_table=11

/product="hypothetical protein"

/translation="MQSRNWNLQFPTFELASEVNIIGDMKLFRIGELAKTAGVSERTI

DYYTKLGLIAPEERTEKNYRLYSNETLTRLERIVHMKQEKYSLDEIKQSLEKWSLVST
EEQVASKLTTLELHVQQLEREVNELKPLLGEMKPVQARKMMAGLLAKSAGTMEALKIL
LENTMM"

CDS 1888091..1889491

/gene="nrgA_2"
/locus_tag="PIKHDJKN_01770"
/inference="ab initio prediction:Prodigal:002006"
/inference="similar to AA sequence:UniProtKB:Q07429"
/codon_start=1
/transl_table=11
/product="Ammonium transporter"
/translation="MRKKWLVSVLIMLTLLAFPVSAFAAAEGPTNIELQSGLNSAFTF
LAVVLVFLMQGGFALLEAGSTRMKNAGHIAGKTILTLGISVIAFWALGFGLGFGNGNS
FFGTTGFFLSGDGMAASFESLAFSDVPLTIKFVFHLAFAAVSLAIACGGMAERAKMSV
YIVFGTLYTIIMYPVVAHWVWGGGWLAELGMQDFAGSTVVHLTGATAALVATILLKPR
IGKYNKDGKPNIIPGHNQVYSVLGVIILWIGWFGFNPGSTLSAMGDGFFGYVALTTNV
AAAAGGVAALLISWAVLGKSDIPSMLNGVLAALVAITGACAFVEPWAALVIGALAGII
TFFTAQFFERKGIDDPIYAFSVHGIAGMWGAISTGLFATPELAENAGVGQAGLFYGGG
FHQLGVQLLGLAGAFAFVLIMSFIILGGMKAVMGIRVTEEEETMGLDLSEHGTYGYPE
QMKNVETKSNGGTFSS"

CDS 1889534..1890619

/locus_tag="PIKHDJKN_01771"
/inference="ab initio prediction:Prodigal:002006"
/codon_start=1
/transl_table=11
/product="hypothetical protein"
/translation="MEPIPYINQSWSYQGIDGAASSQELRQARVILQNELQELLSASL
SPIEWYQTVNDLHDRVARRAVELCIQGMVEEGLGRPPVPYAFIVFGSSGRQEATLWSD
QDNGMIISDIPHKGKETYFAELGLRMTDMLEVLGYAKCEGKVMCSEPLWRKTLESWKE
QLIAWGSDLSWEPVRNLIIASDMRFVAGDPELAEEWISAFYEGFRSVPELSDAVLRNT
VKHKATLNILGRVVTERFGEHAGGFDVKYGLYIPLVNSARFLALQHGIKETSTLKRIQ
RLVSLEAVPLTLLDAAQRSFMTALKLRRSTPIVVKQGLQHSGGFLNEKQLKEKQIHYE

LRDTLGLVRRVHRALQRQLRFAERRRP"

CDS 1890616..1891353

/gene="dinG_1"

/locus_tag="PIKHDJKN_01772"

/EC_number="3.1.-.-"

/inference="ab initio prediction:Prodigal:002006"

/inference="protein motif:HAMAP:MF_02206"

/codon_start=1

/transl_table=11

/product="3'-5' exonuclease DinG"

/translation="MREPARGNTGFWNSLRQGGVPSAIASIMGAPTAQHMAFIRSMMR

EQRRPEVLHTPLNELNAVVFDLETTGFSPQHGDEILSFGAVRINGGVMLENEQFYTLV

QSKVSVPEHITELTGITQEMTMDAPSLLEGLHDFMSFVGGSVLVAHASAHDRAFLNAA

LWRTSKVRLTHRLIDTMMLARWLEPNRPGYGLDELLESKSIPIYGRHHALEDAKMTAQ

LWSCYLEDMSRKNVETLGDLYTQLSHA"

CDS complement(1891339..1891827)

/locus_tag="PIKHDJKN_01773"

/inference="ab initio prediction:Prodigal:002006"

/codon_start=1

/transl_table=11

/product="hypothetical protein"

/translation="MTAPHGQQNTFYIPSSVEQEMSKYMFASLPQETCGVVLGEAAAG

GIRISRFQPIRNVAPDPLHHFALDDAEWIRCVFNEPQLIGLFHSHPRTTPVPSNEDLR

NLPIFAGLLQVYFIGSPDQTTGTQSKMLLKGYQIHASIDSSIGMSHVQSYSLQPAQLC

VT"

CDS 1892012..1892563

/gene="resA_1"

/locus_tag="PIKHDJKN_01774"

/inference="ab initio prediction:Prodigal:002006"

/inference="similar to AA sequence:UniProtKB:Q81SZ9"

/codon_start=1

/transl_table=11

/product="Thiol-disulfide oxidoreductase ResA"

/db_xref="COG:COG0526"

/translation="MKRNIYILLGVVLLVGIALAQNADNGIAAVFKQEEPMPTETGPK

AGLLAPAFSLQAMDGKTYAVGGAKDKAIFVSFWASWCEPCKQEAPALNTLAAKYKDKL

DLYGVNVTTYDKIRDAKAFVDEYKLTFPILLDEDGTAYAKYNGLAFPTNVLIDSRGVV

QEIILGILPEKELERKIKELIED"

CDS complement(1892658..1894277)

/gene="cimA"

/locus_tag="PIKHDJKN_01775"

/EC_number="2.3.1.182"

/inference="ab initio prediction:Prodigal:002006"

/inference="similar to AA sequence:UniProtKB:Q74C76"

/codon_start=1

/transl_table=11

/product="(R)-citramalate synthase"

/db_xref="COG:COG0119"

/translation="MSKAISIFDTTLRDGTQGEGVSLSADDKLKIAKKLDDLGAHYIE

GGIPGSNTKDIEFFKRVKELNLNAKVVAFGSTRRKGSIASEDANLKRMIESGAQAATL

VGKSWDFHVHTALQTTLEENLSMIYDSIAYLKQNGMEVIFDSEHFFDGFKHNPEYAQA

VLTKAHEAGADWLVMCDTNGGTMPHEVYEIVSTLAGRLPQAHLGIHTHNDCELAVANT

LSAVQAGARQVQGTMNGYGERCGNANLASIIPNLQLKLGYECVSEDSMRQLTNVARYV

SEIANVNMPINQPYVGNAAFAHKGGIHVSAILRDSRTYEHIVPELVGNKQRVLVSELA

GQSNIVSKAQELGLEFDPSSANSRQIIEKIKDLEHQGYQFEGADASLELLIREANGDM

KELFTFESFKMLVEKVAGKSVVSEAFVKLNVGGTSAYTAAEGNGPVNALDNALRKALV

QYFPSLANMHLSDYKVRVLDEKDATAAKVRVLIESKNTENTWNTVGVSENVIEASWEA

LVHSFRYALLQEKLQDEPGVVNIPAHGLSNH"

CDS 1894623..1894730

/locus_tag="PIKHDJKN_01776"

/inference="ab initio prediction:Prodigal:002006"

/codon_start=1

/transl_table=11

/product="hypothetical protein"

/translation="MHFVWENTIISFIIIAIIVVAVWGIIRSIVKSLRK"

CDS 1894826..1896130

/gene="dinB_1"

/locus_tag="PIKHDJKN_01777"

/EC_number="2.7.7.7"

/inference="ab initio prediction:Prodigal:002006"

/inference="similar to AA sequence:UniProtKB:Q47155"

/codon_start=1

/transl_table=11

/product="DNA polymerase IV"

/db_xref="COG:COG0389"

/translation="MSSDETHNNINVDRYYPTAGRVILHVDMNAFYCSVHEAEEPELY

RGKATAVAGSSEVRKGVIVTCSYTARNRGISTGMVVHQALKKCPDLIVIRPDFHLYRR

YSKEFMKIAYSYTPLLEATSIDECYLDITGSRQFGTPLEIAESIQTRIRDELGMPCSI

GIAPNKLLAKMASDLKKPNGISILRMRDVPQILWHRPCNEMFGIGKKTAEKLKKLGIE

TIGQLAKSDERMLTDVFGINGSWLKNSANGINHSAVQAEREANKSIGHTTTLPADISE

MDDVHRVLLNISDQVARRLRKHEMLSQGIQITIRTPDMKTITRSRMMEVPTEDASIIY

REACALFAKHWGGGKPVRMLGVTLQTLIPREESVIQLDLFEYEQKPKKENLIRIMDQL

RDKFGENAVVTAGMLGDDPSVLLRNHKVRGTSLQKDNLQSPD"

CDS 1896250..1896492

/locus_tag="PIKHDJKN_01778"

/inference="ab initio prediction:Prodigal:002006"

/inference="similar to AA sequence:UniProtKB:P10245"

/codon_start=1

/transl_table=11

/product="Ferredoxin"

/translation="MSKFTWVEKDTCIACGACGATAPDIYDYDDEGLAEVIFDGDANK

GIKAIPDDLFDDMQDACDGCPTDSIKVADEPFNKEG"

CDS 1896664..1898103

/locus_tag="PIKHDJKN_01779"

/inference="ab initio prediction:Prodigal:002006"

/codon_start=1

/transl_table=11

/product="hypothetical protein"

/translation="MQNTHLRTYVKKHPDNKMAWYLLGKEYLGEGQEAKANYCFQQAG

EVYEAFERSKAPADIWVDYQEKLVEMAEQKEKKHRVRKMWLTLLMLLVLAGLPPADAP

GFNRDAAEALASALEPMDDSADNESGNSPKEDQALITNSNVFTAGAFGGGDQGEAALA

AAWSGSGVKVQTSAVLGMQVAGNWTLWKRNMSVKYIVQTNSSGKLTAQSYDAKQCNCK

PPEVSPEIKRLAQAWTAKQESAAALSSAMITYKKQNGSWPKSVAQLAQPFPNNILGET

APGMTRMFPTLLAMHQQGIMGKGKNTDEGTGESSDSPQGKKAAFVDTLGGKPFLQDRL

EIIVDKSRHKLALISGNTIIRNYEVGLGGDRTPEGSFVISDKVVNPNGRSNGEFGSRG

MQLSDTNYAIHGTNEPNSIGLDESLGCVRMSTEDVEELFALAPQGTPVRIGKDLLPET

AIVPEAKDRYRYTLVPKQNNPNKTYHWLN"

CDS complement(1898190..1898327)

/locus_tag="PIKHDJKN_01780"

/inference="ab initio prediction:Prodigal:002006"

/codon_start=1

/transl_table=11

/product="hypothetical protein"

/translation="MAPKRKPPTLQQKKDEVNRKAIIWTAASVGGLIIIIGALIIIAN

L"

CDS 1898427..1899680

/gene="pyrD"

/locus_tag="PIKHDJKN_01781"

/EC_number="1.3.5.2"

/inference="ab initio prediction:Prodigal:002006"

/inference="similar to AA sequence:UniProtKB:P9WHL1"

/codon_start=1

/transl_table=11

/product="Dihydroorotate dehydrogenase (quinone)"

/db_xref="COG:COG0167"

/translation="MSILHNTIPSVYAQTTFLLKKKRIDLCIECFFTYEKDGKHFFSV

PDQNGVSDLLYRSLAKPLLFKMDPEQAHHLIIGGLSGMGSIRPVPSGLRVMYGVRETS

DLAVDMFGCHFPTPVGLAAGLDKNGQAVTGFSSIGFGFMEVGTVTPLAQPGNDQPRLF

RLPPDEALVNRMGFNNLGAEAMAVELARLKERRIPVAVNIGKNKATPNEEAHLDYAKC

IRALYDYADLFVVNISSPNTPDLRNLQHGNELKELLAAVMNEMETQRNRAGGSVKSVL

VKIAPDVNDQELEYMVSTIADSGVAGIIATNTTISREGLSHQHAKETGGLSGKPLRDR

STEIIRQIYRQTEGKLPIIGSGGIFTSEDAYEKIKAGASLVEIYTALIYEGPEVNRRI

HAGLRELLRKDGYSHISEAVGAEHR"

CDS 1899816..1900631

/locus_tag="PIKHDJKN_01782"

/inference="ab initio prediction:Prodigal:002006"

/codon_start=1

/transl_table=11

/product="hypothetical protein"

/translation="MDGRDWGTFLLPYEQAVEELKVKFKTMRAELKKREEYAPIEFVT

GRVKKISSILEKSRRLNVPLDQVETGIEDIAGIRIMCQFVDDIRRVAEYIRGRKDLTV

LIEKDYITNFKESGYRSFHMIIEYPVQTALGQKNVLAEIQIRTLAMNFWATIEHSLSY

KFRESLPDDMRARLKKTAEAAFVLDNEMSAIRLQILEAQKAFEDDSNIVSRTLNIIHQ

LYFYHLVNEAIEAQKRFNDCWERHDMEGLKDLLDDVKALLNAARKGENPDEQL"

CDS 1900618..1901112

/locus_tag="PIKHDJKN_01783"

/inference="ab initio prediction:Prodigal:002006"

/codon_start=1

/transl_table=11

/product="hypothetical protein"

/translation="MSSYEPLYVDYLIYFNRDQDYFECHEVLEELWLERDRDSLYKGL

LQIAVGLYHFRNDNLRGGRMMLQSSVDLLKSYPETSRGISLGPLVQEVQELVHGLSGP

EPQKFPYRDLHIQIQDEVLAREVRERALELKPNIPQRRSPTRGRIYEEKMKALQQEQN

LQSE"

CDS complement(1901323..1901871)

/locus_tag="PIKHDJKN_01784"

/inference="ab initio prediction:Prodigal:002006"

/codon_start=1

/transl_table=11

/product="hypothetical protein"

/translation="MKSKKKKKSAAILIFLGIFVVLIAALVLVNQQSKKQMDAVGNAY
GIAASKLNPATRELLDDPNYQQIILPDDLKARIDNKDSFFVYFFASDCSHCRATTPQL
MPLVDEEGIQLPQFNLREFDSGWTDYNIEFTPTLVYYQDGVEKERMVGGLQENGSDNG
YTLDDYKQFFEKYKGSTTPSAS"

CDS 1902092..1903780

/gene="rsmF"

/locus_tag="PIKHDJKN_01785"

/EC_number="2.1.1.178"

/inference="ab initio prediction:Prodigal:002006"

/inference="protein motif:HAMAP:MF_01579"

/codon_start=1

/transl_table=11

/product="Ribosomal RNA small subunit methyltransferase F"

/translation="MAVQLPSIFAERMKSLLGDEFEQFMKSYEQSPHAGLRVNTLKIS
LEQFKEIAPFDMRPIPWCGTGFYVPHGVKPGLHPYYHAGLYYIQEPSAMAPVELLNVK
PGERVLDLCAAPGGKTTQIAAKLQGKGVLVTNDIHAERTKALAKNVELYGVRNAVVLN
ESPERIANAFPHYFDKVLIDAPCSGEGMFRKDEDMVKSWENHSVEKCVLMQRDILETA
AKLLAPGGTIVYSTCTFAPEENEAMIAEFLNINPDFVVNDIPESAGFAPGRPEWVRQM
LPEKAEETGSVLDQTRGTARLWPHLLEGEGHYVAVLQHRGESLAEEDGNEEEGSVLQI
GQAVGADFTEPSESVDPFIRISAPISKEERKRERLMRTESRERNDRHTLGGKAQGKQG
KGNERGGRKNERAQGRGTDQANVDPGTVYAQFLQENLEIELAGEPVYYGDRVYQSSVG
AARLEELKVIRPGWFMGTVKNGRFVPSHPLACALNAAEAQRSVNLSSAEGEAVRYLKG
ETLNIEEARVDRQADTVAKGFVLVCVDGFAAGWGKWLDGVLKNEYPAGWRWTSV"

CDS 1903777..1904523

/gene="rluB_1"

/locus_tag="PIKHDJKN_01786"

/EC_number="5.4.99.22"

/inference="ab initio prediction:Prodigal:002006"

/inference="similar to AA sequence:UniProtKB:P35159"

/codon_start=1

/transl_table=11

/product="Ribosomal large subunit pseudouridine synthase

B"

/db_xref="COG:COG1187"

/translation="MSGKGKQTLRLDKILSHMGVGSRSELKKMVKQGRIRVDGKPVKD

SGVQVNPDVNVIEADGERIVYREMIYLMLHKPPGVVSATEDNRDKTVIDLLRKEERVF

DPFPVGRLDKDTEGLLILTNDGPLAHDLLSPRKHVPKTYEARVLGNVDEEDIEKFKAG

IQLDDGYETLPAELTVLHREETEEGVFSSISLIIHEGKFHQVKRMFQAVGKRVVYLRR

VAMGELELDANLEIGSYRELTADELDRLRK"

CDS 1904788..1905591

/gene="ywpJ_2"

/locus_tag="PIKHDJKN_01787"

/EC_number="3.1.3.-"

/inference="ab initio prediction:Prodigal:002006"

/inference="similar to AA sequence:UniProtKB:P94592"

/codon_start=1

/transl_table=11

/product="Phosphatase YwpJ"

/db_xref="COG:COG0561"

/translation="MTIKLIALDVDGTLLNDHHELTEMTQETLIRASRQGAEIVLCSG

RGPANTIPFMEQMGLDGYVITHNGAVTAQVQTREVVHHFAMDGQGLEPFISYCRTQGV

HFDINTAFGLYVDQPEGLELQVREMYQNFMAEPLKLPQWADMTEPLAKFTAFGPIEQM

NAVQQEWSTWNLPYYMTRSGDFFIDLMHPEASKGAALKRLAEAKGILPSEIMAVGNYY

NDITMLTFAGKGVAMDNSPEEVKAAADEVTLSNNDQGVAHAIQKYVLSV"

CDS 1905690..1906745

/locus_tag="PIKHDJKN_01788"

/inference="ab initio prediction:Prodigal:002006"

/codon_start=1

/transl_table=11

/product="hypothetical protein"

/translation="MELKVELIPDLRTSGGEVQDIMVDGQYVGSLLLVFREGDRVSGS

LQIEQNSLPDETEEYIVEQVHEYIRSLADAVSAAEYEVLVSCGTLYSVLQKPESQTDV

FIQSSRSEDKYSHDGKSDRAGVNEVHVISGSQGESFTYEDPEHLLEMELVSAGRSIST

YVFNDANGQELAEASLKQYGADVQGEIHWYDEPAESYQDAAAELLVRELDEEIIDTIT

IRMWHQGQEMESAEWVHRDFADEDEIESEEELEEIEAAEEACYVMLVRKDREFRVYEL

FLQERGGLPVGTATIDTSEADLSGYIDYQVPGTSVQRKSLVEVLMRELDKELEFDTLH

LTMLYRNQIIDEAKIEG"

CDS 1906847..1907401

/locus_tag="PIKHDJKN_01789"

/inference="ab initio prediction:Prodigal:002006"

/codon_start=1

/transl_table=11

/product="hypothetical protein"

/translation="MKHSFSITTDYINDEFKALQAQPEDTEDVMSLLMETAEWLQSQG

SSQWNALLKGEDSHNTAEAIQRGDVFVFKKGSDVAGMVILMSQPSAWDVHLWGSKAHA

GDGALYLHRLAIRRKYAQSGLGRAILQWSSSGIQFEDKHIVRLDCGAENATLNAFYGR

NDYTFVGETNGFSTYQKAVTHLSV"

CDS 1907847..1908977

/gene="mshA_4"

/locus_tag="PIKHDJKN_01790"

/EC_number="2.4.1.250"

/inference="ab initio prediction:Prodigal:002006"

/inference="protein motif:HAMAP:MF_01695"

/codon_start=1

/transl_table=11

/product="D-inositol-3-phosphate glycosyltransferase"

/translation="MRLLQALFFPPEQPGGVSSMIPYMQERFTTPRWEMDLFSLPKRI

RNKGREDIQFETFDWTQYQDSPVVQKYMQTYRDYLWWTKLRIQKPYDLIHAHHPIAGL

AMKKVFPDVPLIQTIHSSYERELILNGRIEPNGPEHQFLLAIYGELEHQAERLLTVSD

SFRRYLAPYVQHPDVIGVIPNGFDEKRFKPIPHENAIPQLVTVCRLVPAKGLDILFKA

CAELKARGQDYVLHIIGDGPIRPDLEELAQRLGIYNETIFYGYTLHPEEFMPFFDIFV

LPSRAEAFGSVFAEAALSCLALVGTDVGGIPEQIENGSNGLLVPSEDPFALAEALEKV

MVDPAYRYELARSACEKAKTHYSLGRSVNELKKMYLQFPSQF"

CDS complement(1909069..1910127)

/locus_tag="PIKHDJKN_01791"

/inference="ab initio prediction:Prodigal:002006"

/codon_start=1

/transl_table=11

/product="hypothetical protein"

/translation="MKQAFRVLQIAFTYIGTVVGAGFATGQEILQFFTQYGKWATITI

GLSTILFVWLGTKMMLIAHDTGSRSYEDLNKHLFGHKAGNWITWVTLLILIGVNSVML

AGAGSVFVEHLGLHYQTGLIVTLIGTYLLLGRGIQAILQMNSIVVPMMLLLSLLMITS

TIHHPGANRFITLTTDSNPIQVWLSPLLYTSFNLALAQAVLVPVGNQIRSRKVLKWGG

VLGGIGVGFMLMAAHFAMSAQMPGITQFEIPMGSIAFQLGWVVQSIYVSLIFMEIFTT

FVADIYGMTLQLRQHVHVHPKLIILAIMLLCYSLSQFGFSSLLSILYPIFGSLALIWG

AKLVMDRWGSFRGRKKHL"

CDS 1910332..1910913

/gene="xpt_2"

/locus_tag="PIKHDJKN_01792"

/EC_number="2.4.2.22"

/inference="ab initio prediction:Prodigal:002006"

/inference="similar to AA sequence:UniProtKB:P42085"

/codon_start=1

/transl_table=11

/product="Xanthine phosphoribosyltransferase"

/db_xref="COG:COG0503"

/translation="MEVLKQRILQEGVVMSDQVLKLDGLLNHQIDPALTMEMGREFAA

RFRESGVTRVITVESSGIPVAFAAAHELGVPLVFARRKKTLLADPDAYCERVPSFTKG

IVTDIMVSREYIHENDRILFIDDIIANGDAARGVIKIIERSGAELVGFGVVVEKCFQA

GARTIREQGIPVEALVRIRSLNDGTVQFDDNEL"

CDS 1911012..1911341

/locus_tag="PIKHDJKN_01793"

/inference="ab initio prediction:Prodigal:002006"

/codon_start=1

/transl_table=11

/product="hypothetical protein"

/translation="MGNQPATEQFFIDKLAESKVHFERALDCKHTEFDDLYPYMIEHP

QFFWYKRYVAWSELLTIVGLCEELSFSWKEQFTPHQVEYLEERVMSAKVLDFWFEKND

SKEHAQR"

CDS 1911465..1911683

/locus_tag="PIKHDJKN_01794"

/inference="ab initio prediction:Prodigal:002006"

/codon_start=1

/transl_table=11

/product="hypothetical protein"

/translation="MISDAQLDALRLSGEKVRVVRDEIQSNDVTGIVVAWDGEQVLIR

RQNRRVVKLDRNYQYQLFSEPRKSPLEE"

CDS 1911801..1912130

/locus_tag="PIKHDJKN_01795"

/inference="ab initio prediction:Prodigal:002006"

/codon_start=1

/transl_table=11

/product="hypothetical protein"

/translation="MTLMNDKVHAYKDQIGELSDVLPAVVKSYHEFTGECFQAGAIDA

KTKQLIALGIGLFANNEVCTFYHVEEARAKGATDQEIMETVAVAGAVGGGHALSQGAM

RVQKALH"

CDS complement(1912230..1912694)

/locus_tag="PIKHDJKN_01796"

/inference="ab initio prediction:Prodigal:002006"

/codon_start=1

/transl_table=11

/product="hypothetical protein"

/translation="MKKLIISIFGAAVLFTSGAASTEASSINTVVNNMTGIPYKWGGT

TMAGFDCSGFMRYIFNKYSIELPRTSQQQAKAGTPVSKANLRTGDLVFFNTMGNGISH

TGVYIGGGQFAHASSSKGVSITQLSNPYFNDRYVTARRVTGQFMYNKMLGKI"

tRNA 1913789..1913872

/locus_tag="PIKHDJKN_01797"

/product="tRNA-Leu"

/inference="COORDINATES:profile:Aragorn:001002"

/note="tRNA-Leu(cag)"

CDS 1914199..1918095

/locus_tag="PIKHDJKN_01798"

/inference="ab initio prediction:Prodigal:002006"

/codon_start=1

/transl_table=11

/product="hypothetical protein"

/translation="MKRIGKRMAMCFMVIMIAAVQFGIIGTTGNVAQAADKSLAQKPY

MGWSSYSMQVYDGAGNWTSAESIKKQSDAMREKLQAHGYEYINIDAGWNGDEDEYGRP

IPSEVLYPNGFQEVIDYVHNNGQKIGIYLIPGLSITAYNKNLEVYGTGGACRMQDIAV

QPLVIMDAWDSYTYKIDFTNPCSQKYIDSIADLLGEWGINFVKFDSVTPGSGINNLSR

DARGDVEAWSKALDRNNIWFELSWALDHNYVDFWKKYANGWRIDWDVEAYDSSKGLTE

WSSISRLFPIAAIWWRDAGPGGWNDFDSLNVGNGSMDGLTKVERQTATTFWSISSAPL

YTGNDLTRLDSYGLELLTNDEVIAVNQAGRPGHPVSMETQQQVWYANNGDGTYSVALF

NLGDQSAEVKVKWSDLGLNGPASVRDLWSRSELGTFNKEFSGGVLEPHASRMLKVTAL

KGTSAVNDDDTGMLYSGDWQRNGGKEQTDGRQDLSIAIKDSSGTGPTNSSQAGALDAE

ADNAANSISVEDATVTNEVYINDDDSSIKYTGSWNSNTGRSYGDYNGDVHFTEKDGDY

FEYSFTGTGIELLTEKDKDQGDMIVTLDNGAPETVSAYTSGEREVQQVLYSKLDLDNG

PHTLKVVKGSGKYMLLDALRVTMEIPPGGQNSTISPASADFDKETEQQKDVTVRLSLN

GNTLTGVENDGVLLSEDDYTVVNDKLTIKKQYLTQLPIGTTSLNVIFSAGDPQSLAIR

ISDTTGIRFIMINNDDPAIKYNGSWSRSTGRGLGDYKDDVQYTEKNDDSFEYTFKGTG

IQLFTEVDQSQGDMDIYVDDQFKETVSAHRNGRLAQQNLYSISGLPDGQHTLKAVKKS

GDYMLLDMLKVELPNLINPVTADFDKSASAQADIDVTLLIQAESFKGITNGSNELVRG

TDYTINGDHVTLSKTYLAAQPVGTLNLSFSFGGDYLNDVHSTTVDGDFFEYTFKGTGI

SLITPTGPEQGEVDIYLDGQLVQTVNAYSPSRQIQQEIYSISGLASGQHTIKVVKKSG

ELMLADQLKFTVPTGNGNGEPTNPSNPGGPSNPGGPSTPVVQPSDTSVPAPTPESGTT

DGTTGTEGGQDIGYKAYIQGYPGGLFKPDNKVTRAEMATILAMVSEKEATGSAVAFSD

VKSEYWGADAIAKVSKMGLMNGYKDGTFKPNQSLTRAEMASLVALLGPDSGTPGNGFS

DIGSHWAKEAILKAQSAGILKGYSDGTFRPNALLTRAEAVVAINRALGRAPLTNVSQP

QWKDVPSTHWAFGDVEAASVDQVVKPRTEGEEPKKK"

CDS 1918418..1920628

/gene="yesS_2"

/locus_tag="PIKHDJKN_01799"

/inference="ab initio prediction:Prodigal:002006"

/inference="similar to AA sequence:UniProtKB:O31522"

/codon_start=1

/transl_table=11

/product="HTH-type transcriptional regulator YesS"

/db_xref="COG:COG2207"

/translation="MSYLTSISVTQSISIENSVTQLQKSQEILEGRMAEVEGFTRQLA

VNQELNVLMNERDNETNVYGIWRTMENVLTFGQTNDFLQNYYIYLANYNLILTPGSSY

RPNHYYTDFRYNDLSLEEWMKDVLGKTHRSEIKPLSSYVSRGKQTSVITYMQSLPLDS

FNDSSPAVAVVIIDEKMILSLLSSLNDRYGGWVHISDAEGNTIALQGSSEPEMVKMAS

DSHFDPSRVSQFYGDDLVITTRSDKNGWVYQAGIPRHVLMENANRIKGMTMLITGGTL

LIGLIVGLVLAYRNSVPINRLLSVMKEQFGKDESAPKNEFDFLSGNIADMLTKNKLLE

SELNRQLPLVRDAFLKRLISGEFKSRDEILSAAEQADINLYQGTGYAGIVQIKGYASM

DSVEILNELNASRLLLKQALAELDVDVLMTDMGSDKVVILFFSKETDSEMDREKEDIT

HIMENLAQYVFNEYRMTIQSGFGNLFTSLTEVSLSFDQANQALEYAVYMNKKGMMWNN

EAQTENTTYYYPLDSEQRLIGTIRAGELEEAKRIVDAIIVQNMEQRELSMEMKHQLVG

EMKGTFLKLLDQKAFTEYASIENVKRRVIDIGSSEPLETIKAEFYEIMEELCSVIANK

KKDAHIQIIKQIKEYTAKMYSDTELTLYRVAEHVERPEKYISQLFKEFTGVNFSDHLI

KVRMDQAVILLKESNYTVDEIASRVGYNSSHSFRRAFKRLNGVSPSVYRQSVDD"

CDS 1920958..1921839

/gene="yteP_10"

/locus_tag="PIKHDJKN_01800"

/inference="ab initio prediction:Prodigal:002006"

/inference="similar to AA sequence:UniProtKB:C0SPB3"

/codon_start=1

/transl_table=11

/product="putative multiple-sugar transport system

permease YteP"

/db_xref="COG:COG4209"

/translation="MKHWQLHLLVIPPLLFFLIFKYYPMINAVLAFKDYNVIKGIWGS

DWVGFQNFRLFFENPLFWTLVKNTILLSGYLVLAGFPIPIVLALMINEVRGAKFKKFV

QLVSFAPYFISTVVMVSMIMLFLAPRLGFVNIALNFFGLDSVNFLGEPGMFRSIYVWS

DIWQTAGYSAVIYLAALAGIDPTLYEAAKVDGASRFQKIRHVDLPGLVPTIVIILILN
VGNVMAIGFEKVYLLQNPLNLANSEIIATYVYRVGLLNANYSFATAVGLFNSLINLVL
LVTVNGLAKRVTKNSIW"

CDS 1921855..1922754

/gene="araQ_10"

/locus_tag="PIKHDJKN_01801"

/inference="ab initio prediction:Prodigal:002006"

/inference="similar to AA sequence:UniProtKB:P94530"

/codon_start=1

/transl_table=11

/product="L-arabinose transport system permease protein
AraQ"

/db_xref="COG:COG0395"

/translation="MAANVQTKIKESAVDKTFLITIYILLSIVALVVIYPLIFIVSSS
ISSPAAVTSGRVWLWPVDISFNGYKALFNTPEILIGYGNSIFYTVAGTLISVVLTIMI
AYPLSRKTFFGRNTLMMIITFTMIFSGGLIPTYMVVKQLHLIDTRWALLIPNAIWVWQ
VIIARSFFQSSIPGELLEASEIDGCSDMRFIWSVVLPLSKPIIAVLVLMYAVGQWNAY
FDALIYLKSADLFPLQLILRSIIIQNNGSGAMDVAKMVERQQLTELLKYSLIVVATLP
VLVIYPFVQRHFVQGMLVGSVKG"

CDS 1922852..1924495

/locus_tag="PIKHDJKN_01802"

/inference="ab initio prediction:Prodigal:002006"

/codon_start=1

/transl_table=11

/product="hypothetical protein"

/translation="MKKSMVTLLMLVVAFTVVMSGCSNGSSSTAQQGSSEEGGKVNIS
VFAVQDSSIDIPTNKFTAFVEDKFNIKFNWEINPSDGAKEKRQISLASGDYPDAYLLT
HYIDQFSQADLLKYGKQGVLVPLNDLIDQYAPNIKAAMEKNPNLKTLNTAPDGNIYGL
VAYTECFHCSYPSKMWINTDWLKKLNLEMPKTTEEFKNVLQAFKTQDPNGNGKADEVP
LSGSIEEFGVRIIPFLMNAFVYDDDRNYLQMENGKVQSAAITPEWKEGLTYIKSLFDA
GLIDPGAFTQNAEAFKKIGENADAEILGAGAAMHPAIFVNIDEGNTRSAHYNPLAPIS
GPQGVSYATHDAGGVSPGAKFVITNKASEEAQIALIKMVDYMFTPEGQTNGASGMKGI

DWTDPVDGDVALGKDVKPVVKQIPMAEGEAPRNAGWSGMAHFYMPKEYRDTFVQGTDI

YASNGYERRLYDASLLYEGHEPKELFPIWSVWIDPNEIDEASLLQTNIRNYIEQNELQ

FITGNKDLNKDWDAYVKGLQNLKLDRYLEILQKAYDTSK"

CDS 1924609..1926372

/gene="glaA"

/locus_tag="PIKHDJKN_01803"

/EC_number="3.2.1.-"

/inference="ab initio prediction:Prodigal:002006"

/inference="similar to AA sequence:UniProtKB:B1V8K7"

/codon_start=1

/transl_table=11

/product="Alpha-1,3-galactosidase A"

/translation="MNALHTKKGGINIPVTEQEHITNSTTIRLTDYGAVPDSELDTQP

AMARAIQAAAEIPGPVVLDCARGRYHFYPEEAIRAPYYISNTTSEEENPDVTKTIAIL

LKGMNDVTLEGNGSLFIFHGKQTLFLLDHCTNVEIRNLRTDYHQPTVTEMTIISSGKH

YFDARIHPDSRYQIRDNKLTWLGEGWSFAKGPMQTYDPLRNTTWRIDNWLELAQSVEE

LEPMTIRLHFDFQPDVVPGYVLQNRDGIRDQVGAFMTECSDITWRDVSLHFLHGLGVV

GQFSTNLTFSRMNLAPRTETERTVAGFADFLHMSSCRGKVAVRDSYFSGSHDDPVNVH

GTYLRIVDQPAVDCVKVRFMHPQTYGLPAFYPGDEIEFVRSGSLTTYASNNVVAVERL

SARELLLTLAHPVPDGIGSHDVIENVTWTPEVEITNNHFARVPTRGILITTRRKVLIT

GNTFERMSMNAILIAVDAVSWYESGRVEDVTITDNHFIECGGSEHPVIFISPENVVVD

ESAPVHRQVVILNNRFETCSDVPILSAKSTRGLVFKSNKIWSSSETGKLSCVEEAVYA

TACSEVDIVDNTFTNVPEGTD"

CDS 1926402..1928777

/locus_tag="PIKHDJKN_01804"

/inference="ab initio prediction:Prodigal:002006"

/codon_start=1

/transl_table=11

/product="hypothetical protein"

/translation="MKLQYDKPAHVVWTEGLPIGNGRLGGMIFGGVEQEKISLNEDTLW

SGYPKNGSNPGAKEALPKVRKLLQEERYTEADTLTKEMLGPYTQSYLPFGDLLLRFEH

GDIYHSYKRTLDVENAVHSLEYQIGHVIYTREMFASHPDQVIVLRLAASVEGALNVHA

SLDSPLRHATSVREGRFVLEGTAPENVDPSYFASDDPIRYGDPGNNKGMVFEGQLAVK
TEDGQVTVDGSGIHVLGATTATFYFSAATSFNGMDAIPGVGGKDASFTASSFLNEAVA
KPYDSLLDSHIADYRLLFDRVKLQLGNSPAPEEMSTEQRITTYGAEDPGLVELLFHYG
RYLLISSSRPGTQAANLQGIWNAVTRPPWSSNYTLNINTEMNYWPAEICNLAECHEPL
LDLISNLAKKGVETAQVNYGTRGWTTHHNTDIWGQTAPVGNYGDGDPSWAFWPMGGIW
LTQHLWEHYAFSGDETYLRNSAYPVMKEAALFALDWLIDDGSGHLVTSPSTSPEHKFR
TAEGVAAVSPGSTMDISLIWELFTNCIEASEILGTDPDFRKELVSIRERLLPLQVGKY
GQLQEWSKDYEDEDIYHRHTSHLVGVYPGRQLSDEETPELFVAARTSLERRGDESTGW
SLGWRVALWSRFREGNRSLRLLSNMLRLVRDGASEQYNHGGVYPNLLGAHPPFQIDGN
FAASAGIAEMLLQSHRPYMELLPALPDDWQQGSVSGLRARGGFEVSIRWDKGQLAESH
ITSHLGRDCVLRGEGPVVVTLDGNVVETEDIDLNIISFPTSAGRTYWVKLS"

CDS 1928910..1930952

/locus_tag="PIKHDJKN_01805"

/inference="ab initio prediction:Prodigal:002006"

/codon_start=1

/transl_table=11

/product="hypothetical protein"

/translation="MLTSDTAVQVQQQNVNLVSVTNGHVSLEINLENGEVSCIGNHSS
HVKGIRSAFRWQGREYSTDHYQNHELTAQEDIVREGFGKGIRLVVLHKASLLPQLEQH
FYMYESSPFVLVQTAIVGDEELKANRMAVIQSKTISLGGDEPSILRVPFDNDKWVRYT
VVKPPLDVESYEATALFAPDSRRGIVMGSLTHKVWKTGIRMQSEKSGHIEGLDLYGGA
VSELTRDSQPHGYVKGKRVESPLVFIGFYEDYREGLEAYGQANTWIEAQLPWEGGVPI
GWNSWSAAMSDLDYDLYTATSDFLKNEVQPLGFENEDTLYINFDAFWDNFTPEEMKSA
LDRVRQNGHRAGTYWTPFAFWGGPDQFGQAVEGTNGKYTYGDILLRDSEGEILPDVDG
GLAIDPTHPGNLQRIDWFTDKFISEGFEYIKLDFLAHGSLEGQHHNSDITTGIAAYHY
GMSYLQHKLSPEVIGRPFFINLSIAPLFPYAFAHSRRISCDVFGTLSDTEYLLNSLTH
GWWMSNTLYRYNDPDHSVLYKSFNQEATGWNEGRSRLTASVIAGTVLLLGDDFRKEDA
AERAREWLGNKDILNVARMGKTFRPVEGDFGKLSSDVFVLESPEEKSFYLAVFNFDAA
QAAVKSISLERAGLNANAIYSLQDLWEGSQGETSGELTVSLEPAESKIFRLTVKGN"

CDS 1931106..1933436

/locus_tag="PIKHDJKN_01806"

/inference="ab initio prediction:Prodigal:002006"

/codon_start=1

/transl_table=11

/product="hypothetical protein"

/translation="MNDFTLWYSTPAADWSQGLPLGNGRIGAVVMASSHREVWSMSEV

TYWSGRIDPEHPSEGGKAALEEMRGYFFAGDYDGGDRLAKQHLQPKKRNFGTHLGLCD

VVIDFGKKNSGSDEAGSFQRELDLTHALAGAFWKDDDITILREVFASHADDIVASRIW

SDSPGGVSFTLGLQAGTESFKVVALDDNTLEFQGQATEDVHSDGTCGVWAKGLVKLVI

SGGTIASRDGRLNVTGADEAWIYFSVSTDYRRSDQDWETENEYTLLKALNKGYALLRE

DHIRDYSEQYDKVDIQLGVKGKSGLATDQRIRLLEQGDGDEDPQLFALFLQYGRYLTI

AGSRENSPLPLHLQGIWNDGEACRMGWSCDYHLDVNTQMNYYPTEITNLGESHLPLFR

YVEDLAQAGRETAHNVYGCDGWVAHVFSNVWGFTLPGWETSWGLNVTGGLWLATHLIE

HYEYSQDHVFLENVAYPVLKESAAFYLDYMCEHPQNGWLVTGPSNSPENHFYSGNSRD

GAQQLSMGTTMDQMLVRRLFEFCLTSVRLLNKDEELGRKLEDAISKLPPLQIGKKGQL

QEWLEDYEEVQPEHRHLSHMYALYPDSQITPDRTPELSAAARVTLENRMVQEELEDVE

FTAALFGLGFARLHDGETAYKHLSHLIGGLCFDNLFTYSKSGIAGAESNIFVVDGNFG

GTAVVAEMLLQSYAGEIYLLPALPQAWSTGSITGLRAKGNAEVDIVWENGKLTSTNIH

TFSPGTFTICWGKQRTILHAEAGQSYYFNSELELVK"

CDS 1933537..1935069

/gene="rhaR_10"

/locus_tag="PIKHDJKN_01807"

/inference="ab initio prediction:Prodigal:002006"

/inference="protein motif:HAMAP:MF_01533"

/codon_start=1

/transl_table=11

/product="HTH-type transcriptional activator RhaR"

/translation="MYSIFLVDDEELGLEMMRDYIRWEEMGIYIMGTASNGREALEKI

EATQPDIVLTDVQMPIMNGIDLARKIHESYDSIQVMFLTGHDEFQYVKSAINVGAVGY

MLKPLDLNEIESVISKVKQRCDEVGIKNRSMEAAKANILKELSYEKNEDRAIDLAFSF

SRLTRQPETNRYAMALFSIDPKEAEGEQQSLEECTGRLVSFLDHFFKAKNLKAIFVDY

KEGETGVFMEAAQQPGHYAWEDLAEAIRSALDFTVTAAVGGQETELSHIHCLYEQTRI

VLNERFYEGTGTIIHAETLSNQFYTEHMPPFEQKEWFEAINRLDFEWAAQKLHGYIEG

LATLRVKKKIICDWSIDLVNELLEQLHKPVPKRAELYHSIYNALTLHEIEDLILGTAE

DAVSMLGERFMDKNAKLIHKVRTLIDQNYNQPITINSLSDQVYLSPNYLRSIFKDKTG
MTIHDYLTRIRLDKARELLADGSLKIHDIAQNVGYESTSYFISLFLKTQGVTPNEYRK
SI"

CDS 1935367..1939935
/locus_tag="PIKHDJKN_01808"
/inference="ab initio prediction:Prodigal:002006"
/codon_start=1
/transl_table=11
/product="hypothetical protein"
/translation="MLGRIMKKGFVFFIIFCLVVGGGQFARTAHADDMNTYEAEAEGN
ILLGNAKISDSPTASGGKKVGGMYQGSSLQFNNVTVSETGNYKISVYYISGDSRPFNI
SANGGEKQFEEPPKTVDWDTVGTYDVTLPLNAGANTILIDDNNWYSPDIDKIAIRGLD
SGHPGEGGGDDWKKNLHGAIIEAEAADNILKGNAKVVESSISSGGKKVTDLNKNSSLQ
FTNVTVPADGTYLIRISYISGDQRPVYMQVNNDTDEMIDLPKTASWNTVGTYDAEASL
HKGANTITFSDHDGYSPDFDRIEVIPYTLSYEAEDSGNTLTGEARVTDSPNASGGKKV
GYLNGGSSLTFNKIIAPMTGNYRVTVAYFSGDPRSFYVHANSGEEQFHDLPKTPDWDT
VGTYDLTLPLTEGENTITFSDNNGYSPDLDRIIVEPAIETEPENPGEGDDDLGTPGDS
QRYGDITVTDYTYGLLVSNGQYEVSYNTQTGFVGYSWAEGQKMRGVFSSIKLGESLVE
TKGYESHASGGTPVEIQDGFGKGIELNFVHTSTGKPTLKQVYRFYEDKSYFLNRLDAF
SDTEIQSNYMSPITVKNTGGVDIGVSSDNRVLTVPFDNDAWIRYKSQTMNRADTSYEM
TTVFNNSTRNGLVIGSVTHDTWKTGIDWKGSANRLNEFAVYGGAASDVTRDTQPHGSL
TGTELSSPLIMVGGFSDYRTGLEEYGKANAIITPPLALNPELPQGVPVGWNSWGAYDS
GLSYQNVVDVSNYFKNNLKKFNNNGNVFINMDSYWDNLSDAELAQAVSVIKDNGQHAG
IYWGPFVYWGNDMSQTVDGTNGQYTYGDIVLKDAEGKPLPTLDGAYALDVTHPGSQMR
MNYFLDKFKSLGFTFIKLDFLTHGSLEGQHYDTTVTTGIQAYNEGMRYVEERLDGKMF
ISASIAPIFPSQYAHSRRISCDTYGKINETEYMLNSLTYGFWQNGTIYSYTDPDHLAL
SSASSLEEARSRVNSGVIAGTVMMDSDDVKDPKAQEYMTALYNNTDVLQVALKGKVFK
PLEGNTNANAADTFVLKDGNDYYLAVFNYSANSSANKTINLGRAGLNESKSYTLKDLW
TGESSSATGSWSISLGPAESKLVKLTEKPVNPENPGNPGNPSNPGNNGNNNNTSGNSG
NSTTPENDTVTLVITADMLKVDSGAGKAIVDIKAGTQELQLSSSVLRQLGNHTLEVKS
GKLTLQVPASLFQQLLSKLPAGQQEESTISLKMVPLGSKAKEVVAAVESSSRATVSLK
GEIYEFSLSATTKSGTTETLSAFDHPVTLSLAAAEGFDPSQGGIYYINENGKLEFLKS

DYVNGVLTTKVSHFSKYAVLELNRTFADVPANHWASSVIKELAVKGYVEGTNKDQFEP

GRAVTRAEFTAMLVHSLGLTVAGEVQFTDVATDAWYAEPISIATQAGIVNGRSAASFQ

PDAQITREEITVILMKAYELNVGKVPVGSTDILFTDMAQVSSWAAVSVKDAARLGLVQ

GQKQGQFVPKGIASRAEAAQVIYNLILK"

CDS 1940102..1942222

/gene="agaA_2"

/locus_tag="PIKHDJKN_01809"

/EC_number="3.2.1.22"

/inference="ab initio prediction:Prodigal:002006"

/inference="similar to AA sequence:UniProtKB:Q9ALJ4"

/codon_start=1

/transl_table=11

/product="Alpha-galactosidase AgaA"

/translation="MINLTIQYNEQLRIFAIQTQNSSYIFGINEREHLQHLYWGDPVE

IEDSAPLLHLQSHSSFDAEVEGEVEEYSFWGGASYTEPSLKLRMPDGVRDLQVRYDRH

EIIEQDGRQTLIITLKDQVYALETQLVYRVIPEFDLVERYANIVNTGQEDIVLESMQS

AAWTLPYLQDYRLTHVTGKWSGEFQLRNTILIEGKKVLESRRGFTDGHANPWFAIDDG

LSTESGGGVWFGALAWSGNWKIVAEKTPFTHVRVTGGINDFDSEWLLGAGETFETPVF

VGGFSREGFGGMSHRLHQYQYDYILPRSEIGKVLYNSWEATYFDVNAQDQMALAERAA

KMGVELFVVDDGWFGQRHSDRAGLGDWNVNKEKFPNGLVELIDRVHGLGMEFGIWVEP

ESVNPDSDLYRAHPDWVYHFETRGRTELRNQLLLNISKPEVKQYIINFMTELLGNHEI

KFIKWDMNRTITEPGMKGHPINRQKEVWVRHVQSLYDIWSQLRAKFPDVEFETCAGGG

SRIDLGIFRYADQSWPSDNTDAFDRLSIQEGFSYTYAPRMMTCWVTESPTGMNGRNVS

LKYRFHSAMMGTLGIGSNLNEWSDEWIEQSGEFIGQYKAIRHLVQFGRQFRLTSLRHK

GVTAVQYSDAAGSDHVLFAFLHSQKLGEPLPRLRLAGLQADKTYLIEELGLSVSGRAL

MNIGVELPLRGDFDSLVYRIREQV"

CDS complement(1942291..1944048)

/locus_tag="PIKHDJKN_01810"

/inference="ab initio prediction:Prodigal:002006"

/codon_start=1

/transl_table=11

/product="hypothetical protein"

/translation="MRQFYRKYIYMPFVNLSFRSKLFMVFVLVTIIPMMLLVYFSYEL
TKTKLTEQIYINMTNSTAQITKNLENKLDSYEHISASIYLDNRLANYLTNEYQDDPSY
LDVYNYIGNRIDTVMAAYPDFDSAFIYSNNPSLPKDNYYIRPITPEVQNTELFHKLKQ
SYGNIIHLSSPQTENGPAMFTLARLLNNNSNQYPYGMLVFQISESVIYSLMEKEAGGK
DIFIINDKGIILSSADKQLINTSLPELLHQNFDETPSGRFDTTYQGVKALAVYNTLKN
GWKTVSIFPYDSIIKDAKSLSQLIIKISLGFIGVALLLIYITASLFSKRIRTLIRMIR
RIERGDFNPTHEEQMGSDEIGQLHFAFEQMTTRLKSLVTEVYQKELQSKEAELDLLQA
QINPHFLYNTLGSISSLAVKHQDPQIQDMVLHLAKFYRISLNKGKSILTINEELKLTQ
SYNAIQLIRFKGKLNIIYTIDQSILPYSTVKLALQPFVENAVIHALWNQDRPLNIHIK
GVIENNSIILSVIDDGMGMRRETLQSLFEEKEGRGYGISNVDRRIKLKFGEYYGVKVY
SKLGMGTTVQIRLPQKEIQ"

CDS 1944272..1945228

/gene="yteP_11"

/locus_tag="PIKHDJKN_01811"

/inference="ab initio prediction:Prodigal:002006"

/inference="similar to AA sequence:UniProtKB:C0SPB3"

/codon_start=1

/transl_table=11

/product="putative multiple-sugar transport system
permease YteP"

/db_xref="COG:COG4209"

/translation="MHKVSAIKTVRSNNLISRLKEQKWLFVLMLPAFIATLLFSYGPM
FGLYMAFTNYQPGGGSFLHQFFHAEFVGFQWFEYFFTTGDFYRVMRNTLATSLLTLFF
GFPAPIILALVLNEARQGFFKRFVQTVSYLPHFISWVIAANIVITLLASDGMLNNILV
LLGIVKEPVAFLQNGPLFWWIIALSNMWKEMGFSAIMYLAAIASINPELYEAARVDGA
SRFKQMWHITLPSMRPTIVILAILAVGGILNAGFEQQYLLQNNTVLEYSEVIDIYAYK
YGLQNSMFSYGAAVGMFKSVVAFILVLIVNRISRKVNDQALF"

CDS 1945257..1946147

/gene="araQ_11"

/locus_tag="PIKHDJKN_01812"

/inference="ab initio prediction:Prodigal:002006"

/inference="similar to AA sequence:UniProtKB:P94530"

/codon_start=1

/transl_table=11

/product="L-arabinose transport system permease protein

AraQ"

/db_xref="COG:COG0395"

/translation="MILNRFGDRIFKIIVYFILILVLLVTFLPFWNILVLSLNSAEDT

VRGGVYLWPRDLTLDSYKQILKDSEILNGLWVTVKRTLIGAPLSVLVITMLAYPLSRR

NLVGRKGWNLYFIFTMYFSGGLIPFYMVLKALNMIDTFSVFILPSLMNVFYMIIVRTF

MEQLPHEIEESARVDGANDLTIFFRIVMPLTTPVLATVGLFQAIGHWNAWFDSYAFTY

SSDLKTLQAVLVKILNQFQTGGMISQSQMLANSAKRNAVSSDTIRMAATMVATLPIVM

VYPFLQKYFVKGMTLGAVKS"

CDS 1946275..1947885

/locus_tag="PIKHDJKN_01813"

/inference="ab initio prediction:Prodigal:002006"

/codon_start=1

/transl_table=11

/product="hypothetical protein"

/translation="MSMTKTKKWMTMGITAALVVGLLAGCSSNDGENNTAGKDGEQGP

ITLTWFDGNTKGEPFTDAVAQEITKKTGIEISIQQPTGNPTEKLSLMLASGDYPDVVV

MSRGDASLDKYISSGAFIPLDELIEKYGSDLKEMYGETLTKTRYTDGKNYYLANWYGL

DSDPVFGMLMRQSLLEEFLPDKADGSQALTTDEFEALLKNFKEKYKTIDGKESIPMTM

NGENMGANLGTFKGMWGLKTYYDNNGRLQYDVKDPKYREMLLYINRLYREGLIEKEFA

ISKTQTWIQKLTTGAVFATPGAYWDPGNGNAALKKDGGEKNQLFAYKVVAPGVDPAQT

TFGPRSSLGWDAIAITKNNKHPEETIKLFNYLASDEGQHLLLWGKEGEQYTTVDGKRE

PDPAFLQSFKDNWDDTVKKSGVRKWLWFVKNGLDPNGQPYDMAVKFQRSEVDELALKS

LGDSVWDTAQYDNLNPDGGTPQALTAQKVKDIMDQSITRAIIAPSEAEANSVFDKMLE

DMKKAGDEKVEDIINEKYAQRMELWSSK"

CDS complement(1947968..1950406)

/locus_tag="PIKHDJKN_01814"

/inference="ab initio prediction:Prodigal:002006"

/codon_start=1

/transl_table=11

/product="hypothetical protein"

/translation="MQKKDMQTKRRNEINKNRGYERAKKHPISFNGPAPDFFEGALLG

NGGLGIVVTTRPDAVMLHFGHNNVWDIRLAEEHRDELMTFQEVYDRFADLPADLVQLT

DHPWYKQYCELAGQNYSKAYPKPMPCGSLLLRYDRRKAEVIGHTIDIANGLCTIDFLI

DGKPAVFELFIEGEHDRVWMKVRDVATGALVPLFEEIKLIPDPETPPEFPQTAVLTSE

ITGRQSFTQILPHVEHPAVPYITHEKDRAFRLAVNSSGLTAMKEHPLHNQTPAVNGSP

LGEPEHGFLACAELWEGLYSQLNNEAHPPELSSFSQAKEESNAMWRDFWSRSSVSLED

EFLERIWYHNLYFFNCSVREGVTCPGLFANWSYRTIGADWHGDYHMNYNTQQPFWLAF

SSNHLDKHLPYVDMVDHVLPVSRAWARDYYGLNGAYFPHSAYPVEMSMMPYPVPHWGW

EICETPWTVQSLWWHYLYSMDETFLRDRAFQPMKEAVLFMIDYMKRPEAQGERWGDDH

YHIFPTVVPELYEISPGFAKNKDCLIDLTLTRFLLNAFTQACGVLERIESEQELLENV

KEILSHFPSYPTAESRNGPVFVSVEGEDPDVVYNVPIPITTVFPGEDHGLHSPEEEYQ

TAVNSYLNHRNEGGNELVFYHLAGARLGVLDLERFKRQIQYCLLPNGTCTDRVLMSGG

RYADTENFDFMSRMGIWFENFSLPAVINECLLQSYNGNLRFFPNWPNGQKAEFHTLRA

VGGFLVSAAIVDGETDWIEIESEAGTSLTFYIPWAEGALCTVSSGEQYMLSGQVGKIS

TVAGDIISIVKATV"

CDS 1950618..1951514

/gene="rhaR_11"

/locus_tag="PIKHDJKN_01815"

/inference="ab initio prediction:Prodigal:002006"

/inference="protein motif:HAMAP:MF_01533"

/codon_start=1

/transl_table=11

/product="HTH-type transcriptional activator RhaR"

/translation="MNFASLHPYVYLATRYPFAKGQSSSPRICYTSSIYLISEGYGVL

STNGKSSRLGPGSLIYIPAGQLHEWVADSDNPMVHLCCYFDWHYVERKDVFDTACPVC

YPPQVLQQAFVGPTFPYALKEISKVESLQIWQDLFQDFYKSGSYTNEQTFMWSLMIQR

NFQTFIDYFLNCCMGNDYVTPDCRISHMLKIIEQDLLHGAPKPLDSYYKSLNMSRGHF

FELFRRSTGYSPIQYMNHFRIGRAKYDLLHTTLNITQIAEKYHFSSVHYFSRLFHQLT

GQAPRQYRLSQSNTAASSAPLE"

CDS 1951591..1952538

/locus_tag="PIKHDJKN_01816"

/inference="ab initio prediction:Prodigal:002006"

/codon_start=1

/transl_table=11

/product="hypothetical protein"

/translation="MNKAGRKGWSCTWKFKMSVVLTGTIFMVYGVGLSVSSAEPQITS

GQTPEQILTEQTSTQVPGSTSGQLSQTPADKSRHKRMAIIIDDAGNDMKGTSEILATP

VKLTLAVMPFLPTTKKDAIAAHEKGMDVIVHMPMEPKKGRPEWLGPGAITSNLTDEEV

RERVEKAIDEVPYAVGMNNHMGSKITSDKRIMSIVLDVCRERGLFFVDSRTNFRSVVG

ELAITKNMPPVGNDIFLDDHNSKQQIRKQMDLAAQRALDKDVCVVIGHVGHTGLNTSA

VVHESVSRLKGQIEFVGISDLVRDVWHWQAEPKLPTDNK"

CDS complement(1952513..1953286)

/locus_tag="PIKHDJKN_01817"

/inference="ab initio prediction:Prodigal:002006"

/codon_start=1

/transl_table=11

/product="hypothetical protein"

/translation="MYKLLAASMALLISISSIPLTSHASAATFALTDLPTLHSQRSPI

SPSMLPVTDGDAYHSTHHAFAAPVILLDVGHGGIDGGTTDHGILEKDINLAISQKVYL

LLRSKGYSVIINRLGDYALSDENRWLNSRSRHRRDLAQRKSLSEEVSTDIVVSIHANW

SPRSSARGPVVLHQNEGRSYMLASSIQNQLNQLYETERDVVWGKPFYLLNHVKQPAVI

VETGFLSNAHDRAMISDPGEQKRIAQSIANGIIYYLSAV"

CDS 1953480..1953704

/locus_tag="PIKHDJKN_01818"

/inference="ab initio prediction:Prodigal:002006"

/codon_start=1

/transl_table=11

/product="hypothetical protein"

/translation="MAKSDELVKYITERVVHYIDTPKDERKGRTKAKEPWTMKWFGMI

PFAVSLWVGKKEKAMNSQGEVQKRSSSGKG"

CDS complement(1953800..1954849)

/locus_tag="PIKHDJKN_01819"

/inference="ab initio prediction:Prodigal:002006"

/codon_start=1

/transl_table=11

/product="hypothetical protein"

/translation="MKRPFHKGWNVIRRITGIVMCLLCVITLLPLLQAEGAGIQNAFR

SILPSTLAKSTAELHSDTLAPDAVQQFAREQAIILSTEHEGNHWDHAQLDFYPLGPGT

RSWLVYANVGESPVGYMIISANDQRQFQLSEYGQGEQRPYNISLLDQALDRQQLNLKK

MINDGGGLQLRYAPPLLAYWKVERPNDDAIYIDATNGDTLPTGILNALDSDSRQATKR

MKFIRSISVQAVVMTPEDSPQVSSLSVDSMDLQPLFDPADNLLWITSKALSISGVKTL

NQQWKEHKKIVFSADQQNVLYGGPLPVTGYHIWRDDRGTVQNQYVAIGGNTSVRRYVP

LQTLLRDGHFYAVKP"

CDS complement(1955033..1956256)

/locus_tag="PIKHDJKN_01820"

/inference="ab initio prediction:Prodigal:002006"

/codon_start=1

/transl_table=11

/product="hypothetical protein"

/translation="MTMSPHEVQAYVLTYLETLDCQIMERSPAHVTVKLSPEADKALT

NRPYYWGFVERTGAPAETMSFTFVFDPDAHQQALEAEEAKAAKLAPPASPGAGPGTPE

APKETILGRYFGIVPSLPQLGPGRILKEDVVYGSRRLQQIFNAAREGGAFVNLFEQAA

KRQLRATAPAVYEPWLGVCFKVEFACDLKKEELHFIGISLRTGEIVEKFGSKLNRRDL

SPRLAENMHVQTAKVSLADAGAALESHLTNRLLELDYSWAEKAKERLDLELAVVDTYY

EAVLREDTPEVDVDTTSASGTDRVSNDQDQAGAGPSAHIHTSRSGSTRIQKAHLQPVE

PTGVMIGSDVELAAEAAVQAEPETDPEQDKVRQAVMDREAMKLQYETRRTEMIWQYEP

KVKVTAISSGMFHLR"

CDS complement(1956259..1958019)

/gene="rapA_2"

/locus_tag="PIKHDJKN_01821"

/EC_number="3.6.4.-"

/inference="ab initio prediction:Prodigal:002006"

/inference="protein motif:HAMAP:MF_01821"

/codon_start=1

/transl_table=11

/product="RNA polymerase-associated protein RapA"

/translation="MNRKNPHQINQPAAELPVPLEFDRSWITQLESRLDKGGPWGDYR

LFQLGIQAEETNLIPNFDEIQCLKHLQGLTPLPHQMDTARRVLFEMSGRAILADEVGL

GKTIEAGLILKEYMVRGLVSKVLILVPASLVLQWVRELNSKFGIPAIAQKKAYSWQNE

IVVASMDTAKRDPHKDMLLNTDYDMIIIDEAHKLKNKKTTNYQFMLKLRKKYCLLLTA

TPVQNDMSELFNLINLLKPGQLGRQGDFAANFVVDKRIPKNQEQLKDELSKVMIRNRR

GEGPVQFTKRNVSNVNLQLSPEEQALYDGVTSFVKDQYQEAGGNLSSMLSLVTLQREV

CSSRDAVFVTLVNLSKKLPLDSPLRDKIWELVAHIKAIKENTKAEKTMELVRDMNEKV

IIFTEYRATQEYLLNYFRNNGLTAVPYRGGMNRGKKDWMMDLFRGRVQAMIATEAGGE

GINLQFCHHMINFDLPWNPMRVEQRIGRVHRLGQQHDVNIYNLSTTGTIEEHILNLLH

EKINMFEMVIGGLDVILERLEKKESIEKSLYKIMLESQNEEDIRRKMDSLGQSLNSIQ

REVADEAPAVSGLDIRKGGL"

CDS 1958150..1958662

/locus_tag="PIKHDJKN_01822"

/inference="ab initio prediction:Prodigal:002006"

/codon_start=1

/transl_table=11

/product="hypothetical protein"

/translation="MIGGVPLPVVWENLLPGYSDQIRQQANEWFHEKLIANIEAGHGA

LYPYVEEFFQYCKEKSIPIYIASNGQIEYLNAIVNYYHLNEWVTETFSIQQISSQNKS

DLVRSIVEKYKITQGMVIGDRLSDIKAAKDNDLISIGCNFDFAQADELAQADRVIDSI

GELKTLVGVL"

CDS complement(1958715..1959233)

/gene="msrA_1"

/locus_tag="PIKHDJKN_01823"

/EC_number="1.8.4.11"

/inference="ab initio prediction:Prodigal:002006"

/inference="similar to AA sequence:UniProtKB:P54154"

/codon_start=1

/transl_table=11

/product="Peptide methionine sulfoxide reductase MsrA"

/db_xref="COG:COG0225"

/translation="MIEKATFAGGCFWCMVSPFEEQPGIHGIISGYMGGTVENPTYEQ

VLTGETGHLEVVQISFDPAIFPYEKLLELYWPQIDPTDATGQGNDRKSQYRAAILYHN

EEQRQLALKSREELGKTGRFDKPVVTAIEEASVFYPAEEYHQNFHRKDPKYYKESRIY

SGRDEIIKKYWS"

CDS complement(1959282..1959971)

/locus_tag="PIKHDJKN_01824"

/inference="ab initio prediction:Prodigal:002006"

/codon_start=1

/transl_table=11

/product="hypothetical protein"

/translation="MKMYRGYEKIQPYMSLFNLEQIFADATSLPIQLREYDSDEYVLT

EGDELDGLYFQVKGLTRVSSSVETGKSLLLRFCHPLSIFGDIELIQKVVVQSQVQAVQ

PSKFLFIDIQTVERNLMQEYRFLNELLKHLSYKLQTCTAASRINSLSSVEERFAIYLM

STRLPKEFGKEISTPHIPEIASVIGTTPRHLNRLIQKLSDLEILCKKRKGFVVLDWER

VDELSNGLRYD"

CDS 1960051..1960857

/gene="yitU"

/locus_tag="PIKHDJKN_01825"

/EC_number="3.1.3.104"

/inference="ab initio prediction:Prodigal:002006"

/inference="similar to AA sequence:UniProtKB:P70947"

/codon_start=1

/transl_table=11

/product="5-amino-6-(5-phospho-D-ribitylamino)uracil

phosphatase YitU"

/db_xref="COG:COG0561"

/translation="MKFVFDLDGTICFQGKPLTREMVQTLDFLVEKGHELIFASARPI

RDLLPILPVHMQQSPLVGGNGGFVYRSGLEISTVYLEASIADHILALLKQHEAEYLID

CQWDYCYSGNPEHPIRRNLDPECRAKNVQLEELTEIVKVVVLSSRNHQEMLEALHNLP

VVTYMHGTENIIDISPKGVNKWTGLQALGVSPHDYIAFGNDANDIPMFEYASRSICVG

QHEKLAQIASEMVANQEKDVIDKIIEIAERLEEESGVFGEAMAEVEDVAT"

CDS 1960997..1961557

/gene="flr"

/locus_tag="PIKHDJKN_01826"

/inference="ab initio prediction:Prodigal:002006"

/inference="similar to AA sequence:UniProtKB:Q9KIT1"

/codon_start=1

/transl_table=11

/product="Flavoredoxin"

/translation="MRKPIDEPMFYSYPGMVAVVTSRHKGMQNVMASGWHTYIGSSPG

IYGISLRKETYSYELIEQSGVFGVHFLPGHHSEWIQAAGTFSGRDTDKFSKFGITYEE

GIKVNIPLLTEAYFAYECKVIDITTYGDHEWIAGEVLQRYQDPEYFLENGMVNLDKLQ

IPMYVGRSSYRILDGSAEEKVHPFYL"

CDS 1962072..1964165

/locus_tag="PIKHDJKN_01827"

/inference="ab initio prediction:Prodigal:002006"

/codon_start=1

/transl_table=11

/product="hypothetical protein"

/translation="MSQSLKSKRHMNGLDGLRAIAVLAVIGYHLNLGIIPGGLLGVGI

FFVLSGYLITDILLTQWQEHGRISLGDFWVRRVRRLLPGMITMTAVVMIWLLCTDPSR

LAALRGDIVSGVLYISNWWYIFHNVSYFESFGPPSPFGHFWSLAVEEQFYIVWPLLLV

AAIVLFKRKGWLVVFIVVAAELSAGAMAIMYNPDLDPSRVYYGTDTRAFALLAGAALA

VVWPSRKLSSSLARINRLVLDVLGLAALAWLIYMMLNSSEYDPSLYRGGMVLQAIATT

FLVAVLAHPSSFLARLIGAKPLRWIGERSYGLYLWHYPVIILTSPTVDTGGAHPVRMV

LQVAATVALASLSLKYIENPIRHNGFRDSWSRLWGRGRNTTGIRNVWWKRAGLLMSIL

LMCFTVSQMMITSAANSDSHSVSMSATLNGDHSVTEEKGQDVLPAAVSTSGADNQPAP

QKNDKPTANAGDPQGKGVQQTTKPGKAPKSEEPTSGEQKGSTPGDSNHAVGKPKGSTD

NPGNPEDNGNEQPEDPVNTSDSSKEQEETAPPAKDGKIQYTVIGDSVILDAKPYLEQT

ISGVHVDGHIGRQMWEAADVLDGLKRNNQMGSQVVLELGTNGSFNSKSLNSVLDYLKD

EEHVYLVTVRVPRPWERTVNKALNEAASDYSNVSLIDWHTASEGHDEYFEKDGVHLTK

EGSEAFAALVKDSMK"

CDS 1964390..1965703

/locus_tag="PIKHDJKN_01828"

/inference="ab initio prediction:Prodigal:002006"

/codon_start=1

/transl_table=11

/product="hypothetical protein"

/translation="MLKKWLSGMLAMVLFSIVLAGCGGGDSSEGSDSKDKVTVTIWHN

WTGQDAKAVAMRKIIEDFRGSHPDIEVVDEGLPTDGLKTRLRTVAAADEMPDLFVMWP

DAMTKEFVKGDLLQPINAELDAKPEWKDNFIPNALDGYTVDGDIYSVPMNLAPTSFIY

YNEALFKQYNVKAPETWAELEQAIATFNENKVIPIALGNKANWVAQSTIFSTLADRVT

GTDWFLKATAQDGASFTDPQFIQALDKMQELGNSKAFQDGFNSIDETQMMQLYFQGKA

AMVMNGGWALANLVNNAPEDILNNTHITILPAVDGGQGEARTTSGVVGTGLGVSKKLS

GAQKDAAMELFYALAGPDGQKATLDSSTLVSYKIDLDKSKAHPLFVELYDLMQEAKIT

PVYDSKLGSATVEVINNGLQELLMGGKAADIANKIQASQANAVEN"

CDS 1965848..1966726

/gene="ngcF_3"

/locus_tag="PIKHDJKN_01829"

/inference="ab initio prediction:Prodigal:002006"

/inference="similar to AA sequence:UniProtKB:O50500"

/codon_start=1

/transl_table=11

/product="Diacetylchitobiose uptake system permease

protein NgcF"

/db_xref="COG:COG1175"

/translation="MNALRSRRFIMLGLAPAVLIYALFVFVPVVWSAYYGFFNWSGIG

ESKYIGLDNYVEIWHDSVFWRALKNNVIFVLASVFGQIPLALMLAVILHKSNPLQRFL

RSAVFLPMVLSTVVIGMIWQYIYHPQIGILNFLLDALGLESWKLQWLSDDKIAIFSLV

PPLLWSFVGLYLIIFISALQNIPGEIHDAAKIDGASGIRKLVSISLPMIWGTVQVAII

LCISGSLKSFDLVYIMTKGGPAHATELLATYMYNSTFTTYRYGFGSAISTTIVLISLL

LIGTSQWATSRKRKEN"

CDS 1966745..1967620

/gene="ngcG_4"

/locus_tag="PIKHDJKN_01830"

/inference="ab initio prediction:Prodigal:002006"

/inference="similar to AA sequence:UniProtKB:O50501"

/codon_start=1

/transl_table=11

/product="Diacetylchitobiose uptake system permease

protein NgcG"

/db_xref="COG:COG0395"

/translation="MSVTPLSMPTPGNTSGHPVRRFSSGIVWTLLTVYGILTLYPFYW

LVISAFKTNEDFYSRPFGLPEKWNVENFSNAWESSKLGTAFGNSLIVSVGSLIITLFI

AALASFILARFQFRWKGLIMTFFVVGMLIPIHSTLVPLFILMKQMSLLNTYWALILPY

TAFALPTAIFVLTAYLTSIPRDIEEAAFIDGTGLWGLFTRIMLPMSVPALSTVTILSF

LHAWNDFSFALVFINKTGLKTLPLAIANFADGYQTDYGLTLAAMTLSVIPTIILYLVF

QEQVMKGMTAGAVKG"

CDS 1967650..1969455

/locus_tag="PIKHDJKN_01831"

/inference="ab initio prediction:Prodigal:002006"

/codon_start=1

/transl_table=11

/product="hypothetical protein"

/translation="MGRWLTSSLQRKLSVVVTASMIVPLLALGLFAFLISSRITEQKT

KLSGMDTLKQVEANLRYMLQDAETLSIFLIGERDIQQYLSSDEDDVQERVDILGRMTN

LAASKKYIANIAIYPERFDTALSTATWYESSNVSYPPAPRGDAVKAWTGVYPVQNYAG

IQNVITLVRPIRSIHDYRPIGWLAISLDEKAISKGWAALGLGMGEGRLELIGPTGEIL

SSMDKSRLGQSLEKIEPEVHTLIQQEGSGTATYGSGNEKRTLLYYPELLTGWTLTGTV

PYDQYKSENGYILILTAVAVSLSAAISAGLVWFTVRRVTRPLRVLTRHLSRMDPDRPL

PLFRSESDDEIGKLGESYNLLGAHIERLKEEVIRGEARKKEADLRALQAQINPHFLYN

TLSSIHWIALMSEEKRIAEMVEGLSDFLRISLNQGRDYCPVGQEIAHIRHYVRVQSIR

FPDKFAVHYIVDPALEQRMMLKLLLQPLVENAMIHGIQPKAGMGTITIMVRQEQERMN

VLVLDDGAGMERDRLEHVRSSLVSSDEEEQHDSNHHWTEKEPMKQTSQGGYGLRNVNE

RLLLHYGAEAQLEVDSRVGGGTRISFSIPILEESP"

CDS 1969452..1971035

/gene="rhaR_12"

/locus_tag="PIKHDJKN_01832"

/inference="ab initio prediction:Prodigal:002006"

/inference="protein motif:HAMAP:MF_01533"

/codon_start=1

/transl_table=11

/product="HTH-type transcriptional activator RhaR"

/translation="MRLLIVDDEVIIRTGLASVIAWHELGIELLSPAASAEEALVRMD

EERPHILMTDIRMTGRTGLELAEEGLHMLPELEVIILSGHDDFSYAQQAIRQGVTDYL

LKTSKPEEIIKTVLQAKQRITERWAEKSREGQLLRENKQRLFAGWIIDGDVESGPCPV

FLQSGINVQSECSSDKEQVQRQILILKAAGWNRSSATLLNFAVQNMLEDVLPGAVAHI

QKDRIICVVELPPDEQDFQFTLGIALTRIEQLLKCILRAAAGQPCMEVQLLHESYRTA

SIAYRYHGLLEDRIWQYEDVFHRIGGKTVLQHEEETTLGTLLMDNDSIMLTTWTRELV

DELLMDPEVTPESFDACLHAAVNAGQRWLIRTMRAIGRDEPERFEPWRPDPDADAGEL

RDLLFHHLYGLMHTYHSQMGQGRTTHVQKAIAYMESGLVQDISLQHVAGQVHLHPGHL

SELFKKETGVTFGDFVTGMRIRRAMDMLVVSPAKVSEVAAISGYEDVKYFSRLFKKHT

GKTPSEYREEAVSYKPSGH"

CDS 1971128..1973704

/gene="lacZ_2"

/locus_tag="PIKHDJKN_01833"

/EC_number="3.2.1.23"

/inference="ab initio prediction:Prodigal:002006"

/inference="protein motif:HAMAP:MF_01687"

/codon_start=1

/transl_table=11

/product="Beta-galactosidase"

/translation="MKMNNSSQDLSGQWKIQHFEVGEKRAMDVAAAALDDRFWIGAEV

PGDVHTALVERSIIDPPYYGHNDARSRWIEQKEWWYRTNFNLVKDGEAEEYFELVFEG

LDTFATVYVNGHEVGQTANMLMSHTFDVTTLVRKGWNAIAVKFDPLYLHHRDRETFDW

SSYTKERPWLRKAAMNFGWDWGPRMVTVGIWGGVRLERRTIAKLENVFARTESVSKQQ

AAVHVTADVKSVLSFRSRQKREKAVVLSCDIRLLDASGQEVARSAEVVGENGLADTIL

KLDSPQLWWTHDLGEPYLYRLEVTLFADGVEVDQYNEPYGVRTIKLALHNEQGEDAFT

FILNGVRVYAKGANWIPADHLIGAIPASRYRELIELTVEGHMNMLRVWAGGIYEKDVF

YDECDRQGVMVWQDFAFANALFPDFNRDFMNNVRDEVENNVLRLRNRASLALWCGNNE

IDWLYDMKSASGDITSPFYGELIYHELIPEVLERLDSSRPYWPSSPYGDSHGNDANDP

AVGDRHNWQVWHGSVYPRKHGEPPLLDYSIEGVTFKNYKKDNALFSSEFGMHASANRY

TLEKNMPAGQFYWGSPEMAYRNKDTNHQKGILLMEGYTGIPQNIEEYMNYSMLTQAEG

LRYGIEHFRRINHRNSGALVWQLNDSWPGTSWSMIDYELLPKASFYYGKVFFHPILLS

LEHEPGEPLTLWVVNDTREILKGQLRLNVYGLNGEKVYSSSHHVVTGSQSSTRIAELS

EAEVLGGRLAEEVMVELVAEGFTAPLNRYFLRDPKDVKLPQSQLSVHVNEEEQSVSVT

AIGAIARLVKLELPLGRVRFSDNYFDLLPGESRTVRLRHPDKLSLPMTELRVSAMNGS

EA"

CDS complement(1973855..1974961)

/gene="oxyE"

/locus_tag="PIKHDJKN_01834"

/EC_number="1.14.13.232"

/inference="ab initio prediction:Prodigal:002006"

/inference="similar to AA sequence:UniProtKB:Q3S8R0"

/codon_start=1

/transl_table=11

/product="6-methylpretetramide 4-monooxygenase"

/translation="MNQNTHLKTDVCIVGAGPGGALLSFLLKRQGISTILIERQPHLL

KSFRGEVLNTDGENVLKKHGLYPSVTQRGVLPLEQIQYWENGQIIHTIFPGEQESHVG

IHVPQDHLLEVIVSQSQHQENEQVLYNTVMTGLLRNKADQTIGINIRQEGRPASIEAA

VIVGADGRYSAVRRNAGLTPDTRKHGYDLLWARIPAPAGWEPAVRMASMDGQQLALFS

QFGGYVQIGWNIPQGSFSKLREQPFAPFVQKLVAAFPCLADSVAEHIQTWSDLVLLSV

ESSFAESWALDNVVLLGDAAHTMTPTGAFGLNAALEDADVLAELLIDMAADQFTSTER

LHELQTRRGEKVKQQLARQLEMESSFQQRYESFQ"

CDS complement(1975213..1975737)

/gene="pnpB"

/locus_tag="PIKHDJKN_01835"

/EC_number="1.6.5.6"

/inference="ab initio prediction:Prodigal:002006"

/inference="similar to AA sequence:UniProtKB:C1I202"

/codon_start=1

/transl_table=11

/product="p-benzoquinone reductase"

/translation="MNIYIVFDSEGGHTRALAESIAAGAASVHGATVHLHSTEEADIY

KLVEMDAIIWGCPGHFGSISSGLKNWIDKLGYLWAEGKLVDKVGAVFCTIATEHGGLE

STLIHLLTPMLHQGMIITGLPGNFADNALYGSYYGVGVTCPVDSDDLLSEQGIALGKA

LGERVARITNRLIT"

CDS 1975927..1976409

/locus_tag="PIKHDJKN_01836"

/inference="ab initio prediction:Prodigal:002006"

/codon_start=1

/transl_table=11

/product="hypothetical protein"

/translation="MSPDTERNSQLANVDQLLEAFFRYKNKVLDQQQRNETNCKLNPT

KSHILGMILREERCMAVDVARQLSLSSGATTIVLNQLESEGLIQRVRSEEDRRIVWLS

LTEDGKQLAKTLINNRGRMTWELLQALTEEEQLQMFGMLKKIELKLLEKMKTLEQTHR

"

CDS complement(1976497..1976796)

/locus_tag="PIKHDJKN_01837"

/inference="ab initio prediction:Prodigal:002006"

/codon_start=1

/transl_table=11

/product="hypothetical protein"

/translation="MAFGIKRQELAEWKERVSRGDLAYLTHFWIDNRFPGITSVTKVG

CADLNRLEDWCRAHELDPRYIHRRQPFPHFDLLGKKQKEILIQEGLTDHIQRFHL"

CDS 1977012..1979036

/gene="nhaK_1"

/locus_tag="PIKHDJKN_01838"

/inference="ab initio prediction:Prodigal:002006"

/inference="similar to AA sequence:UniProtKB:O32212"

/codon_start=1

/transl_table=11

/product="Sodium, potassium, lithium and rubidium/H(+)

antiporter"

/db_xref="COG:COG0025"

/translation="MELFIAVLVLLVLIGLSNILNRFVPFIPVPLIQIVLGVAIALLP

AGVHLPLNPELFFVLFIAPLLYNDGKRTPRHELWNLRAPILLLALGLVFVTVVVAGYA

IHWLIPTIPLPAAFALAAILSPTDAVAVGAMAGRVHLPKSIHRLLEGEALMNDASGLV

AFKFAIAATVTGVFSLAQASFSFILIAIGGLLIGALISFLLIRLGVWIRRLGMEDVTI

HMLLQILTPFVIYLVSEEIGVSGILAVVAGGIIHAIERDRTESVQLKMQVVSASTWSV

ILFILNGLVFVILGVQIPDVLSTIFENVSFDNLQVLGYVGLISVLLLVLRFVWIYLFW

KGNENSGEALSSGKPRLKEITIITLSGVRGAVTLAGAFSIPYVLQDGSPFPERDLIIF

LAAGVILFTLIAASVFLPILAKNEEKAEEGTPQTTERKAQDIMLNAAIRAVKSEMNDQ

NKAAALAVVSDLSKYIRQAAGELTAGKRKDILKQETAINLIGTRAERKEIENMMDENV

IASEAAFRCNSWLDRKEMMLANRTNTQMMFSLSEIGRVFGHVFTNRSRKPDQPFMIEN

ADLFRQVKMRTSEAAIKAIRANMNDSNRIVALSVISRYERVIAKLRTWNQGKNEDPFD

QEKLELQMVAMQEQRNTIQQLYENGEINRDVAAKLRRFINDVEATALKNT"

CDS 1979125..1980063

/gene="iolS_1"

/locus_tag="PIKHDJKN_01839"

/EC_number="1.1.1.-"

/inference="ab initio prediction:Prodigal:002006"

/inference="similar to AA sequence:UniProtKB:P46336"

/codon_start=1

/transl_table=11

/product="Aldo-keto reductase IolS"

/db_xref="COG:COG0667"

/translation="MAEQRTRLGKTDLIVNPIGLGANAVGGHNIYPNMLNDETGKDVV

RTALKQGINFLDTAFIYGPEHSERLIGEVLKETGQRQDTIIATKAAHKFVDGNVVFDN

SPAFLKTSVDEALKRLQTDYIDLFYIHFPDERTPKDEAVGALKRLKDEGKIRAIGVSN

FSIDQLREANRDGDVDVLQSEYNLFKREAEKELLPYTAEHDISFVPYFPLAAGLLGGK

YNRDTTFQDGRAKNPLFTGEAFIQNLDKVEQLRSIAQSKDAEVAHLVLAWYLTQSSID

ALIPGAKKPEQVINNLKTLDVELTTEEIAVIDQIFR"

CDS 1980131..1980907

/locus_tag="PIKHDJKN_01840"

/EC_number="3.5.1.104"

/inference="ab initio prediction:Prodigal:002006"

/inference="similar to AA sequence:UniProtKB:Q81AF4"

/codon_start=1

/transl_table=11

/product="Peptidoglycan-N-acetylglucosamine deacetylase"

/db_xref="COG:COG0726"

/translation="MNLKAARCIGLCTLVFLLGSSSAYAKPVQKNRQYYEERGEIVWE

VPTHDKLIALTFDDGPDPVQTPQILALLEQYQAKGTFFVLGKWAEKFPDLIKQEQREG

HEIANHTYAHTYAVRSTRADKYSHEMDIAEKSIVGAGAQRPTLFRPPGGYYNDMVIQV

AKQQGYTIVLWSWHQDTRDWASPGVSAIVNKVLKNARNGDIVLFHDKVEGKSQTVAAL

KTILPKLQEQGYRFVTVTELLAVKAREAAKDDNSSLPQHP"

CDS complement(1981042..1982592)

/gene="emrB_1"

/locus_tag="PIKHDJKN_01841"

/inference="ab initio prediction:Prodigal:002006"

/inference="similar to AA sequence:UniProtKB:P0AEJ0"

/codon_start=1

/transl_table=11

/product="Multidrug export protein EmrB"

/translation="MDNQKNQLPLGKTIAVLLLGAFIAILNQTLLNVAIPHLMNDFNV

SATTVQWLSTAYLLVNGVLIPITAYLIESYGTRKLFVTAMLLFTVGSLICAISPGFTV

MLIGRIVQASGAGIIMPLVMNVFLTVFPPEKRGTAMGTMGIAMMFAPAIGPTLSGWIV

EHYTWRILFYMVIPLALLDILFAFIWLRNVSKLTSPKFDGFGALFSTIGFGFLLYGFS

SAGDKGWTSATVLLTLIIGVLFIVFFVLRETAMKNPLLEFRVFKYDIFTISTLVSATI

NMALFGGMLLLPIYLQNIRGFSPLQSGLLLLPGALLMGVMSPISGVLFDRIGSRPLAI

VGLIITAISTFEFSKLTGETPYGHIMMLYTFRSFGMSMLMMSVQTEGLNQLPPHLTSH

GTAMSNTVRQVAGSIGTALLITVMATRAGIHLADYSNTVTTTNVALTEQVGILGTQMA

TAAGMPPEQGSSLALQQLYGIAVQTSTIEGINDAFIVATWISIIGLFLSLFLRRARSR

PRKVKPER"

CDS complement(1982601..1983236)

/locus_tag="PIKHDJKN_01842"

/inference="ab initio prediction:Prodigal:002006"

/codon_start=1

/transl_table=11

/product="hypothetical protein"

/translation="MNSRAILINIIVILVILGAGAAGIYYYNQSTSYVKTDNALVTGQ

PISVASAVGGELRSWKGKVGTTFNAGDTVATVTAAGKSTSVTMPVDGTIVQQTAVENS

LVSAGTPLARAYDFNNLYVTANVDETAIDKIKAGQIVDVYVDAFPDTTLTGKVDQIGL

ATASSFSLLPSSNTNANYTKVTQVIPIIITIEGYKGLGVVPGMSATVRVHI"

CDS 1983437..1983907

/locus_tag="PIKHDJKN_01843"

/inference="ab initio prediction:Prodigal:002006"

/codon_start=1

/transl_table=11

/product="hypothetical protein"

/translation="MLIILVVIVVIGVVFLMIYNSRSSRKAAEARASKITEVDEGPVL

LHEEDQARKEDRIYEQAEHKEVLEHEDHRQTDIHQMDSTSEVAPSPQKRETQSTDGDQ

AYRQALLHFRKPAEVEEEAVLTDTNEPEKSADEVYRSALKMMKSKSSDPSSKRK"

CDS 1984033..1984239

/locus_tag="PIKHDJKN_01844"

/inference="ab initio prediction:Prodigal:002006"

/codon_start=1

/transl_table=11

/product="hypothetical protein"

/translation="MNGQQRVNIKPGLEVDIVLKQDQPTGKLTRGIVKDLLTKSPTHP

HGIKVRLTSGQVGRVKQVVTGASE"

CDS 1984261..1984851

/locus_tag="PIKHDJKN_01845"

/inference="ab initio prediction:Prodigal:002006"

/codon_start=1

/transl_table=11

/product="hypothetical protein"

/translation="MITTTDVMAHLASGNERQQDAYKVLQSSGLLSISAAYQPYPAGT

VPIDIDIPGSDLDLLCEAADLGAFETLVHRQLGGMQGFRCDRGDGRAGQRPYVTCCVD

IGNWPIEIFAQSVSVRRQNAYIHMLVEWELLQLWGAPGHREIRKLKLEGLKTEPAFAK

VLGLQGDPYEAMLHLAAMSKDELWQWARLRTSFQFE"

CDS complement(1984866..1986302)

/locus_tag="PIKHDJKN_01846"

/inference="ab initio prediction:Prodigal:002006"

/codon_start=1

/transl_table=11

/product="hypothetical protein"

/translation="MNIQLVPLHYVYLAFIVLIIAVMVRRRDTTIICVAGIMTIGLLA

TETLSGSVSGIFSSFIYAAKELLSTIFIISIIVAMSRVLIRTGINEVMVTPLTRFIRS

PQVAYWGIGFIMMIVSLFFWPSPAVALVGAVLLPVAVRVGLPPIGAAIAMNLFGHGIA

LSGDYIIQGAPKLTADAAGLPVTAVMTASIPLVIVMGTVSTVTAFWMLRKELKSGSPV

KLSDPISNEAVTVLPPSSDSGPISETPAVQHQPMPPRLQKVFALIIPLLFLVDVILMF

LLKLQGGDATALIGGTAVLILIAVSLAAHRHTGPEETTNYLIEGFQFGFKVFGPVIPI

AAFFYLGDAAITELFNNPLPAGSHGIVNDLGVALAGLVPLNNTVGAITMTVTGAVTGM

DGSGFSGISLVGSIASMFAGSADSAPVLTALGQVAAIWVGGGTLIPWALIPAAAICGV

SPFELARRNLKPVLLGLTVTTIVAIFLI"

CDS 1986668..1988467

/locus_tag="PIKHDJKN_01847"

/inference="ab initio prediction:Prodigal:002006"

/codon_start=1

/transl_table=11

/product="hypothetical protein"

/translation="MNFIRSLRFKFIVGLTLIMLPLFILLYYNNVYAMKVVRDQVSLT

NMNHLAKSVEQNERVLQETNRYLYSLGERDPDIISLFFLKYGSGDYIIAKQRIMNKFM

TDIGFYNLIDSFFLYDAVNDDLLLATSGNYDVKMSIVQESMPVQMQQLEGEIQKPEWS

IVRGGTWNALVKTVRINAQFYAGALVDMNALNHPEQFTESGDRGGAVIVGADGGALSD

SALNSAQIKLAAAHITGLNNPYQVISEVTSTGSARQYLMLGIPSAMSEMNYIVLLPEE

DMMRNLPFFQQIIRLLPIGAAVILITAMIFLRQLLFRPMNVLIRGMRRVSRGELDVRL

ETPASSELEFVTHSFNQMTSEIQHLKIDVYEEQLRTREAEFKQLQMQINPHFYLNSLN

IIHSLASLQKHELVQQMAGHLADYFRFSLRAGKRVIRLDEELEHISHYLEIQKLRFPN

KLDFVLDVGPDLGHYVLPPLTIQPFVENAIIHGFQRRTEPFVICITARKSKTLSDPTL

SSVLQLSITDNGVGFEQEILERLQQGQYAEDPGGQHIGIWNVIHRLIVKYGKHAGLQF

SNQTEGGAAVHIHMPAQTEEEEGRAYAEPVDRG"

CDS 1988442..1990061

/gene="rhaR_13"

/locus_tag="PIKHDJKN_01848"

/inference="ab initio prediction:Prodigal:002006"

/inference="protein motif:HAMAP:MF_01533"

/codon_start=1

/transl_table=11

/product="HTH-type transcriptional activator RhaR"

/translation="MRNLLIVDDEVYALQAMVEGVDWSLAGIDQVFSAGDAEEARMLM

KAHPIDIMICDIEMPGETGLDLQSWVLKHDPGLLTIFLTGHALFDYAQTAIKLNSFDY

VLKPAPADQLLKVVSQAIDKIKDGEQRSRTNEVYETVYKRWQTHKPLLTERFWKDAIS

QRVTLTQQKLQELAEVYGAEIDPQVRVLPIVISVEEWVRDFDLRDEEVLEYALRNAAK

EMLLGDKPGEVFQDHSGLNIVLAYEQDGIVPTASEVAQGCQSYIQECGTYFYCRLSCY

VGVPVAVTDLQGMLNELMDMERRNINELEGVFIYDEVGRDTEDRFLPIPWFSELSILF

ETGKFDDLRERVDEIFELLASQERLSPEILRLYYHAMLHVIYPLLHQKNVSVRSLYPG

EREPEENVVTRSLPQLKQWTSDLISRAIPVLYPDDHSPMTIVDQLCIYIENHIGEELM

REELASFAGFNPAYLSRLFRKEKGMSLSEFILQRRVAKAKTLLSQSTVKVTDIAGKVG

YYNYSHFTKMFKKCTGITPQEFRKQSRTVQI"

CDS 1990236..1991231

/gene="yteP_12"

/locus_tag="PIKHDJKN_01849"

/inference="ab initio prediction:Prodigal:002006"

/inference="similar to AA sequence:UniProtKB:C0SPB3"

/codon_start=1

/transl_table=11

/product="putative multiple-sugar transport system

permease YteP"

/db_xref="COG:COG4209"

/translation="MKTQPQVGKAARLSDARDIPDIPVRKRKSVLYNLGKFKVLYLMF

LPGILFLLVNNYMPMFGVLIAFKNVNYADGIWGSPWSGWDNFKYLFSTSDAWEITRNT

LAYNTVFIALNLFVGVGLAILLNEVKNKAMSKLYQSLMLLPYFLSMIVVSYLVLAFLG

KDSGFMNSTILQLFGGQPIDWYSEPKYWPYILPLVNTWKNIGYYAVIYLAAVVGIDEE

YYEAAVLDGASKWHQIRFITVPFLVPLIVIMTLLQIGRIFYADFSLFYQVPLESGALF

PVTNVLDTYVYRTFLIGGDIGMSSAAGLYQAVVGFVLVLVSNTIVRRMDKDNALF"

CDS 1991249..1992157

/locus_tag="PIKHDJKN_01850"

/inference="ab initio prediction:Prodigal:002006"

/codon_start=1

/transl_table=11

/product="hypothetical protein"

/translation="MKSRDPLAVSRRSASIIHAMFIFYAIACIVPILLVFAISFSDET

TVIANGYKLIPEKFSLTAYEFLFKDMDQIIHSYGISIIVTVIGTITSVALTALYAYPL

SRRDLPYRGWFAFFIFFTMLFNGGLVPWYLVYVNVLDLKNSVLALIMPLLLSPFFVLV

MRTFFANSIPVSILESARIDGAGELRTFTRIVLPLSLPVMATVALFSTLNYWNDWYLS

MIFISDNRTISLQYLMYRTLLDIQYLTSNSNVSSQISSQGGLLNLPNKTLQMAMAVVG

IGPIVLAYPFFQRYFIKGLTVGAVKG"

CDS 1992338..1993864

/locus_tag="PIKHDJKN_01851"

/inference="ab initio prediction:Prodigal:002006"

/codon_start=1

/transl_table=11

/product="hypothetical protein"

/translation="MVRTFKVWPRIAAAVMALSLVLAGCSSDKGGTTSGAEGGGAAES

GGKPYEVTLYYPGTPQKDVALVEAEINKKMEPKIGATLKINAIDWGQWDNKLNLMISS

GEKSDIIFTAAWQNYTVNVAKGAFLPLNDLLDKYGQDIEKNLDPAFLEGSQVDGVNYG

VPTNKELAATRGVLVRKDLADKYKLDLTAVKTWADLEPLLKTIKENEPGITPFYMSNT

NGNGLLENLDWDYLGDASVPGVISKTAGGTTVLNEVETPEFKEAAELARKWYQAGYIN

SDAATSNVFPKDQAKAGKAFLWTDGMKPGKDKEEEGYVGYPLTQIEMTQPTITTGDAS

GAMLAISRSSEQPEKAMQVINLLHSDKEINNLLNFGIEGTHYVKKDGEDNIITLPQGV

DANSRTYNPGAQWQLGNQFLNYLWDNEDPQKWEKFKEFNAKGVKSPALGFTFNSQSVK

NEIAAVNNVNKQFKPGMTSGAVDPNEMIPKYLEKLKAAGIDKIIAAKQEQLDAFLSKK

"

CDS 1993987..1994685

/locus_tag="PIKHDJKN_01852"

/inference="ab initio prediction:Prodigal:002006"

/codon_start=1

/transl_table=11

/product="hypothetical protein"

/translation="MSRTRLVFSAMLLLFGIIFTFKHHLGFSVGDTMLSAIGMSPYTT

SYVSGVHITLFFGMGILACSYYLTRKEIMPLYPVLAKRLWLIVLAIVLSYSYMTDKLM

YVAKWGASGINGVSYVQNDSSCTYDVLENGITRVNCDLTLKNYTREPVAAVLLPDLAR

SYKHQDDPLYESLQSVQLKPVHIEIEPHGTFKGQLAFSGLAASPLRVKGKLTDIVMDV

AVDGENTVFDYDIP"

CDS 1995016..1996761

/locus_tag="PIKHDJKN_01853"

/inference="ab initio prediction:Prodigal:002006"

/codon_start=1

/transl_table=11

/product="hypothetical protein"

/translation="MSYRTNLFSKMVILILIMLVPVVLLYWYSNHKTTAVLRDELNRS

NSNQLEFFQNQVNTHIELLSSWPNLLIHDPDIASFRRIYADSKYFDLDTINLVKRIQN

KLSIQESSSNWTTKLYLYSPTLGRVISERDAGYYDQETLRGNISSGWNVRKIQEGEDD

RFIFSWITVSPYGISDPAANAQTVIKLEFDSDNIRDMLDKFKDDGRHDPFYYREETGV

IYNRTSDRALTGQLMDKLAIDKLKDVDNRTVVIDGEPYMVNTVKSSTTGWYLVDYMPL

SAILKPIHQSNITFYSAMICLLLMSFAVAYLLYVQVQVPVKQLIRGFQRLKQEDYSVR

IIPKGRNEFSFLSERFNSMVAQIQQLFEHVYLEQIHVREARLKQLQSQINPHFFYNCF

SFITSMAKLKRVDAVVAMSHNLSRYYRYTTRQERDVVPLTEEIEFVTCYLEIQQMRMD

RIRFKIDLSEDILRQEVPPLIVQPLVENAVIHGIEADAEAGEIRVSGEQRDGMMILTV

DDDGQGMDELARQMLLHKLTGGMDQDMGCGLWNVNQRLQLRYGERAGLSVMESPLGGL

RVTLSWPADEEHLLE"

CDS 1996847..1998469

/gene="rssB_3"

/locus_tag="PIKHDJKN_01854"

/inference="ab initio prediction:Prodigal:002006"

/inference="protein motif:HAMAP:MF_00958"

/codon_start=1

/transl_table=11

/product="Regulator of RpoS"

/translation="MIDILLVDDETYVTESLELTIPWGELGVTTVLRAGSGKEALQIL

EENAVDLVVTDIRMPGMSGLELIEEVSNRWAHIRCILLTGHSDFQYAKKAIQLQAADY

ILKPVNDEEFMASISAAITSLRDDWDEFDKYHRLLYSRKSDYKILRENLMHDLLLGRE

ISARALGEQLEKYEIVLQPDQPAVMMLIRLTGRFSAMDQQSLDLMEFAVGNIAEEVFG

PQLNVWFSRGPHECLVMIIQNQGLTEPEQTNMEELKQPAGTFREHVIRYLQGDLSMVV

TAPFHFADLTTAYRKGLGSLVLSGPEENTIIYMDKELSKRPENDAAQALEELYKPPVL

PQLLETKQWEAAARKLNAVFDAADRVCLSREHVYEMYLSVTNAFMYIAHKQGHLVHEI

DHAGFDLLLAHQLIQSPDKLRRWATEMLAKLQEELSDQEGVQSRRHVIKQVQEMVTRD

AGQDLSVKMIADKVYLHPVYLSKIYKAETGEGLGDYMIRMRMERALYLLKNTNKKIYE

ITSELGYQNPQYFSKMFKKHYGMTPNEYRDQA"

CDS 1998573..2000279

/gene="lipO_4"

/locus_tag="PIKHDJKN_01855"

/inference="ab initio prediction:Prodigal:002006"

/inference="similar to AA sequence:UniProtKB:P37966"

/codon_start=1

/transl_table=11

/product="Lipoprotein LipO"

/db_xref="COG:COG1653"

/translation="MIEGGTTLRAKSTKKRLLSLLATAVCLTGVLAGCTGGNSGGDSE

ASSTPAIENEYKEKYDPAVTLTTAWGIDPELKFKNGESMENNVSTKWAKEKFGIDIKS

LWSVTDTNGAFATKLRLAMSSGQEMPDVVTVGDNLLAQDLIDSGMYQEAGPLFDKYAS

DTWKKAMEQDPNVWNQYSRDGKRMGIPVLDYAYNNDYLLWIRQDWLDKLNLEAPKTID

ELEKVMDAFKNQNPDGLAPDKVIPLSIGFKTSMNTWMGDPSWIFGAYGTLPFQWNVAE

DGKLEYGSINPGMKQGLTKLSEWLKKGYIPQEAALWDENKTAEPAVAGTAGIIPGPYW

MSGWPLLDTVKNVPSAVWKPIEIPSGPEGKAMRHGTQFVNGVILIKKDMEHPEAFFTY

QNYLFDNYADPAPGSPYDNGLFAKYDYQLDANGKQMPIDQIEGGYVNVVRYLLVRDGA

RIPDAQMKALLNLADGKEPETKLEKDVAVNYGKETPAAAKVLLGQEEISYKNMFTGPT

TATMKSKLDYLNKIENQAFNEIIYGKNPVDAFDTFVQTWKSGGGDQITQEVNEWYDSV

KK"

CDS 2000528..2001418

/gene="yteP_13"

/locus_tag="PIKHDJKN_01856"

/inference="ab initio prediction:Prodigal:002006"

/inference="similar to AA sequence:UniProtKB:C0SPB3"

/codon_start=1

/transl_table=11

/product="putative multiple-sugar transport system

permease YteP"

/db_xref="COG:COG4209"

/translation="MRTLKRTWPFHVMLLPAIIFLIIFSYIPMGGIVMAFQNYKPWMG

ISGSEWVGLDNFRYLFEREDSLQVIWNTLIIAVLKLIFNLFIPFVFAILLNEVRKIAV

QRTIQTLVYLPHFLSWVILGGILIDLLSNEGLVNRALGAFGLGPYFFLGDNDWFRFTI

IISDVWKEFGYNTIVFLAALAGINPSLYEAAEIDGAGRWKQTLHITVPSLVPMLMVVG

TLALGNVLNAGFDQIFNLYNPLVYQTGDIIDTFVYRSAMVNGEMGFATAIGLFKSVIS

MILILVSYSLAKKYAGYRIF"

CDS 2001448..2002326

/gene="ycjP_1"

/locus_tag="PIKHDJKN_01857"

/inference="ab initio prediction:Prodigal:002006"

/inference="similar to AA sequence:UniProtKB:P77716"

/codon_start=1

/transl_table=11

/product="Inner membrane ABC transporter permease protein

YcjP"

/db_xref="COG:COG0395"

/translation="MYHKSLPYRVFNIFNTCFLILLAILCIVPLIHVLAVSFSVKSAA

DANLVSLWPVGFSLEAYKKTMNNPIFLHSIWVSLLRTVIGTGITLLITFLAAYPLSKE

NSVFKGRNIYSWLFVFSMIFNGGLVPFYMVIQKIGLMDSFWVLVLPGAVNTFLVILML

NFFRGIPQELEEAALIDGAGHFRTLFSIFLPISMPSIATIALFSMVFHWNSWFDGLLY

LNNAKDYPLATFLQTVIIGRDMSTMSMDPKEMEAISQTTVRAAQIFIGSAPILIVYPF

LQRFFVKGMTLGSVKG"

CDS 2002359..2003468

/gene="ganB_1"

/locus_tag="PIKHDJKN_01858"

/EC_number="3.2.1.89"

/inference="ab initio prediction:Prodigal:002006"

/inference="similar to AA sequence:UniProtKB:Q65CX5"

/codon_start=1

/transl_table=11

/product="Arabinogalactan endo-beta-1,4-galactanase"

/db_xref="COG:COG3867"

/translation="MGNLKEEAFILGMDVSFMDEIEQHGGSYRDEDGQQADLLTLLKL

GDANAIRLRIWNDPVGGFCNLERTVAVAKRIKEQGLQFLLDFHYSDRWADPANQWKPK

AWEKLSYEELQREVCTYTADVLRTLKEHDALPDMVQVGNEITPGMLWDEGRVGGEEHD

TDEQWERFAGLVKYGIAAVKSIDSEIKIMIHIDRGGDNAESRKFYDRFEALGVEFDII

GLSYYPWWHGTLDALRDNLHDLAERYGKPINVVETAYPWTLEQPEGHEWILNQEELLL

PGYPASVEGQTRYLKDLLQIIREVPGGLGAGFYYWEPAWIPSKEEWSVGHPNNWGNLT

MFDFKGQKLQSFSALKAGEENDTALDEQPSAALIK"

CDS 2003505..2005577

/gene="bglY"

/locus_tag="PIKHDJKN_01859"

/EC_number="3.2.1.23"

/inference="ab initio prediction:Prodigal:002006"

/inference="similar to AA sequence:UniProtKB:C8WV58"

/codon_start=1

/transl_table=11

/product="Beta-galactosidase BglY"

/db_xref="COG:COG1874"

/translation="MTYKYPPVSSKAPHMLHGADYNPEQWLRYPEVLEDDIRLMKLAK

CNVMSIGIFSWVSLEPEEGVYTFEWLDQVLDRFAANGIYAFLATPSGARPAWMSAKYP

EVLRVGVNRVRNLHGFRHNHCYTSPVYREKVTAINTKLAERYSDHPAVIGWHISNEFG

GDCHCDYCQDAFRGWVKNKYGTLDELNHSWWTTFWSHTVTDWSQVESPAPHGETQVHA

MNLDWRRFVTDQTADFIVHETKPLKAKNPDLPVTTNLMEFYEGLNYWKFADILDFLSW

DSYPTWHDADEEDTLASRVAMMHDIVRSIKGGQPFLLMESTPSSTNWQETSKLKRPGM

HLLASLQAVAHGSDSVQYFQWRKSRGSSEKLHGAVVDHVGHEHTRVFQDVTDVGVALE

GMQAVVGTSVPAEVAIIFDWENRWAVKDSQGPRNIGVKYEQTVEWHYDAFWKKGVPVD

VIDMDADLSKYKLLIAPMLYLVREGVGERIERFVENGGTFVATYWSGIVNENDLCFLG

GFPGPLRKTLGIWSEEIDGLHDRDLNGVVPVKDNGLQLNAEYDAIELCDLIHLEGAEA

LATYRSDFYAGRPALTVNRLGSGKAYYMATRFKAPFYDDFYGSLMADLGIERALETQL

PAGVTAHIRTDGTADYVFVQNYTPEPKQVQLDNQSYRDLLSGDAVEGSLDLQPYDIRV

LRRAVERK"

CDS 2005857..2007296

/locus_tag="PIKHDJKN_01860"

/EC_number="1.1.1.38"

/inference="ab initio prediction:Prodigal:002006"

/inference="similar to AA sequence:UniProtKB:P16468"

/codon_start=1

/transl_table=11

/product="NAD-dependent malic enzyme"

/translation="MNQRNLDGNSFIIRLEMTTKDIKFGEVASAISEAGGDIIAIDVI

STNQDVSVRDLTVAVTDAQDNSKIIEGVRQLKGASIINVSDRTFLLHLGGKIEVTPKT

PIHNREDLSRVYTPDVARVCAAISEEPGKAFSLTIKRNTVAVVSDGSAVLGLGNIGPR

AAMPVMEGKAMLFKQFAGVDAFPICLDTQDTEEIIRTVKAISPAFGGINLEDISSPRC

FEIERRLNEELDIPVFHDDQHGTAVVLYAGLINALKLVGKSITDVKIVVCGIGAAGVA

CSNILLSAGASRVVGVDRDGALVRTNTYENEVWSDYAARTNPELETGSLRDVIRGADV

FIGLSRGNLLTREDVQTMAADPIVFAMANPVPEIMPSVVEDIVAVMATGRSDYPNQIN

NVLCFPGMFRAVLDCRASEINEEMKLAAAQAIASAISDEERTRYYIIPSVFNDKIVKS

MRNRVIEAAVKTGVARRIPREQTREGGDV"

CDS 2007298..2008023

/locus_tag="PIKHDJKN_01861"

/inference="ab initio prediction:Prodigal:002006"

/codon_start=1

/transl_table=11

/product="hypothetical protein"

/translation="MQENSPQHLNLSTQQLGHGDQVDGTLTGEAILRAARAFVQGDAA

AHTDGHDWPHIERVTALAVELAHRIGADPFICELAALLHDVPDEKLNESLEAGMAKLN

AWLDTQPLEAGTREAVVHIISSISYAGGQRPAVSSLEAQVVQDADRLDALGAIGIART

FAFAGARGREMYDPALPPREQMTREEYRNGRSTTINHFYEKLFKLKDLMNTSYGKELA

EQRHDFMVQFVEQFKREWEGRFS"

CDS 2008275..2009342

/locus_tag="PIKHDJKN_01862"

/inference="ab initio prediction:Prodigal:002006"

/codon_start=1

/transl_table=11

/product="hypothetical protein"

/translation="MSFGARVLKTGIAVTLALYLSSMFLNPQSPVPAAIAAIFAMQPS

IYRSWKYFLDQLQTTTLGAIVALLGGMVLSNEPIAVGLIIVLVIMICLKLNMGETVGL

TLVTVVSIMEASGDWHFALNRFLLTLVGIVSAFLINITVFPPKPKIQFVKQIQSVFSG

MSLLLRTSISDEIKEVVFRDEKNNLGGSIKSLSDKYNLFEEEQKKMKRSKFSETRQMV

VYKQMLLSLQKGFDVLDSVERHYFQASRTPEMDEFFDTHLELVIKFHEHALLKFEDKL

KPNGEEAAQFILDNDSFMEQAISQFDIDQEGMLRLSIVAAAIYDYGYQLERLNRLAEH

VHSASEDKESQDKILNWLKWP"

CDS 2009483..2009944

/locus_tag="PIKHDJKN_01863"

/inference="ab initio prediction:Prodigal:002006"

/codon_start=1

/transl_table=11

/product="hypothetical protein"

/translation="MNLTQLGPEWMQWFNGQDLEQKQHEAMQLLTVSEDGWPHQAMIS

MGEVIAITPNLLRLALWPGTQTSMNMRRTGKATLIAVHQQSLLSIRLDVTSLPQLMEA

VHPRDRFEAQVLNVRVDHVPYAEITSGITFQLKDESGAIKRWRETIEELRQ"

CDS 2010187..2012562

/gene="helD_1"

/locus_tag="PIKHDJKN_01864"

/EC_number="3.6.4.12"

/inference="ab initio prediction:Prodigal:002006"

/inference="similar to AA sequence:UniProtKB:O32215"

/codon_start=1

/transl_table=11

/product="DNA helicase IV"

/db_xref="COG:COG3973"

/translation="MSTDQQWSEEQQRVNIVTEQIDRKIDILEKEVGSFRDEVVGMRK

DFWDEVTMNFSEADDVGETSTSMRQQSQVLSDRERSHLNTAAALDKMKRLHHSPYFGR

IDFKENGDPKAERIYLGIASLLDEKEESFLVYDWRAPISNLYYDGAPGPVTYHTPSGE

IRGDIEMKRQFVIRDGRIRFMFDTGVTIGDELLQAVLSRTSDAQMKSIVATIQKEQNR

IIRNDRTRMLIVQGAAGSGKTSAALQRVAYLLYKYREHLQADQMVLFSPNPMFNSYVS

TVLPELGEENMLQTTFQEYLERRLGREYQLEDPFIQLEYVLTGTEDPQYEARMSGIRF

KSSESFLKVITRYKDSMMTEGMKFKPVRFQGRAVVTAEAMAEKFYSFEPSVKLVNRLE

ILRDLMLKELSAFGKGELEAPWVDQQLDLMEPEDLQRAYQRLKRKQKGKSDTFDDFQQ

EREILARMVVSDRLKPLRKWIKSLRFVDVRQLYAHLFNDRSQMVRLLGEEELPSHWEE

ISEMTLRRLKVQELAYEDITPYLYLRELLLGFHINSNIRHVIIDEAQDYSAFQLAFMK

RLFPRSKITALGDFNQAIYAHSSVLSGTGPLTNLYGPENTEVIELTRSYRSTQEIVEF

TRGMVPGGEEIVPFNRGGEKPKVIVSSNEQEHLDVITADLKHLIQEGYESVAVICKTA

EESKDVHEALSKVLPAAPKLIKKTTLAFERGVHVIPAYLAKGVEFDAVLIYDGSASQY

AQEHERKLFYTACTRAMHLLHVYCMDKPSPFITSQSDGLYDMGKVSAAQAD"

CDS 2012883..2014739

/gene="ade_1"

/locus_tag="PIKHDJKN_01865"

/EC_number="3.5.4.2"

/inference="ab initio prediction:Prodigal:002006"

/inference="similar to AA sequence:UniProtKB:Q72EX7"

/codon_start=1

/transl_table=11

/product="Adenine deaminase"

/db_xref="COG:COG1001"

/translation="MNTPSFERPLMADCVPDLVATARGDLPATLVIRGGTLVNVVSGE

ILPGMSVAVQGARIAYVGKDVSHTIGENTRIIEAEGKYIAPGLLDGHCHIESTQMKVT

EFARAVLPSGTTGGFFDPHEISNVLGLKGLRLMLDEARTTPMAAYMQVASCVPSTHPG
LETTGAYIGPEEVAEALSWGPDMIGLGEVMNFPGVVYGDETMIGEIQATLRAGKVADG
HFTWAADDWRLPAYAASGVTGDHECVTKEDVVERLRLGMYAKMRQGSAWHDVAETIKA
CTELGLDTRRMMLVTDDRSSESLLKEGHMDFVVRLAISQGVKPVTAFQMATINTAERF
GVARDIGAVIPGNIADIILLDGRLADVRVGMTIAAGHVVAENGKMTAVWDSFTYPEEA
LNTVKLEANIQPKDMELSAPITEGTIGAKVIHVTENHVDTKEKHVNVTVEHGNVVIST
SGEICKIAVLERHKQTGNRAVALVGGIGFTGPAAIAMTVAHDSHNLLIIGNDDALMAE
AGNRVIGMQGGVAVVTASGVTEFPLRIAGLMSTESFEVVAVQSAAISEALQSAGCTLN
NAFMTLSLLALVVIPELRLSDKGLVRISAEGIELVSLFDEMVENTPAAPAGN"

CDS 2015130..2016050

/locus_tag="PIKHDJKN_01866"

/inference="ab initio prediction:Prodigal:002006"

/codon_start=1

/transl_table=11

/product="hypothetical protein"

/translation="MKSKKRYQFILLISCFMLFTGNTYVCKATALGSVDSYETIHGTV
QGSVRATWVWDTTQIQNNPQLVLKFATTNKINTIYLQMNRDVKLPYYTSFIRQAREKN
IAVDVMDGRPAWGLTESRGQIADFLDWVEAYQNQVEPNEKFAGIHLDIEPHVHPEWKT
NQASVITQWQGNVQYIVDRAARMKMPVAADLPFWLDGYKIPGSTMNVSSWMIRKFDSI
TIMAYRDTAAAIYNVAKDELEEASLLGKTVSIAVETKQSKEGDFITFYEEGTVYMEAQ
LKLVEKMASVHSSFNGFSVHEYSSWETLKK"

CDS complement(2016203..2017117)

/gene="rhaS_10"

/locus_tag="PIKHDJKN_01867"

/inference="ab initio prediction:Prodigal:002006"

/inference="protein motif:HAMAP:MF_01534"

/codon_start=1

/transl_table=11

/product="HTH-type transcriptional activator RhaS"

/translation="MERERLREDRIHGNAMYPVSVYPDIQQLNGDSILDCHWHDEMEF
TMVTQGSAVFQIDMNTVEVQAGEAIFINRGEIHAGYLKGDVPCVFSSIVFNPELLGSR
TFDAVQEKFIGPLVHKTVLPPYHIQTDEAWGQEIIFHLKRIFADHAAGTPTCEMTTKA

YLYLIFAQMFEHMRPAALKGTVPSGSHDKVERLKSVLSYIHKRYPEPLKLKELADEAN
MSEGHFCRFFKQMVQKSPVDYINYYRIQQACYQLENTDHKIVDIAMDVGFEHLSYFIT
TFKKHKNITPSQYRKMFYEKVAMEPALV"

CDS 2017290..2019602

/gene="yicI"

/locus_tag="PIKHDJKN_01868"

/EC_number="3.2.1.177"

/inference="ab initio prediction:Prodigal:002006"

/inference="similar to AA sequence:UniProtKB:P31434"

/codon_start=1

/transl_table=11

/product="Alpha-xylosidase"

/db_xref="COG:COG1501"

/translation="MKFTDGFWMTREGYHVQNPTDIRDIVQKEDSLTVYAATKYIRTK
GDTLNGTLLKATYSSPMPNVIRVTLNHHKGRVKIGPEFELNNQDAKVDISKNEQGAVL
KSGNLEVHIDKTNGWDVSFLYGGKRITGSGQRAAGYITGPSKEAYFREQLDLGIGEYV
YGLGERFTPFVKNGQVVDTWNEDGGTSSEQSYKNIPFYLSNKGYGVFVNHPERVSYEI
ASENVSKVQFSVEGETLEYFIIGGDNPKDVLDNYTKLTGKPALPPAWTFGLWLTTSFT
TDYDEATVNHFVDGMAERDLPLSVFHFDCFWMKEYQWSDFVWDEAMFPDPEGMLARLK
EKGLKICAWINPYIAEKSYLFDEGMENGYLVKTADGSVWQWDMWQAGMALVDFTNPDA
VNWYKSKLEVLLDQGVDSFKTDFGERIPTDVVYFDGSDPVKMHNYYTQLYNKAVFELL
EEKIGKNEAALFARSATAGGQQFPVHWGGDCSSTYESMAESLRGGLSLGLSGFGFWSH
DISGFESTATPDVYKRWVQFGLLSSHSRLHGSTSYRVPWLFDEESVDVVRDFTKLKIS
LMPYLYNSAVESTVRGIPMMRAMLLDFPEDPTTYTLDTQYMFGDSILVAPIFNKEGDV
RYYLPEGTWTNYLTGAKVEGGRWISENHDFKTLPMMIKPNSLIAVGAVDSKPDYDFAD
NVSLHLFELADGNSAQAAVVNQSAEQELVVNVSRNGSVLEVRAEGAGKPWNLVLRGIE
SVSGVEGGSQASGANGVVVTAAASSLTIQL"

CDS 2019929..2020939

/locus_tag="PIKHDJKN_01869"

/inference="ab initio prediction:Prodigal:002006"

/codon_start=1

/transl_table=11

/product="hypothetical protein"

/translation="MSTTNSGLHQTITAEMLHKLVENTFGTATIVKSFALLQGGLFNT

TYRIQLEHASYADVILRLAPERVEMPLGSAGDPLFSFERTMMSAEPVVYEYYGKAGIP

APNIIACDDSGSIIPRTYMFMEFIPSKQLDHPSISESDKERLYHQLGAYTAVMHQFGG

ASFGWPQGDGTIKGSDHWSEVLHSFAEETALKASIAGYMPGVGEEIADLFKINKDLFD

QVTVPVLVHNDLWEANVLVHEENGQLNIAAIIDGDRSMFADRQFEAILSTESSAGFHE

GYGRPQDMSDEGQARLLAYRILSSYFNAYVHEHQVDQPEAGQKYRERTLDLLKEWKRF

QT"

CDS 2020962..2023874

/gene="xyl3A"

/locus_tag="PIKHDJKN_01870"

/EC_number="3.2.1.37"

/inference="ab initio prediction:Prodigal:002006"

/inference="similar to AA sequence:UniProtKB:D5EY15"

/codon_start=1

/transl_table=11

/product="Xylan 1,4-beta-xylosidase"

/db_xref="COG:COG1472"

/translation="MKEYIQYPMWDATLPLEERLNDLIARLTTEEKIRLIPTREAAVP

RLGIPGYNVGGEAAHGVAWIGEATVFPQPLGLSSTWNTKLMREIGSVIGDEARAYHHR

APERHGLTLWAPTVDLERDPRWGRTEEGYGEDPVLTGEMSAALVKGMQGNHSFYLKMV

ATLKHFFANNNEKDRLNCSSSIDPRNLREYYLKAFETPFVEGGALSMMTAYNSINGTP

AIESPYVNDVVKGEWAMPGFIVCDGGDLSQTVNYHGYHATHAESAAGALKAGVDCLTD

EVDLVVSALEEALERNLLEVEDLDRAIRNIFGVRMRLGQLDQTGLNPYASISESVLCA

PEHAKLSYRAAAESIVLLKNDGLLPLQSERLQKVSVIGPLADVVYTDWYSGTLPYRVT

ALEGLRKQLGGAEVSFADGNDRITLKTGDGKVITLGAEGKLVAVPEGSAEDAEFIHQD

WGWGSHTLRSVKNNRYVSLTEQGIYQANAPEIGGWFVKEVVQIDSQADGNSTLRTWNG

LPLVLNERQGQLVLSPTQEQTAEDLSSSGNPDATEGTQEPAVFKLDVVANGIEQALEA

AQNSDVSVVVVGNSPYINGKEEIDRPSLLLPPEQARLVREVCQVNPNTVVVIVGSYPF

ALQELKDVAPAIVYLTHAGQELGNAVADVLLGNYAPAGRLNMTWYEDESQLPDIMDYD

IIHNNTTYMYHEGPVLYPFGHGLTYSEFEYKAIRVERAQGSFGDDVLKIEVQVENKGE

RASDEVVQLYGHAQTSRVKRPQRQLIGFRRIHLAPGAVENVSFEIPAQKLAFWDVTRD

RYCLETATWSIMAGRSSADIRQTAEIQIEGETIPARALNVPTFAENYDAYAGVLLDEC

LEGRSAVRAIASSEPLYHEGRSSWISFHDNAFRQASEFECRVAAFGEGGELEIRAEGP

EGPLLGTCVVPGSAGTVNGKNLQWNTARCSLKAEREIEQLCIVFRGGAALQQFVLK"

CDS 2024052..2025353

/gene="ymfD"

/locus_tag="PIKHDJKN_01871"

/inference="ab initio prediction:Prodigal:002006"

/inference="similar to AA sequence:UniProtKB:O31762"

/codon_start=1

/transl_table=11

/product="Bacillibactin exporter"

/translation="MKDKKWDLVALASIPVIMTLGNSMLIPILPQIERELKVSSFKVS

MLITVYAVVAILLIPLAGYLSDRFGRKAVIIPSLIIAAAGGAVAGGAAWMLNGNAAYW

SILGGRLLQGIGAAGAFPIVIPLVGDMFDDENEVSKSLGIIETSNTFGKVLSPILGAA

LAVWIWYLPFMAIPVLCVLSLVLVIFLVKTPKRKEEPKSFPEFVASIRSVLKEKGRWL

YAIFAIGGICMFVLFGVLFYLSETLESEFKLKGVLKGLVLAIPLAALCLASFFAGKWI

GKNKTRMKWLGFIGLAVLTVSLGVIGLFDNIYVVVGLFTLGGAGIGATLPCLDAMVTE

GVDKEQRGTITALFSSMRFIGVSLGPPVVSLLISSHHFLLFGILAASGAVGGLLTLLA

VKPDQGNQPTSGSGQNQREHDYDEVKQPGGYLPGRRKKSPL"

CDS complement(2025448..2026770)

/gene="yfcJ"

/locus_tag="PIKHDJKN_01872"

/inference="ab initio prediction:Prodigal:002006"

/inference="protein motif:HAMAP:MF_02091"

/codon_start=1

/transl_table=11

/product="putative MFS-type transporter YfcJ"

/translation="MKSQSSQVKLWTTDFILLMLCNFLLFLQLHMIVSPLPSYVQDRF

HANAFEVSLFTCLFALSAIAARLYSAKALEKGLRNAMIYIGLTVALLATLGYYFAAGI

AVLLLLRMVFGIGFGMSSTAFPTMASDIVPVKRMGEGMGYFGLSTSLAMSMGPIIGVT

VLQGAGFVTLMLCTAGVIVVIYPLSYSLTRKKARRVNDEAIPTPPVGTAAISGTKVKT

PFNRKLILPSVLNCLLSITYGGLVGFIVLYGKEANLANPALFFLFNALAVLLVRPFAG

RIYDSKGPKALLIPGAIFIAAGLIMLSYASSMSILFVAAFLYGIGYGSIQSSMQTWMI

QIVSPSQRGMANGMFLNTLDLGIALGALLLGAIASITSYTDMYRYSTVFMILFLLIYL

IQGKRNGGFAVPVHPLLSHTHIAAQDSDRHEHTETASENNTTTQQKDN"

CDS complement(2026803..2027231)

/gene="slyA_2"

/locus_tag="PIKHDJKN_01873"

/inference="ab initio prediction:Prodigal:002006"

/inference="similar to AA sequence:UniProtKB:B1JJ73"

/codon_start=1

/transl_table=11

/product="Transcriptional regulator SlyA"

/translation="MGNDYSLDQSIGFMLGMTHRKASALLAIRFKPYDITTEQFSVLF

NIGRGEGVNQKEVATRVYKDQPTTARIIDLLEKKGWVERRTSEQDRRAYLLYLTEEGK

KLLDQLVPLEREMNKNLIEGIPEDQMEAFRHTLSLMNSNL"

CDS 2027530..2028003

/gene="fucU"

/locus_tag="PIKHDJKN_01874"

/EC_number="5.1.3.29"

/inference="ab initio prediction:Prodigal:002006"

/inference="similar to AA sequence:UniProtKB:P0AEN8"

/codon_start=1

/transl_table=11

/product="L-fucose mutarotase"

/db_xref="COG:COG4154"

/translation="MLKNIPAIIPPELLKIMSEMGHGDELVLADGNFPAASHAQRLIR

CDALGTVELLDAILSLYPLDTYAERPAAVMQVVKGDQVVPIIWEDYRRLILDHEGITD

AFDHEERFAFYERASRAYVIVATGERAQYANLILKKGVIFPDTDPYPQAKKVELN"

CDS complement(2028012..2029130)

/locus_tag="PIKHDJKN_01875"

/inference="ab initio prediction:Prodigal:002006"

/codon_start=1

/transl_table=11

/product="hypothetical protein"

/translation="MIKLSAGQAYRFMFVYLYSEPIAFLLQRLFKMSGYQGWISILGG

FAISLILLFFTYRLGAVHPDRPWIDFGEEIVGKVIHKLFLGLIVLLCLYLISVDVENF

IVFLQSMYLPETPIWLTATITILCISLSARSGLITIVYLSEGIFLVQVFTSTFLMPAV

MGGGNPDVLLAMVTHHDLKEILTGSISTMPWFSEWMMFLFLAPAIAFRRPLLRSMLFA

GSTLALFIIVFWMFTLMNFGPYEASSLRYPLLEMIRFARYGDFLDNLDPFLIAIWSTT

MFTRSSFLLYVASICLAKLTGVRKDRTVVYLLAGTAASYVLQYAKDAASYELAIRSFA

IATYGVLIECMPILYVVVYWLRFGKGKKTSEASSAPSP"

CDS complement(2029145..2029351)

/locus_tag="PIKHDJKN_01876"

/inference="ab initio prediction:Prodigal:002006"

/codon_start=1

/transl_table=11

/product="hypothetical protein"

/translation="MIAVVLFAVLYILEWPMIKSSKKGMKRTYFVLLGIFLLWNTLAV

SVPSWPNPNDVIRLVFGWADRLFG"

CDS complement(2029395..2030546)

/gene="gerAC"

/locus_tag="PIKHDJKN_01877"

/inference="ab initio prediction:Prodigal:002006"

/inference="similar to AA sequence:UniProtKB:P07870"

/codon_start=1

/transl_table=11

/product="Spore germination protein A3"

/translation="MKTLMVTTKLSVVMICVLLCSGCWSKVEINERTFITAMYVDKGE

KPGEIDISLSMPLPNRLSPEGTGGPGKNPYALVSATASTIADAMEKIQTDLTRKISWG

HTRVVVFGEAYAREGIQDAMEWISREPLFHLSSYVMVSKGRARDVADLTPVFEETPSD

VLREFATEENMLKTQMLSVLMADKMNQGFAAPLLESKGTAMASEDNRRKQWTSQRGGA

LFKQMKMVDSISPDETRTIAWANRKLDTLAVSVETDDEKASMTVYNMNSKIKVRLVNG

QPFFDITLRGQAELNSVIPILKVKDIVGVKQIEQKANEKVSTFLTDAIRTGQRKKADI

LMLGYRLEWAYPRVWEQLRPVWNDYVKNELQFNVHAQINIQFVGSESSY"

CDS complement(2030543..2031988)

/gene="gerAA_1"

/locus_tag="PIKHDJKN_01878"

/inference="ab initio prediction:Prodigal:002006"

/inference="similar to AA sequence:UniProtKB:P07868"

/codon_start=1

/transl_table=11

/product="Spore germination protein A1"

/translation="MEKDNIYEQVKANLLGVADIIYQPFQVGPFHCELIYIQSIVDTP

TMREAIVKPLLEEASRNEIDHQFITRVVTGSFFTLNSHHSNSPEAIVDNIVSGNAALY

IEGISGMIHFSLQNYQRRSVPESTNEVVIVGPQEAFIEDIHVNMSLLRHKIKHPDLKM

IKFTIGKYTKTEVFVVYIQGLCKTDILENVLESLGNIDMDSSLGVSYLSEFLEDHPLS

PFPQYQYTERPDTVAAALVEGRIGVMQDGTPFSLLIPVTFFSLMQSSEDYYQRFYSAS

FIRLIRLLFAFIAFLLPSFYVAVTTFHPEIIPTNLLITIASARENIPFPALIEALIME

VTFEGLREAGIRIPKPLGQTVSIIGGIVIGQAAVQAGIVSAPLVIVVSITGIAAFIIP

HFELGLAFRLLRFPVLFMGGTLGLFGIVISIYLIYWYMVSLRSFGVPYMQPFAPLVLR

DIKDTIVRVPWFLMKKRPAAYEDANKKRQKT"

CDS 2032198..2032695

/locus_tag="PIKHDJKN_01879"

/inference="ab initio prediction:Prodigal:002006"

/codon_start=1

/transl_table=11

/product="hypothetical protein"

/translation="MAYRIRRAELSDVNGVAQLLNDYRMFYEQRTDLEGASEYIKERM

ERNESVILIAESELETEKEEECSLTNRPLAGEFGGFIQLYPSFSSVSMRPIWVLNDLY

VHADHRQRGIARKLLEAARLWGNESGAVRIVLSTAISNKKAQALYESEGYTLDTKFMY

YELHI"

CDS 2032749..2033369

/locus_tag="PIKHDJKN_01880"

/inference="ab initio prediction:Prodigal:002006"

/codon_start=1

/transl_table=11

/product="hypothetical protein"

/translation="MMQWIAMITMLIDHIGAVFYPQVGELRIIGRIAFPIYAFAVYIG
YKHTRNVQKYIWRLFWIAVISQLPFMAAFNHFSLNVVWTLWSALLVLIVLDKLPVRML
SIPIVVGAGLIMEMTHMDYGMYGLLLVLLFRYFQGPVLVVAHLLLNVLYIFLHSSSIQ
MYSVIATAGIAMAQYYNAGFRLKGPRWIWRYFYPAHLAAIALIRLI"

CDS complement(2033476..2037183)

/gene="lgrD"

/locus_tag="PIKHDJKN_01881"

/inference="ab initio prediction:Prodigal:002006"

/inference="similar to AA sequence:UniProtKB:Q70LM4"

/codon_start=1

/transl_table=11

/product="Linear gramicidin synthase subunit D"

/translation="MVENQSSSPQTVESGLPVLQIPLDFGRRQQSFTYESTHISLESS
LGRELKQKFGSEGVYPALLSAYAALLFRLSAEQELAIGTLAPDQAASHVLLQIQGKLT
FKDLIHQISDQLQLHYTLQAGGNPETLFMFNSVQLPKAPQLLNWNIRDDQNMLTLDLF
YDRSLLNESTVTRYAEYYQTLLLAMLRDQDKAIGTVDILSASDRLLYREMNDTAVLEP
EHQTIHGWFEATAAEYPESPAITSSTGRYTYRELNERANQVARVLLSNGLQKGEFVSI
FMERSLETIISLLGILKAGGVYVPVDPEHPQERNSYIVEDTASSFVLTKESSLPEASR
LFSGITTVRQILAVDGRLAGFAASNPNLDIQPDDLAYIIYTSGSTGKPKGALIAHRGV
ANLGSVVQRDCDIQPGDVLTQFATYSFDASVWDTIGALFYGAELYLLSAEERVSVEEF
ASAIERTGTTIITILPTIFFNQLASFLSDEGFHKLAKVKIITVAGEALYGEQVRAFQR
KFGNQIDIVNVYGPTECTVATATHRISEQVPESVVNIPIGRPIHNYKVYIVNEEQQLC
PVGVPGEVYIATPALAKGYLNQPERTAQAFIENPFAIDEKIYKSGDIAKMLDNGQLEY
VGRSDSQLKIRGHRIEIGEIEDHFARLDQVQDVAVIPKKESDGQNMLVGYFTSKDGST
LSVADIKAELTVKLPSYFVPKWICQLDEMPIAPTGKINRKAMVSLPHVERHEDRPDRV
LPETKTEAIILESWKEILQHDDFGVEDSFFAIGGDSLRVIHVLVILKPHYPQLKIADF
FAEKTIRALAQRVETLAQAKEETAHSSVNDGVITQLSEHPVELKSQIGYPEILNPEHV
LLTGATGYLGSHVLQQLILTSNTRIYTLVRRPADGSSAMERLSSVMKGYFGPELLPLL
ASRVEIIEGDLEQPNLGLSVEQTTYVQERIDRVIHCAADVRHFGDVAQFAKTNVDGTT
ALLKLVRSKPGASFHHVSTMGIPEDLALSGQWESSLQYDRFPADLHVDNLYSDSKLEA
EKVLMIAAEEGVPVSIYRAGNLTCHSETGRFQSNIDSNAFYRMIKAMLLLGKAPAADW
MVDFTPIDYASQAIVHLALRQDTAGRVFHICNPEPISYDDLIRSVNRAGYEVETLPFA

EYTSWLFDGSVSKDPEALQLAIAQLEGDGAKDSAYVYASPVTTAYVEPAGITCAKTDD

HFISKMLEHAIEIGYFPKAVRPQFGTSPTME"

CDS complement(2037230..2038996)

/locus_tag="PIKHDJKN_01882"

/inference="ab initio prediction:Prodigal:002006"

/codon_start=1

/transl_table=11

/product="hypothetical protein"

/translation="MKSLSWKSMSFFSKNLLLSFTNIIIIGVALIASSYYFQKTILVD

QLHGQVEQITKKWAEGIDSTEVQAAIAEGNYDGKAQTKLRAYFDEMQQYYPNIAQAYI

FGVELGGDNKRLTSLVAMPTNLKEAFQSENVNIGDMYEQPVVVANALKEMLNTDRPTF

TTFYSDDFGTWTTIAYPIKDSNGKIYSYFAVDADASAVPAGLNKLLKNGIIILVAFLL

LFLIIQYLVVKNTLSPIRHLIKGIDDVSRGNLNVKIPTGKDDLGIVNEKFNTMVRKIN

DTIVKVQLTSQEVNESAKELYEVSERNSENADSINSNVTQITSNIRAQEQATRDSARA

MSEMAIVIQTIASSSASVADEAYEMERRSQQGNSVVRQVSEQMNLITESVKNTASAIQ

VLESRSQEIGDILNIISGISSQTNLLALNASIEAARVGEEGRGFAVVAGEVRKLAEQS

EQSTSQVAVLIQEIQAEIRQAVRAMEQGTSEVDTGLSVADQTGQLFEEILEAAKKVSN

QIQEVSSATEEISAGTEEMTATADDLSASVSKTADNSERISSSVDEQKASLITLVDSS

TRLNSMSEELQELISHFNVSKQ"

CDS 2039604..2040275

/gene="rpiA"

/locus_tag="PIKHDJKN_01883"

/EC_number="5.3.1.6"

/inference="ab initio prediction:Prodigal:002006"

/inference="similar to AA sequence:UniProtKB:Q72J47"

/codon_start=1

/transl_table=11

/product="Ribose-5-phosphate isomerase A"

/db_xref="COG:COG0120"

/translation="MNLKQIAAERAAEYVQDGMKVGLGTGSTAYYAICRIGERVREGL

NIQAVATSEASDKLAKEWGIPIVPFDQIGRLDLTIDGADEVDPEFNLIKGGGGALLRE

KIVAANSDKLIIVADGSKAVQKLGKFPLPVEVVPFASEWTFQALEKLGCHPEWRMDGD

QRYLTDNGNLIADCRMEAIEHAAELNVQLNMLPGVVDNGLFIKMADTVILAKDDGSID

ERHRS"

CDS 2040300..2040899

/locus_tag="PIKHDJKN_01884"

/inference="ab initio prediction:Prodigal:002006"

/codon_start=1

/transl_table=11

/product="hypothetical protein"

/translation="MRENPLVDGEIVLRCVEKEDLTELYELIYSEDVPEWKQWDAPYY

ALEHESYEDFERSMLRRLEATQSNADPESIRIIELNGRIVGTISYYWEHRPSLWLEMG

IVLYRSAQWGRGIGTRVIQMWSSHLFEQLPLARVGLTTWSGNERMMRSASKAGLQVEG

RMRKCRIVDGKHYDSIRMGMLREEWEQIRSSGAGRNVNN"

CDS 2040914..2041372

/gene="mhqR_2"

/locus_tag="PIKHDJKN_01885"

/inference="ab initio prediction:Prodigal:002006"

/inference="similar to AA sequence:UniProtKB:O31672"

/codon_start=1

/transl_table=11

/product="HTH-type transcriptional regulator MhqR"

/db_xref="COG:COG1846"

/translation="MPNNEDNRELSLQLFVVLARAYNSVTSRSNRDIQSHGLNTTEFG

VLDLLYHKGPQALQKIGEKVLMSSGNITYVVDKLQNKNLLIRRASKEDRRVIYAELTE

EGRHLFTQIFPQHHQVIIDAVDGLEPSEKADAIRLLKKLGLAAEGKTDLR"

CDS 2041548..2042066

/gene="rcsC_5"

/locus_tag="PIKHDJKN_01886"

/EC_number="2.7.13.3"

/inference="ab initio prediction:Prodigal:002006"

/inference="protein motif:HAMAP:MF_00979"

/codon_start=1

/transl_table=11

/product="Sensor histidine kinase RcsC"

/translation="MSNSNEHGEMKSSHRTNESLSNQMVHGKENQEQNRTSLPISILL

ADDNVINRQVVQMQLKKLGMTQVDAVSNGEEASEAFLSNKYSMILMDNMMPVMDGLEA

TRHIRAIEQDKLWHPIPIIAMTGNVMDGEKEKCFEAGMDDFIGKPFTLEALNNVIQKW

QQASLSGEITKR"

CDS 2042122..2043084

/locus_tag="PIKHDJKN_01887"

/inference="ab initio prediction:Prodigal:002006"

/inference="similar to AA sequence:UniProtKB:P9WLC7"

/codon_start=1

/transl_table=11

/product="hypothetical protein"

/db_xref="COG:COG1073"

/translation="MLLYSLAVLVLIVLALLWLGGLYFFKTAIRRAPKSFLVDNPNLM

PEPDNEIISSASDLTWLDTQPTKDIYVESHDGLKLHGTWLPSRRGSQQPTVILAHGYS

GKGREMAGFARFYAEKMGYNVLMPDDRGHGQSEGNIIGFGWLDRKDYVQWVHWVLGKL

GTETEIILHGISMGGATVLMTGGEALPDQVKAIVSDCAYTSVKEELTFQLKQLYKLPA

FPFIPVTSLISKWKAGYFFGEASALNQLAKVQVPVLFIHGEADSFVPTEMVYRLYEAC

PTEKELLTIPRAGHGTAFQVDRTRYEQVLESFLTKNQQHSSDSN"

CDS 2043131..2043481

/locus_tag="PIKHDJKN_01888"

/inference="ab initio prediction:Prodigal:002006"

/codon_start=1

/transl_table=11

/product="hypothetical protein"

/translation="MPYTPRVCSYCHEKMAEDEFKCKSCGNLVTTKTVQPEQPYMIEN

DEVILDRYSIVLLVLLTLFLPPVAMTIGGVMLFNDNTYKQDTGKLLVTGAFVVMVILT

IILFAGSGTLTINI"

CDS 2043499..2044212

/gene="COQ3_3"

/locus_tag="PIKHDJKN_01889"

/EC_number="2.1.1.222"

/inference="ab initio prediction:Prodigal:002006"

/inference="protein motif:HAMAP:MF_00472"

/codon_start=1

/transl_table=11

/product="Ubiquinone biosynthesis O-methyltransferase,

mitochondrial"

/translation="MGNEQFEAARKAEAGYHFNFYQNNELFASGTWMARPMPMVMDML

ERLLAHKTELRVLDLGCGVGRHTIPIAQRLGQTNSEVIGVDLLDEAVDGLRKYAKEYQ

VDHIVQVEKADVEHYAVKPDHFDYIAACSCLEHVSNKQALVEAIERLQVGTRTGGIHC

ITMSTSVEEKEINTGREIEPLIELNLPTAEAIALLEKAYANWNILLQEHVTQTIEEEK

YDEPTQFRCELLRFAAQKQ"

CDS 2044335..2045321

/gene="murK_3"

/locus_tag="PIKHDJKN_01890"

/EC_number="2.7.1.-"

/inference="ab initio prediction:Prodigal:002006"

/inference="similar to AA sequence:UniProtKB:Q97ML3"

/codon_start=1

/transl_table=11

/product="N-acetylmuramic acid/N-acetylglucosamine kinase"

/db_xref="COG:COG2971"

/translation="MSTYVIGMDGGGSHTRVALADENGKVLTYVQKGGCNRYHDANAE

QNVLEGIAEAIRGAGLEVKKIASIQAGMAGLDRPEDTVWAEALLAQTGILGRISAVND

THIAHTAAFGGEPGIVAIGGTGSLILGKTEQGIWLRNDQFGHYAPTAARFLAYDAVHS

ILAGRYVPQDQPWIEQILAYWNVTSIPELAALGVSGFAENRQAINRIFGQMAPLVTEA

AAENIPLAVRVCDSAADTAVVGILMLASCFKDQQVRMTLTGSCLSTPYMVEAVRKGLD

AAYKPGGQQIHYITSHVPAVGGALLDAYHLAGIQVPEGITSDLQSELKLHMT"

CDS 2045385..2045822

/locus_tag="PIKHDJKN_01891"

/inference="ab initio prediction:Prodigal:002006"

/codon_start=1

/transl_table=11

/product="hypothetical protein"

/translation="MLIQLKSRIQEPEVQELLSYSVFPDPDHLNRALQQYVNKDELHL

DGYEDEGQLIGLIGYESTGTSEITIHHIAVLPENRFKNYGRGMISQVLAKYNPDRLIA

ETDVEAVEFYRNTGFVVYSLGELYPGVERFRCVLEKDEEQGED"

CDS 2046036..2046494

/locus_tag="PIKHDJKN_01892"

/inference="ab initio prediction:Prodigal:002006"

/codon_start=1

/transl_table=11

/product="hypothetical protein"

/translation="MNTPDARSLVNRYLDASFLVTKRFDTQIRERLGQTITTDQFCAL

RMIEEKPLCTPSDLAELLCVGKSSITALVNKLVDRDLVHRAGDERDRRVVYLTLTDNG

REVYRETEQEIQQLLEPYLVHFKPEEVQNFIESFEKLAALLTHEGGQENE"

CDS 2046491..2049658

/gene="smc_1"

/locus_tag="PIKHDJKN_01893"

/inference="ab initio prediction:Prodigal:002006"

/inference="protein motif:HAMAP:MF_01894"

/codon_start=1

/transl_table=11

/product="Chromosome partition protein Smc"

/translation="MRTILKARWWLMGLWVVVAAVLMFTAPNMSELIREKGQFSVPEG

HSSTQAAAILEEAAAQKGEQQGSQLALVFYNPDGLGAEGKEEAEQAVKKLEAEKEKLG

ILSILEPFSQPELSDKMISADGKTILTSLSIDQGDRTVKEMREDLNEALSSVNVEHYI

TGKGLIDEDTVESSQEGLKKSEYITVVFILLILFLVFRSFVAPFVPLLTVGISYIVSQ

QIVAFLVDGLDFPISTFTQIFMVAVMFGIGTDYCILLISRFKEELAHHENTWDAIIAT

YRTAGKTVFFSALAVLVGFIAIGFAQFMLYRSAVAVAVGIAVMMLALVTIVPFFMAVL

GKRLFWPSKGSLEHAESKIYGAAGRFSLKRPWAALLIVAAVCVPLLATYDGKLSFNSL

DEIGEKYDSVKAFNIISDSFGPGESLPGQVVIQNDEAMDNAKYMALAEKISREVEKVP

GISGVRSMTRPTGDEIKDFEVTQQVGTLSDGLGEGKTGLDKIRDGLSEASSQLSKNEP

QLKEAADGAGELTKGTSQLQSGITQLSEGLQQIEKGIRDGSSGAGDLKAGLQKAKTSA

DQLAQANNQLLQAYRQAGAGVAALGDVVGELQQQLKGVSTALTSLNESFTALEERYPE

LLQDADYQRVKGTLGETGTGTAQLAQGLGQIQTKLGEAAAGINQANVGFASAASGQKA

LADGLGQIVTGISQLQSGLKQAADGQGKVISEIPSIQNGLGQLQGGQEKIQQGFSDLS

GQLTQLTDGLNQSVDGIEQVSGGLDSAQDYLTQLQNSPDSDLAGWYVPEEALNSKDFT

QVFDTYLSKDRKTMTIDVIFAENPYGTEAIDRVPDIEAAVHRAVQGSALEKADIAIGG

VTSTFADLQEVSNNDYTRTVMLMLAGTFIILVLLLRSVIMPLYLIVSLLLAYFTSMAL

TEAIFVNILGFSGISWVTPFFGFVMLIALGVDYSIFLMDRFNENKGMRVQDAMLYAMK

NMGTVILSAAVILSGTFAAMYPSGVLSMMQIATVVLSGLILYSLLFLPFFVPVMVKMF

GRANWWPFPNKEQADSVDSDRNIGM"

CDS 2049853..2050479

/gene="lipC"

/locus_tag="PIKHDJKN_01894"

/EC_number="3.-.-.-"

/inference="ab initio prediction:Prodigal:002006"

/inference="similar to AA sequence:UniProtKB:P42969"

/codon_start=1

/transl_table=11

/product="Spore germination lipase LipC"

/db_xref="COG:COG2755"

/translation="MVYRYVAIGDSLTVGTGALLGTGFVPLYRRMAEMNVRTFVSMDN

LGVNGLTSGEMLQMISNNPRVRQSLREADIITLSIGGNDLIRTFKASGGIPNASKMTQ

VLGDTRSNVSQIMRHIRQLKGNSVYMVRTIGLYNPYPQAAEAAYWVRQYNSFLNGAGS

GNYACAQVYDRFEGRERELLFWDRVHPNAKGYRVIAEQLNRTGYYPFA"

CDS 2050601..2051656

/gene="thrB_4"

/locus_tag="PIKHDJKN_01895"

/EC_number="2.7.1.39"

/inference="ab initio prediction:Prodigal:002006"

/inference="protein motif:HAMAP:MF_00301"

/codon_start=1

/transl_table=11

/product="Homoserine kinase"

/translation="MHIIDSGSLNDSEQQLISTVLSSYGFTSNWKAQRGKGGMNNSTF

MVDVDEECYVLRQYETHNDQMKIAFEHEVLSALSRSEFELDVPAPVSLPDEKHATFLA
VQDRSSGRSKIVTLFHYREGHNPVWNTPDQLSGLGRAAGMLSSVMAHLPISLAPVYPP
YYQIQEAYPLCSPEKLLQLCISPLDTLMACADDLQKLKVVLPDLFNTLRGMELLPHQL
VHGDLNASNVLVDSQDIICAILDFEFATWDLRVMELAVPMSDLLTMDKEQAWMWEALE
GLIQGFRQQVNLEPEELSVIPALILLRSLDVVMHFISRLFEGTDEPEVAVQQIRKLRE
RVDWMKGNEERLRELLI"

CDS complement(2051826..2052503)

/locus_tag="PIKHDJKN_01896"

/inference="ab initio prediction:Prodigal:002006"

/codon_start=1

/transl_table=11

/product="hypothetical protein"

/translation="MLIKFLIFGLLWRIVGNPFLAVLIFLIILYFLDRRYVGVMPSFM
KPLKRMRNISRLRQQLAMSPNEVSSKLDLARLLIERKRYSEAHALLLELERPYEQSAE
YWEELGTTELHLGQTEEGERHILQALDINPRVKYGRPYLTLAGAFKDTHRDKALAYVQ
QFQEIHSSSSEAYYLLGSVYRSLGRTADAKQAYEQSLNVYRSLPKYKKRQERRWAVRS
WFRKRGL"

CDS complement(2052521..2054509)

/gene="fusA_1"

/locus_tag="PIKHDJKN_01897"

/inference="ab initio prediction:Prodigal:002006"

/inference="similar to AA sequence:UniProtKB:P80868"

/codon_start=1

/transl_table=11

/product="Elongation factor G"

/db_xref="COG:COG0480"

/translation="MLNELNRRNIGIFAHVDAGKTTTTEHMLFQSGVVRSPGRVDDGT
TATDSLDIEKERGISVQAAMTSLIWKDTIIDLIDTPGHIDFSSEVERSLRVMDGAVLI
VSAVEGVQSQSETIWHALRSLGIPTLIYINKMDRVGASAETVIDQIRSSLSLFVCEIQ
SFCINEDVFEGIDSLWNESQTQVKGSSSSIPGLFELLAELDEDLMEAYINGETLPVDQ
LDAAFRKQVHQGEVFPICYGASGKGIGVTELLDAIIEFLPAPAQPQEPSVSGVVFKIE
RDKTMGRTAYVRMYGGSVHNRDTIYNSTRKLEEKVTQIRRMDGRKWADTGAVHAGQIA

ALYGLSETHVGDIIGSPEGVPPVPQLAVPLLTVQVHGKDTARYPDLVAALQELTDEDP

LLDLQWLPDERELHLKVMGTIQLEILSSLLMSRFGLDVVFDPPSVIYKETPRSSGEGF

IAYTMPKPCWAILRFSIEPLPRGSGLVYSSTVRTDHLLLRYQNEVERRIPEALSQGLL

GWEVTDLRITLIEGEHHVWHTHPLDFVVATPMGIMDGLASTGTTLLEPILNFRLTVPE

EYGGKALSDLVHMRATFEAPIIGGGRCIVEGRMPLASSMDYPVKLRSETSGRGVLTTS

FAGYQDCPSEVTHTRKRRGVNPLDQSKYILSVRNAITS"

CDS complement(2054646..2055527)

/gene="rhaR_14"

/locus_tag="PIKHDJKN_01898"

/inference="ab initio prediction:Prodigal:002006"

/inference="protein motif:HAMAP:MF_01533"

/codon_start=1

/transl_table=11

/product="HTH-type transcriptional activator RhaR"

/translation="MERSPVRTTIQAESGIPFRLVYADTKSPQNELPDHLHDWHEIIY

VYRGQGTIFIDTGLEDMQEGDLFIIPGSTVHRALPDAGNPVTSSALFFSPGLLASTAG

GNASAMLSLFERCRKRRVYKRHLQAKEQAQVAALIDDIHAEFQLEHHLSAHAALLRLQ

LLLIYLERLEAVGPASPARSVPGPGWLSSTLTHMDEELRSGLSLPELARQAAVSPAHF

SRAFKRYTGMTLTDYALARRVIMAKEHLLRSDETMAVVADACGFESLPHFYRQFKKLT

GTTPAAYRKTMKSQDQT"

CDS 2055731..2057245

/gene="uxaB"

/locus_tag="PIKHDJKN_01899"

/EC_number="1.1.1.58"

/inference="ab initio prediction:Prodigal:002006"

/inference="similar to AA sequence:UniProtKB:O34354"

/codon_start=1

/transl_table=11

/product="Altronate oxidoreductase"

/db_xref="COG:COG0246"

/translation="MSASTKRPRLKLNLLGSDSQRKCKEVMERPVKVLQIGEGNFLRG

FADWMLHESARQGKFHGSVAVTQPRPGGKAKLEQIRDQDGLYTMITRGLSQGKVVERT

EMISIFSQCINPYEEWQDFLKLAELPSLEFVISNTTESGLKYTYSEYTQGEPVQSFPG

KLTVFLHQRYLRFDGDPSRGLIHLPCELLEGNGDVLRSCVLRHSEDFGYSEGFRSWIE

NHNLFLNNLVDRIVTGAPTKEEADSLTNRWGYEDQLVNTAEPYHFWAIQADEALDKKL

PLKQAGLNVHWVKDLKPFQLRKVRILNGAHTLMSSLGILQGKEHVRETMEDPQFGSWV

REAVHQEIVPALDMPDHNLDQYAAEVFERFLNPYINHKLQDIALNTVGKFKVRVLPTL

LAYEQNQGSWPARLVKGFAGLLCLYRPVATPDGYMAQRLNGESVLLRDDVDVLAALAK

HWDSYDPLKRDKQQLEQSVAAVLSDVSIWGENLDDREGLREALVYEIGLLEGES"

CDS 2057245..2058759

/gene="uxaA"

/locus_tag="PIKHDJKN_01900"

/EC_number="4.2.1.7"

/inference="ab initio prediction:Prodigal:002006"

/inference="similar to AA sequence:UniProtKB:P42604"

/codon_start=1

/transl_table=11

/product="Altronate dehydratase"

/db_xref="COG:COG2721"

/translation="MNTTRTVNDWIAIQPQDDVIIVLRDYVKGESITLPDSTAFTLRE

DVPKGHKIAVHTLAPGEDVMKYGFSIGIAKEQIEQGSWIHSHNLKTGLHGLLDYQYQP

GPPVQTDMPPEHLRSFDGYLRPNGEAGIRNEIWIVNTVGCINKVCEALARMGQSQFGS

RVDGVYHFPHPFGCSQLGDDLKYTQQVLASLVEHPNAGGVLVIGLGCENNQVDQFREC

IAPEYQGKVRFLKAQETDDELEEGLRLMEELVELVEHEQRQPLPLSKLKIGLKCGGSD

GLSGITANPLVGSVADMLVAAGGTAILTEVPEMFGAETILMNRAANKQVFDDLVGLVN

GFKQYFVNHGQNIYENPSPGNKAGGITTLEEKSLGCTQKGGRSSVVDVLRYGKRVTQT

GLNIVEAPGNDLVSVTALSAAGAHIVLFTTGRGTPFGGPVPTVKIATQSDLANRKKHW

IDFNAGQLLEGKTMEDVKVQLFSQLIDIASGRAHTLSEQHGFREIAIFKDGVIL"

CDS complement(2058869..2059555)

/locus_tag="PIKHDJKN_01901"

/inference="ab initio prediction:Prodigal:002006"

/codon_start=1

/transl_table=11

/product="hypothetical protein"

/translation="MMNEHESIVTYEEAVQRIEAVGLLPLAPLFPEYPSLSSITPKEN
WHKDNALDPWLWRSRLAEEGIAAYGKFLKNKAILVSREYFPWVHRLLADSRVMKEQYD
AGLLSREAFKLYEIIEKHEGIDGRTLRSQAGFGAKEDKKAFDQGLLDLQGISAIVICG
TKEKEGLAEGKVAWNSSAYETSGCWMKRQAIKPFEGSVADASELLVQQLEKHTSAAAL
TKIKKSFAIH"

CDS complement(2059575..2060546)

/locus_tag="PIKHDJKN_01902"

/inference="ab initio prediction:Prodigal:002006"

/codon_start=1

/transl_table=11

/product="hypothetical protein"

/translation="MNRTNRLAAIVMALQHGHETAHSLAENFEVSRRTILRDIQSLSE
MNVPIIAISGPGGGFRLMEGYVLPPLQLDPVEAATLIFALEGLSHYADTPFQEKRWTL
MDKIKSIIPDDVKARMDPMLKQLQHDVPERNYILHHLEPLLACIPEQGWLRMLYRSAS
RQRWLTICPIRIYASAGFWYCEAYSEEHGEQRLFRADRILDVTPVELLESQQLAEQAK
RQRSKPKSPEPSPTRVTARLSYSGMIEAEQDKHIGERMTEIAPDVWELSFLCPPGEWA
WAVRFFYRLGSEAEVLEPENLRCEIRKHAEEVYQMYLTSNNNRCSTR"

CDS complement(2060959..2062986)

/gene="bgaP"

/locus_tag="PIKHDJKN_01903"

/EC_number="3.2.1.23"

/inference="ab initio prediction:Prodigal:002006"

/inference="similar to AA sequence:UniProtKB:Q09HN2"

/codon_start=1

/transl_table=11

/product="Beta-galactosidase BgaP"

/translation="MISSKLPKMFYGGDYNPEQWDHETHIEDLRMFKLAGIDIATINV
FSWALIQPDEVTYNFEGLDQYINNLYESGVYTCLATSTAAHPAWMAKRYPDVLRVDTN
GRKRKFGGRHNSCPNSPTYRKYSEKIAEKLAEHYKDHPAVLVWHVSNEYSDDCYCENC
EKAFRVWLKERYKTLDNVNKTWNTNFWGHTFYDWDEIVLPSNLSEHSGDNNSTFQGIS
LDYSRFNSDSILDCYRLEYDAIKKHIPDSVVTTNLMGFYKPLDYFKWAKYLDIVSWDS
YPGLATPVSYTAMAHDLMRGVKDGQPFMLMEQTPSQQNWQPYNSLKRPGVMRLWSYQS

VAHGADTIMFFQLRRSIGACEKYHGAVIEHVGHENTRVFREVAELGKELQLLGDKTLD

ATVEAKVAIVFDWDNWWAIEKSSGPTVALNYVDQIHKYYAAFFRRNIQVDIVSVDTDI

SKYDIVLAPVLYMVKPGFAAKLEKFVEAGGTFLTTFFSGIVNESDIVTTGGYPGELRK

LLGIWVEEIDALLPEQRNSIVMKEAYGDLQGEYGCGMLCDLLHSEGAEIIAEYGDDFY

KGMPVVTRNTFGQGEAWYVASDPEDRFLDGLLGQLAATKNIDSLLDTPEGVEVTARTK

DGQQYLFVMNHNATAQSYDLGKAEANDLLTDRSLSGTIEIEGRGVQLLEMK"

CDS 2063120..2063953

/gene="rhaR_15"

/locus_tag="PIKHDJKN_01904"

/inference="ab initio prediction:Prodigal:002006"

/inference="protein motif:HAMAP:MF_01533"

/codon_start=1

/transl_table=11

/product="HTH-type transcriptional activator RhaR"

/translation="MLVSPHYQRYFMTPRDQPLPLYIESIGYNGNQENVYRPAGYPCY

HWLQTVKGTGEFRFAGSTVLLGETSGILLPPNEPHEYVRSQGEWETLYITFGGSQCPA

ITESLGLGEAALHQWDSESPLEYYGREVLGSISSDQDLSGLEASADMYRFLILLKKHG

MTGSRSSISHAVERLAPLISFMNEHYADPEIGLEQMAAIPGITPRHLNTLFKQSFGMT

AYSYFILLRIRKSKEWMTGDSKLTVKETAAKVGFRDASHFVATFRRIEGVTPEQYRTL

Y"

CDS 2064054..2064203

/locus_tag="PIKHDJKN_01905"

/inference="ab initio prediction:Prodigal:002006"

/codon_start=1

/transl_table=11

/product="hypothetical protein"

/translation="MRKGATRYDDIDSLEWKTTIRINVSVQLREDDRSDINYTIVGPC

STLCG"

CDS 2064154..2064942

/gene="axeA1"

/locus_tag="PIKHDJKN_01906"

/EC_number="3.1.1.72"

/inference="ab initio prediction:Prodigal:002006"

/inference="similar to AA sequence:UniProtKB:D5EV35"

/codon_start=1

/transl_table=11

/product="Acetylxylan esterase"

/db_xref="COG:COG0657"

/translation="MTSTIPLWDHAAPYAAKGHEDEMPHLIPFIQPGSESAVIICPGG

GYGFLADHEGAPIAELLNRAGISAFVLKYRVVPHQHPAPMADGQRAIRYVRAHAEQFG

IQPSKIAVLGFSAGGHLTATLGTLYDEGQPDHEDPIERESSRPDRVILCYPVITMESY

GHAGSRENLLGSDVSAEQIKAFSAEQQVKADAPEAFIWHTSDDQAVPVENSLRYALAL

GAHGIPYDLHVFEKGSHGLGLAEEDNAVRAWSDLCLTWLKNQGW"

CDS 2065041..2065703

/gene="axe2_2"

/locus_tag="PIKHDJKN_01907"

/EC_number="3.1.1.72"

/inference="ab initio prediction:Prodigal:002006"

/inference="similar to AA sequence:UniProtKB:Q09LX1"

/codon_start=1

/transl_table=11

/product="Acetylxylan esterase"

/translation="MKLQKNDKLLFIGDSITDCGREHPVGEGSSGLGHGYVAQVYALL

RSIYPELMLRIQNVGNSGNTIRDLKQRWDRDVLDLKPDWLTIMIGINDVWRQFDNPLS

TDSQVFLEEYESTLRDLVASVRPGLKGLVLMSPYYLEANPEEPMRATMDIYGEAVRRV

AAEYDAIFVDTQAAFTPFWDHFYTSVLTYDRVHPDATGHMVLSKAFLDAIGFEWSGGV

KS"

CDS 2065720..2066616

/locus_tag="PIKHDJKN_01908"

/EC_number="3.1.1.99"

/inference="ab initio prediction:Prodigal:002006"

/inference="similar to AA sequence:UniProtKB:P0DOV6"

/codon_start=1

/transl_table=11

/product="6-deoxy-6-sulfogluconolactonase"

/translation="MSNLNIAVHTPALLGEGPSWDAEHNRLLWVDIESYKIHVYVPAT

GEDRTYDVGEHVGAVVPYQDDKVVVALRSGFHTYNLLTGVLQPIEDPEKGKDNNRFND

GKCDALGRFWAGTMSMNNESKAGAFYCLEEGQPVRTLFRDVSTSNGLGWSPDQQKMYY

IDTPTRSIDRFDFDLAAGEITNRTSVIAIPEEFGYPDGMTVDSEGMLWVAHWGGGRVT

RWNPHTAELMQQIEVPADQVTSCCFGGPDLEDLYITTARIGIQEERLTETTNAGSLFV

VRPGVKGQKTYAFGGGAQSDTK"

CDS 2066980..2070063

/locus_tag="PIKHDJKN_01909"

/EC_number="3.2.1.-"

/inference="ab initio prediction:Prodigal:002006"

/inference="similar to AA sequence:UniProtKB:Q3MUH7"

/codon_start=1

/transl_table=11

/product="Xyloglucanase"

/translation="MKTFLGKLRVMSLTVAVAAVSLGVLAGTTDAAPSDSYQWKNVVT

GAGGGFVPGIIFNDAEKDLIYARTDIGGAYRWNPANESWTPLTDFVGWDDWNKNGVDA

LATDPVDPNRVYMAVGTYTNSWDKNNGSILRSTDRGDTWQTTTLPFKVGGNMTGRSMG

ERLTVDPNKNSILYFGARSGNGLWKSTDYGVTWNKVTSFPNPGTYVEDPSYEYTSDIV

GLSWITFDKSTGSAGQATQTIYVGVADKNQSIYRSTNGGATWSAVAGQPTGYLPHHGE

LDANGNLYISYSDGAGPYDGEKGDLWKLNTSTGVWTNISPVPSSSSDNYFGYGGLAVD

AQHPGTLMVATLNSWWPDANLFRSTDSGATWTRIWEFEGYPNRKMRYTQDISAAPWLT

FGTNPAPPEVSPKLGWMIGDLEIDPFDSDRMMYGTGATIYGTKNLTDWDDGNKINISV

MAKGIEEIAVLDLISPPSGAHLLSGVGDVSGFRHNDLDQAPATMFTSPNYSSTESLDF

AELSPNTMVRVGKADYTADPNAKSIGLSSDGGTTWYKANAEPAGTTGGGTVAISANGN

RLVWSTSDKGVHYSTGGNSWTASSGIPAQAKVISDRVNPNKFYGFAAGKVYVSTNGGA

SFTASAAIGLPVEGNAVLDAVPGTEGELWFAGGSEEEGPYGLWHSTDSGATFTKLANV

EEADSIGFGKAAPGQNVVALYTIAKIDGTRGFFRSDDGGANWVRINDNQHQYARVTTI

TGDPRLYGRVYLGTNGRGILYADRIGGNNGGGEGGGETPVTSSTITPVNVEFDKNTGN

QADIPVTLTLNGNTLSAIRNGSTTLVSGTDYTLSGNQVVLSKTYLASLPKGSAALTFH

FSAGNDAILNLTIKDTTAVPAGAIRIEMFNGTTSATGNSINPKIKLTNTGTSAINLSD

VKIRYYYTINGAQAQNFFCDWATAGNDNVTGAFSALSTPVTGADSVLEIGFKTSAGSL

AAGQSTEIQARFSKTDWTNYTQTDDYSFAPSQTAYTDWNKVTGYIAGSLQWGIEP"

CDS 2070348..2071895

/gene="cheB_3"

/locus_tag="PIKHDJKN_01910"

/EC_number="3.5.1.44"

/inference="ab initio prediction:Prodigal:002006"

/inference="protein motif:HAMAP:MF_00099"

/codon_start=1

/transl_table=11

/product="Protein-glutamate

methylesterase/protein-glutamine glutaminase"

/translation="MNRLCKVLIVDDEFLVRQGIKHHMNWEAEGFQIVGEASNGEEGL

EQVHSLQPDIVITDIVMPVMDGETFVRTLKASHPQIEVIVLSSFSEFEYVRSTLQNGA

ADYILKPKLDTNELLQVLQRTAGKIPELQFEPSHDGWRLGQLMEKMLSGFTLDEENEM

HVIRETFPHRCFRLLVYKPQDTQAHVHKLDNEQLESRLRDLLPDVECAIVPAEGLSPV

VLLNVEPSKDEWMVQRIKELASENKDGQDGPCWVLSDSFSSFEEMGDVYRTRLIKLME

YRFYYEDRSILVYGELPPLHPAGYQFNVNMFLQHVKRNRTEAAREYLQEHALTLGRDY

IADVFEIKSFLGNLIFNVTITLADMDVQSAALEESKYAYFKHVDGASSLTEAMNVLDQ

FMAEVQECTTGEGARRSDPNMKMLLEYMHQHFDQPLGLAEVAKHFHFNPSYLSSYFSS

HKKEGFNEYLNKIRIEKAEELLRSDDVTISEISSMVGYSDHSYFCKVFKKFTGLSPSR

YRRKFWA"

CDS 2071915..2073681

/locus_tag="PIKHDJKN_01911"

/inference="ab initio prediction:Prodigal:002006"

/codon_start=1

/transl_table=11

/product="hypothetical protein"

/translation="MKKWMNAFSRLGLFPKLFLVMFVSIILVSVLILWTSIHMSTKLF

TETFSITNSKVLSQIKTNFESFNDAIAAVSNNVSQSGAIRGYLSEGEGDSLTMAKSYY

NMRETMDRIQSITESYEVGITISGINGRTYSTDRSHLRLSADELKEQPITLEAAASPN

RIIFDDYQTNDETAQQMISATKALTDRAENRLYGTLYVTMRENAFRQFYSSFTSRGND

VVILNDAGEIVSSNRVEWIGTTQLELLTYAREMNNESVKGVNARVMDQDSVVLSEYLP

FYRFYIVNVVDKDLAMGQLIDMKTIALICAAIVAGALILVFLITNQITKSLRRLVKQM

SNITKSDLDNYIPVSGSYESRQLGHAYNYMLDELHDYVDQLVLTQREQRNAELAALQS

QINPHFLYNTLASVKVLVQQGNKDRAAETINALISLLQNTISNVSETITVEQEVENLK

NYVFINHVRYGGRIKAAFYVAPDCTHYHVPKLVIQPFIENAFFHGFISKETGTIHVMV

SRAGESLICEIMDNGDGIEGFSMGESLPNPKNNRQLFSGIGIRNVHDRIELLYGAPYG

VTIMSKPGEGTRVTVTLPLITS"

CDS 2073970..2075340

/gene="lacE_1"

/locus_tag="PIKHDJKN_01912"

/inference="ab initio prediction:Prodigal:002006"

/inference="similar to AA sequence:UniProtKB:P29822"

/codon_start=1

/transl_table=11

/product="Lactose-binding protein"

/translation="MNSSRANAFRKRKQITHKEVEGELNRLKKMWVLMLVTVLLLSAC

SSGGGGNKASEGEKTSNEITIWAWDKAFNVAALETAKEAYQKQNPDLKINIIEYAQAD

IIQKLNTGLNSGTSSGLPNIVLIEDYRSQSFLNAYPDAFKDMSSTITASDFADYKLGP

TAFDGKQFGVPFDSGVTGLYYRTDLLEQAGYKAADLQDITWDDYIKIGKDVKAKTGKD

LITLDPNDLGLIRMMIQSAGSWYSKEDGKTPDLAGNAALKEAFVTYKAMMDANIVKLN

SDWSQFVAAFNNGDVATIPTGNWITPSVRQEASQSGKWAVAPLPKMAGQSNSVHASNL

GGSSWYVMNNVAGADQAADFMGKTFGSDKQLYQDLLNNIGAIGTYKPAAEGEAFDKAD

EFFGGQKVFADFAKWTQEIPSVNYGINTYAIEDILVVEMQNFLNGKAIDDVLADAQKQ

AEAQLN"

CDS 2075479..2076375

/gene="lacF_5"

/locus_tag="PIKHDJKN_01913"

/inference="ab initio prediction:Prodigal:002006"

/inference="similar to AA sequence:UniProtKB:P29823"

/codon_start=1

/transl_table=11

/product="Lactose transport system permease protein LacF"

/translation="MKAMNTGDSLQKKNNLTGWAFISLAAIGIVAFYFYPMLQALLLS

FKSGVGANLEFTGLDNYKRLFIDTTFRTAVSNTFLYLIIQVPVMIILGLFISVLLNDS

TLRFRSFFRTAIFLPCVTSLVAYSVVFKYLFSSDGMVNQFLMNLHLIAEPIQWITDPF

WAKITIIIAVTWRWTGYNMIFYLSSLQNIDQSIYEAARIDGANAFTQFFKITVPLLKP

IILFTSITSTIGTLQIFDEIMNITKGGPGNATMSISQYIYNLSFKYSPDFGYAATVSY

SIVLMIIVLSIIQFKVAGERNG"

CDS 2076368..2077198

/gene="lacG_2"

/locus_tag="PIKHDJKN_01914"

/inference="ab initio prediction:Prodigal:002006"

/inference="similar to AA sequence:UniProtKB:P29824"

/codon_start=1

/transl_table=11

/product="Lactose transport system permease protein LacG"

/translation="MAKAKAKRIFTYVFLSIVAFVSIFPFFWMLVSSTNASVDVTKGR

LLPGSVFLDNFNKLLDSTNLVQALGNSAIVSVISTILALLIGSLAGYGFEVYRTKSRD

IVFNILLLSMMIPFAALMVPLYRMFATISGFAPFIGINTMAAVILPTITTAFLIFFFR

QNTKMFPKEMLEAGRIDGLSEIGIFLKIYMPTMKTTYAAAAIITFMSSWNNYLWPLVV

LQTPDQQTIPLLISNLGSSYSPDYGVIMTAIVIATLPTAIVFFIMQKHFVAGMVGSVK

"

CDS 2077374..2080421

/gene="cbgA"

/locus_tag="PIKHDJKN_01915"

/EC_number="3.2.1.23"

/inference="ab initio prediction:Prodigal:002006"

/inference="similar to AA sequence:UniProtKB:P24131"

/codon_start=1

/transl_table=11

/product="Beta-galactosidase"

/translation="MKATQADINWLGDVSVFEVNRLNAYSDHRYYRTMEEAKASGAMA

MRYDLNGTWKFNYAIRPDCRPEAFYKQDFSSDGWDAIEVPGHIQLQGYGQIQYVNTQY

PWDGLNDLRPPALPQDQNPVGSYIRTFHLPTGWQNSPVYISFQGVESAFYVWLNGQFV

GYGEDSFTPSDFDLTPFLQDGENKLAVEVYQRSTGSWLEDQDFWRFSGIFRDVYLYTV

PAAHIRDVRVVTDLDKSYTHGTLNVDLKLEGKAAAGAKVEVELRDAEGNTVKTFGGIE

VTNGLVSFREEIGKVNLWSAEIPYLYRLYIQVYDAAGELLEVVPQAVGFRTFEMIDKV

MHINGKRIVFKGVNRHEFNPRRGRAVTKEDMLWDVQTIKQNNMNAVRTSHYPNQSLWY

ELCDEYGLYVIDEMNLETHGSWQKLGAVEPSWVIPGDLPEWHDIVMDRAVSMVERDKN

HPSILIWSCGNESHGGEVIYKVSQYFKSADPTRLVHYEGVFHDRRFNDTSDMESRMYA

KPADIEEFLNANPEKPYISCEYMHAMGNSIGGMHKYTELEDKYPMYQGGFIWDYIDQA

MYKKDRYGKEFLAYGGDFGDRPSDYSFCGDGIVHADRKVSAKMQEVKFLYQNIKLFPD

REGVKIVNDNLFADTSEFELVYSLDREGHEVLRGTLAVNVGPQSETIVKLPLDVESLA

GGEYAVNTAIVLKAATLWAEKGDEVAFGQFVFAQDKSKIVESDLKLINEIQVVEGDVN

IGVRVGQTHVLFSKAFGTLVSLKLSGQETIAQPPMPLFWRATTDNDKGTAMGFELGAW

YAASLLPRCVNWKAEQNTDQYRIEFTYKLNISADVQVKVAYTVRADGSVHVHNTYKGT

AGLPDLPIHALSFKTSPEFENVEWLAMGPEENYADRAFGARLGIHDSKVVDTVAPYLV

PQESGNRTGVRWAKLTDNAGRGFKIESAAVPVELNVSPYTAFELENAQHAYELPPVHY

TVVTVAGKQMGVGGDDSWGAPVHPEYRIASDGELQFEFVIRAL"

CDS 2080563..2080868

/locus_tag="PIKHDJKN_01916"

/inference="ab initio prediction:Prodigal:002006"

/codon_start=1

/transl_table=11

/product="hypothetical protein"

/translation="MSRKAKTGVWVTILVILGIIVGCFIWYFNTASGERALKTMRSNN

SGGLERVVNVYSNSGELIHTYEGKIDLQDTEYGNKVLFDLNGKRIVIYNATVISEEK"

CDS 2080974..2081273

/locus_tag="PIKHDJKN_01917"

/inference="ab initio prediction:Prodigal:002006"

/codon_start=1

/transl_table=11

/product="hypothetical protein"

/translation="MGMLMVLIAILGMGAFYCLIAYFLIRLISRKAFKRILTKYEAIE

IVVWAAMVFFAIVMIKNQSWNMLLPVVLLMVSMINLRRSNRKYREMSKEMNKEGL"

CDS 2081275..2081505

/locus_tag="PIKHDJKN_01918"

/inference="ab initio prediction:Prodigal:002006"

/codon_start=1

/transl_table=11

/product="hypothetical protein"

/translation="MILDKSTDTSGLTQVAALSDELQPVFDKAERKYQACDIELFFVF

RCLPEEYERKSTVRYAKKENTIYFDMTVSDGQ"

CDS 2082054..2082665

/gene="rlmI_1"

/locus_tag="PIKHDJKN_01919"

/EC_number="2.1.1.191"

/inference="ab initio prediction:Prodigal:002006"

/inference="protein motif:HAMAP:MF_01857"

/codon_start=1

/transl_table=11

/product="Ribosomal RNA large subunit methyltransferase I"

/translation="MRVVSGSAKGRPLKAVPGTGTRPTTDKVKEALFSMIGPYFEGGT

ALDLFAGSGGLGIEALSRGMDKAVFVDLESKSIEVIRANLKATKLEDQAAIYRNDASR

ALKALAKRSTQFDLVFLDPPYRMKNGDELMLTMHELDLLESEATIVLEYESKYSYPEQ

FGPFEQTRKALYGETAVSIYYYAPAATTEDGEPSTAEEEAPHE"

CDS 2082658..2083173

/gene="coaD"

/locus_tag="PIKHDJKN_01920"

/EC_number="2.7.7.3"

/inference="ab initio prediction:Prodigal:002006"

/inference="similar to AA sequence:UniProtKB:O34797"

/codon_start=1

/transl_table=11

/product="Phosphopantetheine adenylyltransferase"

/db_xref="COG:COG0669"

/translation="MSEMIHRQERIAVYPGSFDPVTMGHLDIIARASKQFDRVIVAVL

NNMSKNPLFTVEERKSLITEVTSHLPNVEVDSFRDLTANYVRQKDAQVIVRGIRSVTD

FEYELQLASTNSKLNPDAETIFMMTNPKYSYLSSSIVKEIAHYHGDVTDLVSPEVEVA

LRQKISEKTGG"

CDS complement(2083151..2084407)

/locus_tag="PIKHDJKN_01921"

/inference="ab initio prediction:Prodigal:002006"

/codon_start=1

/transl_table=11

/product="hypothetical protein"

/translation="MTMQSEVTGSGPLRTVLMSTGALLLVIAVVVSPKDAFDASIQGL

DIWWKIIFPAMLPFLMLSQMLTAFGFTHALGVLLGPLMQRWFRLPGKAGLAVVVGMCG

GFPAGADTASRLVQDQQITAKQAGIVASASHFANPLMIILVIGAAFLHQPAAGYFLLI

VHWVSGWIATMIGVRLLPKESKGEKHSTANPSSYKRRSLWSQMMFAAREAQERDGRGF

GKLLGDTVSQAVQTLMMTGGYMIVFAVFVRLLTLYITPGTSVAFWPSLLELHLGTYHL

SQSPLTSVMLMSLLAAVLGWGGLCSHLQVSAVLKAAGLATTSMLYFAGIRLLHALVAF

FISLLLWLPFSRYSSEVWVTFQNNPGNGLSPFLTKQSLVGMNTSDIIDAVWIGFPAAC

IGLALLLTVMICLSGLTFWFNRRFSR"

CDS 2084611..2085651

/gene="ylbL"

/locus_tag="PIKHDJKN_01922"

/inference="ab initio prediction:Prodigal:002006"

/inference="similar to AA sequence:UniProtKB:O34470"

/codon_start=1

/transl_table=11

/product="putative protein YlbL"

/db_xref="COG:COG3480"

/translation="MNRIRKSGGFRASIFVIVVALVVYVAVYMPTPYIIYMPGSADEV

KPMVTVKEGDKEERGVFMMTTVSATYANVFLLGTSLFNRNAQVDKKEDRLRGKSEAEY

SAEQVWFMSDSQSSAMEAAYEQAGVKYSIVPEHIFVFGLSEDPKPKGDIEPGDIILGV

NGTATPDNTVLSEQLKNKKVGDTVEMQLERGGETISRDVQLIEVKDSKTGEVRPALGV

MIGAVQKVKAEDPNKQISFTDTQVGGPSAGLMFTLEIYNQLTPGDLTKGHRIAGTGTI

TKEGVVGAIGGVVHKIVAADRKEAEFFFVPKDNYKEAEAKAEQIGTKMKLIPVSTVDD

ALAYLKTLSVKS"

CDS complement(2085945..2087177)

/gene="tmcAL"

/locus_tag="PIKHDJKN_01923"

/EC_number="6.3.4.-"

/inference="ab initio prediction:Prodigal:002006"

/inference="similar to AA sequence:UniProtKB:O34513"

/codon_start=1

/transl_table=11

/product="tRNA(Met) cytidine acetate ligase"

/db_xref="COG:COG1323"

/translation="MKAVGVIVEYNPLHNGHVYHLSEARRLSGADVVVAVMSGPFLQR

GEPAIVGKRARTEMALHAGADLVLELPVAYAVQPAEWFAFGAVSLLHRTGVVGSLCFG

SESGDLDSLQRIARVLAVEPAVLREDIARRLREGASYPAAYAGAAAALAPGGVDADNA

AVLLGQPNNSLGLHYLIALQRLGSAIKPFTAARTGAAYHEATPGPGAIASATAVRRLL

MADGPEAAAPYMPAATLAILRREWQEGRAPVHWERFAQPLLHIAATRRASELEQIAEV

TEGLEHRLSRTLAQLPEPSVEALLNALKTKRYTRTKLQRMLTHILLNHTKAECSPEKL

AEGPAYLRVLGFNAQGQSLLKQMKKTASLPVLLKPSTFVHNQLELDVQAQAAYALACE

HTDTRMMYSDYYDPPVRL"

CDS 2087662..2088168

/locus_tag="PIKHDJKN_01924"

/inference="ab initio prediction:Prodigal:002006"

/codon_start=1

/transl_table=11

/product="hypothetical protein"

/translation="MLMPFRKVATSDGPLQFNEQWDIKELVSNRQDITAVTPLTADLS

AEFREGDVVDVHGKLTAGVDMLCSRCLKPINEHFHIDFHEQFKKGKQPEELHEDDDTF

YVDGDSVDLKGYAEEAFLLNLPFIPLCSDTCKGLCPKCGHELNEGDCGCDNQVIDPRL

AGLKDFFK"

CDS 2088237..2088410

/gene="rpmF"

/locus_tag="PIKHDJKN_01925"

/inference="ab initio prediction:Prodigal:002006"

/inference="similar to AA sequence:UniProtKB:P07840"

/codon_start=1

/transl_table=11

/product="50S ribosomal protein L32"

/translation="MAVPQRRTSKTRRDKRRTHFKLAVPGMVKCEQCGELKLSHHVCK

VCGTYKAREIISQ"

CDS 2088859..2089407

/gene="fapR"

/locus_tag="PIKHDJKN_01926"

/inference="ab initio prediction:Prodigal:002006"

/inference="similar to AA sequence:UniProtKB:O34835"

/codon_start=1

/transl_table=11

/product="Transcription factor FapR"

/db_xref="COG:COG2050"

/translation="MIEDNPFVTDQELTRQLKVSIQTIRLDRLELGIPELRERMKLMA

ERSYDQVRSLPLHEIIGDIVDLQLDKSGISLFEIKEEHVFSRTGIARGHYVFAQANSL

AVAVINDEIALTASADIRFVRSVHLAEKCIAKAYVRSIPGQKGKAKVEVFTYVGEEMV

FQGNFVIYHSGGEDSVEGGHLE"

CDS 2089404..2090399

/gene="plsX"

/locus_tag="PIKHDJKN_01927"

/EC_number="2.3.1.274"

/inference="ab initio prediction:Prodigal:002006"

/inference="similar to AA sequence:UniProtKB:P71018"

/codon_start=1

/transl_table=11

/product="Phosphate acyltransferase"

/db_xref="COG:COG0416"

/translation="MRIVIDAMGGDNAPASTVEGAIAAAKEWADTEIVLIGDEAKLEP

LLSQSGAKPANLTIRHASEVIGSDDEPVKAVRRKKDASMVVAGRMLKEGEADAMISAG

NTGALMTAGLLVVGRMEGIERPALAPMIPTIDDVGVLALDLGANMDAKPEHLAQYGLM

GSLYRQKVQGIASPRVGLLNVGTEPGKGNELTKHAYPLLEQLPIRFVGNVEARDVLTG

ACDVLVCDGFAGNILLKSLEGTAGAIFALLKEQFSSSLKSKLAAAVLMPELRGLKRKL

DYTEHGGAPLLGLSKLVVKSHGSADGNAIKNAVRQARIAVQNQLVESISKEISGK"

CDS 2090408..2091397

/gene="fabH_2"

/locus_tag="PIKHDJKN_01928"

/EC_number="2.3.1.180"

/inference="ab initio prediction:Prodigal:002006"

/inference="similar to AA sequence:UniProtKB:Q6GIA4"

/codon_start=1

/transl_table=11

/product="3-oxoacyl-[acyl-carrier-protein] synthase 3"

/translation="MNNLRPVGVIGTGKYVPEKILTNSDLEKMVDTNDEWIVSRTGIK

ERHIAAPDQATSDLAYEAAIKALESAGMTGSDLDLIIVATITPDSSFPSTACILQDKL

GAKGAAAFDLSAACSGFVYGLATATSFIQSGMYNNALVIGADCLSRITDYTDRNTCVL

FGDGAGAVIVGEVPEGRGFKSFDLGAEGAGGSLLQMEGGGSRLPASVETVENKKHYIY

MNGREVFKFAVRVMGTATIEVLRKAGLERTDVDLFVPHQANIRIIQSAMQRLELPEEK

VVVNVDKYANTSAASIPLALVEAAEEGRMKAGDTVLMVGFGGGLTWGASVLVW"

CDS 2091425..2092357

/gene="fabD"

/locus_tag="PIKHDJKN_01929"

/EC_number="2.3.1.39"

/inference="ab initio prediction:Prodigal:002006"

/inference="similar to AA sequence:UniProtKB:Q7A5Z3"

/codon_start=1

/transl_table=11

/product="Malonyl CoA-acyl carrier protein transacylase"

/translation="MGKIAFVFPGQGSQTVGMAKDAYESVPAATEIFRTADETLGFSL

SNLVFEGPETELKQTSNTQPALLTASIALLEAFKEKGIQPDYTAGHSLGEYSALVAAG

VLSFADAVSIVRARGQYMEQAVPGGQGAMAAVLGADREALGVLCRDVSESGHAVELAN

INCPGQIVISGVKEGVAAVAERVKEAGGKRAITLEVSGPFHSSLMKDAAEKLAEKLKT

VSFSPGTVPVVANVTARPVEDGKVQDLLTAQVYSPVLWEDSVTWLIEQGVDTFIEIGS

GSVLTGLIKKTDKTVKLYNVNSLETLEATAAALI"

CDS 2092395..2093144

/gene="fabG_2"

/locus_tag="PIKHDJKN_01930"

/EC_number="1.1.1.100"

/inference="ab initio prediction:Prodigal:002006"

/inference="similar to AA sequence:UniProtKB:P99093"

/codon_start=1

/transl_table=11

/product="3-oxoacyl-[acyl-carrier-protein] reductase FabG"

/translation="MSKPLQGKNALVTGASRGIGRSIALALAEAGANVAVNYAGSQAA

AEEVAEAIRAKGVQAITIQANVGQMDEAEQMVKATIEAWGNVDILVNNAGITRDNLIM

RMKEEEFDQVIETNLKGVFNCLKAVTRPMMKQRSGRIINISSVVGVLGNAGQANYVAA

KAGVIGLTKASARELASRGITVNCVAPGFIETDMTKELSQEIVDNMLNGIPLSRLGQP

DEIAGVVTFLASEASSYMTGQTLHVDGGMYM"

CDS 2093296..2093529

/gene="acpA"

/locus_tag="PIKHDJKN_01931"

/inference="ab initio prediction:Prodigal:002006"

/inference="similar to AA sequence:UniProtKB:P80643"

/codon_start=1

/transl_table=11

/product="Acyl carrier protein"

/db_xref="COG:COG0236"

/translation="MSDVLERVKRIVVDRLGADEAEVTLEASFKEDLGADSLDVVELV

MELEDEFDLEISDEDAEKITTVGEVVNYIQSHT"

CDS 2093710..2094948

/gene="fabF_2"

/locus_tag="PIKHDJKN_01932"

/EC_number="2.3.1.179"

/inference="ab initio prediction:Prodigal:002006"

/inference="similar to AA sequence:UniProtKB:O34340"

/codon_start=1

/transl_table=11

/product="3-oxoacyl-[acyl-carrier-protein] synthase 2"

/db_xref="COG:COG0304"

/translation="MKQRVVITGMGVMTSLGKDLETFWGSLMAGKSGISQIEAFDVSE

YTTQIAAEIKDFNPEEYMERKDARKMDRFVQFAVAAGFKAVEDSGLKIDENIDAERFG

VSIGSGIGGLGTWEDQHNALLQKGPKRVSPFFIPMMISNMASGQMSISLGAKGPNINV

VTACATGTHSIGDSFKLIANGDADAMICGGAEATIRPTGLAGFCAMRAMSTRNDDPAK

SSRPFDTERDGFVMGEGAGVLILESLEHAQKRGARIYGEVIGYGLTGDAHHMTEPDPD

GAARCMKMALRNAGIEPEEVDYINAHGTSTPVGDRSETLAIKKAFGDHAYKLAVSSTK

SMTGHMLGAAGGVEAVICGLSLTHQTLAPTINLENQDPECDLDYVPNVPRQTKVNIAM

SNSFGFGGHNATIILKKFEA"

CDS 2094914..2095660

/gene="rnc"

/locus_tag="PIKHDJKN_01933"

/EC_number="3.1.26.3"

/inference="ab initio prediction:Prodigal:002006"

/inference="similar to AA sequence:UniProtKB:P51833"

/codon_start=1

/transl_table=11

/product="Ribonuclease 3"

/db_xref="COG:COG0571"

/translation="MPPLFSKNLKHKGLVRLSEDLKQLQHKLQIKFDNRQLLKQAFTH

ASYVNEHRFSQHQDNERLEFLGDAVLELTVSEYLYHLFPNRPEGELTKLRASIVCEPS

LVKFAEALGFGQYVLLGKGEELTGGRTRPALLADVFESFIGALYLDQGLAPVRTFLDQ

HVFPLIVLGSKLQMSDYKTELQELTQHHNMGALEYRIVEERGPAHEREFVSEVHMGQE

RLGRGTGRSKKEAEQQAASAALDRLKLPEA"

CDS 2096021..2099590

/gene="smc_2"

/locus_tag="PIKHDJKN_01934"

/inference="ab initio prediction:Prodigal:002006"

/inference="similar to AA sequence:UniProtKB:P51834"

/codon_start=1

/transl_table=11

/product="Chromosome partition protein Smc"

/db_xref="COG:COG1196"

/translation="MFLKRIELGGFKSFADKTEMEFVRGITAVVGPNGSGKSNISDGI

RWVLGEQSAKSLRGGKMEDIIFAGSDARKAVNYGEVSLTLDNEDHALALDFGEVTVTR

RVHRSGDSEYFINKQSCRLRDITELFMDTGIGKEAYSIIGQGRIEEILSTRSEDRRGI

FEEASGIVKYKSRKRDATRKLDETEQNLLRIHDLVTELEDQIGPLKEQSEKAIHYKEL

RGQLKSQEISMYVYQIEQIHASWSKANQRLESLKQEEVGLAAIVSTHDAKLESDRNAL

RTLETETEQLQSALLQFSEATEKSEGLGELLKERSRHLQTNQEQLKVTLAASEERHRE

REAELLALREKFAKLELELSDVRNRLSEEEAKLIGVTGGISQQQEESLKGNLLELMNQ

MAQTRNEIRYADQQKETLERRMNRATEESGKWEGQKETLEARKTEIEKKVVRLGKEIS

DLRGGYITESERLQSLQKLLEESRGTVRKWEQKREAQVSRRDTMKEMQDDFDGFMLGV

KEVLKASRKGTLNGVHGAVAELVKVPEKIELAVETAMGASLQHVVMENESVSRQAIAF

LKQRQLGRATFLPLDVIRARTIGAGERSMIEGMDGFVGIGADLVQYESRYAAIIGSLL

GNVIIAEKLEDANKIAARCQYRFRVVTLEGDVVNAGGSMTGGSQHKKNVSLLSRKRQL

DQLDQDILDTENQIVKLHRSVDDVKTQLEQCQDKLDELRQSGDDTRNAEQQASMEMKQ

VEHELRHVLEQVAVAGQEKSGFTEEIKEMDIARDVAVKKLEQLEEEEKATHRAIHAAE

FARKANESAKEELQTQLTELKVREGKLDQERFSNEEQLRRLEREVDSLVKDLRQNRTL

LASMEADLKKTETESVKQIEDLNQYKLKKAESAQELDFKRAARSELSKKLELAENETK

EQRTQLKAVEEQMRQTEISVNRLDVELENILRKLMDEYELGYELAKERYPVPEDVEST

QAEVQKLKRSIAALGDVNLGAIEEFQRVNERYEFLSTQKNDLVEAKTTLYQVIREMED

EMAKRFKATFDAIRREFGTVFTKLFGGGRADLVLMDPERLLDTGIDIVAQPPGKKLQN

LQLLSGGERALTAMALLFAILHVKPVPFCVLDEVEAALDEANVVRFAQYLREFSEQTQ

FIVVTHRKGTMEEADVLYGVTMEEGGVSKLVSVKLEDEEAVIA"

CDS 2099666..2100679

/gene="ftsY_1"

/locus_tag="PIKHDJKN_01935"

/inference="ab initio prediction:Prodigal:002006"

/inference="similar to AA sequence:UniProtKB:P51835"

/codon_start=1

/transl_table=11

/product="Signal recognition particle receptor FtsY"

/db_xref="COG:COG0552"

/translation="MSFFKKLRDSIASKTESVTKQFKDGLEKTRKGLVEKVSDLVIRR

KKIDEEFYEELEEILIGADVGVNTVMKLIEDLREEVKKRKIEDAAELQPVLSEKLTDL

LRGEQNNELKMNPDGITVILFVGVNGVGKTTTIGKLAHRFKQQGKKVIMAAGDTFRAG

AIEQLEVWGQRAGVEVIKQQSGSDPAAVMYDAVQAAKQRGADVLLCDTAGRLQNKSNL

MDELNKIYRVIQREIPEAPHEVLMVLDATTGQNALNQAKLFGEKSGVTGLVLTKLDGT

AKGGIVVAIRQELDLPVKLVGLGEKIDDLQPFDSEQFVHALFAGLIQEQPAESAEEEE

ANS"

CDS 2100824..2101471

/locus_tag="PIKHDJKN_01936"

/inference="ab initio prediction:Prodigal:002006"

/inference="similar to AA sequence:UniProtKB:P67153"

/note="UPF0073 inner membrane protein YqfA"

/codon_start=1

/transl_table=11

/product="hypothetical protein"

/translation="MANTYTYSRREEVANAVTHGIGAALSVAALVLLIVFSSMKGTAW

HVVSFTIYGITMLLLYTSSTLVHAWKDGKVKDLFEIFDHSSIYLFIAGSYTPLLFIAV

RGTLGWTLFGVIWGIALFGVIFKAFFTKRFLFMSTIFYIAMGWLIVIAWQPLMATIPT

GGIVLLVAGGLMYTLGTLFYVWRGFPYHHAIWHLFVLAGSILHFFMVLLYLTPLR"

CDS 2101732..2102082

/locus_tag="PIKHDJKN_01937"

/inference="ab initio prediction:Prodigal:002006"

/codon_start=1

/transl_table=11

/product="hypothetical protein"

/translation="MSQENRLEKTNRINLLFAFYELLLTEKQQTFLKYYFHDDFSLGE

IAAEFEISRQAVYEHIKRAEQVLENYESKLGLLEKHERRNRNLEDLQNALERAGVSID

NNQQINDIIHQLRE"

CDS 2102148..2103524

/gene="ffh"

/locus_tag="PIKHDJKN_01938"

/inference="ab initio prediction:Prodigal:002006"

/inference="similar to AA sequence:UniProtKB:P37105"

/codon_start=1

/transl_table=11

/product="Signal recognition particle protein"

/db_xref="COG:COG0541"

/translation="MAFEGLTTRLQNVFSKLRGKGKVSDEDVAEAMREVRLALLEADV

NFKVVKEFIAKVKEKAVGKEVMESFTPGMVIIDIVNKELTDLMGGSQSKLAKANKPPT

VLMMVGLQGAGKTTTSGKLAKMLQKQNSRPLLVAGDIYRPAAIKQLEVLGEQIKAPVF

TLGDQTSPVEIARQGLQHAKDNGNDYVIIDTAGRLHVDEELMEELRQIHSVVNPDEVL

LVVDSMTGQDAVNVAEHFNQQLNLTGVVLTKLDGDTRGGAALSVKAVTGCPIKFASLG

EKLDALEPFHPERMASRILGMGDMLSLIEKAQSNIDTDKAKEMERKMRNAEFTFEDFL

EQMDQVKKLGPIDQIMDMIPGMGKMKQAKDLKVDDKQMGRIEAIVYSMTTEEKRNPDM

INHSRRKRIATGSGTSLAEVNRLIKQFDEMRRMMKQFSDMMGPKGGKNKAMKQLKGLG

KGMKFPFR"

CDS 2103579..2103851

/gene="rpsP"

/locus_tag="PIKHDJKN_01939"

/inference="ab initio prediction:Prodigal:002006"

/inference="similar to AA sequence:UniProtKB:P21474"

/codon_start=1

/transl_table=11

/product="30S ribosomal protein S16"

/db_xref="COG:COG0228"

/translation="MAVRIRLKRMGAHKAPFYRVVVSDSRSPRDGRFIEEIGYYNPVE

QPAVVKIDEDKALQWLQNGAQASDTVRNLLSKAGVMKKFHESKLTK"

CDS 2103875..2104105

/locus_tag="PIKHDJKN_01940"

/inference="ab initio prediction:Prodigal:002006"

/codon_start=1

/transl_table=11

/product="hypothetical protein"

/translation="MEELVGIIAKALVDHPEDVAVRTVEKDRLVVYELTVHPDDVGKV

IGKQGRIAKSLRTVVTSAAVKMDKRVTVDIIS"

CDS 2104346..2104858

/gene="rimM"

/locus_tag="PIKHDJKN_01941"

/inference="ab initio prediction:Prodigal:002006"

/inference="similar to AA sequence:UniProtKB:P66656"

/codon_start=1

/transl_table=11

/product="Ribosome maturation factor RimM"

/translation="MAEFMNVGKIVNTHGIRGEVRIMPLTDFPEVRFAKNAELFFFTP

DNHPVMVNVESARLHKNMYILRLKEYGNINEVEKFKGGMAKVLKENLAELEENEYYFH

QIVGCSVITDEGETLGTISEILTPGANDVWVVKTPAGKEILLPVIDDVVLDVDIDQQQ

VKVHLMEGLL"

CDS 2104860..2105627

/gene="trmD"

/locus_tag="PIKHDJKN_01942"

/EC_number="2.1.1.228"

/inference="ab initio prediction:Prodigal:002006"

/inference="similar to AA sequence:UniProtKB:Q6GHJ5"

/codon_start=1

/transl_table=11

/product="tRNA (guanine-N(1)-)-methyltransferase"

/translation="MKVDVLTLFPEMFDGVFGTSILGKAQTKGLVSLGATNFRNFATN

KHNTVDDAPYGGGGGMVLKPDPIFAAVEDVLEQRGEAAATMKPPRIILMCPQGETFTQ

KKAEELVQEDHLIFICGHYEGYDERIREFLVTDELSIGDYVLTGGELPAMVAIDSIVR

LIPGVLGNETSAVTDSFSTGLLEYPHYTRPPEFRGMKVPDMLLSGHHLNIDAWRREQS

LLRTLERRPDMLETADLTDKERIWLNKIRSDREKNAE"

CDS 2105842..2107098

/locus_tag="PIKHDJKN_01943"

/inference="ab initio prediction:Prodigal:002006"

/codon_start=1

/transl_table=11

/product="hypothetical protein"

/translation="MNHQPYEVGRVNISEAGERCKMLLGDLNGDGRLEMLLVQADGGI

DDRYVPHQVCCLSAYDLDGTLMWQVGTPDPDAGGPGSDYPAQIADWDGDGNNEVLCVM

NKQFLILDGRTGEIKKRHELPEEQAHDCLILANLTGDQHTMDILLKDRYKTLWALDHD

FNLLWKHEGNIGHFPWVYDIDGDGKDEVMAGYDMLDHNGTLLWSCQNLDDHADCIWFG

DVDEDGEVEIVIGGSVTVMLDRYGNEKWRYEDSIESQHIALGKFYRERQGLQIAGLDR

IIRGDENGKDGLFMLGSDGKELWKEDRKTRGWLTIIEPVRNWDDSGLDYILAYRRGGG

VLPALVNGNMQTVADFGKEGYAVHADLCQSGREQIIIYDAHEAVIYSSSVMDVTTPTS

LDIKRAQLQPKRLYSSTLYPGGEVSI"

CDS complement(2107100..2108065)

/locus_tag="PIKHDJKN_01944"

/inference="ab initio prediction:Prodigal:002006"

/codon_start=1

/transl_table=11

/product="hypothetical protein"

/translation="MKHLKKIALAALAVLTCTITMYAYAVTHPTNAMSHKACTSWDMV

KRKAFLMSHSDYSSKPALDLATFHIAQGSATEVPVLMYHYIQPKNNNHETNNKSIINL

EDFEENMKYLHEEGYNTITLEQLEQYVNGQISLPKKSIVITFDDGYQNNYTLAYPVLK

KYNFHASLFVIGSKIQDQPSAFDPAKNTFISKQEMQAAKDVFEFNSHTYNLHHKGYMR

CGDNVPVGLDTSLLNDDVKLMKETGIDTPYLAYPFGYTSTQMIYQLQQNGYRMAFSVR

SGFVRPGDNPMKLPRLTVTTGTDLAALLHPESDPQEILPVVEISH"

CDS 2108572..2108913

/gene="rplS"

/locus_tag="PIKHDJKN_01945"

/inference="ab initio prediction:Prodigal:002006"

/inference="similar to AA sequence:UniProtKB:O31742"

/codon_start=1

/transl_table=11

/product="50S ribosomal protein L19"

/db_xref="COG:COG0335"

/translation="MNIVQAITQEQLRKDIPSFRPGDTLKVHVKVIEGTRERIQLFEG

VVIKRRGGGISETFTVRKISYGVGVERAFPLHSPKIDRIEVARRGKVRRAKLYYLREL

RGKAARIKEIR"

CDS 2109170..2109790

/gene="sipT"

/locus_tag="PIKHDJKN_01946"

/EC_number="3.4.21.89"

/inference="ab initio prediction:Prodigal:002006"

/inference="similar to AA sequence:UniProtKB:P71013"

/codon_start=1

/transl_table=11

/product="Signal peptidase I T"

/db_xref="COG:COG0681"

/translation="MEQEVQQDQGNTAEEKGSRSKKAKNEIFEWLKAIVIALVLVILI

RWLLFKPFVVDGPSMQPNFHTGERVIVNEILYDIREPQRGEVIVFHVPTEGRDFIKRV

IAVAGDTVEVQDDTVLVNGKKVDETYIQGAIDAAEANGGTYNVKDFPNEQFPDGKVPE

GHVFVMGDNRPNSTDSRMIGYVSLTDIVGRADVIFWPIGDIKWINH"

CDS 2109958..2110821

/gene="rbgA"

/locus_tag="PIKHDJKN_01947"

/inference="ab initio prediction:Prodigal:002006"

/inference="similar to AA sequence:UniProtKB:O31743"

/codon_start=1

/transl_table=11

/product="Ribosome biogenesis GTPase A"

/db_xref="COG:COG1161"

/translation="MTIQWFPGHMTRARRQIQDKLKLIDVVIELLDARLPVSSRNPMI

DEILQGKPRMILLNKSDLADAKVTQEWIEYFKKEGITAFPVDASTGTNVKDIPTQAKL

LLKEKIDRQIAKGINPRAVRGLIVGIPNVGKSTLINRLAGRNIAATGDRPGVTKGQQW

IKVGKEMELLDTPGILWPKFEDQNVGYRLAVTGAIKEEILNAEDIAFFGISYLMRYYW

DSLEERYGLQEFSKDADDSDSVVAIMEQVGRIRGCVVSGGRIDLEKASRAFLRELRAG

KMGRFSMEAPY"

CDS 2111220..2111924

/gene="rnhB"

/locus_tag="PIKHDJKN_01948"

/EC_number="3.1.26.4"

/inference="ab initio prediction:Prodigal:002006"

/inference="similar to AA sequence:UniProtKB:O31744"

/codon_start=1

/transl_table=11

/product="Ribonuclease HII"

/db_xref="COG:COG0164"

/translation="MNEMNELIELTQLDALVEPKKKKKELVEPKDLLIYEREYWESGF

ERIAGIDEVGRGCLFGDVVAAAVILPRDLILEGVNDSKKLTEKKRDALYDIIMEKALS

VGIGYADAETIDQLNIKQAARLAMKRAVEALEGVPDYLLVDAEKVDVNIPQLSIIKGD

ANSQSIAAASIIAKVTRDRLCKEDWDTLYPEYGLSIHKGYATKFHREQIMARGATPMH

RRSFLSNLLGEQHTLF"

CDS 2111955..2113985

/locus_tag="PIKHDJKN_01949"

/inference="ab initio prediction:Prodigal:002006"

/codon_start=1

/transl_table=11

/product="hypothetical protein"

/translation="MNISSVIRGLLGDSKPGNAKPLELKEGQVVRGSVVSVSDDGADA

VLQIQGVQVRAKLETPLRPGETTLLQVQPPGENGVTVMKPLTGTLAELPQASLNNMLQ

EVGLPDTKGNRELLLAMQRSGLPLTKDNVAMVQNMITAKPAQVPVEEWVQATGIAFQR

GLPVTAETVKGLHQAVFGPPLHQLLKGLAEQLESMLTQTAGKTPPTGEQAASVKPAVM

AGLPVSLTGEQTGEALTASGGNRQVGGTTGLSGQPVPGAAAVDAPMADGSEDTAAGGA

VKGNGQNGAGNAENAGFKTGASHVETAGKVGTGIPAGTGIPGESPRTADAGFAGSRPG

MPGAAVDAAAGRVIAGQPEGGAAGRTDGRGETAAAAGPTASAAAPSAAQLAPKLLALL

DALRSASTAAPAQPGAATQAAPASQGGQAAAAAGGAPQLLPAGADARPAGGSAAAPAG

AAAAPVTHEGDPWVGRVLKLLGAEHEQQAVHGAAAQARVSDVASPGTTDTLKGLLLQL

ASSDGAPAALKDAAGQAVQFLTGQQLLLTTDRSATFAQMHWFIPITGPDGEETASVQI

QSRRGQRGELDASNCRLWFDLDMKSLGPTLVDVHVVNNIVSLRVLNDREGMGSILESG

REEIHRALDQLGYQLLTFKAEPWPVGQEPGAERKMTASDYSPEQYKGVDMRV"

CDS 2113982..2114290

/gene="flhB_1"

/locus_tag="PIKHDJKN_01950"

/inference="ab initio prediction:Prodigal:002006"

/inference="similar to AA sequence:UniProtKB:P76299"

/codon_start=1

/transl_table=11

/product="Flagellar biosynthetic protein FlhB"

/db_xref="COG:COG1377"

/translation="MKEDSQPDLISKKAVALKYVPGESEAPVVVAKGRGKVAEAILDK

AKENGVPVQEDAALVEVLSKLDLDEQIPSELYQLVAEVLTYIYRADRLASGREEDESW

"

CDS 2114284..2114682

/locus_tag="PIKHDJKN_01951"

/inference="ab initio prediction:Prodigal:002006"

/inference="similar to AA sequence:UniProtKB:Q6NCZ4"

/note="UPF0102 protein RPA0323"

/codon_start=1

/transl_table=11

/product="hypothetical protein"

/translation="MVERRSEQGPASKLTRQQKGRLGEEAACDCLREHDYRILRQNWR

CRSGEIDIVASHEGMIIFIEVRSRSGTGQYGTPQESVDIRKMQQVRATASVYLQMTGE

TNHQIRFDVIAVILDKAGGTVSVDHIINAF"

CDS 2115058..2115609

/gene="ydjA"

/locus_tag="PIKHDJKN_01952"

/EC_number="1.-.-.-"

/inference="ab initio prediction:Prodigal:002006"

/inference="similar to AA sequence:UniProtKB:P0ACY1"

/codon_start=1

/transl_table=11

/product="Putative NAD(P)H nitroreductase YdjA"

/db_xref="COG:COG0778"

/translation="MSKSTQTGNAVREAIQNRRTVKKFKKEAVPTQQIIDLLDTAVWA

PNHKLREPWRFLLFSGNGLNKLADAIDAEMGEDNKFSANIKQVPAVMIVVMEEDPRQN

IWDEDFAAVSALVQNFLLAAWSEDIGTFWVTKPFLYGPKFRKSLGIQAGEKIIGMVYM

GYPDVIPSAKERTPAKDKLTIFE"

CDS complement(2115833..2117437)

/gene="comM"

/locus_tag="PIKHDJKN_01953"

/inference="ab initio prediction:Prodigal:002006"

/inference="similar to AA sequence:UniProtKB:P45049"

/codon_start=1

/transl_table=11

/product="Competence protein ComM"

/db_xref="COG:COG0606"

/translation="MYGKLHSACLYGIDGVLIEVETDLSNGLPQTSIIGLPDSAIREA

VERVRAAIKNCGYQYPLQRITINLAPADLRKEGSSFDLAIAIGLLTTSGQLVLPVQER

TLVVGELALDGSVRSVPGILSMVDLAKRQGFTSVLLPADNLEEASLIRGIQVYGIRHL

QDIVPESVKATKSKNGTIQNTGPIVLKDYAHLSVRIPDYEDLNNHERSKHISFLEDDY

SDVLGQHHVKRALMIAAAGMHNILLVGPPGTGKTMLIKRLPTILPPLSEDEALEVTKV

LSAAGKLKDTSNGLIADRPFRSPHHTISTSGLIGGGGIPKPGEVSLAHRGILFLDELP

EFQRQVLEVLRQPLEDRKVTISRARAAFTFPAQFMLACSMNPCPCGYLSAQSDDQRCI

CSPARVAAYRAKISGPLLDRIDLQVEVPPPGEWRKAGASPSSAEMQAKVIHAHLIQAA

RYANSSVRWNSQLSGTLLRRTIRLPEEADQLLQQTLQALNLSMRAHDRIIKMAQTIAD

LDHDGVILNAHVAEAIQYRQLDLNLF"

CDS 2117950..2118405

/gene="ohrR"

/locus_tag="PIKHDJKN_01954"

/inference="ab initio prediction:Prodigal:002006"

/inference="similar to AA sequence:UniProtKB:O34777"

/codon_start=1

/transl_table=11

/product="Organic hydroperoxide resistance transcriptional

regulator"

/translation="MQNESLKLDNQLCFAIYACSREITKMYQPYLEVLGVTYSQYLVL

MVLWEREECTVKEIGEALYLDSGTLTPLLKRLQSAGLIHRERSAQDERKVLITLTDAG

RELRHKALSIPEAIQEDACLNSTEFESLLGQFKGLLEKVHQTNTKETRK"

CDS 2118658..2119818

/gene="sucC"

/locus_tag="PIKHDJKN_01955"

/EC_number="6.2.1.5"

/inference="ab initio prediction:Prodigal:002006"

/inference="similar to AA sequence:UniProtKB:P80886"

/codon_start=1

/transl_table=11

/product="Succinate--CoA ligase [ADP-forming] subunit

beta"

/db_xref="COG:COG0045"

/translation="MNIHEYQGKEVLKQYGVTVPNGKVAYTVDEAVAAAEALGSPVTV

VKAQIHAGGRGKAGGVKVAKSIDEVRAYASEILGKVLVTHQTGPEGKEVKRLLIEEGC

DIRKEYYVGVVVDRATGRVVMMASEEGGTEIEEVAEATPEKIFKEIIDPAIGLQVFQA

RKLAYSIRIPNELVNKAVKFMLALYTAFVEKDCSIAEINPLVVTGDGNVIALDAKLNF

DSNALFRHKDILELRDLDEEDEKEIEASKYDLSYIALDGNIGCMVNGAGLAMATMDII

KYYGGDPANFLDVGGGATTEKVTEAFKIILSDAKVAGIFVNIFGGIMRCDVIANGVVE

AAKQLGLTKPLVVRLEGTNVELGKRILGESGLNIVPADSMADGAQKIVALVK"

CDS 2119898..2120827

/gene="sucD"

/locus_tag="PIKHDJKN_01956"

/EC_number="6.2.1.5"

/inference="ab initio prediction:Prodigal:002006"

/inference="similar to AA sequence:UniProtKB:P80865"

/codon_start=1

/transl_table=11

/product="Succinate--CoA ligase [ADP-forming] subunit

alpha"

/db_xref="COG:COG0074"

/translation="MSILIDKNTKVITQGITGSTGMFHTKGALDYGTQMVGGVTPGKG

GTNVDITLEDGKVVSLPVFNTVQEAKEATGATASVIYVPPAFAADSIMEAVDAELDLV

ICITEGIPVLDMVKVDRFMEGKDTVLIGPNCPGVITPGECKIGIMPGYIHMPGHVGVV

SRSGTLTYEAVHQLSARGIGQSSAVGIGGDPVKGSEFIDILKRFNEDPQTHAVIMIGE

IGGTAEEDAAEWVRDNMTKPVVGFIGGVTAPPGKRMGHAGAIISGGKGTAKEKIAKLE

SCGIKVAPTPAEMGSTLVSVLEERGILNLCTTH"

CDS 2121075..2122205

/locus_tag="PIKHDJKN_01957"

/inference="ab initio prediction:Prodigal:002006"

/codon_start=1

/transl_table=11

/product="hypothetical protein"

/translation="MEERWILIGLHETDGIGKKTISKLLSGQHLLTDFLDYKEGDWIA

AGLRRDQATRLTKQFNADWIEQRQESVYKQGIEVVTYLDQDYPILMKETVQPPWVMYG

RGDLNLLHSQSIAMVGTRMPTVYGRKIGEKLAEQLCKAGLTIVSGLARGIDSVCHDAT

LRANGKTIAVFGTGIDNIYPPENTSLAERIAETGLLLSEYPPGTRARQGLFPERNRII

AGLTLGTLVVEADIRSGSLITADAALEAGRDVFAVPGPITSPKSRGSHNLIRQGAKLV

TCATDLLEEFRLDLPNAEQLPYNRGRSAESSESSNEGIFPVVKLSSDEQRVIYLLEQE

EQSLDQLVEQLNWDFGHLHSVLLSLIIKKQISQLPGTKYARV"

CDS 2122326..2124416

/gene="topA"

/locus_tag="PIKHDJKN_01958"

/EC_number="5.6.2.1"

/inference="ab initio prediction:Prodigal:002006"

/inference="similar to AA sequence:UniProtKB:Q7A5Y5"

/codon_start=1

/transl_table=11

/product="DNA topoisomerase 1"

/translation="MADALVIVESPSKAKTIGKYLGSKFIVKASMGHVRDLPKSQIGV

EVENDFNPKYITIRGKGSILKELKDARKKVKKVYLAADPDREGEAIAWHLAHALELDE

TADCRVVFNEITKQAVKDAFKTPRKINMDLVNAQQARRILDRLVGYKISPLLWKKVKK

GLSAGRVQSVAVKIIMDRENEINDFEPEEYWSITAKLTADGNPLEAKFHQLNGTKTEL
GSEAEVQAILKQIEGASFTVKEVKEKERSRNPSAPFTTSSLQQEAARKLNFRASKTMS
VAQQLYEGVDLGKEGTVGLITYMRTDSTRIAASAQEEAKEYIIGKYGEPFAPETPRNY
SKKAANAQDAHEAIRPTSILRDPDSIKSFMSRDQFRLYKLVWERFIASQMSSAVLDTL
SVDIAAGDTIFRAAGSKVRFQGFMKVYVEGNDDGTTEEDRLLPPLKSGDVLENREIEP
KQHFTQPPPRYTEARLVKTLEELGIGRPSTYAPTLETIQKRGYVAIEEKKFMPTELGE
LVIEQMEEFFPEILNVEFTANMEGDLDHVEEGSEDWVKVLAEFYESFEKRLEFAEEEM
KEIEIEDEVSDEICEKCGKPLVYKLGRFGKFLACSGFPDCRNTKPIIKDIGVTCPKCK
EGHVVERRSKKGRIFYGCDKYPECDFVSWDRPSAKPCPSCGSLMIEKRNKQGTRLQCT
SCDHQEPVEEPEED"

CDS 2124446..2125783

/gene="trmFO"

/locus_tag="PIKHDJKN_01959"

/EC_number="2.1.1.74"

/inference="ab initio prediction:Prodigal:002006"

/inference="similar to AA sequence:UniProtKB:P39815"

/codon_start=1

/transl_table=11

/product="Methylenetetrahydrofolate--tRNA-(uracil-5-)-
methyltransferase TrmFO"

/db_xref="COG:COG1206"

/translation="MTNEQQVTVIGAGLAGTEAAWQIASRGVRVKLYEMRPVVKTPAH
HTDKFAELVCSNSLRANGLTNAVGVLKEEMRMLNSLVLGAADRHAVPAGGALAVDRDG
FSGEITSTLHQHPLIEVVNEELTSLPEDGIVVVATGPLTSPALSEQIKALMGEEYFYF
YDAAAPIIEKDSIDMNKVYLASRYDKGEAAYLNCPMTEEEFDVFYEALITAEVAQLKE
FEKEIYFEGCMPIEVMMKRGKQTALFGPMKPVGLVNPHTGELPHAVVQLRQDNAAGTL
YNLVGFQTHLKWGEQKRVFSLIPGLENAEFVRYGVMHRNTFINSPKLLRPTYQFKERP
NLFFAGQMTGVEGYVESAASGLIAGMNAAKAALEQELVVLPVETTLGSMAQYITTADF
KHFQPMNANFGLLPKLETKIRNKKEKNEALAQRALDGIARFAAAEGLTVPERV"

CDS 2125810..2126352

/gene="hslV"

/locus_tag="PIKHDJKN_01960"

/EC_number="3.4.25.2"

/inference="ab initio prediction:Prodigal:002006"

/inference="similar to AA sequence:UniProtKB:P65797"

/codon_start=1

/transl_table=11

/product="ATP-dependent protease subunit HslV"

/translation="MDMSFHATTICAVRHNGKAAIAGDGQVTMGQSVVMKNTAKKVRR

LYRGQVVAGFAGSVADAITLFEKFEGKLEEHHGNLQRAAVELAKDWRQDRILRKLEAL

LIVMDKTGMLLISGGGEIIEPDDDVIAIGSGGNFALSAARALKRHAVGLEAKDIARES

LQIASELCVFTNNNIIVEEL"

CDS 2126491..2127891

/gene="clpY"

/locus_tag="PIKHDJKN_01961"

/inference="ab initio prediction:Prodigal:002006"

/inference="similar to AA sequence:UniProtKB:P39778"

/codon_start=1

/transl_table=11

/product="ATP-dependent protease ATPase subunit ClpY"

/db_xref="COG:COG1220"

/translation="MDTQALTPRQIVAELDKYIVGQKQAKKSVAVALRNRYRRSLLPE

HTQDDIVPKNILMIGPTGVGKTEIARRLAKLVGAPFVKVEATKFTEVGYVGRDVESMI

RDLIETSLRMVKLERTEKVKDKAEEAANERIVHILAPSQSKSKNQRNPFEMIFGNNGN

NTPETDEQEPDTGVTERRRKIKFDLLSGKLEDDIIEIDVEDTTPNMMDMFAGQGNDQM

GMNMQEMFGSLLPRRTKKRKLAIKEARKVLIQEEAGKLIDMDDVTQESIRRAEQTGII

FIDEIDKVASQGRGSGPDVSREGVQRDILPIVEGSTVMTKYGPVKTDYILFMAAGAFH

VAKPSDLIPELQGRFPIRVELNSLTLDEFVSILTEPKNALTKQYVDLLRTENIEIEFS

DDAIREIAKLAESVNQNTENIGARRLHTILEKLLEDLSFEAPELTLERMIITPEYVRE

KLNDIALDRDLSQYIL"

CDS 2128880..2129287

/gene="flgB_2"

/locus_tag="PIKHDJKN_01962"

/inference="ab initio prediction:Prodigal:002006"

/inference="similar to AA sequence:UniProtKB:P24500"

/codon_start=1

/transl_table=11

/product="Flagellar basal body rod protein FlgB"

/db_xref="COG:COG1815"

/translation="MNLLNDISFQRLQGALDASNIRQRTIADNIANADTPYFKRSDVS

FEEMLEDQMNGDMPVLKGKVTDSRHFVIGPSSSIPTPVVNMDQSTSMNNNQNNVDVDK

EMSLLAENQLRYNAYIQQVNEQIKMMRVGVEGR"

CDS 2129293..2129745

/gene="flgC"

/locus_tag="PIKHDJKN_01963"

/inference="ab initio prediction:Prodigal:002006"

/inference="similar to AA sequence:UniProtKB:P0A1I7"

/codon_start=1

/transl_table=11

/product="Flagellar basal-body rod protein FlgC"

/db_xref="COG:COG1558"

/translation="MNISNSFSISASALTAQRLRMDVISSNIANAETTRASVTNGEAV

PYKRKMVVLEPNKTSFNSLLQNQMKSSGSGEGVRVSEIREDQSPLKPVYDPSHPDANA

EGYVYMPNVDIAKEMVDMISASRSYEANVTALNSTKAMISKALEIGRA"

CDS 2129810..2130121

/gene="fliE"

/locus_tag="PIKHDJKN_01964"

/inference="ab initio prediction:Prodigal:002006"

/inference="protein motif:HAMAP:MF_00724"

/codon_start=1

/transl_table=11

/product="Flagellar hook-basal body complex protein FliE"

/translation="MIQNNMFSTQGIQPLQMQSTAQSKPSTPAETIQSFGTYLQDALG

SVAAQETQAHEMSNQFLVGKVNVDQVMIASEQALLSLQLTTQVRNKVVEAYQEIMRTQ

L"

CDS 2130259..2131842

/locus_tag="PIKHDJKN_01965"

/inference="ab initio prediction:Prodigal:002006"

/codon_start=1

/transl_table=11

/product="hypothetical protein"

/translation="MNERIAQYRDKASQYWNSFSKKQKVLFISTFLFLILAAVVLTMQ

LSKTEYEVAFTDLNASDSAGVISYLDSSSIPYKLSADGKTISVPSTDVAIAKVNIGSQ

GIIQNGSLGYKSFEESSSPIGMTDKEFDVKYNNALNGEVEQLLQRMQGIQDAKVLVNM

PKDNIFAGLEEQDKASASVALQFKPGYHPNQAAVDGYFNLVKTAIPNLPIENITITNT

DEAELIPTARGGSGGLSSEVQENMALQKKFENDVRNNVKQFLSQIVGDENVNVLVASK

LNFDKETRKENLVTPVDVDNMKGIEISVQEIQKSYTGASNPTGGVAGTGQEEVPGYPS

SDASGNSTSEETSSTKNYDVNRIVKDIVSSPYTVKDLTINVAVEPPAGQTELQAPVQD

AIENILVNIVRASLADSGTVTSDADLAKKVSVMSQGFQTAAATNTGFQLSTGMMWGIG

ALVAALIAAVVILLVRRRRKQNEIEEEDIPLPVATEFPSITLDSVTNESQVRKQLESL

AKKKPDEFVNLLRTWLADE"

CDS 2131854..2132870

/gene="fliG"

/locus_tag="PIKHDJKN_01966"

/inference="ab initio prediction:Prodigal:002006"

/inference="similar to AA sequence:UniProtKB:Q9WY63"

/codon_start=1

/transl_table=11

/product="Flagellar motor switch protein FliG"

/db_xref="COG:COG1536"

/translation="MAKASSQGLTGRQKAAILLITLGPEVSAQIFKHLRDEEIEQLTL

EIANVRKVDASEKDMIMSEFHQICLAQEYISQGGITYAREILEKALGSQKALEVINRL

TATLQVRPFDFARKADPNQILNFIQNESPQTIALVLSYLQFEQAAAILSSLPQEKQAD

VARRVAVMDSTSPEVISQVERVLEQKLSSTVTQDYTNAGGIESIVQILNGVDRGTERT

ILDSLEIQDPELAEEIKKRMFVFEDIVNVDDRSIQRIIRDIDNGDLQLALKVASEEVR

DAIFRNMSKRMSETFKEEMEFMGPVRLRDVEEAQTRIVGTIRRLEEAGEIIIARGGGD

DIIV"

CDS 2132863..2133723

/locus_tag="PIKHDJKN_01967"

/inference="ab initio prediction:Prodigal:002006"

/codon_start=1

/transl_table=11

/product="hypothetical protein"

/translation="MSNLIKSFQYVPVDDRKRLENHHHYGGPESDAELDGERAEGSEA

EMLQARVDEETQRLTAEMLEDAKEFAEKQVREASEEAERLLQEAREQIDSWWQGQRQQ

DEHLTEALRSQGFQQGFEEGKVQAELDLQVKIEEMMKEARGVLEEAYVAKDQIIQEAE

PFLVDLACGIAEKVIDKQLSVEPEHTLELIRQSLSRKREQGMITLCVAPDQFAFVQAA

REELAMAIDSQAELQILPDATVKDKGCVIRSSFGSVDARIDTQLAEIKKELIRIALED

EGRKNQHEDS"

CDS 2133710..2135029

/gene="yscN"

/locus_tag="PIKHDJKN_01968"

/EC_number="7.1.2.2"

/inference="ab initio prediction:Prodigal:002006"

/inference="similar to AA sequence:UniProtKB:P40290"

/codon_start=1

/transl_table=11

/product="putative ATP synthase YscN"

/translation="MKILSSQRYMEHLKQFDPVRINGKVTQVIGLMVESEGPDASIGD

VCYIYPGKAAKPLQAEVVGFRDNKVLLMPLGELQSIGPGCDVVGTGKPLGVQVGSELL

GKVLDGLGQPLDGSLLPSRMPMYSTSNTPVNPMDRPRVLETMGVGVRAIDGLLTVGKG

QRVGIFAGSGVGKSTLMGMIARNTAADVNVIALVGERGREVRDFIERDLGPEGLERSV

VIVATSDQPALIRIKGAVIATTIAEYFRDRGMNVMLMMDSVTRYAMAQREVGLAVGEP

PAMRGYTPSVFASLPKLLERAGTGPTGSITAFYTVLVDGDDMNEPIADAVRGILDGHI

VLNRSIANKGHFPAIDVLASISRVMKDIAPEEQLEAVNNMKRLMAVYKESEDLINIGA

YQRGSNAAIDESIEQIDSIWNFTRQKVDEKVTLSEVQERLILEFARR"

CDS 2135041..2135493

/locus_tag="PIKHDJKN_01969"

/inference="ab initio prediction:Prodigal:002006"

/codon_start=1

/transl_table=11

/product="hypothetical protein"

/translation="MKFRYHFQKVVDLKSNEKTQAEWMLSTAIGKLQTEEEHLLQLLN

DKKELIDVIQSATESTASVSSLQEIQRYVHHLDECISRKSSDVRHAEVNVQRNQTFLN

GKMMDEKVWLGARDKAKIKFQQEMLLREQNDLDEMATVRFAAKVGRAN"

CDS 2135536..2136471

/locus_tag="PIKHDJKN_01970"

/inference="ab initio prediction:Prodigal:002006"

/codon_start=1

/transl_table=11

/product="hypothetical protein"

/translation="MAVKDTDMEKESSGGWEKFLMISIPIVFTVVLLGVLLTLFNVDI

RNNLLEVANKIPVVKDWVPDPVLDPEKKKLEKSEQQVESAEATIDKLKAQVSEKETEL

KVAKDATTTEAKKASDLQKKLDDAEKAAETAAQNPETESDYQKQIKDLAKMYADMSPS

KAAPILQNMTNEEMVLLLNAMQSAARTKVLEKMDPKTAADVTMMMKDAKPSGDLALDA

LQSRLKKETATTSTASTTNSKNLDKNQLSQTFASMSASSGAKLLLETYKLSPDKTLTI

LNSVDDATRSQLLENMSTENSVETAKILNKLMGNK"

CDS 2136513..2137892

/locus_tag="PIKHDJKN_01971"

/inference="ab initio prediction:Prodigal:002006"

/codon_start=1

/transl_table=11

/product="hypothetical protein"

/translation="MSIVYQMTSTSSAKTTGAAQTAGTGSKGATAGSGDFLQTLAQSL

NGGATAENSSTAAGSLTADPLMFTFAASEDGEQTSITDILSSLFADLDSLDEALENDP

SLLGELQSLIQQMYAQLDANSGIQAEGSDEPTSETGNAASAIDLTEHPAAVRFVLQDV

LTQLTAGMNELDSTVVKNAPEFKHLLQSLQSQLQDAGVDTSLNKGWTELKSILDTLAV

AKDQAVQVVPTNPVQTSKQDSVASQVLVAAASNNGTKVSVETDSTPTANEVGEVDHSS

IITAGELSLRSSGTSAGKPAEPVMQTSQFAKEMTQFVVNKLDIVQQKGFSEATISLRP

EHLGKLDVQITLQNGQLVARFMTEHTMAKDMLEQQMMQLRSSLQAQGIQVERLEVTQN

SSLGSQMYQDGGRQPGSNSQQQRRSREREEQSDDAVTTATLQEELRNWRSEHEEGNDL

RRDTFTAEA"

CDS 2137912..2138457

/locus_tag="PIKHDJKN_01972"

/inference="ab initio prediction:Prodigal:002006"

/codon_start=1

/transl_table=11

/product="hypothetical protein"

/translation="MANEAISTNNTWPNYSAANKATTSAATKELGKDQFLKILITQLQ

NQDPMQPMEDKEFIAQMAQFSSVEQLVNISSQLKTLNQSLGTVSGMIGREISWLSSNK

EDNGTLRQGIVDSIIVRDGVQYAKVGNDEIKLDEIIQVTNPTQAEENETPVQDAADST

VTNETKEPEPSAQPEDNGNML"

CDS 2138454..2138837

/locus_tag="PIKHDJKN_01973"

/inference="ab initio prediction:Prodigal:002006"

/codon_start=1

/transl_table=11

/product="hypothetical protein"

/translation="MSDRITVGQLYTGPITPNLLNRSKAGEASNVPERPFAQVLEDNL

LKLSNHAAKRLEQRGIELKTEQMQQIGTALDKAAAKGAKESLILMKDMAFIVNVKNRT

VVTAMDSESMKNNVFTQIDSAVIIS"

CDS 2138920..2139735

/gene="flgG_1"

/locus_tag="PIKHDJKN_01974"

/inference="ab initio prediction:Prodigal:002006"

/inference="similar to AA sequence:UniProtKB:P0A1J3"

/codon_start=1

/transl_table=11

/product="Flagellar basal-body rod protein FlgG"

/db_xref="COG:COG4786"

/translation="MLKSMYSGVSGMRGFQTKLDVIGNNIANVNTVGFKGSRVMFKDI

MSQTTAGVTAPGDENGGVNAKQIGLGVSVGSIDTLHLAGSPMTTNNPTDLRLNGDGFF

LVTLGGEQEVPFLTRAGDFHVDANRNLVTSDGLFVVDSGGEPITLDEAVVSFTIGQDG

IINQTMDDGTTEAGAQLGIGKVTNPEGLEKIGGNLYRITANANADGEFEIVTANSTEF

GTGSVIAGQLEMSNVDLTGEFTEMIVAQRGFQANSRIITTSDEVLQEVVNLKR"

CDS 2139802..2140023

/gene="swrD"

/locus_tag="PIKHDJKN_01975"

/inference="ab initio prediction:Prodigal:002006"

/inference="similar to AA sequence:UniProtKB:C0H412"

/codon_start=1

/transl_table=11

/product="Swarming motility protein SwrD"

/db_xref="COG:COG1582"

/translation="MISVTRLNGSPMWLNALMVEIVEETPDTYITLVTGKRLIVLEKA

DEVISKIKDYNREIGVQAATIKVQQTEES"

CDS 2140023..2140481

/locus_tag="PIKHDJKN_01976"

/inference="ab initio prediction:Prodigal:002006"

/codon_start=1

/transl_table=11

/product="hypothetical protein"

/translation="MKKMLPWLATSLLAITLIVVVVFVFMQGQNGNKIDTHTASAAEK

KMTADEIVAVSSELGEIKTNLADIDHIVVVSFSFKLSDKKAKEDFEKIKEITVKPIII

QTFADTKSDELATAKGRIQFNKKLTELINEALPEGKLASTSFSAFVMAPM"

CDS 2140521..2141519

/gene="fliM"

/locus_tag="PIKHDJKN_01977"

/inference="ab initio prediction:Prodigal:002006"

/inference="similar to AA sequence:UniProtKB:P23453"

/codon_start=1

/transl_table=11

/product="Flagellar motor switch protein FliM"

/db_xref="COG:COG1868"

/translation="MVDVLSQNEIDALLAALSSGEMDAEELKKEETQKKIRSYDFKRA

VRFSKDHIRSLTRIHENFARFLTTYFSAQLRTFVQINVVQVEQLPYDEFIRSIPKMTI

LNIFEAEPLQGRMVMEVHPNVGYAMLDRLLGGTGNAPTKIASMTEIETTIMERIFSRA

FESLQEAWKTVLDISPRMEALETNPQFMQIVSPNETIALISLSTKIGDTTGMINLCIP

HVVLEPIMSRLSTHQWFVSEKKTRAPEEYDALKERVNKAKLPVVAELGESRISIAEFL

GLSVGDVITLNKPVDEGLSIKVGDRLKYMGSPGTIKDRVAVQIDKIVTEGVEEFDE"

CDS 2141509..2142771

/locus_tag="PIKHDJKN_01978"

/inference="ab initio prediction:Prodigal:002006"

/codon_start=1

/transl_table=11

/product="hypothetical protein"

/translation="MTSKDYLSQEEIDALLRQSESMNSSEPAEKTVDDFLTELEQDAL

GEIGNITFGSAATALSTLLGLKVDITTPKVSIISRTQFDEAFPKPHVAVHVNYVDGFE

GINSLVIKKRDAQVIADLMLGGEGNPVDEELNEIHISAVQEAMNQMMGSSATSMSTIF

NRFVNISPPGIDILNMESGEGVSNLPDDETLIQVSFRLLIGDLIDSNLMQLLPVHFAK

EMVEMLIGGAQESTASAPVASTPEPAPAAVPVAPEQPPVQQQIPPQAQQPPVQDYNGY

GQAPMGMPQGMPPQQPYGMPPQQPYGVPQHYGGVPNRNVNVQPVQFANLQNGAFGQVD

ENNLNLLMDIPLKVTVELGRTQKQIKDILELSQGSIVELDKLAGEPVDILVNNKLIAK

GEVVVIDENFGVRVIDIVSQWDRIQKLQ"

CDS 2142800..2143165

/gene="cheY"

/locus_tag="PIKHDJKN_01979"

/inference="ab initio prediction:Prodigal:002006"

/inference="similar to AA sequence:UniProtKB:P24072"

/codon_start=1

/transl_table=11

/product="Chemotaxis protein CheY"

/db_xref="COG:COG0784"

/translation="MANRILVVDDAAFMRMMIRDILSKNGYEVVGEAQDGSQAIEKFK

ELRPDLITMDITMPEMDGIAALKEIKKIDANAKVIMCSAMGQQAMVIDAIQAGAKDFI

VKPFQSDRVIEAISKTLGV"

CDS 2143174..2143713

/locus_tag="PIKHDJKN_01980"

/inference="ab initio prediction:Prodigal:002006"

/codon_start=1

/transl_table=11

/product="hypothetical protein"

/translation="MMAQDKIPDDVGTGVNYYFQLVWVIVVLAVILVLIVYLIRFLNK

RNQRWFRNGTIRILGGVGLGPNKSLQIIEIGGSVYLLGVGDDIQLVDKISDLEEAQRI

IDSFERDASAQQGSLSPLIAKLAARLRKDEPPREMELEDTTSFHELFESKLRQMPNRK

EKMEKVLEEDNTTDRSRDS"

CDS 2143710..2144465

/gene="fliP"

/locus_tag="PIKHDJKN_01981"

/inference="ab initio prediction:Prodigal:002006"

/inference="similar to AA sequence:UniProtKB:P54700"

/codon_start=1

/transl_table=11

/product="Flagellar biosynthetic protein FliP"

/db_xref="COG:COG1338"

/translation="MKKKIWLACFFIGLISLASVTAAFAEPIPNIDIQIGNGDGGTPS

TSSLSIILLITVLSIAPALLVLMTSFTRIVIVLGFVRTSLGTQQMPPNQVLVGLALFL

TLFIMSPTLSSINQVALQPYLKGDITQTEALEKAADPMKKFMFSHTREKDLLLFMKYN

QTEKPSTYQDIPITVMVPAYAISELKTAFQMGFMIFIPFLVIDIVVASTLMAMGMMML

PPVMISLPFKILLFVLVDGWYLVVKSLLLSFNT"

CDS 2144491..2144760

/gene="fliQ"

/locus_tag="PIKHDJKN_01982"

/inference="ab initio prediction:Prodigal:002006"

/inference="similar to AA sequence:UniProtKB:P0A1L6"

/codon_start=1

/transl_table=11

/product="Flagellar biosynthetic protein FliQ"

/db_xref="COG:COG1987"

/translation="MTSEFIIGLAGKAVYTSLLASAPMLILALVVGLIISVFQATTQI

QEQTLAFVPKIIAVLLAVLLFGPWILNILVDFTYNILDNLYRYIG"

CDS 2144771..2145559

/gene="fliR"

/locus_tag="PIKHDJKN_01983"

/inference="ab initio prediction:Prodigal:002006"

/inference="similar to AA sequence:UniProtKB:P54702"

/codon_start=1

/transl_table=11

/product="Flagellar biosynthetic protein FliR"

/db_xref="COG:COG1684"

/translation="METLLQSFPVALLMFCRIASFFVTAPVFSARNVPNSLKIGLSAF

VTFTVYMVYGIDQVVPTDLSYILLVIREILIGLLLGFVAYLLMTAIQTAGTFIDLQIG

FGMANVYDPMTGASAPLTGNFKYAFAVLLFLTMNGHHYLLDAIVYSYRWIPLSNVFFL

RLADGSIAEFLVRTLGQSFMLAFQMSAPIVVALFLTDVGLGFLAKTAPQFNVFAVGMP

LKVLVGLAILLLLVPSFAFVFSQLFEVMFRSMENLLGTIGQRPG"

CDS 2145583..2146665

/gene="flhB_2"

/locus_tag="PIKHDJKN_01984"

/inference="ab initio prediction:Prodigal:002006"

/inference="similar to AA sequence:UniProtKB:P40727"

/codon_start=1

/transl_table=11

/product="Flagellar biosynthetic protein FlhB"

/db_xref="COG:COG1377"

/translation="MKYQLDLQMFAGEKTEKATPKKRQDTRKKGQVVKSMELSGASIL

LFTFLIMMMFSNFYKERMVRLFTDIFINRLSMEVTGENVVALMMRYGIEVMLLLAPVL

LGAVLVALIVNYMQVGFLLVGEGLKPKLEKLNPIKGFKNIFSLRSLVEFAKSIMKMSI

IGFLVYSTITGYQSDIASLSHFSLDAILHFAASITLNLGIKIAVALLVLAVLDYMYQK

YDYEKNIRMSKQDIKDEYKKMEGDPLIKGKIRERQRRMAVQRMMQEVPKADVIITNPT

HFAVALKYEGSEMEAPQIIAKGQDYVALRIKEIAKEHGVITMENKPLARALFQRAEIG

DAIPADLFQAVAEVLAYVYKLKGRTK"

CDS 2146691..2148724

/gene="flhA"

/locus_tag="PIKHDJKN_01985"

/inference="ab initio prediction:Prodigal:002006"

/inference="similar to AA sequence:UniProtKB:P35620"

/codon_start=1

/transl_table=11

/product="Flagellar biosynthesis protein FlhA"

/db_xref="COG:COG1298"

/translation="MKIKDIAVLAGIIGIVLMMILPIPTWLLDILLVINISLALMILL

VAMNSKEALQFSIFPALLLITTLFRLALNISTTKLILGQGNAGSVVATFGSWIAGGQI

AIGFIVFLILVVVQFIVITKGSERVAEVAARFTLDAMPGKQMSIDADLNAGLINEQQA

RERRSKIEREADFYGAMDGASKFVKGDAIASIIILLINLIGGFIIGMTVHGLDFAESL

STYSVLTIGDGLVSQIPALLISTAAGLIVTRASSEGNLADDITGQLFTYPTLLYIVSF

VIAMLGFFTPIHIITTLPLAGLLAFAGWRMQNNLNQKQEAEEQMEEEQQIEEVRSPES

VINLLQVDPIEFEFGYGLIPLADNQQGGDLLDRIIMIRRQCALELGLVVPVIRIRDNI

QLRPNEYVIKIKGNVVGGGELLLNHYLAMSPGYEEESVTGIETTEPAFGLPALWIDEV

TKDRAELAGYTVVDPPSVVATHLTELIKKHAHELLGRQETKALVDNLRENYAALVDEL

IPSVLSIGDVQKVLAKLLREKVSIRDMVTIFETLADYGTYTKDPDVLTEYVRQSLSRQ

ITQQFSQKGETLRVITVGPGLEKKIAESVQQSEQGSYLALDPASTQSVYQKLTEQVNR

LIQSGQQPVVLTSPTIRMYLRQVIERTMQDIPVLSYSELEPNVEIQSVGVVNL"

CDS 2148721..2150076

/gene="ftsY_2"

/locus_tag="PIKHDJKN_01986"

/inference="ab initio prediction:Prodigal:002006"

/inference="protein motif:HAMAP:MF_00920"

/codon_start=1

/transl_table=11

/product="Signal recognition particle receptor FtsY"

/translation="MRVKQYVVETMPEAMLQIRKDLGSDAVILSTKEIKVGGVLGMFR

KKRIEVVAAVDKEEKKQATNQVQNQFSPVPRAFVPEAYRQTARSFVVASDESAASIAE

KNVQEKTTDATAVSESSKLGSDMSNGRSSLSIEHKPRPQGADFSGLTSARGQQEQATD

PQQGQLMSELQELKLMMTKLSKQGASADPLPEEFYALRERLMEQDIWPEVWESWFDVV

LTKLSEKGLNEVEAVQAVQLEISHFLEKRIDVGILPTTRIVYVAGPTGVGKTTTIAKL
AAEQMFNNQRKVGFITSDTYRISAVEQLRTYASILNVPLEVVQSPGDTQRAISRLEEC
DLIFMDTAGRNYRNELLVSELQSLLAPVENSETFLVLSMTSKSADMVQITEHFSKYGL
DKVIFTKMDETGSYGPMFNLLHRFPLKLAYVANGQNVPDDLLKPDAGSLTKQLLGELV
R"

CDS 2150073..2150960

/gene="ylxH"

/locus_tag="PIKHDJKN_01987"

/inference="ab initio prediction:Prodigal:002006"

/inference="similar to AA sequence:UniProtKB:P40742"

/codon_start=1

/transl_table=11

/product="Flagellum site-determining protein YlxH"

/db_xref="COG:COG0455"

/translation="MKDQAASLRSMVSMPNGVEGRGRDSRSSKIITVASGKGGVGKSN
FTLNFALSLQAMGQKVLVFDADIGMANIDVLMGTSSPYNLYHLLYRQKSIQEIIQLGA
SGLPYIAGGSGMKELFSLSDRDLEFFAGQVEEIAQEMDYVIFDTGAGLSRENMKFIGA
ADECLIITTPEPTSITDAYALVKVMHGQENATPFRMIVNRVEDQQEAERVADKIAAVA
KRFLQIDIPLLGYISEDAQVVKAVKRQVPYSLAYPNAKASRDIEKLAHHYLAAPAAPG
SETLTGIRGFMKKWLKRTT"

CDS 2150994..2152436

/gene="cheB_4"

/locus_tag="PIKHDJKN_01988"

/EC_number="3.5.1.44"

/inference="ab initio prediction:Prodigal:002006"

/inference="protein motif:HAMAP:MF_00099"

/codon_start=1

/transl_table=11

/product="Protein-glutamate
methylesterase/protein-glutamine glutaminase"

/translation="MAVYQVLVVDDSAFMRKIVSDLIEADSEFKVTATASNGKEAIQK
AIDLKPDVITMDVEMPEMNGLDALKSIMKQSFVPVIMLSGINEQGMKETIMALEAGAF

DFIRKPSIVHDQDIAQVGKALVERMRAAMDEIKRKADREASMKKRDEAQSKLLQQSQR
VQVDHPLQDRKGALRKTIEPAQQPGTRLGSGQTESLRAKPKQPAVPLTKQNTGRLDKK
MEPLRDANRASDASTDNLAKPNKLTQPPKENRLSNISRLAEQETAAASAEPSKDIAKV
IPSHKGAGGPEGSFNKLVAIGCSTGGPRALKTLLEQLPGDLPAPVIIVQHMPPNFTRS
LAQRLNTFSELHVVEAEEGMVLRKGTAYIAPGGFHVKVNKTADGRFTIKLTEEEPVNG
HRPSVDTMFESLLPYTSLQRHLVLLTGMGSDGARMMKELYEAGVTSTFAENEETCVVY
GMPRSAVELQCVRHLLPLQEIAPKLVQAVK"

CDS 2152470..2154545

/gene="cheA"

/locus_tag="PIKHDJKN_01989"

/EC_number="2.7.13.3"

/inference="ab initio prediction:Prodigal:002006"

/inference="similar to AA sequence:UniProtKB:P29072"

/codon_start=1

/transl_table=11

/product="Chemotaxis protein CheA"

/db_xref="COG:COG0643"

/translation="MDMNQYLSMFIDESNDHLQSLNENMLQLEGSPEDLGIVQVIFRS
AHTLKGMAATMGFEDLASLTHKMENVLDLVRNEKLKMQDFIFDTLFKSLDALETMVQD
ITEGGEGKADVSSIVASLQAIESGEWTGGTAPVAAVNQSPSTATPSAVELDEFQYSVL
DQSIAEGHRVLYVEVLVSEQSQLKGVRAYMVFDLLERSGEVVKSFPTVQDIEQEKFER
SFSLYYITTKEAQELEKGIMSISEIESAKVIQLDQETLQQMTNQAAATAEVEAAPVPA
PVEASSEPSSSSKEEPKKPIATKTAAPKQAAAPSRTIRVDIERLDVLMNLFSELLIDR
SRLEQLASETGNNDLSDTVAHLSRVSTDLQNIVLKLRMVPVDTVFNRFPRMIRDLAKT
LDKKIDLVITGAETELDRTVIDEIGDPLVHLLRNAVDHGVESIAERVAAGKPELGTVN
LRAFHSGNHVFIEIEDDGKGIYRDNLLRTAVKRGVVTEEQGAKMSDDEVNQLLFAPGF
STADKISDISGRGVGLDVVKSKITSLGGNVTIHSTPGKGTNFSVQLPLTLSIIAAMLV
RLGSEKYAVPLSSIVETAIVQREQVRNIHGNKMITFRESLIPYLSLSEVFGVPDFNDA
DEQETEIVVIRKGERLAAVAVEEFIGQSEIVLKSMGTYLPSIEGISGATILGDGQVAL
ILDPNAFIK"

CDS 2154588..2155049

/gene="cheW"

/locus_tag="PIKHDJKN_01990"

/inference="ab initio prediction:Prodigal:002006"

/inference="similar to AA sequence:UniProtKB:P39802"

/codon_start=1

/transl_table=11

/product="Chemotaxis protein CheW"

/db_xref="COG:COG0835"

/translation="MEEELKVIVFKLGSEEYGIEVDKVQTIERMMPITRVPKTFSFVK

GVINLRGVVIPVIDLRGRFSLPETEYTDQTRIVIVGVDDMQVGFIVDSANDVIDIKRD

AIDSPPEVVGGVKARYLRGVAKLEDARLLIMLNLNEVLNKSEVVQLESIEG"

CDS 2155054..2155680

/gene="cheC"

/locus_tag="PIKHDJKN_01991"

/EC_number="3.-.-.-"

/inference="ab initio prediction:Prodigal:002006"

/inference="similar to AA sequence:UniProtKB:P40403"

/codon_start=1

/transl_table=11

/product="CheY-P phosphatase CheC"

/db_xref="COG:COG1776"

/translation="MELFNRFEGFQMDVLKEVGNIGAGNAATALSQLLNRPIDMGVPT

VQMLPFEEVAEKVGGDERIVVTVFLRVEGEAPGNLFFMMAPEAAKMLLNRLAGFDLKE

GLAFTEMEQSALSEIGNILAGSYLSSLADFTKLSMYPTVPGLAIDMAGAILSYGLLQF

GEMGDDALLIDTSFFEGEDQVEGQFFLIPDPPSFAKIFESLGVPLDHD"

CDS 2155673..2156170

/gene="cheD"

/locus_tag="PIKHDJKN_01992"

/EC_number="3.5.1.44"

/inference="ab initio prediction:Prodigal:002006"

/inference="similar to AA sequence:UniProtKB:P40404"

/codon_start=1

/transl_table=11

/product="Chemoreceptor glutamine deamidase CheD"

/db_xref="COG:COG1871"

/translation="MIEDKSVVKVGMADLNIAHLPGVIRTTGLGSCVGLTMYDPHLKL

AGMAHVMLPTSEIAREGTLNKAKYADTALPELLQRMIQLGASRSRIVSKMAGGSQMFA

FAGAGDTMRIGPRNADSCREWLEKLGVPLLAEDTGGNYGRTIEMDCETGLLTIRSVQM

GVKEL"

CDS 2156174..2156611

/locus_tag="PIKHDJKN_01993"

/inference="ab initio prediction:Prodigal:002006"

/codon_start=1

/transl_table=11

/product="hypothetical protein"

/translation="MIGNYRINLGAGIVGFILTFFVAYSSNVLMTSLIRGLIGFVAWF

VLAYGLRWGLGLLLIPRAEGIGYDSPGAEELRGSQVDIKLEDDGQELNDLLKHGQNAS

SGENLPPSETKAPTGFAPLDPPKLVRTKDPEELAQAVRHLTDK"

CDS 2156627..2157415

/gene="sigD"

/locus_tag="PIKHDJKN_01994"

/inference="ab initio prediction:Prodigal:002006"

/inference="similar to AA sequence:UniProtKB:P10726"

/codon_start=1

/transl_table=11

/product="RNA polymerase sigma-D factor"

/db_xref="COG:COG1191"

/translation="MNERKASHLNHADLWEKWKEQGDLEAKKSLIEKYLHIVNYVSGR

LAVGLPKNVPKDDLESNGVMGLIDALEKFDYERGLQFETYASWRVRGAILDGLRQGDW

VPRSVREKAKRIEDAYQQLEQTYLRSVSDEEMSQYLDVSTKDFQHMLQEVAVMSLCSL

EDPIREEESETRLSLMVDEKAKNPDYKVNEFYLKEALVQGLEKLTVKERTVVSLLYYE

DLSLSEIAEVMSLSPSRISQLHSKAILRLRGTLDKQKDLLMRKD"

CDS 2157499..2158908

/locus_tag="PIKHDJKN_01995"

/inference="ab initio prediction:Prodigal:002006"

/codon_start=1

/transl_table=11

/product="hypothetical protein"

/translation="MTQRTVLEQCLSIVLSDDKSTAYLEFSKQEEGFASTPDELEKYI

ASQGIKYGVLREALLVFVSHPERYVKDRYKIAEGIKPVPGTNGFIKVLVGMDDSNERR

PLEAEDGKVDYKEVTRLNNVRTGQIIAERIPPVDGTVGRAVTGEEIPFRPGKEARFKV

GKNVVVNPDGSAMYAALDGLVTKTDGNKLNVFPVYEINGDVDYNTGNIDFVGTVVIRG

NVLTGFKVKAAGDIRVVGGVEGAELEAGGSVEITGGIIGYNKGLIQAGHNVKCTFIQE

GNVDAAENVLVSQSIMHSTIRAGNAVICEGTKGLIVGGSIQAGENVSARVVGNSMSTV

TSIEVGVLPKLRNELNDLRKEAREQMDALDKTKKALTLLDQLAAAGQLSPDRMSMRIK

LSATQKSALRLSEETKTRIFEIEKVLEDTSRARVDIHKMIYGGSRIVIGRYTKFIKDP

VSRISFYYHDGDISMVPFV"

CDS 2158961..2159275

/locus_tag="PIKHDJKN_01996"

/inference="ab initio prediction:Prodigal:002006"

/codon_start=1

/transl_table=11

/product="hypothetical protein"

/translation="MSFKAVELQIAVPRTSEAGRYQSEAQQRPVIDQNLLAEQTAKEA

NEARQRSEALDETAHTDVRDGQSRNKEQQSSSNENETISALETVKPAEHPYKGKHIDL

SL"

CDS 2159342..2159914

/locus_tag="PIKHDJKN_01997"

/inference="ab initio prediction:Prodigal:002006"

/codon_start=1

/transl_table=11

/product="hypothetical protein"

/translation="MSPWIIIVILGACAIAYALIMPKKNKVQEPGHHLVQEMESTLEH

YMAEIENDNDALIQRVAEMKGEATAADQRMQSQLQELQRRLDQLEESKTTETDIPSNI

IPLHSASGLQAQALVDSVRADSAQQVSEAEQQAAEQESKPARESIKDRYAELFNLYAE

GKSVDAIAKQTGIQRGEVQLILQLAEREES"

CDS 2159918..2160550

/gene="mltG_1"

/locus_tag="PIKHDJKN_01998"

/EC_number="4.2.2.-"

/inference="ab initio prediction:Prodigal:002006"

/inference="protein motif:HAMAP:MF_02065"

/codon_start=1

/transl_table=11

/product="Endolytic murein transglycosylase"

/translation="MDKRSLWIGLGSGMIVGAVLLQLATVGQNALSESNLDPELATAD

LTKEQLEAVAKSMDMKLVASDDELYTEAEWVGKKKQESSELQGKTATAPEAAESAVKP

EQPDDPKQPVTNESDKQDEVDQPKTTEPSTPNSPETAATVSFKVRSGNSLAIVAGNLE

KAGIVDSAQAFIKAGRAERINTKIQVGTYDLEKGESFKSIIAKITKEPPN"

CDS 2160792..2161490

/gene="rpsB"

/locus_tag="PIKHDJKN_01999"

/inference="ab initio prediction:Prodigal:002006"

/inference="similar to AA sequence:UniProtKB:P21464"

/codon_start=1

/transl_table=11

/product="30S ribosomal protein S2"

/db_xref="COG:COG0052"

/translation="MAVISMKQLLEAGVHFGHQTRRWNPKMDRYIFTERNGIYIIDLQ

KTVKKVEEAYNFVKGIAGENGKILFVGTKKQAQDSVKEEAERAGQFYINQRWLGGTLT

NFSTIQKRIDRLKQLEAWEEDGTFAVLPKKEVILLRKEKDRLEKFLGGIKNMKGLPSA

LFIIDPRKERIAVAEARKLGIPIVGIVDTNCDPDEIDYVIPGNDDAIRAVKLLTGKMA

DAVIEANQGEETSA"

CDS 2161606..2162256

/gene="tsf"

/locus_tag="PIKHDJKN_02000"

/inference="ab initio prediction:Prodigal:002006"

/inference="similar to AA sequence:UniProtKB:P74070"

/codon_start=1

/transl_table=11

/product="Elongation factor Ts"

/translation="MAVNASAVKELREKTGAGMLDCKKALEEANGDITKAAELLREKG

LSAAASKAGRAATEGVVESYIHAGGRIGVLVEVNCETDFVGKTDQFKEFVKDIAMQIA

AANPKFVTREEVSTEELEKEKEILKAQALNEGKPEKIVEKMVEGRIGKYYEEFCLLEQ

PFVKDPDKTISQLLNEKVSQIGENISIRRFVRYELGEGLEKKVDNFVEEVMSQVNK"

CDS 2162486..2163214

/gene="pyrH_2"

/locus_tag="PIKHDJKN_02001"

/EC_number="2.7.4.22"

/inference="ab initio prediction:Prodigal:002006"

/inference="similar to AA sequence:UniProtKB:Q831V1"

/codon_start=1

/transl_table=11

/product="Uridylate kinase"

/db_xref="COG:COG0528"

/translation="MEQPVFKRVVLKVSGESLSGQNGYGIDAETISSIAQQVKEVVEL

GVQVAIVCGGGNIWRGIAGSENGIDRATADYMGMLATVMNSLALQDALEQIEVPTRVQ

TSIAMQQIAEPYIRRRAIRHLEKGRVVIFAAGTGNPFFSTDTTAALRAAEIEAEVILM

AKNKVDGVYSADPFKDSTAVKFDQLTYMDILNKDLGVMDSTASSLCKDNNIPLIVFAI

TEQGNIKRVVLGERIGTIVKGSVD"

CDS 2163214..2163768

/gene="frr"

/locus_tag="PIKHDJKN_02002"

/inference="ab initio prediction:Prodigal:002006"

/inference="similar to AA sequence:UniProtKB:P81101"

/codon_start=1

/transl_table=11

/product="Ribosome-recycling factor"

/db_xref="COG:COG0233"

/translation="MPQAVKKHAEERMDKAIQALRRDLATLRAGRATPALLDRIQVEY

YGAMTPLNQLANISTPDSRTLMIQPWDKSSMGDIERAIMKSDLGLTPANDGTMIRLSI

PALTEERRSELVKLTKKFGEEGKVAIRNIRRDANDDIKKMEKSDISEDESRRHQDDIQ
KSTDKFIAEVDKVLAAKEKEIMEV"

CDS 2163858..2164625

/gene="uppS_1"
/locus_tag="PIKHDJKN_02003"
/EC_number="2.5.1.31"
/inference="ab initio prediction:Prodigal:002006"
/inference="similar to AA sequence:UniProtKB:O82827"
/codon_start=1
/transl_table=11
/product="Ditrans,polycis-undecaprenyl-diphosphate
synthase ((2E,6E)-farnesyl-diphosphate specific)"
/translation="MIKRVRSWWNGADKQETLTISEDNIPQHVAIIMDGNGRWAKRLG
LPRIAGHQNGMKAVKRATIAADELGIKYLTMYAFSTENWTRPKEEVDFLMRLPQEFLA
IELDELIEKNVRIRMMGQEEHLPSHTINALREAIRLTEHNTGLVLNFAMNYGSRREMT
DCVKQIALQVKSGELSADDITPELIDRHMLTGDMPDPDLLIRTSGELRLSNFMLWQLA
YSELWFTDIYWPEFGKKHLLEAVAEYQRRTRRYGGLK"

CDS 2164642..2165436

/locus_tag="PIKHDJKN_02004"
/inference="ab initio prediction:Prodigal:002006"
/codon_start=1
/transl_table=11
/product="hypothetical protein"
/translation="MKQRLTTGIIAGVLFLGFCMLGGPWYHGLVLLMALIGYYEFVKM
TGVQPFSGVALIGYAGIFAIVFPWEMVWEARPLTLFQIGWLVMLVLMTASVVTKNKIP
VNTVAMLFLGVMYIGIGFYYIAESRHLHHGLFWTFLLLGSIWASDAGAYFVGKLIGKN
KLWPSISPNKTVEGALGGIVIAVVTSVVFALVSDGLLSWQRAVWIGIACAVVGQMGDL
IQSAYKRVYNIKDSGTLLPGHGGILDRCDSWIVVFPFVHILMLLPY"

CDS 2165504..2166643

/gene="dxr"
/locus_tag="PIKHDJKN_02005"
/EC_number="1.1.1.267"

/inference="ab initio prediction:Prodigal:002006"

/inference="similar to AA sequence:UniProtKB:Q9RCT1"

/codon_start=1

/transl_table=11

/product="1-deoxy-D-xylulose 5-phosphate reductoisomerase"

/db_xref="COG:COG0743"

/translation="MKKIAILGSTGSIGTQTLDVVDMHPERFQVEGLAAGSNIELLIE

QAKRYRPKKVSVGSKELAETVAPHLPVETQLFYGKEGLVEVAAGTDADTVVTAVMGSV

GLESTLAAIDAGKQIGLANKETLITAGHIVTARAAAKGVPILPVDSEHSAIFQCLNGE

PRGRVMGITLTASGGSFRDLTREQLKEVTVEDALKHPNWSMGSKITIDSATMVNKGLE

VIEAHWLFGLSYDQVDVLLHPESIIHSYVEFNDTSIIAQLGNPDMRVPIQYALTYPDR

LPSPAQRLSLAQAGKLHFREMDMERFPCLRMAYECGKMGGTATTAFNAANEVAVARFL

RKEISFLKIEDIIASVLEAHRNVDEPDLEEIARCDQESRQLAFSL"

CDS 2166757..2168028

/gene="rasP"

/locus_tag="PIKHDJKN_02006"

/EC_number="3.4.24.-"

/inference="ab initio prediction:Prodigal:002006"

/inference="similar to AA sequence:UniProtKB:O31754"

/codon_start=1

/transl_table=11

/product="Regulator of sigma-W protease RasP"

/db_xref="COG:COG0750"

/translation="METIQVVFLTVLMFFVIVTVHEWGHYYFAKRAGILVREFAIGFG

PKLFSYKRNETQFTLRLLPFGGYARMAGEDPELVEIQEGQTIAVRSADDQVKMIYLDQ

LDNRKNVIRGEVISIDMENALKLQLDVDGEIQQYRIHPQAMLVSRGKQTQIAPKDRQF

GSKTVGQRAMAIFAGPLMNFILAFVLFAVYAQMAGVPVENPKNLEIGEVLEGGAAYQA

NLQKGDIIETINGTAIGTDSQKMVTMIADSKDKPMEWTLRRGNETFNITITPRAVEGQ

EGGKVGIVPTLPTRSVGFVETFKVSGIAMVDTTRVIFEGFKHLINQFNMDDIGGPVRT

FEVTGQIAKQGIEQLTRWAAILSLYLGIFNLLPIPALDGSRLVFLGIEGLRGRPVDPN

REGMVHFVGFAMLFVLMLAVTYNDILRLING"

CDS 2168070..2169545

/gene="proS_2"

/locus_tag="PIKHDJKN_02007"

/EC_number="6.1.1.15"

/inference="ab initio prediction:Prodigal:002006"

/inference="similar to AA sequence:UniProtKB:Q9L4Q8"

/codon_start=1

/transl_table=11

/product="Proline--tRNA ligase"

/db_xref="COG:COG0442"

/translation="MGGRWSLMSKENDKQFVTEITPQGEDFSRWYIDVIKKADLMDYS

PVRGCIVFKPDGFEIWEHIKDEMDRRFRETGHRNAYFPMFIPESFFQKEKEHVEGFNP

ELPWVTEAGGEKLEERLAIRPTSETIIGHMYSKWIQSYRDLPVLINQWANVVRWEKRT

LPFLRTTEFLWQEGHTAHETEEEAREETMKMLEIYREVVEEYLAIPVITGQKTKSEKF

AGAVDTFSIEAMMKDGRAVQAGTSHYMGTNFAKAFEIQYLSRNNVLELAHTTSWGTST

RLIGALIMVHGDDRGLVLPPKVAPTQVVMIPIGPPKTREAVVGRADELFAELKQAGIR

VKMDDRSDVRPGWKFNEYEMRGVPIRLEIGPRDMENGVCVLVSRITGEKKVVEQANLV

EEIQTMLTQVQADMLQRARTFMSDNFYSVDTLDEMKELMENKRGFTLAGWCGSDACED

KVREVTGATSRNIPFQPAEEKHTCLACGEEAKHTVVFARAY"

CDS 2169698..2174017

/gene="polC_2"

/locus_tag="PIKHDJKN_02008"

/EC_number="2.7.7.7"

/inference="ab initio prediction:Prodigal:002006"

/inference="similar to AA sequence:UniProtKB:P13267"

/codon_start=1

/transl_table=11

/product="DNA polymerase III PolC-type"

/db_xref="COG:COG2176"

/translation="MSGFEEKRKRFELLMKQTELPAGLIEPYFLDGWIERVETSRSNR

DWTIVIAKDTLVPAPIYRTFCLHIQEKMNHISKITFGFKYSEQVEIGDIVSEYWNLFL

EWVTREIPSVNGWMNRTKFECEADLLQLTMSDAMSMELAKKKQIDQAITTFYDKYFQL

PLRVKLLVGEVESNKEAMEQFQLKKREEELQVIEQMMSEVDSEMPEEEDQGDVRLQMG

YEIKEPPVPMQEIQDEEKKVTLQGTVFGLDRKELRNGNTLFTFYLTDFTDSMQMKMFA

KTKEDVKILSLLANGKWVKVRGRVEYDRFMQIPELAMIPSDLVEVKAPPSRKDNAAEK

RVEFHLHSTMSTMDAVTSIDKYVKTAAEWGHKAIAVSDHGGVQVYPEASKAAKKNGIK

MIYGLEANVVNDSVAVVLAPQPLELKSATYIVFDIETTGLSVTQNKIIEIAAVKVEDG

KEIDRFATFVNPHERIPYNIQQLTNINDEMVKDAPELEPVIRDFVKFAEDGVLVAHNA

RFDMGFIQASLKQLGMPELQNPVLDTLELARLLYPKMKNHRLNTLADKYKVALESHHR

AIDDTIALAGILNGLVNDAAQMKGLTMLDRLNDYVGVDLSNTRPFHCGIYALNDVGKK

NLYKLVSLSHTEHFKRVPCIPKSKLINLREGLIILSGCEKGEFFEAVLNKSLEEAEEI

AHFYDILEIQPLTMYMHLVDKGLVATPEELKLAVRKVVDIGQKLGKPVIATGNVHYLE

PRDKLYRDITIHGITGFSPLKDQRKPDAHLRTTDEMLEEFKYLGQDKAYEVVVTNTVE

LADRFEEIKLFPDKLFTPILEGADDEIRNTCYNTAKSIYGEELPEVIVARLEKELNPI

IKYGFSANYLISERLVKKSNQDGYLVGSRGSVGSSVVATFLGISEVNPLPAHYICVNP

ECKHSEWFLDGSVPSGFDLPEKECPDCGGRLKGEGQDIPFETFLGFKGDKVPDIDLNF

SGDYQPHAHNYTKVLFSEKSVFRAGTIGTVAEKTAFGFAKKYEEEHHKKWRGAELNRL

ASGCTGVKRSTGQHPGGIVVVPDYIEVEDVTPVQFPADDVNAEWKTTHFDYHAFEENL

LKLDILGHDDPTMMRMLQDLTGIDPTTIPMNDPKVMSMFNSTEALGVTPEQIRSPVAT

FGVPEMGTKFVRQMLIESQPSSFADLLQISGLSHGTGVWLGNAQDLIKNGTCNIKTVI

GCRDDIMLFLIYKAGMDASLAFKITESVRKGRGLPQEWIDEMKNCKVPQWYIDSCLKI

QYMFPKAHAAAYVISAVRTAFFKLYHPIEYYATYFTVRADEFDIELVCQGYEPIYRKI

VEIEQLGFQAPPKEKNMLPVLEMALEMAARGFSLKPIDLYRSEATKFIVDGNSLIPPF

SALGGIGDNAARNIAAARDHGEFLSIEDFQQKSKASKTIVELLTGMGCFRGLPESNQL

SLF"

CDS 2174277..2174738

/gene="rimP"

/locus_tag="PIKHDJKN_02009"

/inference="ab initio prediction:Prodigal:002006"

/inference="similar to AA sequence:UniProtKB:Q97S61"

/codon_start=1

/transl_table=11

/product="Ribosome maturation factor RimP"

/db_xref="COG:COG0779"

/translation="MSTTNIKSTVEEMIQPYLNEQGFELVDIEYVKEGSNWFLRVYVD

KEGGIDIDDCVLISEKLSAKLDENDPIPTIYFLEVSSPGAERPLKKPEDVSKSVGKNV

FVTTYEPVNGIKEFEGKLLSFDDGELVIEAGKKQHAILYDKVASARLAILF"

CDS 2174862..2175959

/gene="nusA"

/locus_tag="PIKHDJKN_02010"

/inference="ab initio prediction:Prodigal:002006"

/inference="similar to AA sequence:UniProtKB:P32727"

/codon_start=1

/transl_table=11

/product="Transcription termination/antitermination

protein NusA"

/db_xref="COG:COG0195"

/translation="MSMDFIEAMNELEREKGISKDVLFEAIEAALISSYKRNFNTAQN

VRVDMNRNTGVIRVYARKLIVEEVLDSRTEISLPAAREINPHFQLEDIAEIEVTPRDF

GRIAAQTAKQVVTQRIREAERGLIYNAFVDKEEDIVTGVVQRQDLRNIYIDLGKIEAA

LPLTELMPNEKFVHGDRIKAYITKVENTTKGPQIILSRTHPGLLKRLFELEVPEIFDG

VVEIRSVAREAGFRSKIAVHSRNEEVDPVGSCVGPKGIRVQTIVGELRGEKIDIVRFS

DQVDEYVANALSPSKVLEVQVFEEEKMARVIVPDYQLSLAIGIKGQNARLAAKLTGWK

IDIKSESQAEQEFGREKDSSSEMHQDSVSVD"

CDS 2175986..2176294

/locus_tag="PIKHDJKN_02011"

/inference="ab initio prediction:Prodigal:002006"

/codon_start=1

/transl_table=11

/product="hypothetical protein"

/translation="MKTKKVPLRKCVACQEMMPKKQLIRIVKTPEDQVLIDLTGKKSG

RGAYLCGKESCFKLALKNRSLDRALKGKVSPEIYEQLAADFVAVEDEFIAAQEREHDE

"

CDS 2176284..2176613

/gene="rplGA"

/locus_tag="PIKHDJKN_02012"

/inference="ab initio prediction:Prodigal:002006"

/inference="similar to AA sequence:UniProtKB:P32729"

/codon_start=1

/transl_table=11

/product="putative ribosomal protein YlxQ"

/db_xref="COG:COG1358"

/translation="MMNNKTLSYLGLSMRAGKLVTGEEIVLKAIRSSEAKMVIVAGDA

SANTQKKFRDKCGTYKVPLVIGFDRDSLGSSIGKETRVVLAVTDRGFAKMISKQVGIM

SEVEYIE"

CDS 2176606..2179194

/gene="infB"

/locus_tag="PIKHDJKN_02013"

/inference="ab initio prediction:Prodigal:002006"

/inference="protein motif:HAMAP:MF_00100_B"

/codon_start=1

/transl_table=11

/product="Translation initiation factor IF-2"

/translation="MSKQENKDKLRVYEYAKSLNMSSKEIITILKKLDIPVNNHMSVM

ENGSVGKVEQFFKDIKSTAASKQSNEAKSVATSSVRSDKTVDSNKPAGGVNTPNSSNP

SGSPVQTKIQQEKQVGMNNRPNSNNNNGSQRPSGQDSRNRTNSSQGSSQGGQSSNRPR

PAQGGQSSTASRPQSTGQRPSNSGGGQARSAGPNSGGNTGTGGNRTGGQGQSSGQGQR

RGGQGNSGNNNNNNSGNRSNSGGGGRRYDDNRGGNFRGNRGGKNNRNRNQQQYQQREK

IDNTPKKIIVRGDMTVGETAKLLHKDASEVIKKLIAMGVMATINQELDIETILLLSGE

FGVEVEVKIVLEDDRFETLEENDDPAELQSRPPVVTIMGHVDHGKTTLLDAIRSTNVT

GGEAGGITQHIGAYQVEINNKKITFLDTPGHEAFTAMRARGAQVTDMTIIVVAADDGV

MPQTVEAINHAKAAGLPIIVAVNKIDKPGADPDKVKQELTNYELVPEEWGGDTIFVNV

SAKQRMGLEGLLEMILLVAEVNEYKANPDKRARGTVIEAELDKGRGPVARILVQHGTL

KVGDAFVAGNCFGRVRAMVNDKGRRLKEAGPSTPVEITGLTEVPGAGDPFMVFEDERK

ARSIADKRAITQRESDLGTNTRVTLDDLFQHIKDGEIKDLNVIIKGDVQGSVEALKGS

LAKIEVEGVRVKIIHSGAGAITESDIILAAASNAIVIGFNVRPDNQAKATADQEQVDI

RLHRVIYSVIEEIEQAMKGMLDPIYKEKVIGHAEVRSTFSISKVGTIAGCMVTSGKIT

RSAEARLIRDGIVLYEGKLDSLKRYKDDAKEVAQGYECGITLDKYNDLKEGDIIEAFI

METVQR"

CDS 2179220..2179579

/gene="rbfA"

/locus_tag="PIKHDJKN_02014"

/inference="ab initio prediction:Prodigal:002006"

/inference="similar to AA sequence:UniProtKB:P65967"

/codon_start=1

/transl_table=11

/product="Ribosome-binding factor A"

/translation="MAKIRTGRVGEQIKKELSLLIQSELKDPRIGFITVTGVEVTGDL

SQAKVYLSVFGEQEQKDNSLKALAKANGFLRSELGKRIRFRHVPELIFKIDESIAYGS

RIEKLLGDIGTDKNESE"

CDS 2179599..2180576

/gene="nrnA"

/locus_tag="PIKHDJKN_02015"

/EC_number="3.1.-.-"

/inference="ab initio prediction:Prodigal:002006"

/inference="similar to AA sequence:UniProtKB:Q5SM25"

/codon_start=1

/transl_table=11

/product="Bifunctional oligoribonuclease and PAP

phosphatase NrnA"

/db_xref="COG:COG0618"

/translation="MHTYEQALQDGKQFLLEHDDYLVVSHVQPDGDAVSSTVTVGWLL

SCLGKTFTMINEGEIPQRMNFLWEAGKIVNMTEQPPARKYKAIICVDCADFSRVGLTR

HYFEEDAVILNIDHHPTNDAYGTVNIIKPDAAATAEILFDFINLFPVKWNKDVATAVY

TGLLTDTGGFRYANTSPNVMTTASKLLEHGVDGPYLAQILLEQVTLPQVRILNQALSS

LQMTDDGKIAWVVITPEDMIACGAANEDLEGVVNYPRNILGVEVGIFFKVINDNAVKV

SLRSAGKVDVASLAQNFGGGGHVLAAGCRVEGKLEDIVELVLKQVNAQW"

CDS 2180570..2181493

/gene="truB"

/locus_tag="PIKHDJKN_02016"

/EC_number="5.4.99.25"

/inference="ab initio prediction:Prodigal:002006"

/inference="similar to AA sequence:UniProtKB:P65855"

/codon_start=1

/transl_table=11

/product="tRNA pseudouridine synthase B"

/translation="MVKPFEGVLPVYKPAGFTSHDVVAKMRRILKMKRIGHTGTLDPQ

VTGVLPLCLGRATRVVEYMQELPKEYLATLRLGLSTDTEDMTGEVIERSETAVEVTQE

QVQQVLEQFLGTISQVPPMYSAVKVDGKRLYELAREGKTVERKSREVTIYELELTGIE

NQGDTTDISFRALCSKGTYIRTLCVDIGRKLGYPSTMLHLERTISAGISADHCLHFEE

VEQRMIDGTLAEVLIPVDEAIASIPAHTVGADQTKGALQGQKLSARLLEPPAEQPGLL

RLYDQDGTFLGIFERDELKATVRAVKVFLPE"

CDS 2181560..2182498

/gene="ribF"

/locus_tag="PIKHDJKN_02017"

/inference="ab initio prediction:Prodigal:002006"

/inference="similar to AA sequence:UniProtKB:P0AG40"

/codon_start=1

/transl_table=11

/product="Bifunctional riboflavin kinase/FMN

adenylyltransferase"

/db_xref="COG:COG0196"

/translation="MKTVMLTYPQTLQSAVQGTHPQVLAIGQFDGLHLGHASVILSAV

RIARETGMQAAVMTFHPHPKEVMRKGDYEGYLTPLRDKEDILAGMGVDVLYVVEFNEE

FSRLTPQQFAHDLLLPLQTRTAVVGFDFRFGHKGAGDEQLLRTLGEGEMTVETVPPFL

LNGEKVSSSLIRGLLKRGEIDEASQWLGRPYSIRGTVIHGEKRGRTIGFPTANLELTD

HYVTPAKGVYAVRVQYGEQELRGVMNLGVKPTFHENGMKPTFEVHLLDFDGQIYDQEL

KVDLVHYIRAERKFDSIDALISQIREDALTAARLLS"

CDS 2182658..2182927

/gene="rpsO"

/locus_tag="PIKHDJKN_02018"

/inference="ab initio prediction:Prodigal:002006"

/inference="similar to AA sequence:UniProtKB:P21473"

/codon_start=1

/transl_table=11

/product="30S ribosomal protein S15"

/db_xref="COG:COG0184"

/translation="MALTQERKQQLIDEHKTHESDTGSPEVQVAILTENIRSLTDHLR

THKKDHHSRRGLLKMVGQRRKLLAYVKNKDVKRYSALIEKLGLRR"

CDS 2183180..2185282

/gene="pnp_1"

/locus_tag="PIKHDJKN_02019"

/EC_number="2.7.7.8"

/inference="ab initio prediction:Prodigal:002006"

/inference="similar to AA sequence:UniProtKB:P50849"

/codon_start=1

/transl_table=11

/product="Polyribonucleotide nucleotidyltransferase"

/db_xref="COG:COG1185"

/translation="MEQRVEMQLGGRTLTLETGRLAKQANAAVKVTYGDTVVLCTVTA

SNEPKDLDFFPLTVNYEERLYAVGKIPGGFIKREGRPSEKAILSSRLTDRPIRPLFPE

GFRNDVQVLNIVMSVDQDCEPQIAAMIGTSAALSISDVPFSGPIGGVKVGRIDGQFII

NPTIAQLEVSEIELVVAGTKDAIMMVEAEANEVPEEVMLEAIMFGHGEIKNIVAVIEQ

LVQVAGKEKMAVKLHAVNAEVNSSVREFASARLVEAVKIAEKHARQDAIDLVNDETVA

HFEEKYIETPELLKDVKEVLHDIVKEEVRRLITHDKVRPDGRGLAEIRPIECDTSLLP

RTHGSGLFTRGQTQALSICTLGALGDVQILDGISLEETKRFMHHYNFPPFSVGEARPL

RAPGRREIGHGALGERALSKVIPSETDFPYTIRLVSEVLESNGSTSQASICASTLAMM

DAGVPIKAPVAGVAMGLIKDGDHVSILSDIQGMEDHLGDMDFKVAGTPDGVTAIQMDI

KIDGIDRQILSEALAQAKEGRMHILSKMTEVMKTPREQLSQYAPKITTMHINPDKIRD

VIGAGGKIINKIIEETGVKIDIEQDGRVFIASSNQEMNDKAKSIIEGIVREVLVGEIY

VGKVKRVEKFGAFVEVLPNKEGLVHISQLSTERVAKVEDVVAIGDSITVKVTEIDPQG

RINLSRKAVLTAEAPAQS"

CDS 2185506..2186501

/locus_tag="PIKHDJKN_02020"

/inference="ab initio prediction:Prodigal:002006"

/codon_start=1

/transl_table=11

/product="hypothetical protein"

/translation="MGNQSKKLAAVLVGMAAVILVGQVGSVRTYITEIRDGPGTQNAF

DMFKEATGEDQLLSAIRDKAAETKIAPVNARVDRVWKAIPGYNGIEIDVEATYRKALG

GTLNTKIAYVYRQTVPEIQLKDLGAHPIYRGNAEKPMVSFMINVAWGNEFIIPMLDTL

DAEKVKATFFLDGSWLSKNEELAKEIQKRGHELSNHAYSHPNMSRLSTERAKLEISKT

QDLLKDKLGVENHWFAPPSGDFNQQTVDIASGMGLQTVLWTLDTVDWRKPSSDSVVAK

IAKNVEAGTLILMHPTAASSGALKGMIDSIRAKGLTLGTVSETLSSERVKGSAVE"

CDS 2186600..2187883

/locus_tag="PIKHDJKN_02021"

/EC_number="3.4.24.-"

/inference="ab initio prediction:Prodigal:002006"

/inference="similar to AA sequence:UniProtKB:P9WHT5"

/codon_start=1

/transl_table=11

/product="putative zinc protease"

/db_xref="COG:COG0612"

/translation="MKKIQLGNGLRVVMEQIPTCRSVSFGIWVKTGSRNEQPASNGVS

HFIEHMLFKGTDRYDAKAIAEQFDAIGGNVNAFTSKEYTCYYAKVLDEHLPIAVDVLS

DMFFRSKMDDGELLKEKNVILEEISMYEDTPDDMVHDLMALAAYGEHPLAYPILGTEE

RLKAMDSSHLRAYMKEHYTIENTVISISGNIDDSVIDLMEKHFGAFDVNGVAEQVTLP

AFQSGQLFHKKKTEQNHICISFPGCKIGDPLQFAMVVLNNAIGGGMSSRLFQEIREKR

GLAYSVYSYHSSHADSGLFTIYAGTAPKQTKEVLDLTKEVLHDLAVNGLSEDELRKGK

EQLKGSLILSLESTGSRMNRLGKNELMLGRHHTLDEMIAKIEEVTMDDVDAVLNLMFA

EPFALAMVGASDRTIAGLRRDDFVALRSNSKVTGQ"

CDS 2187846..2188289

/gene="dut"

/locus_tag="PIKHDJKN_02022"

/EC_number="3.6.1.23"

/inference="ab initio prediction:Prodigal:002006"

/inference="similar to AA sequence:UniProtKB:Q2YRG4"

/codon_start=1

/transl_table=11

/product="Deoxyuridine 5'-triphosphate

nucleotidohydrolase"

/translation="MHYVQIQKLPGNEDIKLPQKMSELASGFDVVAALQEEVVLQPGQ

RTLIPTGLAMAMPAGLEAQVRPRSGLAFKHGITCLNTPGTIDADYRGEVKVLLINLGQ

EPFTIVRGERIAQIVFQTVPAVELTEVAELSETVRGEGGFGHTGK"

CDS 2188527..2189426

/gene="dpaA"

/locus_tag="PIKHDJKN_02023"

/EC_number="1.3.1.-"

/inference="ab initio prediction:Prodigal:002006"

/inference="similar to AA sequence:UniProtKB:Q04809"

/codon_start=1

/transl_table=11

/product="Dipicolinate synthase subunit A"

/translation="MLTGVRIIVLGGDARQLEVIQKCAELDATVSVVGFDKLERSIPG

IEHQELEDDVFASADVLVLPVVGCDDQGKVSTSFSDTPIFLKKEHIAALPEHCIVFTG

MAKPFLRDLCRENGLRLVEVLDRDDIALYNSIPTAEGAIAMAIRETDFTIHGSECIVL

GLGRTGFTMAKTLQGLGANVRVGIRREEDAARAAIMGWKPFMTTDLAAQTGEVDLLFN

TIPTMIITAQILSKMPQKAVIIDLASAPGGCDFRYAEKRGIKALLAPGLPGIVAPKTA

GGIIADVLIRLLLEEQNAREVEQ"

CDS 2189423..2190019

/gene="dpaB"

/locus_tag="PIKHDJKN_02024"

/EC_number="1.3.1.-"

/inference="ab initio prediction:Prodigal:002006"

/inference="similar to AA sequence:UniProtKB:Q04810"

/codon_start=1

/transl_table=11

/product="Dipicolinate synthase subunit B"

/db_xref="COG:COG0452"

/translation="MNWQGKTVGYAITGSHCTFEEVMPVISRFVAEGANVVPIISNSV
LTTDTRFGTAQNWQKQLKDITGNDIISTIVEAEPLGPSKLLDVLVIAPCTGNTTSKLA
NAMTDSPVLMAAKAQMRNQRPLVLAISTNDGLGLNAANIAKLLVAKYLYFVPFGQDDP
VKKPNSLVAKMELIPEACWSALEGKQLQPMIVQRSAQA"

CDS 2190444..2191661

/gene="lysC_1"

/locus_tag="PIKHDJKN_02025"

/EC_number="2.7.2.4"

/inference="ab initio prediction:Prodigal:002006"

/inference="similar to AA sequence:UniProtKB:Q59229"

/codon_start=1

/transl_table=11

/product="Aspartokinase"

/translation="MRIMVQKFGGTSLSTVQAREHVLRHVKRELEAGLSLVIVVSAMG
RRGEPYATDTLLDWAAQNGNALSAREKDLLLCCGEIISATTLSSLLEHEGIPTTVLTG
AQAGFVTDDNFGNARILDVRPVRVLEQLQSGRVVIVTGFQGQTENGDFTTLGRGGSDT
SATALGAALHAEMVDIYTDVNGILTADPRIVEDARPLTVVSYAEICNMAHQGAKVIHP
RAVEIAMQSQIPVRVRSTFADSEGTLVTHPEGFQDVQTGIVDRYVTGIAYVSNVTQIT
VEVPGGADRLQLKVFKTMAENSISVDFINVTPLGVVYTVFDSDSEKAIHVLQEIGLKP
QSLSGCAKVSVIGGGINGVPGIMARIVESLTLADIQILQSADSNTTIWVLVKKEDMVQ
ALRALHASFELHL"

CDS 2191709..2192584

/gene="dapA_1"

/locus_tag="PIKHDJKN_02026"

/EC_number="4.3.3.7"

/inference="ab initio prediction:Prodigal:002006"

/inference="similar to AA sequence:UniProtKB:Q04796"

/codon_start=1

/transl_table=11

/product="4-hydroxy-tetrahydrodipicolinate synthase"

/db_xref="COG:COG0329"

/translation="MDFGRLITAMVTPFNEQGEIHWEETARLIDYLIEDQKSETLVIS

GTTGESPTLSDKEKVELFEFAVKHTAGRCKIIAGTGSNNTAHSIHLTQDAERAGVDGV

LLVVPYYNKPSQEGMFRHFEAIANATKLPVMLYNVPGRTAASLSAATTLRLAQIPNIV

ATKECVSLEQVTQIAAGAPGHFRVYSGDDASGLPAIAVGAYGIVSVASHVVGAEMKQM

IDAYFGGAPVQAAQIHQKLFPVFKGLFECPQPLPNPVAVKYALTLRGLNVGSVRLPLI

AATEDEQGFIRALFN"

CDS 2193062..2194741

/gene="rnjA_1"

/locus_tag="PIKHDJKN_02027"

/EC_number="3.1.-.-"

/inference="ab initio prediction:Prodigal:002006"

/inference="similar to AA sequence:UniProtKB:Q45493"

/codon_start=1

/transl_table=11

/product="Ribonuclease J1"

/db_xref="COG:COG0595"

/translation="MSKKNNNDKLMIFALGGVGEIGKNMYVIQYANDIVVVDAGLKFP

EEDMLGIDIVIPDISYLTENRDKVRGIILTHGHEDHIGGLPYVLKHLNVPVYGTKLTL

GLVENKLKEANLLGETKRILIDADSEIQLGSVLKATFFATNHSIPDSVGVCVETPEGA

VVHTGDFKFDHTPVNGQYADLQRMAQIGTNGVLALLSDSTNAEKPGFTPSEKNVGIVL

EDIFRKASQRVVVATFASNVHRIQQVINAAEVTGRKVAVIGRSMVNVVGIASELGYLE

IPDGMIIEPEEVGKMAADRVVILCTGSQGEPMSALTRMARSTHRKVDILPGDTVIIAA

TPVPGNEKYVGRTIDELFRLGANVHYSGANSGVHVSGHGSQEELKLMLNLMKPKYFLP

IHGEFRMQRRHAVLAESVGIDPDNIFITDIGEVVEIQGGAARKAGKVTAGNVLIDGLG

VGDVGNIVLRDRKLLSQDGILVVVVTLSKQDGKIVSGPDIISRGFVYVRESEGLLDEA

NRIVSSTLQKLMSENVNEWASLKTNVKDALGRFLYEQTRRRPMILPIIMEV"

CDS 2194957..2195730

/gene="tepA"

/locus_tag="PIKHDJKN_02028"

/inference="ab initio prediction:Prodigal:002006"

/inference="similar to AA sequence:UniProtKB:Q99171"

/codon_start=1

/transl_table=11

/product="Translocation-enhancing protein TepA"

/db_xref="COG:COG0740"

/translation="MSEYMKNKQTPKSEQPGTEAPEVPAQEEKGMSPVTEAIQQFGQT

QSPAAESNIFCMTIIGQVEGHLILPPQNKTTKYEHLIPQLVAAEQNQRIEGILIILNT

VGGDVEAGLAIAEMISSLSKPTVTVVIGGGHSIGVPIAVASTYSLIAGSATMTIHPIR

MNGLVIGVPQTFEYMEKMQERVVRFVTSHSNISEEMFKELMFKTGELNRDIGTAVGSA

DAVKYGLMDAVGGIGQAIAQLNQLIGDKRQTLQAGGYTQ"

CDS 2195727..2195957

/locus_tag="PIKHDJKN_02029"

/inference="ab initio prediction:Prodigal:002006"

/codon_start=1

/transl_table=11

/product="hypothetical protein"

/translation="MTLYTVMPPELLWSGMWNEGEDTREIKMNGLLMQVRPVNENEAV

IVRLLECPLEAYLNPANMPGSIVPLSGNPGPT"

CDS 2196048..2198783

/locus_tag="PIKHDJKN_02030"

/inference="ab initio prediction:Prodigal:002006"

/codon_start=1

/transl_table=11

/product="hypothetical protein"

/translation="MARRKKRKKKAAAFSGVLKYEIYGIVLITLAVIALSGEATVGRS

LSKMFGLMLGKFYFAIPLIGIYYGLMVMIHRKWPSGWTTRKTGLVLLVFALTLMSTVS

AMHQKLIPVGALEPGAVLTQIHNDMQTELLTPAAPGERDSMLNKDISGGYIGAAQFAL

FLWLFGSLGARLIMIVMFVISFMLITNLSYVDLIRIFRTKIWDAGSAMYKKLEARPAA

RATSSSAGKKNGNVRKVVPVPVDDEEDDDDDMQDQQLPKRKAPIFFQLFGKWGSDRER

TKAERGVDEDDSLDTEHIVYRAEQETPADSWHDEPNIASDVMAPAAVPAQSRPNSAPI

IRDFFEHVRSEDASKDDDLDDAYPFPDDLTANMQGEELPIKISDELVDNNEWPGAADA

GTAVIDAVEGSSDVGSTLSNAAQGSVASDMEGQEVQPVKPPSPPPKPYKLPSFRLLSK

PNNGGKGGDQKDYMQTARKLEATLESFGVRAKVLEVVRGPAVTRYEIQPDIGVKVSRI

VSLTDDIALALAAKDIRMEAPIPGKSAIGIEVPNGEVSVVTMREVMETATFQEAESKV

TIAFGRDISGQTIIGNLARMPHLLVAGATGSGKSVCINGIITSILYKAKPDEVKFLMV

DPKMVELNVYNGIPHLMAPVVTDPKRASLALKKIVVEMEKRYELFSKSGTRNVEGYNN

LMKDNPAAVLPYIVVIVDELADLMMVAAGDVEDAIARLAQMARAAGIHLIIATQRPSV

DVITGVIKANIPSRIAFGVSSQVDSRTILDMGGAEKLLGRGDMLFMPMGASKPVRVQG

AFMSDEEVENIVNYVRGQGEAQYDESIVPEVDDSVQAADEVQDELYEQAVQIILEAKQ

ASVSLLQRRMRVGYTRAARLIDSMEARGVIGPYEGSKPREVLISLEQYQQNKISS"

CDS 2198909..2199802

/locus_tag="PIKHDJKN_02031"

/inference="ab initio prediction:Prodigal:002006"

/codon_start=1

/transl_table=11

/product="hypothetical protein"

/translation="MLVIITLAILLIPQEKGRAQSMRKMNIWLFTAILLMSALGIRYL

LPGNTATESPNPSTPQVEEKSLPTFSTNAVKYGSYGQDVYELQGRLKYLGFYNGKIDS

NFGSTTLKSVKWFQSEFGMKADGVVGAKTKLKLYNASKQWSPTETPLHKDQTSGDSGK

NNNTADKEQDNMGSANSMGLSENEIKIMANAVYGESRGEPFEGQVAVAAVILNRVKSP

SFPNTPSGVIFQPGAFTAVADGQIYLEPNAQAKKAVEQAMNGWDPSGGCLYYFNPKTA

TSKWIWTRPQVKTIGQHIFCM"

CDS 2199929..2201209

/locus_tag="PIKHDJKN_02032"

/inference="ab initio prediction:Prodigal:002006"

/codon_start=1

/transl_table=11

/product="hypothetical protein"

/translation="MNTSGFERGNRKEFRIHVLPTKRFKTFAISLYAGVPLQEDTVTK

VALTPFVLRRGTESYPETTQFREQLEHLYGAGFGFDVYKRGDYQIVQFRMDTINDSFV

GGDEQLLDRSFAFLGEVLTRPAQEDGHFRTSYVHAERETVRKMLESIVNDKMRYAAER

SIEEMCKNEPYRLHPLGERKDLSGIDPDTLTASYQEWLQQANMDLYVVGDTTLEEVEN

LVQRYFNVERVTSSDYHTQEAIRGDKEVETVVERLNVNQGKLNMGLRTSITYGDTQYA

AALMYNGILGGYPHSKLFVNVREKESLAYYASSRYDGHKGIATIQSGIEIPNYEKAVM

IIKQQLDEMKNGNITDLEMSQTKAMIRNLLKEMQDSAFELIAYDFNRQLSGKERTAEE

LLAQVEQISKENVREAAEKFRLDTIYFLRDEKGE"

CDS 2201213..2202493

/locus_tag="PIKHDJKN_02033"

/inference="ab initio prediction:Prodigal:002006"

/codon_start=1

/transl_table=11

/product="hypothetical protein"

/translation="MESITYDRLQETLYYEVMDNGLHVYILPKPGFQKTYATFATKYG

SVDNHFRVEGQEEVKVPDGIAHFLEHKMFEEPTGDIFATFASHGASANAFTSFDQTVY

LFSATEHVNENIQTLVDFVQNPYFTDQNVEKEKGIIAQEINMYADNPDWRVYFGLIEA

MYQKHPVHIDIAGTVESIGTITKETLYTCYNSFYHPSNMLLFVVGGVDPQEVIDMVRS

NQAKKNYKPQGKIERIFEPEPKQVGEARRESKLAVSLPKCLFGFKETDVGLTGEELLR

RDMTTKLMMDLLFGSSTPLFQKLYDEDLISDSFGHEYNSSPQYAFSAIGGDTKDPDQL

LARIREEVDAIVEKGFDATDFERARKKKIGGYLRMLNSPENIANEFTRQQFRGGDFFN

MLPLYESLTLEEVNRRLKEHIRWDQLAISLVVSP"

CDS 2202495..2203265

/gene="fabG_3"

/locus_tag="PIKHDJKN_02034"

/EC_number="1.1.1.100"

/inference="ab initio prediction:Prodigal:002006"

/inference="similar to AA sequence:UniProtKB:O67610"

/codon_start=1

/transl_table=11

/product="3-oxoacyl-[acyl-carrier-protein] reductase FabG"

/translation="MERGTGQLKPIGEMTVLVTGASGGIGAAIAERFAKVGMNVVIHY

MKSHEAANEAARRCMEQGSGKVMTVSADLRSREQIERMREKLESHGLLPDILVNNAGI

SHYGMLSDVTEEIWDEVMAINLKGTFLCTQEMMPHMISQRYGRIINVSSIWGLSGASC

EVLYSTTKGGVNAFTKALAKELAPSGVTVNAVAPGAVQTSMLNHLDQDELKMLEEEIP

AGRLAQPDEISSLVYFLALPESGYINGQIISPNGGWLT"

CDS 2203442..2203705

/gene="yflH"

/locus_tag="PIKHDJKN_02035"

/inference="ab initio prediction:Prodigal:002006"

/inference="similar to AA sequence:UniProtKB:O34306"

/codon_start=1

/transl_table=11

/product="putative protein YflH"

/translation="MSTDKTVLTSFDTWKKFLGDRVQQAEKMGMNEETINKLAYEIGD

FLDEKVDPANPSNRALKELWEVGDENERRTIACLMVKLAKQNA"

CDS 2203978..2204670

/locus_tag="PIKHDJKN_02036"

/inference="ab initio prediction:Prodigal:002006"

/codon_start=1

/transl_table=11

/product="hypothetical protein"

/translation="MLGMLEVNILTINGVEGKTRGMLLESDDDEKIRLLGEMLGKVNS

ITVSALRQPKLVDILAVRHGRYIDRDSDDRKTFRFTRDELGLLVDFLGEVFKREGNQV

IGLRGMPRVGKTESIIAGSVCAMKRWTFVSSTLLRQTIRSQMSEDELNPNNVFIIDGI

VSTIRSSERHYNLLQDIMTMPSTKVIEHPDIFVQESEYDFNDFDIIIELRNNPNEEII

YDTFTASYTDEL"

CDS 2204737..2205777

/gene="rodZ"

/locus_tag="PIKHDJKN_02037"

/inference="ab initio prediction:Prodigal:002006"

/inference="protein motif:HAMAP:MF_02017"

/codon_start=1

/transl_table=11

/product="Cytoskeleton protein RodZ"

/translation="MSELGQQLREARLQKGMSLDDVQEMTKIRKRYLEAIEAGDYKVL

PGSFYVRAFIKTYAETVGLNPDELLEGHKKDVPAEETEATMEPVIQKRSSRPVERSNR

WMSVALMWTFPVLILVLLYVYVVMNRDDSDTQGLDQTKITDSQQQPQDKPDEPTDNGQ

ASNPPANESGSGETNEGEAGGNGGGTAEGQTDGQTDGQTTGENEENPTDNPPASVTVA

EDGKSGNITNFKVNGSAGQPVTVTIKATGHSWLEVYKGENSSGEKLEYGNTAEGDSYT

FELDSAGMYIKSGYAAATTIEVAGQVVTDGKATNRIRLRLGEDTSSTGTEGTTTNETG

STTEGNTGTSGE"

CDS 2205851..2206015

/locus_tag="PIKHDJKN_02038"

/inference="ab initio prediction:Prodigal:002006"

/codon_start=1

/transl_table=11

/product="hypothetical protein"

/translation="MTVSWIVTGLGIIVSLLGYYLTPTAWGYGILGFGLAHVLLGVLD

MFRQPNRSRY"

CDS 2206168..2206659

/locus_tag="PIKHDJKN_02039"

/inference="ab initio prediction:Prodigal:002006"

/inference="similar to AA sequence:UniProtKB:P44096"

/note="UPF0234 protein HI_1034"

/codon_start=1

/transl_table=11

/product="hypothetical protein"

/translation="MSSENSFDIVSKMDLQELTNAVTQTEKEIGTRYDFKGSKSSLKL

DKDALTIVSDDETKLKAVIEVLQSKMAKRGLPLKNIDYAKVEPASSGTVRQRLNFKQG

IDQDIAKKINILIRDSKMKVKSQIQGDQLRVTGKSKNDLQAVMQLLNGANLPLDLQYT

NFK"

CDS 2206982..2207569

/gene="pgsA_2"

/locus_tag="PIKHDJKN_02040"

/EC_number="2.7.8.5"

/inference="ab initio prediction:Prodigal:002006"

/inference="similar to AA sequence:UniProtKB:P63756"

/codon_start=1

/transl_table=11

/product="CDP-diacylglycerol--glycerol-3-phosphate

3-phosphatidyltransferase"

/translation="MNLPNRITLARICLIPFLMVFLLVDFPFYPEPLQLGSLSLPYNQ

LIAAIIFIIAASTDGIDGYLARKNNMVTNLGKLLDPLADKLLVTAVLISLVEMGKLDS

WIAVVIISREFAVTGLRQIALLDGSVVAASAWGKLKTVVQIVAIVLLLLNNFPFSFTG

IHMDVIAVWAAAIITIWSGIDYFIKNKNLLHLSKA"

CDS 2207679..2208935

/gene="cinA"

/locus_tag="PIKHDJKN_02041"

/inference="ab initio prediction:Prodigal:002006"

/inference="similar to AA sequence:UniProtKB:Q8DRX2"

/codon_start=1

/transl_table=11

/product="Putative competence-damage inducible protein"

/db_xref="COG:COG1058"

/translation="MKAEIIAVGTELLLGQIVNTNARYLSRELAAIGIDVYFQTVVGD

NLNRLSDAIRIAQGRADVILFSGGIGPTQDDLTKDAIAAVLNRKLHIDRMAMDKIESF

FRDRNVDMTENNRRQAIVIEGGTPLANETGLAAGNAISDNGKHYVVMPGPPKELIPMF

EQEVKPWLFQHVLTEEMPIYSKMLKFAGIGESALEDRLLDLIDTQTDPTIAPYASEGE

VTVRVSTKAPSESEAKLKLDAMEVQIRERLPEHLYANEDVPIEYTIVTMMSDMGLTLS

AAESCTGGLVMQSLTSVPGSASMLKGGIVCYSNEIKEKLLHVPHSYLEGEDAPGAVSP

EVAKVLAEQIRMIGDADFGLSVTGVAGPGYSERKPPGLVFIALAQRGKETEIHELRIN

GNRETVRIRSAKAILYRLWRKLVEMD"

CDS 2209174..2210238

/gene="recA"

/locus_tag="PIKHDJKN_02042"

/inference="ab initio prediction:Prodigal:002006"

/inference="similar to AA sequence:UniProtKB:P16971"

/codon_start=1

/transl_table=11

/product="Protein RecA"

/db_xref="COG:COG0468"

/translation="MSDRRAALDMALRQIEKQFGKGSIMKLGESTHMNVEIIPSGSLA

LDIALGTGGLPKGRIVEIYGPESSGKTTVALHAIAEVQRVGGQAAFIDAEHALDPQYA

SKLGVNIDELLLSQPDTGEQGLEIAEALVRSGAVDIVVIDSVAALVPKAEIEGEMGDS

HVGLQARLMSQALRKLSGAISKSKTIAIFINQLREKVGVMFGNPETTPGGRALKFYST

VRLDVRRIESIKSGNDMIGNRTRIKVVKNKVAPPFKQAEVDIMYGEGISREGSIIDIG

TELDIVNKSGAWYSYEGERLGQGRENAKQFMKEHKDIAEIIEQKIRVASNLTTAVPAP

TSEEQQKEAAEEQELFEINE"

CDS 2210580..2211317

/gene="recX"

/locus_tag="PIKHDJKN_02043"

/inference="ab initio prediction:Prodigal:002006"

/inference="similar to AA sequence:UniProtKB:P66003"

/codon_start=1

/transl_table=11

/product="Regulatory protein RecX"

/translation="MDQHEDDLYEEAELEGISQFPDNEELTITRVERTKSREARYRIT

FGLYSITVLEDVMIKYRMTRGNTFLKKDLEDIIVADERQRTYVQSLRFLEHKPRTRHE

LSQKLRQKEFAAPLIEEALDRLERENLVDDELFAKEWTRQRMEGQRKGKLWIRQELRQ

KGIANELIAEALEGVSTDAEYETALTAGRKKWNQVKGDIQEKKRKTLPFLMRRGFSMD

MVRRVVNCLIEEDEAKDLEDDEALLWD"

CDS 2211628..2213169

/gene="rny"

/locus_tag="PIKHDJKN_02044"

/EC_number="3.1.-.-"

/inference="ab initio prediction:Prodigal:002006"

/inference="similar to AA sequence:UniProtKB:O31774"

/codon_start=1

/transl_table=11

/product="Ribonuclease Y"

/db_xref="COG:COG1418"

/translation="MDIVITIVLVVAALVIGFGVGYFIRKSLAEAKISSAEQAAVQIV

ENAKKEAEALKKETVLEAKDEIHRIRAEAEKDTRERRNEIQRQERRLLQKEESLDKKL

ESLERKEEQVANKEKRIDETQQQIEMIYKNQVTELERISNLTMEDARSIILSNVEQEV

RHETAQMIKDIEQQAKEEADKKSREIITLAIQRCAADHVAETTVSVVTLPNEEMKGRI

IGREGRNIRALETLTGIDLIIDDTPEAVILSGFDPIRREIARTALEKLVADGRIHPAR

IEEMVEKSRKEVDERIREYGEQATFEVGVHGLHPDLIKILGRLKFRTSYGQNVLKHSM

EVAYLAGLMAGELGEDVTLARRAGLLHDIGKALDHEVEGSHVEIGVELAKKYKEHPVV

INSIASHHGDCEATSVIAMLVGAADALSAARPGARRETLETYIKRLEKLEEISESFEG
VEKSYAIQAGREVRVMVQPEKIDDAEAFRLARDITKMIENELDYPGHIKVTVIRETRA
VEYAK"

CDS 2213332..2214126

/gene="ymdB"

/locus_tag="PIKHDJKN_02045"

/EC_number="3.1.4.16"

/inference="ab initio prediction:Prodigal:002006"

/inference="similar to AA sequence:UniProtKB:O31775"

/codon_start=1

/transl_table=11

/product="2',3'-cyclic-nucleotide 2'-phosphodiesterase"

/db_xref="COG:COG1692"

/translation="MKVLFIGDIVGNVGRKALKENLPYLKTKYKPHVVIVNGENAAAG
RGITGAIANEFFNWGVHGITLGNHTWDNKDIFDFIDDEPRMIRPANFPPGTPGRGYTV
VKGEGKELAIVNLQGRTFLPALDCPFRVADEIVDELRQDHKCILVDMHAEATSEKIAM
GWHLDGRASLVVGTHTHVQSNDDRILPGGTAYLTDAGMVGPRDGILGMEREAVLRKFY
TQLPVRFVVDDGKWHFHGVFVEIDEATGAATRIEKIRLMEDEWRME"

CDS 2214244..2214504

/gene="spoVS"

/locus_tag="PIKHDJKN_02046"

/inference="ab initio prediction:Prodigal:002006"

/inference="similar to AA sequence:UniProtKB:P45693"

/codon_start=1

/transl_table=11

/product="Stage V sporulation protein S"

/db_xref="COG:COG2359"

/translation="MDVLKVSAKSNPNSVAGALAGVLRERGNAELQAIGAGALNQAIK
AVAIARGFVAPSGVDLICIPAFTDIVIDGEDRTAIKLIVEPR"

CDS 2214597..2215580

/locus_tag="PIKHDJKN_02047"

/inference="ab initio prediction:Prodigal:002006"

/codon_start=1

/transl_table=11

/product="hypothetical protein"

/translation="MHNWRVADFHCDALSKMLMNPSLSFKNASQLDVNLERLREGNIR

LQAFAIYLPEVLGRGKFEHVMGQLDIYRKRVEITKERSEGTHTLLWREQLTQVEQTDR

PWGLLTLEGVDGLEGNLFYLELCYQMGVRIVGLTWNYANWAADGVLEKRGAGLTEKGR

ELVHLCNQIGMLLDVSHLTEEGFWELADLSERPFIASHSNSYAICPHVRNLKDDQIRA

IIAREGRIGLTFVPWFVKQEGEVCIEDLLPHIERICALGGEHHLMMGSDFDGIATYIH

NLEHTGQYPKLINTLLKHYDESLVRGWMWGNAMSYLGENLPEKNLKMEHLK"

CDS 2215796..2216368

/gene="pduL"

/locus_tag="PIKHDJKN_02048"

/EC_number="2.3.1.222"

/inference="ab initio prediction:Prodigal:002006"

/inference="similar to AA sequence:UniProtKB:Q9XDN5"

/codon_start=1

/transl_table=11

/product="Phosphate propanoyltransferase"

/db_xref="COG:COG4869"

/translation="MSKTVPVGVSARHIHVSQEHVEILFGKGYELTEFKPLSQPGQYA

ANETVAVIGSKGQFDKVRILGPVRPETQLEISMTDSFAIGVKAPVRESGSIEGTPGIT

IKGPAGEVTIDKGVIVAARHIHFHTSDAAKWGIEDKQLLKVRLGGDRGLVLENVLARV

SDSFALDMHIDTDEANAAGARNGDTAEIID"

CDS 2216679..2218235

/gene="miaB"

/locus_tag="PIKHDJKN_02049"

/EC_number="2.8.4.3"

/inference="ab initio prediction:Prodigal:002006"

/inference="similar to AA sequence:UniProtKB:O31778"

/codon_start=1

/transl_table=11

/product="tRNA-2-methylthio-N(6)-dimethylallyladenosine

synthase"

/db_xref="COG:COG0621"

/translation="MAKDSKKDYSQYFDFSDAKVISQDEFSRKIRIRGREINIKSEPN

HRQEKQRGKEDVQVLYETAVPDELKNVGKGKRYIVYTYGCQMNEHDSETIKGLLESMG

YQATEDRKEADIILLNTCAIRENAEDKVFGELGHLKTLKTERPGLLLGVCGCMSQEEG

VVNRIMQKHGFVDMIFGTHNVHRLPHLIQEALFSKEMVVEVWSKEGDIIENLPKKREG

MRGWVNIMYGCDKFCTYCIVPFTRGKERSRRPEDVIAEVRELARQGFKEITLLGQNVN

AYGKDFTDLKYSFGDLMDDIRKIDIPRVRFTTSHPRDFDDHLIEVLAKGGNLVEHIHL

PVQSGSTEVLKRMSRKYSREHYLKLADKIKTAIPDVVLTTDIIVGFPGETEEQFEDTL

SLVKEVGYDFAYTFIYSPREGTPAAVMEDNVPMDVKKERLKRLNETINAYSHSSNEKQ

RGKVVEVLVEGESKRNSNVLAGRTRSNKLVHFEGPKDLIGTFVHVEITDPMTFYIRGN

LLSEPVAANQ"

CDS 2218507..2218956

/gene="ymcA"

/locus_tag="PIKHDJKN_02050"

/inference="ab initio prediction:Prodigal:002006"

/inference="similar to AA sequence:UniProtKB:O31779"

/codon_start=1

/transl_table=11

/product="putative protein YmcA"

/db_xref="COG:COG4550"

/translation="MAQEEVQYNDYGMPTYDTRNLVIRDDIMGKAKELADMLGTSEEV

KQFQQAESKIRDHERIQQLIATIKKKQKEIVAFESFKNADMVSKIEQEIEDLQEEMDS

IPLVTEFQQSQSDINYLLQLVISVIRDTVSEKVNVEAGTDSPPTSCG"

CDS 2219210..2219635

/locus_tag="PIKHDJKN_02051"

/inference="ab initio prediction:Prodigal:002006"

/codon_start=1

/transl_table=11

/product="hypothetical protein"

/translation="MGILDKMVEEGNGRFWGFLGCRFIKGDGKEVQIALTAGEHHTNS

MGIIHGGVLTSLMDQSMGMVATAAMAVDSCVTTNLNVHFLAPMKQGELIVTSTILHQA

GRSVTAQSEVRDASGTLGCMATATFRIARVKAQNTESQG"

CDS 2219694..2220497

/locus_tag="PIKHDJKN_02052"

/inference="ab initio prediction:Prodigal:002006"

/codon_start=1

/transl_table=11

/product="hypothetical protein"

/translation="MSENYEQFNTESDEQAARAYSSKKYRTPDGVPADIVMFTLTKRE

RKTVTKTLPIRELKVMLIKRKGWPFAGRWALPGGFCQENESIYGAAKRELMEETGVDG

GHLEYLNVYSQPGRDPRGWIISHAFFALVEEWMLEHRQAADDAEDVGLFTIQEALQEL

ELAFDHRTIIEDAYRRIQQQMLETTIARQFLPRDFTLSELYQVIQSVVPDFEEPNFIR

KITSTRSRKGIVEEVRDEEGNLLSSNQYSQRPAQLYRFTELVPRLSIYT"

CDS 2220562..2221131

/gene="rutB_1"

/locus_tag="PIKHDJKN_02053"

/EC_number="3.5.1.110"

/inference="ab initio prediction:Prodigal:002006"

/inference="protein motif:HAMAP:MF_00830"

/codon_start=1

/transl_table=11

/product="Peroxyureidoacrylate/ureidoacrylate

amidohydrolase RutB"

/translation="MKALIVIDFTRDFVTGSLPVGQPAVEIDETIAAITEAYCHNKHE

VVMAVDLHEENDPYHPETALFPPHNIRGTEGRQLYGRLAQVMEERESQIRWMDKTRYS

AFCGTDLELRLRERGITDIALIGVCTDICVLHTAVDAYNKGFHITVYEDAVASFNQAG

HDWALGHFRSSLGAQVVKASDTVLAGLNL"

CDS 2221328..2222782

/gene="pncB2"

/locus_tag="PIKHDJKN_02054"

/EC_number="6.3.4.21"

/inference="ab initio prediction:Prodigal:002006"

/inference="similar to AA sequence:UniProtKB:P9WJI7"

/codon_start=1

/transl_table=11

/product="Nicotinate phosphoribosyltransferase pncB2"

/db_xref="COG:COG1488"

/translation="MQTTSLALHTDKYQINMMYAHWVNGTHKRKAVFEAYFRKLPFGN

GYAVFAGLERIVNYISQLRFTMEDIKYLSKQEENYDPVFLEELLQFSFQGTIHSMKEG

ALVFPDEPLIRVEGSIMETQLVETAILNFMNYQTLIATKASRIKQVASQDTLLEFGTR

RAQEADAAIWGARASYVAGFHATSNMLAGERFGIPTAGTHAHSWVQSFMSEQEAFDVY

AKVLPDQVTLLVDTFDTLNSGVPHAIKTAKKLESQGKKMNAIRLDSGDLAYLSIQARQ

MLDDAGLDYVKIVASNDLDENTIFNLKAQGARIDTWGVGTQLITASDQPSLGGVYKLV

EREVDGAMLPTIKISANPEKVSTPGKKEVFRIIDPKHGKAIADYICYPGEEQPLQGGP

LKLFNPLHPYLKKTVTRYEAVNMLEPIFVNGRKVYELPTLDEIRSYHREQMDLFWPEY

LRKLNPEIYRVNISPAAWNMKQKLIAEHVQSTEE"

CDS 2223084..2223731

/locus_tag="PIKHDJKN_02055"

/inference="ab initio prediction:Prodigal:002006"

/codon_start=1

/transl_table=11

/product="hypothetical protein"

/translation="MRWILALIWLAFIVYAWFWVPGQPLGIDPVFKELVTLQSKEPWL

LTVFSWLGIYPAIFACLLLRRSGKARGSRVPAWPFVLLSFGLGAYALLPYFAWSSQTH

RGSGYERLSFGSQRESGIGRVAAHKLTHVVLLLLTLGTGLYAVTQGNPDVYMEAFRES

SFVHIMTIDFVILTLLSVIALFRDSSDNRRFRGWAVAGVIPLIGPLIYLMTSRRD"

CDS 2223832..2224851

/gene="yhfP"

/locus_tag="PIKHDJKN_02056"

/EC_number="1.6.5.-"

/inference="ab initio prediction:Prodigal:002006"

/inference="similar to AA sequence:UniProtKB:O07615"

/codon_start=1

/transl_table=11

/product="Putative quinone oxidoreductase YhfP"

/db_xref="COG:COG0604"

/translation="MKNTFPAYVVRKEEQGGVKAAMEQLTKEDLPNGDVTLQVQYSSV

NFKDGLATTEKGGVVREYPIVPGIDLAGIVEESVSGRFAPGDRVISTGFEPGVSHFGG

YSQYARLRSEWLVPLPHGLSEKEAMAIGTAGFTAALSVDALLRAGVTPEMGNILVTGA

TGGVGTMAVAILAKLGFEVTASTGKKDQQEQLLRNLGASHVISREEADAPAKGAMGKQ

LWAGVIDPAAGPALAERLKQIQYGGAVAVSGLTGGAGFESTVFPFILRGIQLIGIDSV

YCPMERRERIWKLLGGEWKPDRALELGIQEISWAQLPQTLETILKGGAVGRTVVNTLS

ETPAE"

CDS 2224920..2225480

/locus_tag="PIKHDJKN_02057"

/inference="ab initio prediction:Prodigal:002006"

/codon_start=1

/transl_table=11

/product="hypothetical protein"

/translation="MSMRVNRKGLHHRSGLVKSVLVSMIVVLCAISAVACSASNVEQE

RKIYTRQATDEGNVRAKSQGHGLNGPLIQELDEAFAAKGMKLMNNMSADDGEGGISYM

YLLNGSGRHIIKVHIFSDAPTRVAQMNEMYGNDGDEAVMENVLGKTTIRSKDYVSLVY

TASGGEKDDYEEDVMQVFQQVLEQLK"

CDS complement(2225522..2226040)

/gene="moaB"

/locus_tag="PIKHDJKN_02058"

/inference="ab initio prediction:Prodigal:002006"

/inference="similar to AA sequence:UniProtKB:Q816R0"

/codon_start=1

/transl_table=11

/product="Molybdenum cofactor biosynthesis protein B"

/db_xref="COG:COG0521"

/translation="MTNSVEQHRQEAPDTVSCMIVTVSDTRTKDTDTSGQLMHKLLTD

SGYQIVDYIITPDETETIQSILQDAAVRDDIEAVLLSGGTGIAPRDTTYEAVSSLLDK

ELPGFGEIFRFLSYTEDIGSAAILSRAVAGTIGRTAVFSMPGSKGAVKLAMEKLILPE

LRHVMREIYKPV"

CDS 2226210..2227346

/gene="yciC_1"

/locus_tag="PIKHDJKN_02059"

/inference="ab initio prediction:Prodigal:002006"

/inference="similar to AA sequence:UniProtKB:P94400"

/codon_start=1

/transl_table=11

/product="Putative metal chaperone YciC"

/db_xref="COG:COG0523"

/translation="MSELKTNESSIPVYILSGFLGSGKTTLLVQLIEHWQQQGLRPAV

VMNELGEVNLDGQIVDASVPMMEMLGGCICCTVRGDLGLQLADLVQEESPDVIIIEAT

GAANPMEILDAVTETSLYMRLKLKSLITVVDAAHLSGLYQEQKGQTFKLMQEQIRCAS

VLLLNKTDRVNEQQLADLRDLLARWNGFAPVLPTVKCEVDMDMLLRSGDNVHAYQSVE

EASKNAEQAHHVHSEACAEHGCSHGHEYHEHVHHDHKAHQQSQENVHGSPHDHDHNHA

HTHASHEHVMVYTHYFTQPVNSEAFERLIAELPRDVYRAKGILSFSDTSSRFWFQYAY

RESDYMKITPQGDVPNVAVFIGEHFDQSVIRNQLLELEAINQAK"

CDS 2227411..2227746

/gene="yhjQ"

/locus_tag="PIKHDJKN_02060"

/inference="ab initio prediction:Prodigal:002006"

/inference="similar to AA sequence:UniProtKB:O07571"

/codon_start=1

/transl_table=11

/product="putative cysteine-rich protein YhjQ"

/db_xref="COG:COG1145"

/translation="MTQQQYQQCIDACLECMNACNVCYISSLKEYDLAMLRDCIRLDR

ECAEICNFAAQAMTRGSDFIAEICELCVKACEACAAECGKHQHDHCQACAEACRKCAE

ACRMMAAVA"

CDS 2228383..2230119

/gene="argS_1"

/locus_tag="PIKHDJKN_02061"

/EC_number="6.1.1.19"

/inference="ab initio prediction:Prodigal:002006"

/inference="similar to AA sequence:UniProtKB:Q54869"

/codon_start=1

/transl_table=11

/product="Arginine--tRNA ligase"

/db_xref="COG:COG0018"

/translation="MLMNQAAAHIAPLTGLKQDEVLRLLEVPPQVEMGDAAFPCFILA

KSLKKAPAIIAADIAAELQTVGLDASPAGPYVNIRFNREDLAPKLLQELGHKTFGKLQ

LGAGARVVIDMSSPNIAKPFGIGHLRSTVIGAALYRLYNEAGYTSVSVNHLGDWGTQF

GKQIAAYKRWGNDEALQAEPIRTSLELYVRFHDEAESDPSLEIEARDWFRKLEQGDEE

AQRLWTFFVEVSMKEFNRMYERLNVQFDHTLGESFYNDKMGAVVEELKFKRLLEESEG

ALVVRLEDENMPPCLIIKTDGTTIYPTRDLATAVYRHDVMKADKMLYVVGGEQKLHFR

QVFAVLSRMGYAWSEICEHIPFGLMRFEGRKMSTRRGKVVFLQEVLDEAVARALQIIQ

EKNPNLPNSQEVAEAVGVGAIVFGDLRNNRLNDVDFSLEDAVSFEGETGPYVQYTHAR

INSVLAKAAEAERVENENQLDHVSSSSNNAAANSENKPVMVGDTSWALLKLLADYPQH

LEKAIHRNEPSVIAKYAIDVAQAFNRFYHAERIADAPADVKPFRVALTERTAERLANS

LYLLGVQAPERM"

CDS 2230124..2231167

/gene="btuD_10"

/locus_tag="PIKHDJKN_02062"

/EC_number="7.6.2.8"

/inference="ab initio prediction:Prodigal:002006"

/inference="protein motif:HAMAP:MF_01005"

/codon_start=1

/transl_table=11

/product="Vitamin B12 import ATP-binding protein BtuD"

/translation="MGWRASRFFDKAGKAAADSNGRIISLQQVSKRLGDVQVLNDINL

EVVQGECIVLVGRNGSGKSTLLRVLAGMLMPDSGSLRKPRHGRIMYALDGLSRLPFTS

REYLWEMGRIRGMRPEILRQRIGELSELLFLGTALDQMLPQLSKGTLQKVNLIQALLP

GPDGLLLLDEPLSGLDVPAQEAIVALLRQWKQEGSQIVTACHEPLLIEYLADQVIVLQ

NGTLLRRWSQEDLQQAGEPAVSIQSVMEGLEQSVIDALLVDQPGIIESHRIMVRQDYG

REVWDWKVSRSSADRILGMILAAGGSIVSVQPEESRLNMEGLMEGRHPGEAISREVAE

NLSSISTAGGDLQ"

CDS 2231164..2231874

/locus_tag="PIKHDJKN_02063"

/inference="ab initio prediction:Prodigal:002006"

/codon_start=1

/transl_table=11

/product="hypothetical protein"

/translation="MIYLTRYLLKHYIRSQKYVAPVIFYIITMLLIYSYKPNPVADSY

SVTAMLLFFGAAWLGLTVMNTEPAGQYQLLVLHAGSKRKVVFAQLICALIMQFGLTAI

TVLYPVITGMFGSVPSTGEWIMAWAGHLALSLLGLGFSVFFQKSYIPMLSRSMPPFIV

VMLLSFVQGSLSGRLPESIQWVVKILPPAFYLVREMMLFKESELGAVVLTTVWAVLYA

IVLLIIHVWLSGRRDLRS"

CDS 2231977..2232528

/locus_tag="PIKHDJKN_02064"

/inference="ab initio prediction:Prodigal:002006"

/codon_start=1

/transl_table=11

/product="hypothetical protein"

/translation="MQNTDMQHGDMLDSEHSRELFAYYGLAVYYGQALEQQLVNLILL

MKMSQGKVVSEEDLEDLYERKMSSSLGQLIHEVRHHFTFSEEETRQLNELWKQRNSIV

HHYFKERIHETFSPEGRSRMIKELEDFKDRAQELEISLQQYTGAWIAELGLDAESAAA

LQTLERMHAESMHARALEEDESL"

CDS 2232528..2233175

/locus_tag="PIKHDJKN_02065"

/inference="ab initio prediction:Prodigal:002006"

/codon_start=1

/transl_table=11

/product="hypothetical protein"

/translation="MEGKSRIRKIKSRHWLVISIASLVMIGVIWTLTTASLIWTFTDE

ESSRKSDAAIILGAAVEGDNPSPVFRERIEHAILLYQQGTISHLLFTGGSGSAGERAE

AEVGRDYAVAYGVDPSDIQMETESRITEENLVNSLSVGKQAGFNTYTIVSDPLHMKRA

MKLASGLGMDAVPSPTRTTAYQTWRSQFPFLARETILYMGYMVKGWIDNPQQHGE"

CDS complement(2233223..2233396)

/locus_tag="PIKHDJKN_02066"

/inference="ab initio prediction:Prodigal:002006"

/codon_start=1

/transl_table=11

/product="hypothetical protein"

/translation="MSIGWIVGSSLATVVLLTGIISLISSMRKNNNRRWPWWFIGFGV

AALVSAAINYPGT"

CDS complement(2233410..2234204)

/locus_tag="PIKHDJKN_02067"

/inference="ab initio prediction:Prodigal:002006"

/codon_start=1

/transl_table=11

/product="hypothetical protein"

/translation="MKPNRHMICKEWLSEEPLLALSLEWMQGEVHISQGTEAVIRVTQ

WGSARFPRTRCFEANVSHGLFHLIDGRKQVFPLGINFHRTSLHIALPPAVLKRLSING

VGSHFLVDNVSSEQWDCDCTSGKLTLTGHAQHIRLRSVGSRVTATDLHAEHMQLQATS

SPVQLSGQFTHIQSKVTGSQLTIHSSTMLQSLDSRSTGCNVEVRIPPENDGFKLDFKQ

AGGRFNSDLPLQSNHNQHTYRNGTYPFQAEVRGGKLTIGSYSFHSI"

CDS complement(2234210..2234893)

/locus_tag="PIKHDJKN_02068"

/inference="ab initio prediction:Prodigal:002006"

/codon_start=1

/transl_table=11

/product="hypothetical protein"

/translation="MARNKHPEQTVQQILNIAAQLFTDKGFEKTSIQDIIVALGMSKG

AVYHHFRSKEEILDAVMEQQFNYSAQMLDQLVTNTTTTPAREKLVQILEHVVSDQQAH

SLDRILRTQIKNPVFIVRGIQQAVRIDAPVIAAILREGITDGSIHTEDPEACAEVFMM

LVNIWINPTLFGRNSSETKQRLYFLQRLMEMLGADIVSESLIERILDQHAAMGAYPIL

DEEDDRNEG"

CDS complement(2235229..2237319)

/gene="yheH_2"

/locus_tag="PIKHDJKN_02069"

/EC_number="3.6.3.-"

/inference="ab initio prediction:Prodigal:002006"

/inference="similar to AA sequence:UniProtKB:O07549"

/codon_start=1

/transl_table=11

/product="putative multidrug resistance ABC transporter

ATP-binding/permease protein YheH"

/db_xref="COG:COG1132"

/translation="MKSNTGKRLLQYALKAKGTFIAALIMLTIGVAAELAGPFIAKSM

IDNHLLAIEQPFYETTASSEAAVYNGKNYKREGRFATDETKGTQVRVLQAGRSFYFVN

EPVSAPNGDRTYADGTLTVTRAGEVTGQYAAVPLSAGELFAFYNPEMPSIYQLIVYYM

IFLVISVIMEFGKTYWLQSSANKVIQRLRNDVYAHIQRLPVHFFDNLPAGKVVSRVTN

DTEAVKDLFVAVLSNFFSGIITITGVYIALFLLDFRLGLISLFVVPMLVLWIVLYRKI

ATRYNTIIRSRLSEINAIINESIQGMSIIRVFRHQKQTKQEFEDLNDDYMKHQNKMLN

LNAFTSHNLVNVLRNIAFAVVLWYFGSSVLDGTSIISLGVLYAFVDVLGRLFQPITGM

VNQLANLDSSLVSAGRVFELMDEPGEPVTDGSMPRYKGNVVFDDVSFAYKKDYVLNNI

SFKASQGQTVALVGHTGSGKSSIINLLFRFYDPQKGKITIDGQNVKDLPKQWIREHMG

IVLQDPYLFTGTVASNVSLGDEKITRAQIEQALRDVGAERILAHLPQGFDEPVIEKGS

TLSAGQRQLISFARALAFDPAILILDEATANIDTETEALIQNALEVLKKGRTTFIIAH

RLSTIRSADQILVLHRGRIVEQGAHDELMELKGRYYQMYQLQQGAQAAPDTSGNNPLG

EAIRTTSSFAGGGV"

CDS complement(2237332..2239077)

/gene="yheI_2"

/locus_tag="PIKHDJKN_02070"

/EC_number="3.6.3.-"

/inference="ab initio prediction:Prodigal:002006"

/inference="similar to AA sequence:UniProtKB:O07550"

/codon_start=1

/transl_table=11

/product="putative multidrug resistance ABC transporter

ATP-binding/permease protein YheI"

/db_xref="COG:COG1132"

/translation="MFSVLKNLAWFFRLERKRYLIGVTLLIMVGIIELLPPRLLGNAI
DEIVRGSVTGASLTRYILLILGSVIIIYLTTYIWMHKLFGGANLVERLLRSRFMDHLL
RMTPPFFEKNRTGDLMARATNDLRSIATTAGFGMLTLTDSTAFLATVLFAMGFLVSWK
LTLAAILPLPFIAIAMMIYGKAVHQRYTLAQDAFGDMNDQVLESIAGIRVVRAYVQER
MDEKRFADVTEDVYRKNLAVAKMDALFEPTIRLCVGLSYVIALAYGIYLVFHNEITLG
DLVSFNMYLGMMIWPMFAIGELINLMQRGSASLDRVNETLSVEPAVKDVDQPAHITNP
EEIAMQDVTFRYPSSTVDNLSHVSFSLRRGQTLGIVGRTGSGKSTLLKQLLHEYPAGS
GKLSISGHPIQDIAKDDLHSWIGYVPQEQVLFSKSVRQNIQFGKPGADDSVIMEAIRT
AAFDGDLGTLSDGLDTLVGEKGIALSGGQKQRVSLARAFIADPDILILDDALSAVDAR
TEAKIIENIRNKRSSKTTLISTHRLSAIEHADRIIVLEQGKITEEGTHQELLAMNGWY
REQYERQQVESNLST"

CDS 2239609..2239905

/locus_tag="PIKHDJKN_02071"

/inference="ab initio prediction:Prodigal:002006"

/codon_start=1

/transl_table=11

/product="hypothetical protein"

/translation="MSTIHVSDRAARWYKEELNLNEGDSIRFFARYSSGGGLHPGFSL
GIAVEEPRHPADQTKVSGIHFFMEDHDYWYLKGHQLHVDVVEEGQDIEYRYTEI"

CDS 2239977..2240426

/locus_tag="PIKHDJKN_02072"

/inference="ab initio prediction:Prodigal:002006"

/codon_start=1

/transl_table=11

/product="hypothetical protein"

/translation="MNPPARDLAQYIANRSHIVVKAAVRAENEQGELLLIQHAEHGHW
RIPAGQMRPGEAIEDTAHRELWEETGWTTNHMTLEGLHSGPNLRHLHASGDEEYYVIA
MFRAQIVQNSLVESRVNSEAGLKFFDLKSLPRLNDFSRLLLRSSEIE"

CDS complement(2240551..2241066)

/locus_tag="PIKHDJKN_02073"

/inference="ab initio prediction:Prodigal:002006"

/codon_start=1

/transl_table=11

/product="hypothetical protein"

/translation="MSRQFVTEAVMVAIYGQLLAPPAPVEYIVPYTTILELYEFQTSP

DPLMDNPADDQHVKSKINEMISYFEEPLNKKKIERALAVPWAKSPSILFGDQVSIAII

NAVDTAQYGEYFDPIETELLLTSQRLDTPILTDQVELIARIIESASPVQVFDIDDFDF

AMDDEEPLDQV"

CDS complement(2241251..2241703)

/locus_tag="PIKHDJKN_02074"

/inference="ab initio prediction:Prodigal:002006"

/codon_start=1

/transl_table=11

/product="hypothetical protein"

/translation="MKSMDMTSLLLLVFAALGIISSNTPVTVAMVFLLLLRVLNLNQA

FPWLEKYGLTIGIIILTIGVMAPLASGKISLQTIGESFLHWKSLLAIGVGLLVAHLGG

RGATLMSTQPTVVAGLLIGTVLGVALFKGVPVGPLIAAGILSLLLGKS"

CDS complement(2241700..2242698)

/locus_tag="PIKHDJKN_02075"

/EC_number="1.18.1.2"

/inference="ab initio prediction:Prodigal:002006"

/inference="similar to AA sequence:UniProtKB:Q6N2U4"

/codon_start=1

/transl_table=11

/product="Ferredoxin--NADP reductase"

/db_xref="COG:COG0492"

/translation="MEDVIIIGGGPCGLSAAIECERLGLSAVIVEKYNIVQSIYLYPT

HMQFFSTAELLEIGNVPFSTPNDKPFRYEALAYYRKVAERFGLRVHNYEEAQEIKRKD

DGTFEVHTVNRRGQAIVHEGRNVVVATGYFDHPNYLGIPGEDKDKVTHYFREAHPYTR

TRVAIIGGSNSAVDAAMELVRVGAHIDMVYRGSGVSEHIKPWVRPLFESMVQKGRISL

HLGSRVNEILDDSIRLLHSDGSTSELDNDFVLALTGFRPDRKLLSSVGVEMSEDMDKP

VYDPQTMESSVPGIYVGGVIASGRNANEVFIESGRWHGRYIAEHIVGRHRGQTEGE"

CDS 2242875..2243468

/locus_tag="PIKHDJKN_02076"

/inference="ab initio prediction:Prodigal:002006"

/codon_start=1

/transl_table=11

/product="hypothetical protein"

/translation="MQLEKIVAYHKALADPTRLRMLLLLSQGELHGQALAEKLNLSQP

TVTHHASKLREAALIKERREKNTVFFSLNPYFIREHAQASVDFIFRQEEVADMSDVNE

TLKASVMRNFFSKDGRLRQIPAQYKKKLIVLGHLVEQLESGRKYTEKEINAFIQNYHE

DFATIRREFIMHQFMFREDGIYELNPKEMWTRWDQVK"

CDS 2243939..2245030

/gene="rsgA_1"

/locus_tag="PIKHDJKN_02077"

/EC_number="3.6.1.-"

/inference="ab initio prediction:Prodigal:002006"

/inference="protein motif:HAMAP:MF_01820"

/codon_start=1

/transl_table=11

/product="Small ribosomal subunit biogenesis GTPase RsgA"

/translation="MNNTMEKYGWSAKWQELWEQTGMEEQLRKPARIIADHGQLLRLI

TAEGECWGRVSGRMRHDNEETGIVPAVGDWVAVTGQAGEEAIIHSIVPRQSRVSRQAA

GPVTKEQLIAANVDTLLIVAALNHDFNLRRLERYIMMAWNGGVRPVIILSKSDLSEDV

EEQISLVEGIAPGIEVLAISAVQDQGKSLLEKFLSPGTTVALTGSSGSGKSTLVNWMM

GESVQLTQAVREDDSRGRHTTTHREIFVLPQGAVLIDTPGMRELNLWDEGQDGLSHAF

GEIEQLATECRFQDCTHTGEAGCAVKEAIQEGSLDEKRLGNYLKMQKELKFQQRKEEA

ASKRRTASSKPVSSRKPKHVRSTKDWEEA"

CDS 2245087..2245425

/gene="ykuD"

/locus_tag="PIKHDJKN_02078"

/EC_number="2.-.-.-"

/inference="ab initio prediction:Prodigal:002006"

/inference="similar to AA sequence:UniProtKB:O34816"

/codon_start=1

/transl_table=11

/product="Putative L,D-transpeptidase YkuD"

/db_xref="COG:COG1376"

/translation="MPDYSIIVDLSDHMLYLLDGSQVVKGYPVAIGKMLTQTPNGVFT

IINKQENPGGPFGVLWMGLSAPHYGIHGTNDPSSIGKSVSHGCIRMYNSDVLDLSSKV

SVGTQVTIRP"

CDS 2245492..2246025

/locus_tag="PIKHDJKN_02079"

/inference="ab initio prediction:Prodigal:002006"

/codon_start=1

/transl_table=11

/product="hypothetical protein"

/translation="MQIRPAREGDAEAVAHVHTESWKTTYRGIVPDDFLDNLTTESRL

SQWERNIRSGEKDQILVVAEQEDGKIVGFACGGKEREGKLPYDAELYAIYLLKEVQQT

GIGQQLAKHVVRYLHSLDMRNLLIWALEQNSACRFYEKMGGIPVQTQQIRIGGQDLLE

VAYGWEDLTLFGENGSQ"

CDS 2246197..2247672

/gene="yfmT"

/locus_tag="PIKHDJKN_02080"

/EC_number="1.2.1.28"

/inference="ab initio prediction:Prodigal:002006"

/inference="similar to AA sequence:UniProtKB:O06478"

/codon_start=1

/transl_table=11

/product="Benzaldehyde dehydrogenase YfmT"

/db_xref="COG:COG1012"

/translation="MTLMETEHTTWTKQFINGEWVEGSGEKTVENKNPYTGEVIATWR

SSNKEDIDQAYEAARKSSVEWKKSLPAVKEGVLRIVSALMVERKEEIIKLLITESGST

RIKADAEFASAKRIVDEAASFPYRMKGEILPSNTPGKENRVVREPKGVIGVIGPWNFP

LHLCLRSVAPAIALGNGVVIKPASDTPITAGWLIADLFEQAGLPEGVLNVVAGSGSEI

GDYFVAHPVPKVISFTGSTEVGQGIGKLAGEHLKETALELGGNNAMIVLKDADIERAA

ESAVFDKFLHQGQICMALNRIIVHADIYDKFEESFVAKTKKIQAGDPSDSQTLVGPLI

REKEVERLLDLIKGAQAEGARLLLGGTSKGSVLEPTILADVKPEQHIVQQELFGPVAV

LMKARDEQEAVRLVNDTPYGLSGSVFTGDLERGYQVAQQIESGMVHVNDQSVNDEAHV

MFGGEKSSGIGRFGGDWAIEKFTRTRWISIQYQYREYPGVQ"

CDS 2247921..2248073

/locus_tag="PIKHDJKN_02081"

/inference="ab initio prediction:Prodigal:002006"

/codon_start=1

/transl_table=11

/product="hypothetical protein"

/translation="MSIDRFILRKLNTCQEENTRDNLVRLFVIRIQKAEMEEEHENAY

VVSRLH"

CDS complement(2248200..2248439)

/locus_tag="PIKHDJKN_02082"

/inference="ab initio prediction:Prodigal:002006"

/codon_start=1

/transl_table=11

/product="hypothetical protein"

/translation="MRTLITFQNKSIPVYFNQENKQPVQKTLRLLSSALENKISNGKR

ALQKCLQSLISIEIVGGEAILHSRSENDSLALSLY"

CDS 2248863..2249183

/locus_tag="PIKHDJKN_02083"

/inference="ab initio prediction:Prodigal:002006"

/codon_start=1

/transl_table=11

/product="hypothetical protein"

/translation="MILTVYSGREEGEWFDIEVPDECTIEHLKTLLGVRIFGQAPGEG

IQYILEAKFPEGLWFSPQNSQQLMETGLRQGSCVRVQRAFSTTSEEAPVYGRRSLFQQ

DSNE"

CDS 2249240..2253244

/gene="eccC"

/locus_tag="PIKHDJKN_02084"

/inference="ab initio prediction:Prodigal:002006"

/inference="similar to AA sequence:UniProtKB:A4IKE7"

/codon_start=1

/transl_table=11

/product="ESX secretion system protein EccC"

/db_xref="COG:COG1674"

/translation="MNVLYQRSPRMTPVLKEERLEILRPPAEPNKPTFSMISIIVPIM

MTMVSIGFYVYINMSGKMNNPNYMMFQMMTVMMMLTSYTIPFFVYLSNKKSYRKKLEE

RTTMYRAQLEKHREELKEAQAEQVKSLYEIHGDPNVCLQLVKNRNSSLWERSPEDDDF

LQVRIGTGEIPFRIKLQVPRVDGYEKDELIEAAHELAAEFETVPEASITLPLFQSKVM

GLVGNREEVLASLRVIISQLTVRHSPDELKIAAFYEEKESKEWDWLRWLPHIWDEDQG

QRYMADRHSGAHQLADSLFSVLNRRRNNKEDRYKKTVQTPCYVVVLSDTQLIEEEPLL

PLLLESAQEIDVCTIILANRKESLPMHCQLIMEASKGKGLYIKKTEDADVIRQTYTPD

VISRESAEALSRYMSPIRLKRSSASDIPQVLPLFDMLSTSRVEDLDVVTRWSQTRYPD

TLPVPMGVRAGGKKIAINLHDKIERQGHGPHGLIAGTTGSGKSEVIQSIVASLAAEFH

PHDLAFMLIDYKGGGMSNTFVGLPHVVGTITNLDGNLIERANISLRAELVRRQKILND

AGNLQHIDEYYKILRSRHEQPLPHLVIIIDEFAQLKRDQPEFMDELISIAAIGRTLGV

HLILATQKPAGVVDDKIWSNSRFRICLRVQSEGDSRDMIKIPNAAWITKPGRGYFQVG

SDEVFEEMQFAWSGAPYNQQEDTTTVLPVMEVRLNGKREPLLTGERRAVLKGEDVPKQ

LQVFIDYVAEAAAEAGITRLPGPWLPPLPETLEWESLNDWQEEENREVLLDGGASGLK

PVVGLLDDLPNQRQQPLALPVDQGHLVVYGMPGLGKTTFVQTLLMSLARSKRTEPWHG

YIIDMGRMMKDFAGLPQIGAVMMSEEEDRIKRLFRYILKLSAQRKDMISEAGVKTISA

YRRTAHAAVPQIVVVIDGYLSFRNAYPEENELLETILREGGSLGITFILTANRVTDVF

EKFRSNIPNAVSFELSDPSDYYYAVGRPSKAPSQLPPGRGLVKGQVPPLMFQAALPSS

GADEGKRSSALRRTIAEIRENWNGEEAPQIAPLPEEVKLKDVLIRTGTFGQVGDVSSL

TVPVGLLTDDLEPFQLNLREGPHFMVTSPMEGGKTTFLLTWMLSLAYHASPEDVQIYT

VDMRYGSGGLGEISSLPHVRGHVSREEQLAPVIQQLYDEVLKRGELTGGPEIVLVIDD

ADTLSKQLNDFNVKDQLGAIVRQGRDRGIHVILSGVPADFPTFGSDWVNDVKASQSGL

LFGTLDPNDLSFFRIPYSESGGSSTGLKVLPPGQGYYVKRKYSRVKGAIPCDGTWKMT

DWISEIRDRWHVVV"

CDS 2253261..2254091

/locus_tag="PIKHDJKN_02085"

/inference="ab initio prediction:Prodigal:002006"

/codon_start=1

/transl_table=11

/product="hypothetical protein"

/translation="MLTVHDSKQKVVTLQIKELFFLAGILGSDRLLGVEDPFRGYMAE

DIALEWERVKTSLLDKGYLIQDQDSNELIMTPTVFSRVAIAGLSDRACWIRYTHSGKS

YESYVHCTDERVVEVSRVADEPDSFRLSDLGNVREAIDFLIQRMNWSGHSPAEKPALM

CSKKKFYDVMNDLKNKELQAVADELVQETSDPEGSLALARCLVNKESEGELRLLVWNG

EGWKSQSAAFAASTISNWLFRMSTAASDDWLVAALTTREQFHEMLLDWLKQPAGEEER

"

CDS 2254098..2254646

/locus_tag="PIKHDJKN_02086"

/inference="ab initio prediction:Prodigal:002006"

/codon_start=1

/transl_table=11

/product="hypothetical protein"

/translation="MRIRVEPDVLRALSRQIQYAAEQIQQKMTVLDQAIHSLEWDIEA

RAAIMNEWNHSKRLCEAAVLRFMDLSVQLGRKALLFQQVDMEYRTVLSHVNTAYGNAV

NMLNVLQTERAGEIMPAHSATAAVVSDPFSAVAAVYRVQDAAPPDGSPATLIQAMQPE

PVAWRFTDPSFRGRRGTEPVVS"

CDS 2254840..2255115

/locus_tag="PIKHDJKN_02087"

/inference="ab initio prediction:Prodigal:002006"

/codon_start=1

/transl_table=11

/product="hypothetical protein"

/translation="MAGRILVTPEQLDQVSNQFKQSGEQSQQIVSTLTQSITSMEGQW

EGMTKQRFFQEFQEASKQMQSFVQTLNSISAELTAIANKFRTADQAR"

CDS 2255273..2257363

/locus_tag="PIKHDJKN_02088"

/inference="ab initio prediction:Prodigal:002006"

/codon_start=1

/transl_table=11

/product="hypothetical protein"

/translation="MSFQPNPGDEVVINDIAYIIGQHPAAPGLAYAQAGRQGIVYQLL
PRNGSISGAKALKVFFPKFRIPAMVYQSEHMESYSELPGLQVCKRDVLTPERNGALIG
EHPDLLYAVLMPWVHGLTWFDVISDQKQLTAEESLKLARALAGIGSAMEQRGLAHCDL
SAPNVMIPFFSEVENLRGVSAVELVDVEQMYGSRMDRPDALLAGSPGYAAHRTVHSGL
WSAYADRFAGAVIIAEMLSWSDAAIVEKAWGESYFDQHEMQTVNERYYAMRESLQKRW
GSKVSDLFIRAWESHDLSSCPTFGEWFVALAAVDIHLASAASVEESEVMASDQTNAVE
TNQIKGTGAASGISDKQGNTDQNVPAVEPSPGQEAVVNRLFLQARALEDEGKPAAALE
VYRSLHHFIPDNSAMQMEVEAAIQELDAKLNPKHDDAAQPAKIPFYRSKKFMISSAVL
IVLLAGSVPTVKILADQAEVKQKEQEAAAKQAELKAAEEAEAVRVTALKQQEEEKKQK
EADAAKLDAEEKKKLAEAEKKQAEAKKKEEERKALQAKYDKQAKYEAYLAKQKQQKLE
AAKKAEQEKYDKQAKYEAYLVWKKENDAKIAKQEAERKAAAEAQRKQEASQKAALKKI
RAQNVVTLIAHYNKTYNAQKGRKIENAESYARDFKNLYNTDASYFKGVGKVAARMNAI
NKFLSNTDYTLPNL"

CDS 2257397..2258149

/locus_tag="PIKHDJKN_02089"

/inference="ab initio prediction:Prodigal:002006"

/codon_start=1

/transl_table=11

/product="hypothetical protein"

/translation="MNYTIQASQRTPALIIYLIDISASMNMVLDNRRRIDVVYDALSL
AIRQMVFRSTKGNRLTPRYRIAILAYSDDVYDLLNGIKGIDEIAAVGSLPDLTPKRFS
DSAKAFLQAEKILQAEIPNMQDCPAPLVCHMTDGVATGEDPEPIAKRIMGMSVPDGNV
LVENIFISDHLLEGPIPEPRRWKGISQETVLQDEHGEKLRNMSSVLPESYREMLVEAD
YLLSPGALMMLPGTCAELVSIGFQMSAATPVR"

CDS 2258160..2259002

/locus_tag="PIKHDJKN_02090"

/inference="ab initio prediction:Prodigal:002006"

/codon_start=1

/transl_table=11

/product="hypothetical protein"

/translation="MRAMRLATLSAGDKGSRADHYQGNFRYVSVQTGEQPLTRYQGTF
KCRYGYGRAAETVNQGDTGQDFAAVRMNGNVCTFVLCDGVGMSYLGDFAARFLGNALL

EWLETTQHPSLDAMERLLHDLTSPASEQLEKLQPLENSPILLREVLMEKRSKGSQAMY

VCGRIELSAGNRKSKVWLAWQGDSRIRLWRNAQEQSAAFQVNCRTNERWSTLEGPVGG

KPHIYEAKMSANEVIRLQMYTDGLNDLDAIQAYIPDEHIQVLLDARHTGGLEDDAAFI

ELEW"

CDS 2259192..2259998

/gene="bacC_1"

/locus_tag="PIKHDJKN_02091"

/EC_number="1.1.1.385"

/inference="ab initio prediction:Prodigal:002006"

/inference="similar to AA sequence:UniProtKB:P39640"

/codon_start=1

/transl_table=11

/product="Dihydroanticapsin 7-dehydrogenase"

/translation="MTQHNGKSRFEGKVAIITGAGSGIGKAAALKLASEGAHVALLDL

VNDRISETEAEINKLRAGAARAFDVDISDPARVEKAVLETIELFGGLDIVFANAGING

VSAPIEEIQVEDWQQIITTNLNGTFFTIKYALPHMKKRGGGSIIITSSINGNQRFSSF

GMSAYSTSKAGQVAFAKMAALELAKFKIRVNVICPGAIATNIDQSTVKTDELQQIVIP

MEFPEGQQPLADGPGQPEHVADLVAFLASAESRHITGAEIVIDGAESLLS"

CDS 2260177..2262693

/gene="hrpB"

/locus_tag="PIKHDJKN_02092"

/EC_number="3.6.4.13"

/inference="ab initio prediction:Prodigal:002006"

/inference="similar to AA sequence:UniProtKB:P37024"

/codon_start=1

/transl_table=11

/product="ATP-dependent RNA helicase HrpB"

/db_xref="COG:COG1643"

/translation="MKTDLPIQPIIPELRRLFQEKDTGVLIAEPGAGKTTIVPLALLE

EPWVAGRKIIMLEPRRLAARSAAARMAASLGEKAGQTVGYRVRMDTRVSQHTRIEVVT

EGVLTRMLQQDQGLEDTAMIIFDEFHERHLHGDLGLALALESRAMLRPDLKLLVMSAT

LDPVPVCALLGEGTRWIDCPGRTFPVETRYVSKPASEQIETFTAQAVVKALVEQEGDV

LVFLPGAKEIHRTERELSNLMLPAHVEVHALYGSMPVERQDEAVRPADEGKRKVVLST
SIAESSLTLAGVKVVVDAGLSRASVFSPRTGMSRLVTLPVSKASADQRRGRAGRIAPG
VCYRLWSEEAHGVLPDAAKPEIASADLAPLALELAAWGVQSPAELQWLDTPPAAAYGQ
AQALLRQLGGLDDAGRITPFGLRMNTLGVHPRLASMLLRAAELGLASYASMLAALLQE
PAGLRSSGSAGAGTDLRPRVEALLTADSSGMPQAAASVADSAAVRRMLQESRQLRSAL
GPAADPVRPDEDSCGWLLSFAYPDRIGQRREDGRYLLSSGRGVRLAATESLSRSAYLV
AAEADDQGADARILLAAPVQEKRLLDFGPHMLHEEVQVAWDRNTRSVRAHRRLRIGAI
TLKESSIAQPPEDQVLEALLSGIRIEGLDCLPWTKSSRQLADRMRFLHHHLPEWPDLI
EDHLTEELAEYLSPFLTGMRSAADLKRLSMQDVLLGGLSWEQRQQLDREAPTHIQVPS
GSRIPVDYSRPEDPTLAVRLQEMFGQQETPRIGGGRVPLTIHLLSPAQRPVQVTRDLA
NFWRETYFEVMKDLKGRYPKHYWPDDPLEAIATNRAKPRV"

CDS 2263258..2263860

/locus_tag="PIKHDJKN_02093"

/inference="ab initio prediction:Prodigal:002006"

/codon_start=1

/transl_table=11

/product="hypothetical protein"

/translation="MQKKIVGVFNTEREASSAIEGLKAQGFTSDEISVVTQDRDELKA
IREETGTKAPEGVAAGAATGGVLGGVAGLLAGIGALAIPGIGPILAAGPIAAAFTGAA
VGAGAGGLVGGLVGLGIPEEDARQYEEYVQSGKILLLVDSTDRDTDVYDVFSSNSYVN
RDSIDAHREDVPTERPDLDMEERRLEARDKAARFGNNTFL"

CDS complement(2263998..2264408)

/locus_tag="PIKHDJKN_02094"

/inference="ab initio prediction:Prodigal:002006"

/codon_start=1

/transl_table=11

/product="hypothetical protein"

/translation="MNGVIHISDTDQDITTIDSKYVQQHLANERTFLAWVRTGIAMIG
IGFLAAGFGFSSTAYDRMAHIAAIIIGITSLVSGILIILCSAAAYHRKRNQINSQTFQ
ASTKLIRFLTLMLLVIGLALAVLLYVLLFPAWQV"

CDS 2264470..2265615

/locus_tag="PIKHDJKN_02095"

/inference="ab initio prediction:Prodigal:002006"

/codon_start=1

/transl_table=11

/product="hypothetical protein"

/translation="MPRKERITGIESLRGIAFAAVVLQHSIAHYSVVPEAGLEDGVLL

AILLMLSKFAVPLFIFITGMVLFYNTGDKLHYGRFMQKRLTDVIVPYIIWSLIYFTLA

PRGWNGFGWQDIPDLGLKLITGKTTSHFWYIIMLIQFYLLFPLFLRAIRYFYNRYEGK

GRTIALLISGVVYLVLADQLRNIAKFMEWLNIPVLTDAFTTYADRNFLYFFFYFVLGA

AAGLSVQRWNEWIQRLRWGYWTVFIVLGLRFIYKLMQEFQKPEGIQITFYTVSLIRPD

MILFLIASIMVMYQLAGKLHNIRVTRLLAWIGGVSYGGYLMHMLMLRYSYIPDEQFYV

ALGLNPVVRMIITWLLALTLSCVLTWLISRASWGKWIVGTVPKSKPD"

CDS complement(2265644..2265814)

/locus_tag="PIKHDJKN_02096"

/inference="ab initio prediction:Prodigal:002006"

/codon_start=1

/transl_table=11

/product="hypothetical protein"

/translation="MDRQERKVEREFGFSIMNFSCNEIFKFISKSVNHGIGVTFFQVV

AFILLALNSLLF"

CDS 2265937..2266311

/gene="rtp"

/locus_tag="PIKHDJKN_02097"

/inference="ab initio prediction:Prodigal:002006"

/inference="similar to AA sequence:UniProtKB:P0CI76"

/codon_start=1

/transl_table=11

/product="Replication termination protein"

/translation="MAFMIAQRAFIKLYLITMVEQHRGYGYEMLEAMKQEFKAYGYVP

PQSEIYRALHELVQQGVFYRTKKLKGSDPKVDFQEIVLYHFTDDGAEKAELYKKQVKA

DLDRCLGMLHKAEEDNYGVKGR"

CDS 2266314..2267321

/locus_tag="PIKHDJKN_02098"

/EC_number="1.1.1.384"

/inference="ab initio prediction:Prodigal:002006"

/inference="similar to AA sequence:UniProtKB:B3TMR8"

/codon_start=1

/transl_table=11

/product="dTDP-3,4-didehydro-2,6-dideoxy-alpha-D-glucose

3-reductase"

/translation="MDRHLQWGVLGTSTIAKNAVIPAIQQSESGEVLAIASRSKEKAE

ALANELDIARAYGSYDELIADPDIEAVYIPLPNHMHKEWTIKAAQAGKHVLCEKPAAL

NSDEAAEMIEVCQQHGVLFAEAIMYRYHPKHKRVQEIIASGEIGTVRALHGNFTCNTA

DDKDNVRFKREMGGGSLFDLGVYPISAARMYLGQEPEAVTVHALFSEEHDGVDMMASG

LVEFPNSVALTFDCGMWASGRAEMEILGTDGRIELPKVFGWENSDIPPQIIVHTDSVS

REERVSVSNSYVLQVETFAAAVLEGEALPFSPQNTIENMRVIDACLESARTRQRVQLV

D"

CDS 2267612..2268052

/locus_tag="PIKHDJKN_02099"

/EC_number="3.1.27.-"

/inference="ab initio prediction:Prodigal:002006"

/inference="similar to AA sequence:UniProtKB:P00649"

/codon_start=1

/transl_table=11

/product="Ribonuclease"

/translation="MKKFLSTALLAVMLTIVFFTGSQFAPVGQQKAAAATCTIINTFT

GVANYLSANGQLPCNYITKAQATALGWVSSQGNLATVAPGKSIGGDVFSNREGLLPAA

SGRTWREADINYTSGFRNSDRILYSNDGLIYKTTDHYASFTRLK"

CDS 2268070..2268345

/locus_tag="PIKHDJKN_02100"

/inference="ab initio prediction:Prodigal:002006"

/inference="similar to AA sequence:UniProtKB:P11540"

/codon_start=1

/transl_table=11

/product="Barstar"

/db_xref="COG:COG2732"

/translation="MKRVVLNGEDFSSTTELHELLKQKLELPDFYGANLDALWDCLTG

MIELPLELTWTNYQISKERLGSEAEKVYQLMLEAEEESIGFQFKTED"

CDS 2268535..2269821

/locus_tag="PIKHDJKN_02101"

/inference="ab initio prediction:Prodigal:002006"

/codon_start=1

/transl_table=11

/product="hypothetical protein"

/translation="MSEGTVARKQGIGELIRIKPYVQFMVSKVVSRFGDSIDSIAYSW

MVYILTGSKLLMGTLLAVNFLPSIFLGLFVGALVDRMSPKKVIVLTNTGRGLFVGITA

LLFGLGELQVWHLFVITILNSLLECFASPAEVSSVPRLLPKSMLLSGNAMASSATRVA

ELAGLAVAGTLIATAGITWTILIDAGLFALSALLMSRVGYPKTSTSSNNENTSLDSSD

SPPARKSIFSEMTEAFHFMRKHVLLLIVSILFAFVNFCLMPFNVLRTPYVIETLHAGA

GGLSLLSGLIVAGMVLSGLWLSHRGSNYRKSVLVIVGIVMLGLSYAMTALPAYMTSYQ

LPVAAVFCLMMGVGIPLATTPLASYLMEVTPSDMLGRVYALQSMLVVSVAPLGSLVSG

ALADWVAVPILFIVFGVLLAMSAALVLLSKTFRTVL"

CDS 2269932..2270615

/locus_tag="PIKHDJKN_02102"

/inference="ab initio prediction:Prodigal:002006"

/codon_start=1

/transl_table=11

/product="hypothetical protein"

/translation="MQRKVLSTIEEIKVYSDPYRIQIMNMFNKQGRPSTVKEIADQMG

EVPAKVHYHVKKLEKIGLLTIVSTREINGIIAKYYEPFKGEIQLRNEDEENSPLKEVF

RSETFKLLNEMFEQSRRRFMNQAENGDRMFISDMTLYASREEVEQLYNNITKMCEPYL

TKDRHDADQEVFHLFSSLSKDTVNKPVLKNAGKLKKKASPDKGKSVAKTSRPAKKQKE

SSSGGQAEE"

CDS complement(2270774..2271340)

/gene="dksA_1"

/locus_tag="PIKHDJKN_02103"

/inference="ab initio prediction:Prodigal:002006"

/inference="protein motif:HAMAP:MF_00926"

/codon_start=1

/transl_table=11

/product="RNA polymerase-binding transcription factor

DksA"

/translation="MSTLTKDQLQELKNALLEQRENLQRHFESSMEDGAPAESLKDST

GELSSYDNHPADAGTETFERSRDLAIDDTLTDEFNQVNEALERIEQGTYGSCVTCGKD

IPFERLEAIPYTAYCIDDTPNRELSNERPVEEEVMTMPPSGAGEVRQRNAGKFDNADA

WEAVEEYGTSNSPATAAKRDVKDYDENM"

CDS 2271546..2271923

/gene="gpsB"

/locus_tag="PIKHDJKN_02104"

/inference="ab initio prediction:Prodigal:002006"

/inference="protein motif:HAMAP:MF_02011"

/codon_start=1

/transl_table=11

/product="Cell cycle protein GpsB"

/translation="MDEHMKRRLDKQRQLFKQLGVQLDALSIHEKQFNYKLRGYDPDE

VDAYLDLVIKDYERFYANIADLMDKWQEQQLTIRDLKSSAKPVEDPTKIDRKQLDDIV

KQLEYSVRQLKIRARPEQDLFSE"

CDS 2271986..2272420

/gene="ywnA_2"

/locus_tag="PIKHDJKN_02105"

/inference="ab initio prediction:Prodigal:002006"

/inference="similar to AA sequence:UniProtKB:P71036"

/codon_start=1

/transl_table=11

/product="Putative HTH-type transcriptional regulator

YwnA"

/db_xref="COG:COG1959"

/translation="MSTHFSVSVHCLLLLSFSAPERITSALIAGSVNTNPVVVRRILG

GLKKAGLVDSSPGTRGFYLAKPSSEITLAMIYQAAKDEGPLFPIHGNCNPNCDVGLRI

DSLLTNLYQVAESKVEQFFASITLEDMERSCSQLEVLPSHAE"

CDS 2272457..2272954

/locus_tag="PIKHDJKN_02106"

/inference="ab initio prediction:Prodigal:002006"

/codon_start=1

/transl_table=11

/product="hypothetical protein"

/translation="MKIIVISDTHLPRRARKLPDPLVEALSDADLILHAGDWSDWSVY

KLLSAYAPVEGVAGNTDPPEIGRKLGFSRIVEADGLRLGLVHGHLGTKSTEQNAIHTF

AGQQVDAVIFGHSHIPVMHTVNDVLIFNPGSPTDRRFQPQYSFGIMTTNLGGLQAEHV

FFDEK"

CDS 2273186..2274100

/gene="iolE_2"

/locus_tag="PIKHDJKN_02107"

/EC_number="4.2.1.44"

/inference="ab initio prediction:Prodigal:002006"

/inference="protein motif:HAMAP:MF_01672"

/codon_start=1

/transl_table=11

/product="Inosose dehydratase"

/translation="MKLSVFTVATPDLTAEELASAAAAAGIEGIEWRYRGIPEDALAE

EPSYWRHNRCSIDPDQWEEQVPAFREAAKKHGRQSIALVPYLSCGDLLATEQAFQTAQ

ALGASMMRVGVPGYDRKTSYPELYKQAVHYLNEVQEMAQQYKVKAVVETHHQTIAPTA

SLAYRLVQSLDSQHVGVLYDPGNMVHEGYENHRMGLELLGPYLAHVHVKNAAWFKDES

NKNSNSSVNEQNTEILLTSNWHCQWAPLTEGVVNWLQVFRDLKSVGYDGYYGIEDFSG

VLQSKAMLQHFADVFAEIERRVDEEVQV"

CDS 2274097..2275269

/gene="iolX"

/locus_tag="PIKHDJKN_02108"

/EC_number="1.1.1.370"

/inference="ab initio prediction:Prodigal:002006"

/inference="similar to AA sequence:UniProtKB:P40332"

/codon_start=1

/transl_table=11

/product="scyllo-inositol 2-dehydrogenase (NAD(+))"

/db_xref="COG:COG0673"

/translation="MSLVRVAVIGIGNMGAAHARTLVAGEVPGAELVAVCDVRQEMES

WVSSNLPASVTYWQDAEQMMSSGTIDAVIIATPHYDHPEKAIQAFQNGLHVMIEKPAG

VYTKQVRKMNEAAATSGKVFSMMYNQRTNPLYIKLKDLIASGELGEVRRTNWIITNWY

RSQSYYDSGGWRATWAGEGGGVLINQDPHQLDLWQWTIGMMPVRMRAFCSFGKYRNIE

VEDDVTAYVEYENGATGVFVTTTGEAPGTNRFEVNGDRGKIVIEDGQLTFWRLREPEP

EFNQRFTGGFGQPECWKCEVPITGVETGHPGLIRNWIDAILNGTPLIAPGEEGIHGLT

LSNAMLLSTWTDNWVDLPIDEDLFYEHLQQRIASSNTKKDKVGSSSQPADLSKTFK"

CDS 2275364..2276461

/locus_tag="PIKHDJKN_02109"

/inference="ab initio prediction:Prodigal:002006"

/codon_start=1

/transl_table=11

/product="hypothetical protein"

/translation="MAKDGMFYAPKSLKKEIVCGPGEFTIAAVALDHGHIYGMVGGLV

EAGATLKWVYDPDPAKVEAFRKQFPQAQIAASEADVLADDEVQLVAGAAITSERAPLG

MRVMAAGKDYFTDKAPFTTLDQLSLAREEVRRTGKKYMVYYSERLHVESAIYAGQLIE

QGAIGKVVQVMGTGPHRLNAGGRPEWFFQHEKYGGILCDIGSHQIEQFLTFASCTDAE

VAFSRVNNFNHPQFPELEDFGEASLIGDNGASGYFRVDWFTPDGLGTWGDGRTVILGT

DGYIELRKYIDVAREAEGDQVYLVNHEGEYRYSVKGQVGYPFFGELIRDCLDRTENAM

TQEHAFKAAELCLIAQHKAMSERVVWSSGTK"

CDS 2276664..2277107

/locus_tag="PIKHDJKN_02110"

/inference="ab initio prediction:Prodigal:002006"

/codon_start=1

/transl_table=11

/product="hypothetical protein"

/translation="MKTNKAMRNLLNELAKSKESISSNLFNPTFHEWDDCILVDASSL

RETDSIEETIYQDRTDLEASINHYHIEDLRTGIKLISNWRENIKLNYPEKDFILILSS

TIEGEDVVIRFYQFRDHEPEWIKLNDLEGYKEEAILVIEILNAVQ"

CDS 2277278..2277724

/locus_tag="PIKHDJKN_02111"

/inference="ab initio prediction:Prodigal:002006"

/codon_start=1

/transl_table=11

/product="hypothetical protein"

/translation="MKNLFLILSFILILTGCSIQKEQQKKDLASEMDSVINYKLNAVE

AEHAEKVNQWLAKARDTGEVGQYFIYQDGTNKEDLYSYVYRKGYIDYEVSFIYNPSDP

NNKGKIHVTGLNKDLKNDNFVQIKSMNDLSILFILSDESLKSKLKD"

CDS 2277946..2278452

/locus_tag="PIKHDJKN_02112"

/inference="ab initio prediction:Prodigal:002006"

/codon_start=1

/transl_table=11

/product="hypothetical protein"

/translation="MILEYLDKYAQKAYREVTSIITCPTFLFSNDPTLIRNEANYNYV

YIHGFNRLIKNNRISSDQLIEVFSFLEKEIEIYLMKHYTGKNFMYYLKADALVPAFTI

TIVSHYEDKEVQESVSLFELIDWYKENACFDGLEIIARQISDESKEAESKGAFPDSVK

TYTKTINT"

CDS 2278659..2279441

/gene="deoD_2"

/locus_tag="PIKHDJKN_02113"

/EC_number="2.4.2.1"

/inference="ab initio prediction:Prodigal:002006"

/inference="protein motif:HAMAP:MF_01627"

/codon_start=1

/transl_table=11

/product="Purine nucleoside phosphorylase DeoD-type"

/translation="MESRKIDSNEFPILEFDESRVAIIEATNFIKPKEEFEYCVITFF

RDVIEKMKTEGKLKEVASLHCETVDLPIYETYYQGKKVHITLGYLGAAGSAGFLEELI

AYGFQKFIVCGGAGVLQKDIAVGHVIVPVSAVRDEGVSYHYIKPSREVECNLEVLKFI

EDDFNIHNIRYIKAKTWTTDSFYRETKEKVELRKAEGCVTVEMEAAAFMAVSQFRNVK

LGVILYGGDDLSGVEWDSRSWNSRSEIRENLVQISMRICSSL"

CDS 2279706..2280359

/locus_tag="PIKHDJKN_02114"

/inference="ab initio prediction:Prodigal:002006"

/codon_start=1

/transl_table=11

/product="hypothetical protein"

/translation="MKGKILFLFFITIMFSSCQNISQSTTLNSETKSIMNSELPPDVE

SKGNGTESKEITEGRSEPSNNEVLSMRIGNYDLKGEFYDEMLRNPIDHDYEVEFNEFQ

NSKEIITTMGWGALESKYTEIWDKELNQIYKKLLSKLDREPREALIKSQKEWLQYHLR

ETKFVEKTFINNGYLGSKGSVSLGTVIRERIRERTMQLFEYRYLLDSEVEFLYQSKK"

CDS 2280598..2281026

/gene="nudC_1"

/locus_tag="PIKHDJKN_02115"

/EC_number="3.6.1.22"

/inference="ab initio prediction:Prodigal:002006"

/inference="protein motif:HAMAP:MF_00297"

/codon_start=1

/transl_table=11

/product="NADH pyrophosphatase"

/translation="MIRVDVAYSLITDPTNSKILMVRNVDDSSDRWSLPGGAVEREEA

LEQAAIREAKEETGLDIKVHGIVAVNECKFPKKEEHVIFITFKGEVIGGNEEIVRPNE

ISEIAWIDIEKAEQLMPYYKDGLRRLIDGHEITYFNEGNK"

CDS 2281163..2281966

/locus_tag="PIKHDJKN_02116"

/inference="ab initio prediction:Prodigal:002006"

/codon_start=1

/transl_table=11

/product="hypothetical protein"

/translation="MCTSFAVHLDKTFIGMNFDISRRPIKIVLIGEDQLLVLQNDDGQ

FYPAFGLNSSGTFMNLLMVDPIEEGKYRRGKNCVHIMKLFDEILSGRIELSQLSEYLT

NNVIVNVPSHSVHSLIAGRNRKTYIVEPGRKNIDINSSDRDFMVLTNFPVSDNFDNEY
KNVEGPGKDRYIKAYEAITNNKETFNIATGLNLLKDTAQQSGDYPTQFSLIFIPEESK
VHFTLNGDFTKIFEFSFINKQIQSIEGFTSNNSLKLTKKGVLLSELEEW"

CDS 2282256..2282804

/locus_tag="PIKHDJKN_02117"

/inference="ab initio prediction:Prodigal:002006"

/codon_start=1

/transl_table=11

/product="hypothetical protein"

/translation="MDRLVVMTVGKTHSGKTTFAKELESVLQQAIVIDQDNHAEFINE
HYKKLRPIEGPNTLKFSVTDAIVNYAMEQSNFHIILSNSNLNKSGREKVLRYFHNKEF
KSIIVYFDLPNDLLKDRVIRTQRSKAIFRSASSFLEVLERQENLSEIQPSENEANYIF
IIKDPNNTAEVIQQIIAITGTR"

CDS 2283094..2283378

/locus_tag="PIKHDJKN_02118"

/inference="ab initio prediction:Prodigal:002006"

/codon_start=1

/transl_table=11

/product="hypothetical protein"

/translation="MVKTLNNLLYLIARDNECETIIDKLSERSSSFLFLAEEALRYSD
NDARWQFAHYLIQVKDQKTKVIELIKKYSEDYVEYVRRRALAALRIINGK"

CDS 2283586..2284152

/locus_tag="PIKHDJKN_02119"

/inference="ab initio prediction:Prodigal:002006"

/codon_start=1

/transl_table=11

/product="hypothetical protein"

/translation="MKTIIYMVRHGESPYNEGNERTRGLTPKGKIDIEKVTKLLIGEG
IDMIISSPYTRAILSVEGLAEHLKLDIKVFEDLRERNFASYNIENAELMSSIRESFND
YDYTLPGGESNADCQKRALSVLKPILKEHRGKKIVIGTHGLVMTLMMNHFDSAFGLEF
LNQLKKPDIYKMQFEDLELEEVTRMWND"

CDS 2284740..2285849

/locus_tag="PIKHDJKN_02120"

/inference="ab initio prediction:Prodigal:002006"

/codon_start=1

/transl_table=11

/product="hypothetical protein"

/translation="MSNNSQLTRSQNAAIEYISNCARSQKREAQASLKEIFQFSNIPW

NTFEEAVHKIKSHARVALHFHPDRPVLDMKSVAQSLLEHGIYKSQFETFISNGSVSAY

VGGARDLWEEKLFGGAYQLEGATNFERPKYGALHLLLHPDGPSPRFGSCYFLLKPNAS

HRCTFTYGGSQDAPKEKGTYEELDDILSALFTDAFFRDYAIGEKDLTPKKLIDQLLYN

LERPLEDPSKLAPHRNLNHFIETQIHGDIYLEDDVEILVADSSFKGKEIGNYLEQICI

KYSIELFWHMGFAMRVEEVPSDFRGPTMPSLAKRIASNGIIDASVIGKAVNDLKRNPD

LWSDRGNYEEVLQELKYMWHILVRYGKSIEKRYKG"

CDS 2285846..2286274

/locus_tag="PIKHDJKN_02121"

/inference="ab initio prediction:Prodigal:002006"

/codon_start=1

/transl_table=11

/product="hypothetical protein"

/translation="MILLRTSLLIKLISAVLSTIIMSFICATIFFPDEFGSTFLLGII

MFMLLYGIGGIPFSVVASAIIKTSRVSYKFNLIDLIVHLLGGLIITILLLAITGAEHG

IDLFFSHEVIIFYEIGMCSGLIFNLIEQFIKKLKLKEVQF"

CDS 2286339..2286854

/locus_tag="PIKHDJKN_02122"

/EC_number="2.7.1.-"

/inference="ab initio prediction:Prodigal:002006"

/inference="similar to AA sequence:UniProtKB:P9WL55"

/codon_start=1

/transl_table=11

/product="Putative O-phosphotransferase"

/db_xref="COG:COG3896"

/translation="MDKGKIIFLNGVTSSGKTSIVEAMQNYAEPFFYVVANDLFENTI

GDKHLQTDYWKYLSEAIILMYHTAKLFSDHGKNVLIDGVLVERPELKPHYDKVQDIFD

GYPLDIVEVYCPLDLCRKRNIERGDRREDQSDEQNKIMSQHIHYSFSVDTSLNTPEEC

AEMIITSVFKK"

CDS 2286920..2287381

/locus_tag="PIKHDJKN_02123"

/inference="ab initio prediction:Prodigal:002006"

/codon_start=1

/transl_table=11

/product="hypothetical protein"

/translation="MGSRIMHLIVANRIADSLSIVDKTSFLLGNIAPDAVTTKDSSHF

FAGEIQDYSRNVDYKGFLHKYRSHFPDKYYILYANKKRYFQASNDFCPGLVCFIESVD

IESNVKKIRSEMPNCKNAHRYSIKIFEFMVNWLRTNKASHDSKLEEGAIIL"

CDS 2287592..2288614

/locus_tag="PIKHDJKN_02124"

/inference="ab initio prediction:Prodigal:002006"

/codon_start=1

/transl_table=11

/product="hypothetical protein"

/translation="MNKQDIFYNIFFDELESLFSADIMCCQTCIDRFIIQWPGVYNRD

QDFQRNVISLKSFYFSSRVSDYYSENEFNKYSRKLKCPNCGNYLNGYIWPYNMNFEVP

EGFQNNLFEIAEIASETPFLLLKHPFAQQVYSEINNISNTILPVYLSKNLFRARVFEQ

EKNYSDNDFLAPPKRLIKEGRYNHTGYQVLYLAEDPDTCFYEMRLPDEGIMIATVIIE

KPIKILDLMKDSTIENNIVDVIKSSSLLSSPEEGEGWHKPHYIFTRFIADIAKATGFD

AIRYPSVRFNEGYNIVIINFEKLKESIKIIDMYSFNKEDLEINKDTEILSHYLETYLT

NSNRKT"

CDS 2288961..2289743

/locus_tag="PIKHDJKN_02125"

/inference="ab initio prediction:Prodigal:002006"

/codon_start=1

/transl_table=11

/product="hypothetical protein"

/translation="MKIVFRNFDDSYYEQVCDFLIELSKDDRRHINWNWARWEWMFYH

PEFNRDLMDKIGLWYCNDELVGIATYDHYFGEAFFATRQGFEELEEDILEYTTTTFGN

ENGVGIAVNDKDTHTLDLLRSQKFVENDLTENILELTLENVSLEYVIPEGVILKNMDV

KNDLFKYQKLLWKGFNQEGPVPDDEITIGKKKRMLSARNMNSFLHIIAENEDGEYVAY

CGLWYSQKTDYVYVEPVCTIPEYRQKGLGKAVVLEALKEGTP"

CDS 2289921..2290700

/locus_tag="PIKHDJKN_02126"

/inference="ab initio prediction:Prodigal:002006"

/codon_start=1

/transl_table=11

/product="hypothetical protein"

/translation="MNIQDLPNEIIEQIGKVQRITFPRQGHTSIVAILDTCDKKYVIK

KTENDLYNEWLSEEYKVLQYLYHTGLPVPQAYLYHVENQSRWLLMDYIDGISLREFLS

KEPDLKDKEKAIFNFGLCLKKIHECSCPNELLKNDNSWLDTMLNKAEYNLTHFAVDGT

EELLKRLKKVRPKPIDNTLIHGDFTIDNVLVNECNIVGVIDWSGATYGDPRYDLALAI

RPKYNAFDNDRDKEIFFNAYGNLRITDEEYNYFEDGLYSFF"

CDS 2291010..2291564

/locus_tag="PIKHDJKN_02127"

/inference="ab initio prediction:Prodigal:002006"

/codon_start=1

/transl_table=11

/product="hypothetical protein"

/translation="MFMKQTAPIGIQKEEKQIEKATQHLTTCGLSAKLICSPMEEECD

LPVDYFDFVYSIYAIGWTTDLEGTFCRIASYLKKDGIFIFSWSHPIHKCVVEENNRLA

FNKSYFDESWYSVAPDFCQGELTLSDRKLSTYINALAKAGFVIEQVNEETDQEIIQLH

DENNDLVKRAKMFPVTFVIKARKL"

CDS 2291583..2291984

/locus_tag="PIKHDJKN_02128"

/inference="ab initio prediction:Prodigal:002006"

/codon_start=1

/transl_table=11

/product="hypothetical protein"

/translation="MVTLKLHTEKPILQSELMGLYQDAGWWPNRDENGILQLLVSGVA

VGAWKDDILIGFCRSISDGIYRAYVEDVVVLSTYRNKGIGELMVKRLLDELKDIDVIS

LFCTEDLDGFYELSGFKRTKQIVMHYKNQRI"

CDS complement(2292145..2292432)

/locus_tag="PIKHDJKN_02129"

/inference="ab initio prediction:Prodigal:002006"

/codon_start=1

/transl_table=11

/product="hypothetical protein"

/translation="MVNTLDSLVEPLIRMRLELLYTELSEHHNEYSQLSSETDQYFRT

LREALPDQLQHTVFLYEDAQISLQSILERSIYIQGFKDALQLYCELQNSSI"

CDS 2292583..2292807

/locus_tag="PIKHDJKN_02130"

/inference="ab initio prediction:Prodigal:002006"

/codon_start=1

/transl_table=11

/product="hypothetical protein"

/translation="MTVIKCNIRELMAEHRIDDITELMAKSGLSRNSINKLYRETNIE

TTKLETLFKLCDTFNCKLSDLIEYVPGEKI"

CDS 2293127..2293375

/locus_tag="PIKHDJKN_02131"

/inference="ab initio prediction:Prodigal:002006"

/codon_start=1

/transl_table=11

/product="hypothetical protein"

/translation="MFKYEMDDHSYLSLLEIKDAEQLYNLINSNRDHIGEWLKFPSIT

VEADDSKTFIERTRIRYAKEEGYWLGIWSEDKLVGSIG"

CDS 2293834..2294934

/locus_tag="PIKHDJKN_02132"

/inference="ab initio prediction:Prodigal:002006"

/codon_start=1

/transl_table=11

/product="hypothetical protein"

/translation="MISQKKLKRVELVRTLVLETLLTIENTSKKEEALVNLFGVSSFA

SLLAIRREQNSEIAAIAGLLHDFYFYKTGLKYFPGLNSADSVRPILRSTQIFTNEELS

VILRSIFYQNDKHQVHGPYEEIIKDAILIQMYVQNPEDLFSDIEINRLQKGFIELGIP

VEQVEVKCKSNRGTINKGTEDRRFNLAEIAEALAGQGILGIPEDENFREICKYWPDSD

IYKVLESNWCAAFVYHCCMQAGIILPIRYPNHSYRLAGAGAWLDWAQLPETNFLYQDG

YHGFKPKRGDIVIFEKLLSDNSHDHIGIVLACEGNRLLVAEGNKDNKNFSSVFYRDRE

HCIYGYIRIDDSYQFHFDGEYNPIVSKWSKKL"

CDS 2295063..2295416

/locus_tag="PIKHDJKN_02133"

/inference="ab initio prediction:Prodigal:002006"

/codon_start=1

/transl_table=11

/product="hypothetical protein"

/translation="MSKFSPHEFFPYARKFYSNQKDENSELSWKNAWLHHQNHNKHHW

EYWIVNKNTREALPMPKKYLIEMVCDWRAFSRKWGRKVKETNLSERMMTSESIILHPK

TREELENFMKKKQQQ"

CDS 2295543..2296070

/locus_tag="PIKHDJKN_02134"

/inference="ab initio prediction:Prodigal:002006"

/codon_start=1

/transl_table=11

/product="hypothetical protein"

/translation="MSRSVYIISGPAGVGKSTTSRKLVAHLNRSSYISGDDISHLPVN

GRGKPWICQETHKLTWINILSLVKNLIQFNYDVVIDYVTFPSEANWLASELESMNVRI

IYVVFMVDDETIIRRDQLRDPSIQMGERSLILLNEFREVLKDDRHILNTQEYSVDQVD

EVIEEILNNDRFLIS"

CDS 2296283..2296807

/locus_tag="PIKHDJKN_02135"

/inference="ab initio prediction:Prodigal:002006"

/codon_start=1

/transl_table=11

/product="hypothetical protein"

/translation="MEQRNAPEVRIELWDEGDLDLLRQLNSPEMTVHFGGPETEEEIL

ARHKRYYEIAQNGTGKMFKILLLPHLEVVGSVGYWNQTWNEESIYEIGWSVLSEFQGR

GIATEATAKAIASIRSEKKKHKFIHAFPKTKNPASNAICRKLGFSYIGECDFEYPIGT

NIRCNDWRLAVEEN"

CDS 2297054..2297602

/locus_tag="PIKHDJKN_02136"

/inference="ab initio prediction:Prodigal:002006"

/inference="similar to AA sequence:ISfinder:ISSpgl1"

/codon_start=1

/transl_table=11

/product="IS1595 family transposase ISSpgl1"

/translation="MSQFPELETKRLVLRELTENDSTDIFQFFSLDEVTKFYDVESFT

NIKQAEDLFQRWNERYETGQAIRWGITLQSENKVIGTCGFHGWMKKHYKAVIGYELTP

EYWHQGFMTEVVERVIEYGFNTLGLNRIEAFVEPENVGSRKVLEKIGFREEGILKENY

YWKNRFVDNAIYAFLKSDYKVH"

CDS 2297628..2298128

/gene="paiA_1"

/locus_tag="PIKHDJKN_02137"

/EC_number="2.3.1.57"

/inference="ab initio prediction:Prodigal:002006"

/inference="similar to AA sequence:UniProtKB:P21340"

/codon_start=1

/transl_table=11

/product="Spermidine/spermine N(1)-acetyltransferase"

/db_xref="COG:COG0454"

/translation="MITIRFVSNEDIPHIQSIAQETWNYTYKGIYSEGYIQNFLSRAY

SDENLSRSVERDLHSAKRSFLIAEYNGKVVGYAQTRQVKEEEHELQRIYIRPGYHKMG

IGQEFIRRFTQVLKPINQLFAWVGKENYIGRAFYEKSGFKEIEEMIETIEGHSKTQVK

YELEIS"

CDS 2298169..2298591

/gene="rppH_5"

/locus_tag="PIKHDJKN_02138"

/EC_number="3.6.1.-"

/inference="ab initio prediction:Prodigal:002006"

/inference="protein motif:HAMAP:MF_00298"

/codon_start=1

/transl_table=11

/product="RNA pyrophosphohydrolase"

/translation="MKRVNVTYTLITDESKSKVLMVKNVGRSSWSLPGGAVEPDESLE

QAAIREAKEETGFDVKVYGIVAINECKFEHLNEHAIFIVFRAEITGGTEQIVRPNEIS

EIAWKDIEEADKLMPFYKGGIRNLIVGNEITYLNEGTK"

CDS 2298768..2299265

/locus_tag="PIKHDJKN_02139"

/inference="ab initio prediction:Prodigal:002006"

/codon_start=1

/transl_table=11

/product="hypothetical protein"

/translation="MRWGNTIIKIQKTHIITVVLLLVAYFSYQYLNQKNEQLNKEISI

NQIKSTQMTKAVYSHKTPNITFVDIKLTNEQINKIVGWINSVPESSIIELHQTPSNIS

AGIAFRLHSRRDITIQYDLEQIYITRTDIKNKDTKYSIVQNELKSFFDEQLKGFYYGK

DKVNE"

CDS 2299332..2299451

/locus_tag="PIKHDJKN_02140"

/inference="ab initio prediction:Prodigal:002006"

/codon_start=1

/transl_table=11

/product="hypothetical protein"

/translation="MNENNEILLIRGPRRGWEMPGGQVEEGESLQDAAVRETK"

CDS 2299729..2300238

/locus_tag="PIKHDJKN_02141"

/inference="ab initio prediction:Prodigal:002006"

/codon_start=1

/transl_table=11

/product="hypothetical protein"

/translation="MEIELEVIQSMLVKFKSEGKWTLQAISQLSEEDITWSPNQESNS

IANLVAHIRGCVHSRIETIFYDIADSRDRDKEFEHGLKMSIEEAYNMTKESFDIIIQY

LEHLSFNPNLLLSQPFTNRPPLTFSQVNNETTVLNLMIAMVREIHYHTGQIIYAAKVR

KGQLVWQYD"

CDS 2300281..2300730

/locus_tag="PIKHDJKN_02142"

/inference="ab initio prediction:Prodigal:002006"

/codon_start=1

/transl_table=11

/product="hypothetical protein"

/translation="MIQILEEPTGATYKQLVSLAFSLCDEFILVKRDQIKLNVDGKRF

IEEIKPYIKEIKKQEGWPGTQLFGHYADVFYFDCNEELKQIVLKKVDRLYEWLQPELL

EDLCFYKNNKEWLVTIAHEKQGIIKTTDHQEILMIREIEGIMIYYHE"

CDS 2300777..2301199

/locus_tag="PIKHDJKN_02143"

/inference="ab initio prediction:Prodigal:002006"

/codon_start=1

/transl_table=11

/product="hypothetical protein"

/translation="MDFIYGEYLISDNKELMDIHAVKGFLSRSYWANRRSDGKIEKSI

HSSICFGIYKEEVQVGFARVVTDDATMYWLCDVFIHEENRGQGLGKKLIEVITQSDRF

KDLMGLLGTLDAHELYEQYQFNRNSERFMIRLPDYLRA"

CDS 2301199..2301429

/locus_tag="PIKHDJKN_02144"

/inference="ab initio prediction:Prodigal:002006"

/codon_start=1

/transl_table=11

/product="hypothetical protein"

/translation="MTLKRAVYFLSLIIGIVFIALGVLPAIFAYPFSDEPNSGPASFW

ELILIISYEQWILFLIVGLILSLFNVLQLRKI"

CDS 2301445..2301867

/locus_tag="PIKHDJKN_02145"

/inference="ab initio prediction:Prodigal:002006"

/codon_start=1

/transl_table=11

/product="hypothetical protein"

/translation="MYKLKTKETDNSVIEFIESVDSPKKREDAYQLLDIFTETTGYKA

KMWGPSIIGFGAYHYKYESGHEGDAPLVGFSPRKAKISLYFATGDAERENLLKDFGKH

TTGKACVYVNKVADINLDVLQALINQSIKFLKETYPNH"

CDS 2301901..2302332

/locus_tag="PIKHDJKN_02146"

/inference="ab initio prediction:Prodigal:002006"

/codon_start=1

/transl_table=11

/product="hypothetical protein"

/translation="MKEENIHIRWANENDAAKLLKLNDAFNGVGTSIEEVKESLALSN

ELIALAVIDGQTVGFACAQYFKSLCYRGLQGEITEMYIAEVARRRGLATLLIAFIEEE

LRERGVTSVKILTGQRNEMAIKTYVKSNYVRTEEVLLQKNL"

CDS 2302403..2303287

/locus_tag="PIKHDJKN_02147"

/inference="ab initio prediction:Prodigal:002006"

/codon_start=1

/transl_table=11

/product="hypothetical protein"

/translation="MSEYYWDNKIEYLRNTRWLYYNDDYLQFLVQFVWKIDKPVSVID

YGCGFGYLGLKLLPLLPKGSTYTGVDKGETLINEAKTIFSQLPYDTEFITCDIEDVVL

ERKYDIALSHAFLLHMDDSVKTLQRMIESVLEGGRIICFEPHWIGNMANYHVEGVEQS

EIIKLGVLQRLFEQDHIRTGKDGNIGMRLPILMSQLGLTNVECRMSDKVNFLDQNMDS

ARKNILYHSIKEEGIGQDPGDSVEVTECLSTRGLTQNEAKAQYEAELILSKVFNDESW

LTYAASMKISFGTVRREG"

CDS 2303284..2303727

/locus_tag="PIKHDJKN_02148"

/inference="ab initio prediction:Prodigal:002006"

/codon_start=1

/transl_table=11

/product="hypothetical protein"

/translation="MIRTVGSHEGILKNNIVVVVKGVIENKGRILILKRSGADEVAAE

SWETVGGKIDFGEELEEAGLKVSVEKLLFATTFFTDPHRQIVLLTYLCRAIDDQVILS

EEHSEYMWATRSELYDYLPQTILNDFKKYNVIDTVEIIAKQYELK"

CDS 2303781..2304242

/locus_tag="PIKHDJKN_02149"

/inference="ab initio prediction:Prodigal:002006"

/codon_start=1

/transl_table=11

/product="hypothetical protein"

/translation="MNPITIEPIDKMNWEEAIAISLLETQVNLVPTVIESLAYAYVKP

WDAAFDPYLLREGDKAFGFFYLSYTPASTDNYWIGGFQIDKDYQGKGLGSQSLYTILE

SIQEKHPQCQIISLTVERSNAHARVLYEKIGFIDQEMENQDGEVVYKMKLK"

CDS 2304307..2304858

/locus_tag="PIKHDJKN_02150"

/inference="ab initio prediction:Prodigal:002006"

/codon_start=1

/transl_table=11

/product="hypothetical protein"

/translation="MYDDLNKTMTTERLLLRLFIKADAETVTRLCNDYNIYKSTLTLP

YPYTLECAVSWIEHHNENFNADKLYEFAICDRETGDLYGAIALSNQQRYDNGELSYWV

GQPFWGKGYATEAAKAMLDFAFDVKKYHKVYARHFASNPASGQVIQKIGMLKEGVLMD

HVKKEGLYEDLIYYGVVRNKMKE"

CDS 2305014..2305820

/locus_tag="PIKHDJKN_02151"

/inference="ab initio prediction:Prodigal:002006"

/codon_start=1

/transl_table=11

/product="hypothetical protein"

/translation="MNPLIPSLDLIKEIEISEIDYMTDRMLAIQGRDSNPEGIEIQQF

GNAICFYSKTMPWPTFNTVKGLTNNDLEHMDAIIDFYRQRDRKVQFEVIPSVVDQNFL

KRLTDLGMYASGFHSSLIIKPEEKKNHSDHIRIQELEEDQFELYATIHCRGTGLSDDG

IPYVAQNNQVLYHRPGWKFYIAYVNDIPAAVSVLFIKNQKASLTFAATLPEFRNQGVH

QQLLNRRIMEAVNKECNLVVGQCSFLSQSHRNMEHVGMKLGYIRTAWTER"

CDS 2305848..2306126

/locus_tag="PIKHDJKN_02152"

/inference="ab initio prediction:Prodigal:002006"

/codon_start=1

/transl_table=11

/product="hypothetical protein"

/translation="MNNTNKILGAVLMISSIFISTLDRISVRISIAIVEAGYASGGKT

REIIPASDNGFTGFLVYFLFFVGFVLLIAGFPGNSRKDKTNRYPQNRS"

CDS 2306139..2306936

/locus_tag="PIKHDJKN_02153"

/inference="ab initio prediction:Prodigal:002006"

/codon_start=1

/transl_table=11

/product="hypothetical protein"

/translation="MPEYEEALNGGNVNKVVKVGSTVRRDAIPNPYVYELLKHLEKVG

YAHSPRYLGVDEQGREVLSYLDGVVPGNDYPEIESYMWSDETLDKVAKLLKSYHDATV

DFVTSTVSLNNCPKISSKEEVVCHNDFALYNVVFKDRLPVGIIDFDMAGPGSRLWDIV

YSLYTSVPLAGFSPDKDEREVVPYNKQDHASERKRRIALFFNAYGMDVPTDLKEWMIS

RIHFMCTTLSDRAASGEIAFIKMIEEGHLAHYEKEVRFLEKHFGDWS"

CDS 2307149..2307424

/locus_tag="PIKHDJKN_02154"

/inference="ab initio prediction:Prodigal:002006"

/codon_start=1

/transl_table=11

/product="hypothetical protein"

/translation="MGFVMITVDETTDWSYWLNGFMIDVSYQGKGYGRATIDAVISYI

KENYVHSKCLNLTVCAENEVARKLYDKMGFSETGEVYDDEIVYRFVF"

CDS complement(2307444..2307791)

/locus_tag="PIKHDJKN_02155"

/inference="ab initio prediction:Prodigal:002006"

/codon_start=1

/transl_table=11

/product="hypothetical protein"

/translation="MLYLISFVLMFAGLACINIIFSYQSKHIDTQFWSTFKFHLWMLP

LFLAANLSVGYGVKFGMKAVSQLGYVLVIAKCIEVLISIWMGYWFMKEVPSWKTWIGL

VVIIIGVVLVKQK"

CDS 2308097..2308633

/locus_tag="PIKHDJKN_02156"

/inference="ab initio prediction:Prodigal:002006"

/codon_start=1

/transl_table=11

/product="hypothetical protein"

/translation="MLKKRDLHECYSLYDLMVDPAVFPYVRHKCRSYEEYLFATKQVI

DEEEQNTCISRTILNELGHPIGTIDLYHMEHKTGFLATWIGAPYFGKGYNQRAKEAFF

AELFLQREIETVFLKIRKQNIRSKKAVGKLPYVKLANDIYHEVYKLINNTEQIYDLYY

VNRSDFMASEEMKHVVAT"

CDS 2308851..2309405

/locus_tag="PIKHDJKN_02157"

/inference="ab initio prediction:Prodigal:002006"

/codon_start=1

/transl_table=11

/product="hypothetical protein"

/translation="MEIKKIENLSNEDWLQLGSFGYKSTQKYQLTKKETQGSFSMHLT

LINLDDIYEKEEMNSTDDLERYEEIIQLGFSYGIYIDGKIIALAISEPQAWNNTLMIW

HLQVNEKYIRKGYGRLLINKVNEIAQEQGFRAITLETQNTNVPAINFYMQCGYQIEGI

DVSLYSNNHIQNEEIALYMRKKIQ"

CDS 2309490..2309957

/locus_tag="PIKHDJKN_02158"

/inference="ab initio prediction:Prodigal:002006"

/codon_start=1

/transl_table=11

/product="hypothetical protein"

/translation="MTKKRKIVVALSLIVSGIIGVYLILNQYIVLDRTSQELGNYDFV
TSQADVEVLVEGTGKQNILGDRVSTKIKIVNILFNNTSSNLVEGSEIEANEYLVVHHN
RQVSGLQWTPGRSIFSMGTSYKRLEKGEQKTIHLKLNKETNQFWIMPEELIIQ"

CDS 2310050..2311108

/locus_tag="PIKHDJKN_02159"

/inference="ab initio prediction:Prodigal:002006"

/codon_start=1

/transl_table=11

/product="hypothetical protein"

/translation="MTVGRKVFVLKDESRIDPISKETREVLISIYYPSNPVEHESKYI
SLFEPNITLAVDMLSSMGVDKDYICNLNTKIHNDESVDINAKNCPIIFIAPAFGVVRD
MYSFCVEHLVECGFVVITIGATHESIFSIFPDGRFVQQSNEISEIDSLDIHSWKQLLK
LRVEDMSLVMKNVDKVLDSDKDLRAIIDVNEMGIIGHSLGGAAAYELLKREKRIKASI
LLDPSFHLMTTNKEEGVSVPLLIARQEKCSFEELENEISPDLLPLLLTGYESLFNSSD
LNSSFIKLNGAHHMTFSDIPIHYNEDGIEHKHSTINKYISAFLEEHLKKEESKFHELL
KNKINDGIDQVNSKGHVI"

CDS 2311188..2312129

/locus_tag="PIKHDJKN_02160"

/inference="ab initio prediction:Prodigal:002006"

/codon_start=1

/transl_table=11

/product="hypothetical protein"

/translation="MELNNWSEELNFHDLSNEYSIQKAEPEEWGLYCSVYYNMAYTGF
FREEGYFNVSRNNSFWIYKGESKIGGVRMAPNTLYHLFFMPPFNDSFEVLKLLKRILI
KWSDPTQRIKIYEILPDQVHLFTRAGFWPDEFRCRWMQRPTDHFNVRWDHDFIVKSPE
IIENETGAKQYINEDEIAQCDFESFVGGFEALRRKKTSLEDFIPNEEVHYTNEDLTQA
STLVYDKVTGQLIANCRLCLQDNQAAVYSIGVNPAYRGKGLATRMLQRALTELKDKYP
VLRLYVMEGNDAESVYFNLGFVPGVQEIQTMYIPVNE"

CDS 2312174..2312305

/locus_tag="PIKHDJKN_02161"

/inference="ab initio prediction:Prodigal:002006"

/codon_start=1

/transl_table=11

/product="hypothetical protein"

/translation="MGQYQLIARRVMFLVERKVIIGDYINVGSVFVSWYFCCENWNR"

CDS 2312250..2312384

/locus_tag="PIKHDJKN_02162"

/inference="ab initio prediction:Prodigal:002006"

/codon_start=1

/transl_table=11

/product="hypothetical protein"

/translation="MLVVSLFLGIFVVKIGIDNSKSNQYTKEILQELREIKEILRDQR

"

CDS 2312409..2312930

/locus_tag="PIKHDJKN_02163"

/inference="ab initio prediction:Prodigal:002006"

/codon_start=1

/transl_table=11

/product="hypothetical protein"

/translation="MLYDLKGEAKMSPVVGMLFAAVIENSQRLQRITDGMSKEEIDYK

GPNNNFNSTAQLIKNITYVDLNGAYRIKGRPLPQNLREQHGPIIDENGRLPMVQEISL

STLMSTYVGVITMLKDACTQLTDDDLARIVTFGHENEKQASIRWGIWHIADHSRYHQA

HINQLRRWYHEAH"

CDS 2313212..2314453

/gene="amiD"

/locus_tag="PIKHDJKN_02164"

/EC_number="3.5.1.4"

/inference="ab initio prediction:Prodigal:002006"

/inference="similar to AA sequence:UniProtKB:P9WQ93"

/codon_start=1

/transl_table=11

/product="Putative amidase AmiD"

/db_xref="COG:COG0154"

/translation="MGARGVLHGIPVLLKDNIETNDRMHTTAGALALETHISSKDSFL

VSKLREAGAIILGKTNMTEWANGMSSDMWAGYSARGGQVLNPYGDFFPGGSSTGSAAA
VAANFTMVSVGTETSGSILSPSIQNSIVGIKPTVGLISRAGIIPFSHSQDTAGPMART
VTDAAILLGILSGKDEDDPATWKNPYSTIDYTTCLDFNGLKNANIGIFRKAPLTRYRD
NEEYDEVLLENAVIQIKEAGANIIEDIDIPSFDRKWEWNKLNYEFKHSVEHYLQSLPS
HLPVHTLSELIEWNKQNAERALKYGQNHLEYREQLNNPLNNSNYILELITDLYHSQNN
GIDYALHKFGLDAIVFPSYVGADLCARAGYPSIAVPAGYKESGRPFGITFAGKAFSES
VLIRIAFAFEQLTADRKKPNL"

CDS 2314622..2315479

/locus_tag="PIKHDJKN_02165"

/inference="ab initio prediction:Prodigal:002006"

/codon_start=1

/transl_table=11

/product="hypothetical protein"

/translation="MLNQLSESIYYLSNQNDKERPTLGLVCGDKYSLVVDAGNSVQHA
KDFLHEIEKLNVPPVKYVVITHAHWDHFLGMNEYGATIIMNSLTSQKMNDWRNYSYDD
RSLQEYVDSKKMNTLCMETIQNEIPERDTFQLSLPDIIFEKSLHLDLGNKVCVLETIR
STHTDDSTVVYIPDEKVIFLGDSAYNTTTNSLYHIKQDKLLPMIEDIQQYDAVHFLLG
HESICDVEEMDTFWRELISANDAVKSTSLEEAIERFEAANDRTANDNELFFLQGFVND
QILQSKATE"

CDS 2315746..2316387

/locus_tag="PIKHDJKN_02166"

/inference="ab initio prediction:Prodigal:002006"

/codon_start=1

/transl_table=11

/product="hypothetical protein"

/translation="MSTPFIRNHQYNLIKRQAEFVRKTVQSAADRRVLETVRYSAATN
IIDAFTNLTENQQQMLEQISSLGTTDDFQRYMKALEPYLELYPPITLKQIQKLFPKNK
KMKMPDLQSIDFRYVTYLSWVDIATNKLFVVYPFEGQFIGIEGRITPTHKKGYCLFCN
RHQELAFFTVKTKNALPDNYSAIGQYVCMENQGCNHSITNTESLEKFILSIRK"

CDS 2316535..2317038

/locus_tag="PIKHDJKN_02167"

/inference="ab initio prediction:Prodigal:002006"

/codon_start=1

/transl_table=11

/product="hypothetical protein"

/translation="MEIKDRPKITIPKTLLEKILDITTIILFIIGIIYCLQQWMTLPD

QIPIHFNMKGEPDGWGGKWGVWLPLVIGLILWIGLSQLEKYPHTYNYLNLNMENAERQ

YKNARLMINLMKTEISIFFIYISWMIMEFSSADKVTHNNWLPVVIFIIVLFSSIGFFI

YRSFKLK"

CDS 2317104..2317739

/locus_tag="PIKHDJKN_02168"

/inference="ab initio prediction:Prodigal:002006"

/codon_start=1

/transl_table=11

/product="hypothetical protein"

/translation="MTVVGILGTIHNHELREKYGTTLALYRDLITEFNPDIICGEVHP

LSWKKYIKDKQDKGYWGEPASEYWELIFPLCEERNIEFVPIDWFELDVWNDFDPFNGF

SEEQRKALERIDDEWFAKQMNTHDVGAIPFNSDAFDNMTKQKYEWLDQINPKANNFRW

IVRNQIMIQRVKNTYHSNPGKKILCVVGADHNYIFKEELINEPIELVYPLR"

CDS 2317779..2318279

/locus_tag="PIKHDJKN_02169"

/inference="ab initio prediction:Prodigal:002006"

/codon_start=1

/transl_table=11

/product="hypothetical protein"

/translation="MIRLCNSMDADAMVEIINDAAKAYQGVIPEDRYHEPYMPLQELN

QEIRDGVVFWGFEENNRIWGVMGIQDKGDVALIRHAYVRTHQRGSGIGTKLLAHLVAS

TNKPILIGTWDSAEWAIKFYLKNGFTLVSSDEKKQLLTRYWDVPERQIETSVVLCDGK

WGSVGI"

CDS 2318329..2318841

/locus_tag="PIKHDJKN_02170"

/inference="ab initio prediction:Prodigal:002006"

/codon_start=1

/transl_table=11

/product="hypothetical protein"

/translation="MLYHFSEVSDIQVFIPREKQNRAEFPAVVWAIDEEHEYSYYFPR

DCPRIICKKTDTLSDENNDKFFKHSNAAIIVTVESSWYPRMMNQIIYRYHFDNTNFEL

FDPTAGYYISDQVTKPLHIDRMDCLMERLFKKGIELRFTPNLYPLREAILTSDFKEFG

IHRFNNATKV"

CDS 2319012..2319560

/gene="gpx1_2"

/locus_tag="PIKHDJKN_02171"

/EC_number="1.11.1.22"

/inference="ab initio prediction:Prodigal:002006"

/inference="similar to AA sequence:UniProtKB:P74250"

/codon_start=1

/transl_table=11

/product="Hydroperoxy fatty acid reductase gpx1"

/translation="MPTIYDFSVTKTSGERFPLYQYEGKPVLIVNTASKCKYTHQFDD

MQKLYDRYKEQGLQIIGFPCNQFAEQEPGSSEEAESFCQINYGVKFPMFSKMDVNGEA

AHPLYDFLKKSGPFAGFDESDVQAKLLKLMVADKAPEWLHGDAIKWNFTKFLIDAEGH

VVNRFEPVDSIDEIQASIEQLL"

CDS 2319719..2320579

/gene="melR_4"

/locus_tag="PIKHDJKN_02172"

/inference="ab initio prediction:Prodigal:002006"

/inference="similar to AA sequence:UniProtKB:P0ACH8"

/codon_start=1

/transl_table=11

/product="Melibiose operon regulatory protein"

/translation="MPSVMQFSGPLEYSYRSTSTYDPGASDGFHSHPQYEVYYFHDGE

CTYLIGDRVYNLEPGDLVLMHGMTLHRPHPMPGRPYVRTTLHFDPSAIRSSLHPDRVS

EVLKPFEELRNCRINLTGNIRSEFEALLLSLHQLTVNPGDFKEERMNVRLCDLLYVIA

GICRGRVEDHLPSSEKERHVQHIIGYVETHYMEDIGLDDLASELHLSKPYLAGLFKEM

TGSTIFKYVYDRRINQAKLLFQFQPDISVTEASRLAGFKRLSHFSRMFKQSVGCAPDL

YRSRLHRSFD"

CDS 2320636..2321406

/locus_tag="PIKHDJKN_02173"

/inference="ab initio prediction:Prodigal:002006"

/codon_start=1

/transl_table=11

/product="hypothetical protein"

/translation="MQSEQPFKLDYRIGSADADYRSQCRPSALLEHMQLAADRDLAGM

GVTVSQMLDQGLGWMLLTTDVTIMRPARLEEEVSLQSWHKENKGVTWLRDYILTDGEG

THLAEALTAWALVDLKRRRVVRPSALPFEVKSHPDLSLGEAPAKVTVPSELSLQEQYR

LTVQYSSTDSNGHMNNARYADVCCDALTLDELAVGMIRLQISYHREIRMQEQMIVERS

AVTQGAVWIRGRLAEENESIIFEAKLTLGDFIALPSDQ"

CDS 2321586..2321831

/locus_tag="PIKHDJKN_02174"

/inference="ab initio prediction:Prodigal:002006"

/codon_start=1

/transl_table=11

/product="hypothetical protein"

/translation="MKERTPDQNNKDAHKDRFHDNPEQYRTEQESLLDLHEEEQTVDP

IPMEDLNMEKQEEKDKDQTKSNSSSEEKYRADYRESK"

CDS 2321973..2322575

/locus_tag="PIKHDJKN_02175"

/inference="ab initio prediction:Prodigal:002006"

/codon_start=1

/transl_table=11

/product="hypothetical protein"

/translation="MSKSFFEALKNRRSYYGISKESTISDAKIQEIVEEAVKYTPTSF

NSQTSRAVVLLGEQHDKLWNHTEEILREVVGNEEAFQSTAEKMAGFRSGYGTVLFFED

NNVIAQLQQNFAAYADNFPIWANQSNGMLQLVIWTALEQEGLGASLQHYNPLIDEKVK

QEWNIPENWRLIAQMPFGKPTATPGEKEFQPIEQRVKVHK"

CDS 2322726..2324357

/locus_tag="PIKHDJKN_02176"

/inference="ab initio prediction:Prodigal:002006"

/codon_start=1

/transl_table=11

/product="hypothetical protein"

/translation="MNNRIYKRTRRKLWNAVLCGVLTIGSISIGASTAQAAAAQQVFH

DTTSSYAKDAIAHLVKAGIAAGTSENTFEPKKAVTRAEFATFAVRLLGLKPVKNNINP

YNDVSINAWYYGNVAAMTNLSILEGKSQGVFQPNASITREEAAALLVRMLKQSSKSTN

LLSSTYNDAAEISDWARPYVQTVYQLGLMRGSDGKFRPHDQVTREEAAVMLDAILQKK

TWSDQLKSEDQLGIQLGWQYNASTAEFKKQIEQSEANTLVPRWFFLNSSMKVTDLTDT

SLLSWASATDRQVWPLLGNRSNPDLTHQMLSSSANRAAVISQVTSFVKTYKLDGINVD

FENVLPADREGMTAFITSLTTALHALGAVVSVDVSPDLGTDWTDAFDYAQLGAVSDYM

VLMGYEEHWNGDPQAGSVASLPWVEKALDTMLAEVVRAKTILALPLYTRDWSSVNPGA

SSWDITLAEQGTRARASGSVRRWDADLVQYVIGYNSNGTPRYIWAEDSRSLSAKVMMS

EERNIAGLAYWYMGGETADVWNAISNASRFESYIF"

CDS 2324305..2324454

/locus_tag="PIKHDJKN_02177"

/inference="ab initio prediction:Prodigal:002006"

/codon_start=1

/transl_table=11

/product="hypothetical protein"

/translation="MYGMPFRTLHDLNHTFSKFRNNNSFLNRPKRTVIQTDGLGYFIL

VKAKL"

CDS 2324555..2325484

/gene="trxB_1"

/locus_tag="PIKHDJKN_02178"

/EC_number="1.8.1.9"

/inference="ab initio prediction:Prodigal:002006"

/inference="similar to AA sequence:UniProtKB:P52215"

/codon_start=1

/transl_table=11

/product="Thioredoxin reductase"

/db_xref="COG:COG0492"

/translation="MREYDCIIVGGGLAGLQAAIQMGRYSSHRVLVVDAGEGRSTLCR

TYHNILGFPDGVSGEELRSKGRMQAERTGVSFEKDRIIKAGRRGEQIQLFGSSGSEYM

AKTVLLATGLTDRIPAIPGLTPTLGRTVYVCPDCDGYEIQDQRTILLGAGEAGANMAM

VLIQRTNELLYINHEQSPISAELHRSMKEAGVRYLEAAVQEVQQMEDGHITGVLTEDG

QIFESERGFIAFGGNRVHYELAEQLGAVISDNKHVEANARSMQAAPNVWIAGDLGLHA

EQATVAMGEGSIAAIWIHKALQQMKKENKVNQI"

CDS complement(2325607..2325795)

/locus_tag="PIKHDJKN_02179"

/inference="ab initio prediction:Prodigal:002006"

/codon_start=1

/transl_table=11

/product="hypothetical protein"

/translation="MNEYKKERKNKDLEPGVNAIIEPDPNNPGPTDMIEEAVGEIVDN

ITHPHKAKTTEIRNTNSK"

CDS 2325943..2327370

/gene="dprE1"

/locus_tag="PIKHDJKN_02180"

/EC_number="1.1.98.3"

/inference="ab initio prediction:Prodigal:002006"

/inference="similar to AA sequence:UniProtKB:P9WJF1"

/codon_start=1

/transl_table=11

/product="Decaprenylphosphoryl-beta-D-ribose oxidase"

/db_xref="COG:COG0277"

/translation="MLLLLLILCVWTYWRTDSDDQNPYLITDYSRLHPVKVERVVEGH

EEQQLVELLQDARERNLTISIAGQRHSQGGHTYYKDGIVIDMTSYNKVLEVNTDAKKV

RVQAGATWADVQRAINPYGLAVKNMQSQNIFTVGGSISVNAHGRELHQGSLIESVDSF

HLLTADGQIRQVSRTENAELFPLVLGGYGLFGVILDVTLTLTDDEVYSLTTEQVQVGD

YPSYFQKNVLGDPMMRMHLARISVQPGEGYFREMYALNYASDPTASLDDYNHLDERER

GVLTSKLLFNLNREFEWGRAWFWPLQQRYFESQDGKRLSRNNAMASASAFMEYHQPGE

NDLLQEYFIPVDAFPAFVQEMGEIVSQEQLDLLNITVRYVKHDEEAVLSYATQDMFGL

VCLFHASLSEAEQEKFKHSMQQLIDAVLRYNGTYYLPYAGYATLEQFEQAYPRKNEFF

ADKEKVDPNHLFMNYFIEQYGGNHP"

CDS 2327367..2327921

/locus_tag="PIKHDJKN_02181"

/inference="ab initio prediction:Prodigal:002006"

/codon_start=1

/transl_table=11

/product="hypothetical protein"

/translation="MNIKWLVRSQSIVTLASGMIYPYYLLFLKNLGNSFSKYGLAFAV

FTISSAAASQWLAPRMDRHAKQWLIVSSVGMAAAMIAFPWVISYIWVLLLQIMMGICN

AMQRMSERVLLANYTVAGRRGTAIGNYHFWTSAASGFAVIVGGVLIDWLTIDVLFYLS

AMLYMWGGLAVWRLGSISEEAVDG"

CDS 2328038..2328391

/locus_tag="PIKHDJKN_02182"

/inference="ab initio prediction:Prodigal:002006"

/codon_start=1

/transl_table=11

/product="hypothetical protein"

/translation="MNEQNHVIHKDGQVSTDKVDNAIDKIAPEEREQILQNFDAFKGY

LGKRIAMGESIGLGEEQMAKIAEKVADYLAAREEPLNREEKLLQELWNVGKEEERHML

AHMLVRLAQGSTPNH"

CDS 2328626..2329189

/locus_tag="PIKHDJKN_02183"

/inference="ab initio prediction:Prodigal:002006"

/codon_start=1

/transl_table=11

/product="hypothetical protein"

/translation="MKNAAKQWKLRMLGAAVALLGMIVLATYLLTPANAEESTDSVQA

SAKAQAANSEMFTAYVEDIQHENGKLYLIVDKIGWYQGAEADKVFEQRNPGSGIDGAP

DGYYIVNDSKEQEKVEVSANAEVLMQLYDRDGTVSGTTIQWNESVALSKFESLYDNTN

ILDLSVFPYHLTVQDGKVIKIVQQYIP"

CDS 2329347..2330204

/gene="ctaB1_1"

/locus_tag="PIKHDJKN_02184"

/EC_number="2.5.1.141"

/inference="ab initio prediction:Prodigal:002006"

/inference="similar to AA sequence:UniProtKB:O31652"

/codon_start=1

/transl_table=11

/product="Protoheme IX farnesyltransferase 1"

/db_xref="COG:COG0109"

/translation="MLKDMIALTKPGLLRLNVFAVAVGFWVASKWDISWISLLMVLIG

STLVIASACVINNYWDRELDQKMERTKKRIDYINHLSPKFVLGYGIVLGVVGFALLYF

LVNPLSGWMALLGWFAYIVIYTMWLKRSSTWSTSLGGIAGAMPPVIGYCAVTNQIDVG

AWLLFALLFLWQPPHFWSLGIRRVEEYRAAGFPLLPVVKGVKRTKVQMIPYVFLLLPA

VFLLYYYNYVGLVFLLVSVIGGLIWFVHTLSGLKTQDNEKWAKVNFLISVNYLMVVFI

VMVANTTWS"

CDS 2330434..2330871

/locus_tag="PIKHDJKN_02185"

/inference="ab initio prediction:Prodigal:002006"

/codon_start=1

/transl_table=11

/product="hypothetical protein"

/translation="MQEAIELLQKLALEEPDNAQVWYQLAWAHDSLGLEREAVPHYEK

ALSLGLPVEDRTGAILGLGSTYRTLGQYEQAKAWFEKGLSEFPENREFEVFLAMVLYN

LGEHAEAMKRLLVQLADTSRDKGITDYSRAIRFYADQLDRVWD"

CDS complement(2330893..2331276)

/locus_tag="PIKHDJKN_02186"

/inference="ab initio prediction:Prodigal:002006"

/codon_start=1

/transl_table=11

/product="hypothetical protein"

/translation="MCILPLEHPLATEKSVSFDVLLEQPFILPKWGGDNEIERLIRQH

AAKLNVVYEVAEDQAIMAMVRNGLGISLLPEMVLQHHTNELSLVPLTGDPYRTIGIAC

PSLTNLSPASRRFIEAVQEWLTPSL"

CDS complement(2331221..2331748)

/gene="benM_1"

/locus_tag="PIKHDJKN_02187"

/inference="ab initio prediction:Prodigal:002006"

/inference="similar to AA sequence:UniProtKB:O68014"

/codon_start=1

/transl_table=11

/product="HTH-type transcriptional regulator BenM"

/translation="MSLVKYEILNAVVEYGSLTKAAEALNITQSAVSHAIASLETECG

FSLLNRGRSGVRLTAEGERILGYTREILRWTELMNQEISLIRGAEIGTVRIGTFASVS

TQWLPGILKQFRLGHPGIEIKLWEGDYAEIEGWLAGGAIDLGFLSLGDSITKRQDDVH

PASGTSTRYGEVCLI"

CDS 2331914..2332861

/locus_tag="PIKHDJKN_02188"

/inference="ab initio prediction:Prodigal:002006"

/codon_start=1

/transl_table=11

/product="hypothetical protein"

/translation="MNGVRAPQGQASRRADLQMLLATVIWGSSYLFMKSGLESMQELN

LVAFRFGIAFIAAAVLFHRRLFRMDRRTLAAGAIMGTALFAAFVFITYGVQRTTASQA

GFLISLAVIFVPILTTILHRRMPDLRLTISILVAVTGLALLTLQHELSLHMGDILCIL

AALMYAIYIMIAGKYTPKHDPLTLGTVQLGVAALWGLAATFMLETPRLPDTPQSWVAI

LGLGVLCSGIGYILQILAQRHASPTRTSLIFSLEPLFAAAFAFTFQGESLTLQGYAGA

VLMLIGVLITEIRVPYPLFWRRKRTALQTELGDQGAPGV"

CDS 2333083..2334090

/gene="exuR_2"

/locus_tag="PIKHDJKN_02189"

/inference="ab initio prediction:Prodigal:002006"

/inference="similar to AA sequence:UniProtKB:Q9JMQ1"

/codon_start=1

/transl_table=11

/product="putative HTH-type transcriptional repressor

ExuR"

/db_xref="COG:COG1609"

/translation="MQQIADAAGVSKFAVSRALTGKPGVSEHTREMIVRTAGQLGYFK

AEPKRYLVESPAEQEMKMDRQGTILILFPNIRSQNRSSLYWGPIFDGISARLNEKGMD

ILTLTEPSSDRMFSVLNPEAISGIITVGSISTSVLLEIYRLHIPLVMVDHEDPAIHGD

TVFTDNMKCMKELVLMLVGKGYRRLQFAGELPDAASFRERWLGYRSVLEEMQLQVSQQ

ADLLGPDLDHIRESIVGMKMEDIPEVMVCANDHMAAVVIGALQSRGIEVPERCAVTGF

DNTRTEEPILATVHIDKENLGRRAVDQLLWRMEHPDGPFERKLIYSELIIRDEYNAIL

T"

CDS 2334349..2336898

/gene="lacZ_3"

/locus_tag="PIKHDJKN_02190"

/EC_number="3.2.1.23"

/inference="ab initio prediction:Prodigal:002006"

/inference="protein motif:HAMAP:MF_01687"

/codon_start=1

/transl_table=11

/product="Beta-galactosidase"

/translation="MSKIQSQTFQNWMFKACEDQEWMPAHVPGCVHTDLLKLGKIPDP

FYGTNEKEIQWVDKKDWEYRTEFDVNDALLSSQHLELVFDGLDTYADVYVNEQHLLSA

DNMFRVWTVDVKSVVKTSGNVLRIRFRSPINEDLPKLEKLGYALPASNDQSDVGGLGD

KRVSIFARKAPYHYGWDWGPRFVTSGIWREARLEGWTDVRINDVFIQQNEVTAASASL

TAVVEVESTQSIETVIRVGTDEQSWEQAVTLKPGVQTVEVPISIVEPKLWWSRGLGDA

HMYSFHTEVIQGEQAVAESTVKTGIRSIRLVRDSDKAGASFYFELNGVAVFAKGANHI

PNDSFITEVTRERYLHEIVSAAESNMNMLRVWGGGFYEEDVFYELCDEYGLLVWQDFM

FACSMYPGDEAFLNSVKHEAIDNVKRLRNHPSIVLWCGNNEIDSAWAHYIEDGGWGWK

KDYNAEQRERIWADYEAIFHDLLPEVVEAYAPGVDYWPSSPLVSLSGDEKQHAHPSTS

EGDIHYWGVWHNVEPFENYNVHVGRFMSEYGFQSFPEYDSVRKYAEEEDLALESEVML

AHQKNGAGNRLIKQYMDMYMHEPKDFPSFLYMSQVLQAEAMKTAIEAHRRRKPFCMGT

LYWQMNDCWPVASWAGMDYFGRWKALQYYAKRSFSDILVSVDGTKEDVTDIYLISDQL

EPVKGKLQIRLIGFDGTVYREEEHEVALASNSGQQVLSLSQAEWLEGRDAATTLLRID

LKQDGASDITQEHYFAPSKDIALQPAQIKVTEVTENDGVHLVLESDVLAKQVWISTEA

EGVFSDNFYDLIPGIPVKVKFTARGELQGSDGAVSKAGAIQVRSMADFIKL"

CDS complement(2337016..2339010)

/gene="bgaA_1"

/locus_tag="PIKHDJKN_02191"

/EC_number="3.2.1.23"

/inference="ab initio prediction:Prodigal:002006"

/inference="similar to AA sequence:UniProtKB:D9SM34"

/codon_start=1

/transl_table=11

/product="Beta-galactosidase BgaA"

/db_xref="COG:COG1874"

/translation="MNQNRPIQMGVEYYPEHWDPNLWEQDARLMADSGVRIVRVGEFA

WSRMEPADGQFDWTWLDQALDTLHRNGLQVVIGTPTMTPPRWLTEKCPDILPVLASGQ

LYHEGVRGHRCYNSPSMRTYSARIVQKLSERYAGHPAVIGWQTDNEFSFTDCQCTACS

EAFREWVRARYITLGQLNQAWGTVVWSGEYSDWEQVTPPYGGSPFQNPSFLLDYSRFQ

SDSIVAFQSIQIEIIRHNCPDHFVTHNFHSYPQKADQYKIGQDLDFASFDYYPNPSPN

KTDTAPYSGALSLDLTRGIKRRNFWIMEQLSGPPGCWFPMWRTPQPGFLRAYSWQTIA

RGADAVVHFRWRSATIGAEQFWHGLIDHSNVPGRRFNEFQQLCTEVNSLGERLQSSTL

QNEVAILHSHDQLNALRIQPQAEELEYYENIKVWHRALTKGGISTDVIHSSEALDGYK

IIIAPHLYLLDENTAERLQAFAADGGILVLTHRTGVKNDNNVCVMAPLPGMLSACSGV

RVTEYDPIGGDQIQIRDEKGNTYAASQWADVLELDTAQAIAVYANQFYAESAAVTQNN

WGKGAVYYVATQPDEAYLSQLLRTIAEQCGLSEMQSLPDGVQVTTRSGPNGTFRFILN

LSPESVSIPLQASYTSALDGTMKGPRLELGPYDIEILEIQ"

CDS 2339203..2339856

/locus_tag="PIKHDJKN_02192"

/inference="ab initio prediction:Prodigal:002006"

/codon_start=1

/transl_table=11

/product="hypothetical protein"

/translation="MRIHWFMPEPGHAGYTCYPVAYGRYLDPEHREHRPDGIREYNLH

LVFNGQGEVRTEQGSVRLTAGQGFLYAPGQAQQYYANPSDPWDIRWIHLQAHDLERLL

GLRNPDKVWLFSFSGEDRFEELHEQLCQLGAGYESVHEPKLSAVLYELLVELARNSES

IEGTSLLNHQDTIRSTADYIRRHCTQPLSLAEMADTACIILIACSRASWGFRPAGFC"

CDS 2340143..2340820

/locus_tag="PIKHDJKN_02193"

/inference="ab initio prediction:Prodigal:002006"

/codon_start=1

/transl_table=11

/product="hypothetical protein"

/translation="MCNRFSLAADLDEVRDHFKIERVMYYYKNRYNISPTQHTPIILH

QDGERVLDEFRWGFIPFWGRDAVNANLMTVHENPAYYKLVETKRCVIPCNGLYYWRQE

GKKSCAVRVVMPDRGLFGIAGLYEVWKDTRKQPLRTCTMLMAGANMVTREFGSKMPAI

LSEEEINTWLDPANTRVTQLLPLLKSYNSTEMNLYPVTPMVANDEHDCYECVEEMDQK

LAYVRSF"

CDS 2341071..2342201

/locus_tag="PIKHDJKN_02194"

/EC_number="1.-.-.-"

/inference="ab initio prediction:Prodigal:002006"

/inference="similar to AA sequence:UniProtKB:P0DN73"

/codon_start=1

/transl_table=11

/product="putative oxidoreductase"

/translation="MNPKYSPMFEQVSLPSGITLKNRIVLAPMTHMSSNPDGTVSDAE

LAYYARRTGGAGMSVTAVTHVTPNGIGFPAQFGSYDDSFIPGLKRLADTIKQQGSKAV

LQIFHAGRLTPEQAVPAGQVAAPSAVASERPGSPVPRELSDDEITFIIKDFGEATRRA

IEAGFDGVEIHGANGYLIQQFFSPHSNRREDRWGGSIEKRLTFPLAVVDEVQKAVATH

TKLPFIVGYRFSPEEPETPGLTMEETYALVDALKDKNLDYLHVSVNEFWSKPRRGEAD

TRSRIEFILDRVDGKLPIIGVGAIHTADEAAEALQSGVPLLAIGRELIIEPDWVEKIE

SGHEEDIETILTKSDQERLVIPDGLWNAIIHTPGWFPMADDK"

CDS 2342352..2343119

/gene="srlR_1"

/locus_tag="PIKHDJKN_02195"

/inference="ab initio prediction:Prodigal:002006"

/inference="similar to AA sequence:UniProtKB:P15082"

/codon_start=1

/transl_table=11

/product="Glucitol operon repressor"

/db_xref="COG:COG1349"

/translation="MLVAERYEKIVEWVDAQGSMRVTELSERCGVTEETIRRDLDKLE

QAGRLRRSHGGAVSVKYKEEGQSEIPYPERAVTHAEEKRKIADEAVKMVEPGDRIALD

ASTTAWYMAAGLPNIPLTVLTNSIKVAAELSNKEQIRVIATGGQLASKSLSFVGPLAE

RSLDAYHVDKVFLSCKGVHLTKGISESNELQALVKQKMISIADEVILLADSSKFGVQA

FTRVAELSSVAKVITDLGLEEEQVSALNEQSITCVRV"

CDS 2343139..2343597

/locus_tag="PIKHDJKN_02196"

/inference="ab initio prediction:Prodigal:002006"

/codon_start=1

/transl_table=11

/product="hypothetical protein"

/translation="MKHPFHLKATWNGGRNSEGHIDAGGLKTVISIPQEMGGPGTGTN

PDEMLLGAASTCYLITLAAMLERSDITPDELTLESEATVDVTNNVFTYERIIHRPRIV

LKTDASDADVTKAERLAHKAEESCMISRAVAGNVAIETQPVIITAGVNAV"

CDS 2343656..2344246

/locus_tag="PIKHDJKN_02197"

/inference="ab initio prediction:Prodigal:002006"

/codon_start=1

/transl_table=11

/product="hypothetical protein"

/translation="MNLQKSKNSRFARLIRALKLNFLKLLRAPGGAHKVSTGFALGFG

LELIVISTASLVYLVFYPIVRLSGGSLPAAIVGNVVGKLTFLPIILMPLAKQIGSWIL

PAHSMGQGLVHESAFMELFRGNWSALSELLRGGLDILAGMSVFGVILGVISYFVVKFF

YVRALKRRFERRLEKRKQAAMSPTSASVLIRKTSQS"

CDS complement(2344224..2345153)

/locus_tag="PIKHDJKN_02198"

/inference="ab initio prediction:Prodigal:002006"

/codon_start=1

/transl_table=11

/product="hypothetical protein"

/translation="MFNSFLLTLYHVFLPISLPVIGGILLKQFKNWDTRPLSTLSLYI

LSPALIFDTLLHAEITWTDVTGTFWFSIINLIALWALAELLSRVFHLGASEKAGLTLV

STFTNCVNYGLPLVLLAFGQLGLDKASVYVIGQMIIVNTVGIFFAARSEFTVKNAILS

VFRMPSIYAAAIAIILRASNLSLPVALDGGISMLAAGYSPVVLAILGAQMLRPRGSVE

PWKPNVRKAFWTGLVVRLAAAPVLSYLILTALQIEGTLFSVLLILASMPTAVNTVILA

EQFNASPQFVSRCILWTTAASMVILPIMIVMSS"

CDS 2345370..2346452

/gene="pgl_2"

/locus_tag="PIKHDJKN_02199"

/EC_number="3.1.1.31"

/inference="ab initio prediction:Prodigal:002006"

/inference="similar to AA sequence:UniProtKB:O34499"

/codon_start=1

/transl_table=11

/product="6-phosphogluconolactonase"

/db_xref="COG:COG2706"

/translation="MSESKRLLVLVGSYAETENEGIYAYELNEDTGNLSKLDGISGVK

NPTFVNVDTEGNKLYAIGETASAEGNKMSEAVALSIDPSTGKLSLLNRHDSISAPPCH

IQRDPSGRYLILSSYHGGLVGLQALTDNGEVGVLLDEKKHEGKGAHPERQDKPHVHSA

FFSPDGKYMMVQDLGADKIAIYSIDADKNELVLHSETKTHPGAGPRHLAFHPNGQFAY

VINEVDSSITTFRYDVAAGTLTEVSSVSTLPDGYDGSENTTAEIAVSNDGRFVYGSNR

GHDSIVVFAVDAETGHLKLVEHVSAEGEHPRHFALTPNGKLLIAANRDTNNIVSFTVD

QESGRLKYTGHSTGVSKPVCVKPVYL"

CDS 2346856..2347488

/gene="hxlA"

/locus_tag="PIKHDJKN_02200"

/EC_number="4.1.2.43"

/inference="ab initio prediction:Prodigal:002006"

/inference="similar to AA sequence:UniProtKB:P42405"

/codon_start=1

/transl_table=11

/product="3-hexulose-6-phosphate synthase"

/db_xref="COG:COG0269"

/translation="MKLQLALDLVNIPEGIALVKEVEQYIDIVEIGTPIVINEGLHAV

KAMKEAFPNLQVLADLKIMDAGGYEIMKAAEAGADLVTVLGVSNDMTIKGAVEEAKKQ

NKLVLVDMINVSDIETRAREVDALGVDYICVHTGYDLQAVGQSPFEDLRTIKRAVKNA

KTAVAGGIKLETLPEVIKAQPDLVIVGGGITGQADKAKVAAEMQRLVQQG"

CDS 2347494..2348057

/gene="hxlB"

/locus_tag="PIKHDJKN_02201"

/EC_number="5.3.1.27"

/inference="ab initio prediction:Prodigal:002006"

/inference="similar to AA sequence:UniProtKB:P42404"

/codon_start=1

/transl_table=11

/product="3-hexulose-6-phosphate isomerase"

/db_xref="COG:COG0794"

/translation="MSSRQYAVDILKELERTLSQIDDSEMQAMAEHILAAEQVFVAGA

GRSGLMGKAFAMRLMQMGLRVYVVGETVTPGISPKDFLLLCSGSGQTGSLAAMAQKAS

KAGATVGLITIKPESTIGQLADTVVRLPASAKEDTATAGAAVTIQPMGSLFEQGLLLS

MDALVLTMMEMKSMTGADMFGRHANLE"

CDS 2348343..2348702

/gene="hxlR_1"

/locus_tag="PIKHDJKN_02202"

/inference="ab initio prediction:Prodigal:002006"

/inference="similar to AA sequence:UniProtKB:P42406"

/codon_start=1

/transl_table=11

/product="HTH-type transcriptional activator HxlR"

/db_xref="COG:COG1733"

/translation="MAAEVKERINLKEINCEKELTLAVIGGKWKLIILWHLGLEGTKR

FSELKRLIPHITQKMLTNQLRELEEDKLIERKVYAEVPPRVEYTLTDHGQSLMPVLHA

MYDWGKNYGENVIWKSE"

CDS 2349202..2351004

/gene="pepF1_2"

/locus_tag="PIKHDJKN_02203"

/EC_number="3.4.24.-"

/inference="ab initio prediction:Prodigal:002006"

/inference="similar to AA sequence:UniProtKB:P54124"

/codon_start=1

/transl_table=11

/product="Oligoendopeptidase F, plasmid"

/translation="MEKIRTRDEVNQETTWDLRDLFGTDGEWEQELRSLPEAAAHIET

FKGHLGEGAEQLLACLDAREALQERISKTASYARLKQSEDSTNPANIENSAKAGDILS

NLSSSLSFVNSEIVNLPDGTVKRYIEELPGLQPYARSLDRLIQEKAHRLSPETEKVLA

SLGEVLDSPYRIYLRGKLADMTFDDALDGDDNNRPLSWSFYENNYEMSSDTKLRRSAY

AAFSSKLNDYKNTFAEGYATEVKKQVVMSRLRGYDDVTDMLLSPQQVSKEMYNNVLDI

IQQELAPHMRRLAALKKRELGLDKLMFCDLKAPLDPEFSPAITYEEACTLIREALAVM

GPEYGDIVERAFSERWVDYADNAGKSTGAFCSSIYGSHSYILISWANNMRGAFTLAHE

VGHAGHFMLAGHYQRLTNTRPSLYFIEAPSTMNEMLLADHLLKRSDNPRMRRWVILQL

LNTYYHNFVTHLLEGELQRQVYALATKDEPITAKTLSQLKGNILSEFWGPELVIDEGA

KLTWMRQPHYYMGLYPYTYAAGLTASTAAAQLIREEGQPAVDRWLDALKAGGSLDPQE

LMKLAGVDMSGPEPIRAAVAYVGSLVDELERLYS"

CDS 2351114..2351629

/locus_tag="PIKHDJKN_02204"

/inference="ab initio prediction:Prodigal:002006"

/codon_start=1

/transl_table=11

/product="hypothetical protein"

/translation="MTSLSISSSLGWLSMDGFGIFDDMNDVPGFEGNQGGFFAGFNEF

GAMSAFGSIFIGGVFLIIAGVIVYAIISGVRTWSSNNASALLTLHSTIVAKRTRVSGG

SGDSSASTYYYITFELDNGERVELNVDGSDYGMLVEHDRGMLTYQGTRFKHFERDVQP

QSGVSKNSFYT"

CDS 2351876..2353363

/gene="rhaB_1"

/locus_tag="PIKHDJKN_02205"

/EC_number="2.7.1.5"

/inference="ab initio prediction:Prodigal:002006"

/inference="similar to AA sequence:UniProtKB:P32171"

/codon_start=1

/transl_table=11

/product="L-Rhamnulokinase"

/db_xref="COG:COG1070"

/translation="MLAYDLGAGSGRALLGHLNDRGMETMEIHRFKNEPVKAGERLHW

DILRLHHELLEGLNLVKQKGEQPESLGIDSWAVDFGLLGSHGELLGNPYHYRDAQFNG

MMDQVRKELTSQRIFERTGIQFLSFNTLYQLATLKGKGSPLLNEAERFLMIPDLLRYF

LTGEAVNEFTNATTTQLYNPTTGHWDGELLAHIGISETLFGDAAMPGTRVGQLRSSIC

SDLGLSAIPVIAVAEHDTGSAVVAVPATERSFAYLSCGTWSLMGTEIDHPVISSESLG

LNFTNEGGAGGTFRLLKNIMGLWILQECMREWERKGQGISYAELLSRAELAPPFASLF

DPDDERFMPAGDMTSRIQQYCRDTGQAEPQDQGAIARSILESLALKYRRVLEWTEQLS

RQSFNGLHMVGGGIQNRLLCQWTANSIGKPVWAGPAEGSAIGNMAVQWMASGAFKDIW

EARKVIRDSFSVTDYEPKDRPIWEEAYGRFQRLTASPKSQAGSEV"

CDS 2353365..2354126

/gene="ydjF_2"

/locus_tag="PIKHDJKN_02206"

/inference="ab initio prediction:Prodigal:002006"

/inference="similar to AA sequence:UniProtKB:P77721"

/codon_start=1

/transl_table=11

/product="putative HTH-type transcriptional regulator

YdjF"

/db_xref="COG:COG1349"

/translation="MLAAERYDRIVEMVNVKGSMRVSELSEHCRVTEETIRRDLDRLE

QAGRLRRSHGGAVSVKEEQPEIPYRVRETTHAEEKKRIAQAALSMIHPGDRILLDAST

TAGYMAANMPDMPLTVLTNSIQVATELSSRDKIEVISTGGQLAQRSLSFVGPLAERSL

ETYHVDKLFLSCKGVHLEGGGISESNELQARLKQKMVGISDQVILLADTSKFGVRAFA

RVTGLNAVHAVITDQSLDDDLIERLNSYDIKITRV"

CDS 2354194..2354922

/gene="lutA"

/locus_tag="PIKHDJKN_02207"

/inference="ab initio prediction:Prodigal:002006"

/inference="similar to AA sequence:UniProtKB:O07020"

/codon_start=1

/transl_table=11

/product="Lactate utilization protein A"

/db_xref="COG:COG0247"

/translation="MKVSLFITCLSDAIYPRVGEAMVRLLAAHGVRLDFPPVQTCCGQ

PSYNSGYWDETRVAAKTILEAFDDSDFVVCPSGSCTYMIHHYPELFVDEPVWLEKAKR

LETKAYEFTQFLVQVLGITDLGAHFPHKVTYHPSCHGSRLLGVKDEPMALLSAVKGLE

FVPLPYGEDCCGFGGTFAIKMPDISGAMVTEKVDHIKETEAEVLVGLDMACLMNIAGN

LRYRNEPVRVMHLAELLYEGVQTG"

CDS 2354919..2356436

/gene="lutB"

/locus_tag="PIKHDJKN_02208"

/inference="ab initio prediction:Prodigal:002006"

/inference="similar to AA sequence:UniProtKB:O07021"

/codon_start=1

/transl_table=11

/product="Lactate utilization protein B"

/db_xref="COG:COG1139"

/translation="MSQPGVMDVTVKERAELALNDDFLRKAVKFTTERLRNGKKSASE

EHGNWEEWRERGRQIRLHTIAHLDYYLNEFVNNARANGVHIHFADTSVEAAAIALDIA

AHKQASTVVKSKSMVSEEVHLNHVLESAGIEAIETDLGEYIIQLAGEAPSHIVIPAIH

KNRYQIADLLSKEAGETLEPDTTVLAGFVRKKLREKFLEADIGMTGCNFAIAETGSMV

LFENEGNARMVSTVPKTQITLMGMERIIPSWTDLEVMATLLPRSATGQKLTMYMSGIT

GPRRTEDGDGPDEMHIIIVDNGRSLQLGDPEFQELLNCIRCGACLNACPVYRHIGGHA

YGGTYSGPIGAVLTPALNGNIDEWNDIAGASSLCGACYEACPVKIPLHDMLVYLRRRK

VEEGHGNKLESAGMKGFAAVVSNSKRFGAAIRLGQIGQKAVVRNNGISLKLGPLKGWN

SYRVAPSLAKRSFRQQWNKLDQELNQEKKEMDSSVRDRMERILREREGGGGQHEH"

CDS 2356426..2357151

/gene="lutC"

/locus_tag="PIKHDJKN_02209"

/inference="ab initio prediction:Prodigal:002006"

/inference="similar to AA sequence:UniProtKB:O32259"

/codon_start=1

/transl_table=11

/product="Lactate utilization protein C"

/db_xref="COG:COG1556"

/translation="MNTEHQEWLEQLEKKSRAKQEHFMNDIASKLRRPRQTHAPTQPF

RGAPAFWTDLEWDIDKRIQAFTDNFVSVGAHIARVRDISEASDFIANKSHELSAKYII

RQNEQALTDLGLEEQLPDVQISVWNSQTEENWKARAAEADIGVVMADYATAYTGSVTV

LSSPEKGRSVSLLPTVLIIIIPIERLYTRLGETLDRFDEAGRENLPAGIHFISGPSRS

SDIENDLTIGVHGPGIVYGLIMG"

CDS 2357411..2357965

/gene="gpx1_3"

/locus_tag="PIKHDJKN_02210"

/EC_number="1.11.1.22"

/inference="ab initio prediction:Prodigal:002006"

/inference="similar to AA sequence:UniProtKB:P74250"

/codon_start=1

/transl_table=11

/product="Hydroperoxy fatty acid reductase gpx1"

/translation="MSIFTYQVPFMDGHTGDLSVFEGKVLLIVNTASKCSYSRQFTEL

QHLYEQYRDQGLEILAFPCDQFNHKEPGSNDEIAQYCRNHFHISFAIFEKIEVTGQSI

HPLFRYLVDKAPFEGYDMESEEGRWMDQFVKEKHPELYRGDGIKWNFTKFLINRTGEV

SGRYETTVAPIEMESAIRNLLRQA"

CDS 2358345..2361518

/locus_tag="PIKHDJKN_02211"

/inference="ab initio prediction:Prodigal:002006"

/inference="similar to AA sequence:UniProtKB:Q81BF4"

/codon_start=1

/transl_table=11

/product="Bifunctional cytochrome P450/NADPH--P450

reductase"

/db_xref="COG:COG0369"

/translation="MAQISVPQPKTFGPLGNLPQLNTDEPVQSLVKLAEEYGPIFRMD

YPGRSELYISGHELVAEVTDESKFDKRVWAPLEKVRAFAGDGLFTSWTQEPNWKKAHN

VLLPSFSQRAMQGYHNKMIDLAVQLVQKWSRLNPDETVNVPDDMTRLTLDTIGLCGFN

YRFNSFYREEPHPFITSMVRALDESMSSLQRLRLQDKLMITKKKQFEQDIRSMYSLVD

HIIAERKEQPQEGADDLLSHMLSGKDPETGETLDDENIRYQIITFLIAGHETTSGLLS

FAIYYLMKNPDTLAKAQAEADQILKDPVPTYNQVRNLKYIRMVLNEALRLWPTAPAFS

LYAKKDLVLDGKYPLQKGDSVSVLIPKLHRDRDAWGDDVEEFRPERFEDPSKVPHDAY

KPFGNGQRACIGQQFALQEATLVLGMVLKHFEFIDHADYQLKVKETLTLKPDNFTIRV

RARGGQPVMAVPGVAVEEPTPVAKRQEPDTANAHHTPMLVLYGSNLGTAEGIAREIAD

TARYQGFRSEVATLDDRVGKLPKEGVVIIVSASYNGQPPSNAKDFVEWIEHADAGEFQ

GVRFSVLGCGDHNWASTYQRIPRLIDEQLSSKGAERLSPIGESDASGDFEKQVGDWTE

QLWPDLARTLGLKLNTSSGSERSSLSVQFVSGLAVTPLADTYDAHVAEVLENRELHDA

GSERSTRHLEIKLPEGATYKEGDHLGILPQNPSELVERVLKRYGFTGTEHLVLDASGR

SAAHLPLHQPVNLVDLLSHSVELQEAATRAQLRELAAYTVCPPHKKELEALLDESVYM

EEVRKKRISMLDYLVKYEACELPFERFLELLPSLKARYYSISSSPRVQPEQASITVSV

VRAPAWSGQGEYKGIASNYLANLKPGEEVVMFIRTPESGFELPENAEAPIIMVGPGTG

VAPFRGFLQARRVLKEQGQQVGEAHLYFGCRNPEHDYLYKNELETAEQEGLVQLHTAF

SRVDGEEKCYVQHLMRDDAHKLIPLLEQGGHLYVCGDGSKMAPDVEATLKKAYADVSG

NSVEQADAWLERLQQEGRYAKDVWTGI"

CDS complement(2361748..2362149)

/locus_tag="PIKHDJKN_02212"

/inference="ab initio prediction:Prodigal:002006"

/codon_start=1

/transl_table=11

/product="hypothetical protein"

/translation="MNWHQLHTGKTLQQRLAEFQGHLAELNAPLSGLEPGIISKVFPR

NFIKVNRQLFIPLSLFSIRVFDVPRARRGIRVGIRTVFPSGNAFNNIRLVGVGLDYIE

LQGKGKFSSRILFPLTQVESIYRPTNKKKKS"

CDS 2362270..2362614

/locus_tag="PIKHDJKN_02213"

/inference="ab initio prediction:Prodigal:002006"

/codon_start=1

/transl_table=11

/product="hypothetical protein"

/translation="MDVEMYKDLIRDERGNYYIAVQIEGNELTLVNAFVEASFTPELI

YNEEFRNKHKEMEGGFVGKIAMDLLRHDVVMGLKQIDRKLLELSEVEQKYTVNYIDTI

EFYRHPAWERKA"

CDS complement(2362777..2364423)

/locus_tag="PIKHDJKN_02214"

/inference="ab initio prediction:Prodigal:002006"

/codon_start=1

/transl_table=11

/product="hypothetical protein"

/translation="MTEALLNKLRQISIHDHVQPGKPEDQKLIQEWQASGVRFSASGG

RLEATYYSALEKLLTCIVPLNGSEPILQEGGIYLGCWLESTGTINAELLSRLIPSVSE

TTYLAFARHQREDGLLPYKLTENGPSFRQIQLVTPLARSVWNHYQLHGKNRSFLKTMY

KAMVQYDEWIATYRNTRGTGCVEAFSTFDTGHDLSPRFWHVPDTPYMNDAASYHPDSP

VLPFLAPDLTANMYCQRMYLSRMAEELGESGGEWKAKAEASLESLFRYCYDEEDMFFY

DRDRNDEFVRVQSDVLLRVMACEVGDRELFDNMLRRYLLNTSKFFAKYPFTSMSMDDP

RFDPFSSYNSWGGASNFLSLIRAPHAFEYHHRYVELTWVLQPILSSLSKATRFAQALS

PWTGEEGFTETYSPSILCLLDYVERLCGIMSVGSDQLWFTGLLPIDMDHGEEVAGHTA

YSRTVNGLHFELVNTPERVTVYQDGKILMEGPAGIRLITDQGGHLEGVIGMSVRTIEG

ELIYRGKSIPIRIKGNELQRYAEGSLKTERDIGIIYPSYR"

CDS complement(2364447..2365763)

/gene="yesO_4"

/locus_tag="PIKHDJKN_02215"

/inference="ab initio prediction:Prodigal:002006"

/inference="similar to AA sequence:UniProtKB:O31518"

/codon_start=1

/transl_table=11

/product="Putative ABC transporter substrate-binding
protein YesO"
/db_xref="COG:COG1653"
/translation="MFKRLMITVIASLLLLVTACSGTDHTGADEQTGTKTAEDGQVEL
RMMWWGDQKRADITNEALKVFQEKHPNIKIVGEFAPSSGYFDKLNTQLASGTAPDIFF
LGGNVVDYAKKDVLLNLDPYVGSELNLDGMDATMVEYGRLDGKLQHISAGANARGIVV
NQALFDQAGIPLPASDWTWEDYAAISKELSDKLGKGYYGTYNFTVDGMDIFLKQRGKQ
LYDMKNGTLGFAKEDILEWFTYWEQASKSGGVVTPELQVSNPPDDTSKSLLITGKAAM
SLLPSNQLAAFQSLTEDKLILLPVPRGPKGTGVVFESSQGLSGYANTKHAKEVSILMD
FWINDPDAAKILGNDRGVPVTEANRNLLQQEAGPVEEIVYNYTSFVSEATKTEPFDVS
YNPPGFAEFSKLAQTTNQEIGFGRKSVEQAVTDFYNGTVRIFESNQ"
CDS complement(2365843..2366682)
/gene="araQ_12"
/locus_tag="PIKHDJKN_02216"
/inference="ab initio prediction:Prodigal:002006"
/inference="similar to AA sequence:UniProtKB:P94530"
/codon_start=1
/transl_table=11
/product="L-arabinose transport system permease protein
AraQ"
/db_xref="COG:COG0395"
/translation="MPLKRRLVQAGRHAAIILFGLLMLYPVLWLILSSFKPNHLIFTN
SSLIPTSFTLDHFVNGWKGLQGVSFGRFFGNSALISVMSVLGNVISCSLAAYAFSRLK
FKFKGLWFSIMLVTIMLPYHVTLVPQYILYNELHWINTYFPLILPKWLAQDSFFILLM
VQFIRGIPRELDESATIDGCGQSQIFFRIVVPLLVPALITTAIFTFIWSWDDFFSQMI
YLSKIDLFTVQLGIRSLFDPSGQSDWGALLAMSTLSLLPVTIIFLVFQRYFLEGIATT
GLK"
CDS complement(2366688..2367602)
/gene="lacF_6"
/locus_tag="PIKHDJKN_02217"
/inference="ab initio prediction:Prodigal:002006"
/inference="similar to AA sequence:UniProtKB:P29823"

/codon_start=1

/transl_table=11

/product="Lactose transport system permease protein LacF"

/translation="MRKLSKPLYGLQNMTAYLFLLPWLIGLVCLTLGPMVASLYLSLT

KFNLLTSPTWTGLSNYVQIFTEDDTFRRSLGLTFYYVFLSVPLRLAFALLVAMALNKG

IRGLGLYRTVYYIPSLLGGSVAIAIVWRQIFEGSGLVNQFLSWFGITGPAWIAHPDYV

VYTIVTLSVWQFGSAMVIFLAGLKQIPADLYEASDVDGAGKVRQFFGITLPMLSPVIF

FNLIMSMINSFQAFTPAYVIGDGRGGPLDATMFYTLYLYLKGFSFFDMGYASALAWIM

LVIIGVFTAIVFLTSKFWVFYGDNQEGR"

CDS complement(2367726..2369288)

/gene="rhaR_16"

/locus_tag="PIKHDJKN_02218"

/inference="ab initio prediction:Prodigal:002006"

/inference="protein motif:HAMAP:MF_01533"

/codon_start=1

/transl_table=11

/product="HTH-type transcriptional activator RhaR"

/translation="MYNVLIADDEIEVREGLKLKVNWQDLGFVISGEAANGIEAEDLL

KAEHFDLLITDMNMPVMDGVQLLDVCRSLNPSIQIMIITGYEDFHYARAGVRSQAMDY

LLKPVTRDELKTTLNKIKDELDKKTQLHGDSEMMQWRLSQYYKEMKQRFLLDLVRGHR

LPPSSLPERMRLFHLESWQDHSVCFITAGMRNSGIQTASVERVPEKLYLPFELLCHEM

AQSYPDNVQIFHDGAHPGIMHFITLEGIQHEFAQQITAQTRSVLTMDVQVGLGLSVSG

FERWKEGYIHSLLAWNSAGRKGAQDRVPVVDHSPLLPEETSRMLHRCLIRGELDSFRS

VIRKELADSFRISTSHMIRSLFQISLLFEQESSWISPEWVLWLKTPDQAELLLMHWAE

DFVNQQQPSEVGEDTVIESAKRYIEESYMLELTLTSLAEQYNYNSTYFSEIFKEAAGI

SFIQYVTEVRMKHAIRLLKETQLTVWDVTELTGFRSPSYFSSKFKKMFNISPSDYRLL

HSEKIDNHDPKK"

CDS complement(2369263..2371080)

/locus_tag="PIKHDJKN_02219"

/inference="ab initio prediction:Prodigal:002006"

/codon_start=1

/transl_table=11

/product="hypothetical protein"

/translation="MFFSLKSRLIAFIVVLFVLCFGTLSFLLFKESRTVIRSYIESSA

LEKMEEYGSYVDMVQMQIYDVASLVFNSDIAKNWDNAITDPNLPEGEKMLVSLAMSRF

LTQATNSYTSISDVSIYRRNGLRIGSENQVTTDPLFLQESWYKNFFISGNHWLPAHTD

QHENIRDHGNPVVSLLMPLGTFHHATAQNVMKINVSESYFLEPLNRIHLAESSTIFLL

GEQGSPILSQQANQLGAEALAEIDKIRNNSLKSGVVYLNNDEGQRDILVYKKLGRTGW

MLAGLAPEKELYSSLHKLQSTILVVTIVLILVSLFAAAWLSYGVTKPLTRLVLAMRQV

QRGAFDQAESLLPPDKNVKSEVSYVISTFRYMISRLRQHIQNEFELKLLRQQAEYKAL

LMQINPHFMFNTLELVSSLAMQRRTDDTIQVIEDLGKMMRFSMNTSDDRVQLAEELAY

IRHYISILQTRFGHKLDITLTQEGRLDSLVIIKFILQPLIENAVKYSFQHQTTAEVHI

RISRRNNRLHFNISDNGPGIPAEIIQKLHDPIETSQLEPLLRSQSWHIGLGNVVARCR

LHYGSLFAIQIENGEHNGTSIELILPAQEEYDVQRIDRR"

CDS complement(2371189..2372478)

/gene="yteT_2"

/locus_tag="PIKHDJKN_02220"

/EC_number="1.-.-.-"

/inference="ab initio prediction:Prodigal:002006"

/inference="similar to AA sequence:UniProtKB:O34371"

/codon_start=1

/transl_table=11

/product="Putative oxidoreductase YteT"

/db_xref="COG:COG0673"

/translation="MSNKKRYVLVGTGGRAEFFYGALAQNFRETSELAAFCDINQIRM

NYANQLLREKYKYNEVPTYPADQFDQMIENEKPDFVIVTSIDRTHHKYIIRAMELGCD

VVTEKPMTIDEHKCQDILDAVKRTGQNIRVTFNYRYAPHHTKIRELIQNDTIGKVTSV

HFEWLLNTRHGADYFRRWHRDKRNSGGLLVHKSTHHFDLVNFWIGSQPETVFAFGDLM

YYGRENAEERGVTQFYTRATGNPLAQEDPFALHLDSDAHLKAMYLDAEPEDGYQRDQS

VFGDGINIEDTMGVLVQYQNKAILTYSLVAYQPWEGYRIAINGTKGRIEMNIVEQSYV

NSLGDKNLEGALIGKTLRVLPMFDAPYEVQVEEKEGGHGGGDPVLLNDLFGEPAEDPY

HRAANQLDGARSILTGIAANRAIATGLPVRINNLVRF"

CDS 2372604..2373488

/gene="rhaS_11"

/locus_tag="PIKHDJKN_02221"

/inference="ab initio prediction:Prodigal:002006"

/inference="similar to AA sequence:UniProtKB:P09377"

/codon_start=1

/transl_table=11

/product="HTH-type transcriptional activator RhaS"

/translation="MIQTFRADYNDPTGYLNIEYDRRIGYFSMTDDHLHDHYELYYLL

SGERIYFIKDRTYRVQAGDFIFINRNTVHKTMESGRPDHDRIVLYIKPELFTELAILP

ELAEGLKEPFGWNIPILRLPSQECETADRMVSEMIDEMVHTRAGSSLLLRHRSVELLL

FAYRNKHLGTVHSADSEPVLHPKAQAVVRHLNDNYQQALALPEVAALFRISPHYLSRL

FKQTTGFTFSDYLNLLRVKEAQRLLRESEDSITEIALRAGFSNFSHFGKMFKRTVQVS

PRTYRQEYREASSMRVLK"

CDS complement(2373657..2374601)

/locus_tag="PIKHDJKN_02222"

/inference="ab initio prediction:Prodigal:002006"

/codon_start=1

/transl_table=11

/product="hypothetical protein"

/translation="MSAPLFRDPIFDGAADPVVVWNRSEQTWWMIYTNRRATAGGPKF

AWVHGTDLGVASSANGGQTWTYRGTLTGLEHEWGHNTFWAPEIFWHEGTYHMYVSYIQ

GVPHDWAGHDRHILHYTSMDLLSWTFHGKLELSSDRVIDACIYPLPNGKFRMWYKDEA

NSSHTYAADSPDLYHWEVIGPVITGRAHEGPNVFRFNDSYWMIVDEWRGQAVYKSDDL

EHWEWNSLILDQPGNREDDGVIGLHADVVVQDDEAYIFYFTHPDRSEGIDHASEPARR

SSIQAARLDVIDGVMVCPRDEELQMKLRPENDTSQISV"

CDS 2374968..2375414

/locus_tag="PIKHDJKN_02223"

/inference="ab initio prediction:Prodigal:002006"

/codon_start=1

/transl_table=11

/product="hypothetical protein"

/translation="MIQTRLDELGIVLPQASTPAAKYTNAVIVNGIMYVSGKGPDTRE

HGKLGDQFTTEQGYEFARNAAIEVLAVVQEVLGSLDRVKRVVKVQGFINATASYEEHH

KVLNGFSDLMLDVFGEQGVHARSVFGAVSVRDNLPLIIDSIFQVEE"

CDS 2375420..2376010

/locus_tag="PIKHDJKN_02224"

/inference="ab initio prediction:Prodigal:002006"

/codon_start=1

/transl_table=11

/product="hypothetical protein"

/translation="MTNPASINPLMLSFPESFDTPRLTIRAPRWGDGAAVNEAIRESA

EQLRLWLPFAENIPSLEESEANARKASLQFLERTDMMLHLRDRFTDEFVGSSGLHRID

WNARCFEIGYWIRTSRAGEGLMTEAVRGIEQFAISYLEANRLEIRCDARNVRSAKVAE

RAGYTLEGVLRKMRRDSTGTLVDMMVFAKVRGDEFE"

CDS 2376321..2376467

/locus_tag="PIKHDJKN_02225"

/inference="ab initio prediction:Prodigal:002006"

/codon_start=1

/transl_table=11

/product="hypothetical protein"

/translation="MEERVMHLFMPSYKRSQLGQEQITNMQDSHRTIGIQGNECMIAV

GEHQ"

CDS 2376507..2377319

/locus_tag="PIKHDJKN_02226"

/inference="ab initio prediction:Prodigal:002006"

/codon_start=1

/transl_table=11

/product="hypothetical protein"

/translation="MRRIGQGRTAEIYEYPSNQIMKLYHADFPTEAVQNEFRITEAVF

QKGLPVPQARLFMDDVSRKGIIFERIEGNTMLSLMIQEPVLIEELSRQMAVCHHSLHA

QQDDEGAFPTQKQILTGAIRNTSLLSEEDKTQVIAYLSSLPERKQICHGDFHPDNVML

NEMRDQYWVIDWMTGMSGDPAGDVARSWVILMSGTLPEDTDPAVHKGFELARNVMLDH

YIQHYIQISGISRQELDAWILPVAAARLDERLPAVEADQLIKLVQDRIRLLK"

CDS 2377363..2379672

/locus_tag="PIKHDJKN_02227"

/inference="ab initio prediction:Prodigal:002006"

/codon_start=1

/transl_table=11

/product="hypothetical protein"

/translation="MLNALKAIQSYKSGHLPEGEQPLWLQLLAAAEQPNINLERIHSI

AELEGTNPVLDYIERTLQVLEQLQVSFWMREILEDVLVWSETAKAGSPKQRRKWQKQG

VNLFVHNVGSAQLYDMYGSVGNLHGKQGHREEENSSRSESPSDLSNDDPSDSTLIAQT

AYGSPTPRYEIIRTLIATHGLIGQYIRGEIPFAENAPLHSFITQGWLTADELQAILMA

LNECIIAGVDPSLWNQVQAEVQRIVGWIITEPHHTDWNVKERLSRLRSSSIRQGEAMD

EAYAKLQAQLEIEKLLAPLAHRTLWYVESAMHDFSLQEMVKIFLLTLHSEAMVLSVDD

LIMNSTGTVIVADEEAVGLNLTSQEQQMDVVRHISFEPLMNTMYYDYKGVKKLNIYKK

RMIEKYLEQYSWEQIEAGETITYPHLTHRIERHPDLRDTVFVTFEFSPAAEKLIAFCI

EAEKSPLYEKAVLLLFDLFGLRRDAYDRFHNEETYLADMNSSGDYKKVLLDYMVGKRV

LDIGPGGGILLDLIEQERPEMEPIGIDISANVIEALERKKQREAHRWQVLKGDALQLD

QYVQPGTVDTVIFSSILHELYSYIERDGRRFNRDTVVAALRSSFNVLSPGGRILIRDG

IMTEPEAQKRRIRFLEPDGMRWLERYTQDFQGRSIEFERISDNEALLDINDAMEFLYT

YTWGEEAYVHEIQEQFGIFTPSTYEHCIREALGEQAEIITFRHFLQEGYTEALGERII

FMDEQGQPASLPDSTCLIVIEKKKGMADR"

CDS 2379669..2380256

/locus_tag="PIKHDJKN_02228"

/inference="ab initio prediction:Prodigal:002006"

/codon_start=1

/transl_table=11

/product="hypothetical protein"

/translation="MTNEANTALYFVRHAESEYIEGQERERGLTEQGKRDAASVAGLL

HWEQIQSFYSSPYRRAVDTIQGLAEVSGGMVVTEEDLRERELSGPEVKHEHFREAKQR

LYDDPAFAYSGGESGEVAVSRAIAVIQLILDRHPGQKIVIGTHGDIMTLIFNYYDPFY

GYDFWANTTMPDIYKLEFDREGRLVQVTRLWEEQV"

CDS 2380298..2381248

/locus_tag="PIKHDJKN_02229"

/inference="ab initio prediction:Prodigal:002006"

/codon_start=1

/transl_table=11

/product="hypothetical protein"

/translation="MLNLKLIGSVLEQQIRFNDNKSLFYNRGKSTLGPSPRTLCWLLE

YRAQVAKALERIFTDGLLNHFTIKLAEQGIRHFIQVNQFMQFTSKDKLQLAELYKDQF

MRIWSLLQNEGAGEEVVQQFFQKHYQQLRTFLIQTNGEELFKKYTMDPHLFWVPCEEY

SADLQVSWLGLDVEHLHPPVLDLGCGPQANLVQFLRGKKIEAYGMDRQVEESEERPFL

RRMGWMESSFEQEQWGTIISHLAFSNHFTHHHLKADGEPEAYAQKYMELLRALKPGGS

FFYAPSVPFIESFVDQLPQYFVCRTWITPTQQMTRITRLD"

CDS 2381268..2381627

/gene="dinG_2"

/locus_tag="PIKHDJKN_02230"

/EC_number="3.1.-.-"

/inference="ab initio prediction:Prodigal:002006"

/inference="protein motif:HAMAP:MF_02206"

/codon_start=1

/transl_table=11

/product="3'-5' exonuclease DinG"

/translation="MKITFMPDTYTVEELKVSHLQDRTYCIFDLEGTGINPAEESVTQ

FGAMYYKKGESLDSSFSSLVRASKAIPEAVAKLTGISNEDMIQASSFAEVYHAFLELI

GDKVLVTQAGYEYDCPC"

CDS 2381621..2381959

/locus_tag="PIKHDJKN_02231"

/inference="ab initio prediction:Prodigal:002006"

/codon_start=1

/transl_table=11

/product="hypothetical protein"

/translation="MLKRHCDMNGLPMFTNPVLDTKAMFTYIHPEISEVISTDFLIRY

YELNTDGIHRHNALADCGVIARIFEHVLDEYQELQLDHFTADGERVMKRFVIPEMYLT

TPIGGEKPDE"

CDS 2381952..2382326

/locus_tag="PIKHDJKN_02232"

/inference="ab initio prediction:Prodigal:002006"

/codon_start=1

/transl_table=11

/product="hypothetical protein"

/translation="MSRMDADKLLADMNEGRILSQQTFEGLEVETYLDQRDASEFADV

WMQAFERFAQKSDVVEEEVLRACRELAFKRTIALAGDPELAGYVSDDIGLIGAALLQD

EMQDSFVMQLLERYQQGTLPLR"

CDS 2382507..2383508

/gene="yfiY_3"

/locus_tag="PIKHDJKN_02233"

/inference="ab initio prediction:Prodigal:002006"

/inference="similar to AA sequence:UniProtKB:O31567"

/codon_start=1

/transl_table=11

/product="putative siderophore-binding lipoprotein YfiY"

/db_xref="COG:COG0614"

/translation="MVRFSKGFKGWTIILLLMVVVISGCGANSESKAEQAPASETTVS

DGSTSETTEKEAETRVVQDEFGDVTIPVHPQRIAGIYVEDYLKALDITPVVQWYNPMW

GVQEYLDLDVPQFDTTGSIEALLQYDPDLIIVDGGVDMEKYEMYSKVAPTYRLPEDIL

QDSNQILTTIADVVGKPDKGKEVVAAFEAKIADAKAKLQEAVGDETVAVVRLNISNNT

LALFGVKNRFTGFIYSELGLTPHPLVGNMEEYQEILSEEAFPELDADHIIVFPSNGEW

SSPENKEALKVLDSKLWKSLPAVKNNHVYMMERSYWQSGAITANTMKIDDLLQKMTP"

CDS 2383609..2385264

/gene="rhaS_12"

/locus_tag="PIKHDJKN_02234"

/inference="ab initio prediction:Prodigal:002006"

/inference="protein motif:HAMAP:MF_01534"

/codon_start=1

/transl_table=11

/product="HTH-type transcriptional activator RhaS"

/translation="MCPTIVQLIPKPWLCMLQHMKYMDYSQVDIANRQTYHMTSSHAL

FVVTGGHGSIDTLDNRDTRLEKGMIMFVPAGRTIVIEGSSELQYYRLELMVAEVKEDT

SSRLSIKDETVQQGLPVLISDQDRLPLIELNYSPWSSCLKSLEQISRGETAHDWLDQW

EMQLRFQEWFRVLLRQNDPEIESPDDRARLQSSIRYIREHFDQPITVDDLAAEVRLTR
SSYTRQFKGITGKLPLDYVNAVRLEHSKLLLQMTDDRLHDIAQNVGFNNEYYYGRRFK
QYAGVSPGVYRRHYRQEVRVFAPYLEDFVLALGIKPILQTSHRSWGRQQYLGLEDVPE
FDVTQLDVGFNETNVPEFIMLNNGYQRWNLERFEQVAPTFYMEHEGEDWRSILKSTAD
VLGKVNRVQDVIGAYEDKAVEAKGQLARHARGQTVALLRISSSEIILYGDQYGYVGPV
LYQDLGLTPHFRVQQWASQSRRISIGLEQLCQLEADHLFITFDNMYSAYPEEERKLLE
RDEWKRLPAVKSGHVYEVDFMTWMNYGVISHGKKIDDILRFMA"

CDS complement(2385758..2386426)

/gene="yedK"

/locus_tag="PIKHDJKN_02235"

/EC_number="3.4.-.-"

/inference="ab initio prediction:Prodigal:002006"

/inference="similar to AA sequence:UniProtKB:P76318"

/codon_start=1

/transl_table=11

/product="SOS response-associated protein YedK"

/db_xref="COG:COG2135"

/translation="MCGRFTITDPIEEIMDRYYASIAEGFEYKPNYNAAPMQFIPTII
GSKDGNRLGALRWGLVPNWAKDEKIGNKMINARAETLTEKPAFKRLISSKRCIIPCSG
FYEWKKDGSVKQPMRILMKDDSIFSLAGLYDTWIDPEGNKLSTCTIITTEPNSLMADI
HDRMPVILRPQDEADWLNKESDKESVLRLLRPHEADEMIAYKVDSAVGNVRNNNEELI
KEVS"

CDS complement(2386679..2387056)

/locus_tag="PIKHDJKN_02236"

/inference="ab initio prediction:Prodigal:002006"

/codon_start=1

/transl_table=11

/product="hypothetical protein"

/translation="MFTQVGQIMLYVNDQDKALQFWTEKAGFHIVNEVNGNGMRWIEI
APVKDAQTTIILHDKEFVAKMSPELNLGTPSLMLFTDNLDQLYTHLSNKQVTVGEIVT
MPGGRVFNFADDENNYFAVMEKN"

CDS 2387308..2389575

/locus_tag="PIKHDJKN_02237"

/inference="ab initio prediction:Prodigal:002006"

/codon_start=1

/transl_table=11

/product="hypothetical protein"

/translation="MTKRVVLLEEGTAEMKGLMGSKGAGLAALLQAGWPVPAGFIVTK

EGCRAFCTRLAHLSAEGAQEIGRAIQHLEKQSGKFFGDSSVPLLLAVRSSESCSCGTA

SHALLHVGLNDVTVEGFARQTNDRQYALNCYRILLQDYGQLVHDIPYALFEEKLGDIS

ILDEAKLEFAIAEYKRLIEERGHQPFPQDVQLQLKEAMRAVSDSQRVLKPNSQQELRK

PSHFSDETQGAAVLIQVMVNGERSDRSGIGTVYSRHPGTGERGMTGDYVSSAASWRSN

EGLDQLRSEESELYDLLQDACGYLESHTREVQEIQFVVDSGTLYLVEISEARLTSQAT

LRSTVDLVDEGVITKEDAILRIQPWHVTELLKASSNLSPELQLLLEWADEMKGLTILS

NVDHPKDAVKARSLGAEGIGLCRTEQLLLSASRFPFVQKMILADSEAERKRGLDRLLP

MQQADFEQLFEAMDGYPVTIRLLDPPLHELLPDIERLNERREQLALWKLEENNTEVQE

LERVIRRVLELHEQYPLLGQQDCRLGTVFPEIYDMQLEAIFRAAVKGIRQGQWVRPEI

MIPLVGHANELQVMRDLVDHVADQVLGEEKRHCLYRVGARIEVPRAALTAAPIARHAD

FFSFGTDELTQMTFGYSRHNTEKPFLHFIEPQRSLPSNPFQVLDIDGVGQLVEMALVQ

GRIRKPHLKAGICGENAADPASIAFCHRIGLDYVSCLPEQVPLARIAAAQASLLAQKM

DPNENRQDGDISTIA"

CDS 2389680..2391617

/gene="ltaS2_2"

/locus_tag="PIKHDJKN_02238"

/inference="ab initio prediction:Prodigal:002006"

/inference="similar to AA sequence:UniProtKB:O34952"

/codon_start=1

/transl_table=11

/product="Lipoteichoic acid synthase 2"

/db_xref="COG:COG1368"

/translation="MFNSKNERTSSRTLFAVLFILMLLKLSLLRYFFFQGLSGIGFVT

DALGALTVVCILDLIVPKGWKRAVYAGFNVLFSLVLFAATLYNVHFSSVPTYTALSEL

GQVAQVRGSIGPLVRPEHFLFFVDIVLALPVWFILRSRRASVRNSSYRDSGLHFGRSR

RRYWGKLGVALTAAFCIVLSGSFIVKGETIDNELVRAENLGFLNYQVSSAILTSKENE

AIANGNINETIDKINQLVSKYPYQDKSSQGTAIKTKYFGQAKGSNLIVLQLESFQNFP
INASLDGQVLTPVLNDLAKESYYFSHFFQQIGQGNTSDAEFMSNTSIYPTGVVPMSAG
YSDRDLPSLPKLLGKEGYESETFHVNDVTFWNRNKMYPALGFNRYFDKPSFKNDKFND
FGPSDEELYRVGVEKMAAHQAANQPFYAQFITASSHSPFTVPADRARITIPATITNTL
LHDYLQAINYTDYAVGQLIDELKANGLWDNTTLVIYGDHFGLPANDEITQQIQTNLGV
PYDGKVSRFNIPLLIHTPKQAKGQVIEQPGGQLDILPTVMNLMGVSLKEEKFTAFGHD
LLNMDHNSFGIRYYLPTGSFVNNDIMFIPGAGFDDGTAYSLKTYEPVTDLEPYRSDYE
HVLSLMKLSDEYVKLLPKRAP"

CDS 2391800..2392807

/locus_tag="PIKHDJKN_02239"

/inference="ab initio prediction:Prodigal:002006"

/codon_start=1

/transl_table=11

/product="hypothetical protein"

/translation="MKLSVLEHGHINEGRTVQDTLQETVTLAKHADELGFSRFWMSEH
HGSGALSFSSPEVMIAHVAAHTDRIRVGSGGVMLPHYSAYKVAENFRLLEALHPGRID
LGIGRAPGGMPIASRALNEGKSSNVQFFPQQIADLGGYFYEQLPEDHRFASLVAGPSV
PTVPEVWLLGSSSEGARIAAAQGTAYAFAQFFGTPGGEEAMKHYRRHFKPSILNDQPH
SMIAVSAFCAESEEAAAELARSNELFFLRLGRGLEQNSFPSLETVNNYPFTAMEMEQI
RQRRSFSIVGTPDQVKDKITAMAERYEADEVIIASAIHSFEDRLRSFSLIAEAFNLKQ
D"

CDS 2393005..2393652

/gene="apaH"

/locus_tag="PIKHDJKN_02240"

/EC_number="3.6.1.41"

/inference="ab initio prediction:Prodigal:002006"

/inference="protein motif:HAMAP:MF_00199"

/codon_start=1

/transl_table=11

/product="Bis(5'-nucleosyl)-tetraphosphatase, symmetrical"

/translation="MLGDYVDRGPKSKEVVEQIMQMHEQYNIIAIKGNHDAMMVKALN
HDVEEYDKHWIRNGGLQTLASYVEVSLDDEQLDWDVYTEAKRWLRTHYEHHLRFLEQL

PLVYEIPGYIFVHAGINPEVEDWRTQSERDFIWIREPFYSRPTSLEETVVFGHTPVKN

LHNEPGVWFDPSGDKIGIDGGCAYGAQLNLLEISEDGSVQTLFVKRGETRQGPEI"

CDS complement(2393649..2393885)

/locus_tag="PIKHDJKN_02241"

/inference="ab initio prediction:Prodigal:002006"

/codon_start=1

/transl_table=11

/product="hypothetical protein"

/translation="MDFDKVTLHCKLDEILKKHNNMSVLKLSEEIGHRRTTIMELLHN

TNMDKKKISASLIVKLCVYFDITPNDLFEIRRVD"

CDS 2394049..2394693

/locus_tag="PIKHDJKN_02242"

/inference="ab initio prediction:Prodigal:002006"

/codon_start=1

/transl_table=11

/product="hypothetical protein"

/translation="MRDVTPMVNQQAVIHIVRKKPVWTELAAIPDGIHFYHITDSLGR

KWRSIGQHITDAIEVFGFGYREPWTIILEQAPYDSELSVEELLRKLNVTMYDEELICD

MHIILNTLDRRNEFVQRILDINPHSIFQLLYELKAEYLVQDSISEVTFKQKYKLNPIE

ALTFYFLENVDWYTYRQWIEAGGTAELCIRLRNDNPYISLTEAIERAEQNLCSL"

CDS 2394842..2395774

/gene="ppaC"

/locus_tag="PIKHDJKN_02243"

/EC_number="3.6.1.1"

/inference="ab initio prediction:Prodigal:002006"

/inference="similar to AA sequence:UniProtKB:P37487"

/codon_start=1

/transl_table=11

/product="Manganese-dependent inorganic pyrophosphatase"

/db_xref="COG:COG1227"

/translation="MEKALIFGHKNPDTDTICSAIAYADLKTKLGQDVEAVRLGEVNG

ETQFALDQFKVAAPRLIKTAANEVNKVILVDHNERQQSVSDIEDVTVVEVIDHHRIAN

FETSQPLYFRAEPVGCTATILNKLYKENGVEISAPIAGLMLSAIISDSLLFKSPTCTE

QDVAAARELAAIAGVDADSYGLDMLKAGADLSQKTIAELISLDAKEFAMGQAKVEIAQ

VNAVDVNDVLVKQPELEAAIEAIISSKGLDLFVFVVTDILNNDSVALAYGASTKAVEK

AYNVTLSDSRALLKGVVSRKSQIVPVLTEAFNSL"

CDS 2395898..2396389

/gene="infC_2"

/locus_tag="PIKHDJKN_02244"

/inference="ab initio prediction:Prodigal:002006"

/inference="similar to AA sequence:UniProtKB:P03000"

/codon_start=1

/transl_table=11

/product="Translation initiation factor IF-3"

/translation="MIKNEKIKATEVQLTGLNGEDLGIMSTRDALALAKQHKVDLICM

SLMTSPPPCKLIGAGAAKQEKQQEKKKAAKSPDKRKVKEIRLNLQMEDHDRETKQSQA

ERILTKGDSVKLVIQVHGAKEGTAGKEWAEQLCKSLAEYGSKTTGIQVSGKQVVVQLD

PIG"

CDS complement(2396576..2397403)

/gene="tgl"

/locus_tag="PIKHDJKN_02245"

/EC_number="2.3.2.13"

/inference="ab initio prediction:Prodigal:002006"

/inference="similar to AA sequence:UniProtKB:P40746"

/codon_start=1

/transl_table=11

/product="Protein-glutamine gamma-glutamyltransferase"

/translation="MIIVANQPIELVRSDWSAFAWHWLQQLQNSPTVYTYQSMDHLHF

EWTLRESLVDAAEALDRSGVSFASFEKSRCNPAYWNRNAEGGFELRAEVTPAEGIRDI

WKNGHLYAFECATATVIVLYGGVLGSIREDDFNSLFRNLLLYDWHYDSDLRLTEKNGS

DSALPGDVLYFKNPDVSPETPEWQGENTIMLREDLYYGHGIGIASGEEIIRSLNQNRI

PGSTVSAYLMDTVIYPDFLYLSRYAKNSNANSQTASSTPVLPGQLYVRIGGSRYLRT"

CDS 2397678..2400425

/gene="rcsC_6"

/locus_tag="PIKHDJKN_02246"

/EC_number="2.7.13.3"

/inference="ab initio prediction:Prodigal:002006"

/inference="protein motif:HAMAP:MF_00979"

/codon_start=1

/transl_table=11

/product="Sensor histidine kinase RcsC"

/translation="MSNPIVENRSLFEQLYTHAPIGIAVASHVNGHWLQLNPAFCEML

GCSEDELIDSPIVHMIYEEDLEMEEFRSKFWDMTNGLSLMYETELRLKRKDDSLLWAT

LRACIVRDEARGEPLYLLVQAADISKQKDAESRLIVQRKQLEESSRISRLLTESSLDL

IAIHQADPERTFKYVSPASQSMLGYDPEEVVGQSGLFCIHPNDVPLVEAYVAGQIQGL

APDRMSYRLLHKDGSVVWADTITHYLYDKQGNLQEMIAVTRDITASKQQQLSLQEYQS

LFDSNPLGVASLDLHGNLLKANAGQEQLTGHTKEELLSSSFDHLIDPVDLDKTRHHFE

ETVKGTAQSYEVGLIHKDGQRIETSVINVPILLEDRVVGVYGITSDITESKRYVEEIE

NLSYERALILNAMSEGVIGLDQNGNLIFANPIATEMMDFCPSEMNGKPLEEIMLQMQS

EGIPYPSNGTPILQAVREGRSLPRSESVFWKQDGSSFLAEFQLKPIMEQGDNRGAVLV

FRDMTSVKDIIRAKEVAEHADRAKSEFLAIMSHELRTPLNGIMGMAHLLKETELDEEQ

NGFADIIIDSGESLLHILNEILDFSKIEAGKMDLERQPVDIKSVLGGVMELFALRASE

KNIELYCEMSDQIPERVRGDETRIRQILINLVGNAVKFTEKGSIQVHVNVKPAEFYNE

NHLILLVSVKDTGIGIPFNKQHQLFQSFSQLDPAINRKYGGTGLGLAISKKLVELMGG

AIGVQSETGEGAEFQFTLLLERWQDETTDPIEEVADSDLKLDRTSLAAGIKILIAEDQ

SLNSHLLEEMLRKLGGVCDIVENGAEAVKALERETYDLLFMDIQMPVMDGIEATCRIR

QTHPEIPVIAAITAFAGANDREACLKCGMQDFISKPFSSTEISRVLNTWVPYIRSHHE

R"

CDS complement(2400524..2400736)

/gene="khtT_2"

/locus_tag="PIKHDJKN_02247"

/inference="ab initio prediction:Prodigal:002006"

/inference="similar to AA sequence:UniProtKB:O07535"

/codon_start=1

/transl_table=11

/product="K(+)/H(+) antiporter subunit KhtT"

/db_xref="COG:COG0490"

/translation="MNIRECPLPGIGVKYQFDTKSGNKLVIIVHEDGRRELFSVDPND

NEELTLIAELEDDECVTLSGLIGGWS"

CDS complement(2400879..2401619)

/locus_tag="PIKHDJKN_02248"

/inference="ab initio prediction:Prodigal:002006"

/codon_start=1

/transl_table=11

/product="hypothetical protein"

/translation="MSTRIPYYRQLLFACLLTLMLFILSACADGEAHVTVNMDGSSDL

DLNLSVTDSALGMIGQDNLMTLLADTLKQNNFQAEVSDQGDRTQLKATTHYEKSNTVS

TFSTSDLPDGIKVEQSTTPGFFTSKLHIAAEAELLQTIPDGEVKDQISKVPGFLKNLL

LKDVNFDFKLTLPIKAQDSNADLVEDHGKTLIWHVSPLNTNKLDLTVQVPNIRNIIIT

GGIALVLIIALLIWFMVRRRRTRQGRNV"

CDS 2401959..2402051

/locus_tag="PIKHDJKN_02249"

/inference="ab initio prediction:Prodigal:002006"

/codon_start=1

/transl_table=11

/product="hypothetical protein"

/translation="MTYIIIMGIIMVVGMVGMGWFYKIMDNDQH"

CDS 2402253..2403059

/gene="celS"

/locus_tag="PIKHDJKN_02250"

/EC_number="3.2.1.4"

/inference="ab initio prediction:Prodigal:002006"

/inference="similar to AA sequence:UniProtKB:P16630"

/codon_start=1

/transl_table=11

/product="Endoglucanase S"

/translation="MGLKMKKRSGKKAWMLLVMSLLIAAVPITASAASDSTAYAKLNF

SNNKYYIFNNSWGSSSVSGWSQSVYVNSNTDLGYVWNWPTTAGGVKAYPSLVSGWHWT

EGYTPGSGLPTRLSANKSINTGVSYSKASNTNGEYNNAYDIWLHDIGNATWSSTPTEE

IMIWNEWTSGVGPIGSKVFSDVSIGGQTYDLYRGEITDNGVHQWWVYSFLKKSKSPNN

FSINVKNFTDYLISKSYFTNSKYVSSVEYGTEITKGNGQINYSNWYVNVQ"

CDS 2403322..2405016

/locus_tag="PIKHDJKN_02251"

/inference="ab initio prediction:Prodigal:002006"

/codon_start=1

/transl_table=11

/product="hypothetical protein"

/translation="MKFSEYTYTRPDLEQIKTSFRELLSGFEASTTVEEQSGYMDKIN

ALRSDFETQAQLVYIRHSIDTNDTFYKAENEFLDESSPIIQEYITDYYRALVNSKFRA

ELEQKWGSQLFQLADLSLKTFSPEIIEDLQKENKLSTEYNQLIASAKIPFEGEERTLP

QLHPFELSTDRTMRERASEARYTFMAEHEAEFDRIYDDLVKVRTQIAKKLGYPSYVEL

GYDRMNRTDYNAEMVSNFRAQVRDYIVPVATKLRERQRSRIDVDTLYFYDQGFSFKTG

NPTPKGDADWIIDNGKKMYAELSPETDTFFQMMTDNELMDLVSKKGKQGGGYCTFLND

YKVPFIFSNFNGTSGDIDVLTHEAGHAFQVYESRHFEVPEYNWPTYESAEIHSMSMEF

FTWPWMELFFKEDTDKYKFDHLSSGLLFIPYGVAVDEFQHFVYANPDATPAERKQAWR

NIEKTYLPHINYKDNAYLEQGGFWHKQGHIFSSPFYYIDYTLAQICAFQFWKRSNQDM

KSAWADYLTLCKAGGSLSFTGLVELAGLNSPFEDGCVSSVIGDIEAWLDAVDDKAL"

CDS 2405327..2406184

/locus_tag="PIKHDJKN_02252"

/inference="ab initio prediction:Prodigal:002006"

/codon_start=1

/transl_table=11

/product="hypothetical protein"

/translation="MLVLLDQPSQRKQLAPIISGTNGHVVMGGVLAGLQCGRVYADQL

HAPKSALIWAKNEMFYLIGRSDNEQFNRLVSEVLVQELLPEALGAGEDMLNLEVYPEP

GSGWSPVLDHMFNGRLREGLRVPFQFNLAKYEQWLERSSSSLFVPPGYELQVIDQAVM

LEDKSCIIEHEILKFWHSVEDFFRYGAGTCTVCEGQVVGTCISVFVSGIHHETGINTY

DPIHRGKGLATAMASAYVQFVLERDCVPHWTTEDFRHDSISIAGKLGFEQGRAYPVYY

MPIQELLNC"

CDS 2406456..2407607

/gene="adhB"

/locus_tag="PIKHDJKN_02253"

/EC_number="1.1.1.1"

/inference="ab initio prediction:Prodigal:002006"

/inference="similar to AA sequence:UniProtKB:P0DJA2"

/codon_start=1

/transl_table=11

/product="Alcohol dehydrogenase 2"

/translation="MATHAYYVPPVNLMGRGCLHEAGKMIEKMGIRKALVVSDRRLIS

SGVAEQVLSILRKSGLDYVVYDEVQPNPTCQNVHDGLHVFQDHGCDAIISIGGGSPQD

AAKGIGIVATNGGHIRDYEGLHQSKHKSVPLVAINTTAGTSSEVTMNYVITDEERKVK

MVMVDRNSLVDLSVNDPELMLSKPASLTAATGMDALTHAVEAMVTPGGFTVTSATAAA

AVELIFEYLPRAVRDGSDLEAREHMTYACFLGGIAFNNAGLGYVHAMAHQLGGVYDLP

HGVCNAMLLPYVEELNAKHVPGKFRHIAKAIGMDVKGKRDEECSEYVIEAIRQLSKEV

GIPEKLSELGVRDPDVELLADNAMKDACAPGNPYQPSRDEVMELFRKII"

CDS 2407817..2409436

/gene="gsiB_2"

/locus_tag="PIKHDJKN_02254"

/inference="ab initio prediction:Prodigal:002006"

/inference="similar to AA sequence:UniProtKB:P75797"

/codon_start=1

/transl_table=11

/product="Glutathione-binding protein GsiB"

/db_xref="COG:COG0747"

/translation="MSQFKSSTRKSLWVGALAVLLLVIVSGCSGSNNDPAPNATAQPG

TSQGTDENSKTDETATGGTFVYGRPASVTSFDLHNQITSNNAFAIDKVFEPLVAFDNK

GEIVDWLAKSHNISDDGLTYTFVLRDGLKFSNGTDVTAEDAVFSLERHLKVGGPLAIS

AKVDTVKATDSHTLVITLKEPYTPFISELSNFSNGIVPNNFGGVTEEEFFKKPIGTGP

FVVEQWDPAGDVTFTKNTHYWQEGKPSVDKLVYKLIEDDSQAINQLKAGEVDAIESLA

LQNADEIKNGADTTVVTSGSWVTEQLFFNTLDKHFSDVHVRRALALALDRDGLTKALT

FGYAQTANSLLPTTIPYNSNDTLKALNFDVNAAKAELAKSAFPDGFTTKLLVASGNST

RAQEAQIIQAAGKQIGINIEIESIELATFRERFFAYDFAAMLNSGQADSPEANSIIAF

QTDPDGFSKSYWTHYTNDEVTKLLYKGQQTPDGDGRAEIYSKLLQTLADEVPYIPLYY

PDILIGVRSSVDGIVVLPNGSVRFEDVRIQK"

CDS 2409433..2410377

/gene="ddpB"

/locus_tag="PIKHDJKN_02255"

/inference="ab initio prediction:Prodigal:002006"

/inference="similar to AA sequence:UniProtKB:P77308"

/codon_start=1

/transl_table=11

/product="putative D,D-dipeptide transport system permease

protein DdpB"

/db_xref="COG:COG0601"

/translation="MKKFWLISTVGRALAVILCVMTAVFFLIRMVPGDPAKMILGEYS

TPEAVESMHHALGLDLPLGEQFIRFVKTLFTQGDTGNSIINGTSTRELIADRAPVTLL

LIGMACALAIVIALVLATLAATHKDKLLDHLIRIFPTVTLGMPIFWVGILLILLLSVH

LHWFPVGGVGEGWWGTLYSLTLPAITVAFSQIPTLVRSLRAQMLEVLESDFVVTLKAA

RLPSRVILFKHVLRNAALPTLILLGVNISYLIGGTLVVEQVFGIKGIGSLLFSSISKR

DFPVIQGIALYCAVSVVIISLLIELLSRWLDPRTKGKP"

CDS 2410374..2411240

/gene="gsiD_2"

/locus_tag="PIKHDJKN_02256"

/inference="ab initio prediction:Prodigal:002006"

/inference="similar to AA sequence:UniProtKB:P75799"

/codon_start=1

/transl_table=11

/product="Glutathione transport system permease protein

GsiD"

/db_xref="COG:COG1173"

/translation="MKTIRTEPQLYEDSQNTSLMNRILKTPTLLVGSLMFAVLIILAV

IIPWISPYDPSEQNLSAFLQPPSAEHWLGTDQLGRDLFTRLIYAARTDLSIMVLAEIV

PFCMGVFLGMLAGYYGKWIDKVISLVTDTLIAFPFYLIVIIVAFASGAGERGIYITFM

LVGWIVFARVVRGLSASYRKQEWIASAQTLGLPGIRIILRHLLPNVLPQAIVVLMTDM

VGLLVAIVTLGYLGIGIAPPTPDWGTMISDGQSFITTAWWLSAVPGLAVVYTGIALSL
VGDGLADVWRKK"

CDS 2411240..2411992

/gene="oppD_4"
/locus_tag="PIKHDJKN_02257"
/inference="ab initio prediction:Prodigal:002006"
/inference="similar to AA sequence:UniProtKB:P24136"
/codon_start=1
/transl_table=11
/product="Oligopeptide transport ATP-binding protein OppD"
/db_xref="COG:COG0444"
/translation="MSTDPILEVKALSLSTKSNQKLVQDVHFSLGRGESLGLVGESGS
GKSLTLRSILGLLPSGVEQTGGAIRSDVNSSMVFQDPRGALDPLCPVVKQLAEVVYYR
QKLSRKASRIAALELLELLGLPDSLKREDRYPSQLSGGQCQRVVIALALACKPGILLC
DEPTTALDVTVQRQIMETIQCLQRELGFAMVFVTHNLAIAATLCSKLCVMKQGRIVEH
GASLDLLQNPQDPYTQMLINSVLPLPKLEGSE"

CDS 2411995..2412780

/gene="oppF_5"
/locus_tag="PIKHDJKN_02258"
/inference="ab initio prediction:Prodigal:002006"
/inference="similar to AA sequence:UniProtKB:P24137"
/codon_start=1
/transl_table=11
/product="Oligopeptide transport ATP-binding protein OppF"
/db_xref="COG:COG4608"
/translation="MEFSLQVQNVTVQYGDFTALDNIKLNLQKHTTLGLVGESGSGKS
TLARVIAGLITPDEGQILLGDQQLKKKRSREQYKSIQMIFQNPDASLNPKHSIRQILA
EALLFHRIVDRSQVEKRSRELLARVKLESRALDKYPHEFSGGQRQRIAIARALSVEPS
LLIADEPTSALDVSVQRSVLDLFSSLKKELNLTLLFISHDLGVIHAISDTVAVMRQGQ
LVEISPKDQFFTRPETAYSRELLSAVPKMPESSSLGGLYEPTI"

CDS 2412764..2413822

/gene="ppaT"

/locus_tag="PIKHDJKN_02259"

/EC_number="2.6.1.30"

/inference="ab initio prediction:Prodigal:002006"

/inference="similar to AA sequence:UniProtKB:Q988B8"

/codon_start=1

/transl_table=11

/product="Pyridoxamine--pyruvate transaminase"

/db_xref="COG:COG0075"

/translation="MSQQYKGNIPLTLSEYDTLTQLLSRLLSTKYPPVIIPGEAILGI

EAVAAGISAPGRTIVNVVTGPYGSLFGQWLERGGATVIEVKVPFDEVVPVEKVAAAIE

KYQPSALSFVQAEVVTGGSNPAEEIIKIARAHNLITVSDSVSAIGGEPLLVDEWGVDF

VAVGAQKALAGPNGISAVSISPRGWEFLESNAHAPRNSILSLLDLKPSGEGAAPVRVP

PNIPTLEARALILALTAIESEGLEQVIKRHEQAALSAIAGIQALGLEPWQKDNRTYST

LTTTVRIAGEEKLHIDQPIGIVAPGDGELFGQLLRINHFGGNASLAGVEEAVATIAAL

LNEDHEQAVQAVRLVWGE"

CDS 2413819..2414967

/locus_tag="PIKHDJKN_02260"

/inference="ab initio prediction:Prodigal:002006"

/inference="similar to AA sequence:UniProtKB:P9WL23"

/codon_start=1

/transl_table=11

/product="hypothetical protein"

/db_xref="COG:COG1228"

/translation="MNMTQVKSEEQSFKHIFIAGIEGKRFDISIQNGRFASVTESVTQ

NDNPSPPGTDLWISPGVIDLHTHLAWTDFDHADQLKRDSHEVEEMQAQAFAATLRTGV

TTARDAGGMLPSTARHLAQHYQHPLRVETSSEMLGAADARGVKHLEQRLNGIFDTGAG

WIKIMATGGLGAPTEQVLDPVFSEEEFAFIVRHAHAHHKKVLVHTWGGVTIDWSIQAG

VESVEHGMFLSEDQASRLAQSRTAFVPTTSIYRIAADPKGVLALPTVISDRAARATEA

HSTAIGYAKRAGVRFGFGTDYATPSLHGYNLQELDTLIDYGLTRAEAWQSATTNAAEI

LGRGNELGQIAEGYIADAIIFNADPYQTKNADQLRESIVSVIIGAQKP"

CDS 2415460..2416629

/gene="glgC_1"

/locus_tag="PIKHDJKN_02261"

/EC_number="2.7.7.27"

/inference="ab initio prediction:Prodigal:002006"

/inference="similar to AA sequence:UniProtKB:P39122"

/codon_start=1

/transl_table=11

/product="Glucose-1-phosphate adenylyltransferase"

/db_xref="COG:COG0448"

/translation="MAKKEVVAMLLAGGQGKRLKGLTKSLAKPAVYFGGTYRIIDFPL

SNCSNSGIDTVGVLTQYEPLVLHSYIGIGSDWDLDRKNGGVYVLPPHEREDGSNWYRG

TADAIYRNLNFIEQFDPEHVLILSGDHIYKMDYEKMLQYHKEKDADCTISVIDVPLEE

ASRFGLLNTHDDYRIYEFEEKPPEPKSTLASMGIYLFKWDVLKRFLIQDEQQASTSYD

FGKDIIPLLLENEKSLYAYPFEGYWKDVGTIRSLWESNMDLLDEETPFNLNDPDWRIY

TRNPNQPAQYISPSAKVRNCIISEGSVVHGEVNHSVMFYGIEVGENSSVTDSVIMPKV

KIGQNVRIHKAIIAEGLVIPDGVHLSPAPDDESDILLVDQEELERQLLEGMTSKA"

CDS 2416657..2417760

/gene="glgD"

/locus_tag="PIKHDJKN_02262"

/inference="ab initio prediction:Prodigal:002006"

/inference="similar to AA sequence:UniProtKB:P39124"

/codon_start=1

/transl_table=11

/product="Glycogen biosynthesis protein GlgD"

/db_xref="COG:COG0448"

/translation="MKPLIGVINLDHELEELKELTYFRCGAAVPYAGRYRLIDFVLSN

MMNAGIESIGVFVRRKYRSLMDHLGDGKPWDLDRKHGGMFILPPDWNDPTDTSQGDLQ

HFHNNLDFFRRGSGQYVVHAGSRHVTKADLQDVYRYHISKGADVTLVCKKVDQLLPEH

DACVKVEDDGNGNVVDIHQSADHPNIYTEIFIMEKELFLRQVQRCIAHGESHFFRDVI

QKNPDGLNIAAYAYDGYHAVINSIDSYYRNSLELLNSGLYEQLFKEQPVQTKIKYEAP

AKYLDTAEVKHSLLANGCIVGGEVEDSILFRGVHVAKGAKIKGSIIMQKCYIGEGAVL

ENVILDKDVKLSGGQTLIGDPSNPYILAKSTII"

CDS 2418094..2420526

/gene="glgP"

/locus_tag="PIKHDJKN_02263"

/EC_number="2.4.1.1"

/inference="ab initio prediction:Prodigal:002006"

/inference="similar to AA sequence:UniProtKB:P39123"

/codon_start=1

/transl_table=11

/product="Glycogen phosphorylase"

/db_xref="COG:COG0058"

/translation="MFDNKETFKSIFRRNLVSKLGKPLAEATQEDVYHVLGSMIREYA

GQDWAASNQGFKQRQDKQVYYFSLEFLIGRLLGNNLLNVNELELVRDSLAELGFSLEE

VEEQEADAGLGNGGLGRLAACFLDSLASLGYAGHGCGIRYKYGLFEQKIINGNQVELP

DNWLEKGNEWEVRRPDKKVEVQFWGRVEAYEQDGHYQFVTKDAESVVAVPYDVPVIGY

GQPHVNTLRLWSAEPKRETSLDTPSNYYGYLDYSRSVESISEFLYPDDSQYEGKLLRL

KQQYFMCSAGVQSALRTFNKLELSYDRLPDKVAFHINDTHPTLVIPELMRILIDVKGY

GWDEAWDITTRTVSYTNHTTLSEALEKWPVAMISKLLPRIYMIIEEINKRFCGMLLER

YPGDQDRIQHLAIVANDQVRMAHLAIVGSHSVNGVAALHTEILKEREMAPFYALYPER

FNNKTNGITHRRWLMHANPKLSNLITDTIGSEWIREPGRLNELVGVADDASFQQQFHS

IKRNNKERLAAYILDHTGKAVNPDSIFDVQVKRLHGYKRQLLNILHVMHLYNRLKSDA

SFDIVPRTFIFGAKAAPSYYFAKKIIKLINTVADTVNRDVAVKDRLQVFFLENYSVSL

AEKIIPAADVSEQISTAGKEASGTGNMKFMMNGALTIGTMDGANVEMAEQVGQDNMFI

FGLSSDDVLDYYRSGSYLPNEIVQQDERIREVVEQLVHPGAFCERDGEFWDIYDSLLA

HGDEYFVLRDFAAYADAHAAIDSAYRDVAGWTRKAVLNTAQSGIFSSDRTISEYATDI

WGIHPVSGYWKG"

CDS complement(2420638..2421447)

/locus_tag="PIKHDJKN_02264"

/inference="ab initio prediction:Prodigal:002006"

/codon_start=1

/transl_table=11

/product="hypothetical protein"

/translation="MSINRDKLQETLDPTLRVRPRVGWGVLTYFLYSAVFYAIFLLNG

IDYTRVGESERTLLLWYIAPLSGGAVLTITMVTIYGWWRPSLVEGRHLSRWTTTLPII

MMVIAIVNMLTGDFTRVTPLMWVYLIFGSIMVGFNEEMINRGQLMVALRSRFGETGVW

LLSTAMFAVFHLPNMFWGIGAGALFQVIIAFGLGSIFYLARRSTGSLVASIFLHGFWD

LSVFSANGASALPIAGLLAPLLVIAAVITVPIILRREKRRDSAQSVSRPVG"

CDS complement(2421469..2421648)

/locus_tag="PIKHDJKN_02265"

/inference="ab initio prediction:Prodigal:002006"

/codon_start=1

/transl_table=11

/product="hypothetical protein"

/translation="MILTIKELVEGRGDERLDVRNLCIKLRSIIGTLSKDFAGLYLDL

IEVKIIRKQEQMKKY"

CDS 2422078..2422536

/locus_tag="PIKHDJKN_02266"

/inference="ab initio prediction:Prodigal:002006"

/codon_start=1

/transl_table=11

/product="hypothetical protein"

/translation="MNERSWFHSRWMLAIIACMAVLYMLIMGNLLFVSGRTPGVNYQY

NLVPLETIKPLLFEKNRYHTEAWVKNLFGNIVLFLPLGIWIPWLFRRCRKFLPFTGIV

VLLLFAVEITQLITRVGSFDVDDIILNTVGAWIGYIGFALFIYSQGRTRN"

CDS 2422657..2424348

/gene="malL_1"

/locus_tag="PIKHDJKN_02267"

/EC_number="3.2.1.10"

/inference="ab initio prediction:Prodigal:002006"

/inference="similar to AA sequence:UniProtKB:P29094"

/codon_start=1

/transl_table=11

/product="Oligo-1,6-glucosidase"

/db_xref="COG:COG0366"

/translation="MVYIVWWKESVVYQIYPNSFKDSDGDGQGDLQGIYDKLDYLTGL

GVDVIWICPIYDSPGHDNGYDIRDYYSILRKYGTMEDFDRLLAEAHKRGLKIMMDLVL

NHTSDEHAWFAESRSSKVNPKRDYYIWRSGKNGQVPNNWESYFGGSVWKHDPETNEYY
LHLYTEHQPDLNWNNAQMAEEMYGMVHWWLQKGVDGFRFDAVAHIAKAEGLPSAHNPD
NLPVVPAYQLFSNLEQVHSILNKLNDMILKPYGPMTVGETSGLGPEQALAYVGTDRNE
LNMVFQFEHMFVDAKSSGIGKWNYREWKLPELKEIMSRWQTVLHDRGWNANYMGNHDQ
PRPVSRFGDDGRYRVRSAQMLATWMLTLEGTPYIYQGEEIGMTNVAFPDIEDYRDIET
MNYYRQHIGEGRAKHEVMQAIWRKSRDNARTPMQWDDTKHAGFTDGEPWIQVNENYPE
INVEDAKRDPQSILHYYRKLIALRKQHKVLIYGVYELLLPDDPDIYAYTRTLDDEQML
VILNFREHESEMQWPEGWNDENAKTVISNVSRRYSTDKGAILLQPYEARVYRMQR"

CDS 2424438..2425130

/locus_tag="PIKHDJKN_02268"

/inference="ab initio prediction:Prodigal:002006"

/inference="similar to AA sequence:UniProtKB:P99149"

/note="UPF0173 metal-dependent hydrolase SA1529"

/codon_start=1

/transl_table=11

/product="hypothetical protein"

/translation="MLNITYHGHSSVQLGTEEKSLIIDPFLRGNELAVTKPEDIKTDA
VLLTHAHMDHILDAEPIAKANNAKVVAIVELATYMSWKGLDTLGMNMGGTVDLGFAQA
KMIQAFHTSGIVLEEEQRIMYAGLPAGYIINIGGKTILHAGDTSLFGDMKMIGDRHDI
DVALLPIGGHFTMGPEDALQAAEWFNAKLTIPVHYDTFPVIRQDAEKFVEQLASKGLE
GRVLAPGQSLTL"

CDS 2425371..2426723

/gene="dctA"

/locus_tag="PIKHDJKN_02269"

/inference="ab initio prediction:Prodigal:002006"

/inference="similar to AA sequence:UniProtKB:P0A830"

/codon_start=1

/transl_table=11

/product="Aerobic C4-dicarboxylate transport protein"

/db_xref="COG:COG1301"

/translation="MSFVKSLFFQIIVAVIIGIGVGILWPNLGSLLQPLGTGFIKLIK
MIIAPLIFMVIVTGIAKIGDLKSVGRIGFKAILWFEIATTVALVLGLGTANLLRPGAS

MNVDPSTLDASGIEAKTNGSELPHVVDFIMNIIPTSVVDAFAQNALLQVLLVACLFGV

ALAATESKAKENVLALIENVLGILFRMIGYIMKLAPIGAFGAMAYTVGAYGASTLSSF

GLLIIACYGAALLFLVMLALAAWWITGLNFLQFVKYTRSEVMLAIGTGSSEVVMPRMM

DKLTKAGCDRAVVGLVVPTGYSFNLDGASIYLSLATVFLAQAVGIDLTLGQEITILLV

LMLSSKGMAGVPGSAFLALSATAAALNAFPVAVVALLLGADRFMDTMRVFTNLMGNCV

AAFVVAKWEGLLDQKRMRAVLSGEISAEELEREEQASLSSVKLNKREQGKGMVSTDIL

"

CDS 2427036..2428757

/gene="treA"

/locus_tag="PIKHDJKN_02270"

/EC_number="3.2.1.93"

/inference="ab initio prediction:Prodigal:002006"

/inference="similar to AA sequence:UniProtKB:P39795"

/codon_start=1

/transl_table=11

/product="Trehalose-6-phosphate hydrolase"

/db_xref="COG:COG0366"

/translation="MSSTNTTSSSWWKTSTVYQVYPKSFNDTTGSGTGDIRGLTEKLD

YLQHLGIDIVWLQPVYVSPQHDNGYDVADYYRINPDFGTMEDFDELLKGLKARDMKLM

IDIVVNHSSTDHEWFQQSRSSKDNPYRDYYIWKDPAPDGGVPNNWQSKFGGPAWQYDE

QTGQYFLTLFDKTQADLNWENEQVRKAVRDMIKFWAEKGVDGFRMDVINLISKDQRFP

DDDGSVSPGDGRKFYTDGPRVHEYITEMYEEVFGPYNMVTVGEMSSTTLEHCIKYSNP

ASREFSMTFNFHHLKVDYPNGQKWELMPYAFEAMKQLFSEWQTGMQAGGGWNALFLNN

HDQPRALSRFADDGDYRAESAKMLATTIHGMQGTPYVYQGEEIGMPNPVWNDVSEFRD

IESTNMYRLLQEERGKSAEEAFKIVKERSRDNSRTPMQWDGSDNAGFTTGTPWIKVDE

RYPSIHVAQQMADPNSIYYHYRKLIALRKQVSVLTDGLYERLDDAHPDVFAYARTNGS

ETLLVVSNFSKREVKFAFSEAVWNDHIVGKSAELLIGNTEVSPALEQEISLSPYSSYM

WLVPQQD"

CDS 2428787..2430799

/locus_tag="PIKHDJKN_02271"

/inference="ab initio prediction:Prodigal:002006"

/codon_start=1

/transl_table=11

/product="hypothetical protein"

/translation="MAIDKKQVDEIVRAVGGKENIEAATHCVTRLRFALYDESKVDTE

SLEQNDLVKGQFSSQGQFQVVIGPGLVDKVYDEMIQITGGERASKDDVKAVAGKKQNP

IQRAIKTLSDIFIPILPAIITAGLLLGINNILTGPGIFFDGKSLVDVYPAWKDLASII

NTIASTAFTFLPALIGWSAVTRFGGSPLLGIVLGLILVHPDLLSAYGYANAVNEGTVP

TWNLFGWHIEKIGYQGQVLPVLVSAYLLAKLEIFLNKRVHDSIKLLVVAPVTLLITGF

LAFTIIGPVTFAIANGITSGLIYIYDSYAALGGLIYGGLYALLVITGMHHTFLAVDVQ

LIGSQGGTFLWPMLALSNIAQGSAALAMMLVLREKKMRGLAATSSVSAFLGVTEPAIF

GVNIRYRYPFIFGMVGSAIGGVLLTMNNVQATSIGVGGVPGFLSIFPNKWGVFFIGMA

IVLVVPFVLTVLFGRAKLRKEDRNAGQATVAGGQTPTAKSASSTTTTNAVAEPRTRSA

AQVADEPVNTLEIMAPLTGKAVPLEQVPDPAFAEKQMGEGVAIEPSGNQVVAPFDAQV

AHVIKSKHAVILEHASGLQILIHVGINTVSLKGEGFNMHVETGEKVRAGQTLLEFDRK

VIEAAGYPLITPIIIPDGQDMVDRVEVTTGDVTSNQNGVLKVHLKG"

CDS 2431074..2431928

/locus_tag="PIKHDJKN_02272"

/inference="ab initio prediction:Prodigal:002006"

/codon_start=1

/transl_table=11

/product="hypothetical protein"

/translation="MTRVAVMVIHGLGMQKENYADTLISRLHKELDQVMVLPGAAKQM

LDIEPVFWADVFEDREEALFQQLVSSQGLNYQVLRRFVIHYLADAVAYQPVENQGHNY

DAVHYTLNRAMHALAQRNGPEAPLCIIAHSLGAVIASNFFYDLQYPSSRTPSIVDVTS

ALERGDTLTSFYSFGTTLPLWSLRYHDFSRPIQVPSPLAGQYFPGLEGEWINFYDRDD

ILGYPLRPIDPAYEAAVNEDVEINSGGLIGSWNPLSHGGYFSNGAMNRRIAQGLARTW

TWVNRGSD"

CDS 2431958..2432221

/locus_tag="PIKHDJKN_02273"

/inference="ab initio prediction:Prodigal:002006"

/inference="similar to AA sequence:UniProtKB:P0AFT8"

/note="UPF0153 protein YeiW"

/codon_start=1

/transl_table=11

/product="hypothetical protein"

/translation="MECRTGCAACCIAISISSPIPGMADGKPAGVRCVQLTEDNRCGI

FGQKERPAVCSGLQASEEMCGSTDQEAFERLSWLEQETEPSSA"

CDS complement(2432320..2433432)

/locus_tag="PIKHDJKN_02274"

/inference="ab initio prediction:Prodigal:002006"

/codon_start=1

/transl_table=11

/product="hypothetical protein"

/translation="MHKLGSHVVFRFFLSLGVSVLGGLLFTAIHTPIPWLLGPMIFML

VGSQIAKLPLMWPTSIRDYGILIVGYSIGLTLTGEALQGILHQLPMMLLMTLLLIGLC

TLTAYVASKLTDFDFPSLLVGSIPGGLSQMVSLADEMKSINLTLVTFLQVTRLIMIVF

CVPFLLFSPWIGGTTGGGTDQPMIETATWAALFPEILLYSPLCIAGAWVARKLRFPTA

FMLGPMIVMCVIQLSTTMHTPNLPPSLLNISQLMIGSHVGLMLRPQQLQRKMQTVTLA

ITSSVLLIAGSLGLSYILMKMFSLSAATSLLSMAPGGMDQMSIMAHEVNANLSVVSGY

QLFRILFIFFIVSSLLKMILMHLLRKKEASTHSSVT"

CDS complement(2433596..2434927)

/locus_tag="PIKHDJKN_02275"

/inference="ab initio prediction:Prodigal:002006"

/codon_start=1

/transl_table=11

/product="hypothetical protein"

/translation="MRLLQQIHAEIRGWSRNIQLFFLASILYQIGNGMFSVLYNLYIQ

GLGYNDTMNGQIVSIQSLATAIMFVPIGLCGDFFSRKRLLITGALFSGIFLIGRSFDY

SASGLIWFAVFSGLFAGVFQVLAIPFLAENVKKSQRLKMFSYYSCLVLASQVLGSLGG

GVFADLLHTAGLAKVTGLQTVLFVGGAATLVAFIPMLFVSEDKKAPQADTLAQPVTES

ATSATDGKQDVVHSAEQGSKNDTLSQKKDSRLIGQFVITQLLIGFGSGLVVPYLNLYF

TNRFSVSLSAMSLLISLGQIMTIVSMLIGPTLAAKVGSVRAVVIFQVMSLPFLLLTGF

TNLLLIASVSFLFRQALMNAANPIQSAILVDRVSDKRRGIANSLMQTAFMIGWATMGP

VQSYLVTTYGTYWGYAITFSITGSLYVISSLMYYMMFKEPKPSARVLAANR"

CDS complement(2435005..2435709)

/locus_tag="PIKHDJKN_02276"

/EC_number="2.1.1.6"

/inference="ab initio prediction:Prodigal:002006"

/inference="similar to AA sequence:UniProtKB:O07431"

/codon_start=1

/transl_table=11

/product="Catechol O-methyltransferase"

/db_xref="COG:COG4122"

/translation="MNNVNGSAQQKTWTQVDDYLNALLIPSDSLLEQALRTNAEAGLP

AHDVAPNQGKLLQLLLQIQGATRVLEIGALGGYSTIWMARALPENGRIVTLEADPRHA

EMARTNLTRAGLMHKVDLRVGPALTTLPDVQEEYREPFDFIFIDADKPSNPDYLRWAL

RLARPGSLIIGDNIVRDGEVINANSTDNRVQGVQSFLKLIADNPRLEATALQTVGTKG

YDGFVIARVLESSTAK"

CDS 2435848..2436744

/gene="cmpR_2"

/locus_tag="PIKHDJKN_02277"

/inference="ab initio prediction:Prodigal:002006"

/inference="similar to AA sequence:UniProtKB:Q55459"

/codon_start=1

/transl_table=11

/product="HTH-type transcriptional activator CmpR"

/translation="MNLHGLRLFHAIVRYGGVTRAAEELNISQPAVSSQVKKFERELG

IPLFATEGRRLVLTDAGMQLTGYAERLFMLEQDVENFVQDFRAGKKGLIRLTATYLPS

NFLLPGWIARFKQMHEDVELVVSTTNTRMAFDQLLRYEAEIAVYGGSGITHSGVHWDE

LFEDEMWFVVHPDHPYAGKEIELREMVAEPFIMREEGSATRERLVSLCTTNNLAAPRI

ALQFNGLNETISAVKAGYGANFISSLVVKEDVQQGRLARVFVRGVLLKNTVAVCTRAG

EVLSPAAQHLVELIRQEASLMK"

CDS complement(2436774..2437517)

/locus_tag="PIKHDJKN_02278"

/inference="ab initio prediction:Prodigal:002006"

/codon_start=1

/transl_table=11

/product="hypothetical protein"

/translation="MKGRMIMKQNTLQKYAMLLLCMSAGMVDVIGYLGLGHVLTANMT

GNIVLLGIAIARAQEFVVLRALLALIGFIAGNAIAAYMIGHVQMKNGWSSRVTAVFTV

ESILLLLFAIAMISPYSEQLSYLLIAMLATAMGMQTTAARCIGIAGISTTVLTNNLAA

VVEDAVSILQRLRHANIRSLVKALSADAYLRAGAVVIYLGGVILAAMLFHHVPMMAVW

IPVLIVGGVTLYARLYSRGTTKAASTDDI"

CDS complement(2437634..2438365)

/locus_tag="PIKHDJKN_02279"

/inference="ab initio prediction:Prodigal:002006"

/codon_start=1

/transl_table=11

/product="hypothetical protein"

/translation="MQMQKLASRYPPVKQHTMDGIDARWPVLIYCRGGLGNYGGVNTA

WLEQFVHKGYIVFAPSYRGNEGGEGRDEYGGRDAEDVHAVYRLVQEWPFADTKRISMM

GFSRGAINAVHTATTYNEGPQQVHKLVLWSGVADVERTYHERTDLRRTLKRVLGGSPR

SVPEAYLARSPLSKAHKLHCPVLIMHGTSDTQVNYSHGTRMYHWLKRRGADVTFHAYG

GQDHRFQERVHEAAVNNMFDWLAAP"

CDS 2438747..2439493

/locus_tag="PIKHDJKN_02280"

/inference="ab initio prediction:Prodigal:002006"

/codon_start=1

/transl_table=11

/product="hypothetical protein"

/translation="MNAPHPIDQFKKFKYEITRFMMIYKFALDQMETKIEVLKEEFQS

LHDYSPIEHTKSRLKSPESIMNKMFRKNHELTFDSIKENIKDIAGVRITCSFISDIYR

IKDMLCNQSDLRVLEVKDYIQNPKPNGYQSLHLLVEVPVYMSNGQERACVEIQIRTIA

MDFWASLEHKIFYKYNKDVPERLTKELKSAADSANALDQQMERLHREIQEIKDAENER

DEEELRRIIINNQQFTLPSNLLKLLGNGEQ"

CDS 2439535..2440776

/gene="bcr_1"

/locus_tag="PIKHDJKN_02281"

/inference="ab initio prediction:Prodigal:002006"

/inference="similar to AA sequence:UniProtKB:P28246"

/codon_start=1

/transl_table=11

/product="Bicyclomycin resistance protein"

/translation="MKSTPASLSINSTSRVRMALILGTLSAFGPLSLDMYLPALPTLA

DEFQSSTSYAQLSLTACMVGLAVGQLLAGPLSDVRGRRTPLIAGLILYTIASILCLVS

PTMGSFVVLRFIQGAAGAAGIVISRAVVRDVYSGPELTRFFSLLMLINGVAPIAAPII

GGQLLAYTSWRGVFILLSIIGILTLIAVVVGLGETLPKERRSSGGLKQTLVTFRKIAG

DRLFMGYALTQGFVSAGMFAYISGSPFVLQKIYGISPQMFSVCFAINGLGIILASQIA

GRLAGKVSETRLLIAGLITAALGGTSLLIAILAGGNLISVLIPLFLVVSSVGLVNTAS

FALAMANQEKSAGSASALIGVMTFLFGGIVAPLVGLGGEGTAVPMGIVIACADLGALL

IYFVMVGKGKKGIRQDKQPLA"

CDS complement(2440910..2441503)

/locus_tag="PIKHDJKN_02282"

/inference="ab initio prediction:Prodigal:002006"

/codon_start=1

/transl_table=11

/product="hypothetical protein"

/translation="MDYVYNTYSGYDEDRSFQLPPLFPGGAPGGQPMPGFPGAPGSYP

PFFPGGGSGGQPPYFPGGAPGTAPGVPAQLAQGNLPTLLSAYPRPVGQPPAEMVFLYN

LIKSSPGLLPLFIQQQPQNLTQAVQFAQSGVATPRDPEQRVLQNFCYNRWSLVFTYND

VYLMWPAINLFGFVVGYFYPFLTPGILLNTQILFALC"

CDS 2441746..2442759

/gene="yfiY_4"

/locus_tag="PIKHDJKN_02283"

/inference="ab initio prediction:Prodigal:002006"

/inference="similar to AA sequence:UniProtKB:O31567"

/codon_start=1

/transl_table=11

/product="putative siderophore-binding lipoprotein YfiY"

/db_xref="COG:COG0614"

/translation="MKGDSHVFKKNKKMILLLITVMVMSIWLAACGSKPADNGTAAGE

NNTATETETQTEAPTERTLTDAMGHEVKIPANPERIIASYLEDNLVTLGVKPVAQWSV
ANGIQEYLQKDLDGIPTIAFDLPFEAVVSFNPDLIIIGSESVVEGEKYAQYSKIAPTY
VLGDEINSDWRKALLKIGEILNKGDEAQKALDDYEVKATEIKEKINTVTGGTKSAAAI
WLVSGKFFIVSDNVSSGEVMYKELGLAEPEVVKEISANATGNWSSISLEKLAEMDVDY
LFFVNSDEGTGSEALKDPVWQSIPAVKNGNLFEFTRSSSWLYSGVQANLQIMEDIQNS
IVK"

CDS 2442915..2443535

/locus_tag="PIKHDJKN_02284"

/inference="ab initio prediction:Prodigal:002006"

/codon_start=1

/transl_table=11

/product="hypothetical protein"

/translation="MHTLDPRELTGRDNYKLMSGSVVPRPIAFVTTLSHDGSVINAAP
FSFFNVVSSDPPILSISIARKDGVMKDTARNVLSGQELVVHICDESIALDMNETAAML
EPHESELERTNLTTVPSTVVSVPAIQEALIRMECTLYQHIPISNDEGKPVSDLLLVRI
VQYHFSENVYDPTTKYILMDQLKPISRLAGNDYAKLGERFTVVRPD"

CDS 2443752..2444906

/locus_tag="PIKHDJKN_02285"

/EC_number="2.3.1.-"

/inference="ab initio prediction:Prodigal:002006"

/inference="similar to AA sequence:UniProtKB:P9WPF1"

/codon_start=1

/transl_table=11

/product="Alpha-pyrone synthesis polyketide synthase-like
Pks18"

/db_xref="COG:COG3424"

/translation="MNQLDVTVAASIKGIGTALPVHRIEQKDVSARLAQALEDEPDSR
RWAKRIFNQCGVETRYTCEPNLLEPVESCRYLPFTQTENVPTTAERMAKYKAAAFPLG
IQAARQALEDAAVSTSEITHLITVSCTGQFLPGLDVRLILQLELSPRINRIPLVFQGC
AAGLKAVQLANSIVTSDKDATVLIVCVELCTLHFQPSAKRDDLFAASFFGDGASACVV
GLSGLGCKELFRLGSGHSVLLPDCSEEMIWEVGNTGFDLYLSPQIPKLLGLHLGPEVE
RLLNGSDLPEIWAIHPGGRGIVDAVQKLYQLSDEQVAYSRNILRDYGNLSSVTILFVL

QAIRDDYRKREEASSGIALAFGPGLTAELLPFTYVPASVGEREPVNQGAI"

CDS 2444948..2447209

/locus_tag="PIKHDJKN_02286"

/inference="ab initio prediction:Prodigal:002006"

/inference="similar to AA sequence:UniProtKB:P9WJT9"

/codon_start=1

/transl_table=11

/product="Heme uptake protein MmpL11"

/db_xref="COG:COG2409"

/translation="MGYRSLAVLISRYPRFIILCWVFVIGMSAIWAWKLPGIVQDHGL

NLEQGDAHAVELTLENEFHVPADPVILLFEKKTGTTPVEFRGWIKDRLQQVRTLSGVT

SIISPLEARGSRMLRGDVAYALLAFDVPPHKMGEPLEQLRAVLTTDGPGTIRITGKSV

VQQDVNRLSFRDLERAEVVGLPLAFVILCFAFRGFFAALIAVVMGVSGVITAMGVTSM

MGYHLALSNFVINVIPMVGMALSLDFALIILSRYREELQRDRKSKEAGTGEEEVLQRM

LCTAGRAVFFSAACVLLGLLGLLWIRLPMFLSVSLGAIIVLVISVLLNITLLPAILSL

FSARIFRQHSGHSISSKQGQHQQSLPSRAIWAFWSGMIMKRPVRMALLGTGTLLIFVL

PVTRLELAIPDASSLPDRMESRQATEQIQSIFGQKNVSAVEIVIGGTAESLTASHWRI

ALNKMRELQQDSNVVIVESAWGIMLPDKLSNQAQSQIQSQMNILSPSLREALKETKSQ

PPAWVRSYVSDHSIRMVATIHGAPGSDEAASWLEQMRAKDIADSISSIPLRYGGEAVK

QHEIMQEVSGQLPKVLVFVVVSNYLVLLAAFRSLLIPIKAILMNLLSLAASFGVLVLV

FNQGHFGMEPSAIAIMIPVFIAGLVFGISMDYGVFMLTRIQEVYRRTGDSDLAVQQGL

ASTGRLITSAAAILLAVTIPFAFADVAGVKQLGIGITAAVFIDVTIIRLVLVPALMKL

MGRWNWWLPGHRE"

CDS 2447616..2448635

/locus_tag="PIKHDJKN_02287"

/inference="ab initio prediction:Prodigal:002006"

/codon_start=1

/transl_table=11

/product="hypothetical protein"

/translation="MSDIESYLQKQTSPRGRSAYQTGQSLEQWRSQLRARIIVRLGGF

PTLAAELNPVLLERTACDGYIRERVEITTYAGLRMPIYMLIPNIVSATKESRPAIIAC

HGHGYGSREISGMEPDGSPRKGEPGLHKDFAVALVHRGYVVAAPELLGFGDRRLEEDR

DAPPGASSCTKIASHLLMTGQTLAGHRIYETMRVLDYVTTRPEADPLRIGSMGISGGG
LVTAFTAALDERCRAAVVSGYASTFQGSILDRNHCLDNYIPGILREAELPDIMGLIAP
RPLFIEAGSEDQVFPLPTAKEAYARLTEIYEQVGAQKWLDADFFTGGHEISGAKAYDW
LDNVL"

CDS 2448694..2449848

/gene="yxiM"

/locus_tag="PIKHDJKN_02288"

/EC_number="3.1.-.-"

/inference="ab initio prediction:Prodigal:002006"

/inference="similar to AA sequence:UniProtKB:P42304"

/codon_start=1

/transl_table=11

/product="putative esterase YxiM"

/db_xref="COG:COG2755"

/translation="MRYLLCLTLFTSLIGGALAANGGQVAQAAANEYKFDFGAGVVEN
GYTGVSATEAYNSAKGYGFNTPANMRNVSASGTGVASDAVQFVTFGTKSTNTFNVDLS
NGLYEVKVVLGNTARSSVAAEGVYQIINMTGNGATDQFQIPVTDGQLNLLVTEGKEGT
AFTLSALEIRKISNQTVTNRTIYIGGDSTVCNYYPLSSSVQGGWGQLFPSYVNTSTFQ
VRNMASSGQFARGFRDDGQMEAILKYIKPGDYFILQLGINDTNAKNNTTEAEFKEIMR
DMVRQAKNKGATVILSTPQGRATDFNSANVHTAENRWYNASTRALAQEEGVTLVELNK
LSSAYFTSIGPAATLALYMTGDTLHPNREGAAQLARIVAADLRRQGLNGF"

CDS complement(2449988..2450380)

/gene="mrpG"

/locus_tag="PIKHDJKN_02289"

/inference="ab initio prediction:Prodigal:002006"

/inference="similar to AA sequence:UniProtKB:Q9RGY9"

/codon_start=1

/transl_table=11

/product="Na(+)/H(+) antiporter subunit G"

/db_xref="COG:COG1320"

/translation="MKEIVDGTAGIVIGLIVLLGALLSAFSAFGLIRLPDVYLRAHAA
TKSTTLGVLCVLSGTFLFFWYFDNYISARVLLGIVFVFITAPVAGHLNGRAAYRTDVP

LWEQSVQDELEPLLKGKKVNHEAKDMME"

CDS complement(2450364..2450648)

/gene="mrpF"

/locus_tag="PIKHDJKN_02290"

/inference="ab initio prediction:Prodigal:002006"

/inference="similar to AA sequence:UniProtKB:Q9RGZ0"

/codon_start=1

/transl_table=11

/product="Na(+)/H(+) antiporter subunit F"

/db_xref="COG:COG2212"

/translation="MLSSLLFISLLILSLAILGCLYRVLRGPSMADRITALDTIGINV

IAIVAVLSMMLHTQAYLDIILLIGILAFLSTVAFARYIERGAVFKNEGDR"

CDS complement(2450648..2451124)

/gene="mrpE"

/locus_tag="PIKHDJKN_02291"

/inference="ab initio prediction:Prodigal:002006"

/inference="similar to AA sequence:UniProtKB:Q7WY60"

/codon_start=1

/transl_table=11

/product="Na(+)/H(+) antiporter subunit E"

/db_xref="COG:COG1863"

/translation="MAFQIVLNLIIAFVWMFLNNAWNGVGFLTGYLLGLLLIGSMRRF

FPQRFYIVRVWAIIKLIALLIKELVRASIEVIRQIVKPKLDIRPGIFTYQTQLSSDWE

VTLLCLLISLTPGSLPLEISGNQRKLFIHALDIKDEQKMSDDIKNTFEKAIMEVTR"

CDS complement(2451131..2452609)

/gene="mrpD"

/locus_tag="PIKHDJKN_02292"

/inference="ab initio prediction:Prodigal:002006"

/inference="similar to AA sequence:UniProtKB:O05229"

/codon_start=1

/transl_table=11

/product="Na(+)/H(+) antiporter subunit D"

/db_xref="COG:COG0651"

/translation="MNNLVVLPILLPLITGVIALLFFRQINIQRVVSVIGLLLTAAAS

TVLITQVTQSGIQTLNMGGWEPPYGIVLVADMVSALLVVAASIIALACLLYAFRSVNK

EREEHHFYPFFHFLIAGVNGSFLTGDLFNLFVCFELMLISSYALIVLGGTERQLRETI

KYVLINIVSSALFVASIGFLYSVTGTLNMADLSNRIAEVGQSGVITLIAVLFLIVFSI

KAGLFLFFWLSGSYAAPPAVVTALFAGLLTKVGLYAIVRTFTLIFYHDPDFFHALIGW

MAGATMVLGVIGAISYRDVNKILIYNVIAGVGFVAFGMAAASRPALEGLLFYMLHDML

IKTLLFLLGGALIAVAGTSKLDNMGGLIHRYPLLGWMFFISALALAGIPPFSGFPGKL

LLFEGGLQAGLYGLTGIAVLSSLLMLYSVLRIFIQAFWGEPPAGAVRRPYAVNGLLIP

AGILFVFIIAMGVGAEGMFQLTSRAGDILLHPNVYIDAVLKE"

CDS complement(2452602..2452940)

/gene="mrpC"

/locus_tag="PIKHDJKN_02293"

/inference="ab initio prediction:Prodigal:002006"

/inference="similar to AA sequence:UniProtKB:O05260"

/codon_start=1

/transl_table=11

/product="Na(+)/H(+) antiporter subunit C"

/db_xref="COG:COG1006"

/translation="MEILMCVAVGILFAVAVFLILSRSLLRIVLGMSILTHGVHLLLI

TMSRLKTGAPPLLGEQAERYVDPLPQALILTSIVINFGLTAFFFVLSYRSYLKLKTDD

MEEVRGRPYE"

CDS complement(2452942..2455899)

/gene="mrpA"

/locus_tag="PIKHDJKN_02294"

/inference="ab initio prediction:Prodigal:002006"

/inference="similar to AA sequence:UniProtKB:Q9K2S2"

/codon_start=1

/transl_table=11

/product="Na(+)/H(+) antiporter subunit A"

/db_xref="COG:COG1009"

/translation="MLSLLHVAVILPFAAGIVLAMLHRFFRKIHMGWLVLLVPALLFV

YFASLVPAVSQRITVSGYIPWIPSLDIGFNLYLDGLSLLLTLLITGVGTLVVLYSIFY
MDTKEALNRFYLYLLMFIGAMLGVVLSDNMIVLYGFWELTSITSFLLIAFHYKRKAST
SGAQKSFLITVFGGFAMLAGFMLMYLMTGTFSIRATITEWGQIQDSALFLPALVLILI
GAFTKSAQFPFHIWLPDAMEAPTPVSAYLHSATMVKAGLYVVARFTPIFGGQGLWFWL
VTGVGLLTLCYGSFLAVKKNDLKAILAYSTISQLGLIMSLLGVGSAAIYFGYGESSAM
YTVAITAALLHLFNHATFKAALFMVVGIVDHETGTRDIRKLGGLASFMPVTFTVALVG
ALAMAGIPPFNGFLSKELFFQAMVEVTHLRIFGLGSWSILLPVFAWLASVFTLIYALI
IVFKTFLGRTHSEIPAEKLHEAPVGMLIPPVILGILVIIFGLFPNLLAGSLIEPAMAA
VLPSLLNGNEYFDVHFSLWHGITPELLMTIGVIALGITLYKLLPRWRTIYEHYPQGLT
INNMYHVVLDNLQHYARKWTEAYMNGSVRNYLVYIFSFTVALLVYAFFRSGENITWNF
KGNAPFSFYEAVLLIALIAAAVSIPFAKNRLSAVIMTGAVGYLVTLLFVLFRAPDLAL
TQMIVETVSVALFLLCFYHLPELQRGKSSRSYLSVNMIVAIAVGVVMTFVALAASGTA
SLESISDFFIQESYNLAGGKNVVNVLLVDFRGFDTLLEIMVLGVASLSIYSMINLNLE
ARDLGARLKFRKKEQTEETEEDTDDKADNTKKYGNRERSSSTWDTVPLQSNDVLLQTT
TKVVVFIILTFALHLFFAGHHNPGGGFIGGLVTAAGLVLIALAFSTDTVRKALPIDFR
TLTAIGLGIALLTGAGSFLFGVPFLSQTFGYFDLPILGETELATAMLFDLGVYLAVLG
VTMNIILQIGEDR"

CDS 2456200..2457846

/locus_tag="PIKHDJKN_02295"

/inference="ab initio prediction:Prodigal:002006"

/codon_start=1

/transl_table=11

/product="hypothetical protein"

/translation="MSFKLKTVLTMTFAVLSLLVTLTIGYILSQKSSIAVETEIGHSL
ISTASQTSDKLDRFMWSRSGELDLLGRMIALEDRFQPAEMQVLLNQLQDSFPTFSWVG
FMNPKGKVLASTDGILRGENLSERPVYQEGIKGKFIGDVHNAVLLAKLLPNPTGEPLQ
FVDISFPVKYKNGEIAGVLAAHLSWAWAKEVEESVLEPLKHEEKYIEMFIVSQKENTV
LLGPKEWIGRPLKLSGIEGARLNKSSWSIEEWPDGKEYVTGFATSQGHLDYPGLGWTV
VIRQLKSAAFSSVTDMLWFNVWAGLSVTVLFALIGWLISRQISAPIARLTQVANRLGA
GEQVQIPENRGFREIEVLSRSLGDMLTSLTNKDSELVVMQNLAHYDQLTGLPNRTALE
LYLEESLEMESENHILTFLYLDLDGFKSVNDTLGHQTGDVLLQKVAQRLTSLSHEKGI
TVRLGGDEFLIVLRSVGSNPREEARTYAEDIIQSLNKPFIIEYERIHIGCSIGGAEYP

TNSGNPSEIIRMADEALYESKRAGKNRMTFFPELKNTVRE"

CDS 2457899..2458420

/locus_tag="PIKHDJKN_02296"

/inference="ab initio prediction:Prodigal:002006"

/codon_start=1

/transl_table=11

/product="hypothetical protein"

/translation="MSGKYTNVPYGYEPPPATRKGTLIFYDSFEHVTDEQLERAAVIA

DTRSFVQLVLYPLHENTVKRMSKDGVQPFYKREDRLHDWRREQAPSRVRVEGWEGKRK

KYTPIEAALRHMAEEYPAPHFLYLTVEMANQFASFDSFKDWIKEVRLIIDGEPVKLHA

KLEQNAHRWEWAE"

CDS complement(2458517..2459620)

/locus_tag="PIKHDJKN_02297"

/inference="ab initio prediction:Prodigal:002006"

/codon_start=1

/transl_table=11

/product="hypothetical protein"

/translation="MHNSHIPTDYEFKHSKWIKKMLRAYWVVVLTHFVIQIGCFLFLD

YDRTPGDFMIHVLFWPTLASALSILIASWVDRHFSSFSFYSMSMASTVIAWTIIHVNY

DIRIILAICLLPIFASVLFFSKKRVWTVCLMQMIGYVIVLFDPTYRLYLTSFDMVSIP

AFLIVGTYVAQLIVTSGVEVLDDLQASMLAKQDLIVRNAIMSKQSKTDGLTNLYNQSS

FKDYYEKAFEYANNGMSLHLALIDIDNFKSINDTYGHRVGDAILERVSLVIQENITSS

DIAARYGGEEFALLMFEQPFEQAYALVEQIRKKIARLVHLELEGAYITVSVGLKSYSA

HLSKDKLFEEVDACLYAAKRTGENKTMTSLDLV"

CDS 2459875..2466585

/locus_tag="PIKHDJKN_02298"

/inference="ab initio prediction:Prodigal:002006"

/codon_start=1

/transl_table=11

/product="hypothetical protein"

/translation="MRSIPLVVRSTVNATGAITFTGNTLGLSRSETAGVPGTQDSIGA

FTTTNASVQYGTYPLGTTSLYQSNSSAAILVLPVGSTVLYAELIWGGSYINGTVNLSA

AINNPVTFITPAGTSSVTPDPATYNQFDLGNGAAGYVRSANVTSLVQNGGAGTYTTGA

VVGTIVITNDATANHAGWTLGVIYQNPSLPFRNMSLRAGGVLVQSTSAPVVTTLTGFA

TPLSGALGGRALFSAQEGDANRTGDQALFGPTSATSVALSGPNNLAANFFASQINGDT

GALNTTGTFGTRNQTNGAPGTNIIGGRQGWDITNVDVSARLINNQSSAVLTLTTSGDA

YIVNANAIQVDINAPKITVAKASVASGAVAGDSILYTVTVSNAGTASAASVVLSDSLP

AGLTFIPGSVTVAGVSRPTLDVTTGIPLGSLNLSNSIVVTYRALIGQDASILQLVNSA

NAAFTFQSVAGGSVITGVIPSNNSTLPVYSPNLSIVKSASTTNATVGDQVTYTLQVNN

GGNVAANVTLTDNIPSGSSYVAGSFRLNGNVIAGANPATGVNLGSLAAGSTNTVTFQV

LVTSLPTPPTLVDQATASYSFNSPDGRTINGTLASNTLTIPVTLPNVTTVKSASVTDV

AVGETFTYTIVTTNSGIQVINNVVLTDSLPVGTTFVPGSVTVRGTVVPSANPSSGISL

GTLAAGSSASVTFQVNVQSLPASSSLVNRAVVSYSSGSFTGISNSNSITTPVYQPIIG

INKSATQTNATLGDQLAYTLVVTNTGNLGAQITVTDTIPVGLTFVPNSVTVNGAARPG

TSPLTGITLGSLLPGATATVVFRATLTTLPSPPTLENQGTSSYTYQLPSGRNLSGSSQ

SNIVRISASAPNISISKTVNTPDATVGDVLTYTVIATNAGTSAVQNLVISDTPSGSEF

VPGSVTINGTAAGSASPVSGIAVGTLTSSSSVTVTYQTRVTSVPSTGSVSNRASAAFT

SGSFNGVSSSVTVNTPIYQPVIQVAKTASTSNLTVGDSFNYSIQVNNSGNIAATVTLT

DPIPAGAVFSTNSVIINGVPTPGVSPATGISLGSIAAGSSATVTFVATVTSLPDSRQL

TNQAVASFAYTLPSGRTVAGFMSSNTINIPVSLPNVSIVNSDNVEYGVVGDVIRYTSV

IRNNGTVAVNNVVYVNPLPPNTPFVPGSVIVNGSSFPLSNPTAGIPIGTLAPGAEVTV

TFEVTITMPIPSQINNQSTVSFTSGSFSGSSSSNTTQTPVIQPQISLVKTANTVNATV

GDTVVYTVTVSNTGNLEANVTVTDTIPAATTLVANSVVVSGVPQPGATPGTGIPVGIV

AAGATAVVTFGVVVDSLPSPQQLSNFATSSFTFTPPDGRTLTGTATSNTLTFPVSSPN

VAVVKSTASTAAAVGDTVTYSIQVTNSGIEPVNNIHFSDPIPTGASFVAGSVTVNGVV

QSGANPAGGISLGTLAPGTSATVTFSINVNAIPPNGQLSNRSTVSFTSGTFSSTTFSN

TVVTPVFQPILSAQKTASTQNATVGDTVSYTINVNNQGNYSAQINLTDNIPAGTILVP

NSVIVNGQLLPAANPATGIPAGTVAAGATTTITFSVVIDTLPTPQQLVNQASVALSFT

LPDGRNITGSVLSNVLTISVSAPDVDVVKSTTSTAVSVGDVVTYSIAVTNNGIATVSN

VVFTDAVPTSTVLTANSVFVDGVLRPGANPSTGITLGSIAPGVTVTVVFSVQVTALPA

SAVLNNQSTVSFTSGVFSATTFSNTVTTSVYQPILSAVKTGDQAIATVGDTVVYSIAI

SNAGNYGASVTLTDTIPAGTELVPNSVIINGASAPGADPASGIPLGVVSTTTVVMFSV

VIITLPLSQAITNQASATYSYTLPDGRTLGGSLSSNSLNIQVSAPNVSVVKTTPVIDA

VVGDTIVYEMVVTNNGIDPVNNVVLTDPISSAVTFVTGSVLVNGVPRLSANPALGIAL

GSLAPGASVAVSYAVRVNTLPAPPQVSSQSSVSFTSGVFSGAAYSNTVVTPIYQPIIA

VTKTASTSNATIGDIITYSFTISNSGNLAANLTLTDNIPNGAVLLPNSVLIDGVPQPG

ANPETGIVVGTIPPGGSVNVTVTLEVTVDSLPQNQQLVNQAVANYTFSPPDGRQLTGT

VSSNVLVIPVSAPNVVVVKSTSAVDAVVGDVITYTVVVTNSGIEVINNVVMVDPVPTG

TVLIPGSVTVDGVARPTGNPNTGITLGSIAAGASVTVTFRVEVVVI"

CDS 2466582..2468276

/locus_tag="PIKHDJKN_02299"

/inference="ab initio prediction:Prodigal:002006"

/codon_start=1

/transl_table=11

/product="hypothetical protein"

/translation="MSQSSGSLSHTLQNQSLVRFSSGATELEQVAYSNTVVTPWIGPR

LEVHKFCNVAEAALGQTLTYLIEIVNSGNCPAMVLVLDSLSPETSLLPNSVLLGGTPL

PGVSPERGIPAQEVVPGATLRIHFQVIIVGLPPALKLVNQAQARYEFVTPEGRKVEGE

EKSNVVEVNLVSSSLSLVLNADRVQTFVGDIVTYNLLVSNPGFVIGNDARVTIPLPKG

IEFLPGSVIVNEIFVPETTPDSGIMIGEVLPGRSVPIQFRVQVTGGSIAESLTTQAVL

EYTSGDKLETVYSNVATLQVIQPRISIVKSVNPSIATPGDTVQYQITVHNESSFAVDA

IVTDFLPPGLRYVEGSLGWNGVKRRGTSPVKGINLGTLTARSTINIQFEVNLTEQEQD

VPAVFELMNQARLIYTFRLPDSRSVQRIISSNLAVIQLKFPIISVHVEVKPDLIERGG

NVTFHVHVTNTGSWPARVQLADVLPPGAKWIGEAVVRDRAIISEYSSPRYLHLGEMEP

GAERWITYVAQISSAEQLGIQQGSVTAMYTYELNGLKHSGQSISNVYTIIVEDGDE"

CDS 2468440..2468697

/locus_tag="PIKHDJKN_02300"

/inference="ab initio prediction:Prodigal:002006"

/codon_start=1

/transl_table=11

/product="hypothetical protein"

/translation="MAKAKVAKRPTRDEFELEELGNQLVEAYRERSEVLLTVWGKEDQ

VHGMIVKLDSRTRLVHVEYTEEEFTAKVPFLDIMRIDSPRY"

CDS 2468801..2468920

/locus_tag="PIKHDJKN_02301"

/inference="ab initio prediction:Prodigal:002006"

/codon_start=1

/transl_table=11

/product="hypothetical protein"

/translation="MSEVLGGEGRGYGYGGFTSVGAILVLFILLVIISKAFLV"

CDS complement(2468977..2470239)

/gene="yjeH"

/locus_tag="PIKHDJKN_02302"

/inference="ab initio prediction:Prodigal:002006"

/inference="similar to AA sequence:UniProtKB:P39277"

/codon_start=1

/transl_table=11

/product="L-methionine/branched-chain amino acid exporter

YjeH"

/db_xref="COG:COG0531"

/translation="MNTSSSQLKKTIGMPQAVALYISAVLGSGVLIVPGLAAELAGPA

SLLAWGGMVLLILPLALSMGLLSARYPDAGGVSHFVTLAFGQRAGAVVGWFFLMSVPI

GAPVAALTGAGYMTAALGLGEGYRTAIAALMLAAGLLINIVGMQVAGKVQIGVVLAII

IVLVMAFVFAIPNMESIHYTPFMPSGWVSVGQAGAILFWCFIGWEAVSHLSEEFTDPR

KAAIKGVTIAAIIVGVLYFLTALATVGTQSYLHGGADVSLVWIISQALGSWGGLLAGL

TGIFICTATVIAYAGAASRVAYALARQGSAPQWMSRLSSRYHTPIAAIGFLAICFTLV

LGIYGSGLVSLTTLIQLPNATFILTYLGGCAAGIRLLKGSRTGVTISWISFVATAIVF

PFAGWAVLYPVVIVVLYGLLSRKRQHNI"

CDS 2470414..2470917

/gene="ftnA"

/locus_tag="PIKHDJKN_02303"

/EC_number="1.16.3.2"

/inference="ab initio prediction:Prodigal:002006"

/inference="similar to AA sequence:UniProtKB:Q7A4R2"

/codon_start=1

/transl_table=11

/product="Bacterial non-heme ferritin"

/translation="MSKELTEALNEQMNFEFYSAHVYHAMAAYCSGESLDGFANFFIV

QAEEERFHAMKIYKFLNDRGQRATLAALPEPKNEYSSMLDVFEHGYAHEQQNTKKFYN

LADIALNEREHATMYFLKWFIDEQVEEEALFDNIIQKLRRIEKDSNAFYMLDAEFGKR

SFTAPAE"

CDS 2471313..2472053

/locus_tag="PIKHDJKN_02304"

/EC_number="3.5.1.104"

/inference="ab initio prediction:Prodigal:002006"

/inference="similar to AA sequence:UniProtKB:Q81EJ6"

/codon_start=1

/transl_table=11

/product="Peptidoglycan-N-acetylglucosamine deacetylase"

/db_xref="COG:COG0726"

/translation="MLHIRKTFITALAILFIIPLLLPQSASATSSASKNAGNKASSKI

IYLTFDDGPTAHTGQLLDILDQYNAKATFFMLGPQMEKFQKATKRIVADGHGLGLHGV

THVPGKFYKSAYSGLKEMQQANESLNKVAGVKTSLVRTPYGSKPYLKPAYRNVLLGQG

GFHLWDWNVDSEDWKYKKDHQKVYNSVMKQIHNVQKSGTTPVVLMHDQEATLKVLPQV

LKTLKAEGYQFEVLTKKIQPVNFWNDKR"

CDS 2472114..2473022

/locus_tag="PIKHDJKN_02305"

/inference="ab initio prediction:Prodigal:002006"

/codon_start=1

/transl_table=11

/product="hypothetical protein"

/translation="MSKPKLWMTIPVTTAVLLALAGCGGQDTKPSSADNTSPAVGEQA

QSGSGGADFSNGTKDNGTDTTETTGTEGSAGSGTSNSETGSNGQTQQSANDVIKDIRS

QLKMKNASFPTSFSLEKGKHLTASIKSNTSDAFHINFYSTDQPVAVNDVSLTASSSKV

PVLASYEVKTYKDPNSTDIFPETDLKDFPKDMTVDLGHGIKGMTEGAAGSQYLTWTEG

RWTLQIRSISEDNMNNPGIAKRMVEYLESHTLPAPKDKGFVNVEYPSGGESVNVTISW

QDGKQIHQLQTQQVPLEALGMVVSVK"

CDS complement(2473166..2473765)

/gene="desR_1"

/locus_tag="PIKHDJKN_02306"

/inference="ab initio prediction:Prodigal:002006"

/inference="similar to AA sequence:UniProtKB:O34723"

/codon_start=1

/transl_table=11

/product="Transcriptional regulatory protein DesR"

/db_xref="COG:COG2197"

/translation="MIRIVIAEDQRMMLGALSSLLNLEEDMEVVGRASNGQEALALVR

EHTPDICLMDIEMPVKSGLEAAEELKGLNCKVIILTTFARTGYFERALKGGVRGYLLK

DSPIEELAEAIRQVMNGRRIFAPDLVDEAYVQENPLTERENAVLGLMADGKNTKEIAG

HLFITTGTVRNYISIILNKLNASNRIEAITRSKEKGWFK"

CDS complement(2473762..2474898)

/gene="desK_1"

/locus_tag="PIKHDJKN_02307"

/EC_number="2.7.13.3"

/inference="ab initio prediction:Prodigal:002006"

/inference="similar to AA sequence:UniProtKB:O34757"

/codon_start=1

/transl_table=11

/product="Sensor histidine kinase DesK"

/db_xref="COG:COG4585"

/translation="MQLFYKNTGLNPYVWLVFFILPFYYISQYSKLWPMVAGIFIILF

FFVCYLLAFITKGWQVYMWIGLLIAISITMTIAYDYAYFSLFLAFFIGNIKNKAGFFT

LYSVNLAASFLTINYSFIKQSTLLLSQFPFVFISLMASILLPISTYNKNKREQLEGQL

ENANKRLDDLVKMEERQRIARDLHDTLGQKLSLIGLKTDLAKRLLRMNPDQAEIELND

LRQTASTALKEVREMVTTMRGTQLVDELFRAEQILKAASIEFVLEGNPKLQDTSQLNE

NVLGMCLKEAVTNVVKHSQAAVCTIRIEETLSDNIVTVQDNGVGIERTRKHERRGTGI

LGMKERLEFVNGCLDIRSGADMDGTGIVIHVPKLVRKPIKEVEQ"

CDS complement(2475004..2476062)

/gene="des"

/locus_tag="PIKHDJKN_02308"

/EC_number="1.14.19.-"

/inference="ab initio prediction:Prodigal:002006"

/inference="similar to AA sequence:UniProtKB:O34653"

/codon_start=1

/transl_table=11

/product="Fatty acid desaturase"

/db_xref="COG:COG3239"

/translation="MSRTKEMALKKEVTPYEKNNLRLSIQQLVNSILPLLLLWAAAYF

SLSVSYWLTFPIALVASGFVLRTFIIFHDCCHGSFFKSKRANDILGTITGVLTLTPYL

QWKNEHSIHHATSSNLDKRGVGDIWVMTVEEYKAASPWGRLFYRIYRNPFVMFGIGPI

YVFLFAYRFNRKGARRKERINTHLTTVLIVALYASMCWLIGWQAFVLVQAPVFFFSGF

FGIWLFYVQHQFEDTYFEHEDEWSYVKAAVEGSSYYKLPKLLQWISGNIGFHHVHHLS

PRVPNYYLEEAHNATPPLQKATTITLRSSLAALRFRLWDEESKRFISFGEIKTLSRKP

YVQPPIRVNKQAGLTEKP"

CDS 2476450..2477289

/locus_tag="PIKHDJKN_02309"

/inference="ab initio prediction:Prodigal:002006"

/codon_start=1

/transl_table=11

/product="hypothetical protein"

/translation="MKKRITDILFIMAGAFLFALAVNLFVIPNDLAEGGVTGITIILY

YLFEWSPGLMNLLLNGILLLVGYKFLDRTTTVYTIIAVVFNSLFLHLTESWTIASDEL

WINTIFGGLFAGLGIGMIVRVGGTTAGTVILARLANKYLDWNISYGLLFFDLIVAFSS

FFIIGPQGLMCTIVMLFVGTKTMEFIIEGLNPKKAVTIISTKQDAIAQQVIEKMDRGV

TVLSGHGYYTKNPKEILYIVISKQEVPMLKKIVRAEDEIAFITIHDVRDVFGEGFIEL

SKS"

CDS 2477515..2478711

/gene="nagC_1"

/locus_tag="PIKHDJKN_02310"

/inference="ab initio prediction:Prodigal:002006"

/inference="similar to AA sequence:UniProtKB:P0AF20"

/codon_start=1

/transl_table=11

/product="N-acetylglucosamine repressor"

/db_xref="COG:COG1940"

/translation="MAGTPKYIRNLNENLIMDALIATGAMSRADISRQTGLSKPTVSS

AIEHLIERNLVLETGRADNAQGRKATLIRFNETAYYVCGIDIGATRIRIALSDLNGEI

LDYRTYPTLSVQCGDRAGTAILDVLRSHMGELLTENQLNWEQIQCIGFGIPGVVLPDS

GDIDRIVAPLEGLEKLLSLESLSEAFPCEVILENDVNLAALGEYRRGAAADSNLFVFF

SIGTGTGAGIMVNGQLLRGMGGLTGELAEMLLEEDRRLEDVLSAEGLMELAKRQLNAN

LPEGPDSGIEGHREHQTPEELFEAARSGETQAVQILKQYSQMIASALRHICVVLAPHL

IVLGGGIGGNGDVLLPFLRQIVSEQFPVKPALICSELGERAVVTGAVQVALQQTVLNL

QQEALE"

CDS 2478882..2479847

/locus_tag="PIKHDJKN_02311"

/inference="ab initio prediction:Prodigal:002006"

/codon_start=1

/transl_table=11

/product="hypothetical protein"

/translation="MFTDKKEYSFSTCWNIKRHTTGEGMIEEIKSLGFRHVELNYNVT

DELLQTIEPMIERGEIGISSVHNTFPHVADPDYGTDSVLLGFDDEPRRKRAIELLLRS

AEYAHRYGAKAVVVHPGEVPFEYNIDEALKKIYHDQGPDSSDYQSLWQEMLEKREASS

AHYLKRIQESLEEVCELSEQRGYGVNFGIETRSRCYQMPTLQEAGALIRGMKGAPLGL

WYDIGHGMMMDRMGLYDNVREANELIDHVVGVHIHETVGLSDHWCPYIHSKDMAFFDS

FLDIIDRSPVKVYELKAACTPGEIDESHRLITARIAGRQAARQHG"

CDS 2480158..2482308

/locus_tag="PIKHDJKN_02312"

/inference="ab initio prediction:Prodigal:002006"

/codon_start=1

/transl_table=11

/product="hypothetical protein"

/translation="MDLFSIYFSRNLKVIIAERKGKLPVRILATALRNLESLGYTFST

KLIDVLRTWERDRFISWFERLMDELRQIKGINLKETLVYPRFPKQKMNIQESKFYLNA

LMHCWRQQSFEQEQRILLLNKLRLRVIHLGSEADFYQVGKDLLTAKSSLTPVAREQLI

WFATKYEGWAAFEPEAGQIPVRENAAVYCAALLNAGRTSMEQFQRHLRTATDILRLAV

ACSDGDVSLKENTRFRSFTRSERRVILALMESVSDPLEDLFRYKGRWQRLAERLHPGE

YRHRYPGAVAALDQLRGGERPLTFHGKVERAFEQMDNQLIVETLAERPGEMIGQLDRM

IRSGVPVQQVIDKMETVEEKVSTRALLRVLAHFSTRTEFQVRRSFFPKGNTANLYVST

QPLPPLNVEDVHTVISAVENTLLKRFAVLPPLGNVYIDERLQEFSVPWGKRRASESLR

ALSRASWISLPTGDTVRFFLWWREGMVGDEPTGHVDTNLSNVLNDEDWRYMDQTSRNH

LQSNEYIAANSGFMVTALDGAARYMDLHLPSVSRLGARYIVIMVNALRDNTFKDLPEC

YAGWMMRQGPQSDEMFEPSTVQERIDLTADSRICIPAIIDIQERRILWCDLGLKRQPK

AHLPVENYNADLALIARAMTELKRPDLYTLFMLHAKARGTLVETLGMANLVFTAELES

NRVAEVAEYLTEGPPVERTVITPLEQEYIMKQFL"

CDS complement(2482486..2483070)

/locus_tag="PIKHDJKN_02313"

/inference="ab initio prediction:Prodigal:002006"

/codon_start=1

/transl_table=11

/product="hypothetical protein"

/translation="MSKLIFFLGGAGSGKTTLAKALSRKYKAAFFDMDILLRPAAEAI

MTLQGLDPSDRDSPEYKRLCRDLGYRITMDAALDNVQLGIDTVVVGPFTKEIGTPDWI

EQELARIGRYLDDTDVRVAYIYLANEALYRERITARQSPLDEWKLENWDAFTASLARK

QVAWPLPSSAVAYIDNSDENLDIAFAEVEQRMYE"

CDS 2483370..2483705

/locus_tag="PIKHDJKN_02314"

/inference="ab initio prediction:Prodigal:002006"

/codon_start=1

/transl_table=11

/product="hypothetical protein"

/translation="MQVNKQMIKGSTETLILTLLKEQPLYGYELIKELHRQSEGVFNL

KEGTLYPILHAMELEGWVESYWMEVEGRKRKYYSICDKGVEALQHKKAEWLLFRGAVD

RVLGEGGLT"

CDS 2483702..2485051

/gene="ftsW_1"

/locus_tag="PIKHDJKN_02315"

/EC_number="2.4.1.129"

/inference="ab initio prediction:Prodigal:002006"

/inference="similar to AA sequence:UniProtKB:O07639"

/codon_start=1

/transl_table=11

/product="putative peptidoglycan glycosyltransferase FtsW"

/db_xref="COG:COG0772"

/translation="MTDRHEKIKHYLDQLCSQVKAREVHTDLRDELGNHMEEMMLDKE

QEGLSQDEAAAYAIEQMGDPELVGKSMHRLHRYRMHWGLLVGLIGLSTVGLLLMWIFT

MNVTEERYQFLYRGHVVYTVIGVMIMLFFIYFDYRKLKRAAWWIYILLNVMLWISPMI

TTDIHGNSRYWSLFGLTIDLTMASVWILPLAIGAIMLDKLRSNCNMQSILTYIAMVTF

PAVLLFQTLDWVRLFLFGIMCIILFGWLTLKRLYTIVGAVITGSVFVLLLFFADQYGR

LKRLSVVLNLQDDPYGSGYSNNSIIEIIRSSGWWGNGIGTTFDMFKTSYFDYPGVLLI

DVFGWSAGILLLVGVIWFVASMMKILPRIRDDFGRMIIVSITAVFALQIIYSLAMTTG

MAPILSISFPFIGYGNHLMFDYAMLGLLLGVYRRKDTISKRNEKIRQKVDAEPMTSS"

CDS complement(2485118..2486899)

/gene="sgrR_2"

/locus_tag="PIKHDJKN_02316"

/inference="ab initio prediction:Prodigal:002006"

/inference="protein motif:HAMAP:MF_01449"

/codon_start=1

/transl_table=11

/product="HTH-type transcriptional regulator SgrR"

/translation="MKLHQQYLLLHSRFGQLLEHEVTLSDLAELLDCTHRNTLTIVKK

MVAHEWIRWVSQRGRGRRSTLTLLVPADQIAAEYMMQAMNRRELQQAAEQVGAFSSST

TMQDHLNQWLLGYSGHHTEAGSHNERIDTLRLPIRQQLHALDPLYINLLAESFVTSHV

FDGLVQRNENGKIVPCLSHTWDVSSDRKTWFFYLRKGITFHNGHLLTAADVVYTFERL

QSTERRTLYRDVSKQILSIEATDPLTVCFKLKAPHELFLPFLSTSRAAIVASQAPYGQ

HKDEENEPDLPNTMQKPIGTGPFKVTTWDNHLCRLEAFPMYFQGRAHLDRVDILQIPW

STPAHLDEKQDIESPFFHLVHNPSSSEGRDWTQISAGVMVRKLLTCNTQKTRPLSDPD

VRAHVQECITKLYGNGMNESNEGDPGDADKVLMNAEELTYLSQHPTFDLPIPLHITTI

PQYRMDARRLASSLEMYGFTCTVRIGTMEQFKGNLRLESDLILFSLIRDRDEELRRYD

LYLTLSEHLEDSSKKSVQEMLQTVVASQISADRTAELDRIEQFLVQQSLLFHLTEKPV

ETAYLPSVRGLSFNSQGWVNLRHIWFP"

CDS 2487034..2488653

/locus_tag="PIKHDJKN_02317"

/inference="ab initio prediction:Prodigal:002006"

/codon_start=1

/transl_table=11

/product="hypothetical protein"

/translation="MIDKIMASPNRALITLLSALMLAIIHQYLFYGNEIGVSYPIFVI

LFYGFMFLFAGDRMRPLTMIDAFMAGVVLMLALTFLLYDNEPLRILNFLVVPGLIILH

MTYLVGRKQRQWWDIGLIGTAMDHLLPQSIRHWGTVASIVVKTGGRGLGKTQKTAAFK

VFIGLIASVPILIVVVALLSSADGIFNQYLSGFPEWLNQLTLTPGLPRVIWILIAGVL

LFSYVWGFVQPMQYEAEKRENAHWKNEKAIVSASEIEKREDVSVNSIQENQTTNKISP

DTIHSQAHRVPFRLDPVIVGTMLLVINCVYVLFVLVQFSYLFGAGEGQLPSDLSYAEY

ARSGFVELILVTGINFFILIIALQFTRSSGKIGSVVQQVLLFVLVGCSAIMLYSAFIR

LNLYEQAYGYTYIRFLVHAFMIFLALLLLIAALRIRYTSIPLIRWYIVLSLAAYVAVN

YVGMDNRIAELNIARYHQTGIIDASYLASLSADAVPLLREFAQREYPNLKGEMLDRQA

NFYLGEAESSWTSFNTARHRAKLELSTLRME"

CDS 2489033..2489899

/gene="yidC2"

/locus_tag="PIKHDJKN_02318"

/inference="ab initio prediction:Prodigal:002006"

/inference="similar to AA sequence:UniProtKB:Q9KDP2"

/codon_start=1

/transl_table=11

/product="Membrane protein insertase YidC 2"

/db_xref="COG:COG0706"

/translation="MEHKTSKGFTFTSGKGRIYGLIAILFAVMLLAGCSNNVSEINSS

TPGFFNHYVVFPLSYLIQHIATIFNGSYGVAIIVITLVIRLALLPLMMRQAKSQQGTR

VIMNAMKPEMDALKKKYEGKNDPADKQKLSQETMELYKKHKFNPLNIGCLPLLIQLPI

LSGIYTAIRLTPELSSHSFLWFKLGAPDYVLAVVVAIIYLIQAKVSQANMAPEQRKQF

AIMGYISPLMMAFFSLSAPAAMPLYWMIGGSFLVLQTLLFRKLYPVEHPQEPVAVQVK

NKNIKPAKPAKS"

CDS complement(2490044..2491159)

/locus_tag="PIKHDJKN_02319"

/inference="ab initio prediction:Prodigal:002006"

/codon_start=1

/transl_table=11

/product="hypothetical protein"

/translation="MTSRMERNQRLITSLEKGTWTSRTYREAVLKQIQNIVPYEAYCF

TTVDPQTLLSTGAVTEDGIEAIHNRLFVNEYMEEDIHKYADLIRSGEYTAILRASISG

NPVSSARYHNILQPAGFGDELRAVLVSGGACWGYLTLYRKTEQPLFTEEERLSIQAWT

PSIALMLRSTSLTLVDEIKSGSPSDPGIMITSDAFELLSVNAPAQYWLSQLRLLEHVG

PDVLPRPVRAVSSHLQRQIRTHTDSTVPQEPSHPSPSKVCIQLLDGRYLVLYASRMQQ

FSGPDQIAIRLEQAMPQDLLPLLAESHGLSIRERELLGYVLRSYSSKEIAEAMHISAY

TVQDHLKSIFSKTDVSSRRELIWYFVSRFQLSDEPAI"

CDS 2491288..2491995

/gene="COQ3_4"

/locus_tag="PIKHDJKN_02320"

/EC_number="2.1.1.222"

/inference="ab initio prediction:Prodigal:002006"

/inference="protein motif:HAMAP:MF_00472"

/codon_start=1

/transl_table=11

/product="Ubiquinone biosynthesis O-methyltransferase,

mitochondrial"

/translation="MDHNQPSMSWDTADENRYEQSIALKIPGYSHMHDLMERLLAASI

TDNNEAKILIVGAGGGKEIALLGSRHAGWTFTGVDPSQPMLQLAERRVQEAGVDARVQ

LKSITLESLPEEGVYDAATSMLMLHFIQGIESKRRFLSNLAARLKPGAPLIIAAVNAD

LHSSVYPTMMQAWQDHMLSNGIQPEEWERFAASLGRESDPISSERMIELMEECGFSHI

TRYFGAFWVEGYYAKRI"

CDS 2491979..2493076

/locus_tag="PIKHDJKN_02321"

/inference="ab initio prediction:Prodigal:002006"

/codon_start=1

/transl_table=11

/product="hypothetical protein"

/translation="MRNESKVESVKDQIWVVGGYGQVGQMICTQLGRSFPGKVWAAGT

RMNRAEEFSRSTGGIVQPLQLDVRKAIDPVVLQSVKLVIMCVDQTDTRFVEACAKAGT

DYIDISANYDFLGQVEQLHSKMQRSKATAMLSVGLSPGVTNLLVREAGARMEQVDEAD

ITVMLGLGEKHGKAAVEWTVDQMNATYQVMKKGKPAEVQSFHDSKWIDFGADLGRRKA

YRFNFSDQHVVARTLRIPTVSTRLCLDSRWMTRSMAISKRIGLFTLLRIPSLRNGTVK

AFGVIPGGEPMFAVKVDAVGWKKGKQVHVEQLLVGEREADATAAVAAAVAERMYRTEL

PHGVFHIEQCLSLQHIQEALRCPLKVTTKIT"

CDS 2493165..2493458

/locus_tag="PIKHDJKN_02322"

/inference="ab initio prediction:Prodigal:002006"

/codon_start=1

/transl_table=11

/product="hypothetical protein"

/translation="MNCIAVLPYYKVQREVTGLAQKVLQSDRSMILYSDKIVTKYREF

NITEVFDMSFRRMGNEGGFFYLHTSTGVYPYMVNIDPKPFIQSFRTMTTQKLT"

CDS 2493539..2494831

/gene="mtaD_1"

/locus_tag="PIKHDJKN_02323"

/EC_number="3.5.4.28"

/inference="ab initio prediction:Prodigal:002006"

/inference="similar to AA sequence:UniProtKB:Q9X034"

/codon_start=1

/transl_table=11

/product="5-methylthioadenosine/S-adenosylhomocysteine

deaminase"

/db_xref="COG:COG0402"

/translation="MSILIQQATILTMKDTDSPFKGDIRIVGDRIAEIAPHIQAQPGD

EIMDGQHKLAMPGLINAHQHTPMSLLRAFSDDLKLMDWLDRKMLPAEARMTPEDIYWG

AQLSIAEMIRSGTTAYADMYIHMNEIAEAVTETGIRASLTRGMVFVEDDGGRRLQEAV

DLVQRWSGKAEGRITTMYGPHSPYTCPVEPLREVIAMAEQENVPIHIHLAETKEEVVK

IRERYGMTPTEYLEEAGVFEKAHVLLAHGVHLNRRDIGRLKGMRGGVAHNPVSNLKLG

CGIAPITEMIAQGINVGLGTDGAGSATTVDMFEEIKAATWLQKLDYGDPTRLSAREVL
HMSTRGSAGLLNLQDEVGVLKVGRKADLILIDLKKPHLQPVHELESLLAYSVNGADVD
TTIVNGRVLMRGRQLLTIDEEELFRQVEVRAKRIVEGI"

CDS 2495006..2496970

/gene="btuD_11"

/locus_tag="PIKHDJKN_02324"

/EC_number="7.6.2.8"

/inference="ab initio prediction:Prodigal:002006"

/inference="protein motif:HAMAP:MF_01005"

/codon_start=1

/transl_table=11

/product="Vitamin B12 import ATP-binding protein BtuD"

/translation="MTILEVKHVKKSYTLYGKEKVPVLHDVNLSFETGEFVSILGESG
CGKSTLMNIIGGMDSDFEGEILVRGKNLSAMTEKEMDDYRKNNIGFVFQNFNLIPHLS
VLENVTIAMQMTDTSEKERNKRAIDILTEVGLKNHLNKRPNQLSGGQKQRVSIARALS
NNPDIILADEPTGALDKENGDQILALLDSIAQKGMLVIAVTHSQKVADSGTRIVKVEE
GRIKDDIHLKDRSLTTYEGSSGASRNLGLLASFKMALKNMKLNGKRNVLVALGGSIGI
LSVLLMLSLGNGITTYMNDEINSSMDPLLVDITKPSADAKDIQGPQALMAAGEPFTTT
DIETIRKFPNVDHVETITTITGKSTMVNAKQSVALTQLTTLTDAFDPAMITTGTLPVE
NEMLLPLDTANKLSGNDQAESMIGKSVFLYINEMDGNNKPVTLEKEITISGIYEASDQ
RSPMQQSPGYISSQTLEQMYTDKGITIGPIQVNAYATDMKYVNDINAAAVDAGFSGSQ
TAKIMENITTYVNMATIVLSGIAGISLIVSGIMILVVLYISVVERTKEIGILRAIGAR
KKDIKRIFFSESALLGVFSGIIAVVFAIIISYVLNILLDNAFGAKLINLSGYYIVFGI
IVSTVISIIAGLMPSSKAAKLDPMESLRYE"

CDS 2497015..2497683

/gene="hssR_3"

/locus_tag="PIKHDJKN_02325"

/inference="ab initio prediction:Prodigal:002006"

/inference="similar to AA sequence:UniProtKB:A6QJK3"

/codon_start=1

/transl_table=11

/product="Heme response regulator HssR"

/translation="MNTILVVDDDSHIRKLIRIYLETNQFSVVEAADGQEALNILSHT
KIDLAIVDVMMPRVDGIELTEDIRSYMDIPILMVTAKGESKDKVRGFNAGSDDYLVKP
FDPVELILRVKSLMKRYNKISSNVIHIGGATINLSNLTVDAGAQTVELKKKECELLFA
LASLPGQIFTRTQLIEDIWGIDYEGDERTVDVHIKRLRVRLEPIPELIISTIRGLGYR
LELV"

CDS 2497680..2499017

/gene="sasA_11"

/locus_tag="PIKHDJKN_02326"

/EC_number="2.7.-.-"

/inference="ab initio prediction:Prodigal:002006"

/inference="protein motif:HAMAP:MF_01837"

/codon_start=1

/transl_table=11

/product="Adaptive-response sensory-kinase SasA"

/translation="MKIVSKSLRLRIVATFIGIVIVSLIFSFILNMRPQEKTPNHTMV
TIAEDIASMINLIDDPEKVKTGLNLFARYGLNINAVNEQSELLASFPADKVHELFDEN
TTEAFILSNKDETAFVGVPRMNEEKDALLIKINFSSIFHNVKRTLFISLLTVLVIGSL
LIMFMSGYIVRPIKRLTAAAKKMASGDLSVRLKHNNPDEFGELMESFNHMARELQKID
SVRDDFVSNVSHEMQSPLTSIRGFTRALQDGVIPLEEQKEHLDIIYEETLRLSRLSDN
LLRLASLDSEHHPFNLSTFQLDEQLRRTIVLAEPQWARKNIKIELDLLPCEITVDKDL
FEQVWQNLINNAIKYTGPDGVIHIEIEMSMSFVKVSIRDSGQGIPEEALPYIFDRFYM
VDKARSSALRGNGLGLSIVIKILKLHDCTIDVESRVGEGTQFTVTIPRPSTTS"

CDS 2499296..2501131

/locus_tag="PIKHDJKN_02327"

/EC_number="4.1.1.61"

/inference="ab initio prediction:Prodigal:002006"

/inference="similar to AA sequence:UniProtKB:Q9Z8L0"

/codon_start=1

/transl_table=11

/product="4-hydroxybenzoate decarboxylase subunit C"

/db_xref="COG:COG0043"

/translation="MKYRNMEDCINDLEQHGHLIRVKEEVDPHLEMAAIHMKVHEAKG

PALLFENVKGSKFQAVSNLFGTLERSKFMFRGTLEGVQRVMAVRDDPMKALKTPFQHI
STGLAAWQALPKQKSISLPVTAQEIQISDLPLIKHWPMDGGAFVTLPQVYSEDPDKPG
IMNSNLGMYRVQLSGNDYELNKEIGLHYQIHRGIGIHQAKAVKKGEPLKVSIFIGGPP
AHTLSAVMPLPEGLSEMTFAGLLAGRRFRYSYKDGYCISNDADFVITGDIYPDETKPE
GPFGDHLGYYSLTHPFPLMRVHKVYAKPNAIWPFTVVGRPPQEDTAFGDLIHEITGDA
IKQEVPGVKEVHAVDAAGVHPLLFAIGSERYTPYQTVKQPTELLTIASRILGTGQLSL
AKYLFITAEDQQPLDTHREVEFLTYILERMDLQRDIHFHTNTTIDTLDYSGTGLNSGS
KVVFAAYGDKKRELCREVSETLKDIRGYSNPQLVMPGIVAIQGVPFTSYVDTEQEMKA
FTDMLKEKGDLSSCPMIILCDDSSFLSANLSNFLWATFTRSNPSHDMYGVNSSYNYKH
WGCDQIIIDARVKPHQAPPLIPDPSVEKGIERFFVQGASLSSIKI"

CDS 2501408..2502499

/locus_tag="PIKHDJKN_02328"

/inference="ab initio prediction:Prodigal:002006"

/codon_start=1

/transl_table=11

/product="hypothetical protein"

/translation="MRIHIMNLQDGDRLTADTFSEAGLHVLGQGTVIKSEDITLLMQH
RVDYVDIEPREEEITEAEFFAAAAKFASGASTSTKEEPPEEEMKSQFIQTVHNYQSAF
LEALTVGKFNATMVDDALQPMVEGLDEQKDVVHLLMMLERDDVNNYTHSIQVGLLSFY
LANWLGYSQKESYQISRGGYLHDIGKCKVSHRIRNKMEPLTADEQLELQRHTIYGHDI
IKSSMTDEATALVALQHHELEDGTGYPMQLKKDEIHPYTQIVSVADIYIGMRSGSHGS
SNPNLINNLRDIYAMGFGKLNEKPVQALMQHLLPNFIGKQVLLSNGERGVIVMNNTSD
IFKPLIKVESEQYRDLSKERTLAINELII"

CDS 2502673..2503131

/locus_tag="PIKHDJKN_02329"

/inference="ab initio prediction:Prodigal:002006"

/codon_start=1

/transl_table=11

/product="hypothetical protein"

/translation="MIEVVTEITIHAPIERCFDYARDIDIHTRTVWKHTREQAVAGVT
TGRIGEGDTVTFEASHFGIRQKLTSRIAEYNPPYLFVDQMEKGAFKSMRHEHSFNKLG
EQTTSMRDTLRFEAPLGVLGWVAERIVLKRYMQAFLESRNLKLKALLENR"

CDS 2503162..2503320

/locus_tag="PIKHDJKN_02330"

/inference="ab initio prediction:Prodigal:002006"

/codon_start=1

/transl_table=11

/product="hypothetical protein"

/translation="MENEYFVGWGTLALINAGLAQGKNRSGRNWFLISLLFGPLATLF

IVVWNKLD"

CDS complement(2503327..2504547)

/gene="sotB_1"

/locus_tag="PIKHDJKN_02331"

/inference="ab initio prediction:Prodigal:002006"

/inference="protein motif:HAMAP:MF_00517"

/codon_start=1

/transl_table=11

/product="sugar efflux transporter"

/translation="MQNPSYQAHPFSEGRPGMSRLVALLFALCSGLAVANIYYAQPLL

DSIADEFRISHSSIGIVITVTQICYALGLLLLVPLGDLLNRRKLIIIQMLLSVIALVT

VGTSSSSQVLFIGMAAIGLLAVITQTLVAFAASLAVPSERGRIVGLVTSGIVIGILLA

RTIAGTLNDWIGWRSVYLFSASLTLLGIIALFLVLPKHEPKREKLSYTQLIVSVMQLY

RELPILRTRGVLAMLIFTAFSTLWTSMVLPLSAPPLSLSHTAIGAFGLAGAAGALAAA

RAGKLADRGLGQNTTGVALVILLLSWLPIGYVHHSLWLLVAGVIMLDLAVQAVHVTNQ

SLIYEVRPEAQSRLTGAYMIFYSIGSATGSIASTQVYAWAGWTGVCWLGAGVSAAALL

FWTIDRYIHRNSDQ"

CDS 2504793..2505377

/gene="comR_2"

/locus_tag="PIKHDJKN_02332"

/inference="ab initio prediction:Prodigal:002006"

/inference="similar to AA sequence:UniProtKB:P75952"

/codon_start=1

/transl_table=11

/product="HTH-type transcriptional repressor ComR"

/translation="MVRQREFDTDQALEAAMQIFWDKGFEATSLSDLTTAMGIQRPSL
YAAFGDKKELFETALRRYTTLHAAQVRSRLQHTSSVKEAFRALFEHIGAEGDVTDTRH
GCFCINTMVELAPRDPKFAVLTREHQMYLAAIFKETIERGQETGELSNDLDASALSKS
LVVSMIGLTVMMKSEPDRSFVQQSIKVTLSLLEE"

CDS 2505411..2505662

/locus_tag="PIKHDJKN_02333"

/inference="ab initio prediction:Prodigal:002006"

/codon_start=1

/transl_table=11

/product="hypothetical protein"

/translation="MERYFHRIYLVVLYIIGVLLTTYGGMGIIEFSLIVIGMLAFIAI
VGSLTENDQSKLDTIFWKIRSLLQVAMAILMTALLFKLF"

CDS 2505853..2506827

/gene="hprA"

/locus_tag="PIKHDJKN_02334"

/EC_number="1.1.1.29"

/inference="ab initio prediction:Prodigal:002006"

/inference="similar to AA sequence:UniProtKB:Q59516"

/codon_start=1

/transl_table=11

/product="Glycerate dehydrogenase"

/db_xref="COG:COG1052"

/translation="MQTKLRCAILDDYQNVALSSADWTPILNRVEVQTFNNYLGSEEK
VVQELQDFEIIVLMRERTPFPESVITQLPKLKLLITSGMRNASIDLRAAEESGVIVCG
TEGSSNPPTELTWALILGLSRQLVTENNALRSNRNWQSTVGMDLYGKTLGLLGLGKIG
SRMAEIAQAFGMNVIAWSENLTQARAEEQGVQWVETKEELLEQSDIVSIHLVLSDRTR
NLIGRAELQRMKNTALLINTSRAGIVDQEAMVEALQNEWIAGAGLDVYEQEPLPVNHM
LRTLPNLLATPHLGYVTQGNYAIYFNHTVEDIEMFMKGTPIRQLLTKK"

CDS complement(2506975..2508012)

/gene="degA_2"

/locus_tag="PIKHDJKN_02335"

/inference="ab initio prediction:Prodigal:002006"

/inference="similar to AA sequence:UniProtKB:P37947"

/codon_start=1

/transl_table=11

/product="HTH-type transcriptional regulator DegA"

/db_xref="COG:COG1609"

/translation="MSKDQKVTIKDVAELAGVGIATVSRAINNSDGISNKTRDKVMQA

IDELGFVPNTSAQSLKIRQTHQIALVVPDIRNAIIPEISWSVEQTAKQHGYHVVQINT

AGNARTELETIRTIKKLHVDGLIFMPLAYPKTLPGLIDKAPLPISMINYGKKLDPGMK

ADVVSLSQPEGKLVMEHLIKIGRTRIAYAGAPKDIIEERYRAYEQALGHVDISLVYFG

EDFSLNTGANAADYFYGLTHMPDAVYAINDMVAIGLVNRFKELGVRVPEDIAVVGVDN

NQWTTVTSPQISSVSIMGEEVARLATELLLKRIREMSTTDYEHIQFEPRLIVRESSVA

MIRSSSQGPRA"

CDS 2508421..2509470

/gene="gutB_1"

/locus_tag="PIKHDJKN_02336"

/EC_number="1.1.1.-"

/inference="ab initio prediction:Prodigal:002006"

/inference="similar to AA sequence:UniProtKB:Q06004"

/codon_start=1

/transl_table=11

/product="Sorbitol dehydrogenase"

/db_xref="COG:COG1063"

/translation="MKALRWHGVKDLRLENIDEPRPEQGKVKIKVEWCGICGSDLHEY

TAGPIFIPAQAPHPLTGEQAPVVMGHEFSGQVVEVGEGVTRFQAGDRVVVEPIYACGH

CEACKQGKYNLCDQMGFLGLAGGGGGFSEYVTAEEYMVHAIPDSISYEQGALVEPSAV

ALHAVRQSKLKVGDTAAVFGAGPIGLLVIEALKASGASDIYVVELSEERKAKAEELGA

IVIDPTQYKVVEEIHARTQGGVNVAYEVTGVPRVLQQAIDSTRIGGELMIVSIFEQEA

PILPNSIVMKERTVNGIIGYRDVFPAVISLMAKGFFPADKLVTKRISLDEVVEHGFEA

LLKEKNQVKILVKAE"

CDS 2509754..2511067

/locus_tag="PIKHDJKN_02337"

/inference="ab initio prediction:Prodigal:002006"

/codon_start=1

/transl_table=11

/product="hypothetical protein"

/translation="MQSNTKWAAVILTIVAGMGWLLMGNSKPKNNVSTDQQPTVERAL

AKVSNAPITSADVTSKIQLLGRPFKKAPYANNVWDLQLYRGKIYMGHGNSSNLGVSPN

AGPIPVIYYDTANPKFITQSVTNSNPSIVPSTKLFVDEEQIDIYKVLNDKLYIPGNDS

DGEQWDMGNFYRLDGDVWTKYRNLPLGVHVYDLASYQGKLFAALGTEQKPTVLISHDQ

GESWQKFATINTFGFRAYTLFEIKGKLYASGMMYPANKIWGDKTNVLEINEKLEKKDV

VIYGSKMLPGLSYQTGTVPYNKIGKNINFNNKLLYIAGGVFNDGQLLPKSLNVMTDIN

QATRVILPDTQALPTDLLLRDRKVYVLTYTRKSANLYINRVYQSTDLNTWNEILRFNQ

DTYAKSFEEHEGDFYFGLGTDPDVLSTSSGKLLRVNRNDIPVNSN"

CDS 2511387..2512490

/locus_tag="PIKHDJKN_02338"

/EC_number="1.-.-.-"

/inference="ab initio prediction:Prodigal:002006"

/inference="similar to AA sequence:UniProtKB:P32382"

/codon_start=1

/transl_table=11

/product="NADH oxidase"

/translation="MNVPSALFKPFTSDKLTLSNRIVMAPMTRGFSPEGVPGPEVVEY

YRRRAAGGVGLIITEGTGINHPSSISGASIPLFHGEESLRGWANVVKAVHEAGGKIMP

QLWHVGTARRSGDLPNAEAEPVGPSGLSMAGEPEREPLSEDEIKGLIQAFARAAADAQ

RIGFDGIELHGAHGYLIDQFFWEHTNRRTDKYGGDLAQRTRFAVEVIEACRAAVGPDF

PIVLRFSQWKMGNYDARLVETPEQLEQFLAPLTAAGVDIFHCSTRRFWLPEFDGSELN

LAGWTQKISGKPAISVGSVGLEAEFVDRANENQGTGDAHLDQLNEKMENNEFDLIALG

RVLLSDPEWPNKVREGRQSEIVPFTAEVLKTLY"

CDS 2512866..2514206

/gene="nagZ_2"

/locus_tag="PIKHDJKN_02339"

/EC_number="3.2.1.52"

/inference="ab initio prediction:Prodigal:002006"

/inference="protein motif:HAMAP:MF_00364"

/codon_start=1

/transl_table=11

/product="Beta-hexosaminidase"

/translation="MYIHSRKLKNKHINTLQMLFLLLGIVLLLSACGQSQKPSSSEQN

NNNAGTETNANSNAGANDQDNDQDDQQNTVPPQEEKDPVQKQLDQLSLDEKIGQMILA

GVQGTTLDAQAKQMITDQKVGGIIFYANNVSTLQGTAKFVQSIKDANRSNPVPIFMSV

DQEGGKVSRMPETVETIPSNGKVGKTKDAELAETMGKLLARQIQLVGFNVDFAPVLDV

NSNPNNPVIGDRSFGSSASLVSRMGIAEMKGLRSEGIIPVVKHFPGHGDTSADSHLDL

PVVNKTEKQLAQLEWIPFEAAVKEQVEAVMVAHILFPKLDPDHPASLSDVIIGQHLRG

KFKYDGVVITDDLSMGAIAKNYKLNDAAVATVQAGSDILLVAHSYESAKTIFDTLMSA

VKSGKISESRINESAYRILALKQKYNLSDEEQQSGDLKQLNADIVDWRKQVDAR"

CDS 2514280..2514984

/gene="graR_2"

/locus_tag="PIKHDJKN_02340"

/inference="ab initio prediction:Prodigal:002006"

/inference="similar to AA sequence:UniProtKB:Q5HI09"

/codon_start=1

/transl_table=11

/product="Response regulator protein GraR"

/db_xref="COG:COG0745"

/translation="MYTIMIIEDDPKIAGLLQSHIERYGDRAVAVEDFEQVVQQFEQI

KPHVVLLDINLPSYDGFYWCRQIRSISTCPIIFISARSGKMDQVMALENGADDYITKP

FEHEIVMAKIRSQLRRVYGDYAARHEERKVELDGLTVYLERLEMELGDRKIQLTKKET

ILLETLLRRSPKLVSRETILEKLWDDSFVDDNTLSVNVTRVRKRLTELGITDALETVR

GSGYRLNSNWKASSPS"

CDS 2514981..2515967

/gene="graS_2"

/locus_tag="PIKHDJKN_02341"

/EC_number="2.7.13.3"

/inference="ab initio prediction:Prodigal:002006"

/inference="similar to AA sequence:UniProtKB:Q7A6Z3"

/codon_start=1

/transl_table=11

/product="Sensor histidine kinase GraS"

/translation="MKLFIKEHIVLTCWVIVVLLTVVAVFWYDGYDHWLTAAYAVSLG

LVLYAAYLIYRYLSHRMFYSRMSQRMNDLKEFVPLNEPAPLPQALSELLDSQYGHYHA

HLHRLEQRQQEYLTFMNQWVHQMKTPLSVIELTVEDQEDEDPRFISIREEADQMRRGL

ETVLYMARLETFEQDFSVEPVSLKAAGEEAIHELKRFFIRNHVYPEMKIDSALMVQSD

AKWIRFVLVQLLSNAIKYSSGGNGQKVTIQAYESTRSIILEVKDQGVGIPKSDLNRVY

QPFFTGENGRHFKESTGMGLYIVKDVLTRMNHRIDLTSVQGEGTTVTITFET"

CDS 2516246..2517016

/gene="yxdL_1"

/locus_tag="PIKHDJKN_02342"

/inference="ab initio prediction:Prodigal:002006"

/inference="similar to AA sequence:UniProtKB:P42423"

/codon_start=1

/transl_table=11

/product="ABC transporter ATP-binding protein YxdL"

/db_xref="COG:COG1136"

/translation="MEICSVKQISKIYKGIVSYEALSGIDLSIQEGEFVGIMGPSGSG

KTTLLNMISTIDHPTSGELQIAGKNPFELNHDDLALFRRRELGFVFQSFNLLNTLTVK

ENIVLPLTLDGVPVEEMNERVDRLAEKLGIADLLNKRTYEISGGQAQRTAIARALIHS

PKLVLADEPTGNLDSKAARDVMEMLDRRNQEDRATMLLVTHDAVAASYCSRVVFIKDG

RLYNEIHYGDSRSTFYQRIINVLSLMGGSGHEFSPVRH"

CDS 2516991..2518949

/gene="yxdM_1"

/locus_tag="PIKHDJKN_02343"

/inference="ab initio prediction:Prodigal:002006"

/inference="similar to AA sequence:UniProtKB:P42424"

/codon_start=1

/transl_table=11

/product="ABC transporter permease protein YxdM"

/translation="MNFRQFAINNVVRNKRIYLAHFLSSTFSVMIFFTYALLLFHPDL

KDGLKGSSGTVTMLANQGFLSAEIIIFIFSFLFLLYSVGSFLKTRKKEFGIFQIIGMT

RKQMNRLLFMENMCIGLAAIVAGVGLGMIFAKLILLICGSMLAVEESLRFYFPVKAIA
LTVGAFLLLFVVIALSSSLLMRKGSLIDLVKSEEKPKPEPKASRLLALLSVILIGGGY
AGVFAFVWITYSFPLLFGSVVLVIAGTYLLFTQLSVYAIRALKGNKRLFFRKTNLLFL
SELTYRMKDNAVMFFMVSVISASAFTGIGTMLAIADPGLSSMTNPYAFTYQNSWDTSS
AERHIRKIEETLIDNEVPYQKGSYIPLFDDEGNYIIKLSEYNHLAKALGYKERTLENV
NDSFMTPGNLTSRKTLRAEAGQGSTQKNIDMRVGETNKKIRLTTPETEIVIPVEYTRE
VYVVSNELYDKMNKEFKNEIQADENFYFDKKTQFVVTDWMGTRKFAPDLIESIQNDSD
GQGYFEISALVLDWLNSKQTNGIILILSGLIGIVFFTFAASFTYFRLYADLERDEEQY
RMIGKMGLSRPELRNIVTRQLVLMFFLPIVLAIIHSSVAFVALQQLVDFSVMGYTLRI
FLVFASMQILFFLLVRWRYLRHMYAKLI"

CDS 2519134..2520573

/gene="hpaH"

/locus_tag="PIKHDJKN_02344"

/EC_number="1.14.14.8"

/inference="ab initio prediction:Prodigal:002006"

/inference="similar to AA sequence:UniProtKB:A4IT51"

/codon_start=1

/transl_table=11

/product="Anthranilate 3-monooxygenase oxygenase
component"

/db_xref="COG:COG2368"

/translation="MSITMSRGQAYLQRLNDERNVWLDGERVRVTEHPAFRGTLQTIE
SLFNLVDDADTRETVAYWDEQTNGYVHQAFQVPRNAQEISNRAAAFRLWSDRTYGVMS
RLSDYARSRLTGWYATRDAMAVYDPAYGGKIAAYYEEAKRKDAFLTIVQRDPQINRAL
PVGEDEDAMLRIIRTNTEGVVLRGAKMVATAAPYADDVIAYPIQRIPGHRPELAHMVI
VATGSPGLHMVCRESFATSDSDNAHPLSMQYDEMDAVLFFDDVFVPWERVLLHNNPEA
VWQIRCNTASSSLAYHQSVIRLHSKLEFITAITSSIAKEIGVDSFLNVQEQLGEMVSQ
MHTIEGLIIASEAQATPDVHGNWLPAFKYIETARNLGTRYYPRAVEILKTIAAGGLIQ
IPSGKLELEGSISPMMNKYLGGVTMQAPEKIRLFQLAWELTGSPLGARHDLYERFYAG
DPVRNRASQYVQYDKERLYARVEPWLRSK"

CDS 2520794..2521501

/gene="phoP_3"

/locus_tag="PIKHDJKN_02345"

/inference="ab initio prediction:Prodigal:002006"

/inference="similar to AA sequence:UniProtKB:P13792"

/codon_start=1

/transl_table=11

/product="Alkaline phosphatase synthesis transcriptional

regulatory protein PhoP"

/db_xref="COG:COG0745"

/translation="MRYTLLIADDEPEIVELLQLYLEKDYNIVTAVNGVEALECIRST

QIDLVILDIMMPVMDGLQLIKQIRSTHHMPVLFLSAKSQDHDKILGLGLGADDYISKP

FNPLEIVARVEALLRRVNQFDAPDVPAAKELNLVLGELRLDRSQCILFRSGKPVTLTS

TEYKIVELLLEQPGRVFTRKKIYEAVWGDYYAHEDSTIMVHISNIREKIERDSRQPEY

LKTIRGLGYKIEAPMES"

CDS 2521479..2522930

/gene="rcsC_7"

/locus_tag="PIKHDJKN_02346"

/EC_number="2.7.13.3"

/inference="ab initio prediction:Prodigal:002006"

/inference="protein motif:HAMAP:MF_00979"

/codon_start=1

/transl_table=11

/product="Sensor histidine kinase RcsC"

/translation="MKRPWKAREKRLLQTSLTLDFLLFNFFLLLLVAIVYIIINLDVV

DFRISDQVVDPDLNVEAHVYVTELENEWLSGSSDEANNTEIQRLKDSGGWIEVLDAER

RVIRYIGDKQDTFTQYSEAELYAGLENRNEQPYYYSISPLSTEGAAKYVLLKIPRDLV

SVRINDNQLITNLKHPLSFYIMIGIGLVLLLIFVYSYWVARRIKKPLSILSLGLTQMI

QGNYSTRMSISAEREFVQIGETFNYMADVIENTSAEKRYAEESKQRLIVDLSHDLKTP

ITSIQGYAQALVEGRGEDKDRQQRYLGYIYNKSVQVARMIQNMLELLKVDSPDFRMHI

QQREIGEFVREIMADTYGEIEQNHFGLHVLVPDEEIYARYDPELLSRVIQNLITNALS

YNPRGTELRVELIPLDNHVVIEVADTGVGIPQELWATIFDPFVRGDEARTGTGGTGLG

LAIARRNTEKMGGRLTLSRRGRETTVFTIQIPK"

CDS 2523030..2524109

/gene="yhhT"

/locus_tag="PIKHDJKN_02347"

/inference="ab initio prediction:Prodigal:002006"

/inference="similar to AA sequence:UniProtKB:P0AGM0"

/codon_start=1

/transl_table=11

/product="Putative transport protein YhhT"

/db_xref="COG:COG0628"

/translation="MFKVNSFVRFSIALALILINIFLLSRVSFIFQPLVTMLTVITVP

MMLSVFFYYLLRPLVNYMEKKKLNRTLSILLIYLVFGILFALFIIGLWPSLREQLINL

VENAPNLINSLSLQLKELEQNGVITALFPEGSTPFSQITDYINKGFTFVTNYVSGFFS

LISSFAIVLFTFPIILFYMLKQGEKFGRKLVHIAPKRFQKDSRDVVLEIDQALSGFIV

GRVLVNLALGVLMYIGFLIIGLPYALLLTVIAVIMNFVPFIGAILSSVPIVIMGLVVS

PSVAIWSLIIILVAQQIQDNLVAPYVFGKKLDIHPLTTIILVLGAGDLGGIIAILIII

PVYMIVKIILVRIYQLFFKDKWQNA"

CDS 2524143..2524541

/locus_tag="PIKHDJKN_02348"

/inference="ab initio prediction:Prodigal:002006"

/inference="similar to AA sequence:UniProtKB:P0A0P8"

/codon_start=1

/transl_table=11

/product="Alkaline shock protein 23"

/db_xref="COG:COG1302"

/translation="MSEIIPQPYTEPEYIEPQSVQMGAIHISNDVVSKIVGMAAQTTE

GISSMSVGLTEGIAKSISGKSLQKGIDVAIKDDQASIQLRINVQFGKKMHEVCQELQH

NVQQAVEQLTGIMVNEIKVQVVGVTMQEQV"

CDS complement(2524619..2525464)

/gene="araC_3"

/locus_tag="PIKHDJKN_02349"

/inference="ab initio prediction:Prodigal:002006"

/inference="similar to AA sequence:UniProtKB:P0A9E0"

/codon_start=1

/transl_table=11

/product="Arabinose operon regulatory protein"

/translation="MITYMCKNNHFQELQLLHYGTEACAPGHHFGPAMRDYYKIHYIL

DGKGTFEVGGKKVTLRKGQGFLIVPHSVVHYEADQDDPWEYSWVAFQGSTCPSLLQQA

CLSEHHPIFEMGNDDDEIRSCLHRMISSRNTHKGWEISMTGLLFQFFSILIDRASEDR

PIPFQDYTKETYVTQVMDFIEINYANTITVQSIASHVGLQRSYLCSLFKDKMGTSIQS

YLVNYRMRRAAELTLDPALTIGDISRSVGYADQLLFSKMFKKVMGEAPTYYRKHKTAP

SHLCS"

CDS 2525668..2526672

/gene="gpr_2"

/locus_tag="PIKHDJKN_02350"

/EC_number="1.1.1.-"

/inference="ab initio prediction:Prodigal:002006"

/inference="similar to AA sequence:UniProtKB:Q46851"

/codon_start=1

/transl_table=11

/product="L-glyceraldehyde 3-phosphate reductase"

/db_xref="COG:COG0667"

/translation="MPYTASEQRYSEMKYARSGKTGIKLPQIALGLWQNFGGNRTLDI

QEEMILRAFDLGINHFDLANNYGPPPGSAEENFGVILKKHLRPYRDELLISTKAGYHM

WNGPYGEWGSRKNLIASLDQSLSRMGLDYVDIFYHHRPDPETPLEETMTALDHIVRQG

KALYVGLSNYDAEQTKEAVTILRRLGTPCLVHQPNYSMLNRWIEDGLQDVLDEHGVGS

IAFCPLGRGQLTNKYVDKIKEERANPTGNLRKEAYTDERIAKFEALQAVAERRGQTIS

QLALNWILRGNRVTSALIGASRVSQIEENVAALQASDLTTEELNEIESILDGIGNYAW

"

CDS 2526968..2528560

/gene="acsA_1"

/locus_tag="PIKHDJKN_02351"

/EC_number="6.2.1.1"

/inference="ab initio prediction:Prodigal:002006"

/inference="similar to AA sequence:UniProtKB:P39062"

/codon_start=1

/transl_table=11

/product="Acetyl-coenzyme A synthetase"

/db_xref="COG:COG0365"

/translation="MNFQQWISPETYNLTSEMENHPSDRVALRWLSDHKELEEITYGE

LLKQANRLAGGLRDLGLEKGDRVLVMVPRRIIAYVIYIACLKLGIAVIPSSEMLRAKD

LEYRLRHSEARAVIVWSETTAEVEKINADLPALAHRIVASPNGDDSVPGEGWVNVHQL

MQGQSDTMAAVETHRDDMAILAYTSGTTGNPKGVVHSHGWGYAHLRIASSLWLDIQPS

DTVWATAAPGWQKWIWSPFLSVLGRGATGLVYNGSFQPKRYLELLQEHQINVLCCTPT

EYRLMAKTDDLGHHDLSALRSAVSAGEPLNQEVIEIFQRHFDLTIRDGYGQTESTLLI

GSLKEAPVRIGSMGQSITPGLIEIINEEGQPVPAGEVGDIAVHKDMPALFRAYYQDEG

RKEANQRGDYFVTGDRARKDEEGYFWFEGRSDDIIISSGYTIGPFEVEEALMKHASVK

ECAVVASPDEIRGNVVKAFVVLRDESLASPELARELQNHVKEWTAPYKYPRKIEFVAD

LPKTNSGKIRRIELREQEKRKV"

CDS 2528612..2529856

/locus_tag="PIKHDJKN_02352"

/EC_number="3.4.11.-"

/inference="ab initio prediction:Prodigal:002006"

/inference="similar to AA sequence:UniProtKB:P24828"

/codon_start=1

/transl_table=11

/product="Aminopeptidase 2"

/translation="MSNFEQTFEKSLEQYAELVVKVGVNIQKGQDLLVTAPIETLEFT

RLIVQKAYAAGAKYVQVDFDDDDITRSRFEHGSDDSFDYYPAWKAEMMEKFAEAGGAT

LTIKVPDPELYKGINSDNVSRATKAAAHARRGYSKYTRNHEISWCLIKAPTKAWANKV

FADIPEEDRINVMWETIFKMNRVDGGDAVQNWKKHLDTLNAMSDLLNKKNYKSLHYRA

PGTDLKIELVNNHIWGGGGGENKQGVYTVANMPTEEVFTMPKRSGVNGFVSSTMPLNL

NGQLVDQMRITFKDGQVVAFTASSGEEHLKNLFATDEGARYLGEVALVPHDSPISNLN

RIFYNTGIDENASCHLAVGSAYPFNMKDGTTMSNEELLKHECNVSLTHVDFMIGSAEL

DIDGELQDGTVEPVFRKGNWAF"

CDS complement(2529951..2530415)

/gene="sbmC"

/locus_tag="PIKHDJKN_02353"

/inference="ab initio prediction:Prodigal:002006"

/inference="similar to AA sequence:UniProtKB:P33012"

/codon_start=1

/transl_table=11

/product="DNA gyrase inhibitor"

/db_xref="COG:COG3449"

/translation="MEFHIETLPTSRIAYVRQVGPYGPANSHAMETLKTWARSKGLLT

KSTILFGIPQDNPETALPSQCRYDACIVISEDYCLDESTSQLEEAELHGGQYLVCKVK

HTAEDVKQAWTEIFPYLQSNGYIMDNKPILERYAGDLLLLDFCEICVPVKPL"

CDS complement(2530694..2531326)

/gene="mhqN"

/locus_tag="PIKHDJKN_02354"

/EC_number="1.-.-.-"

/inference="ab initio prediction:Prodigal:002006"

/inference="similar to AA sequence:UniProtKB:P96692"

/codon_start=1

/transl_table=11

/product="Putative NAD(P)H nitroreductase MhqN"

/db_xref="COG:COG0778"

/translation="MSELENLVKNRRSAIIFEEGIEIPESELQEMFALNKFAPSAFNL

QHTHYLVLTDPAQKEKVYEASQQYKVKTASAVIVVLGDVNAHHHIRTINEGLLNLGAL

TPFQYEQESKSVIEFYETRGRFFQREDAIRNASLSAMQLMLIAQDRGWDTCPMIGFDA

EELQRCLDIPDHYVPAMLITIGKKSDAKQRPRGYRKPINEYVSFNKMHAE"

CDS 2531457..2531696

/locus_tag="PIKHDJKN_02355"

/inference="ab initio prediction:Prodigal:002006"

/codon_start=1

/transl_table=11

/product="hypothetical protein"

/translation="MKTFITRYRAGKSRNEFQVYVIESSTLKRFLVVEMVLGTLAYNV

ALYLFHNALLAGVGSWAGTESVKRLPVVFRKIVGP"

CDS complement(2531860..2532636)

/locus_tag="PIKHDJKN_02356"

/inference="ab initio prediction:Prodigal:002006"

/codon_start=1

/transl_table=11

/product="hypothetical protein"

/translation="MKSFTRKSIVSTILIGSVVAGSAFTTLPFTKELNPVASAASSSF

TQFLHDNAPGRTIKTSSSGLATVTNLSSTVVLVNKKRNLPSTYAPQDLVVPNIPFSFS

GSSPKKQMRKVAATAIENLFAAAKKDGIDIKAVSGYRSYATQKSIFDRNASIKGEAVA

NKTSARPGQSEHQTGLAMDISSASAGYDLQQSFGNTKEGKWLKANAHKYGFIIRYGKD

QEKLTGYSYEPWHVRYVGVYIAGEITSQNLTLEKYLERAK"

CDS complement(2532882..2533244)

/locus_tag="PIKHDJKN_02357"

/inference="ab initio prediction:Prodigal:002006"

/codon_start=1

/transl_table=11

/product="hypothetical protein"

/translation="MHIQITDLAAARLTERLNDQPGYFKVIYDMEGCGCNGIIAILIV

DEPETLDVQIETNLVPFYVDPKQQLNLEQHMKLDTEPNYPSFKLSSDSGVLSSNVRVR

DARAVAAGSSGNSVSCEL"

CDS 2533412..2533996

/gene="comR_3"

/locus_tag="PIKHDJKN_02358"

/inference="ab initio prediction:Prodigal:002006"

/inference="similar to AA sequence:UniProtKB:P75952"

/codon_start=1

/transl_table=11

/product="HTH-type transcriptional repressor ComR"

/translation="MGRAKEFDTESVLRKATAVFGAYGYEGTSLTVLLNELGIARQSL

YDTYGTKHDLFVSALKFYIQQKTEAGIRLLNECTSVRQGMEQLFNEAVNVLSDEGRRN

ECFIINSAVEQAPQNHEIADFIQANNKRMEDIFLAALIRGNKNGEFNHAEDELPGIAR

FLNYSRLSLTFTAKSGASTEVLRELVRTTLRVMN"

CDS 2534126..2534575

/gene="yesE"

/locus_tag="PIKHDJKN_02359"

/inference="ab initio prediction:Prodigal:002006"

/inference="similar to AA sequence:UniProtKB:O31511"

/codon_start=1

/transl_table=11

/product="putative protein YesE"

/db_xref="COG:COG3631"

/translation="MKHSYQMEAGNDMPQRIMHGFTQLLLDGELDQWVELYATEAVFE

FPYSPPGFPKKLEGKAAIANHVHDLMRMIEIQQFSEPVILADHAKQQFVAEFTCEGRS

LVTGKPYNQTYISVVSHQNGKITHYKDYWNPIVVLESDLGGNENDQR"

CDS 2534562..2535407

/gene="azoB"

/locus_tag="PIKHDJKN_02360"

/EC_number="1.7.-.-"

/inference="ab initio prediction:Prodigal:002006"

/inference="similar to AA sequence:UniProtKB:Q8KU07"

/codon_start=1

/transl_table=11

/product="NAD(P)H azoreductase"

/translation="MINDKPLTLITGANGKTGSRVVAMLQKHHYPVRLAGRKKPSDSG

DNYVYFDWYDSTTYGSALKNVDQVYLVVPVMDMHPEAVMIPFIKEALWSGVKRFVLLG

SASVDEDGPIFGKVHQSLKELAPEWTVLQPSYFMENFTEGPHRETMKQFGKIYSATGD

GKIGFVSADDIAAVAFHALTDVVPHNTAHIITGPETLSYGQVAGMFSRLLDKSIQHES

LSDAELRNSMIQAGMSEDYATALAGLDVFIREEGREDQTTETVLKVTGQSPVSLEQFI

RQHMQ"

CDS 2535642..2536685

/gene="btuD_12"

/locus_tag="PIKHDJKN_02361"

/EC_number="7.6.2.8"

/inference="ab initio prediction:Prodigal:002006"

/inference="protein motif:HAMAP:MF_01005"

/codon_start=1

/transl_table=11

/product="Vitamin B12 import ATP-binding protein BtuD"

/translation="MIQFENVSKQYPDGTHALRQVNLNINKGELFVMIGPSGCGKTTM

LKMINRLIERTDGTVRINGRPIDEYNIHELRWNIGYVLQQIALFPHMTIAENIAVVPE

LRKWKSEQIKERVHTLLDMVGLKGTTYSDRKPAELSGGQQQRIGVLRALAADPEIVLM

DEPFSALDPMSREKLQDDILDIQRQMKKTIVFVTHDIQEAMKLGDRICIMKDGQVLQV

GTPEELIQQPANEFVRDFVGSSGPDRSSQPISGGGDPMYELRAATGEQERLNSIFDLE

SIMSPLSPGHVPKSAKTAVPVSITLPEFVEIMASHDHLLVEKEHQIIGQVSRADLIRY

WSGQLQERGDGHE"

CDS 2536672..2538192

/locus_tag="PIKHDJKN_02362"

/inference="ab initio prediction:Prodigal:002006"

/codon_start=1

/transl_table=11

/product="hypothetical protein"

/translation="MDMSKFTEVFSERKGQLLSALLEHIQISFIALFFAVLIAIPLGI

YLTRKPRVAEPIIGVTAVLQTIPSLALLGLLIPLFGIGTLPAIIALVVYALLPVLRNT

YTGISEVDPSMVEAANAMGMNSRQRLTKVELPLAMPVIMAGIRTAMVLIVGTATLAAL

IGAGGLGELILLGIDRNDTALIILGAIPAALLAILFDVLLRQFQRLSFKKTLVTLGTL

ALVAVLVITIPGLSRGGQKDLVIAGKLGAEPEILINIYKLLIEKDTDLKVELKPGLGK

TAFLFNALKSGDIDIYPEFTGTAISEFMKETAVSTDRTEVYEQARDGMLSQFNMVLLN

PMDYNNTYTLAVPQKVADQYNLKTISDLKSVQQQIKAGFTLEFSDREDGYVGIQKKYG

IKFPNVATMEPKLRYSAVQRGDINLLDAYSTDSELRQYKLVVLEDDQELFPPYQGAPM

LRKEKADQYPQLVELLNQLAGRITDDEMRQMNYEVNVNGAIPEQVATDYLQKAGLL"

CDS 2538232..2539575

/gene="garB"

/locus_tag="PIKHDJKN_02363"

/EC_number="1.8.1.16"

/inference="ab initio prediction:Prodigal:002006"

/inference="similar to AA sequence:UniProtKB:D0VWY5"

/codon_start=1

/transl_table=11

/product="Glutathione amide reductase"

/translation="MTQTYDLIAIGTGSAASSVISRCAEAGWKIAVIDEREFGGTCAL

RGCDPKKVLAGAAELVDWNERMKGKGVDGNASIQWSELMAFKRTFTESIPRASEDKFK

QAGMHTFHGKASFVDEDHIRVGEEVLQGKHILIATGARPAPLPIDGAEHLKYSDDFLD

LEQLPDKLVLVGGGYIAFEFAHIAARAGTEVHIIHRGEEPLEQFDSELVDLLLQKSEE

VGIHVHLNAEVKAIRQQGNAYVVSGTRNGADHQWQCGLVVHGAGRIPNVDGLELEKGN

VSYTKKGISVNEYLQSESNPRVYAAGDVTATEGLPLTPLAGQESRAVFLNLLEGNQNK

PNYKVMPSIVFTVPSLGSVGLTVEQAKKEGYEVKVNDMSKWYTYKRTNEKFAMAKVVI

DKATGRILGAHVLGGKTEELINLFAMAIQFDLTTDQLITMNFAYPTAASDMGSLF"

CDS 2539605..2540213

/locus_tag="PIKHDJKN_02364"

/inference="ab initio prediction:Prodigal:002006"

/codon_start=1

/transl_table=11

/product="hypothetical protein"

/translation="MSANFHRIHDAEWTTEPVQARTEGDRLIVEAQEGSDFWEKTFYG

FCHQNGHAMLAPWDGTKAIEVSFDLSSFTELYDQAGLMLWHGQDQWIKAGVEVNDGVA

HVGAVVTDRFSDWSLSPVPEWGGRIVTIRASYHDEAVVIRARTDEHPWRTIRVARFAY

PTNKQAGPFLCSPKRAGFEVAFTKWKLTEPDQDLHTDPPIAD"

CDS complement(2540413..2540895)

/locus_tag="PIKHDJKN_02365"

/inference="ab initio prediction:Prodigal:002006"

/codon_start=1

/transl_table=11

/product="hypothetical protein"

/translation="MKIMIEVKQSKLGDGEFNRGVFATVDIPKGQLIHQAPVVPYPNE

DHEHVEKTILEDYVFEYGANHTAILLGYGSLINHSYEPNATYDINFENHTFDFYAYTD

IKAGEEVLINYNGEEDSMDPLWFLDDYEERMREFNETNDGEKDADGIESDSKDTDSSK

"

CDS 2541132..2542688

/gene="ybiT_1"

/locus_tag="PIKHDJKN_02366"

/inference="ab initio prediction:Prodigal:002006"

/inference="similar to AA sequence:UniProtKB:P0A9U3"

/codon_start=1

/transl_table=11

/product="putative ABC transporter ATP-binding protein

YbiT"

/db_xref="COG:COG0488"

/translation="MSILNVEKLSHGFGDRAIFNNVSFRLLKGEHIGLIGANGEGKST

FMNIITGKLQPDEGKVEWSKRMRVGYLDQHAVLSKGQSIRDVLRGAFQYLFDMEQEMN

DMYGKMGDVTPEELEQLLEDVGTIQDTLTNQDFYMIDAKVDETARGLGLTDIGLDKDV

NDLSGGQRTKVLLAKLLLEKPDILLLDEPTNYLDELHIEWLKRYLQEYENAFILISHD

IPFLNSVINLIYHMENQDLTRYVGDYSHFQEVHEMKKQQLESAYKRQQQEIADLKDFV

ARNKASVATRNMAMSRQKKLDKMDVIEIAKEKPKPQFNFRDARTSGKLIFETKGLVIG

YNEPLSRPLDLRMERGQKIALVGANGIGKTTLMRSILGEIQALEGTVQRGEHLEIGYF

QQEMKDANYNTCIEEIWQEFPSYTQFEVRAALAKCGLTTKHIESKVAVLSGGEKAKVR

LCKLINNETNLLVLDEPTNHLDVDAKDELKRALKAYKGSILLISHEPEFYRDVVTETW

NCESWTTKVF"

CDS 2543105..2548309

/locus_tag="PIKHDJKN_02367"

/inference="ab initio prediction:Prodigal:002006"

/codon_start=1

/transl_table=11

/product="hypothetical protein"

/translation="MDKLRKPLIFMLSATLALSSGPLMLPSHAYADADRMPATLLQDD

FSDGDYTASPVWDVSSGSWAVMADPTDASNSTLFQSDTGEGIISTGDVMSDVTVSMRF

YTGGGQGFPGILSRLQDKSNFYYFQMQAPNNKLVFSKRVNGTDMTLKSVDYTFTKNTW

YTLKMVLSGSSIRGYIAENGSDRLVFDLVDTTYGSGTVGIRNKWQSVHIDDVIIAEQP

PGNDLQLSNDEQTASSVSLQWTEAAGATTYHLYRSSTPESGYSLITNTGSLGYVDEGL

NADMVYYYKLAYEYGGMTESLWSAPLEVRTTAVAPQAPGELKALALDATSVKLSWPAV

DKATGYRVARAEAGSDQYEQVFEGTALSYTDQGLEPGNSYSYRVTAFNAAGESAFTVA

EANTYSIDSPAEFAATAVTDTSISLGWNVLPGSDVTYTVSRATSAAGTFQQVYSGKES

TFIDSGLTMGTGYFYMIQATVDDIVSPASAPLGVATIRTSFTPGQLWPDQDGKPIDAH

GAGFFYDEQTEKYYWYGEYHTGGWPAVGVRVYSSKDLLNWTDEGMALTMIQSMDDFDK

NLLIAKLYEGRDDKVDIWADIRKGRIIERPKVIYNDKTKKYVMWAHMDGDKDPYNDNA

NYGKARAGYAISDSPTGPFVYQKSYRMDRTPEGEKVYFPSDRGMARDMTLFKDDDGTG

YLIYSSEENLTLYISKLNEDYSDVTGWHKEGRTDDKGNSVRDSTYQAEYGVDYVRVFP

GGQREAPAMFKYQGKYYILTSGASGWAPNENKVTVADNIFGPWSTMTNPFVRTLPSDP

DPGKAFGTQTTSVIPVDPEKGKFIYVGDTWNGGNFSNDGAKYVFLPIEFGIGSDIAIK

WYNSWSPDLLNSMGKVDIADPLPEAVPLGKLPSLPTTVNVRDGGTLVSTPAVWTVDNR

VMTAEDFAKPGPITLQVTTPEFGNKKQSIRVYVIPENAQYFVNSGGYETADYKLMDAY

MKGSLVNSDTADQMYAPVEGRNWGYVSADALASGSSGGDIFSTVRYLNGSNVSNSPKG

SDLTYTFGVSNGTYDIYAGFNDPWTNTSRKANFLINGTNTGAVTYTPASVRAHTGIVV

TGNKLELTVRNTASQDPLISWIIIAKSDAVPPADDSAGLNAVISAATSASLHWHSILG

AASYKLYRSDREKGEYSVVYNGSLREYTDNELSPGTSYYYKVEAFDASGHSMRGLSSA

YHVLTAQQTAADVAASITALEQPSAGAKRLKLPTVPQGFAVEIASSSVPTVIQADGMI

IPPSKETSVTLELEITRTSDDSKAMTIPLTVKVSAYTPSPGGSGSDGSGSDSGGNSGG

NSSSSGGLPGSGTPSTENSSPRPESQKDRAVLELQGIVDQKGVVQTKVDASAIEEALK

VAPASTGHRLVELRQSPVSGANAYELLLPVSALVGQGDANVFNIVTELGTLELPATLS

MKDLAGYETVSVRLIRTQLSQTVADQLGTQHGVRFEIDLDGDAWPAGSELTLRLPYET

LQNVQPDMIVGFAIDANYVATPLPQSRYDQNSREVVFTVTSAAGNYAAVSVQQTFTDL

ADVQWAKNAMEALAARGVFDEGASRDYRQFHPKQEMTRGQYVQWLMTALSLNTLRGNA

FSDVSAEASYYEAVTAARAFGITGGTGDGRFMPEATITRQEMMTLTVRALGVAGLVEP

ESASADSLVQFRDASTIRPYARDSVAMLVKLGIVGGYNGEVKPLAQATRAESATLLYA

MMSKLVWP"

CDS complement(2548476..2549036)

/gene="azoR_1"

/locus_tag="PIKHDJKN_02368"

/EC_number="1.7.1.17"

/inference="ab initio prediction:Prodigal:002006"

/inference="protein motif:HAMAP:MF_01216"

/codon_start=1

/transl_table=11

/product="FMN-dependent NADH-azoreductase"

/translation="MKIKLVVTHPRQDSLTFAVMNRFIEGMKENDHEIDILDLYHDGF
DPLYSVEDERDWQNPDKQHAPVIRKELDRVLAADAIVFIFPVWWYNVPSMLKAYLDKV
WNMGLLNKANSKKALWIALAGGTEASFHKYDYYNMISNYLNNGIAGYAGLQESRVEFL
YETISDSKTHIEGLLEQAYQIGRHYK"

CDS 2549215..2549604

/gene="ytcD_2"

/locus_tag="PIKHDJKN_02369"

/inference="ab initio prediction:Prodigal:002006"

/inference="similar to AA sequence:UniProtKB:O34533"

/codon_start=1

/transl_table=11

/product="putative HTH-type transcriptional regulator
YtcD"

/db_xref="COG:COG1733"

/translation="MEQELKRYESGVQAMLELVGGKWRILILHQLITGKKRTSELRRA
IPGITQKVLTQQLRDLEKNEIIHRIIHPQIPPKVEYELTEYGLTLQAIIDRICLWGEN
HLDRVYGDKNKVLENHFSEYIPLSTSN"

CDS 2549659..2549847

/locus_tag="PIKHDJKN_02370"

/inference="ab initio prediction:Prodigal:002006"

/codon_start=1

/transl_table=11

/product="hypothetical protein"

/translation="MLFFCPLSVKLINTIKSSKLTPTIGYGRFKRVEINMIILVGIKI
DVMREVANGGTSSDKQPF"

CDS 2549813..2551657

/gene="cysJ"

/locus_tag="PIKHDJKN_02371"

/EC_number="1.8.1.2"

/inference="ab initio prediction:Prodigal:002006"

/inference="similar to AA sequence:UniProtKB:O32214"

/codon_start=1

/transl_table=11

/product="Sulfite reductase [NADPH] flavoprotein

alpha-component"

/db_xref="COG:COG0369"

/translation="MVELQVTNSPFNQEQVELLNRLIPTLNDGQKTWLSGYLTAIQGF

AAVAAPAGVVQQPASSAGITPESAPAAVSREVTVLFGSQTGNSSGLSKKVAKKLEEQG

LQVILSSMGDFKPNGLKKVENLLIIVSTHGEGEPPDNAIPLHEFLHSKRAPKLDGLRY

SVLALGDTSYEFFCKTGKDFDIRLQELGGTALVPRVDCDVDFDEAAAEWMNDVLASLS

STPATAGVVTSEAVGAAVSSGESEFNRTNPYKAEVLENLNLNGRGSDRETRHIELSLE

GSDLDYEPGDSLGVFPENHPRLVDELIASMEWNADERVTVNKSGDQVSVREALLRYFE

ITAVTRPVVEQLAKLSPGSGLPALLADDSEFRKVMNSCDLLDLVQDHGLKGIPAASFV

AVLRKIPARLYSIASSSKSFPDEVHLTVRTVRYESRGRERYGVCSVHLAERVELGDTL

PVFIQQNPNFKLPENPDAPIIMVGPGTGVAPFRSFLGEREETGAEGKTWLFYGDQHFS

TDFLYQTEWQRWLKDGVLTKMDVAFSRDAEEKVYVQHRMLEHSKELYQWLQEGAVVYI

CGDEKRMAHDVHAALATILEQEGGLTPEQAAEYLTRLQQEKRYQRDVY"

CDS 2551712..2553433

/gene="cysI"

/locus_tag="PIKHDJKN_02372"

/EC_number="1.8.1.2"

/inference="ab initio prediction:Prodigal:002006"

/inference="similar to AA sequence:UniProtKB:O32213"

/codon_start=1

/transl_table=11

/product="Sulfite reductase [NADPH] hemoprotein

beta-component"

/db_xref="COG:COG0155"

/translation="MAYNNLLNPQRTNSDVEDIKIKSDYLRGSLTETLADRISGAIPE

DDNRLMKHHGSYMQDDRDLRNERNKSKLEPAYQFMLRVRASGGIVTPEQWLMMDRVAH

KYANETIRLTTRQSFQLHGVLKWDLKNTIREVNDSLLSTLAACGDVNRNVMCNPNPDQ

SDIHAEVYEWACQVSNHLDPRTRAYHELWLDGEKIIDSQDTDEEVEPIYGKVYLPRKF

KIGIAVPPSNDVDVFSQDLGFIAIVENGKLQGFNVSVGGGMGMSHGDPKTYPQVSKVI

GFCTPEQMIDVAEKTVMIQRDYGDRAVRKHARFKYTIDDRGLAWFVEELTSRLGWKLD

VAREFHFEHNGDRYGWVKGSNGRWHYTLFIQNGRVKDVDGYPLMTGLREISKIHTGDF

RLTANQNLIIGNISSQKKKKIESLIEQYNLTDGAHYSALRRSSMACVALPTCGLAMAE

SERYLPSLIDKLEPVLDEAGLRDEEIVIRMTGCPNGCARPMLAEISFIGKAPGKYNMY

LGGSFTGHRLNKLYKENIGETEILDTLTPMVNQYARERNDGEHFGDFVIRAGYVPEVL

DGQQFHA"

CDS 2553645..2554361

/locus_tag="PIKHDJKN_02373"

/inference="ab initio prediction:Prodigal:002006"

/inference="similar to AA sequence:UniProtKB:P67182"

/codon_start=1

/transl_table=11

/product="putative transcriptional regulatory protein"

/translation="MGRKWNNIKEKKASKDANTSRVYAKFGVEIYVAAKKGEPDPEAN

RALKVVLERAKTYNVPKAIIDRAMEKAKGSGDENYEELRYEGFGPNGAMVIVDALTNN

VNRTAPEVRSAFNKNAGNMGVSGSVAYMFDPTAVIGVEGKTSEEVLELLLEADVDVRD

IVDEDESVIVYAEPDQFHAVQEAFKAAGVTEFTVAELTMLAQNHIELPEDAQAQFEKL

IDALEDLEDVQQVYHNVEFV"

CDS complement(2554447..2554935)

/locus_tag="PIKHDJKN_02374"

/inference="ab initio prediction:Prodigal:002006"

/codon_start=1

/transl_table=11

/product="hypothetical protein"

/translation="MIITGILLALLAGSLVSLQTIFNSKVNERTGSWSTTTLVLFTGF

IASFLISLMVEGKNTFSFQHMQPWYWLSGAIGVGVVFCLVQGMKLLGPTFAISIVLTS

QLSFALLFDSMGWLGLEQIPFSWNQLLGVLVIVGGIVLFKFGGSKSAKSKKSPGALQS

DS"

CDS complement(2554951..2555649)

/gene="yeiL_1"

/locus_tag="PIKHDJKN_02375"

/inference="ab initio prediction:Prodigal:002006"

/inference="similar to AA sequence:UniProtKB:P0A9E9"

/codon_start=1

/transl_table=11

/product="Regulatory protein YeiL"

/db_xref="COG:COG0664"

/translation="MEEFQNEHQLLHYIKQYQLESVFHEPLRPHMTLCHFEKCELICR

EGETSEYLYVLVEGKVKIFTTSPQDKTLVLCFKTPLEVVGDIEYVRESNVVNTVQAVS

PVVMLRIHYQWLAELASDYAPLLKFLLKIISHKFYIDSNFSNFNLMYPVEVRLASYLL

SISTEEAGTVVHEELDAFNLTDIANLIGTSYRHLNRVIQKLCADGLIMRDQGFIMVKD

RAGLAEVAGHNIYE"

CDS complement(2555684..2556109)

/locus_tag="PIKHDJKN_02376"

/inference="ab initio prediction:Prodigal:002006"

/codon_start=1

/transl_table=11

/product="hypothetical protein"

/translation="MRGIIFALLGGACITLQGVANTRISTDIGTWQAATITQLTGFIL

AALILVFVRDINLQGLKQVKPMYLAGGAFGAVIIFSEVTAIQQIGVTFTISALLIAQL

FLTFLVDSNGWFGVVKQKMKLPQFLGIALMVTGVIIMKL"

CDS complement(2556279..2557076)

/locus_tag="PIKHDJKN_02377"

/inference="ab initio prediction:Prodigal:002006"

/codon_start=1

/transl_table=11

/product="hypothetical protein"

/translation="MNITIQHIMDHLTVEVELPETTVDQLITGSPNQTVTGVFVTFMP

TQHVIEHAIQRGANLIIAHEPPFYNHHSHTDWLADDPVYETKRTLIENGGIAIYRCHD

AIHRYQPDGITEGLVQALGWSLYVEQRRPEADLLSFPEGMTVQLIAEHIKRSLGIEYV

RLAGDPKMICRRAAVLVGFRGNGNQTIPLLQQEELDLIIAGEGFEWETPEYIRDAVQQ

GKEKALLMIGHAESEASGMKLLSERLATAFPEVPVHFIGEQPVFQVL"

CDS 2557348..2557953

/locus_tag="PIKHDJKN_02378"

/inference="ab initio prediction:Prodigal:002006"

/codon_start=1

/transl_table=11

/product="hypothetical protein"

/translation="MTASTIMERLKSETAHYHRQVEQNDYAKAIMNQTVSLGEYKKYL

EKFYGFLKPLEDQAVQQPFWENTGLDIGIRGKANLLENDLRNLGASEEEISQVPLCKD

LPDISTPARLFGYLYVIEGSTNGGQIMTKRLSQFLPIEADRGLEYFNAYGTETRTRWA

EFTELLRQSITAEEDHDIMVHTASETFRLLDQWINTNTVHL"

CDS 2558455..2560038

/locus_tag="PIKHDJKN_02379"

/inference="ab initio prediction:Prodigal:002006"

/codon_start=1

/transl_table=11

/product="hypothetical protein"

/translation="MKEDSRQLEFMEIDLSEEPITAAVPVPDRSSIVRADFDIRLPGG

ILSSEKRFVEEARQLIEVEGEQAPWVPFMSYWPTYGVMNEPQRQWYMYWRTEVRQGRF

PDTDLSYLFVHIYELINGIGWQEQEEGYDQLKELWINYRERLPQLDVYMQEWIIDYDL

VHELNMSLSEMVELSNGNLPPEILDMELQRLLSSNISDISLKLLQRYYDYDITLSKFY

RDGGKKVLEQYIPRVMVLVDSYLLRTRKAGILDQFELNHERTIERTLFRKAVYDESIY

GKSVRITYVPIGDHAEFVQFVTRVFRCTENKCRELLGFRGRLRGKTLELELAKVIERY

LDKAFAVEQMPAVEQRIVRIDAEKLASLQQESEYVRRALMIEENHPSEVEHTNNVGSQ

APMAIQNSTDEAESAKQSNLSEVDVEGALKTDGGQGDGNSEPLSLQWEADFSADLDEE

WIQFSEMLSPQHVQAIYALLDVNPDTELMRVAEQYGTMPALLLDEINDVAMETIGDLL

IDGDRIVSDYMNVFEHVKR"

CDS 2560046..2561386

/locus_tag="PIKHDJKN_02380"

/inference="ab initio prediction:Prodigal:002006"

/codon_start=1

/transl_table=11

/product="hypothetical protein"

/translation="MAELKIPKRLTTALVNSLTAGVVPRIGLEQIAVGRKSEVDAILR

DMDNIAEGGAAFKLITGRYGSGKSFLLQMIRNYAMDREFVVADADLSPERRLVGTKGQ

GLATYRELMTRLSTRTRPDGGALEPILQKWIAGLQQQAMQSQGLRPDDPALPAEVEKQ

IYAVTNEMQNLVHGFDFAKVLASYWNGYKLADDDRKQAALRWLRGEFATKTEAKKELA

VGVIIDDDNWYDYFKLWSEFTARIGYKGLLLFIDEAVNLYKITNSISRQSNYEKLLTM

FNDTMQGKAEHLGIFVGGTPQFVEDERRGLFSYEALRSRLIDGRYAAREYANYTGPIL

KLSMLSHEEILILLQKLRQIHALHFGYSASLSDEDLVDFMQTAVNRLGADELLTTREV

VRDFMDLLHTLHQNPEVTYVQLLGERAVKPQESGKGTDSSSADDLDDFLAEFEL"

CDS 2561383..2563596

/gene="srmB"

/locus_tag="PIKHDJKN_02381"

/EC_number="3.6.4.13"

/inference="ab initio prediction:Prodigal:002006"

/inference="protein motif:HAMAP:MF_00967"

/codon_start=1

/transl_table=11

/product="ATP-dependent RNA helicase SrmB"

/translation="MSENPFYRLAPFVQEFIYKKRWESLRPAQIEACNICFHTPHHML

IAAGTASGKTEAAFFPALTELHERPSKSVGILYIGPLKALINDQFERLKDLLSEGNIP

VWHWHGDVPQAEKTRLMRSPSGVLQITPESLEGLLMNRPNAIPALFHDLRYVIIDEVH

AFMGADRGIQVLSELARIERMAGCKPRRVGLSATLSDYDAATAWLAAGTNQGVDVVSS

PGGRKLRLRVEHFSFPDTRDEEQAEHLHNARKVYYDFIYESTHRKKALIFTNSRTDAE

VTILEMRRVAARRQERDVFHVHHGSISAMLREETEAALRTGSGPAVAAATVTLELGID

LGELERVVQLGAPYSASSFVQRLGRSGRREDMASEMLFVCPEEEDEEAQLPARMPWTL

MRAIAVIELYVKTKWVEPLEARKMPMGVLYHQTMSMLKSMGEAEPKELAEAMLSLAPF

AQIRADQYQLFLNYLIETDHLQWTEDRTLIIGLTGEKTVNNYRFYAVFKDDEEHKVLN

GSEEIGSITTVPPPGYCFSLAGKLWKVEEVDHKHKSVYVKAAKGKVDTLWLGAGGDIH

TTVVQKMREVLSDTVIYPYLSPQAVNRLERARRLARESGLLKQVVIPAGGDSLYVLPW

VGSKSFRTLERLMKHNLSEKLALRSVVPMEPYYFVVSGKVDGRTLLAEIMSECRTAKD

ASALLSEDEAPYLGKYDEFVAPPLIREAFAVDGLDLNGLKEGLQQTLTWDSSSMK"

CDS 2563932..2564885

/locus_tag="PIKHDJKN_02382"

/inference="ab initio prediction:Prodigal:002006"

/codon_start=1

/transl_table=11

/product="hypothetical protein"

/translation="MKYYLSVLAGAMSYGILSTIVVLAYGEGYKLGEVVGTQLITGFI

LSWMLALYSKFRMKRKSQADGKTSAAVAKAFQKLTWKQRLVLMAAGTPTVITGLVYYQ

SLRYIPASLAIILLFQFTWISVLIQAVSKRQRPDKVTFLTLIILFGGTLLAAGFLEQG

LGEFNGLGIALGLMAAVSYSLFVMFSGKAVPSAHPAFRSAWMVTGGLVLLCILFPPTF

LFNGLIWSQLLVFGLLLGFFGAFIPPVLFAVGVPHIGGDMAGILGAVELPIAVLLSSI

VLHEHVSPLQWIGVIVVLIGVALPEIYKLRMRRSRNTPLYS"

CDS 2565089..2565514

/locus_tag="PIKHDJKN_02383"

/inference="ab initio prediction:Prodigal:002006"

/codon_start=1

/transl_table=11

/product="hypothetical protein"

/translation="MKSWISKTWPWLTLGITVVVLLGSFMVYFMGKDMSPGSGSAVTA

KAETPEDLKGYEVVNVNVSNDGFEPDVIEVKSGVPTKINFILTRQVTHVKSVASRKLG

MDLYMQKGDNYYTLDKDLQPGEYEIHCGMYMIYGTVKVI"

CDS 2565612..2566634

/locus_tag="PIKHDJKN_02384"

/inference="ab initio prediction:Prodigal:002006"

/codon_start=1

/transl_table=11

/product="hypothetical protein"

/translation="MKALFIGGTGTISTAITDQLAKQGCELYLINRGNQNGNLPAQIK

VLQADINDEARVAELIADLEFDVVADFIAFVPSQLERDYRLFKDKTKQFIFISSASAY

QTPLADYRITEGTPLSNPYWEYSRNKIACEDYLMKQYREEGFPVTIVRPSHTYSERSV

PLGVHGAEGSWQVLKRIRENKPVLIHGDGTSLWTITHNRDFAKGFIGLMGNIHAIGES

VHITSDESVTWNQIYEIIAGVLGVKLHAVHVSSEFLAACSDQDLRGGLLGDKANTVVF

DNSKLKRLVPEFVATIRADQGIRSTIEYILAHPELQTEDPEFDAWCDKVVGALDEALL

KIRDEK"

CDS 2566644..2567300

/locus_tag="PIKHDJKN_02385"

/inference="ab initio prediction:Prodigal:002006"

/codon_start=1

/transl_table=11

/product="hypothetical protein"

/translation="MSQTGHRLVKEITKTVTLDYLLHIPEKEEPPAEDQKWPVILFLH

GAGERGNDLEMLKAHGVPLIADQDKSFPFIVISPQCPVDEFWGMHREAVMALLEHVLE

NEAADPNRVYLTGLSMGGYGAWDLPLFYPNTFAALAPVCGGADPSKAEELYNIPIWAF

HGAKDDIVYVTESEKIVHALEALNADVKLTIYPEGNHDAWTETYDNPELYKWFLSHTL

"

CDS 2567475..2574965

/locus_tag="PIKHDJKN_02386"

/inference="ab initio prediction:Prodigal:002006"

/codon_start=1

/transl_table=11

/product="hypothetical protein"

/translation="MKKSEKRIKKQAEQLTKVVLAFALLSPQALLVEWGSTAIMAEAV

ETISIVSDTSSMKAVQSSKVKVEMNSDGKYRIVLLPNTNVFYGGDTGNVSTIIDHNGT

PVNFKSLPLNYYRINNNVIEMSRQKDNVEYILRVSIVNATSQGGYMKVELEAVNRSGS

TLNLGGTFYWDTMVNGNDASPFEVIENGWRNYSGGVQVTAFYANTYNVVDADRIFMGQ

YNSPDNAQLTGGSSPSSFTPGQTVTATDTAAQFWWNGKATANQASRKFSTIVGIGPKN

APPSFALSAPSSGQTYYKGEQLQISGTTRDTDVGDLLTVKWSIDGGSENILTQMTATG

SNQSFNTNYTLPDTLVDGAHTLQVWVMDDKGGVSSAGTVNFTVKSFVVPGTPTFTLVN

NNNLTVNWDKKANDASVTYELKNVTTNQSFDTGTSNSRQVTGLTPNTQYSFAVRAKNS

VGSYTGYSSPSSKFTLANAPSDAAVSQSGNSVTASWNNNGNPAGTNYKTEIRNSVGQV

LASGTTSSTRTELALTGLADGLYNVYVAALNGEGIQTAFTSAGQITKDTTGPTTPSIA

VNPSSWTKEDVLVTITAGTDALSGVQKTEYKLGIGGEWKEYTAPITVASEGNTLIIAR

SIDAFGNTGQEASVTARVDRTAPTPPVISLNPTEWTHSAVTVTLTAGTDEASGVGLTQ

YRLGSEGTWVDYREPFTINTEGITEIQARSVDRASNVSASTSATARIDKTGPDEPKIT

LSDDEWTNQDVSFEITSGEDTGSGLAKSQYRLNEQGPWIDYTGEVKVTEEGQTTVYAR

SLDRVGNVSTVAKAIIRLDKTAPTEPVITLSQSGWSKEAVQFTIVGSVDEHAISYEYS

LNGAPYIPGNSGTVSSNGPNVIRARARDAVGNVSKEISRTAYVDQVAPTITLTPNGQG

WSDTNISATVQYEDSDSGINENSRLYSVTNSTASPDNWNEARSNEPVISIESEGIWYI

HAKTMDMAGNTYETVSAPYQIQHKPQQPGNASITQISETSAELTWDLPTGEWYTDGYQ

YEIINHTTGQSWTLDYPKHTLIDHSLSGGQVYDYEVRVRNHTGISDALTVRVLTIPAA

PGSLQVRKVDSQPELAELHFDPVQGANAYRITAATSDGRIVFDQTISDSSNIPYITNL

VPGSIHTISVTALNESGAGESSRAGFLTLPAAPGEFAAVQIREHEISLAWETVTSATY

YSLSRSGAGVYEGLETEYVDAGLDSGTEYSYELLAANETGEGPVTSLQKLLTLPGAIE

SLSIDNATTTSMHLSWDPVRGAERYEVFVNGEKRETLLAGTHEYLLTGLTAGTLVQVD

VQAGNGSGQGQSNRVSGTTLPESPSGLHFVQPTETGAILRWDAVPGATKYRVVLEDQT

YEISDTQLEVHQLSGSRHYTYQVEAGNAAGYGAPTSGELLTLPTRPEGLTVTRTDETS

IGVEWESVNTAESYIVSINGTEMGRTSGLAYTAEELSPGMEYLVEVQAVNTSGVGRSA

SLIRLSKPASPSEIVVEPGVHNANLSWASVAGASEYVIQQGNKEIYRGTELTTLISGL

EDGTMQHYTFVAVNRQGTRSEATNVSVLTLPEKPAEIKALEVSENSLSLDFTKTGVKG

ADTYIIERNGREIARMDASETRFVDENLSPGTKYTYKIRAVNASGSGASLTYGITTQT

LPLSSDGIAVIKGTHAFDLAWEAVKGAAAYEIRNQTTGEVQTVSEPLVHLASLLDGTT

YAFELTVMNEDGHRSSPVQVLLLTKPISPQTASIAVVTDQSATLDLSGSATRGAEQLI

ILRDGIEIDRIPADSISYEDKNLTPGEKYTYTVKTSNASGDSDMGFDVQLRMLPATIT

ETLHPDVIEETEVVIKWKKVQGAEGYIVMIADDEFTTITDSDLTLVTLNGLTSATQYD

QVQMVPYNTAGKGSPMVVAPFFTLPHVDSVEMKLYPETDHARLEWEFPYLNETFVVMM

DGIELYRGKQKEYIVNELQGGTNYSIELYTENEQGDTSHKLEYTLLTKPEAPKQVEYR

STQDSIQLQFEKSLVKGAKQFIIERDGAEIGRVSVDEPYYEDQGLEPGVNYVYTIKSV

NASGKSEIGFDLNAVTLPGKIPSPPQVEERAQHGADIVWDMIPGAAGYRIYQEDELIA

TTMETAIHVTELESAQRYSDFSIVPFNEAGDGETVNVPEFETLPSEEFTITAISQSTS

SIALSWRLESLNETIVLSSSGREIYRGKDRSYIWTGLAGGQRYDVILWTENSAGEKSE

SQQATAVTLPYPPPSGSGAGSSAPSSKPEDVQSGVSPADQNDKVETTAKKEVKFIDIG

QTFNKDQIIWLAEQNIIQGVSETRFEPRRPITRAEFTALIVRLMGLDTSADYQHAFRD

VNDQDWFAPEIEAAVSRGMVHGMGNGKFAPYALVTREQASKIIANVIRKIKPEPATLR

RAFTDQMDVSDWAKEEVEELAGMYMLTGYEDGSFRPMQHLSRSEAAALIFRLNKLIRI

IEEGSNYGRQRNGYRC"

CDS 2575122..2575835

/gene="treR"

/locus_tag="PIKHDJKN_02387"

/inference="ab initio prediction:Prodigal:002006"

/inference="similar to AA sequence:UniProtKB:P39796"

/codon_start=1

/transl_table=11

/product="HTH-type transcriptional regulator TreR"

/db_xref="COG:COG2188"

/translation="MMNNKFIRIYEDIAARIRTGEIEAGTLLPSELDLSESYQTSRET

IRKALKMLSEEGYIQKIQGKGSIVLDIRKIDFPISGLVSFKELAKKMGHRAKTYVKVF

EEKKVDQALFKKINFGLNEKVWEIRRVRKVDGEYVILDKDHISQRLIPGLSKDICNDS

IFEYIEEELGLTISFAKKEILVEEPTAEDRELLDLEGFHNVVVVRSQVYLEDASQFQF

TEARHRPDKFRFVDFARRR"

CDS 2576221..2577201

/locus_tag="PIKHDJKN_02388"

/EC_number="3.2.1.78"

/inference="ab initio prediction:Prodigal:002006"

/inference="similar to AA sequence:UniProtKB:G1K3N4"

/codon_start=1

/transl_table=11

/product="Mannan endo-1,4-beta-mannosidase"

/translation="MVKLQKCTVFTVIAALMLVILGSAAPKASAATGFYVSGGKLYDS

TGKAFVMRGVNHGHSWFKNDLNTAIPAIAKTGANTVRIVLSNGVQYTKDDLNSVKNII

NVVSVNKMIAVLEVHDATGKDDYNSLDAAVNYWISIKEALIGKEDRVIVNIANEWYGT

WNGSAWADGYKKAIPKLRNAGINNALIVDAAGWGQYPQSIVDYGQSVFAADSQKNTVF

SIHMYEYAGKDAATVKSNMESVLNKGLALIIGEFGGYHTNGDVDEYAIMKYGQEKGVG

WLAWSWYGNSSDLNYLDLATGPNGSLTSFGNTVVNDTYGIKNTSKKAGIY"

CDS 2577442..2579511

/gene="hcaB_2"

/locus_tag="PIKHDJKN_02389"

/EC_number="1.3.1.87"

/inference="ab initio prediction:Prodigal:002006"

/inference="protein motif:HAMAP:MF_01647"

/codon_start=1

/transl_table=11

/product="3-phenylpropionate-dihydrodiol/cinnamic

acid-dihydrodiol dehydrogenase"

/translation="MVQSLWNSSQASEKTTGLEQLVYRSNLIGSDRRVCNIFGGNTST
KTTVKDFRGRDIEVMYVKGSGSDLASMQAKHFTGLGLEDIRPLIERESMSDEEMVEYL
GHCMIDSKHPRASIETLLHAFLPYKHVDHTHPDAIISLCCADNGKELAKEIYGDRFVW
VPYVRPGFTLSKMIAESVFSNPNAELVLMEKHGLVTWGETSEECYEQTIKIINEAEAF
IEARVDEGNLFGGEKHPAFEAEVRRQIVSKVMPTIRGAVSDSKKMILSFDDQEDVLAF
VGGVDSLKLSQVGAACPDHLVHTKVVPLFIDWTPDAEDIEGLKSKLVEGVAAYKEQYQ
QYFESNKNDGDVMFEAAPRVILIPGVGMINTGKSWALSQVSGALYHRAIAVMRGATNL
GQFVSLSANESYNVEYWPLELYKLSLAPAETEFSRKVAFITGGAGGIGSETARRLVSE
GAHVVLADLNLEGAQKVAQEINDQYGANRAYALKMDVTDEEAVQSAYAEVAVQYGGVD
IIVNNAGLATSSPFDETSLKEWNLNMNVLGTGYFLVAREAFKLMKQQGIGGSMIFIGS
KNSVYAGKSASAYSSAKALEAHLARCIAAEGGEYGIRVNTILPDAILQGSAIWNGSWR
NERAAAYGIEPDQLEEYYRKRTTLLVNIYPRDIAEGIAFFASSKSEKTTGCMMTIDGG
VPAAFTR"

CDS 2579530..2580801

/gene="rhaA"

/locus_tag="PIKHDJKN_02390"

/EC_number="5.3.1.14"

/inference="ab initio prediction:Prodigal:002006"

/inference="similar to AA sequence:UniProtKB:Q9KCL9"

/codon_start=1

/transl_table=11

/product="L-rhamnose isomerase"

/db_xref="COG:COG4806"

/translation="MENQVKQAYEAAKSLYAQHGIDTDEVLKKLVEIKVSVHCWQGDD
VKGFLNKDGELTGGISVTGQYPGAATTPAELRADLEQAFSMIPGKHKVNLHAIYADTD
EQVELDRIEPKHYENWVKWAKEQGLGLDFNPTCFSHEKSSEGFTLSHSDPAIRKFWID
HCKASRRIGAYFGEQLGQTCVTNVWVPDGFKDNPVDRMTPRKRLKDSLDEVFAEKLDP
KYHLDAVESKLFGLGSEAYVVGSHEFYMGYGLQNDTLICLDAGHFHPTEVISNKLSSL
ALFTNGILLHVSRPMRWDSDHVVIMDDELLEITRELVRHDLLPTTHIGLDFFDGSINR
VAAWVVGTRNTIKALLRAMLEPVDALKSAELEGDYTLRLALTEEFKSYPFGAIWDYYC
AQQQVPVREQWIAEIKNYEQEVLLQRDKSFV"

CDS 2580961..2582634

/gene="rnjA_2"

/locus_tag="PIKHDJKN_02391"

/EC_number="3.1.-.-"

/inference="ab initio prediction:Prodigal:002006"

/inference="similar to AA sequence:UniProtKB:Q45493"

/codon_start=1

/transl_table=11

/product="Ribonuclease J1"

/db_xref="COG:COG0595"

/translation="MNLAHNKLYIAALGGLNEIGKNMYLIQYNQDIIVIDCGSKFPDE

TLPGIDLIIPDVTYLLENQDKVRALVVTHGHEDHIGGIPYLLKQINIPVYASRLTRGL

IELKLKEHGLLRKADLHTVDSKSSITLGGIQVSFFATSHSIPDCLGIFFQTPGGNVVH

TGDFKFDMSPVNGPYPDLHRMAEIGKQGVHVLLSESTNAERPGFTPSERIVGDHILDA

FIRAKQKVFISTFASNVSRVQQIVNAAFETGRKLALLGRSMINVVSVASELGYLDVPE

GLLIEAIDSDQFPPDEVVVLCTGSQGETMAALSRLAASKHPHVKIAPGDTVIIAAGAI

PGNERNLAHVIDNLYVLGARVIYGSSGAAGMHVSGHGSQEELKLMLTLMKPDYLIPVH

GEFRMLYQHRQLAESVGIERDHVFIVNNGDMIQYKDGDASSGPKIASGNSLVDGLIMG

DVGNIVLRDRRQLSSDGMLVIVTTLSKTEKHMVTAPEIISRGFVFVKDSEEFMKEIHE

LVQNKMDELTGSGANQWNVIKRKLKDEIGHYIYAQTKRRPMILPIIIEV"

CDS complement(2582760..2583842)

/gene="aroA_1"

/locus_tag="PIKHDJKN_02392"

/inference="ab initio prediction:Prodigal:002006"

/inference="similar to AA sequence:UniProtKB:P39912"

/codon_start=1

/transl_table=11

/product="Protein AroA(G)"

/db_xref="COG:COG1605"

/translation="MDQSMNLEQLRGRLSEINHNLLHLLSERAQITQEIGQIKEKQGV

PKFDPVREKEMLEELTAANPGPFQDDAIKLLFKQIFQASLNLQVDEHKKQLLVSRKNQ

QEDTVVSIGNVKVGAGKPIMIAGPCSVESYEQVREVAAALKEAGITVMRGGAFKPRTS

PYDFQGLGIEGLKILKEVADEFGLKTISEIVHPSHIELAGQYIDVIQIGARNMQNFEL

LKAAGDVRIPILLKRGLAATIDEFVHAAEYVVSRGNKQVMLIERGIRTYEKATRNTLD

ISAVPILKQETHLPVLVDVTHSTGRKDILAPCAKAALAAGADGVMVEVHPNPAVALSD

SAQQLNIEQFHQFLSTVRQSGLLKDQ"

CDS 2584421..2585404

/gene="ccpA_3"

/locus_tag="PIKHDJKN_02393"

/inference="ab initio prediction:Prodigal:002006"

/inference="similar to AA sequence:UniProtKB:P25144"

/codon_start=1

/transl_table=11

/product="Catabolite control protein A"

/db_xref="COG:COG1609"

/translation="MNKTISDIAQMAGVAKSTVSRFLNGGSVSDDTRQKIERIIKQYN

YVPNTFAQSLKAKKTSIIGTVVPRLDSFATSQTLIGIDEELRNNQYQMLIANTSQDMQ

REIDAIYDFARQKVSGIILLAAEVTDAHLKAVEDIGIPVLLVGQQHEQLHSLVHNDDQ

AGYEMGKHVVEKGHRKIVYIGVTERDQAVGIHRKQGFKRAIDECGGCDVTYYETSFKM

SEAIITAEAILKKSKPTIIVGATDNIALGVMKIAFSNKIQIPQDLSVTGFGGYEITEM

IHPTLTTVKYHYFEAGQVAAQHIIRLVQGDPVEQCTILDVEIIPRESVDKI"

CDS 2585764..2587239

/gene="scrB"

/locus_tag="PIKHDJKN_02394"

/EC_number="3.2.1.26"

/inference="ab initio prediction:Prodigal:002006"

/inference="similar to AA sequence:UniProtKB:P13394"

/codon_start=1

/transl_table=11

/product="Sucrose-6-phosphate hydrolase"

/db_xref="COG:COG1621"

/translation="MKMTREQRYRRIEQAEPGEIGKLEALISACPWRQHYHIQPITGL

LNDPNGFSYYQGYYHLFYQWFPLGTEHGLKYWYHTRSTDLVHWEEVGIGIQPGDTYDS

HGAYSGSAIEKEGQLNLLYTGNTRDEDWIRHPYQCLAIMDESGSITKVQQPVITEVPS

GYTEHFRDPKVWQQGDTYYCVIGAQRVDETGCTVLYRSSDLRNWTFLGEIHTGLPNFG

YMWECPDYLEMQGQGVLIFSPQGIESQEDEYQNIFQSGYLIGEPLDLQTRHFEHGLFQ

ELDRGFDFYAPQTMQSPDGRRILVGWMGLPDLEYPTDKSGWAHCLTIPRQLSLREGKL

IQQPVSEMVKLRGDSEGTHMEFTLDHETRSFADFAGMAYELECEIRDFDAEVVGLELR

ASAEEKTVLQYDRLAQKVILDRSQSGATLADQNGNVRRCALNADVLKFHIFVDSSSVE

IFVNDGEEVFTSRIFPSKESVEIRFFAHGGKAGFQASKWDY"

CDS 2587266..2589233

/locus_tag="PIKHDJKN_02395"

/inference="ab initio prediction:Prodigal:002006"

/codon_start=1

/transl_table=11

/product="hypothetical protein"

/translation="MSDNQQIAQDVIRAIGGKENIASFAHCATRLRIMVNDKDKIDQK

QVENIDKVKGAFFNSGQYQIIFGTGTVNRIFEEVEKLGIEGSSKEDVKSQGKNEGNAF

QRAIRTFGDVFVPIIPVLVATGLFMGLRGLLTQNEILSLFGATPGDISPNFLLFTQIL

TDTAFAFLPALVAWSAFRVFGGSPVLGIVLGLMLVNPALPNAYAVADGSAQPLHMFGF

IPVVGYQGSVLPAFFVGLIGAKFEKVLRRRVPEALDLILTPFITLTVMITLGLFAIGP

VFHSLEEWVLHGTTAVLGLPFGIAGIIIGFFHQIIVVTGVHHIFNFLEIQLLEKTGFN

PFNAIITCAMAAQGAACLAVGLKTKNTKLKALALPSSFSAFLGITEPAIFGVNLRYMK

PFIMGLVGGAVGGFIASLFHLQGTGMAVTVIPGTLLYLNSQLPLYILSNVVAMGIAFA

LTWFFGYKDQPVAEEVPSNAGEESKSEVLSQNSNSSASQAVNHRTKVDMLEIASPITG

TAVALEQVPDPAFSEKHMGEGISIEPSEGKVFAPFDGVIAHVMNKSKHAVILEHETGV

QMLVHIGINTVGMKGEGFTAYVNSGDTVTAGQVLIEFDMEAIQAAGLPLITPVLIPNG

NERIEQVMATMTGPVQANGEALIVVKFTEPQ"

CDS complement(2589339..2589785)

/locus_tag="PIKHDJKN_02396"

/inference="ab initio prediction:Prodigal:002006"

/codon_start=1

/transl_table=11

/product="hypothetical protein"

/translation="MIASCDVEIRSEIRVDSRISSMEIIRYDMRKLPFIAREHSSCYC

GLFKISCGNVHGFAEFELPEGSHPPDLVKWASVFRALKGMELSQVTQYIQQHQELWGN

KRMLFLHEAFSDLLLNLEQGSASDVMLSREEIRIFLMQYALTYYSF"

CDS 2590207..2590602

/locus_tag="PIKHDJKN_02397"

/inference="ab initio prediction:Prodigal:002006"

/codon_start=1

/transl_table=11

/product="hypothetical protein"

/translation="MDEAMMVVSTGHGEAGKNFVDVLLKRGFAVVVLAHSEEEGEQLQ

SLGAVTVRYIQEMADRNQLPLEFVVKNAFVFENSLPQCCQDIELCRSLCPQQLYVVSS

NRAHVGLVYKGLGATYVIHSRSGDISFLL"

CDS complement(2590744..2592468)

/locus_tag="PIKHDJKN_02398"

/inference="ab initio prediction:Prodigal:002006"

/codon_start=1

/transl_table=11

/product="hypothetical protein"

/translation="MNKNRKSALSLTIVTAMILSLFSSAFASAATDDSIQANAAAAPT

NIQSYVTAMEPGWNLGNSLDAVGTDETAWGNPLITQQLIQNIANQGYKSIRIPVTWQA

HIGGAPNYTIDTAYMNRVQQVVNWALDANLYVMINIHHDSWQWISYMENDHANVLARY

NSAWTQIANKFKNSSTKLMFESVNEPRFTEGGTTNTATAYALLDELNVSFHNIVRASG

GNNATRPLVLPTMHTSSAQPDLDALSQTIAKLNDPNIIATVHYYGFWPFSVNIAGYTK

YNAEVQQDITDTFDRVYNAFTAKGIPVIVGEYGLLGFDQHTGVIEQGEKLKFFEFIGQ

YLRQKQMTTMLWDNGQHFGRSSFTWSDQDLYNTMKASWTGRSSTAESDLVYLKKGAAI

QDKTVKLNLNGNTFSSLANGSTTLVQGTDYTISGDVLTLKSSLLTRLTTSGNLGVNAK

LTVKFNKGANWNLNIIKYDTPKLSNVTGTTSSFAIPTTFNGNQLATMEATYTSGGNAG

PQNWTSFKEFSYAFSPNYSSNVIELKQNFFNETNNGEVILKFHFWSGDVITYKITKNG

SSVVGISS"

CDS complement(2592726..2593316)

/locus_tag="PIKHDJKN_02399"

/inference="ab initio prediction:Prodigal:002006"

/codon_start=1

/transl_table=11

/product="hypothetical protein"

/translation="MTDRIDKRILRSKSDIKETFLRLLSQKPFQQISISEIVREANYN

RGTFYANFATKEDLLREIIQEVLVEFVQQIKNPYRYITKVNLKEMHAEDITLFTYLKE

QAALYKLLLSDHIQVDFRYQMARVIEDLFVAEYEYELEEGSVIDPKWLYIYRAHGVAG

LLIRWIEEDFPTPPQYMNTQIVELMLLSTEVFHVKK"

CDS 2593492..2594262

/gene="linC_1"

/locus_tag="PIKHDJKN_02400"

/EC_number="1.1.1.-"

/inference="ab initio prediction:Prodigal:002006"

/inference="similar to AA sequence:UniProtKB:D4YYG1"

/codon_start=1

/transl_table=11

/product="2,5-dichloro-2,5-cyclohexadiene-1,4-diol

dehydrogenase"

/db_xref="COG:COG1028"

/translation="MTFPVLEGKVAIVTGAAMGMGEATAQLFAAAKAKVVVADFNEEK

GQAVVESIRAAGGESAFVKVDISKSEEVQAMVKFAVETYGQLDVAVNNAALTPDDKPA

AEFDEAYWHRLISVDLTGTALCMKYELQQMLKQGKGGSIINISSVSGFRPQPNNIAYV

AAKHGVVGMTKVAAMENGPHNIRVNSVAPGAIDTPMLRGALDQFGFTAEEYAPQLSLL

NRFGQPNEIAQASLWLASDAASYITGTTIHADAGYTSR"

CDS complement(2594436..2595026)

/gene="bioY"

/locus_tag="PIKHDJKN_02401"

/inference="ab initio prediction:Prodigal:002006"

/inference="similar to AA sequence:UniProtKB:A2RMJ9"

/codon_start=1

/transl_table=11

/product="Biotin transporter BioY"

/db_xref="COG:COG1268"

/translation="MKLSLRGIVFSALMAAILVLFGYISIPIGFSPVPITLQTLAVML

AGGLLGPLYGFLSVALVVVLTAIGFPLLHGAGGLAVLLGPTGGYVMMWPFSALLIGLL

LSRIKLNGFVSYFLAFIVFEVFGSMLIYVSGVPWLAYAYKMSLPEAMIQGFYPYIIGD

LIKALFAAIIIAPVRMVFPPQRLTGNMNSTVVKADS"

CDS 2595139..2596002

/gene="ecfA1"

/locus_tag="PIKHDJKN_02402"

/EC_number="7.-.-.-"

/inference="ab initio prediction:Prodigal:002006"

/inference="similar to AA sequence:UniProtKB:Q03PY5"

/codon_start=1

/transl_table=11

/product="Energy-coupling factor transporter ATP-binding

protein EcfA1"

/db_xref="COG:COG1122"

/translation="MQDVTPLIKLENVRVHYASESGRVRKAVDGVSLELAPGEWISIV

GANGSGKSTLAGLLIGFTPLSGGERKASSELVVRGVLQQPDAQVLGDTIEEEFHFALS

SLLESPDEQLRRREHALHTVGLEHPPEAAISMLSGGQKQLLNIAVALAVKPDVLILDE

PTAMLDPGARDRMEAVVQTIVQQGTAVIWITHHLEEATLCDRIIAMEQGRCVYDGAPA

AFFYGREKEETTDDAGKESQQPSLCKKLGLAPPFTVQTALLLKRKGLLHHATPLRPEQ

LVKEVALWQSN"

CDS 2595987..2597741

/gene="ecfT_1"

/locus_tag="PIKHDJKN_02403"

/inference="ab initio prediction:Prodigal:002006"

/inference="protein motif:HAMAP:MF_01461"

/codon_start=1

/transl_table=11

/product="Energy-coupling factor transporter transmembrane

protein EcfT"

/translation="MAIELEHVGIASSQADKRALLQDITVQLNRGEITLLLGCTGSGK

TTLLQTIAGLKPPETGSLKLDGEPLWQNGKVPQSILLQMGLVFQFAEQQLFARSIGRE

FAYSLRPYRLPDEQKKKQISEALERWDSPAGQGGEERFHSDRSPFALSGGERRRLGLA

LGTATQPSWILLDEPSAGLEAQSVIMLLDALNQHRNAGEGAIVATHDLDTFLPHADRV

LLLQEGRLVADLTPRELHTRPDLLELAGIGLPPSMQLAQQFAAAGIHFPCTALTPEEM

AEGIVQSVNEPLEQKVDVIGNKDQLKMDEAKLAFSSPDTSGGEGTQEAIYTHAESGSS
RGLYGAMDPRLKWILYILLVTATMLQHHWLGLSLTLVPVILALVVLPRQALMGCLKLM
KPLVLFFLVSTALSGTTLSTEGGGLQFGFSLMQAEGTLLNVYRLFIVTLASLWFSLTT
PYGRMVEGLNWVLGIGKKIKLPVSSFALAVSLIFRFIPMIWSEWQRFSLIVRARGKAA
LRPNTVRLRDLGPMVIPLLMALFQRAEDMTIAMEMRKVRENSLLGARSSLLVWSKRDT
WICIIGLIVFVFYIWIRD"

CDS 2597839..2598135

/locus_tag="PIKHDJKN_02404"

/inference="ab initio prediction:Prodigal:002006"

/codon_start=1

/transl_table=11

/product="hypothetical protein"

/translation="MKDKFLQEERQEYTDVSTVESQRNDITLEEFPEGPYGSSLMSES
LGKSSPWRVDQRTAHRFDYENHELHEGEVRDYPGQDVYDETVPDNVTKPQYPEE"

CDS 2598323..2598799

/locus_tag="PIKHDJKN_02405"

/inference="ab initio prediction:Prodigal:002006"

/codon_start=1

/transl_table=11

/product="hypothetical protein"

/translation="MDQDYIQYMRSFVGNAKVIMVVSGAIVLDETGRVLLQKRSDNGF
WGFLGGFMEIGETVQETAQREVYEETGLTLNSIELFSIYSGPEYEVVYSNGDEVALVQ
VMFKSNDFCGTIKESEESIETRFFDIHSIPEHFLPTHKQIIEDLRNKEDVFVKLEN"

CDS 2598819..2599394

/locus_tag="PIKHDJKN_02406"

/inference="ab initio prediction:Prodigal:002006"

/codon_start=1

/transl_table=11

/product="hypothetical protein"

/translation="MREINFSAYFWQDNKVRLRALREEDWEDHYYNRFDSPARRLLEC
TVELPPTNAEAKKFTDAFSDFSLDRGRIMFTIENMDGENVGGVNLNSIDERNGTFSIG
IQIDRDHRGKGYGTRAIRILLKYAFFERRLNKFNDSVLEGNEPSAAMMRKLGCVQEGV

RRQVIYTDGKYQDMILFGLTKDEFIDKEGLA"

CDS 2599477..2600025

/locus_tag="PIKHDJKN_02407"

/inference="ab initio prediction:Prodigal:002006"

/codon_start=1

/transl_table=11

/product="hypothetical protein"

/translation="MRKLLWISSCFVVCVLVSVSVFFWNDIFGYKTLERAVQSTWKYP

LQVVNVDQQNELVLSLDQTQYVFAAYEHKNGRYHYDTDSESGWTASSDVGPAFLVQAE

PKNKKGNFIWGALYSDMPVHKFKIEYTNGETQEVESSNNTFIVKMPEAYQSEDKMMLM

ATFTNVYALDEENNVIRAYNLN"

CDS 2600091..2600666

/locus_tag="PIKHDJKN_02408"

/inference="ab initio prediction:Prodigal:002006"

/codon_start=1

/transl_table=11

/product="hypothetical protein"

/translation="MKQTIQSDELIIDCKDIYLREYRSEDLEKLQEITWQPEVYEFLP

GWNATVEDRALWLTQYEIPENQNFKQAVLAGGDIGDLYLRLAIVLKENDEFIGWCCSG

IKDELPAPNREIMYGISKHFRNRGYTTQAVQGITQYLFENTNVEVLNAIALITNQASN

KVIQKCNFEWLNMIEIENEPYNHYQLLKHQR"

CDS complement(2600816..2602342)

/gene="sasA_12"

/locus_tag="PIKHDJKN_02409"

/EC_number="2.7.-.-"

/inference="ab initio prediction:Prodigal:002006"

/inference="protein motif:HAMAP:MF_01837"

/codon_start=1

/transl_table=11

/product="Adaptive-response sensory-kinase SasA"

/translation="MTSLLKKDKHLWTWLHQWISPRSLRSQLLARSLFIMAALLLLIG

ILQFWIMESFLYRNQAKTMEEQLMSMPPVWLGSSSRSGAANNPFGGQSGSDPRGSGNR

FLYIPDRSLALFDNQSGFEDIFGEDGLTAPRLTSVEYQNITDRINDKQHVPYRIVTDN

RGNEQLIVFSSPGPRNRGLPMVQMGTATKPLKDLIVKQLLIFLMLSLLAMVAGLVLYA

KALRRTLIPLSSMVDTVQRIDAGSLAERLPSVQGQQEVDQLALSFNGMLERLEDSFEA

ERESKEQMQRFLSDASHELRTPLTSIHGFIEVLKRGAASNSEQLYSALNSMYGESVRI

NKLVEDLLMLTKLDQAPQQEQEVIQLDSLLLEMQPQLIMMAQERQVHLDLTAEVHVMA

DAHKLKQVVLNLFHNAVQHTDSDRGIISISLYAAQHKAELTVKDNGSGIEPEHLPHIF

ERFYRTSSSRSRKQGGAGLGLAITRSIVESYGGKITVRSQLGAGTEFTILLPLVKSEA

"

CDS complement(2602342..2603025)

/gene="tcrX"

/locus_tag="PIKHDJKN_02410"

/inference="ab initio prediction:Prodigal:002006"

/inference="similar to AA sequence:UniProtKB:O69730"

/codon_start=1

/transl_table=11

/product="putative transcriptional regulatory protein

TcrX"

/db_xref="COG:COG0745"

/translation="MKLSTGIKILLADDEPHILQFLELGLSNEGFDVRTAPDGAAALE

LALDFRPHMAILDVMMPEMDGFEVVERLRKSGAQVGVIMLTAKDEVENRVKGLWLGAD

DYMIKPFAFDELLARIQARLRNQFPLLIGAVTHGPFRIDDQRKEITYHDQILELSPTE

YELLKFIVLNHGIVLSKATILNRVWGYDFGGEENIVEVYVRSLRDKLGDKEHKLIRTL

RGVGYRVDL"

CDS 2603229..2605475

/locus_tag="PIKHDJKN_02411"

/inference="ab initio prediction:Prodigal:002006"

/codon_start=1

/transl_table=11

/product="hypothetical protein"

/translation="MNNTKRRIDLVLLPIVLLAAFLNGYGIWNDQYANSYYTTAVGSM

LRNFHNFFYASLDSAGSVTVDKPPVVFWIQTAFAYVFGLHGWSVILPQALAGIGSVLL

IYFMVKPTFGLAAARISALAMATVPVVAAVSRTNNIDSMLVFTLLLGSWFLFKGSKQG

SAWRILVAFALIGLAFNMKMLQAYMILPAFYLFYLLAFNAKWRRKIVLLIGSTAVLAV
VSLSWAVTVDSIPTDERPYIGSSETNSVLELAFGYNGLSRLTGQQNRSSNAGMPNASD
EGKTRGNAASQTSGSDQGSGSTPTGNAPGNGEMGGMNGMTFPGGQMPNGGEMPNGRNF
GGGNGGGGGMGGMFGTGEKGPLRLFQTELSGQASWLLPIVLLGCIAIFAGLRRRNITD
KHKEALFWLAWLLPVAAFFSVAGFFHQYYLIMLAPPVAALTGAGFATMWTAYRERNDW
QAWLLPLSVLLTTIFGWYIMQVYNETIGAGWSIGELIAGILITVILIVMLNRSHPWKQ
GLIITGFMVMLIGPTYWAFTPITYGGNSMIPAAGPTGSNGIFGGTGMAMGDRQGDRNG
GGMAPSNASTDTDTDQAFPSSDEADPNKAGMGTAPNSGGNMNGGGGMPTTGGRGGAGN
RNEEVDTTTLNYLREHNTGETYLFATTDYNQAAPYIIDENEAVITLGGFSGSDPVYTT
EKLEQLVKSGQVKYFMVGGMGGRGGSSDIGDWIKEHGTEIPTSEWKTGTDSGSGFGGQ
TTLYEVKL"

CDS 2605509..2606495

/locus_tag="PIKHDJKN_02412"
/EC_number="2.4.-.-"
/inference="ab initio prediction:Prodigal:002006"
/inference="similar to AA sequence:UniProtKB:Q55487"
/codon_start=1
/transl_table=11
/product="putative glycosyltransferase"
/translation="MAQKCRYSIIIPMYNEEAVIEETYRRLKKVMGSTGESYELLFVN
DGSVDQSAQMIRDYARWDESVKLIDFARNFGHQIAITAGMDYAAGDAVVIIDADLQDP
PELIVDMIAKWKEGYEVVYARRTRRSGETRFKKWSASLFYRVLRASTDTDIPVDTGDF
RLIDRKVCDEMKRLPEKNRFVRGLVSWVGFRQTAIEYERDERLAGETKYPLKRMLKLS
LDGITSFSHKPLKLAGYVGAVLSIGGFIYMLSVIASAIFTDSTIKGWPSMVSIMLIFN
GFTLIMLGILGEYVGRIYDETKARPLYIVRDVYQLESQSSQARLTGRVAHHD"

CDS 2606488..2606925

/locus_tag="PIKHDJKN_02413"
/inference="ab initio prediction:Prodigal:002006"
/codon_start=1
/transl_table=11
/product="hypothetical protein"
/translation="MTNRLVTIFKFGIVGVLNTVVDAMVFTLLAAAGTPALIAQVISY

SCGVVNSYWWNGRWTFQGAGRQGKKNEIMRFVITNLVVLALSSLILYICNSTLGWSVV

MSKILATLSGMVINYIASRYWVFRSDPSQGSDPSSDANERSVS"

CDS 2606927..2607799

/gene="gtaB_2"

/locus_tag="PIKHDJKN_02414"

/EC_number="2.7.7.9"

/inference="ab initio prediction:Prodigal:002006"

/inference="similar to AA sequence:UniProtKB:Q05852"

/codon_start=1

/transl_table=11

/product="UTP--glucose-1-phosphate uridylyltransferase"

/db_xref="COG:COG1210"

/translation="MRIKKAVIPAAGLGTRFLPATKAQPKEMLPIVDKPAIQYIVEEA

VQSGIENIIIVTGRNKKSIEDHFDKSVELEHSLYAKGKQALLEEVQAISEMASIHFIR

QKEPLGLGHAIGCARQFVGDDPFAVLLGDDIMVSDPPALAQMIHLYEKTGSQIVGVRQ

VQPADVSKYGIIDSQGAEDRVHRITNLVEKPSIAEAPSRTAVMGRYILKPSIFPILDR

IERGAGGEYQLTDALKEVSQVEELLALELEGRRYDIGDQFGYIQAILEIGLMRKELQP

MLTPYLQKLATQWG"

CDS 2607949..2608791

/locus_tag="PIKHDJKN_02415"

/inference="ab initio prediction:Prodigal:002006"

/codon_start=1

/transl_table=11

/product="hypothetical protein"

/translation="MNESIIRDLIKYMDVEDNAAIQYIQTEHRMKLGLFWGIQEGSRV

LEIGCGQGDTTAVLAHLVGEHGYVHGVDIAPEDYGAPLTVGEAAAKLRKSPLGERIRM

DYGFDILSDQAQFAENEFDVIVLSHCSWYLKSFDELAQILSKVRTWGHQLCFAEWDAR

VTDVRQLSHWLSVLIQSQVECYKENSFSNVRTLFTPEDIQELVSAAGWTIKEEASIHS

PELQDGRWETEMTLAEAPVELKMLPIPDKVKTLLLSELKLLRTHHASGPGSPLGTYAI

VAKK"

CDS 2609381..2610466

/gene="asd2"

/locus_tag="PIKHDJKN_02416"

/EC_number="1.2.1.11"

/inference="ab initio prediction:Prodigal:002006"

/inference="similar to AA sequence:UniProtKB:P23247"

/codon_start=1

/transl_table=11

/product="Aspartate-semialdehyde dehydrogenase 2"

/db_xref="COG:COG0136"

/translation="MSAKLKVGIVGGTGMVGQRFVDLLNEHPWFEVTAISASANSAGK

TYEESVQGRWKLAVPIPEAVKNIVVQDASQVEAFASQVDFIFCAVDMKKNEIQALEEA

YAKTGTPVVSNNSAHRWTADVPMVIPEINPGHLDVIEAQRKRLGTKTGFIAVKPNCSI

QSYVPALHALREFNPTQVVASTYQAISGAGKNFTDWPDMLDNVIPYIGGEEEKSEQEP

LRIWGSIQNNEIVKASSPLITTQCIRVPVTDGHLATVFVSFEKKPSKEEILERWLQFK

GRPQELGLPSAPKQFITYFEEENRPQTKLDRDIERGMGVSTGRLREDSLYDYKFVGLS

HNTLRGAAGGAVLIAELLKAEGYIQSK"

CDS 2610653..2611753

/locus_tag="PIKHDJKN_02417"

/EC_number="3.1.1.-"

/inference="ab initio prediction:Prodigal:002006"

/inference="similar to AA sequence:UniProtKB:Q837K0"

/codon_start=1

/transl_table=11

/product="Deacetylase"

/db_xref="COG:COG3964"

/translation="MDGQLILRRVRLTSGEEVDVVINEGLIVKVAPGGTATGDRLWES

SGAYISSGWIDMHVHAFPEFDPYGDDIDEIGVKQGVTTIVDAGSCGADRIGDLAISAH

NAATRLFAFLNISRLGLLRTDELSNLDWIDRDKLLQATRAYSEFIVGLKARISGSVVG

KSGLRPLYLAREFSQETGLPLMVHIGSAPPDIREVMPLLQGGDVVTHYLNGKSNNLFN

ERGAPLEVLKDAIKRGVHLDVGHGTASFSFQVAEAAKKHGIDPDTISTDIYRGNRLNG

PVYSMANLLSKFLALGYSLSETIERVTVRASEWLGRPQLGRIQAGDAANLTLFQVVDK

PVTLVDSEGVSKIASRQIEVKGAIVGDQFIEC"

CDS 2611731..2612843

/gene="dgaE"

/locus_tag="PIKHDJKN_02418"

/EC_number="4.3.1.29"

/inference="ab initio prediction:Prodigal:002006"

/inference="similar to AA sequence:UniProtKB:D0ZLR3"

/codon_start=1

/transl_table=11

/product="D-glucosaminate-6-phosphate ammonia lyase"

/translation="MTNSLNVKYGLKRVINASGRMSILGVSAPTDKVMDAMKQGGQSY

VEIADLVDKSGDYVSRLIGAEAAVVVNSASSGIALAVAAMVTKGNRHASLKLHQEVIT

KNEIVMLKGHNVQYGAPVETMIYLGGGKLIEAGYANEGRAEHIVDAVSDKTAAILYVK

SHHAVQKNMISVEEAWQVAQKCEVPLIVDAAAEEDLHKYVKYSDLAIYSGSKAIEGPT

SGIVAGKRTYIEWLKVQLHGIGRSMKVGKETTFGLLQALDEYAVKADHSEQEKDSLQA

LMSLNQIAGIQVSIVQDEAGRAIYRGRIQVNEAKAGISAKQLNEELRGGPIAIYTRDY

GVRQGYFDIDPRPLMGDDMRVIEDRIRQLTGGEGNV"

CDS 2612836..2613588

/gene="dgaF"

/locus_tag="PIKHDJKN_02419"

/EC_number="4.1.2.14"

/inference="ab initio prediction:Prodigal:002006"

/inference="similar to AA sequence:UniProtKB:D0ZLR2"

/codon_start=1

/transl_table=11

/product="2-dehydro-3-deoxy-phosphogluconate aldolase"

/translation="MSEIAKRFYNNRVALNVLAKDITNAKEVFDAAEGHVLVGVLSKD

YATVSEAVEAMEAYGREIDNAVSIGLGAGDNRQAAVVAEIVKHFPGSHINQVFPAVGA

TRANLGDRDSWINSLVSPTGQPGFVNISTGPVSAAQSELAVVPVHAAIALVRDMGGNA

LKYFPMQGLKREEELRAVAKACAEADFALEPTGGIDMDNFEAILEIALEAGVPQVIPH

VYSSIIDQEGLTIVSDVQSLYASMKTLAKKYG"

CDS 2613581..2614597

/gene="kdgK_1"

/locus_tag="PIKHDJKN_02420"

/EC_number="2.7.1.45"

/inference="ab initio prediction:Prodigal:002006"

/inference="similar to AA sequence:UniProtKB:P45416"

/codon_start=1

/transl_table=11

/product="2-dehydro-3-deoxygluconokinase"

/db_xref="COG:COG0524"

/translation="MVKRVGAFGEVMMRLEVPGHDLLIQSNRLKYSFSGSGVNVVSAM

TRYGYTGLLVSRLPVNPLGDAAESYLRGLGLNTEYVLRGGQYIGMYFLENGYGARPGR

VTYSNRMESSFNIAPEGTYPFATVASGMDAIHFCGITLAMNESVRLAMKHLARAVKEQ

GGTVVFDCNYRPALWGDQGYATAKPHYEEMLHLSDIVLMNERDALHILGLNTDKVQRE

EQLVELIPLVAERYGVRTLAGTHRTIYSDNRHCLQGYMFKEGAFAYSEPLTFSVYDRI

GAGDAFASGIMYGELEGWMPAETISFAATAGMLAHTVAGDTPMASAGDIVQARNSTMR

DLTR"

CDS 2614801..2615472

/locus_tag="PIKHDJKN_02421"

/inference="ab initio prediction:Prodigal:002006"

/codon_start=1

/transl_table=11

/product="hypothetical protein"

/translation="MSLQSSSYEGQKAVFLGDSITFGYGLQEGEHPYPELVSRALGFQ

SYENDGISGSSVADGGFEPMCNRIGQMHAESDVIFFMGGRNDFSSGTTAFGDISTSPN

SKDTFYGALKHIAEELIQRYPTKLIVFLTPPHGSSEAVPEDLPNTVTRRIYCDYVAAI

REVASLYSIPVCDLWNIAGIQPLLPVHKDLYFTGDNGVPDGLHPNQAGHERLASRIVG

FLKSL"

CDS 2615753..2616013

/locus_tag="PIKHDJKN_02422"

/inference="ab initio prediction:Prodigal:002006"

/codon_start=1

/transl_table=11

/product="hypothetical protein"

/translation="MSEVASQRVHKPGPQPVSFGSWMLTLFLMMIPLVNIIMLFVWAF

GDSNPSKANYAKAALLWAAIGIVVYILVFVLIIGAGISLSDY"

CDS 2616284..2617180

/gene="yteP_14"

/locus_tag="PIKHDJKN_02423"

/inference="ab initio prediction:Prodigal:002006"

/inference="similar to AA sequence:UniProtKB:C0SPB3"

/codon_start=1

/transl_table=11

/product="putative multiple-sugar transport system

permease YteP"

/db_xref="COG:COG4209"

/translation="MGQWEFQVMIWPALLFLLIFSYGPMWGIFIAFQDYNLFAGMKNS

PWVGFMHFRMFFESPEFWSVMRNTFAISLLKLCIGFPAPIILALMLNEVAGKFKRVVQ

TVTYVPHFMSWIVISGLVFSMLAVDNGAVNELLLALNLINEPVNWMSTPEYFWGILVS

LNVWKEIGFGSIVYLAAIIGIDPAQYESASIDGASRFQQIFRITIPNIMPIMMIFLIL

GIGNILNAGYEDILVLTKNLSNGIVLPVADVIDTYVYQMGIVNQRFSYATAAGLFKSL

LSVTLLVLANTLARKFGKTSLW"

CDS 2617211..2618098

/gene="araQ_13"

/locus_tag="PIKHDJKN_02424"

/inference="ab initio prediction:Prodigal:002006"

/inference="similar to AA sequence:UniProtKB:P94530"

/codon_start=1

/transl_table=11

/product="L-arabinose transport system permease protein

AraQ"

/db_xref="COG:COG0395"

/translation="MKLSAGDRILKFVIYALLSILAFVTFYPFWNSLVLSLNVGSDTS

SGGVTFWPRQFTLENYAVVFQEKNIYSAYLITILRTVIGTVCALLFTSLFAYAMSKKG

IMFKKTYMILAVITMFFSGGIIPYFILVYHLQLYDTFLFLVLHGMVNVYNMIIFRTFF

MELPDGLEESAKMDGCSNFGVFFRIVLPVSGPVMATLGLFTAVFLWNDWFIPAVFISN

QNLIPLQTLLVQIINAGTTASLMTNLNSYAQSMVQNTVTVKSLQMATMMVATLPILMI

YPFLQKYFVKGVLVGSLKE"

CDS 2618189..2619907

/locus_tag="PIKHDJKN_02425"

/inference="ab initio prediction:Prodigal:002006"

/codon_start=1

/transl_table=11

/product="hypothetical protein"

/translation="MERKQQKRRKSLHWLHLSALSAALMISGCTGGGEGQSPSANESV

NTKEQIKTGETVSPNADPKSKVRPVSFTQFVNYDWYTAPTWEERPHGKWITDELGVVM

EPVQSNGAAATKLNSMIVAKKLPDAIVLDRGKDVERLVSAGQLVALDPYLEKYPEFVK

TIGEETINMLRSADGKLYQIPNWFISGDNGNGNAAYLVNRKIYKDLGSPKVETWNELE

AYLKQVKEKYNGSIVPIDFGEMRDSESQMISMLYSGAAEDRTGGFISPGSGGVFGVPQ

GAQLTSIYTDPAFEQTVMYASRLFREVLTSKDAFTQTRDQILEKLKNGKVAVFSAFDS

VVEGIGREANNHLQAADPEAGYDVIWPVHADGVDKNKVYPSGYNTLGWNVNVITKNAK

DPEAIFAYMNWAISEEGQQVLFFGPKGLFYDEVKDGVPIPNEAYVNRDPKKYDEMKIG

EFNWYGNTSYVDVVKAKREELLPEEAQDWTTVAQSTVSFKTSKNMTEFSNLDPLATSE

EGIILQRLKDYYKQVIPRLVFAGNDEEVKSILAEAQAESVKLGYDKVLKWKTAKWQEN

LKIMGK"

CDS 2619990..2621564

/gene="rhaR_17"

/locus_tag="PIKHDJKN_02426"

/inference="ab initio prediction:Prodigal:002006"

/inference="protein motif:HAMAP:MF_01533"

/codon_start=1

/transl_table=11

/product="HTH-type transcriptional activator RhaR"

/translation="MYKAALVDDENYDLVGMRELIPWDELNIEIAFMGNKPLAALHYL

QNHDVDILVTDIKMPVMSGLELARMALQKQPELKIIFISGYEDFDYAKQAIHLKADGY

ILKPVDDDEIKTTLQKIVHVLKEENARLETKNGSNALEHFDFVKQDFLKHLMEGSIDA

PTLKSFTRLYPLPPRTETAHAVIIEMDHMQHLFTDEEYERWHELTFRQITSWIETKNW

GAWCRLSTDRIGLMYSHDAGDIELNLNELIHYIRDRSRFTITASFGQKTSRVTELPVS

FDEARNLIGCKMFLGKNRLISPGEIHIPLVRDVLDVNNQLDRLFTAMSTYDLVTICDA

VEELFEHVRAFSDSSKVHHFSTHVLSRLEGYLNAEAQHNFPHFARWKAEGFETVQQFD

TVDDIKSWLRRTVFEWSEHIYLRKQTSNWKLFSDIEHYVGQRLSQDVTLKDLAAHFSY

SPNHLGFLFKDKVGKNFSDYVAARRLEHARHLLQTTNLKIYEIADQVGYRSLTYFSRL

FRETFGMKPGDFRKQS"

CDS 2621568..2623316

/locus_tag="PIKHDJKN_02427"

/inference="ab initio prediction:Prodigal:002006"

/codon_start=1

/transl_table=11

/product="hypothetical protein"

/translation="MIKHSNRRRFLPILYKLLISNLALVIIPIIVIGCYAYISSARSV

EEHTRNNLEVAVSQIQSNVEYRVGEIIRSSDEIYQDQGLSTLLSGYYLDWEKYQVTTQ

YVMPRLESATNLPKSKIKLSLYLHAPTISEFYYNETEQSLIAGERQYEILHTSRILDK

AWYQNLRIGYDEMKWQQIENDAEYGYISLLRHAINYETFQSIGLIRASVKLKDIFEDV

DFNQLGKGTKLFVLDQDEQLLYSSSPGGSLPYTAEELNESRDYLKISKPIHNIPVQIV

ALVPNASFKDNSQKVRNVTIIICVISILVMTLVSFIMARMFSSRLSKLVVSLEAFKEG

ELQRRIQYTGNDEFAIIAESFNEMASTTEQLIDEVYTSKLEKKEAELQILHSKINPHF

LYNTFSSISRMAKLGEIDKLHEMIRALAKFYRLTLNKGEMLIQVEKELHIVRTYLDIQ

NIKYAERIHYHLEAEPETLGLETVKFILQPFVENVLEHAWYDDEIEIRIEVKKLAEHV

HIEVHDNGLGMKQEIVDAIFTEGAGGIGYGIRNVDQRIKLHYGKEYGVSIRSEVGTGT

VVTIVFPVKSPTSRQE"

CDS 2623462..2627160

/locus_tag="PIKHDJKN_02428"

/inference="ab initio prediction:Prodigal:002006"

/codon_start=1

/transl_table=11

/product="hypothetical protein"

/translation="MASRSKRRAFAGKGLALALIAALFTTMFPGFVPSKAQAEPASYL

LSSNRPAYASSTEGNNTANLAVDGSSGTRWSSAWGKDPQWLYVDLGDTAAIDKVRIQW

EGAYAKAYQIQVSTDEVNWTDIYNTATGNGGTDELTVNGSGRYVRVLGTERALANYGY

SIIELEVYGTGGTGVPPVTYGPDVARGKQVTVSSYEQSDYLPPGATLPENAVDGNSST

RWGSNHTDDEWIYVDLGSKRNIGRVVLNWEGAAGRAFDLQVSDDANAWTTVYRELHGN

GGKQDISMYASGRYVKMQGIGRATNFGYSLFDFEVYDYVEGTPKPSYPIPELPSSSTV

QVGKGSYLTNDVRMPQPKYPTYKTDNVQAPIASNDWWQSLLIKPLSDSIITLPLKSKF

YPQGLGLLNPGEGWINGDGSAVNADGNPDLYFMSTNINAKNMETRVSGYSDYAVDAIL

SDDASAKMKTTFVKGSPYVFMEFNNPDTAEIFSSLITKVVNGNGETVLQSDGDTFTGD

HIGIQLENQSGDPAGQKEERYYGIFAPEGTVFKKAGAKIKLQLGGGKGYLSAATMPSL

TDLNQFHEHAYAFVTNTEVTYNYNEQTSTVTTNFKAETEVKRPGFSANTLQALYPHQW

KITNSPLTSLTYPSIRGTLKVREGNQFTVEDHFYGMVPQFTEPTNAEYNREEMVAYLE

MLKTETDNNLMAADAYWQGKKLHPLAMGVLAANEMGETVYRDLFLSRMKEILVDWYTY

TEGEPDYFLYYNDDWGTMYYKTSEFGANYGITDHHFTYGYYVFASAVLATFDKQFRAD

YGDMVDHLIRDYGNPSKNDPLYPSFRSFDPYQGHSWAGGYADNDSGNNQEAAGESLFG

WVGQYMWSLLTKDQDFRDAAIYGFTTELKAIEQYWFNYDGDNWLDEWKHASVGQVYGS

SYFFGTFFSGEPVHIYGIHWLPTSEYLTSYGLDKTKAAGLYNGFVTDNGGPEREWQHI

VWPIQALSDPAAVLAKWNASSMQKNEIFNTYWFVNSMATLGERTKDIWATGWSSATVY

KKNGTYTAQIWNPTDESVTVTFKNASGTTGKAVVGPKSLVRVDPTQNTEISDSWIPWN

GENGGNEGGGDGGDTNVNFALNKNVTVSSTEDPFVGTNVVDGNPETRWSSGFGDTPEW

ITIDLGEVKQVKRIKLNWEAAYATAYRIDVSEDGTVWKEVYHTQEGKGGLEELAVNGK

GKYVRLTGEKRATPYGYSLYEVEVYGRD"

CDS complement(2627267..2628235)

/gene="panS"

/locus_tag="PIKHDJKN_02429"

/inference="ab initio prediction:Prodigal:002006"

/inference="similar to AA sequence:UniProtKB:Q8ZKL0"

/codon_start=1

/transl_table=11

/product="Pantothenate precursors transporter PanS"

/db_xref="COG:COG0385"

/translation="MLQALNVRLNRIMPLITPISIIIGVLCGSLLSSYTFLSPWLFAF

MTFAGSISLGIRDFVNVLKKPFPLFVCLFILHLAMPLIALSMGHLVFPTDAYTITGLV

LAAVIPTGVSSFIWVSIYRGNIALTLSIILIDTMLAPFVVPGVLSLLIGTSVSLDIAA

MMSSLFWMIVVPSLLGMLLNEWTKGAIVPVWGPRLNPFSKLFMAAVVAINGSVVAPYL

ADFNWQLAGLAAIIIFLASFGYALSYFVARLLGWNEADQVALVFNGGMRNISAGAVLA

VSYFPPPVAVPVVLGMVFQQMLASLAGYLLGRRSQTLHNADKTSAA"

CDS 2628377..2628853

/locus_tag="PIKHDJKN_02430"

/inference="ab initio prediction:Prodigal:002006"

/codon_start=1

/transl_table=11

/product="hypothetical protein"

/translation="MIKRKRKHTLMMAGLIASSILVISACSVVEQANQSLNYVSGATD

YIEQVSNAGADLQELASGAVSNPEITTQVQEKIDQIQAEASEFSQLTAPAIGESIHEN

LVSYNNQLTEVVGQFENTLAEHGFTAENWEKTGIPELITNINGLKDPLSGLGIEAN"

CDS complement(2628998..2629492)

/locus_tag="PIKHDJKN_02431"

/inference="ab initio prediction:Prodigal:002006"

/codon_start=1

/transl_table=11

/product="hypothetical protein"

/translation="MSRDQQQQPTELQENFTLRMRSLGTRTVLYQQHVAASLGLYNND

FISVDILREKGPITAGELSKLTGLATGSVTTLIDRLEKNGFVRRENDPNDRRKVIIVP

LYENKEEVSSTYLTLHAAMVNLASTYTEEELELITGFLSKAGNVLDGQIEDLSSKIRG

KSSS"

CDS 2629657..2632149

/gene="uvrA_1"

/locus_tag="PIKHDJKN_02432"

/inference="ab initio prediction:Prodigal:002006"

/inference="similar to AA sequence:UniProtKB:P0A698"

/codon_start=1

/transl_table=11

/product="UvrABC system protein A"

/db_xref="COG:COG0178"

/translation="MMIKGARENNLKNISLSIPKYKLVVLTGPSGSGKSTLAMDTLQR

ECQRQYMESMGLTSDSISKPKVESITGLSPSISVGQHITNRNPRSTVGTVTDIYTYIR

FIFSRLGERICPSCNGAISPSWEAAGHLTEEDEYMGHQTIHCPHCNAELEKLGMSHFS

FNKPEGACDVCSGLGHVASINEAAVFNPELSVRDGGVASLNGVHRDIQIRILVAAGKH

FGFGFDPDRPLKDLGEIERDLLYYGVESEAFKQHYSNIKPIQGTKFEGVIPGLWRRYK

EKEGEGGGTEKDGRFFHQQQCPECQGARLKKEILSVSVAGASISDVTNWSFSKVLEWT

VGLQEVISPEAHHLLDPILHDLPTKLKRIMDVGLGYLSMDRQTVTLSGGEAQRLRLAS

LLGSGLTGVLYILDEPTTGLHPKDTIGLIHVLQELRDLGNTILVIEHDIEMMRAADHV

IDIGPGAGVNGGMVVGEGSLEDLMASELSVTGAYLRNESVASPSYNRRTGTGQHLAIR

GAHLRNIDIPVVSFPLGTLISVTGVSGSGKSTLVFDILAQGMESGQKPTGCEEITGLE

HVGDIVIFDQTPMGRMQRSNVATYTDVFTHLRQLFAGLPDAKARKLTAKHFSFNTPGG

RCETCQGLGVLSVDMNFLPDLEVTCHDCKGRRFVDEVLQVQYEGFSISDVLNMSVAES

LPVLKSQTKIADLVEMLCEVGLGYLQWGQSVKTLSGGEGQRIRLAKELSKKTKKHTLY

LLDEPTTGLHPSDIKQLHILLEKLVESGNTVVVVEHSLELIRESDWVIDIGPEGGAAG

GELVAEGTPEQVAEVRASHTGQFLKRILAEGL"

CDS 2632177..2633706

/gene="bmr3_2"

/locus_tag="PIKHDJKN_02433"

/inference="ab initio prediction:Prodigal:002006"

/inference="similar to AA sequence:UniProtKB:P96712"

/codon_start=1

/transl_table=11

/product="Multidrug resistance protein 3"

/translation="MVKRNKLGVILAGLLLAILMASMDNTIVATAIGTIVGDLGGFDK

LMWVTSAYMVAEMAGMPIFGKLSDMYGRKRFFIFGIIVFMIGSALCGTADSIIQLSIY

RAIQGIGGGALMPIAFTIMFDVVPADKRGKVGGLFGAVFGLSSIFGPLLGAYLTDYIH

WSWIFYINLPLGLIAFVMIAFFYKESVEHSKQKIDYFGAFTLVASIVCLMFALELGGK

EYAWNSSMIVGLFAAFAVLMTAFVFIERRVAEPIITFSMFKNRLYSTSNLMAIFSGAA

FITASIYIPIYIQGVLGGSATNSGLVLLPMMLGSVVTAAGGGAMLTKLRYRSIMLPTL

ALLVIGIALLTTLTTDSSRFMVTIFMILVGLGIGSSFSVLSNAAIHGFSARQRGSASS

TLNFNRELGMTLGITVFGIVQSHVFASKLVSFFGNDNASEQGGGVDLSDPSLVLKPDT

RADIPAPMLDKITEALSSSIVHTFAWAIIPAVIALLAAWFMSKEQFDPASESDEYFAS

H"

CDS 2633773..2634528

/gene="linC_2"

/locus_tag="PIKHDJKN_02434"

/EC_number="1.1.1.-"

/inference="ab initio prediction:Prodigal:002006"

/inference="similar to AA sequence:UniProtKB:D4YYG1"

/codon_start=1

/transl_table=11

/product="2,5-dichloro-2,5-cyclohexadiene-1,4-diol

dehydrogenase"

/db_xref="COG:COG1028"

/translation="MSEQNGKVIIITGGASGIGKETALQLSDLGATIVIADYNEEGAK

KLASEIEAGGGTAGAYKVDVSKGDEIKALIDWTVEKYGTLSGIFNNAGIGLVKPFLEM

DPESYHRVIDVDQHSVYYGMYYGAKKMVELNVQGTIVNTASIYGSIAAVGSFNYNAAK

AAVVMMSKSGALELAEHGIRVVGVAPGFIETPILGDDKAMKDALATQHMRGELIQPEK

VASVVTFLFSDAASAVNGTTVAVDDGFLSFKTK"

CDS 2635001..2635798

/locus_tag="PIKHDJKN_02435"

/inference="ab initio prediction:Prodigal:002006"

/codon_start=1

/transl_table=11

/product="hypothetical protein"

/translation="MDTLLFVIMFILGLVGSFFSGLLGIGGAIINYPLLLYVPSLMGL

EPFSAHEVSSISMFQVFFASLAGVIAYIRRTGNGKKSEALIHKGLVAYMGSSILVGSL

IGGFISGHLNGEVINLIYGILAIIAIVLMLIPGKGTEGSAGQLTFNTYVAAGSALAVG

IVSGIVGAGGAFILIPIMLTVLKIPTRTTIASSLAIVFISAIGGVLGKILGGDIPLEP

IIYTVIGSLLGASLGSKVSSMINVRLLRYGLMVLIAITAVKVWSSIL"

CDS 2635988..2638372

/locus_tag="PIKHDJKN_02436"

/inference="ab initio prediction:Prodigal:002006"

/codon_start=1

/transl_table=11

/product="hypothetical protein"

/translation="MNNVLTDQADAGYQLAEQKAAQYFTSLHEQLLNNTYTTALTQDI

HLWQKKHIQPFSWVSFLSPSQRKPDSRDAHRYIHWLNVTGKLDDYLDRSISYIYMRDL

GKALDSSDTKARIQRVAQDTKAYFMRSNTTHRKGSPDYISLAALYRWAQKENVETAVI

WVISKLKNVASNIPKELDAEQAQRKLLKIILGVVLHVNDDMNDETPQAERSERLDAAI

RLGYSYGLTYPFIDDLLDSQALTAQEKEQYSLLIRNALLTGIVPDIGEWKGANLKVIQ

YVHAELRDAFEYIKNYQRPETQRTFFEQSYVFFQSQEIDRAKKLSNANYSNEELYIPI

IIKSSSSRLIVRSVLSESVDEGFDLRTFYYGIYNQLADDFADMFDDMEERAVTPYTYY

LKYRDLRPDLINPYELYWAVISYLIHEVYHSDAKTREVILDRAINGLKRCRERLGQEQ

YNEVMNIFASGQPEFNRLIQQMVRKADDVDFLDKLLRDQVILQLQNDKGEKQDFLDTI

RTVREQINIELQISKPGGLPEMKGILIDAANYSLQGDGKRLRPILTWVMGVREYGLHE

SSIIPLLRSLEYMHTASLIFDDLPTQDNASTRRGRSTLHQVHNSATAELTGLFLIQKA

IGEQSSLKQFDATTVLTLIQYSAEKAEDMCMGQAMDLNAKGKTLTLEQLDMICFYKTG

IAFEAALVMPAILAQVKDVEIASLKKFAYHAGIAFQIKDDLLDLEGDHQVLGKPAGQD

VRNNSSTFVSILGEDGAKKEMWEHYCLATDALNEMPKPISFLRHLLDYIIGRER"

CDS 2638568..2641207

/gene="rcsC_8"

/locus_tag="PIKHDJKN_02437"

/EC_number="2.7.13.3"

/inference="ab initio prediction:Prodigal:002006"

/inference="protein motif:HAMAP:MF_00979"

/codon_start=1

/transl_table=11

/product="Sensor histidine kinase RcsC"

/translation="MQVQKVDHHGLFEQIYNQAPIGIALVAPTGQWMKVNPAFCSMLG

YTAQELILLHYQEITHPDDLAQDLMYESELCEGKSQEYRYEKRYIQKNGDVLWISLHI

SLARNEVSGEPLYFICHVVDITDHKTTELKLLQTEEMFKLISDNAQEIIYLATFDGIC

RYCSPSIYNLLGYSPEEVVGNNNTAVFHPQDLERVSQIDLSTGHMLNMQVRHKDGHSL

WFETTYKVIGDPGQEQQILAIGRDISERKKHEEISAEAERIAMIGSWEWNMLSGHVSL

SDQIFEIFDLDQNKLYKASDIYTFMEPTEGQRLKECIEWVKTGQPLDFEYRHLGSDGK

EKYLHLRGLITFDEDQQPIQLNGTLQDITERKLVEFKLQESVERYTSLKKYNHDAIIS

FDMDGNIINANPVAMKMTGCPVAEMIGTSICRFIGPYHLGLILNSQYEMAEKEINAIQ

HTDGYETEVLATLAPIIINEKNVGFYLIAKDITEQKKLLVAKETAERMNKAKSEFLAM

MSHEIRTPMNGVIGMTDLLLDTPGLSGEQKEYIEIIQKSGDSLLAIINDILDFSKIES

GKTDLVDEPFDLKEIVTETVNIVTPMIREKQLDLRLCLDDAIPVPLYGDAYRLKQVLT

NIIGNAVKFTPEGRVEIEVKVTGQEYNDVQFQFKVKDTGIGIPVEKREHLFEAFYQLE

NFMTRKPQGTGLGLAISKKLVELMQGDIWIEESDDPGTTFIFTASFKASNMEEFNRYD

LQQKKNKADQLRILIAEDNEVNQLVLRRMIEKKGHLVDYVVNGIEAVEAVKRSAYDIV

FMDVHMPRLNGFEATKAIKESMRPEACPYIVAVTANAVRGDMDKCLKAGMDAYVSKPI

KSESIMQVMEEFYKIKKHPTAYN"

CDS complement(2641314..2643083)

/gene="sgrR_3"

/locus_tag="PIKHDJKN_02438"

/inference="ab initio prediction:Prodigal:002006"

/inference="protein motif:HAMAP:MF_01449"

/codon_start=1

/transl_table=11

/product="HTH-type transcriptional regulator SgrR"

/translation="MDTATTHYLRLATAKELSLKFHEAVPVTIESLSAALCCTPRNVK

FILRKLEEQSFIHWQPGRGRGHHSEMTLLRSVNEALEESFLELMGRGKMKEAIELIGS

TEKNDALRERLLNSLNKQMGFHSHAESSSGQDVLRMVRSRRLADLDPAFVYTAFETYL

LGQVCSTLITYDAKTGTFLPDLAHMWEYNEDQRVWVFYLRKGVRFHHGRVMTSRDVQA

TFQRLRTIDSPFIWLYRDIEHAEVVGDYCIRFYLSRPNRFFLHLLSCVSMTILPYDVD

VTQQLIGTGPFRINEINETLLVISAFDAYYGIRPHLDRVDIWFVPNLGPYERHYELPG

TDRLKLATDDTGSNSVDYPATGCRYMLINFRKAGIHHQLEFRQAMRIVFDPVALVREL

GSNRITPANSFLPWKSAEHAWRESSLEDARDLLRSSGYRGEKIILAYTVAKDQKEAEW

LKQRGASIGLCIELQPFVEFPNVRETSEADLIIAEEVLEDDWQFGMINFFINKRNYFH

ICLSPDLQSIFYRKMENFLQQNEEQRSAMLNEAEDLLRDNCWILYGCHMNKKALLNQS

LFGLHTAEFGFMDISKLWIKNHQ"

CDS 2643231..2644451

/gene="mdrP_2"

/locus_tag="PIKHDJKN_02439"

/inference="ab initio prediction:Prodigal:002006"

/inference="similar to AA sequence:UniProtKB:A0A1C7E424"

/codon_start=1

/transl_table=11

/product="Na(+), Li(+), K(+)/H(+) antiporter"

/translation="MKQHLRQIHPLAWTIIVGTMFGRLVTSMSIPFLSIYLTQVLGAT
PTQTGVTVAVSSLAGVMVSFYGGYISDRIGRKVVMMVSVFGWACVFFIFSAAEHLWVF
FVANTLNGLCRAVFEPTSRALLSDITPPQNKLLVFNLRYAAINLGVVFGPIIGLQLGS
AESTFPFMISGLVYIVYGLVLFLQFRLQRANLPEHVQVSAPRLRDALATTGRDRVFLP
VLIGTTFCVLGYGHFSSTLAQYVAQNPLFEHGSQVFSYMLSLNALTVLVIQFPIVRVA
SKFPPVVPLILGNLLVAASLFLFGIAEGVMLLMISVILFTVGEVLMFTMMDVLIDRIA
KPEWKGTYFGTIGFNNLGSVMAPILGGLLLTQFGTENGLFVFVPLALTTALGLPFLIV
AHKRLVIREREVNV"

CDS 2644934..2645665

/gene="stf0"

/locus_tag="PIKHDJKN_02440"

/EC_number="2.8.2.37"

/inference="ab initio prediction:Prodigal:002006"

/inference="similar to AA sequence:UniProtKB:A0QQ53"

/codon_start=1

/transl_table=11

/product="Trehalose 2-sulfotransferase"

/db_xref="COG:COG4424"

/translation="MKRPAQSYTIWFSQRTGSTLLGEALSSTGVAGYPSEWLHYQHKS
PDTLKREDLEQIWDQGTTSNGVFGIKINFEQRWIDAFRSLYCLPTALSRAEVWSTGFP
NCTKHIYMTRRNKARLAVSWWRAIVSGEWHRKHGDSPQLQDIADQYNFDAIKHLLVES
NMCEAAIEDFLSESGIVPLTIVYEDFIQDYEGTVMKVLEFLDVPSDGVAVSPPFFDPI
ADEISEQWVQRFREECQRDWENVKW"

CDS 2645803..2647548

/locus_tag="PIKHDJKN_02441"

/inference="ab initio prediction:Prodigal:002006"

/inference="similar to AA sequence:UniProtKB:Q9WYC4"

/codon_start=1

/transl_table=11

/product="putative ABC transporter ATP-binding protein"

/db_xref="COG:COG1132"

/translation="MPKTEKRSMSWLLQYLKPVKGRLALLMVMLLTSTGLQLLNPQII

KRFIDTAASGGVLSNLFQLAGLFLVVAVVNQLITVAVSYLGNDVAWRATNRLRGDLLK

HCLRLDMRFHNVKTPGEMIERVDGDVTSISNFFAMFIVQVVGSFVLLAGILGFMFSVN

VPIAIVMTVFTLLSILFMVFIRNLGVNSSKNEREASASLFGLIEERIAGIEDVQANGH

VPYVMNRFYRTMRVVFQKGRKAWLLRVIPWNTTVILFALAVTAVLLLGVHYYMQGIIS

IGTLFLIYQYTQMLNDPIEMLGDQVQEFQKAKSGMLRSRELLSLRSEIENGREDKLPE

GPLGLEFKQVNFSYNEDKPVLQNINFAIKPGERLGIIGRTGSGKSSLSRVLLRLYNLD

SGTIRVGGKDISELTLPALYRRVGMVTQDVQLFDGTLRDNLTLFNQDVSDQIIKETTD

RLGLSHWIESQPSGLDTHLKAGGSSLSAGEAQLFALTRVFLTEPSLVILDEPSSRLDA

ATESMLQSAIDQLMKQCTGIIIAHRLATLEKVDQIMVLGDGKVLEFGAREELASDPTS

HYARLLITGREEELA"

CDS 2647545..2649314

/gene="yheI_3"

/locus_tag="PIKHDJKN_02442"

/EC_number="3.6.3.-"

/inference="ab initio prediction:Prodigal:002006"

/inference="similar to AA sequence:UniProtKB:O07550"

/codon_start=1

/transl_table=11

/product="putative multidrug resistance ABC transporter

ATP-binding/permease protein YheI"

/db_xref="COG:COG1132"

/translation="MTVTGFIGRLFRFRPFLFLINGLSWCIFHSLPLAIGIGMQWFFD

RATAGSSDYMWLAAPLIFIALIRATRVGTFFAAFYAWITYIYHIQAILRTNMLAGIMR

WPGRNLPASPGEAMSRFRDDVDEVVEYVESWVDFWGRLVFAIVSIVIMANINWKITLV

AVLPLVVVTLLNNLSGNRARRYAQVNRESTGRITSFIAETFGAVQALKLGQAEEHVST

RFNKLNEERRQAALRDNLFKQWMRSLNQHVMSICTGLILLMCAAEMKSGNFTVGNFAL

FTSYLANIGFSISLFGYMVFQHKRLKVSFDRMRTLFRPGEEDRIIDFRETYLNEDPPE

LPNEQRDPKEKLRELEISNLAYQYPHSANGIQDISFRVKRGQFLVITGRIGSGKSTLV

RTLLGLLPKQEGTIRWNGKDVDPATFLMPPRAAYTPQVPRLFSDTLKENIVQGKQGRT

EVALAKAIRLAVMDKDIQNLDQGLETSVGPRGVMLSGGQIQRAATGRMLMTEADLFIF

DDLSSALDVETEQQVWEGLFQEPDVTCIAVSHRRAALAKADHIIVMKDGRIEAEGSLS

ELLATNEEMQLLWQGEQTPVKVG"

CDS complement(2649419..2650351)

/gene="ctaB2"

/locus_tag="PIKHDJKN_02443"

/EC_number="2.5.1.141"

/inference="ab initio prediction:Prodigal:002006"

/inference="similar to AA sequence:UniProtKB:P24009"

/codon_start=1

/transl_table=11

/product="Protoheme IX farnesyltransferase 2"

/db_xref="COG:COG0109"

/translation="MKTLNKGSRPEINLEYTSSIPKVFFDTIKIGIIKSNLIAMFAGL

SLALYVFDASILANIVPIILSVIGSSLIIGAAGVFNNLYDRDIDAIMERTKTRPTVTG

RVDTKTGLILGIAITIIGGIMLYIASPSAAVFGILGLLLYVFPYTMWTKRTTVYNTEV

GSLSGAVPPLIGWAAISPELGHYEIWALVVTMLLWQMPHFYGIAIRRFDEYKAAGVPM

LPVVKGIRRTYIQTNVYLVLLLFSSFLFWPMSPFIAIVAFLVSLAWLILSVATFDKKE

TKKWSHKMFIFSINHITIIFLVIIAYSLIAQLIK"

CDS 2650740..2651669

/gene="trxB_2"

/locus_tag="PIKHDJKN_02444"

/EC_number="1.8.1.9"

/inference="ab initio prediction:Prodigal:002006"

/inference="similar to AA sequence:UniProtKB:P66011"

/codon_start=1

/transl_table=11

/product="Thioredoxin reductase"

/translation="MNQKMDVLIIGGGPAGLNAALVLGRARKHVVVIDDERPRNWVTR

ETHGFLTRDGASPREFRKVAKEQISAYPSVHFVGDTATSVTGTDGDFQVTTAQGEVHR

TKKVLFTVGKKDLPLDIKGLTEVYGKSAFVCPYCDGWELRDQRLVIIVNGAKAFHMAK

TISGWTSQFTICTNGPDELTDEQREELKQHGVPVWDSPIRSIESSDGMVQKVVLEDGT

EISCTGIFFGPKLTAGSELPQTIGCEVTDAGTVVVDDFGKTTVTGVFSAGDAASELYQ

AITAASLGALTAVTINSELNMEAWDERGKHLNP"

CDS 2651919..2653511

/locus_tag="PIKHDJKN_02445"

/inference="ab initio prediction:Prodigal:002006"

/inference="similar to AA sequence:UniProtKB:Q81BF4"

/codon_start=1

/transl_table=11

/product="Bifunctional cytochrome P450/NADPH--P450

reductase"

/db_xref="COG:COG0369"

/translation="MSIATNVPQPKMYGPLGNLPLIDKEKPTLSLGKLAEEYGPIYRL

SVPGYSGLIVSGPDLVAELCDVSRFDKFIYNELENLRAFGGDGLFTSRTSEPNWKKAH

NILLPTFSKQAMKGYHPMMVDIAEQLINKWARLNSNDTIDVADDMTRLTLDTIGLCGF

NYRFNSFYRQDHSPFIESMVRALNEAMQKSSRLKIQNLLMVKTKRQFEEDIQTMFSLV

DQIIEERKASAAPEEVDLLARMLNGKDPETGETLDDENIRYQIITFLIAGHETTSGLL

SFALYFLLNNPEALQKAYDKVDRVLVSDSPQYEEILQLPYIRMILSESLRLWPTAPGF

DVYAKEDTVIGGKYPLKKGESCSILLPQLHRDKGAWGEDAELFRPERFEDTTQVPHHA

YKPFGNGERACIGMQFALYEATLVLGMVLKHFELVDYSDYRLDVKQTLTLKPGDFRIQ

VRARTVSPILSTSVAHLNEKPNQVIHTNVAGHGAMKESMTTTEDVNPTILVLLGDGRR

HCQSVCRESPFLWNSKQSRNIE"

CDS 2653862..2654086

/locus_tag="PIKHDJKN_02446"

/inference="ab initio prediction:Prodigal:002006"

/inference="similar to AA sequence:UniProtKB:Q81BF4"

/codon_start=1

/transl_table=11

/product="Bifunctional cytochrome P450/NADPH--P450

reductase"

/db_xref="COG:COG0369"

/translation="MEYSSRSTLQMEFVREKADLPLARTYNASYATIVVNEELQAMDS

GRSTRHIEMLLPEGMTYKEGDHLGVLPQYK"

CDS 2654061..2655152

/gene="cypD"

/locus_tag="PIKHDJKN_02447"

/inference="ab initio prediction:Prodigal:002006"

/inference="similar to AA sequence:UniProtKB:O08394"

/codon_start=1

/transl_table=11

/product="Bifunctional cytochrome P450/NADPH--P450

reductase 1"

/db_xref="COG:COG0369"

/translation="MAYCHNTNEILNRILHRFELKGEVQIQLTSNETQLTHLPLNRPV

QVNEMLRSCVELQAPVTRAQLQELANHTVCPPHKRELEAMLEENNYREHILENRTTML

ELLEKYEACELPFERFLELLPPLKPRYYSISSSPNFNAKQASITVSVVREPAWSGNGE

FRGVASSYLADCQTGESILMFVRTPESGFELSEEPDVPIIMVGPGTGVAPFRGFLQAR

SVLKERGLPLSEAHLFFGCRNDSDFIYRQQLEQYEQAGIVKLHTAFSRVEGKPKNYVQ

HLLIEEAEHLLHLLDAKGRIYVCGDGSQMAPAVEETLRQAYQNAKGATLQEAKDWLNQ

LQSEGRYVQDVWAGKRAVGSAKALYVETN"

CDS 2655372..2655686

/locus_tag="PIKHDJKN_02448"

/inference="ab initio prediction:Prodigal:002006"

/codon_start=1

/transl_table=11

/product="hypothetical protein"

/translation="MKKKLMVGLLTVVACFTLGSTSFAASVPSTSTSSLPESTQVVPY

AQKYITWQTILPKSQFPTLADIPMTASYNVDPYWGTLTLKSVDGQTGYTWTVTYEGYV

NY"

CDS 2655913..2657130

/locus_tag="PIKHDJKN_02449"

/inference="ab initio prediction:Prodigal:002006"

/codon_start=1

/transl_table=11

/product="hypothetical protein"

/translation="MATWFLIIIYLAFISLGIPDSLLGSAWPVMWPELGASFGSAGIL

SMVVAGGTIVSSLASGTVIQKWGTGKVTLISCCLTAGALLGFSFAPSLVWLVILAIPL

GLGAGAVDAALNHYVAEHYKAHHMNWLHCFWGVGATTGPIIMSYYIAEHHSWRSGYTA

VAVVQCCLVLILFATLPLWKRVAASRESASLNNESDHVQADNTLVQSGNRPERNVLKI

KGVKNSLIAFLFYCGVETTVGLWGASYLVGARNISAETAAGWISLYYGGITIGRLITG

FITLKIKNRVLILAGQVTAIVGGIILLLPLSISFALAGFMLIGLGLAPIYPGLLHETP

ARFGKANSARLMGYQMALAYTGTTLLPPLFGVLAAKTDIGFFPLVVLVFLILMLVSSE

QVNRILKKRILNG"

CDS 2657201..2657320

/locus_tag="PIKHDJKN_02450"

/inference="ab initio prediction:Prodigal:002006"

/codon_start=1

/transl_table=11

/product="hypothetical protein"

/translation="MLSLILLCLIVYVGAYGCLAASKQPMDVKPVRSFDDFFE"

CDS complement(2657400..2658902)

/gene="ypwA_1"

/locus_tag="PIKHDJKN_02451"

/EC_number="3.4.17.19"

/inference="ab initio prediction:Prodigal:002006"

/inference="similar to AA sequence:UniProtKB:P50848"

/codon_start=1

/transl_table=11

/product="Carboxypeptidase 1"

/db_xref="COG:COG2317"

/translation="MTTIQRLDAKLEAHIEKVEHYKQVQTLLAWDARTGAPRKGASYA

ARAKATLAGELFKLQTDADFGLRLEEAAQQEDLDELSKRIIAEHWKTYKRWSAIPIEE

YQAFVQQQGLANAAWLEARAANDFSIFAPHLKQIFDASLKFAGYWGYDQHAYDPLVQQ

FDPDLDKVTLDRIFSELKSALIPLIRRVTSSAKQPDTSIFTHPFPLEQQRQLGIELLQ

AIRYDFDAGQFGATVHPFSSAINPYDARLAAKFVENDVRVSLWSALHEGGHSLYTQNI

DPNLIHTGLGIFTSYGIHESQSIFFEKFIGRHKGFWENNYFILQKIKTETFGNVSLDD

FYFALNAVKPTFNRFEADELTYNLHLIIRYELEQAIIAGEVDYEGLPEAWNDKYEEYL

GIRPPTHLEGILQDGHWSAGFGLFPSYTLGFVYAAQIHKVIRRELPDFDHLIESDHIE

TITGWLTTHIHQFGKSRSADELIKSITGKSIDTAPLIRYLTDKYEQLYEL"

CDS 2659159..2660766

/gene="gsiB_3"

/locus_tag="PIKHDJKN_02452"

/inference="ab initio prediction:Prodigal:002006"

/inference="similar to AA sequence:UniProtKB:P75797"

/codon_start=1

/transl_table=11

/product="Glutathione-binding protein GsiB"

/db_xref="COG:COG0747"

/translation="MMRKHTKPALMMILAFLLLAGCASGGEGQQSTQASSTTGESSSQ

QTSAEGKTGGTLNIGYPTQPVTLDAHVSSNTAVKDISREIYEQLVTLDKDAQVIPLLA

ESYEISDDKLSYTFHLRQGVKFHNGKELQAEDVVASLNRWIKLSSHGKANFVGAEAKE

TDPYTVVLQLKTPFLLTLQLLADPIPAAAIYPKEVVDEADDKGIKSFIGTGPFQLEEW

KQDQYIHLKKYDDYSARTEPSDGAGGKKEALVDDLYFRFVTDESTRLAGITSGEYDIA

LNISVDNEQQIADNLDLQTFLSPGGMIGYFYNSNDGLFKNVKLRQAVNAIQNVDDILT

SAFRDPQFYVKTSSLVLPEYPNWYSEAGKESYSQANADKAKQLLQEAGYNGEEIKIFT

TRDYVDQYNVAVVLQQELESIGVKVKLDVYDWPTLQEKLGTGTGWDIYPMSYAFRPTF

YQYAFWGGGAGLLKSEKLSELLQGIRTSDSVEAARPLIDELQQFVWTEVPFSQIGHTK

QVTALSKNISGFQNILGPIFWNTTNNK"

CDS 2660857..2661807

/gene="gsiC_2"

/locus_tag="PIKHDJKN_02453"

/inference="ab initio prediction:Prodigal:002006"

/inference="similar to AA sequence:UniProtKB:P75798"

/codon_start=1

/transl_table=11

/product="Glutathione transport system permease protein

GsiC"

/db_xref="COG:COG0601"

/translation="MGGYIVRRLLSLIPVLFVVAVVVFSIIHVTPGDPAAILLGEEAS

KADIDELRSKLGLDRPIYEQFGIWLLGLLSGNLGESIFLDKPVTEAFFERLAPTLSLA

IIAQVIGVFLALFLGITAVKRRGTIVDQSVMGFSMLGISIPSFLLGSLLVMFFSVKLR

WLPVAGYQPLTEGLWQHIKYLLLPGIVLGIIQAALITRMTRSSILDVMSGNFIKTAKA

KGVKEKTIVYTHALRVAFIPILTVIGESFGGLVTGAAVIETLFNIPGVGQLIVNSISR

RDYSVIQGSVLLVTVAYVFVNLIVDLLYGLVDPRVRLSKK"

CDS 2661824..2662747

/gene="ddpC_2"

/locus_tag="PIKHDJKN_02454"

/inference="ab initio prediction:Prodigal:002006"

/inference="similar to AA sequence:UniProtKB:P77463"

/codon_start=1

/transl_table=11

/product="putative D,D-dipeptide transport system permease

protein DdpC"

/db_xref="COG:COG1173"

/translation="MITNPDTTIATSGLHKRKNRRIVFRRFMHNQLLTTGAGILLVVV

LLVFIGPMLTPYGPLQIDPVNRLKGPNGQHIFGTDNFGRDVFTRAVYGLRISVAVGFA

VTVVSSLIGMIVGLLSASYSVLDHIFMRICDGLFAFPSILLAISIMAALGPNPGNVII

ALSIIFIPSMARIIRSKALVIRELTYIEALHAQGASSWRIIWIHMAPNTLSPLIIQSS

FIFAVSILTEASLSFLGAGIPSPSPSLGNMLFDGKTVIYNAWWMTVFPGIFLVLVVLG

LNLFGDGLRDLLDPHANKVKRGRRRGKTTAA"

CDS 2662725..2663741

/gene="oppD_5"

/locus_tag="PIKHDJKN_02455"

/inference="ab initio prediction:Prodigal:002006"

/inference="similar to AA sequence:UniProtKB:P24136"

/codon_start=1

/transl_table=11

/product="Oligopeptide transport ATP-binding protein OppD"

/db_xref="COG:COG0444"

/translation="MGKQPLLEVKDLKVSISTEKGTIHPVEGVSFHVLAGETLGIVGE

SGCGKSVTAESILRLFDEDYVDYSGEVLYEGTDLLSLSKRQMERIRGNELSMIFQDPM

SSLNPVQTIGKQIAESLKLHQGMNGRQARLAAIDLLRMTGIPSPEKRVDEHPHEISGG

MRQRVMIAMALACNPKLLIADEPTTALDVTIQAQILELIESLQAKTGLGVILITHDLG

VVAETCTRVAVMYLGQVVEEASVDDLFFNPQHPYTSGLMKSIPRIDGEQHSKLHVIEG

TVPALHQVPTGCRFAPRCAYANEQCLRAAPSLKQVDENHSVRCWYPGIATIQEETHEL

EIIG"

CDS 2663719..2664708

/gene="oppF_6"

/locus_tag="PIKHDJKN_02456"

/inference="ab initio prediction:Prodigal:002006"

/inference="similar to AA sequence:UniProtKB:P24137"

/codon_start=1

/transl_table=11

/product="Oligopeptide transport ATP-binding protein OppF"

/db_xref="COG:COG4608"

/translation="MNSKSLVESKPLLQVSNLNKHFPVRSSFGRTRGQVKAVNGVSFD

IHTGQTFGLVGESGCGKSTLGRSILRLVEPTSGEAFYEGTDILGLSKKELRSYRQHMQ

MVFQDPYTSLDPRKRIGYSIEEPLLIHKKGNKRDRMETAMSLLRKVGFSEEHYERFPH

EFSGGQRQRLGIAKALALGPKLIICDEPVSALDVSIQSQVINLLEEVQEELGISYLFI

AHDLSVVRHIADRIGVMYLGEMVEQGPAEALFADPLHPYTKSLLSAVPIPNPRAKRER

VVLSGEIPSPLHPPAGCVFHTRCPYVMETCRQVVPQKRFVTPDREVQCHLYDE"

CDS 2664724..2665977

/gene="scmP_1"

/locus_tag="PIKHDJKN_02457"

/EC_number="3.5.1.-"

/inference="ab initio prediction:Prodigal:002006"

/inference="similar to AA sequence:UniProtKB:P54955"

/codon_start=1

/transl_table=11

/product="N-acetylcysteine deacetylase"

/db_xref="COG:COG1473"

/translation="MSFHSSAKKWKDEAIRLRRHIHQNPELGHQEFETAKLVAQFLED

VGLEVQTGVGGTGVVGLLKGGREGRTIGIRADMDALPIEEKTGLPFASVHEGVMHACG

HDVHTAILLGTAAALSEHRDELQGTVKFIFQPAEEVQGGATDLIDAGVLHNPEVNAII

ALHVWPDLPAGKIGLRKGAMLAAADAFDIRVTGKGGHAAYPHRTIDPVIIAAQIITAL

QTVVSRNLSPLDSSVISITKLQSNSNAYNVIAGEVQLRGTIRTHNPETREAVPVYMKR

LIETIAEGFGGKAEFDYYRGGSPVINDDHIVDVIEQAAADTIGQEAVVYLKEPSMGAE
DFGAYLEYVPGAIFRLGTITEEDARSELPLHSDSIIFDEQAIVTGISVLGEAAIRYLN
KSSEDESRIALGVAAGNEDRGDRHD"

CDS 2665970..2667676

/locus_tag="PIKHDJKN_02458"

/inference="ab initio prediction:Prodigal:002006"

/codon_start=1

/transl_table=11

/product="hypothetical protein"

/translation="MTELQQQSEVDSILHEIDRDLLLEYNRQIAKEIRLSGSEEELRA
FHYIKEQLEDFGLTTELTFNKAYISIPVRAELKVGGISFAAITHSMSAQAERLSTLLR
FIELKDTDQSVWANQVTGHAVIVHGLANGPTIYRLQQLGAAAVIFINSGAYTHEMIVS
SVWGNPTPEDDGYPSIPVLSVNHSDGEQIKSLIAAEERENVEITLTAEVRTDWVNIPT
LIAEIQGTEEPESFVLFSGHVDSWHYGAMDNGSANAVMVETARVLSLHRDTLRRSVRF
AFWSGHSHGRYAGSALYSDLHWEDLHENGVLHINIDSVAAKGASHLGAANTMAETRTL
CADAIRIVTDEPFCGSPFGRSGDQSFWGAGLPSAFMGLSYQPEGWFGWWWHTTEDTLD
KIDGDYLARDCKVYVAAVYNTASAPIIPIDQRQAIRTLSERIGYYRELIGDKLDLSRL
TSRVQQLQNATDQFYDRIGSTSLPDASVNEANRTILSLSRSLVPINYVRGSRYDHDAA
GSHTLVPALSDIELLAVAEVGSEAFFRLQTSLIRQVNRIGYEIKQAERLVVEAENNLV
WN"

CDS 2667735..2668499

/gene="fabG_4"

/locus_tag="PIKHDJKN_02459"

/EC_number="1.1.1.100"

/inference="ab initio prediction:Prodigal:002006"

/inference="similar to AA sequence:UniProtKB:O67610"

/codon_start=1

/transl_table=11

/product="3-oxoacyl-[acyl-carrier-protein] reductase FabG"

/translation="MPERTFAGKKVVITGAAGVFGQWIAQAYAAEGASLYLSDIREDA
LRNTVDKLKQEGVDVHWHVTNLVDEHSINELVQAVEQQWKAPDIVINNAGIYPYRTLM
NMTLQHWNETMDLNLAAPFLLTQHFANQMIASEVEGSFINISSGAASTAGVGMGNYSV

SKVGIEMLTKTFALELSPYRIRVNAVVPGYAPGSEVSVMTKEHTEHMIKITPLGRTSG

PDDAPQAILYLTSDQASFITGSILTVDGGRTAGRLK"

CDS complement(2668548..2669477)

/gene="gltC_4"

/locus_tag="PIKHDJKN_02460"

/inference="ab initio prediction:Prodigal:002006"

/inference="similar to AA sequence:UniProtKB:P20668"

/codon_start=1

/transl_table=11

/product="HTH-type transcriptional regulator GltC"

/translation="MEMRMIKTFQTIVKLGSFQQAAEALQYSQPAITLHMQKLEADLG

MKLLDRGKKIKLTEAGKIFYARADQLLKEYEYLTNTITDIQQGQAGFVRIGVSEPTAS

HRLPAILSSFVKQNPKIKLSVRIGDSKLLNELLIDEQLDFAVCPSPETSMDTEFQPLL

FEPMALLLYDSHPFAQGDEIHLADLKGQQFLFTPPNCPIRIRIEQLLAEKIDSDYQGI

EISNIRSHKYYVQAKLGITIAPIATICPAIPGTVIQKIADLKVGPIAGILRKRNIPIG

SASEKLMLDIRAALLQSNLYVPEFELPSQLAMP"

CDS 2669750..2670337

/gene="yxaF_1"

/locus_tag="PIKHDJKN_02461"

/inference="ab initio prediction:Prodigal:002006"

/inference="similar to AA sequence:UniProtKB:P42105"

/codon_start=1

/transl_table=11

/product="putative HTH-type transcriptional regulator

YxaF"

/translation="MSEKSNARELIVSTAARLFFSQGYHATGLNQIIKESSTPKGSLY

HYFPHGKEELAHECIQKANENILQKFEEIFAAHDNTGDAIQQFIHDLAIETEAAGFTG

FLPFSFWAAVETSCISQQLRIACQDVFAGWQQIITKHLILDGIGEKKAQEMGLLVISL

MEGALIISLTNQDKQPLLTAADYLSVVAKNAKEKQ"

CDS 2670403..2671884

/gene="emrY_1"

/locus_tag="PIKHDJKN_02462"

/inference="ab initio prediction:Prodigal:002006"

/inference="similar to AA sequence:UniProtKB:P52600"

/codon_start=1

/transl_table=11

/product="putative multidrug resistance protein EmrY"

/translation="MKAGSVQDQQETKQYKVFPILFAMLLSGFIGLFGETALNVALTP

LMGLLEVGPTTIQWLTTGYLLVLGILVPVSGMLLQWFTTRQLFTTSLIFSIAGTFVAA

LAPSFEILLVARVLQAVGTALLLPLMFNTILVIFPIEKRGAAMGLIGLVIMFAPASGP

SISGLILANLSWHWIFWISLPFFIISLVCGLLFLPNISKLTKPKIDVLSIVLSTLGFG

GIVYGFSSAGGHGESGGGWTSPIVVATLVIGVLSLLLFSIRQLRMKQPMMDLRAFKYP

MFTVGLILIFLCMMMMLSSMLILPMYLQQGMAVTALTAGLVLLPGSLLNGFLSPVMGR

LFDKFGPKWLVIIGLAIVTVVLFMYTGITPTTTLSKIITLHVFMMIGISMIMMPAQTN

GLNQLPPDFYPHGTAIMNTLQQVSGAIGTAVAVSILSAGQTRFLSGVTDPGSPENQLA

GFTSGVQNAFIFALVLAVIGLITSLFVKRVKVGAQQGQQGPMH"

CDS 2672162..2672815

/locus_tag="PIKHDJKN_02463"

/inference="ab initio prediction:Prodigal:002006"

/codon_start=1

/transl_table=11

/product="hypothetical protein"

/translation="MRIVKSIPANIEQLLDRYEKNGHLTMQASLMGKQSVVYRLQEYC

LKVYTPRGKVDGELECEALLSLQNNHHVPELYAYAPGNFLLTEWIEGFNLRQYRAAYG

HIPHNLIYDMFSTELQQIQASYLDWDVIRYENLLWTATGEVKRTDFWLCEPVSCLRIR

ERLQQEIIRKIERIYSGDGADLEEVVHYFDRHGLTTAEVQEALAHFRSQTPRTALAQ"

CDS complement(2673108..2674082)

/gene="rutD_3"

/locus_tag="PIKHDJKN_02464"

/EC_number="3.5.1.-"

/inference="ab initio prediction:Prodigal:002006"

/inference="protein motif:HAMAP:MF_00832"

/codon_start=1

/transl_table=11

/product="Putative aminoacrylate hydrolase RutD"

/translation="MKKLRRRLFKTFIFGAVALLLLLVTGLIYQQVSTRQDQKTFIPP

GKLYSIHGDNMHLYTGGQGDTTVILASGWGTADPYVDYYPLYEKLAPYTKFAVYDRFG

YGYSDMTDKKRDVDTVADELHELLEVSGQKPPYVLVGHSLGSLETLRFAQKYPDEVQG

IVMLDGGSPEYYANDTDDMASVIGGFMNQFRIKTGLFRLTLQSDAVVEASNANRNGLK

FVPDDLKKIDTTALLLNYGNANTVDELREMTANAKVVVDNKKPFPFPLTIVTADYFGA

SEPIWDKTQAEFTSWSEHSKHVTVKDTEHYIHQYHPDLVADEILELVK"

CDS 2674358..2675866

/gene="rhaR_18"

/locus_tag="PIKHDJKN_02465"

/inference="ab initio prediction:Prodigal:002006"

/inference="protein motif:HAMAP:MF_01533"

/codon_start=1

/transl_table=11

/product="HTH-type transcriptional activator RhaR"

/translation="MKVLIADDDDYTREGLVEAIDWKSFGVHEVLQVEDGAKALRIAT

AQRPEIVITDIRMPKLNGIEFAEQFVAVCPDSKLLFMSGYMDVAYLKSAIQLSAVDYV

EKPIKLPEMENAIQKTVHYIAERRAQHLLLDQKMNLERVKLANALRTKSHPADELRRK

AQEIGYPVHASYVCLIVSQFNKEMLVDAVNIQNYWEQHNLATIGEQLDYDRLLLVVAL

PRPNEEMVRVLAETLCREYSELRIAAGQVVAGLEEVSRSFREAALAMERVFFKPDLCY

LKYDPVNYSPQGLRSDIYPEFYRLLKQEPVGLINWMETFCNRVLEEGDLARERVLSMC

ITFANALFKERNGVLIHMGKMVQIQDVEKQLNECCTMLEVKDHMLELSHAYQEEFTRI

SPYSGLIRSVMNYISDHCSDVDLDVREIADYVHLSTAHLGVLFKQETGTTIKQYLNDY

RLELAKRLILEEHFKMIDISKMSGYASAGYFAKVFKAATGVTPVEYRKKFSQ"

CDS 2675863..2677629

/locus_tag="PIKHDJKN_02466"

/inference="ab initio prediction:Prodigal:002006"

/codon_start=1

/transl_table=11

/product="hypothetical protein"

/translation="MRVLKWLGNLSLQKKLVILLALAGIIPLMITFSATYGELRNSVI

TGQNYAANQSFDQTLSLLSSKFSHIEEVSSMITIDEQINAVFSKDPERMQVWEQLSAF

ERITDYTRILQTNSEINGIVYYINDDFVITGPHATMYRPYAQVSSLPWVRKIVAGGGE

AVWVMMKDESSTSSVSYLALGRVLWNINDYSKPVGIVTLRVELPQLQPYIMNLAMEQL

VYLDTDEGEVVISNGHAELEQRNGYRSQMSRNQFEEVRVGDSSYIVRDSRVGSSNLFL

HSVISPDTAATAVNQVRNKVLFVYLLVCLALLVCIIPVTKSITSRIFLLMNRMSQVRQ

GRLNTLDIPNREDEVGHLVSSYNYMINSVRELMDNQYRLGQDKKGAELRALQSQINPH

FLYNTLDMIVWMAQKEEKQNVQQVVYALSDYYKLILNKGEDFVTLRDELRLSEIYVSI

QQKRFKGKIQFDVHVDEAVMECMLPKITLQPLVENAIVHGILEKPESRGIITITGGIE

EGRVRIVVTDDGPGLEQSPSRLHRHQGSGYGVHNIRKRLEFYYNETDGIHYESVQGRG

TSVIINVPMVFRDIHELKQAST"

CDS 2677844..2678806

/gene="yteP_15"

/locus_tag="PIKHDJKN_02467"

/inference="ab initio prediction:Prodigal:002006"

/inference="similar to AA sequence:UniProtKB:C0SPB3"

/codon_start=1

/transl_table=11

/product="putative multiple-sugar transport system

permease YteP"

/db_xref="COG:COG4209"

/translation="MAEPNIAMTKALKQKKRRFADLREHKTYYIMLIPLVVFFTLFNY

LPMAGLYNAFTDYDFSKGLFSPFVGLKNFEFLFKGGWDSIVWKLTRNTLLYNVAFIVL

GNICQIAMAVLLRELTFKKFMKTSQTLMFMPYFVSMVVVGSIVYNLFNYKYGVFNNVF

TSLGFERFDFYQSPEVWPFIIVIIEIWKGLGYGTVIYLASIMGIDESVYEAAYVDGAS

KWQRIRYITLPLLKPTVIILVLFSIGGIMRGQFDLFWNIIGNNGLLLNATDIIDTYVY

RSLTVNFSLGLGTAAGLYQSIFGLVLVVTVNAIVRKVDRENSLF"

CDS 2678833..2679747

/gene="araQ_14"

/locus_tag="PIKHDJKN_02468"

/inference="ab initio prediction:Prodigal:002006"

/inference="similar to AA sequence:UniProtKB:P94530"

/codon_start=1

/transl_table=11

/product="L-arabinose transport system permease protein
AraQ"
/db_xref="COG:COG0395"
/translation="MAQLAATSDKKKLKVDASSKIFDVMAVVIVGLVALICVAPFILM
LSGSLSDESKIVVNGYSLLPQDFTLDAYKIVFTTLGGEIGRAYLVTIYVTLLGTLLGL
LTTSMSGFILSRPDFKHRDKVAFFIYFTTLFSGGLIPTYLVVANVLHLNHTLWAIILP
GMISPFNVFLFRNYVKSIPNSLMESARIDGAGDFRIYWQIVLPLSKPVSATIGLFLGL
GYWNNWFGYSLYLISDQEKWGLQYLLYRMLSRVTDAVRDAQIVVDQLPSETMKLATTM
LVTGPIILLYPYMQRHFVSGLIVGGVKG"

CDS 2679837..2681393

/locus_tag="PIKHDJKN_02469"
/inference="ab initio prediction:Prodigal:002006"
/codon_start=1
/transl_table=11
/product="hypothetical protein"
/translation="MKALHIRKSLLSCLMVFTVILSACSNGGSDKGATEGSGTPTSDG
KTIVQLKGITMGNAPASGMDNFYKQLDELTIKDLGATIRFDFVPWGDEKNQISRAIAA
KEYDVYVGGAWSDFASFATKNAFANLTPLLGEVPDLVEHYKGTLDTVKINGSLYGIPQ
YNKPGGGGEGLLFREDLRKAWGLPEITDLDTVEQYLYKTKEAYPDTPMINDKRFADNV
WTLVAGSKYLDLVKGYVVATLDEPYKAISMYDTPEYKQVLERSKKWYEDGIVSHDILA
AQGNATSETLELMKADKKPLEFNNHFGAVSSSYVGALKELHPDFEYGWFDYYFNNIPA
YMPFMAPDNITMISIGAHSKHVETALKLIEKAHTDRTYYNLLQYGVEGENYKLAGEFV
SYEGIKSENKKPGWTGLYDGYMNLQEKYPDEWQAINDKLQNEGAKKAEENGQSPLAGF
GLATSNVAAELASMETVKSQYIVPLSVGITKDIDADLANVKKQLEGAGFDKFMAELQV
QLDAFAASKK"

CDS 2681515..2682924

/locus_tag="PIKHDJKN_02470"
/inference="ab initio prediction:Prodigal:002006"
/codon_start=1
/transl_table=11
/product="hypothetical protein"
/translation="MKPIICYPLIQEIPQSQDYIIRIREAGSDGPWSDVNVYGVKVDM

HHVRQASMAYFDMGVPVELEIECKYTEVQTVDIRPLSYKIQSQIQGNVISFELKEPLK

LSIEVNGERFRNLHLFGNVPETDAPSMQDPRVHVIQPGIHRTEWLETEAEVLYFAPGM

HYIEETILRIPSGKTVYIDGGAAVAGSFVCDRVQNVTIRGRGMVYLADFHRFSAFRGI

RLIHASRIVIEGIMIIDVPHYSVYIGSSQYVSVKNIKSFSTRGWSDGIDIMSSSDIEI

QHVFMRNSDDCIAIYGSRWGYRGDSRRIRVRHAVLWADVAHPLMIGTHGDHEANGDVI

EDIRYEDIDILEHHEPQPNYWGAMAINAGDHNTVRNVVYSRIRVETFEQGQLFDLRVI

RNKDYNPAPGRRIEHIVFEDIEYAGGTLYPSRIYGYDEERSIEGVTFNNLTIAGQRVH

SPDHPAIEMNEYVRGISFH"

CDS 2683047..2687354

/locus_tag="PIKHDJKN_02471"

/inference="ab initio prediction:Prodigal:002006"

/codon_start=1

/transl_table=11

/product="hypothetical protein"

/translation="MGMNSGFHEGKRWRRVIALLAAISLLVTLLPVGTHSIAAAADSN

DVTVSGYEVQINETITGGFTHPGVGLTKEVLENMREQVLLQQEPWYSYYVAMTSSAAA

SKTVTSSNASQTDPTKPASDAFNSQGFNSRFISDGLKAYTQALMYYISGDETYRSNAM

RIIQIWSQMDPSKYVYFNDAHIHTGIPLNRMVTAAEILRYTSYQTEELGWTAQDTTNF

TTNLINPVIETFQHDNNRFMNQHNYPLLGAMAGYIFTNNQSRYDEAVEWFTVNSTAQD

QGFNGSLKQLFRLMDTDDLTGEPLVEPVIQHVEMGRDQAHGGGDLTNAAILSRMLLAQ

GTKVDPVDGTVSTAANALGPYEFLDDRILAAADFFWQFMLGYDTPWTPVAYAISPDGT

IRDTYNHISNAYKGRMNTANFWDMYYYYTYVRGINIAEKAPHYYEAFTKRLPSNFYYG

GTLNKNWDNVDGGGDFWLYMPEEAASEGAEYLPREQTSGSLIDIEDRYTKLGGNTTTI

KEGDTSFVRFQATQVGSRIAFLNGSSSEKIIGFRIRTTGPARLELTPGINDTLTLPDT

HGKWRYVTYTLDSFQSLNDILYMKVTGSDTTVDMDHIHIKASEQLTPPVFKTGNSGLS

IFAYPGSPIRVDFEATDAGGLDVVTYSSPNLPQGAALNEATGEMLWQPGVSQTGTHSF

HVAASDGTTITTRDVKIVVSANRASAVEQAIATYDEDRIYVAATLNSFHAVYEDILDQ

LDTASDEVFYGQLQELAQAAEAMKLVTPLLEQGGGIDYHNIVASSTFGTDIANMVDGD

NQTGVSYTRAPNLYHMIDFGPDFKVSATSFGFQSNIFADRLAKSAVFGSNDGENWVRL

TPGLTAFTQSFQTLDVASEYQDDSFRFIKVQLLHPQPDVLYGIVRNLFEMTEFRIYGE

RHETGNKLESVSIFSDQSVSGKIATGDTVQLKIQAKEAIQNVTATIQGIAATVTTQDQ

ILWTALATMGDDVPTGTVKFTVDYEQSDGTTGERTVLTTDDSKLFLVDGGRYIDVSMM

AKVKASDKQWPGNGLSQDEVGYLLFDGDTSTYGDLNTSSGSFYTVDFGEGLSVRLDEI

VLMPRASNSSRMNGLVIQGSNDNSNWTDLTQPLKGSQEGTWSDIRQDQLLNDGYYRYL

KLYNNSAWSGNVAEVEFYGDVDYDAEVVDSKVVAPEGYTQGSYYLYMQEVNRIKASLT

EPDANKMALLKELIAAKTMLISVADLFPPIHVDTAMVLASSISWDGKVDAMTNGYRAF

DGDPATSPDTKTAAGWTRVDLGVDHTKVVGSIRFIPRTGNAARMNGALIQGSNDGVSF

DTLYTVGGVSEIKWYKQNLSSRNAYRYLRYYTPNGFANIGELEFYEKNVDRTLLTLLM

ERANAVNEEDYTGESYAQLHAAVTTADAVDTDLEANQNDVDSASNSLKMALGSLVSI"

CDS complement(2687577..2688110)

/locus_tag="PIKHDJKN_02472"

/inference="ab initio prediction:Prodigal:002006"

/codon_start=1

/transl_table=11

/product="hypothetical protein"

/translation="MIKLIQDVVEDMNKQLERIETSLSRLNEEQIWKRLQPGMNSVGN

YCLHLAGNEYQNLVTGIGNQPLIRERSAEFNINDGLSREELLAKLRHVRTESTAILSS

LEEEDLFREVTIPYELTDWNRMNRSEDEAKDAHEHKIVRSLLIKVATHYGYHTGQIVL

LSKILQPSDEHLTGLYH"

CDS 2688283..2689254

/locus_tag="PIKHDJKN_02473"

/inference="ab initio prediction:Prodigal:002006"

/inference="similar to AA sequence:UniProtKB:P9WKP3"

/codon_start=1

/transl_table=11

/product="hypothetical protein"

/db_xref="COG:COG2220"

/translation="MTKYENQIPTSAGMSFSSLVSMLKDSMRRSIERRPAGNIPMEKY

EPAESLNVSDNPQVTWFGHSAFLLEIEGHRLLFDPMLGNRPSPVSWAGTKRYSTNLPI

QPEDFPALDAIIISHDHYDHLDYSSIRRLKNKTKRFIVPLGVRRRLIQLGVPSEQITE

HNWWDELSFKGLTLACTPARHFSGRGLLDRNSTLWCSWVIAGQETKVFFSGDSGYGPH

FKEIGSKYGPFDLTLMECGQYDERWSNIHMMPEETVQAHLDVRGGLLIPIHWAAFTLA

YHAWNEPVERITKAAHALNVTIATPKIGEKVVLHTGAYPSQPWWRSN"

CDS complement(2689320..2689901)

/gene="eamB_1"

/locus_tag="PIKHDJKN_02474"

/inference="ab initio prediction:Prodigal:002006"

/inference="similar to AA sequence:UniProtKB:P38101"

/codon_start=1

/transl_table=11

/product="Cysteine/O-acetylserine efflux protein"

/db_xref="COG:COG1280"

/translation="MNIIPLLTYAIVASFTPGPNNIIAMTHARNEGFKKILPFIAGVA

AGCLLIMFLSSYFNLILHQYIPRIKPALNVLGCVYMIYLAIKIMRSKPANTKDPKVNR

FTFLFGFTLQFINPKVILYGLTAISVFVMPLGESHVHMIVFSLLLTIIGISANMTWGL

CGMLFQSFLLKYERPFNILMGVLLIYSALSILK"

CDS 2690036..2690590

/gene="sutR"

/locus_tag="PIKHDJKN_02475"

/inference="ab initio prediction:Prodigal:002006"

/inference="similar to AA sequence:UniProtKB:P77626"

/codon_start=1

/transl_table=11

/product="HTH-type transcriptional regulator SutR"

/db_xref="COG:COG1396"

/translation="MKNINVVLAQNLKQLREQRKLSLDKVAEMSGISKTMLGQIERGE

SNPTIATVWKIANGLKISFTALIHEPKSDTTVVTGSDIQALMEDEGKIRIYPHFPFEE

GRRFEMYMMEMDEESSLSAEPHIEGAEEFITVFEGEVTIRVGAEGHTVKKGESIRFRA

DKPHGYKNSGASANKLSMVIHYSK"

CDS 2690685..2690927

/locus_tag="PIKHDJKN_02476"

/inference="ab initio prediction:Prodigal:002006"

/codon_start=1

/transl_table=11

/product="hypothetical protein"

/translation="MNRILIVDDDVYIRELVSTILKQEGFLTIEAVDGNDALQKVAGQ

MNVQTSEGAGSISILLPLELMDVKQYNVLYKVAIKE"

CDS 2690933..2691370

/locus_tag="PIKHDJKN_02477"

/inference="ab initio prediction:Prodigal:002006"

/codon_start=1

/transl_table=11

/product="hypothetical protein"

/translation="MSTANRGVNIGPPRFKIAVHSIVWLAKSGSVLSSAMIASQVNSH

ATFMRRVMQALSNAGFVESKGGREGGYSLLKSADEITLGDIYSAVNAAAGELEFDVDC

GGETGEQLDLELEKILYEAELRTIEYLRNYTIRDVMGRVDFCS"

CDS 2691590..2691964

/locus_tag="PIKHDJKN_02478"

/inference="ab initio prediction:Prodigal:002006"

/codon_start=1

/transl_table=11

/product="hypothetical protein"

/translation="MNILSIVLQSLLVLIFLMAGFGKITGSKMHVEGFQKWGLPQWFR

VVTGLVEIVGAAALIVGYWEPSWTAAGALLLGVTGVGGTLTHLRAKDSMKETFPIVLI

GVLAFVVLIIQRSELMNFPGFN"

CDS complement(2692106..2694520)

/locus_tag="PIKHDJKN_02479"

/inference="ab initio prediction:Prodigal:002006"

/codon_start=1

/transl_table=11

/product="hypothetical protein"

/translation="MKRYWVSILNISLLSSALLTTTALAPVSVFADSPGAISSNPQVS

TSIENAVPAFRNVSVHDPSVIKVGDTFYIFGSHLQVAKSKDLMNWDSVASGVTDDNPV

VPNVTKEFAEALQWAQTDTLWAADVIQLADGKFYMYYNACKGDSPRSALGVAVADNIE

GPYKDQGILLKSGMWDEISEDGTIYDATIHPNVVDPDVFFDKNGKLWMVYGSYSGGIF

IMEMDETTGKPLPNQGYGNKLTGGNHSRIEAPYMLYSPETDYYYLYLSYGGLGADGGY

NIRVARSKTPDGPFLDAEGNDMINVKADKDKPLFDDLSIEPFGVKLLGNFLFQRQIGD

PGTGQGIGYVSPGHNSAYYDAETGKQFLIFHSRFPGHGEEHEVRVHEMHMNSEGWPVV

SPYRYGGSEEDTAPLTTQDIAGQYKWVNHGKEITAEIKSSQTVQFTADGQISGAVTGT
WSLGSDNQVQITSNNVVYKGFFTHEWEPDSQKTVLTFSALSSSGVAIWGSQMAALKDQ
DIVNAVKNDLSIGDTGNVFFNLSLPTKGTRDAEITWKSSNASALSATGVVNRPRTGKG
DAKVALTATIRKGSAVSSKTFNVIIPQQAVSPLLGEYTFDQKKLAKIAQDFSKNEYHG
QAFNVTTSAISSKNQAAAFNGTDSYIQLPGIITDTTDFTFSAWVNWSGGGTWQRIFDF
GNGLTRHMFLTPAQHTGSLQFTIHDQGRDQSLIAAEPLPSNQWVHVAVTLQGDTGTLY
VNGKAVASSTEITFNPKDLQVTEAYLGKSRYTADPFYKGSMDNVKVYDKALTSTEIQR
QAKEKP"

CDS 2694908..2698759

/locus_tag="PIKHDJKN_02480"

/inference="ab initio prediction:Prodigal:002006"

/codon_start=1

/transl_table=11

/product="hypothetical protein"

/translation="MTTYHNQLIAHYKFEDAANIGKDSSGNGHVGAAAGEKIPDISEV
NGRWAVTFTGGSNGTSYLELPSNLLQNVSDNTGVTVTAWVHLGKGSNVWERIFDFGKG
EKGPYLFLTRQMRGTLFAGDDLVVDPGRGFASGEWMHIALSVAGSQGGTRSSAGPIVY
VNGEKVADGSISQTSSGNYAKLRRWFDSFVDPTNYSRNYIGRSQYAADVDFAGSLSDF
RIYQAALSMDEVIEVMCESLTDEEIVKLAGDKYLSAPARIITKDVSLPVDLLGGKVNV
QWNSSQPEVLSANGEVQPLSSAQKIHLNALLSKGGSMLNKSFDVSVVPEHLPPYTVTI
HGDQQVTDISEVMYGLFYEDINNAADGGIYAELVQNRSFESFAFDTYSHDSGECGCST
GRNRDPLFSWSGDIEKMIVQHTDGINTHLNVGDPEVNAYYVTVQTGATIRNRGFSDSN
QNCAMSIKKGEAYDFTVWAKAVSAGTITIQLQDGNGDAISDSVMLQVEGGNTWKKYGI
AYSDTRVLVANAEFSSHASGSHVLTHPGSPHITHSNVTLTGMETALGQLALTFEGDIS
IDMVSLTPQDVWGADPKEQGVSASAHANYTGNPNYRLRKDLVQALVDLHPKFLRFPGG
CISEGSFIWDNVYDWKDSVGTVELRKENYNVWGYMMTMGLGYMEYFQLAEDLNAAPVP
VMACGVLCQARSDYAHPAGGALRDYYIRNFTDLIDFALSTDIEHNEWAAIRSSMGHPE
PFDLRYLGVGNENWGTEFFANFEVFKTSIDDYMKRNYPDHELHIISTVGAQADDDAYQ
QGWKFLSGNLTGSAQVAFADGKEVIEETVTWYENQDNYMDTIADEHYYRSNDYLLNNV
DRYNYYERAYHEDGSMDWTETSKVFVGEYASTDKNTLAGAIAEAAVMTGFENNADVVR
LAAYAPLFNKVLTDGTYRWTPDCIWFDDETVWYTPNYYVQQLFAKYVGEQVLGTSFST
YSKGQPMELIPHGGIEIATGNADIVVKRITVTSNQDGSVLFTEDFREQTALNEAWNWI

PGSSGYTLETGKGLILKAQASGLNGLYVLNDEWSNYKVEVEAQRIAGEDGFTIGVGLT

DISSDNKDVIEYAIGYGGNATGVKVYKQGVEGYTLGDYSSSSAAGNLRAANYEPLENE

TNYTITVNYGGDTGKNLICSYTDGYTTSRILDYKLDAYNRDVFHSVTRDEQHVYVKLV

NADGVDKATQIRLQNLNVTSVAKLITLSGDEELLHVPNVNQKNDEKIVPQEQMIHLQE

DSVVLQLPAYSVNVLVMDVQK"

CDS 2699116..2700021

/gene="yteP_16"

/locus_tag="PIKHDJKN_02481"

/inference="ab initio prediction:Prodigal:002006"

/inference="similar to AA sequence:UniProtKB:C0SPB3"

/codon_start=1

/transl_table=11

/product="putative multiple-sugar transport system

permease YteP"

/db_xref="COG:COG4209"

/translation="MRSNYKQFIRNVPLHLMILPGLIIIIVFGYIPMAGLSIAFQNFS

PIAGFKNMNWVGLDNFRYLFDLPGFGQVVWNTVFISAMKIVSGLVIPVLVALLLNEVR

KTGFKRTIQTVIYMPHFFSWVILAGIIVDVLSPSTGIVNMLLKAIGVEPVQFLASNEW

FPYILVITDQWKEFGFGTIIYLAALTNIDKSLYEASVMDGAGRWKQTWHITLPGIRPI

VILMVTLSLGNVLNGGFDQVFNLYNPLVYESGDILDTMIYRIGLQDAQYSVSTALGLI

KSVVSFIFIGLGYFLAYRLANYRIF"

CDS 2700037..2700927

/gene="dasC_3"

/locus_tag="PIKHDJKN_02482"

/inference="ab initio prediction:Prodigal:002006"

/inference="similar to AA sequence:UniProtKB:Q9K489"

/codon_start=1

/transl_table=11

/product="Diacetylchitobiose uptake system permease

protein DasC"

/db_xref="COG:COG0395"

/translation="MHHASRAYRWFLGLNYVILTLLALICLFPIVNILAISFSSSDAV

KAGSVTFWPVDFTFSSYKYILENQQFLNSFGTSLLRVVLGVATNLIFTILVAYPLSKE

AAKFRSRTFYAWIFVFTMLFSGGLIPGYLVVKEVGLLDSIWALILPGAVPIFNVLLML

NFFRGLPKELEEAAWMDGAGHFRTLWSIYLPISLPSIATITLFAMVGHWNAWFDGMIY

MKSPEGYPLATYLQSMLQQVTMIQSEMMTLEDATLLSQVSDRTTQASQIFLSVIPILL

VYPFLQRYFVHGLVVGSVKG"

CDS 2701003..2702754

/locus_tag="PIKHDJKN_02483"

/inference="ab initio prediction:Prodigal:002006"

/codon_start=1

/transl_table=11

/product="hypothetical protein"

/translation="MRFAVGTWKNASIVVKLMIAFVLVILPLYGISIMITHTSSKQMQ

SEVEKAHESKLYFYHNHLQFELERMSGLVTEFSFDETMSTLSTRAAIMSRYEVTSSLN

NIHNKLGQIMVTSPYISDVVYYVPALHKRVSAVDGIRNVEDTEWKDLLATMGNLNGEL

SYSDNNLYFLKSNPYNMNHEEPPNFILGIRLSAEELKHSLQQLAETGGSDITLSFGEQ

HNVAISSSDEPASPAQLQQVLPESSVSMQVTRFQSDPYLYYSLYDPDHSFTLTASIPN

DVLQAPLEQYNLWLWTLTLISIVIIMIFSFSIYRSIHKPLSVLIRGFRMAEQGQTNIH

IPQSRRDEFGYLYSRFNHMIERLHILIDENYVARIRTSEAELKHLQSQIQPHFLYNSL

FSIKQMAEVENVELIQEFTDYLGQYFRYMSRDSHSEVSLGEEVDHALVYASIQKIRFG

SRVQLQLEELSREYRDIAIPRVIIQPIVENVFEHALAKRSQGGILKLSYQVEHHKLSI

RIEDDGDQLTDEVLSSLQESFQESANQRHGNEEITGLMNVNQRLRIKFGTGFGLCAQR

SELGGLCMILNIPWRRE"

CDS 2702759..2704417

/gene="rhaR_19"

/locus_tag="PIKHDJKN_02484"

/inference="ab initio prediction:Prodigal:002006"

/inference="protein motif:HAMAP:MF_01533"

/codon_start=1

/transl_table=11

/product="HTH-type transcriptional activator RhaR"

/translation="MQRMLIVDDEPVILDGLYAFFQKADFQDMEIIKAYSAFEAMEWL

NSVKIDIVLSDICMPGMDGMELIEKIMDRWPRCKVLLLTGHNEFDYAHQAIRNPCVVD

YLLKTEGMSHIRAAVERALTQISEENDFRYQHAWFRSKLPRALPQLQRQLLLDLLKRT

ERHDIASLQEELDAVQLPFMSNEPLIPVIIRVEEWNNYQSQADRSLIRYAVANVAEEL

LQDKAKVKAIDLDYQVIACFVQPKVELSSLHTGRQEKWEQTLRFVYGTLESVQQSCAD

CLNLSVSIMVSEQAVEWMELSQAIAQLRLSIHEGPGLGMEKLLRVNVQHDSPYTPNIL

NQQSVATLHLDQIRQRVTAGDREWMESFHKWTEASKEGLQDPFFRMKIYSGAGNGLIE

LLHELGLYESAMEEIALSRMLHFDIHTPWSDLVVFYQSAYEWIISKRSVTRIHDQSQI

LITIHHYIKHHLEDDLSLTRIAQEVSLNPSYLSRWYKRITGKGISDYIHDRRIERSKE

LLLGSTCKMHEISAKVGFSDQHYFYRFFKKATGCTPQEYRDQKS"

CDS 2704537..2706180

/gene="lipO_5"

/locus_tag="PIKHDJKN_02485"

/inference="ab initio prediction:Prodigal:002006"

/inference="similar to AA sequence:UniProtKB:P37966"

/codon_start=1

/transl_table=11

/product="Lipoprotein LipO"

/db_xref="COG:COG1653"

/translation="MNMSLKKFSLLTLTMVLATTIAACSSGTGSAENEAQPNTQQDAG

GPLTKYDPPIKLTSTMNETGKESLAEGDTHANNIWTRGYKDDLGIDVTYDWIVPDANY

NDKMNVTLASGDLPDVLKVSAVQFEQLHDAGMLEDLTEVYDKYASDLVKEFMSAEDGA

GLKPVTKDGKIYAMVSFPGSLDSSDMIWIRQDWLKKVGLEAPKSMQDVIKIAEAFTFE

DPDGNGKDDTYGIALNKDLPINAFLLGYHGYLETWIKDTSGQVVNGTIQPEVKEGLRE

LQNLYSKGVLDPEFGVKDFTKMMEDVNAGKSGMFFLPQWAPFQVSSMIKKDKNVDWLP

YPVQSIDDQPAKTQNHLSLGGIFAVRKGYEHPEALIKLLNFQAEKMFGESAKEERAAY

LNGLTGLGFHNATVSNLPANKNVKAQDEVEQALKTGDTSGLELEAKLFYDDIMDYRNG

NLDKWHMERIFGPESSQGVIKYYRDNDLIVMNEFIYAPTRTMNTKQATLDKLRAETFL

KIIYGNLSIDEFDNFVANWKKLGGDQITQEVNEVINANK"

CDS complement(2706253..2707863)

/gene="rhaS_13"

/locus_tag="PIKHDJKN_02486"

/inference="ab initio prediction:Prodigal:002006"

/inference="protein motif:HAMAP:MF_01534"

/codon_start=1

/transl_table=11

/product="HTH-type transcriptional activator RhaS"

/translation="MNALLVDDDYFVVMALEKKIDWKALGINTIFTAYNIAQAKEILQ

NHPVQILICDIEMPQGSGLELLAWVREESHNVQTIFLTNYADFNYAQKAIELQSFDYF

LKPIEFDKLTLIIQKAVAKARDQQFIEKAIHEGELWQKNRSKLIEDSWRRLISGKAFP

SGPADVSAFLTEQNLPYETTDLFLPILVNLFPYDHTLGKTDKNLFDYACLNVMVERFQ

DTLFSIEAITEIKYHNWMVILKWNGAPDAQLIESMCGSFIPEVNNYLKSDACCSIGFS

LMLGQIRHTINELLLMNEERIKHRNQTFLLENYNRPELNYVPPDLGLLEQLLNENRFQ

AFLDETGRYMHQLVREGVVGTAVLSLLRLDLVQLVYAQLKSKEIEVHKLYMGKTNDQL

FMQSLHSIEDMQSYIAYLVNTAAEYLNVAEQPKSVVHEITHYIRTHYGEDLTRNNLGE

MVYLNPDYLARLFKKEMGISLGHYVIHTRLVAARHLLETTAQSVHTIAGNVGYTNYSH

FTKLFKQEMGCSPNEYRKKQKESSHETG"

CDS complement(2707844..2709595)

/locus_tag="PIKHDJKN_02487"

/inference="ab initio prediction:Prodigal:002006"

/codon_start=1

/transl_table=11

/product="hypothetical protein"

/translation="MANLNTIKPYPIRHYIRMMFVISFVVLILDLVISIASISMVKQQ

STQYLQDAADLYINRINHDFAYINHFMGWTLANDENLDNMNTYGVNSIPFLKANEKLH

LLFAELQRNYGQSYNFFYYLENSEYFLNCAPISISYSDYSEVKKQIITLTRDKGVYEK

FYSHWTPIHVNGTSYLINIVPYYNRYLIALISADELIAPLQQINLGANGYASLVDESG

VQLSGPAGGDHIPEERHSFLDLFQSHNIVSSDFSNAAFSAHMVIKFGAFEKIMVAQLL

IMLLFLIVTSTLSAIMMFFRRNLLVPIQRFSKNLARINEGDEATDFKSSRIMELEQVN

AQFKELVGQIKRFKIDRYEQELEKQKIRLDYMKLQIKPHFFLNCLTSMYSMAQMQMYE

EIENMAMATSRYFRYIFQSGENFVLLENEIEHVRTFLDIQKSRYRDAFSYRIEQPEVI

TGIAVPPLVIQTFIENAVKYGVSRDRELCITLSVTEQRQENGDHVLIRISDTGPGFSP

DVLDALMRGKALEQTGGNRIGIMNTIQRLELLYRDEANITFANDASGARITLSLPKMI

LNSDTPGEVTNHECIAG"

CDS 2709931..2710854

/gene="yteP_17"

/locus_tag="PIKHDJKN_02488"

/inference="ab initio prediction:Prodigal:002006"

/inference="similar to AA sequence:UniProtKB:C0SPB3"

/codon_start=1

/transl_table=11

/product="putative multiple-sugar transport system

permease YteP"

/db_xref="COG:COG4209"

/translation="MRTNPLYIGRTLERIRRNWGLYVLLFPAVLLTLLFAYKPMYGVL

IAFKDYSPALGIGDSPWAGFKYFEKFFHSYQFTNTIRNTLVISLYSLATFPIPIMLAL

LVNQMRSGRFKRLFQTVSYMPHFISTVVMVGLMLILFSPSTGLVGNMYQLFGAQAPDL

MGSSALFSSVYVWSDVWQHVGWDSIIYIAALSAVDPSLYEAATVDGASRWHKVRYIDI

PMLLPTAITLLILRVGGLLGVGFEKVYLMQNDLNILSSEILSTYVYKIGLLSSQYSFS

SAINLFNTVINFILLILVNQLSKKYSENSLW"

CDS 2710871..2711791

/gene="dasC_4"

/locus_tag="PIKHDJKN_02489"

/inference="ab initio prediction:Prodigal:002006"

/inference="similar to AA sequence:UniProtKB:Q9K489"

/codon_start=1

/transl_table=11

/product="Diacetylchitobiose uptake system permease

protein DasC"

/db_xref="COG:COG0395"

/translation="MSVMEPAAMTRTPRTKRRSRVTVAEVVLYAFAVLFLIAIIYPIY

FIVIASFSDPSSVANGQVWLFPKGFTLEGYRELLRHENIWIGYRNTILYTVVGTLFGL

VVNISAAYALSRKDLVGRKFFSLFFIFTMFFSGGLIPTFLTIRDFHLYNTFLVMVLPF

SVVVFDMIVARTFFQTSIPGDLWEAAQIDGCGNLRYFVLIVLPLSKAIIAVLGLWIAV

GYWNSYFNALIYLKDPNLYPLQLILRNILITNQMQSGMGTGEAAQVALRLANLMRYSV

IIIATIPIMCVYPFIQKYFNQGVMIGAVKE"

CDS 2711831..2713444

/locus_tag="PIKHDJKN_02490"

/inference="ab initio prediction:Prodigal:002006"

/codon_start=1

/transl_table=11

/product="hypothetical protein"

/translation="MGKKIGKKLFTKMSSLLLASAMLVSVVGCSSGNSSEHAETTISS

DFNQEGLPIVNKPVTLKVLTVRWGNMGDTFTQNQWLKDLEKDSNVKIEWQVMSSNDWG

EQKSIMLASGTLPDIILGDQVFSDSDIVNNLSYFRPLDEYIDSYMPNLKAAMEETPDM

KKISTFPDGKIYSMPTRLPARPKSRNQPVINKAWLDKLGLKAPTTTEELYQVLKAFKE

KDPNGNGKQDEIPYTETGLNVDFLNPFGITDINASSMIVQDGKPVFFPTTDAYKEALI

YTHKLYSEGVIDQELFTQDNTMTSAKWQNADIPIVGFSNQWTPDAVFGKWSDQYEAIA

PIAGPDGKRYQPGDPGGMNLARNELLITSSCTVPEVAARWADQFYSSEASIQNFWGAI

GTVIEKNDDGTYTLMDPPAGTSADAWYWDQSLRDFGPKYVSPSFEEKIKLNPKAGDGL

KLQIDQLGSADVTTPYPKVMYNAEEFQELPTLTTDIDGYVATMRAQWVTKGGIEDGWD

AYIKKLNDMGLEQLLTIRNNAFERYMNVK"

CDS complement(2713523..2713786)

/locus_tag="PIKHDJKN_02491"

/inference="ab initio prediction:Prodigal:002006"

/codon_start=1

/transl_table=11

/product="hypothetical protein"

/translation="MASVKAVPQPDLVLIYWSRNPLVPGSARRIRSVRVIGNTSPCTF

TLVRGALLINALNCLLDNDIGFKVVYSKKTSNISGVLLLKRRS"

CDS 2714020..2716062

/gene="katE"

/locus_tag="PIKHDJKN_02492"

/EC_number="1.11.1.6"

/inference="ab initio prediction:Prodigal:002006"

/inference="similar to AA sequence:UniProtKB:P21179"

/codon_start=1

/transl_table=11

/product="Catalase HPII"

/db_xref="COG:COG0753"

/translation="MSKEPIDENKKIEQRNTFLRDNNGKEMSSNEGVKISDDSNSLKA

GDRGPTLLEDFLMREKLSHFDRERIPERVVHARGYGAYGEFELYESLEELTMAHFLQD

PGVKTPLFVRFSEVAGSKGVNETNRDVRGFSVKFYTDEGNFDLVGNNIPIFFIQDGIK

FPDLIHALKPEPNNEIPQGSTAHDTFWDFIANNQESAAMVMWIMSDRTIPRSFRMMQG

FGVHTFRLVNKEGKSRFVKFHWRPVLGMHSFVWDEAQKLGGADPDFHRRDLWENINAG

NFAEFELGIQVLEEADEFKFDFDILDATKLWPEEDIPVRIIGKMTINQNVENVFAETE

QSAFHPGNIVRGIDFSNDPLLQGRLFSYTDTQLARVGTNYQELPINRPVCPVHNNQRD

GASRYTIDRGRVAYHENSLANNSPYTVPGTKGGFVTYPSTVQGLKERKTASSFLDHFS

QARLFWNSMTGVERSHIIQALTFELGKVKDVSIRQQVVEMLGNISTELATILARELGV

AVPNVHVHESSVTKSSPALSMANTVFSPKTLRVGVIVGKGFDGPSTQYILDELKGAGL

RPVIVHERQGVVHGTGGVELKVDDSFLTGSPLVYDGLFIVGGQKEDDYFQKYTRSYAI

ETYNHFKPIGATPTGALLIQPVGIIGKPGVIVEQQPGTFAQQFIQAMTQQRFWDRT"

CDS complement(2716181..2716309)

/locus_tag="PIKHDJKN_02493"

/inference="ab initio prediction:Prodigal:002006"

/codon_start=1

/transl_table=11

/product="hypothetical protein"

/translation="MESTHEFVKKVNENAEKARHNKNNGKGTPTDKLPSKQHSTNK"

CDS 2716518..2717276

/locus_tag="PIKHDJKN_02494"

/inference="ab initio prediction:Prodigal:002006"

/codon_start=1

/transl_table=11

/product="hypothetical protein"

/translation="MLQKFGFTQYESQVYEAIFAQDAPLDATSIVNYSNVPKAKIYEV

LNRLIDKGTVLTTMHGKKKLYMAVDLQSIILKIRADFEKDIEELKTYKIKRTFTDEHI

WTLKDASSIASNIEQLIEEADSSILFLAWNEQMEKYRELLERKEAEGVHVEVLAVGGL

QTSLTHKYSLIPMLEDSNLEPSQLIIVDHGYLLFAGIEHDSWKAIKTTSKPIVKAMTD

YFYHDVALTQITKKYGDQLLQDEEIARLLARLIY"

CDS 2717382..2718542

/gene="pbuE_1"

/locus_tag="PIKHDJKN_02495"

/inference="ab initio prediction:Prodigal:002006"

/inference="similar to AA sequence:UniProtKB:Q797E3"

/codon_start=1

/transl_table=11

/product="Purine efflux pump PbuE"

/db_xref="COG:COG2814"

/translation="MNKKVYVLAIAAFVVGTVELILGGILDLIATDLHLTLAKAGYLI

SIFSLVYALSAPILLNMTARFERKKVYMCTLFVFLISNLISAFSANFYMLLAGRALGA

ATGSLIFVLSLTLAARIVEPQYKGRAVGIITMGGSASLILGVPIGIFVGNLAGWREVF

MLIAILTVVVMVAIGIAMDRVQPIPAVSLKKQLTALWNPKMLAIHVTTLLVLAGHLTL

YAYFTPFLQEALGASSTMVTFIYMMFGIAAVAGGGIGGILSDRLHPAKAIIIVLIPFI

ISMAVIPFSTGLPLIAFLILLSIWSALSWTVTPVQNSLIIKTSPATAETLISTNSGIS

HAGIALGTYIGGVVIDHSSIMYTGWVGSVLILMGLVSAVYAIRRKEQTVQAA"

CDS 2718793..2719368

/gene="maa"

/locus_tag="PIKHDJKN_02496"

/EC_number="2.3.1.79"

/inference="ab initio prediction:Prodigal:002006"

/inference="similar to AA sequence:UniProtKB:P77791"

/codon_start=1

/transl_table=11

/product="Maltose O-acetyltransferase"

/db_xref="COG:COG0110"

/translation="MSNITTKSEKQKMIDGELYLASDPQLSQEREHARRMTRLYNQTT

ETDGEIRTKFLKELLGSTGEHLSMEPNIHFDYGYNIHVGEHFYTNFNCTILDVCEVHI

GDNCLMGPDVHIYTATHPLNPFERITGAEYGKPVNIGNNVWIGGRAVINPGVTIGNNV

VIASGSVVTKDVPDNMIVGGNPARIIREIEL"

CDS 2719410..2719997

/locus_tag="PIKHDJKN_02497"

/EC_number="2.3.1.-"

/inference="ab initio prediction:Prodigal:002006"

/inference="similar to AA sequence:UniProtKB:Q5HCZ5"

/codon_start=1

/transl_table=11

/product="Putative acetyltransferase"

/db_xref="COG:COG0110"

/translation="MTEKEKSQLGLLYNANYDKELIDERLYAKGLCYEYNQLHPGKIS

AREALLKKLLGKTTDRFLIEQPFVCDYGYNIEIGENFYSNHNMVMLDGAKISFGDNVF

VAPNCGFYTAGHPYDVEQRNEGLEIVGPITVGNNVWIGGGVTVLAGVTIGDNTIIGAG

SVVTRDIPSDVIAAGNPCKVIRKITEADKTKYRRS"

CDS 2720116..2721120

/gene="nagK"

/locus_tag="PIKHDJKN_02498"

/EC_number="2.7.1.59"

/inference="ab initio prediction:Prodigal:002006"

/inference="protein motif:HAMAP:MF_01271"

/codon_start=1

/transl_table=11

/product="N-acetyl-D-glucosamine kinase"

/translation="MKIANAYLMKEININHVRQVMKRVETATKPQLASLTKLSVVTVN

SLVKELCDLGELFEDEMVPSNGGRPALTYRYNYDFSLALVMYINEKQGQDLITATVIN

LEDNMVFREEYTMPTFDQKHFYEIIGNVLTQHASIKVIGIGIPGQTVNGEIKVSSHQQ

LEGIRMIEDLEAQFGLPVMVENDVNAAISGYCAKKELDEDQCVIGIYFPTQYPPGMGI

YMDGKVIKGKLGMAGEVKYLPMGIDWYKLVEKETFVATVCKMIQTVNAILAPDQVVIY

QKMVEEDAVVQAWQKYQADLSMPSYPDVILIDSFREDFEAGMRWLTLKSLEPDLVQFN

"

CDS complement(2721252..2722685)

/gene="bglH_1"

/locus_tag="PIKHDJKN_02499"

/EC_number="3.2.1.86"

/inference="ab initio prediction:Prodigal:002006"

/inference="similar to AA sequence:UniProtKB:P40740"

/codon_start=1

/transl_table=11

/product="Aryl-phospho-beta-D-glucosidase BglH"

/db_xref="COG:COG2723"

/translation="MTHSQHPSFHFPENFLWGGAIAANQAEGAYNLGGKGLSTQDVAP

KGIMGPITEEPTEDNMKLIGIDLYHRYKEDVKLFAEMGFKVFRTSIAWSRIFPKGDEL

EPNEEGLQFYDDLFDECHKYGIEPLVTLSHYETPLHLSKEYDGWVNRKMVGFYERYVT

AVFKRYQNKVKYWLTFNEINSILEAPFMSGGIYTPKEKLSKQDLYQAIHHEFVASASA

VKLCHEIIPNAQIGCMMLSMPTYPLTPNPDDMIKVMEFEHSNYFFGDVHVRGRYPGYM

KRYFRENGIQIQMEDGDEDLLLHTVDFISFSYYMSICQTADPEKQVVGEGNLLGGIPN

PYLPASEWGWQIDPQGLRYVLNMFYDRYQKPLFIVENGLGAVDELITGPDGEKTVEDD

YRIQYLNDHLVQVGEAIEDGVEVMGYTSWGCIDVVSASSAQLKKRYGYIYVDRHDDGS

GTLKRYRKKSFHWYKDVIDSNGKSLKR"

CDS 2722852..2723820

/gene="ccpB"

/locus_tag="PIKHDJKN_02500"

/inference="ab initio prediction:Prodigal:002006"

/inference="similar to AA sequence:UniProtKB:P37517"

/codon_start=1

/transl_table=11

/product="Catabolite control protein B"

/translation="MSKFDEIMKLSGYSKATVSRVINHSPHVSDEARQKITQIMKQLN

YIPNRNAVSLSTGQTKQIGIVTSATNEIILTFMNQFIDTAMDYGFQTLIYTSRGDKEI

ELQAFEDLRSKRVDGLVIMTCVNHPDKLKAYCEYGPIVSWQRMEDDEIPSVAMDQAQG

YRLALEHLVSKGYTRIANAFGRAESLNTQSRREAYESFMADKGLPVLREAYQYSVFSS

SDGEEAMRRMASGPELPQAVLCSNDYAAIGIWSEARKQNIQVPEQLAIVGFDDIELSR

VLGITTIHNPIAEQATQAFHQLWAVLGKQELHPQQLEFQLIERATT"

CDS 2723902..2725110

/gene="tylCV_1"

/locus_tag="PIKHDJKN_02501"

/EC_number="2.4.1.318"

/inference="ab initio prediction:Prodigal:002006"

/inference="similar to AA sequence:UniProtKB:Q9XC67"

/codon_start=1

/transl_table=11

/product="Demethyllactenocin mycarosyltransferase"

/translation="MARVLFINGGSEGHVNPTIGVVQELISRGEEVVYFTIETYRERI

VKTGAIVRTFDGQKFIKAFVSGGRTYLLERVNGLLLTADIVIPSVLEQIEGEHFDYII

HDSMFGCGRLLAQILKLPAINSCTSFAQTEESFNHLMQHFFLNVPAEIVQPINDTFHS

LTIRLQEKYDVEIASPYEVFCNPAPLTIVYTTREFQPYGEEFDHTYQFVGPSIFSGLT

HEDFDMEAIKGKTLIYISLGTVFNQALDFYKLCLEALGNTNHTIVMSVGDRTQITDLG

AIPENFIVKRYVPQMDVLQYAKLFITHGGMNSTNEGLYYGVSLIILPQSADQPIIAEQ

VVSRGAGLRLQMQTLTASQLREAVDTVLSVSSFEKAAANLRESFRKSGGYHYAVDAIF

EFKTQHFIHG"

CDS 2725058..2725690

/locus_tag="PIKHDJKN_02502"

/inference="ab initio prediction:Prodigal:002006"

/codon_start=1

/transl_table=11

/product="hypothetical protein"

/translation="MLLMRFSNLRLNISFTDSSVINYSKLKHFRTRTMRYTEQEVSRE

AALLQMMWPQVATSCNKLHISQTSRWKGEIEQDEDSHRSIQNCRHMGRCIITAVDFYS

HAAKIFCRYVFPSNHYGYDSRYSFSAISDCTCAFGSCEEVESFTLVHGIGSRFIAFRN

ICIRILSSQERLEVRIAEKNNDNDSRRPTPDVIRTGRLLVSMQILFLERV"

CDS complement(2725695..2725832)

/locus_tag="PIKHDJKN_02503"

/inference="ab initio prediction:Prodigal:002006"

/codon_start=1

/transl_table=11

/product="hypothetical protein"

/translation="MKKSFVFLLTALVAVASFSAYAPVSDASADIGVSLLSDRQQGTV

K"

CDS 2725999..2726340

/locus_tag="PIKHDJKN_02504"

/inference="ab initio prediction:Prodigal:002006"

/codon_start=1

/transl_table=11

/product="hypothetical protein"

/translation="MKELHTLTSIEMIEEMIKQHELVFLYVSRPECSVCHALLPKIRE

LLEPYPMVHLAHINANQVEEVASRFLIFTVPTMIMLVEQKEYIRADRFVRFDRLEEQV

QQIYHMYTQNG"

CDS 2726500..2727522

/gene="exuR_3"

/locus_tag="PIKHDJKN_02505"

/inference="ab initio prediction:Prodigal:002006"

/inference="similar to AA sequence:UniProtKB:Q9JMQ1"

/codon_start=1

/transl_table=11

/product="putative HTH-type transcriptional repressor

ExuR"

/db_xref="COG:COG1609"

/translation="MGKITIKDVAREAGVSISTVSNALNGVDVLNPETKSHVLKVAER

LNYVPNLNGKLLKSGQTKMLGFFTTSVSGPYFYKLVESMSRECDRLGYGLNVFVTKDK

QVIMSNILGRRVDGVIIYEELRIDEQDIVAMEKDKIKAVFLDRVFQSDTMGSVIFDSY

VAGYEATKYLIGLGHKKIAYISGVDTMFDSNQRRDGYLAALREYQLPLDDDYILQGYF

EEEGTYSAVKSFLHLHPAKLPDAFLAGNDVSAIGCIHALKSHGFEVPQDVSVVGFDDI

DIAQYFSPPLSTVRNQIARQGILAINHLVRMIKKKEHGVSQKLAGELIIRGSSHVKMD

RSDFLT"

CDS 2727641..2728633

/gene="yteP_18"

/locus_tag="PIKHDJKN_02506"

/inference="ab initio prediction:Prodigal:002006"

/inference="similar to AA sequence:UniProtKB:C0SPB3"

/codon_start=1

/transl_table=11

/product="putative multiple-sugar transport system

permease YteP"

/db_xref="COG:COG4209"

/translation="MSKLAVETSSTGKKPVRPGGRKPIGQRVKEFVIDYRRQWEIQSM

IIPGIIFMIIFCYIPIYGLTIAFKNYTVIDTLSSAPWVGLENFRIIMSDKYFWDAVVN

TLGISFLKLGIGFVIPIILAIMIYELNSGRFKKFVQTVSYLPHFLSWIVLGGMLITWF

STTGLFNQLLLSLGVISQPQNILLDAGKYWWIAVLSDIWKEAGWGTILYLAIMSKIDP

TYYEAAKIDGASRLRQIWNITLPNMKSIISLNLILTVSGLLGSNLDQTLVLMNSQNRD

KAEVINSYVYRMGMSQGDFSYATAVGLGVSIVSVILLVTANKITSKLNDNQSVL"

CDS 2728649..2729536

/gene="ngcG_5"

/locus_tag="PIKHDJKN_02507"

/inference="ab initio prediction:Prodigal:002006"

/inference="similar to AA sequence:UniProtKB:O50501"

/codon_start=1

/transl_table=11

/product="Diacetylchitobiose uptake system permease

protein NgcG"

/db_xref="COG:COG0395"

/translation="MNGKVAKEDLDSRIFDTLNIILLAICTVIIVVPLWNVIISSFSS

GKALAEGGFIFWSPEFSLENYRAVFNDSSIWQAFFISISKTTIGVVTHVFFCAMVGYG

LSKKHIRGRKLYVAMGVITMFFSGGMIPTYLLIKSLGLLNSFWVYIIPALFSFYDVVI

LMNFFRNVPDSLEESAKIDGAGDWHIFLKIFIPLSMPAMATIALFNGVGQWNDFMTTK

LYITDQSLYPLQMMLYEIIVQSQTQSMQNVGGSAVIETTTKGVQLATIVITTLPIILI

YPILQRYFISGMMLGAVKE"

CDS 2729607..2731250

/locus_tag="PIKHDJKN_02508"

/inference="ab initio prediction:Prodigal:002006"

/codon_start=1

/transl_table=11

/product="hypothetical protein"

/translation="MFKMNKAAGKKGVKLCAALLTAVIMITGCSSGSGGSSEGNWVSI

EDRYTVDPEKPAWQLDKKEEATDLTWYVNADWWNTDFGKDIVTKKIKEDLNINIKFIT

GDDTKLNTFFAGGDMPDLLTVFDSNSPVVQKAATWAMPLNDLAEKYDPYFNKVAAADT

LNWFQLADGKTYGYPNYSNTQADYDSGNIPAKTAFIIRKDVYEALGSPVMGTPEEFES
VMKQIKEKFPTLIPFGFNSIGEGTGSLGDTLQDFIGVPLETENGEFYDRNQDEDYLTW
LKTLNKVYRDGNISDDSFADDGTAFEEKVKSGKYATMLLDGTPQQGGNLQIYLSANAG
KEYVAIDGPQSTKGNAPTLNQSGITGWMINFISKDAKDPAKAIQIFTYLLSEEGQLLM
NYGIEGETYKKNADGTVELLPAVKDLQLHNADKFKKEYRLGEFMFFGHDRHKALSADA
FPEAIKQMQEWGKGKLKPHFILENISPNQGTPEARALSAINAKWNSTLVSMVRAKDDA
SFDNALAAYKSFLGENRWDDILKVRSEKMKQNKEKLGIQ"

CDS 2731273..2732622

/locus_tag="PIKHDJKN_02509"

/inference="ab initio prediction:Prodigal:002006"

/codon_start=1

/transl_table=11

/product="hypothetical protein"

/translation="MTKFKMYKSTGEDALFVSVSQDQLKSQDSSMETTTILLDDQQTY
QEMDGFGASFTDSSAYLINQILSEEQRAEVMTRLFHPEEGIGLSVIRNPMGASDYART
VYSYNDLPENQTDPELSGFSIAHDEADVIPLTQKALELNPDLKLFASPWSAPGWMKTS
GSMITGQLKNEWYPAYADYFVKYIQAYASHGLPIHAVTPQNEALYEPGHYPGMLMPAE
AQADFIKNHLKPALVRNDIQSKILCYDHNWDRPDYPLTVLDEAAEEVDGVAWHWYGGE
ASAQTKVYEAFAGKEVHFTEGSGGEWIPPFEQAFSNVIRTGIEILRNYSKSFVLWNMA
LDENNGPAVPGFGKSTCRGIVKVNQQTKELTYTLDYYALAHFSALIRPKAVRIDSSSN
EASIRSVAFKNTDGSIAVVLFNDGEETGNVQVQLGQEELLSFQLESKSALSILINQE"

CDS complement(2732780..2733724)

/gene="chbG"

/locus_tag="PIKHDJKN_02510"

/EC_number="3.5.1.105"

/inference="ab initio prediction:Prodigal:002006"

/inference="protein motif:HAMAP:MF_01246"

/codon_start=1

/transl_table=11

/product="Chitooligosaccharide deacetylase"

/translation="MSRQVIINADDFGLSPAVNEGIIKAYHAGGITSTSMMVNMPGFN
HAVRSAWSLPDFGIGLHFNLTYGRSISDPDSVPSLVRPDGTFHNGIRTSVRDSDDVAR

ELDAQWNRFVGTSLYPAHLDSHHLLHQNDPLIYKVMAEKAIRENIPLRRSQIVHSIPN

VPNPLMSNTILLDTYGDQEGLQRLLHHLFSLPEGITEIVCHPGYVDDVLRGISEWTDI

REDELSVFINEEVPQTIQALGIVPISYKAMMDTPDEPCELSELPEIPRPSQPIKKKIL

LAGPRPHRELKRKRKTRRWFKKRGTKPRPSATRIRKRV"

CDS 2733987..2735222

/locus_tag="PIKHDJKN_02511"

/inference="ab initio prediction:Prodigal:002006"

/codon_start=1

/transl_table=11

/product="hypothetical protein"

/translation="MRKRKIVSMLVLFSFLIALLPGTTTNAATNWNLVWSDEFNGSSL

NTSNWSAEIGTGSGGWGNNELQYYTNRTENLQVTGGNLVITARKENYNGSSYTSARIK

TQGLKDFTYGKVEARIKLPSGQGLWPAFWMLGSNINSVGWPKSGEIDIMERVNNNAFV

NGTVHWDANGHADYGKISENLDFSQFHVYSIEWDSKYIRWFVDGKQFNEFYIENGTGN

TEEFQRPFFLLLNMAVGGNWPGSPNNATPFPSQMLVDYVRVYQTASTPNIVSGGLYTI

GSKASGKVLDIVDVSTASGAKVQQWTNYSASNQTFRVDSTGDGFYKLTAVHSGKVMDV

PNSTASPGVQLQQWDDNGSNAQRWSIQDAGNGYYKIISKVNGLAVDVSSSSTTDGAAI

QQWNDNGTDAQRWSFNKLN"

CDS complement(2735139..2735333)

/locus_tag="PIKHDJKN_02512"

/inference="ab initio prediction:Prodigal:002006"

/codon_start=1

/transl_table=11

/product="hypothetical protein"

/translation="MSPFEHFPPGKHHLLDVNNILSRRNFVIDSPKSIVFELIQFVKR

PSLCICTIIIPLLNCSPVRR"

CDS complement(2735373..2735996)

/gene="ytaF_2"

/locus_tag="PIKHDJKN_02513"

/inference="ab initio prediction:Prodigal:002006"

/inference="similar to AA sequence:UniProtKB:C0SP79"

/codon_start=1

/transl_table=11

/product="putative sporulation protein YtaF"

/translation="MAWMLILALAVSSSLDNLGVGLSYGIRKIRISLFSNFLIAVVCL

LFSYSGILFGKWISIVLPGVFPILLGTFILLVIGFRIILLSVPRKRKDHEETDSQTLG

IGSILQNPERVDLDKSNHIGVLEALILGVALSTNALTNGLSAGLMGLSPLYISLTAAI

GSFVTVWFGVWMGSKAANVRIGSFTLGQFGTLVSGVLLLLIAANNLF"

CDS 2736168..2736821

/locus_tag="PIKHDJKN_02514"

/inference="ab initio prediction:Prodigal:002006"

/codon_start=1

/transl_table=11

/product="hypothetical protein"

/translation="MRANDQTKVCHIGDLIKPLLARRSLSMRKFSTLCGMDTATISRI

LNGKQQPKLAHLQIFSEHLDISLDHLIEAAGYKINHAKRGAEQGILDSLESIKTVLQH

TKLFDLDVTTERIEQEMVKYEQYARTDEGHRIICKEFDVKINQINGAGPFIEHLKEMH

IRYCDEITPENDRSLIGSALLYFISSADLIPDYLFPIGYLDDAIVVYLVLDRLKQRT"

CDS 2737506..2742704

/locus_tag="PIKHDJKN_02515"

/inference="ab initio prediction:Prodigal:002006"

/codon_start=1

/transl_table=11

/product="hypothetical protein"

/translation="MIKMNRLRKPLSMGLSLLLALSIIHPVSAENAASQKIDMKSSSG

KITTSKVNAKLSKEFESNDYVTYLVKMKEQVDTASVSKQALQKATIEKQTASATKLSV

RSTVVSSLRETASRSQYTLESFLEQELDLGKVKDYKSYFIVNALAVTSTKEVMEQIAL

LPEVDKILPNEQRYMQKTEISSENATAAPATESAKAPAKEAESKESAAANDKSLQTEN

VEWNIDSINAPAVWDRGIDGTGIVVANLDSGVDYTHPALRSKWRGLDASGNIVDPELS

WYDPHSHASLPADGDGHGTHTMGTMVGSEANGTNKIGVAPGAKWIAVRIFNPSTTDAI

ILDGGQWLIAPVDAEGNLHPELAPDVVNNSWGGGAGLDEWFRPMVQAWRDAQIFPEFS

AGNVTLTNPGGPGSVANPANYPESFATGATDINGNLASFSLLGPSPYEEIKPEVSAPG

VNIRSSVPGGVYEGGWNGTSMAGPHTTALAALLLQANHSLTVDQLEQIIMDTATPRTD

SRYPTSPNNGYGHGIINALDAVGSVLEGIGTVSGRVVTAGDDLEAPVLEHTPVNSAFT

GLDIPLTARVTDNVGVTTVEAFAKTTGTSQYVYLPMNRVAGDAKDGTYTATIPAFLIE

SQGLEYYIRVNDYGNNGFESEVYKVTVSNGVQPGYLQDFEEDNLGFTSGGVGNTWAWG

APTSGPGSAYSGDKVIATNLTGTYAANSNAYLLAPPIDLTQSSEGALLSFKHWYDLES

NIDFGKVYIATEDTDFVFEEALSFTGAGGDWKTQYIDLRDYAGQQVFIKFNLTSDNSV

QKAGWYIDDFSVQAPDDIAPGAPSGLSASADILGNVNLSWTGSDEEDLKSYNVYRSTN

SGSGYELLGNTSATTFTDTATVTDSTYFYTVAALDYSGNESDKSNEVSVTVEVPQDIF

IDHFDGNSDNGWTHSGTKDEWERGVPVVGPASAVSPPNVWATDLDSTYENGSNYSLVS

PVVDLTQADQATLTFNHWYEIESGYDYGYVEATKDGGNTWSELGKFSHSSNGKQWTPV

FYDLAALKGNEVQFRFRLTSDSSVVKQGWYIDDFRILGIAAETVTKDTAVVLNSDKPK

PEYDNSWYKISSTDKAEFNKTKQEQPEVQKPETGSVTPQSLPASATVTVLETGRSVKT

DPTTGKYSFTHVAGDYTLKAEAYGYYPQTKTVTITDGSGATANFNLETIPQGKIEGTV

SDERTGKPIAGASVLVLEDAQVGAVRTGTDGKFVLDVLEGSYTLSIIATDYYSKTVNV

TVPGNGTVEAKVALKPFIGFPGEIAYDDGTAENARAFVAADNAWAVRMTPELETAQLT

GASLRFWNTQWPVPGGTAFKYAVYDASGSGGAPGRLLAGPFDGTALRNDQWTTVEFPE

PVVVQGDFYIVYVQTAVGTSAPGLATDENGVNAGRSWQRVGGVWSTAPAEEGNYMIRA

VVKYPVNAPVLTQPTNTYTNQPTFDVSGTSPASGAQIKIYNGKEVAGTTTVENGKFKL

NVKLRAGVNAISAEAIVNGKTTDRSLPVVIILDQTAPVLTVLTPEEGSRTNTEVVHVT

GDVQDQFLDKVTINGQKVKLEQDRSFNHRVLVNEGKNTITIIATDIAGNKTTVTRTVY

VETSLPELTNISPAEDVRIVAGETVTVSFDSVPKLQASFRIELPSNLSTQAGAEIPLV

ETEPGHYTGTYTTASSLVLEGGVIVIRAQDAAGNKVEVEAPGRLFVSAAEVPDTNEPS

DENLLP"

CDS 2742933..2743253

/locus_tag="PIKHDJKN_02516"

/inference="ab initio prediction:Prodigal:002006"

/codon_start=1

/transl_table=11

/product="hypothetical protein"

/translation="MGIEKSQPLRSKWLKTKQLINNDSIKPFIPDTQKFNKVNLKSMI

ARYGMLYIKPEIGTHGVGVIKAEMENQQNFAYQMNKKRLTFNSFDSFHKSLAHVVNQR

SYLI"

CDS 2743250..2743432

/locus_tag="PIKHDJKN_02517"

/inference="ab initio prediction:Prodigal:002006"

/codon_start=1

/transl_table=11

/product="hypothetical protein"

/translation="MNKKYKRLKGIGVDIGLDQSLKPWVIEVNAKPDPYIFNQLKDKT

MYRKVIRYFRHASQKQ"

CDS 2743760..2744764

/gene="iolG_7"

/locus_tag="PIKHDJKN_02518"

/EC_number="1.1.1.18"

/inference="ab initio prediction:Prodigal:002006"

/inference="similar to AA sequence:UniProtKB:Q9WYP5"

/codon_start=1

/transl_table=11

/product="Myo-inositol 2-dehydrogenase"

/db_xref="COG:COG0673"

/translation="MTIRIGKISLWHVHAWDYIKQAQEHEDTVIAAVWDEDAERGQEA

AERLNVPFYASLEDMLAQDNIDAVIVDAPTRLHEEVITAAAKAGKHIFTEKVIASTLK

EVNTIISDVQDNGVKMTVSLPRLNDGYTLTIQDILNQGLLGKVTYVRVRLSHNGAIAN

WLPEHFYHLEDCQGGALIDLGCHPMYLTNLFLGQEVTGVNANFGYVTGKEVEDNAVAT

LFTDSGAIGVVEAGFVNSHSPFAIEVHGTEGTLLYGTPEDKLLIRTNVGSDQQKEWTE

IPLLDRRESAFSQWVSHIQKDTIATENLQTATQLTRLMEAANLSAKEGRKISLKELQG

"

CDS 2744784..2745707

/gene="rhaR_20"

/locus_tag="PIKHDJKN_02519"

/inference="ab initio prediction:Prodigal:002006"

/inference="protein motif:HAMAP:MF_01533"

/codon_start=1

/transl_table=11

/product="HTH-type transcriptional activator RhaR"

/translation="MSRYPFEKMLERQDILERLDVSILWGHYEIRVLRFHLTSFPAGR

IVDFHNHAEFELHFIPRGKGKVILGDEMHPLSEGMLYLTGPGLVHYQEADSQEAMDEL
CLHVDIIEHPREGVDPWEAAESEETIEKLRTLPLAPVHDYHRAMNCFLEAYEACDRKL
IGYYTSIKQQVISILLKTVRAYDIGGNRAEAPVRDMSVYRYDYAIQYMEANHPSAVTL
ENVAEKLHISSRQLQRIFYQVQPEMPFSRVLEDIRLRAVCRNLEESHVSIEQIALASG
FTNANYLHAVFRKRLGMTPSAFRKMKQPNQK"

CDS 2745720..2746817

/gene="iolG_8"

/locus_tag="PIKHDJKN_02520"

/EC_number="1.1.1.369"

/inference="ab initio prediction:Prodigal:002006"

/inference="protein motif:HAMAP:MF_01671"

/codon_start=1

/transl_table=11

/product="Inositol 2-dehydrogenase/D-chiro-inositol
3-dehydrogenase"

/translation="MSKTYRVGIIGCGGIANGKHLPSLSKLDNVELVAFCDIVQERAD
EAKEKYGSSEAQVYTDYQELLQDDTIDIVHVLTPNISHAEISIAALEAGKHVMCEKPM
AKTSAEAQEMLEAAERTGKKLTIGYNNRFREDSLYLKQMCEAGELGSIYFAKAHAIRR
RAVPTWGVFLDEEKQGGGPLIDIGTHALDLTLWMMDNYQPKVVLGTTHHELSQRENAA
NAWGPWDPKKFSVEDSAFGMIVMENGATIMLESSWALNSLDVDEAKCSLSGSEAGADM
KNGLRINGEKFSRLYTNEIELSAGGVAFYDGKSESAPDVEMRKWIEAIDKDLEPIVTP
KQALVVSQILEALYESARTGKAVYMNESNQA"

CDS 2746958..2747671

/locus_tag="PIKHDJKN_02521"

/inference="ab initio prediction:Prodigal:002006"

/codon_start=1

/transl_table=11

/product="hypothetical protein"

/translation="MITKKAGNGVSIIVCTNRPQFFDNILQNYNRQRYKVKELIIILN
HDSMNLQLYQNRVRKHANVSVYQVPESISLGQSLNAGITRARFPLIAKFDDDDYYSPF
YLKEQVNGLRRTKSDVVGKHSCLVYLGASKTLLVRSPKEKNKHVEFIQGGTLLFKREI
LKKVRFTDRSIGEDVTFLRQCRKKGFKTYATSPFNYVYHRRQNKQSHTWRADDHFYLE

GSKRLAITENFRSYADKEL"

CDS 2747852..2748601

/gene="nanR"

/locus_tag="PIKHDJKN_02522"

/inference="ab initio prediction:Prodigal:002006"

/inference="similar to AA sequence:UniProtKB:P0A8W0"

/codon_start=1

/transl_table=11

/product="HTH-type transcriptional repressor NanR"

/db_xref="COG:COG2186"

/translation="MSSTFSFRFEKVSTKKVSEFIREQLEEAIILKELMSEEQLPSER

ELAEIFNASRITVREALSTLEDKGLIEKRVGAKGGTFVLPLTANSHKRTREEIKRDWE

QMLKVFEYRTIIEPEAAFLAAERITAGELELLEAYMEQSIEPHCTREWFRALDVKFHL

TIAKASGNPYCEAAVRQIRTKINPALDLMPYDDRIRSVNHGIHMEILEALKAHDSIKS

RETMKRHIEFSADAIYARLVSESDDKEGNEN"

CDS 2748601..2749812

/gene="scmP_2"

/locus_tag="PIKHDJKN_02523"

/EC_number="3.5.1.-"

/inference="ab initio prediction:Prodigal:002006"

/inference="similar to AA sequence:UniProtKB:P54955"

/codon_start=1

/transl_table=11

/product="N-acetylcysteine deacetylase"

/db_xref="COG:COG1473"

/translation="MDQSQLYQRAQALQPQLIAWRRDFHRHPEIGYEEFRTSEIVAKH

LESLGLEVTRNVGKTGVVGLLRGEAEGPTFALRADMDALPIQDQKSAEYSSQVEGKAH

LCGHDAHTTILMGAAELLTSLGRPKAGNIKFVFQPAEEGLAGARAMIQDGVLENPKVD

AIAGLHMTPGQDTGTVGVSKGVAFASADRLVIRILGKGGHAARPHEGIDAIAVSAQVI

TALQNLASRMLDPLEPVVVTIGKITGGYMGTAIAPEVEMIGTVRTLSPAIRERMPALI

EQVVSGVCSSFGAGHEVIYGDGYPVVVNDLGMVDLLTETVDGLEWAKGWSSIPPSTGG

EDFAFYCEQIPGVFFRLGSGNEEERTRYPLHHPMFDLDETVMSYGVGMLSAVALEYLA

RHTTSEGEHGK"

CDS 2749809..2751374

/gene="gsiB_4"

/locus_tag="PIKHDJKN_02524"

/inference="ab initio prediction:Prodigal:002006"

/inference="similar to AA sequence:UniProtKB:P75797"

/codon_start=1

/transl_table=11

/product="Glutathione-binding protein GsiB"

/db_xref="COG:COG0747"

/translation="MKKRQWTSMWITLLAVVLVLSACGGKSTGTDSNSASSESGRSTS

ASTKLTIAAATDIESFDPHNNNNTSSEAVLVNVFDYLIKNDSEQKKVPGLATSWEQVN

DTTWRFKLREGVTFHNGDPFTSADVKYTLERVAKDSTLKQNSYFKNIVDVKAVDEYTV

DIITDGPDPLLLNRLSKMGAGILPAKYIEDNGFDQFLKQPVGTGPYKFSKWTKDDRVE

LVKNEKYFDGEPKWNEVVFRVIPEASTRVSELLAGGVDIAASIPSTDIARIEGEADKK

IVKAPIQRVLQLIFRQTEGSVTADPKVREAIDLAIDKQGIVDSIAGGAGIVTRTSVTP

GNFGADHSLYKTSLYDQEKAKQLLQEAGYAEGEAEMTISVSSQYKEPAEVVAAMLEQV

GFKINLDVLEPSAFSERYSSKSFKEIFMIGIGNSLFDASNNYNRYMLDEAKGESDYNN

PEVEKLLQSALVNMDPASREKEYQQVQQILAEDRPAVYLYQMEGVYGTDSRVKFEPRS

DEMFYADEITPAQ"

CDS 2751473..2752390

/gene="dppB_5"

/locus_tag="PIKHDJKN_02525"

/inference="ab initio prediction:Prodigal:002006"

/inference="similar to AA sequence:UniProtKB:P0AEF8"

/codon_start=1

/transl_table=11

/product="Dipeptide transport system permease protein

DppB"

/db_xref="COG:COG0601"

/translation="MGKYVLKSLLQVIPVLFIVSLIVFILVRVTGDPVALMLPETATA

EDRAVLTQALGLDQPLYTQYMKFLGSALQGDFGQSFRYGEPALRLVLERLPASFELAV

AAMFFAIVMAVPLGVASAVKRNTFTDLIISGISVIGKAMPNFWMGIMLILLFSVMWGV

LPVSGRGGVSHLILPAFTLGVGLAAQMTRLIRSSMLEILSQDYIRTARSKGLGRMVVI

GKHAFRNGLIPVVTIMSLQFTSLIGGTLITETVFSWPGLGQLLVVAVNTHDMAIVQAA

VFVIAFIVVVTNILTDVAYRLLDPRIKYD"

CDS 2752406..2753335

/gene="gsiD_3"

/locus_tag="PIKHDJKN_02526"

/inference="ab initio prediction:Prodigal:002006"

/inference="similar to AA sequence:UniProtKB:P75799"

/codon_start=1

/transl_table=11

/product="Glutathione transport system permease protein

GsiD"

/db_xref="COG:COG1173"

/translation="MTGSNEMPMPGAEQEHENVRTPTGLHYIWKQLIISKTGIFGAVL

VLLVVLIAICAPLLTSHDPGAVNPLNRLKPPAWLDGGTAEYWLGTDNLGRDMWSRIVY

GARVSLIVGMGAVIVSGIIGAILGLLSGFYGKWLDAVIMRVGDAFMAIPTILFMLVVM

AIVGQGITTLILVIGVTNWVPFTRVVRSEVLSIKERDFVHAARSIGAKNGRLILKHIL

PNILSSFIVICGMNVGTTIIMEASLSFLGLGIKPPDISWGGMLSDGRQYVATSWWVAT

FPGLAITLTVLGVIFLGDWLRDVLDPRTDTNKK"

CDS 2753356..2755395

/gene="dpp5_2"

/locus_tag="PIKHDJKN_02527"

/EC_number="3.4.14.-"

/inference="ab initio prediction:Prodigal:002006"

/inference="similar to AA sequence:UniProtKB:B2RIT0"

/codon_start=1

/transl_table=11

/product="Dipeptidyl-peptidase 5"

/db_xref="COG:COG1506"

/translation="MDMNKRPILPEDLVHYRWVSQPVINQHGQVAYVEQTIDQQKNEY

HTQIRGVFLDGCEDKPLTDGVKDSSPAWSPDGTQLAFLRLESGGKGLWTLASDDTKAI

NLIPPERKLLQFIWSPDGQYIAFTSKVVEGKDEAMADKTSKTDESPKVTPLRGKVFER

TTPKAEGSGWWDGLYSHMFVYEIGSGTIAQVTTGPWNVAAPVWSPDGQAISFISKQVQ

DEGLDADLLYFADVHTVNLASGEQCRVTDSSLQVSQFAYSPDGEQLILIASDREYGSG

SHNRLYEVPTSGGIPKPLAPDVDMQLGNAALGDMKSASPSPSPLMDYHHAQRTAYVLG

THGGNVDVYQITEDGHCQAVTGSGEKDIYQYTLSPDGKTLIIGALTEDHPGELYRVDI

NSGEMFRLTRRNDELMAGLEVNIPERVEFTSSDGWPIQGWLVKPVMQESDTKVPMILQ

IHGGPHAMYTGIFSHEMQTLAAQGYAVLWVNPRGSMGYGQEFARACRGDFAGGDCRDL

LEAVDYALATYDFLDETRLGIGGGSYGGVMTNWIVAHTHRFKAAVTQRCISNWLSLYG

TSDIGISYVEGVIGSNPTEHADMLWSRSPLAHAHKIETPLLIMHGEQDYRTPIGQAEE

LYTTLKRYGKTTKLIRYPGSNHTLLKSGKPSLRVDSFEQVNAWFNQYLRDGEGGG"

CDS 2755392..2755868

/locus_tag="PIKHDJKN_02528"

/inference="ab initio prediction:Prodigal:002006"

/codon_start=1

/transl_table=11

/product="hypothetical protein"

/translation="MSRVQLSIPVALLVENVLTSGEPSELIIDCLQRKDFSSLLSKIK

EPTMDLVERLDTAAEVRDNWEEAIRSRVYEFKFLHINALKRLLYFRFGLKVDQDYIQE

DLSLKHIHLTLQEIELLQLLVGRQWIVQEEEQNKDEHRDKGRDASFPIPVHIRLKY"

CDS 2755993..2756664

/locus_tag="PIKHDJKN_02529"

/inference="ab initio prediction:Prodigal:002006"

/codon_start=1

/transl_table=11

/product="hypothetical protein"

/translation="MVYYKKLFNGEKMIIIQDGVYVMFQYKDQEYEGVHFEGRDLRYG

ELISCVFKQCTFMNASMEEIETSNCRFIECDFKGASMNGSIHRESAFENCLFGGANLF

ASKFTSCKMTGSDFSGAQMDGITLSQGDWSYTNLRHTRLGKQDLRGIRFFEADFTDTD

FTKADLRDCDLTRVVLSRAKLQGADLRGANLEGIDLKSLDIKGVRLDREQAVLFVRSY

GAKVD"

CDS 2756847..2757230

/locus_tag="PIKHDJKN_02530"

/inference="ab initio prediction:Prodigal:002006"

/codon_start=1

/transl_table=11

/product="hypothetical protein"

/translation="MSTTLIAIVAVIGFGLITGTITMIMIFKLLHNNKQKQHLRKSIL

MEGIPAEAIIHDITQTSSSMDGRPGVRLDLTVTQVDGRTFPTIVETYIPVTHIPQFQK

GSVVHVRYIANGNERKVEVEDAYVP"

CDS complement(2757239..2757331)

/locus_tag="PIKHDJKN_02531"

/inference="ab initio prediction:Prodigal:002006"

/codon_start=1

/transl_table=11

/product="hypothetical protein"

/translation="MWFPIINGIIILIGMFAIFLVVRNLSKEED"

CDS complement(2757377..2758882)

/gene="smvA"

/locus_tag="PIKHDJKN_02532"

/inference="ab initio prediction:Prodigal:002006"

/inference="similar to AA sequence:UniProtKB:D0ZXQ3"

/codon_start=1

/transl_table=11

/product="Methyl viologen resistance protein SmvA"

/translation="MSKLKRWMILVIVSSALLLIVMDMTILYTALPSLTHDLGASASE

KLWILNGYSLVMAGLLPAMGTLGDRLGHKIIFTLGLLVFSVASLTAAFSPVPAVLVMS

RILLAVGASMMMPATLSIIRVTFTNERELALAIGIWGSIASGGAGLGPIVGGLLLQHF

WWGSVFLINLPIAILAFVFALKMIPKHQGDASKKWDFTSSIQIMIAMVGIIYSIKEFT

RREGSLTLASIAAIIGVLSLIIFIRRQNNSTNPLLDLSLFKIPRFSTGFITALVGLFA

QMGVQYMVTQRLQLVEGMSPLQAGLFTLSIPVAALIAGPIVGSIMHRVDVVYIKSLSL

LIAGLGIGIYLMQFNAGFTGQILGLALLGAGLGAGMTAASHSIMSYAPPHKAGMAASI

EEVGYELGGASGIAIIGSMSTLVYTLAMRIPVGINVPANVKDSLDEALLAAESLPTES

AESLKLAAFAAFDQSFFVVIAGITLFLLVAALIMSWVALRLKRSKRNGVET"

CDS 2758995..2759831

/locus_tag="PIKHDJKN_02533"

/inference="ab initio prediction:Prodigal:002006"

/codon_start=1

/transl_table=11

/product="hypothetical protein"

/translation="MSGNHTQQFTTSEFAKICGVTKHTLFHYDEIGLLKPEFTNSNGY

RYYGIQQTYVLDVIHVLKKAGSSLEEIKSFIQNQNTPLLIELFQQKVKELEREQLRIK

RMQKLLNGAIVMTRQAMSASYEGLAFEECEKEYFIAVQLERGDGDKEFSRKFVEYRNH

CEERMIEYEFPVWTVVCQESFETGHFYPDYFCNQIKAPIPGEAMLVKPKGLYAVMNHR

GSYESMPKTYLIIKEYIEREGFEICGNVYAMDLLSYFAENNPDEYIIKIYVEVCKVAE

TK"

CDS 2759867..2760403

/locus_tag="PIKHDJKN_02534"

/inference="ab initio prediction:Prodigal:002006"

/codon_start=1

/transl_table=11

/product="hypothetical protein"

/translation="MKLIKAYDMDLNEEWSTLLQELLVASFPEVYPKDRLYFKQIPHC

RILAFTSDNQLIGHVGLDYRIMNLNGNAIKVLGIIDLCVSITHRSQGIGSFLLAEVEK

LSKGHIDFVLLFADQEDLYSKNGFKTVNNTCKWLKIDHETLTTIGVGIQKVEGLMVRE

VGTMSWSEGELDFLGYLY"

CDS 2760734..2762434

/gene="maeA"

/locus_tag="PIKHDJKN_02535"

/EC_number="1.1.1.38"

/inference="ab initio prediction:Prodigal:002006"

/inference="similar to AA sequence:UniProtKB:P45868"

/codon_start=1

/transl_table=11

/product="putative NAD-dependent malic enzyme 2"

/db_xref="COG:COG0281"

/translation="MNSFFVDSNGNLKTSLKGKDVLSNPMLNKGTAFNEEERRELNLN

GILPPMVLNIEEQVSRVYQQLQNESDNLRKSIMLSDLFNRNIVLFYRLMKDHLSEMLP

IVYTPTVGQAIQEYSNQYHRPGGLYLSINDMDGLEEAFRNSGLHAGNVDLIVVTDSES

ILGIGDWGVGGINIAIGKLAVYTAAVGVDPSRVLPVVLDVGTNNPKLLNDPLYVGNRH

ERIRGEKYDQFIEAFVKETTKWFPDILLHWEDLGSVNARNIIQKYGEELLTFNDDIQG

TGAVTLAAIMSGVQVTGVPLREHRVLVFGPGAAGIGNADQILDAMMEEGLEREEAENR

FWAFDYRGILTDETEGLFEFQKPYVRKAAEVKDWALNEQGQVSLLEAVKQIKPTILIG

TSGVTGAFNEQVIKEMAKHVERPIIMPMSNPTSLAEAAPRSLIEWTDGKALIATGSPF

ESVMFNDTLYEIGQSNNAFVFPGLGLGAIVAKASKFTKNMFTAAAVAVANSVDSSAPG

APLLPRVKELHAVSYKVAVAVAKAAVEDGVARVELKDVEEAVKAAMWHPVYKPLYAAV

"

CDS 2762602..2762730

/locus_tag="PIKHDJKN_02536"

/inference="ab initio prediction:Prodigal:002006"

/codon_start=1

/transl_table=11

/product="hypothetical protein"

/translation="MTELEVVDVYVNLSTNMNTDMVHNITNQNPALYPSMHRSSWR"

CDS 2762977..2764455

/gene="ybiT_2"

/locus_tag="PIKHDJKN_02537"

/inference="ab initio prediction:Prodigal:002006"

/inference="similar to AA sequence:UniProtKB:P0A9U3"

/codon_start=1

/transl_table=11

/product="putative ABC transporter ATP-binding protein

YbiT"

/db_xref="COG:COG0488"

/translation="MSLINVTNLTFAYDGSYDNIFENVSFQLDSDWKLGFTGRNGRGK

TTFLNLLLGKYEYSGTISANVSFEYFPFQVENKGAMTLDVIEEVYPEYLHWQLVRELN

LLKVSEDILYRPFDSLSNGEQTKLMLAALFLKDNRFLLIDEPTNHLDIHARKLVSNYL

RSKSGFILVSHDRSFLDNSVDHILSINKNNIEIQRGNFSAWWENKQRQDQFELASNEK

LIKDIKRLSDSSKRTGGWSHEVERTKNGTRNSGSKVDKGYIGHKAAKMMKRSKNIEQR

QQSAIQEKSKLLKNIENAERLQIHQLDFHKKELVELEHVSIAYDSNTVCKDVSFTVEK

GDRIALYGKNGSGKSSILKLICDENIPYTGLFRKDHQVKISYVSQDTSDLGGHLSDYA

VTQGIDESLFKSILRKLDFSRLQLEKDISTFSAGQKKKVLIAKSLSEKAHLHVWDEPL

NFVDVISRMQIEELLLEYSPTLLFVEHDSEFCANIATRIIEL"

CDS 2764680..2765246

/locus_tag="PIKHDJKN_02538"

/inference="ab initio prediction:Prodigal:002006"

/codon_start=1

/transl_table=11

/product="hypothetical protein"

/translation="MKKEMTSMKQTRLNSILDEATKLLIEKPNASMNDIADSAKIGIA

TLHRYVESREQLMVYLGLRAIEVVSEAMQNIQLDEENCETYIPQLIEVLIPLGDKIFF

LTHDAAIHYNPEIEGADLKLREPILHAVSLLQQKNYFRQDVNKNWIVDVLYSILFLTW

QQVVSGDIARKSAASLVVDTFYHGFRAR"

CDS 2765596..2766750

/locus_tag="PIKHDJKN_02539"

/inference="ab initio prediction:Prodigal:002006"

/codon_start=1

/transl_table=11

/product="hypothetical protein"

/translation="MLYTIIVPVNQDYNILNLFTDSLLRTVSPSTQIIFINDGSGSAV

LQHLEKLKQEVREGVTIEILQHDFPLGCAVSINSALRRAEGDYIFFLDSDTILQPNWQ

SMMKETMDSDITIGMIGGVLLYPQTGGVQHCGIAFADTIGRHLFLNASPEDIPKETFS

VQLVVFAMFGMKREVYETVGELDEKFFNGYEDFDYQMRARAAGYDTVINPNILAYHWE

RSSGIHRNFNRKNNLARFWKKWGGEIEADVWPFVFGHLKEQLDSQSEYQHLPIVGIDL

AEVRSDADTFWTKLEEADIAPVAEVRDYSNRFNSNGAIWLPQVLGKELIHSENRLLFL

VDNFARLLENRYWIEMRHAHRAKDLIIDLYGNVITMDRIYDGCWPGSKVR"

CDS 2766784..2768064

/gene="kat"

/locus_tag="PIKHDJKN_02540"

/EC_number="2.6.1.111"

/inference="ab initio prediction:Prodigal:002006"

/inference="similar to AA sequence:UniProtKB:B0VH76"

/codon_start=1

/transl_table=11

/product="3-aminobutyryl-CoA aminotransferase"

/translation="MKSKESTLDRAVRFTRNLEWLEHVEKVIPSGCSTLAKAPERLFP

GYTPVCCAEARNSRFIDIDGNEWLDCEMAMGTAPWGHARHEVQLTVIHQLKKGTSYSI

PADIELECAELILERFEGRYPSLRFTKSGADAVSGAVRLARAGSGKSRVIATAYHGWH

DWSAYGYYGRETKERGIPVDIERTTIWIDKPSPERIEAQIQSSPEDIACIVLCPNEWK

RDPLAQVVSLCRSLDIIVIFDEVTSGIRMGKQATAGEYDIWPDLLCISKGMANGLPLA

AVMGPEHLMSLSGQVRFSNAHSSETTALAAAIACERLMKSAKVWPSWREATIRIMDLI

EAELVLLGLTDQLVLKGTYASFSISSISEENFHTDPFREFMVKRMAHFGIFTKGYFIF

SDAHTRDELLWVEEALLQTLSDWKEMLKQEHLSN"

CDS 2768125..2769156

/gene="gutB_2"

/locus_tag="PIKHDJKN_02541"

/EC_number="1.1.1.-"

/inference="ab initio prediction:Prodigal:002006"

/inference="similar to AA sequence:UniProtKB:Q06004"

/codon_start=1

/transl_table=11

/product="Sorbitol dehydrogenase"

/db_xref="COG:COG1063"

/translation="MKALVYQDLKKVTYQDIEEPTIQKPNQVKIKIYGSGICGTDLNI

VKGKVPANKGTIIGHEGVGTVVEVGDEVRGFKIGDRVLVDPTQSCGTCSYCREGLFIY

CENFDDYQVGMTTHGTFAEFFVGDEKYIYAIPDSMSWETAMMIEPLGCVLQTFMKAGV

KPSDSVLVLGSGAIGSLCQLVSKRLARLTVGTEVDPYRKEFASEIADHVFYPQELTLD

KVYEINNKKKFDIVVDAVGNQLHVGFEMIAKGGKIIPMGYDDSYEVTFKSTAVINDGI

SIIGAVANHSMISTALKFAQSIPELEQMVTSTELLSDYEQAFNGTIGYHLHTGEKLPM

NSVKTALIL"

CDS 2769241..2770485

/locus_tag="PIKHDJKN_02542"

/inference="ab initio prediction:Prodigal:002006"

/codon_start=1

/transl_table=11

/product="hypothetical protein"

/translation="MKLLQDLPRNPRYSILLEPVWAIPGTIVLFYAPLYMKEAGLSDI

EIGLINSVNLYFAFIFQLFAGSITNKLGRKRTTLIFDLIAWSVPMFIWAFSQNFWLFL

IAYLLNATSKFVTVSFNCLIIEDVEEHKRSKVFAILNMIITGAGVLTPIAGVVIADFG

IVPTLASIYFVGGILMTVMFFIRNRYTDETAVGKELMGMHSQTRVIQSLASSLRLFGK

SFYKRRLLPIILITVLSNLILQLNFFQVIFFKEQLKFNDQIISFIPVVTAVTVMLLYL

VIIPRLKRRSEEKYVSSSIILSTVGAILFLLIPAGNIAMLFLTLIVLAAGNFILQTYR

DSLLMNRLGTHEKADMFSAVQTVMTLTAIPSGYLTGLLYHYNPVLLFSVILALYIVLM

VIMLFLPDPQKHSQAISTYKNM"

CDS 2770551..2771144

/locus_tag="PIKHDJKN_02543"

/inference="ab initio prediction:Prodigal:002006"

/codon_start=1

/transl_table=11

/product="hypothetical protein"

/translation="MTNVFATETRNQMNWINEPKSWSYTDQGVLIVEAQADTDFFRDP

AGKHIRATAPFLSMPVPNDFEMTTQLTVDMKNQYDSGCLMVMADERNWCKLCFEYDGK

AATIVSVVTKEGSSDDCNSVEVPVTNPYLRIRKVEDCISFFYSPDGVEWKLIRYFGMP

IEGEIRAGVVGQSPAGTGCTCHFLSMNVTRPDVTARF"

CDS 2771192..2771833

/gene="cbbY"

/locus_tag="PIKHDJKN_02544"

/EC_number="3.1.3.-"

/inference="ab initio prediction:Prodigal:002006"

/inference="similar to AA sequence:UniProtKB:P95649"

/codon_start=1

/transl_table=11

/product="Protein CbbY"

/db_xref="COG:COG0637"

/translation="MVKAFILDFDGLIVDTETPWFYAFRDIYKEHGLELGLELWSKNV

GTSFEEFHPFLYLEQALQKPIDHDHIKLLSEQKYEAYLGQAAILPGVHELLQSARKKG

IQLAVASSSTRDWVHGYLRTLGIFELFDVIHTAEDVKRVKPDPELYLLALESLGIGAS

EAVVFEDSPNGLKAAKAAGIRCVIVPNEVTRNLEFSKYDLRLTSLAEITMEIL"

CDS complement(2771830..2772645)

/gene="rhaS_14"

/locus_tag="PIKHDJKN_02545"

/inference="ab initio prediction:Prodigal:002006"

/inference="protein motif:HAMAP:MF_01534"

/codon_start=1

/transl_table=11

/product="HTH-type transcriptional activator RhaS"

/translation="MPKPSMGNSLDTDMPLMITGQNQLTVDELMNWNGHSRDYSIVQC

IGGTGKIAAKNHEFSIREGTALIMFPEVTYRYDNLSDSLLFDCLSFNGYLLPRFLHTL

QIGELRAFKPDGMLHILLHEITLALQSRHKDQIWHVSALLYMLLVRLTIDGDRYGAYE

RSDARLKLGGLVTFIKQNYDQDISLSMLAQQMDVTEQHLNRIFKKEFQMTPLDYLTRY

RLLKAKEMLVHDAGITAHEIARAVGFNSASYFGSVFKKYEGISPIELRKQYQG"

CDS 2772887..2774017

/gene="thiO_1"

/locus_tag="PIKHDJKN_02546"

/EC_number="1.4.3.19"

/inference="ab initio prediction:Prodigal:002006"

/inference="similar to AA sequence:UniProtKB:Q88Q83"

/codon_start=1

/transl_table=11

/product="Glycine oxidase"

/translation="MKKVIVVGSGILGASTAYQLAKRDAEVLIIDRKDRGQATDAAAG

IICPWLSQRRNQDWYQLAKAGARFYPGLIEELKSEGETETGYAQVGALSIHTDVEKIN

KMQERAHLRKEDAPEIADITRLDAKETHERFPLLEENVQSVYISGAARIDGRALRDAL

IRSAQRNGAVLMDGEATLQYESDRVTGVVVDGKVISADEVIVCAGAWANELLKPLGID

FKVHYQKAQIMHLQVHDREDTGNWPVVMPPSDQYLLAFDQQKIVIGATHENDIEGYDT

RVTAGGMQEILNKGLELAPGLADSTFLEVRVGFRPFTPGFLPVMGAVPGWNGLITANG

LGASGLTMGPFIGSQLAKLALGIDLDIDIQPYDLGKAMDDQK"

CDS 2774026..2774316

/locus_tag="PIKHDJKN_02547"

/inference="ab initio prediction:Prodigal:002006"

/codon_start=1

/transl_table=11

/product="hypothetical protein"

/translation="MTYSQRLTHGNSSDIIYLEHQIGIAEEELAKAEEERRTYESDLD

QLRTSPAYRSTSATNISNEHKLVDELNKVQSMIKTIRTRLKNLQEDLGELED"

CDS 2774579..2775250

/gene="tenA"

/locus_tag="PIKHDJKN_02548"

/EC_number="3.5.99.2"

/inference="ab initio prediction:Prodigal:002006"

/inference="similar to AA sequence:UniProtKB:Q9K9G8"

/codon_start=1

/transl_table=11

/product="Aminopyrimidine aminohydrolase"

/db_xref="COG:COG0819"

/translation="MSFSRTLKDKSITVWEDGYNHPFLHEMGKGTLDQEAFKFYLLQD

YTYLIEYAKIFAMGAVKATDEKLMTNFTVIQHAILYEEMDLHRNYMNEFGITLDENHA

VKPSLYNKAYTSNMMSVAQTGGVAEIIAVIFPCAWTYYDYATRLKEQYRDSLEGNFYQ

TWIENYASDEFRDSFEWFYDTLDALCEHKTEQELEAIEDIFRRSVEFEYLFWDMAYKR

QLSYF"

CDS 2775294..2775827

/gene="rimJ"

/locus_tag="PIKHDJKN_02549"

/EC_number="2.3.1.267"

/inference="ab initio prediction:Prodigal:002006"

/inference="similar to AA sequence:UniProtKB:P0A948"

/codon_start=1

/transl_table=11

/product="[Ribosomal protein S5]-alanine

N-acetyltransferase"

/db_xref="COG:COG1670"

/translation="MGNHGERIYLRFLNVEDAASLLDLQFRNRAVFEEISASERSDTF

YTLEGQVALLEGWNKAREEGKRYSFGIFLNHTHELIGEISLFELELDSTTKWIVGYVL

DQVQNGKGYMSEALQLALDFAMNEAGIKRVEAGALPDNAGSIRVLRKAGFQETDRQNI

KIKGIWKEHMMFAVDLQ"

CDS 2775877..2776776

/locus_tag="PIKHDJKN_02550"

/inference="ab initio prediction:Prodigal:002006"

/codon_start=1

/transl_table=11

/product="hypothetical protein"

/translation="MNKRKQMLLDSALVLFGKHGVTNTSIQMILDESGVSKGTFYKFF

GSKDDCILAILEQRVQEDMMIRKDLESQSYASDFDLLVDQIMVPMILPEKKLVVELFW

TGFYSGECDLKNLTRMQLNWLSERLVQVFGKELKPFAYEGAIFGFGMIHQISNMWRNF

HLDQPAWNHLVPKILNYIEILLRSMLERQEHIIDFSSLSMIAPDSKEIALDKNNLLEG

LQKFSHSILHSEVSIKAKELTKGLLALVEQKEINISMLEVTLLAFQTEFESGSVQYEA

STIVKDCSSYLEQIRQIPMESTK"

CDS complement(2776943..2778442)

/gene="emrB_2"

/locus_tag="PIKHDJKN_02551"

/inference="ab initio prediction:Prodigal:002006"

/inference="similar to AA sequence:UniProtKB:P0AEJ0"

/codon_start=1

/transl_table=11

/product="Multidrug export protein EmrB"

/translation="MSQARLKERDDVKKGPILFVMILGAFLATLNQTVMSVATPELMG

DFNISAATAQWFTTGYMLVNGVLIPITAYFMQRFSTRQLFQASMFIFLIGTIISALAS

NFGTLLTGRMVQAAGAGIIMPLLMHVILTLFTPEKRGAAMGMVGFAIIFAPAIGPTLA

GYILENYTWQTMFYGMIPLTVIVIGFAFVYLKNVSERVNLKFDTLSVLLSTIGFGALL

YGFSRAGSLGWSSAEVIICIAAGVIALGLFTWRQLASANPLLDLRAFKYNMFSLTTII

NIAITMIMYADMMLLPLYLQNARGYTALESGLLLLPGALVMGFLMPITGRLFDRFGAK

WLAIIGMVITIVTTIGFIDLTDSTSYTYLVLMSTGRRIGMALLMMPIQTAGLNQLPPR

LGPHGTAISNTVRQVAGAVGTSLLVSVMTSRTTAHVQDMVATGAAKGLTQQQLGMESM

IQGINDAYVVIIGIAVLGLLLSFFIKRTKQATEEDSKQPVRQKVSMNTN"

CDS 2778664..2779269

/locus_tag="PIKHDJKN_02552"

/inference="ab initio prediction:Prodigal:002006"

/codon_start=1

/transl_table=11

/product="hypothetical protein"

/translation="MKNEKKSSTDPRILRTRQLIRDAFVDLLQEMDIEKLSVNRIAER

ATINRVTFYLHYRDITDMMEKMADEMIEHIERIVDEYAPQFEDAAKDDGWPVLVKLLE

HFAEHSKFYRVVLASRRTPIFTERLMKLLTTLVSAKIESLELGSSLSQAGIHKEIAIW

YSSSALIGTIVSWLRNDMPYAPHFLAKQFSLIRAYSYNDLI"

CDS complement(2779338..2779997)

/locus_tag="PIKHDJKN_02553"

/inference="ab initio prediction:Prodigal:002006"

/codon_start=1

/transl_table=11

/product="hypothetical protein"

/translation="MNWIRPVNRGESVFKVKNNRIIYFAKLSIPLNALIAVGKILLGI

FTLSFFLCINAFYNIGIAVAKYYAAKAHEDSNLDDSSPEEILKKQYAAYRFIGNIVLA

SSTVYIIYCSKLLFVTGSKTHYPHYVAFIIAAVTFTEIAVSLGGAIMARRNNEPVIEA

IKLTNFSSSLISLSLTQTAIMSFSYKGDPTFYNGLSGIIFGALAALIGLYMILRTPTG

D"

CDS 2780449..2781681

/locus_tag="PIKHDJKN_02554"

/EC_number="1.14.14.-"

/inference="ab initio prediction:Prodigal:002006"

/inference="similar to AA sequence:UniProtKB:P14762"

/codon_start=1

/transl_table=11

/product="Cytochrome P450(BM-1)"

/db_xref="COG:COG2124"

/translation="MYKEVIRVHDITNFQSRSEEFFPLHWFRKMLSEKPVYYHEDTDT

WNVFRYDDVKQVLSNHEYFSSEGSRTTISVGAKNKEGTPPDKMNISSIDPPRHQKSRS

LLSAAFTPRSLKNWEPRIRNIAKQLVEDIELNTTIDIVQALAAPLPSMVMSDLLGIPL

TDSHRFKSWVDILFQPANRENAEEMELKKQVAAQEYYQFLYPIVVQKRTQPGDDIITD

LLKVDVDGEKFSDDEIVRTTMLLLGAGIETTSHMVSNTFYSFLYDDPNLYVQLRENPE

LIPLAVEEMLRYRFHNAKRHRTVKKDNDLLGVELKKGDVVISWMSAANMDETIFENPF

DLNIHRPNNKKHLTFGNGPHFCLGAPLARMELIVALTAFVERVPFIESVESFDLENNL

TVSAPGQSLTYLPVKIVQ"

CDS complement(2781911..2782186)

/locus_tag="PIKHDJKN_02555"

/inference="ab initio prediction:Prodigal:002006"

/codon_start=1

/transl_table=11

/product="hypothetical protein"

/translation="MISEINSWIRHIASNYILIIAAAVIFFAIKAVIGFYTYKHYNKQ

LDHLHHKVDLLIKEQQLLAQQFSVPNGNRDMSQKEDHFESSSQTPLM"

CDS 2782374..2783333

/locus_tag="PIKHDJKN_02556"

/inference="ab initio prediction:Prodigal:002006"

/codon_start=1

/transl_table=11

/product="hypothetical protein"

/translation="MLELSFNDPEKLVTVTHALSTRSRIDILQLLNANKLNIIEIAEA

LNLPVSTVANHIKVLEAANLIHTEMLPASRGAMKVCSRNYDDIHIALDRVSSFPTRDT

NVYEVQMPIGHYSDCEVAPTCGMASTEDMILKEDDPASFYHPKHIDAQIIWLAKGYLE

YLLPMDIPQGATIEALELSMEICSEVATYNNDWPSDISVWVNGTEIGMWTSPGDLGDR

RGKLNPAWWSDGSTQYGILKKWRVDDNMTTLDKEKISDVSLSDLHLEDKPKLRLRIGI

HPDARHQGGINLFGDEFGDHEQNIIMQVRYTMNAGDKDARYAK"

CDS 2783323..2784171

/gene="rhaR_21"

/locus_tag="PIKHDJKN_02557"

/inference="ab initio prediction:Prodigal:002006"

/inference="similar to AA sequence:UniProtKB:P09378"

/codon_start=1

/transl_table=11

/product="HTH-type transcriptional activator RhaR"

/translation="MPNNGTSCIVSIERFKPNLFHLEKGEGIFKSHSHAYDELTLILD

GEGYYSSPEQNVKVVAGDLIMIPLGLYHGYVCTEPWQGISVHYYHDYLPVHCRYLFNG

QDHQRNRIQLAHLNQDSLRWADISLSELEKEWRSEDKRSIDSNHLIRLALETALLLFQ

RNCSIEAPHPVIDPTGQGIIQEVLKEIHSGYYTPITVSELASRHFLSESNLRKKFTES

VGVSPKQYIINLRVMEAKRLLQQTNKAVEMISSEVGFTSSSRFYDYFVRSVGVTPLEW

RMQSIQ"

CDS 2784335..2785285

/locus_tag="PIKHDJKN_02558"

/EC_number="3.2.1.55"

/inference="ab initio prediction:Prodigal:002006"

/inference="similar to AA sequence:UniProtKB:Q82P90"

/codon_start=1

/transl_table=11

/product="Extracellular

exo-alpha-(1->5)-L-arabinofuranosidase"

/db_xref="COG:COG3940"

/translation="MTTSTFCNPVALQSADPWVYRHSDGHYYFMRTRSNYLELIQSTR

LSHIDEGNRKIIWSPEPGGRFSHHLWAPEIHFLNGKWYIYYTANDGGGDDSRRICVLE

NESSNPMDGDWVWKGALDTPVPGLDGTVLKLAGQLYFLYAGYGHFPDYGSAIYIMRMS

NPYSLTGDHVLLTAPTLSWEKQGGMAINEGPVTLHRNGRVFLIFSASTTWSEDYALGM

LTMNEEDDPILASSWTKSPEPIFRKSMENGVYATGHNSFTTSPDGLEDWIVFHALPAP

GADTGLRATRIQKFEWNRDGTPDFGVPFSDDHALQIPSGE"

CDS 2785725..2787479

/gene="lacZ_4"

/locus_tag="PIKHDJKN_02559"

/EC_number="3.2.1.23"

/inference="ab initio prediction:Prodigal:002006"

/inference="protein motif:HAMAP:MF_01687"

/codon_start=1

/transl_table=11

/product="Beta-galactosidase"

/translation="MNKDKMTLTPRPEYPRPQWVRNHWINLNGTWQFEIDHGKSGKER

GYEQSEHHLSGIITVPFCPESKLSGVAYTDFMAAVWYKREFSIPDAWMNGRILLHFGA

VDYEAEIWVNGKAVGKHRGGYSPFHFDITSHVSLGTNVVTVYAEDDVRSGLQPRGKQS

GRFHSHGCDYTRTTGIWQTVWLEQVPEVYLSDMKVAADPDNACVHLEVKICGNAAGGE

LAATTRFDGKDVGWATAVVHGPAVKLTIPLSEIHLWEPGNAKLYDLELKLHGQDQTSD

TVASYFGLRTIRLDGMAFRINEKSVFQRLVLDQGFYPDGIYTAPSDEDLRKDIEISMG

LGFNGARLHEKMFEPRFLYWADQLGYLVWGEHANWGQDITTTESLARFLPEWMEGMER

DFNHPSLIGWCPFNETWDRDGTRQDNDVLRIVYEMTKRMDPTRPVIDTSGNFHVVTDI

FDLHDYDQNPETFRARYEPMKSGGEVFNTFPERQTYEGQPYFISEYGGIWWNPDQQDE

KAWGYGDRPQSEEAFIARYEGLTNVLLDHPKMFGFCYTQLYDVEQEVNGLYTYDRRPK

FDPEMIRRINSRKAAIED"

CDS 2787536..2788936

/locus_tag="PIKHDJKN_02560"

/inference="ab initio prediction:Prodigal:002006"

/codon_start=1

/transl_table=11

/product="hypothetical protein"

/translation="MKIMNYRNKLLLALCAFLIFPMGQTTHAATVQNNFYNVVMQDGA

DPWVYKHTDGFYYFTKTTGGNVTIWKSAQLTTIDAAPTTVVNTGCCGIWAPELHYING

AWYIYYAKDSGDNVNHRMYVMENTSADPTQGTWQYKGQITDPNNKWAIDGTVLQVNGQ

LYFIWSGWEGDTNVRQNLYIAQMSNPWTIDSNRVEIARPTYAWETNHSPNVNEGPQVI

VRNGIISLVYSASGSWTNDYCLGLITANASSNLLNPASWSKRTQPIFQSGNGLYGPGH

HSFTKSPDGTEDWIMYHTAKFNNSGWNREIRLQKFTWNADNTPNLGTPVNPNVPIALP

SGEKSIVRYEGEEGTFGGIAYASQSPSGSGGMKAGHIDTADSYVEFNVNAATAGTYIL

RARTANGTAGGNWSNLLLSVNGGTASPFYITNKGWENWGLSTARVQLKAGVNKIRFSK

GEGYGELDFFDIKPAN"

CDS 2789090..2790121

/gene="drrA_1"

/locus_tag="PIKHDJKN_02561"

/EC_number="3.6.3.-"

/inference="ab initio prediction:Prodigal:002006"

/inference="similar to AA sequence:UniProtKB:P32010"

/codon_start=1

/transl_table=11

/product="Daunorubicin/doxorubicin resistance ATP-binding

protein DrrA"

/translation="MTNTQQRTTGNELGDWAIEAEGLVKIFGDKRAVDGVNLNVKAGT

IYGVLGPNGAGKTTTIRMLGTLLRPDGGSAKIFGHDVVKESHIVRQLIGLTGQYASVD

ESLSANENLMIFSRLLGLGRAESKRKTAELLEEFGLTEAAKRPIKGFSGGMRRRLDLA

ASLIAQPPLIFLDEPTTGLDPRTRAQMWNTIRQLVKTGSTVLLTTQYLDEADQLADRV

AVIDQGHVVAEGTVDDLKASVGTASLQLRIQEPTRIEHARQIVEQILRTETSVSAEAG

KITAPMANANIAADLLIAFRSAGIDLAEMSVQKPTLDEVFLTITGQDASGNASHTSQE

SKAVEELQV"

CDS 2790118..2790933

/gene="drrB_1"

/locus_tag="PIKHDJKN_02562"

/inference="ab initio prediction:Prodigal:002006"

/inference="similar to AA sequence:UniProtKB:P32011"

/codon_start=1

/transl_table=11

/product="Daunorubicin/doxorubicin resistance ABC

transporter permease protein DrrB"

/translation="MNSTIAKQNPSRKLRKYTSFGQTVRNSLTMAYRGILKVRRTPEQ

LFDVTLQPIIFTLMFTYIFGGAISGNIQAYLLVIIPGILVQTVITSSVVTGVQLREDM

DKGVFDRFKSLPIARIAPLAGALLADTIRYTIATFLTFTIGYLLGYSPAGGLGHVALA

GLLVIVCAWAISWIFAFLGVIARTASSVQGISMLVLFPLTFASNAFVPVDTMPNWLQW

VVNVNPISHLVSAVRELANQGTMGTDLVTSLAGAAVIVAIFAPLTVRAYMRRT"

CDS 2791066..2791740

/gene="hssR_4"

/locus_tag="PIKHDJKN_02563"

/inference="ab initio prediction:Prodigal:002006"

/inference="similar to AA sequence:UniProtKB:A6QJK3"

/codon_start=1

/transl_table=11

/product="Heme response regulator HssR"

/translation="MTSILVVDDDPHIRELVGHFLQQEGLHVIEAVDGLDALRLLADH

KVDLVVLDIMMPGMDGWELCRQLRQQTDLPLLMLTAKGETSQIIKGFTLGTDDYLVKP

FDPLVLVARVKALLKRYRIMTSQVILLGELVLRGDTFECKKGDQDIVLPRKEFELLFT

LASYPGKTFTRDQLIEKIWGYDYEGDERTIDVHIKRLRERFPEEEHAFVIRTMRGLGY

RLEVRQ"

CDS 2791737..2792810

/gene="sasA_13"

/locus_tag="PIKHDJKN_02564"

/EC_number="2.7.-.-"

/inference="ab initio prediction:Prodigal:002006"

/inference="protein motif:HAMAP:MF_01837"

/codon_start=1

/transl_table=11

/product="Adaptive-response sensory-kinase SasA"

/translation="MKQEGGFLHIARNVILVSLALFACWTAAFYLTRYVYSFIPWEPH

ELLAFLINAMLGFIFFGICITIIGPLVKGRQYEFFNEMIQALKSISRGDFRVNLDAEF

ANRNGRQRNPHPFVQLVESINDMAANLKVMEDLRQEFISNVSHEIGSPLTSISGFARA

LKNKDMDQEKREKYLTIIETECVRLSRLSDNLMKLAVLDSSEHASHKTSYRLDRQLVT

LVLACEPQWDDKKIDMNVELEEVEIFADEDLMIQLWINLIHNAIKFTPEGGKIDVFLT

QGEGKVSVRITDSGPGITKQDQLRIFERFYKADQSRTRALGGSGLGLSIVHKIVEMHE

GTVSVFSEPEEGATFTVQLPVHS"

CDS 2792952..2793695

/locus_tag="PIKHDJKN_02565"

/inference="ab initio prediction:Prodigal:002006"

/codon_start=1

/transl_table=11

/product="hypothetical protein"

/translation="MLISPTQQQRLIDVMNSFPLQPKYSKAELLMNDLKLSSSGEVEM
FYSPHNEYINPSAKVMLVGITPGWQQMEIAYRTPIQALKAHKSYEQACKEAKIAARMA
GSMRTNLILMLQEIGLHEYVRVPHVQRLFEADCTLLHTTSLIRYPVLVNKQNYNGHQP
SIPQSEFLYQAVLESFLPELEQLKHPLIIPLGKSVEAVLHQLLDEQRIDEGKILWGFP
HPSGANGSRVKQFLHTKSGMQKILREWRD"

CDS 2793778..2794452

/gene="hssR_5"

/locus_tag="PIKHDJKN_02566"

/inference="ab initio prediction:Prodigal:002006"

/inference="similar to AA sequence:UniProtKB:A6QJK3"

/codon_start=1

/transl_table=11

/product="Heme response regulator HssR"

/translation="MPTILVADDDPNIRELVCLFLRNDGFETAEAADGKEALTVYGST
HVDLVVLDIMMPIMDGWTLCKELRRANPDLPLLMLTARGETWEKVKGFELGTDDYLTK
PFDPLELTARVRALLKRYKIGSTHTIHFGNVILDRQTYKVTSGTESLTLPLKEFELLY
KLAGTPGQVYTREQLIDQIWGIDYAGDDRTIDVHIKRLRERFATTPDFRIETVRGLGY
RLEVYE"

CDS 2794449..2795816

/gene="sasA_14"

/locus_tag="PIKHDJKN_02567"

/EC_number="2.7.-.-"

/inference="ab initio prediction:Prodigal:002006"

/inference="protein motif:HAMAP:MF_01837"

/codon_start=1

/transl_table=11

/product="Adaptive-response sensory-kinase SasA"

/translation="MIRSLYIRVVLTFLVSVIAGTIISFFVSTMIFEDQLNENAQINL
RNFGQDVVQIYKILPLNEAESFVSGMKLLNSYHIRIYEATGQFKSYGKLNGHKSATVT
KEQLKKVIDGGVVQDNPNGIATVLLGLPVKTEMGTKAMFLETLSPPSASFVVNWASIF
ATCSLIAGSIIILVASIYLVRPIKKLTKATKRIAAGDFNVKLNIKQTGELGTLARSFE
EMMHDLQQLEQMRREFVTNVSHEVQSPLTSISGYAQALKQVNLSEQERSRYLEIIIAE

AKRMSKMSDNLLKLSMLESQSQQPRLSTLSLDEQIRRVIVTLQPQWSARNIHFELDLQ

AVKVLADHDQLNQVWTNILSNGIKFSKDGGVIHVSTKQDIKNVTIRISDTGIGIPLED

QKRIFERFFKSDRSHSRKYDGSGMGLAIVKQIVSLHQGDIRVESEPGQGTTFIVTLPI

TPPTE"

CDS 2796037..2797023

/gene="rutD_4"

/locus_tag="PIKHDJKN_02568"

/EC_number="3.5.1.-"

/inference="ab initio prediction:Prodigal:002006"

/inference="protein motif:HAMAP:MF_00832"

/codon_start=1

/transl_table=11

/product="Putative aminoacrylate hydrolase RutD"

/translation="MSVTQETEKEQWGGTKTKKVLNILLKVLGAIVIAILLFVATVYS

VNKFSSHSEQKRMETYGQHVAVDGKQMNISIQGEGEETVVILPGYGTASPALDFKPLI

SELSPYYKVVVVEPFGYGLSDQTEKERSTENIVSEIHEALQSLHIDRYILMGHSISGI

YSLDYVNKYPNEVRAFVGLDSSAPALSEKKIDSSMTRPLKILRELGFGRLQLKLGADP

YDGLPYDDETKEQLKILINKNMYNTTQLNEAESMYSNFKAAELLTFPVNLPVLFFIQK

NHPATDRWIPEHEKLIKDSLHGEVVLLEANHYLYRSHAKEIAEKFRGFMTSQ"

CDS 2797154..2798134

/gene="rutD_5"

/locus_tag="PIKHDJKN_02569"

/EC_number="3.5.1.-"

/inference="ab initio prediction:Prodigal:002006"

/inference="protein motif:HAMAP:MF_00832"

/codon_start=1

/transl_table=11

/product="Putative aminoacrylate hydrolase RutD"

/translation="MIQPEEKKAKNGSKAKKVRNIILKILGAIVIAIVLFLGIVYITN

VISTNSEAKKIETYGQHVSVDGKNMNVLIQGEGKETIVLLPGYGTAAPALDFKLLIDE

LSPYYKVVAVEPFGYGLSDGTEKERTAENIVSEVHEALQQLNINQYILMGHSIAGIYG

IDYVNKYPNEVTAFVGIDSSVSTQPGMDAKLPTKMFGFLKKSGLQRLVVKFGGDPYDG

LPFDEHTVEQMKMLSNKNSNSSTMLNEMDHISSNFKEAQGLTFPKDLPLLLFIQANNE

GVKGWIPLHEGQIKDSVHGKVITMDGEHYLHHTLYKEIAEDFRAFMNEAK"

CDS 2798264..2799718

/locus_tag="PIKHDJKN_02570"

/inference="ab initio prediction:Prodigal:002006"

/codon_start=1

/transl_table=11

/product="hypothetical protein"

/translation="MRLFEILLVLSCFALLVDLLFIKRSAKKIGLGLGIGSSVILLVQ

LFVEGYRWQLLLVYIMTALFILIVLFRHSEKMVNLKIGKFLKYSLSSLIVILLVVSTG

LSVYLPVFHLPKPDGPEKVGTQTFHFTDQNRDEVLTEDQSDKRELMVQVWYPTENRNN

NKRDTLFPKDKERFKKYIQSFSTSLKLPEFVLDYWKYSQTNSYENVEILPSASPYPVV

LLSHGMGTSRVLHASQAENLASHGFIVVTIDHTYSTFATIFPDGRVTDYTAKTTTVEN

RTKIGNVWTQDVEFVINQIEKLNSGAIESQFKGKIDLNNIGVMGHSFGGATAFNTTYL

DHRIKAGVNMDGSLYEVENRDDINKPFMFIRSGNFKDWLANFDNDRNSDDEVTKSLSD

ELHIMKNVINQGGSVIYIEGTQHFNFTDLQFYSELIKLTGITGDINGKRGSSIVNQYV

LDFFNKQLKGTGGNLIQGPSDKYPEVKFVGPEEL"

CDS 2799916..2802006

/gene="yfeW_2"

/locus_tag="PIKHDJKN_02571"

/EC_number="3.4.16.4"

/inference="ab initio prediction:Prodigal:002006"

/inference="protein motif:HAMAP:MF_01034"

/codon_start=1

/transl_table=11

/product="Putative D-alanyl-D-alanine carboxypeptidase"

/translation="MGRQKGKRTSIAALTLVLTMLAPMSVMAAPTADNRSNFSYEPTK

KTVAEKAKILTETFGTTSVQYALIDGGEIVVSGQTGKNDINNKVPLTSNTIYGIGSTS

KMVLTTAVMKLVDDGKIDLDVPVVNYIPDFKMKDNRYKQITPRMLLNHSSGLLGSSGS

NATLYGDNDTYSHDTFLDQLANQNLKADPGEYSVYCNDGFTLAEIMVERVSGMSFTAF

IHKYITEPLDMKHTKTPQDVVDPVAMAGIYSPLYEGQLPQENYNIIATGGIYSTAEDL

VKFSQIFTGEVEGILSSKSVEAMEQEEYKRGMWPEDSDTSISYGLGWDSVNLFPFSEY

GIKAVTKGGDTISYHSSLIVLPEYNLAAAVTSSGGTSAKDQFIASELLLSALEEKGII

TERKPEKSFGVPVKADIPKEISTYAGMYGANNLVKKIEMNNAGQMTVSTLTAASDSAQ

KYTYTADGTFVNGKGTEKLKFVTEQNGSTYLWSRSYISLPGLGQLAFSEYTAEKLEAN

ELSQDITASWEKREGKRYYVVNQKYTSTVYLHSSPILPIHSNKETPGYMSNNKVVGAN

EAVTELQIPGMAGRDTKEIYFSKKNGVEYITAVGSVFASEELVQPLYSGKQSVTMIQA

DGYAKWFSVPANVKGKVMTVKMPANGAFAVYDQNGVCINHTVVSGKNEVVLPENGRIV

FAGEVNSKFEISLK"

CDS 2802298..2804421

/gene="yfeW_3"

/locus_tag="PIKHDJKN_02572"

/EC_number="3.4.16.4"

/inference="ab initio prediction:Prodigal:002006"

/inference="protein motif:HAMAP:MF_01034"

/codon_start=1

/transl_table=11

/product="Putative D-alanyl-D-alanine carboxypeptidase"

/translation="MIQQEKMRVVKRTVKKRTSIAALTLVLTMLAPLSAIAAPAAMNN

NSNLTYEKTKKTVIEKAKLLTETYGTTSLQYALIDGGEIVVSGQTGKNDINDKVPLTS

NTIFGIGSTSKMMLTAAVMKLVDEGKIDLDVPVVNYIPNFKMKDNRYKQITPRMLLNH

SSGLLGSSFSNATLYGDNDTYSHDTFLDQLANQTLKADPGAYSVYSNDGFTLAEILVE

RVSGIGYTAFIHKYFTEPLDMNHTKTPQDVVNPAEMAGIYSRFYEGQLPKENYNIIAT

GGLYSTAEDLVKFSRIFTGEVNGILSSKSVEAMAQEEYKRGMWPEDSDTLMSYGLGWD

SVNLFPFNEYGIKAVMKGGDTLSYQSSLTVLPEHNMAAAVISSGGSSLTNQFIANELL

LSALEEKGVITERKPEKSFGVPVKADMPKGISKFAGIYGGNNSVTKININTTGQMTVS

SLTAPSNPAQEYTYTADGTFVNNEGTDKLKFIVEKNGKTYLWSRSYISVPGLGQLAFS

EYNAEKLEANKLSKEINAAWAKRDGKKYYLVNEKYTSMVYLNASSILPIHMNKENPGY

MSNNKIIGANEAANPLQIPGTAGRDTMDIHFSQKNGGEYLTALGYLYASEELVKPIYP

GKQSATTIQADGYAKWFSVPATAKGKVMTVKIPANSAFAVYDQTGICINYTVVSGKNE

VVLPENGRIVFAGEVGSRFEISLKK"

CDS 2804475..2805416

/gene="rutD_6"

/locus_tag="PIKHDJKN_02573"

/EC_number="3.5.1.-"

/inference="ab initio prediction:Prodigal:002006"

/inference="protein motif:HAMAP:MF_00832"

/codon_start=1

/transl_table=11

/product="Putative aminoacrylate hydrolase RutD"

/translation="MMKKIFIILLKIFGAIVIAFALFIATVFIVNVFSNKSEEGKIKA

YGQSVAVDGKNMNVLIQGKGEETVVLLPGYGTAAPALDFKPLIDELSPFYKVVVIEPF

GYGLSDVSEKERTIDNMVSEIHEALQQLNVTHYTLMGHSISGIYGLDYVNKYPNEVNA

FVGIDSSVPTQGGTDDPFPTDVYKLLKKSGFYRLLMKLAPDQLVAPAVDDETREQIRM

LSLKNTFNPNNLSEGENFDPNFKAAENLTFPKDLPVIFFLQANDTETEGWIPLHEEQV

KNSVHGKVMTFEGGHYLHHTRSKEIAENFRKFMNEVK"

CDS complement(2805520..2807613)

/gene="yfeW_4"

/locus_tag="PIKHDJKN_02574"

/EC_number="3.4.16.4"

/inference="ab initio prediction:Prodigal:002006"

/inference="protein motif:HAMAP:MF_01034"

/codon_start=1

/transl_table=11

/product="Putative D-alanyl-D-alanine carboxypeptidase"

/translation="MTLKKRTSFAAVTLVLTMLAPMSALAAPATSNSSNLTYDVTQKA

VMEKAKLLTETYGTTSVQYALMDEGEIVISGQVGKNDPENRIPLSSNTMYGIGSTSKM

VLTAAVMKLVDEGKIDLDVPVVNYIPDFKMKDNRYTQITPRMLLNHSSGILGTSSGSA

ILYGDNDTYAHDTFLDQLAEQNLKADPGAYSVYSNDSFTLAEILVEKVSGMSFSHFIQ

QNFTEPLGMSHTKTPQDLVDPDQMAVTYSQLNKEQLPLENTNMIGSGGIYSTAEDLVK

FSQIFTGEVKGILSSESAKAMTQEEYKKGVWPEDSDSSISYGLGWDSVDLFPFSEYGI

KAVTKGGNTIAYHSSLIVLPEYNMAAAVTSSGGSSTTDQLIASELLLSALEEKDIIKE

RKPEKSFGVPVKTTMPKELTQYAGIYGAVGSSLLKLEVNDEGQLTMSLLLSPNSTEQK

YTYTSDGSFVNEQGTEKLKFAQGDNGNTYLWSRSYQSVAGLGQIASSEYKAEKLETNT

LPEDVTAAWQKREGNIYYLVNEKYTSTVYDSATPIIPIHMFDEAPGYVYTNKIIDANS

ALNQLQIPGLAGRDTMEYKFFEKDGVEYVSAGGKVYVSQELVRPLYSGKQSKTTIQTN

GYATWYSIPAAAAGKVMTVKMPSKGAFTVYDQTGISTNHTVVSGENEVTLPENGTIVF

AGEAGSRFKISLTNQ"

CDS 2807818..2808186

/locus_tag="PIKHDJKN_02575"

/inference="ab initio prediction:Prodigal:002006"

/codon_start=1

/transl_table=11

/product="hypothetical protein"

/translation="MRERHINVNEQPDRHGERSRHGRGEDEQQGERSRRGRGHGDHHG

KRGNGAQTFRRGRILVFLEQMQNRRATLARQLGQEEFRNIHQMISGELKAIDQVIDEY

IQLFELQNEEESPNQDLESE"

CDS 2808189..2809907

/locus_tag="PIKHDJKN_02576"

/EC_number="3.6.3.-"

/inference="ab initio prediction:Prodigal:002006"

/inference="similar to AA sequence:UniProtKB:Q99T13"

/codon_start=1

/transl_table=11

/product="Putative multidrug export ATP-binding/permease

protein"

/db_xref="COG:COG1132"

/translation="MVRRFFSYYRPYKKLFLIDFGCAVLAGLLELAFPLAVSKFINEL

LPGQDWPLIILACIVLLSIYALNTVLNYVVTYWGHMLGINIETNMRSKMFAHLQKLSF

RFFDNRKTGHLIGHLTNDLNDIGEVAHHGPEDVFIAIMTLIGSFWLMANINLELALLT

FIIIPIMAWVIIVFGGRMTKTYRRLFGDVGNFNSRIEDNVGGIRVVQSFANEEHEKKL

FSVDNENFRKTKLLAYKTMAKSISVSYMMMRLVTVFVMISGAWFFIDGRIDMGDFMAF

LLLSNIFFRPIEKINAVIESYPKGIAGFKRYLEIIDTEPEIADAKNAVELKSVRGDIR

FENVSFGYEENRRILNNISLSINPGETVAFVGPSGAGKTTICSLLPRFYEVEEGRITV

DGVDIREVQLESLRRHIGIVQQDVFLFSGTIKENIAYGDLTATDEQIWDAARRASLEE

LLLTLPEGIDTIIGERGVKLSGGQKQRLSIARMFLKNPPILILDEATSALDTETEALI

QKSLAELSVGRTTLVIAHRLTTIKNADRILVVNSDGIAEQGNHEELVAAGGIYSRLHQ

VQYSHS"

CDS 2810099..2810533

/locus_tag="PIKHDJKN_02577"

/inference="ab initio prediction:Prodigal:002006"

/codon_start=1

/transl_table=11

/product="hypothetical protein"

/translation="MLTEMRRFNRFYTNILGVLDKHILGTGYSFAEARVIIEIGIQGE

SIANNLVDTLTIDRSYMSRIVSKLTREGLLMKVDSAADSRVSLIRLTEKGQELYGELN

ERSDQQIVKLMQGLDEEEIREVYASMMNIQDKLNKRAGETRR"

CDS 2810530..2811594

/gene="ltaE"

/locus_tag="PIKHDJKN_02578"

/EC_number="4.1.2.48"

/inference="ab initio prediction:Prodigal:002006"

/inference="similar to AA sequence:UniProtKB:O50584"

/codon_start=1

/transl_table=11

/product="Low specificity L-threonine aldolase"

/translation="MIRFECDYSEGAHERILQRLISTNMEQTSGYGKDAHCDRARSLI

RQACENEQADVHFLVGGTQTNTTVIASILRPYQGVIAATSGHIAVHETGAIEATGHKV

ITVPSKDGKITSDQVKAVYDAHWNDAAPEHCVQPGMVYISQPTENGTMYSKAELQALF

DVSQQCGLPFFIDGARLGYALASRNCDMTLADLARLCDVFYIGGTKIGALMGEAVVIL

NDKLKPDFRYVIKQKGGLLAKGRLLGIQFETLFEDGLYLEISQHAVDMAMLIHDSLAE

QGIAFLYDSPTNQQFPILPDDLLQDLRSRYSVTFWEKVDDTHSVVRFCTSWATQRQNV

DSLIRDISQGLNKLKLVCNY"

CDS complement(2811701..2813677)

/locus_tag="PIKHDJKN_02579"

/inference="ab initio prediction:Prodigal:002006"

/codon_start=1

/transl_table=11

/product="hypothetical protein"

/translation="MKFRQVHLDFHTSEAIEGIGRDFSKQQFQEMLKTGYVDSITIFS

KCHHGWAYHPTTANQMHPHLNFDLLGAQIEAAHEIGVKTPVYLSAGLDERLARKHPQW
LIRNQQEQISWTADFMKPGYHQFCMNTPYLDVLTLQVEEVVKKYDADGIFLDIVGVRE
CYCQYCVAEIRSQGSDPRNIQDMRKLWEQTYARYANRMNETVHAVKPGLPVFHNSSHV
DRGRRDLAHVNTHLELESLPTGGWGYDHFPLSARYAQTLGMDFLGMTGKFHTSWGEFG
GYKHPNALRYETALSLANGARCSIGDQLHPGGQMDIATYSLIGEAYREVEAKEEWCRD
TTAKADIALLSVEATLWEAGGDPHLQHSHHDTGAVRVLLEGHYLFDVVDLQADLSKYK
VVILPDAISITESIKTKLTAFLAGGGKILATGRSGLSPDGTDFEFDLGVEFQEVSPIR
PSYFRPNFELPSLRTASFVMYTEGQNTVPTTDGIVLGSKENSYFNRDLFTFCSHQHTP
SDLQESGTGMAEGQDGIYIAWNVFEDYATKGSLAVKEIICYALDRLLSNNKSLMTNLP
AQGVTTLQMQTNSSRWVNHLLYASPVRRGQGVEVIEDLLPLNNTEVTIAAAEKVQDVY
LAPQRLSLPFSQVNGLVTYTIPEWSCHQMVVIQF"

CDS 2813812..2814690

/gene="rhaR_22"

/locus_tag="PIKHDJKN_02580"

/inference="ab initio prediction:Prodigal:002006"

/inference="protein motif:HAMAP:MF_01533"

/codon_start=1

/transl_table=11

/product="HTH-type transcriptional activator RhaR"

/translation="MLVLELQVPPFPLLAAIGHTEWQPGMQHAQRCFDVFDLIICAKG
ALYMEEDGLRYEIAEGMMLVLEPGKEHRGYRPTDVQTEVYWIHFQLPSVPKMILKEKT
TWEQPLLQQTDQDIEAPPGVIEIPKFAAVDLRNLEPIMKEMLELHHVLTRQRSFELHV
LLGQFLLQLQKGMRQNSPQARSYFLSEKVATYLGQRLEQPFDSGQMERDLLYHFDYLA
RCLKQYTGMSPLQYRHHLQMEKAKRLLAHSELPLKRIGELCGLQDHNYFTRLFKRQTS
LTPGEYRKKHQLYFME"

CDS complement(2814765..2815421)

/locus_tag="PIKHDJKN_02581"

/inference="ab initio prediction:Prodigal:002006"

/codon_start=1

/transl_table=11

/product="hypothetical protein"

/translation="MKIAIIGTGNIGGNLTRRLAKLGHDVSIANSRGPHTLTDLAEET

GAKPLEVTEVAHGAELVIVTIPQKNIPDLPDGILDQIASGAAIIDTGNYYPRQRDGFI

AEIEGGLPESRWVEQYLKHPVNKAFNGISAINLLERGLPAGSPDRLALPVAGDDPDTK

KIVLQLVEELGFDAIDAGGLGESWRQQPGTPSYQEHGADSLRQALSNASPVRSEDFKA

"

CDS 2815567..2816157

/locus_tag="PIKHDJKN_02582"

/inference="ab initio prediction:Prodigal:002006"

/codon_start=1

/transl_table=11

/product="hypothetical protein"

/translation="MTRGGRPREFKDTAVIDAAMDAFWLNGYEGCSTEDLCKQTGLGK

GSLYNAFGSKHDLYEKVLQRYHEEGIREQSEILQSSLPLKERLRGLLEWGIQEDFSES

GRRGCLLINAAMERARVDPMVEQVVTRHIGFLEDALCDLMEQGSQTGEITSGRPAKEL

ASLFLSSYYGLRVLNSSSQNRDLAKSIVEGTLAAIY"

CDS 2816249..2817418

/gene="sotB_2"

/locus_tag="PIKHDJKN_02583"

/inference="ab initio prediction:Prodigal:002006"

/inference="protein motif:HAMAP:MF_00517"

/codon_start=1

/transl_table=11

/product="sugar efflux transporter"

/translation="MPLAIYVLSLAIFCMTTSEFMVAGMMNTLAGEFGVSVSQVGYLI

TAFSAGMVVGGPLLTAALLNMPRKKAFIMLTGVFLAGQLLGAIAGSYEVMMASRIIAG

IASSAAFGVSISISASLVEENQRGRAVSIIFGGLMIATVLGTPAATWVADAYGWRFSF

WAVAILVLLSGVTALRLIPVLSRPEPTSLRHEMKSFRNRKLWAAYLTSFLIIGATFAA

FSYFAPIFTELTGFSSASVPLLFVVYGAATVVGNLITGRLADRYTMLILTIGLVLLTF

GLLIFAIGVHTAAVVIPAVIIIGLAGVPMNPPMVARVIRTGGSGLMVNTVHTAIVTFG

VVVGSSVGGLAISAGYGLVAPIWVGVVLALVGVISLLPYMRGIFKVKVVDMDIKH"

CDS complement(2817583..2818764)

/gene="mdtL"

/locus_tag="PIKHDJKN_02584"

/inference="ab initio prediction:Prodigal:002006"

/inference="protein motif:HAMAP:MF_01530"

/codon_start=1

/transl_table=11

/product="Multidrug resistance protein MdtL"

/translation="MKKYAVPSLLLMIVLIAFPQISETIYTPSLPDIAAALGVSNSSV

QFTLSIYFIGFALGVFCWGWLSDLIGRRPAMLGGLIVYGIGSLMCFYSESIQWLLVGR

FVQAFGAATGSIITQTMLRESVSGDRRHAMFAQISAVIAFTPAVGPLIGGWIDQALGF

RWVFLALVLMSVLLFIYAFLKLPETTNVALRSKVAILPVVKKMLAMPRVIVFGVLIGG

INGVLFSYYAEAPFIFIEHFDLSPGVYGFLGIIVALASVVGAMISKRLLTVYAPEKII

RLGCLVMTSGALLLTLGSSVSLVPNLLQIICMLTAMFVLLIGAGIALPNCLSLALVHF

QDVIGTAGAIFSLGYYLLVSLTTWGMGALHNGSLLVMPLYFLIISGVMWLLGKRFISE

E"

CDS complement(2818989..2819474)

/locus_tag="PIKHDJKN_02585"

/inference="ab initio prediction:Prodigal:002006"

/codon_start=1

/transl_table=11

/product="hypothetical protein"

/translation="MNPISLTVSLLIVVLILRGVIQGSKKPLHESGRKLLIPILYIST

SLFELFDPTLVLTSGRMISSIGIGAILSIPLILVTQFERKSDGKMYYKRNVSLYVLII

VIFSIRFFDFMFISGIDPKTLGFLNNVVALSYIAIWRMASFIKFHNSKSSTFSAWDKT

A"

CDS complement(2819792..2821579)

/locus_tag="PIKHDJKN_02586"

/inference="ab initio prediction:Prodigal:002006"

/codon_start=1

/transl_table=11

/product="hypothetical protein"

/translation="MKTLINYDCPTVIINSNSTLFDSFTNIETITENLFLVHIRLSAV

KKEQPPVCRVEWSIPAVDIHSLWHPGTDRNKALQVDWGDGFQSKSTSLAPVASLYNLE

GRNRMTFAFTDALNNVSIKAGIHEEDSTFLCSVTLFEEAMPKLDHYEATLRIDRRDIP

FYKAIEDVTEWWECLPGYEPAAVPEVALGPMYSTWYSYHQKLTAAELEEQCRLAKELG

CDTIIVDDGWQTDDNNRGYAYTGDWEVYAGKIPDMKAHVDTVHRLGMKYMLWYSVPFV

GKRSKVWSKFENKLLRVDEKLNTGVLDPRYPDVREYLINTYEQAVSGWDIDGLKLDFV

DSFRLSPDQKEGTAEGRDYESVPEAVDRLLSDVMERLRAIKPDILIEFRQTYIGPLMR

KYGNMFRANDCPNDSIQNRIRTLDLRLLSGNTAVHSDMIMFNPKESVESAAMQLINVL

FSVPQISVRLDEIGEAYVGMLRFWLSFCKENSDILLKGKLVPEQPELLYPLVSSQRGG

TKIVAVYANIVVPMDATYDRFIVANGTYNEQVVVRCHHQGRYDLTVKNCCGQIVLLRQ

IELQDGLYELPIARAGLVILQRIDDSEGI"

CDS 2821897..2822952

/gene="melR_5"

/locus_tag="PIKHDJKN_02587"

/inference="ab initio prediction:Prodigal:002006"

/inference="similar to AA sequence:UniProtKB:O34829"

/codon_start=1

/transl_table=11

/product="HTH-type transcriptional repressor MelR"

/db_xref="COG:COG1609"

/translation="MPTIRDIASLAGVSPSTVSRVLNGDPSLSVLEETRRKILSAAEQ

LQYKVTQRRHTNQAAQLKDYKIGLVTFCSEQFESEDPYYLTIRLQIEKEFNLLGLHIT

RTIRWVNHDSYESLSGLDGLIVIGKFEFDPYNLYFSRIHNIVFVDYSPDEDRFDSVVV

DFDKVTRMALDHLTGLGYTKIGLISSRDFINRFGSNSGIDSVDQRQSSFESYMKQKNL

YLPEHVHIGNNFSMSTGYQLMKEVIEKQDLPEAFLIGSDPMAIAAGRALKEAGLKVPK

DIAMVGIDDIEMASYANPPLTTVKIYTEQMGQSAVKMLLERIGGREVPLKIVIPSKLI

IRSSCGAEIRSIGDAEG"

CDS 2822991..2824238

/gene="yesO_5"

/locus_tag="PIKHDJKN_02588"

/inference="ab initio prediction:Prodigal:002006"

/inference="similar to AA sequence:UniProtKB:O31518"

/codon_start=1

/transl_table=11

/product="Putative ABC transporter substrate-binding

protein YesO"

/db_xref="COG:COG1653"

/translation="MKKWSLIILSLLVVIALSACSGNGGNSSESQSGTKDKGKITFAF

WGAQSEADAIKQAIDNFETNYPDIQVESQWIQKDYLTKLQTMIAGGTTPDVMLISGGD

LPGFANAFQELKLDESLFSSPSLVDSMSVDGKAYAAPFIIKPKVMAINVDLFEKKNIP

LPSKTEPMTIEQFNDIAKNLTSGEGATKIFGSEPLWLGNWIYAFGGQFYSDDLSKSAL

DSPQAIAAAEYIVSSKQAGIVPNDSEKQGQSMMNWYLGGRIGMFTDFGPWYLPQMADV

QKFKWDIVPFPGNGGSKEVNGLALSKDSKNVEAAETFINHLTQNEEVQKIIGGNKNAY

GVPVISSATSAFETVSPDKNLKAFVLAAEKQHTQEAQKGTNEINNEMKAIDDTTPIGI

GNKDVKEVFPQVAEKINKILQQN"

CDS 2824251..2825309

/locus_tag="PIKHDJKN_02589"

/inference="ab initio prediction:Prodigal:002006"

/codon_start=1

/transl_table=11

/product="hypothetical protein"

/translation="MNGIIEGIYNFYEGIGAPAAVLPLLPFLTVIVLVFAIIFTITRV

LVKKRIIHTKTASFYLFVSVWIIGFLLFTVGPMIFSFYISFNKWEVVSDPKWIGLDNY

RMLFKDTMFYKSLSVTFYYTLVSVPLQVILSLAIALLMNLKLRGIYFFRTIYYLPTLV

QGVAQMVLFIWIFNPNVGLVNSLLRLIGIEGPGWFSSPEWSMPAVIIMSLWTVGGNMI

IYLAGLSDIPQSLYEAAEIDGATAARKAWHVTLPQISPILFFNTVTSMIGAFQTFTQG

FMVNGGPDNSLLFYAYYLYQNAFMWFKMGYGSALAWVLFVIILLFTALVFRSSALWVY

YETEQTGRRKRRVRKKRA"

CDS 2825287..2826138

/gene="araQ_15"

/locus_tag="PIKHDJKN_02590"

/inference="ab initio prediction:Prodigal:002006"

/inference="similar to AA sequence:UniProtKB:P94530"

/codon_start=1

/transl_table=11

/product="L-arabinose transport system permease protein

AraQ"

/db_xref="COG:COG0395"

/translation="MSVKKERKSAHKKVHWLAYILLLIGSVMFLYPFLWMVSASLRSL

EEVAVSGMSIWPDNWRWDNYAKALTAFPFGRYLINTVITTVFPIIGTVLSSSIVGYAF

ARLKVRGSGILFVLVLSTMLLPGEVTMIPQFILFKNLGMLDTLYPLIVPAFFGSAFYI

FLLRQFYSRLPVALEEAAIIDGCGYFKIWWKIFLPLSKPALMAVGVMVFMGSWNNFMG

PLIYINSDKWKTLTLGLAGFQGTYATDTNLLMAAAVVITLPCILLFFTAQKAFMEGLT

FSGSKES"

CDS 2826167..2827555

/locus_tag="PIKHDJKN_02591"

/inference="ab initio prediction:Prodigal:002006"

/codon_start=1

/transl_table=11

/product="hypothetical protein"

/translation="MSYEREIESQAGAVTFQSSDKELNEGFVWAKRQALEYAHFGEDP

VGLWYEAALPAREAFCIRDVMHHSTGAAVLGLRLHTKNMIMHFAENIAESRDWCTYWE

INRNNVPAPVDYESDEDFWYNLPANFDIIDASLRQYLWTGDTYYLNNSKLLDFFNLTL

NEYTSTWDKDGDGVLEYYPEYGRRGLATFNEAGHQPLMGGDMIAAQIAGYRAFVHLAS

LRGQTEIAEVYTTKAQDLKRRYEQEWWNEEEGRFFGAKLQDHSFLTGYNAEGTFLPLY

YGSVSDPEKLHKALEDVKINRVANVEGKTYLPDIFYQYGQNEEAFSELKELVNPSLER

RDYPEVSYCVIGSMAAGLMGIKGNGASVISTLPRLTHSLEWSEMGNIPILDRNIMIKH

TGNTESEVTLQSGKPFVWKAAFPVNTETLYHNGIAVAAKQETNEGGEQISFISIKVAE

DEMHRVSIAQEG"

CDS complement(2827713..2829239)

/gene="cotA"

/locus_tag="PIKHDJKN_02592"

/inference="ab initio prediction:Prodigal:002006"

/inference="similar to AA sequence:UniProtKB:P07788"

/codon_start=1

/transl_table=11

/product="Spore coat protein A"

/db_xref="COG:COG2132"

/translation="MPEKIKLQKFVDELPIPNTLSPKKRSSNSSYYEVTMKEFSQKLH

RDLGPTRLWGYDGTFPGPTFEVMKNETVFVKWMNDLPNKHFLPVDTTIHGSEITVPEG
RSVVHLHGGRTPAHSDGYPEAWFTRNFEQTGPLFEREVYTYPNIERATMLWYHDHTVG
ITRLNVYAGLAGFYFIRDQNEQSLNLPKGKYEIPLLIVDRSFNPDGSLYYPSKPDQND
AGLPHPSLVPPFDADTILINGKVYPFLEVEPRKYRFRILNASNSRILTLRMESNQPFY
QLGTDSGFLETTVRINEFVLLSAERIDVIIDFSQYYGQTIILKNVTKSASPETIDIMQ
FRISVPLKSIDTSSLPFYLGAFERLPKSMAKRTRYLTLVRVKDQYGRSLNLLDGKMWM
DRISERIELNTVEIWRIINIIDDDPHPIHIHLVDFQILDRQPFDVSHFQKTGKLIFTG
PPTPPNPYEMGLKDTVATPFGYVTTIIARFGPFTGRYVWHCHMLEHEDYEMMRPFDIF
"

CDS 2829487..2830251

/gene="iclR"

/locus_tag="PIKHDJKN_02593"

/inference="ab initio prediction:Prodigal:002006"

/inference="similar to AA sequence:UniProtKB:P16528"

/codon_start=1

/transl_table=11

/product="Transcriptional repressor IclR"

/db_xref="COG:COG1414"

/translation="MKGSVALDRKSGTNTLGRALDILFALSEANGTLSVSEIAEKVNI
PESSTYRFLQTLEKNGIVERRGKNQIGLGLRILDLARSLNQQIHRELLSLALPIMINL
MESTQETSLLFVRTGNDAVCIQHVKGRGLIQFVIENGRRLPLHQGASGKSILAFENEK
TIEQVLGNITNDRNRSELIDSLKAVKEQGFACTEGEYDPDVFAIGAPVVDSHGLVVAS
LSIAGPLFRGTKERIPEMIDSVMESSKHLSNLLGGG"

CDS 2830551..2831690

/gene="opuCA_1"

/locus_tag="PIKHDJKN_02594"

/EC_number="7.6.2.9"

/inference="ab initio prediction:Prodigal:002006"

/inference="similar to AA sequence:UniProtKB:Q9KHT9"

/codon_start=1

/transl_table=11

/product="Carnitine transport ATP-binding protein OpuCA"

/db_xref="COG:COG1125"

/translation="MTEAVAVEFQHVNKDYGSGSVLHDFNLTVPKGQIVTIIGPSGCG

KTTLLKMINRLEEPSQGKVLVEGRDVGAADAVKLRRDIGYVIQQIGLFPHMTIEENIG

IVPRLKGARKEERKERVDLLLNLIGLPAEQYSKRYPHELSGGQQQRIGVARALASDPS

IILMDEPFSALDPISRVQLQKELIHLNQRLHKTIIFVTHDIEEALKIADRIILLNEGE

VVQNSTPAELLAHPANDFVIEFIGQERFRPSFPSGMAQHIMIDGLSISNISTIVEALD

LLRLNQADALAVTGNWGELLGIVGIEEISEHLQHDSKMKVGDIVKNGTRALFENTPIS

DVFELMQSQRILPVVDDQNRLIGTVTRSGVIEAIGRHFHREETSA"

CDS 2831793..2832332

/gene="opuCB_2"

/locus_tag="PIKHDJKN_02595"

/inference="ab initio prediction:Prodigal:002006"

/inference="similar to AA sequence:UniProtKB:Q9KHT8"

/codon_start=1

/transl_table=11

/product="Carnitine transport permease protein OpuCB"

/db_xref="COG:COG1174"

/translation="MQYPLVFTCRKRMADTIITVVSAIQTIPSLALFGFMIPLVGIGT

TPAIIALTLYAVLPMLQNTYVGINGVHPSLIEAGTGMGMTRWQMMKMIQLPIACSVIM

AGVRLVAVQTISLTTIATYIAAGGLGDIITRGIAMINTVTIMEGAIPVSLLVISVNFI

LLLLNRALTPKGLRHLNKF"

CDS 2832362..2833300

/gene="opuBC"

/locus_tag="PIKHDJKN_02596"

/inference="ab initio prediction:Prodigal:002006"

/inference="similar to AA sequence:UniProtKB:Q45462"

/codon_start=1

/transl_table=11

/product="Choline-binding protein"

/db_xref="COG:COG1732"

/translation="MTTTNNKRWNVIKGGRLLITLGLVLTTILTVAGCSNSNASGRND

TIVLATKGFAESDILANAFKLLVENDTKLKTEIKKLDNTLLWNAIDSGDVDAYVEYSG

TALINILKQEPEFDADEAFKTVVTQLKEKNKLIALEPLGFNNTYVFTVRRETAEKYGL

KTASQLAEKSGELVFGASEEFLNRPDAWPYVESIYKPKFKETKSIQDPSLQYQAIKQK

LIDVKLSYSTDSQILANDLVALEDDKHVFIPYDAFPVVREQTLDEHPELKEVLNKLAG

KLDEKAMQKLNAEVEQDQKPALDVARDWLKAQGLIK"

CDS 2833377..2834738

/gene="ntaA_1"

/locus_tag="PIKHDJKN_02597"

/EC_number="1.14.14.10"

/inference="ab initio prediction:Prodigal:002006"

/inference="similar to AA sequence:UniProtKB:P54989"

/codon_start=1

/transl_table=11

/product="Nitrilotriacetate monooxygenase component A"

/translation="MSNGKRQLALGAMMFFPAGEHISSWRHPGSEAEGLLDFNYYKRI

AQTAERGRFDMFFYADELYVWDRFESGISHANSIRPEPFTLLSALAAVTSHIGLAATI

STTYNDPYHIARKLATLDHFSGGRAAWNIVTSQTDEEAWNFGKERHLQHELRYERAKE

FVEATQGLWDSWEEEALLFDKESGYFADKNKLHNLDYEGTHFKVKGPLNISRPPQGYP

VLIQAGASEAGKEVAAATAEIVFAPGGTFSEGKALYEDIKGRLAKYGRSPDEIKILPA

LMPIIGATAEEAAEQLAIVENLTPDRLALDLLSHYIGVDVSGYPLDEPLPFLPEEEGF

NQSKSGLQRIRRILKEEDLTLIQLAKRIVSKRAFAGTPEQIADHIEEWFNNGAADGFN

IAFTHLPGALDDFVAEVVPILQRRGLLRLEYEGTTLRDHFGLSKPENRYSQEKITLGT

AER"

CDS 2834806..2836476

/gene="hutU"

/locus_tag="PIKHDJKN_02598"

/EC_number="4.2.1.49"

/inference="ab initio prediction:Prodigal:002006"

/inference="similar to AA sequence:UniProtKB:P67417"

/codon_start=1

/transl_table=11

/product="Urocanate hydratase"

/translation="MKRIVAERGGVLRCKGWRQEALLRMLENVLENGENQKELIVYAS

LGKAARNWPSYLAIVDTLKQLEEDETLVIQSGKPIGVFRTHRQAPLVVMANCNLVGRW

ATSENFYELQDKGLIIWGGLTAAAWQYIGSQGVIQGTYEIFQSIARLHFGGDLRGRFI

LTAGLGGMGGAQPLAGYMANAAILCVEVAEERIDKRIASGYLQRKTDSLDEALLWITE

AVSSGTSLSVGLLGNAADVYPELLRRGVQPDIVTDQTSAHDLLYGYIPSGYSPASAGV

ARREHPTELQAAAGASIANEVKAMLEFKQRGAVVFDNGNNIRSQAVQYGVTNAFEIDI

FTEAFLRPLFARAIGPFRWIALSGEAEDIRIIDDYILKEFSDNDIVSNWIKLANTYVP

FEGLPARIGWFGHKDRSKLALAVNQLVREGKLAGPVAFSRDHLDAAAMAHPNIMTERM

KDGSDAISDWPLLNAMLNCSSMADLVAIHSGGGGYSGYMTSAGVTIVADGSLSSDARL

RTALDNDTGLGVLRYADAGYEQSLAEVKTKGIARIDTHQYETISSTVK"

CDS 2836522..2837628

/locus_tag="PIKHDJKN_02599"

/inference="ab initio prediction:Prodigal:002006"

/codon_start=1

/transl_table=11

/product="hypothetical protein"

/translation="MRELTRDDVNAAVKGGSVFASGGGGWVDHGLEIGHAALSIGRPK

LLSVDELPDDAIILTCTAIGAPAGRDWQMLGKDYIKAVQLIIENYDGKIAGVMTPQNG

MSSTINGWLPAAALGLAVIDATGDIRAHPTGKMGSLGLASSIDYETIQAVAGGKPEIG

SYMELVVKGTPARTSNILRTASDMAGGFIAAARHPLPIKHIKKHAAIGGISIAIELGR

VIMQAEPEGPEAVLEAVVHKTNGRILGRGTVTTKNIRYSGAFDVGTIEVSIPNRRLTL

HVMNEYMAVEDDEGARLTTFPDVITTFDLKKGLPVSVGDVEEGMEIAVFAIDKSKVPL

SSSVKDPSVYPEVEEMLGIDLQSYAFDKEKTIYA"

CDS 2837621..2839165

/gene="hutH"

/locus_tag="PIKHDJKN_02600"

/EC_number="4.3.1.3"

/inference="ab initio prediction:Prodigal:002006"

/inference="similar to AA sequence:UniProtKB:P10944"

/codon_start=1

/transl_table=11

/product="Histidine ammonia-lyase"

/db_xref="COG:COG2986"

/translation="MLEESILSLSSNQIVLDGSHMTIEDVERVARSRRSVVIADEAWK
RLVTARKVIYELAAEGVPVYGLNRGVGWNKDKVIGDDYFEDYNRNLLLSHSAGVKPEA
TEDVVRAVMLARLNSLLVGATGAQPDIAKRYADFLNMGIHPVLPLRGSVGAADITSIS
HIGLAMIGKGEVHMAGNRIPASDALMVMGLEPLRLGPKDGLAIVSSNALSAGNGALAL
FDIEELLDTADSVYALSLEAIRGNISPLDEAVHFKRPFPGQLKSLAMVRRSLRGSGLW
NGFNPDSLQDPLSFRDACQIHGAARDAYAYARQQLEVHLNSSDDNPCVLTEERRILSC
ANYDPITWTLGFEMLGSALHHVSKSSCYRMIKLGDPDFTGLSRFLTADPSRSVGFCTV
QKTATSLDAEIRHLSNPVSSDYFSLAGDMEDHAANSPYVVSKVREIVDRVTYILAIEA
LHAAQAIDLRMDMTLGEGTRAGYELLRSEVPFLSEDRNQTIDIERAYLLLKEGKWLKA
VRGKMN"

CDS 2839201..2840028

/locus_tag="PIKHDJKN_02601"

/inference="ab initio prediction:Prodigal:002006"

/codon_start=1

/transl_table=11

/product="hypothetical protein"

/translation="MAHVDPQWSLRDHSQEGWQIRAEVSPKWIRVKFDGEFIADSKEV
LVVTETGRLPVYYFPKKDVNLDVFKPSEKLVNDPYKGNQLFWHISSGGRTVENAVWSY
SGAQQEEVSLQDHYSFSWKKVDAWYEEEEEVFVHARDPFSRVDAIPASRHVQIELDGV
IVADSKRPVILYETGLTPRYYLHQEDVRLDLLKPVDITTTCPYKGIASYWTASVNGRE
YNNLVWSYKDPLPEVHEVEGLLCFYHESVDALYVDGVKWSLSADDRLPYRNIGWGIK"

CDS 2840030..2840872

/locus_tag="PIKHDJKN_02602"

/inference="ab initio prediction:Prodigal:002006"

/codon_start=1

/transl_table=11

/product="hypothetical protein"

/translation="MVNHLDSSIACSLRQEDKLMLKADLNPRRVHVTFAGETIADSTR
VITLHERGKLPVYYFPQEDVTQQFLVKTELQTHCPIKGDASYWTIQVGERISENAVWG
YENPLKDIESIKGFVAFYWNKVDAWYEEEEQIFVHPRDPYKRVDAIQSSRHVQIVLDG
IKLADSRRPVIVFETGVPVRYYLPVEDVDQNVLTANDKQTSCPYKGDASYWTAVLNGQ
TYENIVWSYLNPIPEIPKIAGLMSFYNEQVDVYVDGELQPKPKWYYSALEFFNETEVT

VRRQ"

CDS 2841007..2841129

/locus_tag="PIKHDJKN_02603"

/inference="ab initio prediction:Prodigal:002006"

/codon_start=1

/transl_table=11

/product="hypothetical protein"

/translation="MSEVGYGNVGGLGGYGGFTSIGAILVLFILLVIISKAFLV"

CDS 2841288..2843000

/locus_tag="PIKHDJKN_02604"

/inference="ab initio prediction:Prodigal:002006"

/codon_start=1

/transl_table=11

/product="hypothetical protein"

/translation="MTLKKRIFLLFFLSAFIPFISIFAISYYTIDSIFVNKIDDGIRS

NLQQVTSSLENSITNLNHVTQQLSYSGTLGKKLDEVLKPSSNIFELIETRDELKSELN

VVTFTNPNIGLTLYYFQKDGSTQFGNFPIKDRFSPESLPVLSKAYGITYYGPHVSMNR

FDDQLVLSAMRKVKLPQRDDVYIYVESGFHLAQDILGYNQYKGDLSHLILDGEGNIVY

SEIPEAMKVGENFSNLSNGAASDGIARGHHWFRQDSNQNWRVVSVISQAKYQQEKNQW

LLQILLVALFFLGFTVFLAWLLWKMVYKPLGLFHSQINGMSQNPQTAGSQTRTQIPEF

DFLLGEFSNMQHQIGDLFKEVQQKEKIRADLEVEKLLYQINPHFLMNTLDTVHWLAVM

NGQGEIDRLVQSLNKLLYYNLGKLGQVSTMAEEIDALRQYLILQQIRYDFEFDVRISA

DEQVLQIPVPRFILQPLVENSLYHGLSDEGFIQIEVTRKETLNIMIQDNGAGMTEEAI

ERLLNNRVAEHQKVGMGIGLNYVHRMLQAQYGDQAQLVIESELGKGTSILLILPIKGE

DISA"

CDS 2842997..2844403

/gene="cheB_5"

/locus_tag="PIKHDJKN_02605"

/EC_number="3.5.1.44"

/inference="ab initio prediction:Prodigal:002006"

/inference="protein motif:HAMAP:MF_00099"

/codon_start=1

/transl_table=11

/product="Protein-glutamate

methylesterase/protein-glutamine glutaminase"

/translation="MIKVLIVDDDKLVRKGISSAMPWNEFDMEVVGEASNGAKALEFL

ESNSVDLMLTDLAMPVMSGIELMRAARQLYPELHIVVLTLHQDFDYIQEALRLGAIDY

IAKVQLEKEQFEHVLNRIHTRIGELGNTRRKLPLLGETNVHYRKVYALVSLDRKSGQI

WPIGLESNVDEVRFEVERNSWMWAAPLDGEDQLFDRLKGYLDQVPQGALLVLSDVQER

TWSQIQNWIINYTETSLFYAYEPQDPVIAVSLNEEDTSPTEPQDEDMDRIKRSWFLSP

WTHNDNYYNQLVEEFKSLRLQKGQLMGLLYSLVMEWNHLFSQTTLGRISMIHSFQSWY

EVEQWIKQTSESIRKADEQTSYSQEMIDGVKKAVMIMQNDLDQAFTASGLSQQLNISR

SYFSQCFKDIMGKTFNDYSRYIRMEKSKEYLLNTNNTIFWIAERVGYTDEKYFSRIFR

DLTGVLPSEYRQLGRADK"

CDS 2844453..2846111

/locus_tag="PIKHDJKN_02606"

/inference="ab initio prediction:Prodigal:002006"

/codon_start=1

/transl_table=11

/product="hypothetical protein"

/translation="MIKKMAACLVVVSLIAGIVFFSISYFRESPIGPATTIPDTAATD

PFGKYEHPVSIRLGYVVDPTGTDLSEGETLEKNMWKTAIKQNLNIDVEVMWQVSKENF

GQKIDLAIASNDLPDAMIVNQVQLNEMVKAGEIEDLTNAYASSASPEMKKIIDSTNGL

AREQVTFEGKMMAVPSVTAEDFSMLWIRQDWLDRLGLKPPKTVDELEAIAKAFVEQDP

DGNGKRDTIGLASSTSLFNDFNNSAFAFDLTPIFSAYGAFPGYWLEKDGKPVYGSILP

ETRNALVRLRDMYAKGLLDPDLGIRKEPEETVISGQAGMFFQGFFAGYWPLPSAWLND

PQANWQAYALPLDANGAYRVKVDNPSSSFLVVRKGYAHPEAIIKINNLYLRDEFKYGT

SFMLSRNFFAPADEARYESKAVQEILSGTKSPADFKDKSEYKLLENTVSTIKQTKLKP

YDQLGISYWDQHSENFMRGYSLLVGGRNFFDPNIHKVRSLTYKRTPAVESSWVKLSKL

EHDTFLKIILGAAPIDSFDQFVQEWKEQGGDKVTAEVAASQNHR"

CDS 2846447..2848183

/locus_tag="PIKHDJKN_02607"

/inference="ab initio prediction:Prodigal:002006"

/codon_start=1

/transl_table=11

/product="hypothetical protein"

/translation="MRNNWSIKSKLLTKRLAVLSMSALMVSVLAACGGSSNPPTAEDA

GQYEVTPGDPFSAYKDEVTVTMGRVTTANPKLPAGDTYENNAYTRLVKESFNAQIKDQ

FEANGEDYSRQVSLAIASGELPDMMRVDSKDELKELVDNDLIEDLTEVYKQYATDNIK

KTYDSYDGRALDNATIDGRLMGLPATSLDSAPTMVWVRQDWLDVLGIKLDADGDGAIK

LEDVEKTAEEFLKKDPGQTGKPVGIPFVNTLNTTDYNGSAYTMLGVASTENAFPQYWM

KGEDGSIVYGSTTEETKQMLGIMADWFKRGIIDPQFGTRTFDDITALYTNGQSGIAFG

PWHIPDWGLSNVKQMDKHAKFTAYTLEDADGKVNVAHANPSGQFIVVRKGYEHPELAI

KIINLFYDKLANDKDVETSMPEAAKYLESGVDGSTRPFNIEVNSATSLLDDYSDVVRG

IKGEIGLDQVRTTESKNNIESIQTYLKDQDTDDVTAWSKYHSRMNGVGLIDKLTQESK

FNWMTPAFSGTTPSMKQTWANLNKLEQESFIKIITGAEPLDYFETFVSNWKKQGGDQV

IQEIEAETTSQK"

CDS 2848245..2849159

/gene="yteP_19"

/locus_tag="PIKHDJKN_02608"

/inference="ab initio prediction:Prodigal:002006"

/inference="similar to AA sequence:UniProtKB:C0SPB3"

/codon_start=1

/transl_table=11

/product="putative multiple-sugar transport system

permease YteP"

/db_xref="COG:COG4209"

/translation="MKQENYKARNRLGTGAMYHMMMLPGILFLLVFSYIPMVGVITAF

QDYVPAKGMLGSEFVGLKHFIYMFKLPDIGQVISNTLVIAIAKILLGTLMAIIFSILL

NEIRFKFVKKSVQTIVYLPHFLSWVVLASVVVNMFSLDGSVNQILAVFGLENINFLGS

NTWFQPLIIGTDVWKEFGYSSIVYLAAITSIDPGLYEASGIDGASWWRKVWHITLPGM

LPIILLMGVMSLTNILSAGFDQIYNLYNPVVYESGDILDTYVYRIGLVGRQYSFGTAV

GLFKSVIGIVLLLSANELAKKYTDRKIF"

CDS 2849173..2850066

/locus_tag="PIKHDJKN_02609"

/inference="ab initio prediction:Prodigal:002006"

/codon_start=1

/transl_table=11

/product="hypothetical protein"

/translation="MSYSASLKDRMGRFVIYAIVILLALICLLPLWNIVAISFSSSEA

VSANAVGLLPVKFTTAAYSKIIEDAQFWRSFGISVLRVALSLVLNMILIVLMAYPLSK

SKREFKGRNIYMNVMIFAMLFSGGMIPSYLLIKNLDMLNTIWSLVLPGAVPIFSVILV

MNFFAAVPKALEEAAFIDGANALQVLFKVYVPVSIPALATVSLFSIVGTWNDFFSGLI

YMTKVSNYPLMTYIQSLNVNIAELLQSGTNSAQLSNLTEISNKNLNAAKIVVAVIPLL

LIYPLLQKYFVTGIVVGSVKE"

CDS 2850126..2851748

/gene="malL_2"

/locus_tag="PIKHDJKN_02610"

/EC_number="3.2.1.10"

/inference="ab initio prediction:Prodigal:002006"

/inference="similar to AA sequence:UniProtKB:P29094"

/codon_start=1

/transl_table=11

/product="Oligo-1,6-glucosidase"

/db_xref="COG:COG0366"

/translation="MGNKNWWKEIVVYQIYPRSFQDSNGDGIGDLKGILSRLDYLKDL

GIGAIWLSPVCKSPQDDNGYDISDYQDIDPMFGSLEDMELLIQEAGKRDIRIIMDLVL

NHSSDEHPWFKEARKSKDNPYHDYYVWRDGVEGTPPNDLGSTFGGSAWEWVPELGQYY

LHLFSVKQPDLNWENPKVRQEIYDMIQWWMDKGVGGFRLDVIDLIGKQPDLKITGNGP

NLHQYMRELSTETFQKGDLLTVGETWGATPESAKLYSNPDGSELSMVFQFEHISLDEQ

EGKGKWDLQPLDLLQLKKVLSKWQTELKGEAWNSLFWNNHDLPRIVSRWGNDGEYRVQ

SAKMLATLLHGMQGTPYVYQGEELGMTNVRYPIEEYRDIELLNLYKERVENGYPEEEV

MKSIYAKGRDNARTPMQWDASENAGFTEGEPWILVNPNYAEINAEESINDPDSIYHYY

KTLIQLRKEHEVIIYGDYELLYPEDSNLFAYTRKLDGTKLLVLCNFYGNTVTFDIPEE

FREDKTLLISNYSDVVSENALRPYEARMYLQV"

CDS complement(2851854..2852747)

/gene="cysL_1"

/locus_tag="PIKHDJKN_02611"

/inference="ab initio prediction:Prodigal:002006"

/inference="similar to AA sequence:UniProtKB:P39647"

/codon_start=1

/transl_table=11

/product="HTH-type transcriptional regulator CysL"

/db_xref="COG:COG0583"

/translation="MITDALKVFVTVVEQSHFSKAAELLNLSQPGVSLHIKNLENELG

AKLLHRSPKQVCLTEAGTILYKHAKVIQARYDEARQEIQMLRDEVTGSIQIGASFTIG

EYILPKKLAEFAAQYPQVNIQVTIGNTQEILSAVRGNQLDIGFVEGEAQASADLDVIP

YMKDEMIVVAPSGHPLSGSVVVEQNMLQDQVWVLRELGSGTRAFSDHFMKQTAIRPKR

SYVFTSSQGVKEAVAAGLGIAMVSRWIVHKELATGEVEELRIRQLHLERSFSIIRHKE

HSHSMALDVFLQKLLFVKKEG"

CDS 2852930..2853985

/locus_tag="PIKHDJKN_02612"

/inference="ab initio prediction:Prodigal:002006"

/inference="similar to AA sequence:UniProtKB:P62723"

/note="UPF0324 inner membrane protein YeiH"

/codon_start=1

/transl_table=11

/product="hypothetical protein"

/translation="MILQTQLKFVKNIKVVRNRGFLQGIGLTLLLSFIAKLLGYLPLL

NIMGQLVIAILLGILYRAVRGVPLTAQSGISFSGKRLLRFGIILLGLKLNLLDIVHAG

YKVVALAAINVVVTIFVVYGLSKWLKIDKRIGLLTACGTAICGAAAVVAIAPQIKAKD

EETAVGAATVAILGTIFTLAYTLLYPVLGLSANGYGLFTGATLHEVAHVIAGAAPGGQ

AALDMAVIVKLTRVAMLVPIALIIGLWSGRQARGGEQKLSKGIRWRELPVPWFIFGFL

AMSGLNTLGIIPQEMITGLLVLSYMLLAMAMAGLGLGVDIATFRRLGKKPFLAGLLGS

VVLSIIGFMLVRGFGLN"

CDS 2854659..2858042

/locus_tag="PIKHDJKN_02613"

/inference="ab initio prediction:Prodigal:002006"

/codon_start=1

/transl_table=11

/product="hypothetical protein"

/translation="MRNKYIVWSLVLTMLISNVFLTVGFPSPVSAAERPDLAQGKQVT

ASGYNQTYSPTNVIDSNQATYWESTNSEFPEWIQVDLGTNTNIDQIVLKIPTVWEKRT

QTITVQGSTNGSSFTDIVGSADYVFNPSVGENSVTIDFPAVETRYVRLSVTGNSEWPA

AQLSTFEIYGPGSEGPTLPGPDPVDPPIIPTEGSNIAIGKSITASSSTLSFVAANAND

NNINSYWEGGSNPSSLTLDLGSNHKITSIVLKLNPDPVWSTRTQTIQVLGHNQDTTTF

SNLVSAQSYTFNPASGNTVTIPVAATVKRLQLNITTNSGAPAGQIAEFQVFGTPAPNP

DLTITGMSWSPSSPVENSPITLNAIVKNIGSAASPASSVNFYLNNELAGSSPVAALQA

GASTTVSLNAGNKAAASYTLSAKVDENNQIIEENEGNNNYTHTSSLVVAPITSSDLVG

TVSWSPGTPTANSTVTFTVNLKNQGNMASAGGAHGVTVVLKNAAGATLQTYSGSYTGT

LAPGASVNVNVGTWTAATGNYNVTTTVAVDNNEAPVKQTNNVVTTSLNVYSARGASMP

YTRYDTDDATRGGSATLKSAPTFDQALTASEASGQRYIALPSNGSYAQWTVRQGEGGA

GVTMRFTMPDSADGMGLDGALDVYVNGSKAKTVPLTSYYNWQYFSSDHPGDTPSAGRP

LFRFDEVHWKLDTPLKAGDTIRIQKNNGDNLEYGVDFLEIEPVQAVIPRPANSVSVTD

FGAIANDGKDDLAAFEAAVQSAVSTGKTLYIPEGTFHLGNMWKIGTPTNMINNLTIVG

AGIWHTNIQFTNPNAASGGISFRVQGKLDFSNIYMNSMLRSRYNENAVYKGFMDNFGK

NSKVSNVWVEHFECGFWVGDYAHTPAIIADGLVIENSRIRNNLADGVNFAQGTSNSAV

RNSSVRNNGDDGLAVWTSNVNGAPAGVNNTFSFNTIENNWRAAGIAFFGGSGHKATNN

LIVDTVGGSAIRMNTVFPGYHFQDNTGILFSDTTIINSGTSKDLYNGERGAIDLEASN

DSIKNVTFTNIDILNTQRSAVQFGYGGGFQNIVFNNININGTGLDGIETSRFTTPHKG

AAIYTYTGNGSATFNNLTTSNIANPNVNQIQNGFNLIIQ"

CDS complement(2858166..2860061)

/gene="sqhC"

/locus_tag="PIKHDJKN_02614"

/EC_number="4.2.1.137"

/inference="ab initio prediction:Prodigal:002006"

/inference="similar to AA sequence:UniProtKB:Q796C3"

/codon_start=1

/transl_table=11

/product="Sporulenol synthase"

/db_xref="COG:COG1657"

/translation="MSHVLSAIDEEISRMTTSLLQQQQQDGSWHFCFENGTVIDAYVI

ILFRILEVPNEAFIRELHDRILNEQQQDGCWRLYTDEEDGNLSASVEAYYALLYSGYS

QSTDEPIQRAKQYIHSKGGIGKVSSILTKAILAATGQIKWPLSISMIPLEILMFPTYF

PINYFEFSGYSRVHLTPMLVMADLRFALTVPHAPDLSDLTDLRNVEDESTTRGYQEMQ

DDILSGRSRLLGNPRHIHDTARTKAEQFMIERIESDGTLYSYASSTILMIFALLALKY

DKQHPLITKAITGLTAMQCRSSGGKTTIQNSPSTVWDTALITYALQEASITDDHKAIQ

RAASYLLSRQQDKTADWSIHNPNTVPGGWGFSESNTINPDVDDTTAVLRAIRSLSTTL

PTVQQSWNRGLNWVLSMQNKDGGWPAFEKNTNKEMLTWLAIDGAKSAATDPSEADLTG

RTLEYLGNFAGLDTRHEWIKRGTKWLIRNQEKDGSWYGRWGVCYIYGTWAALTGLTAV

GLPSGHDTVQKGAQWLLSIQNSDGGWGESCQSDRLLHYVPLGESTPSQTAWALDALIA

VQPKPTPEMNQGVLRLIASMHENDWRTSYPTGAGLPGNFYSHYHSYTYIWPLLTLSHY

RRKYGAS"

CDS 2860415..2861926

/locus_tag="PIKHDJKN_02615"

/inference="ab initio prediction:Prodigal:002006"

/codon_start=1

/transl_table=11

/product="hypothetical protein"

/translation="MKIWKKSALVLLSTFLVTAGSYSSYMPKTAHAAGEQVEVWISTS

DPNSEPAVGLRTDARLTKIASKNFSSNSGNADVTITVNENQTYQQMDGFGVSLTDASA

WLMNYKLDNNKRAEVMERLFGNTGIGLSLLRQPIGSSDFAWAAYTYADTPNDTSLNQF

TIDRDKTYILPMVKAAIAKNPNIKVMGSPWSAPAWMKYSNNLNGGKLKAEHYGTYANY

FKKYIEAYQAEGVPIYAVTLQNEPMYEPSHYPSMGMNVQDQTGFIGDYLGPTLRNAGI

NTKIIAFDHNFLDWNFPNQVITNLKNAGKGSYVSGSAFHHYDSGDGSTMTSMNNSHPD

KEVWFTEGGFGNWNDPQNGTSSGFDNMMNEFINITRNWSKSIILWNAALDQKDGPALL

SPNNTNKGMITIRNSDNRNDAPENNVTYHKQYYLLGHFSKFVVPNAYRIDSNTGSEVK

NVAFRNPDGSKVVVAYNSSSSSKNVKIQWGSQSFVVTIPGKSAMTYKWYGNVS"

CDS 2862135..2866223

/locus_tag="PIKHDJKN_02616"

/inference="ab initio prediction:Prodigal:002006"

/codon_start=1

/transl_table=11

/product="hypothetical protein"

/translation="MGEGLDNLKDINQRWVAMILVAVMVIQSFWTFAGIGHAATSENL
NTSNLAKNVVITHFYGGKKDDVLDVYGNDFVELYNPTAEPVSLNDWSIQFADTQKNNW
KVAQLGTTTAQIPAYGYYLIALGSNGNVGEQLPQPDATEASFKLGNNSGKLVLLNTTQ
LLTANDPTADVLQEHVIDFVGYSADAYWGSPAPEAGKQSTMQRLVYDPLNPEKSTTTA
AFPNHGNNWDTRNNGNDFEKVKGAKARNSSTTSAYVILESNQRLEMQSASSVDPVHNK
ITLNAFGGLVKQGPWSSDDFSVLGLPAGFTAEANANGDQIEVTITGDGTGNMVSDSLL
TFEIHPGAWDQLHKPASSLTVYQGSNTVTFAQFTPSNKIVATLDSSSSSIHMDSAKDL
NTTIQLNLSAGVPVDGVLDSNAYIVTGLPEGDWSIKAEGRSSDHTITLSISGTSASAV
MNSVMLNVTLMAEAVQDKDWLASEMISVPLLRYSQPLLADENRKHVVEDSIIADNTGF
NDPATKQYKYGSDAMGANEYTFLRGTHSLFKADLAAGLIPSPNSVIPGWNEKDILTYT
QGDAHIQNVGTFNDSTNQMVFSLNDVDSSGIGSFYDDLIRFVTSIYVVKYEKDSSAIT
NLQDADFRDVSRTFLDTYKDTLIEINNEINPKNTKLTKSNVTAYTKEVMDKVSKVSYE
EALQKLLGKRAIDGKLNIASNSDKFEAATDAEKSSLQAAWEAYKLQVRSNFPNLSDDE
FDAYFKIKDIVRRIHQGIGSIGVERYNVLIEGASEANTDDILLDVKEQTRNANLSKDA
YLKTSVDPDSYLGVLEAMNRSYLIKEVSPFKGDYTDKPFKNKQELEQYLVDAAKAYAY
ASSRLDKVSDELNYRFEERFVTNIIPIWDDLQNFILNAAEDYSHQVVADFVLVKEDMR
TGKLIDVSTLDNLSVDQGSLTPAFNLAITAYTLNVEQATDSISITAKATDSQATLSVK
GEPYANGINQSFGLSVGKNVIPITVQARNGSSKIYSINVVRAGDSSSVPPTSSTPSTP
TGGTNGDQKGTTANSGTITVNGGSLSLDGVKINMPAGAMESSIVISVNKVSQTSNLFT
DSTLILLGDVYEITASKSGNFTKPVTVTLPFDKNKVDFDKSIVGLYWFNEQAQKWVSL
DNLKVDQMNGTVSGTINHFTKFAVLVSDKTVTSTPEGTKNFTDLVGHWASGSIMDLVE
LGVIQGYPDNSFRADKKITRAEFVTLIIKALHLQDSADKGFADTTTHWAQDAISTAAG
LGIVNGYSDNTFGPDELVTREQIATMVVRAAQIADSDSNISFSDGAEISGWARSALAA
AITNGLVNGYPDGTLKPKGNTTRAEAATLIERTIQLTKQLTK"

CDS complement(2866326..2866784)

/locus_tag="PIKHDJKN_02617"

/inference="ab initio prediction:Prodigal:002006"

/codon_start=1

/transl_table=11

/product="hypothetical protein"

/translation="MANVQTFKATAHLQDGVKVITKARQFELIIDEPTNLGGTDTGMN
PVEALLASLGACQSIVARVYASKFDVVLDDFRVDVEGDLDLDGFFNRSDVRPGYSDIR

YTFYIKTSSSEEKVESFVQFLESKCPVGDTISNPVNTKLNRVIIENGISL"

CDS complement(2866967..2867884)

/gene="rhaS_15"

/locus_tag="PIKHDJKN_02618"

/inference="ab initio prediction:Prodigal:002006"

/inference="protein motif:HAMAP:MF_01534"

/codon_start=1

/transl_table=11

/product="HTH-type transcriptional activator RhaS"

/translation="MSDNAVQQKQELTELIKRHSLHNSSKETEIPSLYVYQHSSISEP

AYRVYKPSFCVIVQGLKEILLAQERFEYGPSNYLIASMNLPVIGQIIKASTDTPYLSL

KLEFTQNQILEVLNDCNIKVTSKENARRALFVGQMEPSIQDAVLRLVRLLDTPGEIPF

LAPLYTREILYRLLQGPYGAELAQIAVEGSSSYRIREAIEHIVRHWEQPFRIEDLAET

ANMSVSSFHRHFKEITAMSPLQFQKQLRLQEARRLLMAESADAADVAFRVGYESASQF

SREYSRMFGAPPRADIKRLKEKYDHVMSE"

CDS 2868129..2868716

/locus_tag="PIKHDJKN_02619"

/inference="ab initio prediction:Prodigal:002006"

/codon_start=1

/transl_table=11

/product="hypothetical protein"

/translation="MSHVLILGANGQIAREAIDLFLKETDVRLTLYLRKSSRLGNIDS

NRARVIDGDVMDQVKLKDAMNGVDVVYVNLAGDLEQYARTIVETMKSAGVKRLIFISS

MGIYDEVPGQKYSSILDPYRKAASIVEASDLDYTILRPAWFTRANEIDYEITQKGQPF

KGSEVSCKSVAALVVKLTESPELEIRRSLGVSKPH"

CDS 2868799..2869788

/gene="iolS_2"

/locus_tag="PIKHDJKN_02620"

/EC_number="1.1.1.-"

/inference="ab initio prediction:Prodigal:002006"

/inference="similar to AA sequence:UniProtKB:P46336"

/codon_start=1

/transl_table=11

/product="Aldo-keto reductase IolS"

/db_xref="COG:COG0667"

/translation="MMQKRTLGNSGLEVSAIGLGCMGMSYGYGPASDKTEMIAVIREA

VERGVTFFDTAEVYGPYINEELVGEALSPVRDQVVIATKFGFDIQGGKQSGMDSRPEQ

IRKVAEESLKRLKIEAIDLFYQHRVDPDVPIEEVAGAVQDLIREGKVKHWGLSEAGVN

TIRRAHAVQPVAAVQSEYSLWWRRPEEELIPALEELGIGFVPFSPLGKGYLTGSFNAK

TTFDQGDLRNILPRFTPEALEANQVLVDLLKEKAEKIQATPAQIALAWLLAQKPWIVP

IPGTRKSSRLKENMGAADIELTPEDVQSINDAATKITLMGSRYTEELEKRTGL"

CDS 2870048..2871025

/gene="yajO_2"

/locus_tag="PIKHDJKN_02621"

/EC_number="1.1.-.-"

/inference="ab initio prediction:Prodigal:002006"

/inference="similar to AA sequence:UniProtKB:P77735"

/codon_start=1

/transl_table=11

/product="1-deoxyxylulose-5-phosphate synthase YajO"

/db_xref="COG:COG0667"

/translation="MEYVKLGRTGLDVSRLCLGCMSYGVPERGIAPWSLNEEESRPFI

QKALDLGINFFDTANVYSDGTSEEIVGRALKDFARRDEVVIATKVYFRMKPGPNGAGL

SRKAIMNEIDNSLKRLGTDYVDLYQIHRWDSSTPIEETMEALHDVVKAGKARYIGASS

MYAWQFMKAQHTAERNGWTPFVSMQNLLNLMYREEEREMLPLCREEGVGVIPWSPLAK

GRLTRDWDEKTARSSNDEAGDRLAAEMVDADRKIVGTVADIANKRGVSRATVALAWVL

QKEGVTAPIVGATKEHHLEDAVAALSVKLDAQEVASLEEYYVPHKIYGM"

CDS 2871301..2871642

/gene="gdnC_1"

/locus_tag="PIKHDJKN_02622"

/inference="ab initio prediction:Prodigal:002006"

/inference="similar to AA sequence:UniProtKB:P49856"

/codon_start=1

/transl_table=11

/product="putative guanidinium efflux system subunit GdnC"

/db_xref="COG:COG2076"

/translation="MNKTWLSVVIAALFEVGWVIGLKHASGFLEWGATLIAIIVSFTL

MIAASRSLDVGTVYAVFVGLGTAGTVLAEIILFDAPIQSGKMLLIGVLLLGVIGLKML

SKGKTKEVHES"

CDS 2871639..2871953

/gene="gdnD_1"

/locus_tag="PIKHDJKN_02623"

/inference="ab initio prediction:Prodigal:002006"

/inference="similar to AA sequence:UniProtKB:P49857"

/codon_start=1

/transl_table=11

/product="putative guanidinium efflux system subunit GdnD"

/db_xref="COG:COG2076"

/translation="MNWVFLIFAGVFEMIGVLMINKLHKDRNLMSIILLVAGFGLSFL

FLSLAMKTLPMGTAYAVWTGIGASGGAILGMIFYGEPRNALRILFIAMVLGSAVGLKL

VS"

CDS 2872085..2873464

/gene="rhlE"

/locus_tag="PIKHDJKN_02624"

/EC_number="3.6.4.13"

/inference="ab initio prediction:Prodigal:002006"

/inference="similar to AA sequence:UniProtKB:P25888"

/codon_start=1

/transl_table=11

/product="ATP-dependent RNA helicase RhlE"

/db_xref="COG:COG0513"

/translation="MTFKDLNIIPAILEGLSKANYTAPTPIQEQAIPAVLAGRDLLGC

AQTGTGKTAAFSVPIIQLLSENSNGPKAARHIRSLILTPTRELAIQISDNIKAYSRFT

DIRCAAIVGGVSQKVQERALNQGADIIIATPGRLIDLVNQKRIDLKHVQILVLDEADR

MLDMGFIHDVKRIIAKMPSKKQTLFFSATMPPEITKMVKTLLVDPVKVEITPVSSTVD

RIEQSVYLLENGKKQSLLNHILQDKSIVSALVFTRTKRGADRVTRDLSKVNITAQAIH

GNKSQNERQRALNNFKSGVTRVLVATDIAARGIDVEELSHVINFNLPNIPETYVHRIG

RTGRAGHSGMAISFCEKEELPFLKDIEKVIKKTIPEVKNHPYPMTSVPAFVKSNQTSK

SAPHKSAADKPAKSKANPARKPKSEWFKKSGKANHSKPNSSKPNSSKPNHNSTSHSKP

KSSKVNKAL"

CDS 2873641..2873970

/gene="hmoA"

/locus_tag="PIKHDJKN_02625"

/EC_number="1.14.14.18"

/inference="ab initio prediction:Prodigal:002006"

/inference="similar to AA sequence:UniProtKB:O31534"

/codon_start=1

/transl_table=11

/product="Heme-degrading monooxygenase HmoA"

/db_xref="COG:COG2329"

/translation="MFIQTRVMVVEKGHSHKLVERFSAPSPVEEMSGLIDFSVMVNKK

SKEHEEVMVLIRWESEEAWKNWEKSDVHIKGHKEKRGQEKPEYLISTTVNMYEVQKVK

TARVSNA"

CDS 2874169..2874945

/gene="tam_2"

/locus_tag="PIKHDJKN_02626"

/EC_number="2.1.1.144"

/inference="ab initio prediction:Prodigal:002006"

/inference="protein motif:HAMAP:MF_00560"

/codon_start=1

/transl_table=11

/product="Trans-aconitate 2-methyltransferase"

/translation="MSGEQFKNEHTSVPYIHSTDFLSNLGVDVLSLLQPQTGERILDI

GSGNGDMTAKIAAAGGIPTGIDLSERMVKLAQQKYPDLNVQVKDASQYRTNIPFDAVF

SHAAIHWINNPAALTRSIWLALREGGRFVAEFAGSGNVAILTTAMKQALEEHGYTWAG

RNPWYHPTLGEYATLLEQNGFRVTFAQHFDKPAPLKGEEGLRNWLNSFADYFFKDVSS

ADMESIYQAIVTEVKPHFEREGQWFLDTTRLRIVAIKEPV"

CDS 2875070..2875420

/gene="yjbR"

/locus_tag="PIKHDJKN_02627"

/inference="ab initio prediction:Prodigal:002006"

/inference="similar to AA sequence:UniProtKB:P0AF50"

/codon_start=1

/transl_table=11

/product="putative protein YjbR"

/db_xref="COG:COG2315"

/translation="MMDKIIEYCLKKKGATKEYPFGPDPVVIKIAGKMFALMFEDKAD

NVRLNLKCDPIIAENLREQHESVQPGYHMNKKHWNTIILDGSLSEQDIFVMIDHSYDL

VVKTLSKSLLESIS"

CDS complement(2875578..2876126)

/locus_tag="PIKHDJKN_02628"

/inference="ab initio prediction:Prodigal:002006"

/codon_start=1

/transl_table=11

/product="hypothetical protein"

/translation="MTKVDRRIRKSQEAIKAAVIELMSEKNFDDITLQDISDRADVSR

GTIYLHYVDKYDLLDKLIDGHIKELSEICHSVADLEWKESIIPWFTYLEQNYLFFSTM

MASKGAVQFRNRLVEFLNEDFKEEVHLSQARKSGLSEDIILQYVVMSYVGVVEWWIKN

EMPHPPEVMSEQLGELIKMNLG"

CDS 2876331..2877506

/gene="cntE_1"

/locus_tag="PIKHDJKN_02629"

/inference="ab initio prediction:Prodigal:002006"

/inference="similar to AA sequence:UniProtKB:A0A0H3JTK0"

/codon_start=1

/transl_table=11

/product="Staphylopine export protein"

/translation="MQRLWTKSFILMTLGLLFLFTAFYMLYPTLPLFIKEMGGNEKQV

GLAMGALMLSSVILRPFVGGLLDRFGRRPFMIGGLLLFILAMYMYNWVGGIIMLIGLR

IVHGMSWAVSTTSMMTAVTDMIPSARRGEGMGWFSTSMTLAMAVGPMIGLGIMQGTSY

QTMFLFAVGLSVVALLLTLGAKMPFQPHTDRKKIQIFEKSVLPVMAPIFFLFIAYGGI

TTFVPLFAEQIKVNSGTFFLVYAATLALSRPIAGKLSDKKGETAVIVPALIITIIALI

VLSFATSLLGVLASAVLYGIGFGSAQPALQAITLRLAPEDRNGAANAFFSTATDLGIG

LGAMVLGLVSEYMSYHILFVVSALSVMVSFLLFMIYVRRLLKIKQTLPGKTSDSVSL"

CDS 2877593..2878063

/locus_tag="PIKHDJKN_02630"

/inference="ab initio prediction:Prodigal:002006"

/codon_start=1

/transl_table=11

/product="hypothetical protein"

/translation="MFIREIEEKDNQEVESLIRSCLIEFGANKSGTAWADPNLGDFYH

LYQREGSRYWVVEHNSTVVAGCGIGPVEGFSHICELQKMYALKEARGTGISYELLRIS

LEFAKEHYEQCYLETLNNMVAANKFYIKNGFIPLDEPLNATEHFACDAWYIKTL"

CDS complement(2878154..2878750)

/locus_tag="PIKHDJKN_02631"

/inference="ab initio prediction:Prodigal:002006"

/codon_start=1

/transl_table=11

/product="hypothetical protein"

/translation="MDKTNTTSRSERRDAAENRQRILNMASKLFELHGVERVSMNQIA

SEAQIGAGTLYRRYRNKSELCMDLIKDNAALFFDDVEIYVQENAQHPPSERLRGLLTL

FIQFREKNAELLSGIENAVYQGSESRTSSPLYDQLHQQLFGLFEEMATESDESQAISL

FKTDMLLTAMSNDSYLFQKNVRGNSPDRIVEQLCLTFL"

CDS 2878880..2879461

/gene="rutB_2"

/locus_tag="PIKHDJKN_02632"

/EC_number="3.5.1.110"

/inference="ab initio prediction:Prodigal:002006"

/inference="protein motif:HAMAP:MF_00830"

/codon_start=1

/transl_table=11

/product="Peroxyureidoacrylate/ureidoacrylate

amidohydrolase RutB"

/translation="MESHHNQSALLVMDMQNIIVSQYAKHEDALFPFQKAIEAARRHN

VPVIFVKVGHRQDSPPISERNKLFGKKSSRGGGNEANPAMKVAMDIHESVKPLPGELI

VDKFRVSGFSGSNLENLLQSHGINTLILSGIATSGVVLSTLCEAADKDYALTVLSDAC

LDRDPEVHRVLVESVFPQQADVLAVDDWINALI"

CDS 2879497..2880759

/gene="pksS_1"

/locus_tag="PIKHDJKN_02633"

/EC_number="1.14.-.-"

/inference="ab initio prediction:Prodigal:002006"

/inference="similar to AA sequence:UniProtKB:O31785"

/codon_start=1

/transl_table=11

/product="Polyketide biosynthesis cytochrome P450 PksS"

/db_xref="COG:COG2124"

/translation="MSELDLNVSQKAALSLFSGDSGENPFSLIKQLRETGPVIPVSLP

MGGTDRGQWLVTRMEEASQVLKDHAHFTVDPLSIDGNENILKAYVENVDPDAPPTFFT

GKSMLSVDDPDHRRLRRLVSKAFTPKYMESLRPRVQQIADELLDQVQSQVEMDLVKDY

AFPLPIHVISDMLGVPKADRPQIQTWSSAIAHGLGLGVREPGVEEHLKAFGDYTAQLV

AEKRKHPADDLISQLIAIEEEGDRLSEPELLSMITLLIFAGHETTSNLIATGAMMLLD

HPEQLEKLKADLSLAPSAVEELLRFNGPATIAGPRFATQDIELAGQQIKKGDMVIPVL

KSANRDELQFEQSEDLDVARHINRHLAFGHGIHSCLGAPLARVEGDIAFTTLLKRMPN

LRLNVPRENIEWHLALSSQSLAALPVAF"

CDS 2881308..2882237

/locus_tag="PIKHDJKN_02634"

/inference="ab initio prediction:Prodigal:002006"

/codon_start=1

/transl_table=11

/product="hypothetical protein"

/translation="MLELSFDNPNELVMVTHALSTRSRVDILRLLISQNLNIVEIAEA

LKLPVSTVASNIKVLEAARLINTELLPASRGAMKVCSRNYDDIHIALNLEKAIPKGDI

QVYEVDMPIGHYSDCEVSPTCGMANSEGMIIREDEPASFYHPKHVGAQIMWFRKGYVE

YLMPLEIPAEAVIESLELSMEMCSEAPNYDHNWPSDISVWINGVEIGMWTCPGDFGDR

RGKLNPAWWFEWSTQYGLLKTWRVDKNKTTLDMEKISAVDLAQLNLHNSHKLRVRIGI

KPDAVHQGGVNLFGRQFGDYDQNIKMTIHYAIH"

CDS 2882436..2883728

/locus_tag="PIKHDJKN_02635"

/inference="ab initio prediction:Prodigal:002006"

/codon_start=1

/transl_table=11

/product="hypothetical protein"

/translation="MKKKIGWLALTLLLSFSTVLSACSGGSNGDGDSGSGDGKVTLSF

WTLFDGGDGANMQTLVDEFNKTHPEIEVKNTKLAWGEYYTKLVTAVGNGNGPDIGISH

SSRLPDLINQGVVTELDTPATDAGVDWSTYNQNLLDAVTVEGQHYAVPIDTHPFIMFY

NKKLLKDAGLLGEDGKPILEQSADGFVSFLKTLKEKLPAKTTPFALSNNNDDPYRLWW

SLYSQLGGNDVVSDDLKSAAIDKEKGIKAADYIQKLYTEGYIKKNDPDFYKNFQSGTA

AIIMTGVWTTGTWESTKGLEFGAMPIPKFYDKEATWGDSHTIVLPLTKDEDPAKRKAA

MIFADWVADNGQVWAKAGHIPSKPSVLEKQEFKDLPYRSDYSEVASVVKFSKHSTKNA

QIRDEAAFKFLNEVWMNKMTPADAMNNMETAIQKILSE"

CDS 2883816..2884706

/gene="melD_2"

/locus_tag="PIKHDJKN_02636"

/inference="ab initio prediction:Prodigal:002006"

/inference="similar to AA sequence:UniProtKB:O34706"

/codon_start=1

/transl_table=11

/product="Melibiose/raffinose/stachyose import permease

protein MelD"

/db_xref="COG:COG1175"

/translation="MKIRKMRADLQALVFLLPFLIVYGLFTIWPMIKGVEMTFYKWTL

IKKMDFVGLDNFRKVLQDREVWEAIWHSTFFVFLSTPTMIILAILLALIANRKSSLQK

FYRIIFFIPSVLSVAVAAYLGLFVFQPYTGFVNSVLHLVRLLPQNSEIFWLTETGLAW

VVITVITLWWTVGFNFILYLSAMQEIPDEIYEAARLDGASDTQIFWRIILPYLNPLTK

TITMLQIIASYKVFMQIYVITGGGPLDQTRPIIQLIYQTGFKNNNLGYAATMSYILFV

ILLVLSALQYWMNNRKGAGN"

CDS 2884706..2885512

/gene="araQ_16"

/locus_tag="PIKHDJKN_02637"

/inference="ab initio prediction:Prodigal:002006"

/inference="similar to AA sequence:UniProtKB:P94530"

/codon_start=1

/transl_table=11

/product="L-arabinose transport system permease protein

AraQ"

/db_xref="COG:COG0395"

/translation="MKWLYRIGSALMAVMFAAPLVWMLMVSIKEEGMKIITVLDWFKP

PYSLAVYNEILTTTKLSQWVVNSLFVAVVVTVLTVIFAAMAGFALSKVPFRFRTFIFF

FILGGLLIPGEATIIPLYQVAKDLHLLNSYSGLIIPALASPVAVIVLKSFFDGIPNDL

LESVQIDGGGFWRIFTSIVLPLTRPAMASMAILTFIGSWNNFLWPFLSITDDNLFTLP

MGIPTLMGQYSEDYVKPMVINAVASVPIIILFIIFEKQIVKGISMSGIKG"

CDS 2885563..2887329

/gene="uidA_1"

/locus_tag="PIKHDJKN_02638"

/EC_number="3.2.1.31"

/inference="ab initio prediction:Prodigal:002006"

/inference="similar to AA sequence:UniProtKB:P05804"

/codon_start=1

/transl_table=11

/product="Beta-glucuronidase"

/db_xref="COG:COG3250"

/translation="MTTKFYNKDYPRPQFVRNEWLDLNGEWDFSFDDNQAGESEHWYD

QNQFPEGTKIKVPFTYETKASGIGIETFHPLVWYRRSVQIPQEAKGKRSILHFQGVDY

RAKCWVNGTVVGEHEGGYAAFSFDITPYITYGSENNIVLEVEDSQNAMQPRGKQRWVD

DNFECFYVQSTGIWKSVWLEHVSEARVEAVKMTPDIDRHMIRLDFQLNGIEGKNNLRL

ETRIELKGQHVQTISLSPDRPWMTVEASVKHEAGGPWKQSLWSPDSPNLYDIEFVMYE

DEKEIDRVHSYFGMRKISIENGQILLNNAPLYQRMILDQGYWTESHLTPPSVEALIED

IDKIAEMGYNGIRKHMKLEDPRFLYWCDVKGMLVWSEMAATFEFNDEAVSRFTKEWLE

IVPQQYNHPSIITWVPFNESWGIPTIGHEVRQQQFTQSIYHLTKAIDPYRPVITNDGW

EHTVSDILTLHDYVESGEAFLERYQDKDLIVNNKIASNRWKFAFAEGYHYKGQPIIIS

EFGGIAFQSDKGWGYGNQVDSVEAFIERFRSITEAIKAIPYISGYCYTQVTDVQQEIN

GLLTEDRKPKVPLEVIRKINLG"

CDS 2887379..2889163

/gene="uidA_2"

/locus_tag="PIKHDJKN_02639"

/EC_number="3.2.1.31"

/inference="ab initio prediction:Prodigal:002006"

/inference="similar to AA sequence:UniProtKB:P05804"

/codon_start=1

/transl_table=11

/product="Beta-glucuronidase"

/db_xref="COG:COG3250"

/translation="MLRQDYPRPQFVRKDWLSLNGEWEFEFDDDNKGRSEKWSEGKQA

FSRRIQVPFAFQSQLSGIADPNFHDNVWYKRTFDTPENFTGKRLLLHFGAVDYEASVW

VNGQFVVRHEGGHTPFHADITDALLEGSNVLTVQAIDYSRDVTLPRGKQFWQEQSAQI

FYTRTTGIWQSVWLEAVAPVHLQKVRLTPDVDRNEIEFTAHLHREQTEPRDWDAQDVR

LRVQIAYQGELLSEDEYRISGRTETRTIGLHTFNEHGLGRLWAPEHPNLYDIHFTVLV

DGVPVDEVDSYFGMRKISIEQGKLFLNNRPYYMKLVLDQGYFPDGNLTPPSDEAIRRD

VELTKELGFNGARKHQKIEDPRYLYWCDKLGLLVWGEMANAYDFSEQYVAQVTREWQE

ALERDYNHPCVVVWVPLNESWGVPNILTSKEQQYHALSMYYLTKSLDSTRPVISNDGW

EMVQTDLYNIHDYEWRREVLEERYRTRENAVNALPGSRKLSVGNYRYEGQPILITEFG

GIGYKKSDWEGWGYSGAENDEDFTQRLHAVVQPLLDSPVVEGFCYTQLTDVEQEINGL

LTYDRQPKMPIEQIRAIIEGHYPSSAGE"

CDS 2889176..2890186

/gene="galM_1"

/locus_tag="PIKHDJKN_02640"

/EC_number="5.1.3.3"

/inference="ab initio prediction:Prodigal:002006"

/inference="similar to AA sequence:UniProtKB:P39840"

/codon_start=1

/transl_table=11

/product="Aldose 1-epimerase"

/db_xref="COG:COG2017"

/translation="MLNTKETSRSWEGDFQGEKAIYLRFGSSEAVMLPEIGGNLVAYR

DLERGLRFLHEPSPEEMESFKARPMIHGIPVLFPPNRYEDGRFAWNGQKLQFPVNEEA

TGNHLHGFLYNTSWVVDEYGHTEKESHVTVSITVDETHPVYVSFPFQFSITLRYSLSS

DGLMQHVRIRNHGATCMPCLLAFHTSINAPFTADGSARDYSLKLTIGDRWELDERMLP

TTRFQPLVPAEEQMRDSGVYPFFAEMDNHYTAVPQNGRNRMELQDTKNGMTLIYDVGT

SYKQWMIWNNNAEEGFFCPEPQINLVNAPNLDMQAEEAGLFSLQPGEIWEETSRLYLR

HS"

CDS complement(2890290..2890820)

/gene="chrA"

/locus_tag="PIKHDJKN_02641"

/inference="ab initio prediction:Prodigal:002006"

/inference="similar to AA sequence:UniProtKB:P14285"

/codon_start=1

/transl_table=11

/product="Chromate transport protein"

/translation="MYWDLFMMFIRVGLVSFGGGYAVIPIIQHEVTEKGWLTSAEFQQ

TVALAGMAPGSIATNAATLIGYRTAGYIGAAVSTAGMIFPSLVVIVLLSALFYRIHDN

SWVKSSLYGLRPVTTGLIIYAAIHFGYPSEDAGISWVWIGTLLISGVCLFLLLKYKLH

PLLILTVAGAAGIILF"

CDS complement(2890845..2891378)

/locus_tag="PIKHDJKN_02642"

/inference="ab initio prediction:Prodigal:002006"

/codon_start=1

/transl_table=11

/product="hypothetical protein"

/translation="MLPIIEREVVNKKKWMQEDEIGELISLAGSAPGGVGVNAAAMIG

HRQAGIAGAVSAVIGVTCPTFLIVILISVFAILFRNQPKVEAALKGMNGGIIALISMA

AYRTARYSILDTTTAIAAIVTVAVLLFAGIHPLYLIISGLICGMAVIRIKKQLGLQVK

MEKKSVTGSYPELEYYI"

CDS complement(2891491..2893029)

/locus_tag="PIKHDJKN_02643"

/EC_number="3.1.6.1"

/inference="ab initio prediction:Prodigal:002006"

/inference="similar to AA sequence:UniProtKB:Q0TUK6"

/codon_start=1

/transl_table=11

/product="Arylsulfatase"

/db_xref="COG:COG3119"

/translation="MSRTPNILLITSDQQHWNTIGAFQSEISTPNLDRLARQGTTFDR

AYCPNPTCTPSRASIITGMYPSQHGAWTLGTKLLEDRHTVGDSFKEAGYRTALVGKAH

FQPLKSTEAYPSAEAYPILQDLEYWRHFHGPFYGFDHVELTRNHTNEAHVGQHYALWL

EEQGCTNWRDYFLPPTGTMDPSVTYTWPIPEKYHYNTWIAERTNSLLEQYTHADDPFF

LWASFFDPHPEYLVPEPWDSMYDPDDLTIPSITPGEHDRNPPHFQLTQEENPNFEHLV

ETGFGIHGYRSHHYYEYGDKSRLTAYDKKKLVGVYYGMISMMDKYIGKILDKLDELGI

ADDTIIVFTTDHGHFFGQHGLQAKGGFHYEDLIKIPFIVRYPGQVPAAKKSNALQSLV

DLAPTFLSLCNIPIPHAMTGMDQKKVWLGAKEAVRNHAICEFRHEPTTIHQKTYIEQR

YKITVYYNQTYGELFDLEKDPDERFNLWNEPEYTHLKADLLLKYIWAELGKESMPMPR

IHHA"

CDS 2893153..2894007

/gene="rhaS_16"

/locus_tag="PIKHDJKN_02644"

/inference="ab initio prediction:Prodigal:002006"

/inference="protein motif:HAMAP:MF_01534"

/codon_start=1

/transl_table=11

/product="HTH-type transcriptional activator RhaS"

/translation="MQEYEYEYAEFIYYKPGYLDKEEHIWPVRAGRSIAKSNYRVGPK

RIECYSLHFVHEGMVRFEFDGKRVDLQKNDLFCLFPGRTYYYHMLPSESPLQMNWLAL

DGVKVKPLLELAGLTPESPFGKQMLSPQVQETSERLIHALACVERWKPAVSLELHGLL

YGLLASLVPDTNYAQPTELAGWIHECMDYMELHATEGISVQQVADFAGVHRSYFSNMF

TTQVGLPPLKFMQRIRMDKAKQLLKDTDATVTEIALSLGYPNLYSFTRAFKIYYKVPP

IMMRRTSS"

CDS 2894083..2895882

/locus_tag="PIKHDJKN_02645"

/inference="ab initio prediction:Prodigal:002006"

/codon_start=1

/transl_table=11

/product="hypothetical protein"

/translation="MKLSTLQRWISPKRSIQGKIFIAFIAVTLVSIILITVIVYMSMR

ETITQNAITSVSDSIRQADESLNMMLEEIDRLNTVVVTNKNTVIDTLLSPNEEISYEW

FQEQKRMDEFLSSLIDYKAYISRIAVVGLNGKVFFSGGPWLDRTILGTPMMNYMLQNG

SRHAYFKQTGVSDAITIGREIRYNRASIGVVMVDLNYEFIQKTYDVRPTTDSMIYVLD

KGNQFVYETRATSSLTPSYQQIKEINQQFKTDGEAVRRYIDGKEYIVVCRQSDYTGWS

TISLVPMDSLLTESVKLRKLLAEVSIIVFVVVLIVSLQVSSRITLNIRKLKSLMMLVK

DGNLTLPQNEIKSEDETGQLYHVFNGMVEELKGLLEGIRVSEKEKREAELTALQAQIH

PHFLYNSLNTIKYLARLNGVPNIEAVSGSLIELMRSVLGNSSEFLTVREELAYVEHYI

SIMKYRYMKPIRMITEIEDDVLLDCRVLKLMLQPLVENAIIHGIGPLEQEGFVLIRVY

EEGKNLNIDVVDNGKGITEEQREYLLKGTGTNGKTSRFSGMGVRNVHERIVRTYGDSY

GVYLCSEPGLYTKAEIRLPKLEKSMDNTKQEVI"

CDS 2895886..2897577

/gene="cheB_6"

/locus_tag="PIKHDJKN_02646"

/EC_number="3.5.1.44"

/inference="ab initio prediction:Prodigal:002006"

/inference="protein motif:HAMAP:MF_00099"

/codon_start=1

/transl_table=11

/product="Protein-glutamate

methylesterase/protein-glutamine glutaminase"

/translation="MFQVLLVDDEPLVRHNLWTLLDWADYGFELCGQAHNGLIALTMI

EQSPPHIVIIDVDMPEMNGVELNRAINERFPCVKTIMLSSYDDYDYVRDCLNNGSIDY

LLKHRLDESTLLNILNKAVSELQQEDRLREKQVAESAIAEKVSPEVMRDYLLYLLKRQ

TKTTYGFEDFSQKDNLYPHASRYATAALQIVPFLLLTESYSDVRTNGLVQQVVDMMQQ
SLGDIHERTAVYVEEGRFVIVFSFRERSEHIMQREVDGQLRKIQHALELFLNLQSVTA
VGPICTSLSQLASSYCSAEYELDGSSSVKTFRSHTLQIETPCSDKHSHENDQSQRVSL
TIEEHKQLLLSIERFDEQGVHQLISSIIASIDHLPIHSHTVQMIVSELMQTGDKAWRT
CLPSTDPAVANLPSRSELGRMKNVRELEQGLQSFFSSLFHLLNQHRVIGSYSRHVTQT
IHFILERYPGYVTLELAANVIGLNASYLSRIFKEETQRTFSEYLNQIRIDAGCKLLDS
GRYSIKEISMQVGFTTHNYFFKVFKEITGLTPQAYLSNPGKRKNDTKLPSLHLGN"

CDS 2897736..2899304

/gene="lipO_6"

/locus_tag="PIKHDJKN_02647"

/inference="ab initio prediction:Prodigal:002006"

/inference="similar to AA sequence:UniProtKB:P37966"

/codon_start=1

/transl_table=11

/product="Lipoprotein LipO"

/db_xref="COG:COG1653"

/translation="MRKRWGRNMIMMMIASSLMLAGCNSGGSNNEASPEVSKNDVHTT
GFPIVDEPVNLKMFTRIAPVNGPFKEMPVFQDYEKLSHVKVEFIEAQTDGFQEKKNLL
FASNELPDALFRSGISPLEAIRYGSAGQLIPLEGLIDEYAPNLKKLMEQYPEIRSGIT
TPEGHIYTIPGIVTLSAARTDKKWINQMWLDKLNLKVPETTEELYNVLLAFRDGDPNG
NGKQDELPMTARVGLAVVNAMSGSFGLDSQLGYNINIENNKVSIWMGSDQNKEMLMYL
NKLYKEKLLDQEIFSHTEAQYLAKQGSGNTGFFFDQTNNNFLSIKDQYTGIAPLEGPQ
GDRLQSQALPIARDFGAFAITSVNKYPEVTMRWIDYFYGDEGSTLLRFGREGEHYEMK
DGIPYYKEDFLKEISNQAKITPYAGGGAPHLISEQVASFINPPQVQEAQQKLDPFMPK
VRYAAPMFDEQTAQEVNILRNDIDKYYEEQSTKFIVGAIGFDKWEQFQTTLKKMRVEE
LEKLYQDAFDKMQK"

CDS 2899338..2900300

/gene="yteP_20"

/locus_tag="PIKHDJKN_02648"

/inference="ab initio prediction:Prodigal:002006"

/inference="similar to AA sequence:UniProtKB:C0SPB3"

/codon_start=1

/transl_table=11

/product="putative multiple-sugar transport system

permease YteP"

/db_xref="COG:COG4209"

/translation="MKSVKTETTITMPRSHPKKSSGLLRQMGKRLDLYLMLLLPVTWY

VVFHYAPLYGLQIAFKNFNPAKGILGSNWEGFGHFERFFDSYYFWRLLWNTLSINLFS

LLIAFPIPILLALIINEIRNKTFSKWLQNITYIPHFISVVVIVGILNVFLSPHTGPVN

LLIEAFGGTPIRFLEEAGWFKTIFISSNIWQNMGWQSIIYIAALSGVNPHLYEAAKMD

GASRLRRIWHISLPGIAPVVIILLILDIGHFMNVGFEKILLMQNNLNLESSDVISTFV

YTTGILKGEYSYTAAIGLFNSIINLLLLLLVNRIARKTSETSLW"

CDS 2900316..2901209

/gene="araQ_17"

/locus_tag="PIKHDJKN_02649"

/inference="ab initio prediction:Prodigal:002006"

/inference="similar to AA sequence:UniProtKB:P94530"

/codon_start=1

/transl_table=11

/product="L-arabinose transport system permease protein

AraQ"

/db_xref="COG:COG0395"

/translation="MSKQATLQASSNERIFDVIIYIIAAIIIIAVLYPLIFIVSASFS

DPTKVLNGEVWLLPKGLTLDAYTNILHNEKIWLGYRNTIFYTVVGTVINIIMTVLAAY

PLSRPDLPGRNVIMVFITLTMFFSGGLIPTYLLIKNLGMVDTMWALIIPGAIATYNLI

VMRTYFQSSIPWELQEAAHMDGCSNWRLLWSIILPLSKPILAVMVLFYAVGHWNSFFN

ALIYIRNENLHPLQLVLREILLISQSDAVDGSLGLEDKILLAESIKYAVIIVSSLPVL

LMYPFVQRHFVKGVMIGSIKG"

CDS 2901240..2903282

/locus_tag="PIKHDJKN_02650"

/inference="ab initio prediction:Prodigal:002006"

/codon_start=1

/transl_table=11

/product="hypothetical protein"

/translation="MLKSNARISIDCSRASEHTVNPYLFGHFVEDIRDHMEAMLAFPL
KDMDFESEAESRTSVSGNWHAHTNGRNTQYAMEAPALRHSGRAQRIRILSDDEAYAGI
AQRSALKGPMDYTVRIVARASIELQYLMVEAVDRRTEEVLGQLKVELDSHNWREYEGR
LTILRACPDAEVRVYIPAEHPRWIDHVSTGMLWLDHVSLLPIDHIALVKHEVIEMTRA
LNAGMMRLAGNYISAYHWEHGIGPVLERPVMYNEAWGGWTSKYFGTDEFIRFCHELQV
EPLICVNDGSGTPEEAAQWIEYCNGSVDTPMGALRAKNGSPEPYHVKYWEIGNEVWGQ
WQVGACSAEQFAERTVQFAKTMKEADPSIVLLACGHYEQDWNKTVLALAGEHIDYLTL
HLYHGYGPFGMNPDTPAEDRYKAMASYPEWTRHYIRQTMEVIHSDIKHQHVKLAITEY
NTMYYPNTVRKGLPDEHTLGAAVANAANLNEMIRCSDSVHIGSFSDLVNGWLGGCIRV
GDFYADQYRGKQAGWSGHPLTIYGTPTYEVLKLYANRDIHHILPVEAECGTFSVASNK
RTPVELDALPDLDVVAGTNADGSRVTLFVVNRSLEEVPIELDLQAFHASEEGTLYEIT
SDSHNNINSVFQPNHIKCTERSVLTEHWQRGYVLHPSSIYVLEFGKADQLKRRPGV"

CDS complement(2903391..2904632)

/gene="tetA_2"

/locus_tag="PIKHDJKN_02651"

/inference="ab initio prediction:Prodigal:002006"

/inference="similar to AA sequence:UniProtKB:P02981"

/codon_start=1

/transl_table=11

/product="Tetracycline resistance protein, class C"

/translation="MFKFRSRSHHIQNTEQTIDKHALLFGLISVFLCGIGFSIIAPVV
PFLVQPYISNPGQQAIVVTLLTSVYAFCVFFAAPVLGALSDKYGRRPLLLVCLLGSAI
GYLVFGIGGALWVLFAGRIIEGITGGSIGTIFAYFADIIPPEQRTKYFGWVSAVVGVG
TVIGPTVGGLLAKIDYSAPMYFGAIITLVNVIYGSFFMPESLDKKHRLKEITLVRLNP
FIQLANLLSMKNLNRLLISAFLLWIPNGSLQAVFSQFSMDTFNWKPAIIGLMFSIMGV
QDIISQSFIMPKLLKRLSDKQIAILGMISEIIGYSLIALSTLFSFYPLFIAGMFIFGF
GDSIFGPSFNGMLSKSVDSSEQGRIQGGSQSIQALARMIGPIIGGQIYVSFGHAAPAV
MGMILIAAAIPVLYKRTQVTI"

CDS complement(2904787..2905260)

/gene="pchR_1"

/locus_tag="PIKHDJKN_02652"

/inference="ab initio prediction:Prodigal:002006"

/inference="similar to AA sequence:UniProtKB:P40762"

/codon_start=1

/transl_table=11

/product="HTH-type transcriptional regulator PchR"

/translation="MNKEEQVLLGFRDLYNKLVWLNKDKMEESLKGYKPSEVHCIEYI

EKNVDSNVTKLADSFYMTRGAISKLTKKLIEKGLIESYQKSDNKKEIYFRLTEQGKVI

YQIHEDLHNEFQERDKAVFEQVTDEQFESMLNFVDKYNRHLDAEIKKLGVNLKSE"

CDS 2905466..2906812

/gene="gerBA_2"

/locus_tag="PIKHDJKN_02653"

/inference="ab initio prediction:Prodigal:002006"

/inference="similar to AA sequence:UniProtKB:P39569"

/codon_start=1

/transl_table=11

/product="Spore germination protein B1"

/translation="MQRIHEQDLHDIEKQLYEHFKKSSDFLSKNVVIADQEFRAYYME

TLVDLPTTLSAVNQAASGNKNGSFQNMFRPFEEEAALSMDEICGHLLHGKLVLFSTEG

TAAVMEPEHTDISRSVSAPESENPLQSAYDAFTEDINKNIGLLRKKMISDELVIERRE

TGTRSTKKIAMVYLEGVAQAKVIASIRDKLEQNKDKELTTVRDLLSILGHSKFSITPT

YISSELPGEMVQNLMNGKVVILIDQFSFAFAFPAIITDLWSTTLDVNYPYLFQVFLRT

VRAIGALLAVTLPGMYIVLNSVNPELLRIQLAITVAKSREGVPYPSLIEMLLVMLLLE

MIIEATIRLPKNIGPTITMIGGILLGQAIVQAKLVSNLLIIILVASAIANFVLAGYMN

TTGIRMYKYVVMLISSFFGIWGLEAAMIWIILYFASLNTFSVPYLSLSVKGKAPDE"

CDS 2906805..2907875

/locus_tag="PIKHDJKN_02654"

/inference="ab initio prediction:Prodigal:002006"

/codon_start=1

/transl_table=11

/product="hypothetical protein"

/translation="MNKAQILTMFVLLHLASIFAIFPERIISATSKGHWEPITILFLA

ELLVLWLYLKALSQFPGKTVVDICNDSLGKWVTRLIVLPMMIFLYIELLLLMYFQSVE

IKAVLLQRTPPAATSALFIILCFYAVWKGLGVMIRASIGLCILLMPFIFFSMMISIQN

FRISYIFPIWDSGMSFFSHSDFYVCTVISAGFLFLGMIPSKQRLSFGKAAAAVGIIFV

FALGSVYVPLLVFGQETVIHLQYPMLMASDTIDLEWVVFDWLPSFYVVASSALGVLKV

SVLLWILVSLLHQLFMPKINRLWILSVVCFSLYMISLMIPDVNALNSYLYLNSFFCVY

SVIGFPIIVFLAAQWQRKKVST"

CDS 2907872..2908966

/locus_tag="PIKHDJKN_02655"

/inference="ab initio prediction:Prodigal:002006"

/codon_start=1

/transl_table=11

/product="hypothetical protein"

/translation="MNMRTIRTLIVALSMMMLLPGCWDTKDINKQYLPVVMGVGKGKT

EKYRIILQIPDASGKTQILDKEAKSISKAIDLIRTDSEKSIELVHLRLLLIDRQVAEQ

GIDNIINFAVRANDISIKGLVGVINGDFEKTMYHQISPTPEISSYDFFSQDAGWTPNQ

SIVRIWEAYQNQNSYTEDMAIPMLKNGDRTLFTFKGTAVMRDDRMVGTLNKEETLLFN

LFKGKYSGGTIEVAQNTSVLIQDTKIKHKTSWSENGPSILTDILLNVVITESPEGKGN

VVIEKEMKEQLSRQFNETVRKIHSLKSDVLGIGMIFRPKMSEEKIKEWKSKWFPKLEQ

KINVRVNVLNEIYFKENANDNDSQGQMLKK"

CDS 2909014..2909460

/locus_tag="PIKHDJKN_02656"

/inference="ab initio prediction:Prodigal:002006"

/codon_start=1

/transl_table=11

/product="hypothetical protein"

/translation="MKSPWWRLFYKIFLHVILFPSLHVHCCPRGSHYSGFLHPFYCSL

ALTVASTPVSREEAPQAVSEIMLGGHHGNGIWRTVNRGKTWLAIAAVILMASSTESVN

SYIAEYLGSITHISGKTLSIVLFIYGLASLVGNVVGACRLSNFKQP"

CDS 2909533..2910444

/gene="yajO_3"

/locus_tag="PIKHDJKN_02657"

/EC_number="1.1.-.-"

/inference="ab initio prediction:Prodigal:002006"

/inference="similar to AA sequence:UniProtKB:P77735"

/codon_start=1

/transl_table=11

/product="1-deoxyxylulose-5-phosphate synthase YajO"

/db_xref="COG:COG0667"

/translation="MSAVPGGHQWVINEENSRPIIKKALDLGINFFDTANVYSAGTSE

EVIGRTLRDYANRDEIVLATKINGRMHQGPNGAGLSRKAIMSEIDKSLKRLETDYVDL

YQIHRWDYTTPIEETMEALHDVVKAGKVRYIGASAMYAWQFLKALHVAEQNGWTRFVS

MQDHYNLIYREEEREMLPLCKAEKIGVIPYSPLAGGRLTRDPQEKTHRSETDQIAKQK

YDATAVTDRLIIDQVAAIAEKHGVPRVQIALAWLLQKEPVTAPIIGATKISHLEDGVA

ALSIKLTPEETASLEEPYVPHRIVGHQ"

CDS 2910725..2911450

/locus_tag="PIKHDJKN_02658"

/inference="ab initio prediction:Prodigal:002006"

/codon_start=1

/transl_table=11

/product="hypothetical protein"

/translation="MSFFRTLSVRAREDELMDDFSIGGEELSEALKHLRRLNKIFAAP

APTRAGVEKLWKAIGSPTTLTLLDVGAGSGDVNQKLLRWADQKGVKLEITLVDLTEEA

CEEARNLFRNEPRIKVRRADVRELPDASADIVTGSQFVHHFEGKQLVEMVSHMLRASK

YGVVINDIHRHPISYAAVWITTRMISRNRYIRHDGPLSVAKGFTGRDWRELKQQLNHD

TLTYEWKPLFRYSVVIPHHGTLS"

CDS 2911507..2912661

/locus_tag="PIKHDJKN_02659"

/inference="ab initio prediction:Prodigal:002006"

/codon_start=1

/transl_table=11

/product="hypothetical protein"

/translation="MSKPMDVIVIGAGIAGSTCALQLARQGHRTLLLDRQEFPRHKTC

GEFMSPETTEMLDYLGIQLCEQEIKPSTLDHAKIIMPQGGEIEAPLPGLAYGISRYEL

DRILHEQATAAGVEIVTRATITNIQQLDDYSYEVEAKQGNERIQYRAKTVIGAYGSKK

PRGMTSPAKMRDKTAYVGVKSHYRGITIPPRVELYFCKGGYVGVSPIEDGIANVAALL

TLDTVRGSGKSVKDILQAASLTNERLAARLAEGTPVPGTQVSIAPLHLSNVPEPWSQY

PHIGDAMLMIPPLCGDGMSIALRSSLLCAEWTDRYLQRKISFTQWQQEYSREANLEFS

RLLRRARRIQKLAFAKTNKFYPGLARMVPGLATYLVKATRLSEINLVHRS"

CDS 2912853..2914037

/gene="lysN_2"

/locus_tag="PIKHDJKN_02660"

/EC_number="2.6.1.39"

/inference="ab initio prediction:Prodigal:002006"

/inference="similar to AA sequence:UniProtKB:Q72LL6"

/codon_start=1

/transl_table=11

/product="2-aminoadipate transaminase"

/db_xref="COG:COG1167"

/translation="MNYSFSNRIAALQPSIIREILKASSGQNVIPFSAGNPAPETFPI

EAIRTFTQSILEQDPVTALQYGITEGYAPLRDTLTTHLKTGFDTGKASDELFIVSGAQ

QGIELACKVFCNEGDTIICESPSFIGSLNSFRASGANLVGVPMETDGMDIGKLEHALQ

TEQNVKLIYVIPSFQNPTGFTTSLEKRKAIYDLAKKYGVMILEDNPYGELRFRGEDVA

TIKSMDDEGLVIYVGSFSKILSAGLRVGYVLAPSEVVQKMVVAKQGEDVHTAMLPQIL

AHKFMTEYNYAEHISSIRAIYRRKSALMIDKLQEHMGESITFTQPDGGLFLWCDLPAH

IPMLDYAKTAAAEGVAVVPGTAFLVDENEPCNAIRLNFSTPSDEQIVKGIEILGQVLH

KF"

CDS 2914053..2914361

/locus_tag="PIKHDJKN_02661"

/inference="ab initio prediction:Prodigal:002006"

/codon_start=1

/transl_table=11

/product="hypothetical protein"

/translation="MNQRFRITRSMQENNEEQSISLEECKQYFASKPGFTYSPVLTVQ

GPESVMSIDGDFFMWTYENLQIPFRLYMGDLYVAIVNEAVVPQMIEVATDLRADVVEG

"

CDS 2914575..2915075

/locus_tag="PIKHDJKN_02662"

/inference="ab initio prediction:Prodigal:002006"

/codon_start=1

/transl_table=11

/product="hypothetical protein"

/translation="MSSIVLAAAEDIRSEDSELLIKELSEELGLLYGGDGTAGFQPSD

VETPRAAFIVARLDGYPVGCGAIRPLDDTSVEVKRMYTRSDFRRKGVAQAILAEAERL

ALEFGYTNLKLQTGPKQPEAAALYERVGYYRIPIFHGDWDRVLAYQKDLVLSATHRNE

HSSLNA"

CDS complement(2915182..2916456)

/locus_tag="PIKHDJKN_02663"

/inference="ab initio prediction:Prodigal:002006"

/codon_start=1

/transl_table=11

/product="hypothetical protein"

/translation="MLHTRIGKTAAATILAISLLGTTANLNDAYAAPAISVLLDDVPL

KFDAAPLIDKGVTYVPFRTVGEALGISITWNSKAQTVKAIGNVKGQATEVLLQVGSTT

ATVNGEKVKLAAPPVQREGRVLIPLSSFSSQFGVNVGWNQSTRTVSLVSPQREMHLRA

FYALQSYKEIDLVPSMNSVAFGWSRIDREGQFTLQGNEYQIPAAAGDVTPQSIVADAA

NQSIKPYLMVYSLDGNGELTKVLSDSSMRQKSIEGITAAIAENGFGGVVLDFEGLGFK

LDAVEQQKLLNNYVKQLKDSLPKDIALSLAVPPLNSAYKGYDYKTLASLADDIIIMAY

QYNPVGTKSQVPEPNSLVDQAIQLALDAGVSKQKLLLGISLNSETSSSIDDKLGLAKR

YDLKGAAFWRLGLFRTYNNQMEDAVHASVIKE"

CDS complement(2916687..2916827)

/locus_tag="PIKHDJKN_02664"

/inference="ab initio prediction:Prodigal:002006"

/codon_start=1

/transl_table=11

/product="hypothetical protein"

/translation="MELAKIGLTLNVIGDILGYFSILKAEEEAANLAADTNKMGNTTA

VK"

CDS 2917126..2918361

/locus_tag="PIKHDJKN_02665"

/EC_number="3.4.11.-"

/inference="ab initio prediction:Prodigal:002006"

/inference="similar to AA sequence:UniProtKB:P24828"

/codon_start=1

/transl_table=11

/product="Aminopeptidase 2"

/translation="MLVDFHEKLQRYAALVVNVGVHVQRGQTLVITAPISAAPFVRLV

VKNAYEAGAHDVYIEWSDDEITRLKYELAPDSVFHEYPAFRAAGWETFAENNAAFLTI

TAPNPDLLQGIDPQRILNLNASKGKALAKFRSYGMSNKISWSIVTVPSTDWAAKVFPD

VAEEHLIDTMWEAIFNVTRINSADPVESWKTHLQLLRNKTEQLNRKKYRALRYRGPGT

DLRIELSPKHIWVSDADNINEKGVSFLANIPSEEVWTVPLKQGVNGTVRSTKPLSYEG

YLIDDFSLTFENGKIINVQAAQGLEVLKRLISIDEGAAYLGEIALVPHHSPISESGLV

FYNSLFDENASNHLAIGNAYPFCLEGGNEMTESEKAANGVNSSLIHVDFMIGSANMDI

DGELSNGGLEPVFRNGNWV"

CDS 2918412..2919086

/locus_tag="PIKHDJKN_02666"

/inference="ab initio prediction:Prodigal:002006"

/codon_start=1

/transl_table=11

/product="hypothetical protein"

/translation="MSPRNILKDQQMRKERIEHILNSALQTMAKRGIDSTNIKDIAKE

AKLSVGNIYTYFTSKDEIFSEVLRRGQTMYGRTIAQIADMDIDPREKLFEISKGWLSN

ESNWAFTIMLQSIRTNHAVSTEIKQAATQRFTDNLEPLAEIFRQGQESGGIIQGDTRQ

LAFYFVSLIQGLTLQLAPGYEIQVDIDPQKIVRLFMEPETVVSTAGLFEGNNDLSYIN

KKMFDR"

CDS 2919269..2920045

/gene="ssuB_3"

/locus_tag="PIKHDJKN_02667"

/EC_number="3.6.3.-"

/inference="ab initio prediction:Prodigal:002006"

/inference="similar to AA sequence:UniProtKB:P97027"

/codon_start=1

/transl_table=11

/product="Aliphatic sulfonates import ATP-binding protein

SsuB"

/db_xref="COG:COG1116"

/translation="MSADMLMISDLNKTFVAPVGEIIALNNIQLNVRKGELVTIIGPS

GCGKSTLLKIVAGLDTQYTGTVLLNGKPIKGPSIEKGFIFQEPRLFPWLTVEKNIAAN

LSLRDLNVRRKVEELIELVRLKGFEKSYPRELSGGMAQRVSIARALLRNPDVLLLDEP

FGALDAFTRSHLQEVLLDIWQRNGTTMLFVTHDLDEAVFLGERVVIMNPRPGHIRSIL

SIDLPFPRKRSSSAFQEMRRLVLNEFEKVDEPLYDVGGGI"

CDS 2920100..2921110

/gene="ssuA_3"

/locus_tag="PIKHDJKN_02668"

/inference="ab initio prediction:Prodigal:002006"

/inference="similar to AA sequence:UniProtKB:P40400"

/codon_start=1

/transl_table=11

/product="Putative aliphatic sulfonates-binding protein"

/db_xref="COG:COG0715"

/translation="MKKSHLVLVMVLALSLLTMACASKNSNDNASGSGTSQNVTVKIG

IQGSGGLFGKAREAKWFEQEFEKTGAKVEWIEFQSGPPMTEAIASNKLDIASLGNMPI

IAAQAANIPFKIVSQVLDGKNNVAIIVPAGSTAQSLSDLKGKKIAVTKGSNAYNFLYR

GLEKSGLTDKDFEIIQLQPDETQSAFETGSVDAWATWDPYITLNTLTGKGKVLSDGEQ

LGVLSPSFTIAGTGFAEEHPELVTLYLKVVNKALKWESEHDTEAIDRYAAERNVPASV

ISGTFERSRMINTPVNEDIVDEMQQTADFQYRMKNIRNQVVIKTITDNQYIDAALKDP

LK"

CDS 2921147..2921998

/gene="ssuC_5"

/locus_tag="PIKHDJKN_02669"

/inference="ab initio prediction:Prodigal:002006"

/inference="similar to AA sequence:UniProtKB:P40401"

/codon_start=1

/transl_table=11

/product="Putative aliphatic sulfonates transport permease

protein SsuC"

/db_xref="COG:COG0600"

/translation="MQMRAEKAVIPPLIQKKNSVKRRKLYLRASNMVLGLIVPVALLI

VWEVAGAMGYLNPVLLPTPSSIWKELVSMTETGELVKHLGISTWRALLGFLIGGGLGL

TMGLWVGFSYKTERLLDPSFQMLRTLPHLAIAPLFILWFGFGETSKLLLIAKGAFFPL

YVNTFLGIRSVDSKLFDVGKVLQFSKWQMITKLILPASLPNIFLGIRLSVSVAWLGLV

VAELMGSSAGLGYLINDARSFSLTTVVFVGIIVFAVVGKLSDSIIKLLEARALRWQDG

FKGEEAK"

CDS 2921995..2922807

/gene="ssuC_6"

/locus_tag="PIKHDJKN_02670"

/inference="ab initio prediction:Prodigal:002006"

/inference="similar to AA sequence:UniProtKB:P40401"

/codon_start=1

/transl_table=11

/product="Putative aliphatic sulfonates transport permease

protein SsuC"

/db_xref="COG:COG0600"

/translation="MSKLEIGKQTERAGDWLSRIPGRLHGWIIPLAILLIWELASALQ

LVSATMLPAPSKIAEQCIRLILNGELLEHLGISSYRAGLGFLLGTTVGLMLGLSTGLG

KLAERTLDPSLQMLRMIPLLSLIPLFILWFGVGEFSKILMISLGAFFPVYVNTFLGIR

NVDMKLIEVSRIYQYSRWELLGKLVIPAALPNILLGVRLSLGAAWLVLVVAEMMGTSA

GVGYMIQDARAYSQTDIVFVGIVVFAAVGKLSDSAVRLLEDRWLRWRDTFKG"

CDS 2922840..2924201

/gene="ntaA_2"

/locus_tag="PIKHDJKN_02671"

/EC_number="1.14.14.10"

/inference="ab initio prediction:Prodigal:002006"

/inference="similar to AA sequence:UniProtKB:P54989"

/codon_start=1

/transl_table=11

/product="Nitrilotriacetate monooxygenase component A"

/translation="MGTRQEQLHLGAFIYYTGHHHYGWRRSDSGAEQIFDYKFYRSIA
QTAERGKFDMMFLADLLYVMGADQSAAGMLDPLTLLSALATDTEKLGLTATVSTTYNE
PYNVARKFSTLDHISEGRAGWNIVTSQLDVEANNYGRTEHPEHGLRYKMAREFVEVAT
LLWDSWEDQSLIVDRQAGLFADPSKLKEVSYKGQWYSTQGTINVPRSPQGYPVLIQAG
SSEPGQDFAANYGEVIFTAQQSLNAAKEFYNNVKSKLALYGREQGSMKIMPGLSPILG
KTEEEAQRKYEELQELIPPAAAVGMLSGMLKFDLSEYPVEGQLPDIPDPVEASNGMKS
RVQLIMDMARQDKLSILELGRRLIGARGHMQFVGTYEQLADLMQQWFEEYGCDGFNIM
PPVLPGDLNEFVDHVVPILQARGLFRTDYTGRTLRDHLGLDRPGISHFQKEKSVDKTS
ITI"

CDS 2924438..2924962

/gene="sigV_2"
/locus_tag="PIKHDJKN_02672"
/inference="ab initio prediction:Prodigal:002006"
/inference="similar to AA sequence:UniProtKB:O05404"
/codon_start=1
/transl_table=11
/product="RNA polymerase sigma factor SigV"
/db_xref="COG:COG1595"
/translation="MDKLSKVSTALTEQQFSERLEACKEKLYRFAFSYVKNEQEALEI
ISEASFKGFLSYEKVKSPDYFETWMTRIVINCSLDYIRRKKKYTYMEDSTIPFAAEES
SMGLEEQWDLYEALDHLHPEDRAFIVLKFFQDQRFKDMAEVLSLPESTVKTRFYKILN
KLKKHLTKEEVDFT"

CDS 2924959..2925810

/locus_tag="PIKHDJKN_02673"
/inference="ab initio prediction:Prodigal:002006"
/codon_start=1
/transl_table=11
/product="hypothetical protein"
/translation="MTGKERYEQINIPSGLSGVIQQARQRARNQKRRKMMIWRSSSLV
AACAAVMITANVPGVAKALSDVPVIGSVVKILQVGGGGERTDGVSVTTTKESDTLNIN
FQIDGEQITSAPSYTVDHKEGPNRLIFTFNGVRHLDYDKLEKDIKALKNVKDVYQNII
LDDSAIGFVVELNDDVDYSVSEYQQPGYIQLKLFSDGKKTQSHELFFVRSEDMEQGEA

LAMLAEQYSADGSSVVHTQNGKFTVVMGAFDNRADAEKHLKEFTDREDYSEPLHVDSW

MSNENPQ"

CDS 2926063..2927217

/gene="alr"

/locus_tag="PIKHDJKN_02674"

/EC_number="5.1.1.1"

/inference="ab initio prediction:Prodigal:002006"

/inference="similar to AA sequence:UniProtKB:P10724"

/codon_start=1

/transl_table=11

/product="Alanine racemase"

/translation="MYRDTWAEIDLTQIRENVIEIRKFLPRSTKLMAVVKANAYGHGD

IETAKEAVEAGADYLACAFIEEAIRLRNAGLRQPILILTPIRPELVPLALEHDLMLTV

TQASWFHEMRKYKPVHTPHKLSVHVKMDTGLGRIGIRTKEEWLEMVPWLRAPDVVVDG

FYTHFATAGEADNRYLQLQIQRFLEMKEWSSMSRIPINHYHCAGSVAALRFPELCMDM

ARIGAAIYGFYPEKLVPNIQLKPAFSLHSRLLQTKGLKKGEYVGYNNAYQTDSDQWIG

TVPIGYADGWSQRMQNTEVLVEGYRAEIIGKISMDQLMVKLPMYFPEGSKVTFIGYSG

EREIPIQALANHIGGVSQEITSSITDRVLRIYKEGGEVHHEITETRTKTY"

CDS 2927183..2927989

/gene="vanY_2"

/locus_tag="PIKHDJKN_02675"

/EC_number="3.4.17.-"

/inference="ab initio prediction:Prodigal:002006"

/inference="similar to AA sequence:UniProtKB:P37711"

/codon_start=1

/transl_table=11

/product="D-alanyl-D-alanine carboxypeptidase"

/translation="MKSRKLEQKHIEISVSNEERFQGHLVLINDQNPIRRQVQVHQLE

SLDSLPSIKKLNKEMLLEKQCFNQFHALLKACGGTDEIVAVSAYRTKEDQVQIYQDCL

IEQGSEYTSKYVALPDHSEHQTGLAIDVGKLESNIDFIAPSFPDTGIYKPFRQHAIQF

GFILRYKQEKESITRIAYEPWHFRYVGYPHSKIMEENNLCLEEYIDFVQQYRYSGEQL

TIKEKDMTIQIYYVPTDKGTSSHIPILSCDSYRISGTNREGFIVTTFSKN"

CDS 2928042..2929094

/gene="vanB"

/locus_tag="PIKHDJKN_02676"

/EC_number="6.1.2.1"

/inference="ab initio prediction:Prodigal:002006"

/inference="similar to AA sequence:UniProtKB:Q06893"

/codon_start=1

/transl_table=11

/product="Vancomycin B-type resistance protein VanB"

/db_xref="COG:COG1181"

/translation="MQKKSIAVLFGGCSGEYNVSLSSAASVIENLDTEKYNLVLIGIT

QQGSWLRYSGTVEDIRNDRWHLHPSCIPSFFSPSREVKGLIELVHTEYHVTTIDVVFP

VLHGKYGEDGTLQGLLELSGIPFVGCGMLSSALCMDKELAHKLVQAAGIDTPRSLTIH

RSERMEEVLSAIEALGFPLFVKPARSGSSLGISKVNNMQELITGIEHAFIHDSKVVIE

QNINGFEVGCAVLGNSDPMIGVIDEIELQGQFFDFSEKYSLISSKIHLPARIDDDTEM

KVKESALTIYKTLGCRGFARVDMFLTTDGRIVFNEVNTIPGFTSNSRYPNMLQASGMT

YANILNQLIELAIDEN"

CDS 2929218..2929847

/locus_tag="PIKHDJKN_02677"

/inference="ab initio prediction:Prodigal:002006"

/codon_start=1

/transl_table=11

/product="hypothetical protein"

/translation="MRHLVMNKPSLRDIKREATTKALSSSAFELAVERGLDGFIIDDL

VQRAGYSRKTFANHFSCKEEAIAMAAILTTDHYDEEPLNEITQQNILPLDTLYVLIKR

SLTAEYVSKMRQLVSLSKQYPTLEPYLLSVLSRLQATAQETLTDLSQGRYPQGYSHLL

SGAVYGAVLAIFDGSINVLLPGQGATDSPGEMTLDQYLDDMFNYLRTGF"

CDS 2929875..2930858

/gene="aes_2"

/locus_tag="PIKHDJKN_02678"

/EC_number="3.1.1.-"

/inference="ab initio prediction:Prodigal:002006"

/inference="protein motif:HAMAP:MF_01958"

/codon_start=1

/transl_table=11

/product="Acetyl esterase"

/translation="MRVTTKMIDKQLRVKGKLIDLLMKSSHEEKWLNSMRKIKKRSNA

TAGKDIDGLQCSEEWIPRTSDGSKIRVRIYKPLHPQGKAPGVLWIHGGGYAMGNPEQF

GATYKKLIDAGNCIVVAPDYQLSIDAPYPAALNDCYDTLLWMKSHVKELGIREDQLMV

GGESAGGGLTAALTLYARDKGEVNIAFQMPLYPMIDDRMITESAKDNNAPIWNSDYNQ

WAWNLYLGELAGKDVPPYAAAARATDYSNLPPTVTFVGDLEPFRDETIQYVENLRKAG

VHVDFELYKGCYHGFDIIQPNAEVSKKAVSVFIQSFKYAVDHYFAEQTAKR"

CDS complement(2930937..2933228)

/gene="metE_1"

/locus_tag="PIKHDJKN_02679"

/EC_number="2.1.1.14"

/inference="ab initio prediction:Prodigal:002006"

/inference="similar to AA sequence:UniProtKB:P80877"

/codon_start=1

/transl_table=11

/product="5-methyltetrahydropteroyltriglutamate--

homocysteine methyltransferase"

/db_xref="COG:COG0620"

/translation="MAKSSVLGYPRIGADREWKKALEAFWSGKLEETEFQARLQEIRL

DHLRKQQEKGIDLIPVNDFSYYDHILDTAVMFGIVPKRFAYDGGAVPLSVYYGIARGT

KDAAASEMTKWFNTNYHYIVPELDGASPALTENKPLIAYREAKEKLGIEGKPVLVGPL

TFLKLSKGYDKSETNAWLDRLLPLYVQVLQELANEGVQWVQIDEPILVTKTSDADLKL

LSKIYETFAAAVPGLNIMLQTYFESVENYSNIVALPVQGIGLDFVHGATGNIQSIQAS

GFPADKVLGAGVIDGRGIWKASLPGKLKLLDELAELVTPERLFVQSSCSLLHVPVTTE

RETKLTSELKNALAFADEKLDEIVLLTKALSSRNAEIIAEIENSDRTIQALQQSEERN

RVAVQEAVASLSVQQPERSRPFAERHIAQQDKWKLPLFPTTTIGSFPQSAEVRKARQL

WRKGELNNEQYAAFIRDQIDIWIKIQEEIEIDVLVHGEFERTDMVEFFGEKLAGFAFT

QFGWVQSYGSRCVKPPIIFGDVAFTGEMTVEETKYAQSQTQRPVKGMLTGPITIMNWS

FVREDIPREHIAYQLAYALRQEVEALEQAGIGMIQVDEPAVREGLPLKENQQADYLAW

AVKAFRISTCTVQDTTQIHTHMCYCEFHDMIDSIEAMDADVISIETSRSHGELIHSFE
LNTYKLGIGLGVYDIHSPRVPSVDEMTSMIERALRVLDPKLFWINPDCGLKTRGLEET
VASLRNMVDATKIAREKHVATTV"

CDS 2933879..2934757

/gene="rhaS_17"

/locus_tag="PIKHDJKN_02680"

/inference="ab initio prediction:Prodigal:002006"

/inference="protein motif:HAMAP:MF_01534"

/codon_start=1

/transl_table=11

/product="HTH-type transcriptional activator RhaS"

/translation="MGVPTFLETGHMTGGFPIKVIHTEEQFNFAAHWHEEVELVVVNG
KSARIGVNNHVVDLEHGDVLLIKPGDIHCFLPGTDHLTIILFRLELLTGSFTSEKETH
DLGELFNKTTVIPSNPCNEKNLGKYIDALVNEEKDQQAGYRWLMVARLYDLIVHMLRT
KAPITEELTSGWPCTPSKKFEFLESVCEYLEEHYAEPIKLDQVAEHIKFSKFHVCKLF
KEIKGITLMEYLNHFRIIKSEWALLFREESILDIAIGHGFNNVNSYNRLFKKYNDCTP
SEFRKKHRTNITTYEG"

CDS 2934843..2936117

/gene="lacE_2"

/locus_tag="PIKHDJKN_02681"

/inference="ab initio prediction:Prodigal:002006"

/inference="similar to AA sequence:UniProtKB:P29822"

/codon_start=1

/transl_table=11

/product="Lactose-binding protein"

/translation="MKKFKLKYISMIALVSAVIMVSGCQPSGANQSKQEEITVWSFTD
EAGYAIEKFEEKYPDIKVNFVNIPGNFYITKLKSALQTTSKAPDVFMIENANIRELID
VPYLENLSEAPYNANELITEQYAFVQANEKDSEGNVRAIGYQGTPGGIYYRRDLAKEY
LGTDDPEKVAPQIDTWEKIFEIGEKVEKRSGNKVHALANWNAISNSYDGIPWVKDGKL
VIDPTYMKVLDLVREARQRHVLAELDDGSAGYAASMQKGQVMFYPGATWALQYTFKAN
APDTEGLWGLAPGPSAFSAGGTYIAMYSKSDKKGLAWKFIEFYNFNHDFLSGLAKEQD
YFTSNMVVNDQLAGSVSSAYLGGQKHFEFFSEAAKQVPVYERTKYDSIINNDIFKNVL

QLYLNNDIQTKEEAVKRINRDVKLRFPELEVD"

CDS 2936170..2937060

/gene="lacF_7"

/locus_tag="PIKHDJKN_02682"

/inference="ab initio prediction:Prodigal:002006"

/inference="similar to AA sequence:UniProtKB:P29823"

/codon_start=1

/transl_table=11

/product="Lactose transport system permease protein LacF"

/translation="MFAKSIRKDHYGYYFIAPFFILFAIFGLYPILYSLYISFTNFDG

ITTPDFVGMGNYIAVLQDPLFYKTLFNTLFIWGVSVVPQLTVSLVLAFILNDKLLKGR

DIFRAVYFFPNIVTAASLGLLVSLIFDWQSGGLNHFLVQVGIIDDPINWKNDPWFMRL

IVSSILFFQYFGYSMVIYLAGLQGIDPALQEAAQMDGAKKKHIFIHIIVPMLRPIILF

QMITSIIGGIQIFDQPFTLTNGTGGPDRAAMTSIMYLYNVAFQSTRFGYGAAIAFCLF

IIIILLSVVSFMMTKRKSRA"

CDS 2937076..2937957

/locus_tag="PIKHDJKN_02683"

/inference="ab initio prediction:Prodigal:002006"

/codon_start=1

/transl_table=11

/product="hypothetical protein"

/translation="MQTVKPVEQQKVILQQYDSARRLTLGKVIIYLCLISLAVICIIP

FYLMLIYSTHNNATIASTFTFLPGPFLLDNYTNMASKINIWRGFANSFFIAGTSTVLS

LYIGSLTAYGFAKFKFKGRNWLFLFLLATMMVPGQLALIGMYRLFSTLGLLDSYAAII

LPAAANAFNVFFIKQFMESSIPDEIIESARVDSAGEFRTFNQIVLPILGPAISALGIF

TFIGSWNNFLTPLVLFFSLDKYPLPVLVALVQGYYGMDYGLLYLGVAISILPIIIVFA

IFSRQIIGSVALGAVKG"

CDS 2938185..2938937

/locus_tag="PIKHDJKN_02684"

/inference="ab initio prediction:Prodigal:002006"

/inference="similar to AA sequence:UniProtKB:Q5HJF7"

/codon_start=1

/transl_table=11

/product="putative response regulatory protein"

/db_xref="COG:COG4753"

/translation="MFNVLVVDDECVQREGIKDLISHYGFPFQVLEAENGKSAERILV

QNAIDILITDVKMPLMDGLELSKQARKTQQNIKIVICSGYDEFEYARSAIRLGVVNYL

LKPLVREEFLQVMEDITQIRAHAGGPEPESVESKVIRQVKDYVKDNHQRDISLSEAAH

DVYLSPGYLSILFKKETGENFSKYLTDYRLRRASHLLAHSNMKINDIAGAVGIDNHSY

FAKLFRNRFGVSPLQFRECGVLHDRMDQEQIQ"

CDS 2938906..2940558

/locus_tag="PIKHDJKN_02685"

/inference="ab initio prediction:Prodigal:002006"

/codon_start=1

/transl_table=11

/product="hypothetical protein"

/translation="MIGWIKSKFNDIRFRNKLLLSHLVIALIPILLLGLLSFIQSYRI

QMKELRSEAEASLEQVANIMDYKINRYNTLSEFMVLNRDLSQIFTKDYEENYYNMYLD

FRDILEPLISNIRLMDDDIEGITFYTSGELLGIRNNILPIGDLKSKAWYDGFRGRRWI

VDGEKLFLVQELISNPKDSNFMVISIQHDSIFADLDNLSEHVAVHIEDAKQHVIFQEN

HERAAVAAESKVGKSGDLRLSRPLANNGWTIHYNLLNTNAYANAMSIFQITLFVIILS

LIITFLLIYVISNNFTQRIIHIKNKVDKVERNNLDVIIRSSSRDEFGELTNGIGKMLT

RINSLISQVYHAEIAKKESEYNKLISQINPHFLYNTLSFIRWRAEKRADIETSYMVSA

LARFYRTTLNQGKNMATIEAEVQHIKAYLDLQLIMNDGRFDVDYHINDEVLHTEVIHF

ILQPIVENAIKHGFVEADAIDYRIHITVNRVGEGIKLTVRDNGVGMTPVQTAGLLTGD

NGGCGVRNVNDRIKLYYGRDYGLVIHSEPGSGTEVSIVLPAT"

CDS 2940777..2942414

/locus_tag="PIKHDJKN_02686"

/inference="ab initio prediction:Prodigal:002006"

/codon_start=1

/transl_table=11

/product="hypothetical protein"

/translation="MKKRSKMATLLIGSMMMAVISGCTGGNNESAQPQISQQDYEPYG

KYETPVEFTIGRNTNHVNNLPAGDTIENNLATRYVEDRVNVKAKVAWETDDMDQKLSL

SMTTGDLPDVMLVSREIFNQLVDNDLIADMTEVYEKTASEGVKNIYGSYGDLLLNQVK

VDGKIMGLPMTNIGNQHQLLWVRKDWVDKVGAKLPESVEDVWNLARTFVEKDVSGTGK

TVGMVMDSNAMNFTPIFAAHGAFPMNWIQKNGQVEYGSVQPEVKQALTELSAMYKEGL

IDKQFAVRSSEEKEALVINGQAGMVFNPWWIGYTNYKDSIKQDPEAVWVAVSAPVDKN

GKFKTIRQDPVGGGIVVVKKDFAHPEAIMKSVNLTTDFLYSLTEDAINYKTEHPDELL

TDASRWNNDPTQIPMQVDYDDVLKRYYDDLIKADEAGDASAVQEDRVVSFKAYQEFKE

KGNAIDANTYGEYLSRIEGQREANNPNLEVIHAGFYGTTETMKLKWANLQKLEEETML

KIIMGEAPVDEFDKFVDTWKRTGGDEITEEVNEMSNR"

CDS 2942517..2943467

/gene="yteP_21"

/locus_tag="PIKHDJKN_02687"

/inference="ab initio prediction:Prodigal:002006"

/inference="similar to AA sequence:UniProtKB:C0SPB3"

/codon_start=1

/transl_table=11

/product="putative multiple-sugar transport system

permease YteP"

/db_xref="COG:COG4209"

/translation="MAQMETTVNQEREVRYRRKKKRKTFDEMTYHFMLLPGMIMLFIF

SIVPMFGVVMAFQKFIPAKGIFGSKWAGLSNFTYMFQLPDAKQIFINTLVIAVGKIAL

GLIVPIVFALLLNEVRLKVFKSTIQTIVYLPHFMSWVVLGTMLTMIFSFDGMVNNFLE

FLGLERIMFLASNDWFRPLLIVTDTWKEFGYGTIVYLAALTAINPALYESAAMDGASR

WKQTLNITLPGMFPTIILLGTLSLGNVLNAGFDQVFNLYNPLVYETGDIIDTFVYRMG

LINMQYSFATAIGLMKSVISFVLIVISYRLASRYAGYRIF"

CDS 2943478..2944365

/locus_tag="PIKHDJKN_02688"

/inference="ab initio prediction:Prodigal:002006"

/codon_start=1

/transl_table=11

/product="hypothetical protein"

/translation="MIQSKSLGSRLSRLLIMFALILITFMSLAPIVNTIMVSVSSSTA

VNAGRVYFFPVELNFSSYGQILNDTKFWKAFFISVERVVLGGAINMILTILMAYPLSR

SVTQFRSRNTYMWIIVFTMLFSGGIVPWYMVISKLGLINSIWALVLPGAVPVFNVILL
MNFFKGIPKELEEAAFIDGASPLKILLKIFIPISMPSLATIMLFVIVGHWNNFFDGII
LINDSSKIPLQTYLQQLSLTRDQMQNLSVEQLQQFNKISNTTLNSAKILVSMIPILLI
YPFLQRYLIHGIVLGAVKE"

CDS 2944529..2945677

/locus_tag="PIKHDJKN_02689"

/inference="ab initio prediction:Prodigal:002006"

/codon_start=1

/transl_table=11

/product="hypothetical protein"

/translation="MSKRKMVSLTLVLSIILVTTMGATSGGKNRYADQAQWAQEKLHE
YYWNDASKMMNNAYPSTPEGEHALNYWWKAHAVDALVDGYERTGDKAYTERAEELVRS
IIARNGSLHNEFYDDMEWLALAGLRLYDATGSEEMKGYVLELWDDIKTAWWEDELGGM
AWKKDQRYNRNACSNGPAAILAARLYERFGDARDLEWAKKIYDWEKQHLVNPKTGLVA
DGLVLKEDGTLDVNEAWIFTYNQGTFIGAGVELYRITGKKTYLKDAEKTAAGSLKTLT
DEKTGIFKEDGDGDGALFKGILIRYMVELYEIGHNKSLKKAVYRNADALLKGSRETGL
FGQAWGKSAQNPLDLTAQLSGVFLLEGAAKLEAGKADKLGKPEKPVKK"

CDS 2945841..2947124

/locus_tag="PIKHDJKN_02690"

/inference="ab initio prediction:Prodigal:002006"

/codon_start=1

/transl_table=11

/product="hypothetical protein"

/translation="MHSITELPEAMRRLTEIVREKQSERTADMLEQCLANTWNTTLKR
MEDGSTFVITGDIPAMWLRDSAAQVRPFLVLSEDPEIGDMIQGVVRRQFDYILQDPYA
NAFNELADGKGHQNDATDMSLWIWERKYEIDSLCYPIQLSYLLWKNSGRIDHLDETFR
AGVRQIIELWKREQRHETDSAYTFERSNCPPSDTLIRDGRGSETGYTGMTWSGFRPSD
DACTYGYLVPSNMFAVVVLRYVTEIAKEILMDEELAEAAENLATQINAGIHQYGVVED
DQCGSIYAYEVDGLGSTNLMDDANVPSLLSIPYLGYSNADDPVYVNTRQFILSERNPY
FYRGAAASGIGSPHTPDRYIWHIALAMQGMTAKDTAEQQRLLELLLATDGGTGFMHEG
FHVDRPEEFTRPWFSWANMMFCELVLLCSGVTVKR"

CDS complement(2947221..2949275)

/locus_tag="PIKHDJKN_02691"

/inference="ab initio prediction:Prodigal:002006"

/codon_start=1

/transl_table=11

/product="hypothetical protein"

/translation="MARRFRMLCLAFICAFSLTAGAASASETDVQTAKPDGSKHRELP

SFKTPKHLDAADIYDAPGDIKLLLGTLQGIVNREQPRIYLIESQEEGKLTWLKDIKVP

YTLHQDYWEVFSAYRSEVKGMIVYDPKVPDTINVATTLAGLKNAVVASPELAVKLSKA

PYKLKIMDDLRGKFNSGIDAYTWQYENLWKKTNHQMLIGLDPDTAIRIPSGMPESFVT

IAEEKQQIRDASNRNTYSLDLSSLLGKSSVYLRFDDAFTQDGWGPAVHEITVKADGKE

IANFIAGTPEEEQFLYDRQNSKVAEGEGGHRFADNGNYFVYEFSPPAGTQNLTAEVDM

WNQYKVSAGNIRPLSAEQREPYAYLRDYAVANKAMVFWLDSNVPEQKALFEKIMSDVK

PGTPYLGWFSNDVEGEFSSVEIASRYGVYVLAADWFSNLTVFSGTKPKLTPVKKSPRP

ALENKIYVTYTFTEGDNFQYNQHRMRVMWDDPARGKVPINWTSSPLLYEGAPAILNYY

VNTATPNDLLIAGPSGAGYFYPGAWPDSSFRQFLQQTEKYMKKTGMTIPYVLNRVDSE

NVPLTPFKAKAYAQDYNAPGLFISYEDNYGVEIVDDSLPVSTIRGISTVQDGLQVLNE

AKANWDGKSPLFVSLGLLAWSTTPSDALAITEELGTEFKVVRADDYFSLIRESYHLPV

SK"

CDS 2949697..2950731

/gene="araR_3"

/locus_tag="PIKHDJKN_02692"

/inference="ab initio prediction:Prodigal:002006"

/inference="similar to AA sequence:UniProtKB:P96711"

/codon_start=1

/transl_table=11

/product="Arabinose metabolism transcriptional repressor"

/db_xref="COG:COG1609"

/translation="MDQFQVSKITVANALTGLANEKLIARVPGKGSFVAEEADSASVT

STQATTMRGREGTLSTGMIGIIVPSIHDYFAIRLVEGIEQTLREEGYRSMIMFTHGKV

DKEKDVIKEMKALGAEGLLIFPVDEENYNEEILGMKLSGFPFVLMDRYLPGVETHYIA

ADGRRGTRLAVEHLWELGHRDIAICSDSPLQTVTVQERIEGYIEALKGKGALINPAHM

ITDFQPLSELKEAEAHPLYRYIQNRMVTAYVSLNGRLGVQIYQMAKQAGLRVPEDVSI

VSFDDPTSIVEEFSIFTHVKQFERDMGYRAAAKLLEVLRGHGEAKSYSKMLIEPELVV

RQTTGTVPQA"

CDS 2950900..2951151

/locus_tag="PIKHDJKN_02693"

/inference="ab initio prediction:Prodigal:002006"

/codon_start=1

/transl_table=11

/product="hypothetical protein"

/translation="MARDRKEERTGFQESAAYHPDYDLQTDFVMVYGIDESMQERILK

WKEKGYVVHLMTGVAWGTYNSYLDGAKNSIAYWFPPTQT"

CDS 2951180..2951533

/locus_tag="PIKHDJKN_02694"

/inference="ab initio prediction:Prodigal:002006"

/codon_start=1

/transl_table=11

/product="hypothetical protein"

/translation="MKALWPSLWQDIHYAGVDITNHLVKGENELEVLTVSLLKPMPAI

SFPAYLIGSFAIEDQTTLTVESEQVTGVWNVTSYMVRGNNVIDIHVANTLENLYGKSS

LPSGMNGSVKLVTKI"

CDS 2951796..2953370

/gene="ptsG_1"

/locus_tag="PIKHDJKN_02695"

/EC_number="2.7.1.199"

/inference="ab initio prediction:Prodigal:002006"

/inference="similar to AA sequence:UniProtKB:Q7A807"

/codon_start=1

/transl_table=11

/product="PTS system glucose-specific EIICBA component"

/translation="MKQYFEKAQKFGKSFMLPIAVLPAAGLLLGIGGALSNANTINSY

PFLQITWLQQVFTVMSSAGSIVFDNLALIFAIGVAVGLARADKGTAGLAAGLAYLVMN

ASINAMLVNSGKLAADHLASAGQGVVLGIQTLQTGVLGGIVVGLVTAWLHNRYNKIEL

PQFLGFFGGSRFIPIVSSFAAIFIGIALFLIWPTVQLGIAQLGSLVDRTGYIGTLFYG

FVLRMLGPLGLHHIFYLPFWQTGVGGTMEVAGKVYEGTQNIFFAQLGDPATEKFFSGT

SRFMSGRFITMMFGLLGAALAIYHTAKPERKKIVGGLMLSAALTSFLTGITEPLEFSF

LFVAPILYVIHAVFDGLAFMLSHIFEITIGQTFSGGLIDFILFGVLQGNAKTNWIIVP

IIGVVWFFLYYFTFRILIVKLNLKTSGREEEVTSEPDAKTPSQHSNTPVSTKGEERSI

AILNGLGGADNINELDCCATRLRVSVKDIEKVQKDALTATGAKGVVLVGNGVQVIYGP

QVTVIKNEIEEIMETE"

CDS 2953376..2954092

/gene="nanE_1"

/locus_tag="PIKHDJKN_02696"

/EC_number="5.1.3.9"

/inference="ab initio prediction:Prodigal:002006"

/inference="similar to AA sequence:UniProtKB:P65517"

/codon_start=1

/transl_table=11

/product="Putative N-acetylmannosamine-6-phosphate

2-epimerase"

/translation="MNLDKLGLQGGLVVSCQALEYEPLHSSLIMGRMAIAAKEGGAIG

IRANTAADVKEIKEQVDLPVIGIVKRNYGTNPVFITPTILEVDELAEVHAEIVAVDAT

LRARPDGKSLDEFVHEIRTKHPQLLLMGDISTKEEAVNAERLGFDLISSTLVGYTEET

AGLKLYDNQFAALKEILTSVQTPVVAEGNIMTPEMAATCLEAGVYCVVVGGAITRPQQ

ITERFVSEIAKLKKPSPSVQ"

CDS complement(2954140..2954994)

/gene="ybbH_2"

/locus_tag="PIKHDJKN_02697"

/inference="ab initio prediction:Prodigal:002006"

/inference="similar to AA sequence:UniProtKB:Q45581"

/codon_start=1

/transl_table=11

/product="putative HTH-type transcriptional regulator

YbbH"

/db_xref="COG:COG1737"

/translation="MRIDYTKVNMFWERGIIMSSSVIDMLQLEYQRFSVKEKEIADYV

MRNKTSINNINIRDLAAYTNASMSTITRFCKKVGCSTFVDFKIRLNREVDQPRNQTDF
FIRTQQIHNDIINATAEMLDTDRIEQVVQYIKKARRIYVYGLGSSGLSALEFKYRLMR
MDIVIDAVTDSHMMIMSASLLGEDDLVIGLSNSGRTLEVSDALNAAKKRGSKVVGITN
FDHTPLSEVSDLCIFTPDIGRTGDVNFINSQLAIIYILDVISLLLLEDEHLLNARQQT
LKALYSAE"

CDS 2955241..2959302

/gene="cpdA_2"

/locus_tag="PIKHDJKN_02698"

/EC_number="3.1.4.53"

/inference="ab initio prediction:Prodigal:002006"

/inference="protein motif:HAMAP:MF_00905"

/codon_start=1

/transl_table=11

/product="3',5'-cyclic adenosine monophosphate
phosphodiesterase CpdA"

/translation="MISHLYKKMLSILLSIALVFGWLAGFGGVNSAQAASLSEPEMLL
ERNANWHYFDQGQDLQSEWRASYDDSSWNSGQAPFGYKDNGNGVSTSYFGSLKTEVNY
GQDKKNKPRTTYFRTNLTVNKDQIDDYGQILGTFGIDDGAVIYVNGVEVIRFGMPDGE
IGYSTQATSNKDLPVMYENIDLTEPMKANLHEGSNEIAVEVHQQSDSSSDLYFDMELK
ALANAPSLDISKVTVTFYGDATSSKGFTWYTPLASAQSTVQVVPNQGGVADFSQAQSF
NGRATVASNSPGEMVHKAEATGLSPDTSYSFRVGDQDLGIWSEVGTFKTAPVDGAFTF
IDLTDTQAKEEDEAILSGSTLSKALATVPNAEFVVHNGDVVENGTSEQEWNWLLGHSQ
ASLMNTTIAPSAGNHENKNYAFYDHFNVKQPDGAATETGAYYSYNYSKAHFIVLNSNE
NSTEYANFSNEQVAWMKQDVAEAKAAGAKWIIVNIHKGPYTTSNHATDSDIIGTNGER
KKIAPLMNELGIDLVLQGHDHIYARTKPIKSDGTAEDVSKITETFNGQSIEYSVKPDG
TIYMIPATAGAKVYFKNVKPELGEAYFSLFERAEENHARQYGDTTSAARGQVQNFVSL
TIDGDKLSAVTYEIDQNNNAEPFIVDTFGIIKESDTVPVPEQVNKVTVTFHGDPTKSK
GFTWYTSDQVTNSDLQVVEKTSAAPDFTQAKSVQGRSAQPSNAPTELMHKAEVTDLKA
NTTYYFRVGDASQQVWSETGTFRTAPEKGKFTFIDLADTQAKEEDEAILSSETLAKAL
ATVPDAQFVVHNGDIVDNGVKEEQWNWLLGHSQESLLNTTIVPSAGNHEDENYAFIDH
FNIQSPVNSATETGAYYSYDYSNAHFVVLNSNEDSDEYDNFSLEQVEWLKKDVQAAKK
NGSQWIIVNIHKGPYTTSNHATDSDIMGANGVRTKIAPIMAELGIDFVLQGHDHIYAR

TKPIDQKGKARQPEMITEMQNGQKIEYSVNPDGTIYLIPATAGPKVYYKNPSTSLGDA

YYNLFELAEENHAAKYGPDPNDNRRPMRSQVQNFVGITIDGSKLTAITYEIDQNQNGA

APFIVDRFGIVKKTEPSNPGPGTSNPGTSNPGSGSGSGNGSGSGNGSSGSGGDTTTGN

GSNSGSGSGTDSGNKPDTGSGNNGNGGTAPALKDTAGHWAKSAIDKALAAGFVNGYGD

QTFRPNQKVTRAEFITMLGRALNLNTVSESIHYTDDNKIPSWAKQYITAAASAGIVNG

YENGSFGPSKTLSRAEMVTMIARAGGIQTDSNAKLDFKDAKDIPAWAVSYVASAVEAG

LVGGVGGNRFAPMQTATRAEAVTLIVGLLEQSK"

CDS 2959461..2960507

/locus_tag="PIKHDJKN_02699"

/inference="ab initio prediction:Prodigal:002006"

/codon_start=1

/transl_table=11

/product="hypothetical protein"

/translation="MKSHTSERIKFPAGFWTGLHQLGIAAHDVARKAQLPLTIITEPA

VTTAQYFAIWQAYSDLVGDTAEAIIKLATVFETGKYPPSVLATYHARDYRDALHRMAR

YKQLCPPESLRITEEGDDCSIELEWSKEQPGPSLLVGVTLAFLLELGRRGTGQPLTAR

LVEFSHTMGDVRTLETYFGCPIRIGATCNRLTLHRKDLDRPFLSYNEELLEILTPALD

RSLDEQQGSRSITEMVKWIMKRSLTGGRPDIQVVAKELGMSDRTLQRRLTEENTSFKH

LLTQARHEQARAYLADPTLDIKEVAFLIGYEDQNSFYRAFRSWEGDTPTNWRTEYLGA

NSLTEGSSESTTLH"

CDS 2960676..2961470

/gene="bdhA"

/locus_tag="PIKHDJKN_02700"

/EC_number="1.1.1.30"

/inference="ab initio prediction:Prodigal:002006"

/inference="similar to AA sequence:UniProtKB:O86034"

/codon_start=1

/transl_table=11

/product="D-beta-hydroxybutyrate dehydrogenase"

/translation="MDMGLYGKTALVTGSTKGIGKAIAIELAKEGVNVLINGRNDTEV

ERTVHEIKSDFPATSPEKATADLVDIGQREALFEKYPQIDILVNNMGIYEIMRYEDIS

DEVWEKYFRTNVLAANALSKFYMPKMLKNEYGRIIFIASEEAIMPSGQMPQYCMTKSM

LLSLSKSLSKLTIGTEVTVNTIMPGPTLSENVQQIIESIYPDESMTFLEKEKDFMQKN

LPQSEIQRFIKPVEIGRLATFVCSPYASAFKGAPIRMDGGMVPTIF"

CDS complement(2961587..2962249)

/locus_tag="PIKHDJKN_02701"

/inference="ab initio prediction:Prodigal:002006"

/codon_start=1

/transl_table=11

/product="hypothetical protein"

/translation="MSRDQTKKDASTSTRKAIINLLKHQGGMDVVALSSQFALSGMAI

RQHLNALKEEGLVTFEEEARPMGRPTKLWRLTPAADRFFPSGYSELSVSLINSMKEAF

GNEGLDKLLSVRNKNMQEQYLQYIGDASGVKERLEKLAEIRTNEGYMAEVKDQGDGSL

LFIEKHCPICEAAAVCTGLCKNELHLFKTVLGDQVHIERGEYIMAGGRNCIYTVREHH

SL"

CDS 2962415..2963818

/gene="ycaD_1"

/locus_tag="PIKHDJKN_02702"

/inference="ab initio prediction:Prodigal:002006"

/inference="protein motif:HAMAP:MF_01149"

/codon_start=1

/transl_table=11

/product="putative MFS-type transporter YcaD"

/translation="MSSHSQSIFSPRYFALSIGIILSVMAVGFEGLSVTTIAPSIAGD

LKGLNLFGWIFSTYLLAQIIGTLVVGRIIDRRGPAVPFTLALLLFIVGLVAAATAGDM

YTMIASRAMQGLGAGAMMTCVYTAISLSYPDELRAKILGAFGTAYVLPSMLGPYVAGL

IADQWSWRFVFWGILPILLISALLSLPAFTKLKSQPAQGENGSAATWMALLLTIGTGT

LLVGLGKLPSIIGFVIVLIGGVLMVYPLRKLLPEGTLALRRGMPAILATRGLFFAAYT

STQNFLVLALIEVKGITPSQAGLIVASAALSWCVIAYLQGRWDSADQGRGRHTRIIIG

VLLLAIGITIVFWVPVVTVTVAVIGQIIAGIGIGLAHPISGVVAFSQTGEEGVGKTSA

NLQFADSFTPGVVIGIGSSILVVCQAGGMSLQSGLIVTMGFHLLLIVLSLIASTRIAP

VTDQIRTGLQSLGLKNK"

CDS 2963852..2964157

/gene="yodB_1"

/locus_tag="PIKHDJKN_02703"

/inference="ab initio prediction:Prodigal:002006"

/inference="similar to AA sequence:UniProtKB:O34844"

/codon_start=1

/transl_table=11

/product="HTH-type transcriptional regulator YodB"

/db_xref="COG:COG1733"

/translation="MLTSDTEKLFTAYRIIGTKWTIHILCTLSQGPKKFSEIYENVPS

ISEAILSKRLKDLQNDQLVKRKALTHPIQIMYELTPKGAALAAFIPCLMEWFTTNMN"

CDS 2964208..2964546

/locus_tag="PIKHDJKN_02704"

/inference="ab initio prediction:Prodigal:002006"

/codon_start=1

/transl_table=11

/product="hypothetical protein"

/translation="MNHVLILSDTHHIVKSLSLLIQTEPSLHVLDATRDVIGNMDQLP

DNSVIIVDMNVDNIELLIEQFPEKYRVILYSGSLELMDIPIHLQSTGCRYFNAYTSPE

EIIKILMGCV"

CDS 2964592..2964948

/locus_tag="PIKHDJKN_02705"

/inference="ab initio prediction:Prodigal:002006"

/inference="similar to AA sequence:UniProtKB:O67709"

/codon_start=1

/transl_table=11

/product="hypothetical protein"

/db_xref="COG:COG0316"

/translation="MIQVSEAAAEKIVEILVSADTQSSFLRVGVDEGGCSGLSYTLIV

DEQQAEGDILLDKGVFRILVHTNTVPYIEGLEIDYEESGMLGGFTMNNPNAKVSCGCG

ASFRMANYRGEVKKCD"

CDS 2965062..2965952

/gene="murB_1"

/locus_tag="PIKHDJKN_02706"

/EC_number="1.3.1.98"

/inference="ab initio prediction:Prodigal:002006"

/inference="similar to AA sequence:UniProtKB:P65463"

/codon_start=1

/transl_table=11

/product="UDP-N-acetylenolpyruvoylglucosamine reductase"

/translation="MNIFENHIPLEKLKWNEPLKDHTYIKLGGKADILIHPTTKEEIV

NAVNIAKTYQIPLTVIGKGSNVIIKDQGVRGITLSLSQFDQINVYGDKIVAQSGSNII

DVSRTALHNSLTGLEFACGIPGSTGGALYMNAGAYGGQMDEVVERALVVTKNGELLNM

VRDDMQLGYRNSIFRTDQYIILEVEFKLKPGNKDVISSTMQDLTLKRESKQPLEFPSC

GSVFKRPEGHYVGKLIQECNLQGTRIGGAEISKKHAGFIINADDATAEDYLELIKLIK

KTVRDQFNIELETEVIILGE"

CDS 2965959..2966387

/locus_tag="PIKHDJKN_02707"

/inference="ab initio prediction:Prodigal:002006"

/codon_start=1

/transl_table=11

/product="hypothetical protein"

/translation="MNSELSVVVYILVLLSSHENEKLTSTQISERIRVNPARIRRVLS

SIKKVGYVKAKEGIDGGYVLSKDPYEITLKDVYTLVSSKPLQIPKPSIMKKGNLTAEV

DIQLASIFIESEQQLMQQLEKITLGSMLHVMKECALETVE"

CDS 2966425..2966973

/locus_tag="PIKHDJKN_02708"

/EC_number="1.5.1.45"

/inference="ab initio prediction:Prodigal:002006"

/inference="similar to AA sequence:UniProtKB:A4IT49"

/codon_start=1

/transl_table=11

/product="NAD(P)H-dependent FAD/FMN reductase"

/db_xref="COG:COG0431"

/translation="MKLIGLSGSLIGSKTPVGVDAVLQFVKNNHPEIEVELIDLRDYK

RIEFCDGRKLEDYNEDTQLVVQKLIDADFYVIGTPIYQSSLTGVLKNVFDLLPVQSIY

NKVMGFIATGGTYQHYLVVENQLKPIAGFFRSYVAPSYVYLNSDHFDAENNIKDTEAL

SRLEKLAEELVFMQTQLKASSR"

CDS 2967154..2968560

/gene="bamB_2"

/locus_tag="PIKHDJKN_02709"

/inference="ab initio prediction:Prodigal:002006"

/inference="protein motif:HAMAP:MF_00923"

/codon_start=1

/transl_table=11

/product="Outer membrane protein assembly factor BamB"

/translation="MLKTKKKQQFTNLKGQLFRCIVASFTAVGLLALGGNAVILSQAH

ADAPDVSVRNWYSYMGISVPELKPSWTAQVDNYLNMNEPYIGHQAVAEEGKVFTFADS

KLISLDAKTGKRLWSYGKGLTPYVVYHNNVIYGLTGDHKPYALNAKTGKAIWQSGSST

WIDTRYRTEMLVPTGDTLYIIQGSTTFAFDMKTGKMKWRADEPLGEGNGTDYLEESNG

VVLRTFYVQGALTSIQLNAYDKKTGKKLWDHFGQGEALQIKDGLVYSVDYHSSKLTEY

QSKPERKVSVNAYNLNTGVQKGSLEYNWKMNGDPPYDYGYGSVFASNGKLYIEQGERV

AEYNFDKYTANADPLRTYQRPFYKENGQSLGIVQERLIYKNETTGELAGIKLVNGQEI

KWYGDAPVAQISVYGKGMYRTQRNGTMFGINMLTATPVFRVTTGADLHEATLKTDGMI

IIQAEGKLLGVKLPASLR"

CDS 2968768..2969991

/locus_tag="PIKHDJKN_02710"

/inference="ab initio prediction:Prodigal:002006"

/codon_start=1

/transl_table=11

/product="hypothetical protein"

/translation="MSGAHDTKPVYPDEQESIAIFENTYEQYRQRISKYFSLKLNPLV

ADDLTQQVFLKAVENIHNFKGSSNLFTWIFKIAQNTVKNEYRRLSRQKESPYDFTDYE

SQSISLEFTKHVDIRIDIGSALQKLDEIDQEIIALRFFVDCTLPEISKIVGRRESAVK

NRLYRALEKLRKELKEWGEIAIMSIQDLISIVNKGENQDTNDRTKKVHQDLFDELNNS

VERVSTKYKHHPSQKVVIEIYPDLPTFHQAVGETDAPNWFMGTYEGRTLEIVSPLEPG

PEHTYQSILKGTVHLFAMWLISDINPAAPKWIRQGIGGYEAKQMSQDFIKGSTEDSIR

NHSIPSFDELNNDTWDFETMKGFQFSYMMVEFVVERYGVDALNKLIRSPQDFEGIFQC

SETELHEQWVKYIKI"

CDS 2970179..2971207

/gene="srkA_1"

/locus_tag="PIKHDJKN_02711"

/EC_number="2.7.11.1"

/inference="ab initio prediction:Prodigal:002006"

/inference="protein motif:HAMAP:MF_01497"

/codon_start=1

/transl_table=11

/product="Stress response kinase A"

/translation="MTFFQNGTHEELQCLLVQARSVCLRALDQYELPWDCIHFIQLSD

TITYKIDTAPSKSYLLRIHSNRLSKEAIRSELTLLQSLGTSDDLHVPQGVASRGGSYV

LSMDTEEEGQYFDVTMMNWVDGEHMDGNEVNERCAYRMGSMAAKLHEATARFVPSLDF

ARPTWGEESFKVDMTKLRRYYTRFLSNQAWKTYQKASHKILSQLADLDQNPQHYGIIH

GDLHIGNIVFNDEQPNPIDFGRCGYGYYLYDIANTILGLFPAQRLEFIRGYESVRTLP

TDYVEVLESFFIMCMIENYSHHSSDPRETSRIIDEQPYAQACIREYLMNAPFLFNGIE

VQEVTYIN"

CDS 2971570..2976234

/locus_tag="PIKHDJKN_02712"

/inference="ab initio prediction:Prodigal:002006"

/codon_start=1

/transl_table=11

/product="hypothetical protein"

/translation="MKSRLALITAILMTVTSLIMPGTMKPIHAASSHNFYVSHEGNDD

NAGTAESPFRTIEKVKQAVRGINGAMTEDIYVYLQDGEYMLDDTLVFNSEDSGSNGYN

IIYTADTGAKPVISGGVDISDGWELYDTEKNIYKRTGVNWNFRQLYIGEDRGIRARQP

NLTEADADLQGPYFRAKNGEYPYVIGEDAREWGNILNGTNAEMVVLQSWSQVRGRIDT

INTTTGQIDFKFPESGFSYNHHGQGNSPFFFENALSLLDAEGEWFLDTDDQTVYYKPR

SNETMNTTRIIAPRLETIVKIEGTEDSKVRNLEFSGLTFKHSNWLAPSSYGYVDVQAG

FRYQSVTGGNNTEIRNTARYTAANAMLELKYSGDIRIARNTFQHAGSWGIMGYEGTEH

TDITWNTFTQNAGGGIAMGIVGDLWDDDEGQNPSYTLPDGQSQHDSITHNTIDRVALD

YGDMVGIGAMLPQHMTIANNEISNLPYSAISIGWNWSDLDHGMTNNQVHNNYLHDSVK

LIQDGAGIYTLGRMDGKSNIYHNYIKNMRQGKYAAWNHIMGVYLDGRTSYRMVEQNVI

DNTEHAFSGSDHDNIFRHNYYNVDFGNNNASNTAYGNVEVAGSNWPQEALDIIAAAGP

GKEPLPAPKEPTNLALMKPVTASSQSKDPAFAVDGDPTTRWAQEEGRADPQWLMVDLG

DVYPIASTSTMFELENGYTYKIEYSTDGDVWDMYADKTANVTTQQTNLDLKAVEARYI

KITMTHDWGGSIYELSVFPGEGTKLPESNSLLKINEATFEKNPTKQDDIRVKMIFNGG

ALNGIKLGDTYLVADRDYKLFWDQVIIQKAYLDTLPIGENHLTFDFATGVDPVFKVTV

MENDGATVISLNKPAMASSQSLDPALAVDGNPRTRWAQQEGLWHTEGWLMVDLQGVYA

IKSVTTMFELSSGYKYKIEYSMDGQNWQTYADRTESETTKQTYQDHHSSEIISRYMRI

KALYSMGPSIYEFSVYGVPVGSNSSINLATAAFNKHPNKQDDIIVEMNLQGNTLTGIK

NRSLHLKPDTDYTVAGNKVTIHRDYLTKLGEGKNHLTFEFSSGENSLLELNVTDVSGE

EINIALSKVVVASSQNKDPLFTVDGDPDTRWAQQEGLTTSQWLKVDLGDVYSISGTKT

IFELSSGYKYNIEYSIDGFNWTKYVDKTSVATTEQVNEDRKDAEARYVRLNITDSSGN

GASVHEFSVYTVKETTVTDATINPVAVTFDKNPSQQQDVVTNVQLNGNVLKSVNNGNQ

LLNLNSDYTFAGEKLTVHKSYLASLGIGTATLTLVFDQGNDAKLEVKVMNTQGTGNNG

GNSGGNHGSGNGNSAGNPNNGGTESTTKSGNENNGNNGNTEGKSDGEAGGENGVVVPT

FSDVANHWAEANIYKAAKLGIVNGYNNGTFKPNRTVTRAEFTVMLMKALKLADSESEL

SFTDAGKIGSWAQQAVAQAVQAGIVNGYKDGSFRPSASVSRAELAVMVARAGKVKIAD

VETTGFADDNDIPAWAKSSVFALKEADILKGSSGNKFMPNALATRAEVITIIMNQLSV

DQ"

CDS complement(2976279..2977514)

/gene="rssB_4"

/locus_tag="PIKHDJKN_02713"

/inference="ab initio prediction:Prodigal:002006"

/inference="protein motif:HAMAP:MF_00958"

/codon_start=1

/transl_table=11

/product="Regulator of RpoS"

/translation="MTTLLIVDDDQFIREGLKRLINWKALNIDVKLEAGGSYEALELM

ELHKPHIVLTDIRMPKGNGLHLIEQIRLRKWKTFIVVLSGYNEYAYVRQAMKFQVEDY

LLKPIDASELTQIIVSCTERVEGQFIAEQMQRESFQLLKNNILLRWAQNRIQNDQLRE

KLKFLQLPLLMNENYQICLIDWSNPQEGELSRMEEQFRSFAILNSMEEALHETGRGIT

FLNEQRQIIVLFADEGTETEKFNIINQRWMDDIAQRYGEILKTPWNNYFGKVITLPQQ

LHESYSEALASCNHVVLDDAGQDAGILSQNPITRKVESYILKNYSEELSLQLLSMRFN

VNNVYLGRLFKEETGQYFNDYLNRIRLGNAKKMLVSTALKASEIANAVGFLDPNYFFR

KFKQVHGISPSDYRMSEKK"

CDS complement(2977538..2979319)

/locus_tag="PIKHDJKN_02714"

/inference="ab initio prediction:Prodigal:002006"

/codon_start=1

/transl_table=11

/product="hypothetical protein"

/translation="MTLNEHQRGRWMSIVSFYRKWIGRYIFRKLNIAIGTALLLVFLL

LGMAIYNNFYSILEKKEIEMLSIRTEKLKIQLVDMIERFQYDTLSIYQGMGSNMQTSV

SEMFLPGNIPTDMEDREAITQQNYIKNVLTHMLSRNPYTSAVLLYRLQDGQLFIESQQ

QKYRINEAFALQPFFETFPKAYQHPYFGSSDELLTPAEHILYIANPIYNPLRIRPEDV

YGYQLAVLNTGVITNEFDSQNSDYRLIIKQRNNLLLDSKPNEPFDLSTSTDLISRITI

DKNHLEIIGISSKSNIESKLANTMIRILITLGISWLVCIMIIHIIQRLIVGRFNQMSK

HFKKVQRNPFTTLMPVNGDDEISDLIMRFNRMTEELQNHINQVYVAEIRKRDAEFIAL

KTQIHPHFLYNTLESLRMQAVISEQPFLAEKIYHLGLLYRWMLQPTDDLISVQEELAH

TNYYLELLMLGKSNRIELRVISELNLENCFMLKFTLQPVIENAIQHGRLEQYDEPIIS

IEIKKETNLLTIEICNNGQCITAEDYSRLQELLQTPNTFPDQHLGLKNIHERIKHYYG

NEYGLSLPEMQQDTELFRLIMIFPHKR"

CDS 2979539..2980828

/gene="msmE_3"

/locus_tag="PIKHDJKN_02715"

/inference="ab initio prediction:Prodigal:002006"

/inference="similar to AA sequence:UniProtKB:Q00749"

/codon_start=1

/transl_table=11

/product="Multiple sugar-binding protein"

/db_xref="COG:COG1653"

/translation="MKKWFTMCMTLLILGTVLAACSGSEGSKGNQTNKEAGKDIEIVF

QSWVTPNLTVDYYDQLIQRFESENPGIKVKRIQPPANEGSPDSYLKALLASGDFPDVV

QNATIQMFVDADALLEVPIDDDIKNINNYEAEMIDGKLYNISAIRQPQSLIFYNKKLF

DEAGIEATPKTWAELETAAEKLKAKNITPILTAGDWTAGFTFSVMTSSNIYKENRNWY

ADRFDNKVKFTDADWIESASYYTDLVKKGYFNKGALSIDYTAAEQEFLKGKGAMYPMG

SWFSAAEATADKDFEVGVFATPTKDGTQYLLGGENAGGYAVAKNSNHPEEALKFAKFM

MLDPEAHKGYLQADALFSNLKVPLKWDMSPLQTEIYELLLNNPPMVGHMNNKVGKVPV

AGIQDQYNKVAQSILLGDTDVENNMKTLDEYWDKNNK"

CDS 2980903..2981775

/gene="melD_3"

/locus_tag="PIKHDJKN_02716"

/inference="ab initio prediction:Prodigal:002006"

/inference="similar to AA sequence:UniProtKB:O34706"

/codon_start=1

/transl_table=11

/product="Melibiose/raffinose/stachyose import permease

protein MelD"

/db_xref="COG:COG1175"

/translation="MNKNDRWSYTLMFSPGIIIYFCFLFIPIILLVYYSFYNWDGVSP

SYQFAGLSNYIEAFHDKAFGRALVVTLFITVVATTVVNILGIGFAVLLNKKGRMTNFY

RSIFFFPLLLSTVSVGFIWKSILSYNGLLNNLLERIGVERIEFFGNPNLAVLTITFLA

IWQSTGFVIVIYLAGLQSISSSLYEAVKIDGANRWQQFRHITFPMLAPAFTMSIIFMF

TGSMREYDRVAVLTYGGPAGATETIAYQVVKIGLNANRLSYSASLAVYMLVIVSILAV

SLTVYLRRREERLL"

CDS 2981772..2982605

/gene="melC_3"

/locus_tag="PIKHDJKN_02717"

/inference="ab initio prediction:Prodigal:002006"

/inference="similar to AA sequence:UniProtKB:O34518"

/codon_start=1

/transl_table=11

/product="Melibiose/raffinose/stachyose import permease

protein MelC"

/db_xref="COG:COG0395"

/translation="MKNNLSQYIRHVFLIPLSLVLLVPFYIAVVNAFKKKADILSNPF

SFPLSNMTFDNFIRSAVTPSFNIFKAYWTSIMITSVSVVLLIFLCSMMSFVFARVNNR

FLSVCYLLLLSGLMIPPQVILIPLVQVLSKLGLMFTPQGLILYNIGFYVPFTVFIFVG

FIRTLSKELDESAKMDGASQLTIFWRIIFPLLRPATASAVIFLCLWIWNDFINPVIIL

GSTKSYTVTTGIFQAVGQYSTNWEDIFALVVLASAPIFLLYLFMQRQFISGLTEGSVK

G"

CDS 2982852..2983766

/locus_tag="PIKHDJKN_02718"

/inference="ab initio prediction:Prodigal:002006"

/codon_start=1

/transl_table=11

/product="hypothetical protein"

/translation="MPQVKRVIRSKWFLWMTGVVVILIAAFCVLSFILVNSVSATFET

GSVEEKYALPGTYKVDMLEIKDEQGEKLYRIYSPQSTDVLHPLIAWGNGTGALPDNYD

QLLRHLAGWGFIVIDTYSQTTGTGKEIMAAIQYMLSENEATASPFYHRIQKDRVGAAG

HSQGSTGVINAHTNFENGALIKTVVSIALPELKYCDPEDVYDTSALKVPFFVIGGTRD

FIISPSESNKLALNNTNSDLPVLMAMAKGAAHTAIEQDGGQHRGYLTAWMRYQLMNDS

EARMAFVGKHAEIGTNKHWKDVTSANLQ"

CDS complement(2983919..2985034)

/gene="araR_4"

/locus_tag="PIKHDJKN_02719"

/inference="ab initio prediction:Prodigal:002006"

/inference="similar to AA sequence:UniProtKB:P96711"

/codon_start=1

/transl_table=11

/product="Arabinose metabolism transcriptional repressor"

/db_xref="COG:COG1609"

/translation="MVRTFKPMYKKIETYIIDQIRSGNWKPGSRIPSENELAEQFSVS

RITVKNALTALVDKGVVYRLQGKGTFVNDHSPEEIRNITSAVKEQLTAPTIGFLLPRL

DNRFTANLLSGIEDTLSENGYQLLFAKTNDSQETEILKIQEMKQAGVRGLIIYPVEGE

HYNNEILALTLNRFPLVLLDRNLKGVETNSISSDNHEAAIQAVKHLHELGHSRIGFIS

TLAEGTSSIEDRLHGYEKALEERHILIDRSIQMTRLAMSDSNEEVIMMIQKFLQANPQ

MTALLSSNFSPHVIQAAMQMGISVPEDLSVVFFDDVEFPEFCIIPPTAVVQQELELGR

EAGRVILKQVENPSSEYLQVKLPTMLIPRQSTAKAKS"

CDS 2985442..2986821

/locus_tag="PIKHDJKN_02720"

/inference="ab initio prediction:Prodigal:002006"

/codon_start=1

/transl_table=11

/product="hypothetical protein"

/translation="MKKRAGMIVTLLTVFSLIVSACGDAQSGGTEPKASKELTMWTWK

VAYTPGIEAAAKLYEEKTGIKVKLETFTPDDTYRQKFQAAANSKNLPDIVNWWATAGD

SIENSVLELSGEVSDDLLNSYYSAAMDPIIVTQSQVDSWKEDKNATTIQKSLKTGQFY

GLPLDIGGFFTFYGNKKLIEEAGLTAEAPKTWEEFVTMMETVKEKTGTPGLVFGAKLP

DLWENWAGSALSIMLNEPQGYIDLLERKSKLSDPSNLPVVKAMETLANKDLFMPGILS

TDIDGADQAFAAGKAAFDLGGSFTMSTLLAMGMSPDDIFTFPVPPLEGSKINSWTTDP

FTLTMLSVNKNSQNKAEALDFIKFLTGDPDAAVAFANAAYTVPALNLGDRANDLDPNL

KSISDAFAAEPGPYSQASPAINTYRGKHKEWEVYAQSMQSMIEKKMTAEQVAKKFDDT

MESLRASGN"

CDS 2986894..2987787

/gene="lacF_8"

/locus_tag="PIKHDJKN_02721"

/inference="ab initio prediction:Prodigal:002006"

/inference="similar to AA sequence:UniProtKB:P29823"

/codon_start=1

/transl_table=11

/product="Lactose transport system permease protein LacF"

/translation="MIKSRNKWMPYLFLLPGVLLFLAIGVYSVGFSIMLSFYNWSGID

FSTARFEGLANFRQFLLGDDPIVTRNFYNAVLHNLIIAVSQVVVVVPIALVLAFVLQN

TKAATWYRTIYFLPMIASGVAIFYVWKGLFEPSGAFNSLFLWMGLDFLAVPGGMLGSS

STSLLGIILTSIWGGLPGTLILYYAGLTSIDPTLYEAASVDGAKKTTMILKITWPLLK

PITLIAVIQMVNGAFQAFENVFIMTGGGPAGSSEVIGTLVYRTAFLNNDYGLASAIGW

VSFLITGTIAIFSIRSFKADI"

CDS 2987800..2988618

/gene="ngcG_6"

/locus_tag="PIKHDJKN_02722"

/inference="ab initio prediction:Prodigal:002006"

/inference="similar to AA sequence:UniProtKB:O50501"

/codon_start=1

/transl_table=11

/product="Diacetylchitobiose uptake system permease

protein NgcG"

/db_xref="COG:COG0395"

/translation="MVKYIKHIILILYGLTCLYPFVWMIGTSLKTSQDALANPQSPFP

QAAPMWSTFGDVWNKLNFYQFFINSVIVSAVVIIGVILIYTMMAYSFAKFVYRGKKFI

YYTFIALLLVPGVTTLIPLYINMTNLGLQNTYIGMILPMINGAAPFAIFLFTSYFRTI

SHELYESAVLDGCNNFKIYYRIYLPLALPAIGTIAILNFIGSWNNILWPMIIVDSRDM

FTLPMGLMYLDSSSFKKWNELMAGALITVIPILLAFPFMQKMYVKGMTVGSVKM"

CDS 2988659..2991418

/gene="mngB_1"

/locus_tag="PIKHDJKN_02723"

/EC_number="3.2.1.-"

/inference="ab initio prediction:Prodigal:002006"

/inference="similar to AA sequence:UniProtKB:P54746"

/codon_start=1

/transl_table=11

/product="Mannosylglycerate hydrolase"

/db_xref="COG:COG0383"

/translation="MTTKTAHIVAHSHWDREWYLSFEKHRMLLVQLMEDLIETFENDP

EFTNFHLDGQFIVLEDYLEIMPHRSNQVRKLIEEGKLIVGPWYILQDEFLVSSEANAR

NLLIGIQASEQMGGYAKIGYFPDSFGNMGQAPQLISQAGIEVAVYGRGVKPVGFNNEI

QSGNEHTSKYSEMYWESPDGTRVLAILFANWYNNGMEIPVEPEKAKAYWAEKLAAAEE

FASSSELLFMNGCDHQPLQKDLTQALKTATEIMPDVTFRHSSFPEYIQALQKAKPQSL

DVIRGELRSQWTDGWITLVNTASARVYIKQQNVKSQTLLEKVAEPLAVMASLSTGIYP

RHELLYAWKTLLKNHPHDSICGCSVDEVHREMMTRFAKSQEVAEAISVQSARTIAESV

DTSGFDADSALPFVVFNTSGHRRSNVIVTRMDVERRYFDHQPPHEKYQEFKREYETFD

SNDWVVLNAEGKQVEAVITPVGPTFGYDLPDDRFRQPYWAYQVDVELHAQNIPGFGYH

TFALMPQSVIGESVLSELNTPELVTEQQVMENDYVRIEIAGDGSFTMLDKVNGRNYTG
LGIYEDTGDIGNEYMYRQPDQEAPLTTQGLPAQIKLVHHTSLKAVYEIVHNWELPIGA
DELLDEEIRSMVPFRYRKSRRLDETREVSIRTELSLEKTGRSVGIKSYIDNTVQDHRI
RMLFQTGLEASHVTVDSIYELAQRPIQPEAEWRNPSNAQHQNAFVSISNGHEGLTIAN
KGLNEYEVLANQNTIAVTLLRGVRELGDWGVFATPEAQCLGQHILEMELISHDLDVIQ
SDAYVQAYQFPVPWTVQQTGVHGGTLPATHEFLQWEGDHLAFSAIKRGEANDDMLFRV
FNVSSETTSLKVTPSFEAKKVYESTIVEDQGEAMKVADEGSYVLNVGPAKIQTIGIQP
TTRLK"

CDS 2991542..2997097

/locus_tag="PIKHDJKN_02724"

/inference="ab initio prediction:Prodigal:002006"

/codon_start=1

/transl_table=11

/product="hypothetical protein"

/translation="MNKRLWLGARCTISVLLTIVMLCSGLYITPATTHAAAEGEDITE
QGDVHLNDVSPDSVSITLGETPVQQGITARPGDGPPAGLQVGTFDGKGYWKTNQTVNP
TNPADNILYFYFNVADEYLFENTDQDVFVTVEYYDSDSGSMSLQYDSKQSNFKDAPLF
RYENTGTWKTHTFKLSDANFGNRTHDADFRIGVSGAGAPANIPELHLAKVTVRKQPQQ
QAGTQTKVYDTEFPTSDIVIADRSVTDYGAIGDGVSDDTQAFKEALAIAGNQGGGVVF
VPSGTYKLTGELVIPTGVTLRGDWGNPEEHDGLVKGTILAVYSGKGQANGNSFIKMEP
SSGVTHLSIWYPEQNMNNPVAYPWTMEQLPGDSATVKNVTLVNAYQGIKIGPEWNELH
YVKDVYGTTLKTGIFLDFTTDIGRLEELHLSPDVWANSGLPGAPEQSQLREYMIQQSE
GIVMGRSDWEYMSNIHISGYKTGIRITTRTGSLETANAQLYKIQIDDCNVALKIEGVN
DYGLLVTDSQFEASAGPNPVAIYATEGFHSIAQFNSVTVGGEPKHAVVNEGRGTLSFE
SSSFENWGEGSGDYAIVAKSGSLILGSSTFAKPDRHLFLQGDVATINAIHSGHEGNLA
VNDESTSAELNIHQEEGLVLETIPDVPSVDIVVQPKPGTRELFNVVSQPYLADNTGNT
DSSQAIQQALNDAEAAGGGTVYMPAGIYRVEQALVIPTAVELRGNYDVPHHTIGNGTV
IFTNYGENAPDADAFIQLEEAAGLRGLSVYYDQQTWSDANPVKPYAWTVQGQGKNVYL
IDTTLVNPYQAVDFGTHDTSGHYIDYVAGSPLKEGIYLGGGSDGGYMRNVQFNPHYYG
RNNYPNHPSNDEEFNQIWNYQKENLDAFRIGDVTNQVIFNTFVYGSKYGIHFEEQNGR
GPEAVVIGHGTDGSKKGAVLQHAGTAGLSFINTELVSMSTTDKVYVEVGPDFGSKAVF
FNTSMWGDTTRSFDIHGGDIRIQQGNFTNVGEIGINAIGGDITLYNSYFQQPRTNHVV

AGPNVEKMVISNNLFNGGFQIDNEAPPGKVTGTNLFPLALKLNQLGYDPSRPGEPGST
LTLTNVAGAPLLKGQIELIEPSVYHAIFKPVRFEGLGIGESMDVALPYISSDLLKFRV
TLESGFRYETSVKLSQSFAAQKDAVGTLPFIEVSSPEHYNSVGGSWGGPNDLSIQAKA
SWDAENLILKVDVKDDIHHQSNTNGDIWQGDSLQLGIDLGKEDGAASKQVNELGFALN
QEGVITKWRWRAPEGVATGLLESASANITRDETTHITHYQITLPFAALHQEGYSFQPD
KPIGFALLVNENDGEGRSGFMEFNGGIGTSKDARLFGNLYLLTSGYVTLLEQSAEASV
RTAEQKKDVTSIDTATNFVNLLPGGTLKSSLLVRLAALTSGSNTNPEGPNPHPGSGSG
SEPSAGGGSTTGGKNEATPAAPVATMQLQPKLDKAAALATAELTNKMMEDAFNRITPD
QDGKKKLIVDIESVDGAEGYALTLPSSFLQETPSTHWLEIRTAGGVLLVPSNLLANTQ
GTIGERSTLVLSKADRSTWNAKMKSDLGERPAISLSVISDGKSVIWQNSITPITVSLP
YQPNVTELLDPEKLAVRLIADDGQIISIPNGKFNKKSEQMIFKTRQNGVYAINQDNRT
FQDIRQLSWARHAIEVLVSKGVIRGMSKAEYQPQSMIRRADFVTLLTNALELQPDQGT
KVERFADVPEHVYYEQSVNTARTLGIIQGAGADLFSPASPITREEAAVMIYRAMQVIG
MQPLPEGESSLDDYKDTHLIKAYARDAIDSLTRSTIMEGSNDSFHPEATLTRAETAVL
LYRLYNRLP"

CDS 2997143..2998366

/locus_tag="PIKHDJKN_02725"

/EC_number="4.2.1.-"

/inference="ab initio prediction:Prodigal:002006"

/inference="similar to AA sequence:UniProtKB:C6CBG9"

/codon_start=1

/transl_table=11

/product="D-galactonate dehydratase family member"

/db_xref="COG:COG4948"

/translation="MAKITRIECLRTRHDGSWTIVKISTDQDGLYGLGSASDLYNPEA
VVQVIEQLLAPLLMGRDASQIEDLWHLMHMSGYWRNGAMLNTAIGGIDMALWDIKGKE
ANLPVYQLLGGACRSAVPCYGHAGGADISELKEDVSRFMEEGYTVIRVQMGGYGGGGF
VSGKDANLPREPWSSGPVFDEHAYLHAIPDMFEKLRLEFGNGIQFTHDVHEHLSPIHA
IQLSKRLEPYHLFFLEDALAPEQIGWYRQLRQQSATPQAVGELFVNPQEWTDLIQEKL
IDFIRVRVSKAGGISACRKIATLGEAYGVRTAWQEGGENDPVNQAAAVHLDMALWNFG
IQEINHFKPHELEAFPGHIVREGGYLYPSNKPGLGIELDEVKAKSLLNDSWDPNKYHR
PYPLDRKADGTLVRP"

CDS 2998401..2999243

/gene="fcl"

/locus_tag="PIKHDJKN_02726"

/EC_number="1.1.1.271"

/inference="ab initio prediction:Prodigal:002006"

/inference="protein motif:HAMAP:MF_00956"

/codon_start=1

/transl_table=11

/product="GDP-L-fucose synthase"

/translation="MKTIAVTGGGGKLGSQVIEKLQLQGHDVVSLDKHLSDRVRCRQI

IVDLNDYGQVVGALAGVDAIIHLAAIPAPLHYPHSYIFANNTLSGYHVLEAASLLGIK

KVVMGSSESSYGFAWANTPFSPDYFPVDEQHPQQPQECYGLSKIVNEVTAEMFHRRSG

MQVISLRFSMIVGPGEYRHLAIAKAETFKHILWSYIDIRDAVDACLAALHADYNGAVH

VNVTANDTLSDRQTTDLLATFYTDVKDLRTRFSNREAIVSNGRVKEVLSWGPKHSWTD

EEQE"

CDS 2999279..3000970

/locus_tag="PIKHDJKN_02727"

/inference="ab initio prediction:Prodigal:002006"

/codon_start=1

/transl_table=11

/product="hypothetical protein"

/translation="MLNFMQKNKRVLAFASAALLILTLCLSISPSIVSAASAFNQTQA

SSYNSQSGVQLESSSEGGQNVAFIDNGDYIVFNNVDFGSGANSIDVRAASNNSGGTIE

VRLDSLNGTLAGTVVVPGTGGWQSWQTKSGSISGATGVHTVYLKFTGGTGNLFNLLWF

KFSASAAGSGGDVVGKLYAGYQGWFNAAGDGSPNGGWVHWSKNSSAPSANGNVNFELY

PDLREYSKLYQTSLANLGNGSPAKLFSSYDQETVNKHFEWMQTYNIDGAALQRFGADE

SDTPNNWKSNRDSVAVKVKNAAESYNRKFYVMYDITGMNSSNWVQAVKHDWTTNVVNN

MHLPSSSAYAKQNGKMVVCIWGIGFTDRPGTAAEAADLIGWFKNQGIYVIGGVPTYWR

TGNNDSRSDFMNVYKSLDMISPWSVARFGNIQQADSFKTNQLQPDLAFTQQNGIDYQP

VIWPGSAWSNMTGGPRNENPRLHGDFMWRQAYNLKSIGINTGYIAMFDEYDEGTAIAK

MSENSSMIPTNQYFLTLDADGVAVSSDFYLRLAGDINRMFKNQIPVTANHPTSHQ"

CDS 3001022..3002350

/locus_tag="PIKHDJKN_02728"

/inference="ab initio prediction:Prodigal:002006"

/codon_start=1

/transl_table=11

/product="hypothetical protein"

/translation="MTTNDIDFETTRQKTTSMNPVITSIYTADPSAHVWNDGKIYIYA

SHDMDPARGCDLMDRYHVFSSEDMVHWLDEGEILRSDDVSWGRPEGGFMWAPDCAYKN

GTYYFYYPHPSDSNWNDSWKIGVATSRYPAHGFTDQGYIEGLGGFALIDPCVLVDDDN

SAYIYYGGGNVCLGGKLNEDMMSLENGMTEMQGLEDFHEAAWVFKRNNLYYLTYADNL

ENNNRMRYATSENPLGPWTYRGIFLEPTGCSTTHGSVVEFKGQWYLFYHNQAISGEGT

LRSVCIDYLEFDEDGSIKPVVQTREGVSSVGPAPVPVQQRKVYAGAASQVFGGAEIRE

LQSGQTVVGNLQSSEAFALFKNIDGGEGGRASVELHYATGERLAKLHLTINDQDYSLL

NALSTGGLQEFNGITNITVPLEPGNVNTIKISGGHGEINLLCLTVIPLLD"

CDS 3002612..3005083

/locus_tag="PIKHDJKN_02729"

/inference="ab initio prediction:Prodigal:002006"

/codon_start=1

/transl_table=11

/product="hypothetical protein"

/translation="MRKSRRFMFTLLVILLMVSSVPLPAKAYQNPQTLPDEWGQYGLG

DPYVLKFNGYYYLYVSTRDTDDGVKVWSSKDLVNWSYRGLAATDPITHGAYAPEVVYW

NGMFYMYTSPAGKGHYVLSSTSPTGPFNVVTGNLGKSIDGNVFIDDDGSFSFYHAAQG

RIDAAPMSSPTEIGVSASTGVGMGGWTEGSTVFKRNGKYYMTYTGNHVFSKGYRVDVA

VSTTGPRSGYVTDSTNPVLIRTEGPTVGLGHNTVVTGPNLDTHYMMYHNLEGNGIIGP

LRHLNMDRIVWNNDKMIVQGPTSTDQPNPEMPVFYDYFDRSAIGSNWSNVNGGNWGIY

NQELMWQNSMGSSTWYKQVTASSSASDYTAEFNMKEMNRGTSTNPRYGATFSYTNENN

YGVALISSKNKRLETFFRINGVDQAWQTFDLPADFNYSKWHNIRIEKSGSTFKYYVDG

MFKATRTANVGAGKIGVLTEDTHADFGYTAFNNDVNGSSAWNASKPIPGTIEAVHYMK

GGEGVAYHDTTAGNLGGAYRTDAGDIRLNNDGLFNLGWNQTGEWYKYKVNVESSGYYD

IDFRMATSLDGAQIRVWDGATDLTGIVNVPNTGGWDNWKNISKKKVYLNAGQRELKVE

FVTGEADFSRMTFQKNVDVTSVSDNFNDGDDNGWTRFEGTWAVENGEYSAASTGPAKS

TFGDSGWADYTVEADIKMIDSGGDGGLLVRVSNPSHGTERGQNNYDALQGYYAYLTTS

GVSLGKMNYNWALLKSNAQSYSTNTWYHMKVAVNGTNIKVYVDDMVTPKIDYTDNSLN

PFTHGKVGVRTANKHTHFDNVSVTP"

CDS 3005301..3006218

/gene="nfo_2"

/locus_tag="PIKHDJKN_02730"

/EC_number="3.1.21.2"

/inference="ab initio prediction:Prodigal:002006"

/inference="similar to AA sequence:UniProtKB:Q5KX27"

/codon_start=1

/transl_table=11

/product="putative endonuclease 4"

/db_xref="COG:COG0648"

/translation="MKSSDLLIGSHVSFGGKEMLLQASKEAASYGASTFLMYTGAPQN

SKRKPISQYNIEAGQLHMKHQGISNIVVHAPFLINLANSYKREVFDFSVQFLRDEIER

TASLGSNQLVLHPGAHVNAGLDIGIRQIIRGLNEVLQDDNHPEVQISLETMAGKGTEC

GTSFEELAQIVDGVQRNERISICLDTCHVHDAGYDIVHNFDGVLEKFDKVLGLDRLKV

LHINDSKNVRGSRKDRHENIGHGHIGLQALDYIVHHPELVSHPKLLETPSIGKIKYKS

APYRHEIALLRRECSEPVQKEEDSLENSK"

CDS 3006320..3006664

/gene="yojF"

/locus_tag="PIKHDJKN_02731"

/inference="ab initio prediction:Prodigal:002006"

/inference="similar to AA sequence:UniProtKB:O31858"

/codon_start=1

/transl_table=11

/product="putative protein YojF"

/translation="MQPIEPQDIQHRINQLLDQDLYLHLELTSGAYASHFDSTRHPGS

AFISNAVIRYTQGSISGTGPYRVGLKTTHGWIYAEGLTHIDELEQERLILAGHDSQGK

LVVALQLSRELF"

CDS 3006688..3007362

/gene="bshB2"

/locus_tag="PIKHDJKN_02732"

/EC_number="3.5.1.-"

/inference="ab initio prediction:Prodigal:002006"

/inference="similar to AA sequence:UniProtKB:Q81WT0"

/codon_start=1

/transl_table=11

/product="putative N-acetyl-alpha-D-glucosaminyl L-malate

deacetylase 2"

/db_xref="COG:COG2120"

/translation="MNNKQIEHQRILVVFPHPDDEAFTASGTLAKYIEGGAQVTYACL

TLGEMGRNMGIPPFANRVTLPIIRKDELIESSNAIGIQDLRMLGFHDKMIEFEDPELL

DGHILALIKELNPSLVITFYPGYSVHPDHDATGAAVTRTIGRLPAEERPIVYCCAFSN

NHEESIGKADVIEDVSGFLAKKMASIRAHRSQFQAPELVGKREMDDEQIRNRFGREAF

WTYKFD"

CDS 3007566..3007868

/locus_tag="PIKHDJKN_02733"

/inference="ab initio prediction:Prodigal:002006"

/inference="similar to AA sequence:ISfinder:ISDha13"

/codon_start=1

/transl_table=11

/product="IS200/IS605 family transposase ISDha13"

/translation="MANKSYSLAHTKWMCKYHIVFTPKYRRKEIYNQVRKDLIEIFKR

LCKYKGVEIIEGHMMPDHVHMLVAIPPKIAVSTFMGYLKGKSSLMIFEKHAQLKYK"

CDS 3007902..3008039

/locus_tag="PIKHDJKN_02734"

/inference="ab initio prediction:Prodigal:002006"

/codon_start=1

/transl_table=11

/product="hypothetical protein"

/translation="MSTVGLNEATVRKYIREQEAHDQAVDKLSVKEYEDPFSSNRSKK

K"

CDS 3008300..3008932

/locus_tag="PIKHDJKN_02735"

/inference="ab initio prediction:Prodigal:002006"

/codon_start=1

/transl_table=11

/product="hypothetical protein"

/translation="MKELRSVFIKIPIFVMFLAITACSGETEPPATSEPSAPTNESVT

APTTENVETSETTRPQTESFDLMTGEGNSSQTATLHQGEGFTLYVFEKFSFDAAKGRL

SLTSNPDYYVDIEPLPSDYDLKKLETAGREELEKFSAVTDYSGELIEHPLGFAELYLQ

ASSEKGINDYMVWKSEKGDAFLFRLHNPKGDEASDFAGPVLVSLSTVQDE"

CDS 3008967..3009668

/locus_tag="PIKHDJKN_02736"

/inference="ab initio prediction:Prodigal:002006"

/codon_start=1

/transl_table=11

/product="hypothetical protein"

/translation="MPKMRSKQFIRITAIVILLLLITCIAFVYVNDRPGGLADRRMSK

AMGMSETMHIPIGKTAEEAIQKFRNNDDSLQIIHQEPVDGGIILFKQKTGQENNSNLQ

MEYARKIILGWKWAWGGGYSIGESSQFTSAMDYMSIPGLSHISTPFPMLFGHILDPAI

QRVTVEFEGDEKPVVTEAKLVEVGPESIIWFVYLPSSATIPYEMKGFNDKGELVTHKQ

IDDPNGMGSMVLGER"

CDS complement(3009787..3010599)

/gene="uppP_1"

/locus_tag="PIKHDJKN_02737"

/EC_number="3.6.1.27"

/inference="ab initio prediction:Prodigal:002006"

/inference="similar to AA sequence:UniProtKB:P9WFF9"

/codon_start=1

/transl_table=11

/product="Undecaprenyl-diphosphatase"

/db_xref="COG:COG1968"

/translation="MEDFILWLKYVLLGVVQGATEPIPVSSSGHLIIVQRLLGMEQNG

LSFEILTNTASLIAIMFIFREDIKKLIVGGWRFLVSRNAEYRADFMFCLYIIIGTLPA

AIGAVLFKDKIEEIFTSVYTVAISLLITGIALWLIRNLRGRKRDGDLSAKDAVLVGLA

QAVALIPGISRSGATVIASIAVGMKQETALKFSFMLYIPISIGGVILGASDIVTDPHR

AQLAIPYILAFITTLIVTYFSMRWFMGIMARGNLIYFSYYCFVVGILLLIFL"

CDS complement(3010616..3011380)

/locus_tag="PIKHDJKN_02738"

/inference="ab initio prediction:Prodigal:002006"

/codon_start=1

/transl_table=11

/product="hypothetical protein"

/translation="MKPLVQFLHFGYHQAMSCIFPVAIFGTLALSSVIQIPFLYRYDA

ILLVLLAVQYLMYRSGLETRDEIKVICIFHVIGLVLEMYKVWMGSWSYPEPGYTKLFG

VPLYSGFMYASVASFMCQIWRRLRMDMTGWPGFASSVLLGGAIYINFFTHHFIPDFRW

WLTALVFIVFRKTWIIYRVRLTTYRMPLTLAFIIVGFFIWTAENIATFFNGWKYPDQH

DSWQLVSFSKISSWFLLVIISVIIVAQLKYVKANRK"

CDS complement(3011548..3012399)

/gene="folD_1"

/locus_tag="PIKHDJKN_02739"

/inference="ab initio prediction:Prodigal:002006"

/inference="similar to AA sequence:UniProtKB:Q0PA35"

/codon_start=1

/transl_table=11

/product="Bifunctional protein FolD protein"

/db_xref="COG:COG0190"

/translation="MSTDKLILNGQTVADFMKEDMASKVAILKEKGIQPSLATILVGD

DPSSATYVRMKGNACARLGVHSIKVVLSADTTTDELIQEINKLNDDPSVHGILLQHPV

PNHIDERAAFDAISIEKDVDGVTTLGFALNAFGDAEFPSCTPQAIMRILDFYNIQIEG

KHAVVIGRSPILGKPVSAMLLNRNATVTTCHSKTVNLEEIVKLGDIIIAAVGKPLFVK

GDWIKLGAVVIDAGYNKGNVGDVDYEACFVPSSAITPVPGGVGPVTIATLIEHTIKSA

YKLSENA"

CDS complement(3012614..3013426)

/locus_tag="PIKHDJKN_02740"

/inference="ab initio prediction:Prodigal:002006"

/codon_start=1

/transl_table=11

/product="hypothetical protein"

/translation="MFKKITISLLTTTLLILPSFAATGEAANQYITMDTGMLKNNRVL

IPLRAVSENLGAEVNWNKELKNITITKGETTILLTINSKKILVNQEEVSLDVPAELND

NTTYVPARFISQTLGADVQWNQQTSQATITLDGKQLQVTMAKPKVQVPNTKRITAKQQ

QIFVDKLNEATNLSSIKQIRTYFSAYFTDRFINQIIRDKGLEYDFKFKIISQSGLTYP

DSNTARLSQYSGSSSIYGNKETLYRSATLVYTNKGWKVDHVEFSLIEEILNP"

CDS 3013710..3014192

/gene="pchR_2"

/locus_tag="PIKHDJKN_02741"

/inference="ab initio prediction:Prodigal:002006"

/inference="similar to AA sequence:UniProtKB:P40762"

/codon_start=1

/transl_table=11

/product="HTH-type transcriptional regulator PchR"

/translation="MDSNDRTKQLLYEQFIRFLHVYENHKDTEIEHFLSIAQRESMDK

IPQHLTAVHMIDCIGKHEPINNTGIADAMNLSKASITKIGNKLLEEGFVKRTKMNDNK

KESYFRLSPQGKKIFELHERLHVLEAERFYRSLDKYSEPELKIIHQFLQDSSMDMESR

"

CDS 3014213..3014788

/locus_tag="PIKHDJKN_02742"

/inference="ab initio prediction:Prodigal:002006"

/codon_start=1

/transl_table=11

/product="hypothetical protein"

/translation="MSLAELIRERRSIRKCNSTPVEQELVVELLRKATRLQPSVEAGS

WRVVYAGTPEARKRLVDCMLEQMSQSKLGKLIPGKLLDVFKKRFTDIPAHVIVMSTVG

PDRLTNDRNYAAACGMMQSFQLLGWEHGLGMLWDTESMIQNEGFFKGIGLREDERFVG

ILHMGYYDKAPRSRKRTPAEQKWTVFQGQTT"

CDS 3014806..3015291

/locus_tag="PIKHDJKN_02743"

/inference="ab initio prediction:Prodigal:002006"

/codon_start=1

/transl_table=11

/product="hypothetical protein"

/translation="MELSDLQGGIHSPKDFLDRPVSQELILELLNHAVWAPNDGLREP

WRFIFADNRNGKLMQGLQEHAPAYLLILVKEEADHHKREEDFAAVCCLIQNFRLLAHE

QGLGVRSTLHDWMYDRSRTETLGVLGNERIAAVLELGYGAEQPEGKAEIAEPQLQFEL

L"

CDS complement(3015443..3016105)

/gene="yflN_3"

/locus_tag="PIKHDJKN_02744"

/EC_number="3.-.-.-"

/inference="ab initio prediction:Prodigal:002006"

/inference="similar to AA sequence:UniProtKB:O34409"

/codon_start=1

/transl_table=11

/product="putative metallo-hydrolase YflN"

/db_xref="COG:COG0491"

/translation="MEISKGVEMLHLDYHGNVIHPILLKDQEMTILIDTGFPGQYEDL

RVAMEKVGVSLDKLKAVILTHQDVDHIGNLPEILQTRGNAIKVYAHELDKPYIQGELP

LIKDGHLETTPKGTVNHTLIDGQELPFCGGIRVIHTPGHTPGHISLYLKESKTLIAGD

SMYSVDGMLGGIHAPTTPDMNAAKLSLRKYADLDITSVVCYHGGLSDVNVKDQIAALI

QD"

CDS 3016271..3016798

/gene="sigV_3"

/locus_tag="PIKHDJKN_02745"

/inference="ab initio prediction:Prodigal:002006"

/inference="similar to AA sequence:UniProtKB:O05404"

/codon_start=1

/transl_table=11

/product="RNA polymerase sigma factor SigV"

/db_xref="COG:COG1595"

/translation="MDLSEKAELARQGDKEAFVFLIRAVQQSLFVIGRSIVKTDEDCA

DAIQETIAKAFSNVHTLKEPTYFKTWIIRILINECNRIIKKKKRVSLVPYDMRKTSYK

GDFEQIELFEVIDELEEQLQTIVTLFYIEDLSVKEISRVLEVSEGTVKSRLFRARRQL

SELVLGEGEGKYEFS"

CDS 3016785..3018257

/locus_tag="PIKHDJKN_02746"

/inference="ab initio prediction:Prodigal:002006"

/codon_start=1

/transl_table=11

/product="hypothetical protein"

/translation="MNFHDPVDRELKQLFDAKRKDLIIPDTVQLAMENALSSISEQNV

KKRYPHKRWRWAAAVIALFIILGAVSIYSVPTFGEMIRSLFAKDNPDIGLLRAQELRL

VHNPHIKVKDKGYTLVIDEAVADPTRVTMALQLFDKKGKHDRDKLHLGDPNNITIKDA

NGKELDTMYDMGYTNDFYYMVAFFNEPLQTDKITIEGNIGTLGNRNEPSIKGDWNFSF

DIDMREANQQTKIEELSGSYSTPHGMTVTLKRLTRMVQGVRFELETELNDAAMARSPG

DLSEKQMLSFHFETIENEEIHSVNSRKNGDMVSLMSTDYAVIGDGKIRWSYIFKYLPE

HEPYRFILDGYSVAEIDGSKISFSPSELIEPKSFKILTDRIELVGTSLEDSQNPDATY

ETAVSFYGEMDNEINNEEWKAYDSAGKQYDVSKRGVSTLKNTLTDNWREGFISMGNRD

TQQPYEFRITGLDHIPEELILVRRVVDKRYKDPDWSVLLK"

CDS 3018575..3019210

/locus_tag="PIKHDJKN_02747"

/EC_number="3.2.1.8"

/inference="ab initio prediction:Prodigal:002006"

/inference="similar to AA sequence:UniProtKB:V9TXH2"

/codon_start=1

/transl_table=11

/product="Endo-1,4-beta-xylanase Xyn11E"

/translation="MFKFSKKMLTVLLAASMSFGVIAATSSAATDYWQNWTDGGGTVN

AVNGSGGNYSVTWQNSGNFVVGKGWNVGSPNRTINYNAGVWSPSGNGYLTLYGWTRNA

LIEYYVVDSWGTYRPTGTLKGTVTSDGGTYDIYTTMRYNAPSIDGTQTFAQYWSVRQS

KRATGVNSAITFSNHVNAWASKGMNLGSSWSYQVLATEGYQSSGSSNVTVW"

CDS 3019415..3020254

/gene="pkn1"

/locus_tag="PIKHDJKN_02748"

/EC_number="2.7.11.1"

/inference="ab initio prediction:Prodigal:002006"

/inference="similar to AA sequence:UniProtKB:A0A0H3MBJ2"

/codon_start=1

/transl_table=11

/product="Serine/threonine-protein kinase Pkn1"

/translation="MRKLLMFFLIATMLVISACSQDKPEPKDNLVFVQGGTFKNNKSN

FAGRNATLSNFYIGKYEVTQKEWEDVMGNKPSGFKGDQLPVEMVSWYDAVEYCNQRSI

QEGLKPHYNIDKDTQDLNNKNDNDNIKWTVTINEGANGYRLPTEAEWEYAASGGQESK

NYTYSGSNNADEVAWYWKNAGDKILTGDWNWPAIESNKNKTKSIGTQKPNELGIYDMS

GNVREWCWDWYSDAESQIASFRVVKGGGWIGGVNNNEIAFRGKFDANGFGPDQGFRVV

RGE"

CDS 3020597..3022027

/locus_tag="PIKHDJKN_02749"

/inference="ab initio prediction:Prodigal:002006"

/codon_start=1

/transl_table=11

/product="hypothetical protein"

/translation="MPEYQLHLKDKRIVWGNAIDVETLIGTYPQDSIRPGENAALDWK

LPDGIYRAKEVVIELDKLLEAILVQLGDPIDGNPSVLLDSLQANLAISGDLSSLPLGP

LALKDKAGVELTAQAARIGEQLVLWAREINSDKKALAQYGPETLGELEFRSHCYGHTL

IPEAIAQVWGPAGGPRVMQLYNEYLHQFVLLRDALLPFANWEQVPFEVKEYTQSKGLR

FLEPARKVFLTQLLGKELTHKAIVQYAQSVLSSGLTSAGYGFQYSLGTVLPAGLGESA

GTAARYLLKWHPVQTLEIEDTKDIVAISFEYEYDDYYAAPRDEAGTGIPGSKDYLHIS

EDHSDLPFIARLLPNTESDRTTVRFSVEREGHASSTVDLGQLFRGHRFSYRPEKNNHS

KDATVKRTSTSIHRVADILSHSGLVTNTDNGVHVIPTGGNELVQWALLGKLYPENVVL

LAKGDQEELETALVSGKGYGTQFLVL"

CDS complement(3022108..3022491)

/gene="hxlR_2"

/locus_tag="PIKHDJKN_02750"

/inference="ab initio prediction:Prodigal:002006"

/inference="similar to AA sequence:UniProtKB:P42406"

/codon_start=1

/transl_table=11

/product="HTH-type transcriptional activator HxlR"

/db_xref="COG:COG1733"

/translation="MINFRNKTYRCTSEIVMSLLSGKWKISILNHLTKGPIRFNELQR

LLPDATQRMITMQLRMLESDGLIIRKVYPVAPAKVEYYLSDLGEELAPMLQMLCKFGT

TYIEKFPDEVSCDNDDSRRDIVNVN"

CDS complement(3022484..3022846)

/locus_tag="PIKHDJKN_02751"

/inference="ab initio prediction:Prodigal:002006"

/codon_start=1

/transl_table=11

/product="hypothetical protein"

/translation="MSMAEYQGKVKHIQDTPFGYTLSVIGGKWKMVIIYLLAENQPVR

FNELKRQIGAITFKTLSSQLKELEADGMVERKEYPQVPPKVEYRLTDKAETLLPVLEG

LCEWGVQNQGERDVLEDD"

CDS 3022952..3023866

/locus_tag="PIKHDJKN_02752"

/inference="ab initio prediction:Prodigal:002006"

/codon_start=1

/transl_table=11

/product="hypothetical protein"

/translation="MNQKTIRLLMPQWQGGNNPNYSFGAELLAWLAPDNDQPLIQVPV

QAYDGTPLENENGVNGRKQLIEQLEAAQHIIHAHKPDRIVMFGGDCLVEQAPFAYLNE

RYNGELGLIWIDAHSDLVGYVGYDNGHTLPLGNLLGEGDEEFAKHVKIPLKPENVFIA

GLATPTEQEGELISEAFERLGITTAESDTEMILRLGIKTAGAEELTKSTESIKEWIKE

SGIKHLAIHLDLDVLDPKAFRSLLFANPEGPYTFSPAGSLQIPQLLHLLKELSEETDV

VGLGITEHMPWDSINLKNLLGEIPILNK"

CDS 3024134..3024589

/locus_tag="PIKHDJKN_02753"

/inference="ab initio prediction:Prodigal:002006"

/codon_start=1

/transl_table=11

/product="hypothetical protein"

/translation="MSINHHSNTTHPYWIENVGVREREPMIPHPVVVEVRQVASNQIV

VTYDQPADLASATNISNYWIRSNMSSPSDIASVGMGEAITKENTIRADRGMIAPINNS

KTRFVITFNVNATMGVLYILLPCFVNLEGRSGFTGGNWGPFSRNMFIGL"

CDS complement(3024659..3025510)

/gene="yvgN_2"

/locus_tag="PIKHDJKN_02754"

/EC_number="1.1.1.-"

/inference="ab initio prediction:Prodigal:002006"

/inference="similar to AA sequence:UniProtKB:O32210"

/codon_start=1

/transl_table=11

/product="Glyoxal reductase"

/db_xref="COG:COG0656"

/translation="MEKVILNNGVEMPIMGFGVYQIADQNECEQSVYDAIMAGYSLID

TAASYLNEEAVGRAIKRSGVAREELFITTKLWVQDTGYENTKNAFEKSLERLQLDVLD

LYLIHQPFGDVYGSWRAMEELYREGKVRAIGVSNFQMDRLIDLIIHNEVVPAVNQVET

HPFNQQIESAAFMKENNVQIQSWAPFAEGRNDLFQNEVLVSIAEKVNKSVAQVVLRWL

TQREVVVIPKSVRKERIVENFNIFDFELSQNDMESITALDTKQSLFFSHNDPEMVKWL

GTRKLDI"

CDS complement(3025736..3026779)

/gene="glmS_1"

/locus_tag="PIKHDJKN_02755"

/EC_number="2.6.1.16"

/inference="ab initio prediction:Prodigal:002006"

/inference="similar to AA sequence:UniProtKB:Q5NHQ9"

/codon_start=1

/transl_table=11

/product="Glutamine--fructose-6-phosphate aminotransferase

[isomerizing]"

/db_xref="COG:COG0449"

/translation="MSLTYREVKEQYKALQQTYNYLLAKRTEFTAFVQAQGITSVTFV

GAGSSYCLSEAAAFSFRLRAGLPAMSLAAGDLMLHADRYRPMLEGSLIIAPSRSGSTS

EVVEALRLLQSDQRIPLLTISCVTDSPLSKSADFPLELPWAFDHSVCQTRTVTNLYAA

NLLLAAFLADDEELIIDMQEAIHQGEAYMARVEDSIRHVSDFNWTNAVLLADGELQGL

AAEGAIALTEIAKTQAHAYHLLDVRHGPMVTVGSDTLVIAALSDSGIEHQRNLLRDIK

ERGATIITFADRAMGIPQEFIDLEITTSHPLNTAVRGIPFIFISQIAALANAERNGIN

PDQPDGLVAWVKL"

CDS 3027109..3027975

/gene="fbaA"

/locus_tag="PIKHDJKN_02756"

/EC_number="4.1.2.13"

/inference="ab initio prediction:Prodigal:002006"

/inference="similar to AA sequence:UniProtKB:P13243"

/codon_start=1

/transl_table=11

/product="putative fructose-bisphosphate aldolase"

/db_xref="COG:COG0191"

/translation="MPLISSTEMLQTARRDQYAIAAFNVHTLEMLQAVVEAAVETESP

LIIQTTVGTVNHLGPDYIAHAAKTAADLTDIPIALHLDHCTDFSTIIRCIRAGYTSVM

IDASMYPFEENVNLTRKVMEVAQASGVNVEAELGKVGGVEDDIVVEEQHASLADPEDC

VHFVERTGVTTLAPAIGTAHGIYKGQPNIDFERIETIANRVSVPLVLHGGSGIPAEQI

HRAVALGMSKVNIATELRIAFSKAIKDVFLIHPEENDPRKYMAPAKEAVKRLAMEKIR

LCGSTGKAGVVR"

CDS 3027972..3028901

/gene="lacC_2"

/locus_tag="PIKHDJKN_02757"

/EC_number="2.7.1.144"

/inference="ab initio prediction:Prodigal:002006"

/inference="similar to AA sequence:UniProtKB:P23391"

/codon_start=1

/transl_table=11

/product="Tagatose-6-phosphate kinase"

/translation="MITTVTLHAAIDRTLYVDKFGVGQVHRVTRQVNEPGGKGNNVAK

VIRQLGGQVTATGIIAGNNGEFIESSLTERGIRTAFIQSAGDSRVCLNIVDDSSGSST

ELLGQGPTMLEAEVEAIKKKVQELATQSSAVVMSGSLPPGAPDTLYAELICIVRSAGA

KAFLDTGGTSFSIGLSATPHFVKPNEQELAEWLGQKPRNVSEWARAAHRLADQGIDEV

CITLGSDGALAILKGNAYMVKPPSIQPVNTVGCGDAFVAGMAYAGERGDSAESRLRTA

VAAAATNAMSSKAGDIDYVLFEEYEHQVQIIPL"

CDS 3029086..3029886

/gene="srlR_2"

/locus_tag="PIKHDJKN_02758"

/inference="ab initio prediction:Prodigal:002006"

/inference="similar to AA sequence:UniProtKB:P15082"

/codon_start=1

/transl_table=11

/product="Glucitol operon repressor"

/db_xref="COG:COG1349"

/translation="MQYSKGELRRAQTLELIKSHGKISLQEIVQTVGCSEATARRDLD

ALEKAGEIIRTNGGAIYEGGLTSAVSELPFAAKRDFRRMDKERIAATAAALVKAGDII

CLTGGTTTFFIAKALKKHRNITIVTNAVNIAYELADAEDIQVVVIGGVMRSKSYELCG

PLAESVIDKINITKMFLGVDGVTQNFGFTMHSELEARIAQLTMERSSEVYAVFDQSKI

EKSALFTIAPLTQVTGVITNKQPEGWFEQACREHQIAIHTTTDHVASI"

CDS 3029957..3031249

/locus_tag="PIKHDJKN_02759"

/inference="ab initio prediction:Prodigal:002006"

/codon_start=1

/transl_table=11

/product="hypothetical protein"

/translation="MHFSKFMLIIAMVCVVFAEAGCSSSSSSGGDTSADPQGKVNLVF

WSHQEDAIVGAYKKLISKYQTLHPDITIEYQTFPYDVYSQKLKASFSAKKPPDIAEFF

GTWVPEYSKNDLLVEIPDSENVKKAYYDAPLGGYLRDKKLYGLPLEYNIENGGMLIHP

QMLKDQGFDHTPSNWTELVDYAKKLTVRDNNVIKVKGFDFVSADNITFTFLSLILQQG

ASFMGEDGHVNFHTPEADKAMSTLVSLVADDKVADLTTFGGELDTSDYFFQGKSAMTY

RGPWTIAAGQNNYKVDDFEYVSVPSFTENPPVFAAESGWGLAVSKKSKQQEAALDFIQ

FISEKDNLSTWNTDTFTVPAKKEIAENPEFLKNNPHLKPSLDILALGQWIGPIADRDF

FFKQVNDNFQLMASGQQSVEDGLANIEQTINDNQDQHK"

CDS 3031265..3032200

/gene="lacF_9"

/locus_tag="PIKHDJKN_02760"

/inference="ab initio prediction:Prodigal:002006"

/inference="similar to AA sequence:UniProtKB:P29823"

/codon_start=1

/transl_table=11

/product="Lactose transport system permease protein LacF"

/translation="MSTAISTNKAGKVNPAVKSTAFRAKRRFIIISLVPILLLFGVFA

FLPIAWSLGLSAFKYDPLSSNAIFVGAENYIRMFQDSIFLKSLWVTFKFVFIAVLINI

VITLLIASAIQRVRIPWLKNGFRTVFFLPTIAPLAGTAIVWNTMFNYNDGLFNMILAK

LNMAPIQWLSDPHYALYSVIMMTLWADIGYNIVLFIAGLDSIPDMYYEAATLEGAGRW

HVFLHITLPLLRRTLLFVSITTVVSYFQAFPQFQIMTKGGPFNETRVLSLQIYEQAFS

SSNMGYASAMATVFLMIILLVTLLQLRWGRTQWEH"

CDS 3032223..3033047

/gene="araQ_18"

/locus_tag="PIKHDJKN_02761"

/inference="ab initio prediction:Prodigal:002006"

/inference="similar to AA sequence:UniProtKB:P94530"

/codon_start=1

/transl_table=11

/product="L-arabinose transport system permease protein

AraQ"

/db_xref="COG:COG0395"

/translation="MPRKFSLWEGFTIAVLAVIACVMLLPYVWMVLSSLKSNMDIVSG

SGGFFPSKPSLDGYRTVFSEAPFVTWLLNSLVTSVIITVVTLFTSALAGYVFAKHQFK

GKKLLFILILATMMIPFQVIMIPTYLITAELGLVNHLLAIILPNLVSSYGVFLAKQFI

EEIPQELLEAARMDGSGEFRLMLRIVMPLILPMLSALGIFTFMNSWNNYLWPLIVLND

ESKMTVPLALVYFNGTHQVNYNVVMSAAVLITIPVIIMFLIFQKQFIKGLAMTGMK"

CDS 3033066..3034367

/gene="melA_2"

/locus_tag="PIKHDJKN_02762"

/EC_number="3.2.1.22"

/inference="ab initio prediction:Prodigal:002006"

/inference="similar to AA sequence:UniProtKB:O34645"

/codon_start=1

/transl_table=11

/product="Alpha-galactosidase"

/db_xref="COG:COG1486"

/translation="MTKISIIGAGSAFTREIAMDILLIEGLEGGTIALVDIDERRLDL

ARRLVLQIVERTGKKWEVIASTDRREVIGGSQFVINQIEVGGLETVRYEYEIPLKYGV

KQCIGDTLGPGGLFKTLRTLPSWMEIVRDIEALCPDCIILNYTNPMSAVTLLTSRITD

IPVVGLCHSIQNTSAQLAEYAGVPYEEMSWKAGGINHMSWFVELSHAGRDLYPVLLEK

IQTPELLRQDPVRFDAMKYLGAFVSESSGHFSEYIPYYRKRQSLIDQHCNSGYNGATG

FYADNWPIWRRENDEQIVSQLEGTAPLELKSSNEYAAIIIEAVLKNEPKVIYGNVPNQ

GLIENLQPDGVVEVACLVDGKGVQPCRFGRLPEHLAALCRSNMAFFDLAVEAVRRSDR

EMARHALMLDPLTAAVCSLEEIGSMFDELYEAEQGFVPVLK"

CDS 3034364..3035320

/locus_tag="PIKHDJKN_02763"

/EC_number="2.7.1.101"

/inference="ab initio prediction:Prodigal:002006"

/inference="similar to AA sequence:UniProtKB:A9CES5"

/codon_start=1

/transl_table=11

/product="Tagatose kinase"

/db_xref="COG:COG0524"

/translation="MMQRQETSPIVVIAGELNVDAIVSGKDVMPEWNREKMVDGFEIV

LGSSSAITACALASLGADVRFVSVVGDDDFGKFCIAELQRMGVNTEHVTRLSGIKTGV

TLSLSTPDDRGLLTYAGSIPLLTPDYIPESLLQQATHLHFGSYYLQDGMRPHWRELFT

RLRANGISTSFDTGWDVSNQWDHEGISALLTVTDLFIPSEDELLHIFPADGVADVLDS

MLPAVAGIVAVKQGSKGATLRELDESTISAGPYTLTPVDTTGAGDCFNAGIIYGYLLG

MRSEELLRFANACGALSTLGIGGTGSLPTMEAVLLLQAEHVQ"

CDS 3035371..3036918

/locus_tag="PIKHDJKN_02764"

/inference="ab initio prediction:Prodigal:002006"

/codon_start=1

/transl_table=11

/product="hypothetical protein"

/translation="MLKFGKTLLVGLSMVVGIGLVSGIEWSSSAAAAGTDYYVATNGN

DSNAGTSSAPWKTLQHAADAVPAGSTVHVRGGVYNQKLKITRSGSASQGPIVFTNDGT

ETPIIDGTGLSVSGIEGLIDLTDVNYVTLQGFEIRNYTTATKDVMPVGIYVHGSGSFI

NLSGNKVHDIKNTATPTGSDLLGRDAHGIAVYGTKAPESIHDITINGNELYNLVLGSS

ESLVLNGNVDTFSVTNNVIHDNDNIGIDLIGFEGKAPNTAYDQVRNGLVKGNRVYNIS

SNNNPSYGKSLPNNSNAADGIYVDGGKDSIIEQNYSYNNDIGIEIASEHAGKSTSNIT

VRSNAVYNNRLTGIAMGGYDTKRGSTVNCKIVNNTVYKNDTLGDGSGQLYVQYDTQNN

VIKNNIFVASSTDVLIYNEYTKNSGNVVDYNLYFAPAGSNGANWTWKNKDYTGFSAYK

TGTGNDAHSLFIDPKFVNASGYDFHLQSTSPAIDAGNTDNAIIGTLDIEGKPRVQGAA

VNIGAYE"

CDS 3037169..3038830

/locus_tag="PIKHDJKN_02765"

/inference="ab initio prediction:Prodigal:002006"

/codon_start=1

/transl_table=11

/product="hypothetical protein"

/translation="MKPGSIKHCMVIYWTIILMLVLAACSRSEQNENPSLPQPAHLIQ

QPVEYKPNPDEPAWKLDNMPVDLTWFVGANWYGHTWGESMTSKVVTEKTGVNIKFEVP

SGEASEQIALMMTSGKLPDLMTIGSWESAINKLWEGDQVYALNELAEKYDPYFFKVAG

DGALKWYRQENGNTYGIPNDSYSPSLMQATGLTAANQTFLVRKDLYEAMGRPDLSNPE

GFLNALQLLKDQYSVYKGQLISPFFAQGNVSYGMTEYLQNLLAIPHEKDGKFNDRMTD

PDYITWLKTFRTAYERGLINVDFMVDSNAQVEEKTSNARYFMMIREWTGMAAINPILA

AGAYPNSYYIAVDGPQNSHGDSARLFPGNMDGWMVTMISKSTKNPERAIRFLTYLASE

EGQRDIFLGKEGETWAMGNGKPYLNTEMVQLLDTDRERLEKEYGIMDTYWMLRNPAFV

NQWRPEKAPSVKQMEEFANKQADMDSGIYKGIDPVGDSDIALSWSRISQNWEEVLPEL

ITAKDEAAFDKIFENFLARRVNYGFNQVMEYRQTELEARKAKMAE"

CDS 3038906..3040741

/locus_tag="PIKHDJKN_02766"

/inference="ab initio prediction:Prodigal:002006"

/codon_start=1

/transl_table=11

/product="hypothetical protein"

/translation="MNYLLTRLRKLYRNARISQKLFLAFSLMIAIPVILVSFLYIRMQ

ETQLYKDAMAAGNSHVSWLNEQLRSRMDIIENASNTALTQKSFVDFIHSNMLVDGLRL

VKFKQNQFEQMQNIIQSNAMISELSFYVDNPNLYEIWPEIYHYQKFWPQDYWAKLRDE

GGAAYRLFSFKDGEHTLSYYRLVRLQGQEKKRPSIMEIRVPHSMFFSDLLQESKGDFF

SVLMNESDPPQYVYNPQHEFSRKAGEGLDEILGGIHRQLDVLPQESPVKIKVGDQSYY

ALYRYIAPLNTYVVDIASHQALMKGPRSWYAFVVTITLCVLLLIMLLVSHTTRRIFRR

LDSVLASMRQVRKGELDATIDTGLDENERGDEIDEVAVSYNKMLHEVKRLMTQVVDKQ

LIAKNAQLHSLHSQINSHFLYNALESIRMLAEVQRQPAIANSLVSLGSQLRYSMQWRS

DTVALREELANIQSYIQFINLMEGGSFMLTADLPQEVLRYAIPKMCMQPIVENAVHHA

APSGGSVCIQITVSVENDRLLIKIQDDGEGLDPEMLVRLQAALRGDSDSPIVTSKNGL

GLENVNKRLQLHYGEDCGLWIDSVQGAYTCVTIRLPWENVNLGGW"

CDS 3040745..3042316

/gene="rssB_5"

/locus_tag="PIKHDJKN_02767"

/inference="ab initio prediction:Prodigal:002006"

/inference="protein motif:HAMAP:MF_00958"

/codon_start=1

/transl_table=11

/product="Regulator of RpoS"

/translation="MINILVVDDQKHIRDGLQAMLHQFPLELNNIYCAASGIEALILL

RQHNIHIVITDIMMPDMDGLALMAQTKEERIEVEYLIISGYSDFTYAQKAIELGAKGY

LLKPLKREDLQTSLEHVWQEIQTRQALTHNMQHVSRLARETDRKELRMFMQGALNDEA

WILQIQEQNPALWHNYRLCLLREKCWINQPGASSVHSMEAIAYGVFGRQGCICLQHSP

YLILAVNAALDPDALPASLKEGMIDAITVMTDPQQGLKMLPGSYTQVHELYHHSYLFP

DQYIILPAHIEHMEQQWQLPYEELYELFQSVGTDDSGRIAQGLSAIFHKNVLQRYHIR

YTQQLCGATVQMMEEYERVIRPYMGEEALDLESLRNLFDYPGMRDYIQALQQQLLRLN

QFYFEYKHSYRNSQDLNEAIRFIHESYHKPLDLAMVSNHVSLNYAYFSNLFKKNIGKG

FAEYLRDVRMDKARRLLAETDHKIVEVAAMVGYESYKSFTRAFRLVMEIQPTEYRQMM

RQKVEREVQYHSTNI"

CDS 3042475..3044523

/gene="glcA"

/locus_tag="PIKHDJKN_02768"

/EC_number="3.2.1.39"

/inference="ab initio prediction:Prodigal:002006"

/inference="similar to AA sequence:UniProtKB:P23903"

/codon_start=1

/transl_table=11

/product="Glucan endo-1,3-beta-glucosidase A1"

/translation="MKPSHFTEKRFMKKVLGLFLVVVMLASVGVLPTSKVQAAGTTVT

SMEYFSPADGPVISKSGVGKASYGFVMPKFNGGSATWNDVYSDVGVNVKVGNNWVDID

QAGGYIYNQNWGHWSDGGFNGYWFTLSATTEIQLYSKANGVKLEYQLVFQNINKTTIT

AMNPTQGPQITASFTGGAGFTYPTFNNDSAVTYEAVADDLKVYVKPVNSSSWIDIDNN

AASGWIYDHNFGQFTDGGGGYWFNVTESINVKLESKTSSANLVYTITFNEATRNSYVI

TPYEGTTFTADANGSIGIPLPKIDGGAPIAKELGNFVYQINLNGQWVDLSNSSQSKFA

YSANGYNNMSDANQWGYWADYIYGLWFQPIQENMQIRIGYPLNGQAGGNIGNNFVNYT

FIGNPNAPRPDVSDQEDIAIGTPTDPAIAGMNLIWQDEFNGTTLDTSKWNYETGYYLN

NDPATWGWGNAELQHYTNSTQNVYVQDGKLNIKAMNDSKSFPQDPNRYAQYSSGKINT

KDKLSLKYGRVDFRAKLPTGDGVWPALWMLPKDSVYGTWAASGEIDVMEARGRLPGSV

SGTIHFGGQWPVNQSSGGDYHFPEGQTFANDYHVYSVVWEEDNIKWYVDGKFFYKVTN

QQWYSTAAPNNPNAPFDEPFYLIMNLAVGGNFDGGRTPNASDIPATMQVDYVRVYKEQ

"

CDS complement(3044791..3046143)

/locus_tag="PIKHDJKN_02769"

/inference="ab initio prediction:Prodigal:002006"

/codon_start=1

/transl_table=11

/product="hypothetical protein"

/translation="MNTILVLLSTLTVAGSVVVAFILSLRLAPFQVIPAKWRFGIGKM
ALVLYLLPIFLVFNWIFSLFTSSTTVNELPLNVQQVLPGPFNPAPAIQVQTISTNVAL
ALICLWVIGAIAFAAWQTYSYLRFLKTLKLTQTIVPKTSEVAMLLPVVKENLGLKSEV
RLACSALIRSPFLIGLRKPTIYLPMKNSANVDMGMVFHHELIHLKRKDLWIKALILVA
RALHWFNPLVHMLSKDIHTWSELSCDEEVVKGMSLADRKRYGETILNVVAGSRLLPIQ
FCSSLSGDGKQLKRRLTFMLNVKNVKKKTFVVAISTLFVVAAISTVTTAWASSNTPRV
VASEEASTVTRPSQQTAVREEAPAVTRPSAQVTAREEVPVVTRPSAQAAAREEAARVT
RPSAQAVAREEAPEVTRSAVQAVVREEAAGVTRPSVQAVVREEAPVVSRPSAQAASRH
"

CDS complement(3046153..3046527)

/gene="blaI"

/locus_tag="PIKHDJKN_02770"

/inference="ab initio prediction:Prodigal:002006"

/inference="similar to AA sequence:UniProtKB:P06555"

/codon_start=1

/transl_table=11

/product="Penicillinase repressor"

/db_xref="COG:COG3682"

/translation="MNHLQKLSDTEMELMEVIWECDPPVTSTELLNMFSQKGKAWKAQ
TISTFLSRLVDKGALTATRDGRTNKYVPRISPEDYKLLEAQQVLDGLYQGSVKNLISA
LYDGDKLSDQDIAELRQWFSEK"

CDS 3046955..3047581

/locus_tag="PIKHDJKN_02771"

/inference="ab initio prediction:Prodigal:002006"

/codon_start=1

/transl_table=11

/product="hypothetical protein"

/translation="MNLFHIQSTLQETDWTASFLKNNFIGIGWPATGDLEHEPAEEWT
EQLVQRYRIGKTELSDVLDTLHTFVYVMQDGDYVLIDDEEWAYVGDLGDYFYDDSNGT
GEEYICHRRGVTWLGRIPLAELNDKVLALVSHYSIIAKFEHPISQAQLEPWVSTISAT
TEGDNHSSVRVDEGTIQEALDVLKKALQSEEEDLRIRAAAAILHYAKS"

CDS 3047830..3048423

/locus_tag="PIKHDJKN_02772"

/inference="ab initio prediction:Prodigal:002006"

/codon_start=1

/transl_table=11

/product="hypothetical protein"

/translation="MTTNKTNPKVDEYLKKAKKWKEESEKLREIVLSCGLTEDFKWMH

PCYTLENKNVVIIHGFKEYCALLFHKGALLKDEQGILIQQTENVQAARQIRFTNLQQI

VDMETILKEYIVEAIEVEKAGLKVALKKNTEYDVPEEFQTKLDENPALKTAFEALTRG

RQRAYLFYFSQPKQSKTRVSRIEKSTQQILDGKGLND"

CDS 3048606..3049637

/gene="fhuD_2"

/locus_tag="PIKHDJKN_02773"

/inference="ab initio prediction:Prodigal:002006"

/inference="similar to AA sequence:UniProtKB:P37580"

/codon_start=1

/transl_table=11

/product="Iron(3+)-hydroxamate-binding protein FhuD"

/db_xref="COG:COG0614"

/translation="MLKRRNSIWLLSALIILLVLSACGQAATTSDTTETTNTTGAADT

ESSTDSTSASVADTTTGAEGETVTYQSDAGEVEVPADPKRIIDLTAFSTGYFVALDAP

VVGATSGAINNEYIKDQLKSEGTTDLGEQPTAEQLISLKPDLIIAYTGMEGLDKLSQI

APVVQLQYGKRNFKDLMLEMGKLTNREAEAKDWITQWEAKINELKPKVLAAVGDRTVS

ILNPYSKGLFVFGHNYGRGGEILYDEFGLKAPKRAQTEAIDSGTGWASISMEVLPEYA

GDIIFTSPWSGDTTDPKIVYDNTVWKNLTAVKEGYVFQLDPTSDTYNDPVSLEGQLKF

ISDSLLSVK"

CDS 3049953..3050324

/gene="fra"

/locus_tag="PIKHDJKN_02774"

/inference="ab initio prediction:Prodigal:002006"

/inference="similar to AA sequence:UniProtKB:Q797E6"

/codon_start=1

/transl_table=11

/product="Intracellular iron chaperone frataxin"

/db_xref="COG:COG5646"

/translation="MDVFAEYLARIDNLGHRDRTEEILEWIIKKYPNLVPKIAWNQPM

FTDHGTFIIGFSVSKHHLAVAPEKAGINHFSDEIVKAGYEHTKELVRIRWDGPVDFSL

LERMIDFNITEKADCSTFWRK"

CDS complement(3050394..3051284)

/gene="gltC_5"

/locus_tag="PIKHDJKN_02775"

/inference="ab initio prediction:Prodigal:002006"

/inference="similar to AA sequence:UniProtKB:P20668"

/codon_start=1

/transl_table=11

/product="HTH-type transcriptional regulator GltC"

/translation="MELLQLRYFLTLARCEHVTEAAGKLHVTQSSLSKTIQRLEDDLG

APLFDRLGRKLRLNEFGRTFLRRTEKALFELEQGKQEIADLSNPEHGTLQLAVTTAST

LPGILRAFRKNKPNVQFHVQMVTLEDMSTLLHRGEIDFCLSSPPIQGDDIECQMLFQD

PIVVAVPLGHRYADRSSITLAELKEEWFVGVKKGYGIRDLVDSKCQSVGFVPKYVYEG

DEPARLTALVEAEIGLAFIPNTARNSQERIVYLRIEDQNLVREIALLSHKNRYISKAA

IAFRNVVIDYFGAMSKPFAE"

CDS 3051396..3052781

/gene="ribZ_1"

/locus_tag="PIKHDJKN_02776"

/inference="ab initio prediction:Prodigal:002006"

/inference="similar to AA sequence:UniProtKB:Q180E3"

/codon_start=1

/transl_table=11

/product="Riboflavin transporter RibZ"

/translation="MNTRNIWLMISVGLGIMLNPLNSSMISIAIARLQQEFLLDVTAV

SWIIFSFYIASAVAQPVMGKCSDLFGRRKIFLIGLVVVFVSSMLAPLSPDFSWLIVFR

IIQSVGTSMMVSVGMAIVRIHVTEKQAAALSILAMFLSGAAAVGPFIGGVLIHWWGWP

SIFLVNIPFVIASFIFAWKAIPKEGSFKSTSGDMSIRKWFLLIDAPGILLFTIALVTL

LMSVLSVKSTGHLSTWHLMAGGIGLIALAIFIRHELKTPSPFIPLRVFAKYPEMTWVN

VQFMLVNTLFYALFFGIPSYLQQVRHLNEVHTGIIMLTLGLCSLISSPLAGRWIDKSG

TRPALIVSAVLMTLGSVLIVALDNASPVVNVILPLAVFGISNGLNSVGMQAALFKSTP

KEMIGVASGIFNTSRYLGTILSSLLIGIVMGDTFSFIGFQTLGIILTVLAASLIMMTL

RRQRSSDTQTL"

CDS complement(3052797..3053660)

/gene="rhaS_18"

/locus_tag="PIKHDJKN_02777"

/inference="ab initio prediction:Prodigal:002006"

/inference="protein motif:HAMAP:MF_01534"

/codon_start=1

/transl_table=11

/product="HTH-type transcriptional activator RhaS"

/translation="MNEDGIIELKVPQLPYYLGAGRSEYEIGDHHPNRKKLGIYDVLI

VVKGELYIGENDQQWTLEKGDSLILLPEGEHYSVRPCTQKTVFYWLHFEHVERYESLL

TSSVLEPELQDSSRPFGNPYTLRLPKHSKLANSQAAFNLLEQLLTPEMDSSFWQEQRL

LGELLSMLEEGSLRVMDSVQNRLAELAAIYIQQNYKKKVTNETLAAALHFHPNYVIRC

MKMKYGKTPIEYLNELRLERAKRLLVTTDWLIDRIAEEVGFRYAPYFSACFKRYIGLS

PLQFRKQYMTT"

CDS 3053787..3055730

/gene="hypBA1"

/locus_tag="PIKHDJKN_02778"

/EC_number="3.2.1.185"

/inference="ab initio prediction:Prodigal:002006"

/inference="similar to AA sequence:UniProtKB:E8MGH8"

/codon_start=1

/transl_table=11

/product="Non-reducing end beta-L-arabinofuranosidase"

/translation="MPKTKVAIQDTFWKEYSELVRQTVIPYQWEALNDRIEGAEPSYA

IRNFRIAAGLEKGEFGGWVFQDSDLYKWLEAVAYSIGNHPDPKLESIADEVIDLIGQA

QHENGYVNTYFTIKEPGRQWTNLYEAHELYCAGHMIEAAVAYYQATGKRRFLDIACRF

ADLIDSLFGTEQGKIQAYCGHQEIELALVKLYNVTGEQRYLDLSSYFINERGNKPSYF

VEEWERRGRKNIWSQGTPNLEVYQSHLPVREQSVAVGHSVRAVYMYTAMADLARLTND

SSLREACERLWNNTTRKQMYITGGIGATHLGEAFTFDYDLPNDTVYAETCASIGLIFW

ARRMLQLEAKSDYADVMERALYNNILGSMSKDGQHFFYVNPLEVHPDASEKNPDKHHV

KPVRQKWFGCSCCPPNVARLLSSLHDYIYDVSDDGKTVYVHLFIGSQLQWVTGDGLQV

TLHQRSELPWHGKVEFSISFAGNHAKAAPFEIALRIPQWFQSQDPILKINGEPQHYRK

HNGYAKMERAWSDGDTVEWILPLETQLIAAHPLIRPDAGKAAIQRGPLVYCLEQVDNG

SPLSSLSFAEDPELREHETPELLGGCITVEVNGLLVDESVWLDNQPYQPLHKNVKPVQ

LTAIPYYLWGNRQAGEMNVWLRT"

CDS 3056013..3058154

/locus_tag="PIKHDJKN_02779"

/inference="ab initio prediction:Prodigal:002006"

/codon_start=1

/transl_table=11

/product="hypothetical protein"

/translation="MILIACPIISTGSAAANAAPVSAISQATKDIKAQAPIQYRIQAR

LDEKKMTIEGSEFITYRNTSKDTLKQLVFHTYADANLSESTQTTMFKHSNEEISKNNP

DKKPEDFLGGIDIEKVMTGGQVLDFSNKDQAMSVKLEQPLQPGESVSVQVHFNLNIPY

GSQRLSYYKDIINGAHWFPVMSVYDEAKHEWDSKPYSKTFETDYYTSADYEVQFNVPD

EYQVVMPGTITTRDDAETGRKVVSTVAENTREFAFFASPNFKVESATRNGLTVEYYYF

DNQPGKKKIIDGYIDQAFKAIDFFSGKYGKYPYPEFRIVESYVEGVAIEYSRLIQMGQ

IGINSASEQDTVFVHEIAHQWFHALIGNNSETESFLDEGFADFSKVYFSEKQGDKMNG

FKSIQFDDSTVDKAIASTNDEVGDWASPVYYDKGRQAIYQLYRSVGEEKFDTFMKEYF

KRYVYQNATIDGLLQTIEDVLGKEERDDMKTALFEPNFALKPEYQLSNEEKTAYLHDQ

FQSLYGSALTQVPNLPFETMSRVMDKALQGEPLAIVLSDQVSKAANKQQEAMVNQLTT

LLDLTGVKYDLIQDRQELKQKMKKELATSNLIVLGNAKSNGFVQALKSSIIDRATHIG

FQWKTTMNQPSAAGGYIIKHPYNQNRLMLHYFWNEDHLSGGSLESFMMKMQESIGFTN

AYCQYYVLDKTGKVTLDKKVENPLSKFFAEE"

CDS 3058501..3059946

/gene="mltF_1"

/locus_tag="PIKHDJKN_02780"

/EC_number="4.2.2.-"

/inference="ab initio prediction:Prodigal:002006"

/inference="protein motif:HAMAP:MF_02016"

/codon_start=1

/transl_table=11

/product="Membrane-bound lytic murein transglycosylase F"

/translation="MKTTKVSFYVLSLILLLVAGLSGWSGDANANSNSGKTYVIGTDI

TFAPFEYQDENGDYVGIDMDLLDAIAKDQNFNYQIKALGFNAAVQALESNQVDGVIAG

MSITDERKQKFDFSEPYYESGVVMGISANNDTVKSYEDLRGKKVAVKTGTEGYSFAES

IASKYGFTIVPFDDSSQMYDDVKTGNSVACFEDEPVLRFGVNQNNGLKIVTKKEDGAS

YGFAVSKGQNQELLKMFNDGLTNIKVSGEYDRIKENYLGENAVVANQGRWELVQKSLP

ALFKGLGNTLLYTIISLFFAFIIGLIFGFMKVGQNKFLRGVATVFVDIFRGIPLIVLA

FFIYFGIPQAMGFTMPLFLAAILTLSLNAGAYVTEIIRGGIQSIDRGQMEAARSLGLP

YRKAMIKIVIPQAIRVMIPSFINQMVITLKDTSILSVIGLVELTQSGKIVIARTFASF

DIWLTVAIMYLIVIITLTKIADYLEVKVRRG"

CDS 3059939..3060667

/gene="artM_2"

/locus_tag="PIKHDJKN_02781"

/inference="ab initio prediction:Prodigal:002006"

/inference="similar to AA sequence:UniProtKB:P54537"

/codon_start=1

/transl_table=11

/product="Arginine transport ATP-binding protein ArtM"

/db_xref="COG:COG1126"

/translation="MGKIKVEGLKKSFGTNQVLKGIDISVNEGEVVCVIGPSGSGKST

FLRCINQLDEITAGRVIVDDRDLNDPKTNINKARENIGMVFQHFNLFPHFSVLKNIMF

APRELGILKEQEARDTALRLLDRVGLSDKADSYPNQLSGGQKQRVAIARALAMNPDVM

LFDEPTSALDPEMVGEVLGVMKDLASEGMTMIIVTHEMGFAREVADRVVFMDGGYVVE

QGTPVEIFGNPKNERTISFLEKVL"

CDS complement(3060780..3062060)

/locus_tag="PIKHDJKN_02782"

/inference="ab initio prediction:Prodigal:002006"

/codon_start=1

/transl_table=11

/product="hypothetical protein"

/translation="MKSLFRNPTFCKLFIAAFASQLGTVIGNMAFAFYLVDRYSERPS

LATMAELMYSLPTLAVFWIVGVIADRFDRKHIAAYSDWIRAGLTLLLLLFVHYDMLFF

CFLMLFLRSSISKFFGPAEMGLLQGSIDQDQYVQASGLNQMIMGMFMLFGMSLGAAAY

HFIGIKGAFIIDGVSFLISGLLLAWGDFPERARIPNGRTSIRELRLSLLFKDFWHGLR

YIAGNRLLLVLISGFLFFGIVNGVFSVLPIFTMKYKLSPDNYVVQSSLITVFLGIGFL

IGSIIAPHFIRRFTRTPILISGLFISSLLIGGLGTTVRIEVYLFLILLAGIAIAPINI

ALGSWMPELVPPQNMGRVNSLIEPIMMLGHSLALGGIALAFPVWLSVTWLHYMLAFCT

IAVSFFYLKFLPSLVRNRLVYATTEEAIADGKEG"

CDS complement(3062078..3062734)

/gene="yfiR"

/locus_tag="PIKHDJKN_02783"

/inference="ab initio prediction:Prodigal:002006"

/inference="similar to AA sequence:UniProtKB:O31560"

/codon_start=1

/transl_table=11

/product="putative HTH-type transcriptional regulator

YfiR"

/translation="MKREGSGEMSPRLTDTQKERRNLQILEAAKRVFTEKGYGAATLK

DIIQETGMSRGWIYLYYQTKEDIFEALLDYQDADYEQYINQLMESSSSIWEMVTTLYS

QQLQELHRSPHGGLMPAFYEYFLVGWRDEPRHKLLLERYEKGIALFAELLRIGVEREE

FSPVMDIMDISRLAASFQEGIMTHYITIGPEKANTRMQYDALMLYLKSLLHPVSIKET

"

CDS 3063093..3064274

/gene="pbuE_2"

/locus_tag="PIKHDJKN_02784"

/inference="ab initio prediction:Prodigal:002006"

/inference="similar to AA sequence:UniProtKB:Q797E3"

/codon_start=1

/transl_table=11

/product="Purine efflux pump PbuE"

/db_xref="COG:COG2814"

/translation="MEKQKSLFIFILTIGVFGILNTEMGIVGILPLVADHFHISVSKA

GLLVSLFAIAVAISGPTLPLLFSGFNRKKVMLLVLGVFVLGNMVSIFATNFNVALIAR

VIPAFLHPVYVSIALTAAAASVSREDAPRATSKVFKGVTGGMVLGAPISSFIGSSTSI

EMAMIFFTIVNAIAFIAVLLFVPSMPVQERLSYGSQLSVLKKSITWLSIVGVIFINAA

MYGVSSYLAEYLETITNVNGGTISLMLFIFGGASILGNIVAGKVLTTNPLKSVALFPF

ILGVVYIVLFLMGEFTGPMAFIVFVFGIMVAVGNNISQYWITSAAPEAPEFSNGLFLA

SGNLGVATGTSVGGLVISGMGTQYVVLGGLLFLILSLVFILLRNSLHITHPTGNSSTN

M"

CDS complement(3064237..3064989)

/gene="murR"

/locus_tag="PIKHDJKN_02785"

/inference="ab initio prediction:Prodigal:002006"

/inference="protein motif:HAMAP:MF_02108"

/codon_start=1

/transl_table=11

/product="HTH-type transcriptional regulator MurR"

/translation="MNIQELAKATYTSHSAINRLTHKLGLSGFKEFSIKLAREFQQDA

QNISNIDPNYPFALGESSLQVAKDIAELMKETIEKNFAFMDEASLTEAAHLLDQAKRI

FIYALGDSQIRAKSFQNKLIKINKYVVITTELSEWAYHTINLTAQDCAIFLTYHEKSP

IFLKAAQHFSREKIPFITITANHQSELAKMSTICIQVPNDETKHAKIGTFSSQIAFDY

VLNVIYSCIFQIDYAKNMQMTTYSLKNFQLDE"

CDS 3065269..3066288

/gene="yhhX"

/locus_tag="PIKHDJKN_02786"

/EC_number="1.-.-.-"

/inference="ab initio prediction:Prodigal:002006"

/inference="similar to AA sequence:UniProtKB:P46853"

/codon_start=1

/transl_table=11

/product="putative oxidoreductase YhhX"

/db_xref="COG:COG0673"

/translation="MLTIGYIGNGKSTNRYHLPFSLNRDHLKVKTIYARHPEKTEWVK

APGVLYTDDLAALMNDDDIQLVVVCTHTESHYEYAKMALDHGKHVLVEKPFMLTKEEA

ESIFQYAKEKNLLIQCYQNRRYDSDFLTTKKVIESGKLGDLLEVEMHYDYYRPEIPNA

TTHFSKYKSYLYGHGVHTIDQVLSYFGQPHKIHYDVRQLLGPGRMNDYFDLDFYYASL

KVSVKSSFFRLKPRPSFVVYGKKGVFVKQTEDRQEEHLKLFYLPKGHPDFGKDLPQHY

GVLTYLDDAGIYHEEKVISEQGDYARVYDDIYEAINHGKEKVIQDKETIAVMEILEKG

MEECN"

CDS 3066290..3067276

/gene="iolU_2"

/locus_tag="PIKHDJKN_02787"

/EC_number="1.1.1.371"

/inference="ab initio prediction:Prodigal:002006"

/inference="similar to AA sequence:UniProtKB:O05265"

/codon_start=1

/transl_table=11

/product="scyllo-inositol 2-dehydrogenase (NADP(+)) IolU"

/db_xref="COG:COG0673"

/translation="MNLGIVGAGMIVWDLLSFIHEIPAITLEAICSRPAHLDKLLTLQ

EQYGIARIHSEYSEMIANDEVDTIYIGLPNHLHYAYAKEALLGGKHVICEKPFTSNLQ

EFLELKEIAREKQLVLVEAITNQYLKNYLSMKENLRKLGDIKIVECNYSQYSSRYAAF

QAGEVLPAFNPEMSGGALMDINLYNIHLVVGLFGSPKKVEYWANMERGIDTSGMLLLD

YGEFKCVCIGSKDSTAPNAMNIQGNKGYIHMTSSANKCESFDLALHKETPIRVDDKDH

PHRMYDEFVEFERMIREQDLEKVTVMLEHSEKVMDVIEQAKQSANLIFGPDR"

CDS 3067406..3068407

/gene="rbsR_2"

/locus_tag="PIKHDJKN_02788"

/inference="ab initio prediction:Prodigal:002006"

/inference="similar to AA sequence:UniProtKB:P0ACQ0"

/codon_start=1

/transl_table=11

/product="Ribose operon repressor"

/db_xref="COG:COG1609"

/translation="MITIKDVAKLAGVASSTVSYVLNGKKHVSEQTRQKVLAAASELN

YIKHGAASELKRKNTQTIGVIVHDMSVPYFSDLVSGIESSAISQGYDLIVCSSLGGER

STAARFIRERRLDGVIVIAENIEDELLLQAAETGFPIVVMDRELHSEHIVNVLMDDEQ

GGYMATRFLLDKGHRAIAYISGPLSSDCNLMRYQGYLRAMHEAGVEENENWRLGGQFL

KTGGYNAAKLLMEGELPTAVFFANDEMAIGGLEAFKEHQVSIPEDLSIIGFDNIHVTE

YLNPPLTTFRQPKTDAGSFAGHVLIQMLKGEAVESTYRINIQCVERDSVSQMILSEG"

CDS 3068605..3069513

/gene="exsA"

/locus_tag="PIKHDJKN_02789"

/inference="ab initio prediction:Prodigal:002006"

/inference="similar to AA sequence:UniProtKB:P26993"

/codon_start=1

/transl_table=11

/product="Exoenzyme S synthesis regulatory protein ExsA"

/db_xref="COG:COG2207"

/translation="MSMRNGKIIKAPQELERAPDKQGSLKQNGLSVIQFCLHTQGTTG

SFFLKDHMLLFVISGVYTVRFKDQEYTVRSDEMVFLHKSIRIDYIKSGEPGSEFMLDY

MMFFLNESMLEEFVQFSGFKQTNSVNEITPVSIIPVNELTRSYIASLKPYFENPDKVQ

DGLIRLKFMELLFHLAQANEQFLHQLLQPNHNQNSNFGKIMEENITNPISISDLAYLS

GRSLSTFKRDFQTLYHTSPLKWIRNQRLDKAKKLLAETSLSVTDVCFSTGFENVAHFS

KVFKLQFGLPPSEFRQQCKRSEGKTT"

CDS 3069745..3070740

/gene="yajO_4"

/locus_tag="PIKHDJKN_02790"

/EC_number="1.1.-.-"

/inference="ab initio prediction:Prodigal:002006"

/inference="similar to AA sequence:UniProtKB:P77735"

/codon_start=1

/transl_table=11

/product="1-deoxyxylulose-5-phosphate synthase YajO"

/db_xref="COG:COG0667"

/translation="MEYVKLGNTGMDVSRLCLGCMGFGEPGRGFHQWVLDEENSRPVI

QKALELGINFFDTANVYAGGTSEEILGRALKDYANRDEIVLATKVFSRMHDGPNGAGL

SRKAIMSEIDKSLKRLGTDYVDLYQIHRWDNHTPIEETMEALHDVVKAGKARYIGASA

MSAWQFQKALYVAERNGWTRFVSMQNHYNLIYREEEREMMPLCQEEKIGVIPYSPLAS
GRLTRDWQETTQRSETDQVQKAKYDATASTDRLVVERVAEIAEKRGVPRVQIALAWLL
HKNPVTAPIIGATKTSHLENAAEAISIALTSEEMACLEEPYVPHPVFGLNVGFSS"

CDS 3070939..3071916

/locus_tag="PIKHDJKN_02791"

/inference="ab initio prediction:Prodigal:002006"

/codon_start=1

/transl_table=11

/product="hypothetical protein"

/translation="MTLNIVNSEVHSLFDQFTDLIQGQTRLQTWEQHITIPHEMGQGS
IHRTRIRPGMEIVITDVTYVQDMKLRIQEACPLFEISYCLNGDIYCEWEGKESYTHHW
TGNVLFFEDELVYEEKKADVRHHMLEIRLSPQELLYYAADLTEEQKMQSWLQRYKGNI
DNYPNTPEIHRCVSNMLNCTYNGALKRLYMESKSMELIALFGEVEGYQALNEKSFLSR
DDLANLDYARELVIRHFEQPLSIQELSRKVGLNEFKLKKGFRERFGMTVFELVRKQRM
EMALYYMEVQRMNVGEAAVSVGYSNVSNFATAFRKIYGYNPSEHLKMLP"

CDS 3072099..3073550

/gene="jefA"

/locus_tag="PIKHDJKN_02792"

/inference="ab initio prediction:Prodigal:002006"

/inference="similar to AA sequence:UniProtKB:P9WJW9"

/codon_start=1

/transl_table=11

/product="Drug efflux pump JefA"

/translation="MIMHKSTATATREVYTAPQVDPRRWMALVLILLPTLLISLNNYM
IQVALPMMQSSIKASFGEAQLIFSGYSLGLAVALILGGKMGDLYGRKRMLWIGVLGFT
LMGLLGSVGSNPTLLIVIRIIQGLSAAMIQPQVLSIIQSSFPASEKGFVFAIYGAVIG
VGFTLGIILGGILVDWNLFNLGWRIVFLFNVPIGLLVLAGLHIIPESRGHYKQSIDWL
GTAMIMLGLLMLIYPLTIGQKLGWPIWTWGCVFGSLLLQVLFVLRQKRREEEGRTPLM
ELSIFKIDSFRIGIITEIVVYLSMFSFFFILNYYVQAGLQFDVQSTSLVFLPLGLGFF
ATSLLSSRMVKRWGETVLKAGTLTMGGSIFLLMWSLHADAVHLFQFHNILILLVYGLG
LGMATTPLANTILNRVPTASAGTGSGLFTTFMYLANTLGVAMISILFSAALRSPLADA
GLGDYVRAFSISSAASGVLAFAACGCIYLLKEK"

CDS complement(3073575..3074471)

/gene="rhaS_19"

/locus_tag="PIKHDJKN_02793"

/inference="ab initio prediction:Prodigal:002006"

/inference="protein motif:HAMAP:MF_01534"

/codon_start=1

/transl_table=11

/product="HTH-type transcriptional activator RhaS"

/translation="MSKQLHETILFPDASFPYIMYTANSEKIIPEGRGFNDLHWHEEL

QITLVTQGRLVIQVNGVDHELLAGQAILINKGVLHVTTHLTHNGQYVSFNFPEKLLAF

YADSAMEKNYVLPYTDSSLVSLVIKGDVEWQQIVLDMLWDMKKRFDMKKKWGWEYEVS

IKIVQLWLILIHNIILPSEEAPKYVKLQQERLQLMLSFIHQQYSMNITLQEIADTAHL

SISECTRSFKKTIHMTPYDYLIKYRIKKSMELLTSTDYTVTEIAHRVGFNHVNNFIQS

FKKQQNQTPKSFRNSSNEIKNQ"

CDS 3074627..3075535

/locus_tag="PIKHDJKN_02794"

/inference="ab initio prediction:Prodigal:002006"

/codon_start=1

/transl_table=11

/product="hypothetical protein"

/translation="MKMNKSLYPPHPYLLLFISILSISISSILIKSSETPTSVAGMYR

LFITVILMLPFVPWKSLRNLKMSKKDWSVVLLAGLFLGLHFLFWMESLVHTSVASSMV

ILSLQPLFVMVGSYFIFKERANMVTILCLATALAGSVIIAWGDIGVSREALIGDGLSL

LGTISVSAYMLAGQKVSHKIDANVYSVIVFFLAGSVLLIYSLFNHISLTGYNTSDWMY

FLLLAIIPTIFGQLLINLLLKSLGATTVSVGIIGEPVLAIILAFVFLGETISLFQFIG

GMMTVIGMGVYFWAKSWRCTVSEVQT"

CDS 3075593..3075925

/gene="csaA_1"

/locus_tag="PIKHDJKN_02795"

/inference="ab initio prediction:Prodigal:002006"

/inference="similar to AA sequence:UniProtKB:P37584"

/codon_start=1

/transl_table=11

/product="putative chaperone CsaA"

/db_xref="COG:COG0073"

/translation="MATIDDFLKLDIRIGTVTKAEFFAEAKVPAIKLEIDFGEEIGIK

KSSAQITKRYAAEKLIGKQVIGIVNFPPRRIAGFKSEVLVLGGIPEKGDVVLLKPDIE

LPDGTPIG"

CDS 3075989..3077308

/locus_tag="PIKHDJKN_02796"

/inference="ab initio prediction:Prodigal:002006"

/codon_start=1

/transl_table=11

/product="hypothetical protein"

/translation="MKRISAKSSWPIGRNVFLSAAILVALAGCSSNSASPKPLEISGM

NSSQEEKEDVPSEKMSYWNEQRKGTNFMNSTSLPDNYEAAKEQGIEFVRLAPDKWSKD

RDFLFNDKPDANINADFLIGSSDKYEGIVEADFARLKADLDAAEAKEMKVIITVLSLP

GDRWRQFNGAKNDDRIWNDTEYHKQAAQFWKDLAQRLKNHPAVIGYNLINEPHPETGT

GFHDFWTQDYSDWYAKVEHTTADLNSLYDTITKEIRTVDQTTPIILDSGLYATPWAFT

YLKPIDDPNVLYAFHMYEPYEMTSQNKKKGFTYSYPGSVKVGDDEREKEFNRQALKQF

VEPVAKWASDHNIPANRIIASEFGTNRMVQGADRYMSDLISIFNEYGWHWAFYAFRED

TWDGMDYELGSEKPNWKYWDAIEKGEHPLRGTVDNPIWNEIKESLTK"

CDS 3077305..3078156

/gene="lpxG_1"

/locus_tag="PIKHDJKN_02797"

/EC_number="3.6.1.-"

/inference="ab initio prediction:Prodigal:002006"

/inference="similar to AA sequence:UniProtKB:O84467"

/codon_start=1

/transl_table=11

/product="UDP-2,3-diacylglucosamine pyrophosphatase LpxG"

/db_xref="COG:COG1408"

/translation="MKWRKITVIFLLVLLGIGCYAREIEPKALAVTHLDIVNEFVPIS

MNGIQIVQFSDTHLGPDYSVNQLQSLVNRMNRLKPDIVVFTGDLIDHYTHFGSENRKR

AQRVLAKINAPLGKYAVYGNHDRGGGGSQKYKQYMDDAGFSVLVNEVHAIRTTTGDKI

NIAGLDDFLLGKPQVKRTLQSLRKQDFNLLLVHEPDVADQLSAYPIDLQLSGHSHGGQ

VRIPLIKPLITTALAHKYVAGLYSLQGKSNPLKLYVNRGIGTTRMPLRFFAKPELTVI

RLIAAVS"

CDS complement(3078223..3078864)

/locus_tag="PIKHDJKN_02798"

/inference="ab initio prediction:Prodigal:002006"

/codon_start=1

/transl_table=11

/product="hypothetical protein"

/translation="MKKRGTLYGWLACVSIFFIVYALVKNYWIDPEASSFLSHKTGSK

RELHLPIWLNVMYVHVAFACLAMASGLLNFSNRIFKKSRRFHRINGYVYIVSVALVVL

TSGYMAPYATGGKISSIGFNALNIIWLFITITALIQIKQKRMVQHRNWMIRSYAFCFT

NMLIHLITSLFHLGFGLAYVTCYTISIYGSIALLLVIPEFIIRMNFKLRTAPE"

CDS complement(3078914..3079948)

/locus_tag="PIKHDJKN_02799"

/inference="ab initio prediction:Prodigal:002006"

/codon_start=1

/transl_table=11

/product="hypothetical protein"

/translation="MLTVQIVLFDGFDLLDAVAPYEVFCAASMHTDNAVRVEMVTAEG

PRPVTSGMNGLTIEANNTLDPERGGIILVPGAAGNVEGDGPDTIPAILGKAMNTELTT

MIKTALDHKDILVATVCGGSLLLAMGGLLEGRSAVTHRLGMDLLGATGAIPVPARVVD

DGNLVTGGGVTSGLDVALYLVERELGPRIAHDIEQLFEYERRGTVWRAKGIAPTTHKS

LTENEPNIVMNQTVVTSGILLNEKTQTTVFDGNWDTTIATPVGKLEVKLYIKTISDVI

QGSATQGNETIPFLNPVLCDNKLTWSLSITKPMRLNLKFEVAVDGDQMNGIAKAGLLP

ASKLTGKRIN"

CDS 3080208..3080894

/gene="ompR"

/locus_tag="PIKHDJKN_02800"

/inference="ab initio prediction:Prodigal:002006"

/inference="similar to AA sequence:UniProtKB:P0AA16"

/codon_start=1

/transl_table=11

/product="Transcriptional regulatory protein OmpR"

/translation="MSMYHTILVVDDDQSIVELLRDFLENDQFHVKTACDTEQAWAIL

QQHSIDCIVLDIMMPGQNGFELCRRIRGDSNVPVLFLSARSDDVDKIRGLTLGGDDYI

VKTASPGEIVARVKAVLRRTTSRQPMEGKILDYGRIQLNLTAREVLVEGGEVELTPKE

YELLRLFAEHPRQVFSYEQLLAKFWEGVGDRHTIRVHLSRLREKIESDPNHPQFLINV

WGVGYRFEGV"

CDS 3080896..3082227

/gene="sasA_15"

/locus_tag="PIKHDJKN_02801"

/EC_number="2.7.-.-"

/inference="ab initio prediction:Prodigal:002006"

/inference="protein motif:HAMAP:MF_01837"

/codon_start=1

/transl_table=11

/product="Adaptive-response sensory-kinase SasA"

/translation="MRKLRIRTFTMLCLFFLLTLPWIFFVTAHFMDTQSFSISTSQQQ

NETLQRHLSEMIHMIEVGTNKWTDTGWQNELHTELSKAKMDVEIESASNSEIYRSSQK

HHSRLSSTEQFSVIKDGQLLGRVVLYLPKSNVVPMLAAFFGLLLAFFVVGVEMRRFLL

KPLEKMSKAARQITAGHWEVQLPKSRIMEIAEVRDGFEVMVKGLEQSHRKQVELEEER

RFVTAAVAHDLRTPLFALRGYLDGLEQGIAQSPEKVTRYIAVCKEKSAQLDRLVEDLF

TFTKMEYLESELNTRTLDLTQILRKSIESVSPQAEQKHISILNQLTDGCFINGDSYVL

ERAMNNILENAVRYTPSGGAITVQCCKDGNKIRFTIRDTGPGFSFEELERVFEPLYRG

ETSRSRATGGSGLGLTISQRMMKQHGGELMVSNHPDGGAVLAGCLPLTDPN"

CDS 3082431..3083432

/locus_tag="PIKHDJKN_02802"

/inference="ab initio prediction:Prodigal:002006"

/inference="similar to AA sequence:UniProtKB:P99173"

/codon_start=1

/transl_table=11

/product="Zinc-type alcohol dehydrogenase-like protein"

/translation="MQAMVIEKYGKNVPLIRKEMPTPQIGDYDVLVEIHAASLNPIDF
KIKEGKVKLLLNYKMPLILGNDFSGVVVKVGKQVNAYKPGDEVYGRPRKDRIGTLAEY
IAVHEKDISLKPQNLNFEEAASIPLVGLTTYQAFYDILNLQKGQKILIHAGSGGVGTF
AIQLAKLMGAYVATTASDRGYELVQSLGADLIINYRKENFEEMLSGYDAVYDTIGGEV
LEKSFQVLKPNGKIVSISGLPNARFGREANLGWLKTTLLSIVSHKITTLEKKNQARYH
FLFMKPSGVQLERIKGFLEEGLIKPVIDKIYDFKDAGQAFHYLEGGSAKGKVIVKMK"

CDS complement(3083500..3084345)

/gene="rhaR_23"

/locus_tag="PIKHDJKN_02803"

/inference="ab initio prediction:Prodigal:002006"

/inference="protein motif:HAMAP:MF_01533"

/codon_start=1

/transl_table=11

/product="HTH-type transcriptional activator RhaR"

/translation="MNWIESLQLAIAYMEEHLLENMTMEQISAQAHISPFHFQRTFAL
LTDVTVAEYIRRRRLTLAANELIQSDHKIIDLALKYGYDTPESFSKAFRRQHGIAPSE
ARKNSTSVKSYNRLVIQLSLKGAEPMNHRIVEQAAFTLVGIKQAFSYIDGENLRGIAK
MWQDAYTSGTEDRLFELNNGVIQGLLGVCVDQNEIQEKQMEYWIATAYDGEIPEGLSS
FTIPASKWSVFEVEGPMPESMQRLWKQIISEWFPSNPYEHAGIPELEVYPGPHQPPQI
WIPIK"

CDS 3084676..3087105

/gene="helD_2"

/locus_tag="PIKHDJKN_02804"

/EC_number="3.6.4.12"

/inference="ab initio prediction:Prodigal:002006"

/inference="similar to AA sequence:UniProtKB:O32215"

/codon_start=1

/transl_table=11

/product="DNA helicase IV"

/db_xref="COG:COG3973"

/translation="MQTKDWQLEQERLESVRSKLQARITELEPEVAGMHDQATEIRKR
FWEEVTINTSTHEDFEETFYTINQQSAVLAERERGHMRLTQQWNNMKRLLPSPYFGRI

DFQEKGLALTDQIYIGVSSFVDEDGLSFLIYDWRTPIASMYYDSSPGVASYVTPIGRI

HGTMELKRQFQIHNGQIRNMFDANETIGDQLLQQVLGKGADSQMKSIVATIQKEQNAI

IRNDKSRMLIVQGAAGSGKTSAALQRVAYLLYKHRQTISADQIVLFSPNPMFGSYIST

VLPELGEDNMQQTTFQQFLDYWLGSSLCPEDVFDQIEYVLTAHGAPRYEARLQGIEYK

ASDAFLQALQNYGNWLGQEGMRFNSIRFRNRDLITAEQMRAKFYGYDRSLPLLNRVLL

LQEWLLKELASLERMEREEPWVQEELNYLDTDQYADIFEMLHKDKEVFDIAEQYAQIR

EKISKKRREDEGDFDFAQKEEELLGRKIVKERFSPLRKGIKKFSFVDIKGIYGELFVD

EAAYREKTNAAVIPSLWPEICSQTKESLLRNELFHEDATPYLYVKELIEGVRTNTEIK

YVFVDEGQDYSTFQYEYLKKLFPRARMTVLGDFGQAIFTQATNLAAADSPLVRLYGEA

ETSLFSLVRSYRSTKEIVEFTRAMLPDGDEIVPFERKGPKPLLTRSNDREKLDAQMVA

DIAALRAEGFDSIAIITKTAAESHKAHESLQIQGGEALQLVTKETFNFEKGVMVIPVY

LAKGVEFDAVLVYDASSGAYGRDNERKLLYTACTRAMHRLHLYTTGEWSPFVQALPAD

LYEKSDVNTTE"

CDS 3087372..3090221

/gene="lacZ_5"

/locus_tag="PIKHDJKN_02805"

/EC_number="3.2.1.23"

/inference="ab initio prediction:Prodigal:002006"

/inference="protein motif:HAMAP:MF_01687"

/codon_start=1

/transl_table=11

/product="Beta-galactosidase"

/translation="MLNKINLQGSWELQLDEHKQGAQGPFDDTITLPNTTSHARKGRK

SDVSHIGSLTDEYSYEGWVWLKREIDIPATLAGKRCSLYLERTRITKLWVDGEEYGAQ

DSLNTPHVYSLPEGLSEGKHTLTIRVDNTDYPTKGGHLTSVDTQTNWNGITGKLELHF

YHDVHMGEVQIYPDAKERTFKITYTLSKSIPGTVLSVTAQTFKPLLKETHGYIALDRN

HVVNEQTYPCNQDEVNVLYKMGDDALLWSDSDPHLYVLTLTLLGEHGDVLDTTEIITG

LRDFEANGDAFTINGRKTFLRGKHDGLIFPLTGYAPTDVDEWLRILSISKSYGINHYR

FHTCCPPEAAFTAADMLGIYMEPELPFWGTITDELDPQHNQAEQDYLIREGFAMLKAF

GNHPSFVMMSLGNELWGSKERLNAIIKAYRAFDPRHLYTQGSNNFQFTPLILEEEDFY

CGVRFSKDRLFRGSYAMCDAPLGHVQTDVPGTLKDYDDMILPVGTVSENERIQAEESE

IQIQYGTEAKTVKAASVEQQLIPHLPVISHEIGQYAMYPNYEEIEKYTGSLKAKNFEV

FRERLQSKGMGHLAEPFFKSSGKLAVACYKEELEAAFRSNHLAGFQLLDLQDFSGQGT

ALVGILDAFMDSKGLVTPEEWRTFCSDAVLLARFERFNYISGEDFNAHIQLRYYRTDS

LKGRTVEWEMRDSNQLLAQGKDEIRFDSEETWFEISQIKFKLPDVKKMTPITLSLHIP

GTDIRKQYDLWVYPDHDVTAIEHDHIYKELTDEVIHLLEKGESVLLLPTLEDNDSSVE

GTYCTDFWCYPMFRSISESMNKQVPVGTLGLLINNSHPALKHFPSETHSTYPWWHIVM

NSRSIILDKLSEKVQPFVQTIDNFERNHKLGLLFECQIGKGKLLVGALNYDKLISQTE

GRQLLYSLSQYVKSEDFRPEAKIELDELQQLLGKK"

CDS 3090549..3091895

/gene="gabP_2"

/locus_tag="PIKHDJKN_02806"

/inference="ab initio prediction:Prodigal:002006"

/inference="similar to AA sequence:UniProtKB:P46349"

/codon_start=1

/transl_table=11

/product="GABA permease"

/db_xref="COG:COG1113"

/translation="MKSHVHRKEEDKLSWISLSLFGAGCTLGTGFFLGTSLAIHWSGL

FVLVLLVLAAAGTYFVFGALAQMTADDPKEGAFRTYAGEAFGPWAGFGSGWMYWSSEM

LIMGSSLTALGLFSQFWFPKFPLWVFVAVYACIGLFIAALGAKGLTQTENVLAVIKLA

ATVAFIAIAAAACLGWLDVKITPQKIMNEWFPHKIKGVCTGFIYAFFAFAGIEVMGLM

AAKLKEPKDAVKSGRVMLLTVSTLYVVAIALILLLVPAAKLTPDESPLVKAMSSIGFT

VVVHILNGILIIAGFSTMVASIYAITSMLVKLGEDGDAPKMFTKTWGKKSMPLYALGC

TAAGLILSTLLALWMPKSLFEYVTTAGSLVLMYTWLLICITYIKKMKPRKWNRIKVWT

AMVLILAAVAGTGMEKTSRRGLLASIGLVVLIALITYIINKVRKPRPSPSTKPSKS"

CDS complement(3091981..3092742)

/locus_tag="PIKHDJKN_02807"

/inference="ab initio prediction:Prodigal:002006"

/codon_start=1

/transl_table=11

/product="hypothetical protein"

/translation="MADLYSADEINASFKGLPEWQQDAYSQFANMIADEGNTYPCVPG

RMGFLSGHLRYGFTSDPRTQASAEDMAELLSQYGAVSRDTGHYASLVVICDTSDDLRK

NTTVEQYQSLFWEMLNRVSSLDAASWPEHISTDPHEATWEFCFGGEPYFCFCATPAHE

LRASRHFPYLMFAFQPRWVFESINDNTPLGRKMKTLIRKRLAAYDAVPAHPSLMWYGE

QDNLEWKQYFLPDDEQTPSKCPFMRMKQAISKLTK"

CDS complement(3092826..3093452)

/locus_tag="PIKHDJKN_02808"

/inference="ab initio prediction:Prodigal:002006"

/codon_start=1

/transl_table=11

/product="hypothetical protein"

/translation="MSVLFSYIILGISLSAPIGPINAAQLDRGARHGFMHAWILGLGA

MFADLVYMLLIYFGVAHFLDTPFMRSFLWSFGSFILVYTGVETLSKLKDGIQASFTED

ARVGKSFISGFLMALSNPLNILFWLGIYGSILATTVKHTDTAHLLLYSSGIFIGILVW

DVIMAGMASRFHKRSNETVLRWISIISGICLIGFGLYFGFEAVRSIFF"

CDS complement(3093572..3094189)

/locus_tag="PIKHDJKN_02809"

/inference="ab initio prediction:Prodigal:002006"

/codon_start=1

/transl_table=11

/product="hypothetical protein"

/translation="MNSTKTHKQSIHIPAKTGYALRVKKGSLIRVTDLEGQQVVDFVA

YDAQNINNRLDPGVTLDVLRTYRIKPGQCLYSNRYEPLISIVQETVGIHDFFNSACRP

EMYEVLYDKKDHPSCYHNLNTALEPFGIAPPDQHYPFNLFMNSVVDGEGKIDVIAPES

SAGDYVEMEALMDLIIGLSACPCEESSCNGYHCTPVQLDVESTTV"

CDS complement(3094328..3095833)

/gene="nylA"

/locus_tag="PIKHDJKN_02810"

/EC_number="3.5.2.12"

/inference="ab initio prediction:Prodigal:002006"

/inference="similar to AA sequence:UniProtKB:P13398"

/codon_start=1

/transl_table=11

/product="6-aminohexanoate-cyclic-dimer hydrolase"

/translation="MSSSSSFSYTSYDAIGLAELVRAREISPVELLEAAYARLEEVNP

QLNAVIRTYETRARQEASMVRPGEQPFAGVPLLLKDISQSLEGEILTSGSRLLSEHRA

LRNSNFVSRLRDAGFIIIGHTNTPEFGLKNITEPRLHGPSRNPWNTNHSPGGSSGGAA

AAVASGIVPLAGASDGGGSIRIPASFSGLFGLKPTRGRTPVGPGVGRQWQGASIDFAL

SRSVRDSAALLDTLQVIQPEAAFHAPLFPGSYLSDMHYPHQRKLRVAYTTASPVGTPV

SDEAIEAVYKMVRWLEEQGHEVEEKLSPVNGVKLMENYYMMNSGEMAAMISSMERSMG

RALTSDDMEIESWVLAEAGKKVSAAEFVHSLAEWDVAAAQMSTLFERYDFYVTPVNAF

PAPKIGELTPHDEQIQHLMRISELDKIQQQQLIYEMFEPSLTYTPFTQLANLTGQPAM

SVPVHLTTEGLPMGVQIMATKGREDWLLHLAGQLETSELWVGMKGNPLFPV"

CDS complement(3095970..3096950)

/gene="impX"

/locus_tag="PIKHDJKN_02811"

/inference="ab initio prediction:Prodigal:002006"

/inference="similar to AA sequence:UniProtKB:D5RAW5"

/codon_start=1

/transl_table=11

/product="Riboflavin transporter ImpX"

/translation="MEKTQPASTVYRKERSNTGFWLVVLGAALWGVDPLFRIILLKTM

TSTQIVLVEHIIVSLVAIPVLWKFRADLKNLRARHWIAVIFISWGGSALATVLFTLAL

THNDPNTVLLLQKMQPLFAIVLAKLLLKETLPRRFGGLFIIALAGTYLLTFGFTLPLG

NWDNWVHAGSLLSLGAAALWGGSTVMGRLMLGQTRYETVTSLRFVVALPLLVFMTWNE

GAAWTFPSATGEQAAVALNILGQALLPGLLSLLLYYKGLSSTKASVATLAELSFPMAG

VLVNWIAFRTLITWEQLVGFILIWIALFAISKQQERSATPADGAPKLRTD"

CDS complement(3097332..3097829)

/locus_tag="PIKHDJKN_02812"

/inference="ab initio prediction:Prodigal:002006"

/codon_start=1

/transl_table=11

/product="hypothetical protein"

/translation="MTTTATELLLIQKDDTWDQCNWIVPLSKALEGLTAEQAAWVPSS

GGLSIWQLVNHMYYYNQRLLSRIQRTTPSGTPAENNESTFGDPGDPSDSTGWSTLMQN

TTQLAGQLRHQLASLEESDLEAPFMDTDEKWAHELARWVLHDAYHAGQIVLLRRQQGS

WNIIF"

CDS 3097967..3098527

/locus_tag="PIKHDJKN_02813"

/inference="ab initio prediction:Prodigal:002006"

/codon_start=1

/transl_table=11

/product="hypothetical protein"

/translation="MDRLWTDFVNSDYHDWRGGDRSEDRLGKAEWQQAFIKQWQLNAS

IPASSEEEGALRNFRNELQALATRLSGGNPLSNKDQEWLNAMMEEGQVIRRLKAGDEG

LKISLIPVGTQWQQVMAEVAADFAITVIEGDGGRIRICDNTDCRWFFYDDTRSRTQKY

CDDKMCGNLMKVRRFRAKQKKDQQQE"

CDS 3098737..3100104

/locus_tag="PIKHDJKN_02814"

/EC_number="3.5.4.32"

/inference="ab initio prediction:Prodigal:002006"

/inference="similar to AA sequence:UniProtKB:Q9I6Z0"

/codon_start=1

/transl_table=11

/product="8-oxoguanine deaminase"

/db_xref="COG:COG0402"

/translation="MGTSYLLKNGCVLSMDSSVGQYKQADVLIEDSLITAIQPDLSCI

EAEVIDASEMIILPGLVDTHRHVWESLVKTAGTNWSLPVYLQNLYYGAMGSKLRPQDS

YIANLLGSLEALNAGVTTVLDWSMPYSPEHTDELIRGLQDAGIRSVFAYGLPGETPYW

NRESQLTYSNDVRRVKEQYFSSRDQLLTFGLAIRGPEFSHWDITVKEIQLAQELDVIC

SMHVGFGSWGSVDRSISKMYEAGLLSPGLNVVHGNTMDTDEYKMLADSGASLSVTPEV

EMMMGHGYPATGYFLENGGTPTLGVDVVTSTGGDMFSQMKFALQAERSRENEQLLLQG

EMPGELNLQSSQVLRFATSAGAKALGLERKIGTLTPGKEADLIMIRTTDLNLFPVHDP

VGAVVQFANPSNVDTVFVAGRPVKREGRLLHVDLNEVRRKALDSKEYLLSQYRMSDAE

RIVFS"

CDS 3100648..3102087

/gene="yhdG"

/locus_tag="PIKHDJKN_02815"

/inference="ab initio prediction:Prodigal:002006"

/inference="similar to AA sequence:UniProtKB:O07576"

/codon_start=1

/transl_table=11

/product="putative amino acid permease YhdG"

/db_xref="COG:COG0531"

/translation="MNQNQNRSSLFRKKPIATGDNESNMLKRVLGPFDLMTLGVGAII

GTGIFVLTGVAAAKYAGPGLVLSFLLAGIICAFAALCYSEFASSVPASGSAYTYSYTA

FGEVIAWILGWDLILEYGFASAAVASGWSGYFQTLLSGFGLQLPHALTSAFSPEKGTY

FDITAAIITLIITFLLTRGVKEAARANGIMVAIKIIVVLIFIGVGVFYVQPDNWQPFL

PFGISGVTAGAATVFFAYIGFDAVSTAAEEVKRPQRDLPIGIIASLAICTVLYIVVSL

ILTGIVPYHMLNVSDPVAFAFEFVQLKGLSWIVSLGAITGITTVLLVMMYGQTRLLYS

MSRDGLLSPVFSKVSGKSQTPATGTWVAGIIVALFSGFISLGHLAELTNIGTLFAFAV

VSLGIIVLRKNNPELKRGFRVPFVPLIPILSAVGCMYLMTRLAALTWITFFGWLIIGL

IIYFAYGRHHSHLNPARRISDTFKKTKGK"

CDS 3102400..3103473

/locus_tag="PIKHDJKN_02816"

/inference="ab initio prediction:Prodigal:002006"

/codon_start=1

/transl_table=11

/product="hypothetical protein"

/translation="MNTPLHTNQHHQNSNFGFALADSAVLAETKLIISHPGDTNEFQL

DIDPQRRLKDGRKVSVVAQQMDAPPDRQDAIIIYGEELGFVQYAVTLRPDSTCSLTPI

EGIDHPIVLNLGDFAEGEYELRISLHVKTPRIAEGPLEPEQHAMVKYAQVVTVAICLF

PAEVVQMNTLPETVWTRDNHVFDSYGSGGFILADLPRMAKRVEDLIGSGNHNLIEQFS

EGDLSDTLLEEGLMAIAWGVTPWCYSIYSAPDEHSRTILSVDKLGDEPQTTGIYRMHP

ESKQLSIVPVNELAYWPSCAEKAWPVIDVAGEGETLRMDLYVQICESVNGLHENPLPS

FVLTRSEGQPEAIIPLIDVVIVD"

CDS 3103501..3104433

/gene="xerC_2"

/locus_tag="PIKHDJKN_02817"

/inference="ab initio prediction:Prodigal:002006"

/inference="similar to AA sequence:UniProtKB:P39776"

/codon_start=1

/transl_table=11

/product="Tyrosine recombinase XerC"

/db_xref="COG:COG4974"

/translation="MMTEEIQEQYADELEAFHIWMKDAGYTGHTVKSYTGDVAEFLIS

INGKSLEQVKKLHVLSFLSRARERGVSDSTRNRKHAAVNCFYKSLIELELVTNNPAFG

IKKSKTEQNRAPVFLDESGLERFLQSVEGKYRTRNLAIFLLMGYMGLRVGEVHALNCK

NYNAERRTLDVFGKGRKWRTLPVPETVAELLSQALQERLDPWRAKEEALFVSQKGKRL

SIRSIQLISTETFERFQQESASNQRVNYSSHKLRHSFATMMLRRGADLRTVQELLGHS

SIQTTTVYTHVTSREKEEAMALLDVKLPAFVADM"

CDS complement(3104776..3105711)

/gene="ldh1"

/locus_tag="PIKHDJKN_02818"

/EC_number="1.1.1.27"

/inference="ab initio prediction:Prodigal:002006"

/inference="similar to AA sequence:UniProtKB:P14561"

/codon_start=1

/transl_table=11

/product="L-lactate dehydrogenase 1"

/translation="MKMVNSAALKPSRVVIVGMGAVGTTTGYTLMLRQRSSELVFVDV

NHDKATGEMLDMNHGLPFTGGVKVWAGDYSDCKDADIIIITAGASQKPGETRIDLLKK

NASIFKEIIERITEVNSHGILLIATNPVDILSYTSWKQSGWPASRVIGSGTLLDSARF

RYLIGKNKGIDPRSIHAHIIGEHGDSEVPVWSLANVAGTDLELDEETQQDIFDRTKNA

AYEIINAKGATSYAIALALDRIVAAILGNEGSVLNVSTLLEDYNGVSDVYLGVPCVVD

RNGVREILPLPLNETEKVAFQASANKLKEQIAGLE"

CDS 3105979..3107295

/gene="hcf136_1"

/locus_tag="PIKHDJKN_02819"

/inference="ab initio prediction:Prodigal:002006"

/inference="protein motif:HAMAP:MF_01348"

/codon_start=1

/transl_table=11

/product="Ycf48-like protein"

/translation="MRACLLDERGGNHLRSQWIKIAQTALLSIGIAALLAACTDSGET

PVAEPPQEREDNGNMGQTLTVVPPANSTQAADTAKYQIQTRLTDFQLLNGNGGLAWGV

TRNALRLYYTQDQGSTWTNISPSENVQFPANPQYGQSIYFVDSTHGWIVREGMGGTDT

MVLRTNNGGASWSLSSLSKTDKVTAITFVSPEKGWILTTVDTSIGKQDKKLYLTEDGG

ITWNQMTSSDEDGKPASAEIPTRGYSTGMTFSDSDHGFLTALEFGTPKLYVTSDGGQH

WKAGPSFFDRNKFNGCGNFNISSPQFFGREAQTAWMSISCSQGESTKFNGFFTTNGGE

SWQLSTFSLNKQTGINRGLTPTFLNPLEGWVMQKGKTYYTKDAGKTWKALPVSEVLEN

ILKEYPEIVKLQMVSSKLGWILVENTDTKRSLLLQTVDGGVRWKVL"

CDS 3107431..3107640

/locus_tag="PIKHDJKN_02820"

/inference="ab initio prediction:Prodigal:002006"

/codon_start=1

/transl_table=11

/product="hypothetical protein"

/translation="MRTNEQAQFTQADELTRYMLQLCYTCDDANRCTTEEEVKACMAK

YEQADEPVQTDGGEVTRGFMELMYA"

CDS 3108192..3108314

/locus_tag="PIKHDJKN_02821"

/inference="ab initio prediction:Prodigal:002006"

/codon_start=1

/transl_table=11

/product="hypothetical protein"

/translation="MSEVGYEERRYGGGYGGFTSIGAILVLFILLVIISKAFLV"

CDS complement(3108396..3108665)

/locus_tag="PIKHDJKN_02822"

/inference="ab initio prediction:Prodigal:002006"

/codon_start=1

/transl_table=11

/product="hypothetical protein"

/translation="MKKRVTTAFASILLFAALAVPASAQNVNEATVDGRDNNTYHYNS

SNYNPNSVRANSTDDGKRMDWGWLGLLGLLGLAGMRKRVSDHSDR"

CDS 3108816..3109334

/locus_tag="PIKHDJKN_02823"

/inference="ab initio prediction:Prodigal:002006"

/codon_start=1

/transl_table=11

/product="hypothetical protein"

/translation="MGILSGNPKNEPMHYGEIFSVWQCSTVAKGALSFYQAFLNHAGD

KDLKKTLDALIDQAKLEIKECDTLLTDNGIAPTPALPDRPPVKLEDIPVGARFTDPEI

AAKIASDTSMGLVACSQVMGQSIREDIGALFAKYHVTKTALGLRILQMSKEKGWLVPP

PLQVKRPEAVEA"

CDS 3109850..3110989

/locus_tag="PIKHDJKN_02824"

/inference="ab initio prediction:Prodigal:002006"

/codon_start=1

/transl_table=11

/product="hypothetical protein"

/translation="MSCPDEKKRCKKKIVTVHSPKGKKKNRRVKVIKKIVQLKCPSPE

VNVTTPTVVGPTGPQGLQGIQGKQGVEGVPGPIGPQGPAGGPPGPQGPQGQPGPAGAV

GPQGEAGPAGPMGAQGIPGEQGLVGPQGPQGLPGPAGAIGPQGFPGPAGATGPAGATG

AAGATGVAGPTGVTGAAGVAGVAGPTGATGAAGVAGVAGPTGATGAAGVAGVAGPTGV

TGAAGVAGVAGPTGVTGAAGGVLGFADFFALMPPDNAATVAPGTDVSFPQDGPTSGTT

ISRTGPSSFNLAAIGTYQVLFQVSINEAGQLILTLNGADLAPTVVGRATGTSQIVEVA

LVQTTVINSILTVRNPAGNSTALTITPLAGGTRPVSAHLVITQLA"

CDS 3111362..3111754

/locus_tag="PIKHDJKN_02825"

/inference="ab initio prediction:Prodigal:002006"

/codon_start=1

/transl_table=11

/product="hypothetical protein"

/translation="MGIESSWMFDFFGTVFPVVFVLIIGIVLLSVGKEVWRWGRNNSE

PLLTVPSRITSRRMQMSQSHSEPGSTARTHYYITFEVESGDRLEFKVNGDEYGMCAEG

DEGRLSFKGTRYVGFERYNRLYSERVRG"

CDS 3111938..3112687

/gene="gdhIV_1"

/locus_tag="PIKHDJKN_02826"

/EC_number="1.1.1.47"

/inference="ab initio prediction:Prodigal:002006"

/inference="similar to AA sequence:UniProtKB:P39485"

/codon_start=1

/transl_table=11

/product="Glucose 1-dehydrogenase 4"

/db_xref="COG:COG1028"

/translation="MSFTDQVVVVTGAAQGIGRSVAEAYAVAGAKVVLADYQEAEGAA

AAASIRNEGGEAIFVQCDVRQEKDITNLIQTTMDEFKQIDVLVNNAGVSRWKSPYELT

LEEWDDILNTNVRSCFLASREAAKHMKHNGNGGAIVNMASTRAFMSEPETEAYAASKG

AIVALTHAMAVSLGKDKIRVNCISPGWIETGNVEKLKPEDHEQHPAGRVGVPSDISRA

CLYLSDPSNTFVTGTNLIIDGGMTRKMIYED"

CDS 3112832..3114580

/locus_tag="PIKHDJKN_02827"

/inference="ab initio prediction:Prodigal:002006"

/codon_start=1

/transl_table=11

/product="hypothetical protein"

/translation="MFGNSSRKVFWLLLLIGGIIMTGFESTAEADADIRAAWVWQAKS

VSDGGNELLENAAKHKINRLYVNVDMNLSEEIYHTFISKASKAGIAVEALGGDPSWAV

SGREGPMLKLASWVSNYNLAAKQEERFDALHLDIKPYVLPQWKEDAKPLVQSWVVNMK

LLLEQVKQAGAVEVNVDLPFWLDSYTVTGSRTTDDADNESLSHWFIGQFDHITLLAYR

DSAQGNNGIIRLVEQEMIWADARNGSITVGVNTKPMPGEEFTTFAGKGAAQLESVIKE

VASAFTDHASYSGSAVHDIVYWGQLEPEEQPPLPENPSNQPEIRGTYIWQASQVTNDG

GDHILEFAKEQNVNWLYVRLDLDQPYSSYRSFVKRAQAQGIEVHAMGGHPIWGKEENR

PRIKRLIDYVKNYNAESEPDERFKGIHLDIEPYTLPEWENDRDKLLTEWAANIAYFHE

ETKKDSDLETSADLAVWLDTSPLPGRDMSVSEYMITTLDHVSLMAFRNIAEGSNGIAD

VVSQEMKIADRLGKPLLISVEMKENFEGKHISFYDKGAAEMERQLALLPDLLSEYKAY

KGNIVHAYDYWIQAKP"

CDS 3114740..3115162

/gene="cwlJ"

/locus_tag="PIKHDJKN_02828"

/inference="ab initio prediction:Prodigal:002006"

/inference="similar to AA sequence:UniProtKB:P42249"

/codon_start=1

/transl_table=11

/product="Cell wall hydrolase CwlJ"

/db_xref="COG:COG3773"

/translation="MAVIKANSEDVKLLARLMRAEAEGDGEQGMLLVGNVGVNRVLGD

CLDFGDIRDINRMVFQNPGGFESTQKGYFYQRARQSEIRLAQRVINGERFWPASNSLW

FFRPVGECPPTWYDQQNTGRFKAHCFFSPTGEDCPTVY"

CDS 3115181..3115672

/locus_tag="PIKHDJKN_02829"

/inference="ab initio prediction:Prodigal:002006"

/codon_start=1

/transl_table=11

/product="hypothetical protein"

/translation="MINQPYQPTQVMGQQANSQVQGTSYKIGNGMPMMSPTPGMVSPS

STSVPPLVSSGAPMTPTGGVVTTTAPQFEQSYIENILRLNLGKCGTFYMTYEGNKEWN

ARIFQGILEAAGRDHIIISDPNTGKRIILLMVNFDYATFDEPLLYQYPGVIGNYPQQP

SRR"

CDS 3115888..3117459

/locus_tag="PIKHDJKN_02830"

/inference="ab initio prediction:Prodigal:002006"

/codon_start=1

/transl_table=11

/product="hypothetical protein"

/translation="MKTWIDGAIQGEAEAYEQLVKQFRGMALAVAYQKLRDTFLAEDV

VQEAFTEAFANLSKLDNPDAFPGWFKVIVERQCYRLMRRKQHGTIPVLEIQDVIQEED

QAHNPEEQALKGEVHRLLRDSISILPTSMQLAVELFYFQGYSLKEMSEFLGVNVPALK

KRLYDARSRLRRSLPVADVISVFSDLYEGGKGMLHIMNGDHAADRLRQSGIQGEILVW

RELYTFGPITRNMRNEKDRSGRAQYLEQHLGIPQTEYLKIKELEQTFRSFQQYEEIVL

WFEYDLYDQAMLSYLLYYFKEQDLKNTKLNLLCIDTYPDIEHFRGLGQLTASQIGRLS

GTWHVMGAEEIQAGSEFWAAYASSDIQDHKDYLLKNTSVLPFAHAAFKAHLSRLPSLS

NGLGIIEQSTLEAIREGVDSPYPLFKHVGDQLHILGMGDLEYWAHLKRLTEEPHALLR

LSGVQAFPNFQQHHDEFRHGVLSLTELGIQVLNGEADWALLRHDNSWIGGLQIGEGKK

VNWRWDTVNENVVIL"

CDS complement(3117629..3119485)

/locus_tag="PIKHDJKN_02831"

/inference="ab initio prediction:Prodigal:002006"

/codon_start=1

/transl_table=11

/product="hypothetical protein"

/translation="MKSHHAMKWNALLLSAAVAITAVPWQVHAEPVSAQQQTAPAAFT

ILPIDRAQILAGQKLDFRVELNGAKLKSKDIKIQVNGLSPDKYFGKSGTWTTSTDLST

EYTIRDVAFKENDSIVVTAETTATGKRTYSRVVYQVFDPSKDTATLTNGEQAKNVVLM

IGDGMSANIRTAARVVSKGINEGKFKGWLEMEQMDAMGMVTTSGMDSIVTDSANSASA

YATGHKTAVNAEGVYPDNTPDPFDDPKVENIVELVKRTRGMATGLVTTADVTDATPAA

MAVHTRKRGEQEAIASMYLNEANRPDVILGGGLEWFLPAADGGKRKDGRNIVEEFKKE

GYTYVTDATNLDKSASTKSLLGLFRKGNMNVYYDREYTQPNNAEIVGKDTDQPTLMEM

TDTALQVLEQNKNGFFLMVEGASIDKQAHTMDFERSVWDAIEFDQTVGRVKEYAKEHP

DTLVIVTSDHGHSLTLNGTYNTEAAKGKSGDELRELIGTYADSKFPTYVDANGDGFPD

NPDSEWKIAVGWGNMPDNNENYLANPVPISPTIQDPNVKDKTWYIANPDKDPDGVHYT

GNLPHDEGQEVHTFDDVPINASGPGSRLFSGYLDNTDVFRKMVTALKLDASK"

CDS 3119866..3121182

/locus_tag="PIKHDJKN_02832"

/inference="ab initio prediction:Prodigal:002006"

/inference="similar to AA sequence:UniProtKB:P9WFP3"

/note="UPF0053 protein Rv1842c"

/codon_start=1

/transl_table=11

/product="hypothetical protein"

/translation="MSDPLPSILNLFLIALLVLMNGFFVSAEFAMVKVRGSRIEALVE
TGNKNAIYASNIVRNMDAYLSACQLGITLASLGLGWLGEPAIAHLLEPMFTAFGMGPV
YVHGISIAIAFVIITILHIVLGELAPKTMAIRKSETITLWSAALLTFFYKLMYPFIWA
LNGMANSLLRVFRMAPASEHDSAHSEDEIRILMKESNKSGLIDNTELALVDNIFDFTA
TTAREIMIPRTEMICLNANQSMLENLEIASESMRTRYPVYNGDKDHIIGFIHIKDLMR
SQLTDTISVIRPILAVPDSTPISDLLKRMQRSKTQIAILIDEYGGTSGLVTLEDIMEE
IVGEIQDEFDQERPAIEKVDEMEYSIDGLMLIEEVSERFGLDMDRADYDTIGGWLYSR
VEAIPPEVGQSVEYDGYVFVIQETEHKRISRVNVIKLDLMVEGEGA"

CDS 3121341..3122249

/locus_tag="PIKHDJKN_02833"

/inference="ab initio prediction:Prodigal:002006"

/codon_start=1

/transl_table=11

/product="hypothetical protein"

/translation="MSYRIEVGFTPVFEFMASLHTYICRKSHKKIDLSIAWAQETEGQ
LTPELATVLQDMNVDDDWKWMYLLVCLRVEAEEPEDFIEWFSKRTVLELRDIFARYSF
PYTDDIAAFQTKMTYVLTGWNEQYFRLLDPAILQHLRQEVLNRKEQLSHSPQEEVLDD
ATNGLMFRDMKGLDRLLLVPQFHFQPLNVILHFGDTLLCHYSSRLYMGDHEYIPTHHL
RVIRSLGEKNRLKIMRFLHQGPRSFIEIVRHLKLSKGITHDHLSKLRGAGLVYAHFEG
ESLVEYSLRLHALEQILPNVMNYLEQ"

CDS 3122572..3124296

/locus_tag="PIKHDJKN_02834"

/inference="ab initio prediction:Prodigal:002006"

/codon_start=1

/transl_table=11

/product="hypothetical protein"

/translation="MRLKGISVASLIISSAIVLSACAGGGGAETTTAGSGTTNSGTQA
TPVSAALDAPFQATDLNQLPETAKKRTDTVIVGLTDPSGAFTPYFHQSGYDGNVASLL
FASLVTVDEKGLPVPDLAESWDVSEDQRTYTFHLRKDSKFSDGSPLTADDIAFTWTIL
HDKSYDGGFDIFSTKVKGGKAYTEGKADHIEGIKVIDPLTISVTLEQPNATALLTLGS
EVLSKAYYGKDYQFGKLEYIKNLHATPVGNGPYKLEKFIPGQEVRFVANEFYYKGKPK
TEHFIYKTSEGDTWQFIETGEIDFTSFTATQENIDKLKNIPYLNLLPYTPSTYGYLQL

NLEHEQLQEKEVRQAITYGLDRQSIYVDANQGAGAVANIPTSPISWSYTEEGINPYAY

DPDKANQLLDDAGWVPGADGIREKNGKKLSLHFLGTKSPATDIFIAVAKENFEAIGVQ

FQPEVFADFNSLVSKVEGGDYDLASFSTPMLTDPSDGVLQFVDGELKGYDNPKVKELY

NKGLATTEIEERKAVYKELYQLLNDELPVIFTSYKKTVYAYNGRIDGLSVSPYRGIAT

SLPEWSLK"

CDS 3124398..3125354

/gene="dppB_6"

/locus_tag="PIKHDJKN_02835"

/inference="ab initio prediction:Prodigal:002006"

/inference="similar to AA sequence:UniProtKB:P0AEF8"

/codon_start=1

/transl_table=11

/product="Dipeptide transport system permease protein

DppB"

/db_xref="COG:COG0601"

/translation="MSTYIVKRMTYMVLILLAASVLIFSLYALTPGDYISSNIKLSPE

RKAELRELYGLDKPVIERYLIWMKNAVHGDFGYSLAQQKPVLALFNEYIWNSFLLAAV

TTFLTWFIAVIIGVIAAYKQYSWFDTFVMIGIFAAMSVPSFFIGLFLIKLLAVDLKWL

PPGGMMTTGSNATGLAYVKEVIQHMTLPVIVMTLLGVGSLTRYFRSNMIDVIKQDYIR

TARAKGLKESKVLYTHALRNALLPAITLIGFELPSLFGGSLIIEKIFSWPGIGQLYMQ

SFSLRDYPLLMGFTMFIAILTVIGTLLSDVLYHIADPRVRLQ"

CDS 3125368..3126309

/gene="gsiD_4"

/locus_tag="PIKHDJKN_02836"

/inference="ab initio prediction:Prodigal:002006"

/inference="similar to AA sequence:UniProtKB:P75799"

/codon_start=1

/transl_table=11

/product="Glutathione transport system permease protein

GsiD"

/db_xref="COG:COG1173"

/translation="MTISSSSVTRNAAESALPLKVPAKSSLWKLSLRRLLKNKLAVGG

FIVVLLMFVACFIGPLFSPYTDNKINMTAMKQGPGIHHWLGTDALGRDVLTRVLMAGR

ISLTVGLASMVLSVFIGSLLGAIAGYYRGIVDQVIMRIADLLLTIPGLPLLFIFGALL

SDWKVPPDSRMYIVMLMLSFVNWPGMARMVRGQMLSLREREFMQAAVVLGLRDRRKLF

HHLLPNIVPLLIVMATLNIGGSILSESVLSFFGLGVMPPTPTWGNMIDAANNMIDFQQ

HPWLWIPPGLSIFVTVIAINIFGDGLRDVLDPKQKRR"

CDS 3126319..3127302

/gene="oppD_6"

/locus_tag="PIKHDJKN_02837"

/inference="ab initio prediction:Prodigal:002006"

/inference="similar to AA sequence:UniProtKB:P24136"

/codon_start=1

/transl_table=11

/product="Oligopeptide transport ATP-binding protein OppD"

/db_xref="COG:COG0444"

/translation="MQNMIEIEQLETHFYSEEGNVKAVDGVSLNVREGETVCIVGESG

CGKSVTAMSIMGLIEEPAGKVVGGAIRFGGRDLLRESKSSLRAIRGNDISMIFQEPMS

SLNPVLKIGEQLMEPLIVHLKMSKKQARARAVELIAEVGISRPEQIANSYPHELSGGM

LQRIMIAIAISCNPKLLIADEPTTALDVTIQAQILDMLQHIKSQSGTSILMITHDLGV

VAEMADYVVVMYAGKVVEEGEVVQLFESPKHPYTQGLLRSKPIINQRQDELYSIPGHV

PDPLSLKDSCHFADRCEHCMSICTTQMPMLKQLGPGQKAACWLYEEALSHV"

CDS 3127295..3128260

/gene="oppF_7"

/locus_tag="PIKHDJKN_02838"

/inference="ab initio prediction:Prodigal:002006"

/inference="similar to AA sequence:UniProtKB:P24137"

/codon_start=1

/transl_table=11

/product="Oligopeptide transport ATP-binding protein OppF"

/db_xref="COG:COG4608"

/translation="MSEPLLEIEHLKTYFPVRKGLLNRTVAHVKAVDDISITIHEGET

FGLVGESGSGKSTVGRTIVRLTEKTEGQIRFQGVDLHHLSMQEVRKIRPQLQLIFQDP

YSSLNPRIRIGDAIGEAVLDHRLATASEVRDLVKEVLGACGLSSYHIDRFPHEFSGGQ

RQRIGIARALILNPKLIIADEPVSALDVSIQAQIINLFRRLQQERGLAYLFISHDLSV

VEHLCDRIGVMYLGSMVETASRDELFGNPLHPYTKALLSAVPVPIPKLKRERIVLKGD

IPSPVNPPTGCKFHTRCPWAEDICKQQIPAYRNVGADHFVACHLV"

CDS 3128485..3128976

/locus_tag="PIKHDJKN_02839"

/inference="ab initio prediction:Prodigal:002006"

/codon_start=1

/transl_table=11

/product="hypothetical protein"

/translation="MRKIRITSMLKVMIVAALSAPIILAIPTAGVAVASPSIQPHALT

PPPPPLEPSIHALTSPKYIYQAEGTVTPGGGLTAVVNTTTTTFSEMKSLQVDYRLERW

TGSAWILYQSGSKKKTQTQTLNAQSNWTVITGYYYRVVSLHTADDGVKPETTTHISSN

VLF"

CDS complement(3128978..3129949)

/locus_tag="PIKHDJKN_02840"

/inference="ab initio prediction:Prodigal:002006"

/codon_start=1

/transl_table=11

/product="hypothetical protein"

/translation="MSKGKSDKHIDSFDSELEDDFFDAAFDMAFDEAFHQAVSATPAS

NTEPMRQSWLRVQKEIVRVGVRKKRMRTIRLSVIVAASVTIGAVIFSMPTGTQAVSPF

VQSIKDWGNGMKSIVIEDRTTHLGADPSTAKTPPPPERGINEAPTFSSGDEQPEEEGE

IKLPFYEESHLLEPIVVTKDIARSGFMGDFLLSKAIPERFTKIKFELMLDTLNPVNPE

DYYESQRMRVRYSGEGKNGEEEVIQFDYAYVSPGQIIEGPMLRETSIVKLADGSDAFM

YLGPPYNTFQWMMGSVNMSLFGTVSEEEMTAIANDLQEQKFPGSTRK"

CDS complement(3129927..3130517)

/locus_tag="PIKHDJKN_02841"

/inference="ab initio prediction:Prodigal:002006"

/codon_start=1

/transl_table=11

/product="hypothetical protein"

/translation="MANRLQLLLASDFHNLGPQLQEEVYYEYYNMVHGLIVYIIKERA

AAEDIIQEAFIKIIKNKPLFDNEVKLKAWLKVVTRNTAINYLRKNKNNRNQLDTDSVF

IDMETINQTAASVESTVETQMMQESIEFYLEQLKPEYRVLVELRWKEGLSYREMADLL

DTSEEIVKQRLFRARGSIKKKLHKEWGGSIEQRQVR"

CDS complement(3130731..3131963)

/locus_tag="PIKHDJKN_02842"

/EC_number="3.4.11.-"

/inference="ab initio prediction:Prodigal:002006"

/inference="similar to AA sequence:UniProtKB:P24828"

/codon_start=1

/transl_table=11

/product="Aminopeptidase 2"

/translation="MLSFEQKLDRYAELAVKVGANVQPGQIFVISAMIDTAEFVRLLV

RKGYEAGAKKVIVKYGDETVNRLRFEMAPEDSFQDPPKWHAAELEELAANNAAFLTIL

SSSPDLLKGIDPERISTHQRTYGQAMSKYRQYQQADKMSWTGVACPSPDWAAKVFPDL

PPAEQVKQLWEAIFAAVRADLEDPVAAWEQHIERLEHKAVALNSKKYKTLHFVSPGTD

LTVDLPEGHIWAQAGSINEQGTRFVANIPTEEVFTAPAKYGVNGKVSSTKPLSYGGSI

IDRFSLTFENGRIIDFHAEEGQDTLERLINMDEGSHYLGEVALVPFHSPISESGILYY

TTLYDENASCHLAIGSSYAFNIDGGKTMSIEELAARGMNSSITHVDFMMGSPETNIYG

VTANGDREAIFLNGDWAF"

CDS complement(3132060..3133292)

/locus_tag="PIKHDJKN_02843"

/EC_number="3.4.11.-"

/inference="ab initio prediction:Prodigal:002006"

/inference="similar to AA sequence:UniProtKB:P24828"

/codon_start=1

/transl_table=11

/product="Aminopeptidase 2"

/translation="MNNFETNLQQYAELAVKVGVNVHPGQTLVVNAPISAAHFVRLIV

KAAYGTGAKLVKVNWSDEVVTRLHYDLAPEESFSIEPKWFAGEMTELVEEGAAILHVI

AENPDLLAGVPQERIVTSQKVRGKALEKYRSYQMADKFSWSIVAVPSPEWAAKVFPDL

PAEQQIDKLWDVIFKTVRIGEQDAVAEWKTHLLNLDSRADLLNEKKYKKLHYTAPGTD

LTIELPEGHLWVSGGSINEQGHVFVANMPTEEVFTAPLKTGVNGTVRSTKPLSYGGNL

IDGFSITFENGRIVDYTAEQGLDSLKSLIEMDEGAHYLGEVALVPHQSPISDTNILFY
NTLFDENASNHLAIGNAYAFCLEGGKTMSKDELISHGMNSSLTHVDFMIGSGEMNIHG
VTSEGSEEPIFLQGNWAF"

CDS 3133373..3133492

/locus_tag="PIKHDJKN_02844"

/inference="ab initio prediction:Prodigal:002006"

/codon_start=1

/transl_table=11

/product="hypothetical protein"

/translation="MVPSLNKVYVATIYIVQLTNNNYQTRFTINCGDLQLKRD"

CDS 3133507..3133773

/locus_tag="PIKHDJKN_02845"

/inference="ab initio prediction:Prodigal:002006"

/codon_start=1

/transl_table=11

/product="hypothetical protein"

/translation="MNIEIIRAEMRREDEKNYVGNTVFRAEGEKSVYEITFMSKNGKD
WDYSLHFTEQSGDEEELLRMDELLENDDDMYNQLLDAALDAYPA"

CDS 3133958..3134767

/gene="yflN_4"

/locus_tag="PIKHDJKN_02846"

/EC_number="3.-.-.-"

/inference="ab initio prediction:Prodigal:002006"

/inference="similar to AA sequence:UniProtKB:O34409"

/codon_start=1

/transl_table=11

/product="putative metallo-hydrolase YflN"

/db_xref="COG:COG0491"

/translation="MASDNLITAGMTGLEEVAHDILSLRTLFVNVVFIGEPGSRNWVL
VDTGMASFTDQILHVATERFPGPPSAIILTHGHFDHVGTVIELEQFWGVPVYAHPLEL
PYLTGVKDYPPADPTVGGGLMARLSFAYPNEAINLDDRIFSLPDDHSIPGVSGWEWLH
TPGHTPGHVSLFRKDDRTLIAGDAIVTVKQESLWSVLLQDKQLHGPPAYFTTDWEAAH

RSVQHIRRLEPIVAVTGHGHSLTGEMLKDSLERLDLDFEQTAVPNHGKYVD"

CDS 3134982..3135992

/gene="galM_2"

/locus_tag="PIKHDJKN_02847"

/EC_number="5.1.3.3"

/inference="ab initio prediction:Prodigal:002006"

/inference="similar to AA sequence:UniProtKB:P39840"

/codon_start=1

/transl_table=11

/product="Aldose 1-epimerase"

/db_xref="COG:COG2017"

/translation="MTQQNAAFEEQFGGIPAVWLRFNQFEAAVIPSVGANLVAFRDTE

QGFRYLREPDQEHMDEFMAAPAVYGIPILSPPNRYDDGRFPWNGEVYQLPINEPATGN

HLHGFLHDAEWKVEGYGSDELESYVVLSQEVKEGHVFYKYLPFTFTVTLRYSLSSLGL

QQQLNVHNNGKERMPNLFAFHTAISVPFAPESQASDYTAKITIGQRRELNERSLPTGQ

FQPLTPEEEQLKGEGVSPFFAAMDNHYTAEPQNGRNYMELTDHRTGDKLVYDVGTSYK

HWMIWNNNMTGGFFCPEPQMNLVNAPNVQGIPAEEIGLIGLEPGEVFEQTSRLYPIKP

QK"

CDS 3136217..3137605

/gene="fumC"

/locus_tag="PIKHDJKN_02848"

/EC_number="4.2.1.2"

/inference="ab initio prediction:Prodigal:002006"

/inference="similar to AA sequence:UniProtKB:P64173"

/codon_start=1

/transl_table=11

/product="Fumarate hydratase class II"

/translation="MEYRIERDTLGEMKVPADRLWGAQTQRSKENFPIGREHMPMEVV

RALAILKKSAAASNHKLGKLSAAKSDAIAYAADEIIAGRIDDHFPLVVWQTGSGTQSN

MNVNEVIANLGNQLLEQKGKEERLHPNDDVNMSQSSNDTFPTALHVAGVLAVEDQLLP

AIAVLKATFADKSEKFKDIIKIGRTHLQDATPITLGQEISGWEAMLDKSERMIRDSVN

YMKELAIGGTAVGTGINAHPDFGDYTAKEIGKHTGKEFVSAPNKFHALTSHDEVVYAH

GAVKALAADLMKIANDVRWLASGPRSGLGEIRIPENEPGSSIMPGKVNPTQSEAMTMV

VTQVMGNDAAIGFAASQGNFELNVFKPVIIYNFLQSVQLLADSIVAFNDKCAVGIEPN

LSQIEHNLNNSLMLVTALNPHIGYENAAKIAKLAHKEGLSLKEATLQTGLLTEEQFDQ

YVDPAKMIAPKA"

CDS 3137789..3140533

/gene="rcsC_9"

/locus_tag="PIKHDJKN_02849"

/EC_number="2.7.13.3"

/inference="ab initio prediction:Prodigal:002006"

/inference="protein motif:HAMAP:MF_00979"

/codon_start=1

/transl_table=11

/product="Sensor histidine kinase RcsC"

/translation="MLKKRRFTIRSKILLGYLVVVLLFGAVLLVLSAQIDASQKEIDF

ISHHDLEVHNLTNAIEKNVLNMETGQRGFMITGEESYLEPYNQALTQWNSNYAELYEL

VSDNPSQQMTLETIKTHIVRWIDVAGAASVALKRQGDQEKVIEFFHSDPGKSEIDLLR

SQFENFRNTELRLTESRVTELAARSSTLLTIMYTLWAAVAGLSVAAALVISGNIVKTL

REVKHTISDISQGGNLTQRILVRTQDEVGDLGKETNILLDTVQEQNRLKDRVASIATL

LQNPTSLEGLSRLFLNQVAILLEAPYGVLYAMKENRLIRVAAYAADGEKERSLGKVSV

APGEGLVGQSAIEKRVLQMNDLPQNYIRISSGLGDASAASLTVAPVVFEGKTIAVIEL

ASMKQIEPKETKLLTELIEIFGVSLHSTVTRMELQHLYDESQVLNEELQTQSEKLQAQ

TEEMVSQTEELHMQTEELYMLNERLEMQKNVAETSVSELAEVADQLRTSSGYKSEFLA

NMSHELRTPLNSMLILSEILAENKHQHLNSEEQKYASVIHASGKDLLNLINDILDLSK

VEAGEMEVDLDDVYLGSLPEAMNQYFLKTAEQKGIDFRIQLQSPLPETIFSDEMRLHQ

ILRNLLSNAFKFTNGGEVALTISRVSLSNAEANGAETDVIAFSVSDTGIGISDNKLLQ

IFDAFKQADGATARKYGGTGLGLSISQSLANLLGGSISATSREGQGSVFTLFLPLRRE

EPEVMQNSRLFLNEVAVTAPEINSASGMNSTKIEMLLTPLEESLLNGRQVLVVDDDIR

NVYALANALEQYDMKVITAQNGYECLEMLESGEANPDIILMDIMMPELDGYETTRQIR

ERLNLKKLPIIALTAKAMKEDRDKCIAAGASDYISKPLNMKEVLSRMKLWLSHETLGI

"

CDS 3140790..3142412

/gene="pucR_2"

/locus_tag="PIKHDJKN_02850"

/inference="ab initio prediction:Prodigal:002006"

/inference="similar to AA sequence:UniProtKB:O32138"

/codon_start=1

/transl_table=11

/product="Purine catabolism regulatory protein"

/db_xref="COG:COG2508"

/translation="MQLTVKEALQVYPLSEARLVAGGEGTSRMMKSVNVMDAPDIADW

IKSGELLFTTAFIMKDNETDALRLMRRLNERGCAGLGIKLGRFWQAIPQGIIEEADRL

RLPLLELPFQFTFSDQMNALFKAEHERSNRLLHEVVQKQKKLMHFALQQQQHRNVFAE

LATVLNYPLAVIGSRGHVLYGSEGISGDAVVQGWPWKSVMHRVKWNQGSCHRVPIKQN

DEEYGFLLVFTESALSLRAEEELFQQAADVLAFYMDMTYREHINPTVQDEMRTLLTQY

LDNKMTVDELTKLSENKGVHLFQGTYQCVLITLEPSVFAEGKLLKQIHRELQYNPLMQ

FTASQHFQIEDGILSIYTCPTGRDYGEELSAFLLNRFGDVLAAQEAKGASAPRFWISK

MKHEPKSLREAYQECLDTRQLARRFGMKDRALQFEMLEFAYVFQHVPDNIMENYCNKV

LEPLLSRDGDPNQVLMNTLESFIENDGLINEAAKQLFVHRNTVTYRMEKVGSLLQMDF

KKTNDLLKLKLVFTFRKFVRDKTAARPYSQHH"

CDS 3142709..3144214

/gene="uao"

/locus_tag="PIKHDJKN_02851"

/inference="ab initio prediction:Prodigal:002006"

/inference="similar to AA sequence:UniProtKB:Q45697"

/codon_start=1

/transl_table=11

/product="Uric acid degradation bifunctional protein"

/translation="MGDFIKLVNNWSITQFVHTFGGLFEESPWVAERSWPSRPFDSFG

QMMNVMKNAVQTSDEKVKLQLLCNHPDLGARISMSSNSVQEQAGAGLNSLSPEQYNEL

SKLNKEYTSQFGFPFILAVKGHTAQSILESMRRRNRKGREEEFQTALKEVFKIASIRL

EQWLVQIGHEHEFEFKSSEVEQRTMYYGKGDVWMYRSYAKPLTGIQPIPESPFTGRNN

ILFGLNIKVAVQGDEFLPSFIEGDNSHVVATDSMKNFILKHAADYYGATVEGFLAYVS

RRFLETYPQMTKVQMTADQIPFEDVPVRREGSLRASELVFRYSQNDRATAAIEAQRKG

NQVELSNHFSGVADIRLIKVKGSEFAGFVKDGYTTLPETWDRPLFIFLNINWRYEDPR

DGMDDQRGRYVAAEQVKDVAAAVFHACRSASIQHLIYQIGLRLLRRFGQLSEVSFESN

NRTWETVLEEVKEGEGKVFTEARPPYGFQGFSMTRDDLEADHRGSKKDREA"

CDS 3144214..3144591

/gene="pucM"

/locus_tag="PIKHDJKN_02852"

/EC_number="3.5.2.17"

/inference="ab initio prediction:Prodigal:002006"

/inference="similar to AA sequence:UniProtKB:O32142"

/codon_start=1

/transl_table=11

/product="5-hydroxyisourate hydrolase"

/db_xref="COG:COG2351"

/translation="MSVAGGRITTHVLDTSKGVPAEGVRVELFVLHRDGEKENKMKVA

ESVTNADGRLDASLLEGGKLEQAVYELQFNVEGYYAQRSLEELGQALWTVVPIRFAVS

DAASHYHIPLLIAPGGYSTYRGS"

CDS 3144631..3146037

/gene="allB"

/locus_tag="PIKHDJKN_02853"

/EC_number="3.5.2.5"

/inference="ab initio prediction:Prodigal:002006"

/inference="similar to AA sequence:UniProtKB:Q9KAH8"

/codon_start=1

/transl_table=11

/product="Allantoinase"

/db_xref="COG:COG0044"

/translation="MTRLDTIIRGARVVLRDSVEELDIGITGEKISALSSHLVGDEDT

EIIEANGLTVMPGAVDIHVHFNEPGLASWEGFKSGSASLAAGGITTYVDMPLNGVPPT

IRPEAWESKMKAADGQSYVDYAFWGGLVPGNRVELAPLAGMGAAGFKAFMSEPGGEGE

DIFARADDDTLLNGMYEIAKLNRVLALHAEDEAMVAELGARSIAGGRIGPMDYVQSRP

VEAEVRAVFRALQYGEQTGCALHFVHISTREALDLIAEAKQRGLDVTSETCPHYLTLT

DEDVVRLGAVAKCAPPLRSQAEQDQLWDALEAGLIDVIASDHSPCPPSMKQADNFFEI

WGGISGAQSTLLLMLEEGHLQRNVALPLLGKVLALQPARRLGLESKGEIAIGKDADLV

LIDWVKNTTLNTDDLHYTHKQSPYVGRTFSSRIADVFCRGTRVYSSKTGLSDEPVGRF

IRANSSVPVGVEGTEGIS"

CDS 3146034..3147335

/gene="pucF"

/locus_tag="PIKHDJKN_02854"

/EC_number="3.5.3.9"

/inference="ab initio prediction:Prodigal:002006"

/inference="similar to AA sequence:UniProtKB:O32149"

/codon_start=1

/transl_table=11

/product="Allantoate amidohydrolase"

/db_xref="COG:COG0624"

/translation="MTDFEVHTPSGTERPIQPLPQLEQVELKGMLDWLSAYGADPQGG

VTRLLYDQAWCEAQHALAAKMQEKGLAPEFDQSGNLYGTLYGTGLGTGMEEAPIVTGS

HIDTVVHGGKYDGAYGVVAGVLALEYLQKSFGAPKRTLQVVSLCEEEGSRFPFAYWGS

RSITGVSGLEEVQHLKDQEGITFAQAIRDAGFGPDSSYRSASHTYGAFIELHIEQGQV

LERLGHSIGIVSDIVGQKRFSITVSGEANHAGTTPMAWRKDALAGAAEMITAVRHIAL

EAGEPLVATVGRITADPGVGNVVAYRAVFSLDIRHIRQESIDRCWQNIIQAFSRIAAE

QQLELNWEEHLSVTPIPMNEQITSDIRDICEVHQLSYIHMPSGAGHDSQIFQPACATA

MIFVPSQDGISHNPLEYTAEEDLMKGFRVLVQLLYKYGYGS"

CDS 3147342..3148583

/gene="pucG"

/locus_tag="PIKHDJKN_02855"

/EC_number="2.6.1.112"

/inference="ab initio prediction:Prodigal:002006"

/inference="similar to AA sequence:UniProtKB:O32148"

/codon_start=1

/transl_table=11

/product="(S)-ureidoglycine--glyoxylate transaminase"

/db_xref="COG:COG0075"

/translation="MSNYKELSPSLRTIMTPGPVEVDPRVLRALSFPILGQFDPEFTS

LMNETMAMLRELYMTDNEWCYPVDGTSRSGIEAVLVSLIQPGDKVLVPIYGRFGHLLV

EISERCGAEVIFFETEWGTVFDPEEVIKAIHAHKPSLVAMVHGETSTGQMQPLTEIGK

ACRELDILLVVDAVATIGGTPVETDAWHLDAVMGGTQKCLSVPSGMAPITYNSRVEKK

LMSRKTVERGLRDATSARAEGRTIASNYFDLSQLQDYWSSARLNHHTEATSMLYGLHE

GLRILLQEGLDARFQRHRINERALVAGIQGMGLKLYGELSSKLPMVTCIEIPDDIDGE

SVRSMLLNDFSIEIASSFGPLKGKIWRIGTMGFSCQRKNVLHVLGALEAVLLRHRHAL

PAGEAVQVALDVYAGKEGVLC"

CDS 3148577..3150172

/gene="ywrD_2"

/locus_tag="PIKHDJKN_02856"

/EC_number="2.3.2.2"

/inference="ab initio prediction:Prodigal:002006"

/inference="similar to AA sequence:UniProtKB:O05218"

/codon_start=1

/transl_table=11

/product="Glutathione hydrolase-like YwrD proenzyme"

/db_xref="COG:COG0405"

/translation="MLNQMPISREVMVTSPHYLASAVGSSILQQGGNAYDAAVAVSAA

LGVVYPHMTGLGGDAFFLIHDGTSGDITAYNGSGRSAAGIHADTFKAMGMSAIPQRGV

LSAITVPGMVDAWWEVWSRYGKLKWEQLLEPAAQYAEKGCPVSRNLRLWLERDEDFIM

GHTPLRAVFAPFGTLLQEGDLLLQPDLASSIRLIQAGGRDVFYTGELAEQLTSAIRED

GGMLALADFAAHKGEWVKPITTDYRGYQVHQMPPNSQGFSMLMMLNILEHIDLSSVAR

TSPEFYHLMAEVVKKAFRDRDRYLTDPEFRDIPLENLLSKVYGDALWNEIQSAPPVAQ

PFLSKMMGQDTAYAAVVDNEGNAVSFIQSLYFDFGAAYVPGDTGVIMQNRGSFFSLDP

KDANVLEPNKRSFHTLMPGLVTRNGKPYMLVGTQGGEGQPQTQLSVLTGVLDYGLNIQ

EAIGLPRWVYGRTWGEEGDTMRVENRYHDDVCDTLARWGHNVEARAPWDGIMGQSQGI

VIREDGMISGAADPRGDGMAIGW"

CDS 3150281..3151603

/gene="triA"

/locus_tag="PIKHDJKN_02857"

/EC_number="3.5.4.45"

/inference="ab initio prediction:Prodigal:002006"

/inference="similar to AA sequence:UniProtKB:Q9EYU0"

/codon_start=1

/transl_table=11

/product="Melamine deaminase"

/translation="MGTILLKGAQLVTMNAEEDVFTGDLLIEDNKIKEIAEHIDVQAD

QVIDARGKVLLPGFIQTHIHLCQTLFRGRADDLELMDWLRQRIWPLEAAHDEESVYYS

AMLGLGELISSGTTTILDMETVHHTDSAFQAMAQSGIRVISGKVMMDHGDEVPEPLRE

NTATSLQQSVDLLEKWNGFGGGRIQYAFCPRFVVSCTEELLVEVRDLSNKYHVKVHTH

ASENRGEIELVEHERGMRNIVYLDHIGLATPRLVLAHCVWLSEEEKEIIRKRGVKVTH

CPGSNMKLSSGIADIPDLLNRQIAVGIGADGAPCNNNLDMFQEMRLTALIQKVPHGPT

VMDARTVLRMATMGGAEVLGMSKEIGSLEVGKKADMLLLDLEDFHTYPSYETDVYSRV

VYSATRSCVDTVIIDGSIVLKNRKIQTIDRGIVLRESDKSIARLMKRI"

CDS 3151736..3152914

/locus_tag="PIKHDJKN_02858"

/inference="ab initio prediction:Prodigal:002006"

/codon_start=1

/transl_table=11

/product="hypothetical protein"

/translation="MEMHDLCAIAAREPRCILATAVKVEGHAYRKQGVSMLLTEEGAM

FGSISPGCLESDLLARVDRVLDTQQLEFVEYDMRPEDDLSWGETIGCGGLIIVLLEPV

CGELRSILSHMHTYFQSGSAVELTRSFYDQYTRIHYVTQLAEGAESQTSLSPSPVARG

ASLGPINTMITDDTPRLSLVVIHSSEADAVAFNSGENDLQRHEGLTDTERWDVPLKIS

ARYEPKPRLIIVGAGNDVIPVAHLGKSAGFRVVVADWRETLCTAERFPGVELIRGFPR

EIMPVLGIQSGDYIVLMSHQFPREREFLELLEDRNYAYLGIMGSRTRTARLLNDLPPL

KHLHSPVGLTIGADGPEEIAISIAAELIACKRAAVTAERRNVQRGIAHATDGHRASGR

"

CDS 3152880..3153524

/gene="pucB"

/locus_tag="PIKHDJKN_02859"

/inference="ab initio prediction:Prodigal:002006"

/inference="similar to AA sequence:UniProtKB:O32146"

/codon_start=1

/transl_table=11

/product="Purine catabolism protein PucB"

/db_xref="COG:COG2068"

/translation="MRLTGIVLAAGKSSRMGRDKLSIIMPDGRCLAAWTLEAALNSDL

ERVICVVKPEDSLAWIPSMWLNAASYGCSLDMRAKLQIAICTEYAYGMAMSLHCGILS

AMAYRPEGVMMILADQPLLQAQDINRVAASLAANKLCDYAAATDGGGGKPPVAFREHM

LGPLLSLNGDEGARKIMRNAKYTGTHVSLSEFSFWDADTEPELERILDYVNGIR"

CDS 3153833..3154747

/gene="pucC"

/locus_tag="PIKHDJKN_02860"

/EC_number="1.17.1.4"

/inference="ab initio prediction:Prodigal:002006"

/inference="similar to AA sequence:UniProtKB:O32145"

/codon_start=1

/transl_table=11

/product="putative xanthine dehydrogenase subunit C"

/db_xref="COG:COG1319"

/translation="MVTPAYGSGAQPTVWQPVNLDELQALKNELSGKYCFAAGATLLR

TQWEGGLVPVPEHMISLSRIPGASGVYVDGDDLVIGALTRLSHCATDVHLQQLPLLQS

AMNAIAAPSIRNVATIGGNIVSGVGDSIPALLVYDAQLYWMTDNGMEIRSLSSWLNGG

RDGSRNPYDVLIAIHIPMKHTPFLDFTSERTSAEREISFYRKLGRRETFTASLVTIAF

HGEVGADGRWNKIAIAAGGGSGMAMRLTESEKLLLGVEASVMQASSLAATAASEFVTY

SDAFATEQYRKQTAGNMLGVGLWEALRS"

CDS 3154816..3157149

/gene="pucD"

/locus_tag="PIKHDJKN_02861"

/EC_number="1.17.1.4"

/inference="ab initio prediction:Prodigal:002006"

/inference="similar to AA sequence:UniProtKB:O32144"

/codon_start=1

/transl_table=11

/product="putative xanthine dehydrogenase subunit D"

/db_xref="COG:COG1529"

/translation="MLLNRNSSGKRWHLRPDGVGKVTGKLQYLTDMTLPDMIHGRVLR
SSYPYARILSIDITGAEAMEGVFAVLTSKDVPGLNLFGIATPDQPVFCEHVVRYVGDA
IAAVAADSPERAALALDAIRVEYEEWTPLDSPEAALAPSAPELHEHGPGNVLHCTEIK
RGNVDEGFAACEHIVSETYYTPRQMHAYMETEGGLFVPDESGRLHVYAATQHGYKDRM
QLARIIGCSEEDIRVVSSPIGGSFGGKDELNVQPYGALLALRSGRPVKMHNSRKESVR
AGLKRHPMKIEMQTGISREGMIQAHRVRITADTGAYATLGAPVLNFCTEHCLGPYSIP
HVDVEGVSVYTNNGVSGEFRGFGGNQAIFAMEGQMDRLAEIMNMDPWEFRRRNMREKS
DPGPLNQRILVTDGLSQVWEAMDRSELWQKHQHPPADLALPPWIKRGVGAAIAMHGAG
LGYGIPDPAGGRLSLNAEGKIEVAFSYEEFGQGLIATLEMMLCDLFHCSTSDLSIIIG
DTDRVPHSGSSTASRSTTMAWMALQRLQTPFRSKILSVASALSGIPADELITGIEGVW
RKGQLSPKITANEPAMKSRVFVSYVDLAKHGEAEEWIFDTQFEYPTTPDNVVGGHYLY
TYAAVAAEVEVNTLTGEAKLLDTRHVVAAGPVINPMGFIGQIEGGSVMALGFTLTEDA
VMQDSRYMTTNLDTYLIPTIRDIHTNLEVEAIEDLPEGDPFGPRGIGEIGSVALAPAI
TAAIHQAIGVWVNRLPVPRDQLLKPLDVPLQEGVNPT"

CDS 3157146..3157649

/gene="pucE"

/locus_tag="PIKHDJKN_02862"

/EC_number="1.17.1.4"

/inference="ab initio prediction:Prodigal:002006"

/inference="similar to AA sequence:UniProtKB:O32143"

/codon_start=1

/transl_table=11

/product="putative xanthine dehydrogenase subunit E"

/db_xref="COG:COG2080"

/translation="MSEPLENPWTAVVNGEKKQLQIAQTTRLVDVLRSHLNLTGTKIS
CEIGRCGACMVLMDGGPVNSCLVMAYQCIGSEITTIEGLHGEKEGELHPIQQAFVEEG
GFQCGYCTPGMVISTKALLDRNPQPSQEQIEMGLCGNLCRCTGYGGILRAVNKVIDEH
DRMGSTQ"

CDS 3157880..3158827

/locus_tag="PIKHDJKN_02863"

/inference="ab initio prediction:Prodigal:002006"

/codon_start=1

/transl_table=11

/product="hypothetical protein"

/translation="MNKSDRMLAIVLELQRGKVQRAEDLALLFETSVRTIYRDIQALC

EAGVPVVGEPGVGYSLMEGYFLPPISFTAEEAVTLLIGLDFVEQRFDNYYKSTSLTSR

SKIEAILPVPLREEVGQIQQGIRLLRSPDSHAGEREIEHMGIIRKAMQHKLKIRFHYH

KPGTINENVQKTIRMASPYGMLWMQGSWMLIARCDLRQEIRHFRISRMSDLEISTDPF

DLPPGFNIHSYSPPDNRDVHVRVWFHGRIADRVIEANNYYMEKAELQTDGLYADFRVR

HPEDVLRWILGWGASALVLEPESLRIRIREEASNLLKHY"

CDS 3158894..3159460

/locus_tag="PIKHDJKN_02864"

/inference="ab initio prediction:Prodigal:002006"

/codon_start=1

/transl_table=11

/product="hypothetical protein"

/translation="MDTPRTRNNDEIRRHFELTLERYLQELELMSDAQLSYKPSEIEW

SVGQMYQHLIQSAMKMHLANVQLCISADEDLGKKKNQGKTETGEAVFAQASFPPIRIH

VPASPEYTPLQPDGRESIIQGFNEVRDEMRRMEVLLEETTQECTLAHPSFGGLNAEEW

FALVEMHYRHHFLQLERLKKAWEEQVQR"

CDS 3159457..3160002

/locus_tag="PIKHDJKN_02865"

/inference="ab initio prediction:Prodigal:002006"

/codon_start=1

/transl_table=11

/product="hypothetical protein"

/translation="MNEQAKHDLMANSKEIQQTHTREFLSHQLDVEAESEEGNRYWIG

VVSASHVEKGVEGSFAQLCHGKVASLRKMNAGDWLIYYSPRTSMQGGKVLQAFTAIGR

VIDNQVYTYRMSDSFVPYRRNVQYYPCQQVKIADLLDQLILTRGQARWGYHFRYGHLQ

IQREDFLKIAVAMLRAEVETC"

CDS 3160672..3161748

/gene="glpX"

/locus_tag="PIKHDJKN_02866"

/EC_number="3.1.3.11"

/inference="ab initio prediction:Prodigal:002006"

/inference="similar to AA sequence:UniProtKB:Q03224"

/codon_start=1

/transl_table=11

/product="Fructose-1,6-bisphosphatase class 2"

/db_xref="COG:COG1494"

/translation="MERELALEIVRVTELAALASAPWMGRGDKNSADEAATLAMRAMF

DSVSIRGTVVIGEGEMDEAPMLYIGEEVGNAEGPEVDVAVDPLEGTEIVAKGLNNALS

VIAVAGKGNLLHAPDMYMEKLAVGPALVGKVSIEDPVEVTLEKAAEALNKNISDLTVM

ILDRVRHESTIKTLRKVGVRIKFLSDGDVAGAMAPAFPEAGIDLYVGSGGAPEGVLAA

AALSCLGGEIQGRLMPANADEFQRCLQMGIDNPYKVLTMQDMIGTEDVIFAATGVTPG

EILGGVRYLADDRAETDSIVMRAKTKTIRFIRSQHFLPNKEVLHKVRQLQSTPEPSDR

IQSHVTILEQAEFSQSSVGNGVTT"

CDS 3162238..3163284

/gene="degA_3"

/locus_tag="PIKHDJKN_02867"

/inference="ab initio prediction:Prodigal:002006"

/inference="similar to AA sequence:UniProtKB:P37947"

/codon_start=1

/transl_table=11

/product="HTH-type transcriptional regulator DegA"

/db_xref="COG:COG1609"

/translation="MASRKEVADLAGVSEATVSRVLNGVGPIKEETRRKVIEASEQLG

YVPSALARSFARSKSGNLGVVLPYVPKAHLFSAYFFSEMLSGIGSKARDSGMDLLVMF

RTPGEVMNYTDLFRRQKVDACIILGARDDHGELAAMQQLQQEGHPFCIMSQHFAGESF

MEVDADHVEGSRLAIRHLTDQGYRKIAFLNGPDSYSNSQERLQGVQIGLNEAGMNMDS

SLLLEGNYGRRSGVEAAATIAARLDEIDAVFAANDRMAIGVMHGLRERGIKVQDFPAF

VGYDDSDAAEMAVPPLSSVRVPFYEMGELAASKLIHGSLGATASSVNTEVPSETARQL

LPTELIIRASSIRN"

CDS 3163322..3164494

/gene="iolG_9"

/locus_tag="PIKHDJKN_02868"

/EC_number="1.1.1.18"

/inference="ab initio prediction:Prodigal:002006"

/inference="similar to AA sequence:UniProtKB:Q9WYP5"

/codon_start=1

/transl_table=11

/product="Myo-inositol 2-dehydrogenase"

/db_xref="COG:COG0673"

/translation="MSNRLRVGMVGYKFMGKAHSNAYRSLPMFFPSAPLQPEMSVICG

RNEQRVQEAASQFGWSESVTDWRELVRRDDIDLIDINAPSDAHKEIALEAARQGKHLF

CEKPLALSLADSREMLQAAEEAGVAHMVGFNYRFSPAVQLAKELVQSGRLGKIYHFRA

FFLQDWIMDPSFPLVWRLQKEVAGSGSHGDLGAHLIDLARFLVGEFKEVIGMSETFIK

ERPLASEMTGLSAKGSSNADAPKGEVTVDDATLFLARFADGALGSFEATRFAAGHRST

NSFEINGSLGSVRFDFERMNELEVYFTKDDEDVQGFRRVLATDPAHKYAEAWWPAGHT

IGFEHTFTHEMLELVTAISEGRQPEPSFHDGVACQAVLEAVERSVTERRWVSLEEM"

CDS 3164657..3165385

/locus_tag="PIKHDJKN_02869"

/inference="ab initio prediction:Prodigal:002006"

/codon_start=1

/transl_table=11

/product="hypothetical protein"

/translation="MSKALIVWGGWDGHEPEQVAAIFERILKEEQFEVEVSDTLEAYA

DAEKLLGLDLIVPLWTMGQIEQELVNNVSAAVQSGVGLAGLHGGMCDAFRNNVDWQFM

TGGQWVAHPGNDGVEYTVNMKRGSSPLLDHIEDFQVKSEQYYLHVDPAVEVLATTRFP

IVNGPHAANGPVDMPVVWTKRWGAGRVFYNSLGHHADIVEMKQVTEMMRSGFKWTAAG

KALAQSRKDAVTEVYTGMADNQNH"

CDS 3165500..3166606

/gene="iolG_10"

/locus_tag="PIKHDJKN_02870"

/EC_number="1.1.1.369"

/inference="ab initio prediction:Prodigal:002006"

/inference="protein motif:HAMAP:MF_01671"

/codon_start=1

/transl_table=11

/product="Inositol 2-dehydrogenase/D-chiro-inositol

3-dehydrogenase"

/translation="MKTMKVGIIGCGKISGIYMENCHRFEVLDLVAVADLDRNRAEEQ

AAAYNVPNVYTVEEILADPEIDLIINLTIPAVHADVCLRALEAGKHVYVEKPLAVTRE

EGQAVLETAKRKGLLVGCAPETFFGSGIQTALKVVEDGVIGKPVAATAFMMSRGHEHW

HPDPEFYYAAGGGPMFDMGPYYLTALVQLLGPIKSIAGMTGKAMEQRTITSEKKRGQS

IPVEIPTHVAGLLQFEQGAIGTLITSFDVFGGSVLPPIEVYGTHGTLQVPDPNTFGGP

VRYRLLGDEEWTEVPLLPGYQENTRGIGVADMAYAVGSGRAHRASGELAYHVLEAMWA

FHDSSDEQTFYKMDSTCQRPAALPADLPLYTLDQ"

CDS complement(3166684..3167502)

/locus_tag="PIKHDJKN_02871"

/inference="ab initio prediction:Prodigal:002006"

/codon_start=1

/transl_table=11

/product="hypothetical protein"

/translation="MKTSLVYKVPKTLLNKGSGFLNGYTHTLNPYTGCAFSCSYCYVR

QMPVSLFRKEPWGSWVDVKQEAANVLAKELQRAKSKGKVTLFMSSSTDPYQPVEYKEQ

ITRSLLEVMAQHPPDFILVQTRSPLVTRDIDLLQKLGDRVRVSMTVETDLDDIRKQFS

PAAPPIAGRLRALEQLRDAGIPTQAAIAPVLPSSESFAAILRPLVDRICIDDYFMGDG

SEGKRTRRLGMKPLYEQVDKEDWFSPEAYKMVVERMKAHFEEDEIWISQAGFEP"

CDS complement(3167499..3168107)

/gene="adaA"

/locus_tag="PIKHDJKN_02872"

/EC_number="2.1.1.-"

/inference="ab initio prediction:Prodigal:002006"

/inference="similar to AA sequence:UniProtKB:P19219"

/codon_start=1

/transl_table=11

/product="Bifunctional transcriptional activator/DNA

repair enzyme AdaA"

/db_xref="COG:COG2169"

/translation="MRLEVILLESHSPYQTDSVPIDDKQWNAIIHNDSSFDGKFYYGV

KTTGIFCRPSCKSKAPKYENVSIFNNLEEALARHFRPCKRCKPTGERVPDQEWISVVT

DYIEHHFAEPLTLDILANVSHGSPYHLHRVFKRIIGLTPVEYIQEKRVTEARKLLEST

RLTVTDIGRQVGIANPAYFITVFRKHTGCTPANYREQRRDQS"

CDS 3168270..3169250

/gene="iolU_3"

/locus_tag="PIKHDJKN_02873"

/EC_number="1.1.1.371"

/inference="ab initio prediction:Prodigal:002006"

/inference="similar to AA sequence:UniProtKB:O05265"

/codon_start=1

/transl_table=11

/product="scyllo-inositol 2-dehydrogenase (NADP(+)) IolU"

/db_xref="COG:COG0673"

/translation="MVRFGVVGTNWITERLLEAAAQVEGFELTAVYSRTEDKANAFAD

QYQAALRFTNLEEMATSDGLDAVYIATPNTFHAEQAELFLRNGKHVLCEKPLAANGAE

VHRMIDTAKEHGVLLMEAMKSTLVPSFKMVQSHLHKIGPVRKYVASYCQYSSRYDKYK

EGIVLNAFKPDLANGALMDLGVYCLYPLITLFGEPKQVQSQALMLESGVDGQGSVILN

YDEMEAVVTYSKISNSHVPSEIMGERGSIIIDKIGSPEHAEIRYNDGTVEKLTAEQIY

PSMYYEVEEFVNLIQQGKKESDMNTYERSYVTMQVMDQIRQQIGLVFPND"

CDS 3169275..3169991

/locus_tag="PIKHDJKN_02874"

/inference="ab initio prediction:Prodigal:002006"

/codon_start=1

/transl_table=11

/product="hypothetical protein"

/translation="MSRDEQKSRVAGLELAQIIFIGRTFEEYMKMFNLTVEEIKGKSI

LDCPGGACSFSSHARECGAEPMAADIAYEHEIGQLEVKGLQDIEHTMKQMKQVQDKYR

WDDFGSIDGLKEERRRAITDCVADMRRFPDRYVASVLPELPFADEQFDLTLSAHFLFT

YADRLDAEFHLQTILELLRVTKRELRVFPTVDLSGKRYEHMDELKAILEKRGYITSEE

KTSYEFQRGAHTMLHIVKSR"

CDS 3170085..3170978

/locus_tag="PIKHDJKN_02875"

/inference="ab initio prediction:Prodigal:002006"

/codon_start=1

/transl_table=11

/product="hypothetical protein"

/translation="MKKVLILGGTRFFGKRLVDHLLWEGKSQITVATRGKTDVDFGPE

VNRIKMDREDPKSLAEVAQTDMWDIVYDNICYSPDAAKSACEAFAGRTQRYILTSTLS

VYGDPKPGFTEVDFDPYTYPVQYGSHENFSYGEGKRLAEAVFFQEADFPVAAMRIPIV

LGIDDYTRRLHFHIEHVQKGKPIGMPNPNAEIGFINSTEAARFLAWLGHSSITGPVNA

ASKGAITLSAMMHLIETVTGMQSQVLTETIKEDMSPFGISESWTMDTSKAEQAGYTFE

ALMDWFPGLVREVALALQSEG"

CDS complement(3171308..3172318)

/gene="degA_4"

/locus_tag="PIKHDJKN_02876"

/inference="ab initio prediction:Prodigal:002006"

/inference="similar to AA sequence:UniProtKB:P37947"

/codon_start=1

/transl_table=11

/product="HTH-type transcriptional regulator DegA"

/db_xref="COG:COG1609"

/translation="MITIYDIAKKANVSAMTVSKVINQTGRISPATRERVQQVIEELG

YIPNSNARSLVLQRTQMLSLLITDITNPFYTTLARGAEDAANLRGYRLLFSNSDEDYD

KEKNYVETILSTRVDGVLFAPAGDRSLNHLKQLQERNIPFVILDRTVPGITSDVIAGD

SRDGALQLIRYLTNLGHRCIALVNGSSEVSTARLREKGYMEGLREAELPFDEGLILRT

GYRDFSDEVGIDSLLAHQHKPTAIFAANNMLAIGVIRLLRKRGLRVPEDISVVCFDDL

DLASAFDPFLTVAAQPAYDFGAIGMQMLIDRIEGKAPEEPQTVIMPSELRIRASASVP

CE"

CDS 3172728..3174518

/gene="fucI"

/locus_tag="PIKHDJKN_02877"

/EC_number="5.3.1.25"

/inference="ab initio prediction:Prodigal:002006"

/inference="similar to AA sequence:UniProtKB:Q97N97"

/codon_start=1

/transl_table=11

/product="L-fucose isomerase"

/db_xref="COG:COG2407"

/translation="MTHQDYRYKQAFPKIGIRPTIDGRRKGVRESLEEQTMRMATSVA

ELLAAELRYPDGSPIECVIAESCIGGVNEATAAAELFSRSNVGVTITVTPCWCYGTET

MDMSPSIPTAIWGFNGTERPGAVYLAAVLSAHAQKGIPAFGIYGEDVQDGGDTTIPDD

VREKLLRFSRAGLAAATLKGRAYLSIGSVSMGIAGSIVNDSFFQEYLGMRNEYVDMSE

LTRRIEEEIYDPEEYKLALAWVKENCSEGPDNNAAHLQTDRKRKEYEWETVVKMTQIV

RDLMAGNPKLAELGFTEESMGHHAIVSGFQGQRQWTDHSPNGDFLESILNSSFDWNGK

RSPYLVATENDSLNGVSMLFGSLLTHTAQIFADVRTYWSPDSVKRVTGHQLEGNAKDG

ILHLINSGSAALDGTGQQSRAGEPVLKPFWEITDEEVQDCLKATSWRPASVEYFRGGG

YSADFLTKGGMPVTMTRLNLVKGLGPVLQVAQGYTVDLPEDVHDTLDQRTDPTWPSTW

FAPVLTGSGAFTSVYEVMNQWGANHGSISYGHIGADLLTLASMLRIPVSMHNVPESEI

FRPRAWGLFGTSEPESADYRACSVFGPLYR"

CDS 3174560..3176062

/gene="rhaB_2"

/locus_tag="PIKHDJKN_02878"

/EC_number="2.7.1.5"

/inference="ab initio prediction:Prodigal:002006"

/inference="similar to AA sequence:UniProtKB:Q1R415"

/codon_start=1

/transl_table=11

/product="Rhamnulokinase"

/translation="MGTQATHVLAADYGAGSGRVVRGTFDGSKLSLEEVHRFSNDPVH

LGDGLYWDFLRLFHELKQGIVKGMQGISQPVRSIAVDTWGVDYGLVDGAGRLCFNPRH

YRESRNAKWMEEALRMVNEEELYSLSGVLPQQINTVFQLLGSVREHSEGLRPDVRMLL

MPDLFNYYLSGQQACEYTIASTSGLLHAGEARWNEQLIGRLGIPESLFPPLVQPGTVL

GQLTDELRSELKTGPMQVISVGSHDTASALAAIPATDSEFAFISCGTWSLMGVERDSP

VLDNRSRQLGFTNEGTVNGKVRTLKNRSGLWLLQECKRQWERENQRFTHEELVLLAAS

ATAHQCYVSPGDKVYLAPGDMPERIRQQCSASGQAIPETIGAIARCILESLALEFRQT

LDELELITGTRPRVIHMVGGGVHNRLLCQFTANAAGIPVIAGPAEATSAGNCMLQFLA

HGEVSSLSEVREVMASSFSPETYEPEDTARWQEAYVRYQNLNQTLASRSN"

CDS 3176116..3176778

/gene="drdA"

/locus_tag="PIKHDJKN_02879"

/EC_number="4.1.2.-"

/inference="ab initio prediction:Prodigal:002006"

/inference="similar to AA sequence:UniProtKB:P0DTQ0"

/codon_start=1

/transl_table=11

/product="5-deoxy-D-ribulose 1-phosphate aldolase"

/translation="MSEQHVREELTKYARRAVAQGLVVGPGGNLSARSEGTMLLSPSG

YALEDLEPDEWIAIDIATGETRAGSTRPSSEVLMHLYSYRVNPDIQAIVHTHPAYTIA

LSLVFDELPHLFPDQSALVGDIGFVPYVLPTTKLLADAVAAKVEEHTALILVNHGLVT

TGKNLREAYYRTQVVEESAKVYMIAKAAGEPKVLTAEEYKEIQSLESEAYRVQLLQQL

KS"

CDS 3176986..3177336

/locus_tag="PIKHDJKN_02880"

/inference="ab initio prediction:Prodigal:002006"

/codon_start=1

/transl_table=11

/product="hypothetical protein"

/translation="MKTHSELNSLLVDDYLDLLNIAKQLGDEEWKEAILQALKEEVDA

NHHLDSTEVRQDLWTQFEGINEQMHDLLMQLKNADGSEEKEQIIEAIWGLKVDRHAIT

RKLETMSRASAGHQ"

CDS complement(3177453..3178079)

/gene="azoR2_1"

/locus_tag="PIKHDJKN_02881"

/EC_number="1.7.1.17"

/inference="ab initio prediction:Prodigal:002006"

/inference="similar to AA sequence:UniProtKB:O32224"

/codon_start=1

/transl_table=11

/product="FMN-dependent NADH-azoreductase 2"

/db_xref="COG:COG1182"

/translation="MSTLLYITAHPHDHETSFSMAAGKAFVDAYRESHPSDEVIHLDL

YRSDIPHIDADVFSGWGKLQSGSELTSEEQAKVQRLNELSDQFASADKYVFVTPMWNF

SFPPIMKAYIDSICVAGKTFRYTEQGPVGLLSNKKALHIQARGGIYSEGPAAQMESGH

RYLSIIMSFLGVPKLEGIFVEGHNQYKERADEIKQQAIEEARTLAKQF"

CDS 3178261..3179205

/gene="deoR"

/locus_tag="PIKHDJKN_02882"

/inference="ab initio prediction:Prodigal:002006"

/inference="similar to AA sequence:UniProtKB:P39140"

/codon_start=1

/transl_table=11

/product="Deoxyribonucleoside regulator"

/db_xref="COG:COG2390"

/translation="MGMDLEKQRLSIEAAKLYYQSDYSQQDIAVRLGVSRPTVSRLLQ

YAKDRGYVRIEIMDPLEDIDIIAGELKAKYNLDTALVCFAPLKSDEEILKHISKRAAD

YLQDTVQDADIIGVTWGTTMNAVARQLRPKQVKGVEVVQLKGGVSHSHVNTYAAEIVH

LFAEAFHTVPRYLPLPVIFDNIEVKKMVEADRHIGRIVELGRQANIAMFTVGTVKEDA

LLFRLGYFNEEEQQLLMHSGAGDICSRFFDAEGRLISEEINNRTVGIDLAELRNKEKS

ILVAGGKRKIEAIHAALKGHYANILVTDQYTAQALLRL"

CDS 3179435..3180109

/gene="deoC"

/locus_tag="PIKHDJKN_02883"

/EC_number="4.1.2.4"

/inference="ab initio prediction:Prodigal:002006"

/inference="similar to AA sequence:UniProtKB:P39121"

/codon_start=1

/transl_table=11

/product="Deoxyribose-phosphate aldolase"

/db_xref="COG:COG0274"

/translation="MTTPQLAKMIDHTLLRADATQSEMAKLTEEAKQYQFASVCVNPG

WVAYAAEQLQGTGVDICTVIGFPLGASTSETKAFETKDAIAKGATEVDMVINISALKD

GKDDVVEQDIRAVVEAAAGKALVKVIIETCLLTDEEKVRACQAAVKAGTDFVKTSTGF

STGGATPEDIALMRRTVGPDVGVKASGGVRSLEDMQKMIDAGATRIGASSGVKIMQGE

QSTSNY"

CDS 3180383..3181573

/gene="nupC_1"

/locus_tag="PIKHDJKN_02884"

/inference="ab initio prediction:Prodigal:002006"

/inference="similar to AA sequence:UniProtKB:P39141"

/codon_start=1

/transl_table=11

/product="Nucleoside permease NupC"

/db_xref="COG:COG1972"

/translation="MKFLIALLGILVVFGLAYLVSNGKKKIRYRPLIIMIVLQVILGY

ALLNTEIGTFLIGGFATVFESLLGYANDGISFVFGGLTTVGADSGGAPFFLSVLMPIV

VISALIGILQYIRILPFVIKYIGLVLSKVNGMGKLESYNAVASAVLGQSEVFISVKKQ

IGLLPKHRLYTLCASAMSTVSMSIVGAYMSMIDPKYVVTALVLNLFGGFIIASIVNPY

EVSEEEDILEVQEEEKQSFFEMLGEYILDGFKVAIVVAAMLIGFVALIALVNGIFSAV

LGISFQELLGYVFAPFAFIMGVPWKEAIQAGSIMATKMVSNEFVAMLDLTKQTTLSAR

TTGIVSVFLVSFANFSSIGIIAGAVKGLHEKQGNVVARFGLKLLYGASLVSVLSAIIA

GLFL"

CDS 3181604..3182782

/gene="deoB"

/locus_tag="PIKHDJKN_02885"

/EC_number="5.4.2.7"

/inference="ab initio prediction:Prodigal:002006"

/inference="similar to AA sequence:UniProtKB:Q818Z9"

/codon_start=1

/transl_table=11

/product="Phosphopentomutase"

/db_xref="COG:COG1015"

/translation="MSTFKRVHLIVMDSVGIGEAPDAAEFDDFDVDTFGHIARERGGL

NMPHMASLGLSNIKEIEGVPVADTPKAFYTKMQEASRGKDTMTGHWELMGLYIDTPFR

VFADGFPDELIQRIEEKTGRKVIGNKPASGTEIIDELGEEHVKTGALIIYTSADSVLQ

IAAHEDVVPLKELYEICEFCREITLDDPYMLGRIIARPFVGEVGNFKRTANRHDYALK

PFGRTVMNELKDGGFDVIALGKIADIYDGEGVTKSVRTVSNMDGMDKLSETMDEAFTG

LSFLNLVDFDALFGHRRDPQGYAQALEEYDARLPEIFSKMTDDDLLLITADHGNDPTY

RGTDHTREYVPLLVYSPRFSGGKKLELRSTFADVGATVAENFGVKLPEYGTSFLKDLK

"

CDS 3182929..3183636

/gene="deoD_3"

/locus_tag="PIKHDJKN_02886"

/EC_number="2.4.2.1"

/inference="ab initio prediction:Prodigal:002006"

/inference="similar to AA sequence:UniProtKB:Q5EEL8"

/codon_start=1

/transl_table=11

/product="Purine nucleoside phosphorylase DeoD-type"

/db_xref="COG:COG0813"

/translation="MSTHIGAKPGDIAETILLPGDPLRAKFIADTYLEDVVCYNEVRG

MLGFTGTYQGHRISVQGSGMGIPSFSIYANELISEYGVKNLIRVGTCGGMQEHVRVRD

VILAQASCTDSSMNKLVFGGYDFSPIATFSLLKEAYDRATAKGMKIHVGNVFSSDSFY

RDDRSVTEKLMQHGVLGVEMETTALYTLAAKFGVNALTILTVSDHLLTGEETTAEERQ

KTFNDMMVVALETAITL"

CDS 3183868..3185169

/gene="pdp"

/locus_tag="PIKHDJKN_02887"

/EC_number="2.4.2.2"

/inference="ab initio prediction:Prodigal:002006"

/inference="similar to AA sequence:UniProtKB:P39142"

/codon_start=1

/transl_table=11

/product="Pyrimidine-nucleoside phosphorylase"

/db_xref="COG:COG0213"

/translation="MRMVDIIAKKRDGKELTTAEIDFVVQGYTQGEIPDYQVSAWAMA

VFFKDMTDKERADLTMSMVNSGDTIDLSAIEGIKVDKHSTGGVGDTTTLVLAPLVAAL

DVPVAKMSGRGLGHTGGTTDKLESVAGFHVELEKEEFIRLVNEHKVAVIGQSGNLTPA

DKKLYALRDVTATVNSIPLIASSIMSKKIAAGADAIVLDVKTGAGAFMKTTEDAKELA

HAMVSIGNNVGRKTMAVISDMSQPLGLAIGNALEVKEAILTLQGKGPKDLEELCLALG

RQMVFLAGKADSLEHAEEKLKEVIQNGKALEKFKDFLANQGGDASVVDHPDRLPQAKY

LVEVPADKDGYVAGIVADEIGTAAMLLGAGRATKESEIDLAVGLMLNKKVGDQVKAGE

SLVTIHANREDVADVIAKIKENITIADHADAPVLVHDIVTE"

CDS complement(3185255..3186238)

/locus_tag="PIKHDJKN_02888"

/inference="ab initio prediction:Prodigal:002006"

/codon_start=1

/transl_table=11

/product="hypothetical protein"

/translation="MNHPGYTMTKAPVTKSHPIQWHNLIRALWIGGLAVYIIHLNTTD

SLHYYLAPTMQRLLLCCPVPFLFIAAIMAWHGLFGNSQLHCDCEHPPPSGWVRSLLIY

GLIAIPLVLGFLLPDQALGSSMASQKGMSLSYGPPEIRRKEPLPDATDLDIKDLSKKT

ATVQSSVSAIKVQFVPPDEYSRELAELAEKLYAESVIKVYPEIFSETLGSIDMFQRQF

AGKAISLTGFVYRDKSMDHESHFALGRFLVMCCPADAAPFGLMIHVPNADSFPTDSWV

QIDGTIGSAQVNGEDTIEIRASKVTPVDQPSTPYIYTSADSVVTYDKLQNK"

CDS complement(3186279..3187313)

/locus_tag="PIKHDJKN_02889"

/inference="ab initio prediction:Prodigal:002006"

/inference="similar to AA sequence:UniProtKB:Q8DUY3"

/codon_start=1

/transl_table=11

/product="Putative two-component membrane permease complex

subunit SMU_747c"

/db_xref="COG:COG0701"

/translation="MKMAANLKLLSFLIPCAFLVPVLITMAPNMKEAWNSEALQNMKT

VFISIFLEAAPFLLIGVLLSSLMQWFVSEEMIRKLTPKNPVGGVLVAGLLGIIFPICE

CGMIPVVRRLMHKGMPPYIAVTFILSGPVVNPIVFTATLLAFPSHPEITIARMGLAFA

VAASIGGLVYMFVRQNPLRKPKATVTEVKTHPGFKMAQPHSHSTDHDHAHDKNWHSFF

IHAGDEFVDMSKYLVIGALITACIQTFISRSDLISLGNGPVASYAFMMGFAYVLSLCS

TSDAFVASAFSHTFALGPLLSFLVLGPMLDFKSTLMLLSTFRTRFVIGLSLAIITLVF

AGSWLINLLA"

CDS 3187595..3188797

/gene="yciC_2"

/locus_tag="PIKHDJKN_02890"

/inference="ab initio prediction:Prodigal:002006"

/inference="similar to AA sequence:UniProtKB:P94400"

/codon_start=1

/transl_table=11

/product="Putative metal chaperone YciC"

/db_xref="COG:COG0523"

/translation="MTEQQQVLPRVPVTVLSGYLGAGKTTLLNHVLHNREGMRVAVIV

NDLSEVNIDAGLIRDGGGLSRIDEKLVEMSNGCICCTLREDLLKEVERLALDGSFDYI

LIESTGIGEPVPVAQTFTYIDEELGIDLTKFTRLDTMVTVVDAAQFWRDFYSKETLKD

RGQEAGEDDVRGIVHLLTDQVEFCDVLILNKCDLVSEDELLKLEKALHAMQPEARLIR

TTHGQIDPREILNTGRFDFEKASQSAGWIRELMKEEHTPETEEYGIHSFVYHRKRPFH

PDRLLRWIAKWPDSIVRSKGLIWLATRNHMAVSFSHAGTSKQLGPAGLWVGAMSDEER

QMHFGDTLPPIPDWDEQWGDRVTKLVFIGIDMDRAEIERTLDDTLLTEEEMQMNWREL

KDPFPAWS"

CDS 3188872..3189021

/gene="rpmG2"

/locus_tag="PIKHDJKN_02891"

/inference="ab initio prediction:Prodigal:002006"

/inference="similar to AA sequence:UniProtKB:P66231"

/codon_start=1

/transl_table=11

/product="50S ribosomal protein L33 2"

/translation="MRVIVTLACTECGDRNYTTTKNKRNHLERIELKKYSPRLKKMTI

HRETR"

CDS 3189071..3189778

/gene="znuC_1"

/locus_tag="PIKHDJKN_02892"

/EC_number="7.2.2.-"

/inference="ab initio prediction:Prodigal:002006"

/inference="similar to AA sequence:UniProtKB:O34946"

/codon_start=1

/transl_table=11

/product="High-affinity zinc uptake system ATP-binding

protein ZnuC"

/db_xref="COG:COG1121"

/translation="MILSSMRDVVFGYSKEPVIDQLSLDIHVGEFVGITGPNGSAKTT

MLKLLLGLLQPWSGTVHMNAQSVRGSKLNIGYVPQQVASFNSGFPSTVIELVRSGCYR

KLGLFRRFTSEQEAIVERSLREVGMWEYRNARVGELSGGQKQRICIARALAGQPQVLV

LDEPTTGMDRRSREGFYDLMRHYADVHGLTIIMVTHGLEEMGNRLDRTITLERQESEE

WQCLSTNSCSVLSGQAD"

CDS 3189730..3190617

/gene="znuB_1"

/locus_tag="PIKHDJKN_02893"

/inference="ab initio prediction:Prodigal:002006"

/inference="similar to AA sequence:UniProtKB:O34610"

/codon_start=1

/transl_table=11

/product="High-affinity zinc uptake system membrane

protein ZnuB"

/db_xref="COG:COG1108"

/translation="MFEYEFMQRAFWAGGLIGVIAPILGVYLMLRRQVLMADTLSHVS

LAGVALGSVMNLDPVICGFAIAIIGALLVEQLRRNYLTYSEVPVAIIMTSGLALAVVL

MSLKTNLSKTFSSYLFGSIVAVSDIQLWIMTAVCVIGLIFFIVLRRPLYNLTFDEETA

SIGGIQVKGLSFAFAILTGMTVASAMPIVGVLLVSALIVLPAALALRLSRSFAAAILI

AVVTGLIGIFSGLTTSYHLNTPPGGTIALILLVFLLIGISAQKLIGRYNRKRHRKERK

QQRATLFIHSRENTPHEIQ"

CDS 3190604..3191683

/gene="znuA_1"

/locus_tag="PIKHDJKN_02894"

/inference="ab initio prediction:Prodigal:002006"

/inference="similar to AA sequence:UniProtKB:O34966"

/codon_start=1

/transl_table=11

/product="High-affinity zinc uptake system binding-protein

ZnuA"

/db_xref="COG:COG0803"

/translation="MKFNKKSALALLFSFTLIVAGCGQNQSASDSSTSSANTPVPAET

EEAKLNVEVSFYPMYEFTKNVAGDLANVHTLVPAGMEPHDWEPTPQDIASIEKADVLV

YNGAGMESWIDQVTDSLSSAKLIQVEASHGIDLLEGSEEDEHDHEHADTTDTHDHADE

ATTEEHDHDHDAEAEEGHTHDHNHDGLDPHVWLSPALAVKEVRNIEAGLAQAAPEHAE

QFKQNADAYVAKLEALDQDFKAAVADSKRKDFITQHAAFGYLAQEYGLQQVPIAGLSP

EQEPSAAQMAAVIDFAKEHQVKTIFFETLVSSKVSETIASEVGAKTAVLNPIEGLTEE

EIAAGMDYIGVMRQNLEALKLALNE"

CDS 3191885..3192361

/locus_tag="PIKHDJKN_02895"

/inference="ab initio prediction:Prodigal:002006"

/codon_start=1

/transl_table=11

/product="hypothetical protein"

/translation="MDLNEEQEVLLSQQPAHLWRRRKLELMQWTEREKQTVAAKRTEI

WNDVEVDAELVDALTLLHRAGVRTEFSCAGVSPLDEPVDHSLYAYVTLVQNEAADRFV

TYALSQMRNRLLATLETGTGRYDLSSFFIGHNRSFCWWMQYCAAHFMDRNDYTVAT"

CDS 3192409..3193116

/locus_tag="PIKHDJKN_02896"

/inference="ab initio prediction:Prodigal:002006"

/codon_start=1

/transl_table=11

/product="hypothetical protein"

/translation="MNKWLKTILFLVGSAILTRFIPFSSLFRNLDTMIHEFGHALVTL

LLSGKVLRIELYADHSGVTYSSMLTPGRSILVSLAGYVTASLFAWLLFYLYRKGRHMW

GLGIMTGVALVSLLFYVRGEFGMLWLTGFSALNVVIMIFGAKIAKFYYLLLAFLTLEE

SVVSAMYVGLMSWTQPSRAGDAANLAQQTFLPALFWGTLFALFALWCAKGALTLFFRK

ESTARTSKSRGLRGRNV"

CDS complement(3193177..3193548)

/locus_tag="PIKHDJKN_02897"

/inference="ab initio prediction:Prodigal:002006"

/codon_start=1

/transl_table=11

/product="hypothetical protein"

/translation="MKSRFWLLYVLPICLILVFVAIMASGALLKKPFGADDRLLESVQ

TLQKQVEGKKWTEARSQIDYAMKAWDKIVNRIQFSVERETIYDILGTLARMKGGVAAE

DDKAIMEEIYYFYVLWDNLGD"

CDS complement(3193572..3194300)

/locus_tag="PIKHDJKN_02898"

/inference="ab initio prediction:Prodigal:002006"

/inference="similar to AA sequence:UniProtKB:O31533"

/note="UPF0702 transmembrane protein YetF"

/codon_start=1

/transl_table=11

/product="hypothetical protein"

/translation="MMEETWVVAVRSIIAFLTLIIYTRVLGKQQMGNLTYFDYINGIT

IGSIAGTFATDLSSKAWIHFVALTIFTIITIIFQYITLKNRTISKLMDSDPTLVIQNG

KILEQNLHKMRVKFDELTMMLRQKDVFDITTLDYAILEPDGSLSVVLKPENQPVTAKD

MHMHPAKSKLMTEIIIDGVLIKQNLEERNKDTNWLSEQLKKQKVTIQDIAFAAILPND

KLYIDLFKDKITEKIDMGDYEGPF"

CDS complement(3194536..3195399)

/gene="uxuB"

/locus_tag="PIKHDJKN_02899"

/EC_number="1.-.-.-"

/inference="ab initio prediction:Prodigal:002006"

/inference="similar to AA sequence:UniProtKB:O34896"

/codon_start=1

/transl_table=11

/product="putative oxidoreductase UxuB"

/db_xref="COG:COG1028"

/translation="MNDFAKERSSRLEGRTAVITGGAGVLCRSMAEELARHGASVAIL

NRTVSKGEEVVAAIEAQGGKAIAVACDVTQADSVIAAAAVVQEKLGPCDILINGAGGN

HASANTTNEIFRQEDLEQPDITTFFDVSVDGFRQVMDLNFVGSLIPTQIFARQMIERD

VPTTVINISSMSAPSPMTKVPAYSAAKAAINNFTQWLAVHLAESGIRVNAIAPGFFLT

EQNRNLLLKPDGSPTERSSKIIAHTPMRRFGKPEDLLGTLMWLADERQSGFVTGTVIP

VDGGFMAYSGV"

CDS complement(3195396..3196463)

/gene="uxuA"

/locus_tag="PIKHDJKN_02900"

/EC_number="4.2.1.8"

/inference="ab initio prediction:Prodigal:002006"

/inference="similar to AA sequence:UniProtKB:O34346"

/codon_start=1

/transl_table=11

/product="Mannonate dehydratase"

/db_xref="COG:COG1312"

/translation="MRMVFRWFGEGNDTVTLDHIRQIPGVEGIVWALHDVPAGEEWPM

DKILAVKAAADKAGLHLDVVESVNIHEDIKLGLPTRDQYIENYKKTMEKLAQVGVKVI

CYNFMPIFDWLRTDLHKEMEDGSTALFYENSKIADIEPLELVRRITENSALTMPGWEP

ERLQYLTGLFEAYKDVTEEDMWEHARYFLQEIIPTAERLGIRMAIHPDDPPWPIFGLP

KIITSQDNVRRYLNLYDSPYNSVTLCSGSLGANPNNDIIGMIHEFKDRIPFAHIRNVK

IYDNGDFIETSHRSQDGNVDIAGVVEAYHDIGFEGYARPDHGRHLWGEQCRPGYGLYD

RALGIMYLWGVWDSLQRRSKA"

CDS complement(3196511..3197242)

/gene="rspR_1"

/locus_tag="PIKHDJKN_02901"

/inference="ab initio prediction:Prodigal:002006"

/inference="similar to AA sequence:UniProtKB:P0ACM2"

/codon_start=1

/transl_table=11

/product="HTH-type transcriptional repressor RspR"

/db_xref="COG:COG1802"

/translation="MEFPPRSSSKAELLSSLINPSSLNKQAFSSREAVYTTLKQLILS

LKLPPGTAISEKEISLEFNVSRTPVRESFVRLAQEGLLEVYPQRGTYVSLIQVDLVEE

ARFMREQLERAVIRLACVQFPDEQMKILEQNLAQQQECILEPDDERMFGLDEEFHSTL

FAGCNKKNTWSVIQHMNVHLNRSRMLRLSSDHHWDHLLEQHRSMVQAIQQHDAQTAEH

IMQEHLHLTVTDLGVLRDTYPSYFQ"

CDS 3197438..3197740

/locus_tag="PIKHDJKN_02902"

/inference="ab initio prediction:Prodigal:002006"

/codon_start=1

/transl_table=11

/product="hypothetical protein"

/translation="MGQKSISIFQTCIPLFQVLSDNHRQAILLALSEKGSMTVNEITE

QFSLSRPAISHHLKLLLEKGLISVEQKGTQRYYSASLRSSVELLKELVGALEEECL"

CDS 3197829..3199229

/gene="aldH1_1"

/locus_tag="PIKHDJKN_02903"

/EC_number="1.2.1.-"

/inference="ab initio prediction:Prodigal:002006"

/inference="similar to AA sequence:UniProtKB:Q2FWX9"

/codon_start=1

/transl_table=11

/product="4,4'-diaponeurosporen-aldehyde dehydrogenase"

/db_xref="COG:COG1012"

/translation="MSVMQELSEHEVEEILQNQRQFFRSGVTRSAESRIAHLTRLKQA

IQKYESRLTEALYQDLGKSEFESYTTEIGFMLDSITHTIRQVKKWVKPVKVKTQFALV

GSKSYIIPEPYGAVLIIGPFNYPFQLLIEPLVGALAAGNTAVLKASENTPAVSAVIRE

MITSVFEPAYVHVVEGAKDTTTALINAPFDYIFFTGSVPVGKIVMEAAAKNLVPVTLE

LGGKSPVIVDKHADLKVAAQRIIWGKLLNTGQTCIAPDYLLVHEQVKDTLITEMKAAV

ESFFGTNIQHNRDYGRIVNKAHFKRLTTLIERDQALVIYGGYSDEEDRFIEPTFIDAE

SWEAATMEDEIFGPILPIISYSHIDEAIAGIVKRPKPLALYLFTSDTQLQDKVMREVS

FGGGCINDTITHVANPRLPFGGVGHSGLGSYHGRYSLETFSHMKSVLKKSIRLNLSIL

YPPYDNKLKTIKRLLK"

CDS complement(3199295..3200206)

/gene="mtrA"

/locus_tag="PIKHDJKN_02904"

/inference="ab initio prediction:Prodigal:002006"

/inference="similar to AA sequence:UniProtKB:Q9WW32"

/codon_start=1

/transl_table=11

/product="HTH-type transcriptional regulator MtrA"

/translation="MSSTYLPPALGESVHEVFYPDVQTTVNLFAIHLRSVGADWDYPA

HEHPQYELNYVTEGEQYMTVDGKLYIQKAGELLLIPPGSIHSSLSHNGKGFTYFCMHF

DIDDQLFLSLLARIKQVLFDVDSPVTRQIEPVLRKLMSSADNRAASTMVQRMQLQSAV

FELFGHLWEAVSQEAAQLFTDGYEKIELAHQIRNRLQGLVSQQFKQGRESDSHYGIDD

IAAELGISSSHCNRVFRQVYGQSPRVYLSEMVLHEAKVLLGNPKLSVQNIAAMLGYKD

IAHFSRQFKRWSGISPTRYRQEQPAPE"

CDS 3200386..3202617

/gene="xylA_1"

/locus_tag="PIKHDJKN_02905"

/EC_number="3.2.1.37"

/inference="ab initio prediction:Prodigal:002006"

/inference="similar to AA sequence:UniProtKB:P45702"

/codon_start=1

/transl_table=11

/product="Beta-xylosidase"

/translation="MSTSKSIFYNAHHSPIGAFASFTLGYKGAKGGLGLELGKPADQN

VYIGLQSRDSENYEALPFFEAVEDASVRYDVEKVENSDSDQVSSASTESGASTPAAVA

AQGTRPLISAFRDEEITREFTSGIDTWTAGDLTFRIYSPVRPVPEPLSGDREAIMDAL

VPAVLVEMTIDNTQGRQSRKAYFGYQGNDPYSAMRIIGGPEGGALTGVGQGRLTAILS

ADEGLWPARGFTLDKILQEKHRENLAFGLGETAALLMDVPAGEKRTYQFAVCFYRGGI
VTSGMDTTYWYSRYFADILEAGKYALDRFNELTASCKEVEQRLGVSALSEDQSFMLAH
SIHSYYASTQLLDADGQPFWIVNEGEYRMMNTLDLTADQLYFELALNPWTVRNELEWF
VKRYSYTDQVRFPGEEKEYPGGITFTHDIGVANVFSRPGHSAYELVGIDDCFSQMSHE
ELVNWLCCATVYIEQTQDRDFVKEMLPVIRDCFESMLNRDHPDEQQRNGLMGLDSSRT
KGGAEITTYDSLDVSLGQSRNNIYLAGKCWAVYVALEKLFAAEKLAELSHHAGRQADR
CAASIAAQLTDGGYIPAVIAENNDSRIIPAIEGLVFPYFTGCEGALDVNGRFGPYLQA
LRTHFKTVLVPGTCLFEDGGWKLSSTSNNSWLSKIYLSQFIARELLGVEWDETGKASD
VAHVNWLLHPEESYWCWSDQILAGVAVGSKYYPRGVTSILWLLEGKGNRLEQIYASKE
AVQ"

CDS 3202614..3204764

/gene="aguA"

/locus_tag="PIKHDJKN_02906"

/EC_number="3.2.1.131"

/inference="ab initio prediction:Prodigal:002006"

/inference="similar to AA sequence:UniProtKB:Q09LY5"

/codon_start=1

/transl_table=11

/product="Xylan alpha-(1->2)-glucuronosidase"

/translation="MSNTTVQSNISGPQYDAWLGYLQSQSAVKGNAKKASPAWSHTVS
VAEDQEIISTAIAELTQGLDKLFGIKPKVNHEVASQASEPDLANQEAQGIWLGTWTGS
EAVAQAFSDTERSTVQGEGYIIREDKERGRLIIGSETPQGVLYGTFHLLRELVLEQEN
SNDVHPAAGRLSYISEQPVNALRMINQWDNVDGSIERGYAGKSIFYDNGEFTRDLGRI
RDYARLLASTGINAISINNVNVHKRESLFLTERFLGDVAKVAAQFRAYGIRLFLSANY
ASPIEIGGLLTADPLDEQVREWWNIQTAKVYAAIPDFGGYLIKADSENRPGPFTYNRD
HADGANMLAEALCPFGGLVIWRCFVYNCKQDWRDRSTDRARAAYDHFKPLDGRFADNV
ILQIKNGPMDFQVREAVSPLFGAMKTTNQVLEFQITQEYTGQQRHLCYLVPQWKEVLD
FDTFAKGPGSEIKRIADGSLYNRPYSGFAAVSNIGADACWTGHPLAQANLYGYGRLAW
NPELSSEEIAEEWVRLTFGHDEEVVRLISGMLLNSLDIYESYTAPLGVGWMVNPEHHY
GPNVDGYEYSKWGTYHFADCDGIGVDRTVSSGTGYTSQYHRENAERYESLDSCPDELL
LFFHHVPYTHVLHSGKTVIQHIYDTHFEGSEQAAALAQTWGQLEGKIDSTVFDKVSAL
QAGQAEHAKEWRDMINTYFYRKSGIADKQGRTIY"

CDS 3204844..3205911

/gene="gutB_3"

/locus_tag="PIKHDJKN_02907"

/EC_number="1.1.1.-"

/inference="ab initio prediction:Prodigal:002006"

/inference="similar to AA sequence:UniProtKB:Q06004"

/codon_start=1

/transl_table=11

/product="Sorbitol dehydrogenase"

/db_xref="COG:COG1063"

/translation="MGHHQLPETMKAAVMTEPGHIIIEEQAVPQPAEDEVLIQVMAVG

VCGSDVHYFEHGRIGRFVVEKPIILGHECAGIVAAVGSKVSRLKEGDRVAIEPGVTCG

RCTACKEGRYNLCPDVQFLATPPVDGAFVQYMTIREDMVFPIPDYLSYEAAAMNEPFS

VGIHAARRSKLAPGTTLAIMGMGPVGLMAVAAAKSFGVERIIVTDLEEVRLEAARRMG

ATHTINVRNEDAQAVIRELTNGVGVDTAWETAGNPKALQSALYSLRRGGKLAIVGLPA

QDEIALNVPFIADNEVDIYGIFRYANTYPAGIEFLSSGQHDVMSLITDRYSLEETQQA

MERALHNKSGSLKVMVYPNGK"

CDS 3206079..3206345

/locus_tag="PIKHDJKN_02908"

/inference="ab initio prediction:Prodigal:002006"

/codon_start=1

/transl_table=11

/product="hypothetical protein"

/translation="MRTHEFMIHSDFQRDDLMSVSSQASRFASDISLSFMDANHEHRV

DVKSLLGMALLPIRHGSVVRLQTRGRDELEALEYMLNVLEKGLT"

CDS 3206490..3206738

/gene="rpmE2"

/locus_tag="PIKHDJKN_02909"

/inference="ab initio prediction:Prodigal:002006"

/inference="similar to AA sequence:UniProtKB:O34967"

/codon_start=1

/transl_table=11

/product="50S ribosomal protein L31 type B"

/db_xref="COG:COG0254"

/translation="MPKADIHPKTQTVIFFDASADYKFLSSSTKFSNETMEWEDGNSY

PVIRVDTSSASHPFFTGKQRNVDIGGRVDKFNRKYNIK"

CDS 3206954..3207259

/locus_tag="PIKHDJKN_02910"

/inference="ab initio prediction:Prodigal:002006"

/codon_start=1

/transl_table=11

/product="hypothetical protein"

/translation="MSSINHMVTFTLYAGKDTPEAEAFLKESADALANIPGVEQFQVL

RQVSEKNEFDYSFSMVFANQAAYDAYNDHPVHRKYVEKRWEKEVSRFQEIDLIQHGK"

CDS 3207419..3208180

/gene="fabG_5"

/locus_tag="PIKHDJKN_02911"

/EC_number="1.1.1.100"

/inference="ab initio prediction:Prodigal:002006"

/inference="similar to AA sequence:UniProtKB:O54438"

/codon_start=1

/transl_table=11

/product="3-oxoacyl-[acyl-carrier-protein] reductase FabG"

/translation="MKLDLTGKTALVTGATGQLGRVISRTLAYCGADLALHYINNETK

ARELQAEIEAMGRKTVIVQGDITKQDTAFRIRDEIQSSLGDVDIVVANAVIQYAWTTV

LEQSPDDYISQFESCVMQSVYLAKAFIPYMKEARAGRFIGINTECAMQNFATQSAYTA

GKRGMDGLYRVLAKEVGEYQITVNQVAPGWTISERDRNSESGHDEGYIQTVPLKRRGE

DQEIANAVAFLASDLSSFITGAYIPVSGGNVMPAI"

CDS 3208406..3210133

/gene="poxB"

/locus_tag="PIKHDJKN_02912"

/EC_number="1.2.5.1"

/inference="ab initio prediction:Prodigal:002006"

/inference="similar to AA sequence:UniProtKB:P07003"

/codon_start=1

/transl_table=11

/product="Pyruvate dehydrogenase [ubiquinone]"

/db_xref="COG:COG0028"

/translation="MKKTIADVLVEALLNAGIKRIYGIVGDSLNAVLDSIRRSGKIEW

IHVRHEEVAAFAAGADAQVSGSIAVCAGSSGPGNMHLINGLYDCHRNRVPVLAIAAHI

PSDEIGSEYFQATHPEYLFQECSHYCEVITTAKQMPRSFTMAMQTAVARSGVSVIILP

GDVAALEAADLPVPEHVYHATKPVVHPSEPELRKLAEFLNQGKKITLLCGAGCAGARE

PLMQLCDRLKSPMVIALRGKEYLEYDNPYSVGLTGLIGYSSGYHAMMDCDVLLMLGTD

FPYRQFFPEDAVVLQVDIQSSHLGRRTKLDYGVCGDVKATIETLLPYLTEEHSDKHLH

KSVERYEKVRKELDELAVGKPGKTPIHPQYLTKVISNAAAENAIFTCDVGTPTVWAAR

YIEMSRNRRLLGSFSHGTMANALPQAIGAQVSDPGRQVISLSGDGGIAMLMGDLLTLK

QHNLPIKVVVFNNGALSFVELEMKAAGLLESGTELVNPNFAMVAQAMGLEGIRVEDPA

DLEGAVERALQHDGPVLIDVVVNRQELSLPPKINIKQAEGFTLWMMKAVLNGRGDELI

ELAKTNLLR"

CDS 3210472..3211227

/gene="lvr_2"

/locus_tag="PIKHDJKN_02913"

/EC_number="1.1.1.-"

/inference="ab initio prediction:Prodigal:002006"

/inference="similar to AA sequence:UniProtKB:Q9LBG2"

/codon_start=1

/transl_table=11

/product="Levodione reductase"

/translation="MSHKGKVAIITGAGSGLGQATALKLAEKGASIVVVDLVAETGQE

TVKQIEKLGGKAIFVQTDVSKANEVENYVNKTIEEFGRIDMFFNNAGIAGPGIKLIEH

TIEQFDQIIDINLRSVFYGLKYVITEMLKTGGGSIVNTASTAGIVGVQAVAPYAATKH

GVVGLTRTAAIEYGKENIRVNAIAPGTIETPMVVQFGKDNPEVFKATLDSIPSGRLGK

PEEIANLVSFLLGDEAPYINGAVYPIDGAVTAQ"

CDS complement(3211325..3212506)

/gene="pbuE_3"

/locus_tag="PIKHDJKN_02914"

/inference="ab initio prediction:Prodigal:002006"

/inference="similar to AA sequence:UniProtKB:Q797E3"

/codon_start=1

/transl_table=11

/product="Purine efflux pump PbuE"

/db_xref="COG:COG2814"

/translation="MSNTWKIYVLALVSFLVGTSEYVIAGILDRISDTMNISLIAAGQ

LITIFSLVYALGTPIIIALTSRMDRRKLLLYFLGLFVVGNVLAYLLPGYGLFIAARVL

MALGAGVVVVTALTVAAKIAPAGKQASSIATVITGFTASLIVGVPLGRMVAAAWDWKL

IFAGIAILGVIAMVVIASTIPPSEGEAPVPLKKQLALLKQPRIGFALLVSFFWLGGYS

IAYTYISPYLVSVAGMSEMLLSSALLAFGIASLIGSKSGGYSADRMGVKRTLILGMSL

HVVSLLMLNITAHSGVAIFIVLILWSFAAWSSGPTQQYNLVTMAPESSGIMLSLNSSV

MQLAMAAGAGIGGIAVSAVSLSSITWIGAAGVVIAMIIIILAYRQGNSTAVQTESSNV

A"

CDS complement(3212753..3213037)

/locus_tag="PIKHDJKN_02915"

/inference="ab initio prediction:Prodigal:002006"

/codon_start=1

/transl_table=11

/product="hypothetical protein"

/translation="MQSKSIDTEQVVKIHKALGEQTRYKIIQILSGESNLCPADLENR

LVTVALSTLSHHLKQLSDCGLLTSQKKGTYIYYSLNADTVQRFAPYLLER"

CDS complement(3213210..3213554)

/locus_tag="PIKHDJKN_02916"

/inference="ab initio prediction:Prodigal:002006"

/codon_start=1

/transl_table=11

/product="hypothetical protein"

/translation="MEINTIVLVVFLLLILILLVRVISLQTQLNELKRDVERLENGSG

STAHSPLSYNTSSVNVSPPPPTHADLTELDRELQALAQQGKKIIAIKKAREARNLSLK

DAKEYVEYLERL"

CDS complement(3213594..3214784)

/gene="pksS_2"

/locus_tag="PIKHDJKN_02917"

/EC_number="1.14.-.-"

/inference="ab initio prediction:Prodigal:002006"

/inference="similar to AA sequence:UniProtKB:O31785"

/codon_start=1

/transl_table=11

/product="Polyketide biosynthesis cytochrome P450 PksS"

/db_xref="COG:COG2124"

/translation="MSTNETSQPEFFTREFTQNPYPVYEKLRQNDPIYRVLFPHGDFG

WIITQYEDAVEVLKDNRFSKDVVKRYGPDQQNIFVHNMLFSDPPDHRRLRGLVQKAFT

PRLIEGMRSHIQDIADDLLDNLDSQDKMNLIDEFAFPLPIIVISEILGVPLEDQDKFR

LWSNSIIDATSAENSEVFEKHAQEFIDYLNAWFAKVREQPGDDLISQLVVAEESGEQL

SEKELLGVVALLIIAGHETTVNLIGNGVLALLEHPEQRDLLIKKPELIHNAVEEMLRY

NGPVEFSTSRWASEDIEFHGHHIAEGELVIVALDSANRDEEKFKDADVFDITREKSAH

LAFGKGIHLCLGAPLARLEGEIAINTLLRRFPDMQLQTGVNELEWRPGMIVRGVKEIP

VQLK"

CDS 3215221..3216321

/gene="yehL"

/locus_tag="PIKHDJKN_02918"

/inference="ab initio prediction:Prodigal:002006"

/inference="similar to AA sequence:UniProtKB:P33348"

/codon_start=1

/transl_table=11

/product="putative protein YehL"

/db_xref="COG:COG0714"

/translation="MEMLKPPAETLYETELRALREEDTGKRPPNWLLSPAYVRDFIIG

RDKPAMLDGEEVPITRKFYGNDVLIERSVVTLAGNRGLMLVGEPGTAKTMLSELLTAA

ISGTSLNTIQGTAGTTEDMIKYSWNYAMLLDKGPSEAALVPSPLYNGMKKGILTRFEE

ITRCPAEAQDSLISILSDKVMSIPELDGGVLFAKPGFNVIATANIRDKGVNEMSGALK

RRFNFETIKPISSVKMEAKIIESQAQSLLLHSGIDTEINSDVVELLATTFMELRTGMT

REGYKLDTPQASMSTAEAVSVYVQSAMTSYYYEDKGITLDRLVQNMLGTIAKESDKDL

SILKTYFSKVVKERSREEGIWKDYYEEKKWIG"

CDS 3216309..3218711

/locus_tag="PIKHDJKN_02919"

/inference="ab initio prediction:Prodigal:002006"

/codon_start=1

/transl_table=11

/product="hypothetical protein"

/translation="MDRLTGKADLDVAQLQTLFESQVYNLNNGTLYFPIRHHSPACSY

HLLRLIEEYKPDIILIEGPESGNPLLQVLADEATLPPVSLYYTYESEDERAACYYPML

RYSPEYVALKEAARLSIPATFIDLDYHHFSKPNKAEQREISIQDETLLAGSEFIHRLC

QKTNCRSFDELWENVFEIGGLEKSTQAFVQDVFTYCTLSRMCYSAERLHSTGDLAREA

HMRSRIKQAGQEHERVLVITGGFHTYGLLMPENHETDSEEMTMQEHAKAQNSVNQQMY

PMVYTFAEADRLNGYASGMPYVNYYEQIWTQLLRQKDSVYNLTALDLLSRLTRKLREG

HEHVSTSDAIEAYSMVQGLAGLRGKREGGVYELMDATLSSFVKGELTLASDKPLQELH

RLLTGNVIGKVAPNPFSIPIVEDFKERCTASKLQIRTTGQHKKILDLYAKPDHRRQSQ

LFQCMVYLVPDFAKRQSGPDWIAQRDMNLVRETWVYTYSSRIEARLIENSLYGGTIRE

AATRKMEEEMQGIPDHHSGDLAKSMLQALLMGLQDTAMKLYEHVKSALRKDGNFLSLC

GSLHILNRIQQHRRLLGLSEEQQLLILVSEAYNNAVDKLMQLSRTNPDEHEAIVQGLK

LLAMLAESSEEHFRDEPFRAHLDELLSDSQLPAQLEGVCIAISSGLGDRHREEIVDRA

RAYIRGSQEQTRQTALYLQGVFSVARDAFLYEDQLLSELNYMIEQLDYEEFIRMIPEL

RLAFTYFTPMETGLIADRVASLHQVENEEMSWPSVDERLLIQTKTWDEALRKEFDAWK

LI"

CDS 3218696..3219886

/locus_tag="PIKHDJKN_02920"

/inference="ab initio prediction:Prodigal:002006"

/codon_start=1

/transl_table=11

/product="hypothetical protein"

/translation="MEADLNELNTTKEIVKDNAELNRAETINRWRLILGESAEEGLSN

AEDYSPDDFKYTEVDEILGYLYNREYGEEQGYRKGGGRGPSDLTVPKWLHKVRELFPK

PTVEILEKQALDRYGLTELLTDKKLLESLEPNMNLLKNIMQFKGRMKGDVLKSAKEIV

RVVVEELRRKLESQTKASIMGKRSRYASSSVRSLRNLNFKQTITKNLKNYDKNNRRFV

IDRLYFDGNIQSHNKWDIIIGVDESGSMLDSVIYSSVMASIFYRLNALRTKLFIFDTQ

VVDLSDRLEDPVDVLMNVQLGGGTHISKALRYGETLIENPGKTIYILVSDLEEGYPIQ

HMYKACKDILDTGCKLLVLTALDFNGDTVYNKHAAQTLSNMGAHVAAITPNELADWIG

EIIT"

CDS 3219938..3223453

/locus_tag="PIKHDJKN_02921"

/inference="ab initio prediction:Prodigal:002006"

/codon_start=1

/transl_table=11

/product="hypothetical protein"

/translation="MLTADRAVEALVEKFAEVCSNEYSLKTDRSSEIIDYVMGTTDKF

PDMMGGSSHYAYQTIEVLMKIAKKDDGDIFFRAGAIVIHMESNYSRRNSWRTDGFTLC

LGNDSEAYGLSGKSKNNDRMGGLLSRLEKINRYAGEKEILDELKSRLKGNFQDVINGM

ILLRLYSDLNNSPSHLTQVQQFTQSLPALFQHVMTHDTSKLRNELHALIEPLVVCNFQ

TKHNRSAYELNDEFLREKHNQVIAENYSDTSLSKKEQLSYSEFDKTMILLSNALLYLV

NISGGKQRFLENNSETGLILLEAINQLHEIYPLEVRRYLLSMDPRTKNANELLSGLVP

LDEPYQLIEMLRNELEVYTIPWQFLQSAIVNEPQQAIRAYHILKPPFLKLCIQKMLEG

HGIAVPDVDETVEQAVFSALRHPLDGGRQGIAIARYLNGESSLEEYWEDPNSRGLFNN

LNRDKKRVHNLISISFLPLDSEAMRRFVILTTYPEWTLDIVSDMYQSYAFSGEKLLEL

YGQDPEVKQEKLLSSLISLNGMTDYKYKSMPQDEYRRIIQQNLDFALGQYKKLPTDTR

ILILEITFEQRHELSSKQVAEAIRAGLQDSSKKANGVALSEFNRIPDQDLYTTIYRSE

KKTGVKEMALSAIRSLDNSKDVYLELLKGEKSAEWKNLIQILLDTADQSPEYAHAALA

DQADAKKLTRLSWLSLNDLPSLIRSEDNQPLDDRIKQYILVQSIDHTSAPNERLNELR

EYVSEDSLTRFASELLQLWIQESASAKEKWVMYLSALFGDTQIVNILAPQIKEWTENS

RGAIAADAVKVLAYLKEPSALMAIDKIKRSVKNRQVKGAAEEALQLAAENMGLSSEQL

EDRLVTTLGFDKNGTMQLSYGERSFLIKVNGDLQVVVVNEETGKSVKSLPAPAQKDDT

ELAAQSKARFTQLKKDLKTMVNIQAQRLEESLSKQRLWSADEWTALFVQNVIMQKFAV

GLIWGTYEDGQLVTTFRYMEDGTFNTVDEDEYELPSGSLVGLIHPLELDQSTLEGWKT

QLEDYEIKQPFEQLNRQIHVPEDEDKSKVEYDRLPESDFSPTAFPKALEKYGWIKGPA

QDGGWYHEFYKEYGDLVAELRFSGTSISYYEGLEDITLESLHFFKPGSQKHYYYSDHK

SIALGEIAGRVFSETIYDILRASGR"

CDS 3223515..3225188

/locus_tag="PIKHDJKN_02922"

/inference="ab initio prediction:Prodigal:002006"

/codon_start=1

/transl_table=11

/product="hypothetical protein"

/translation="MIRYANKGLYKRSLKDLDNGVSVQYTWNEASVNCQLTDGTHCTL

ENTLDHWECSCPADNICKHILISILYYQREHQTEDTFNQGEEASSAELEPASGMVMES

RFRWMVESDLAPLIKSYSSSLIEEVLFRLRYPEQIEVTEDSLLTVRLVKQELEVSFTE

EASLAKALCKVKQTVGNMAKLEALLRYRMQQGLDDTEAISGRVHDAKFSLQTVRECRS

MLAGLLNTGLARLPQSYMAQLETLAIAAHSGHLPDVERGLRGIQGELQMFFNRHVRFS

MQSMLDRVSKLYLALQVLEQEKVSVFKQSQLIGSFRSKYYAVPALHLYGLGADAWETR

SGYRGITYYFYCFDDAEIYTYSDVRAVYYEDNQFSYTEHYGSFTPWQSNLTFRQFAGE

EVQFRSVKVNEEHRLSSGEGAKLTLLPRTGVEEVNLGKFMQDIPSVLAEDARSFELFS

VPQERLAMIKVTRVVEHRFDKQSQDLILTMESDHGETLDLTLSYSTEWETMIKRLESG

YGAGTLEDFYAFVRIKQQRIYPISFLKGPTVISLKLDTGYGRTGRVGRL"

CDS 3225157..3225453

/locus_tag="PIKHDJKN_02923"

/inference="ab initio prediction:Prodigal:002006"

/codon_start=1

/transl_table=11

/product="hypothetical protein"

/translation="MEGRGELEDYSQLMQKISSCSEELLLRGLSQFTLKDIEVLEQLI

VETSRFQMTFLREILELMIEEGRKTALGSSDEELMLLHYCRLTQYVQLSTQESS"

CDS 3225564..3226106

/locus_tag="PIKHDJKN_02924"

/inference="ab initio prediction:Prodigal:002006"

/codon_start=1

/transl_table=11

/product="hypothetical protein"

/translation="MKEEQIRKAVARRDHEAMEWVMNNYAGLLWKIIYSILHNASTEE

IEECVADTFFSFWQNPLSFQPERSSLKNYLATIAKHKASDRYRKWNRRSEISYEESIH

FVQTEDTLSKLISKERQLELEQAIESFPEPDREILKRRFYEAQKPLEISEALSLHVRQ

VNNKIYRSKQWLRKWWLQKK"

CDS 3226085..3227494

/locus_tag="PIKHDJKN_02925"

/inference="ab initio prediction:Prodigal:002006"

/codon_start=1

/transl_table=11

/product="hypothetical protein"

/translation="MVVAEEIEEVIPISNPGDHFTEQNLTHIKKIFHEKARLEANLQF

NSQKKSKQLNRRRTRKLIYIPIAAILSLTIVTGAAAAMGVIDLSSILQFLSGDRINIL

RPVNKTSEDQGIRMQTLGAVRDGSTTEIYVALKDLTEDRVNASLDVYDFHVSGGRSHN

AKLVQYDPAEKTAIIRFLVQGKRLSNRMSVQISSFLSGANKEENYDSGLNLSELLSKK

QQTEYDTLDPLKDSISGWGGESALELSEQEFIHVLRQDQTHIKLPGINWMYISNIGFV

DGKLHIQINPDDEMGRYNHGYFFFTDDQGNKQDIPMNSVSYGSYIKGGTRYGGDYEEY

IFDISNISQLNKLQLKGDFTSYDQYVTGKWETTFDLKQDLLSKTGKATLTLNDELRTE

IVVSSLGVTLMGKGVNQITPENLHMVLYLNDGTSIYPKSGFLNIDDNLMKWISPQTIP

VNEVNYLMLNGQKVLLQDP"

CDS 3227667..3230177

/locus_tag="PIKHDJKN_02926"

/inference="ab initio prediction:Prodigal:002006"

/codon_start=1

/transl_table=11

/product="hypothetical protein"

/translation="MSMSEPLLTQIVNQHIPADATVVTIDKPVKHPAIYASDLTGDGM

PEIAAVYEQGGELMLLVLKFYQGHWIKMSLTKGLGYGVTLLTAASVTSTARNNLIVGW

QIGAIWSKLAVYEWTESGLTDQAPEDLYYSHAEIIDMPGHHGRDGKVEIALWIHDTGE

AYRVEIIRWQNGKWVPATDVYPYYFPKVVKYYEQLAEQHPDYIFYWYYLADAQYKAGL

FPAALVSVQKALSFSDPYPSRKVLLELEKKIRQAMGTEGPRETTWLFPISVRTTTGTR

WGYMDAQGRIRLEPILDDAQDFQRNGLAVVVKDGKTGIINLNGQFVIQPVYDSINSFT

EGRAIVIDSQGFKMIDEQGAVLTKRAYPYIADVKDGRALFYLSDSSQAGGEVSRYGYL

DTSGNEVIRAQFASANDFQDGQAVVQIKENEYALINRNGQRLATYPYAYVGTLGDGLL

PFQKEASGKYGYIDESGHIRIQPSFTSAFPFSGGYAIVNTAEDYNSIYGVINTQGIFT

IQPAYNDIRDLGEHRLALGQAIDPKQSFIGSVYAIADVNGRKLSDFVYTDVSNYKGGL

ASATNGTQTFFLDLNGRPATGYPRVEGSGTLELVAPDLIKAYVDQRVSYVNRTGQIIW

RQNTIVPLHPPYRVREEKYKPNPDYLVYYPQVEGLTDQAVQLAVNAKLKALSQVKPIP

ADQKLDYTYTGDFDITFYQQQLLQLKLTGYNYPAGAAHGMPTMIYAIINLSSGQMYEL

KDLFKPNSDYVKVLSKIVGDQIKNDPQYSYVFPDTYEGISPDQPFFVTADALHLYFSP

YEIAPYVAGFPTFTIPFVQIKDIINTEGSFWKAFHEQP"

CDS 3230412..3232211

/gene="btuD_13"

/locus_tag="PIKHDJKN_02927"

/EC_number="7.6.2.8"

/inference="ab initio prediction:Prodigal:002006"

/inference="protein motif:HAMAP:MF_01005"

/codon_start=1

/transl_table=11

/product="Vitamin B12 import ATP-binding protein BtuD"

/translation="MNTTSEVSGLMEVTKERKGLLITASIFSVLSSLLQMVPFIGVYK

IVEELLIHAREPSALDRDLLMYWGFVSFGALIAGLIALYIGGMCSHIAAFNILYQLRV

KLADHVAKVPMGYHTRTATGELKKIIEVSVEKIEKFIAHQLPDLVSAIVIPLVLLCYL

FWLDYRMALVLLVPMIIGFWLQSSIFRNERGRQAYRDFQHAIEEMNSTGVEYVRGMPA

VKVFGITADSFLTFKQAVTRYRDISLRITDLCKTSYGMFFIIIISLVTFIVPIGILLS

SGNAGNQSFAITFILFLIITPSLSAPLLKLMYLGSGMREIVEGQNRIKAVFAESVVQE

PLSPQVPDTYEVAFRQVSFAYEQKDSEAFKPVLQEVDFVAKAGEMTALVGPSGGGKST

IANLLLRFWDVQQGEITIGGVPIQEMGTEKLVETVSFVFQDVHLFYDTIEENIRMGNT

AASRDDVIAAARTACCHEFIEKLDEGYDTKIGEGGTYLSGGEAQRIAIARALLKDAPI

LVLDEATAYADAENEHKIQQGLAQLVQGKTVLIIAHRLTTIRAAEQILVIRQGAIVER

GTHDQLREANGLYEKMWQAHIHAASWKLGGAKQ"

CDS 3232208..3233950

/gene="msbA"

/locus_tag="PIKHDJKN_02928"

/EC_number="7.5.2.6"

/inference="ab initio prediction:Prodigal:002006"

/inference="similar to AA sequence:UniProtKB:Q9KQW9"

/codon_start=1

/transl_table=11

/product="Lipid A export ATP-binding/permease protein

MsbA"

/db_xref="COG:COG1132"

/translation="MRNYLYNISGGNPRSLLPSIIAAILDGIAKIVPAALLVDIFNTI

YKSFAEQEKELDIQRMWIVCCILFAWLLVEYAAYSSLYDKTYRAAYSLAATGRLKLAE

HIRHLSLGFFGKRDPGDLTNLILSDYGQVEHTISHNLSQLISAIVLPVLAFAGLLFVD

WRMAVAMFIAVPLGVALLWMTDSIQARLSENHVRAKNEAASRLQEYLTGIREIKAHNM

GGERFERLRRSFDELKSASIRLEGIMGPMIMGAMLLARSGMTWMILAGSYLLAGGELT

LPIFLLFLLVGTKVFEPLTVALMSYGEMRYSAYSARRIMEVQQEAPMTGTGSVQPNQA

ISFDRVTFGYDDELPVLKDLSFTIPPHSITALVGPSGSGKSTVTRLIARFWDVQSGSI

TIGGQPINRMDPEKLLEDISVVFQDVYLFQDTIGNNIRVGKKEATQAEIEEAARRACC

HEFIASLPQGYDTLVGEGGSTLSGGEKQRISIARALLKNASIVLLDEATASLDPENEA

AVQQAINELVANKTVILIAHRLKTIQNADQILVLDQGQLVEQGTHEQLLLTSGIYSRL

WQLQHDAEGWQVKV"

CDS 3233974..3234936

/gene="yfiY_5"

/locus_tag="PIKHDJKN_02929"

/inference="ab initio prediction:Prodigal:002006"

/inference="similar to AA sequence:UniProtKB:O31567"

/codon_start=1

/transl_table=11

/product="putative siderophore-binding lipoprotein YfiY"

/db_xref="COG:COG0614"

/translation="MLKRIHIWIALCSMLMLAACGTEQTSSQEVRSSEGTTQETTKVI

TNPNGEEVTIPLNPERIVDLSGSTEELLIVGKTPVATTSADYGQQDQITPPLKDKLDA

NTVVLGWLGSKFSIEAVTASNPDLIILGKDFNTDQYETLSLIAPTIVLPYSYYDWRER

LTFLADTFGEETKKDEFLAKYDAKSAEWKQKLESAVNGESFAVIETYPNNLVIYSNKG

AAEMLYGEWGLKRTEGIPEPEGWGGKQISIEALISVNPDELVLMENSENKMEDSKVWN

NMNAVKDGKIYKISSVDNYNYSYTAMGRMELMDRLGNMILEGQK"

CDS 3235226..3235513

/locus_tag="PIKHDJKN_02930"

/inference="ab initio prediction:Prodigal:002006"

/codon_start=1

/transl_table=11

/product="hypothetical protein"

/translation="MYKAGFWIRLGASILDGIIIALPLMVIAGLITGNYDSDEPIAKI

LSALYSILLPAFWYGRTIGKRICGIRIRKYDTHEPPGIGTMLMRVIVAGLV"

CDS 3235944..3237569

/gene="lipO_7"

/locus_tag="PIKHDJKN_02931"

/inference="ab initio prediction:Prodigal:002006"

/inference="similar to AA sequence:UniProtKB:P37966"

/codon_start=1

/transl_table=11

/product="Lipoprotein LipO"

/db_xref="COG:COG1653"

/translation="MRRKVLSTSLMILLLGTTILGCSSGDSKTGDADKGTPSGSTEAT

TYPIQTDKTLTYWGEINGNLIGVKNSHSEIPFFQKWQGKTGVPLKFTAPPTGQVRESF

NVMLASGDLPDMLEYNFIGDFPGGPEKAINDGYILKLNDLIDQYAPNLKKYLQENPDI

DKMVKTDSGSYYVFPFVRGDEYLRVFQGPIIRQDWLDELGLPVPETIDEWTTTLRAFK

EKKGAAAPFSVVSKPRFFNDSGNGAFLGAFGVNRGFYQEDGQVKFGPAEEGFKEYLTL

FQEWYKEGLIDKNISTADTKSVDGNLASGATGASVGNAGGGIGKWQPLVEANDPDAVL

VAAPYPVLNKGDIPKFGQLNQGYASEGTVAITTAAKDPELAVRMLDYGYSEEGHMFFN

FGEEGVSYNMEGDYPKFTDLLLKNPDKLAPAQAISLYARSSYNGPFVQDKRYAEQFFA

LQTQRDAIDVWKNTQAAKYNLPSITPTPEESSEYATIMNDINTLVDEMTIKIILGNEP

VDQFESYVAKMKSLHLDRAIEIQTAALERYNNR"

CDS 3237626..3238549

/gene="yteP_22"

/locus_tag="PIKHDJKN_02932"

/inference="ab initio prediction:Prodigal:002006"

/inference="similar to AA sequence:UniProtKB:C0SPB3"

/codon_start=1

/transl_table=11

/product="putative multiple-sugar transport system
permease YteP"
/db_xref="COG:COG4209"
/translation="MSNRQSFRSRFVRDFMMNKYLYIMMIPVIGYYLIFHYGPMYGAI
IAFKDYSPMKGILGSDWVGLKHFEEFFNSYYFLRVLKNTLLISLYTLVFEFPAPIILA
LLINEVRKRAFKRVVQTITYMPYFISLVVICGIITDFTNADGLINRLIMMLGYDGQAM
LQKPELFRPIYVLSEIWQRIGWESIIYIAALMSIDLEQYEAAKIDGASRLKQMFHITL
PGLLPIITIMFILRMGNMLNVGFEKIILLYNPVTYETADVISSFVYRKGLLEFGWSYS
SAVGLFNSVINLVLLISANYISRRVSQNSLW"

CDS 3238560..3239441
/gene="araQ_19"
/locus_tag="PIKHDJKN_02933"
/inference="ab initio prediction:Prodigal:002006"
/inference="similar to AA sequence:UniProtKB:P94530"
/codon_start=1
/transl_table=11
/product="L-arabinose transport system permease protein
AraQ"
/db_xref="COG:COG0395"
/translation="MIKESGWFDRIFTIFNYTFLILLVIVTLYPLLYVLFASLSDSAQ
LLANKGLLWKPVGFSLEAYKSVLANPGIATGFRNTLFILVFGVIVNLFMTALGAYVLS
RKNVMWNKVFMFFIVFTMFFGGGLIPLYLVVKGVGLLDTLWSTILPFAISTFNLIIMR
TSFMGIPDSLEESAKIDGANHFTILFRIIIPLSMPVIAVMILYYAVDKWNGWFYASVF
IKSRELFPLQLVLREILIANSTESMSAGASAGDRFQIGETIKYATIMVATIPILCIYP
FLQRFFVKGVMVGSLKG"

CDS 3239481..3240602
/locus_tag="PIKHDJKN_02934"
/EC_number="3.2.1.180"
/inference="ab initio prediction:Prodigal:002006"
/inference="similar to AA sequence:UniProtKB:Q8E372"
/codon_start=1
/transl_table=11

/product="Unsaturated chondroitin disaccharide hydrolase"

/translation="MSVLNEVNKDVWDQIKSKADQMLAAIGEKSPHVAGTNGIYDDMR

IDWWTSGFWPGILWIVYDMTGDEKYKEAAWNWDERLSERFLENNYFDHDVGFHFLPTA

VIKYKLTGDADAARRALFAANFLAGRFNTKGNFIRAWNGDMLGWSIIDTMMNLSLLFW

ASEESGDPRFNHIAKAHADMVMKQFVREDGSVHHIIRFDPETGERAEAIGGQGAAPDS

AWSRGAAWALYGLTNTYRYTEDPAYLDAAQRVANFFISSLPEDSVPHWDFRAEPEEGE

EIPRDSSAGAIAASGLLELADVLPDPEGRLYATVAKRILSSLRDHYGTWNLPEHEGML

LEGTGHKPAGQNVNVSLIYGDYFYVEASAKLNGWKHRIF"

CDS 3240831..3243179

/gene="rhaR_24"

/locus_tag="PIKHDJKN_02935"

/inference="ab initio prediction:Prodigal:002006"

/inference="protein motif:HAMAP:MF_01533"

/codon_start=1

/transl_table=11

/product="HTH-type transcriptional activator RhaR"

/translation="MLLNIRYLWQKRESIIVTWLVSYSAVLIVPILISLVIYMQANET

LKSEIHRANDSLLKQMRYTIDTQVDLMKRLNMEMTWSPNLQTLMYSNQPAKEAPYTAY

QLVKELRLYKTSYASIDEFYVVWKKDQSILRSGNIRDMQTAFHTIHNTGAMSFEAWRD

QILGAETNQFVILPHQNAAPAESSIAYITRLPDDLNGQETGTVVVMADTRRFQEAIES

ISGFSDATLLILNQNNEILMSNRPGSEELKPFMNGNQVQLDNAKVGKSEMFYMDSAVS

DLKYALIIPSSLYWEKAVYVRNFTYISIVVSLISAGVLTWFFMRRNYSPIQQLVESLK

DKNTQNEPADRNELRFIQKVIMNTRSEKNEIAQQLQKHQQVLRSNMINRLLKGKQDTL

VPYEDAFRSFHMPLYSSEFAVILFVIENEENLYGKLPGIDINERNKLIHLIISNVVEE

LTSEHQHIGYVAEVDDMMVCLVNMKADSSDWNQDLHHIAAEAQRFLERYDMELTISIS

GRHTSWIGIAEAYQEAVDAMEYKMVLGKKGIITYGDVRSDAMADDPFGYYYPLQVEQQ

LLNFIKAGDTEQASAYMNEITERNFDKTIMSITLARCLIFNLVGTMIKAINDLGDRDN

NTLTHYPHRIDDIIAGDTIQEMQEALQGLLEEVCAYAGEKRATNVSQEREDSLRHLST

QVTQYIESNYTDVNLNVNAIGEHFELKGSYLSKLFKNQTGEGLLDCIHKCRIRQAKEM

IEHKQESITEISRMVGYNDAATFIRVFKKYEGITPGKYKEIC"

CDS 3243199..3244848

/locus_tag="PIKHDJKN_02936"

/inference="ab initio prediction:Prodigal:002006"

/codon_start=1

/transl_table=11

/product="hypothetical protein"

/translation="MNVKRKLAFIAKLTGFILLLGMLTACDQNGTDSDAKLDERSGSS

EYQTVSIASPNDAGKLTYWAELNGNAASIKSSFNEVPFFQEWQRRTGVNLQFIRPPAN

QAKEAINVLLTSGELPDMIEYEWSNYPGGPEKAIKDGYILRLNDVIDQYAPHLKQYLT

EHPDIDMQIRTANGSYYAFPFIQGDDKLRTYQGPIIRKDWLDELGLDVPTTIDEWHTM

LQAFKDKKGADAPLTFLGVPNPLFGIEGGGFIGAFGIKKGFYVEDGQIKFGAQEPEYK

AFLSLFREWYAEGLIDKNLAAVDSETQDTNMTTGRSGASIWNAGAGIGTWLPILQETD

PHAKLVPAPYPVMNKGDRPKFGQLAPAIGSSGVAISSNSHHVEEAARMLDYGYGPEGH

LLFNFGIEGVSFEMKDGYPTYTETILKNPDKWSPAQALAMYTRASYFGPFVQDTRYME

QYYILPEQKEAVQLWSNTDAVLHQVPTLPKTEKESTEMSVIMQEVNKVVDEMSLKIIF

GIEPVDAFDTYVEQIKSLQIDRAIEIQQQALERYNRAVSSK"

CDS 3245044..3247710

/locus_tag="PIKHDJKN_02937"

/inference="ab initio prediction:Prodigal:002006"

/codon_start=1

/transl_table=11

/product="hypothetical protein"

/translation="MPRKWPFLLLAFTLTVGLSVPGTQEARASEGDSQPSIDRVGENL

SLSTDGRSRLYPNDWYPGFKDEQGRFLHDFSYAGYRRGETELPKTAKNKRIDVTKAPY

FADPSGSRDATQAIQKAIDAAVALGGGVVYLPKGTYQVNPQDGKDYSLNIPASRVVLK

GDGMNKTHIYNAQQNMKNKDIIRIGNGDWKKTGISTKLRKSVTDPTVLLPVENTSGFA

VNDYVVISFETTPGFLRELGMQNKWSSRLGKVEPLFYRQIVGVDPENKTITLDIPTRY

PMKLRDDITISQTADPIVEVGLEDFSIANIQNAKSGLGEDDFKVVGTAGYEADNAKAV

NVIAVANSWIRNINTYKPAGNTDYHLLSKGIILDRTKNVTVDHVTMQYPQYRGANGNG

YLYQFIGNDNLIKNSKAIGARHSFTYANFSANGNVLQGSYSEKPSLLTDFHMYLSMAN

LIDNLVVNGDGISAITRDYGSSETNRHGVVTTESVFWNTTGQATHPSKSGVIVESEQF

GNGYVIGTKGKDTGVNVNIVGSIPDANTQPFDMAEGIGEGDRLSPQSLYQDQSKKRIK

DIHLGLQSLLVNGEAIGGMQFLRTDYVHTLPYGTTETPIISAKAYAKDAKVKIKQPQG

TNGTGEITVSYRGHNQNVRVKFKVADTPVLPENISISPNKTVPGWRVAGNAISAGGSG

ELSSFVTLDNGEIVNIAELNVPVTYTSSDETIGYTEGTTFHAVKAGKVDIIVSCVFNG

VTVEAREKFEVKEPMAEPEGPFAVVTKVTASADDGNLPIHTIDRDPDSRWSADGKGQY

LQLELEQQTQVGQVSIQFYNGHTRSNYFDLEISLDGINYQKVLSNVASQKQATYETFE

FEPVQAKFIRYVGQGNESNTWNSIIEFWVHGN"

CDS complement(3248252..3249256)

/locus_tag="PIKHDJKN_02938"

/inference="ab initio prediction:Prodigal:002006"

/codon_start=1

/transl_table=11

/product="hypothetical protein"

/translation="MRQLPHQIVIENVLQTKMRGKIAYQSAFPSSIAMHSTYVKPYND

PTGKGYQRPVDPKRVDEFALYLSKGDNALFTPILLNASGMWEFTAYDSSRPMFGRLLC

KSKASVLDGQHRLGGIKRYTQETHSEINVPFMVFHYLDDDEEIEFFDTINTKAKGLGT

SLSKYLKREHDELSWMSTELLIFNDSPFYHIGSITGKRSKGRHITLQNLHRMLKYFFK

DTETTNLSKQEKVDLVLEFFNLMRQEYPHEWMNYKEYKLTHIVCLDALAMAASKALSN

CKKENSTQIDHQKFIKSVTRLKRIDWSTDGPFKYLKGISSSKTLASELFDIMNLEKES

"

CDS 3249399..3249767

/locus_tag="PIKHDJKN_02939"

/inference="ab initio prediction:Prodigal:002006"

/codon_start=1

/transl_table=11

/product="hypothetical protein"

/translation="MIKINAFVGERIKSLRKSKGLTQANLGEQVDLPQPYIGGIEKGE

RNISLDTLQKLIEALDITPEELFRNYDNDHLSNNKEVHEILDRINELLVKRDIEEIKI

VEKFVYDILKTIDNLKTSKK"

CDS 3249776..3250471

/locus_tag="PIKHDJKN_02940"

/inference="ab initio prediction:Prodigal:002006"

/codon_start=1

/transl_table=11

/product="hypothetical protein"

/translation="MKNRLLDYYTNKVRARVLIYKNMPFLFWGYMITCILTVPISIFL
LILFLTENWTLITIISATVFFVLFVCIHKQFNNKSRIILNREFSVKTEKGKWIDGFHE
VQIHLITEYLIEHNLYSKWKIERLMDSYKTDNSKDKMPALIAPSILIAVLAPNLNYLL
KHIYGMNIYQSTEGQFAIFFGVLTISIFIVTAITYSKNLFEEFTEIIVYKKVGQRNNL
ISILDDILITIES"

CDS 3250493..3254266

/locus_tag="PIKHDJKN_02941"

/inference="ab initio prediction:Prodigal:002006"

/codon_start=1

/transl_table=11

/product="hypothetical protein"

/translation="MLEITGDEIAQLNDSDLRSLIGLLCEADLSSMQLQTAGVTWGGN
QNAKDGGIDVRVDANSLLMNDGFIPRQHTAFQVKKPDMPRSEILLEMRPNKLLRQVIK
DLADNKGAYIIISSQGSISDSSLKSRKSAMLEAISDYSNASQLKVDFYDRERVASWVR
SHPSIVLWVRDKIGHPIQGWQAFSNWANHSESTEEEYLLDGTSRLSNSASVSTESTDL
LQGINELRTILGRPGSSVRLIGLSGVGKTRLVQALFDNRIGEEALNKSLAFYTDLGDA
PIPDPRSFAARLKTLRKRGILIVDNCPPDLHNHLTKLYANDNYISLLTIEYDVRDDQP
EQTAVFRLEPSSTELIEKVIRRKFKHINSVTVRQIAKISGGNARVAIAIARTISIGDN
ISHLRDAELFNRLFIQRNNSDSKLLKAAEICSLVYSFSSETVGPNSIEIKLLAELSGM
SVLELYQNIAELKRRDLVQQRSSWRAVLPHAIANKLAQNALENLPKQLIRSTFEDHAN
PRLLQSFSRRLGYLHNSQLAREIAKDWLSSGGLLEDFRKLNKAALNILRNVAPLHPQL
VLSRMEEINDQANESIFFSRENSDFYYYTNLLRSLAYDSDLFERSTRLLCNYALTENV
NENNNSIRTTLKSLFYIYLSGTHASVEQRLRIISCLIESDLDNEVELGIYLLDASLET
SKFSAINSFNFGAHVRDYGYYPKSKEEIQTWYETFIEYAVSTAISDKLIGKKIKRVLC
NKFRGLWTRAKMYDALESAVNRILANETWYEAWYAVKKTKRYDSDRMSDEASTRLDSL
YTLLEPKCLYDEVMLLTLQGQGYPYDIFETEEAFTKVRALSNELGHKLCYQEDILNSL
LIHLLSTNNRNLYELGQGLSQGASDAKRLWTHMIEKLSEITPSNRKYHLLQGFLYYLV
LTDKSSAEEILDEAIDHELLSEVFPSLQSVLIDTDGLGRLEKSLQFGKAPISSFASLA
LIQTHDIDFNEKFFELIRLISSKEDGLAVAVELFYMRISRDNKGLSQDIISLGQELIL
NYDFSQRSFDNHHFVSEIIKICFTDVKSTRNAKRIATKLYFSITENNVFFSDLDHIFL
SLVEVQPLEFLEVFVRDTEEMDYRIAQIFSGQLNLNPLNLISDELIINWCDVKKTERY
PLMASIIDAYQETSPSQQIEWTSLAKYLIDNYFEPTVILEQLMHVFHPNQWSNSLANV

MLKRLPLLESLMEYENPIVVKWAKNKEPILKKEINRIRNREQAQEQNQNEHFE"

CDS 3254583..3255182

/locus_tag="PIKHDJKN_02942"

/inference="ab initio prediction:Prodigal:002006"

/codon_start=1

/transl_table=11

/product="hypothetical protein"

/translation="MSWESHDKYKPCRCGLGFIVETTRSDDWNRFEYSTRLECPECRK

NYVTYIYDYISSGMYEQATCWVDRQLYDTFQKKEERLKETKSRLTQQFNEHMRKEYLD

KWVALFEDSPYTKKAVWEKFKKYKLTSYSYKTFCSKTDKQSLSQFVENQVLYENRYLI

LSAIGVESDSFLESYSANLMEHQKARDNARREMMQSSFR"

CDS 3255483..3256400

/locus_tag="PIKHDJKN_02943"

/inference="ab initio prediction:Prodigal:002006"

/codon_start=1

/transl_table=11

/product="hypothetical protein"

/translation="MIGIDEEAGVPVSLVGVECLDTDKEKLRLLEIFRSRIEPRITNI

DVEFIEMEESKYIILIRTQRSWLRPHRGWHNSKFYARQSNGKYELDVQQLRQIFLGSS

EFNTKYADFLAERAAYHIEKYGFKPFILLHFIPVSAFENPNVVDLTGIFNQIRSTPIG

TSNDNRRINFTGVYSESEDKSCKFQLFRNGIIEHATTRIIEEKEISGKRLHDCLKKTF

AYALHNYRSLEIMEPVYAMLSFYGVEGLTAVDIDTFFTGHDVVPYESDKMIFPEVLIE

DVENVNLPELLRPIMNALWNAYGFSSYSR"

CDS 3256861..3260796

/locus_tag="PIKHDJKN_02944"

/inference="ab initio prediction:Prodigal:002006"

/codon_start=1

/transl_table=11

/product="hypothetical protein"

/translation="MTVEIAGVRGYEYQYLVSAYVALKLLDQENVGIYIECADGEDLR

ITFQVAGEPYILDVQVKDRSGQVDLAEFSSWISHFDKHSRDMNLLEKLKQDANRFVLF

ITNARCQDDVSLFVDSNEVYLPLTAGMGNDLLDRVKTNVLSHYTNATTAGQGRRRFLE

QFFKDNSNNQLRTILKKNRVWESMDWDIVHERLAQLLNRKYAVPQSETEGVISKLDEL

VRQGRDSGESITEDVVRLLTAYNGNRIFPYDEKLVLRAEMPLCSEALEASHVLLLTGV

SFCGKTYLAKEIAQRYQESGFKVKITSELDGDSGALAFLRHVSPEERLLVLEDPFGSM

TAHPQRAELIAKVERLLDACGPHRKLIVTTRSDILLHALRRDSIAECRMKAHVWHDLT

ADEVATGEQIWSRYYGESDPSKALFKRLQDWLERNERTSVLQPGQIVHLYYAQPDLGV

LEQWEESAIVNEARIDSTRLARQIDEQGQYSKRVFMALGLSCNTYRAAKLDDLAFLLS

ESTEEPALNPQRNEPVTIAYHFGGKVAETPPYPAYQTTYEITVDNREALRDLRDRGYI

KVDSVYKTIVFTHPIYYHASKILFQTNLEDVFADKESIFRLLGRSLASPSKEGNLCAL

MMAEWQYAETQDATLKQRVKILLLRSLHSLFPSVKDRVIMYFNSRLDKLDDQEQKAFV

RAVKVQNSMNDDHILWHKGEPYFNTATERRSSFNWLNWDEDRHVASLQKLAASEPLTA

EEIRDMLAAQVADKMKPADLLHVIRLALDYDESFIRAKAVKLLFQQFAYTFEDIAGYL

EPQEHPDYIYNLFRGALSSWSSYTSQAKSAILDYFRNSLSIMSVALRSKRFLEKFEDQ

YARDGLDWDGLTDEEARELWLVWYDVFIALLNHFPSQYIRMNEAHMVKVARESLAHVD

DLERISQLASSWHRWLHHYSEHHLPEDYGMNVADYLLKGTGQAADVRAELFQELLSVR

QTSLLTVTIGTLVDHWPSLSIGERTELIAVLQAKREDVRWLRGVALNRRTVPDEVQIA

IVGEVLFGQEIDAVVDRLMAEGLLECCLNVHCGYPQPLWWNGYHHHNEKVWNRVILEV

LGRPPEGRCFDIALREFIDALYNHDTHPFPDGMAVYTRLVANPAQRQAVFRQLLTVTA

TNNQSNKQMWKLLFACSSEEERQKFFTIIAENIEAVQYYCDDEDDLLELFEQEVVFGQ

LYPRLVMDHEVVAFCFRYMDYVGQQVTASLTDEKQGEFKAFINSVYEHTPPRMTLTNK

IISNTLCNLRISDDEIDQLIEASRCQLIDLGHEQSQSFDDHYELEHWVQ"

CDS 3261649..3264036

/locus_tag="PIKHDJKN_02945"

/inference="ab initio prediction:Prodigal:002006"

/codon_start=1

/transl_table=11

/product="hypothetical protein"

/translation="MKDLAIKREGLCLSIEYTNSNTKLKWRCNCGYVWEATPNNIKRG

KWCPSCAHTKNANSKRKTIQDMQVMAERNHGRCISSEYSNMNSKLVWECSKGHQWEAY

PSNIYSGKWCPTCAGRNKSLTDMQAFAEEYNGKCLSTQFFNMTRKLKWQCNEGHIWDA

TPNSILNQKSWCPVCAGNNKKSIKDMEQLAGRRGGKCLSKKYINIDYPLLWECTNGHD

FIATPYSIEKGSWCPKCNNISVFFNEEKCRFILESLTQKKFYKTRKEFGNGYELDGYN

EELRLAFEYHGRQHFSYTPYFHNQSEELFFEQQLRDLKKEQLCKENSIHLIVIPYNVE

SDTDKVGFLHKKLWELGYDVLDNDLDWRSFYKGFNPLEELHTLAGLKNGICLSNEYNG
SDEKLSWECSEGHVWEASPYYIRKGSWCPQCLGRGRTIEDMKILAQEKGGECLSDSYT
NNSTKLTWKCESGHVWDATPLSISNGAWCPRCSGRLITIKDMHLLAESKNGKCLSTQY
SNNITPLLWECHMGHQWKAATRSIQQGSWCPSCNSKTINNMKQIAAKYGGECISEIYS
GIDGKLLWKCDHGHQWESSPNYVQRGRWCPQCKKDQKQRKTINKLKLLKELAQQREGR
CLSRIYYNVDTKLRWECKVGHQWESTPYSVKNGSWCPTCASKHSGMYRKNNIRDMKRL
AEKNDGICISDTYVNSQTKLRWKCKEGHEWFANPNNIQQGSWCPTCRRNEISIKQRKS
IEDMIELARKRNGKCLSTEYINAHTKLMWKCVHGHEWEAKPNNIQQGKWCPRCIK"

CDS 3264167..3265237

/gene="xerS_1"

/locus_tag="PIKHDJKN_02946"

/inference="ab initio prediction:Prodigal:002006"

/inference="similar to AA sequence:UniProtKB:A2RKP9"

/codon_start=1

/transl_table=11

/product="Tyrosine recombinase XerS"

/db_xref="COG:COG0582"

/translation="MDKQMKMHYMQRIDDKLSDLPWYVTEFIDSKKRKLSPTTLLNYC
HDYIIFFDWLITEDITSSTRKEIHLETLERLTIREAENFMSFLNYQLGNKKLTINRKL
SSLKSLFDYLQNKAETRDLKPYIQRNVMAKMDLNTIKESQETIANRIEGKILREDDFE
LFRQFVAYDFGEKHKDNKRIFSFHQFNRERDTAIVSLILGSGLRLSEVSGINTDDLDM
NKALARVIRKGDKEQYVYFSRQALVDLESYLQIRETKYVPEKHENYLFIAAPVGRKGK
SRRLTQRSIEKLVEKYATAFGKPSLTVHSLRHSFATRYHLENNDVPRLKNQLGHSSIQ
TTMIYTHLTDEEMRNAVNNMDR"

CDS complement(3266450..3267133)

/locus_tag="PIKHDJKN_02947"

/inference="ab initio prediction:Prodigal:002006"

/codon_start=1

/transl_table=11

/product="hypothetical protein"

/translation="MWLSPKVFQWRAEIDGSYCNEPTLYKRPYEIWKHAERILNRQAN
HFQRVDAICALKRAVNSRLNTLQNEYKFNLIPLSDVPKKPLQQLEYFGIIRPFILNKL

IDIRNQIEHEDMNPPTLVQCREFLDVSWYFLKATDSYVHYITDGYCLILESNDETEAV

SLRMETSPEKNWDLEISIWLPDESPYISKELRDGWLQLSNASKCGLYTIAPGGFHKGI

YEAYFTAIH"

CDS complement(3267573..3267836)

/locus_tag="PIKHDJKN_02948"

/inference="ab initio prediction:Prodigal:002006"

/codon_start=1

/transl_table=11

/product="hypothetical protein"

/translation="MSMWCWSVLSETVGARYPQGVNHRTDSAIFNTHAAYLIIWYLAV

GASNSRGYPKSIRTFKQLILDTVEFAGLIEGIAVGASTSQLQE"

CDS complement(3267903..3268010)

/locus_tag="PIKHDJKN_02949"

/inference="ab initio prediction:Prodigal:002006"

/codon_start=1

/transl_table=11

/product="hypothetical protein"

/translation="MSVYHSESQSEKDCDTKTNGDYTIEIDGNIMKILK"

CDS complement(3268347..3270167)

/locus_tag="PIKHDJKN_02950"

/inference="ab initio prediction:Prodigal:002006"

/codon_start=1

/transl_table=11

/product="hypothetical protein"

/translation="MSIKIFNDVIDVAVVGITNSGKSTFIASMIDQQKFGNSLTKLHQ

NDGGLTKVTTFYELSDCDNPSVSEVKFDLDNLTLNIDSKDVVGMNAKLRNMDYKGFKI

NPVTASEDGSFKKSLEDNLKIVSDKIKNDLNYAISMINSSKADKLVSYIKIKVPASKK

ALEIMRKYGFKTVILRDTRGFLDLELSESSKKPPSLAEAGLDGIQACIFMNGTNLTMP

SLGREMYGEFVKSIFEAVPTFIIERSAILGSKLEDCMNDEINISSDIYNDLVKNKSVI

KLNFKEIHKFLKSLSIIDENGEATNQLIKTHKRELLLPEVGVLTDDEINYDEEEYQRY

EFCSLEVFDRLLSSLSDFRTLLNNIILYFDDQNKVNDVHVEFFNVFYKKLFTRIVKEY

CAYEKHSNYIVRPVTEGYSKDKLLNDLVHGSLLGKRDGITTPAVDRPYEYGATGVFSV

TAWKAINMLILDLSESEEIIGSIKKHVENPEDNGIFNKYILEIQQCLKYVLQNSFTDV

RANFAGYPIVDRFIVVNAIENAKNKWKTEYSLKFGEEAIDNVFIIKVKEELDRISVSD

YVRFSQLYQFFYNTIDQYFGYVSTNHNYVETENKHHYSMT"

CDS complement(3271407..3272390)

/locus_tag="PIKHDJKN_02951"

/inference="ab initio prediction:Prodigal:002006"

/codon_start=1

/transl_table=11

/product="hypothetical protein"

/translation="MKPFIYLPDYQKEPYIERIHSYVKTYKERLATQFNNIDEESEQE

TEKYYSEIGSYFNPDRDDPADFAERARDFGFDYWQTLNLMKYNTYMMTLSTLYQVWEQ

QVRELSFKEITRYHHFVDKKGMKLEFKNFCTTFGEIKTLFLNSDWQPTSITSWDTINE

LRLVQNVIKHGDGAAADQLKSIQPGYFRDVNGKLIMDLYSSTLNEIALSIPDTAIDRY

REALVEFWEEMPERVHFTIETAPVRIKGPITLEEETVTDIWGHYVENHKLIPGCTIQI

RFQKKWISGTYQWTGNPADKPELHNKNSSEILTFDLNHQITLEVQNLGSQS"

CDS complement(3272834..3273652)

/locus_tag="PIKHDJKN_02952"

/inference="ab initio prediction:Prodigal:002006"

/codon_start=1

/transl_table=11

/product="hypothetical protein"

/translation="MAMAKRLQPTSAVLRELYLRSGNQCAFPDCIHTIVNSDGNYVGQ

ICHIEAAEEGGQRFNPNQTNEERRALSNLMLFCYDHHIKTNDVQTYTVEVLQRMKREH

EDKYTDIVGQLQRSISDLTGQQTFLYSESGERFFQYFGWNYREDEAKSVVEDINGWVD

KLKIFSPDTRQVFCLIIQRSSKLTWGTGAIIHEITEITGKTTSEIVKHIILLNKYGFV

YEPERDEDLHVDVVLMYGTSKLDWHLWLDIKEFCEKNNIDLDDIIIDLNFTKLD"

CDS complement(3273673..3273891)

/locus_tag="PIKHDJKN_02953"

/inference="ab initio prediction:Prodigal:002006"

/codon_start=1

/transl_table=11

/product="hypothetical protein"

/translation="MEPTEETDRAQEIQKSTKQMKQHSLLNSHMTIWQITKVTEVLSI

LEYEFSENRAAKQSSLRLVSHFNLSADT"

CDS complement(3274097..3274651)

/locus_tag="PIKHDJKN_02954"

/inference="ab initio prediction:Prodigal:002006"

/codon_start=1

/transl_table=11

/product="hypothetical protein"

/translation="MGLNTEHVTTSKEHIHLDEFVPKPSQFFQEKVYAHGLDAYHQGK

EDKRAGVIFDSLVEGIPSIQICCSGNGIYIGTIGLYVVGEIFVVSNRDIATGIDSANR

RYTDIHTFSLAKMLVNPNEEIFEAIVVPREVRSIWITTDCDEDKRETAQRLSMKYQLP

IVVLEPNIAGDDSKIRRLREVTKS"

CDS complement(3275835..3276002)

/locus_tag="PIKHDJKN_02955"

/inference="ab initio prediction:Prodigal:002006"

/codon_start=1

/transl_table=11

/product="hypothetical protein"

/translation="MSSIELLNNEDSATVEEYIGSSKKAIQISYNYLSKGEFIEEKDK

VFLRYNDDMLI"

CDS complement(3276183..3276545)

/locus_tag="PIKHDJKN_02956"

/inference="ab initio prediction:Prodigal:002006"

/codon_start=1

/transl_table=11

/product="hypothetical protein"

/translation="MDETDYADLAQDLLIGTIRSMENVLEVIAKNPKNVDGVPHYYLV

MQKLQATYESCLILLRNDYYIEASPLFRLIFEQVAWAYSIADKDLAFIEKSIKTKSVK

DLNDLIQGSSKIYKKIYK"

CDS complement(3276819..3277625)

/locus_tag="PIKHDJKN_02957"

/inference="ab initio prediction:Prodigal:002006"

/codon_start=1

/transl_table=11

/product="hypothetical protein"

/translation="MKATDRDYSVLTPDEELEYARMLEEEKVEKRGKLNIRVNLFRKY

PKAARHFLSLFPNNYLDIVDLENERGIKIFLNQLSDQLDDGTLSDERDIARYIKENGA

YYFIGSILNYYDFGHHEAYIFPEFQLSSTFKVDYLLIGRNSDGYSFIFVEIEHPIKQI

TLADGELGNAFRKGIKQVKDWKNWINANSSTFTTTIKGYKHAEKQLPDEFYSFDPTRC

QYVVVAGRRDDLNDTTRRIRREHIEEQKIRLLHYDNIIDSTLEVVKKKNY"

CDS complement(3278300..3279139)

/locus_tag="PIKHDJKN_02958"

/inference="ab initio prediction:Prodigal:002006"

/codon_start=1

/transl_table=11

/product="hypothetical protein"

/translation="MHSFLFNCLTNSIAFRVHEDSKLHGKIYIAHKNGVPIRGIITSA

NFTDNGLNQNHEWGVLVEDQEALKKVIADIGKVSSHALTREELQGIIKNIDSYAKNEE

VHKVPKFDLEVGHLFKKKTVELKPDVRYFIKPVGSSDRPFETTRKLNSDFEKMHFSRR

PTPVRIGDILICNGVGTTKLLGYFEVISEPYIWDSTSQWSWELEVKNLCPEYSESWNT

FNNTIASIVASYDHVHPVTNVGGSTLGALQFGRGRIQLTEEFAKHVIELMEGSVKAKK

ENK"

CDS complement(3279793..3280266)

/locus_tag="PIKHDJKN_02959"

/inference="ab initio prediction:Prodigal:002006"

/inference="similar to AA sequence:ISfinder:ISDha13"

/codon_start=1

/transl_table=11

/product="IS200/IS605 family transposase ISDha13"

/translation="MANKSYSLAHTKWMCKYHIVFTPKYRRKEIYNQVRKDLIEIFKR

LCKYKGVEIIEGHMMPDHVHMLVAIPPKIAVSTFMGYLKGKSSLMIFEKHAKLKYKYG

NRKFWAEGYYVSTVGLNEATVRKYIREQEAHDQAVDKLSIKEYEDPFSSNRSKKK"

CDS complement(3280704..3281033)

/locus_tag="PIKHDJKN_02960"

/inference="ab initio prediction:Prodigal:002006"

/codon_start=1

/transl_table=11

/product="hypothetical protein"

/translation="MKNELEVLVMFEEDRAEQNVTAYIPCLKLGAHGDTVEEARMNAV

DLVEIEIKKGCLREVSMHSDAQVETLQYNGGHLTVMFESQTESREVTAYIPALRLGVR

GINTDAA"

CDS 3281421..3282911

/locus_tag="PIKHDJKN_02961"

/inference="ab initio prediction:Prodigal:002006"

/codon_start=1

/transl_table=11

/product="hypothetical protein"

/translation="MLDNFDNFKKVWDSDPLIVLDTNVFLRLYSQPPKVINQYIEILR

SFKDKIWLPAQVVQEFEKNHKKEHGRNFTKYEVFLKQIQSTIEKNKNTLEGEFIKHLE

LKYPLIDELKVQITGLIDEMQKATVHYKDNIKQQIEENTKVLREDAVKKFVHELVESG

ATGKRFSLVETISLYQEGEIRFKHLIPPGYEDISKDADDPTKQRKFGDLILWKQTLSN

AKLENKSVIFVTNDVKEDWWVLVDNQPACARYELSQEFSEIVGEKFIMSTGQNLFRYI

SSINQIAIDTHLMFLEENSDDLIEELFDGEDWSIILNNKNDLDFYLVHSGDLQPYIDD

AISDVEIIGYPEDITDVELTVESFDYHHEKAFIQGSFATDIEIAVSSFFYGEEITSTE

GKCSINGSFSIEFTLDDTDDELSIKEGISKFTVGGFGISGYKETKSYLNDYNDSCSHC

NSDVVYYTDSGDGVCEDHKDLYDYCPGCNTLFEKGSLPGGLCNSCD"

CDS complement(3282969..3283370)

/locus_tag="PIKHDJKN_02962"

/inference="ab initio prediction:Prodigal:002006"

/codon_start=1

/transl_table=11

/product="hypothetical protein"

/translation="MHDYFKNTDDLIYIDAGIEGVYVPEGESWSQKDREESSESGYSG

QVVVGMKKNGKEILAPLDSIYPNDKNDLIPPSHNCGLEPHQPQRMIANEFLVFHISTI

LNELFSNHSIQVNYANFNARTGACRPVYVEV"

CDS complement(3283741..3288303)

/locus_tag="PIKHDJKN_02963"

/inference="ab initio prediction:Prodigal:002006"

/codon_start=1

/transl_table=11

/product="hypothetical protein"

/translation="MAIAESGGPTTQAGISYQNAIAALYLGRMLDSRSFSESEKVAKV

RVEAPESVDDIVITFSNEVKWYIQAKESISKGNGAESTWGKLWKDFEIQFIHPSFKKG

KDRLQLICGTYKDEIYDAKDLVERASSCESYEEWQGRLTTNQLVFFEHLSKFLSLELK

ENNDLILQFFMSIDVKIEPRQTVEKDKMRYWIPESNLSPTVLFSILRDKVGGDARIRG

SHTLKSLQEWLQDNQIVISESVSLSELRSAVYSCSSIIRHQKNTIGNTGKKLLRDNYK

DISKWLLETSDKEPVGILLDQAGTGKTIVIQEVLEELESRQVDVLAIKADQQLLDAQS

LKDVQSKLDLPVRVERLADTLAKQGRFVVLIDQIDALSLSMAHDQGSLNLALDLISRL

RSIPGVRIIISCRKFDFDSDPRLKNLENCKAFSLEEFSNEEVGNFLCDVGVDYKQLLP

TTQNLLRIPLHLNLFLMAYESDGSKELLQDRSLSSLQELYQLVWVCVIMKQHADAPPA

WERERALFLIAQYMDHKQRITVPKFLLSQKETNALERAFHWLSSEGIILNEGKELSFF

HQTFFDYCYARNFVQQGYSLVDVLVASPQGLFQRPQLVQVLSFLRGQDDKRSYFMQLN

ALLKSDDLRYHLKNHLYRWFAALASPTEQEWRLMKRTSADLNMRSQLLRFSYLNPGWF

EYWRTQVIPEIMDGQDERFLEEVFGYLTSMIDLRQSEIIELVLPYYNKSQCLEQLVEN

LVLQIRSWKCEEAVKLFEVVMEKSELDNRVSYLGWKELAQYDSKAFCRILRNFMNQAI

KNYRRKKEERNQIQTESYQFNFISLNKEMNKLGSTLMDEALQIVVNKSALYFLEIIIP

WLIEAVKIESYEVPETEEDFIPDSLFRNWYDRIQVEFDVVLNDTLVKGFILLAKEHPQ

IFVENINYLSNIPHDSIQFLIAHILRQVPEQHSITALNFILADPRRLTIGNEDFESRY

LIKSIMPYLQESERQELEDFIITRLPLNKKWKLDGYMYHDLSQYRILNSISSEYLSSH

GKQRLMELERKFPEYQASDNPRLIRGGVVGSPIDAEAVRKMTNQQWKKAMAKYQRGVR

HSDWSKGGAESLARLLQEQVRKEPERFINFFSVNKDGFDHNYVQAVLNGLTESTISGS

EFFAAISLFEDYEDRSIRRTIAWAVEKRADEEVPERIIGLLISYFYDTSLIKESYTED

PVSGYHNSVRGAAMNALMKIYREKEKEKWKILDYAVGCEDISLRAGAIYELIYMIRID

RERSLDLFEKAIEGNPQLYRIAYTNDFCYWAMGQPFERLVSKLDAFITDEKDEIQQRG

AELVALSLISENLFESQYVKEQVMTLLTKTINGNPYHRRGVARILSYNFPDNVSSILV

DGLLQLMNDEDEEVQRQISFLLLDIKKNCKELRQEIRTILMAFAGSRSMTYGGKLGAI

LWQHATDYPEWTLLVISEIMGNEHLRNRDNWYLDSKNLILAIINIYTDPLSDEVLQNR

AMDIFDILMNSFSMHAQGVLAEWDRR"

CDS complement(3288407..3292480)

/locus_tag="PIKHDJKN_02964"

/inference="ab initio prediction:Prodigal:002006"

/codon_start=1

/transl_table=11

/product="hypothetical protein"

/translation="MVTKPENGKKLVGSYIKKTVIPQGMTVKDAAELLGVGRPALSNL

LNGNASLSADMAARIERAFGINAHELMDMQAMNDTKHAQEKGVTASTKAYIPPFMQFK

ANDIEQWASTIKARTRFAVFLRTLINSTGQKIKRIDFPGNDDAERPGWDGFLEAEDGT

PWIPTGLSGWEFGINNDPKQKADKDYKKSVEQTAIEERMNITFVFVTPRRWDKKNAWR

KERLNEKNWKDVLVFDASDLEQWLEQSISGQAWFSGEIAVPAQGVKSLDKCWEQWVVD

TEPALTESLFEEAVEKSKSTVLKKLKDFEPITIWSDSTVEALAFLNCLFAVNNSQLAQ

FRDRVAIFTTPDTLPKLATKPADFIAVIPNREVEQEFAPYKKDLRSIIIYPSNSTNTE

PDIILEPLSYESFRKSLNAMGYNDDEINKLSHESGRSLTVLRRRLSSLEGVRTPEWAS

DKKITSMLSCFLFAGVWDESNEADRAILSLLGDDLEYPELMREFADLLALNDSPVWSI

GRMRGLVSKLDALYATNKRLVWEDIKRFLEIAKLVLSEDDPSLDLPEEQQWAASIYGK

TREISSRLRKGISESLVLLAVHGEALFRGRLGHDLESEVCQLINSLLSPLTVRKLEAH

SSDLPTYAEAAPDIFLKILENDLRSEDPQSLRLMKPVNSGFFGRHSRTGLLWALESVA

WSPEYLIRTVDILGKLSEPELNDNLANKPIASLGAIFRSWMPQTTAPIVARIAAFKFL

VRKYPKVAWPLCMEQISSYSKIGSYSYKPRWRTGAHGAGQPVQYHESNEFVLISLECA

IGWESHSVDTLGDLVSRSGHLPHEYQEQIWNLVEAWSGGANEEEKAILREKIRTTTFT

RRGKMNKNNLELRARKAYHILEPKDVVVRHEWLFKNQWVEESYAEMESDEFDYNTYST

RIEALRLEAIKTVMCERGLDGVLELAGRGETAYLIGDILFKIVCDHRKALDLCKNILA

SGILDDSHVRQLIISGLMLGLVNEGQADLIHELIDPKDNEMSVSLLLQAPFSKEIWDI

IETVGSEVKESYWKKITPSFSFRNEKKDYIQYALDHLISVNRPRAAFRSIQYCLDIVP

PELLYRVVAAIATNDEATGSYLVDSYHLKKAFKVLTASGEIPVDQMAGLEFQFIEALS

GEEASIPNLEKYVEAHPELFVQALVMAYRRGDGGEDPKELQAPDQRTGEQRAKGGYRL

LEKLSSIPGHDNNGELDPELIEQWVQKVRELAGELSRKDICDNCLGTLFSKAPVGKDD

IWPCEPVREVIERIWTEKFAKGICTGLYNSRGVHWSSSEGGDAERDLAEKYLQWASAL

EFSYPYVAEIHKQMAKSYERDAIWEDERAGVSRRLLE"

CDS complement(3292560..3292772)

/locus_tag="PIKHDJKN_02965"

/inference="ab initio prediction:Prodigal:002006"

/codon_start=1

/transl_table=11

/product="hypothetical protein"

/translation="MLVLVLLLGEGLLGTHPIAFVTLLQKAIHCEFPDWEPNSFEPLK

QQGETMKSNGVLWINDIKYEEQYPNK"

CDS complement(3293489..3293836)

/locus_tag="PIKHDJKN_02966"

/inference="ab initio prediction:Prodigal:002006"

/inference="similar to AA sequence:ISfinder:ISStin10"

/codon_start=1

/transl_table=11

/product="IS200/IS605 family transposase ISStin10"

/translation="MCKYHIVCTPKYRRKEIYNQVRRDLIEIFKRLCKYKGVEIIEGH

MMPDHVHMLVAIPPKMAVSTLMGYLKGKSSLMIFEKHEQLKYKYGNRKFWSESYYVST

VGLNESNIKKVYS"

CDS complement(3294279..3294764)

/locus_tag="PIKHDJKN_02967"

/inference="ab initio prediction:Prodigal:002006"

/codon_start=1

/transl_table=11

/product="hypothetical protein"

/translation="MSFKDLFSNKIELYTVQHPSVLGILNNEKKYIATGYLATDLVEN

YKNMAMYLSKKIKNEVEKPIFCFVATKPLQQHIQNKDFCGFNFNDGDIIFHLKVPLKY

CTVYKFDWFDYFDNNNMLLEHIIKRRADMKKDGLHCCLPYIKSQWIVNSTSLGNFQKI

I"

CDS complement(3295187..3295663)

/locus_tag="PIKHDJKN_02968"

/inference="ab initio prediction:Prodigal:002006"

/codon_start=1

/transl_table=11

/product="hypothetical protein"

/translation="MSQTFNSRKKTKKPWLDSIYLQRKNRTFELGKRSIDELIKQDIK
ISYRNIAELSKKIDTENKGIHPNSIKSNPELYEYYKENAKTKTTVRKSLNSSFKSDLS
AKNYNLIKPDRDIQALRRRFAKYKKNELIELLVNAEQYIAENNQKWIVSQFENFKE"

CDS complement(3295629..3297689)

/locus_tag="PIKHDJKN_02969"

/inference="ab initio prediction:Prodigal:002006"

/codon_start=1

/transl_table=11

/product="hypothetical protein"

/translation="MASENIVEKDWYKKLFGIVDKEILHLKNVNDDYLWDILNKQSLN
YIYKYCLESPWLNQFSLAVLCATDRELSPASINNMMSILNKRLKEIFDAFDLVHIEDL
NYTHFHQYLSGQIYEDHSNRQRQMLISYYKSFLYNVSKWLKNRIELEHQEHFSKFLFP
EFPFDNRDYKARDLAVSSAQKKRKEETSAVTPLLPDIRAQSHFRWNQIKRLREITNKM
IAKVENEKIFLPFSFDYEESEYLNERLYFTLNKNSDDEYYLEFIKSINLVDGSNGEGL
WFFEILNNRLLGAWSNQASDERKTEGISFLEKWGHDISANIHPFQSRNDGVLTQGFQL
TRKQQYNSSRLFINVEPLYIACMFAILSLDIQSYSGARINEILQVSYDPECCIITEDK
SIKPTKRNYILRLIPKGRDSLENYYMPESVFQTILMIIKELKSHYNSNILPEVDYSIS
SRKHLMNKKRKFVFQYNHQHINQFTLNSVIRFLTHGLIIQTEEGKQVILKTHLLRHAF
ATHAAQTEKLPIDIVRKLLHQKDESVTSYYAAPTNIQISGTIESLHDNWMSYIDIQQG
IQRSPEEIKDIYEEYKEKVGTVSKVVGGICTIDSVCPTKMACMGCAAKVPQPEFKYEI
EQYHAWAQESEKRFVELGLEVEAKKMKLAMRRAKIELKEIEGIEMYIKDESYEPNIQF
QKEN"

CDS complement(3297693..3298964)

/gene="xerC_3"

/locus_tag="PIKHDJKN_02970"

/inference="ab initio prediction:Prodigal:002006"

/inference="protein motif:HAMAP:MF_01808"

/codon_start=1

/transl_table=11

/product="Tyrosine recombinase XerC"

/translation="MTEFYYYQQKRISESSALSYLITLERFFSWLAKSSNYQGTRVKW
SDSHMAIRTAVENYLIYQMGCKLRSKENFQYVIMTKKSPQTINHFLAAIKSFYKTALH

LKLYEFQNPLVDADYSISIGSFSPTSMRENKPRLPKIAGTEIPSDSSYRSQSDSYFKL

VANDWKPNIIADHDLPYIIYRAGSEIGWSLRDEVVIRMLFETGARINEILELTLGDYR

QRINQYEIATLNKGSEKRRIKFLRFSQDTLKLLIRYVNGERKTYAHDKQSFTKLSDSE

VIFLTRRGDPYNYNAFYSQWSKIITQAKIKMNIHKTRHWFVTNMIRNIYDTANDGGEI

EMKKNELINYMKWKDPETIKVYEHYFNEALFREIHENMAKKMQEYEQEYLKNSHSSSE

GEQLSNSVLPTKAILPKEEIEWINEFYKGME"

CDS complement(3299374..3299877)

/locus_tag="PIKHDJKN_02971"

/inference="ab initio prediction:Prodigal:002006"

/codon_start=1

/transl_table=11

/product="hypothetical protein"

/translation="MRKNSYKLVLIASIFLNVLLVILSYNNHLNKQRDYASGDPSAYR

TFIHGLTYFSESIGTLKAENSKSENTNILINAKERLNLAMNSYIKFENSMASTNLESS

ILDDKVAMVNESMFKVLSSYVRGEYDLQQINQFKKQVDELLNNLPKSYDTKMKEDFIK

TINNLSY"

CDS complement(3299901..3300752)

/locus_tag="PIKHDJKN_02972"

/inference="ab initio prediction:Prodigal:002006"

/codon_start=1

/transl_table=11

/product="hypothetical protein"

/translation="MNNFRKLSFLGLSVALVSALFLPFSNISHAASSGSDGEYWYKGE

SYANLLHWDNSNVNSNDTSLSTAVVTAKANYNPKIDRGKESITAWSRLDTINRLYPSP

LYYPAQVNEIEVKFRVMDLSTDFDIYSTVPKHNITEYTVNPYLTGGVGMMFTILNYAI

PGSDIIAAPFDMVMNNIKVQKGTDTSGGTGTIQSVKFKLPDSDKSSLPNSISYKEADY

KTKGVGKEKGVTASFSYDMFSSSKRVIVTPQTKIKYSIHPVPFGGSYFPTIYASSNTA

YFNHTIN"

CDS complement(3300755..3301375)

/locus_tag="PIKHDJKN_02973"

/inference="ab initio prediction:Prodigal:002006"

/codon_start=1

/transl_table=11

/product="hypothetical protein"

/translation="MKKKEFLGALIFLSFMISFAVIIGTGFSDETGTKVQSNGEITMN

ATEEYVDALPDDLKNDLNQQLNRETIDNQRTFLRTMVTNNNDINLQLTMTPEGEFFSV

KGSGSITLLGETSKLTLNESIISKAKLESGETFVYGPLDAKMTLGTGETKDIIIGIGA

IVETGQRYFSLTMGNLGDKELTYLTFGDTSFLNEEIQKIQLENAGG"

CDS complement(3302222..3302575)

/locus_tag="PIKHDJKN_02974"

/inference="ab initio prediction:Prodigal:002006"

/codon_start=1

/transl_table=11

/product="hypothetical protein"

/translation="MERKIQLMETYVNIDFGNSPFLKLDKGLQIPYSSIKGITTGRPI

PNAFKLYGALLGEMRNGLFRSNSGYQLHFFENELKTLHLDLNNFKVGKFEISKLIIGV

DNPEEISENISERVK"

CDS complement(3303123..3303596)

/locus_tag="PIKHDJKN_02975"

/inference="ab initio prediction:Prodigal:002006"

/inference="similar to AA sequence:ISfinder:ISDha13"

/codon_start=1

/transl_table=11

/product="IS200/IS605 family transposase ISDha13"

/translation="MANKSYSLAHTKWMCKYHIVFTPKYRRKEIYNQVRKDLIEIFKR

LCKYKGVEIIEGHMMPDHVHMLVAIPPKIAVSTFMGYLKGKSSLMIFEKHAKLKYKYG

NRKFWAEGYYVSTVGLNEATVRKYIREQEAHDQAVDKLSIKEYEDPFSSNRSKKK"

CDS complement(3303811..3304119)

/gene="gatD"

/locus_tag="PIKHDJKN_02976"

/EC_number="1.1.1.251"

/inference="ab initio prediction:Prodigal:002006"

/inference="similar to AA sequence:UniProtKB:P0A9S3"

/codon_start=1

/transl_table=11

/product="Galactitol 1-phosphate 5-dehydrogenase"

/db_xref="COG:COG1063"

/translation="MNADIVVEAAGTPQTAATVFGYAKKGGGVVFLGIPYADVAMERF

YFEKVLRSELTVWGSWSCVSAPFPGKELEEGPAMFELLMERKELIGKVIMYPEKVYSI

"

CDS complement(3304712..3305116)

/locus_tag="PIKHDJKN_02977"

/inference="ab initio prediction:Prodigal:002006"

/codon_start=1

/transl_table=11

/product="hypothetical protein"

/translation="MNFASVRIITDDVDRLVEFYERVMGVSAERPAPAFAELVVPSCT

LAIGHSHTVPLFGIGSAVAADNRTVILEFLVHDVDAEYERLKPFVDEWVKEPTTMPWG

NRAVLFRDPDGNLINLFKPVTEEAIKRFSGRG"

CDS complement(3305346..3305792)

/gene="xerD_3"

/locus_tag="PIKHDJKN_02978"

/inference="ab initio prediction:Prodigal:002006"

/inference="similar to AA sequence:UniProtKB:P0A0P0"

/codon_start=1

/transl_table=11

/product="Tyrosine recombinase XerD"

/translation="MSFYIVQCEVEKLNVEDLNWENCSAIVNGKGAKQREVYFTTECK

VWLKKYLDSREDSCKALFVTDTHPVKRMAIPTLRWALKRLASRGAVEANVYPHRFRHT

YACQLLDNGAPLDFIQGMLGHEKASTTQIYAQLRGERRRELYRRFF"

CDS complement(3305767..3306150)

/gene="xerC_4"

/locus_tag="PIKHDJKN_02979"

/inference="ab initio prediction:Prodigal:002006"

/inference="protein motif:HAMAP:MF_01808"

/codon_start=1

/transl_table=11

/product="Tyrosine recombinase XerC"

/translation="MISSELWHHYEADKRIQGFSPNTLRAYALQLKMLVTDLGDVGIS

RVTQYLLKEYLVKQSDRLKPSSLGHRIRFVRSLFRFAYEETYLPNNPSLKLRESKMEK

RVPKFLMEEDVIHLRELLEFLYSTV"

CDS complement(3306253..3306615)

/locus_tag="PIKHDJKN_02980"

/inference="ab initio prediction:Prodigal:002006"

/codon_start=1

/transl_table=11

/product="hypothetical protein"

/translation="MIKLILTIVLALGLTSLIKIPASGEETLSVTHTTQCSLIEQELI

IELNPQIMSTLRQKYHDNFLYANVHVLPIQGSDNLEANFILEMTVMKGERPETVQMTF

RRDGLNGYKVDDITVITT"

CDS complement(3306768..3307169)

/locus_tag="PIKHDJKN_02981"

/inference="ab initio prediction:Prodigal:002006"

/codon_start=1

/transl_table=11

/product="hypothetical protein"

/translation="MKIFIGSLLTVIIFLMTIQPVCARPNDEPLKRLVLTLLAPKIQE

QINQYYTNKLTVSPTFTPFLDGTDVDVKYHSSHIDVQVITIPYVGPHLDVGLDSMKFS

LDNSGSVMVLEYKHIRDYDLPPNWQGIIKTR"

CDS complement(3308296..3309060)

/gene="yheD_5"

/locus_tag="PIKHDJKN_02982"

/inference="ab initio prediction:Prodigal:002006"

/inference="similar to AA sequence:UniProtKB:O07545"

/codon_start=1

/transl_table=11

/product="Endospore coat-associated protein YheD"

/translation="MGVDKLQPLRSKWLKTKLIINNESIKPFIPDTQKYNKTNLKSMI

SKYGMVYVKPERGTFGMGVIKAEMDSHQHFVYQIEQKRLTFDSFESFYSSLTRLVHKR
SYLIQRGIHLLKHNNRRFDIRVMIQLSPTKHWEATGIIGRLGHPKKIVTNYHSGGTPM
DIHKLLQSHASTKRRNELVQEMNELSLRIARHMKKKYPYLMQIGVDIGLDHSLKPWII
EVNVKPDPFIFNQLKDKTMYRKVIRYYRRAMPKKTE"

CDS 3309264..3310073

/locus_tag="PIKHDJKN_02983"

/inference="ab initio prediction:Prodigal:002006"

/codon_start=1

/transl_table=11

/product="hypothetical protein"

/translation="MSVQRGLRLNNAPKIDVLIPAIEKDLATLPLVIDNIRRYVKHPI
GSIYIVSPVSTKIRKLCSRKNCIFVNERSVLPITKNEIHYQSSRWNRSGWLYQQLLKM
NGDSIVKAKYFLVMDADTVLIKPHSFNVEGKTVFYCRDWSQPEYFNTYSKLLGMKAPR
PRSFVTHYMLFDKSKLSALKQKIEAVHKLPWYKAIISNINKKKQFGFSEYETYANFMY
TKNPGSMVLRSSMNKSLNVNASSLKEQQIRNLALKYRSLSFHKRKIYSKAP"

CDS 3310091..3311470

/gene="rfbM"

/locus_tag="PIKHDJKN_02984"

/EC_number="2.7.7.13"

/inference="ab initio prediction:Prodigal:002006"

/inference="similar to AA sequence:UniProtKB:P26404"

/codon_start=1

/transl_table=11

/product="Mannose-1-phosphate guanylyltransferase RfbM"

/db_xref="COG:COG0662"

/translation="MHIVLLCGGSGKRLWPLSNELRSKLFIDILPLPAGGRESMISRV
CRQLDSSSLTDSTLIISHQDQANITARHTQGKIPVIGEPYKRGTFTAAALATLYLQSF
RMAKDDDVICIAPADVFAGEDFFGNFQLLPDILKHSQADIALIGTRPTHPSEQYGYIL
PGGEERNAYVPITQFIEKPEATIAETLLQRGALWNCGVYAFTVAFMISHIKKMGLPVH
YDQLSSLYEALPERSFDKHVAEKAQRAVVLPYKGVWQDIGSWDTLCAQLDSHVFGHGG
ISGSSSDSYIINELPYPIQVIGVPGIIAAASPDGILIANKNNSNEIKAQLGSLPLKPM
YGEATWGSYRVIESSVEDENTTVTTLNITVLPGKHIGLKWHRAGCRAWTILAGSGQLL

INGQVTQMTLGTQFAMISESVYSIFAETRLVFLEVQIGEPEDENNILFEEEDWNIIVE

RAGSYNYGV"

CDS complement(3311941..3312825)

/gene="bepA_1"

/locus_tag="PIKHDJKN_02985"

/EC_number="3.4.-.-"

/inference="ab initio prediction:Prodigal:002006"

/inference="protein motif:HAMAP:MF_00997"

/codon_start=1

/transl_table=11

/product="Beta-barrel assembly-enhancing protease"

/translation="MTSSTKTHYDEALVYFKKALNDNIDIQDTLNYMGRCLTELKEYN

AAIEVYDRAIRLFPTWERLYFNKGYVYLQLGNTEDALIFCKRAVMINSNSEDALYYLA

LCYQKKEEYKKAIEYYQKSLALNTNQEEAHINLGLIFHELGNNQEALDEFQWALQIDP

SSTNAMYNKALVLKHMEKKEEAIIDFKKILISEPEDYDVRLQIAKIYLEENQFELTAY

YLDSILSFDPNNTVALYFKGKLLERQGNYEASIKLLEHASSLDPIDIDILLLLAQIYS

LIHLKEKERGLLHPHLKN"

CDS complement(3313470..3314192)

/locus_tag="PIKHDJKN_02986"

/inference="ab initio prediction:Prodigal:002006"

/codon_start=1

/transl_table=11

/product="hypothetical protein"

/translation="MNSKETNGASYESVTDQQLQDQLKYYKERAPEYDDWWYRRGTFN

QGDEANQIWFDEISTIKDAFKAEHFHGDFLELAAGTGTWSSLFLKEALILTIVDGSEE

MLSHNPVVSELNVRTIIADLFSWSPDQLYDSVAFTFWISHLPRERLASFFTMVSRCLK

PGGKMFFIDDREVAGTRESHVVGTSGQTMVRKLNNGDHATIVKNFFGDKEIEQIAASV

GIELHVHCTPKYFQYGIGSKTV"

CDS complement(3315538..3316368)

/locus_tag="PIKHDJKN_02987"

/inference="ab initio prediction:Prodigal:002006"

/codon_start=1

/transl_table=11

/product="hypothetical protein"

/translation="MNDSERRLALGSFLRNLRSSISPRDVGLPTGSRRRTRGLRREEV

AQLASIGPSWYTRLEQGRDSSPSVSVLESLASALKLTPNERRHLFLLSGESLPPQLNG

AEEIISPSVQMMLDELNPNPAYVIGRKLDFLAWNRAAEAVFTISEPSPPHDYNLMWRQ

FTDPVWREGLENWEYVSQRIVAEFRTSRARYLQDVSFKRLIDDLKRESTDFVRMWQHH

EASSTLDGYKKLCHPTLGAMEFEHITLQFPNDPDRKIMIYMPHTATKSRLENYLILSI

"

CDS 3316495..3317268

/gene="barS1"

/locus_tag="PIKHDJKN_02988"

/EC_number="1.1.1.413"

/inference="ab initio prediction:Prodigal:002006"

/inference="similar to AA sequence:UniProtKB:Q9LBV3"

/codon_start=1

/transl_table=11

/product="A-factor type gamma-butyrolactone 1'-reductase

(1S-forming)"

/translation="MENISGQLLMDKVVMVTGAGRGIGAAAAKLFAEEGASVMLVSRT

ESELRTVRDEITATGGNADYFTADLSDASSVEEAVKATVRRYGRLNAAFNNAGIVVTV

ASLVDEKEEDFDLIQSVNYKGVWLSMKAQIKAMLETSGSGAIVNSSSVGSLKGNPGLG

AYGAAKRGVNSLTQTAAIEYGPAGIRVNAIAPGTTMTDMIQQWVSINPKIIEQISAKT

PLRRPAEPIEVANAAAWLLSDQASFITGVILPVDGGLSA"

CDS 3317327..3317665

/locus_tag="PIKHDJKN_02989"

/inference="ab initio prediction:Prodigal:002006"

/codon_start=1

/transl_table=11

/product="hypothetical protein"

/translation="MPIWKPHLFYRCILQPLFLLATGSNENANGLLREFIPKGTDFAQ

MTDEALCRVLYLISHRPRKCLGWRMKKEAATKGPGNAKPLDAASFVFLLVLHSEFIIT

LSLAKHLSLG"

CDS complement(3317633..3318592)

/locus_tag="PIKHDJKN_02990"

/inference="ab initio prediction:Prodigal:002006"

/inference="similar to AA sequence:UniProtKB:Q7A788"

/codon_start=1

/transl_table=11

/product="putative epimerase/dehydratase"

/translation="MNKILVTGALGQIGSELVVKLREIYGADHVVATDLRSSAHEITR

SGPFELLDVTNDKAMFEIAKRYEVDTVIHLAALLSATAEEKPLLAWNLNMGGLMNALE

ISRELGCQFFTPSSIGSFGPSTPKDNTPQDTIQRPNTMYGVNKVSGELLCDYYFHKYG

LDTRGLRFPGLISYMTPPGGGTTDYAVEIYYAAVTAGRYTSYIAKDSNMDMMYMPDAL

NAIIELMEADSSRLMHRNSFNVTAMSVDPEAIAAAIRDEFPGFILDYDVDPKRQAIAD

SWPHSIDATAAKTEWGFHARYDLKAMTKDMLSQLTERQMLRKA"

CDS complement(3318630..3319820)

/locus_tag="PIKHDJKN_02991"

/EC_number="2.3.1.-"

/inference="ab initio prediction:Prodigal:002006"

/inference="similar to AA sequence:UniProtKB:P60120"

/codon_start=1

/transl_table=11

/product="Putative pyridoxal phosphate-dependent

acyltransferase"

/translation="MSSQALDQFLSSNIEDLKTKGLYNVIDTLQGANGPVIRIDGKSL

INLSSNNYLGLATDYRLIDASVKASQKYGAGAGAVRTINGTMDLHIELEEKLARFKKT

EAVIVYQSGFNCNMAAISAVMDQHDAILSDELNHASIIDGCRLSRAKVIRFKHSDMND

LRQQAKDAKESGQYHKIMVITDGVFSMDGDIAKLPEIVKIAEEFDLITYVDDAHGSGV

LGAGAGTVKHFGLSDRIDFQIGTLSKAIGVVGGYVAGKKQLIEWLKLRSRPFLFSTSL

PPAAIASCSASVDILMNDKVLIEKLWENGNDLKNGLSRLGYDVGQSETPITPCIIGDE

VTTQQFSKRLYEEGVYAKAIVFPTVPKGTGRIRNMPTAAHTKEMLDQVISVYEKVGKE

MKLI"

CDS complement(3321343..3322248)

/gene="gltR_2"

/locus_tag="PIKHDJKN_02992"

/inference="ab initio prediction:Prodigal:002006"

/inference="similar to AA sequence:UniProtKB:P94501"

/codon_start=1

/transl_table=11

/product="HTH-type transcriptional regulator GltR"

/db_xref="COG:COG0583"

/translation="MELRQLNTFCTVATTLNFTRAAEVLSYVPSNVTMQIKALEDELG

VRLFDRLGKQLALTTAGKRFLTHAQGVLEKMDEARSAVHDNEKLTGTLTISANEVICS

YRLPPVFQRFRSQHPGVRLIFRSVPNQELKQTLFEGTTDIVYMLDEPIRSSGLSVEPL

LEENFRLLAAPDHPLAKRTVLQLEDFHEEVFLTNEKGCPYRTMFDRSFEKEGIDSITY

LEFQSAEAIKQCAISGIGIGFLPEIVVEAEVERGELVVLPWQIPDLRVYTQMLWHKDK

WLSPIMLSFIETAREVFRLQNENKL"

CDS 3322393..3323412

/gene="metXA_1"

/locus_tag="PIKHDJKN_02993"

/EC_number="2.3.1.31"

/inference="ab initio prediction:Prodigal:002006"

/inference="similar to AA sequence:UniProtKB:Q3M5Q6"

/codon_start=1

/transl_table=11

/product="Homoserine O-acetyltransferase"

/db_xref="COG:COG2021"

/translation="MDYEIFNLGDVLLQSGVTLTNAFLAYKTYGKLNEQKDNVIVYPT

AFGDQHVQNEWLIGSEMALDPEKYFIIVPNLLGNGLSSSPSNTPPPFDRANFPQVTIY

DNVRFQHQLLTEKFGIQKIALVVGWSMGGIQAFQWGASYPEMVKRIAPFGGIAKPWPH

TYVVLEGVKASLLSAVGFDSSKLNQLSSTDMRAVGRVYAGWGLSHAFYREELYREMGF

DTLEDFVAGVWENSFMNMDPHNVLSMLWTGQHADISANPSYNGDFDKALQSIKALACI

MPGSTDLFCTADDNEYEAKRIPNAVLKPIQSIWGHFAGRGINSADNQFIDDNLKHLLS

LSTKE"

CDS complement(3324122..3325183)

/locus_tag="PIKHDJKN_02994"

/inference="ab initio prediction:Prodigal:002006"

/codon_start=1

/transl_table=11

/product="hypothetical protein"

/translation="MKRKAMHLITILCLVVMFLPTVVFAEDKNETSIIVATGKDVPTG

GILGSLPEKYPANAVEFVTQDGVKLTGYVLGNSNKGITLGHENGWMVDSWLPFAERLV

KEGYMVILWAFRNDEPSDSVSGEATYRRDLDVLAAAQVLRERGATKILAMGASIGGTS

TAVAAPNIPELVGLGILSSPRQFGVSPPISALEAVKKVEVPSFFAVSEEDPTGSYYDE

VKALYEASASKQKQFNILHSSEHGTDMLSEEGSYSKRSKEDSTVKLKQERIALADKLL

LFVNNSFGQTTRTDGDRNETSKNTTDYADSSKSSDNTSDYIDKNKTEKSAFMNPAIIG

VGILFLLAIVASIIVKKKR"

CDS complement(3327086..3328102)

/locus_tag="PIKHDJKN_02995"

/inference="ab initio prediction:Prodigal:002006"

/codon_start=1

/transl_table=11

/product="hypothetical protein"

/translation="MHTSTMLPAPRGGGFRMEGYWVWCGSVVQGEDGKFHMFASRWPK

TLPMHPGWMTASEIVRADSDTPEGPYRFCEVALPARGAEYWDGRCTHNPHIVKHNNKY

LLYYTGSTHPLSDPEPPGNFGLTDPQCIVARANKRVGLAIADSVLGPWRRADEPILPT

RPGRFDSFLTSNPAPCVREDGSVLLIYKARHYEGNLHGGMTIGVAYADHYEGLYRVLS

DEPVFPPESFHIEDPFIWQTNEGYHLIAKDMEGHLCGEKYGGIFAESPDGLKWSLHGQ

PQGYSRTVRWDDGTVQTLGNLERPFLLFQNGKPTHLFAAVSDGSDGFKDASDTWNMVI

PLKQ"

CDS complement(3328263..3330557)

/locus_tag="PIKHDJKN_02996"

/inference="ab initio prediction:Prodigal:002006"

/codon_start=1

/transl_table=11

/product="hypothetical protein"

/translation="MGTKSMTCIILFCMLVALMPGVVGAADAKFTIGSSDVTASGHDG

NVPANTVDGDFVTRWSANGDGQWIQYDLGVNRKVSSIKIAIAFLNGASRTSTFDILTSTD

GSTFTTVSSGVVSSLVEGLQTFDFPDVDSTRYVRIVGHGNSSNLWNSYTEVELYGTAS

GPPPGASKLAITVPQLMASGDDGNIVAYTIDGDLNTRWSASGEGEWVQYDLGSSKRVE

YVKIAFTNGVERTFAFDIQTSYDGYNFSTVLSGAVSSLSNSLQTFDFADAAPVRYVRI

VGHGNSVNAWNSFAEVEIYGSDSSGSGSEGTVVEVSTSTQLAAELATATAGKTIVLAN

GTYSRTSPFAVQNKNGTANAPIVIKAKNRGQAIISGPSGFRVENSSHVVLDGLKFTNT

SNGAVVLEGSHHVRLTRNTFALPSSGSGLMWLQVRGTNSHHNRIDRNDFGLKSDTEPL

IAYEGQNGSGQISQYDIIEYNYFHDVGPWVANGKETIRLGLSGLTLSHGYNTIQYNVF

QNCDGEPEIISVKSSSNSVRFNTFRTSKGSLTLRHGHNNSVYGNFFLGDGVESDQEGI

RMFGNDHKIYNNYFENLTGEAIYLPNGDFDGGTGGSPPSPTVEELRKQWKVYRALIVN

NTIVNSKTGIVIGSGKAYAPQDSVVANNIVYNSTGTLYYEAATTNTLFQGNIGFGSTI

SNISRSSEQIRNINPLLITVSGIQKLSASSPAIDAAMGTYAFVLADMDGQMRATADGG

ADEYSGAPLLNRPLAADDVGLNTH"

CDS complement(3330692..3331822)

/locus_tag="PIKHDJKN_02997"

/EC_number="3.2.1.-"

/inference="ab initio prediction:Prodigal:002006"

/inference="similar to AA sequence:UniProtKB:T2KLZ3"

/codon_start=1

/transl_table=11

/product="Unsaturated glucuronyl hydrolase"

/translation="MSNENVKLKPSELTAWWDKVKIKVDHMIEADWTEAPHVSAAGKY

DQIKVDQWISGFWPGILWILYDMTGEQRYREEAEPWDERMEKCYLRDNHFHHDVGFQF

LPTSVIRYKLTGDPDARRRGLFAANYLAGRYNPAGQFIRAWPRNQTGWSIIDSMMNLP

LLFWASEESDDPRFKHIAVAHADMVLRSFIRGDGSVHHIVIFDPETGEVERYDGGQGF

APQSSWSRGQAWALYGMSCAYRYTGEARYLNAAKGVAHYFISALAEDDVPHWDFRAAT

DLTDEPRDTSAASCAASGLIDIASQVAPEEATLYQRAATRILRSLSNNYSALDQPEYE

GILLCATGHKPVNTNINVSLIYGDYYYIEALAKSKGWSQHIF"

CDS complement(3331844..3332719)

/gene="araQ_20"

/locus_tag="PIKHDJKN_02998"

/inference="ab initio prediction:Prodigal:002006"

/inference="similar to AA sequence:UniProtKB:P94530"

/codon_start=1

/transl_table=11

/product="L-arabinose transport system permease protein

AraQ"

/db_xref="COG:COG0395"

/translation="MRVSLGEHIFRVCNTLFLSALMIVTMYPILYVAFASFSEPALMM

AHKGILWKPLGFSLETYGAVFSNPMILLGYRNTLFIVIVGVALNLLLTSFAAYALSRK

TLQYRKQLTLFIVFTMFFSGGLIPFYLLVRGLGITDTLWALILPTAISAFNFIIMRTS

FEAVPDALEESAKIDGANDFGILFRIFLPLCKPALAVVGLYYGVSHWNSWFNAMIFLQ

DRSLYPLQLILREILIIGDANSMAEGASQDEIIILGETLKYATIMVATLPIFLVYPLL

QKYFVKGALIGAIKG"

CDS complement(3332732..3333685)

/gene="yteP_23"

/locus_tag="PIKHDJKN_02999"

/inference="ab initio prediction:Prodigal:002006"

/inference="similar to AA sequence:UniProtKB:C0SPB3"

/codon_start=1

/transl_table=11

/product="putative multiple-sugar transport system

permease YteP"

/db_xref="COG:COG4209"

/translation="MAQLKLGRSKVTHVGRVPLNIAKDMVKNKYLYMMLTPVLLYFII

FHYFPMYGSIIAFKDFNPRLGIGGSAWVGLEHFKDFFGGMYFWRVLKNTLMISLYDLI

FGFPAPLILALLLNEVKNTLFKRSVQTITYLPHFISLVIICGMIKDFTSSDGVINDII

AFFGGDRVTMLLEPSFFRTIFVSSNIWQHIGWGTIIYLAALSAIDLEQYEACKIDGGG

RWKQMLHVTLPGLLPVFVILLILDIGRIMSVGFEKVILLYNPTTYVTADVISSYVYRI

GLQDFQFSFSSAVGLFNSVINFALVIGSNWISRKLNNTGLW"

CDS complement(3333788..3335458)

/locus_tag="PIKHDJKN_03000"

/inference="ab initio prediction:Prodigal:002006"

/codon_start=1

/transl_table=11

/product="hypothetical protein"

/translation="MAENRNGKTRKRLQLTLGIVMVLSLLLTACSSNGGSGNKDKTEP

TQSAVSNKGGDGVSKGNEGSDTPVYPLDTKDTFSYWAPINGNLITFTTNLAEAPIGKA

IAEATGVKVNYIHPTDGQAEEQFNLLLASSKLPDVLEYDWSIYPGGPEKAISDGVIVP

LNDLIDQYAPNLKKMLEANPELDKMVKTDSGQYYIFPMVRNAEGLVFRGPMLRKDWLD

ELGLQLPVTIDDWHKVLTAFKEKKGAVAPLTALYANKMNLQDAFFGAYKTSQGFYIDD

QGKVKYGPLDPQYKDVMTLLRQWYSEGLIDKDFPIVERNTLDKRLLNGESGATVFLLG

GGMGKWLESGKAQNPAFDLVAAPYPVLKAGEKPFTGQRDFKYNPKASAAVTTSAKDLE

TIVKWLDFAFSEQGTMMYNFGVEGESYTMDNGVPKFTDMIVKNDKYTSQQMLAQYTVP

NGPFPMHEQKTTNTFPQQDEAVQIWSKTDAAKHILPAFITPTVDESKKVAQTMTAINT

YLEEMFIKFVMGNEPLDKFDSFVKQLEDMGIRDVIDIYQGALERYNNR"

CDS complement(3335633..3337987)

/gene="yesS_3"

/locus_tag="PIKHDJKN_03001"

/inference="ab initio prediction:Prodigal:002006"

/inference="similar to AA sequence:UniProtKB:O31522"

/codon_start=1

/transl_table=11

/product="HTH-type transcriptional regulator YesS"

/db_xref="COG:COG2207"

/translation="MAQTRNRWTIYLKQIRHKNRSVFLTWMLSYVSVLLVPILISGVI

YGATLNVVKAEINRANQSILLQMEQAIDSQFKGIENLSLEVSLNRRIAAFISADTNLS

DSDHYDIYQIANDLRVYEIANDFLEGIYIYYKNSDTVIARNNRMNIEALYDHIRLEEH

MPFQSFREYFDQWYIKGYHSVVVPEENEWRPTVMYAQNVNSTIMNQPGAVLLFMIDRS

KLLEQASAPEKGTIFILDEDNRFIASTGDSLSEPALHYSDMPEESGQLEIETAGRRLV

ASYTTSSITGWKYVMTLPANVYHQKMNVVEQLTIASILLCLIVGGGVTYWLLKRNYLP

IHALIQSLSFKAGFTFDRGSNEYSFLEQALSSTFHEKEELHYKLNQHNNTLRSHFLQR

LLKGQLDSHIPLHESFSAYDLRFPLGGFSVILLRIEHFGKFHRKDEDQVHNKRLLHFL

IANVVEEVARKSVNAYAVELDQELLACIVNHQLASPAEQRGELGHIAEETMSFIGSHA

HVQLTAAISAQQEGEYGIFQAFQEAIEAMEYRIIAGSGGIIHYGDLSGNDNLGAAPGG

YFYPLKAEQQLIVMVREGQLDKAKAFVGEIIQKNVTAEPFPFHTAKCLMFDLMATLQK

VIGEIRITGKPDMPDGVRAIERLMHCQTVGEMRNKLYDMLELLCSYVNNNREYCSNPL

IDKVMKYVNEYYQDENLNISMIGEAFSMTPSYVSKLFKDLAGESLLDYINKTRLVQAK

LLLTDGKYTVSEISLKVGYNDVNTFHRIFKKFEGITPGRYREVK"

CDS complement(3338009..3339190)

/locus_tag="PIKHDJKN_03002"

/inference="ab initio prediction:Prodigal:002006"

/codon_start=1

/transl_table=11

/product="hypothetical protein"

/translation="MTTIYWVSSSDELAAAIDRAEPGSSILLADGVYDRNEDYIIEGK

VGSDTAILSIRAVNKGKATIAGESSIRILNSAYVEVWGLHFQVEQREYIILDGSRHVR

IARNRFEPRQVGAEYSLLSVMGSGSGWNRIERNEFGPKADLGPLVIFDGDGEQISQHD

VIEYNYFHHIGPRVVNGLEAIRLGLSGISLSNGYITIQHNLFENCDGEPEIISVKSCY

NTVRYNTFLNSAGQVTARHGHGNRFHNNVFIGDGVKEEMGGFRIYGNDHHIFDNYMEN

LTLDALLIDGGNYDGGPDGYPEAPTQEELRKHWRVYRASVVNNTIVNCRHGITLGKRI

PYAPEQCVIAYNRVCGKEQAAIYEYRPSPAVCINNHKADVADIRCPAILTRIDVGPDS

D"

CDS complement(3339473..3340183)

/locus_tag="PIKHDJKN_03003"

/inference="ab initio prediction:Prodigal:002006"

/codon_start=1

/transl_table=11

/product="hypothetical protein"

/translation="MKALIISDIHSNVKTLESICKAESDCDVIYCAGDLVDYGPFPRE

VIEWVHKNDANCVRGNHDDIIIDLYRNGNSGHDVAEDEIKWAHYNARLLGEAEISFLE

SIPVYRTFEMDGYSYTMQHLYENYLTIDSLPKYDAFWAERNGRTIGAEEDRRIIFGHT

HRRCIHYLSDDSLWLNPGSVSYRRRDDPSKEAHYFTITDGKIAMKSLVYDRTSLLEAA

QKLPLKEEELKVAYFFFG"

CDS complement(3341184..3341315)

/locus_tag="PIKHDJKN_03004"

/inference="ab initio prediction:Prodigal:002006"

/codon_start=1

/transl_table=11

/product="hypothetical protein"

/translation="MYLKEFDLDLPYIVDDENIESIIKKQKCEYVSTTKPQNELNWK"

CDS complement(3341877..3342917)

/locus_tag="PIKHDJKN_03005"

/EC_number="1.-.-.-"

/inference="ab initio prediction:Prodigal:002006"

/inference="similar to AA sequence:UniProtKB:P32382"

/codon_start=1

/transl_table=11

/product="NADH oxidase"

/translation="MNFSQSVEALFQPIELGHLKLSNRIVMAPMTRQFSPDGIPGSNV

AGYYRRRAENAVGLIVTEGTIINHPDASNQANVPHFYGEAAMNGWAHVVSEVHEAGGR

IIPQIWHMGAKGHVNDYSEAEIATIVQEFAQAASEAKRVGFDGVEIHGAHGYLIDQFL

YEKTNSRTDRYGGDMMARTRFAVEVIEACREVVGPEFPIVLRLSQWKTDDYQAKLAET

PELLEQLLAPLVQAGVDIFHCSTRRFWEPEFEGSDLNFAGWTKKLTGKPTITVGSIGL

DGDFTSLFTEGKGASNVGIDGLVQRLQNDEFDLVAVGRALLVDPEWAKKIQEGRTDDL

VPFTREAMTVLT"

CDS complement(3343126..3343701)

/gene="comR_4"

/locus_tag="PIKHDJKN_03006"

/inference="ab initio prediction:Prodigal:002006"

/inference="similar to AA sequence:UniProtKB:P75952"

/codon_start=1

/transl_table=11

/product="HTH-type transcriptional repressor ComR"

/translation="MSRPREFDVDRVLHQSMEVFWNQGFKATSYEDLTRTTGVKKQSL

YCVFKDKRSLFLKALALYREQVIAKLKEIEALDSSPGDKLDALRYSLLDDETGCQGCL

IVNASLEFGTDDEQVTREAELMVEEVQLVLEKIISSGQKQQLISNRYTSIELASYLNN

TILGVRVMEKSGSSREQIETVLRTSFGMIMS"

CDS complement(3343798..3344361)

/locus_tag="PIKHDJKN_03007"

/inference="ab initio prediction:Prodigal:002006"

/codon_start=1

/transl_table=11

/product="hypothetical protein"

/translation="MLELEFTTIEEWDEALWARMERIYHEAFQSGAKTKAILRSMLYR

GIGYLHTCVHHGEVVAMAVTGLEGKAADRILIIDYLAVEQKLRGLGIGTWMLEQLRAW

ALKKHGIKGMIIEAESGTTEAHQERIQFWQRNGFILTSYVHQYRMVPEPYQAMMLPLD

KSTHVPDDGEALFRYINAFHKVAYRKS"

CDS complement(3344522..3346495)

/gene="tetO"

/locus_tag="PIKHDJKN_03008"

/inference="ab initio prediction:Prodigal:002006"

/inference="similar to AA sequence:UniProtKB:P10952"

/codon_start=1

/transl_table=11

/product="Tetracycline resistance protein TetO"

/translation="MRNINIGILAHVDAGKTSLTERLLFETGIISEIGSVDKGNTQTD

SMEMERQRGITIQSAVVSFTVNNMKVNLIDTPGHPEFISEVERALRVLDGVILVISPV

EGIQTQTRVLMKTLAKLKIPTIFFINKIDRVGARYESLLTEIHDKLTPHALAMGFVYN

IGTSAAHMELFTENNQKFMEGLANRLADNNERFLERYLYSAEDLAPVDFESEFLEQFK

QAQLYPVLFGSAITGEGIPHLITAIQNCHSSVHYENTSFTRGRVFKIERGKNDEKIAY

VRVFSGEIKIRESLSYYRMNQTGEIIKFTNKVTGLKHFFSGKTTDGTIAASGDIVKIM

GLTDCQVGDDIGVPSNETTETLFSPPSLVTTIKASDENETIHLFRALKKLAEQDPFIH

IKQNSLQRELSIHLYGEIQKEVIRDQLKKDAQIDIEFLATQPIYIEKPIFTGEAVERM

GDKGNPFYSTVGFRIDPNPRSTGIEYHLEVELGSLPLSFHKAIEETVYETLKQGLYGW

EVRDLIVTLTDTAYASAVSTASDFRKLTPLVLMRALAEATTQIYEPIHRFELEVPANY

LSRILFKLTQASSTYQDPIQSDQVFMIYGMIPIKNIQSFKNQLPGWTQGEGVLLSEFY

GYQPFDGEVPSCTRYDHNPLNRKEYLLHVLNSV"

CDS complement(3346789..3347871)

/locus_tag="PIKHDJKN_03009"

/inference="ab initio prediction:Prodigal:002006"

/codon_start=1

/transl_table=11

/product="hypothetical protein"

/translation="MNEIMKKKLLGKNELLINMVIERAKRDFLDDIAIIGLTGSFSTG

DFHEKSDLDLIIINNTERGWEISDCFILDDVGYDIYCTPWDSRIQEQSTLESPNVSSL

TELKILYYSKPEDLEKLNDFRQKALDALASPLGEACLNRAKKWIDLAKQAYSDTMLGE

DIGSVRHASADVLYNLVNSLVSMNNTCIKRGIKRYLEEIRSFRYVPDDLESLYMSIIE

AHTIEDIRIASFNMLNSVTRLHSTMCDNFIIKPAPNFDNLRGTYEELWCNYRNKILNS

VTTNDASYVFLSVFGAQGYLDEMAVEKGTKKFDLMQYFDASDLPVMKEKFLEVMDEYL

DEYNKVGREVKRFTNFEQLYDYYMNH"

CDS complement(3348028..3348294)

/locus_tag="PIKHDJKN_03010"

/inference="ab initio prediction:Prodigal:002006"

/codon_start=1

/transl_table=11

/product="hypothetical protein"

/translation="MKYSNAEFVFPEELLRIIQEYVQGELIYIPKPKEAHLKWGERTH

SKSRVSARNAEIKSLFRNGVSIEELAGQYFLSYESIKKIVYSKN"

CDS complement(3348600..3349265)

/gene="glaR_2"

/locus_tag="PIKHDJKN_03011"

/inference="ab initio prediction:Prodigal:002006"

/inference="similar to AA sequence:UniProtKB:P37338"

/codon_start=1

/transl_table=11

/product="HTH-type transcriptional repressor GlaR"

/db_xref="COG:COG1802"

/translation="MAKRNGSSTLSENVRKKLQSDILTGQYEPGSPLKVSELAKGFEV

SVAVVREALTRLATQGLVQQNPNHGFSVKTASEEELNNIIQARLINESEALRLSIAYG

DLTWESNVIAVHHKLSQTAEYKEVNEKLVVREEWSEIHRQFHYELIAACPNPVLLDIC

SNLWNLSEFYRHCSPPQQQVRDGLAEHKALTDAVLSRDGERATQIFRAHIQLTAEVLF

DNQ"

CDS 3349445..3349978

/locus_tag="PIKHDJKN_03012"

/inference="ab initio prediction:Prodigal:002006"

/inference="similar to AA sequence:UniProtKB:P9WFN1"

/note="UPF0098 protein Rv2140c"

/codon_start=1

/transl_table=11

/product="hypothetical protein"

/translation="MNNNPFSKLPEVAVFQLESNDIMEGHPLPTQHYSEMSTLEGSKD

LSPHLKWSGAPDGTKSFVVTAYDPDAPTGSGLWHWAVINIPASVTELPTGAGDEHSSG

LPQGSLQLPNDLRLERFIGAAPPAGHGPHRYYFVVHALDVENISVAPNSTPALLGFTM

LSHTLGRAVLMATGEIK"

CDS 3350013..3350279

/locus_tag="PIKHDJKN_03013"

/inference="ab initio prediction:Prodigal:002006"

/codon_start=1

/transl_table=11

/product="hypothetical protein"

/translation="MKNSEYLMRVYDFFCTQLGLKSKVLHSYAESSTEEVVKLNDQPH

HHVCPNCQEPIIILKHTVICGCGSKIKNKVKFKIYPEKLKSPHN"

CDS 3350434..3351837

/gene="mdtD_1"

/locus_tag="PIKHDJKN_03014"

/inference="ab initio prediction:Prodigal:002006"

/inference="protein motif:HAMAP:MF_01577"

/codon_start=1

/transl_table=11

/product="Putative multidrug resistance protein MdtD"

/translation="MQTNHSVQKNKGELLLGLLVFTLIISVMNATMFNIVLPNISKQF

QISSSQASWIITGYMIVYAIGSVTYGKLTDRYSLKNLLTFGLLLFAIGSVIGLVASEY

WMVIVGRIVQAAGASVIPAISMIVPVRYFTSERRGRALGTVAIGFSLGLAIGPIIAGV

VSSTWNWRVLFVISLLSLLTLPMYRKYLDDEKGVSRKMDYIGGILLAGTVAILLLALT

NGSVWLALGGTLTFIFFLLRIRYTPNPFINPAIFRNKPFSFGLIITFVMIGIGFAFPF

ITPQLLSSVNHLSPVFIGIVMLPSSLMAALLGRQGGKLADEKGDRFLVFTAAALLIIG

FISLSSVVGMSPVFIAIFLVFGILGQSFMQIAMSNTVSRTLSKENIGVGMGLFSMVTF

IATATSTAVIGKILDLGTSTISLNPIPQNSATFVYSNIFLTLPILIVAVAALYYVQFR

RTGKMAKDEKMNSEPVT"

CDS complement(3352108..3353082)

/gene="srkA_2"

/locus_tag="PIKHDJKN_03015"

/EC_number="2.7.11.1"

/inference="ab initio prediction:Prodigal:002006"

/inference="protein motif:HAMAP:MF_01497"

/codon_start=1

/transl_table=11

/product="Stress response kinase A"

/translation="MDKKIKLLYTQDILRQSAQCFGIREDSILELNGFQNFVYSGAVG

DREVILRIAHVTHRNELLTRAELDFIMFLADRGMKVARPIQSLSGDLIHIIDESFVVT

AFEKASGRNAVIAEESNTFYENIGQMVGKIHKFSLNYSSQHSRFEWNDNALLASFKKC

CPLELHDNFDRLITEVSALPKEKDTYGLIHGDISPGNYLNDGDKAFIIDYDDAEYSWF

VSDIATPLFYEIPIPWVVSGDVRKEIGKRYYCNFLNGYCKENTVSQGWLNKIPLFINL

RQARVLAAFYRSRDFNAPDWSEWDEQARRFYYENLLNNTPYIDMDYLC"

CDS complement(3353209..3353475)

/locus_tag="PIKHDJKN_03016"

/inference="ab initio prediction:Prodigal:002006"

/codon_start=1

/transl_table=11

/product="hypothetical protein"

/translation="MKYVNADTIFPEELLKEIQKYIHGRMVYVPTPEKLHKKWGEKSG

SREQLSLRNETIRQTYFNGKSIDELSNEFGLSYESIKKIIYSRK"

CDS complement(3354690..3355283)

/locus_tag="PIKHDJKN_03017"

/inference="ab initio prediction:Prodigal:002006"

/codon_start=1

/transl_table=11

/product="hypothetical protein"

/translation="MVNQEDPRVLRTRQLIRTAFRQLLRRRGFDAITIKDIAQEATIN
RATFYAHFEDKYALLDEVTEQAFHEMIPEHVANAQAFTDEICDQLILLTHQYIVDFYR
ICRIDSNSMAKLVDEKIKKMLQQTIESIFLKGDTHHGADKHHIKITAAMTGSAIYGGA
HYWLNVEEKDRTDELVDIVRLYVMNGLGRIVNGDEYR"

CDS 3355444..3356337

/locus_tag="PIKHDJKN_03018"

/inference="ab initio prediction:Prodigal:002006"

/codon_start=1

/transl_table=11

/product="hypothetical protein"

/translation="MSTRPHRLLIFGAGVIGSVYALRFAQSGLDVTLLARGKRLEALK
RDGLKYNDNGTIKQISIKSIEKLDNDDIYDFILVPVRYDQAESALSAIKHNRSKTIVT
LTNTIGYDSWLEIVGDRLLPGFPGAGGDIKEDVLYAQFGSEKHQGTIFGEINGLTTER
VKELAQIFESVDLHYEIQKDIQAFHISHTALAIVNKHFYTNDGMVDLDRARSVSTLSK
IAADIKQNIHLVEQAGIPVIPPETKEMGKLTDNDIISRYRQMLSNDFIIDVKLGNHAI
SQKAEIMLLDEMFHKMISSQR"

CDS complement(3356482..3358413)

/gene="fbp"

/locus_tag="PIKHDJKN_03019"

/EC_number="3.1.3.11"

/inference="ab initio prediction:Prodigal:002006"

/inference="similar to AA sequence:UniProtKB:Q45597"

/codon_start=1

/transl_table=11

/product="Fructose-1,6-bisphosphatase class 3"

/db_xref="COG:COG3855"

/translation="MDEQFLDLLAQKYDTEEKIITEIINLEAISNLPKGTEHFVSDLH
GEFQAFQHVLRNGSGTVKEKIKELFREVWTDQEINDFAALVYYPEEKIQLVIGELCNK
QALNQWYRQTIEQMLKLISYASSKYTRSKLRKALPEQYVYIVEELLYKTDSTNNKDPY
YEEIYRQIISLGQADKLIIGLAYTTQRLVVDHLHVVGDIYDRGQDPDKIMDTLINYHS
VDIQWGNHDVLWIGAYAGSLVCLANIIRICARYDNLDIIEDVYGINLRPLLNLAEKYY
EDNSPFRPKLQAGHNVSEQEILQITKIHQAIAMIQFKLEIPIIKRRPYFNMSERLLLE

QIDYDKNEINICGKTYLLENTCFATVNPEKPEQLLEEERQVMEKLLFSVQHSEKLARH
MNFLMKKGSLYLKYNGNLLIHGCIPLDEEGNMEEMQIEDKTYAGRQLLDVFEEYLRYA
YAHPEETEDLATDMVWYIWTGECSSLFGKREMTTFERYFIQDKEAHKERKNPYYHLRE
NEEICRKILLEFDLNPDHGHVINGHTPVKESRGENPVKANGKMIVIDGGFSKAYQSTT
GIAGYTLLYNSFGMQLVAHQKFNSKEDVLCNGTDVLSVKRLVDKELARKLVRETNVGE
KLLQKISNLKDLLEYRYMK"

CDS complement(3359408..3360658)

/locus_tag="PIKHDJKN_03020"

/inference="ab initio prediction:Prodigal:002006"

/codon_start=1

/transl_table=11

/product="hypothetical protein"

/translation="MNRHEKTMNAPSLFRNRFLQTILLSSVLLQVGIWVRNFAILLYV
ADRTNNDPYAISLISVAEFAPIFVFSFIGGTFADRWRPKRTMIWCDLLSAVSVFVVLL
TIHYGSWHSVYLVAFISAILSQFSQPSSMRLFKYHVPEEQLQQGMALFQSLMAIFMVL
GPMLGTFVYSTFGLEISIAVMGLVFLLSALVLIRLPEDHMQTQTVPVKGQFLKEFIEG
FRYVWQSQVLRMLGLAFILAGLAVGVAQALNLFIVTERLGRSEEFLQYLLMVNGAAML
IGGGIVAVFAKRVPPQILLAIGMIAGAVCTAIVGYSTSVSLTLTVQFLNGLVFPCIHI
GISTMILKWSEASIVGRVNGVLNPMFVGMMVVSMSFAGALKGAFSLGTIYGGAGLLFL
LGALVMVPIMNHKAPDNTHIAQET"

CDS complement(3361001..3361147)

/locus_tag="PIKHDJKN_03021"

/inference="ab initio prediction:Prodigal:002006"

/codon_start=1

/transl_table=11

/product="hypothetical protein"

/translation="MVKKKISKALSAALIVSMLATGVIVAIQGNTTLEVAKSAGDKKV
EVIK"

CDS complement(3361815..3362468)

/locus_tag="PIKHDJKN_03022"

/inference="ab initio prediction:Prodigal:002006"

/codon_start=1

/transl_table=11

/product="hypothetical protein"

/translation="MSTHYYFSWFNNFFPEKLVHCLHEDIIDRKSLVMISADPSEQIN

FDDVSEWTWLNQANIIFDKYHFIDYRMQKEEARRFIQNASVIFLCGGYPVLQNDFLTE

YELSDVIKNSNAVIIGASAGALNMAAKWLSLNNTDEVATSTIYNGIGFDQFAYESHSQ

RDFATFVQGYLFPLSEEIDVYAAEQESAVRVKDGKIEIVGPVYLISHSKIQKLVETL"

CDS complement(3362709..3363194)

/gene="pchR_3"

/locus_tag="PIKHDJKN_03023"

/inference="ab initio prediction:Prodigal:002006"

/inference="similar to AA sequence:UniProtKB:P40762"

/codon_start=1

/transl_table=11

/product="HTH-type transcriptional regulator PchR"

/translation="MQPMDHSKQWIFKSFIHLMSQQDVRESKLSVRMSEELHKFEKEF

GEKVNLTLSEIHLIACIGDFSPINVTSISEKTGLTKGSITRISKKLLKLALIKRQQIN

DNKKEVYYSLTSKGQKVYVIHHRIHQEIEERFMNFLDKYTADELVFASKFIQDLKEWD

Y"

CDS complement(3363543..3365042)

/gene="oxyS"

/locus_tag="PIKHDJKN_03024"

/EC_number="1.14.13.234"

/inference="ab initio prediction:Prodigal:002006"

/inference="similar to AA sequence:UniProtKB:L8EUQ6"

/codon_start=1

/transl_table=11

/product="12-dehydrotetracycline

5-monooxygenase/anhydrotetracycline 6-monooxygenase"

/translation="MNYDVIIIGGGPVGMMLAGELALANVKVCILEKLEDTTPYSRAL

SIHPRTLEMLDLRGLKTELLKIGKPLPTGHFAGLETRLDFTVIDSSSNYSLFIAQSET

EKELERRAKELGAEIRRGTEVSSISPNSEYVEVVATGTEGEMTLTAAYVVGADGAGSI

VRKQANIPFNGTDETLTSVQGDVVLKRPPATSVVSSFDENGMIMIVPVSKELHRVVFI

DPDRKSIPKDTPVTLEELRSGMLRMLGDDYGISDPYWMTRFGNATRQADRYREGRLFL

AGDAAHIHFPAGGQGMNVGLQEAMNLGWKLAAEVKGWAPEGLLDSYHEERFPVNSALL

KNTRVQTLLFGNDFSSTTIDLRRLISNLLEIPEANYRLASQIAAVDVGYKVLDTVSEH

PLNGRRLAEIKLRKDNGDIFSSYELFRDGKYILLNLVNDQLVEGIIRRLPNQHFTCVS

AILAEPALDWNNVHTAMIRPDGYIAWAISDQDSKLEQCIKEGITSISKG"

CDS complement(3365103..3365264)

/locus_tag="PIKHDJKN_03025"

/inference="ab initio prediction:Prodigal:002006"

/codon_start=1

/transl_table=11

/product="hypothetical protein"

/translation="MKRINPSTFTQTVADLISKIVITASPLAFISGQVALNEKGVLLE

KDDYLFQAK"

CDS 3365603..3366124

/locus_tag="PIKHDJKN_03026"

/inference="ab initio prediction:Prodigal:002006"

/inference="similar to AA sequence:ISfinder:ISBth10"

/codon_start=1

/transl_table=11

/product="IS3 family transposase ISBth10"

/translation="MSKFNLDLKIEAIHQYLSGNEGIKSIAKSLGINHEVLRMWIKQY

EYHGLKAFQKTYTAYSPPYKLDVLNYMNENGTSPNEAAVIFNISSPGVIRRWRSQFNE

SGIDALKPKKKGRHRMTDKNKKRLKKQEPAEGSVEALQAELERLRMENAYLKKLNALV

QSKEKSPNKTKHK"

CDS 3366160..3366963

/locus_tag="PIKHDJKN_03027"

/inference="ab initio prediction:Prodigal:002006"

/inference="similar to AA sequence:ISfinder:ISBth10"

/codon_start=1

/transl_table=11

/product="IS3 family transposase ISBth10"

/translation="MLAFAEVPRSTFYYWVKQFDKPSLDTDLKLLIQSIYEEHQGRYG

YRRIRDELVNRGHRVNHKKVQRIMKELGLKSVVRMKKYRSYKGTVGNIAPNVLGRNFQ

AEKPNEKWVTDITEMKLFGEKLYLSPVMDLFNGEIIAYTVGSRPTYSLVSEMLNKAFK

RLSNGDELLLHSDQGWHYQMKQYRQALKGQAITQSMSRKGNCYDNAVMENFFGIMKSE

FFYLHEFESVDHFKQELARYMDYYNHKRIKSKLKGMSPVQYRAHAQQVA"

CDS complement(3367136..3368110)

/locus_tag="PIKHDJKN_03028"

/inference="ab initio prediction:Prodigal:002006"

/codon_start=1

/transl_table=11

/product="hypothetical protein"

/translation="MVTAGVILALISSIIFICATSPWMLYLARAVEWIALGAFMGTSN

ALLLHHTSQQYIKRSLAYSSMATLLGFSLGPAICGVIVQYSSFSPETFPYVVLLVLLL

FALGLLFSLPPTNEKRNKQTPIRIRLGIPSKSRRLFVFFVCTAVFVMLALNGVVISLI

PTFVHTILNLNNLAWSGLLLLIFLGGGAIAQQIAWPSHTNSRIQVGIILLLIGAWFMI

TAGFTSSMFLFIVGMILLAIGNGWTFQASMQLAGSLGETFERPTIISTYYLAGYTGMA

IPTIGVGSLSTIVGLLPALIIFGTIVTLIGAGIIAVPKISERKKVSSM"

CDS complement(3368172..3368342)

/locus_tag="PIKHDJKN_03029"

/inference="ab initio prediction:Prodigal:002006"

/codon_start=1

/transl_table=11

/product="hypothetical protein"

/translation="MEALHDLVEAGKVRYIGASSMLAWQFAKAACREVQWLGTFRFHR

KEIIFSFPIITS"

CDS 3368537..3369862

/locus_tag="PIKHDJKN_03030"

/inference="ab initio prediction:Prodigal:002006"

/codon_start=1

/transl_table=11

/product="hypothetical protein"

/translation="MNVLTKSIIGLLCVTTLAGTYYSYREHQNNRQWEQQQQRNTQYW

SHMQSIYLREFDYALAESLAAPTFTVRQEALQSAIESANDILQYVRSASATMNFPSWS

SYESFITDGSRYLAYPAREKGISLNPDQLEQIQRLRTFARTALPYLDQMRKKAVDSSL

SDIQQLSTEMEEALSQLDSVSTHGNKAFNEFLYNQNPYIPPKPGTVFAGETTYTATEL

AAKAKIFMGEAWNKGAGKQIVHMASGGSSPQFGEVMDFKLSDTDGKPTSSYTATLSKS

GHVLQVKRKGSISEKGAVEQSMDLKQVIAVADTWMARWSDERLTLVAKEIKETSIYLT

YIPVREQVSIPQMKVVWQFDRRTGALKSFDASNYYSHYNKTFPIHPKLTAEQASAKLG

PKVNASGKPKLELHNDKLVYAFPVTGIEGVSHVYVNARTGAGEGIDYNL"

CDS complement(3370532..3370762)

/locus_tag="PIKHDJKN_03031"

/inference="ab initio prediction:Prodigal:002006"

/codon_start=1

/transl_table=11

/product="hypothetical protein"

/translation="MWAHINLEWACCIHFHRGVECIVLEEALLDFWNVSGRWVGVMIS

GCDITPEELRCKAQLRTATHADGIYIAGVLDS"

CDS 3370926..3371300

/locus_tag="PIKHDJKN_03032"

/inference="ab initio prediction:Prodigal:002006"

/codon_start=1

/transl_table=11

/product="hypothetical protein"

/translation="MTFLPPFGPGGGFGGPGGPGGPGGMPGPPSFPGGGGGGPQGMQA

PTGPPPSFIPQQSVSLFAVDPRAISGCLFRYTYIWPNRGPGFWMFPVFVGRNSVAGFR

WNGFFWLYSGIDLQRISSFSCF"

CDS complement(3371683..3371781)

/locus_tag="PIKHDJKN_03033"

/inference="ab initio prediction:Prodigal:002006"

/codon_start=1

/transl_table=11

/product="hypothetical protein"

/translation="MDRRIEIEQKSIKLQKYEMRPDLILEVYQLKL"

CDS complement(3372158..3373078)

/locus_tag="PIKHDJKN_03034"

/inference="ab initio prediction:Prodigal:002006"

/codon_start=1

/transl_table=11

/product="hypothetical protein"

/translation="MSAKQDRILIFGAGVIGSMYAIKLIEAGFDVTLFAHSNRFKSLR

ENGLQYKEKDTVRSIQVNVIDTLENDDIYDFIFVTVRYDRSESALLALKDNQSKNIIT

MTSNSSGFSSWLDIVGDRLLPAFPGFGGQIKDGVLHARFLPKIIAATAFGEINGVVTE

RIENLAKLFKTAKLPYVIKKDMQAYLITHSVSDIAMLSFLHSENKIIDKKTARTRKTA

RKITVTLKAHLRAIQKAGVSIDPPMLKMVLKFPNLFLDLFFMTWLRTKMVRDMMLPDY

ANNANNEIVQLSNDLMKFLSQNDIKSEIHD"

CDS 3373228..3373779

/locus_tag="PIKHDJKN_03035"

/inference="ab initio prediction:Prodigal:002006"

/codon_start=1

/transl_table=11

/product="hypothetical protein"

/translation="MKKQPQITEKTRQQFIEVFCELYSQKPIEKISVQEIANKSGYNR

STFYQYFTDIYELLDSVENDLLNDMKKEMANKELSMNMVQDTLYCLDNREHLLVLNAL

LGDYGSARFLKRLKKEITLHQLELNVPQNHSLTPYFIEFYLTTSLSLFRLWIQRQKDL

SSEEFFKLVENLYSRGITPYSKE"

CDS 3374366..3374941

/locus_tag="PIKHDJKN_03036"

/inference="ab initio prediction:Prodigal:002006"

/codon_start=1

/transl_table=11

/product="hypothetical protein"

/translation="MRDAAEGQQKHGQQKHIETMPLFSTMDRNGRMTMLRSVRSTARS

GADTRDQHFPRSSGHGEHCRGAAACRDRCNRWSVLASGRAVRTDQGRRPFRDTCDRRR

AGYRGGSPPALDADKQSGAALRHRSHSDVADDPARARRHRRSWLRPYGGYHARTVTRL

NRPRRATSGGHPAPPLGGFVIRRDIDCRELH"

CDS complement(3375398..3375601)

/locus_tag="PIKHDJKN_03037"

/inference="ab initio prediction:Prodigal:002006"

/codon_start=1

/transl_table=11

/product="hypothetical protein"

/translation="MKNRNVTGIVVAIIYCVVLYVFLTDAPPGEAPNNPFWVYLMIPL

GSIVITSLFDYVIKFDFFRKKKK"

CDS 3375941..3376675

/gene="phoP_4"

/locus_tag="PIKHDJKN_03038"

/inference="ab initio prediction:Prodigal:002006"

/inference="similar to AA sequence:UniProtKB:P13792"

/codon_start=1

/transl_table=11

/product="Alkaline phosphatase synthesis transcriptional

regulatory protein PhoP"

/db_xref="COG:COG0745"

/translation="MLEVFCYAECKHLDMGDRALMPKILIVDDEADLRKLLTDYFEIN

GYSVMTAKDSREALRKVEQQPDLVLLDINMPEQNGLQLCEKIRDFVSCPILFLTARIE

DADKIAGFRAGGDDYILKPFSIHELGARVEAHMRREQRIQSKSSVRFNEDLVIDYTAR

ELYYREKRISLAKKEFDIIELLSTHPGMVFEKERMYETIWGMDNQGDSSVIAEHIRRI

RSKLKEYSCDNRIETVWGVGYKWPSS"

CDS 3376660..3377760

/gene="sasA_16"

/locus_tag="PIKHDJKN_03039"

/EC_number="2.7.-.-"

/inference="ab initio prediction:Prodigal:002006"

/inference="protein motif:HAMAP:MF_01837"

/codon_start=1

/transl_table=11

/product="Adaptive-response sensory-kinase SasA"

/translation="MAKLMNRLLLKRFTLLQSFILLCVVTLIAVLVVITIEFSWAENL

RLRFGETEWVLPSLVAAVLLTVAAGIVTMAVLFYRWKLKRPLELLKQASESISANDLS

FRISYDSRDEMGELIAVFENMRSQLEKNVKTLWRSVEERKQLNAIFAHDLRTPLSVLK

GNAELLATYLPEKKLSEEKVLDLIHTMNLHILRLESYAEAMNSIQRLEDVLLNFKSMD

TISLTTLLDSSGEQIARQFSIRFVSSLKYDNASIHVHVDPYLVMQIFENVVANGVRYA

SSQVNVCYDLQEGTMKITVSDDGPGFSDEALYKAVLPFYRGEVWDSSEHRGLGLYICK

VFCEKHEGSLQVANGPHGGGSVTVSIGTQVDK"

CDS 3377812..3379275

/gene="flp"

/locus_tag="PIKHDJKN_03040"

/inference="ab initio prediction:Prodigal:002006"

/inference="similar to AA sequence:UniProtKB:Q7A3Q5"

/codon_start=1

/transl_table=11

/product="Protein flp"

/translation="MLKLRKRMYITISCLLLLSTMWGTLVSSNEARDRTEVLQEIDEY

MNRSMKANHIKAASLAIANNDEVFYAKGYGTFADGQNVTGDTPFPIASLSKSFTALAV

LQLADKGMIDLDATYASYFPELSPQDPRVLDITIRHLLNQTSGLNDKINPDMTRNPQF

QSLNETNRLLNKVQLAHNPGTAYSYHNPNYVLLANLVESVSGEFFSDYLKKHIFEPLG

MNHTFSVTTTQQIYENKTIPLGHYLSLGRSIGQSEPLWFIDGPAGIVSTAEDMTLWML

AQYHGSLLSPALMKQYHTAGAIGPYGIGWLADQDESGRRTISHSGIFWTYKSEEMVYL

DDQLGIAVMFNSGLNAFVNYSAFIDGIANIMRGEKPTISFLNGRNMEIIMIALILATL

IWGVHAYFRIRRRNKRLTISKLILITVGRLIPILVLLSLSPLVTFIGGGRVLPWFGIW

TTLSSPIIWLVVWSLVNFVHLACYYYIYVENKKNGYA"

CDS complement(3379405..3385788)

/locus_tag="PIKHDJKN_03041"

/inference="ab initio prediction:Prodigal:002006"

/codon_start=1

/transl_table=11

/product="hypothetical protein"

/translation="MDRRTVSIQKSMFVSLLALLMVISLVIPPAPVRAQSSSSLPGRQ

AEYLNRGLVAVLVDNGVFLSWRYLNNDPDDIAFNIYKNGTKVNASPISDVTNYVDTTG

FDSSQYQISTIVAGKEQMLPEVVSVWHNNYLPIPLDKPADGRTKDGGTYTYYAGDASV

ADLDGDGEYEIVFLWSPSNSKDNSQAGYTGNVYIDAIKLDGTKLWRIDLGVNIRAGAH

YTQLMVYDLDGNGKAEVVVKTADGTKDGQGTVIGDGTKDYRNESGYILSGPEYLTLFD

GETGAAVSTVNYDPPRGNVSDWGDGYGNRLDRFLGAIAYLDGTKPSVVMSRGYYTRTV

LVAYDYVGGELVKRWTFDTNEAGSQYQSQGNHNLSVLDVDNDGKDEIMFGALAIDDDG

SLMYSTGLGHGDAMHAGQLDPNREGYQVVSVHEHSDAAYGLEMRDAATGEILWGEKTG

FDTGRGMSADIDPNYPGYESWATTITNGQSVPVTNTYSAAGEAIYTVDEGPKTANFAI

WWDGDLQRELFDHEWDNSTAKGIPLIYKWDYENKKLNEIFRATGTLTNNHTKGNPALQ

ADILGDWREEILLRSEDSSEYRLYTTTIPTDYRIPTLMQDPIYRLGVAWQNVAYNQPP

HSGFYLGAEATAFPKADLALTREAEMPEPVYRFDFGTETASGNTAIQDTLYTQETGYG

FKDTTGVTVGENEVSVATGTTFAVDLPNANYKMTLRLGNDAHDSNVGVKSEFVQKLAV

TNVKAGEPLEYSYDIALVDGQLDFLFTGTAIDIQDILIEKYPEKAAGAATTIYMAGDS

TMQSYSEMQAPQEGWGQQFGRYFSNGAVIVNDAIGGRSSKSFMVDGRLDTILQRIKPG

DFFFISFGHNDASVGIPDRYASPEDYKIYLARYVNGAKQRGATPVLLTPVGRRDFNTV

TQEFNVSFPAYVQAAKEVAEELDIPLIDLSKLSIAYYDKIGKTATEKVFLYANPGEYP

KYPNGINDNTHFSSYGAQKIAGLVAGAVKDMELSISSLVIDPDISEPEPEPEIQLYEE

NFEGDAADYQYAMVNATGIAGTMTGTVVEQSGNKVLSVAGSGSGNRAKVFRLFDAVNG

DQVKVNFDWQSGNVSAYPSEGHLTVQDSNENALFTLYTKTGSTKIHYFVGAYNPDYGT

GDTAIPEGGTATEISKNQWVDVDATVNFAKKTLDLTLTNLADPTVTQTISDIPLSSGV

YADNVRAIRFLGTRKGGGGTLNWTTQIDNVRIEGSQLPSESGDQAALIALYDEIKALD

LSEYTEASKAVVNRALAAAEAIFNTEATQAQVDHAFNKLTVAKDSLTSEPGEEISEYQ

FDFGSGGATEGYTKVDAKRAYIEGNGYGFADTASTKDENRETGDALKEDFTRVNGTSF

LVEMEPANYRVTMTIGDAEESTSANVVVEQMTKLPLTTIAKGEFKEISYDLALIDGVF

NFDFTGNAPKINALKLERLPDNSASDEPVIYLASDSTVANYAENYRPQAGWGEMLGRY

FDQNEVSIDNRAVGGLSSKTFLVGGYLNDILLDIHEGDYLFMQWSHNDSTPSRPERYL

TPEQFKVYLKEYIDGALQRGAIPVLVTPVNRRDFTGEVLNKSFPEYVQAMKETAQETE

TLIIDLNQASWEHFQELGTEGTKSIFMWVGTTEDNTHLQMNGAIKVAELVARLVKDLN

IPLSDFVTLEGESPDQEAPHTTATVEGESQNDWYTTPVQVTFTATDEDSEIEGTYYKI

NGGETVSGTQLSLTEEGTHTITYWSVDVDGNKESEQSLSISIDLVPPTIEIHGQTEYT

IDQHVEIGYTASDTISGVAEPTGVLVDIPAYTLEPGLNQVTATVSDLAGWEQTIEYSF

GVIATFESLINLTNSFADESIDPNAGVLAGQLANTLQKANQAANDREGANARQLLSSY

GNDVQAARGTVFTDEQATVLLKWEEWLKQATPLANGAPGTPVLSDNNGYDTGLKDGDY

TVTMNLWWGNNGNQFKLYENGELIKEISLVDQSPSAQSVKVDITGKKNGTYIYTGELI

NALGTTTSVPLTVIVTDASPGQSVLSNDNWDGDGNFNVTMNLWWGTNATEYELYENGV

LIDTQSLQAHTPSAQSAVTAISGRVPGIYEYEAVLRNSSGETKSSKLNVTVRE"

CDS 3385993..3386865

/gene="araC_4"

/locus_tag="PIKHDJKN_03042"

/inference="ab initio prediction:Prodigal:002006"

/inference="similar to AA sequence:UniProtKB:P0A9E0"

/codon_start=1

/transl_table=11

/product="Arabinose operon regulatory protein"

/translation="MVHTVSYAFRNDDTSIMTLDSIGWQIVSSEEYRCPSDNRPDPGH

VVFQYTLNGQGYLDIENQTIPLPKGHALLVKISGEHCYYYKQENNEPWEFIWINIRGD

EANRIWDMIHDNEGHVILRNADSPLIQELWQIINLIHQEKVTDKYRLSMQVYRWLLIL

VQTSRDAEKDIGALSITTIEKCKKFIRENYASPLTLDLLASHCDINKHYLCRLFQKSE

KTSPLAYLKDRRIEVAIRLLRTTELPISLIGQQCGFESPSYFGKVFRQYMSMSPKEYR

MNKLEFPYEAIYYE"

CDS complement(3387486..3387956)

/locus_tag="PIKHDJKN_03043"

/inference="ab initio prediction:Prodigal:002006"

/codon_start=1

/transl_table=11

/product="hypothetical protein"

/translation="MAIMTDSRGNIFLEFINIEYDKITTTTLDAPLTHALIVVKYQGK

YLFMHNKWRKNWELPGGIIETGENAQQCVIRELFEETNQNIDTAEFKGLMKFRLQPSF

HGPERTEYGALFVGELYQLDDFVENDEASSIILWDGTSEIGDIAEIDRKLIEFV"

CDS complement(3388271..3388549)

/locus_tag="PIKHDJKN_03044"

/inference="ab initio prediction:Prodigal:002006"

/codon_start=1

/transl_table=11

/product="hypothetical protein"

/translation="MNTFNGGARLNKYVLHIVASVICILTPAVGLLYGLWDSHQPKIG

PVGDGKPNYPTIPQWISIVSSFICGIVNLPLAIIRYRQNIKPNKDIKS"

CDS complement(3388726..3388962)

/locus_tag="PIKHDJKN_03045"

/inference="ab initio prediction:Prodigal:002006"

/codon_start=1

/transl_table=11

/product="hypothetical protein"

/translation="MYSYDEFTEDLNLGHEIEFILNDTHFLISYNESGWYCIKENEEA

QIKYESVEQLLNQVKIKGKTLKSLWGNIIVSDVF"

CDS complement(3389086..3389523)

/locus_tag="PIKHDJKN_03046"

/inference="ab initio prediction:Prodigal:002006"

/codon_start=1

/transl_table=11

/product="hypothetical protein"

/translation="MKNTKLKRIKLGDLYAIPLPNGKFAFGRRFKDANIAIYNYMGNT

FEDKPQQENYQFIVGVYDDVLKSGDWPVVENRPFVNEAEAWPPPACVIDKLTGEHSIY

YMGEIKPADIKECEGLEIAAVWEAQHIVDRIMGNNTWHKDLSK"

CDS complement(3389723..3389950)

/locus_tag="PIKHDJKN_03047"

/inference="ab initio prediction:Prodigal:002006"

/codon_start=1

/transl_table=11

/product="hypothetical protein"

/translation="MKIDTVDNPGWSVEIDLTDTYLEDVPFESIEEERNEEDWFYCVV

RDGVFHFAGGVTNLEEILDSFKTWASSLERS"

CDS complement(3390023..3391765)

/gene="ettA_1"

/locus_tag="PIKHDJKN_03048"

/inference="ab initio prediction:Prodigal:002006"

/inference="similar to AA sequence:UniProtKB:P0A9W3"

/codon_start=1

/transl_table=11

/product="Energy-dependent translational throttle protein

EttA"

/db_xref="COG:COG0488"

/translation="MIKVDNLSFSFPQKELYNNISFTFEEAQHCAFIGTSGSGKSTLI

DILMDPERYLFEGKLEIDPDCRIGYVSQFSQVDKTKEMTVFEYIGEEFIKIQDEISAL

YAEMATTSDMDSLLEKVQLALDAFEAMDGDNFENTINKQLNLANLMKLKDLNVSELSG

GEFKLIQVMKEMLSRPDLMIMDEPDVFLDFENLNALKKLINSHKGMLLVVTHNRYLLN

HCFNKIIHLENTEIQEFDGRYIDYNFSLLQTKIELQEIAVAEAEEIERYDHIIDNLRE

IATYNSEASRGRALKARVKFQERLEARRIKEPFVDIKQPNISFGIDKELEDTTVINVN

NYSVSFDELLLENVNFEIKSTDKVALIGPNGTGKTTLLREIFKNNQDSIEINADVVNV

AYLSQVQGEMLKDSNTILDEFIEAGFETYDEIRSFISNYGFEGEILDQKIESLSGGEK

NMLQLAKVSASQANVLLLDEPTSHLDIYTQIALEKAIEDYKGAILMISHDFYSVVNGM

DYVLIIEDKTISKMSILEFRQMIYASHFNEDYLETEQKKKSVEMKIELALKDTNFELA

KVLVDELEELIKLL"

CDS complement(3392425..3393204)

/locus_tag="PIKHDJKN_03049"

/inference="ab initio prediction:Prodigal:002006"

/codon_start=1

/transl_table=11

/product="hypothetical protein"

/translation="MTSKNQNELVRKAVAVLDSFESGNPEAITAHVHPDQYIQHNQGL

SDGRAAMLGALDHLKEIGTKVSIKRALVDGDYVVLHSVYEFFGPKIIIDIFRFKDGLI

VEHWDNMQEMEGETPSNHTMIDGPVTIKDLDKTDANKEFVKSYVENILLGKNSGLLAS

YFDGDNYIQHSPHIADGLSGLHVALQALKEKNIKFEYTHVHQVIGQGDFVLTVSEGLL

DGQPTAFYDLFRVEIGKIAEHWDVVEAILPPEKRKNSNSRF"

CDS 3393397..3394002

/locus_tag="PIKHDJKN_03050"

/inference="ab initio prediction:Prodigal:002006"

/codon_start=1

/transl_table=11

/product="hypothetical protein"

/translation="MNKKTDLRILRTKQSVRKAFYELIQDKGYEAITIQDIADRAMIN

RNTFYLHYQNKPDLLDTCINELLSELKDAVILCPISMNPFSISILETVMQTVLEHISL
NTTFYNAMLIEENRIYQFRAKMENIIKDKLNEGWSPIQGQSHLVISKELLLEYLVSAF
MGIVIWWIKNDQPLPADEAASQFSRIVTYGHLKAAGIHLEE"

CDS 3394433..3399472

/locus_tag="PIKHDJKN_03051"

/inference="ab initio prediction:Prodigal:002006"

/codon_start=1

/transl_table=11

/product="hypothetical protein"

/translation="MAFIERYSINENGAITFTGNTLGLSRSGTPGVPGTVDSIGAYIT
TDTTQQFGTYPPGTTSVFLNNSSAAVLQIPAGSTVLYAELIWGGTYIDGTENNSAFIN
NPVLFTTPLGQTSVLPDTATRFEVILSTQAGFPTTYAYVRSAEVTNLVSAAGAGAYTT
GGVVGTLSIPDPTSNHAGWTLGVVYQNPALPLRNMSIRVTADVILSTSGPVNMVIDGF
ATPFMGPLAGRALFSSQEGDAIKTGDQARFDPTIGTVTNLSGPNNFATNFFASQINND
AGLLDTSGTFGTRNQINGTPGTNIIGGRQGWDITNVDVGFTLHNTQTQATFQLTTNGD
GYLVDGNGLQIDINTPVLTAVKSANVADAIVGDTVTYSIVISNTSLVDATNVMFFDNI
PDSTTFVMNSITIDGVPQAALDPITGAPIGILVSGASVTVSFQVVVTSLPAPPRLFNQ
AKVGYTAPTVPGGPVINTAVPTNAIEIPVFQPNVAIVKSAETSNVQVGENVTYTLVVT
NTGNISATTTVLDNIPDGSVFVPGSVTINSVVQPGADPVTGIPIGPVAPNTVFTVTFQ
VNVVSQPAGFQLVNQGTASYTYQPPDGRVIPGSVVSNTVIIPVTLPFVSVVKSTPVLD
AVVGDTVTYSIVVTNQDTIPVTDILLTDAIPPGSVFAVGSVTVDGVSQPTSDPMTGIA
VGTLLGGASSTITFSVNVTSLPEPAELTDQAQVTFTSGTFSGASFSDVVTIPVFQAII
GITKTAVPTFAVVGQIISFQFIVNNTGNIDATTFLTDIIPAETAFVPGTVLVDNISIP
AADPNSGFSLGIVPAGGSVLVSFQIVINSLPESFQIINQATTSFTYTPPDGRVIPGAA
DSNVVVIPISADNVTVIKSANELQAIVGDIVTYTLVVQNNDAIDLTSVILTDPVAPAS
VFVTDSVTIDGVSIPGADPDTGIAIGTITANGTVTITFQVTVVGTAPPVPFPLFLTDQ
ATLVFSTGDFTGVALSNIVNVRVFEPIINLVKSADTLAAIVGDIIAYTVVVSNLGNYN
ADVVLLDPLPAGETFVTNSVLINGNRWSSADPITGINLGTVQPQFPVTVTFLATVTSL
LDPPELVNQATGNFTYILPDGRVIPGTSQSNIVTIPVSDPGTITVTKLSNQTIVTSGN
IVTYTFSVLNGSSAIATNVILTDPIPDGSTFVPDSLTLNGVVQIDADPSDGFLIGSLA
GNSSNPLTFQVSVDNLSVQPVPNTLINQANLTFILNGLGGSTSSNLVEIPILAPVITI
LKSASPAEVDLGDTVQYVLSVQNSGNTSANVTLVDPIPLEAVFIPDSLTVNGVPIISD

PVDGIPIGTLPPGDITTVAFAVNILNFPANNQVVNEAIATYTYTLPDGSVTNNSVNSN

SVVTIISDPLVDIIKTASPTQVAPNQIIDFSFTLVNGNNLSLSDVLFFDQIPQGTTFV

SGSVTSRGVNIPGANPATGVGIENVEAGATISIRFQVTVNSGITLTQITNQASVEYRL

GSIPFRVDSNIVIIPVTAITPTVPIIPSGPVPSTPPVASEAGSGVSQTSSAPAIPVQK

TANKTVVEVGETINYAILIQNTSPYTATQLVLKDKVQSEAHIVSGSIHAPNLPQTGTN

LEQGLILNQLLPGESIQIHYQVLVKGQPANSKLVNQASLSIVYVIPGAGTATITLASN

TTVVTIVESEE"

CDS complement(3399477..3400322)

/locus_tag="PIKHDJKN_03052"

/inference="ab initio prediction:Prodigal:002006"

/codon_start=1

/transl_table=11

/product="hypothetical protein"

/translation="MGSGINKFKDCIGIDRIRYEMTDIVHDYNLPIPLKNDTVDFIMC

SHSLQYVDNLILVMKDIYRICRHKAIVCIVAPYAHASVHMVNPQFKQQFNEHSPKYWT

TYPGRMEDDSELSLYPSVAFPVEEDLSQNRIDFRMLKMEFFYFPAYHGLYNEVELGLL

RQSQFNVAHQIMYHLLVVKEPMPEAEIAGLQGTVRLEEPDYVMEQRIRTTEKEEQEQS

FNLDHFRSLSSGEEESLNKSVEEKPPILKKSSKGGKPKSSRTKTRAKSNRKTSLGKKK

VGTKR"

CDS complement(3400682..3401266)

/locus_tag="PIKHDJKN_03053"

/inference="ab initio prediction:Prodigal:002006"

/codon_start=1

/transl_table=11

/product="hypothetical protein"

/translation="MKTNPRFIFTYRQMGGLLFILKHIRQLDLNDINNKYRNGGDRGH

TKTYTYKANSGLQELAYTTGTGASKVNFEYTGLEQVKSIAKDNQSLILYQYNDTGAMQ

QVDRANGIITKATYDQGDQLLTYSDYFGTGSALREHNYTYDSNGNVKIIQTDAGCTAY

LYDTLDQLTQETLPDGRTIRYSYDEQGRRIQSIG"

CDS 3401348..3403111

/locus_tag="PIKHDJKN_03054"

/inference="ab initio prediction:Prodigal:002006"

/codon_start=1

/transl_table=11

/product="hypothetical protein"

/translation="MRQTRKKKMDLRFKLYLIILVPLLAMGGLIMWTTIDSSENASLM

TMQHNNQQLAVNTASQLGQESELIKTLSSTASEESNEYKSLRDELVKVRLQSGALYVY

MYNKTSDGWIYTVDGADWDDREYSPYGTAMEFNSQIQERLLRGEVVTTGIVDDPTWGQ

LLSSFTPIKDSQGTVIGYLGIDISAGAVNQVSATSLNNAYRTIIPIFVVVLILSLVMM

LFVVRGILRQVHDIKSSLEQVADGNLKVVSTHMTNDQLGDISDLINVMVAQLTNILMG

IQQGSNTLQQSSKNIAETAQTNQRQTEELSKAIEEIAIGSVKQAEETEQSVQHSENLG

KIMDEVGSYVQQFTHTSEQLSAVQVQVTREHEVLLEKSRENAKRVEHQQKISSSLTTQ

SELASSISGQIHNILKQTQILSLNASIEAARAGEAGRGFAVVAGEMGQLAQQSERSIQ

EIDEILSSFVQEIHRMGSHFDANMVAVKEQEEQIADCLQTFRQVSRLSTEVQELAQRL

ESRTVDMHSIREEVEQHLSYIASATEETSAMAEEVTASAVEQQRSANELSNISGQLAS

LAGNLKSYSDQFQIEVRKSDS"

CDS complement(3403059..3403298)

/locus_tag="PIKHDJKN_03055"

/inference="ab initio prediction:Prodigal:002006"

/codon_start=1

/transl_table=11

/product="hypothetical protein"

/translation="MIRSKIHNGNKSEYTHDPLKLHWLNALIRWEIRTEWYRFKYISQ

LEKVKTPPSDSTGNGVFIIMNLIYGLRSEIGRNMI"

CDS complement(3403432..3404934)

/locus_tag="PIKHDJKN_03056"

/inference="ab initio prediction:Prodigal:002006"

/codon_start=1

/transl_table=11

/product="hypothetical protein"

/translation="MRLFELLLFLSNIGLFVLTFLLKKGRRRIPVFVASGIATLLLVI

HWMVEGYRVQLFFPYFITIIFLAILSYSYFKKNGQKKIPRFMLGSAYTAIATTLVVTA

GLMYAFPVFKLPEPTGEFKVGTQTFHLVDTNREEIFAKARSGKRELMVQVWYPVQAAT

DKYTSFIPNPQILRYMAASYGLPGFTFQHLKYVSSHAYSGAEVSSAQTSYPLILANPG

FSSSRFLHTSQAENLASHGYIVAVIDHTYNTFATEFPDGRITTSTTNDLFSPDDDYRT

GRENRDKLGKVLTGDVVFALDQFELIQSGQIPSHLKGRIDLGHVGVFGHSIGGATAYD

ASYDPRITVGIDLDGGLYRLRDREDLRKPFLFINSESYFEELKMVMNNRVYTDAELNR

MGSTREWMDQVTEDKKLELERMRETFDEGGQILYIENTEHLNFTDVQFISPVFNMLGI

TGKIAPERANSVINAYMLDFFDMYLKNQGGALMKGPDSRFPEVKFITSLL"

CDS complement(3405370..3405606)

/locus_tag="PIKHDJKN_03057"

/inference="ab initio prediction:Prodigal:002006"

/codon_start=1

/transl_table=11

/product="hypothetical protein"

/translation="MYSYDEFTEDLNLGHEIEFILNDTHFLISYNESGWYCIKENEEA

QIKYESVEQLLNEFKINGKTLKSLWGNIIVSDIC"

CDS complement(3406074..3406388)

/gene="trxA_1"

/locus_tag="PIKHDJKN_03058"

/inference="ab initio prediction:Prodigal:002006"

/inference="similar to AA sequence:UniProtKB:P14949"

/codon_start=1

/transl_table=11

/product="Thioredoxin"

/db_xref="COG:COG0526"

/translation="MAIQHAKDSTFKEMVQTEGITVVNFWAPWCGPCRMFAPVLEEFE

KEANDSIKVVKVNIDENPETSSQFQIMSIPATIIFKDGKPLYKEIGILPKGAMQQLVA

SE"

CDS complement(3407522..3408106)

/locus_tag="PIKHDJKN_03059"

/inference="ab initio prediction:Prodigal:002006"

/codon_start=1

/transl_table=11

/product="hypothetical protein"

/translation="MLASNLALTGTYAWFASMTLSLYTVLYGVTVWISAARGMSALEA

GLLILPMSVLSDLVVGPISKRNLVRGPLIVVAGSSIVDSIGVFFLKTRTPMIWIVLIT

LIFGIMKGTMSSGNQTALYMQVSEDQIGTASGFLRTFGYIGSIASSALIGIVFHTKIT

DSGLHHIAIIMIVVSIIALILTIADRRLKTTSSS"

CDS complement(3408322..3408597)

/locus_tag="PIKHDJKN_03060"

/inference="ab initio prediction:Prodigal:002006"

/codon_start=1

/transl_table=11

/product="hypothetical protein"

/translation="MPTENPYSWRFVVPLYMGSTLNPINSSMIATTSADRCIYTDFSG

SNHNTSDGSISSDQLAAWLWIRGDGEQRFSLTSLFTHHVCHDHVLDS"

CDS complement(3409108..3409722)

/locus_tag="PIKHDJKN_03061"

/inference="ab initio prediction:Prodigal:002006"

/codon_start=1

/transl_table=11

/product="hypothetical protein"

/translation="MRMYSKNNRILIGVVFIVCVVGGVTVFQSTYAKSTTDQSHTNSP

NYPENEQGQTYGSAAEATSVEMLPNLIYAGSVNGIKGYLLKKDYLGEPGDVPLYDVYG

KTIIGSFHISPNIINYPKNKNGQTYGSSADATSPETEPELISAIGEDGTAGYVLKIDL

DGELPKSPEEAIAIQNSRSPDGRDIPMFDVDGATVIGVFHVGGK"

CDS complement(3410135..3410647)

/gene="sttH"

/locus_tag="PIKHDJKN_03062"

/EC_number="3.5.2.19"

/inference="ab initio prediction:Prodigal:002006"

/inference="similar to AA sequence:UniProtKB:C5NU54"

/codon_start=1

/transl_table=11

/product="Streptothricin hydrolase"

/translation="MIIDIQAGPFENPLFIHHGEKLLTNIKQLITWAHISKIPVILTQ

HNGKPGTTTSKLSPGWSIHPTLPLIADDVKIEKNYPDSFQETNLDSHLTRLGVNQLVI

AGIQTEICVDATCRRAFSMGYDVILIKDGHSSADTSILKADQIISHHNTVLQDWFVSL

SDTQNIIMSF"

CDS complement(3411372..3412550)

/gene="pbuE_4"

/locus_tag="PIKHDJKN_03063"

/inference="ab initio prediction:Prodigal:002006"

/inference="similar to AA sequence:UniProtKB:Q797E3"

/codon_start=1

/transl_table=11

/product="Purine efflux pump PbuE"

/db_xref="COG:COG2814"

/translation="MKNYKWIIYLLALGATVVGTAESIITGILDMVAADINVTIGLAG

QLVAMYSIAYAVGTPILIAITSKIDRKKLLFIALLIFIIGNVIAVLSPNFIILMISRA

ILGVSAGVFAVVALGIAAQIAPPEKRGSAIATILMGTSLALVVGLPLGTLIGEYMGWR

WIFAILGIITIIPIIAIVKAIPKMSGQETIPLSKQLAILKDFKVISGLLASLFFITGY

SVVSTYLAPLLRENAGLNSNTISLVLLGMGLMAVVGARVGGYGADKWGIAPTLIVSLL

IHVIALFFLPFAAVTFVGAMFILALWMLGAWTTAPAQQFYLVTMAPKSTEIILSLNSS

AFHLGVALGAALGGVVVERLTLSSIGWVGGLVVILATVFAVISILMGKKTASGVLESE

"

CDS complement(3412734..3413306)

/gene="betI_1"

/locus_tag="PIKHDJKN_03064"

/inference="ab initio prediction:Prodigal:002006"

/inference="protein motif:HAMAP:MF_00768"

/codon_start=1

/transl_table=11

/product="HTH-type transcriptional regulator BetI"

/translation="MKTKSENKVNLIYQATIELLNESGLSEISISKIAKRAKISSSII

YFYFESKEDMLIKLYFLLKDQMHQLMFHGVTESTPVKVGFENILRNYSNYILNNKDSF

LLMEQFIDSPFIRKKCKDHNGGVFKPMYPLFERGIKEGYFKDLETNLLVTYSCLPFVE

MGKEYLNGAYEFSPINIEKMIQMSWDAIKV"

CDS complement(3413602..3414087)

/locus_tag="PIKHDJKN_03065"

/inference="ab initio prediction:Prodigal:002006"

/codon_start=1

/transl_table=11

/product="hypothetical protein"

/translation="MITPFVGIGPFRLGMSREEVEQIFENLNHWRADDGISVNPSYIE

MLFMREHFEYDSNARVKFIQLINPRYSGQFYIPCLYQGVDVFTTKVEDLIHSLRQEYS

MVGDDDPRTGFSFAIQDLELSFWREGIMNDEIMEDPFFLEMSEENQEIEKHISILWIT

I"

CDS complement(3414335..3414808)

/locus_tag="PIKHDJKN_03066"

/inference="ab initio prediction:Prodigal:002006"

/codon_start=1

/transl_table=11

/product="hypothetical protein"

/translation="MAEKKRVKVKLGDVFAIRLNQDRYCYGQVVSEGRISDCMIVYDL

VSMEHLTVSEITSKPIIFLIQTVNTRIEDGIWNVIGNGPIPSMTFPLYKVETEDGYML

VDHKGDVVNEDPSASEIEEVPKLESWSPVSLEKAVNARFVTGEWDPYYNELIYTN"

CDS complement(3415380..3416351)

/gene="yajO_5"

/locus_tag="PIKHDJKN_03067"

/EC_number="1.1.-.-"

/inference="ab initio prediction:Prodigal:002006"

/inference="similar to AA sequence:UniProtKB:P77735"

/codon_start=1

/transl_table=11

/product="1-deoxyxylulose-5-phosphate synthase YajO"

/db_xref="COG:COG0667"

/translation="MEYVKLGQSGLDVSKLSLGTMSFGVPERGNTPWSLDEEDSRAII

KEAIEMGINFFSTANMYSDGTSEEFLGRALKDFARRDETVIATKVFVPMRKGPNAMGL

SRKAIMTEIDNSLKRLGTDYVDLYQIHRWDPNTPIEETMEALHDLVKAGKVRYIGASS

MLAWQFAKAQHVAERNGWTRFVSMENRLNLLYREEEREMLPLCKDEGVGITPYLPLAA

GRLTRDWNEQTSRSENDQIAKTLFIKTEEADRKVAERVAEVAANRGIPRAQVALAWLL

QKEEVTAPIIGSTKISHLEDAVSALSVKLTPEEITSLEELYIPHPLV"

CDS complement(3416435..3417184)

/gene="fabG_6"

/locus_tag="PIKHDJKN_03068"

/EC_number="1.1.1.100"

/inference="ab initio prediction:Prodigal:002006"

/inference="similar to AA sequence:UniProtKB:Q9KQH7"

/codon_start=1

/transl_table=11

/product="3-oxoacyl-[acyl-carrier-protein] reductase FabG"

/translation="MHKPVALVTGANKGIGLQIAKDLASQGFTVLIGSRNLEKGEAAA

KSIGEDARAIQLDVTNQDSISAAAARIRSELGRLDVLVNNAGISHAGLPGRPLEEVGR

SGRPSVASLDEIRTVFETNVFGVIAVTQAMLPLLHEVAVARIVNVSSGSSSLTLNADP

ANSHRVMFGAVYSPSKTALNAITLAFAIELESTGIKVNAVCPGFIATDLNNFEGTGTV

QQAARHPVRLAMLGEDGPTGTFSNEKRQLPW"

CDS 3417313..3417810

/locus_tag="PIKHDJKN_03069"

/inference="ab initio prediction:Prodigal:002006"

/codon_start=1

/transl_table=11

/product="hypothetical protein"

/translation="MCQPWYYIRMDINELNEDEMRLWNTWKGSFKRVFGRVVKEMSEY

TGLSEGDFGVLDRLVQFGDGKLRQQELADSMDWDKSRLSHHLTRMEKRGLVLRKPLDT

DRGVQVIITSVGKSALDEALPIVSKAIRKYFLDLLTDQDIKSITELAERTKKSDISPS

NDSSP"

CDS complement(3418199..3418495)

/locus_tag="PIKHDJKN_03070"

/inference="ab initio prediction:Prodigal:002006"

/codon_start=1

/transl_table=11

/product="hypothetical protein"

/translation="MNTLKWLQNWYYENCNGNWEHSYGVKIDTVDNPGWSVEINLAET

YLEHTPFDSIEEERSDEDWFYCNVRDGVFHGAGGATNLEEILIHFKNWASSIKR"

CDS complement(3418660..3419775)

/locus_tag="PIKHDJKN_03071"

/inference="ab initio prediction:Prodigal:002006"

/codon_start=1

/transl_table=11

/product="hypothetical protein"

/translation="MKSSPKESYVSHDIRLTRYAIVSTAVTLLSDEQLRERVEDATGL

DAGIGGATALMQIEETPIFVKIIPLTEKERLTENTMSTENVFKLPPYCQYGIGSPGFS

VWREVAAHTMTTNWVLANHCASFPLMYHWRVLKSPERRTPISDELSDVSRMVEFWDTS

KMRDRIEALEQSEYSVVLFCEYIPYNLHDWLSEQIAIGESKAASELSMVDLNLRSAVS

FMNANKLHHFDLHFKNVLTDGNRVYISDFGLATSSRFELSDSELNFLEANKTHDGCYV

VTTLVNWLVTALVGMVNRTERIDYIRRCTERDELPEMMDSAAKIIMRYAPIAVVMNDF

YTNLVSMSRTTIFPVEEIERICTKTGFDPFYSDAKLV"

CDS complement(3419930..3420112)

/locus_tag="PIKHDJKN_03072"

/inference="ab initio prediction:Prodigal:002006"

/codon_start=1

/transl_table=11

/product="hypothetical protein"

/translation="MMLKGILLTIEKQKQSINPDEMTIEVSKYRANEGSISLDMKLAY

QQGQWNVVEYAMIRES"

CDS complement(3420605..3422107)

/gene="guaB_1"

/locus_tag="PIKHDJKN_03073"

/EC_number="1.1.1.205"

/inference="ab initio prediction:Prodigal:002006"

/inference="similar to AA sequence:UniProtKB:P21879"

/codon_start=1

/transl_table=11

/product="Inosine-5'-monophosphate dehydrogenase"

/db_xref="COG:COG0516"

/translation="MAVYLNEVSRTFGEYLLIPGYSSVECIPANVSLQTPLVKFKKGN

KPPMSLKIPMASAIMQSVSDDTMAIYLAKEGGLSFIYGSQSIESQAQMVARVKSYRAG

FVVSDSNISPEATLDELLMLKKKTNHSTIAVTIDGSSRGELVGIVTSRDYRVSRMTLD

TKVKDFMTPFEELICGNKDTTLKEANNIIWEHKLNCLPIIEDEQRLISMVFRKDYNTN

KKNENELIDESKRYMVGAGINTRDYAERIPALLQAGVDVLCIDSSEGFTEWQKIVLEY

VRKNYGDTVKIGAGNIVDKEGFRFLAEAGADFVKVGIGGGAICITREQKGIGRGQATA

LIEVAKARDEYFNETGIYVPICSDGGTVHDYHITLALAMGADFLMLGRYFARFDESPT

KKVNINGNYMKEYWGEGSTRAKNWQRYDLGGDEKLSFEEGVDSFVPYAGSLEDNVGLS

LSIVRSTMCNCGYLTISELQKNAKITLVSSTSIVEGSAHDVLLKDSRQIL"

CDS complement(3422109..3422570)

/locus_tag="PIKHDJKN_03074"

/inference="ab initio prediction:Prodigal:002006"

/codon_start=1

/transl_table=11

/product="hypothetical protein"

/translation="MEFPIEKKITPKAYVYGSIFTLSNRLQLLGDRRYKDLSLKQAFV

LWNISLYEDYFLNLKDIAQIVGTSSQNVKKIIDILEKRGFATVGKDETDRRSLRVALT

SKGKKYYLERGKEEEQYIEELFTDFDDEMLMGLYQGLYKLMEKTAQEQGGK"

CDS complement(3422813..3423448)

/gene="rhaS_20"

/locus_tag="PIKHDJKN_03075"

/inference="ab initio prediction:Prodigal:002006"

/inference="protein motif:HAMAP:MF_01534"

/codon_start=1

/transl_table=11

/product="HTH-type transcriptional activator RhaS"

/translation="MPSIIREEAVPKEKVKMNKNYPVYVGDTIGVTIPYQKLHWHNVL

EINYIKSGTGYYIINGQKFDFQQGDVLLINSNDLHCAYETKDLVMTVITFDSTLFIHN

LRFDPELFSPFREIDKHYMNLIHRDHPAMDRLRSLLLELQVEHEREQRSYATVVYSLI

LQFLTIVNRECRIEGICGSQPIISERQYEKIRQVIMVMIELKYRVNNTGDI"

CDS complement(3423736..3424506)

/locus_tag="PIKHDJKN_03076"

/EC_number="3.2.1.73"

/inference="ab initio prediction:Prodigal:002006"

/inference="similar to AA sequence:UniProtKB:P23904"

/codon_start=1

/transl_table=11

/product="Beta-glucanase"

/translation="MKKLMDFRKKMSAVLMASGLICAMLLPIQASAADIAFNEPLNGV

NPNLFYTSDGWANGPDFGVGWKAVNQEFVNGIMALRLDNTGCPASCSGKNYASAEYST

HVKYGYGRVEARLKAASGVGLVTSLFTYSGQGTGTSNDEIDIEILGKDTTKMETNYFT

NGVGQHSTIINLGFDASLDFHNYAFEWSPSSIKWYVDGKLVHTENGSRGTLPTSPGYI

MVNLWSGSGPAEIWTGKFNYPGMPVRAYFDWIKFKPAN"

CDS complement(3425035..3425925)

/locus_tag="PIKHDJKN_03077"

/inference="ab initio prediction:Prodigal:002006"

/codon_start=1

/transl_table=11

/product="hypothetical protein"

/translation="MTMTFRKLSELSIEENVRIWNLGFEGYFIQMVMTIEALMTRIVN

EGLSLENSLAIYVDGEPAGFVMNGFREVDGKKLAWNGGTGIASAFRGQGIGRALMLRN

LELYREHGVEIAILEALTQNESAIKLYQYVGYEITDRLMILQHTGTIDPQLLKRSVEH

PFSITKGLPRDVYALDFYRGLSAWQTQWPSMKEGESIIIRQDGEPVCYALYKRTFDQE

DKLVTISLYQCEVLPGREDAEEIYKLALSELFAPLDYVCRRGTSNLRKSNQSLNDLLI

KLGFTTNVEQVFMMRNMDLN"

CDS complement(3426135..3426470)

/locus_tag="PIKHDJKN_03078"

/inference="ab initio prediction:Prodigal:002006"

/codon_start=1

/transl_table=11

/product="hypothetical protein"

/translation="MEFKFGDAAILLPPLHITIIAIIIILFLIRWSKQLEIRRFTVFF

YFLISTFITPIYSRATTEGVFQLWIPLGFIVVFFYLFRSKRNHPSKMKASILGLSIAI

YQLILQYFG"

CDS complement(3426620..3427192)

/locus_tag="PIKHDJKN_03079"

/inference="ab initio prediction:Prodigal:002006"

/codon_start=1

/transl_table=11

/product="hypothetical protein"

/translation="MYHINRDKRSIQSSEWIFEALLALTDEMDYEKISVTDIVNRAKV

GRSTFYRNFDQKDDVFKFKCNQTCLRLREYLTDYQTQYDVPPGPASSKPFFTFWSSNT

LLIEALIKINRIDILSDSYVEVLRQYYDDYTYLLDGIPSDYVEYVMAANVGSSIGVLV

QWIKNNKRESPEDIAEIVMHQLLSNYNRLI"

CDS 3427311..3428510

/locus_tag="PIKHDJKN_03080"

/inference="ab initio prediction:Prodigal:002006"

/codon_start=1

/transl_table=11

/product="hypothetical protein"

/translation="MSELEVKKNKNLIIWFWAPFMVFALITALLLFTRESRLLEWVLF

AVVGTIAIFMRKPLVRLRTLFKFASWILTSAFLGLIVILTGPILVNRSVWNDEMESMP

TEPVETQDGLISGLYNEDKSVRAFAAIPYATPPVGELRWKAPQPVQPWKGVRELKRFP

NAAMQKKTPSILTNFINLRLGTSAFMDNFSIKHNEKISEDSLYLNVWSGAKSDQEKQP

VLVYIHGGAFKNGSGSMDLYNGENMAKKGVIFVTINYRLGIFGFMAHPELTEESDYGA

SGNYGILDQIAALKWVKRNIAAFGGDPDNVTIAGESAGAGSVNILTASPLAKGLFNKA

IAESGAFFDMERERTGGIYTQSWPKPNFTEMNSRSRLIKLLLAIYGRCPQRTYFVLLQ

TRRSPQL"

CDS 3428453..3429187

/gene="fumD"

/locus_tag="PIKHDJKN_03081"

/EC_number="3.1.1.87"

/inference="ab initio prediction:Prodigal:002006"

/inference="similar to AA sequence:UniProtKB:D2D3B6"

/codon_start=1

/transl_table=11

/product="Fumonisin B1 esterase"

/translation="MSAADLLRASADETIAPAIDGYVLPNTVYNIFETGKQNDVRTLI

GSNSDEGTLLTMPWPIYAIKDVDEFQASVRKRFGDKADEVLELYRSDSDNESKQAQVD

LSTDKLFTWQMHTWAQLQSTTGKSNVYYYIFDKGQPGPSRFETLGAYHSGEIVYAYNN

LDKVNLPYTPEDYELADRMSSYWVNFAKSGDPNDDKLPFWAPYDAETDQVMTLGTKLG

MIEMPRRTYMNFFDDYESELRSSFKE"

CDS complement(3429557..3430126)

/gene="COQ5_3"

/locus_tag="PIKHDJKN_03082"

/EC_number="2.1.1.163"

/inference="ab initio prediction:Prodigal:002006"

/inference="protein motif:HAMAP:MF_01813"

/codon_start=1

/transl_table=11

/product="2-methoxy-6-polyprenyl-1,4-benzoquinol

methylase, mitochondrial"

/translation="MNPKLQRKIEHLENIERFPAEELFEYIQLNKEDELLDLGAGTGY

ISFSFAKYVKKVVAFDYDNDILKYLEVKAKEKGITNIETTAANFKEMPLENERFEKAV

ASISLHEVQPLSLVLDEIHRVLKDKGIFLCIELEKSKMSEMSNAPRVSSKEMEGEIVK

AGFSVIDMFHSPTQIANQPVYIIIAQKNK"

CDS 3430650..3431135

/locus_tag="PIKHDJKN_03083"

/inference="ab initio prediction:Prodigal:002006"

/codon_start=1

/transl_table=11

/product="hypothetical protein"

/translation="MKKVFLSCLAMLMFLISFSPVTSAASGDRLIYDTMYNVAGGGLV

SNNIRFSFQFDNYGDAQIWDVKSRKMIWNSKTKDTRIHKLQINYFSGKLVISDPQNTP

YWTSDNLAWAKTYYGADNVPANLRGNVLIMQNDGNLVLYNNENPALGWYPVWASDTGG

H"

CDS complement(3431406..3432278)

/locus_tag="PIKHDJKN_03084"

/inference="ab initio prediction:Prodigal:002006"

/codon_start=1

/transl_table=11

/product="hypothetical protein"

/translation="MNTLARLNEGLSYIEENLMDSVDMKEVARIACCSEYHFTRMFSF

LSGITLSEYIRRRRLTLAALELSRNDSKVIDVALKFGYTSPDSFARAFQSMHGVTPSE

AKNHGPSLKAFPRMTFQLTIQGGSEMNYRIEDKEAFRIVGISKRVPIVFNGVNPDIAA

MYQHLTPELIETIKGLSNVQPLGIISASTHFSEERMEGKGELDHYIGAATTQEAPEGL

AQLEVPASTWAVFTAVGPFPSTLQDVWGRIYSEWFPSTDYELSEGPEILWNEHKDVTS

LQYRSEIWIPIRKR"

CDS complement(3432601..3437673)

/locus_tag="PIKHDJKN_03085"

/inference="ab initio prediction:Prodigal:002006"

/codon_start=1

/transl_table=11

/product="hypothetical protein"

/translation="MSCMVKRKLFFLIVFMLTIGVPSSLIGIRAAYAETLPYQTIIID

DGSIKINDVVTPDAGNENNGYSAPNWTTSTGVKGYDLSSTKYTSTAGRTITWNPRLEA

GTAKISFYKLDWADNADSNVKIEIVHNGTTDVVFMDLRPSSGAPAGWVDLGEYYFSGV

GEEFVKLTRSTGTTNTILTRADAVKFEGNIQQKEPHKTIIIDDGSLTIDNVVTVDSGN

VNNGFSAPYWTTSAGVKGYNNSSSKYTDAVGRTITWNPRLEAGTVRISFYKLDWADKA

DSNVKIEIVHNGITDVKFMDLRPSSGPSVGWVDLGEYEFSGDDSEFVRLTRTQPTTGT

IITRADAVKFEGNIRQQAPPLPPLRSRTLANLSYTEKGSIENANYKATFYEAAWDGGK

SIVRDMFYKNTDTGNWMPINNVSERLEEQWVLLDGNAGSRTNYYDTMNKHWITFDGVH

FPDSHTAVLTDSTHGSDYDFEVNWSMAGEKPDVSFAFTPRRDGNYVIGYQSFTTEAVS

GINEVLNGFRSHAKMVGTVESTSLRELSAPMSLVEKNDGSGNPLTYGVFVPSAELPVE

FEPTGGVTKQRLGMSLVNNEGSVQPILYAPQLGTYSQMTAESTYQFHMGLIAQKSNLY

ESYADILRNEYGYSAYRENVSDQSLTDAMFNMIDLLKIEPQGDDSVNYVPSPSGWWSR

AKGFIDIENEDSVRTSSNAVLLGAYYLTGDDQLYDTRALPSVQYGVSRNGIGWSPTQK

KVYGVPSLWKMATLPFDVSSVAAVNQMMGTSAGIGALAQEEYLMRDPDQKDRGPVIQP

LMMYRMTGESQYLQAAKDAADSYIMQYIDTPASVDVSKNEFIYYYSKLWMEILELYEE

TQDPKYLNAAYKEAKRYATMFVARPVPEGNVTIPQPETYNYAESFHWPESGKFQYPRL

KLPEDIAGGVQADSWLVSPNGLTFEAGSTTGYYRMNAQEAPFMLRLAQYTGDKLLQDI

AHNAVIGRYSSYPGYYYKGFAVSQLEPDFPLEGPSGATSIYYHHIPGQLGQTMDYLIS

EQSLKSNGSITFPSVFETNFLWFKYHLYGNKPGQFYGNSDVWLWMPKGIIKTNHPQLN

WITGESGDKFYIGLSNASSEEVQTPIELNEQIIGFNPAQDYTVTIIRDNGTPEQAVMS

GGIIQATVSGKGITAIIVEGLDIDVPLHQVRTADTSDASYFFDIHSPIDAVKGMLMVK

PDETAYDAYVQAKTTKPATIHYSLDGGATYTTIPDTIYPMEWSIRVNDLSQTFTYYVE

SEGKQTRKRTLYLPDQVTETPVQPDGQDSSSIIVDNTEAETEGVWIRDTTADNYYYDN

YVYAKSTAGAATSKMRWRPELPESVTYSVYYKIPQITAASENWATNASFTVYYSGGSE

TVTVDETTANGTWVHLGDYPFAAGDSGYVELTNKANKSRVVADAIMWVNPNRIPQLES

AVILSDRNELQMTQTAQLSVTGYLDNGLIGDLTQANVQYFVDRTDLAEVDSSGLLTLL

NLDGDTDHIEVWATVTIDGVTLTTPPLNITIKDLTVIVDSTNTTGLYTTEGSWSQSNL

AGYKIGVKSRYSTVQGSSATWKGQFPEGKYTVSIYKLVHTTANDNNVKVEVKHKTGTE

VTYIDATVGSSGWVNLGTFDFTGDGSEYVRLTRVTPTTVDPPTLPADMIYTRVDAVMF

ERHSDGSELLANEAPGEPTLSE"

CDS complement(3437927..3439135)

/locus_tag="PIKHDJKN_03086"

/inference="ab initio prediction:Prodigal:002006"

/codon_start=1

/transl_table=11

/product="hypothetical protein"

/translation="MKEFFPRRGLPNVIQKLENGETVTIVYFGGSNTRSEGYRVMTAD

WLRGQYPNADIRSVNAGIDGTGSDLGCARLETDVLRHQPDLVFVEFVGNDGGVPESKA

RIEGIVRQIRKRSRFTDILFVYTVKERDLTSFQSGQYQKGARMQEEVADYYDIPSIHL

GVAVSQLVLDGKLIFTLRADVSIPGAVIFTHDSIHPTIPEGHQIYTDTITRSFEKMSE

LRDHVGKVEHHLPQDPLVPANPWEYATMLPLDSLTHFSAGWSYMIPDDFALVREYEWL

FPGLWRAVDPGETITVEFEGTHIGLFDIGGPDSGRLKVTVDGGEPFLIDRFTSYNDHN

RNQYVFLPELPNGKHTVCFEIDHKKTDKAAVFEASGNERSMEHVRQHPAWYDQTVIQL

GKLLLVQPPL"

CDS complement(3439153..3441384)

/locus_tag="PIKHDJKN_03087"

/inference="ab initio prediction:Prodigal:002006"

/codon_start=1

/transl_table=11

/product="hypothetical protein"

/translation="MEVIKMELMKADVTVVGGGIAGICAAIAAARQGLQVSLINDRPV

LGGNASSEVRVHINGSAYLGNSPSYYAREGGLVEELKLKIFHYNPLYNKKLMLSLSDT

VLLDMVYDEPNISLFLNTCVHETGMENGRIQWVEGLQLASERKFRFESPTYIDCSGDG

IVGYQAGADFRWGREAKHEYKEELAPEVADHYTMGDTILFQARDVNYSVPYKRPGFAY

DITKLEFFDSIRKGLNHRSFPRKINGLGGLWWLEYGGHMDIIKNNEDIALELRKLVYG

IWDYIKNSGEFDDVDNLILDYVCPIPGKRESRRFIGEHMLSQNDLTAKSHFEDAVSVG

GWYMDLHANKGIYDEGPATAWNFVPGLYNIPFRSLFSRNIPNLMFAGRNISATHVAFG

STRVMATCGCMGQAVGTAAALCVKYDTDPADIVKAHMGELQTQLLRDGQTIVGLQEQL

DSYFADGLTIRASSQRSYEHLHPTEEISLEKALCLVLPIQTSVAESVQIKIKNRSEHS

ETLQVKLFGGERKENYIPASELKGYNLVISAGHDDWITLDLSCKKPADDKIYIVLEGT

ASLAVYGNEEKMTGAVSFYYRPEEPSRLKKFSRSICFKDLIPSQDMYNPANVVNGFSR

PYGLPNGWISERTEGQEWLEFGFASPRNLEEIHLVFNSQLNLEHFDDPIEPLIQDYDV

ILTLEDGTESEINIRDNYLSLNKHQVHEKGVTRIRFDFCATYGSPYYEVFAVKFFAPK

NAK"

CDS complement(3441472..3443094)

/gene="cheB_7"

/locus_tag="PIKHDJKN_03088"

/EC_number="3.5.1.44"

/inference="ab initio prediction:Prodigal:002006"

/inference="protein motif:HAMAP:MF_00099"

/codon_start=1

/transl_table=11

/product="Protein-glutamate

methylesterase/protein-glutamine glutaminase"

/translation="MVRDYTCFIVDDEDLIIQRLELFFNELSHRDKRFVLVGKANNGL

NGIEEIIKLKPDIVISDIVMPRMDGISMIEQLKAELPHTQYILLTAYSSFEYAQRAIQ

ANVLEYIVKVPLREADLNRALDKAAGILNEFKKKEAEFQSLNVSVLENKYRVRKQFFN

ELIRGEIPSHRASDFANRMQFHFFQANYCCFIVEMNTYESFRNEYAATDQNILKYAIT

NIIEETVMNGGSGVAADLSDNRFIGFLSWENNRSDMETEYACLSLGGQIVSHLHQYLN

QRVSVAFGGTHRGWESIKQAYTEAKNVSEDFYYHSEKVVKTPMHRFQYHNDKKADFQQ
KLADFLIRMKRKISKEELENALADLSQFVSDHKIHKSIMAPMIRDLYRDITVKFKSGN
KMATEVPDFPMEFMAFQEQLAYIGDFTFEYVQAGQLLHRAEIMSAMHFIEKNLKQRLT
LEAIAEEVNLAPSYFSSLFKKTMSEGVISYINRKKIHLALELLNVRDYSLLELCEEVG
IVNEGYFCKLFKEYTGDTPKQYRIKMTRYESK"

CDS complement(3443094..3444833)

/locus_tag="PIKHDJKN_03089"
/inference="ab initio prediction:Prodigal:002006"
/codon_start=1
/transl_table=11
/product="hypothetical protein"
/translation="MRRRISLPLKLFFLVFAFVLGCIILISQLSYRYVQKEIRTNDIF
YTNQILDKVDQYFTVNFSSFQTILFSVETSVKANIDNAEVIKKQLRELYELNSNYVSN
IYLIKCDLSILGGSTATRIFDEPLSEREPLFDAADKNRRTTFVSEPYKSKYSGWTVTM
VRYLNGAPFPMAIAVDLDLNAIEETLFKINKQEQMNLALITASGKIIAGFSENKGLLN
IQDHTFSIGETSAEQILDTTETSLQLHTMDGIPVSLLKKPTEKFNWTIISINDESRLK
AALSRLETYYIELLAAGLLLSLIISFLVAKYIRKPLYTLKTKMKQVEQGLLTTTITVN
RNDEFGDLSRAFDRMLQQIVELIRGAELHNELERKLEIQVLQSQINPHFLYNTLGSIS
NVIRLGQIEKVDVVIGSLISLLEYGIDDASEKVSLSQELRNVADYIEIQNVRYNRNFH
LIENIEAGLMDFPVFRMLLQPLVENSIFHGYNGGGIEGPITIHAYREDSIVIIEVVDQ
GEGIPADKIKHILISEPSEMELKRKRIGLNNIHGRIRLHYGNQFGLQIISIPKEITRI
RALFPAELQKGDA"

CDS complement(3444928..3446514)

/locus_tag="PIKHDJKN_03090"
/inference="ab initio prediction:Prodigal:002006"
/codon_start=1
/transl_table=11
/product="hypothetical protein"
/translation="MQRKKISFAILSAILGLGTLLSGCGGNEEVTSTASSNSQGQSGQ
FATKMKISMFNQGTFNAAAPVPPRDEDVQRQMLEKEMNIDLDMMIPQAGQATTKLNTL
IAGGDIPDLIFLKSRADLAQYYDQGVLADLTPYLDQFPELQKRFGNDSWEAMSYQGKT
IGVPGYDNVNGISRSFFIRNDWLKKLNMEVPTTPDELFEVMKAFTEKDPDGNGKNDTY

GFIGGMNKEGNLQTYGFDSLMWMFGVNPPSAIEIKDNEPIFLFIDPKMKEALAYINKM

MAANVVDPDWVTMNSPDLLDQKMFKGKVGFMIRDARRLEPDYQQKMKEISGEVPEWIV

IPPMKGPYGDQIVERKSFQGNSWAISAKADKDKIIRILSMLNYLFTDEEAYPNFAYGI

KGIHWDVVDGKIKNKTSELSKEMKEKYLWVDHYRMPRRGDDAEYFSFQNPKTAEAFKD

NQQYVGPTLPGNLLTPDPSDTLDADRTRFINESLVKFMTGKEPLSNWDNFLQTLDTKF

DMQKYKETAIKQFKEAGLIK"

CDS complement(3446580..3447449)

/gene="dasC_5"

/locus_tag="PIKHDJKN_03091"

/inference="ab initio prediction:Prodigal:002006"

/inference="similar to AA sequence:UniProtKB:Q9K489"

/codon_start=1

/transl_table=11

/product="Diacetylchitobiose uptake system permease

protein DasC"

/db_xref="COG:COG0395"

/translation="MINLTIGEKVWQSVVYVILIFLSLLCLLPFLYVIAVSVTPESEV

LRRGIVIIPESFTFLAYKEVFISHGIWQAYKITLFRTIVGTMLNVFFTVLAAYPLSKK

YLPGRSPFLLFIVFTMMFSGGLIPTYLLIRSLGLLNSPWVLIIPNLISAFNLVIIKGF

FEQLPGEIEESARVDGASELQSLWRIILPLSLPVISTISLFYAVGHWNSYFDAIVYIN

DSNFMPLQVVLRNILLNVATQSAESLANSGAVSTFAVQMATVVVTTIPILIVYPFLQK

HFTKGVLLGSVKG"

CDS complement(3447463..3448410)

/gene="yteP_24"

/locus_tag="PIKHDJKN_03092"

/inference="ab initio prediction:Prodigal:002006"

/inference="similar to AA sequence:UniProtKB:C0SPB3"

/codon_start=1

/transl_table=11

/product="putative multiple-sugar transport system

permease YteP"

/db_xref="COG:COG4209"

/translation="MQAKLTADAIPLSKKRKSWIRTIQKYKVMYALLLPALIYFAVFK

YIPMAGILIAFKNYNLALGVWDSPWVGFKNFTDFMNGVYFWDIMKNTIVISLYKLLFG

FSAPILLALLLNEVHTHWFKKIVQTITYLPHFLSWVIVYGLMVALLAPGDGLFNMILK

ESGFQPISFLTEPAWGRLLVIASEIWKDIGWGAILYLAALAGIDPSLYEAARMDGASK

WRQLWHITLPGIRGVIILMLILKLSHILDAGFDQIFMFANTFNQEKIDIIDTWVYREG

LERLKIGLATAVGLFKAVIGFGLVLAANKLAKKFDGQIW"

CDS complement(3449010..3449780)

/locus_tag="PIKHDJKN_03093"

/inference="ab initio prediction:Prodigal:002006"

/codon_start=1

/transl_table=11

/product="hypothetical protein"

/translation="MIALNRSNFPADDRFIQNHLKDGWLRAYKVKDLTDYGSLRHSLE

EIKGKEQRIDILFNNAGGGLSELRYSKQGCEMHYELLTVVPYIILMELKELLKNGSLK

TVINTSSQALRFTKVFTIDNLEHPQTFRKMFGPYATSKLALSLWTQAVAPKLAKEGIK

IRSVDQGINNTLRKGKDSGLTAWFELFMRFFSSPPTHGPNLLFEGALGIHNNETGVFL

LKDRVADLKFTEHAQRVLDKISDTYNHEFLGGNVPSYL"

CDS complement(3449927..3450925)

/gene="tdh_2"

/locus_tag="PIKHDJKN_03094"

/EC_number="1.1.1.103"

/inference="ab initio prediction:Prodigal:002006"

/inference="protein motif:HAMAP:MF_00627"

/codon_start=1

/transl_table=11

/product="L-threonine 3-dehydrogenase"

/translation="MENLDNSIKDLKNQASSSITMRSIRFHDYGEPTDVLRLEEVAIP

EPGPGRIRVSVRACGLNPVDWGMCRGHFAGQLPLPRGIGLDVSGIVDAIGEGVTDVAI

GDAVLGAADFMSGSSAGASDRAIMYYWFRIPAGLDFVQAAALPMAVETAYRGIDSLGV

GSGQTILVHGAGTTVGFAAVQIALMRGARVVATAGTTYADKLRAMGAMVTSYDDGMVE

RVTELAKQPVDLALDTAPISGSLIDLIQIVNGHPQRVLTVSDFAAAAEFGVRASYGEL

HTLRYDVVGDFAQYAADGNFSIPVGKTFALDAWRSAVEISQSGRAHGKLILLPATD"

CDS complement(3451089..3451847)

/locus_tag="PIKHDJKN_03095"

/EC_number="1.1.1.119"

/inference="ab initio prediction:Prodigal:002006"

/inference="similar to AA sequence:UniProtKB:Q5FPE5"

/codon_start=1

/transl_table=11

/product="Glucose 1-dehydrogenase"

/db_xref="COG:COG1028"

/translation="MEQFSKGKFSGKKVVITGGSSGLGLATAKLLVDEGARVLITGRT

QAKLDSAREELGNNAVTVLSDAASLTDIVELADRVKAEFGMIDALFVNAGITRYVPFE

SMSEDVYDELHTINAKGPYFTVQKLAPLLNAGSGVVLTTSITNVLGIPMISAYSASKA

ALRSMTRSLARELLPRKIRVNAVSPGPIDTGILERSMPKEAAEQIGAQTKQQIPMLRF

GDPVEVAKAVSFLAFEATFTTGAEFPVDGGGSQI"

CDS 3452029..3452667

/locus_tag="PIKHDJKN_03096"

/inference="ab initio prediction:Prodigal:002006"

/codon_start=1

/transl_table=11

/product="hypothetical protein"

/translation="MRKAVSVDTYVEKIKPIIRRTKFSQLKVDDLAKYMDISKVTLYK

HFSSKDDIIEQVVDYYIEYAKNADTVVEDDSVSYLDRFQITFLQSFMCATYISDLFLQ

DLKEFYPHHFENLSVALQNRNKSLQTFFESGMEQQIFNRMNAVLFMVQDDAVLRRFIE

PSFSIQYDITLKQAIMDFYRMKQYQLLKPEYLKMIDDSAVERKIVNILQTIS"

CDS complement(3453142..3453972)

/locus_tag="PIKHDJKN_03097"

/inference="ab initio prediction:Prodigal:002006"

/codon_start=1

/transl_table=11

/product="hypothetical protein"

/translation="MRIFVTGAAGYIGTAVVRELIGAGHQVVGLTRSESGAHVLESLG

AEVARGVLEDLDLLRSCAATSDGVIHLAFNHDFSNFDASLATDLRAIEAMGEALASSG

KPLVITAHANGKTSEDAALAFIEQGVRASIVSLAPSVHGEGDKGFVPELIRIAQERGS

SAYIGDGSNRWPAIHRLDAAVLFRLAVESAPAGSRLDGVHDEGIAFSDIAAAIGRHVD

VPVVSIPPEEAQAIFGFLGMVASLDLARSSEGTKELLGWKPVQHGLLADLEQGHYFTK

"

CDS 3454070..3454483

/locus_tag="PIKHDJKN_03098"

/inference="ab initio prediction:Prodigal:002006"

/codon_start=1

/transl_table=11

/product="hypothetical protein"

/translation="MKIQELADLMGLTPHTIRFYEKEGLLDSRHIQREKNNYRNYSDE

AIERLRLIKKFQGIGCSLAELKTILQDHDTNARTNHEVIEWILQKINEIERKKEEYDH

MLVTLNWMLTYRKLLNEDPQQAEAMMAELRLKINI"

CDS complement(3454571..3455326)

/gene="COQ5_4"

/locus_tag="PIKHDJKN_03099"

/EC_number="2.1.1.163"

/inference="ab initio prediction:Prodigal:002006"

/inference="protein motif:HAMAP:MF_01813"

/codon_start=1

/transl_table=11

/product="2-methoxy-6-polyprenyl-1,4-benzoquinol

methylase, mitochondrial"

/translation="MNSRERFSSRVDSYLKYRPSYPKEAIDYLYDVVGLRANSNIADI

GSGTGIISKLLLERGSYVIAVEPNQAMREAAEQMLESNLKFQSTSGSAESTGLPDQSV

EFIVCAQAFHWFDRSAAQIEFRRILRPGGRVILIWNSRLTNGTPFREEYNHLLHTYGT

DYEKVNHKNISQTMLLSFFKEGSMHKMRFRMSQEFDFEGLKGRLLSSSYSPVPGHANY

DPMMTELRNLFDRNNQDGIVEFDYETEIFWGEV"

CDS complement(3455344..3457632)

/gene="secA_1"

/locus_tag="PIKHDJKN_03100"

/inference="ab initio prediction:Prodigal:002006"

/inference="similar to AA sequence:UniProtKB:P28366"

/codon_start=1

/transl_table=11

/product="Protein translocase subunit SecA"

/db_xref="COG:COG0653"

/translation="MNVVAKLIREFKERDTRRLLKDYRDKVELIRKRNLEAWDDQRLQ

AESLRLQEEARLGTHLDELLVDAYALVCEAAKRTLGLQPYDVQVMAAIALHERFLIEQ

HTGEGKTLSAVMPAYLNALTGEGVHVLTFNDYLANRDAEWMGPIYRYLGLTVSSVQAG

MRLLEKQEAYAKDITYVTAKEAGFDYLRDTIAFNVADTVHRPFHYVIVDEADSLLLDE

ARVPLVISGDSSASKSDGVLFAEVARQLQPAEHYDFDEFQRNVYLNDAGAAKAELLLG

CGNLYDSHNSHLLTSLNCALHVESLLKKDVDYIVRDGEIELIEEHTGRVAENRYLPDG

LQAALVAKEGLQWKAGGRILGTITIQHFISLYSGICGMTATAHASAMEFEDIYALQVV

QIPPNQPNIRIDHKHRIYTHKEAKYKALVQEISSVHRVGRPILIGTSSVEESDILAEA

LAVAGVPCQVLNAKNDAKEAEIIAKAGEIGAVTVSTNMAGRGVDIRLGGGNPAQAEVV

AELGGLYVIGTYVNESMRIDNQLRGRSGRQGDPGASVFYISLEDELMFRFGIHRLFRA

PRQDEVLDDPALRSKIEHIQRVIIGQNFDIQRELNCYSDMVEDQRRILYEERLGILKG

ERPMSPSEQRVRLFYIDEFWADHLAYVSYIRESIHLESITSRNPIDEFHTQITQAFEQ

IPAKIDHESANMLRKLGGSNDPAKWEQFGLKSPISTRTYMINDQYSQDKRSSWTGTTV

FAFWGREILRLVLWPVHRLSKY"

CDS complement(3457818..3460121)

/locus_tag="PIKHDJKN_03101"

/inference="ab initio prediction:Prodigal:002006"

/codon_start=1

/transl_table=11

/product="hypothetical protein"

/translation="MNMKKKLSIFTALAVFQAFAVGSVNAQSAPQDDTASVNTAKVES

VEVLKKEQPIAESEVKTGSSNASSSTPSTKVKESGTLTDTEATPKTDIEKPAIDGGET

TTNKTSTGSTTIQEGSTEDVETPATPAQIEQPSIDGNSAVAAGGNLTLYMNSNKMEQD

GKTYLAGQPMTVKNGVSYVAIRALVDRVGYGVKYDNKTKETIIISGDNELRFKTNSTV

YTVNGESRTMKGPAYQQKNTFMVPLTSITQALNITYKVNQSAKTVVLNLNTKPIASFV

ISQKEIFAGDKVDYVTSSSSPNGLDILDERWTGRQESFDQAGTYTISYQVQDSNGQWS

DPYSMTIQVLSPNLPPVAMFTTDKEQYKMGEKIIYIDQSTDDENSIDKTVWENNSLAF

FEPGPKTVTLTVTDNHGATNTYSKVITITNETLYSFSDFNLLFTPVGQKFTFNGGEVT

SMEKLPYTYTDEPSLLIRSNSPETVNTEGIVYKESSSGQTRFMIHHVNNTGKRVKMYV

IATNNNPNPVVFEQQNMGFAGPTPYATVAGKLSIDKWFKSIQTGADQKKEVIQPGESK

LILTELNKTPMKEGQVISLYSDAYSDYSLDYNVILVEENKDPFEALPMLPVLDRDGVH

NRGTYPNATRIITYDQQVGAKPARLPLGDNASDPNLVGTDPMAYTDASNAGNFGVLYK

ITLSNVAPRTLISFNPRGGKYSGVALVNNQVVSIAEGNVAVSNSSEQSVLYRTGAYGE

SVTILFSAAPGSNLPVNLLFTPLPSEK"

CDS complement(3460422..3461177)

/gene="rutD_7"

/locus_tag="PIKHDJKN_03102"

/EC_number="3.5.1.-"

/inference="ab initio prediction:Prodigal:002006"

/inference="protein motif:HAMAP:MF_00832"

/codon_start=1

/transl_table=11

/product="Putative aminoacrylate hydrolase RutD"

/translation="MVELFYEIEGNGVPIVMIHSGGVDSRVWRYMVPELAKSFKIITF

DGRGTGRSPAIQEPNSLVEDLRKLLEHLGLDRVTLVGHSIGGQVATDFTLSYPSMVER

LVVIAPSLTGFAYSQEFTTYIASVNAAAPDISKLVELSLAGPIYRVTMASPHRSFLSE

MHAHYMARVFTEWRSFEILWPEPPSIERLEKIAASTLFIQGTIEWPDMYKIAEHFKRV

PAIRFAEIKGADHMLVLTHAEKLANNIRQFCHR"

CDS 3461566..3461856

/locus_tag="PIKHDJKN_03103"

/inference="ab initio prediction:Prodigal:002006"

/codon_start=1

/transl_table=11

/product="hypothetical protein"

/translation="MISNTILELLHQYGNLIFYFAFSLGPFGIPIPNEITMISGAILS

HTGVINSWITYFCILAGTYFGGPFYLRFIYYMSTGFDNIPSHIWRNHKNEFQ"

CDS 3461843..3462409

/gene="olsG_1"

/locus_tag="PIKHDJKN_03104"

/EC_number="2.1.1.344"

/inference="ab initio prediction:Prodigal:002006"

/inference="similar to AA sequence:UniProtKB:L0D9B6"

/codon_start=1

/transl_table=11

/product="Ornithine lipid N-methyltransferase"

/translation="MNSNEPILFLKSFLQSPKHVGSIIPSSRFLANKMVNQAPWLEVK

AVAELGSGTGAITRYIHQQAKDWTKVLLFEMNETMRNNLKTEYPTFPCYPDASRLVES

MKQEGVEQLDCVFSGLPFFNFESELRNTLVDQIHKALKPGGLFIAFQYSLQMKKTLSE

HFIIEKIELVPLNIPPAFVYVCRKKETI"

CDS 3462500..3463180

/gene="phoP_5"

/locus_tag="PIKHDJKN_03105"

/inference="ab initio prediction:Prodigal:002006"

/inference="similar to AA sequence:UniProtKB:P13792"

/codon_start=1

/transl_table=11

/product="Alkaline phosphatase synthesis transcriptional

regulatory protein PhoP"

/db_xref="COG:COG0745"

/translation="MSTVLVVDDEPDIRDVIHVYLRNEGYQVIEAANGEEALNIIKTT

SVQLVILDVMMPIMDGIKACFKIREVSTTPIIMLSAKEEDIDKITGLTTGADDYMVKP

FNPLELLARVKAQLRRQTLIGKAEFNSLILIKDLVIDTSKHSVKLKENDIPLTPLEFS

ILVLLASHPGQVFSSEKIYETVWKEPFGYSDNTVMVHIRNLREKLEVNPREPQYIKTV

WGVGYKID"

CDS 3463173..3464273

/gene="rcsC_10"

/locus_tag="PIKHDJKN_03106"

/EC_number="2.7.13.3"

/inference="ab initio prediction:Prodigal:002006"

/inference="protein motif:HAMAP:MF_00979"

/codon_start=1

/transl_table=11

/product="Sensor histidine kinase RcsC"

/translation="MTDLSNPSHRNRKKKIQINILIRIILSTIAAAGFNNMLIFLSID

VFKAWEQAWFRNSLPYVITPIFIVIFIITFLLLTRRMVKDIIHLEQGLQFISEGNLDY

RVPVVRQDELGRVASNINHMTEQLQLQMFKERELEKSKMDMITGISHDLRTPLTSIIG

YIELLRTDSFQDKAEYDRFIQNTYNKATHLKKLLDDLFEYTRLTSVDTQLDLKKIDLY

QLLDQLLFEFEPLAQENGIHIEKEIGDAPIMACVDSDKIARAIDNLLINALKYSFKPG

TIHIRMNMRHEHITIEVENKGTPLTIEQKNKLFDRFYKVDYSRSSEGIQSGSGLGLSI

ARNIAELHQGTLTLQHTHNVFTFQLSLPSNIQ"

CDS 3464308..3464904

/gene="olsG_2"

/locus_tag="PIKHDJKN_03107"

/EC_number="2.1.1.344"

/inference="ab initio prediction:Prodigal:002006"

/inference="similar to AA sequence:UniProtKB:L0D9B6"

/codon_start=1

/transl_table=11

/product="Ornithine lipid N-methyltransferase"

/translation="MKTSEPLLFLQGFLKNPKRVGSVLPSSKFLANKIVQSVRWDEVR

TVAELGPGTGAITRLMRAHLPKSATVFLFERDPKMRSNLKKKYPEFMFHSNASYLLKR

INQEHVHQLDCIICGLPFFNFSREMRQNLLSQINTALRPGGMLVLYQHSLHMKKRLAE

LFDIEYIQFEPFSLPPVFVYICRKKEDEGHSETRELGI"

CDS complement(3465287..3465682)

/locus_tag="PIKHDJKN_03108"

/inference="ab initio prediction:Prodigal:002006"

/codon_start=1

/transl_table=11

/product="hypothetical protein"

/translation="MKNLGSMNVDVITLFVEDLQRAWAFYQEVFGVYAVYEDDVSTVF

NFGNMSINLLNISEAYGLIHPGTVASRESGSRFQFTIRVDDVDAVCKELNIRGVALLH

GPVDRPWGVRTATFTDPAGHVWEIAQQLS"

CDS complement(3466088..3467458)

/gene="mepA_2"

/locus_tag="PIKHDJKN_03109"

/inference="ab initio prediction:Prodigal:002006"

/inference="similar to AA sequence:UniProtKB:Q7A7N0"

/codon_start=1

/transl_table=11

/product="Multidrug export protein MepA"

/translation="MKNRINYRFANESIPKLIFWLATPAVIAQLINAMYSVVDRMFLG

RMEESGTLALSAIGVSFPIIMLISAFAALFGTGGAPLASIKMGEGEHKKAEELLGSCF

SMLLMASIIITTVFLLFKSPFLTLFGASHETLPFANDFLDIYLFGTLGVLISLGLNPF

IAAQGYAKTAMLTVCVGAVVNIILDAIFIFGMNLGIKGAAWATVIAQFISAIWVLGFL

TSKRAFLRLRLSNMRINLKQATSVLMLGLSPFIMLSTESLIQIVFNTSLARYGGDMYV

AAMGILGTLMQIFGLMLSSFAQGAQPIISYNYGARDFSRVKSTILYCSIICATIGLLM

WGIAIFIPQLPINMFTNDLELIVLTARLMKIFFLGTCIYGIQLAFQQVFIALGQARVS

IFIAILRKIILLIPLVLLLPNFISPKTDAVIIAEPIADLCAALTCCVLFGVTIKKLLK

DKSTKS"

CDS complement(3468730..3470028)

/locus_tag="PIKHDJKN_03110"

/inference="ab initio prediction:Prodigal:002006"

/inference="similar to AA sequence:UniProtKB:A0A2A5K5H4"

/codon_start=1

/transl_table=11

/product="Nucleobase transporter PlAzg1"

/translation="MDNWFKLKERGTTVSTEIIAGITTFFTMVYIVIVNPGILSSTGM

DFNGVFIATVLASIVATLIMGIGSNYPIVIAPGMGLNAFFAYSVVAGYGVSWQVALGA

VFIAGLLFIILSLTSFRYMLLDAIPASLKHAITAGIGLFITTVGLQNAGIIADSESNL

ITIGNLAEPMTYLTIIGLIITVVLMSYNVKGYLFIGMVVTAILAWIMGLFQMPESIVS

MPQGLTTTAFQLDLAGVFSNGLYTIIFTFLLITLFDTTGTMLGVAEQAGLLKEGKFPR

SRGALLADAVGTTTGALLGTSPTSAYIESSTGVAAGGRTGLTAVTVSVLLGLTLFFTP

IVSVISGIPAITSPALIIVGYFMISVISKINWKDLEETFPAFLIIILTPLTHSIATGI

GVGFIFYPVLKLLRGKGKDVHPIFYIFAVLFFIQLVFLDH"

CDS complement(3470475..3471275)

/locus_tag="PIKHDJKN_03111"

/inference="ab initio prediction:Prodigal:002006"

/codon_start=1

/transl_table=11

/product="hypothetical protein"

/translation="MLSHRLLIHDTIIKLLPHINTEQNLLFYSYLTQRKDKAQYICQF

FNSQLTAVLAYFSELSFPAFSFFRVEDRDIFFPELIAFTRETIKLDESAVCGTILCQR

DLELFQSFGLIKGIPQRFLTMKHQDESKLLESNIAKKINENDYSKVIDFLHTGEMRFF

TRSELENYPFLGIKEGEDFIAVGGYHFYDPQLVELGNIVTRLDYRGRGLAKRLTSELT

HLGKKNSPDVYLGVLANNLPAVHLYEGLGYQTTMELFIVNFTLSKTKI"

CDS 3471442..3471843

/locus_tag="PIKHDJKN_03112"

/inference="ab initio prediction:Prodigal:002006"

/codon_start=1

/transl_table=11

/product="hypothetical protein"

/translation="MPQDIWINLPVKDVERSTAFFNEIGFHAVSVGNERAKLSIGQTT

ILLFPESAFEKFTGSKTADTSHSAEVIFSIGAESKEEVDAFIQKVEFAGGNIFGKPSE

IDGWMYGAGFADLDGHRWNLLYMDESKMPRK"

CDS 3472279..3473646

/gene="hsrA_1"

/locus_tag="PIKHDJKN_03113"

/inference="ab initio prediction:Prodigal:002006"

/inference="similar to AA sequence:UniProtKB:P31474"

/codon_start=1

/transl_table=11

/product="putative transport protein HsrA"

/translation="MEVLHRNEVHASRFILWLSILTFFSVMNETMFNVTLPDIAAYFD

IPPSAANWTNTCFSLSFAIGIAVYGKISEHIGMRKLLLFGILIYGFGSIAGLLCHTWY

PGVLIARFFQGVGASAVPSLIMVIIVKIVEPGKQGKAFGLIGSVVAFGEGIGPVIGGA

ISDYIHWSLLFALPLLSLLALPFVLRTLPDETSERKSFDITGAILLSVGILSFALFTT

LYHWIYLTASLVLFGLLALHIRRQQAPFLEPFLFRKKRYIAGVLTGALLLGTVAGFFS

MVPYLMRAVYHMPTGLIGSAILFPGTLSVILFGMLGGMLADRRGHIAGMLSGLGMIVA
GFLIVLFYTDRAPWLITGSMILTFGGLSFVKTVISASVAKTLEPDEAGSGMGMLNFAC
FLAEGIGIAGVGGLLTKSWLNSPLIATVTSQAAVLYSNMMLIFIALVTFGGLLFALSY
KRNRQ"

CDS complement(3474076..3476196)
/locus_tag="PIKHDJKN_03114"
/inference="ab initio prediction:Prodigal:002006"
/codon_start=1
/transl_table=11
/product="hypothetical protein"
/translation="MTMIRKFLCSTLGLMLVATIFTLTSNAQSGLGTPEGTRLAPATI
HTYQPSVRYSNSANYTLKANGVVIPVVKGFSDYDYAHFSMSEGPVTYELTILNTDKVH
EYSISPKKLGIQADKIEGKTITFTTQSDEYLIVTMNNRQTRMVIAADPMETDVPASSG
DGIFNVTTAPYHVVTSGTPGSGVSTRTASIQQAIDDASQYGTLQGDGRQGIVYIPAGT
YYIGNLVLKSNTAVYMEPGATFIGTGKTVDYKEHWFKDSMGRPATWWISTAFQSENIR
IYGRGTLDGNGQALHDNKTTNGKGMINNLVVPIATSNFVMDGILIRESSGWAVVTVRS
NDLTFTNLKLFNSLGMGENDGIDICESQDVVVQNAIGISLDDPFSTKAWKEDTDIASG
KVPWPGNPEPVQNVLFEDTIAWTLCYGYKIGQGVMQDQSNIVFRNGVVYKAAVGFAIH
HKYGTGIVSDVRFESMDVEDISGSNEDNSAWMTLFTVNGGNHGVGPIRGVTVKDIMVR
DAGESFAKIKGLEGAMISDLTFENVYMPGSSTPARTLQEMNFLNKEHYSGVTIKPVQN
NEPLPLTNLALHRPAVASSNDGVENTAPYVTDGNLTTRFGSKRGVDPGWIYVDLGETK
KINEVRLYWEAAYGKSYRIQVTDDPNQEANWRDVYSTTNGKGGLERITFNEVEARYVR
MYGTARATIYGYSIWEFEVYGPAQ"

CDS complement(3476512..3479565)
/locus_tag="PIKHDJKN_03115"
/inference="ab initio prediction:Prodigal:002006"
/codon_start=1
/transl_table=11
/product="hypothetical protein"
/translation="MTIWDLLQHPPANYRTVPFWSWNDDLDKNELERQIEEMHKARIG
GFFIHARGGLTVPYMGKEWMEAIQICIEKSQELGMETWLYDENGWPSGFADGKVPALG
VAYQQKRLAYERAPFNTGNKVTIASYVQSEEELHLLTADDTRNPDFRVYYELNPYYID

TLSKKAVAAFIDSTYNAYWAEFGDQFGSVLKGIFTDEPQFGRGDLPWSLEIEDAFQNQ

HGYSLRKVLPGLFLETKDCRRIRHDYWSCVTTLFTESYAKQIGEWCEQRGWSATGHVV

DEQTLMSQATSVGDPLLFYEYLQIPGCDWLGRSVSEEPVVPKQVSSVVRQLGKKQAIT

ESYGCSGWNVSFQDLKRIGEWQFVHGMNLMCQHLQAYSLRGLRKRDYPPSLFYQQPWW

RYYYHFNDYFARLSMLLVEGARQAEVLLLHPIRTAWVELEGSNSSEVEQYHESFAQLS

RWLCQTFIEHDYGSEGIIERHGKVEGNLFFVGEAGYRVVVVPPSVTLSEMTVKLLNQF

VLQGGSLVVFEPVPFLINGQPSEEISRLVNAAVNPDWNRSALVESVSRTVSPSISIVD

KEGLPIVSDMVNIQTLKLEDSYLYYLVNSGEKCYPDVEVTLPRKGTLSLIDLESGDIR

PLNYTGAGNGVKLKLTLHPAQSYVIRLVPMEDQSQVDQVTELRVDHSKVNNQQLNELW

KIDHIDHNSLTLDTCRYRLENGEWSESQPLIFIQEKLLKIGYPVQVELEFEFQIAYDL

MITREMHLVLENPEKARIIINGSKIASHSCDWWRDVSFKKIDLSGFLQHGRNTISLTL

DFWNSPETYEAMERAQSFESEGNKLTFDTELESVYLVGHFAVKSESPYKNGEHGVIYT

DGPFVLEELPHQYVLTTDLGCQGLPFFAGSIRLMQTVAINLEDSASWLWQYGSHPDAI

VTRLSINGQEVRTFLWEPYEADISGYLQQGENLIELELTSSCRNLLGPHHHVKGEPMK

VGPDSFKNTVGWTDRDLDPDINLYQSHYAFVRFGLSTLPRIVMMT"

CDS complement(3479583..3480584)

/locus_tag="PIKHDJKN_03116"

/EC_number="2.4.1.320"

/inference="ab initio prediction:Prodigal:002006"

/inference="similar to AA sequence:UniProtKB:Q8A8Y4"

/codon_start=1

/transl_table=11

/product="1,4-beta-mannosyl-N-acetylglucosamine

phosphorylase"

/db_xref="COG:COG2152"

/translation="MNSPYIGKLASSSVIKRYLNNPILDASCVPYPTALVFNAGVTKF

NGKYVMVFRNDYGSLEQQTIEAHHTTDLGVAFSDDGIHWEVQPKPCFKLHDQEIIRAY

DPRLTVIDGRCYMCFAVDTHHGIRGGVAVTDDFEEFEILSLSTPDLRNMVLFPEKIGG

KFMRLERPFTVYSRGGVDRFDMWISESPDLRYWGNSDLLLAVEDVPFANDKIGPAAPP

IKTEQGWLTTFHAVDIDPARGKNGWETSWQKRYTAGLMLLDLDNPKKIIGMYQNPLLA

PEASYEIEGGFRNNVIFPGGMVLEDDGEVKIYYGAADTVECLATAHIDDLIQLCLKG"

CDS complement(3480617..3481444)

/gene="araQ_21"

/locus_tag="PIKHDJKN_03117"

/inference="ab initio prediction:Prodigal:002006"

/inference="similar to AA sequence:UniProtKB:P94530"

/codon_start=1

/transl_table=11

/product="L-arabinose transport system permease protein

AraQ"

/db_xref="COG:COG0395"

/translation="MRIAWNTVLWAFLLVVAFLTLFPIIITLLGSFKTNAELTAGATI

LPAIWQFTNYIEAWKQANFSTYTLNSVFVAVASTIGTLIVSSMAAYVVNRMDFIGKKL

YVSIQAFTMFVAIGAVVLRPQFELMIKLHLHNTLWGVILILISTHASAFFILLSFMNG

IPKELDEAARIDGCSPGRTFWRIIIPLLTPGLGVCALFAFRGAWNEYLIPFVFTLSKP

ELQTLTVGLANLKYGISAASQTHFMMAGACLSILPILIAYIFANKSFMQMTAGSLKG"

CDS complement(3481458..3482360)

/gene="lacF_10"

/locus_tag="PIKHDJKN_03118"

/inference="ab initio prediction:Prodigal:002006"

/inference="similar to AA sequence:UniProtKB:P29823"

/codon_start=1

/transl_table=11

/product="Lactose transport system permease protein LacF"

/translation="MNKAKNLAFSYSFLLPSYILTIVLGIYPIMWAMRYMFYDYKGFG

TAQFVGLANLERVMTDHQFWDSVANTFVYAGGKLLLSIPLALILAVILNWKIKGRNLL

RAIIFMPTIISTAVMAVVFFTIFNSYNGILNQFLLKYKLIGSAIDWLGVDYAMLTIIL

VAVWGAVGNYMLLFLAGLQNIPEDVYESSALDGANKFQQFRYITIPMLGPVMQMIIML

AIINALKGYESIMVMTDGGPIGKTNVMFLYVYKLFFPPAGSTASTQIQEFGYGSAVAF

VTAVIVGIITILYFYGSKRMSHNE"

CDS complement(3482434..3483798)

/locus_tag="PIKHDJKN_03119"

/inference="ab initio prediction:Prodigal:002006"

/codon_start=1

/transl_table=11

/product="hypothetical protein"

/translation="MKLSKKFLLTALCIVLTGSLAACSSPESTEKGTGTSAKTKITYW

TGDRHDSEFVKETIDKFNETNKDNIEVELVIKGDDFDQALDMSFQTSEPPDIIRVKEN

TIQTFYKKGFLAPIDEFLTEEIKDTFPVMEDLNSFDGKRYSLPNYGTTMRLIYNKELF

AKAGIEKPPTTLNELVEAAKKITEVGKSNGAYGFAQNFKSPSSALGRSARVIAEVSGY

GGYGYDFKTARYDYSGFKSIMEAFKQIKDDGSMLPGVESLDIDPLRAQFAEGKIGMYL

SFSAEAGVYQSQFPAKIDWAAAPVPSIDGNFNGASGFLGGQWLAISSETENKEAAWKF

LEYMYNESVLQQYQENGFGISMVPSVSAVAATPEVKGIEGFLPNKYDGVWPVYPTVPV

HGMKSDDAFVKYILSGGNLDSIINDLNTRYNAALDTAIASGDVKAEPIADFDPAELAG

KYVK"

CDS complement(3483972..3485633)

/gene="cheB_8"

/locus_tag="PIKHDJKN_03120"

/EC_number="3.5.1.44"

/inference="ab initio prediction:Prodigal:002006"

/inference="protein motif:HAMAP:MF_00099"

/codon_start=1

/transl_table=11

/product="Protein-glutamate

methylesterase/protein-glutamine glutaminase"

/translation="MINEPQRIKLCIIDDIRSVVDMISQKPPWGEHHIEVVGTALDGE

EGIRLVKETKPDIVLTDIRMPKKDGLAMTQEILVSAPSTKIVILSAYTDFSFTRQAIR

LGAFDFVKKPFSIDEIIQVVLKAKAAFEEERQGQSKAIEMEQQVRKSLPVLQQEYLSL

LVHHRTNADAAFQKWQTLGIQLAPQNFNLFIVELDRFMDKYKAQPAREVELIRFSLRN

ILEETIAAFISGGVVFSEALNRFVCLINCEDMIMAEQISEACRLNIYQFTNSTISIGI

GSCVTSIDELPDSYEQALSALAYHFYSDGNGVNSYSGISEDEKTLLNYTASTEHEFLM

ALRSGNREKCRLILEQIFNDLLQRDPLPKPQQVDNLCYELSSKICRVMLEQFPPDRVK

RLEEQWNHVKRTGVITFQIMRDTVRELCLEACSWIEQERSDESTKLIYQAKDYICANL

HLNLSLDYCAKQFNLSPGYFSNLFKKVLNISFQQFVIHQRMEKAKEMLIDDYQVQEIA

RELGYEHRRYFSEIFKKHTNMTPSEFKLYSTGKISQPLDLAHKQS"

CDS complement(3485630..3487435)

/locus_tag="PIKHDJKN_03121"

/inference="ab initio prediction:Prodigal:002006"

/codon_start=1

/transl_table=11

/product="hypothetical protein"

/translation="MRRLWADRITDKFNRLIYSSSFKQRIRLSIITMITLAIGAVGMT

TYWIASKELQKNAFETSQETVNKTTQILDDKLTGISNSIRSLTFSYAFKDMMLDVQSN

DISNYYVHLSELQYVFSQVYFNEPLIESILIATPIGDFYPISQRRSQTQSFYESSMYY

DMIENYGGKWIKGHTDKLFTGNKRAISFVTKGTQENVPNTNVFIVVNVNESGLIGLVN

KHSSNDDSDYFVIDSKGEEVIRTSWSSSHNLMKDKPFLYNVSESNQGSFFHKFDETDY

LVNYTRSTVVKDWSLFGIQQKDTLLEQMNGVKRTTLYIMLFFLLFTSLLTNKLTALLL

NPLYKLQRLMREVENNRLNVRFTSRSSDEVAQVGFQFNRMLDEINRLIDDVKLSESGK

RKAEMRALTAQMEPHFLYNTLNTIYCKSVLGENEEVNEMILSLSQMFQLGLSGGKDWN

TLQDELLHAEQYCLIQQKCYEGLFELEVTVLDESVLNCLLPKILLQPIVENSIQHGFK

DITFGGQIHLQVDRQNNLVHLIIEDNGVGMDAAMVSQAMLQPPVSKKGYALNNIVNRL

NLFYGEEARIELCSLPGKGSRTDIWIPIREGEVEP"

CDS complement(3487864..3488301)

/locus_tag="PIKHDJKN_03122"

/inference="ab initio prediction:Prodigal:002006"

/codon_start=1

/transl_table=11

/product="hypothetical protein"

/translation="MRKNVYLSGSVLTSEERRKLEQLNTIMEAHFLTTHLPHRDIETQ

SVHSKSGDEMFKFAVKAIEECDMFVAVLNNNDLNLSDAFWEIGYAYAHKKPIFCLLEG

EFNINQWGQLMLMNSSVVCHNVEELKGQLDQAQNYLKSKTKTV"

CDS complement(3488368..3489168)

/gene="ybiV"

/locus_tag="PIKHDJKN_03123"

/EC_number="3.1.3.23"

/inference="ab initio prediction:Prodigal:002006"

/inference="similar to AA sequence:UniProtKB:P75792"

/codon_start=1

/transl_table=11

/product="Sugar phosphatase YbiV"

/db_xref="COG:COG0561"

/translation="MIRLVVTDLDGTFLNNQGSFDIELFNEVYEEMQKKGITFVACTG

KQCERVEKLFGDHGKGIWILGDSAARIKKDGKVVKEFSIDRTLVLRAIEEIQGFDPQM

TIIVCASDAAYVHSSISDDMYNVVKGSYEQVIKMESFSEIDSDFIKITVFDTQGRSTM

LRKHVEQTLNGQIYIVDSEARWLDITALHTHKGETVKKLQDMLGVTPEETMSFGDGEN

DVELMAIAKYSFAVSNACENTKKAASFITKSNEENGVLTTIQKMMDLQ"

CDS complement(3489296..3489412)

/locus_tag="PIKHDJKN_03124"

/inference="ab initio prediction:Prodigal:002006"

/codon_start=1

/transl_table=11

/product="hypothetical protein"

/translation="MVYIILAAIIWIGVYATLKLSSQPIEVKPIRSINDLFE"

CDS complement(3489747..3490490)

/gene="gdhIV_2"

/locus_tag="PIKHDJKN_03125"

/EC_number="1.1.1.47"

/inference="ab initio prediction:Prodigal:002006"

/inference="similar to AA sequence:UniProtKB:P39485"

/codon_start=1

/transl_table=11

/product="Glucose 1-dehydrogenase 4"

/db_xref="COG:COG1028"

/translation="MKLHEKVAIVTGGASGIGEATVRLFAKEGAKVVIADFSEHGKEL

AAELNGEGHDTLFVKTDVTKEEDIKHMINETVEKYGKLDIMYANAGVADDETADVLSY

DKWKRTIDINLSGVFLSDKYAIEQFLKQGNGGVIVNAGSIHSFVALPKTTAYASAKGG

VKLLTQNLCTAYASKGIRVNAVCPGYIETPLLKNVDEKSKEYLTSLHPQGRLGRPEEV

AKAVLFLASDDASFVNGTTLLVDGGYTAH"

CDS 3490707..3491840

/locus_tag="PIKHDJKN_03126"

/inference="ab initio prediction:Prodigal:002006"

/codon_start=1

/transl_table=11

/product="hypothetical protein"

/translation="MTQNFHPYTEMTFQEFVLFFKTHSKQLEENTNIYLIAEANISET

HRNETEVLMRSFFQMFTYSDLDHMDNHYSSYLDTWVQSQLTILNMASLQWLQIILEKA

LLTLLIQLKHQRMMDPLMFLLSVFSYLMYSFHKWADTESGNHETEENDELKKLRLLDQ

LNQLLVSSSGASDLAFILKKCEKYFNYKRCVFYAYVPWSNQFYGVIGEELPKIQSMKG

QLTEQNLIFNSKRPIYLKNPKPYLRDEHIQLFQLSSIIFIPIAHEHQLYGWVTFDQMG

DSFDCTKGELDLLEQVGKRLGLFLSRKEKENTAKTMAIALTERESTVLELLAEGYDNK

KIASLLFLSEYTVRDYVSSLMTKLRAKNRTQVVAYAFRWGLLS"

CDS complement(3491903..3493207)

/gene="sasA_17"

/locus_tag="PIKHDJKN_03127"

/EC_number="2.7.-.-"

/inference="ab initio prediction:Prodigal:002006"

/inference="protein motif:HAMAP:MF_01837"

/codon_start=1

/transl_table=11

/product="Adaptive-response sensory-kinase SasA"

/translation="MDTKSRKSKADRTIGIDPLVLIIAVILGIFLLINEYSYLGVGLR

FSTSRFILELLSIIILGLLVARLFRYFKAPHNKRTFWDGSVLLDYFYIWRTGFRASLQ

NGRLTFGLIIMFLLIALTGICFWEAVTYYGEGWLILYLLLFVLFVVPYMLSKLRRFIA

IINGSKKISEGNIQNSIQDTAGNDSLSELAGYINNMKAGYQSALEHQMKSERLKTELI

TNVSHDLKTPLTSIINYVDLLKKENHSSETTRGYIETLERKSLRLKLLIEDLFDISKM

ASGTIELDMEYVDVATLLTQAIAESNTHMGQESLEMRERIAKFPIHAHLDGNKIWRVF

ENLISNAQKYSLPGTRIYIYLDETDDLVRFKIQNTSAYEIDFDAEELFERFKRADESR

QTEGSGLGLAIVKSIVELHGGQIKVEIHGDQFNVIVHLPKKR"

CDS complement(3493182..3493895)

/gene="srrA_1"

/locus_tag="PIKHDJKN_03128"

/inference="ab initio prediction:Prodigal:002006"

/inference="similar to AA sequence:UniProtKB:Q9L524"

/codon_start=1

/transl_table=11

/product="Transcriptional regulatory protein SrrA"

/db_xref="COG:COG0745"

/translation="MKVRNMEINKILVVDDEPEIREALIIYLKSNDVDVFTASNGLEA

LEILEHETIHLIIMDIMMPQLDGIKTTFKIREDKNIPIIMLSAKSEDSDKILGLNVGA

DDYITKPFNPLELVARVRSQLRRFTNLGSFHSIGEEIIQVKGLVLNKSSKTVEVDGAD

VRLTAKEYKILELLMENKGRVFSIEEIYERVWNEPVFASENTVAVHVRNIREKIEINP

KDPKYLKVVWGIGYKIEKK"

CDS 3494229..3494729

/locus_tag="PIKHDJKN_03129"

/inference="ab initio prediction:Prodigal:002006"

/codon_start=1

/transl_table=11

/product="hypothetical protein"

/translation="MIVHNVLDLVHQYGYLFFFLAFSLGPFGIPIPNEVTILTSAVLS

RSGFINHWAAYISILIGLITAITIAYFTGKLFGHKFKNKFSHNKHYLIAESILRKRGH

VAMCIGMFIPVVRYFMPLLIGVSGIHYTKFALITYSSALLWTITFFCLGNFFGIPILS

MLEDIQ"

CDS complement(3495316..3496524)

/gene="yycB_1"

/locus_tag="PIKHDJKN_03130"

/inference="ab initio prediction:Prodigal:002006"

/inference="similar to AA sequence:UniProtKB:P37482"

/codon_start=1

/transl_table=11

/product="putative transporter YycB"

/db_xref="COG:COG2807"

/translation="MSVLSDTKSTIGKKPLSLAWPIILGIVLIAMIMRSPLTSVGPLI

GSIRESLGLSNATAGLLTTLPLLAFALLSPFAPILARRFGVERTLLYALVLLIVGIAI

RSLAATASLFAGTALIGLSIAVCNVLIPSLVKRDFAHQVGLMTGVYSVSMNLSGAIAS

GVSIPVARDLGFGWNGALGIWGVLVLLAALFWLPQARASRGSAVAATSGASKRRSLWR

SPLAWMVTIFMGMQSLFFYVLVAWLPEIMTSRGLSEDSAGWMLSLFQTAALPVAFIVP

IVAGRMKNQRLLVIIMTILLFVSLGGLYFGGNALISLWTVLLGIGGGCAFSLAMIFLS

LRTKDAHDTVSLSGMSQSVGYLLAATGPILFGLLHDITASWNSPLLLLLVVSALLLLS

GLGASRNRTV"

CDS complement(3496521..3497213)

/gene="lldR"

/locus_tag="PIKHDJKN_03131"

/inference="ab initio prediction:Prodigal:002006"

/inference="similar to AA sequence:UniProtKB:P0ACL7"

/codon_start=1

/transl_table=11

/product="Putative L-lactate dehydrogenase operon

regulatory protein"

/db_xref="COG:COG2186"

/translation="MLQKTNRLTLVEQVALQMERQIESGKWAVGMRIPAEPELMVELN

VSRNTLREAIRALVHSGLLKTKQGDGTYVCSSSVLGVVLHKRIMRSDTMQTLEVRHAL

EREGAHLAAQRRTDDEARELLLQLERWEGAAARGDMDGIVTEDIHLHQGIIAASHNEI

LIELYNHIADASQDTIISLTRYEIEASFKEMHRQLVEAIVDQDADRAVAAVHRHIAES

KAVLEVKGDEQP"

CDS complement(3497453..3499441)

/gene="nhaK_2"

/locus_tag="PIKHDJKN_03132"

/inference="ab initio prediction:Prodigal:002006"

/inference="similar to AA sequence:UniProtKB:O32212"

/codon_start=1

/transl_table=11

/product="Sodium, potassium, lithium and rubidium/H(+)

antiporter"

/db_xref="COG:COG0025"

/translation="MELFEFILLMLVAVSLSNLVNRFIPSISVPIIQIVLGMLITLLP

LHYELKLNPELFLLLFIAPLLFNDGRHADKEALWKLKKPILLLALGLVFVTVAVMGYF

LNWLLPVIPLAAAFALAAALAPTDAVAVGALEQKVKIPHQTMQILEGESLINDASGLV

SFQFAVAAMVTGVFSFKTASISFIAISLGGVVLGLLLTLAKYGVVKWLRKLGMENVTL

HMLIEILTPFLIFMAAEELGVNGILAVVAAGIAHSFGYKKMNPEVAKLIVVSKSTWSV

IIFVLNGLVFLLLGTQLPDIIQTIWSNPEIGNFKIIMYTLLLTSAVLGLRLIWAMLMD

ISEGTKPRDPWALRLKKALILSLSGVRGTITLASTLSLPFLLNDGTAFPARDLIIFLA

AGVILWTLLASNYLLPLLLGKETQTEQEEDEIQAKIEILRNVVTGLNELTTDQNRLAT

AQIINTYTSRIRTLKKDEKKDKAQRSLGLSVLKWERENTLHALEKKEVNPYTAYRYLN

RINKLLFMHTRDARYKKEFFPVKHWDEVLGILQQVRLSFRERRAELNSLRIKNIAYVI

KQLNVLLAEGDWDMEMVSSLILQYERMLLGLTRNEPDTGTRKEFGISMQELARVGIQL

ERDNIQSMFETGRISRQGVKEMKRDILFMEHDLREETS"

CDS complement(3499714..3500427)

/locus_tag="PIKHDJKN_03133"

/EC_number="3.2.1.73"

/inference="ab initio prediction:Prodigal:002006"

/inference="similar to AA sequence:UniProtKB:P23904"

/codon_start=1

/transl_table=11

/product="Beta-glucanase"

/translation="MKKKSWYTLVVAGVVSLFISVSAFASYVFWEPLTYFNPETWQKA

DGYSNGDMFNCTWRANNVNFTNDGKLKLGLTSSAYNKFDCGEYRSTNNYGYGLYEVSM

KPAKNTGIVSSFFTYTGPAHGTQWDEIDIEFLGKDTTKVQFNYFTNGVGGHEKAINLG

FDASQGFHTYAFDWQPGYIKWYVDGVLKHTATSNIPITPGKIMMNIWNGTGVDSWLGA

YNGANPLYAEYDWVKYTSN"

CDS 3500732..3501838

/locus_tag="PIKHDJKN_03134"

/inference="ab initio prediction:Prodigal:002006"

/inference="similar to AA sequence:UniProtKB:P9WM51"

/codon_start=1

/transl_table=11

/product="hypothetical protein"

/db_xref="COG:COG0654"

/translation="MKKNILVSGASFAGISTAYWMKQFGYNVTVVEIAEGLKKGGTPV

NIRENTADIAKRMGLLDQIQSNKIDMELMEFKNSDDVTERVEIREQLVEDEYEIERDV

LLDIMFEAVQDDVEFIFGDSITSLKEEANGVEVAFKGGQKRSYDLVFGCDGIHSAVRK

LWFGEEAEFSHFLKTYFSITIVDKLLIRENTTQMYNEPGKAVMLNSYNNKTDIVLCFS

SEKELPYDYRNEEQQRDIILNQFSGLGWRTPELLEEVKNSKTFYFDKLCQMKMPSWTK

GRVALVGDAGYCASPAAGMGGSLAIDGAAALADAFQKNHGNFELAFQEYNQSFRPFIE

EVQANAELMVDFLVPRTEEDIRERISQTASIKDI"

CDS complement(3502025..3502513)

/locus_tag="PIKHDJKN_03135"

/inference="ab initio prediction:Prodigal:002006"

/codon_start=1

/transl_table=11

/product="hypothetical protein"

/translation="MAFPTHIVSSGGIVEDGNGNILLVKAHDDGWVYPGGITEVGENL

IDGVIREIKEESGIDAEVNHLINVVSNTAIHKWYDGVTDVPTKVMFDFVCKAVGGELA

TSDETSECRWVPKENVLDWITLPAIRMRYEAYLNFNGSVNYIEYVTGTMSECDIKLQR

RV"

CDS complement(3502643..3503824)

/gene="nupC_2"

/locus_tag="PIKHDJKN_03136"

/inference="ab initio prediction:Prodigal:002006"

/inference="similar to AA sequence:UniProtKB:P39141"

/codon_start=1

/transl_table=11

/product="Nucleoside permease NupC"

/db_xref="COG:COG1972"

/translation="MHYLISVVGLVIVLLLAWLASNNKRKIKYRPIIIMIVIQLGLGL

IILKTSIGEFLIKGFADSFSKLLGYANEGTNFVFGGIANEGAHTFFLFVLMPIVFMSA

LIGILQHYKILPFIIKYIGLVLSKVNGMGKLESYNAVAAAIVGQNEVFISVKNQLGLL

PKHRLYTLCASAMSTVSMSIVGSYMTMLEPKYVVAALILNLFGGFIITSIINPYEVTA

EEDIVEVKEEGKQTFFEMLGEYIMDGFKVAIVVGVMLVGFVGIIALINGVFSGIFGIS

FQGVLGYVFAPIAFMIGVPWHEAVHAGSIMATKLVANEFVAMLDFVKIQDGLSGRTTA

IVSVFLISFANFGSIGTIVGAVKGLNEKQGNIVARFGLKLLYGATLVSVLSAIIIGII

F"

CDS complement(3503840..3505144)

/gene="ywdJ"

/locus_tag="PIKHDJKN_03137"

/inference="ab initio prediction:Prodigal:002006"

/inference="similar to AA sequence:UniProtKB:P39618"

/codon_start=1

/transl_table=11

/product="Putative purine permease YwdJ"

/db_xref="COG:COG2233"

/translation="MKGILSGLQWMMFMIAGAIAAPIAIADLYNLTPVETSGLIQSTL

ITLGIAGFLQGVIGHKFPIHEGPAGLWWSIFTIYASLVGVLYSSNIAALQALSGGMIC

SGVLFILISAFKLMDKIARLFTPTVTFIYLLLLIFQLSGSFMKGMMGITSSHTLLDVK

VFLLSVIVICITFGLGNSRFVLLRQFSVIISISIGCLLFIICDKMPVISPADTLFKLP

KVFPFGSPRFDSGTVTTAFLLTLLLITNALASIRLMETVMKQKKLDHNRYLRAGFTSG

ITHFIGGSFGAIGSVPISGAAGYVEQTGMRERKSFLIGCILVISVSLFPPIMNVISGI

PAPIGYAVTFVIFVKMVGLSLKELKKVLHEERTFVVSGVSLLVGVGLMFIPTSATSHL

SPIVIAILNNGLICGSLLAIILEQGLMWKDANRYEDSKKMYR"

CDS complement(3505190..3505897)

/gene="deoD_4"

/locus_tag="PIKHDJKN_03138"

/EC_number="2.4.2.1"

/inference="ab initio prediction:Prodigal:002006"

/inference="similar to AA sequence:UniProtKB:Q5EEL8"

/codon_start=1

/transl_table=11

/product="Purine nucleoside phosphorylase DeoD-type"

/db_xref="COG:COG0813"

/translation="MSRHLGAKKGEIADIVLLPGDPLRAKFVAEHFLDEANCYNEVRG

MYGYTGLYKGIPVSVQGTGMGNPSMSIYATELIVDYEVKKLIRIGTCGAMQKEINIRD

IVIAQSVSSDSNMTDKIFHGCNYAPTADFSLLMKAYQQAQAKQVSVFVGNIYNSDEFY

RESLDRLHKFMDFGVLGVEMESTALYTLASKYGVKALSILTVGSQLLTQEHLTHQESE

QSFYEMVEIALNTAIES"

CDS complement(3506057..3507493)

/gene="acpB"

/locus_tag="PIKHDJKN_03139"

/inference="ab initio prediction:Prodigal:002006"

/inference="similar to AA sequence:UniProtKB:Q9RMX9"

/codon_start=1

/transl_table=11

/product="Capsule synthesis positive regulator AcpB"

/translation="MDKGFTLASQLLTLLDTEQRWFTLAEVEKSLGISDKTIRKMVEE

ISKQLPPTVTIEVSRGKGIVLRRDGRSETMSEVISTMFRQTIFYRLMNVLFMNVDRLS

VEELAGVMFMSTSSLKKLIVQLNNNDLKAYKLRITYSTPMIKGNEMNIRYFYWKLYCD

AYEFTGWPFANVDFAYINQLITITENEKNIVYFINSKRRLSFLLAIVVERVAQGKCVK

IDESGYPWEKGMFYMPVKTLAKSLGEKLSINFPNSEICFMQSQVSLSQYHYYEGSEIT

PMKEVELHKDKEEYQMGNLLLKLLANVYPNLEMEERFLLEIYAFFDKLLIDNAIPEWM

MISKSNLTAYVQKECQQLYQELQTCMQTWSKAYPAVLYNNFHLTKLTLIVRSSLRYKR

KRAFLVIGEEFSIRHYIADLIKKEIGDQLIINTSIMKGLSDEMMQQHQIDFVISNIPV

ALQTVPVVIISTIPSKRDLDNIRKELLL"

CDS complement(3507826..3507984)

/locus_tag="PIKHDJKN_03140"

/inference="ab initio prediction:Prodigal:002006"

/codon_start=1

/transl_table=11

/product="hypothetical protein"

/translation="MMPLIVEIIISVCILIGLYFVVGSLLESPRVVSYVKHLEEQFEK

NLYEDKAN"

CDS complement(3508059..3508688)

/gene="lacA_2"

/locus_tag="PIKHDJKN_03141"

/EC_number="2.3.1.18"

/inference="ab initio prediction:Prodigal:002006"

/inference="similar to AA sequence:UniProtKB:P07464"

/codon_start=1

/transl_table=11

/product="Galactoside O-acetyltransferase"

/db_xref="COG:COG0110"

/translation="MNITERMANRQLFTDHDANYPQEASDLARARQRGKALCYEINRL

HPDDIEMRKTLMEQLFGSIGENVWMEPPIRMSYGSNTTVGNNVYINFNLTVVDDYKVT

IGNDVMFGPNVTIAVTNHPVHPEKRKEGGMFALPVVIEDGAWIGSCAVIVPGVTIGAG

SVIGAGSVVTKDIPSNVIAVGNPCRVLRDITDHDREYYYKNMRFDQVEW"

CDS complement(3508761..3510206)

/gene="yeeO_1"

/locus_tag="PIKHDJKN_03142"

/inference="ab initio prediction:Prodigal:002006"

/inference="similar to AA sequence:UniProtKB:P76352"

/codon_start=1

/transl_table=11

/product="putative FMN/FAD exporter YeeO"

/db_xref="COG:COG0534"

/translation="MNLSYLDNMDTNREEWMYVKKKKPIQLTTPMTRDFNKELITLVI

PIALQNLISATVISADVVMLGMISQSTMSAVSLAGQITFALTLFYMGMATGAGVLTAQ

YWGKKDVQTIQRILSTACLFSFSISILFFLVSFLYPETLMRFFTNDSELIRFGSIFLK

MNSFSYLAMGISQMYLSVIRSMENAKLSAWISSLCLILNILFNALCIFLLFPDRPEMA

IMAVALATVLARFIELGCCVIYSVTRGSIRFRFPIWDGIQRNLLKDFLKYTMPIQGNY

IVWGGALTATAAIIGHVSADMVAANSVASVVKNLTIVLCGGIASGGSILVGKYLGKGE

IEKAKQVGNRMNLYALLFGIIAGCTIILIKPLVFSVVNLNVISQNYLDGMLYISAFYC

IAKSINATNISGIFTAGGDSKFGFWCDSIVMWGIILPLGYFCAFVWNVSPIVLYIVIS

LDEIIKLPLAFYRYRQYKWLNNITRTFSQAS"

CDS 3510274..3511173

/gene="melR_6"

/locus_tag="PIKHDJKN_03143"

/inference="ab initio prediction:Prodigal:002006"

/inference="similar to AA sequence:UniProtKB:P0ACH8"

/codon_start=1

/transl_table=11

/product="Melibiose operon regulatory protein"

/translation="MKNLEVFSDLSERLNYNLPDLPLYVRRGSLNEFDNFTAAAHWHS

DVEFIYILEGSMEYSVNGQTIKLEQGNGVFVNSHRLHFGFSHTHCNCSFIVIVIHSSL

IGEGSFIRAYWEEKFGPNMDDFVLLAEQISWQHEILLSIQSIYAEMHNSFAPNPFRLT

SQALSLCAGIGDHLQQKVGDIHDVQLWTNIQKMTRFIHQNYEQKITLEDIASSGAVCR

SRCCTLFNKYVGQTPNNYLTRYRLHKSCEMLRETKRSVSEIALACGFQSVSYFSSLFR

KQIGLVPLAYRKSGMNGLSKTEN"

CDS complement(3511170..3512963)

/gene="spoIIAB_1"

/locus_tag="PIKHDJKN_03144"

/EC_number="2.7.11.1"

/inference="ab initio prediction:Prodigal:002006"

/inference="protein motif:HAMAP:MF_00637"

/codon_start=1

/transl_table=11

/product="Anti-sigma F factor"

/translation="MINAFLRLASSFRNRMILIFLSITIIPFVLFSAYAHTKSIEGIQ

QANIAFSMSYLEQAKINVETYLAQLNEGINSLIGDKTLQYLLERPPLSPKEEETISVD

MLSIVYEKKKQMDALKLRVYPLIPADYPTYMRALEESASVGEEEWFRQAADSNVPYWQ

LSVSDGPNRGPLLSYIKTFTSLHSRTPRGIVVTDLADNHLSRFFSPTKRLPRQKFLII

DDKGAILFDSAGNEWTGDVLPSPQLLAKRNLYNEGVEKLSIYDQEHLITFVRMDSLPW

TIISLTPMGALVEPVTEMNRLLLVIIIVYLLVSIGVVIYLTRNFTQPVLRLVRLMRKL

EEGDFGMYIPHQERKDEIGWLFRGFGSIVRKIEDLIAQSTRAERSKKELEFQVLSHQI

NPHFLYNTLESIRWKAENHGRNDIGEMVSALGNLLRLSLNQGKDITTVGREIDQVKAY

VQIEQARIGMPLRVVYFFDEDMLNLTFMRLLLQPLVENAIQHSIRGNFEHGKVILSGR

IEEQDLVIDITDNGKGIPDSVIQALEEESEDQKMNGRSGVGLRNVNERLKIYFGLEYK

LQIESKEGIGTRITIRHPILKEKLENGHKSV"

CDS complement(3512956..3514587)

/gene="rssB_6"

/locus_tag="PIKHDJKN_03145"

/inference="ab initio prediction:Prodigal:002006"

/inference="protein motif:HAMAP:MF_00958"

/codon_start=1

/transl_table=11

/product="Regulator of RpoS"

/translation="MISVLIVDDEYEIREGLRKRFPWHEYGIGTVRSASDGDEALQIA

QEYKPDLIITDIKMNRMSGLELLSELQMREPKDWKAILISGYDDFSLVKQAIHLGAID

YILKPINMDEMERIVRKATDHIRQERLERQNKTNIAYQVQFAVPILQEELLREVVENE

YNPYRETRITHRLKSLNLDWMKESALLLMVVEGDDLKALVNMQTDEKDLVLFGIGNVV

SQTLAEELCVPAALFRDSTKRWVGVISCLDQEILELCKSVAEICLQRINQFVKVKASI

AVSPVPKELKHLNALYVECVESLEQKVIYGGNRLFTESVDGMDTEHEKMSIREPAAVL

DLVRYGTETDITESMKGFEDIVKSWELLSLKDIQQQIFQWLMGIYRIAASAGWPDRSW

ERDPIGLWERLEQYDTLESLKKHVEKNLHLLATDFHKMDNSSSRIVQEAAKLIRTRYS

ENLSLQYVASMVHVTPIWLSKLYKKERGHTFLEDLTSIRMIRAKELLGDIKYKIYHVS

YEVGYKDPVHFSKLFKKHVGVTPKEFRRLRGILDD"

CDS complement(3514607..3516148)

/locus_tag="PIKHDJKN_03146"

/inference="ab initio prediction:Prodigal:002006"

/codon_start=1

/transl_table=11

/product="hypothetical protein"

/translation="MKSSVFRFQRWLVPYLLCMALALSGCMGNPGADINDFFTSSNHP

SEPEVTLKFYFGGEKKAATDEVWTAVSEYVKDKGLNVKFSIHFISWPEYPEKLLVMAA

AGDRWDLNFDSDSSFREMASRGSYMALDKLLPEYAPNVYKKYQMDGNLRSTTMNGDIV

GLPWSIKMNQRPYAGWRVDLAEKAGIYREPDSVQTVQDVDILLHELKHAYPNSKVTRI

TALPFYLVREEWLDLGFHGLGINLNDPDHKVQAIEQQPFYVEAAAMSKKWYEDKILSP

ESLIDRADSSDQWRNGKVLFTLTSHEWAHAADQGFVDPNYRQQMSLLYPDKKQVNRSP

ISNVVAINRNSEHAELVLQFLDMMETDRKLYDLVIYGIEGKTYQMDGEQVVYPHNLND

STSNYMGWGGQWALWNSRFIRSTETYPGDFWGEEARFAKIPVNLDSPLEGLLLSDYAI

SKEVEKRVQLYEDMGRSIEFGLVSDVKEAVLDYRIKQQNNGLDKIIVEVQRQVDLNLA

SNHPD"

CDS complement(3516383..3517543)

/gene="iolG_11"

/locus_tag="PIKHDJKN_03147"

/EC_number="1.1.1.18"

/inference="ab initio prediction:Prodigal:002006"

/inference="similar to AA sequence:UniProtKB:Q9WYP5"

/codon_start=1

/transl_table=11

/product="Myo-inositol 2-dehydrogenase"

/db_xref="COG:COG0673"

/translation="MKKLRIGIIGMGNMGSDHAKKLINGTVTEVELTAVSDMDEKRIT

ASKTYLPETLGYFNSPEELIDSGLCDAVIIATPHYDHPRLAIQAFSKGLHVLCEKPAG

VYTKQVREMNEKAQESGVVFCMMFNQRTNPMYRKMRELVQGGELGAIKRVNWIITDWY

RTQAYYDSGSWRATWAGEGGGVLINQCPHNIDLLQWICGMPSKVRAFCHEGKWHDIEV

EDDVTAYLEFPNGSTGVFVTTTADLPGTNRLEITLEMGKLVCEHGKLTLHKLSQNERD

YCFESKEGFTTPSVEVSVVPAEGDNSQHMGIIASFANAILHGTPLIADGEEGIHSLML

SNAMHLSSWIGETVEFPIDEDLYLSELKKRISTSKRKIGESVLFDTTGSFGT"

CDS complement(3517562..3518308)

/locus_tag="PIKHDJKN_03148"

/inference="ab initio prediction:Prodigal:002006"

/codon_start=1

/transl_table=11

/product="hypothetical protein"

/translation="MILGAQLYTVRSFLQTEKDIKRTLQKIAEMGYTSIQVSAIGKIK

PETLRAMCDELSLQIALTHTSPERILYETDQVIEEHNILGCDYIGIGAMPDKYRSLDW

YGYFASDFREAAGRIARSGKLFMYHNHHFEFEKSNGRRMIERLIEDFSPEELGITLDT

YWVHAAGADVCEWIDKLKDRLHCVHLKDMGSDKQMEPVMEGNMNFTGILKALENANTK

YLLVEQDVCQTSPFDCLQTSYNNLASLGYR"

CDS 3518463..3519296

/gene="rhaR_25"

/locus_tag="PIKHDJKN_03149"

/inference="ab initio prediction:Prodigal:002006"

/inference="protein motif:HAMAP:MF_01533"

/codon_start=1

/transl_table=11

/product="HTH-type transcriptional activator RhaR"

/translation="MNTVNIHTDSLILHHAIDHRKTEQYALHCHNFYEVFYFVAGDVS

YLVEGKHYVPKPHSLLLIAPHVFHGVKIESDIPYERISLHFVPGFLPLENQSVLLSPF

HPDFGSADIYYMDNGDQVMFPFFKQLIDARLADEAIRELSLRVRLEALLSQILISSYS

LKGASESSSSSTKSVSKIIAYLNDHITESIKLDDLSAAFYISKHHLNKMFRRATGTTV

GNYVIHKRVTMAQNLISQGQTANSAASAVGFHDYSSFFRAYKKVLGYAPSVKQNTMLL

K"

CDS complement(3519349..3522501)

/locus_tag="PIKHDJKN_03150"

/inference="ab initio prediction:Prodigal:002006"

/codon_start=1

/transl_table=11

/product="hypothetical protein"

/translation="MLEVTMNASLPNHVMEGTHMMPDRQLERMLGKLVRFTKTIEPLM

FTKVDEVPMKKYHTTESLYSIPDESNFKDCKKGDIWEGEKTYCWFKGSYKVLSNLAGQ

NLYLYPHIGGYEMMMWVDGVPSGIFCSKIIVNSHGNHYCDMLKHQVKGGENIEIALEV

YAHHDFPGVAPLETESQGNFTYVYESVDICTKNDEICEFYFDLKTLNQVVAKLDSNNF

VRSNIVAQLVEVHKVLYYDYDNVDYDTFMGAIRKAHPILQNLLKNQNSISAPSAGIIG

HSHMDTAWLWHKDETLKKCTRTYANQLNLMKQYDEYKFVQSSAYHTAMVQRHYPEVFE

GIKEQVINGRYEPNGGVWIESDCNLPNGEYLIRQFIWGQRYTQENFNYRSDCFWLPDT

FGYSASLPQIMKGCGIKYFLTTKMDWNDTNVFPYDTFIWKGLDGSRVFVHFNRTHIWP

DAASLVDYVAGGDMRGIKEKSVINQRLLAYGFGDGGGGPQFEMIEMARRVRDLEGIPK

AKHTTVSEFMKELEETAIDPSIFSGELYLELHRGTLTNQHNIKRNNRLAEISLRNLEY

VTVREAIRKNIIASDEKIRPLMEDMLVNQFHDILPGTSIARVHDESLAETSAIIEKSA

EYIQELIKPKNRPLSRTVINTLSFDRSEVVYLDYTDGLKVQGDYLQQVTADIHGNKKL

AVKVMIPAFSSIVLKLEEGAPDGEAAFSLDGNQLETPLIKVSFNEKGYIESFFDKKAK

RELRGEGYAFNTFLIAEDVPTAWDNWDLDVDSQYKFADTAAMISRELISNGPLEIRIR

SKYQITAKTTIIQDMVIYSDSLQVRFDTLIDWQDEHRFLKTAFDTSIFTNTAKQEIQF

GYIERPTTRNNSMEKAKFEVVNHKFTDLSEARYGATLLNDCKYGISVENSSMQLSLHK

GGNRPDPRGDKGLHECSYSFLPHDGSFCAKNVVHPAYQFNMKPLILAGEFQQNSLLSI

EADNILVEAIKPLENNDRGFVIRMYEAEGAYTITSWNLNISFDKMEITNMLEETLESL

DSNMNLVFKPFEIKTIKVSY"

CDS complement(3522614..3523975)

/locus_tag="PIKHDJKN_03151"

/inference="ab initio prediction:Prodigal:002006"

/codon_start=1

/transl_table=11

/product="hypothetical protein"

/translation="MKKKRLGTSILAAVLSLTFMLAGCSGNSGTSGNKESSPPAGNDT

VVTEPGQDGDREPVTLKFISWKEGTYDDLYELFHQKYPWITIEQIPVNKQPIMEIIAS

LEAAGTPADVTEIDTDLVAFEQGGLIEDLTPYIEESEIFQNAKFPEGFLDTMTFNSKR

LAIPMVDVPMWILVNKDLLAKHGVDMPSNDWTYDDFRNIAKQITDSNAGEYGVTSQPE

IQMRMLSSKAIADGYAANHQYMNEDMTQSLLSTPEIMNEVKWFQELVTKDGSMQSNAA

AEADGNVTKDFINGKTGFAIGGDWVLPTLKEKAPFQWDVLPFPKGKVSQPGYSIYGPL

AMLAGSKQKEAAFLWLSFQFTPEAQKWKIDQGANASVNDPEITAYYDEAPIWEGKNIE

AVKIAQQNARIQSGLTVVGWREYNYNNLLNDIIFGDRDINDIIPETEAWNKKTLELRE

SLK"

CDS complement(3524110..3525087)

/gene="lacG_3"

/locus_tag="PIKHDJKN_03152"

/inference="ab initio prediction:Prodigal:002006"

/inference="similar to AA sequence:UniProtKB:P29824"

/codon_start=1

/transl_table=11

/product="Lactose transport system permease protein LacG"

/translation="MKEELWFDRAKNYARDLLWRRESQRLKALLMGRNLSDGLLAKLI

IYVLLSIVAYLYLQPLLYMITTMLKTVSDLLDPSVKWIPRVVTWENMTKAYEGLQYPE

ALRNTAVIALTCSLVQVLICSMTGYALARLAIPFKGLITALVLLTFLIPPQVIIIPLY

VIFSKLGLLNSLFVFLVPAIFGQGLKGALFILIFRQFFKSQPPSLEEAAKLDGASAAR

LYFRIMLPLAQSACLVVFLFSFIWYWNMYYEPSMFLAKGFTPLSIRLDNLEEVLNPSL

LSSQESVNNPVTEGTKMAAAFLIIFPPMLIFMFLQRWFITGIERTGIVE"

CDS complement(3525084..3525980)

/locus_tag="PIKHDJKN_03153"

/inference="ab initio prediction:Prodigal:002006"

/codon_start=1

/transl_table=11

/product="hypothetical protein"

/translation="MSKLQEHKKKAVQYHFKRYGVGLLFMLPWMIGFSVFVAIPIGWS

MFMSLHKVAVVPGGFKYDWIGIQNYRDAFLKDNVFPIEMITYFQQMLLMVPIIVIFAL

LISLLLNQKFPGRFIYRALFFLPVIFATGQVLSELFTQGAGDIPFLDQYNLEPLVKQY

VGGEMAKMIMEVLGRVVLILWYSGVQILIFIAGFQTISPSIYEAVRIDGASPWDSFWK

ITLPASIPFISLNVLYTIIDLFTFPLNPVLKHIRDNMFKVDTGYGYASALAWIYFGFI

FVLIAIVLGMFSHSMRQRGRRS"

CDS complement(3525977..3528148)

/locus_tag="PIKHDJKN_03154"

/inference="ab initio prediction:Prodigal:002006"

/codon_start=1

/transl_table=11

/product="hypothetical protein"

/translation="MLVLGLMLVMLVIKPWQQSTTESFAATGMQMLLPEKDPLYQGER

WEPATQADGYAIAVDNDQYTLYVNPLNTQIYVLDKSSGYRWTSNPTEVELADETVKGQ

LLDNLQSPFVLTHVRTQGADQTVRETLNSKAPGLETTMIRGMKGLQIQYWFPDKRIGF

AIQYELTESGLKVKVPAAGIREEGDHVLFSLDVLPHFGAAMASEDGYLFVPDGPGGLI

RYDTAKASLSRGYIHQVYGQELTNAGNINLMVQNREDIAYPVFGLKRGDHAYVAVMRK

GEDSANVVAMAPGQRSSFYNVYSSQIYREEYLYRMSRLATPSKAIQKQKLDLDREVEY

RFLNGEQASYVGMANAYRDYLQESGQLGEALQPVEHIPLYLKLMGGNYEKAFGRVQYV

ATTTFDQATEIAERLQQQGVASLKTIFYGWQNEGDYNMEKRFPIESSLGGEAGAKSFV

ASMKKSGIDVAFQEDFLWVDSESSFSGKTDAIRGIDGTAYNIDGWYLAKPVYTAVSAA

KTIDKLKDIGVHGIHYSGLGEILVHDYELSGIRTREYTKTVYNGLLDYTRKELGSASV

ERGNAYVLGSTDFIDGMASSSSYDFMIDETVPFYPIALHGNVPYTFEAGNLRDDEDTE

FLKAIEYGALPSFFVTHDDSRKLKNTPVYGLYSSRFDKWESRIVEEYGRFDSLSDLYS

LRIVDHEQISDERYMTTYENGTRVIVDYAAKSFKVEKGAAQ"

CDS complement(3528195..3530210)

/locus_tag="PIKHDJKN_03155"

/inference="ab initio prediction:Prodigal:002006"

/codon_start=1

/transl_table=11

/product="hypothetical protein"

/translation="MKKWLSMALIGLIALTVLPRAASASLPYWTAYYDSNQSNWFQIQ

PIYRPAGAYSADFGEPVDLDVASDDKVYIADKKQNRIVVLDQDGSLLRTIGEEEGSGQ

LSSPEGVFVTPEHAVYVADSGNQRIAVFDQNGVFQRDFKKPDTPLMGSEHFVPVKLIV

DRRGVMYIQLNSSYQGLVRLNPQGEFMGYFGANKADQSMLNWLKKLVLNKDQLAKEKA

ALPKPITNVSLDKDGFIFTSTAGGEGKGAIRKLNAGGVDIFKNKTLENGHGIVDVAID

ADAFLYNIDLDSMHINLYDRNGEAMFEFGLVSKDTQQYGILGYPTGIGVDSHHAIWIS

DSGTNTVHKYVRTDLGRDVLKALALYAEGKYEQSKPYWEAVYARNDMYNGTFQGLGKV

LLHEGRNDDALMFMKAAFDTEGYSKAFWKLRLDWLQNHFMILIYSLIGGGLFLHFGIR

GLRAYLRKQPLSERWQQPLADLKNFSYVMFHPYEGFYRIKEARIAPWITIALLFLVLA

IKLCTIYWTGFLFHPVELSEVNLLKEMGFFILPWITWIVANYLVCSVKDGEGKFREVI

QGSVYALSPYVFFSIPLLLLSNIVSLDEAIIIHAMNMVMVLWLLSMFFVMTQVIHNFE

FVETIKNSAVTVFAILTIWMFGFIIFGLSFNLYDFFYELYKEVNINL"

CDS complement(3530239..3531105)

/gene="araQ_22"

/locus_tag="PIKHDJKN_03156"

/inference="ab initio prediction:Prodigal:002006"

/inference="similar to AA sequence:UniProtKB:P94530"

/codon_start=1

/transl_table=11

/product="L-arabinose transport system permease protein

AraQ"

/db_xref="COG:COG0395"

/translation="MIAKLRKLRSVSSFDVILILLFTCLAVFMSLPIVFIFNHAFKPQ

NELFLFPPQFFVQQPTLNNFETLFLHASNAMVPFTRYLFNSMIVVSFTLISVIMVSTM

AGYVLAKYQFHFKKLIMGMITLSLMFAPETVSIPRYLVISALGLNNTYFGHILPSLAS

PVAVFMLVGFISQIPNDLMEAAKIDGASHFGIFRRIILPLSVPAIATISILTFQNVWG

DVETSTLFMQKEAMRTLPFYVNSLLSGLQNNVAGQNMAAAAGLLIFIPNLIIFLLFQR

KVLETMVHSGIK"

CDS complement(3531108..3532079)

/locus_tag="PIKHDJKN_03157"

/inference="ab initio prediction:Prodigal:002006"

/codon_start=1

/transl_table=11

/product="hypothetical protein"

/translation="MKTEVFPVLHPAKRPSLLVSKWMRFLRELRKDAFCYLFLAPFLI

CFLVFIVIPVVMAATLSFTSYDGFGSLNFIGFDNFIALFTQDIIFLTKALPNTFLFAL

IVGPGGYILSFFFAWLIHQLPLRVRDYYTLVFYAPSMAGGVALSVVWIAAFSGDRVGY

LNNFLLRMGWIDSQITWLQDPKYLLPIMIMVSLWMSFSVGFLAMLAGLQTVNRELYEA

GRIDGLSNRLQEVFYITIPSMKPQMLFSAVMTIVSTLKAGSISTQLTGMAVTPQYAGH

LIINHVDDYAFIRFELGYASAVSVMLLLFSYFVMRFAYRLFNSKEEL"

CDS complement(3532076..3535033)

/locus_tag="PIKHDJKN_03158"

/inference="ab initio prediction:Prodigal:002006"

/codon_start=1

/transl_table=11

/product="hypothetical protein"

/translation="MKKRPFPRYLRIPIQILIVAALVVGVIRWMTSEEQVVYSGSVTA

DQLLQFQPAGDKNPYAILMQGVSEQERSGEKGNEIRINPSQYDTAAPITKLSQSKEDG

KTVLNWHNEAGSVDWTFHVAKAGWYELHLDYKPLAGSNSSVIRGVMIDGSYPFEESER

VELERMWKDAKYPYDRNELGMQVRPQQTELNEWTSKAVSDYSVSSRPLLFRLERGEHT

LRLVGERGGVALQQLSFQSQEHVKPYEEYKGAYPSASDASDWYMVREAEQFERKSHLS

IQTDHWAEPYISPDPKGRITYNVLGGTHWNLPGEWVEWDIEVPTNGWYEIDLKNFQNY

RSGFKAYRTISIDGKVPFKEMLNYAIDYHKEFEITTLSNISGEPFKFYLEQGKHSIRM

TADSSSMQPILIALKDTLLQIASFDREMRLITGNYSKSASDSNIDSTRTWEMKKYDPE

VENKLQAITDRLNMIRLYTNSVNERDSDLSQAIKASVTILEDMLGDVNEIPSKIKEFS

TIQANIGTWMSTLTKQPLLLDYLVVRKPDAKTGLKEPTALSRIPYSLADFSRSFYVDY

DLGGPEEEGALTIWVGRGRDYVDLLRELVSQDFTPKTGIEVNINLMPNPNMLILGNAA

GEVPDIALGVGESTPADYAMRNAVEDLSQYPGFDDVMNQFIPGVTRALMYDGGTYGLP

EVQNFQLLFYRSDILERLDLEVPDTWDDVFDILPTLQENGMTMNVPKGDYSTLFLEHG

TDPYTKDGLKSNIFSDTGQQAFKLWTEMFTKYNLPIDIPAFFQHFRDGDIPIGISDFN

TYVQLQVAAPEITGHWKVAPLPGIKQENGEVVRWSPQGLTGAMIMKKSEQKDKAWEFL

KWWTSGEVQSRYAADMESFYGIEYRWNTANVNAMESLNWPSDDLKAIREQASWALNMP

NVPGYYFLGREMDFAWNRTVFDGIPADESLEEAHLSLQREMDRRQRNFGIMGRSLNIP

QITEPYVWEDTES"

CDS complement(3535030..3536148)

/gene="msmX_2"

/locus_tag="PIKHDJKN_03159"

/EC_number="7.5.2.-"

/inference="ab initio prediction:Prodigal:002006"

/inference="similar to AA sequence:UniProtKB:P94360"

/codon_start=1

/transl_table=11

/product="Oligosaccharides import ATP-binding protein

MsmX"

/db_xref="COG:COG3839"

/translation="MGGITFDHVFKRYKGESRPAVNDFHLTIDEGEFLVLVGPSGCGK

STTLRMLAGLEEISSGELYLDGKFINYVSPKDRDIAMVFQNYALYPNLSVLENIALGL

KLRKTAKHEIDTRVKRVASILEISHLLDRKPSQLSGGQKQRVALGRAIAREPQVFLMD

EPLSNLDAKLRAQTRAELIKLQQELRRTTVYVTHDQVEAMTMGSRIVVMKDGVIQQVA

PPRKLYDEPANVFVAGFIGSPQMNFIKGTIEVSDGFYYFSNKRMRLLLPENGVSRLER

KSGAVRNVILGVRPEHISLRRGRIDDPSFLPGIVQMTEETGADSYVYIQLGHIRLIVR

TEPVTRYETDEIVSVGLQMNKIHFFDETTELVFGRDET"

CDS complement(3536436..3537110)

/gene="yodC_3"

/locus_tag="PIKHDJKN_03160"

/EC_number="1.-.-.-"

/inference="ab initio prediction:Prodigal:002006"

/inference="similar to AA sequence:UniProtKB:P81102"

/codon_start=1

/transl_table=11

/product="Putative NAD(P)H nitroreductase YodC"

/db_xref="COG:COG0778"

/translation="MSASQPSLTMEQQLFFDVIGERRSVRSYDPEIKISREELTEILR

QATLAPSAANLQPSRFLVIDSPELKQKLLPIAFNQQQVVEASAVIAVLGDLECYKLAE

KIYGMTVDAGYMPAETAKSFVDRYTGMYSSMPPEAIREKLYTDGGLVSMQLMLVARAK

GYDTVSMGGYDKQKFMEAFCISERYVPIMLIAIGKAAKPGHPTVRLAVDDVAFFNEMP

KEDHDG"

CDS 3537300..3537896

/locus_tag="PIKHDJKN_03161"

/inference="ab initio prediction:Prodigal:002006"

/codon_start=1

/transl_table=11

/product="hypothetical protein"

/translation="MTNKKNEADPRVIRTRRLLQDAFASLIQEKDFESITVRDIAERA

TVNRATFYAHFVDKFEILETKLMESFMTIIDRRIDGHEVLNEETIQSIFLGVCDFHEG

LSTMCKRRYTSLGSVFEIQIKEKIQSILLSFIDKDKMPSSPNAHLLINTASIMLSWGM

YGAAYVWNNEGRPSSAESFVKQTMPLIMNGAKELKSLI"

CDS complement(3538191..3539096)

/gene="gltR_3"

/locus_tag="PIKHDJKN_03162"

/inference="ab initio prediction:Prodigal:002006"

/inference="similar to AA sequence:UniProtKB:P94501"

/codon_start=1

/transl_table=11

/product="HTH-type transcriptional regulator GltR"

/db_xref="COG:COG0583"

/translation="MEIRQLKTFWTLASTRSFSRTAELLSYVPFTITMQIKSLEEELG

VKLLDRLGKNVMLTDAGQQFLPYATKILNDVEEAKSVSSQHGETTGTVTIGADEVLCA

YFLPALFKCFREDFPDVRLLFRPLSGQGLRNSLREGLADVIFVLDEFVGAKDLHSEFL

KDEYFKMVVSPDHMLATRSSLIIDDFHKQHFLLTEKNCSYRTHFNQSLLKKGADALTE

LEFHSVEAIKQCAVAGLGIALLPKMALKEELREGRLVALPWDLSDVSFSAQMLWHREK

WISPSMAAFIHTAKSELKTEEVSGS"

CDS 3539227..3540252

/gene="metXA_2"

/locus_tag="PIKHDJKN_03163"

/EC_number="2.3.1.31"

/inference="ab initio prediction:Prodigal:002006"

/inference="similar to AA sequence:UniProtKB:Q3M5Q6"

/codon_start=1

/transl_table=11

/product="Homoserine O-acetyltransferase"

/db_xref="COG:COG2021"

/translation="MNDYETYPLGDISLQSGQQLPQAFIAYKTYGTLNAAKDNAIVYP

TWFAGLHTDNEWLIGENKALDPNRYFIIVPNMLGNGLSSSPSNTPAPFGKSNFPLISM

YDNVRFQHQLITEKFGISKIKLVVGWSLGAMQVFQWGTSYPEMVERIAPFGGTAKTRP

LAQMVFQSMIAALQADSKWEKGLYKHPPVDGLAAMGRVYAPWGFSNSYYLEKLYQSEG

YSSLKQYVEEYWDQVFLSFDANDLIAMLRTGISGDISNNHVDCYNFEQALRKISSPAL

VMPGSSDLFFTPEDSAYDVQHMPNAVFQPIESKWGHCFGIGANEADSLVIDSHLKRFL

KLGFQWD"

CDS complement(3540375..3540803)

/gene="slyA_3"

/locus_tag="PIKHDJKN_03164"

/inference="ab initio prediction:Prodigal:002006"

/inference="protein motif:HAMAP:MF_01819"

/codon_start=1

/transl_table=11

/product="Transcriptional regulator SlyA"

/translation="MKSRKETPYLDLFQIIGLKLKKRADESIKELGLNAQQGKVIDYI

YENQNSHIIQMDLADRFHLSRASITSMLQALEQKGLIERKIPANNERQKNIYVLPKAV

ELIEDFQDSFQKVEEEIVQVLTDEEKQVLKKLLIKINETI"

CDS 3540962..3542272

/gene="ade_2"

/locus_tag="PIKHDJKN_03165"

/EC_number="3.5.4.2"

/inference="ab initio prediction:Prodigal:002006"

/inference="protein motif:HAMAP:MF_01518"

/codon_start=1

/transl_table=11

/product="Adenine deaminase"

/translation="MEKVTQNAAIEQVTAITNVRIFDGEQIIAPRHVVIKGESIVSVG

GDLPADATIINGENATLMPGLIDAHVHTSIGGLRDALKFGVTTELEMNGDFTKRGREI

QLKNVNDIADVRSAGTAITAPGGHPDELLPDGDEIPEFVLKELEKLSEEDREAMLAAY

AHDHDEIPQVTTVEEAIKHVHTQVENGADYIKIMIEEGTVMGAPGLPVLSDEILKTAV

TEAHKSGKLVIAHVLTALSSQQAIDFGVDGLGHLFIDRPEYTSELVKSIAGSGAFVTP

CLVLNSSIIGNTASELANDPRVHSKLSPDWIDILNSSFNTFPQGNMENSFKNVMDLHR

AGVDILVGTDVAPVPVPNLGGLAHGASVHHEMQLLVKAGFTPIEALQSATSKPARCFG

LHDRGRITEGARADLVLVDGDPTTNISDTLSIKSVWFNGSQQLG"

CDS complement(3542602..3543588)

/gene="xsa"

/locus_tag="PIKHDJKN_03166"

/inference="ab initio prediction:Prodigal:002006"

/inference="similar to AA sequence:UniProtKB:P49943"

/codon_start=1

/transl_table=11

/product="Xylosidase/arabinosidase"

/translation="MSKFPKPHQPIVTHIFTADPSAHVFEDRIYIYPSHDLDHDGPVN

DNGDQYKMEDYHVLSMADTDSPVTDHGEVLHVKDVPWASSQMWAPDAAYRNGNYYLFF

PARDHDGIFRIGVATSSSPAGPFKPEEQYIQGSFSIDPAVYVDEDQQVYMLFGGLWGG

QLEKWQTGSYVEHTEGPAQDAPAIGPRVAKLSEDMLSMVSEPLEISIVDQEGKPLTAG

DEDRRYFEGPWMHKYNEKYYLSYSTGTTHKLVYAIGDNPMGPFTFQGTILPPVMGWTT

HHSIVQFREKWYLFYHDCSLSGGVDYKRCVKFAELTYNPNGTIRMIDPYPEK"

CDS complement(3543672..3544694)

/gene="xynB"

/locus_tag="PIKHDJKN_03167"

/EC_number="3.2.1.8"

/inference="ab initio prediction:Prodigal:002006"

/inference="similar to AA sequence:UniProtKB:O69231"

/codon_start=1

/transl_table=11

/product="Endo-1,4-beta-xylanase B"

/translation="MGSFREEAPLKELYKDAFHIGAAVSPKTIESQRSLLAYHFNSLT

AENEMKFSSVHPQQELYTFEDADAIASFAREQGMALRGHTLVWHNQTPDWLFENETGG

LVERGVLLERMKAHIQTVVGRYKDVIYAWDVVNEVISDDADDENALLRPSKWLDIAGE

DFIAKAFEFAHEADPQALLFYNDYNESNPHKRERIFRLVRSLLDQGVPIHGVGLQAHW

NLYDPSLDDMRAAIERYAQLGLQLQLTELDVSVFRFEDKRTDLVRPSQEMLELQAERY

ESIFRLLHEFKEHISAVTFWGAADDYTWLDNFPVRGRKNWPFLFDESHRPKEAYQRVA

RIAVPK"

CDS complement(3544870..3545823)

/gene="araQ_23"

/locus_tag="PIKHDJKN_03168"

/inference="ab initio prediction:Prodigal:002006"

/inference="similar to AA sequence:UniProtKB:P94530"

/codon_start=1

/transl_table=11

/product="L-arabinose transport system permease protein

AraQ"

/db_xref="COG:COG0395"

/translation="MTKKTPLSGSGNTAALSVRRPGGRGMEPLLFNTFNTIFMILLVI

VTLYPFLNTIVVSFNAGNDTIRGGLYLWPRDFTMQNYKAVFASGTIYNAFLVSVARTV

LSTVLNIFLTTMLAYTLSRREYIFRKLITTIFVLTMYFNAGLIPGYFLMKDLHLINSF

WVYVLPSLISAFNLIVIRTYIYTIPESLIESAKIDGAGDFRIFWKIIFPLCKPVLATI

ALFVAVGAWNSWFDAFLYTSSRQELSTLQYELMKLLSSSMNANSNPSVANGVGMEHST

QVTPISIRAAITIVASVPILLVYPFMQKYFVVGLNVGSVKE"

CDS complement(3545823..3546788)

/gene="yteP_25"

/locus_tag="PIKHDJKN_03169"

/inference="ab initio prediction:Prodigal:002006"

/inference="similar to AA sequence:UniProtKB:C0SPB3"

/codon_start=1

/transl_table=11

/product="putative multiple-sugar transport system

permease YteP"

/db_xref="COG:COG4209"

/translation="MEEIVVGNQPLSPKKKPKIRRLTWQNIKAQRQLIWMSVPLLAYI

VTFAYVPVWGWTMAFQDYKPARSFSQQTWVGFKHFQFLFTDDNFLRVLRNTLAMSVIN

LILGFVTAIILALLLNEIKKVMWKRTVQTISYLPHFLSWIIVTGIVATSLASDGIVND

ILMKLHLINEPILWLTEGKYFWGVVAGSHVWKEVGWNTIIYLAAIASIDPALYEASDI

DGANRYQKMWNITLPGIKPTIVILLIMSIGHILEAGFEVQYLLGNGLVVDWSETIDIF

VLKYGIAQGNYSLATAGGIFKTVVSITMLLIANWTAKRLGEERLL"

CDS complement(3546930..3548603)

/locus_tag="PIKHDJKN_03170"

/inference="ab initio prediction:Prodigal:002006"

/codon_start=1

/transl_table=11

/product="hypothetical protein"

/translation="MGDSKKSFFRMGATLLLSLSVVLAGCSGGNSGSGEESSKGSEVG

ADNPLEISVFLNEAGQQPTADNKIYKKIKDELGVTFKFEFLAGDKNQKLGVMIAGGDY

PDLISADTKLTAAGSVIPLEDLIEEHAPNLKKHYEKYWNQMKDPNDGHIYYLPNYGAY

NGEVSDTYYSGPAFWIQKAVLKEFGYPTPKTLDEYFDLIAKYKEKYPTIDGKPTIGFE

VLNYDWKNWGLLNPPQHLIGHPNDGGVVVNDGKAEIFADKDYAKTYYEKLNEINGQGL

LDKEAFAQNYDQYMAKLSSGAVLGMFDQHWNFGSAEDSLRTQDKIERTYVGFPLVYDS

STKDYYRDRAALNLNNGYGITVSAKDPVKIIKALDKLMEEDWQKTLTWGIQDEDYYVN

DEGRFMKTQEQRDNSADAAWKLANKADAFYGTGPKMEGYFSDGNATSASNQPEEYQAS

LKPFDKEVLDAYGFNSFVDFFSDPPENPIYYPAWSVDLVEGSPAKIANTKLNELSTKY

LPKAILANTSEFSSVWSEYVSEIQKLDIKAFEGRINEVLQWRIDNWSVK"

CDS complement(3548828..3550366)

/gene="rssB_7"

/locus_tag="PIKHDJKN_03171"

/inference="ab initio prediction:Prodigal:002006"

/inference="protein motif:HAMAP:MF_00958"

/codon_start=1

/transl_table=11

/product="Regulator of RpoS"

/translation="MAFKVLLIDDEPWALEGLQLWIPWGSLGFEVCAMCGNGAEGLKR
MEELKPDLVMVDIQMPIMNGLEMIEEWRRRGNWSTKFIILTGYSDFEFARKALKFKVS
GYLLKPLDEEQAESEIRTMGMELLKEQEQLYIGQIALREQEILTMKQALLGKVLSTEG
TQLMQSLSRSAQSWIVCLVQVSEKEFGRWSSLASEILNGPHVIYIIRMRNDLVSIVFG
DVSPGPTRTSDNLRNQIETLARRLAGFRTYMAIGSTESSLYHISNSRITAEKALLHAF
YEAEGNSILDYADIQNHDFQRHNKQVELIERVLGALQLIDYANYQSAVEFMEQSFRQM
RVHPDEARKFVIYLLHEIRAYMRAQMAENSENYRMFEIPDLDEALLTFDDLIGMLHSC
GRVCFEFLLKESVFEAQGIVQDINDYIHTHFREGLTIKLLSERFFLHPAYLGQLLIRK
NGIGFNELLHNLRVEEACRLLQMNQHKNSEVSEKVGYANYHHFLKQFEKRMGMSPNEY
KKNV"

CDS complement(3550341..3552179)

/locus_tag="PIKHDJKN_03172"

/inference="ab initio prediction:Prodigal:002006"

/codon_start=1

/transl_table=11

/product="hypothetical protein"

/translation="MFKKFRFRGIVNDIPLNYKFMLIYVIGILLPIIASNYLFMDRMS
GLIKEREEQNLQISLERARKDIHTMIDGGIAVSHALITDKLLSESMDRVYNGQLDFYN
TFDEQLRHRVTSYMPVNNQIQRIGIYTDNPTIVSGGDYYYLDSSKNASAWFREWEAAG
ESLNVLAYRDQAANDRAATASYFSVIEKMDYYNSDNSYKKLVRIDFYLNRFYDVIARE
KSSLDLYLVNAKNQIIVSTDDKYQGEVDSDYALFDTSKAADKDLHIVPIGKASYVKGW
KLIGVPQETRVKQAMLSMKLYFGMLAGIITLVTSIFIYVMLRSYNYRVKRLSRHMQKV
GNEKFDLINIDGGQDEIGHLIRNFNMMTAQIHSLINNVYKLEIQQRSQEAERIRAELN
LLQSQMNPHFLFNTLNALLVVSTKNNYVDVKYIIKDLSKLLRRLLNWKDDVVTLEEEM
SFTVMYLGIEKFRFRDKFEYYIDISDEARQYRIPKMSIQQLAENACKHGIQAIEGLGY
VKIKAEVVSERLRVVVSDNGKGMDKERLQEVLYHMRSKEESGGSNIGIRNVYRRLELY
YDDKVNFNLVSRIGEGTEVSFEIPLQLLEHQGEEGGRDNGIQSAAD"

CDS complement(3552223..3553749)

/gene="rhaR_26"

/locus_tag="PIKHDJKN_03173"

/inference="ab initio prediction:Prodigal:002006"

/inference="protein motif:HAMAP:MF_01533"

/codon_start=1

/transl_table=11

/product="HTH-type transcriptional activator RhaR"

/translation="MMNVLLVDDEPWVLEGLRTMVNWNKYGFQICGEAENGNAAWSII

EDLQPDLVFTDIHMPSVSGLELIDRSIQKLAKPPRFVILSGYDSFDYAKTALEQRVED

YLLKPIDEVEIENVLEQMSRKIQNELVAEKLHQHERTLYVNCLFNRLFQGEDSSELQA

EVESILQMEGHENMACLLIETDTCNEEIKLQVQRFASAHHYEFFMDAEGRMGVFAAEI

EDSLSWVERLGRTLFEMYSGHGSIIVAFGYHSGGVVAMRPAYEKAFSALKWKRYQKGS

GIIRDQDLPRDGNMAIVNQKALTNLFKTLLTGEEDGLEREVHELLTNPDSGLPVSDIE

YVRVQLLALEMGVLKQLKELDGDVDRFVLLLQHMLGGLTDIDSYPAFREYALILSLNA

LRALQEQRKEKECSTIFRVVQYVNLEFREKLQLQEISQKFHMNANYLGQAFKQQTGKS

FREYLNDKRIEEAKRLLRQSCCSIAEVAIHSGYPNADYFVSQFKRMTGMAPSAYRKQP

"

CDS complement(3554072..3554425)

/locus_tag="PIKHDJKN_03174"

/inference="ab initio prediction:Prodigal:002006"

/codon_start=1

/transl_table=11

/product="hypothetical protein"

/translation="MRIKRIVTNINTQNVPAAKSFYQDVLELDLLMNHGWIETYGLLG

EENVQISIATQGGSNTPTPDLSIEVDDVDVVYERMKSAGYTIEYELTDEPWGVRRFYV

RDPFGKLINILAHDH"

CDS complement(3554752..3556044)

/locus_tag="PIKHDJKN_03175"

/inference="ab initio prediction:Prodigal:002006"

/codon_start=1

/transl_table=11

/product="hypothetical protein"

/translation="MIMDVVLSKLPRKLTITMWDFSWYTMTLPGEPYSDLEARFKEAV

ERGYNTIRICAMPFLLFTAAGKRPGPLHFGSLGEIGQRTRWYNCRGGAQLDGHAHLLE

LFRQAKAHGCYIILSSWEYQQSPSFLAFPGLRDELAAIAPEERFMAIARSMDQLIRYI

KSEGYDKQIVYAELHNEVEFGQLTDIGTAQGIAPSNVPALVETMQPYIEEAVGYLRSV

HPDILMTASYTLNEAYPKSYVAGNLQVAHFHLYIKGVLNELTKAAGLDDEQAPFPGAF

VQSLLREDAPPFEEYTLPRGQEWRMEGNPVGLRLLYLHDWVEPDQWDLFLYDNYSKHK

LAMLQKADMRFEEANEWAAHSGLPVVIGEGYVGYTPLLAGFEEGPVGKFIAEYALRKG

IALGFWGMTLCSNCAPHHPFWDDVAWQQKWNRYILESQ"

CDS complement(3556135..3559530)

/locus_tag="PIKHDJKN_03176"

/inference="ab initio prediction:Prodigal:002006"

/codon_start=1

/transl_table=11

/product="hypothetical protein"

/translation="MKHEAVDHKLEADPTGSSQVQVWETSVLIPTYEAGEADPNPMFL

EKRVYQGSSGRVYPHPVIESISDVKHDKNYKLVILENQYIRIEIMPEIGGRIYRALDK

TNNYDFVYYNRVIKPALVGLAGPWISGGIEFNWPQHHRPNTFGPVEYKYEATNDGSAT

VWVSEIDRMYGTKVTVAFKLYPDKAYLEINAQLYNRTPEPQTFLWWANPAVAVNEYTQ

SVFPPDVTAVFDHGKRDVSRFPIATGTYYKQDYSEGVDISRYKNIPVPTSYMAYKSDY

NFVGAYDHGVEAGLLHVANHHISPGKKQWTWGNGEFGQAWDRQLTDEDGPYIELMTGV

YTDNQPDFTWLQPYEEKTFTQYFMPYKNIGVVKNASIEAAINLEVDAEAREAVIKVYA

TSKLEHAVVELSGAATRYLQETVELSPVDVYQKAIPLESGEQEHDLKLLVRNREGRLL

ISYSPKRPAIEQIPEAAKPLVAPEELRSTEELYLAGQHLEQYRHATFEPEAYYLEGLK

RDNGDIRLNVAYGTLLLRRGLYIDSEQYFRKAIERLTWRNPNPYDSEAYYQLGVALRG

QGRLDEAFTAFHKSVWSAAWQDAGYLSLAQISSLKGQYTEALEQVDRSLIRNSRNYKA

RNLKAALLRKLGLIDNAKACALETLELDVADFGAYNEWALAHTAMGDKDAAQGIQTEL

QQLMRNDAHNYLNVIADYMDSGFYEEAIAVGERMMNSENSVYPMLHYALAKLYERIGQ

HEHAQEALRNGQMANPTYCFPNTLFELELLVSAVHANPQDDKAHYYLGNFYYDKKRPI

EAIASWEKSRELRDDFPTVHRNLGLAYYNKQNNPQAALASLEQAFACAPDEGRIFFEL

DQLRKKLAWSTDERLHILEERRDLVEKRDDLYVEYVTLLNNLERYQEAIAALSLRNFH

PWEGGEGKVTGQYKLAHTELGKQALQNGHYEAAAQYLQQALVYPLNLGEGKLEGTQEN

NIYYYLGMAYEGLQRETEAISSYTIASQGLDEPTSALFYNDQPPEMIFYQGLAWLKLR

NGKEAKRSFNKLIDYAEKHIFDDIKMDYFAVSLPDFLVFEDDLNRRNVIHCRYMRGLG

LLGLGRDEEAGTELKLALEMEPNHQGAMVHRRYSRQLHEGFQP"

CDS 3559658..3560554

/gene="araC_5"

/locus_tag="PIKHDJKN_03177"

/inference="ab initio prediction:Prodigal:002006"

/inference="similar to AA sequence:UniProtKB:P0A9E0"

/codon_start=1

/transl_table=11

/product="Arabinose operon regulatory protein"

/translation="MGPDHIRKREGFVEEKLYVLPEYWMKELEQEELTSTLFITDIGY

FPNAQYHFRERLEGSPSHIFIFCEAGEGWVELNYGERIIMQQGNMMIIPPETAHRYGA

MQENPWSIYWFHFKGSHANRLVKMFGLSNASLALSPSGKARLMEWFVPTYEMLAERTY

ALTTHVHVAQTTRQLLSGIGLNAVKSAQEKKRETYLEQAIQYMNVHMSGSISLTELAN

HVGVSKQHLIFLFNSETGVAPIEYFLRLKIQRAGHLLDLTEMSIKEVSSTVGISDPYY

FSRLFKKISGFSPSSYRNIPKG"

CDS complement(3560674..3561903)

/gene="bcr_2"

/locus_tag="PIKHDJKN_03178"

/inference="ab initio prediction:Prodigal:002006"

/inference="similar to AA sequence:UniProtKB:P28246"

/codon_start=1

/transl_table=11

/product="Bicyclomycin resistance protein"

/translation="MIKSKNWNVFGLALLLGLFSTLGPFTIDMYLPAFPEIAKNFNTS

ASLVQFSLTACLLGLGVGQLVMGPLSDAHGRRKPLLISMAAYIICSLACAFAPNIGLL

ILFRFTQGFAASAGIVISRAIARDLYSGHELTKFFSLLLLVGNLGPLAAPIAGSGILS

FTTWIGVFISLSFLGVFLLIMTKWGLKESNPPERRMAPNFKQQLGNYRTLLRDRKFVG

YMLAQGIMTAGVFAYVAGTPFIYQNIYGVTPTVFAILFASNGISLIIGSQIVGRLAQR

IPEQTLLLSGLWLALAASVAALVVTLVHGPLFALVIPLFFFVCSIGITSTAAFPLAME

SQAKMAGSAAALLGVVPFLLGALVAPLVGIAGEDTAVPLGLTLLMTSLVAILTYFLLV

KNISHHTSQHAQSDSDF"

CDS complement(3562266..3564524)

/locus_tag="PIKHDJKN_03179"

/inference="ab initio prediction:Prodigal:002006"

/codon_start=1

/transl_table=11

/product="hypothetical protein"

/translation="MRREKLLFDVTVIGGGLSGICAAIAAARLGQKVALVHNRPVLGG

NSSSEVRVWVCGATGHGVNRYARETGIMGELFIQNQQRNPEGNPYLWDVTLLEAVRAE

PNLTLFMNTDVREVEADGEKDARIIRSVKGWMMGSEREITFESEIFLDCTGDGLVGFL

AGAAYRIGREARHEFGEEWAPEVADDITLGSTLLFYTKDTGKPVRFVAPSFAKDITTT

PIPLKRVIRSGDSGCHYWWIEWGGELDTVHENEQIRDELWSVIYGVWDYIKNSGKFQA

EEMTLEWVGSIPGKREYRRFLGDTILTQNDVLSQRPFEDRVAFGGWSIDLHPPQGMYA

AESGSKHLHADGIYHIPFGCLYSRNVSNLLFAGRNISASHVAFGTTRVMATCAVLGEA

AGVAAALSVIKGITPRELREQHLTELQQTMLRTDASIIGLRNIDSADLARKGTITASS

TMTRILINEPSEEYRLVADVGWLFPAEGGIEGFTLLVSAREATSLTIELWDTGRPENY

VPANFKQALDIQIDVGKNQWLNVPADSAMFGGDPCNVFIIVRANEAVSLYTSAVPISG

VLAFERGVKPIVSSDLEDHQPNQPIIEWSMKRLVRKPFCFAVETGAYSPDKVINGYVR

PFGGPQLWVSEPIANGREEWIEVKLPELVDIREIHLTFNDDVNEDLINLHHHETPFLI

IPELVKSYEVHAYINGKWVSFAGDSHNLTRKKIHRLENPIQSKRIRVIIKETHGGDRA

EVYEIRLYDSHR"

CDS complement(3564544..3565371)

/gene="araQ_24"

/locus_tag="PIKHDJKN_03180"

/inference="ab initio prediction:Prodigal:002006"

/inference="similar to AA sequence:UniProtKB:P94530"

/codon_start=1

/transl_table=11

/product="L-arabinose transport system permease protein

AraQ"

/db_xref="COG:COG0395"

/translation="MLRTTKTRSVKLSVTILMLIAGLFMIVPFLWMLSTSFQTPSEVF

RQWLPSKLDWTSHKRIWTGNYNFLPYYLNSIKIAVIGTAGAVFLSAFAAYGFARTEFK

GRNTLFIVYLSMMMIPPQVVFVPKFILFNWLHIYNTHWALILPAMFSIFGVFMLRQFF

LSIPNEITESALLDGAGHFRIFFKLVLPLAKPALATFAIIDFSWQWNDYENALVFLQS

PKLYTIPLGLQNFVLENNVDYNGMMAASSAAIIPMILIFLLGQKYIIQGVSSSAVKG"

CDS complement(3565387..3566271)

/gene="lacF_11"

/locus_tag="PIKHDJKN_03181"

/inference="ab initio prediction:Prodigal:002006"

/inference="similar to AA sequence:UniProtKB:P29823"

/codon_start=1

/transl_table=11

/product="Lactose transport system permease protein LacF"

/translation="MKRNSWIRKQGWVGYVFILPNMLGILAFFILPALYSFFMMFTDY

QFANPNWTFTGLDNIKRLFGDDQFYASIKYTIIFLMSVPISLAIAFLVALVLNRSVYL

KGLLRGMFFLPYISSGVAVAFVWMLLFQPSQGPINEVLRFIGIVSPPGWFADSSSAMY

AIDVVQIWFMLGYNMIIYLAALQEISTEQLEAARIDGAGKATITWRIVWPLVSPTTFM

LLITGLIMTVKSFSLIQAITGGGPSGSTTILSLFVYKTAFSYYEMGYASTISWFLFAV

ILVITIIQWVGQKRWVHY"

CDS complement(3566378..3567685)

/locus_tag="PIKHDJKN_03182"

/inference="ab initio prediction:Prodigal:002006"

/codon_start=1

/transl_table=11

/product="hypothetical protein"

/translation="MKYRNWLKVALITTLIASMLSACGGSNNEGASAGSANQNSGETI

NLTLWGAVPAEAGPQEVIDSWNKENPDIQVEYFRYVNDDPGNLKLDTALMTGQDADLF

VNYTINRLQKRVDAGVALDLSSRTDYNIDEQMGVDAAQWKIEDKYYGIPTKKNMLFIW

LNKSMLDEAGLPIPPVDWTWDDLREYAKALTKSNVYGLMQHDSAITAAIDGTIAGLGV

TTPEGTSNFNNELWKKQLQIVHDMMFVDKSTPEYGEQVTSKMPVDTMFLKGEAAMLAA

GEFIFRNANNLKEFPHDFKIAFATIPKVSADQKDYKYAGGLGDMLSVNAKSKHQDAAW

KFAKWYADGGMLPMASGGRLPSSKSVDNKQAMDLLLKGVEDKYDTESMNSVVFGEFPS

FQLNVPQQALDARKEEYEKYFLNEQDIDTTLKSMAKRHQEYIK"

CDS complement(3567779..3568546)

/locus_tag="PIKHDJKN_03183"

/inference="ab initio prediction:Prodigal:002006"

/inference="similar to AA sequence:UniProtKB:Q5HJF7"

/codon_start=1

/transl_table=11

/product="putative response regulatory protein"

/db_xref="COG:COG4753"

/translation="MWRTLIVEDEQYARASLRKLFAKANIPFEIIGEAVNGEEGLKLI

RELQPDVVISDIFMPLMDGIRLLQLTRKEGHECRFVMLTAVSEFEYARQAVEYGASGY

LMKLSLDLKELKQTMDKVAGELTRMDKLRLADKWFPDKTAQEPTDHPELNRMITYIEE

HFAEDVTLKRLAEYVRMDASYISDLFKKKMGITMTHFIQNRRVQAAKMLLTETEKTVS

EIGRLVGFENDNYFIKIFKRWCGLTPNEYRKEQKNVL"

CDS complement(3568524..3570347)

/locus_tag="PIKHDJKN_03184"

/inference="ab initio prediction:Prodigal:002006"

/codon_start=1

/transl_table=11

/product="hypothetical protein"

/translation="MLTRWLRRITPGTFKHRILFSFLLFLLTPIAILFLYNFRETEVM

LQRDASDINVKQLEGIKADLIDLMSLVMKTGMLLDQDSVLREVMQKPELYDAIHRKRI

VENKFASIENSFFLTGATVFYTLIDLKGNAYTSYTPENSLNYAHISSEPWVQELKRTE

GQRYVWKSNDLTIGVRERKGNRMLSLYEVLRDDGLKPFAYGRFSIDYEAWFQEKTEEN

TFGSDREGAYFLMDGYGNIMLQSKSGDNVPSTVSSAITAASQSQGQDTSSQSIVNNHM

MYTYSKIQELDGYLVKKIPLSLLFEEVNKQKLRFYTVYGAILLLFVLLTYFISSTVTG

PLKHLQRKMETTVKLNLKTKLPEQGRGELLALTRSFNFMIADINNLLEQLKLEERQKQ

FVRFQVLLGQMNPHFLLNTLNTIKSIALDKDEDEIYEICVSLGKILETTLNLEVDLIL

LKEEIVLIESYMDIQRKRFGHGIELHYDVSRDLEYALVPKFCLQPLVENSLIHGFGQT

QLEGRIDIRASSHHSLLILEVADNGIGIKKSQANQSYRKRKSIGVQNLRESLELMFKN

QPTGLRIESTDEGTQVIMHFPLLLSKPYLKEGSTFVAHIDR"

CDS complement(3570507..3571337)

/locus_tag="PIKHDJKN_03185"

/inference="ab initio prediction:Prodigal:002006"

/codon_start=1

/transl_table=11

/product="hypothetical protein"

/translation="MNKLNRSEIMDKFRGEVKQGKILLGVGAGTGITAKSSEAGGADM

LIVYNSGRYRMAGRGSLAGLLSYGDANQIVVEMGAEVLPVVKHTPVLAGVCGTDPFRI

MEVYLKQLKEQGFSGVQNFPTVGLIDGVFRQNLEETGMGYDLEVEMIRIAHELDLLTT

PYVFDPAQAKAMAEAGADILVAHMGLTTKGSIGAKTALTLDDCVQRIEAIIEAGRAVN

PEIMIICHGGPIAEPEDAAYVIERTKGIDGFFGASSIERFAAEKGITQQTESFKSIRK

"

CDS complement(3571364..3572578)

/locus_tag="PIKHDJKN_03186"

/inference="ab initio prediction:Prodigal:002006"

/codon_start=1

/transl_table=11

/product="hypothetical protein"

/translation="MKTVAIIGTFDTKGDEYLYIKSIAEELGLGTLMIHTGVFEPSFV

PDVSNQEVAHAAGMVIEELATKKDRALATEVLSIGLKKLVPQLYKQGKFDGIISFGGT

GGTSLVTPAMRALPIGVPKVMVSTVASGNTAPYVSTSDIVMIPSVVDVAGLNSISTKI

FTNALFAMAGMLKFEHTHKPDKKPLVAATMFGVTTPCVTAARKYLEERGYEVLVFHAT

GIGGQSMEALIEAGFIEGVLDLTTTEWADEIIGGVLNAGPNRLESAGHNRIPQVVSVG

ALDMCNFGPNDTVPEKFKDHKFYQHNPTVTLMRTTVEENEQIGRKLAEKLNMAKERTV

LMLPLRGISAIDVEGEPFYGPEEDRMLFDTLRQHVDRSIVEVIEMDCAINDPAFAEAA

AQKLIALMTSNS"

CDS complement(3572723..3573922)

/gene="rhaR_27"

/locus_tag="PIKHDJKN_03187"

/inference="ab initio prediction:Prodigal:002006"

/inference="protein motif:HAMAP:MF_01533"

/codon_start=1

/transl_table=11

/product="HTH-type transcriptional activator RhaR"

/translation="MNRTVILERLQSQLYEGNHIIGVSTGTGITAKYAAQSGADFILM

LNSGKFRQMGRSSLAGFLPFCNSNDMVMDFASREIVPLVRHSPILFGLNANDPTIEMV

DYIEEIKRRGFSGVNNYPTVGLIDGLFREALEDDGISYDVEVEAIRLAHRQDLFTVAF

VFDELQAAQMIEAGADVICAHLGLTEGGLLGARKVVSLEAAKSKALRIFDTCNQLKPD

LIKMIYGGPVKTPVDVQYMYSDNTDIMGYIGGSAFERIPSEKSITTITRDFKRLGKLD

EDDFMVKMLKGITQHYDYVEFVKEYVAQNYSSEIVFSDLAKVAHVSRSYLSSLFKKEV

GCSFQTYLVSFRINKAVTLLHAPQLQLSEVAGMVGYPNYAQFSKMFKKLKGCSPKKYR

SNLNTKT"

CDS complement(3574096..3574836)

/gene="map_3"

/locus_tag="PIKHDJKN_03188"

/EC_number="3.4.11.18"

/inference="ab initio prediction:Prodigal:002006"

/inference="similar to AA sequence:UniProtKB:P99121"

/codon_start=1

/transl_table=11

/product="Methionine aminopeptidase"

/translation="MIAKTEEDFNGLKEIGRIVAFIRDELVQRTVPGITTKELDDIAG

ELFEKEGAVSAPKSAYNFPGYTCISVNEEVAHGIPGQRVIQEGDLVNIDVSGSKNSYF

ADTGISFVVGEGEEVLTKICDVAKLAFEAGLKKAKPGSKKSGIGKAVFLTAKQHGLTV

IKNLTGHGVGRNIHEAPDHIYNYNDTSDDELLKEGMVIAFEPFISTLEEQVYQKEDEW

TFATEKSYVAQVEHTIILTKNGPIIVTQ"

CDS complement(3575088..3575681)

/locus_tag="PIKHDJKN_03189"

/inference="ab initio prediction:Prodigal:002006"

/codon_start=1

/transl_table=11

/product="hypothetical protein"

/translation="MSVNPQDPRVKRTRQLFVQAFNDLIGKKRNVYSISVHDITNQAT

VNRTTFYAHFQDKYDFLEYWMTEKFQMTIRDRLPEEARCNAENLRILMQSIYDFLLQF

QQCSTPGDKQFEAMFENAMHQDLHQLLLKRINEVRTQGFSQENLEALALVVSWGIFGS

ALQWSRNPQDRSFETMFEQVIEVLSINLAPLWEQTTR"

CDS 3575730..3576761

/locus_tag="PIKHDJKN_03190"

/inference="ab initio prediction:Prodigal:002006"

/codon_start=1

/transl_table=11

/product="hypothetical protein"

/translation="MLGTQQKQPFACCSHNSILISFAYATFSTRMYTIMMAQKKLYNK

KLFIGFANERMSVEMIVVTGANGKLGRKVVEELLKRVPAEDVGVSVRDVNTAQDLKER

GIRVRQGHFDNSASLLHAFEGASQVLIISSHSLGEVAIGQHQTAIDSAKKAGVSRLLY

TSQMFSSSTSHFPPMDVHAATEEMLKASGVPFTSLRNGFYAASAMMWMGDALNTGELI

APEDGPVAWTTHSDLAEATAVILTEQRYDGITPHLTASEAIDMDKIASIASKVLDRPI

RRIVVSDDEYQNHLTSRGLPEAMISMLTGMFQASRLGIFSQTHTALENLIGRSPVNFT

NFLKDSIYQ"

CDS complement(3576816..3577334)

/locus_tag="PIKHDJKN_03191"

/inference="ab initio prediction:Prodigal:002006"

/codon_start=1

/transl_table=11

/product="hypothetical protein"

/translation="MAKTNEIRLKAKRYKARPVCVTLHSGETYIGYISDVNNGGVVLT

GGGKISPKATTGDSSQRSNRKVVSRSKSGRSQNQKRANGTRKPSTRSRTVSRAQVSSF

LPMMGSLFGGLGGAASAGGMLGGGMRIFGMIQQFMPVMKMGYGMIKSIHPFMGAVKGL

MAPPSQAEEEEV"

CDS complement(3577601..3578911)

/gene="ugpB_2"

/locus_tag="PIKHDJKN_03192"

/inference="ab initio prediction:Prodigal:002006"

/inference="similar to AA sequence:UniProtKB:P0AG80"

/codon_start=1

/transl_table=11

/product="sn-glycerol-3-phosphate-binding periplasmic

protein UgpB"

/db_xref="COG:COG1653"

/translation="MKKFGSVLLAATLALTITACGASNSDGSTGAASGSNSNDTAKKP

IEITYWYSWGDKIAENNENLVKMFNESQDEVIVKAEYQGTYADQHSKAQAAFAAGNAP

EVWQNEIASVGVFAESGMTQELTSFVEKDQLDMEDFIQGLMGNSYVNGKLYALPYLRS

TPILYLNRTMLKEASLDPAGPKNWAEFEDYARKLTIKDQRYGISMPVDIWLYEAFVRQ

SGGTMISEDGKSAAFDNETGIEPVKLWLKMKDEGIMSIGTTDDAGTQAKTDFHNQRSA

MLFSSTADLTNNMKVAEEQGFELGTTFMPANDTYGVPTGGANLVMTSGLSPEKQEAAW

KFIKWMTSKEQTIYASSFTGYLPSRISAVESDEMKKLYEEKPLFKVAVDQLEYAGPRP

MQKVYPEIGEIIKNEILRAVVDSSVSAEVAVAEAAKKANVLLNK"

CDS complement(3578936..3579760)

/gene="araQ_25"

/locus_tag="PIKHDJKN_03193"

/inference="ab initio prediction:Prodigal:002006"

/inference="similar to AA sequence:UniProtKB:P94530"

/codon_start=1

/transl_table=11

/product="L-arabinose transport system permease protein

AraQ"

/db_xref="COG:COG0395"

/translation="MARKSLLRMLNITATIALVAIFAIPFLWMISTSLKSYPETVIFP

PKWIPSKVMWSNFQAAWESGPFLKYLMNSVVVSVSILVLQFTTIILAAYAFARYSFKG

DKLLFGITMVTLMIPGQLIFLPVYLLMSKWGLINNLLALILPFSSSAFGIFMLRQSFK

QVQEELIEAARLDQAPEWKIILKIMVPMARPTLVTFALFSFIAHWNDYFWPLVMTTSD

AARTLPIGISKLREVDGGAAWHILMAGNMILVVPILIVFLFGQRQIIKAFVYSGVK"

CDS complement(3579781..3580653)

/gene="ugpA_2"

/locus_tag="PIKHDJKN_03194"

/inference="ab initio prediction:Prodigal:002006"

/inference="similar to AA sequence:UniProtKB:P10905"

/codon_start=1

/transl_table=11

/product="sn-glycerol-3-phosphate transport system

permease protein UgpA"

/db_xref="COG:COG1175"

/translation="MTVWSKLRPYVLVAPSITIFSIFFLYPIFYMIFLSFYNWDFINP

VKEFVGFNNFVELFKDSAFLQVLSNSLNYTLLSVSLSIVISLLLAIWLNREGAWYGFV

QGALFSPHIVSLVSVSLVWMWLMDPKFGLLNGLLDMVGLPKLAWLSSPDSSLLSLVLV

SVWKGVGYNALIFIAGLQSIPRDVYEASALDEANWWRKFYKITLPMLSPTLFFLIIIN

LISSFQVFETIAIMTNGGPINSTNTLVFYIYEYGFRYFKIGYASAAGVILLVVVGLLT

LVYFKLLSRKVHYQ"

CDS complement(3580650..3581738)

/gene="msmX_3"

/locus_tag="PIKHDJKN_03195"

/EC_number="7.5.2.-"

/inference="ab initio prediction:Prodigal:002006"

/inference="similar to AA sequence:UniProtKB:P94360"

/codon_start=1

/transl_table=11

/product="Oligosaccharides import ATP-binding protein

MsmX"

/db_xref="COG:COG3839"

/translation="MAQIVLKQIEKSFKQDKVIEGLDLTIEDGSFTVLVGPSGCGKST

TLRMIAGLEKETGGEIWIGDHRVNGVEPGKRNVAMVFQNYALYPMMTVKENIEFGLVN

RKVPRPERERLVKEITEVVGLAEYLHKKPQALSGGQRQRVALARAMVKSPAVFLMDEP

LSNLDAKLRHQMRTELIQLHKRLGATFVFVTHDQVEAMSMGDRIVIMDKGRIQQADSP

MKIYDDPDNVFTAQFIGTPPMNVIDRERILPFLNGQLHDDIGHIGFRPEKATMTLENR

PQTGHIQFSAHIMTRETLGAEIIYQLDSALGPIAVKSFLKPLESASEVNIIVSMQDLY

YFDRNGVRARQHELAEAGELIVLGGGYS"

CDS complement(3581763..3582599)

/gene="cpdA_3"

/locus_tag="PIKHDJKN_03196"

/EC_number="3.1.4.53"

/inference="ab initio prediction:Prodigal:002006"

/inference="protein motif:HAMAP:MF_00905"

/codon_start=1

/transl_table=11

/product="3',5'-cyclic adenosine monophosphate

phosphodiesterase CpdA"

/translation="MRLALLGDLHYSSMSHGTEEMRAARVRAYEHMLKAFLKEDADYH

ISIGDLTHEGTAQELRSIFSILAKSERKFIHVLGNHDTYSLPKHEISTLTGQARYRAI

DTSHARLIFLDTTQEMNREDWGGELDEEQLTWIKGQIADSHEKQVLVFGHHPVFDTTA

RSTLDKLYIRPQDELRDVFNSRPGGGIYFCGHNHMNSIVKQGEWHYVQTAACLDVPAY

RVIEVDEGEVVITHQRMLGAVLMNDIQSFCHDTPGFGPVANAVGEESDLILSVRIASA

AQ"

CDS complement(3582638..3583471)

/gene="yutF_1"

/locus_tag="PIKHDJKN_03197"

/EC_number="3.1.3.-"

/inference="ab initio prediction:Prodigal:002006"

/inference="similar to AA sequence:UniProtKB:O32125"

/codon_start=1

/transl_table=11

/product="Acid sugar phosphatase"

/db_xref="COG:COG0647"

/translation="MEDVYAYDAYCFDLDGTIYIGQMRLPGVNSFINSLRTRGKRLLF

LTNTPVHTKEDCYKRLLSLGISCQPDEILTAGYLSGMYFAEHAPDAKVLVVGEEALRR

ELRALGVAITEDPYRSTHVLVGMDRKFNYDKLHLAAKAVRHGAALLATNPDNCCPVQG

DIIPDTGAVLSSIEAASQQKASVVIGKPSRYYAEKALQLLDVDRSRCLMIGDRLETDI

LFGQQNGMKTALVLTGISSREDIGKTKIVPDYIWPSFNEFIMEQQAHPKPSNVLQVEK

A"

CDS complement(3583490..3584203)

/gene="glpQ_2"

/locus_tag="PIKHDJKN_03198"

/EC_number="3.1.4.46"

/inference="ab initio prediction:Prodigal:002006"

/inference="similar to AA sequence:UniProtKB:P37965"

/codon_start=1

/transl_table=11

/product="Glycerophosphodiester phosphodiesterase"

/db_xref="COG:COG0584"

/translation="MIIQAVAHRGYPSKYPENTMSSFLKAVELGFTHLELDVHLSKDG

IPVVIHDHTLDRMTNGTGRVADYTASELQSLKIKQDEHIPTLEEVLRALKGKIQLNIE

LKQMGSYYPHLEERVLEVIEEFNMLDQVVITSFDFDALEKIRERSGDVRIGLITFGCS

KAILDWSLEMGATFLSIHHAFVTEHYIHKCQELGIQLMAWTINETSVMKKFQEYPSVW

VCTDELECFIDVLELSSSL"

CDS complement(3584348..3584887)

/gene="glpP_1"

/locus_tag="PIKHDJKN_03199"

/inference="ab initio prediction:Prodigal:002006"

/inference="similar to AA sequence:UniProtKB:P30300"

/codon_start=1

/transl_table=11

/product="Glycerol uptake operon antiterminator regulatory

protein"

/db_xref="COG:COG1954"

/translation="MKNYPIIASITTDEQISQAIASDVQRVILMTGNITNLGSIIESL

HRSGKQAYVHTEMVSGIGRDVSAIQYLADVFKIDGIVTTKSNAVSAAKQAGILSIQRI

FAIDTAAITTAVRMIMSYKPDEVELMPGLMPRVIQELKERISQPLIVGGLIRYEEEIQ

QALKSGADFVSVGHEKFWN"

CDS complement(3585073..3585912)

/locus_tag="PIKHDJKN_03200"

/inference="ab initio prediction:Prodigal:002006"

/codon_start=1

/transl_table=11

/product="hypothetical protein"

/translation="MSKEARQSITGLAMVLPSFAILLIVVIIPIVQSFIMSLSNGSGG

YDLSRYTYLFTDKGMRSNIVFTLGVTAITCVAVILVSYTLAIYMRFNQGPIVNLIRKT

YMIPLFIPGVIATYGLINLLGNHGWLARMLEVVGITLPRIIFDEKGIIIANLWFNIPF

TTMLLSSALSGIPSSIIESAKDVGVGRLTLFTRFIFPLSYKTFLVALTFVFMGVIGSF

TAPYLIGANSPQMLGVSMQQVFSVFQEREQAAAIAFFSFLLCSVLGAFYIRSMAEEEK

AKI"

CDS complement(3585909..3587075)

/locus_tag="PIKHDJKN_03201"

/inference="ab initio prediction:Prodigal:002006"

/codon_start=1

/transl_table=11

/product="hypothetical protein"

/translation="MRMKKPFLSLLTVVGLSMSLLAGCGNGSEIKSTSNTEGNAAASS

NPVSFNFYFTGSQNVKDLWDSLEPMFEKQHPDIDVNLVYIPSGTGAEPTYDRIVAAKK

AGKGSGDIDLYEDGITSVAQGTKDGIWEQLNEKDIPNLSKVNTDTMKDVDNFAVPYRS

SAVVLAYDSSKISNPPTTLDELLQWIKANPEQFAYNDPSTGGSGSSFVQTIVYKDLPE

EDIHNSDPAIMEKWDGGFDTLKEIGPSVYGQGIYPKKNQGTLDLLMNGEVSLIPAWSD

MVLQQLNEGLLPDTIKMQQITPGFNGGPTYLMVNQESDKKQAINEFLNFVLSPEAQEV

IVDKMNGFPGIELSNMSQEMQDKFDGVAEGFRTFNIGDLGTEINKRWQSDVAAK"

CDS complement(3587090..3588130)

/gene="potA_1"

/locus_tag="PIKHDJKN_03202"

/EC_number="7.6.2.11"

/inference="ab initio prediction:Prodigal:002006"

/inference="similar to AA sequence:UniProtKB:Q7A679"

/codon_start=1

/transl_table=11

/product="Spermidine/putrescine import ATP-binding protein

PotA"

/translation="MQLSVRELAKRYKTGDGVSGINIDIEKGELVTLLGPSGCGKTTV

LRSIGGFLEPDSGDILIEGKSVIQLPPEKRPTSMVFQSYNLWSHMSVYDNLAFGLKIR

KMPKAEISTAVEDALKLVRLSGFGKKYPAELSGGQQQRVALARSLLLNPAVLLLDEPF

SALDAKLRHEMREELREIQMKTGLTMVFVTHDQEEALSLSDRIIVMNHGRIEQIAPPQ

QIYDEPASLYVAGFIGKMNFLKGVAEGENIRIGHLRLPNEKGLSGQVTAAVRPEDVQV

SSTVMGEGTLVGKVKQVMILGHYAEVTVELVEFGIIRAFLNKEFVEQLQVGKDVGVSI

SKMTGFPVDESN"

CDS complement(3588175..3588999)

/gene="sugB"

/locus_tag="PIKHDJKN_03203"

/inference="ab initio prediction:Prodigal:002006"

/inference="similar to AA sequence:UniProtKB:P9WG01"

/codon_start=1

/transl_table=11

/product="Trehalose transport system permease protein

SugB"

/db_xref="COG:COG0395"

/translation="MSWYWKHVGPRRIIEFVLLTVFAVFFAGPLVNLLVLAFSEKWNY

PDILPQVWGFKWWEFVLAKDDIVSSISLSFLIATLVTLLSIVVCIPAAYAFARLQFPL

KRMFLFSFLLTHAFPKMGLYVSIAVLFYRIGLMNTLLGVVLIHMINVLMFMTWIPTSA

FRNVHRAQEESARDVGASPFKVFRSITLPMAMPGIMVASIFTFLNSLDEAQGTFLVGI

PDYKTMPIVMYSIISDFPSSAGAVFSIILTLPTIILLVAAQRLVSADVLASGFQMK"

CDS complement(3588975..3590039)

/gene="rbsR_3"

/locus_tag="PIKHDJKN_03204"

/inference="ab initio prediction:Prodigal:002006"

/inference="similar to AA sequence:UniProtKB:P0ACQ0"

/codon_start=1

/transl_table=11

/product="Ribose operon repressor"

/db_xref="COG:COG1609"

/translation="MDNKLNHSLRELAQLAGVSKSTASRVISGNGYASPEVRDRVLKA

VKQLRYKPSAVARAMVAKRTYNIGVIVFRDRLPIVSHTLYGRIIDEILMSAEEMGYSI

FLKTDREMSLRSTDYMLEQRVDGLILVSRLQKNVIDYVKNFNIPYLMVNGSTEDPDVV

HLVSNDEAGGERAAQHLYDCGHRKFFIIAGPGEHRSHSLRLEGFRKRLESLGVSSEQD

GFTVVESSESSFDSGRKLMEEHYAAFREGQYSALFSTNDMLALGAMKVLQERGVRVPE

EVSVMGYDNTEVAGMYHPSLTTVSVDASGIGRDAVTVLDRLISGEGSVPRLIEYESEV

IVRQSTNGREDGTCLGTGNT"

CDS complement(3590024..3590776)

/gene="ugpQ"

/locus_tag="PIKHDJKN_03205"

/EC_number="3.1.4.46"

/inference="ab initio prediction:Prodigal:002006"

/inference="similar to AA sequence:UniProtKB:P10908"

/codon_start=1

/transl_table=11

/product="Glycerophosphodiester phosphodiesterase,

cytoplasmic"

/db_xref="COG:COG0584"

/translation="MNKFPLITAHTGCMGHPDHSFESLQAALKLGVDIYEDDIRSTQD

GVPVLAHDDDIVLADGSRGSLAEMTLKELNTARLAPIPTLQDVLEQIRQAGKIMNLDI

KTDSALGPVSALVDRMGMEEMVFLSGCEYGSAVKASRNAPSIRRLLNVNMQSFGSLSY

EEAMFQACANAREAGCFGINVPYQLVQPELMEAVQRSGLAIYVWTVSEADDMRRLAQL

GVDSITTRDPAKLIAVREETDFAKENRDWTIS"

CDS complement(3590773..3591849)

/locus_tag="PIKHDJKN_03206"

/inference="ab initio prediction:Prodigal:002006"

/codon_start=1

/transl_table=11

/product="hypothetical protein"

/translation="MKWFACELHTHTLHSDGSQSLGELAAGAANLGFDAIALTDHNTM

SGLSGKEEIEQRYGLRIIPGMEWTTFYGHMVTIGLSAFADWRQADRDGIDEGIENVHR

LGGIAGLAHPFRIGSPACTGCFWEYEIGNWSAVDYIEVWSGTFPSIQTNNRRAFDLWT

YKLNEGYRIAATSGRDWHAQGETDDPISVTYLGIEDDAVAGNEDSTALWLHEETLIQA

LREGRASVTIGPLLTLTLEAGGAFYAIGSTVPTVVERREAAEEPIRANLTLDFSVRTG

LWSLPKQVCRLKLCSNLGEELNLEIPSSSDEQIMQFEAQLRESALDTPRRWIRAELWG

TVRGVHVLIAFTNAIYFEEGGYSQ"

CDS complement(3591846..3592313)

/locus_tag="PIKHDJKN_03207"

/inference="ab initio prediction:Prodigal:002006"

/codon_start=1

/transl_table=11

/product="hypothetical protein"

/translation="MKLLDLQGYASPEDSQTHIRIPFELDEGCGKLNLRLQYTPKTLE

NREKALRLLKDSYDLYILPDNREYAIANADRHLPLKNLMTLSLDDARGYRGACHRQDE

VQDLYVSEREASPGLMAGELVPGPWSVTLSLHCIVTDTCDYQLEIWTTEEDSI"

CDS complement(3592644..3592973)

/gene="csoR"

/locus_tag="PIKHDJKN_03208"

/inference="ab initio prediction:Prodigal:002006"

/inference="similar to AA sequence:UniProtKB:O32222"

/codon_start=1

/transl_table=11

/product="Copper-sensing transcriptional repressor CsoR"

/db_xref="COG:COG1937"

/translation="MDKTTHEATSVDHTSSTERKSHHSEKTKLSLIHRLNRIEGQVRG

VKGLIEKDTYCDDVLHQIASIQSALNGLGKQLFEHHLKSCVIERIQEGDREVLDELMT

TVNKLIK"

CDS complement(3593123..3593368)

/locus_tag="PIKHDJKN_03209"

/inference="ab initio prediction:Prodigal:002006"

/codon_start=1

/transl_table=11

/product="hypothetical protein"

/translation="MEKTIKIYTIPTCSDCSYAKRFFKENELPYTDFDCTENPKYPEE

VWNLTGKQIVPTIVIGDKVFIGFAENLSEISKLLNKE"

CDS complement(3593391..3594218)

/locus_tag="PIKHDJKN_03210"

/inference="ab initio prediction:Prodigal:002006"

/codon_start=1

/transl_table=11

/product="hypothetical protein"

/translation="MIRNILAFGVLPHYGNPKESNHSDGHGGTSHSHIGHGDHDSSVQ

TASNLKASFTFASGVAKTNTDVELNIQIKDNEGNLVKEFERSHEKLLHLIVVNKDLSY

FSHIHPEYDGNGNFKINTTFLNGGGYKFFADILPKGGSSTTLSEWVKVEGEEKALEPI

EADTKLVKVVDGKEIELTISNTTAKDEITLTFNIVDAQTKKGISNLEQYLGAVGHVVI

LSDDAEQYLHVHPMDEKATGPKAEFMTSFPKSGTYKIWGQFQHQGEVFTVPFAVDIK"

CDS complement(3594246..3596678)

/gene="copA_2"

/locus_tag="PIKHDJKN_03211"

/EC_number="7.2.2.8"

/inference="ab initio prediction:Prodigal:002006"

/inference="similar to AA sequence:UniProtKB:O32220"

/codon_start=1

/transl_table=11

/product="Copper-exporting P-type ATPase"

/db_xref="COG:COG2217"

/translation="MESTAAQNKQSTLQITGMTCAACANRIEKGLNKMEGVSSANVNF

ALEKANVTYDPVKVDRNTLEETIKKLGYGTVTEVAQFNLEGMTCAACANKIEKGLSKL

PGVTNATVNFAMETARVEYSSGDVTIEDMQNKVKKLGYKAMIKQENQNADDHRLNEIR

QQKRKLLISAIFSFPLLWTMVAHFSFTSWMYMPDFLMNPWVQLILATPVQFYIGKQFY

VGAYKALRNRSANMDVLVSLGTSAAYFYSLYLTLDWYAMGDSMHHGPSLYYETSAVLI

TLVIMGKLFEANAKGRTSEAIKSLMGLQAKTALVIRDGKELTIPVDEVIVGDVVLVRP

GDKLPVDGEVLEGVSSVDESMLTGESLPVEKKAGDAVIGATINKNGILRIKATKVGKE

TALAQIIKVVEEAQGSKAPIQRVADIISGIFVPIVVGIAVVAFLVWYFLVTPNDFAGA

LEKAIAILVIACPCALGLATPTSIMAGSGRAAELGILFKGGEHLEQTHKIDAIILDKT

GTVTKGKPELTDVLAEGNESEFLRLVGAAEKNSEHPLAEAIVVGIQERNIELPRTESF

EAIPGFGIKAVVEGKQLLIGTRRLMEKYKVNAKAAYEPMSRLEESGKTAMLVAIDDRY

AGMVAVADTIKETSKAAVSRLKEMGIQVIMITGDNERTAKAIAAQVGIDHVRAEVLPE

GKAEEVKKLQAQGKKVAMVGDGINDAPALATADIGMAIGTGTDVAMEAADVTLMRGDL

SSIPDAIYMSRKTMRNIKQNLFWALGYNTLGIPIAAIGLLAPWVAGAAMAMSSVSVVL

NALRLQRVKIRN"

CDS complement(3596708..3596908)

/gene="copZ_2"

/locus_tag="PIKHDJKN_03212"

/inference="ab initio prediction:Prodigal:002006"

/inference="similar to AA sequence:UniProtKB:O32221"

/codon_start=1

/transl_table=11

/product="Copper chaperone CopZ"

/translation="MKNITLQVEGMSCQHCVNSIEGALKEIGAKGKVDLKKNTVDVSF

DEIQITIDRIKETVEDQGYDVV"

CDS complement(3597276..3597479)

/gene="copZ_3"

/locus_tag="PIKHDJKN_03213"

/inference="ab initio prediction:Prodigal:002006"

/inference="similar to AA sequence:UniProtKB:O32221"

/codon_start=1

/transl_table=11

/product="Copper chaperone CopZ"

/translation="MKEASLQVQGMTCRSCVPRIESAINEIGAEGQVNFEQGTIDVRF

DEEKIQISEIEEAIRKKGYNVLV"

CDS complement(3597485..3598123)

/locus_tag="PIKHDJKN_03214"

/EC_number="2.1.1.272"

/inference="ab initio prediction:Prodigal:002006"

/inference="similar to AA sequence:UniProtKB:O87689"

/codon_start=1

/transl_table=11

/product="Cobalt-factor III methyltransferase"

/db_xref="COG:COG1010"

/translation="MVEKIKIAISPVIQLGGSLFTPNQLVKIGNSVGTDAKVEMTTFK

QLYAEVPIEQRETIKEELERIGLEVNPAGFVTKSLIACNFCKGAEEAGLKTAKTLNKA

IAGIETPAPLKIGYAGCALGTSEPLIKDIGVIKMRDKFDIYVGGESKGLKTSFAKLLR

SEITEDQLIWAVTGIIDFYKEQAKGKEKFSKFVKRISLEQLQQIAEESIERR"

CDS complement(3598157..3598996)

/locus_tag="PIKHDJKN_03215"

/inference="ab initio prediction:Prodigal:002006"

/codon_start=1

/transl_table=11

/product="hypothetical protein"

/translation="MQRKMMLILTVVLVLGVLSACGTSKKNENSATPSTGHGEHGDHD

TKEQPANSLKASFSFASGTVKATENSDLNIQIMDSDGNSVNEFEWSHEKLMHLIVVSK

DLSYFNHIHPDYNTNGNFAIKTSFPNGGEYKVFSDFVPKGGASTTLSEWVEVEGEEKV

QEPIEADTKLVKVADGKEVELTLSSTTAKDEVTLTFNMVDAQTKKDISDLEQYLGAVG

HVVILSDDAEQYLHVHPVDEKGTGPKAEFMTSFPKSGTYKIWGQFQHQGQVFTVPFVV

DIE"

CDS complement(3599055..3599396)

/locus_tag="PIKHDJKN_03216"

/inference="ab initio prediction:Prodigal:002006"

/codon_start=1

/transl_table=11

/product="hypothetical protein"

/translation="MILDTKESIYSFPKNQESNYLTLKDRLSIQMRLNRIEGQVRGVQ

RQMERDDYCDQILNQVEAIHSALSAVGILLMSNHLKSYAASADSKHSESIAQEMLKTM

SILVNRKEEEK"

CDS complement(3599778..3599882)

/locus_tag="PIKHDJKN_03217"

/inference="ab initio prediction:Prodigal:002006"

/codon_start=1

/transl_table=11

/product="hypothetical protein"

/translation="MCTWIQKCLNMKAEMIRVAALLMEHQEKVIIRIK"

CDS complement(3599938..3601368)

/gene="efpA"

/locus_tag="PIKHDJKN_03218"

/inference="ab initio prediction:Prodigal:002006"

/inference="similar to AA sequence:UniProtKB:P9WJY5"

/codon_start=1

/transl_table=11

/product="putative MFS-type transporter EfpA"

/translation="MMNQALSLPDPRRWKALALLCIANFMVIMDTSIIGVALPAIKEA

LGYTQANLQWVFNAYVIFFGGLLLLGGRLSDLFGQRRMFMWGFSILTLASLLAGVAWN

EESLNIGRALQGFGSALIAPSALTIVMMLFSGNPKELGKALGFWGASAAAGGSAGVFL

GGVITEWLSWEWTFLINVPIGIVAMLVAPRILSKGVRRKGSIDVIGSISVTASLVLVV

YGIVTAEHNGWSAPTTVWTLVLGAVLFILFLIIQAVKKEPLIPLRIFKAPNLAAGNIA

LIMLSAAWIPLWFFLNLYLQQVLKFSAFAGGLGLLPMTILIAICMIFVSGKLIGKIGV

KANVVIGLIALGGSLLLFATNTPVDGNFATHVLPASLLGALGMSLAYIPATMSAMSGA

KPEESGLASGLANTSYQIGSAISLAIMVAIAASRTASQAGADQLIALNEGFQQAFLWS

GIIAIAGAILAILFIRSPKSGKQTGV"

CDS complement(3601701..3602825)

/gene="soxA_1"

/locus_tag="PIKHDJKN_03219"

/EC_number="1.5.3.1"

/inference="ab initio prediction:Prodigal:002006"

/inference="similar to AA sequence:UniProtKB:P23342"

/codon_start=1

/transl_table=11

/product="Monomeric sarcosine oxidase"

/translation="MHYDVIVIGAGSMGMAAGYFLSKSGKSTLLIDSFNPPHNKGSHH

GETRIIRHAYGEGENYVPLALKAQELWNDLEKVSGKQLFLPTGVLNVGYEKTDFIQNI

ISSSKTYSLPLEILDSIEVNKRWPGITLPNNYIGCYEPTSGVLKCEECIEAYRELTEQ

NGATIITNSKVKEIMVHGKRVTIKTDNQTFSANALVVSAGAWSGSLLSMLDLNLPLNP

IRKTFAWFDTNEDLYNHKEFPAFSFETSHGHYYGFPSIDGSGLKVGRHDGGDTINPDE

AILGFGELEEDTTDPKQFLHHYMPSAQDLKFGKTCMYTMTPDEDFIIDTHPNYPNVAI

ASGFSGHGFKFSSVVGQILSELIISGKTEHNISPFSINRF"

CDS complement(3603133..3603879)

/gene="map_4"

/locus_tag="PIKHDJKN_03220"

/EC_number="3.4.11.18"

/inference="ab initio prediction:Prodigal:002006"

/inference="similar to AA sequence:UniProtKB:P19994"

/codon_start=1

/transl_table=11

/product="Methionine aminopeptidase 1"

/db_xref="COG:COG0024"

/translation="MIIIKSPREIEEMKPASQIVADCYRKVTKLIEPGITTLEINDFV

ASHITKMGGKQFTKGYNGFPAETCISINDVVAHGIPSNRVLMDGDLLKLDIVVEYGGW

FGDSCMSYAVGNIRPEVKKLMKVTKECLDLGIARAIPGGRLGDITSAIQQHAEAHGYS

VVRDLLAHGIGRSLHEEPTYEHIGVAGKGIRLKEGMVFTIEPMINEGTFRITIDDDRW

TARTADGKLSAQYEHTIAITLDGPLILTAQ"

CDS complement(3603907..3604602)

/locus_tag="PIKHDJKN_03221"

/inference="ab initio prediction:Prodigal:002006"

/codon_start=1

/transl_table=11

/product="hypothetical protein"

/translation="MTPRTKEQNEEIRLRRLAQIRKAAADVFLNKGPLLEIRDVAVQA

GLGYGTVYHYYSNKGDLLHDLLWDALERAGEWPEAPRTSALGEELAGGHFDLAAVDSR

SRSHETPASAELGTVAAASVRLLQLWAEDHALYLLYKLALEGFASLPEARSTKLTAAF

QDEVLAPFAALVDTGRAADEDAAFGGNAAEQPYRLQRAEMLVAALVGCASLSLRRKKL

HEEARDIVRFLKL"

CDS 3605271..3605600

/locus_tag="PIKHDJKN_03222"

/inference="ab initio prediction:Prodigal:002006"

/codon_start=1

/transl_table=11

/product="hypothetical protein"

/translation="MSQNKITSDLLRGHTDTMILRLLSEADRYGYEIVKMIAERSGGE

YELKEATMYSSVRRLEAEDDIEWYWGDESQGGRRKYFRITEKGKTTYDSNKDNWEYAK

RVLENLL"

CDS 3605615..3606478

/locus_tag="PIKHDJKN_03223"

/inference="ab initio prediction:Prodigal:002006"

/codon_start=1

/transl_table=11

/product="hypothetical protein"

/translation="MNEKLTSYLNGVFAPYDGVKSVTELKADLHSDLLERFRDLKAEG
KDDETAFEMTIESIGDIEQTVQEVANLSRSLERQVVTNFSASNLPTSDFAGVIAHKGQ
FKSSALRGSNFTNADLTGSSFASSDVRDANFDGANLTDCILSTLDLSNVSFNQSILVR
TTFSKSGLDRAKFTAVRLTDVTFSLTDLRKTIFENCIFNGVEFKSSDLGGLCFDGQTF
IGVKFDKVALNEVSFKNATLKNVSFPTSAWSKKYYRIIKTVCFDGAMMDKLTYAALKG
MDANLSKVTVI"

CDS 3606490..3607251

/gene="drrA_2"

/locus_tag="PIKHDJKN_03224"

/EC_number="3.6.3.-"

/inference="ab initio prediction:Prodigal:002006"

/inference="similar to AA sequence:UniProtKB:P32010"

/codon_start=1

/transl_table=11

/product="Daunorubicin/doxorubicin resistance ATP-binding
protein DrrA"

/translation="MPNKSIQVIDLQKSYKQLHVLKGVDFEVEKGSIFALLGSNGAGK
TTVVKILTTLLQKDSGTATVNGFDVATKPDNVRQAISLTGQFAAVDEILTGRENLIMI
AKLRYLQNPRQVADDLLKRFGLTEAADRRVSTYSGGMRRRLDIALSLVGKPQIIFLDE
PTTGLDPEARIEVWKIVKELADGGTTVFLTTQYLEEAEQLADRIAILHEGRIIASGTL
SDLKKLIPSAKVEYIEKQPTLEDIFLAIISKKEEN"

CDS 3607252..3608019

/gene="drrB_2"

/locus_tag="PIKHDJKN_03225"

/inference="ab initio prediction:Prodigal:002006"

/inference="similar to AA sequence:UniProtKB:P32011"

/codon_start=1

/transl_table=11

/product="Daunorubicin/doxorubicin resistance ABC
transporter permease protein DrrB"

/translation="METVKKHFFSDMSVMLGRSLRHIFRSMDTIITVTIMPIAFMLLF
VYVFGGAIQTGTDNYVNYLLPGILLIAIASGISYTAYRLFIDVQRGIFERFHSMPISR

STLLWGHVLTSLVSNVISVVVILLVALIMGFRSSAVVLSWLGVAGILALFTLALTWVA

VIAGLSAKSVDGASAFSYPIIFLPFISSAFVPTESMPSVVRAFAENQPVTSIVEAIRA

LMLGQPVGDDIWIALAWCVGILLVAYILAMRVYKKRV"

CDS complement(3608450..3609160)

/gene="fabG_7"

/locus_tag="PIKHDJKN_03226"

/EC_number="1.1.1.100"

/inference="ab initio prediction:Prodigal:002006"

/inference="similar to AA sequence:UniProtKB:P99093"

/codon_start=1

/transl_table=11

/product="3-oxoacyl-[acyl-carrier-protein] reductase FabG"

/translation="MKKTAFVTGGNKGIGFEIVKQLGQAGWKVVLGARSAERGEAAVS

ELTSKGLDIEFVHIDMSDLKTIERAADTIAKDYPDLKLLINNAGMPGAFSRSFTDTKE

EDLRNAFEVNFFGTFRLNQRLFPLIKDNEGTIVNVSTDMASLDHMQNAGFALNAFDYN

SSKTANNAMTVSMAYELKSSRAQVFAVTPGFTSTDLNGNAEGGKSKEAAAAIIVGYAT

DGEHHNGEFLNEQGVYAW"

CDS 3609262..3609621

/gene="adhR_1"

/locus_tag="PIKHDJKN_03227"

/inference="ab initio prediction:Prodigal:002006"

/inference="similar to AA sequence:UniProtKB:O06008"

/codon_start=1

/transl_table=11

/product="HTH-type transcriptional regulator AdhR"

/db_xref="COG:COG0789"

/translation="MLTIGEVAKKVEITIGAIRFYERKGLLKPAARSEQNNRLYTEDD

LNWLVFIKCLRETGMSVEDIKTYYDKVNEGTSTLPERTKLIQDQRQKLLEDIEEKKAQ

LVHLDNKLERYYRGENY"

CDS complement(3609780..3611558)

/locus_tag="PIKHDJKN_03228"

/inference="ab initio prediction:Prodigal:002006"

/codon_start=1

/transl_table=11

/product="hypothetical protein"

/translation="MDRKLQLQQSPEWTDDLIIYEIPTKSFTSPNGPESGTFASLKEK

LEYLKDLGITGIWLTGHSLSHPDHFYNIWNQYAVIQHDKLDPSLGGEEEFLALIEEAH

HNGIKVFLDVITHGVMSDSPLIEAHPEWFKGGSWGMTDFDWFGDHEDLDDWWVKVWVD

YVQKFGIDGFRLDVATYRFDLWARIRSQCSALGHPIVIIPEKGPAVQGVTDFLQHGFR

ISNNHYGLQEQSRVLSDLPSYLQLVNRLQPNMFQVKVLYEDDSEAGSGPEHDYVLTLA

VEGKETKPVPSDGRHEGYEREYFLLSIRGIDPSKQIKQLFVMDYDSNEWQLENTLFPD

YRIHRSERQGEVILLSLPMRLPTGQKISFQLSCHDDGWEGYPEEKTPYVAQGKRSVFG

YSFIFAPAIPVFMAGEEFDAQYRPLPQLSPHLFGGQDAGCGKWLYGSWIDWSQLEQEK

HRAMLEDVKQMIRLRKKYRSVIKPLQLGKDNGNSQPQSAQHQSLDNLPVPYYYQTPTG

YLLIASNPYHDKDSDISVTIPDFSDNRIYKVTDEWNGVYSFRLTGKQIKERQWRIKRD

QTPRGGLMVLFIEPLESESESRAATE"

CDS 3611719..3612624

/gene="rhaR_28"

/locus_tag="PIKHDJKN_03229"

/inference="ab initio prediction:Prodigal:002006"

/inference="protein motif:HAMAP:MF_01533"

/codon_start=1

/transl_table=11

/product="HTH-type transcriptional activator RhaR"

/translation="MQHVFHIPNLYNDILLDTFHYGCFEKGWSNSNCRHFFYEFIYCA

KGSIVQWINGATYSLQRGDFLIIKQGLQHHTPPLGEETELFVFHFDLTNQQVRSIFQM

IEDPVVRSGQLQAVSLEKWVDQFMKEYKELFLQAGEKDTVTKTRLTSSVALLRMQSKV

LDLIGQIAEYFLQECNWNHLSIATPAQIEIAHNAAYQFELQALDGVRIHDLASQLGVS

RSYLCECFKLIYGISPKEYLSKTLMRHAKNELQNSTLSIEKIAEKLHYSSAAHFSKAF

RSNEGITPSHYRNLTRSNFINFSEI"

CDS complement(3613161..3614069)

/gene="ydaD_3"

/locus_tag="PIKHDJKN_03230"

/EC_number="1.-.-.-"

/inference="ab initio prediction:Prodigal:002006"

/inference="similar to AA sequence:UniProtKB:P80873"

/codon_start=1

/transl_table=11

/product="General stress protein 39"

/translation="MLKGYPMYPYIGWRTKTVMEPVAFPPQHQDQIPGLEYLMEPRPI

FDNPGYVGSGKLRGKTAIITGGDSGFGRAIAVAYAKEGANLTLVYLNEHRDAEETQRY

VEQFGVRCLILPRDLRNPAASSEIVAETIHHYQRLDILVSNAAVQPYTPSILDVSDEQ

LENTFRTNVFPMFYLTKAAIPFLNPASAIICTTSRVAYEGNKNAIDYSATKGAIVSFI

RTMAISLADKGIRVNGVAPGPGWTPLTVSSWPAEMVSTWGVDSPFGRAVQPFEVAPAY

VYLAASDSMFVTGQVLHVNGGVIRYS"

CDS complement(3614707..3616311)

/locus_tag="PIKHDJKN_03231"

/inference="ab initio prediction:Prodigal:002006"

/codon_start=1

/transl_table=11

/product="hypothetical protein"

/translation="MRKRKKWMVMMMLGAWIVGLGSVVVPLTTAKAAAGPQTAQLKLM

TFNVRNLHGDDGTVNSWDNRKDRAVNVINSFGPDIIGMQEAYAVQIDYFVSHLNGNYQ

SVGTSRYGNKTDEYSNIVYRADKFQVLESGQFWLSETPDVVGSRSSYDTKWPRICTWA

KFQAKDDPRTVFYYFNTHFSLNSSARALSSQLILNRVSEYVTSPDTPVFIGGDLNSPQ

TESAYSIIEQSDFSDVWTQSGHNFNNASTVSNYNGNTTDHHIDWIFQRGAGTVESMEI

NRYNENGLYPSDHYPVQLTIQIPRTGKALPSGYVNVAAKGTASADGYVQGEAPNKAID

GTVLGNSKWCSTGPEPHWLQIDLGKKYNLYRFVVKHAAAGGETPSINTRDFKIQVSDN

GSTWSDAASVTGNTRDVTLHDVNTFGRYIRVYITDAGVLSTDTSARIYELEAYGLNKG

AVFYKDGSYGGYAVTLAPGSYTLAALREAGIQNDDITSLRVFGGVTVELYEHDNFQGA

KLVRTEDDSSLTPEGWSDRTPSIKIN"

CDS complement(3616490..3619789)

/locus_tag="PIKHDJKN_03232"

/inference="ab initio prediction:Prodigal:002006"

/codon_start=1

/transl_table=11

/product="hypothetical protein"

/translation="MDLFRIQREIIIVRKSKYKGGAKLSMRFVLQKLESRIRELAGLR

YRESRTLGDVYIVPDVRDLHEARQQLEGGVAVHVGDRWGGRDRYAWFITDVEFPPYWK

GQDVIGLIKLGETSGGNTRGYEAQVYLKGEPLQALDRNHAELFFPKDAVEEGRVELAI

YAWSGLKVPDGRTPFDHRIDLLSIAVLDAAVDDLYYTSLHMLQTVEQLDERQLERHEL

TDALEAAFRQLDWSRPGSEVFYASAVQACQTLRKRLEAVPRGPRPQITAVGHCHIDVA

WLWRLHHTREKSGRSFSTVLMLMERFPEYVFLQSQPQLYAYLKKDFPALYAKIKDRVQ

SGNWEATGAMWLESDCNIPSGESLVRQLLIGKRFFRDEFGVDNRVLWLPDVFGYSWAL

PQILKKSGVDYFMTTKISWSQYNRFPYDTFTWRGIDGTEILTHYITTPDNGDNSYYTY

NGNVTGASVQGLWNNYRQKDMNNELLLAYGWGDGGGGPTRDMLEAVRRYEDMPGAPQV

KTGGAEDFFVKLEKQVKDQPQLHLWDGELYLESHRGTYTSQAYNKRMNRRMEMLLHHA

EFLNMFALVLNPEHAYLSVQLNDSWEIVLRNQFHDIIPGSSIYEVYEDSRIEYEESER

LALAAIEQGLGSFGLNRTMDCEAGEKGIPVRIFNALGWKRSFHAEVDINDAVKYTWYT

AEGFKLDAQIVASETADLTALNRVLVAVRDVPAYGYTTIYGRDEAEQEQEDLALDNWY

EAPVLESWSINVDHLETPYYSIRLNDLGQIASLYDKQARREVLKKGQPANILQVFEDK

PMAHDAWDIDIYYQDKMTVVDRLQELTVLEDGPLRGVLKLLYSYESSTIEQYLIVYRD

HPRIDFKTQVDWHEHQQLLKTAFPVDIRSTKATYEIQFGNVERPTHWNTSWDYARFES

VAQRWVDLSEGGYGVSLLNDCKYGHDIRNHVIRLSLIKSAIYPDNNADQGLHEFTYSL

FPHNRGWYEAGTHQTACELNAPLIAAVVQGSSHTPEELGMIKTDARNVMIDTVKKAED

GKTLIVRLYEFGGIRTKTSVTVEPYLGQLCSVEETDLMEEHPTALPFDCQQIELSLAP

YEIKTLSIAIS"

CDS complement(3619969..3620229)

/locus_tag="PIKHDJKN_03233"

/inference="ab initio prediction:Prodigal:002006"

/codon_start=1

/transl_table=11

/product="hypothetical protein"

/translation="MTTQTQEQAIQAYEQWLSSQVIDEASKDELHGIRNNEHEIVDRF

YKELEFGTGGLRVYILNISKGSSAPRSTLESELFSLAHNLLK"

CDS complement(3620359..3621111)

/gene="ycsE_1"

/locus_tag="PIKHDJKN_03234"

/EC_number="3.1.3.104"

/inference="ab initio prediction:Prodigal:002006"

/inference="similar to AA sequence:UniProtKB:P42962"

/codon_start=1

/transl_table=11

/product="5-amino-6-(5-phospho-D-ribitylamino)uracil

phosphatase YcsE"

/translation="MAKYKLLALDMDGTVLNSNHEISEHTCEWIHRAIQNGVAVMFAT

GREVQSIEPYVQQLGLKSPLVSVNGSEVWHAPGNLMARHSLEEDWVKEMHELAVEKKC

RYWAYTVKDVYTTANWPERIERLHGDPWLKFGFHCDDPECLLDVHSTLDATGRYELTN

SSPRNIEVNPKGINKASGLREVCQLLGIRMEEVVACGDSLNDLEMIRAAGLGIAMGNA

QDAVKRAADGVTGTNDEDGVAQLIQTYLVNSI"

CDS complement(3621124..3621819)

/locus_tag="PIKHDJKN_03235"

/inference="ab initio prediction:Prodigal:002006"

/codon_start=1

/transl_table=11

/product="hypothetical protein"

/translation="MTVNLTVWHIDHLEISIRVGVSLLVGGIIGLEREHGQHHAGFRT

HILVCLGSTLITLISIYGFSQFAGELNVRMDPARLTAQIISGIGFLGAGTILRNGNRI

SGLTTAASLWVVAAMGIGVGAGFYYGTLLTFVSVLISLFVLQRVENRIIPRNKTTTYS

VTVGRDGDPGMFLNFFRDEGIRVKSFKMESLEEGTVRLDFVLLNKNSEIKQKISQFAM

QRDSEVKQMEVKM"

CDS complement(3621835..3622785)

/locus_tag="PIKHDJKN_03236"

/inference="ab initio prediction:Prodigal:002006"

/codon_start=1

/transl_table=11

/product="hypothetical protein"

/translation="MTLLDQRIQFEEKKKIYDSSILTFNEVEGFDVYNISIPFERSGK

RYLFGRVERREEWARSWVRLFEETGHDEWTLVPGSMIYTLEDPYISLIGNEIVMGGTH

VQYEAGRVKTYFGYFHRGTEFLNLYYFTTGPNKMKDIRLVELSDGKIGVFSRPRGAET

RGEYGSESMIGFTIIDCLDQLTAEVIENAPYIDGIFGEGEWGGVNQAYLLDSGKIGII

GHNCYRDRDAEGGEVKVYLNMAFVFDPVTREATDLHVIGSRTCYPAGPSKTRHLVDCA

FTAGIVTRPDGKADLYSGIGDCQAGRITIDYPFEGHGQLV"

CDS complement(3622823..3624358)

/locus_tag="PIKHDJKN_03237"

/inference="ab initio prediction:Prodigal:002006"

/codon_start=1

/transl_table=11

/product="hypothetical protein"

/translation="MKKIVTLMLVAMLACITLFGCSDKTASTGGNQENEKKGGIVKIK

YVVPGTEPKDYKEVFDKINEKLAADGVGVEVEKIYIPSDAWDQKLNLMLSTGEDFDLF

HIMQDRTPFSSYYNRGALADISGAIEQYGQNLKKYIPGDIFDGAKIDGKYYAVPSYWV

EMASEGEFNIRRDILRQNNLEEPSTPEELISSWETVMKNWKGGKKPYFSNKADFDPVS

LHTTVLHRTYDTFPFTVKDKFFYVNQNGDVKSWIETEEFKKDAEFMRQLYTKGLTSPD

ILVMKGEQLKAQIDSGDWFVNLGTAGSLSSLKKNNPEATVEDVGVVWFNPEKEYLRPL

TFKNGNAVPVNSKHPEEAVKFMDWMLASQENYDLVQYGLEGTHYTKDGDKGMKAINDP

GNNDKPGYRGSESQNGNVNFMRFDLENSIPENNKVLFEANPEAVNSIAANFAFDPTNV

RTEYTNIASEAAASITPIYMGVQDYDKAFPAALDKMKKAGLDIVVAEYQRQFQEYQAN

QGN"

CDS complement(3624413..3625285)

/gene="araQ_26"

/locus_tag="PIKHDJKN_03238"

/inference="ab initio prediction:Prodigal:002006"

/inference="similar to AA sequence:UniProtKB:P94530"

/codon_start=1

/transl_table=11

/product="L-arabinose transport system permease protein

AraQ"

/db_xref="COG:COG0395"

/translation="MKKGLISQTIIYTCITLYSAMCLLPMILVLMISITDEDAILKNG

YSLFPEKFSLYAYKLIFTGGSQVLQSYMISIFVTVIGTILAVLITAMAGYTLANKSVK

YRNTLALYFFITMIFSAGIVPWYLMNRSLGLTNNIFALIIPSLLFSPFNLFLVRNFMN

GVPDSLRESATIDGANDLTIAFRIYLPLCTPVLATIALFYGLDYWNNWWNAIMLIDNK

DLYPLQFMLLQMKSEISMLNEMTMLGGANDVTLPAESVKMATAIVTIGPIIFLYPYLQ

KYFVKGLVIGSVKG"

CDS complement(3625335..3626276)

/gene="yteP_26"

/locus_tag="PIKHDJKN_03239"

/inference="ab initio prediction:Prodigal:002006"

/inference="similar to AA sequence:UniProtKB:C0SPB3"

/codon_start=1

/transl_table=11

/product="putative multiple-sugar transport system

permease YteP"

/db_xref="COG:COG4209"

/translation="MEKKLRWHSRIGILSDIRRNGTSYLLILPAMIYTFIFGYMTYPY

MLIAFQRFNYAKGIFNSEWVGMQNFEFFFRSNKALIVTFNTIFLNLLFIFFGTLTALA

IALLLNELRKKLFIKISQSVMLFPNFISWIVISYVIYAFFSMDMGIVNQLLNKFGLPS

VNWYTEAKAWPAILTLMHVWKGAGMSAVIYLATITGIDDTLYEAAQIDGANRWQMCKK

ITLPLMMPTVVILTLLSIGKIMYGDFGMIYALIGDNGTLYQTTDIIDTYVFRSLRQIG

DPSEAMAVGLFQSVVGFILVFGTNAITRRFFKDGALY"

CDS 3626600..3628954

/gene="yesS_4"

/locus_tag="PIKHDJKN_03240"

/inference="ab initio prediction:Prodigal:002006"

/inference="similar to AA sequence:UniProtKB:O31522"

/codon_start=1

/transl_table=11

/product="HTH-type transcriptional regulator YesS"

/db_xref="COG:COG2207"

/translation="MRTTLSRLIRYKTFQKLTGSYFLLILVTISLLSSVLFSLFSDSA

VKEIDRNSKAMLSQVSYASDVVYNQVMTIGSALINDPKIKGFLNSKKEDKTENYHILQ

QLSQIKSAYPFIHSIGIYRSSTDTNVETSGFSFDKSLYAKSAKQYMEFYPRKLTVAGF

NNNNPFRFLTFLLYPDYSFLSSANPLISINVEEQSILTTIRKIARADSMSNVFVMNSQ

GQVLSHTDSELFLKDLSQEEYVQRILSEGKSEHSFTTAILKKKHLVTYVKSAEMDWYF

VSVAPYETLISNIHKLRNVTLIVTAALVLAGLLISIFLTKNMYKPMSSLFDTIKQKSP

PMPNSHSIDEYKILNEVFTTLEEKERSMKFVLHRSSQTVREHYLQALLKGNIFNIAIP

REIVNDIDEQLPGPYFCTMVFKIDEIEAVKERVSIDQQSLLRFALGNIAKEILEVFGA

CDLLITDENEVVAISQYEIDELPDGLKEALRSVQGFLKRYFKLTVSIGIGDMVYGRKN

IQQSYISAQQYVKYRLIYGKESILDFAMTRHHFMTTFSYPIAFEKKLVEAVQMRKQSV

IQERIEEFTELVTSGSSNQVTTYSIQLLLSLLKHFDYLKSLPDTNFNEYLDAITDIEA

AETLEEIKRILSDYCAKLCALIDEKNHWLNAQKHNKVIEEVQYYIRDHYPEPNLSLEL

VSSSAGLSPSYLGKLFKGSTGQSFSEYLNHTRLEMARELLLTTKDTAAKISEAVGMYN

ITYFSTLFKKKYGASPSAYREQAAIRRVEGDLYIGNEKNEVNRG"

CDS 3629124..3629951

/gene="rhaS_21"

/locus_tag="PIKHDJKN_03241"

/inference="ab initio prediction:Prodigal:002006"

/inference="protein motif:HAMAP:MF_01534"

/codon_start=1

/transl_table=11

/product="HTH-type transcriptional activator RhaS"

/translation="MKINVNQLAEHFAHIPFQVEGIYRYATNPGVPHADYTDSFPGFV

FPLTGKIQFQFNGTPYILSPGKVVHGGAKMKLDEKTFDNSNWEYILVLYRICSSELDE

SSFSHQHFELSTGQSPRLIELIMQLWHVYNQGGGISMFQTEMLFRDVLNEALISVANR

QMSSESQALFERVSNYIHEYYYHSLTIASLAEQYNVNRNRLSYIFRKHAGMGPAEYLL

KHRLNMAQNMLFTSDVPVQQIAQTVGIADPFYFSKVFKKQFGFSPTEYRRKFINNPC"

CDS 3630158..3630418

/locus_tag="PIKHDJKN_03242"

/inference="ab initio prediction:Prodigal:002006"

/codon_start=1

/transl_table=11

/product="hypothetical protein"

/translation="MNDYQFKIVKITGDYEWHVHPETDKLFFVIEGEMIIDFRDGQVR

ISKDEMFIVPRGLEIKPSAEKECHIMLMEPKSEGNTRGTESA"

CDS complement(3630557..3633052)

/gene="cdr"

/locus_tag="PIKHDJKN_03243"

/EC_number="1.8.1.14"

/inference="ab initio prediction:Prodigal:002006"

/inference="protein motif:HAMAP:MF_01608"

/codon_start=1

/transl_table=11

/product="Coenzyme A disulfide reductase"

/translation="MSKKVLIVGGVAGGASAAARLRRLDEHAEIIIFEKGPYISFANC

GLPYYIGGSIEDRERLLVQTPKGMADRFNIDVRTRSEVVAIDPHTRTVQVQSQERGQY

EESYDELILSPGAKPIIPDLPGTDNPLIYTVRSIPDIDRIKKQISSSNNVSSIVIGGG

FIGVEMAENLKEAGLDVTLVEGNGQLLTPFDPELAAALAQEMEQNGVKLLFSQRVQGF

QVLEQGIGVELADGHVLTADLVILAIGVTPDTAFLRNSGVSLGTRGHIIVNEALESSV

PHIYAVGDAIEVTETIHGTKTAIPLAGPANKQGRIVADRIAGLSSIYKGTQGTAIIKV

FGMTGATTGSNEKTLKRLGIDYRTVIVHPASHASYYPGSSAITLKLLFTPAGKILGAQ

AVGYDGVDKRIDDIAVAIHFGGHISDLTELELSYAPPYSSAKDPVNMAGYAAENITAG

RVETFTYDQLSERQPEQSILLDVRSELEHRNGNIPDSINISVDELRQRLDELDSTKDI

WVYCQVGLRGYTASQILRQHGFNVKNLSGGYKTYRQAQFKPAPFSTENHDNNGRQDQS

KPIHALKETELSNPISAEPQRIDNELNVCGLSCPGPLIQLKQKMDQLSNGETLRVKAS

DPGFYEDVKAWATMSGSSLIHLERSKGGIIEAVIAKNTMQTASSAMEIPKEEPASTLV

VFSGDLDKAIASLIIANGAAASGRKVTMFFTFWGLNIIRKHQPQPVSKTMIGRMFDIM

LPRGSHKLGMSKMNMLGVGPKIIRGIMKNNNVASLEELMQTATAQGVEFVACQMSMDL

MGIQREELMDSVNIGGVGYYLGQAAHANHNLFI"

CDS complement(3633049..3633363)

/gene="pagR"

/locus_tag="PIKHDJKN_03244"

/inference="ab initio prediction:Prodigal:002006"

/inference="similar to AA sequence:UniProtKB:O31178"

/codon_start=1

/transl_table=11

/product="Transcriptional repressor PagR"

/translation="MNTEAFDSTNLKQFDEPANLLKALSHPIRLCIVRGLMRKKKCNV

SYMQECLDLPQSTVSQHLQKLRSAGIVATERNGLEVNYVLADQRIEQMIPILFGEEDC

ES"

CDS complement(3633603..3633845)

/locus_tag="PIKHDJKN_03245"

/inference="ab initio prediction:Prodigal:002006"

/codon_start=1

/transl_table=11

/product="hypothetical protein"

/translation="MSKRLNKNIPIWFLFVGIVVFILLCINFFEMNTRINMLALVILN

IANTIRLWKNERQSAMMFLLLGVLCTFFLIRSLILY"

CDS 3634359..3634973

/locus_tag="PIKHDJKN_03246"

/inference="ab initio prediction:Prodigal:002006"

/codon_start=1

/transl_table=11

/product="hypothetical protein"

/translation="MPKKTFFHLSKDKQDTLIQCAKREFSRVPLHEASIANIIKDAGI

SRGSFYQYFEEKEDLYYYLLNQSSQKNSDRFIAALKENNGDIFEAFIDSFQFMIQNHT

NQEHKNFFRNAFLNMNYKTEKTFTKNIYEENRDSHLLPIIGLMNTETLNIQGDRELQH

ILKIIIAVTFDNLIQIFVKDLTYDEALKNYGEQMELLKKGFKKI"

CDS 3635049..3636563

/gene="emrB_3"

/locus_tag="PIKHDJKN_03247"

/inference="ab initio prediction:Prodigal:002006"

/inference="similar to AA sequence:UniProtKB:P0AEJ0"

/codon_start=1

/transl_table=11

/product="Multidrug export protein EmrB"

/translation="MDSALQEKKPPYLMIAILFIGAFVSFLNNSLLNVALPSIMVDLN

IKDYSTIQWLSTGYMLVSGILIPASAFLLTRFSNRSLFITSMTIFTLGTAIAAVAPNF

GILLTGRMVQAAGSSVMGPLLMNIMLVSFPREKRGTAMGIFGLVMITAPAIGPTLSGF

IVEYYDWRLLFEMILPLAVISLLLGIWKSKNVMEQNKNATLDYFSIILSSIGFGGLLY

GFSSASSDGWTDQVVLITLIVGAISLITFVVRQLKMETPLLDLRVYKYPMFALGSVIA

IVNAVAMFSGMILTPAYVQNVRGITPLDSGLMMLPGAIIMGIMSPITGRLFDKYGPRV

LGVIGLTITAISTYMLANLQIDSTHTYIILVYTLRMFGMAMVMMPIMTNGLNQLPTRL

NPHGTAVNNTAQQVSGSIGTAILITIMNSVTKTKAEDLMTGVDPTTLTAANKALLTQE

ALLAGIQYSFYIALGINIVALILVFFIKRAEVSDEPVEALKNQQGNTKSKTAVN"

CDS 3636750..3637460

/locus_tag="PIKHDJKN_03248"

/inference="ab initio prediction:Prodigal:002006"

/inference="similar to AA sequence:UniProtKB:O31533"

/note="UPF0702 transmembrane protein YetF"

/codon_start=1

/transl_table=11

/product="hypothetical protein"

/translation="MFMELTIKLVISFFGLWAITFITGRKTLSQLTPLDFLSSLVLSE

IVGNTLYDDQVNISQLIYALAVWSALSYLFEKVTTRFVKFGYVAEGRVVLLIDNGEVN

QSLLKKYDIEFKQLLSMLRKQNVFSLQEVKHAVLETDGSLSVLRKPEYEPPSAQDLGL

EAHSDSFAITVIDKGELLKMSMRGKEIDTDKVRIEMQKQGYDNIKGIAYAEYREDGTL

YIIPRKRKKNRTIIRNMT"

CDS complement(3637659..3638135)

/gene="crr"

/locus_tag="PIKHDJKN_03249"

/inference="ab initio prediction:Prodigal:002006"

/inference="similar to AA sequence:UniProtKB:P69783"

/codon_start=1

/transl_table=11

/product="PTS system glucose-specific EIIA component"

/db_xref="COG:COG2190"

/translation="MFGFKRKQEHTMEIFSPIQGTTRSLDEVEDEAFSSRALGDGFAV

EPSEGKVTAPFSGKIAHIMEKSKHALILEHDSGVQILIHVGMNTVSLKGEGFTSHVQT

GDRVVQGQVLLEFDIPLIKTAGYDLLSPVIVPNGQEMVKGVQVLGKGPDLMIEVKY"

CDS complement(3638188..3639651)

/gene="bglH_2"

/locus_tag="PIKHDJKN_03250"

/EC_number="3.2.1.86"

/inference="ab initio prediction:Prodigal:002006"

/inference="similar to AA sequence:UniProtKB:P40740"

/codon_start=1

/transl_table=11

/product="Aryl-phospho-beta-D-glucosidase BglH"

/db_xref="COG:COG2723"

/translation="MRTGFSEDFLWGGAISASQADGAYDVGGKGLDTQDLRYFDSNWT

KEERDHHRNINMTGERFKQALLDKDMTHYPFRWGIDFYSNYKEDLRLMEELGLKIFRT

SINWARIFPNGDDAEPNETGIQFYIDLFDECRKRGMKVFATILHYNIPVNLVTKYGGW

KNRQTIEFYLRYARVLFERLGDRVDYWLPFNEINAGKFNPYNGVCLIKDQEEDYENAI

YQCLHHQFIANALTVKLAHEIIPGSKVGGMIARFTTYPATCKPEDVMQSILDEQYANY

FYTDIMARGKYPAYMERYFNDNDINIHKEADDDNILREYTVDFLSFSYYMSMVATTDK

NWDRTGGNLVTGNKNPYLKASDWGWQIDPIGLRVSLNQMYDRYQLPIFIAENGIGALD

ELNEGEIHDPYRIDYLKEHFKQMKEAIIDGVELIGYTMWGIIDIVSCGTIEMRKRYGV

IYVDRDDRGQGTNKRYKKDSFYWYQNCIKSNGEHLED"

CDS complement(3639796..3641175)

/gene="bglF_2"

/locus_tag="PIKHDJKN_03251"

/inference="ab initio prediction:Prodigal:002006"

/inference="similar to AA sequence:UniProtKB:P08722"

/codon_start=1

/transl_table=11

/product="PTS system beta-glucoside-specific EIIBCA

component"

/db_xref="COG:COG1263"

/translation="MDYKRTGSNIIKEIGGRENVNQLTHCATRLRFTLQDSAKADQEK

MKAIPGVLGVVNSGGQFQVIVGQEVARIYNEIVPLLSDNTSDQKEIKTEDSNVSARKE

SVISRLLGTISGIFTPILPAITGAAMLKTLLIILTMTHLMSKETQTYAILNFVTDAAF

YFLPVMLAFSAANKFKMNPMMAVSLGGVLLHPSFVAMVEAKDPVYFLGIPVSLVSYGS

SVIPIILVIWAASYVERFADRVTPKSVRFFMKPLITLLVMAPVALIFIAPLGNIVGTY

VAVGINFLNNHLVWILPFIFGIFGPVFIMLGMHYAVTIPIVIQSITTYGFDMIGAGFL

VANVAQGAAAFAVGRHVKNDPDFKVLANSAAFTALLGITEPAIYGVNLKLKKPFYAVL

VAGGISGLFVGLMGVKRLALAPTGLLTLPIFADPNHAMNLVYAIIGAIISFVLSYFLT

TVLLKKSKH"

CDS 3641326..3642318

/locus_tag="PIKHDJKN_03252"

/inference="ab initio prediction:Prodigal:002006"

/codon_start=1

/transl_table=11

/product="hypothetical protein"

/translation="MDYNQLDFFLKSYTDSEKKYLANPDAVSPIYQNMIQVDVRGHMV

YRFKPLNVIETGIAISKDSRFTSVPLYVHTNVNMNYIYSGECTYVVDDKTITLKEGDV

CIFDKDVIRMKNKIGENDLVINISMSDQFLSSNFLSRLAKQSIVSNLILSAISNSQNH

DNFLIFRTKGNHKIKRLFADLLCEYYDLRMYSSEIINSYLVIIFTELLRIYQDDSENQ

KIQLSNDSSGNIVDILRYIEKEYLNCTLTSLSKQFGYHPKYLSYLIKEKSGNNFKELQ

VSLKLKLACYYLANTSLSIQEIAEKVQISNLNSFYQKFGVTYNMTPKAYRQAHS"

CDS complement(3642607..3644370)

/gene="arsA"

/locus_tag="PIKHDJKN_03253"

/EC_number="7.3.2.7"

/inference="ab initio prediction:Prodigal:002006"

/inference="similar to AA sequence:UniProtKB:P08690"

/codon_start=1

/transl_table=11

/product="Arsenical pump-driving ATPase"

/translation="MLNRFEPRSITLTPYLFFTGKGGVGKTSTACATAVSLADSGKRV

LLVSTDPASNLQDVFGMALSNHPVPVTGINNLDAANLDPEIAAGEYKAEVVDPYRGVL

SDAVIATMEEQLSGACTVEIAAFNEFSSMLTNKELRERYDHILFDTAPTGHTLRLLQL

PTAWSGFLSESTHGASCLGPLAGLEAKKNLYQEAVKTLSDRNETTLILVTRPDDSPLK

EAARASEELREIGIQNQILVVNGLLSSVVEQDMVSSAFYGRQVGALEKLPEAVKQLPT

YQIPLAPFNITGIEHLRRFFDTPKIRLVEEEIINTSSALPLQHLVEDMERKGIRVIFT

MGKGGVGKTTVASAIAVGLAERGHQVHLSTTDPASHLGHVLEKETDNISVSRIDPKIE

LERYKQEVLEQNKDSLDEDALAYLEEDLRSPCTEEIAVFRAFANLVERSSDEIVVIDT
APTGHTLLLLDATEEYHKEIARSAGAIPAAVQNLLPRLRDPNETSVVIVTLAEATPYY
EATRLEQDLQRANISTKWWAINQSFAATDTTDPILRGRAIAERDWIKKIQKASDDHTV
IIPWEPKEAVGYDSLKKWTEV"
CDS complement(3644388..3644753)
/gene="arsD"
/locus_tag="PIKHDJKN_03254"
/inference="ab initio prediction:Prodigal:002006"
/inference="similar to AA sequence:UniProtKB:P46003"
/codon_start=1
/transl_table=11
/product="Arsenical resistance operon trans-acting
repressor ArsD"
/translation="MKTMEIYDPAMCCSSGVCGPGVDPELIRVSAVVHNLQKKQFQVS
RYNLTSEPDAFVTSPLVGQLLAEKGPQVLPIIIVDGQVVKEKSYPTNEELVEWTGLTK
EELVAKPRIRLEVNTKSEH"
CDS complement(3644809..3645234)
/gene="arsC"
/locus_tag="PIKHDJKN_03255"
/EC_number="1.20.4.4"
/inference="ab initio prediction:Prodigal:002006"
/inference="similar to AA sequence:UniProtKB:P45947"
/codon_start=1
/transl_table=11
/product="Arsenate reductase"
/db_xref="COG:COG0394"
/translation="MNMSKKTLYFLCTGNSCRSQMAEGWAKKYLSEDWNIYSAGIEAH
GLNPKAVEAMNEVGIDISSQTSDIIDSELLNSADFVVTLCGDAADKCPMTPPQVKREH
WGFDDPAKAQGTEEEKWAVFQRVRDQIGERIKTFAETGK"
CDS complement(3645258..3646553)
/gene="arsB_2"
/locus_tag="PIKHDJKN_03256"

/inference="ab initio prediction:Prodigal:002006"

/inference="similar to AA sequence:UniProtKB:P0AB93"

/codon_start=1

/transl_table=11

/product="Arsenical pump membrane protein"

/db_xref="COG:COG1055"

/translation="MFSTFLACIIFLITLVLVIWQPKNLSIGWSACGGAILALLVGVV

HFQDVWDVTQMVWNATLAFVAIIIISLILDRIGFFEWAALHMAKAAHGRGVRMFVYVS

ILGAVVSAFFANDGGALILTPIVLAMVRALNFDEKKVFPFIIASGFIADTTSLPLVVS

NLVNIVSADFFGITFMEYVGRMFIPNLFSLAASIGVLYLFFRKSIPKRFDASELKAPV

TAIKDLKMFRLSWIVLGILLVGYFGSEFVGVPVSIVAGVVAIFFLIMASRSSAVPTKV

VVKEAPWAIVFFSIGMYVVVYGLRNVGLTDLLAVAIQAVADQGLFVATIGMGFIAAII

SSLMNNMPTVLIDALAIDATTATGTVREALIYANIIGSDLGPKITPIGSLATLLWLHV

LSTKGVKISWGTYFKTGIIITVPTLLITLVGLYLWLSIL"

CDS complement(3646627..3646953)

/locus_tag="PIKHDJKN_03257"

/inference="ab initio prediction:Prodigal:002006"

/codon_start=1

/transl_table=11

/product="hypothetical protein"

/translation="MDSIQEASEKLKLLGDKTRLTILTLLKEREWCVCEFVDILDMSQ

PGVSQHLRKLKDQGLVKENKRGQWVYYSLNVEDTPYIKSVLELMPDSAAILKSLNKEN

ISSVCN"

CDS complement(3647098..3648498)

/locus_tag="PIKHDJKN_03258"

/EC_number="1.18.1.2"

/inference="ab initio prediction:Prodigal:002006"

/inference="protein motif:HAMAP:MF_01685"

/codon_start=1

/transl_table=11

/product="Ferredoxin--NADP reductase"

/translation="MKNVEFKQVDSCCAPTLEKKKVSPSESLIQGELTHLPVVIIGAG

PIGLAAAAHLIEAGQSFIILEAGTKVGENISQWGHVHLFSPWQYNVDKAAGRLLSKKG

WIAPAPDDLPTGHELRDLYLLPLAELPEIQSNLHLNSRVVGISRNNTDKMKTASREKQ

PFMIYVEEEGKAKRYEARAIIDASGTWGHANPVLASGVLTPGEQALRNHIFYGIPDVF

GVDRERYTDRHIAVVGSGHSALNTLLDLAKLQKLNSGMTLTWIMRKDHVEEAYGGEAS

DQLEARGELGRRIHELVDSGKITVVTPFHIQELKQEGKQITISGEQHGELQVIAHIDE

IIVNAGSRPDLSFLRELRLSIDPTTESVATLAPLIDPNVHSCGTVRPHGEKELRHMEQ

GLYIVGMKSYGRAPTFLMATGYEQVRSVVAFLAGDLDAATKVELDLPETGVCSLKRPK

VQVAEQTSDCSSSCSN"

CDS complement(3648577..3649035)

/locus_tag="PIKHDJKN_03259"

/inference="ab initio prediction:Prodigal:002006"

/codon_start=1

/transl_table=11

/product="hypothetical protein"

/translation="MLSDIQNPREPLQIMVRRFGLLDKNCCTVGEHEISLVQSHILYE

IEHQHEPSMQQIAETLAMDITTFSRQVQTLIKKNLVQKTPLPEDRRVYILSLTDEGEF

VTTAIDHRMNTHLDEVFSFMTDTEKETVIQSIKLLNESMLKSKMCCTPLG"

CDS complement(3649102..3649719)

/gene="azoR_2"

/locus_tag="PIKHDJKN_03260"

/EC_number="1.7.1.17"

/inference="ab initio prediction:Prodigal:002006"

/inference="protein motif:HAMAP:MF_01216"

/codon_start=1

/transl_table=11

/product="FMN-dependent NADH-azoreductase"

/translation="MKVLIIFDHPYTANSSKNVPHHRSYSAALLASVTKGLQSSGHNV

DLIDLHADGFNPVMSGEELSAWRQKKTLDPLVKDYQKRLLAADHIIFIFPIWWESMPA

STKGFLDKVFAKGIIYEEPKKGRPFVSQLKKLKGVSLLTVMATPGFFYRWIFGNPITK

ILFRGTFRKMGFHKLKWYNYAGMDGRTLEVRTKALEKTEKRFASF"

CDS complement(3649738..3650835)

/locus_tag="PIKHDJKN_03261"

/inference="ab initio prediction:Prodigal:002006"

/codon_start=1

/transl_table=11

/product="hypothetical protein"

/translation="MNKLVTLAHTNEDYKRLGITKEPANWEDGMRTDGQAGSYEWWYF

DAEFTDGTKIVVVFYTKYEFDLKGEACPTIRFDLTMPDGRNISKKLSEEKGSTIRASK

ETCDVKIDRSSIQYSNGDYIIHFVDDDIEYRCTMRSIVPMWRPKTGHIFFGEKQKDFF

AWFVAQPSADLEAVLTVNGQRRELVGNGYHDHNWGNAEMNKLINHWYWCRANIGPYTV

ISCDIIAEKKYGYTRMPIIMIARDGVILEDREENTRIERMDTIKHPFTKKFMDNQLKF

IQSSGNNTSYQIEYHREADIVASSLLEGMKLSLPMRVAVKVLKINPTYVRSIGTVKLT

VNENGKQEVHQQEGLWEQMFFSNIKDAIICE"

CDS 3650960..3651499

/locus_tag="PIKHDJKN_03262"

/inference="ab initio prediction:Prodigal:002006"

/codon_start=1

/transl_table=11

/product="hypothetical protein"

/translation="MSTRVNPIAQRSKRWLCDALIELLQEKPYSAITITEMCDKAGLV

RKTFYRNFTSKESVLVEIIDLMFQDFFEYISDQNLGPHEMPLAYFTFWEKHKVFLHIL

IENQLFGLLNDQYVLYLEAMEHIIGTKREYQSEIEKDYMRTMVAAGLWSILKKWIIRS

CTESKEEMAKMTLDFFDID"

CDS 3651736..3652599

/locus_tag="PIKHDJKN_03263"

/inference="ab initio prediction:Prodigal:002006"

/codon_start=1

/transl_table=11

/product="hypothetical protein"

/translation="MLLAILSIVMVVLFTAFIVLKTYKRKEKLTGMPGMMIAMTIGMM

SSLVLGVQLGIVFPRDLTVPSIAAILFGLGAGYFTGKPISILAALDGMLAGIMGGMMG

AMLGVMIGPSYIMMIFVDILFIFILSVALQLSNQETDTSDKTNKSGIGMPFIGGILTV

VFGIIAVGATLFYQYQPNDTSAIKSQESQSEQANSIQENSGYQIVSVDVKSDGFSQEN

IEVKAGVPTKINFIKHTGYTCIRSVQSKDLGIDVYLEKGDNFITLKDLKPGTYNFNCG

MYMYYGTITVV"

CDS complement(3652741..3652866)

/locus_tag="PIKHDJKN_03264"

/inference="ab initio prediction:Prodigal:002006"

/codon_start=1

/transl_table=11

/product="hypothetical protein"

/translation="MSEVGYGNVGGLGGGYGGFTSIGAILVLFILLVIISKAFLV"

CDS 3653070..3654518

/gene="ade_3"

/locus_tag="PIKHDJKN_03265"

/EC_number="3.5.4.2"

/inference="ab initio prediction:Prodigal:002006"

/inference="protein motif:HAMAP:MF_01518"

/codon_start=1

/transl_table=11

/product="Adenine deaminase"

/translation="MSLTMEIRSFRKYIVGFMALWIALAGGTGTGYASIEPAARQTLV

LEHATIVDVKSGKLTSDQTIIIKDNKISYIGNSSQTAIPDYAQVRDAKGQYVIPGLWD

MHVHLEDNYKDAFPLFLANGVTGVREMGAALTNLDIWKTSIQAGMPAPRLVYAGSTLN

GGPADEAPHMFYLSTEEQARDAVRLNVAKGADHIKVYSWLPRPLFLAVLDEAQKYGLP

VVGHLPVEVRASEAIQLGFKSIEHLHGLFIATSSIEDELFKNADMSDLLGYSLAEIEA

SESYDSVKASKLFRKFKEKGVWPVPTMVTYLNMAKTEIDSRSKYVPTAVQKQWAEVIR

ATPPEQTELMTAINSTTPGMVKKLNDAGVPMLAGTDSSFEMTNFIYGVSLHDELEMLV

KSGLSPLQALQTATLNPARYLEREQELGTVEKGKLADLVLLDKNPLEDIRNTTSISAV

VLDGKLMEKQTLDRAVKTYPVIKVADMKDATQ"

CDS complement(3654681..3656162)

/gene="sacA"

/locus_tag="PIKHDJKN_03266"

/EC_number="3.2.1.26"

/inference="ab initio prediction:Prodigal:002006"

/inference="similar to AA sequence:UniProtKB:P0DJA7"

/codon_start=1

/transl_table=11

/product="Sucrose-6-phosphate hydrolase"

/translation="MNTYHNNTKQAEEVLKQAEGKMNKRYRLGYHIMAPANWINDPNG

LIQYKGEYHAFYQHHPYDENWGPMHWGHVKSKDLVHWEHSPIALAPGDACDLDGCFSG

SAVDNNGELTLIYTGHHYIDQPNNIFFQNQNVAVSTDGIHFTKLQQNPVIAEPPADSS

QHFRDPKVWKHEDTWYMILGNSTKEDIPRVILYSSPDLRAWSYQGVLLQGDKDMGFMW

ECPDFFELGGKYVFMFSPQGIDAEGDQYNNLFQTGYYVGEYDYEKNEYKHGEFIELDN

GHDFYAVQTFLDDQGRRIAIGWMDMWEADMPTKADGWCGALTIPRVLTLGENDKVLMN

PVDEMKLLRQSEHVVYHDHVISGSSLREVEADLLEVKVVFDLTKTSAERVGLKLRGTG

HEETVITFSIPDQKLVLDCSKTGKKTDGVRRAALKAEGQLTLHVFLDRSSIEMFANEG

QATMTSRIYPSEKLLGIELITEGGEAQVKELSYWKLNDIWQAK"

CDS complement(3656174..3657418)

/gene="cscB"

/locus_tag="PIKHDJKN_03267"

/inference="ab initio prediction:Prodigal:002006"

/inference="similar to AA sequence:UniProtKB:P30000"

/codon_start=1

/transl_table=11

/product="Sucrose permease"

/translation="MRYSKSMYWKLSAYFFFFFFTWSASYSLFSIWLGQEISLNGAET

GIIFTVNAIFALCMQPLYGYISDKIGLKKHILFLISGLLLLVGPFYIFIYGPLLQYNV

FLGAVVGGLYLGVSFLAGIGAIESYVEKVSRKYDFEYGKSRMWGSLGWAAATFFAGQF

FNINPNINFWIASVSAIVLFIIMMTIKVEMTESEMERAESINFKQVGNLFADKHFWFF

ILYVLGVTCIYNVYDQQFPIYFSSTFSSTALGNQVYGYLNSFQVFLEAGMMFLAPFIV

NKTGAKKGLILTGFLMAFRIMGSGLVIGPYGISAMKLIHALELPIMLISVFKYLAANF

DTRLSSILYLVGFQFTSQVGASIFSPIAGTLYDIRGFSNTYVFMGIFVFAFTTISIFT

LKRDPKNLNKLDPKPNSHIETA"

CDS complement(3657621..3658604)

/gene="degA_5"

/locus_tag="PIKHDJKN_03268"

/inference="ab initio prediction:Prodigal:002006"

/inference="similar to AA sequence:UniProtKB:P37947"

/codon_start=1

/transl_table=11

/product="HTH-type transcriptional regulator DegA"

/db_xref="COG:COG1609"

/translation="MKPKLNDVADLAGVSPTTVSRVLNNRGYISEETKNRVHDAMQQL

NYFPNDVARSLFSKRTNLIGLIIPTTSNPFFGELTFHIENICASSGFKLLICNSLNQL

DKEESYVDMLMRNQVDGIIVGTHNRGIVNYDQKNIAVVAIDRFLSDTIPVVGSDNYMG

GKMASELLIEKGCKHILHINGPVELETPASLRRKAYEDVMEQHGREAITYEVYDPFHQ

QNHDEPIGRLLDEHPEVDGLFASNDLVAASFIAKAKQRGKRVPEDIRVIGYDGTETTR

NLLPELTTIQQPIQEIARSAVELVIKEIEGEFSKVPRETYLPVKLIEAGTV"

CDS complement(3658804..3659886)

/locus_tag="PIKHDJKN_03269"

/inference="ab initio prediction:Prodigal:002006"

/codon_start=1

/transl_table=11

/product="hypothetical protein"

/translation="MFLPVKKGAILCLITALVFVLAACSSSNGSSKDANISSQEAVMD

SATNASTDEQKTDTDATGAAQTTYPFVVSNYTTENGTWVAKEQTFDKAPERVVANTQG

AAELLIRLGLTDKIVGVAALFGNVPDDIAEDFKKIPVLSEGYVGKEVTIGASPDLVMG

RGGLFEDADWGVGTVNSLNEMSIKTFVQSTSVTDAKLDNLYQDITELGEIFNVQANAA

AYIEQLKAREKALSARASAETINYASFFDNGDGTIGVYNGNGDTFIESAMSLINMHNM

LFNEEGTLSLEKLIEINPDAMIISKYAGGIDPEQTIEKLLANKQLQSINAIKNKKIYV

IDFNNFWGYGDSIFTGVEALADDLNL"

CDS complement(3660219..3660785)

/locus_tag="PIKHDJKN_03270"

/EC_number="3.2.2.-"

/inference="ab initio prediction:Prodigal:002006"

/inference="similar to AA sequence:UniProtKB:P48636"

/codon_start=1

/transl_table=11

/product="Putative cytokinin riboside 5'-monophosphate

phosphoribohydrolase"

/db_xref="COG:COG1611"

/translation="MRRIAVYCGASKGNNAIYEKAAVQIGNWIVDQEHELVYGGGKAG

LMGVLADTVIQRSGIVTGVIPTFLQARELAHEQLNKIIIVSNMHERKAKMIELADCYI

AIPGGPGTLEEITEVISWGRIGQHVNPCIFYNVNGYYDLIGQFYDKMVEEGFLTKTDR

QKILVTDSLDEINTFISTYVPPDIRQYN"

CDS complement(3660950..3661828)

/locus_tag="PIKHDJKN_03271"

/inference="ab initio prediction:Prodigal:002006"

/codon_start=1

/transl_table=11

/product="hypothetical protein"

/translation="MDYTSPIEAALKVKRPKYTAIAAGRRHTIGLRSDRTVTAVGDNK

VGQCDVSGWRDIVAIAAGNVHMATNTGNAHTIGLTSQGTVVAVGWNKHDQCNVEYWRD

IVTIAAGWRRTVGLKSDGTVIAVGRNNEGECDVSSWRDMVAVAAGDWHTVGLKSDGTV

TIVGNNQYGQCDVSDWSDIVEVAAGYLHTVGLTSDGKAVAVGRNKHGQCDISDWRDIT

AIAVGSKHTIGLKSDGTVVAVGWNKHDQCNVGDWSDIVAIAAGCAHTLGIKSDGTVVA

VGDNEYGQCDVSSWCG"

CDS complement(3661986..3663338)

/gene="yvdP"

/locus_tag="PIKHDJKN_03272"

/EC_number="1.21.-.-"

/inference="ab initio prediction:Prodigal:002006"

/inference="similar to AA sequence:UniProtKB:O06997"

/codon_start=1

/transl_table=11

/product="putative FAD-linked oxidoreductase YvdP"

/db_xref="COG:COG0277"

/translation="MISKVKLTGRVVFKGDPGYEVARKNWDPHTDRFPKVFVFAQKTQ

DVANAIRWARQNKVPIRPRSGRHALEPNLSQVNGGIVIDVSEMKRIKLNKKSGTAVVE

TGNRVGRIVDTLARQGFMAPFGDSPTVGIGGITPGGGIGPLQRTTGLISDNLIELEMV

DAKGRIIRANKKQNSDLLWATRGGGGGNFGVYTKYKFKVRRSPAKATVFSITWPWEQF

EKVVKKWQVWAPKASNKLGSELSVGPKKGGNVSMLGVYLGSKSEALRQLEPILSVGTP

TKKSILYLPYRAATKFLLAPDPVLTQRYSNQFSSGFGKQPFPDKAFKVMREFLENAEG

GTPAGFYFLNWGGAISRVAPQATAFYWRKAEFYVEWNSSWVKPSRAAKNIALTRNTRK

KLQPYIVGSYINVPDQGIKNPGPTYYGKNYARLRKVKAKYDPKNVFNNPQSIPPAKLK

"

CDS complement(3663522..3665573)

/gene="dap_4"

/locus_tag="PIKHDJKN_03273"

/EC_number="3.4.11.19"

/inference="ab initio prediction:Prodigal:002006"

/inference="protein motif:HAMAP:MF_01960"

/codon_start=1

/transl_table=11

/product="D-aminopeptidase"

/translation="MKKLISMLCTAVLVVIPMTDVSAETNKMGAIEDQAGRLATEMVQ

NYGVTGLQYAIMDEGKIILSDSVGFQDKASKTPVDKDTMFGIGSVSKMVVTAATMMLV

DSGKVDLDQPLTSYIKDFEMADARYTQITPRMLMNHSSGLYGTHYGNSMLFDDTDTRN

HDELLLKLKSEKLKSNPGAYSVYSNDGFQLLEILVERVSGLSYSEFIAKFISEPLQLQ

ATKTPLDSFDRKQLSETYWPSFETKLPVENANIIGAGGVYSTAEELSKFAEVLIGNKP

DILSKSAAESMQNHEYRRGIWVSEERNTFNYGLGWDAVALAPFSDYGITALAKGGDTM

MYHASLITIPEHDISMAVLSSGGSSIYNQMFATKVLLEVLKDQGIIDEIEPDQTFSPP

VKTKMPLELTAYSGLYGTVGSTLDIEVKNGEIALPAMQGGLIPEQNYVYTGEGEFTSN

DGSVKVSFEKESNDKVYFKVQANITLPGLGQTVMNTYDYQKLENNSLDTASKEKWKAR

NGSKYYALDEKITSIFYLVPSMLIKNLSVDVENGYANGTRVVDPDNAENVAQIPVMNG

RDAFDLHFRTKNEAEYLTQDGQEYIAEKAITPIFGGKTGITTIPSNGQARWYAIDSSS

ANKTLTVDSPESGGYAVYNANGEVVQFSKATNQQSTLLPKGGFIVFGGEAGDVFKLQL

K"

CDS complement(3665692..3666990)

/locus_tag="PIKHDJKN_03274"

/inference="ab initio prediction:Prodigal:002006"

/codon_start=1

/transl_table=11

/product="hypothetical protein"

/translation="MDMYVILSIFVVPLLMAFQVNFYFNSVLGKSKRKPYRVIYFIVF

IVLGYFYLVSSFSPVVSSAFALLFIFSLAQSYEVEFIKKIAFTILYAVLLTTVNYIAV

YIMSAIDSTDYSIWDHFNVDYHWVFSKVMLLGCGIMFIVIQIVRLIAKRRSFAMHYRY

YFLFLIVPTITIYQINVASTYSEKNIYYVVSVLGSLFLNVFIVYLFDNMVEKVQLAHE

NVQLQRQMDYQDANYEKTVHSFKNVKSIIHDTNQQFLYIEECIKQNKLDAAGEHIKLT

LNKIEGAYQRVNSGNLVIDALVTNTIAVGQANGIKVDTRIQLHSQHLHIDRYDLCVVL

GNMLDNAIEASKKVRHAEDRYILVAIHSTASALVIQILNHVEQKLTHFKSEKPNPEFH

GIGLTNISRMCDKYGGHMSIENHHRTFNNMVVLPFDMNSL"

CDS complement(3666992..3667732)

/locus_tag="PIKHDJKN_03275"

/inference="ab initio prediction:Prodigal:002006"

/codon_start=1

/transl_table=11

/product="hypothetical protein"

/translation="MFNVAICDDEEKQRELVKTMLISLSLKTNIDFQITLFASGEQLV

SHYKNVGDAFHILIMDVEMSGMNGIQTAQEIRNMKYLDVQIMFLTSYPEYMVESFDVV

TFQYLIKPILPDIFEEKMIKLCKYFQALDKKYVLIKSDYEELLLKYDDILWIEVMKSL

TIKNKLIFVTQENSHESKGILSGYATALKDHGFLQIHRSIIINLMHVQKFAGTQVVML

NGTELPIGRSKVKEVKDAYTKYMIMRIQ"

CDS complement(3667982..3668863)

/gene="pdxS_1"

/locus_tag="PIKHDJKN_03276"

/EC_number="4.3.3.6"

/inference="ab initio prediction:Prodigal:002006"

/inference="similar to AA sequence:UniProtKB:P37527"

/codon_start=1

/transl_table=11

/product="Pyridoxal 5'-phosphate synthase subunit PdxS"

/db_xref="COG:COG0214"

/translation="MQTGTDRVKRGMAEMQKGGVIMDVMNAEQAKIAEAAGATAVMAL

ERVPSDIRAAGGVARMADPTIVEEVMKVVSIPVMAKARIGHYIEAKVLESLGVDYLDE

SEVLTPADEVFHIDKHEFTVPLVCGAKDLGEALRRIGEGASMIRTKGEPGTGNIVEAV

RHMRLINSQIRKVKNMSKDELYAEAKNLGVAYELLRDVHENGKLPVVNFAAGGVATPA

DAALMMHLGADGVFVGSGIFKSDNPEKFARAIVEATTHYTDYKLIAEVSKNLGAPMKG

IEISKLAPEERMSNRGW"

CDS 3668999..3670489

/gene="gabR_1"

/locus_tag="PIKHDJKN_03277"

/inference="ab initio prediction:Prodigal:002006"

/inference="similar to AA sequence:UniProtKB:P94426"

/codon_start=1

/transl_table=11

/product="HTH-type transcriptional regulatory protein

GabR"

/db_xref="COG:COG1167"

/translation="MQFHVAYSSYLNRKYTKMKALYHAIRDAIHEGNLVHGEKLPSTR

ELAATYHISRGTVNQVYDTLTAQGYIHSEHGRGTFVAYQLDLSNDASTVKACAHHLSA

WGNRIQQFEQHTTQRNSQTIAQKSDSRLEGDEVIDFSKYQPDFSKFPYDEWNNRLYAE

IRNREHHIETTSALVSSTGEPKLREAIAAYLRRMRGIQVDADHIAVTAGSMQAIALLT

QLLADPGDHVVTESPCYIGISQAILAAGAKLIEANLDGQGVVPQNWDARMLFVTPSRQ

FPTGEMLSLERRQTLLDWAHRHDAMIVEDDYDSEFRYRGMHVEPLKTLDKAGRVIYLG

SFTKTLPLEVRLGYVVLPPTLADTFRKAQALYEPRPVNLIEQRALAAFMTSGQYERHL

RRMNRLYSRKFHLLLKLLNQQLSIWFDWVENEAGLHVFGWWRGDASTYEVFRTSARSE

GVVYSEVSSSTPDGTKYGIYLSFAHLSDVQLQEGVSKLKNAVTASL"

CDS complement(3670656..3671294)

/gene="codA"

/locus_tag="PIKHDJKN_03278"

/EC_number="3.5.4.1"

/inference="ab initio prediction:Prodigal:002006"

/inference="similar to AA sequence:UniProtKB:P25524"

/codon_start=1

/transl_table=11

/product="Cytosine deaminase"

/db_xref="COG:COG0402"

/translation="MVLLRNPSVEQSRFVEVVAKEAYERGLGSRTTAKGSYNDAYTYK

LFRLLKMADLNFVSNPLVNIHLQGRFDTYPKRRGLTRVKELQETGLNVCFGHDDIFDP

WYPLGTGNMLQVLHMGIHASQLLDYDQIVNSVDLITKNSARTLHIEDVYGIEMGKPAN

FIVLEAENEYEAVRKQAGVLYSYRGGRKIAETKPRDTSIILEGGSEKDTFNK"

CDS complement(3671351..3672757)

/gene="melA_3"

/locus_tag="PIKHDJKN_03279"

/EC_number="3.2.1.22"

/inference="ab initio prediction:Prodigal:002006"

/inference="similar to AA sequence:UniProtKB:O34645"

/codon_start=1

/transl_table=11

/product="Alpha-galactosidase"

/db_xref="COG:COG1486"

/translation="MNGTNIQHPKVVVIGAGSLFFGRQSIWQMVHSPYLNQGTLALVD

TDEERLSKMVTLAERVARENNVSLKIEGSVDRRQVLPGADFVVLSFAEQSVKYRGIDC

QVSLKYGIRMCSGDTIGPGGIFRAMRELPVIMECARDIEELCPDAWVINYINPSTVHG

IALHRYAPKLKSFALCDSHHMPHKKAYYAVRAGIIEKTDEFTNEIDQKFDFRIAGVNH

FTWLLKAEFEGKNVMPLIAEAMRKMAGDENNGGDRGAKAIFNDAITYELYDIFGIIPT

CTAHTKEYVRYWQGLGKTTDAIPPLSIWETEDRYQRHDEMWRQVDDFLAGNIPIADYM

STFGPDHATDIIENMVGNLGKKFFINTLNQGAVTNMNADSFLEVLCDVGMDGVKPVHV

GEMPRGVRGMQELVLDTHELTVEAVLEQSYEKLRRAMLTDPLVNSISDADKIIHELLE

LERDMIPDVWYKDRLQYS"

CDS 3672934..3673785

/locus_tag="PIKHDJKN_03280"

/inference="ab initio prediction:Prodigal:002006"

/codon_start=1

/transl_table=11

/product="hypothetical protein"

/translation="MHARTVNMSIIDELSEYITLRMSSYLEQTHDSNWIEHKSHSDYD

LWFITAGSVKITIDGIEHMADPGDVVFFYPDMPYIASTTGELCRFIYMHFDFSIAEQK

RILGEFQLPGIVPSNLVREESTLFTSSYRRFKQDSGASGSPLYLKASLLLVIAKILEL
HGQGLYNGEFIKDQKPRKTEGSLEVLQNVFPYVDANLHRVIRVNELAAIAGVSEKYFI
SLFKKILGITPGQYINQIKMNRARDYLYEKKYTVQQIAGFLGYPDPFTFSKAFKKFYN
VPPSKFE"

CDS complement(3673886..3675001)
/locus_tag="PIKHDJKN_03281"
/inference="ab initio prediction:Prodigal:002006"
/codon_start=1
/transl_table=11
/product="hypothetical protein"
/translation="MWTLQCGKPSRQCLLEEIIINFNEAVNISNTHVLELVSELFHLE
GYNIQLIPPHEGGRNVVYTCEQEGCESLILRISFLPDRNREDYIAELEYVQYLFDHGA
SVSNVVSSKKGNLLEEITYDHHTFFVSLFNKAKGKMLVENNYQYREGVPITEHYYNCG
KVLGKMHQVSKEYSPVHRRHHFFDNYKGEYINNLIPESFSLLKEKMVELLNTLKGLDT
NQETFGMTHFDYNDGNYSIDFDTGQITVYDFDNSCFGWYMYDLADLWTHGVGWIQFEP
DAGKRKAFMRDYFETALAGYTSETKIEDSMLEKLPLFIQVTLLENILGQFEEMQHTGE
EPEDNEEVLYLIKCLEEDIPYRGFFHDMYSSEAPFTL"

CDS 3675384..3676988
/gene="lipO_8"
/locus_tag="PIKHDJKN_03282"
/inference="ab initio prediction:Prodigal:002006"
/inference="similar to AA sequence:UniProtKB:P37966"
/codon_start=1
/transl_table=11
/product="Lipoprotein LipO"
/db_xref="COG:COG1653"
/translation="MNKKTKTLMLSLCLSTLLVAACTNGSSTEKSTSNTDSDANEGGI
TTIHTVTSEYTSAKYPKGDDITNNVWIKRYKEQFNIDVKTDWVSDEYDTKLNLAIASN
DLPDVFRVNPSQLRQLVEADMVMDLTDVFEQHASERLKGYMETDADSYESGKKDGKLY
GLPQMHWGLIEQPDFIWIRNDWKEELGLQDPKSVKDIKNIALKFIEKHGGYGIAVDQS
LDYLNLLAIAWNAHPDMWMEDNSGKLVYGSVQPEMKNALAEWSEWYKQGIIDPEFAIK
DFNAMNADIVAGKVGVQPYYQWWGYNPGVDTVANLGKDAIFYPYIIPTIDGKEAKQSI

FFANNNYIVMKKGFKSPQEVIKILNDYAYIVDEGNGKESKETLSALLDNDIAHVVGAF

RVLNPNSDYEQFEAVSAALQSKDTSGLTTSGMWQKYNNSVEFMKNATPGAVGDYLQQG

APKNAYGLAKKVLDSENYTKTALWGVSPEVLSSYGTTLDDILTEGFTKIIMGSESIDY

FDVVVQNWRAAGGDQVTQAVNDAYGR"

CDS 3677020..3677910

/gene="yteP_27"

/locus_tag="PIKHDJKN_03283"

/inference="ab initio prediction:Prodigal:002006"

/inference="similar to AA sequence:UniProtKB:C0SPB3"

/codon_start=1

/transl_table=11

/product="putative multiple-sugar transport system

permease YteP"

/db_xref="COG:COG4209"

/translation="MLRKWKQQSPYHLMLIPSLVLVFIFSYIPFYGLIIAFQKYNPGL

GFNSPWVGWDNFSHVFSQPNFVRTIWNTLYMSVFKIIGGIIVPVIFALLLNEVLNSGV

KRTFQTLVYIPNFLSWVIMAGIMLDILSSDGIINTFLGVFGIAPISFLGTPSIFPWTM

IVSDIWKGFGFGTVVYLAALTSIDPGLYEAAVIDGAKRWKQTIYITLPLLMPTIVLMT

VLSLGNVLNAGFDQIYNLYSPVVYQTGDIIDTYVYRLGIQQAQYSIGTAVGLFKSIIS

SVLVAVSYFLAYRVAGYRIF"

CDS 3677926..3678816

/gene="dasC_6"

/locus_tag="PIKHDJKN_03284"

/inference="ab initio prediction:Prodigal:002006"

/inference="similar to AA sequence:UniProtKB:Q9K489"

/codon_start=1

/transl_table=11

/product="Diacetylchitobiose uptake system permease

protein DasC"

/db_xref="COG:COG0395"

/translation="MIYDKGMSRRLFHILNYTILLLISLLCILPFINLLAVSFSSSSA

VSAGSVTFWPVEFTTKAYEFALTGGSFFSSLWVAIQRTVLGTFVNLVLIVLTAYPLSK

SKQKLMGRNIYMGFFIVTMLFSGGLIPTYLVVVKMGLIDSIWSLILPGALPVFSMIIL

MNFIRGLPEEIEESAIIDGAGPIQVLLRILLPLLKPALATVGLFSIVGHWNSWFDGII

YMNNPANYPLQSYLQTLLQSFEQIMLKSGSDYTQLLSMMNARTGRAAQMFLGAIPILL

VYPFLQKYFTKGLVLGSVKG"

CDS complement(3678924..3680666)

/locus_tag="PIKHDJKN_03285"

/inference="ab initio prediction:Prodigal:002006"

/codon_start=1

/transl_table=11

/product="hypothetical protein"

/translation="MKHARLLRIKYKIMVICITVIILPVLVMTISSYYSSERLLAQNY

TNLLTDLAKQTNIRIDEFLKEIEKITLLAGNGLSNNLSATHEGSFPIQDFLREGDEQN

EIAAYNILMNYIMMKDRVFSIYLYNLNGGQDLFVSPNQPIDPDFKVANELWFKKFMHD

NDRTITLTTRIDEQSENKILAVSHARKIHDVASGKLLGVIVVSIDIKFIEIVNRNLQE

GLRSRFMIVDENDKIVYNVNDRLIGTLFRDNVRPPESLNVVVTSPLSQQKWTTYLYMP

LDELTADGKILGHNLVTLAIVLVLFAAILSIFLSHVITTPIKKLLRNIALVEKGQFEQ

VEYIRSHDEIGHLSVRFNKMSHELKRMVERIQQEEIEKAKAEMRALHDQIKPHFLYNT

LGSVKWIASMQQADKIVEMTDALISMLRYATRSDGTLVTIREEIDNIANYVTIQNVRY

YDCIQMKYEIEDRLLDYHMPKMILQPIVENAIFHGLAELEEDGVITIRIQPRGRDIVI

EVCDNGIGMDQHTIQNFMEDKLEADKGTGIGLHNVQRRIQLHFGKPYGIQVASQIGEG

TTFTILLPAIIEIT"

CDS complement(3680689..3682311)

/gene="cheB_9"

/locus_tag="PIKHDJKN_03286"

/EC_number="3.5.1.44"

/inference="ab initio prediction:Prodigal:002006"

/inference="protein motif:HAMAP:MF_00099"

/codon_start=1

/transl_table=11

/product="Protein-glutamate

methylesterase/protein-glutamine glutaminase"

/translation="MYKLMIVDDELLMRVGIRSMLNWEEHNFYVAGEAGNGKEALELA

LEVSPDLIITDIKMPIMDGLELIHEASSLLKACKYVILSNFDEFHYVKEAMKLGAVDY

LIKSEITETALIDLLSTIEEKLQSERGSQTNVRFMTPDYSKSLRHLKDSFFQDIVSGF

INEKDMVSKAEEMHFRIRSDKLVVIQFIVNYYEEAKRKYVEKGEKLLRFSILNIMEEI

IPSKWEKEIFVESSSAYWVIVNVLPGSESVQADLNKLCNKLLSSIKDFMNLSLSAGVS

TIVSGFHGIRKACQEAEFSLKQSFFQGSNQILYYADVIQAPARKEVNGMLSPEQERDF

LKLWVSKDNKKAEEFLEEIRSDLEVQRADESSIRKQYIMVMETIHSHLSRATERGKPS

FTQRSPYEVVLKGEYWEDIHQDMLAHIAYYFKTDSLIMQERSYTDLAIELMDKYYAED

ISLQSIASQISVNPSYLSRLFKQEKGENFITYLTRVRIEHAKAYLLSKELRVYEIANK

VGYHNYTYFSKIFKKSVGFTPEEYRERQQGLM"

CDS 3682452..3682913

/locus_tag="PIKHDJKN_03287"

/inference="ab initio prediction:Prodigal:002006"

/codon_start=1

/transl_table=11

/product="hypothetical protein"

/translation="MINQELARTDAVYVFGVFRKEDDTHPGMIDIATLARSDFQWGSF

GYTIQNPYWRKGYGKEAVHEAFHIAFEHLKFHRVEAHINLDNTPSMKLAERVGMEFEC

VRKGFIHKTMSGQTIWFITRISINLFQPLIKKSEQSTGVPCSLLCDHPVSR"

CDS complement(3682965..3685229)

/gene="nagZ_3"

/locus_tag="PIKHDJKN_03288"

/EC_number="3.2.1.52"

/inference="ab initio prediction:Prodigal:002006"

/inference="protein motif:HAMAP:MF_00364"

/codon_start=1

/transl_table=11

/product="Beta-hexosaminidase"

/translation="MQLKMMTICGAVLGSLLIWAGSADAKERFNDLQHSKWAEDGIEY

MAKRGTVAGYGNGIFKPQGLVTRAQAVTFMVRELYPQQLEKSVEDTTYSDVPTTHPFY

REIAIASKNGLASGFPGGSFRPDAPLSRAETAAFLTRAYSLLEGKQPANWSDTKQHWA

EAPILILSSNGLVGGYSDGTYRPDQTVTRAEYAVFMSRVIRFEREAAIRTQDWDKLIS

YMTVSEQVGQMLMPDIRQWNGKVTTTVNEGLKRSIHDQDLGGLILFDKNIVDIRQLTT

FTHDLQREVGDIPLFLSIDQEGGVVKRIPGGTNLPGQMALGATGDTALAEAAGRLTGE

ELKALGLQVNFAPVLDINSNPDNPIIGIRSFGSDADLVMRLGLATIKGLQQSGVIAAV

KHFPGHGDTTVDSHLGMPVLTHNRARLDAVELKPFRDAIENGVEMIMTAHIAFPAIDN

EHVTSLKDGKSVPIPATLSKKVLTGLLRGELGYEGLIISDAFTMNAIAEHFGENKAVE

RAVSAGVDIILMPKDSAAAHQTLVNAVKSGKIPDETIHASVKRILELKAKYGLFERSQ

TLAQQLNALNGVIGSKQHRAVEQEIAERAVTVLASREGMPPDQIQQGDRVVILAAEQE

QAKQLEKQLIQAANNLSLKTDISLMGQGKTNEALQAIGQADYVILASYQFRNVASEFS

WSDYQTLIDTMNRRNQRYSLFSLGNPYEMIYLQNVRSGLAVYGKQEPNTTAGIKVLVD

QRKPGGKLPVRTGT"

CDS 3685434..3687455

/gene="yoaA_1"

/locus_tag="PIKHDJKN_03289"

/EC_number="3.6.4.12"

/inference="ab initio prediction:Prodigal:002006"

/inference="similar to AA sequence:UniProtKB:P76257"

/codon_start=1

/transl_table=11

/product="putative ATP-dependent DNA helicase YoaA"

/db_xref="COG:COG1199"

/translation="MNWLNDLYKINKRESKKEIERSLVNAIFRIYDRFPRIGLRERIG

QQEMSLDIADAYIHGHHAMIEAGVGIGKSFGYLIPSLLMNQMSQKPVIIATSSIQLSE

QIHKDLRVIGSRLGFTTVRSVVGKGMGQYACRSRAAEWIRPDDPNSSLSTLAQRILDF

EIDERADIKVGISDAEWSNVSVNDCKFERCHHKNACLFYDMRAKLNAKAHEIDFIIVN

QDLLIRDLMKKKEGTRSIISEQPALIVIDEAHNLETKVRDARTLEFTYRGICRILDNT

VQLLTKQSGDKSLFSQSKFIKKCVERIFKQVNADLLHHAKQDSDRIKVSEIKGIPLNQ

VSNDLKDLSLRLSILTSRHEREIDDAFESINGLITLINVLAEIEDNYLIWASSPLGVA

TISICPKDISQFLKYALFNGKAPVILTSATLCQGGHTPEEQYSYLTNSLGYKGDFMDR

KSSPFDYTNHAMMYISNKVPYYHHDQRENYLEAAYKELVQLCNLTQGRTIVLFSAKED

MKYIHKKLISRTNEYTWAVHVQKEGSSQDSVITEFRKSKGVLLSTGVFWEGVNIEGSD

LSQVIIFRLPFPVPSDPIYEYKASVTKNPFMEVFVPDMLLRLRQGTGRLIRSETDLGI

LSILDSRLSAAAKKNYREQVLDTLPFKEVTEDFSVLEKFVQQKGIRRTE"

CDS 3687601..3688128

/locus_tag="PIKHDJKN_03290"

/inference="ab initio prediction:Prodigal:002006"

/codon_start=1

/transl_table=11

/product="hypothetical protein"

/translation="MVERPTTEEYAAFYEGYIQLVPEGNIIERLELQANIVQTLLSSL

TEEQANYRYAEGKWSVKEVIGHLLDNERIMSSRLLRIARGDKANHPGYNQDVLMQTHP

FNAYTVADLNEEYAVTRRSTILMLRHLTPEAWLFRGIVSDNPASARSIAFIMVGHELH

HLSVFRDRYSLDMKL"

CDS complement(3688135..3689208)

/gene="nmoA"

/locus_tag="PIKHDJKN_03291"

/EC_number="1.13.12.-"

/inference="ab initio prediction:Prodigal:002006"

/inference="similar to AA sequence:UniProtKB:Q9HWH9"

/codon_start=1

/transl_table=11

/product="Nitronate monooxygenase"

/db_xref="COG:COG2070"

/translation="MSIQLNSKFCESFNIRYPIVLAGMAGIANMAPLVAAVSNAGGLG

TLGAAYMSPEELRHAIHEIKKRTHASFAINIFAHVGPDHYTNFDAVNEELTPIRESLG

VKQRAQDEIRTPNHFEEQFAILLDEKVSIISTTFGLLSESHMQKAKSLGIKIMSKVTT

VEEAIQAEKRGCDVIVAQGSEAGGHRGTFDVSSQPSGANIGTMALVPQIVDHVHIPVL

AAGGIMDGRGLVASLALGAQGVQMGTRFLTSIESGAHEVYKRALLDSTEESTVITNAF

SGRPARGIKNDFIQHWEASSIKPLTFPTQNTLTREIRNASAEQNNPEYMALWAGQGTR

MLTDNQSAGDIINQIVEEARNII"

CDS 3689341..3690216

/gene="gltR_4"

/locus_tag="PIKHDJKN_03292"

/inference="ab initio prediction:Prodigal:002006"

/inference="similar to AA sequence:UniProtKB:P94501"

/codon_start=1

/transl_table=11

/product="HTH-type transcriptional regulator GltR"

/db_xref="COG:COG0583"

/translation="MDIHLLEIFIQTAHEGSISKTAKKLNYAQSNVTHKIQQLEADLQ

TVLFYRHKKGVTLTPSGHVLVAYAEKILNTINEAKAAVGSSSIPSGPLVIGSMETTAA

VRLPSVFAQYHLKYPNVDFSLVTGPTEKLLHGILHYELHGAFVSGPIEHPDLTQDKVI

EERLVLVTSSKHPLIQSIHDLQNHTMLVFPKGCSYRAKLNTLLQEEGLLPVKLMEFGT

LETIIACVSAGMGITLLPYSIIADYEVRGKVTCHSLPDKYSSVTTMFVKRKETLITPA

LAAFLDETKKEILQS"

CDS complement(3690304..3690768)

/locus_tag="PIKHDJKN_03293"

/inference="ab initio prediction:Prodigal:002006"

/codon_start=1

/transl_table=11

/product="hypothetical protein"

/translation="MSYIVDFKNVSTVGLESSPVAAALAGLRANEARYFMNKYKHEFT

VVSASESQDTLDYVKRILKEERDIEFAAKPLETSAFQVENIRFSYVFYEDGLGLNVMY

TVDDPKKRAVGFKLSEGMEVPGELEGKFKFARQKSKLAGTIRGSYFVIKREY"

CDS complement(3690788..3691267)

/locus_tag="PIKHDJKN_03294"

/inference="ab initio prediction:Prodigal:002006"

/codon_start=1

/transl_table=11

/product="hypothetical protein"

/translation="MSNYNFEEKDSFIVLGIGTELKSDYTDYAGINKEKADFWSAVKE

DGSLDNLKEIATNDYIFAVNEAVNNKMMHYAGVMTEETLPEATRVIQFPKGEYLVVKG

EGTTSDELSNNLTGVAFGQVLPTETNYAYVGGPNTTVEMGQRNGLLFGEIWIPVVKK"

CDS 3691375..3692337

/locus_tag="PIKHDJKN_03295"

/inference="ab initio prediction:Prodigal:002006"

/codon_start=1

/transl_table=11

/product="hypothetical protein"

/translation="MKKVERINIIMRYINNRAHFTISEIMREFNISRSTAIRDIREIE

AMGMPLVAEVGRDGGYFVMNNSVLPTVRFTDNEIKALFIAFMATRNQQLPYLKSRQSL

TEKLLGLISENQQDALVILNQILLFEGTNPHNPDLLDLSDLPHPMLEKLIQLLLIESY

LLVTVQEEREIKSYPIYLLHLYREKSLWLIEGFDLEEEKRRILPVDNLTHIEPFPTKK

KVSKKKILEKLSKQEEMINLVLELGPKAIAQFKKYHPLKLSLSYTNPYQTTAVIKTFI

NVHHAEELTEITNWLLFLGGDIKVKEMPEEILAGVQERLSLYCP"

CDS complement(3692526..3693557)

/locus_tag="PIKHDJKN_03296"

/EC_number="1.1.1.169"

/inference="ab initio prediction:Prodigal:002006"

/inference="similar to AA sequence:UniProtKB:P9WIL1"

/codon_start=1

/transl_table=11

/product="2-dehydropantoate 2-reductase"

/db_xref="COG:COG1893"

/translation="MRIAILGAGSLGTIAGAYIAAGGQDVELIDVYQAHVDALNQTGA

QITGTTDFQAKVKAITPDDMSGTYDLILLLTKQLYNESVLKDLLPYLDDQSVVLSLQN

GIPEEKVASIVGRERVIAGSVEFGATFIEPGVSRLTTEFNRFKQYAFQIGELNGETTE

RIQQIKSILDLVGGTHISDNLVGTKWSKLLINNAFSGLSAALNVEYGVVLDHDISIVS

AVRIADETIKVGHANGVKFATMSGFDIESLEIQSDKDIPERIETLRVLMESSRLLKAS

MLQDLEKQRKTEIDYINGVVPRSASGKQISTPYNDMVVRLVKQAEESQTVPDFDTNIK

FFEELNNNR"

CDS complement(3693602..3694345)

/gene="adc"

/locus_tag="PIKHDJKN_03297"

/EC_number="4.1.1.4"

/inference="ab initio prediction:Prodigal:002006"

/inference="similar to AA sequence:UniProtKB:P23670"

/codon_start=1

/transl_table=11

/product="Acetoacetate decarboxylase"

/translation="MRKEEVVKQFTTPLDAGAFPLGPYRFYNREYLNILYRTDLERLR

KIVPEPLEVTSPLVKFEIMKMPDVTGLGSYTECGQVIPVSFEGEEGDYLHAMYVDSFP

AIASGREIGAYPKKMGKPSLYVDSDTLVGLMDYGSLRVATATMGYKHFPLDHSEALQE

IVKPNYMLKKMPGYDGYPRICELVRSQITDIKIQGAWSGPARLELFAHALAPMADLPV

LEVVSASHIITELSLPSPTVIYDYLADEK"

CDS 3694522..3695391

/gene="gltC_6"

/locus_tag="PIKHDJKN_03298"

/inference="ab initio prediction:Prodigal:002006"

/inference="similar to AA sequence:UniProtKB:P20668"

/codon_start=1

/transl_table=11

/product="HTH-type transcriptional regulator GltC"

/translation="MKYFQTVAYTEHISKAAEQLNIAQPSLSLTIKRLEDELGTTLFD

RKGRNIQLNASGKILLNHVNRIFTEIENARMEIQTKDMAIDHTIKISISNPRFLSGLI

SRYITQWPDTNIQQGLGARNDILASLKKGDIDLGIAGPPIADDEIESCVLINEDIVLV

LPSTHKFATKSEISLSKVADESFISLANNEEYSFFTTELCKKAGFTPKNIFEVDSHLL

AEIIKVNQGIALLPISVCRKLDLNYVRVADINPTYPVGLSWVKDRHLSSSARNFRDFI

IAYFNDHYALYKV"

CDS complement(3695450..3695965)

/locus_tag="PIKHDJKN_03299"

/inference="ab initio prediction:Prodigal:002006"

/codon_start=1

/transl_table=11

/product="hypothetical protein"

/translation="MEKVIYLITGVMASGKSTVSELLASKLGKAVHLRGDIFRSMIVS

GREDMSAEPSEEAVHQLYLRYRLAANAAKTYYENGFSVVLQDNYYGAELVYMMEQLKD

YPVQLVVLSPKVEVVKEREKSRGKVGYTGFTVENLYSDFLKETPQIGFWLDSSDQSPE

QSVNDILQHYR"

CDS complement(3695995..3696906)

/locus_tag="PIKHDJKN_03300"

/inference="ab initio prediction:Prodigal:002006"

/codon_start=1

/transl_table=11

/product="hypothetical protein"

/translation="MNKIERLISIIMILLQKNVVSATEFAKLFNVSKRTILRDMETLG

LSNIPIYSINGVNGGYGIMDEYKIDKRLLSSKDLENILTALGGLGKILFSDEVESTLK

KIESMIGSTTSGGTIQLSFYNWDGRPEIVQILKTCQEAIEQGRLLTFDYIDQSGVKSQ

RRVEPYQLHFSEMSWYLKGFCLDRMEYRTFKLSRTDNLNIDIKTFVARDYLTEQKTEQ

YYQPKLITIKTLISHRIKDQFIERYGQKRIVAYNSELLIAEIDMPQNHFGFRFLASFG

TDLEIVEPKAYVEEFREFLNAMINKYV"

CDS 3697016..3697480

/locus_tag="PIKHDJKN_03301"

/inference="ab initio prediction:Prodigal:002006"

/codon_start=1

/transl_table=11

/product="hypothetical protein"

/translation="MSVIAYLNFDGNTEQVIAFYSEALNASEVKKVKFKDFSQDPNYP

LPENELNMIMESSLEFAGGKIMMSDLLPSMKNVTGELVKGNNVIISIIIDDKQAMENY

FSNLSVGGYVIMPLSEMPWSSCFGMLVDKFGIVWKFNSDADTFLDIVISNAT"

CDS 3697470..3698051

/locus_tag="PIKHDJKN_03302"

/inference="ab initio prediction:Prodigal:002006"

/codon_start=1

/transl_table=11

/product="hypothetical protein"

/translation="MPLNELLPAKSKSELRSWFQEHCLTENSCWVVVSMKSTPSTLMY

LDAVEEALCFGWIDGQKKKISETQLAQRLSPRSKKSSWTELNKERARRLESLGLMTDE

GRKALPDLSYESFKIDPIIEQRLKEETDVYQNFAAFPDLYKRIRIDTIQSYKNQPELF

NKRLDKFITNTKQNKMYGNWNDNGRLYTQNTSP"

CDS complement(3698078..3699061)

/gene="iolS_3"

/locus_tag="PIKHDJKN_03303"

/EC_number="1.1.1.-"

/inference="ab initio prediction:Prodigal:002006"

/inference="similar to AA sequence:UniProtKB:P46336"

/codon_start=1

/transl_table=11

/product="Aldo-keto reductase IolS"

/db_xref="COG:COG0667"

/translation="MKKRILGINGLETSEVGFGCMGLNYHRGPAKDRSEMMSVVHSAI

DAGITMFDTAEVYGPYTNEELVGEALTGHRHKVQIATKGGHKIASTTNELDSRPESII

RSVEGSLKRLKTDYIDLYYIHRVDPKVPIEDVALTMQKLKKEGKILHWGLSEASATTI

RRAHSVEPLTAVESEYSMWWRELERDIFPVLDELGIGLVAYSPLGRGFLTGKLDKNAS

FTENDNRGELPRFTKEAMEANQVVVQYLSKLAEKKNVTTAQIALAWILAQKPSIVPIP

GTTNVSRISENVAATDIVFTEQELMTINDEISKIQIVGDRYPESEKKKTGN"

CDS 3699249..3700130

/gene="hcaR_2"

/locus_tag="PIKHDJKN_03304"

/inference="ab initio prediction:Prodigal:002006"

/inference="similar to AA sequence:UniProtKB:Q47141"

/codon_start=1

/transl_table=11

/product="Hca operon transcriptional activator HcaR"

/translation="MDIRVLKYFLTVANVGNITKAAEILHITQPTLSRQLMDLEEELG

SRLFIRGKRQITLTDSGLLFQQRVKEIIFLLDKTERDLAEQKDLIGGVVSIGCVESTV

SRALPELLEEFSNRHPRVQYELYSADGDDIREKLDRGNIDIGIFLEPIEAAKYEFIRL

PYEEKWGILMRRDDPLAQKKSIGIEDILALPLIPPRRTIVQNEIASWLGVENDSLSIF

ASHNLLTNAMLLVERKLGYAICVSGSYTIRESSHTCFVPFEPERTTSHVLAWKKNKIF

SSATAHFIQFIKETYQT"

CDS complement(3700306..3701274)

/locus_tag="PIKHDJKN_03305"

/inference="ab initio prediction:Prodigal:002006"

/codon_start=1

/transl_table=11

/product="hypothetical protein"

/translation="MNEEQFKRNYKKAVDHMKTNDQMKKRIEETLNTQRHEQKRQRKP

LYIAASVVIAASVGLAAPSIWQQVSGPTAPATQVAQVTPGASFDPVVIPKTELPDSKN

GVKADMLQLVVYQGNVYTQSATSLDGADALALRGEKLGTTTGGIHELSDKGEYTELAS

NIGEADIYAVNGYDTNFRIMSYTEIDGQGYAQLFDQSNGITIGSGEDLIGKLHLKANV

ASAQWETFASWNNGLQQMQPLAADKALTSFILALYAAKPVATSPELEERLYGQEDRKI

IYLTLEDKTQVQLVLFGEGLVRYGNVPVFFEIEKGAFQAFWNNLGE"

CDS complement(3701271..3701765)

/gene="sigW_3"

/locus_tag="PIKHDJKN_03306"

/inference="ab initio prediction:Prodigal:002006"

/inference="similar to AA sequence:UniProtKB:Q45585"

/codon_start=1

/transl_table=11

/product="ECF RNA polymerase sigma factor SigW"

/db_xref="COG:COG1595"

/translation="MQRSVPQRGDHVMKVYETYADTLFRIAMVHLGRREDAEEATQDT

FIKLMEKAPTFNDAEHQKAWLIRVITNHCKSLLGRGWRKREVKLEGEDPLTTDAPEDQ

ALIELVLSLPVKYRSVVHLYYYEDYPIREIGEILQISESAVKMRLKRGRQLLKLELEG

EEPQ"

CDS complement(3702097..3703155)

/gene="iolG_12"

/locus_tag="PIKHDJKN_03307"

/EC_number="1.1.1.369"

/inference="ab initio prediction:Prodigal:002006"

/inference="protein motif:HAMAP:MF_01671"

/codon_start=1

/transl_table=11

/product="Inositol 2-dehydrogenase/D-chiro-inositol

3-dehydrogenase"

/translation="MNNRIYNIAIVGFGGMGSYHCQLIEPVSQISVIGVYDTVEYRME

LGKEAGYKTYANLDAVLTDELVDIVLIATPNDVHKDIAIQALQAGKHVICEKPVTITS

KDFNDIVKVANEEKRVFTVHQNRRWDEDFRIAKDIIDKKTFGELFHLESRVQGANGIP

GDWRQLKDYGGGMLLDWGVHLLDQLLQITDSQIESVAANLSYILGTEVDDGFTSYITF

KDGLTALIEVGTTNYVKLPRWYLKGTEGTAVIRDWDLSGEIITRNPDVAHIEPKPIKA

GQGLTKTMAPPSEQSTLKFAIEKPDLKEQSFYENFVSVLEGESEIAIKNDEVHRVLVL

IETIFEAAETKSVIHRSI"

CDS complement(3703319..3704209)

/gene="rhaR_29"

/locus_tag="PIKHDJKN_03308"

/inference="ab initio prediction:Prodigal:002006"

/inference="protein motif:HAMAP:MF_01533"

/codon_start=1

/transl_table=11

/product="HTH-type transcriptional activator RhaR"

/translation="MSLYLEIPELDKRLPFRSFIHKGDVLCYPHWHKEIEIIYVTQGS

LDLGLNDKVIRLKQGEVQFINGGDVHFFLSSPDSERIVIQFDLSLFQDVSTLIYDDRS

LRDIFTQIEQSSRNWPRETATKLLPLLQNISTEDYERKEGYAYMIKAKLYEMLAFILR

EVPRGSEEHRPQISENILTPTKEMLLKLERIFEYVESHYQESIILKDVADYIGFSPYY

FTKFFKKNTGMTFVTFLNEYRLNKAKWILLNENYSITEIAEQAGFNSVKTFHHFFKEA

TGTSPLKYRMTISGNKTARK"

CDS complement(3704486..3705166)

/gene="tetR"

/locus_tag="PIKHDJKN_03309"

/inference="ab initio prediction:Prodigal:002006"

/inference="similar to AA sequence:UniProtKB:P04483"

/codon_start=1

/transl_table=11

/product="Tetracycline repressor protein class B from

transposon Tn10"

/translation="MKKQQPQISEDKILETSWELLGDEGIEKFSMRRLADRLGIQAPS

LYWYFKSKQHLYQHLANQISKMILNEFQSDGDWKVQMEGLAISIRSVLRRYPCSTQLM

MQTLPHEPDMIRFTNRMLLCMESTPLEQEQKLQAVLTLVNYVFFFVLDNYEHQRTVSV

MIKDQGELPGGEMIQLLDSMSETEAGLFRRMHKGGLFEMMGTDGSFEFGLKLILSGIE

QVIKEQEN"

CDS 3705224..3706690

/gene="mepA_3"

/locus_tag="PIKHDJKN_03310"

/inference="ab initio prediction:Prodigal:002006"

/inference="similar to AA sequence:UniProtKB:Q7A7N0"

/codon_start=1

/transl_table=11

/product="Multidrug export protein MepA"

/translation="MITSNTVRYKLTLLDWKLEYRSNSKSFQKINEESSDSHMDAENL

HYFEKAPIAKAVAHFAVPMMLGTSMSVIYSILNAYFLGTLGNTAMLTALALTLPLFAV

IMALGNLIGMGSGTFISRLLGEKRYDDLKHVSSFAFYSSLVLGLIVMAVGLPLIDPIV

HSLGATPESFGFTKDYVTIMLIGSPFVILFFTLENIVRSEGAAITSMIGMILSVVVNI

ILDAIVIFIFHWGVIGVASATVISNLVASVFYAYHMGYKSQFLTVSVKWFKASKEIMS

NVFKIGVPVFIMSVFLGAMSLILNHFLVEYGDQAIAGYGISSRLLQFPEFILMGLCEG

VVPLIAFSFTANKLRMKHTIGFTIKAILALAVVFGVVVYLISDHLIGLFTNDPQLIEM

GSYILHVTFLSLFITGMTSLFVGIFQATAQGTAAFVMSVIQGITLIPVLYIANRLNGF

HGVVWSLVIADVVAFLVGAIMLYILRNKLQPDLENLVH"

CDS complement(3706861..3707685)

/gene="gpmA_2"

/locus_tag="PIKHDJKN_03311"

/EC_number="5.4.2.11"

/inference="ab initio prediction:Prodigal:002006"

/inference="protein motif:HAMAP:MF_01039"

/codon_start=1

/transl_table=11

/product="2,3-bisphosphoglycerate-dependent

phosphoglycerate mutase"

/translation="MKKNLIFSLLMIMMVFNFAACVSSNQNASGNSDEDNQVAEEQKE

PGKVTIYLVRHGKTILNNTDRAQGWADGPLIPEGEELIKNVGKGLSEIPFTAAYSSDS

GRAIQTANLILAENQTDAKNLELIQKQGIREGYFGSYEGEKNEVMWGDAAKHLGFDSQ

EELLASGGNIIEKIMNAVSEIDPSGEAESYEELNERAFSEFKSIADEAYQNGGGDVLV

VSHGITMGTILAQIDVNKVPAGGFANGSVTKFEYDGKDLNVLEVNELKYVEEGKSK"

CDS complement(3708041..3709210)

/gene="dnaN_1"

/locus_tag="PIKHDJKN_03312"

/inference="ab initio prediction:Prodigal:002006"

/inference="similar to AA sequence:UniProtKB:P05649"

/codon_start=1

/transl_table=11

/product="Beta sliding clamp"

/db_xref="COG:COG0592"

/translation="MNKLLVHLSRDLLAKSIQHVFKALSNNSQGVLSELYIYAGSKEV

VFSGSNASLTVQHRACITDNNKIHIVKEGSFVCPAKYLYEIIRKLNDGNVVMEVNEQL

ILSITSGNTRVCLSGMSPIEYPFIPKTYHQYTGKSIDVSSSLLKSLIKQVAGAVSTSE

AKPILTGVSLEVQGNLLTMIATDGVIRMASRSICIENGREFFGSAVIPGVNLMEIVKL

LDDEGDGMVTIQLSHHEIRFIAQNQIIESVLIEGLYPSTRNLVPTTYVSEIIMNIKSF

RQVIDRVFVLADHHLITIHVSEKKLELISSTAEIGDVRDELLLEDKNGENFSITINGK

YLISTLRCIESDSIRIRYAGTKSPLLLLPASDNSTWLFLLNPVMTRINDRFKNII"

CDS complement(3709207..3709458)

/locus_tag="PIKHDJKN_03313"

/inference="ab initio prediction:Prodigal:002006"

/codon_start=1

/transl_table=11

/product="hypothetical protein"

/translation="MEDKQFKIGLSKKDGQLGFVFTRGGPREGAGRKGIGVTKKVSLT

LTEDIWEEVDRECEDLEISRSSFLRNLIEAYYDANKKGR"

CDS complement(3709691..3710329)

/gene="lacA_3"

/locus_tag="PIKHDJKN_03314"

/EC_number="2.3.1.18"

/inference="ab initio prediction:Prodigal:002006"

/inference="similar to AA sequence:UniProtKB:P07464"

/codon_start=1

/transl_table=11

/product="Galactoside O-acetyltransferase"

/db_xref="COG:COG0110"

/translation="MTEEERIFNGILFSPGHPDLKAIKLRSHNLSSQYSRTFEDQTEE

REALLMQILGRKGERCFIQGPIFFHYGVHTEIGNHFFANYNLTVQDDAKVTIGDHVSF

GPNVTIVTPVHPFIASERRQMLDQNGEPKSLCYAKPVTIGNDVWISANVTVCGGVTIG

DGCVIGAGSVVTQDIPEGSFAAGVPCRVIRPITESDSMRYKPEVLADCSVIE"

CDS complement(3710548..3710769)

/locus_tag="PIKHDJKN_03315"

/inference="ab initio prediction:Prodigal:002006"

/inference="similar to AA sequence:UniProtKB:O31818"

/note="UPF0291 protein YnzC"

/codon_start=1

/transl_table=11

/product="hypothetical protein"

/translation="MIKNLGRINELAKKQREEGLTNAERVEQQVLREDYLREIRGQVL

NTFSVLTILDPDGNDVTPDKVRNEKGISL"

CDS complement(3711146..3712780)

/locus_tag="PIKHDJKN_03316"

/inference="ab initio prediction:Prodigal:002006"

/codon_start=1

/transl_table=11

/product="hypothetical protein"

/translation="MSLRQQKGRKLALLSLALLMLFGITACSSSTESGSSSADSFKLW

LGWNATINNQSMVQMYWKEKEPGIDVRLEATQGDVMTELNLKLNTGGFDDAALFSRSD

VVTTAMTRSGSVLPLEQYFNMPDKYPGLASIPKIYLDQMKASDGHIYSIPTWFDQNPD

DPWPGWASYGWFVRTDVLEKTGMTMDDLKSLDGIEKYLRLSAKQKDENGKPLIPLSFL

SDASDENVILSTFGVTVSTAGGVIPVEKQGNDYQFIYDNPQYKAAYQWMNHMFREGLL

DHEAITDKKERYKEKNKSGRVALNAGGFFNMDAQLWEVLDGPTDPAWYYDVIPYPKVD

GVSEIGANQIINPYPANDVFINKNTKNLDSILAFFDYTLQPKPEQQQVVNEGPPGEFW

DWVDKPLGKWVYTDEKYKALHDSGDQAKKASTTPELYAASSYSNDWYPWWNYGVTDPK

GRFKTIEFTEKIGKMGGTRVAENYDMVKAKPGGLWEKYLPELDNVRKEYKAKLIMAKD

DSQFEQAWSDFQAALEKRAHWSELKQEWNEELQGKQ"

CDS complement(3712857..3713753)

/gene="melC_4"

/locus_tag="PIKHDJKN_03317"

/inference="ab initio prediction:Prodigal:002006"

/inference="similar to AA sequence:UniProtKB:O34518"

/codon_start=1

/transl_table=11

/product="Melibiose/raffinose/stachyose import permease

protein MelC"

/db_xref="COG:COG0395"

/translation="MRTSMRERVGQTIIVFLLALLCISVIYPFTYMLAVSLNVGSDAA

KGGVYLWPREFTLYNYEVVLGNSVIQHAYLITISRTIVGTFVGLLITLLAAYGLSYRQ

LPFRKSLLGYVLITMLFSGGLIPLYIQLNHLSLLNSFWVYIIPSAFSAWNMFVMMKFI

QGIPEALIESAEIDGANPVRILFVIIMPLSKPMLAAIGLFTAVGHWNDWFSGAFYVSD

QNLIPVQTFLQQLLSAQDISTVLGSNNNQEALARGTMLSNVTLMSIKMATVMVSALPI

LCVYPFLQKYFVKGVLIGSVKG"

CDS complement(3713755..3714648)

/gene="yteP_28"

/locus_tag="PIKHDJKN_03318"

/inference="ab initio prediction:Prodigal:002006"

/inference="similar to AA sequence:UniProtKB:C0SPB3"

/codon_start=1

/transl_table=11

/product="putative multiple-sugar transport system

permease YteP"

/db_xref="COG:COG4209"

/translation="MIERLAKYRWQYVMILPGAILLLLFSYIPMVGIQVAFKDFHIGS

TMWSSEWVGLENFSFLQDEQFWIVVKNTIYIAILKFVFGFPAPIILALLINEVKNNKY

KRFVQSVSYLPHFFSWIVVAYILQSLLTLDGGLVNQLIEKLGGDSVFFLGSTEWFRPM

IVVSGLWKEVGWNTILYLAAITTIDPQLYEAARVEGAGRLAQIRNITLPGIMPTISIV

LILSMPGLIAVGMDQIYPLMNPANQPVADVLDTYILRNGLQQGYFGMATAVGLLSSVI

SLVLVLCTNQMARKFNGEGLW"

CDS complement(3714758..3717223)

/gene="lacZ_6"

/locus_tag="PIKHDJKN_03319"

/EC_number="3.2.1.23"

/inference="ab initio prediction:Prodigal:002006"

/inference="protein motif:HAMAP:MF_01687"

/codon_start=1

/transl_table=11

/product="Beta-galactosidase"

/translation="MLLIDLNGRWQMKRVDHAEWLPATVPGSVFNDLLQAGQMEDPYY

REQEQAALELCNYDYEYKHSFEVNEADLKHDRIILLCEGLDTIGELFLNGMNIANVSN

MHSTYEIDITSFIKQGENTIHIILRSPVEYVLRKQAERPLINCSDAVEGISHLRKAHS

MFGWDWGPQLPDLGIWRNISIQGYDHARMEDVYITQMHSEGKVTLDVRVRASGWVEEE

REITVTLDTPSGETLKGCTSVVNDGDHHIWIEVDHPELWWPNGLGNQPLYQLSVALME

QGVELDRDDKRIGLRTLTVKQEQDQWGESFEFEVNGVSFFSMGADYIPEDNILPRCNP

DRTERLIKSCAQAHFNTIRVWGGGHYPENYFYDLCDEYGLIVWQDLMYACGVYELTEE

FQQSITKETIDNMKRLRHHASLGIWCGNNEQEMAWVEWDWAKKTSLQLQADYIKQYEV

LLPAIAKEYDPNTFYWLASPSSKGSFDNPNDENYGDMHYWDVWHGKKPFTEFRTLFPR

YMSEFGLQSFPNHKTIETFTLPEDRNIFSPVMESHQKNGTGNEKILYYIGETYRLPKD

FSSLLYASQLIQAEGISCGVEHWRRHRGRCMGALYWQLNDCWPVASWSSIDYFGRWKA

LHYAAKRFFAPVLVSAREDGSKVELHVSNESRSAVKGELIWRLMDSQSNEIVASSKVI

DLKALSTSLFEQLDFTEMLDTTAKKRNTYLQFSFVVNGEVVSEGTVLFVKPKHFGFID

PAIETMMTEEEERFVITVDSKAFARFVELDFSELDGIFSNNYFDLSAGERKTISLRKD

DLNRSASLEELQSQLIVRSVFDL"

CDS 3717351..3718211

/gene="chbR"

/locus_tag="PIKHDJKN_03320"

/inference="ab initio prediction:Prodigal:002006"

/inference="similar to AA sequence:UniProtKB:P17410"

/codon_start=1

/transl_table=11

/product="HTH-type transcriptional regulator ChbR"

/translation="MIKYTASTHIHPDLLVDPFWVDSLSKVSPEHTHDFYEFFILSEG
QCQHIVNGTTQHLRPGCLVFIRPHDIHRYEPEGTQDCRFLNSPCRSAVIEEAFAYLNE
QKYAQGLLQAPVPQIAMLSQLEMTEMIRSFERIMMLSTVDKKKARVYAKGLIIQILTQ
HFFALETIEQPILPLWFEHAISKMQLKENMNRGLHALYELSGRSVGHVNRAFRQYLNQ
TPTEYINQLRLNVAKNLLLTTELRVLEIALEAGFENVSHFYHQFKKFYNQAPLDFRKN
ATMNKVSLLP"

CDS complement(3718433..3719896)

/gene="sasA_18"

/locus_tag="PIKHDJKN_03321"

/EC_number="2.7.-.-"

/inference="ab initio prediction:Prodigal:002006"

/inference="protein motif:HAMAP:MF_01837"

/codon_start=1

/transl_table=11

/product="Adaptive-response sensory-kinase SasA"

/translation="MKLVHQINLAFGLSLVLILSVTAVLLHYVLLDHFIGTEKNDLKT
LSAAMSASITKAGDITMSPVLSTGAAVTMPNSNITGMQSVQLVTGEVTSAPSTLIPSD
VEAFVTDFNGNVLSGTLSVSGTTGMASSDTLTEAVPANLSKYTAVTSSSIKDLWKGTD
GRYVMDVSPIPQGTLTLLTPMSKIKAIEQALLGRLILVICIVGAIMFLLSLFITKRLI
QPLMNLKQELKKVKERHFTDVQLVKAGGEIGAVAQTVYEMAGELNRFNEVQKQFFQNA
SHELKTPLMSIAGYAEGIRDGIFEGENVRKGLDVILGESGRLGKIVTEMTLLAKLDSE
EDIFKPSKVSLNELLTETSERVNPLLVKKGLTLHIVCPENKELFVMADQDKLLQALLN
VVTNAARYAKEEIHITANLEKGKIALSVSDDGPGFPQELLPTLFHRFVKGKDGESGLG
LAIARAIVERCGGLIQATNRKEGGAVISFGFPAAQRV"

CDS complement(3719898..3720614)

/gene="cssR"

/locus_tag="PIKHDJKN_03322"

/inference="ab initio prediction:Prodigal:002006"

/inference="similar to AA sequence:UniProtKB:O32192"

/codon_start=1

/transl_table=11

/product="Transcriptional regulatory protein CssR"

/translation="MNNDYLIAVVDDDENIRTLIQAYLHKENYRTIGLANAEDAWSLY
RISPPSMWVMDIMLPGMDGYELCKRIRNEGEVPIIMISAKDNEVDKILGLELGSDDYL
VKPFSPRELVARIKRQLERWIRLTGAEERAVVSEAATPRIDTGELQLMLEERRAIWRG
EEVDLTSKEFTMLKVLAEHPNRAFTRDELLSFVWGEDYFGSDRAVDHLIKRIRKKIEE
LPVESVWGHGYRMRTDRSEV"

CDS 3720776..3721540

/locus_tag="PIKHDJKN_03323"

/inference="ab initio prediction:Prodigal:002006"

/codon_start=1

/transl_table=11

/product="hypothetical protein"

/translation="MNYSFKFRNVALSALLLTAISAPIVANADSAANANAVTNGTATL
KEAKSAIATFTLANPVEQAKKYAPETVSEWEKTLARYNKLLSQNYTSQVMATLVAVDV
DSDEWKKVSENAKPGEIKEMQALKVEEGKLTSVESVKAEKLPNSENKNTLSFSITTSS
SDLKGLVENAESGVAIALSPLNLDEKSTGTTSAVIANAESLQFSGTTAASAFFLAQSN
LNNAVTAKDEAAIKTSLADLLKQYKAQITELEAAQE"

CDS complement(3721743..3724259)

/locus_tag="PIKHDJKN_03324"

/inference="ab initio prediction:Prodigal:002006"

/codon_start=1

/transl_table=11

/product="hypothetical protein"

/translation="MTLEQQIKQAVIPTLERKWKIYVIHHSHTDIGYTDRQEKIEQYH
IDFIRQALRIVNDAHNGVNPEWKGFRWTCETFWAVEQFLKQAGDEEKGAFAEAVRRGD
IELSGTYLNMTELPDLQLLNKIHSKAQTYAGHIGHPIDSAMTADINGYSWGYADSLLN
NGIGHLFSCIHTHHGMYALGRKQAPFWWEAPSGERLLVWNGDHYMLGNELGFCPGALG
KYMIRDEFGHRLVEPANIHDQIANIRIHRYLAQLEAEQYPYDFVPMMLSGLPTDNGSP
NSAIMEWIDAWNRQNGEGISVEMSTLSGFFARLKEEGTDGLPVHKGDWPDWWSDGVSS
TPMHTQIYRDAQRTLRKVEKLDPEQKSVSLQEIEAVEQALTLYAEHTWGYHSSVYEPW
HKNVQMLEVRKTAHAAEASRLAYRALDQVLLADNAATLYPGRPYRFKVTNLSEREVTE
LVQLKLEGWEPDELLNGVEVIREDTGQVLIQQSSHPQTIFAELKLQGMESCILILRPL
LAAQKSLSSLTSTSNSKLIGADQVYDMEDMYSLTTGGQQAPISVCQTGLESPFVRLTW

SKERGIVSWIDKETGRELLKADDLYGAFTPIYEVTNPANPADASQVWSVRSKMGRNRK

GLNVERSVGQLISVRAVDNGPLYATVELGYQLKGIAYFSLHLRIYTRQNRMDISARFH

KESIWNPENVYLALPFTPGGTGPVTLFADKPGGLVRPWKDQIPGTCLDYSAVQSGIAW

QDNDRSLLLAVPDTPLVQWGTLDYETRKVHTQQTPDAQPEAYAWLMTNYWETNFKATL

GGFYEFQYHISAGGRLSVEDMAAALQALNEPFVVTRVNEK"

CDS complement(3724284..3725252)

/locus_tag="PIKHDJKN_03325"

/inference="ab initio prediction:Prodigal:002006"

/codon_start=1

/transl_table=11

/product="hypothetical protein"

/translation="MNPDVTIAIDAGGTFLKGAVVLSDGNLLPQLYVKRSSHSDSSAY

EIASNLAGVIQELAAAYVAYMERLSTDKGTRATFANFHIGFAFPGPFEYDTGVSRIQG

LNKYEQLYEFNLKTLLRCELTALAAERTAPTWMTQLAAADIRFGNDAALFALGVSRLF

PQDRLLCLTLGTGLGSAFVENRSIVSGKWGIPDSGMLYAEQYNGGTVDDLFGSRGILA

LADSHDARRQGEDVYHLAIAARQGNSSAIRVWQLYGQRLGEMLRPYVAEFRPARLILG

GQIAETHDLFGGALSEALLPEKIPLHNEKQMQEHVFHGIFQLVKGT"

CDS complement(3725249..3727153)

/locus_tag="PIKHDJKN_03326"

/inference="ab initio prediction:Prodigal:002006"

/codon_start=1

/transl_table=11

/product="hypothetical protein"

/translation="MPRIAPLAFQTNPVNFIRLQPDQEAEPGIWNAYEEWLDQIWTEC

VQNDLLPFYMVIDGTHGAAFSECLEILRSRCHLESRVLIEIDSADYLKSSADLLQQFH

PYLTDNRAFGVVASDVSVKDYFRPHAQSVWLSDVDDQLRLYHSAEHAEAQSPNVDQLA

PIVVTFGPGSGFLSTASGLTNVSLFCDLSREAQQKLHQQGMGSLGLGPCRDTVETYKQ

ALFLEWPVWEEYRKRHLYDFDYYVDTNNRERPVLVTVPMLRRLLASAAQQPFRVKPFF

APGIWGGQYLKELCDLPKDWNNCAWGFEPIAPENTILIGYRDFIVELPFPLLLSAHPE

AIMGERNVQLFGDYFPIRFDYLDTIDGDHLSLQVHPQQAYIEQTFNETMTQQESYYIM

EQKEGAKPFVGLGFTEGTTGEDLLQAVESAHTLGKPLKIPEYVNIWEASKGDLFLIPP

GAVHFSGKNNLVLEISSTTWWFTFKIYDHLRLDRDGLPRPINGDHARANMQEEFDTDY

VQKHLIAVPKESRVQGDSREELLGEREDLLFQIKRLKLDGEWIDDTAGEFVMFNLVEG

VRVRLTPLDDEAAAVEWGYAEAYIVPASVGGFRLESLGDRSCTLIRAQVSPDWKMPLL

PHHWKSGEPL"

CDS complement(3727248..3728174)

/gene="rhaR_30"

/locus_tag="PIKHDJKN_03327"

/inference="ab initio prediction:Prodigal:002006"

/inference="protein motif:HAMAP:MF_01533"

/codon_start=1

/transl_table=11

/product="HTH-type transcriptional activator RhaR"

/translation="MTIEASFRRANQICNRYITKVEQTGLSLSILYWGFMPDHFDNAF

HRHSFFEACYVVDGEGSYIEQNQHYALNKGTAFLSLPGVWHQIRSQTGLTLSYVAFEL

DEAHTDVYYTESFRRLAELGQSVAQQADLTPTGHLWQALITSFDQPGATLPLAVKQIS

ASLLLSIADLHVPTRSDSADMGEQPVEPNTLFRQAKRYIDDNLINELTLMTVSRHLHI

SSRHLTRLFQENLGQSFVHYIQERRVQQAAGLLLNEQTPIKDIAIQCGFQSVHYFTRI

FTRELGVSPAKFRRNQFAEGRSGKMHYPPRMS"

CDS complement(3728273..3735520)

/locus_tag="PIKHDJKN_03328"

/inference="ab initio prediction:Prodigal:002006"

/codon_start=1

/transl_table=11

/product="hypothetical protein"

/translation="MRKKNNKMIFKLWIWMMVLLVASSQLMVLPSNTAYATSKEFGDL

PIEPQEKTIESIPSIKVETYVDEQPAIPSVIQAVYSDSTTAEVMVSWDSIESSDYAQV

GTFTVKGSVEGTSIKAEAGITVLEKDMETTGNVDIGTFLDELKNNVPSNEEVVSYTNN

YQSMEPEAMTFVKGNGTTSVNNGLTVSISNLGGSGAIATWDKAPWLSGGIIDTTMRYT

STNQSNIGFVLGSNDSNQGMAIRYDAGTSWVIQSPDGTSKWETFDGPELKMDTDYHIQ

IGFNGTKLLIIVDDVTYYNKNTDLLSSLSGLGQIGLYKRYATGEIAIKSVRIEGVGTK

EKPSNIVNYVQDYEDTNYIPHWSGLKATVVTDITGNKVLSLKKGSSERGVDLDSPQIQ

QGTLSLDFKLVNPDKLVSGQGFAFGFRMNDSASIFNELGVDPSSWIPESNTGWGDKQE

IPYPIQGRWNNLMFNFNGKKITVFLNKKRIGDITFEQYSEAAGRFGLRIRSTVELQID

NVQYTNEIIEPKQIAQYSNDFEDGIKGTWTGADSTIITEGSNRVLRLSNIKDNVMNLD

APSLQSATYMLNVKPATTDIGFVIGDGAIVKYEGTKWVLLNKDGTSTDFVISDAMEFN

PLPNTWNKVGLQYSDHSVILSINGATLQANLPTGQQLGEGSFGVVAQNYIYIDNVVFT

EEFLDMNTSSSHVGKLRYEEYYEGDSIVNWEGFKDTPQVSSGYLQGMLDAGTAAINKD

VTPVANGIYQVKMQSDGATGITLGNIVIYEESPGNWKYKLEGSNDAFAIGATTALASG

KDYTLRVEMIENELGLYVNGIRAGSVNVTGYTPGSFGIYNSVTHAIQAKIDAITAEEV

RVYAPDYNAQNWGALDNSKPEVLANGDSQIRLNMPGVSLAVDKDSPKFADQVLTFDFN

TNVSAGADGGRYGFIVRGSAADKYVGVVHDINGKWKVTTSNGNEISFPNTYQMQANTN

YHIELRLVGTTVSLNITGPDGVATDMGSISEESMAVVPGWFGLRSWYGTKQMTVSNLK

MIELESLPKLKLAQETDTIGNDGMTVTVYKDFPGIVEYQVDHHTLQADVEQTNSVKIN

NKDYVPRTVSKKESASTYQYTMTFDEIGVVIDGHLEAKANNVVRFEIDKISDGPDFLV

RSIQMNNALIHVNSSMKDASYAWSKSNGEWHGVSEELVDDMSLMKQSGSTGVTMAMVS

GDGLAASAEDNVMSGGNKLIINTEKKPLVNKITVKPGSWTYRHLQSSETEELPWYEVV

VTGERNNDDKTDWQDAAVAYRTEIYNEPFGAQDMKNNMMYIAFNFASQANDPFLNSLD

TGKVLYNFTDGFGQMILHKGYQAEGHDDDIPSYSNIGVRQGGLDDFNYLIDEGDKYNL

HVGVHLNATEYHLDANELDYSNLTGATANGPKTDRLSKGWDWIDTAYYVDQTKDVLSG

ELKSRLTNLYNLTKDANNPKDPALDFYYVDVYTGNDYNAYKLLQYANGLGVKVGTEFS

GPIEPGVDFVHWGPDLGYPNKGNKSILSRMVKNNLDIFVGNALFKGQKIPGVTTWGDS

KPDVQQGVTVFFNEVLPTKYMQHFGVLKYEEDQVTFDNDVVSKRNKSTGMIELTKNAK

LISSWKDTGTTTDESVRHTGEANSLIPWVWDIKANRTLDVNDGAKLYHWNTTGNSTTW

QLTDEFKDVKQFNMYELTQQGKVLVDTLVARNESLTITKAKKNTPYVLYPASADALTL

VPNAGNWGEGSLIKDFAFNSEKFNVPDSWTVDDPANITIKTVQGDTEYDITKEMNKSN

WNRYAEVGSNSGVISQEITGLQPDQDYTVGVWTQTEKGRKSSLQVTVNGKTYSNNVTG

QDGIHKSSFKFVDTKWQRMNVEFKVPAGINSATVKLVADPGAGTVQFDDVRIWEHTTV

EKDPTNKGYVVYEDFENVYEGWGPFEYGGGDRKIHIATDQSNKQDNNPVVSASENKVG

PVMTWVLDGENSLKVNETDVGKLIKTNESSVKMEPNTEYDLGFIYTLEKYAGYEVSVQ

SRSTGEIVLKETLSTLPNPGNKAGEDGGYTRFQKKFATGDQNDYQVIFKMVSKGAGGP

TSDYALILDDFYIKEKLAVNIVSIENVQVETEAGSTPILPAQVTVTLSNDTTANAAVV

WETIDPSLYAQAGTFQVKGSVNGTDIKAIATVKVKPKNSSNPGTNPGTGSNPDTGSNP

STGTKTDTHPNPGTGTNNGSDQDNQTGAKPDTGNTKPKAFLDTVGHWANKEIEALVSK

GIIKGMNDQKFAPDLTINRSEIAAMLTRAFGLKADGSSSFKDVTEDKWYKDDVRAATD

AGLFKGYAGNLFEPMKPLSREEMAVIIVRTLEMKGRLPEVSNTTQMVLSKYKDADKAG

IWSKEALAICIELGLIQGTPDQKINPKRNLTRAEAAVLFSRLLTYCEKGII"

CDS complement(3735555..3737729)

/locus_tag="PIKHDJKN_03329"

/EC_number="2.4.1.211"

/inference="ab initio prediction:Prodigal:002006"

/inference="similar to AA sequence:UniProtKB:A9KIW5"

/codon_start=1

/transl_table=11

/product="1,3-beta-galactosyl-N-acetylhexosamine

phosphorylase"

/translation="MSTKRGRVTIPTDLDVIPQTLEIMKRWGADAVRDCDGTDFPAEL

RDIDAKVYSTYYTTRKDNVWANANPEEIQQMYLMTPIHTAEGESISISLMKGIYPDML

KVNCRDDIQRWWEVIDRTTGEVVPTEQWSYDDKTSDVNITTQPFHDYTVSFLAYIMWD

PVHMYNAVTNEWKDVEHQITFDVRQPKTREYTMERIRKFIEDHPYVNVIRYTTFFHQF

TLVFDELAREKYVDWYGYSASVSPYILEQFEEEIGYRFRPEFIIDQGYYNNTYRIPTK

EFRDFQAFQRREVARLAKEMVDITHECGKEAMMFLGDHWIGTEPFMEEFASIGLDAVV

GSVGNGSTLRLISDIPGVQYTEGRFLPYFFPDTFHTGGDPVKEAKVNWVTARRAILRK

PIDRIGYGGYLKLALDFPEFIEYVESVTDEFRTLYDNVQGTSPYCVKSVAVLNCWGKM

RAWGNHMVHHALYYKQNYSYAGIIEALSGAPFEVKFISFDDIKENPSVLDDIDVILNV

GDADTAYTGGEWWLDTDIVTAVKRFVYEGGGFIGVGEPTAHQANGRFFQLANILGVEE

ERGFTLGYDKYNWDEHEHFITADCKTNPDGSKEINFGEGKKNIFALDGTTILRQTDKE

VQLAVNEFGDGRSVYISGLPYSFENSRLLYRAILWSAHSDQELNSWFSENYNVEVHAY

VEKGKFCVVNNTYEPQETVVYRGDGSSFSLKLDANEIKWYEI"

CDS complement(3737744..3737926)

/locus_tag="PIKHDJKN_03330"

/inference="ab initio prediction:Prodigal:002006"

/codon_start=1

/transl_table=11

/product="hypothetical protein"

/translation="MMEDFTKNYLRNLLMLIFFIVGIGLVIVGQKNIGAPGLGLMLLG

LAMLIGLLWLYNRKYK"

CDS complement(3737933..3738826)

/gene="ngcG_7"

/locus_tag="PIKHDJKN_03331"

/inference="ab initio prediction:Prodigal:002006"

/inference="similar to AA sequence:UniProtKB:O50501"

/codon_start=1

/transl_table=11

/product="Diacetylchitobiose uptake system permease protein NgcG"

/db_xref="COG:COG0395"

/translation="MNTNNRSRRGVSDKLYKGFIYVILIALAILILIPVLWAFMASIK
QDSEFYGSPWALPAGMHLQNFADAWLKAKMGSFMLNSVLVTVIALVLLLIVALPASYV
LSRFKFRGNGFWNMMFMAGLFINVNYIIVPIFLMLNNGDKMLRGILGHPVLLNNLFIL
AVVYAAMALPFTIYLMSGYFKSLPKEFEEAASVDGAGYFRTMVQVIMPMAKPSIVTVI
LFNFLSYWNEYIISMTLLTKPTLKTLPVGLMNLMAAQKSAVQYGQMYAGLVLVMLPTL
IIYILVQKKLTQGMTMGGLKG"

CDS complement(3738838..3739731)

/gene="ngcF_4"

/locus_tag="PIKHDJKN_03332"

/inference="ab initio prediction:Prodigal:002006"

/inference="similar to AA sequence:UniProtKB:O50500"

/codon_start=1

/transl_table=11

/product="Diacetylchitobiose uptake system permease protein NgcF"

/db_xref="COG:COG1175"

/translation="MDMKKGRGRFIFFCLAPAVLLFAIFLLIPTIDVFRMSLYKWGGY
TDNKTFVGLQNFNKLFHSDKFYQTFQNSVLLIVLVTIVTFALALLFASMLTREKLKGQ
NVLRVIFYIPNILSVVVISAIFSAIYDPNSGLLNSLLKLFRGLQAEPVLWLGDQKVVI
FSLAGAMIWQAIGYYMVMYMASMANIPESLYESAGLEGAGRMAQFFQITIPLIWTNIR
TTLTFFIISTINMSFLFVMTMTSGGPDGATEVFLSYMYKEAYTNSSYGYGMAIGVIVF
LFSFALAGILNAVTRREHLEF"

CDS complement(3739825..3741183)

/gene="ngcE"

/locus_tag="PIKHDJKN_03333"

/inference="ab initio prediction:Prodigal:002006"

/inference="similar to AA sequence:UniProtKB:O50499"

/codon_start=1

/transl_table=11

/product="Diacetylchitobiose binding protein NgcE"

/translation="MKKILSLAITAALTLSLAACGSSSENGSNSTKDSSNTNSATSTE

QRTLKLAALETAYGADVWKEITAAFESANPGVKVETTIDKNIEDVIGPGMKSGEFPDV

IHLATGQPKGLTETFVKDNNLTDLSDVLSMTIPGESVTVKDKLIPGFTDTTITNPYND

GKTYLMPMFYSPTGLFYNEGLFEQKGWKVPTTWEEMWALGDQAKAEGISLFAYPTSGY

FDSFFYALLNEVGGADFFTKVTHYNEGIWQTPEAQQTVELIGKLATYTEKTVPANANK

DNFTKNQQLILDNKALFMPNGTWVVGEMSKAPRAEGFKWGFTALPAIKDGGDRYSYTF

FEQIWIPAKAENADLAKQFIAYLYSDEAAAIFAKVGAIQPIKGMSDKLDGDNKLFYSI

YDNGAKAAMGAFATTDPVEGVNISDAVFASIDSLVTGAKTQQQWVEGIIKASDALRGA

LK"

CDS 3741401..3742234

/gene="rhaR_31"

/locus_tag="PIKHDJKN_03334"

/inference="ab initio prediction:Prodigal:002006"

/inference="protein motif:HAMAP:MF_01533"

/codon_start=1

/transl_table=11

/product="HTH-type transcriptional activator RhaR"

/translation="MGNVRYWLPGKGRGEIPAKLIYVSSSIYEGDWFSIRHSHNFAEL

FYVRSGRGNFIVEDEIFSVQQDDLVIVNPNVEHTEVSIGSDPLEYVVLGVEGMSFDFG

KQSGTRNHEIINYSKQRNELLLYFNAMLAETENKNENYEDVCQNLLEIVIISLMRRSG

HPFSVVTTQRADKICSRIKRHIDSNYAEEISLHSLAEKAHISKYYLAHTFAKYYGLSP

INYLNEVRIRASKELLETTDLSIAQVAESTGFSSQSYFSQNFRRCCGLTPSGYRLQTK

K"

CDS complement(3742345..3743292)

/locus_tag="PIKHDJKN_03335"

/inference="ab initio prediction:Prodigal:002006"

/codon_start=1

/transl_table=11

/product="hypothetical protein"

/translation="MKRLTGLILSVIILAGGILAGCSEKPVAVTPEASKIEGEAWLRT

IKDASDNEVVLEKRPERIAVLHPLYLDYFFALDTPPIASISAAEAMEKFATLQPYAES

ADIIDLGSDSANLEKIIEAQPDVIVTFKGSVDTIYDELNKIAPVILIDYTDTWENTTM

LLAEVVGKEELAEQLIKETQEMIAQTKEQLGTLEDKTFALLRVDGKANFNAQGSKNTM

YYNQTAGFGLLAPKGYPEGGEALTLEGLYDMNPDYIIIQHDIDVAKAAVKEKESSKVW

NSLEAVKNNHVLIFDNSLNSASVLAVRLASAYFLKMAEQ"

CDS complement(3743325..3744626)

/gene="ntaA_3"

/locus_tag="PIKHDJKN_03336"

/EC_number="1.14.14.10"

/inference="ab initio prediction:Prodigal:002006"

/inference="similar to AA sequence:UniProtKB:P54989"

/codon_start=1

/transl_table=11

/product="Nitrilotriacetate monooxygenase component A"

/translation="MTSNRQLCIGLSLNATWNKGDGWRHPDSGVEQTGNIDYYIHWAK

MAENSKLDFLFRADYLYISPQMLGDSSSFGSPDPTLFFAAIARETNRIGLVTTISTTF

NPPYVVARQLQSLNWLSNGRAGWNIVTSIEGADNFGNSPMPSPEERYAKAIEFTDVVC

KLWDSFPNEAIVIDRESGMFSDKEKVTAIHHSGEFFNVKGPLTLPSHPSGSIPLFQAG

ASDIGRNFASSVADAIFAAMPDLESGVELRSDLRERAAEHGRDTIKVLPGLYFFLADT

HEEALELHKAAHAHLSLERRHTSLQSLMGLDLSSFPLDQRVTEDMLPDPDHAVRSRTH

AELLRRYITKYQPTVKDILNRPEVVGSAHWVSVGTVETVMNDIIERVEAGAIDGFIAL

PGGSEKSMEIFFEKLIPKLVERGLFRSEYTGETLRDHLGNS"

CDS complement(3744957..3745928)

/gene="nlhH_2"

/locus_tag="PIKHDJKN_03337"

/EC_number="3.1.1.1"

/inference="ab initio prediction:Prodigal:002006"

/inference="similar to AA sequence:UniProtKB:P9WK87"

/codon_start=1

/transl_table=11

/product="Carboxylesterase NlhH"

/db_xref="COG:COG0657"

/translation="MNEPKSRSRHLIDQEIISAIDLIPATDLSAATLMEARKSQTKMM

QQIDVTQLFPVTFEERAVPSYFGGPDIRIEIIKPRQPKNSKLPLYYSIHGGGMVMGSP

VGERQGNAALAAQHGFCCVSVYYRLAPEHIQPSQLQDCYSGLKWCIEHAEELGIDTEK

VALAGSSAGSGLAAGLALYIRDQKEFDIHHLRLRSPMLDDRTSILPDHPYAGEFVWTK

KSNYFGWKSVLGHEPGQEETSEYYVPARAKSLAGLPPTYMSIGSIDLFIDETLLFAKQ

LAFDGVLIELQVLPGYHHLAPMFPNAHFSQIGATSSEYALLKALELL"

CDS complement(3746166..3746432)

/locus_tag="PIKHDJKN_03338"

/inference="ab initio prediction:Prodigal:002006"

/codon_start=1

/transl_table=11

/product="hypothetical protein"

/translation="MKYTNAKKILPEKLIIMIQEYVQGETIYIPKQEKEYNHWGSLSG

GRQWLDHRNATIRQAFKSNSSVEQLAKDYFLSIETIKKIVYSKK"

CDS complement(3746668..3747054)

/locus_tag="PIKHDJKN_03339"

/inference="ab initio prediction:Prodigal:002006"

/codon_start=1

/transl_table=11

/product="hypothetical protein"

/translation="MRRVLNIFVSCTIFCILMLNLSGLAYAEPNTVNKEEFKPLSLTL

LNPSIQLAITQYYQKIQKPQPSYGLYDMKVLELQRKSPGSYAFRIVVEVSTYYGPHNP

PNAKEFITFDIDTRKVKLIKYIHRSD"

CDS 3747190..3748293

/locus_tag="PIKHDJKN_03340"

/inference="ab initio prediction:Prodigal:002006"

/codon_start=1

/transl_table=11

/product="hypothetical protein"

/translation="MNEINLASVLVAKRRKKGLTQDAIAAFIGVSKASVSKWETGQSY

PDITFLPRLAAYFNISIDELMGYSPQMEHSDIAKLYTRLANDFAALPFDEVIAECTAI

IKKYYACFPLLLQMVRLYTNHFTLARTQEQKETLLNETVTLCERIQGESGDMQLSNDA

ILYQSVCCLALRDPQRVLDLLGETVRPMMPKGVLISQAYQLMGNIDKAKETLQAELYQ

HVMAVFQGLLNVLQLNIEHIEKAEEAFLRAFDFARIFDMKRLNPNNVVLLYALGAHLY

CVNKSPEKSIEFLEKYVDTCVEGFFPFELRGDSFFDLIDPWLKDFDVSAPRSEQVIKN

SMMRDVLMNPAFNSLREVPEYQRLVQKMNRFIE"

CDS 3748318..3749268

/gene="estD"

/locus_tag="PIKHDJKN_03341"

/EC_number="3.1.1.1"

/inference="ab initio prediction:Prodigal:002006"

/inference="similar to AA sequence:UniProtKB:Q9WYH1"

/codon_start=1

/transl_table=11

/product="Esterase EstD"

/db_xref="COG:COG1073"

/translation="MNEKLIIGKGTKYPLNGILSLPEDCSSKVPAVVLVHGSGPADMD

EKVGNNFPFKDLAEELSDKGIAVLRYDKRTLVYGKEMKNDTGLSVKEETIEDAILAAD

LLRKDPRIDTNQIFIIGHSLGGMLAPRIDAEGGHFAGIIIMGGSPRKLEEILMDQNND

VLDSLNMFLKMIAKKQIAALSSKFDKIYKLSDAEAKSTVILGKYSRAFYFKEMGEHPS

ISYLNVLDKPVLILQGDQDFHVSVDKDFKVYKDLLGEKPNVTFKLYPNLNHLFMPAVY

GEILKMKKEYKVAQHVDKNVINDISEWIHSVWKNPLVISN"

CDS complement(3749258..3750139)

/locus_tag="PIKHDJKN_03342"

/inference="ab initio prediction:Prodigal:002006"

/codon_start=1

/transl_table=11

/product="hypothetical protein"

/translation="MSSILNVIEKLKLNVLNIENVPESFSSDVYKLTLVSGEDVYVKI

PFNKDKLFREFQMLEILKDVIPVPKVLDIWYGDDSNTGALLLSAIQGVPCTENMDEKL

SFQMGVYHAMLHEVKTPGYGYHSTDGFNILDQNNWRLHIKNNFEKWKEPCKELLEPAL

YERCILHFDGVFSALPDPDGPCFVHMDFRPGNILVNGNKVTGIIDFESARGGSSEIDF

TKINRYIWEVNPRTKSPYFEGYQTIRTMLNLEIVLPFYNFYDAFSAVVWCKNRGVERN

QSFLQESIEVLKKSVSY"

CDS 3750414..3750815

/locus_tag="PIKHDJKN_03343"

/inference="ab initio prediction:Prodigal:002006"

/codon_start=1

/transl_table=11

/product="hypothetical protein"

/translation="MHYVDKYDLLDEIVNEQLEELEEICEQKKEKGFVEGTVIWFQYF

EKHKVFFSALFTSRSTVSFRERLLDFMMNQLSYKIDKSDSENDDEILLKFLGMAVLGI

MESFVLDQLNGTTEQVATQVGGLLTRTISIK"

CDS complement(3750939..3751811)

/gene="cysL_2"

/locus_tag="PIKHDJKN_03344"

/inference="ab initio prediction:Prodigal:002006"

/inference="similar to AA sequence:UniProtKB:P39647"

/codon_start=1

/transl_table=11

/product="HTH-type transcriptional regulator CysL"

/db_xref="COG:COG0583"

/translation="MDLIYLQTFREVAIRESFTRAAEVLGYAQSSVTTQIQKLEKAYQ

VKLFERYSNNKIRLTSAGEELFKLAGQMLELFEQSKEKMAKQGGGSLTIGTMDSIASY

FLPPYMQATRKEYPDLNIRLHTNREGLILHQVREGEADIGLILADDSSDPALQWITIR

QEPLLLIVHPQHPLIAKTQIELNDLKDMEWIMPEDTCNYRQLLERVLRANSIPYRIGL

ELGNPEAIKRSIKSGEGISILPQMAVMEEIERGELKALPLEHAELQLEIQLVLHPKKW

VSNALHDFMESLKK"

CDS 3751923..3752819

/gene="dapA_2"

/locus_tag="PIKHDJKN_03345"

/EC_number="4.3.3.7"

/inference="ab initio prediction:Prodigal:002006"

/inference="similar to AA sequence:UniProtKB:Q9X1K9"

/codon_start=1

/transl_table=11

/product="4-hydroxy-tetrahydrodipicolinate synthase"

/db_xref="COG:COG0329"

/translation="MMNEQHVQGIYIPVIAPFSDDNKLDIESYRKYVTHLAKHDIQGL

VVNGTTGESPTVSWDEVKQMVEVTKEILISLNKTIPLVVGTGTNDTYSTVHRTEMAAE

LGADAALVVTPYYNRPTQEGILEHFSNASRTGLPILVYEIPARTGSRMTLDTMRKVMD

LDGVIGLKDSTGGLDLLLALTQYETKPVLCGEDAYFHAMLCQGAAGGMVASANIHTQR

YVDVYQRFTSGDVNASRDSFKQLQPLISDVFAEPAPASIKWWLAQQQLIATAKLRLPL

TAASEKAKQRLSDTLAEMVVPS"

CDS complement(3753016..3754230)

/gene="eis_1"

/locus_tag="PIKHDJKN_03346"

/EC_number="2.3.1.-"

/inference="ab initio prediction:Prodigal:002006"

/inference="protein motif:HAMAP:MF_01812"

/codon_start=1

/transl_table=11

/product="N-acetyltransferase Eis"

/translation="MHRKQNLKMKKVDLSHLEQYSQLLRYVFQVTHNDLHKHGWKESE

ILHEKSPVLQQADVLGWFDGEKLISQVSIYPFEVRIFNQTYAMGGVTGVGTFPEYSGQ

GLMNKLLLQALTNMREKKQSISYLYPYSIPYYRRKGWEIISDKIVFEVQDFQLPKNKP

VPGDVARVEPESEDVKVAYERFSMQTHGAMLRKELAWSEYWRWDSDDLTAAVYYGEDG

QPDGYILYWIEEEVFHVKDMIFIHEEARSGLWNFISAHFSMISKVVGNIHTDEQLAFL

LEDADIKETISPYFMARIVDIEQFISKYPFKPAAMDREWTFTLQDPLLSWNQGTFTLQ

IEPNGSGRVTRSQSRRSEDSISIQTMTTMLLGYKRPEYLHKIGRIKCDPETLDLLEDA

IEQQAPYFSDYF"

CDS complement(3754465..3754740)

/locus_tag="PIKHDJKN_03347"

/inference="ab initio prediction:Prodigal:002006"

/inference="similar to AA sequence:UniProtKB:P11540"

/codon_start=1

/transl_table=11

/product="Barstar"

/db_xref="COG:COG2732"

/translation="MKTVVINGKDIHGKEELHDVLQTSLELGQTYGRNLDALWDCLTG

LIAMPLTIQWTDFDATRMSLGEYADHLLDLMHEAEKELEGFTFESRN"

CDS complement(3754756..3755193)

/locus_tag="PIKHDJKN_03348"

/EC_number="3.1.27.-"

/inference="ab initio prediction:Prodigal:002006"

/inference="similar to AA sequence:UniProtKB:P00648"

/codon_start=1

/transl_table=11

/product="Ribonuclease"

/translation="MVVKKWLGALLFILATLLFTGCSLETVSLNGSSQESAALTQFDE

VAKYISEHNELPENYITKKEARELGWEPSKGNLEKVAPGKSIGGDVFQNREGLLPKKK

GRTWYEVDINYSGGTRGSDRILYSNDGLIYKTTDHYRTFEQIE"

CDS complement(3755356..3757938)

/locus_tag="PIKHDJKN_03349"

/inference="ab initio prediction:Prodigal:002006"

/codon_start=1

/transl_table=11

/product="hypothetical protein"

/translation="MMKILLKKTGVLMLALTLLLGLMTVPVHADSPLFTIESENALLS

SDLQVTTEIYGQPKPGFSGSGFVWMQNSGTITFTVTVPETGMYAISTRYMQELSADGR

LQYLSVNGVTKGSYMLPYTTTWSDFNFGFQKLNQGSNTIQLKAGWGFAYFDTFTVDYA

NLDPLDVQPVLTDSQATPESQLLMNYLTEVYGNHIISGQQEIYGGGNDGNTELEFDWI

HNLTGKYPAIRGFDFMNYNPLYGWEDGTTDRMIDWVNNRDGIATASWHINVPRNFTTY

QLGDHVDWKEATYKPTETNFNTANAVIPGTKEYQYVMSTIDDLAEQLLILQDNNVPVL

FRPYHEAEGNGGLNGEGAWFWWASAGADVYKQLWDMLYTELTETYGLHNLIWTYNSYV

YNTSPAWYPGDDQVDIVGYDKYNTIYNRNDGLSGVPNEDAITSIFYQLVDLTDGKKMV
AMTENDTVPSVQNLTEEKSGWLYFCPWYGEHLMSSAFNYPATLKTLYQSDYVITLDEL
PNLKANNPTPNASLTPTTVEFDKHTPNPSDKAITVNFNSNTLTALRVGTTLLTANQDY
TLSGSTLLLKKEFLTSLPVGEHSIVFDFNQGQDPVLKVKIVDSTPSTSAVISPVNAAF
DKAANLAQDVSLSLTLNGKQLTSITNGNYTLVSGQDYTASSTAIVLRKSYLSTLPLSQ
NTITFHFNGGNNAVLTVNVVDSSVSVPSGDLTIQAFNGNTSASTNGISPKFKVVNTGN
SAIQLSDVKLRYYYTIDGEEAQSFWSDWASIGSANVTSKFVKLATPVAGADYYLEVGF
TSSAGTLNAGQSAEIQARFSKNNWSNYNQANDYSFKASSTQFANHEQVTGYINGQLVW
GIEP"

CDS complement(3758114..3758851)

/gene="bacC_2"

/locus_tag="PIKHDJKN_03350"

/EC_number="1.1.1.385"

/inference="ab initio prediction:Prodigal:002006"

/inference="similar to AA sequence:UniProtKB:Q8KWT4"

/codon_start=1

/transl_table=11

/product="Dihydroanticapsin 7-dehydrogenase"

/translation="MGKLQDKVAIITGGASGIGAATARLFVSEGAKVVLVDLNEEKGK
AFEQELKALKADALFIKANITSEQEVAEIFKKTVEVFGKVDIVFNNAGIGRVHPSHEL
EYAEWRNTVNVDLDGVFLVAREAIREMLKIGGGTIVNTASMYGWVGSPGSAAYNAAKG
GVVNLTRSLALEYAEKNIRVNALCPGFIDTPIIPEESKQALSSATPMKRLGQADEMAK
AVLFLASDDSSYMTGNSLIVDGGYTAQ"

CDS 3759029..3759640

/locus_tag="PIKHDJKN_03351"

/inference="ab initio prediction:Prodigal:002006"

/codon_start=1

/transl_table=11

/product="hypothetical protein"

/translation="MNDEQILKSQQRNRTEEHLKSALIQLIKKKGYHAISVKDIVDQA
GYNRSTFYLHYQDKFVLAEELLNTKLEGLRLAVGKPYSHGQKVYTNKLNFESFQIIDY
IYENRDFFELIRYDDTLPGLHTGFPQTILKIYEEQFIFETINDFPVNMEYFKYYTAYG

FFGILNNWILSGFRESREVFIKNVIELSKTHIHSFHYVGNKLD"

CDS complement(3759780..3759908)

/locus_tag="PIKHDJKN_03352"

/inference="ab initio prediction:Prodigal:002006"

/codon_start=1

/transl_table=11

/product="hypothetical protein"

/translation="MARQKKRQEAAWKSRKQEQHPHGKIKSLKQLSSEYDEKHTTI"

CDS 3760356..3760976

/locus_tag="PIKHDJKN_03353"

/inference="ab initio prediction:Prodigal:002006"

/codon_start=1

/transl_table=11

/product="hypothetical protein"

/translation="MAKQTLRHLKKEATEKALASAAFELALEHGLDGFVVEDIVQKAG

YSRRTFANYFTCKEEAVARGATSVNNTSEFENLLTKIPSDASLLDVLYQLIQMQLTTD

LIGRLRELLLLSQKYPVLEPHFLGVVHQMQTTAQETLLDLSNGNDDEVYTYLLINAAY

GTILPLIDGRLNVLLPGQNISDHQRTGAVSFDEFLENMFGHLRKGF"

CDS 3761024..3763324

/gene="ydfJ"

/locus_tag="PIKHDJKN_03354"

/inference="ab initio prediction:Prodigal:002006"

/inference="similar to AA sequence:UniProtKB:P96687"

/codon_start=1

/transl_table=11

/product="Membrane protein YdfJ"

/db_xref="COG:COG2409"

/translation="MSTLLYRVGKTAFRKPGYFILGWILILGIVVSMIGVNGVHISSE

MKIEGTESQKVLDQLAKELPAASGGQGSVVFEAPPNERLDTPERLAAITKGVNDVYGI

KDVINPADYAAEASGATAEMAQAINTEQSATVAAPSYGPLMVDGVPVSGVLLSPDGRI

ALFQFQFTIEQSAITQDVFDSVIDSVMTVEKGTNITVLPGETLKTVSIGVGSAEIVGL

VIAALVLLATLGSVVAAGLPLVTALLGVGIGVGGAFSISKFIEMPSVTSVLALMVGLA

VGIDYALFIVNRQRRMIIDQRLSAQEAAARAIGTSGSAVFFAGLTVIVALCGMLVIGL

TFLSTMALVAAATVLINVFVALTLLPALLGLVGERICSPKAREKSAKSNKAGGRGVAD

RWVKFVIKYRWATIVAIIVLLGVAAIPITKMEMGIPGAASANLDTTARQSYEAISEGF

GEGFNGPLILVAEPKDSSTQVSPELLGNLVKELQSQKNVAQVSPLGMTEDMAIFSLIP

ETGPNDKTTKNLVNDLRSSESSIAQTYDVKLGVTGFTAVNIDMSTKLAEVFPIYVGII

ILLSLIILLLVFRSIIVPIKATIGFLLSILATFGITTAVFQWGWLHSLFGFDTGGPLL

SFMPIIVTGILYGLAMDYQVFLVSSMRESYVHGHRGTESVVHGYKQVSRVVVGAAVIM

VSVFAGFIFTDDVMIKQIGFTLAVGILIDAFIIRMGLVPAIMAIFGNKAWALPKWLDR

ILPNLDVEGEKLIASLNAKDNEHHKS"

CDS complement(3763639..3764169)

/locus_tag="PIKHDJKN_03355"

/inference="ab initio prediction:Prodigal:002006"

/codon_start=1

/transl_table=11

/product="hypothetical protein"

/translation="MEYVRDYAMYASVFGMFSFIWFGWAQESPRPSWRLYIGIASCIA

LLVCLMGVYLSVTNWGAPSALREASAFRNYLISFYAEFILAGAGAFVLIRLKRKDYIA

PWIAFIVGIHFISLKSVFNDPSLWILAALMVAISIIAVYLSTKLQVANSAITGIGAGT

VLFGFALLGLIRFITV"

CDS 3764507..3764704

/locus_tag="PIKHDJKN_03356"

/inference="ab initio prediction:Prodigal:002006"

/codon_start=1

/transl_table=11

/product="hypothetical protein"

/translation="MQIMLYIKLDFMRFAERSGYINLQFNSSIRPYHRDPIVFNDFIH

VSRYQHDGSLVPEVLPLGHFR"

CDS complement(3764631..3765893)

/locus_tag="PIKHDJKN_03357"

/inference="ab initio prediction:Prodigal:002006"

/codon_start=1

/transl_table=11

/product="hypothetical protein"

/translation="MNLVQKLTPSAKQLAFQNWEFGLFLHFGLRTFYEGYVDFDERQM

DPAAFNPVDLDCEQWIRSAKEAGMSYAVLTAKHHDGFSNWPSNYSRFSVAASQWKDGQ

GDIISEFVDACRKYDVKPGLYYSPYDGSADFYTQDAKAYDDYFVNQITELLTQYGDID

ILWFDGCGSEGHSYDWKRIIGEIRRLQPHILIFNMGDPDFRWVGNEDGIAPIPCWNEV

DSTEFSILTEKKEQLGEQLWLPAECDVQMRGNWFYSDSDEHTLKSTEELMGIYYHSVG

RGANLLLNIGPDRNGLLPEKDVERLLEFGSEIRRFERPFATIEQCRRIEKSWVYETEV

PQLLDHVIIQEDLTSGENIRSFKIDIITQKSRRPVTIYAGQHIGHKAIIRIPAIKIRG

VIVEITESDQEAVLQELSFHHVGSGKRE"

CDS complement(3765952..3767667)

/locus_tag="PIKHDJKN_03358"

/inference="ab initio prediction:Prodigal:002006"

/codon_start=1

/transl_table=11

/product="hypothetical protein"

/translation="MNCGLQNLFYKYVWSSNWWNQGREDTQLKHLELRCCKNDSAAFQ

ALVSSDQEDMLVTINRDALLWKGGPIKIVRCEVETDAPCKIDLQLIGLIEDDNRVMTS

DVLLDDPYQWLRKRQIQPLWVELHADDSVVSGQYEGKLKFYTHTMFEDEVLEKELTFR

LTISEQVLPEPQNYTFVLDLWQHNCNIARKYDVSYWSDEHFTLLDSYLHSLSQLGQKA

LTLVVSEEPWSGQRSFIDAEPSDLNEYSIVQIVRRSDGRFDYDFTHLDRYVEIGEKHG

ICKEIEVFGLINIWKHEDEGFGGVVDDYPDAIRVRYYDEETGSYKFIRNKSDLSAYIV

ALEQHFISKNWIDRVRVVADEPADICLFEESLACLRDMAPSFRYKAAINHTEFIRKHI

KGIVDYVPVLDCAAEEFEQLKQLREDVKGTFTFYVCCSPEKPNTFLSSPSIESRVIPW

LAEKLKLDGFLRWNYTVWPDKPLEKLSYRPSIWKAGDTNFVYPGKRGKPLLSLRYKWL

QRGIRDYEYMQIAKQNGQEEQVEKILQQVFRFDHPRELAPSSGKTAMQLYSFNIEDYD

RLYEV"

CDS complement(3767758..3769449)

/locus_tag="PIKHDJKN_03359"

/inference="ab initio prediction:Prodigal:002006"

/codon_start=1

/transl_table=11

/product="hypothetical protein"

/translation="MKTWKKSFSCVLGAALCLSLVLSACSGDSSTTTNTPSASAPEAS

DVFTPVGEYPIVNTPMTIKMFAPQYAVIENMETNTFTKYLQDKTNIQIKWDLVPEKAL

NDRKQLMLASGDYPEVILHGSLTKEEQMKYGKEGVFIPLNDLIDKYAPNFKKAMAELP

YLESSITSPDGNIYALPQVNECYHCNYAQKLWINQSWLDKLGLKMPSTTDEFYEVLKA

FKENDPNGNGIKDEIPLTGADDMWAGNVASFLMNSFIIDDQVDKDNGTFLQVKDGKVD

FVANKEEWKQGLLYLKKLYTEGLIDPASFTQNSDAIQQLGNRQDANVIGGITTALISY

AHSPDDKHPRHKEYVTVPPLKGPNGVQQAGYFAGVSVSQMAITNKATDEQKIAAIRLA

DYLYTEEAIVNQEHGPEGEGWRKAEEGELGMDGKPATYAIISDGETKITYNDRWDQLG

PSLRTYQYRDSWAALQDPLAEGAYEVRLKRESAKYEPFQSKENYPSSVFIALEDTQVA

AQLKTSIRDYVKSNMAQFITGSKDIEKEWDSYVKGLDGLQLGQYIEIYQKAIDTK"

CDS complement(3769560..3770459)

/gene="araQ_27"

/locus_tag="PIKHDJKN_03360"

/inference="ab initio prediction:Prodigal:002006"

/inference="similar to AA sequence:UniProtKB:P94530"

/codon_start=1

/transl_table=11

/product="L-arabinose transport system permease protein

AraQ"

/db_xref="COG:COG0395"

/translation="MFKASRIRESYGDRLFLLGVYAFLIVVTIVVLFPLIFIISSSFS

SPQAVVSGKVWLLPVDFTLEGYKAVFRNPQIVFGYLNSLYYTVIGTAVNVALTVMLAY

PLARKTFYGRNFIMVLLVITMMFEGGLIPLYLVVKNLHILDTRWAMILPGAMAVFQVI

VARTFFQTTIPEEIAEAAELDGCSDIRFITSMVIPLSKPILAVLTLMYAVGHWNAYFD

ALIFLKSPDLFPLQIVLRNILILNSIDPTMVTNVDQMLAQQGLKDLLKYSLIVVASAP

VLIIYPFVQKHFVKGVMIGSLKG"

CDS complement(3770489..3771484)

/gene="yteP_29"

/locus_tag="PIKHDJKN_03361"

/inference="ab initio prediction:Prodigal:002006"

/inference="similar to AA sequence:UniProtKB:C0SPB3"

/codon_start=1

/transl_table=11

/product="putative multiple-sugar transport system

permease YteP"

/db_xref="COG:COG4209"

/translation="MSSMQKRIRKPETSMQGQPHEPALPDSKSSLRFIGKKMKRHWQL

YLVVFLPLLYLVIFKYIPMSGVVIAFKEYNVIQGIWGSPWVGLKYFEQFFESPNFWLY

LKNTLGINMYGLLVGFPAPILLALALNEIRNGFFKSSVQMITYAPYFISTVIMVSILI

VTLSPSVGIISKFTQLLGIENTNFMGIPSLFKSIYVWSDVWQFSGYGAIIYIAALAGV

NPELYESAKVDGASRMQKIINIDLPSLIPVSVILLILNLGNFMKVGFEKVYLMQNPLN

ISTSEVISTYVYKVGLLSSSFSFSTAIGFFNSIINLILLVSVNYIAQKLSNSSLW"

CDS complement(3771711..3773933)

/gene="rhaR_32"

/locus_tag="PIKHDJKN_03362"

/inference="ab initio prediction:Prodigal:002006"

/inference="protein motif:HAMAP:MF_01533"

/codon_start=1

/transl_table=11

/product="HTH-type transcriptional activator RhaR"

/translation="MRKTLFNRLLFSYMPIFIVVITFTFFVFFQILSEQSRKEALNAN

KMLSLQAMRLIDTSLKAIDNMIMSETINNKQLIDFFNNEEKNNIFINISAVKKMQDMI

SYYPMIDSIYIVRYEDNFVLSNSTSDQLSNFRDEPFIKKAMDPLASKQWSDVRSFEQF

STKGSKPVVSLVRGAPFITGEKGMVVVNVATDSLQNSIADLYDSKTSFIGIRDAAGND

LFSELHSKEQSKIFSNYVSSYTGWSYQSGLVNGKLFHVISSLYNVWFIIGIVTIGGGF

VWLLYVTRRNSRPLEQIVARIRGYRLPLSKELGDEFSFIESALSNIIEQSNQYQQKHD

EDLHLRKRYLFHQLIEGDMGLTHEEWIGETNSLQLPYSFNKQVVLVIEMDKYSDFCCQ

YSRRDQNLLKFALRSVIQELAPKYELALWSEWTSAFQLSVMVFVDSDEEADTSITLKL

FESVRVWVQENLKFTVTVGIGEQVAELSSVANSYKEALRALKYKIVLGENRLITSEHI

ANHGQVEVFSQLNVIRSIIQSFRLLEDEWKTKYDKLMNEMKLGLLTKDEITNLMNYLI

YYLGLEISGMTKEVQGIWMRDGLPRLSKIIDECHSLEQMWEESLIVLNSFFTDLKEAQ

NRRLHAATIRDMRKMIEEEYANPNMSLEYLSSIFNINGKYVSKLFREETGQKFVDFLI

DIRLQAAKRLLTETQGSMQEVAEKVGYTSAISFSRVFKKVVGYSPSEFREEAARRQIG

"

CDS complement(3774203..3776113)

/locus_tag="PIKHDJKN_03363"

/inference="ab initio prediction:Prodigal:002006"

/codon_start=1

/transl_table=11

/product="hypothetical protein"

/translation="MSNARFARLGLMMVVFCLLGSIMFINFKPNHAYASGEWQAQTYD

PNKAGLELNPLKGFLPFSKLPSESVDPNNSFPHSMEWFYVSLRDLMTDWNTYNWAPLD

AYLNDIASRGNQAAFRVYVDYPGRTSGIPQFLINGGLMTRDYDQNGNHDTSTHSLAPD

WNDANLNQALRQFVSALGTRYDGDPRIGFLTTGLYGFWGEWHTWPYNTDPTNWEMNQS

NRDGLLSAFKGAFQKTQVLARYPYSASTADLKNAFGYHDDSFSYETLFANDWGFWNLI

TQNGLQDIWKTHPIGGELYPPLQQDTSFWQHWPNQNGEDFQTSVETTHASWLMYDRVY

KNSLPDETAKLNALRAHKMLGYTLYNSSVQLSDSSASSLQVNVKLQNKGVAPLYYNWE

VEFGVLNSSDTWVKSLGTKSWNLKSILPGDTDYSFSYSANHNLPAGDYRLVMRIVNPL

TGGKTIRFANTRQDANRDGWLTLSGFHVSASATTSTLLADPPVTPVTPPNSGVSAYEA

EAVGNTLAGSAVIANCTTCSGGAKVGYLGNGSGTLKFNNVTVPSAGNYKLTIQYLNGE

ASRSAILSVNGATGTSLNFPSSGAWTTVASQETTVHLQAGFNTLLFSQPTGYAPDIDR

ILVSVNPTGSGG"

CDS complement(3776317..3777153)

/gene="araQ_28"

/locus_tag="PIKHDJKN_03364"

/inference="ab initio prediction:Prodigal:002006"

/inference="similar to AA sequence:UniProtKB:P94530"

/codon_start=1

/transl_table=11

/product="L-arabinose transport system permease protein

AraQ"

/db_xref="COG:COG0395"

/translation="MSRVVSSKHRNNLIIHLVLIAGAAVMVLPFLWMILTSLKTTTEA

TQIPITILPAEPSLRGYVDALRQSAFLHYYANTLMAAFMKTLFPVVFSAMAAFAFARI

SFPGRGLCFFLIISVMMIPNPVFYTPQYLMMSKLGLVNTTTALWITSLVSPFATFLLR

QFFLGISKELEEAAVLDGCNPFQIFRYIMLPLVKSALVAVVIIQLLWSWNELQWPLII

NSSPEKLTLSAGLATLVSMFGTDYPVLMAGAFMAVIPMLILFFIFQRRFIEGVAFSAS

KG"

CDS complement(3777166..3778035)

/gene="ugpA_3"

/locus_tag="PIKHDJKN_03365"

/inference="ab initio prediction:Prodigal:002006"

/inference="similar to AA sequence:UniProtKB:P10905"

/codon_start=1

/transl_table=11

/product="sn-glycerol-3-phosphate transport system

permease protein UgpA"

/db_xref="COG:COG1175"

/translation="MHSKSRYIWAYMMIAPTLIGTLVFWSWPAVYSIYLSFLKSENFG

LVNHWVGWANYERLFMDEEFWQNLRNTILYVVLFVPTTIILSTFFAVLLNAKIKGQSV

YRVIYFLPQVTMAAAAAMVWVIIFAQDYGLLNSVLGTHIAWISDKRFAMYALVMVGVW

GAIGFNMLLILAGLQGIPRTLYEAAEIDGAVGFRKFRSITLPLLTPTLFFTSIVLVIG

ATQIFDSIYLIIGKTNVALPAVRSLVYAYYQNSFIYYDQNYGAAIVNILLVINLVLTG

IQFMLQKKWVIYD"

CDS complement(3778111..3779406)

/gene="yesO_6"

/locus_tag="PIKHDJKN_03366"

/inference="ab initio prediction:Prodigal:002006"

/inference="similar to AA sequence:UniProtKB:O31518"

/codon_start=1

/transl_table=11

/product="Putative ABC transporter substrate-binding

protein YesO"

/db_xref="COG:COG1653"

/translation="MTMNIRKKMAAGIASLLLVSLMAGCNSSNDGGGATDSGAQKSVT

ISYTIWDKNQQPAMEAIADAFTEQHPNIKVKVDAIPWGEYWTKMSAAAPAGMLPDVFW

MHGGQFMKYAEGQFLEPITDKVQANEIDLNNYASNLVSIYTLDGQNYGIPKDFDTIGL

AYNKELFDQANIPYPDDTWDYAKLAEVAKQLSQPDKGIYGFGAKLDTQAGYWNDMLAN

GGFILNDDMTKSGYDELASIEALKARYQMILDKASPTHQQMTDTDSVEMFKSGKLAMI

FDGSWDNGNLASSEIIKGKYDWAPLPKGKVKRGNIINGIGNVMSTKGKHKEEAWQFLK

FLGSKEAAEITAQMGAAIPAFNNTQDAWVKNTPDLNLQVFIDQAADATPYPSGSKSYP

VWFSKEAEIMSQAWGGAISIEEGAKKVAEMMNQAIEDAK"

CDS 3779714..3781252

/locus_tag="PIKHDJKN_03367"

/inference="ab initio prediction:Prodigal:002006"

/codon_start=1

/transl_table=11

/product="hypothetical protein"

/translation="MNNISIARLPGEYWWGGVINDGSSMPFGQTAYTRDLRVTDDNQA

SPLLLSNKGRYIWSEEPFAFHVDENGLSVFDNAGEVVVEDEHSSLQGAYRHAAGTHFP

PSKQMPDALVFTAPQYCTWVEMFYNPTQDRIIRYAESILANGLPPGVLIIDDNWMMDY

GMWDFDKYRFPNPREMIDTLHGMGFKVMLWMCPYVSADCTMFKELRAGDLLLTGVDKA

PIIRHWWNGYSAVVDYTKEEGAAWFKRQMDRLITDYGVDGFKLDAGEPFLPGGADTTQ

PIAWTRPLRQLEDCEAYANLGVGYSLVELRMCWKLGGQGLIQRQRDKTHTWDATGLSG

LIPNAIAQGLLGYAFNCPDMIGGGMDGDINSPEFHFDSELFVRYTQCAALFPVMQFSM

APWRVLKGEELTWCLDAVRLRSELGPELLTLARQAAADGLPILRSLEFEYPDCGYELV

NDQFLLGQNLLVAPVVVKGLTSRNVVFPEGQWQDEDGNIINGPTELQVDAPLSRLPWY

RKIQ"

CDS 3781266..3782546

/gene="iolG_13"

/locus_tag="PIKHDJKN_03368"

/EC_number="1.1.1.369"

/inference="ab initio prediction:Prodigal:002006"

/inference="protein motif:HAMAP:MF_01671"

/codon_start=1

/transl_table=11

/product="Inositol 2-dehydrogenase/D-chiro-inositol

3-dehydrogenase"

/translation="MKKIKLALIGAGARGLLSYAPYVLAHSDEAEFVAVAEPDAARRN

EAAKQFGLASSQLYESWEHLLAQPKLCDAVLICTQDRMHYGPTMAALDRGYHILLEKP

MSNDPLECLKMAEAADQAGLLLTICHVSRYASFWQRLKHLLAGGAIGEIVSIQHNENV

GYLHYAHSFVRGNWRNDQLASPMILAKSCHDMDLIRWLIDDECVRLSSFGSLKHFQIS

EAPEGSTERCTDGCAVEASCPYSAPRFYMQPLEDNGFAALIATPPTETNRMNAILHGP

YGRCVYRTDNNVVDHQIVNMEFAGGSTVMFSMCGFTRDINRIVQIMGTGGEIRGDLLK

EEIDLFEFSSGKRSIITTSAGAAGHQGGDEGIMSRFLADLREGNHSGTLTSAQASLDS

HLMAFAAEASRLRQGLVVEMSEFKATILASSACN"

CDS 3782630..3784267

/gene="rhaR_33"

/locus_tag="PIKHDJKN_03369"

/inference="ab initio prediction:Prodigal:002006"

/inference="protein motif:HAMAP:MF_01533"

/codon_start=1

/transl_table=11

/product="HTH-type transcriptional activator RhaR"

/translation="MYELLIVDDHAHLVDSLERTMPWEALGIGKVHKAFSGEEALEHC

KSYPVDIIITDIRMPGISGLELIEHIKKYDKHFKSILLSGYGEWDYAQEAIRQQVDGY

LLKPVDQRELADMLRQLTEALHLEWDERGKQVKAMSMLSEHMPLFREKLLMDLLQGRK

LPPKLLEEKMAYYRLPFCPSDETAICLLRIESSAEELNWKDQSLLSSAVANIAEETFS

ELFHVWHGRDAYEHEVFVLKVREESWLAREKEPELNRQRSLEHLAARLQQHVRHFLKS

SISIVVSHWQVMAHGLRETYQDSLAILTRHLGGETDLLIRLQDALVPEKPYRVHSLYE

SPTMIQLFEIYQWEAISAKWDAIFLEVTNKRMDSRETLLELYYHAMGAFSFFAHKNGL

SLLEIIPPELDKATEANRFRHMYQLQDWIVLTLDRLREMTKSEWNNEQVALIRKVQQF

ITAHLADATLQTAADHVGLHPAYLSALFKSKASENISEYLYKVRMQRAREILIEQPEL

KVSDVATAIGYHKPQYFIKLFKEHFGLTPQDFRHQSS"

CDS 3784286..3786016

/locus_tag="PIKHDJKN_03370"

/inference="ab initio prediction:Prodigal:002006"

/codon_start=1

/transl_table=11

/product="hypothetical protein"

/translation="MKLTIFTKIVLVIFLLLVSTLLLYYLTNRSSVRLIKQEIQRVKL

NELTFLAGDIDNNLSKVSSFSDFLVLDKDVRLLATPSFLEGDFELNKLKLRVSDKLNL

LHALNNWNAEFMVIYDEDSSSFSSTSSSSKLSELYLETHFRPFWQYEQTGDGTYLTYY
LAHPYTFRFDPEEVSYVVRAKIDTKNVLAYLDRAKGTNDNSAILLSAAGDVLASDTEK
LPFIRELTSQLEVGKEVSGNRLIKLNGEQYLVNFTALSNFKDWYIADYTPISEVLNPI
YANQRLYYFSSVFLLLFSILLALLLYRDVQIPIRKLLWGVRKITSGQYSITLHQESND
EFMYLFERFNEMSKEIKELIERVYMEQIRSKEAKLRQLHSQIQPHFLYNSLAFIKSMT
ELDEKQAVIDMSISLSRYFRQTIKFENTYTTVRDELELVRLYLTIQSLQMDRFVFTID
VEEQLLDIQVPRLLLQPLAENAILHGIESLRMVGDIRISGYSNGEYCELIVADNGCGV
EPGQLDTLRNAIYYGTDANTAWALHNVYERMRALLGSEADMELANGELGGLQVTFRWK
VVREQEATEI"

CDS 3786013..3787728

/locus_tag="PIKHDJKN_03371"

/inference="ab initio prediction:Prodigal:002006"

/codon_start=1

/transl_table=11

/product="hypothetical protein"

/translation="MRSWKWLKQTGLFGLAIILFLVVMIFSFIFWEDPTGPSQLEEKE
NRSVGPAPSFIDGKYESPVVLTTVGHLYPDIEFKNGEDLQNNVLSRLFEDKLGIKVNY
LWTTTGDEDVYTNKLNTLIATGQSLPDIVTFKGGQNMYPLLRSGAFRDVGELFEQYAS
PKWKAAMAQAPESWDRYIINGKKMAIPILDGKMNSNEVLYIRQDWLDKLGLKAPTTLE
EMESVMDAFVHLDPDGNGKDDTYGLSISLKNSFFTWMSDAGFVFGAYGEMPDVWSTDA
DGMLSYGSVQPSVRQGLTTLKRWMERGYIPKDAEILDEVQATESWKLGKAGMIVAPLW
AAGWPLGNVTANEPDARFEAYPIPLGPSGLSGTRGTPPEYGAIAINADMANPEIFFNY
MNFVYDNFIDPPAGSPYRWGIAEGYDYGYEDGKVTFLESQVPGGFADPQKYLLSLSLP
SVPNLLSESIARINSGASDQTSYDIKNKLLRTPQELRGWQIVESNPSVYKTDAFTGAP
TPTMEKIGDALDELLHDAMLKIVYGQEPLSSFDLVVVRWHELGGDEITEEVNDWYTSL
GKINQ"

CDS complement(3787901..3788656)

/locus_tag="PIKHDJKN_03372"

/inference="ab initio prediction:Prodigal:002006"

/codon_start=1

/transl_table=11

/product="hypothetical protein"

/translation="MKYTVITGASSGIGYETALAFAARGKNLILVARRLDKLKELKSA
IQNIDPAVHVIAHTSDLSDTSQVYTLYNTLKEYQIETWINNAGLGEGSFVAEQNLEKV
ETMLRVNIESLTILSTLYVRDYADVEGAQLINVASALGYAIAVGSVAYSATKFYVSAF
TEGLAKELEIKGANLKAKVLAPAITETEFVKKSLDTEDFDYKANMSKYHTAKEMAGFM
LDLYDNSEVVGIVDLNYNFNLRSPIYPIIGALH"

CDS complement(3788969..3789544)

/gene="comR_5"

/locus_tag="PIKHDJKN_03373"

/inference="ab initio prediction:Prodigal:002006"

/inference="similar to AA sequence:UniProtKB:P75952"

/codon_start=1

/transl_table=11

/product="HTH-type transcriptional repressor ComR"

/translation="MMARSKEFEVNEVLDKAIHLFWTQGYEKTSMQDLVEFMGIHRRS
IYDTFGDKHALFMKALERYETKQTNKMSFLVDTQKPIKEIIRALFDSTIRNEGQPLGC
FLVNSGVELGVLDPEVASLVNESYLRTEELLRNLVLEGQQTGEIKADVDPVVISHYLM
NAWVGIRTLVKTTTDQQKLKNIMEMTLSILD"

CDS 3789782..3790228

/locus_tag="PIKHDJKN_03374"

/inference="ab initio prediction:Prodigal:002006"

/codon_start=1

/transl_table=11

/product="hypothetical protein"

/translation="MVRTHDARTSLGSNGVAPFNVPLAPNTPTLVGVLGLNIVNPGLV
IRTQFNGTYRISVANLSSQPSSIRFEIFRGSTYTGTPIYVVSQPIFPSVGSILASFDG
SDYNVPPPPSGQLIYSCFVTFVPASPSEQASRVGPECFNAVAYSDD"

CDS 3790275..3790727

/locus_tag="PIKHDJKN_03375"

/inference="ab initio prediction:Prodigal:002006"

/codon_start=1

/transl_table=11

/product="hypothetical protein"

/translation="MVKTLDARTSQGSNGQHVLTPSFTNGVPLLFAQVGLQISNPGMW
IRTFFSGIVEVTASQLPVNGNLVIEVYRGLGGSQQLVYRATTTVLANPFFSPCILSFT
GSEYNVPPPGNGQLVYSAYATFSYNPIYNVSNVRLGPESFNATVYSDD"

CDS complement(3790980..3791345)

/locus_tag="PIKHDJKN_03376"

/inference="ab initio prediction:Prodigal:002006"

/codon_start=1

/transl_table=11

/product="hypothetical protein"

/translation="MENVSTEVKMESFQAFQSTIRKSENALANMSQKGSNTTLLDKRL
NALYIGLAMLETAWNLKPHHYTEEDLTEARNVLAGLLPLLDNMYDKSKTGSPQKTLIE
RRIKAFELAVQAMDDVVEK"

CDS complement(3791533..3792462)

/gene="rbsB"

/locus_tag="PIKHDJKN_03377"

/inference="ab initio prediction:Prodigal:002006"

/inference="similar to AA sequence:UniProtKB:P0A2C5"

/codon_start=1

/transl_table=11

/product="Ribose import binding protein RbsB"

/db_xref="COG:COG1879"

/translation="MKKLTLLLVSVMMIVLAGCSLEPPGWAKPDSAGSNGQKKIGLSV
STLNNPFFVSLKDGVVAEAKKQGIQVIVVDAQNDSAKQTNDVDDLIQQGVEALLINPA
DSAAISTAVQSANSVGIPVITLDRSADKGDVAALVASDNVKGGQMAAEYFVEHLGEGA
KVIELEGVPGASATRERGKGFHEVADQKLNVVAKQSADFDRSKGLNVMENLLQGNPDV
QAVFAHNDEMALGAIEAIQSSGKDIPVIGFDGNDDAIKSIQDGKLTATVAQQPVLIGQ
LAVQAALDVLDGKQVEKSIPAELKLVTKENANN"

CDS complement(3792477..3793448)

/gene="rbsC_1"

/locus_tag="PIKHDJKN_03378"

/inference="ab initio prediction:Prodigal:002006"

/inference="similar to AA sequence:UniProtKB:P0AGI1"

/codon_start=1

/transl_table=11

/product="Ribose import permease protein RbsC"

/db_xref="COG:COG1172"

/translation="MTTLQENQPAKSGFRLTNLTQKLGPLLGLIILIIIVSVLNPSFL

EPLNILNLLRQVSINALIAFGMTFVILTGGIDLSVGSILALSSAFVANMMLSGLDPIL

SIIIGVALGGVMGMVNGLMITKGKMAPFIATLATMTVFRGLTLVYTNGNPITGLGDSL

LFQLFGRGYLLGIPVPAITMLITFAILWMILHKTAFGRKTYALGGNEKASIISGIKVH

RVKIMIYSLVGMLSALAGAILTSRLNSAQPTAGTSYELDAIAAVVLGGTSLSGGRGRI

VGTLIGVLIIGVLNNGLNLLGVNSFYQMVVKGVVIAIAVLLDRKKTA"

CDS complement(3793450..3794931)

/gene="rbsA_2"

/locus_tag="PIKHDJKN_03379"

/EC_number="7.5.2.7"

/inference="ab initio prediction:Prodigal:002006"

/inference="similar to AA sequence:UniProtKB:P04983"

/codon_start=1

/transl_table=11

/product="Ribose import ATP-binding protein RbsA"

/db_xref="COG:COG1129"

/translation="MHIQMQDIYKAFGTNQVLSGVNFDLKEGEVHALMGENGAGKSTL

MNILIGLHGRDQGTILIDGKETYFANPKEAEKMGLAFIHQELNVWPEMTVLDNLFIGK

EITSSFGLLNTRQMKALATEQFAKLSVQIPLDRPAGECSVGQQQMIEIAKALMTDAKV

IIMDEPTAALTEREIQKLFGVIASLKKNGVSIVYISHRMEEIFTICDRITIMRDGKTV

DTKAIPETSFDEVVRKMVGRELTERYPARNPSYGEVVLEVRDASSKELFQNISFTVRA

GEILGFSGLMGSGRTEIMRAIFGLEPLDGGEIMIRGKKAHIRKPADAVRHGIGFITED

RKDEGLVLDFSIRENMALPNLFSFSSKGFISTQKEQEFVDTLIKRLQIKTQSSETAVR

NLSGGNQQKVVIAKWVGIGPSVLILDEPTRGVDVGAKREIYQLMNELTDRGVAIIMVS

SELPEVLGMSDRIAVVHEGRISGELAKEEATQENIMTLATGGQ"

CDS complement(3794946..3795347)

/gene="rbsD"

/locus_tag="PIKHDJKN_03380"

/EC_number="5.4.99.62"

/inference="ab initio prediction:Prodigal:002006"

/inference="similar to AA sequence:UniProtKB:P36946"

/codon_start=1

/transl_table=11

/product="D-ribose pyranase"

/db_xref="COG:COG1869"

/translation="MKKQGILNSHISKVLSDLGHTDTIVIADAGLPVPEGVPKIDLSL

KLGTPSFQEVVELIADDMVVERVILATEIKAGNPKALQFITDKFGDEAIDVSISHEQF

KALTRNAKVVIRTGEATPYANCILQSGVIFG"

CDS complement(3795344..3796225)

/gene="rbsK_1"

/locus_tag="PIKHDJKN_03381"

/EC_number="2.7.1.15"

/inference="ab initio prediction:Prodigal:002006"

/inference="similar to AA sequence:UniProtKB:A0A0H2WZY4"

/codon_start=1

/transl_table=11

/product="Ribokinase"

/translation="MAKICVIGSSSMDLVVTSSRRPGAGETVLGDSFKTVPGGKGANQ

AVAAARLGADVSMIGRVGDDAFGTAILNNFKANAVNMQNVKPVTHMESGTAHIVLAEG

DNSIVVVEAANREVTPAYVDEAAEVIQRADIVLIQQEIPEETVVRVSEICAQSGTPLL

LNPAPARKVPDEVIDKAAYITPNEHEAEILFQGMTPAEALRKYPNKLFITEGSNGVRY

FDGEQEIVVPTYKVEPVDTTGAGDTFNGAFAVALAEGKPLQDSIRFANRAASLSVTKF

GAQGGMPTRDEVEESLK"

CDS complement(3796218..3797207)

/gene="rbsR_4"

/locus_tag="PIKHDJKN_03382"

/inference="ab initio prediction:Prodigal:002006"

/inference="similar to AA sequence:UniProtKB:P0ACQ0"

/codon_start=1

/transl_table=11

/product="Ribose operon repressor"

/db_xref="COG:COG1609"

/translation="MTTIKDVANLAGVSVATVSRVINERGYVHADTRKKVEEAVKQLN

FSPNEVARSLYKRKSKLIGLLLPDIANPYFPQLARGVEDRMQEQDFRLIFGNSDEDEQ

KEKDYIQTFIQNNVVGVISSTNYPHSSIYENLKIPVVFLDRTPLDSPSVYADGREGGR

LAAREIIKRGSRRITVMQGPAHIKPAQDRFEGAIESIRQAGLDYRVIQTTSFSFNDVG

LWADELFSKYGDTDGVIASNDIAAMAVLHEALRIGKKVPEDMQIIGFDDIPMSSLLSP

ALSTIRQPAYEMGREAAGLLIKLVEQAPIENKHIQLPVSFIERGTTRKVNSDG"

CDS complement(3797655..3799118)

/gene="emrB_4"

/locus_tag="PIKHDJKN_03383"

/inference="ab initio prediction:Prodigal:002006"

/inference="similar to AA sequence:UniProtKB:P0AEJ0"

/codon_start=1

/transl_table=11

/product="Multidrug export protein EmrB"

/translation="MQASTATSGAGFKAAKKDIKVFPIVLAFLMSGFIGLFGETALNV

ALTDLMQIFNIGSATIQWLTTGYLLTMGVLVPLSGLLIQRFTTRQLFVTSLLFSCLGA

LIGAVASSFGVLMVSRIIQAVGTALLLPLMFNTILLIFPIEKRGAAMGLVGLVIMFAP

AVGPTIAGLLIEALNWHYIFWLSFVFLAIALIIGIITMQNVSETTRPRIDLLSMLLST

LGFGGIVYGFSSAGEGDGGWTSSTVVISLAIGLVALILFTIRQLKISNPLLDLRAFKY

PMFAIGALMVALCMMVILSSMLVLPMYLQVGLAFSTLSAGLILLPASALNGLLAPIMG

KLFDKYGPKWIVLPGIVLVAVALWLFSGVTLASSVAFIITVHAIMMIGVSMIMMPAQT

NGLNQLPPQLYPHGTAIINTMQQVFGAIGTAVAVSVMSNGQAKYMSQATNPNDPSLIP

FSLTAGVQDAFIFGMIVALVGFVLSLFIKRVVVNRLH"

CDS complement(3799493..3799582)

/locus_tag="PIKHDJKN_03384"

/inference="ab initio prediction:Prodigal:002006"

/codon_start=1

/transl_table=11

/product="hypothetical protein"

/translation="MDLRFYYTHAEVAQKLLNSLVPKMSTKQI"

CDS 3799651..3800076

/locus_tag="PIKHDJKN_03385"

/inference="ab initio prediction:Prodigal:002006"

/codon_start=1

/transl_table=11

/product="hypothetical protein"

/translation="MKFNEYVIGETYRTESIQLSKKDIIDFAKIYDPQYMHIDEAKAK

EGRFGSLIASGMQTMSTSFKLWVELGIYGEDVVAGTGMNNIKFIKPVFPDDEISVLSE

VIELAPRRSSGGIVTVLLTTFNQNNEKVFQAELSALISN"

CDS complement(3800178..3802793)

/gene="rphC"

/locus_tag="PIKHDJKN_03386"

/inference="ab initio prediction:Prodigal:002006"

/inference="similar to AA sequence:BARRGD:NG_063825.1"

/codon_start=1

/transl_table=11

/product="rifamycin-inactivating phosphotransferase RphC"

/translation="MSSLVIRFQEMEKLQLALVGGKGLNLAKLSKVEGVQVPDGFCVT

TVGYQKAIEQNETYHALLDQLTLLKVEEREQIGEISRKIRELIIGVEIPSDVEKEVAH

HLSKFGEEHAYAVRSSATAEDLPHASFAGQQDTYLNIIGKEAIMQHISKCWASLFTDR

AVIYRIQNGFDHVQVHLSVIVQKMVSPTASGILFTADPITSNRKLLSIDASFGLGEAL

VSGLVSADCYKVREGEIVDKRIAAKTLAIYGRKDGGTYTHQIDPILQKTQTLTDQQIL

ELARIGRQIEAHFGFPQDIEWCLADDTFYIVQSRPITTLYPIPEASDLENHVYLSVGH

QQMMTDPMKPLGLSFFLLTTPAPMRIAGGRLFVDITPRLASPVMRENFINTMGQSDPL

LKDALTTVIERGNFIKSLPIDQPAPTPRTNNLQSTSEGFQAHIENESTIVSDLIKSNQ

ASVEMLKRSIQNKSGLDLFEFILEDLQELKKFLSDPQSSNVFMTAMNASAWINENMKE

WLGEKNAADTLSQSVPNNITSEMGLALLDVADVIRPYPEVIAYLQHVKEDDFMDELVK

VEGGQETRDAIQDYLDKYGMRCAGEIDLTRTRWMEKPITLVPLILGNIKNFEPNASKR

KFEQGRQEALKKEQEILDRLKLLPDGEQKAKETKRMIDLVRNFIGYREYPKYGMINRY

FVYKQALLKEAERLAQAGVIAEKEDIYLLTFEELREVVRTNKLDGKIITQRKDDYKIY

EKLTPPRVLTSDGEIITGQYKRENMSAEAIVGLPVSSGVIEGRARVIFNIEDADLEDG

DILVTPYTDPSWTPLFVSIKGLVTEVGGLMTHGAVIAREYGLPAIVGVENATQRIKDG

QRIRVHGTEGYIEIL"

CDS complement(3803153..3804892)

/gene="sasA_19"

/locus_tag="PIKHDJKN_03387"

/EC_number="2.7.-.-"

/inference="ab initio prediction:Prodigal:002006"

/inference="protein motif:HAMAP:MF_01837"

/codon_start=1

/transl_table=11

/product="Adaptive-response sensory-kinase SasA"

/translation="MKIQQRMSFHFTYQLIFYALIILAITLIACIIFFQNTTNSELRR

NFPVGALQLIAQEASSKDGTIQISPKWDKLIEEKGMWMQVVNAEGEVIYEVNTNSHDG

LSPSYSITKLLDIQETRTLGTYAVQTHLNFAFNVPVLYILGYQSSDLDQLMAWFDEYG

QQGMVSSEKVSFLDQRLRETGSYLQVINSTGHIEQAIGDGTYLQKGYRPLDILAIQQS

PSNYDTNIVAHRDQLSGMTWILYTPNPTGGPLKHPAMDTATRGLMWFAGLALLLAVPL

SIWHGYRYGQPLILFAGWFERMGRGQYDQVLTAKDKRKIFRSNGTMRNRYRLYKEVIE

SFYQMADQLAQTEKERERLEKDQREWMSGISHDLRTPLSTIQGYGYMLESLPEQWSQE

EMRTMGATIREKGDYMLELISDFSMIHQLKQGDSIMELQKIDLVELVRCSVLKYVNDA

TLSEYQFHYIGEEHPLWVQADETWMLRLMDNLLSNAVKHNPPGVNVTVSIGRMNNKAC

IRVFDNGKGMDEETLQHLFNRYYRGTNTEESTAGSGLGMSIAKTIVEAHGGEIHVQSK

VRQGTKIEIFLPC"

CDS complement(3804889..3805641)

/gene="regX3_3"

/locus_tag="PIKHDJKN_03388"

/inference="ab initio prediction:Prodigal:002006"

/inference="similar to AA sequence:UniProtKB:P9WGL9"

/codon_start=1

/transl_table=11

/product="Sensory transduction protein regX3"

/db_xref="COG:COG0745"

/translation="MKWVKMMTNQTDIKIAIVDDEPSIVTMLQMVLRRAGFHQLYDAS

TCAEALNLVQRVSPDIVLLDITLPDGSGLELCSKLREYGNPHILFLTAKASDLDVLRG

FAMGGDDYITKPFNPLEVAARIQARIRRLEPEAPTVSQVKRPEPGIYQFGRFTVNEAE
GELIVEGQPVPCPAQVFHLLLHFCKFPGIVFSKTQLYDQVWGINGQGDDSTVMVHIRR
IREKIEIDPGNPKLLLTVRGLGYKLSKEFQEQ"

CDS 3805794..3806114

/locus_tag="PIKHDJKN_03389"

/inference="ab initio prediction:Prodigal:002006"

/inference="similar to AA sequence:UniProtKB:Q63F05"

/note="UPF0145 protein BCE33L0904"

/codon_start=1

/transl_table=11

/product="hypothetical protein"

/translation="MIITTTAIVEGSPVRQYIGVATGEVIIAANVGRDFLASFRDLVG
GRSRTYENKLKEARDAAFEEMTQQASQMGANALIGVDLNYEVVRQGMLMVAVSGTAVI
INSL"

CDS 3806124..3807389

/locus_tag="PIKHDJKN_03390"

/inference="ab initio prediction:Prodigal:002006"

/codon_start=1

/transl_table=11

/product="hypothetical protein"

/translation="MQMKKITASILSLCLLTSGLSSVHATEKAISVSLNGEPIQFNQS
SAVVDKGVTLVPLRLLLEKMNVHVKWDEANHTVTGVKDGLSLSLKLGSTNAIANGKAV
NLDAAPKQIRNVTYVPLRFVAETAGYQVAWDQSLRQVSLTSKHQSDESRGFLWKVEHK
GNTVYLLGSIHYVQEGMYPLRPEIENALKAADYLGVEVDLSKIAPEELQKHILDLGVY
KDGTTLKDHISADTYTKVTTLLKANGLPENSFDSFKPWFAQQNILGIIVGNEGYQSEI
GIDQYLIDKANQANKPVISLETLDSQFQTNNNYSDALQEKLLLQTVDPSAPVTLAPSG
GLDDLSKMWTEGDDNALNAYTNSSDWDAEYHKALLTDRNVEMAEKIKGYLNSDKKETY
MVVVGMLHMLGDQGVVPLLEKEGFNLERK"

CDS complement(3807487..3807978)

/locus_tag="PIKHDJKN_03391"

/inference="ab initio prediction:Prodigal:002006"

/codon_start=1

/transl_table=11

/product="hypothetical protein"

/translation="MFKSKELIDSILANFNNIEITNVIEEKHNREYEGMIIYIGDYKY

RSRLAKLTPKKKGFFVVFWKKDEKNQNQPYSFFESPDKLIISIINGNMKGQFVFPKSK

LLEKGILKNEDTKGKMAIRVYPSGEKELNKSAAQTQKWQQEYFIDLSDEIDDQKLIDL

YFG"

CDS 3808145..3809110

/locus_tag="PIKHDJKN_03392"

/inference="ab initio prediction:Prodigal:002006"

/codon_start=1

/transl_table=11

/product="hypothetical protein"

/translation="MNEIKALENPNKFSDPLFVTLVASLCCLLWGSAYPFIKLGYSAF

DILAEDIPSKFVFAGYRFILAGLLLLFLFRVISKRKFELSGRQFSSLILLGMFQTGLQ

YMFFYVGVANTTGVKGSIMNATTTFFSVVLAHFLYKNDKLSKNKIVGCLLGFIGVIIV

NFHSNLLEFSFSLTGEGFVIMAALIFSITAIYAKHLTGSIDVLVITGVSLFVGGLALT

ILGLLLGGRVTHFTLESTGILIYLVSLSSAAFCLWNLLLKYNKVGKVSVYNFLIPVFG

ALLSALFLGETILEFKNLIALLFVSIGIYMVNRVTSRKFSNTLEP"

CDS complement(3809243..3810376)

/locus_tag="PIKHDJKN_03393"

/inference="ab initio prediction:Prodigal:002006"

/inference="similar to AA sequence:UniProtKB:P9WLZ3"

/codon_start=1

/transl_table=11

/product="hypothetical protein"

/db_xref="COG:COG1680"

/translation="MDFKPLASFIDRITSWRIPWAEVLVMHQNDTVFRYRNGYANLEE

QTPIGDGAIFNLYSMTKIMSCVAALQLVERGQMLLSDPVSDYLPEYAEMTVKKTLSSG

EVRLEKATKAITVRDLFTMTAGFSYDIGSPSIQEAVQSTNGTLSTRDFAKALAKEPLL

FEPGTHWNYSMCHDVLGALVEVVSGRRFGKYLQEEIIGPLGMNDTTFDLNEEQQTRLI

PQYAYNDELEKPVRMDGNGFRVGTDLESGGAGLLSTVSDYALFLSALTGKGTSPKGVR

ILSQASVELMRTDHLNDLTRGDYSWDHMGGYGYGLGVRTHISKAGSGSLSPLGEFGWS

GAAGCMAIIDPDSELTVMYAQHLLNNQEPYVHRRLRNVVYSCL"

CDS complement(3810543..3811058)

/gene="paiA_2"

/locus_tag="PIKHDJKN_03394"

/EC_number="2.3.1.57"

/inference="ab initio prediction:Prodigal:002006"

/inference="similar to AA sequence:UniProtKB:P21340"

/codon_start=1

/transl_table=11

/product="Spermidine/spermine N(1)-acetyltransferase"

/db_xref="COG:COG0454"

/translation="MTVEIRACSHEDLKILQEISIETFNDTFKDQNSPDNMRAYLERA

FNAKQLEAELSNSCSYIFFVYYNDELAGYLKVNRDGAQSEQMSDVSLEIERIYIRKKF

QKHGLGKYLLNKSMETALEHKKKEIWLGVWEKNDNAIAFYKKMGFVQTGSHSFYMGDE

EQIDFIMVKTL"

CDS complement(3811253..3812761)

/gene="rssB_8"

/locus_tag="PIKHDJKN_03395"

/inference="ab initio prediction:Prodigal:002006"

/inference="protein motif:HAMAP:MF_00958"

/codon_start=1

/transl_table=11

/product="Regulator of RpoS"

/translation="MYNIMIVEDSKPILRNIKMLMDKLDFPIHVAVTATNGEEALVAL

QKQPIDLLLTDIRMPKMDGLSLIEQAKLAQPELKVILISGYSDFEYTRKALNLQVFDY

LLKPVEPEALEEVMGRVIKQLDQSRSRNFHELQEIVDPHSYAELKPGEHAAFLAQMMI

ILRRQPFTSERERWGQKTLQASLEDFFAPRPCKVFLSQTPQQFIVFVNRGILDLYSSV

HECLESLRRHLVVQGKDTTIAGQLVWSESANLSELYHQISSILSMHQRLNNGLVLDSG

NPVSITRTEAGCLDSTLESAFVHMIQARQKEHLTLKLSELMNRWTEDNVHVKELERFI

NLVVDTFAHLVEEQGAGIRLGLELRAKKLFDKETYGDFCSELTEWIGQCFEMLQSHGR

KSREDLFVQMDEYIKRNKYTQISINDIAMKFHVSPSYVSRVIKNVTQVTFVQYYTKLR

IKEACRLMECQPDMKFKELSDLLSFSDQHYFSKVFKEYTGLSPTEYKEILLK"

CDS complement(3812780..3814543)

/locus_tag="PIKHDJKN_03396"

/inference="ab initio prediction:Prodigal:002006"

/codon_start=1

/transl_table=11

/product="hypothetical protein"

/translation="MAKFIHRATQFSLQTKVLLTFLALLLFVLGCFIVYVNLVVIRPL

TQKTEEDTLVTATKVREQVDLYVEQQNQMSQRILSNKDIFATMDKSSLAPSNYERLKQ

IRKLKDIMFQAIGPSMNIKDMSIYDKQGVLLTSYLGSGHPPTILSTLTEKSRSGREWT

GNGFILLREADTISFVRTVNDQNGKLYGYLSIQMDQAYIQNLTEGITAGDVFILNKQG

EQMTGSEARKNALVWKEPLSDEGLAVDEQNNYVTHSTSHHTGWITFIITPKNSVLGSI

RSVQSMSVLLITVLMLISFVYIFFSTRNLLLPIRKLRSQIWRINYSNMKLKVDDRPKN

NDLILLNEAFQDLMERLQQSIDREKKALHEEVTARNSALQAQIAPHFLHNVLYLISIA

AQEGRTQVVSDMCKHLSGSLRYIVSSPYAHVTMTDELEHTRHYLSLVQQKYEEDLEWN

IDADELTSEIRLPRLVIQPFVENCIEHAFFNTTPPWRIQITVKQYNGLWALEIKDNGE

GFQPNKIEEILSNIQKSGSGMNQTPDRQTALGNMGIVNSVNRLKLMYSNRLLFNIYNN

HSDNEKGATIQIIGSMTRDFY"

CDS 3814750..3816063

/gene="msmE_4"

/locus_tag="PIKHDJKN_03397"

/inference="ab initio prediction:Prodigal:002006"

/inference="similar to AA sequence:UniProtKB:Q00749"

/codon_start=1

/transl_table=11

/product="Multiple sugar-binding protein"

/db_xref="COG:COG1653"

/translation="MNMHKRKKAASILLASLLSISLTACGSGGTDSDTSASNGNKNSK

VTLELAISKSSQDSAFIQQDILDEFEKQTHISVNLQLIPAEQTTTVLQTKLAVDETPD

IIQYNLASAITDLNLERNFEILDNESWASRIVNKDVLSAAGHIYSFHVSQDTGMQGVV

YNKQIFADLGLSIPTSYEQFLAICEKIKAGGITPVFMPYKDAWAANIWPAAAFADFVA

KNDPSFFDELNSNKKKWSDIPEFKTFLQQQYEVYTKGYTNTDVLSDSYDMAVGKFLNK

EVAMMFMGDWLIEGVAEQDPSMELGVFPIPSSDDAKLGASPLGGQLFIPKKAKHLDEA

KQFLDFIASKEIAQQIVDSKGYVSNFSDVTTPELPAYKQDIVDNYILPKKTVLTTDAY

MLVDRSELYRLLQDQFAGGLTPEEVLKSWDEKFSQLMQDKGVSGF"

CDS 3816132..3817004

/gene="melD_4"

/locus_tag="PIKHDJKN_03398"

/inference="ab initio prediction:Prodigal:002006"

/inference="similar to AA sequence:UniProtKB:O34706"

/codon_start=1

/transl_table=11

/product="Melibiose/raffinose/stachyose import permease

protein MelD"

/db_xref="COG:COG1175"

/translation="MKRAKNLYSYYMVFPALLIYSIFFVIPALAAFYYSFTDWRLDRI

DLKFIGWDNFEKIFSDKTLILALKNTAIFAVVTVAGKNIIGLLLAVALNMRLKTKNLL

RAIFYSPSILSILVISILFTPMLRTEGTINHVLEAVGLHSLSQAWLTNPSLVIWTIAI

VSIWQSAGFQMAIYLAGLQSISQEYYEAATIDGASSWKSFFKITLPLLLPAININLML

TLIGGLKVFSEVYVMTGGGPGNASQVVGTIILRSFGEGNWGLGTAVNTLLFVVVTIIA

IPLLIFMRRKEVTE"

CDS 3817006..3817836

/gene="melC_5"

/locus_tag="PIKHDJKN_03399"

/inference="ab initio prediction:Prodigal:002006"

/inference="similar to AA sequence:UniProtKB:O34518"

/codon_start=1

/transl_table=11

/product="Melibiose/raffinose/stachyose import permease

protein MelC"

/db_xref="COG:COG0395"

/translation="MGYTRKLAIRNYVVEGFLLLASLLILLPLVILILGSFKTSAEVL

SFSLSLPDKWQFSNYVRVFEEGGLSRAFLNSIWITGISSIINIVASSAASFILARRDT

KLSNTIYMYFFMGLIAPMSIITTIRVVQGLGFYGSISSVILIYAALNTAFSVFLYSGF

IKTIPKALDEVAFLEGASVFGVFFRIVTPLILPVNATVAIMVFMSVWNDITIPVYFLT

NSSTWTMPLSIYNFYGKYSRDWNLIFANLVLTSLPVFILYLFGQKYIVSGLTAGAVKG

"

CDS 3818117..3818848

/locus_tag="PIKHDJKN_03400"

/inference="ab initio prediction:Prodigal:002006"

/codon_start=1

/transl_table=11

/product="hypothetical protein"

/translation="MDRPYMICHMLSSIDGKIIGDFLEDDRSTYFINEYEHIHDRYGC

DAWMCGRVTMQEHFAHEYIPPSNQDKQSPIPRTDYIAQKDVETYAIAVDPHGKLAWTH

NYISEENGNRTKDHIIEVLTEEVPDAYLAYLQELGISYIFGGTKELNVVTVVKKLKAL

FSIDRLLLEGGGVLNGSFLNEGLIDELSLVFVPFAEASSPALTIIENGSSSKETFPVH

FELIRVEQMEHNGLWMTYRVEGNSK"

CDS 3818885..3819202

/locus_tag="PIKHDJKN_03401"

/inference="ab initio prediction:Prodigal:002006"

/codon_start=1

/transl_table=11

/product="hypothetical protein"

/translation="MEKKQTAGRDILGDFAPKFAELNDDILFGEVWSRVEELSSRDRS

MITVSALIAGGNFEQLTPHLHKAKENGVTRNEVAEMITHLSFYVGWPKAWSAFNIAKD

IYK"

CDS 3819224..3819619

/locus_tag="PIKHDJKN_03402"

/inference="ab initio prediction:Prodigal:002006"

/codon_start=1

/transl_table=11

/product="hypothetical protein"

/translation="MTNEHLSNSTIFPLGKKVEANFIGDAYLEMVFTDETPLNTSIGN

VTFAPGARNNWHSHVVGQVLLVTGGEGWYQEEGKPSRMLKTGDVVNIAPNVKHWHGAT

ADSWFVHLAMTPGQTEWLEPVNDEWYNAL"

CDS 3819658..3820404

/locus_tag="PIKHDJKN_03403"

/EC_number="1.-.-.-"

/inference="ab initio prediction:Prodigal:002006"

/inference="similar to AA sequence:UniProtKB:Q7A3L9"

/codon_start=1

/transl_table=11

/product="putative oxidoreductase"

/translation="MSNIEGKVVILTGASSGIGEATAKVLASKGAKLVLAARREERLV

QLQKEIQNNGGHAIVKPTDVTSQTQMEDLAEYALSEFGKIDVLINNAGIMPQAFLFKK

KIDEWDNMIDVNIKGVLYAIAAVLPTMREQKSGHILNLSSVAGHNIYPGGTVYCGTKH

AVTAISEGLRQEEAMSGTNIRVTNISPGAVSSELLETTSDPESKAGLDEFYKTAIDAD

NIARAIAFAIEQPSDVAINEMIIRPTVQVG"

CDS 3820781..3821203

/locus_tag="PIKHDJKN_03404"

/inference="ab initio prediction:Prodigal:002006"

/codon_start=1

/transl_table=11

/product="hypothetical protein"

/translation="MTTFIDARTSQNASLANSIAIPILVINTPQLFGQLGLQTVGAGT

NPRVQFKGTISVQVPLALAGITITIVRGTLSTDPVIYSATSTFNLSVLAPQIITFSAD

DFNPPITPQLTYTAFVSSNLLGTIRVGPENFDGFIVSD"

CDS complement(3821349..3822389)

/gene="adhA"

/locus_tag="PIKHDJKN_03405"

/EC_number="1.1.1.-"

/inference="ab initio prediction:Prodigal:002006"

/inference="similar to AA sequence:UniProtKB:C0SPA5"

/codon_start=1

/transl_table=11

/product="putative formaldehyde dehydrogenase AdhA"

/db_xref="COG:COG1064"

/translation="MITAKARAVDGPDQSFRAAEIKRRDLDLHDVLIEIKYAGICHSD

IHTAHGEWGPVNYPLVPGHEIAGIVTDVGSEVSKYKVGDRVGVGCMVDSCGECENCRQ

GEEQYCLKGNVPTYAGVDKYGEPTQGGYSTHIVVTEEFIVRIPDNIELDVAAPLLCAG

ITTYSPLNHWGAGPGKKVAVVGLGGLGHMAVKIAHAMGAEVTVLSQSLKKKEDGLQFG

ADHYYATSEPDTFAKLAGSFDLIINTVSANIDINAYFSLLTLDGTLVNVGAPGEPLAV

NVMSLIGHRRSFAGSMIGGIRETQEMLDFCAEHSIVPMIEVISADQIDEAYKRVLASD

VKYRFVIDTSTI"

CDS 3822569..3822982

/locus_tag="PIKHDJKN_03406"

/inference="ab initio prediction:Prodigal:002006"

/inference="similar to AA sequence:UniProtKB:P44558"

/codon_start=1

/transl_table=11

/product="putative HTH-type transcriptional regulator"

/db_xref="COG:COG0789"

/translation="MTYSISEVAKELKLTVYTLRYYDKEGLMPFVERTPGGARNFKES

DIDFLKIIQCLKSTGMPIKDIKDFIAWCSEGDSTLQERYDMFTERKASVEAQMEELRK

TMEVIEHKRAYYRTALDAGTEEIHKSNRMGNFIAN"

CDS complement(3823075..3823554)

/locus_tag="PIKHDJKN_03407"

/inference="ab initio prediction:Prodigal:002006"

/codon_start=1

/transl_table=11

/product="hypothetical protein"

/translation="MKIPTTLKHKPVVVSDNYEKVDGRMAGNTDAKGLSLGLAQWNDR

GKVDISAKVWRYTGEKWSRQSEELPLHRVLDLSILICRSLEHFREAYRYENLYDPEQP

VIDRVGLQGDAMSVEICTDNERINEDIKLFSQALSDDDEMIGERLRTLSKILKDMGY"

CDS complement(3823657..3824016)

/locus_tag="PIKHDJKN_03408"

/inference="ab initio prediction:Prodigal:002006"

/codon_start=1

/transl_table=11

/product="hypothetical protein"

/translation="MDMNKRRELLEEYKQIKTYMGVAQIKNKVNGKIYIDSYPNLKNK
WFTLQMQLDQGRFANAQLQKEWREFGEDTFTYEVLEQKEIDKVTDIRWEQKQILKPWL
QKLQPYGDKGYNRPPKN"

CDS complement(3824003..3825232)

/locus_tag="PIKHDJKN_03409"

/inference="ab initio prediction:Prodigal:002006"

/codon_start=1

/transl_table=11

/product="hypothetical protein"

/translation="MFANPYVRTIILSRVLLQLGIWIRNYAVLLYVSELTHNNPVDIS
LISVAEFSPIFIFGLIGGTFADRWRPKKTMVWSDLLSGLSVGVVLLAVMNGGWIALLI
GSFVSASLSQFSQPSAMKLYKRHVPAEQLQGVMAMSQTLVAVFMVLGPVIGTFIFIKF
GISVSLILTAVMFLGSSLVLSWLPRDVDEARSGNTGGFVKELTDGLRYIRANKSLRTL
GLTFSAVGLASGLTQPLQLFLVIENLGQDKEFLQWLVMANGAAMLVGGAVIMTIAKKV
KPQLLLLVGLLVSANCTVGMGASTQIWLTIIFLVISGLFFPCIQGGIQTLLVRNTEGA
FIGRVSGTIMPIFMGMMVIGMLTSGYLKDTFSLLSVFIASGVFVMIGALLLLPIVIER
RNKFISNNKERERHGYE"

CDS complement(3825657..3826877)

/gene="sotB_3"

/locus_tag="PIKHDJKN_03410"

/inference="ab initio prediction:Prodigal:002006"

/inference="protein motif:HAMAP:MF_00517"

/codon_start=1

/transl_table=11

/product="sugar efflux transporter"

/translation="MTRKRENLLILTLTLVSFVLGTTEYVIVGVLKEIETYMKVSLAA
AGALVSGFAIAYAIGTPFAVAFLAKIPRRNSILIGFAVVLALNVLTIFSTTFYSLMVI
RIVSAVACGLTISLSISIASDAVNRERRGEAIAWILGGFSIANVLGVPLGTFIGQHLN
WSMTFVVTACIGVVPFIFMFRILPRQTMTIAGSFSDQMALFMKPRILLACLIPVLGNS
CIFVIFTYITPLLGQSMDVPARWISAVLLIYGACSILSNWIGAKIAKGDFLPKLKWLF
VIQAAIFLGLSFTVSHLWLGLAFLFLIGCLSSSMSAASQLYLFDVSGAIAPNSRTFAS
TLLPVAANVGIALGSGIGGLAVNLGGVKWVPPVAVILALMAFVITAICQRSIQLKPEG

ATAPKVDYSIPSPN"

CDS 3827045..3827974

/gene="rhaR_34"

/locus_tag="PIKHDJKN_03411"

/inference="ab initio prediction:Prodigal:002006"

/inference="protein motif:HAMAP:MF_01533"

/codon_start=1

/transl_table=11

/product="HTH-type transcriptional activator RhaR"

/translation="MIFIQNIMYIVISPQNLEGKTLMNTRHEPDRLHEDQDCIIIYVG

KLADNPHWSFPTHKHDDLHEIIYVNEGQGSFTIDGTKHSAQTGDLLIYNKGTLHEEKS

DPEFPLSTFYCGFRFKEGTRANNDWVVPPGSEPVVRANRYSEEIRSLMQTMFNEFSIR

EEGYNSISHHLLNAVLLIIDRMSHHQRTANEIVRSNTLAESIKDYLDTNYRQNIKLKD

LADQFHIDFYYLIHMYKNCYGTSPYHYLIQRRMGEATRLLVSTKKKVWEISKLVGYEN

PNYFTILFTKTVGESPRSFRKRNQKDMFGDPSN"

CDS complement(3828074..3828976)

/gene="mneS_1"

/locus_tag="PIKHDJKN_03412"

/inference="ab initio prediction:Prodigal:002006"

/inference="similar to AA sequence:UniProtKB:P46348"

/codon_start=1

/transl_table=11

/product="Manganese efflux system protein MneS"

/db_xref="COG:COG0053"

/translation="MEQIKYDNLKLGEKGAIISIIAYICLTAIKMIIGYMSNSEALKA

DGLNNATDIIASITVLIGLRLAQRPADKDHTYGHWKAETVASLVASFIMMVVGLQVLY

GAITSVFQGKSESPDIIAAWTGIFCAAVMYLVYRYNKRLALKIKSQAVMAAAKDNISD

AWVSIGTVIGIIGSQFGLPWLDPLTAVAVGFLICKTAWDIFREATHHLTDGFDEELIK

EYRSTIANVDGVEAVKDLRARNYGNNAVVDVVILVRSDLDLQKAHDISTNVEDELLKE

HEVYTVHVHVEPDSDEAPNEALQL"

CDS 3829291..3829830

/locus_tag="PIKHDJKN_03413"

/inference="ab initio prediction:Prodigal:002006"

/codon_start=1

/transl_table=11

/product="hypothetical protein"

/translation="MMLQKQFDQVFEIMKQSFPEAEYREYSEQKKLLSHSCYRLLTEE

NEQNEVIGFLTGWEFESFRYVEHLAVSPNIRGGGIGKRLMERFMKQSTKAVILEVELP

EDELKRRRIGFYERLGFCLGDHSYVQPPLRAVRQQTLSLQIMSYPEKLTPTQFEMVRK

LLYKEVYGATSAYAHAYNS"

CDS 3829853..3830671

/locus_tag="PIKHDJKN_03414"

/inference="ab initio prediction:Prodigal:002006"

/codon_start=1

/transl_table=11

/product="hypothetical protein"

/translation="MKNDYKINEISKLYGIGVDALRYYEKLGLLEPRRDGNGYRLYSL

QDMYKLNIIRDLRQLDFTMQQIKAYLDQQSVENTLELLFTEQELIKEQIAKLETTQRI

IQGRIKVLTDASKIQTGVFSIKTYPDRLCLQLNTRITRDEEMDYAIKKLHQKHKSHIP

DFGNQLYGASVSLEELKHGVRDVFNSVFFIFNPGDAERIDYDYSLPAGDYLSLFYRGN

YRQSHEKMLEVVAYASEKGVRVMGDPFEIYEIDNRDTIITEQFLTEIQLRICKE"

CDS complement(3830815..3831687)

/locus_tag="PIKHDJKN_03415"

/inference="ab initio prediction:Prodigal:002006"

/codon_start=1

/transl_table=11

/product="hypothetical protein"

/translation="MNQRNVQKSNKLKIISKVLLIMIGAFITSYGLEAILIPNNVSDG

GVTGLSIVGSQLFGLPLGMLIGIINIPFVWLGYKQIGKSFAIYSIIGIASLAVGTSLM

HHVPTIIQGDTLLITVVGGIIIGFGMGLALRNGGALDGIDMLAVLLSRKLPFGTSDLI

LFLNMFVFIVVSTVFGLQGAILSALAYYIASKVIHIVEEGLSGSKTFKIITNQPEIMV

ETIRDRLGRGATYTDAYGGYSNEQFKEITCVINRMEESKIKDIIHEIDPNAFIVVYDV

AEVKGGNFKKKDIH"

CDS complement(3831799..3832926)

/locus_tag="PIKHDJKN_03416"

/inference="ab initio prediction:Prodigal:002006"

/codon_start=1

/transl_table=11

/product="hypothetical protein"

/translation="MRWGGKFIISSVSCILLAALAGCANDKTNLEDATVPLALGLDVI

DKKLHYYISAPVFSKDIQKKSREGGGIAEGLRQSRNQQDAQFPGSLAGRNFQVIIVGK

QLLQYKDWFKVLDVTFRDPRNTITDRIVAFDGSIADIFQFQPKDQPPLPLFVKAIVDS

GSRSSATVKTTAQELHRQLYDRAMTPSISEIKIENNKIMLKGTTLLSQKGQYRTSLSY

QETTLLLHILKNEAEPGVSLTFQVDDLQQSLPFNIDKVSFSLGDISVKTKSFHENGRFQ

YKIKVKSIVSITEKFLEFELLGNSEEMENKLSKEMKKSIEALIKKCQDYDIDPFGFGF

YARAYHYPLYKEAQANWGEELSQAHFDVEVDLRIGATGAVE"

CDS complement(3832923..3834017)

/locus_tag="PIKHDJKN_03417"

/inference="ab initio prediction:Prodigal:002006"

/codon_start=1

/transl_table=11

/product="hypothetical protein"

/translation="MKTFRPVTMLQFILIISTFQISVAFLSLPRELARHAGTDGWLVV

ILGWGLATLASVPIVKVMKYNPNSSILELIQRYIGVWAAKVAAFIFLAYYLLLAYDGY

MIASLVIKLWLLPSTYIYILVLLLLIPTFQIAQHGFQAIGRYSEIVTMLSIWIPFIYV

STLSHAHWLYLLPIFKEGWFPVLSSIKFMIYPMLGIGLVLFLYPHLVNKEKALPAMVI

SNTLTCMVYLGITLICYVYFSPDEIGEYNDPVISIMKSIEFQFIERVEVLFIAFYLFV

FSCIWIPSMYLVSRCSASLVGKGQERYHLAVWCIVIEISFFLYRPSFIESAQINYALN

YIGFMMEFALPAGVLLYVAIRKKMGGGLRT"

CDS complement(3834021..3835514)

/gene="gerBA_3"

/locus_tag="PIKHDJKN_03418"

/inference="ab initio prediction:Prodigal:002006"

/inference="similar to AA sequence:UniProtKB:P39569"

/codon_start=1

/transl_table=11

/product="Spore germination protein B1"

/translation="MDEQSAMNRKEESITKSLDESIRRIEEIFTDTPDLIIRKLCIKQ

TGEQATIIYMEELTDKTSLNHDVLAPLQLETGNPSGDFITTVGYMKPYSKWDDLKLSI

ILGYSVLFIEGRSVAYALDTKGFPQRAIEDPQQEPSLKGAHQGFIETGSQNIAMIRRY

IADKELKLKRYIVGKRGQTEVSLLYLEDVAKPEMIKVLEERIQSIDVDAIINTGELAE

FIEDQPLALLPQIITTERPDTAASQILQGRCVVVVDGSPSVLVAPVTFMSFFQTVDDY

SSRWSISSFLRILRIFAFFIAIFLPGFFISVISFHFEIIPMKLLLTLGESRGQVPFPP

FVEAIIMEITLEMLREAGVRLPAPVGQTVGIVGGIVIGQAIVQAGLISNVMVIVVAFT

AISSFIIPNQDMMAAVRIMRFMMMVLATWFGFIGLVVGMMTIVGRLITLNSLNTSYST

PIAPFRAADWKDTLLRVPLWMMNNRPVSTQSRQSRRQGKSRGWKEKN"

CDS complement(3835820..3836947)

/gene="nspC"

/locus_tag="PIKHDJKN_03419"

/EC_number="4.1.1.96"

/inference="ab initio prediction:Prodigal:002006"

/inference="similar to AA sequence:UniProtKB:A8FNH9"

/codon_start=1

/transl_table=11

/product="Carboxynorspermidine/carboxyspermidine

decarboxylase"

/translation="MRFEQLPTPCFVVDEGLIERNLKILNGVMQRTGAKIVLAQKAFS

MTTMYPLIGEYLSGATASGLYEARLGHEEMGKENHVFAPAYREDEIDEILSISDHIIF

NSFSQLEKFKGKALQAGRKVGLRINPECSTQEGHEIYDPCAPGSRFGAKQEDFRAELL

EGVSGLHFHTLCQQNSDDLETTLNAVEEKFGQWLPQMEWINFGGGHHITREDYDIPRL

EACIKRMQEKYDLEVYLEPGEAVALNAGFLVTSVLDFHKNGMDIAILDTSATCHMPDV

LEMPYRPPLIGSGEAGEKPHLYRLGGQTCLSGDVIGDYSFDQPLKTGDRLVFEDMAIY

SMVKTNTFNGMPLPAIAVKRKDGDCEVVREFGYQDFKMRLA"

CDS complement(3836949..3838187)

/locus_tag="PIKHDJKN_03420"

/EC_number="1.5.1.43"

/inference="ab initio prediction:Prodigal:002006"

/inference="similar to AA sequence:UniProtKB:P0DPE4"

/codon_start=1

/transl_table=11

/product="Carboxynorspermidine synthase"

/translation="MGKALIIGAGGVASVAVHKCVQNSEVFEEICIASRTKSKCDDLK

AKLDGGKTKITTAQVDADNVDELIALINEVKPDLVMNLALPYQDLTIMDACLATKTNY

MDTANYEPEDTAKFEYKWQWDYKERFEKAGITALLGSGFDPGVTGVFSAYALKHYFDE

IEYIDILDCNGGDHGYPFATNFNPEINIREVSANGRYWEKGEWIETKPMEIKRVYDFK

EVGEKDMYLLYHEELESLAKNMPGLKRIRFFMTFGQSYLTHLKALENVGMTSIEPIEY

EGKQIIPLQFLKAVLPDPASLGPRTVGKTNIGCIFKGKKDGKDKTYYVYNICDHQECY

KEVGSQAISYTTGVPAMIGAAMVMTGKWNKPGVYNVEEFNPDPFMEELNKWGLPWVED

FNPVLVDELPEEVKESELVR"

CDS complement(3838869..3839990)

/locus_tag="PIKHDJKN_03421"

/EC_number="1.-.-.-"

/inference="ab initio prediction:Prodigal:002006"

/inference="similar to AA sequence:UniProtKB:P0DN73"

/codon_start=1

/transl_table=11

/product="putative oxidoreductase"

/translation="MNSKYQPIMESFLFKNGVEVKNRVVLAPMTISYSQQDGTLSEAM

LAYYESRSKCVGMAVTGSTLVAPNGTVFGGELAADRDEMIPSLRRLATAIKKGGAKAI

LQIFHGGRESLPEVVANGEIVSASAVAKEGLSVMPRALEEQEILEMIHAFGASTRRAI

EAGYDGVEIHGANRFLIQQFFSPYTNRRDDRWGGSVDKRMAFPLAIVDEVKKVVNQHA

KRPFLVGYRCSPEEEMTPGITMADTLELVDALANKNLDYLHVSLMDFWSKPRRGVEDN

RSRMEIIYELVSQRVPVIGVGGIKTPDEALRALQSGAPFIALGRELIIDPNWVRKIAQ

GRESEIRTEITLNDQGQLGISDHLWKKIISTPGWFPIKN"

CDS 3840200..3840709

/locus_tag="PIKHDJKN_03422"

/inference="ab initio prediction:Prodigal:002006"

/codon_start=1

/transl_table=11

/product="hypothetical protein"

/translation="MITEQTFTIKQTAELTGFSEDTIRYYEKIQLLRQADRKDNGHRV

YHQQDIHTIKLIGCLKKTGISLEAMKPFLKLSTNADPKEYDELVEELRSQRNNILGQI

SSLQQIVDFIDIKLEEGRPQPDCSDQSLDNVLEERDEKTNIKPISVAEMNYFSGTASK

MGKIPSLIR"

CDS complement(3840858..3842591)

/gene="hepA"

/locus_tag="PIKHDJKN_03423"

/inference="ab initio prediction:Prodigal:002006"

/inference="similar to AA sequence:UniProtKB:P22638"

/codon_start=1

/transl_table=11

/product="Heterocyst differentiation ATP-binding protein

HepA"

/db_xref="COG:COG1132"

/translation="MNELAILSKAMIQERKDIILSILGGFIAGIAGVALFSASGYLIS

QTVFAPPLYTLIVLTSLVKLLGLLRAASRYGERLYSHRATFSMLSRLRTSFFARLIPL

TPGILNKKRSGDLLARIVGDVESLQHYFLRVAYPPIIVVMVFLATVLFTSAFSFWIAG

LFVLGMLITALVVPGLVLLGQRKIHGHVREQRALLSTEVTEVLYGFRDLKVYGQLAQR

EQQLQQASAALTVQQQRAAGHLLRGQSMHALVTFLISWGVLTLGAYLIIDGALAGVFL

AMLVMASLTVFEEAAAMATLPLYKQDSEHAAKRLTETIQTSDRQSVSTQPKGALSGNQ

AVSLDLSNVTFQYEGEWRPALKDISLHILPGSKTAIVGPSGSGKSTIIELLLKLRTPT

TGEIRLNDISVKELDEASIWQTANVVLQQSHFFRGTIRDNLLLNDEGHSDEQLLDVLA

KVQLPNTSLTDVIYEKGENLSDGEKQRLALARAMLRKGRLWLLDEPTSSLDYVTEERV

MKHLYSQAAEDTLLLVCHRLTGLEEMDRIVVMEQGRVVEAGSYTELMEQKGYFYEMKK

IERQMIGEVGV"

CDS complement(3842588..3844426)

/gene="cydD_1"

/locus_tag="PIKHDJKN_03424"

/inference="ab initio prediction:Prodigal:002006"

/inference="similar to AA sequence:UniProtKB:P29018"

/codon_start=1

/transl_table=11

/product="ATP-binding/permease protein CydD"

/db_xref="COG:COG4988"

/translation="MKKKTSLISQQMSGQRKRLVLLAVISLALGAAIVSQAALLAEAV

QRIFVEKASLSSVVMLLGILLAVMVVRTGLSYGNGKVGLHMASSAKTNMRAAVLQHLT

HASMPSTLRGQTGGKVSVALDAVDEADSYFSLYMPRMMEAAIIPILILVVTFMQHANS

GFIMLFTAPFIPLFMILVGLKTKNKSEEKYAQLAEFSGTFLDSLQGLVSLKIFGRAHR

QQQEIERTSLGYRDATMGILRIAFTNTFMLESIVMLSIGIVALELAIQLLVFKSMSFH

TAFLVLLLVPEFYSLLKNTGTAFHSGRTSMGAVRKVEQMLEETAGGSTSVDGKEGLGT

SDGIDEVDNMDRMERENRIGTLQMDRSNDSSMPPSMILNHLRFRYAPESFELETGRVQ

LDPGEHIAIVGKSGSGKTTLLHLIAGLLKPASGEVMVNGQPLSKYDEASWFEQVSYIT

QHPYIFAGTFAENIAIGAGRNVSRTEIEQAAEDAGLAEVAAQLEHGFDTLVGEGGRGL

SGGEKQRLALARAFLKRPSIILFDEPTVGLDLHTEQILQHSIATLADTATMITVAHRL

YTIQHADRILFMDQGVLVDTGRHEELMARLPQYAEMIDVQRKGGLA"

CDS complement(3844491..3845504)

/gene="ythB_1"

/locus_tag="PIKHDJKN_03425"

/inference="ab initio prediction:Prodigal:002006"

/inference="similar to AA sequence:UniProtKB:O34505"

/codon_start=1

/transl_table=11

/product="Putative cytochrome bd menaquinol oxidase

subunit II"

/db_xref="COG:COG1294"

/translation="MSYELIGISVLWLFLYGYLIVASIDFGAGFFAFYARLTKQDHLI

NRLISRYLSPVWEITNVFFVFFYIGIVGFFPDTAYYYGSALLVPGSIAVILLAIRGSF

YAFENYGSKNNIVYLFLYGASGLLIPASLSVALTLSEGGFIVKQGSTVSLDYWTLFTN

PLSWSIVGLAIVSVLFISSSFLAFYASRAEDHSALKLVRTYALFWSTPTIIIALTAFI

YLGQHNERHFQNMMDLWWLLALSVAFFMIAMWLIYNGRRYGLAFICIMLQFFCAFFAY

GIGQYPYILDPFITIQNSVTSPAMGFALVVVFIGGMCMLIPSLILVFKLFLFDADYVK

GKK"

CDS complement(3845501..3846865)

/gene="ythA_1"

/locus_tag="PIKHDJKN_03426"

/EC_number="1.10.3.-"

/inference="ab initio prediction:Prodigal:002006"

/inference="similar to AA sequence:UniProtKB:C0SP90"

/codon_start=1

/transl_table=11

/product="Putative cytochrome bd menaquinol oxidase

subunit I"

/db_xref="COG:COG1271"

/translation="MAIDTVMWSKLVTGMTLGFHAIFATLGVGIPLMIAIAEFMGIRK

KDPHYTLMAKRWSRGFVISVAVGVVTGTAISLQLALVWPNFMKLAGNVIALPLFMEVF

AFFFEAIFLGIYLYTWDRFKNPYIHWLLTIPIVTGAGMSAVFITTVNGFMNQPGGFVM

EAGQFTAVNPVEAMLNTATFSKVFHVLSSAYLTGAALLAGIAAFALLRKGASAYHKKA

LNLMMAVVLLFGLLNTLAGDVSAKFLAEHQPEKLAAAEWHFETESGADLILLGWLNAE

HEIIGALHLPKLLSFLAFSDFNAEVTGLEEFPKDEWPPLLVHYLFDLMAGIGFTLLAI

SVLYFLFVFWKKRNELNKWLLGIIALGAPMAFLGVELGWFYAELGRQPWIIRGYMRVE

EAATSSPNIRMIFFFFLLLYIVLGVMCVLVLRRLFNNNPAEVEMEKWMKEEHNQSIEK

GERA"

CDS complement(3847486..3848067)

/gene="resA_2"

/locus_tag="PIKHDJKN_03427"

/inference="ab initio prediction:Prodigal:002006"

/inference="similar to AA sequence:UniProtKB:P35160"

/codon_start=1

/transl_table=11

/product="Thiol-disulfide oxidoreductase ResA"

/translation="MKVNRLEIKSKKMSAKMRRLITFLIGMAALLAAAWAIYQGTSSS

EPKRTGAIEAGAIVPEFTAINSAGEQVKLSDYRGKVVMINFWASWCTPCVREMPMINQ

IAQTYQNDVETLFVNVGEAKGTIREFMEKQQFDFPVIIDVTGKISSKYRITGLPATMI

IDQEGIFSHILLGELTKDIPLQQWLEETVQQKN"

CDS complement(3848064..3848777)

/gene="yxlF"

/locus_tag="PIKHDJKN_03428"

/EC_number="3.6.3.-"

/inference="ab initio prediction:Prodigal:002006"

/inference="similar to AA sequence:UniProtKB:P94374"

/codon_start=1

/transl_table=11

/product="putative ABC transporter ATP-binding protein

YxlF"

/db_xref="COG:COG1131"

/translation="MDNVETEIVGLCKSIKKQQIVEDISFRVSSGHVLALCGGNGAGK

STVLRMLAGILQPTSGEIIVNKMRWSKERKQYSEQIGYMPDDYHFNQGLTAEEVLQFW

AALRKVPKERVTEVLTMVGLADQKKKRVTTFSKGMRQRVLFAQALLSKPPLLIMDEPT

NGLDPFWMQEFANLIKQIKEEGHMVVFSTHQLEIADDVADQIVFMNQGRNVGNGSTED

FRQQFGSLHAAFHHSLGLK"

CDS complement(3848770..3849552)

/locus_tag="PIKHDJKN_03429"

/inference="ab initio prediction:Prodigal:002006"

/codon_start=1

/transl_table=11

/product="hypothetical protein"

/translation="MGFRNPWSYSFLILFCVFSLSLLIINSQNFVAGYSGTTGSMLNL

ILYLLPLMTLFLGSFSLTSEKEEGSWQLLSTYPIGTMSFIVGKYLGLSVVLMTIVTFG

YGLMGLLSGWVGSPLDVKTYLLFLIFSCGLVLLFLTIALFIGSLSKNRWQALTISVSV

WFFAVIGWPTLLISVLGLMPYQWVKPLLTVLTFINPAELVRLFVVIKLGGGSILGPEY

YQWVDWVHPSSGSWIFMGICMLWITCSIVAVYGIWERGRSHG"

CDS complement(3849617..3850192)

/locus_tag="PIKHDJKN_03430"

/inference="ab initio prediction:Prodigal:002006"

/codon_start=1

/transl_table=11

/product="hypothetical protein"

/translation="MRIKTHWKTVMVLLFGVLLLTACGKKYEALPINEDVDICAICKM

QVKDDAYATQLTTKDGKNYKFDDIGCMHQWKEENGTDNIGMDFVRDYNDKEWIEYSKA

TYVYDASLRTPMAYGILNFKDKPSAEAFVAEQGVGTIMTAEDLASHDWKQNTETMDMM

GEHGHSEEGMGEGMHEETHGESESTEESGHK"

CDS complement(3850189..3851544)

/gene="nosD"

/locus_tag="PIKHDJKN_03431"

/inference="ab initio prediction:Prodigal:002006"

/inference="similar to AA sequence:UniProtKB:P19843"

/codon_start=1

/transl_table=11

/product="putative ABC transporter binding protein NosD"

/translation="MFRERIVLGMLLFIGLLLIPLSYPLDTANAAPEAKDAIPLQPII

DGSRPGEVLVLEPGIYSGPISIDKNISIRGDSSVMVLNREAESTITIRSDGVTLQGFT

VRHLTNEPTAAIQVEGDRVEITGLDIQSQSFGIVLRGSVDGLIHSNTILWAGPESASS

GQKGNGIDLYNSHRTHIEDNKITGMRDGIYLENSRNLTVKANTLLHTRYGIHCMYIDG

SSVVDNVGENNSTGAMVMGVKNTVVSGNSFRKQSENVHSQGILLYDVQQSSIHNNVVE

GNRVGMNIAESSGNEIRGNAVLRNFIGIQLVLTEDNQFTRNQFVSNVIEASAMDSRNN

EIKENYWDSFQGLDLNEDGISELSYGINPFYEQVVNRNSAYQLFFQSPGMIFMSNLFT

EGKAEWTSDRSPLMSMEAESQTTKTGTQAVKDTTGVWIIGCILLCLATFIMINMGVLR

K"

CDS complement(3851733..3852386)

/gene="degU_2"

/locus_tag="PIKHDJKN_03432"

/inference="ab initio prediction:Prodigal:002006"

/inference="similar to AA sequence:UniProtKB:P13800"

/codon_start=1

/transl_table=11

/product="Transcriptional regulatory protein DegU"

/db_xref="COG:COG2197"

/translation="MSHTRVLVVDDHAHAREAICEILSMDPGFEVIGVVSDGQQAIDY

TEQWLPDLILMDIQMPIMNGLEATQRIKLSFPYVKIVMITVSDDVLHLLEALKRGAQG

YLLKNLEPSMWLEYLHSIVTEEAPLSREVAYQILKDVSLTEKKEPDVPLTTREKDILY

GVAAGWTNKEIAVNYKISEYTVKNHLKNILQKLQVQNRVQLTRYALEQGLITDHNRL"

CDS complement(3852383..3853228)

/locus_tag="PIKHDJKN_03433"

/inference="ab initio prediction:Prodigal:002006"

/codon_start=1

/transl_table=11

/product="hypothetical protein"

/translation="MSYKQIKWMILLIPTLTVGLWEYVRHQFLLPYISMDLGNWLTPV

IVYVVSITLLTRLFLMLERIQEELEHEREAKAALESREKMAKELHDGIAQSLFLLSVK

VGRLEEQAKQDQQLDDVYSIKKTVHEVNRYVRQAIADLRYDPNANYALAMNESATLLG

QVRRIAKDIPVRIELDWSIPDDAFTDKEKVELLACIREALFNIQKHASASQGWIQGNG

HKKCWNITIRDNGIGFEMNPFERKDRYGLKIMRERMNELNFNMQFRREQQLTVVEFSK

DGEML"

CDS complement(3853257..3853985)

/gene="srrA_2"

/locus_tag="PIKHDJKN_03434"

/inference="ab initio prediction:Prodigal:002006"

/inference="similar to AA sequence:UniProtKB:Q9L524"

/codon_start=1

/transl_table=11

/product="Transcriptional regulatory protein SrrA"

/db_xref="COG:COG0745"

/translation="MNGTLQQNRILIVDDDERVRRLLRMYLEKEQFIVEEASLGWQAL

GQLHHGSYALIILDWILPDIKGIEICHYLRTNLSIPIMFLTAKSEETDRLEAFKAGAD

DFVMKPFSPREVVLRIKTMMARYNGLIEPAPKDHSASEVLISSILINPTSRRVTVEQE

EIHMTPKEFDLLYHLATHSEIAFSRQDLLRIVWKVEHEEDLDYRTVDTHVKRLRDKLS

EYVPNREDLIQTLWGFGYILRLPH"

CDS complement(3854176..3855258)

/gene="fhuD_3"

/locus_tag="PIKHDJKN_03435"

/inference="ab initio prediction:Prodigal:002006"

/inference="similar to AA sequence:UniProtKB:P37580"

/codon_start=1

/transl_table=11

/product="Iron(3+)-hydroxamate-binding protein FhuD"

/db_xref="COG:COG0614"

/translation="MSHTENNDLFRRGRSKKATLGRKRGFAGLMLLLLVLSLMVVACG

NRTNTAGEQATTETTKTNASAATQTAEEPQTQVIEHAMGKTEVPLHPKRVAVFGLEDL

TLSLGVPMVYAYDFDGYYLDDQLKALNIPLSGNADFNPNLEAILATQPDLIVVQRNAI

DDKAYEELSKIAPTIAYSPDDWETTIVELGKATGLEDKAQEVLQANTDKLKQAKEDII

SAVGPDKTVVFMRPSDKDLQVFFPSFAPLIYTELGLNPDESVTAFQKDTTDDWGVTTS

LEKLPSIGADYVFAIYGGSISTEEEFNENSVVATEVEKLHVWKTMPAVKDNHVFKVSA

RHWMMSGPIADGKVADDVVAAVTGKK"

CDS complement(3855360..3856973)

/gene="btr_2"

/locus_tag="PIKHDJKN_03436"

/inference="ab initio prediction:Prodigal:002006"

/inference="similar to AA sequence:UniProtKB:P40408"

/codon_start=1

/transl_table=11

/product="HTH-type transcriptional activator Btr"

/db_xref="COG:COG0614"

/translation="MMTQLQQNHPQGEGQTAADLAWYGLKNVFKDSVDKAYRLIYEET

PDHVILVAANRHGKIVIDGIPHPFVPNSAHLVLPGQRVETVYLTDGEQIVYRIHIGII

PYDGQKVSSECLLTELGGSITVPDAQVQQLCEKIVGYWHTGDSTDRFASQAGFQDLLH

LLFKKQNQHENALERAREYMLHHYQDTITVEDLADEAGMSRYYFMRSFKEKFGQSSMD

YLAEIRTNEAKRLMEAGQPLKDIALLAGFKDQHYFSSQFKKQVGLPPRTYMANRKCKT

AAYSWPNIGHLLTLQIIPFAAPIDQFWTDDYRRKYRYDVKVALSHDYDFNLKALMRAR

PDRIVALDEMIPEEEKEKLRHIAPVLFLSWHGEDWRQHLRRTARFLDRENESERWLLR

YEEKVETVLHHIPDSFQQGKLIALSVSPRGIQIWGRRAGTVFYDDLKIAAASGVDQIE

FTRYVEAEQLAAFDADVIIVSVMKDHHSQMVWERLQHAESWKQLHAVRNRHVYLTQGG

HWLSEPILEHTANRHELLLQEVNLLFSAR"

CDS 3857255..3857614

/gene="nrdI"

/locus_tag="PIKHDJKN_03437"

/inference="ab initio prediction:Prodigal:002006"

/inference="similar to AA sequence:UniProtKB:P50618"

/codon_start=1

/transl_table=11

/product="Protein NrdI"

/db_xref="COG:COG1780"

/translation="MLIVYDSITGNVKRFISKLQLPTVQIQNQMTIDEPYVLVTYTTG

FGQIPDKVSSFLQKNSEYLIGVAASGNRNWGERFAKSADLISASYNVPVISKFELSGS

KRDVEYFEQEVSRIASY"

CDS 3857601..3859685

/gene="nrdE_2"

/locus_tag="PIKHDJKN_03438"

/EC_number="1.17.4.1"

/inference="ab initio prediction:Prodigal:002006"

/inference="similar to AA sequence:UniProtKB:P50620"

/codon_start=1

/transl_table=11

/product="Ribonucleoside-diphosphate reductase subunit

alpha"

/db_xref="COG:COG0209"

/translation="MRHIELNNMLLKRDSDGFFQLHKDKEAVEEFMKEIKDKSMAFAN

IDDQVKFMIDHDYYEDFYASYTTDEVRTVFEITRGYQYNFPSYMAASKFYTDYALRTY

DRKTYLEQYADRVAAVALHLGQGHFDTACLLSRSMMEQRLQPATPTFLNAGKSRRGEL

VSCFLLEMDDSLNSINYVLNTCMQLSKIGGGVAVNLSKLRARGEVIKGVEGAAKGITP

VLKLMEDGFSYADQMGQRKGSGAAYYNIFGWDVMELLDSKKINADEKIRLKTLSIGLI

VPNRFYKLAQDNEPLYLFAPYSVYKAYGTHLDDMDLDVMYDTLLADDRVKKKKSLSAR

DMLTKIAMTQLESGYPYIINKTNANQAHALKNVGQVKMSNLCTEIFQLQETSEITDYG

QPDIIRKDISCNLASLNIVNVMESGKIKESVHEGMIALTSVSDMTHVANAPGVAKANS

EMHSVGLGAMNLHGYLAKNRIAYESSEAKDFVRTFFMTMNYHSLEKSMEFAEQTGKRF

YGFEQSDYASGVYFDRYVTTDYRPTTSKAQELFNGIAVPSPEDWNLLKSKVMTNGLYH

AYRMAIAPTASISYIQNATSSVMPIVEQIETRTYANSTTYYPMPYLQKDNVFYYKSAY

QMDQFKVIDLIAEIQPHVDQGISTILHVNSDVSTRELARCYLYAAHKGLKSLYYTRTN

KLTMEECIACSI"

CDS 3859728..3860690

/gene="nrdF"

/locus_tag="PIKHDJKN_03439"

/EC_number="1.17.4.1"

/inference="ab initio prediction:Prodigal:002006"

/inference="similar to AA sequence:UniProtKB:P50621"

/codon_start=1

/transl_table=11

/product="Ribonucleoside-diphosphate reductase subunit

beta"

/db_xref="COG:COG0208"

/translation="MPAIQAVNWNRADDDFTLMFWNQNIMQFWTDDEIPLSDDKMTWG

MLSEIEKDAYMKVLGGLTLLDTIQGGVGMPQIMEHVDGLQRKAVLSFMAMMEQIHAKS

YSSIFTTLASTEEIDSVFKWVEENPYLQTKATVIRQYYMNIETSKDLYMAMAASVLLE

SYLFYSGFFYPLYLAGQGKLTCSGEIIDLILRDESIHGVYVGVLAQEIYASFDETQRN

DVYQGLVDLVQLLHENELKYTEELYTPIHLTEEVEAFLRYNANKAMMNLGFEPLFDDE

EVNPIVLNGISTHTKQHDFFSKKGNGYIRTLNVEPLTDDDFKFE"

CDS complement(3860778..3861821)

/locus_tag="PIKHDJKN_03440"

/inference="ab initio prediction:Prodigal:002006"

/codon_start=1

/transl_table=11

/product="hypothetical protein"

/translation="MNAVQLPVLNPPLKGFLRWAYTLSITSAHEETIPWYYSNFIQLS

CKKTFLEDGRQFYIDFFRGKPNELNFNNPFLLTCSVNYTILEHLALDAWPQFFADQIR

NGYYCVVFLDEAKLPPAAAYQKEPFPHHLFLYGFHWERRVFDASMFDHTGVYRNVQIP

FTEFLDAIHSMRHLLREGLTADHHTYFYKYEPHSPYPFDRIVALDQLYDYLNGETHLN

RINYNPDDEQAFGINVYDYLQMYYDAVESNDPRLGDRNDVRHLHVLWEHKKMMSERIA

YLIDERTIPYDEELVNGYQDITTRAMKLRDQYIRHEIREDKAVFAKVRLRLEQFRKEE

PALLQRLISLLEK"

CDS complement(3861834..3862547)

/locus_tag="PIKHDJKN_03441"

/inference="ab initio prediction:Prodigal:002006"

/codon_start=1

/transl_table=11

/product="hypothetical protein"

/translation="MMSLLVESVYLLAKLLYVLVVFRTDLHVDGIPPEGLLLFIGMHT

VVTGIYVGLFFTNFTKIPEYIKDGSLDLMLTKPVSLQFMASLRYVDLALPIPDILVGF

VMIGIGWHAMDIPLTFLQLAGFMLLLFVSVIITYCLMIIPALLSFRFVQTGSASEIAH

SAWDANNFPMAIYPTWVRRIGTFVIPLFLITNFGPMFLLGQLSWLYTGLALVASLILF

TVIRLLWKQAVKGYSSASS"

CDS complement(3862650..3863459)

/locus_tag="PIKHDJKN_03442"

/inference="ab initio prediction:Prodigal:002006"

/codon_start=1

/transl_table=11

/product="hypothetical protein"

/translation="MNGRKLYTAVFSMGVQQSMEYRFHFFLGLLGAAFPILVQYFIWT

AVYQHSGESALFSYSYNQIILYTILAGLVSKLIATQFEHQIVDDIKNGGLNKYLIKPV

SYFGYRLVSFFGQKAIYYGVTAVLLVGIIWISSAGGVLEFQVIRMILFVVTLSGALLL

NFLISYCICASAFYLNEISYFFVITSLLVNILSGGMFPLEIFGDSIVEALQYTPFPYT

IYFPVNVLSGKTEVVAMYQGLLIQCGWILLFLWLSRLTWRISMKKYSAVGG"

CDS complement(3863456..3864439)

/gene="btuD_14"

/locus_tag="PIKHDJKN_03443"

/EC_number="7.6.2.8"

/inference="ab initio prediction:Prodigal:002006"

/inference="protein motif:HAMAP:MF_01005"

/codon_start=1

/transl_table=11

/product="Vitamin B12 import ATP-binding protein BtuD"

/translation="MDFIRVRELHKSFVYYRKEAGLKHSLKNLFARKSLVKEAVKSVS

FDIGPGECVGFLGPNGAGKTTTLKMLSGILYPTSGEADVLGYVPWERKNEFKRLFSIV

MGQKNQLWWDLPASDSIYLNKCIYDVEDDLYRRSLAELSEMLDVQDLLDVQVRRLSLG

ERMKMELIAALIHRPKLLYLDEPTIGLDFPSQKRVREFLKYYNEQFGATILLTSHYMK

DVEDLCKRTIIINEGSLLYDGDLHKINDLFGEHKIVKLQFSEPVAEQRLAKFGKMISA

DDYSVVLELPKHRLKEVSRAMLDSFPIVDWTIEDVPIEESISMLYQKESVG"

CDS complement(3864499..3865674)

/gene="moaA_3"

/locus_tag="PIKHDJKN_03444"

/inference="ab initio prediction:Prodigal:002006"

/inference="protein motif:HAMAP:MF_01225_B"

/codon_start=1

/transl_table=11

/product="GTP 3',8-cyclase"

/translation="MQPKTAVTFDKLTFQNMKRTLVPLAETGRKRREDPTYAAPVPYE

IGIKLNNGCNLRCKHCFEWNPDGFHHGFASEVKRDEIEIPVVEKLLAETRERKSRVFL

WGGEPLFYSKFDELAELLAKEDRHTTICTNAILIEQKLDSILKMSKNLVMLVSLEGFE

KENDAIRGKGTFKKVIHAIQLLLDLQKQGLYKGKVSVALTVNDQMVDQLYSLMEFFEG

LGVDSVYFCFPWYIPSDTAEKMDTYFDAHFPHLASRFASDHKNSWHSFTFHISPDRFD

TLIEEFKRVNSRVWNVRIRYQPALEPEEVEGFIRGSEKPAMNRTKCFSISNRMDVMPD

GKVNPCKFFPEFSVGNLYEESVADIFHGEDLRKQREILACGLTPICSKCVLLYNNGV"

CDS complement(3865646..3866752)

/locus_tag="PIKHDJKN_03445"

/inference="ab initio prediction:Prodigal:002006"

/codon_start=1

/transl_table=11

/product="hypothetical protein"

/translation="MNSSSVTLLASGNSLGAYIPAMHLNTYLQDRGVRTDVHVLENLY

QEEIRCKINSTKKAFHADFSVALMGHKLAKPVDSSLNEEEVQFLLDAWIRDGVSHFAV

FTGFWLPILERYKAIADKPLDIQIIRLDACDTPSFKVHKSLYPDYDNVWLYEPSRLSP

NCYIASDEKVLLPYAQREGRILIHGGGWGIGTYVSTIAELRQLGYLLDVIVYDSSEVE

EDDGSTRYFGTEASWNPWSRDAQGRHSFPPMNQYVREEGILRATPLRDYSAYTDLMRN

CAAIISKPGGATLNDSLSYGIPFVMLEPFGDHELQNSNYWKSCGFGIRFAEWKAMDYS

EQHLEEMYRRLLKQRSKIKHLGRKTHAAENSSYV"

CDS complement(3866776..3868167)

/locus_tag="PIKHDJKN_03446"

/inference="ab initio prediction:Prodigal:002006"

/codon_start=1

/transl_table=11

/product="hypothetical protein"

/translation="MSRSEAGVIPTIVMGTFDPETRWRDPDLSKLPAMPDKDRESIVL

CMDELLFPFCGPDDILYTRCKIDPKLYEYLNGLGFSFRSRYHFDLNDVESSLPEPDVF

KQCMFSLASDDQQREAMLTHNVKHLSPYAITADTEEYLHAEGSLGSLPDLETVKHVNS

KFYSNRLLTRIGEKCYGTEVNSVVEVQAVGNTLLKRGAYLLKDPFGVSGKGNMLIENE

AMQRRIAEHLDKQERRGLRSQFLLEPLLRKEIDFSSHWFIHKSGERDFLSVQRMLNQQ

QNYGGSIRADEALLLLLENAGYFDTIERALRVLFEDGYHGFVCFDSMILKDGDVVPIV

EINARKSMGLINAYLDKGWETYGYSGLLTFLSLGLPEGFTFESLLDALHGSGLLFRGQ

GEYGIMPISSNTIMVNEILTSRRISEGIQSHRGMPKGRLYISVVGRDDEHRSELIVSL

RQVLADLSIKVYN"

CDS complement(3868154..3868429)

/locus_tag="PIKHDJKN_03447"

/inference="ab initio prediction:Prodigal:002006"

/codon_start=1

/transl_table=11

/product="hypothetical protein"

/translation="MYDKIVDILCAIKEDQPELRQSLSPATELNNDIGLDSLQMIQFM

LKVEDQLNVIIDYDEFDYEHLQSIDTFITFLKSCQSQEQSLYEDVQK"

CDS 3868780..3869658

/locus_tag="PIKHDJKN_03448"

/inference="ab initio prediction:Prodigal:002006"

/codon_start=1

/transl_table=11

/product="hypothetical protein"

/translation="MIEITIELVRQLIDSQFPKWKGLTITPVEKSGHDNRTYRFGDEM

TIRLPSHERYASAVEKELKWLPVFKPLLSLPVPAPIAKGEPSDEYPLPWSINRWIKGE

TVSPANVLDLGAFAEDLAKFLRELEAIDASHGIPAGIQNFHRGGNLAVYDTDTRTVIE

KVSGQYDPKLMTEIWELALATKYQASPLWLHGDVAVGNLLVQDGRLCGVIDFGTMGVG

DPSSDLVMAWNFFDDVSREIFLSCMKVDQDTVDRARGWALWKALISYDWNEQGSEASN

WGKRVIDVIIHDYKSL"

CDS complement(3870165..3871058)

/locus_tag="PIKHDJKN_03449"

/inference="ab initio prediction:Prodigal:002006"

/codon_start=1

/transl_table=11

/product="hypothetical protein"

/translation="MELRNYGNTGMKVSTLGFGGAEIGKNVSKADVETLLNSALDAGL

NVIDTAECYGDSEELIGDVLSHRRDDYYLFTKCGHAAGVDGPDWDAKVLEQTIDRSLR

RLKTEYVDVIHLHSCSEEVLRQGAVIEVLQRAKEAGKTRFIGYSGDTTDALYAIQTGV

FDSFETSLNIADQEAIDLTLPEARKRNMGVIAKRPIANAAWTYDSLPEEAYPFVYWKR

LNELGYGFLGEDAQAAVETALRFTLSTEGVDTAIVGTAKPNRWQQNADLVAKGALSKE

LYDEIRERWKEIAGADWTGRT"

CDS complement(3871212..3871700)

/locus_tag="PIKHDJKN_03450"

/inference="ab initio prediction:Prodigal:002006"

/codon_start=1

/transl_table=11

/product="hypothetical protein"

/translation="MNIKIELVPRERSQVIRHLMQFYLYDFTKYLNIDVDSNGIFPEY

PGLDAFWSEEDRKFPFLITSDEAPAGFALVERCEPGSKDNDYYLTEFFVMQKYRRSGV

GTRAAYELFERFPGRWKVTQVRNNVIAQAFWRKIIGEYTGGRFREKFHPELGNPSQFF

VT"

CDS 3871875..3872966

/gene="xerS_2"

/locus_tag="PIKHDJKN_03451"

/inference="ab initio prediction:Prodigal:002006"

/inference="similar to AA sequence:UniProtKB:A2RKP9"

/codon_start=1

/transl_table=11

/product="Tyrosine recombinase XerS"

/db_xref="COG:COG0582"

/translation="MTNVQKEIDRRKLDDKLPSMPWFVQQFIDYKLPDLSPSTLLEYL

RDYEAFFGWLRAEGLSEASSNKEVTLLELEVLRMESVTSYRLFLATKREGANSRITVS

RKLSSLRSLFHYLSQIAEDEDFYPLLKRNIMAKIEIKRTHKPKDTAAKLKGKLLEEEE

LLEFIGYILEGYAVDMEKNKQAIYSHELNKERDACIASLILNSGLRVSEVVNLNVDDL

DLNNKLLYVYRKGNNDETFKTPVYFREQAKDELATYINLRQSRYRTPKREKGLFIALR

NGDSEGSRMTKRAIQAMIMKYAKQFGKPYLTVHKLRHSFATDYYLQNDIYKTKEQLGH

ASTETTEIYAHLTDKTMSEAIERRTDDGI"

CDS complement(3873154..3873699)

/locus_tag="PIKHDJKN_03452"

/inference="ab initio prediction:Prodigal:002006"

/codon_start=1

/transl_table=11

/product="hypothetical protein"

/translation="MSEISSTSPHIMQIVGYKNTGKSTLIAALTRYLTSAGRRVAVIK

HDGHDHFEMDHEGTDSYSFAQAGADAVVVMSDKRTALIERQATPLEDMINYLSAYDWI

LIEGYKQAPYPKLVMVREVKDLSLIETLQDVVGIVSWLTDERRECVEARGSLVWHSIH

ETEDIARWLVSLERHEEKRKL"

CDS complement(3873707..3875002)

/gene="moeA"

/locus_tag="PIKHDJKN_03453"

/EC_number="2.10.1.1"

/inference="ab initio prediction:Prodigal:002006"

/inference="similar to AA sequence:UniProtKB:P99139"

/codon_start=1

/transl_table=11

/product="Molybdopterin molybdenumtransferase"

/translation="MKINSHSTDMTTAKFIRKAIQVPDAQAKVAAHVSSGSIEKVTLE

QAHGRTLAETIHAPHPYPFFRRSGMDGFAILSSDTIGASSEQQIWFRVIDEIPCGYTS

DLTITSGTAARIMTGAQVPEGADAVVMMEMTESKEEHGEQWIALKRHIQPDANITPIG

LEVHEGQLLLEAGTIIQAGEQSVLATFGVGQVPVYKRPKVAIFATGTELLEVDEPLQP

GRIRNSNSYMLRSLVVEAGGEPVMYGSIADDVDTARTKLAEALEHNDMVVTTGGVSVG

DYDIMGDLVREGNMEMLFNKVTMRPGSVTTAAVVNDKLLFALSGNPGACFVGFGLFVR

PTIRTMQADAHPYLEEWTAILDDDYTKVNNFTRFVRGRTEIRNGMVYAKPSASRVDES

SVMITIKDSDCLIVVPPEKKGIPAGEQVRILRLPTGHVR"

CDS 3875148..3875438

/locus_tag="PIKHDJKN_03454"

/inference="ab initio prediction:Prodigal:002006"

/codon_start=1

/transl_table=11

/product="hypothetical protein"

/translation="MNQRCELCGREPVETTVHHLTPKEMGGTHMPTADLCIPCHKQIH

SLYTNRHIITLGLTEIQALRQDKRIAPYIRWIRKQPATTIPRVRKSHHVRKS"

CDS complement(3875552..3876298)

/locus_tag="PIKHDJKN_03455"

/inference="ab initio prediction:Prodigal:002006"

/codon_start=1

/transl_table=11

/product="hypothetical protein"

/translation="MKKRLAHGLKLMLVLSLVMSLLSACGAPSVQDTYPLESVSGSGN

TTSYVVYRAADRTVPEVAQELSEQRKPDQISAENTERMFLVYQDQYYHLQQDPNKAEDT

LVEVDSKEYVRQNYDSSFLQGYLTATLIGNLFDSFGGRGSGTYRGYTNKDTYKPSEGS

YRTPTSKDKKVAPPITVDRKGSITRRGSDKDSSVGSGGGLFSRNKEQSKGTIDRNKSS

GGLGGLFDSPKSSYKKPKTRVGGGRIMRRR"

CDS complement(3876310..3876816)

/locus_tag="PIKHDJKN_03456"

/inference="ab initio prediction:Prodigal:002006"

/codon_start=1

/transl_table=11

/product="hypothetical protein"

/translation="MSVWKRIKGILAKPEPPQAEKTMLQLAPGDICEVSLVTYEVVGR

VHHRARNAAVLTLQDGTAIRHLHIEEREMTRYALYTPIDGRLDAPDEVPTLLDLDGRA

YHLEEEYGGMVTTAGRTPYGQAGEQLVWQYQSDDNMLLRVEWQDRRFTLYEGESILPA
DIKVIRSS"

CDS complement(3876840..3877241)
/locus_tag="PIKHDJKN_03457"
/inference="ab initio prediction:Prodigal:002006"
/codon_start=1
/transl_table=11
/product="hypothetical protein"
/translation="MDLNILAMLVWTLSGSVLLFVLMYVDSLFTKYKDFAEVKAGNMA
VTTRMVMKLFAQGYVLATSISTAGHLGEALLVSVVSFIILLILESVVHFMIRRWANLD
LDTGIQQGKTGYGLFSGALHIVGALIIAACL"

CDS complement(3877337..3878008)
/gene="liaH_1"
/locus_tag="PIKHDJKN_03458"
/inference="ab initio prediction:Prodigal:002006"
/inference="similar to AA sequence:UniProtKB:O32201"
/codon_start=1
/transl_table=11
/product="Protein LiaH"
/db_xref="COG:COG1842"
/translation="MSIFKRLRDLTMSNINSIIDKAEDPIKMTDQYIRDMQEDLEDAE
KAVAAQIAIEKKFKQLFEEQEALVKKREEQAHTAARAQNMDLARRALEEKKVAEEKMA
EYKTSYDQNKASADNLRGKLDEMRKQLTQMKNKRETLVARYNAAKAQTEINKALNGFG
SDTASAGMKRMEEKMMQMEAQAEASNEMSSKGKSLDDEFEKLGKDQAVEDELAALMKQ
YENKN"

CDS complement(3878138..3878482)
/locus_tag="PIKHDJKN_03459"
/inference="ab initio prediction:Prodigal:002006"
/codon_start=1
/transl_table=11
/product="hypothetical protein"
/translation="MSTYDLNSVRLQVIEAGEDKVFMVTGGRSHIGAVATFYPDRERV

SGATVHIPGHKEQELCERLARKAALHLKVTVTVIMGIHFDAITRMQIDEIVQTAEKLL

DEELCQTGRLIQ"

CDS 3878630..3879418

/locus_tag="PIKHDJKN_03460"

/inference="ab initio prediction:Prodigal:002006"

/codon_start=1

/transl_table=11

/product="hypothetical protein"

/translation="MRIRLIMLSIMVILLGGSYAPAQLWASSSPQAEPASKSDSPEEE

QLTLGQLRQKYADTFKTNGPSTKKVALTFDDVPDPRFTPQVLDILKKYKVRATFFIVG

NRAEKHPDLVKRMVKEGHIVGNHSYNHPEFSKLSMNAFRKQILHTGDIIRNLAGYTPK

MIRPPYGDINEQQLQWAAKQHYSIVNWNVDSLDWKGLSKEEVKQNILSAVKPGSIVLQ

HAGGGVGSKLSGTIEALPEVIEELRNRGYELVTLDEMLELPKGK"

CDS complement(3879550..3880206)

/gene="yocH"

/locus_tag="PIKHDJKN_03461"

/inference="ab initio prediction:Prodigal:002006"

/inference="similar to AA sequence:UniProtKB:O34669"

/codon_start=1

/transl_table=11

/product="Cell wall-binding protein YocH"

/db_xref="COG:COG1388"

/translation="MINKKRIAKTATALLTAAMLIQAVPAHADSVHMAKEGDTFYTLS

KQYGVGLNALIKANNDISAYNIYGGLKITIPGKTTNTASAAAAKTVTAASLDVNADSK

VVQAWGKTFDYSKAVDVKATAYSADASENGGWGAVDYFGNPLELGTIAVDPSIIPLGT

KVLVTGHTHPGLPKQAFVATARDVGSAIKGHKIDIFIPGSKQSVSTFGIQDVELYILK

"

CDS complement(3880691..3881419)

/gene="artM_3"

/locus_tag="PIKHDJKN_03462"

/inference="ab initio prediction:Prodigal:002006"

/inference="similar to AA sequence:UniProtKB:P54537"

/codon_start=1

/transl_table=11

/product="Arginine transport ATP-binding protein ArtM"

/db_xref="COG:COG1126"

/translation="MQVIDVRQLYKSYDKNEVLKGINVTIGKGEVVVVIGPSGSGKST

FLRCLNLLEQPTSGEIDFEGVSITDPKHNINATREKMGMVFQHFNLFPHKTVQQNITI

APIKVKKQSAHEAEQICADLLKTVGLSDKKDAFPNQLSGGQKQRIAIARALAMQPHVM

LFDEPTSALDPEMVGEVLDVMKRLAEGGMTMVIVTHEMGFAREVGDRILFMDGGVIVE

EGTPNEVFGEPKHARTRDFLAKVL"

CDS complement(3881400..3882866)

/gene="mltF_2"

/locus_tag="PIKHDJKN_03463"

/EC_number="4.2.2.-"

/inference="ab initio prediction:Prodigal:002006"

/inference="protein motif:HAMAP:MF_02016"

/codon_start=1

/transl_table=11

/product="Membrane-bound lytic murein transglycosylase F"

/translation="MKLISRYTMMLLALVVLLTTVAPVASAAGTTGSSGSSGSKKLVL

GTSADFAPYEFHKVIDGKDQIVGFDISIAKEIAADLGAELVIEDMGFDGLLPALQSGR

VDMVISGMTPTDERRQSIDFSDTYYKSKQVIMIRNADKDKYPTMADLENEKIGVQKGS

IQETIGQGIPGAKLTALDKISDIVLQLQTNRVNAAIVEDTVAAGYLDDVIGLAPAIPD

EEQAEAAIGIRKGNTELLNAVNGTLERLKSENKIDQLVTEASQMMAEKSKQNIFEVFW

QYKSFYATGLGYTLLLSALGVFFGVIIGLIICLFRLHDIAILRWIGTAYVELIRGTPM

LVQLMIIYYGVSLTFGINFSALQAGIITLSINSGAYLAEIFRAGIQGVDRGQLEAARS

LGMGRGAAMRFIVLPQAFKAVLPAIGNEFVTIIKESSIISVIGMVDIMYQASVVKNIT

YQGMNPFLIAAAIYFVLTFILSKLLGRLERKLSASDRR"

CDS 3883048..3884223

/gene="abgB_1"

/locus_tag="PIKHDJKN_03464"

/EC_number="3.5.1.-"

/inference="ab initio prediction:Prodigal:002006"

/inference="similar to AA sequence:UniProtKB:P76052"

/codon_start=1

/transl_table=11

/product="p-aminobenzoyl-glutamate hydrolase subunit B"

/db_xref="COG:COG1473"

/translation="MTHTKTQISTVIDQYASRFKEISSYIGANPELGNEEYLASARLK

EELAYHGFSVEAPVLGLDTAFIGTYAASKPGPTIALLCEYDALPEIGHACGHHLICMM

SLGAAVGLKSILDEVGGTLKVFGTPAEETRGAKVPMAEAGLFDDCDVALMAHPYYAYE

KSGSSLAIDAVQFEFHGKSSHAAASPHEGINALDAVIQTFNGINAFRQQVKSTVRIHG

VINSGGQAANIIPDYASAQFYVRASTRKELNILTQRVIQIAEGSALQTGCRLVTSNYE

TSYDEMVTNESLSAAFSANLLELGISQEEIVSGNDHGSMDIGNVSLRCPAIHPYIRVV

DEVHTLHSIEFRDLALQERALDGMILGAKALAATAYDVLTQPELLQTIQTEFKQASR"

CDS 3884469..3886220

/locus_tag="PIKHDJKN_03465"

/EC_number="3.2.1.54"

/inference="ab initio prediction:Prodigal:002006"

/inference="similar to AA sequence:UniProtKB:Q08341"

/codon_start=1

/transl_table=11

/product="Cyclomaltodextrinase"

/translation="MLLEAMYHVPRDKWAYAYNPSTIHLRVRTKRDDVQYVTALTGDK

YDWNGTYKEIQLEKAASDSMFDYWETAVKPKFKRLTYIFRITAGTENIYLADNGIHYA

PPYPTGGYYEFSYIHEIDVFKVPEWAKEAVFYQIMTERFANGNPDLNPEGIQEWGGRP

ELDNYFGGDLQGVLNHLDDLTKLGVNAIYFTPLFQANSYHKYDTIDYKKVDPQFGDNE

MLKKVAEQCHRRGIRVMLDAVFNHCSEDFPPFQDVLKNGKNSKYADWFHINEYPVQIK

DGIPTYDTFGFYGNMPKFNTANPEVKDYLLDVAEYWIKEIKLDGWRLDVANEVDNHFW

RDFRKVVKTANPEAYIVGEVWSDSLTWLMGDQFDSVMNYPFADKVLEFFCGSMDGYNF

ANEMGSLIMRYPQQTNEVIFNMLCSHDTPRLLTRAGEDKRRLKLSVVFLFTYIGTPCI

FYGDEVGIRGEGDPDCRKCMQWDPAKQDQELYDFYRLMIDLRKSNEALRKGRFRFLKA

DHNDPCIIYERMDDTLHFTVWMNNTPQERTLSHPMETKDWKDALTEDAVVPTNGMMNI

KLDPYGYRILYRQLEPS"

CDS complement(3886310..3888016)

/locus_tag="PIKHDJKN_03466"

/inference="ab initio prediction:Prodigal:002006"

/codon_start=1

/transl_table=11

/product="hypothetical protein"

/translation="MNNYINFTSTYNPNLQALSSADGYNAVKMNEIFSQIMGNIAAVQ

STLTGNIIDVRSFAPGDGSDQTVQIKAFFDAAQEGDVLLFTPGHYKVVKMGWFYMFSG

RSVIIKALPGAILEVSDQGIRIEGSNHVEISGVTLVRTTQAAWSKNKTGLYIENSSNV

LLQNNDISKFTDGIGVNGSSSQDNPARNVVIRNNKLHHLGEEPIAVRSHLVFVVIREN

DCYRYLGDGILVKGTWHVSITDNFLHTPVLSSDSDYAAFSGGQQVSNMPAVGGGITCN

NEGGETGAKNLHIHGNSLIGTAFGVGLIGFEGAFVTSNVFKDIKITSVISVSFFPSRY

NPNGLSNHLFVIQGNLIDTISRSSATAAIEAKTSIGAEGMDIGIISENIIIPRGKHWG

IVADGHIIVKGNYIAQAGIAMDIANGVIASSNIIEPGFETANPDRMVSLHNDCTFSHN

SVAGKGTCIQLRGSNNIVTGNRLKYEGTWWAVHLDIVNNTVEGNIIRDNMLIVAASAT

GRYLFGASPFTNGKNVVVDLVKDNNGTLYQLVNEIVMLGPNSTRWRITIDAKGDLKTA

KL"

CDS complement(3888090..3889532)

/gene="glgA"

/locus_tag="PIKHDJKN_03467"

/EC_number="2.4.1.21"

/inference="ab initio prediction:Prodigal:002006"

/inference="similar to AA sequence:UniProtKB:P39125"

/codon_start=1

/transl_table=11

/product="Glycogen synthase"

/db_xref="COG:COG0297"

/translation="MNILFAAAEVHPFIKTGGLADVIGALPQALKKSGADVRVILPKY

KDIPDEYKEAMEPVITTDVPLGWRRPYCGIEMLVHDGIPVYFVDNEYYFGRDGVYGYM

DDGERFAFFNRAVLEVLPQIEFKPDVLHSHDWHAGMIPLLLEAHFAHDPFYSEMKTVF

TIHNLLYQGVFPHDLFSDLLELDDRYFTVDGAEYYGNVNFLKAGVVFADHVTTVSPTY

AKEVQTSYYGYGLDGLLSSLGDRFSGIVNGIDTKSYNPAADNKIAVKYRTSLSKKIEN

KIELQKELGLPVRPDAPLMVMVTRLVDSKGLDLVCRVLDELLYYDDIQFAVLGTGDPA

YEHWFREAANRYPLKMSAQITFNDGLSRRFYAGSDLFLMPSRFEPCGISQLLALRYGS
VPLVRETGGLNDTVQAYNEFSGEGNGFSFTSFNAHDMMNTIRRAEEFYRQPEHWKKIV
KNAMSGDYSWNVSAEEYMDIYRRITLESVK"

CDS complement(3889529..3891550)

/gene="glgB"

/locus_tag="PIKHDJKN_03468"

/EC_number="2.4.1.18"

/inference="ab initio prediction:Prodigal:002006"

/inference="similar to AA sequence:UniProtKB:P30538"

/codon_start=1

/transl_table=11

/product="1,4-alpha-glucan branching enzyme GlgB"

/translation="MAIQPLANPEISPEHIYLFHEGNLHHSYRILGAQPAIEDHRQGY
RFTVWAPNAKEVGLALDRNDWKGEQEPLYKIPDSGFWTRFFPEISEGTLYKFRILTEE
GTELLKADPYAFQAEVRPRTASVTSSIEGYKWNDGAWRRKQRGVYNKPLHIYEMHAGT
WKRKEDGTLYSYRELADLLVPYLLEMKYTHVEMMPLSEHPYDLSWGYQNTGFFAPTSR
YGQPKDLMYLVDTLHQAGIGVLLDWVPAHFAKDAHGLRLFDGTPLYEYADPLLAERPG
WGTLSFDYSKPEIRSFLISNALYWMEMYHFDGLRVDAVTSMLKLDFEKQPGQYRTNNE
GGLENKEAVAFLQQLNETVFKYFPYALMMAEESSAWPMVTSPVDQGGLGFNYKWNMGW
MNDTLDYMESDFHERPSKHHLLTFPVVYSFTENYVLPLSHDEVVHGKKSLLDKMPGTY
EQKFAGMRAFMGYFMTFPGKKLLFMGGEFGQFIEWKDEDQLDWFLLNYDSHRKLHKFE
RDLSELYLGEKALWELDHSFEGYEWITPDDQDQSVISYVRKGKKSADTLLVLINFQPV
KRERYRIGVMRPGMYTEVLNSDHSDYGGSGIINDMQLTTEKIPFHGQPHSLEVVLPPL
SVVILKKSTRRKTDALPLEVQSVRAKVSTTKTSKTKDSTTVKPKRRTKA"

CDS 3891746..3892972

/gene="glgC_2"

/locus_tag="PIKHDJKN_03469"

/EC_number="2.7.7.27"

/inference="ab initio prediction:Prodigal:002006"

/inference="similar to AA sequence:UniProtKB:P39122"

/codon_start=1

/transl_table=11

/product="Glucose-1-phosphate adenylyltransferase"

/db_xref="COG:COG0448"

/translation="MFNKDCIAMLLAGGEGKRLAPLTSSIAKPAVPFGGHYRIIDFPL

SNCVNSGIDTVGVLTQYQADSLHDHIGGGEPWGHGTSSKAGISLLPSYHTGNDEYLGT

ADAIYKNIDYIDQQNPENVLILSGDHIYHMNYREMLEAHTANEAVATISVMEVPWEEA

HRFGIMAADENLKVTEFAEKPAEPKSNLASMGIYMFKWEYLKRHLLEDAANPESSHDF

GKDVIPQMLNENTPLFVYNFNGYWKDVGTVKSLWDAHMDLLHNDDDWSLQKEDWPMFT

RDWRTKPSAYKARHTRAELLQSMIHESCNMDGRAERSVIFCGAEVGKGSEVKDSVIMP

NARVGRGVHIERAIIGEGAIIKDGAIVKGTADEIVVVGPNEIVSAKPAVRTQPVRMLK

DVYEKSGRLRAGELSS"

CDS complement(3893079..3894575)

/gene="rcsC_11"

/locus_tag="PIKHDJKN_03470"

/EC_number="2.7.13.3"

/inference="ab initio prediction:Prodigal:002006"

/inference="protein motif:HAMAP:MF_00979"

/codon_start=1

/transl_table=11

/product="Sensor histidine kinase RcsC"

/translation="MIKKGITRQIVLHYFIVVFLALLLVEFVFLIAVQRYYYESIYNT

INQQITSTNDFKETLSRTDEANNLSYLLANLQLGSTELQILDMDGRVLASSTAFESEH

AVLQTSDISQAMNGSIGRWVGRQPLTGEQVMAVSHKLDLGGENTYIVRYLTSLDMVNS

KLLVMGLLAVAVGVAVLVIVLIISIGMANSIVRPLNNITAVSAQMARGRLDVRVKGNY

KHELGELASTLNFMAHEIVRSNQIKDDFISSISHELRTPLTSIKGWSETLDSGGYDPD

ETRIGMSIISKETERLIGLVEEMLDFSKLQQNQMKLVKGSVSIREIVQETMLNVWAKA

EQKQVHLKLDTDETRAFNVFGDGNRLKQVFLNLVDNAIKFSHENSWIFLSVKEEKGQI

IAAVQDTGIGISEEHLIKVRDRFFQVNHQNGGTGLGLAITQELVELHEGTITIQSELG

SGTTVTVSLPALPEEEAVISEAPKHNSDSEEVQETTQAQQTNTDKEET"

CDS complement(3894572..3895270)

/gene="phoP_6"

/locus_tag="PIKHDJKN_03471"

/inference="ab initio prediction:Prodigal:002006"

/inference="similar to AA sequence:UniProtKB:P13792"

/codon_start=1

/transl_table=11

/product="Alkaline phosphatase synthesis transcriptional

regulatory protein PhoP"

/db_xref="COG:COG0745"

/translation="MSKVLILEDEESIRSFIVINLKRNGFEVLEAGDGHEALRILQSV

PDIDLALLDVMVPGIDGFEVCRRIRETNERLGIIFLTAKVQEQDKVYALSVGADDHVS

KPFSPTELIARIQSLLRRVNVHRETAAKVTFQSGPFSLDLISKQFKKQNELIELTPTE

FSLIQFFLEKENTPLSRDVLLDHVWGKEYMGDPKIVDVNIRRLRQKIENNPSEPEYLQ

TVWGHGYKWKGRDQ"

CDS complement(3895263..3896540)

/locus_tag="PIKHDJKN_03472"

/inference="ab initio prediction:Prodigal:002006"

/codon_start=1

/transl_table=11

/product="hypothetical protein"

/translation="MIVLGSVLSGCTVLSDPKLLMKQPMMSMDKERLLTIVQKEQPPE

SQLIRPKDLNNTSMIRVADLDGDGTREAIVFYETPSENVRIHGMLLEEQGDTWVKKLT

FDVPGNTLQSFKLLDITNDGNPDIVLGVSLEAQKALMAYSYKGGALEQVMGGVPYNEL

IIDDLQGNPLDLNGDKKNDFAIVSLNTSGFSSIALYQYDDGSFKEVDRIQTDYTVKEF

YNALGGQIAKDKVGFVLDAELDDRSTFTQIVYVKDNKLVYAFNSPDQTYRDDKILSGD

FNNDGIIEFGLKETPRGWEHYVFDARMWFYTFYQWNGKDGKDFVSFQYRDDANAFHLN

LKTDWYGKVTIDTKSEKGKYIRFKMIDTDETVGEIKYFTLTQWEQDKGKGWKEITKSN

DRVIAYRGAQSANNDGGLLDNMSHLNRKEELNE"

CDS complement(3896608..3897333)

/gene="glnQ_3"

/locus_tag="PIKHDJKN_03473"

/inference="ab initio prediction:Prodigal:002006"

/inference="similar to AA sequence:UniProtKB:P10346"

/codon_start=1

/transl_table=11

/product="Glutamine transport ATP-binding protein GlnQ"

/db_xref="COG:COG1126"

/translation="MIRVEGLSKSVGADRVPVLRDIRFDMQQGEMIGVVGSSGSGKSM

LLKCLAMMEKWDSGRFTVDGAEIIKEGWSGKRKIKREWAYLEQNPQLYPRRTALKNVL

IGRSGQTPLWRMVTGMVRSDDYMGAMDYLEGLGLLDKAHQTAEKLSGGEKQRVAIARA

LAHGAKVVLADEPVIGLDPHTADTVLETLRKLCEEERATVIAVLPIEMAEKHATRIWG

LAEGKIAFDIRGRRLTQQEKNMI"

CDS complement(3897417..3898607)

/gene="ycaD_2"

/locus_tag="PIKHDJKN_03474"

/inference="ab initio prediction:Prodigal:002006"

/inference="protein motif:HAMAP:MF_01149"

/codon_start=1

/transl_table=11

/product="putative MFS-type transporter YcaD"

/translation="MSSIPTPKLSHSTSNYLILITVIVVAGISQGLLLPVLSIFLEQK

GVSPGLNGMNAAALYVGSFAMTLVAERLLGAIGFKKLIIGGLLFVMIPLILFPYFPDI

KIWFILRLVVGIGDSALHYSAQLWVLLVTAPEKRGRYISLYGMSYGLGFSIGPLGIKL

LGFGDAVPFWVLFVCMAAVLILVLMKLPDTKPEKAEHGQLPERRFRRSLAWAWYALLP

ALLYGYMEAGMNSNFPVYGLRIGLDANQISSLLPFVGIGGLFLQLPLGMLSDRYGRKQ

TLMFAGITGGIIFMLVPMAGTHFWWTLVLLTIAGGLVGSFFSLGLAYAADILPKALLP

AANVVASFHFTIGSIIGPNLGGQVINWISPGSMFILLGFMYLLFGAAGVLFRRKPLLE

SVVK"

CDS 3898818..3899738

/locus_tag="PIKHDJKN_03475"

/inference="ab initio prediction:Prodigal:002006"

/codon_start=1

/transl_table=11

/product="hypothetical protein"

/translation="MAYHVKIDVSPIYEMLNSFLVYYVTKKWIQHLDVGPEWIIEVEGK

LSSNVRAALAPAATWPFDDFDVLFAWAAYRNASNENIDFLDMLAGLTAEELFARVSVL

LPHLTIEESTRIRDSYVPLLRLWDQHYCQNMSEDYRTWLEEDAEEKRVLLDKMGPELL

IEYATAGVLVEPMPGLDEVILFPTVHNRPINMYCFYEGMMIMQYPVDAPEEDEDQPPT
CLLRFTHALADPERLRLLRYVSGEPKSLAEMCEELGKDEDMVKDQVMALRIAGLLRTH
LLGSNRKEKYSIRPDGVSELNMFLESYIRI"

CDS 3899784..3900548

/locus_tag="PIKHDJKN_03476"

/inference="ab initio prediction:Prodigal:002006"

/codon_start=1

/transl_table=11

/product="hypothetical protein"

/translation="MKIIKQHILFDLDDTLVYCNKYFNLVLGEFFENMQEWFDGHALT
VQQIREKQLEIDVTGVNKLGFASHHFPQSLIDTYRYFSQKFVKPTSHQEESYLTKLGM
SVYDQEVEPYPHMVETLENLKSAGHALYLYTGGETVIQQRKIDQMKLSAYFDDRIYIR
QHKNVEALEGILSTGPFERRATWMIGNSLRTDIMPAVTAGIHSIYIKQPNEWQYNMVE
LQPNPETSMYTITALEEVPKVIHENIQQQQQKRTLG"

CDS complement(3900641..3902989)

/gene="topB_2"

/locus_tag="PIKHDJKN_03477"

/EC_number="5.6.2.1"

/inference="ab initio prediction:Prodigal:002006"

/inference="similar to AA sequence:UniProtKB:P14294"

/codon_start=1

/transl_table=11

/product="DNA topoisomerase 3"

/db_xref="COG:COG0550"

/translation="MKTLVIAEKPDMGRTIAAVIEPRAKNNRNYLEGEHYIITWAIGH
LLGLAEPDAYDSKYKRWNIGDLPIIPDQFKIVPNPRTKDQLKMIGELAKRASAIVNAC
DAGREGQYIFALIQQQLKLRQPVKRLWISDLTAESIKRGFDGLKDASEFENLTHAARA
RSEADWLIGMNASRAFTTRHNALLSVGRVQTPVLALIYDREIEIEAFQSQTFYEVAAW
FRQEGVEYRGLRQGDKLTDPAVAESIATSVKGKTGQITKYEAKQTKEYPYRLYDLTLL
QREANAKYGYGAKKTLDIAQALYEKHKVISYPRTNSNYVTEQNIEGMHKTLNLLKNGP
YSELAQGAKPELVHKKNKGVCNPSRVEDHHAILPTLKRPGTLSKDEQNIYDLIVRRFL
SHFYPPAEYKQHTVLTDVEKHQFKTSVKELLSLGWKVVLGSSDQEQTGKKKTKKNSNE

EEEAEEWTDKAFAVQPELPVQCTKSEFKEKATQPPKSYTEGTLLKAMESAGKQIENEE
LRDAMKDSGLGTPATRAATIERLKNVGYITMQGKKMQLTLKGRTAIELIRKAGVDLLT
SPEMTGQWERRLYQISKGEAGQDKFMENVKKFTMSIIDKVRVQAPAPADAFGEDTRGG
RGKGKGAKAQRSSTRTTSAKISSAGAGTSVGSKTSRQTSGASSRTASVANATAQTRSS
TSSHSGVREMLASCPSPGCTGQIIEGKKGYGCTRFKEGCGFVIWKEYVGKKITSTMLK
ALIEKGSTQVLAFKRKDGSTVKARIILQDKATGKLSAEKQDA"

CDS complement(3903115..3903606)

/gene="fur_1"

/locus_tag="PIKHDJKN_03478"

/inference="ab initio prediction:Prodigal:002006"

/inference="similar to AA sequence:UniProtKB:P54574"

/codon_start=1

/transl_table=11

/product="Ferric uptake regulation protein"

/db_xref="COG:COG0735"

/translation="MASNRDKSISEQIDDIKKQLVAKGYKLTQQREVTVRVLLEHEKD
HFSAEEVFLLVKEQFPEIGLATVYRTLELLSDLQVVEKINFGDGAARFDLRSTDGSHH
HHHLICTECGTVEEIMEDGLLRLEQQIERQYGFAVTDHRLDFQGVCKECRAKQALNEQ
AAG"

CDS complement(3903888..3905375)

/gene="gltB_2"

/locus_tag="PIKHDJKN_03479"

/EC_number="1.4.1.13"

/inference="ab initio prediction:Prodigal:002006"

/inference="similar to AA sequence:UniProtKB:O34399"

/codon_start=1

/transl_table=11

/product="Glutamate synthase [NADPH] small chain"

/db_xref="COG:COG0493"

/translation="MSTPTGFMEYKRQLPADRKPAERVKDWEEFHKHMAEEELRTQGA
RCMDCGTPYCHTGIDMTGGTSGCPVHNLIPEWNNLVYRGLWREALDRLHKTNNFPEFT
GRVCPAPCEGSCTVGLIGEPVTIKTIEEAIIEKGFEEGWVVPQPPEKRTGKRVAVVGS

GPAGLATAAQLNKAGHSVTVYERSDRVGGLLMYGIPTMKLDKNVVQRRVDLLEAEGIQ
[truncated: 324,628 more chars]
