# Supplementary material for: Phenotypic and Complete Reference Whole Genome Sequence Analyses of Two Paenibacillus spp. Isolates from a Gray Wolf (Canis lupus) Gastrointestinal Tract
Source: Vet Sci. 2025 Jan 13;12(1):51. doi: 10.3390/vetsci12010051 (PMC11769508; doi:10.3390/vetsci12010051)
Supplement: Supplementary file 1 [file Supplementary Table S7 ClWae19_GenBankFile.pdf › Supplementary Table S7 ClWae19_GenBankFile.pdf]

LOCUS: contig_1 ClWae19 7032512 bp DNA linear 20-MAR-2024

DEFINITION as Genus species strain: *Paenibacillus amylolyticus* ClWae19

ACCESSION: The data is retrievable at the National Center for Biotechnology (NCBI) as SUB14651639, BioProject PRJNA1145425, BioSample SAMN43065443 with a Localid as ClWae19_contig_1 with Accession CP168019 as *Paenibacillus amylolyticus* ClWae19

KEYWORDS: genomics; probiotic; canine; antimicrobial; *Paenibacillaceae*

SOURCE: North American Gray wolf (*Canis lupus*) GI tract

ORGANISM: *Paenibacillus amylolyticus*

COMMENT: Annotated using prokka 1.14.6 from https://github.com/tseemann/prokka

FEATURES: Location/Qualifiers

source 1..7032512

/organism= "*Paenibacillus amylolyticus*"

/mol_type="genomic DNA"

/strain="strain ClWae19"

rRNA 159..1709

/locus_tag="EFAGFIKM_00001"

/product="16S ribosomal RNA"

rRNA 2018..4939

/locus_tag="EFAGFIKM_00002"

/product="23S ribosomal RNA"

tRNA 5009..5084

/locus_tag="EFAGFIKM_00003"

/product="tRNA-Asn"

/inference="COORDINATES:profile:Aragorn:001002"

/note="tRNA-Asn(gtt)"

tRNA 5088..5179

/locus_tag="EFAGFIKM_00004"

/product="tRNA-Ser"

/inference="COORDINATES:profile:Aragorn:001002"

/note="tRNA-Ser(gga)"

tRNA 5189..5260

/locus_tag="EFAGFIKM_00005"

/product="tRNA-Glu"

/inference="COORDINATES:profile:Aragorn:001002"
/note="tRNA-Glu(ttc)"
tRNA 5328..5403
/locus_tag="EFAGFIKM_00006"
/product="tRNA-Val"
/inference="COORDINATES:profile:Aragorn:001002"
/note="tRNA-Val(tac)"
tRNA 5421..5494
/locus_tag="EFAGFIKM_00007"
/product="tRNA-Met"
/inference="COORDINATES:profile:Aragorn:001002"
/note="tRNA-Met(cat)"
tRNA 5522..5599
/locus_tag="EFAGFIKM_00008"
/product="tRNA-Asp"
/inference="COORDINATES:profile:Aragorn:001002"
/note="tRNA-Asp(gtc)"
tRNA 5695..5770
/locus_tag="EFAGFIKM_00009"
/product="tRNA-Phe"
/inference="COORDINATES:profile:Aragorn:001002"
/note="tRNA-Phe(gaa)"
tRNA 5791..5866
/locus_tag="EFAGFIKM_00010"
/product="tRNA-Thr"
/inference="COORDINATES:profile:Aragorn:001002"
/note="tRNA-Thr(tgt)"
tRNA 5876..5961
/locus_tag="EFAGFIKM_00011"
/product="tRNA-Tyr"
/inference="COORDINATES:profile:Aragorn:001002"
/note="tRNA-Tyr(gta)"

tRNA 5968..6041

/locus_tag="EFAGFIKM_00012"

/product="tRNA-Trp"

/inference="COORDINATES:profile:Aragorn:001002"

/note="tRNA-Trp(cca)"

tRNA 6071..6145

/locus_tag="EFAGFIKM_00013"

/product="tRNA-His"

/inference="COORDINATES:profile:Aragorn:001002"

/note="tRNA-His(gtg)"

tRNA 6167..6241

/locus_tag="EFAGFIKM_00014"

/product="tRNA-Gln"

/inference="COORDINATES:profile:Aragorn:001002"

/note="tRNA-Gln(ttg)"

tRNA 6246..6320

/locus_tag="EFAGFIKM_00015"

/product="tRNA-Gly"

/inference="COORDINATES:profile:Aragorn:001002"

/note="tRNA-Gly(gcc)"

tRNA 6327..6400

/locus_tag="EFAGFIKM_00016"

/product="tRNA-Cys"

/inference="COORDINATES:profile:Aragorn:001002"

/note="tRNA-Cys(gca)"

tRNA 6406..6486

/locus_tag="EFAGFIKM_00017"

/product="tRNA-Leu"

/inference="COORDINATES:profile:Aragorn:001002"

/note="tRNA-Leu(caa)"

CDS 6669..7349

/locus_tag="EFAGFIKM_00018"

/inference="ab initio prediction:Prodigal:002006"

/codon_start=1

/transl_table=11

/product="hypothetical protein"

/translation="MTNGKNVSDTDLLFDLSVWEEAWKNRPRSSKYKKKSVPSNTEEA

FERWARDYHAQSFTEEGKQRSERIMGWIENQGVEFAGLSILDIGAASGIFTIPFAEKG

ASVTAVEPSELLVSLMKETIPSALESKIEIVNERYEEISIQDKGWEKKYDFAFASMCP

AMSDWEAIEQAINCARKYVVYISTMAGQKEHTLMHELKDVLGVTSSYKAGDMGYIQQLL

YLKGILIR"

CDS 7346..7594

/locus_tag="EFAGFIKM_00019"

/inference="ab initio prediction:Prodigal:002006"

/codon_start=1

/transl_table=11

/product="hypothetical protein"

/translation="MIITREVNSFEVPVEEIIEKLPEWLNTYELPSDEDSLRLAEDYI

SDTYKDSTVTFSRGGRFGKILIQLEQPNMKTVGTKDKR"

CDS complement(8029..10419)

/gene="yesS_1"

/locus_tag="EFAGFIKM_00020"

/inference="ab initio prediction:Prodigal:002006"

/inference="similar to AA sequence:UniProtKB:O31522"

/codon_start=1

/transl_table=11

/product="HTH-type transcriptional regulator YesS"

/db_xref="COG:COG2207"

/translation="MRPISYLHKMIIFGILLSTLPILLTGIAAFIYSSNQTELHRTQA

NRQLLEQMQSNVEHKLATVSYMLDQAVRSPVTLRSLSDSSSVQGNGPLLADKLQSEFQ

NMRSWEPLLDITLVNQSQNWLIDHAGLYRNTDFPLALPMKELISDTLTSGWQLSPSSV

FSNDERSPGTGCIYHIALARSIPNLNTSSDAALIAGIPACSLQNTLGEASLGNAASGL

IILNREQRILVHPDPVYIGQPLSAIGLQDRDTEANITKLSPITFSTGFEKREVKIADT

HYSLTYSPSTLKGWTYVLISPTNILTQEYIDIGLHTLYISIFLLLLSLLLAWLGSKRM

HVPIRKLLTQLGDNHLSKEGNTLAQQSPPFSDEFEQIRAGVSQLSASRSQLENTLNVH

LLQIRNHFMMNLFQGKIPVHTLHDTLNEYGYTHQVREWQQIAVITLQADLVNHTKYRA

SDRDLLLFAIQNILEETVVHNQQLLPIVLEHTVVTVIGTVEQDQFIFSQQLYVLTDQL

QQQMDKILALQVSIGFSQSHSSLHHIPQAYTEALEALRHRMKLGTGIIFQYENIDHYT

PTWSMTYPESLEYSLIQAIQNADEVQASALLKQLLEVIFGMEVTPEEYEVALTRLLTH

ILQMTQESGIRLGQISQSRGSMFHELHGLQYAAEVEDWFKYQVILPVIQVFKARQYAQ

YQHISEKMIAMIHQDYDKDLTLEECAVKLHYNANYLSSVFRKETGCAFSEYLTKYRFN

IAKKWLDESDLTVKDIAARLRYNNPQNFIRSFRKWEGITPGQYRERKQKAEVSNRN"

CDS 10975..11283

/locus_tag="EFAGFIKM_00021"

/inference="ab initio prediction:Prodigal:002006"

/codon_start=1

/transl_table=11

/product="hypothetical protein"

/translation="MRLNKGNGWAIVLIALGALLLFGKLTPLLGHLMGYLIPVLMIAL

GYYGVKRGNVMLGWIVLIIGIISLMGKLAWLIGPIIAIGLIIFGISMLSNNRSRRGRY

"

CDS 11319..11990

/gene="liaH_1"

/locus_tag="EFAGFIKM_00022"

/inference="ab initio prediction:Prodigal:002006"

/inference="similar to AA sequence:UniProtKB:O32201"

/codon_start=1

/transl_table=11

/product="Protein LiaH"

/db_xref="COG:COG1842"

/translation="MSVFRRMRDITVANLNEHLEQSQDPVKLIDQFLVSTRQDIGEAE

KLRHQYASHTRQMKQQADQAAGMVNKREEQASMALKAGEEHLAKLALQEKILHEEKME

QYNELYAQSYAALQELDEQIDQLKVEYQNVYSKRQYYYARMQTIQLQQRMNQRGNHNG

QNVPRMFNRLDDQVSDLEYEAQSLRDIRRMNQDGSGVTGSMSSSTLDKELERLRQKLN

NDRKE"

CDS 11996..12229

/locus_tag="EFAGFIKM_00023"

/inference="ab initio prediction:Prodigal:002006"

/codon_start=1

/transl_table=11

/product="hypothetical protein"

/translation="MSKLYRSARNRMLTGLIGGISENLGLSTTLLRIIFFISIFATGG

TSLLIYFIAALVVPKESNHVDPYAYDSNVRGYK"

CDS 12247..12756

/locus_tag="EFAGFIKM_00024"

/inference="ab initio prediction:Prodigal:002006"

/codon_start=1

/transl_table=11

/product="hypothetical protein"

/translation="MSKLYRSTRDRMLTGLCGGISETIGMDSTLLRIIFVISIFVTGG

TSLLIYFIAALVVPKEPYPPYDPYGYGPGQGPGPGRGYDNYDHQPPRGPFQNNHNQQG

PGNFGPGPGFNGRPQSGPRSYDNNAYGAGAPQESELDSMMKDIEKKALKKEVEELRQK

LSRYEKGEK"

CDS 12758..13465

/gene="liaH_2"

/locus_tag="EFAGFIKM_00025"

/inference="ab initio prediction:Prodigal:002006"

/inference="similar to AA sequence:UniProtKB:O32201"

/codon_start=1

/transl_table=11

/product="Protein LiaH"

/db_xref="COG:COG1842"

/translation="MGVFKRIKDMTKASVNDMLDKVEDPIVMLNQYLRDMEAEIHEAE

VTVAKQMANERRMKQRLDEAERTSVQRESQAEAALSNGQEEVARKLLEEKIYFDQKIT

EYSELHAQSEAQAKDLLQQLHDMKDEFYKMRNKRNELVSRAQMAKAKKQMSSINSLHS

IESGGASLGFHRMEEKIMQLEAEADVARAPYRNTSSTYTNPVDVEKQFKVDQQLQALK

NKMGNGSKNEANGTSKE"

CDS 13616..14263

/locus_tag="EFAGFIKM_00026"

/inference="ab initio prediction:Prodigal:002006"

/codon_start=1

/transl_table=11

/product="hypothetical protein"

/translation="MNQKSKWLAVVIMIIGTMMLLNDNINFLTAAALILLTVGMLQVR

NGDTRKGHRYLGIGAALLLLDNLMIVFLIVLASLAYFYSKNKKIHLDEGVMKKQNFMA

RYYWDQSSWTLRSMSLWHAIGEVNADLSLSIPEEKQTIVLLQGVMGNVRLTLPEDYGV

EIEASVLFGRINLLGEQDSGMMNKLVWRTPGYESSEHKAKFVISYIVGDLNISNP"

CDS 14295..15410

/gene="liaS_1"

/locus_tag="EFAGFIKM_00027"

/EC_number="2.7.13.3"

/inference="ab initio prediction:Prodigal:002006"

/inference="similar to AA sequence:UniProtKB:O32198"

/codon_start=1

/transl_table=11

/product="Sensor histidine kinase LiaS"

/db_xref="COG:COG4585"

/translation="MKTKRHTDMVTRSMGEGILLVFIVLAVVLYVLYTYGYLAPFTGW

RHLIQSGLALLLLLIGIGAVFGFYQSYRVKRRIELLRETLLLWEKGSLSRTVPDLGDD

DVGRLSEQLGRIGKKWEDQISSLQRLSTHNAQLAEQARITAIVEERQRLARELHDAVS

QQLFAISMTATAVGRKMEKDFERAQRQVALIEEMASVAQSEMRALLLHLRPVYLEGKH

LEQGLRDLVMELKTKVPMDIVLEMDEDIDLIKGIENHLFRIVQEAMSNTLRHAKAEKM

EIRIQRRTDAIRVLIRDDGQGFDLDENKQASYGLANMRERVTEIGGAIQFVTAPGKGT

RIEITIPLMNDESEEEHVNGTGSRNGDGNVDQSIARG"

CDS 15352..16020

/gene="liaR_1"

/locus_tag="EFAGFIKM_00028"

/inference="ab initio prediction:Prodigal:002006"

/inference="similar to AA sequence:UniProtKB:O32197"

/codon_start=1

/transl_table=11
/product="Transcriptional regulatory protein LiaR"
/db_xref="COG:COG2197"
/translation="MEPEVETETETSIKVLLVDDHEMVRIGLAAVLDTEDGIEVVGEA
GSGEEGIRLAQEYKPDVVLMDLVMEGMDGIEATRQVLKMYPECKVIVLTSYLDDEKMY
PVIEAGAFSYLLKTSRANEVADAIRAAARGQSVLESQVASKMMNRFRQPQAAAPLHDE
LTDREMDVLRLLAKGKSNQDIADDLIIGIKTVKFHVTNLLAKLGVDDRTQAAIYAYKN
GLAE"
CDS 16470..17219
/locus_tag="EFAGFIKM_00029"
/inference="ab initio prediction:Prodigal:002006"
/codon_start=1
/transl_table=11
/product="hypothetical protein"
/translation="MLNRWMTAGILAIAIIILIGVTQVYAEKNESKVAQLEQLIYTAD
SVIDNVDRLVIKWQGEGKGDAQEQAVLLASHLGLEPPMQVRQTSHNVYRSEVVVGTAN
GGVGMLVNVVDTGANGYYAIVQLSGDAHTDRKALMTLHEEVDQLLVDSGMKATWNMSV
QGTAVTATEHDASQQLELIEEQLSTKVDIASVERYTDAGTASVSYKAAELPLSIKSGS
HMLNMQLAVHQVGDEVDNRITVGFPVITIEY"
CDS 17435..18985
/gene="zwf_1"
/locus_tag="EFAGFIKM_00030"
/EC_number="1.1.1.49"
/inference="ab initio prediction:Prodigal:002006"
/inference="similar to AA sequence:UniProtKB:P54547"
/codon_start=1
/transl_table=11
/product="Glucose-6-phosphate 1-dehydrogenase"
/db_xref="COG:COG0364"
/translation="MVEKQLQDEVQTPGAVFFIFGATGDLARRKLFPAIYSLYREGKL
AEDFAVIGVARRPRSPEEFREDIYESIKEFCRYPAGEPDEWNAFVEHFEYKALDINNV
EGFKELRAQTEHLESKFNTPGNRLFYLALAPELFGSVSYSLRDGGMLESQGWNRLVIE

KPFGYDLQSAEHLNEQIREVFREEEIYRIDHYLGKEMVQNIEVIRFGNAFFEPLWNNK

HIANVQITLGETVGVEERGGYYDHSGALRDMGQNHMLQMLTMIAMEPPSRLFPEDIRD

EKVKVLRSLRAFTSDEEVSNNVVRGQYSEGEYRGKQLPGYRQEDKVDPQSNTETYFAA

RVFVDNFRWAGVPFYIRTGKRLPVKTTEIVVEFKSMPNNVYLGKKYKLEPNLLVIRVN

PMEGIYIKINAKKPGSDSEIQPLAMDFCQSCMIGINSPEAYERLLHDAAEGDSTYFTR

WDEVATAWSFVDRIAAAWGQNQGELHTYPAGTWGPEATDKLLEQDGFHWWPVNGQDED

SVIWQVNG"

CDS 19355..19756

/gene="mhqP_1"

/locus_tag="EFAGFIKM_00031"

/EC_number="1.-.-.-"

/inference="ab initio prediction:Prodigal:002006"

/inference="similar to AA sequence:UniProtKB:P96694"

/codon_start=1

/transl_table=11

/product="Putative oxidoreductase MhqP"

/db_xref="COG:COG2259"

/translation="MMLDVGLLLIRLVIGLSFMAHGAQKLFGWFGGYGIKGTGGWFES

MGMKPGALVALLAGLAEFGGGLLLALGLLTPVGGILIALTMVIAIVKVHGANGYWSTQ

NGFEYNLAILVIGVALALTGGGQYALDALIF"

CDS 20078..21691

/gene="prfC"

/locus_tag="EFAGFIKM_00032"

/inference="ab initio prediction:Prodigal:002006"

/inference="similar to AA sequence:UniProtKB:Q99V72"

/codon_start=1

/transl_table=11

/product="Peptide chain release factor 3"

/translation="MNERTKGIKPMSKAANDILQQEVDKRRTFAIISHPDAGKTTLTE

KLLLFGGAIRLAGTVKARKASKHATSDWMEIEKQRGISVTSSVMQFDYLDHRVNILDT

PGHQDFSEDTYRTLTAADSAVMLIDVAKGVEAQTIKLFQVCAKRGIPIFTFINKLDRE

GKSPFDLMEELENVLGIRSVPMNWPIGTGRELCGVYDRMKNQVELFQGDDHSTIKVQK

VDGYKDPIIREMAGEYLHDQLCQDLELLDIAGDQFDMEKVQRGELTPIFFGSAINNFG

VQTFLENFLELAPKPEPRRSTAGEIQPTNEKFSGYVFKIQANMNPAHRDRIAFLRIVS

GKFQRGMSVKHTRVGKEIKLSQPQQFLAQDRDIVEEAYAGDIIGLFDPGIFRIGDSLS

QGSEVVFEELPTFSPEIFAKVTVKNALKHKQYQKGIDQLTEEGTIQVFQTVSFDETIL

GVIGQLQFEVFEYRMKGEYGVDVQLQRMQYQFARWIVDENLDPSKFRINSALVKDKKG

NYVVLFENEYAMRTAMDKNPTAQFLETAP"

CDS complement(21920..22153)

/gene="sspI"

/locus_tag="EFAGFIKM_00033"

/inference="ab initio prediction:Prodigal:002006"

/inference="protein motif:HAMAP:MF_00669"

/codon_start=1

/transl_table=11

/product="Small, acid-soluble spore protein I"

/translation="MPITLSLREAIVHKVHDKSDDQLREMIEGSVDGPEAALPGLGAI

FEMIWKNTEPAKQEELIQIAQEHLHTIPVQPLI"

CDS 22321..22983

/gene="ktrA_1"

/locus_tag="EFAGFIKM_00034"

/inference="ab initio prediction:Prodigal:002006"

/inference="similar to AA sequence:UniProtKB:O32080"

/codon_start=1

/transl_table=11

/product="Ktr system potassium uptake protein A"

/db_xref="COG:COG0569"

/translation="MAKKQYAVIGMGRFGSSVANALSGMGFDVLAIDADEQRTQEMSN

VVTHAVSADSTDEEALRALGIRNFDVVVVAIGEDIQASILTTLILKDMGVPVLIVKAQ

NELHGKVLQKIGADKVIYPERDMGLRVAHHLTSPNILDYIELSEDYSILEMRASEQMI

GKNLMELNIRARFGCNVMAIRSGNSMNISPYAEDRIEAGDVLIIVGHKDHLTKMELAY

PK"

CDS 22983..23798

/gene="aviRb"

/locus_tag="EFAGFIKM_00035"

/EC_number="2.1.1.208"

/inference="ab initio prediction:Prodigal:002006"

/inference="similar to AA sequence:UniProtKB:Q9F5K6"

/codon_start=1

/transl_table=11

/product="23S rRNA (uridine(2479)-2'-O)-methyltransferase"

/translation="MDADGKDGKMDIVSPQNTRVKEWAQLLEKKHRTRQHKYIIEGIH

LVQEALRAGADLECIVYDGEQGVPSELGGLENPLQRVEWVSVSPAVIAKCTDTMTPQP

VFAIVRKGSEPLESLITGARGLVVVLDGVQDPGNVGTIIRSADAAGAAGVVLGAGCAD

VYNPKTIRSTMGSLFHLPIVEGQLESLLPEAKAAGVKLVSTSLQAEHSCYSYDFTQSV

WLVIGNEGKGISDATARLVDDAITIPMQGQAESLNAAMAATILLFEAMRQRMV"

CDS 23934..24278

/locus_tag="EFAGFIKM_00036"

/inference="ab initio prediction:Prodigal:002006"

/codon_start=1

/transl_table=11

/product="hypothetical protein"

/translation="MILGGDIFMAKKGQTFQTYTEEFKLNAVRSYVEGSSSYKVVAER

EGIRNCSQLKVVWVKKWKNGEAFDERKNSVPNPLKGRPRTAFGSVEEERDYLQAQVDYL

KKRYPNLVKEKR"

CDS 24443..25138

/locus_tag="EFAGFIKM_00037"

/inference="ab initio prediction:Prodigal:002006"

/inference="similar to AA sequence:ISfinder:ISBth8"

/codon_start=1

/transl_table=11

/product="IS3 family transposase ISBth8"

/translation="MMAIHFANREFGYPRMTTALWEAGLNVNHKKVWRIMRELSIQSV

IRKKRKKSSYTPSVIYPNRLKRQFHATAPQQKMVTDITYISDGSAFVYLSVIQDLFNN

EIVAWQLSKRNDVQLVLDTVEQWTQKRDVSEAVLHSDQGFQYTSQAYNSRLEAFGVKG

SHSRKATCLDNACIESFFSHLKTEKLYLNQCNSEAEIRQAVEDYMYNYNYRRFQAKLK

QRAPIEYRCALAA"

CDS 25406..26101

/gene="walR_1"

/locus_tag="EFAGFIKM_00038"

/inference="ab initio prediction:Prodigal:002006"

/inference="similar to AA sequence:UniProtKB:P37478"

/codon_start=1

/transl_table=11

/product="Transcriptional regulatory protein WalR"

/db_xref="COG:COG0745"

/translation="MNNAKILIIEDEAPIADLLSYGLSLEGFQTKVATNGAEGLNEIR

LFQPDLLLLDWMLPDQSGLDICKKVTSEYNIPIFMITAKSDITDKVLGLEFGADDYIT

KPFDLREVIARIRTILRRVEQASNVDPSEAIATIHFQDIEIYLEERVVKHKNNSIELT

PKEFDLLITLARNPGRTFTRSELLDLVWGYHFAGDTRTVDTHIQRLRKKLDAADYITT

VFGIGYKFKKTGG"

CDS 26109..27569

/gene="sasA_1"

/locus_tag="EFAGFIKM_00039"

/EC_number="2.7.-.-"

/inference="ab initio prediction:Prodigal:002006"

/inference="protein motif:HAMAP:MF_01837"

/codon_start=1

/transl_table=11

/product="Adaptive-response sensory-kinase SasA"

/translation="MVNKISTKFLLGFLLIFSFSFVLLNQTVKEFTRTGNQNLITSEL

VGLKNNSNVYYVRQAFLINHFSNNELYFGEMAEELGNTLNHSTGNSVGVYTVNGELLYA

SDQSLFSEAKENDIQEAIEGKTAYSINYNDNKTSVLFSYPVVMDGSKVGILRFYKDFT

LLYQQSGKILEIILYITLAIFSVAFLFSYILSRHVTLPLGKLARASSEVKDGNLDVRI

HFKRKDEIGRLAENFNDMINQIGLQIRIIEKDRDQLRELHKQEKLFFDNITHELKTPL

TSILGYAELIKANGERDREFFEKGTTHIIEESRRLHAMVIKLLEVSRQHVNELERVEV

GAILRDVCESMSIRAQRYKKNIVVNIESELYLLAQTDSIRQLFINLLDNAIKYSLAFA

EISVRGVQEEGKVRIYIGNPSDPIDADELTKLFQPFYLAHPKDSEAGSVGLGLSIVKS

IVEELEGSISIVSQNNYMVVTVEFDSLKSDRGEEQL"

CDS 27569..28693

/locus_tag="EFAGFIKM_00040"

/inference="ab initio prediction:Prodigal:002006"

/codon_start=1

/transl_table=11

/product="hypothetical protein"

/translation="MKNYLQRVPWTLLFMLFLLTSCSVGHQSETIIISSNDAEDIQHP

GSSPVQVQKIYRLPDEYMGQWLGWSSSHSIIGSYKTASIPERSILTRLDQPFEQSNNI

KELSTNNVQTILSPDGKYITEMYNTNLDVNIKILSLADGQEATVDTVNTKDKFLQDVS

WSNNGKYLSYLVLDSSGSDKASLRLYNMESRTLQVYEFQDIDEGGTLLGMNVSEDGRS

VLFESMHSGKTNVLLGKINNKTIEKRYVRQIGREQNPAWISNDQFVFLGTDGTLYEYD

QRNSELSIILERVSIFEISQDKKFIAYSLRDEDVVYVGKMQGRNILFNEPVYHGIVPT

TMYWNTDSSRLLIQNQKSYTHSEPGQVDFAAGPSFIIEFK"

CDS 28861..30645

/gene="oatA"

/locus_tag="EFAGFIKM_00041"

/EC_number="2.3.1.-"

/inference="ab initio prediction:Prodigal:002006"

/inference="similar to AA sequence:UniProtKB:Q7A3D6"

/codon_start=1

/transl_table=11

/product="O-acetyltransferase OatA"

/translation="MNGKNNLTIKRYMPGIDGLRAISVLAVIAYHFDFKWAQGGLLGV

GIFFVLSGYLITDQILLEWKMHKNIFLWNFWIRRLRRLLPAMISMLMFVALWLIVTDP

TRLLSLSGDFISSVFYVNNWYLIFHNVSYFDSFGPPSPIGHLWSLSIEEQFYLVWPVV

LLLAIRFAPRRGRLMVYIAVLASISATAMAVMYEPGTDPSRVYYGTDTRAFAIFIGAA

LAVGWPSWKLNRRISPTARTLLDCVGGLGLLILLILIYRTNEYDDSLYRFGFLYLSII

SAIVISVLAQPASRLAKLLGCKPLAWIGKRSYSLYIWHYPVITLMHPTGNERLNILQI

MIQLIITFILSILSYTWIEEPIRKVGVKAQLRFIRSHHWFKPAIIFTFTLLALFRVES

WITNETKVRSESVQPVIVQEVTSNNQQELNTDNTEEIPAPVPMVKGEGITVIGDSLAL

NVASVLEEKLPGIVVDGKVGRQMSQAQSVIEDLRAKDKLGDRIVIALGTNGAFNQSSL

SSLLDSLQGEKKIYLVTTRVPKNWQNTVNEALKDVTREYPNVKIIDWYAASENKNDLF

YHDGVHLTPEGAQFYASVLVEHIQEDQG"

CDS 30684..31382

/locus_tag="EFAGFIKM_00042"

/inference="ab initio prediction:Prodigal:002006"

/codon_start=1

/transl_table=11

/product="hypothetical protein"

/translation="MNKNKARKAWMVWGLALLIMILGYMGFRSLDTTNAQQEESDAQA

MGATKYISNQTQPEQSNNASSNDSQKTDVLSTEGEGVTIIGDSVTVGIEPYLKEKLPK

INVDGKVGRQMSQAESTVSELKAQGKLGDRIIIELGTNGPFSKKHLQHLLTALSDAKQ

VIVVTTRVPKGWQDSVNSTINDVAQEFDNAQVVDWYAASEGKGDYFYKDGVHLKPDGG

RFYADLLIQALEQK"

CDS 31425..32444

/gene="tagU_1"

/locus_tag="EFAGFIKM_00043"

/EC_number="2.7.8.-"

/inference="ab initio prediction:Prodigal:002006"

/inference="protein motif:HAMAP:MF_01140"

/codon_start=1

/transl_table=11

/product="Polyisoprenyl-teichoic acid--peptidoglycan

teichoic acid transferase TagU"

/translation="MSLKTKKIAIWTSVMIFLMIIALGLYYVVAIYKGLDSFSKEQED

SPFYEVKASEEHVIEPPKWAGKEPVNILLMGVDARGLKKGEVPRSDSMMVVSLDPVRE

SIHVLSILRDTYTDIPEHGKNRINTAITHGPKTAMNTVSELLGIPIQFYVYTDFQGFI

KLVDAVGGVDFYVEKDMNYESKADNYEYDINLKEGQQHLDGKTALQYVRFRHDAMSDY

SRTKRQRELMNEIVEQMKNTTSLVKLPVILEEVSPYIDTNLSVNDMWKLASVGYQSKI

ESNEQIPPMKLLKETYVGRAAVLAVTSNLHLKEYVRDLLESNPVDTVETEQKAMTNEE

IHQDK"

CDS 32460..35849

/locus_tag="EFAGFIKM_00044"

/inference="ab initio prediction:Prodigal:002006"

/codon_start=1

/transl_table=11

/product="hypothetical protein"

/translation="MFKKITKKRAWAVFASLSILMSTIAHGTAFAASDSNTEQNSPTG

EPKAEKALQKHSEVQPLVMNHNIASMTEKRTIQVKFTLPEGVDAKKIEWTYGGKPLSE

WKTFEQQDYTGPSFIRVSHEKVSGRQFTADITFDLVYGTTNLAEPQLQRPLFASLMGT

YDLTVKANGKAIAQAPVKLTPYASYLTYDELKPAIDEVTSQAAQKNDRYIQTTSIGKS

VEGRDIYLTIVAKDKNTIDKYENVTHPAMLNDPKRLQADIKSGAFGDYAVPIWVNNIH

PNESPGVDAIFNYFKSTSLDEKVTFRTISTDGKPDKVSFNVDDALNNVFFIFVYTNNP

DGRVHQTRGNANDFDLNRDNSYQTQPETRSVTEQIAKWSPLSFLDLHGYDRNFLIEPA

TPPHDPNMEYDLLIDRMLEQAKVMGEAGIANTKYDYYHIPYEENRKTAEDPTYVSRGT

SSGWDDASPAYTAVFAMHHGALGHTLEIPEFNEESTKALYYSTAAATAYVMDNKEKLF

LNQLHIYERGIDNIDDHAVDPYLINAKHEEIGRPREGNSNFFPEYYVLPVDESVQKNA

LETYHMIEYFLRNGVKVERSTEAITVDGVTYPQGSFVVNMHQALRGMANLVLYDGIDV

SDYEFVAGEIVQNFPNLRGFDRYVIREPHVFANKTIPVTSVSVPATSMPANTAYILIR

NSNNDAIRAVNELLTAGKSVTMLTSSDTGDQAGDFVVSTTDLSPLASKYFLDVKAFGD

SKPSGKELQSLTVSALGESAFVLEGLGFNVTQDQDKADILVNTFESSAFVEKGKPYIA

YGNMGMTNVKDLIPDFTFAGPQWERYEGVFASDVNQDEMITAPYNKEEYFYTVSGSYI

TSVPKTATILAKISEKDNFYKAGWWPGHASAKGQIMAFTYRGNHKNITVFSNDLTNVG

HSQHQYRLLANSIFSAVHETSESSGSDVKFSDLKNVESWAGREIRELVDADVLQGTGK

NRFEPLKPVTRAEFVAMIVRAFDIPESKGKVTFKDVPPSSWYHTYVTAAVESKVAEGV

GGDRFEPGLSITREEMAQIVANTLKLKQEVSTSNTTEVLEQFTDKDLIAPYARDSVAY

LTEARLIQGVSVSNYGPKQLANRAQAAVMISRMKHFINHDL"

CDS complement(36284..37492)

/locus_tag="EFAGFIKM_00045"

/inference="ab initio prediction:Prodigal:002006"

/codon_start=1

/transl_table=11

/product="hypothetical protein"

/translation="MSLKFQSPDESGHENEDKVDQIQSRSQKKNKDTQIKKGDSRGSG

RLIALDAVRGLAVIGMFIQHFAMNQWNGNIVSGNTTLLFVLCGGISYSIMAQRMKERG

AADSSFRGKMLARAVFVDVIGYILIMLNIPYGIILPAYAAMFVLGLILVGRSIRTLAT

TAVSLTVLAPPLMLLGQSAFSGSFLLADITGGPMSSIALAPAFVAGMAIGRLDLTRKR

VSLSLAFGGLVMLVISKSLASKVLPELGRSFETWLVSVQGVAAQPDPYAIWPLNVDSP

MWHMLLWTAPHSASTFQTMTGLGIALLVIGLAFLIPKKVNVLLQPFAAVGRVALTMYA

IQFVFVWILSLSGINYSYGELPFGDLLVVIVTLILGWLISRLGTGPLESLMRYFDRIF

SPSSPKINVS"

CDS 37786..38544

/locus_tag="EFAGFIKM_00046"

/inference="ab initio prediction:Prodigal:002006"

/codon_start=1

/transl_table=11

/product="hypothetical protein"

/translation="MKYTVITGASSGIGYETALAFAARGKNLILVARRLDKLEDLKST

IQGIDPKVNVIVHTSDLSVTAEAYALYNDLKEYEIETWINNAGLGEGSFIAEQNLDKV

ETMLRVNIESLTILSTLYVRDYANVEGTQLINVSSALGYAIAVGSVAYSASKYYVSAF

TEGLAKELELKGAKLKAKILAPAITETEFVQKSIDAEGFDYKANMPKYHTAKEMAGFM

IDLYDHNEVVVGIVDQNYEFQLTGAIYPVISELN"

CDS 38605..39690

/gene="nemA_1"

/locus_tag="EFAGFIKM_00047"

/EC_number="1.3.1.-"

/inference="ab initio prediction:Prodigal:002006"

/inference="similar to AA sequence:UniProtKB:P77258"

/codon_start=1

/transl_table=11

/product="N-ethylmaleimide reductase"

/db_xref="COG:COG1902"

/translation="MEKIWSKTKIGNMELPHRLAMAPMTRSRAQEDGTPGELAALYYA

QRATMGLLITEGTQPSDDGQGYLWSPGIYTDKHIDGWKKVTDAVHEAGGYVYIQLMHA

GRMSHPENTPHHRQPVAPSAIAPGVEMFTVTGMQDIPVPRELSQEDIQTNIADFRKAA

AAIEAGADGVEIHGANGYLINQFLGENSNTRTDEYGGSIENRARFAIEVTKAIVEEI

GAERTGFRISPGTPLGGIQDGEQGPELYRYLVKELAQLDLAYLHVMHLRDEQLLQDIR

SIWQNPLLVNRSGRTLEELSVDLDRGLADVVPVGVWSLANPDLVERLQQGAPLNEADP

PTFFGLGSKGYTDYPTLEELKSNEGKH"

CDS 39866..40864

/gene="tdh_1"

/locus_tag="EFAGFIKM_00048"

/EC_number="1.1.1.103"

/inference="ab initio prediction:Prodigal:002006"

/inference="protein motif:HAMAP:MF_00627"

/codon_start=1

/transl_table=11

/product="L-threonine 3-dehydrogenase"

/translation="MRAAQIQKYSKKIQVEINNIEIPQIHSREVLVRVKAAGVNPLDI

LNMNGSVRMIADYTLPLTLGNELSGVIEAVGDDVLNFKVGDSVYTRLPLNKIGAFAEY

AAVHEDALSMMPENLSFIEAAAVPLTALTAYQALHDVLRAEPNKKLFIPGGTGGFGAM

AIPIAKSMGLTVITSGSERGRSRTLSIGADQFINYKKENYADILSDIDYVIDTLGAEE

IKAELSILKPQGKLVSLKAGPNYRFAADSHFPMWKKALFGLVGARLDSMARKNQNEYR

FMFVHSSGSQLENITTLIEKNDIKPSIDSTYRFDEIEKALIKVSTGHSQGKVILTF"

CDS 40920..41498

/gene="comR_1"

/locus_tag="EFAGFIKM_00049"

/inference="ab initio prediction:Prodigal:002006"

/inference="similar to AA sequence:UniProtKB:P75952"

/codon_start=1

/transl_table=11

/product="HTH-type transcriptional repressor ComR"

/translation="MARSKEFEESVVLDKAMRLFWEQGYEKTSMRDLVNHMGIHRKSL

YDTFGDKHTLFLKTVDLYDHKISSALAAGVERSKTATEALQFIFCSLIHGEESPASGC

FIVNSTVELAARDKDMNNRSTEMFGNTEKLIKDIMVWGQQEGEFTTEYNAEVLAEYLH

NVSIGLRGMAKTSMTKEKLLRIAMLSMDLILR"

CDS 41588..42370

/locus_tag="EFAGFIKM_00050"

/inference="ab initio prediction:Prodigal:002006"

/codon_start=1

/transl_table=11

/product="hypothetical protein"

/translation="MRKTVLITGVSGGIGKELADRFAKGGHHIVLVARSEGKIQDLAQ

EYRKKYGIQATVFAKDVAAPGVPQEIYHELKERGIVVDYLVNNAGFGLFGTFMETDLE

QEVNMIDVNIKALTVMTKLFLPDMIQRGHGGVMNVASLVGFFPGPMMSVYYATKAYVL

SFTEALENEVSGTGVTVTALCPGLTSTGFVDRSGMGVSKMLQGPIMEAGQVAQEGYQG

FLRGKTLIMPGARNRFIAFMPRLLPRKMMTRMIRSSQDKTGH"

CDS complement(42375..42953)

/gene="olsG_1"

/locus_tag="EFAGFIKM_00051"

/EC_number="2.1.1.344"

/inference="ab initio prediction:Prodigal:002006"

/inference="similar to AA sequence:UniProtKB:L0D9B6"

/codon_start=1

/transl_table=11

/product="Ornithine lipid N-methyltransferase"

/translation="MKTSEPLLFLQGFLRNPKRVGSLLPSSKFLARKIVQSVRWDEVR

SIAELGPGTGAVTRLMRAHLPKSATVFLFERDPKMRSNLKRIYPEFMFHSNASYLLKR

INQEYIHQLDSIICGLPFFNFSREMRQNILSQIHTALRPEGTLVLYQYSLHMKQRLAE

LFEIEKIQFVPLSFPPVFVYVCRKRQDEGHTL"

CDS complement(42988..44088)

/gene="rcsC_1"

/locus_tag="EFAGFIKM_00052"

/EC_number="2.7.13.3"

/inference="ab initio prediction:Prodigal:002006"

/inference="protein motif:HAMAP:MF_00979"

/codon_start=1

/transl_table=11

/product="Sensor histidine kinase RcsC"

/translation="MIKRLPQFKKKIQINILYQMLISLLISFVGSVGVNNMLIIAAAK

ISERFNWPSLLYIFPYVLTPIFIVIFTLIFLFFTRKIVRDLITLEQGLQIISEGNLNY

RVPVNRQDELGRVASNINHMTEQLKQQIAKERELEQSKMDLITGISHDLRTPLTSIIG

YIELLKSESFQDKAEYDRFIQNTYNKATHLKKLLDDLFEYTRLNEVNTQLDLKNVDLC

QLLDQLLFEFEPLAQEHGIRIEKTLGNAPIMVSLDSDKIARAIDNLLMNALKYSFKPG

TIHVRMSVQHNHVTIEVENKGIPLTIEQKNRLFDRFYKVDYSRNSEGIQSGSGLGLSI

ARNIADLHQGTLTLKHTRNVFIFQLSLPSNIQ"

CDS complement(44081..44761)

/gene="srrA_1"

/locus_tag="EFAGFIKM_00053"

/inference="ab initio prediction:Prodigal:002006"

/inference="similar to AA sequence:UniProtKB:Q9L524"

/codon_start=1

/transl_table=11

/product="Transcriptional regulatory protein SrrA"

/db_xref="COG:COG0745"

/translation="MNTVLVVDDEPDIRDVIHVYLRNEGYQVIEAANGEEALNIIKTT

SVQLVILDVMMPIMDGIKACFKIREVSSTPIIMLSAKEEDIDKITGLTTGADDYMVKP

FNPLELLARVKAQLRRQTLIGKPESNSLILIKDLVIDTSKHSVKLKDNDISLTPLEFS

ILVLLASHPGQVFSSEKIYETVWKEPYGYSDNTVMVHIRNLREKLELNPREPQYIKTV

WGVGYKID"

CDS complement(44819..45385)

/gene="olsG_2"

/locus_tag="EFAGFIKM_00054"

/EC_number="2.1.1.344"

/inference="ab initio prediction:Prodigal:002006"

/inference="similar to AA sequence:UniProtKB:L0D9B6"

/codon_start=1

/transl_table=11

/product="Ornithine lipid N-methyltransferase"

/translation="MNSNEPILFLKSFLQSPKHVGSIIPSSRFLASKMVKQASWLEAK

AVAELGSGTGPITRYIHQQVQDSTKVLLFEMNETMRNTLKTAYQEFSCYPDAARLVES

MNQEGVQQLDYIFSGLPFFNFEPELRNTLVDQIYKALKPGGLFIAFQYSLQMKKTLSE

HFIIEKIELVPLNIPPAFVYVCRKKETI"

CDS complement(45430..45933)

/locus_tag="EFAGFIKM_00055"

/inference="ab initio prediction:Prodigal:002006"

/codon_start=1

/transl_table=11

/product="hypothetical protein"

/translation="MISNTILELLHQYGYLIFYFAFSLGPFGIPIPNEITIISGAILS

HTGVINSWITYFCILSGLLTAITFAYFAGKLFGSKIRHKFQHHKHFVKAELILNKSGN

WAMCIGLFIPIVRYVLPLLIGMSGVQYRKFALISYSSALLWTITYFTAGTYFGGPILS

TLQLFHF"

CDS 46798..47385

/gene="sigW_1"

/locus_tag="EFAGFIKM_00056"

/inference="ab initio prediction:Prodigal:002006"

/inference="similar to AA sequence:UniProtKB:Q45585"

/codon_start=1

/transl_table=11

/product="ECF RNA polymerase sigma factor SigW"

/db_xref="COG:COG1595"

/translation="MSEQDEYIELVTLIRTGHEEAYGELYEKTVTRIYQTVRFLIKDK

SDAEDVVQEIYIQAYRSLARYDAERAFRPWLMGVTMRQVQSYRRKRLMQFRFGKRIEK

SDVGMEYDFSTDLINKLANRPLLEQVHRLPYKLQQVVTLHYLNEYTQEEIAGILEIPL

GTVKSRIHAALAKLRQKEKMNPRLRGKAEDLHETR"

CDS 47372..48121

/locus_tag="EFAGFIKM_00057"

/inference="ab initio prediction:Prodigal:002006"

/codon_start=1

/transl_table=11

/product="hypothetical protein"

/translation="MKLDEQLRTAYQEETKDWSVPARIKHKMMDGIRSDSHIRKNRKK

WLVTGILAAVLIIPTGAYAGYTYLADGIYGSQENITAMGGTAEDYMRLEAKLQTAKAH

FSEEEFVKYMDLLKQLGQMAVKYADSQGNMHPEQWNTVEQERYNLLVAELEPFFEKLE

AVSVGSSKKLMDEQQFWTEQLEQAEKTFTKEQYLEFKSVYEQMKKYKVMVMDKDGSIQ

EERLSAEQKDDLRQLERRLIPYLKRLGLDVR"

CDS complement(48196..49071)

/gene="rhaR_1"

/locus_tag="EFAGFIKM_00058"

/inference="ab initio prediction:Prodigal:002006"

/inference="protein motif:HAMAP:MF_01533"

/codon_start=1

/transl_table=11

/product="HTH-type transcriptional activator RhaR"

/translation="MKSIFLGEQFNLVCRRTSNTTSREVFHAHSQLEITYIHEGYGQL

ITEGQVFSLEPGMLMIFRPFQLHHIQIQVSRQQPFIRNVLMVELDLLKAHWPQFIVTH

DFIQDLLEEQSPIQPIRLAATSPLVQRMEQYADTYPGLLPHEIEEDTLLFLLDLLAQL

RYLWQDGQQRCSEDVFSSSANVLHPHAEAIMQWIEQHYQEPFRLEHIADTLHLSPYHL

SHVFKKATGTTIVAYAQATRIRHACVLLTRSSLTVPEIGHRVGMTSPSYFCKVFRTTT

GSTPHQYRLKVQGRK"

CDS 49180..50337

/gene="gfo_1"

/locus_tag="EFAGFIKM_00059"

/EC_number="1.1.99.28"

/inference="ab initio prediction:Prodigal:002006"

/inference="similar to AA sequence:UniProtKB:Q07982"

/codon_start=1

/transl_table=11

/product="Glucose--fructose oxidoreductase"

/translation="MLKVAIIGAGAISGAHISAYLAFPERCQIVAVVDIYVEKAQKRI

NEYGLEGAQAVMDYTDLLAQNIDLVSVCTPPYTHAPIACDFMQAGAHVLVEKPMASSL

EEADLMLRAAQASGKLLSVVAQNRFTTPMMKLKGVLDSKRMGPIVHVQVDSFWWRGHN

YYDLWWRGTWEKEGGGCTLNHAVHHIDAMLWMMGPPVELQAMMANTAHDNAEVEDISM

AMLRFQEGELGMITSSVVHHGEEQQLIFQGKEARVSAPWKVVASTARNNGFPEPNREL

EHQIQKLADELPDVTHVGHAGQVENVLNAIETGSPLLVDGQSGRNTLELIVGIYKSAS

TGEKVVFPLGAEDAFYTREGIMQHAVHFYEKKTTVDNFEDSSITLGRKLDF"

CDS 50949..51881

/locus_tag="EFAGFIKM_00060"

/inference="ab initio prediction:Prodigal:002006"

/codon_start=1

/transl_table=11

/product="hypothetical protein"

/translation="MLFANAGMALMPHKLMSAYMFFISLYAAGMIIAQIGIFRLYYNK

RTDKLYVHLGGTAVTLITAAMSLFLPVQMSSFLVFLSVVGVTLYTLIKIFPDVGLNPQ

YFMVTILFAMQLLLHLSGAFTGYELLAELSLILSVFVSFIVFTMIFEKIIGVMQAATY

TSTRDEVTGLFTQKHFIHQANQALSKGKTVGVLYIEIQNPTHISEEESFKRAGRAVGR

ALEGSGFAGRYQSDVIVALITSSNLPLAELANSIRMKIDVEASSTVLIGYIDTKPEFT

LDDHIREAKEGAIRTKQSGLNRIFDLDQSKILAD"

CDS 51896..53155

/locus_tag="EFAGFIKM_00061"

/inference="ab initio prediction:Prodigal:002006"

/codon_start=1

/transl_table=11

/product="hypothetical protein"

/translation="MNAVIISSKASTLVHEAIGNTDVSFEKVGRFESGQFIEFCEAVA

RININAFITDLDCTDSNTFLRGISQYRTVRSNTRIIVIALDRYEDDGVVHELADMGIN

VVTTSPGTKPKSIIPALRNLLPNQSEVTGNGQEDWESTDSDLEKIKDRIYRYTAKLTS

AGSFDSSDVMDLELPDVPIQERIILKDRIIGTILVGVMGVESKVGSTHQSILIANYLK

RKGYSVGLVEANSSSDFTSIENAYEGTQDFKSNNIHFSIEGVEYYKNNNKLDINHLIT

AGHDYLILDIGSFEESDWSKEFYRASVNIVLGAGSEWRQGKIRRFRDLHKKMDQSNWV

YCVPFVENLSIQDIRKDLPGNLVYRIPPHADPYKHQGDTDSVLNKLMKSYMGDSKKIS

SKALLYGVLGLCIVIIIVLIILLVVNN"

CDS 53188..54222

/locus_tag="EFAGFIKM_00062"

/inference="ab initio prediction:Prodigal:002006"

/codon_start=1

/transl_table=11

/product="hypothetical protein"

/translation="MSKIRFRQKQLLLSASIGGAIILFVCVIAGYFIIDYIQGRYVER
TLQIEQQLQDAQKKLNEDKMKVIVLNDDHKAGDLITEDDVSSLVVPVSTVPQNTVEQA
EIVGKRIKIDVQKNSIITTPMLFEDGITPNDLRNQEFSLVQLPLKLKKDEFVDVRIRF
ATGQDYIVLSKKKVEDLNNGTIWYKMTEKEILTMSSAIVDAYINDASIYALSYVDPYM
QDGAIINYPSNPKVLDLIESNPNIVGLATTALERQVRAKLEQDLQSMSDEDRQKYISG
RNTTEMNSREYITETEGQGNGTTSQQDNSLMNENSTDMNQQSNTGNTPNATSDSGSIF
KDSNTREPAQ"

CDS 54237..54920

/locus_tag="EFAGFIKM_00063"

/inference="ab initio prediction:Prodigal:002006"

/codon_start=1

/transl_table=11

/product="hypothetical protein"

/translation="MKQILVMGHYDKTDFMMYLGKLLSMDHRVLIVDATQNKDYEFVF
PKIDHSLTMNTHDQFDVMEDIHSYEDLMHNLEEQKYDFVLIDCNDEASLVDWPEVDFV
YVVTSYENPVIQKNVNLIRAYFQGEKLSSVNLPVTKVIYEVSNHLDEKYMDSLLDELP
LSWKESLVYYPDERDQTLKIINQYKNTVMVKRMSSSYKLVIKQAIEFILGLETSKVNA
LWKRAERSN"

CDS 54922..55761

/locus_tag="EFAGFIKM_00064"

/inference="ab initio prediction:Prodigal:002006"

/codon_start=1

/transl_table=11

/product="hypothetical protein"

/translation="MGKIALWGMQHGLGVTSATAALAAFIGLEYDVRTLVSQPQRTDG
DMERFFNKTINQYNRQFFAIGGLGLDSLERVAKSSKLERNSVKNYTLMVERNRLDLLW
GSERLNRPQQGSIEAVNMVFRMAAEYYGISLLDARSGQDNEVSNSLIRDADLVVICVN
QNISVLEKYFDNQRKHWPEVLEEKPHLLLMTQYDPMSKYKIKNIANKYKLKAPILTIP
YNTSFKDHLNDGDIKGFFARNRFVGSSHENHYFIQEVGRAAKTILHEIGINTKLKRIE
RGN"

CDS 55763..57670

/locus_tag="EFAGFIKM_00065"

/inference="ab initio prediction:Prodigal:002006"

/codon_start=1

/transl_table=11

/product="hypothetical protein"

/translation="MGLNFTINFLAIILIVAVLIAYLFLKLNRKPGQVGTRQKNENEE

RFQFDYLLEYVKTMINEYVNSNLLDMGMTSEEYNRRLGVVAELRSAMKNATSGDIQEK

IYLKQFISDLLERNYGFNDDNIDRIIPFEDEDQLTQQDKFDILLHLYKKKHGFQGLSK

LIDKYQLDQPKKVIEDGTVDSYVITGDEIDSIFVKEARGSLEFDDKLAIVVQRVYQHY

KGFGVIDEIRDMSIDGVSGGVSGVPTNMQIVEDEISLLQGMKQYKYPGYQSVWIFFRG

KSIHLSFLSFETDLELKRVCQNIYKYDNPGQLTETNGFKVNEMKDGSRVVVVRPPFAE

SWAFFVRKFDLPNMKLDHLISSKDAVNAELARDMIKYLMKGAQVTAFTGPQGSGKTSL

VMASVKYIYATLTLRIQEMTFELHLRRLYPQRNSLALRETENIAGQAGLDLQKKTDGS

VNILGEVATDPVAAWMIQMSQVASLFTIFTHHATSARELVWSLRNSLLKTGVFQNERI

AEEQVANAVQWDVHMRKEMNGKRYIERITEITPVRFESDTLEHEEVLSKGDSIESKLD

ALVNLQREVYRRNSGRVWNARNIIEYRNGEYVAVSPISPEKIEKMLFNMNEEDRENFG

QFLNAHWGQAQ"

CDS 57667..59739

/locus_tag="EFAGFIKM_00066"

/inference="ab initio prediction:Prodigal:002006"

/codon_start=1

/transl_table=11

/product="hypothetical protein"

/translation="MNKWIWIFAIAGGALLISGGILLYLILKDRKRKKNRENVWPSSK

NVTGKTPRQLLVFYQKSYHQLIKVPIVKSQILKIRKRLTAINTYDEYSLRKETMRITY

LTLGSIIIGLIVLAIISKSWMALFFGALAAIVINELCIDIFVNRVEDRLLKQFSNVLE

DDRHHYQEVRMVDEALYQAAQSSPHDIKLQTERIHEVLTSKDPARELEKYYAVAPNRY

LKVFSGVSHLIMEYGDRNVQKGSMYLNAINKFVQEIRYDLMRRQKLRYKLGGLTLLAL

IPILLSFPLQRWAENYFPITTEFYDSRIGFLVRMFIYASAVICYLLIRKLSENDEAKY

VAKGPRKEWEKKAYSWSWVKWIVDRLVPSPNTRKHHRTNMLLKEANSHLTLEWLYIHR

IVISVSAFIGVIVFSVFLHYNSVHHIFSKPTSEATNILGYMNADELERANQVTTMDNQ

IIEDLRTNKDLTREQVVQSIETHSNEPLTDVAMNNTVNRIIGKVTVVNNEYLKWWELL

IAIGFGVTGYYITIWILQFQRRLRALEMQNEVDQFHVLISILCEFERISVETVLEWME

RYSIIFRPALQKCLLNYDSGAEEALKMLKSDAPFDSFGRIVKRLMQAVDKLSLREAFD

DLEMEQEYYAEQKKERLERLIRKKTSYGKLFGWTPGALLIALYLVFPFLYMSFKQLSD

ISTQLQTI"

CDS 59758..60243

/locus_tag="EFAGFIKM_00067"

/inference="ab initio prediction:Prodigal:002006"

/codon_start=1

/transl_table=11

/product="hypothetical protein"

/translation="MEENTGVGIRVAAGIFLTIMLITIVVIITISSQDAAKQGQTKMA

AITTQLSDTEYQTYSNTTLSGSQVLNAVRQYMNQEQFGVQVITGKQRLSGDADKRKGV

FYGNTFDAYGEVSEKSKNNSLTNAEKQSEDAYINPSGKFKSSIVRDKNNMIRGIIFEQ

E"

CDS 60277..60657

/locus_tag="EFAGFIKM_00068"

/inference="ab initio prediction:Prodigal:002006"

/codon_start=1

/transl_table=11

/product="hypothetical protein"

/translation="MSETVEKFILVAAEISILIVACMFALGGIQSQDAAVNITHKSAV

NEERRLFTSLEITEQVTYTGAEVLQSIRQMKTLDANIRVVNKYFYSSDDIESIDVSGV

DLNRTYVVSYIRDSQGKVQTIVFT"

CDS 60671..61219

/locus_tag="EFAGFIKM_00069"

/inference="ab initio prediction:Prodigal:002006"

/codon_start=1

/transl_table=11

/product="hypothetical protein"

/translation="MENSFSKILGIFIAIVLLFLYPLFQQAQRQDDLSQIVIHSAVTT

FVDSARTKGYITPEMYLEFNQKLGATGNQYDIQMEHLHKKYNPEYTDPANPATFRDSF

TTYFDGHYNQEIMQRLFPNSPASDLNRRYVMAVGDFFTVKVKNVNRTMATILSDVFTG

GITDGNVKVYVPYGGMVINEDY"

CDS 61206..62189

/locus_tag="EFAGFIKM_00070"

/inference="ab initio prediction:Prodigal:002006"

/codon_start=1

/transl_table=11

/product="hypothetical protein"

/translation="MKITNLAILFICIFVPFYLVMDFRTGDQKTAQALSDQYSASLHT

AVQDASQMLNMNVLQEYEAGYQSRKFFFANKERALDTFFRTLYLNFDVVNDPVRQGAL

AGYIPAVAVIDYDGYDLYAVDEYRDANSERVFKHMWRPKKPYSYSDDRGNSINFTLDS

YVYAYDSYAKAWVEGFREDLEGTTNIPLLDNAANFEAMRKSVIVKSIQQDLAYYINKH

NEYATRYGVHYTFSLPQISQEEWINSIDDIGIMAFIQGIPIGDQFYNNYALGGGRLVK

KTEIKGAVDLTTGIKYYYPSTCSYGYREDETFSSERDAAAAGYYPKGCMNR"

CDS 62221..66225

/gene="essC_1"

/locus_tag="EFAGFIKM_00071"

/inference="ab initio prediction:Prodigal:002006"

/inference="similar to AA sequence:UniProtKB:Q2G184"

/codon_start=1

/transl_table=11

/product="Type VII secretion system protein EssC"

/db_xref="COG:COG1674"

/translation="MSHFFYKRSPRIIKETSGEEVEIFSPPSLPQPPQLNIISIMVPI

MVTVAGGAAMMTFYQSRGNGQFVTVQMISLCMMVVSYFVPVLVHFQNKAKHRKKIKDR

ERLYDNHINENRETLQNWKNELILNWHQTHLDPQFCINMVKERSSSVWERIPQDFDFL

KVRVGIGTVPSNFDITIPKQNGVEKDPMLEKAREMTEKFTTITNAPALLDLSKYRIIG

LVGDEEELNMFCRTVVTQLAAHHAPDELKMAVFMNKIQRENWDWMRWIPHVWNDTRSG

RYLFEERGYQPQLMEQLFTMLQRRQWLNKGEKALPFYVCFLPFIEMLEHEPILPLMMK

SSEVIGVCSIVLAPSKDVLPKECELVIDLHSAEGVMRSTNVTGGAAGDATYAKPKDEI

PFVQPFKSDQMTLEQADQFARQIAPYRIKRNSADEIVNVLTLFELFGIQNIEQFHVEN

NWQKSRYPNTLPFPVGVRGGKKPVLLNLHDKIERKGHGPHGLMAGTTGSGKSEVIQSI

IASLAVEYHPHDMAFMLIDYKGGGMSNTFQELPHVIATVTNLEEEGLIERSKVSLKAE

LKRRQRLFIAAGNVQHIDEYYLTEWKDRDPLPHLFIVIDEFAQLKKDQPEFMSELVSI

AAIGRTLGVHLLLATQKPGGVVDDKIWSNSRYKVCLRVQDESDSREMIRIPDAAHITN

PGRGFLQVGSNEVFESVQFAWSGAPYRPDQSITQSDRNMYAYELDGRKTKLRDQLELI

QETKPSEEKTLSKKQLNVLIEHIAMKAEREGIQRLPGPWLEPLPNHLTLEDLVISSSE

SGKILNPQVGLIDDVVNQRQFPLRIDLESGHWLIYGMPGTGKTTFIQTLLYSLAMETT

PNDVNIYALDFGRMLKDYTLLPHVADVIQDDQEEKMNRLFNYLEETISKRKTLFAETG

VKSRLSYCSETGNTLPAILVIIDSYVSFRGQFEKLHEKLDPLLREGPGLGIYFVATIN

RISDMLERIRSNFPNVVSYMLADAGDYNYAVGRLTKAPTQLPEGRAFVKGAIPPYEFQ

TALSINAVNESSRAKLLREQFAELDATCTTAKPESIRVLPEVIAIPEVLKDINIVSDP

TLPVHMSIDSLRMLGWDMEADGPYFIVAGRMESGKTSLLVTIGLISAMKYSTDELELY

LCDFRRTPQGLGALKELNHTVAFATNEATMEEMIENLRTKLEERVADPDEFAPKMVLM

MDDVDFLAKRISRTITGHLEYITRNGREHGVYIIVSGQANAINRNYDEWMKEIKSGQI

GWLLGTAESTDTELFNIKVPFVSGSRVLPDGEGYYIKRKFVSVKAVHAFANGVAQVEE

QVEEINRNAVLVVN"

CDS 66354..66623

/locus_tag="EFAGFIKM_00072"

/inference="ab initio prediction:Prodigal:002006"

/codon_start=1

/transl_table=11

/product="hypothetical protein"

/translation="MAKIQITPEEMDNAASKFDQGAQESEQLAQQLNSLMQSLQGGWE

GMTSQGFYNRYESDKKNMTVYVEMLRAVGTELKSIADRFRQADQQ"

CDS 66698..67447

/locus_tag="EFAGFIKM_00073"

/inference="ab initio prediction:Prodigal:002006"

/codon_start=1

/transl_table=11

/product="hypothetical protein"

/translation="MSYSVHATGSTPALLIYLIDISGSMTLELEGQRRMDIVNEGLQT

IIRQIVYRSTKGSKVSPRYRIAILAYSDEVYDLLDGVKSIDRIANRGSLPPLYPKRFT

DTAKAFKQAEKIIQSELRNMEHCPAPLICHMTDGIYTGEDPEPIVERIKTMSVPDGQV

LVENIFITEDASTSRIRDTRAWKGMMPDSFVVDPYSEKLKRMSSVVPESYREMMMESG

YSIQKGALMMLPGTSKDLISLGLQMSASTPM"

CDS 67466..67747

/locus_tag="EFAGFIKM_00074"

/inference="ab initio prediction:Prodigal:002006"

/codon_start=1

/transl_table=11

/product="hypothetical protein"

/translation="MDRTLLTLIIDATGKKTDIELPNTIPLHELLEPMLKGLNEGMAL

TFQASNYRYYLSSDGSEWNSMDLQQSLEDVKAMDGSYLRIEQVNSFSTI"

CDS 67786..69144

/locus_tag="EFAGFIKM_00075"

/inference="ab initio prediction:Prodigal:002006"

/codon_start=1

/transl_table=11

/product="hypothetical protein"

/translation="MFPPDINTELRIRNQVYRLVQHPQFEGMPYGQEGREGTVYKLIN

SSGENSALKVFRPAFRQPSVMKLTELMAGLTVHSGLSVWSRTILNPIEDMELLGHYPE

LLYGAHMPWIDGVTWSELMLGREALSREQSMKLAMVLVSTMAVLEQAGIAHADLSASN

LLIVNSNQEAEDMSVELVDVEGMYAKQLEEPDNLPEGATGYTATFLREHGDWSPLADR

FAGGILLAEMLAWSSTDICKQAWGDSYFDPQEIQQHSERYVLLQATLEQQYGPEVAAL

LDRLWNAEQLQQCPTFGEWQIALASAKQFVEQEAISTSEMEIELEQPAEESIHLPSEY

AVNALSHAQENQLARARQLERQGNLTSAFWEYGKLIEQFNDRSPFHVEISIAMEQVQE

GIDQTVFNPEPKHTLVKKGWNQLIQQNKQSTYDRMWIYVIGASVGALVFALIIYVIGL

FK"

CDS 69166..69975

/locus_tag="EFAGFIKM_00076"

/inference="ab initio prediction:Prodigal:002006"

/codon_start=1

/transl_table=11

/product="hypothetical protein"

/translation="MENELTEEWSFQLSPEEFFFLTQLVGVRHIANFDDPYRGYLAKE

LEAEYKVIEKTFVEKGYLVPKQEGTGYDMNEMLAYCIAACGSADVMHVSKKIEGTGKF

AGHYYFTPRIVVEQTADELGGIVLVPVADAQLTLEAMKGFFPLTLQYKSAAKKAVIKD

CTWDQWNQLTEVDRLNRLVQAGHTEEQAELALDIFSNGERSGSMMFMKRHGLMWHQED

YHYAQWGDKLYLAVELTEGTLQFQPYKPAILKKALLRFADQIEKLDGKGVK"

CDS 69976..70251

/locus_tag="EFAGFIKM_00077"

/inference="ab initio prediction:Prodigal:002006"

/codon_start=1

/transl_table=11

/product="hypothetical protein"

/translation="MADRIKIEPERIKEIATRFLASSQESIALSRELKSLIDGITGEW

EGKSQERFYSSYKDAHVQLESVSTVLKDVGDELKAISERFSKADSIK"

CDS 70264..70623

/locus_tag="EFAGFIKM_00078"

/inference="ab initio prediction:Prodigal:002006"

/codon_start=1

/transl_table=11

/product="hypothetical protein"

/translation="MPIRPDVGKLRSIASKLRSNSNKLENERSSINSNVQSMIWKGRV

YQHFMDDFRDTTQRMRQTADEMEQFARRLEALANQFMQEDLEEERRERERQERERQER

ERQRAAAAAAAAAAKKK"

CDS 70636..75453

/locus_tag="EFAGFIKM_00079"

/inference="ab initio prediction:Prodigal:002006"

/codon_start=1

/transl_table=11

/product="hypothetical protein"

/translation="MKLEVSLSDLKRTSKSLNGAADEMGDLRVDLNRAMNSLSYKTLS

QGGVQSTFTHLMRELDGLQSKLELLSELTRRKREEYEQADREGKKFNWGKLASFLTFA

LGTVLDFLPVVGNVKGIIEAITGRDLLTGEKLAWYERGLSVLGPLGKGANKAASLFKY

ADEAMDLTGKAVKHVDDAADAAKTVGRNADEAIDAIPTSPNKAGVPGNSTREAGEDIA

GATPKSTKAGEAPAGSRDTGDAPMTSNKPTGKADDVPSDAGSTRHDTGLTDAEKAAAA

AGTAAGTGSAAAASMIKKTPGGSAPKGVDAPGAPKSKDVNSSPHQPSTGRTTETQNPQ

KSHAESKVEDPIHAATGHQFMNHRLLTLHGAADWPFTLIYHSGLLQRGELGPAWTHQY

GLRLDIIPKDSQTEVEASDAFQPLVDVCWTPGRKNRFTHQGDGLYRSDDLDVNHDELI

YTEQCWLYTARSPREMYQFDLNGVLLRHTNKDGLSLDLVHDEEGKLQQLQDRVMGRVL

TLDYTAEGNLAAVRDALREATFGYDSAGFLVRLTEPDGTITEMTHDGEGRLMSLSEAG

VVQFTNTYDEKNRIIAQTDPSGAAGRMAYDTESHPGQTWTTVTNRLGQTRTLVHDDAL

QLLEVHAEDGGITRYTYTPQGQMESVTNPLGETTTYVYDEEGHLIREIDPLGHETEYH

YTAHHQVARETDAEGGVTRYQYDEQQRLAEVSRPDGSTAKWTYTETGQTATYEDFTGA

IWTYAYSETGELQHTLDPEGRRLQIGWDEAGRLVELTDAAGSVSRRSYDGADRLTSIT

DPLGLMWQTVYDASGQRIRETRPSGASMQYTYTPNGEVASITDALGHTRQYTYDAENR

LITETNALGEATQLSYDTAGRLQTVTDALGHVTRYYYDGAGRLVSVIDGEGRTVQQLT

YDEAGRPVAWQDGLGHMTRLRFNALHQRVEDTDADGHTRHYRYDAAHRLTEVIQGEGA

DEVTYRQTWDGEDRNTSYTDASGNETRLTYDRSGRLLQETNAAGARTAYTYDDRGWLS

AKLDAKGQETRYAYDAAGRLIRATDEAGEIVLTYTPDGQVARVLEAEQHEERTYDAAG

RLLTRTDGFGHLLQYAYDAAGRMTAMTYPDGKVVQYRYQATGELAEVKDWNGRLTRYR

YDASGKLIETRRPNGSQEQREYDAAGQLTRLTDTSGQGIRLQQFKYEYSPSGLLLKEE

NKQYTYDTLKRLRSGAEPGRVVHYTYDPSGNLTTEQMGTSETEYTSTSPMQHLHYTWD

NRLQRVGDYPVEIDANGNLLYATDGSTASAYEYDARNRLVKAGKLKYRYNPQGDRIEL

AQRGQLTRYVIDDAHELSRVLMELDGEGHVKTRYIYGLGLIGCEDADGTYLSYHYDLR

GSTMLLTDEQNRVTDRYTYGLYGELEQHEGVTQQPFAYNGRDGVMTDANGLYYMRARY

YDPKLKRFLNRDVIRGEIQDGQTFNRYAYVNGNPVSYIDPLGLMKCETADPYGGYYER

KAQRTNDYNNKTGDWTPSAKTTPPNGPYRTDKEAEVAANILGFEKTTGGRAKAAVFYS

KEKGVYITRDIPRQSTGSTDNGGVWKVAKTIEDLMSRETRSGTYDKNLNRIGD"

CDS 75456..76013

/locus_tag="EFAGFIKM_00080"

/inference="ab initio prediction:Prodigal:002006"

/codon_start=1

/transl_table=11

/product="hypothetical protein"

/translation="MDYWQVRKYPPIKKGEEKKSKIPTWTSIIDIGQYYEGKEFTAED

YLFTEDKYVEAVKIYTSSIQVHELEIYLINKLKEELFLGHTLKYPECYSAEIISLYYS

LVDGAILSGKQIEHIVRLGMREDITCQLRCGNEFFLNFTYDYYMDIGSSIICEDAINQ

IKETGLYIDLWEWPYQDNVWDPSPN"

CDS 76963..77499

/gene="syd"

/locus_tag="EFAGFIKM_00081"

/inference="ab initio prediction:Prodigal:002006"

/inference="protein motif:HAMAP:MF_01104"

/codon_start=1

/transl_table=11

/product="Protein Syd"

/translation="MSIKEAMNQYFLLRKSLAEGGLDFLFKTPFDEDVDSLIYSGKVD

EDDYISWRPVEKKEIYDFSSLEEKLGIRMHVSVIEYFNSYWFADLDGFITDHYIKLES

VLQTTDIESYFNLLVGYKRNHDNKLEHIPIGIEGNGLIVVVDNQNGLVKLEDFERKSF

EVISEGIEQLIVTLRLQK"

CDS 78339..78641

/locus_tag="EFAGFIKM_00082"

/inference="ab initio prediction:Prodigal:002006"

/codon_start=1

/transl_table=11

/product="hypothetical protein"

/translation="MKTILEALYRGQINPDGVIVPSQPEYRSISRQVAAQTEQWRERL

GEEVFRELEEYFDLCDSADSMHVEAAFLHGFRLGANLEVMGNKEALVPDATLGMPL"

CDS 78616..78765

/locus_tag="EFAGFIKM_00083"

/inference="ab initio prediction:Prodigal:002006"

/codon_start=1

/transl_table=11

/product="hypothetical protein"

/translation="MQLWACHYDDNNDILTVVDSDGIIYRYPCYEIGNSIEMHPAARP

CLRWM"

CDS 79502..80758

/locus_tag="EFAGFIKM_00084"

/inference="ab initio prediction:Prodigal:002006"

/codon_start=1

/transl_table=11

/product="hypothetical protein"

/translation="MKKLTQLILSGALVIGIFPFTGNTSQAAVVSFKDVPTTHWAKAS

IDAAVEKGYFKGYSDGTFKPGATVTRAEFAALLARVAKGTPDTEQTNVFKDLTGHWSE

AEVNRAVSFGFLKPSDYPNGFKPGTALTREEMAKWLSSGLAAQDEDYKQALKDTENTL

VPVAEYYKGGLKKAAYPYVSVVLGTGLMAGYPDGTFGPGKTTTRAEAAVILSRYEQVQ

KSKATSYKDLNEMREVGLTGTNLTSATPYVYGLKAGTNIVNSFDKVKEKPVQLRNNLG

VATLHQMIVVDALSKTDINNLYGKMFIDKEYSYPVLNDYYTVFIELSVTPSGDNLKNL

TFSQAIADKLTPGNNFSSGTVGQYGIVNLPERDFITSGFFKKGVTRKFWMMTMLNRKH

GGPAGSNGKTIVIGEDIASFHLPIKF"

CDS 80771..84265

/locus_tag="EFAGFIKM_00085"

/inference="ab initio prediction:Prodigal:002006"

/codon_start=1

/transl_table=11

/product="hypothetical protein"

/translation="MSLMKGMYKVSILFCCWILVFQPVTVFAADAESQRVVAVSSDAE

DQEDFNLLPKPVASPDGPTGYQKVVNIVEGTKQATPGVYWFQVDNGDWVAEASNDRIV

ADNQGTRADPAPVVTEVPDQIEMSGKLEYLPNTYTDQYGNSVNQSKIIDPHIAKVQST

ELQGIKSVIKSLQLTSSKTGVKIVSKTGRLNSELYKAEYNDKYGENTDHEPKHKTLYH

TPLDITWRGKLVEQKKIRITGTQRLAINEEASLKGEVATMNSGDTDYGNWIDISTRSG

VVWKSDQQGIVDVNSSGKIKGVSPGTAKISVRWKSGDPYQYDLIAYLNVTVGDGPTQP

EPGGVAGCTYTINPPNEVASPQTAFMDPGAQGHILADDPTNGILFDATRGIPTSEHLY

ANTWAMNYLFQHTFGKQQGTITYDCTADITYVRTWKVEQPDLPGPNGTSIPQEDTEDS

DEVPVQYRFSFDRNYSYWKINNVELYSIEQAEMENYALPDGSRIILRPQGYAPPDLEM

EHDEDVEIHVMPKETGEISYVPEVLATEGYEPLDVPDDTELLKGMAEDQTGPPDVKND

ALDFTWEGSTTNVMDGSTVVENGPDPTQIPAPTKIGSYKNTGEQVLYEDRLLIPRTLL

NKADTESTGEIQYALHPTTLGGSGGKTFPVDPINTVTVHTPVVNYSLVSDDQPHNQKT

VPNMSRSALILERPFTVRIPTSGQHLDARSYPGYGDRDYAKYYRIKQVRFPFDVYSAD

RTQFYPRNTWIDVQVPVLDTTFYLPVWVDEGDYQVEFRNIAENAPSHFSTEPEIDAQP

DANTDLYYHAASDEVSVEVIGRLYDFEITDIADYNWELVFRRFKNSLAPTWISYWTGT

QDIDGDKRGNKPQFTVPIRPGSHPLQGYQNVAVKTGYHFKFDFKTKGNMFGPRDGIRL

TPTFDFVSKDGRTRVPVDLYYSTNQRNFIRIGSTEDQVKRFVILNDRLRQVPSEQLRD

TATYKYNRYGEIHPGMMSERAYQEYYRDKFTKMKTPVGGYSLLLMPEQLRTFIGPKTN
IPTTASADVLRANAAIQQWYGEYSLPAEPYVVQAGTNLAEYGRTHGGLDAKSPIFLKD
GYIVVNFNFESIREGNLAAPHLQYIHAPLMNQWLLEGFQREVEDSYGNSFTLRDGDVV
FYHADRSSRDDFSAQVPH"

CDS 84450..89357

/locus_tag="EFAGFIKM_00086"

/inference="ab initio prediction:Prodigal:002006"

/codon_start=1

/transl_table=11

/product="hypothetical protein"

/translation="MKLEVSLSDLKRTSKSLNGAADEMGDLRTDLNRSMNSLSYKTLS
QGGVQSTFTHLMRELDGLQSKLELLSELTRRKREEYEQADREGKKFNWGKLASFLTFA
LGTVLDFLPVVGNVKGIIEAITGRDLLTGEKLAWYERGLAVLGPLGKGANKAASLFKY
ADEALELTGKTVKHADDAADAAKTVGRNADEAIDAIPTSPNKAGVPGNSTREAGEDIA
GATPKSTKAGEAPAGSRDTGDAPMTSNKPTGKADDAPSDAGSTRHDTGLTDAEKAAAA
AGTAAGTGSAAAASMIKKTPGGSAPKGVDAPGAPKSKDVNSSPHQPSTGRTTETQNPQ
KSHAESKVEDPIHAATGHQFMNHRLLTLHGAADWPFTLIYHSGLLQRGELGPAWTHQY
GLRLDITPKDSQTEVEASDAFQPLVDVCWTPGRKNRFTHQGDGLYRSDDLDVNHDELS
YTEQGWLYTARSPREMYQFDLNGVLLRHTNKDGLSLDLVYDEEGKLQQLQDRVMGRVL
TLDYTAEGNLAAVRDALREATFGYDSAGFLVRLTEPDGTITEMTHDGEGRLMSLSEAG
VVQFTNTYDEKNRIIAQTDPSGVAGRMAYDTESHPGQTWTTVTNRLGQTRTLVHDDAL
QLLEVHAEDGGITRYTYMLQGQMASVTNPLGETTTYVYDEEGHLIREIDPLGHETEYH
YTAHHQVARETDAEGGVTRYQYDEQQRLTEVTRPDGSIAKWTYTETGQTATYQDFTGA
IWTYAYSETGELQHTLDPEGRRLQIGWDGAGRLIELKDAAGSVSRRSYDGADRLTSVT
DPLGLTWQTVYDASGQRIRETRPSGASMQYTYTPNGEVASITDALGHTRQYTYDAENR
LIAETNALGETTQLSYEAAGRLQTVTDALGHITKYHYDGAGRLVSVIDGEGRTVQQLT
YDDAGRPVAWQDGLGHMTRLRFNALHQRVEDTDADGHTRHYRYDAAHRLTEVIQGEGV
DEVTYRQTWDGEDRHTSYTDASGNETRLTYDRSGRLLQETNAAGARTAYTYDERGWLS
AKLDAKGQETRYAYDAAGRLIRATDEAGEIVLTYTPDGQVARVLEAEQHEERTYDAAG
RVLTRTDGFGHLLQYAYDAAGRMTALTYPDGKVVQYRYQATGELAEVKDWNGRLTRYR
YDASGKLIDTRRPNGSQEQREYDAAGQLTRLTDTSGQGIRLQQFKYAYSPSGLLLKEE
NKQYTYDTLKRLRSGAEPGRIVHYTYDPSGNLTTEQVGTSETEFASTSPTQHLHYTWD

NRLQRVGDYPVEIDANGNLLYATDGNTASAYEYDARNRLVKAGKLKYRYNPQGDRIEL
AQRGQVTRYVIDDAHELSRVLMEMDGEGNVKSRYIYGLGLIGCEDADGTYLSYHYDLR
GSTTLLTDEQNRVTDRYTYGLYGELEQHEGVTQQPFAYNGRDGVMTDANGLYYMRARY
YDPKLKRFLNRDVIRGEIQDGQTFNRYAYVNGNPVSYIDPLGLMKCETEGTGRGHRTV
ISDEMKEKILFGQRKDPAKNELIGGHSPKIANYNPNYAVEDVLINADGTKKVKFTTQF
SDGNLAKIKTSTLFPEAWSDRKILDSIVKMGDTPKIGERTRDGATLHRGIVDGVQVEV
IKIGDDVVSGYPTGGGATSLLSGFH"

CDS 89434..89850

/locus_tag="EFAGFIKM_00087"
/inference="ab initio prediction:Prodigal:002006"
/codon_start=1
/transl_table=11
/product="hypothetical protein"
/translation="MYEELDEYLSGEFTTDYWYDEGFSIAREMLEEFEDNDWEELLNA
ILSKSIEWQIRYAYCVDSDVNDGAVVKSLLLLSSVDDEELFTTCVDSLRVILNSTNIE
LVSNDEVIMRKIKEILPKCGMATRKMYEDFIENFPK"

CDS 90619..91461

/locus_tag="EFAGFIKM_00088"
/inference="ab initio prediction:Prodigal:002006"
/codon_start=1
/transl_table=11
/product="hypothetical protein"
/translation="MIDDAHELSRVLMEMDGEGHVKSRYIYGLGLIGREDADGTYLSY
HYDLRGSTTLLTDEQNRVTDRYTYGLYGELERHEGLTQQPFAYNGRDGVMMDANGLYY
MRARYYDPKLKRFLNRDVIRGEIQDGQTFNRYAYVNGNPASYIDPLGLTKWETDGSSE
PSTPRTGSLKLDLQQFAEDASKGKKLSDPNPVPKVIRDQYEEIILNKGVPRVDPRTGK
QTVFEGREGQGRAWIGSLEFDVPGTNNRILLRPDGKSGYVLKHDYTKPRLFPSPWYPD
GGKK"

CDS 91481..92266

/locus_tag="EFAGFIKM_00089"
/inference="ab initio prediction:Prodigal:002006"
/codon_start=1

/transl_table=11

/product="hypothetical protein"

/translation="MVAKFQIKDDESDMIIGEFKDLINLPGDNIANDVENFTCKTKLI

DFTVTDEFKQYCLQTKVYVNNLHFIILNMNGDAIGSYYFTIDEPMFSHKSIFLSGDKN

IELVGTFSEHPFVTALELWEKWRTCPPIKKNKWAHLASSERAGWLEVARLHQEYKGDI

ETLNQERVYELDGTYVEDSTSFFCALGEAMHGPGGYYGFNLSSLEDCLSGNFGIVSPF

SIVWSNFSFEIQSDNSIRMEPDFFYDVIKLMTSFQLTLTILPK"

CDS 92448..93704

/locus_tag="EFAGFIKM_00090"

/inference="ab initio prediction:Prodigal:002006"

/codon_start=1

/transl_table=11

/product="hypothetical protein"

/translation="MKKVTQLILSGALVIGMLPFTGNTSQAAVVSFKDVPTTHWAKAS

IDAAVAKGYFKGYSDGTFKPGATVTRAEFAALLARVAKGTPETEQDNVFKDLTGHWSE

VEVNHALSLGFLNSSDYPNGFKPGMALTREEMAKWLSSGLAAQDEDYKQALKDTENTL

VPVAEYYKGGLKKAAYPYVSVVLGTGLMGGYPDGTFGPGKTTTRAEAAVILSRYEQVQ

KSKATSYRDLNEMREVGLTGTNLTSATPFVYSKYENANYLNSFDLITEKPFSLRNNLG

EMIVHRMIIADAPTASTVKNMYGKMFIDKDFDRFIDEDRYMVFMEVTVTPNGNNLSNL

ALANSTGYRLTSGAASNFTSGAIKKYGIINLPRVDYVKSGFFKKGVSKRYWMAQSINR

YRSEDVALNGSSIRVGNSLTSFYIPN"

CDS 93720..94061

/locus_tag="EFAGFIKM_00091"

/inference="ab initio prediction:Prodigal:002006"

/codon_start=1

/transl_table=11

/product="hypothetical protein"

/translation="MKFRGLRKCIVILSVFIVCSNYQMMLVFSAPGNSTKVIAVSSNP

EDREPLTASVASPVGPTGYQKVVRKVDGVRTASPGVYWVQRDEESGVLDHLEEMRLLT

ALVLLKHLGQH"

CDS 94505..95041

/locus_tag="EFAGFIKM_00092"

/inference="ab initio prediction:Prodigal:002006"

/codon_start=1

/transl_table=11

/product="hypothetical protein"

/translation="MLWIWSVPENFPNKRIGEYVKGSGTDRFVFREGRELTGQIVSQK

IVFECLEESLTDVLPNSGLLLIVSKKVVEILQEICPEDIQIFEANVYIGEKKIPDYYL

LNIVSAIEVIDKTESEFTTIKGTDSILNFKKIVYKFDGLPNNHHLVRNLDYKSHVLIS

DDLKEVFERNNIKGVQFT"

CDS 95250..96104

/locus_tag="EFAGFIKM_00093"

/inference="ab initio prediction:Prodigal:002006"

/codon_start=1

/transl_table=11

/product="hypothetical protein"

/translation="MTRYVIDDAHELSRVLMELDGEGHVKSRYIYGLGLIGREDADGT

YLSYHYDLLGSTTLLTDEQNRMTDRYTYGLYGELEQHEGVTSQPFAYNGRDGVMTDAN

GLYYMRTRYYDPKLKRFLNRDVIRGEIQDGQTFNRYAYVNGNPVSYIDPLGLTKFETK

SEGTDNASDIYKHQQYKQSLLKEDVLSNSDVIIKGEDLWDDKARKMLTSDGSKVEDWA

KMTSKQQYETPYGKGEIHFYQNQKTGDISSFDIKLKVSKPKKLRENESDLFWIIDLDV

DFNIKGVR"

CDS 96106..96741

/locus_tag="EFAGFIKM_00094"

/inference="ab initio prediction:Prodigal:002006"

/codon_start=1

/transl_table=11

/product="hypothetical protein"

/translation="MRVCLKKDVEKQENISMNKKYIVYSVETSKNGEEFYRIENDARQ

VVPYSISLFEIVSDKVNSDWILWNKPNNSSALLPKQFAYLSFWEDYYNDELEALKKFN

MVKEQLLQEELEEKEVREIIELENEDNIRFVLNFLSKSEDNRFIDQVIQYVKSNLEKN

YGVDNTTLLAFKYLNLFNQNEVEDFFLYYLTNIEFGDDQLTAIVNEYFSKM"

CDS 97938..99173

/locus_tag="EFAGFIKM_00095"

/inference="ab initio prediction:Prodigal:002006"

/codon_start=1

/transl_table=11

/product="hypothetical protein"

/translation="MKKVTQLILSGALVFGMLPFTGNTSQAAVASFKDVPTSHWAKAS

IDAAVEKGYFKGFSDGTFKPGSTVTRAEFAALLARVAKGTPETEQGNVFKDLTGHWSE

IEVNRAVSLGFINVKDYPNGFKPSTIITREEMAKWISSGLAAADKDFEQALEDTKTSL

IPVKEAFSPGISQSKAPYIAVAMGTGIMTGFPDHSFGLSKTTTRAEASVILLRIANME

GKKASSFEALNEIRSVGVAKSNFKEITPFEFTTGRDFSDASEKKVTFSNKSGSLVLHR

TIAVDASDWNNKKGVYAPFFISESEKDWYQSTAKRKNIVAVFQEITIYPSKKGFNLDD

YRGGLANSFFGQRLDQDMTRQFGYKTLPFSNAENFFSNYSSNNGVRLWIGKWYDKDYK

GLVARFKTDDGSGVSIYGR"

CDS 99170..99790

/locus_tag="EFAGFIKM_00096"

/inference="ab initio prediction:Prodigal:002006"

/codon_start=1

/transl_table=11

/product="hypothetical protein"

/translation="MSERRFMKNKILHIILIMTVLMGLYPHGVARASDYHFNKGYPSN

LEKLIATDAQGRSRGKQKIKVLVTGEKDPNPKVIWTQGDGEIWSATGELPSSRVTTTT

HWNQGGTRGYPVSERFIDKIDATVYAPDSLEDINGNAFDRRFMDDLGVKDINYLDGPA

YEPGNEKPRFTQGDKFIEINVFTGYPKPFSEEKEYGTRPDDKKNGK"

CDS 99805..102552

/locus_tag="EFAGFIKM_00097"

/inference="ab initio prediction:Prodigal:002006"

/codon_start=1

/transl_table=11

/product="hypothetical protein"

/translation="MHIDYIAHVYETKEIRVNSDNILQEGETVQLAAEVREIDYQGNT

TDWVNVSTREQTEWKTDKEGVATVNSNGVVKAEGRGTAKITALWKKGPYYIWENVTIT

VGEDNEIPEEPVTACSQPQPGRTLEGQIMDPVVTAMIRADQRGNEPFDVLKGIPTSES

LYGNVFSRDYLFEHTFVQMTGTCTYQFEVEKIWTLKWDPKKKETDAEGNEREVPDPQE

AEEPVSKQYTVERPYAYWTIDTLSVYSLDEATLINYAFGSGQITLQPEGYVPPDFQAE
TTGDYYPPPNPVKVTAPPGTKTGGKDRPSPPDEDLQSLAEEAVPDVEVENDAFYFNGG
TVMDPQRSPESGPQPGEIPDPVQIDENVLYSPYNYIPISKANKQDTISEGTIRYTLMD
NSVNGGDDQEFDIYGINTVTVHTPVVNYSLVSDDQPHNQKTVPNMNRSALILERPFTV
RIPTSGQHLDAGSYPGYGDRDYAKYYRIKQVRFPFDVYSADRSQFYPRNTWIDVQVPV
LDTTFYLPVWVDEGDYQVEFRNIAENVPTHFSSEPEIDAQPDANTDLYYHAASDEVSV
EVIGRLYDFEITDIADYNWELVFRRFKNSLAPTWISYWTGTQDIDGDKRGNKPQFTVP
IRPGSHPLQGYQNVAVKTGYPFKFDFKTKGNMFGPRDGIRLTPTFDFVSKDGSTRVPV
ELYYSTNQRNFIRIGSTEDQVKRFVILNDRLRQVPSEQLRDTATYKYNRYGEIHPGMM
SERAYQEYYRDKFTKMKTPVGGYSLLLMPEQLRTFIGPKTNIPTTASADVLRANAAIQ
QWYGEYSLPAEPYVVQAGTNLVEYGRTHGGLDAKSPIFLKDGYIVVNFNFESIREGNL
AAPHLQYIHAPLMNQWLLEGFQREVEDSYGNSFTLRDGDVVFYHADRSSRDDFSAQVP
H"

CDS 102727..103014

/locus_tag="EFAGFIKM_00098"

/inference="ab initio prediction:Prodigal:002006"

/codon_start=1

/transl_table=11

/product="hypothetical protein"

/translation="MDTKDKDQEIIDRSEAIAIQYLKDTYDLDVKITRKQMQPKMAMS
WVTFYGYVSQHEEQSFNVSINYEENRTESFSFSPELEEVLLNEGYDPYKLE"

CDS 103036..104331

/locus_tag="EFAGFIKM_00099"

/inference="ab initio prediction:Prodigal:002006"

/codon_start=1

/transl_table=11

/product="hypothetical protein"

/translation="MNKESSNNREITDLVYKKMSQEAYKDYETGKKIGVLPGWEVLDQ
KHNSSGMDVVTFYNPDTKQAVIAFRGTEGSSTWDRKAPDLKADVLNIGLPEAGATIAR
IIDPLSEVGKENVQGIEDTLGITWLNNLFGDTGKSIDKEFGPDNQLYEAEDYVKEMQS
KYPDADFTLTGHSLGGANAQYAAVYTGLDAVTFSAPTVVASLTPEMRRKAEEGAFDRQ
VTNFIHPGDFIASGMLGGYERHVGSTLVIDKNYDDFNASYGAVDKIKDTLGGPSYHDM

KHYSFDDDGYIDNTLYDEITGERVSFSPRKPSDHNILDHAREAWDTLSKGFKSVVNVV

GGYSAGTIELTSEELKEIAGKWSRQAQDMSRTFERIQRNFFEYTESSHSQRLVPIVWD

LQMSIKQLDEWHLEHTSDLVDYIQNKADLFIQADSGTVA"

CDS 104363..104638

/locus_tag="EFAGFIKM_00100"

/inference="ab initio prediction:Prodigal:002006"

/codon_start=1

/transl_table=11

/product="hypothetical protein"

/translation="MSGSSIAMDLDQLLQAEQELDLILSELRENEREARVLYGKLNTW

KGQSADKLRIKVEVFFYQLDTRTQLLLKQKQEMLDAIKRIKDADGSY"

CDS complement(104825..105874)

/gene="ugl_1"

/locus_tag="EFAGFIKM_00101"

/EC_number="3.2.1.179"

/inference="ab initio prediction:Prodigal:002006"

/inference="similar to AA sequence:UniProtKB:Q9RC92"

/codon_start=1

/transl_table=11

/product="Unsaturated glucuronyl hydrolase"

/translation="MKEHIGDKCPHFAGKDGKYDNINTDWWASGFWPGMLWIMYDMTG

EQSYRDAAWSWDETLEQWFVKPTVELHHDVGFQFLSTAVIKHKITGDEDALRRGLEAA

NFLASRYNPAGKFIRAWNEDKYGWAIIDCMLNISLLFWASEVTGDPRYRHIAINHAET

TMQYGVREDGSTKHIISFDPEDGRYIECFGGQGYAPDSSWSRGTSCGLYGFANTYRYT

QDERFLNTAKRIAPYFIAALPEDHVPYWDFRLPTLEGQSRDSSAAAIAASGLLELAAV

VPEGEKRLYTDAAERILRSLTENYAVWDQPEHESILLHATGSGNSFIDVSLIYGDYYY

LEAIAKLNGWKHRVY"

CDS 106093..106458

/locus_tag="EFAGFIKM_00102"

/inference="ab initio prediction:Prodigal:002006"

/codon_start=1

/transl_table=11

/product="hypothetical protein"

/translation="MLERVEFGGSTLAWDYQIKSVDHFKGYYHWHQCGEFLYVHQGEG

IVVVNNHTYPIGQGMLFFFQPYQLHHVYVEVHPNTPYERNIFYVEPIVMDRYLEAFPH

RRSFFRGCGMGAISSKFLT"

CDS 106467..107024

/locus_tag="EFAGFIKM_00103"

/inference="ab initio prediction:Prodigal:002006"

/codon_start=1

/transl_table=11

/product="hypothetical protein"

/translation="MDQVDWMYESYNQSRSRVGGEWEESVLFLLQLLSVLDQQKEGAD

EALSQLTDSRHFRHLEKMMWWIKKHLDYEIRLDQIAADVHLYKSYASRIFHEETGRSI

TDYVTARRLKKAYLLLETTSLWVKRRGRRARFPNGCISSSYFANLPGRARCIIGNNLN

GAASIILVCKIIALLRLYTQLVFTV"

CDS complement(107140..107460)

/gene="yodB_1"

/locus_tag="EFAGFIKM_00104"

/inference="ab initio prediction:Prodigal:002006"

/inference="similar to AA sequence:UniProtKB:O34844"

/codon_start=1

/transl_table=11

/product="HTH-type transcriptional regulator YodB"

/db_xref="COG:COG1733"

/translation="MSDDENAKQICTKVEQSYQIIGRKWVALIIHALMEEPKRFSEIH

AYIPDLSKRVLNERMKELEEEGLVVRHVVTERPVRTEYMLSRKGTELGRALSAVERWA

DKWL"

CDS 107876..109330

/gene="zwf_2"

/locus_tag="EFAGFIKM_00105"

/EC_number="1.1.1.49"

/inference="ab initio prediction:Prodigal:002006"

/inference="similar to AA sequence:UniProtKB:P54547"

/codon_start=1

/transl_table=11

/product="Glucose-6-phosphate 1-dehydrogenase"

/db_xref="COG:COG0364"

/translation="MDAMTFVLFGATGDLAKRKIYPALYNLYMDQKMPKSFSVIGLGR

RELSDTDFQANVEKSLHEFSRQTPEEASQVRDFIGAFRYCSLNNTKLEDYTKLLELVQ

QREQELNIPENRMFYMSVAPEFFEPIALNIQESGLGNTKGWKKLIIEKPFGHDLQSAR

DLNEKLSNTFAEEEIYRIDHFLGKPMVQNIETLTYANPVIQALWSNRYIANVQITASE

TVGVEERAAYYDQSGALRDMFQNHMLQLLMMIGLHLPKRCTPEEIQFKKQKIAEALRP

LTKENIASEVVRAQYAAGELKGSSVVGYLDEPGIPAGSQNETYVAARLWIDDPFWSEV

PFYIRTGKRLAEKSTRIVVEFKAPLKTGHESENTTEPNLLTIEIGPGESISLQLNAKN

PLNHDEVEPMHMTFNSGKRNIPEAYENLIFDAMRGDSTFFAHWNEVELAWQWVQPIQE

AFEAGSVPLDTYSAGSHGPESADRLTAADGFRWW"

CDS 109915..110808

/locus_tag="EFAGFIKM_00106"

/EC_number="1.1.1.343"

/inference="ab initio prediction:Prodigal:002006"

/inference="similar to AA sequence:UniProtKB:G5EBD7"

/codon_start=1

/transl_table=11

/product="6-phosphogluconate dehydrogenase,

NAD(+)-dependent, decarboxylating"

/db_xref="COG:COG1023"

/translation="MKLGLVGLGKMGLNLGRNLIDHKHEVVAFDLNAEAVNEMKEYGA

EGVSSYAEMVASLESPRVLWIMVPHNVVDAVLAEVSPLLSKGDIIIEAGNSHYKESIR

RYEEMKPKGIHYMDAGTSGGMEGARNGACYMIGGDPEAWAIVEPAFKDTSVENGYLYA

GKAGSGHFLKMVHNGIEYGMMASIGEGFDVLEKSGFDFDFEQVARVWNNGSVIRSWLM

ELTERAFSKDANLDEIKGVMHSSGEGRWTVETAFDLQTATPVIALSLLMRYRSLETDT

FTGKVVAALRNEFGGHAVEKN"

CDS 110895..111539

/gene="tal_1"

/locus_tag="EFAGFIKM_00107"

/EC_number="2.2.1.2"

/inference="ab initio prediction:Prodigal:002006"

/inference="similar to AA sequence:UniProtKB:P19669"

/codon_start=1

/transl_table=11

/product="Transaldolase"

/db_xref="COG:COG0176"

/translation="MKFFLDTGNVEEIKRIERLGLVDGVTTNPSLIAKEGRVFKEVIQ

EICGVVKGPVSAEVIGLKAEDMLKEAYEIAEWAPNVVIKLPMTEDGLYACHELTQKGI

KTNVTLIFSAAQGLMAAKAGATYISPFVGRLDDIAVDGMKLIRDLRLILDMYDLPSEI

IAASIRNIKHVEDAALSGAHIATIPGSLLPTLWKHPLTDSGIERFLKDWESVPK"

CDS 111804..112682

/gene="yunB"

/locus_tag="EFAGFIKM_00108"

/inference="ab initio prediction:Prodigal:002006"

/inference="similar to AA sequence:UniProtKB:O32131"

/codon_start=1

/transl_table=11

/product="Sporulation protein YunB"

/translation="MMRRKWRSRRRRKPPSGKRKMWLIILLVTAFCLMQGFAYVDKKM

KPPIMHLAKIRVKQIATEAINKAITAQVADGKTNEGLIDWKTDTTGKVSGFMLNYNEH

MRITASTMNIVQSTLQNVHMLKEKIPLGQALGSPVLASFGPSIPVRIEPQGAVKVDLN

TRQQNAGINMILVEVYIHIIAEVAVVVPFDMEPETVDTEIPISYLLVVGDVPMYYYDN

QGKPVGSNGSNAPAIALPSGHTGVSNGNGVTTNPPSQNQQQTPSDHLQGNELEIEPDE

LPDVNGGLQLNKDAHP"

CDS complement(112770..113840)

/gene="lytH"

/locus_tag="EFAGFIKM_00109"

/EC_number="3.4.-.-"

/inference="ab initio prediction:Prodigal:002006"

/inference="similar to AA sequence:UniProtKB:O32130"

/codon_start=1

/transl_table=11

/product="L-Ala--D-Glu endopeptidase"

/db_xref="COG:COG0739"

/translation="MQQRLTFRHTYRFWIKTLLAGTLLLPFLPVEVYSEPAQAAPKPQ

ATELKPAEIFAARRHLYETIGQMTQIPWYRLAAIDQYERTITRAHPKDRKHPERLTGI

FMTPPAWRGWLNPDETDQHPESILFFKGYGRDGSGDGIADANNDQDVLYSMASVIQGY

GNKQEDFNIALWEYYHNSRAVQRIQQFAKLYEHFDNLDLFGHAFPVPLGTNYSYRSTW

GTKRSWGGYRIHEGTDIFAPHGLPVRSTCYGVVEIKGWNPFGGWRIGIRDLNNHYHYY

AHLSGFDQSARIGEVVIPGQVVGWVGSSGYGKPGTQGKFPPHLHYGIYRDSGLHEWSF

DPYPQLKHWEQDERKQKNKKSK"

CDS 114081..114980

/gene="lipA"

/locus_tag="EFAGFIKM_00110"

/EC_number="2.8.1.8"

/inference="ab initio prediction:Prodigal:002006"

/inference="similar to AA sequence:UniProtKB:O32129"

/codon_start=1

/transl_table=11

/product="Lipoyl synthase"

/db_xref="COG:COG0320"

/translation="MAKRVKEPKPDWIRIKLTTGDNYQEMKTMMRSKTLHTVCEEARC

PNIYECWANRTATFMILGDICTRACRFCAVNTGLPTELDLQEPERVAEAAEQMNLQHC

VITSVARDDLKDGGATIFAETVKAVRRRLPLCSVEVLIPDFLGDLESLQIVMDAKPDI

LNHNIETVERLSDKVRAKAKYKRSLELLARAKEMQPNIPTKSSIMLGVGEEYNEILST

MDDLRAVNCDIMTIGQYLQPSEKHLYVEKYYPPEEFAALKQEGLKRGFSHVESGPMVR

SSYHAHEQVKSATKHAEQAATHA"

CDS 114977..115402

/locus_tag="EFAGFIKM_00111"

/inference="ab initio prediction:Prodigal:002006"

/codon_start=1

/transl_table=11

/product="hypothetical protein"

/translation="MMGDTGLEEEKITEQIQDQLQEPVQDSVVESVEEKPKEPVIVQI
GGKNYEIVQNHKEGWNPEVFRDRYSEVLERYDYIIGDWGYSQLRLKGFYRDNHPKATK
DSTIASMVDYINEYCNFGCAYFVLQKSKDQPQAKAKSGS"

CDS complement(115430..116233)

/gene="ppnKA"

/locus_tag="EFAGFIKM_00112"

/EC_number="2.7.1.23"

/inference="ab initio prediction:Prodigal:002006"

/inference="similar to AA sequence:UniProtKB:O31612"

/codon_start=1

/transl_table=11

/product="NAD kinase 1"

/db_xref="COG:COG0061"

/translation="MRYYVQDRGDQLSIDLSQQFHALAKEEGFKLDAESPEIVISIGG
DGTMLQAFHNFIDRIPDIAFVGVHTGHLGFYADWKKEELRELVRLMSGKGDPERLKPR
IVQYPLLELEIRKKSGNSSYIALNEFTLKGVDGTVVAQVDINDVTFEMFRGDGICVST
PSGSTAYNKALGGAMVHPTIEAIQIAEIASINNRVYRTLGSPVILPKHHHCDIFSRKD
QRLLMTIDHVNVMVEDLISVRCQVSSHKVSFARFRPYPFWNRVRTAFLD"

CDS complement(116338..117591)

/gene="ylbJ_1"

/locus_tag="EFAGFIKM_00113"

/inference="ab initio prediction:Prodigal:002006"

/inference="similar to AA sequence:UniProtKB:O34765"

/codon_start=1

/transl_table=11

/product="Sporulation integral membrane protein YlbJ"

/db_xref="COG:COG3314"

/translation="MAASQRLTHVLVTLALLILCVLMVLYPAETWHAGVRGLSIWWDV
LFPSLFPFLVLSELLLGFGIVHFLGTLLNPLMRPLFRVPGSGGFVFAVSCASGYPTGA
KLTAQLWAQKLVTREEGERLVAFTTSSDPIFMIGAVSVGFFHNVAIAPVLVASHYAAA
FLVGMLMRFHGGTAKGSHQDISSASPSEGIPRNRLVRAIYAMHEARKADGRAFGELLR
QAVSSSLRLIIIVGGLVVFFSVMMELLVQTGWLGGLYGITEQLLRHSGLPPSLSPSLV

GGLFEVTLGNKEAGSAGASIPLVFKVAAAAFVLSWGGLSVHAQIMSVLSNTPMRYGPF

LFARAIHALIAPVLVLLLWTPMMGRSSSPVLMEPGFAPSLSTYTPDWGLIFLCGMIVF

VSLIVLLLFLAILSSILKPRRHAKK"

CDS 118152..118529

/gene="yjbI"

/locus_tag="EFAGFIKM_00114"

/inference="ab initio prediction:Prodigal:002006"

/inference="similar to AA sequence:UniProtKB:O31607"

/codon_start=1

/transl_table=11

/product="Group 2 truncated hemoglobin YjbI"

/db_xref="COG:COG2346"

/translation="MNPSLSIYDNLGGEKGVRALVEAFYPIVQQNEQLAPLFPEDIQP

VIDKQYMFLSQFFGGPGLFSEAFGHPMMRARHMHFEVTVERAEAWLACMDQALTQIGV

EEPLHAFILQRLSGPAHHFVNTP"

CDS 118573..119304

/locus_tag="EFAGFIKM_00115"

/inference="ab initio prediction:Prodigal:002006"

/codon_start=1

/transl_table=11

/product="hypothetical protein"

/translation="MELEPLYKVKVTCHYCETEYETSRVRPSLKRPYRTDSDFCAYYK

LENPDFYVVRICPQCGFATTENATEHLNDVQRKAFKEQIGNRWVKRDYSGARTLEQAL

ATYKLALLCAQVIQEKDRVVAGLLHHIAWLYRYMEDHTQEHRFLEFSLEAYVKVFERE

GTGGNEAKLLYLLGELNRRVGRFNEAVQWFSKVIHDKRITDAAMIRASREQWAVLREQ

MISSKLELPEEMLETDKEAAKRSPL"

CDS complement(119317..119550)

/locus_tag="EFAGFIKM_00116"

/inference="ab initio prediction:Prodigal:002006"

/codon_start=1

/transl_table=11

/product="hypothetical protein"

/translation="MIPFENSWPYDIVMGDIYVQQCPYCHASNVLLPLKPKELVLIRE

GKKKLLVFPCCNTSMTVIDNDSDYLLSSRPVRN"

CDS complement(119547..119717)

/gene="yycC"

/locus_tag="EFAGFIKM_00117"

/inference="ab initio prediction:Prodigal:002006"

/inference="similar to AA sequence:UniProtKB:P37481"

/codon_start=1

/transl_table=11

/product="putative protein YycC"

/translation="MKPLQVSADTAVKLAESLGVPLEHLMHMPQHILMQKIAELAKQE

ASKPSAPEGEQE"

CDS 119836..121635

/gene="pepF1_1"

/locus_tag="EFAGFIKM_00118"

/EC_number="3.4.24.-"

/inference="ab initio prediction:Prodigal:002006"

/inference="similar to AA sequence:UniProtKB:P54124"

/codon_start=1

/transl_table=11

/product="Oligoendopeptidase F, plasmid"

/translation="MKTPLHPVWDLESIFSGGSSSETFAAYLIELEEDVRKLQHLLNE

TPAPTSLEETAAFDPILELLQSCYMRVSEGSAFVSCLSSQNQKDKKATQLQGAISSLG

AMLNGSKSKFDNTLSQTSDLVWDAWIAREDIQPLAFVLNESRTLAREKLSPELEGLAL

DLGVDGYHGWGKFYNTIVSKVNIPFEQDGETVMLSAGQAANKLSDSDRNVRETVFANW

EQAWTDVEDFCADTLNHLAGFRLKLYEKRGWDDILKEPLAINRMSRQTLDTMWDVING

AKPALVQYLERKAELLGVDKLSWSDVDAPVGKSSGKITYDEAAINIVEQFAKFSPKLS

SFAEMAFEKRWIEAEDRPGKRPGGFCTSLPLSKATRIFMTFSGTPSNVSTLAHELGHG

YHQHIMEELPALNQRYAMNVAETASTFAELIVADALVQAATDEQEKLALLEDKIQRSV

AFFMNIHARFLFENRFYEQRKKGLVNADELSKLMVEAQQEAFCGVLASDHPHFWASKL

HFYLTGVPFYNFPYTFGYMFSAGIYARAQQEGTAFADKYDDLLRDTGRMTVEELAQKH

LGTDLTQPEFWQNAADLVIADIEQFLQMTATEK"

CDS 121910..122320

/locus_tag="EFAGFIKM_00119"

/inference="ab initio prediction:Prodigal:002006"

/codon_start=1

/transl_table=11

/product="hypothetical protein"

/translation="MKIDELSLARQLDLVFKELDHELSGLDSGVVFVQIRNNVIGKFG

IRHNPIAGRDGQMAVEEGGLNETQRTSFRAMALETLKFKRNWTHGEISYDFTVRQGMI

LVDATMESNYNMASLMIRYPRTNTYKDSDMESTS"

CDS complement(122406..122639)

/locus_tag="EFAGFIKM_00120"

/inference="ab initio prediction:Prodigal:002006"

/inference="similar to AA sequence:UniProtKB:P22065"

/codon_start=1

/transl_table=11

/product="Small, acid-soluble spore protein alpha"

/translation="MYGNQNQGSGSRSNNLVVPQATAALQQLKIEAAQELGVTIPQDG

YYGNYTSRETGSLGGYITKRLVQIAEQQLSGRS"

CDS complement(122845..123819)

/gene="galM_1"

/locus_tag="EFAGFIKM_00121"

/EC_number="5.1.3.3"

/inference="ab initio prediction:Prodigal:002006"

/inference="similar to AA sequence:UniProtKB:P39840"

/codon_start=1

/transl_table=11

/product="Aldose 1-epimerase"

/db_xref="COG:COG2017"

/translation="MKQVTKGQWNGYDTYILHSRELEITLLPRLGNNIISIRDLVQDR

DVVRRPDEDDLAFYLQKPYHFGVPLLIPPGRIHRGQFEYEGVRYQFDQNTANDNHIHG

LHRTQSWCVSDIEEDEDGCAITTELLTENEEHWMAQFPIPLKLEMTFSLQNAVFSQRL

RVTNLSSTPAPFGMGYHTWFLLDGKPADWTLQLPVSGIYGQNEEQLPTGELESLGECS

ALNEGINLQGLNWDTLLKATEGELATAYLRRQDGYTLKYSADEAFFKHWVLFTKGESD

QFLCIEPYTWLPDAPNLALSDEQTGLIRLEPEQPVELFSRIEVIPPTD"

CDS 124409..125401

/gene="trpS"

/locus_tag="EFAGFIKM_00122"

/EC_number="6.1.1.2"

/inference="ab initio prediction:Prodigal:002006"

/inference="similar to AA sequence:UniProtKB:P00953"

/codon_start=1

/transl_table=11

/product="Tryptophan--tRNA ligase"

/translation="MKTVLSGIQPSGKLTLGNYIGAIKNFVKLQHDYQCHFMVVDLHA

ITVAQEPAALREQSEAVAALFIAAGIDPSKSNVFLQSHVPQHAELGWLMTTLTSMGEL

ERMTQFKDKSSGKDSVGAGLFVYPSLMAADILLYNADLVPVGEDQKQHLELTRDLAGR

FNHRYGEYFTIPDPYIPQVGARVMSLDDASSKMSKSNPNAGSYIALLDPPDVIRKKIS

RATTDSGREVVYDPANKPEVSNLMSIYAECAGMTLKEVAERYEGKMYGPFKKELAEVV

VSVIEPLQQRYNEIRESGELADVLDTSARRAEEVAAQTLDAVKERMGFVPRRKQ"

CDS complement(125516..125716)

/locus_tag="EFAGFIKM_00123"

/inference="ab initio prediction:Prodigal:002006"

/codon_start=1

/transl_table=11

/product="hypothetical protein"

/translation="MSKGLSLWFAFSSIVLLTVSAIMISYSGWFAALLFVLSIANIGW

GFVIRAKKERQEVRSAASEPTS"

CDS 125837..126055

/locus_tag="EFAGFIKM_00124"

/inference="ab initio prediction:Prodigal:002006"

/inference="similar to AA sequence:UniProtKB:P22065"

/codon_start=1

/transl_table=11

/product="Small, acid-soluble spore protein alpha"

/translation="MAQGSRSNNLVVPQANSALQQLKIEAAQELGVTIPQDGYYGNYT
SRETGSLGGYITKRLVQIAEQSLAGSGK"

CDS complement(126173..127153)

/gene="yfhP"

/locus_tag="EFAGFIKM_00125"

/inference="ab initio prediction:Prodigal:002006"

/inference="similar to AA sequence:UniProtKB:O31583"

/codon_start=1

/transl_table=11

/product="putative protein YfhP"

/db_xref="COG:COG1988"

/translation="MDTSTHFVMGIGLAGLAYVDPVVAASPMLAAAVMIGTIAGSQAP
DIDTALRLKSNSLYIRNHRGLSHSLPFLLLWVLLITGVIALIFPGVPIGHVATWTAVA
VGFHVFTDLFNTYGTQAARPFTERWIAWNIIHIFDPFLFTTHVIAILLWAFDLIAPAP
LFVTLYSLTGLYYIWRTIARAQAVRKVRRLDNSPEQARYIVIPTISWNRWHVVKRVED
GSYVIGKMDGSNLVWSLHASSSTHAAVAASRKSPEVSAFLYFTSYAVAEVEELPAGYK
VRWADVRYRHRKQYPFVAVIVMDRNFETIDTYVGWLSDEKMDKKLLSARP"

CDS 127576..128187

/gene="ypmQ"

/locus_tag="EFAGFIKM_00126"

/inference="ab initio prediction:Prodigal:002006"

/inference="similar to AA sequence:UniProtKB:P54178"

/codon_start=1

/transl_table=11

/product="SCO1 protein"

/db_xref="COG:COG1999"

/translation="MLKKYKWTWMLLGLALIMAVYLMWGTVFASKEKLPEIREIQSFS
MENVDGSTVSLEDTQGKVRLFYFYFTSCPDVCPITTFTLSQVQDLLKEDDSFGKDVSF
VSISFDPKVDTREKIKTFADRFHADYSGWYFLRGDMDKTKQLARDSFQILIEGENKDD
FAHMNMIGLVDENNQLRKVYNAFNTEDVVPAVIAEDIRNLIKE"

CDS complement(128256..128603)

/gene="rnmV_1"

/locus_tag="EFAGFIKM_00127"

/EC_number="3.1.26.8"

/inference="ab initio prediction:Prodigal:002006"

/inference="protein motif:HAMAP:MF_01469"

/codon_start=1

/transl_table=11

/product="Ribonuclease M5"

/translation="MPIHVIVEGKNDRSKLKRLVGPEINILCTFGTLNSLKLETLRKQ

IGYDEVFLFMDNDSSGKKIRGVLRDAFPDAVQMYTRRGYAGVEGTPDEYIIAQLEKAG

LETYIQYPEHPSF"

CDS complement(129135..130310)

/gene="mdtG_1"

/locus_tag="EFAGFIKM_00128"

/inference="ab initio prediction:Prodigal:002006"

/inference="protein motif:HAMAP:MF_01528"

/codon_start=1

/transl_table=11

/product="Multidrug resistance protein MdtG"

/translation="MKTAIWLYLFLFLAVFDLHAQYPILTPFAISLGAAPTFIGWMMG

IYSLTHLPGNLIAGTQIDKHGSRRYIVFSLLGAGFILLLQAYVQTPWQLLALRSISGF

VLAFLSPACLALLAQLSSDPVKQGKYMSGHGVVHTLASVVSPAAGAIIVGSMGFSATF

SGLGYLLILTGVIAFMTMPRGIVKQHEKVTVPAKPDASPADATSAFLPVSWRYFALPL

VIACAQGILFFELPLRGGGQSSIMSTGLLFSIISIGALFTLSMLFLNRYSPKLRLVAG

VLLMSLCFFVMAAIPQVPLSTVLFVLGMSKGIIFPAMATLFIRLSGGSKLGRIFSLQS

IATSIGSFIGPITAGQLRIGLSPYFIAFVLLMIGMLLLPYYTSRREASLSDPKSILN"

CDS 130501..130983

/locus_tag="EFAGFIKM_00129"

/inference="ab initio prediction:Prodigal:002006"

/codon_start=1

/transl_table=11

/product="hypothetical protein"

/translation="MNSNTEGVVMNIEVIKEFVMQNWLVIVVALIILFFVLNVVKTVL

KWAIAIIIIAALLIYSGISIDQIKQTVTDVQSSTMETLKKEATSMMLKEASKAKYVKG

QDGAFTITSPNVEIKGRTQSDKVDVTFRGISLGEWKLDNETIRTFVEQAQENKTAPAS

"

CDS 131002..132147

/locus_tag="EFAGFIKM_00130"

/inference="ab initio prediction:Prodigal:002006"

/codon_start=1

/transl_table=11

/product="hypothetical protein"

/translation="MLQNWLDSLKELNGITIMLLLIVAASLLQGWSRGASRSAGRLFG

YLMDGIMAVIGILLSIGLTLWLAPYVQQWLSEYASAMPNRELNRWEQMYYTLVTAIAD

FPLMRFAVLFVLSYGLIRLILGFLSSFIFSSRQRSAEESAPKGMFSRLTGAFIGTIIG

SVRGMIVIAVLFMIVSLYPGSMFSRYVEASPIYMQGAKSVIEPLSGTFIKDKLPVFTQ

AVQQELGGILQRKYEVIDHNIPTDIESAASEIVKGQSTDEAKARALYDWVGSRIQYDF

GKVDDYEQKGIWHEQNPQNTFDTRKGVCIDYARLYAVMARSQGLEVKVVTGLGYNGQG

GYGPHAWNEVYLSGSESWVPLDPTWAISGDWFNPPNFADTHLKDQSA"

CDS 132253..134292

/gene="mrdA_1"

/locus_tag="EFAGFIKM_00131"

/EC_number="3.4.16.4"

/inference="ab initio prediction:Prodigal:002006"

/inference="similar to AA sequence:UniProtKB:P0AD65"

/codon_start=1

/transl_table=11

/product="Peptidoglycan D,D-transpeptidase MrdA"

/db_xref="COG:COG0768"

/translation="MKKHSNEKDELTDKRRFSYRMNVFFFASFVIFSVIIVRLAFLQF

VEGPELSQEEASNITKDVPLPPVRGTIYDSTGEVKLAYSKPIQSLYLTLYKNYGDVGG

QPSPNIGEVQDIATRLHDVFEQYKLKDSEPLTVEKIIEEMDLNSRKANGFMPRLIKSD

LSEEEVAYFLQHKDEFKGIQIVEESVRFYDPDTVAVQTIGYLKKFRSSKSLNKYKEVD

EANKTQTDPGLVYTENEFVGFDGLELQYQDALRGKSGYTSVDVDLRNLPEGVAGSTPP

QKGYDLISSINKNVQVKTEQAILDQLNWLHRNPVSGKLHLNAKTGFAVAMEVDTGKIV

SAASMPDYDTNVWRTGGISSEDYDNIKYIYQNGTIRGFPPDDSGKRAESVVLLGSTIK

PLSVLIGLKEGFFTTNTVYSDRGSTTFGGDNRRVQNSSGHVYGAMYPRDAIRHSSNVF

MIDEIGKKLYSKYGAKGVDVWDEYMEKFGLGISTGVDLPNEFLGRKEYGEDSIESSLT

KLVYASFGQQGKYTTMQLAQYTTMLANKGKRMEPQLVREFRDSEGNVVEKVKPKVLST

VEFNDAYWNEVQRGMATEVSAFSGFPYDFARKTGTSTQVVGGKPVDNGVFIAYAPRNN

PKLAVAVVIPEGGFGSSSAAPVARAIFDAYDEEFGLDGVPKKDKNKDSDSESGTQ"

CDS 134661..136634

/gene="mrdA_2"

/locus_tag="EFAGFIKM_00132"

/EC_number="3.4.16.4"

/inference="ab initio prediction:Prodigal:002006"

/inference="similar to AA sequence:UniProtKB:P0AD65"

/codon_start=1

/transl_table=11

/product="Peptidoglycan D,D-transpeptidase MrdA"

/db_xref="COG:COG0768"

/translation="MFNRLRKKPMTEEDTPVNKPSTARLNLFFFAAFVIFSILIFRLA

FVQFVEGPELTYMETSRNTKDIPLAPVRGPIYDATGEVALAYSEPVQSLYVLLYEDYR

NDERRQEAEELAHDLAAVFKQFNPGDKEQPDGEEIIKRLDLDYQKTFGYVPRLVKSDL

STKEIAFFMEKKTEYPGVMVLEENIRKYDPDGVAVQVVGYTREFKRAPDSIAKYKAIR

EGASTQRDPGLVYHEEEKVGFDGLELQYQEELRGRSGYQSIDIDARNLPDGTMLQTPP

EKGYSLVSTINKEIQMAAQEAITDELRRLPKAITGYAVAMEVDTGNVVAMASMPDYDP

NDWDYDKIKYVFRNGTTESFPPNDAKPSRAESVILLGSVIKPLSVLIGLKEGLFTAGQ

TYHDQGYAILGKDGRQVKNSHSAYNGSITARRAIEKSSNAFMIDMVGKRLLSKYGSEG

IDIWDKHMKEFGLGVSTGVDLPNEFLGRLEYTNKDESALTRLAFASFGQQAKYTTMQL

AQYTTMLANKGKRMEPHLVKEIRDADGNVVKEIKPKVLNEVDFADAHWNEVHKGMVTK

VSAFDGFPYDYARKTGTSEQGTGPNKKENGVFIAFAPRDNPKLAVAVVVPEGGFGSVS

ASPIARKIFDAYDEVYGLDGTPKGKKGEGKDKE"

CDS 136873..137817

/gene="mntA"

/locus_tag="EFAGFIKM_00133"

/inference="ab initio prediction:Prodigal:002006"

/inference="similar to AA sequence:UniProtKB:O34385"

/codon_start=1

/transl_table=11

/product="Manganese-binding lipoprotein MntA"

/db_xref="COG:COG0803"

/translation="MVKMRSIQRGFITLAALVLVVVLTACSSDAETSSGGKLQVTATT

GMIADVAREVGGAYVDVTGLMGPGVDPHLYKASQGDIRKLEQAKVIFYNGLHLEGKMT

DILEKMSSSKLVTAVTETIPVEELHSGKDTGGTEYDPHVWFNVSHWMHAAEAVRDTLV

EADPDHAEEYKAQAEAYLAKLEALDAEVREKILEIPEASRVLVTAHDAFGYFGQAYGM

KVMGLQGISTAAEYGAKDVSELRDYLVDNHIKAVFVESSVPAKAMEAIIAGAAQKGHT

VSIGGELFSDAMGAEGTEEGTYIGMIRHNVETIVEALK"

CDS 137848..138669

/gene="mntB"

/locus_tag="EFAGFIKM_00134"

/inference="ab initio prediction:Prodigal:002006"

/inference="similar to AA sequence:UniProtKB:O34338"

/codon_start=1

/transl_table=11

/product="Manganese transport system ATP-binding protein

MntB"

/db_xref="COG:COG1121"

/translation="MEDTTLLKQTPTKLNNSHLQGTQPTSAPLSVRDLAVAYHKKPVL

SSVSFDIPEGQLIGILGPNGAGKSTLIKAVLGLVPKMHGEVRIFGQSYREQRRRIGYV

PQRESVDWDFPTHALDVVMMGRYGHLGWFRRPGKKERDLAAHCLEQVGMGDYMYRQIS

QLSGGQQQRVFLARALVQDADLYFMDEPFAGVDATTEKAIISLLEQLKKQGKTVLVVH

HDLATVEEYFDHVLLLNGRLVAGGPTSEVFVPDTLQETYGGRIAMIGSRTEKGQV"

CDS 138673..139632

/gene="mntC"

/locus_tag="EFAGFIKM_00135"

/inference="ab initio prediction:Prodigal:002006"

/inference="similar to AA sequence:UniProtKB:O35024"

/codon_start=1

/transl_table=11

/product="Manganese transport system membrane protein

MntC"

/db_xref="COG:COG1108"

/translation="MWNWIVAILSDPNTRWILLGCLLLGFSSGIIGSFTFLRKQSLMG

DTLAHAALPGICIAFMLTETKSVGLFLFGALVAGIVATFGISWITRYSRIKQDAAMGI

VLTVFFGVGVVMLTRIQHGASGSQSGLDKYLFGQAASMVMTDVYVMGGVCLVLLIACL

AWFKEFKLVSFDPGFARGMGLPVAMLEQLILLLTVIAVVAGIQAVGVVLVAALLVTPA

AAARCWTDSLALMVVLAGIFGALSGATGTIFSTLVPNLPTGPVTVLAATVLFAGSALL

APRRGLLARRLRSIQAKSAYMREERATLQTLATQRNQQERGEM"

CDS 139632..140504

/gene="mntD"

/locus_tag="EFAGFIKM_00136"

/inference="ab initio prediction:Prodigal:002006"

/inference="similar to AA sequence:UniProtKB:O34500"

/codon_start=1

/transl_table=11

/product="Manganese transport system membrane protein

MntD"

/db_xref="COG:COG1108"

/translation="MATFWIILTAVLVSSACAILGCFLILRRMALVGDAISHAVLPGI

AIAFLWSGSRDSLWMLLGATVFGLLTVFFIQSLQAGGLSSDASIGIVFTALFAVGVIL

ISLNAQHIDLDLDCVLFGEIAYVQWDTLTLGGTDVGPRAVWMLGITLLVILVVIGLFY

KQFKLCAFDPALAAACGIPVVLFHYLLMGLVSMTSVASFESVGSILVVGMLIVPAATA

YLLTDRLGKMILYAVLIGAASSVGGYIMAYALDASIAGCMVAVAGILFVLALLLSPKH

GIVFRYARRKFAAR"

CDS 140816..141565

/gene="ycsE"

/locus_tag="EFAGFIKM_00137"

/EC_number="3.1.3.104"

/inference="ab initio prediction:Prodigal:002006"

/inference="similar to AA sequence:UniProtKB:P42962"

/codon_start=1

/transl_table=11

/product="5-amino-6-(5-phospho-D-ribitylamino)uracil

phosphatase YcsE"

/translation="MSELKYKLLALDMDGTLLNDNHEITQETAKWIQIAIRRGVHVCL

STGRAVFHAMPYAVQLGLETPMVTVNGSEVWKAPHDLYMRHLMDPALIRKMQEIGEKY

NSWYWAYSVEELFNRDRWTDNIEGLEWLKFGFNTEVDEVRHQIMMELQQMGGLQMTNS

SPVNIEINPAGVSKASGVAEVCKLLGIEMSEVVAVGDSLNDLAVIEAVGLGVAMGNAQ

EQVKEAADLIVASNNEDGIVEVIREHILKGE"

CDS 141569..142060

/locus_tag="EFAGFIKM_00138"

/inference="ab initio prediction:Prodigal:002006"

/codon_start=1

/transl_table=11

/product="hypothetical protein"

/translation="MLATVAWILIVLLFAVGMAGTVYPILPGAVAIFFAFLVYGWFFS

FDPFGVWFWIIQILIVVVLFVADYVVSAWGVKKFGGSKLSTTLSTIGVIIGPFVIPAF

GLVLGPFIGAFIGELIGGSSPSKASKVGFGSVVGLFTSTVMKIILQIVMIVLFIIWVV

RFA"

CDS 142122..143750

/gene="murJ_1"

/locus_tag="EFAGFIKM_00139"

/inference="ab initio prediction:Prodigal:002006"

/inference="protein motif:HAMAP:MF_02078"

/codon_start=1

/transl_table=11

/product="lipid II flippase MurJ"

/translation="MSKKETFIRGTLILAAAALIARVLGLVQRVPLEHILGDIGNASF

TISNTVYLMLLTVATAGIPSTLSKMVSERYALGRASEAQQIYRAALIFAAVAGVVMSA

LLWFAAPYYATYVSKVPESVSAIRALAPALLLFPAIAMMRGYFQGRGNMTAGGISQIV

EQFARVGTAIIVAYVMLQWNYDDQTIAAGASFGGVLGSVGAFGVMLYFTLKLRRSDRA

AQLNYERAEQLPMRGIYSDIFKLSIPIVLSSLAVPAINFIDSSLVVPLLSGQIGLEEA

TGVLAILGAKAQSIAGIPPILAIALSQSLVPVISAAFARKDEAHLKSQVTLALRISIL

TGMPIVIALCAAAYSVNGLLFTNLDGTPIIALLTFGTIFQITMMTTNSILLGVGKPRI

TMISVAAGVVIKLIASLILAPIFGIYGIIIATALCFLVITYLNLRVLRKIVDFSIMGD

RWKGFIITVLLAAGVGFATNWIGNAIFGLFLPARVSFLFTCLVVGVLVVVVYLVLMVV

LRVLRKDELGSYPRILQKILRPLMRLQRGAGQRG"

CDS complement(143949..145235)

/locus_tag="EFAGFIKM_00140"

/inference="ab initio prediction:Prodigal:002006"

/codon_start=1

/transl_table=11

/product="hypothetical protein"

/translation="MTSTRTDSSSEQHHGSLLQMVRSIPGAAREIWRGKQAAWHASAQ

LRHPLREIEWDTDTAAALQSEVERLLPASNKSFIRTDQVQSALVCEEDCIILEEIKTL

TQENNRSNITRTAAYLECYEQYPELHWALLAHMVSRNGGYHMTDLQSDLMHNLQNQSD

REHMYRLLERCNALIFQDAYPQLLLYMNSRRIGRSCFHLLSHFHVSAFMTPFWERFWL

ERCSSLLSVALIINEQNYIESRVVQHPYFQKEVLSKPAFHLHNLAGLNHIVFPLGRGA

GLAGRVIEHFGKLDERIMFGKGLYALLFGVEQVHTQVLEFARSVPHRGSRAEYWPDLF

TNREDEAGDHTLYAQGLLDEEWLPEGQRLYSPELLAVWGDTPYEPITRQDWLQTRDSL

GYLTAPRRPWLFEMSHEHRYGMLKTALAHDAKAITH"

CDS 145378..145722

/gene="ytpP"

/locus_tag="EFAGFIKM_00141"

/inference="ab initio prediction:Prodigal:002006"

/inference="similar to AA sequence:UniProtKB:O34357"

/codon_start=1

/transl_table=11

/product="Thioredoxin-like protein YtpP"

/translation="MEQITSKPAFDVAIQSPRLTIAVFKADWCGDCKYIDPFMPEVEE

KYTRELTLIEVDVDQVGTVSEEQNILGIPSFVAYTDGRELVRYVNKLRKSREEIEQFL

DRAVEVYNTIHK"

CDS 146274..147212

/gene="ctaA"

/locus_tag="EFAGFIKM_00142"

/EC_number="1.3.-.-"

/inference="ab initio prediction:Prodigal:002006"

/inference="similar to AA sequence:UniProtKB:P12946"

/codon_start=1

/transl_table=11

/product="Heme A synthase"

/db_xref="COG:COG1612"

/translation="MKHLTLFKWLTVLTCLVMFLATFGGGIVTKTESGLGCGTEWPLC

NGKLVPAHTVASLIEYSHRAVSALAGLLSIASFVAFLRFGKSRRDLQLFSLLTLVFVI

VQGIMGAFAVVFSQSSAVMALHFGFALIAFASSLMMALGIRQEAKDGGLERLNKYPRV

SKKFRNLVWFSTIYTYLVVYTGAFVSHTDSAGGCSGFPLCNGQIIPELSGGVAVAFAH

RAAAASLVIVIAILGHFAYRNHPDNKEMRTLGVVSVVLILMQVVIGIFMMVTMNRPEV

YMFVALAHMLDIAILFGVLTYMSFLVYKLHRPANRF"

CDS 147378..148094

/locus_tag="EFAGFIKM_00143"

/inference="ab initio prediction:Prodigal:002006"

/codon_start=1

/transl_table=11

/product="hypothetical protein"

/translation="MLRTLLGEKPRINEGKLKVAMDSMAHTLELFQRQMHVDHDPTHD

YRKLYVWTQGLISSLDELEQSCFAAAHFRKKVVAGSTDDMTATEKAEYARYVYFYKDG

FIRCFAILDKVGTVLNEIFQLNTSNTKAHFSYFTVLRQFDYAREHHDLALQLIQIKDS

YREPMSKLRKRRNMEIHYMNSEMHDDLWQLHQTLQGKVKLEDLNQHVQEMRQGVDMVC

ETLTASYSYINKIWKRNNSL"

CDS 148486..149526

/gene="metN2"

/locus_tag="EFAGFIKM_00144"

/EC_number="3.6.3.-"

/inference="ab initio prediction:Prodigal:002006"

/inference="similar to AA sequence:UniProtKB:Q831K6"

/codon_start=1

/transl_table=11

/product="Methionine import ATP-binding protein MetN 2"

/db_xref="COG:COG1135"

/translation="MRLIELKGLTKTYGKGAKSTTALSELNLSIQKGEIYGVIGHSGA

GKSTLIRCINLLERPTEGEVWVDGINLTELSKTELQQQRRKIGMIFQHFNLLSSATVY

DNVAFPLKLVNTPKEAIDRKVKEMLALVGLEHHSSKYPAQLSGGQKQRVGIARALASD

PNVLLCDEATSALDPQTTNSILKLLLDINEKYNLTIVLITHEMHVIQNICDKVAVIHQ

GGIVEQGPVTDVFLKPQHAITRDFMMRDHEAGLALEETALANAGVELQAGAASKLVKI

SFLGNKTYEAILSRTVRKTGVDFAILQGTISTIKQVPYGQLTVRFEGDSNAIDRTISE

LTAEGLDVEVLR"

CDS 149527..150186

/gene="metI_1"

/locus_tag="EFAGFIKM_00145"

/inference="ab initio prediction:Prodigal:002006"

/inference="similar to AA sequence:UniProtKB:P31547"

/codon_start=1

/transl_table=11

/product="D-methionine transport system permease protein

MetI"

/db_xref="COG:COG2011"

/translation="MDFSTVRWEEVGKASIETLQILGVSGLFTVILGLPLGVLLFMTA

RSASIKSRAVYTILSLIVNILRSVPFIILIVALIPFTRILVGTATGVLGVIPPLVISA

APYFARLVETTLREVDRGVIEAAQSMGASTGQIVRRVLLPEALPGLLAGITITIVTLV

SYTAMAGMVGGGGLGTLAINYGYFRYQNEIMIISVVSMIILVQILQMAGDRLVTFFTR

K"

CDS 150262..151101

/gene="metQ_1"

/locus_tag="EFAGFIKM_00146"

/inference="ab initio prediction:Prodigal:002006"

/inference="similar to AA sequence:UniProtKB:O32167"

/codon_start=1

/transl_table=11

/product="Methionine-binding lipoprotein MetQ"

/db_xref="COG:COG1464"

/translation="MKKWSFALLSLMLIAVLAACGNNKDADSGADNSAGAPRTVELKV

GASPTPHAEILESIKPELEAQGIRLQVVTFNDYVQPNQQLADKKLDANFFQHQPYLDT

ENKERGFNLVSVTPVHVEPFGGYSKKIKSLDELADGAKVAIPNDPSNGGRALLLLAKE

GIITLKDNTNITSTIQDITANPKNLDIIELDAAMMPRQLDEADLVFINANYALEANLN

PANDALLIEDLQGNPYANILVSREDNKDADAIQKLAAALHSEEVKTFIKERYKGAVEP

ATE"

CDS complement(151229..152227)

/locus_tag="EFAGFIKM_00147"

/inference="ab initio prediction:Prodigal:002006"

/codon_start=1

/transl_table=11

/product="hypothetical protein"

/translation="MKNKRWISFLTLLMLSLFTLSSALQVSAASENEQAPDEYDWITG

PASVSLDGKATLEVPESHSYLDKPNTQRSILNSDGKPNGNEIGSLTSNSEFGSWYVVF

EYVKTGHIHDDDQNLNAEELLSSYIRGTEEDNRELDPEYRTYITGWEIEPAYDSTKHQ

LVYSLGFKNADQQSMVNYNVKLLTREGYITAILVTDTANFQQSRQEFEETVLNQLSIN

AGYTYEEYNASTDKTSTIGLNSLLMGGIGYTASQKFSALLLLKKGWALILVVVLGLIG

WIRYRIKSSHGEEEKLSPSERTYLQEADEQQYADQNELSYYRQSGHPSDVDKPGKL"

CDS 152401..153180

/gene="fabG_1"

/locus_tag="EFAGFIKM_00148"

/EC_number="1.1.1.100"

/inference="ab initio prediction:Prodigal:002006"

/inference="similar to AA sequence:UniProtKB:P99093"

/codon_start=1

/transl_table=11

/product="3-oxoacyl-[acyl-carrier-protein] reductase FabG"

/translation="MKGKIALITGSAKGLGKMTALSLADQGCHIALNYVHSRTEAEAL

KAQIIAKGVRCIAIQADISKVEDISSLVEQVEGNLGSIDILVNNAGPFVRERRLFADY

SEAEVQMLVQGNLLGPMLLDQRVLPEMRRKQWGRIIHFGFSHAGEARSWPHRAVYAAA

KVGLVSFTKTLAVEEAPYGITVNMVCPGDIRGANKEKTIDEMAGITDEETPRGRPGSG

EDIARVITYLCLDHSDFITGNIMDVSGGLDPIRPTIQREDT"

CDS complement(153319..153564)

/gene="nfuA"

/locus_tag="EFAGFIKM_00149"

/inference="ab initio prediction:Prodigal:002006"

/inference="protein motif:HAMAP:MF_01637"

/codon_start=1

/transl_table=11

/product="Fe/S biogenesis protein NfuA"

/translation="MSENAQSTMYDEVIDVLDKLRPFLQRDGGDVELVDVEDGIIKLK

LVGACGSCPSSTITLKAGIERALLEEVEGVQEVVQVF"

CDS 153643..153897

/locus_tag="EFAGFIKM_00150"

/inference="ab initio prediction:Prodigal:002006"

/codon_start=1

/transl_table=11

/product="hypothetical protein"

/translation="MRPIIEFCANNMHFGTDEVMDQLEENPDYDVIEYGCLTNCGQCS

MTPFALVNGEVVITDKVEDLYNAILAKIGEADAWDELDLD"

CDS complement(153894..155027)

/gene="yjlD_1"

/locus_tag="EFAGFIKM_00151"

/EC_number="1.6.99.-"

/inference="ab initio prediction:Prodigal:002006"

/inference="similar to AA sequence:UniProtKB:P80861"

/codon_start=1

/transl_table=11

/product="NADH dehydrogenase-like protein YjlD"

/db_xref="COG:COG1252"

/translation="MLLHSCSTVYSPISVGLMQKGSRVMKNFVILGGGYGGLTIIKEL

LEGKIPSDTQIILVDRSPFQGLKTEYYALAAGTVSDYDLRIQFPVSDKVTYRYGEVTS

INLEQRQIEFEGQDPLVYDKLVIGLGCTDRFHNTPGAENYSCTIQSFSKTRETYLRLN

EVKAYGNVHIVGGGLSGVEIAAELRESRPDLNISILDRGERVLSAFPQRLSVYVHEWF

NEHQVETRGHIAISRVEPNAIYNRDEQILTDAVVWTAGIQPVKVVQDLDVTKDPQGRV

VLNEYYQIPEYTDVYVVGDCASVPYAPSGQAAEVQGEQIAHIQHALWKGEKPNPHPLK

LRGTLGALGKKSGFGLMGKTSMMGRVPRILKSGVLWMSKRHLG"

CDS complement(155190..156296)

/gene="mqnE"

/locus_tag="EFAGFIKM_00152"

/EC_number="2.5.1.120"

/inference="ab initio prediction:Prodigal:002006"

/inference="similar to AA sequence:UniProtKB:Q5SK48"

/codon_start=1

/transl_table=11

/product="Aminodeoxyfutalosine synthase"

/db_xref="COG:COG1060"

/translation="MSTLVTPFTDKRMAEIVEKVQNGVRLNVEDGVYLYETDDLLTLG

QLANEANLRKNGKKVYFIENMSLYFTNVCEAHCAFCNFRKDQGEEGSYTLSGQEMIDY

VEQHIHPGVREFHIVGGHNNHVPFQYYVDSLRALNEKYPDVTLKAYTAAEIDFFTRIS

GLSIKEVLQELQKAGLKTLTGGGAEILSDEYRKKMRVDKANVDRYLEVHRTAHNLGMR

THTTMLYGSIESYEDRVNHMVQIRELQDETNGFMVFIPLSMQPKSKNASIMRRNSAYE

DLKTIAISRLMLDNIDHIKAYFINIGPQLAQVALGFGASDAHGTIVRERISHAAGALT

PEGLTRKELIWLIKGAGRIPVERDTFYNEIQVYE"

CDS 156926..157282

/locus_tag="EFAGFIKM_00153"

/inference="ab initio prediction:Prodigal:002006"

/inference="similar to AA sequence:UniProtKB:O67709"

/codon_start=1

/transl_table=11

/product="hypothetical protein"

/db_xref="COG:COG0316"

/translation="MINISETAADRLKEMLAQQETPGMFLRLGVAPGGCTGFSYAMGF

DDKESDEDLYMDIQSMKVVVEKENLKYLDGLEIDFEESGMTGGFTIHNPNAVATCGCG

SSFRTKEDAGVPDKDC"

CDS 157502..158146

/locus_tag="EFAGFIKM_00154"

/inference="ab initio prediction:Prodigal:002006"

/codon_start=1

/transl_table=11

/product="hypothetical protein"

/translation="MMNIAIIGATGKAGSVILKEAADRGHKVTAIVRNASKLEDKSLN

TLEKDVFDLTAEDLKAFDVVVNAFGAPAGKENLHVEVGQALINILKDAPNTRLIVVGG

AGSLFTDESKTLRVFESPGFPDAYKATATNQGQNLQDLQASSGIQWTFLSPAGFFNPE

GVRTGKYQAGNDVILVNSEGNSYISYADYAIALVDEIENPQHKNERFTVVGEVK"

CDS 158402..158797

/locus_tag="EFAGFIKM_00155"

/inference="ab initio prediction:Prodigal:002006"

/codon_start=1

/transl_table=11

/product="hypothetical protein"

/translation="MKMYSKGVILLIFAVFMISVSSGSGLSQYTNVKNKMNQFHITSL

YPKNKNGQTYGSAAYATSPETEPDLILATGVDGTDGYLLKKDLDGEQPKTPEEAIAIQ

NSRSPDGRDIPLYDKDGETVIGVFHVGGK"

CDS 159143..159721

/locus_tag="EFAGFIKM_00156"

/inference="ab initio prediction:Prodigal:002006"

/codon_start=1

/transl_table=11

/product="hypothetical protein"

/translation="MEKLKLRIITLIFFISTVFFISNAQDVNALSCAESGGPQEQLEI

YDGAVYGEVKQVKVDMKQEGFTGTKEKIRYILVEVERSWNTEVDSQLIIATNFTWGFD

FKEGNKYLIYFSEADGELSSSPCSLTIEMNHLNQATELFGEGFPPKQQVNVEHKMWFM

FEQDNDLFIVGVAVFAAIFIFFMRVRKKKRKA"

CDS 159874..160752

/gene="rarD_1"

/locus_tag="EFAGFIKM_00157"

/inference="ab initio prediction:Prodigal:002006"

/inference="similar to AA sequence:UniProtKB:P27844"

/codon_start=1

/transl_table=11

/product="Protein RarD"

/db_xref="COG:COG2962"

/translation="MNSGLINAIIAYIMWGVLPLYWKLFENVPAGEILSHRVVWSFVF

MGIFVAVQRRWSDMKRILTSRSTLLSLTVSGLLIAINWLIFIWAVNNGHVVETSLGYY

LNPLLNVLLAVVFLHEKPNRGQWLAIAIAGVAVLIIAIDYGRFPWVAISLAVSFGLYG

LAKKKIKQDASVGLFSETAVVLPVALGYWIYLAVVGKATAWTLPAPMFFELLLSGVVT

ALPLLFFARAAARMSLSTLGFVQYIGPTIMLILSVFVFKETVSPVLLVGFALIWTALI

VYAAASIRATRLAKVS"

CDS 160947..161288

/locus_tag="EFAGFIKM_00158"

/inference="ab initio prediction:Prodigal:002006"

/codon_start=1

/transl_table=11

/product="hypothetical protein"

/translation="MMVNNRYRKRDPFFVKKAVVQILFQFAVSAIIGSIYGFMMVNET

IAGYINKLLPSTDGPDLHLYLPVVITVFIYSCLIQKRQRILLEKISFSCLHILVALLG

CISSVVVLLVI"

CDS complement(161297..161752)

/locus_tag="EFAGFIKM_00159"

/inference="ab initio prediction:Prodigal:002006"

/codon_start=1

/transl_table=11

/product="hypothetical protein"

/translation="MSFLKGFGQLAGEVTGKVLGGSVRVVGEIAGSPFIKEIGNGVEK

ATINTGKTVGQLASGTYVLASGAIRKDDTAIDAGLADIGGAVSNTAKGVAISAKYVYN

SGKDVVVGMKEEDKDRVKLGAKNLLAAAAVTTLAVGVIDAVDGVEGVDT"

CDS complement(162507..163556)

/gene="ganB_1"

/locus_tag="EFAGFIKM_00160"

/EC_number="3.2.1.89"

/inference="ab initio prediction:Prodigal:002006"

/inference="similar to AA sequence:UniProtKB:Q65CX5"

/codon_start=1

/transl_table=11

/product="Arabinogalactan endo-beta-1,4-galactanase"

/db_xref="COG:COG3867"

/translation="MFKNVRGFKTSIMLAFVLLFTSIMLPAGQHASAAPSFAKGADIS

WVPGMEAQGYKWKDKNGVQRDIIDILKKDYQINSVRIRVFVNPSNDYGNGYMNKDRAA

ALAQRAKNAGMSVMLTLHYSDSWADPGQQTKPAAWKNYTFQQLMDAVWNHTRDVMTAM

QSKGVTPDWVQIGNETSNGMLWEDGKASTNMKNYAWLVNTGHNAVKSLSSGTKTIVHL

AGGDDNALYVWNIGGLINNGANFDMIAMSLYPSASGWNTAVTNTVNNAKDMINRYGKE

IIISEIGMDNNQAAAGKSFVAAMKNQIRNLPNGKGKGVFYWEPQATPGYNSGYGKGAW

QSNMMPTVVMEGFID"

CDS complement(163904..164131)

/locus_tag="EFAGFIKM_00161"

/inference="ab initio prediction:Prodigal:002006"

/codon_start=1

/transl_table=11

/product="hypothetical protein"

/translation="MKSNKPRTLQKNIEFFTAALSQCMVSAWQEDPAGVYCEVGCGIV

ERISEDSVRIRNNDGTKSHYARDITMFQTEK"

CDS 164380..166461

/gene="ppk"

/locus_tag="EFAGFIKM_00162"

/EC_number="2.7.4.1"

/inference="ab initio prediction:Prodigal:002006"

/inference="similar to AA sequence:UniProtKB:P9WHV9"

/codon_start=1

/transl_table=11

/product="Polyphosphate kinase"

/db_xref="COG:COG0855"

/translation="MHRDVKTGNYVNRDLSWVEFNRRVLQEAQDPTTPLLERMRFLGI

VASNLDEFVSVRVAETKEKIKAGFTQKDFTGYTPSGLYRRLIKRTGTMVAEQYKTYRE

LIRLLAKKGVNIQEYTDLNVTQQRAMDQYFHEIIFPVLTPMAIDQSRPFPLVHNKSVY

LSVMLQKEGEQEGEPFMAIVQVPSNLPRVVQAPIRANSKKKTFILIEDLIKHHIHTLF

SGYISLAAQEFRVTRNADLFINEEEAGDLLEAIEKELRRRRRGAPVRLEVCKDFRPDA

LLELQDEFDIHDPVYEIDGPLDLSFLAGFVDSLESFSHLKYSPVKPVYPLEFLPRESH

FELLRKRDVLVHHPYESFEPVTDFILEASEDPRVLAIKMTLYRVNGDSRLIPALALAA

ESGKQVTVVVELKARFDEERNIAWARKLEKAGCHVVYGLVGLKTHAKIILVVRREQQG

LRRYVHVGTGNYNESTAKVYTDVGLFTSNPIIGEDASELFNEITGYSGPKALQAFRVA

PDGMKDELFALIRRETEHALKGKPSRIIAKINSLSHQEMIDELYEASQAGVQIDLIVR

GVCCLRPGVEGLSENIRVISIVDRFLEHSRLFYFENSGNPDVYISSADWMTRNLNRRI

ELMCPVFDPELKKMLVDILNLSLMDNVKARELMPGGNYVFVENEKPPLRSQTEAMGII

PWKPAEWSALT"

CDS complement(166421..167950)

/gene="gppA"

/locus_tag="EFAGFIKM_00163"

/EC_number="3.6.1.40"

/inference="ab initio prediction:Prodigal:002006"

/inference="similar to AA sequence:UniProtKB:P25552"

/codon_start=1

/transl_table=11

/product="Guanosine-5'-triphosphate,3'-diphosphate

pyrophosphatase"

/db_xref="COG:COG0248"

/translation="MTHTNETLGIIDIGSNSIRLVIYELDQNEAYRIIHEDKYAARLS

SVVESDGTILRHSLDKAITILSQFKATCEAYQTKLIRAAATAAIRNASNVLEIIEWLE

TETGLTIECVSGDREAYYGFLGVTQSIDLADGYVVDIGGGSTEITVFRNRKRLHSISL

PIGAVNSHARYGGEDQWTEANANALCNEVIEALRGQDWIREHPGLPLIGLGGTMRTLA

KVEQKRTQYSLPVSHHYEISEEAMENIARSLPHLTSAQRKKVPGLAKDRADIIVPGVL

ILRTVFQLIQGDRYVVSGAGLRDGLLRDYMAGGQPVVPDALKDSIRNFIHFGPPIPEK

RLQRIHQDMVTLYTALQGTPPDQADARILYAASMLHMAGKQINYFRYTQHSAYWTMNS

SIYGLSHRETILSASAADYHPKKRTPQLLNKHQDILKNSDERHAHRIGSLLRVAEAIN

RSESIAAIEATKENDSLQVQFTCTAEPLLELDGLEEAVKDLKEAWGVTLTHSIQQASK

G"

CDS complement(167977..168231)

/locus_tag="EFAGFIKM_00164"

/inference="ab initio prediction:Prodigal:002006"

/codon_start=1

/transl_table=11

/product="hypothetical protein"

/translation="MEAFFKSLYGVAYFAMSIVLVAVTVLLFVTGIRLFMQKRNRGFA

VSCLIFACFIVFIIVVMLTTPFSATPPGSPEAMAALLHFG"

CDS 168470..169129

/gene="yheD_1"

/locus_tag="EFAGFIKM_00165"

/inference="ab initio prediction:Prodigal:002006"

/inference="similar to AA sequence:UniProtKB:O07545"

/codon_start=1

/transl_table=11

/product="Endospore coat-associated protein YheD"

/translation="MSRQLASKWRKTAALMKYPVAAVHIPQTKAFNSGNLLQMLSRYG

MVYVKPIVGGGGYGVIRVSASGGAYRYTHMKTTRSFASFSQMYRSLVRVKARRKYLIQ

QGIHLATIQGRPVDYRVKVVKTQRGWVFRSLVGRLARPGLCVTNLSKGGTMLSGRRAL

GLSLPHISGRHKRREMRSLTLTCTYIMESQFPGVGQLGFDYGLDYSGKIWILEVNTRP

Q"

CDS 169490..169627

/gene="sda"

/locus_tag="EFAGFIKM_00166"

/inference="ab initio prediction:Prodigal:002006"

/inference="similar to AA sequence:UniProtKB:Q7WY62"

/codon_start=1

/transl_table=11

/product="Sporulation inhibitor sda"

/translation="MLSDEMLLDSYHKAIELNLERDFIALLLAEIHKRKLGTDVSAIL

H"

CDS complement(169977..170978)

/locus_tag="EFAGFIKM_00167"

/EC_number="1.18.1.2"

/inference="ab initio prediction:Prodigal:002006"

/inference="similar to AA sequence:UniProtKB:Q816D9"

/codon_start=1

/transl_table=11

/product="Ferredoxin--NADP reductase"

/db_xref="COG:COG0492"

/translation="MTAQNSSHDMTDLLIIGGGPAGLFAAFYGGMRQASVTLVESMPQ

LGGQLAALYPEKYIYDVAGFPKVTAQELVNNLVEQMSHFNPNIRLEEKVVSVEKKDEQ

HFIVKTDENEYHAKAVIITAGVGAFEPRRLELEGAAKFEKSNLHYFISDLNAFAGKKV

LISGGGDSAVDWALMLEPIAEQVTLIHRRDKFRAHEHSVENLMNSKVNVVTPTEITEL

HGDDTITKVTLSHVKTKETQEIEVDDVIVNFGFVSSLGPIAEWGIEIDSNSIVVDSRM

ETSIPGIFAAGDITTYPGKLKLIAVGFGEAPTAVNNAKVYFDPDAKLSPGHSSNMKR"

CDS complement(171320..171511)

/locus_tag="EFAGFIKM_00168"

/inference="ab initio prediction:Prodigal:002006"

/codon_start=1

/transl_table=11

/product="hypothetical protein"

/translation="MTTNVNFANLFKRLHQFHNSLKMDFMKLDIDVSNRFDLYLDLIE

ATKGFMKLIRLHASYRFKL"

CDS 171606..172799

/locus_tag="EFAGFIKM_00169"

/EC_number="1.6.99.-"

/inference="ab initio prediction:Prodigal:002006"

/inference="similar to AA sequence:UniProtKB:Q2FZV7"

/codon_start=1

/transl_table=11

/product="NADH dehydrogenase-like protein"

/db_xref="COG:COG1252"

/translation="MSSIPKIVILGAGYGGILTAQRLQKELNYNEADVTLVNRHDYHY

ITTHLHMPAAGTDTIEHARVPISKLIDEFKIDLVKSSVKEIRLQDRKIILEDGTLSYD

YLIIGLGGEPETFGIPGMLDHAMTIRSINSVRLIREHIEYQFAMYKNDNKRNRIRFVV

GGAGFSGVEFVAELADRIPQLCKEFDVNPKDVHIYNVEAAPSALPGFDPELVEHAMNV

LKKKGVTFKIGVPIKQCLPDGVIVGEGEKLDAATVVWTGGIRGNSLLEQAGLEVMRGR

VKVDEYLRAPGHEHVYVIGDNSLVFNAEGRPYPPTAQIAMQQGVNCAKNVVASIRKKQ

PQPFVFTSKGTVASLGKGEAIAVVGGKKYKGWKAAQLKKLVDLRYLFIIGGIPLVLKK

GRFFG"

CDS 172796..173050

/locus_tag="EFAGFIKM_00170"

/inference="ab initio prediction:Prodigal:002006"

/codon_start=1

/transl_table=11

/product="hypothetical protein"

/translation="MRHCSVQVRGLLTREELDRYNALMQVGGYLEEQDQYDLAYIVQK

EVDILIMPAIERLKEKGRDRDRATAEFLESLKAVDDEDEE"

CDS complement(173202..173948)

/locus_tag="EFAGFIKM_00171"

/EC_number="1.11.1.-"

/inference="ab initio prediction:Prodigal:002006"

/inference="similar to AA sequence:UniProtKB:Q5KUD5"

/codon_start=1

/transl_table=11

/product="Putative heme-dependent peroxidase"

/db_xref="COG:COG3253"

/translation="MNEAASTLEGWYALHDFRSINWAAWKAADDEERAVALDELEEFW

KEWKEVENSSQGSTVVYTVVGQKADLCMMHLRETLEDLKAVENAFNKTMFAQYTTKSY

SYVSVVELSNYLGKEGEDPMQNPEIIARLKPVLPQRQYICFYPMNKKRELNDNWYMLS

MDERRTMMRSHGMIGRSYAGKVKQIITGSVGFDDWEWGVTLFADDALQFKKLVYEMRF

DEVSARYGEFGSFYVGSLLNEETLEDMLKL"

CDS 174223..174516

/locus_tag="EFAGFIKM_00172"

/inference="ab initio prediction:Prodigal:002006"

/codon_start=1

/transl_table=11

/product="hypothetical protein"

/translation="MQFIPVLVLMVLFFVMMFGIGFILNMLMKTTWFPSYLFIIVILP

VVVYSLWDHSSSLMSHLGSFQLVDYMTGIAGLAGAVISGWTIQKLRLGGYRMF"

CDS 174899..176434

/gene="dnaB"

/locus_tag="EFAGFIKM_00173"

/inference="ab initio prediction:Prodigal:002006"

/inference="similar to AA sequence:UniProtKB:P07908"

/codon_start=1

/transl_table=11

/product="Replication initiation and membrane attachment

protein"

/db_xref="COG:COG3611"

/translation="MRMKNLHHYTEHHRYCVYREFGLSALDDRMLTGAYQPMVGAFAV

GLYRLLFQHLPGEQVGYSPLEQQRRLFMTLGLEPSEKGRKYLLEQTSKLEAVGLLQTS

RLYIPENDDYIYEYELQPPLSPAEFFRTQHLTLLLRDKIGKFAVLSLRSGFSAVENGD

APYPAANKENISVPFYDIFELNTHVIDYELEQALSEVSTTAQRTGTNDAAEENSLNYA

DIILRFPRESVNRRHVEQLRFDHEQLGIVNYVVNKFNLSVQDVCRLLDEDDIFSPQGQ

LILDDLQHKASMQFRQTKKRHEQQTVQAAKVVALRQHMEEPERKEDSGEPPVEHGVQM

EYYVEVPPQFMTKCDIHQYNMMLRNEPYTRLLQTFFPGAVPDNLIDIFEKIDLSYKLP

GEVINVLIHYLMALLVSGGEQRINRNFVEAIASNMLLKQVNSYEKAVQYIRDQAKVKG

KQAAGAAGTRTRTYGKGTKAKPEIPIVQDISTDGDAVSEEEFEEMMRFAQQMQASKQK

GTS"

CDS 176681..176947

/locus_tag="EFAGFIKM_00174"

/inference="ab initio prediction:Prodigal:002006"

/codon_start=1

/transl_table=11

/product="hypothetical protein"

/translation="MTQDFIEGTTEEAIHNLEIPTFEQLDNDTWDFETMKDFQFSFLF

VEFVVQQYGIDHLNKLIRDPQDFNGIFQCSEKELHQMMVEFISK"

CDS 177071..177631

/gene="ydaF_1"

/locus_tag="EFAGFIKM_00175"

/EC_number="2.3.1.-"

/inference="ab initio prediction:Prodigal:002006"

/inference="similar to AA sequence:UniProtKB:P96579"

/codon_start=1

/transl_table=11

/product="Putative ribosomal N-acetyltransferase YdaF"

/db_xref="COG:COG1670"

/translation="MNPIMIDFPDEFFTERLLIRMPKPGDGKVVCEAVIASVDELKPW

MKWAQDKHTEYNSELGIREAHIKFLSRENLRLLVFLKDTGQLIASASLHSIDWEVRKF

EIGYWIDTRYSGKGYMTEAVQGITDFAFNHLNANRIEIRCDTKNTKSKMIPERLRFDL

EGVIRNEDLSVDGSELRDTNIYALVK"

CDS 177769..178434

/locus_tag="EFAGFIKM_00176"

/inference="ab initio prediction:Prodigal:002006"

/codon_start=1

/transl_table=11

/product="hypothetical protein"

/translation="MKTHYYFSWFNHFFPEKLVHCLQEDIENRKSLVMISADPSGYKD

EQVNFDDVSEWRWLNQANIIFDEYHFIDFRMQKEDAQRFIQNASVIFLCGGYPVLQND

FLAEYELSDVIKNSNAIIMGASAGALNMAAKWLSLNNTDEVETSTIYDGIGFDHFAYE

SHSQRDYATFVQGYLFPLSEEIDIYAAEQESAIRVKDGKIEIMGPVYLISHSKIQKLV

ETL"

CDS 178849..179388

/gene="tadA_1"

/locus_tag="EFAGFIKM_00177"

/EC_number="3.5.4.33"

/inference="ab initio prediction:Prodigal:002006"

/inference="protein motif:HAMAP:MF_00972"

/codon_start=1

/transl_table=11

/product="tRNA-specific adenosine deaminase"

/translation="MDKHYQNLLLAFEEAEKARDEGTFPIGAIIVDADGSVVSRGRNR

VFSSCDSTAHAEVDAIRKAGNVILHLESKKFIPKNELTLYTTCEPCPMCTGTIVLSLI

KKVVWAANDKDIGAFKKFKELNSELPIYNDLFHDIEFVAAPYRDLELRQRKMLAEWNN

SRGYTDNHWNDELVNEIVQ"

CDS complement(179791..181437)

/locus_tag="EFAGFIKM_00178"

/inference="ab initio prediction:Prodigal:002006"

/codon_start=1

/transl_table=11

/product="hypothetical protein"

/translation="MDSIGKNGKEAAEAIRSYVKPIFGFALNRVKHRAEAEDLAQEIM

LQLLKSFSGVRDIRSLDAYVWTVARYTWVNWLKKRAHAPQAIEINGMSELSAAPSREP

LDRLLVTEAYRELRREVAFLSDIHRRIVVMHYYDELKIGDIAIALNIPVNTVKWHLSE

AKKKLRKGMKRMRATGTLSVNPVSMGEMGHSGSAGSLGETNDFLGRALAQNIVYAAYH

KARTVHQIAEELGMPPSLLEGEVRHLADYNFLIQTSPGKYQSNTIVWDLFELAVAGHQ

FWQDCAAEVADVHFDALMEVRRQVVDSGVYVPEGDYNFLLWTLLPKNIEEQSWRSMPA

GENFDAIAPMRKDGGQYIAYAALNRSHYADPGFDMSSYVTFGPSLRYVEDTPLYLWQF

NTYWSDRQMDWRFLEYRNVEICHAFQQGELPDNEENSEQYSFLLEKGYIRKTEEGYKF

NAVWMDSPQTLDRLNKAMPDLSAVYAPAVGKLYDKMLNLFLQNQPKHLEPQLAYMVRG

NTGGGRLVAYILKHLIDNGKLKPPLPHQQKTISTWMGPVK"

CDS complement(181939..182454)

/locus_tag="EFAGFIKM_00179"

/inference="ab initio prediction:Prodigal:002006"

/codon_start=1

/transl_table=11

/product="hypothetical protein"

/translation="MEVIIIKVNKMYFIFSIVALFLLSACAGGVTGSSQQNELGEPYR

MALEELISTDTALNENMEYISLDFDKGIPLKDSDKKSIEDYLHSKYNVKIYNLTYEEL

IEEGLSEQSENTLKGILLKIEKQEKQSDNNIAIEVSKYRANEGSLSAKITLTYEEGKW

KVATHTPTRES"

CDS 182717..184312

/gene="lcfB"

/locus_tag="EFAGFIKM_00180"

/EC_number="6.2.1.3"

/inference="ab initio prediction:Prodigal:002006"

/inference="similar to AA sequence:UniProtKB:O07610"

/codon_start=1

/transl_table=11

/product="Long-chain-fatty-acid--CoA ligase"

/db_xref="COG:COG0318"

/translation="MLLSERLPIAASMYPEEPALMRYGQTMSYGELYGLVERLSHGLY

DEGLRAGDRFALLGDPDPQLVVAFYAAVGIGAIPLVPSPMLTVPELAAIFQDAEPHMV

IHDNQHAKAAIATVKLLGYCPKLFTTDEEGTISSSLAALLQQEPAFSPKDHFKRNVDD

TAVLIYTGGTTGRPKGVMHSHRGMAAWNQLTPSAGFGHDFKRRVLVLNLSHLVGQFQL

WATMAAGGCLVFLDEYPTDVHRIMEAVERDQITQLSTVGQLLRDLTREASVGGRNLKS

LSLIGCGGSIISPDTLQEVVSQFPEALIVNNYSQAECGMSISRLFPAQHMGDPHRLRS

VGRPADLAAQGEQGFEVRILDTDGSETVTGEAGEIVVRGAQTMLGYWRQLEATAETMP

DGWIRTGDVGCLDVEGYLYVFDRLKDMVIVGGSNVFCAEVEHVIVSHESVSEAAVIGH

PHPDEGEELVAFIVLRDGENMDLTRVRTFCEPHLAPYKWPTRLFIVDTLPRTAVDKID

KKHLRKHLSSISSEDYANGNAVK"

CDS complement(184437..187265)

/locus_tag="EFAGFIKM_00181"

/inference="ab initio prediction:Prodigal:002006"

/codon_start=1

/transl_table=11

/product="hypothetical protein"

/translation="MTRKLKPKGIIPKVTAMVLTTALLGQVVASSVYAGDTLPYTGES

AKGVNQPYQHGYTSAQILNWTPESDIHGDLLRAHVPLQPRNEAFAATQAYPELSPDTQ

LFTMSGDYGNAFFDSTPYTNEFSQYLFNYWQYTDYYSYWHGMASAGVPEELYDPSKEW

TEKYFEFGILNIPNPAYTNAAHKNGVKSIANIFFSDNDRGPQTYKQMLIQDENGNFPV

AEKLAEMAEYYNYDGYFFNQEEVARGVAPEDIASYKKFMKYLRDKGLYVQWYDSTINT

TGKIQYQNQFNGLNSPFVQDSVLGRVSDSIFLNYVWNHKMLRDSRDHALSLGLDPLET

VFAGVEGGHDKFGRWKQSYDLRHNLDENGHPMNSIATLGADFTHNALDEEMGDGSTNH

RAEDEYQWMTFVRDRAWWSGPNQDPTDARRNASADLSDVYASGANWDGIAAYLTERSV

INGSNFATSFNTGHGLKYYEDGAVSNDKEWSNINIQDIPVTWQWWMDSKGDKLSVDFD

YGPSYEKGVRYNYDSIGAFNGGSSLVVNGTLNAENFLRLYKTDLSVNGQSKLELTYNK

PSTSDASSLHVGLIFEDDPSNVVNVNVPNAGQHTTGWKTTSLDLSAYQGKTIAAMGLS

FDPHGMEIKNYQMNIGEIRISDGSAAVPDAPTGFHIAKALTNTDELVVAWDMKDYSEV

KQYNLYENGAYVGGVYDSTFYIKSLKQPSGELSIRAVGADGTESEATVLPYDLNAAVQ

NMDVKFKKNGDAIVSWKKPKKAKESGKDKDKDQNTDKDKDKNKDKGKGNESIQLTLQT

EYTKEPFTKSLQVKKGKQSAVLTGLPTNGEHYVLNIAIGGQNPVTHTGQLADLQITPY

AKEKVTVKDGKYTLALPDLEDWYKIYVYENGVPREFGVTYVSQKFPYIIRGRTKLSEL

TFTPSSNNSSLKLVIEDYAGNQASTILR"

CDS 187478..189307

/locus_tag="EFAGFIKM_00182"

/inference="ab initio prediction:Prodigal:002006"

/codon_start=1

/transl_table=11

/product="hypothetical protein"

/translation="MKIVSVYNNFFKNNMFMKMLLIFSMISIVTIITLSYIIFLSVSD

STIRRELAIQKAAMEHVDRYIHQQYESVQNMVRDMHQNEALSANITYLMNHSYAEYVQ

HMTNGYYANQDDYSADVLKHFQNIMDRNSQINQLMLYSAEQQDLSTFNQHKQFRKLDA

NVAHSYIPDVMAMETPNISAPNYWIRKEINQWDPALYSIRVPINNTQTLRNLGQFLVF

FDSEGIGNALDNYESNLKGEIVVLSAKGTVLFDSNNHYYGKKYPYVNVADSLFDQTDM

DSMKKEQNMYVNKFVSADQGYVVIGTVPVEEMAETYAGIRNTIISISIVCILFAVLVP

AFFIINFAKRTRRIIRFTQKVKYGNLTARIDDPRDDELGQISHSFNDMLDELNLYIER

VYKAEIKQKETELVALQARINPHFLYNTLEVIRMRAISQGARDVGEMIYSLSVLFKSL

VQQKKNYTLKDEMEACRLYLELFRIRYKDIFIYTIQIDAAYYQHPVVKLSLQPIIENY

VVHGIQTERSDNRLSIVVEETDDVVQVEVKDNGKGIDPARLTEILEELERPEESGQMF

GLRSVHSRLRFLYGPEFGITIESTLGEGTRIRVRYPHTEGTGV"

CDS 189300..190823

/gene="rssB_1"

/locus_tag="EFAGFIKM_00183"

/inference="ab initio prediction:Prodigal:002006"

/inference="protein motif:HAMAP:MF_00958"

/codon_start=1

/transl_table=11

/product="Regulator of RpoS"

/translation="MCNMYKVFIVDDEPFIIEGLYDIVDWSSYGMEIVSHAGNGQAAL

QALSSQPVDILITDISMPLMNGLDLIREARRVQPELKVIILSGFNEFEYLKEGMQLGI

ENYLLKPINVEELESTLSNTASKLDHTVSPQADDAYGIQILKDNILHRWLTGQIAATE

FEERAQFMNLSLDAPDVAVAILRDKGQVPGMFERVEEMLRDRRHLNVFHDIDGDIVII

ANIQGTSDEEQKLMDGLQALSDEITVTHPAARIGAGRLMKGLYHAPTSYAEAKKALQY

VMIYPDLKVIDHAMLERCGNSTATFFIEWREYAKLILSRNTEQLADRIRMDFEQHRDG

LTPGELQNTALEVVIRFKMELESIKHSDESAIYQQGLQLVLDSGTFDELVRALQQVGS

QTIQSLLQDLKNPIVNQVLQHIDKHYAEELSLKLLGAHYHLHPVYLGQLFHKETGETF

AEYINKYRIERAKEQLRNSNLKVHEIARNVGYWETGYFYKQFRKYVGISPTDFKVFG"

CDS 191105..192577

/locus_tag="EFAGFIKM_00184"

/inference="ab initio prediction:Prodigal:002006"

/codon_start=1

/transl_table=11

/product="hypothetical protein"

/translation="MSKQRKRFSLVLALLMAVTLVLSACGGSKDASESGGAGDDAVEL

IWYTIGTPQKDVNKVMEEVSKYTKEKINATVTMKMVDWGDYPQKMQVNVASGEPMDIL

FTSSGGFDYVQNARKGAFLELDDLLAEYGQDLTKTIDPAFLSGSKVDGHNYGIPANKE

LPQQEVWRFNQMLVDKYKLDISGVRTLDSLEPLLKTIKENEASVTPFAMDKNYVPYVP

YDYVIQNLPMAIKLDTTDYKLVNILETPEMKEALTTMHKYYKAGYVSAEAATTGSTND

LTTSGNWFLDRAQTQPLADNQWSASYGYQVVSTPASEAIITNTSVQGSITAISANSEY

PEKAMEFLNLLNTDPVLRNMVDSGIEGTHYKKVDDTHMENLPESKNYDMPSYSLGNNM

LLYLNSNDPDNKWDEFKKFNAEGVNSPILSFNFDASNVSSELTAVQNVKEQYWAALMT

GTLDPAANLDQVIEKFNQAGLEKVMAEAQSQLDAWRAENK"

CDS 192655..193584

/gene="yteP_1"

/locus_tag="EFAGFIKM_00185"

/inference="ab initio prediction:Prodigal:002006"

/inference="similar to AA sequence:UniProtKB:C0SPB3"

/codon_start=1

/transl_table=11

/product="putative multiple-sugar transport system

permease YteP"

/db_xref="COG:COG4209"

/translation="MTTFLKNLIRNRVMLLMVLPGAIWFFFFSYLPLVGTVAAFKEYR

FSREGFWASLMKSEWVGWDNFKFLFSTSDAWLITRNTLFYNIAIIFLGLVFAVALAIL

LSELVNKRLAKLYQTGMFLPYFLSWVIVGYFAFSFLSMDRGMLNQIIGWFGMEPIQWY

SEAKYWPYILVFVALWKTIGYSSVVYLASILGIDKSLYEAAMIDGASKWQQIRNITIP

LLSPIITIMTLLAVGRIFYADFGLFYQVPRDSGTLYSVTNVIDTYVYRGLKTSGEIGM

STAAGLYQSVVGFVLVIISNYVVRKVDKDSSLF"

CDS 193608..194528

/gene="araQ_1"

/locus_tag="EFAGFIKM_00186"

/inference="ab initio prediction:Prodigal:002006"

/inference="similar to AA sequence:UniProtKB:P94530"

/codon_start=1

/transl_table=11

/product="L-arabinose transport system permease protein

AraQ"

/db_xref="COG:COG0395"

/translation="MAQAGLVKKRDFHHVSNGWNVVMNIIAGLFALICVFPFIFVVII

SFTDEKALARDGYRLIPAEWSLAAYQFVWQSGDTLLRAYGVTILVTVLGTIISLILMS

LYAYAVSRKSFRYRRFFSIFAILTMLFNGGMIPTYMVVSQLLGLKDTLWALILPLAMN

AFYIMILRTFYSTSVPDAVIESAKIDGAGEFYTFMKIVVPLSLPGLATIGLFSTLGYW

NDWFNALLYIDNPNLVPLQSMLMRIESSIQFIQQNSANSSMSLAAMQSIPQDTSRMAM

VVLATLPIIFAYPFFQRYFVQGLTVGAVKE"

CDS 194562..196919

/locus_tag="EFAGFIKM_00187"

/EC_number="3.2.1.-"

/inference="ab initio prediction:Prodigal:002006"

/inference="similar to AA sequence:UniProtKB:T2KMH0"

/codon_start=1

/transl_table=11

/product="Beta-xylosidase"

/translation="MMTYKDQSKSVEERVQHLLSLMTTEEKVGQLTQPFGWQTYTNDQ

GNITLNESFKQQVESGGIGSLYGALRADPWTGVTLENGLSAREGAEAVNLIQRYAVEH

SRLGIPILIGEECSHGHMAIDGTVYPVPLLLGSTWNVDLYREMCQAVARETRAQGGAV

TYSPVLDVVRDPRWGRTEECFGEDPFLIGELAVASVEGLQGESLDRGDTVAATLKHFV

GYGSSEGGRNAGPVHMGWRELLEVDLYPFRKAVEAGAASIMPAYNEIDGTPCTVNTEL

LEDVLRKDWGFDGLVITDCGAINMLASGHDVAADGMEASVQAIEAGIDMEMSGEMFGQ

YLVQAVAEGKLDMQVLDQAASRVLALKFRLGLFEQPYVDADRAAEVIGSEAHVQLARQ

LAAEGIVLLKNEAETLPLSIVNVGRIAVIGPNADQAYNQLGDYTSPQPKSKVATVLDG

IRSKLANHVDGQTEKSSVGALTGDSSTDQVLYAPGCRIKGDSKEGFERALEVASQADT

VIMVVGGSSARDFGEGTIDLKTGASNVSDNSWNDMECGEGIDRMTLGLAGVQLELIQE

IHKLGKKLVVVYINGRPITEPWVDEHADAILEAWYPGQEGGRAIADILFGDVNPSGKL

TISIPKHVGQLPIYYNGKRSRGKRYLEEDLEPRYAFGYGLSYTTYEYSEPQLSAASMT

TDDSVTVSVNVNNTGRYAGAEVVQMYISDVVSSLTRPAKELKGFAKVNLEPGETQTVE

FHIGAEQLQYIGRDLKPVVEPGQFHIHIGSSVNDTQIAELTVREA"

CDS 196924..200148

/locus_tag="EFAGFIKM_00188"

/inference="ab initio prediction:Prodigal:002006"

/codon_start=1

/transl_table=11

/product="hypothetical protein"

/translation="MERIRRFIRELSEHQWLEQLQLRSWDITRAYYHVPGQYEDQGEY

PEGQDFERFPSKQGTTYFFRTRLEIPATWQQAPYGLVFETGGEGLLRVNGHSYQGLDR

NHTYVTLNPSKVGTNPELEIEMFDPVPEPVDPLNQQAVIQPPITSITSLLVRPNEAVR

SLMYTVIIVRDSAVLLPESDFRRVRLLELVYRAMDQFVGMSTEEIEQGEDIRQIENNL

KHHVREIGGNAEGLEHMVGQSHIDIAWLWPVRETVRKTSRTFSTVDALMNEYPDYVYS

QSQPLLYAFLKEHDPELYARVKQRIVEGRWELVGGMWVEPDLNIPSGESLIRQMLYGQ

RFYMEEFGKTSQIEWLPDTFGYCASLPQILKHGNVEYFMTTKLGWNDTNVFPYDLFHW

VGIDGTPILSYLNHGVNEHTLPKDIHDHWQSYREKAAHPEHMLLYGHGDGGGGVTREM

LEYVDRAELMVGQPASRYSTAGAFFAGIEKEQPVLPKWHGDLYLELHRGTYTTHARNK

RNNRKAEVLYREAELWNTLALPDMEANTEAEVRSALHDGWKLILLNQFHDIIPGSAIT

ESYVTSNEEYVQVFELGRTGLDQGIAALTTGINTEGPEGSVAYVVFNSLGWKRSAVVQ

LPVQDGLDRYGIDEKGQRLRMDWEDGSMSILVTDIPAFGYKTVWLVPENTRETNVREM

TTLAAQPTFNDTWDTTFYHVQFNERGEITRLWDKTAEREMLKPGERGNQLHFFHDRPT

LWDAWDIDSRYEEQIAGEVELLEKKLVLAGSTKDVLRFRWKLHQSEITQDIVFYHDSR

RIDFQTQVNWNENHKLLKVGFPIDVVTSKATFEIPFGTLERPTHRNTSWEQAQYEVCG

HRFADVSEYGYGVSLLNDCKYGYDVQGSTIRLSLLRAPKWPDRTADLGEHEFTYSLYP

HDGDWRTAHTVRQAAELNHEVTIQQVNQVQQKGQIQQEQQIEQVQQREEVQQSTGTEV

APTADATANAAPVRPATGSWINFNSNHVILDTVKMAEDGQGTVLRFYESSGKRETITL

QWPHAFEQAYHSNALEEPIKPMAHTNGQITLSFKPFQIQTVLLR"

CDS 200406..201725

/locus_tag="EFAGFIKM_00189"

/inference="ab initio prediction:Prodigal:002006"

/codon_start=1

/transl_table=11

/product="hypothetical protein"

/translation="MEQFRLPKIPMPPVALPQSIQAVLEEAEQKLAHRPKLLQLFKNC

FPNTIETTTKLMEDGTTFVITGDIPASWLRDSVEQVVHYIPFAKEDEDLQRIIGGLIK

RHIQYVHIDPYANAFNETANDWHWNTTDETEMSPWVWERKFEIDSLCFVVRLAYLYWK

ETELTDIFDSSFKAAMRKIVDLFKVEQHHMEQSPYRFTRNNGIPTDSLRNHGKGMPVN

YTGMIWSGFRSSDDACDFHYNIPGNMFAVVALRQMQEFAEWVFRDMELLQELKDLEQD

VDHGIQLYGIYRHPEFGPIYAYETDGYGNHCLMDDAGTPGLMSIPYLGYVTADDPIYQ

NTRRFALSKENPFYYEGKVAKGIGSPHTPPNYIWHMGLSMQGLTAQSAEEKLEIIRML

EATDADTGYMHEGFHADDPTIFTRKWFAWSNSLFSQLVYRSMKDGLL"

CDS 201722..204901

/locus_tag="EFAGFIKM_00190"

/inference="ab initio prediction:Prodigal:002006"

/codon_start=1

/transl_table=11

/product="hypothetical protein"

/translation="MSIQPNDKKLIIFADPAFPMEGASPTHEALNTWKAVEEITVVNA

EELAEALKTTAGEGCLVNLHAPYFPKSAWTEIAAYLHQGGSLISVGGAPFKRPVRYEN

GTWVVESEQTAYHQELYIHEALNVSSANVQSYTSSEQIPLLTGKEGLFEKADTWNLVP

HTTKTSDLPHQMGSSGPMSTQIYPLLCGMSKDGRSIAAPVVLWENSRGSFVGSRWIFV

HLPLTTSFWTQDGAAEIVKWAQFCAQGVTELSLKTNYASYEPDERAVLTLQGQILQRA

GNRLTESELWTFDITVEHEDRTSGTTEKVWSHRLEMELSGEQRFERILLPVSIESGLY

RIICHAQAPDGEVRTLRQGFWGQDAGLLAEGEVITRSRDYFVKDGRPLPVVGMTYMTS

DVARKFLFLPNADVWDRDMAQMAKAGINWIRTGIWTAYRNMMQVDGHMAEDVLRAIDA

FILTAKRHGLQVTFTFFSFTPETWEGTNPYLDPQSVEAQKRFISSIVSRHTHTTHVDW

DLINEPSMFDPVRIFLAGPRSARDSYEQQAYIAWLQQRHETIEALQEAWNMSPKQLPN

FAAAVIPEAEEINFDVQDMHKAKKGTRWLDYCLFSMEMHNVWARELVGTIKDLVPDHL

VTVGQDEALGAQRPSPFFYEREVDYTTVHSWWLNDDLIWDGIFAKTPHKPNLIQETGI

MYVETPDGRAKRTEEELRGILERKYAYAFSTGGAGAVQWIWNTNFYMDNANESHIGAL

RADGTEKPEADVSYDFGRFMDSIRDLFEDRDLEDIAVVFPYSNDFSNRALAYDATTKL

TRVMSYDLKLPFRALSEYHLEALEQQPPKLIIVPSPHNMESDALQQLLTFAEKEGTSL

LITGPLGLDAYWKATDRADHIVGKRSLGNVQREELLNINGVNHRVTYGRRRIAEVAKE

TLLHGDKGTADEVHVLPLGKGKLIWTPLPLELNGRDEPLAELYRYATEISGIEQELEW

ISGGDIAGIYGRKLSFPKGNLYVFVSEFAFNHEVHVRDQRTGVSYSFQLEKNRSVLFA

TDASGKLTAVYRPDDVEIVQQDERGGITR"

CDS 204898..207681

/gene="mngB_1"

/locus_tag="EFAGFIKM_00191"

/EC_number="3.2.1.-"

/inference="ab initio prediction:Prodigal:002006"

/inference="similar to AA sequence:UniProtKB:P54746"

/codon_start=1

/transl_table=11

/product="Mannosylglycerate hydrolase"

/db_xref="COG:COG0383"

/translation="MTKKKRAHIISHTHWDREWYLPYEKHHMRLIQLVDSLLDQLDEG

SDYKSFYLDGQTIIIDDYLQVRPEQKERLEKHIRNGRIVIGPWYILQDAFLTSGEANV

RNMQVGHKDAKRYGTPSKIGYFPDTFGLVGQTPQLMQQSGIDNVFFGRGVKPTGFNNT

VSDAGYESSFSELMWEGPDGSKVLGVLFANWYSNGNEVPVDEESARKFWETKLADAEK

YASTNELLYMNGCDHQPIQRDLPEAIRMAEQLHPDIEFIHSNFPDYLEALRASSEHEL

STVKGELRSQRTDGWGTLVNTASARVYLKQMNQLGQTMLEKVAEPLASYAYLLGHAYP

HDQLTYAWKTLMQNHPHDSICGCSVDEVHREMVTRFDKSHHVAEALIEESTSQIATAV

DTSIFERYGENARPVVVFNTTGWERSGVVQMELDAARLYFRDGYTLEEMATQMKAVDL

SDRVLVDEKGNHIPCTVEDLGLQFGYDLPDDRFRQPYSCRRVRIRFEAEHVTALGLKT

YALVNSADYAKSGSPSKTTTLLRGERGMENQHMIVTIADNGSFTLTDKRTGRTYKDLG

VYENVGDIGNEYMFKQPENEVALTTHALQAEIRVLEDTSYLASFEVIHHWEIPESADE

MLDQEQRELIYYPYRKAQRSAKMIPLQIRTVISLSRSGKGVQMETTFNNQAKDHRVRA

LFPTDLVSGVHHVDSMFEIATRDNTPAPEWQNPSNTQHQQSFVDVSEQNAGLVVANLG

LNEYEVLQDGRNTIAVTLLRAVGELGDWGLFPTPEAQCLGEHTFQIEIIPHDGNGAAS

GAYIEAYQFQVPWTLAQTEVHTGYLTPNNTPFAWQGDGLAFSSLKVNEDSGDVMLRWF

NMTTDSATLNLAASQANPQPFETAYKSNILEEEGERLHARSIEEASTLSFANKEWNIQ

TGSCEIITVGLRR"

CDS 207857..208894

/gene="dnaI"

/locus_tag="EFAGFIKM_00192"

/inference="ab initio prediction:Prodigal:002006"

/inference="similar to AA sequence:UniProtKB:P06567"

/codon_start=1

/transl_table=11

/product="Primosomal protein DnaI"

/db_xref="COG:COG1484"

/translation="MIESAYAIKMAMDTLHCSFYIETLRGLEAMESLGGLLQQLNPAF

REQSRRIAAELMENPYVREFRAGHPELQDAQLMTDLSKLFQYAKDSKNCANCPGLDNC

PNDFQGHFCKLEVEHFNGKPEIIDIKAPCSKHIARQNEHIIKQRIRSFYVDERALSAG

YNDVEIMGKDRMRAPAVNQVLRYISETKENGLSPQGLFLEGTFGTGKTFLMCYLLHEL

AVAGHTGVIIYMPDFVEDLKSMISEGQKLKEMTDILKSCDLLIFDDIGAENLNPWVRD

HVMGSILNYRMNRKPTFYTSNYNLDGLEKHLSFTNRDGEEMNKGQRLMDRIRPFVDVI

SVRGENQRGKR"

CDS complement(209075..209230)

/locus_tag="EFAGFIKM_00193"

/inference="ab initio prediction:Prodigal:002006"

/codon_start=1

/transl_table=11

/product="hypothetical protein"

/translation="MDANVRISDPREHVNEEPRNDLFDLIAGVAGMAGLMTVIFFGMV

IFKFLTE"

CDS 209493..209810

/gene="trxA"

/locus_tag="EFAGFIKM_00194"

/inference="ab initio prediction:Prodigal:002006"

/inference="similar to AA sequence:UniProtKB:P14949"

/codon_start=1

/transl_table=11

/product="Thioredoxin"

/db_xref="COG:COG0526"

/translation="MAIVNVSDQSFNAEVEGEGTVLVDFWAPWCGPCKMLAPILEELS

TEVGDAVKIAKVNVDENPESASRFGVMSIPTLIFFKDGQPVDKVVGLNSKDALKGIIA

KHQ"

CDS 210284..212371

/gene="uvrC_1"

/locus_tag="EFAGFIKM_00195"

/inference="ab initio prediction:Prodigal:002006"

/inference="protein motif:HAMAP:MF_00203"

/codon_start=1

/transl_table=11

/product="UvrABC system protein C"

/translation="MDQFMNVLQDQEKALEQIRHKLALLPDMSGCYLMKNSEGTIIYV

GKAKVLKNRVRSYFIGSHNGKTQRLVSEIRDFEYIVTGSNMEALILECNLIKKHMPRY

NVLLKDDKTFPYLKITNEKHPRLEVTRRVLKDKAKYFGPYPNSYAAHQTKKLLDRMYP
LRKCGVMPKEVCLYYHMGQCLAPCVKEVEKEQYDQISQEIGSFLSGGHEEIKKDLQRK
MQEAAEDLYFERAKELRDQVIAIDAMMEKQKITMADARDRDVFGFAIDKGWMCVQILY
MRQGKMIERHVSTFPFYGEAYSDFMSYVTQYYSDNPALPQEILLPEMPKDMSTGDDST
DTVSDADGTASAAVTVEFEQTGQVEHSNASQPLVAETRAAYGGSTEAEGEQPDSMQED
APITDTTDSTEKLPSGLEDPTQVAAALQEWLEIKVLIPQRGLKRQMITMAVDNARVAL
EEKFRLIERNEERTSKAAEGLGRFIGLDQLHRIEAFDNSNIQGTNPVSAMIVFTDGKP
DKKEYRKYKVRSVEGPDDYETMREVIRRRYERVLKENLTQPDLIVVDGGKGQISAAVD
ILENELGLFIPVCGLVKDAKHKTSQLMIGNPPEVISLPRDSQEFYLLQRIQEEVHRFA
ISFHREQRGKSMVTSRLDAIPGIGEKRRKLLLKHFGSLRKIKEASVEDFRPLSIGDKL
ANQIIAALRDEES"

CDS complement(212340..214037)

/locus_tag="EFAGFIKM_00196"

/inference="ab initio prediction:Prodigal:002006"

/codon_start=1

/transl_table=11

/product="hypothetical protein"

/translation="MNPLGQPLQLKANFKRLGLLAAIGLILFFVFQIFPATSSQTTEI
SSTSFITKEQAKQSARSFAASVNDYTLPNDDEKTLVTYQTHSDIYGYMAKTKQLDTYN
QKWETNYPYDVFRVRLPDQHKGGYLNVDVHMKTGKVVGFKRELPSSLYTAIEAEQVSG
RVTTSQVLAEGNISLDEKQQLVAGVLTEFGYEVPALQLETRDGEAGLKYVDSEKAIGD
SKLELNFTFEEGAVRSFDPGFSVPESHTDYVKDQTRQANWMTYGGYAFLTFVLGVLAI
IYSILTRAHTSFKRGIILSLVYFAASVIGTLNMIPLLQAQGFSNFMLAFLMLFQIGIT
LVMSATIYLSLVAGDGMWRKIGLNPWPRAKEPGYGKYVLHSMYAGYLWAFILLGVQSI
LFFILERSIGTWSTTSADQSTFNMSYAWIFPIMAWMAGIGEEAVYRLFGIRMMQKIVR
NTFIACLIPTIIWALGHTLYPIYPVITRPIELMVIGLLFSLIMLRHGFIAVVFAHVIF
DSLLMGLSLVFMGDALNMTAGLFWIVLPAIVGYVVYKFNPKQKEKPYVTTPHPEVLQ"

CDS 214645..215985

/gene="ktrB_1"

/locus_tag="EFAGFIKM_00197"

/inference="ab initio prediction:Prodigal:002006"

/inference="similar to AA sequence:UniProtKB:O32081"

/codon_start=1

/transl_table=11

/product="Ktr system potassium uptake protein B"

/db_xref="COG:COG0168"

/translation="MKLNVQWMRLSPPRILVLGFAGIILVGTLLLMLPASNRNGNSLA

FIDALFTATSAVCVTGLVVVDTGTHFSVLGQVVIAILIQIGGLGFMTMSTLVAIAFKR

RISLRERLILQEAMNQSTMEGIVRLIRKVVIYSLIIEGICGTLFAIRWSFDMPVGQAI

YYGYWHAISMFNNAGFDMFGDFRSLTGYVYDPLVNFTAMFLIVSGGIGFVVLSDLVDY

RKTRKLSLHSKVVLLMTGLLIVFGALVIFVFEFSNPRTLGGLNWGGKILGSFFQSVTP

RTAGANTVDIAGLRQATQFFMIILMFIGASPGSTGGGIKTTTFMIMAGAVIAMMRGRE

DIVFFRYRLVQERIFKALTITLLALLFIIAVTMALSTTEDASFMMILFETTSAFGTVG

LSLGLTLKLTTLGKLLICFTMFAGRLGPITLAYALGQKKGKELYRYPEGKMIIG"

CDS 216040..216711

/gene="ktrA_2"

/locus_tag="EFAGFIKM_00198"

/inference="ab initio prediction:Prodigal:002006"

/inference="similar to AA sequence:UniProtKB:O32080"

/codon_start=1

/transl_table=11

/product="Ktr system potassium uptake protein A"

/db_xref="COG:COG0569"

/translation="MKTQQFAVIGLGRFGSSLAQELMELGYEVLGIDKNEEVVEDISE

LVTHAVVADATDEEVLRSLGIRNFDCCIVAIGDDIQTSILTAILLKELGVKTVVAKAI

SVLHGRALDKLGIDRVVYPERDMGIRVAHQLVTPNLLDYIELSNDYSIVEMKVPACLH

NKTLSTLNARVRFGCSIVALQKEAGVIIAPTALDSLQMGDIMVIIGNNADIDRFEEEV

ISQES"

CDS complement(216772..217695)

/gene="cysL"

/locus_tag="EFAGFIKM_00199"

/inference="ab initio prediction:Prodigal:002006"

/inference="similar to AA sequence:UniProtKB:P39647"

/codon_start=1

/transl_table=11
/product="HTH-type transcriptional regulator CysL"
/db_xref="COG:COG0583"
/translation="MFEELNAFAAVVEQSSLNRASKLLNLSQPALSRKIAKLEDELGV
ALFHRRGKRLELTSVGQFTYTYAVEQKQQQQKFLTMLAQYKDEEQSTITLGASLTTLQ
TTLPPLVNAFMEKHPNAELKLLTGKTHEIVSFVREKKADVGIVASSISEVGLNCVPLF
DDHLELVVPLTHPLSGKEAGMEHLQDLPMITFSKGTWYRKLTDDLFQRCAVMPDIRME
IDSFEAIIRLLPSAKAAALLPKSYLRPQLLADNDLVSVHLPQLQQTRRTTCMIYGEKE
DLSETSRQWVKETAALFTAKAPLPSRRTTTP"
CDS 217940..218659
/gene="sdhC"
/locus_tag="EFAGFIKM_00200"
/inference="ab initio prediction:Prodigal:002006"
/inference="similar to AA sequence:UniProtKB:P08064"
/codon_start=1
/transl_table=11
/product="Succinate dehydrogenase cytochrome b558 subunit"
/db_xref="COG:COG2009"
/translation="MKSFMKTLYLAKGDRHFMKGFYSRKLHSLLGVIPLSLFFVEHLV
TNFTAVEGGKEAFYGAVAFLNGLPLVIVIEALLIWLPLFYHGVYGLYIAYQAKPNVGR
YGNERNWRYTLQRVSGIITFVFVIWHVWETRVQIALGNVSHEEIGGVIHDAVTNPITF
AIYMISVVAASYHFANGLWSFLVSWGITVGPRAQRVSSYVCMSLFAIVSIMFIASLFA
FRSIDFQTATSMIDAVKTVLI"
CDS 218703..220445
/gene="frdA"
/locus_tag="EFAGFIKM_00201"
/EC_number="1.3.5.4"
/inference="ab initio prediction:Prodigal:002006"
/inference="similar to AA sequence:UniProtKB:V3TQ67"
/codon_start=1
/transl_table=11
/product="Fumarate reductase flavoprotein subunit"

/translation="MASTNVIIVGGGLAGLMAAIKSAEAGVHVHLFSLVPVKRSHSVC
AQGGINGAVNTKGEGDSPWVHFDDTVYGGDFLANQPPVKAMCEAAPGIIHLMDRMGVM
FNRTPEGLLDFRRFGGTKHHRTAFAGATTGQQLLYALDEQVRRWEAAGLVTKYENWEF
LQAVLDDEGVCRGIVAQDLKTMKVQTFPAEAVILASGGPGIIFGKTTNSVINTGTAAS
AVYQQGVNYANGEFIQIHPTAIPGDDKLRLMSESARGEGGRIWTYKDGKPWYFLEEKY
PSYGNLVPRDIATREIFSVCVDMGLGVNGENMVYLDLSHKDPKELDVKLGGIIEIYEK
FMGDDPRKIPMKIFPAVHYSMGGMWVDYNQMTNIPGLFAAGECEYQYHGANRLGANSL
VSAIYGGMVAGPKAAEYIKGLKKSSADIPSSVFEKHTKQQSDKYEGILKMQGTENAYV
IHKELGEWMTANMTVVRYNDKLEATIGKIKELKERYGKINMYDSSGWNNPGAAFTRQL
WNMIELAEAMTLGALLRNESRGAHYKPDFTERNDEEFLKTTIASWSKEGPKISYEPVD
VSLIPPRIRDYSKD"

CDS 220900..221664

/gene="frdB"

/locus_tag="EFAGFIKM_00202"

/EC_number="1.3.5.1"

/inference="ab initio prediction:Prodigal:002006"

/inference="similar to AA sequence:UniProtKB:P17596"

/codon_start=1

/transl_table=11

/product="Fumarate reductase iron-sulfur subunit"

/db_xref="COG:COG0479"

/translation="MAEQATANKTVKFIITRQDSPESSSYNEEFELPYRPNMNVISAL
MEIQRNPVNTDGKSIAPVCWDSNCLEEVCGACSMVINGKPRQACAALIDKLEQPVRIE
PMKTFPVVRDLVIDRSRMFSALKRVKAWIPIDGTYDLGPGPRMPEKKRQWAYELSKCM
TCGVCLEACPNVNEKTDFIGPAALSQVRLFNAHPTGEMNADDRLEALMEDGGIEGCGN
SQNCVQSCPKGIPLTTSIAEMNKQTTKHMFKRWLGV"

CDS 221840..222814

/gene="cpdA_1"

/locus_tag="EFAGFIKM_00203"

/EC_number="3.1.4.53"

/inference="ab initio prediction:Prodigal:002006"

/inference="protein motif:HAMAP:MF_00905"

/codon_start=1

/transl_table=11

/product="3',5'-cyclic adenosine monophosphate

phosphodiesterase CpdA"

/translation="MAETPRQGSNNSPSQRSHKASGAEESVFPGEEKDHDPSRRSFLL

RMVLMTAGASLLTGGYAWLWEPRRLEIKQVELKLPKFPKAFDGLRVVQFSDAHLGFHT

GVKELKKLAATIEEQQPDLICFTGDIVEREAEPMRECIPVLASMQAKYGKFAVLGNHD

YRGGQQNEVTTMFREAGFTLLRNEHVVIEQGGERLAIAGLDDALTGRPDPAQAIKGLD

HDVWKLLLMHEPDYADIATPYGFGLQLSGHSHGGQVRFPWIGALTTPRGSHKYVQGLY

YTDVQGVYYTTQTQMPVYVNRGFGMTQLPIRFLCRPELTVFELKGSTT"

CDS 222873..223604

/locus_tag="EFAGFIKM_00204"

/inference="ab initio prediction:Prodigal:002006"

/codon_start=1

/transl_table=11

/product="hypothetical protein"

/translation="MERIAIVSDIHGNMTAWEAVLKDIRSRGVERIFCLGDLVGKGPE

PVQVVDQVRATCELVIRGNWDELVAVNQDNENFTWQAERLGEERLAYLAGLPFSHQFQ

LSGRTIRLVHASPQSVYHRVQPWDAMEKRLAMFDPPADEPEQGIADVVGYGDVHNAYL

QYMNGKLLFNAGSVGNPLDLPQASYCILEGEDCNDNNTPFNVQFVRVPYDIEREVQVA

QSANVPALDFYIREIRTGIYRGLQE"

CDS 223773..224405

/locus_tag="EFAGFIKM_00205"

/inference="ab initio prediction:Prodigal:002006"

/codon_start=1

/transl_table=11

/product="hypothetical protein"

/translation="MLTRKMLVQGLPALWTERLVLRSLRQSDYSTLSELFSDPQVIRY

VNRGSQPTPIRARRLLNQIRSSSAKLDSLHYGICWKGKEQVIGITSFQHWNDQNGTAQ

IGYILNRSCWGRGVATEAVQRLLQFGFDDLHLGRVEARCYEANVSSQRVLSKIGMSYE

RSLPSFGLHDEDDEGSESLMDVKVYSMYREQYVRPLQEKDLQTLVSDKPQ"

CDS complement(224855..225664)

/gene="hisK_1"

/locus_tag="EFAGFIKM_00206"

/EC_number="3.1.3.15"

/inference="ab initio prediction:Prodigal:002006"

/inference="similar to AA sequence:UniProtKB:O34411"

/codon_start=1

/transl_table=11

/product="Histidinol-phosphatase"

/db_xref="COG:COG1387"

/translation="MKFDLHTHHFRCGHADGNIRDYLEAGIKVGLQAIGISDHTPYFG

SELEQAFPRIAMGKSELQHYVEEVLALKEEYAGKIDVLLGIESDYFPAHAELYRTTLG

QYPFDYIIGSVHHTEDVSIFNKTRWNGLSNARKVEVKESYYSLIRESARSGMFQILGH

IDAMKGNYPPFSEIIADQAIDETLQVIAESNVAIEINTSGKTKLSGGWYPSDAILERA

HHYGVKVTFGSDAHKPQRVADELDDVRTRLKEIGFTDWVYFKQKQMQIVSL"

CDS complement(225695..226597)

/gene="cmpR_1"

/locus_tag="EFAGFIKM_00207"

/inference="ab initio prediction:Prodigal:002006"

/inference="similar to AA sequence:UniProtKB:Q55459"

/codon_start=1

/transl_table=11

/product="HTH-type transcriptional activator CmpR"

/translation="MNISQLETLITISKTMSFRKAGELLNLTQPAVSAQIKSLEDEFS

TVLVDRNQPVTLTDRGQVFLEHAERMLDIVDELKQKLSDLDETPQGRIVLGTTTSIAI

QILPRVLSYFQDQFPLIKTSIQSLPSSQIYTQVENGLVDIGIGYLTERNPNLNTSVLY

YDTFELVVSPSHPLAKKKHAAVDVLRSTPLILLSPDTVGRRFTETIFKKHNIEPNIVM

ELSSSEEVKRMVEIDLGAAVISKQSVAHELRQGTLRMIPLSELEVSHPVGVIYKSSRY

LNSAMQQFISDLKGMPETQFISSE"

CDS 227054..227512

/gene="cheX"

/locus_tag="EFAGFIKM_00208"

/EC_number="3.-.-.-"

/inference="ab initio prediction:Prodigal:002006"

/inference="similar to AA sequence:UniProtKB:Q9X1V3"

/codon_start=1

/transl_table=11

/product="CheY-P phosphatase CheX"

/db_xref="COG:COG1406"

/translation="MKAEVINPFLESARNVFEQLIQVSPSTGSLGVKNVEYIADHVWI

VIGMTGQLSGNIVFGINEQVALKIVSAMMGGFVITEMDEMSKSAISELGNMISGNAST

ILSNQGVVVDITPPQVMKSEHLTTFSATKALSIPLLMDGIGEMDIQVMIS"

CDS 227569..228342

/locus_tag="EFAGFIKM_00209"

/EC_number="1.-.-.-"

/inference="ab initio prediction:Prodigal:002006"

/inference="similar to AA sequence:UniProtKB:Q7A3L9"

/codon_start=1

/transl_table=11

/product="putative oxidoreductase"

/translation="MLKDQVVFITGASSGIGALCAQMLIEEGAIPILAARSRDKLEEI

GASLKGPHELLTLDVTDSEQVQAAVDAILHKYGRIDILLNNAGYGKFAAMTEMSVQEF

DEMMDVNYMGIVRCTKAVLPQMLERGKGQIVNVASMAGKIGTAKSASYTATKHAVLGF

SNALRQELRKTGVMVTTINPGPIDTPFFHRADPSGNYVNNVRWMMLKPEGVAGHMIRA

MKKRKEEVNLPRLASAGIWLYQLFPRLADRLSHGVMNQK"

CDS 228738..230111

/gene="deaD"

/locus_tag="EFAGFIKM_00210"

/EC_number="3.6.4.13"

/inference="ab initio prediction:Prodigal:002006"

/inference="protein motif:HAMAP:MF_00964"

/codon_start=1

/transl_table=11

/product="ATP-dependent RNA helicase DeaD"

/translation="MTNQTFASIGVEQDLEAVLAKHGINEPSPVQAQTIPVILEGRDV

VSKSQTGTGKTLAYLLPLLQSIKMDIKGTQKLIIAPTQELAMQIVREAQRYAEERKIG
VLGLIGGAAAKRQIEKLREHPQLVVGTPGRLKELITLKKLKMHNVSTIVIDEADQVFQ
LGGVSDVNFVLKSALRDRQLIFLSATIDEHTAGLAKREMKEPVQIGIEPDRATAAGLE
HYYFVEENRNKIDMLRRLVRQYNPDRAIVFVNATEDIGEVEAKMNHLGLSAAALYGDA
DKVTRSNVLSAFRNGKLQLLIASEVAARGLDIEGLPMVINYDPAFDSEHYVHRAGRTG
RMGRSGIVLSIVDETQIFIMRKFARELGIELSERVLFGGKVLEADPRPDTRPEGHSFS
RKPGQSRDRKPGQGNRNGGTKATISNSRPAGSKPTGNTGRTERDQDRKNKGAPKWSKD
KTPRSEE"

CDS 230139..230891

/gene="ecsA_1"
/locus_tag="EFAGFIKM_00211"
/inference="ab initio prediction:Prodigal:002006"
/inference="similar to AA sequence:UniProtKB:P55339"
/codon_start=1
/transl_table=11
/product="ABC-type transporter ATP-binding protein EcsA"
/db_xref="COG:COG1131"
/translation="MENVQSPVLQISGLSGGYSAKRPVLHGIDLEVGRGEMVGLIGLN
GAGKSTTMKHILGLMTPQQGEVRVMGKKRDEDAQIYQSAMAFVPESPELYDEMTVMEH
LEFTARAYNVSEADFKQRTEKLLELFRMNEKSTSLSTHLSKGMRQKVMIMCAFVAGPP
LYIIDEPFLGLDPLGIRSLLDFMLEMKASGSSILLSSHILSTIENYCDRFIVLHRGQV
IAQGTLNELRSQFGESDATLEHMFYSLVQGRD"

CDS 230894..232111

/locus_tag="EFAGFIKM_00212"
/inference="ab initio prediction:Prodigal:002006"
/codon_start=1
/transl_table=11
/product="hypothetical protein"
/translation="MDLKLLWKQRRTGFWNGILPYLGYVIQSGVAMVFLFLVIAFSAW
YTSFVQNIPAEFPIRWIALLLLAPLVLFSSYRTYLLPADIVFLRPQEYRMQEYLKNSF
ARGIIYKTLGLLLVFVTLWPLYVRADLDARPFGWFIVFLLLWKGLSSYGAWQELRMVQ
VGAARGYRLFRWALSVLAVGAWLWQPPQRSVWFLLLLAVVYIVALRIPVKHRVAWERL

IQVEQGQAGRVMRTLGWFVDVPSSGQKVSSRRWLSKWGSGLPWNAGKAYRYLITKTFI

RTEVFSIVLRLVVLGMLLSWWTAGSYFGVGVYLFFLLLAGVQLGALRRSHSESFWIMI

YPISGESRRSQVLGFIFHLHALAALLMWLPMLAAGASGLTVTGAALILGILVIVIMRR

SQGNKWLKEEEDE"

CDS complement(232675..233973)

/gene="hisC_1"

/locus_tag="EFAGFIKM_00213"

/EC_number="2.6.1.9"

/inference="ab initio prediction:Prodigal:002006"

/inference="protein motif:HAMAP:MF_01023"

/codon_start=1

/transl_table=11

/product="Histidinol-phosphate aminotransferase"

/translation="MNPLAEQLNESIQTGSSHVYSMLSQLGKEIYFPKEGILSQSAEA

ASLAKTHNATIGIALEGGVPMHLQVIQEKLSAFQPKDLYPYAPPAGKPELRTVWRDKM

LKETPSLKGKTFGNPIVTNALTHGLSIVADLFADEGDAVIYPDKNWENYELTFGIRRH

GQLVHYPLFDDQLNFNSDGLLQALLDQKDKGKAIVLLNFPNNPTGYTPGAEEADAIVN

TILQAAEAGVNVVAVTDDAYFGLFFEDSIHESLFGKLANIHPRVLTVKVDGATKEEFV

WGFRVGFITYAHEDAAVLHALEQKTLGIIRATISSGPHPSQTFVLDALKAPEFEAQKQ

EKFEIMKGRANKVKAILDSGKYGDAWDYYPFNSGYFMCLKLKEVGAEELRSHLLHKYG

VGTIALGESDLRIAFSCIEESGLEDLYETIYRGVQDLRTT"

CDS complement(234152..234769)

/locus_tag="EFAGFIKM_00214"

/inference="ab initio prediction:Prodigal:002006"

/codon_start=1

/transl_table=11

/product="hypothetical protein"

/translation="MRRKEFTVDEEQEITAFLDQCSFGFLGTVSPDGQPRVTPLNFVY

MDGCFYFHGSLAGEKMKQIKKDSSVSFTVAEEFSLIPSYFSDPELACPATSFFKSVMA

FGQAEPVKDLDIKGKVLQRFMEKLQPQGGYVSIDATDSRYTGNLKAVAVVRIVPERIS

AKFKFGQNLSSERFDHISSQLEQRNEGRDAETAEMMRKYCPFHQQ"

CDS 234925..236382

/gene="gabR_1"

/locus_tag="EFAGFIKM_00215"

/inference="ab initio prediction:Prodigal:002006"

/inference="similar to AA sequence:UniProtKB:P94426"

/codon_start=1

/transl_table=11

/product="HTH-type transcriptional regulatory protein

GabR"

/db_xref="COG:COG1167"

/translation="MELWLPMDTYELQHRYKYEALYHALRDAIHAGTLVGGTRLPSTR

ELAKQYEMSRGSVAQVYDMLLADGYVHAHRGRGTFVTETLSTQENEKQEAVLKLSPWG

ERVQRLNTQHEILRTQMSSPKASVINFQMQRMPAEHFPLGEWKSALAAVHRSGWRESS

GVAGDPELREAIASHLRWTRGIQAEPSQIVLFSGSMQGITLLSQLLIAEGAAVVLENP

GYPGIAHAVKSCGGHIIPAELDAAGIIPQPWEAQTLFVTPTRQFPTGAVLGLDRRRAL

LAWASKRNAVIIEDDYDSEFRWGGRPIEPLKVLDREQRVVYVGSFSQTMVASFRLGYA

VLPPGLVEPLLAAKALYEPVPPALLEQRALAKFMTRGGYLRHLRRLTRLYGERHDFFV

REMELQLPEAFTMQLGDAGLHIYATWNGDVDSYQRFKKLAEEDGILFRDAERYRLTSG

HPAACFAFAHLEKEEITEGIRRMRLAWEKCTFSFR"

CDS 236561..237346

/gene="rhaR_2"

/locus_tag="EFAGFIKM_00216"

/inference="ab initio prediction:Prodigal:002006"

/inference="protein motif:HAMAP:MF_01533"

/codon_start=1

/transl_table=11

/product="HTH-type transcriptional activator RhaR"

/translation="MLQASPSSFVILPAVAKIVCEPGWKWQKREKPMQNYDLFYVWSG

EGTLVLNDQSYEVGKGSCFLFRPGDHPTATHNKQKPLVLTYIHFDVDVPVADVPQSYR

EVQETVEFEHLLARYVRLFLSDVYGRDEESRLILKQLMIHLLRADTEEPVEKKVSNQL

SDVIQEVANYVRQHPGITHRVEDLAARAGLSPRYFSIKFKELVGSSVQSYIIRMRIER

AEHLLVHTGMNVTEVADALGYRDIFFFSRQFKQYTGKSPSEIR"

CDS complement(237173..237694)

/locus_tag="EFAGFIKM_00217"

/inference="ab initio prediction:Prodigal:002006"

/codon_start=1

/transl_table=11

/product="hypothetical protein"

/translation="MSPYANKEDGAAPVTRYCAILFMNSYSSIYTCNTKLRSVVIGLL

SSPQNHTSCKSSAGLKRRRRFRKILHPFVEHPRLRTRRFPFITSSPFWSSGMLDNYPC

IPDLKRFPVFLIRILTNLGRAFAGILLELTTEEENIPVAQSIRHFGHIHAGMHQQMFR

PFNTHADNIRLHR"

CDS complement(237694..238485)

/gene="psd"

/locus_tag="EFAGFIKM_00218"

/EC_number="4.1.1.65"

/inference="ab initio prediction:Prodigal:002006"

/inference="similar to AA sequence:UniProtKB:P39822"

/codon_start=1

/transl_table=11

/product="Phosphatidylserine decarboxylase proenzyme"

/db_xref="COG:COG0688"

/translation="MAKTLLRLMTELSSRKWISRTVGAFSKSRGSKAFIPYFVRTYDI

PVQEAEKDWKEYRSLNDFFTRKLKPGMRPLELSEHALISPVDARITAAGPISAGTLLN

VKGQNYTLAELLNHSPHLEKYKHGYGFVLYLSPRDYHRIHAPVSGRKIESEHIKGKVY

PVNDFGLTHMRSVLSRNERLITYIAHDYGEVAVVKVGAMNVSSIQYADTDTSTWAQGD

DLAYFEFGSTVVLLTQSGTFEPNPGLQLGDSVKMGALLGRLKPKT"

CDS complement(238571..239701)

/gene="yheD_2"

/locus_tag="EFAGFIKM_00219"

/inference="ab initio prediction:Prodigal:002006"

/inference="similar to AA sequence:UniProtKB:O07545"

/codon_start=1

/transl_table=11

/product="Endospore coat-associated protein YheD"

/translation="MLQETIGIMFDSRMYRGIPAGRTGQESLANYEQAAASYGLTPCF
LRLEDIDSDQKTCLAYVKKEGRYVRERMPLPSVIHNRSLQLRRTEQHQITKLMLQGIQ
VFNVRNRYRKDHIHDMLHQDLALREHLPRAVKATPESLAYMMEQYDDLVIKPCSGSIG
HGIMRVFERDGQWKLTCETKAARKGWATFRLSKGQLPSATLRRIFRHAYLIEERIPLV
RYEGRPVDLRVSVQRGSDGLWGITGMFAKAAPAHTFVSNIAQGGKVMKLAEALGLGES
GFDLARLEYRIGLVALRIAQNLAASLPHLADLGLDLGITRSGQIYFIECNGRDQRYGF
RKAGMSEHWKATYSKPMAYGRLLLEQNSRIPRQPQTYDRRLY"

CDS 240218..245431

/locus_tag="EFAGFIKM_00220"
/inference="ab initio prediction:Prodigal:002006"
/codon_start=1
/transl_table=11
/product="hypothetical protein"
/translation="MSKNGKKKVPTPKLRHVSPKYKELELTDRRADKMNSGFTVNREE
RLNTNKRDSRAVQESEEFLFDSMELPASTNELEELEIEAEIKPEPVESDSKEIETEDS
SDKQIQVSSVAPLVLEEEVGIIKQETTSKDRLQILNQNQMPGHTGHYIYTDDLSEFAV
TESKLAPFSVDASKLKPDAVGSEALQDYAVTSIHIADGAIVSAKLAEASVSEEHLIDG
SVSGHKIRNASIAGEKIRDGSITSQKLGNQAIDSAKIADGSISTRHLSRMLVTEELIK
NHAVTGDKIAISGVDTRHLTGGAVNTSKLADDAVTTTKIREAAVTGSKIEEHSIESHH
IQAGAVKQTHLAEGSVGRSQLLNGSIGSDQIEDGSIQTRHLAEGSLSGRHLLDGSIGS
SQIRAHSVGKEQIGNGEVGSEHLTEGAVTSSKLADQSVGTAKLLEQSITASKISDQSV
ISSKIADEAVQGKHLAKGSIRAEHIANRGITPVHVDNSAIHSIHIASGSIEAAHLSAG
SVSGDALADDVVCERHLAESAVGTYELQDAAVTDVKLADGSVTTEKLGTASVSSRALA
PGSVISSHLASGGVTGTHLAPGSVGSEALRPYAVKSEHLTEHAVGVPHLQPGSVETDA
IARAAVTTDKLAIDSVTSAQLAGGSIFPPHLTDHAVTSPKLSPESVATDKLADFAVTS
AKLADGSVTSSKIMAESINAKHIPAGTIRGYHLKQHAVSLEHLSEEIRSPELFADGSI
TGNKLRHGSISADHLTADSVSGTELQQAAVGSEHLQPSVVQSVHLAEGSVKSDHLGTQ
VVSSQHLKPDIIHEEHIAEQVVTSHHLAPGSVETDHLAPLSITAAHLQPGLISGLHLQ
AESTSAVHLQQGAVHSRHIQDGEILPHHIHERSIGTSHLEEEAVSTIILQDESVTRSK
LATGSVDGSKLAAESVSATHIANESVQTRHIQEGAILADHIQECSIGSVHLDEESVSE
VHLQNGSVTSAKLADGSINGSKLLEGTVSGIHIASESVQSGHIQADAIHADHIQERSI
GTTHLEEEAVSAIHLQNGSVISTKLADGSVTGSKLIEDAVSGIHIASESVQSRHIQAG

VILSDHIQEHSIGMLHLEQEAVSAIHLQNGAVTSTKLADGSVTGSKLLEGAVSDIHIA

DDSVRSSKIQAGAIHADHIQERSIGTAHLEEESVSAIHLQNGSVTSAKLADSSITGSK

LLEGTVSGIHIASESVQSEHIQAGAIQADHIQERSIGTTHLEEEAVSAVHLQNGSVTS

AKLADGSISGSKLLEDAVLGSHIASESVQTRHIQAGSILADHIQEHSISTFHLEEEAI

SAVHLQNESVTGAKLADGSVSSSKLLEDAVLDVHIANGSVQSRHIQERAIHADHIQER

SIGISHLKAESVSAIHLHNGSITSAKMADGSVNGNKLLEGAVSAIHMADKSVQSRHIQ

DGTIHADHIQERSIRTTHLEEEAVSGVHLQNGSVTSAKLADGSVNGSKLSEQSITSNH

LNAGIVGPAHLSEEIWNAIRQFSGETLEQLAAIKRQEGLLQPENEGVQQSQQVIVEAI

PSAGLEQQQELVSQTDQLGELPAALFTTEQAEQEVQEDQAQQPQLERSQEIAGTTEFM

LSEQSITQEHLCDDAVGSNQLQSGAVQAQHLAFQPVRSVSKQPVVQQFGMEAFVLPES

EVSTEVTVVFEESFASEHYVIVGMSNDRGFQISLLSQSEDEAVLEVSRTADCKHTYGL

LSWIAAGPSQG"

CDS complement(245602..247167)

/locus_tag="EFAGFIKM_00221"

/inference="ab initio prediction:Prodigal:002006"

/codon_start=1

/transl_table=11

/product="hypothetical protein"

/translation="MKFAKVMPTILGGALLLASVSSATAAPVSDQSIPLQAPYASEEG

IPLNSGTDDTIFNYLGQQEQFLNTDVKSQLKIVKRNTDTSGVRHFRLKQYIKGIPVYG

AEQTVHLDKTGAVSSALGDLPPIEEQAIPNDGVAEISGEDAIQIATEEATSRIGELGA

AEITPQAELNIYHHEEDGQTYLVYITEVNVLEPAPLRTKYFINAVDGSIVSQFDLINF

ATGTGTGVLGDTKTLTTTQSGSTFQLKDTTRGNGIQTYTANNGSSLPGSLLTDSDNVW

TDRAGVDAHAHAAATYDFYKNKFNRNGINGSGLLIRSTVHYGSNYNNAFWNGAQIVFG

DGDGTTFRSLSGDLDVVGHELTHGVIEYTANLEYRNEPGALNEAFADIFGNTIQSKNW

LLGDDIYTPNIPGDALRSLSNPSLYGQPDKYSDRYTGTQDNGGVHTNSGIINKAYYLA

AQGGTHNGVTVTGIGRDKAIQIFYSTLVNYLTPTSKFAAAKTATIQAAKDLYGATSAE

ATAITKAYQAVGL"

CDS 247936..248892

/locus_tag="EFAGFIKM_00222"

/inference="ab initio prediction:Prodigal:002006"

/codon_start=1

/transl_table=11

/product="hypothetical protein"

/translation="MRKTRTTARYATRSNVTRRKTVLKKRRWKSGHKKGTKAYTFVIP

SKQLMSQAKDGVENGAESGMSDAPSHRDVSQRTMLSVIIPAMNEEKRIGAVVREALRI

CRDAEVLVIVNGSTDGTANAAKRAGARVLTYAEALGHDGGRKVGAAEARGQVLLFTDA

DIPIPAEHLAPYVKAVLKGTDVALNDYHGPVTHHPVHPVVEAKHMLNSILARSDLRGA

SMTAIPHAISRKGLEVMGISSLEVPPLAQAKAVIGGLRVRAVHHVPVGRMNAVRIKKR

SGPDPLSQVVLNDHMAAIKWITDTLGPRGGYTDLQRVRNLAR"

CDS 248895..250046

/locus_tag="EFAGFIKM_00223"

/inference="ab initio prediction:Prodigal:002006"

/codon_start=1

/transl_table=11

/product="hypothetical protein"

/translation="MSHRMMQHAGGEEMKGRSGVVHRRRTTNRRTTARRTVMNKRHRN

KKHVDIQKTRRSRSKSGQVDLHLMWKAGQVAGSEAKANNTVSEKHARLVWNAHAANVA

ELSNFELALKAGKAFMQGYAQATGHSFQIVPLPLRHSAAAVVCACNEEHTLGQVLLQL

KRLPLTDIIVVLNGTTDDSLKQVLRQPGLTVIYEPDRAGHDVGRALGAKMTDAETVLF

VDGDMVVSAEQLAPFLFAVDRGQDVALNNLTSLLPAFVGQDEVSRIKAYLNRTLGRAD

LGSNSMTAVPHALSRRMIQTVKPASLAVPPKAQSLAIQHGMNVSAPSQVDVIRSNRLR

PGNTGSGNDVAKLIIGDHLEALATLLGTGWNPAQAHTLSRVEVAYRRNA"

CDS 250046..250819

/locus_tag="EFAGFIKM_00224"

/inference="ab initio prediction:Prodigal:002006"

/codon_start=1

/transl_table=11

/product="hypothetical protein"

/translation="MTLTSMIIPTYNGLDLIRPCIDAIRQYSGDPASYEIIVVDNGST

DGTAEYCALERIRFVRFPDNRGFPVACNAGLRAACGDELLLLNNDVTVTPRWLENLRT

ALYSDASIGITGPVTNYASGIQQIKLQFRDMEHFQELANSNNVADSSRWREVRRIVGL

CMLMKRDVVESVGLLDEAYSPGHYEDDDYCYRTRLQGYRLLVCGDVLVHHQGSASFQK

TDPVAWKQLLERNRSIFMNKWHVDPLEYMDISDEGGNVE"

CDS 250816..251547

/gene="rmlA"

/locus_tag="EFAGFIKM_00225"

/EC_number="2.7.7.24"

/inference="ab initio prediction:Prodigal:002006"

/inference="similar to AA sequence:UniProtKB:P39629"

/codon_start=1

/transl_table=11

/product="Glucose-1-phosphate thymidylyltransferase"

/db_xref="COG:COG1209"

/translation="MKGVILAGGTGTRLYPLTRLINKHLLPVGKHPMIMYGIDRLRQA

GIDELLIVINKHSAGLYTEYLGGGADHGVKLTYRIQEKAGGIAEALDLATTFILPGEK

FVVLLGDNLFSDDLKPYVDRYMQQPAGTARVLLKEVDDARRYGVPVFDPKHPERISYI

EEKPSQPKTSYCVTGIYMYDDHVFNLIRNIAPSARGELEITDVNNHYASAGGLEYDIL

QKYWSDAGTFESLQEAAVRMKGQLP"

CDS 251676..253094

/locus_tag="EFAGFIKM_00226"

/inference="ab initio prediction:Prodigal:002006"

/codon_start=1

/transl_table=11

/product="hypothetical protein"

/translation="MRVVTRTGAARASVSGKPRGIHPAVFMGSGTPTAAGTGSAGVRG

VKSGKHSTPTKAGTSAGSSHKVRARKASSTGSVVGRAGKAGSASASKAARASRTGKGK

AGLRRATAGRRPASARGRRAAARAKQRTAMRRAPMPAPQASTPQAMPASGAGPAPRQA

THPKGVAPAGRGGAAGAKAGSAKGLPPPPAEHTVQAEAVPGGYAEGYRDGVFAGGEAL

VAQHIPPDHILPAVAAADLIAAGFRQYAPSLARLSSPHEMAGHITAALDAQRPLSVVR

LGDGELLTLAADTVLPGQEVQELAPFLPYAGVPCSTPDIRAMLAEAIQAADWVGVPIS

RAPTFQGLLFPVLRHFGIDWSRLKLTSSTINYSLHQSGLVLPLLQGRRVLLIGSQAPE

LAALLHNRGIHVTGIIDSVAGVMDIPRVMQQTAEHAFDIALVAAGIPAVILCRRIAGE

LGKVALDFGHLADKLVTGELQL"

CDS 253161..254888

/locus_tag="EFAGFIKM_00227"

/inference="ab initio prediction:Prodigal:002006"

/codon_start=1

/transl_table=11

/product="hypothetical protein"

/translation="MNTPDQQRGEGRHGRNNKVNARNVIRRNGNIHHNITPNISTHSD

NSRSENNRNGSISSEKKAARRATQQDVSRSDMAVLHAAGRHSIRKSRRKGKSRRTPKG

ARLYKQGYTRGYNEGVRQGQSSFGLVFEGVSIIIPTYNQREYVLQCVSSIEKHTPPPF

EIIVVDNASKDGTAEAMLRKGGMVRVAALDQNRGFAGGVNHGLMMARGRHIIVLNNDT

LVTPGWLDNMMTCLAGDPKIGVVGPVTNYIGGDQQIEVPYREVEEMWPFAATHNRPDA

DKHRITDRLVGFCWLFSRELLERVGYLDEGYAVGNFEDDDWIIRVKLAGYQLAVAGDA

FIHHFGSVSMKALGEQDFAVVNKDNEQFYSQKWGDPHALVAETSRLARQQTSGTPSGQ

QEEGDTNPMDPRQRISTQGSQDPTLQFRHSYDFYPEGSFLSDAKGDVYTLTGGRRRKL

NIPVPRGISPVQVAKPDLLAIPAGEALISAGDVQGWPRESQQVHTTAVHGSHHGLAEG

SIVAAVDAPEIRYQISGDKRRRFVSVYAAERWGVHSVHIISVSADQLNAMEEGWPIIA

PPQLLNPDL"

CDS 255240..255518

/locus_tag="EFAGFIKM_00228"

/inference="ab initio prediction:Prodigal:002006"

/codon_start=1

/transl_table=11

/product="hypothetical protein"

/translation="MSSSTKKVVLWSKTGCHFCGEVKAFLTERNQPFENIEVQGNDVL

RDVLEAKYGIRHVPVIEVGGDGKFEALLEPDLEKLAELLARVDEAAAV"

CDS 255841..257184

/gene="moxC_1"

/locus_tag="EFAGFIKM_00229"

/EC_number="1.14.-.-"

/inference="ab initio prediction:Prodigal:002006"

/inference="similar to AA sequence:UniProtKB:O34974"

/codon_start=1

/transl_table=11

/product="Putative monooxygenase MoxC"

/db_xref="COG:COG2141"

/translation="MTAKRSLKFGAIIHGVGGSNTTWRHPEVLSDASVNFGFYKRQAL

KAEEGKFDLVFIADGLYITEKSIPHFLNRFEPISILSALAAVTSRIGLVGTLSTSYSD

PFTVARQFGSLDQISDGRAGWNVVTSPLEGTAKNYSKSSHPSHPERYRIAAEYLQVTK

GLWDSWEDDAFVRDKERGVFFDPSKLHTLNHEGEFFSVQGPLNIARSRQGQPVIFQAG

SSEDGKTLAAQEADAVFTGHDTIEDAQAFYKDVKTRAASYGRSAQDIVILPGINPIVG

RTEEEAEQKYQEIASLVTIDKALDYLGRFFEHHDFSQYPLDEPFPELNGIGSNSFRSG

TDKIKKDAKEQGLTLREVALRAATPRSKFLGTPEQVADKIQEWFETEAADGFIIASEL

PSGLSDFVELVVPILQERGLYRTDYEHDTLRGNLGVQIPVNRYTAAKKQVVSDLA"

CDS 257354..258997

/gene="gsiB_1"

/locus_tag="EFAGFIKM_00230"

/inference="ab initio prediction:Prodigal:002006"

/inference="similar to AA sequence:UniProtKB:P75797"

/codon_start=1

/transl_table=11

/product="Glutathione-binding protein GsiB"

/db_xref="COG:COG0747"

/translation="MNRSISWMKYMALAAVLVMVLSGCGAAGGSTNSTAQASGGGQAG

QAEGGNLTFALATSPDTLDPHRSGLAVTVRAIRTIYDNLVVQLPDGSIKPWLATEWSV

SKDGKSYTFKLREGVKFHDGTPFNAEAVKYNLDRVIDPATKAANSLALIRPYSSSEVI

DEYTIKVNLESPSQAFLGNLSQALLGIVSPTAAQKYGDQLGKNPVGTGPYTFVKWDEN

ADIVVAKNKDYNWGPETVENKAAPHVDTITFKIVPEEATRIGSVQSSQVLAAETVPPQ

NIAALKNDPNQQLLQANTVGLPYTLFFNLRKAPWDDVKVRQAVQSAVDVESIVKTLYL

GNYERAWSALSPGILGYDASLEGSINPDINKANQLLDEQGWVKGADGIRAKDGQKLTL

RYVDGSPNREKRNDIAAIIQQQLKQVGIAVEVEITKDIATVIYQNWDYDLYGNSQVNS

DPNALYAFYHTSAEGERPTLSGLSDPKIDELLEQGAVETDPDKRVQIYNQIQQYLIEQ

AVILPIYVFPYTVAASKKVEGIKFDSLGYPLFNDVRIQP"

CDS 259259..260191

/gene="gsiC_1"

/locus_tag="EFAGFIKM_00231"

/inference="ab initio prediction:Prodigal:002006"

/inference="similar to AA sequence:UniProtKB:P75798"

/codon_start=1

/transl_table=11

/product="Glutathione transport system permease protein

GsiC"

/db_xref="COG:COG0601"

/translation="MVQTILSRLATSLLVIFGASVLVYCIMYLLPGDPVLLMLDPSSA

TPEMIENLRVQLGLDQPFYIQFANYFGDMLRGDFGKSMINSDPVLPKILEHFPATLAL

TALSSIIAITIGITLGVLSAIHRNGVIDFVARLVGLFGISMPTFWTGILLILLFSVQL

GWFPAMGSDGFSSLVLPAATLGLVGAGFIVRMVRNSMLEVINEPFIVALRAKGLSERA

IMYGHALRNALIPAVTVIGMLIGDLLAGTVVVETVFSRQGIGRIIADALMAKDLPVVQ

GVVFFTSIIYVVLNLLVDISYSYIDPRVRRAVRT"

CDS 260252..261169

/gene="gsiD_1"

/locus_tag="EFAGFIKM_00232"

/inference="ab initio prediction:Prodigal:002006"

/inference="similar to AA sequence:UniProtKB:P75799"

/codon_start=1

/transl_table=11

/product="Glutathione transport system permease protein

GsiD"

/db_xref="COG:COG1173"

/translation="MSMKPLAGDKKAWAGSVGRIHLWNRRPWRYRVSFAVSRLFFYAA

LLVVVFTVACAIVPGWIAPYDPTQMMTDVILQAPSAAHLFGTDYFGRDIFSVVVHGSR

DSLLIGFASVLVGGIVGSALGIISGYAGGVVDTITMRAVDILMAVPGVLLALSVAAAL

GPGLMNIALAVAVSSIPGYARVMRGQVISVKGLPFITATRSLGGSNARIFWKHVLPHS

LSPLLVMATLGVGTSILTGSGLSFLGLGVLKEIPDWGALLSQGRGYLTVAWWICTFPG

LAITLFVLAVNLIGDDIRDRLDPKVKGAA"

CDS 261292..261840

/gene="ssuE"

/locus_tag="EFAGFIKM_00233"

/EC_number="1.5.1.38"

/inference="ab initio prediction:Prodigal:002006"

/inference="similar to AA sequence:UniProtKB:P80644"

/codon_start=1

/transl_table=11

/product="FMN reductase (NADPH)"

/db_xref="COG:COG0431"

/translation="MSHVVIIAGSPSKRSRLTGLTDYSSQKLTEAGITVEVIHVVDLP

AEDLVQARFDSSFIRDALAVVEAADAVIVATPVYKASYSGVLKLFLDLIPQEGLRGKL

TLPLVIGGSIAHLLAIDYALKPVLAALGGRHILGGVYAVDQGIERLDDGKFVLSADIT

TRLDRSLDELLLTLEKDGITIS"

CDS 261910..262782

/gene="cmpR_2"

/locus_tag="EFAGFIKM_00234"

/inference="ab initio prediction:Prodigal:002006"

/inference="similar to AA sequence:UniProtKB:Q55459"

/codon_start=1

/transl_table=11

/product="HTH-type transcriptional activator CmpR"

/translation="MNIENIEAFVYINHYGSFNKAAEVLFLSQPSVTARIQSLERELG

CKLFDRLGKQIVLTEEGRKFLPYAQQVLQVIQKGKQKIQQRRSTPDALRLGSTVSVSN

YVIPDFLPKIKEAYPEINIKLTTATTDQLIAKLLGQEIDLAFVRKVMHPAIRTVAFYE

DPIQLYVYKGHPFIESGHVSMEAIRNEQLVFFECGSLDWLRIHRAFDSLEHPPNITYH

VDNSETAKKLVMQGAGIAFLPGLTVKKEVQNQELFPIQVHEVAGVSLQISVVTLKEEY

SPLAEPFGELLRKL"

CDS 263023..264081

/locus_tag="EFAGFIKM_00235"

/inference="ab initio prediction:Prodigal:002006"

/codon_start=1

/transl_table=11

/product="hypothetical protein"

/translation="MSIRVGILDQTPIYEGETAVDAFRHTIELAQRAEQLGFHRFWVS

EHHDSGHVAGSSPEVLISHLLAHTKRIRLGSGGVMLQHYSPYKVAENFNILAALGPGR

VDLGIGRAPGGLPRSTQALQEGIQEASSLKEKIVQVKRYIHNEPLEDPSHPLAGLSAS
PLSDIPAELYVLGASVDSAGMAAELGLPYVFSLFINSNIEVALQAIRVYREQFDRSQG
REPYAALAISLIVAESEEEAEGLASEHMLVKIHLESGKVLTVGSVEQAEEFGRQSNET
YRIEILEPSVTRGTKESVGQALLKFQQEFAVEEFIVTTATRDFTKRIRSFELLREILA
EQGLSEFALESKEEAAIG"

CDS 264132..265358

/gene="scmP_1"

/locus_tag="EFAGFIKM_00236"

/EC_number="3.5.1.-"

/inference="ab initio prediction:Prodigal:002006"

/inference="similar to AA sequence:UniProtKB:P54955"

/codon_start=1

/transl_table=11

/product="N-acetylcysteine deacetylase"

/db_xref="COG:COG1473"

/translation="MKSDLEQPKQHSDQQVQGPEHELHGERAEEFVERLITIRRHLHR
NPELSGEEKETTAAIRSWLEEEGVHIADEYALRTGLVAEVGQGNGPVVALRADIDALP
IQEETKLEFASQVDGKMHACGHDAHTAILIGAARLLKQRESILPGKVRLIFQPSEEKA
TGARQVIQSGALSDVRAVFGLHNKPDLQVGTVGIREGALMAAADGFVVKVEGVGTHAA
VPEAGIDPIVVAAHIVTALQAIVSRNVGAQESAVISVTKLHSGTAWNVIPDEAILDGT
VRTFDEKVRARIRERFNQVVAGVAAAYGTRATVRWIQGPPAVVNDESLASAAEQVASQ
IGLNSIRPLPSPAGEDFSFYQKEVPGLFLFLGTSGPHEWHHPGFDVDERALPLGAHLL
AALAEKALHNVQAHQE"

CDS 265687..266547

/gene="cntK"

/locus_tag="EFAGFIKM_00237"

/EC_number="5.1.1.24"

/inference="ab initio prediction:Prodigal:002006"

/inference="similar to AA sequence:UniProtKB:A0A0H3JU78"

/codon_start=1

/transl_table=11

/product="Histidine racemase"

/translation="MKQEIDFIKFSPTQNMTILVKTDHAAEKYNHIATRLMSYDNVYA
EQVGFIEPTRRPEAVARLEMAGGEFCGNACMALAAHHASEAGMAQQESMEIMLEVSGT
DQLIMCHVKKQQNEYDCQVTMPIPKQIEQRTIRYEGIELDMVIIRYAEFIHIVIEVDD
FDDTMKKRAQTLARLLGLTQGDKLIGILLYQSHLEEMAPLIYVPELDSLIWERGCGSG
TASVGAYLAWSQQREITQYIKQPGGAIKVAAQWNGAELGSITIEGSVGIVAQGKAFIE
APAEWSVVNA"

CDS 266591..267385

/gene="cntL"

/locus_tag="EFAGFIKM_00238"

/EC_number="2.5.1.152"

/inference="ab initio prediction:Prodigal:002006"

/inference="similar to AA sequence:UniProtKB:A0A0H3JXA8"

/codon_start=1

/transl_table=11

/product="D-histidine 2-aminobutanoyltransferase"

/translation="MIILVHFQTCLSEFAEKFDKLASKYDHTIQHSAELEAVINDYSR
FVTDQDNKAAWEQLERSELEELDSLVWALRNKSAQCVAIMEKYRALKLENGDVKIADY
FKNIEECIDKEFGSFHVTSDSKVLLVGSGSFPMTPLLIAKRTGAQVVGIDIDEESIRL
GQKVVETLGAGLKIRLEPTLLENLEYTKEATHIIFSSTVAPKYELLDQLHALTNAQVV
VAMRYGDQLKSLFNYPMRATDSSKWKLVDQILRPDDVFDIALYQKA"

CDS 267452..268753

/gene="cntM"

/locus_tag="EFAGFIKM_00239"

/EC_number="1.5.1.52"

/inference="ab initio prediction:Prodigal:002006"

/inference="similar to AA sequence:UniProtKB:A0A0H3JT80"

/codon_start=1

/transl_table=11

/product="Staphylopine synthase"

/translation="MSNLQRILILGTGPASIQLAVTLKDHLNCCIGIAGRQSVRSASV
FSALEESNQRIQASIQNEKHQSLAGECYVDHVFKGYDTVDGEWDTLILAVTTDAYIDV
LEQMNDEFIKQQKCIILISPTFGSNRLVTHYMRERNAAPEVISFSTYLGDTRWMHEHP

SNHVITTGVKKKVYIGSTRYPSEQVSKLCQVYERLDVALEVMRSPLEAETRNISLYVH

PPLFMNDFSLEAIFGASDTQRYVYKMYPEGPITEVLIREMRAQWQEIMNITERLHIQG

VNLLQFMTDDNYPVRLESISRQDIENFNQLQVIHQEYLLYIRYTSLLIDPFSEPNSEG

KYFDFSAVPFRQTFMNREGELDIPRMPKEDYYRIKIIQGIARHLDVSCPTIDQFIAKY

EAKIQEVADANPDQRLSDAFVIQSFDEDIRMICIGLVSSRV"

CDS 268971..270608

/gene="cntA"

/locus_tag="EFAGFIKM_00240"

/inference="ab initio prediction:Prodigal:002006"

/inference="similar to AA sequence:UniProtKB:Q2FVE7"

/codon_start=1

/transl_table=11

/product="Metal-staphylopine-binding protein CntA"

/db_xref="COG:COG0747"

/translation="MKKGLTFTSILLLVSAIALFAGCAQSTTEKESAQTEPANSQVQT

ELIYASAKDINDMNPHLYTGSMPAQGMVYESLVENTPDGIKPLLAESWDISEDGKTYT

FHLRQDVKFRDGEPFDAEAVKQNIDAIQANAEKHAWIKLSTKITSVKVIDEHTVELTL

SEAYYPALVELSMTRPYVFISPKDFKDGGTKDGVSGFHGTGPYKLTDHKVEENATFEA

NEDYWGGAPAIKKITSKVLPAGETTFLALQKGEINFVFTDDRGADSIDVEAIDQLAES

GGYQVVRSEAMNTNMIVANSSRENSPVQETAVREALWVGIDRETISKDIFNGTQTVAD

TLFSANVNYAKVDLKKREYDPEMAKKLLDQAGWTLADGEGVRTKNGQPLAMKLYYDSN

SSSQKIQAELIQYSMKELGIQLEILGEESTSIANRRATGEYDLLFNQTWGLAYDPQST

IAAFTSDSAYKHTTSGIAEADELYKKIDAVMISTDEASRQSLYADIMKIVHDEAVFIP

ITNGRVTVVAPENLDGISFKQTQYELPFEKMNFKYIN"

CDS 270800..271732

/gene="cntB"

/locus_tag="EFAGFIKM_00241"

/inference="ab initio prediction:Prodigal:002006"

/inference="similar to AA sequence:UniProtKB:Q2FVE8"

/codon_start=1

/transl_table=11

/product="Metal-staphylopine import system permease

protein CntB"

/db_xref="COG:COG0601"

/translation="MGSYIVKRILLAIPLLIIISFITFVLINLSPLDPAVVVLQAQEV

PQITDELIAQTNKALGFDQPFMIQYANWIMAVVQLDFGNSYVSGEPVWSLMGPAFMNT

LKLTLVSSVFIIALSILLGVICAMREGKLLDRSVRGISFFLTAMPSYWLAAMMIWYFS

VKLDLLPTSGMDSYRSYILPVIVITVSYTGIYFRTVRSSMLSNMNEDYVLYARASGLS

EKKVTLHILRNSLQVAVSIFCMAIPIVLGSTVVIENVFAWPGLGRLSVKSILSRDFPI

IQAYVLMLAVTFVLFNTLSDIINAAMNPRLRKEF"

CDS 271732..272628

/gene="cntC"

/locus_tag="EFAGFIKM_00242"

/inference="ab initio prediction:Prodigal:002006"

/inference="similar to AA sequence:UniProtKB:Q2FVE9"

/codon_start=1

/transl_table=11

/product="Metal-staphylopine import system permease

protein CntC"

/db_xref="COG:COG1173"

/translation="MRILKNLSKDKLALTSLVVIMVAIMAGVLAPLIAPHDPGQVNMK

LRYASSSWDYLLGNDHLGRCVLSRLIYGIRPSVLWVLVALSLSVLIGAIVGFVAGYFK

GKVDAWIMRVCDIMLSFPGYVMTLAVVGILGAGLENILIAFVCMKWAWFARVIRTSVM

QFSDMDYVKFAKASGTPNLRIITKHIVPVTFADIAVIASGSMCSMMLQISGLSFLGLG

IQAPHAEWGMMLNEAREVMFSRPELMLAPGLAIVVVVSAFNFLSDALQVALDPKLMTS

QGKSDKSYESEVRKAAYEYSRG"

CDS 272609..273418

/gene="cntD_1"

/locus_tag="EFAGFIKM_00243"

/EC_number="7.2.2.-"

/inference="ab initio prediction:Prodigal:002006"

/inference="similar to AA sequence:UniProtKB:Q2FVF0"

/codon_start=1

/transl_table=11

/product="Metal-staphylopine import system ATP-binding
protein CntD"
/db_xref="COG:COG0444"
/translation="MNIVEVEHLKVWDTNTDKVIIHNSSFEVKQGSCMAIVGESGSGK
SVTCRAIMRLNKPWIRQTGTMLFQGEDLNQLSEPEMRRKRGKHLCMILQNGMSAFDPS
CVIGVHIRETLQQHFGWNTREIERKIINAMESVMLRHPCEILNQYPHQLSGGMLQRIM
IALALVLEPDLIIADEPTTALDTISQYEVVEQLIQLRERMGCSMMFISHDLGVVQKVA
DDVMVMKDGNIVERGNMHSVLTEPKHAYTQYLVSTRLELSNHFKALMQGDL"

CDS 273419..274171
/gene="cntF"
/locus_tag="EFAGFIKM_00244"
/EC_number="7.2.2.-"
/inference="ab initio prediction:Prodigal:002006"
/inference="similar to AA sequence:UniProtKB:Q2FVF1"
/codon_start=1
/transl_table=11
/product="Metal-staphylopine import system ATP-binding
protein CntF"
/db_xref="COG:COG1124"
/translation="MLKVESIEKSYNQGGLFSKHRQQVLKQVTFECQQGECLGIIGES
GSGKSTLGRLILGIERPDLGTILLDGQDVQNRRVRMGNLSAVFQNYTSSINPFLTVET
AIMEPMQAQQKLRKHQGNSEKVDLLLNQVGLGPSYRSKYPHELSGGEAQRVCIARAIS
TAPKLIVFDEAISSLDVSVQIQVLQLLKELKEIYKLSYVFITHDIQAAAYICDRVIIF
REGQVVEIVPTKQLKDVQSDYAKRLLKHLITF"

CDS 274186..275379
/gene="cntE_1"
/locus_tag="EFAGFIKM_00245"
/inference="ab initio prediction:Prodigal:002006"
/inference="similar to AA sequence:UniProtKB:A0A0H3JTK0"
/codon_start=1
/transl_table=11
/product="Staphylopine export protein"

/translation="MSGAMSWPFLRLYILVLLYFSANAILNVIIPLQGESLGASGTTI
GLIMGAYMFTTMFFRPWAGRIIQKHGPIKILRLILIINGFALILYTFTGLGGYLVARI
LQGVSTAFFSMALQIGIIDALPEKDRSQGISYYSLFSYIPGIVGPVIALGIWQTGGMD
YFTVVLIGIAVCTGVFGYTAKMEPSKEQPAVNPSEQNVSMWESFGQLVKNPYLFRCSV
LMLAASVVFGAITTFIPLYASQVPNGNAGVYLMLQAGTVVLARIMLRKRIPSDGSWHS
PFIMGTMCLLAVAAQCVSFAVTGGVVFFYVGAVFMGIGQAILYPTLTTYLSFVLPKLN
RNVLIGLFIAMADLGISLGGVIMGPLADFSSYSFMYMMCAILGAVMIVFAYERRRPVT
GTSVS"

CDS 275376..276641

/gene="cntE_2"

/locus_tag="EFAGFIKM_00246"

/inference="ab initio prediction:Prodigal:002006"

/inference="similar to AA sequence:UniProtKB:A0A0H3JTK0"

/codon_start=1

/transl_table=11

/product="Staphylopine export protein"

/translation="MTARRESGDERLKPSMDATSNGKLNPVSFSFIRFYMLAFLFFAA
NSALTIILPLRSEAAGLNQAEIGLMMGAYMFTCMLLRPFAAQLLGKYGPLRVMQWLLL
LHAGTLLLFVVFGVETYLWLRALQGVATAFFSMTMQAGIVEKLEDKDRAQGLSMYTLF
TMVPSLVIPILAIQIWENASDLAFTLLMIGLAALPLLIGYNVDLPRSTVQNKFYTLGD
MFRSFSGIWRSTPLLISSVVMLFASCVFGATATFLPLYMVSTGMASAGIFLTIQGLVV
ILCRFILRKKIPSDGSWNTWLMAGLMLCAALGTQLLSLVEIIGPLVYLSAVFSGFALA
LLYPTLTTYLSFVLPVDSRYVLMGIFMSSYDLGFSLGGLAMGFIVQVSSYSTMFMICT
LLSVAATILVLVFRQRMEAGNKARSMVSV"

CDS 277216..278412

/gene="yciC_1"

/locus_tag="EFAGFIKM_00247"

/inference="ab initio prediction:Prodigal:002006"

/inference="similar to AA sequence:UniProtKB:P94400"

/codon_start=1

/transl_table=11

/product="Putative metal chaperone YciC"

/db_xref="COG:COG0523"
/translation="MTQKQVPVTVLSGYLGSGKTTVLNHVLNNRQGLKVAVIVNDMSE
VNIDAALVKGEATLSRTEEKLVELSNGCICCTLRDDLMQEIEKLVNEGKYDYILIEST
GISEPVPVAQTFTYADEESGIDLTRLARLDCLVTVVDANRFWHDFGSEQSLLDRNQAT
GDEDTRDVVDLLIDQIETCDVLLLNKCDLVDETELNKLEGIIRKLQPNAKIIRTENGQ
VNPSEILNTGRFDFEKVSMSAGWIQELEKESHTPETEEYGIGSFVYRRRKPFHPSRLA
EFMSYWPEEVVRAKGLVWLAAEGDVAASLSQAGPSIQFGPAGHWVAALPEADKEEILR
NEPDVLEKWDVQWGDRQTELVMIGIEMERASIEDELDQCLLSDEEMLADWGHFDNPLP
WPVEAV"

CDS 278693..278962
/gene="rpsN2"
/locus_tag="EFAGFIKM_00248"
/inference="ab initio prediction:Prodigal:002006"
/inference="similar to AA sequence:UniProtKB:O31587"
/codon_start=1
/transl_table=11
/product="Alternate 30S ribosomal protein S14"
/db_xref="COG:COG0199"
/translation="MAKKSKVVREKQRQAIVAKYADLRRELKEKGDYEALQKLPRNSS
PTRLKNRCEVTGRPRGYLRKFKVSRIKFRELAHQGHIPGVTKSSW"

CDS 279078..279452
/locus_tag="EFAGFIKM_00249"
/inference="ab initio prediction:Prodigal:002006"
/codon_start=1
/transl_table=11
/product="hypothetical protein"
/translation="MLDSFTLRRIQSVMNGYIHEKVPAPLRTMVKLTYELNNNELILT
EERPAEERYQWDKMHIARFYWEENQWKVYARDEQSSWNPVDVITPCSDFENVLEQVER
DEAGCSGVRERDRLRVHFIREN"

CDS 279691..280167
/locus_tag="EFAGFIKM_00250"
/inference="ab initio prediction:Prodigal:002006"

/codon_start=1

/transl_table=11

/product="hypothetical protein"

/translation="MKRGTTIFLKLAVLLIGVPILALCIFGIPWLANNPVNPNYAGAL

YPIVIIMYVSVIPFIVALYQAFRLLSYIDKNEAFSLMSVKSLKTIKYCAIVISSLYFV

MLPFVYVVAEKDDAPGLIIMGMVPVFASLVIAVFSAVLQRLLQEAIDIKSENDLVV"

CDS 280177..280410

/locus_tag="EFAGFIKM_00251"

/inference="ab initio prediction:Prodigal:002006"

/codon_start=1

/transl_table=11

/product="hypothetical protein"

/translation="MAIIINIDVMLAKRKMTVTELAERVEITIANISILKTGKAKAIR

LSTLDAICKALDCQPGDILEYKADEDEDTNEHI"

CDS complement(280490..281341)

/gene="btr_1"

/locus_tag="EFAGFIKM_00252"

/inference="ab initio prediction:Prodigal:002006"

/inference="similar to AA sequence:UniProtKB:P40408"

/codon_start=1

/transl_table=11

/product="HTH-type transcriptional activator Btr"

/db_xref="COG:COG0614"

/translation="MKEHIFLPKPVFPRHVCFPDFIGGYSDFPKHHVNREYRTKEINL

DQCYNLHLVLEGKGFLDTGTTRYELTRGQGFLYGPGLRQTYYSDLDDPWSIRWIHFYG

IRLEELLNGKGVDEPWLFQCSNFPVVTALMDRLLELGRGYQVEDEHSVAATLYELLTR

LQSAASQINVSLNHTSERIREAGNYIRSHSNEHITLDHAAGIAGYSTTYFSRKFSQTF

GISFPEFLLESRLLHSKQLLATTNLSIKQITLETGFSQSSYFIRCFKTQENVTPMQFR

MMHNHSL"

CDS 281523..283595

/gene="bgaA_1"

/locus_tag="EFAGFIKM_00253"

/EC_number="3.2.1.23"

/inference="ab initio prediction:Prodigal:002006"

/inference="similar to AA sequence:UniProtKB:D9SM34"

/codon_start=1

/transl_table=11

/product="Beta-galactosidase BgaA"

/db_xref="COG:COG1874"

/translation="MAKSIQMDELKMGVCYYPEHWSEALWEDDFRRMKEMNITVIRMA

EFAWSIFEPEEDQFDFSFFQKALDLAHQYGLSVILGTPTATPPAWLTSKYPEVLNANK

DGVLYQHGMRRHTNYSSPIFRQQCDKIVRNMVIAYKDHPALIGWQIDNEFNCHMDVFY

AEADHVAFREWLKDRYESLENLNQAWGTIFWSQTYTDWEQVHLTRNMVSQSPNPHLAL

DEKRFISANTISFAKLQVDIIRELDPKHWITTNGTFGHLDNHEMTEDLLDFFSYDSYP

QFATIFPGTDGNSLQDRAWSMKLSNVRNMSPNFCVMEQQSGPGGWVDSIGMGTPRPGQ

IRLWSYQSVLHGTDLLVYFRWRTATFGTEMYWHGINDYHNQPNRRVREVAQVGEEFAK

IGRVIAGTTYQADVAILQDYDNIWDGELDEWHGPLQQQSVRSWYKQLQYRHIPTDMVT

LQPTTTLEELSEYKVIIYAHPAIMTDETAELLQAYVSQGGTLFFGARTGYKDIKGHCY

MRPFPGAVAELCGVTVEDFTLIKGTVPAAKLQWSGASERLQEGTSTAGFNEVLHVEHA

DVQVVAEYASEYYSGSPALTKRTVGQGQVWYYGAAYNEPVVDALLDEIGLSSPVGDLV

EVPSEVELGIRSGKDKAYVFLLNYSDQQVPIKLKKQAKELLSGTTLQNEINMPAYGVF

IFEIDLPH"

CDS complement(283740..284783)

/locus_tag="EFAGFIKM_00254"

/inference="ab initio prediction:Prodigal:002006"

/codon_start=1

/transl_table=11

/product="hypothetical protein"

/translation="MTEEQSYTTEEISKLLKISKLTVYDLIKKGDLVAYRVGKQMRID

ATDLEAYKRRSKQLQSSGQRIPTPHQTSASGTQPGQGDFQATSLLSTTPVGSAHAITP

ASSAHAMTAVPRHLVITGQDVSLDILMRHMEKQTRDIRPLRSFMGSLDGLISMYRGES

DLVSTHLLDGDTGEYNLPYIRKILTGRSYVVVNLLSRPAGLYVQRGNPQNLQNWTDLS

KPELRLANREIGSGARVLLDEQLRLHGIPAAGLIGYETEETSHMGVAAKVSSGEADVG

VGIEKAARLVGQVDFIPLTQERYDLVMLRKQGNEAWTGSVLRILQSQEFRQELQSFEG

YDVSRTGEILYEA"

CDS 285021..285893

/gene="modA"

/locus_tag="EFAGFIKM_00255"

/inference="ab initio prediction:Prodigal:002006"

/inference="similar to AA sequence:UniProtKB:P37329"

/codon_start=1

/transl_table=11

/product="Molybdate-binding protein ModA"

/db_xref="COG:COG0725"

/translation="MRKKIGYVLGSMSLGLALVLAGCGANTGTTDTSTGAEQTTSTPA

ASGETSSAGNTDPQETVDLTISAAASLTDAMKEIETNYELANPYIELNFNFGASGALQ

QQIEQGAPADIFVSAATKNMNALVDENLIASGDQKNLLQNSLVAIVPADGTNTVASET

DLTSDSIKTVAIGIPESVPAGTYAKEALTNAKLWDELESKLVQGKDVRQVLQYVETGN

ADAGFVYKTDALTSDQVKIAFEVDKNSYTPANYPIGIIEGTKHRTEAEQFYAYLQTPE

VLDIFAKYGFTIPK"

CDS 285890..286576

/gene="modB"

/locus_tag="EFAGFIKM_00256"

/inference="ab initio prediction:Prodigal:002006"

/inference="similar to AA sequence:UniProtKB:P0AF01"

/codon_start=1

/transl_table=11

/product="Molybdenum transport system permease protein

ModB"

/db_xref="COG:COG4149"

/translation="MNVNAIDWSVFWSPVRLSLQVALLSSVVATVLGIAIAWKMSRSS

FRGKILLETAFMLPLVLPPTVVGFLLLVILGRKSLFGQWIETIFSAPVIFTWWAAVIA

SVVVAFPLVYQTMKSGFSGVDRDLEDAGRSIGANEWQVFRYISLPLAGRALMTAFILG

FARALGEFGATLMIAGNIPGKTQTVPTAIYVAVDSGNQTMAWAWTVSIIIISFLMLLM

TRQQRDGKND"

CDS 286850..287182

/locus_tag="EFAGFIKM_00257"

/inference="ab initio prediction:Prodigal:002006"

/codon_start=1

/transl_table=11

/product="hypothetical protein"

/translation="MNTKSDCGVSLQIDDYLDLLHFAIQIQDQEWQQSLIRQLKIFQT

ATEQQRTPEEELWTRFDYINSKLTGLCHQIHAAHSMDERKKFEERIGLLQLQRVEVAR

KIKWASRR"

CDS 287481..288320

/gene="tcyK"

/locus_tag="EFAGFIKM_00258"

/inference="ab initio prediction:Prodigal:002006"

/inference="similar to AA sequence:UniProtKB:O34852"

/codon_start=1

/transl_table=11

/product="L-cystine-binding protein TcyK"

/db_xref="COG:COG0834"

/translation="MVKKRGIQKFRTALLFITMLAVLAGCSTGTASNDSESASAAGDT

KVKKIIVGTGTQFPNVCFIDENGKLTGYDVELIREIDKRLPEYEFEFSTMDFKNLLLS

LETKKIDLIAHQMEVNDERQAKFLFNDEAYNIFPNKIVVSQKNEEVKSIEDLKGKKLI

VGATSNAAVLAEKWNAANGTGIDIVYSGAGEDTITQIKTGRVDATISTQFAIDYQNKA

VDAQLKTVGDALSNSKVYFILNKDEQELKTKVDEALKAIKEDGTLGKLSTEWLGADYT

VEE"

CDS 288344..289087

/gene="tcyL"

/locus_tag="EFAGFIKM_00259"

/inference="ab initio prediction:Prodigal:002006"

/inference="similar to AA sequence:UniProtKB:O34315"

/codon_start=1

/transl_table=11

/product="L-cystine transport system permease protein

TcyL"

/db_xref="COG:COG0765"

/translation="MGKSFDLSLVLDFIPELLRYLHITLIVLGGSIVLGLVGGVLLAV

PRLYRIPVLSQLATLYVSFMRGTPILIKLFLVYYGLPELLKPIGIDLSRTDPLLFVIV

TYALSDAASFAEIFRGAVRSVDKGQTEAAYAAGMTTFQSFRRIVVPQALIVAFPNMAN

TLIGSLKDTSLAFSIGVMDMVGRGQTLISATSHALEVYISLSVVYYVIVIVLEKGFAF

AERRLQRHERKRVVHKPAIRAKRLKRIAG"

CDS 289084..289803

/gene="tcyM"

/locus_tag="EFAGFIKM_00260"

/inference="ab initio prediction:Prodigal:002006"

/inference="similar to AA sequence:UniProtKB:O34931"

/codon_start=1

/transl_table=11

/product="L-cystine transport system permease protein

TcyM"

/db_xref="COG:COG0765"

/translation="MSNMSIDLQFIYTSFFQILKALPLTLVITIVPLIAGFGIGLATA

LIRIYRVRWIHRIADFYVSFLRGTPMLMHLFLIYYGIPMIIDKLAERYGWAFQSSSIP

ILVFVLIAFSLTAGAYMSEIIRSGILAVDIGQMEAAHAVGMSTFQALRRIIIPQAIGA

VLPNLCSMFVGFLHGSTLAFTVSQMDILGKADVVASVSLKFLEAFIAAAFIYWGLTII

AERITALLERRVAVYSKGGVS"

CDS 289800..290552

/gene="tcyN"

/locus_tag="EFAGFIKM_00261"

/EC_number="3.6.3.-"

/inference="ab initio prediction:Prodigal:002006"

/inference="similar to AA sequence:UniProtKB:O34900"

/codon_start=1

/transl_table=11

/product="L-cystine import ATP-binding protein TcyN"

/db_xref="COG:COG1126"

/translation="MITLTNIHKTFGKQEVLKGIDLTVEQGDVVAILGPSGSGKTTLL

RCVNFLERADEGEVQISGLTVDCKHARKHDIVQLRRKTAMVFQHYNLFKHKTVLDNVT

EGLIIAQKMSKADARTRALGVLEQVGLSAKINEYPSMLSGGQQQRVGIARALALNPEV

ILFDEPTSALDPELVGEVLSVIRSIAQEGITMIVVTHEMGFAREVANRVVFMDGGSVV

EEGTPEEVFVRPKQERTRQFLSRYSSDWSYVI"

CDS 290674..290961

/locus_tag="EFAGFIKM_00262"

/inference="ab initio prediction:Prodigal:002006"

/codon_start=1

/transl_table=11

/product="hypothetical protein"

/translation="MQSKIDEIITHIAHSHQQIARVLDAKRQVAVRMSEIINHLPDIE

PELDGVDGLLDSSGQINKSIISYLGGLADLEEAVAETLTQVMREIAVQEEE"

CDS 290985..291326

/locus_tag="EFAGFIKM_00263"

/inference="ab initio prediction:Prodigal:002006"

/codon_start=1

/transl_table=11

/product="hypothetical protein"

/translation="MSREQSLILMLDAAAKMQWNIALILEAKAIEAEKVRNWTLNHLN

GDTFLTHGDQVGEPLKMHDQLVELLEGLTRMETGLCNNLKAVMVQNDSEDGGGMDGGM

FGGMDLGDMGK"

CDS 291323..291622

/locus_tag="EFAGFIKM_00264"

/inference="ab initio prediction:Prodigal:002006"

/codon_start=1

/transl_table=11

/product="hypothetical protein"

/translation="MSLMRREQEAKLALIESIAHSQQALARILDSVASVTAHSEVSAR

SLAENIRLLSRYQAEMSRMVTGIRLARIQHGEPGLPWLKDPGCAIRIVRDIQEEC"

CDS 291624..291965

/locus_tag="EFAGFIKM_00265"

/inference="ab initio prediction:Prodigal:002006"

/codon_start=1

/transl_table=11

/product="hypothetical protein"

/translation="MLKKKKIRLRTKRALLIRGARLRKIRKLRALRAKRVLHKRPLKG

RSAWLKKKKRAVRRHRSPKPVQPVVPATPTDPNPDGAYSQGYNEAYNEGFNAGFAKGF

EDGHQLAYKAQ"

CDS 292219..293313

/locus_tag="EFAGFIKM_00266"

/inference="ab initio prediction:Prodigal:002006"

/codon_start=1

/transl_table=11

/product="hypothetical protein"

/translation="MKNVAKRKHRPLTEAETAYRGGYAEGRRFGGCQAMMERVQMFEP

TLRDMKVLYIPQGFDAIDEGVTLALQQSVRECVVGSPAAMLQEASQHRPDVVLVMNGL

HVFPADHVEQVNGIRALGIRTAVWFVDDPYFTEDTTSLCQHYDVVFTHEEAAVPFYLG

HGANQVIYMPLAVNPGMFQPRRAAPQHQHDICFIGTGFWNRIALFDELAPFLADKKVF

IAGSQWNRLARFDVLGRFIHEGWIAPGETVDYYNGAKIVINIHRTCENGEDNRNTHHL

EGHSINPRTYEISACGTMQITDARADLPRYYKPGYDIETFTNAAELQRKIHYYLKHEE

ERQAMAWRGLLTTMNQHTFTRRIGQLLEHL"

CDS 293374..294513

/locus_tag="EFAGFIKM_00267"

/inference="ab initio prediction:Prodigal:002006"

/codon_start=1

/transl_table=11

/product="hypothetical protein"

/translation="MSLKHRKTKKIHAPVLSLADQARKNGQHAGYDAGKEEGYLRGRA

NYIVNCAQEPLPFRQLHVLYVSSGKGFPYSPLDEAIMATLQGMVAQVTLSDPRQPISE

IALQTRPDLVLVLDGMDIPIEHIDAIRQAGIQTAIWLTDDPYYTDMTLEIVTHFDHVF

TLELNCIDLYRQIGCASVHYLPFAAFTNHYFPITTPSPLKRDVSFIGSAYWNRVYFFN

PIMPQLMSHNTVFNGIWWDRLPDYTAYGEKIELGRWMSPPETNDVYNGTKIVINLHRS

HEDDSVNNNHLKIPPASPNPRTFEIAASTTLQLTDARDDIARFYKPGVEIETYSSPQE

LLDKVEYYLTHEKERREIALRGLERTLKDHTYGKRINEMLTIIFP"

CDS 294532..296205

/gene="mshA_1"

/locus_tag="EFAGFIKM_00268"

/EC_number="2.4.1.250"

/inference="ab initio prediction:Prodigal:002006"

/inference="protein motif:HAMAP:MF_01695"

/codon_start=1

/transl_table=11

/product="D-inositol-3-phosphate glycosyltransferase"

/translation="MHSTLYRIIVYRVRAAMATKPKMMLFSHVCNTRSITGAEKLLLH

FMREIGTIFECVLVAPQEGKLAGLARRFGIQVKICTLPMLHGVYTPYLGIADDAEHLR

HTPAYQEAVSLIRETAPDIVLTNTCVNVMPAVAAKSLQIPIIWKITEIIHTNEHTTEA

IQMIGRYADWIIGISETAVAPFQEAGMGDKVTIISPTWEPALPAPDRWVHLRERKRKE

LGFKSSQTCIGYISSFIYDAKGLKPFVDMALRICETHSRCRFWIIGAPSDKKYYDECV

SRVKKSGYSRRFTFTTFEENVSLAYTAMDILVIPSMVKEGFGMTALEGLYFAKPVIAF

AQGGLKELMESVGSDAFLAPPGDTEALVTLATSLLNDAELASNTGWRNRTEAERLYGV

ETYRTKLHTMVTQWLLRFPGWFAYIQPPNGPVYTHGEGGLRTVLVLEPTTVRALLFPL

TVIQALPHSSLPPIALGHDAPVASGTVPAVKPIQQRGKTRKRRRKPLAPHSRRDREGL

KRTSGTGRRKGRTRTTKGPRPHMGKSASRRRKSAKAGRSRAGRKGSNTR"

CDS 296202..297311

/gene="wbpI"

/locus_tag="EFAGFIKM_00269"

/EC_number="5.1.3.23"

/inference="ab initio prediction:Prodigal:002006"

/inference="similar to AA sequence:UniProtKB:G3XD61"

/codon_start=1

/transl_table=11

/product="UDP-2,3-diacetamido-2,3-dideoxy-D-glucuronate

2-epimerase"

/db_xref="COG:COG0381"

/translation="MKAGITMKIMTVLGTRPEIIRLSLIISKLDQYASKHILVHTGQN

FTESLSGLFFKEMGLRAPDYVLQDEAATLGRQLSSMFTQMEDLILQEKPDKLLLLGDT

NSALCAILAERMGVPVIHMEAGNRCFDLDVPEEKNRKVIDAISTVNMPYTEQSKKHLV

SEGVPSRRIVLTGNPIYEVMQHYDAQVSSSKILKKLKLKSGQYFLVTAHRAENVDHAP

HLLEIMKGLNQVAEEHGLRVICSIHPRTAIRIAEHLHLEMNPLVEFHEPFGFFDFVML

ERHARCALTDSGTVQEECCIMGVPTVTMRRTTERPETVDCGSNVVSGLDAARIADCVK

VMTKMSSDWDCPQGYKATDVSSKVVKFLLGGKMHV"

CDS 297304..298290

/gene="pseB_1"

/locus_tag="EFAGFIKM_00270"

/EC_number="4.2.1.115"

/inference="ab initio prediction:Prodigal:002006"

/inference="similar to AA sequence:UniProtKB:O25511"

/codon_start=1

/transl_table=11

/product="UDP-N-acetylglucosamine 4,6-dehydratase

(inverting)"

/db_xref="COG:COG1086"

/translation="MFENKRILVTGGTGSWGYELVAQLLPQQPSEIIVYSRNESSQVA

MSREFEDPRLHFRIGDIRDKDALTAACQHVDYVFHLAALKHVPVCEDQPYEALKTNVI

GTQNVIEAAIENKVEKVIYISTDKAANPSNFYGMTKAIGEKLIVYANLLHSDTKFVTV

RGGNVLGTNGSVVHLFKNQIRQKGQVSITDMSMTRFFLTLKDAITLLFKASVESVGGE

IFVMTMPTCKIVDLAEVLIEDSGVENVSIVERGIRPGEKIHEILMSEFESMTTVVYDE

QYLVILPTLGIPGLREHYTNCPPVSFNSFSSEHQLMTKEEIREILKRGGFLS"

CDS 298287..299114

/locus_tag="EFAGFIKM_00271"

/inference="ab initio prediction:Prodigal:002006"

/codon_start=1

/transl_table=11

/product="hypothetical protein"

/translation="MKLLILGGNGMAGHILVDYFRRQGVHSVFYTSRDVTDPNGLLLD

VNDSFMVDRLVEAVHPDVIINAVGVLNNFADEDKITAYHINGFLPHRLRRVADTIGAR

LIHISTDCVFSGERGAYREDDVTDGTSAYAITKALGEVQDEGHLTIRTSIIGPEIRQG

GIGLMQWFMSSTGEVGGYTRVFWNGVTTLELAKWIDHYLASSVSGLIHLAHPAPVSKH

DLLVLFKQTWDKQDVTIMRDDSVVQDRTLVSTREDVKTDLPDYSTMLKELALWMEQS"

CDS 299096..300037

/gene="rmd"

/locus_tag="EFAGFIKM_00272"

/EC_number="1.1.1.281"

/inference="ab initio prediction:Prodigal:002006"

/inference="similar to AA sequence:UniProtKB:Q6T1X6"

/codon_start=1

/transl_table=11

/product="GDP-6-deoxy-D-mannose reductase"

/translation="MDGAELRGKKVLITGASGFTGRHAVSYFREAGAVVAAVVRRPDV

YSFGKGAQVHVCDLNDKQQVRHLIGEVQPDYVLHLAGKNSVPDSWSDPLLVLETNVMA

VLYLLDALRSCPTARTVIVGSRLKYTPEPGRIPQPPHPYSLSKALEEMVSLSWMSLFG

QQIMLAEPGNLIGAGPSTGICSLLVRHVVACEQAGKTEAFRLSGRDNTRDFLDVRDAV

RAYATLLIHGSSGTVYPVVSGVERSLGEIADLLLSMTEAEVPVRWDGASSGPDGSGKQ

EELSLLRKLGWQPLIPFATSLQDILSDVRVQQGRTTS"

CDS 300034..300750

/locus_tag="EFAGFIKM_00273"

/inference="ab initio prediction:Prodigal:002006"

/codon_start=1

/transl_table=11

/product="hypothetical protein"

/translation="MNPRVSIVIPFYNCPYVPQAIQSALNQTYPDVEIVVVNDGSTRH

AELLQPYLPYIHVLGKSNGGTASALNHGIRHASGDYVAWLSSDDYLYPDKIRYQLEFM

QRENVLVSHTNFHYINEHSAVTRMHGGPEPMSDMDLLRRLVNGNPVNGCTVMIRKDLF

SGVGLFDELLPYTHDLDLWMRIVLNGHRFPYLNEPLTAYRWHGAMGSVRHADVIGREA

SMVWSRYREPLLQRIATLGG"

CDS 300847..301563

/gene="wfgD"

/locus_tag="EFAGFIKM_00274"

/EC_number="2.4.1.305"

/inference="ab initio prediction:Prodigal:002006"

/inference="similar to AA sequence:UniProtKB:B5L3F2"

/codon_start=1

/transl_table=11

/product="UDP-Glc:alpha-D-GlcNAc-diphosphoundecaprenol

beta-1,3-glucosyltransferase WfgD"

/translation="MEPKVSIVIPFYNCAYIEQAVHSALHQTYPHIEVIVVDDGSTEH

VEKLQPFMDSICYIHKENGGTATALNEGIKHATGDYFVWLSSDDVMLLDRVEKQLKFM

REVKASFCHGAYHYVNEKSEWLDTVHPEVGSRLEMLQVLLEGCPINGCTVMLEMEAFE

RFGLFDTDFRYTHDYEMWMRLFPMYELFYYNEPLMCYRLHESMGTKRHFKALVAEMEQ

VQAKHRPILFNLLQIGGYWK"

CDS 301623..301760

/locus_tag="EFAGFIKM_00275"

/inference="ab initio prediction:Prodigal:002006"

/codon_start=1

/transl_table=11

/product="hypothetical protein"

/translation="MKHFLSVISGSSFFALWTAMINLLNDLSQENKAIRFSILAQRNV

Q"

CDS complement(302131..302301)

/locus_tag="EFAGFIKM_00276"

/inference="ab initio prediction:Prodigal:002006"

/codon_start=1

/transl_table=11

/product="hypothetical protein"

/translation="MTLLVPSDLYNRWFSTPVSTPHIDVDYAVMNELMKKLPKGYVFP

DPASMAIMNSKD"

CDS complement(302298..303536)

/locus_tag="EFAGFIKM_00277"

/EC_number="2.4.1.11"

/inference="ab initio prediction:Prodigal:002006"

/inference="similar to AA sequence:UniProtKB:P9WMY9"

/codon_start=1

/transl_table=11

/product="Glycogen synthase"

/db_xref="COG:COG0438"

/translation="MKVLLVTYWELTHMGGIWTYVKQLADRLIALGVEVDIMGTNGAH

NEVYYVRNLNRAFSKDKVWPMLQAKLNPTDLPQFTADPLLAYYELNRYAFEMAAAYLGV

DHYDVIHAQDPVAAVAIKRILNRNIPLVTSYHGALAREAFYDAQNSNPQLTLPSYLQS

KRGRYFLSLEERSAAQSELILVSSHWIKSTLTELAVPESKFRQIPYAVDLPAYRASAA

VKFRQRPPAGKKVIAFTGRLEYIKGVHVLIKALASLKNKRSDWVCWIAGEGNLTDELR

SQAASTGIGADVVFWGKLDNIPSFLRHADIYVQPSLQDTQPFSVTEAQLAGVPVIVSG

TAGMPEMVDPGRTGWVVPPQDVDSLRELLDALLEDNVTRKTVGAAAKAWAEQHRSLEV

MGFRTFQVYQEAIYRGGLTT"

CDS 303723..304082

/locus_tag="EFAGFIKM_00278"

/inference="ab initio prediction:Prodigal:002006"

/codon_start=1

/transl_table=11

/product="hypothetical protein"

/translation="MAVYLLEIHENLLQTFAPVKYMDVELAYVGGEEGTDVFVGGASM

HGELYRHLFHLTSNSGGAGGNQVIHNLDVTSEPYELRVISNSSSPNHLVIRIYCRSIH

GQTLGILSEYQMMKLAD"

CDS 304301..305830

/gene="hsrA"

/locus_tag="EFAGFIKM_00279"

/inference="ab initio prediction:Prodigal:002006"

/inference="similar to AA sequence:UniProtKB:P31474"

/codon_start=1

/transl_table=11

/product="putative transport protein HsrA"

/translation="MDKPAIYWLEKSVENVKEQTAVPQEPAEFSLKAIILPLLAIIVG

MIMVILDSTVVNVAIPNLVQYFETDLKTIQWTVTGYTLALSAVIPLAGWLTDRFGAKR

VFLFTIAMFTLGSVLCSIAQSPEQLIIYRIIQGLGGGMVAPIGMAMVFRLAPPERRGS

IMGMLGIPMLLAPALGPVLSGWFIESLSWHWIFLINLPIGIAAFILCIKFLPDTDRGR

TPALDLLGMILAPIAFSMLAYGVSEGGTSWTSATTLTGVIVGGVALILFIIVELRHQN

PLLELRVFKSSDFSRGIILAWVSQVALFGAMILIPLYLQQIKGYTALETGLILLPQAL

ASGVGMPLGGRLFDKIGARPLAFVGLGIISGALFILSSITAETGLGLIITALVMMGLG

MGLSMMPLNTHVLNAAPRKLVGRVTPLTAAAQQVVVSFAVAGLTGFLTSRIADNTTST

EPTAIVHGLVAGFNDTFFLAACIALFGCLLSLILRKPKPMPEEELQNGDKPDPAMMMG

H"

CDS complement(305895..306968)

/locus_tag="EFAGFIKM_00280"

/inference="ab initio prediction:Prodigal:002006"

/codon_start=1

/transl_table=11

/product="hypothetical protein"

/translation="MKVLFTFYVPSGGVDTLNRLRTAVLKRHGIEAHLLYLSTGSGLQ

NNSDTPIFTASSDEDIHHIIHTHHYDAIIATSDIAMPGRLRGLGYTGRIIFEAQGLGT

RDQALETIQMGVPYLQAHCDAAVIPPTDHLLDMFIHICPWLHRFVIPNMLDTDTFAPI

LVDTPPYPVLAWVGRLEHNKNWREYLIISSELVKKNPKARLWLFHDPTLANPEDEVMF

RHMLAEYGLEDKIGIFINVPHFQMPTYYSMIAASGGIMLSTSLLEGFGYAVAEAISCG

CPVLSTDSDGVRSFITHNKTGKFYPIGDVKAAVAEAKDLMKNKKLREYIRIQGRQHMS

LSFSPDRYAISFREMMTALRIFH"

CDS 307214..308347

/gene="btuD_1"

/locus_tag="EFAGFIKM_00281"

/EC_number="7.6.2.8"

/inference="ab initio prediction:Prodigal:002006"

/inference="protein motif:HAMAP:MF_01005"

/codon_start=1

/transl_table=11

/product="Vitamin B12 import ATP-binding protein BtuD"

/translation="MLEVKQVSKVYEGQRGVHQLDFTMERGEIVGFLGPNGAGKTTTM

RMITGYVHPTAGSIMVDGVSVHEQGQRVRSKIGYLPETPPLYPDMTVQSYLKFVANLR

DVPAREVKLRVSEMVSRLGLQGRERQMVRGLSKGYKQRLGLAGAIIHKPDLLVLDEPT

SGLDPNQIIEIRDLIRELGENHTVLLSTHILPEVSMLCNRMLIINQGQLVLDGSPQHF

GSAMGDQFKVSIEVKATAEQLHNVLTPWEKVRSEVIQASDANTAKDTSLNSDSANTVK

MLLTGENSEDFREELFYLLSGAGLPILEMKKENLSLEQIFLKLTTTEATDTDANSADV

DKVAGAEMDQDVTSDIDSSSTSGTSVSTAADALSQARKGEDSK"

CDS 308344..309060

/locus_tag="EFAGFIKM_00282"

/inference="ab initio prediction:Prodigal:002006"

/codon_start=1

/transl_table=11

/product="hypothetical protein"

/translation="MRRMMAVCNKELQAYFLSPTSYFAFAVYVLMTSLLFYSSFVYYQ

PSIVDYRLVLGDTLSMLLFVVPLLTMRLVAEEFRQGTDELLLTSPARVTEIIFGKYLA

SLAILVVLILCSLVYPFIMSFYGTLDMTTVWMSALGLFFLGGSMMAIGLFASTLSQHQ

MVSAVAGFIILLVLWMLDSFAGNTGSALQQWLDPFALTNRFDSFMKGVLSGPDILYYV

TLSSVFLLLSIQIVERKRWR"

CDS 309065..310459

/locus_tag="EFAGFIKM_00283"

/inference="ab initio prediction:Prodigal:002006"

/codon_start=1

/transl_table=11

/product="hypothetical protein"

/translation="MKKWLSHTNSTVLSVAVIGIFILLTLFLNSLGGFQLDLTSNKQY

TLSDQSLTAIKNLKDDVNILVLTVENANNTVLNREVTDMVEEYTKRNSKLKIKQYNLT

QEPALASKYGITGSSIVLEQGDQHKVIDIASLFTATGDGSDGSYQFTGEEKLTQALMN

MSSTEMHKMVFLTGHEELSLAQMTTLQSSLEQNNVQTEELQLNQAGKVPEDADVLAII

GPQRDLSDTEMKAIRTYLSNGGKLLLSLGFVEDMKSSWKNIDALMADYGVVDEHAVMV

DNQQASTMGPLWVVPEYGTHAITDKLAASQLYPMLSLSIALTSKEQDKYTLSPLIHSS

NDSYGETNIGGLLQNETTNDAEEDIQGPVELGYAADTTDGKPKAVILGSSIFMQDSEI

ANGGNRDFILNTVNYLSEKENGLTIRPRVQAGYEMAYLNGEQARTIFFVAIVAFPLIF

VIIGVLLWWRRRRV"

CDS 310456..311472

/locus_tag="EFAGFIKM_00284"

/inference="ab initio prediction:Prodigal:002006"

/codon_start=1

/transl_table=11

/product="hypothetical protein"

/translation="MRKWVPTILVVIVLIVGWAYAASQNYFREEEAVQAKLLGIQSGD

IQSITIHDTTEATSDAAATSTLTLENGVWQMIEPKAYPLNGYSVSSWLDALSGANQEL

VVEEAPTDLDKYGLGTDATRMDIKLKDNREIKLTIGGQLPADDARYVRVDSGPVVAVQ

TEAITSIALSRRDLLDTTPFNMNETNVGSLEWEGEAATWMLTSTSENGVAEQTWTLNG

KTIEATDAVSLIGKIKNLSTADDVRKASELKGTVPRFTLSVEQTVSGQQVRDVYRGLT

VPSEPDQIWVITPDGQWAYGMDATSLTETEKFPDTIKASATSSEESSSSANDETTSSS

TDGK"

CDS 311746..312735

/locus_tag="EFAGFIKM_00285"

/inference="ab initio prediction:Prodigal:002006"

/codon_start=1

/transl_table=11

/product="hypothetical protein"

/translation="MPAVKKATAITLSLSTAIFVMAGLTGCGTNRDTNNMHTQSVRQQ

ANGINRYGVETNGMDGIRAKSYRMHNVTDLKSSEELAKRITELKEVKSARVMLTDRNA

YVAVRLADGHAGKLESKSNGRTSMLNGTMRNHASDTMRGGNMNHDMGGMRVNGGTGTM

SPYSTSGIAPGLNTNSATDRSHMGNDRSIYGTMGTGAVGMMRGLTNSGKARGTDDGHY

GMKSEGQRVDSTDDNTSAEIKGKISAKIKQFAPNIENVYVSANPDFVEHVENYATDIR

NGKPVSGMIDTFQSMVERIFPTNGTDTNHRDDILDDGLMNRNHNGGVMNRMNR"

CDS 313196..313804

/locus_tag="EFAGFIKM_00286"

/inference="ab initio prediction:Prodigal:002006"

/codon_start=1

/transl_table=11

/product="hypothetical protein"

/translation="MNEKIVVAAMRVFEEQHGFARTKDFIEAGVSPYYIKKLESAGEI

ERVKQGIYRQSGQFNEPPNEMVEVSKLVPKGVICLLSALSFYELTTYNPWEYQIAIHR

GGKKPKLPDYPPIKVIYLADVQYKIGIDEVCIDGSAVKIYDREKTICDMVRYREKIGI

DLMKEGLRNYLQSPYKNITRLVSYADKLRIRTVLQKYLEVLI"

CDS 313804..314202

/locus_tag="EFAGFIKM_00287"

/inference="ab initio prediction:Prodigal:002006"

/codon_start=1

/transl_table=11

/product="hypothetical protein"

/translation="MSEIKNIPASVSERLKNIAKKSGKAFDTLLLLYFQERFLYRLSI

SEYRDKFILKGGLFLFSQTRFKARPTKDVVFLARQIANELDLLRELFVSICSIAVPTD

GIVFQLDELTTERIKEDADYEGVRIKVTAL"

CDS 314221..314724

/locus_tag="EFAGFIKM_00288"

/inference="ab initio prediction:Prodigal:002006"

/codon_start=1

/transl_table=11

/product="hypothetical protein"

/translation="MQFDIGFGDVVVPKPQLIEYPVLLDMAAPQVQAYSKESVISEKF

EAMITLSVMNSRMKDFYDIYTLLTTNNFDGRILYEAVSETFQQRGTIFEKEHPLFHEE

FAGNEGRTKQWQAFLRRLGVEEQLAFQDVMDALVSFLEPVYHSIIDEKEFFRCWSYTE

RAWNNNN"

CDS 314897..315121

/gene="gerE"

/locus_tag="EFAGFIKM_00289"

/inference="ab initio prediction:Prodigal:002006"

/inference="similar to AA sequence:UniProtKB:P11470"

/codon_start=1

/transl_table=11

/product="Spore germination protein GerE"

/db_xref="COG:COG2771"

/translation="MKGIDHKSKFLLTHREREVFELLVQDKTTRDIAGQLFISEKTVR

NHISNVMQKLNVKGRSQAVVELIKLGELKI"

CDS 315590..316129

/locus_tag="EFAGFIKM_00290"

/inference="ab initio prediction:Prodigal:002006"

/codon_start=1

/transl_table=11

/product="hypothetical protein"

/translation="MDRFLNEFENQFRAAFLTVLEQTMVKVEHEKVYACAFGTDSDWV

TLFMAVNTEESLSKHITNMKEQGLCDSEQDEIYYRWGCSEYQYGEDTHFNHISRLLYA

TEEVQEYKEEIIRIIAKVVNETADDVFARYGQAKADITFFVSLTDDDLAEEIENQSVN

QMTVLSLASKFLKRYDGIN"

CDS complement(316270..316617)

/locus_tag="EFAGFIKM_00291"

/inference="ab initio prediction:Prodigal:002006"

/codon_start=1

/transl_table=11

/product="hypothetical protein"

/translation="MNRRQRKKFIPFTWIIATKQTEAHAYYALYAIDWKRGGRLSWEG

WNQLEDLLQFHIPIKRKAGGRKSFSQPAAKIAKRALHLHLNEVQFEQLEQLFYQSLSK

KRWRMFIQTNRNL"

CDS 316841..317704

/gene="ybbH_1"

/locus_tag="EFAGFIKM_00292"

/inference="ab initio prediction:Prodigal:002006"

/inference="similar to AA sequence:UniProtKB:Q45581"

/codon_start=1

/transl_table=11

/product="putative HTH-type transcriptional regulator

YbbH"

/db_xref="COG:COG1737"

/translation="MAAILRALQQELNNLPSQERRIAEVIMQSPSDIPGWTISHLAEQ

SGTSAATVTRFCKSFHFKGFPDFKMKLAAELSRASDETAYQDIVAGNPLSKIVEAIEA

NHLASIADTTRLLDLGRLEQAVQLLCQARRIDLYGVATSSIVTQDFYQKLVRIGKSCT

ALSDSHMQITSASSLAEGDVAMAVSYSGETPETIDALHCAKQAGASTISLTSYGSNKL

ATVSDIPLFTSSLEEGMRRGDMASRIALLHVIDILFTGMVSADFDRFIPRLEQSYHNV

QSYRVQHNGGA"

CDS 317708..318442

/gene="nagB"

/locus_tag="EFAGFIKM_00293"

/EC_number="3.5.99.6"

/inference="ab initio prediction:Prodigal:002006"

/inference="similar to AA sequence:UniProtKB:O35000"

/codon_start=1

/transl_table=11

/product="Glucosamine-6-phosphate deaminase 1"

/db_xref="COG:COG0363"

/translation="MKMNIRIFENEEDLNATGAGLIASLLQTKPRAVLGLATGSSPVG

IYKQLITLYQKGLVSFSQASSFNLDEYVGLPTEHRESYRSFMNEQLFNHIDVDIARTQ

VPDGQAADLEQECNSYEQRLDDRGPVDLQLLGIGHNGHIGFNEPGTELTGRTHVVDLK

EETRKANARFFDSIDEVPAQAITMGVGTILKAKQILLIARGEEKAEIIREAFMGPITT

ACPASLLQCHPNVVVLLDRAAGRLVR"

CDS 318439..319659

/gene="agaA_1"

/locus_tag="EFAGFIKM_00294"

/EC_number="3.5.1.-"

/inference="ab initio prediction:Prodigal:002006"

/inference="similar to AA sequence:UniProtKB:Q8XAC3"

/codon_start=1

/transl_table=11

/product="N-acetylgalactosamine-6-phosphate deacetylase"

/db_xref="COG:COG1820"

/translation="MTGVNSHEPGGQRDENISLLRGKLLLADEILEDGVIAWRDGKIH

YAGIPEGLPEQIRREALPLSVPERGLIVPGFIDIHVHGGNGEDFMDASPEVLDKITSF

HSTQGTTAMLATSMTAPKERLDSVLAEVERYRSGEMPYAQLEGVHLEGPFFSPKWPGA

QNPEHIILPDVSWLEAWEKQYPGLIRQVTLAPEREGALEVISWLREQRITAALGHTDA

TYEEVERAVEAGLHHAVHTFNAMTPLHHRLPGAAGAVLSDPRISAEVIADGIHVHPAA

ISILAQLKQHNDQLVLITDAMSAAGLDDGEYKIGDLPVIVKHGEARLKDGGALAGSTL

TMIRGFRYLIQEVGLSLNAASRAASLTPARLLGIDHRTGSLTQGKQADIVLLNAELDI

EGVWVKGRRIGESY"

CDS 319824..320195

/locus_tag="EFAGFIKM_00295"

/inference="ab initio prediction:Prodigal:002006"

/codon_start=1

/transl_table=11

/product="hypothetical protein"

/translation="MERNAMLEHDPFITVLAEKLHIHGYYAFYGEHYNETDMELYRRH

LFTSFSNIVWVELDARKKYMIVDHRGRNTVMKLIEGMLNTRRTLRANQAMAGTDTAGV

QQEIAHLSKLVHMLKFTTFRT"

CDS complement(320815..322170)

/gene="dapE"

/locus_tag="EFAGFIKM_00296"

/EC_number="3.5.1.18"

/inference="ab initio prediction:Prodigal:002006"

/inference="protein motif:HAMAP:MF_01690"

/codon_start=1

/transl_table=11

/product="Succinyl-diaminopimelate desuccinylase"

/translation="MKEQTYFEQNREKHLAELNEWLSIPSISAISEHKEDINRAAQWA

ADALTRAGMENVEVIQTAGHPIVYADHLHAPGKPTALIYGHYDVQPVDPLNLWETPPF

EPTVRDGKLYARGATDDKGQIFLHIKAVEAILAENKELPVNIKFCIEGEEEISSPNLP

IYLNEHTDKLRADMILISDTSLLEKGKPAISTGLRGLCSMHVDLNTANTDLHSGSFGG

GVPNALHALVSLLASLHDEQGRVSVDGFYDGILPLSPEMREEFVKQGFNEEQLRQDLG

LEQLYGEEGYSFVERVGARPTLELNGVWGGFQGEGSKTVIPKEAHAKITCRLVADQDP

QHVLDRIEAHLRAHVQPGATLHVKQIEKAFAFNTDPSNPIMQKAADAYEHVYGVRALF

TKDGGSIPIVEKLSRVLEIPAVMMGFGLPDENLHAPNEHFNLENFDKGLLTIVQFLKS

L"

CDS 322386..324791

/gene="yesS_2"

/locus_tag="EFAGFIKM_00297"

/inference="ab initio prediction:Prodigal:002006"

/inference="similar to AA sequence:UniProtKB:O31522"

/codon_start=1

/transl_table=11

/product="HTH-type transcriptional regulator YesS"

/db_xref="COG:COG2207"

/translation="MEPTRSDRIFGRFKRLSDLKAGRHKGRFYRNSLMLILLIASIPG

LITGIVMYQQVVGRMETEFNQMHQNQIENRARNVDDQLAYLEMNLSHWAFEPRFGNAL

RTLDFVYYFNETQEIVTTLYVLQGSHPLIKSAQLYLQEPKPILFNRDYTELNDEAKVQ

AYDRYLSMGNHVYWTDWVSSSNRDTEPSTNDSPLHTDAGNALVLVHKIPGESINPFGA

LIITLDNEKVASLLKTLTPYDEGLTFLMDQEGNTLVTGNPGTAGETSAFEQQLKEEVA

LHVGSRSFLFRYEDQTYSVSYGSLSRIDSDWTYVSAAPLTSVTSPVKLVSKIIVIASA

GSLILGLLLSWFASRRIYSPVARMLHLLTPGRNETTPTDAKLDEFELLEQQWNELTSR

SVTAHRQLQEQLPHLRDSFVLQLVQGHLYAYNEQDLQQRMRNLGFELEGQQFLLVQMY

FTGYEQLQGRFGSKDTGLVTFAAVNITQEVAKNYFRQISVMNFHDLSSAMLVIAPQDE

PVKSQALLWGQELVEVIGRTLKMKVTLMVSRPAASLQELPGRFVEMEQAVAYRSVEEG

SQILDLEDEACFHKNEAASYPLGLERELLQAVRLGKQTEAERVLEQFMSEITRTGSTE

FQVQQMMLQLLGSIQHMMLQTGVTPYKLFGGYNMYEQLSGIREPVQMRQWMMNEVFIP

YIQEVEVRSQEPLKLVVERTMLYIDTHYRSDISLENCADEEQMTPYALSKAFKQVSGI

NFIDYLTRVRMDAAKQLLRETTMKINDVAVAVGYQHSYFNRIFKKQEGVTPSQYRDQW

FGQ"

CDS 325070..326053

/gene="yteP_2"

/locus_tag="EFAGFIKM_00298"

/inference="ab initio prediction:Prodigal:002006"

/inference="similar to AA sequence:UniProtKB:C0SPB3"

/codon_start=1

/transl_table=11

/product="putative multiple-sugar transport system

permease YteP"

/db_xref="COG:COG4209"

/translation="MNGKTDWNTNGTATAQADMATRPLKQESNWKRQIKRNKWLYVLV

LPGFLYFVIFKYLPMWGIIIAFQDYQPFLGIRESNWVGLENFTNFFSNPDFFRLLRNT

LVLALYDLIFFFPAPIIIALLLNEIRVAFFKRTIQTLVYVPHFVSMVIIASITYVFLT

PQGGVLYDLIAWITGKPIDVLSSPGSFRPLIIVQMMWKEMGWGTIIFLAALAGVDTEQ

YEASIVDGAGRLRRMWHITLPAIRTTIVILLILRLGNFLDTGFEQIYLMTNSLNRDVA

DVFDTYVYTVGITQGAFSYSTAVGLFKSVVGIILVLGSNKLAKKFGHPGIY"

CDS 326066..326953

/gene="araQ_2"

/locus_tag="EFAGFIKM_00299"

/inference="ab initio prediction:Prodigal:002006"

/inference="similar to AA sequence:UniProtKB:P94530"

/codon_start=1

/transl_table=11

/product="L-arabinose transport system permease protein

AraQ"

/db_xref="COG:COG0395"

/translation="MNSRLYNSPAGKVFDIFNYVMLGILGILTVLPFLYIIGNSFATE

AEITERSFFLIPKVFSFSAYEYIFSSSTIFRSIGVSIFITVAGTLVNLFFTLTMAYPL

SRSDFWGRNVMMNMVIFSMLFGGGMIPTYLVIRGLGLLDSYWALMLPGAISAFNLIVV

KNFFQQMPPGLEEAARIDGCSDLGVLWRIVLPLSKPVIATFALFYAVGHWNNFFSALL

YISDSDKWPLQVMLRQIVLLSQASVGDMANMDPNFVQPPEQSIKMAVIVVGTIPILLV

YPFLQKHFAKGVMLGSIKG"

CDS 326972..328477

/gene="lipO_1"

/locus_tag="EFAGFIKM_00300"

/inference="ab initio prediction:Prodigal:002006"

/inference="similar to AA sequence:UniProtKB:P37966"

/codon_start=1

/transl_table=11

/product="Lipoprotein LipO"

/db_xref="COG:COG1653"

/translation="MGQKTGFKKGALLLSASLVLSTILGACSTDKAGSGTAAGGTDEI

TIMLPNFEAENPPENSPVIQKLEELTKVDVNLQWVPSSSYEDKFNITLASGKLPQIMV

VLGKSPSFINAARTGAFWELGPYLKDYPNLSQMNEIITNNASIDGKTYGIYRARALGR

NGVTIRKDWLENLGLEEPKTIDEFYNLLKAFTNDDPDGNGKDDTYGLVASKFTGPWDN

MQVWFGAPNKWGDDGSGGLIPAHETPEYMEALKFFRQIYSEGLVNKDFAVMDATKLPD

PFVNGQAGVMVDVADNAQRMDQKILDKDPNATGRVDVLQAMEGPKGLRDMPTSGYSGL

IAISKSSVKTEEDLKKVLSFLDQLNEPELQALLYNGLEGKQYEKKEDYIVPSTDKLAL

RDLQGLNQILMFVPEDRTLRVQQTPVREKVAQVQKANEEIVIANPGEPLISDVYAQKG

PQLDNIINDARIKYIVGQIDEKGFEDAVALWKNNGGDDYVKEVNELYAALK"

CDS 328610..332002

/locus_tag="EFAGFIKM_00301"

/inference="ab initio prediction:Prodigal:002006"

/codon_start=1

/transl_table=11

/product="hypothetical protein"

/translation="MWNGDAFENPEAQYRVHPFWFWNGDMEEEQVKRQVAEMATQGVG

GFFICPRQGLQIPYLSEAWFDKVRIAVEAAAAHGMQVWLYDEYPYPSGMAGGEVTLDF

PEAKQRQLVHHQLVVQGGEAVDLELPWGRVLVAKAIPKDEQGKRLWQESIDLRSKIGN

IQTHQVYQETGLTSYNRKRFFTYRTAFRLLWDVPAGDWELIIFQEKEIDDFKYYGNFV

DPCHKEAMSRFIGLTHERYKSAVGDRFESVIKGMFSDEIAPLGRIPWSPQLPRYFSER

CGYSLIDSLPALLYSDVPDAHRIRYDYYQSLHLLLRESYHKQVHDWCEEAGIQYAAEA

PGVRMTTQLFSHMPGGDSAHEKIGRSLSWILERYGQKMRDNPKMVSSLARQLGRSRNL

IECFHSVGWSMTLQDAKWMIDRMAAMGTNFYNFHAFFYTIGGLAKHDAPPSQFLQNPY

WKHFRQLGDYTGRLSYLMSTGAADIRIAVLDPTTTFWSLMGNPLHGFEYSGEDESERV

QLERLTKDWMRITTHLLENRRDYDHLDPELLAEADLTDGIIQIGVARYDVLILPPMLN

LEAAAWEQVKRFVQQGGTVISVGMPPHIAIQPGSPEGDEAAQFFGAGEQPQEVYWGRS

VTTDHDNRETMWHQGEGNAYFLPAGTGQGDDFSLELISLLLDQVLPEPICWIMEEESA

SLLMHTRELSAGEHMVFLTNQEGQDLKGQLKLVARQLWDGEELAEGACIVAERLDLET

GRLQILPYTSSGGEWCISLTLAPYEAHAVRLTLQPAKEEALTTKVDVADKTGSTFGAD

VSAGVNQACKIMVPSEGPWRLETVQSNILRMGQFHLEASNANGIILESQHVAVKPFID

QAAELSEQQHLPVQFNQVFGTPKKASVAYPIECRYTTTFELRTALPACLLFMDRAAIS

GTWSMEINGQPLGKEQFDPIEITDHNNIACDITELLHSGLNEMTVIVQVTKDEDGIVD

PLYLQGRFGVEFHYEGMASLVREPDTALCIGPEPQPLYPHFAGEMSYKCTFWLDELGE

DVASFELEFSDWRVQDVTEVRVNGHSLGVRCWSPYRWKGDASWLRPGENDIEVRITNT

LIGLLEGTYFDSEHHRLQDAGHVLLKNSDKGVEAGDGTSYSV"

CDS 331980..332801

/locus_tag="EFAGFIKM_00302"

/inference="ab initio prediction:Prodigal:002006"

/codon_start=1

/transl_table=11

/product="hypothetical protein"

/translation="MGHRIRFDRYPGGVMKALTLSYDDGVVHDRRLVEICNRYGLRGT

FNLNSGTLGNPGRIEAGEVAELYTGHEVAVHTVTHPTLPYVPNELLTEEIIEDRKALE

QLVGYPVRGMAYPNGGYDRALSTKLEWLGIDYARTVESHGRYTLPERPLEWHPTCHHN

HDLAGHAKRFIQHSKTGTPLLLYVWGHSYEFNDNDNWEIIEQFGEQIGGRDDIWYATN

IEVIAYWKAVQRLECSADRSIIHNPSAIPVWFTADQQVVEVQGGETLRLSERIKV"

CDS 332859..335036

/locus_tag="EFAGFIKM_00303"

/inference="ab initio prediction:Prodigal:002006"

/codon_start=1

/transl_table=11

/product="hypothetical protein"

/translation="MYDSIPDILTTVAQRYIGDHPKHSFLFRAHNQGGFRRLGDYRYD

LNLNEKSIEARAGQYVYVWGKIWSEQNTTLNTGLNCYSPVTIYVNGEEVFKSELLQEL

FPERRTQIPISVNYGWNDVLLCFVKTEVGFGGIYGTASFKNFPLHFLTPGADRQGQEG

WLYSEPVDEPVLSLPRDGMMEEETGLTWYPRRGWTDEEQGAGSFARIFGTEQLAVAYA

WSRLDVTLPGSTPVILNGRHDGALTLYVDGTEVYSAGSSGNFRIELPRRYGSSDVVVK

GEVQAVGGSWGFTLEDAERSGSAGWRLKPSHPVAGCSDPWLYTGVFAPGAEPTHVDVV

RTDTVFDDGAQGVYWRVDLPDTHVRPYLENTHYGKWNYPLGVTLLGLLQTGQELGRDD

YIQYVRQHIELSTSFDRYGLWDREGYGAAGINNQLSAIDSLDDCGSFGSTLLAAMEFG

DIRGGRDTADRIAGYITDEQERLEDGAFYRVRGSGEMRQETMWCDDLYMSTPFLMRYG

QLTGDATYWEDAVNQFLMFRKYLYLPEQQIMSHVYDFVRGRATGIPWGRGNGWVLFSL

TELLSILPHDHVKRSELLTFYRDLSQGYLALQGENGLWHQVLNRPDSYEETSCTSMFI

YAYARGIREGWLEQPEPYLEAVRKGWEGMTRLSIDYKGNIYGVCRGSGYSFTALYYRD

DLGWNLNDTHGIGIVLLAGIELYKMNQQLCKTSIDSSVTAAQR"

CDS complement(335227..336102)

/locus_tag="EFAGFIKM_00304"

/inference="ab initio prediction:Prodigal:002006"

/codon_start=1

/transl_table=11

/product="hypothetical protein"

/translation="MIHIGLTGWGDQEDLYPNRTKAKDKLSLYGQYFSTVEVDSSFYA

VQPRDRMARWAAETPESFAFIIKAYQGMTGHLRGKPYFTSTSEMYKAFQESLEPVIEA

GKMQAALFQYPPWFECNKDNVKELREVKLRMEGIPCALEFRHRSWYEGDMRERTLSFL

KEQGWIHSVCDEPQAGLGSIPIVPQATDSEMTLVRLHGRNVSGWHQNGAPNWRETRYL

YRYNKEELLEWKGYLEQLQEQSKDVYVIFNNNSGGDAAANAQMMMDLLDQPVKPFPDR

SEPEKEEGPEQLELF"

CDS 336335..337078

/gene="cypM"

/locus_tag="EFAGFIKM_00305"

/EC_number="2.1.1.301"

/inference="ab initio prediction:Prodigal:002006"

/inference="similar to AA sequence:UniProtKB:E5KIC0"

/codon_start=1

/transl_table=11

/product="Cypemycin N-terminal methyltransferase"

/translation="MTEWYEKSFGEDYLLVYKHRDVQGAHQEVHKMINWLKLEPNAEV

LDLCCGMGRHSMALADAGLRVTGVDLSDVLLREARLMDTEHRVSWCHADMRELPLKGG

FDAVVNLFTSFGYFLEDGEQLKVLRAIHRMLRPGGSYIIDFMNTAYVTRHLVQHSVRE

DEGQRIEEYRKIEEGFVQKEIRITDTTSGHEPRVYQERVKLYTREQLSQMLSAAGLRV

DQVHGGYDEEKYDEQTSPRMIFVGRRPVE"

CDS 337106..338089

/gene="gloB_1"

/locus_tag="EFAGFIKM_00306"

/EC_number="3.1.2.6"

/inference="ab initio prediction:Prodigal:002006"

/inference="protein motif:HAMAP:MF_01374"

/codon_start=1

/transl_table=11

/product="Hydroxyacylglutathione hydrolase"

/translation="MKRTEEISMTEGIHRVKISMSFPLRWVNSYVLSEPDGKITIIDP

GPRNSETEQEWSEALEDLGLTFSDIRQIVLTHQHPDHLGLSGWMQQMTGAPVRMSTRS

RQEADYMWGPDSRINTILPDYYGRHGMPEDKTEQIHEHMKAFASQITPLPNVTPIEDG

EWLNMGGKRWVAVETGGHAPGHLSFYAPESMEILCGDAVLPQISPNISLQPGSDPQPL

LSYMEGLQRLGELNVKQAYPGHRNPFAQFTERTVQLLAHHEERLQKMTERIQESSANA

YDICVHMFGNRLGTHQLRFAMSETLAHLQELIRRELVKQEQQPDGTFYFQI"

CDS 338288..338704

/locus_tag="EFAGFIKM_00307"

/inference="ab initio prediction:Prodigal:002006"

/codon_start=1

/transl_table=11

/product="hypothetical protein"

/translation="MTRRKRNQWRKWQASAAATLTVAALFQYVRTSDAFDVAYAAANG

TDTSVTASSQVDTHDDVMDEWINSTTDSNNWNTQDNDNNDSQYNGTSDSTQAARPDQS

TSNEGNYSDSQRIGQNNQGTDSGYSSGNYQSRTGAS"

CDS 338701..339744

/locus_tag="EFAGFIKM_00308"

/inference="ab initio prediction:Prodigal:002006"

/codon_start=1

/transl_table=11

/product="hypothetical protein"

/translation="MSRTEQVARPLRFQFRAMNTDVEVQLSAGREEAEHAATVVKNWF

ETQEQRFSRFVADSELNRLNGSTGWMPISAAMDEVLSLAYGYIGLTEGIFQPGISQAL

QASGYDLSFEKVQQRQPQRPLLERILRTKTPESSGWMEDRYDHSRPSWIQREGSRMVH

LDPGAELDLGGIVKGWAVERIADWLQRTLHIPAGLINAGGDIQVWGSPDHSQWTLQVA

DPLHDQENVSGVVRLQRGAVATSGVSRRQWQNTDGSLAHHLIDPRTMEPADTDVLQCT

VMGQHASQCEITAKTVCILGSAEAVGWLNRHYDRHDVLWMTRDGSIYFRGNTDTLAEH

WPGFDPDHRFSLI"

CDS 339777..340430

/locus_tag="EFAGFIKM_00309"

/inference="ab initio prediction:Prodigal:002006"

/codon_start=1

/transl_table=11

/product="hypothetical protein"

/translation="MAQWIVDFLPTWNIIRISGIAAYVLLFAGVFLGIAQGMPMAKGK

PKAAMFKWHTRTTWLAFGLGLVHALTLYIDHYSPFTWGELLIPFTASVHPIGSGLGTL

AFYGLVIVLLSSDLRSKLGRKWWFMFHMLSYPVFIGLLVHGMVTGSDSGNIIMRMMYV

FTGLSILGITVLRGMLRERKGPEITIGPKRATSGSPSEQNWVEGFGLVGTVRPKHLK"

CDS complement(340592..341158)

/gene="ypjQ_1"

/locus_tag="EFAGFIKM_00310"

/inference="ab initio prediction:Prodigal:002006"

/inference="similar to AA sequence:UniProtKB:P54173"

/codon_start=1

/transl_table=11

/product="putative protein YpjQ"

/db_xref="COG:COG1267"

/translation="MEHPEQEKIPYSLNSRKVAEATREWLHKRGVTIPEIAELVMLLQ

QKYYPGLTMEECIENVEMVLRKREVQNAVLTGIQLDILAEQGQLISPLQEMIENDEGL

YGVDEILAFSIVNVYGSIGFTNYGYVDKLKPGVLERLNDKSLGPVHTFFDDIVGAIAS

AASSRIAHRKQSELEEALGEKPVSDDAV"

CDS 341431..342486

/gene="gerM"

/locus_tag="EFAGFIKM_00311"

/inference="ab initio prediction:Prodigal:002006"

/inference="similar to AA sequence:UniProtKB:P39072"

/codon_start=1

/transl_table=11

/product="Spore germination protein GerM"

/db_xref="COG:COG5401"

/translation="MRKIHSLRTVSAAALLSIPMVLSGCGMFGAQSSEAVDPPPPIQE

AAMIQAAEGNGALAMLPLTTVYLQDQQGLLAPVSLTLPSGTDASSPKTALDTLVTGGA

YAGMLPEGFQGVLPQGTVVQNVTIHADDKLAVVEFSGNFAKYDAKEERKMLEAVTWTL

TGTPDVENVQIWVDGKKLTQMPVNSTPLPEPLNRAVGINLDLGDTFVTNSSPVTVYFS

AASPAGIQYYVPVTRLVTPGEDRVQAALNELIKGPDKGGELEEVMTGGTELQSVKTAE

DGTVTVALKDDMFAEGDIVPSELLQSVVLTTVENTASKDAKVQIEWNGQKTVMGDDNR

DYSAPVSKPEYINEIPI"

CDS 342814..343569

/gene="rph"

/locus_tag="EFAGFIKM_00312"

/EC_number="2.7.7.56"

/inference="ab initio prediction:Prodigal:002006"

/inference="similar to AA sequence:UniProtKB:Q81LA9"

/codon_start=1

/transl_table=11

/product="Ribonuclease PH"

/db_xref="COG:COG0689"

/translation="MRSNGRTSEQLRPLNLTVNTNKYAEGSVLIEVGDTKVICTATVE

ERVPPFMKGQGKGWVTAEYSMLPRATHTRNQREANRGKLTGRTMEIQRLIGRALRSVV

NLQALGERTITLDCDVIQADGGTRTTSITGAFVALALAVNKISQQHKLQVFPITDFIA

AVSVGVVGEQPVLDLNYDEDSKAKVDMNLVMTGGGKYVELQGTGEEAPFDRRELNAML

ELGEQGILEMIERQKEVLGPIALKIGARGLGEA"

CDS 343571..344194

/gene="rdgB"

/locus_tag="EFAGFIKM_00313"

/EC_number="3.6.1.66"

/inference="ab initio prediction:Prodigal:002006"

/inference="similar to AA sequence:UniProtKB:P52061"

/codon_start=1

/transl_table=11

/product="dITP/XTP pyrophosphatase"

/db_xref="COG:COG0127"

/translation="MSLDSPILIVATRNAGKVREFAHAFAPLGKEVKSMFDYPELPDV

VEDGVTFAENAWKKAKAVGDALGLPVLADDSGLCVDLLDGEPGVYSARYAGEGATDAQ

NNAKLLETLESLKSGEDTEQPLLSPARFVCALVLYDPTTGDKYESEGTAEGWITAQAA
GAGGFGYDPLFYVPEYEMTMAELTLEQKQAISHRGHALRALVSRLEG"
CDS 344545..345387
/locus_tag="EFAGFIKM_00314"
/inference="ab initio prediction:Prodigal:002006"
/codon_start=1
/transl_table=11
/product="hypothetical protein"
/translation="MQEVKLVVSYDDYEDGIRMEKLIKELAAKPEASSLESLVIGDWG
QAYENSPDEFMGTLVESAPSFPSLKKLFIGDMGFEECEVSWIIQTNLTPLLGAFPELK
SFSVMGSSGLSLESLQHAKLEELIIICGGLPKEVLSSITHAELPELRKLELYLGVDNY
GFNGSLEDVLPLLEKGLFPKLVYLGLKDSEIQDEIAKAASEAPILDQLEVLDLSQGTL
SDEGAEALLASDKIKKLKHLDLSYHYMTNEMIIRWNQSGISVDVSDQQQSDEDDWRYP
SLTE"
CDS 345391..346656
/locus_tag="EFAGFIKM_00315"
/inference="ab initio prediction:Prodigal:002006"
/codon_start=1
/transl_table=11
/product="hypothetical protein"
/translation="MSGVKEMNESLHQPIDVPDLTQDQPFLLIGNPDNRRTQGLQEAR
HRLGLKPAVVLPYAQLLQNWRHGRTIADTVDSNLPVPLIRIDAPGEDWEVECELLFLG
AMNDTLALTDGIGAEAFSAEQALALEQDWGRIYAPAQWFRGWKACLDRIGHEAREVWP
EVRFMNDPADIQLMFDKRTCQQHLSSHGVDVPPALQTSQPIRNYMELRSSMKVDEMHR
VFVKLACGSGASGVVAYQVNPRTGDEIAVTTMGMEQRQGKTIFFNEGRLRKYTRSEEI
ATLMNWLCAEGAQIERWMPKATLDQRAYDIRQLVAGGQAGHAIMRLSRTPITNLHLRN
ERMLPAEAGLDEQRMSLVRSAAQAAMSAFPNSWSAGIDVMLTSGSNPRAYVLDVNPFG
DLLYRVEHHGLGTYEWEMELLRKEPIQHA"
CDS 346649..347434
/locus_tag="EFAGFIKM_00316"
/inference="ab initio prediction:Prodigal:002006"
/codon_start=1

/transl_table=11

/product="hypothetical protein"

/translation="MPDMNTIVGSHDLLMITLDTLRYDVAKLEEENCPNLCGSGPWEK

RHTPGSFTYAAHHAFFGGFMPTPANTDKASHVRLFHSRNTGLKTHPHTWLFDTPDIVS

GFAAEGYRTICIGGVIFFTKKNPLAKVLPGYFQHSYWRMNFGVTNPKSTEHQVQHALK

LLEQTAPDEKIFMFLNVSAIHGPNRYFVQGAKEDSVETQRAALRYVDEALGPLFEAMR

KRARPAYCLAFSDHGTAYGEDGYQGHRLAHDVVWTVPYREFLV"

CDS 347482..348870

/locus_tag="EFAGFIKM_00317"

/inference="ab initio prediction:Prodigal:002006"

/codon_start=1

/transl_table=11

/product="hypothetical protein"

/translation="MEKHIQHTTTGHSTQSGSNIATSLSEVLAFPYRSYLYSYPHKTA

YRELDPPLPLGPLWERENTDTYFLYMHIPFCAARCGFCNLFTLPDRRDDTHERYVDAL

ERQAKQWAPITSRRPYSRFAIGGGTPTLLNEVQLNRLFDIAEHVMGLDPAQASISVET

SPDTVTEAKLAIMKERSVDRVSMGIQSFIEAEASAIYRPQKPQEVERALEKLTRYDFP

LLNLDLIYGLPGQTVESWIYSLERVLAYEPGEIFIYPLYTRENTIVKPDDIRRQGPDI

RMELYKAARETLKSKGYVQYSMRRFAKEQSSSKALLPYSCQEEGMVGLGCGARSYTSE

VHYASKYGVSYKATQSIIADYVATERYDVADYGIVLSREEQRRRFILKALLHREGLTL

SDYQQRFGTDVMSDYVWLAELLTEGMAELESDEDKQVLRLTEEGLGYSDAIGDWLISA

EIREQMEGFVFS"

CDS 348867..349730

/locus_tag="EFAGFIKM_00318"

/inference="ab initio prediction:Prodigal:002006"

/codon_start=1

/transl_table=11

/product="hypothetical protein"

/translation="MRATLYYRGKLSSCNYDCPYCPFSKTVDSKETLEVDEQQLRQFV

NWVREQEVEGHQFSIFFNPYGEALVRRWYREAMVELSHMPHVDKVAIQTNLSVKLDWA

RELDASKAAFWATYHPRETKEASFVKKCLTLRQMGLAFSVGTVGLRSAFPAIESMRQA

LPDDVYMWVNAFKDRPKYYTPEEIQFLRGLDPLFEGNLQDYESLGKRCAAGSEVFYVQ

GSGHVKRCYKDRRIIGHLYRDGLQALSADRPCRMKKCGCYIGYIHMEDSPFQETFGSG

LLERNPVLIDR"

CDS 349862..350179

/locus_tag="EFAGFIKM_00319"

/inference="ab initio prediction:Prodigal:002006"

/codon_start=1

/transl_table=11

/product="hypothetical protein"

/translation="MAIKGQKYKTYSEKIKKEAIRLHTVEGWTYRKINEHLGIHDPGR

MKRWMRKHREQGEFGLMDQRGRRKEYLDQERYVQKLKRENELLKKCLVIWKEEANKKD

FRS"

CDS 350128..351012

/locus_tag="EFAGFIKM_00320"

/inference="ab initio prediction:Prodigal:002006"

/inference="similar to AA sequence:ISfinder:ISBth8"

/codon_start=1

/transl_table=11

/product="IS3 family transposase ISBth8"

/translation="MLGNLEGGSKQERFQIIEKVAAYGDIQKLCDVFGVSRSGFYAYV

KRKRFDRDAKAKKQVLQTYQRYEGKYGYRQLQLFLWQDQGIWMNHKKVLRLMQMLGIQ

SRIRRKRRSNSSYAPAQRVAENRLKRDFSAEKPNQKWVTDITQYRVGERWIYLSAIKD

LFNNEIVAYEIGERNDNELVLRTFSKAFAKQTDVTGLVVHSDQGFQYTSHAYHDMLPK

VGAQISMSRRGNCYDNASMESFFSHLKTEGLYPYHIRTLTEAQSKIEKYIRFYNRKRP

QRKLKKLTPVEYRRQFAA"

CDS 351336..353030

/locus_tag="EFAGFIKM_00321"

/inference="ab initio prediction:Prodigal:002006"

/codon_start=1

/transl_table=11

/product="hypothetical protein"

/translation="MKRVSISRKLLLGFGSVLLLLVAVVVISYTQFISVEKTFTDLIR

ERTAKLLTIKNMIIDVKSQQVALRNFVTEENDQSEQQFKSFYEDYRKTSEELRSGIQT

PTMIDFLDRSDRSAEEYYAFGQSVIELKKQGRTSEITRMLLDTGPTIISGFEETVTAM
EQHQQNSLDTGIVNANQLIQKVLRLIMIIGIISIVLGVVIALIMGRIISKPVAHVARA
ASRIADGDLTGEAIVVRNRDEIGELAESFNKMMDNLRHLIHQVGQNADRVAASSEELT
ASTEQTATATEQVATTMEEIATGMDTQVSMVGDGFHTINELSTGFQQMTVNTQNMSDE
ATNASAKTISGNDAVQSAVGQMNSIHQTVRVLAKVIEELGNHSDEIGSMVESISEISA
QTNLLSLNAAIEAARAGEHGRGFEVVATEVRKLSDQSAKSAEQISVLVAAIRNGMNNA
SQSMGEVNAEVQEGIELVRKAGGTFEEIREAVSNVAGQTQEVSASIEQMAAGVEQINV
SMKTIMEVTENAAAGTEEVSATSEEQLSAMQEIASAANDLSSMAEELQESVSRFKV"

CDS complement(353173..355017)

/gene="asnO"

/locus_tag="EFAGFIKM_00322"

/EC_number="6.3.5.4"

/inference="ab initio prediction:Prodigal:002006"

/inference="similar to AA sequence:UniProtKB:O05272"

/codon_start=1

/transl_table=11

/product="Asparagine synthetase [glutamine-hydrolyzing] 3"

/db_xref="COG:COG0367"

/translation="MCGITGFIQWNRDLTQESELLVRMTDSLSNRGPDASGTWISNPC
AFGHRRLSVMDPENGAQPMHALQGDTSYTVVYNGELYNAPELKKELLQRGHQFRTQCD
TEVLLASYIEWGPACVDRFNGIFAFAIWDGGREQVFIARDRLGVKPLFYSNNKDTLVF
GSEPKALLIHPDVEAAVGPEGLAEVFIVGPARTPGHGVYSSLSELKPAHALIYNRNGI
RTYAYWKLESQNHEHNLEETAAEVRSLLQDTLERQLASDVPVCSLLSGGLDSSALSAL
AVDYYKRTGQGQVSTYSVDYVDNAKHFQAHSFQPGADGPWIKRMVDELKTDHHWIEIE
NGELVHALTQAMLVRDLPGMADVDSSLYLFCKEIKKGATVAISGEAADEVFGGYPWFH
REDMLNSGTFPWSVAPDMRAALLSPDIREWIRPLDYLADRYSDAVAEVPLLDGETGKA
AQMRVMSYLNITRFMPTLLDRKDRMSMGAGLEVRVPYCDHRLIQYVFNIPWEMKITGG
REKGILRKALEGVLPDDVLYRKKSPYPKTHNPQYLAAVKQQVLDILDDPTSPILPLID
KAQIRNLASSPDAASNLPWFGQLMSGPQLFAYLTQINTWLRTYKVAIR"

CDS 355210..355509

/locus_tag="EFAGFIKM_00323"

/inference="ab initio prediction:Prodigal:002006"

/codon_start=1

/transl_table=11

/product="hypothetical protein"

/translation="MDSMKYYVSVMGRSVIPDPSVTSYEWIIQATPQEAEQLLGLLSL

MQEKEEEAFPGMVFPWPDTPEESVNRAYETVLQQVYREIYRLGTPETRYQIEQST"

CDS 355541..355897

/locus_tag="EFAGFIKM_00324"

/inference="ab initio prediction:Prodigal:002006"

/codon_start=1

/transl_table=11

/product="hypothetical protein"

/translation="MYAIQDAKIRRLTEVDGLSCAEVEVQPGNAGDPSLLVYVASTQP

GNSLEVVRIVRNHSDSAIDWYNNNMHTAFEEATDNAFENSEGMTVEEDKASFQSELFS

FGLLGPTLQRYLIGQA"

CDS complement(356073..356366)

/locus_tag="EFAGFIKM_00325"

/inference="ab initio prediction:Prodigal:002006"

/codon_start=1

/transl_table=11

/product="hypothetical protein"

/translation="MRQLLSALSYFSIFFAPFLFPIIIWIVAKDDYIEGHAKRALFSH

VFPFLAAIPLFYFFVTAHSLGSAVGFVILFFVIYGLSFVYNVVKGIQVLREYA"

tRNA 356656..356731

/locus_tag="EFAGFIKM_00326"

/product="tRNA-Arg"

/inference="COORDINATES:profile:Aragorn:001002"

/note="tRNA-Arg(tct)"

CDS 357023..357793

/gene="tam_1"

/locus_tag="EFAGFIKM_00327"

/EC_number="2.1.1.144"

/inference="ab initio prediction:Prodigal:002006"

/inference="similar to AA sequence:UniProtKB:P76145"

/codon_start=1

/transl_table=11

/product="Trans-aconitate 2-methyltransferase"

/db_xref="COG:COG4106"

/translation="MKSLNQWQADEYDDKLAFVSGYGKNLISWLQPQKGEYILDLGCG

TGDLTHEISLAEAEVIGMDASPDMIRRAREKFPELEFVEGDGHQFETNRLYDAVFSNA

ALHWMRSPRLVVDSIWKSLKPGGRFVAEFGGKGNVQTIVNALEQVMERNTGIRASERN

PWYFPSIGEYSHILEQVGFVVRHAYHYDRPTRLEDGEQGIVGWLTHFGGDYFEGLSHE

EIQQICHETSQIVMSHLWKDDAMYADYKRLRIEAVKPE"

CDS 358176..362135

/locus_tag="EFAGFIKM_00328"

/inference="ab initio prediction:Prodigal:002006"

/codon_start=1

/transl_table=11

/product="hypothetical protein"

/translation="MSKRLISTLLSVLMVFSIILPAAGAAPADSVIQLDNLPSTAEAK

QWREILAGREAKLAADPFLDKSLEGLGGENTRVIVELSNKPVAVAQGESTLSGKSFTS

SMETKAVTQVEQQQQSFVHSLTQKKIKHEVLEEYAYALNGVAIELKGNQVGQLLQIPG

VVSVYPDLEVTIAPETDEVNPYMKDTAPFIGAPEVWDLGYKGNGIKVGVIDTGIDYEH

PNLQEAYKGGWDFVGNDNDPYEATYQEWKASGEPEFNATGSAYYTSHGTHVAGTVAAR

EAGDYGIVGVAPEADIYAYRVLGPYGSGQTSWVLGGIDRSVEDGMDVINLSLGNAAND

PSYITSVALNNAMLSGVTAVVASGNNGPNRYTLGSPGASAMAITVGNSTGPSQLITAD

THFWIGEEGEEAQPEQQPVPEVEQSPELGTAPGTEEPEGSEQDVPATADEESATAVED

TVSNEAEVPNVGEATTEENTPSASPSAPIEASPEAAPVEEESVIAPTEAPLAITESVY

RLDLMGWSLSADPETVLTGKYELEYAALGKPTDFEGKDFTGKVALIQRGEIAFVEKIA

NAKAAGAVAVIVYNNIPGPIGVSLGDNFELIPTLSMSKEDGDVIKAELDAGQAVEVSF

SDFERSQTPGDEMSSSSSRGPAKVTLDIKPDIVAPGTSILSTIPAYGKDEPDADYAQA

YDRKSGTSMATPHVAGLIALLIEKHPEWTPFDIKVALMNNSKVLDTTKFDVFDQGAGR

IQAVNTIDPAAFVKVLDVTQYTESGSVVEKENITGSINYGNFLSTDEKTITKTIKVEA

LNGSGGDYTVNVNPTRNVAGVSVAVDKNSFTLNGEEELAVTITVPRGVTATTEAQGYI

EVSNGTNTFTVPYVAHFNVTLSGVKYIETVKGTEKNPFHYPLKADGTLDTLNVAMEFY

NPMTFALIEIYDGLNPEGGYYQDGYIGSIFGNYFNFNANARYNLAWDGSYADYTTDEV

TEIPDGLYTVDITTIGTDGKTYKEDTSPFLVKKTAPAVTAPEALEFKEDESAVLNGSV

EDLYFRVAPALASGWDITFDPAASLEATYVVKKEDGSVYKEGVFEVQADGSFSLPLEG

IEAGEYAVELTVKDQQGLSGTANTALKLEAVETEPETPATKAPGTPVLSHNNWVGNGL

PEGAYIVTMNLWWGENATSYKLYEDGVLVDTQKLTYNAPWAQFAQTKITGKANGTYTY

VAELTNDKGTTRSQTLTVQVTNATPGKAVLSQDNWDGDGHYKVTMNLWWGTNASEYRL

YENDKLIDTQELRSATPYAQSAVTTLSGKASGTYTYRAELINAAGVTSTDTIKVKVK"

CDS 362290..362979

/locus_tag="EFAGFIKM_00329"

/inference="ab initio prediction:Prodigal:002006"

/inference="similar to AA sequence:ISfinder:ISSth1b"

/codon_start=1

/transl_table=11

/product="IS3 family transposase ISSth1b"

/translation="MSKRSPISYEIKIHVVRRCLQHESNPNHEAKQLGVHKNTVTDWI

RKYKVDGEDGLRESKRWKSYSKELKRSAIQDVLSGEYSVRAVAKMYHISSKSVLESWI

SKYTEGVKMKPTRQRIESPHMNKGRKTTYEERIEIVQFTIAHDLDYQKAIDKYGVSYQ

QVYAWVRKYQANGHEALKDLRGRKKLVEELNEQERLKLRIKELEARNEHLEMENALAK

KLAEIRRRNTR"

CDS 363108..363506

/locus_tag="EFAGFIKM_00330"

/inference="ab initio prediction:Prodigal:002006"

/inference="similar to AA sequence:ISfinder:ISSth1b"

/codon_start=1

/transl_table=11

/product="IS3 family transposase ISSth1b"

/translation="MPSNRELENFALAKEVKRRYNKRNGILGYRQMRTQLNRKLKKSY

NRKRYYRIMRALELRAVIRKKRSKYLKVPALHIAENIMNRKFQAVAPNQKWCTDVTEL

KYGNGRKAYLSAIIDGYDNSIVSWVLQPFQ"

CDS 363520..363855

/locus_tag="EFAGFIKM_00331"

/inference="ab initio prediction:Prodigal:002006"

/inference="similar to AA sequence:ISfinder:ISSth1b"

/codon_start=1

/transl_table=11

/product="IS3 family transposase ISSth1b"

/translation="MNTVKKAYKGNPNATPLLHSDRGFQYTSLEYNQLEKRYKFTKSM

SRVSRCLDNQPIERFWGTFKAESFYLEKYDTYDSLVRSVRTYIHYYNNFRYTERLNGL

SPNEYRRAA"

CDS complement(364003..365232)

/gene="yycB_1"

/locus_tag="EFAGFIKM_00332"

/inference="ab initio prediction:Prodigal:002006"

/inference="similar to AA sequence:UniProtKB:P37482"

/codon_start=1

/transl_table=11

/product="putative transporter YycB"

/db_xref="COG:COG2807"

/translation="MSSSLQQHSSIHTEDEHSSTSKRFGLLLAGIIVIAATMRSPITA

TGPVVEMIRSDTGIGHTMVGLLTSLPLLAFAAVSPFAPRLAKRLGLESALLLAVMIVT

IGVALRLLPSVLSLYAGTAILGCGIALSNVLLPSLIKRDFPLRVGIVTGLYSVSMNIF

GAIASGVSVPVAGATSMGWRASLGMWALLSILALLLWLPQIAAGRQRMLFIATQSEGT

PVRLRTSSLAWFITLFMGLQSLIFYTTITWLPEILAEQGFSPTSAGWMLSLMQMVSVP

ATFIVPILAGRTRDQRVLTAITCSSLIVGYALLLSGIASLVTIGVTLAGIGAGASFGM

VTMFFVLRTKDARQAASLSGMAQSFGYMLAAVGPLLFGMLHDWTKGWTLPLLIQVILA

IALLIAGIQASKNRMIG"

tRNA complement(365730..365805)

/locus_tag="EFAGFIKM_00333"

/product="tRNA-Arg"

/inference="COORDINATES:profile:Aragorn:001002"

/note="tRNA-Arg(tct)"

CDS 365994..366974

/locus_tag="EFAGFIKM_00334"

/inference="ab initio prediction:Prodigal:002006"

/codon_start=1

/transl_table=11

/product="hypothetical protein"

/translation="MLIPTGDSGYRGGAPIKESTTPETEHNNNIVLFPKTLDYYQIQL

TVMLENERYGEAMKLLHFLLQCQGQEERHYDEWRALLEWLQAAFPQYEETSPAVLEER

EEEEEISEEDMARQHARMKLEQDDGYADKMLRTVMEEPLSEQTMLALEQLAYLDLPKI

DDALTSWLSEKSLHPLLQFRVLQTLRRRGVQGMILFTRGEEQVEVEIDTVPLKPEEFP

LQIVQILERVADQTEVHEPTLFYFAQELWIQYVMAVYGTRDYVSMLEENDSMTDIWAA

ALHMTVADSLGGSHDEENTRSMYAVTGAMRFRLEQAYRSMKQFVSAGLDG"

CDS 367113..368453

/gene="tig"

/locus_tag="EFAGFIKM_00335"

/EC_number="5.2.1.8"

/inference="ab initio prediction:Prodigal:002006"

/inference="similar to AA sequence:UniProtKB:P80698"

/codon_start=1

/transl_table=11

/product="Trigger factor"

/db_xref="COG:COG0544"

/translation="MKATWEKIEKNLGVLEVEVDADRVTAALDKAFNKVVKQANVPGF

RKGKVPRSIFEARFGVESLYQEAIDILLPEAYTEAIDQTDIFPVDRPDVDVEQFAKGE

TFKFKAKVTVKPEVTLGEYKGIEVAVAKGEVTDAEVAEELERLQQRHAELVVIDEGSA

ANGDVAVIDFDGSVDGVPFEGGQAERYSLELGSNTFIPGFEEQVVGLSTGDFKDVEVT

FPESYHAAELAGKQAVFKVKIHEIKRKQLPALDDEFAKDVSEFDTLEEYKADLKTQLE

SRKADEAKAAQENAVVEKVAENAEVDIPAAMVDSEVQNMMRDFDNRLKNQGMNLEMFL

SFSGQTQADLRGQMQEDASKRVRNNLVLEAVGKAENIEVSDEEVNQELEKMAESYKRP

ADEIRGILEGNGSLDSLRDEVKLRKTIDVLVENSTVVEPVEAPAEEVVAEADEK"

CDS 368833..369423

/gene="clpP_1"

/locus_tag="EFAGFIKM_00336"

/EC_number="3.4.21.92"

/inference="ab initio prediction:Prodigal:002006"

/inference="similar to AA sequence:UniProtKB:P80244"

/codon_start=1

/transl_table=11

/product="ATP-dependent Clp protease proteolytic subunit"

/db_xref="COG:COG0740"

/translation="MSLVPMVIEQTNRGERSYDIYSRLLKDRIIFLTSAIDDDVANLV

IAQLLFLAADDPEKDISLYINSPGGSVTAGMGIYDTMQFIKPDVSTICVGMAASMGSL

LLTAGAPGKRYSLTNSEVMIHQPLGGIQGQAADIKIHAEWIIKTRQKLNQIYVDRTGQ

PLEKIERDTDRDFFMSAEEAKTYGIIDQVLSRPINS"

CDS 369436..370689

/gene="clpX"

/locus_tag="EFAGFIKM_00337"

/inference="ab initio prediction:Prodigal:002006"

/inference="similar to AA sequence:UniProtKB:P50866"

/codon_start=1

/transl_table=11

/product="ATP-dependent Clp protease ATP-binding subunit

ClpX"

/db_xref="COG:COG1219"

/translation="MFKFNDEKGQLKCSFCGKSQEQVRKLVAGPGVYICDECIELCTE

IVEEELGHDEEVDLKDIPKPKEIRNILDQYVIGQDQAKKSLSVAVYNHYKRINTQSKI

EDVELSKSNILLLGPTGSGKTLLAQTMAKILNVPFAIADATSLTEAGYVGEDVENILL

KLIQAADYDVEKAERGIIYIDEIDKVARKSENPSITRDVSGEGVQQALLKILEGTVAS

VPPQGGRKHPHQEFIQIDTTNILFICGGAFDGLEQMIKRRIGKKVIGFNTVSEQKDLK

PGEYLGMVLPEDLLKFGLIPEFVGRLPVISTLEPLDEDTLVRILSEPKNALTKQYQKL

LELDNVNLVFEPAALLAIAKEAIKRNTGARGLRAIIEGIMLEIMYEVPSREDVTNCVI

TEEVVEKRVVPELSQSKDDKQEESA"

CDS 370789..371907

/gene="ispG"

/locus_tag="EFAGFIKM_00338"

/EC_number="1.17.7.3"

/inference="ab initio prediction:Prodigal:002006"

/inference="similar to AA sequence:UniProtKB:Q81LV7"

/codon_start=1

/transl_table=11

/product="4-hydroxy-3-methylbut-2-en-1-yl diphosphate

synthase (flavodoxin)"

/db_xref="COG:COG0821"

/translation="MWGEKSLMYLRQDTRPVKVGNVTIGGSNEVIIQSMCTTKTADVE

ATVAEILRLEEAGCQVVRVTVNNEEAAAAIKEIKKRINIPLVADIHFNYKLALLAIEN

GIDKIRINPGNIGKRAKVEEVVKACKDRGIPIRIGVNAGSLENHLLEKYGYPTADAMV

ESALYHIGILEELDFHDIIVSLKASDVPMAIEAYTKAAQIIPYPLHLGITEAGTLFSG

TIKSSAGMGALLSMGIGSTMRISLSADPVEEIKVARDLLKTFGLITNAATLISCPTCG

RLDIDLFSIANEVEEYISKLKVPIKVSVLGCAVNGPGEAKEADIGIAGARGEGLLFRH

GKMIRKVPEEIMVEELKKEIDKIVEAYEETGVIPGRSH"

CDS 372075..373829

/gene="lon2"

/locus_tag="EFAGFIKM_00339"

/EC_number="3.4.21.53"

/inference="ab initio prediction:Prodigal:002006"

/inference="similar to AA sequence:UniProtKB:P42425"

/codon_start=1

/transl_table=11

/product="Lon protease 2"

/db_xref="COG:COG1067"

/translation="MEFGMVIMLIQLFFGVVIGLYFWNLLRGQKTNRSAVERESRKEL

EKLRKLRSISLTKPLSEKTRPATINDIVGQKDGLRALKAALCSANPQHVIIYGPPGVG

KTAAARVVLEEAKKNPTSPFKPDAKFTELDATTARFDERGIADPLIGSVHDPIYQGAG

AMGVAGIPQPKPGAVTKAHGGMLFIDEIGELHSIQMNKLLKVLEDRKVFLESAYYNSE

DTHTPAYIHDIFQNGLPADFRLVGATTRSPHELPPALRSRCMEVYFRPLLPEEIGRIA

EDAVQKIGFSPCPDAVDVVKRYATNGREAVNIIQLAAGLALTEKRETLQASDVEWVAG

SSQIQPRPDRKVPTQPQVGFVNGLAVYGPNMGTILEIEVSAVPALKDQGRINITGVVD

EEEIGGGSRTLRRKSMAKGSVENVLTVLKAMGIRPNDYDLHVNFPGGTPIDGPSAGIA

MATAITSAIQRRPVDHETAMTGEISIHGRVKPIGGVLAKVEAAFQAGAKTVIIPAENW

QSIFENLDGLRVIPVDTVQDVFREVFAWVPESQQMEQTKLAEETVAPAPEIFPPASAS

YLRADVPRPGQVTDSGSC"

CDS 373916..376255

/gene="lon1"

/locus_tag="EFAGFIKM_00340"

/EC_number="3.4.21.53"

/inference="ab initio prediction:Prodigal:002006"

/inference="similar to AA sequence:UniProtKB:P37945"

/codon_start=1

/transl_table=11

/product="Lon protease 1"

/db_xref="COG:COG0466"

/translation="MGPSKAKGRRFPLLPLRGLLVYPSMVLHLDVGREKSVKALEKAM

VEEHLILLCSQAEVNIEEPTQEDIFRIGTVAKVRQMLKLPNGTIRVLVEGLERAEIIE

YTDNEEYYEVLAKELPEEENTHPETDALMRTVLNQFENYINLSKKVTPETLAAVSDIE

EPGRLADVITSHLSLKIKDKQEILETIDVRERLEKLLDILNNEREVLELERKISQRVK

KQMEKTQKEYYLREQMKAIQKELGEKEGRAGEVEELRTQMEELGLPDKVKEKVEKEID

RLEKMPASSAEGGVIRNYVDWLLGLPWSQMTADDLDIQKAEDVLNADHYGLEKPKERV

LEYLAVQKLVKKLKGPILCLVGPPGVGKTSLARSIARSLGREFVRISLGGVRDEAEIR

GHRRTYVGAMPGRIIQGMKTAGSLNPVFLLDEIDKMASDFRGDPSAALLEVLDPEQNN

TFSDHFVELPFDLSNVMFITTANTVHNIPRPLLDRMEMLNIPGYTELEKQQIAKNYLL

PKQKQDHGLEEEQLEIPDDALLRVIREYTRESGVRNLEQQMASLCRKAAKNIVSGVEG

QINISSDEIKDYLGPGKFRYGMAELEDQIGTVTGLAWTEVGGDTLIIEVTVVPGTGKL

TLTGKLGDVMKESAQAAFSYTRSKATQLGISPDFHEKNDIHIHVPEGAIPKDGPSAGI

TMATALISALTNKHVSKDIAMTGEITLRGRVLPIGGLKEKSLAAHRAGYKKILLPKDN

ERDLRDIPDSIKEDVEFVPVAHMDQVLKHALVEQAGIHL"

CDS 376412..377068

/gene="engB"

/locus_tag="EFAGFIKM_00341"

/inference="ab initio prediction:Prodigal:002006"

/inference="similar to AA sequence:UniProtKB:P38424"

/codon_start=1

/transl_table=11

/product="putative GTP-binding protein EngB"

/db_xref="COG:COG0218"

/translation="MKVNQSEFIISAVRPEQYPEDGLPEIALAGRSNVGKSSLINRLI

NRKNLARTSSTPGKTQQLNYYKINQDLYFVDFPGYGYAKVSKEQRFAWGKMMEKYLLG

REELKLVMQMVDMRHEPSKEDKMMNEWLRHNGLPLVVVATKMDKIPKTRRAKYIKVIK

ESLGLRSGDLFVPLSSEEGLGKEELWDIITRHARIDKYAPVLDAEGASDEEETNGING

"

CDS 377336..377923

/locus_tag="EFAGFIKM_00342"

/inference="ab initio prediction:Prodigal:002006"

/codon_start=1

/transl_table=11

/product="hypothetical protein"

/translation="MAQRLATEYVKATMKLSEPQMHQFLRMTEDGRLHHRVKVLDNGC

QEVVLGDVSGEEVHFPFDRIEGFYICELSCRLVNLHLTNVVRKLFVTFKGDGVVHRIY

KGFTMTYVYAQGTVRKIVEKTGENTRVIYEYKNTLLELQHLFQARDVEREINRVYAEI

DSLLDTRKDATADQLHQIDETLARHQKRLFELEAY"

CDS 378593..379978

/gene="hemA"

/locus_tag="EFAGFIKM_00343"

/EC_number="1.2.1.70"

/inference="ab initio prediction:Prodigal:002006"

/inference="similar to AA sequence:UniProtKB:P16618"

/codon_start=1

/transl_table=11

/product="Glutamyl-tRNA reductase"

/db_xref="COG:COG0373"

/translation="MHIVVVGLNYRTAPVEVRERFTFAEKDLSEALQQLKLTKSVLEG

VIVATCNRTELYVVVDRLHMCGYFIRTFMEQWFNVPREEFTQHLYIYEDDQAMRHLFR

VICGLDSMVIGETQILGQVKQAFLKAQEEKSTGTWFNKLFKQAVTLGKRAHSETSIGE

SAVSVSYAAVELGKRIFGMFTDKKVLILGAGKMSELTVKHLYANGASEVIVANRTLAR

AEELAAKFRGTPCTMEQALDRLNEVDIVISSTGAERYVMDAAVVRESMKRRQSRPLFL

IDIAVPRDIDPAIGELSNVFLYDIDDLEGIVESNLEMRKVEAAKIERMIELEMEDYYH

WLKTLGVRPVIRALQEKGVSIHEETMDSLFNKLPELDEHQRKVIRRLTKSIVNQMTTD

PINRIKEMAGTKQGDEALRMFTQIFALEDAVEMAAEKVSQSDATALAKPAAHLNENRQ

DPVPAYAPAGV"

CDS 379993..380820

/locus_tag="EFAGFIKM_00344"

/inference="ab initio prediction:Prodigal:002006"

/codon_start=1

/transl_table=11

/product="hypothetical protein"

/translation="MTLAEQVYEALIYMYALSLLFYFSDCIRRNAGAKRTGTGFLVVV

WGLQVTHVILRMWTEGHFPIYTTFDFLFIFSFSIVLMSLVMTRIQRSEFVILLLNVVG

FSVTVLNRLWFTAGEISLHNWQTVHGLLIMHITLANLGFAALTVATVFAMLYLFLHRK

LKKKKWNDTMRRLPSLEVIGKYMDGATLIGTPLLGVSVMLAVLSIVAERRWILLLDLK

VIATGLAIAIYVGYFVSKRRKQFSTIIMARWTLIGYGFVIISFLSNAYSAFHRWTGE"

CDS 380826..381476

/gene="cysG_1"

/locus_tag="EFAGFIKM_00345"

/EC_number="4.99.1.4"

/inference="ab initio prediction:Prodigal:002006"

/inference="protein motif:HAMAP:MF_01646"

/codon_start=1

/transl_table=11

/product="Siroheme synthase"

/translation="MDRYTPIFVNTSGKKCCVVGGGRVAERKIKGLLHAEAQITVISP

ELTPVLKQLHHESRIQWIDRSYRNGDLRGAFLVYAATDCSEVNSTVVRDAEAAGILVN

NAMSSEHSSFITPSVVRRGRLSIAISTAGAGPAAAAEIRATLERQFGDEYETYLEFLH

QMRTEVKSRVSSSSLRSTLLRQLTEMHILEDIRTGHFRWWTEEEIGDWISRNQEEV"

CDS 381479..382435

/gene="hemC"

/locus_tag="EFAGFIKM_00346"

/EC_number="2.5.1.61"

/inference="ab initio prediction:Prodigal:002006"

/inference="similar to AA sequence:UniProtKB:P64341"

/codon_start=1

/transl_table=11

/product="Porphobilinogen deaminase"

/translation="MRTIKVGSRQSALALTQTGHVIQDLRDICEREGLAFDFEVHKIV

TKGDLILDVTLSKVGGKGLFVKEIEQAMLDRTIDMAVHSMKDMPSELPEGLINGAIPR

RADPRDALISNGGLTLDQLPEGARVGTSSLRRSSQLKAYRPDLQLESIRGNIDSRLRK

LETEGFDAIILAAAGLYRMGWEDRITEYLTETACLPAVGQGALGIECREDDEELLHLL

QLYNDPETAFPVQAERRFLSVLNGGCQVPIGAHAVWVPQQDADSLNGENTLQLTGMVG

TPDGGLILKEAMIGKDPVRLGEEVAWRLIERGAEQILAEVRG"

CDS 382440..384002

/gene="cysG_2"

/locus_tag="EFAGFIKM_00347"

/EC_number="4.99.1.4"

/inference="ab initio prediction:Prodigal:002006"

/inference="protein motif:HAMAP:MF_01646"

/codon_start=1

/transl_table=11

/product="Siroheme synthase"

/translation="MVGKVFLVGAGPGDAKLITVKGWESIGKADAVVYDRLASPRLLK

QMKPGAVKIYVGKRPDRHTMKQEEINQLLVDLALEGKVVVRLKGGDPTIFGRVGEEAG

LLHKNGIPFEIVPGVTAAISVPAYAGIPVTHRDYASSISIITGHESPDKLDRSIHWDK

VTNATGTLVFMMGVAKIGYISEQLIRHGRPAQTPVALIRWGTRAEQDTLTGTLEDIEA

KVIAANFQPPAVIVVGDVVNQREQLKWAEALPLFGKRILVTRARSQASELVNRIEELG

GEPYEFPVIETVMPSSESAQQSVKDAFSALNTYDWVFFTSVNGVEFFFRHLEQEGKDI

RSIHQARIAAVGPSTADALRKHGIVAEVVKGPFQAEGMLEAFESELKEGQRVLLPHGD

LARTWLRDQLRERGLQVTEAITYDTILAGEDDDELLKLLEEGGIHAVTFTSSSTVTNF

MSMLKRMGLQDPLPLLKNVEVACIGPVTAKTAEAAGLKVTLMAEEATMDSLITVLCDW

KRTGTKEGALRN"

CDS 384105..385106

/gene="hemB"

/locus_tag="EFAGFIKM_00348"

/EC_number="4.2.1.24"

/inference="ab initio prediction:Prodigal:002006"

/inference="similar to AA sequence:UniProtKB:P64334"

/codon_start=1

/transl_table=11

/product="Delta-aminolevulinic acid dehydratase"

/translation="MSFPIVRHRRLRQSTGIRNMVRETHLTVDDFIQPIYVTYGENVK

SEIKSMPGVFRFSLDRLQEEVTEIAELGIPAVLLFGIPETKDSVGSSGFAEDGIVQEA

TRLIKSWYPELLVVADTCLCEFTDHGHCGMVHTVEIDGHICGDVLNDESLDLLVKTAV

SQAKAGADIIAPSNMMDGFVQAIRAGLDENGFSHIPIMSYSVKYASAFYGPFREAADS

TPQFGDRKSYQMDPANAREALREAETDVLEGADMLMVKPSLSYLDVMRTIKDQFDLPL

VAYNVSGEYAMVKAAAIQGWIDEKKVAMEILLSMKRAGADMIITYYGKDASRWLAEK"

CDS 385135..386439

/gene="hemL_1"

/locus_tag="EFAGFIKM_00349"

/EC_number="5.4.3.8"

/inference="ab initio prediction:Prodigal:002006"

/inference="similar to AA sequence:UniProtKB:P30949"

/codon_start=1

/transl_table=11

/product="Glutamate-1-semialdehyde 2,1-aminomutase"

/db_xref="COG:COG0001"

/translation="MTAQLGNRRNDERSRSAIEEAKQYIPGGVNSPVRAFKSVGLTPI

YVERGEGSKIYDIDGNVFIDYVGSWGPLIMGHAHPDVVEALRETAVKGTSFGAPTLLE

TEMAKLVCERVPSIDIVRMVNSGTEATMSAIRLARGVTGRSKILKFEGSYHGHADSLL

IKAGSGVATLGLPDSPGVPEGVAVNTITVPYNDLESVTLAFERYGEELAAVIVEPVAG

NMGVVPPASGFLEGLRSLTTQYGSLLIFDEVMTGFRVGLNCAQGRYGVTPDLTCLGKV

IGGGLPVGAYGGRRDLMEQIAPTGPIYQAGTLSGNPLAMAAGYTTLKLLTPEVYDRLE

TLSARLQAGFEKNAAETGIAITINRVGSMVCPFFSAVPVTNYDIAKESNLDQFRRYFA

AMIDQGVSVAPSQYEGMFVSGVHTEQDIDDTIEANRKALQSL"

CDS 386436..387341

/gene="rluD_1"

/locus_tag="EFAGFIKM_00350"

/EC_number="5.4.99.23"

/inference="ab initio prediction:Prodigal:002006"

/inference="similar to AA sequence:UniProtKB:P33643"

/codon_start=1

/transl_table=11

/product="Ribosomal large subunit pseudouridine synthase

D"

/db_xref="COG:COG0564"

/translation="MTIKSWKRRGEWLELMPGKAVTGSLDKPMAAEQWLLSELQFPEK

LLRQLKANRGIQLAGDRLRLALFASQPIDVDPRWADVDVLYEDDFCLVMHKPAGMKLH

PDGSRADQAITLDHVVASYYEMNGIQASVRHVHRLDEDTTGPVLYAKNAFALAKLDEA

MRRKEIGRHYVAIAGGQIPLELRKIDAPIGKDRHHKQRRRVSEGGQEAVTHVEIVEVW

ERATLVRLKLDTGRTHQIRVHLSYAGHPLIGDALYGGRADVIGRQALHGEMIKFGHPL

TGAMIEVNDPWPPDFKQLAEREVAY"

CDS 387714..389297

/locus_tag="EFAGFIKM_00351"

/inference="ab initio prediction:Prodigal:002006"

/codon_start=1

/transl_table=11

/product="hypothetical protein"

/translation="MLNQPYGLRFDIYERVHLSEGVPAIEELEEIELYPRIQVIGQDD

HATLRGHLLLTGAYRGENEASEELKHFIPVEITVPLNRVRSIEDISIEIENFDVDLLS

NRSLNITGVLSLRGIEGFPVEEPQVWSADEFTVVHSPDAQQSNRSDEAAQAGSDPNTF

AQEYLLQRSEEERLANAAELAYGAPEFADLSIREETLGNSEEWQQEYANSSLAEPLQS

EEQSAEYANENRQPDANEDPASLTANGDELASHEQYSEPSPISSFMAETADRLASYPE

SEPLLPLEDESSGWSEPSVDALPANSRSADQAVSEPTAHSSNELAAPDVPDAPEVWHF

ESVRSVPQQENQAVADVPVESQAENWQGVFASSEPGNAEEDRPPFEATGIDEFVQNEA

FVPEPVAETEDKPELKVAFGSKKESAPRQEEGVGISSLLSSGRAARDAEVERGDEVPA

GVVQEETYPADDVEWKNLFLGTIVDQTPFRKVKLCIVQREDTLDAIADRYQLSTRELQ

LYNRLSEQVVEEGQILYIP"

CDS 389484..389687

/locus_tag="EFAGFIKM_00352"

/inference="ab initio prediction:Prodigal:002006"

/codon_start=1

/transl_table=11

/product="hypothetical protein"

/translation="MTFQRVELQRCEIPQEPTDRSRPGVACLNTRDERDLSSFISEVG

WAGSSRYLLEVSRHVQRVKSILL"

CDS 389796..392462

/gene="valS"

/locus_tag="EFAGFIKM_00353"

/EC_number="6.1.1.9"

/inference="ab initio prediction:Prodigal:002006"

/inference="similar to AA sequence:UniProtKB:P11931"

/codon_start=1

/transl_table=11

/product="Valine--tRNA ligase"

/translation="MSEEKKSAATEMPTTYDPKAAEDKWYATWMERGYFKAGQRKDAE

PYTIVIPPPNVTGMLHIGHALDFTLQDILIRTKRMQGYDALWLPGSDHAGIATQTKVE

QKLREEGLTRYDLGREKFLEKVWDWKDQYATTIRQQWGKMGLSLDYSRERFTLDEGLS

QAVRKVFVQLYEKGLIYRGKRIINWDPVNRTALSDIEVEYKEVQGHLYHLRYPLKDGS

GYVTVATTRPETMLGDTAVAVHPKDERYADMIGKVLVLPIIGREIPIIADDYVDKEFG

SGAVKITPAHDPNDFEVGLRHDLPQITVMDESGTMNAEAGKYQGLDRSDCRKQIVADL

KEQGVLINIEDHTHQVGHSERTGAVVEPYLSTQWFVEMKPLAERAIKKQQSGEGVNFV

PDRFEKTYLNWIENVRDWCISRQLWWGHRIPAWYDEETGEIIVSAEDPTTLPEYAGRK

LRQDEDVLDTWFSSGLWPFSTLGWPEDTEDLQRYYPTSVLVTGYDIIYFWVARMIFTA

LEFTDEIPFKDVLMHGLVRDADGRKMSKSLGNGVDPLDVIEKYGADAMRYMISTSSTP

GQDLRFRWERVEQARNFANKIWNASRFALMNLEGFTYEERDISGELGTADYWILHRLN

ETSRDITRLIEAYEFGETGRVLYNFIWDDLCDWYIEFAKLSFYGEDPVAKKKTQSVLA

YVLDQTMRLIHPFMPYISEEIWQHLPHEGETITLASWPVYDPALENPEAVAEMNLLMD

TIRAVRNIRAEVNVPMSKKIELMVKANSAETSSIIARNSHYIKRFCNTSEFDSGLDLN

SPDKAMTAIITGAELYLPLAGLIDIEQEVARLEKELENLEGEVLRVEKKLANEGFVAK

APAKVIEEERAKQADYSDKRDKVIARIKELKG"

CDS 392490..393917

/gene="fpgS"

/locus_tag="EFAGFIKM_00354"

/EC_number="6.3.2.17"

/inference="ab initio prediction:Prodigal:002006"

/inference="similar to AA sequence:UniProtKB:P15925"

/codon_start=1

/transl_table=11

/product="Folylpolyglutamate synthase"

/translation="MPSGRFFHSNSGPKVNQMTELDGTDAAAPLLTYNEAVDWINGLI

PFGIRPGLERIESLMSMLGNPHQRLKFIHVAGTNGKGSTCAFLTSVLLQAGYDVGTFT

SPYITKFTNRFQYNGEDIPEEILLKLSNRLHPLVMEMASTPLGSPTMFEVSTALALLY

YAEECYPDVVVWETGLGGRMDVTNIVAPVVSVITNIGMDHTDVLGDTIEQIAGEKAGI

IKPGVPVVTCATQPEAVKVIQEKAQQLQSSVYLAGDQFSYHRLDSNENGQSFHFTGPF

RDLDVRIRMQGSHQCDNAAAALMVLELLRQYMAFMLDDNDIALGLENAFWAGRFEKVV

DEPRIVLDGAHNPEGAESLAKSIMDVYPHNKLILMMGMLANKHHEAYLQHILPLVDTL

ILTEPDFRRKMDAAELLQIVERVRPAIAKQELEIIVEPEWAKALDLLKSRTEAEDLGV

VSGTLYLIADVRAALLHQTDSEKGW"

CDS 393974..395293

/gene="murC"

/locus_tag="EFAGFIKM_00355"

/EC_number="6.3.2.8"

/inference="ab initio prediction:Prodigal:002006"

/inference="similar to AA sequence:UniProtKB:Q9HW02"

/codon_start=1

/transl_table=11

/product="UDP-N-acetylmuramate--L-alanine ligase"

/db_xref="COG:COG0773"

/translation="MSAIARVMLEMGYTVTGSDVASQELTEKLAAKGAKIYIGHTAEH

VTGADLVVYSTAAPADNVERIAAAELNIPILHRSQMLARLLNERKGVAVAGAHGKTTT

SSMIALVMDKCDTDPTYIIGGEIMNVGTNAKAGQGDWVVAEADESDGSFLQYHPWLGI

VTNIEADHLENYNSDFEELKRAYVQFLSQIRPEGTAIVCSDDENVQAILPELKSRITT

YGIDRAADYTATDIVLGDRRISFTMNHQGAAMGTVELSVPGKHNVYNAMATVITCLEA

GIPFEKIVAAIIQFHGAKRRFQVLGEARDMLIIDDYAHHPTEIEATISAAKATGKRII

AVFQPQRYTRTFFLLDAFSRAFAEADEVLITDIYSPAGEKQIEGVTSARLVELIVQNS

NASARYLPTKEEVVADLQHRLQPGDLVITMGAGDIWKVGDTLAKGLK"

CDS 395410..396639

/locus_tag="EFAGFIKM_00356"

/inference="ab initio prediction:Prodigal:002006"

/codon_start=1

/transl_table=11

/product="hypothetical protein"

/translation="MSNARMTFRFGDHESDKPENKRISASSPLVTLSEELPHNQPLTP

KQMDTTPSWTPEDIPGDWGETVLTSSTVPEPDERVSHDSNLNLDEAHYFGNRSGYGYV

NHTDNPEEERRDLPNTHDDQGHDWLADSENYSYKRNRPPRGWKMIGSVTGALVTGALF

GMVILSFFNKEGAVKPGDLLPVNQAVSTVTGQEGTAGTEQQVQTATTGSTYYALQYGV

FSSPERAEQAKLELAQAGIAAGSDPEDGNRVYAGISADREEAKLLSSRLKAQGVELYV

KEIVNPEINPAIFGVKQEDVQFFFTNSSALVEQLSTLSIQQLGQSAPAAVSTETMTAI

QNKHQSWMTELNSLIPGLTADVQPIIGGMEKSMNSAITAIAEYNKNPDDVHMWSVQSD

LIEYVLQQKKWLEAIKQ"

CDS 396785..397027

/locus_tag="EFAGFIKM_00357"

/inference="ab initio prediction:Prodigal:002006"

/codon_start=1

/transl_table=11

/product="hypothetical protein"

/translation="MKKNFGMLLLFLLLGWMAGAWIAKALQPVKAVAFLTKATTIRWS

PQADLDIISYDISLQFQMSLLSLIGMIAAVWLYRRL"

CDS 397122..397733

/gene="maf"

/locus_tag="EFAGFIKM_00358"

/EC_number="3.6.1.9"

/inference="ab initio prediction:Prodigal:002006"

/inference="similar to AA sequence:UniProtKB:Q02169"

/codon_start=1

/transl_table=11

/product="dTTP/UTP pyrophosphatase"

/db_xref="COG:COG0424"

/translation="MDNTQQRPIILASTSPRRKELIASLHLAFDVIPSHANEDTPPEW

TPEQTVQELALRKALAVYRGLEGREQEAIIVGSDTVVVLDGEILGKPLDEADAARMLS

RLQGRVHRVFTGVACIDAGNGQSLVHYRQTDVTMKELSDATIRAYVQTGEPSDKAGSY

AIQGIGASLIDRIEGCYFNVVGLPLSLLSDMLDGFGVHVLPRT"

CDS 397771..398460

/locus_tag="EFAGFIKM_00359"

/inference="ab initio prediction:Prodigal:002006"

/inference="similar to AA sequence:UniProtKB:Q02170"

/note="UPF0758 protein YsxA"

/codon_start=1

/transl_table=11

/product="hypothetical protein"

/translation="MESPQYMMRDIPQEERPRERMMEYGAGALSHAELLAILLRTGTR

QESAVHMAQRILAETGGIRSLMDLSLEELTAMKGIGNAKAVQLKAGIELGHRIAKSRL

TQSTSIRTPRDAADILMEQLRYLQKEHFVCLFLNSKNHIIAQETLSMGSLNASIVHPR

EVFRAAIKCSSASIVCAHNHPSGDPTPSPEDIQITKRLIEAGAIVGIDVLDHIIIGDG

TYVSLKEKGLV"

CDS 398644..399678

/gene="mreB"

/locus_tag="EFAGFIKM_00360"

/inference="ab initio prediction:Prodigal:002006"

/inference="similar to AA sequence:UniProtKB:Q01465"

/codon_start=1

/transl_table=11

/product="Cell shape-determining protein MreB"

/db_xref="COG:COG1077"

/translation="MFGGFTKDLGIDLGTANTLVYVRGKGIVVREPSVVAIRTDTNSI
EAVGEAAKKMIGRTPGNIRAIRPMKDGVIADFDTTATMIKYFIREAQKQRSMFQRHPN
VMVCVPSGITAVEQRAVEDATKQAGAREAYTIEEPFAAAIGADLPVWEPTGSMVVDIG
GGTTEVAVISLGGIVTSRSVRVAGDEMDESVIQYIKRQYNLMIGERTSEQLKMDIGSA
MPLEQVETMEIRGRDLVTGLPKTITITSDEISEALADTVNAIVEAVKVTLEKCPPELA
ADIMDRGIVLTGGGALLRNLDKLLAGETGMPVIVAENPLDCVAIGTGKALENIHLFKS
RSSSAVRSKR"

CDS 399865..400737

/gene="mreC"

/locus_tag="EFAGFIKM_00361"

/inference="ab initio prediction:Prodigal:002006"

/inference="similar to AA sequence:UniProtKB:Q8Y6Y4"

/codon_start=1

/transl_table=11

/product="Cell shape-determining protein MreC"

/db_xref="COG:COG1792"

/translation="MFELFKLLGNKRLFVLLVGLVTFIALMGFTLSPRTTLSWPEKFL
KDSVGFVQYVFYKPASYVAGFFKDVANMRTVYDENEELRIAMGHYTRDRLKYNDMEQV
NEQLQKALNFTEEQKNRYNYGSRIAQVISANSDPNSRTINIDIGENAGIKPGMAVTSQ
EGLVGVISHVSSYTSTVNLLTSMDANDPTSNAIAATAVGKEKVFGMIESYDPKTGMLK
MTKISEDSNIKEGDEIISSGIINNFPKYMRIGEVKSVEKSEYGLTKTATIDPYASFLD
WKELIVIIPPEVKE"

CDS 400737..401270

/locus_tag="EFAGFIKM_00362"

/inference="ab initio prediction:Prodigal:002006"

/codon_start=1

/transl_table=11

/product="hypothetical protein"

/translation="MVTRKQVLFLLLFVLFIAEGTILPLLIPSGWHMRISANLVYIVI
LFIAVYHHRHTALVLGIFFGLLHDVVFYGEMIGPYGFSMGLSAYMMGLIFQAPRAPLP
VMVSVVILGSLLNDTMLFFLYKLFQLNHVTFDWALIEYMIPNLFIHFVFALIIYVPLR
KQLERIGKRRSKTEEAS"

CDS 401346..402008

/gene="minC"

/locus_tag="EFAGFIKM_00363"

/inference="ab initio prediction:Prodigal:002006"

/inference="similar to AA sequence:UniProtKB:Q01463"

/codon_start=1

/transl_table=11

/product="Septum site-determining protein MinC"

/db_xref="COG:COG0850"

/translation="MTVKSNHVTIKGIRDGLVFLLDDQCEFEELLYELRYKLEHSHQN

ILTGPIVHVDIKLGAREVTEDQKEAILDILKQKGNLLIRSIDSPALQPEVKGPPPIVT

MCGMVRSGQVLHHEGNLLFLGDINPGGTVTCTGDIYVLGSLRGMAHAGIGGDEEAIIA

ASVFAPTQLRIADIISRPPDEWESRETGMEFAYLQDNQMQIDKMSNIVRLRRDFNVFK

GV"

CDS 402012..402806

/gene="minD"

/locus_tag="EFAGFIKM_00364"

/inference="ab initio prediction:Prodigal:002006"

/inference="similar to AA sequence:UniProtKB:Q01464"

/codon_start=1

/transl_table=11

/product="Septum site-determining protein MinD"

/db_xref="COG:COG2894"

/translation="MGEAIVITSGKGGVGKTTTSANIGTALALLGKKVCLVDTDIGLR

NLDVVMGLENRIIYDLCDVADGRCRLNQALVKDKRFEELYMLPAAQTRDKNSVSPEQV

RDIILELKKDFEYVIIDCPAGIEQGFKNAVAGADQAIVVTTPENAAVRDADRVIGLLE

SSHIQSPKLVVNRIRNNMVKSGDMLDIDGILQVLNIDLIGIVPDDELVIKAANSGEPT

VMNPDSLAAIAYRNIARRILGDTVPLMQLEQKKGAFTRFKKFLGMG"

CDS 402956..403804

/locus_tag="EFAGFIKM_00365"

/inference="ab initio prediction:Prodigal:002006"

/codon_start=1

/transl_table=11

/product="hypothetical protein"

/translation="MNTKLRIKQRREERIRRLMEGATVEVLQEQKLGSLLDKNEIIHP

KPFTPSEGIQERDPEWLWKKENGHFVPGGHSRFNLFKSLFKRTVISALIFGGVWGLFQ

LDTSWTTSPKTVIADALHRDMDFASAAAWYERHFGGTPSFLPVLGHTTDAVNGLKVRQ

LLGKPISGTVVQPFALSMKGIEIVPDAAGAELIQVASSDAGRVMEVIGDAASGFTVVI

QHTGNVTAIYGRLNESEVSVNDWVEAGNPVGSLKATGGEQPATLYFAVKEGEEYVDPA

EVVALD"

CDS 403791..404657

/gene="spoIVFB"

/locus_tag="EFAGFIKM_00366"

/EC_number="3.4.24.-"

/inference="ab initio prediction:Prodigal:002006"

/inference="similar to AA sequence:UniProtKB:P26937"

/codon_start=1

/transl_table=11

/product="Stage IV sporulation protein FB"

/db_xref="COG:COG1994"

/translation="MPLIRVWGVRITFHPFFVIIMMASLLTGHFIELITLFAIVFIHE

CGHAAAAALLGYRVLSIQMLPFGGVAVIEDGGTITAYREIIIALAGPLQNILMVGVVM

LLQYGNLGDPVFLNYIIQGNLLIALFNLLPVLPLDGGKIVQALVSLWAPYYTTLMWTY

RISILCSVGVILYAISRWFTGDYGLPLNILLIGLFLFYSNVTDYRNVPYRFIRFLMNR

EGVFARHAATGSLAQPIISFPAKPLDTILRLLKRERYHMVYVMNRQGRIMAVLPEQRI

IGSYFQQNGDKL"

CDS 404764..406014

/gene="rng"

/locus_tag="EFAGFIKM_00367"

/EC_number="3.1.26.-"

/inference="ab initio prediction:Prodigal:002006"

/inference="similar to AA sequence:UniProtKB:P0A9J0"

/codon_start=1

/transl_table=11

/product="Ribonuclease G"

/db_xref="COG:COG1530"

/translation="MKQMIVHNEHNLMQMALLEEGKAVEFTAERTRDRGLLGSFFKGR

VVNVLPGMQAAFVDIGQKKNAFLYVDDVLHPHLEKQPKVKPSISELLRPGQEVIVQVL

KEPVGGKGARVTTHYSLPGRWLVYMPVADYVAVSKKIAREGDRSRLKALGEQLRRDEE

GLIIRTVSGEEQHEAIKSDLETLRAQWYLIREKADSLPSPSLLHRDHSMVQRIIRDVY

TPGSDEVITDSEGQAREVKALLEEISPGHQPKVQVYRGTESIFAAYGVQEQLNKDFAR

KVWLPGGGYIVIDHTEALTVVDVNTGKYTGAGGDSLEETVTETNIQAAVEIARLMRLR

DIGGMIIVDFIDMEEASNRHEVAATLEGELKKDRTKAFVMGWTKLGLLELTRKKVREE

STLPYEEPCSSCHGTGKRYISPLH"

CDS 406170..406481

/gene="rplU"

/locus_tag="EFAGFIKM_00368"

/inference="ab initio prediction:Prodigal:002006"

/inference="similar to AA sequence:UniProtKB:P26908"

/codon_start=1

/transl_table=11

/product="50S ribosomal protein L21"

/db_xref="COG:COG0261"

/translation="MYAIIETGGKQYKVQEGDVLFIEKLTANDGESVTFDRVLAVSND

QGLTAGTPLISGATVTAKVEKHGKGQKVIVYKYKPKKNYHVKQGHRQPYTKVTIETIK

A"

CDS 406496..406825

/locus_tag="EFAGFIKM_00369"

/inference="ab initio prediction:Prodigal:002006"

/codon_start=1

/transl_table=11

/product="hypothetical protein"

/translation="MIIVQIFRDEDGNIERFSIEGHANFAKRGEDIVCAGVSAVTVGT

VNSIETLTGVEMDAKMKNGFLSGSLPLLERGETWSQVQLLLESMVVMLSNIAESYGKY

IKIQQFK"

CDS 406849..407160

/gene="rpmA"

/locus_tag="EFAGFIKM_00370"

/inference="ab initio prediction:Prodigal:002006"

/inference="similar to AA sequence:UniProtKB:P05657"

/codon_start=1

/transl_table=11

/product="50S ribosomal protein L27"

/db_xref="COG:COG0211"

/translation="MLKLNLQLFASKKGVGSTKNGRDSNAQRLGVKRADGQTVTGGSI

LVRQRGTKIHPGTNVGIGKDDTLFAKIDGVVKFERWGRDRKKVSIYPVNVAPVAAAVE

A"

CDS 407311..408048

/locus_tag="EFAGFIKM_00371"

/inference="ab initio prediction:Prodigal:002006"

/codon_start=1

/transl_table=11

/product="hypothetical protein"

/translation="MKSWKRVPWIAACSLLIPLVFVMLYPAMISSVVCALWSVAVLLI

SVNAMKKQAEAERRAIIQSMEKTAIASLNHHRHDWMNDLQVLYGYLRLGKLDKSLQCV

ERIKERVTEESRISRLGIPSLVFYLQSFRASGIALDLHVEIEDELQLSALVSPEDGES

LTGAIADAIRAYQYGGGRSSWGEVRKLTLNFGQDHGDVVVRLDGDQTPDPETLRQLTA

VLKGKKVRTEQIPSEDTFIQFRMPCGI"

CDS 408066..409376

/gene="obg"

/locus_tag="EFAGFIKM_00372"

/EC_number="3.6.5.-"

/inference="ab initio prediction:Prodigal:002006"

/inference="similar to AA sequence:UniProtKB:P20964"

/codon_start=1

/transl_table=11

/product="GTPase Obg"

/db_xref="COG:COG0536"

/translation="MFVDKAKIYVKAGDGGDGIISFRREKYVPNGGPAGGDGGRGADI
IFRVDEGLRTLMDFRYQRHFKAPRGEKGRNKSQHGANAENMIVRIPPGTIILDEDSGE
VLADMTRHGQQVVIARGGRGGRGNIRFATPNNPAPELAENGEEGQERYIVLELKVMAD
VGLVGFPSVGKSTLLSVVSSAKPKIGAYHFTTITPNLGVVGVGEGRSFVMADLPGLIE
GAHEGVGLGHEFLRHVERTRIIVHVVDMSGSEGRDPFEDWQKINDELKLYNPLLAERT
QIVAANKMDMPDSEANLEQFLQQVREAQPDIEVMPISSLTRKGIQELLYRAADLLDQV
PDERVVEEVADVSERKVYSLDKKEDDGFKIVRENEMFVVESAKIDRMMKRMQLNSHEA
ILKLARTLRYMGVDAELRKRGAVEGTIVRIGDFEFEFVEGSSYY"

CDS 409605..410042

/locus_tag="EFAGFIKM_00373"

/inference="ab initio prediction:Prodigal:002006"

/codon_start=1

/transl_table=11

/product="hypothetical protein"

/translation="MNERYYLVREDILPEAVVKTMQVKELLASGDVKTVHEAVEKVGL
SRSAFYKYKDGIHLINQLERERIVTISIDLEHQSGILSRVLGHVAGYGANVLTINQSI
PLQGRANVVISVETTHLHGEIGEMLDRMQDMPGVRRTRIVGQG"

CDS 410101..411387

/gene="hom"

/locus_tag="EFAGFIKM_00374"

/EC_number="1.1.1.3"

/inference="ab initio prediction:Prodigal:002006"

/inference="similar to AA sequence:UniProtKB:P19582"

/codon_start=1

/transl_table=11

/product="Homoserine dehydrogenase"

/db_xref="COG:COG0460"

/translation="MKPVKVGLLGLGTVGTGVVRIVEGNQEDLSSQVGSPIVIEKIAV
KNTEKERVIAVDRAKLTEDPWEVIRHPDIDVIVEVMGGIDQTKEYILEALERGKHIVT
ANKDLMALHGTEILAKAQEKQCDVFYEASVAGGIPIIRTLIEGFSSDRITRIMGIVNG
TTNFILTKMSQEGASYEEVLAEAQALGYAESDPTSDVEGLDAARKMAILSTLGFRTNV
ELKDVTVKGISSVTREDIMYAKRLGYEMKLLGIADRIGDEITISVQPTMVRQNHPIAS

VNGVFNAVYVHGEAVGETMFYGAGAGELPTATSVVADIVAVTKNLKLGVNGLKAIVPY

KIKRLQSDEQIVSKNFILLHVDDKAGVLAQITQIFAEYDVSLASVVQQPNEHNPDAEI

IIVTHNASKASMDKVLKHFESLSVIRRIKSVYRVEG"

CDS 411503..412573

/gene="thrC"

/locus_tag="EFAGFIKM_00375"

/EC_number="4.2.3.1"

/inference="ab initio prediction:Prodigal:002006"

/inference="similar to AA sequence:UniProtKB:A0R220"

/codon_start=1

/transl_table=11

/product="Threonine synthase"

/db_xref="COG:COG0498"

/translation="MRYQGLLQTYREHLPVNENTPLLTLQEGNTPLIHAENLSEELGL

NVYFKYEGLNPTGSFKDRGMVMAVAKALEEGSRTIMCASTGNTSAAAAAYAARGGLNC

IVLIPNNNIALGKLAQAMIYGAKVIAINGNFDRALEIVREITAKHPITLVNSVNPFRI

EGQKTAAFEVIEQLGEAPDVLAIPVGNAGNISAYWKGFKEYKEAGKSSTLPRMVGFEA

EGAMAIVKGEPILEPETVATAIRIGNPASWKTAVAAAEESGGQINYVTDEQILTAYRT

LASREGIFAEPASAASLAGVYKLKSEGYFKGGETVVCVLTGNGLKDPNIAIKTVATEP

LVVEDTEEAVMAAIAQLEQQSV"

CDS 412570..413538

/gene="thrB_1"

/locus_tag="EFAGFIKM_00376"

/EC_number="2.7.1.39"

/inference="ab initio prediction:Prodigal:002006"

/inference="similar to AA sequence:UniProtKB:Q8Y4A6"

/codon_start=1

/transl_table=11

/product="Homoserine kinase"

/db_xref="COG:COG0083"

/translation="MSLREKVTVKIPASTANLGPGFDTLGMALSLYAWLEMKPAEQTT

FHLHGNHLTGLPTDKSNLIYEVAQMVFNEAGVSLPELEISMYSDIPLTRGLGSSASAI

VGALAAANVLIGAPLSDAKLLDMATSLEKHPDNVGASLYGGIITAAWNGQQVDHIRIE
PHQDLQALVIVPEFQLSTSKARNVIPEQFGMSDVVHNISRSSLLVAALASGRLDMIQK
AMSDRIHQPYRASLVPGMAEILEHAVDHGALGAALSGAGPTVLTLVDRHDTRKEGLEQ
YLLDTMEREGISASALWLDPDLDGVTVLPDQDERPFLDRIKGEVNA"

CDS 413535..414407

/gene="pheA"

/locus_tag="EFAGFIKM_00377"

/inference="ab initio prediction:Prodigal:002006"

/inference="similar to AA sequence:UniProtKB:P27603"

/codon_start=1

/transl_table=11

/product="Bifunctional chorismate mutase/prephenate
dehydratase"

/translation="MKRIAVLPEGSVSHEAIDFLFNGEPLNLLHSKLISDVFRATDSG
NAEYSVIPIENTIEGSVSLHMDWLVNEVDIPMQAEWVYPSIQNVIGHGSEFKTESGEY
DFGRITKIMSHPVAIPQCQNFIRLHSPGAELEGVNSTAEAVEIVKKNPGKGWVAIGTK
LAAQKHGLDIMAERVTDHDNNYTRFVLIGHEPVNIPREPDHVKTSLLVTLPEDAPGAL
HQVLSAFAWRKLNLTRLESRPTKKRLGSYYFYIDVVETVDSVLLTAAMAEIEALNCQV
RVLGSYPCYTYPSR"

CDS 414556..415440

/gene="ilvE_1"

/locus_tag="EFAGFIKM_00378"

/EC_number="2.6.1.42"

/inference="ab initio prediction:Prodigal:002006"

/inference="similar to AA sequence:UniProtKB:O86428"

/codon_start=1

/transl_table=11

/product="Branched-chain-amino-acid aminotransferase"

/db_xref="COG:COG0115"

/translation="MAEQWIYLDGQYVTKENATVSVYDHGFLYGDGIFEGIRIYNGNI
FRCKAHLDRLYDSAKSISLNIPLSIDEMLEVMAETVRRNDMRNGYIRLIVSRGPGNLG
LDPLRCPKASVIIIVEQLAIYPEEAYLTGLKAVSVSQRRNIPDALNPKIKSLNYLNNI

LVKIQSNYSGAGEAIMLNSQGYVTEGSSDNIFIIKNGVVYTPPCYLGALEGITRQAII

DLCGELELELKEVPFTLHDVYIADEVFFTGTAAEVIAAYEVDGRTIGTGVAGPVTLKL

LEAFRQIVDKDGYKVWES"

CDS 415636..417258

/locus_tag="EFAGFIKM_00379"

/inference="ab initio prediction:Prodigal:002006"

/codon_start=1

/transl_table=11

/product="hypothetical protein"

/translation="MKIHMVKKGDTLYLLSQKYNVALDKLIAANPQITNPDKLDIGMK

VKIPAEPVTPKPEGVLHSHKVQQGDSLWKLAQAWGVPLKDMVNANPQLKNPNALLVGE

TVYIPSIHAQGNSTSNSGASNIAAHEKLSPEGKEYTGVKEPEPPAPVTPTPPAPVAEV

PVPVPAPPPANTEKPNVKPELEVLPQLPELPEVKPEKETHKKEEVKPEVYVKPVAESK

KYTMPNLSPEIMPLPVMPNTKSPTEVAPATKKPCGCGNKLHHAPAEHPYAQIQVPVQE

VYGAQQDMYTAGASNNASFPGIPEVSPYSGSDLPNSPWTGAEQGYNHNNVMPNLSADM

QNNSFPIAPAGEVNSPFPTYAAPSHMNHQPPFISPYSMLPYPPCGCGSHTHMPQYTYP

SHGYQDPAWNMYGPSYGMPPEMSTSTMPNQPIEYAYQNPYPTQNMVPPSPLGAFGEMY

PPQGQGKGGKKGGREDANLSQVAIEEVGSDSEGKSKQAGSKPAAAKRRTSKAPLKNKS

KVSVSGKTSREGAAASNQRKSDKKRRNPWIQN"

rRNA 417809..419359

/locus_tag="EFAGFIKM_00380"

/product="16S ribosomal RNA"

rRNA 419670..422590

/locus_tag="EFAGFIKM_00381"

/product="23S ribosomal RNA"

rRNA 422670..422781

/locus_tag="EFAGFIKM_00382"

/product="5S ribosomal RNA"

tRNA 422868..422943

/locus_tag="EFAGFIKM_00383"

/product="tRNA-Asn"

/inference="COORDINATES:profile:Aragorn:001002"

/note="tRNA-Asn(gtt)"

tRNA 422948..423019

/locus_tag="EFAGFIKM_00384"

/product="tRNA-Glu"

/inference="COORDINATES:profile:Aragorn:001002"

/note="tRNA-Glu(ttc)"

tRNA 423026..423101

/locus_tag="EFAGFIKM_00385"

/product="tRNA-Val"

/inference="COORDINATES:profile:Aragorn:001002"

/note="tRNA-Val(tac)"

tRNA 423119..423192

/locus_tag="EFAGFIKM_00386"

/product="tRNA-Met"

/inference="COORDINATES:profile:Aragorn:001002"

/note="tRNA-Met(cat)"

tRNA 423220..423297

/locus_tag="EFAGFIKM_00387"

/product="tRNA-Asp"

/inference="COORDINATES:profile:Aragorn:001002"

/note="tRNA-Asp(gtc)"

tRNA 423321..423395

/locus_tag="EFAGFIKM_00388"

/product="tRNA-Gly"

/inference="COORDINATES:profile:Aragorn:001002"

/note="tRNA-Gly(gcc)"

tRNA 423402..423478

/locus_tag="EFAGFIKM_00389"

/product="tRNA-Arg"

/inference="COORDINATES:profile:Aragorn:001002"

/note="tRNA-Arg(acg)"

tRNA 423494..423574

/locus_tag="EFAGFIKM_00390"

/product="tRNA-Leu"

/inference="COORDINATES:profile:Aragorn:001002"

/note="tRNA-Leu(caa)"

tRNA 423581..423654

/locus_tag="EFAGFIKM_00391"

/product="tRNA-Pro"

/inference="COORDINATES:profile:Aragorn:001002"

/note="tRNA-Pro(tgg)"

CDS 423856..424548

/locus_tag="EFAGFIKM_00392"

/inference="ab initio prediction:Prodigal:002006"

/codon_start=1

/transl_table=11

/product="hypothetical protein"

/translation="MNTFNFRKQFKRRWRRWKRTVWMTAALLAVTILAYSGLSISSAI

ERLLTTNFSEATSVMGPVTQETRSEQEIQALVEQLDSDPDHLTSVVLETQYICGVETE

QLGKMARPQLKMLLAQHPEWDATVGTSDELHLKQRVDDLSPLCKQQAYISIDAVGNLN

LYEGKPAEEKVIRTFFQLDVGTLESSLPEGVLEQLQQGIRIQDKDEYDSVISTFSDYA

VDEDHHLIRNGG"

CDS 424851..425360

/gene="ruvC"

/locus_tag="EFAGFIKM_00393"

/EC_number="3.1.22.4"

/inference="ab initio prediction:Prodigal:002006"

/inference="similar to AA sequence:UniProtKB:P0A814"

/codon_start=1

/transl_table=11

/product="Crossover junction endodeoxyribonuclease RuvC"

/db_xref="COG:COG0817"

/translation="MVLRFLGIDPGIAIVGFGFVDKIGSKVVPVQYGCIQTEAHTPEE

ERLLHVYEGMVQLIDKYKPDAVALEKLFFNRNVTTAMSVSQARGVMVLAAAQKGLPIA

EYTPMQIKQAVVGYGKAEKKQVQEMVKMFLRLQVVPKPDDVADALAVAVCHAHSYTLN

SKLNEVLRK"

CDS 425357..425971

/gene="ruvA"

/locus_tag="EFAGFIKM_00394"

/EC_number="3.6.4.12"

/inference="ab initio prediction:Prodigal:002006"

/inference="similar to AA sequence:UniProtKB:P0A809"

/codon_start=1

/transl_table=11

/product="Holliday junction ATP-dependent DNA helicase

RuvA"

/db_xref="COG:COG0632"

/translation="MIDFLRGQFIHVENDYIVLDVHGVGYRVFCPNPFAFAKQEGEIT

VYTHHHVREDAMLLFGFVTRDEQRLFRKLIEVSGIGPKVALGILAGGTPEHVVTAIYQ

ENLTFLTKLPGIGKKTAQRMILDLKDKLDSFGAAAYATGLFAPPSEESGSGSAWDEAR

EGLKALGYTDSELDKVWLKLKKDVTTADSVDVLMKKALQMLFTG"

CDS 426027..427037

/gene="ruvB"

/locus_tag="EFAGFIKM_00395"

/EC_number="3.6.4.12"

/inference="ab initio prediction:Prodigal:002006"

/inference="similar to AA sequence:UniProtKB:O32055"

/codon_start=1

/transl_table=11

/product="Holliday junction ATP-dependent DNA helicase

RuvB"

/db_xref="COG:COG2255"

/translation="MDDRIISANLMMEDQNVELSLRPRYLNEYIGQNQVKENLKIYIE

AAKFRNEALDHVLLYGPPGLGKTTLANIIANELGVNLRTTSGPAIERPGDLAALLTNL

QEGDVLFIDEIHRLHRTVEEVMYPAMEDSALDIMIGKGPSARSVRLDLPSFTLIGATT

RAGLLSAPLRDRFGVISRLEFYTVDELAYIVSRSTEILGVEIVGDAAEEIALRSRGTP

RIANRLLKRVRDFAQVRGDGIITQTLAEEALQRLQIDPRGLDDIDHKMLKSMINSFRG
GPVGLDTIAATIGEESQTIEDVYEPYLLQIGMIQRTPRGRIVTDLAYHHLGLPVPPRD
GK"
CDS 427143..429230
/locus_tag="EFAGFIKM_00396"
/inference="ab initio prediction:Prodigal:002006"
/codon_start=1
/transl_table=11
/product="hypothetical protein"
/translation="MGKAIRTKKWSRRLKVLAAALLTVGCLQVPAYAAGNDGQTIRVA
MFANLGSTYKSTTPLITLESTGQWSIQSESGANVSLPAGQVRFSADGFRVKVLETADF
KAASAAAKLLQATADKPLLFTTSVNGNAIYQLYTGNYATEALAGQAVTRVQKVAAAQL
AGQKPAVTGSKRLSAGTYGSIQEAEAAQANLLSAGVSSAYPVLQLSGGSQAYAVWVGE
ASTDAELTALKNSVEAKAPGVSLSPVNTNAGLIIRQDAGLSTDALKTAPHYTIAGTES
KALVQGNGNGIKVVERSQRTYRGDMEISIVSGDLALVNVVPLEQYLYSVVGAEVYSSW
PAEALKVQAVAARSYALQQGERFKIANVVDTTLSQAYNGIGSENDKVTSAVDATAGEV
VKSGGKIIEAVFSSNAGGQTAHPSEVWNGGAGVFTNVVSSGDTSAQAGLHTWYHVLLG
TGVSGYIREDNIKELTTKTNAGLAKVTVTAQNTNVRAVPLIQSTVEPVAKMNPGNEAV
VLAKVAQSNDYAWVRGPFTSAQLVKSLQGKTTASVPASISTLEVTKRGPSGRALEVTA
NGQAMTVKYADTYRSALGGLPSTLFDIAGTGSYTVLGADGKTASKTGSNGASVLSSSG
AGTSSGNALVVMSGDGQARAVTQGQTFMFIGQGNGHGLGLSQWGAKGMADEGYDYQAI
LKHYYQNATIVKE"
CDS 429237..430265
/gene="queA"
/locus_tag="EFAGFIKM_00397"
/EC_number="2.4.99.17"
/inference="ab initio prediction:Prodigal:002006"
/inference="similar to AA sequence:UniProtKB:O32054"
/codon_start=1
/transl_table=11
/product="S-adenosylmethionine:tRNA
ribosyltransferase-isomerase"

/db_xref="COG:COG0809"

/translation="MNVNLYDFELPEQLIAQTPLLDRTASRLLTLNKDSGEINHQTFP

DIIDFLNPGDTLILNDTRVLPARLFGTKEDTGAKAEVLLLKNVEGDKWEALVKPGKKL

KAGSVIVFGEELKAIIEEEGEMGARTLTFTYDGIFQEILDRLGEMPLPPYIKETLDDR

ERYQTVYAKHEGSAAAPTAGLHFTDELLDQIRAKGVNVAFITLHVGLGTFRPMSVDVV

EDHVMHEEYYSLSQETADLINQTKANGHRVFAVGTTSCRTLETVGSKFENGILQASSG

WTSIFIYPGYSFKVIDGMLTNFHLPKSTLVMLVSALAGREHIMQAYEEAIQEKYRFFS

FGDAMLIY"

CDS 430309..431445

/gene="tgt"

/locus_tag="EFAGFIKM_00398"

/EC_number="2.4.2.29"

/inference="ab initio prediction:Prodigal:002006"

/inference="similar to AA sequence:UniProtKB:P66905"

/codon_start=1

/transl_table=11

/product="Queuine tRNA-ribosyltransferase"

/translation="MAAITYEHIKTCKQSGARLGRVHTPHGIIETPTFMPVGTQATVK

TMSPEELKEMDAQIILSNTYHLFLRPGHEIIREAGGLHKFMNWDRPILTDSGGFQVFS

LSEMRKITEEGVNFRSHLNGDKKFLSPEVAMEIQNALGSDIMMAFDECPPYPAEYEYV

KKSLERTSRWAERCLESHDRPHDQGLFAIVQGGMHEDLRRQSAADLTSMDFPGYAIGG

LSVGEPKHLMYGVLDYTLPLLPSNKPRYLMGVGSPDALIEGSIRGVDMFDCVLPTRIA

RNGTTMTSQGRLVIRNAKFATDFGPLDPECDCYTCRNYSRAYLRHLIKADETFGLRLT

TIHNLHFLQNLMRNVRKAIMEDRLLDFRDEFFDQYGLHDNDKGF"

CDS 431513..431824

/gene="yajC"

/locus_tag="EFAGFIKM_00399"

/inference="ab initio prediction:Prodigal:002006"

/inference="similar to AA sequence:UniProtKB:P0ADZ7"

/codon_start=1

/transl_table=11

/product="Sec translocon accessory complex subunit YajC"

/db_xref="COG:COG1862"

/translation="MFQGMTLAGAQGGGIVSLIVPLVLMVAIFYFLMIRPQNKKQKQR

NSMLSQLKKGDKIVTIGGLHGTIAEITDDVVVLRVNDVTKLTFDRNAISTAVARDTAV

E"

CDS complement(431948..432340)

/locus_tag="EFAGFIKM_00400"

/inference="ab initio prediction:Prodigal:002006"

/codon_start=1

/transl_table=11

/product="hypothetical protein"

/translation="MQLIRRLVSFRVSNPILSGLCQAFVWMFIGAFILSLFLWMSGMR

EQDLSLYTYVVHGLSLLVGGFVAGKRSGEKGWYYGGITGIVYGLLVLLIGFLALDASF

NWKDSLQLLSAFFISALGGMFGVNTHRS"

CDS 432524..433282

/locus_tag="EFAGFIKM_00401"

/inference="ab initio prediction:Prodigal:002006"

/inference="similar to AA sequence:UniProtKB:O31533"

/note="UPF0702 transmembrane protein YetF"

/codon_start=1

/transl_table=11

/product="hypothetical protein"

/translation="MNFPDVGSHIFRTILMYFLVYCAMRVMGKREIGKLSMFDLVVSI

MLAEMAAFVIEDIDKPLSYGIAPMLTLIIVQIGMAFIGLKSRRLRLIIDGKPTVLISK

GVLHRDEMRKQRYNLDDLLQQLREQNVDSIGEVDFAILETTGKLTVFPKDKNTSNDSN

SSGSDSEDSSSSKSKTGKNQIDGFPNIKYEGLPLPLIMDGKVQDQNLEIIQKTRFWLK

NQIQHNGILDFKDVFICSIDHNGKIYVSPKDDKK"

CDS complement(433290..434993)

/gene="spoVB"

/locus_tag="EFAGFIKM_00402"

/inference="ab initio prediction:Prodigal:002006"

/inference="similar to AA sequence:UniProtKB:Q00758"

/codon_start=1

/transl_table=11

/product="Stage V sporulation protein B"

/db_xref="COG:COG2244"

/translation="MKNNGFVPFMLIACTFNRERGVVLKKQTFIQGAMILLAAGIINR

ILGFIPRIALPRIIGAEGVGIYQLSYPFFIVLVTLITGGIPLAVAKLVAEADTGANRY

SPQRILQVSLSFTLTLGVVFMFLCILFAPWVTKYVLTDERVYHTFVSMSPMIAIIAVS

AVYRGYFQGKQNMMPTAISSIVETIIRIVCVIWFAWLLLPHGIAQAAAGAMLGALVGE

FGGMLVLLWKYNRQKKELPLAMQQNMSDLTTKPSAKPLNPIPSVLKETGTPQPGLIRR

LLAISIPVTAGRLVGSLSYLAETIVTAQSLALAGISKGLATAQYGALQGMIIPLLLLP

GALTSSLATSLVPSLSEASAQGDRALIHKRMHQALRLALVTGAPFSVFMYVLAEPMCL

VLYNDASIGSMLKLMAPFALFIYIQAPLQAALQALDRPGKALLNTFIGAVIKITLILT

LASRPEYGIFGAVIAICVNSTVVTLLHAQSVRSLLQFRFKMMDWVKTGAGMFIMGAAT

LLVYEETAMILPFWVRMLLAPFVGLIVYFIVMGWTKMIDFHDLSRAPLIGSWFKRRSG

K"

CDS 435143..435391

/locus_tag="EFAGFIKM_00403"

/inference="ab initio prediction:Prodigal:002006"

/codon_start=1

/transl_table=11

/product="hypothetical protein"

/translation="MEEMNDVELEQSIEMLCRSKAEELRLVGYEYVTSKDVWNCVSHK

YEKQGIPPLHQLVNDILSLKATSFMNFMTVSAYRGSSF"

CDS 435483..436733

/gene="secDF_1"

/locus_tag="EFAGFIKM_00404"

/inference="ab initio prediction:Prodigal:002006"

/inference="similar to AA sequence:UniProtKB:O32047"

/codon_start=1

/transl_table=11

/product="Protein translocase subunit SecDF"

/db_xref="COG:COG0341"

/translation="MKRIISFILVVLVTTGVMVGTSPELLKNIRLGLDLKGGYEILYE

AEPLEAGQQVTKASLVQTAKSLESRANALGTSEPEVTTEGSNRIRLKLAGVTDEAEVR

AKMKEPAVLTFRSKAENDKEGEYTKIELRGNEFVENAAKVVFTQLNEPMIDIQVKDKA

KFSEITKRLIGQPLAIYLDDELLSAPTVRQQLTDGSAQISGAYTRDEANQLRDTINLG

ALPLKLTEKYSQSVGATLGKLSLDQTIKAGLIGSVIILVFMIGLYRIPGIIASFALIT

HTWLVLAIFYLGEFVLTLPGIAAFILGIGMAVDANIITYERIKEEMRSGKSILSSVKA

GGKHSFRTIIDSNITTIIAAAVMFFLGTGAVKGFALVLIFDIVTSIITNIFFSRFLLT

LLVRGNVLKKPKYFGVKESEIRAL"

CDS 436723..437631

/gene="secDF_2"

/locus_tag="EFAGFIKM_00405"

/inference="ab initio prediction:Prodigal:002006"

/inference="similar to AA sequence:UniProtKB:O32047"

/codon_start=1

/transl_table=11

/product="Protein translocase subunit SecDF"

/db_xref="COG:COG0341"

/translation="MRFNWNFDYVKGSKIAYIFSIILTVTGIISILALGLNYAVDFRA

GSNVDISVSKAITAEQIKPIVKDIGVDEGDVTITPGADRVNVRFSNELDETQESKFKQ

EFTKLDASASYEVNTVDPEMAKELERNAIYAVLIASIGIMIYVAIRFEWRFGLAAVIA

LFHDAFVVISVFSIFRLEVDLTFITAVLTIVGFSINDTIVIFDRIRENLRFAKKTTKA

DLREVVNRSLAQTMTRSLNTTFTVFAASICLFIFGGESIRMFSLAMVIGTLFGAYSSI

YIAAPLWLMLKGKQVDKPKTVAKESN"

CDS 437705..438664

/gene="mneP"

/locus_tag="EFAGFIKM_00406"

/inference="ab initio prediction:Prodigal:002006"

/inference="similar to AA sequence:UniProtKB:C0SP78"

/codon_start=1

/transl_table=11

/product="Manganese efflux system protein MneP"

/db_xref="COG:COG0053"

/translation="MTREHIPSAQAATWSGIAGNMGLAVIKAGVGYMANSKSLLADGL

YSASEAGSALAGLFPSKSVRDKSKARRGRFKEHSRVARPGISVLLSVLILMGGLQLAI
SALGSLSGSEPEAPGKSVLLVAVAALAVKEAIFQFQYRYFRKRNQSQALTYAQQHRRA
LYCSLAVLIGIIGSMAGEETGLSMLLYLDPTAALIASCFVLHKGYRMIVDTVYGSLVQ
ELEQEEASDFKETVQRVYGIITIEHLVAREQEHDVTIELVISVNPRISILEAQEIANR
AKTLLLTRFSHVKEVQIQAIPYDPGYPYKSNHELPDHEATLIQ"

CDS 438705..441113

/locus_tag="EFAGFIKM_00407"

/inference="ab initio prediction:Prodigal:002006"

/codon_start=1

/transl_table=11

/product="hypothetical protein"

/translation="MLYSQYRWNTPNIDLEAAAGLSQALSVSPLVGRLLVSRGVHNEK
EAERFLHPDLNQMHDPYLLLGMNEAVPRIKLALEREEHILIYGDYDADGVSSTSLMIH
LMRHLGASYDIYIPHRSNEGYGLHNHALDWAHQQGVSLIITVDTGISAVEQIAYAATL
GIEVIVTDHHEPPEILPEAFTLINPKLPGCPYPFKGLAGAGVALKLAQALIGEVPGEW
MEIAAIGTVADLMPLEGENRVIVSYGIESMRGSRLPGVSALLEISGVDQSQVTSINIA
FAMAPRINASGRLDHAGRAVSLLTTENLDEAHTLAGQLDLLNRERQQVVEGILQEATA
QLEQKIQLRSGSVPDVIVLAGEGWNVGVVGIVASKLLERYYRPTLILGIDPESGVCKG
SARSIEGLDIYQALTVNKHTMDHYGGHPAAAGMTLHRDQLEELEAGLNEFAASVLTEE
DFVPQRLADDECRISDVPLKVIQEIDRLQPFGMSNPSPRFVLRSVTVKETRTMGREKR
HLKLVLEQDGQQLETVAFGKGALAEFLLPGSVIDVMGELSINEWNGMRKPQLMLQDIH
VPQVQVFDLRGNAEPLNAMKHFLNSLEVHLRYKSGSVGAIVRKETDVSEGNSLYEPCL
WVYDRKVGLFPCNAAAQHYGQEQVRTLFVFDTPETPEQLNAMLALFSGAENIILLHGS
GNRRDRLQMPSRDLFKRIYMQVLKWKAEPVEEQEITPVLSRQCGCSPRMITMMLDVFE
ELSFITRDNGKIAFVDNPPKRELTASRHYQALENMAEIEQVMLDAATPQLTQWMISRM
KGVS"

CDS 441151..441663

/gene="apt"

/locus_tag="EFAGFIKM_00408"

/EC_number="2.4.2.7"

/inference="ab initio prediction:Prodigal:002006"

/inference="similar to AA sequence:UniProtKB:P68779"

/codon_start=1

/transl_table=11

/product="Adenine phosphoribosyltransferase"

/translation="MDFKDYIRVIPDFPQPGISFKDITTLLKDGEMYRNAINELKVMV

SDLKIDVIAGPEARGFVVGAPLAYALGVGFAPIRKSGKLPGETIEVGYDLEYGKDTLA

MHTDAIEKGQNVLIADDLLATGGTIATSINLIEQLGGKVVGAAFLIELSDLNGRAKLP

EIDVFTLMNY"

CDS complement(441817..443133)

/gene="uraA"

/locus_tag="EFAGFIKM_00409"

/inference="ab initio prediction:Prodigal:002006"

/inference="similar to AA sequence:UniProtKB:P0AGM7"

/codon_start=1

/transl_table=11

/product="Uracil permease"

/db_xref="COG:COG2233"

/translation="MQREIQVNQKMPLGSGSLLSLQHLFAMFGSTVLVPNLFGVDPSM

ILLMNGIGTLLYILMCKGKIPAYLGSSFAFIAPVTMVLKQHPENGYSMALGAFIITGL

VFCFVALIIKFAGTGWINVVFPPAVMGAIVALIGLELVPVAAGMAGLINSDPVANPNW

VPQAKPIILSMATLGITVIGAVTFRGFPKIIHILIGIVVGYILGYSMGLVDKQAIANA

DFISLPTVTTPTFDWSVIFTILPVALVVIVEHIGHLLVTSSIVGKDLSKDPGLHRSLL

GNGVSTILSGFVGSTPNTTYGENIGVMALTRVYSTYVIGGAAVIAIVLSFSGTFSALV

ANIPVPVMGGVSLLLFGVIAASGLRILVEQKVDFAKPTNLLLTTLVLVIGLSGTEVTF

YGIHLKGMALATIVGILMSLLFKLFEVLGWLNDDSRKQPITEKTPD"

CDS 443507..445684

/gene="relA"

/locus_tag="EFAGFIKM_00410"

/EC_number="2.7.6.5"

/inference="ab initio prediction:Prodigal:002006"

/inference="similar to AA sequence:UniProtKB:O54408"

/codon_start=1

/transl_table=11

/product="GTP pyrophosphokinase"

/db_xref="COG:COG0317"

/translation="MGIEQLLEKAGAYIKEPDLVRIREAYEFADQAHHGQTRKSGEPY

ILHPLAVADIVVNMQMDTISIIAALLHDVVEDTTVSLEEIRNHFGNTCAMLVDGLTKL

ERIQFRSKEEQQNENYRKMFIAMAQDIRVIVIKLADRLHNMRTLKFQSEESQRRISYE

TLEIFCPIANRLGISAIKWEMEDIALRYLNPQQYYRIANLMHKKRAEREQYIDTVMDG

ITNKLDEMGIQADLSGRPKHIYSVFKKMTTKNKQFNEIYDLLAIRIIVDNIKDCYATL

GIIHTLWKPMPGRFKDYIAMPKANMYQSLHTTVVGPNGEPTEVQIRTWDMHRTAEFGI

AAHWAYKEGAANGNHFEDKITFFREILELQNEAQDASEFVESLKMDFFSDLVFVFTPK

GEVIELPIGSVPLDFAYRIHTEVGNRTIGAKVNGRIVPLDYHLKTGDIIEILTSKHSY

GPSQDWMKIAKSSHARAKIKQWFKKERREENVEKGRESCERELKRMGLDPSAWMTDDK

LMEAAKKYAFNDIDDMLAAVGFGGITAAQIVTKATEKLRKEQEESSLLELNSEMRELK

PAPERRNRPTNGIRVKGIDNLLVRFARCCNPVPGDDIIGYVTRGRGVSVHRSDCPNIP

TSADGEEAARVIEVEWEENIEASYSVDIEITGHDRNGLLNEVLQAVSESKTNISAVTG

RTDKNKLALVHVTILIRNTDHLQSVVERIKRVKDVYSVHRIMQ"

CDS 445697..446158

/gene="dtd"

/locus_tag="EFAGFIKM_00411"

/EC_number="3.1.1.96"

/inference="ab initio prediction:Prodigal:002006"

/inference="similar to AA sequence:UniProtKB:A0A023W421"

/codon_start=1

/transl_table=11

/product="D-aminoacyl-tRNA deacylase"

/db_xref="COG:COG1490"

/translation="MRVLVQRCKEAQVTVGDELTGKIESGLMLLVGITHEDTAKDAQY

LADKVSGLRIFEDDQEKMNLSLLDVGGAVLSVSQFTLYGDCRKGKRPSFAAAARPEAA

ELLYETFNQLLRDKGIQVETGRFGAMMDVTFTNWGPVTLMLESPVRQEPSA"

CDS 446235..446402

/locus_tag="EFAGFIKM_00412"

/inference="ab initio prediction:Prodigal:002006"

/codon_start=1

/transl_table=11

/product="hypothetical protein"

/translation="MRQSAKETRTRTEPLLLPGNLYESLHLARSAAEYVATIWALQDS

DKGKQSHLPRS"

CDS 446670..447176

/gene="yhbO"

/locus_tag="EFAGFIKM_00413"

/EC_number="3.1.2.-"

/inference="ab initio prediction:Prodigal:002006"

/inference="similar to AA sequence:UniProtKB:P45470"

/codon_start=1

/transl_table=11

/product="Protein/nucleic acid deglycase 2"

/db_xref="COG:COG0693"

/translation="MSKVAFLLANDFEDSEMQVPYDEVKKAGHEVEIIGLKAGETLKG

KGGKASYTTDKAIGDASASDYDAVVIPGGSSPENLRSDAHILKFVTEINSAKKPIAAI

CHGPQILASAGLLKGRTITSYPPLKDDMVNAGAEFKDHEAVVDGNYITSRTPEDEPAF

VRELLKVI"

CDS 447281..447727

/locus_tag="EFAGFIKM_00414"

/inference="ab initio prediction:Prodigal:002006"

/codon_start=1

/transl_table=11

/product="hypothetical protein"

/translation="MTKHIQAYFRTEDEAEGARTSLQTFRTEHLEVGQLDSAVGRDTR

VMVPLVPYNTAGGVGTNGAMGVGAAPGVPASENVIPVVANVDRDAERDTRDRVNEDGD

LLNAADVSSADYDDLHYVLSAKVRDEDYDEIVHKLRSNHAYVEDLN"

CDS 448319..449626

/locus_tag="EFAGFIKM_00415"

/inference="ab initio prediction:Prodigal:002006"

/codon_start=1

/transl_table=11

/product="hypothetical protein"

/translation="MRMARDRFKGELVLSKVGRNDLCPCGSGKKYKKCCLNKESSAVE

SILGIISTEQPVREASIQPESKLTLTKLKKMVASDLKWEHPAHEQLALQTTENMRNQY

EPEVILEALVLWNGYSRQTRPTVKKTGSFSAAIEYLLSEEYGFNVSKAELADKYEVTT

GTISRKVKEMNSYIEEYGMGGETDELLMLNGPGTSKDKAQALLSKAREASSSKRRVHL

AETALEMYPDSSDAYLILAEESDNENDARSYLKEGIAAGKRELGELFFEENKGDFWGL

HETRPYIRICKSYAESCWFGGDTKEAAQILEHMLELNTEDNTGARYLLAAVYLYSNQL

DQAEQLLEKYGKGDAATAFAYDQIILEYKKNGITSQLKMLYRVARGVNKHVPDYLLGL

KRLPHNLPDFVGLGDSDEAIEYVIMHSRLWASAPDLLKWMLKQ"

CDS complement(449656..450780)

/gene="cheB_1"

/locus_tag="EFAGFIKM_00416"

/EC_number="3.5.1.44"

/inference="ab initio prediction:Prodigal:002006"

/inference="protein motif:HAMAP:MF_00099"

/codon_start=1

/transl_table=11

/product="Protein-glutamate

methylesterase/protein-glutamine glutaminase"

/translation="MLNLARRSLKEVRGYLHQIESPQEILPIIQSLQQLVEDFQAQAR

VNVHFRTFGEEYPLPKLAKMAFYRCLQESLTNAVRHGESTEITATLKFEQRQARLEIQ

DNGKGMEEWQDGFGLNAMKERTMNLQGQVSVYSEPGEGTLVTCTLPRQVELSDEVIRL

LIVDDQPFIRESLRTILEGHNDLQVVGMSEDGLQAIERCEQDQPQVVLLDLDMPNMDG

ISAAKVIKQKWPHIRILVLTTFQDTEQALEVLRSGADGYLLKSIEPRELAETIRLVYR

GGTLIDQEMSHKLFEKLEMDKTGNKMLEPNTSENFGLTSREMEILQLVCKGLRYKTIA

AKLYLSDGTVRNYASAIYMKLGVRNREEAVQKAIEAGFIR"

CDS complement(450834..451466)

/locus_tag="EFAGFIKM_00417"

/inference="ab initio prediction:Prodigal:002006"

/codon_start=1

/transl_table=11

/product="hypothetical protein"

/translation="MRNLFETVKQWFWYDWIMFIFRLIFSISLMFTTVQLHEQIPLPL
WALIFWQVFAFSVPWLCLQLSYKYYLLTEIIFSGGLCLYLSSLFPEAYLAFLTYAFLI
AVNSARQSYRWTGPITILLLPILINVLSHQSNYWVMMIQIGLAYAIGFAFHLLVVNHR
QSEIIREQNTVLQQYFSQIERITLAEERDRLSKDLHDTIGHSYLPLSSWG"

CDS complement(451575..452162)

/locus_tag="EFAGFIKM_00418"

/inference="ab initio prediction:Prodigal:002006"

/codon_start=1

/transl_table=11

/product="hypothetical protein"

/translation="MNKTIRCFFQNGYFLLLTFATGLFYFCFYLITLTLGLGLSFTVA
GIPLLTQVLRTTLTFVQFERIQTKIYTDITTQPYERRIRSEQSLWAQAKEELMNIRNW
VAIYWLMMKLWIGLLCLLCAALLCLMPLLLMLTPLFFPIFDIQFFGIQIDTFMKSLFI
MAMGVIFAFISSWILKVLVLLIGGYTRQMTKSLNR"

CDS 452367..454319

/gene="dap_1"

/locus_tag="EFAGFIKM_00419"

/EC_number="3.4.11.19"

/inference="ab initio prediction:Prodigal:002006"

/inference="protein motif:HAMAP:MF_01960"

/codon_start=1

/transl_table=11

/product="D-aminopeptidase"

/translation="MRKAIRNTSLAVLLISASIVTGCTATNGAVASDQKSTEKVSLEG
PKDSKEVEAFANSVFADKMKKYNTVGSNFVVVKDGKVLLSKGYGYADKEKKVPVDKDT
VFQIGSVTKSFTALAAMQLVDQGKIDLKHDIQEYLGEMKVPNKTGKPLTMFDLLTYTS
GVDLPDIVTDFSVEYLNKDIPMKGYLNQNIPTVVRPPGEAYTYDNYGFMLAGYAVENV
SGMPYSQYMEKNVFKPLGMNKTSVRLTPEVLSNVAAHYGPSGELQPTMGFAPSEKPEG
GMTSTGGDMAKYLIMHLNKGEFEGKEIVSPKSIDLMHTYQVYADPTIPITTVGFEGYF
NNVMNGQHVILKGGNVPGHSSLIAILPEKNTAIYLSYNNDSMMSLDVYEAFMNHYFPK
TVKTQPSTYSNVSKQAAQAYVGLYKNTRLHSIRTKISYANGNLLMETGTTGKHTLKMV
HPLLFEDEAGNKMAFKKDKSGDITYFYYTNPKGMDFVAHAQKVKMKTAFSDVPNNSVY

KSYIDNLNGLDIMGAQSGNRFDPKGKMTQGEFSDVLLRAHGMHTFPDEFRENKKRMIE

GIPNFQPKSLITRQMAAVMIQNLKHAAPGTNVKLIGKTDSWAVDAITALVSQGIVDPD

TKINSNQTVDFRSKQTLSRQEARCPA"

CDS 455055..456308

/gene="hisS"

/locus_tag="EFAGFIKM_00420"

/EC_number="6.1.1.21"

/inference="ab initio prediction:Prodigal:002006"

/inference="similar to AA sequence:UniProtKB:P60911"

/codon_start=1

/transl_table=11

/product="Histidine--tRNA ligase"

/db_xref="COG:COG0124"

/translation="MAFQKPTGTQDLLPGVVEKWQYVEEKARDLCRRFNYREIRTPIF

EQTSLFVRGVGETTDIVEKEMYTFDDKGNRSMTLRPEGTAGVVRAYVENKIYGEPDVS

KLYYIGPMFRYERPQAGRQRQFHQFGVEAIGALDPAIDAEVIALGYQLCVELGLKDVK

VEINSVGNSTSRAEYRETLLGFLRPMKDSLCKDCQSRMERNPLRVLDCKVDQDKFVGA

PSILDSLDEESLNHFAKLQAYLDDFGVDYAVNNRLVRGLDYYTLTAFELKAQGIGAID

TVGGGGRYNGLVGDIGGPDQPGIGFGIGLERIQLILEHQNIEVTTLAPLDVYFVALGE

AADREVNRLLFKLRQSGLSGERDYLGRKMKAQMKSADRFKSRYTAILGDDELERGEIA

LKSMDTGEQQTVKLDDLVAAIREGK"

CDS 456523..458301

/gene="aspS"

/locus_tag="EFAGFIKM_00421"

/EC_number="6.1.1.12"

/inference="ab initio prediction:Prodigal:002006"

/inference="similar to AA sequence:UniProtKB:P67015"

/codon_start=1

/transl_table=11

/product="Aspartate--tRNA ligase"

/translation="MKRSHHCGALTPAHIGETVTLNGWVQTRRDLGGVLFIDLRDRSG

IVQVVFNPAYSGEALQIADRVRSEYVIAVTGKVVKRDAETVNKNLPTGEIEVQITEIE

VLNAAKTPPFFIEDGVEVDESLRLKYRYLDLRRPEMFETLKLRSKASKVFRDYLDGEE
FVEVETPILTKSSPEGARDYLVPSRVHEGEFFALPQSPQIYKQLLMVGGVERYYQIAR
CFRDEDLRADRQPEFTQVDIETSFLQQDDLLPMMEELMAKLLRETKGIELELPFQRIT
YADAMGKYGSDKPDLRFGMELVEMNDIVANSGVKVFASVIEKGGEVKVLNAKGCGTWS
RKEIDDLGPFAARYGAKGLAWIQVKDGEFKGPIVKFFTPEEIEAVKERTGAEDGDLLL
FSADTKKVVADVLGALRLKIGRQLGLIDDSKFKFAWVVDFPLLGYDEDAKRYVAEHHP
FTRPKDEDVHLFDTDPGAIRAQAYDLVLNGYEVGGGSMRIYKRDVQEKMFAALGLSTE
VANEKFGYLLEAFEYGTPPHGGIAFGLDRLVMLLAGRTNLRETIAFPKTASATDLLMN
APSEVDDSQLEQLHIRVARKPVAEKK"

CDS 458320..459078

/gene="tcdA"

/locus_tag="EFAGFIKM_00422"

/EC_number="6.1.-.-"

/inference="ab initio prediction:Prodigal:002006"

/inference="similar to AA sequence:UniProtKB:Q46927"

/codon_start=1

/transl_table=11

/product="tRNA threonylcarbamoyladenosine dehydratase"

/db_xref="COG:COG1179"

/translation="MLHQFSRTELAIGPEGLDTLKNSTVAVLGIGGVGGIAVEALARS
GVGRIILIDKDVVDITNINRQIHALTTTVGQKKADLMVERVKLINPECDAIALNMFYT
EETYEELFKYELDYVLDASDTIIYKIHLIKECLKRKIPMISSMGAANKMDPTKFQVAD
ISKTTMDPIARVVRTTLRKDGIKKGVKVVFSTEKPMKPREDVTQKIVPENAPEIRKAK
QPPASNSFVPPVAGLIMVSVAIKDLLEIAENKQQ"

CDS 459554..460852

/locus_tag="EFAGFIKM_00423"

/inference="ab initio prediction:Prodigal:002006"

/inference="similar to AA sequence:UniProtKB:A0A2A5K485"

/codon_start=1

/transl_table=11

/product="Nucleobase transporter PlAzg2"

/translation="MKHGWMTRRLGMEPGDQWKKEVVAGAISYFAVVYIVMVNATILS

DAGMPLQAAMVGTLLTSIAGCLLMAFGGKSPIIVVPGMGINAFFTYTLVHSMKLSWQE
ALAVVTVTGILFAIVAFTSLYKMISEAIPKNLQHGITVGIGLFLTFIGLQKSGIVIAH
QTTFVAIGHFNDPNVITACVTLVLALILFIRNVQGGLLISILVGTGLAYVLGAVNPGE
SVSAMDALRQYGGLFGELSLMKLADVAFWIAVFLLLLIVVFENIGLITAQTQMIGRPE
RFKGSLRALSISTVLAGIFGSSPPVAAAETTAGIAAGGRTGLTPLVTAVLFGATFFFI
PLLGYIPDSAIAPILIIIGGLMVQGVKDMDFGDFTEAFPAFLIMVMIPFTYSIVDGMA
FGFIAYPLAKLAAGQGKQVPVVMYGISILFLANFVLHGVL"

CDS 460926..462725

/gene="bmrA"

/locus_tag="EFAGFIKM_00424"

/EC_number="3.6.3.-"

/inference="ab initio prediction:Prodigal:002006"

/inference="similar to AA sequence:UniProtKB:O06967"

/codon_start=1

/transl_table=11

/product="Multidrug resistance ABC transporter
ATP-binding/permease protein BmrA"

/db_xref="COG:COG1132"

/translation="MEEQQQAGQNEPKVGKKGFKDFVKLIASTNPPKLILILALILTL
IQTTAGLIVPLMTKGLIDGLTTSALNKSVILLLLGAFVIQAIASGISIYMLNYAGQRV
VATLRTKLWNKVLSLRMPYFDRNRTGDTMSRITNDTSQIMTLIADYLVSFVSNIVAVV
GGVALLFYLDWVMSLIILSLVPLTLLILLPVGKKMYKISKKQQDEMAGLTSVLSQVIG
EIRLVKAYGTEKQEVEAGDSRIRKMFAFGLQEARILALIGPLFTFVMTAVLVVILGVG
GMRVASGLLSPGELVAFILLLFQVIMPMGQFTTLYSRLQKVVGATERIQAILADEEEP
HDSTKVAPKGNETIAFHDVHFSYVTGEEVLHGINLTVPTQKVTAIVGPSGSGKSTLFS
LMEQFYMPDSGHISYGGQAITDYSLASWRSKIGYVSQESPLMAGTIKDNITYGLGREA
TMDEIRRAAEMAYSADFIDKLPQGYDTEVGERGIKLSGGQRQRIAIARALLRSPDILM
LDEATSSLDSTSEYEVQQALSNLMEGRTTIVIAHRLSTVVDSDQIVVLEHGHVTGTGT
HTELISNHPVYRELAQKQFVAQEGNAATVQNEE"

CDS 462872..463537

/locus_tag="EFAGFIKM_00425"

/inference="ab initio prediction:Prodigal:002006"

/codon_start=1

/transl_table=11

/product="hypothetical protein"

/translation="MARNKYPEQTLDLILAVSTQMFTEKGYEKTSIQDIIDELGMSKG

AIYHHFKSKEDILSAVMEKELGRAEDMFMELIQNTHAPNARQKLISILENIIVDPGIQ

SNSIDQVLSTQIKNPQFVLGGIRKGVLKDARIIANIMEQGKSDGSISVEYPLECAEIF

MLLVNIWINPLLFGRDRAQTLERLKFLQHMMKLLGADIVSEQLIQRITNRHASTGGYA

EAE"

CDS 463574..464365

/locus_tag="EFAGFIKM_00426"

/inference="ab initio prediction:Prodigal:002006"

/codon_start=1

/transl_table=11

/product="hypothetical protein"

/translation="MLKSSELAICKSIDIDQPIQDIQLNWLSGDIRVLPSVDELIHVI

QYADTWFSKLRLMQVNIQGGTFSIADGRKRRGLVGINIGRTALEIQLPDRVFDRILLN

SSGGHLRLNRLLATRCQCKITSGRVDLSGRMDELELCALASVVKGQHLSADKFNLQSS

SAKIDISGEFRHLQIITTGRKLNMDCLKMPEKFHSVSTGAKAVVSIPDNEGFRILLKR

RSGALKSEFDLTPLHGDSNCLIHKSGKSEFQVDIRGGAFHLKSRK"

CDS 464411..464581

/locus_tag="EFAGFIKM_00427"

/inference="ab initio prediction:Prodigal:002006"

/codon_start=1

/transl_table=11

/product="hypothetical protein"

/translation="MAGWIVGISFSTIVLLIGIYGALNSRSRQARNKWAWWFILFGCC

ALVSAIINYQFR"

CDS 464718..466898

/gene="helD_1"

/locus_tag="EFAGFIKM_00428"

/EC_number="3.6.4.12"

/inference="ab initio prediction:Prodigal:002006"

/inference="similar to AA sequence:UniProtKB:O32215"

/codon_start=1

/transl_table=11

/product="DNA helicase IV"

/db_xref="COG:COG3973"

/translation="MSDSFQSAYQEEEYRLERTMEEVDSQLERLQNIPVYTGHDFTEQ

VLEAGREERRTALSKSAQQPYFGRLDFEEQGSGARKPLYIGKIGVDREEVGEHPLVID

WRAPVASLFYSFTGGEASATYEAPEGLIEGLVYLKRNVVIRQRILERVADTYNRDSDQ

PAVSDEFLVYRLGENKDNKLRDIVSTIQAEQDLIIRAARNTALIIQGVAGSGKTTVAL

HRLAFLLYQYKEQVSAEKMVIFAPNHMFLDYISDVLPELGVGDIQQRTFPDWAMNLLG

LEMPLANTSDTLNTWFEKPDARPELNDDVPGRYKGSIRFMELIREWLSGMEADSVPDM

DFSPWDGTVLSKTEIESWFGEEYKHYPLAKRKERVMARVHRWIEMELKKPMPEAVRKE

RKKKASTREKAYAKKWPQYEPLTLYKQLFKAAKGGSVPASLSGLIPKLVMKQTAADLK

QDIVREEDLPALVYIHTLVHDIEGSERFDHIVIDEAQDFSPFHVALLDRFVKGHSFTI

LGDLSQGIHEYRGVHAWEEMSSLFPEEQNAYFALTRSYRSTMEIIDYANTILERGVKS

GITAIPVFRSGDPVRTLAYGGEGRTASLEQALKVLTVKDYRTVSILTRTLHEAEEIHR

ELQAAGWDLNLIDGGKKQYTGGHSVLPVYLSKGLEFDAVIVADVDQKHYLPNAGDAKL

LYVGCTRALHELWLFHDGVLPIYAAATHNPDGEPVTIQGWPELG"

CDS complement(467021..467923)

/gene="yijE"

/locus_tag="EFAGFIKM_00429"

/inference="ab initio prediction:Prodigal:002006"

/inference="similar to AA sequence:UniProtKB:P0ABT8"

/codon_start=1

/transl_table=11

/product="putative cystine transporter YijE"

/translation="MSSSHDKDTRIIKPLKYTLLIILTTLIMGTSFPVGKIGMLYAPP

FLLMGIRYILAGGLMALFLRKRPLPTGWQSWLKIILIGLFQSAGVMGCAYYSMNWITS

GESAIITFTNPLLVIILSALLYRVTYRFSQWIGVILGFIGIMLTFGWQMSFSPGTWIG

FGGAISFAVSTLLIKRWGNGYDTFVLTAYQMLAGGAALLLLSVWTEQPHFMVNTTSVM

ALLWLTLVCSIIQFSLWFYLLQNSDPAQTSSYLFLAPFFGVLSSWILLGEHVQWFALA

GGLFIGIGIFLVNRRSSRMRNSVT"

CDS complement(467920..468663)

/gene="fabG_2"

/locus_tag="EFAGFIKM_00430"

/EC_number="1.1.1.100"

/inference="ab initio prediction:Prodigal:002006"

/inference="similar to AA sequence:UniProtKB:P99093"

/codon_start=1

/transl_table=11

/product="3-oxoacyl-[acyl-carrier-protein] reductase FabG"

/translation="MTYWKNRVALITGGGTGIGRAVSEMLAERGALVAVNYSRSQDAA

RETVQHILDTGGHAFAVQANVANDLDVRRMVTTVTETYGPITALVNNAGITRHIPMHD

LESVTDDVWNELVDVNVKGMFHCARAVTEGMRQAGAGSIVNLGSIAGSTGSGSSLPYA

VSKAAVHGLTLSLAHALSPHIRVNAIVPGAVATRWWAGNEERMYRLGGEVLLQHIASP

EDIAHMICAALEQQSMTGQLITVDSGQTL"

CDS 468800..469690

/gene="yofA_1"

/locus_tag="EFAGFIKM_00431"

/inference="ab initio prediction:Prodigal:002006"

/inference="similar to AA sequence:UniProtKB:O34685"

/codon_start=1

/transl_table=11

/product="HTH-type transcriptional regulator YofA"

/db_xref="COG:COG0583"

/translation="MESGDLRIFQCVAQEGNLTKAASKLGYVQSNVTARIRHLEAEVG

TTLFIRHNRGMTLSPAGEMLLTYADKIIGLLNDASKALRATSTPSGPMRIGSTQTAAA

VRLPELFVRYYKQYPDVSLSLVTGNSQILMDQVIGYELEGAFIGCPCDHPDIISIPVF

DEELFVVYSGMGPTDEDLKSKPILVYSMGCSYRQILEEWLEVGGVTHPVIMEFGTLEA

IISGVTSGMGISLLPEIVIRHQIENGLLRTHTLPVGMNRMMTHFITRKDAFVSSALHA

FMAMLPREYDMIESGEPSEV"

CDS 469733..471040

/gene="rarA"

/locus_tag="EFAGFIKM_00432"

/inference="ab initio prediction:Prodigal:002006"

/inference="similar to AA sequence:UniProtKB:P0AAZ4"

/codon_start=1

/transl_table=11

/product="Replication-associated recombination protein A"

/db_xref="COG:COG2256"

/translation="MDLFSFQQDSKPQARLLADRLRPEHLDEYIGQEHIIGPGKLLRR

AIEADQISSILLYGPPGCGKTTLAHIISQQTQGQFVRLNAVEASVKDVREVIEQAQTN

KQLYGTKTILFLDEVHRFNSSRQDALLPAVEKSTIIFIGATTENPFHYVNGALMSRST

LFQLESLNKDHSLIAMRRALSDADKGLGFMELQADDEALEHIATMANGDIRRALNALE

LAALTTPPEKDGSIHITLGVAEESIRRPIVKADESTQYDVLSAFHKSIRGSSDAALFW

FLYAVEKLGMDPMTFIRRLIAASSEDIGLANPQAMTQAIGALDAYRNNGWPEAKLNIA

QAILFAVESPKSNAVYTAISKAMNAIDEVKSAEVPLHLRDTHYSGAVKLGHEGYQYPH

NYPGHYVKQEYLPKQLSRRVFYEATEQGNESKIRLNQQRRREL"

CDS 471073..471474

/gene="crcB_1"

/locus_tag="EFAGFIKM_00433"

/inference="ab initio prediction:Prodigal:002006"

/inference="protein motif:HAMAP:MF_00454"

/codon_start=1

/transl_table=11

/product="Putative fluoride ion transporter CrcB"

/translation="MKELLYIGAGGFLGTLTRYAIQLAIPTVHGGFPWAVLLINAMGS

LFLGWFFTIAVPGKITPQLRLAIGTGFTGAFTTFSTFTLDIVRLSEGSEWISAAIYMI

VSLLAGLLLCALGMSLGQRMLGAQRQEGDAS"

CDS 471471..471830

/gene="crcB_2"

/locus_tag="EFAGFIKM_00434"

/inference="ab initio prediction:Prodigal:002006"

/inference="protein motif:HAMAP:MF_00454"

/codon_start=1

/transl_table=11

/product="Putative fluoride ion transporter CrcB"

/translation="MILWIGFAGVLGAVLRYSLGKWVSGLLGTAFPWGTWIINISGSL

LLGLLYGWHQSAMISDGIWVIWGTGFCGAYTTFSTFGYETLGLMSRQRYASGALYVIS

SVVVGVLACLAGVWLTA"

CDS complement(471935..472630)

/locus_tag="EFAGFIKM_00435"

/inference="ab initio prediction:Prodigal:002006"

/inference="similar to AA sequence:ISfinder:ISBth8"

/codon_start=1

/transl_table=11

/product="IS3 family transposase ISBth8"

/translation="MMAIHFENREFGYPRMTTALWEAGLNVNHKKVWRIMRELSIQSV

IRKKRKKSSYTPSVIYPNRLKRKIHATAPQQKMVTDITYISDGNSFVYLSVIQDLFNN

EIVAWQLSKRNDVQLVLDTVEQWTQKRDVSEAVLHSDQGFQYTSQAYNTRLEAFGVKG

SHSRKATCLDNACIESFFSHLKTEKLYLNQCNSEAEIRQAVEDYMYNYNYRRFQAKLK

QRAPIEYRCALAA"

CDS complement(472795..473139)

/locus_tag="EFAGFIKM_00436"

/inference="ab initio prediction:Prodigal:002006"

/codon_start=1

/transl_table=11

/product="hypothetical protein"

/translation="MILGGDIFMAKKGQTFQTYTEEFKLNAVRSYVEGSSSYKVVAER

EGIRNCSQLKVWVKKWKNGEAFDERKNSVPNPLKGRPRTAFGSVEEERDYLQAQVDYL

KKRYPNLVKEKR"

CDS complement(473267..474556)

/gene="mnmA"

/locus_tag="EFAGFIKM_00437"

/EC_number="2.8.1.13"

/inference="ab initio prediction:Prodigal:002006"

/inference="similar to AA sequence:UniProtKB:Q99TM8"

/codon_start=1

/transl_table=11

/product="tRNA-specific 2-thiouridylase MnmA"

/translation="MLTYLHALWKRKIDDIPKCAPVVDTIQNWPGVSYLESGYARIRN

ETHLYIEMVITMSKTIENTRVVVGMSGGVDSSVTALLLKEQGYDVIGIFMKNWDDTDE

FGHCTAEEDSEDVRRVCEQIGIPYYTVNFEKEYFDKVFTYFLDEYKSGRTPNPDVMCN

REIKFGEFLNKALDLGADYVATGHYARLIEEDGTLKLLRGVDNNKDQTYFLNALNQNQ

LSKAMFPIGHLPKPEVRKIAEAAGLYTAKKKDSTGVCFIGERNFKEFLSNYLPAKGGD

MVDIATGEVKGRHDGLMYYTLGQRQGLGIGGSGNGEPWFVADKNLEKNQLLVVQGDAH

ASLYSTGLTATGVNWIAGAEHMPNVPYRCTAKFRYRQPDQGVTLTWQVDGSVDVQFDQ

QQKAITPGQAVVFYDGEVCLGGGTIDQVQKVPVPAMQ"

CDS 474713..475132

/gene="cymR"

/locus_tag="EFAGFIKM_00438"

/inference="ab initio prediction:Prodigal:002006"

/inference="similar to AA sequence:UniProtKB:O34527"

/codon_start=1

/transl_table=11

/product="HTH-type transcriptional regulator CymR"

/translation="MKISTKGRYGLTIMMELAARTGEGPTSLKSIAERNQLSEHYLEQ

LIAPLRNAGLVKSIRGAYGGYILAGDPATVTAGDVIRVLEGPISPVDFTEEDDPAKRD

LWLRIRDGIAEVLDSTTLKDLITFQDQDKKDSYMFYI"

CDS 475307..476458

/gene="iscS_1"

/locus_tag="EFAGFIKM_00439"

/EC_number="2.8.1.7"

/inference="ab initio prediction:Prodigal:002006"

/inference="similar to AA sequence:UniProtKB:P0A6B7"

/codon_start=1

/transl_table=11

/product="Cysteine desulfurase IscS"

/db_xref="COG:COG1104"

/translation="MKRIYLDHAASTPMHPQVAEAMMNVMTGQFGNASSIHAFGREAK

RTVSGARDVIAASLGCFPDELVFTGGGTESDNLAIFGAVVSRQEKGKHVITTAIEHHA
VLHTCQELERQGYEVTYLSVDRHGRINLDELQEAIRPDTVLITMMYANNEVGTIQPIR
EVGELARQHNILFHTDAVQALGSQNISCKELPVDLISFSAHKINGPQGVGALYVRRGI
VLEARAHGGLQERQRRAGTENIAGITGFAEALKIASAQTEVHRQHDLELRKLLLEQLE
IHVGTEHFHVNGHLEHTLPNILNISFPEVSTETMLMNLDMEGIAVASGSACTSGSLEV
SHVLKSMKLPETFLHSAIRFSWGLGNTTEEIMITAEKIGTILGRLRNRP"

CDS 476623..477144

/locus_tag="EFAGFIKM_00440"

/inference="ab initio prediction:Prodigal:002006"

/codon_start=1

/transl_table=11

/product="hypothetical protein"

/translation="MKLQEMIGLAVFDVEDGKQVGKIQDFIVNDDWEIEGIELENKGL
FTNHVKIVQWQDIVAYGEDAVMIRNQQAVRKTGADDIKYTYLLGRSKLKEMSVLTEEG
LLLGRVSDVYFDQELGNTIIGIEITDGFVSDLIEGRKWLPCTSDMSIGESAIMVPSLS
EQRLENAIHSVNG"

CDS 477149..477364

/locus_tag="EFAGFIKM_00441"

/inference="ab initio prediction:Prodigal:002006"

/codon_start=1

/transl_table=11

/product="hypothetical protein"

/translation="MNMKCPNCSSKDIGKIGSHQFYCWGCFIELTVNGEKMSVYQVEE
DGTLSSLDDLFFGDELPQDFPHLHASS"

CDS 477661..478728

/locus_tag="EFAGFIKM_00442"

/inference="ab initio prediction:Prodigal:002006"

/inference="similar to AA sequence:UniProtKB:P9WFM5"

/codon_start=1

/transl_table=11

/product="Putative transport protein"

/db_xref="COG:COG0628"

/translation="MEQLTKNKLFRYAIWLLLGLIILYFIWLLRPLLLHIYAFLKTVL
APFIVALIISYVLNPIVSMLGGRKVPRTIAVLLIYAFFLTCLGVILMNVIPVLIEQLG
ELNEHMPELSMRAQSLMNNMDHKLMPPSVRTGMNSWFFQMEDRLTQGITVLMDNIGAT
INVLFNVFIVPFLIFYMLKDFEVFERTIVAYLPRSRRKAIVSVMKEIDTALGNYIRGQ
FIVCVIVGIFAYIGYIVIDMPYALLLASIVAVFNIVPYLGPFLGAAPAVVMASTVSFK
MVLLVVIVNTLCQVLESNVISPQVVGRTLHLHPLSIIFALLVGGELAGIVGLILAVPV
FAVLKVIVQHFFAYYIKRRTV"

CDS 479164..481794

/gene="alaS_1"

/locus_tag="EFAGFIKM_00443"

/EC_number="6.1.1.7"

/inference="ab initio prediction:Prodigal:002006"

/inference="similar to AA sequence:UniProtKB:P67011"

/codon_start=1

/transl_table=11

/product="Alanine--tRNA ligase"

/translation="MKASEIRSKWIEFFASKGHKIEPSASLVPHNDPSLLWINAGMAP
LKPYFDGREKPENPRLANSQKCIRTNDIENVGKTRRHHTFFEMLGNFSIGDYFKEETV
TWAWEFLTSKEWIGFDPERLSVTVYPEDEEAFKLWNEKVGLPAERIIKLDENFWDIGE
GPCGPCTEIFYDRGEAYGNDMSDPEMYPGGENERYLEVWNLVFSQFNHNKDGSYTPLP
NKNIDTGAGLERFASILQNVDSNFDTDLFQPMIQRTAALAGVKYNDSVEIDVALKVIA
DHIRTVAFAVGDGVLPSNEGRGYVIRRLLRRAVRYGKVLGLDRPFLYELTTTVGEVMG
MYYPEVVDKQEFIAKVIKTEEERFHETLTDGLAILADISGTAKSEGRTVISGPEAFKL
YDTYGFPFDLTEDYAAEHGLTVDREGFDASMQKQRELGRAGRQENESMKVQGGPLADL
EVKSEFVGYTDLLTEAKVVAIVAGDALVESVGEGQTCQVVLDKTPFYAESGGQVSDQG
LLRGAGVTAKVQGLFKAPLGQHVHLVTVESGELRVGDVINAEVDSAKRGDIIKNHTAT
HLLHKALKDVLGTHVNQAGSLVEPQRLRFDFSHFGSITPEELTEIERQVNEQIWNRLN
VNIELKAIDEAKEMGAMALFGEKYGDIVRVVQVGDYSLELCGGCHVNNTSEIGIFKLV
SESGIGSGVRRIEAVTGRGAYLYVESQLELLKQSAALLKANVADVPKRIEGLNQQLKE
AARETESLQSKLSAMEAGQLTDQVVQAGNTQLLAARVDAPNMDALRTVADELKVKLPN
AVLVLGAPADGKVNFVVAVPAEQVKQGLHAGKIVKEVAAICGGGGGGRPDMAQAGGKD
ATKLDEALKLAVSLVSGHNA"

CDS 482037..482297

/locus_tag="EFAGFIKM_00444"

/inference="ab initio prediction:Prodigal:002006"

/inference="similar to AA sequence:UniProtKB:Q836B3"

/note="UPF0297 protein EF_1202"

/codon_start=1

/transl_table=11

/product="hypothetical protein"

/translation="MDSMDKTVKFNVKGDEQEASSKEILLTVYDALVDKEYNPINQIV

GYLISGDPAYIPRHNNARSLVRKKERDELIEELVRSYLANHR"

CDS 482426..482842

/gene="yrrK"

/locus_tag="EFAGFIKM_00445"

/EC_number="3.1.-.-"

/inference="ab initio prediction:Prodigal:002006"

/inference="similar to AA sequence:UniProtKB:O34634"

/codon_start=1

/transl_table=11

/product="Putative pre-16S rRNA nuclease"

/db_xref="COG:COG0816"

/translation="MKILGLDYGDRRIGVAASDAFGWTAQGLEVLERRRDEGEFTRIA

DLVREHEISEIVVGLPKNMNGTVGPRGEICIAFADRLRDELNLPVHLWDERLTTMAAE

RTLIEADVSRKKRKQVVDKMAASLILQNYLDANSRR"

CDS 482855..483154

/locus_tag="EFAGFIKM_00446"

/inference="ab initio prediction:Prodigal:002006"

/inference="similar to AA sequence:UniProtKB:Q97SX0"

/note="UPF0473 protein SP_0194"

/codon_start=1

/transl_table=11

/product="hypothetical protein"

/translation="MAEDQLGMEEEAEIIYIADDEGNEEEFEVIMKFEVDGSEAKYMM

VAPVEPEDGETDVYAFRYEEEGDDIKLFVIQDDAEWDIVEETFNTFLAEDEEEAN"

CDS 483155..483469

/locus_tag="EFAGFIKM_00447"

/inference="ab initio prediction:Prodigal:002006"

/codon_start=1

/transl_table=11

/product="hypothetical protein"

/translation="MTEYSRKDLKWTDSLRLAFGAHVELEEENGKSQPYDLLAEFEVN

GQQYAVLRSSLRPYDEVELLRVSPGSEDQIMPELVTIDDDDEWENISELYDECTLPID

ED"

CDS 483546..484586

/gene="mltG_1"

/locus_tag="EFAGFIKM_00448"

/EC_number="4.2.2.-"

/inference="ab initio prediction:Prodigal:002006"

/inference="similar to AA sequence:UniProtKB:A0A0H2ZLQ1"

/codon_start=1

/transl_table=11

/product="Endolytic murein transglycosylase"

/translation="MKGKAIVVILLIIVVLAGGAGGYVWSMIRPVEASTEPVVFEIKS

GSGTSKIADQLQEEGLIRSGLTFKGYLKWKKLGSNFMAGTYSMNPGVTYDEIVSKLSS

GEVVPEEMVKFTIPEGYNVLQMAGKLSYEHVVDRDEFIKLANDPSAFDVDIIKDIPVD

EELRYVLEGYLFPETYELKKGSSTHDVMQRMLEEFQTKINSIPDLEAKLQEKNLSLHE

LLTIASLVEKEVVVDEERALVAGVIYNRINQDMKLEIDATVQYLLDRPKERLFFKDLK

VKSPYNTYLNKGLPPGPIASPSLPSIEAALNPEASEYLFYVTKKDGSSGHLFAKTYKE

HQQNIAKSKAAQ"

CDS 484601..485530

/gene="rlhA"

/locus_tag="EFAGFIKM_00449"

/inference="ab initio prediction:Prodigal:002006"

/inference="similar to AA sequence:UniProtKB:P76104"

/codon_start=1

/transl_table=11

/product="23S rRNA 5-hydroxycytidine synthase"

/db_xref="COG:COG0826"

/translation="MSKKHELLVTAANVKEAEVLLQAGADALVIGDDRFGMRLPGSFS

VEETAEVVAIAAKHQARVYVSMTNLMSNELLKELPEYVQALGRIGIDGVEFNDPSVLA

TMKEYAPHVKLHWNAEMTSTNYATANYWGTKGASRVVLARELNMDELTEMVPFLKVEA

QVQVHGMTNIYHSKRSLVQSYMAHQGRPVEGHLGKERGLFLIEAERRDEKFPIYEDIN

GTHIMSSEDICILEDLHLLMEAGVHSFKIEGMLKSLTYNEAVVRAYRTAIDSYVADAD

AYAFYEEWLAEVRKLQDPERELSFGFFYKEQVY"

CDS 485548..486876

/locus_tag="EFAGFIKM_00450"

/inference="ab initio prediction:Prodigal:002006"

/codon_start=1

/transl_table=11

/product="hypothetical protein"

/translation="METVAVQRKFSGKRNRLDKPELLAPAGNLEKLKFAIHYGADAVY

IGGQAYGLRSNADNFSFEEMREGVEFAKKYGAKVFVATNIYAHNEDVEGIETYLQNLY

NAGISAIIVADPAIIEVALRAVPGLEVHLSTQQSTLNWQAVKFWKDEGLPRVVLGRET

SFEEIEEIKANVDIEIEAFIHGAMCSSYSGRCVLSNHFTDRDSNRGGCCQSCRWKYDL

FEDAREDTVWVSEEDMQMKAPAPFKLGENQLPLFQEQDNSFSMGSKDLCMIGHIPELI

DVGVDSFKIEGRMKSIHYVATVVNVYRQAIDAYMADPENYVLKPEWVEEMNKAANRPL

NTGFFYDTPDHEDHIYEPEEKAVPFDFAGLVMGYDAETGMATIQQRNHFKPGQEIEFF

GPGGHFFKQVVGEIQDEEGNVLDVARHPLQLIKMKVDQPVSYFDMMRKKK"

CDS 487117..489399

/locus_tag="EFAGFIKM_00451"

/inference="ab initio prediction:Prodigal:002006"

/codon_start=1

/transl_table=11

/product="hypothetical protein"

/translation="MVQKKQKDSGEQVIKSEQVNPEQVQKVKKEKVGKSKITGNGKKL

LKFDRSKLKLGWIKTDWVKKQFKNRDWKNLGGSSFQQIKKANPVKSVGVKLFLIFFAG

IMIFVVSLGLLSYSKAKGTIEKNASRANQETIDQTKQKMDIILERFVDTSTQIFFDPE

MQSLLQKMSDQNLTAYDTFINSSSINKQLSNVAFTNKSMEAIYLVPTDDTKSVMGTGS
NSSSMGNIRQEAWYSELIEAGGYRWLPTEVKEDGSTPTFKIARSMKNLQGTTQSYVLV
IELKLEVLEEQLKSLDLGPGAVLQLIAPDNKVVASSISDRTGQDTELAFVKELTEPAG
STNTEYMVDGKSTEMLAVYSTMDTSNWKLVGMIPTSVLVQDAKGILTLTLWMALIDAG
IAVLIGIWMVRMIARPLGKLKDLMQEGAKGNLKVRTPYSSQDEIGQLSSAFNLMMEQI
TKLVEQTNRSAQEVLDTASELSSASKKTAVSASEIAVATEEIAGGAGSLATEAERGNE
LTDNISRQMQSVIAANEQMGDSARHVEKSSQTGTQHLNQLMTKTQKTEEMIGALVNKV
DSLKESTSSVLKVLEVLQNITKQTNILSLNATIEAARAGAAGRGFMVVADEVRQLAAQ
SRQSIEMVGEITDKIMTEMNETVDALSAAYPLFKEQMDAVKDTNVIFASVQEQMGAFV
ERLGMVTGSIGDLNKSQGTLSEAMSNVSAVAEESSATSQEVASLSSEQQNISNQLVNL
SGKLENVSTELKETLSRFTV"

CDS 489415..491334

/gene="pbpI"

/locus_tag="EFAGFIKM_00452"

/inference="ab initio prediction:Prodigal:002006"

/inference="similar to AA sequence:UniProtKB:O32032"

/codon_start=1

/transl_table=11

/product="Penicillin-binding protein 4B"

/db_xref="COG:COG0768"

/translation="MRKTCPAHGTGFLFVYRIIPSHISNNMVKTTKGEIGMHLLAKRR
IYITCILLTLVFGLLILRLGWVQIVQVNQTMPGSHRTVREMSVLQRERGVMLDPGRGQ
FTDYKGEALTGKLQWGLVLFPQASPLEKLRKQGALEGSEMIQLAAILHTDPDQLKKEW
NQESIPHFWTAGTHQPVHLTAAQVERLRSLHLQGAGIYPMMTRYLQGHTGMQWMGYLA
EQPESSKVLTRHQDEVKQPFAMKSGAAGLERTLEPLLRGIGPTLVSRMVSGGGEIIPD
IQPHVIAPTNSHYPLRVETTVDAELQRGLEELTQESGLQEGAIVVLDAANADVRAMIS
RPFYQPQHVDPKQSAWGNRAIQGAVPGSIFKIVTAAAALEYHAVSNGEEFHCGGEYGR
YGLSCWKEHGHGDLNLEQGFAESCNIVFAETARRLSMEQLENTADRLGLARPIGWEGK
QMAGMPVLRHFDHEDHGRVRTEAVSSADEGAKIQTAIGQRDVLVTPLQAANLIVTLLH
DGKVSSPRLVKRIRYADGGTMLELPLHDSPSASGQIAPATAHKLLSWMNKVVREGTGK
SLQRARWHVAGKSGTAQVQKHGEKRNNQWFIGYGPVEQPQYAVAVLVQNVSPDSQHQA
TALFRKVMDYLAESS"

CDS complement(491313..492509)

/locus_tag="EFAGFIKM_00453"

/inference="ab initio prediction:Prodigal:002006"

/inference="similar to AA sequence:UniProtKB:A0A1W5X0D5"

/codon_start=1

/transl_table=11

/product="Sodium-lithium/proton antiporter"

/translation="MLPLYKKYGRTVFDIALLVLTVYVIMYGFSQLYHVAAPVFLSFI

VYWMIEPLAKFLHRKGLPKTLGAAISVLLFLALIVAAFFGVGLIIISQISNLQDNFPV

YIEMIQREFTNLVLFIQDKSDALPDGVMEKANDYFATLTGFLSKWVTSGAQFVVGFLS

SFSSFITNFGIAIILAFFLSIEIESWRKFARAKTPKTLKSAIEFMRNHVFKTIRSYLK

AQMIMMLITFVLIYAGLLILGTSNAFTIAAVCAVFDLVPLLGVPVVFIPWIVYLFIIG

NSSLAIGLIVILAVTMLTRQLLEPKISGNSIGVSSAYLMLSFMLISLSIFGLAGVVLS

PVLLILLKELLQQGYLQKWIHLPKDEFESSPLVMDPPVNKDSSNSAESTVQRPIPAKD

HEDSAK"

CDS 492684..494087

/locus_tag="EFAGFIKM_00454"

/inference="ab initio prediction:Prodigal:002006"

/codon_start=1

/transl_table=11

/product="hypothetical protein"

/translation="MTTILHSILLWLLYLSSFYAFIPSLISRFFGFRVFRRGRSDTQF

ALTFDDGPDPHYTPRLLDLLRQHQAKATFFVVGEHAASHPELIQRIHEEGHLIGIHNY

IHKTNWLMRPRTVRDQIQRTGQIIHEVTGVKTCYYRPPWGIMNLFDFFSKKERKIVLW

SSMFEDWRSRVGAQRLTERMLKELRGGEVMLLHDRGTTLGADAHAPEQMLQALEVVLQ

EAERLGLQSVRVDTLMGGITVSESQNKMQLQTPLKLWKRIVVALWLGWEKLFHWVYHL

RTASPEDPMLHFRSRVYHGARVEMNDGHVIQNGDPVIELHFDNQKLFELGVTSRSSMH

LAIRMIRTMEQQLPDLARMVALEPELRSAKAIYGVSMINRGPEKFGFTIQELPPGPFS

AASKVYLKLLLSVIHPAGTKRLKQRTEQLVPKMIAMPLDLLLERYGQHMMQVAATVEE

SRDESLLIEQEILPERN"

CDS complement(494173..495858)

/gene="ilvD_1"

/locus_tag="EFAGFIKM_00455"

/EC_number="4.2.1.9"

/inference="ab initio prediction:Prodigal:002006"

/inference="similar to AA sequence:UniProtKB:P51785"

/codon_start=1

/transl_table=11

/product="Dihydroxy-acid dehydratase"

/db_xref="COG:COG0129"

/translation="MSAKKMRSDMIKKGFDRAPHRSLLRAAGVKEEDFGKPFIAVCNS

YIDIVPGHVHLQEFGKIVKDAIREAGGVPFEFNTIGVDDGIAMGHIGMRYSLPSRDII

ADSVETVVSAHWFDGMVCIPNCDKITPGMMMGALRVNIPTLFVSGGPMKAGRDSNGKA

LSLTSVFEGVGAYQAGKIDDKSLLELEQFGCPTCGSCSGMFTANSMNCLAEAMGLAMP

GNGTILAVAPERREFVKQSATQLMELIKMDLKPRDIVTVEAIDNAFALDMAMGGSTNT

VLHTLALAHEAGIEYPIERINEVANRVPHLAKLAPASDLHIEDVHNAGGVSAVLNELL

KKPGAIHGDCITVTGKTIRENVEGKEIQDTNVIHHLDNPHSEKGGLAVLFGNLAPQGA

IIKVGAVDASVGGYHKGPAICFDSQEQALEGIANGKVKEGHVVVIRYEGPKGGPGMPE

MLAPTSQIVGMGLGAKVGLITDGRFSGASRGISIGHISPEAAEGGPIAFVEEGDIIEL

DLNNRIIELHISDEEFERRRAGWKGFEPKVKTGYLARYSKLVTNASNGGVLSI"

CDS complement(496167..496418)

/gene="gsiB_2"

/locus_tag="EFAGFIKM_00456"

/inference="ab initio prediction:Prodigal:002006"

/inference="similar to AA sequence:UniProtKB:P26907"

/codon_start=1

/transl_table=11

/product="Glucose starvation-inducible protein B"

/db_xref="COG:COG3729"

/translation="MARQPTSEHKMSREEAGRLGGKATSKNHDRSFYQMIGKKGGEAT

SDAHDTDFYKQIGRKGGEATSETHNKEFYRKIGRKGGSN"

CDS complement(496542..497582)

/locus_tag="EFAGFIKM_00457"

/inference="ab initio prediction:Prodigal:002006"

/codon_start=1

/transl_table=11

/product="hypothetical protein"

/translation="MKNQTLIRDDQESFFYNLRFMLIVCVLAGNALEPLITRFAGAEA

LFLWIYTFHMPLFVWVTGYFATHSLQGASGRNVLKQIALQYVLFQSLYALMDFTVFHT

PHMRLSFFAPYLLLWFLASHFCWRLLLRLTISWKPIYRLIGSIALGVIAGYLPIDGFW

LSFSRTFVFLPFFVIGYDYGATIRSRLLSGWGRKTAAILSAALLVWIGYGGLNISSGW

LLGSMTYAELGHHEWYAGIFRLGVYLLEIASAALFLAWVPSLTSRLTDLGRRTLYVFL

LHGFLVRLAIWSGIYSYMGSSAYIPVILVIAILFAVTLALPAVRHTFKPVIEPDISRF

SFNRHEVFKRSA"

CDS 497805..498770

/gene="kdgK_1"

/locus_tag="EFAGFIKM_00458"

/EC_number="2.7.1.45"

/inference="ab initio prediction:Prodigal:002006"

/inference="similar to AA sequence:UniProtKB:P50845"

/codon_start=1

/transl_table=11

/product="2-dehydro-3-deoxygluconokinase"

/db_xref="COG:COG0524"

/translation="MSTSPYPSPEIITFGESMGLLTAKDTRGLEYAATLDKSFGGAES

NLAIGVSRLGHTSGWFGRLGNDPIGSMILKAIRGEGVDVSRVRLSDQEPTGLMIRENA

SGKASVHYYRKLSAASAITPDDLDPDYIAGAKILHVTGITAAISPSGLATVEAAIHIA

KQAGVKVSFDPNLRLKLWSADEARPVILRLAELADYFLPGLDEMKLLYNEENDQKVLE

RLSAMKAVCIVKGGPDLTYVLANGTLTEVPYFKADNVLDTVGAGDGFCAGFLSGLLKG

YSPQEATRLGNLTGSMVIQAVGDWEALPTWGQVEAKLNNVAHVER"

CDS 498859..499524

/gene="kdgA_1"

/locus_tag="EFAGFIKM_00459"

/inference="ab initio prediction:Prodigal:002006"

/inference="similar to AA sequence:UniProtKB:P50846"

/codon_start=1

/transl_table=11

/product="KHG/KDPG aldolase"

/db_xref="COG:COG0800"

/translation="MKKLQLLQKITDNGVVAVLRADSADQVIAMAEQAIAGGIKVIEI

TMTVPSALKAIEKLSRVYHWNTQDPEKFAIIGAGTVLEPQTARAAIMSGAEFVVGPSL

NPDTVQICNLYRIPILPGVMTIADVQRALELGVDIVKLFPGNLYDPSIIKTMKGPMPQ

ANFMPTGGVSLSNLGDWIKGGAVAVGIGSDLTSEAVKTGDLSHVRRKAEQYMDAYRKA

KAE"

CDS 499619..500383

/locus_tag="EFAGFIKM_00460"

/inference="ab initio prediction:Prodigal:002006"

/codon_start=1

/transl_table=11

/product="hypothetical protein"

/translation="MRNRYNTGRKKRGIGKFLLVLLALIVIGCGFVAWKLFTPYGPQE

TAQTAMETANPVTVSEQENWIDFVPDKPTGKSVLFYPGGLVKPESYAPLAHELAAAGH

HTIIAKMPVNLAVLKPNLADEILSAYPDEQFVMGGHSLGGSMAARYVAAHPDALHGIF

FLASYPDEKGSVKSLGIPALSILGTKDEVVNATKYQSGRMYLPEDTVYYTIEGGNHAQ

FGDYGHQKGDGEAEVSGEEQLNQTVKTVLAWLSTMK"

CDS 500448..501215

/gene="gph_1"

/locus_tag="EFAGFIKM_00461"

/EC_number="3.1.3.18"

/inference="ab initio prediction:Prodigal:002006"

/inference="protein motif:HAMAP:MF_00495"

/codon_start=1

/transl_table=11

/product="Phosphoglycolate phosphatase"

/translation="MTMLQVNGVQIPCKGILFDKDGTLLEFLQLWGPWAETLLSQLQA

RMNELGASFTVEREHVLGTIHNAEGHIVGYDPQGPLAIATVDECTGLLAGQLYAAGIP

WNEAITTIRQFSSVAMRSVRERKSAEPMSGLLDFLQKCRAADIPLAVVTSDSTAAAET

HLDWMGIRSFFTSIVGCDRVTQGKPDREAALLACRELHIDPAEAVVIGDSNGDMQMGR

HAEVSYTLGYCPHLDQGSHLVDAHAIIRHYNEISIIL"

CDS 501488..502228

/gene="cobB"

/locus_tag="EFAGFIKM_00462"

/EC_number="2.3.1.286"

/inference="ab initio prediction:Prodigal:002006"

/inference="similar to AA sequence:UniProtKB:P66815"

/codon_start=1

/transl_table=11

/product="NAD-dependent protein deacetylase"

/db_xref="COG:COG0846"

/translation="MNATEQLAAWIQESSRIVFFGGAGTSTESGIPDFRSAAGLYQTE

QHSPYPPEELLSRHFFDQHADIFYGFYRGKMLHPDAEPNGCHQLLARLEQEGKLQAVI

TQNIDGLHQKAGSSNVFELHGSIHRNACMDCKQFYTLNDIIQSQDTVPRCTTCGGVIK

PDVVLYEEELDQTTLYRSIDALSSADLLLVGGTSLTVYPAAQLITYFQGKHTVLLNAT

PTAYDRRADLLITEPIGEVMNNVDQLLG"

CDS 502307..503314

/gene="gpr_1"

/locus_tag="EFAGFIKM_00463"

/EC_number="1.1.1.-"

/inference="ab initio prediction:Prodigal:002006"

/inference="similar to AA sequence:UniProtKB:Q46851"

/codon_start=1

/transl_table=11

/product="L-glyceraldehyde 3-phosphate reductase"

/db_xref="COG:COG0667"

/translation="MVYVASDTRYETMKYNRVGRSGLKLPAISLGLWHNFGGINNAEN

GRNMITRSFDLGITHFDLANNYGPPAGSAEQLFGQVLAQDLKPYRDELVISTKAGYYM

WPGPYGEWGSRKNLVSSLNQSLKRMGLDYVDIFYSHRYDPETPLEETMMALDHIVRSG

KALYVGISNYPAEQTRQAAEILKGLGTPLLIHQPKYSLLDRWIEDGLQDVLDEYGTGS

IAFCPLAQGVLTNKYLNGIPEDSRAKGPSVFLNENNISPETLRKVRALNQIAAARGQS

LAQFSLSWVLRNGRVTSALIGASRPSQIEENVAALSQLDFSTEELERIESILSPEPDD

K"

CDS complement(503395..504369)

/gene="rhaS_1"

/locus_tag="EFAGFIKM_00464"

/inference="ab initio prediction:Prodigal:002006"

/inference="protein motif:HAMAP:MF_01534"

/codon_start=1

/transl_table=11

/product="HTH-type transcriptional activator RhaS"

/translation="MSDQSNPKKESLASSNEKIQESPSGKSGALSYSVASNPVYYEQG

ALHVLFAGASQTLPGHALGPKLFDYYLLHYVEKGAGTFRTELHTYELSAGDCFLIHPG

QLVSYQSHARNPWQYRWIAFTGSQAAQHTEEAGFRPEKSVFHAGPSCGISDWLSVMQD

AFAERKESSHFTSLGTLYMILAEAQNHLSQGQTLIPGESSIRRTVKQMIQYMSTQYAY

PVSIEQMSASLGYNRAYLSRIFKQETGLSPVTYLLKLRIDKSRQLLRERPDLSIEQVS

ASVGLPDALYFSKQFKRFHGEAPSLYREKILSRPLHQGLQQNAQQIKR"

CDS 504523..505704

/gene="galK_1"

/locus_tag="EFAGFIKM_00465"

/EC_number="2.7.1.6"

/inference="ab initio prediction:Prodigal:002006"

/inference="similar to AA sequence:UniProtKB:Q9R7D7"

/codon_start=1

/transl_table=11

/product="Galactokinase"

/db_xref="COG:COG0153"

/translation="MNVTELKQKFIEKYGESGADIRVFHAPGRVNLIGEHIDYNGGYV

LPAALEFGTTLIIRERQDNKLQLASTNMSYEGVLDTSSIGKEKTGEWADYPVGVMVEL

QGKGVNVKKGYDFLYHGEIPNGAGLSSSASLEVLTGFAIQSLEGVADIDTVQLALLSQ

KAENEFVGVNCGIMDQFAVANGAQDHAILLMCDTLEYEKVPFRTGAYKLVIGNTNKRR

GLVDSAYNERRSQCEQALAILKEQLPALNYLAQLTPEQFVTLQDQIKDEKVRQRAQHV

VEENARVLASVEALQGNDLETFGKLMNASHESLRDLYEVSCDELDVMVEEAQRIPGTL

GARMTGAGFGGCTVSLVHENDVERFVSEVGAAYEARTSLKGDFYVCGVGDGVKELKEA

K"

CDS 505706..506695

/gene="galE"

/locus_tag="EFAGFIKM_00466"

/EC_number="5.1.3.2"

/inference="ab initio prediction:Prodigal:002006"

/inference="similar to AA sequence:UniProtKB:Q7WTB1"

/codon_start=1

/transl_table=11

/product="UDP-glucose 4-epimerase"

/db_xref="COG:COG1087"

/translation="MAILVTGGAGYIGSHTVAALLERGEEVVVLDNLQTGHREALLGG

KLYEGDLRDKEILAKLFAENSIDAVIHFAANSLVGESMKDPVKYYDNNVFGTLCLLEA

MNAANVRRIVFSSTAATYGEPEKVPIEESDRTEPTNVYGETKLMMERMMSWFDKVQDI

KYVSLRYFNAAGAHDSGKIGEDHQPESHLIPLVLQTALKQRPHIAVFGDDYATEDGTC

IRDYIHVSDLADAHLRAVDYLRKGENSNVFNLGNGTGFSVKQVIETAKKVTGLDIPVV

QEPRRAGDPAVLVASSAKARSVLGWNPKWTNLEDVIQSAWSWHQSRPDGYGKN"

CDS 506710..508308

/gene="galT"

/locus_tag="EFAGFIKM_00467"

/EC_number="2.7.7.12"

/inference="ab initio prediction:Prodigal:002006"

/inference="similar to AA sequence:UniProtKB:E8MF11"

/codon_start=1

/transl_table=11

/product="Galactose-1-phosphate uridylyltransferase"

/translation="MSQTHIAAGATERTPEQQEALHAIERLVAFALQKQLIEEADWDY

SRNLLLEQFGFSEPYAGELDTNVPDGPQAMLDTLIDYGFSIGLIPENSDTFRDLLDAK

IMGHLMARPSEVVRAFRHTEQTEGIEAATSQFYDLSINSNYIRMDRISKNVYWTQDTA

YGDMEITINLSKPEKSPKEIAMAKLLPPPVYPKCQLCRENVGYAGRVNHPARQNLRVI

PLELNNEPWLFQYSPYVYYNEHCIIFHHDHVPMKLTKDTLRRLLAFVGEYPHYFIGSN

ADLPIVGGSILTHDHFQGGRHTFAIQNAQPEAVFRHADAPGLTLSLVKWPMSVMRLAS

HDPAELLEAGNAVYEAWKVYSDSTVDIEAFSEVDGEQVPHNTVTPIVRRSADGGYEMD
LVLRNNRTNDVHPEGIFHPHREMHHLKKENIGLIEVMGLAILPGRLKEELDSIADILA
GDAKLAEAAKASDHVLNKHLGWAEELIERFGPKLDKEQAVAIVQQEVGLKFAEILEHA
GVYKYDEAGRQAFRRFVSSMGYTE"

CDS 508464..509036

/locus_tag="EFAGFIKM_00468"

/inference="ab initio prediction:Prodigal:002006"

/codon_start=1

/transl_table=11

/product="hypothetical protein"

/translation="MNIPLITDAEELQGMVGEPHEHVRNKAISFVDSHVQNFISMSPL
FFLSTSDRDGKSDVSPRGDGAGFVKVYDTYRLVYPERPGNRRIDSLLNMLSNPGIGML
FLIPGMNEVLRINGTASITKDEEFIASMGWSGKTIGAAVIVDVEECFIHCPRAFKQAG
LWASETWVDAEDLPSTSEMFRAHLEINGLL"

CDS 509130..510293

/gene="adh2_1"

/locus_tag="EFAGFIKM_00469"

/EC_number="1.1.1.192"

/inference="ab initio prediction:Prodigal:002006"

/inference="similar to AA sequence:UniProtKB:A4ISB9"

/codon_start=1

/transl_table=11

/product="Long-chain-alcohol dehydrogenase 2"

/db_xref="COG:COG1979"

/translation="MRSFQFYNPTRLIFGKGQLEALKTEVPKYGKRVLLVYGGGSIKR
SGLYNQVIGLLKETGAEVTELAGVEPNPRLSTVHKGVDLCKTNNIDLILAVGGGSVLD
CSKAIAVGAKYDGDMWDFAQRKAVAQDALPLGTVLTMAATGSEMNSGSVITNQDTQEK
LGWGSAYSFPAFSILDPVNTYTVPLDQTVYGMVDMMSHVLEHYFHLDANTPVQLGFCE
TILRTVMEAAPRLVEDLENYELRETILYCGTMALNGVLNMGLAGDWATHNIEHAVSAV
YDIPHGGGLAILFPHWMKHNLDVNVDRFKRLAINVFEVSPEGKSDRQIAEEGIDALSK
FWTSIGAPNRLADYDIDDSQIDVMADKAMLFGPFGNFKKLQREDVVSIYKASL"

CDS 510573..511088

/gene="yuaF"

/locus_tag="EFAGFIKM_00470"

/inference="ab initio prediction:Prodigal:002006"

/inference="similar to AA sequence:UniProtKB:O32077"

/codon_start=1

/transl_table=11

/product="putative membrane protein YuaF"

/translation="METIYWGCLIGGAIFAVVSLVLGDLIDGLLDGAFELPGLDFFKP

VVLAGSITTFGGAGIMLTRYSSLSAMSGLILSLLIGIAAAMLVFFAYIKPMRNSDVSI

AFSMKELSGKIGEITIPVPEKGFGEVMIRFASGSTIQTASSFEHLPIAAGARVVVVDV

VDGVLRVSEWD"

CDS 511188..512738

/gene="yqiK"

/locus_tag="EFAGFIKM_00471"

/inference="ab initio prediction:Prodigal:002006"

/inference="similar to AA sequence:UniProtKB:P77306"

/codon_start=1

/transl_table=11

/product="Inner membrane protein YqiK"

/db_xref="COG:COG2268"

/translation="MENLDWDVLLIPIVVVGVILILGLAFWARYKTVGPDEAMIVTGS

FLGSKNISDDDSGRKIKIVRGGGAFILPIFQQSEFISLLSHKLDVSTPEVYTEQGVPV

IADGVAIIKVGGAVEDVATAAEQFIGKPVEALRSEAQEVLEGHLRAILGTMTVEEVYR

NRDRFAQEVQGVAARDLKKMGLQIVSFTIKDVRDKQGYLDALGKPRIAAVKRDAEIAE

AEAMRDARIQKANAEEQGQKAELLRDTNIAEAAKEKELKVATFKRDQDTAKAEADQAY

HIHEARARQTVVEEEMKVELVRKEREIDLQEKEIVVREKQYDAEVKKKAEADRYAVEQ

AAEADKAKRMREADAVQYSIETHAKATAEQKRLEGQAMADAELAKGTADAEVIRLRGL

AEAEAKEKLAEAFQKFGEAAVLDIIVKMLPELAGKIAEPIASIDKLTVVDTGKGEGAA

RVSNYVTELMATAPEMLKSVSGIDVEQLIKGLTKSKTPAPVAIQQSEAATTPSIIDKI

VERAGVDE"

CDS 512904..513758

/gene="glcT"

/locus_tag="EFAGFIKM_00472"

/inference="ab initio prediction:Prodigal:002006"

/inference="similar to AA sequence:UniProtKB:O31691"

/codon_start=1

/transl_table=11

/product="PtsGHI operon antiterminator"

/db_xref="COG:COG3711"

/translation="MSSLHVDKALNNNVIIAQHPEHGEVVVIGKGIGFNRKPSDHIPL

MAVEKMFILKNQQEQEQYKQLLPQVDEALIEIINEVITYIAERTDVPLNEHIHIALTD

HISFALKRKEQGIIIQNPFLYETREIYPEEYRMGEYAVRLIKEKMGVDLGMDEIGFVA

LHIYSAMTNQNISQVREHSQLITDLVNLVSDQLDYSFETESLDYSRLLTHLRFALERV

RRGDKVEELHKLDSLLKLEYPEMYSLAWKLTKVMEKRLKLPVYPAEVGYLTIHLQRLN

QRKEEENK"

CDS 514046..516097

/gene="ptsG"

/locus_tag="EFAGFIKM_00473"

/EC_number="2.7.1.199"

/inference="ab initio prediction:Prodigal:002006"

/inference="similar to AA sequence:UniProtKB:P20166"

/codon_start=1

/transl_table=11

/product="PTS system glucose-specific EIICBA component"

/db_xref="COG:COG1263"

/translation="MFKKLFGVLQRVGKALMLPVAILPAAGLLLGIGNMLVNPDFLQY

VTALDTPWVNSIATIMMNAGQIVFDNLALLFAVGVAVGLAGGEGVAGLAAIIGYLVMN

VTLGTAVGVTPAMIGEVPGYASILGIPTLSTGVFGGIIIGIAAALCYNRFFKIELPSY

LGFFAGKRFVPIITSVVSLLIGLLLVIIWPPIQNGLNTVSHFMVDTSPTLSAFIFGVV

ERSLIPFGLHHIFYSPFWFEFGEYVNKAGDVIRGDQQIFFNQLRDGVNLTAGTFQVGK

FPFMMFGLPAAALAMYHEARPEHKKYVAGIMGSAALTSFLTGITEPLEFSFLFVAPIL

FAVHCIFAGLSFMTMQILGVKIGMTFSGGFIDFLIFGIIPNRTPWWDVIIVGLILAVI

YYFGFRFIIRKFNLKTPGREDATPETESGGGSGSTDDLPHNILEAFGGKENIKHLDAC

ITRLRIEVNEKSNVKKDRLKQLGASGVLEVGNNVQAIFGTRSDTIKSQMQDIIAGRTP

APTPAAAKPTPEEEKAQGEQGERIVAEDIVMPVNGELLDITNVPDPVFAEKMTGDGFA

ILPHDGTITSPVYGKVFNVFPSKHAVGIMSDGGKEVLVHIGVNTVKLKGQGFNVLVQE

GDLVSAGQPIMEVDLEYVKANAPSIISPVIFTNLPEGSTVTLKKSGVLKVGEQPIIEI

K"

CDS 516112..516450

/locus_tag="EFAGFIKM_00474"

/inference="ab initio prediction:Prodigal:002006"

/codon_start=1

/transl_table=11

/product="hypothetical protein"

/translation="MASKCKYMQCLNQTTESEMIVMQQTFRITDEDGIHARPATALVN

TANKFKGAESFAEANGKKVTLKSILGVLSLGLEQGDTISIIVEGEGEAEALQALTDVM

VNEGLGEINA"

CDS 516443..518155

/gene="ptsI_1"

/locus_tag="EFAGFIKM_00475"

/EC_number="2.7.3.9"

/inference="ab initio prediction:Prodigal:002006"

/inference="similar to AA sequence:UniProtKB:P08838"

/codon_start=1

/transl_table=11

/product="Phosphoenolpyruvate-protein phosphotransferase"

/db_xref="COG:COG1080"

/translation="MLNVSGIAASAGIAIAKAFILEHPDYSVEKRQINDVDAEIAKLD

SALGKSQAELEAIKERTLQELGEKKAEIFASHLLILNDPELIDPVKARIADDMINAEF

ALNETASQFISMFENMKSAYLQERAADMRDVTKRVLNHLLGIDFMSPAEINEEVIVLA

EDLTPSDTAQLNRQFVKGFATNIGGRTSHSAIMARSLEIPAVVGTKDILAQAKQGDMI

IVDGLDGHVLVNPTDEVIAEYRAKQEQYDAQRAEWRKLRDEPTVTVDNVHVELAANIG

TPNDVTGVLENGGEAVGLYRTEFLYMGRDKLPSEDIQYNAYKAVLEKMEGKPVVVRTL

DIGGDKELPYLDLPKEMNPFLGFRAVRLCLDRLDIFRTQLRALLRASVHGNLRVMFPM

IATLGEFREAKAVLLEEKEKLVAEGIAVSDSIQLGIMVEIPSTAVLADQFAKEVDFFS

IGTNDLIQYTMAADRMNERVAYLYQPYNPAILRLVKMVIDAAHREGKWVGMCGEMAGD

ETAIPLLLGLGLDEFSMSATSILPARSQITKLSRADMQELAAKALDMQTAEQVVELVQ

SIQA"

CDS complement(518398..519978)

/gene="gcvPB"

/locus_tag="EFAGFIKM_00476"

/EC_number="1.4.4.2"

/inference="ab initio prediction:Prodigal:002006"

/inference="similar to AA sequence:UniProtKB:P99168"

/codon_start=1

/transl_table=11

/product="putative glycine dehydrogenase (decarboxylating)

subunit 2"

/translation="MTQDTTTTTTTSVQGQSVTGTSVSTSVQPETDTTFSAQTLSPAP

EQSLIFELSSPGRVAYSLPECDVPRQSADTLIPREMLRSEAAALPEVFEVDVIRHYTA

LSRRNFGVDNGFYPLGSCTMKYNPKINEDVARYNGFAKIHPYQHESSIQGALELLYTL

QNDLAGLTGMDAVTLQPAAGAHGEWTGLMMIRAYHEGRGEKRTKVIVPDSSHGTNPAS

ATVAGFETVTIPSRADGLVDLDALRAAVGSDTAALMLTNPNTLGLFEKDIQEIASIVH

QAGGLLYYDGANSNAIMGITRPGDMGFDVVHLNLHKTMSTPHGGGGPGAGPVGVKNRL

IPFLPKPMVIKNDDGMYAFDREGDQSIGRVKAYYGNFGILVRAYAYIRTYGPEGLRRV

SECAVLNANYMMARLAPYYEIPYPGVCKHEFVMSGRGLKQYGVRTLDVAKRLLDFGYH

PPTVYFPLNVEECIMIEPTETESRETLDGFIDTMIRIAKEAEETPELVLNAPYGTPVT

RLDETTAARKPVLNCACS"

CDS complement(519975..521330)

/gene="gcvPA"

/locus_tag="EFAGFIKM_00477"

/EC_number="1.4.4.2"

/inference="ab initio prediction:Prodigal:002006"

/inference="similar to AA sequence:UniProtKB:P64218"

/codon_start=1

/transl_table=11

/product="putative glycine dehydrogenase (decarboxylating)

subunit 1"

/translation="MSKHRYIPMTEQDQSAMLATIGVDTLEDLFQDIPKEIRYQGELP
VSSKLDEYALTRHMSKQAGANANFETHASFLGAGIYDHHVPSVINHVISRSEFYTAYT
PYQPEISQGELQAIFEFQSYICELTGMAVANASMYDGATAFAEAGNLAAAATRRKQLI
VSRTVHPEARQVLQAYAHGLSLEIVEIGYKDGVTDWDALQAAISDDTAAVMIQSPNFF
GAVENVKQAADLVHAHKGLLVVSANPLSLGLLEAPGKLGADIVVGDAQPLGIAASLGG
PTCGYFAVSQPHMRRIPGRIVGQTTDRNGKRGFVLTLQAREQHIRREKATSNICSNQA
LLALSASVYMSIMGKQGMIDVADLNLQKSHYTLNTLTAIPGVSLTFNAPTFNEFVIKL
PEGTDVDTLQLKLLDAGFIGGYELGRDYPELAGQMLIAVTERRTKEEIDEFARALEGS
L"

CDS complement(521327..522463)

/gene="gcvT"

/locus_tag="EFAGFIKM_00478"

/EC_number="2.1.2.10"

/inference="ab initio prediction:Prodigal:002006"

/inference="similar to AA sequence:UniProtKB:P54378"

/codon_start=1

/transl_table=11

/product="Aminomethyltransferase"

/db_xref="COG:COG0404"

/translation="MSDLLRTPLFPLYQQYEGVRCIDFGGWELPVQFSGIQKEHEAVR
ERAGLFDVSHMGEFTVQGEQAEAFLQQMTTNDVTTLVPGQAQYTLMCYPDGGVVDDLL
VYKLEDQHYMLVVNASNIDKDWAWLQEHLIPGVSMTNDSEQTALLALQGPLAVDIIRK
VTDTDVSSIEPFRFVQDAEVCGVKLLLSRTGYTGEDGFELYVPADQAAVVWNGLMQAG
DGHGLVPTGLGARDTLRFEAKLPLYGQELSATISPLEAGVGMFVKLNAGPFIGHEALL
QQKTDGLARKLVGIEVLERGIPRPHYPIYAEGVQIGEVTTGTQSPTLKRNLGLALIDS
KYAALGTPLEIEIRGKKLKAEVVKTPFHKRTRTSKTPTQGADQA"

CDS 523251..523643

/gene="gcvH"

/locus_tag="EFAGFIKM_00479"

/inference="ab initio prediction:Prodigal:002006"

/inference="similar to AA sequence:UniProtKB:A0QYG3"

/codon_start=1

/transl_table=11

/product="Glycine cleavage system H protein"

/db_xref="COG:COG0509"

/translation="MSELKSDFLYSEEHEWVQTVGEDTVRIGITEFAQHQLGDIVFVE

LPDLETNVKAEDSIGTIESVKTVSDLFSPVTGSIIAVNDSLQDSPELVNSSPYEEGWM

IEIRVEGDLTAALSTLMNADAYRKHTEE"

CDS 524063..525517

/gene="mtlA"

/locus_tag="EFAGFIKM_00480"

/inference="ab initio prediction:Prodigal:002006"

/inference="similar to AA sequence:UniProtKB:P50852"

/codon_start=1

/transl_table=11

/product="PTS system mannitol-specific EIICB component"

/translation="MSNLDQSSNSKGGLRVGVQRFGRFLSGMVMPNMGAFIAWGLITA

LFIPTGWFPNETLAQLVDPMIKYLLPLLIGYTGGTMVHGQRGGVVGAIMTIGVIVGSD

IPMFLGAMIAGPLAAWIIKKFDKSIEGKIRAGFEMLVNNFSAGIIGGILGIFALLGIG

PAVEAISKVLSAGVQGLMNLGLLPLVNLIIEPGKVLFLNNAINHGILTPIATDQAREL

GQSVLYMLESNPGPGLGILLAYCFFGRGMAKSSAPGAVIIHFFGGIHEIYFPYILMRP

ILIFAAIAGGVAGTLTFMLTGAGLVSAPSPGSIIAYSLLTPKGGYLPMLAGVLVAAVV

SFLVAAVLLKTGKQKDEDLESASSRMKDMKSQGTVPNAGITANQHEADRAPDMASSTV

KTDVKKIVFSCDAGMGSSAMGASILRKKMKAEGIDVTVTNTAISDIPPDADVVITQQV

LTDRARSVAPNAEHISIDNFLKSPEYDALVERLK"

CDS 525556..527658

/gene="mtlR"

/locus_tag="EFAGFIKM_00481"

/inference="ab initio prediction:Prodigal:002006"

/inference="similar to AA sequence:UniProtKB:P96574"

/codon_start=1

/transl_table=11

/product="Transcriptional regulator MtlR"

/translation="MSITKRQREIVEFLLEHPHEVTAGEIAVEVKVSTRTVHRELQMI

EQWLEPLGMRLEKKSGTGIRIDASSDDLAVLRKQLEGKEYVEFTPEERKLFMLCILLD

EPEPVKLLALASDLKVTVSTVTTDLDDLESRIRQAGLKLVRRRGYGVKINGSETIHRT

AIAALALEFLDESDLFGRQPEQGGSIVNQKLLDMIGHGDVLTVENALWQPDIEWLENI

PERQYMKLLIQLSVAVVRIRKGIGIGRMSTREKTGDAVAEQDEMKVPPYMASRLCGVL

STQLGLTFSQDEQAYFHRLLVETEQRIHSSRLLPIDDLILLDRVHSLIDQMQARTHYA

FHEDRLLREGLLGHMQPVMERIEGQQMIRNPLLQQIRKDYDSLFEEVKQSVHQAWPGT

EVPDEEVGFLVMHFGASIERLRALKREIRAIIVCTSGIGSSRMLSSRLSKEIPEIRIM

DSVSWYEAARIPTDQYDLVLSTVDLPMDEHQYYKVSPLLTAEESERLRHFIRTTTLQR

QHNKPREAEVQTTSRYSDPDGMEAILVEIVRIIGKFQVYPLDNQDIGFYKTVYAMCNV

LHGSGVLKEPGEIAKRLEAREAVGSQKIPGTRLALFHTRSEGIYRPSISLFQLTEPLL

CTPDDPAGVTHVLLMLGPRELSKESLEVLSEISALLLQEEMITLLEKGIRDELIHYLS

QELVGFYRSKTEIGGQPL"

CDS 527655..528089

/gene="mtlF"

/locus_tag="EFAGFIKM_00482"

/inference="ab initio prediction:Prodigal:002006"

/inference="similar to AA sequence:UniProtKB:C0H3V2"

/codon_start=1

/transl_table=11

/product="Mannitol-specific phosphotransferase enzyme IIA

component"

/db_xref="COG:COG2213"

/translation="MSMLTTDKVIMNATAQDKYEAIRMAGQILKDAGHITADYIEKMI

EREEIVSTYVGNGLAIPHGTKESKAFILSTGISVIQYPQGVDFGEEKAYMVIGIAAQG

GEHMEILTSIAVICAEDENMEALRLAKTAEEVIAILESEMEL"

CDS 528086..529231

/gene="mtlD_1"

/locus_tag="EFAGFIKM_00483"

/EC_number="1.1.1.17"

/inference="ab initio prediction:Prodigal:002006"

/inference="similar to AA sequence:UniProtKB:P09424"

/codon_start=1

/transl_table=11

/product="Mannitol-1-phosphate 5-dehydrogenase"

/db_xref="COG:COG0246"

/translation="MKALHFGAGNIGRGFIGLILSRAGYEVVFSDVNQTLVTALQQQG

QYTVELANESKDQETVTGVNAIDGTQLETVAQTVVEADLITTAVGVGVLKHIAPGIAK

GLEKRLNSGPAQNPLHIIACENAIGASTQLKEHVYALLNEDVRSLADQYIYFPDSAVD

RIVPIQHHEDPLHVQVEPFYEWVVDRSQMAPAFKPVEGIVYVDDLEPYIERKLFTVNT

GHCVAAYIGNVHGYDTIQKAIADEKVKSIVYGALQETGKVLVKRFAFNPEEHEQYIVK

ILGRFVNPYLTDEVTRVGRSPLRKLSPNDRLVRPALQAYADGSETTYLAMGMAAACKF

DVSDDPEAVELQDMIRQKGITATLHHYTSMDEHHPVLEQAVAQYNQM"

CDS 529374..529691

/locus_tag="EFAGFIKM_00484"

/inference="ab initio prediction:Prodigal:002006"

/codon_start=1

/transl_table=11

/product="hypothetical protein"

/translation="MAIKGQKYKTYSEKIKKEAIRLHTVEGWTYRKINEHLGIHDPGR

MKRWMRKHREQGEFGLMDQRGRRKEYLDQERYVQKLKRENELLKKCLVIWKEEANKKD

FRS"

CDS 529640..530524

/locus_tag="EFAGFIKM_00485"

/inference="ab initio prediction:Prodigal:002006"

/inference="similar to AA sequence:ISfinder:ISBth8"

/codon_start=1

/transl_table=11

/product="IS3 family transposase ISBth8"

/translation="MLGNLEGGSKQERFQIIEKVAAYGDIQKLCDVFGVSRSGFYAYV

KRKRFDRDAKAKKQVLQTYQRYEGKYGYRQLQLFLWQDQGIWMNHKKVLRLMQMLGIQ

SRIRRKRRSNSSYAPAQRVAENRLKRDFSAEKPNQKWVTDITQYRVGERWIYLSAIKD

LFNNEIVAYEIGERNDNELVLRTFSKAFAKQTDVTGLVVHSDQGFQYTSHAYHDMLPK

VGAQISMSRRGNCYDNASMESFFSHLKTEGLYPYHIRTLTEAQSKIEKYIRFYNRKRP

QRKLKKLTPVEYRRQFAA"

CDS complement(530565..530816)

/locus_tag="EFAGFIKM_00486"

/inference="ab initio prediction:Prodigal:002006"

/codon_start=1

/transl_table=11

/product="hypothetical protein"

/translation="MSAEQDDQEHIHIDVLVCPLCGEANRCSYAAGHPHSECWCNRAT

FPEGVFDRIPAEQRRKSCICQRCLDDYADKSQPKEEPHS"

CDS complement(530955..532958)

/gene="mutS2_1"

/locus_tag="EFAGFIKM_00487"

/EC_number="3.1.-.-"

/inference="ab initio prediction:Prodigal:002006"

/inference="protein motif:HAMAP:MF_00092"

/codon_start=1

/transl_table=11

/product="Endonuclease MutS2"

/translation="MNENTLNSLGYPQIQKNVADCALSYLGKRYARELKPMVDAHLIQ

LRLEETAEAAALIRFGASIPLPSLDGMETIMDLLGTGYLFSERDFSHLAQFLRSCAQL

MKYMEGKSGAAPTVSRYAASMIMIEPLLSEIERCIHSGRIQDQASKELIRIRKKMTVN

EERMKRKLDSLVSKHRSIMQEHVISQRGGRTVLPIKKEFRKQVKGSVLDESGSGQTVY

IEPVELVSLQMELAALQAEESREEMRILGDLTSLTESYSREIALNTETVGVLDFLFAK

AKYAATMDGRTVRVNTQGHISLQRARHPFMGSSMVPLDFAIGRTYSSLIITGPNTGGK

TVALKTLGLLTLMMQSGLLIPVEEGGEMAVYNEVAVDIGDGQSLEQALSTFSAHIRNM

IGILEQADTSTLVLIDEMASGTDPGEGVGLSIAMLEELHSRGATVVATTHFGEIKHFA

AATPGFENARMEFDTVSLQPLYRLRIGEAGESYAYSIALKLGMPQRIIERSKSLSDQS

ISRDRTLGFTSPPLETPVLTPDRSVAEADGPAELSKRNQSKQSKYSIGLHKNTTPRES

APPAPAKNFRKGDRVYVAYLNQSGIVCDVEDSRGNIGVMLRGRKVKIHKKRLTLHISA

DELYPGDDYDLDIVLETKENRKKRKLMGRKHVEGLQIELPPEE"

CDS 533487..535823

/gene="yesS_3"

/locus_tag="EFAGFIKM_00488"

/inference="ab initio prediction:Prodigal:002006"

/inference="similar to AA sequence:UniProtKB:O31522"

/codon_start=1

/transl_table=11

/product="HTH-type transcriptional regulator YesS"

/db_xref="COG:COG2207"

/translation="MPKYLLRLLCFTLILGALPVIVIGSVSYTIASRDIEQKVRESNL

QILHQTQMRVEQVLRSLQLSSIQYVNSPLVLQAMKKPLDSSEFQEIRDLTSGFNNLQA

VTNIDQAYLVNLDEDWVVSMRSFGKLDDFSIRDRIGSYLSYTNSLFWVTQNASSAKES

VPVSAGMGELTPEQAPALISSDNVVSMVFKIPMIPTNVKPKGFLVIDIADTELSTYLS

RNTNSGDMYVLDREQKYFLNDMQQGGKYDALNKEIHEMVQTTGQPEGFFSAEVEGNQV

AVSYRQSPLNGWLYVSVVSLGQITAQSQKIALVTGIATLVMLCVTGLFAIYGSRRMYS

PISRLLQFTKGLDSPVTPSGRRQDEFIYIEEQLSTLFSSEKTMREQMKGQHVHLQEFF

MTKLLTGKISEEDFRYQGELYDFPKGWASLGVLMLQIDTLEGTRYEEQDRDLLLFAVN

NMVGELLPSAVRFTPVMLDDAQVTVLASELTDEVKLKEWMHTQADWIRERVVTYLNLP

VSIGISRSYACIGDTPRAVQESREALQGRVSLGSRIILHYEDIQPRGQMEAALYTQLR

MIEDQLASALKQGDEEKTDAYFKQYLGLLADKKLHFSEYPVIMVQLLSRVYQLVQEQG

GDVAEVLGEKASMSHLLKLSTLDEMTNWFRKRLFLPVIRFWREQEESQYMNIARRMIR

LIEERYDRELSLEACAAELNFHPVYLSRVFKKEAGVNFTEYLAEYRMEKAKTWLQTTN

LKISEIAEKLNYTNPTAFIRTFRKITGTTPGKYREQQR"

CDS 536106..537059

/gene="yteP_3"

/locus_tag="EFAGFIKM_00489"

/inference="ab initio prediction:Prodigal:002006"

/inference="similar to AA sequence:UniProtKB:C0SPB3"

/codon_start=1

/transl_table=11

/product="putative multiple-sugar transport system

permease YteP"

/db_xref="COG:COG4209"

/translation="MKAETAARTRPATRSDKNLLWRDIIKNRWLYIMLIPGVLYFVIF

KYIPMYGITMAFQDYTPYKGILGSDWVGFKHFQRFFGEPQFWTLFRNTFLLAIYNIVF

FFPLPIVLALMMNEVRRERFKRFVQTLVYVPHFVSWVVVVGVFYMLFTTEGGAINELL
YNLTGQKVAFLLEPGWFRTMIVGQSIWKEVGWGTIIFLAALSGVDTQLYEAARIDGAN
RWRQTWHITLPAIRSTIVILLILRLGNFLDTGFEQIFLMLTPTNRDVGEVFDTYVYTK
GLTQAQYSYSAAVGLFKSVVGLALVLGANTLAKKFGEEGVY"

CDS 537105..537977

/gene="araQ_3"

/locus_tag="EFAGFIKM_00490"

/inference="ab initio prediction:Prodigal:002006"

/inference="similar to AA sequence:UniProtKB:P94530"

/codon_start=1

/transl_table=11

/product="L-arabinose transport system permease protein
AraQ"

/db_xref="COG:COG0395"

/translation="MQQDKTWGNRIFDFLNHGLLLLIGIVTVIPFMYILAVSFTSPHE
VAKGGFILFPKEFSLAAYRYIFSTDTLIRSLGVSVYITVIGTFINLLFTSLMAYPLSR
RYLRGRQPILLGVLFTMLFSGGMIPTYFVVKSLHLTDTLWSLMLPTAISAFNLIVLKN
FFQAIPDELEDAAKIDGCNDVSVLFRIVLPLSMPAMATFSLFYAVAHWNSFFSAVIYI
NDSEKWPVQVWLREIVILAQSRIGDTSIEETEIQPLTIRMAVIVFSTIPIMLVYPFLQ
KHFAKGVMLGSVKG"

CDS 538040..539539

/gene="lipO_2"

/locus_tag="EFAGFIKM_00491"

/inference="ab initio prediction:Prodigal:002006"

/inference="similar to AA sequence:UniProtKB:P37966"

/codon_start=1

/transl_table=11

/product="Lipoprotein LipO"

/db_xref="COG:COG1653"

/translation="MKATKKKAVAVLSTLALVTGLLAGCGSDEGQAAEGGVQNVSIAI
AQVGDVPSKGNEVQQMIEASTNTKLDIQWIPASAYNDKINVMIASSDMPKIVKVQYNP
TVTSAMRNDVFWEVGPLLKDYKNLSAQNERFFDNIKVEGKIYGVPVFSDIARATVIYR

KDWFEKLNLKVPATPNEWYETIKTLATSDPDGDGQDNTFGLMLFKKYNEDQYSFTTRL
GVSFGAPNKWKVEDDGSFTPEFMTPEYMQVLDLLKRLYDEKLLNQDFAVFDSTEAEKK
YDSGVVGIRVGVAQNGKSQQERLSKNNPDGVVDIAGLLGANGDRVAGQTGNSGILAFP
KSTVKSEEELKNLLSFLDKLMDPEMATLLMRGMEDKHYTKVGEDQVEMSDFDAFQREV
KPYRDNLPYVEGYNVPKLKDTELGEKGTALAKELAEHAVPNPALTLYSPTYGDRGADL
DQVIADAQTKYIMGKIDQSGWEKEIENWGNAGGNKIREEYAEDYKKQAQ"

CDS 539564..540667

/gene="yteR_1"

/locus_tag="EFAGFIKM_00492"

/EC_number="3.2.1.172"

/inference="ab initio prediction:Prodigal:002006"

/inference="similar to AA sequence:UniProtKB:O34559"

/codon_start=1

/transl_table=11

/product="Unsaturated rhamnogalacturonyl hydrolase YteR"

/db_xref="COG:COG4225"

/translation="MKETLQLTSVRMAQQFMESYRNHELYATWHYENGCLLKALEELY
THTGEQKYFDYIRELMDHFVQEDGFIRSYTIEEYNLDQINQGKSLFLLHEKTGEEKYR
KAAELLMTQLKGQPHTSEGGFWHKKIYPFQMWLDGLYMATPYLTQYGAVTGEEKWFDK
AALQLLLVEQRTRDPRSGLLYHAWDESKEQRWSSGETGCSPHVWSRAMGWYVMAVVDT
LDYLPVDHPQRGQIVGIFERVANALVHVQDQQTGLWPHLLDQPGRERNYLEASGTSMF
VYALAKGVRKGYLSGKFKAVAEKGYQGLVQHLLQTDREGVLSLTQCNGGAGLGGSPYR
DGSYEYYVTESIRINDPKSVAPFILAGVEIELA"

CDS 540776..542287

/gene="lipO_3"

/locus_tag="EFAGFIKM_00493"

/inference="ab initio prediction:Prodigal:002006"

/inference="similar to AA sequence:UniProtKB:P37966"

/codon_start=1

/transl_table=11

/product="Lipoprotein LipO"

/db_xref="COG:COG1653"

/translation="MSPKGPMSRLGGIVVIGAMSAGLLAGCGGEKAPAAGEGKLPISI
SLMQVGDIPAKENGIEKKIEEYTNTDVNVQWIPQSAFDDKVNVMVASGEMPTIMRVNY
VPTTFNAAKTGLFWELGPYLKDYKNLTAQSEAYFNNIKIEGKVYGIPNFRDIGRTAIV
YRKDWFDTLKLDIPKTLDDWYEVMRSIRKDDPDGNGKEDTYGALLFKKYNEGVSSPLT
RIAVSIGGVNKWGVDDAGKLTPEFLTTEYVDTMKLFKRLFSEGLINSDFPALDPSDAD
KKMDSGLVGMKLNGVAQNGKSSQQRLTPNAPDGEIDVAPFQGTDGIRIAGEPGNYGML
VIPKASVTDEEQLKQVLTFLDQLMDEELSALQLRGLLDVHYTKTADGKTELKDFDAYQ
REVKPYRDNLLSIEGYNVPELVDVPIGMKGTKMARENEQYAIANPALTLSSAIYTERG
QELDQMIWDAQTKYIMGKIDDAGWEQEIASWRKAGGDQLITELEASYKELNGK"

CDS 542635..543903

/locus_tag="EFAGFIKM_00494"

/inference="ab initio prediction:Prodigal:002006"

/codon_start=1

/transl_table=11

/product="hypothetical protein"

/translation="MKKPIKGHEKKEKRNVWIVGLLTTAVLAGTLLPVGPVHLTSTAN
AASGTSDSALSKFKDIKGHWAQATIAKAYEKNLISGYQDGTFRPNGKITRGEYATILA
RATGLEKVEGQNPFADLKGHWSEAAVSQLVGQGFINAGDYAKGFNPNAELTRYEMMKW
IANGLIKSGASFQDAFNDTKNTLLPTPEVNRGTIRAEQIPYLALVRGTGIVGGFQDGT
LKPADSTTRAEVSAILLRYMDVEGTDAGKYSDLNEMREVGTTGTNLTTVSNYKYIKGN
FGDIVNTEYTLKNNVGVVRYHRFIIVDTRGTKPKGLYATLFVDQNNLTRAQKGRFSTY
AEITFTSKLDKSNLLTIARANDNIAIPIQRLFNVSYLKEKGFITLPDDSNAMLKQGVS
TKFWSSHTLDPVKGGYMLKNDAGKEVQIYQ"

CDS 543912..547595

/locus_tag="EFAGFIKM_00495"

/inference="ab initio prediction:Prodigal:002006"

/codon_start=1

/transl_table=11

/product="hypothetical protein"

/translation="MVFKFNIKRYLLGGFKSLYRILVIIFMMLPVYYCTFDTLKANAV
EEQYGSKSMKGPAVTVDANPTQGTPGVYWYQLDSGEFRAEYQGTHKDVDNGGTDYDAY
PVKTEIPDNVDMSRWPPLSWKPYRGIGIDKEMVTDIKLKNDPDGVKYQQVNSYEGIGS

PRVTSEKNADLRTYTGKGFEFFEREPYGTRPGNKPKYKMVYHTPVSIFWEGKIHEEKE

IDVTPDSTLTIGKTQQMEAKVKTKGYGATVFGEGIDVSRRETEIKWFSSDETIASIEL

KTGMLKAESPGTVTVRAIWNNGTYLISDTATITVTSEPGLVVNLPNACKANTTPLQAE

AVLTKPDRSVHKLTAHPKLTWQSSNPAVATIGADGKITTKGIVGSTTIKAHFLDSAQQ

LDEQGTQVLEVKDCTDNGEGGNDGDPGGDPANACPVTISPPSRGTVIEASVMDPSVRG

VLKADDRGSEKFDVTHGIPTSEDLYANVLAKEYLFQHRWVNMSGTVTYTVKVKRVYHK

TWTIPGRASSGEGDPGTPPQPRERDVPGDKTMQVTRTYSYWQIDNLEVYKLNEAKVSN

YALGGYGDTLTLTPNAYTSPTLQSAMDTAVTNHVKPAPCREIDLGIKGVPGGSDEPPT

PDETSLFQSKAEAEVRENTVNNDKVTFNGATIMDSAPVEKTAPQPGSIPQPGMIGDDV

LYQNRLTIKNTLMNKANQPTTGEITYGLLPGNVNGGQDQKFPILGINSVTVHTPVVNY

AWVSDDQPHNQKTTPDPTRAALILERPFIVRIPTSGQHLDVASYPGYGNHDYAKYFRI

KQVRFPFDVYNATRSQFIPALTWVDIPVNQLDTPFYLPVWVDEGNYQIEFRNIAENAP

ANFTEQQDANTNLTHHVAADTVAVEVIGRLYDFHITDISDYNWENVFRKRIGSPEPTG

VSYWTGENSIDGDPRGNLAPYVLPIRPGSHPVQGFRNAAVKTGYHFKFDLKTKGNMFG

KQDGIRITPTFSFVSKDGTTRQEVDLYYHRGQERLIRIGSAQDLEKRFVVLNSRLRNV

PGTELGDTARYQYTYELSAEERNQGTMAEHMVRFVDQTSHHKTWVGRYDWMILPSQIR

TLIGPKTDIPSSVNVDRANAAIQRWYGEYSLPADVYAVPKGTDLESLARQNQLDEKAM

VFLRDGYIVVNFNIETLRSGNMSAPHLQYIHAPLMNQWQMEGFDNSPVDGQGRSWLMQ

DGDVVLYHADQSSRNDFQSQVPH"

CDS 547767..549815

/gene="pulA"

/locus_tag="EFAGFIKM_00496"

/EC_number="3.2.1.41"

/inference="ab initio prediction:Prodigal:002006"

/inference="similar to AA sequence:UniProtKB:O33840"

/codon_start=1

/transl_table=11

/product="Pullulanase"

/db_xref="COG:COG1523"

/translation="MIEEDKQELLISEDTYKGRDLGVTLEAQTCHFKVWSPLAVQMEL

LLYPPLTGGSPEEEPNQLKQQALPMQKKGQGIWVLELEGDLNSYRYMYKPTFEDGHST

NAVDPYARAVTMNGEMGVIVRLEQTHPNGWSEDIRPELTSPVDAVLYELHVRDFSIQP

SSGITNKGKYMAFTETGLRDSEGNTLGIDHLVELGITHVHLMPVFDFATVDESKVDGD
TSDDSNYNWGYDPLHYNVPEGSYASRADDPETRIREFKSMVLALHNKGIGVIMDVVYN
HTFDTAGSSFEKLVPGYYYRQNADGTYSNGSGTGNEVATERTMVRKFIIESVRYWAEE
YHVDGFRFDLMGLIDTTTMKQLAAELHTEVSPSILLYGEPWGALDSPLGDDMTQKGTQ
RGAGFAVFNDNFRGVIKGDSDGMGTGFATGADGKEDELWTGIRGAIQDFTDGPSETIN
YVTVHDNLNLWDKVARTQGLHDTLNFLTYDEDGSIRGCQSVEEAVEKAKPYLQIDPEH
ILENETVRRSLLANGIVLTSQGVPLIAAGDELLRSKYGDANSHQSGDVVNAIHWEQKK
LFKPIFDYYRGLIRLRREHPAFRLRTREEIENHVCLIEKGNGLLAYELNGTAASDSWE
RIVVIYNAAKENRDISVPTGTWNVIVEKGQAGIDTIRTIQDGQVHVNAISLTVMYSNS
"

CDS 549969..550187

/locus_tag="EFAGFIKM_00497"

/inference="ab initio prediction:Prodigal:002006"

/codon_start=1

/transl_table=11

/product="hypothetical protein"

/translation="MRQKPLPWSQGYTLSCLELFYAVHLLVHFNLNPLTSCNLFLYIP
NFFCQDRYKCCQSMALVFISYILKAIAN"

CDS 550303..553179

/gene="glgX"

/locus_tag="EFAGFIKM_00498"

/EC_number="3.2.1.196"

/inference="ab initio prediction:Prodigal:002006"

/inference="protein motif:HAMAP:MF_01248"

/codon_start=1

/transl_table=11

/product="Glycogen debranching enzyme"

/translation="MPEWTSFCYEGNDLGLTYTRAASTFKLWAPTAQQVYILIFDDEG
HYNDSGKVQEHENGQEHTLKCDADGIWSLKLSGDWTGHYYMYRIIHDDQRIEVVVDPY
ARAVTANGQRTAIIDPETTNPVDWDLDVKPAFLRPVDAVLYELHVRDFSSDPHADIPY
KGKYLAFTASGLTDRAGNSIGVDHLAELGVTHVHLLPVADYQTVNELATADVGSNVRA
PYNWGYDPQHYNVPEGSYATDPRDPAVRIRELKSLVQSLHRQGIRVVLDVVYNHTYSV

EEGPFERIVPGYYYRYREDGTLSNGSGVGNELATERPMVRKYILDSLRYWAEEYHVDG

FRFDLMALIDTETMNELTRELKEQIDPAFLIYGEPWMGGDSPLDRKTLKGTQRGQGFA

VFNDHFRSAIKGDSDGSGRGFVTGAGGQEYELIRGLAGALDDFTSSPVEVVNYVTAHD

NLNLWDKIATTMNLRHELGFPVWKDGQPVGGGSAESAVKAADPYRYVDADDVLNHEMV

RRSLLSSGILLTSQGIPFLHSGDEMLRSKAGDHNSYRSGDAVNAITWANKSRFRPVFD

YYRGLIHLRRTHPAFRMIDAEQVRNHLRVIRADGNVVAFILQNGANQDTWNQIMVIYN

GTEHEQHVDLGEGDWHVVVNDHQAGTDLIETVRGTAQVERWSLMVMYDTEHAGGAEPS

TVEISFPRQVYSPKETTQLRAIVRDSIGNVVEKAPVTWTSSDPDIIQIQPDGTIHALE

RGEASITVHCGDITASCMLQVDYRHAERIEIVGEPVMYMTRISRFRALVLDQFGQPLQ

DSNIRWSSSHPELAAVSAAGIVRAMTPGVTHIIAEAGGIRATLGVTIHKQISRTVTLR

YEREDQHYEGWDVWVWGTGMEDGAVRLEQVGNAAEARFRVAPGLHHLGLIIRLNEWEA

KDTCGDRYVDIVPEDGDVHIVVRSGSDEMQIIRESEKTEDRNSA"

CDS 553460..554089

/gene="rplY"

/locus_tag="EFAGFIKM_00499"

/inference="ab initio prediction:Prodigal:002006"

/inference="similar to AA sequence:UniProtKB:Q7A7B3"

/codon_start=1

/transl_table=11

/product="50S ribosomal protein L25"

/translation="MKSNGKMAQLTATPRTEKKGAALRLLRQGGRVPAVVYGPGLEGA

SIHVDEKEMLKVARTGRSEMFNLNVEGGKTIPVLIKDQQERNGRLLHVDFLQISKNKP

ISVSVSIDFQGTAAGSKAGGVFQTQETQLEVEGLPADLPTSIEVDVSGLDIGDRLTAA

DIKLDKGLTLVTSPESIIASVMPPQAAEEEPTASAEEAEPATEEKATEE"

CDS complement(554545..556317)

/locus_tag="EFAGFIKM_00500"

/inference="ab initio prediction:Prodigal:002006"

/codon_start=1

/transl_table=11

/product="hypothetical protein"

/translation="MITTTVRFFTKNLLTFLLPMLIPLVVLGALSAFLIQQYVVHGIN

SNNMNMLKQTKENIELLFNEQNSLNLHIIASTTQFMNLKGMLNKPLPSAKDYQQLAVL

KNFIDSPSIASPYIDSIYIYLNNDNQRYISSVTGGFIELGEDPDRLWYDSYLKNEHHE

GIWTESREVPRYNLNEEIEYSDVITMYRNFKVSDDTRGVIVLNIKREYIERRLNDLAT

AKGQLLAVVDQQGSVIFRNNARYELSAEELEQVISSKSTSITTQAPNNEALINMIYSK

TYDWTFISVTPKQSLYTAINRLLRIGAILMVMALVVALLLAFQLTKKNYSDLKIIMSI

LGAAEQGKPLPLHRTKGNSVYSQIIRSLLQNFIEHNYMRVQLSESQYKTQAAQFAALQ

SQLNPHFLYNTLETIHWKAAGYTEGPNELTSIVEHLSDILRYSLDGQNGLVPLNVEIA

NTKSYIAIQKTRYGERFDIWWEYDAELEKYQVLKLIFQPLIENSLQHGLSRSDQKLAV

KIKIQRSGNCLRLAVIDNGNGISEERMVELRTRLFSDSTDANHIGLINTQRRIQLAYG

HRGELRIKSKPGRGTLIDIKLPIN"

CDS complement(556321..557061)

/locus_tag="EFAGFIKM_00501"

/inference="ab initio prediction:Prodigal:002006"

/inference="similar to AA sequence:UniProtKB:Q5HJF7"

/codon_start=1

/transl_table=11

/product="putative response regulatory protein"

/db_xref="COG:COG4753"

/translation="MYNLLITDDESEIRNGLSNYFPWHEFGFQVVGQACDGEGALAFI

QEQPVDVLLCDIRMPSLSGIDVAERLYRQGSEVKVVLLSGFKEFEYAQLAVQYGVKRY

LTKPTKYTEIAEVFGHLRSEMDNARMPDPPHSASTAESSLADQTIEKVKAYLQDNLSA

ATLENAAKHVFLNPFYLSKYFKTKTGENFSDYVTKLRMHRAAELLKTTYCKTYEISEI

VGYSNAKNFTRMFRSFFGMTPREYRNEA"

CDS 557444..558442

/gene="oppD_1"

/locus_tag="EFAGFIKM_00502"

/inference="ab initio prediction:Prodigal:002006"

/inference="similar to AA sequence:UniProtKB:P24136"

/codon_start=1

/transl_table=11

/product="Oligopeptide transport ATP-binding protein OppD"

/db_xref="COG:COG0444"

/translation="MLLELKDVRTVFKTEKGEVTSVDRVSLKLEKGETIGIVGESGCG

KSVTSLSIMRLLGKNGSIREGEIWFMDKDLAKLPESELRLIRGGDIAMIFQEPMTSLN

PVFTIGNQLTEAIRLHTDLRGKAAEGYAVEMLEKVGIPRPEAIMKSYPHSLSGGMRQR

VMIAMALSCKPKLLIADEPTTALDVTIQAQILNLMKELREEMGASIMLITHDLGVVAE

MADKVVVMYAGQVVEETDVFTLFEKPLHPYTKGLMRSIPHIETDAEEMLASIPGAVPP

LTNMPKGCRFHTRCEFAEGKCLSEAPTLLPVQEGHQVRCWMAQDPDAWKTREEEAI"

CDS 558439..559437

/gene="btuD_2"

/locus_tag="EFAGFIKM_00503"

/EC_number="7.6.2.8"

/inference="ab initio prediction:Prodigal:002006"

/inference="protein motif:HAMAP:MF_01005"

/codon_start=1

/transl_table=11

/product="Vitamin B12 import ATP-binding protein BtuD"

/translation="MTIAERKVAQEPLLRIEGLKTYYPIKRGLLSRTVGNVKAVDDIS

LELYQGETLGLVGESGCGKSTIGRSIIRLENPTDGRIWFEGQEITTSTMSDLRGERTK

MQMIFQDPYSSLNPRMRVQELLAEPMRVHGLAGGAELEARIDNLLDTVGIPRSYKQRF

PHEFSGGQRQRIGIARALSLNPKLIVCDEPVSALDVSIQAQILNLLKELQRELGLTYL

FIAHGLGAVKYISTRIAVMYLGKVVEIGTKERIFASPRHPYTQALLNAYPVPDPRKRG

QERIVLQGDVPSPASPPSGCRFHTRCPFVQAKCKEEEPLLQGGEHAAACHYPLTTG"

CDS 559460..560410

/gene="gsiC_2"

/locus_tag="EFAGFIKM_00504"

/inference="ab initio prediction:Prodigal:002006"

/inference="similar to AA sequence:UniProtKB:P75798"

/codon_start=1

/transl_table=11

/product="Glutathione transport system permease protein

GsiC"

/db_xref="COG:COG0601"

/translation="MFTYVIRRILIAIPVLFGITIINFFIINLAPGNPIDMYMTPDTP

PELLELRKEQLGLNDPTIIQYFKWLGQLLSGNLGYSFSTSESVVSLIGERLGQTVLLG

ICALIIGLLIALPIGIMSAVRQNSKFDYLMTGLSFVGTSIPQFFLGLLCIYIFAVQLG

WLPVGGTEDLGGGGGFGDRISHLVLPSIAMAVGIAGRKVRYIRASMLDVLKQDYLRTA

RAKGLKEFFVTNKHALRNALIPIITVVGMEIPMLFGGAVIVEQLFQWPGIGQLTIQSI

MSRDYSTIMGLNLVAAIIVLVANLLTDIFYSVADPRIQYH"

CDS 560463..561362

/gene="gsiD_2"

/locus_tag="EFAGFIKM_00505"

/inference="ab initio prediction:Prodigal:002006"

/inference="similar to AA sequence:UniProtKB:P75799"

/codon_start=1

/transl_table=11

/product="Glutathione transport system permease protein

GsiD"

/db_xref="COG:COG1173"

/translation="MSAVQTPVAGGVILPIQQEEPEGVFRQMARRFLKHRLAVAGLVV

TLLLILIAIFAPWLSPHSPYAVTAEFSAAPSSSHWLGTDQVGRDVLSRLLYATRVAVT

VGFGTVALYVVFGTVIGLWSAYVGGWVDMTVMRITDMFMAFPYMMVILVVVSVLGPTL

WTLVLVLALFSWPSIARLVRGSVLTLKQMDYIKAGVALGLSTPRIVFRHILPNALAPI

IVNATFGVAAAIMAESGLSFLGMGVQPPTASWGNMLTDAQSISVMTDQPWLWLPPGIM

ILVTVLAINFVGDGLRDALDPKQ"

CDS 561421..563070

/gene="appA_1"

/locus_tag="EFAGFIKM_00506"

/inference="ab initio prediction:Prodigal:002006"

/inference="similar to AA sequence:UniProtKB:P42061"

/codon_start=1

/transl_table=11

/product="Oligopeptide-binding protein AppA"

/translation="MKRMRKAWICGVLTLLMVLVTACGSNSDSNTTSSPGSDGESASA

SGGSGQPLYIGLVNPVTTFDVFNLDNAGVFVNSLMSDTLVAMNSEYGFDPKLAESIET

TDNQNFTIKLNKNAKWSDGEPFTADDVEFTFRMFTDVNVKTLLNMTFVAGLNEAGKRE

EGQTELAFFEKVDDYTFVIKTKNPMETNQFKSQFGTYFRFLPEHVLKDVAPVDLNKNE

FMMNPTVTNGAYKFVKFAKDQYVQLTASEQYYEGAPKIKDIYIKMMPATNLAAQLQTG

DIQLTSPGVGLIPVQDFDKVANMANVTTGYGDANNPNLMFINTKKFPDPKVRQAIAYA

MNRQLIVDQLLKGQGELVDGMLGQNHPYYNKDIPLYGYDPEKAKALLEGAGWDFNQTI

EFVVPTGNKIREQASDIIAENLKAVGLKVQVTKLDFPTTYQRALKHDYDLTIINLGFI

LDPNSVLGLFKTGVSFNIADYSSTEVDELLVQGAEELEPSKRFEIYKQVQQMLHDDVP

VIALFADKQMYAAAKNLDLGKGLETSTVGLTNNVAKWTFTP"

CDS 563110..564093

/gene="oppF_1"

/locus_tag="EFAGFIKM_00507"

/inference="ab initio prediction:Prodigal:002006"

/inference="similar to AA sequence:UniProtKB:P24137"

/codon_start=1

/transl_table=11

/product="Oligopeptide transport ATP-binding protein OppF"

/db_xref="COG:COG4608"

/translation="MRGLKKERPPLLSVESLKTYYPIRQGIFSQVTEYVKAVDGVSFS

IREGETLGLVGESGCGKSSVGRTLLQLETPYEGRILYAGRELAELGKRELRRTRASLG

LIFQDPYSSLNPRMRIGDILAEPLLAHRLESRSSVWNRVDMLLETVGISIEDKSRYPY

EFSGGQRQRIGIARALSLNPKLIVCDEPVSALDVSIQAQILNLLKKLQKENGLAYLFI

GHGLGAVRYISDRIAVMYLGKIVEIASREKLFRAPKHPYTNALLSANPVTDPKLRSQK

RIIIQGDVPNPVRPPGGCRFHTRCPIAKAICREFEPALKGHDHAVACFAFD"

CDS 564180..565658

/gene="abgB_1"

/locus_tag="EFAGFIKM_00508"

/EC_number="3.5.1.-"

/inference="ab initio prediction:Prodigal:002006"

/inference="similar to AA sequence:UniProtKB:P76052"

/codon_start=1

/transl_table=11

/product="p-aminobenzoyl-glutamate hydrolase subunit B"

/db_xref="COG:COG1473"

/translation="MNEKIIEEVTTYIEGKRDLFIDVSDSIWGFAETRFEEFESADLL

CKALEQEGFSVERGVAGLATGFIGSFGDGSPVIAILGEFDALASLSQQAGAAVFDPVV

PGANGHGCGHNLLGAGSLAAAFAVKDYLQEHGISGTVRYYGCPAEESGSGKAFMARAG

LFKDVDIAFCWHPATMNVVMHMSSLANLHVHFKFAGRSAHAAAAPHLGRSALDAVELM

NVGVNYLREHMISDARIHYAVTNTGGFAPNVVQAEAEVNYLVRAPKSSQVAELFERVL

DVARGAALMTGTKMSYHYEGGASNLIPNATLEGKMHEFLSGLELPVYTEDELAFAQEI

FDVIPEEDKTSASRQNGKGVHDMLSAHPLAGFVAPLMETMRVMPASTDVGDVSWNVPT

AQCGTATWAYATPVHAWQTVAQGKSGYAHKGMLLAGKAMACTAIAALLDPALIEKAKE

ELAERLEGESYVSPFPDEVMPPNLSFSQSTGGAANEQTPVNM"

CDS 566301..566819

/locus_tag="EFAGFIKM_00509"

/inference="ab initio prediction:Prodigal:002006"

/codon_start=1

/transl_table=11

/product="hypothetical protein"

/translation="MFDPTVYDNLKVGFENYLYDMDNLDERIQITGRKDRLEMAVMSR

EFMLQFCLRDLPEVTGEVVLSSSLEELAAEILETPGASPGCRLELRFSMVVKDPEKQC

PVIRPILQKSWPEQRMCQTIQYIFDEQPIMYNVTAHVYFDRSVNEDQMGDIPELCEHM

ANVMNQLLQLHN"

CDS 567095..568066

/locus_tag="EFAGFIKM_00510"

/inference="ab initio prediction:Prodigal:002006"

/codon_start=1

/transl_table=11

/product="hypothetical protein"

/translation="MTEKLIRLLRILQAIQANPGISAKELALKCGTTVRTIYRDLRIL

DRVAPIMNEGYGKGYRFIGEFAMYPLDFTEQEAMVFSLLPSVVDTSKLPAEFDSAFDK

VMSAHTKLKSRNSDIVENIAGIIRMGTPAYREEGKDPNLLIPIIEAILSQQTIRTQYH

TLTRNEITVRDIDPYYLIPRDQRFYLIGYCHLLQKVRLFRISRFLDVTRTNAGFDKGD

FNITQYMKNTWSIDRGDENIHFKVRFSEKVAPYIKEEEMFVRPRMTNMSDGGLLFEVT

LNSSREFLKWLYQFGPEAEVLEPREFRREIRRQLVEWLEHYETDVSL"

CDS 568109..568471

/locus_tag="EFAGFIKM_00511"

/inference="ab initio prediction:Prodigal:002006"

/codon_start=1

/transl_table=11

/product="hypothetical protein"

/translation="MSTKLFAAMYGMDHYHGVQALHVGDTVYLVKDPDNRLDHQAIKV

VIPPIGAVGYIVNDPLVVPHGCWTGTAFYDVFHQLTCAKVRFMMRDMVIMELIEMSHI

PVPQMDSLIKGWQFREKA"

CDS complement(568493..569596)

/gene="cotSA"

/locus_tag="EFAGFIKM_00512"

/EC_number="2.4.-.-"

/inference="ab initio prediction:Prodigal:002006"

/inference="similar to AA sequence:UniProtKB:P46915"

/codon_start=1

/transl_table=11

/product="Spore coat protein SA"

/db_xref="COG:COG0438"

/translation="MKIAMVAPEKLPLPGNGSVEICILGIARELAQRHQVTIISRQMQ

GLPATEQIDGITIQRVPASSPVMYTKAVIRLLSQQPYDVIQVDNRPRSMATIKRAFPR

TPVVLYLHSLTFAQPGPSKLTLMKKADWIAVNSHSLRQRLGRRFPLVKHRMSVVPLGA

DLSRFRPVESSEEQIHLRTQFGVTKPFSILYVGRLIPGKGVDVLIRATALLQQQVPVQ

LVVAGKGPPYYMRKLRELAQKQKVHVSFRGQINHEHIDQLYRAVDCLVCPSQEHEAFG

LVNVEAMASGLPVIASDNGGIREIIESGINGYLVTAYKRPQRFTSYLYKLASNSTFAE

KLGKAGRDSARAYFSWARTAMHLEATYTRLIRK"

CDS 569812..570123

/locus_tag="EFAGFIKM_00513"

/inference="ab initio prediction:Prodigal:002006"

/codon_start=1

/transl_table=11

/product="hypothetical protein"

/translation="MLHSTLGRFRLMLWLQGISYVLLLFVAFPLRDAGVMPQAVTWFG

NLYGFLFLMYLLFMVTMYTSQKWRLRRPIALFFVSFIPLGNMIYDVVVFRKLYRQLNK

A"

CDS 570398..571219

/locus_tag="EFAGFIKM_00514"

/inference="ab initio prediction:Prodigal:002006"

/codon_start=1

/transl_table=11

/product="hypothetical protein"

/translation="MLDVSLLLEYGWVLAVLVVLEGLLAADNALVLAIMVKHLPEDKR

KKALFYGLMGAFVFRLGSLFLISFLVDVWQVQAIGALYLLYISINHIVKKILGSRNKT

DEAADSTPKKPKKQSSFWMTVFKVEVADIAFAVDSILAAVALAVALPPSGLPPIGGLD

GGQFIVIFLGGFIGLVIMRFAASYFVKLLHSRPGLEVAAFVIVGWVGVKLAVITLAHP

SLGVLDEHFPENKIWKFGFYIVLITIAVCGWFLSSKVPEKEDENPVEELEKQAGL"

CDS 571402..571497

/locus_tag="EFAGFIKM_00515"

/inference="ab initio prediction:Prodigal:002006"

/codon_start=1

/transl_table=11

/product="hypothetical protein"

/translation="MMLGYDLIQTGRNVDTIAYPAHRFIGLIRST"

CDS 571722..573185

/gene="rtcB_1"

/locus_tag="EFAGFIKM_00516"

/EC_number="6.5.1.-"

/inference="ab initio prediction:Prodigal:002006"

/inference="similar to AA sequence:UniProtKB:P9WGW4"

/codon_start=1

/transl_table=11

/product="RNA-splicing ligase RtcB"

/translation="MNTEFNLFTNPNELTGAYRHEVKLPAGDLTVYAAQQLFSSLDYK

VFEMANNNLQIPGISYMSYTPDVHVGVGTCIGTTAVWDAQSGYVSPSIVGSDIGCGMR

VHMTNLHKDELKDIKLRRKLVKAIEKVLPMEANQRGHYLDIRLEHIVRKGLHGLPKKY

IPDSYTPKKSTALTHVESSKFSYDEDVLNHVPDMTWHRSHRQLGTLGGGNHFAEIQAI

EIAEENREIAEAWGLYDGQVVVMIHSGSRAWGGYVSQTSSSAIAKVMQRLNLGTSDPR

LVFAPLEHAEGRHYVNMMYSALNYAVVNRHLIAYAIREAFRDVFGSKCEFRTLYDLMH

NYAWEESHADQGSFFVHRKGATRALPAGHPDNPKPYQATGHPALIPGSMGTASYVMVG

QPQGADNYYSICHGAGRIRSRTATKRLISVEDFAAALGVGTEDEIVVNQRSLESIIDE

SPQAYKNVDEIIDSVTGAGLAQVVAKCKPLVAVKGAK"

CDS 573185..574489

/gene="yjoB"

/locus_tag="EFAGFIKM_00517"

/EC_number="3.-.-.-"

/inference="ab initio prediction:Prodigal:002006"

/inference="similar to AA sequence:UniProtKB:O34703"

/codon_start=1

/transl_table=11

/product="putative ATPase YjoB"

/db_xref="COG:COG0465"

/translation="MTINEKQTDPTQAIYTYDEQHDTRIRVEGYAIYSRLIRGISEAL

HQRYGVEYKLYASSDPNNEYWELLREDLQNGSDEVELVARIFEDLELQTLHYDDDGDV

PTYGVHYSIRNNVFAYPKWGVALVRVPFFRENGIYSEDFVFAIGDEEMKQFLGSVRER

ERQQNMKKVTVYTDARNGSDRHVESITRSVGREDVVLSAQIKQDIFRSLDQFFEADRS

FYRDYDIPYKRGILLYGHPGNGKTTLVKSIAGSIPGPAAYWQITEYTNSESVREVFEA

AKRLAPMVLIIEDIDSMPDEVRSFFLNTLDGATSKEGIFLIGTTNYPEKIDPGLMNRA

GRFDRAYEIPLPDEALRLQYLRQRGFTIFAGEEGTVEAARLTDTFSLAQLGELYVSAA

LEWHQNGQTDIVKVIQSMRGELDKSHKHTWLAQPGKGRAGFY"

CDS 574798..575055

/locus_tag="EFAGFIKM_00518"

/inference="ab initio prediction:Prodigal:002006"

/codon_start=1

/transl_table=11

/product="hypothetical protein"

/translation="MGWLWSLIIGGIIGWLAGLIVGRDIPGGVIGNIIAGFIGGWLGG

VILGDMGPEMGGFYIVPALIGAIVLVAIVSLIFRSMGRSRG"

CDS 575234..575749

/gene="msrA_1"

/locus_tag="EFAGFIKM_00519"

/EC_number="1.8.4.11"

/inference="ab initio prediction:Prodigal:002006"

/inference="similar to AA sequence:UniProtKB:P54154"

/codon_start=1

/transl_table=11

/product="Peptide methionine sulfoxide reductase MsrA"

/db_xref="COG:COG0225"

/translation="MEKATFAGGCFWCMVTPFEEQPGIHGIISGYAGGHVENPTYEQV

KTGETGHVEVVEITFDPDVFPYERLLELYWPQIDPTDDGGQFQDRGTQYRTAIFVHNE

RQRELAEHSKQELAASGRFTQPIVTEIRDAAIFYPAEDYHQDYHKKNEKHYKEDRALS

GRDEFIDENWK"

CDS complement(575832..577103)

/locus_tag="EFAGFIKM_00520"

/inference="ab initio prediction:Prodigal:002006"

/codon_start=1

/transl_table=11

/product="hypothetical protein"

/translation="MHTLTKHKSKTWAALILSGMLLFTMNTTGYAAASTESMPKPAWT

SSSLMLSEANTEKDVLIKGIHAVPSKNLVYVHASQSVTKTSSKTTLDWQLDSLQAFDV

TTGQLKWNTIFHEKSGPYTTYSDSVYASNGTAYVYMEYSDKTKKLYSFNTSGKTNWVK

TVNSPASISLLDNDTLMVASSQGVQSNGSVRSAISLYDKKGKLITEKTINGNVLKADH

GRIVVDASKQTKVGNFWQQAANPKVEIYDRTLNRLSFYQFPANANTLGDGGGESLAIL

DDGSIIMRANFENTGNRLMGFGIDGKLAWGRAITGDAYIQTAGNGYTVFTGQKLELYT

MKGKVTERTFKDAQPALMRVDQTQDGQYQLDFAKTGYILDPQTLEDVHIYTTSASVPS

LEGVSTYVDDVFYSMNNETLSKYVLKSNSAK"

CDS 577366..577962

/locus_tag="EFAGFIKM_00521"

/inference="ab initio prediction:Prodigal:002006"

/codon_start=1

/transl_table=11

/product="hypothetical protein"

/translation="MNWSENATVWVIGCIIILLLLAWIIRRVIGRGQRIRSEANPRKI

TIKDIDKMEDGSEFELYLYHLFEELGYDEVHKTTRSRDFGADLVFVDRLGRRSVIQAK

RYGANHPVGLSAVQEIYTSMRYYEADRSIVLTSARYTEACRTLAAVNGVKLLDREDLM

ELILLFKARRVEEALELIEEDDHEPIETWQSRRKRQSR"

CDS 578082..578309

/locus_tag="EFAGFIKM_00522"

/inference="ab initio prediction:Prodigal:002006"

/codon_start=1

/transl_table=11

/product="hypothetical protein"

/translation="MARTQTQKALRKAERSGQWCSEQSRRVNGDYGALSQHVRLMPTK

QEKLQKVKHKKQIIQDGGASFYFFYEVPIAG"

CDS 578359..579606

/locus_tag="EFAGFIKM_00523"

/inference="ab initio prediction:Prodigal:002006"

/codon_start=1

/transl_table=11

/product="hypothetical protein"

/translation="MTTALHMNKKIVRRFLLQTQALLGRWPAVSLPSGPDQVLSLIRS

LGCVQIDPVAAVTGNQHLVLGARDPGYTADSLNTLLSDHKVFEYFANAACVIPIEDYA

LFEPVRARLRERLAPSLQGLEKTVQHVLQRLKEEGPLPSRAFRAVERVSGYWDSADVP

KTKDTTLALNLLLDSAVIRVVARQGNERYFHITESELQQQGLTMKEDIDVLAQRQALL

DKYIYAYRVVDARDPRLGWLKSTAAERRADIAARVSDGRMIPLKVEDVATPYYIRAED

EDLLLHMEREEPHYDPSGPVRFLPPLDNLLWRRERIVDLFDFHYKWEIYTPEVKRTYG

YYAMPILHGDRLIGRMDPRLDRKTGVLTVRLLSMEETAPPVEEWITDFREGLQFFASM

HGARSITIEKTVPKELKKLLKLI"

CDS complement(579812..581005)

/gene="fabV"

/locus_tag="EFAGFIKM_00524"

/EC_number="1.3.1.9"

/inference="ab initio prediction:Prodigal:002006"

/inference="similar to AA sequence:UniProtKB:Q9HZP8"

/codon_start=1

/transl_table=11

/product="Enoyl-[acyl-carrier-protein] reductase [NADH]"

/db_xref="COG:COG3007"

/translation="MIIKPRTRGFICTTSHPVGCAAQVQEQIDYVKSQPELKGPRNVL

VIGASTGYGLASRVVSAFGAGANTVGIYRPSSSTEKRTASAGWYNSAAFEKAAEEAGL

KSYSVTGDAFANETRDKAVELIRSELGQVDLVVYSVASARRTDPNTGEVFNSVLKPIG

QSYTNKTVNFHTGEISSVTLEPANEEEIRQTVTVMGGDDWELWMDALQQGGVLADDAT

TIAFSYIGPELTHAIYRDGSIGQAKNHLEATAHKLNDRLSAKGGRAYLTVAKALVTQS

SSAIPVVPLYISALYKVMKEKGLHEGCIEQLQRLFADRLYAGGEVPTDAEGRIRIDDW

EMRADVQEEVAKLWNELTTENIYDLSDLEGYRREFFQLFGFETDGVDYEADVDPNVEM

PHLVN"

CDS 581209..582489

/locus_tag="EFAGFIKM_00525"

/inference="ab initio prediction:Prodigal:002006"

/codon_start=1

/transl_table=11

/product="hypothetical protein"

/translation="MLKERIELLSKRKQIFRKDLVEGLVTQAHFANILADRYPLPEDL

AEAIAGRLGVSPSYILKAAAQDEETLERAEAIFEQMSVPASAISEDMVHALADRDDTL

TVELTTALMKAVYYQQLNDATAHEYIHTSYLNFYLEKYGRPDDIDLPRPLVKALLFYK

IQYYRSKMAFVDVLTHATRLSELALPGSEFWLSVQNIKMEAYIQVKQYEEAKQVFELT

MRHVYDQRMFHRLSGLYVAYSGYCFAMGLVQEALSALTMAEANLVYAGNQGDLVTAIA

NNRIVMLTMTGELDQAQAEIERFESLLQQEPEETQQAMQPLIGVYRCEVALARKNWGV

LAQSVDQLLKCATTQDQQMSATFYQSHLALAHGDREVFMERALACLPYFESAQHVMRL

EPLYEGMAVVSEDQRKYKEAAMYYRKLVYLLRKK"

CDS 582767..583183

/locus_tag="EFAGFIKM_00526"

/inference="ab initio prediction:Prodigal:002006"

/codon_start=1

/transl_table=11

/product="hypothetical protein"

/translation="MGWFGKKNKQEDAIAKADKIMNKGLTGMMMKGFVSKEHREAINQ

SLDSAKQAQLAASGSLPLTATATVVTITDTGKLINFDPIVVLVLDVTETNGNQYQRTL

ETLVSKMQIPRVGDRVGLGQNPANPSELIYMGLMSI"

CDS 583295..584089

/locus_tag="EFAGFIKM_00527"

/inference="ab initio prediction:Prodigal:002006"

/codon_start=1

/transl_table=11

/product="hypothetical protein"

/translation="MVGFLRFIGVLTVFGVGFFPFLAEQWWNDSFKFVQAWWYAPAIF

AGFALIFVPAILSNIFGVGRVKTGLPAVGVIKTIQQTGTYINEQPEVRLGLTVSRKGR

EKYDTEMKAVIPLTSMAQFQTGSFIPLVVSVKDERKVGIDMQGQLSQEDMQQLLDEKM

VQQGVSPEMMDIARTGEKAMAKILDVTPLGSGGSNKIKLQLKLSITKTNGETFNVTTQ

KEILSSALPQVQQGHIINVIYSRQDPTKLVLALNLQERQLSDLFSS"

CDS complement(584188..585003)

/gene="COQ5_1"

/locus_tag="EFAGFIKM_00528"

/EC_number="2.1.1.163"

/inference="ab initio prediction:Prodigal:002006"

/inference="protein motif:HAMAP:MF_01813"

/codon_start=1

/transl_table=11

/product="2-methoxy-6-polyprenyl-1,4-benzoquinol

methylase, mitochondrial"

/translation="MQDIRRNNVERFKGFGTLYDQNRPAAPTEVVDILTTYLGSTPRM

VADIGCGTGLSSWIWLNEAERIIGFEPSDDMRSVAESKWESAGKPDNLRFVSGLSHDL

GLPDGSVDVLTCSQSFHWMEPQPTLREFARVLRTGGVFAAYDCDWPPMLDWQLEQAYL

QLNKEADQRAASLAPQDSQAHKWSKDGHLQQIQQSGLFRYVREIVFHHHETFTADRYV

NLALSQGGLQTALKLGADDLLTAADEFRALANLVFDGESRKVLFSYRMRLGIV"

CDS complement(585077..585994)

/locus_tag="EFAGFIKM_00529"

/inference="ab initio prediction:Prodigal:002006"

/codon_start=1

/transl_table=11

/product="hypothetical protein"

/translation="MLLLLLEKKKATAPELARMFEISVRTVYRDIDRLSAAGIPVYTT

TGKHGGIHLMDNYVMDKSLLSEEDQNEILMGLYSISAIPHLNSAHMLQRLTALFDHKL

DWIEFNFSPWGSIPLQERELFNQVKQAILTNQLITFHYVNSDGEKSIPTVEPQKLIFK

NSTWYFKGYIHDDPDRKEFETFKMKRITQLTFLARKHDHSVTAGSNDPESEAAPVLTS

LTLSFSSNIAYRVYDFFEPSLIKKEHDGRLRVSLELNVGEWLYSFLLSFGSELTVIEP

VHVGQELLRRHIKAVEHLQNVMNKLPNNE"

CDS 586107..587540

/gene="bglH_1"

/locus_tag="EFAGFIKM_00530"

/EC_number="3.2.1.86"

/inference="ab initio prediction:Prodigal:002006"

/inference="similar to AA sequence:UniProtKB:P40740"

/codon_start=1

/transl_table=11

/product="Aryl-phospho-beta-D-glucosidase BglH"

/db_xref="COG:COG2723"

/translation="MTMKEGFYWGGATAANQFEGGWNQGGKGPSTSDMMTGGTHTIPR

RITPVLEEGTYYPSHEAVDFYGHYKEDIAMMAEMGFKMFRMSINWSRIYPNGYDLEPN

EEGLQFYDNVFAELKKYNIEPLVTISHYETPFGLTQKYNGWASREVIDCYIRYCTTLF

NRYKDQVKYWLTFNEINCLTMPMGAYMAAGILFEGKETLVDGVDDPQTRFQALHHQFV

ASAKAVKLGHEINPEFKIGCMVAFMTTYPNTCNPNDMLLAQKKDQLSNMICGDVQVRG

AYPGFAKRFFAEEGIQIEMQPGDEQTLREGCVDFYSFSYYMSLVESADESLERAEGNL

LGGIKNPYLEASDWGWQIDPKGLRYTLNHLYDRYQIPLMVVENGLGAVDVVEEDGSIQ

DDYRIDYLKGHIEQMKEAVADGVDLIAYTMWGCIDLVSASTGEMKKRYGFIHVNKDND

GNGDLSRTPKKSFHWYKKVIESNGEEL"

CDS complement(587708..588376)

/locus_tag="EFAGFIKM_00531"

/inference="ab initio prediction:Prodigal:002006"

/codon_start=1

/transl_table=11

/product="hypothetical protein"

/translation="MIHNWKKNISFPLLVAALCMIAFISIALSISDNQIHRFDDTLIG

WIQGLESPGMTQFMHLFTWIGSEMPVVFITIIAMIVLYVCLRHKRELLFLACVLAGST

LLNALLKLVFQRARPTINRIIEVSGYSFPSGHSMAAFSLYGGLAFLIWKHVPTVTGRV

LMIVASAVFILTIGISRIYLGVHYPSDIVGGYFLSGCWLSTCIWFYRRHLERMSQLQS

RRLA"

CDS 588546..589118

/locus_tag="EFAGFIKM_00532"

/inference="ab initio prediction:Prodigal:002006"

/codon_start=1

/transl_table=11

/product="hypothetical protein"

/translation="MKRSWVMCMLILLLVTSGLALFPGDKVFACSCVESDVQKRLETY

TTVFTGKVVKKERSQQSKHDGLLREYTFDIDTAWKGVNAKKMKILASDGGSESCGIQF

EKNQSYLIYAYQYEVDHKLHTNLCSRNVPIEQAGDDLKLLGAGKVVSKEDFGETTDGR

SEYFQLILYGGVILLAALAIVWIWKRKKRI"

CDS complement(589247..590023)

/locus_tag="EFAGFIKM_00533"

/inference="ab initio prediction:Prodigal:002006"

/codon_start=1

/transl_table=11

/product="hypothetical protein"

/translation="MERFNYKKSQDVLALSASFTDFAYKKHCHEEYAVGVTLRGIQQY

NLDGQYQASHQNGVMLFNREQSHDGSSYDKAGIDYVMLYLKPELVEEIIGKKELRFGS

PIVYDPELARNILMLNDAVQLRQNEAECSERVFDLVHLLAQKEMDTKLWLPQDNLVRK

AKEIMFCSTEDVLKLDNLSSEFGMSKFQFIREFKAHAGISPYQFFLNCKVERARQSIE

KQKDVYTAVADCGFVDLTHLNRHFKRVFGVTAYEYMLQLN"

CDS complement(590043..590624)

/gene="eamB_1"

/locus_tag="EFAGFIKM_00534"

/inference="ab initio prediction:Prodigal:002006"

/inference="similar to AA sequence:UniProtKB:P38101"

/codon_start=1

/transl_table=11

/product="Cysteine/O-acetylserine efflux protein"

/db_xref="COG:COG1280"

/translation="MNLASFLIYCIVVTFTPGPSNIVILSSVQQVGARQTMHYVWGAT

LAFGLLLTASAFLNQLLAGVLPGILKVMQIVGGLYMVYLAYKIYKMGTTEDAPKQVTG

FWNGFIMQFVNPKVVLFTFTVIPSYVLPFYQSSFSSFLFVLLITFIGFLAYSSWVVFG

KVFKTLLNRHQKLLSILMALFLLYSAIIVSGLI"

CDS 590831..591838

/locus_tag="EFAGFIKM_00535"

/inference="ab initio prediction:Prodigal:002006"

/codon_start=1

/transl_table=11

/product="hypothetical protein"

/translation="MKKMLLVYILLISAFALYVLRFEYTSQVNSSWEDRGLRGDIGET

YIMITFQSGLEYWKSPLKGFEDAADALGVTVEYRGATRYDAKEQTMVIEQAIARKPAG

IAISAIDPQSLVPAINKAIDADIPVVLFDADAPGSQAYSFLGTDNYKSGVMAADKMAE

LLGREGEVAVLTLPGQQNHEERTKGFRDTIKERYPSMQIVEVADGHGDAMVSRDETQR

MMKEHPELAGIFVTEATGGAGAGEAVQTAGDRHPLQIISFDTNKATLDMIKNGTISAT

IAQGTWNMGYWSLQYLFHLHHQLTVPAPSSSGENAPLPVMVDTGISVVTRSDVDDYYA

K"

CDS 591828..593798

/locus_tag="EFAGFIKM_00536"

/inference="ab initio prediction:Prodigal:002006"

/codon_start=1

/transl_table=11

/product="hypothetical protein"

/translation="MPNNKEDFKSPGHWSRRAILSTFRNGIRRLRLRNMPLRYQLMLL

FLLFAIVPSVGLGLLVNWTVERVVERQVEGHTMQLIGKVNEALNSKMENLQNMTYLIA

FDPDIDAFMNDKMPQNDDAGIEPMNMDTNAETEQNRLYGIKQTLQGFTTLYPEIAGIV

LVNANGDYISNEMYPRAEQSLIQENWYQKASANPGIFMVLGQPKERNLTTHVRYKDDE
IVSVARSITDEASGRVRGVIMIDLKLRSVSQAARNVTLGKSGYVMVTDAEGQSVYKPE
HPLIEHIPTDWFPSGESGTFTADTEGGTLLFMYQSSTFTGWRTVGVFPTRDSISEVRQ
IQFYVVSFVFVVCLFGLSASLWFSRSIAQPIFRLMSYMRRAETGNLRPGRWSDRADEI
GMLGNSYNRMLAQIRQLISLNELRERQKRDAEMRSLQEHIKPHFLYNTLDTIHWMARK
EGAEDVSGMVGALSRLFRIGLSKGQDYIPLHSEIEHMTSYMQIQQTRYRDRLQYTLNI
PEELRDLFVLKLLLQPLIENAIYHGIKGRRGPGHIRVEARLEHNRLLLTIHDNGAGMS
NERLAEMQQLLEAPLASLEASSPGMTGKSYGMLNVQARLRLSFGDEYGIVLESQEGEG
TSVTIIHPLMRELPPTKQINNEERQESEWENS"

CDS 593783..595105

/gene="rssB_2"

/locus_tag="EFAGFIKM_00537"

/inference="ab initio prediction:Prodigal:002006"

/inference="protein motif:HAMAP:MF_00958"

/codon_start=1

/transl_table=11

/product="Regulator of RpoS"

/translation="MGKFMKSTVTTSTETDETSALTTVMEITPDSHVAKKYRVLIADD
EPIIREGIRDAIDWTTLGMEVVGEAEDGEEALERAADLGVDIVLVDMNMPFLNGIELI
RALQQKCPGCRYLIISGHDEFAYAQEAVRLGVEDYILKPVQAEQLYAALERLHQRLTE
EHQRTAYVQQAAHQIERNIPLLRQRFMLEWLEGQAEGRDLTEQLVFLRLPAVSPVQIG
VVRWPAAEARQTILRENDRQLFLYAVENIISELLGDLPHVLFRDVSGLIGMCLWQEAP
EEIESLLEQQISSCLNIAIHAHVETHAGTLEETPDTYRHCRERVYGEVQLSPLVRRAR
QLIHEEYAERELTLESLASRLQVSAVYLSRVLKKELNDSFVTLVTHARIRKAIQLLDS
TTLPIHTIAERVGYDTQHYFSTAFKKTMGISPVQYRKNGGTAYQPSAN"

CDS 595358..596509

/gene="chvE"

/locus_tag="EFAGFIKM_00538"

/inference="ab initio prediction:Prodigal:002006"

/inference="similar to AA sequence:UniProtKB:P25548"

/codon_start=1

/transl_table=11

/product="Multiple sugar-binding periplasmic receptor

ChvE"

/db_xref="COG:COG4213"

/translation="MYCKRYLITHSEPYDVRKGELHMKKGAMLIWLLVMTLMLSACNL

AESGPGSKEKGYVGISMPTKSSERWVGDGENMVRLFQEQGYKTDLQYAEDVVENQISQ

IENMITKGVDVMVIASVDGNTLTDVIKKAHDEGIQVISYDRLIRNTPYLTYYATFDNF

KVGVLQASYIEQKLGLKDGKGPYNIELFGGSPDDNNAYFFFDGAMSVLKPYIDSGKLV

VRSKQMTMAQIATLRWDGALAQSRMDNLLSAYYSGDHLDAVLSPYDGISIGIISSLKG

IGYGTSNKPLPVITGQDAELASIKSIVAGEQTQTVFKDTRKLAEKTVEMANSILQGKQ

AEVNDKTSYNNGIEVVPAYLLDPISVDRTNVEQDIVGTQYYTKEEIGLK"

CDS 596531..598096

/gene="xylG"

/locus_tag="EFAGFIKM_00539"

/EC_number="7.5.2.10"

/inference="ab initio prediction:Prodigal:002006"

/inference="similar to AA sequence:UniProtKB:P37388"

/codon_start=1

/transl_table=11

/product="Xylose import ATP-binding protein XylG"

/db_xref="COG:COG1129"

/translation="MAGIILEMKNMTKTFPGVKALENVNLKVREGEIHSICGENGAGK

STLMKVLSGVYPHGTYEGDILFQGKNCEFKDIKQSEDLGIVIIHQELALIPYLSIAEN

IYLGNERASRGIVNWKETFVGTRELLSKVGLSENPNTLVSSIGVGKQQLVEIAKALSK

KVRLLILDEPTAALNEDDSENLLQLMLEFKKQGIACILISHKLNEVSKVSDSVTILRD

GKTIEMLDMKKEKVTEDRIISGMVGRDLTSRYPERHATIGEVILEVKDWTVYHEHHAE

RKVLDQIHMNIRRGEIVGIAGLMGAGRTELAMSIFGKSYGRNISGQLIKNGKPIHNNT

VTEAIQNGFAYVTEDRKEYGLILMDDIKRNISLTGLSKLTRNAVVNEREEVLVAEKMK

KSMNIKAPSILQKTGNLSGGNQQKVVLSKWIFAGPDILILDEPTRGIDVGAKFEIYTI

IHRLAAEGKGVLVISSELPEVLGLCDRIYVMNAGRITGEVSREQASQETLMRYMTKSG

GGKHGNDNKAFQK"

CDS 598065..599228

/gene="xylH"

/locus_tag="EFAGFIKM_00540"

/inference="ab initio prediction:Prodigal:002006"

/inference="similar to AA sequence:UniProtKB:P0AGI4"

/codon_start=1

/transl_table=11

/product="Xylose transport system permease protein XylH"

/db_xref="COG:COG4214"

/translation="MEMITKLFKNNIRQYGMIIALVVIMLLFEVLTGGLLLKPINITN

LILQNSYILVLAIGMVLVIITGHIDLSVGSIAAFVGAVAAIMMVDWQLPAWLAVIASL

LVGALIGAWQGFWIAYVRIPAFIVTLAGMLLFRGLTMIVLEGQSISPFPGGFQKISSG

FLADIQFSGFGLVSIIVGLVLTVWYIVNELRERRSQRKYGFEVVSQGLFLLKLIVVAV

VTNLFTFVLASYAGIPNILVLLFVLIVVYSFVMNRTVMGRHVYALGGNEKAAGLSGVK

TKKVTFWVFVNMGTLAAVSGLIFAARLNAATPRAGTNFELDAIAACFIGGASASGGIG

TVFGAIIGGLVMGVLNNGMSLIGLGIDWQQGIKGLVLLLAVAFDIYNKNKRMA"

CDS complement(599353..600723)

/gene="mgtE"

/locus_tag="EFAGFIKM_00541"

/inference="ab initio prediction:Prodigal:002006"

/inference="similar to AA sequence:UniProtKB:Q830V1"

/codon_start=1

/transl_table=11

/product="Magnesium transporter MgtE"

/db_xref="COG:COG2239"

/translation="MNGPHTKKDFSVEEITEQLVQHVQSRNLPDFYKLVKELHPYDLS

LVYKKFPEEETNRFLLLFKPDALADLAETLKLHEQIQLFERLGPERTLEVMQQMDKSD

LIRFMHDLPAKRREELLSNMNLDHSAIIRSVLNYPPETAGRIMTDQYRTLLAHETAKE

ALRQSQGTMHISPSSYLYVTDNEGKLVGVVSYRSLALADDKTQVEELMTRRVIHATVD

MDQEEAAQLLQRYEFIALPVVDENHRLCGIIQMDDVIDIIMDEASEDLAKMGGGSKDI

DFDTKPLVAVRRRLPWLILLLFIGLISGSIVDFFEDTLNQVVALAFFMPMIAGMTGNT

GTQSLAVVVRGLIGRKLDKATVLALIGREIKVGTMIGLVCGLLITVIAYFWQGDWLLG

AIIGVSLFLTLVIGTLTGTCIPLLLSRFKVDPAVASGPLITTLNDILSLFIYFGIATR

FLDALM"

CDS 600914..601618

/locus_tag="EFAGFIKM_00542"

/inference="ab initio prediction:Prodigal:002006"

/codon_start=1

/transl_table=11

/product="hypothetical protein"

/translation="MEVDIRTQLLSMVEPEYQKFSAALIPNITNVLGVRLPALRKIAK

QLAAGDWRTYLETAEDEYFEEVMLQAMVIGHVQADLDELLKAIETFVPKIDNWSVCDS

FCAGLKYTKVHSEPMWAFLQPYLRSDQEYEIRFGVVMLLNFYLNERYIDQVLSALDQI

RHEAYYVKMAVAWAISMAYVKQPEVTMRYLNRNTLDDFTYNKALQKITESYRVDPESK

QMIRSMKRKVKKQITS"

CDS 601661..602215

/gene="ogt"

/locus_tag="EFAGFIKM_00543"

/EC_number="2.1.1.63"

/inference="ab initio prediction:Prodigal:002006"

/inference="protein motif:HAMAP:MF_00772"

/codon_start=1

/transl_table=11

/product="Methylated-DNA--protein-cysteine

methyltransferase"

/translation="MRKKLMYTTMQLDGRPWVLLATEQGLCRVVMPNETLEDWNGWIQ

KVAPGVELEENEDALKQTGMMDWLQSYFAGELIAYTDAIPLDLIGTNFQQEVWRELGR

VPYGEIRTYGDIAAAIGRPSAVRAVGAANGANPIPVLLPCHRIIGANRKLTGFRGGLE

MKRTLLDIEQIEGVTDGGHARFHF"

CDS 602453..602758

/locus_tag="EFAGFIKM_00544"

/inference="ab initio prediction:Prodigal:002006"

/codon_start=1

/transl_table=11

/product="hypothetical protein"

/translation="MSYAPQKRHSNRAKRWIITIGIFIVVQIIFIAIDGTFLEPSIND

SDNVLAKTVRGVLESKLLTERIAPYSFPFFNLFLIVHVVAILIQALGDSILAIFAKR"

CDS 603257..604531

/locus_tag="EFAGFIKM_00545"

/inference="ab initio prediction:Prodigal:002006"

/codon_start=1

/transl_table=11

/product="hypothetical protein"

/translation="MEDIHISSEELNGVHLHLYNTNKFKTVSIMLTMKSPLQESLITA

RALIPHILNGGSINYPNRKLIQQKLDKLYGATLLLDVQKKGEDHSIVFRMEIPADKFL

DNSASLLKSSLELLHEIVYNPLIENGGFHSIIVEQEKRSLKQRIDSIHNEKMLYANVR

LLEEMYKGEPYQIPAFGRKEDINSLDAISIYQAYENMLQTDRFDLFIVGAFNEDQVKL

MVQNIFCEQHNNEFMKGFSSASVTVNDIKVIQDKMDVKQGILLLGYRISSTIQDEDYE

AARVANAIFGRFPSSKLFRNIREKESLAYFSHSQMESNKGLLIAMAGIDFIHYERVVE

MIREQERAMKQGKFTEAEVEQGKGMLINLLLEAHDSPVGIMDIFIQSVDSGLSIDIHD

QIQRISKISKADVICAAKKWELDTIYFLNREE"

CDS 604537..605820

/locus_tag="EFAGFIKM_00546"

/inference="ab initio prediction:Prodigal:002006"

/codon_start=1

/transl_table=11

/product="hypothetical protein"

/translation="MHPIIYEGLEETIYYEKLPNGLEVYIHPKKDFHKTVATFTTKYG

SIDNKFSPEGKDYSVQFPDGIAHFLEHKMFECEKWDAFHKFAEQGAFVNAFTSFTRTA

YTLSATSRIKENLTTLLDFVQEPYFTDETVEKEKGIIEQEIKMYDDNPDWRARFGVLD

NLYKSHPVKIDLAGTISSIQQITKEDLYTCYNTFYHPSNMLLFVIGPVVPEEILRLVR

SNQEQKEFPETKKIRRFFPEEGAAVNKSSSVIKLLVPIPKVFIGYKESFPIKQGQEIL

KYELSLNVLLELMFGIGSEAYEKMYENGYVDNSFNFEYTHEKNFGFSIIGGNSHSPED

VIKIISETIEKFKKDSINKDDAERVINKEIGAFLSAINSPQFIANQFTRYRFNDLDLF

DVLPILESLTASDLEDVLHVHFSSDSRTTLIVKDK"

CDS 605847..606698

/gene="lnrL_1"

/locus_tag="EFAGFIKM_00547"

/EC_number="3.6.3.-"

/inference="ab initio prediction:Prodigal:002006"

/inference="similar to AA sequence:UniProtKB:P94440"

/codon_start=1

/transl_table=11

/product="Linearmycin resistance ATP-binding protein LnrL"

/db_xref="COG:COG1131"

/translation="MSNLLEIKNVRKSYGNKVVLKGISFNVPYGSIVGFIGDNGAGKS

TTFKIVLGLISNDDGMVNIFGEENINKNPKIKEKIGVVFDAANLPAHLTIKQLNKVFG

KLFDSWDQDNFHRLVSSFSLPVDKKVNEFSRGMSMKLSVAVALSHNAQLLLLDEATGG

LDSSSREEVLNELKSFASKENGGILLSSHIMSDIEKIATHLVIIKEGEILLNAEKDEV

FEKYTIVDMDMEKLNSLNQDIVVAKRNFDTYYSVLVSDKKQLPHDLVTKPISMDDISL

LLTRSNE"

CDS 606699..607340

/locus_tag="EFAGFIKM_00548"

/inference="ab initio prediction:Prodigal:002006"

/codon_start=1

/transl_table=11

/product="hypothetical protein"

/translation="MNGLLLTSYYLVYRSSFLYTGLAILLSVIILTFADASMHRLVAL

LNILLMVMSALDVIKFEGKSGYDKYVLTLPVSRSTIVQSHYLFYFLVVIYGVILSYSI

FYVYDLVSGTPINEMSNSIFFGIFAVLLTGAINFPLYYIFGPEKSDSITLGSVGGAIL

VTIVSQSLVGYVASFFNPINPDLLAPIIYLIFGVIVYIVSLWAAILIYRRKEF"

CDS 607464..610076

/gene="ramC_1"

/locus_tag="EFAGFIKM_00549"

/EC_number="2.7.-.-"

/inference="ab initio prediction:Prodigal:002006"

/inference="similar to AA sequence:UniProtKB:O88037"

/codon_start=1

/transl_table=11

/product="putative SapB synthase"

/db_xref="COG:COG0515"

/translation="MEGNMLYNLYLKPNSEYYGKLENDEKKQNRYELGDIPDTYAVIS

DRESVWKHYHVKGSRLPDQGWKIHVTALFEESQNLLDRVSKLCIDERIEFKHLKDRQS

FIKTNSKNANRASSGKFITIYPVNNNVFVKLLDMLCLATQGFKKGAYILSDKRWKNSN

VFYRYGGFRSIYNEYGEHCIKDGQGNLVKDERTPFYQVPDFVKDFDDYLNSLNYSDDL

ETKGDGNLERYEIESALSYSNAGGVYRATRKKDNLNVIIKEARPNAGLDGSAHDSLSR

QIKEYDALAKLKDVTGVVSLIEYFQEWEHYFLVEEFIEGGDLRQWLAQHFPFFRDDER

MRNHAANVKKVLLQLFTLIDQMHKKGIAMGDFQPANIMVADDLTVRLIDFETAMHVTS

KDKPTMMTIGFASQEMKISGARDWFGLKKLTKYLALPVLSSEVLDEYLQYNHFRWIKE

IYEDSFYQFIVDLQEKCDQKIRNYQEYFPKEMVLYDKTSDFNISSITKKLIEGMQDNL

TNDERFVHGDIRQFEMNGGKFNFLTGGTGAAFTLIKNNTNSLEVDKWIQDFLLGNLNT

IESNGMFDGKTGILALLYETGYEAVVLNELKNIRANMNESNISLRSGLSGIGLFVISL

YIETENDEYLQFAKEIEESIERNRNNDARFSTNDWMAVGIGVIDGLSGVSLFYSAMYS

ITDDKQYLERARQLIKEDLEKTIFDEVTGSLQTLDNRNRQLPYLSGGSIGIGIAIWFL

NHVSGEDAYQEEINAITKLSQICSTISGGLFDGAGSFLLIPSLMKNIGGREVIIKEVL

SLLNLFLIDKNGYYVYPGQFSYRLADDVYTGSAGIILALMGIVKDNPLYWLPLVNLDE

FLTRTKAHDVSVTS"

CDS 610142..610270

/locus_tag="EFAGFIKM_00550"

/inference="ab initio prediction:Prodigal:002006"

/codon_start=1

/transl_table=11

/product="hypothetical protein"

/translation="MNAVLELQKLAKDKEGKGQAVDGTITTTWTVTTFLSTISNSC"

CDS 610307..610435

/locus_tag="EFAGFIKM_00551"

/inference="ab initio prediction:Prodigal:002006"

/codon_start=1

/transl_table=11

/product="hypothetical protein"

/translation="MKAVLELQKLAKDKEGKGQAVDGTITTTWTVTTFLSTISNSC"

CDS 610581..611414

/gene="COQ3_1"

/locus_tag="EFAGFIKM_00552"

/EC_number="2.1.1.222"

/inference="ab initio prediction:Prodigal:002006"

/inference="protein motif:HAMAP:MF_00472"

/codon_start=1

/transl_table=11

/product="Ubiquinone biosynthesis O-methyltransferase,

mitochondrial"

/translation="MEKLLDIAKIPEPFEEGSVQIWLDSNRADFVLKAHFDENIPGGS

KGNYFIDETVNFISEIAPLTKYKKIIDLGCGPGLYSQNLAARGYDVVGVDFNEKSIEY

AVHEAQKKGLIIDYRIEDMTNIEIEQEFDLALLIYEIYSVFNPENRKKILSNIHRGLK

SGGLVLLDVLSENSYEKFEQNFMWGLTRKDNPFSDKKHLSLFASIKYPDHVTLSKNVL

VFGDGELVNYHYWNQHFTIESLEKEVNDAGFTLEKIYADVNGGEYQAEGESFAAVLKK

K"

CDS 612033..613259

/gene="atzC"

/locus_tag="EFAGFIKM_00553"

/EC_number="3.5.4.42"

/inference="ab initio prediction:Prodigal:002006"

/inference="similar to AA sequence:UniProtKB:O52063"

/codon_start=1

/transl_table=11

/product="N-isopropylammelide isopropyl amidohydrolase"

/translation="MEKVYWLTNVTLERDYVREGKQVTGTRTSPAHLRIEDGVIAEIV

EGNNPLSSDLPQVDGKGLLLLPSFEEAHIHLDKTYYDGPWTAVKRISSIFERIEEEKV

LLPKLLPDAKRQAESILTLIQGYGSTHVRSHCNIEPVSGLKRLEATWQALESFSGKIS

SEIVAFPQHGLLRSHSVELMGQAMKEGATHVGGLDPHTVDEQIEKSLNAMVELAVQHQ

AGIDIHLHDGGQAGKQTLLELANLTEAAGLQGKVTVSHAFWFASAEQQEAEDMAQRMA

SLGMSIASTVPIGRTMMPLPMLHRMGVNVKLATDSLTDHWSPFGNGDLLEKAGRFAEL

YGYSDELSLSQSLAFVTGGITPLDSQGEQVWPKVGDTASFVLVHASCSAEAVARRSAR

QAVWYKGQLVSGSTAD"

CDS 613387..613797

/locus_tag="EFAGFIKM_00554"

/inference="ab initio prediction:Prodigal:002006"

/codon_start=1

/transl_table=11

/product="hypothetical protein"

/translation="MKIEYATEADYDYIIERDRHIHQPLVKTKISEKEIFILWDEDEG

SRVGWMRYGYFWDNLPFMNMIWIDEPYRNGGYGKKVVHYWENLMKQQGFDTVMTSTQS

DEHAQHFYRKLGYVDAGALLLDTQPLEIILIKKI"

CDS 613830..614279

/gene="bltD_1"

/locus_tag="EFAGFIKM_00555"

/EC_number="2.3.1.57"

/inference="ab initio prediction:Prodigal:002006"

/inference="similar to AA sequence:UniProtKB:P39909"

/codon_start=1

/transl_table=11

/product="Spermine/spermidine acetyltransferase"

/db_xref="COG:COG0454"

/translation="MEQVTLQPITKENELECIHLKPREDQLDLVASNADSLIHATKEI

TSKPYGILAEDQMVGFILFDNEIYNDGYYWILRFMIDEKYQGRGYGKLAIQEVIHMLQ

ERSDCQQIRVSHVPHNIAANALYKRCGFQETGEFEDNGDIILSYQVR"

CDS 614297..614725

/locus_tag="EFAGFIKM_00556"

/inference="ab initio prediction:Prodigal:002006"

/codon_start=1

/transl_table=11

/product="hypothetical protein"

/translation="MIREAEARDAAVIERLYKELLPNNLNTKVLAERIEEVRNNPNSF

LFVYEMDDQVIGTAHLHICLDALVENRPFGVVERVIITEHVQSKGYGSELMKHIEHVC

VQKNCVKVFLTSGSSRNEAHHFYTKLGYDGESSKAFKKYL"

CDS 614767..615495

/locus_tag="EFAGFIKM_00557"

/inference="ab initio prediction:Prodigal:002006"

/codon_start=1

/transl_table=11

/product="hypothetical protein"

/translation="MKDNKHPVQELQLIVTLLVATLCCLGVVMYLRAQTDTNMYRFLS

WDMFLAWVPFIISSCIRYIFNKKLTTTSMICMGLMCGVWLFFLPNAAYLFTEILHSFR

YFDAQGEVRFWVNIDFWYGLTLTFAVAIIGLLLSTCSIIQIHGMLNKLVNKYISMIVV

GVILLLSSIGVYIGRFNRWNSWDVLSRPGKIFVDLVNDFNAANSIMVEFVAIVFTVQL

FGYVILSLLTARSSSSSSLERIRV"

CDS 615618..616562

/locus_tag="EFAGFIKM_00558"

/inference="ab initio prediction:Prodigal:002006"

/codon_start=1

/transl_table=11

/product="hypothetical protein"

/translation="MSLTEWSTDILQYDLSDEYSIRKADFAEWGLYCTVYYDMRYVGF

FREEEFSSSKISAYWIYKGESKIGGVRMEPNRMYHLFFIPPFQQCFEVLKLLKHKLLQ

WSDRTKRILTFEILPDQVDLYARAGFWPVEFRCRWMQRPTDHFEIEWDSRVTVRSPKI

IESETGTRKYVNEDEIAQCDLESFAGSLEATRRKHTTLADFIPSDDPNYTNEILTQAS

TFVYDKETGQLIANCRLCLQDNQAAVYSIGVRPAYRGKGLATLMLQRALNELKGKYPV

LRLYVMQGNDAESVYLNLGFIPGVQEIQTMYIPEEESQ"

CDS 616559..617299

/locus_tag="EFAGFIKM_00559"

/inference="ab initio prediction:Prodigal:002006"

/codon_start=1

/transl_table=11

/product="hypothetical protein"

/translation="MKIAILTDIHGNVPALQAVLADIDLRGDVEHTFCLGDMIGIGPY

SNEVIEMLYSRKNMIAISGNHEEAVLSLLQNKGPLEGHKGMAEHHEWIGQHLSVESIK

RIQALPKFVEKEVEGHRFLLTHYHMSNDKLDVIDKEPSGTKLDAKYHGTGHDLVCFGH

YHILHYYKTDQRIYLNLGALGCNDLAIARYGIVDLQEGKVDVKFIGVPYDNREYLKSY

ERLNVPDRAFILQAFHGNQLEREDSERS"

CDS 617334..617483

/locus_tag="EFAGFIKM_00560"

/inference="ab initio prediction:Prodigal:002006"

/codon_start=1

/transl_table=11

/product="hypothetical protein"

/translation="MGMIIMLVVYLFLGIYVVKVGIDNSKNSQHTKEILSELREIKEL

LKERG"

CDS 617501..617959

/locus_tag="EFAGFIKM_00561"

/inference="ab initio prediction:Prodigal:002006"

/codon_start=1

/transl_table=11

/product="hypothetical protein"

/translation="MSRAPFQILVFPYIRLKDSNDYAYAIFRRSREGYWQGIAGGGED

EEKPLEAAMREAFEEAGIPLNSKYRSLNSRSTHSVVDVVGYFMWGPDTFVIPEYCFGV

ELDEMDLSLSNEHSEYKWLAYEEAREILKWDSNRVAIWELNQRLIRESSK"

CDS 617987..618514

/locus_tag="EFAGFIKM_00562"

/inference="ab initio prediction:Prodigal:002006"

/codon_start=1

/transl_table=11

/product="hypothetical protein"

/translation="MIYDLQGEANMEPIVGMLFSAVRENSERLKSITEGTSQEEVDYK

GEKNQFNSTAQLIRHIMFVDMNWVYRIKGVVLPQSLIEQYGPMTDENNRLPLVQDVSR

DTLISQYEGVLTLLKETCTQVADADLDRVVTFGHQNEKQATVRWRLWHMADHNRYHQA

HINQLRKWFQTQNDC"

CDS complement(618581..619486)

/locus_tag="EFAGFIKM_00563"

/inference="ab initio prediction:Prodigal:002006"

/codon_start=1

/transl_table=11

/product="hypothetical protein"

/translation="MIKANGKPQFLPLYEALASEVRWRIMDMIADREMNVKDIAAVLE

LSPSIVTMHIRKLEDAGLIGSRRVRINGGTHKLCYLKQNQIEIELPSASQTSRTREQT

IAVGHYTAFDIHPTCGLGTLEKEIGVWDDPRYFLDPERVHAAILWFGKGYVEYKTPNF

VLPDQTTDAIEISMELASEAPGLRDHWPSDIRFTFNGVSLGTWTSPADFGRAARGKYT

PEWWHRNVNQYGLLKTIRIDASGTYMDGERMSDITLADIKLSEPFWTLRFTVDEESPN

VGGLTIYGAGFGNHDQDIVIRVLQI"

CDS 619667..621157

/locus_tag="EFAGFIKM_00564"

/EC_number="3.2.1.55"

/inference="ab initio prediction:Prodigal:002006"

/inference="similar to AA sequence:UniProtKB:A7LXT8"

/codon_start=1

/transl_table=11

/product="Non-reducing end alpha-L-arabinofuranosidase

BoGH43A"

/db_xref="COG:COG3507"

/translation="MKYNNPVVKGFYPDPSVIKVHDTYYMVCSSFQYFPCVPLFESED

LLNWKQIGHCLTRKSQIQLETVNSSGGVFAPTIRHNDGRFYMVTTNDTTHQNFYVWTD

DIYGEWSEPIYVDQGGIDPDLYFEDGKTLFMSNGVDDEGVGGIVQCELEIENGRKLTP

SRSIWNGTGGRYLESPHLYKMNGYYYLLAAEGGTEYGHMVTYARGTSSSGPFEAYAHN

PVLTNRNLGGYELQGVGHGDLVQDDLGNWWLFHLGFRQIGRWATYHHLGREVFLTPIT

FDEDGWFTAGHEGTTLTSFETNRIPDTVIQQDKKHYTFENTDWNLDWCYLRHPNTEHY

QLELDKLTLTGTEVTLDVPASPTFIGLRQKDFNATISVDVSLRSGEAGITIYMDENHH

YEVAIRGQQDGYKVIERLNIGDIKSREHEVDLGNKQHATLVIRSSQERYSFLFQADGE

EILLGTAQTRYLSSEVAGGFTGVLIGLYASGQDTSAEFTNFKCDYH"

CDS 621256..621828

/locus_tag="EFAGFIKM_00565"

/inference="ab initio prediction:Prodigal:002006"

/codon_start=1

/transl_table=11

/product="hypothetical protein"

/translation="MEVHYKMPESLEGIVRIDKELRKEHDATLHDYVGFYISFNNDED

RYYCTPDDAIIFGRTGADGDHFAFFTFNRSISDLEEAPVIFIQPTAFGNQVTLVARNL

KDFVALFINLKEVYVLERFRFYKNKLDFMNDYNDNYMEDIRMRESDYHLIIELLKENI

KGIAEIDDVYEYIIESRKQIELGINNDDFG"

CDS 621929..622474

/gene="coaE_1"

/locus_tag="EFAGFIKM_00566"

/EC_number="2.7.1.24"

/inference="ab initio prediction:Prodigal:002006"

/inference="similar to AA sequence:UniProtKB:P9WPA3"

/codon_start=1

/transl_table=11

/product="Dephospho-CoA kinase"

/db_xref="COG:COG0237"

/translation="MEDQWRIAKYDPAWRDLFLETGSKLREALGEKAVRIDHVGSTSI

VGIDAKPIIDIQISVDNYENLSDYKREIETVGFVFRAENPDKTKRYFREEPGNRRIHI

HVRQAGSFSEQMTLLFRDYLREHPEDCLKYAEEKHRLMSLYKDQRPKYVEGKGPTVWS

ILQRAHRWSQEIGWQPAKSDA"

CDS 622515..622937

/locus_tag="EFAGFIKM_00567"

/inference="ab initio prediction:Prodigal:002006"

/codon_start=1

/transl_table=11

/product="hypothetical protein"

/translation="MNMGRKLMTTVLTIICFTLAASIISMNESYSFDFYILIYLVYAT

PLILLIGLPLSILLDYLLSRIQFPNHVLYLSTRILGYACAGVLGMYIYYLVMGAGEAI

LDFKETLVLTLFGVLAAILFVLIDMGLEVLLKKQRRKS"

CDS 622968..623489

/gene="sttH"

/locus_tag="EFAGFIKM_00568"

/EC_number="3.5.2.19"

/inference="ab initio prediction:Prodigal:002006"

/inference="similar to AA sequence:UniProtKB:Q1MW86"

/codon_start=1

/transl_table=11

/product="Streptothricin hydrolase"

/translation="MTTHCALLIIDVQVAMFDEADPVYQGERLLQKIQVLIARAREAG

HSIIYIQHSEGAGSPLERGTPGWDIHPSIAPIAGDLVIEKETPDSFHKTNLHLKLQED

GVTELILAGMQTEVCVDTTCRRAFSLGYTVNLVQDAHSTWNSKTLQAEQIIAHHNTVL

RLFANVMDTENAL"

CDS 623489..623971

/locus_tag="EFAGFIKM_00569"

/inference="ab initio prediction:Prodigal:002006"

/codon_start=1

/transl_table=11

/product="hypothetical protein"

/translation="MLGKRRRFWFFIGAAIMFLYFLITSNPDPNKPISRNQVISTEMS

VVVYTRTGDGGAHVSIDNVTLSKEDTSRIVGWLNAAPESAKIPVDDVTGSISAGIALK

LKHNAKVTIQYNRKQIIVSTKSRFNRDSKYILDQKDLRDFLDQKLEGTYFGEDTVSVE

"

CDS 623984..624262

/locus_tag="EFAGFIKM_00570"

/inference="ab initio prediction:Prodigal:002006"

/codon_start=1

/transl_table=11

/product="hypothetical protein"

/translation="MTINKRIMNLSAWLAVLAIICIPGTLHTEDGPLRTEYGFPLRFF

TDYHYANSEGTIWFISGIYLNVLLYLFNVLFIYAGIHVILYIKKKLTK"

CDS 624499..624978

/gene="yizA"

/locus_tag="EFAGFIKM_00571"

/inference="ab initio prediction:Prodigal:002006"

/inference="similar to AA sequence:UniProtKB:Q7WY73"

/codon_start=1

/transl_table=11

/product="putative protein YizA"

/db_xref="COG:COG2318"

/translation="METFFRYNWMVREQWYVWCEDVPLEELLRPRTGGVGNILQTLFH

IIDVEWSWLQLLQGKPDDAENFANYQNLEAVRRHDRKLRPDVEAFVTAWESRMEKRAY

IDHRSAGKASTDSWGEVMRHVIVHEIHHMGQLSVWARELGKQPVSANVIGKGLIIPD"

CDS 625021..625449

/locus_tag="EFAGFIKM_00572"

/inference="ab initio prediction:Prodigal:002006"

/codon_start=1

/transl_table=11

/product="hypothetical protein"

/translation="MIHCRRATIQDGEVMSQLSHQLGYVSTTEEIKERLTLLSSLPDH

YVCVAEIDSRVVGWGHVQGRHSIESPSFAEIGGLVVDGIHQGMGIGKIIMSECEEWAR

VNGFDKIRVRCNGKRESAHRFYIAIGYEEKKWQKVFDKRL"

CDS 625483..626355

/locus_tag="EFAGFIKM_00573"

/inference="ab initio prediction:Prodigal:002006"

/codon_start=1

/transl_table=11

/product="hypothetical protein"

/translation="MTIQLSQMNWVELDERIQQVMEQEYQIIPLPPGLEASVMRIEME

DQRYVLKVWDKDSKPDIAKQYRLLSKLYQSGIRVSKPYGWGLDEQENQVLLTSYNGKP

VTQLTPTKMTHLAERLMEVHRYPVNEVSTGEDEEYIFRYDFVQYFFHQIELHDDINDL

LANLIQQVQIRQDRLIHGDYYLGNILEMDDHYTIVDWTNGQYGDPRYDMAWSVFLITV

YNGESYGEMYRAQFARSASYTYTLEEEQVFEAVACLRWILLKRVGDVPMGPNVMERVH

SMAIHNPYLNEHLL"

CDS 626492..626839

/locus_tag="EFAGFIKM_00574"

/inference="ab initio prediction:Prodigal:002006"

/codon_start=1

/transl_table=11

/product="hypothetical protein"

/translation="MEQKVQTYFELKQQQKDIEQQLSVLRDEIVAYCAEQGVYETQVG

GYTVKTVLQNRKEYDDQKLYASLPDPDIWRLLSKVDSHKLKSLIKLNILSEEQINPAC

TVKQVTLLQVDKL"

CDS 627079..627894

/gene="xynB_1"

/locus_tag="EFAGFIKM_00575"

/EC_number="3.2.1.8"

/inference="ab initio prediction:Prodigal:002006"

/inference="similar to AA sequence:UniProtKB:O69231"

/codon_start=1

/transl_table=11

/product="Endo-1,4-beta-xylanase B"

/translation="MPTEIPSLHAAYANTFKIGAAVHTRMLQSEADFIAKHFNSITAE

NQMKFEEIHPEENRYDFEAADKIVDFAVAQGIGVRGHTLVWHNQTSKWVFEDTSGAPA

SRELLLSRLKQHIDAVVGRYKGQIYAWDVVNEAVEDKTDLFMRDTKWLELVGEDYLLQ

AFSMAHEADPNALLFYNDYNETDPVKREKIYNLVRSLLDKGAPVHGIGLQGHWNIHGP

SIEEIRMAIERYASLDVQLHVTELDMSVFRHEDRRTDFTAPTSEMAELQERRY"

CDS 628284..628454

/locus_tag="EFAGFIKM_00576"

/inference="ab initio prediction:Prodigal:002006"

/codon_start=1

/transl_table=11

/product="hypothetical protein"

/translation="MERTRNYLLIFAGNLVAAYYIFEEGTFAKPLMFATLMLLLIMTI

DYMKSRNKYTLE"

CDS 628620..629438

/gene="punA_1"

/locus_tag="EFAGFIKM_00577"

/EC_number="2.4.2.1"

/inference="ab initio prediction:Prodigal:002006"

/inference="similar to AA sequence:UniProtKB:P77834"

/codon_start=1

/transl_table=11

/product="Purine nucleoside phosphorylase 1"

/translation="MHQSAHIQEARDYILNRIHTKPAVGMILGSGLGALADEIENATV

IPYTDIPYFAQSEAIGHANELVIGELMGKTVVAMKGRLHYYEGFTLDEVTFPVRIMKA

LGVEQLLITNACGAINTSFEPGQLMLITDHINLVGNNPLMGPNNAELGVRFPDVSQVY

NRELRSIALKVAEEQNVGLQQGVYAWWSGPAYETPAEIRMIRTMGADAVGMSTVPEAI

VAIHGGMKVLGISCLTNMACGILDQPLSHDEVIEVAAQVKTTFIGLVKGILKEI"

CDS complement(629556..630632)

/gene="degA_1"

/locus_tag="EFAGFIKM_00578"

/inference="ab initio prediction:Prodigal:002006"

/inference="similar to AA sequence:UniProtKB:P37947"

/codon_start=1

/transl_table=11

/product="HTH-type transcriptional regulator DegA"

/db_xref="COG:COG1609"

/translation="MAKEKVTIQDIADALGISRNTASKALNDSGNIPDETRNRVIKKA

IELKYKQFAYMDNEHVLTKAPGNIALLTENLPNTSHFGSLLISGLEKRISAEGYNLSI

HIVREDDQDTLTLPNNFDIAKVDGIICIELFDLEYTQLITDLGIPTIFIDCASNICYP

EFKADLLLMENEHSIYQLTTKLIESGYTSIGFVGDYNHCKSFNERWVGYQRAMLEAGL

QVDLSHCIVDNDRLCFSKPGWLNQRVAELASLPSAYVCANDFIAVDLIRALKDRNVAV

PQDIAICGFDNAPQSRIIEPALTTVHIYSNEMGIKTAEMLLSRINSPTQPYQVSHIVT

KPIIRESTPAIMADHSQMVHSSAK"

CDS 631068..632033

/gene="yteP_4"

/locus_tag="EFAGFIKM_00579"

/inference="ab initio prediction:Prodigal:002006"

/inference="similar to AA sequence:UniProtKB:C0SPB3"

/codon_start=1

/transl_table=11

/product="putative multiple-sugar transport system
permease YteP"
/db_xref="COG:COG4209"
/translation="MDNIADSKKGEGRVLKVRTTSGHRLQQLKKNYFLYMLLAPALIL
TLIFKYIPMYGAIIAFKDFSPIKGIMGSDWVGLKHFEKFIASPNFDIILMNTLKLSFL
GLIFSFPVPILLALMLNQVRKAGIKKNIQLFLYAPNFISVVVVVGMLFIFLSPTGPVN
QLATWITGQPIMFMSEPEYFRWIYILSDIWTGAGWASIIYVAALANVDPELHNAANLD
GANLLQRIRHIDLPTIRPIMAIVFILAAGGIMSIGFEKAYLMQTSMNLPSSEIIATYV
YKVGLQSGDYAYSAAVGLFNSVINVILLVTVNLIVKKLNEGEGLY"
CDS 632050..632931
/gene="araQ_4"
/locus_tag="EFAGFIKM_00580"
/inference="ab initio prediction:Prodigal:002006"
/inference="similar to AA sequence:UniProtKB:P94530"
/codon_start=1
/transl_table=11
/product="L-arabinose transport system permease protein
AraQ"
/db_xref="COG:COG0395"
/translation="MVVKHTGMDRLILTLNAIFLTCAVLVVVVPLIYIVIASFMDPTV
LLNRGLSFNVSDWSLDGYQMILSNPAMIRGFANAVLYSVSFALITVTVSIFAGYALSD
DRLAGRGFFMIIFIITMFFGGGLIPTYLLIRNLGMLDTVWAIIIPGAVNVWNIILSRT
FFKGVPRELKEAANVDGASEMKIFFQIVIPLSKPIIFVLALYAFVGQWNSYFDAMIYL
DNPNLHPLQLVLRSILIQNQAAPGMISDQLAMAELKRLSEMIKYSAIVISSLPLIIMY
PFFQKYFEKGVMVGSLK"
CDS 632987..634594
/locus_tag="EFAGFIKM_00581"
/inference="ab initio prediction:Prodigal:002006"
/codon_start=1
/transl_table=11
/product="hypothetical protein"
/translation="MKKVNKSRKAVTTASISMLSAMIFLTACGGGGGGAASEAQSPDG

KVTLNFITQSSPLAPADPNDKLINKRLEEKTNVHINWKNYTSDVFAEKRNLAVASGDL
PDAIFDAGYGDYDLLKLAKDGAIIPLEDMIEQHMPNLKKVLEEAPEYKSMITAPDGHI
YSFPWIEELGSGKQRIQSVDNLPWINVEWLNKLGLKMPTTTEELKEVLIAFKTQDPNG
NGKADEIPLSFINKPGGEDLTFLFAAFGLGENWDHTVVTNDGKVVFTAADEGYKEAVK
YIHELVKEGLVDVESYQQDWNTYLAKGKDNKYGMYFTWDKANITGMNDTYDVLPPVAG
PNGEVNVTRTNGIGLDRGRMVITSSNKNLESTAKWVDQLYDPLQSVQNNWGTYGDESQ
QNIFEFDEAKGMLKHLPLEGSAPVELRQKTSIAGPLAILDSYYDKYTTKPEDAAWRME
LLDKVMVPHMKAENVYPSVFFSIDELDRLSTIETDLFAYVLRLRTEWYQNGKIDQEWD
AYLKELDRLGLQEWLQIKQAGYDRNNK"

CDS 634620..636092

/gene="sacC_1"

/locus_tag="EFAGFIKM_00582"

/EC_number="3.2.1.80"

/inference="ab initio prediction:Prodigal:002006"

/inference="similar to AA sequence:UniProtKB:P05656"

/codon_start=1

/transl_table=11

/product="Levanase"

/db_xref="COG:COG1621"

/translation="MSTILKQDYRNEYHFSPKEKWMNDPNGMVFFNGEYHLFYQYHPF
GTTWGPMHWGHAVTRDLVTWEELPVALAPDEHGMIFSGSAVVDWNNTSGFFEDEPGLV
AIFTHHLEVPNEHPIQRQSLAYSKDNGRTWTKYEGNPVLTHESFVDFRDPKVFWHEQT
KEWIMIIACGQTVCLYRSPNLKDWTLGSEFGEGIGSHDGVWECPDLFPLAVDGDSGQV
KWVMLVSIGADPAFKEGSRTQYFTGDFDGSTFVPDEASHTIRWIDHGRDNYAGVSWSD
IPAEDGRRLFMGWMSNWMYANQTPTNDYRGAMTIARELSLETRSGEVILIQRPARELE
QARTPVLSLQDASIQQVSEQLNALQLVNYEIHAEWTPNQSFHFALRSGADNETLVGVD
ANRGEVYIDRSGSGIGDFHEHFLSRHAAELKDVNANQSLRIFVDQSSVEVFANDGQAV
ITDLIYPDVDSKGISAHSENTDLVFSSLHIYEISPTKAKG"

CDS 636121..637008

/gene="gmuE_1"

/locus_tag="EFAGFIKM_00583"

/EC_number="2.7.1.4"

/inference="ab initio prediction:Prodigal:002006"

/inference="similar to AA sequence:UniProtKB:O05510"

/codon_start=1

/transl_table=11

/product="Putative fructokinase"

/db_xref="COG:COG1940"

/translation="MRIGAIEAGGTKFVCGVGNEHGQIEDQISFPTEHPETTLAKVIE

YFRDKDVEALGIGSFGPIDLQPDSPTYGYITTTPKPGWENCNVVGTLKHEFPVPFGWD

TDVNAAALGEVTWGAAQGLDNCVYYTIGTGVGVGLVAGGQRVHGLLHPEGGHIRTRRH

PDDHFAGLCPYHGDCLEGMAAGPAIEARWQIPGSELPADHPAWEIESFYIAESITTAI

LLHSPQKIILGGGVMQQDHLFPMIREQVVRNLNGYVNAAPITQHIDQYIVQPGLGQHA

GLCGALALGLEALQHAAQA"

CDS 637263..637700

/locus_tag="EFAGFIKM_00584"

/inference="ab initio prediction:Prodigal:002006"

/inference="similar to AA sequence:ISfinder:ISPaen4"

/codon_start=1

/transl_table=11

/product="IS1595 family transposase ISPaen4"

/translation="MNPITMRQAVQADQEQLADLRALVLYDDLTRLGRYDDVKVRERF

RNTFNPAQTQIIEVEGSMVGCVALKPKSEAYLLEHFYIHPDYQGQRIGTQVLNMLLEQ

DEVQGKRIILNVLQGSPARRLYERFGFILDSEDEVDVFMSKHL"

CDS 637976..638257

/locus_tag="EFAGFIKM_00585"

/inference="ab initio prediction:Prodigal:002006"

/codon_start=1

/transl_table=11

/product="hypothetical protein"

/translation="MQEYPVAYDQFDVHFYQVVEMFSEQKLDVSALDEDLRVLGLNSI

SFIKLIIALENEFNIEMDDEYLELENFTTLQHLKDSLRKCINYEIPNES"

CDS 638350..639546

/gene="acrC"

/locus_tag="EFAGFIKM_00586"

/EC_number="1.3.1.95"

/inference="ab initio prediction:Prodigal:002006"

/inference="similar to AA sequence:UniProtKB:G3KIM8"

/codon_start=1

/transl_table=11

/product="Acryloyl-CoA reductase (NADH)"

/translation="MKVELNAEQLMWQEQFKDFVDSEIIPYASLNDSEERIHPELLAK

ITEAGYLGSMLPKEYGGMELDNITIGILNEEVGRGCSSVRSLLTVQGMVGLAILRWGT

EQQRQYWLPALATGTTLGSFGLTEPSVGSDAKSIETTAVLDGDEYILNGHKKWITMGQ

LADVFLILAQCENKPTAFIVERDSIGFSVEPMSGLLGARASMIAELKMDSCRIPKENL

LGQVGTGLSHVALPCLDYGRYTIACGCVGLAKACLDASVHYANSRIQFGRAIRENQMI

QKMITEMSVNMKAARMLCYRAGYLRDVGDPESIMETWTAKYFASTMVNKVASDAVQIH

GANGCHRDYPVERYYRDARINEIIEGTTQMHEILIATQEVVTHRREMRRSNKAEKVKD

SANRNV"

CDS 639690..640496

/locus_tag="EFAGFIKM_00587"

/inference="ab initio prediction:Prodigal:002006"

/codon_start=1

/transl_table=11

/product="hypothetical protein"

/translation="MKRIGQGQTAEIFRHGETSILKLFREDFSAEAIQHEFDNTRIVL

ELGVKCPQTIDMVTINNRKGIIFELASGRTLLEEMIIKPWSIERRARQMAALHHQIHQ

QSGERIQQKHKTVLEKNISNASDLSDMEKQQIMDYVHSLPEGGSLCHGDFHPNNVITG

KESWIIDWMTGASGCPAADVARTLVLLKYGNLPKGAPLMMKVLLKIIKSRMSKAYIEY

YLSLSGMDIREIHRWVLPIAAARLTEWIPPEEKKDLLDYIRNQLKKFNTT"

CDS 640571..641377

/gene="rhaR_3"

/locus_tag="EFAGFIKM_00588"

/inference="ab initio prediction:Prodigal:002006"

/inference="protein motif:HAMAP:MF_01533"

/codon_start=1

/transl_table=11

/product="HTH-type transcriptional activator RhaR"

/translation="MDFQLRSVVSYDMVVREKKHLFDLSLRRQIAIFEVMDALEISVF

SKTVQVKAGAVLLANKIRLQNYDKPVLKMRGIIFGSDLLERLPSCHILSENGSKHYDL

AVSLLNRTVESREDIEIAERDFLEVYSSYYQTNLTNLMRPETDREDSVKRNLVMICKY

IQNNYEKPITLQFLADMVGYNPVYLCNLFSKVFNVSPLKYLQQIRVDKAQEYVTKTDL

PISEITTKLGYSSSTQFSAMYKKKVGKSPTEHRNQFRSNKGDLPLSIQST"

CDS 641466..642173

/gene="lgrE"

/locus_tag="EFAGFIKM_00589"

/EC_number="1.1.-.-"

/inference="ab initio prediction:Prodigal:002006"

/inference="similar to AA sequence:UniProtKB:Q70LM8"

/codon_start=1

/transl_table=11

/product="Linear gramicidin dehydrogenase LgrE"

/translation="MKKIQLFCLPHAGGSAMIFHRWKSSLSPFIDVIPIELKGRGARV

GEPFYESFEEAIEDIYPTVSSLIHGPYAIFGHSMGSWMALELYYRLAQSTKQLPEHMM

LSGNRAPHIFKDENIHALPDEEFRETIQNMGGTSDEVFTNKELFSLFAPVLRADFRIV

ELYRFQPKPFKVQSDITVLTGRSDTRVKSSDLIGWKKYAGSQCDIVKLDGGHFYIQEN

IQETTRIINEKLQPYVI"

CDS 642197..644761

/gene="menE_1"

/locus_tag="EFAGFIKM_00590"

/EC_number="6.2.1.26"

/inference="ab initio prediction:Prodigal:002006"

/inference="protein motif:HAMAP:MF_00731"

/codon_start=1

/transl_table=11

/product="2-succinylbenzoate--CoA ligase"

/translation="MSMDNLAQLFNSLGSVTDRGITFLNGSKEEKVVSYQSLLQSAKI

RLGELQAFGMQPGNELIFQLNSEEEFIVTFWACTLGKIIPVPITVGGNQEQRMKLYNV

WRTLNHPFIAADQDLTESLDKFARKNGLEEVVQEMHARIFIVKDISNLREEPIVEGIE
ASIQSDDIAFIQFSSGSTGQTKGVVITHRNVMINIQAMNIATQISSNDRSLSWLPLTH
DMGLIAFHLTSTFQGLQQFIMPTSLFIRHPTLWLTKTSEHRVTQLYAPNFAYKYFLDS
YNPLTFSSTDLSTVRFIMNGAEPISPALCFKFLEAMSPYGLASNTMLTAYGLAEATVG
VSFGNVGDLTCYVLDRRYLETGKPFIEVEPGSEHAVSFVEVGKPVQYCDVRICDDYDK
PVEELVLGSIQIKGLSVTSGYYNNAAATEKASTVDGWIRTGDIGFMNDGAIVITGRTK
DIIFINGQNVYPHDIERVTEELEQFDLGKVAVCGVSNPLTGSESVVMFVLYKKDLPSF
IPRVGEAKAHIQQRMGIELKSVLPIKQIPKTTSGKFQRYRLQERYESGEFSEMEQSIQ
SMLSHMQEIKEVQPARDHIEQKLIEIVESVAGLRNVGVTDNLAEAGFDSLKVTQIHQA
IDDAFPAKMAISSLYSHTSIVSWANLIRQDRVELEPVRMDRSFFHTDRGYEALSYQFT
LPASLVQDMRLIAQSEAISIHVLASAMYAYLLHTLSEQPFIDMHVYYGTARMITPVRL
NFDQYKTMNELFQNVHQQIITMPSDRAFPVEQMSQIRISSTEWAINPLYGEQHLFKAS
DDILNYYDLIILVSDEGDILQCNCRFNGRKLKQSRVKRLIANYVRGLQLLVQPELK"

CDS 644782..648633

/gene="pksM"

/locus_tag="EFAGFIKM_00591"

/inference="ab initio prediction:Prodigal:002006"

/inference="similar to AA sequence:UniProtKB:P40872"

/codon_start=1

/transl_table=11

/product="Polyketide synthase PksM"

/translation="MLDQLFDREQWEEEDDQDTTNELDITTDNRHDIAVIGIGAKLPT
GESLDEFWKMLDYGIDCIRELPNRRKGQLDQYLLHKQGSLEHVRYLNGAYLDEIDEFD
YSFFRMSAQEAKLMSPVQRLFLQTVWRAVEDAGYGGDRLAGSSTGVYVGVIGDQEGYK
YKQMVEELSPDMLPIATVGNLSSIIPARIAYLLNLTGPAMVVDTACSSSLVALDMAVR
ALREGSCDMAIAGGIRLNMIPLDKEYYKIGIESSDGTTRTFDEDADGSGMGEGVVSVV
LKPVEQALRDHDHVYAVIKGTAVNQNGYSAGITTPNAVAHEKVLTHAWRDAGIHPESL
SYLEAHGTGTLIGDPIELEGIQSAFRRFTDKKQFCAIGSVKSNVGHLYDSAGLAGLLK
AIMALRSKRIPSTLHFNKPNPKFEFDKSPVYVNVISREWEAATPRRCGVSSFGLSGTN
CHVVLEEAPNISVELGRDDHYPFLISSVSSGNLSRQVQSYVKWINENPKADVRDVCCT
AATGRQHHAHRAVWVVSSLDQLRQHLNDFLAEPKTGKYEQDTFRKSNQIFTTEELISK
WHIEGCDRAPVLEQLCTHYQGGAQINWTRIYPDVTYNKLSLPTYSFEESKCWIDTNPD

NSVQSEEEAMYYSLSWKPWSEIGKTNSSIIHNVERVLLIRNDTQLHATQLVNQIREAG
VAVIEVILGTSYLQQDAQTYVIRNNESDYIKLLQEVSFGGPYRIVHIASLDHLENKEN
RLDKGLMSVWRLTKACSHVIAEPVEMILVTSFANDVTGDEKVLYPENSAMLAFGNTVQ
REIQNLECFCIDVDQDWDTSQYLRYILGPLEKKSLAIRNGQAYQAEFAPADVGSMPDA
EMNWKEDGVYIITGGLGGLGMEIAERLLSFGRMNLALIARTPLPKRHNWDIILNAKNT
SPDLKSKLLRLLDMEKRAGQMMYLGADVADADQLSQAIEQLRIRFGCIRGIIHAAGVG
STQQADQMTEEEFTAMLAPKVKGTPLLDQLTADDQPDFLVMFSSVATLFGSTGQAAYA
AANAYQDAYAPMRNRKGMRTITLNWTTWKEVGMASRAGFDFDTLFKAMPKEWALDRLF
EIMGKQTDRLLVGEMNWNSPYLNILNNYHFHLDANIGQRLQKLMRMPERAKQISMKKE
SASTVQIPTEEYELELESSVEMDRTIANIVGQYLGIKEMSVHDSFFELGADSIMIKQI
FLKLDKTYPGKLILTDLFEYPSISRLTTYMSTTEKQKPHQSPQKETFEEERDTDLENL
FDEIERGAMDLDEAIINLQKM"

CDS 648647..660589

/gene="dltA_1"

/locus_tag="EFAGFIKM_00592"

/EC_number="6.2.1.54"

/inference="ab initio prediction:Prodigal:002006"

/inference="protein motif:HAMAP:MF_00593"

/codon_start=1

/transl_table=11

/product="D-alanine--D-alanyl carrier protein ligase"

/translation="MEKILKLLVESVKDQKMNKTTAAQIVKMLKEGEKKPVKDIAIIG
ISAKLPNMETLDDYWDNIQSGVDCIRPIPKTRKSDMDAILSYMDIPEGDIQYYEAAYL
DNIDQFDHEFFRLPPKEASLTDVNQRLFMETAWDAMEDAGYGNDKLSGTNTGVYVGFS
NNIKDMYMRYITETMQTSALSIVGNLASILSNRISYLLNLKGPSIVVDTACSSSLVAV
AMACQAIRNKECDMAIAGGVKLHLMTRENQNEKIGMESPDYRTKTFDDSANGAGMGEG
VTALLLKPLDAAMRDGDQIYAVIKGLAVNQDGSSGGLTVPNPSTQAELIVKAWEDGNI
DPETISYFEPHGTGTELGDPIEIKGIQNAFKKYTSKKQFCAIGSVKTNIGHLYEAAGT
AGLIKAILALKNKKLPPTLNFEKPNRAIDFSGSPVYYVNTTLREWTTDGTTPRRCGISS
FGIGGTNCHLVLEEAPDRATAEHKANTSVTGPSILTLSAKSEESLRQMISKYIYFLEK
NELPTLQDICYTANTGRGKFPYRVAIIMNSNIDLHQKLTQLERSKQLFGTSHGLHIYT
GFHKLVQDEEAADQEFRMSLSSRHELSVSLGQALGEFMNSGQQDINALHQAASFYVSG

GDADWEALYANQNVLKVSLPSYAFQTHRCWIDIPKRNRRSEKVQAEGMFYQAIWQRYT

PVSRDSVDSKMKGTVLLFRGNDQVSVALTVQLEREPEARVIQVSWGDRYEKYDDSHYV

IRGDESDYIQLLNDMKDQGLTRIIHGFTLAENDSDDYLQSEEQLKLSQDRGIQSAFYL

TRAILHHHMTEGLQLQFISSLANAVTGYESQVRPENATLNGFAKVIEQEYPEISCRFI

DVDSDTDADTIYAELNSSYDRFQVAYRHNQSYVEELDIANILEASHEEVTIRENGLYL

IAGGTGGIGLQFAKHLADERCIRLALISRTAMPDRTVWDKLIQEEDPKIISMEMIERM

KTIMELEAGGSSVELISADISDLEQMTQVVQRLRTKYGQIYGVINSAGVANDGLIIRK

EETAFQEVLRPKVQGTWVLNQVTAQEPLDFFVMFSSGISMLGEPGQSDYVAANCFLDS

FASYRAKGSGRTLTINWPAWKETGMAARFGLNVDTAFKTLATHQAISAFDMVLSRRIR

RVLIGEPNYASELLPLLESSSVIQFSEAMTARIQQERNHLNYHNNHLTRIEYEQKVEL

HGRDNGIYTTTEKELAQLVGHVLGFTGLDVYDNLFELGVDSISMMRIITEIRVRYHVH

LNYHTFFEGMNLAKLAIHLIGALKETGAISEYPQQEPEPATLYEDFPLTEVQMAYLLG

RDERFEMGGTSTHYYLELETKLNMAHFQTALQKVIQRHPMLRAVILPEGIQRILPQVP

EYQLEIVDVCHLNEQEKWKHIEVERDRMSHHVFEPSQWPLFEFKAFKIDDTRHYLLFG

YDLLICDSASIQILGEDLVHYCREPEKELPALKFSFKDYMMAYGELKKGEMYARDKKY

WMDQLDTFPSAPALPLKQEPRHIKKPQFSRLSWILEQDPWEQLKHKAQQKNVTPSALL

CTAFARVLARWSNQDRLAINLAAFNRFPFHEETNQIIGDFTSIMLLEINEQKNKPFWE

QARDVQSGMLNMLEHRHYDGVEFIRELARHREAGTSAIMPIVYTSTLFVDAIGSWQHI

GDLKMGISQTSQVYLDNQVMETDGRLTINWDYIQDLFHYDTLSAMFDDYTAMITHLCE

QENIEFLPGLPEQDNAIWNAFNATAQSFPISTLHGSFMEQATKTPYQTALIYGNQQLT

YQQLDERSNQLAHYLTQQGITRGDRVGLLAKRGMETIVNTIGILKAGAVYVPVDPDYP

EDRQQYILQNSNCKMLLLPELYTFEGLAELPVEPLENNAAPDDLAYIIYTSGSTGTPK

GVATAHGPAMNTIRDINMKFEVTDQDRMLGISSMCFDLSVYDIFGALATGAALVIIAD

QRDIHDMYDAIKQHKVTIWNSVPAIMQLMADYVTDQTNEQLRLILLSGDWIPKKLPEQ

VHGRFPSAQVISLGGATEASIWSIYYPIDTVKSDWKSIPYGRPLANQTFYTLDYTGCL

SPVDVIGELCIGGAGLAQGYFNDPVRTDNAFFNHPELGRLYRTGDFGVLRREGYIEFL

GRRDSQVKIRGYRVELGEIESRLSTVPSVKQAVVIDKVDMQGTKVLVAYYSAERSYDE

LEMKRLLAKALPDYMVPQHLIQLPEFPLTPNGKIDRKALPEPTFGQQSSALAGTESPT

EQKLIELCESVFGMNGIGTHDSFFQMGINSILMVQLVNRIGQEFNQRLSFKEFMALRD

IYELAALLESSVENEESVGTIHYPQHDPCPDPEGRPFPLTEVQMAYVAGRSQAFEMGG

TSTHGYLEFETQLEMTRFNEALIKLIEHHPMLRAIILPTGEQRILPHVPVYRMDIEDI

SGLSPDEMERVIIAERERMSHHIFELEQWPLFEIRAKKISKDTHLLLFGYDLLIADGA

SIQIFNNDLLKFYHHPEINPEPNPFTFRDYILAYNEFRKSNTYLADKQYWLARVEEFP
AAPALPLKTDPAQISEPKFKRLNEVFEPQRWEILKKVANGHGLTPSALLCAAYAQILS
YWSNQQKIALNLTVFNRYPFHPSIQDIIGDFTSMILIDVDMASGNDFMGHVRHVQDSI
LEALEHRHYEGIEFIRDLSKHHQMGNKTVMPIVFTSMIFNERETSEATQGLIGKVRTG
VSQTSQVYLDHQASDAGGQLTLTWDYVDDLFDQQVVETMFAQYIALLDGLIVGETITG
PDIPSVDRSILERYNQSEENIMPTTLQHLFDRMAKHYPDQDALLFEDERMTYSELDRR
SGQLAGYLFEQEIGRGDRVGVVARRRMETIVNVMAILRTGAAYVPLDPDYPEDRQAYM
LKNSDCQIVLDVDAYDQVSASGYPLLEGELSGTPDDIAYVIYTSGSTGRPKGVAITHG
AVTNTILDMNRKFGVHEKDRMLGVSSMCFDLSVYDVFGALSSGAALVIVSDQRDIRMM
KEAVDRHQVTIWNSVPAIMDMFMDRLESDAGRSYRWASDEEIQASTQLAVEKESVESV
YHWSFSTLWRMDGKHIYVDEFKCPAYATGMFPELYFLAQKGIRQSEIASFFPNVPVSE
VNSLMELLIRKRALVSGLMEPAEIFKKQTQLFRNPYDEKVAYDAQAYEAFKKIQLHRN
FGENTTLEVSLERGVEYPDFISKRRSHRSFKEDQPVTMKQFSDLMGVLGQYDQGQEIR
YYYPSAGGLYPIDVFVYVKEGRVAGMEAGLYYFSPVDYTVRLVHGNQSFTDEMHYYTN
RSIYQSSAFTVFFIYNAEATMPKYGAQGYLYSLIDTGIMVSTLNSVGEMLGIGMCSIG
DMPFEKVKPLFNLNENQIWLHTVEAGLKPDDEMASPEEWSSRNYIRQSPSVSVSYGEN
PMAADPSLLSERSVAVLGAQTAPLANLNIQSSDIERAVNITNEGNHQSSLRLILLSGD
WIPLSLPGKVRNLCPDASIVSLGGATEASIWSIYYPINSISDAWNSIPYGYPLANQTF
HVLDYKLRPCPVDVPGEMFIGGVGLANQYVNDLDKTEKAFIQHPLLGRLYRTGDHGVM
RKEGYIEFLGRKDNQIKIRGHRVELGEIEAIFIQHHALKRVYVMDRQNEQKKKYLCAY
YVSDQEVSIAELREFLISRLPEYMVPSFFIRLEAIPLTANGKINRNALPEPTALHTAQ
VQYKAPANDIEKLLVQIWSEVLGNAHIGVNQHFFEIGGDSVLMIKAHARLDDQYPGIV
KITDMFNLPTISDITAFIENQLDEMDVIHVSRSRIPLSMDYFVEGDSTSNETEELQFA
LSAELYDRVVNATSRWDVEVDDILFAGFAYLLAQTSNSDRISIHAAIGQSDYIQVFDL
DFGSLEQFNELFQLIHQSKGQDGRENFHTKYVSTLLQEPGTGTLPLFYNAGAVASGVP
MSELYDLTLQVVHQEDSLHFVCGFRSMKWRKSKVESLLQLYFKLLNLILE"

CDS 660618..668363

/locus_tag="EFAGFIKM_00593"

/inference="ab initio prediction:Prodigal:002006"

/codon_start=1

/transl_table=11

/product="hypothetical protein"

/translation="MRKGLMGSIKLDDLLESGAESVDIESVSTKDIAIIGMSGRFPGA
EELEAYWSNLVNGVDSISDFPAERQSFTDYYMKTRGESVAYTKAGFLNHIDRFDYRFF
NLSPREASLMDPNQRLFLETAWHAIENSGYGGGKLKGSNTGVYVGFRNDEVYDYKRLI
TDLEPQADPSAVPGNLASILASRISYLLDLKGPSLCLDTACSSSLVAIHLASQAIRNG
DCDLAVAGGVKIRLFPLDEGNKLGVEAPDGKVKAFDADSDGTVWGEGAAAIILKPLNK
ALRDRDHIYAVIKGSAVNQDGTSVGITAPNTASQADLLVKAWKNAGINPETLNYIETH
GTGTDMGDPIEFEAIRRAFQKFTNKKQFCAIGSVKTNVGHLDCVAGMASLFKVIMGMR
EGMMPPTLHFQRQNSKINFEDSPVYLNTRPRIWHAEGAPRRCGVSAFGLSGTNSHIVV
EEPPRYHRAVTAVTPGMQILTISAKSAASLTKLVQAYQALLVSDPELSLANLSFSANT
GRGHYTQRLAITYSDRKDLERKLKLVSDANGALDLEINGVHTGIHRIITAGAGESTRS
TGEITEQEVRLLGQKAQAWLSEPHLHPREVLLEAICDLYVQGADVSWEDLYREKSVRR
IPLPSYQFEPNICWLDWKSDMDQRQTDDMYRSLYRTRWIADLLEPSIPDEEQQPIDDG
ITLILRKDKDLEGERLETTLRNLGRKLVSVTFDGDVAVMEGLSGLVQLSSEDVYDHLL
GALPFNDITRVIHLLSSTPRMQVHNLADLSQAEIEGPLSLFRLVKSLVQRAVSGPVDI
SIATTTAYQVTGDEVELYPEHAALIGWAKVIGKEYPHIRCQCIDRDEHSSLESIPELV
SGAGISNENFAYRNGIRYVEQLDEWNTETQENRDWQLVEDGVYIITGGAGGIGSEIGR
WMAGQAKVRIVLLGRTELPNREQWPLLRKKGKDQTAVQQIIREIDAMESLGAMVTYFS
VDVSDEMRLTHTLDVIRKTFGSIHGIVHCAGTGSDLSTDQKSEDDVKRTLAPKIRGTW
LLDQLTRQDQPDFMLLCSSIATTFSTLTMGDYVAANAYLDTFAAQRTKQGLHTLAINW
VTWRERGMAARSGFTADTIFKAILPEQAIHTLADVVTQKVDRVIIGELNIDGGGIRLL
ERSGIRVAPHLLERLKRMKSTTGSIAEMASTRSLSQHGTGQVILMGKEKEDYTEAERR
VADVCRQVLGFEEINIHENFFELGADSLLLKTMHARLELLDPGSLMITDLFEHATVYK
LARFLVDKRQGSNNMTHHTPMKGTEQDIAIIGISVKLPNANNTDEFWDNIRNGLDCRT
ALSAERKDAIDRYIRYKQFPEGSVALVEGSYIDEIDKFDSTFFRLSPKEASLMDPHQR
LFLQACWEAVEDAGCVENISGSYTGVFVGYSPNIRDMYSRLIYETNPTLLSSAMVGNT
AAVTSGRVSYMLDLKGPSMVVDTACSSSLVAVDAACQSIRSGKCEMAIAGGIKIHTMP
IHHTNTRMGIGMESMDGITRAFDEESEGTGFGEGIGVIMLKPLEKAKQDGDQIYAVIK
GSASNQDGSSAGMTAPNPAAQEDVIRKAWDDAGIDPATIGYVEAHGTGTPLGDPIEVN
SLTNAFRMFTDKRNFCAIGSVKANMGHLLEAAGIVGLIKAALALKNKELPATIHFNRP
NENIPFYDSAIYVNTVTRKWEVEGHPRRAGVSAFGMSGTNAHIVLEEPPADYTHKELP
DHVPHGGPQLFMLSAKSRESLQQLVTRYVGYGPQLLQMNLQDICYTVQAGRSHYPHRL
IFAVNTTMELVSRLNELNRLYFWESELGDTGKVFYRYAEHRLVSGTDGIRRPDDLTLV

QKESLNAEIQEVALAWRNGLYRDESMQIKISELYSAGADLPPLLWNDGQAPRKVRLPV

YAFERKTHWLDIPDVAPVKVERMEQPFHVIRWVNTPSNDGTSWMSSESGDVLLFTGKT

ALSASIYERLQAAGRKVVRVEIGLQKDSTPGDGWYGVDSNKQAYEDLLTDLQSEYQFT

HIIHMSTTEQAVFPESIEQLEVTQQRGARSLMYLTQALAHVGYERGLSLTLVSDYVNE

VTGNERVLQPQQAPFMGMGRVVGQELMGISCKFLDVDDVVSAEDVLQEIGRKSDLYLV

AYREGTRYEEQFETLNDTTVIGHSPAMQNDGVYVITGGTKGIGLEVAQYLADKGAGHV

ALLARSPFPPRDQWTEWLDQNRDREVCETINRVMDLEKKGTSCSFYQVDIGHLDRLRE

VFDELRQNYGPIRGVVHSAGVPGEGMLMRKTEEQFSAVIGPKVHGTWALDQVTLEDNL

DFFLLFSTISSVFAAPGQGDYAAANAYLDAFAAYRSKLGKRTLTINWNTWRDTGMGVR

YGINVDGVFSAIPTEQGIAWLDRAMGMTLNRVIVGEINRESKLLGMMSSVAVRLSDDL

RSLATNEKTVFPETQNQTGYAEVTLDGRTGEGAYSDAEDLIAKIFGSVLGFSEINIYD

SFFELGGDSIMLGQMHELLDRHFPNKIKLVDLFEYTTVDKLSNYIAGNEQHTLQAAPI

TSASEEQDGDWNDSLSHMLDKLQDGEVSVEDILHRLDEIRPGE"

CDS 668368..677334

/locus_tag="EFAGFIKM_00594"

/inference="ab initio prediction:Prodigal:002006"

/codon_start=1

/transl_table=11

/product="hypothetical protein"

/translation="MEKVYKHVVENIASGKIDKVTGIQVINLLKQEEANHQEDIAVIG

MSLKFPHANRPEEYWHIVEQGIDCIGDYPSDRKSVTDEYLRLSGYSDEFLQYLDGAFL

DEVDQFDYKFFRLSPKEASLMDPCHRLFLQTAWHAIEDAGYGGQRLAGSNTGVFTGFA

NSNNYKEMIKDTNPEELSIAMTGNIASMLPTRISYLLNLKGPTMVVDTACSSSLVSVY

LACKSLMNGDCNMAIAGGVKLDILPVDNDFLKIGIESTDYRTRAFDKEADGSGMGEGV

AAVILKPLRQAQKDGDHIYGVIKGIALNQDGTSMGITAPNPVAQSEVIEEAWKRAGIH

PESLAYIETHGTGTNLGDPIEIQGLERAFRKYTNKKQFCAIGSVKTNVGHLYETAGMA

SLIKGLLSLYHRKLPPSLHFNYPNLNIDFGNSPVYVNTRLREWERGADPRRCGISTFG

LSGTNCHMVLEEAPVQATLTVTHDGHTGVHPFTLSAGSDHGIQVLMKQYLDGVSTFPD

RYSLLDVCRTANACRGHYAHRVIILAEHLEDLRIKLTQVISGDIHTFTAPWFHYGEHR

LISDNREVAGVLDITQKNVRALSEEVRQELNQVNEATFDWGRLCTLYVQGASVDWWRL

YEGKSTKTVPLAGYPFDSQKCWIDMGSRPAEESVHHDNGLFSLTWHELEGDYEDNEVH

SSERVVLFTADDKGGADLALFLQQSGKDVIKVCPGDYYEQRNDTDFRIGSKEGDFDSL

VNALDSKSWSQVIYAPTFASDTSVQNLVELNESQERGVYSLLYLVRSLTRHGIDYDMD

MVILTQCVHHVTGKEQVLRPELAPLEGLAKVIRKEHLNIQCRVIDLEDMLWNKGSAES

VLRKTDHFLTAIRDGKRYIQSFGTLQPVAESDSNNGQFPALHSEGIYLVSGGAGGIGI

EVSKYLAGRDKVHLAWINRTAFPPRRTWEAILEAQQDQSLCRKIQGVLDIENMGATVE

IYSADISKKEEVVPIVEELRQRHGKIRGVIHGAGIGRSDLLMNRTDDQFKSIFNPKVM

GTRILDEVTHSDDLDFFLLFSSVATMFSGAVQGDYTAANAYLDAYGEYRNKLGRRTHV

VNWTTWKETGMALEGGFTIDTIFKTLPTGKALEGFEQVLSHNVPRALVGYLNYENGGV

FLLEKSGVMLSPVLTAQIELFKEKKKQEKANKAKSVTTNPAGEVSLTGSLNEEFSDTE

KRVAECCRKILGFDQIDIHDNFFELGADSILLMKIQSEIERIFPGAINVTDLFEYSNV

HHLAKFIGGKNKKEEQRPSPAMHLKKRTAAERDGDIAIIGMAVNVPLASTPEELWAHL

VNGTDLVRSFPESRKVDIDKHFAFTGHDQTQMKYNENAYLEDIDKFDAGFFRLSPKEA

SLMDPNQRLFLEAAWTAIEDAGYGGTRLSGSKTGVYLGYAATLRDLYARLLYEVDPGS

GSSSMVGNLTAMMSARISYLLDLKGPSMVIDTACSSSLVSVDMACKAIRSGQCDYALA

GGIKINLCPVDDENMKLGIESTDHRSKAFDDEADGAGIGEGVAVVLLKSLQQALDDHD

SIYAVIKGGAVNQDGSSIGITAPNPASQTEVIISALEDAGVEPETITYIETHGTGTTL

GDPIEIKGISDALSSYTDRKQFCAVSSIKTNLGHSSEAAGVISLVKAVMALREKKLPP

TLYFNRPNRMIEFSNSPVFVNTRLRTWHSEEGPRRCGISAFGISGTNCHVILEEAPVQ

TFVQAEERRPYILPLSAKSKDSLSRLIATYHDYLDSYDELDLLSLCGTAAQGRGHYKL

RIAIPFKDKEELVSRLDKLHGCPFSEIKPPVAYASEHRMVPETKEPGVGELTEQKLRE

LNEQADRVLKLINEHGWEDQHLEEISILYTQGADIDWSRLYAEEETFRLHLPTYPYER

NRCWIDIPERVAVQPSVVQKEEPTMHYTIGWVEATNPERLIPNPQTVLLLKGTIPLCD

SLSRELTERGAHVIEVTLGATYAKISPVHYQIDGSEDQYLQLLKATKNHRIDQIIHTF

TINGHTAPESPKELEDALCRGVHSLFYLTKAIVGAGLRNRMRIVLLSQYVHEVSGHES

HLAPEHATMFGLGKAIGQEHPQLECKVMDIDDDTAIPILLNAMNADDVFYSTAYRKGQ

RYVEEFRKTDLLGAELSDVTIKEQGVYLITGGMGGIGRAFAEHFANRHNVTLILAGRS

DFPDRETWPAILEDNKDLEQCNKIKWIHSIENRGSTVGLYSVDTGDLQQVSRLMENIR

EQHGKVDGIVHGAGVAGEGFLASKDAETFNRVLLPKVQGTWNMDQATAHDSLDFLVLF

STTSTLFSSPGQGDYSAANSYLDAYAAYRNRRGGRTLTINWVAWKETGMAKDFGVNDD

MVFKSVLTKSALKAFDQAFQRKVHRSIIGELNYDSEYMAYVGDMPMMMSEDVYNTIQD

QSASVIAEVEMRKATLNKEVSLHGRNDGQYTAMEQMLSRIWSLHLGLTEMDLYDDFYE

YGGDSIIGLKMASDIYRETGIQINPSDVLQYPTIWGLGVHLQSLVDVENATVGKQSIP

HAEVKEEYPLSSAQKRIFYLCTQDPDNISYNLNKALIMEGNFDPFTLNGYMNTLIQRH

ESFRTSFHWEQGEPIQRIHENVDFSIETLPDVIRGTEAVEDVVKQFVRPFDLEAAPLI
RMIVGSLDDGRHLLMFDVHHLVTDGMSMDNMMHEITSMYSGSPLPNIPYQYRDYSEWS
NQYQQSDSIKSQEAFWLETLKGPLPTLQLPTDFSRPDRKTYDGAKMTFYADEALTARI
GKLGARTGTTLYMLLLSALNVLLHKYTNQSDILIGSPVTGRQQGNFDATIGMFVNTIV
LRNEPVPDDSFRELLLDVKQRTLSAFAHQDCQFDRIVQLVNSTRDPSRNPLFDVYFAL
QNVGMYTGEIEAFKGTLIDYDSGVSRFDLALDAIERDGTIEFILEYNTSLFLPDTAEY
IAKDFVKILEIISEQPDTLIYDIEVDRVTEITDEAVDEEVNFNF"

CDS 677392..678213

/gene="mmgB_1"

/locus_tag="EFAGFIKM_00595"

/EC_number="1.1.1.157"

/inference="ab initio prediction:Prodigal:002006"

/inference="similar to AA sequence:UniProtKB:P45856"

/codon_start=1

/transl_table=11

/product="putative 3-hydroxybutyryl-CoA dehydrogenase"

/db_xref="COG:COG1250"

/translation="MGKGVAQSLAQSRHNVVLIDVSDEVLDQARTALFTNIRFSGFYN
KDTEAEDPETVLSRVEFTTHYDSLSDADFIIENVPEQWDVKKEVYAQLENYCPDSCIY
LVNTSCISITKVGSITKRPDKVIGVHFMNPVPLKPVVEAIQGYHTSDTTIEATRSLLH
TMGKEAILVQDLPGFVSNRISHLLMNEAAFVVQDQVASPEEVDDIFVKCFGHKMGPLH
TADLIGLDTVVHSLDVLYESYQDPKYRVCPLLRKMVDAGLLGAKSGQGFFEHVIL"

CDS 678249..678482

/gene="dltC"

/locus_tag="EFAGFIKM_00596"

/inference="ab initio prediction:Prodigal:002006"

/inference="protein motif:HAMAP:MF_00565"

/codon_start=1

/transl_table=11

/product="D-alanyl carrier protein"

/translation="MESKVKVKTFLSRFYRKREIQDDEDIFELGFANSLFAMQMVMFL
EKEFNIRIETQDMDLSNFRTLNKINELIERKTA"

CDS 678541..679611

/locus_tag="EFAGFIKM_00597"

/inference="ab initio prediction:Prodigal:002006"

/codon_start=1

/transl_table=11

/product="hypothetical protein"

/translation="MGEPSVSSVMDKQKQEKVKLVVWDLDNTIWEGVLLEDENVVLRE

GIIDVIRTLDERGVLQSIASKNDHQTAMEKLEEFGIHEYFLYPQIHWNSKSSSIQSII

TSINIGANTVAFVDDQMFELEEVQFEHAEVRCIDAAQYLDIPDMEMMNPRFITEDSKL

RRLMYKSDEIRNKAEDEFTGPKDDFLASLGMTFTISPLKGDDLQRAEELTVRTHQLNT

TGYTYSYEDLEQLSQSPNHKLLITGLEDKYGAYGKIGLALLEMEEEYWTVKLLLMSCR

VMSRGVGTILMNYILEQAKEAGVKIRAEFKMTDRNRMMYLTYKFGGFKEIHQEEDLVI

FEHDMSYIQKIPSYVELNIINN"

CDS 679642..680916

/gene="fenF_1"

/locus_tag="EFAGFIKM_00598"

/EC_number="2.3.1.39"

/inference="ab initio prediction:Prodigal:002006"

/inference="similar to AA sequence:UniProtKB:Q9R9J2"

/codon_start=1

/transl_table=11

/product="Malonyl CoA-acyl carrier protein transacylase"

/translation="MEKIALLFPGQGSQYVGMGKSFYDQYSIAKQTYEEASDVAGVDL

AKLCFEGSLSELNQNENMQPALLATNVAAFRVYMEEVGIRPQFCAGHSMGEYSALVCS

GAMTFSDAVRITKLRGAMMQIHIDRGTGSMTIMDGIDASIVEQLCEQYSGEQGVAVVS

CYNSPTQAAVSGHQYVLEEIEAAVLDADGQVTPLMSSAPIHSSLMAEDAERLGQELAK

YSYNMFKWPVLSNVTGQPYGDVERIPGLLTDQMVKPVQWTRILQYLERFGVTLAIEMG

PKNVLNKLTKTNCPDINSLCFGEKAERQKLISMFQSDGKKKKGVPTVITKCLAIAVAT

PNQNWDEAEYQQGVVQRYKRIQAIQEQVENAGDKPSKEQMIEALELLKGIFETKKLPV

KERNQWMNHIIDDTGHHYELAEYLVHEAVGVK"

CDS 681004..681984

/gene="lnrL_2"

/locus_tag="EFAGFIKM_00599"

/EC_number="3.6.3.-"

/inference="ab initio prediction:Prodigal:002006"

/inference="similar to AA sequence:UniProtKB:P94440"

/codon_start=1

/transl_table=11

/product="Linearmycin resistance ATP-binding protein LnrL"

/db_xref="COG:COG1131"

/translation="MRVLSAENVTKSYGKHLALDNMSFELRKGEIHTILGTNGSGKTT

FIKVLATLLTKDSGIVNIGGYDLDTDANIIRRLIGYVGQDTERSAYARLTVRENLIFF

GRLTGLSKQEISNQIDKLSTYLDFSEHLNKQFMQLSGGQKQTVVIMRALLHDPEVIYL

DEPTKGLDPVISRRVREFIKSYVRDEGKSIILTSHILTEVEDMTDRVSLMRKGTLLKT

GTIAELKSNLGAVEFIDIPRADLPPSIQERITALPEVTSTIIREEEDTVSFGITNLYD

GMGAVISELKEGNIHTTIQHRQLTLEDLFVQLYVKSDENLEYTLAGGREQ"

CDS 681981..682853

/locus_tag="EFAGFIKM_00600"

/inference="ab initio prediction:Prodigal:002006"

/codon_start=1

/transl_table=11

/product="hypothetical protein"

/translation="MSIEYAKTGTGQETLKLQAIGKPTGFFAVVWATTVKELQIAVRY

LPNFFGNFLQLGLRVLFFLLMSTFVVYEGENVLQGDNLFIFYLGAILVWLFYGVALNA

PLGAVTNDLHNGTLEYLYCNPISRYAYYVGTVVASALMNVIVFIPMYILLVLYAGLNF

AESMMILVTCLVVIVALMALGVMIALLGILYRQVSSIVGILFIMFEFVAGAYFPVDQF

PAVVRWIAYLLPYTWGYDMVRYYSFKTDWVPLAPIWMEWCILIFFAISYTIISIVMLR

KVEKMAKKKGLNLI"

CDS 682891..686577

/gene="dltA_2"

/locus_tag="EFAGFIKM_00601"

/EC_number="6.2.1.54"

/inference="ab initio prediction:Prodigal:002006"

/inference="protein motif:HAMAP:MF_00593"

/codon_start=1

/transl_table=11

/product="D-alanine--D-alanyl carrier protein ligase"

/translation="MSAEPSAFMQNLLLSSRKFEEEKQYWLNHLSGDLQFSMFPSEKT

RGTWGEQVFEEMQIAFPQPVSEQIFRISGNTEAGAFILLLAGVTYVLHRYLDNDDITV

GIPASSGAANSSQLRAIRINFGEECSFKELVLQLRTLVGAAKKFNHLSLEKILELLGH

EGTDLPRVATVVRMDNMQENLQETGPLADMVFSLQMSKMGLALRMNYNAALYSRETIE

QIVDHVMHVLSMCTAHPDEGLHHIKMLSCKEDQVIQHLNDTEIRFPEARSIIQMTQSW

SAQQPDIPAVICGDHILTYRSLDCKSDQLAKYLLAQGVRSGAIVGILLTRSEDMLLAM

LGVLKAGAAYMPMDPAYANNRLQFMLEDSQAAILLTKPDLIGSLIYTGQIIDISRMEE

WEDQHISLPVVSSEQLAYLIYTSGSTGRPKGVMIEHRALRNFVEGVSRSIPFEAGKRI

LALTTFSFDIFVLETLGALANGLTLIMAVEEDQLSPSRIAHLIVTHNIELIQITPSRL

RMLQIGLTVDSPVWSVIQTLMIGGEALPYALYEEIAGMTSARIYNMYGPTETTIWSSI

KELSPTTTISIGHPIANTRIYILDKRYRHMPIGATGELCIAGDGVARGYWNRSELTAE

RFVKDPFHEGAYMYKTGDLARLNANGDLEYLGRTDFQVKIRGYRIELGEIENRLLQYP

AISAAAVTVRSDQDGQSLLCAYYTVTESQSLNQEQLRSFLLTELPEYMLPSRYTMLDH

MPYTANGKLDRKALPDPLLLQGEGTDITLPRNAFEQVLAELWSDLLQLKEIDVHSRFF

EIGGHSLNVIKLEANMERQSIPLTADEVMRHETIASLAEVLRNKGYEADEHNSEKEIF

LEEQDKPETDGAMEQFTEEVGATAEADHLILHHSHRPIVIIAGIEPFNDVFYKNCFYN

SLFPVLNFYSIGVLPVMINDRMHYRFDEHQGILRLEYQAVLSDDQLIERMGLTYETKT

YSEDVRKEIMEAVNLNRPVIIRVDCYYESLRKDMYLRTHWPHSLLIYGYDLQRQVFHV

IEHDHRDNLTYQPMEMPMNDIVQAYEGFVRLFMEEEEVPTFYAFSACETVSHKRNNIM

LGELRDMFIDHVRVTPGEINQELSHLLSFIQKTYQFNEEEDSPDTAYAELVLQSIVDI

VNAKKVETYRMQRLYEHHTDLVSAAQAIQDHWSELRNVLAKAVYRENMTHNQWRKFTS

KLEAIHELEKHNLINCSGLEISHD"

CDS 686570..687595

/locus_tag="EFAGFIKM_00602"

/inference="ab initio prediction:Prodigal:002006"

/codon_start=1

/transl_table=11

/product="hypothetical protein"

/translation="MIKKLQMNEPLMTTYPHHASLFSMLDLDKRSRSWIYHNFLLVIL

SHNDEGGYGLDVCSQYYPWHKFKMSTCPMFITRVYDRDMILKQWELQDFIVELIQKNN
YIYFLRNIPGGGKHEVFISGFDSDRKEFLCHDFWDGFYGEKWLPYDEITVIQDSNFDA
EWSTDYLNGIWAIEKTDEYKEPSEYYYETVLQFSKENLMNILKEYVGETNQVRSILRK
DHRYLGSEIYDKMIDMLGMQKEGMQNQPFAIHPFHLLYEHKQLLAQAILDFLPDPSLK
IRLEELVSEALKLRNLVMYMNEFIMEQGVYKKYDVMLDKIIELKNMELAIMKHVFEYE
YQSQLGQ"

CDS 687750..688979

/gene="sbnD_1"

/locus_tag="EFAGFIKM_00603"

/inference="ab initio prediction:Prodigal:002006"

/inference="similar to AA sequence:UniProtKB:Q2G1N0"

/codon_start=1

/transl_table=11

/product="Staphyloferrin B transporter"

/translation="MPQWKRNLYILWFGLFFNHMAYSLSVPFFPIFLQNDLGIQSGLE
AWSGISISISFLISGLCAPFWGSLADKYGSKLMLVRSGVGLGIAHLANFFVYDPYTFI
AVRVFQGLMAGFHPASITLIGTNTPEKHVGYALGVISTASAAGGILGPLAGGVLSHWI
GLRGCFTASAVITLLAALMVLGVKESRQARTIFPTRIIGDLKRAASTPGLMRIYGLIL
LVSTSVLIIEPVLTLYVVQIGGDVGSATLSAGIVFSAIGVATVIMGPRWGKIGGRIGY
EKTLFIGLIGGGIGSLLQMTATNLIYFGSLRFVYGLFFAAVYPALNALIIKYADHDFR
GRAVSLSQTASQFGIVLGPLIGGLLGGWIGIPFIFLFTGIVLLGAAWAVRVSDVKKAD
KTELRMAVDSGSTNMKK"

CDS 689010..690332

/gene="moxC_2"

/locus_tag="EFAGFIKM_00604"

/EC_number="1.14.-.-"

/inference="ab initio prediction:Prodigal:002006"

/inference="similar to AA sequence:UniProtKB:O34974"

/codon_start=1

/transl_table=11

/product="Putative monooxygenase MoxC"

/db_xref="COG:COG2141"

/translation="MSQRQLKLGANLNGVGNSISFWRHTDIPINASVSLEFYKKQVRI
AEKGKFDLLFIADGLFINEKSNPHFLNRFEPLTLLSALAGATSHIGLVATLSTSYSEP
FTVARQFASLDQLSGGRAGWNVVTSPLEGSALNFGKGEHPNHALRYEIAEEHLNVVKG
LWDSWEDDAFVGDKEQGVFFDPAKLHTLNHKGTHFSVQGPLNVGRSKQGYPVIFQAGS
SESGKDLAARSADAVYTGHETLKEAREFYRDVKARAVAYGRKAEDILIFPGIGPIVGR
TAEEAEQKYQEIAELVNIDQALNYLGRYFDHYDFSQFPLDEPFPEIGEIGSNSFRSTT
DKIKQQAREQGLTLRQVALLASTPRTSFIGTPDQIADQIQEWFEGEAADGFNVRTVVP
NGLEDFVELVVPVLQERGLFRADYEHETLRENLGLEIPRNRYTLETVK"

CDS complement(690496..693645)

/locus_tag="EFAGFIKM_00605"

/inference="ab initio prediction:Prodigal:002006"

/codon_start=1

/transl_table=11

/product="hypothetical protein"

/translation="MFLTENKLHARIYELSELRYRDRLPLNHFLACEDHREEINPVLP
GDDSHWKPMHTGESWSGRDLYLWLRTKVDFPSSWEDKRIVGRFDFGDTGGGNNSGFES
LFYWDGKPFQGVDSNHKEVFFPASAAGSTNELVFRLWSGLEGGGQPCNQTHTFRQAEL
CWLDEAVDDLVFTGTAILETVNILDAHAPERTKLLHALQQSFRLIDWSQPKSDIFYSS
VHDARDDLSVRLNAMDKASDVTVTCIGHTHIDVAWLWRLKHTREKCARSFSTVMRLME
MFPEYVFLQTQPQLYEYVKEDYPELYESIKERVAEGRWEAGGGMWLEADCNLTSGESL
VRQLLIGTRFLKEEFRTDCRYLWLPDVFGYSWSLPQILKKSGIHTFMTTKISWNQYNR
MPFDTFHWRGIDGSEVLTHFITTPEPWSEPGSWFYTYNGKIIPKTVKGIWDAYRDKEM
NQDLLLSYGYGDGGGGVNREMLEMRRRLDQLPGLPKVKTGRADDYFEKLQETVEKTDS
YIHTWDGELYLEYHRGTYTSQAYNKRMNRKLELAYREAEWLNALQSIVHNDWNLYQAK
SLTAGWKIILRNQFHDIIPGSSITEVYEDSRIEYAEAEQIGSHVDTQARLAIAGSGES
PYTYTIWNSSPWVVTEIVSISVASDELETGTWLSDSGEILNAQYQDARWAVEVKDIPS
LGYTTIHFQSSTVNAISEQSPFIHTANGIETPYYLLEWNESGQLNRLYDIELHREVLA
KGARGNVLQVFEDKPLAHEAWDIDIFYQEDMREITKCTSIEVVETGPLQAVIRFAWEY
LGSTITQNMTVYTGHRRIDYATEVDWHEQQQLLKVAFPVGIRSTEATYDIQFGNVKRP
THWNTSWDWARFESVGHQWADLSDRGYGVSLLNDSKYGYDIKDNVMRLTLIKSATHPD
PHADQGLHSFTYALLPHQGDWLVGGTVQQAWLLNAPARYTQGVSEQPSCSMFSLSGST
AMISAVKKAEDSKRVIVRIHDYSGSMNTLELTSDLRIHAWRECNLMELPEGELLYDTP

ITFALEPYEIKTFEIDLRV"

CDS 693895..695709

/locus_tag="EFAGFIKM_00606"

/inference="ab initio prediction:Prodigal:002006"

/codon_start=1

/transl_table=11

/product="hypothetical protein"

/translation="MYKNYLKKKMFNKILLFYSMVMVLLFISIAVLAYRYYEQRVVQE

QMDASLQELDIVSINLNQQYERLYNAVQQIYTDAMIGDDLKYFLTHEYEDFLNWRLNQ

YAYSYRTERGNFDYQLRLLLKDEASIANVVLYSTDQEFFYVLNRETQHFYYHPKLSAG

HQGWFQRLRNAPWQYMGSEPLFEGKMADEPTPYSYANVLKDSVTLKQYGAIMFELNTD

RIKGLLRDRPGGKNSRIMLMTGEGEVIFDSQGKYDHTVYPYWKKMNLPEDWVDLEERS

KVQLLNIGNTGMVVASIIPESHIKQSLQAIRFSLIGIVILCIMVSIAVTFTIIRRYSK

KIHRLVLYMRRWQEGDLSKRIEMEGEDELQQISQRFNHMCDRLESYIETVYVSEIKQK

NAQLVALQAQINPHFLYNTLESIRMKAISLGARDVGQMIYILATMFRHLIKKQTHVTL

VEEIELCGMYLALVQYRYEDKLHVETHIENAAAGSMVVKLLVQPIIENYIVHGFRTYD

DDNRISITAEEENGVIRIRVKDNGKGISAERLLELQQGLHKGDEAAETSDRSLGLKNI

HDRIRLNYGSDYGLHLNSVEGEGCEVTISIPVTREEMG"

CDS 695706..697265

/gene="rssB_3"

/locus_tag="EFAGFIKM_00607"

/inference="ab initio prediction:Prodigal:002006"

/inference="protein motif:HAMAP:MF_00958"

/codon_start=1

/transl_table=11

/product="Regulator of RpoS"

/translation="MRSVMLVDDEPLIVKGMQAIIDWDQNNMQIAGTACNGLEALAIM

DEVPVDLIITDIMMPQMTGLELIRETKARNPYTKFIILSGYEEFQYVREGISLGVENY

LLKPVNMDELEATIKHMQRDWEHEEMRQIQMDQSWRILRNNILQRWANETIDPKEFRE

RAQLLEIPMNKSTYCAASLRIVGDDKGDDPQMYLPGIAEQCEQIGNKGLPDGCGIICF

PDQDGDIMLLFVSAGTEGNDEWQLQALTRMKQAVTEDTDARVWGIIGQSESTYLGFPK

SCKSVKLWLQNHLFKDCNTFIMQVENQEATNQHQEKPGPVMEQFHKLLRDGEGTEVDR

FIDEFFNVEGKEVCLPRTPFSNSAIQLMLAAKELEKTPDYGDIFGPLSRIHTLNKLKQ
LVRKVVHRTLDRYHTVEKTYSPHIMAVLEVVKNHYPDELSLKTLSQRLELHPNYLGHL
FQQEVGTTFSDYVNQYRIERATHLLLYSDKKTAEIAGEVGYWDSTYFYRQFKKYAGVS
PTELRSIYMKK"

CDS 697527..698456

/gene="yteP_5"

/locus_tag="EFAGFIKM_00608"

/inference="ab initio prediction:Prodigal:002006"

/inference="similar to AA sequence:UniProtKB:C0SPB3"

/codon_start=1

/transl_table=11

/product="putative multiple-sugar transport system
permease YteP"

/db_xref="COG:COG4209"

/translation="MQDIFKSLMKNKAFLLLVLPGAAWFIIFAYLPMFGTIIAFKDFR
IHPGGFLESVLKSEWVGFKNFEFLFSTSDAYIITRNTILYNLGLIFLGLVLAVTLAII
MNELLNKRLAKIYQTAMFMPYFLSWVIISYFVFSFLSVEKGVFNQIITYFGGDPVSWY
SETGYWPYFLILLGLWKGAGYGSVVYLAAIAGIDRSYYEAAMIDGATKWKQIRYITLP
LLRPLMIILTILAIGGIFRSDFGLFYQVPRDSGALYPVTNVIDTFVYRGLTIMGDTGM
STATGLYQSVVGLILVLLANYAVRKIEKDYAVF"

CDS 698479..699435

/gene="araQ_5"

/locus_tag="EFAGFIKM_00609"

/inference="ab initio prediction:Prodigal:002006"

/inference="similar to AA sequence:UniProtKB:P94530"

/codon_start=1

/transl_table=11

/product="L-arabinose transport system permease protein
AraQ"

/db_xref="COG:COG0395"

/translation="MSNLSQPVKRASAHAKRTGKKSRDLNAISPLSNVIFNIAIGLFA
LSCIFPFLFIVIISFTDEKTLAINGFKLFPEVWTLDAYRFLMESGQQMAQSFGVTLTV

AVVGTALTLYLVTTYAYAISRKNFAQRRFFSFLAFFTMLFSGGLVPSYIVVTQVLHLR

DSIWALILPSIMNAFYIIVMRTFFTTTVPDAVIESAKIDGATEFGIYTRIVLPISLPG

IATIGLFSTLGFWNDWFNALLYIDNANLIPLQTLLIRIQNNMDFVVQNSSRVVSYDIA

ATLPTETVRMAMVVLATLPVALAYPFFQKYFIQGLTIGSVKE"

CDS 699494..700993

/locus_tag="EFAGFIKM_00610"

/inference="ab initio prediction:Prodigal:002006"

/codon_start=1

/transl_table=11

/product="hypothetical protein"

/translation="MKTKKIALLMVTLLLAFATVLAGCGGSNTNGTSGNAASGGESGD

KDTVKLKVYLIGGPQRDLPMVQEEMNKYLMEKINTTVDITMIDWGDYSKRMPVITASG

ENYDIAFTSSWAYDYLPNATRGAFLPINDLLDKYGQGIKEQLDPRFLTGSQIDGQNYA

VPVNKELASQWVWRFNKQYVDKYNMDITKIRTLEDLEPYLQQIKDNEPADITPLAVPK

GFKPYLAFDFLLGDEFPVGINMNGDTGKYVNMLESEELNSSLKTIRKYYQAGYLRKDV

ATLEGIDNIKTGKWFVDREQTQPYAELGWSRSAGYDIVTTPMQDPVIFTGSATGAMHA

ISANSKNPERAMMFLNLLNTDPYLRNLINYGIEGTHYKKISDNVIEDLPAMKDGFQMP

GFALGNLFLTYMHKEDPADKWEAFKEFNNSAKVAPSFGFNFNPDPVKTEVASISAIVK

EFYPSIMTGAVDPDEHLPKAIEKLKAAGLDKVIAEAQKQYDEWKAANPK"

CDS 701092..703749

/locus_tag="EFAGFIKM_00611"

/inference="ab initio prediction:Prodigal:002006"

/codon_start=1

/transl_table=11

/product="hypothetical protein"

/translation="MKKLGASLLFVILVCAALSSAALAKQPYSSYWLPEQLLNWNPAS

DPDAAFNRSNIPLQSRFTGEAVNPNATKDPKVMALSALNKGTSGVPSQGSDIFSANTF

SYWQYVDKLVYWGGSAGEGIIVPPSADTIDAAHRNGVPIMGTVFFPPSVYGGKYEWVK

QMLQQNSDGSFPAADKLIEVAEYYGFDGWFINQETEGGTAADAQLMKAFLRYLQDHKP

AGMEIIWYDSMTKEGNISWQNALTDRNTMFLQDNGSKVSDSMFLNFWWNDLGSSAAKA

RSLGRSPFELFAGIDVEAKGYDTSLKWNLLFPEEKPAVTSLGIYRPDWSFNSAESVTD

FFARENKFWVGQNGNPANTATSQAWKGIANNVVESSPVDQLPFTTNFNTGSGEKFYVD

GTQVRETGWNNRSLQDVLPTWRWLTESKGKALTPSLDWSDAYYGGSSLKVAGTLSSAN
ATHLKLYQTDLKIEPATKLSITHKTQNKPSMKVGLAFADRPDQFVFLDVKDKKASGWT
TDILNLTPYKGKRIVALSLYFDSKEIISDYDIHIGQISIQNNANPVKPLEAVQALNVI
QSDFRGGIYGDARLQWTALDEEVQQYEIYRILPDGKETWVGATANNVFYVPEMKRINA
EQATVLKVVAVNAKYEHGQAASVTIQWPAYPRPEAGFKADKTLITPGQQVHFTDLSSE
VTEGWSWTFDNGSPAVSTEQNPVVTYDKEGTYNVTLTATNSSGQDTVTKESLITVSKA
ASGIQNVALGKKATADHACGSAEGAEKAIDGKVTGNSKWCALGNQPHWLQVDLGKEHQ
ISGFVIKHAESGGEWSGFNTSDYSIQVSADGVNWSDVVHIQGNTAAETSDAIALVKAR
YVKLNVLKPTQGGDTAARIYEFEVYGVRP"

CDS 704364..705191

/locus_tag="EFAGFIKM_00612"

/inference="ab initio prediction:Prodigal:002006"

/codon_start=1

/transl_table=11

/product="hypothetical protein"

/translation="MIRGLTSAGLGNIESNEQYITNAAKYGFQSVDLNPLSFIEEVGK
EQAIHLLESNQVIIGSSGLTVDWRTTDEQFREGLQSLVQMAEASASLNCYSCCTYILP
STDQPAAAFMAAATKRLRLCADIMDAYGIRLGLEFVGCHHLRTQWKNPFIWSVEDTLD
WIETIHAPNVGLLVDSYHWHTNGLPMEDLLKLKKEQVVHVHINDAYDLPIEELKDGER
LYPGEGVIDLGGFLRNLQAIGYNGVVAQEVLAPAPTLSSSELFAKTKAGFDKVFAAI"

CDS complement(705262..706140)

/locus_tag="EFAGFIKM_00613"

/inference="ab initio prediction:Prodigal:002006"

/codon_start=1

/transl_table=11

/product="hypothetical protein"

/translation="MRTKHPIIVITGATSGLGQLAAMELAKRGAHLILTARSKERADA
TRYKIKEIIPSAKIEFFYGDLSLLSDVKRVGLEIAAAYPCIDVLLNNAGLHAFEQRVT
SEGYAEMIAVNYLAPWLLTHTLQSSLIQSGHARVVNVASEASRNHGTLVLPDDLTDIT
PFTARGSSPIYGKTKLLNIMFTGELARQWCGTGICVNALNPGFNVTGLGRELWFASVL
EHILTFLHIGDPRRGADIITRLAVESKYGEVSGGYFNVGTGASIVPMHPGGDEAMQNR
LWIFTTQLMKDHGVLD"

CDS 706277..706750

/locus_tag="EFAGFIKM_00614"

/inference="ab initio prediction:Prodigal:002006"

/codon_start=1

/transl_table=11

/product="hypothetical protein"

/translation="MSENPVKTKDRAALELALGEQIHALISASHALNVRSAERFDANL

QPAAFQLVRWLYSYGPTSAAALAEANAMDRSSVSRLIKQLEKSGYVSKEQDPKDRRGV

LLSLTELGQQSTVAALKEKESAFYNRISRWDDKELERFTAMLRQFNGLDDETVEA"

CDS 706754..707701

/locus_tag="EFAGFIKM_00615"

/inference="ab initio prediction:Prodigal:002006"

/codon_start=1

/transl_table=11

/product="hypothetical protein"

/translation="MNKTERQLAITLELQRRKMLRAEDLAAQFETSVRTIYRDIQALS

EAGVPIMGAPGHGYSLMEGYFLPPVSFTAEEAVSLLMGADFIEQRLDTEYAMEAKSAQ

RKIEAILPESVRDKSTRVRETMRLLHTVEPLTRARVKTYLNQIRNAILDQRKISFMYL

KKMPGTDGNRYNMREVSPYGLSLVQENWMLIARCDMRQDIRHFRLSRMTELSVLEDQF

LLPPDFDLNSYRPPDDRNEQVLIRAKPEIADKVMEALHFYMDAFEEQEEGVIFHFRVR

YPEEILHYLLGWGGDIEVLEPKSLRFRMQEVAKNILKHY"

CDS 707769..708305

/locus_tag="EFAGFIKM_00616"

/inference="ab initio prediction:Prodigal:002006"

/codon_start=1

/transl_table=11

/product="hypothetical protein"

/translation="MKSTTEVLKSFETAVERYLAELNKLDMGSLHKKQSEEEWSIGQM

YVHLIQSALFLHLHNIEQCLSSKDSTLSSGEEKTELGRQVFELGQFPPVPIKVPASPQ

YTPQPPESMESLIDGLHKVVDRMRSTASVLHQASVNNKIHHPRLGALNAQEWFLLIEM

HYRHHFLQLDRLKSNLEM"

CDS 708332..708808

/locus_tag="EFAGFIKM_00617"

/inference="ab initio prediction:Prodigal:002006"

/codon_start=1

/transl_table=11

/product="hypothetical protein"

/translation="MKPNQDSRYWVGVVSASHVNVAVEGGFAQLCHGKSALLRQMSAG

DWMIYYSPRTDISTGQPLQAFTAIGQITDDRIYQYQMSESFVPFRRDLRYLPCREVKI

ATLLDQLSFTRGNRNWGFPFRKGHFEIGAEDFRMIASSMLEDETQSLLDIILSQRC"

CDS complement(708851..709357)

/gene="yidD_1"

/locus_tag="EFAGFIKM_00618"

/inference="ab initio prediction:Prodigal:002006"

/inference="protein motif:HAMAP:MF_00386"

/codon_start=1

/transl_table=11

/product="Putative membrane protein insertion efficiency

factor"

/translation="MRNSESEELIFNNMPSEEELIRLLHTHHEENDPRSSFYIRTHVI

PEIDWLKSLLNVTLALFAGLIISMICFYLLNPFIPVYALLSAQIVFIASMLFIVLRRV

RVILKWSIRIYQRFAPIEVRNKCRFEPSCSVYMIQAIEKYGAIKGLSLGIQRLRKCNI

NGGGYDYP"

CDS complement(709374..709595)

/locus_tag="EFAGFIKM_00619"

/inference="ab initio prediction:Prodigal:002006"

/codon_start=1

/transl_table=11

/product="hypothetical protein"

/translation="MREYTVVPGPKNVQVNRGDTQAAVNLFADIINHKAQSGWTYQSM

ESLSVTEKPGCLQQPVTTHHYMLIFYREI"

CDS 709861..710454

/locus_tag="EFAGFIKM_00620"

/inference="ab initio prediction:Prodigal:002006"

/codon_start=1

/transl_table=11

/product="hypothetical protein"

/translation="MTDTSGNRKVDGYLKKLKTWKEESTKLREIIRDFELTEDMKWMH

PCYMLDGKNIVLIHGFKEYVAILFFKGALLKDTHGILVQQMENVQAERQLRFTSLEQI

VEQEAMIKAYIQQAIEVEQAGLQVEMKKTAEYSVPEELQQQFDENPAFQAAFEALTPG

RQRAYLYYFSQPKQSKTKVSRIEKYMQPILEGKGLND"

CDS 710576..710833

/locus_tag="EFAGFIKM_00621"

/inference="ab initio prediction:Prodigal:002006"

/codon_start=1

/transl_table=11

/product="hypothetical protein"

/translation="MIRTYQYYAIFNFAEDGINVTFPDLPGCITCGDSVEEALLMAKE

ALELFLDGEPVEDIPPPQRIMPELLNSRDKAYLIEANITYY"

CDS complement(710989..711735)

/locus_tag="EFAGFIKM_00622"

/inference="ab initio prediction:Prodigal:002006"

/codon_start=1

/transl_table=11

/product="hypothetical protein"

/translation="MLVSGFFLSVTGGHGAPFLALVGDSPWEARKNVRINVKHGVDLI

KICVTGGVTDAKMVGEAGRLQMTVEEIAAICDEAHKIGRRVAAHVESTEGVRVALQGG

VDTIEHGSEMDDEIIRLYRNNPNALNGYTALFPTLQAAYPSASLDTNVTKLSATVKEN

SRLVYDSMLKGVKQAIENDITIGIGTDAAMPYVTHYDMWREMDHYMRQANLNNKQVID

MVTRTNAKILGVDDVTGTVDIGKHADLIII"

CDS complement(711729..712184)

/locus_tag="EFAGFIKM_00623"

/inference="ab initio prediction:Prodigal:002006"

/codon_start=1

/transl_table=11

/product="hypothetical protein"

/translation="MNTAYALKNVHLIHGDLNRNLQKNMTILVNEQGLIQDIGRDHAL
NIPSHYTTIDLSGKYVMPGLINAHVHLFADGKPFSLSVSEGLLQFAYDRILNTKFGRN
VLKKRMKRNALTALHAGVTTMRSVGEFFYTDVQSYGMRLTTVTLSVPIC"

CDS complement(712529..713140)

/locus_tag="EFAGFIKM_00624"

/inference="ab initio prediction:Prodigal:002006"

/codon_start=1

/transl_table=11

/product="hypothetical protein"

/translation="MVQAKKDEVRKEIEYAALKVFFEKGYVDAKMRDIADEIKISVGN
IYTYFKNKKELFYAVVPPDLVDYLKNVLVETIHFDNQTLFEETDSERKSALLQEQVDV
LRKYRNQIVIIFEKNKGTIYTNAKNELVELMIETKKAYLKNQYKRYEIGTEENLILLN
ILAHNVIDMNLDLLKRDMSEDSRKQIFEALYVYRLYGMKSLNQ"

CDS complement(713560..714006)

/locus_tag="EFAGFIKM_00625"

/inference="ab initio prediction:Prodigal:002006"

/codon_start=1

/transl_table=11

/product="hypothetical protein"

/translation="MFNYKEQHIYLETCSSKKKLQELRKNTSLLLKQIDEKISIIPTK
SIPINCPQCRSSNTKKNGRLQKRSSCNDCGQSFSNVYMPNIFQNTYYPEKKLKLICLI
YMTEKSTVQIIKEINISRLTYNKWREEITYAFPFLLQFFEKRIKTR"

CDS 714174..714608

/locus_tag="EFAGFIKM_00626"

/inference="ab initio prediction:Prodigal:002006"

/codon_start=1

/transl_table=11

/product="hypothetical protein"

/translation="MVKIFLGSADNPSALDDLRKIAALIEHVGHFVIPWNSPGVFPLG
KHMSDSLDNICKQVDAAILIFGEEHQVLFGNEFSLHPKPNLLYEYGLFKGYLGLERTI
ICRRGSSSIPSDLAGVIYCNLDKEFKAERELLAWLRLLSPKN"

CDS 714642..716177

/locus_tag="EFAGFIKM_00627"

/inference="ab initio prediction:Prodigal:002006"

/codon_start=1

/transl_table=11

/product="hypothetical protein"

/translation="MDKIPSMQSLRGITELKYVNADNVTRYRAIMRYIYQQYQKLNYW

LKPEQIHEALMEWDLLKNYTLEQCQLDLTQLAEWGNLTAQHDGERAVTVEEYLRKKFR

YLLTPYSIEIERMLESLETIRGYGGSLEPTLFDTIANTLFKIRNNAGEYEQGTALEYW

KSLFGAFQTLHEASVDYIASLQTGMAEDLMATDSFLLYKDSITHYLQDFIQALQRRSY

KIEGNLSQITIGVRDLFLNEVLNDEWRIPKLEQLVTKSEYMDGLLEKWSSLVRWFIGD

DNSISDSFLLERATKEAIVKVVRSAVRIQERKRSIVSRKKDLDYLGKCFYRISDLDNA

HKLSANVFGLFSARHLQGEDLRESDSKELSMWKEAPIPRKIRSRSRKRSERQGILAAI

NNSKLKEQFREAYVIRQKHELEFLIRMVNSEEISISEFEPVKESTRLQMLGWISRCNA

SSTYSFQNSEGIRIELGQPPDNKRALLQCEDGTLELLDYRLKFVVVNRKAWMDQVAWF

NEL"

CDS 716208..717437

/locus_tag="EFAGFIKM_00628"

/inference="ab initio prediction:Prodigal:002006"

/codon_start=1

/transl_table=11

/product="hypothetical protein"

/translation="MVKIKVGKLSARRQRMNRPDRNEILERKQICMNALLNRPWVDKE

KNAEIYYWIKEQYFEIRDWFMEYTGYTLIMNRNFAKLNKFPEKSFPWMGFQEFREPLD

YALFTYSLWYLESKIEGEQFLLTQLVIDIRQYMVEQEMNVDWGNYYHRLSMARALKKL

KNLNVIRAVDGEEANWASNHEYHDVLYESTLYSRYILRNFNAELSSYIHIDKFNDTKT

TYSNETAGVDVRNRHELYRRILLEPILLNYQWSGDPIYLKQELNLLIRNIGEMFGWQG

SRYREGIFLFESNTITESEVFPTQLALSDLAMLVCGEVRKMVINSESNLEVDINGLLT

LTKNEIERILIRLKSSFSEFWTNEHKKKNSSELAEQICNHLAEWGFGYWEDHIFFVLN

GAAGRWEVEYGLAEQDD"

CDS 717449..721558

/locus_tag="EFAGFIKM_00629"

/inference="ab initio prediction:Prodigal:002006"

/codon_start=1

/transl_table=11

/product="hypothetical protein"

/translation="MERWQMKRAGILNFWYYDEEEFLFEDGRLILRGTNGSGKSVTMQ

SLIPLVLDGDKRPERLDPFGSRDRRLEYYLLGDANQGHTDRTGYLWLEFYHPSKNLYK

TIGIGIRARRGATQLGFWGFLIEDSRRINEDFYLYDRNLWIEEGNKIPLNKKVLEEKI

GSGGQVVQEQTAYRDMVNKAVFGFRETESYKDLLKLLLELRSPKLSKDMKPSAIYEIL

NKSLPPLMEEDLSSLSDVLEDMDQITDRLDELQLHVAELTNLEKNYARYNEFLLYDAS

TDVIHRWKVHNQIISQLKQAEKHLEDLEESKTNIIQKFLECKQRLGIVEADLEVLTQG

EAIGKQKELEIYEEQLREVRQQVQTLEEKVFSNQKKVTKLTDDISVLSETLHQLTKEQ

EQKIDEMEDMARIIEFHRHDIYHGVWDRSIPEENNWYRSWRGDLDSHRKKLIFAQQIA

RKEGEASRVAKDVEIQLGEVLQQRHQVEEEQNICEQELEKIKENMRLDIVSWYRGLEF

VPVQEELLRDSLRALTEISTDNRQFEPVKQPAIQAFEREKELYAQERARLNQEVVSFK

KKQLRLEEELEEWKVAKEPEPLKTEKRQSARRNRGPNSGAPLYAVCDFNDTLTQIQQA

QLEATLEQAGLLDAWILPGGKVELMEQQNTEEVWIQIQSSNKKNGKTLCSVLHATPTE

KSGLSMEDINQALNSISWREQTELLINEYEKNRAYVTETGHFSLGPLSGTNISKSRSE

FIGEANRLLTKQLEMARIEEEIKHLNEQIDDINQQLEELNINQKKMESEINSFPPDDH

LQIQLELFSKTTVRLDNLMEQEKNIEERYKLKVSEWRALQMSLLEQTAEWSLLKNESQ

ISEAIEQCSVYNSYISELYSIWIQYRNSSENKRSNEGLLADLLLDVEEDQMELVAKKN

LIIKRAEQVKLLNQIIKELGIDDIHRQISNFKKERELLGNEISRIQDLREDRSTQIGE

AKRDVENCSANLRESEDLLNTKLEKWTKEAHLGLIPLSKEFIELVSKETSIANIVFFC

GRVEKEYGKLFSNVNQDRIVSDLTKEFHTSKPNLIEYAIEMEYDTQNSSRIVILSKMD

RLHPVTPGLLLSELKQQIEEQQILLTARDRELYEEIIIGSVGKAIRKKIHRARAWVDQ

MKDLMRQRNTSSGLKLSLNWLPLQRNNENELDTEKLVDLLMLDQHRMDDEQVEQVITH

FRVRILQAKQEAQREDGVLRNYIFSQLDYRSWFEFKLEHIKGDRTNYTELTDPKFNVL

SGGEKAMAMYIPLFAATYSRYSEANLDAPKIISLDEAFAGVDDANMRDMFHLLTDMGF

DYIMTSQVLWGCYDTVPSLAIYEIYRPKDTEFVTLFHYRWDGESKVLIN"

CDS 721567..722976

/locus_tag="EFAGFIKM_00630"

/inference="ab initio prediction:Prodigal:002006"

/codon_start=1

/transl_table=11

/product="hypothetical protein"

/translation="MELLNRKTAKQYYSDPLFENLLNAVFNKYKGQNGVRGNILIIIK

SNEEAERLQDYFGGRVKQLIHVDSKVEIHLSFFAEELERGYDLTIPDLYEVLYNTQLL

TNCEQKELKLSVWIQLFEQVKRDFINEFNILSVEYEEFTQKTFNWFNRLKNGDGGGYR

VLKNVVEKGENPYSKLLVCMSALWHLLMRKEEMFVERGISTGKIRLPTFAAHVTKDPH

AFDKKKASGRLLWHALHDIDNHTSIINKIEKNERTVVQEYLLERQIYRNFDVLDDDLS

SISHIFVPNLIRGTSPRTINLREVEEIQQLPKYKAVYIIENPSIISYLADETIHFLNV

NKLSMEQLPENFPVLLCTTGQARAASKLFIEKCLASNPDCVIYYSGDLDVPGVQMLYG

MKEQFTAKFKAWRMDSYIYRKYVSPKSRPLSKEDLKFLIENEENLHRVMVQLGAKVYQ

ESFNKELGKDWIEVICEAI"

CDS 723084..723353

/locus_tag="EFAGFIKM_00631"

/inference="ab initio prediction:Prodigal:002006"

/codon_start=1

/transl_table=11

/product="hypothetical protein"

/translation="MTSTNSEDFASQYAQLVRQEHDYVNQLVTCNKLILDAMDIIAKQ

AGVLDMGTVKQAAYHLHAMEQDLNRKLFKVRLEKSILANQMSQST"

CDS 723550..724587

/locus_tag="EFAGFIKM_00632"

/inference="ab initio prediction:Prodigal:002006"

/codon_start=1

/transl_table=11

/product="hypothetical protein"

/translation="MLPTSHNTQQVKRINVELVKNTLRSMGVGTKASIANLTKLSVAT

CGTILNELLQTGEIIDLGPDESSGGRPASRYQFNADYASVLCLIIRTEGGIHSIMHTY

ANLNGEMADEQTLFLDEINVPVVEGLIANLIEQHHNVQAIGIGIPGVAHNGVIGICDV

PELMYQPLGPRLKEQYEDVEVVIGNDMNLTVYGLYNQQQFEEEKNFAVVTFPENHFPG

AGFIIDGRPLTGNTQFGGEVSFLPFGVSREEQLRMLKSTEGLQELVVQTLVSIIAIIN

PATIVVTGDTMDPAMRDGLTQGCLDRMIPLEHMPELIIQRDTRREYVTGLVAVTLESL

TYRIQVIEKQW"

CDS 724836..726092

/locus_tag="EFAGFIKM_00633"

/inference="ab initio prediction:Prodigal:002006"

/codon_start=1

/transl_table=11

/product="hypothetical protein"

/translation="MNQQAGTQGNKRKFNKLYAMQLATIFLGFIVFGISENIKGPAIP

RIQFDFNLDEKQLGTLLSLNALGYLIACAFTAILVRKWGIKAVSIISFASMILSGVFI

YVSHTYPLFASSYFFMYIGNGMLEIALAILGARIFVKNTGTMMNLSHFFYGLSSTVAP

LLATGVMSLSVFGHELDWRGMYLVMLSLCLLPIIAALRSKFPGDDLPHEDRTSLKTLT

RDPALWLMVLILSFGVVSELAVGGWLVNFLEKAYTWDTVKASGLLSAFFLTFSLGRLL

LGPLTDRIGFVLSLILFSAFSGVCTFVALAGGERLAIFFAISGAGIAMIYPTVMAFIA

RRYPNGSDTAITFTVTLMGVGSVIGNYVIGWVIEGVKNLYGSTTQLGLLRGLQAGYGF

IGLCAVICAASGVVLYVYLKRRQELI"

CDS 726197..726580

/locus_tag="EFAGFIKM_00634"

/inference="ab initio prediction:Prodigal:002006"

/codon_start=1

/transl_table=11

/product="hypothetical protein"

/translation="MKRIGLLFLICLCPLIIVACNQKNNDDVVAEIEYHKEGNVIKIT

QRDTVNQILNILESAQKNKKVIDVGKTDEKIVVNYSSNDIKIFDLYIDFDKSVGMVVM

ESDSNYAYELSSQSIKDLKTILKGT"

CDS complement(726751..727356)

/locus_tag="EFAGFIKM_00635"

/inference="ab initio prediction:Prodigal:002006"

/codon_start=1

/transl_table=11

/product="hypothetical protein"

/translation="MTEWITQFILFFKDLSYAGLVIALSFEFVPAELVLPMAGYWVYL

GDMKLWLAILAGTVGGTFGPLTLYALGRYGGRPMVEKFGKYFLIRPHHLDASDKFFEK

YGSGVAFYGRFVPGIRTVISIPCGMAKMNVFKFSIFTFLAMLPITSLYIYLGFKLGSQ

WEHVDEIVKPYIVPAAVVFLGAFGLYVLLKRWKRRTALNKS"

CDS 727645..727755

/locus_tag="EFAGFIKM_00636"

/inference="ab initio prediction:Prodigal:002006"

/codon_start=1

/transl_table=11

/product="hypothetical protein"

/translation="MTVTSIIDQIHIIEYAPSYAAVRKTVEAGWPTGVWF"

CDS 728193..728495

/locus_tag="EFAGFIKM_00637"

/inference="ab initio prediction:Prodigal:002006"

/codon_start=1

/transl_table=11

/product="hypothetical protein"

/translation="MEHQPNERRGIAMTTATLSKWGNSSAIRIPNQLLKRLNLEEGSE

IEILVTEDNELLLRPKTKPVESNEELRNHLKTLLSKVKQDTRHEEIDFGTEGNELL"

CDS 728492..728827

/gene="mazF"

/locus_tag="EFAGFIKM_00638"

/EC_number="3.1.27.-"

/inference="ab initio prediction:Prodigal:002006"

/inference="similar to AA sequence:UniProtKB:P0AE70"

/codon_start=1

/transl_table=11

/product="Endoribonuclease toxin MazF"

/db_xref="COG:COG2337"

/translation="MIIPKRGDLIWLDFDPQAGHEQAGRRPAIVLSELEFNEITGFAV

VCPITSQAKDYPFEVTLPEELPFSGVVLTDQLKSLDVRQRRIQIAGHAEPTSEFMKSV

LRNVRSILA"

CDS 729127..732294

/locus_tag="EFAGFIKM_00639"

/inference="ab initio prediction:Prodigal:002006"

/codon_start=1

/transl_table=11

/product="hypothetical protein"

/translation="MTVTSIIDQIHIIEYDPSYAAALADMWNRSNESWGGGTNLRTEE

TVRREMESSSNLYVFLAVHEKEVVGFCSFAHYRYDERALYVPLLNVRPDYHGYKVGRN

LILNAVRKTVEAGWPRLDLFTWAGNTKAVPMYKKCGFFWEKKDDNVHLMNFIPTILQT

EALTPYFEELDWYADSTRELVVEPDGRRERGFDFFDYTWKKGDVSLRAEFEKSGRGLT

ALETPDYEISTEVDDHDLVFGSSYKVRYRIKNNSASELAVEIEGEDNKNIRFALDVAR

AVASGETIIVEGEFHVDPITEEQNNNKTHPVVASTWLIGGKKAEFRLGIAPKFPAKIK

TILPVKELYPGIPDELVLNVENNVDAETEFSLDLPAGEFLEWAERSVRFTVPAKGKVS

VPVPFTLRSYGLYSQEVEVTAIPAGNHAISFTSKLSVLMKGIEGKYGGQIEDQWVAVN

GAFSLHMNKQDNNMWIEYPGSRHSFWWTYPKLGKPFAEELSKKQAKEVNIYPEGERQV

LEALYDSEDFPGLQIKSVVKLSANGVAEFHHEVENTRDTAWEEDLYLMNNFGFYGSRL

ILPYQDRFVDMGDSYAGDPSHWDSAQITENWLFCKEEYGASGIYWDLSLKLLRPEYTL

GFQHELGRIPAGAVVRTKATVYALNTFAKWQDFRAFARKQRNPVIPLLDSHLDLTLSE

GNPFAPDVLQATLTEHKLVPLAGKLELYVQQGGEPEVMANAKEFSPEQNLNSAKLTFS

PDEADRDQHVSGWKVRGLYRGEDRIQERTALWFPQSGTEVKQKIEEGSAGPLYTVDNG

VLSIAVAPVFGSVVHSLQYAGEEWLDSSYPEPAPRSWWNPWYGGLGVGVPGMNGFSRQ

LEQRAASWTEQKDQYGNVWKGIKLTTRIEKHEANRGITIHQHYLMLPSVPVLCTLLAV

TNESGLTLTDYSLAEERFLQPSSVFAEGWIEQPGKDRFPLGKVDVGLPLEDLLRVGSV

SRENMLHAVHRYPNQSAWGFANNQVTGLHVNHHLTLQHGETVWTEPTYLVLGQIPLSS

ADVHDLLKLNFATSTDEKEASHADH"

CDS 732281..733159

/locus_tag="EFAGFIKM_00640"

/inference="ab initio prediction:Prodigal:002006"

/codon_start=1

/transl_table=11

/product="hypothetical protein"

/translation="MPIIDIHIHLSDIDSFHQTAIDLSKVDYSAAGLKAEFDKNDVIL

GIGMGVTEQTKGAFPDSSSPNPMGLDLEEKVPSFLMECVGINPNALDGKNAQDELDRI

EARLQSPEVAGIKLYAGYYHHYVYDKIYTPVYELAAKYNLPVVIHTGDTYSMNGLLKY

SHPLTVDELALQQRGVNFMICHLGDPWVMDAAEVVAKNPNVYADLSGLVVGDRPHFER

FMNEPLFMDHFRRALVYSDHYEKMLFGTDWPLAPIDLYAEFVRRLVPEQHHDKVFYEN

AFGLFPRIGQRIADLG"

CDS 733417..734580

/gene="adh2_2"

/locus_tag="EFAGFIKM_00641"

/EC_number="1.1.1.192"

/inference="ab initio prediction:Prodigal:002006"

/inference="similar to AA sequence:UniProtKB:A4ISB9"

/codon_start=1

/transl_table=11

/product="Long-chain-alcohol dehydrogenase 2"

/db_xref="COG:COG1979"

/translation="MRSFQFYNPTRLIFGKGQLQALQTEVPKYGKRILLVYGGGSIKR

SGLYDQVISQLNEIGAEVTELAGVEPNPRLSTVHKGVELCRTHNIDLVLAVGGGSVLD

CGKAIAVGAKYDGDMWDVVVREATPEGGLPLGTILTMAATGSEMNNGSVITNQDMQEK

WAWFSEYSFPAFSILDPEYTYTVPLDQTVYGMVDMMSHVFEHYFHLDTNTPVQLGFCE

TILRTVIDTAPRLIKDPENYELRETILYCGTMALNDVLNMGLAGDWASHNIEHAVSAV

YDIPHGGGLAILFPHWMKHNLDVDVDRFKRMAVNVFDINPTGKSDKQVAEEGIEALRT

FWTSIGAPSRLADYDIDDSQIDVMADKAMRFGPFGFFKNLQREDVVQIYQAAL"

CDS complement(734737..735162)

/locus_tag="EFAGFIKM_00642"

/inference="ab initio prediction:Prodigal:002006"

/codon_start=1

/transl_table=11

/product="hypothetical protein"

/translation="MAKTALPTLLNVVRILLSVKLIYVIVSFIVFLIDFNQNMEAYLG

FLRKGDDLAYASGVILARMLFIIGPSLLAVIFITKRKFKLTVTFLSLALFVSIPNESN

LFTLIHLFALLIVLLHRPSKMYLKRKDTPVNEAVVEPKN"

CDS 735439..737496

/locus_tag="EFAGFIKM_00643"

/inference="ab initio prediction:Prodigal:002006"

/codon_start=1

/transl_table=11

/product="hypothetical protein"

/translation="MVFSKARERMRNMSFRVKLPLMISLLVCIVLAVTAVVSYTVAAV

ITLDKSKDEIKATSDRVGAGLYASLSLEEQSTFLITTHRTFQDLLEIRNTDGQEGENF

FSENNELLDKASGILADSLAGMEGNNSLILLDRNGIIIAANDPAVIKAERGDRAYFQE

AIQGKNVISEGIVAKSSGQLGTAFAMPIYNENQEVQGVLVSAASTSFFVNQLQNININ

DEGKVIILDRVGTVIYDSADEKLAGQKMESGEYQSLIDLVPGAELQQGDISTDEKVAY

YSKIPRSDWTVVVEDKLSDVQKPLRAMAKQMYIVLFSAIAVSVVAGILISLLVTRPIT

RLTVLFRQLAGGDLTVQAQGKYSGEFKQLVDSFNTMAAGNKQLISSMNHSIGILKTST

SELEQSTQQTSTTIAETTTTAFEISRAMESQANDTGAIVDKFMNVGNKIANVNGMSQA

VKFKADEITDAFKNNHEVIDALIAVNGQNEIEVGNISKITVQLAESSSGIHQITGTIA

EIANQTKLLALNASIEAARAGDQGRGFAVVASEIRKLAEQTTAQSEDINRIVMQTIDH

VEQNNRSVQAIEAIAEQHKSSVSQTKETFNFVTENMNEMMSQVQAIATEIESIERDKD

DVIGAAQNLSASGEEVSASVEEVTATMQEQSGMTEHLTDMVQRIDELTKQLAGESSRF

KTK"

CDS complement(737597..737995)

/gene="yabJ"

/locus_tag="EFAGFIKM_00644"

/EC_number="3.5.99.10"

/inference="ab initio prediction:Prodigal:002006"

/inference="similar to AA sequence:UniProtKB:P37552"

/codon_start=1

/transl_table=11

/product="2-iminobutanoate/2-iminopropanoate deaminase"

/db_xref="COG:COG0251"

/translation="MTATMSTIIHASTAPKFPLPFSHAVRAGDFLYVAGQVGVDPQTL

EPIGGIKEQTEQCIRNIEVILQEAGLTLDHIVKVTTHLARVEDQSEYNEVYARMIKQP

YPARITVFSGLGPYLIEMEVLAYAPSIRGE"

CDS complement(738003..739379)

/gene="gatA_1"

/locus_tag="EFAGFIKM_00645"

/EC_number="6.3.5.7"

/inference="ab initio prediction:Prodigal:002006"

/inference="similar to AA sequence:UniProtKB:Q9HVT8"

/codon_start=1

/transl_table=11

/product="Glutamyl-tRNA(Gln) amidotransferase subunit A"

/db_xref="COG:COG0154"

/translation="MITKPLNELTIAEAATLIKNKLISPVELTKSYLDRIEKVEPAVQ

AFVTVTAEQALQAARESEEKLMNGEYLGPLHGIPYGAKDIIHTAGIRTSAGSSTFPDF

VPGTNATVINKLQAAGAILLGKTTTTEYAFQGGEPPTRNPWNLEHTPGGSSSGSAAAV

AAGMASFTLGTQTFGSLLRPAAYNGLTCMKPTYGRVSRNGVITASWSLDHIGAFTRSA

QDNAIVLEAMAGQDELDPFTLPHGKPDLTNALEHPISEMVIGLPSNFFQTDEPAILLA

VESAIAVLEKLGMQWKKVDLPSYMGETIAAHRTVMRAEAAAFHQERFAEASDRYGHTM

REQLELGYQTSAVDYLQAQRIRTIFRSEMMMLFDEVDVLLTPSTPYVAPHGYKTGSPI

FNGPFTNTGLPSITVPIGFDTTSGKQLPIGMQLAGPLMREDRLLSITHHYQQATNWHQ

LTPFIHTH"

CDS complement(739573..740604)

/locus_tag="EFAGFIKM_00646"

/inference="ab initio prediction:Prodigal:002006"

/codon_start=1

/transl_table=11

/product="hypothetical protein"

/translation="MRTFKKIAILTAMTVILTTLLSACGSNATTPASGAGASNSGGQA

NTQVDKIMVSEVYHNLLYLPLYVANNQGFLEENRIELSSIRAAGSGPTALSSVISGES

AFSFHGPEHVAFANAKGGDARSLVLLSGSAPVWAVAREGVTVNSTADFKGKTIVVGLA

PTSSNSLLRKLFADNNIDIDKDVTITEVQNGSELGAVLAGKADIAIVYEPQLDQGIAE

GLHIVHDFTKDYPDFAFATMNTTASFIEKNPDLTQRFVTSIEQALDYIQANPEGAKAV

AVKEFPNLDKNVVEQAVQRMIDSKVYPAEGLINESAFKTAIDMQRFIGNLKEDLAYED

IIDSSFTTK"

CDS complement(740639..741505)

/gene="ribX_1"

/locus_tag="EFAGFIKM_00647"

/inference="ab initio prediction:Prodigal:002006"

/inference="similar to AA sequence:UniProtKB:A9WGD1"

/codon_start=1

/transl_table=11

/product="Riboflavin transport system permease protein

RibX"

/translation="MKSTSPEITLAGKPARVVKSKRRQSTFSKEWLMTMLWRFIIVAV

ILVIWESAVRLKLVNGFLMGSPSAILDAAITMASSGQLLTDAFATVNATVIGFVAGSL

IGSLAGLLMWYSKSVARVLDPFIVAMNGIPKIALAPMIIIWFGSGIFSKIALASVATF

IVALLSAYQATHQIDESQINLMKSFGAKKSQIFRKIIVPSSLPWIISAFRINIGLALV

SVVGGEFISSDKGLGHMVFVEGNLFNLPAVWVGVFMLMLVAMLLYTCVGYIESRLLPW

NDNKTSSKSTSV"

CDS complement(741505..742401)

/gene="btuD_3"

/locus_tag="EFAGFIKM_00648"

/EC_number="7.6.2.8"

/inference="ab initio prediction:Prodigal:002006"

/inference="protein motif:HAMAP:MF_01005"

/codon_start=1

/transl_table=11

/product="Vitamin B12 import ATP-binding protein BtuD"

/translation="MTIMQTVYMIRNIFINVMLINIKLHQETKKHQAVGKGLPIMAIQ

IQGVSKSFVSATGEDIQVLAEVSMQIKKQEFFSIVGPSGCGKSTIFNIIAGLLKPTNG

KVIVCGEEIDQTTGHVGYMMQKDLLLPWRSILDNVTLGLEVKGMSKKDRGDIAMDYLD

RYGLASFAEAYPSTLSGGMRQRVALIRTLVTQPDIILLDEPFSALDYQTRLILEDEIL

SILKSEGKTGVLITHDIEEAIAISDRIAVMSKRPTTVKQVYDIGLASQYGSALKARSD

QKFKQYFESIWSELDIQMGRIS"

CDS complement(742513..744192)

/gene="pucR_1"

/locus_tag="EFAGFIKM_00649"

/inference="ab initio prediction:Prodigal:002006"

/inference="similar to AA sequence:UniProtKB:O32138"

/codon_start=1

/transl_table=11

/product="Purine catabolism regulatory protein"

/db_xref="COG:COG2508"

/translation="MDLQQLLAIPILSKAKVIAGHRGLDRLVQSINIMDAPDIVQFLK

PGDMLLTNGYILKDRPDAHLGFITDMHAIGCAALAVKTQRFSLELSPQLLETADRLSF

PIIELSEIDNTLGEIFQHSISAILQNKTHELHYALSIHKQFSTMVMQGKGIPSIVDTL

SQLLSSPVLLLGSKKQITASSNYVQQLDDQSLAAPILTFIDENPSFHTAISICLLTAD

KYRHAELHPIFTDRHEGYLIALYDSSAISKLSTLALEQAVNVIGLELTKKQAVKERSR

RYKNEYFSDLIQGFIRSEQEALHRGKKYGLQAKGSSVLIIAKKDESLAGIPKQNSTSS

GEERFISERDANYELIKQEFARLDLSFVMFTKNDQFGILVFLAESSWDEHTVVQQLER

MASNLYSESQLSLSFGIGNPYTNVLDIGLSYKEAVKALQSGYQMRKTRFAHSYQTMDI

SRLLRMIPHDEMLQFHQETFKPFNDRDPNERSELMKTLSSFYENHCQIVDTAKELFVH

RNTVIYRLEKCEKLTGRNIKDPMESLRFRLAFALESLLNVNPAPSEANHTS"

CDS 744411..744887

/locus_tag="EFAGFIKM_00650"

/inference="ab initio prediction:Prodigal:002006"

/codon_start=1

/transl_table=11

/product="hypothetical protein"

/translation="MKGKGLVSLLLASVVAGSILVASTTAAERVVAQTPMEAATEYFQ

AYIARDVEGMMFYSKDTNYLDEKSREEGYVKDFKTNPTTGYEIVETRQINTDEVQLYV

VQKYAGQGYEQSPPLPYKIFKSENGWKVLVEPLEINTVTGKVTKGTPIHALQYTDH"

CDS 745044..745688

/locus_tag="EFAGFIKM_00651"

/inference="ab initio prediction:Prodigal:002006"

/codon_start=1

/transl_table=11

/product="hypothetical protein"

/translation="MKISDRLLRQYLDHVYWICGGPCGGKSTMTKLLSSKWDMNYYSS

DDHTFDYQKKANPQDHPAILRHFIDWEWFFLGRGNDRSHWLNSVFDESVEFIILDLLQ

MGKEKPIIVDTFMEPAYLREIVNPNRVVYMFAEDDLIRAEYLDRDHLRGMEGLFPSLS

DPQRAREETLNTVVRASNHYLQQAEQYQLKWFMRTSQSNKDQMLLEVEKHFGFR"

CDS 745930..746442

/locus_tag="EFAGFIKM_00652"

/inference="ab initio prediction:Prodigal:002006"

/codon_start=1

/transl_table=11

/product="hypothetical protein"

/translation="MKKKRLFATVISLALATATVTACDSTSQTQENMSNPKTGVLSSE

SESTTLSNSLSEQESKPSHFIASGGATVQQPFSVNPEFVHIKLLMKNNSSHEVNVSLT

HLDTSKLYLARTIAPGESLDWIDSKEGFTQGMSTGDYLLQWSGGGYRVEGELWGSAGS

DPDDTVQLTY"

CDS 746528..747061

/locus_tag="EFAGFIKM_00653"

/inference="ab initio prediction:Prodigal:002006"

/codon_start=1

/transl_table=11

/product="hypothetical protein"

/translation="MHNMMNKRQFIEMTVKEQHSVVFKVNNRDEFFRGVAKGLKEGYL

VVETEEGKNFKFNMDDIIEIYAIKSSSPKHSAIQSHSFMKDSKDDLENLVLQFESLND

NVKKQITSSLSKVLSKQVIISKMDITSYNVSEIEMITNTINGYLLTIKTRDIHSEFFE

SYNQKTCFGKGLDNNRE"

CDS 747135..747338

/locus_tag="EFAGFIKM_00654"

/inference="ab initio prediction:Prodigal:002006"

/codon_start=1

/transl_table=11

/product="hypothetical protein"

/translation="MKEGIKNVLLGTVVFVVGFSIILLLTSSDIEAELTYYAAIAYAL

LYLASVISVCTAVLLKKMTQLKK"

CDS complement(747479..748084)

/locus_tag="EFAGFIKM_00655"

/inference="ab initio prediction:Prodigal:002006"

/codon_start=1

/transl_table=11

/product="hypothetical protein"

/translation="MNQNVTLIGKTIKLLPLESKHKEDLLETLMNPNIWEFTWRKNAT

YDDIKHLIEQAIQNQLYGTQLPFVIVELMSEKIIGTTRIGDIDMINQNAEIGWTWLTP

TYWRTGVNTECKFLLLNYCFEQLALVRVQFSISGYNIRSQRAIERIGATKEGIFRKHR

RDTQGRFHDNVFYSITDNDWPEVKSTLKHMIHIKYSPKNTR"

CDS 748355..748843

/locus_tag="EFAGFIKM_00656"

/inference="ab initio prediction:Prodigal:002006"

/codon_start=1

/transl_table=11

/product="hypothetical protein"

/translation="MTFPTHIVSAGGIVEDGTGNILLVKAHDDGWVYPGGITEVGENL

IDGVMREIKEESGIDATVSYLISVISNTAIHKWYDGVTDVPTKVMFDFVCKAVGGELA

TSNETSECRWVPKENVLDLITLPGIRMRYEAYLNFNGSVNYMEYVTASTTEFNVKLQR

SV"

CDS 749595..749792

/locus_tag="EFAGFIKM_00657"

/inference="ab initio prediction:Prodigal:002006"

/codon_start=1

/transl_table=11

/product="hypothetical protein"

/translation="MAIMTDSRGNIFLEFIRIEYDKVITSTLDAPLTHALIVVKYQGK

YLFMHNKWRNNWELPGGIIEA"

CDS 749847..750065

/locus_tag="EFAGFIKM_00658"

/inference="ab initio prediction:Prodigal:002006"

/codon_start=1

/transl_table=11

/product="hypothetical protein"

/translation="MDSAEFKGLMKFRLQPSFHGPERTEYGALFMGELCQLDDFVEND

EASSIILWDGTSEIGDIAEIDRKLIEFV"

CDS 750198..750605

/locus_tag="EFAGFIKM_00659"

/inference="ab initio prediction:Prodigal:002006"

/codon_start=1

/transl_table=11

/product="hypothetical protein"

/translation="MLVLALSMIFAGCSPKEKNEDTITERIVTSDFVLEVTTPTVLGT

GETMKVKGTLKYRGMKPVEVSHGKPIIRFFFSGSDEQRNYGDVGYVTEFKSGQVVEVE

DEFIVTKKGKQNLIVDMTADISMTMNPIEIVVK"

CDS complement(750757..751119)

/locus_tag="EFAGFIKM_00660"

/inference="ab initio prediction:Prodigal:002006"

/codon_start=1

/transl_table=11

/product="hypothetical protein"

/translation="MIIQVSPLAEARLTEKLGDRPGYFKLFYDTDGCGCDGIAVLLIL

NEPDSDDVTVEAGSLPFVINKQQQIYFEPSLRLQSEHSFPSFRLSSDSMIYGSNVKVH

DLRDTADVAPQPTGWFVR"

CDS 751407..752435

/gene="degA_2"

/locus_tag="EFAGFIKM_00661"

/inference="ab initio prediction:Prodigal:002006"

/inference="similar to AA sequence:UniProtKB:P37947"

/codon_start=1

/transl_table=11

/product="HTH-type transcriptional regulator DegA"

/db_xref="COG:COG1609"

/translation="MTPITIYDIAKEANVSVATVSRVLNDTAPVRASTREKIMSIIEK

HQFQPNAQARSLIKKETGTIAIILPDITNPFFPEVFWGAENEARGLGYTFFLCNTAGD

YNRESEYLSILREKRVDGIIFLGGRINMQVCPDDMAQELIDMGKRMPIVLVNGNIAKG

GFHRVYTDEGAGAALAAEHLLELGHRDIAFVGGLKELSTTMVKVRAVQKKLREHGLEI

PKERQLLGSFSIDDGKREMSKLLDRDNPPTAVICVNDNTAIGAIKSTIEHGLSIPRDI

SIVGFDDTPLASAVIPELTTVSQNTYQLGKQAVDVLHELINQGKPKKQIILQPELIVR

QSTGPAAR"

CDS 752617..753888

/locus_tag="EFAGFIKM_00662"

/inference="ab initio prediction:Prodigal:002006"

/codon_start=1

/transl_table=11

/product="hypothetical protein"

/translation="MRRSLKVISLLMLVMVMGLTACSSGSKNGSSGESGGKVDLSMTI

WGSEDEKKIYQERLDIVKQTYPDINVKLNVVAGDYDQKVQTMIAGGTAPDIMMIAENY

QAYASKNQIIPLDDMIQANNVNMSERYSDDIANLMKYDGKQFGLPDRAGAMVLFYNKD

LFDKAGVEYPTKDWTQDDLMAAAHKLTVKENGKTVQWGYYPGSWWPQWMQLIYQNGGS

LFDESGKPTFNTDPVRKALQFMNDLTFTHGAGPTPTEIADMGNIGADPLFAQGKIAME

TTGFWNIGSLAKVEGINWDISPVWGETNAFFNGLTITNASKHKEEAFKVIEALTTPEA

QMPMVKAGQDAPATKAGLSSDEFLNAEYGGKKINMAAFSESTIYAEPFNPQWNEMMKL

INDKLGVYFNNKASLDDTVTEIQSGLERLYK"

CDS 753948..754877

/gene="lacF_1"

/locus_tag="EFAGFIKM_00663"

/inference="ab initio prediction:Prodigal:002006"

/inference="similar to AA sequence:UniProtKB:P29823"

/codon_start=1

/transl_table=11

/product="Lactose transport system permease protein LacF"

/translation="MRVPSALCVMGGLNVIKKDGKWFYIFISPWLIGFLGLTLGPILF

SIYMSFTNWDLFQSPEFIGIDNYKTLLTDDPIFWKSVGNTFFYALISIPLGMSISLWI

AYYLNKKIKGITFFRILFYLPSVVPVVASSLLFIHLLAPTEGLINQALAIFGIQGPAW

LLDPNWVKPALILMSLWGVGGGVVLLLAGMKGIPQELYEAAAIDGAKSTQSFFHITFP

MLTPVIFFNLVTGMIGALQTFAQVFIVTAGGPDNSSQMVVPYLFQNAFQFYKMGYASA

IAWVLFIIIMVLTLVVFRSSALWVHYEEGKANE"

CDS 754870..755715

/gene="araQ_6"

/locus_tag="EFAGFIKM_00664"

/inference="ab initio prediction:Prodigal:002006"

/inference="similar to AA sequence:UniProtKB:P94530"

/codon_start=1

/transl_table=11

/product="L-arabinose transport system permease protein

AraQ"

/db_xref="COG:COG0395"

/translation="MSNPSTVEKTISYIFLILVGVLLASPFLYMISIALASDATTVKS

AFTFVPMEFQWSNFYTIFTNNNLGTYLKNSVIITVFTIVGSVLSASIVSYGFARIKAK

GSRFLFIVLLSTMMIPGEVTMVPQFIIFRHLDWINTFYPLIVPSFFAGAFNVFLIRQF

VMSIPKSLDEAAMIDGMGHPGIYWKIIMPLTYPILAAIAIFSFSYNWGNFMGPLIYIN

DPEKMPLALGVQLLTTVGGGQMPPWNLVMIASLFLTIPMVLVYLFGQRYVVYEANISGG

SSGIK"

CDS 755806..757302

/locus_tag="EFAGFIKM_00665"

/inference="ab initio prediction:Prodigal:002006"

/codon_start=1

/transl_table=11

/product="hypothetical protein"

/translation="MAHKEQRSRGKRSYILMGVALIALLAGGGIVINHFANESSKTLE

FQGEPVRLKLDQSYDHFEGWGTSLAWWANNLGGWKDQAKVSEVMDLVFDPEKGLGLNI

VRYNIGGEENPEMKALRPGGDVPGFQPEPGEWDWDADAGQRAVLQGSMERGVNIAEAF

SNSPPYWMTISGSVTGAVDGSNNLRDDQYDAFADYLTEVVKHYRDEWGITFRTLNPLN

EPSSDWWKKGNMQEGSHFTNEKQAEIIKKVAASLKSKGLDGTVISAADDNSIDETVFN

FNLYDQDTLDVIQQINTHSYNGSKMEELRTLAERQGKKLWMSEYGTGGSEPHSHEDMT

SVQELAERIMFDLKIMQPSAWVYWQAVEDEGANNNWGFIHANFNGEEQYEMTKQYYGM

AQFTKFIRPGATIIPTDGGRTLAAYDAEHQRLVLVIRNELSAGTTAFELEGYAYGKSS

TAQVYQTSPERNMEQLEDIPVYANGLEVPTADNSITTVVLEGVTLQGN"

CDS 757567..760608

/locus_tag="EFAGFIKM_00666"

/inference="ab initio prediction:Prodigal:002006"

/codon_start=1

/transl_table=11

/product="hypothetical protein"

/translation="MTEKASLLLQEQEKNIARAENGVLSELTYDARSFFLNGERVFLN

SATVHYFRMPKEEWREALLKAKLAGMNCIDTYFAWNVHEPEEGQWSFEGDNDCGAFLD

LCAELGMWVIARPGPFICAEWDFGGFPYWLGTKEGVKFRENNETYLHYVNLYFDQLVP

IIRERQLSAGGTVILVQVENEYGYLMDDAAASEHMNTLRDELLQRGIDATLITCVGGA

EGTIEGANFWSGADGHYEKLRVKQPDTPKMVTEFWTGWFENWGGPSAIQKTAPLLERR

IMEILRAGYTGISHYMFYGGTNFGGYGGRTMGSSDIFMITSYDYDAPLNEYGRVTPKY

AVTKNLSYFVHAFGALLMKTEGIPEEQIVVRHPEGISVRGRQADNQKIWFLESHKDER

ETMHITLEEGRTLQVSVNPGQIVPLLDRVQIAEHVYLTAGTMITGNEMINGDLTLFIV

AAAGQRSVIELEAEALNVTDSTVQVLVEHDSARNLHRFDLFHFQEPGMIQLEANGTPI

RFVILDQETMNRTWRLEATDQKGLRYVIGFDDVDVLPSGQVKGMITDPDRTIMLLGDW

TDGERTLTGHEFYASVKPIAGKQQQLPWTLPSAPTLSDSPEMSRITLTAKQRSASGQP

EDFSRYGQDFGYLLYECDFERPTEGVTNLILPDIQDTARIIVNGVQQALVRQVGAAGV

QLQVAQGQNTLQILVQHMGRLNFSPYLGESKGLAGAVYLGGKVQDIRRDWRMESGLVH

LDEVNSLQGSPLLSRSFTLDSMDRAILVGALSKGLRINGIEVPMEGYQDWFAFQTLDI

SLYIKPGETNTLEMPYSRSSLNRLELITYPSQGELKDWRMAGTDALLPQQQWESYEVG

QESIHLGAQGSIQRVGQPGIAAGGNCTANLVPDTTNESVNRATLSEKSHPVSNHTPVR

GSYGQPVWYRWRFAKPTISEHHKVNLMLRLTGMSKGTIHLNGHHLGRYWQIGPQEDYK

IPVAWLQEENELLLFDEEGRTPERVRFLLDALSRYPWVVVE"

CDS 761053..762402

/locus_tag="EFAGFIKM_00667"

/inference="ab initio prediction:Prodigal:002006"

/codon_start=1

/transl_table=11

/product="hypothetical protein"

/translation="MLTKKEEGIGGIGVGKSKGFKAGMILLLGTLLLLTILFPNTARA

ATSVYTISAFTNTSESNMYIYESYNATHYGLLKGPAYTPPADLIRDPSIMKHTDGLYY

VIYTTNWSGNTIGIASSPDKVNWTFVRNITLSTPTTIAHTWAPEWFKDSNGSLNIIVS

LSPGNYQNFKPYVLTASGSNLTSTSWSAPTELAGIAPNYIDTFIVKTGSTYHAFTKNE

TTKYIEYATSTSLTGPYTFKGTGDWAGWGSWVEGPALVQLDNGSWRIYFDGYATQKYY

YSDSADNFQTWGVKQEIAGLTGLVRHMTVMKESGQPGDIRKLESFNVPGSFIRHYNYQ

ARIDANVSPAEDAQFRIVPGLSNATGISFESMNYPGYFLRNNNGNITLVKNDGSTTFR
NDATFKRVNGLANASWTSYTSFSNPNLYLRHYNNMLKLEAVVTALDKSDATFREVAQ"

CDS 762640..764484

/locus_tag="EFAGFIKM_00668"

/inference="ab initio prediction:Prodigal:002006"

/codon_start=1

/transl_table=11

/product="hypothetical protein"

/translation="MKWLKRMSSLLLAGSLLTGSLGVGTEVSAAGAYTAVADPSKQYQ
TMEGWGTSLAWWGKVVGEYSNRDEYVEKMFNANTGLGLNILRYNIGGGDNPSSNILEY
RKAVPGYQPSPGVYDWNADANQRYMLQAARAQGVNVIEAFANSAPYWMTISGNVSGSA
NGGNNLKPDYYDDFADYLTEVVKHFRDNWGITFDSVTPLNEPISTWWKQGNDQEGMHF
DRADQNTILSQVQASLNAKGLSTKLSAPEEYSIDDTNVSFNSYSNAVKSAITQINTHT
YGGSDRTALRNTATSAGKHLWTSEYGDGDASGLTMSRTILKDIRNMGASGWVYWQAVD
SAEGWGFFKNVLNNTQTTSYTVNQKYYVMGNYSKFIRPGYKIIGMSDANTLAAYDAAS
GKVVLVTTNSESTDTTVNYDLSRFTNTGTSAQVYRTSSTEKLQQLTNISVQNKTFTAT
AKANSVTTYVISGATYNGGTGYESGAIYKLINRNSGLALDVNGASSTGGATIIQWNDN
GAANQQWKLESAGNGYYNIRNVGSGLLLDVNSGSTQGGAALIQWQDNGGNNQQWLPID
VGGYVVLGNRNSGLTVDINQGSLTAGASTIQWADNGGANQQWSLVKVN"

CDS complement(764615..765706)

/gene="soxA"

/locus_tag="EFAGFIKM_00669"

/EC_number="1.5.3.1"

/inference="ab initio prediction:Prodigal:002006"

/inference="similar to AA sequence:UniProtKB:P40859"

/codon_start=1

/transl_table=11

/product="Monomeric sarcosine oxidase"

/translation="MGMSAGYYLSRSGCKTLLIDAFDPPHTEGSHHGDSRLMRHVYSG
GPDYIAMALLAQKLWHELEETVGDQLFVPSGVINVVDPEIHSFHNRLSHADDAGIRYE
TLHAAEVMKRWPGITIPEHYEGMYEPDAGYLYSEPCIRAFRKAAETHGATLLTHTLVE
HIQYGPHSVAVQTSGGDTYHANQIILSAGAWFQTLKPFVDLPIQAVRKTVGWFDAPEA

LYGEAHFPGFTLAGKEGTYYGFPSIGGSGLKMGRHDTGQVWTPGSTMAPFGTEETDEG

DLRRLLELHMPQAAGALKRGSVCKYEFTSDEDFIIDRHPAHERVWLAGGFSGHGFKFA

SAIGQILSDLVQTGHTDRDISRFALSRFR"

CDS 765879..766436

/locus_tag="EFAGFIKM_00670"

/inference="ab initio prediction:Prodigal:002006"

/inference="similar to AA sequence:ISfinder:ISSpgl1"

/codon_start=1

/transl_table=11

/product="IS1595 family transposase ISSpgl1"

/translation="MSEQFRFDLFPLIQTERFILRSAEAGDSHDLLALYSDEAVVQYM

PFTPFESVEDAQGEMNWYTKIFKEHTGLRWMIEDRETRKVIGTCGFLNYEDVHNRAEI

GYDLRSSFWGRGVMTEVARAVLHFGFTSMQLNKIEAKVEPENEASIRLLHKLDFQQEG

VLRQHEFEKGRYIDLAVFSKLRSEI"

CDS 766546..767301

/gene="exoA"

/locus_tag="EFAGFIKM_00671"

/EC_number="3.1.11.2"

/inference="ab initio prediction:Prodigal:002006"

/inference="similar to AA sequence:UniProtKB:P37454"

/codon_start=1

/transl_table=11

/product="Exodeoxyribonuclease"

/db_xref="COG:COG0708"

/translation="MKLVSWNVNGLRACVTKGFMDYYQESQADIFCLQETKLQAGQIE

MDLGEDVYQYWNYAIKKGYSGTAVFSRIKPLSVRYGMEEVSEPEGRMITLEYDEFYLV

NVYTPNAKRDLTRLPYRLEWEDRFRGYILQLEQTKPVIVCGDLNVAHQEIDLKNPKPN

LGNSGFTLEERGKMTDLLASGYVDSFRHLYPDRTDVYSWWSYMPKVRERNVGWRIDYF

LVSNQLAPKVADAHIDCHVMGSDHCPVGLTLQL"

CDS 767579..768517

/gene="rutD_1"

/locus_tag="EFAGFIKM_00672"

/EC_number="3.5.1.-"

/inference="ab initio prediction:Prodigal:002006"

/inference="protein motif:HAMAP:MF_00832"

/codon_start=1

/transl_table=11

/product="Putative aminoacrylate hydrolase RutD"

/translation="MLATGLLLLAGTAYEAYQSTQDMKSYPPPGKYYEVSGRNMHLYT

AGKGEVTVVFASGWGTPNPYVDFSPLYDKLKSQVKIAVYDRFGYGYSDYTDDPRDVDT

ISEEIHQLLRTSGQRPPYIMVGHSLGALETLRFAQRYPDEVAGMVMIDGGSPEYYSRT

DLNTPEWMMDWSRFLVKTGIARTLLQSDRMMASLVIDPNLVTEPMKKAVTISTLRHAY

NDNVMEEVRNSRTNAACVLENKTKFGFPLTILTAGSEESDEDSHAWQDDQANFASWSR

HGTQLTVPQATHSIHNSQPDIVAAEIMKLVTPSSDL"

CDS 768514..768711

/locus_tag="EFAGFIKM_00673"

/inference="ab initio prediction:Prodigal:002006"

/codon_start=1

/transl_table=11

/product="hypothetical protein"

/translation="MSNMIPQTHDEQEEEGWMKKFKVITRFLSILVASCLLSLSVLSV

SVIWVKNNVHDVPVIQSFERD"

CDS 768734..768961

/locus_tag="EFAGFIKM_00674"

/inference="ab initio prediction:Prodigal:002006"

/codon_start=1

/transl_table=11

/product="hypothetical protein"

/translation="MVTDGEEKELCIGLCGTGDDGVLAKKLTDAGQTIDDKNIIIQGT

DPETKDVWSLIGGTLAGQEGVIELTLTWTES"

CDS 769175..770152

/gene="ddh"

/locus_tag="EFAGFIKM_00675"

/EC_number="1.4.1.16"

/inference="ab initio prediction:Prodigal:002006"

/inference="similar to AA sequence:UniProtKB:G1UII1"

/codon_start=1

/transl_table=11

/product="Meso-diaminopimelate D-dehydrogenase"

/translation="MIRVGIVGYGNLGKGVEKAISQNEDMELIAVFTRRNPEQMVAES

TEVRFEHISAAEQYIGKVDVMILCGGSATDLPEQTPAIAKLFNTVDSFDTHAKIPEFY

KEVNAAAEQGGHVSVISTGWDPGLFSMNRLLAQSILPEGKEYTFWGKGVSQGHSDAIR

RVPGVKAGVQYTVPVEEVINRIRAGETPELSTREKHLRQCYVVAEDGANQDEIRETIV

SMPNYFADYDTTVTFISEEELKSEHEGMPHGGFVIRSGVTGAGQKQIIEFGLKLDSNP

EFTASVLVAYARAAQRLSVEGHKGAKTVFDIPLGHLSPKSAEDLRRDLL"

CDS complement(770311..771165)

/locus_tag="EFAGFIKM_00676"

/inference="ab initio prediction:Prodigal:002006"

/codon_start=1

/transl_table=11

/product="hypothetical protein"

/translation="MSNQPFASTASPHLSADCEQCFGLCCVALPYGKSSDFAFDKASG

TPCPNLRTDNRCGIHTQLRQKGFKGCTVYDCFGAGQKLSQVTYAGKDWRDHPETANEM

FDCLPALRQLHELLSYMKEMMMRPETSSLHSAFGELYEEVERLSNLEPAALLRLDIAN

YRSSVNQLLVQASEMVRANVPPTAHGQGKSKKSKKRTGSDFFGANLKGTDLRGASFRG

ALMIAADLRNADTRNADWIGADLRDTDLGDADLRGGIFLTQSQINAAKGNVNTKLPDH

LNIPSHWV"

CDS complement(771321..772706)

/gene="gdh"

/locus_tag="EFAGFIKM_00677"

/EC_number="1.4.1.4"

/inference="ab initio prediction:Prodigal:002006"

/inference="similar to AA sequence:UniProtKB:P31026"

/codon_start=1

/transl_table=11

/product="NADP-specific glutamate dehydrogenase"

/db_xref="COG:COG0334"

/translation="MSTITKETTESVQVTAAEYVHSVYESVVARNPHEDEFHQAVKEI

LDSLIPVIEAHPKYKNHSLLEQLVEPERVITFRVPWVDDQGKVQVNRGFRVQFNSAIG

PYKGGLRFHPSVYSGIIKFLGFEQIFKNALTGLPIGGGKGGSDFDPKGKSDLEVMRFT

QSFMTELYKYIGSDTDVPAGDIGVGAREIGYMFGQYKRIHGGHEAGVLTGKGLLYGGS

LARKEATGFGCVYFVKEMLQSKGLSFQDSTVVVSGSGNVSIYAIQKAQELGATVVACS

DSGGYIHDPQGINLDTVKRLKEVDRLRISEYIKEHPHATYTEGCEGIWSIPCDIALPC

ATQNEIDEEAAALLIKGGVKAIGEGANMPSTLAAIDVFHGAGVLFGPAKAANAGGVAV

SALEMSQNSMRLSWSFEEVDAKLHDIMKNIYTSCVEAAEEYGCEGNLVAGANIAGFLK

VADSMIAQGVV"

CDS 773095..773568

/gene="greA_1"

/locus_tag="EFAGFIKM_00678"

/inference="ab initio prediction:Prodigal:002006"

/inference="protein motif:HAMAP:MF_00105"

/codon_start=1

/transl_table=11

/product="Transcription elongation factor GreA"

/translation="MNHRTSRHNCREKLVSQLFTFGEEKRTFLDAYFDARDPERIQLE

KQLSAYTEYVEKLLLGPDEDLDSAVLIGSHIDFEYIDFHTSDSFMIVMPEDTNPDEGR

ISFLSPVGSQLLLGSVGEIRSVHTPSGSMRIRITGIELRSETLNEPALGGADHAL"

CDS 773558..774064

/gene="khtT_1"

/locus_tag="EFAGFIKM_00679"

/inference="ab initio prediction:Prodigal:002006"

/inference="similar to AA sequence:UniProtKB:O07535"

/codon_start=1

/transl_table=11

/product="K(+)/H(+) antiporter subunit KhtT"

/db_xref="COG:COG0490"

/translation="MHFKETDLPGIGRKYWLHTRSGEHLVIVIHNDERRDLFHMESGD

TPEEVGDMVSLVTLDDDEARAVAAIVGGMTYKPKFQDEREVMLEGLLIEWLRIEPHAA

SVGMTIGELDIRQATGAVILAVVTKDKEKHFNPGPDYAFTAGATIVVAGERNQIKHLK

QLLTDGRT"

CDS 774068..775273

/gene="khtU_1"

/locus_tag="EFAGFIKM_00680"

/inference="ab initio prediction:Prodigal:002006"

/inference="similar to AA sequence:UniProtKB:O07536"

/codon_start=1

/transl_table=11

/product="K(+)/H(+) antiporter subunit KhtU"

/db_xref="COG:COG0475"

/translation="MDMLIFEVGIAVALITLTGLISSRLRFSVIPFYILIGMAVGPHA

PQIGIVDLRFIESSTFIEFMGRLGILFLLFYLGLEFSVSRLLKSGKAILTGGMFYVGL

NFVSGLLLGWFMDLPLQETLVVCGIMTSSSTAIVAKVLVDLKRTANPETEIIMGMIMF

DDLFIAIHISILTGLVLSGATSFLSVLLVSLSALLFIVLFLIIGRKSIKYIDKALNIK

SSELFLLTVMTLLFLVAGFSETLHVAEAIGALMMGLVLGESRHVSRIEHQIMPFKDFF

GAIFFFSFGLTIEPASLGGAVGMTIIAVILTIASNYGAGMIAGRLAGMSPKASLNVGF

TLVSRGEFSIIMANIGKAGGLMASIQSFAVLYVLILAVLGPILTKESPRIYAQYARLR

KRMKRRETG"

CDS 775617..776333

/gene="yoaJ"

/locus_tag="EFAGFIKM_00681"

/inference="ab initio prediction:Prodigal:002006"

/inference="similar to AA sequence:UniProtKB:O34918"

/codon_start=1

/transl_table=11

/product="Expansin-YoaJ"

/db_xref="COG:COG4305"

/translation="MNKKSRFQRLKWAGTGMLLSLVLFALPASAAWNDTYQGYATYTG

SGYSGGAVLLDPIPADMKITALNPFQLNYNGVKAALAGAYLEVQGPKGKTTVYVTDLY

PEGASGALDLSPNAFNLIGDPKAGKINISWKVVKAPIQGNVSYRIKEGSSQWWAAIQV

RNHKYPVLKFEVKQKDGTWLNLEKQDYNHFLGTGLGKEKLNVRITDIRGQVLNDTIPA

LPNDGSGGAYIVPGNVQFPD"

CDS 776443..777390

/locus_tag="EFAGFIKM_00682"

/inference="ab initio prediction:Prodigal:002006"

/codon_start=1

/transl_table=11

/product="hypothetical protein"

/translation="MKKSLPSYQTTHPYWKQYQAFFPEEVRLGENTVPEEEWWESNGV

HLHIDRLPASESSIKILFIHGAGGNGRLLAPYARMLQRHGYEVVSPDLPPYGLSYAMS

GTSINYELWIELLVSLIEREYKNDGKPIVVLGSSIGGMLAYHTSARSKRVKGLIATTF

VDTSNQEMRDQLAPNRLVSRAGKRMMDLFPGLLDHVRISVKQVSRMQLITNNKDLTRL

IMDDPQAAATRIPLELLRTFLNKEPEVSPEQFSQCPVLLVHPELDPMTPIRFSQSFFD

RLMVDKECVILEGAGHFPIEQPGLDQLEVAVLRFLQQIQ"

CDS complement(777456..778334)

/gene="yddE_1"

/locus_tag="EFAGFIKM_00683"

/EC_number="5.1.-.-"

/inference="ab initio prediction:Prodigal:002006"

/inference="similar to AA sequence:UniProtKB:P37757"

/codon_start=1

/transl_table=11

/product="putative isomerase YddE"

/db_xref="COG:COG0384"

/translation="MEVEVNTLHAFSDKAQGGNPAGVVLQAAHLSESQMQEIARQVGF

SETAFVMPSDQANFKVRYFTPSDQVDLCGHATIALFYLMKTQHLVNVGIYTLETLAGI

LKVDIEVNGEVYLSQTLPEFGEIVDRQQIADSLRISMEDLHYELPVQIVSTGLPDIMI

AVKDVDVLTKIDPDFQRITEISKAHHAIGYHVFTLESDVMDVLAECRNFAPLYDIPEE

SATGTSNGAMLCYLSQYNQLTDPHLHTYTIRQGYTMNRPSEIRARLTLDSSNEITQIQ

VGGSAIHIENIQIHLP"

CDS 778531..779457

/gene="pikAII"

/locus_tag="EFAGFIKM_00684"

/EC_number="2.3.1.239"

/inference="ab initio prediction:Prodigal:002006"

/inference="similar to AA sequence:UniProtKB:Q9ZGI4"

/codon_start=1

/transl_table=11

/product="Narbonolide/10-deoxymethynolide synthase PikA2,

modules 3 and 4"

/translation="MKAIVIEEFGGAEQLKEQEVAKPACGVNQVLIRTHATSVNPVDF

KIRQGYMKEAAENFPLILGGDVAGIVAEAGSNVTRFREGDRVFARPRQFGTYAEYVAV

DADIIARIPEQLSFEEAAAIPLAAMTAWQALVDHGRLGKGQKVLIHAGAGGVGTYAIQ

IAKSLGAEVASTASESNEALLRSLGVDHFINYKKEDFSEILSGYDVVLDTMGGDIQRN

SFKVLKSGGHLVSLVEQPDEKLAKEAGVTANVFMMEPKGDQLDQLAELAAEGKLKSII

DGTYPLTQQGVREAHEKSETHHTRGKLVIQVQ"

CDS complement(779441..780079)

/gene="ydeA"

/locus_tag="EFAGFIKM_00685"

/EC_number="3.2.-.-"

/inference="ab initio prediction:Prodigal:002006"

/inference="similar to AA sequence:UniProtKB:P96658"

/codon_start=1

/transl_table=11

/product="putative protease YdeA"

/db_xref="COG:COG0693"

/translation="MMQTKNVYLYVFDTMADWEVGYLTAELNSGRYFRKEIKPLQVVT

VGVDKHPVTTMGGLNILPHISIDECTLKGDDVIILPGGNTWMDTIHDPLLKKVAASIE

EGTVIAAICGATVALAKIGILDSRQHTSNDLEYLKMICPDYTGETYYVTEPAVTDGNL

VTASGIAPLEFTMHVLKLLNVFAPETLQAWFNLYQTHESKYFYQLMNSIAPE"

CDS 780277..781014

/locus_tag="EFAGFIKM_00686"

/EC_number="3.5.2.20"

/inference="ab initio prediction:Prodigal:002006"

/inference="similar to AA sequence:UniProtKB:A0NLY7"

/codon_start=1

/transl_table=11

/product="Isatin hydrolase"

/db_xref="COG:COG1878"

/translation="MSNELIQAIQLLKEKKWVDLTHTFGPGSPHFSAFEAAQFDTLFD

HDQGFFAQSFKFPGQYGTHLDAPIHFVRDTRYLDELGLKELVLPLVVIDQSAEVENNP

DFTLDVEHILEFEREHGVIEAGSFVALRTDWSKRWPKHETFDNKDDKGNSHAPGWSVR

ALMFLFEERKVQAIGHETFDTDSAVDYQKNGALLAEYYVLAQDTYQVELLTNLDQVPA

KGAVIFNIVPKAEKASGFPVRSFAILP"

CDS 781630..782088

/locus_tag="EFAGFIKM_00687"

/inference="ab initio prediction:Prodigal:002006"

/codon_start=1

/transl_table=11

/product="hypothetical protein"

/translation="MQIRAFQESDKDFIVSLALRFMDFELMSWRDPGKMEESQLSLAQ

ESVDSLSPGTEIFVAEDETGELLGFLEVQPHKDKLNDIEQGYIVAIAVSPQGEGKGVG

KGLMTKAEEWSRQKGYRQLILNVFNNNLRAVNFYKHLNYEIEVVKMVKEL"

CDS complement(782352..782552)

/locus_tag="EFAGFIKM_00688"

/inference="ab initio prediction:Prodigal:002006"

/codon_start=1

/transl_table=11

/product="hypothetical protein"

/translation="MDDKFIKELREISRDDRRRSEFMIQGLKETLQERKEEGILKRWI

RRKKTEKKISQRFNQDPHSDQK"

CDS 782763..783215

/locus_tag="EFAGFIKM_00689"

/inference="ab initio prediction:Prodigal:002006"

/codon_start=1

/transl_table=11

/product="hypothetical protein"

/translation="MKQRMGRSWLLMLMHGILGIGAIVGGVILIIDPSGSMVNLPASL
LGNSPFASFLFPGIILLLVLGVMPTMIAISLIRYVHSGLGERLNLYPDRYWAWTFSLY
TGFALIIWIMAQVYWIQDVSIIHLVYFAWGVGIQVVTLLPGVQRIYSK"

CDS 783501..783617

/locus_tag="EFAGFIKM_00690"

/inference="ab initio prediction:Prodigal:002006"

/codon_start=1

/transl_table=11

/product="hypothetical protein"

/translation="MVYVIMAVIIWAGTYASMALSKQPMEVKPVKSMDDLFE"

CDS 783762..784769

/locus_tag="EFAGFIKM_00691"

/inference="ab initio prediction:Prodigal:002006"

/codon_start=1

/transl_table=11

/product="hypothetical protein"

/translation="MKKANATMMKYINLNNVREVMQQIETATKPQLALLTNLSVVTIN
ALIQELCDGGELFQDKVVPSNGGRPAQAYRYNYNFKLALVLYIKEMKGQELISATVMN
LENKVVLKEERILPAFDKQHMLQLIARFIKEYPSIDMIGIGIPGQAVDGDITVSSHEE
LIHSHLIREIESEFQLNVLVENDVNAAISGYCTQHADMKEQSVAGIYFPNRYPPGMGM
MLNGQMIRGKNGMFGEIKYLPYSPDWHRDMSKDDFVVNVCHILQTINAVVAPHQIVIY
QERVEREELDMAWKQYGKDHPMPSLPEIVHQDSFQHDFDAGLRGMVLQALKSGILSEA
L"

CDS 784831..785472

/gene="lacA_1"

/locus_tag="EFAGFIKM_00692"

/EC_number="2.3.1.18"

/inference="ab initio prediction:Prodigal:002006"

/inference="similar to AA sequence:UniProtKB:P07464"

/codon_start=1

/transl_table=11

/product="Galactoside O-acetyltransferase"

/db_xref="COG:COG0110"

/translation="MREEERIMKGVLFSPSDPELKIIKRRAHNLSQRYSQTFEEQTEE

RDQILQQLLGQIGEGGFMQGPIFFHYGVHTRIGEHFFGNYNLTIQDDAQVTIGDYTSI

GPNVTIVTPIHPMIASERRQMVDQNGDVKSLCYAKPVTIGNDVWISANVTVCGGVTIG

DGCVIGAGSVVTRDIPPHSFAAGVPCRVIRKITDADSMRNYPDVLADCRVLEE"

CDS complement(785535..787355)

/locus_tag="EFAGFIKM_00693"

/inference="ab initio prediction:Prodigal:002006"

/codon_start=1

/transl_table=11

/product="hypothetical protein"

/translation="MKKRTLISLLLILVIVISGCSVKTKTESQAETTPADTTETAQKP

ADIELLAMSSSENDVNIVRDQLTKNGFNVKLNLQPDYGSFKSQQDAGNYDIALSSWTT

VTGNPDYAVRSLFKTGGDYSILADGELDKLIDQAATQTPDEYKDTYKQLEDRLVTDQA

YIAPLYISLKSQAVNQDILNVDTVRLSKSRAMAWEPIEFKDSSKNAKDPLILTQSASV

LTSLDPIKGNDGSINQLNTNMYVRLVNLTDDDQLTADGSLSHNFSIAEGNSDYYFILR

DDINFAKIDNKKAVDTGERVGADDVIFSLDRAKNKDSVPDHRTYSLHEHIKEAEVVTD

LSALQSIKQSSGNGTILEALEQGLGSKITELVTDKTKADNSAGKYQVVKLTTTEPFPQ

VLNYLAHQSAGIVSKKQVESINTYDVASFDVNKDIPYGDQNTVTEGAAYNNTLYTSGP

YILSYKNDYEGVFLKNPGYRKGTEDEPKIAQVNVRFIADADSALSALRSSEIHLYYGV

PETKYDIIENDSKLKLQSLPSNAVSYLLFNTANREVAKSSDLRKAVLYSINQDEILSF

YKNNKLKAYSTVSPLVQTGNELKADPAKVKEFLSKYNASK"

CDS complement(787429..788940)

/locus_tag="EFAGFIKM_00694"

/inference="ab initio prediction:Prodigal:002006"

/codon_start=1

/transl_table=11

/product="hypothetical protein"

/translation="MTNSSLTQEMRFRLKSSREYSQASFAWITSLLLTALLLFNSYDW

SGQTFKPFLLTILGIYVFFTLVQSVITLRIRKDLIRTGTISTLTRRLAWVQLLAIISG

NIFIVTAAFHLIRKARNVEYTFAVYMLLTQLFVIGVSALNVFKPYVADNFLPAMAVLI

FILVIDLVVLIIVSRYNATSILPRWMIGVSVVLILTSITGNVFALLLGISIIGRIRRQ

GKQKSNFWNDLWERLAPNMTAMSGLFFIIFLFSISICSFFTFDYSMAVENNYSALLQP
PSLAYPLGTDDFGRCLFSRIVFGARISLIVGCMSTIIPVLIGGVLGAFSGFYGRHTDN
IIMRLLDILYAIPGILLAIAIIAAFGANTVNLILALSLGSIPTYARTMRASVLYVSTF
EFVEAARALGYNNRTIIFKHIIPNSLAPMIIKSTLTIGGAVIATSSLSYLGLGVEPHI
PEWGNILKLGSTYLETHSYLAIYPGLAIILLVLSFNFLGDGLRDALDPKLEKA"

CDS complement(788977..790419)
/locus_tag="EFAGFIKM_00695"
/inference="ab initio prediction:Prodigal:002006"
/codon_start=1
/transl_table=11
/product="hypothetical protein"
/translation="MNLAEGSRLTRLVHNLSFIYGKMLLHPSYRYVLFILGLPINLVV
FLIWRSNRKNDGWVAARQTAEQELLASSYRNKLKLEVEEQLRRKHRFFQQQVTEGEFR
RQTEEWLEETVQKELKERTYRILQEQGSQRLTMADTFHTLIDKPWFFAISIIPGCLMY
GILFLYGNPYLKYIFERILMTVFVILGVATLVFTILYMSPFNPAANILGETATQEQIA
AFNQVYGLDQPYLTQLWNNIKGVALFDLGKSFAGNEDVTATIARKFPITLTLAVISLL
LALVIALPIGIISAIKPNSWFDYTFMFIALIGLSIPNFWQGLIFILNFSIKLQWLPAT
FNPQNWLSIIMPTIVLGTGLTAAVARMTRSSTLEVIHEDYVMTARAKGLSERQVMLKH
AVRNALIPIVTVVGLQFGAMLGGAAVTEKVFNISGLGSYIVDKQFIPDIPSIMGGVIY
TAITISIINVAVDLLYAFIDPRVRSKMKQY"

CDS 790552..792321
/gene="gsiA_1"
/locus_tag="EFAGFIKM_00696"
/EC_number="7.4.2.10"
/inference="ab initio prediction:Prodigal:002006"
/inference="similar to AA sequence:UniProtKB:P75796"
/codon_start=1
/transl_table=11
/product="Glutathione import ATP-binding protein GsiA"
/db_xref="COG:COG1123"
/translation="MRAGDNMEPLLKVNDLSVSFHSGESEFQAVRGVSFEVRKGETLG
IVGESGSGKSVTARSIMRLLASPPSQMKKGEILFKGVDLANKTQKEMESIRGRDIGMI

FQDPMSSLNPTIKVGKQISESLIKHQKVSKREAKKQAIAMMERVGITRSEIRYNQYPH

EFSGGMRQRVMIGIALACRPELLIADEPTTALDVTIQAQILNLMKDMQDQLGTSIILI

THDLGVVAGMCDRVVVMKEGQIVETGTTTEIFANPKHPYTIRLLNALPRLDQKKKPKP

VSLVPRDLEDDQPLLEVKSLRQHFNLGKGNTLKAVNDISFHIRQGETLGVVGESGSGK

STTGRAILRLHEPTGGDVLFKGVPLNRLSASEMKTMRRHMQIIFQDPYASLNPKMRIM

DIIGEALDIHQLAGNAAQREKRVEELLEMVGLDPTHAQRYPHEFSGGQRQRIGIARAL

AVEPEFIVCDEPLSALDVSIQAQIVQLLEELQQRLGLTYLFIAHDLSMVKHISDRVAV

MYNGKIVELAESEELYSNPQHAYTKALLSAIPVPDPAVEAKKKRHVVKETTRENVEVE

DRYNLEHSKWVEVTDGHWVAISS"

CDS complement(792436..793215)

/locus_tag="EFAGFIKM_00697"

/inference="ab initio prediction:Prodigal:002006"

/codon_start=1

/transl_table=11

/product="hypothetical protein"

/translation="MTIPKKASLFAMLIMIMLVAAACGDKADNTAATEEPTPTETTTG

TDSDTANTEETENTEPASEESKETDNSQDVELGTTEKGSYTNDYFGVSLKFPEAWEFQ

DAAGMNELTSASSEAIAGDDETKKKQIELSQTKTLNLLMASKYPLDGGQVGPSAMAIA

EKVSLLQGIRTGKDYLEATKKFMVDSQFPYDYKEMTTETIGGKEMDLMQITMDPGDGS

TVTQDYYSTIIEGYAFNFIFTYMDDKTKAEIDTIKKSVQFK"

CDS complement(793326..794087)

/locus_tag="EFAGFIKM_00698"

/inference="ab initio prediction:Prodigal:002006"

/codon_start=1

/transl_table=11

/product="hypothetical protein"

/translation="MPAITFATRHEESPTCSHTAGTAPFDYLPKSSLDRLHKINHFLI

HAFQNSISDIKENLTGTYLFLLTDTEGVLLSMDYSSDLKAVVEHSPICPGMIFTAKSC

GVNAISETMDNNKPVLLLPEQHESPYFQSWHCYAAPLFMGSQHVGYLDVSTINADMQG

ELIAIAKLIPAYMQNCYQSQQTAEVCDKPAVEFTERQLTILEMIAGGLTVKAIALKLK

IKECTVNHHKKVIFNKLGVQSSTEAVSIASRMSYV"

CDS complement(794114..795178)

/locus_tag="EFAGFIKM_00699"

/inference="ab initio prediction:Prodigal:002006"

/codon_start=1

/transl_table=11

/product="hypothetical protein"

/translation="MTQEENTSGWDAIDQSMRELYGEQEPKHYGTALPYMLGGPDPLD

GISVYEVNTPMPHWHFVTYGFSELYEKEMQDASKSGYGFELTFRLTRSEAETDPPAWA

LNLLQNVGRYVFNSGNIFQPGDYMDANGPICLESDTLLTALSFIEDPDLPAISTPNGS

VQFIQMVGITGRELEMIQSWNARGFLSASSMFMPKYVTDLMRNSYADIPSVIQAVEDG

MEQDGSSTAFLFIQQLGWESPRKKLLQKSVPAKLQLGAKQAVLVGTILRSRISKGASL

SLIGPDTNILFEAGEQPAVLESDRQVTLTVNKQIVNELAENLRPVEGTFEITSLDHII

VQIVRTEIKDQEGHVIQTIG"

CDS 795386..795610

/locus_tag="EFAGFIKM_00700"

/inference="ab initio prediction:Prodigal:002006"

/codon_start=1

/transl_table=11

/product="hypothetical protein"

/translation="MDGERYPGFRGPKSRCLPGNGGGSAGIKGSDYAYATLIFSMVRE

CERSYDPVKLILQNRYVTLTLQEQTQQNLQ"

CDS 795789..797246

/gene="bglH_2"

/locus_tag="EFAGFIKM_00701"

/EC_number="3.2.1.86"

/inference="ab initio prediction:Prodigal:002006"

/inference="similar to AA sequence:UniProtKB:P40740"

/codon_start=1

/transl_table=11

/product="Aryl-phospho-beta-D-glucosidase BglH"

/db_xref="COG:COG2723"

/translation="MTTAKKGFPENFLWGGATAANQLEGAFDKDGKGLSTADMIAHVP

KEKRTGGHAMEISSSRIEEILSGKIEERFPKRFGIDFYHHFREDIALFAEMGFKVFRL

SIHWARIFPNGYDQEPNEAGLKFYDEVFDELLKYGIEPLVTLSHYETPLGLTQKYNGW
AGREVIQHYVRYAETVFNRYKNKVKYWLTFNEINVMLFSPYTGGGILIDKVDNKLQTT
YQALHHQFVASALVTKLAHEIMPGSQVGCMLARMETYAATCNPVDVRLAQHENQINLF
FTDMHARGKYPNYMARYFEENDIVIQKEAGDDEILLNNTVDFISFSYYMSVTKSASAD
KEETSGNLTGGVKNPYLEASDWGWEIDPIGLRVTLNNFWDRYQKPLFIVENGLGAYDR
VEEDGSIHDSYRVDYLKKHIEQMKEAIKDGVDLIGFTAWGPIDLVSMSTSEMSKRYGF
IYVDLDDEGNGTLKRSKKDSFDWYKNVISSNGEQL"

CDS 797382..799070

/locus_tag="EFAGFIKM_00702"

/inference="ab initio prediction:Prodigal:002006"

/codon_start=1

/transl_table=11

/product="hypothetical protein"

/translation="MKRWSMNTKSMGWMLALTLSLSVIWPATVGAESTQVTRIQANTY
VGDPGEMVESFDITVANPEKYQNLKASDFEITGNYDGYPLNEAEEIIQNEYEDDGIKL
TITDHTIHMEVEPFKYPGGMISKFKVTNKTYPELSFDETNVDKVVTRTVDDFEKGTYT
GSNGEALSYRLKRSPSTNAQPLVVWLHGGGEVGTDGRSHLTANRGAVVWTESGYDTSV
LAVQYPENYSFKIYDNPEQLAKMQAYFVAQYELIQKLVSEGEVDPDRIYLAGVSSGGG
GALRFMMQYPDLFAGAVVIAAKDTVADYTGSVDGFKDALKDIVNVPIWLHHAKGDPTT
DSRTSILAYEALLELGSDPDIFVYDDDFMEGNNLYGGLKHWSWVPALASYQTPDFLFN
QDKGISDVDIYTRMDRRIKAHEIVDRTQLAVWIADFLKLPNPQYEESYSDTFERWSQK
AISQVTEAGVMQGTGNNKFSPLGDITRAQLAVILDKMKKNLGIEPDQSEAAMFDDVPP
THWAYEAIRNSSQYDIMGGKEAQLFAPNDVVDGEEFHDIMDRLYLTKANRHLED"

CDS 799092..800516

/gene="bglH_3"

/locus_tag="EFAGFIKM_00703"

/EC_number="3.2.1.86"

/inference="ab initio prediction:Prodigal:002006"

/inference="similar to AA sequence:UniProtKB:P40740"

/codon_start=1

/transl_table=11

/product="Aryl-phospho-beta-D-glucosidase BglH"

/db_xref="COG:COG2723"

/translation="MTTPFPKDFLWGGAVAANQLEGAYNTDGKGLSVQDVMPHGITTP

RTEVPTEDNLKLIGIDFYNRYKEDVKLFAEMGFKVFRTSIAWSRIFPKGDELEPNEKG

LQFYDDLFDVCHKYGIEPLVTISHYETPLHLSKTYDGWVNRKMIDFYERYVTVLFNRF

KGKVKYWLTFNEINSILEEPFMSGGIYTPKAELSKQDLYQAIHHELVASALAVKLGHE

IMPEAKIGCMVLSMPTYPLTPNPDDVVAAMHAEQRNDIFADIHARGYYPKYINRYFKA

NNINIKFEDGDAEILKHTVDFISFSYYVSICETGDPEKRIEGKGNLFAGVQNPYLKAS

EWGWQIDPQGLRVTLNKYWDRYQKPLFIVENGLGAVDELITDENGNKTVNDDYRIQYL

NDHLVQVGEALEDGVEVMGYTSWGCIDLVSASTAEMKKRYGFIYVDRNNDGSGTLDRY

KKKSFHWYKEVISTNGASLKNNNE"

CDS 800750..801601

/gene="licT_1"

/locus_tag="EFAGFIKM_00704"

/inference="ab initio prediction:Prodigal:002006"

/inference="similar to AA sequence:UniProtKB:P39805"

/codon_start=1

/transl_table=11

/product="Transcription antiterminator LicT"

/db_xref="COG:COG3711"

/translation="MKIEKVLNNNVVTVIDPGGNELVVMGRGIAFKKHTGESIDESLV

EKIFSLESKEVSQKLKTLLSDIPVEYVECSDEIIRYAETVLGEKLHESIYISLTDHIH

FAIDRHRQGLQIRNALFWEIKRMYRKEYAIGLKALQIIEETLGVLLPEDECAFIAMHL

VNAQMNGEMRETISITNIVKDILNIVRRSFVIELDEDSLSYYRFLTHLKFFAQRVLQG

TAIEDKEVDNPLHDLVSKQYPEAHACAVRISDYTRKIYNRVLSKEEILYLTIHIERIV

RNEQTIE"

CDS 801730..803631

/gene="bglF_1"

/locus_tag="EFAGFIKM_00705"

/inference="ab initio prediction:Prodigal:002006"

/inference="similar to AA sequence:UniProtKB:P08722"

/codon_start=1

/transl_table=11

/product="PTS system beta-glucoside-specific EIIBCA

component"

/db_xref="COG:COG1263"

/translation="MDKQQLSKDILKLVGGEENIDQVTHCMTRLRFNLNDNQKADKAT

LKNTPGVMGVMENGGQFQVIIGNDVPVVYNALVGNMSKSPTTDNASSGASTGEKKKRN

PISALFDFISGVFTPILPAITGAGMIKGIVAILVALGWLSDTSSTYIILSAIGDGAFY

FLPIILAISAARKLGSNMYIAAALAAGIMHPTITALLANGDTTFVGIKVIAATYSSTV

IPIILAIWIASYVEKAVDRVTHASLKLLIVPTVTLLIMVPLTLMTVGPLGTVLGNGLS

GGISWLFDNMSIFASILIGGTMSLLIITGMHYALLPIIVGSMTTLGYDFIIPLMFAAN

LAQGGAAFGVGLRSRNSKTKSLAYSTGLTAIMGITEPAMYGINMKFKKPFIAALIGGA

IAGGFMGIVNVKAYVLTGLVGLPSIAAFISPAISTLLYALAGGLIAIVAAAVLTYILG

FQEENATEPAPAAEPATPAATTSAVVTEEAKAQDEQIFSPITGEVKPLSEVPDPAFSE

EIMGKGFAIQPSEGRVVSPINGTVFSLSKSGHAIGLVSDTGAEMLIHIGIDTVKLKGQ

FFSPKVQAGAKVAVGDVLMEFDREQIEKAGYTTITPVIITNMHQYESIESAGRTTIKE

KDLLFTAKA"

CDS complement(803698..804600)

/gene="benM"

/locus_tag="EFAGFIKM_00706"

/inference="ab initio prediction:Prodigal:002006"

/inference="similar to AA sequence:UniProtKB:O68014"

/codon_start=1

/transl_table=11

/product="HTH-type transcriptional regulator BenM"

/translation="MDIKQCRYFIAIAEEKQITAAARRLHMAQPPLSQQLKLMEEELG

VILFERKGRIMELTQAGRSFYDYAVTLTKYMEEAVMEMQSFRHGIRGKLAIGINTISD

RLIPQALQQFRTTHPQVTYKIQQNESAQLCRLLEDGKVELACVRMPVQTERYEVLHLP

QEPLFYISSTPLDCPTEMGPGVEMGTYFEQLTGIPLLLPSTEGLGMFELILDKFREHQ

VTPSIMGECSDINMLLELVRLGFASSIVPHTVLQLYHEHPFHVYRIQDQHSTVGSALV

WLQKRYLSKPAQHFVQLVQGMLPV"

CDS 804785..805372

/locus_tag="EFAGFIKM_00707"

/inference="ab initio prediction:Prodigal:002006"

/codon_start=1

/transl_table=11

/product="hypothetical protein"

/translation="MVRTIHDAAQAGHTDEVIRFITQQGNRLNERDTLGRTPLLAVVH

GNKIDTARMLVDAGADINLRDARLDNLLLYASAEGMYDMVELAITAGADTRLTNRFGG

TALIPAADRGHVDIVELLLTRSDVDVNHINNLGWTALLEAVILGDGGPRHQQIVALLL

QYGADPKIADRDGITPLAHASRHRYAEMERLLTQV"

CDS 805423..806766

/gene="ybbY"

/locus_tag="EFAGFIKM_00708"

/inference="ab initio prediction:Prodigal:002006"

/inference="similar to AA sequence:UniProtKB:P77328"

/codon_start=1

/transl_table=11

/product="Putative purine permease YbbY"

/db_xref="COG:COG2233"

/translation="MNRHQEHFTQKNMKDVSSLILAGIQWFFFLFTNTVVVPLSIGHN

FHLSPDAIAASMQHAFILTGAVCILQAVFGHRYAIMDGPSGLWWGLTLSLTVSASSAG

MSLERIGGGLAAGFLLAGLTMVILGWLGAAQVLQKLFTPMVKSAMLFLMTIQLTMNFF

KGMIGYTEFGRFNLPVAALSVVIAFLVAWIQLKGRGKLGNYAILIGIVAGWIAYSLLF

PGQHSGQPNQAPAGLELFPWGAPRWEPGIVITAFFVGLVNMTNSITTLSTVEKLYRTE

TTSRQYRHSYALTGLFTMLSACVGVLPFGLFASSIGFLESTRILRRAALIIGAGMLCV

IGLTPSVTAFFAQIPPSVGSAVLFVAYLQMFGTALRTLEGTTFNSKTIYRVALPVLTG

VAVMNIPAEAFQTLPMYLIPIISNGLVIGVLVSLVLEKTVNWSKMEQPATVSKAA"

CDS complement(806842..807219)

/locus_tag="EFAGFIKM_00709"

/inference="ab initio prediction:Prodigal:002006"

/codon_start=1

/transl_table=11

/product="hypothetical protein"

/translation="MSINNRKEPFRYTLKEPIHFEIYILSINGVNAPAKPIQAELCDI

SRSGCQLSFPLSLPVENNDIRIGMNMLLFEDPLYMEGTLRWGQEKNTSWHYGVQLEMQ

DGNQDRLSREMRMLAGQGKIIVK"

CDS 807426..807875

/locus_tag="EFAGFIKM_00710"

/inference="ab initio prediction:Prodigal:002006"

/codon_start=1

/transl_table=11

/product="hypothetical protein"

/translation="MVTDTATLCLYDLAALKHRLEDTSDWWSIPVDELAEVNAGHCLF

LNLGADGVYEVEWSLEDVEEDSDPDHAAEREKVYHLQVPSGHVYLGAADDVTGGELEP

DEACEGVLFQLKPGNYACIVSREASRITIVMTPSIQGNNTLDELIRI"

CDS 807904..808584

/locus_tag="EFAGFIKM_00711"

/inference="ab initio prediction:Prodigal:002006"

/codon_start=1

/transl_table=11

/product="hypothetical protein"

/translation="MQDWNEITMMNADALRKRMRILAALDIIFSEEEWLRVHHYEDKL

QPGLAWGNINNGAGDHLHVLFTNSGTLIKGFDHESPLSPHAREDGEIYPGMYDEVPEA

LMAVLRDHEETLDLEDVTFCLWQEGNDLPWKVGNWIQMAMAEEDKADARGGAEFLLGY

LEKNPEDYADWAKGYYDLPDLPLEAVTEVYEEKPVTASLIKQLCPERDVAATLGELQQ

RGYAVEQT"

CDS 808614..809261

/locus_tag="EFAGFIKM_00712"

/inference="ab initio prediction:Prodigal:002006"

/codon_start=1

/transl_table=11

/product="hypothetical protein"

/translation="MYTVQLERMREKLITLRSLDPDLDLFGAENHEYEMEPVWTQEDI

THFEQKWRIELPEEYKVWLLHMGTGGAGPYYGLENPDDGVYAVLGYDDELNAVSDPFQ

FTEAWNWNYDWFEDSKEEEEWEALEHEYFDPKWSAGMLRISDFGCGISMNLIVKGASY

GEIWVDDRANRNGIYPDQYWGNTNRLHFLDWYELWLDRSIHQIHEQKQQSGANEV"

CDS 809333..809734

/locus_tag="EFAGFIKM_00713"

/inference="ab initio prediction:Prodigal:002006"

/codon_start=1

/transl_table=11

/product="hypothetical protein"

/translation="MVKKSKKTIPALMYQYQGPQRNVGFVLSPLYIEESVKVEMEDML

FIYNEDFDYIRPSLDRTFPLIDPRTGEEMKALDPCWDNPIMKDVWVGILSELDQQVVA

EPELRNFLNQFSTWVRNHLQLADGIEVTGNL"

CDS 809908..810456

/gene="sigV_1"

/locus_tag="EFAGFIKM_00714"

/inference="ab initio prediction:Prodigal:002006"

/inference="similar to AA sequence:UniProtKB:O05404"

/codon_start=1

/transl_table=11

/product="RNA polymerase sigma factor SigV"

/db_xref="COG:COG1595"

/translation="METKAEMNQRIYVQTEDETEFVQSIMEHQDTLISIAYSYLRNRQ

DALEAVQEMTCRAWIKRRTLNNEKAFKSWIIRILIYVCIDEQRRRKRATPLAKEDMED

SIPASWMISVDDNRIAMESLLQKVKPKYRHVLLLKYYNDMTLTDIASILGKPEGTIKT

WQHKGLQQLRKLMNNRRDWHDH"

CDS 810446..811588

/locus_tag="EFAGFIKM_00715"

/inference="ab initio prediction:Prodigal:002006"

/codon_start=1

/transl_table=11

/product="hypothetical protein"

/translation="MTTNPEEKALLADAYQVKREKQQWNRADTTVAIQRGLAKARTRR

SGRTIRVKWITAVLVTTLAAGWFLMGPLGNYGQQRATYTEPVNHWGVLEPFRGLLTSD

MDRATITSTLNNGYVQLIDRTVTSGIYQFTVNAVMADENRITVLYTARTDASQEIYSI

VNTKMTDARTGYVLDNGNIGGMHSSNDKNTIHGRSTIDRNQNEPLPEQVNFQFQLSSV

VPGVVKYSENGEKERKYQFSQKMNVSFALDPKFSIPKTEVINVNRTFTLGGYEVFLSE

VEVSPLVTRVKFVHAPDQEIDYKTKLSLNEIVRPIEIISTSRNGKQTKLSMVGGSGSE

DGMMYSFSSNLLDHPESMVLKLSGKPDKVYDDLQEAKNDELEIKIK"

CDS complement(811712..813697)

/gene="bgaA_2"

/locus_tag="EFAGFIKM_00716"

/EC_number="3.2.1.23"

/inference="ab initio prediction:Prodigal:002006"

/inference="similar to AA sequence:UniProtKB:D9SM34"

/codon_start=1

/transl_table=11

/product="Beta-galactosidase BgaA"

/db_xref="COG:COG1874"

/translation="MTVKIGIDYYPEQWTPELWEKDAILMQQTGVAVVRMAEFAWSRM

EPTEGNYQFEWLDAAIEVFASRGMEIVLCTPTNTPPNWMTTRYPDVLPMDEQRHIIRP

GVRGHRCNNSPSLRMLGSKFVEAISQRYGKHPAVIGWQIDNEMHYQECHCDTCNRSFV

AWLQKRYSNLEELNREWGTVVWSGEYSSWAEVTTPLGASKPMNPSYLLEYKRFCSDSV

GYLLGWQLEILRRNCPGQFVTHNMWQYPNSLDYYDMHNELDFVSVDYYPNELYRDYGD

RFPRNGALTLDLVRGIKRRNFWVMEQLSGAQGAWMPIQRTPYPGLIRARSWQTIARGA

DMVVHFRWRSATVGAEQFWHGLIDHSNVPGRRFREFAELTREVNQLGELLADSTIVTQ

VAILHSHDQHTALSLQPQAEGGMHYLDNLSSMHNAFLKMGVGTDVINWTEELDGYKLV

IVPSLFLLSEEVAQRLERFAAKGGTVVLTNRTGVKNMRNVCEMLPLPGLLGEAAGVVV

SEYDAIGNSEHRIRNAEGKTFAAKQWCDLLELRGAEPIAWYDNDFYEGVPAVTINKFG

EGEVYYVGTWPEPSYFYQLFEGILKERDLAAKIQLPEGVELSIRTKGEQNFLFLINLT

NEQREITLDVPYRSLLTSERLDQKLTLPALGVDILTV"

CDS 813812..814654

/gene="rhaS_2"

/locus_tag="EFAGFIKM_00717"

/inference="ab initio prediction:Prodigal:002006"

/inference="protein motif:HAMAP:MF_01534"

/codon_start=1

/transl_table=11

/product="HTH-type transcriptional activator RhaS"

/translation="MPHPNRNFPVLSSRDKLLPFYLLGIGLHHEQEHIRRDQGIEDYQ
WIQCRSGVGKLKLSETEHIVKPGMGMLLFPGQKHEYYALSGSWEVDWIIFDGNGTASL
FETVGIVDTCVYTLAQPEYLLSRMRELLQAALTPQEQTLSNYACSAVLYTLLTEIIQR
VSVEGSDTVGQQYERIKPVLDYIEQHYAEEITLPTLAGLICVTPEYFCHLFKKTTGIR
PISYVNQVRVNKSKELLLDNVQRGMEDIARQVGFESTSYYGAIFKKLERITPGAFRRS
YQKS"

CDS 814945..815988

/gene="pelB_1"

/locus_tag="EFAGFIKM_00718"

/EC_number="4.2.2.10"

/inference="ab initio prediction:Prodigal:002006"

/inference="similar to AA sequence:UniProtKB:P94449"

/codon_start=1

/transl_table=11

/product="Pectin lyase"

/db_xref="COG:COG3866"

/translation="MKKFSMLLLAVTLLLLTVSTAPSTYGAASYPNTGTNGLTGFAGS
AKNENGVSKSATTGGKNGQVVYVSNLNDLRTHMAGSTAKIVVVEQNISSSTLQKVEFG
ANKTLVGSFGKHTLTNIHFRSTSSSSNVIFQNLTFEHASNINANDDIQMYITSGSNYW
IDHVTFAGHSYSSGGSDLDKLLYIGDRADYITISNSKFANHKYGVILGHPNDGNSSYN
GVPHVTMSNNYFENLYVRGPGLLRYGYFHLKNNYANNFNQAITIGEKARIYSENNYFG
AGAEKGGILDDKGKGEFTDTGSTPALNSPTSPKTNWRPSSNYSYQVEGASYAREFVTK
YAGSSNTTLVFGK"

CDS 816291..817769

/locus_tag="EFAGFIKM_00719"

/inference="ab initio prediction:Prodigal:002006"

/codon_start=1

/transl_table=11

/product="hypothetical protein"

/translation="MDRMDEIIWKRNKLIGIILWIIIAIGMGLAFTMPKLLVSNGVAL
VFVVWITYANAKKKHIHLIPWLITVLLTICGVYVGWGTVNFALSLVITAVLLLFPNKK
FFVVGFSVLLVNSVLQLFVMNTVDSVELIGNIVNVGLFALTGAILMLVSQLNQRLFHE

SEVRQNEVEHSKQRVETMLERVKESVEGLSRYTDQLKQKVNATGSITNEVTLGFSEVA
KGVEFQATSVAEISESLSVSDQHIKDVASYSQQMKELSASMATSTQTGSTQMDQLNIQ
MQDLYETIDTTANDMRKFNEESESMTLMLNSIADIASQTNLLALNAAIEAARAGEHGR
GFAVVSEEVRKLAENSGQSASDIGTILSRLKGQTQALTDRFERIRQSLQQGRDSVHTA
EEVFRTINSNSQHVLNQATDIETSSATMKASSTRVVNEVSEISSVTEQSSAATEEILA
SMEEQRNLTQKMVESFGELEQLIVNLNELVSDHQASSVANKL"

CDS 817968..819176

/locus_tag="EFAGFIKM_00720"

/inference="ab initio prediction:Prodigal:002006"

/codon_start=1

/transl_table=11

/product="hypothetical protein"

/translation="MKDRLDCDDSTYEVKNDNRAARVRFYDQQSFKDMDWPDTEDGRY
ARVYLEPMMLDGTQSFMSNVETTLLLARIDNLVIPLTVNDTEYDNAYVCSPYTHYVSY
AKQELTMLQKPVLEKGLSVLLSLIGWGMKQSQINKVLHVNNWLLSTNLYPAMSGEQAE
CLLAAVQERYPEHVIVFRSLCQGLHPDLTARLTEVGCRLIPSRQIYLYQAHDPNFGNS
KSRWLLKRDFELLAKHDYKFVSESDMTDADIPRIVELYKLLYLEKYSYHNPQFTERFI
AAAMASGTLRLYGLRKEGRLDAVMGYFCRNGVMTTPLFGYDTTVPQSVGLYRMLSACL
IGQARENGHLLHESAGAAQFKRNRGAVADFEYSAVYERHLPWGRRWCWLLLDRLLNRV
GLPLMRRMKL"

CDS complement(819301..819666)

/locus_tag="EFAGFIKM_00721"

/inference="ab initio prediction:Prodigal:002006"

/codon_start=1

/transl_table=11

/product="hypothetical protein"

/translation="MLIKLNWITLRVSNLEASLGFYHDMLGLPIQRRFESRGRQIAML
GIENEAKLELIEGSESILKPEAGVSIGYEVNSLDEAMERLAELNIPIVRGPIQPNPHL
RFIYITDPDGFEVQLAEHV"

CDS complement(819830..821482)

/gene="phoA"

/locus_tag="EFAGFIKM_00722"

/EC_number="3.1.3.1"

/inference="ab initio prediction:Prodigal:002006"

/inference="similar to AA sequence:UniProtKB:P19406"

/codon_start=1

/transl_table=11

/product="Alkaline phosphatase 4"

/db_xref="COG:COG1785"

/translation="MLNRFNVRAAKMMLAVTTIVTATLGGSSAAWAVEDTTSTTSASP

IKNVIILIPDGMANDATALARWYKGSSLTLDSMASGMVRTHSADAPIADSAPAGTAFA

TGYKSHTGFVGVLPDKATMPGQKAIAPGDERKPIASVLEASKLAGKATGIIATSEIMH

ATPADFSSHYPDRKNYDALSKQQVYNGMDVVLGGGSKYLEPAGRKDGENLISSIKALG

YDYVTTPAAMKASDSNKLWGMFAPAGMAYNMDRDPAKQPSLAEMTSKAIEVLSKDKDG

FFLMVEGSKVDWAAHANDPIGIISDVLAFDDAVKVAMDFAKADEETAVVAVTDHGNGG

LTIGNNATSGTYDKEPLSTFIGPLKKAKLTGEGVEAKLNAKRTNIKAVMKQYYGIIDL

TSEEIATIKAAEPGSLNYAIGPMISKRAGIGWTTGGHTGGDVVLYTYAPNNDRPFGVI

DNTDVAKYMARVLDLDLDSVSKQLFVPAKQAFTAKGATYKLDLTDAKNPKILVTKGNT

KLELQIYKNIALVNGKKTTLEGVIVYNGVEAFVPQTAVDLIQ"

CDS 822205..824448

/locus_tag="EFAGFIKM_00723"

/inference="ab initio prediction:Prodigal:002006"

/codon_start=1

/transl_table=11

/product="hypothetical protein"

/translation="MKTIKRSIAVATILGLCVSALPAYAAGNTASSSSAAMDKLIQSE

LIRGSGQGVNATYLNKKATRIQAAVLYLRLSGLEKEALAYQGAETYDDAAQAGKVLQP

VVGYLKQHPYVSEIVTGTEKFEPNAPLTAEGYAEMLLKVLGYEAGTDYEQGAASTYAA

GKGIKALQGKGASQLTNRLMAEATAQALQIQLKNGSGTLEQSWLKHKEAGAFRPQTVQ

QTKYGAIDGKTYEQYGTLGWLGVPYAAPPVGELRWKAPQEPDSWTGTRSAKEFAANSL

QISGKNTVGSEDSLYLNIWRPDTTSSKLPVMVFLHGGGNMTGSGKDFQGEQLARSTNS

IIISVNYRLGALGFFENAALKTGNALDDSGNYGLLDAFRALEWVQDNIEGFGGDTGNV

TLAGQSAGARDVLATLISPLSKGLYQKAVAFSGGLTTASPEEGEQKSEDVLVKLLVQE

GKAANAEEAKAWIGKQSQAQLESYLRALPADKLVTAFGATAIRMDPFPHLFRDGTVIP

KEGFDAINNGNYTKVPVLLGSLETEFSGFAFGDPNFSPSITDETLFTDKTKAEQYAAA
LKYGSEAYAGFNAERVAEQLTSEAGQPPVYAYRFAWGTQPGVISERLLTLLGAPHGAD
MDFYTGHADGIAAYFPDGYFSDTNKPGRDQLSAAMAAYLKQFLYTGNPGTGGSPDLAA
WTPWTKDAQAPIMRLDASNTTAEIGMSTQYNQGKDAVMAKMKKELPEETYKLLTEKVF
AGRFFWE"

CDS 824596..824802
/locus_tag="EFAGFIKM_00724"
/inference="ab initio prediction:Prodigal:002006"
/codon_start=1
/transl_table=11
/product="hypothetical protein"
/translation="MISSIGIAGIITLLIFFIMIGLVLYLVFYLVMKKSGVKEEVSAL
RMEVRVLQEEVERNRRELENMKRR"

CDS complement(824939..826525)
/locus_tag="EFAGFIKM_00725"
/inference="ab initio prediction:Prodigal:002006"
/codon_start=1
/transl_table=11
/product="hypothetical protein"
/translation="MKKRNQWLAVLSMTAILTAGATSYTSMIHAADATKPANDHVVLR
TAIQNMQGTVTWNGSSRSVDIQIADTKVQLPVGTSSATINGVKTPLDSKSYITKGTTY
VSSQTLKLITDQLILKQNKTGFEQTASFQMPGGKAEISASTPDGQRLLVTEADNGSIS
VLDIADLKNINVLKKVSFQDLSAKAEVTSVTVSKDGKYGLAVIRTGDTDSEANKGLLA
IVDLTTYKTVKTYELGIGPDSIALSPDGLHAVIAIEDEELNKAADEIDYANTKRPGSI
MVVSFAGGNVLEGEITNLPVSLDNVEGAIYPHDPQPEYVAISPDSKTAAITLQENNVV
AIVDLETKKISSIFALGTTSHQADLKDDGIVQFKETLTARYEPDGIAFSADGKYLLTA
NEGDLGKNEFEDGVKAGGRNIAVWDLQGQRMYDSQNLIDEETTMVGLYPDDRSPNKGS
EVENLTVATVEGKTYAVVASERADAILFFDLADPVNPDYLGLIPTAGESPEGIHRVNG
RNLFVSADESTGTLSFFAKK"

CDS 826847..827806
/gene="rhaR_4"
/locus_tag="EFAGFIKM_00726"

/inference="ab initio prediction:Prodigal:002006"

/inference="protein motif:HAMAP:MF_01533"

/codon_start=1

/transl_table=11

/product="HTH-type transcriptional activator RhaR"

/translation="MRTDDALGVVYQHYLTQEPYSQQDEHTYLLSPKAGSGNIKRVTT

YSGIEILYSQVEYHQPYPTYFASETPIVELQFALSGERNVDISGQDYSLSIGQGALIF

MHDFEAWFHPPARELYTSFSLGIPVSLFNYAAAQLGGFKSIAFNQVLGRKVFKPIVFQ

LDNRIRTMIDSLIVELNNSYRSSLMMEATALEILNRFMIQLFDLAPIPAGFSREDIRK

LHKAREIMEACMVDPPSLLALSRQVGLNDFKLKKGFKTLFGSTVFEYLRQVRLDNAMK

LLRNQDNNVTEAAIAVGYSNVSAFSQQFYRKFGVKPSEMKKIY"

CDS 828120..829322

/gene="tetA_1"

/locus_tag="EFAGFIKM_00727"

/inference="ab initio prediction:Prodigal:002006"

/inference="similar to AA sequence:UniProtKB:P02980"

/codon_start=1

/transl_table=11

/product="Tetracycline resistance protein, class B"

/translation="MNRIRGSIFFSVFAAMLSLMLIAPIMPPLIRELGMKESHSGLII

SLGSIMMACMAPVWGRWSDIRGRKPIILIGFIGMSVSCALFALALYGGLNAWIGGGVL

LTLLIVTRSLIGMFIPAVLSSAQAYMGDVTEGEERGSGMAIISAANGLGLVFGPAIAG

AFTLIGLLWPLYFGIFIAIATFIIALLLIPSAKPVIQAKPPKVNPFQRGLRMYLAAGL

VTMMGIMTLQVVGGFYFQDQLGLSSAATARMVSFGLMFSGAAMLIMQVVQMKWLKWQP

RPMILLGSLFLIASMILFLVFNVLSMYYFSFFLFGMGSGLMMPGFMAGASLAVSKEQQ

GGAAGLVAAIQGISAVITPLLTTTLYQVDKHVPFMLVAVLVVLLAIMMLGMRKNQSNT

VADSIHTL"

CDS 829514..829957

/locus_tag="EFAGFIKM_00728"

/inference="ab initio prediction:Prodigal:002006"

/inference="similar to AA sequence:UniProtKB:Q03928"

/codon_start=1

/transl_table=11

/product="18 kDa heat shock protein"

/db_xref="COG:COG0071"

/translation="MFDLIPFRKRNEDPFGHMLKSFNDMVENSFLSPFGTGSQPFRTD

IREEEDKYSVEAQLPGIAKEDIDIQFQGNELIIRAKRNDIVEQKDDSNRIIRQERRSG

EFIRRFYVDHIDDEHIKAKLEEGVLKLEIPKRPGDDQSRRRIQID"

CDS complement(830093..830650)

/locus_tag="EFAGFIKM_00729"

/inference="ab initio prediction:Prodigal:002006"

/codon_start=1

/transl_table=11

/product="hypothetical protein"

/translation="MIYVALLRGINVGGNNKINMKQLKETFEQAGMLDVVTYINSGNI

IFADHQEHANANVEISHVLEQAIAADFGLQIRVMVRNMDEIQSVIQALPEEWVNDDTA

KSDVMFLWDEINDPSVLNQLPIKPEIGTLIYVPGAILYSVSREDASKSGMNKLVGSKV

YAYMTVRNVNTTRKIYALMQAAAEK"

CDS complement(830685..831335)

/locus_tag="EFAGFIKM_00730"

/inference="ab initio prediction:Prodigal:002006"

/codon_start=1

/transl_table=11

/product="hypothetical protein"

/translation="MQQLDAALSRLVEQQDLYNEAYGLFKNHDLRPREQHHQPEVDTR

IPLSPELQYLYSHYEMMDADAEGTLKMKNAAVEIGDAALIFFVAPEHLLRQQLGFRWI

GMNEPYEESSTWPKNHVVIANFNDDPLIVDVEAPNSPVYAAFTGGEPNRVADSLTDFF

SALAILIEAARTYAGEVKDEETYETKPEYLEQVEPLLQDLLGEEYTAHLFEYLSFC"

CDS 831671..833305

/locus_tag="EFAGFIKM_00731"

/inference="ab initio prediction:Prodigal:002006"

/codon_start=1

/transl_table=11

/product="hypothetical protein"

/translation="MNKRKWLNGFAAGCLSIILVISGCASKAPQTASDTPNQATEQTE

NETQEPANNQAAKGRTVITTDGEVDDMNSVIRFLLYSNEMDLAGIVLTSSVYHYAGDE

EAGIQPFRWTGTQWVYDMIDAYGEIYPNLSKHAEGYPEPEQLRAMTKIGNISDKGEME

KETEGSEFLETLFLDDDSRDLIVQTWGGTNTTARALKSIEEQYKDTAEWDAIRKKVSD

KLVLYIILDQDDSYNEYIAKNWPSIRILNDQSNFWHFAYAWKMHAEEVNSKLHGDWMV

KNILNGHGKLMDMYASMGDGKMIEGELAEEQRGSAEYLKNNPQYDKYDFISEGDSPSF

FYLIDNGLRSMEDPSYGGWGGRFGVVNDKLFRNNVLDYDPYTKRYEAEYSLMRWFDDV

QDDFAARADWAVADTYEGANHNPSLTIKEGVDLSVSPGEEVTLHAEGEDPDGDQLTYT

WWRYFEADTYEDSKVTPNKVQPEMAGDLQLGLHRELAKDEQVDTIKLQGSDTEAVTFT

VPDDAKSGDTLHIVAEVQDDGEHQLKHYQRVILTVK"

CDS complement(833391..833954)

/locus_tag="EFAGFIKM_00732"

/inference="ab initio prediction:Prodigal:002006"

/codon_start=1

/transl_table=11

/product="hypothetical protein"

/translation="MSKKRIKEIAIQHFNRLGYEGTKMAQIAEEAGIRKQSLAYHYSS

KKALLLELYEEVVQEEQQFVRQFFSDSVTQTLDQQLYAFLHEHKNRFLTNPNVSFMYI

LSFITPLEVHDFVLAQYRTYLGTLKEELAAVFTRHSDIRLSPEEATVAFVTVMDGLDV

QLVYETRQSYEQALAITWNVFWSGIQR"

CDS complement(833947..835161)

/gene="cntE_3"

/locus_tag="EFAGFIKM_00733"

/inference="ab initio prediction:Prodigal:002006"

/inference="similar to AA sequence:UniProtKB:A0A0H3JTK0"

/codon_start=1

/transl_table=11

/product="Staphylopine export protein"

/translation="MSTSTHSSIPQQLWGRSFLFIMLANALLFMAFEMLLPTLPLFVS

SLGGEASQIGLVTGVFMFSAILIRPFTSVLAARIDKKYLLIIGITICSLMTGAYYLSS

GIWMILVFRVIHGFGFGLATTYFATIATENIPRDRRGEGMGYFGVGETVAISIGPLIG

TSLLFRYDYQSLFMGGMCILLLALLMTVFVSRKPKTGGSSESMQTHVPSVKLIEKKVL

FPSLLIMLVGIAAGSIMSFVALFAAERGFENIAWFFFIVAIASFAVRLFSGKMFDRWG

PGSVLLPSAVFAIAGLLVLIIAQSDVQFLIAGALYGFGFGAIFPAIQTWCVNLVEEHE

HESAMASFFNFFDLGIGGGSLILGVVASAFSYTVVYAISIGIFVVYILLYLVYSQKQQ

RYTARQLEVDIE"

CDS 835313..835783

/locus_tag="EFAGFIKM_00734"

/inference="ab initio prediction:Prodigal:002006"

/codon_start=1

/transl_table=11

/product="hypothetical protein"

/translation="MHTKTKLQELYQATQEANKELERLHEEYKIKEQALIKVNDQLET

LASTDLLTGLKNRRFFQEKMLESLMMFQEKQRYFSLLVVDIDHFKSINDTYGHPIGDL

VLGNLAGLLQSLSRSTDVVARYGGEEFVVILNALYASKTGGRNRVTHAAQMVKS"

CDS complement(835914..837401)

/locus_tag="EFAGFIKM_00735"

/inference="ab initio prediction:Prodigal:002006"

/codon_start=1

/transl_table=11

/product="hypothetical protein"

/translation="MIRVVILMIPILMVLVSGCQNHTSAMKEPASVQKSDPQVIHYIS

PQGNDSNKGTIQSPWKTLQHAADHASPGSTIYLREGVYHQKVKITQSGNSSANPTLFS

SYPQEQVIIDGTGLSVRGIEGLIEIENASYITIQNLEIRNFTTTLGGQVPTGIYVHGA

GEHIQLLGNTIHAIASYATPEGPDLRGRDAHGIAIYGTEHPQALRDIIIKDNELYDLT

LGSSESLAVNGNVDTFAIQDNIIHDSDNIGIDLIGYEGTSEDDTYDQARNGIVRGNEV

YDITSNNNPSYGTDLPNDTNSAGGIYVDGGKDHIIDQNRVYRSDIGIEIASEHAGRST

SNITVRDNLIYSNRLTGIAMGGYDEERGATEDSKIMYNTLVGNDTLNAGNGQLFMQSR

TKNNTFIRNILVSGSSEVLIYNEYTSNTGNVFDHNVYYSPGEQEDALWVWKNKAYSGI

AAYIKGSGNDAHSIYVNPAFMDEPSEDFRPQANSPAKAYGYLAPR"

CDS 837504..838160

/locus_tag="EFAGFIKM_00736"

/inference="ab initio prediction:Prodigal:002006"

/codon_start=1

/transl_table=11

/product="hypothetical protein"

/translation="MNTLTIAELVDRENHAWEELKELLNSGQNTYVYVPAKQEAGEDT

LYRLQISTKSYLGAVAYETEGIVLDHGWITLLGAGGDSTSGSLTSWNGVSEQPWVDVL

EGMMVVAYDAAGGFFGLDTGKYGRTGHIYYFAPDTLEWESTELAYSGFINWLANGDLE

QFYQTFRWKGWQEDMSQLQTGQVFAYYPPLWTQEGDGKSSSKTPISIMEAWKFALDGK

"

CDS complement(838317..839657)

/gene="mdtD_1"

/locus_tag="EFAGFIKM_00737"

/inference="ab initio prediction:Prodigal:002006"

/inference="protein motif:HAMAP:MF_01577"

/codon_start=1

/transl_table=11

/product="Putative multidrug resistance protein MdtD"

/translation="MKDKIVMPLWTCCLFIVVMNTTMFNVSLPVIIHDLQITSDLGSW

VISSYSIGYALSTVIYSRLSDRIPVRKLLTVGLLILGLSSLLGLFAHNFAILLLTRIL

QSAGAGVMAGLGLVIASRYIPVERRGAAIALISSGSAMAFGLGPIVGGLISEYWGWNG

LFAITVLVLLALPVLLYFLPRESVKTDQPFDVIGAILTVINATTLLVAITQQSWLWFA

IGVISLVVHLLYIRKASLPFVNPQVFRTPGYTRLILIGFCVLVVNLGNLFLMPLVLAD

LYGRSSLAIGLLIAPGAIVAAFCTRFVGRWIDRYGNMRFMIIGHILLAAVLALFMLGL

DQSALIITSGYLFFSPALSASMASLNNEASRVLPKAQIGSGMGMLQLIQFFGGSVSVA

VCGLLLHSIPGVSVEEAYHVVYACLLIICVVSLGLTIWHSKASRPSIKPVTLDN"

CDS complement(839913..842564)

/locus_tag="EFAGFIKM_00738"

/EC_number="7.2.2.10"

/inference="ab initio prediction:Prodigal:002006"

/inference="similar to AA sequence:UniProtKB:Q8Y8Q5"

/codon_start=1

/transl_table=11

/product="Calcium-transporting ATPase"

/db_xref="COG:COG0474"

/translation="MQHYRHSAEETLQDVQSSSKGLTTSEAAKRIETEGYNELKGKDA
TPVWKLFLENFKDPMVIVLLIAAAVQVVLGHLIESLIIFLVILLNAVISVVQTKKAES
SLDALKQMSAPEAKVIRDGQKKTIPARELVPGDIVMLDAGDYVPADGRILESGSLKIN
EGMLTGESEAAEKHADTIPDEAPIGDRRNMAFSGSLVVYGRGMLVITGTALKTEIGKI
AELIENAEAKNTPLQRKLESFSKKLGFFILGLSILIFGIEAGRVWLTEGTENIGPSIV
NALMFAVAVAVAAIPEALSSIVTIVLSLGTNKMAKQHAIIRRLPAVEALGSASIICTD
KTGTLTQNKMTVVDYYIPHGTKDEFPDDPDQWSEEERRLLHIAVLCNDSNINQEGKEL
GDPTEVALIAFSNRVNKDYNEIRDQFPREAELPFDSDRKLMSTVHTFEGQTALLTKGG
PDVLFSRCSHVFINGEVQPLTPEIRTQFEEKNEAFSKRAFRVLAYAYKKFDDKNQVTI
DDEHDLTLVGLTAMIDPPREAVYGSIEESKKAGIRTIMITGDHKTTAQAIGVDIGLAE
PDDLAITGAELDKMSDEELDQQLEHISVYARVSPENKIRIVRAWQRKGKVAAMTGDGV
NDAPALKQADIGVAMGSGTDVAKDAAAMILTDDNFVSIVNAVSVGRMVFDNIKKAIAY
LFAGNLGAIIAILFALIVGWVNPFTALQLLFINLVNDSLPAIALGTEKPEPDVMRRKP
RDINEGIFAGGTLQAVITRGVLIGAAVIVSQYIGLGISEEISVAMAFTTLILARSLQT
FAARSNTQTIFQVGFMTNKFVLGSILVCLCLYAITLIPGVRGVFAIPDTFGWNEFLIA
GGLALAAVILMDVIKWIRNRGKQVTVA"

CDS 842874..844166

/gene="hcp"
/locus_tag="EFAGFIKM_00739"
/EC_number="1.7.99.1"
/inference="ab initio prediction:Prodigal:002006"
/inference="similar to AA sequence:UniProtKB:Q01770"
/codon_start=1
/transl_table=11
/product="Hydroxylamine reductase"
/db_xref="COG:COG1151"
/translation="MFCYQCEQTPSGGCTVVGVCGKNETIASLQDTMIFALKGIAAYA
THARQLGYSDPEVDRITHEALYMTLTNSNFNVQEHLEMAMKVGNAAIRIMDVLDRAHT
DRFGVPQPITVSQNQIEGQCIVVTGHNLYALEELLRQTEGKGINIYTHSEMLPAHGYP
ALKKYAHLKGNIGKAWYDQRRLFEQFPGAILATTNCVMPIKGTYADRFFSYEVAGLEG
VAKITEDDFSPLIERALSLPAANVQSEQVLTTGYHHETVIGLAPEIIQAVKDGHIRRF
FVIAGCDAPGKGGNYYRELATSLPNDTVILTTSCGKFRFNDVDYGTVGDTGIPRYIDL

GQCNNSGSTVKIAMALADAFGCTVNELPVSIVLSWFEQKAVAILLGLFSLGIQDIRIG
PKPPEFISEGVLDVLVDMFGLKLITTAEEDMNAMLALS"

CDS 844464..845867

/gene="bglF_2"
/locus_tag="EFAGFIKM_00740"
/inference="ab initio prediction:Prodigal:002006"
/inference="similar to AA sequence:UniProtKB:P08722"
/codon_start=1
/transl_table=11
/product="PTS system beta-glucoside-specific EIIBCA
component"
/db_xref="COG:COG1263"
/translation="MHNTQIENWLKLAGGKENIKLVEHDKGTTTLALKDSTRLNMSVL
ASTEYVVDIRVTSEQCHLVIRDESSLIYNALQGVGEEEGTALQEAGHAKKNRFSILHF
VSDVFRPLMPAILGAAVIKILLAIITLTYSSADSSALLDSQTFTIFSIIGNSVIYLLP
VLVAISTAYRLKSNIYVAASIGGLMFHPEMTMLMSGEKEVHFLGVPLVSQPAFYSTPI
WVILTICAAAYLEKAVQRISPKFSKWMVAPFLTLLILVPLVLMILGPIGTWIDEYLPE
ALDSLVANAPVLSVMLLGAIFSLMLIMGLHYWLIAIIINEMVMNGGSMVIAAMLVAIV
GQAGAALANGIRSKEPASRRLAFWATGTAILGVVEPALYAVNMRKRSSFYAALLGGAA
GGLYFGLLSVKAFAMVGNPSLVSLPLFIEEGSLNLLHTCIGVVIAFGVSGGLTYWMAG
MDRNGDRGHRNPALSNT"

CDS 846091..847716

/gene="rhaS_3"
/locus_tag="EFAGFIKM_00741"
/inference="ab initio prediction:Prodigal:002006"
/inference="protein motif:HAMAP:MF_01534"
/codon_start=1
/transl_table=11
/product="HTH-type transcriptional activator RhaS"
/translation="MSDNMEWIAQWKSTNALSNAWITKGELHHFLSHTLIFIIDGKAI
WNINGHRVHVSYGELIALEENSVMEVIEGGNLDLAGWHVQFDTYSVLHDRREAEKFEW
RLPSGEAYQKVQLSGGGLASIIQHWSEVHTQDQSAERVGHQHLLYELLRNLYRKQPDD

ELKPEHGILRSIDYMQQHYDQVITRTQLAQIAGISPWHYSRKFSERYGKPPLDYLAHY

RIYRAQEELLLTAATSQDIAKKSGFEDAHYFSRRFKQLTGVSPKIYRQTMTQRKMVSL

SPICAEIMIHLGVIPHAVVVTPILLSPHHHEQFIAHGVQMLEVTQYEVEIELVRQVQP

EMLIGNVLTEEVKRELRTIAPILTGLHQDVEPMLNQLAAWFYKEDEAYRLHQQMKHEV

SVAKRQLQPFIQSASTVMLLRVEAFGYRYLGGHSHGVSHLLYEQLGLTLPQALDSGTA

WFNPCSLELLTQANPDYLFVEKRIMQHFSAEENMRKLWESPQWNELKAVKNNRVFYVD

THLWVDGHGITGHTLILNQIIRHFTESFHERAQ"

CDS 847819..848835

/gene="yclQ_1"

/locus_tag="EFAGFIKM_00742"

/inference="ab initio prediction:Prodigal:002006"

/inference="similar to AA sequence:UniProtKB:P94421"

/codon_start=1

/transl_table=11

/product="Petrobactin-binding protein YclQ"

/db_xref="COG:COG4607"

/translation="MSRRFKGLGIMLLVISMMLAACSGGTKEENATAGTAGASTEGAA

STAEETATESGTKVVKDQFGEVTIPTHPQNLVVFDSIYAEYLIEMGVTPQIVLLTPEV

EAEYRPDYLKEHRVQIIEVEQYQYNYEQLLALSPDMILTAGEGMEQGVYDELSKIAPT

VALDANSEMKRAMPKLAAIFDKTEESAKVLAEFDEKASQAKEKIAAALGEKTVLVLRV

EHNRYRFMGPKGGSSSVFFYDILGLNSPEVIKDSTDWFSQFSLEFLPEMNPDYIFLED

RTLVGYDTKKSMEDLKASQVWSNLEAVKNDHVFPLKTSQFVSGVGPIGSVKLMDYVVE

KLVP"

CDS 848854..849861

/gene="ycgT_1"

/locus_tag="EFAGFIKM_00743"

/EC_number="1.18.1.2"

/inference="ab initio prediction:Prodigal:002006"

/inference="similar to AA sequence:UniProtKB:O31475"

/codon_start=1

/transl_table=11

/product="Ferredoxin--NADP reductase 1"

/db_xref="COG:COG0492"

/translation="MDIYDITIVGGGPAGMYASFYSGMRAMRTKIIEAKQELGGFMRT

YPEKLIWDVGGIDPIRCEKLIESLERQARTFDPTIVFGQEIAELERREDGVFVLMSKT

GERHYTRTILLCAGRGMTQVQKLDVEGANRYELTNLHYTVTDLSRFAGKRVLISGGGD

SAVDWANEMGKIASEVTVAHRRHEFTGHESPVAQMKAQANVMTPFNISRLYGTGDKID

RVELAHVETGEITHVEVDEVVVSHGYDRDFGNLVQWGLAREDYGVSVDQRMRTNIPGI

FGAGDFITYGSKVRLIAGAFNDAVLAVNSAKTYLEPAASDMAGVSSHNARFYEKNKAL

L"

CDS 850114..850902

/locus_tag="EFAGFIKM_00744"

/inference="ab initio prediction:Prodigal:002006"

/codon_start=1

/transl_table=11

/product="hypothetical protein"

/translation="MLNRHGKRICRRKFKQSMHCRVKVKVVKVRPKVVVNAPQGAQGI

SGPVGPIGPKGATGAQGIPGPQGPIGLKGATGAQGIPGPQGLQGIPGIQGPVGPAGAL

GAVGPVGPVGPVGPAGATGPAGPVGAGISDFLSVFRADPGTGTDTVPGNAPITLTGVL

ANVGGAFTFVPPSSTITINEIGSYLISFSIHNQGNADFNLTVNGTPITPVPFTGNGGG

PISAEVIVTVTTVPTTIQLVNANATPAQLHNNLNTTVTILKLTP"

CDS 851216..851758

/locus_tag="EFAGFIKM_00745"

/inference="ab initio prediction:Prodigal:002006"

/codon_start=1

/transl_table=11

/product="hypothetical protein"

/translation="MKKQMWIIASVAVVIVIGTYLFASNTGEEEAGTANAPNIRKLVE

DISTGKETPESASINAKQLIVTDQDKKTTTYDLPENEFFLSIAPYVDQTHPCAIHSLT

GCQGEMSNKEFSVTIHDSQGNTLMKDEVMKSGANGFMDFWVPRDRTYLIRIVRDGKVA

ETQLSTYENDNTCITTMQLS"

CDS 851935..852936

/gene="fpuA_1"

/locus_tag="EFAGFIKM_00746"

/inference="ab initio prediction:Prodigal:002006"

/inference="similar to AA sequence:UniProtKB:Q81L65"

/codon_start=1

/transl_table=11

/product="Petrobactin-binding protein FpuA"

/db_xref="COG:COG0614"

/translation="MKKGLNGLLIMLAFVLVLAGCGKTTTTTDTSQGSDSGSAPAEET

AGPVTVKHKRGELKLDKPAERVVTLEWTYTEDVVALGVQPVGNADNANYKVWVTSEAA

LDDSVTDIGTRSEPNLEAIAALKPDLIIANADNNTAVYDQLNAIAPTIEYDPYDGDGY

DYDKMTEIFNNIATALGKEDKAKEVLSNLDQHYVEAKEKLAAAGKENFNFALTQAFTY

QNAVSLRMFTDNSVVIGTLDKIGLVNDWQPDKVENYGFSTVGIESLTDVKDSNFIYIT

QPDDDVFGTAMKDNSVWNGLDFVKEKRTYQLDSTTWTFGGPISSKVLVDGVVEAITK"

CDS 852933..854981

/gene="fpuB"

/locus_tag="EFAGFIKM_00747"

/inference="ab initio prediction:Prodigal:002006"

/inference="similar to AA sequence:UniProtKB:Q81L64"

/codon_start=1

/transl_table=11

/product="Petrobactin import system permease protein FpuB"

/db_xref="COG:COG0609"

/translation="MSSVQGSKPAMNWRTISIYGGGLTALIVLFFVSLCYGEASIPLH

VVWDALTGRQDSLEHNMVWDLRMPRTVIGILAGGALAVAGALLQTITRNPLAASDTLG

INAGAYFVVVLGAIAFPGLLSQSPFLFAALGGLLAAFAAYFMGGGRTSSPVRLALSGM

IVSMVLGSFTSALHIFFSMETQGLFLWGSGTLVQNDWSGVFYALPWVVGITLLALVLS

RQWDMLELDESTASSLGQKVGLARAGGLILAVLLAAVIVSVIGPIGFVGLVAPHLVRL

SGVRSNRLLLPGVFIWGAALLVGADVLAKMVHNSSMELPTGAVMAIIGAPWLIWLVLT

RMKAANGSGMSTSMSTGGAARRFAFGPMAVLFSVITVVLILLSTMFGGMRIPLADWLP

SLFQSDGLFSALIQLRIPRTLVAAGAGAALAISGVLIQMAVRNPLADASIVGVSSGAG

LGAMMVIILWPGLPIYLLPIAAIIGAAIAAVVVFSLAWKKGLNPSAVVLLGIAMSAIA

GAGIQILIVRGAVYGSSGYIWLTGSTYARTWEQVRVIGLFLVILVPVAWWLARRFELL

VFDDNSASGLGLGVRRTRLLAMTVGVLLAAGAVACVGTVGFIGLIAPHMVRLLTGNKL

RRSMFLSALAGAVMLVLADTIGRTVMAPTEIPSGILIAIIGTPYFLYLMYRSNWRKSV

"

CDS complement(855269..856441)

/gene="dapL_1"

/locus_tag="EFAGFIKM_00748"

/EC_number="2.6.1.83"

/inference="ab initio prediction:Prodigal:002006"

/inference="similar to AA sequence:UniProtKB:A0LEA5"

/codon_start=1

/transl_table=11

/product="LL-diaminopimelate aminotransferase"

/db_xref="COG:COG0436"

/translation="MNFAKRMDHFNEGIFTRLLEIKRRRLENGQPVIDLSVGTPNIPP

AQHIITALCEAAADPANYIYAVNDQSELLQATSDWYKQRYQVELDPATQICSLLGSQE

GLAHISLSIVDEGDLVLVPDPCYPVFADGPLLAGAELYYMPQKEENGYVIQLQDIPED

IAHRAKFMLVSYPNNPTTAMAPDQFYLDLIAFAKKYDIIVLHDNAYSELVFDEKSCGS

FLAFPGAMGVGVEFNSLSKTYGLAGARIGFCVGNATMVSMLKKLKSNMDYGMFLPIQK

AAIAAITGDQSEVERVRAIYEQRRDILCEGFSSLGWPIAKPEATMFVWSRIPAHYDSS

EQFAMDLVTHAGVIATPGSAFGPSGEGYVRFALVQDNEILQQAVQSVDDSGILKSS"

CDS complement(856536..858614)

/locus_tag="EFAGFIKM_00749"

/inference="ab initio prediction:Prodigal:002006"

/codon_start=1

/transl_table=11

/product="hypothetical protein"

/translation="MSNMLNKWGKTGARTSSMANTLSIVLLVIIVVVFAVLGTFMFAS

TRNILVKQQESMLQTKTEAIVSEFDALFKEKGALVKQMSTNTLFQQYIETTASAADAA

SSTYAAQTQATLAAIVKAEPSFADAWVAGLDGKGFWIQNDGVVSAPDFDIQSRPYYKP

VTDEDGLYFSDPYIDIAAGDVLMGIFYPIKDENNTTIGFAAADIAFKDIPAIMESYSL

GSTGYSMLLSKSGEILYHPDQEKVLKENILNSTGDMGDIGKKMLAGENGVQLMNDNGE

RRYIGYATSKDTGWSVGLTISEEEVLAELKTFTWITLGGFAAATLLLVIICYITLRRL

LRSIPQLLAKIKQIEQGDLTVKVDIKSNNEIGQIATGLNSMVQKIQAMLQMVGSSAQI

LNQSSNDLQAISSRTAVTMNDTSTAINEIANATNYQSIETENILRKTGSLSSQIDEIA

TDAQAMGTVVQTSADQSSQGLAVVEQLSKWSEENHQSTQAMSSIIQEIDLSRNEISSF

VDTVKQIASQTNLLALNASIEAARAGEHGRGFAVVAEEVRKLAEQTAQATEEINKKVN

VIEEKTTLSVEHTMRGMKIAEENAKSVEDTKHVFFSINKDLKDLQQRMMQITTNTANV

HKHKDEIFQALEIISSTTEENSASTEEVSASTQDQLDSIEQVADLSKQLNQLSGKLQD

ELSQFKVKSS"

CDS 858871..859899

/locus_tag="EFAGFIKM_00750"

/inference="ab initio prediction:Prodigal:002006"

/codon_start=1

/transl_table=11

/product="hypothetical protein"

/translation="MSELWHASIQGVPWNAEKMHQAVHLLRRRATVHFRVGADTYEDL

AELDAKVFFARPDLILHISHMDPKGVYSSGFLGRLAQLKHVSALQLDLSQAQDLTILG

AMEQMQFLRLNSPAKATRLDFIEHYRKLTFLELRGKFNGFDSIAACLQLHTLILNCTV

ESVDVLAELPMLQYLSLDQCELKGGLDVLTQSTISMLKLSSVRKLTSIQGLEQMRGLE

YLHLSLPKLEQLCDFSHMTSLRQLELDYMKSLRDTECLWTARALESIELKEINTALKA

DSFARLTGLEHLRQIDFRFIDVNKGRIAAMRDHLDKAGKSHLLYENIPEHERMNSIGI

AHLSQVLM"

CDS complement(859976..860695)

/gene="rhlG"

/locus_tag="EFAGFIKM_00751"

/EC_number="1.1.1.100"

/inference="ab initio prediction:Prodigal:002006"

/inference="similar to AA sequence:UniProtKB:Q9RPT1"

/codon_start=1

/transl_table=11

/product="Rhamnolipids biosynthesis

3-oxoacyl-[acyl-carrier-protein] reductase"

/translation="MKNIIVFGASKGLGDAFVRGVPQSGDKVWVVSRSKPESLSVEDG

VERIWIQADLSELQAAEQVASQLQGETLDVLIYNVGIWEKEGFEEHYTFDKDEPADIS

QLIHVNVTSTIVCIQALLPQLRQSEAGKIILIGSTAGLSNAGSSQVSFVASKFAIRGI

TEALREHTRNDRIAVTCINPGELAAEIPYEDGMERALAEYDGTRIPLQDMVAIVRCII

SLSKAACVKEINIPALTDTNT"

CDS complement(860941..863004)

/locus_tag="EFAGFIKM_00752"

/inference="ab initio prediction:Prodigal:002006"

/codon_start=1

/transl_table=11

/product="hypothetical protein"

/translation="MWKKISALLLALPLLITSFPLTSPAASAATFSNPVIYADVPDSD

VIRVGNAFYMTSTTMHMNPGVPVMKSYDLVNWSIVNYVYDTLGNGDIQNLNNGQNEYG

RGSWASSLRYNNGIYYVAFGSLSTGKTYIYQTSNIETGPWTPYTLNSYYHDPSLLFDG

NRTFLVHGSDNISIVELTPDAKAVKSGGLNQVLIPNASSVAGSNMIVKAEGAHIQKIN

GMYYVFLIAWPSGDGRIQLAYRANTLTGPYTGKVVLRDSGIAQGGIVDTASGAWYGLL

FRDSGAIGRIPYLVPVTWSDNWPVFGVNGKVPLNMNMPVEGQPAAKIYGSDEFNTTTS

GTPSVTQLLVNGGFEESNIQPWTSNNTATINLTNAEAFSGTKSLLVSGRQQTAGGAKQ

MVTGKLVPGGTYSFSAKIKYTTGPASKTFNLSIQNGASYTGITIMGSATLTRGQWGTI

QGTYTVPSNADLSQSFIFVETPYNSNPDPTNDLMAFYVDDVSLTSNSSSGTSSLATFW

QWNHNPDAANWSLLQRPGFMRLTAGKISKNLLEARNTLTQRTFGPKSTGMTALDTAGM

KDGDYAGLAAFQSKYGFVGVKMTGSTKSIVMANASSGTMTEVATVPFNQNRIYLRVIC

DFTNQTDKAYFAYSLNGSNWTTIGNTLQMSYTLPHFMGYRFALFNYATKSTGGYADFD

YFRVE"

CDS complement(863038..864972)

/gene="xynD_1"

/locus_tag="EFAGFIKM_00753"

/EC_number="3.2.1.55"

/inference="ab initio prediction:Prodigal:002006"

/inference="similar to AA sequence:UniProtKB:P45796"

/codon_start=1

/transl_table=11

/product="Arabinoxylan arabinofuranohydrolase"

/translation="MIRKPLVLLLSCLLIFVTLPVESSHAANAAVAKLPGNSNPLIDH

KLGADPYVLVHNNRVYVYMTGDAYLYNSDGSVRENQYSTIGKINVISSADMVNWTDHG

SIPVAGANNANNGQGIAKWATQSWAPAMAKKTIAGKEKFFLYFANSGGGIGVLTADSP

IGPWSDPIGRALLTHGTPGMSGVTWLFDPAVLVDDDGSAYLYAGGGIPNDTNAASIAN

PKTARVIRLGADMTSVVGSAVTIDSPYMFEDSGIHKYNGKYYYSYCINFAGTHPTAYP

KGEIGYMVSDSPMGPFTYKGHFLKNPGTFFGVGGNNHHAVFQFNNEWYVAYHAQTVSK

AFLGDGKGYRSTHINKLTHNADGTIQEVQGNMTGVPQITNLNPYVRVEAETIGWQAGI

NTEPSQASGGPVANQNVTNIHNGDWIAVGNVDFGSAGASKFKANVASTGNGNIEVRLD

SPTGPLAGTLNVSSTGGSQTWREMETNVTNATGVHRLYLVFTGSGNGNLFKLDYWQFT

SGSGGNPNPNPNPNPNVTRYEAENMTLGGQYAGVVSSPFNGVALYGNNDYAAYNQYFA

FPTHQFSVRGASNNNNTARVDLVIGGVTAGSFYFTGTEPTVQTLTGITHAIGNQEIKL

VVTTDNGTWDAYIDYLEYSL"

CDS 865876..866235

/locus_tag="EFAGFIKM_00754"

/inference="ab initio prediction:Prodigal:002006"

/codon_start=1

/transl_table=11

/product="hypothetical protein"

/translation="MAIFSTGPIENNPVSGVRPTQQVTIRLASRAEVDSVTVSIQGYV

LSTTRTLYVSEVISIAPNEAVTRNYFADLDAFEFVFGTDVEGVEEVEISVWGKQASGQ

LVDAHRVVEHEKTVNNI"

CDS 866270..868054

/locus_tag="EFAGFIKM_00755"

/inference="ab initio prediction:Prodigal:002006"

/codon_start=1

/transl_table=11

/product="hypothetical protein"

/translation="MSFLSTGPIENNAVSGVRPTQTVTVRIDNRSDVSTSTIQIQGYY

MSGGMRVLYVSESFDIAPNQVITNNYFANLDAFEFTFTTTATVNDPVQVSVWGKSSTG

ALVTAHRLVSSELLGETPSINGATGATGATGATGTPGTAGVTGATGAQGTAGTTGATG

TTGVTGATGTAGTTGVTGATGTAGTTGVTGATGTAGTTGVTGATGTAGTTGVTGATGT

AGTTGVTGATGTAGTTGVTGATGTAGTTGVTGATGTAGTTGVTGATGTAGTTGVTGAT

GTAGTTGVTGATGTAGTTGVTGATGASGTTGVTGATGTAGTTGVTGATGTAGTTGVTG

ATGTSGTTGVTGATGTAGTTGVTGATGASGTTGVTGATGATGTAGTTGVTGATGTTGT

TGVTGATGASGTTGVTGATGTTGATGASGSGTIIPYASGLPVVLTTVLGGLLNTSSLV
GFGNSSTGVSVNGGIIDLTGAAGTLLNFAFSASRAGTITSLAAYFSTTAGLSLVGSTV
TLTAQLFRSTTPNNSFTAVPGALVTLAPPLTGILALGTISSGVTTGLSIPVSAGDRLL
MVFSASVTAGIDVATTVAGYASGGLTIT"

CDS complement(868121..868216)

/locus_tag="EFAGFIKM_00756"

/inference="ab initio prediction:Prodigal:002006"

/codon_start=1

/transl_table=11

/product="hypothetical protein"

/translation="MYEARSNPRLFMFLYSPYLSFEESLKTKAHT"

CDS complement(868235..870196)

/gene="rhaR_5"

/locus_tag="EFAGFIKM_00757"

/inference="ab initio prediction:Prodigal:002006"

/inference="protein motif:HAMAP:MF_01533"

/codon_start=1

/transl_table=11

/product="HTH-type transcriptional activator RhaR"

/translation="MKATLNVQEHILIWNCASLKIMDVRHIVIQADECVRPYLFPASA
FLYVTNGNATVILDGNEHATKPFYMLHGGKNTRLDIIPTNESFHYYLIFYKASLPLPG
KQHTLQQPDQPLPFHLQYGFIPYHPAILYEITERMLGEWNRPGRLERLHTKTLFYQFV
YELLRQMDQQQVSIVRPNLASQLIRYMQEHYAQPLTRETLARTFHYSVPYLSKYFRRE
TGASLIDYLIGIRMNKAVTLLQKTELSLQKVAVSVGYADVSYFIRMFKKHTGVTPKQF
KDESGQVIRGSYRPILRLPSSIAPRKLRRYIDIRDDNHYQHSEKGDLRMYRSKLPVGI
TLLLCLTLLVSACSRPANTNGSTGSTQNPTVGAESNISNTTTGSSEMVTYSAVNGEVQ
IPKKPQSVVMVAGAFTGHLLALGLKPVGTGDESFNSYTEGKLHDVVNIGNDVPYEKIM
ELQPDLIVVWNDPETIEKLTKIAPTVAVEYGVPLRQQLMDFGKMTGREEQAKAWIAEW
DAKIAEYKPLVEQAVGDRTVSIFDTGSAKEFYAYGSFGRGGDIIYGEFGLKAPPIIQK
EAIDSGKGWAKLSLELLPQYAGDYIFISGWTGDESGDSVFEGPIWDNLPAVQNNHVYR
EESRGFVFSDPISLEAQLKFVVDSLTKTE"

CDS 870411..871340

/locus_tag="EFAGFIKM_00758"

/inference="ab initio prediction:Prodigal:002006"

/codon_start=1

/transl_table=11

/product="hypothetical protein"

/translation="MGKRLLIALLSMIMLFGVVPAYGAGTSGNSLIWEGYNDNGRMVG

INYTTFTGKKSTTKSIYNKQASQQKIIGNWIYFLVEDEEDHYSIAKIKTDGSAFAYVS

QDDNFASFDVAGDTIYFVKSEYNGNLYDDLSYGSMKTDGTKKQIILPVSPFYNFEINK

EYVYYTERHTDLFYRMKKDGTATKLLSSKGVSVYKFYGNVLFFSEMIDGGYSSRGVFA

ELDGARKKTFASDGYIHPLAYLNNKFYYRKNTIDKVGNTRSIIYVYNRYTGKKNKFAS

IDYYDRFLGQVGNSLAFLTFPKGDVYKIGLDGK"

CDS complement(871568..871921)

/locus_tag="EFAGFIKM_00759"

/inference="ab initio prediction:Prodigal:002006"

/codon_start=1

/transl_table=11

/product="hypothetical protein"

/translation="MNVTLWIVQLILALGFIYSGWMKTIRIESSKKTWAWVNDVPKSL

VIWIGIAELLGALGLVLPWALNITPVLTPIAAIALATVTLFGMLFHIRRKEYRELGVN

IFFIVLALIIAFGRL"

CDS 872062..872454

/locus_tag="EFAGFIKM_00760"

/inference="ab initio prediction:Prodigal:002006"

/codon_start=1

/transl_table=11

/product="hypothetical protein"

/translation="MTVYCKFGTALDILTGKWKSLILLRLLSNGTMRFSELQKAIPDI

SKKMLTQQLKELEYHDIVHREVYAQIPPKVEYSITEYGQLMKPVLQTMSDWGAGHMQH

MEKLYSQEDEKGAHPSEPRKIDSLGGIG"

CDS 872471..873508

/gene="dadA"

/locus_tag="EFAGFIKM_00761"

/EC_number="1.4.99.-"

/inference="ab initio prediction:Prodigal:002006"

/inference="protein motif:HAMAP:MF_01202"

/codon_start=1

/transl_table=11

/product="D-amino acid dehydrogenase"

/translation="MNKQKIVIIGAGIVGACMAYHLAKRNQDVTVIERHSAAAREVTE

KSFAWIHTTHRVAPEYWHLYDAAVEEYHILQQELSELEIHWHGALTWDIPPLREQHFQ

KLNQEQLEALEPNLKEYPDEAMFVSEEGAVDPIAVTELLLSKAQEYGAKIQFDTNVTQ

LKQEGSRLVGVHTSKGFVESDVVVLAAGTGTPELCNPLGCHVPVTSSPSILIRMKTAN

KLIHTLISNAQFEARQLTDYTLLAAEDYIDESEANGPQAVGKRAYDTLRRSLKHGNQL

ELESIKVGWRPMPEDGYPIVGFHDHMNGLYLTLMHSAITLAPLIANLAASEIIDGKSR

NELDPCRLSRF"

CDS complement(873667..874461)

/locus_tag="EFAGFIKM_00762"

/inference="ab initio prediction:Prodigal:002006"

/codon_start=1

/transl_table=11

/product="hypothetical protein"

/translation="MEFIHEDEKLAVSVIQAIHTGDIPTLEQLLAENPGLANIRILDR

KPDNVDADSNISRTLLHVVTDWPGHFPNGADTVRVLTKFGAEVNAPFVGPNIETPLHW

AASSNDVQVLDALLDAGADIETPGAVIAGGTPLDDAIAFAQWDAARRLVERGAIFALW

HAAALGDIHAIQEHFKGAQLPERYPWGTSTSPSPPNSVTVAFWCACHGGQRETAEYLL

DQGAELNWIASWDGMTPLDTAKRNFSSELIPWLESQGAKSASEIHR"

CDS complement(874495..875466)

/locus_tag="EFAGFIKM_00763"

/inference="ab initio prediction:Prodigal:002006"

/codon_start=1

/transl_table=11

/product="hypothetical protein"

/translation="MMSNNHLTREELQDYVQHVFGASYLITQSARIHGGAQKVIYKID

CTNGFTCMLYVWDISSNYFQEEILNEPTFHSSYGSELFSLNTRFLTQHGIRTPLLYDL

NNDRDQHSYDFALVEYVDGQKAEAYFQHKNHKDRDELFQRIRDMLSNMHNIQRNVYGN
ANDNSKKAGPCYQVQRLDAETALSYASKHMTDIRKNDERLLEKLYELEAAIQPRDLYS
FIHGELGPDHIWIDHAMQPCLIDIEGAGFFDLEHEHSFLEFRFGDFYHYLKNNDLDPE
RMTFYRFCHHLSLISGGLKLLHRQFPDQQFARGLSEYHARCAVQMLS"

CDS 875766..877652

/gene="rhaS_4"

/locus_tag="EFAGFIKM_00764"

/inference="ab initio prediction:Prodigal:002006"

/inference="protein motif:HAMAP:MF_01534"

/codon_start=1

/transl_table=11

/product="HTH-type transcriptional activator RhaS"

/translation="MDIRHITMQRKEVLHSYRFPASCFLYMVRGNAVIRLDGKDHQVS
RFHVLHGGKGCCLNILPVEEFLEYYMIYYKAIIPLPERQDILALMEHRNPFKLQYGFA
PDQPVSLLSKVQYMMTAWEEHEALEQFHVKSLFYQFVYELLWQIHNGRVPTIKPDLVA
QMKYYMQEHYAEPITMDLLAELMDSSPRHLSRLFKRQTGYSPIDFVIQIRMNKAKELL
LATDATLQEIAKAVGYPDGYYLAKIFKKYMGIAPIRYRKQHESPNVPSTVARLGIVQD
LTSLYIDNDYHNQHIFEEELLMYRSRKTPLAITLLLCLSLLLSACSTGGAGSPSAKEG
SQKPTNQVAANNAHNTDTQSQMKTVTTLKGDIEVPVNPQRVVVLYLMGDLLALGVNPV
GISSVEEGAAFASELEGATSLGAFNPDPEAVTALNPDLIIVTNEEIYEGLKMIAPTVY
IPYDLPVEERMNLLGEILGKEGQAQEMLDNLHKKVEQSKEKLEQAGILDKTVTIMESN
KGSMAVMTGLGYGRGSQIIYQYLGMKAPEVLQREIENARNDATSKDVSYEVLPEYVGD
YIFRSSFEGMVDLSDNSIWNNIPAVKEGRLIDMSFGLFFYNDIYSLDKQLDFIVDNLL
KTAE"

CDS 877720..878307

/locus_tag="EFAGFIKM_00765"

/inference="ab initio prediction:Prodigal:002006"

/codon_start=1

/transl_table=11

/product="hypothetical protein"

/translation="MNYYFLESQYPRRGFISGGTTFTPQLDMNYLAIENTLPEGTTAE
VELLSTVRNLNVDYFETITGTRHVSDRFKKLLEETKTNTQFIPTTVCYHDGRAVEKTY

WTAHQLDRLDVFDYERSEYGRKAVIAASVQQPPRKIVKVVSQICLHEERIGKHEFFML

DYINIFKPIVSKDFYEVCRKHKLNLNVTEVGNVSN"

CDS 878764..880323

/locus_tag="EFAGFIKM_00766"

/inference="ab initio prediction:Prodigal:002006"

/codon_start=1

/transl_table=11

/product="hypothetical protein"

/translation="MFINRTIRKFGVSVGIISMFTFALPAVGFSMPLQPTTIDKVEGD

YYVTSAIENIRWGSLPNRDSSPILTVPSGSAITFDTVSHEGMIEDQGRNPVEYFGKFG

ISPDGVLDDAKVIAASQIKHDYVKDGPHIITGPVAVEGAEPGDVLKVEVLSLQTRVPY

GVISNRHGKGALPNEFPENTGLQEGASATKPELYNNVSIFTPIEEINGNWYGVLPTKA

GKNVRFPINPFLGVMGVAPNTSEVVSSIPPIETGGNMDINELGVGATIYYPIQVKGGL

FYTGDPHFAQGDGEVALTALEASLRGTVRLTVLKKGDPSLPHSGEFTQPFAETEDYWI

PIGLDPDLDEAMKESVRESIQFLSDKLDMDRSVAYAYLSAATDYEVSQVVDRTKGVHG

LIRKTDFLEHVDVAMNVGGTLIKPVVHNNEFYVPIRTISELLGGTVVWDNKTRTTQIK

LGTKNISAQIGSDVYSINDKLVFNSNVPKLLNGETVVPVSVINEILGAYVNWTTIDKT

LTANVSLSKKQ"

CDS complement(880535..881383)

/gene="sseB"

/locus_tag="EFAGFIKM_00767"

/EC_number="2.8.1.1"

/inference="ab initio prediction:Prodigal:002006"

/inference="similar to AA sequence:UniProtKB:P9WHF5"

/codon_start=1

/transl_table=11

/product="Putative thiosulfate sulfurtransferase SseB"

/db_xref="COG:COG2897"

/translation="MKNIVSMRWLLARMYEPDVVIADCRFLLGQPEAGRQAYEAGHIP

GAVYLDLEKDLSSPVSAHGGRHPLPDPAVLASRLSKAGIGSNSRIVAYDDQGGMNASR

LWWLLRYIGHEQVYIMDEGFSAWKNAKFPVTTDVPVQIPSSFEVNVQPAMLASAQDVQ

QASASGIAVLIDSRDARRYAGLEEPIDAKAGHIPGAVNYFWKDVLDWDGRWTDTGVLE

ERFSKLDKDGAIIVYCGSGVSACPNVIALEEAGYTNVKLYSGSWSDWISYEENPVATG
EAHKDV"

CDS complement(881447..882391)
/locus_tag="EFAGFIKM_00768"
/inference="ab initio prediction:Prodigal:002006"
/codon_start=1
/transl_table=11
/product="hypothetical protein"
/translation="MTSLNRAGRSIYLLFFVGIIAISFSSIFVRWSTADVAVIAMYRL
FLTNLLMLPFVWKYRHEMMRLSLRQWGLLLASGVMLALHFLLWMGSLRLTSVASSTVI
LALEPILILAGSVWLFKAKINRMMIIGMGIALLGSIVIGAGDFQLAGTALQGDVLSLL
GTIAVAVHMLLGQFLRAGLSAFSYNFWVFFVAACTLAVYNLIMGHPFGGYAASEWGIF
LLLAIVPTIFGHYLFNWLLQYMNATTVSMGVLGEPVFSSLLAWMLLGESLSALQMSAG
VVIIFGVWIFIRYGKTKPQIASAEASVAGKGPIEPTTV"

CDS 882614..883522
/locus_tag="EFAGFIKM_00769"
/EC_number="3.5.1.104"
/inference="ab initio prediction:Prodigal:002006"
/inference="similar to AA sequence:UniProtKB:Q81EJ6"
/codon_start=1
/transl_table=11
/product="Peptidoglycan-N-acetylglucosamine deacetylase"
/db_xref="COG:COG0726"
/translation="MTVRVQQERAGERGSIPPSTSRTKTHKRRKIRYGRVIIALMLLV
LFVTGITYVFFGMTHWIKSVVAPPPITVIEQPAKLGMIQVTPDAKEEPARFQGQVRKL
AYITFDDGPTEYTEQLLDILKQHEAKATFFMIGRQLNQHPEAVKRLLKEGSYPGLHSM
THNYKKLYKSGNSANFVKEFKKEQKMVQDLIGFTPHLIRAPYGSSPQIGEKFRGDIAA
AGFKMWDWTTDSLDWNLPGQPDKIVARVSKSVHRDKEVILMHEREQTVQALPRILKLL
EDRGYEFEVYDPDAHWVANFSGDTRL"

CDS 883560..884213
/locus_tag="EFAGFIKM_00770"
/inference="ab initio prediction:Prodigal:002006"

/codon_start=1

/transl_table=11

/product="hypothetical protein"

/translation="MLTSKKVALAAVSVLLILLASGCGDPQEPAANQINQLVLSGQEI

DQSLKTLTSHEQEDMKLYKSILDKGKNKNSDLEALLDQAADHIHERRTLLKQAEEAMK

QTNEKTVALRSSLKELSFEKEETLAQAEKVLDQYEARARALENFVVSYQLSLSADEQL

YDLMRESAEPNLVKIKRAIRVRNSEYAKLAEFRKQFNLQTKAFNGANAKLVQMDQAS"

CDS 884356..885246

/locus_tag="EFAGFIKM_00771"

/inference="ab initio prediction:Prodigal:002006"

/codon_start=1

/transl_table=11

/product="hypothetical protein"

/translation="MRKGNTMKVSASLLVLLMGFSLVACGNSNPAAQPTVNNTEEQSP

APEQQPNESAAIEAEGILTGWADPHTVEIRVNEKPMSFQVSEDLQKSLDDIDDEDSVR

FKYVEKTIDATTKQLVLTEIAKLESSDDNNGSNAGGTVTSDRPKTSELEVTVEGMTEK

RPVTLAQAKGYSLYVFEPMAFDAEDHELTMNIDPDYEVEIEKLPANYNLDALKMDAKE

ELSETGEVKQLTAEQLSNGPMKDAKLYMMSQNDKKTEYFIVKELDGNGFIFHIQAPIG

EPSEGFLPMAYASLNSIMNE"

CDS complement(885289..885429)

/locus_tag="EFAGFIKM_00772"

/inference="ab initio prediction:Prodigal:002006"

/codon_start=1

/transl_table=11

/product="hypothetical protein"

/translation="MQQHRSFRLFVYARRGTARYIYLYCSNVNKTAYLLSQGNTLFRE

SF"

CDS complement(885501..886673)

/gene="mtlD_2"

/locus_tag="EFAGFIKM_00773"

/EC_number="1.1.1.17"

/inference="ab initio prediction:Prodigal:002006"

/inference="similar to AA sequence:UniProtKB:P09424"

/codon_start=1

/transl_table=11

/product="Mannitol-1-phosphate 5-dehydrogenase"

/db_xref="COG:COG0246"

/translation="MKAVHFGAGNIGRGFIGHMLSASDYKVCFVARNPKKISMLQERQ

EYPITLANRDQDTTIVNNVTAINVSEQNLVAEEIASADLITTAVGVSALGDIAEPIAK

GIQLRMKNNNQAPLHIIACENAIGGSTRLKKRIYPFLDEQTRKKAERYVSFPNAAVDR

IVPAQNHKDPLQVTVEPFYEWVVHRPALLDGFKKIDGVHYVDSLEPYIERKMFTVNTG

HCVAAYFGYLEGFKTIRQVMSNSTLRAKVRHVMEETGQMLIQKHGFNAQKHNKYIDTI

LERFANPNLTDQVTRVGRSPLRKLSPHDRLVRPAMQASEFGIKIPHLTSAMAAAMLFN

DKRDEEAMKLQHMLREDGVSAFIRERMGIPDEHPVHQNVIARYQELRGCKESTILT"

CDS complement(886721..887389)

/locus_tag="EFAGFIKM_00774"

/EC_number="3.1.3.-"

/inference="ab initio prediction:Prodigal:002006"

/inference="similar to AA sequence:UniProtKB:Q9X0Y1"

/codon_start=1

/transl_table=11

/product="Phosphorylated carbohydrates phosphatase"

/db_xref="COG:COG0637"

/translation="MIKALVFDFDGTIIDTETAWYIAFRDAYKEHGVDLTLEMYSQCI

GTSLKTFNPYEYLITDLNLPIDREAFRESVQLQHAALMNKEKVRPGIQEYLEQAREAG

LKLAVASSSKREWVEQHLEQLKLKDYFEVIRTADDVANVKPDPELYTQALEALGVTAD

EAVAIEDSPNGARAAAAAGMHCVVISNTITGTLEFDMPHQRLSCLTDLTFNDLISKPL

VTTV"

CDS 887589..887732

/locus_tag="EFAGFIKM_00775"

/inference="ab initio prediction:Prodigal:002006"

/codon_start=1

/transl_table=11

/product="hypothetical protein"

/translation="MIVYIENFNMYDLFSENDERCKPLTLLHVSGVLNKLFLQLKKIK
LFL"

CDS 887729..887944

/locus_tag="EFAGFIKM_00776"

/inference="ab initio prediction:Prodigal:002006"

/codon_start=1

/transl_table=11

/product="hypothetical protein"

/translation="MKICYSDRSTAFDTFIIEEMELNLLDFISFTPDQALWENGRKIL
KVYGVREEDKHVAKGPFQWLLHRACSG"

CDS 887892..888818

/gene="tri1"

/locus_tag="EFAGFIKM_00777"

/EC_number="3.2.2.19"

/inference="ab initio prediction:Prodigal:002006"

/inference="similar to AA sequence:UniProtKB:B0KTG8"

/codon_start=1

/transl_table=11

/product="ADP-ribosylarginine hydrolase Tri1"

/db_xref="COG:COG1397"

/translation="MLQKDRFNGCFIGLAAGDALGTTVEFSSPGTFEPVTDIVGGGVF
GLEAGQWTDDTSMALCLAESLVRKADFDPADQMRRYTNWYKVGYMSSTGDCFDIGGAT
RSALERFEITGEAYSGSTDPMTAGNGSIMRLAPVVMAYANQPNEAVHYAGLSSRTTHA
ATESVEACEVLAAIIVAGLRGANKSVMLMPETCRQWREEPTFSPAIEEVVLGSYQRKE
PPEIKGSGYVVRSLEAALWAFHKSSSFEEGALLAVNLGDDADTTGAVYGQIAGAYYGL
SGIPAHWRDKLAMRGTFEQLTDDLWLKATENR"

CDS 888844..890091

/gene="ydhP_1"

/locus_tag="EFAGFIKM_00778"

/inference="ab initio prediction:Prodigal:002006"

/inference="similar to AA sequence:UniProtKB:P77389"

/codon_start=1

/transl_table=11

/product="Inner membrane transport protein YdhP"

/db_xref="COG:COG2814"

/translation="MSTTGHTIQPQVSPSGKRGAFPLSLLCLTIGAFAIGMTEFIIMG

LLPNVAIDLNVSIPQAGQLITGYALGVAVGAPILTVFTHKIPQKKLLVLLMCIFIIGN

ALSVIAPTYGLLISARILTAFSHGTFLGVGSIMATRLVAPERRAGAVSVVLAGLTIAN

IIGVPFGTFIGQQHGWRSSFGAITILGIISLMGIIRFIPVIPQGPPANLGQQFRNLVR

PQVLLVLLIGALGCGSLFTVFTYITPMLVDISGFAEQSVTWILVLFGFGVTLGNLVGG

RLADWKLMPSLIVNFGILAVLLAALTITLENPYLAVITIFFWGVAAFGIMPGLQIRIM

NMTREAPLLATTSSHSAFNLGNAAGAYMGGYAITHTGLISVPLYAAVIAGLGLLGLLF

SLFMERKHHGPEIAQVAEAASAS"

CDS 890493..891440

/gene="xynZ_1"

/locus_tag="EFAGFIKM_00779"

/EC_number="3.2.1.8"

/inference="ab initio prediction:Prodigal:002006"

/inference="similar to AA sequence:UniProtKB:P10478"

/codon_start=1

/transl_table=11

/product="Endo-1,4-beta-xylanase Z"

/db_xref="COG:COG2382"

/translation="MKKRLSKILSLSITAALLVPTMASATDMQETPAPAPIQSVIDQG

ATGTMDNTGQNALANTPVVPAPPGYDGYRNNIPHGNTNLISYYSTTVGNTRKATVYTP

PGYSPNKKYNVLYLLHGIGGDEYEWVNAMKPKNILDNLYSEGKLSQMIVVMPNGRAMK

DDRPVGDIFAPDKVAAFERFEQDLLKDLIPHIEANYPVYKDRNSRALAGLSMGGGQSL

NFGLKNLNTFAWVGAFSAAPNTQSVSQLVSNPGQVASQLKLLWISCGSSDGLLWVSQN

FKNGLSSMNIPHTFYLDVGGHEPSVWNSGLYQFSQRIFK"

CDS 891660..891839

/locus_tag="EFAGFIKM_00780"

/inference="ab initio prediction:Prodigal:002006"

/codon_start=1

/transl_table=11

/product="hypothetical protein"

/translation="MFYYKGAFRETDNVIYFKSEVPLTENDVVIFGEKTYFIESVEKE

FLLLQRVGSIVSVSL"

CDS 891970..892668

/gene="sigK_1"

/locus_tag="EFAGFIKM_00781"

/inference="ab initio prediction:Prodigal:002006"

/inference="similar to AA sequence:UniProtKB:P62181"

/codon_start=1

/transl_table=11

/product="RNA polymerase sigma-28 factor"

/translation="MPGLFTAIALFIKELTLLVSYVKNNAFPQPLAEGDEAKHLRLMA

EGNAHSRNLLIEHNLRLVAHIVKKFDNTGEDQEDLISIGTIGLIKAIESFQQGKGTKL

ATFAARCIENEILMHLRSLKKTRKDVSLHDPIGTDKEGNEITLIDILGTEADDVVDKV

QLKIEKSKIYRNLDILDDREKEVVIGRFGLEAGGEERTQREIAKELGISRSYVSRIEK

RALMKLYHEFYKQK"

CDS 892912..893103

/locus_tag="EFAGFIKM_00782"

/inference="ab initio prediction:Prodigal:002006"

/codon_start=1

/transl_table=11

/product="hypothetical protein"

/translation="MIVFNSFQSYSSIINFIALLMISVANAIRVWKTERSLAIAFLVV

VALCLAYIFKYFYRYVLSA"

CDS 893423..894784

/gene="cycA"

/locus_tag="EFAGFIKM_00783"

/inference="ab initio prediction:Prodigal:002006"

/inference="similar to AA sequence:UniProtKB:P0AAE0"

/codon_start=1

/transl_table=11

/product="D-serine/D-alanine/glycine transporter"

/db_xref="COG:COG1113"

/translation="MQQETLTRGLKNRHVQLMAIGGAIGTGLFLGAGKTIQLTGPSIL

LAYIITGVVLFLIMRALGELLLSNLQYHSFVDFVRDYLGNMAAFITGWTYWFCWISIA

MADITAVGLYTQFWFPNVPQWMPGLIALVILLIMNLATVKLFGEMEFWFALIKVVAIL

ALIIVGLYMIFKGFTTDQGPASFTNLWSHGGWFPNGLHGFIISFQMVVFAFVGMELVG

LTAGETENPEKVIPRAINQIPIRVLLFYVGALLIIMSIYPWNAIVPTESPFVQVFAAV

GIAAAAGIVNFVVLTSAASACNSAIFSTSRMVFSMAKDHNAPESFARLNKRKVPSNAL

FFSTIVILIAIVLNYIMPEGVFTLITSVSTVCFIFVWGIMVICHLRYRRTQPELASRS

RFKLPLYPFSNYLILAFLAFVLVVLALAEDTRVALFVTPVWFILVAGIYLLKKKNLSN

NRQ"

CDS complement(894840..896024)

/gene="estB_1"

/locus_tag="EFAGFIKM_00784"

/EC_number="3.1.1.-"

/inference="ab initio prediction:Prodigal:002006"

/inference="similar to AA sequence:UniProtKB:Q9KX40"

/codon_start=1

/transl_table=11

/product="Esterase EstB"

/db_xref="COG:COG1680"

/translation="MRKKDMTTLHNYVDEAIDRALSEKRIVGTVVQVALRGELIYSRA

AGFADREQKRSMSENALFRLASVTKPIVSTAALALISQGKLSVHDPVTRWLPAFRPKL

ANGQDALISVHQLMTHTAGLTYRFFQEDQGTYELAGVSDGMDWSEMSLEENLQRLASA

PLLYEPGNRWRYSIATDVLGAVIEKVTGMSLREAVQTLVTHPLHMTDTDFVAVDSERL

TAAYANGSEEPRLLHQELEQIPFIEGTAGFRLSPNRANRTSAYLSGGAGMVGSTGDFL

TLLEALRQGGQPLLTEAVAAEMSTNQIGDLVMPYWPGRGFGLGFTVLKDPVAAGTPES

AGTWRMGGTYGHSWFVDPKEELSVAAFTNTALEGMSGQYTTDICDAIYAGIREGKEAV

RI"

CDS 896195..896647

/gene="lrpC_1"

/locus_tag="EFAGFIKM_00785"

/inference="ab initio prediction:Prodigal:002006"

/inference="similar to AA sequence:UniProtKB:P96582"

/codon_start=1

/transl_table=11

/product="HTH-type transcriptional regulator LrpC"

/db_xref="COG:COG1522"

/translation="MDEIDQHILYHLENQARLSMTELGKLVGLSQPAVTERVKRMEEK

GIIEEYRTVISPTKLGKQSTAYVLFRTRDCYPFLDFCRASPEVVECYRISGEYNYLLK

ILTDTIQGLEEFENKCDPYGTYMTLITMSSPIAHKNLVGGPNLLAQAE"

CDS complement(896742..897947)

/gene="nepI"

/locus_tag="EFAGFIKM_00786"

/inference="ab initio prediction:Prodigal:002006"

/inference="similar to AA sequence:UniProtKB:P0ADL1"

/codon_start=1

/transl_table=11

/product="Purine ribonucleoside efflux pump NepI"

/db_xref="COG:COG2814"

/translation="MTAHTPLPKQRELPSISSERLPWAGLLALAMAGFICILTESLPA

GLLPQIAKDLGITEALTGQLVTLYAIGSLVAAIPLTAATRGWRRRPLLLVCISGFLVF

NTVTALSSDYILTLAARFMAGVSAGVLWGMTAGYARRMVPDSLKGKAMAVAMVGTPVA

LALGVPIGSFLSSYVGWRLIFGMVSLLTVTLIVWVLWRMPDFAGEPTGERLPLHKVFL

IPGVRPILFVVLAWMLAHNILYTYISPYLAQTVFANRVDLILLIFGFTSIVGIWLTGM

LIDRFLRKLVIISLASFALASVVMGIATHQPVIIILGVMIWGLTFGGAATLLQTAIAQ

AGGKSTDVAQSMLVTAWNLAIGGGGIIGGILLEVFGAGYLSGALFILLIPALLVAMRA

YKYGFTRAN"

CDS complement(898168..899232)

/locus_tag="EFAGFIKM_00787"

/inference="ab initio prediction:Prodigal:002006"

/codon_start=1

/transl_table=11

/product="hypothetical protein"

/translation="MKFSSRTIGIAALSLLLMLLAACSNTSTSSPAEETTSTANTTET

SAPAENTNKTTYPLTIENFTISGEGGEWKPKVQVFDKAPERVVANTQSAAEMLIKLGL

TDKMVGVAALYGSVTPDVADDFAQIPVLSKDYVGKELVVGASPDLVLGRGDLFADADW

GVGTVDGLNDMNIRTFLQTTNHKGSLDSLYSDIAQLGQIFDVQENAATFTESLKTRVE

AIKASVADQPDHTFAYIVPATEDTITVSSMQNDTYQLDALGLLKLKNTFDGVQGEVSV

EQLITANPDYLLLSAYAGSPDIDKLIENLYANPALQSMNAIKNKQIYVTDFSQFWGYG

YQILDGIEKMGQEMKANPIK"

CDS 899366..900475

/gene="btuC"

/locus_tag="EFAGFIKM_00788"

/inference="ab initio prediction:Prodigal:002006"

/inference="protein motif:HAMAP:MF_01004"

/codon_start=1

/transl_table=11

/product="Vitamin B12 import system permease protein BtuC"

/translation="MAQTVQVQTQRMGKRESLIHSKSGFALLIAALIMITLISVGIAV

SIGQVQIPLAESYRILLYKLTGFQWGTTPIEAGSFTDIIWQIRFPRVLMAMFIGAGLA

LCGAVMQAAVQNPLADPYILGISSGASLGATFAILIGFGAIGWLGQTGVAFWAFAGAM

GASLLVLTLAGIRGKMTSVKLVLAGMVINALCSAFSNFIIYFANNAEGIKTVTFWTMG

SLAASGWNKLPLVGIVVLVAILFFLLQSRVLNTMLLGDEAAVTLGINLSVYRRLYMLL

TALVTGVMVASCGMIGFVGLIIPHIVRGLVGSDHRKVMPVSVLFGAIFLIWTDVIARS

LISSVELPIGIITAMIGAPMFMYMLVKKGYGFGGN"

CDS 900478..901245

/gene="fepC_1"

/locus_tag="EFAGFIKM_00789"

/inference="ab initio prediction:Prodigal:002006"

/inference="similar to AA sequence:UniProtKB:P23878"

/codon_start=1

/transl_table=11

/product="Ferric enterobactin transport ATP-binding

protein FepC"

/db_xref="COG:COG1120"

/translation="MKLNVDNVSISVLNTDIIKDISLQVNGKQFVGLIGPNGCGKSTL

LKSIYKVIKPQQGKVFLDNTDILKSSPKLVSRHMGVVGQFNELSFDFTVREMVMMGRT

PHKKLLETDNERDHEIVEQALEKVHLTEHADRNYVSLSGGEKQRVVLARVLAQQPEFL

ILDEPTNHLDIKYQLQILNIVRGLGIGILAALHDLELAAEYCDYLYVVKKGQIVVHGK

PADILTREMIGEVFDVDCEIYTNPVTGGLGIAYLSTR"

CDS complement(901382..902014)

/locus_tag="EFAGFIKM_00790"

/inference="ab initio prediction:Prodigal:002006"

/codon_start=1

/transl_table=11

/product="hypothetical protein"

/translation="MDWIWKSVLLVLIGMILLRIAGRKSISQMSVATTVIMISIGTTI

VQPIANHELGKAIGSASVFILTLLVVEQLQLKFNIFERLMSGSSKIVVEDGKVIIANL

KRMRYTMDQLEMHMRQNGITSVDDLETATVEPNGQLGYVLKRHARPVTIGDLEEILRS

YAITGSSPATGEHSQQDNNSKSSTHSGNSSDVFQEVSKGAHAHPVDPKLQ"

CDS 902169..902822

/gene="rnhA"

/locus_tag="EFAGFIKM_00791"

/EC_number="3.1.26.4"

/inference="ab initio prediction:Prodigal:002006"

/inference="similar to AA sequence:UniProtKB:Q9KEI9"

/codon_start=1

/transl_table=11

/product="Ribonuclease H"

/db_xref="COG:COG3341"

/translation="MAKQKYYVVWEGKKPGVYNTWADCKAQTDHYTGAKYKSYESKAA

AEEAYRAGWKGNWGLSAGGASKTKSAGSGRSAGMETSEEVDYDSLSVDVGTRGNPGPV

EYKGVDTRTGEIIFSVGPISKGTNNLGEFLAIVHALAHLKKEGSTKTVYTDSVNAMKW

LKQKKVATTLARDNSTEEIWLMIDRAEQWLQTNTYSNKVLKWQTKQWGEIKADYGRK"

CDS 903155..904024

/gene="nap"

/locus_tag="EFAGFIKM_00792"

/EC_number="3.1.1.1"

/inference="ab initio prediction:Prodigal:002006"

/inference="similar to AA sequence:UniProtKB:P96688"

/codon_start=1

/transl_table=11

/product="putative carboxylesterase nap"

/db_xref="COG:COG0596"

/translation="MSSIYRSEEGKSSILEEYEIYLNELGEKFTREYVETRFGKTHVL

LTGPLEGKPLFILQGGNCVNPMTLSWFSSLFKEYRIIAPDTIGHPGYSEEASISTRFD

SLALWVSDLLDHYDIEKSAFIGPSFGGGIILRLATYIPERISCAVLVAPAGLAVNSKI

KTAQDITLPLVKYRMTSSPSSLQKITDNMSCNCMKEMDKNIIGKLFKYVSIDQDLPKL

TEREELVNYTSPTLLLVGEKDVFFPADKCIERAQKIITNVEAIKYDTGHFPSQDVLVQ

MNEEIGRFLQNNY"

CDS 904324..905313

/gene="pknD_1"

/locus_tag="EFAGFIKM_00793"

/EC_number="2.7.11.1"

/inference="ab initio prediction:Prodigal:002006"

/inference="protein motif:HAMAP:MF_01957"

/codon_start=1

/transl_table=11

/product="Serine/threonine-protein kinase PknD"

/translation="MRKDNCKMALQRNVIINDTYQVRQVLASSELAIVYAGRDRNTGA

KVAIKEFFPQRMAERQADKRGVFCSSRGYGGQYQELLAAFLLEGELLSKLDHPHIVSY

LDHFEANGTGYLVMEYCTGITLTEYLNEHNHALDAAFITETLLPLVDTLDYIHKQGIL

HRDVKPSNIMVMEDGTPKLLDFGSATRWPMQSGEKQVIFTSAGYSPLEFYSEKSSQGP

MSDIYSLAALLHYWTCGQPPMDVKQRLFQDELPSVRSHNEYVNPWLARVIHWGLTVRS

EKRCASLAWVRSSLRVQAWVWKVRKPGTVEWSLAENEHTQVYEVESKKQHETA"

CDS 905869..911595

/locus_tag="EFAGFIKM_00794"

/inference="ab initio prediction:Prodigal:002006"

/codon_start=1

/transl_table=11

/product="hypothetical protein"

/translation="MKRKSTRWLSGLVATGVMLSSMFSLPAYSAPSYGPLQQSESAKS

ILQSLTPEERQALNQLQVMDGEVIDPKVNTKSNDLVQVIIQFKSEPAAVALKRAGLNN

EKLSELTAASRVKQNHQQFKQHIKSLQQKRGLSYAAASIQITQEYETALNGVALTIPG

VAVEDLMRSGVVKKVWADQEVVLDLPETDTSSEFASASGMQPRMADSVPYLGIDNLHE

EGITGKGIKVGVLDTGIDYNHPDLTGAYKGYRAHAAEDPKQVDPATVKGWDFVDNDAD

PMETTYEDWENSGYKEQGVLAEAYYTSHGTHVSGTIAGQAKNDVGYAVKGVAPDVDLY

VYRVLGPGGSGEMSGIIAGIDKAIEDELDVINLSLGAATNDPLDPSSIALNNAMLQGV

VSMVAAGNDGPNEGTLGTPGASALAITIGASDVPLTIPTATVTLDVYNNPGEGETEET

PVMDEGTSAEEEAPAEGEETSVVEEEAPEEEEAPPTAAEPSAADVEASATEEETPAAE

EAPVVDEEAAAEEDAPDVEVEAPPAAEEDETPVAGEEAPTKEAPASTFTELLPLFGKD

FADDLTTLEGKTYELVYAGLGNAFDFEGIDVQDKVALIARGDIALDAKIVNARKAGAA

AAIIYNNVEGTIPNYLGEMNGYIPTFQMVRELGEKLKELNGVTVTFGAAGQTVTEGNK

LADFSSRGPASVNDDIKPDIVAPGVAVFSTYPVFVNDHTGENYNIAYARISGTSMATP

HMAGIAALMLEAHEEYTPFDVKTALMNTAVDLNGNYSVYEVGAGRVDPYRAVHTSVFA

KVQDKTIHFSNDESILIDEITGSIVFDSAYLEENDQGIVSDSRTVKIENKGDGAKTYN

TSMKLLNDMNLNVHLPESITVSAGSTEEMTVSVDIPQDTPSGRYEGYVVYLDTENAADH

IQIPFSIRISEKGITTVEMIPPSVTNDTPFHQFLNRMTLSAIQLGSPIDSVDVLLKDR

ETGEEIGFVGTLNSQSMLPGVEYLILNVFRGYAFPIENGEISNVARFVPEGSYTLEFI

GTDSDSQQYSYERLVVVDNTAPEIDLGIEPGVYELNDSMLTEREGGQGVWYDGTAIDS

TVQTFVDNGITAYDQSSNRVIFYENGSPFYSGDLPVSKEGNISFGITKEEYDNGYELR

LFGLDVGTAANAVKDHRYGFISEGKPYLTSTYDKDEVRKGDTITQTIQLNNVEKLNGI

SFTLESMYDYKQFNRIEPTPELKALLDDTGAEVNFSEPAVDQRELSFEAFISGGSFEG

ITGDLDLFHVIFDVPKDEFYFKKSDVPFLEAKYTTVGMKENEEKLALAAFNTDSYDVI

SKSSRTYGFVRPEAFMVDGEYLDFENDYSAMGIEVYAETASGEKYEGTVQNYGEFLID

GIPATGEMTTIYTRAPGHLPSRLAVVLSKTIDGEYYGINQLNSSDFTYAGDINGDGAI

DIYDMELVTKYYRTSDETADIDQDGYVGDTEVSYITNNFGMTANYSTKKPVLQIGEKD

LGYFLNKIQEMNNGGPGNNGGGSGNNGGGGSSTIPAPVPTPAPPTDPDPSQNQVITEK

ELKAVTNGTANVDITVGKKIELPVNAGDLLGANSLTISQQGVLSLTIPSSVLKELQTK

GGSGAAGGKIILDIMKQDVKAPETKQGLTWTTAGAAYEVELYLQVSSEKLSLSSLPED

FSLTLHYDRTLNSDLIGMYHEHSSADQWDYAGGIVDKEKGTVTSQLSDMGTYAPFVYD

KSFEDVAPTHWASDAIKSLSAKHVLTGKTETAFDPQGQTTRAEFAALIVRTLGLEVKG

NELPFGDVSKDAWYADSVAAAYQAGLVQGIANDKFAPTQTISREEMAVLMMRAYEYQS
QSNHIQSAVAGYTDEANIASWAKEAVAEASELGVMNGSTGGQFKPKNSATRAETAQTV
HNLLSLLK"

CDS 911778..913019

/locus_tag="EFAGFIKM_00795"

/inference="ab initio prediction:Prodigal:002006"

/codon_start=1

/transl_table=11

/product="hypothetical protein"

/translation="MIEGKIIKFYREKNRRTQGELVKGICSVTHLSKIERGMTEYSQE
IMDMLCDRLCIDMAREVARYHQMNDLLDSWQEAIIMQRTVEAIRLKDKLEVEPLKGIP
DLRLSYDLLQIRFYLFMNNIVEAERLIAEVEIPDESEHSYEHYFYHHVRGIYYFLTGH
YRKSIQTLTSINQSKYTNHEYFYHLSLAYHSIYSNITSYYYAEKAIQHFRKTLNMVRI
IDTETIMLVQLNSREYHDFPETKRRYENLIRISDDCGLSDRKAKLLNNLGYEYNRRKQ
YKDAAYWYRQALDIIPEKQPLYLMVLNNYIESNRLGNLIPANKLLDMSLETLHNSKLV
SDASYMELELQSVILQGQEQAYYEMIEERILPYYRELGSQYLAERYEGILFQYYMDHG
KKDKALRLAKNRFESQLTSLI"

CDS 913111..913551

/locus_tag="EFAGFIKM_00796"

/inference="ab initio prediction:Prodigal:002006"

/codon_start=1

/transl_table=11

/product="hypothetical protein"

/translation="MTDFINADDINDVILAAAANELEQMVDKMCELIGTPLEQTTELE
RQVMAAFGFGAVYGITHRDQLAEPQAHALSIRMLIKPFNYSEQQAVDFADDLIRVASD
REVHPVMNTIIQRGIDGHRQFNQEDDEGLERNIQEILTAVQSQK"

CDS 913583..914137

/gene="kptA"

/locus_tag="EFAGFIKM_00797"

/EC_number="2.7.1.-"

/inference="ab initio prediction:Prodigal:002006"

/inference="similar to AA sequence:UniProtKB:A3DJX6"

/codon_start=1

/transl_table=11

/product="putative RNA 2'-phosphotransferase"

/db_xref="COG:COG1859"

/translation="MDLMKLSKELSYALRHAPWEYELELDEEGWVEISQLLVALHESP

QWKEVTQADLEQMIQASEKKRHEIRSGRIRALYGHSTPHKISKISAEPPETLYHGTPA

RAVNSIMEHGLQPRQRQYVHLSADIDTANQVGRRRDDQPVILRINAAQASTDGILFYH

GNENIWLADHIPARYILEQVSSEQ"

CDS complement(914326..915183)

/gene="qorB"

/locus_tag="EFAGFIKM_00798"

/EC_number="1.6.5.2"

/inference="ab initio prediction:Prodigal:002006"

/inference="similar to AA sequence:UniProtKB:P39315"

/codon_start=1

/transl_table=11

/product="Quinone oxidoreductase 2"

/db_xref="COG:COG0702"

/translation="MKILVTGATGHLGSLVVEAFLKTDSAKDVAVSVRNPEKAEALRT

QGVDVRHGDFDQPETLKKAFAGVDRLLLISTDGDNETRIRQHQAAIDAAKSAGVGFIA

YTSVVNAEKNTLSLAEVHRATEKAIRESGIPYSFLRNNWYLENEAGSVQAATQGAPWV

HATNDSQVGWATRSDYAHAAAAVLAGEGHENTVYELSGKLRTQAELAAIVGEVLGQEI

NVQNVDGAAYADIMKGAGLPDFVVSMLVDMQSAIREGALAVESDTLEKLLGRPAQPLS

EGIKAIVGK"

CDS complement(915295..915720)

/gene="ywnA_1"

/locus_tag="EFAGFIKM_00799"

/inference="ab initio prediction:Prodigal:002006"

/inference="similar to AA sequence:UniProtKB:P71036"

/codon_start=1

/transl_table=11

/product="Putative HTH-type transcriptional regulator

YwnA"

/db_xref="COG:COG1959"

/translation="MRQISSRFSIAVHTLSLIAVMPNECTGDVIAQSVNTNPVIIRRI

MSKLKQAGLIDVRPGVGGASLLKDPANITLLDVYRALEVVEDGELFNFHKHPNPKCPV

GNMIEHTLRAELIEAQTAMEQRLNRVTIQQMMDQIHVSE"

CDS 916144..916620

/locus_tag="EFAGFIKM_00800"

/inference="ab initio prediction:Prodigal:002006"

/codon_start=1

/transl_table=11

/product="hypothetical protein"

/translation="MWFLVLWLLGLISGGVHLYVLGFPGEMSEISRVLLLHQFVVTFG

LVGVIGYVVNIARADQTAKMLGWPGGPFQVKYGFSQVGLGVMGIMAIWFQGNFWVGVL

VTMYIYGLSGLWSHTYVMIKNRKADGDSIGNLIMDVVYQTFITVLSVLAGGIWVMH"

CDS 916684..933462

/gene="dltA_3"

/locus_tag="EFAGFIKM_00801"

/EC_number="6.2.1.54"

/inference="ab initio prediction:Prodigal:002006"

/inference="protein motif:HAMAP:MF_00593"

/codon_start=1

/transl_table=11

/product="D-alanine--D-alanyl carrier protein ligase"

/translation="MVQKTVELSETQKGIYFDCQVNDPTSYNISASMQFDHLQMEIFR

RAIELLIYEQVVLRTRIEIQNDVPVLSVHEDMDYSLTLWDLSSENQENSKEQQLEALV

DECIGTPFDLEKGPLFRIMIVQMDKSRYILTLCIHHLICDGLSLELMKNKLIQSYTCL

LEDQPVDVKLDEGFISYVEAENKKLHENKYSKEREYWLNKMKGAEPLSLKHDFPAGSV

QEGIGHELRFDISESLMADITQMSKDNEVTVFMFFMGAFGILMSKYVGTDDITFASPY

SHRPGMDLEETIGCFVHMLPMRFHIDLESTLKPLLQQVATTWMDTYRNIGYPNNLIVR

DSMLQAQPGSPSLFDISFVYDSYEEDMLGTRILDQDKVTFPGDLMVVLSRLPQGAQLK

LQYKPSLFREDSMERMGQRFLYLLNQLVTNSDQKISEFSLLMQDEKENLLYRFNQTSY

FPYTPQHIMDIFHDKVARFPERVALMEGKQHQTYAQVNAKANQLARQIVASKKSDNAA

VGVQMPRSSDMVIALLAVLKAGCAFVPVDPSYPLSRKDYIISDADISIVLAMQPEKDD
ETLDVQYIYVEGDQAYTGDVSNLEEALNPHSLAYIMYTSGSTGKPKGVMVENHSVVNT
LLDLERRFPMQAQDVYLLKTAYTFDVSATELFGWFMGEGALSILEPGAEKDPARIVST
VASHNVTHINFVPTLFRLFLETMELRSDLSALETLKWMFVGGEAVTPDIIEKFNRLGI
RASLENVYGPTECTIWVSHYPLSHYEGLGNVPIGYPLNESRWYVVGNHDELQPLGIPG
ELCLSGVGLARGYLNLEQMTREKFVPNPFFQEGQDAEHFRYMYRTGDLVRSLPSGTIE
YLGRIDFQVKIQGARLETGEIENTLSSYPGVIQTVVVMKSFEGRSGLLYAYYLSEQEL
PSVELREHLSRLLPAYMIPSVFVHKSEFPLNSSGKIDRKALMADITHAHASSTFYTAP
SSELEKVIAEVWAEVLGVERVGRDDHFFELGGNSFSVIQAHNKLKHYTDVEFPVPRFF
ECPTLRQFAALGPKDEGVRIPTEREQLFQRKDGIMREDIAIVGMAVNVPGAEDIREFW
SNLVERRESIHFYSDDELRELGVEEALLHSPRYVKAKGRANGIDEFDAGFFDYTPGEA
KMMSPQFRLLYQGIWEALEDAGCRPDEAGKVGVFLGGSDDFEWYRQVLFNDANYSSKY
EAFTLSTNHFLATRMAYKLNLKGPAYTALTGCSTSLVTPHLACQSLIVGECDVAVAGG
ITVELPNEGGYIFEDGMMFSTDGHCRPFDASASGTVFSNGMGIVVLKRVTDALRDGDH
IYGVIKGSAINNDGQEKLSFLAPSVSGQAEVIQDAYKVAGVDPETVSYIEAHGTGTLL
GDPIEVNSLTRAFASDQKQFCYLGSVKGNVGHMDTAAGVVGLIKVALSLGQGYIPGTA
NYEQPNPKIDFLNTPFKVNAQGVDWKQGNVGVMRAGINSFGVGGTNAHMVLEEAPEME
KCSPDDDVNILLFSAKTEYSLNRTAERIMEHLTQQPNQSVSNAAWTLQSGRSVFAYRK
ALILDKTWKDNPDKLIQQLGKARVYHSAQGTRPVYFMFPGQGSQYQGMGAELYRSTEQ
AGLAPIFRSYIQEILDLLPAEEREDMLSVVYGDQHPERINQTEYSQFALFMTSYALAK
SLLDLGIKPDGMIGHSIGELAAAAVAGVFELGDAVKLVRLRGRLMQQQQPGVMLAIMA
DAQQVKSQLEEDMWLALVNTTGSSVIGGTAEAISRMEAKAEGLGWKCIRVKTSHAFHT
PMMNQAAEEFRRYLAEHSLHTPQIPLLSNTSGTWALGDEITQPDYWSRHILQPVLFES
NLAEVLKDERAVLIEVGAGRTLSSFTRQHSLSRDSQTVLNMLRHVRETEQDSIYVNRR
LAELWCEGMEPDWKVLKGQTVRRKCSLPTYVFNKQSYPVSVSLSRSSAVDGMGTGDDR
QHAEQGKGSSPTLIKGTVYTGGSDQLKQIVLDAYRTIFGIDDLDEEVNFFAVGGDSLK
AVSLSAWIRSHLGIQPEVADIFNHSTPIQLAEHLYVMAKPKVDQQTIQSAPTMDAYPI
SSAQMRMFTQAMLDPGNTAYNLPSATIIEGELDRIRVEAALQKLMQHHEALRTSFEIQ
GGQIVQIIQPSVRLSMGYAEQHVADDQELPAMIETLIRPFDLGNAPLMRVELIKIGER
KHLLFFDIHHIIADGTSVEIITRDFNDLYFGKEQAVRLQYKDYAVWQQRRLASQEYIS
HQTYWLDHLGPSLPVLELPLDQERPSQRSMQGGRLHFTLNRSLTQDLHELASSSEASM
YMVMLSVWNLLLAHRSGQQDIVVGTPVAGRMQEEWKETVGMFVNMIAMRNFPTFDRTY

TDFLRELKQHTLDAFAYQEYPFNVLVAQLGVTRELNRNPVFDVCFDYQNMDLHGLELH

GLQFSSFPVETGTSTYDLLMTCQENREKQVIEGYIEYSLDLFRQDTVEGMMQEFIGLC

QEVVKQPDLSVSELFERNERSHEHPVNRLAGPRLDYNPAVGLHRLFELQAEHTPDKKA

LLISSGASFTYRELNEKANTLAWKLIDQGVRRDEPVGILSRRDESLIIMLLAVMKAGA

AYVPLDPAFPPQRIVNMIEDSRMNVLLCTPDAEGTVTFSGERFVYDPTSQDVRASVNP

DRSFLSSDLSCVIFTSGSTGRPKGVMITHEAMVNFVEDIRHRHIFAHETDRIISVTTI

SFDIFGFEVWTPLCTGYSLYLANEQEQLDPALASRRIREHGITHILSTVSRIKAFVEN

PEFAQALGNLRCVLTGGENVPVGLLTDLKRLTPARVFNMYGPTETTIWSTTKELNDTA

AITIGKPITNTEIYILNDEGEILPPGSYGELCIAGKGLSRGYLHNAAETDARFIANPN

VLGGRLYRTGDLARILPDGDVELMGRLDQQVKIRGYRIELNEIEQAAMKYERVREAVV

KVEGAEQHSLQLILFYSLKPGMGPSVMEDEDVQLRTWLKQRLPQYMMPARLIRLENLP

VLPNGKLNRNALRLANFETGEKANPALVYGSSIQGMERAKVDLEQMLIAAWKEILNLD

TVNIHDHFFDIGGNSLGLILINNRLNAYLDKSVPLVQLFEHSSIASLVNHLVAEQMLP

IVTQQVQEESGHIASAEYPVTNPEPTTAPKEYVRSTVEHSEVEQGRPEQSIPFASEHS

TRIPSSSDIAVIGMAGHFPGSRNIEEFWDNLMAGKDGITRLNEEDLTAAGVRPEDFKH

PDYVRAKGVLQDTEYFDSGFFEYPHQESNMMDPQIRLLHQCSWEALEHAGCDPYSYEG

AIGLFAGSGLSLPWMVQFLGRSGNLLQAFEAMTLNEKDYITTRVSHKLNLRGPSMAVQ

TACSTSLVAIHQAAESLIRGECDVALAGGVSISYPLKEGYHWHEGMIYSKDGHCRPFD

EQASGTVSGNGCGMVVLKSLRDAQRDGDHIYAVIKGSAINNDGMDKIGYTAPSVAGQV

KVIQSALHRSGVSPKEISYLEAHGTGTKLGDPIEIEALRQSWGTDRRNYCALGSVKAN

IGHLDAAAGVAGFIKTVLTLYHRVVPPQIHMNRANPMLELEHSPFYINREPQPLEDAK

RILRAGVSSFGIGGTNAHVILEQPPVQESQANYESLLLLPFSAKSESALERTYDSVLQ

AIHALPEQLPNAAWTLQKGRSRFIHRKALIVVDGYIPPSEQSVEFETQASVMNGKPKV

MFYLSGNANWEARKIRELYASVYRSQVSEIFKQHFEEILNLFESKEQYAIRQGIGRSL

TDSRLERMQCFVVQYALYKTVMQLGIKPDAVCGQGVGELVGLVISDALDPVHAVGLLY

NLGHWSLEEGALSDALTGRITDATVTDHASSIAPLLSPNFLFTLLNRKALSESAGHTL

WIVSNQLQDTEQITPDSFSEPNIIALTGDRQSLLEADVYLMKVLAFCWCSGMELDWEQ

LNGTGRRQKISLPTYSFDPIVHQHDVALYQLAGMSQVAAGSIAALDQSPSGKVRVQRT

DDEIRHVLSQLWLELLGNGAVGLEDDFFELGGHSLKAISLASRIKEMFGLNLGLEEVF

DHSVFEHMVQWIAEHQVTSDSDSVNPILPVTKKEKYKTTSAQRRMYVVHEWMKGESTA

YNLASSYMVRGKLDPTQFRVIMDALVERHEAFRTRFEMIDGELFQIVEGQVPSVVELL

ELETAGLEQVMEWIKPFDLTSAPLVRVKLIRLSQEEHILFIDMHHIISDQSSIAILMQ

DFARMYSGETLQSLDIQYKDYAEWRDEAASHGQSVKDAEFWIREFEDKPAPVELLTDF

PRHGASSYSGDRRSIELGESLSTQVNDFCTQQGITPYMLFMATLNLLIWKYTGQDDLV

VGTAVAGREKPELNNVMGMFVNMLAIRTSMDRECSTEAYLNEMRGKLLSCYEHQQYPY

EALVEQLGLFGDLSRNPLFDVVLNYINMGTDDPEAGELMLEPWVEGKVDAKFDLTWTL

VEHKQCYTVELDYRSDLFKAESMELMLDKFRYILSQITTGDNERIGQIKLTTPEEEEW

LLYGVNDNAAQYPRDATISQLFEEQVHVHAQKTALVWEDGEWSYADLYERVQWLAFRL

LGQGVKSGSSIALILDRGPIQMISILATLQVGCSYVPIDPASPSSRIEFILQDCDAAV

LLTETAYASTWQSVVPCIVPADELSAFDSGDMQEPVEHMNKTALDPAYIIYTSGSTGT

PKGTVMSHRNVTKVMKNSNFVTVVPEDRILQISNYAFDGSIFDIFASLINGATLVLIS

KETILDMARLADVIRQQRISVFYIPTSLFNMLVDWDADCLKNVRRVMFGGEAASVSHA

NKALACVGPGRLLNGYGPTEATFFASYHLLQQTEPYTGSLPIGYPLSNTALYVLDDEL

KPVPPNVPGELYISGDAVGIGYLNREDLTQKHFLDDPFRKGERMYRTGDIVKRLIDGS

LIFIERSDFQVKIRGFRIELSEIGNCMERLSGVRDAFVMTVTEPSGSLYIAAFYTTDT

GADTEYIRRELHQQLPEYMVPARIIRTDHLPINANGKVDRRALSEHLVEEQQRSLEVS

SPRTKTERIILAAMRQILGNPDIGVEDHFFQNGGHSLKAIAFTQILSKEGIAINVNEL

FQYPTVRGLAKLVDINPPASVWSEDSLHIQSPIVIPQDITLHEKQLDSLVLHVLNSCN

QVSSFMAGMTPMGQFPLSPIQQAHASTTSRASGFTTTLEGELNEQAVKHLILGVISRH

QLLHSMIYEEEQLYWHPCDISLAADMLQQLIPYLDIRHYASATIEQLEYKLVQSLLHK

TYTLKELPWRLCILRTGATEHKLIWSFDHLAFDGMSAQIIKQQLESKALYRPQGIEIE

SDLLYQVRNYEDYVSFLSQGPVGVSEDEMEKQFSLQAWNRCNTIVMNKLKETETLSPS

AMTVELSLAGKEPADVWWTCFETFSNIIAAYLNISQLPIALVHYGREYGEESYYDYVG

EFLDLIPVLVDSEFTQEDLLERLEFTKRHSINFMALTHDSSLSAQYGGIQEQLGTAYR

QAEQEPRQMILFNYQGFISSSEAIPMSSQSSNDQDSNSGLAQFEWVIRYDEECLYVHL

ACQAGLDIKRMKQLIDDKLGSDVKVVVHNEAMEVQHVQ"

CDS 933452..937651

/gene="menE_2"

/locus_tag="EFAGFIKM_00802"

/EC_number="6.2.1.26"

/inference="ab initio prediction:Prodigal:002006"

/inference="protein motif:HAMAP:MF_00731"

/codon_start=1

/transl_table=11

/product="2-succinylbenzoate--CoA ligase"

/translation="MSSKSIQRGPELIVPEHYSTVAEMLIYIADIHPEKGMTYISSSG

DEQFESYPELLMQARKYLQSLQEQGIQQGQVVILEIDQSQEFYRTFWACMLGGIIVAP

VSPPAAWEPGSAGLEKFTKVWEVLGRPVVIVEEAYMPKYKQLAESPVFKGLMLISVNQ

LQVGQQMADLCPTSPDDIVFLQFSSGSTGIPKGVQLTNHNIIANSIACKTFLGAEEGD

NVFTWLPHTHDMGIFGQHLTPVLSGANIMVMSPYTFVRSPYLFLRKITEHQGKWFCCT

NFGFEWMVQKIPDSKLSTLDLSSLRMTLNGAEPISTSVIERFVKKFSSCGYRPNMMFP

AYGMAEATVGVCTSRLGEEPRIERISRTRLVQEGLAVPVQLPSSDSHTSDVIEFVHEG

YPMTGIQIRIADEQGEVLDERVVGEIQIRGESVTSGYYSLPEINREMYVDGWLRTGDL

GYMADGSLVVSGRIKDIVFIRGQNHYAHDLEEILYQNSSIPRGNLTVVGHFNSLKQVE

ELLVFVKYKSGTPQFLDVRQDLIQRMRAGLGIEITHVLPIKVIPKTTSGKVQRFMLRN

EYERGVFTKDIEAIEELIRENTQQQGHETVSASSLEYTVRKAWADILQLPAGKIEMDT

SFLALGGNSILSYQLLDKLSAHMGRELGQELLVFCSTVREMVEYLQELPAETKDFEQA

ETTDNHLNVHRAIAITGLSLRLPDADTQEQFWSNLVNGLDSVDRVSLERATRSKQPGW

DDWIGELKQPDTFDHEFFEIPSEQAAFMDPQHRKLLEVAHEALEDAGMLVDHEDKRDV

GVYVGINPSTYFDLVIDYMNRSLGKAVHSHAMVGNMSNIAAASISHMYNFTGPALSID

TACSSFLVALTQAVAAIQDKRISGAVVGGANILATPHVHQLSRNAGICSSTQHTKVFD

READGSVLGEGTVVVYLEPLTEAIRNNKHVYGVIRGTAVNNDGYSLGIMAPNPQGQYE

VLRAAYRDANLNPSEISYIEAHGSGTRIGDPIEVNALLRLFREKGYPSDNSIGLGSVK

TNIGHLFAASSGASLAKVLLSMQHGELAPSLHMDNPNPALRLEQSPFHIVSEHKTWDV

PEGKTRKAGISSFGLGGTNAHIVLEEWKAPVRAAAPARGQLLTFSAKSEQALKKLIDN

TEQYVKKHADVAPEDLCFTRNRYRKHYRYRAAVAVSSTGEWNIEEMEFGFKPRNRAAR

VNIVVGGHEIRGEDEAQARQVIRPDDFNEDKVSTLNYWVTLLETLVRLVPSIQEIRGI

AAGEELVSSLHLDGEEHHSAHASQSIPVNNRVTLDSLQDTPGKMANTDIVIMIAADQT

VQQLEYSGKESFIRLSAEPTLTMEDQLALLLGQLYVRGAALDWEQLHPNGSGQIVHLP

AHPFEPYVHWIDVNQGTEVMI"

CDS 937648..937905

/locus_tag="EFAGFIKM_00803"

/inference="ab initio prediction:Prodigal:002006"

/codon_start=1

/transl_table=11

/product="hypothetical protein"

/translation="MTKEEIELFIKSEIAAALNQEPEDIDEEMNFLKIGVSSVQALKI

INRARKHLQVDISPVALFEYKTIAEFAAYLNESLLQEGVAE"

CDS 937902..939053

/gene="mmgC"

/locus_tag="EFAGFIKM_00804"

/EC_number="1.3.99.-"

/inference="ab initio prediction:Prodigal:002006"

/inference="similar to AA sequence:UniProtKB:P45857"

/codon_start=1

/transl_table=11

/product="Acyl-CoA dehydrogenase"

/db_xref="COG:COG1960"

/translation="MIEVGVEQEIIHEAELFAAREIRPYAQQIQEQQAIPRTLIRAMA

EKGYLAASIPAEYGGMGLGPKAYGLLTEVFGKALPAVRSLLTVHTSLVSETLVRFGTP

EQKSSWLPKLVKGEWIAAFGLTEPEVGSDAKSVKTEWREESDCYVLNGKKKWITFGHL

ADVFIIIASNQGRSTAFWVERQFEGVQTKPIEGVMVAGETGIAEIDLHEVRVPKNHII

GRLNEGFEYIVTGALDQGRYSIAWAGLAIAQEALDAMVTYSRKRKQFDQKLSHFQLIR

GMIGDAVTKVHAARALCLRAAELREAKDPQAAMETTIAKYFTSKVAVEVASDALQVHG

ANGISNQYPVARLYKEAKILEIIEGSSQMQQEMISHYGLKAYYKRGGSV"

CDS 939050..940030

/gene="fabD_1"

/locus_tag="EFAGFIKM_00805"

/EC_number="2.3.1.39"

/inference="ab initio prediction:Prodigal:002006"

/inference="similar to AA sequence:UniProtKB:O85140"

/codon_start=1

/transl_table=11

/product="Malonyl CoA-acyl carrier protein transacylase"

/db_xref="COG:COG0331"

/translation="MTFCALFPGQGSQYMGMCSELLDSEPEANQIFAEASEVIGFDLK

ALVMEGTLDQLTVSEYAQPAVLTASYVLFDSFVRRTGMTPDYAVGHSLGEISALVAAG

ALRFADGIRFTAQRGALMHRSIQEQKGRAGIVVDITQEALEELVENIRQNEYVTISGY

NSPGQFVVAGTAKGLQLLDDQVDGYGGEFIPFRMMPMKADAPYHSELMQFIQPELEEM

LAGISFSAPVFPICSTVHGEFISGAEDIPKLLSSQLLQPIRWNQALARVQNLGATVWI

DIGPGQSVRNMLAENKALPAAYSLDDPQDRSRLLDRYEAVNQTSGGGDKG"

CDS 940027..941346

/gene="hemL_2"

/locus_tag="EFAGFIKM_00806"

/EC_number="5.4.3.8"

/inference="ab initio prediction:Prodigal:002006"

/inference="protein motif:HAMAP:MF_00375"

/codon_start=1

/transl_table=11

/product="Glutamate-1-semialdehyde 2,1-aminomutase"

/translation="MITEMMQYNQRVKQWLPGGVHYNFHLPWEETPLHFKHASRSRVT

DMNGNEYLDLYARFGALIVGHGNEEYNECLKDTIDRVLSVSHCDLDAEALELIHQYVP

SAEMIRFGLSGTEIVQNALRLARAWTGKNRFVRFEGHYHGNADNIMGGKTARTGLPVP

SDYPGDMKGTKGRARDAMESQSYLLPWNDAPRLEELFRQYGDDIAAVIMEPVCVNGGG

VMPAPGYLQKVRQLCDQYQVVLIFDEIITGFRMGLGGAQQLFGVTPDLTTLGKAIAGG

GVPVSALVGRADIMKLLVDKKVIHAGTFNGYPLGTAAVKATLEMLGRNGGQAMHSMNR

HAEMMHDILCQEAVKVGLPLIVQGPSGCASYHCCTEPLTDTADYTFELMSFDILLNSK

LAEHGVLVSTLSRMYPNILLDMQDVAWFGERVPAALAEMKEIYDELI"

CDS 941375..942226

/gene="mmgB_2"

/locus_tag="EFAGFIKM_00807"

/EC_number="1.1.1.157"

/inference="ab initio prediction:Prodigal:002006"

/inference="similar to AA sequence:UniProtKB:P45856"

/codon_start=1

/transl_table=11

/product="putative 3-hydroxybutyryl-CoA dehydrogenase"

/db_xref="COG:COG1250"

/translation="MHNRLIAIIGAGVMGCATALDVARAGYRVILQDISASVIEHAPD

VIRREYRSACFLKQGYGQVPLDQIMERISFQTQDEGVEEASIIIENVTENLELKKEVY

ARLYHKVRPDALFALNTSCISVTKLASFLPDPGRVIGVHLMNPVPVKSMVEVIKGHHT
TQGTEHEMVAFLETLDKSPVVIEDLPGFVSNRLSHLLMNEAAYIVQDGIATPEQVDAI
MKKGFNYQMGPLETADLIGLDTVVHSLKVLYDSYQDPKFRCCPMLQKMVDAGQWGRKT
GQGFYAY"

CDS 942246..943295

/locus_tag="EFAGFIKM_00808"

/inference="ab initio prediction:Prodigal:002006"

/codon_start=1

/transl_table=11

/product="hypothetical protein"

/translation="MEKLKDIKCIVWDLDHTIWDGVLLESADVQLKPGLKEVIEKLDQ
RGILHSVASKNDAELAWAKLNELGIAEYFLYPEIHWDAKSVSVQRIQQNINIGMDAIL
FVDDQPFELEEVQSVHADIQVLHADQYETMLDDPRLMPRFITADSACRRQMYQADTLR
KQAEEQYEGPSEQFLATLGMKFSIYEATEDDLQRAEELTVRTNQLNATGITYSYEELH
QLMISQDHMLLVCELEDKYGTYGKIGLALIHMQQKVWRLNMLLMSCRVMSRGAGSVLL
TYIMREAQKQGKQMLADFKDTGRNRMMNISYRFAGFKQQSADQEGRVLFYHDLQQAVE
FPDYIEVFVPEGQHQ"

CDS 943311..943574

/locus_tag="EFAGFIKM_00809"

/inference="ab initio prediction:Prodigal:002006"

/codon_start=1

/transl_table=11

/product="hypothetical protein"

/translation="MDYRQEIREFIGNNLTMDHEDTQINDNDNYFEQRFVNSLFAMRL
VDFVERRFQIEIANEDLDLANFCTINRLHDLIERKLTEKEAAL"

CDS 943571..944455

/locus_tag="EFAGFIKM_00810"

/inference="ab initio prediction:Prodigal:002006"

/codon_start=1

/transl_table=11

/product="hypothetical protein"

/translation="MITANQASIRPVTSPATSPSNKYMYLFLGISFGTCYGIASLFMF

FADFIVPITGELTLMHPLAIVALYSPSIAGLITCYVMGGKEALKGMFMKLVPRRQDLF
WIPLIIGISIIFASTMHFGSLLFGIPVPELTYSVPEMIWLGIVNLIKETGLIGGLFGW
IGFLLPFLQGKLKNNITSGLITGLLFGLWVLPGYLISSFGTSTTYIYYVVQLMAFIVF
QSYIFNATKGTLLFYLLAFWLVATGSQIDLYYFNPQVQIMQFFFFAISAIVIHVIFKR
KNVDTSLQTFPAYVLERK"

CDS 944481..945221

/gene="srfAD"

/locus_tag="EFAGFIKM_00811"

/EC_number="3.1.2.-"

/inference="ab initio prediction:Prodigal:002006"

/inference="similar to AA sequence:UniProtKB:Q08788"

/codon_start=1

/transl_table=11

/product="Surfactin synthase thioesterase subunit"

/db_xref="COG:COG3208"

/translation="MEPIYLEQLQKGEGNRQMVCFPYLGGNSNSFQPLVPYLHDTEIW
AFTPPGHGLNTETPLEHMQQLVELYTSKLLDVIQPGSLLFGHSLGGITAYFVAQRLCQ
ERPDVAKTLRLVLSACNTPDECGMQNYDRMSDENLIDHLFSYEALPQELIHEKELLNY
FLPVIRADFRMLESAATLEYEPLSLPVLYLWGERDRTVTLQSALRWGRYFGTSMKLQT
IKEGAHMFMMHQPSTVAHCLTSFMTPAK"

CDS 945257..947590

/locus_tag="EFAGFIKM_00812"

/inference="ab initio prediction:Prodigal:002006"

/codon_start=1

/transl_table=11

/product="hypothetical protein"

/translation="MLLRMLRNDITRKKGITAALFIFVLLAALLVSSGSRMIMELTSS
IQYLFSESKTPHFVQMHAGELDQAKVNTWAEENAMVQQHQIVEMVNIDGSNLFLGAES
EKNSVMDIDFVTQNQGFDYLLNLNSEIIQVNDGEIAVPVYYMQQKKLSVGDQVRIDNG
QFERSYTIVDFVRDAQMNPSVVHSKRFIVSAGNLQELKQSIGEMEYLIEFRLTDPGLT
SEFTQAYQNAGLPNAGPVVTYGLFQVLNAMTDGVIAAVIILVSLVLILIAMLCIRFTM
LATIEEDYREISVMKAIGIAEKDIKRLYLMKYVFMAGFASVLGYIASLGVNRLFVSNI

MLYMGKAPATLLHFAIPLLAAGIIFAMVVLFCRTVLRRFRSISAVDALRTGSLGDTQI

IRNRLSLSRNRWSSVPVFLGLKEVIQRIKMFRLLLFVFVVSSFIMIVPVNFLNTLQAP

SFISYMGVGQSDIRIDLRHTDDVEQRYDNLVAQIQNDKDIKAYSPLVTSQFKIKNAEG

SYDNLSVETGDFSIFPLSYVSGNAPTTENEIALSDANSSELRLKTGEQLTLLVNGKDQ

MMTVSGIYQDVTNGGKTAKALLPYNKDSVLWYVVSLDLNNRGDMATKIAEYEAAFSPA

KVTDLQGYLDQTLGGTIEQLKLVTMLALVIGVFISILITALFLQMLVAKDNNDIAVLR

SLGFALSKIKFKYVVMSLVILILGVVTGTILSNTLGPLLVSAIMSTFGASNIVFVVNP

LQAYVLCPLLLAGTIVLTAWISIQSIKETSISKMIVE"

CDS 947603..948370

/gene="bceA_1"

/locus_tag="EFAGFIKM_00813"

/inference="ab initio prediction:Prodigal:002006"

/inference="similar to AA sequence:UniProtKB:O34697"

/codon_start=1

/transl_table=11

/product="Bacitracin export ATP-binding protein BceA"

/db_xref="COG:COG1136"

/translation="MTTILEAKDVNKSVAIGENEEHNILKDINLQLKKGEFVSIMGPS

GSGKSTLLYNISGMDQISAGSVYFNGKKISAFEERDLASLRLTKMGFIFQNIHLLKNL

NLLDNIVLSAYLAKNSSRETINTRAMSLMKKMGIDELAGHNITQASGGQLQRIAICRA

LINNPDILFGDEPTGALNSKSTYEIMDILGDINATGTTILLVTHDVKVAARSERVLFM

MDGKLVADRNIGKYARERQDLKTRESHLAQWLTEMGF"

CDS 948431..949066

/locus_tag="EFAGFIKM_00814"

/inference="ab initio prediction:Prodigal:002006"

/codon_start=1

/transl_table=11

/product="hypothetical protein"

/translation="MRLIKNPEERRNEILDAAEILFITKGYTKATVMDILQACNIAKG

TFYYYFQSKEEVMNAIVMRFILSGEASARHVVSNPKLNAHDKIFRIMMAQNQPDGRKH

DLIEQLHSVHNVEMHQKSLVETVIRLSPILAEVVEQGIQEGVFHTPNPKESIEFLLVS

SQFLLDRGIFQWEEEELQKKVEAFTHIMERVLGAEKGSFAYVTRLYFPNQG"

CDS 949087..950415

/gene="entS_1"

/locus_tag="EFAGFIKM_00815"

/inference="ab initio prediction:Prodigal:002006"

/inference="protein motif:HAMAP:MF_01436"

/codon_start=1

/transl_table=11

/product="Enterobactin exporter EntS"

/translation="MEHTVQKSFKKFLVVWFGQLISMIGIGLTAFSLGVYAFEKTNTA

TSVALITLFTFLPNILLRPIGGVLADRFDRRTMMIIGDLGSAAGLIFILSIMLTGDIQ

LWHIYVGVAFSSVFSALQSPAYKASATDLLDKDQFSKGSGLVQLAESSKFLFSPIIAG

ILLSITTIEVILVINILTFLVAILAVLVIRKSMKVEREDREGKNWITDIQEGWREVVT

NKGVLLLVVIISLVTFYLGFLETLIGPMLLSFTDAKTLGTFQSVSAIGMLISSLCIGI

FTITKRYASVLVMGLILCGLSFSLLGISTNIYFIIFAGFLFLSSLPFVNMSADVLVRN

NIANDKQGRVWGIIGILSQLGFIIAYSLAGFLADHVFNPLLMEGGALASSVGQIIGTG

PGRGIAFLFIIAGLFVILIAIITSRLKMIKSLEQSPDITAPSDVGGLAHD"

CDS complement(950577..951401)

/gene="melC_1"

/locus_tag="EFAGFIKM_00816"

/inference="ab initio prediction:Prodigal:002006"

/inference="similar to AA sequence:UniProtKB:O34518"

/codon_start=1

/transl_table=11

/product="Melibiose/raffinose/stachyose import permease

protein MelC"

/db_xref="COG:COG0395"

/translation="MRLRKRLASIGLNALAWLLSLVVLIPFVVIVLNSFKSDAEAKVL

KLTLPEKFIFENYKIVYEQGHLGGSFFNSLLHSGASSLLLVFVVAFSAFTLSRNHSKL

SKFLYFFLILGITLPLNYIPLMEVMKSMGMINSHVGMILLYTAMGIPISLFITYAFVS

NIPKELDEAAIMDGCNGIKLFLRIIVPLLTSVLVTVFVLNFLSVWNEFTAPLYMLNTV

EMWPMTLAVYNFFGQFSAQWNLVSADIVLTSLPVLIVFLIGQKYIVGGLTSGAVKG"

CDS complement(951403..952287)

/gene="melD_1"

/locus_tag="EFAGFIKM_00817"

/inference="ab initio prediction:Prodigal:002006"

/inference="similar to AA sequence:UniProtKB:O34706"

/codon_start=1

/transl_table=11

/product="Melibiose/raffinose/stachyose import permease

protein MelD"

/db_xref="COG:COG1175"

/translation="MLRIMNANKIYPWYFSSGAIVLYLLFIVAPALLGIYYSFTDWNS

YSSEKNFIGLEHFRTILAGDPTYLLFIKNTVLFTLVTSIAKTVLGLFLALLLVSGVKA

ANLHRMIIFSPQVLSFLIVGLVFKSLLDPNNGFVNVTLRSMGLDVLAQNWLGSLTWAM

PSIMAVDTWKGMGYIMVLFIAGLLAIPRDYYEAASIDGAGFGQKLFRITIPMLMPTIT

IATVLNITYGLRVFDAVYVLTNGGPGNATDVINTAVYSSFAKGYWGLGSALSTILFVI

MAIISFFIIRLMNRKVEY"

CDS complement(952376..953677)

/locus_tag="EFAGFIKM_00818"

/inference="ab initio prediction:Prodigal:002006"

/codon_start=1

/transl_table=11

/product="hypothetical protein"

/translation="MRKRFLFSLASVLLLSTLLLAGCNSGKSTEGSGKVTLTFGTSQS

GIPRTGIMQTMAKEYEQETGVKIDFQVVPDAQWRDLIKVKLASGEAPDIFNVDVDPLS

MPANVRPEENAIDLTNEEFTGRMSEEILPTVSHNDKVYGVSFAPTKIWYVYYNKRIFQ

ELGIEPPTSYAEFKAISQKIKDKDIIPFYQAPASGWYQVLPLFETGPNYEQTTAGTYE

KLNNNEMKVADMTQLKTVIEQLKEFADLGYFGKDFLSNTVEAGIEAFGQEKAAMLLRV

PGTEKEVSEAYPEMEDNMGFFVMPWGDNQTIGVNPGGSAAMFGNKNSKHPEEVLKFFR

WITEHDHLQRVFDEGEGNLTICWPEIEPKLTQDYIDYEKNHEKGTVMQAAVKYIDPQW

MDIGKDLSGMFAGAMTPDQILQNIDKRRAEQAKVLKDEKWQ"

CDS 953858..955735

/locus_tag="EFAGFIKM_00819"

/inference="ab initio prediction:Prodigal:002006"

/codon_start=1

/transl_table=11

/product="hypothetical protein"

/translation="MRYRFGTLQQSLFAYYSAVFIIFSLIVAGLLYVFLASDIRQRSE

EQQRQLGVSIVSNLDQEVVKMNNFSMNIVYSNLVKEHFSHYLAPAEDSEQTMTSDPAL

YKDTTTLIDVILAIISSSNTAKQANIYDVNGKMLGAGAFNGQLSVDLTQRPWYKETWE

RSGWRVIQLLEGGALPMPTGEGKSDQPYISLTRVYKDGNYVSQGVVEILQDAGTLFKH

LNELQTNNDDLRIYVVDEQNTRLFPLSSDADASPAGHFDDVDLIQNLSFPPDTMQETT

DPLSQSKQMMTYMTSQETGWTVIVMQSKQSLFASLNRMAVMFIGATFATLMLILILSY

LISKRVTLPLHRLQRIILKTGENDLLSGQDSGQLFKLEHPGSIREMDELNDTFIRLNQ

QLVQSFQDRLAMKSQETEARLLALQSQMNPHFLYNNITNISIMAEEGMNEQIVAFCAN

IASMLRYISTPGKNGVPLSQELDYCERYLECMKIRFEDDLHYEFHIPEEMKYIQVPML

MVQPLLENAMKYGLGDTPPWKLNITGDMDAVQQTWQIHVEDNGPGMEPEALAKIMHYI

EQSQEWERMPDLEINGMGLKNIWVRLKLWYGTAAHMQITNKPSGGVQITIGGSIRKEH

D"

CDS 955740..956549

/locus_tag="EFAGFIKM_00820"

/inference="ab initio prediction:Prodigal:002006"

/inference="similar to AA sequence:UniProtKB:Q5HJF7"

/codon_start=1

/transl_table=11

/product="putative response regulatory protein"

/db_xref="COG:COG4753"

/translation="MTIKYRTIIVEDEALIRRNVSRKFRELDTRFEVIGEARNGQEAL

QLIEQSVPDLVVTDIQMPVMNGLELAKHLYFAYPHVKIVILSGYHEFEYARQAISYKV

EDYLLKPLSEEQLRTLLDAMELKLGSAVDSLAHVSAVLDEQVKPEDIAEAIKLYLKQN

YMHEITLQDMAGQLHFSVDYLGKCFKKVTGETPLKYMTGLRINEAKRMLGTHENMDIK

TIGGAVGYSDSHYFSRIFKNKTGMYPSEYRLQLQERKKALSMDDENHVDLS"

CDS complement(956550..957179)

/gene="yfbR"

/locus_tag="EFAGFIKM_00821"

/EC_number="3.1.3.89"

/inference="ab initio prediction:Prodigal:002006"

/inference="protein motif:HAMAP:MF_01100"

/codon_start=1

/transl_table=11

/product="5'-deoxynucleotidase YfbR"

/translation="MGIHTYFRSLNDLERIIRTPGKFKFEEHSVSAHSWKVVQYAKTL

ADIEEQHGTTIDWKKLYEITSSHDYGEIFIGDIKTPVKHYSLELRSMLQKVEEGMVEH

FINENIPEEFQSIFRRQLREGKDQSVEGLILEVADKMDQVYEAFAELQRGNTEKEFIV

MYRYALVKIKNIDLHCVQYFLEQILPDMIEEGIRSPFDIRKITEEALAQ"

CDS complement(957388..958170)

/gene="nirC_1"

/locus_tag="EFAGFIKM_00822"

/inference="ab initio prediction:Prodigal:002006"

/inference="similar to AA sequence:UniProtKB:P0AC26"

/codon_start=1

/transl_table=11

/product="Nitrite transporter NirC"

/db_xref="COG:COG2116"

/translation="MYTPSVEGIIEAAVKKRDQMNSNLSRYMVAALMAGAYVGLGIVL

IFSIGAPLLAAQSPLQTMLMGMSFGLALTLVIFAGSELFTGNNMFFTMSTLAGRTTVN

ETLKNWGLVFVGNLLGAILLSLLIVGSGLFKTATPEHLLFVVSAKKMAAPVSELFFRG

ILCNWLVCLAIWMAARSKEDIAKIALIWWCLYAFIASGYEHSVANMTLLSLSWLLPNH

PDTITLAGWFHNMIPVTLGNIIGGALFVGMAYWYTSPVRKKA"

CDS complement(958220..958546)

/gene="nasE_1"

/locus_tag="EFAGFIKM_00823"

/EC_number="1.7.1.4"

/inference="ab initio prediction:Prodigal:002006"

/inference="similar to AA sequence:UniProtKB:P42436"

/codon_start=1

/transl_table=11

/product="Assimilatory nitrite reductase [NAD(P)H] small

subunit"

/db_xref="COG:COG2146"

/translation="MTTKQSGTYFPAGVVEEFLPRIGRVVEIKDHQLAVFRASDGTIF

AADNHNPHPKGGPLAEGIVSGHYLYDPLYDWKIDLTTGVVQAPDNGQVQMYPVKVENG

QVWIEI"

CDS complement(958604..961033)

/gene="nasD_1"

/locus_tag="EFAGFIKM_00824"

/EC_number="1.7.1.4"

/inference="ab initio prediction:Prodigal:002006"

/inference="similar to AA sequence:UniProtKB:P42435"

/codon_start=1

/transl_table=11

/product="Nitrite reductase [NAD(P)H]"

/db_xref="COG:COG1251"

/translation="MNGKKEKLVIIGNGMAGISTVEQILKLTTRFDITVFGTEPYPNY

NRIMLSYVLEGSKTLDDIVLNDLHWYEDYGITLHTGTTVTRIDAETHEVVTEDGTLVP

YDKIIIATGSNSFILPVPGHDKEGVVGFRDTADCNVMLEAAKQYKRAAVIGGGLLGLE

AAKGLVQLGMEVTVVHLMEDLMERQLDPQASAMLKAELERQGIRFKMGAQTSELLGGE

RVEGIRFADDTVLNVDFVVMAVGIKPNTAVARESGMEVNRGIVVNDYMQTSLENVYSV

GECTEHRGVCYGLVAPLFEQGMILAKHICGVETAPYEGSVVSTKLKISGVDVFSTGEF

IDSPEHTVISHKDDWKRTYKKILLRDNKMVGAVLFGDITDSAELQKLIKNQTEMTEEL

YGSLMGTGCGGHKKTTSVEMMAEDEIVCGCNGVTKGTIVDVITNQGLTSVDEIKACTG

ATRSCGGCKPVVEQILQYVLGDSFSTGAKQGICGCTSMGRDEIVAEIREKGLQTTKEV

MNVLGWSQPEGCSKCRPAINYYLGMIAPDTHEDEKESRFVNERMNANIQKDGTYTVVP

RMYGGVTTPADLKRIADVSVKYDVKAVKVTGGQRLDLIGVKKEDLTKVWAELDMPSGY

AYAKSLRTVKTCVGSQFCRFGTQDSMAMGARIERKFERLDLPAKFKYAVNGCPRNCAE

ACTKDIGIVGNDGGWEIFIGGNGGIKARLADSLCKVKTDEELIELCGAIMQYYREAGN

YLERTSEWVERMGLEHIRSVVVDNLEERKALMQRIEFALEHVEEPWQKAIRNEEGQSK

MFHGIEVSARP"

CDS 961309..962016

/locus_tag="EFAGFIKM_00825"

/inference="ab initio prediction:Prodigal:002006"

/codon_start=1

/transl_table=11

/product="hypothetical protein"

/translation="MMGRSHLIIGTGVSLSVLQLAGMPVTAPAVTVALIGSLLPDIDE

PNSLLVSRALPNSLIRLLQTILLPTAVFVYFYVHAKPWNLLLAILIALVSFLPSRSLR

KVLMFAIGLGLVFYGHAFAPWNLIAGSLLMLCTTLTHRGLTHTFYGTAIWAGLLYSTT

QQQGPEIWVAGGTAYVMHLLADSLTNRGIRPLPPFKFRIRANLMSTGTKHGAVVENVC

IVLTLILAWFAFSSLFL"

CDS complement(962100..962219)

/locus_tag="EFAGFIKM_00826"

/inference="ab initio prediction:Prodigal:002006"

/codon_start=1

/transl_table=11

/product="hypothetical protein"

/translation="MSDFDGIGFAELLIGVILVLLFVYGSTDIEGLSSTPTTS"

CDS complement(962244..962564)

/locus_tag="EFAGFIKM_00827"

/inference="ab initio prediction:Prodigal:002006"

/codon_start=1

/transl_table=11

/product="hypothetical protein"

/translation="MAVRRNGKSNKGSKKTNAANTLLNGNLKGRNLELIVAALLVSGK

LRVDAVTLFREATLVVELVGQYKTLQKVTPSNSDKLVQFLDETGADMTLDDVIRAFQQ

RINS"

CDS complement(962793..963866)

/gene="ysdC_1"

/locus_tag="EFAGFIKM_00828"

/EC_number="3.4.11.-"

/inference="ab initio prediction:Prodigal:002006"

/inference="similar to AA sequence:UniProtKB:P94521"

/codon_start=1

/transl_table=11

/product="Putative aminopeptidase YsdC"

/db_xref="COG:COG1363"

/translation="MNEKTMDMFRTLTEFPSASGFERELRGWMKDKLSAYTDEFVQDR

LGSLFGVLRGEESGPKVMVAGHFDEVGFMTTGITETGMVKFRPLGGWWSQAVLSQRLE

IITPDRRITGVVGSTPTHLLDESQRSKPVDLNTMYLDIGADNRAEAESWGIHPGMQIV

PICEFTPMANPKKIMAKAWDNRYGVGLALELVEALHKEKLPNTLYAGATVQEELGLRG

ARTAANLIQPDIFFALDCSAANDMTGDKQSFGHIGEGALLRIFDPGMFTHRGMVEYVQ

DTASSNQIKMQYFISPGGTDAGQVHLSGIGVPSTVIGICARYIHTSSSIIHTDDYDAA

KELIVKLVKGLDRTTMNTIINNA"

CDS 964512..965015

/locus_tag="EFAGFIKM_00829"

/inference="ab initio prediction:Prodigal:002006"

/codon_start=1

/transl_table=11

/product="hypothetical protein"

/translation="MPAQSASGSEKKSSSLSMDEKEYKKVAKKHEPPRPILKNCLKAF

LVGGTVCLIGQAIQEAFMAGFDMTSKEASSPTVAVMILISVILTCLGVYDKMAQWAGA

GTAVPVTGFANSMCSAALEHRAEGLVLGVGANMFKLAGSVIVFGVVAAFVVGIVYAFL

GFGGGHL"

CDS 965012..966028

/gene="spoVAD"

/locus_tag="EFAGFIKM_00830"

/inference="ab initio prediction:Prodigal:002006"

/inference="similar to AA sequence:UniProtKB:P40869"

/codon_start=1

/transl_table=11

/product="Stage V sporulation protein AD"

/translation="MKRLGRQTWQFENRPRIIGKAAVVGPEEGEGPLSSDFDYVYDNL

EIGEKTWEKGERKLLEQASQLALVNANITKEELHFFVGGDLMNQIISSSFSARKLGVP

YLGVFGACSTSMETLALASMIVDSGAGDYVLAGTVSHNCTVEKQFRYPTEYGSQKPPY

AQYTVTGSGCGVVSRTGDGPVVTKATIGRIMDLGIKDPFNMGSAMAPAAADTIISHFR

DTGLEPGYYDLIVTGDLASVGLPITKELLQKEGIPMEQTVFNDCGLMIYDREKQPYVV
AGGSGCGCSATVTYGHILNRMQKGDLKRVLVVATGALLSPLSYQQGESIPCIAHAVAI
EKEG"

CDS 966034..966384

/locus_tag="EFAGFIKM_00831"

/inference="ab initio prediction:Prodigal:002006"

/codon_start=1

/transl_table=11

/product="hypothetical protein"

/translation="MQFLWAFIVGGLICVVGQLLMDGVKLTPAHTMSTLVVAGAVADA
FGLYDPLVKFAGAGASIPITSFGNSLVHGALTELEKEGWLGVITGIFDLTAAGISSAI
IFSFLAALVVRPKG"

CDS 966593..967549

/locus_tag="EFAGFIKM_00832"

/inference="ab initio prediction:Prodigal:002006"

/codon_start=1

/transl_table=11

/product="hypothetical protein"

/translation="MPVRKESIQIISAVRSNLESCIMGKSFEIQLLLTALLAGGHVLI
EDVPGTGKTQLIKALSKSMRGEYRRIQCNPDILPSDITGVSVFHPKDERFYFRPGPVM
TNILLADEINRATTKTQSALLEVMEERSVTVDGDTYDLPHPFMLCATQNPIDFEGTYT
LPEAQLDRFMLKISLGYPDKEIEKILLKQHQLGQPVDRLESVTHMDQISAIQQEIKEV
FIGDPVMDYLLDVVRQTRSHPSVLLGASPRAAISFMMAVKAFAFLQERDYVLPDDVKT
MAPYVISHRIVLRPESRLDSMSSEAVLNAVLQQVRVPVSMGQ"

CDS 967616..968800

/locus_tag="EFAGFIKM_00833"

/inference="ab initio prediction:Prodigal:002006"

/codon_start=1

/transl_table=11

/product="hypothetical protein"

/translation="MVWMCCLAYVLFQGGKTSLMLLSMVTLLCVYLAIAGFSGVRRAQ
GVRRLSSGPDHEELLHAGDQVQVQLSLTIPGFLPLPYVVVREMLHRHNGESWSFKESL

IPNMRGNGELSFQTPPLERGKYVFSETECASEDIFGLIEHRGKFKAKGEFRVLPRTVF
IPYWQLYDRKSRLSGPQTALTRSRRETTQINGVRDYVYGDRLSRIHWNATAKTGNWKS
KEFEHESVPKTVLVLDALASSYEHGDAFEIAVSTAASLLEYGVRERMGMGLLTLSDQT
SFMAPSESLMERQKMMHHLVDIQYNGQDTHLLPGVERIARQLPQGAYFVVISPQKDEK
IMELLRWADTRGMTPCHILIDTSESRRSAEWNAMLRGRGTRSFTVSHLQELPTVMGGG
SV"

CDS 968797..971010

/locus_tag="EFAGFIKM_00834"

/inference="ab initio prediction:Prodigal:002006"

/codon_start=1

/transl_table=11

/product="hypothetical protein"

/translation="MSSKGNESVTGKRSWYHAASLLWIFLIGMQWISFTQESWYTETT
SLVLWTLAAVSVLEVILPFKTIYRAIIKAVVVGYILHKTLIDYTVYIPYGSLTDRAEQ
FILHMTPYIWFSLCAWVMLEAALRLVTTTRRILVFLGVNIISMGILDSFTQIPLWIEI
AWVMFAGMGWLVCQHFRNYQLQYPQGWKRIIRYPYKVLANIAIIFSLIIVASVNMPEI
PPTLTDPYTAWRNYTGTSTSQAGNGNLDIPAATESGYSREDNQLGGGFNFDYTPVMSV
TTNERSYWRGETRAEYTGTGWDDRGRGATENVEAGQPLENQESGNVNTKQVTQNVTML
NDNVYPILFGAYSISEVSSINGEDRTERMLWNAEQAELHVVTDRQQPQYPKTYTIVSE
APVIVEDELRTKSFEDLYNSNPADDMYLQLPSRFPDRVSDLAAEITASANTPYEKVAL
LQNYLQTNFEYTNNPDLSRKVSSDFVEGFLFDIMEGYCDYFSTALVMMARSEGIPARW
VKGYAPGQLSLNSDMQAPRQPGAEIETTYTVTNADAHSWAEVYFGEYGWIPVEATPGF
DMPLLTEQPDVQPVDEPEEEPEEEPVQEEEQTPAPEQTSSAIPTFVIWAAGAIIVLWV
AYMFWRNRFSMRFFLLRLRTGGPLTPEQKVVAETERWIQYVKRKGLTRSGDETLRESV
ARWSQAKPAAEATLYELLTKFEQTRYSPASVTADDWKGVYQTALKLRKELKAERA"

CDS 971121..971648

/locus_tag="EFAGFIKM_00835"

/inference="ab initio prediction:Prodigal:002006"

/codon_start=1

/transl_table=11

/product="hypothetical protein"

/translation="MFKMLMPKLRVDTVFDINLEELYAQGYRGIITDLDNTLVGAKAP

DATPELIEWFARVKEAGFKLMIVSNNNLNRVSLFATPLDIQFVHSARKPSNVPFRRAM

KMMELSPEKTIVVGDQMLTDVYGGNRMGLYTVLVLPISIGDEGFMTRFNRRVERIALT

SLRKKGLWLEEEKKK"

CDS 971645..972772

/gene="yqeH"

/locus_tag="EFAGFIKM_00836"

/inference="ab initio prediction:Prodigal:002006"

/inference="similar to AA sequence:UniProtKB:P54453"

/codon_start=1

/transl_table=11

/product="putative protein YqeH"

/db_xref="COG:COG1161"

/translation="MTEPHNGNLAVRCSGCGVHLQTENSELPGFIPEKALGREPVICQ

RCFRIKNYNESSSVTVDQDEFLALLSKIGDKDALVIHIVDLFDFDGSIISGLQRFVGN

NPVLLAVNKTDLLPKVTNWNKIRNWVQKQAKEQGLRTVDVVLCSAKQNQGFDRLLELV

GTYREDRDVYVVGGTNVGKSTLINRLIRDYSDLEQELTTSRYPGTTLDMVNIPLDDGK

YIIDTPGIVYPWRFSEIVSRKDLAAIMPEKPLKPAVYQLNSGQTLFFGGMARFDFVEG

DRQSFTCFISTALDIHRTKLERADDLYRDHLGELLSPPTREDAAEMAEWTRHEFRIKR

GSQSDVFISGLGWIKVNGDIGALVAVHAPKGIRVQIRPSLI"

CDS 972815..973684

/gene="aroE_1"

/locus_tag="EFAGFIKM_00837"

/EC_number="1.1.1.25"

/inference="ab initio prediction:Prodigal:002006"

/inference="similar to AA sequence:UniProtKB:Q5KWX7"

/codon_start=1

/transl_table=11

/product="Shikimate dehydrogenase (NADP(+))"

/db_xref="COG:COG0169"

/translation="MSEQNKFNSSLPVLLGVMGDPIAHSKSPAMHNAALQAAGVNGMY

MPLHVHPDQLEAAVRGIVALGYRGVNVTIPHKEQVMQYLDVIDESARLIGAVNTIVNE

GGTLTGYNTDGIGYVRSLKEEAVPELAGKRIAVLGAGGAARGVIYALALEKPERIHIL

NRTADRAIELASDLRGHGLGEISGSGMEDAATVLATADIVINTTAAGMHPHVDDVPVD
PALIREGAAVSDLIYNPLETRLLRESRKCGCTVHGGLGMFVYQGAVAFEHWLGIPAPV
ETMRRAVLNSFEV"

CDS 973767..974060

/gene="yhbY"

/locus_tag="EFAGFIKM_00838"

/inference="ab initio prediction:Prodigal:002006"

/inference="similar to AA sequence:UniProtKB:P0AGK4"

/codon_start=1

/transl_table=11

/product="RNA-binding protein YhbY"

/db_xref="COG:COG1534"

/translation="MLNGKQKRFLRSQAHHLTPVFQIGKGGTNEHLFRHIEDAIEKRE
LMKVQVLNNNDEDRKEMAEEVARETGSELVQLIGNTIILYKESRDNKQIELPR"

CDS 974076..974666

/gene="nadD"

/locus_tag="EFAGFIKM_00839"

/EC_number="2.7.7.18"

/inference="ab initio prediction:Prodigal:002006"

/inference="similar to AA sequence:UniProtKB:P54455"

/codon_start=1

/transl_table=11

/product="Nicotinate-nucleotide adenylyltransferase"

/db_xref="COG:COG1057"

/translation="MKIGIMGGTFDPIHMGHLLAAEAARDSHALDEIWFMPSHVPPHK
RGAGASGQQRLDMTEAAVKDVQQYEVLGIEMELGGVSYTIDTMKELWRRHPEHEFYFI
IGADMVNYLPKWEQIEELAERLTFIGVGRPGFQLHLDDLPDELQDRVLLAEMPLVDIS
STAVRRRLAKGHSVRFMIPDEVHQYIVRSGLYGTKP"

CDS 974650..975216

/locus_tag="EFAGFIKM_00840"

/inference="ab initio prediction:Prodigal:002006"

/codon_start=1

/transl_table=11

/product="hypothetical protein"

/translation="MALSREELIQAVSGQMPEKRWKHTLGVMESSVILAEKYGADPVK

ADLAAILHDVAKYWPVAEMEAVIRDNGLNQELLQHDKQLWHSEVGAFVAKRDYAVEDS

EVINAIRWHTSGRVGMSLLDKVVCLADYIEPGRDFPGVDHIREQAERSLEEGLIAGFD

STISLLISQRRVIYPLTMLSRNDLIAQL"

CDS 975234..975581

/gene="rsfS"

/locus_tag="EFAGFIKM_00841"

/inference="ab initio prediction:Prodigal:002006"

/inference="similar to AA sequence:UniProtKB:Q9KD89"

/codon_start=1

/transl_table=11

/product="Ribosomal silencing factor RsfS"

/db_xref="COG:COG0799"

/translation="MTVSSKELMNMAVAAADDKKASNIVALDLINVSLVADYFVICHG

NSDTQVQAIATEIRKQAHAAGATIKGIEGMDSARWVLMDMGDVVVHIFHRDEREYYNI

ERLWSDAKVVETV"

CDS 975578..976483

/gene="cvfB"

/locus_tag="EFAGFIKM_00842"

/inference="ab initio prediction:Prodigal:002006"

/inference="similar to AA sequence:UniProtKB:Q7A5Q1"

/codon_start=1

/transl_table=11

/product="Conserved virulence factor B"

/translation="MSLIAGTVVSLPVSREVSPFGFFLTTGSEDVLLHYTELTRDIKI

GETLEVFVFFDTEDRLAVTMKKPYLMLGEMARLVVADVHPRLGCFLEMGLGRQLLLPI

RELPELEELRPQVGDEVFVIMEHDKQGRLRAKLAGERELSPLSFHAPTSWLNEWVEAT

VYKPLQMGTFVLVEGGVLGFGAIGMIHSSERSHMLRLGEKVKCRVTLVREDGRVNLAM

TQLKQVGRNEDADKLLAFMKERPMGGMPYSDATPPDIIKQRFGISKSAFKRALGKLMK

DGLVVQKESWTYLSESAPADQSENK"

CDS 976614..977384

/locus_tag="EFAGFIKM_00843"

/EC_number="2.1.1.156"

/inference="ab initio prediction:Prodigal:002006"

/inference="similar to AA sequence:UniProtKB:Q83WC4"

/codon_start=1

/transl_table=11

/product="Glycine/sarcosine N-methyltransferase"

/translation="MSYRKFAYVYDELMEDMPYPDWIRFARTAWEKHGMPKSVAELGC

GTGSITIPLVNSGFEVTGIDLSSDMLSVARSKMEATPQGHRLYREGSVRWVQQDMREW

RVPEPVDSVISFCDCVNYLLEQEDVIRTFQRTYEMLKPGGTFLFDVHHPNTLIRYDEE

QPFVLDERSVSYIWTCDLDQDRCEIEHHLSIFSRVDDGRKDIYQRFEEVHVQRAYNPD

WMKLELSKAGFRDVKVYADFEWKEAGDSAQRLFYVAVK"

CDS 977610..978566

/locus_tag="EFAGFIKM_00844"

/inference="ab initio prediction:Prodigal:002006"

/codon_start=1

/transl_table=11

/product="hypothetical protein"

/translation="MESTYKTRLTSEQLNAIIEQHFSASIQNYKEMVDGWANHAYLIT

LSDAQRVVLKIAPSASTNLMRCEQDLMIAEVEALRLITELQDVPVPQMLAYDDSLTMA

SARYFIMEYMSGTPYNQVKDQYSAEEQEAIEHELGRYNHRINEIKGEKFGYFSEQKQR

YSTWKEAFMNLMEDILADGEEAGITFSIDYPELRRLIEEKSNVLDDVKEPVLVSWDLW

DGNVLVDAGRITAIIDFERSLWADPLMEHYFSHFNYTPGFVKGYGHAITTESELKRRS

LYDLYFDLVLRIECAYRQYDNQEHVNWTIHNLEEGIERFRLS"

CDS 979026..981470

/gene="leuS"

/locus_tag="EFAGFIKM_00845"

/EC_number="6.1.1.4"

/inference="ab initio prediction:Prodigal:002006"

/inference="similar to AA sequence:UniProtKB:P67513"

/codon_start=1

/transl_table=11

/product="Leucine--tRNA ligase"

/translation="MSENNQPKHGYQPQVMEKSWQQYWDANKTFKTGEDPAKPKFYAL

DMFPYPSGSGLHVGHPEGYTATDIVSRYKRMRGYNVLHPMGWDAFGLPAEQHALDTGE

HPRDITFRNIDNFRRQIKSLGFSYDWDREISTTDPEYYKWTQWIFIQLYNKGLAYVDE

VAVNWCEALGTVLANEEVIDGKSERGGHPVVRKPMRQWVLRITEYAERLLEDLEELDW

SESIKDMQRNWIGKSTGAEVVFAIEGREEVIKVFTTRADTLFGASFAVLAPEHELVEA

ITTDDQREAIQAYQEQASRKSDLERTDLAKDKTGVFTGAYAINPVNGAKLPIWIADYV

LAGYGTGAVMAVPGHDARDWEFAKQFGLDIIEVIQGGDVTQEAYSGDGAHVNSDFLNG

LNNEEAVVKMIAWLEENGKGQGKVTYRLRDWLFSRQRYWGEPIPILHLEDGTMKTVPE

DQLPLLLPDIDQIKPSGTGESPLANVTEWVNTVDPETGMKARRETNTMPQWAGSCWYY

LRFIDPRNDKELISKEKQQQWLPVDLYIGGAEHAVLHLLYARFWHKVLYDLGVVETKE

PFHKLVNQGMILGTNNEKMSKSRGNVINPDEIVNEFGADTLRLYEMFMGPLEATKPWN

ANGVEGMHRFLSRVWRLFINEDTGAINDKITVDGGTDEFKRTAHKTIKKVTEDLEHLR

FNTSISQLMIFINDAYKADTLPREAMEKFVQLLSPLAPHMAEELWSRLGHEGGISYVA

WPEYDESMTVDAEVEIVVQVNGKIVTRATIAKDLDSQGMQDYTMELAPVKQALEGKTI

RKVIAVPGKLVNIVAG"

CDS complement(981451..982296)

/gene="proC_1"

/locus_tag="EFAGFIKM_00846"

/EC_number="1.5.1.2"

/inference="ab initio prediction:Prodigal:002006"

/inference="similar to AA sequence:UniProtKB:P0A9L8"

/codon_start=1

/transl_table=11

/product="Pyrroline-5-carboxylate reductase"

/db_xref="COG:COG0345"

/translation="MKVGFIGTGSMGSLLIYALIQSGALEPRQIAASNRTPSKVRQLS

LRYPGLHESQSNRETVIRSNIIFLCVKPLEFKHVIDDILPVVNPNHIIVSITSPVQLR

HLESSLPCKVSKVIPSVTHQVGSGASLCIHGERMTSEDRAVLESLLSHIGRPYQVDEA

CTRITSDFSSCGPAFISFFLEQWIESAVKLTGIKRADACALAGEMLLGTGKLLTEGGY

TPQELQARVAVPGGITAQALALLKVSLDGVFDSLIHTTHDKYDEDLLKLDELFRAGEI

NRQQY"

CDS 982482..983120

/locus_tag="EFAGFIKM_00847"

/inference="ab initio prediction:Prodigal:002006"

/codon_start=1

/transl_table=11

/product="hypothetical protein"

/translation="MRWNKGMTIAAAVVGSILILWSGKSEQPPSGWEPLQLGTEKPTI

EQELPAVQSANSVQGNTGASSSGTEASGLQTGNDAKADESKELASVGVEGDSVVQTTS

QTNDNPSDAATSIEPVAQSTNSNPSVTSETGSNSNPPVVREEASDNGKIDVNTAPVSK

LIELPGIGEKKAQAIVDYRNAHGPFAKVSDLTKVKGIGMKMLEKMAPHVQIR"

CDS 983161..983679

/gene="tadA_2"

/locus_tag="EFAGFIKM_00848"

/EC_number="3.5.4.33"

/inference="ab initio prediction:Prodigal:002006"

/inference="protein motif:HAMAP:MF_00972"

/codon_start=1

/transl_table=11

/product="tRNA-specific adenosine deaminase"

/translation="MSTEVRKDWDTYFMDIAYMVSTRSRCSRRHVGAVLVQGKKLLGT

AYNGAPTGVPDCSEAGCMISEEYELVVTDGQEEMVKKQRCIRTIHAEQNLLLFTDRID

REGSSVYVTDEPCWTCANMLANSGITEIVFHRSYPKDTGKVTNMMQQKGITFRRLENY

QPPRETMMTVSN"

CDS 983782..986658

/locus_tag="EFAGFIKM_00849"

/inference="ab initio prediction:Prodigal:002006"

/codon_start=1

/transl_table=11

/product="hypothetical protein"

/translation="MKGRPLLSFTICWLCGSGMACALTGWTLIWGFIGALACLPLLLH

FMDVRGWSVLFLLASFIGGAAHWEWNDARNHSSLPETMHISADALDGWSAEIQGELVS

DVRIDGDRADFKIQMSSILSISNSSTDTDLATSSAFKIDEQLMVQVRLLEEEEQQVAA

GWKRGDLITLNGSFVLPGEARNFGGFDYRSYLRTLHIHWLFKVKGASSVTAVAPEGLG

QYNVLRWTDWTRHKLGSAVEQLFPEPHAGYMKGLIIGMANDINPGTYGQFSQLGLTHI

LAISGTHVAVYVASLLLILSWLRLTRETALTIVLILVPAYVLLSGGSPSVIRAGIMSM

IGLYMARRGLARDGLQMISAAALLMMWWEPYFLLSVSFQLSFLVTAGLMIYMPLINRL

FSSWPKSLAATASVTVTAQLISFPVTILYFNQFSLLSFVANFLLVSLISAIVLPLGTV

AMLLSFIWIPLAKPLAWIAMKLNQLTFVSVEWMNSLPGFVLIWATPPLLWIAAYYAVL

YALLYLLHRGNGEQQETAFVEEETAPLVRRQPSVSMGNVGIRSSMSTVAQDQGRNRVD

GKETSLTTLGASIAWPEYTSAFTARGAGYYSNERMSKAQQWLCGLLAISFIAGLWWAY

QTPKPAGTGIVQFLDIGQGDSTLITTPEGKHILVDGGGTVSFGNTEQSWKTRRDPYEV

GAKVVVPLLKKRGIHQLDAVIVTHADQDHAGGLHAVLEQIPVKRFMFNGTTSGKENFD

KLLDTAMERKIPLYAIQQGMSYKVDKETSLHFIAPDLAHMDVNVSGNLPVSEHQNHDS

VVFLLEMAGASMLFTGDMDAAAEQDLLYMIQDGSLAAQFEQKGADIGVTEGVVQRVLT

RPLQVDSKDASAASASVSIDVLKVAHHGSKTSSTEAWLQYWNARTAVISAGVNNTYGH

PNPGVMERLEATGSEIYRTDQMGEVQMRVKDGKIDIRYKLVGVE"

CDS complement(986674..987123)

/locus_tag="EFAGFIKM_00850"

/inference="ab initio prediction:Prodigal:002006"

/codon_start=1

/transl_table=11

/product="hypothetical protein"

/translation="MTKTHRINRPRGKILAAVLTAVLISLAMGIYHYVPIDERLENTY

YYSFGYKFAVGLLINLAILLFLITPLSILVDGLLLYRKKDNTYLRLLVAIAAYVLLGF

IGGIIYSIFTLNFNTFSAQTIHIIAGSLVFLAFQLILNKLPSTSANR"

CDS 987354..987620

/locus_tag="EFAGFIKM_00851"

/inference="ab initio prediction:Prodigal:002006"

/codon_start=1

/transl_table=11

/product="hypothetical protein"

/translation="MVPPAQIRRGRIKNMKYRCYRKSTTTKALQTTKRLIDPIGFSLV

RLSAFTAHSHKEDGTGFYCFYSGEGLTTEEELKYFMLEMAGYRQ"

CDS 987880..988392

/gene="sigV_2"

/locus_tag="EFAGFIKM_00852"

/inference="ab initio prediction:Prodigal:002006"

/inference="similar to AA sequence:UniProtKB:O05404"

/codon_start=1

/transl_table=11

/product="RNA polymerase sigma factor SigV"

/db_xref="COG:COG1595"

/translation="MFNRKTEKILTHCITEHKENVYRLAYSYVKNKDDALDIVQDSIY

KAMTNLERLKNPDAVKSWFYRIVVNTALDFLRRNKKVQAMDHEMIETYSLGAEDTYTD

IDLKLTLESLPDKYKSVIILRYFEDMKIEEVAAVLDENVNTIKTRLYQAHQLLRIKMN

DETTKGDRVR"

CDS 988389..989279

/gene="rsiV"

/locus_tag="EFAGFIKM_00853"

/inference="ab initio prediction:Prodigal:002006"

/inference="similar to AA sequence:UniProtKB:O05403"

/codon_start=1

/transl_table=11

/product="Anti-sigma-V factor RsiV"

/translation="MNNNMEQLRKEYHEIPIPDELDNIVNQSIHKFSKEGRRGRGSKY

RWLAVSSAAAFMFLVAINVSPPVAKALSSIPGVDRIIQVFTFKEYLVDESNYNANIKV

PSITDMENEQLQSGLNEKYMKENKALYEKFQAEISELKKQGGGHLGLDVGYEVKTDND

EILSIQRYVVKTAASSSEELQFDTIDKKNQILITLPMLFKDDRYVRLISDNVKEQMQG

QMKADPNKIYWAPGATDVPVTSEFQSIAKDQGFYINNDGKLVIAFNEYDVAPGYMGSV

EFVIPTDVLKDVLINNKYIK"

CDS 989450..990226

/locus_tag="EFAGFIKM_00854"

/inference="ab initio prediction:Prodigal:002006"

/codon_start=1

/transl_table=11

/product="hypothetical protein"

/translation="MKKKTIKYMKMGAMGCVAMLGVGAASLSAYALASAPVSANVTVS

SKVQATVPSATGVKIVEKVLSTTSKNLIVNIKVPQLDGMLDTRYQNELNGILLFRAQK

DLAQWEKDAAKTAKKAKVNHQKFHPYEFYVSYKLKSDGTGNPAGVVSLEVITEGYRGG

TSMPRIDTYNLKNASAAQRVTLEDLLGSNYKEKLDANILAQMRKDPDQYFLEEFKGTF

TEQSFYIEKGELVIVFPKYSIAPGYVGSPEFRFNLKNASL"

CDS 990942..992381

/gene="pdaC"

/locus_tag="EFAGFIKM_00855"

/EC_number="3.5.1.-"

/inference="ab initio prediction:Prodigal:002006"

/inference="similar to AA sequence:UniProtKB:O34798"

/codon_start=1

/transl_table=11

/product="Peptidoglycan-N-acetylmuramic acid deacetylase

PdaC"

/db_xref="COG:COG0726"

/translation="MNKRKKTRWPVWLVGFIVLAGGILGINLMVQNVLAGNHSVTENV

EVSPSAYPGLNIETRTKNTKHYILSISNVLSNSKEINTPIQTWVNDQEKKFLAQVGTG

AKTLQGDHRAELNITVDTNKINDHLYSLVFTSYQITTGGANGQSIIKSFNIDTAENKI

LNLSDIMTYDKAAVNHIMSIVKEELKKQKNVHSYVFEAELQKSIKNPSGWKWSIGNGV

FTLYFNKYEIAAGAAGTVQVNIPLKSLHSYLKKDLTKKWNITYENNDEPKGKRPTQPP

LDPKGKYVALTFDDGPHPKVTPRVLKTLKEYNAKATFFMLGVQVEYYPDMAKKVAEAG

HEIGNHSKSHPNLANMSLSEVRKQIKESSDRIEDATGEKPTLFRPPYGAMNGSVKKVT

KEQKTPIILWSVDSLDWKSRNAQAVNKEVAKNIRSGSIVLMHDIHASTADALPQMLKS

LKKQGYQFVTVSQLLSLNESKGNGPYYNR"

CDS complement(992628..992930)

/locus_tag="EFAGFIKM_00856"

/inference="ab initio prediction:Prodigal:002006"

/codon_start=1

/transl_table=11

/product="hypothetical protein"

/translation="MPALAKIQALKEQMPKEYQSISHFVEHALDSIDTLVEEHRKYVA

AQALYGDKIVGSEERLYRETVLDVRAQLLMTLEKTVEDLLHKGDKHWNKHFKDGVE"

CDS 993282..993830

/gene="sigW_2"

/locus_tag="EFAGFIKM_00857"

/inference="ab initio prediction:Prodigal:002006"

/inference="similar to AA sequence:UniProtKB:Q45585"

/codon_start=1

/transl_table=11

/product="ECF RNA polymerase sigma factor SigW"

/db_xref="COG:COG1595"

/translation="MVEPELIRAAQSGDRDALITLLREIEQHVYRTAYYILNNEQDAL

DAAQEALIRIYTKINSYEEKAQFKTWVQRIVTNICIDKFRRTKPSVSIDEHDMVFQDN

QDVEYEVMSTYVAQDIQDAINKLPEHHRTVIVLRYLQDLSYNEIADCLDLPLNTVKSY

LFRARQQLQNLLQEYQKGGVTG"

CDS 993827..995110

/locus_tag="EFAGFIKM_00858"

/inference="ab initio prediction:Prodigal:002006"

/codon_start=1

/transl_table=11

/product="hypothetical protein"

/translation="MKCEEVVEWMHRYLDHDLGEAETAQMLQHVAKCPECAENFSLLR

ALSRELEELPQVSPKFSLVDAIMPQLDAIDEARKEQSSTIQEMNPVPATFENLHRSSE

RKTKQKWLNSMAGRMSMGAAAAAVILGFAIWGNPPEQIENADSMLMSSGASQESGNVD

ENPLSKNIENYDPATSSQPSSNDVFVDQSNVSPTKPDNQDVETMPEENGGDEVQEPEV

QVDLTPKQPATESTPAPNKGPQDNSSKTPPTDNQKNPVQNQDSEQRIQGSEDSPKSSA

DGEESGVTETENGDAQSFTSQDTTPNITEDVPVEGNTVPDSSAGADASGGNKGFAAGP

DQGMTTTAAPKEWKSPDGSYVVMLIGDQISIYSKPVSEPDVLSLVEQRSIEGTLKSAS

WSKDGTLFNYETDKDGTTAKNSFNVPAVSGGAPAK"

CDS 995367..996383

/gene="yqeN"

/locus_tag="EFAGFIKM_00859"

/inference="ab initio prediction:Prodigal:002006"

/inference="similar to AA sequence:UniProtKB:P54459"

/codon_start=1

/transl_table=11

/product="putative protein YqeN"

/db_xref="COG:COG1466"

/translation="MDVKSATKAIRNGETAPIYVLYGTEKYQIQQFTDMLKEQVIEEE

HRDFAIIPYDLSETAVEVVIEEAETVPFLVPRKLIIVRDASLFTAGKESKIEHQVDRL

LTYMENPADYSTIVFLAQGDKLDERKKLVKAVKKQAVVLAFAPLSGEELLNWIVKLAK

QRDVSLETGAAETLISYAGTGLQTLSAEVDKLCLFAGNGGTIKRADIESLVARSTEQN

VFSLVEELANLRLEKALALFYELLKQREEPIKIAALIARQFRIMIQVKELGQQSYSQQ

QIASQLGLHPYAVKIAGEQARKFQPERLRMILSHLSELDYQMKTGAVDKVLGLELFLL

RLGA"

CDS complement(996573..996845)

/gene="rpsT"

/locus_tag="EFAGFIKM_00860"

/inference="ab initio prediction:Prodigal:002006"

/inference="similar to AA sequence:UniProtKB:P21477"

/codon_start=1

/transl_table=11

/product="30S ribosomal protein S20"

/db_xref="COG:COG0268"

/translation="MPNIKSAVKRVKTSDKRRALNASQKSALRTAVKAADAALVSNEV

DTAKAAIQAASKKLDKAVTKGLVHKNAAARKKSRLAKKLNALSAQA"

CDS 997021..998022

/gene="gpr_2"

/locus_tag="EFAGFIKM_00861"

/EC_number="3.4.24.78"

/inference="ab initio prediction:Prodigal:002006"

/inference="similar to AA sequence:UniProtKB:P22322"

/codon_start=1

/transl_table=11

/product="Germination protease"

/translation="MTLDLQNYTVRTDLAIDSKEMAQGGQKQTIPGLREDVEKKEGIT

ITRIDVLTEEAATAIGRVKGHYVTLEVPSLRNGDTELQERVAAEFTREMEDFMVKAGI

NKTSKVLIVGLGNLNVTPDSLGPLVVENLMVTRQYFELVPDQVAPGYREVSAIAPGVL

GTTGIESSDIVQGIVDRTKPDAIIAIDALASRSLERVNTTIQVADIGIHPGSGIGNKR

RGLTREILGVPCIAIGVPTVCYASTIVNNVIEMMRSHFRQETDQTKQIMGMLDDIGEQ

ERLSLVKEVLEPLGHDLIVTPKEIDEFIEGIANVIATGLNAALHDAVNPDNVAAYTH"

CDS 998169..999464

/locus_tag="EFAGFIKM_00862"

/inference="ab initio prediction:Prodigal:002006"

/codon_start=1

/transl_table=11

/product="hypothetical protein"

/translation="MNKILAWNIGKWKKRCLHVLAMGRTLLLLMIISALFFVVLGLGG

MAEKRLNNSPVSSMKGFASAVSSRFFVDMLGMEMPHLTQKEQTSAISGENLTSFVFQL

LTNVNPQDPKSLIAREMPGMGSNQPVLLRPGSGNEKAEAPEDYQPGPGLTDTASAGGG

KPGSELHVPPGQDNTDPPESSEPGDGEGKEDPPAKNGEKDNSGSPTTGKKSILIYHSH

PREGYNPLLGTKSDNPSSGKSTGNVFQVGTYLSDSLKKLGIGVEHAKDDYPTKVKDYN

WNYSYKYSRQTVKAALAQNDDLTYLIDIHRDSQRHGKTTTTINDLGYAKVYFIIGHEN

PNWRQNEAFAAKIHKKLEAKYPGVSRGVWGKDGGGANNGEYNQTLSPNSILIEIGGID

NTGAELQRTSKILADMIAQVYWEDQKVDKASAETSSQGG"

CDS 999519..999878

/locus_tag="EFAGFIKM_00863"

/inference="ab initio prediction:Prodigal:002006"

/codon_start=1

/transl_table=11

/product="hypothetical protein"

/translation="MSRFGKRLLSIAVLMLLGVLFGMQLAGSNFHMGNSSGGTSTVVT

TEPTPSVTESVPTAPVQKEIKEPPFVPVQSPSQVLGADQNKAPVDVLADKTAGLLQNL

SYSGIKWVVSLFSGVAE"

CDS 1000024..1001838

/gene="lepA"

/locus_tag="EFAGFIKM_00864"

/EC_number="3.6.5.-"

/inference="ab initio prediction:Prodigal:002006"

/inference="similar to AA sequence:UniProtKB:Q2FXY7"

/codon_start=1

/transl_table=11

/product="Elongation factor 4"

/db_xref="COG:COG0481"

/translation="MTDIRARQRKIRNFSIIAHIDHGKSTLADRILEHTGALTSREMQ

EQVLDKMDLERERGITIKLQAVALTYKADDGEEYLLNLIDTPGHVDFTYEVSRSLAAC

EGALLVVDAAQGIEAQTLANVYLALDNNLEILPVINKIDLPSADPERVKQEVEDVIGL

DTSNAVLASAKSGIGIKEILEQVVKSVPAPTGDPEQPLKALIFDSHYDPYKGVIVYVR

VVDGKIKSGSKIKMMATGKSFEVIEVGAFKPRMTIVDELNVGDVGFIVAGIRHVGDTQ

VGDTVTDAKNPTPEPLPGYRKINPMVYCGLYPIETSDYVDLREALEKLQLNDASLSFE

PETSSALGFGFRCGFLGLLHMDVIQERIEREFNIPLITTAPSVIYHVTLTNGEVMKID

NPSNYPEVGRIDYVEEPYVKADIIVPNDYVGTIMELCQTKRGEYVNMEYLDTTRVTIT

YQIPLSEIVYDFFDQLKSGTKGYASLDYELSGYRQSNLAKMDIVLNGEQVDALSFIVH

RDRAYNRGRIVCEKLRELIPRQMFEVPIQASVGTKVVARETVKAMRKNVLAKCYGGDI

SRKRKLLEKQKEGKKRMKQVGNVEVPQEAFMAVLKIDD"

CDS 1001987..1003171

/gene="hemW"

/locus_tag="EFAGFIKM_00865"

/inference="ab initio prediction:Prodigal:002006"

/inference="similar to AA sequence:UniProtKB:P54304"

/codon_start=1

/transl_table=11

/product="Heme chaperone HemW"

/db_xref="COG:COG0635"

/translation="MTLAAHSRKTGAPQAVYLHIPFCTNKCFYCDFNSYVLKDQPVMQ

YLEALEREMEHTVKANPPGEIKTIFVGGGTPTALKPDEMAVFLRSVKTYFPNWADDIE

FSMEANPGTTDAEKLAAMKEGGVNRVSFGVQAFQNDLLTGIGRIHNTDDVYRSLENAR

KAGLNNLSIDLMFGLPNQTVEMLNESIDKALELDLPHYSIYSLKVEENTLFHTLYQKN

QLPLPHEDDELEMYLLLMRRMKEAGYGQYEISNFAKPGFESRHNITYWRNEDYYGLGA
GAHGYVGRERHMNIKGINPYVEASRAGLPRLDHFEIARPEAMEDYLMVGLRMLEGASA
SRFSEQFGESMEEVFAKPLGKMLNAGLLERTADGFRLSEQGILFGNDVFAEFIGSISL
NS"

CDS 1003290..1003760

/gene="argA"

/locus_tag="EFAGFIKM_00866"

/EC_number="2.3.1.1"

/inference="ab initio prediction:Prodigal:002006"

/inference="similar to AA sequence:UniProtKB:O33289"

/codon_start=1

/transl_table=11

/product="Amino-acid acetyltransferase"

/db_xref="COG:COG1246"

/translation="MSVICRKAVPEDVEPLYEMIKGYAERGIMLPRSREILHRQLEHF
VVAEVDGEVVGCGSLCRLGNDLVEVRSLGISEGHKGLGIGSRLLDRLVEEAEKQQIPK
VMALTYEVSFFLKNGFAVVEKEIFPEKVWTDCVHCSKQDCCDEIAVLKELNVSA"

CDS 1004015..1005046

/gene="hrcA"

/locus_tag="EFAGFIKM_00867"

/inference="ab initio prediction:Prodigal:002006"

/inference="similar to AA sequence:UniProtKB:P30727"

/codon_start=1

/transl_table=11

/product="Heat-inducible transcription repressor HrcA"

/db_xref="COG:COG1420"

/translation="MLTERQRMILNAIVDDYIRSAEPVGSRSISKRGDVGYSPATIRN
EMADLEDMGFLEQPHTSAGRIPSHKGYRYYVDHLVPWNQVEPQELDDLKSFFAEKLNV
MEQVIQHASNILSHMTNYTSILLGPEVFHTSLRHFQLLPLNESEAVAIIVTNTGQVEN
KTVQIPPEISVAEMENVVRLLNTKLVGVPIYKLKSRLYTELGQEMERHITRYEEVMQV
LNSAFDNEHDNRLFLSGATNMLTQPEFKDIEKVKDILDLLEETPTLMKLMMPVPGGSG
IQVRIGTENDHEAFANCSLITASYSLDGEALGSIGILGPTRMDYARVIHILNTLSRDL

TAMLTHRFK"

CDS 1005091..1005654

/gene="grpE"

/locus_tag="EFAGFIKM_00868"

/inference="ab initio prediction:Prodigal:002006"

/inference="similar to AA sequence:UniProtKB:Q5KWZ6"

/codon_start=1

/transl_table=11

/product="Protein GrpE"

/db_xref="COG:COG0576"

/translation="MKEEQSFTEEQEVTATEQEPINEAGAAEAQAEEMAGQEQDELAR

LKAEAEEQQQRYVRAQADFDNFRRRTQKEKEELAKYASMKLVTELVPVIDNFERAMAT

VPEGAEVESFSKGIQMIFRQLETVMNNEGLTAMESVGQPFNPEFHQAIMQVESDEYEE

GIVVEEVQKGYMLKDKVLRPAMVKVSS"

CDS 1005815..1007659

/gene="dnaK_1"

/locus_tag="EFAGFIKM_00869"

/inference="ab initio prediction:Prodigal:002006"

/inference="similar to AA sequence:UniProtKB:Q5KWZ7"

/codon_start=1

/transl_table=11

/product="Chaperone protein DnaK"

/db_xref="COG:COG0443"

/translation="MSKVIGIDLGTTNSCVAVMEGGEAVVIPNPEGARTTPSVVGFKK

DGERVVGETAKRQAITNPDRTIMSIKRHMGTSHKETIDSKDYSAQEISAIILQKLKSD

AEAYLGQTVTQAVITVPAYFNDSQRQATKDAGKIAGLEVLRIVNEPTAAALAYGMEKS

EDQTILVYDLGGGTFDVSILELGDGFFEVKATSGDNKLGGDDFDQVIIDYLVSEFKKD

QGIDLSKDKAAVQRLKDAAEKAKKELSGVLTTTISLPFITVADGVPQHLELNLSRAKF

EEISEGLVERTLEPTRRALSDAGMTPNDIDKIVLVGGSTRIPAVQEAIKKLTGKEPHK

GVNPDEVVALGAAVQAGVLTGDVKDVVLLDVTPLSLGIETAGGVFTKMIERNTTIPTS

KSQVFSTYADNQPSVEIHVLQGEREMAAGNKSLGRFMLGDIPPAPRGVPQIEVSFDID

ANGIVNVSATDKGTNKTQKITITSSSGLSDAEVEQMMKDAELHAEEDKKRRELVEAKN

SADQLIYSVDKTIKDLGEKADAGEVEKANAAKEKLQGVLASDNLEDIKAATEELTEIV

QQLSVKLYEQAQAQEQEAQGTEEQQGSAKRDNVVDADYEVVDEDKKQN"

CDS 1007789..1008913

/gene="dnaJ"

/locus_tag="EFAGFIKM_00870"

/inference="ab initio prediction:Prodigal:002006"

/inference="similar to AA sequence:UniProtKB:P17631"

/codon_start=1

/transl_table=11

/product="Chaperone protein DnaJ"

/translation="MAEKRDYYEVLGVSKTANDDEIKKAYRKLARQYHPDVNKAADAE

AKFKEVKEAYDVVSDGQKRAQYDQYGHVDPNQGGFGGGGDFGGGGFGDIFDMFFGGGG

GGRRDPNAPQRGNDLQYTMTIEFKEAVFGKETDITIPRTEGCDTCHGSGAKPGTKPET

CSVCQGSGQQEVVQNTPFGRMVNRRACSNCSGSGQIIKEKCTTCSGSGKVRKQRKIHV

RIPAGVDDGAQLRMTGEGEGGLRGGPAGDLYIVIRVKSHDFFEREGDDIYCEIPLTFA

QAALGDEVEIPTLTEKVKLKVPAGTQTGTYFRLKGKGVPRLRGMGQGDQHVKVVVVTP

SKLNDEQKDLLRQIASLDGEQTHEHEQSFFDRVKRAFRGD"

CDS 1009154..1009522

/locus_tag="EFAGFIKM_00871"

/inference="ab initio prediction:Prodigal:002006"

/codon_start=1

/transl_table=11

/product="hypothetical protein"

/translation="MTEKNILAYFHSPEQAQGAAKKLEALRVEDLSINRFSRYAGVGP

SGASYKPGVSGSFSSWVPDSMQTNASAGIMAAADPSASGMSDGGQGGPTGRDVLLTVI

VDESVHHRAMSIIEQAGGMI"

CDS 1009782..1009961

/locus_tag="EFAGFIKM_00872"

/inference="ab initio prediction:Prodigal:002006"

/codon_start=1

/transl_table=11

/product="hypothetical protein"

/translation="MAEHERPGKILTKTEKMKRMQSAKNEDVEFSAEAADHEDIEALR
RSEAADRRQGRELHD"
CDS 1009954..1010346
/locus_tag="EFAGFIKM_00873"
/inference="ab initio prediction:Prodigal:002006"
/codon_start=1
/transl_table=11
/product="hypothetical protein"
/translation="MTEDRSAVTLEAVFTSWTEAESARRALELLHPNQLSIESLGNEA
SALDSNDVTGSDNSPTAVVGGFSGELNVLALISDETEMLSNGQVYDTPKEDSANRLKQ
EILLTVSVSNDSLPQAAEMITQNGGRFC"
CDS 1010537..1011502
/gene="prmA"
/locus_tag="EFAGFIKM_00874"
/EC_number="2.1.1.-"
/inference="ab initio prediction:Prodigal:002006"
/inference="similar to AA sequence:UniProtKB:P0A0P5"
/codon_start=1
/transl_table=11
/product="Ribosomal protein L11 methyltransferase"
/db_xref="COG:COG2264"
/translation="MLWHELTIHTTEESVEMISNFLHEAGAGGVSIEESGTLNKKRDT
TYGELYDVPLNDIPEGFAVIKGYFSEGTDMVALQSEVHPRIEELSEFGIDTGEVRYET
RTVDEDDWATAWKQYFKPVRVSERLTIKPTWEEYTPESPDEKIIELDPGMAFGTGTHP
TTSLCLRTLETVIQGGEEVIDVGTGSGILAIGAIKLGAKHVLALDLDLVAVISARENV
ELNGLEQQITVKESDLLSVLGSQDPALGVQLPVKVVVANILAEIILLFVDDVYNALES
GGTYIVSGIWKNKEQVVHDALVASGFEVSAIHRDEDWLAFVARRG"
CDS 1011506..1012186
/locus_tag="EFAGFIKM_00875"
/inference="ab initio prediction:Prodigal:002006"
/codon_start=1
/transl_table=11

/product="hypothetical protein"

/translation="MDFWNSFFRYPIDQLPFFLLTILIAFTVHEFSHAYFANKFGDPT

AKLLGRVTLNPVVHFDLLGVLLLIIAGFGWARPVPVNRANFDKPRLMGIIVSAAGPLS

NLLLAIIGTIIYASLVGSGALGGIDNERLLTAITTFFAIFNLTNFFLFLFNLIPLPPL

DGYRILEDVVPPSISRKLQGVEQWSIFIFLLILVIPPLQNVTIQPLYEFAQTMYVDLA

KVIIELYR"

CDS 1012324..1013088

/gene="rsmE"

/locus_tag="EFAGFIKM_00876"

/EC_number="2.1.1.193"

/inference="ab initio prediction:Prodigal:002006"

/inference="similar to AA sequence:UniProtKB:P54461"

/codon_start=1

/transl_table=11

/product="Ribosomal RNA small subunit methyltransferase E"

/db_xref="COG:COG1385"

/translation="MQRYFVSAEQFNEHAVIITGDDARHIGKVMRGKPGDKLIVSDGN

SREALVEIESIEVGQVTANIMEPLEMDHEARIRVTVAQSLPKGDKMETVIQRCTEIGA

VSFVPFLSERTIVQYDAKKEGKRLERWRKIAKEAAEQSHRNRIPSVEQPLSWKGLLSS

FEGYSLVCYCYEKENGKQLRDALKPFIEQLAPDASAEVLIVVGPEGGFSEKETAEADQ

AGAVSVGLGKRILRAETAGMAALTCVLYESGEMGGM"

CDS 1013091..1014437

/gene="mtaB"

/locus_tag="EFAGFIKM_00877"

/EC_number="2.8.4.5"

/inference="ab initio prediction:Prodigal:002006"

/inference="similar to AA sequence:UniProtKB:P54462"

/codon_start=1

/transl_table=11

/product="Threonylcarbamoyladenosine tRNA

methylthiotransferase MtaB"

/db_xref="COG:COG0621"

/translation="MPSVAFYTLGCKVNFYDTEAIWQLFKNEGYDQVDFDEQTADVYL

INTCTVTNTGDKKSRQIIRRAIRRNPEAIVAVTGCYAQTSPAEILDIPGVDLVIGTQD

RDKILPYVNEIQESRQPVNAVRNIMKTRVFEEMDVPDFADRTRAFLKIQDGCNNFCTF

CIIPWSRGLSRSREANSIIQQAHQLVHAGYKEIVLTGIHTGGYGDDMENYDLTDLLWD

LDKVEGLERIRISSIEASQIDDRMLDVIKRSDKLVRHFHIPLQAGDDTVLKRMRRKYT

TEEFYNKMLRIREAMPDVAITTDIIVGFPGETDEMYRNGYKLMRKIGFSEMHVFPYSK

RTGTPAARMEDQVDEEVKNARVHELIDLSEQMQLEYAEQFVGQVLDVIPEGEAKGREG

SGKLHGYSDNYIQLVFDGTMDMVGKVCRVKVVEAGVNESQATLVRVLEENLKSAAM"

CDS 1014551..1014985

/gene="ndx1_1"

/locus_tag="EFAGFIKM_00878"

/EC_number="3.6.1.61"

/inference="ab initio prediction:Prodigal:002006"

/inference="similar to AA sequence:UniProtKB:Q75UV1"

/codon_start=1

/transl_table=11

/product="Diadenosine hexaphosphate hydrolase"

/db_xref="COG:COG0494"

/translation="MNKKEISAGGVVYRTGEDGRLQIQLIVDRYGKTTLAKGKMEEGE

TIEQTALREILEETGMVGRIVEPVDIIAYTYQHADFGPVDKEVHYYLVEAESGDLQPQ

IEEIKGVDWYAPEEAWSLQQQSGYDNNDDILRVALNKLGINV"

CDS complement(1015043..1016044)

/locus_tag="EFAGFIKM_00879"

/inference="ab initio prediction:Prodigal:002006"

/codon_start=1

/transl_table=11

/product="hypothetical protein"

/translation="MFRDVILPLLYGLIIFFGGMKLMETALQRMAGPMLAGWLNRATS

APWKGMAFSAGATALLQSSTAVTVLTIGLANARLITYGRTLGIILGTNIGTCLTTELV

GLQIGRASLPLLVLSLTGWAMFVVLGERNLAAPERTRQTLFNIQYSFLAAAGFALVMT

GIQVMQSIALPLRSMGVFQWFLDRSADSLWWGFLAGACLTALIHSSAAVISMAITLAA

TGVLPVEIGIAIVIGSNVGTCITAVIAAAGGASAGKFVAASHVVLNIAGALLFMPLIG

QLHAASAWLSADNGAQIAHAQTLFNITSSLLALPFCYLPIWHKKPPVHAPAAKSPVA"

CDS 1016604..1017980

/gene="rlmI_1"

/locus_tag="EFAGFIKM_00880"

/EC_number="2.1.1.191"

/inference="ab initio prediction:Prodigal:002006"

/inference="similar to AA sequence:UniProtKB:P75876"

/codon_start=1

/transl_table=11

/product="Ribosomal RNA large subunit methyltransferase I"

/db_xref="COG:COG1092"

/translation="MPSVTLERSRKKRLEHAHPWIFNNEIASVDGNPEPGDLVNVLNH

QGRYLATGYYNPASQITVRVVAYQPLESEQMDMAFFAARFRDCLRHRERFIQDGEAYR

LVYGEADFLPGLIVDRFGSILVVQLLTLGMDRCREAIVQALIEVMQPEGIYERSDVSI

RELEGLEQTKGPLYGECPRHVTVTENGLLIKVDIVEGQKTGYFFDQRENRAAIEPLMK

GWGYKSGITLQTTEQDGTEQLLPVNKSGKVVTFPYWDGATVLECFSHTGSFTLNACKY

GAKKVTCLDISEHAIESARTNVELNGFTDRVEFVVADAFQYLREQVKGLDERTTRARA

GEQKVDTSKALAAGGKTFDVVILDPPAFAKTKSAVKGACRGYKDINLQGMKLVNEGGY

LVTASCSYHMRPDLFLDTIADAAEDAGKVLRLIDWKAAGKDHPQILGVDEGHYLKFAI

FEVRSKKN"

CDS 1018180..1021683

/gene="addB"

/locus_tag="EFAGFIKM_00881"

/EC_number="3.1.-.-"

/inference="ab initio prediction:Prodigal:002006"

/inference="similar to AA sequence:UniProtKB:P23477"

/codon_start=1

/transl_table=11

/product="ATP-dependent helicase/deoxyribonuclease subunit

B"

/db_xref="COG:COG3857"

/translation="MSVRFVIGRAGSGKSSLITREITSLLQKEPQGTPLILLVPEQSS

FRTEQALVSSGAIKGTMRAEVLGFRRLAYRVMQEAGGSARIPIGAEGKKMLLYKVIQR
RKEELKLFGASGSQLGFIGELNDLYSEFKRYEVDPSLLEEGLSGGITSSTSPILEDKL
HDLNLIYRDYEQELTHLYIDDEDTLTELTDRLSESALLQDAQIWIDGFQGFTPQEMSV
IGRLMLQSSSVTIALTLDRPYDHGALPGELELFYPTASAYTRLKGMADELGVPSDITL
LDSEIPPRYKDRPGLAHLEAGFDRRIRWKSEGLDSGIRLVAAENRRAEMEGALREMRR
LAQNEGARYRDMAVLVRQLDTYADIAEPLFRDYDVPVFLDRRRNELHHPLSEFIRSAL
DIVRRHWRYEDVFRCVKTDLLLPRDGSITREDMDQLENYVLACGIHGYRWTDGKPWKY
VPSLSLEDNGQDGRSRGRDQMLSLMESCRTVITDSLGAFEKRMKKAKTAKAQCEALYR
LLEDAEIPWKLENMSAEAKAQGDPERSREHRQMWGAVLDLLDQMVDMMGNERLDITLF
AGMIETGLTELKLGLVPPALDQVLVGSMDRTRLQDIKYVFILGAVDGELPAVPQDDGV
LTELERALLTDRGVALGPGATRQMLDERFLIYTALTAASSQLWLSYPVADDEGKTLLP
SEIVRHVRKMFGLQEQPLLAQPPVANSEEAHWSYVTHPGQSLSTLIGQLRKWRRGEDI
PEMWWAVYNWHVSRETSRPQLERLMGSIFYRNRALPLRTATSRRLYGTEVRTSVSRME
RFVACPFSHFASHGLRLKERQLYRLQAPDIGQLFHAALSQLAMRLREENRSWGSLTPD
QCRQEAEQTVEQIAPQLQGEILLSTKRYGYIFRKLKDIVSRASVILGEQSRRGSFEPI
GLELDFGPDKTLPPLRFELENGCVMEIVGRIDRVDVAEGENGLLLRVIDYKSSQTDLK
LHEVYYGLSLQMLTYLEVLLSAAEEWLGETAMPGGTLYFHVHNPLLQSANGMTSEQAG
QELLKRFKMKGLLLADRDAIAQMDNTLDKGYSAIIPVALKADGSFYSSAAVATPEQWD
TLLASVRSNIREIGTRITDGDVAIEPYRIQQEVACTFCPYKPVCQFDENIEGNEYNLL
SKPGKQQIWDMLSHTKGGETS"

CDS 1021680..1026107

/gene="addA"

/locus_tag="EFAGFIKM_00882"

/EC_number="3.6.4.12"

/inference="ab initio prediction:Prodigal:002006"

/inference="protein motif:HAMAP:MF_01451"

/codon_start=1

/transl_table=11

/product="ATP-dependent helicase/nuclease subunit A"

/translation="MTNMPKPEGSFWSDDQWSAISESGKDILVAAAAGSGKTAVLVER
IIRKIADPSQGYSVDRLLVATFTKAAAAEMKQRIREALERALEEQPGEEHLRKQLSLL
GRASITTLHSFCMEVIRRYYQQIPLNPAFRILNENEAEIMRQELLEELFEEKYGEEDE

GSTFRELVDWFSGERNDDAMHRLVQRLYDFSRSHSWPDHWLSEMASAFQVESVEALGH
SAWVQSILRDAALALSGAAGLLRQGIHISMQPEGPAPYADTLKEDLVMVEELLSAAQV
MPWERLPEVFQPATFGKLKPCKKDQTDPALQDQVKELREAAKKAVTDLKGSLFGRSAF
AFWQELEQAAPLMQELSRLVSTFGERYRQAKQERGQVDFSDLEHYCLHILRHEDSTPE
LSMPSDAAMEYRSRFDEVLLDEYQDTNTVQEDIVKLISRENPGNRFMVGDVKQSIYRF
RLAEPGLFMNKYRQYGASTREDGMVEDRLNGTGQRIDLARNFRSRAEVVHSVNMIFRQ
LMNEGVAEIAYDERAQLAYGATFPSETLGDEAYTPELMLIDRQGGGPDLTESTDENGD
ASVSAELESAELETAQLEARAIARRIREMVGDTDKPALHVYDKALQTMRPARYGDMVI
LLRSTLMWAPLMIEEFRQQGIPAGGEQSKGYFQATEVEIMMSLLQLVDNPRQDIPLAS
VLRSPIVGLDEEELAQIRLGDKRQSFYDAVISAAGDWSDSSTNAVQQSWQDNHPDSNM
VQLQWPEAWSEMEQGQRETAASVEAEILDDVHEFDVHRVQTDGMQDAGVAGHASEDLG
ITEISGSELQQKLIRFMRQLEQWRLEARQGSLSELIWRMYRETGYLDWVGGLPGGLQR
QSNLKALYDRARQYEESTANRGLFRFLTYVSRLRENGGDLGTVASSSGEQDNAVRIMT
IHRSKGLEFPIVFVGGISKMFNQQDLNAPFLMHKELGFGPRFVDRENRVAYPTLANLA
IRRRAQFELLAEEMRVLYVALTRPKEKMILLGTVKDAAKKALGWSQVKDSPELALPDY
LLAAGRSYLDWIGPSLMRHPDAAVLRELAGSNDSFSACLADDQSRWAISIISADQVSR
DQVVDQTLGEEDGMTEVRKERIAALKAVQPVKLVTSSTVEELTKSAQDSANQNIGDEG
ADTVALHQEETRLINEVDQRLSWTYPYEAATQVAASTSVTELKTLLVMQDIQSVEVME
ELGEQSEKLLDYNTPGINNIKDETSHAHRDASSDSIKHEAGIGSDSNLTVNVPDAGSA
GTSFKLHLRRPKFMEATQLTGAERGNVYHTLMQHLPLDGSAIDSQLIEQTTQRLVELQ
ILLPHHVEAIELEELVEFFDTEPGKELLRAAWVKREIPFVYGLPAHQSPVEWLHERAA
NSDTQMLKEDSKMQATIENETVLVQGIIDCLYEVDGELVLLDYKTDRVLEHRGGLDEL
TKNYRFQLDLYGRAIEDILGRKVDRKWLYFFDGGYAVKL"

CDS 1026167..1027351

/gene="sbcD"

/locus_tag="EFAGFIKM_00883"

/inference="ab initio prediction:Prodigal:002006"

/inference="similar to AA sequence:UniProtKB:P0AG76"

/codon_start=1

/transl_table=11

/product="Nuclease SbcCD subunit D"

/db_xref="COG:COG0420"

/translation="MRILHTGDWHLGKTLEGRSRLREQEDFVEELVRVADEQQADAIL
MAGDVYDSVNPPAAAEQLFYEAAARLTEHGRPLVVIAGNHDQPERVASVTPLVNRQGI
TLVGMPISEAVTIHAKRTGEIAQIAALPYPSEARLNELLTTDGDENVLRQAYSHRVGM
LMQRLAASFRPDTVNLAMSHIYVLGGLESDSERPIQIGGAYTVDPSSLSTGAQYTALG
HLHRAQAVKGDGVIRYSGSPLAYSFSEAGQSKSVTMVDLAPGGAAQVEEVLLTSGRPL
VRWQARGGLAEVYRWLDEGRDPQAFIDMEVWLDEAMSLKEIQQLRKSHEGIIHIRPVY
PEMAAAGLLEQRSELPVHELFRKFYQRQTGGAQPEDSLVQLFLELVDEDEPGVREEVE
ER"

CDS 1027348..1030767

/locus_tag="EFAGFIKM_00884"

/inference="ab initio prediction:Prodigal:002006"

/codon_start=1

/transl_table=11

/product="hypothetical protein"

/translation="MKPILLKVAGLQSYREMQEIDFTVLTETGLFGIFGPTGSGKSSL
LDAITLAMYGKVERAVNGTQGIMNHAEDSLSVAFTFELMSAEGTRRFRVERRFKRNNE
VSVSNTISKFIETVNGEEQVLADKLADVNRCVEDVIGLKMDDFTRAVVLPQGKFAEFL
SLKGSERRQMLQRLFHLEKYGDQLAIKLSRRVKDNDMVYQSLAAEQQGLGNASKETLE
ETKRLLQTAVQQSELSRKALDEAAKEAEQLGKIRELSNERQARTDEQQKLKALEPQIE
AGEQRLKQSVVADAILPALQTVRDATATQKQRQDIAESAHQQALEHERLAVQEAEKAE
TARKQMTEEEPKLLLRLEQLEQAKELQRERDGLQADCSRLAQLLEQGQGEQTALGQQL
EKEKELQQRGRKRREELQESLKPNEVKSEERRQLQAALELKHRVDTAAEQLQQNKADM
QAYKQSAEQGADKLKAAAEEEKRLSDQRQHLVEQASAGLEALLACEQDISREQSLLAL
AEEQLRGTMREQELHRLSSALRAELHDGEPCPVCGSPHHPLPAAPPGEGPHAGDADLE
LLRSLHAELQELRFGLRQQLHERRSLLTQLGAAAGEAPAAAEAAPAAAEPEPAGSPEH
GRSPAGWASRAAALREAAQALAAAAAPLQAEAAALQQATVGAQQRRMEAAAAQEAGRA
GLAQAERKLAESEAAAAALQGRWATELSGIAQEEAKALYQAMQERDARAEDIKERLNK
SVTFLEEKDQIIQSLQQKLVELDKQLIQWQTQWQGNSKQLTEKEERLRQWVGEQRVED
LISAAQARLGGMRKTASDSAQRFKEVDTLKQESAKKDVMAQQAAASATEHLQQAQERW
KQLLEKSPFDSDDAVVSAAIPSQEAERLAGEIQQHRERERELASQLRELEQKLGGAVV
SEEQWLACTERLQQVRIEDEAALRAKARAERDLEDVEQRHVRWTELENKRVEVSRQGE
LLSKLQSAFRGNAFVEYIAEEQLMQVSHAASQRLRFLTKQRYSLEVDSGGGFVICDDA

NGGVKRPVSTLSGGETFLTSLSLALALSAQIQLRGQYPLQFFFLDEGFGTLDPELLDT

VITSLEKLHDDRLAVGVISHVPELRARLPRKLVVIPAGEAGNGSRVVLETL"

CDS 1031356..1031700

/locus_tag="EFAGFIKM_00885"

/inference="ab initio prediction:Prodigal:002006"

/codon_start=1

/transl_table=11

/product="hypothetical protein"

/translation="MEKVEAKKICKEHMHRYVCVQMNDGMHYDGIMENVDDEMIYLAV

PVGPEAMVNHANWAPNAMPGANYPIGHTRGFYPGYGYPTPYPYYGYGRRRFNRLVLPL

FALTALTLLPYY"

CDS complement(1032404..1033537)

/gene="mccB"

/locus_tag="EFAGFIKM_00886"

/EC_number="4.4.1.1"

/inference="ab initio prediction:Prodigal:002006"

/inference="similar to AA sequence:UniProtKB:O05394"

/codon_start=1

/transl_table=11

/product="Cystathionine gamma-lyase"

/db_xref="COG:COG0626"

/translation="MRPKTKLIHAGIVGDPHTGAVSVPIYQVSTYEQESVGVHKGYEY

SRTGNPTRHALEEVIKELEDGVRGFAFSSGMAAIHAVLSLLKTGDHVILTDDVYGGTY

RIFTKVLNRLGIESTFVDTTSLQALEKALQSNTKAIYVETPTNPLLKVTDIAAVAIWS

KQHELLFIVDNTFSTPYWQTPLALGADIVLHSATKYIGGHSDVVAGLAVVNGEQLGED

LHFIQNAIGAVLGPMDSWLLMRGLKTLGLRMEAQERNTEQLVIFLNQHPAVSKVYYPG

LPDHPQHKLASTQARGYGGMVSFDVGSAEKVDDMLSKVRYFTLAESLGAVESLISVPA

RMTHASIPYERRQELGITDGLIRISVGIEDVEDLLEDLKSALS"

CDS complement(1033539..1034462)

/gene="mccA"

/locus_tag="EFAGFIKM_00887"

/EC_number="2.5.1.134"

/inference="ab initio prediction:Prodigal:002006"

/inference="similar to AA sequence:UniProtKB:O05393"

/codon_start=1

/transl_table=11

/product="O-acetylserine dependent cystathionine

beta-synthase"

/db_xref="COG:COG0031"

/translation="MTVYQHVQELIGNTPLLELTRYPLPEGIRLFAKLEFMNPGGSVK

DRIGKFLLEQALARGDVKPGGTVIEATAGNTGIGLAIAAVGLNLKVIFTVPQKFSVEK

QQLMKALGATVVNTPTSEGITGAIRKAESLAKEIPGSYIPGQFSNADNPLAHYEHLGP

EIWRDLNGQVDVYVAGAGSGGTFMGVSRYLKEQNPLIKTCIVEPEGSILAGGPSGPHR

TEGIGVETLSSFMDVSYFDAIHTISDEDAYERVKDLALLEGLLVGSSSGAAMQAALNE

AVQAAPGSNIVVIFPDSSERYLSQNIYNGGQ"

CDS 1034710..1035069

/locus_tag="EFAGFIKM_00888"

/EC_number="3.9.1.-"

/inference="ab initio prediction:Prodigal:002006"

/inference="similar to AA sequence:UniProtKB:P44956"

/codon_start=1

/transl_table=11

/product="Purine nucleoside phosphoramidase"

/db_xref="COG:COG0537"

/translation="MDCLFCKIVEGSIPSNKVLENDHVIVFHDIQPAAPTHVLVIPKK

HIASMNEVTAEDLPLIGEIHLAAQEAAKRLGVEETGYRLINNCGKDGEQTVHHLHYHL

LGGTRLGVLTSLSDSHK"

CDS 1035303..1035476

/gene="rpsU"

/locus_tag="EFAGFIKM_00889"

/inference="ab initio prediction:Prodigal:002006"

/inference="similar to AA sequence:UniProtKB:A2RHW9"

/codon_start=1

/transl_table=11

/product="30S ribosomal protein S21"

/db_xref="COG:COG0828"

/translation="MSETKVRKNETIDAALRRFKRSIAKDGVLAEVKKRKHYEKPSVK

RKKKSEAARKRKF"

CDS 1035492..1035935

/gene="yqeY"

/locus_tag="EFAGFIKM_00890"

/inference="ab initio prediction:Prodigal:002006"

/inference="similar to AA sequence:UniProtKB:P54464"

/codon_start=1

/transl_table=11

/product="putative protein YqeY"

/db_xref="COG:COG1610"

/translation="MNLSERLNEDMKQAMKSKDKFRLSNIRLIRSTIKNLEIDLKRDL

DDNEVLDILSREIKQRKDALQEFDKAGREDLAANAKAEIEVLIQYLPTQLTEEEIKVI

VQQTIQETGASSKAEMGKVMAALMPKVKGKADGKLVNQAVQQFLQ"

CDS 1036179..1037540

/locus_tag="EFAGFIKM_00891"

/inference="ab initio prediction:Prodigal:002006"

/codon_start=1

/transl_table=11

/product="hypothetical protein"

/translation="MKGKRRKKLTGPLMLLMLLTLLLPVWIGSPSAHAAEKSGAVYII

PVDKPIEQGLGKFMERGFKQAEEMNAGLIVLDINTPGGRVDTAEALGTLIKDSPIETV

AFVRGDAASAGSFLALNADKIVMSPGSMIGAAAMVDSTGKHVDDPKLVAFWKSKMQGA

AEISGRDGKIAAGMTDVNIVVEMPEINKTKQKGEIIALSAEEALKVGYADHISNTPEE

AAAWLGYSQDDVFKVERTTAENISSFLTNPVVMTVLLFLGIAGVIIELIVPGFGVPGI

VGIVCFVLYFSGNYIAGFAGAETWVLFTVGLIMIILEMFIPSFGILGILGSIALVAGV

VRAAYDTSDAFVSLGIAFGAALVVIAIISIIFKDRGIWNRFILSDSMSADRGYSSATE

RKELVGLQGISLTPLRPSGTAMFEGERIDVVTDGDFIPIDTPIIVIKAEGTRIVVQQA

LPV"

CDS 1037702..1038712

/locus_tag="EFAGFIKM_00892"

/inference="ab initio prediction:Prodigal:002006"

/inference="similar to AA sequence:UniProtKB:Q7A5C5"

/note="UPF0365 protein SA1402"

/codon_start=1

/transl_table=11

/product="hypothetical protein"

/translation="MDTSMITILLIAVVGIIVLSVFFSFFPVMLWVSAIASGVRVSII

TLVAMRLRRVTPSRIVNPLIKATKAGLKLTMNQLESHFLAGGNVDRVVNALIAAQRAN

IPLEFERAAAIDLAGRDVLQAVQMSVNPRVIETPIVSAVAKDGIEVKVRARVTVRANI

DRLVGGAGEETIIARVGEGIVSTNGSSNSHKDVLENPDLISRTVLSKGLDAGTAFEIL

SIDIADVDVGKNIGAFLQTEQAEADKRIAQAKAEERRAMAVAQEQEMKARVVEMRARV

VESESQVPLAMAEALRNGKIGVMDYMNLKNIEADTQMRNTLGKPGEGSNSNDQGDSKN

GR"

CDS 1038726..1039325

/locus_tag="EFAGFIKM_00893"

/inference="ab initio prediction:Prodigal:002006"

/codon_start=1

/transl_table=11

/product="hypothetical protein"

/translation="MGLFEWIFDNLYIVAVIGFALFSFLGKAAKSADPNKKRPANGMP

TFGGSGDSGRDERSRDNSSPQQSSGPTDPRYGEQYDDRYDDVQYENQYDEPYSEPAAS

SRPATDYRSEGTMSAMANSLEEQMRGMEEQQRLIEERLNRISSANRPVVSDSSWDETS

TDGSGASPLRPADIRTGVLWAEVLGSPRSKQPFGTRGKL"

CDS 1039505..1039798

/locus_tag="EFAGFIKM_00894"

/inference="ab initio prediction:Prodigal:002006"

/codon_start=1

/transl_table=11

/product="hypothetical protein"

/translation="MTRISRKLRRWTSEVLDLPQDVLYDMPRLTLIGSKQLYIENHRG

VIHFTPDRIVLALSQGQLEIKGTALVIRNILPDEVAVEGTILDIHMNGVEGNG"

CDS 1039795..1040988

/locus_tag="EFAGFIKM_00895"

/inference="ab initio prediction:Prodigal:002006"

/codon_start=1

/transl_table=11

/product="hypothetical protein"

/translation="MKQPSLYKLRGAVRITVTGGDIETLINTVAEQGLEVWDLRAHDG
RVAEMNILLPHFFRLRPVLKRTGCRVKVTHRSGFPFFAARLLRRKFFLGGMLFFVAAL
FALSSMVWSVEVKGNVTIPTDEVLAAAKKEGIYPLQWGFRLQSQDKLSRQLALALPDV
TWIGVSKEGTTITIQVVESAQPKREPLLNPRHLISKSDAVVTQIYAEQGRPVVQKDMR
VKKGQVLISGILGDEENTKTIVAKGEVRGLVWREYQVEAPLVQKHNTMTGESKERFYM
VLGNWAIQLWGYGSTPFTSFDTESNHKPLTWRSFTLPMGWLTEKDLETQEHEQQQTIE
WARTKGLEGARNDIIAKNGKGTKIISEKILHEKKENGKVYMKVLFEVEESIAEELPLV
HSQGE"

CDS 1040995..1041966

/locus_tag="EFAGFIKM_00896"

/inference="ab initio prediction:Prodigal:002006"

/inference="similar to AA sequence:UniProtKB:P9WIA3"

/codon_start=1

/transl_table=11

/product="PhoH-like protein"

/db_xref="COG:COG1702"

/translation="MSEQTRSIQISLQSAGEGQSLFGPQDTFLKLIESEIPAQIASRE
AEIVIFGNAQQVESLEQLFDVLLQLIRNGYVLTERDVKYAIELAKDMRADQLLDLFKG
EITTTYRGKPIRVKTIGQKHYVTTIKKRDIVFGIGPAGTGKTYLAVVLAVAAIKEGSV
KRIVLTRPAVEAGESLGFLPGDLQEKVDPYLRPLYDALYDVMGQEQTAKALERGLIEI
APLAYMRGRTLDDSFIILDEAQNTTPEQMKMFLTRLGFGSKMVITGDVTQIDLPRGKK
SGLVEANTILNEVNEIGFVYFAEQDVVRHSLVQKIIVAYNHAAENQA"

CDS 1041983..1044232

/gene="pgpH"

/locus_tag="EFAGFIKM_00897"

/EC_number="3.1.4.59"

/inference="ab initio prediction:Prodigal:002006"

/inference="similar to AA sequence:UniProtKB:A0A0H3GGY3"

/codon_start=1

/transl_table=11

/product="Cyclic-di-AMP phosphodiesterase PgpH"

/translation="MTSKEPSKGKSFQNKATGWKYSVWARYLLFLFLVILFYVSLASK

LLPERYDIQEGTRSEVDIAAPMQIPNNKATLKAQEEAAERVQPIFQIVQLRNENLMTT

LLDRVDRLNQDDQISSQDKIDIYRDEIPQRQKDFVTNYINNNRKAGTYSETLLEEIRN

VVQEQSYRIPEETYIKISRLTSDDIQEMKPVARDIVARLMTDQISDATTARAKVAEMV

SVSSLGKRTQREVVQELARLVVTANRFYDEEGTKEAKVQARENTQTVFIKQGDTLVAK

GEMITPEMYTLLDENDLLKNEVNYWPQLGLLMFSCLLSAAILMYIQQGSGTHFKYNNA

QLLMLVLIFIITIVVMHVTAIIQTNERSYVGFLAPVAVGAMLIALLLDTSLAFVCSIL

IGMLSSIILNTHQGQLFDFELGFFAVLVSFVAIFATHKASQRSTILKGAIMVCLFGSI

AVFTLALIDSGDWNRTTTLYGVGFAFAGGVLTAILVIGLMPFFETSFGILSALKLVEL

SNPNHPLLRKLLTETPGTYHHSVMVGNLSEAAAEAIGANGLLCRVGSYYHDIGKTKRP

IYFIENQNNMENPHDSIDPKLSKSIIVAHARDGVEMQKDYKLPRPIRDIAEQHHGTTF

LHYFYHKALRQAEEAGVEPDFTEEDFRYPGPKAQSKESAIVGIADSVEAAVRSLRKPT

VEQVESMIEKIIKGRLDDHQFNDCDLTMRELDIVARTLKETVMGIFHSRIEYPEEIKK

PKPTSPEAG"

CDS 1044267..1044764

/gene="ybeY"

/locus_tag="EFAGFIKM_00898"

/EC_number="3.1.-.-"

/inference="ab initio prediction:Prodigal:002006"

/inference="protein motif:HAMAP:MF_00009"

/codon_start=1

/transl_table=11

/product="Endoribonuclease YbeY"

/translation="MSLNLAWNNEQQDKEITEPMIAMLEQLLNLAGEAEGVADGEVAL

TFVNDEQIHELNRDYRGIDRPTDVLSFAMNETVDEELDIIYELDEDEEMEEMPDVLGD

IIISVPRTILQSEEYGHSFERELGFLFVHGFLHLLGYDHQDEASEAEMMGKQEAVLAQ

AGLTR"

CDS 1044764..1045135

/gene="dgkA"

/locus_tag="EFAGFIKM_00899"

/EC_number="2.7.1.66"

/inference="ab initio prediction:Prodigal:002006"

/inference="similar to AA sequence:UniProtKB:Q05888"

/codon_start=1

/transl_table=11

/product="Undecaprenol kinase"

/db_xref="COG:COG0818"

/translation="MKRRSWGLVFRNAAEGIAYGLRTQRNVRVHTGVAILMCAAGFFF

RISRTDWMFVLTAVFLVLVTELMNTAVEAAVDLAHPHIHPLAKAAKDTAAGAVLLAAV

FAVIIGCIVFIKPVMSWLGLY"

CDS 1045437..1045850

/gene="cdd"

/locus_tag="EFAGFIKM_00900"

/EC_number="3.5.4.5"

/inference="ab initio prediction:Prodigal:002006"

/inference="similar to AA sequence:UniProtKB:P19079"

/codon_start=1

/transl_table=11

/product="Cytidine deaminase"

/db_xref="COG:COG0295"

/translation="MDNGLLMQEAIKARTKAYTPYSHFGVGAALLDSEGHVHHGCNIE

NAAYTPGNCAERTAMFSAIAGGQKPRSFKAIAIVGDTDGPIAPCGVCRQVMYELCEPD

MKVILGNLKGDLQETTVAELLPWAFGPSDLNSAKK"

CDS 1045888..1046787

/gene="era"

/locus_tag="EFAGFIKM_00901"

/inference="ab initio prediction:Prodigal:002006"

/inference="similar to AA sequence:UniProtKB:P42182"

/codon_start=1

/transl_table=11

/product="GTPase Era"

/db_xref="COG:COG1159"

/translation="MKKQKFKSGFVAIIGRPNVGKSTLMNQVIGQKIAIMSDKPQTTR

NKIHGVYTSDNQQIVFLDTPGIHKRQSKLGDYMNQTALNTLSEVEAALFLIDASEGMG

GGDRYIAEQLKNIRTPVILVMNKIDKIEPEALLPLIEEYRKLHDFAEIVPVSAKHGNN

VSTLLEQLGKYLPEGPQYYPDDQVTDHPEQFVCAELIREKILQMTREEVPHSIAVTIE

DMKVQDNGVVYISAVIFVERDSQKGIIIGKQGALLKEVGKRARHDIQNLLGSKIFMDL

WVKVKKDWRNQDRVLRDLGFGRD"

CDS 1046892..1047032

/locus_tag="EFAGFIKM_00902"

/inference="ab initio prediction:Prodigal:002006"

/codon_start=1

/transl_table=11

/product="hypothetical protein"

/translation="MRDFSWKVFAMTGDVESYLLYTEACNSLGQETDNAREVIEDEEA

EG"

CDS 1047066..1047818

/gene="recO"

/locus_tag="EFAGFIKM_00903"

/inference="ab initio prediction:Prodigal:002006"

/inference="similar to AA sequence:UniProtKB:P42095"

/codon_start=1

/transl_table=11

/product="DNA repair protein RecO"

/db_xref="COG:COG1381"

/translation="MLYRVEGIVIRSMDYGEGNKIITLCTESGGKVGVLVRGAKKPKS

RHAALVQPFTYGQYVYFRNTGLGTLNAGEIVESYHELREDLVKASYASYACELLDRVL

QDEETGTFWFKQLKACLQALKEEKDPVVITSLYEMKILQASGYGPQFDECISCNQERP

DEQLFISPRLGGVLCRACKHFDPPAMSVSPKALKLLRLFAQLDLQRLGNISVSESTRD

EIKKLMRAFMDHQLGLNLKSRSFLDQMEKYGI"

CDS 1048229..1049116

/gene="glyQ"

/locus_tag="EFAGFIKM_00904"

/EC_number="6.1.1.14"

/inference="ab initio prediction:Prodigal:002006"

/inference="similar to AA sequence:UniProtKB:O67081"

/codon_start=1

/transl_table=11

/product="Glycine--tRNA ligase alpha subunit"

/db_xref="COG:COG0752"

/translation="MNFQQMILTLQQFWAEHNCIIVQPYDTEKGAGTMNPMTFLRSLG

PEPWKVAYVEPSRRPSDGRYGENPNRLYQHHQFQVIIKPSPDNIQEIYLESLKRLGID

PLRHDIRFVEDNWENPSLGCAGLGWEVWLDGMEITQFTYFQQVGGIETNPVAVEITYG

MERLASYIQDKENVFDLEWVEGITYGDVFRQPEFEHSKYTFEVSDVKMLFTLFNMHEE

EANKAMAQHLVFPAYDYVLKCSHTFNLLDARGAISVTERTGYITRVRNLARQVAATYM

EEREKLGFPLIKKGGAEHV"

CDS 1049109..1051184

/gene="glyS"

/locus_tag="EFAGFIKM_00905"

/EC_number="6.1.1.14"

/inference="ab initio prediction:Prodigal:002006"

/inference="similar to AA sequence:UniProtKB:P00961"

/codon_start=1

/transl_table=11

/product="Glycine--tRNA ligase beta subunit"

/db_xref="COG:COG0751"

/translation="MSKDLLFEIGLEEVPARFMRAAIAQLQERVVKWLDASRIAYGEV

NAYATPRRLAVLIQNVAEKQEDIEEEVKGPSRKIALDDSGNWSKAALGFARSQGVEPD

QFTFKELNGVEYIYATKSSKGVETASVVSEGLLSVLHAMTFPKFMRWASYDFKFVRPI

RWIVAMLGSEVIDLEVTGIKSGNVTRGHRFLGKEAVISTPSSYVEVLRSEHVIADIQE

REQMIVSQIQALAAEKKWDIAIKEDLLKEVLFLVETPNVLFGTFDSSFLNIPQEVLIT

SMREHQRYFPVLDNEGQLLPFFVTVRNGGSDSLDVIARGNEKVLRARLSDAKFFYEED

QKLQIKDALSKLESIVFQEELGTVGDKVRRIRKIADGLAAKLQVSGDVAESVSRSADI

CKFDLVTLMVGEFPELQGVMGEDYARKAGEKEEVAKAVFEHYQPRFAGDQSPASLVGA
IVSAADKMDTIVGCFSINIIPTGSQDPYALRRQAAGIVQILLDHKLPLTLSDVFGVAL
QVHAQMNLLKRADEEVRKDLQDFFGLRVKKLLSETVRYDVVDAVISSGFDDISAVVPK
GEALMAAVLTGDAFKTTVESFNRVGNLAAKASNASVHPELFTEDGERQLHEAWSKTNA
EYRQALTQHHAAEALAIASAWKDGITSFFDSVMVMAEDEAVRANRLALLAAIDRDLKG
FADFSKLVW"

CDS 1051414..1051881

/locus_tag="EFAGFIKM_00906"

/inference="ab initio prediction:Prodigal:002006"

/inference="similar to AA sequence:UniProtKB:P0A8D3"

/note="UPF0178 protein YaiI"

/codon_start=1

/transl_table=11

/product="hypothetical protein"

/translation="MSELNIRHIVVDGDACPVKTEIAQTARLFNIPVLLVSSFDHLLQ
GGEGVRTVQVDRSDQSADLYIANHIKPYDVVITQDYGLAALALGKRCYVLSFRGREFN
DRDIDFMLDSRHTAAKARKRGHYGKGPKPFTEQDREMFQHKLTKLLKDLQENV"

CDS 1051929..1053749

/gene="dnaG"

/locus_tag="EFAGFIKM_00907"

/EC_number="2.7.7.-"

/inference="ab initio prediction:Prodigal:002006"

/inference="similar to AA sequence:UniProtKB:P05096"

/codon_start=1

/transl_table=11

/product="DNA primase"

/db_xref="COG:COG0358"

/translation="MSTGQGGIPESIIESVLQQNDIVDTVSRFVHLTKQGKYMKGLCP
FHSEKTPSFTVTPEKQIFYCYGCGTGGNAIKFRMEIEGLSFPEAVKTMAEESHISMGD
WQGRESAHVNPETERLLEAYELTAKLYHFLLKNTEHGKSAMEYLRSRGFGDKLIDQFQ
IGFAPNRWDTLVQFLEKRNYPLEEMEKGGLLSPRNEGQGYVDRFRDRIMFPINGRSGK
PIAFAGRILGDGQPKYLNSPETRLFNKSRVLYNLHHAKNAIRKQRQAILFEGYGDVIS

AWDQDIQNGVAAMGTALTENQALMLKGMCDEVIICYDGDRAGQAAALKNFPILEEAGL

QVKVALIPEGLDPDDFIRKHGGERFRNQIVDGAVTTTKFKLINLKKSHILLEGGGQIA

YSKEAVKLIAPLPSPTEREVYLRELAAEVDVSFETLKQECNEEREAMKNNLQFGDNNP

KRWNNGRQQNRQVPTPNLLPAYHAAERKLIAWMLQDDEAAQYVNEHLGEAFNLDDHAA

IAAYLYAYYAQGKPSDTSRFMSSLHDDRLEKTVSSISMMDGPGEWSIQMLDDCIREVL

KHPRKKEYDLKKEEMIAAERAGDSVRAAQIAIEMIALERQ"

CDS 1053793..1054932

/gene="sigA"

/locus_tag="EFAGFIKM_00908"

/inference="ab initio prediction:Prodigal:002006"

/inference="similar to AA sequence:UniProtKB:P06224"

/codon_start=1

/transl_table=11

/product="RNA polymerase sigma factor SigA"

/db_xref="COG:COG0568"

/translation="MANDQHTELETELTLDQVKDQLIESGKKRASLNYKEIIEKLSPF

EQDAEQMDEFYEQLSDLGIDVVNENNEEVTLRPSEDSENNTREGEDEFHFDDDLSLPP

GIKINDPVRMYLKEIGRVPLLSADDEVQLAKRIENGDEEAKRRLAEANLRLVVSIAKR

YVGRGMLFLDLIQEGNMGLIKAVEKFDHKKGYKFSTYATWWIRQAITRAIADQARTIR

IPVHMVETINKLIRVSRQLLQELGREPTPEEIAAEMDLSVEKVREITKIAQEPVSLET

PIGEEDDSHLGDFIEDQEALAPADAAAYELLKEQLEDVLDTLTEREENVLRLRFGLDD

GRTRTLEEVGKVFGVTRERIRQIEAKALRKLRHPSRSKRLKDFLE"

CDS 1055117..1055875

/gene="trmK"

/locus_tag="EFAGFIKM_00909"

/EC_number="2.1.1.217"

/inference="ab initio prediction:Prodigal:002006"

/inference="similar to AA sequence:UniProtKB:P54471"

/codon_start=1

/transl_table=11

/product="tRNA (adenine(22)-N(1))-methyltransferase"

/db_xref="COG:COG2384"

/translation="MKLSNRLQRIHDQIPDGSCMADIGSDHALLPVAAIRSGKAASAV

AGEVNPGPYDAACKQVSDAGLKEKITVRRGDGLDVISAGEVDVITIAGMGGALIASIL

DRGLSKLEGVQLLILQPNVGEDILRRWLLEHHWVVVAEQLLEEDGKIYEIITAMPQSV

SPIANVEVYRARPLQGGAELTEDLLLRMGPYLVDRPTDVFFAKWESEIVKLQGVVNSI

SKSDQDSSRDKAAEVERLIANLKEVLACLPKVKL"

CDS 1055851..1056966

/locus_tag="EFAGFIKM_00910"

/inference="ab initio prediction:Prodigal:002006"

/inference="similar to AA sequence:UniProtKB:P67272"

/codon_start=1

/transl_table=11

/product="GTP cyclohydrolase 1 type 2"

/db_xref="COG:COG0327"

/translation="MFAKGQTVIQLMEQLAPKHLAVPDDRIGLQLGSLQKEITHVLVA

LDVTDEVVDEAIRIGANLIIAHHAIIFRPVKSLSTDTPMGKLYEKLIKHDIAVYISHT

NLDVAEGGMNDWMAEALGIESKESLEDVHTDHLYKLVVFVPRTHHEQVLQAILEAGAG

SIGQYNKCSFNTEGTGTFVPGEGTQPFIGTQGQMERVEEIRIETIVPQSLRSKVVQAM

LKAHPYEEVAYDLYAMDLKGRTLGLGRLGPLREPKTLGELVEVVKTQFNVPHVRVVGD

LNKQIKKAAVLGGSGSRYALTARFKGADVIVTGDIDYHTAHDALMAGMCIIDPGHNSE

KIMKPKTADWLRSRLEEKRYDTQVTASEVNTEVFQFI"

CDS complement(1057714..1058943)

/gene="lysN_1"

/locus_tag="EFAGFIKM_00911"

/EC_number="2.6.1.39"

/inference="ab initio prediction:Prodigal:002006"

/inference="similar to AA sequence:UniProtKB:Q72LL6"

/codon_start=1

/transl_table=11

/product="2-aminoadipate transaminase"

/db_xref="COG:COG1167"

/translation="MSIPWSKMAQNTPSSVVRDMLQAAQAPGMISLAGGLPAQTSFPL

EAIRVAYEKVFMSGAAALQYAETEGYRPLRAKIAERLESKGIPASPDHMLLTTGSQQS

IDLVCRILLDPGDRVLVESPTYLAALQVIHSYQAESHGVACDDHGMLPESLEEQLQLH
RPKLVYINPTFSNPTGKVWSRERRQQAVDLCRKYGVLILEDDPYGEIRFNPEQLDVPA
LAELDAVSYDGPSNVIYTSTFSKTVAPGLRTGWILAAPDIVKMAARAKQGADLHSSSI
DQRALHALLECFDLDAHIRHISEDYEQRMKTLTTLMAAKAWEGISWNSPQGGMFLWLQ
LPEGMLASNLFTYGIQEKVCIVPGDSFYAGTPELNRMRINFTHTDPELLPEAVERMDR
AIQRWHASLTSDSVVTL"

CDS 1059051..1060571

/gene="dapL_2"

/locus_tag="EFAGFIKM_00912"

/EC_number="2.6.1.83"

/inference="ab initio prediction:Prodigal:002006"

/inference="protein motif:HAMAP:MF_01642"

/codon_start=1

/transl_table=11

/product="LL-diaminopimelate aminotransferase"

/translation="MHIELKRGSSTKLYVQIALTIADRIRSGLIEPGTRLPSVRKMTA
DLGVSLVTVSKAYAELEAIQLITCSQGKGCYVRGTLNVDQMEDVDRTQRNNANGESST
SWNWQMALVDYLPRAQLWRHFDTSPQVRYELHMSAIQPELLPTREIIDSAYRLSSDHT
ERMAAYGSFQGDRELRQIFAEHFAARGLQAAPERMLITSGAQQGIDLVARTFVGPGDV
VYMEAPSYTGAIDVFTSRGAKIITVPMDDEGMRIDLLTRLCDTYPPKLIYTIPTYHNP
TGITMSARRRAQLLNLAQSYHCLILEDDPFADLYFREPPPASIKSMDGTGHVVYIKSF
SKVLSPGCRIACAIADGSVLTRLVAAKSTADLGSPLLTQKALQSFIQNQYGAYVSRLR
DELYSRLCAASEVLEEHATMGMQWRLPEGGLNLWLQLPSNLDMRELHHQSLAAGVSFL
PGSACYVGEMDTPSLRICFTVTSVELLCEGLRVLCGVIGKVTPQEGGTVADRLPLI"

CDS 1060658..1060915

/locus_tag="EFAGFIKM_00913"

/inference="ab initio prediction:Prodigal:002006"

/codon_start=1

/transl_table=11

/product="hypothetical protein"

/translation="MNHYCPPLCPIVCDPIQVVEDYYIPQIVPVIHPIEVIKKHHCVP
IHHHMYPVVVKEEDPCYVSSHNPKKKSVKKSSKARTTSRKR"

CDS complement(1061018..1062892)

/locus_tag="EFAGFIKM_00914"

/inference="ab initio prediction:Prodigal:002006"

/codon_start=1

/transl_table=11

/product="hypothetical protein"

/translation="MSRPKWINWALAAGAGALALTLLLPTSNRQEPKPSALSDSAHEE

HTSKQRLKVQDVKATDLLTRMDAKQHLSIMLEKTATMNAAQVSRYVNDLQSSHEHIKS

IHLMNTNGSFNKPFDHASTQGSKLEQQKLQHSLNLAKKAVNKRQSFESSSFPLGKEKY

FVMGQPSKDGKRAVIALFSQNVLNAVEQHQRKNLRMIPYPREGKFKIESVHPDTLNEI

TVKTGHDNANASHFYENEIVIRFRQDPGERDMRIIKSDLRAQSARKLGYTYVFRSEHM

SYKQLHSYFESKWNPLYMEPHYMYLTNDAVTEQTDVTIPNDILFSDYQWNLPAIETNR

GWNITKGNKDVIVAVVDTGVDMNHPDLKGKLLEGYNVVEPGSQPMDDVGHGTHVAGII

GATVNNNEGVAGMSWYNKVLPVKVLDNSGSGTTYAVAEGIIWAADHGAKVINMSLGNY

ADAQFLHDAIKYAFDRDIVLIAATGNDNTERPGYPAAYPEVFAVSATDPDMSKASYSN

YGDYVDVMAPGSSIASTYPNNQYAALSGTSMASPHVAALAGLIRSLNPDLTNTEVMDL

MRQSVIDLGDPGHDKYFGYGQIDVYKALQAASGNSAPLQFWPQHVRQQMDNTMKKYTQ

"

CDS 1063176..1063682

/locus_tag="EFAGFIKM_00915"

/inference="ab initio prediction:Prodigal:002006"

/codon_start=1

/transl_table=11

/product="hypothetical protein"

/translation="MSAANVQKTCESTREKLKPAIDRIEKFLNENALPELDQNQTEES

TAFYKGFLSDLRHLLVFSEVSYEKLGVVLRRANFDVDFAEKALYNTYHQCVNSFFYPK

NECYSEDGRYAYTGQDAIRFRDKPIRAVRDVILEVSKTYEELRDDLAYYESDYLTQRR

MQNQRNHA"

CDS complement(1063779..1064018)

/locus_tag="EFAGFIKM_00916"

/inference="ab initio prediction:Prodigal:002006"

/codon_start=1

/transl_table=11

/product="hypothetical protein"

/translation="MIMVIGVKCLTGKFLMQTTGGMLVDVNHDGISVAEVVLSPKVAV

GDAAFDNWFVLAHFLSPRLLLSPRHKPDIPRQNAR"

CDS 1064102..1064515

/locus_tag="EFAGFIKM_00917"

/inference="ab initio prediction:Prodigal:002006"

/codon_start=1

/transl_table=11

/product="hypothetical protein"

/translation="MKNDDNKFNFQDRPKINVDGEKQRMRVDYPRKAHREEYGAEVAP

PTVVRTGGSERAEPVPEKHEADRVMESTGKVAGYMGLAFGIASLFMWSIVLGPAAAVL

GYYAYVNGRKTAGAWSIGLGVVATLSYFFMIPFAR"

CDS 1064705..1065457

/gene="mltC_1"

/locus_tag="EFAGFIKM_00918"

/EC_number="4.2.2.-"

/inference="ab initio prediction:Prodigal:002006"

/inference="protein motif:HAMAP:MF_01616"

/codon_start=1

/transl_table=11

/product="Membrane-bound lytic murein transglycosylase C"

/translation="MQIDPNGSRQLLELQLSNVNNQTSGSQSDTGSTVDFANVMDGLL

GMGANSTSNTDSTATVSKRSSDGLLWLQLGSTYNPDINTAGSSSVSNNIVESLLSSSN

TGIVDSGASVPTDYESLIAEASAKYGVPESLIKAVIDTESNFNPNVVSSAGAKGLMQL

MDGTAAGLGVSNSFDPAQNIDAGTKYLSLQLQRFGGEVKMALAAYNAGPGRVSRLGVS

SDSELMSVLNRLPSETQNYISKVEKAQSKYVI"

CDS 1065508..1065600

/locus_tag="EFAGFIKM_00919"

/inference="ab initio prediction:Prodigal:002006"

/codon_start=1

/transl_table=11

/product="hypothetical protein"

/translation="MWEQKRLKRLFPVGLFVLYGYRVTMLTVDS"

CDS 1065631..1066779

/gene="iscS_2"

/locus_tag="EFAGFIKM_00920"

/EC_number="2.8.1.7"

/inference="ab initio prediction:Prodigal:002006"

/inference="similar to AA sequence:UniProtKB:P0A6B7"

/codon_start=1

/transl_table=11

/product="Cysteine desulfurase IscS"

/db_xref="COG:COG1104"

/translation="MKYFDYAATTPPHPDVIRTMAEIMETQFGNPSSIHGYGERAHQL

LRRARSGCAAAIGVKPEEIVFTSGATESNNLAIKGAALRYQSRGRHIITTATEHASVY

ESFMQLQQWGWEVTLVPVNSDGVVDTQQVVDAIRPDTVLVSLMHVNNETGAIHPIAEI

GKQLKKKAPRVLFHVDGVQGFGKMKATPAAWGADLYSLSAHKIRGPKGAGLLYVRSGV

ELTPLLSGGSQEQGLRAGTENVALLVGMAKAMRMAAEDQVDFARRTTVLRDRLMETIR

VIPEFELNSSPEGAPHIVHFSYPGMKAEVALHTLEQLGVTISTQSACSSRSAEPSRAL

LAMGRDAACAGGGLRISLGDEHTEEDVALLEQALHQMVAQLRPLERRM"

CDS 1066779..1068026

/gene="thiI"

/locus_tag="EFAGFIKM_00921"

/EC_number="2.8.1.4"

/inference="ab initio prediction:Prodigal:002006"

/inference="similar to AA sequence:UniProtKB:Q81KU0"

/codon_start=1

/transl_table=11

/product="putative tRNA sulfurtransferase"

/db_xref="COG:COG0301"

/translation="MDMNYDMLLLRFGEFMLKGKNRARFEKTIITQVKALLKPYPGAS

LRKEYGRVYVDLGGESHTELIKVLKRVFGVMSISPVKVTPSELDEIVKTAVAFMDERE

DEFKEGTTFKVNVRRVWKEFPNSSHEMNHLVGSPILRKFQQLSVDVRQPDIELRVEIR

DQGTYIFNETIPAVGGFPLGTNGKAMVLLSGGIDSPVAAWSSMRRGLEVECVHFYSYP

FTSERAKEKVIDLARALADHAGKIKLHLVPFTEIQTAFTQLGQDNLIITLMRRSMLRI

ASKLAERERALALITGDSLGQVASQTLPSMNVIGRATDLPLLRPLVMMDKQEIITLSK

QIGTYDISILPYEDCCTLFVPKSPSTNPNLRIVDKIESTMSHLSEWVDQAVEQTETIT

LHAGETSVVTNPTSDNGMKDDWF"

CDS complement(1068149..1068859)

/locus_tag="EFAGFIKM_00922"

/inference="ab initio prediction:Prodigal:002006"

/codon_start=1

/transl_table=11

/product="hypothetical protein"

/translation="MELFSPTFWLALLNVVFIDLILAGDNAIVIGLAARNLHPSVQKK

AILYGTGGALLIRILATVVVLWLLKVPWLLLVGGILLIWIAYKLLADQGDEHDDVQAG

TSLWTAIRTIVIADAAMGLDNVIAVAGAAQQHLVLVILGLLISVPIIVWGSTLFIKLI

NHFPWIIYVGAIVLGYTASNMITEEQRLLPYFTEHPALRILFIVVVIGGVVFAGYRKR

SSSSSHKGSGGEQQRSYS"

CDS 1068947..1069606

/locus_tag="EFAGFIKM_00923"

/inference="ab initio prediction:Prodigal:002006"

/codon_start=1

/transl_table=11

/product="hypothetical protein"

/translation="MDTLWLLTEILMINLVLSGDNAVVIALASKDLPPVQRKKAVWWG

AFGAVVLRCVLTFVAVLLLGIPFIQAAGGLLLLWIAVKLLLQNDDEVHIREVSTTWKA

IQTILIADFVMSLDNVLAIAALADGDLALIVIGIAISIPIVVWGSGLIVGLLKRFPIL

VFVGSGILAFTAGEMVMKDPKLGEWLGGMASEVHTLLPVAMACLVVAIGGAHKFVRKN

A"

CDS 1069904..1070392

/locus_tag="EFAGFIKM_00924"

/inference="ab initio prediction:Prodigal:002006"

/codon_start=1

/transl_table=11

/product="hypothetical protein"

/translation="MRNEKAIGLFIVAAGIIILLGKFGVFGFIGRNFWPLLILLPGIA

LHALYYARISPSWSLVPAGILTVYGVLFSITNTWGAGLMSRLWPAFLLGIAIGLLEYG

MAERRRPEYVLPAALILGAVSIILFGFTLLQTGIIYVLAVLLILGGVWLLLGRGRQGR

GW"

CDS 1070570..1072414

/gene="typA"

/locus_tag="EFAGFIKM_00925"

/inference="ab initio prediction:Prodigal:002006"

/inference="similar to AA sequence:UniProtKB:P44910"

/codon_start=1

/transl_table=11

/product="GTP-binding protein TypA/BipA"

/db_xref="COG:COG1217"

/translation="MHSREHIRNIAIIAHVDHGKTTLVDKLLQQSGTYRDHETVQERV

MDSNDLERERGITILAKNTAITYKDYLINIVDTPGHADFGGEVERIMKMVDGVLLVVD

AYEGCMPQTKFVLRKALEHNLTPIVVVNKIDRPAARPTEVIDEVLDLFIELGANDQQL

EFPVVYASALNGTSSMDNDPAKQDDNMMAIYNTIVSHIPHPTENVEEPLQFLVTLMDY

NEYLGRIAIGRVNRGVIRQGQSVTVIMRDGKSKTARIEKLFGFQGLKRIETEEAGAGD

IVAIAGIKDINIGETIADPNNPEALPVLKIDEPTLQMTFLVNNSPFAGREGKWVTSRK

LRERLFKELETDVSLRVDETDSPDAFIVSGRGELHLGILIENMRREGYELQVSKPEVI

VREVDGKKMEPVERLLIDIPEESMGSVMESLGARKAEMVNMVNTGSGQVRLEFLIPAR

GLIGYSTNFLTLTRGYGVMNHAFDSYAPVVSGQVGGRHQGVLISTETGTSTFYGMMGV

EDRGTLFLEPGTEIYEGMIVGEHTRDNDIVVNICKEKQLTNVRSSGKDDTVKIKTPII

FSLEQALEYLNDDEYCEITPKSIRLRKKILNKSERERAEKQRKMASKA"

CDS 1072719..1073015

/locus_tag="EFAGFIKM_00926"

/inference="ab initio prediction:Prodigal:002006"

/codon_start=1

/transl_table=11

/product="hypothetical protein"

/translation="MQAWFASHPIVAYIVIFVLITYVVYNKVFRVRQKLPLGKEIVLYI

LMAMGTFMLLIFQIDKLPIIQCLLVAVGLMLLVRVRYFIEGRQKKKAEAAARNS"

CDS 1073106..1074167

/gene="tagU_2"

/locus_tag="EFAGFIKM_00927"

/EC_number="2.7.8.-"

/inference="ab initio prediction:Prodigal:002006"

/inference="protein motif:HAMAP:MF_01140"

/codon_start=1

/transl_table=11

/product="Polyisoprenyl-teichoic acid--peptidoglycan

teichoic acid transferase TagU"

/translation="MNSNSNGLPPRRQAPTQSGASGSKNGKGKQPKKKKRMNAFGRIL

LSLLVIAIVVGGGYAYWVYNQVVDTGIDKPVPPGMSATTKPITMLLLGTDNRPETGTY

LSDVVMVASLNPETKTATIVSLPRDTRIQLDGYKSNKLNSYYPRFKAQEKTTGKNAED

QMKEMMGKYLDVDINYTTVLNFQAFRDAVNAVGGVDVTVDKNMCYRDTADGTDINLVA

GAQHLDGKAALDFVRYRKSNCNPKTDESNDFDRNKRQNQVLNSMLDQMKSLGGITKIS

KVIGAVDKNMTTDVESEQMKNFISTYWNISTSDVHYTPVTGEWRSPYVYINETELANA

KQALQDTLSGKVTASPSGE"

CDS 1074266..1074718

/locus_tag="EFAGFIKM_00928"

/inference="ab initio prediction:Prodigal:002006"

/codon_start=1

/transl_table=11

/product="hypothetical protein"

/translation="MSEAVTQLTESLLQQFKNETFVLLSTVDVESGGPTSTAISWIYA

ENASTFRLAIDHRSRLVNNMITNPLITVTVFGEETVYAVNGRAAVRQDPLLDVPFNMC

CFDITIEAVRNALFYGAQLSSVPQYVKIYDQRAAEKLDEQVFAAMKKA"

CDS complement(1074726..1075421)

/locus_tag="EFAGFIKM_00929"

/inference="ab initio prediction:Prodigal:002006"

/codon_start=1

/transl_table=11

/product="hypothetical protein"

/translation="MKIWVCTLLLIFILTGCNSSTRSASPNQGTHAKGYGGNVTTQQD

QGKGSHMLNAQEDRMNPSRLDRLNENTGEVHDTNIADDAKSGRMPNEKRINHLKALAK

QVEGVEDANCVILGNTAVVGIDVDGELERARVGTIKYAVAEALRKDPEGIDSIVTADA

DVTERIKEIGEHIRQGHPISGFASELADMVGRIIPQLPKDVKVRQNPDEHVNQKQQMQ

QLHSSDKRQQKAQ"

CDS 1075574..1076908

/locus_tag="EFAGFIKM_00930"

/inference="ab initio prediction:Prodigal:002006"

/codon_start=1

/transl_table=11

/product="hypothetical protein"

/translation="MKKIFVLDTNVLLHDPNAIFAFEEHEVIIPAVVLEEIDSKKRNA

DEIGRNARNVSRLLDGLRELGHLHSGVPLANGGNLKVELNHRSFVKVQEMFGEVSNDN

RILAVALNYQIEENEKEVVERQVVLVSKDVLVRIKADVLGLFTQDYLSDRTAGLSELY

PGYTALKVHPSVIDEFYTYRFLPIKPLQLSYSLYPNEFVILKDEMGTNKSALLKVNTE

GTKLEPLFLSNDNVWGISARNAQQRMALELLLNDDIPLVTITGKAGTGKTLLALAAGL

LKVEDDHKYKKLLIARPVVPMGKDIGYLPGEKEEKLRPWMQPIYDNLEFLFDTKKAGD

IDKILMGLGSIQVEALTYIRGRSIPGQFIIIDEAQNLSRHEVKTIVSRVGEGSKIILM

GDPEQIDHPYLDSASNGLTYIVERFKQEGISGHIMLEKGERSKLAQLAADLL"

CDS 1077130..1078401

/locus_tag="EFAGFIKM_00931"

/inference="ab initio prediction:Prodigal:002006"

/codon_start=1

/transl_table=11

/product="hypothetical protein"

/translation="MKRRHQVVLFAVLLLSLTILSPSFDFRGSQLNQERDQEKDAFHG

SSSRNEDAQAPLEIVVAMSDEEFQDFQKIAKEVAASQLVEINLRNEKPDTYKHVLDLE

FSLGENGDVVLLDSEEVQYYAQKGYLYPLNGTTLSKSLGDTVVGLRGMTEWNGYQWGM

PFDFDPYILAAQSSFLKRAGLESLPRNKDQWVKLTQQAIAEGTPLITMNINDTYGTNA

WLNHLEPRMAPDQVKRTQIDSQTGDLQQGIQLMNQIQPLIRLESTNPDFSPTADQNDT

PMVVSLLSQLLSADQASKVGKDPLERISFEPLETVNSRSLVITAGSVEAEEASRWIEG

MTSATTQLKWYQQVKRLPAKQQELDMESEKLVGPYDMTKGQSWFPTTQDAPAATTESH

ALITRFHKKIQQFLKGEITANQYVDSIRTTN"

CDS complement(1078425..1079504)

/locus_tag="EFAGFIKM_00932"

/inference="ab initio prediction:Prodigal:002006"

/codon_start=1

/transl_table=11

/product="hypothetical protein"

/translation="MKRQYGISAFILCIILFLQLPLAHAYGESSPEDTREILQKSLSI

VEIDHEIERITERQKQLAQQYETLSVQLQEQEEQIHIQQDRAGAVVRSYYTGERDSLL

MTVLGARSFKDLFVLYDYYQIIIGRDQAVLDKYQDRYRNLQQTSTQIRQTTTELTEMK

TNLQNQRERVVALQKEVDAQIATSGDAAAIQKLMDELTLYWENIGIYEVKRYFKALAS

AMQNLPDFIQKQNGGISTTGTTYTIRIGQDELNAFLRSQNPIFEDFAFQFNQDKIIAS

GQRDQLQLSIEGHYTVENEPQNSIRFHVDKLVFNQLELPDTTRRMLEKEFDLGFYPQK

ILSFVKATDVSTSEGILEVQLAISF"

CDS complement(1079689..1079925)

/locus_tag="EFAGFIKM_00933"

/inference="ab initio prediction:Prodigal:002006"

/codon_start=1

/transl_table=11

/product="hypothetical protein"

/translation="MARMFRVLGFFTLAIGLMAFAGDLVEMALLFFLQTAFFVILGYL

KFTERTYILLFWGYMIVTFTGFSYWTVFEMGLPL"

CDS 1080241..1080891

/gene="rsfA"

/locus_tag="EFAGFIKM_00934"

/inference="ab initio prediction:Prodigal:002006"

/inference="similar to AA sequence:UniProtKB:P39650"

/codon_start=1

/transl_table=11

/product="Prespore-specific transcriptional regulator

RsfA"

/translation="MTAVRQDAWSTEDDLILAEVTLRHIREGSTQLAAFEEVGEKIGR

TSAACGFRWNSCVRKKYDSAISNAKAQRQKRSYLRKQPAMLGPQVAALSTLDTEEHLY

KSDGISDDSLSMDAVIRFLRQWKGTVQESNRQLKMLEKELREKEDELLELRLENDRLS

KEVNEVQTDYRVVNDDYKALIQIMDRARRLAFLSEEEEELKTRFKMDANGNLERIE"

CDS 1081042..1082001

/gene="panE"

/locus_tag="EFAGFIKM_00935"

/EC_number="1.1.1.169"

/inference="ab initio prediction:Prodigal:002006"

/inference="similar to AA sequence:UniProtKB:P0A9J4"

/codon_start=1

/transl_table=11

/product="2-dehydropantoate 2-reductase"

/db_xref="COG:COG1893"

/translation="MIIDIVGAGALGLLYGGKLLASGNQVRFWTRTTAQADLLSHNGV

TIVEQDEEIHILPDQIQAKPISKLTDTWKNTPGEWLLLMVKQTSIDDFIHEISPLQDH

VLNVACFQNGMGHLTKLQAALPKSLLCSAITTEGAKRTQSQVTRAGTGETRLGRYNIH

IGASTSIEEKRTFTLSGSLQQAGFDCTVSNEIDKLIYRKLLINSVINPLTALWRIPNG

ELIVKEERKRLMRQLYDEVLLIYSANGICLDQDMWDQLISVCRSTATNTSSMLADVLA

GRGTEVRSINGHIVKMAQKLDLVAPTHEILLHLIEGMQPEGVN"

CDS 1082001..1082375

/locus_tag="EFAGFIKM_00936"

/inference="ab initio prediction:Prodigal:002006"

/codon_start=1

/transl_table=11

/product="hypothetical protein"

/translation="MGLFIVLSILPFFPFFLVYWGMYVWKKDKRKALRMAMDVTTFFL

VFSVSALFNLTFDSNFGFYLTLILILVALGFIGGAQNRLKGKVDRGRMFRAVWRMSFV

IMSFGYVLFTLFGLFRYFMQQM"

CDS 1082529..1084226

/gene="oppA"

/locus_tag="EFAGFIKM_00937"

/inference="ab initio prediction:Prodigal:002006"

/inference="similar to AA sequence:UniProtKB:P24141"

/codon_start=1

/transl_table=11

/product="Oligopeptide-binding protein OppA"

/db_xref="COG:COG4166"

/translation="MKRKSLLVLLTLILAFGTVLAACGSKNEGTGNTDTGSANEGNGL

AKDQILKINLSAEPPTLDPAQAKDSQTNTVLKFLYEGLVRIDADGKEQAGVAKDWTIS

EDGLKYVFNLNPDAKWSNGDAITAEDFVRSWERALKPETASPYAYQLYYIKGAEGYNL

SKDETYKGTKVTDFSQVGVKATDEHTLEVTLENPTPYFLGLTAFYTYYPVHASADTND

KFFTDYKNMIVNGPFVMDQYSKGQKIVVKKNDGYHAAADIKLAQIDMSLTNSSASELQ

AYKSGQLDYTGAPNGEIPSDQIPSVKAELPDEFKATGIASTYYYQFNVNEAPFNNVKI

RKAFAMAIQRQLIVDKVTQGGQIPAFGFVPPGIRGENGEFRDEHKDDYFTENVEEAKA

LLAEGMKEEGYTTLPAVTLIYNTSDGHAKIALAVADMWKQNLGVDVKTENQEWGVFLE

NRQNQNFQVARAGWSADYNDPYNFLEMWTTGNTNNDSKFSNEQYDKDVKETVKSADPA

ARMAAFADAEKILIQDEMGVMPIYYYTNVSLTKPYLKGVQLDFSGAIDYTRAYLEEK"

CDS 1084416..1085348

/gene="oppB"

/locus_tag="EFAGFIKM_00938"

/inference="ab initio prediction:Prodigal:002006"

/inference="similar to AA sequence:UniProtKB:P24138"

/codon_start=1

/transl_table=11

/product="Oligopeptide transport system permease protein

OppB"

/db_xref="COG:COG0601"

/translation="MVKYVLKKLLFMLLSLFILASATFFLMKAIPGDPFTSEKKVSPE

IRVLLEQKYGLDKPMYEQYLKYMGGIIKGDFGVSMKYLNRDVAGMIGETFTASLKLGV

FAIVISIIVGVLLGLIAAVYHRKLIDDITMILAVIGIAVPSFLLASLLQYLFATKLGW

FNVMGFDGPLDYVLPVAALSASPIAFIARLTRSSMLEVLHADYIKTAKAKGLKWPAIM

FKHVVRNGILPVVTYVGPMTANIITGSVVIEQIFNIGGIGKVFVESITNRDYTMIMGI

TIFYGILLMLARFFTDIAYVLIDPRIKLESRKGA"

CDS 1085351..1086277

/gene="oppC"

/locus_tag="EFAGFIKM_00939"

/inference="ab initio prediction:Prodigal:002006"

/inference="similar to AA sequence:UniProtKB:P24139"

/codon_start=1

/transl_table=11

/product="Oligopeptide transport system permease protein

OppC"

/db_xref="COG:COG1173"

/translation="MSGTNNKKNETANTNLTSQAAVKPQESVSLFKDAMYRLATNKAA

MISLSVLVLVVIFSLIGPTSLFTSYNYYSNDLLNANAAPSAEHWFGTDELGRDVWVRT

WVGARVSLTVGLAAALIDLVIGVIYGAIMGFYGGRVDGIMNKFSEILYSLPYMLVVIL

LLVVLEPSLTTIIIALTITGWISMSWIVRGEIMQLKNRDFILAARSMGASTGRQLFRH

LLPNAIGPILVTLTLSIPNAIFAEAFLSFLGLGVSAPRSSLGSMINDALTGWTLFPWR

MWFPAGLMVLTMLAFNLLGDGLRDALDPKLRK"

CDS 1086295..1088064

/gene="gsiA_2"

/locus_tag="EFAGFIKM_00940"

/EC_number="7.4.2.10"

/inference="ab initio prediction:Prodigal:002006"

/inference="similar to AA sequence:UniProtKB:P75796"

/codon_start=1

/transl_table=11

/product="Glutathione import ATP-binding protein GsiA"

/db_xref="COG:COG1123"

/translation="MEPILTVKDLSVSFSTRSGEFDAVKHVSFELGKGETLGIVGESG

SGKSVTAQTIMKLIPSPPSKVKSGEITFHGQDLLNKTDKQMEAIRGKDIGMIFQDPMT

SLNPTIKIGKQITEVLRKHQNMSKQAANKAALEMLELVGIKNAAIRMNHYPHQFSGGM

RQRAMIAIALACRPSLLIADEPTTALDVTIQAQILDVMKDMQQKLGTSIMLITHDLGV

VAGMCDRVVVMKEGEVVETGTTAEIFSNPQHPYTIKLLNALPRLDEPKKEKPAPAGII

KGSNKPLVQVKNLKQYFNLGKGNILKAVNDVSFDIFEGETLGVVGESGCGKSTTGRTI

LRLYEPTGGNVNFNGTDIYKLSPRKMKEMRKDMQMIFQDPYASLNPRFNVMDIIGESL

DIHGLASSSKERKRRVEELLDLVGLNPSHALRYPHEFSGGQRQRIGIARALAVDPKFI

ICDEPLSALDVSIQAQVVKLLEELQQRLGLTYLFIAHDLSMVKHISDRVAVMYMGKVV

ELAESEELYANPVHPYTKTLLSAIPVPDPEVEANKRRILLPEEHMSPIQNGSGGPAND

PYNLENSQLIEVSKGHWVAEPYV"

CDS 1088222..1089853

/gene="bshC"

/locus_tag="EFAGFIKM_00941"

/EC_number="6.-.-.-"

/inference="ab initio prediction:Prodigal:002006"

/inference="similar to AA sequence:UniProtKB:P55342"

/codon_start=1

/transl_table=11

/product="Putative cysteine ligase BshC"

/db_xref="COG:COG4365"

/translation="MNGITEALRSGSRLAEDYICSRDAARGLYEYDIRWESGLQARAE

WLDQSENARIDRRDLAEYLRIYNKRVNDHGEVHASITRLAEQDALVVTGGQQSGLLTG

PLFVIYKAASVVAAAREAEERLQRPVVPVFWIAGEDHDWDEVNHTYLPDHQGDMKKVK

LQGRFAGRDSVSNVHVDSQQWVNVLEQVEHLLPDTIHKPGLMKCITEIHQSSSNLSDA

FARMISVLFASSGLVLMDASDPDLRRLEQPVFERLIRKNEVLRSAYTQGASLVQQAGY

AMPAEVAEDGANLFYIHEGTRLLLLLKDGLYSDRKGLVSFTEERLLQELGEHPERFSN

NVLTRPLMQDSVLPVAAVILGQGEIAYWGLTREAFRQFGLQMPILLPRLSFTIMEDIH

HKHMKQYGLSFQDVQYHMEEKKEQWLAQQETFQVDEQFDKVQEAFLDLYGPLLDEIAE

IHPGLERIGDTNLSKINEQMQYLKQQTHKAIEDKHNVSLRHWNGIQNSLFPTGKPQER

VHNALFYLNRYGTEWIDELIKASSEFLGEHKVIAL"

CDS 1089898..1091163

/gene="ahcY"

/locus_tag="EFAGFIKM_00942"

/EC_number="3.3.1.1"

/inference="ab initio prediction:Prodigal:002006"

/inference="similar to AA sequence:UniProtKB:O51933"

/codon_start=1

/transl_table=11

/product="Adenosylhomocysteinase"

/db_xref="COG:COG0499"

/translation="MTTPALQNSIVKDMGLASEGHLKIDWVEAHMPVLNRIRRQFEQD

LPFKGLKVAICLHLEAKTAYLAKVIQAGGAEVTITHSNPLSTQDDVCAALVEDGVTVY

AKYNPEPAEFKSLQLRALEVKPDLIIDDGGDFATIIASERPDLAATIRGGAEETTTGI

IRLKALAKEGQLKFPMVAVNDAYCKYLFDNRYGTGQSAFDGIIRTTNLVIAGKNVVVA

GYGWCGKGVAMRAKGLGANVIVTEIDPIKAVEAHMDGFRVMTMVEAAKLGDFFIAVTG

NKDVITGEHYDVMKDGAILSNAGHFDVEVNKPELAKRSESIRTVRRNIEEYRFKDGRK

MYLLAEGRLVNLGAADGHPAEIMDTTFALQALGLRYVSENYKDLGKNVVNVPYDIDQQ

VASYKLESLNIGIDSLSAEQEKYLDSWKF"

CDS 1091437..1091874

/gene="mraZ"

/locus_tag="EFAGFIKM_00943"

/inference="ab initio prediction:Prodigal:002006"

/inference="similar to AA sequence:UniProtKB:P65439"

/codon_start=1

/transl_table=11

/product="Transcriptional regulator MraZ"

/translation="MFMGEFQHSIDDKGRVIIPAKFRESLGPSFVVTRGLDQCLFVYP

MEEWGVMEQKLKALPLMKSDARAFTRFFFSGATECELDKQGRVNLPGNLREYAKLDKD

CVVLGVSNRVEIWSKGIWESYFNQSEEAFNDIAEKLVDFNFDL"

CDS 1091893..1092843

/gene="rsmH"

/locus_tag="EFAGFIKM_00944"

/EC_number="2.1.1.199"

/inference="ab initio prediction:Prodigal:002006"

/inference="similar to AA sequence:UniProtKB:P60392"

/codon_start=1

/transl_table=11

/product="Ribosomal RNA small subunit methyltransferase H"

/translation="MFHHITVLKEEATEGLNIKQDGIYVDCTLGGGGHSSVIASKLGP

GGRLIALDQDDWALDNAREKLAAYGERVTLVKTNFRDLEQVLKDLDVPMKDGAPQVDG

ILYDLGVSSPQFDEGERGFSYNHDAPLDMRMDQDAMLTAKEIVNEWPEEEIARILYRY

GEEKFSRRIARVIVGKRAQSTIETTGELVELIKEGIPAAARRTGGHPAKRSFQALRIA

VNDELGAFEEGLHQAVRCLAPGGRVSVITFHSLEDRICKQIFSSYLEKCTCPPDFPLC

VCGGKGTLRLVNRKPLSPTETELAENSRARSAKLRVAEKL"

CDS 1092871..1093299

/gene="ftsL_1"

/locus_tag="EFAGFIKM_00945"

/inference="ab initio prediction:Prodigal:002006"

/inference="protein motif:HAMAP:MF_00910"

/codon_start=1

/transl_table=11

/product="Cell division protein FtsL"

/translation="MAYTRGNLAVKEKASQERVTQQRYKETTKVVTRRTGLPAREKIL

YLITLVAVILVGGALMSRYAHIYDLNKQAQSTISDIKVYEKTIADLQVEKETLNNQVI

ENAKQLGYVEASGKDVIYVPRSTSTKSTGTESSSNTASSK"

CDS 1093312..1095654

/gene="spoVD_1"

/locus_tag="EFAGFIKM_00946"

/inference="ab initio prediction:Prodigal:002006"

/inference="similar to AA sequence:UniProtKB:Q03524"

/codon_start=1

/transl_table=11

/product="Stage V sporulation protein D"

/db_xref="COG:COG0768"

/translation="MIKRIKMRTLLIGGCITLFFLVLVTRIFFIQVVNGGVWQERAAG

LVEREQTIKAARGTITDRNGNILATDAPAYTVSVNPLLINELGIQDVVTEKLSNLLGK

NESEMRALVTAKNAKGKFFQQREVRSEGYKISPELAAQVNELREELQDEYKARNAIVM

AQESKRFYPEETLASHLLGYMSRDGKAVNGLEVSYDEALTGTDGYLNYQKDAKGIKLP

DSQDNYLPPQNGKNLTLTIDDTIQFYIEDAMKEAVAKYNPLSMTVIAADPNTMEILGM

ANWPTFNPNTYGSTPDQKNFINHATQSIYEPGSTFKIVTLAGAVEEKLFDPNASFESK

RMYIGGFPISDNGHSYGWISYLEGVKRSSNIAFVNLGYNMLGGERLRHYIDEFGFGKK

TGIDLPNEAASPIKPLVYKSEIATAAYGHGLVQVTPIQQVAAISAIANGGKLLEPHLV
KEIKNPNDGTTEVIKPKVVRQVISKESSKLVSGYLEQVVADQTIGTGRNAYIEGYRVA
GKTGTARKVVNGAYSKSKDVVSFIGFAPVNNPKIAILVVIDQPDGTNIGGGTAAAPVF
KKIVSQTLQYMGIPKDTAKTPDKKSKEVSVVQAKAPDLSGKTAKQARSQLLSAGIAYV
TLGQGDNVIRQYPVAGASMNPGQRIYLLTEESSKMKIPDLTGESLRDALEVLSLMKVG
VTVKGEGYVVKQTEQVAGEQRTVQLNLQTAKAAVTGIADEAPISSDPSSEAEVKEQEA
SAGEAKTDETDGTDETDNNKAPANESESSDDPATNGASLP"

CDS 1095749..1097701

/gene="spoVD_2"

/locus_tag="EFAGFIKM_00947"

/inference="ab initio prediction:Prodigal:002006"

/inference="similar to AA sequence:UniProtKB:Q03524"

/codon_start=1

/transl_table=11

/product="Stage V sporulation protein D"

/db_xref="COG:COG0768"

/translation="MKGSSVNLRRRLLWSLMILVLLFSALMIRLAYVQLGEGPELSAK
AEESWRRNIPYSAKRGEILDRNGTSLAYNVTTPTIMAIPAQVKEAETTAKALAPLLGM
TEEKVLTTIKKRELIVRLQPGGRKITMEKAQRIRDLKLPGIVVAEDNKRFYPYDDLAA
HILGFTGIDNQGLTGVEKKYDDKLNGLNGSVSYLSDAAGRLMPGSSEKYVEPKDGLNL
KLTIDKSIQSIMERELDQAMVKFQANSALAIAMNPKTGEILGMSSRPGYEPADYQQYP
AEIYNRNLPIWMTYEPGSTFKIITLAAALEEKKVNLQQDQFFDPGYVEVGGARLRCWK
KGGHGSQTFLQVVENSCNPGFVALGQRLGKESLFSYIKDFGFGTKTGIDLSGEASGIL
FKLSRVGPVELATTAFGQGVSVTPIQQVAAVSAAINGGKLYKPYVTKAWVHPVTGEVM
EEAKPELVRQVISENTSKQVREALESVVAKGTGRPAFIDGYRVGGKTGTAQKVINGRY
SSTEHIVSFIGFAPADDPQIVVYTAVDNPKGIQFGGVVAAPIVQNILEDALHYMNVPV
RKDQVAKEYKYGETKIVTVPDLTGATVEDLYEDLNMNFMLAKSGSGKYVINQAPKPGA
RVDQGSTIRIYMGNVLNDAHDHTKDE"

CDS 1097842..1099329

/gene="murE"

/locus_tag="EFAGFIKM_00948"

/EC_number="6.3.2.13"

/inference="ab initio prediction:Prodigal:002006"

/inference="similar to AA sequence:UniProtKB:Q819Q0"

/codon_start=1

/transl_table=11

/product="UDP-N-acetylmuramoyl-L-alanyl-D-glutamate--2,

6-diaminopimelate ligase"

/db_xref="COG:COG0769"

/translation="MLLKQFASMLTTARLVGESNTECQNLQTDSRQVKPGDLFICLPG

HTVDGHDYAEKAVNAGAVALVVERQLDLPVPQLLVKDSRFAMAVLADFFFDSPSQKMN

MIGVTGTNGKTTTTYLIEKIMSDYGRKTGLIGTIQMRYDGRTYPMSGTTPEALDLQRS

LHDMVQKGTDCCVMEVSSHALEQGRVKGTNFRTAVFTNLTQDHLDYHHSMEEYRGAKG

LFFARLGNGYTEDVSQRKFAVINADDPAADYFISVTAAEVITYGMGEKADVRASQISI

TSQGTSFHVDTFAGSTDIRLRMVGKFNVYNAMAAISAALVEGIPLEEIKRSLETVPGV

DGRVEGVDEGQPFAVIVDYAHTPDGLENVLRTVKEFAEGRVICVFGCGGDRDRTKRPL

MGKIAANYSDFVLVTSDNPRTEDPDLILKDIEQGLIEESVPAERYTMIVDRRQAIHEA

IEMASPADVVLIAGKGHETYQIIGTTKTDFDDRIIAKEAIRGKSN"

CDS 1099326..1100741

/gene="murF"

/locus_tag="EFAGFIKM_00949"

/EC_number="6.3.2.10"

/inference="ab initio prediction:Prodigal:002006"

/inference="similar to AA sequence:UniProtKB:P11880"

/codon_start=1

/transl_table=11

/product="UDP-N-acetylmuramoyl-tripeptide--D-alanyl-D-

alanine ligase"

/db_xref="COG:COG0770"

/translation="MIKRTLAQLAEMCGGTLSDVAAHGNVMVEGVFTDSRKPLEGSLF

IPLVGERFDGHEFVQACLEKGASGAIWQKDHGIPPQGAVIVVDDTLVALQALASAYLT

ENKAAVVGITGSNGKTTTKDIVDAILSTTFKVHKTQGNFNNHIGLPLTVLSMDPDTEI

IILEMGMSGRGEINDLSVIAQPDVAVITNIGESHLLQLGSRLEIARAKAEIAAGLKPG

GLLIYNGDEPLIAQVLEEPATKQPDGLQRFTFGLQTDNDDYPTGLMNAQNGVVFTTKQ

SGEHAFTLPLLGTHNVVNCLAALAVARHFIVTTEQIAAGLSRLKLTGMRIEVTQGVSG
LTLLNDAYNASPTSMKAAIDVLEGLKGYRMKVAVLGDMLELGPQEQDLHYGIGEYITS
AKMDMVLVYGPLSAKIAEGASKHMPTEAVHAFIDKEEMTRYLLEKLHPRDVVLFKASR
GMKLEDVVEALQIAPLQNRVD"

CDS 1100754..1101722

/gene="mraY"
/locus_tag="EFAGFIKM_00950"
/EC_number="2.7.8.13"
/inference="ab initio prediction:Prodigal:002006"
/inference="similar to AA sequence:UniProtKB:Q2FZ93"
/codon_start=1
/transl_table=11
/product="Phospho-N-acetylmuramoyl-pentapeptide-
transferase"
/db_xref="COG:COG0472"
/translation="MDFQVLLLTIGVSFILAVIAAPLLIPLLRRMKFGQQVREDGPQS
HLKKSGTPTMGGVVILVAFTLAFLKFSAVKNTDFYVLLVATLGFGLIGFLDDYIKIVF
KRSLGLTARQKMLGQLFFSAVMCFLLIQNGHSTAISIPGTSFSFDWTGWFYYPFVVFM
MLAITNAVNFTDGLDGLLSGVSAIAFGAFAIVAMQATSMPAAVCAAAMIGAVLGFLVY
NAHPAKVFMGDTGSLGIGGAIGAVAIVTKTELLFVIIGGIFVIEILSVIIQVVSFKTR
GKRVFKMSPIHHHFELSGWSEWRVVITFWAVGAILAALGLYLNKGL"

CDS 1101726..1103147

/gene="murD"
/locus_tag="EFAGFIKM_00951"
/EC_number="6.3.2.9"
/inference="ab initio prediction:Prodigal:002006"
/inference="similar to AA sequence:UniProtKB:Q8E186"
/codon_start=1
/transl_table=11
/product="UDP-N-acetylmuramoylalanine--D-glutamate ligase"
/translation="MNHPESYRGQQVVVLGLAKSGVQVAKVLDRAGAKVTVNDKKERD
QCPEASELEALGISVVCGGHPDDLIHSGVKLVVKNPGIPYHAAPVQQALALGIEVVTE

VEVAYHLCAAPMIGITGSNGKTTTTTWVGKMLEHAGLKPIVAGNIGTPLCEAAEQASP

DNWMVVELSSFQLKGTVDFRPRIASLLNVAETHLDYHGNMDDYVASKAKLFANQQPDD

VAILNWDDAVCRGLVPYIKGRLIPFSVTEKLDTGVYADPPYVDGEEDDVKRQVIYADE

NGDHHIIIDVEDIGLPGRFNVENALAAVAIAVAAGADPALLAAPLADFKGVEHRLEYV

LEYNGSAYYNNSKATNSKATVMALNSFKEPVVLIAGGLDRGSDMMELLPLFQERVKAV

VAIGETRTKIAKIAELAGLKQIKVVDNEEDAARTLTVAVQEASKLATAGDVVLLSPAC

ASWDMFASYEERGRIFKEAAHNL"

CDS 1103196..1104293

/gene="ftsW_1"

/locus_tag="EFAGFIKM_00952"

/EC_number="2.4.1.129"

/inference="ab initio prediction:Prodigal:002006"

/inference="similar to AA sequence:UniProtKB:P0ABG4"

/codon_start=1

/transl_table=11

/product="putative peptidoglycan glycosyltransferase FtsW"

/db_xref="COG:COG0772"

/translation="MKQTRPAPDIWLLICILALLAIGIIMVYSAGSVLAFHDYGDSFY

FVKRQALFAVLGLVAMFVTANVDYRVWRKYAKPILIACFIMLIAVLIPGIGVVRGGAR

SWLGIGSFGIQPSEFMKLGMILFLAHWLSKEPGKIKTFTTGLLPPLGLIGLAFGIIML

QPDLGTGTVMMGAAMLIIFTAGARMKHLSLLALGGIAGFAALIAAAPYRLQRITAFLD

PWSDPLGAGYQIIQSLYAIGPGGLAGLGLGMSRQKYSYVPEPQTDFIFSILAEELGFI

GGMIVLLLFLVLVWRGMRVAMTIPDAFGSLLGVGIVGMVAVQVIINIGVVIGMMPVTG

ITLPLISYGGSSLTLMLTALGILVNLSRYAR"

CDS 1104301..1105410

/gene="murG"

/locus_tag="EFAGFIKM_00953"

/EC_number="2.4.1.227"

/inference="ab initio prediction:Prodigal:002006"

/inference="similar to AA sequence:UniProtKB:P17443"

/codon_start=1

/transl_table=11

/product="UDP-N-acetylglucosamine--N-acetylmuramyl-
(pentapeptide) pyrophosphoryl-undecaprenol
N-acetylglucosamine transferase"
/db_xref="COG:COG0707"
/translation="MRVVLSGGGTGGHIYPAVAIARQCEAENPDSTFLYIGGTRGLES
KLVPQENIPFKSIDITGFRRKLSIENLKTVMRFIQGVRKSKKMLKEFKPDVVIGTGGY
VCGPVVYAATKLGIPSIIHEQNAIPGLTNKFLMRYVDTVAVSFEGSEKSFSGAKKVIY
TGNPRATTVAQASRDRGFATLGVPMNSRVVLVVGGSRGAKAINQAMVDMAPLLEQLDD
VHVVYVTGDTYFDETREAIRSSLGTMPNHLHVLPYVHNMPEVLACTSLIVNRAGASFL
AEITSLGIPSILIPSPNVTNNHQEANARTLEGGGASLTMLEKDLTGKVLYEAIAKIMN
DESGRKRMAEASRKLGKPDAAEVLVHEIQRLAARR"

CDS 1105560..1106465

/gene="murB_1"
/locus_tag="EFAGFIKM_00954"
/EC_number="1.3.1.98"
/inference="ab initio prediction:Prodigal:002006"
/inference="similar to AA sequence:UniProtKB:Q65JX9"
/codon_start=1
/transl_table=11
/product="UDP-N-acetylenolpyruvoylglucosamine reductase"
/db_xref="COG:COG0812"
/translation="MHQWISLLSQNNVGRVLENESLAKYTTWKIGGPADALVIPENKE
QMMNLVQLLHTYQIPWMQLGRGSNMLVSDKGIRGVVVKLGEGFDYAEFHEGGVTAGAA
HSFVKLSVVAAKKEWAGLEFGSGIPGTVGGAVYMNAGAHGSDVSRIFKFAEIVLETGE
LVRYSKEDMDFAYRHSVLHDRRGIVLEAAFELQHGERNVISETMAAYKDRRRRTQPLQ
MACAGSVFRNPPGDHAARLIEAAGLKGMTQGGAQVSTMHANFIVNTGQATAEDVITLM
QQIQSTISSQNGINLVPEVFVVGER"

CDS 1106483..1107766

/gene="murA"
/locus_tag="EFAGFIKM_00955"
/EC_number="2.5.1.7"
/inference="ab initio prediction:Prodigal:002006"

/inference="similar to AA sequence:UniProtKB:Q88P88"

/codon_start=1

/transl_table=11

/product="UDP-N-acetylglucosamine

1-carboxyvinyltransferase"

/db_xref="COG:COG0766"

/translation="MDKLVIEGGKPLSGSIRIHGAKNAALPIMAASLLADGEVTLHNV

PHLLDIEVMLYILERLGCTCRHEQGTVTINTSSIRSYDVPEDLMKQMRSSIFLMGPLL

AKFGQVSVYQPGGCAIGERKIDLHLRGLEALGALIEEQDQQIICHGQNLVGTDIHLDF

PSVGATENIMMAAAMAKGTTTICNAAREPEIQDLQHFLNAMGASIIGAGTDTITINGV

EKLKPCSYEIIPDRIVAGTVMIAAAATRGNVTLTHCNPAHLTSLIHVLKRTGVQITVC

NDIMTVSCMSRPKSVDRIVTSPYPSFPTDLQSQIMVLLSLADGFSVMKETVFEGRFKH

VDELNVMGADISVDLNAAFIRGVPRLYGATVEATDLRAGAALVIAGLAAQGKTVVEQV

HHIDRGYDQIEKLFQSLGASVERQSPVSKQLDFAN"

CDS 1107824..1108591

/gene="divIB"

/locus_tag="EFAGFIKM_00956"

/inference="ab initio prediction:Prodigal:002006"

/inference="similar to AA sequence:UniProtKB:Q5L0X5"

/codon_start=1

/transl_table=11

/product="Cell division protein DivIB"

/db_xref="COG:COG1589"

/translation="MPKSQIPVLKKNRPKGKTSRKIVIILLLLFIVLLAVLFFRSSMS

RISAIEITGNVYTATSELLEKSGLKEGEQFFGTSTAEVIERLKTIKAISTVTVDKQFP

GIIHIKVQEYATVAYELGSDGTLKAILASGTSLTVPATIGVAVEKPILTQWKADDPLK

AKLSQTLAKIPNELTTDISEIIPNPTPSFPDQIRMYTKSQFEVITTVSLLSDKVEYLN

QVIETERPGKITMLEADTYVPFIPDDPEDDAEPGASP"

CDS 1108885..1110168

/gene="ftsA"

/locus_tag="EFAGFIKM_00957"

/inference="ab initio prediction:Prodigal:002006"

/inference="similar to AA sequence:UniProtKB:P28264"

/codon_start=1

/transl_table=11

/product="Cell division protein FtsA"

/db_xref="COG:COG0849"

/translation="MSNNDIIVSLDIGTSKIRAIIGEMNNGTFNIIGVGSADSEGIRK

GVIVDIDQTVQSIRNAVDHAERMVGIQISEVYVGISGNHIGLMSSHGVVAVSNEDREI

GEEDMERVLKAAEVIAVPPEREIIDVVAKQYVVDGLEGIQDPRGMIGVRLEVEATIIT

GAKTAIHNLLRCVEKAGLKVSDLVLMSLGAGQLALSKDEKTMGSVLVDIGAGATTIAI

FEEDSLVATSTLPIGGEFVTNDIAYGLRTLTDQAEKVKLKYGCAWLDDAAADVMFKVT

RIGSNVDKEFSQEDLAAIIEPRVQEIFQMISQEVKRLGYNELPGGYILTGGTVSMPGV

LQVAQHELAASVRVAVPDYIGVRDPGFTSGVGILHSVIRSLRIRPSINNGGGNNNNNN

NNKKPTNRPKPNTAQESEQKPGLFERLKNMFSEFI"

CDS 1110319..1111434

/gene="ftsZ"

/locus_tag="EFAGFIKM_00958"

/inference="ab initio prediction:Prodigal:002006"

/inference="similar to AA sequence:UniProtKB:P17865"

/codon_start=1

/transl_table=11

/product="Cell division protein FtsZ"

/db_xref="COG:COG0206"

/translation="MLEFDFEMESLAQIKVIGVGGGGSNAVNRMIENGVQGVEFITVN

TDAQALHLAKSEHKLQIGDKLTRGLGAGANPDVGKKAAEESRDLIMNTLKGADMVFVT

AGMGGGTGTGAAPVIAEIAKECGALTVGVVTRPFTFEGRKRSSHAEQGIEALKEKVDT

LIVIPNDRLLEIVDKKTPMLEAFRQADNVLRQAVQGISDLIAVPGLINLDFADVKTIM

HERGSALMGIGESTGENRAAEAARKAIMSPLLETSIEGARGVIMNITGGVNLSLYEVN

EAAEIVTSASDPEVNMIFGAIIDEDLKEEIKVTVIATGFEDKPAPPPPGRKPAPATAE

TTDTRSPNLRPFGNQPSNDQLDIPTFLRNRSRNNNND"

CDS 1111696..1112676

/gene="spoIIGA"

/locus_tag="EFAGFIKM_00959"

/EC_number="3.4.23.-"

/inference="ab initio prediction:Prodigal:002006"

/inference="similar to AA sequence:UniProtKB:P13801"

/codon_start=1

/transl_table=11

/product="Sporulation sigma-E factor-processing peptidase"

/translation="MVVYVDLIFLTNLCIDGALIGMTAWMRKTKLVWWRWLLSAIVGA

LYVVMMFVPEFDFMFTFLIKFGFSLVMLTIAFGFKGLQAFARTLGTFYVINFVAAGGI

LGVHYMLQSSGELFNGIWFTASGGMSFDLKIAFWFTFIVFFAVLFLFKAVQSSKRKTD

RMTTYLGKVEVCIDEVVISCTGLLDTGNQLTDPLSRMPVMVMEVSLWQDMLPASWKGR

LKDEAPDNLILELDQESFQWQDRLRLVPYRGINKGTAFMLAMKPDRVKVTMEETCYET

TRVLIGLDGGVLSSDGKYQAVIHPELVQDAASAQSTALSAGATEKPLNVV"

CDS 1112688..1113410

/gene="sigE_1"

/locus_tag="EFAGFIKM_00960"

/inference="ab initio prediction:Prodigal:002006"

/inference="similar to AA sequence:UniProtKB:P06222"

/codon_start=1

/transl_table=11

/product="RNA polymerase sigma-E factor"

/db_xref="COG:COG1191"

/translation="MLVKWKLVAQLQYYRLLFLLGLKSEEIYYIGGSEALPPPLTREE

EEFLLQKLSSGDSAIRAMLIERNLRLVVYIARKFENTGINIEDLVSIGAIGLIKAVNT

FDPEKKIKLATYASRCIENEILMYLRRNSKIRTEVSFDEPLNIDWDGNELLLSDVLGT

ENDTIYRNIEEQVDRKLLHKALEKLTERERMIMELRFGLTDGEEKTQKDVADLLGISQ

SYISRLEKRIIKRLRKEFNKMV"

CDS 1113522..1114304

/gene="sigG"

/locus_tag="EFAGFIKM_00961"

/inference="ab initio prediction:Prodigal:002006"

/inference="similar to AA sequence:UniProtKB:P19940"

/codon_start=1

/transl_table=11

/product="RNA polymerase sigma-G factor"

/db_xref="COG:COG1191"

/translation="MTRNKVEICGVDTAKLPVLTNTEMRELFHSLQQHHDRSAREKLV

NGNLRLVLSVIQRFNNRGEFVDDLFQVGCIGLMKAIDNFDLSQNVKFSTYAVPMIIGE

IRRYLRDNNPIRVSRSLRDIAYKALQVRDSLTNKNSREPTIFEIAEVLNVPKEDVVFA

LDAIQDPVSLFEPIYHDGGDPIYVMDQISDDRNKDVSWIEEIALREAMHRLGQREKMI

LSMRFFEGKTQMEVADEIGISQAQVSRLEKSAIQQMQKHVKS"

CDS 1114479..1114871

/locus_tag="EFAGFIKM_00962"

/inference="ab initio prediction:Prodigal:002006"

/codon_start=1

/transl_table=11

/product="hypothetical protein"

/translation="MKVNTSEVAARGMKISDFQTKDVINITDGKRLGQISDLELDLKQ

GRIEAIVVPGYSRFMGLFGGGTDLVIPWRNIVKIGSDVILVKMDEVKENTYDERDREA

RLYDEQQHNRTERVERIERSERNQRRTI"

CDS 1114923..1115822

/locus_tag="EFAGFIKM_00963"

/EC_number="1.10.3.-"

/inference="ab initio prediction:Prodigal:002006"

/inference="similar to AA sequence:UniProtKB:Q89ZI8"

/codon_start=1

/transl_table=11

/product="Polyphenol oxidase"

/db_xref="COG:COG1496"

/translation="MEPFVLDKELLERTKNPNSDFGPDPLLLYVEPWTQQFEQLSVGF

TTRHGGVGKIPYATLDCAYHVGDDPEVVLNNRRLVTEKLGFAAEAWTCGEQVHGKHVA

VITAEDRSRGLLDRESALQDTDGLVTNVPGVLLTSFYADCVPLYFYDPVQQAVGLAHA

GWKGTVAGIAVSMVETMEREYGSRRQDIRAAIGPSIGDCCYEVDEAVMQYVRVWFDDS

PVNDKYKDSASKQAYRAVNNGKTMLNLKECNRHIMMKAGIMPDHIECTTWCTSCHPEL

FFSYRKENGVTGRMASWIGLEER"

CDS 1115829..1116530

/locus_tag="EFAGFIKM_00964"

/inference="ab initio prediction:Prodigal:002006"

/inference="similar to AA sequence:UniProtKB:P44506"

/codon_start=1

/transl_table=11

/product="Pyridoxal phosphate homeostasis protein"

/db_xref="COG:COG0325"

/translation="MSLEERIQQVNQKIEDACRRSNRHRDDVNVIAVTKYVSLETTGS

VLDHGLEHIGENRWQDAQAKWEAFGQQGTWHFIGHLQTNKVKDVIGKFRYIHSLDRLS

LAKELDKKAASLGTQVETFLQVNISGEESKYGLQPEQASSFLRDIRSFNNLKVVGLMT

MAPHEEDPELTRPVFRGLRELRDQLNGQALTAEPLTELSMGMSNDFEVAIEEGATWVR

LGSILVGKEEGSRWA"

CDS 1116521..1116988

/gene="sepF"

/locus_tag="EFAGFIKM_00965"

/inference="ab initio prediction:Prodigal:002006"

/inference="similar to AA sequence:UniProtKB:O31728"

/codon_start=1

/transl_table=11

/product="Cell division protein SepF"

/db_xref="COG:COG1799"

/translation="MGVMNKFMNFLGLQEEEEIVERERMAAQEENESEHQEAETSSLD

KRRNQRGNNVVSIHSQKNVKVVLYEPRSYDEAQEIADHLRSHRTVVVNLQRIRQDQAL

RVIDFLSGTVYALGGGISKIGGNIFLCTPDTVEIQGSITEILADSEQDYNRMR"

CDS 1116996..1117265

/locus_tag="EFAGFIKM_00966"

/inference="ab initio prediction:Prodigal:002006"

/codon_start=1

/transl_table=11

/product="hypothetical protein"

/translation="MYQIESVLYTLYQIYFYMVIVYILMSWLPNARESFIGEWLGKLV

EPYLRPFRRFIPPLFGVLDISPIVALIVLQLALNGLISILRYFAY"

CDS 1117504..1118286

/locus_tag="EFAGFIKM_00967"

/inference="ab initio prediction:Prodigal:002006"

/codon_start=1

/transl_table=11

/product="hypothetical protein"

/translation="MSGEIYEHFSHDERDFVDKASDWVEQAGKYHDMKLTDFLDPRQV

FILQTLANRRNDVQIRLDGGYEAAERKRALVAPDYMYLDDEDMGMQVLSITSDDQKIS

ELEHGDYMGSLLGLGMKRGKIGDIQVLEDGCHTVVAAETGAFLSLQLNQVHRLHVFTE

LLPLDQMRWSESKLETMDITVASLRLDGICADVYRLSRSKVLAPIKAGRCRVNWKVEE

DPSKSLKAGDVVSIQGFGRFKVMEQDGMTKKGRCRVKIGKFA"

CDS 1118527..1119036

/gene="divIVA"

/locus_tag="EFAGFIKM_00968"

/inference="ab initio prediction:Prodigal:002006"

/inference="similar to AA sequence:UniProtKB:P71021"

/codon_start=1

/transl_table=11

/product="Septum site-determining protein DivIVA"

/db_xref="COG:COG3599"

/translation="MPLTPLDIHNKEFSRRLRGYDEDEVNEFLDQVIKDYEGVIRENK

ELSNQLLSVQEKLDHFSTIEETLSKTIIIAQEAADDVKGNAKKEAQLIVKEAEKNADR

IVNESLGKSRKIALEVEELKKQASIYRARFRTLVEAQLELLTQDGWEVLESREQEVRD

REREMKEIY"

CDS 1119720..1122824

/gene="mupB"

/locus_tag="EFAGFIKM_00969"

/inference="ab initio prediction:Prodigal:002006"

/inference="similar to AA sequence:BARRGD:NG_048009.1"

/codon_start=1

/transl_table=11

/product="mupirocin-resistant isoleucine--tRNA ligase

MupB"

/translation="MIMQRVDVKEKARARELRVLDKWKTENTFKRSIENREGKPNFVF

YEGPPTANGKPHIGHVLGRVIKDFVGRYNTMKGYRVVRKAGWDTHGLPVELGVQKKLG

ISHKWEIEDYGVEKFINECKASVFEYEQQWRDLTEGIGYWTDMDNPYITLDNNYIESV

WNILATIHEKGLLYRGHRVSPYCPSCQTTLSSHEVAQGYKDVKDLSATAKFKLNDSGE

FVLAWTTTPWTLPSHVALAVNPDMDYSRVRQGDEVYIMATNLVEKVMKDTKGEYEIIG

ALKGADLVGKTYDPPFNYVQAEKANIILGAGFVTDASGTGIVHMAPAHGEDDYRVCRE

NGISFVNMVDLEGKFVAEVTDFAGRFVKDCDIDIVRYLSEHGRLFSKEKYEHSYPFCW

RCDTPLLYYAMDSWFIQTTAIKDQLIANNSEVDWYPGHVREGRFGKFLEDLVDWNISR

DRYWGTPLNIWVCEETGEQFAPHSIAELRARAVGDVPENLELHKPYVDDVKVMSSCGK

YEMKRTPEVIDVWFDSGSMPFAQQHYPFENKEVFEQQYPADMICEGIDQTRGWFYSLL

AVSTLLTGKAPYKAVMATGHVLDENGQKMSKSKGNVIDPWEVIEEYGTDAFRWALLSD

SAPWNSKRFSKGIVGEAKSKMVDTLVNTHAFLTLYATIDGFDPQEHPFQLSAHKLDRW

ILSRLNSLILVVEKALLVNDYLNSSKAIEAFVDELSNWYIRRSRDRFWGSGLTEDKLD

AYRTLTEVLVTTAKLVAPFTPMLAEDIYLNLATGESVHMEDYPVANEALIDAGLEQDM

ETARRVVELARNVRNETGIKTRQPLSELIVSLDKGFDLASYEEIIKEEINVKGIRTEH

NDAEFVDFTLKLNLKVAGKKYGKNVGFLQNFFKGMTADETRKVVSEGVLNIVSPEGEE

LQVTSEELLVDKQAKSGFASASGYGLTVALNTEITPALEQEGWVREVVRAVQDTRKRL

DLPIEKRVRLTLDVDASLQEAIQAFDDVLRENVLVTEVTFGSNESMERVEAGGKSIGI

YIEA"

CDS 1122990..1123349

/locus_tag="EFAGFIKM_00970"

/inference="ab initio prediction:Prodigal:002006"

/codon_start=1

/transl_table=11

/product="hypothetical protein"

/translation="MSEHDKQTASTSQQSTSDNLNSHDKIEELHTLTNRLANELERSR

IAQYTELLNRPWKLIGLNLLSGAARGVGIAIGFTFFAATIIYVLQLLGALNLPIVGDY

IADIVRIVQRQLDMNTY"

CDS complement(1123391..1124128)

/gene="dksA_1"

/locus_tag="EFAGFIKM_00971"

/inference="ab initio prediction:Prodigal:002006"

/inference="protein motif:HAMAP:MF_00926"

/codon_start=1

/transl_table=11

/product="RNA polymerase-binding transcription factor

DksA"

/translation="MSHFTNEQLQSLRSQLMSDKRDIEHRLSENEHYGLGDSLKLQTG

ELSPIDNHPGDLATEVYEREKDISLLEHDEFQLERIDSALHSIEEGHYGTCAVCQQPI

PYERMEAVPYTKYCKKHQPETVVSENRPVEEEFLAPAFGRTSLDERDDQNGFDGEDTW

QIVESWGTSNSPAMAEGRDIDSYDVMAIEATDEVEGCVEAYESFVATDIYGHDVSIVR

NRQYRQYLENREGEGLLEPDMETDDTY"

CDS 1124350..1124844

/gene="lspA_1"

/locus_tag="EFAGFIKM_00972"

/EC_number="3.4.23.36"

/inference="ab initio prediction:Prodigal:002006"

/inference="similar to AA sequence:UniProtKB:Q9HVM5"

/codon_start=1

/transl_table=11

/product="Lipoprotein signal peptidase"

/db_xref="COG:COG0597"

/translation="MVYYILAFIVFLLDQGTKYLIATRMELREEIPVIGNFFVITSHR

NSGAAFGILQDQRWFFIVVTLIVVVALIWYLQKVKDTPHKLLPVALSLVLGGAIGNFL

DRALTGEVVDFVQLNFGSYTFPIFNIADSAICIGVGLIIVETLLEGRREKAAAKIEGN

EHHE"

CDS 1124786..1125799

/gene="rluD_2"

/locus_tag="EFAGFIKM_00973"

/EC_number="5.4.99.23"

/inference="ab initio prediction:Prodigal:002006"

/inference="similar to AA sequence:UniProtKB:P33643"

/codon_start=1

/transl_table=11

/product="Ribosomal large subunit pseudouridine synthase

D"

/db_xref="COG:COG0564"

/translation="MKDGAKKQPRRLKGMNIMSNPNKEQINDEELMNGNERMEWTVAA

EHKKERIDKYITEAVDNVSRSQVQLWIGDGMVTVNGAVVKANAKLSEGDLVELQIPEP

AAVEIVAEDIPLEVVYEDSDLIVINKQRGLVVHPAPGHTSGTLVNALMHHCKDLSGIN

GELRPGIVHRIDKDTSGLIMAAKNDRAHASLAAQLKDHTVNRRYIALVHGHLNHDQGT

VDAPIGRDTNDRKMYTVTERNSKHAVTHFTVSERINDYTLLDLKLETGRTHQIRVHMK

FIGHPLVGDPTYGRNKGIKMQGQALHAAILGFVHPTTGEYLEFSTPIPQDMEDVLASL

RSR"

CDS 1125910..1127163

/gene="dapL_3"

/locus_tag="EFAGFIKM_00974"

/EC_number="2.6.1.83"

/inference="ab initio prediction:Prodigal:002006"

/inference="similar to AA sequence:UniProtKB:A0LEA5"

/codon_start=1

/transl_table=11

/product="LL-diaminopimelate aminotransferase"

/db_xref="COG:COG0436"

/translation="MSIDKYQETYIQTNFADRIGGSNYGKDTNIYKFEKIKRAKASAK

KDFPDVELIDLGVGEPDEMADAGIVAALAEEASRPENRGYADNGIPEFKAAAASYLKN

VFNVEGINADTEIVHSIGSKPALAMMPSCFINPGDVTIMTVPGYPVMGTHTKYLGGEV

FNIQLTKENNFLPDLTAIPEDIAKRAKLLYLNYPNNPTGASATVEFFTEVVEWAKKYN

VVVVHDAPYAALTYDGKKPFSFLSVPGAKDVGVELHSLSKSYNMTGWRIGFVAGNPLV

VKAFSDVKDNNDSGQFIAIQKAAAYGLNHPEITEKIAEKYSRRHDMLVAALNELGFQA

EKPKGSFFLYVEAPKGVVGGRRFESGEDFSQFLIREKLISSVPWDDAGNFVRFSVTFE

AKGEEEEKRVIAEIKRRLSDVQFEF"

CDS 1127343..1128257

/locus_tag="EFAGFIKM_00975"

/EC_number="2.4.2.45"

/inference="ab initio prediction:Prodigal:002006"

/inference="similar to AA sequence:UniProtKB:P9WFR5"

/codon_start=1

/transl_table=11

/product="Decaprenyl-phosphate phosphoribosyltransferase"

/db_xref="COG:COG0382"

/translation="MLSSRTSTVSSPATGTGSTVSGLIRLLRPKQWTKNLLLFAALLF

SFEEIRTETILATLLGFILFSLVAGCVYILNDFVDRDRDRQHPVKKYRPMASGQVNPS

HALMFGIILLIFSVGTAFMMNPLFGVLCIVYFLLNVSYSFVLKHLVILDMMTIAAGFV

LRAIAGGVLIHVPFTPWFLICTMLLSLFLAIGKRRNELTLLEGNTGSHRKVLDNYSIT

LLDQFNTIVTTATIISYSLFTFTSDRTIHLMWTIPLVIYGMFRYLYLIHMKNQGGSPD

RVLFEDKPILITVMLYVISVITIFAIFE"

CDS 1128276..1128908

/locus_tag="EFAGFIKM_00976"

/inference="ab initio prediction:Prodigal:002006"

/codon_start=1

/transl_table=11

/product="hypothetical protein"

/translation="MKSTKVAIFDIDKTIIRSDSMFQFVHYGVRRYPWQVWRLPVIAL

HTVLFKAGFMTVEQVKRSYFQEIERMSEKDLEHFFDTRLRTSIFAEASVEMQHRKEAG

YHVLLVTASPHAYMKYFNNFPWVDHVIGTELVRHENGYTCRVDGSNCKGEEKVRRIQA

YLSKKNMVIDYDQSCSYSDSLSDLPVMQLVSQRYFINKRVPDMEALTWGK"

CDS 1128896..1129225

/locus_tag="EFAGFIKM_00977"

/inference="ab initio prediction:Prodigal:002006"

/codon_start=1

/transl_table=11

/product="hypothetical protein"

/translation="MGEIKSRHTGKWLMLVSAFLTATGQLFWKWGLTEWIYLGVGFVC

YGLGAILMIKAFALEKLSVAYPLMCASYVFALIYGYFLLGEEITMQKLAAVVLLGIGV

TLTSVDR"

CDS 1129230..1129532

/gene="arnE"

/locus_tag="EFAGFIKM_00978"

/inference="ab initio prediction:Prodigal:002006"

/inference="protein motif:HAMAP:MF_01869"

/codon_start=1

/transl_table=11

/product="4-amino-4-deoxy-L-arabinose-phosphoundecaprenol

flippase subunit ArnE"

/translation="MVAVLIVMTLCGALGGAGLKAYASSRNRLHVLMGLGFYGTGALL

NIVLLKFLPLTVVLPANALTYVWTLIIARLVFKETVGPLRWIGVACIMGGLLLLVF"

CDS 1129564..1130868

/locus_tag="EFAGFIKM_00979"

/inference="ab initio prediction:Prodigal:002006"

/codon_start=1

/transl_table=11

/product="hypothetical protein"

/translation="MNFLDYLFYNRRGNWTAFYLFAGFALFYGLMNGSYVLYIENNAE

MLGAYSPFNTTLFPINLFNFDPSMYYGDNSSSVIHPLISFLAVTLAAVAKLLGGNWFF

LILQSLVNAGSVVLAYLFLSQKEDKPTIIPLLFALLFGFSSYLMYTALIPDSYPYVQF

VILLSVVYMQYTRERQDVRYVPNALLASINFGLTSTNIVPFAAATFFNMHAWRNKANL

KKYIGIMALAVLIIVVLTGIQYVAFGGRSWISNWLLGIQNGGTSYATPFQFAVHWKAL

NMLTINPMLTPKMHLLDPGMAAFVTDLSRSNPIYVQMTGIFILLLAFMGFIKGIRERE

VWTLVPYILFAFLLHVVVGFGLAVFQYDMYLYAGHYLFAFFLLGGGFVISLRPGLGKK

LVIGLIMLCVIVTASNNIYRHVETLTTIKQSYNQLEQERSVN"

CDS complement(1130960..1133635)

/locus_tag="EFAGFIKM_00980"

/inference="ab initio prediction:Prodigal:002006"

/codon_start=1

/transl_table=11

/product="hypothetical protein"

/translation="MRHIWHVYKTDWLHILKVPTGVFLIVAIILLPGVYDWVNVKSVW

DPYSNTQGIKIAVTSEDAGATVEGTHVNIGDELVSSLKHNEKLGWTFVDQAEANRGVQ
TGEYYASLLIPGDFSSKITGIVDGKLERPEVIYTVNEKVNAIAPKITGSGVSAITTQI
NENFTEAVSEAVLTKLNEAGVEINAQLPTLRKMENGIFTLEKNLPAIQAAGQKVLEVE
KAMPEIVKDAQKIVEIEKKLPEINKAAQYVLKVQEYWPQINDAASEVLTIQGRIPDIQ
KAVERIREVDENFGQVSGVIQTALDKTNKALSIVTAAEQDLDKVSQIAGNGIELAEGL
NQFVDSSEEAFQTIGPTIRQNLLLVQQITNAAGDVFAQLQNSDLNNLPTVEDLDRIAS
RLGIAVKLVDSMAELLGNINNLLPDQPLADKITQLNSISDNLQLQIRLAGIISDAMRR
NTTPPTDVIAQLNTLSKDISSGIGNILNTYESEISPALAAGADKLRSILSTSAETLQG
AKDRIPDIADILASAKEGITFGQTELMKIQSELPQIQSKIHEISETLTNKSEGFIQAL
DTVSSLIRNDLPKLGNKLNEAANFVRNDLPNAEKQIGKASDFVQNQLPEVEKGVHRVA
TLVRDDLPALESAISKAADKLREVEGNNQFAELAKLLRGDIEEESAFLSSPVQIKEQQ
LYPIPNYGSAMSPFYGVLSLWVGSTLLISLLRAEAENPGGKFRGYELYLGRLATFLTI
GLLQAICVTLGDILILGTYVADKLWFVLFAMLVSAVFVTITYTLLSVFGNIGKGIAII
FMVFQFSSSGGTFPISMTSPFFQALNPFMPFTYAISLLRESVGGILWSTAIKDILWLC
MFIALSLIVALALKRPLSSLTKRSAENAKKTKIIA"

CDS 1134344..1134907

/gene="pyrR"

/locus_tag="EFAGFIKM_00981"

/inference="ab initio prediction:Prodigal:002006"

/inference="similar to AA sequence:UniProtKB:P41007"

/codon_start=1

/transl_table=11

/product="Bifunctional protein PyrR"

/translation="MSTETHVIMDETAIRRALTRIAHEILEKNKGIDDCVLVGIRTRG
VYLAERIAAKIEEIEGAKVPWGELDVTPYRDDRLDENKANRKEMLIMTPESLSIHNKK
VILFDDVLYTGRTIRAAMDALMDCGRPQNIQLAVLADRGHRELPIRPDFIGKNVPTSK
SEEIEVALMETDGQDEVKITQNRGEQA"

CDS 1134907..1135818

/gene="pyrB"

/locus_tag="EFAGFIKM_00982"

/EC_number="2.1.3.2"

/inference="ab initio prediction:Prodigal:002006"

/inference="similar to AA sequence:UniProtKB:P05654"

/codon_start=1

/transl_table=11

/product="Aspartate carbamoyltransferase"

/db_xref="COG:COG0540"

/translation="MITQTALRDRSLLGLKELSRGEIESILNRAAHWEAQKEKLVPVL

ESRFVANMFFENSTRTRFSFEMAEKRLGVQVLNFTAAASSVEKGESIYDTVRTLESMG

IDAGVIRLKPAGVLQQLAQKVNVPLVNAGDGNNEHPTQALLDLYTMRKAFGELKGLRV

SIIGDILHSRVARSNLWALQKFGADVRFCAPQTMQAPELAEYAPYVGLEEALDADVVM

MLRVQLERHQHGLITSAEDYREHYGLTEERASRLKPSTIIMHPAPVNRNVEVDDAVVE

SEASRIFPQMANGVPIRMAVMERAMKL"

CDS 1135945..1137249

/gene="pyrC"

/locus_tag="EFAGFIKM_00983"

/EC_number="3.5.2.3"

/inference="ab initio prediction:Prodigal:002006"

/inference="similar to AA sequence:UniProtKB:Q81WF0"

/codon_start=1

/transl_table=11

/product="Dihydroorotase"

/db_xref="COG:COG0044"

/translation="MLQIIKNANVLNQQGELERKTIIIDEGKIKKIAGLEDQAVLDAE

KSAQSVTDASGKLVIPGLIDMHVHLREPGFEHKETIETGARSAAQGGFTTIACMPNTR

PVTDTAEVVKLVLDKAKEADLVKVLPYAAITKNELGRELTDFAALKEAGAIGFTDDGV

GVQNAQMMKDAMNLAASMDMPVIAHCEDDSLVVGGYVTEGEFSKRHGIKGIPNESEAI

HVGRDILLAEATGVHYHVCHVSTEQSVRLIRLAKSIGIKVTAEVCPHHLVLSDEDIPG

MDANWKMNPPLRSPRDVQACIEGLLDGTLDMIVTDHAPHSEEEKAKGMELAPFGIVGF

ETAFPLLYTKFVETGLWSLDFLVKRMTADPARVFRLDTGKLEEGAPADITMIDLNEEK

AVDPATFATKGRNTPFTGWKLKGWAVQTWVDGKSVWHNTVQQ"

CDS 1137678..1138820

/gene="carA"

/locus_tag="EFAGFIKM_00984"

/EC_number="6.3.5.5"

/inference="ab initio prediction:Prodigal:002006"

/inference="similar to AA sequence:UniProtKB:P99147"

/codon_start=1

/transl_table=11

/product="Carbamoyl-phosphate synthase small chain"

/translation="MQAQARLLLEDGTLFTGKAFGAEGETTGEVVFNTGITGYQEVLS
DPSYCGQIVTMTYPLIGNYGITRDDFESIRPYVHGFVVRRHEPTPSNWRAEYSVDNLL
KEYGIVGISEIDTRMLTRRIRHHGTMKGILTTGSKPVEELLEMMGDTTIAELRNQVPM
TSTEHVYNSPGTAERIVLVDYGAKTGILRELSKRNCDVVVVPHDVTADEIRRLNPDGI
QLSNGPGDPKDVPHAVNMISELLGEYPIFGICLGHQLFALAAGADTEKLKFGHRGGNH
PVKELESGRCFITSQNHGFTVNEESVKSTDLEVTHINNNDKTIEGLKHKSFPAFSVQY
HPEAAPGPYDNSYLFDRFIEMIREHKITNPQKPRQAVLAAAVKGAQ"

CDS 1138822..1142040

/gene="carB"

/locus_tag="EFAGFIKM_00985"

/EC_number="6.3.5.5"

/inference="ab initio prediction:Prodigal:002006"

/inference="similar to AA sequence:UniProtKB:P63740"

/codon_start=1

/transl_table=11

/product="Carbamoyl-phosphate synthase large chain"

/translation="MPINKDLKKILVIGSGPIVIGQAAEFDYAGTQACQALKEEGVEV
VLINSNPATIMTDTNMADKVYIEPITLDFVTQIIRQERPDGLLPTLGGQTGLNMAVEL
ARAGVLERENVKLLGTQLTSIEKAEDRDLFRDLMRELEQPVPESVIVTTLEESLEFAN
EIGYPIIVRPAYTLGGTGGGICANEEELRETVAAGIRYSPIGQCLVEKSIAGMKEVEY
EVMRDKNDNCIVVCNMENFDPVGIHTGDSIVVAPSQTLSDREYQMLRSASLKIIRALN
IEGGCNVQFALDPHSFQYYVIEVNPRVSRSSALASKATGYPIAKMAAKIAMGYTLDEI
VNPVTGQTYACFEPTLDYIVSKIPRWPFDKFISANRKLGTQMKATGEVMAIGRTFEES
IHKAVRSLEIGVHRLYLKDAETLDEATLNERLIKADDERIFLIAEAFRRGYTLQQLQD
LTKIDWWFLDKIEGLIAFEDRIREESELSSDILYQAKRLGFTDRAIAELRAQGQPGGT
LTTEAEVRTRREAENLRPVYKMVDTCAAEFEATTPYYYSTYETENEVIPSDKKKVVVL

GSGPIRIGQGIEFDYSTVHAVWALQKAGYEAVIINNNPETVSTDFNTSDRLYFEPLFF

EDVMNVIEQEKPVGVIVQFGGQTAINLAAPLRNAGVTILGTDLESIDEAEDRKKFERL

LSRLEIAQPKGKTVISVDDAVETAQSLGYPVLVRPSYVLGGRAMEIVYSDAELLTYME

QAVKINPEHPVLIDRYMMGKEVEVDAICDGETVLIPGIMEHIERAGVHSGDSIAVYPP

QHLSQDLKEKIVEITIKIAKELKTVGLVNIQFVIHDGQVYIIEVNPRSSRTVPFLSKV

TNIPMANLATQAILGVKLKDLGYVDGLWPESDHVSVKVPVFSFAKLRRVEPTLGPEMK

STGEVMGRDPNYAKALFKGLIGAGMKIPATGAIVVTVADKDKDEAVPLLEGFYRLGYK

IMATGGTAAALEAANIPVTTVNKLSEGSPNILDMIRSGEANFVFNTLTKGKTPQRDGF

RIRREAVENGIVCMTSLDTIRALLIMLQTINFSSEAMPVSVK"

CDS 1142143..1142883

/gene="pyrF"

/locus_tag="EFAGFIKM_00986"

/EC_number="4.1.1.23"

/inference="ab initio prediction:Prodigal:002006"

/inference="similar to AA sequence:UniProtKB:P0CB75"

/codon_start=1

/transl_table=11

/product="Orotidine 5'-phosphate decarboxylase"

/db_xref="COG:COG0284"

/translation="MNHTNFNEMASRLMVALDYPGAEEAQELVQALEGIPCYLKVGMQ

LFYAAGPDFIRELKTKGYSVFLDVKMHDIPNTVRGGAESITRLGVDMFNVHAAGGALM

MRAAREGAEAAIAADPSLSKPEIIAVTQLTSTSLETMNNEIGIPGSVEAAVVHYAGLA

QEAGLDGVVASPLEVPAIRAACGSAFHTVTPGIRPAGSGLGDQTRVLTPGEAIARGSH

YIVVGRPITGAPNPREAVETILKEMLNA"

CDS 1142880..1143521

/gene="pyrE"

/locus_tag="EFAGFIKM_00987"

/EC_number="2.4.2.10"

/inference="ab initio prediction:Prodigal:002006"

/inference="similar to AA sequence:UniProtKB:Q81WF6"

/codon_start=1

/transl_table=11

/product="Orotate phosphoribosyltransferase"

/db_xref="COG:COG0461"

/translation="MIELNEIPNHIASQLLKIKAVALRPQQPFTWTSGIKSPIYCDNR

LTMSYPEIRNDIAEAFATIIRNQYPDAEVIAGTATAGIPHAAWVAQKLNLPMAYIRDK

AKGHGKENLIEGLITEGQKVVVIEDLISTGGSSIKAAEAVRVAGATPLAVLAIFSYQL

DKGVKAFEDAGIPLQTLSNYTALMDVALAQGTIQESDFELLKSWREDPSSFGK"

CDS 1143800..1144741

/gene="btuD_4"

/locus_tag="EFAGFIKM_00988"

/EC_number="7.6.2.8"

/inference="ab initio prediction:Prodigal:002006"

/inference="protein motif:HAMAP:MF_01005"

/codon_start=1

/transl_table=11

/product="Vitamin B12 import ATP-binding protein BtuD"

/translation="MIAGMKNLFLRKRPAKSQSGDEHQQGETPLEASTESGENTYNNE

HDYLTSKPPLPESEIASDEVAATAVTTVDEPQKKIKPKKDMLPPYDGPVLEVRNVHRS

FQTGSRIIHVLKGIDMEVNPQQLVMLKGRSGSGKTTLLNMLGGLDQPSSGDILFSGQP

LQDWGDRRRTALRRKEIGFIFQAYALMPLLSAWENVELSLRMADVPRAEWKDRVGHCL

DLVGLSKRVKHRPFEMSGGEQQRVAIAKAIAHRPRLLLADEPTAELDSKMGAQVMAVF

RNIIEVEQVTICMTTHDPTILEVADHVYEMADGRFIK"

CDS 1144704..1145813

/gene="macA"

/locus_tag="EFAGFIKM_00989"

/inference="ab initio prediction:Prodigal:002006"

/inference="similar to AA sequence:UniProtKB:P75830"

/codon_start=1

/transl_table=11

/product="Macrolide export protein MacA"

/db_xref="COG:COG0845"

/translation="MFMKWRTADLSSKGAAPKRGKRAALIVLGAIMVATMSGCSLLPS

ETEEEVLPPITPPTISKKPEYEVRTETLEKKVSGSGKMMSQREEKVYFTLDGMHVKEL

NVKPGDKVKKGQLLAVLDVESVEKEIRGKKLAIRKSEVQMKETLRKKDEMDPVEFEEA

TIAFEELRQELADLEEQLGKATLTAPFGGTVIAVQVEKGAAVKAYDPIATIADTSNLV

VAATFAKEDLEKFSAGMKAEVDINGAGKVAGKIKVMPIAEASGSGSGEGAGEGGTPPT

KETLDKYVIVTLAKMPKGVERGTPLSVSIVTQRTENAIVIPVSALRSIGSRTYVQVVE

SDGSKREVDVEVGQQTSTDVEILKGLTVGEKVVGR"

CDS 1145813..1148734

/locus_tag="EFAGFIKM_00990"

/inference="ab initio prediction:Prodigal:002006"

/codon_start=1

/transl_table=11

/product="hypothetical protein"

/translation="MGLPLLRLLFRKMWNTRWMTFSTLIGLIVAVAFTVSIPMYADGA

LKRVVAQTLQDNSEGLPAGSLLMSYQAPGGVKTDTRGLDEVDRYIREDVPRDIGFPFH

TYVNSRSIRSTEVSPEDPTKVDASRARSMSLGTMSGLDAQVNYSAGVKPGNQVKDDTI

EAVMLEEGMYRNDLHIGDILEYPVYSGLDITLRVKITGSFKADDPNSPYWVQGFDGMM

NGLYVDESVFNDVLLKEKGIPLQNSRWYYAFDLKEIQTSQLSGLTSMLERLDIDLYQR

LKDTKVDITFGDLLKQFRSQSLQLQTMLFTLAAPMIAMVFYFIAMNARQSLQKQESDI

AVLRSRGASARQIFSLYLLEGIVLGAIALVIGPLLGWFMAKSIGSASGFLSFVDRKSI

PIGVSKEAILLGVAAVLVAIIASLIPAITYARATIVSAKRRQARTDRAPVWQRWFLDV

VLLGLAGYGYYLFYERQMLTFQTGMTTDQLQVQPFLFFVPALAIFALGLFFLRLFPWI

LKLIQLIGRKFLPVPLYLTLTQLSRSSSSYYPLMILLVLTLGLGVYNSAAARTIDLNS

TERTLYRYGSDVIMQTVWEGTPEVKPGGSGQNGGTGGGQQGGGNGGGGSAGGGSGGGN

GGGGAPGGGGGGSSQPSKVIYSEPPFEVFRRLDGVEHAARVLQTKGNIIVSGKSGGQGM

LVGIDNVDFAQVAWFRNDLFPAHPYKYLDLLGKYEGAVLISSKFADKFKLKTGDLVSM

GVQGQAIEFVVFGIIPYWPAQYPDQMPFFVANLDYVYDQVPLIPYEVWLKMEPDAKVA

PLMEKLATEGIELSSVRDVRTELVSQAKHPSRGGVFGILSLGFLVSVIISLIGYVLYW

FFNLSGRVVQFGVLRAMGLSRAQLSGMLLLEQVFTAGLSILLGIGIGQVSSRLFLPFL

QTTDNVSAQVPPFRIVFEQQDMLQLYGVTVVMLVIGATMLLWQIRRLRVHQAVKMGEE

R"

CDS 1148737..1149600

/gene="btuD_5"

/locus_tag="EFAGFIKM_00991"

/EC_number="7.6.2.8"

/inference="ab initio prediction:Prodigal:002006"

/inference="protein motif:HAMAP:MF_01005"

/codon_start=1

/transl_table=11

/product="Vitamin B12 import ATP-binding protein BtuD"

/translation="MIQCEGLVKIFKSSDVEVVALQGLNLTVNQGEMMAIIGNSGSGK

STLLNILGGLDRPTAGTAVVGDWDLLKMTDAQLVEYKRHTVGFIWQNNGRNLLPYLTA

LENVETPMILGGKRDRAYAKQLLEWVGLKDRMHNKLHQLSGGEQQRVAIAISLSNRPK

ILLADEPTGSVDSETCDTIMGIFRRMNKELGVTIVIVTHDLTLAGKVDRIVAIRDGLT

STEFVKRNPNLDDEHNLSEAGMPDIHEAFVIIDRAGRLQVPKEYLEALSIDNRATLEF

DGERIVITPPR"

CDS 1149621..1151072

/locus_tag="EFAGFIKM_00992"

/inference="ab initio prediction:Prodigal:002006"

/codon_start=1

/transl_table=11

/product="hypothetical protein"

/translation="MKNSMWGKRVLAVIATATLALPLIAGCTASEAKDTEQRVLRVAT

LWGGQDDSYFRQQFTDAFELTHPNVTIEIVAAVDQSSMYGYGNTEEQPEVPDTMESLK

KIMTGDNPVDVIVADTATVKSLIQENLVKQLDPLMQEDKFDTSDIVPSVLEGIKDLGD

QSIYALTPTFSSSALFYNKGMFEKAGVEPPTDNMTWDDIFNLGTRLTKGEGKDHVFGF

SFTTYQGGSPYYSMQQYYSSLQLKTFDDKAEKMTVDSPQWEKVWSTISKLAIDKVIPK

GDEPQDQNPNGRYDPVQGDLFLSGKSAMVIGDYSYINQLIDANKNADKMKDFTKVDWD

VVTPPVHPEAPEIGGSIYLSNLMAINSAAQNPDDAWELIKYMNSEDWAKIKARSSYEM

VSRKSFIKPKDGLDYNIQAFYALKPIPPTNTNLDKMYQKSPGLWQVNEKGMEYFNQVL

ENKKTPKEALGEWAAKGNEMLEKLKKDPKATFQ"

CDS 1151179..1151370

/locus_tag="EFAGFIKM_00993"

/inference="ab initio prediction:Prodigal:002006"

/codon_start=1

/transl_table=11

/product="hypothetical protein"

/translation="MTTPERTSGMHWVYFSKLYATKFQAGCLAKRMEQDGWIYGHNEP

AEVEVYRSRKGRYGVRFIP"

tRNA 1151442..1151515

/locus_tag="EFAGFIKM_00994"

/product="tRNA-Met"

/inference="COORDINATES:profile:Aragorn:001002"

/note="tRNA-Met(cat)"

tRNA 1151572..1151645

/locus_tag="EFAGFIKM_00995"

/product="tRNA-Met"

/inference="COORDINATES:profile:Aragorn:001002"

/note="tRNA-Met(cat)"

CDS 1151825..1152250

/locus_tag="EFAGFIKM_00996"

/inference="ab initio prediction:Prodigal:002006"

/codon_start=1

/transl_table=11

/product="hypothetical protein"

/translation="MSQFIDARTSQNASLANSIAIPILVINTPQLFGQIGLVTGGSIG

ANPRVQFKGTVSVQLPLALAGITITIVRGTLATDPVIYSVTSTFSLSVLAPQIITFSA

DDFNPPITPQLTYTAFVSSNLLGTIRVGPENFDGVLYSD"

CDS 1152488..1154044

/locus_tag="EFAGFIKM_00997"

/inference="ab initio prediction:Prodigal:002006"

/codon_start=1

/transl_table=11

/product="hypothetical protein"

/translation="MTSNESQRFRYSEAPVWDWQRAYYEQKGLQAWTENQVPQYITSN

PLIATAYAEMIFGFLQDLSNKGKIRETVTILELGAGVGRLAHQVLLKLLELKEFAGIP

LPPFRYVMTDLVAENVLGWQEHPSMQSFIQQGIVDFARFDAVADTELNLVVAGTVIRP

GDLKQPLLLIANYFFDSIPQELIYIGDGEIYECDLLVQSPDRRLDLEPAEMLKNMTLS

YEYRRAPEYSAGNYPYQELITLYKEELEDSHILFPAIGLSCLERLNKLSQSGYVLITA

DKGDHRLDNWKFAEPPEFVLHGSFSLTANYHAIQYVLEQQGAHTRFTTHHYKDLNVGC

MLMVDEPISYVNTRLAYHRFVERFGPDDFFSMKQWVDSRIESMELKHILPFWRLGGYD

AEFLIHSATHISSLLPDASDEEMLDIQSGIHTMWSSYYVMEQQGGLAFLAGQLLYEMY

MYEDAKRFLEISLVADPSNHNSAVLYDLAVCCYELELEEETLSYTHKVLALEPDHEEA

AALLQSFELI"

CDS 1154183..1154350

/locus_tag="EFAGFIKM_00998"

/inference="ab initio prediction:Prodigal:002006"

/codon_start=1

/transl_table=11

/product="hypothetical protein"

/translation="MKKMLSIALYPLAWVLGALSILSGNENSVLLVKDIATDRSYDGQ

AIYDSIERRNA"

CDS 1154527..1155225

/locus_tag="EFAGFIKM_00999"

/inference="ab initio prediction:Prodigal:002006"

/codon_start=1

/transl_table=11

/product="hypothetical protein"

/translation="MKIIIGQPRLEQHLLQLEAELKQHPDADVVFFPEGYLNQNVEDA

CRLAAEYGTMIVSGHRRLHERPKDRSIIISTAGEIVLEKAKYTPAETVEEQEWAISTL

LCDELVLQGFRNENIGNVDIVMHSIGVGMFSEEQYSEWVEEARQIALKHQCIVMGTSH

ADGSYRDSEISIPIAYCITADGEVVLASRSDTRTRTILLDRGNTDPSPQESQEKQLKI

SITHPVKLPGMNPR"

CDS 1155317..1155439

/locus_tag="EFAGFIKM_01000"

/inference="ab initio prediction:Prodigal:002006"

/codon_start=1

/transl_table=11

/product="hypothetical protein"

/translation="MSQVGYGCGNVGGFGGGWTSTSAILVLFILLVIITKSFWL"

CDS 1155600..1155929

/locus_tag="EFAGFIKM_01001"

/inference="ab initio prediction:Prodigal:002006"

/codon_start=1

/transl_table=11

/product="hypothetical protein"

/translation="MPFIRFKGFTGPQLEEVVPQITEQMALITHIPRERMKAERHDVQ

ALTPSPASIEILMFQRDQEIHNRIASSVQAILEEAYMPDVHIFFNILSPTLYYKKGRP

LTDYRLD"

CDS complement(1155971..1157194)

/locus_tag="EFAGFIKM_01002"

/inference="ab initio prediction:Prodigal:002006"

/codon_start=1

/transl_table=11

/product="hypothetical protein"

/translation="MNIAFYPYWAAKTLLSLINVIYIMVITTFIYGLTGSVLSAAIFP

LIQIVARIIAGFTLPLLVNRSPFSRLLISISIAKTLIMTCIAISLNHLISQLPLLLIG

VVILSFLDGWETPLLKTLTPRLVQGEDLIKANSLLSFSNQTVTIVGYAMTGFAVMNWG

ASQTFWAATSLSWAVLIFMIAMGSLTRDVEQPQKSAVSWNVLSEGWSILWNNRSLRLI

TLMDIAQALAGSIWIGAVTLAFAKEALGRGEGWWGLMNSSYAAGTMLGGILALALAKR

IQKHLIASMTIGSLLFSLLTVVYGLNNLPWLALVLCILMGPVHQIRDVAQQTALQNSV

PVESLTKVYATHGVLISTVMSVSIVIFGLVADQLGVRWVYLIAGALFILSAICSLSLS

RVHRNQPIAKHTKNM"

CDS complement(1157335..1157523)

/locus_tag="EFAGFIKM_01003"

/inference="ab initio prediction:Prodigal:002006"

/codon_start=1

/transl_table=11

/product="hypothetical protein"

/translation="MRTLFFIFMVLCIINLCFPKFGWYLRYGWISRGEAEPSRPYMTM

VRTTSLLMLLIAFTLAMA"

CDS 1157837..1158985

/gene="rcsC_2"

/locus_tag="EFAGFIKM_01004"

/EC_number="2.7.13.3"

/inference="ab initio prediction:Prodigal:002006"

/inference="protein motif:HAMAP:MF_00979"

/codon_start=1

/transl_table=11

/product="Sensor histidine kinase RcsC"

/translation="MSQHNQLSLQWEFIISKVKSSVTVVDATLPELPLMYVNEHFTQL

TGYTYEESVGHNCRFLQGQDTDPETVMQIRDALKKQQSIKIDILNYTKSGQKFWNELN

IDPIFNESGECLYFVGIQYDISERKYAEQQLKFATSMAEMNSRGQLEFIGKLNHELRT

PLNGIMGMIELASMGETTDEQKEYLELARQSGEALLNIINNSLDMAKLGRGKMDVENI

EFQPLKLIQQIVKTHEPAARNKQIRLLCHADLNVPDVLIGDPLRLRQVLDNLLSNAIK

FTEQGEVQLQVDVRQQLMDTVVLVFSVRDTGIGIPQHQIEQLFDAFTQTDISHARRFG

GSGLGLTICKELLELMNGQISVESTEGKGTQFEVTLPLLRQQAIPNVG"

CDS 1159095..1159577

/gene="nucB"

/locus_tag="EFAGFIKM_01005"

/EC_number="3.-.-.-"

/inference="ab initio prediction:Prodigal:002006"

/inference="similar to AA sequence:UniProtKB:P42983"

/codon_start=1

/transl_table=11

/product="Sporulation-specific extracellular nuclease"

/translation="MSKRRTRSKRKGKQGFKKQVLTLVAMLLVALYAWAGGEWPEEIP

NPFVGTNKSVDHTITFPSERYPETAKHIKAAIKAGHSDVCTIDRNGAEGNRDLSLKGV

PVKKGKDRDEWPMAMCAEGGTGADIQYITPKDNRGAGSWVGNQLSTYPDGTRVKFVVK

"

CDS complement(1159772..1159996)

/locus_tag="EFAGFIKM_01006"

/inference="ab initio prediction:Prodigal:002006"

/codon_start=1

/transl_table=11

/product="hypothetical protein"

/translation="MKKEVEIAIRRKQQIMVRNTSGQTVTGFPERTADTSILSLRTVH

GSLWIPFDEVEHVTRLLSIRSHHLSGMQLV"

CDS 1160204..1160653

/locus_tag="EFAGFIKM_01007"

/inference="ab initio prediction:Prodigal:002006"

/codon_start=1

/transl_table=11

/product="hypothetical protein"

/translation="MTQLIIGLARTENEAILMLNQVKEEGIKDKYLGAVAKEQLNLEL

VSEKTGLPKPLKGAGTDGAFGALKGILAGLGKRMDQTMSIGNAVRRLAGNEIGSETDD

LVLTLTEAGISEEDARYYEDWLLKDHILVIVECSEEEAARVRPILLF"

tRNA 1160889..1160977

/locus_tag="EFAGFIKM_01008"

/product="tRNA-Leu"

/inference="COORDINATES:profile:Aragorn:001002"

/note="tRNA-Leu(taa)"

CDS 1161348..1164269

/locus_tag="EFAGFIKM_01009"

/inference="ab initio prediction:Prodigal:002006"

/codon_start=1

/transl_table=11

/product="hypothetical protein"

/translation="MLKQLKPYKVLQERRIEELKSDAYLLEHQKSGAKIVLLSNQDDN

KVFSIGFRTPPEDNTGVAHILEHSVLCGSKKFPAKDSFVELLKGSLNTFLNAMTYPDK

TIYPIASRNDHDFHNLMDIYLDAVLNTNIYENEKIFLQEGWNYNLTSADDELTYNGVV

YNEMKGAFSSPERMVRREVLNSLFPDTTYSSESGGFPEAILDLTYQGLLDFHTRYYHP

SNSYIYLYGDMDMEEKLQWLDENYLSAYDRIEIDSAIQLQPAFAERVDTVKTYSAGST

ESEVDNSFLTYNAVIGTSLDKELNISFQILLYALLNAPGAVLKQALLDKGIAKDVYGS

YDDSLYQPVFTVGLKKSNLASKEDFLGTVKEVLERVVKEGFDPKALLAGINSYEFNHR

EADYGRMPKGLIYGFSSLQSWLYDEEAPFTHLEANDVFAELRTKMNEGYFEQLIEKYI

LQNTHTSFVALTPDKGLNSRKEEALKERLKTIQAGLSEEEVQTLIQKTEALAEYQNTP

STKEQQQVIPTLSIEDIEPKASTLHQTVNQVDGTTILHHNLYTNGIGYLRLLFDIKEV

PRHLLPYAGLLKNVLGYVDTQNYSFNELSNEIHIHSGGIQSGIGSYANAHKHNEFKAT

YEFNAKVLYDKLGFAFDMIKEIVFTSQFDNSKRLYEIISQMKGNLQRNLINSGHSAGI

GRSSSKHSAVADFREAVSGIAFYQWLEDLQANFEARKEELSSSLQELTGYIFRPENLL

VSYTADEQGYEGLEQQVSDLKAKLFTHEVAKEEFTFTPATHKEGFRSPSEVQYVVQTG

NYIDKGYQYTGSLRVLQGILSLDYLWTNIRAKGGAYGCMSGFRRNGDSYVASYRDPNL

DKTYKVYEEIPQYLNDFRADEREMTRYIIGAIQDLDTPRTPYGEGAFSLECYLSNVTE

ADLQQERDEVLSTKESDIIGFAELLSTVLKQQQRCVIGNENKIEEQKQMFDETLDLIK

N"

CDS complement(1164415..1165122)

/locus_tag="EFAGFIKM_01010"

/inference="ab initio prediction:Prodigal:002006"

/codon_start=1

/transl_table=11

/product="hypothetical protein"

/translation="MNNINQEVGKKIRNFRKWRGLTIQQLADQIFKSKGTLSKYESGD

ITLDLVTLHHIANALNIQVEQLLYQEPRHASPLMNAVPSSFFKNSTRFYSYFYDGRNN

SLIRCVIDMMAQSDANRYRTVMYMNVKDFENYQECENMYWGHTEHYDTLTTLILKNQA

TPLENLYINILASFQESEKKWGLMAGVSFRPFMPIALKMLFSRTPLPENQELYNELKI

SKEDLRTLKIYNMLAVT"

CDS 1165374..1166594

/gene="patB_1"

/locus_tag="EFAGFIKM_01011"

/EC_number="4.4.1.13"

/inference="ab initio prediction:Prodigal:002006"

/inference="similar to AA sequence:UniProtKB:Q08432"

/codon_start=1

/transl_table=11

/product="Cystathionine beta-lyase PatB"

/db_xref="COG:COG1168"

/translation="MKYNFDEIIDRTGTNAMNTDGFRQYIFNATEDMTFPFKDEEFIR

MWIADMEFATPPEILDAVKERLDRRIMGYSQVFDPAYYEAVSNWMKRYYDWSFPKEQL
ETSNGIIPALYELVEYICQSDEKVLIVTPSYGFFKSAAEHNNLELVCSDMLNEQGRYS
IDFEDFEAKAKDEKVRVCIFCNPHNPSGRVWTTEELQRVGEICLRNNVWIISDEIHCD
LLRTGKRHTPLAKLFPDTDRIITCMSPSKTFNMAGLMFSNVIIPNKALRDIWQARHYS
FKNPLSIAATQAAYETGDEWLKQLKAYLDANFACVEQYVKQHLPLAVFHIPEATYLAW
IDISAYAPQHVSLPLFFAEQAGVLLEDGHMFVANGEGCIRLNLACPRSVLQEGLRRIS
SVLVKEVEEVPVQV"

CDS 1166837..1167505

/gene="ung"

/locus_tag="EFAGFIKM_01012"

/EC_number="3.2.2.27"

/inference="ab initio prediction:Prodigal:002006"

/inference="similar to AA sequence:UniProtKB:P39615"

/codon_start=1

/transl_table=11

/product="Uracil-DNA glycosylase"

/db_xref="COG:COG0692"

/translation="MFGNDWDKVLQEETEAEYFNKIRYTLAAEYKTQTVFPPKEDLFS
ALKLTPYHQVKAVIIGQDPYHGAGQAHGLSFSVRPGVRVPPSLKNIYKELHADLDLPI
PNHGSLVHWAQQGVLLLNAVLTVREGQPNSHQGLGWQTFTDAVIRALNERSEPMVYML
WGSHAQKKGAFINRDKHLVLESTHPSPLAAHRGFLGSRPFSKANDFLTSKGIEPIDWK
IPEN"

CDS 1167525..1167944

/locus_tag="EFAGFIKM_01013"

/inference="ab initio prediction:Prodigal:002006"

/codon_start=1

/transl_table=11

/product="hypothetical protein"

/translation="MRHWIGRKVAVYRATGIREPLKGTLVEWDEEAECVRIGPKRIKV
SFDNIAMIRPLPEESLPLKKARSEVHKVGYVMKKAIQFENAIYFKSQVMIWRRAKIVA
LSTTILRHDEDQVELADGRVLRKDKHMFVVRSRRGIR"

CDS 1168366..1168950

/gene="yvdD"

/locus_tag="EFAGFIKM_01014"

/inference="ab initio prediction:Prodigal:002006"

/inference="similar to AA sequence:UniProtKB:O06986"

/codon_start=1

/transl_table=11

/product="LOG family protein YvdD"

/db_xref="COG:COG1611"

/translation="MKRIAVFAGSNPGNHPDYTEKAIQLGKQIADSGYALVYGGSCMG

LMGAVADAALEQGGEVIGVMPTGLFRGEVVHGGLTQLIEVGTMHERKATMAELSDGFI

ALPGGMGTFEELFEVLCWAQIGIHRKPVGLLNVNGYYGPLMKMVEHSVQEGFSNTSHL

SLWSLEADPAELIKQMSSYIPAELTQKWSQLNGE"

CDS 1169071..1169199

/locus_tag="EFAGFIKM_01015"

/inference="ab initio prediction:Prodigal:002006"

/codon_start=1

/transl_table=11

/product="hypothetical protein"

/translation="MSELKGTGYGYGGGFSGGAWTSTGAILVLFILLVIISRTFIL"

CDS complement(1169418..1169684)

/locus_tag="EFAGFIKM_01016"

/inference="ab initio prediction:Prodigal:002006"

/codon_start=1

/transl_table=11

/product="hypothetical protein"

/translation="MNPSKKKSIPTRPHTSQLQSASRLSTSSVHIKNKSGKTANQRSL

NSSSPNTLDQLAIVAAILSLIAAAIGLYIAWKSLSVPDQTAVVV"

CDS 1169916..1170653

/gene="glpQ_1"

/locus_tag="EFAGFIKM_01017"

/EC_number="3.1.4.46"

/inference="ab initio prediction:Prodigal:002006"

/inference="similar to AA sequence:UniProtKB:P37965"

/codon_start=1

/transl_table=11

/product="Glycerophosphodiester phosphodiesterase"

/db_xref="COG:COG0584"

/translation="MRNMEIIAHRGASAVCPENTMSAFERSLELGATGIETDVQMTSD

GRLVLIHDETLSRTGGAEGWVKDATYDQLRTRDAGSWFHADFTGERIPSLEELFKLVQ

GKGTLLNLELKNGIVSYKGMEEKVIQAIRDWNLEQQVVLSSFNHASLVRCKRLAPEIR

TALLYMEKLYRPYDYAAKLEATGLHPYKLAVTQEDVAAALAHGVVTYPFTVNDPAEMQ

AMIDMGVQGIITDVPDVLASLLTVHAR"

CDS 1170926..1171981

/gene="feuB_1"

/locus_tag="EFAGFIKM_01018"

/inference="ab initio prediction:Prodigal:002006"

/inference="similar to AA sequence:UniProtKB:P40410"

/codon_start=1

/transl_table=11

/product="Iron-uptake system permease protein FeuB"

/db_xref="COG:COG0609"

/translation="MSSQASPEKHNPDSPTAKIHTRPWAATLILTGGVLLLALGMALS

ISFGAADIKLSVVWKAIFDFNPELTPHQIIWEIRLPRILGGAMVGACFAVAGAIMQGM

TRNPLADSGLLGLNAGAGFALAVCFAFFPGLPFMYIIMYSFLGAGLGVLLVYGFGAAS

KSGLTPLRLVLAGAAVSAMLSALSEGIALYFRIGQDLAFWTAGGVAGTKWSQLEVMFP

WVLAALIAGLLISRSITLLSLGEDIAVGLGQRTGLIKLIGLIVVLILAGTAVSVVGAV

GFVGLIIPHLTRKLVGVDYRWIIPCSAVMGSLLLVFADLAARMINPPYETPIGALVAL

IGVPFFLYLARKERRTL"

CDS 1171984..1173015

/gene="feuC_1"

/locus_tag="EFAGFIKM_01019"

/inference="ab initio prediction:Prodigal:002006"

/inference="similar to AA sequence:UniProtKB:P40411"

/codon_start=1

/transl_table=11

/product="Iron-uptake system permease protein FeuC"

/db_xref="COG:COG0609"

/translation="MESSTIVGAERKKRTKSTIVLIVLALLIITAFVVSMNTGFTKLS

PLEVLRTLFGGGTAKQELILFEFRLPRIVISVLVGAGLALSGCILQGVSRNALADPGI

LGINAGAGLVVMLFVSFFPTTTAAPVFLLPILALIGSGFAAFLIYILSYKKGEGILPT

RMLLTGIGVAAGISSAMIVLTLRLSPEKYQFVATWMAGSIWGSNWKFVTALLPFLIIL

VPLVLYKARVLNVLNLGDQTASGLGTPVERERLILLAAAVGLAGSCVSVSGGIGFVGL

IGPHLARRLVGPKHQFLLPASALIGSLLVLVADTLGRVILQPSEIPAGILVAIIGAPY

FLYLLSKTK"

CDS 1173060..1174775

/locus_tag="EFAGFIKM_01020"

/EC_number="3.6.3.-"

/inference="ab initio prediction:Prodigal:002006"

/inference="similar to AA sequence:UniProtKB:Q99T13"

/codon_start=1

/transl_table=11

/product="Putative multidrug export ATP-binding/permease

protein"

/db_xref="COG:COG1132"

/translation="MLRRFFAYYRPYKKLFILDFSCAILVALLELAFPLAVNRVVDDL

LPGGRWDWILWACLALLGIYLLNSFLNFVVTYWGHKLGINIETDMRKSLFNHVQKLSF

RFFDNTKTGHLVSRMTNDLMDIGEIAHHGPEDVFIAVMTLIGAFSIMMSINGNLAVLT

FIIVPLIIYLSLYFGSKMSKAFSRMFGDIADFNARVENNITGIRVVQAFANEEHEKAQ

FAVNNGRFRQTKLIAYKIMAWNSSVSYMLMKLVSLFVLVCGTWFVIQGSMTYGQFIAF

IMLSNVFLTPIQKINSVIETYPKGIAGFKRYTELLDMEPDVEDRPGAVTVSHLRGDIR

YEQVTFGYSDQEPVLKGIDLNVRAGETVALVGPSGAGKTTLCSLLPRFYDVLEGRITI

DGQEIQDMTLDSLRSHIGIVQQDVFLFDGSIRENIAYGKLDASEEEIWMAARRAQMEP

LIQSMPEGLDTLIGERGVKLSGGQKQRLSIARMFLKNPPILILDEATSALDTETEAAI

QQSLAELSEGRTTLVIAHRLATIKNADRIIVVAEQGITEQGRHEELLAAGGVYSRLHY

AQFGA"

CDS 1175008..1175337

/locus_tag="EFAGFIKM_01021"

/inference="ab initio prediction:Prodigal:002006"

/codon_start=1

/transl_table=11

/product="hypothetical protein"

/translation="MQLNDMLKILITCLLFILIWWFLIQGIRKLSSTRAGSGFQRLGG

WSIGLFIVGAVPLGFSLYTEIFDKRLDANIGLGLAMFFVWAYCVILLCAALVVWGRYV

YKRDMKK"

CDS 1175463..1176032

/locus_tag="EFAGFIKM_01022"

/inference="ab initio prediction:Prodigal:002006"

/codon_start=1

/transl_table=11

/product="hypothetical protein"

/translation="MSNLRRVLILLSILIVCLTGCSGHIECNSVQTQANAPSEDGFTG

YVVERKDNSILVVDPAYEDFSANGGGNRYYPAKWFSNAPDPQIGSYVEVWTDGGPENQ

PYPGQARAEKIAVSCPNTPDGAHLSEADAIRGGLYSPDGEKVRVPVIEDVQFYQDAGT

WAIRIRDAMSTTKNQDEIEIRVEDVEPVE"

CDS 1176077..1176331

/locus_tag="EFAGFIKM_01023"

/inference="ab initio prediction:Prodigal:002006"

/codon_start=1

/transl_table=11

/product="hypothetical protein"

/translation="MDIRECLPRDKHDFEAVRKLNQFSDVELKVIIPELMEWLQDGNW

PISKPVENLLLRLGEDLVPHIKDVLQTQDSTWEYFIFWWG"

CDS 1176564..1176833

/locus_tag="EFAGFIKM_01024"

/inference="ab initio prediction:Prodigal:002006"

/codon_start=1

/transl_table=11

/product="hypothetical protein"

/translation="MLKYQLLFPTFLSSEIYCLHIVQLLIELFDISEKEAFLRINSFW
EGNDFLSEEDLIFHESPEFWAKTIYYEESNWWNFPENELIPRKID"

CDS 1176926..1178011

/locus_tag="EFAGFIKM_01025"

/inference="ab initio prediction:Prodigal:002006"

/codon_start=1

/transl_table=11

/product="hypothetical protein"

/translation="MELSRSQQLAQEHVTNYARSRKNEAEQTIREILRMSNIELKTFE
KAIKKLKSHTSIALHFHPDRLDPNMKSVAEALLEQGFYKSQFETFLSNGSVSAFSGGE
RDVWENKMFGGAYQINGSTNSERPKYGALNVMLHPDGPAPRFGSCYFLLSTEVSQRCT
YTYLDSHQDPEEKGTYEEFDLILAALMRDVFNSGFAIGERNLTVQKLIDHMLVNLEKP
FQNPSNQEPNRNLDYYIEAQIHGDISLEKDVKMLVADPSFKGTHTGRILEQICLKYSI
DLHWHMGFTLLVNDVPIDFRGPSMPSLAKRIAKSDLIDANIIGSAAMDLKQNPSHWSD
RGTYKEVLQELKLLWHVLVRYGKPMEE"

CDS 1178319..1178498

/locus_tag="EFAGFIKM_01026"

/inference="ab initio prediction:Prodigal:002006"

/codon_start=1

/transl_table=11

/product="hypothetical protein"

/translation="MSAKKVGRPKSDKPKSRTIEIRVDEEIMNKLDISAEKLNTNRSD
IVRKGIEMIYDELQK"

CDS 1178641..1178934

/locus_tag="EFAGFIKM_01027"

/inference="ab initio prediction:Prodigal:002006"

/codon_start=1

/transl_table=11

/product="hypothetical protein"

/translation="MNSILEALYNGRLRPDEMMMPTHPEYQALGRQIAALTEQWKNRL
SEEEFLELEQLFDLCGRCEGMHTEAAFAQGFRLGANMLIEVMSQREESVLEFN"

CDS complement(1179155..1180057)

/gene="ydbD_1"

/locus_tag="EFAGFIKM_01028"

/EC_number="1.11.1.6"

/inference="ab initio prediction:Prodigal:002006"

/inference="similar to AA sequence:UniProtKB:P80878"

/codon_start=1

/transl_table=11

/product="putative manganese catalase"

/db_xref="COG:COG3546"

/translation="MFKRLDEILIEIPNVEKPDPNAAAAIQELLGGKFGEMSTLNNYL

YQSFNFRSKEKLKPFYDLVMSITAEELGHVELVSHGINKCLRGSTEYREPDDTPLGSV

KDARLSYHYLAGAQGAMPFDSMGNPWTGANVFNSGNLVEDLLHNFFLECGARTHKMKV

YEMTDHPAAREVVGYLLVRGGVHVVAYAKALEIATGVNVTKLVPIPSLNNKAFNEARK

YEEKGVHTKLYTYSDKDFNTIGQIWKGTHPEDGQPLEVIQGIPEGFPIPEAPAVEEEF

APGISQEDFKEIARRLKMAGNIAD"

CDS 1180320..1180628

/locus_tag="EFAGFIKM_01029"

/inference="ab initio prediction:Prodigal:002006"

/codon_start=1

/transl_table=11

/product="hypothetical protein"

/translation="MNKRWIRITGIVMFLMIQMYISSEPATAYVVTNEVQVDEQTRQR

LEKKYGLDRPEEPKTQQHVFSGDWGMSFRTEPPDSFDRLVHVVPFSLRPLAWIFRLFV

"

CDS 1180685..1181005

/locus_tag="EFAGFIKM_01030"

/inference="ab initio prediction:Prodigal:002006"

/codon_start=1

/transl_table=11

/product="hypothetical protein"

/translation="MRMLIVTVMSVVLSLAGITSGTGAAIPDYAKWGIIAVKETQTKY

NVDILDYKHIGRTSLTADQSREQFKLWVRGKDGKQFAVYVNVDFNPSTQQLKKVQFSE

SDRH"

CDS 1181152..1181373

/locus_tag="EFAGFIKM_01031"

/inference="ab initio prediction:Prodigal:002006"

/codon_start=1

/transl_table=11

/product="hypothetical protein"

/translation="MLGKYIGQMVELVYMDRAGQITQRQIRINSVRGGMIRASAGKEG

SPRTFLEQGILAWRPVTDLNGEVKYAERL"

CDS 1181357..1181638

/locus_tag="EFAGFIKM_01032"

/inference="ab initio prediction:Prodigal:002006"

/codon_start=1

/transl_table=11

/product="hypothetical protein"

/translation="MLNDFERKILRIMVNYAGQRRRLPRMDELETKTGRSRAYIHESL

LELERTGHIAWENRSTLEGIQILQAWEQVQQPIRKSQPSGDSVKYFTDY"

CDS complement(1181714..1181950)

/locus_tag="EFAGFIKM_01033"

/inference="ab initio prediction:Prodigal:002006"

/codon_start=1

/transl_table=11

/product="hypothetical protein"

/translation="MDQTTRESFTAVQKNGDGDLTAFQTSAGRVLDYQQALAEVKAGA

IAGVNVFKGKDGEMYIRGDADGDPTNNLDQLPHF"

CDS complement(1182063..1182506)

/locus_tag="EFAGFIKM_01034"

/EC_number="2.3.1.-"

/inference="ab initio prediction:Prodigal:002006"

/inference="similar to AA sequence:UniProtKB:Q5HH30"

/codon_start=1

/transl_table=11

/product="Acetyltransferase"

/translation="MAAEISYVSTEEQLEQALGIRHHVFVIEQQVPAEIEIDQYDVIS

PDVHHVLLSTDGQAVATGRLIYYSKDTAKMQRIAVLESHRSFGYGRVLLLAMEELARE

LGLSYSVLDAQCQAQKFYEKLGYEVISEEPFYDADILHVRMRKSL"

CDS 1182616..1182729

/locus_tag="EFAGFIKM_01035"

/inference="ab initio prediction:Prodigal:002006"

/codon_start=1

/transl_table=11

/product="hypothetical protein"

/translation="MGMTGIMVITLLMSAGMITLLYYVTKKAYSRKWDEDE"

CDS complement(1182745..1183002)

/locus_tag="EFAGFIKM_01036"

/inference="ab initio prediction:Prodigal:002006"

/codon_start=1

/transl_table=11

/product="hypothetical protein"

/translation="MRNLTKRSKSNKYTFTKCFFLFAGLVILSTNVTGCGLEKDEHTV

QFQQLQQPHENSESLPVWLDVYSKSVIQDVYSPRGSSEVLP"

tRNA 1183205..1183276

/locus_tag="EFAGFIKM_01037"

/product="tRNA-Val"

/inference="COORDINATES:profile:Aragorn:001002"

/note="tRNA-Val(gac)"

CDS 1183512..1185725

/locus_tag="EFAGFIKM_01038"

/inference="ab initio prediction:Prodigal:002006"

/codon_start=1

/transl_table=11

/product="hypothetical protein"

/translation="MNYSNPFTSKLARVGIAGTLALILATSPLANMGVVAAEADPAPV

STKIYNTQKDSLEQVPIQYHIQARLNEKDMTLRGNEKITYRNTSNDTLNQLVLHTYAD

ANLSKSTQASMFKQANENISKNSPDKKPEDFLGGMDIQGVTTAGGQSLDFKNENQALT
VQLNEPVQPGKSVSFQVSFQLNIPYGSQRISYYQDIINGAHWFPVMSVYDEGKHQWDT
APYSQTFESDYYTSADYEVDLNVPDDYQVAMPGVITTREDTEHGRKIVSALAENTREF
VFFASPKFQVESVTRNGLTVEYYYFDNQPGKKEIVEQYIDQAFKAIDFFSDKYGKYPY
PEFRIVETYVQGVAVEYARLIQMGQIQNGAVPEEDTTFVHEIAHQWFHALIGNNSETE
SFLDEGFADFSMVYFAEKQGDKLNGFRSLQFDDSSVEVAIASTNDEVEDLASLVFYQK
GRQAIYQLYRSVGEEKFDQFMKEYFNRYVYQNATIDGLLHTIEDVLGKEQRMEMETAL
YKPNFVLKPEYQLSLEERTAYLHDVFQLQYQTVMKLIPDLPFEVMNRMMDKVLQGERL
TIVLSDRVSKIADKQQEAIVSQLTSFLDMSGVKYDVVRDRKELKQKMKKEIGNSNLIV
IGNAKSNGVVQALKSSIMDRANQIGFDWKQTMKQPLAAGAYITKHPYNQNRLMLHYFW
NEDHLSEARFEPFMMKIQQTIGFTNDFYQYYVLDNQGKEKSNKKVVNPISSLFAE"

CDS 1185908..1186180

/locus_tag="EFAGFIKM_01039"

/inference="ab initio prediction:Prodigal:002006"

/codon_start=1

/transl_table=11

/product="hypothetical protein"

/translation="MKDQHYPGVPMKGTLADYNCKGIKGMNKMKAYSYNARIRPIICN
TQEPAANEGNVVDFSILLKDTSAVLYSRPVNGNALLTKELSSHVYD"

CDS 1186167..1187165

/locus_tag="EFAGFIKM_01040"

/inference="ab initio prediction:Prodigal:002006"

/codon_start=1

/transl_table=11

/product="hypothetical protein"

/translation="MSMTDNIAQAALDAAMKANRAVAPYDYCDAESRRMYTPQFDKKM
DNPMYTAAMAGLQSIYWFRAIKVKGLIPDAKADYYATYSTNHNHIGGIGLAYSNSPTG
PWTKHGQVYLDTVVGNSTETPFVIWNDEEQLFFMYYQQNGLGINQSTALATSVDMINW
TRVGLILDKPPGFPGDGHTGYFSCFRNGGKWIGFSVMGGGDFGRKCIWHSHDGRSWLA
DPRPLMGGNDATLSAGENISRPNAGVFMYKGSMWWLGMLSAYASGGVTTVARPAIAKI
SADFRQLLDKPTDIYFPALTWETTNMQSLMVSVMDGVVYVYYNTDHNIGVAYSVGV"

CDS 1187168..1187854

/locus_tag="EFAGFIKM_01041"

/inference="ab initio prediction:Prodigal:002006"

/codon_start=1

/transl_table=11

/product="hypothetical protein"

/translation="MLIRDAAGQIVPFGKVHKQLVADFLYGTLPDWVEVVGTPTFEPL

PDRGTITVTTPTTAGATAELKMKHLIDSSKVTAIAITLEALQLNGNIGISVQVGIKSK

DSKAGITFFQNVNQKYGMVRAYKADGTYTDFNQNMVFFTTHGVAGDEAQNRKNLTVLL

CTGLQFSAGFERPSVWLGADDQYGGVAADLSGGLFQHGELQCLLKLTTSKAEAKYFKC

AQMKVDIWSN"

CDS complement(1187979..1189190)

/gene="chrA"

/locus_tag="EFAGFIKM_01042"

/inference="ab initio prediction:Prodigal:002006"

/inference="similar to AA sequence:UniProtKB:P14285"

/codon_start=1

/transl_table=11

/product="Chromate transport protein"

/translation="MENNSFLTPDKPKHTVRALSEVLAVSTKLGLTSFGGPIAHLGYF

HEEYVRRRKWMDERSYADLVALCQFLPGPASSQVGIGIGIIRSGLLGGLMAWLGFTLP

SVIALVLFAFLLQGFDISSTGWIHGLKIVAVAIVAQAILGMGQKLTPDRYRATIAIFT

AAATLVWQTAFTPILFIVIAGIIGMIYFRKMTGIKTADLSVPVSRNLAIVCLATFFGI

LILLPLITPFDRSGWLLFFDSFYRSGSLVFGGGHVVLPLLEREFVPTGLMNKSDFLAG

YGAAQAVPGPLFTFASYLGAMMRGIPGALVATIAIFLPAFLLIVGALPFWNSLCKSSK

IQGALIGINAAVVGILLASLYDPLWTTAILAPVDFALVCMLFLMLVFWKVPPWIVVVA

GAAGGTIMNLL"

CDS 1189408..1189827

/locus_tag="EFAGFIKM_01043"

/inference="ab initio prediction:Prodigal:002006"

/inference="similar to AA sequence:UniProtKB:Q7A6M9"

/codon_start=1

/transl_table=11

/product="Organic hydroperoxide resistance protein-like
protein"
/translation="MKTLYETTVINTGGRQGIVQSPDNVFMLDVAAPPELGGQVTTAT
NPEQLFAAGYSACFNSALEFQLKKHKVEIERSTVAATVMLVTDSEDNGVKLQVDLEVK
ILGLDEETAQKFVKLAHDYCPYSKGIKGNVNVNVELA"

CDS 1189973..1190152

/locus_tag="EFAGFIKM_01044"
/inference="ab initio prediction:Prodigal:002006"
/codon_start=1
/transl_table=11
/product="hypothetical protein"
/translation="MMDFSKPPHGSETDTHRQRHQENEQKYLSGGKSIWYEISNWLIL
GGILLVIFLLFKYVF"

CDS 1190627..1191181

/locus_tag="EFAGFIKM_01045"
/inference="ab initio prediction:Prodigal:002006"
/codon_start=1
/transl_table=11
/product="hypothetical protein"
/translation="MKWKRILLCVTVFSLLGGSLLFADSVNEKIRVLINGKEAADGGY
LIDGTTYVPVREAGGVVKWDSSNKRVTVIKPNVHIFLFKGDTVFGNVNVGKLKFNVFS
QVDSLTADVAAVKVTITNPSGQVKDIQSQELTTQKDNFWFRTYDFTYDFSRAGKYQVG
FHIKENANSSYVLVAEKIITALND"

CDS 1191279..1191596

/locus_tag="EFAGFIKM_01046"
/inference="ab initio prediction:Prodigal:002006"
/codon_start=1
/transl_table=11
/product="hypothetical protein"
/translation="MSDHTHEHGDGCGCGQDHDHDHEHEEVLLTLTDENGQDVEMVLV
ETFDVEKHVYALLLERNNPEADGIILRMEEEDEEMVLYNIEDEEEWNRVEAAYNELVA
SQE"

CDS complement(1191803..1193290)

/gene="speA_1"

/locus_tag="EFAGFIKM_01047"

/EC_number="4.1.1.19"

/inference="ab initio prediction:Prodigal:002006"

/inference="similar to AA sequence:UniProtKB:P21885"

/codon_start=1

/transl_table=11

/product="Arginine decarboxylase"

/db_xref="COG:COG1982"

/translation="MTALMNHHRTPLFTALKNHAALNPVQFHIPGHKKGLGADTEFRE

FIGDNAFSIDLINIAPLDDLHQPTGVIQEAQILAADAFGADYTYFSVQGTSSAIMTMI

LSVCSPGDKIIVPRNVHKSVLSAIIFSGAKPVFVSPSQDANLGIDHGVTTQSIRRALE

RHPDAKALLVINPTYYGVVTDLKEIVELAHSYQVPVLVDEAHGVLIHFHEDLPLSAMA

AGADMAATSVHKLGGSMTQSSVLNLNTKNGFVNPQRVQTILSLLTSTSTSYILLASLD

TSRRNLALHGREIAQKAIELAEFARRSINDMDGLYCFGRELLGTEATFNYDPTKVTIH

VRHLGITGYETENWLREHYNIEVELSDMYNILCLITPGDTDNSVDILLNALQDLSRTY

YQVNPAHELVVKVPDIPQLMLTPRDAFYGDTEVIPFKESAGRIISEFIYVYPPGIPIL

LPGEVITQENIDYIIDHVEVGLPVKGPEDRSVTNVKVIVEADAIF"

CDS 1193456..1194748

/gene="ltaA"

/locus_tag="EFAGFIKM_01048"

/inference="ab initio prediction:Prodigal:002006"

/inference="similar to AA sequence:UniProtKB:Q2FZP8"

/codon_start=1

/transl_table=11

/product="putative glycolipid permease LtaA"

/translation="MQTDTKPAKILRSPFFIAMWLTLFLVEIIKGALLVAVLPVYMDN

ILGLSAGVIGVAFALQYLGDNLFRAPSGWAAERIGFRATMVTALICTLIAVIMILFLK

SAVGLAMACLILGIGTSPLWPCAMTGVTAMSGPQNKNGTAMGALEMAALGGTGLGPIG

MNWLLERTHHDYRTIFLVLMGCAILVILVAMILPGRVIVEGEHAEQAAKNSDVKYPAK

PNLLTPFIRLQKSVKDTLQRVRSTLNVNPLVYPALFMQSFVIGLLSPVITLYTRTDLH

ISPNLYSLLLIAGGGITVIALLPVGKMVDRFGTKPFLNIGFLMAAASLFAFSSITSIP
VVFGVVMLVGISYAMILPAWNAFVATLIPKGERGAIWGFFLTLQGSGMVVGPIVSGLL
WDHVSHPAPFIGSAIVMAGLAVVHFVLSRNPFRTAPAK"

CDS 1195002..1195601
/locus_tag="EFAGFIKM_01049"
/inference="ab initio prediction:Prodigal:002006"
/codon_start=1
/transl_table=11
/product="hypothetical protein"
/translation="MNKSYEVEYCNLELRFNRRHIQNLIRDLIREGYSLYWSEDESFF
ILSVRTGRKLVKLRFQQTRTGNYKMTGDYIIKDAKLAEWLEKLIGDTRGHAVVKRFKD
QQMTVENILFGEVIRLVEVSGFKQRVIFQKEPAPTAEEMEEMFLSREGEERLMLLRME
VDDELERLYTAMQMDNIDKANACRQVLKQLSGHLLMLEG"

CDS 1195774..1197183
/gene="gndA_1"
/locus_tag="EFAGFIKM_01050"
/EC_number="1.1.1.44"
/inference="ab initio prediction:Prodigal:002006"
/inference="similar to AA sequence:UniProtKB:P80859"
/codon_start=1
/transl_table=11
/product="6-phosphogluconate dehydrogenase,
NADP(+)-dependent, decarboxylating"
/db_xref="COG:COG0362"
/translation="MTKQQIGVIGLAVMGKNLALNIESKGFSVSVYNRSPEKTNDLLK
EAEGKNLTGSFSIEEFVASLESPRKILIMVQAGKATDATIEQLLPHLDEGDIIIDGGN
AYFPDTQRRSKELEDKGIRFIGTGVSGGEEGALKGPSIMPGGQESAYKLVEPILTAIS
AKVGDDPCCTYIGPDGAGHYVKMVHNGIEYGDMQLIGEAYHLLKSVLNVSVEELHAIF
TEWNQGELDSYLIEITADIFSKYDPETGKPMVDVILDAAGQKGTGKWTSQSALDLGVP
LSMITESVFSRFLSAMKDERVAASKILNGPATEAFSGDKKAFIESVRKALFASKIVSY
AQGFAQMRAASDEYGWDLKYGNIAMIFRGGCIIRSQFLQNIKEAYDKDAALKNLLLDP
YFQNIVESYQGAWREVVASAVKQGVPVPGFSSALSYYDSYRTERLPANLLQAQRDYFG

AHTFKRVDKEGSFHHNWME"

CDS 1197410..1198066

/gene="aroK"

/locus_tag="EFAGFIKM_01051"

/EC_number="2.7.1.71"

/inference="ab initio prediction:Prodigal:002006"

/inference="protein motif:HAMAP:MF_00109"

/codon_start=1

/transl_table=11

/product="Shikimate kinase"

/translation="MPGVTRSVLEVLADGRKEVGEEMVFPSPQGCVMIGQKCSRIALI

KGVYTLSKSNNIILIGMMGTGKSTVADMLARELGYRLIDVDTAVEKEEGCTIPELFTG

KGETYFRDAESRMLCSVLEKKSQVIATGGGVVLRSDNCDVMSKNGWVVALTADPAVIV

ERVSGCDNRPLLAGNAEERIKTIMEERKDAYRFAHYTVDTTELSAAEVTRLILAHYRV

"

CDS complement(1198203..1198508)

/locus_tag="EFAGFIKM_01052"

/EC_number="2.8.1.-"

/inference="ab initio prediction:Prodigal:002006"

/inference="similar to AA sequence:UniProtKB:D3RPB9"

/codon_start=1

/transl_table=11

/product="Sulfurtransferase"

/db_xref="COG:COG0607"

/translation="MTQIAEIETSELRRRLQAGEKLQMIDVREDDEVAQGMIEGAKHI

PLGQIPDRLSEIEKSGEIVIICRSGYRSERACEYLQQLGYEGCTNMVGGMLQWQQED"

CDS 1198614..1199909

/gene="aroA"

/locus_tag="EFAGFIKM_01053"

/EC_number="2.5.1.19"

/inference="ab initio prediction:Prodigal:002006"

/inference="similar to AA sequence:UniProtKB:Q482G5"

/codon_start=1

/transl_table=11

/product="3-phosphoshikimate 1-carboxyvinyltransferase"

/db_xref="COG:COG0128"

/translation="MDVIVKPTPTLNGEIGALSSKNYTTRYLLAAALAEGTSTIHFPA

HSEDSDAMRRCIRDLGAVLEEDDSKIVIQGFGSHPRDVRELNVGNAGAVLRFLMGVTA

LCPDVTFVNTYPDSLGKRPHDDLIDALGQLGVDVQHEQGRLPITIKGGNAKGGHIRVS

GSVSSQYLSALLFVTPLLAEDSTIEVLNDLKSKVVIGQTLEVLEQAGIVIHASDDYMS

FRVPGGQAYQPTTYTVQGDYPGSAAVLAAAAVTQSDVKILRLMEQSKQGERAIVDVLR

MMEVPLTHENDVVHVQGNGRLKAIEFDGDAATDAVLAMVAAAVFAEGTSRFYNVENLR

YKECDRITDYLNELRKAGANVEERQAEIIVHGRPEGVEGGVEINAHYDHRVIMALTVV

GLRSKEPLRIRDAHHVAKSYPQYFDHLQALGASVQWVKE"

CDS 1199969..1200403

/gene="yccU"

/locus_tag="EFAGFIKM_01054"

/inference="ab initio prediction:Prodigal:002006"

/inference="similar to AA sequence:UniProtKB:P75874"

/codon_start=1

/transl_table=11

/product="putative protein YccU"

/db_xref="COG:COG1832"

/translation="MEFNNPTREEIGQLLRKAGNIAVVGLSDKSDRTSYMVAEAMQSR

GYRIIPVNPQVQGEILGETVYATLADIPEPVDIVNVFRREEFCADVARDAAAIKANVL

WLQLGIHSDEAVQIAAENGMTAVTNRCIKVEDSIVLRGAGRG"

CDS 1200600..1202480

/gene="gamP"

/locus_tag="EFAGFIKM_01055"

/inference="ab initio prediction:Prodigal:002006"

/inference="similar to AA sequence:UniProtKB:P39816"

/codon_start=1

/transl_table=11

/product="Putative PTS system glucosamine-specific EIICBA

component"

/db_xref="COG:COG2190"

/translation="MNWLGSLQQLGRAVMLPTMVLPAAAILLSVGSLPWDAWGLDIVG

EVSTVAGHGIFYFLPYLFAVGVALGLSNQAGQAGLAALAGIVIYDQVTRNFGDGTVQP

ASLIGIILGALAGGMHNRFKSIKLPEILQFFGGSRFVLLIVGLCSALFSCFMLWLAPI

LQHGLNGIATWEYSLGGFGLFIYGVLYRVLVAFGLHHLLNNVFWFQLGTFETPDGNIV

QGDLPRFFAGDPTAGFFMAGLFPIMMFAIPAIAFAIIQEAREDLKPKIKKTFLTSALV

CFLTGVSEQIEFAFLFAAPYLFIVHAVMSGVAMWISYWLDIRHGFSYSAGIIDYILNF

HLSENAWKLIPIGILYGLVYYFLFRWAIRTFKIPTPGREEGSMLEDWVGNIPYQAPLI

LEALGGKSNIVQVEACITRLRLTVHDDRLIDTGAMKSMGSAGLIKLGGGNVQVVFGTY

SELIREEIAKLLERDLQQVLFCAPVQGKMLPIEEVPDQIFAAKLVGDGVAFVPEKGEL

VSPVYGTIMHLYPTMHALGISTREGLEVLLHIGIDTSQLKGHFEAFVQEGDTVEPGQL

LIKFDLAVLREQAASLTTPMVITNPDRVKSWSFAPFKQVKKGQASVMSVVLYDRNVGG

VE"

CDS 1202477..1204237

/gene="ptsI_2"

/locus_tag="EFAGFIKM_01056"

/EC_number="2.7.3.9"

/inference="ab initio prediction:Prodigal:002006"

/inference="similar to AA sequence:UniProtKB:P08838"

/codon_start=1

/transl_table=11

/product="Phosphoenolpyruvate-protein phosphotransferase"

/db_xref="COG:COG1080"

/translation="MKIQGINAAAGIAIGKAFVLPSWEWDLPDQKMDSVDLAQEFERL

YEGIRTSKDEIEYIKNEFKEVVGPEESSIFDAHLAILEDPVFMNEIRGIIERQYKAAE

VAVKEAIDHFVTMFDLLEDEYMKERAIDIKDVGNRLLKHLLGAPEITLPSDTQPYVLV

AKELSPSQLAHLNPTHVLGIVTLIGGKTSHSAIMARALGIPLVSGLEASLGMPVETGD

MLVVDGDNGLVFTDPDRKTIERYTMLRSKQLKKKEQLQVLATVDAVTKDGAVLHLASN

ISSVKELDLALKQGAKGVGLFRTEFLYMDRATFPGEQEQYEVYRLVAEKTAGQSVVIR

TLDIGGDKQLDYFELPEEDNPFLGYRAIRISLDRKDLFKTQLTAILRASAAGNVKIMY

PMISSVEELQQANEILREAMSDLDERGLSYDPHIQVGIMIEVPAAVMIADLLAEEADF

FSIGTNDLVQYVLAVDRMNEQIAHMYHPYHPAVLRMLRATADAAHTAGIDVSVCGEMA

GDERAIPLWLELGIRHLSMSPQSLLRVKHRVLNTTASAAKEEARKCFALRTSGDIEER

LQVFNDSTGSPDEQSGSNAS"

CDS complement(1204358..1204882)

/gene="yfkM"

/locus_tag="EFAGFIKM_01057"

/EC_number="3.2.-.-"

/inference="ab initio prediction:Prodigal:002006"

/inference="similar to AA sequence:UniProtKB:P80876"

/codon_start=1

/transl_table=11

/product="General stress protein 18"

/db_xref="COG:COG0693"

/translation="MRLTGKKVIALVDEEFEDLELWYPVHRVREEGAEVHLAGEKKGK

TYIGKYGVPAEAEYSFDELDSSDYDGILVPGGWAPDKLRRYPKVLELVKEMNADRKPI

GQICHAGWVLISAKILDGVTVTSTPGIRDDMENAGAIWKDEAVVVDGHIISARRPPDL

PPYGKAFCDALADK"

CDS 1205126..1205572

/locus_tag="EFAGFIKM_01058"

/inference="ab initio prediction:Prodigal:002006"

/codon_start=1

/transl_table=11

/product="hypothetical protein"

/translation="MVRKSCRCGHVMELELRTVVYAKKVEIRNVPVFACTDCVSYEVL

PPVKPDLTECINTLGEQPVKQDVSFGERNELADLFHELLLTWPDATDREMEHRMQQAV

EERINLLLDIMGYATKSGDAAWMETTERRLSQLSGFVWQEYFSNAV"

CDS 1205695..1206042

/locus_tag="EFAGFIKM_01059"

/inference="ab initio prediction:Prodigal:002006"

/codon_start=1

/transl_table=11

/product="hypothetical protein"

/translation="MADMTKMTSIEQLNSAVEATKDQPLLLFKHSTRCPISSNAYQEM
NDYLLNNANEQVKYGIIYVVEDRPVSNEAADKLGVKHESPQAILIKKGIPVWHTSHSD
ITRTTLTNVLSES"

CDS 1206135..1207148

/gene="ccpA_1"

/locus_tag="EFAGFIKM_01060"

/inference="ab initio prediction:Prodigal:002006"

/inference="similar to AA sequence:UniProtKB:P25144"

/codon_start=1

/transl_table=11

/product="Catabolite control protein A"

/db_xref="COG:COG1609"

/translation="MTVTIYDVAREAGVSMATVSRVVNNNPNVKPQTRKKVYEAIERL
GYRPNAVARGLASKKTTTVGVVIPDISNSTFAEIARGIEDIANMYHYNIILCNADKKK
EKEIRVINTLLEKQVDGLLFMGGTVTDEHIQAFQSAAVPIVLCATSDEKGTFPSVDID
HEAAAFDAVNTLIRHGHKEIAMISGTLQDPANGYARFQGYKKALEAAGIEYQEDLVRI
GNYRYESGVEAMKYFLGLKKKPSAIFAATDEMAIGAIHSIQDDGLKVPDDFSIISVDN
IRMASMVRPQLTTVAQPMYDLGAVAMRLLTKLMKKENVENPRVILPHETILRLSVSHA
DVV"

CDS 1207268..1207963

/gene="mtnN"

/locus_tag="EFAGFIKM_01061"

/EC_number="3.2.2.9"

/inference="ab initio prediction:Prodigal:002006"

/inference="similar to AA sequence:UniProtKB:Q7A5B0"

/codon_start=1

/transl_table=11

/product="5'-methylthioadenosine/S-adenosylhomocysteine
nucleosidase"

/translation="MSETIGIIGAMDEEVELLLAGMKQLETVKQTGITYVAGEWLGKH
IVVCKSGVGKVNAAVTTQILIDRFQVNRIIFTGVAGAVNPELNIGDMVISSVCVQHDM
DVTPLGFARGVIPYQETSAFPAEASLITLAEEACKAQGANYLIGKVLSGDQFIANKDT

VNMLYTEMDGACAEMEGAAVAQVCYMNRVPFVVLRGMSDKADGSAHVNFAEFTVESSQ

RSHRIVEHMLENM"

CDS complement(1208073..1208705)

/gene="acuA"

/locus_tag="EFAGFIKM_01062"

/EC_number="2.3.1.-"

/inference="ab initio prediction:Prodigal:002006"

/inference="similar to AA sequence:UniProtKB:P39065"

/codon_start=1

/transl_table=11

/product="Acetoin utilization protein AcuA"

/translation="MEHIKEHHMRSIFKSGHRITIEGPVQAEEITRLAFHPDLDAFRR

PTEQQEALAEIAALPEGRIIIAHENETIIGYVTFHYADDCERWSEGNMTDLIELGAIE

VADDYRSLGIGREMLLTALEDKYMENYIIFTTEYYWHWDLKGSGLNVWDYRKMMERLM

ETIDMTWYATDDPEICSHPANCLMVRIGSQVPITSEEQFDRVRFRHRFMY"

CDS 1208975..1210699

/gene="acsA_1"

/locus_tag="EFAGFIKM_01063"

/EC_number="6.2.1.1"

/inference="ab initio prediction:Prodigal:002006"

/inference="similar to AA sequence:UniProtKB:P39062"

/codon_start=1

/transl_table=11

/product="Acetyl-coenzyme A synthetase"

/db_xref="COG:COG0365"

/translation="MSQAHGEMLQTVVSESNLGDYTEARSRFDWESVERHFTWHASGK

VNMAHEAIDRHVLEGRGGKTALLYSDASREEAYTFADLSEQSSRFGNVLRKYGIAKGE

RVFIFMPRSPELYFSLLGILKAGAIAGPLFEAFMETAVKDRLEDSGAVALVTTPELLK

RIKRSELPELKHIFIVGGGAQTEEGLIDFDAEMSAASDEMEVEWLNREDGLLIHYTSG

STGKPKGVYHVQNAMIQHYYTGKVVLDLREDDVYWCTADPGWVTGTSYGIFAPWLNGV

TNVIRGGRFSPQDWYSTIQKNKVTIWYSAPTAFRMLMGAGEETIAQHDLSSLRHVMSV

GEPLNPEVVRWGWKAYGQRIHDTWWMTETGAQLICNYPGMPIKPGSMGRPLPGIEAAI

IDDRGQELPPYSMGNLAIRTPWPSMMAKIWNNPVKYEEYFRLTGWYVSGDSAYMDEEG
YFWFQGRIDDVINSSGERIGPFEVESKLVEHPAVAEAGVIGKPDVTRGEIIKAFVALR
EGYEATPELKEEIYRFVKEGLSAHAAPREIEFKDKLPKTRSGKIMRRVLKAWELDLPT
GDLSTIED"

CDS complement(1210938..1214033)

/gene="mtgA_1"

/locus_tag="EFAGFIKM_01064"

/EC_number="2.4.1.129"

/inference="ab initio prediction:Prodigal:002006"

/inference="protein motif:HAMAP:MF_00766"

/codon_start=1

/transl_table=11

/product="Biosynthetic peptidoglycan transglycosylase"

/translation="MVDVNKKKPDEQPVRKRSFARRLGSFVKWMVVLGFMGALFVGGA
LMGYVSSIVKDEPVRSRALIEQKVSENSITGFAYFADGSPIGQLRTEEDRRPVTTDQI
PQKVIDAVISIEDNHFYEHKGVDMSGTLRAVKQKVLKESVQTGGSTLTQQLARRVFLN
LDRTEDRKVKEILLSLRLERFLTKDEIMTAYLNKVPFGNGSSGYNVYGIKAAAKGLFN
INDLEKINIAQAAYLAGLPQLPSSYSAFNGKGDFVEENFDRAINRQHLVLRRMLELGK
INQSEYDKALAFDIKSALAPKTIKAYNTYPYLMMETERQAAQILMKQLNSDTAESTDK
ATDADTPQKESSALLEEAQTQLRTGGYRIYTTINKSIYKTMRTIAEDDSNFSADDPVK
GKEQTAAMLINHKTGAILGMIEGRDFQDEQMNYATQMIRQPGSTMKPIAAYLPALDEG
LVQPASIIDDSPIILKNGPSGYHIAKNANNRYQGLVTARRALNYSLNIPALKLFNEEV
GIEKAWTFAKKLGITTIQKEDYQAQTGVLGGLQYGVTVEELTSAYGAIANNGVYNDSY
MISKIVDSKGNIVYKHDTEPVQAFSEQTAYLMTDMLRTVITEGTADKVRENYKYSKSV
PIVGKTGSTQNYADVWFEGYTPDVTLGVWVGYKQPVNTLESKSQRKRAQQLWSKILNE
VIDTQKDLFVTDSFKKPSGIETRTVSAYSGKLPTSMTDKFVTDIFNSKFVPKDSDDGV
AKAKYITYNGVNYIPRDGTPSDMLKEKTVIKRKKPISDLIKELQNAFSRMSRHESLAY
YLPEDAGADMPTQIDPRTDNGKAPDAPGNVRISTSGERAVITFNATPENDVVGYRLYR
SVNGGGFQNQGQVVLTGESRSFSAYAAQGGNFAFYVTAVDVAGRESAPSATVNSAGVA
PPVEEEIDEPIEVPGTVVTPGETQTDNTTTTAPGTPGQVSVSALTQGLRIQWAASPNA
DSYTVYYSETGSAPYTKIGTTAGNTMDYGVPASTSGWFKVSASNSAGESEPSAALHFQ
P"

CDS complement(1214263..1215381)

/gene="mcpB_1"

/locus_tag="EFAGFIKM_01065"

/inference="ab initio prediction:Prodigal:002006"

/inference="similar to AA sequence:UniProtKB:P39215"

/codon_start=1

/transl_table=11

/product="Methyl-accepting chemotaxis protein McpB"

/db_xref="COG:COG0840"

/translation="MAVLLLASVVTYTIISIVRQLNQVNIVAGQLAGGDLTQKLPVRS

KDEFGIMAGHMNQMMDTLRHTISVISEHALSVGSTSQQLTAGAEETGKAAELIALTGV

ELADKAGKQVQELQESSRSMNEISAGIGRIAKAASDVSDSSRAVAERTTVGTDKIQSA

MRMVDSATSSVQTSMTALENFRQRSEEIGHITGMITEVSRQTNLLALNASIEASRAGE

NGRGFGVVASEIRNLAEQSRVSAAQIAALIHSVQQEVLSLVENMEHGNAEVSHIAEVI

NESGELFLSISSQISDVNEQIEHVSAIAQQMSAGSQQVDATLVQLKTIGHETADHATR

VASASEEQLASMEEITAASASLANLTQELLELIQRFKT"

CDS complement(1215519..1216367)

/gene="mcpU"

/locus_tag="EFAGFIKM_01066"

/inference="ab initio prediction:Prodigal:002006"

/inference="similar to AA sequence:UniProtKB:Q88NI1"

/codon_start=1

/transl_table=11

/product="Methyl-accepting chemotaxis protein McpU"

/db_xref="COG:COG0840"

/translation="MHFFRNRKLAVKLGLLLGIVLLCCIGALIAFNTKSIYDKSLQYG

ETVAGQAANRATNEFMTDINQVKNTLDTMSTTLLDAAQNGSLNREEVVRLLEQYLKKD

EKVFGFYTGWEPNAFDGNDAEHINKKEYDDATGRFVPYVIRDGNSLHFEPMPTYEGSG

ETSTYYQQPKKTKSIYWSEPVTYTVGGKETLLVSIVLPLLDENKNFLGIVGADFTIDR

FQQNIASLNPDQGYAMLITSEGQIAAHGSKPELANEGAVIPSEMRQSFSVYSLASLSF

TLQTLK"

CDS complement(1216556..1217155)

/gene="rpsD"

/locus_tag="EFAGFIKM_01067"

/inference="ab initio prediction:Prodigal:002006"

/inference="similar to AA sequence:UniProtKB:P81288"

/codon_start=1

/transl_table=11

/product="30S ribosomal protein S4"

/translation="MARYTGPKFKLSRRLGISLSGTGKELKRPFPPGQHGANQRRKIS

NYGMQLQEKQKLRHMYGLGEKQFRTLFSKAQKMQGIAGENFMFLLECRLDNLVYRLGF

ANSRAGARQLVAHGHVTVNGKKVDIASYQVSTGDVIGLREKSRALSSIKEALEGRSHL

PAYVEYNEAAVEGKFIRLPERAELSQDIDEKQIVEFYNR"

CDS 1217621..1219612

/locus_tag="EFAGFIKM_01068"

/inference="ab initio prediction:Prodigal:002006"

/codon_start=1

/transl_table=11

/product="hypothetical protein"

/translation="MLEHLRSLPASLNSRSSGDSASFAAPHSHEEHVLWMQKMDITPF

DFSYLGSLLEQAYSDWNSRVGSRLARPSTFYSVWNTEGDCIGYDSDHEADPGVNARRL

VLECLDKRQVLSLKGTSEHGEYLIITHPLFSRTNKDMFAVFTAVIYESNGYETSEAVV

QSEALHYRTCFYRRFEYIFMTDLLHAHEQTAREEHRRSILFQIVQRMHDKMDVDAILD

EVFDSMDYLYPATYIKLYMSQDQSNSDPRIKPLLVHEQGEDICVRSFMEGKLIVARSH

DAENRIVDVGIPLKGKQGIYGVFHIEMNEEIMEESDLQLITMMVDTAGTAFENAKLHE

QSNMLIQELRLINDLTQRLNKSLHLSEIYQLSEQELKEIFQAETCCILQLNDSTNDFE

VMSSNVKDVFHQSFSVDYGIAGLVYRTEEPLIIANYAQFDKVSSFFMEDTGSMSLIAS

PIRVNGEVKGAILLGHSREHYFSYDNYRLLQMLSIHIGLALSNATLHAEVRRLANLDM

LTGLYVRHYLDSVIHERQAHEFCGSLIVVDIDQFKQVNDTFGHQTGDQVLKQVSEIVT

SSVRSEDVCARWGGEELAIYMPQVSVRQALDYAEVIRKRVAEETRPPVTVSSGIAEWN

WMDEKVSVESLFYRADMALYSAKNGGRNRIVVEEQDVTR"

CDS 1219714..1220604

/locus_tag="EFAGFIKM_01069"

/inference="ab initio prediction:Prodigal:002006"

/codon_start=1

/transl_table=11

/product="hypothetical protein"

/translation="MEKVHGRVKIDMFNRHSVRTLWLFVSIVIIGVPGCGLIQRDQTP

EEVFSLALSGIAGKETLSFEGEAGLRRENSGLFENQFKFEGKLENHDRLTLQTRLPGE

VTAVGTGIQTTAVKNNGQPSGFSASFERKQGQWMALSAQHEPLRGSLSRFNPIAQLEK

IDGMHKTIQSEYGAGRRTRTLRIELAPEDAKSWAVTQLNDEMNAIRAEYMHKAATVKG

ARKEKLEKDLEKVWKQGETTMQQMMKQASVQTVYHLTVDRKSSLPLRLSSESQMSYTD

NNRKSNKEALVTDVNFKSYQ"

CDS 1220716..1221831

/gene="pepS"

/locus_tag="EFAGFIKM_01070"

/EC_number="3.4.11.-"

/inference="ab initio prediction:Prodigal:002006"

/inference="similar to AA sequence:UniProtKB:Q9X4A7"

/codon_start=1

/transl_table=11

/product="Aminopeptidase PepS"

/db_xref="COG:COG2309"

/translation="MKDPRIQKLAANLVGYSVDVQPGENVLVEMIGSERDLINAIIEE

VGKKGGNVFVQLTDKTVQRAMLKNATEEMMKTWAEIDLNRMKQMDCYIGIRAGENVND

LSDVPEEKMKMYNSLYSHPVHSEQRVKHTKWVVLRYPNASMAQLANTSTEAFEDFYFD

VCNLDYAKMDKAQDSLANLMKRTDKVRITGPGTELSFSIKDIGAEKCSGQKNIPDGEV

YSAPVRNSVNGTISYNSPTLYNGVTFENIKFTFKDGKIVEATSNDTERLNEILNSDEG

ARHIGEFAIGFNPHILHPMKDILFDEKIAGSLHFTPGQAYEETDNGNRSSIHWDLVLI

QRPDYGGGEIYFDDVLIRKDGIFVIPELECLNPDRLK"

CDS 1221928..1222173

/locus_tag="EFAGFIKM_01071"

/inference="ab initio prediction:Prodigal:002006"

/codon_start=1

/transl_table=11

/product="hypothetical protein"

/translation="MSSNNAAVVEIAQTAGKFTSSIVLHSENKYIDVKSILGLFTTLI

STHSYELHVHGPDAVEAKAAMSEVFAKHGLNVSIASE"

CDS 1222399..1222692

/locus_tag="EFAGFIKM_01072"

/inference="ab initio prediction:Prodigal:002006"

/inference="similar to AA sequence:UniProtKB:O07638"

/note="UPF0358 protein YlaN"

/codon_start=1

/transl_table=11

/product="hypothetical protein"

/translation="MTSSDLQDQLSLKAISLLQEDADKIQKLIEVQMENLATRYCPLY

EEVLDTQMYGFSKEVDFAVRAGLLPEGAGKQLVSALERNLAILYEALNKKNEQ"

CDS complement(1222875..1223945)

/gene="chaA"

/locus_tag="EFAGFIKM_01073"

/inference="ab initio prediction:Prodigal:002006"

/inference="similar to AA sequence:UniProtKB:O34840"

/codon_start=1

/transl_table=11

/product="Ca(2+)/H(+) antiporter ChaA"

/db_xref="COG:COG0387"

/translation="MRNRISSILLIAFFALSAIAHYLKWDSILQFVISAISVIFVAGF

LGKATENVAHYAGQRLGGFLNATFGNAAELIIAIFLVKEGLFDMVKASLTGSIIGNLL

LVLGLSIFAGGLKFKIQNYNVSLAGLNGSLMIVAIIALFIPAVFLNTHSITQKDTNTL

SLIVAGLLILAYIAWLLFSMVTHKNYLADVTEDRDEELPHEHAPAWSKKKSILYLVLA

TVMVAFVSEWLVGTLEVFTSEFGLSELFVGAFLVAIIGNAAEHSAAIMLAMKNKIGAA

VEIAVGSSLQIALFVAPVLIFVSYFTGRTMDIVFTTIELVAIGVSVFIAKSITQDGST

NWYEGLLLLVVYIILGVSFFLV"

CDS 1224129..1224491

/locus_tag="EFAGFIKM_01074"

/inference="ab initio prediction:Prodigal:002006"

/inference="similar to AA sequence:UniProtKB:P0A0P8"

/codon_start=1

/transl_table=11

/product="Alkaline shock protein 23"

/db_xref="COG:COG1302"

/translation="MAEQLQLESGNIRIADDVVAKIAGMAAMETPGIAAMSGGLSEGW

AKRLSGKNVQKGVSVEVGQLEAAIDLRIIVLYETPIHEVSRMLQQNVREAVETMTGLR

VVEVNVKVEGVSFKGDDL"

CDS complement(1224620..1225900)

/gene="ftsW_2"

/locus_tag="EFAGFIKM_01075"

/EC_number="2.4.1.129"

/inference="ab initio prediction:Prodigal:002006"

/inference="similar to AA sequence:UniProtKB:O07639"

/codon_start=1

/transl_table=11

/product="putative peptidoglycan glycosyltransferase FtsW"

/db_xref="COG:COG0772"

/translation="MKQQTAQTKTKRGTPDFQLLILTLLLVGFGLVMVFSSSSSIAIA

SDKFNNDALYFTKKQLMWAIIGLFGMFFAMNIRFNKYKKLYAPFFLLTTVMLVIVLIS

GAVLNGARSWIHIFGFSLQPAEFAKIAIILYLSALITKKGEKFRVFKTGYVPVLFIVG

FIAGLIMLQPDFGTTFILVSTCGLLIYAGGASMKHILGSILLVVLGGALAFGANSLFS

SMSPSDTTTAATAEQNYKIGRIQAFLNPLSDINGGSLNLYRSLVAIGDGGMTGSGIGQ

GTMKLHYLPNAYNDFIFSVIGEELGFIGSALFLLVYLYFIWRGIIVSLRCPDPFGTLV

GIGIMGLIAIQAFINIGGVTQTIPVTGVTLPFISYGGTSLFVMMVAMGILLSISRTNN

LDVIKEEKTKSVTVQTQTRTSPALRSRESIRRIR"

CDS complement(1225914..1226261)

/locus_tag="EFAGFIKM_01076"

/inference="ab initio prediction:Prodigal:002006"

/codon_start=1

/transl_table=11

/product="hypothetical protein"

/translation="MIFENTGLDGLKSDLAYLDESAEKVGFVRWQWEYYRATYDYKIE

DEQTNSEYFVRINTRAVGGKLEKPDTVLAVEAVYLGKATFPHGLDYDSSVPQPVVKLA
AEKLQQLKELLEA"

CDS 1226472..1227641

/locus_tag="EFAGFIKM_01077"

/EC_number="3.5.1.47"

/inference="ab initio prediction:Prodigal:002006"

/inference="protein motif:HAMAP:MF_01692"

/codon_start=1

/transl_table=11

/product="N-acetyldiaminopimelate deacetylase"

/translation="MTNNIWWEDLQIHMVEWRRHLHRNPEVSFHEEKTSSFVADMLES
FGVEVRRHVGGHGVIGTIRGDKPGPVVMLRADMDALPIQDEKNIEYASQQAGAMHACG
HDGHISILLGTALYFSRHKQEIQGEIRFLFQPAEELLPGGAVQVIADGALEGVDVIYG
IHLWTPLPVGVVASKAGPMMAAADDFYIEIKGKGGHGGMPQSTIDSLIAGSALVMQLQ
TVVSRSVDPLQPAVLTIGTMQAGSAQNVIAEQCKMSGTVRTFDEETRNGMKERVLTMV
AQTGAAYGAETQVKYIMGYPPVVNDEQETARFFREATELFGAERVQASPMLMPAEDFA
YYLQKVPGCFMFVGAGNPDKNAIYPHHHPMFDFDEDAMQTAVRLFIAMAKGYTAE"

CDS 1227831..1229525

/locus_tag="EFAGFIKM_01078"

/inference="ab initio prediction:Prodigal:002006"

/codon_start=1

/transl_table=11

/product="hypothetical protein"

/translation="MEGTEHMVSTDACIVQRDFTVLLEVGHPGFERAREQLGMYSELV
KTPAAFHTYRITPLSLWNAAALGWNADQVIASLELVSRWNVPAALIQDVRRIMDQYGK
LKLHSEADHTRMRLYSEDERLLDELSGLKTIAAFRMERKDAHELLLPREQRGLLKREL
TRLGYPVLDYVGYREGTKLSFDWRSNDPGDHSERFALRSYQREAVDAFEGSEGMGGSG
LLVLPCGAGKTVIGMAVLERLQCECLILTSNTTSVRQWIQEIQDKTTITSEQIGEYSG
QKKQVKPVTVATYQILTHRKSKDADFTHIKLLSERQWGLIIYDEVHLLPAPVFRATAD
IQATRRLGLTATLVREDGCEQDVFSLIGPKLYDMPWKQLEQQGWIADVQCQEIRIPFS
PELRSNYLRAEVKHQFRLAAENPAKVRVVKRLLERHRDLPALVIGQYLDQLELIAREI
EAPLISGTMSQQERIKWFAAFRRGEIKTIVVSKVANFAVDLPDAAVALEISGSFGSRQ

EEAQRLGRILRPKAGENKAYFYALVSEDSKEQEFALNRQMFLVEQGYEYAIVHENE"

CDS 1229562..1231280

/locus_tag="EFAGFIKM_01079"

/inference="ab initio prediction:Prodigal:002006"

/codon_start=1

/transl_table=11

/product="hypothetical protein"

/translation="MSSFEPIDEPVVLNLDSWSKLASVEQKVLGAIFHKYAGQPFSSL

LPREQWAIEGLSVAEANFSFANLRQQRWIESVYKSWGEQLFYISASLMDTLTIAYAQK

VGITVKQMAQLAHVLQEGKPDIAAELLHLIAWIEREGLPLTGKGTIHKKSVKKLSVIT

VLSSTDFEDLGIRYEHSDLYPTHVAILLDLLLSLNLVQKSDGQIQIVDHRLQTWLKLS

WRQMQREIYQACMERYGEAEPALQHFRYQLAVFAPERNIWCHIENSELNPRIRGWLCA

LAGWGYGEVGGDQSGALAFRWLIEPQSLLYQEQEMVSETEPCGFYMQPDFEVLVPPEA

GPDVIWMLEQYAERVTRDRMSIYRLTRERFISAIARGYALHEVIEFLDQYALTGIPEN

VRIALADWGKEADAAPLTVIRSMKVMEASTGSADDPKLLEKDMLSGVYSSFYTPDNLG

LVDVPASLHGLERDHSVIEKKFSLLGFEEIPETWYKEWRRYHSSTARQIAAKAIEWQT

KLGIQQENRTQYLIPHQVEGHEQWTLSGWCMLDSSENTSETEWRTFSPSEWDTMRLIL

PDDVTT"

CDS 1231433..1231870

/locus_tag="EFAGFIKM_01080"

/inference="ab initio prediction:Prodigal:002006"

/codon_start=1

/transl_table=11

/product="hypothetical protein"

/translation="MSVAEMNTVDMAQVLTGAYELGDMINQSAEVSDYLYWKQQVETT

PEIQMGIRKLNAKKELFEETQRFGHFHPDYHAAKDQVKALEQELENFEAVIRFKQAEK

SLDDMLFQMSETIAFAVSTTIKVPSNDPNPKGGCGSGGKCGCS"

CDS 1231898..1232164

/locus_tag="EFAGFIKM_01081"

/inference="ab initio prediction:Prodigal:002006"

/codon_start=1

/transl_table=11

/product="hypothetical protein"

/translation="MMFAERTGFIIWVSDLKAARNLEKYGTVHYISRRMHYVVMYVNA

ERADDTMKNVKRLSYVRKIERSYRNEIKTEYASKTMDKTSFYGI"

CDS 1232330..1232767

/locus_tag="EFAGFIKM_01082"

/inference="ab initio prediction:Prodigal:002006"

/codon_start=1

/transl_table=11

/product="hypothetical protein"

/translation="MRDKVMQRWSTYEPFFVALGDKKVADIVITHHAKMRYADRIEPA

RSDDDHIAAWLWECLKQDRITPYYRNEQDVYLIDEDLVVVAEFSELEGEYDIAGNPLH

KMIVVTFLGRMSETIELRDLKSYYSWLRHSRRMTLVKNSRKHK"

CDS 1232900..1233823

/gene="cmpR_3"

/locus_tag="EFAGFIKM_01083"

/inference="ab initio prediction:Prodigal:002006"

/inference="similar to AA sequence:UniProtKB:Q55459"

/codon_start=1

/transl_table=11

/product="HTH-type transcriptional activator CmpR"

/translation="MNFHQLHIFYTVAEKGSFSAAAQALHMTQPAVTMQIQSLEDYFG

SKLLHRSTKKIELSEAGRTLLPHAKRSVELVRQTDEAMSAFTQMLQGRLQLGASLTIG

EYVLPRMLGPFALQYPDISIVMKVMNTTQIMDDILKHQLNFGLIEAPVHHPDMIVEPV

MQDELKLIVPAGHDLAKRSKVNIEDVMNYPFVLREKGSGTRQVMEDQLQKKKIDPQDM

NVVMELGSTGAVKSAVEAGVGITMLSPSSVQHELALGLVHIVDIRGLEFKRQFYAIHL

KSSLLPLSAVAFLNYLRQQEQPKSVLKTGKA"

CDS 1233891..1234760

/locus_tag="EFAGFIKM_01084"

/EC_number="3.1.3.97"

/inference="ab initio prediction:Prodigal:002006"

/inference="similar to AA sequence:UniProtKB:Q7NXD4"

/codon_start=1

/transl_table=11

/product="3',5'-nucleoside bisphosphate phosphatase"

/db_xref="COG:COG0613"

/translation="MDQRQGRCDLHTHSQASDGMQPPADNVKLAKQKGLSAVALTDHD

TVAGVAEAQQAGKEYDINVVAGVEISTRAGGKDIHVLGYYVNTEDEIFLQRLAGLREA

RDERNHLIIAKLQELGLDISWQEVLDELGRPLEPDESIGRPHMADVLVKKGYAVDMRD

AFDQYLAEGKPGFVSVPRVAPEDACKWIRAAGGAAVIAHPGLYNDDELVRRIIEDALP

DGIEVFHSDHGPEEERRYGKLAKEYDLIETGGSDYHGKRQGVVFHGDLGSKTVTVDVL

ERLKAAASQQRGE"

CDS complement(1234903..1235349)

/locus_tag="EFAGFIKM_01085"

/inference="ab initio prediction:Prodigal:002006"

/codon_start=1

/transl_table=11

/product="hypothetical protein"

/translation="MTVDIRETQPNDLKPLMALMHDYVVGFYNNPWPGDQSIQLLINN

LLNQQIGAQFVAEEDGKLIGFSTLFFTYSTMKAERVAIMNDLFVTETFREGEAEAKLF

AHCQQYTRDHGCAYMSWITAVTNERAQQLFERLGATRGAWVNYSIT"

CDS complement(1235728..1237500)

/gene="rqcH"

/locus_tag="EFAGFIKM_01086"

/inference="ab initio prediction:Prodigal:002006"

/inference="similar to AA sequence:UniProtKB:O34693"

/codon_start=1

/transl_table=11

/product="Rqc2 RqcH"

/db_xref="COG:COG1293"

/translation="MALDGIVTRAIVHELQGCIGGRISKIHQPNGHDVVLTLRAQRGN

SKLLVSASPTYPRVHYTEKTFLNPTEAPMFCMLLRKHCEGAIIEEIRQIGMERIIHIN

VRQRDELGDVSVKRIIIELMGRHSNIILLDPVTGTILDGIHHVTPSISSYRVIMPGFA

YTAPPEQHKSNPLEVSRTAFEDSYHAAEEDASRWLVNSFSGLSPLIAGEIAARGIAAE

GPVSTQAVVEGESQVEAGALWNAFEAVMGPVRDHIYTPVTGMNAKGKMIFSAVHLQSI

QDVEKTYDTMSKCMEDYYGDKAERDTVKQRVSDLLRFLQNERSKNIKKLDNLNKDLLE
ADDADKFRLWGELLFASLHQVHKGDKSVELVNFYDEDQANITIQLDPLLTPSDNAQRY
FKRYNKYKNSLAVIHEQLGKTKDEIDYLDNLLQQLSIASMNDIEEIRDELVQQGYLRD
RNKKGKKKKKSDRPTVHQFTSSEGIDILVGKNNLQNEYVTNRLASSNDTWLHTKDIPG
SHVVIRSTDFGEATLEEAAQLAAYFSQAKESSSVPVDYTFIRHVRKPSGAKPGFVIYD
HQKTLFVTPNDELIKSLPSTIKNG"

CDS 1237856..1240714

/gene="yloB"

/locus_tag="EFAGFIKM_01087"

/EC_number="7.2.2.10"

/inference="ab initio prediction:Prodigal:002006"

/inference="similar to AA sequence:UniProtKB:O34431"

/codon_start=1

/transl_table=11

/product="Calcium-transporting ATPase"

/db_xref="COG:COG0474"

/translation="MEHTKWHQLSDEELRNTLGVSPQEGLTDEAVAEKRKVVGSNELS
EGKRISPITLLLNQFKDFMVLVLMGATLVSGLLGEYLDAVTIVAIIVLNAILGFVQEF
RAERSLRALKQLSAPLAKVLRSGQEVHLAAKQLVPGDIIMVESGDRIPADVRWLQTNS
LDVEESALTGESVPVSKHCLPIADEDVPLGDQKNIGFMGTMVTRGTAKGVVIRTGMET
EMGKIADLIQNTEEQETPLQHRLEQLGKILIFVALGLTIMVVVAGILHGQPAVGMFLA
GVSLAVAAIPEGLPAIVTIALALGVQRMIKRKAIVRKLPSVETLGCASVICSDKTGTL
TQNKMTVTDVWLEGRNIKVTGDGYAPEGQMLENGRTVELKSDQSLRRMLQISALCNNA
SIVESAASEVGSKKKGKDTKKESKKNTKKGDLQEETVQRDNVFWELKGDPTEGALVTL
ASKMGLTPAGLKELYARQQEFPFDSERKRMSVLVHHQGGRMIYTKGAPDVLIGHCSYI
LWEGKVVPFTGTLRQKVMAANESMAGAALRVLGMAYREVRPEEKVADEHAAESQLVFV
GLAGMIDPPRREVRDAIATCRKAGIRTVMITGDHGTTAEAIAHQLGILPRGGASLSGQ
QLAGMTDEQLDKQVEGIYVFSRVSPEHKLRIVKSLQRKGHVVAMTGDGVNDAPAIKAA
DIGIAMGITGTDVTKEASALILSDDNFSTIVAAIEEGRNIYENIRKFIRYLLASNVGE
ILTMFFAMMMGLPLPLVPIQILWVNLVTDGLPAMALGVDQPEKDLMEHKPRGAKENIF
ARRLGWKIVSRGVLIGLCTLGAFWLTLQAAPDNPGQLIKAQSVAFATLVLAQLIHVFD
CRSSRSIFHRNPLQNKYLVLAVISSVVLMLVVMYVEPLQPIFKTVPLGLREWAICIVA

AGIPTFLMGAGSVWGGRRNRRRMGGSGRFVPKSTKFSA"

CDS 1240897..1241730

/gene="dapF"

/locus_tag="EFAGFIKM_01088"

/EC_number="5.1.1.7"

/inference="ab initio prediction:Prodigal:002006"

/inference="similar to AA sequence:UniProtKB:Q81XR2"

/codon_start=1

/transl_table=11

/product="Diaminopimelate epimerase"

/db_xref="COG:COG0253"

/translation="MEFTKMHGLGNDFIVVFGEKELPADAAELAVKWCNRFFGIGADG

LVYILPSKKADFQMRIMNSDGSEAEQCGNAIRCVSKYVYDHGHVNQEQITIETIGAGV

QPVSLNIRDGKVETVRVDMGEPILNGLQVPTTVDANPVVDHYIEANGHAFKFTAVSMG

NPHAVIYVDDAVNFDLTTWGPLLEVHPMFPKKINVEFATVRDRGYVDMRVWERGAGPT

LACGTGACATLVSSVLNGHTDRTAVISLKGGDLHIEWNEADNHVYMTGPAEVVFKGIT

S"

CDS 1241945..1243834

/gene="yitJ"

/locus_tag="EFAGFIKM_01089"

/inference="ab initio prediction:Prodigal:002006"

/inference="similar to AA sequence:UniProtKB:O06745"

/codon_start=1

/transl_table=11

/product="Bifunctional homocysteine

S-methyltransferase/5,10-methylenetetrahydrofolate

reductase"

/db_xref="COG:COG0646"

/translation="MKADLRKVMQERVLVGDGAMGTFLYQMGFPVGISYEELNLISPE

VVADVHRRYRDAGTEIFETNTYSANYDKLSKFGLESKVEDVNRAGVRIAKEVAGDIGY

VLGAVGSIRGGKRTNVSTSELKRFYQQQISALLDEGVDGILLETFYDIEEMDIALLQA

RKLSDLPVIGQFAVEDVGHTLDGYTMPEAFRIMREQGADVIGFNCRSGPNGIMRAMET

VSGRIGIPMSVYPNAGAADYVDGQFRYGATPEYFGQTAVQFAELGARIIGGCCGTTPD

HITAMKEALADYVPSPILEPDPSESKPRIVLHENVDERSGRGGQPTIVDLVKQRHTVI

VELDPPRDLDIAKFMKGAETLKAAGADALTLADNSLAVTRMSNMALGHLVQDRTGLRP

LVHIACRDRNLIGTQSHMMGFDALGINHVLAVTGDPARFGDLPGSSSVYDLTSFEIIR

MIKQLNDGVAFSGKPLKQRAGFVIGAAFNPNVKYLEKAVQRLEKKIASGADYIMTQPI

YDPELIVAMHEATKHLDVPIFVGVMPLASGRNAEYLHNEVPGIQLSDEVRSRMAGLEG

EEGRAMGVKIAKELLDVATAHFKGIYLMTPFMFYGMTAELTQYVWEKSEHQCPTCFNP

NNQLQ"

CDS 1243854..1244753

/locus_tag="EFAGFIKM_01090"

/inference="ab initio prediction:Prodigal:002006"

/inference="similar to AA sequence:UniProtKB:P44726"

/note="UPF0701 protein HI_0467"

/codon_start=1

/transl_table=11

/product="hypothetical protein"

/translation="MSFSMTGYGQSAFHFGGYKVQLEIKSVNHRYCEVMMRLPREWTY

YEDCLRKIVQSRLKRGRIDVYVMKEKEEDQVLPAVLNEQTVRAYLQAAEQLETQYGLQ

GKPSIVDMLGLPDVMVHSDGTSSIPEEQKGEWERVLQEGLKEALSSLEQMRAREGLHL

ASDLERRITRLESLHTEMLDLAPTVVSDYRNKLRQRLTEMQEEGSFPFDEHKLGMEIA

MFADRSNIEEELTRLQSHFGQSRELLKSDEPVGRKLDFLIQEMNREVNTIGSKANHLA

LVNRVVEMKAELEKIREQAANIE"

CDS 1244805..1245065

/gene="remA"

/locus_tag="EFAGFIKM_01091"

/inference="ab initio prediction:Prodigal:002006"

/inference="similar to AA sequence:UniProtKB:Q7WY72"

/codon_start=1

/transl_table=11

/product="Extracellular matrix regulatory protein A"

/db_xref="COG:COG2052"

/translation="MAIKLINIGFGNIVSANRIISIVSPESAPIKRIIQEARDRHMLI

DATYGRRTRAVIITDSDHVILSAVQPETVAHRLSSKDDDNDE"

CDS 1245079..1245651

/gene="gmk"

/locus_tag="EFAGFIKM_01092"

/EC_number="2.7.4.8"

/inference="ab initio prediction:Prodigal:002006"

/inference="similar to AA sequence:UniProtKB:P99176"

/codon_start=1

/transl_table=11

/product="Guanylate kinase"

/translation="MSKGLLVVLSGPSGVGKGTVCSALRKRVPELIYSVSATTRQPRL

GEEHGVNYFFRSHEEFQNMIAEDQLLEHAEYVGNYYGTPRDFVEKTINEGRDIILEIE

VQGALKVKEKFPEGIFVFLLPPSLDELKDRIQGRGTESQATIDHRMSVAVDEISLLEQ

YDYAVVNDEIDLACKRIESIIIAEHCKINK"

CDS 1245809..1246021

/gene="rpoZ"

/locus_tag="EFAGFIKM_01093"

/EC_number="2.7.7.6"

/inference="ab initio prediction:Prodigal:002006"

/inference="protein motif:HAMAP:MF_00366"

/codon_start=1

/transl_table=11

/product="DNA-directed RNA polymerase subunit omega"

/translation="MLYPSIDEMMNKVDSKYSLVVAASRRARQLREGEKSDLRGARSH

KQVGVALEEIYGDLLVVIKGQDEEEE"

CDS 1246160..1247395

/gene="coaBC"

/locus_tag="EFAGFIKM_01094"

/inference="ab initio prediction:Prodigal:002006"

/inference="similar to AA sequence:UniProtKB:P0ABQ0"

/codon_start=1

/transl_table=11

/product="Coenzyme A biosynthesis bifunctional protein
CoaBC"
/db_xref="COG:COG0452"
/translation="MLNGKKIVLGVTGGIAAYKAATLCSRLVQKGADVHVIMTASATQ
FITELTLQTLTRNTVYTDTFDEREPAVVSHIHLADLADLVLVAPATANVIAKMAHGMA
DDMLSTTLLATTAPVMIAPAMNVHMYDHPAVKHNMNLLVERGAMMIEPGEGLLACGYV
GKGRLEEPESIVDVVERFFEQRESADISRQGQAPLLQGKKVVVTAGGTIERIDPVRYI
TNDSSGKMGFAIAAAARDLGADVKLVMGSTQAKPPENVELIPVQSAQDMYEAVTREWD
DADIVVKAAAVADYRPKEVYTEKIKKKGDTLSLELIKNIDILETLGKQKTHQFLIGFA
AETQSVEMYAREKLERKNCDLIVANDVTRTGAGFGTDTNAVHIYDREGLVEELPVIAK
DDVAHRLLRIAAERIAGRN"
CDS 1247399..1249948
/gene="priA"
/locus_tag="EFAGFIKM_01095"
/EC_number="3.6.4.-"
/inference="ab initio prediction:Prodigal:002006"
/inference="protein motif:HAMAP:MF_00983"
/codon_start=1
/transl_table=11
/product="primosomal protein N'"
/translation="MEIAKVIVDVPVKETDRPFDYLVPESMREWIEIGSRVGVPFGHR
TVQGFVIDLVPRTGEETFKLKQIQELLDIVPPLSKDLVELAEWMSGRYASNRILSLQV
MVPTALKGKAERYISLGDALDGQMAGAQPDDEVLFVWGEESTTEKVQQDIIRFVKSRG
QVPLQQLSRKYPNHAALIKKLLLGGVLLESQAIKDKLNKKTMKSVDLAVDIAAAQEAL
ASFPAKAQRQKEILAFLLEMKELLPMPMKEVLSTLQVSAATIKGLEEKGLIVTEDVEV
FRDPYQGRTFKATEPLVLTDEQKLVYDNINGRLQEQRHGVFLLHGVTGSGKTEVYLQT
IQQCIEQDRQAIVLVPEIALTPQMVERFKGRFGDQVAVMHSRLSGGERYDEWRKIREG
QVKVAIGARSAVFAPFSRLGLIIMDEEHETSYKQEETPKYHARDVAVKRAQQHQAVVV
LGSATPSLESYYAARSQSNDDFAPLLLEMPTRALGNKLPEVRIVDMREELKDGNRSMF
SRALHKGLEERLERGEQTVLLLNRRGYSTFVMCRSCGYVAGCPECDISLTYHQRSNNL
RCHYCGYAEAAPEVCPDCGSEHIRYFGTGTQRVEEELAKLFPGIRVIRMDVDTTTEKG
AHEKLLKQFREKKADVLLGTQMVAKGLDFPDVTLVGVITADSALNLPDFRAAEKTFQL

LTQVAGRAGRHQLPGEVFVQSYTPEHYSIGHASQHDYVSFVREELLHRRNLQYPPYCR

LILVTFSHEQLPVLIRLAENYTRILKEKANAAGWLGSLDRFSNDAFDVLGPVASPIPR

IKNRYRFQCMIKWRGDVDAIGLALATARRMDDDVQAQKLLISLDVDPQMLM"

CDS 1250065..1250553

/gene="def"

/locus_tag="EFAGFIKM_01096"

/EC_number="3.5.1.88"

/inference="ab initio prediction:Prodigal:002006"

/inference="similar to AA sequence:UniProtKB:Q4QMV6"

/codon_start=1

/transl_table=11

/product="Peptide deformylase"

/translation="MSIRIIVQEPDEVLHKKAKEVTKITPNVQKLLDDMADTMYDAEG

VGLAAPQVGILKRLIVIDAGDEQGLIKMINPEIIASEGEQFGPEGCLSIPGINGDVRR

FETVTVKGLDREGKELIITGSGLLSRAFQHEIDHLDGVLFTDVAEKVYEIAADQTGPR

RN"

CDS 1250566..1251519

/gene="fmt"

/locus_tag="EFAGFIKM_01097"

/EC_number="2.1.2.9"

/inference="ab initio prediction:Prodigal:002006"

/inference="similar to AA sequence:UniProtKB:Q81WH2"

/codon_start=1

/transl_table=11

/product="Methionyl-tRNA formyltransferase"

/db_xref="COG:COG0223"

/translation="MNIVFMGTPEFAVPSLDMLMAEGYNVVGVVTQPDKPQGRKKVLT

PTPVKAAAERHGLPVFQPVKLRDPEAVARLAEWKPDLIVTAAFGQILPKAVLDMPVRG

CVNVHGSLLPKYRGGAPIQRSIINGESVTGVTLMYMAEGLDTGDMISRVELPITDEDT

SGSMFDKLSEAGSKLLQTEMPRLIAGETTAVPQDDAEASYARNLTRDDEKMDWSRTSR

ELFNQIRGLVPFSGAFTMWDDQVFKVWATANPDQVDLATSSDAGQAEPGTVLQLNKAG

IEVRTGNGSLWLTEVQPAGKKVMQAADFARGGTLKPGTVLR"

CDS 1251516..1252994

/gene="rsmB"

/locus_tag="EFAGFIKM_01098"

/EC_number="2.1.1.176"

/inference="ab initio prediction:Prodigal:002006"

/inference="similar to AA sequence:UniProtKB:P36929"

/codon_start=1

/transl_table=11

/product="Ribosomal RNA small subunit methyltransferase B"

/db_xref="COG:COG0144"

/translation="MSGNIPGRSSGKGQKANGNSSGREGNRRPNSGKSGGASRSQQPK

TSARTLAVKVLSAVEQDGAYSNLELNRRLKEADLSPADAGLATELVYGTIARLNTLDY

FLERYVAKGVSKLQPWVRSLLRISVYQMIYLDRIPEHAVVSEAVNLAKKLGHQGISGM

VNGVLRNMIRNRDELRIPEHLPVAERISLEHSHPLWMVERWIAQYGEETAEAICRANN

EPPAVSVRVNTTMTTREKLMHEMTSTGAVVEASQLSSDGILVRSGGNMALTSWYRDGL

FSVQDESSMLVAEAVAPEVDQLVLDCCAAPGGKTAHMAEKMQDRGRIVANDVHAHKRH

LILDQAERLGLTCIDAVTGDALDLNERYPEASFDRILLDAPCSGLGVIRRKPDVKWTK

TVKDIEDIAGLQRELLDHVAPLLKPGGILVYSTCTIEPAENEDMVADFLNRHPEYSPA

EASVWSESETANLKVVNGGIQILPQYAHSDGFFIARLTKTVE"

CDS 1253248..1254288

/gene="rlmN_1"

/locus_tag="EFAGFIKM_01099"

/EC_number="2.1.1.192"

/inference="ab initio prediction:Prodigal:002006"

/inference="similar to AA sequence:UniProtKB:Q7A600"

/codon_start=1

/transl_table=11

/product="putative dual-specificity RNA methyltransferase

RlmN"

/translation="MKPFIYDYSLEQLQQWAVENGEPAFRGGQIFDWIYVKRVNDFSE

MTNLSKPLREKLTEQFEFVTLKEITKFESKDGTVKFLFGLHDDHAIETVIMKHNYGNS

ICVTTQVGCRIGCTFCASTLGGLKRNLTAGEIVAQVVQAQKILDERGERVSSIVIMGS

GEPFENYEATMTFLRIMIHEKGLNIGQRHITVSTSGIVPNIYKFADEDTQINLAISIH

APNDALRSKLMPVNRRFPFEDVMESLRYYLAKTGRRITFEYALIGGVNDQPEHAAELA

SVLKNMLCHVNLIPVNHVPERKYVRTSRSDIFNFQKILSEQGVNVTIRREQGHDIAAA

CGQLRAKHMELR"

CDS 1254296..1255072

/gene="prpC"

/locus_tag="EFAGFIKM_01100"

/EC_number="3.1.3.16"

/inference="ab initio prediction:Prodigal:002006"

/inference="similar to AA sequence:UniProtKB:O34779"

/codon_start=1

/transl_table=11

/product="Protein phosphatase PrpC"

/db_xref="COG:COG0631"

/translation="MIKTVHVSHIGRVRSVNEDSAWIRNLDTGYILGIVADGMGGHLA

GDTASRLAVETLVKDLGTLEPGLSHASLSAALSDAILHANEVIFRTASTDDKYHNMGT

TVVAALLNDTEGVIGHIGDSRAYKIANKAVIQLTEDHTLVNELFKNGQISKEDVSHHP

RRNVLTRALGTDAEVKVDLDTVKLEEGEVLLLCSDGLSNLVSNEQIIQVAGNLELALE

DRADRLLQLALLAGGDDNITLALFELQREGSVDTETGCES"

CDS 1255069..1257285

/gene="prkC"

/locus_tag="EFAGFIKM_01101"

/EC_number="2.7.11.1"

/inference="ab initio prediction:Prodigal:002006"

/inference="similar to AA sequence:UniProtKB:O34507"

/codon_start=1

/transl_table=11

/product="Serine/threonine-protein kinase PrkC"

/db_xref="COG:COG0515"

/translation="MIGHQLGGRYEVIERVGGGGMALVYKAQDLLLNRNVAIKVLRQQ

FVHDEEFIRRFRREAQSAASLSHPNVVSIYDVGQEDDVHYIVMEYVEGKNLNEIIKER

APLQVDESVRIASQIADALDHAHHNQIIHRDIKPHNILIGRNGRVKVTDFGIARAVTS

TTITQTGSVVGSVHYFSPEHAKGIVTGEKSDLYSLGIVLYQMLTGQLPFLGESPISVA

LKHLQEEFDEPRKFNPLIPQSVENVILKSMRKNPQERYQSAKEMQTDLETCLMPERRN

ETKIDFPDEDDIDQTRVMPAIKPEPRGVTSTGAVPVMESDEETGKGKAKSKSWKKPAL

LISLTVLILIAMVGVVWYVKGMLVVPEVTVPNVITQTEEKAREMLEEKGLVVSDEVIR

LYQEGVEPGIVYDQSRKEGDVVKEGSEVQISVGAEKELLKMIDVKQVTYDEAVKKLTA

LGIKEDQIQRKDEFSNDVASGSVISQTPGVNEEFDPAIVQIELIVSKGTETVKMPDLK

NLTRSEAEKKLKSAGLELAQVQEEPSYTIEKGKVTQQWPVEAGTEVSPGDKITIFIST

GYPPEALQYPFNINVSPKEEGKNSKIRITYEDARGKNQEWGTRTVNSTQTLTIPLVLA

PNENGAVSVYRDGQFLDTYLVSYSDAKNGTVNVPSIDPEQSTETPPENEPDPGEGSVD

EGSESVDPNQEGEPESTPADGEGDSVDEDTSAMNNGKGHGKEKEKKKEVINASSRP"

CDS 1257347..1258255

/gene="rsgA_1"

/locus_tag="EFAGFIKM_01102"

/EC_number="3.6.1.-"

/inference="ab initio prediction:Prodigal:002006"

/inference="similar to AA sequence:UniProtKB:O34530"

/codon_start=1

/transl_table=11

/product="Small ribosomal subunit biogenesis GTPase RsgA"

/db_xref="COG:COG1162"

/translation="MPEGIIVKALSGYYYVMPVEDNGVPSVEGSAVQCRARGIFRKRG

TSPLVGDRVSYMLTENGEGTVDEIRKRETELIRPPVANVSLAVLVFSVKEPDMNLNLL

DKFLVHIEQAGLDALIVLTKLDLADPAKDTVAEVKALYEQVGYEVISTSSRTGEGSEL

LRDRLAGKISVFSGQSGVGKSSMLNALMPGLTLETNAISMRLGRGKHTTRHVELIPLD

NGGFVADTPGFSQLDFLEIGVEELSTCFREFAQFADQCKFRGCTHTHEPGCRVLAAKE

EGLISESRYQHYVLFLNEMKDKKRRY"

CDS 1258257..1258916

/gene="rpe"

/locus_tag="EFAGFIKM_01103"

/EC_number="5.1.3.1"

/inference="ab initio prediction:Prodigal:002006"

/inference="similar to AA sequence:UniProtKB:P74061"

/codon_start=1

/transl_table=11

/product="Ribulose-phosphate 3-epimerase"

/translation="MIKIAPSILSADFARLGAEVAEAQAAGGDWIHVDVMDGHFVPNI

TLGPAIVKAIAPHTSLPLDVHLMIENPERYVEEFAKAGAAVITVHAEACVHLHRVIHL

IKEQGVKAGVALNPGTPASAILEVLDDVDMVLVMTVNPGFGGQAFISGTMNKIKQIRT

WLNEKGRHDVHIEVDGGIAADTAPLVVEAGADVLVAGSAVFGREDRAAAITEIRRSYG

G"

CDS 1258920..1259171

/locus_tag="EFAGFIKM_01104"

/inference="ab initio prediction:Prodigal:002006"

/codon_start=1

/transl_table=11

/product="hypothetical protein"

/translation="MTLKETLWTMAASLVTGLVLALFAVIQSPFNAITSLIGVGVVIM

YFRKFDRTGHRVTFVIFGILYYLLSVFMIAAYQYIPAQT"

CDS complement(1259614..1259802)

/gene="rpmB"

/locus_tag="EFAGFIKM_01105"

/inference="ab initio prediction:Prodigal:002006"

/inference="similar to AA sequence:UniProtKB:P37807"

/codon_start=1

/transl_table=11

/product="50S ribosomal protein L28"

/db_xref="COG:COG0227"

/translation="MSRKCYVTGKKPGTGNHVSHANNRNRRTWGVNVQKVRILVDGKP

KRVYVSTRALKAGKVTRV"

CDS 1260479..1262251

/locus_tag="EFAGFIKM_01106"

/inference="ab initio prediction:Prodigal:002006"

/inference="similar to AA sequence:UniProtKB:Q7A5Z4"

/codon_start=1

/transl_table=11

/product="hypothetical protein"

/translation="MVLAGAEQLGQHAEHVNSLNVFPVPDGDTGTNMNLTMSAGVAEI

KRKSSASIGEAAGILSKGLLMGARGNSGVILSQLFRGFSRSAAPYEELNTLQFAAALQ

NGVDAAYKAVVKPVEGTILTVAKEAAKHANYYARRTNDITELMNEVLLKAKEALAMTP

ELLPVLKQVGVVDSGGQGLVYIYEGFMEVLLQSDGGSRTSLNKEVTPSVAASALKPAA

PTEVDIKKPSQQQVIAPEMPLSAQARLVTEDIEFLYDMEFFINRELGENAGVAFDDEA

FRKALSVNGDSIIIIADDEVIKVHVHSKTPGDVLNLALHYGEITQIHILNMREQHRDL

LTAGMDIAPSPELFAEIPPEAARSMEEAVLPADEMAPYGFIAVSSGDGIAEIFQSLGV

DVVLSGGQTMNPSTEDFVKAVRSIAAEQVFILPNNSNIVLAAEQARELLEDERRITVI

PSKTIPQGMAAAFAFQEDESAETNRDQMLEAISRVQSGQVTHAVRDTQYDELDIKAGH

YIGIHNSKIVATDETMLQACEGLLKQMMESGDEVVTILEGDEADPEVTAALAAWLEEQ

YPDAEVEVHLGGQPVYYYLFSVES"

CDS 1262280..1263143

/gene="degV_1"

/locus_tag="EFAGFIKM_01107"

/inference="ab initio prediction:Prodigal:002006"

/inference="similar to AA sequence:UniProtKB:P32436"

/codon_start=1

/transl_table=11

/product="Protein DegV"

/db_xref="COG:COG1307"

/translation="MSHKVAIVTDSTADIPEELIRKYGIHIVPLRLLFGEETYADGVD

LTSEQFYEKLKKVSVLPTTSQPSPTDFMNVYQSLLDENPERPIVSIHLSSGMSGTYQS

AMLGKSLLEREGDITVLDSKSASYGYGLMVVQAAELAEQGKSAVEIAAAIEGMQQSRK

LFFLVDTLEYLQKGGRIGKASAILGTLLNIKPILSIDEEGVIYAVEKVRGHKKAMARI

IELFQKDLAGKRVNLAVGHTADPGSAIACAEQLRGHFTLNEVVYTNIGAVVGSHVGPG

VIAIFMWPVPE"

CDS 1263154..1265199

/gene="recG"

/locus_tag="EFAGFIKM_01108"

/EC_number="3.6.4.12"

/inference="ab initio prediction:Prodigal:002006"

/inference="similar to AA sequence:UniProtKB:P64325"

/codon_start=1

/transl_table=11

/product="ATP-dependent DNA helicase RecG"

/translation="MKLEEISVKQINGVSALKEGELHAFGISTVKDLLEYYPFRYEDY

RLRSLSEVKDGDKITVQGQVMGIPVLQRYGKKSRLTCKIMTEEWMITATWFNRHFLKE

QLTPNREIVITGKWEQKRMQMTVTDSEFPDKGDGRSGTLQPVYSVTGKLTQQWMRKTI

NQGLIQFGDMIPEILPQSLMKKYSLMPRKQAIAGIHRPQDNREGQQARQRMVYEELFL

FQLKMQAYRALNRNRMDGVVHTTDNTTIREFVRSLPFELTDAQKKVELEILHDMRSPY

AMNRLLQGDVGSGKTVIAAIALYTTVRSGFQGALMVPTEILAEQHMRSLQKLFEPFGV

TVGLLTGSVNGRKRKDLIASLQMGMIDIVVGTHALIQEDVFFRDLGLVVTDEQHRFGV

NQRSVLRRKGYNPDVLTMTATPIPRTLAITAFGDIEVSTISERPKGRIPISTYWVKHD

MMERVLGFISREVDQGRQAYLICPLIEESEKLDVQNAIDLHVQMQQNFPKYRVGLLHG

RMTAAEKEEMMRDFYSNDIQLLVSTTVVEVGVDVPNATLMVIMDADRFGLSQLHQLRG

RVGRGAHASYCVLIADPKTEVGQERMKVMTETEDGFEVSRRDLDLRGPGDFFGTKQSG

LPEFRLADMVADFAVLEQARDDVTHLIEDATFWTSMDYAPLRDFLQQQQVFKGDLID"

CDS 1265315..1265602

/locus_tag="EFAGFIKM_01109"

/inference="ab initio prediction:Prodigal:002006"

/codon_start=1

/transl_table=11

/product="hypothetical protein"

/translation="MGYQQYGISPQLVERIKLKMKNPAVKERIKKLIDGVTKSDLQDK

AKVRRLVKSSAVIMNENFSPAQEEQFVAFVLAQKIDPNNTFHLIKLWGMFR"

CDS 1266225..1270682

/locus_tag="EFAGFIKM_01110"

/inference="ab initio prediction:Prodigal:002006"

/codon_start=1

/transl_table=11

/product="hypothetical protein"

/translation="MTLTRGFKQKIGGKVIAVITVVSLTWGSWGAMPLSASTTNTLKF

QTQQGGIFDGMPLFDGVPAHLDAYVDTYFEYTGLEGPAVYVTGSRNHYTLKQGKHVGK
VIPGALSAADNVQGVSTDFPALIGMGQSWNKELLSDIGEVMGNEKISKIKVKQGESNI
HGGNDPSLSVAFSVVSDMRINPLSGRFDEGFSEDPNMAGVLIDKMASGLSGIDQSTSE
DGFWMRAAVGTKHYSVYNAQWFRQTANNSASARSLFEYQTRAALPGFESGALAGTMTS
YGRTNGIPNILSPLQLLANLDAKYGVYSSPDFNGDAIVSNANQLGNGYDDRYAVDRGH
ATVLMALAQANAGRPSGTTPADVTDLVALVEQGEYGITEEELIEAARPHVNQLVRLGI
FNETDEQGIPKNYPFAQEARDVRTSPPSDYNQVNHQEIALQAAQESVVLLKNDGTLPL
EKGNDAAVSGVYADARFKTTYSVGATPTLENSGESPLLSIIKMNGSSNVNYAPGNEVI
ALTSKANQQTVTADVYSPNAGTEGAQLTTTSDAPDEASFSHLFRVYDWGQAGSSLLSL
YNQRWVTSPATNDAAVGNTDRTSLNLTNNDWNLPEMMGDTSAIPPTLRMETNEDGSMS
IVTNGYRTGFSGDFTNWYYANGRFIKVDDAGKLVTTSSPLGSKDNAANRPDEVKFEKA
VVKNVGEEAVARATDDDYAIVFVGAIPRHSAGEGNDRSTLDMGEADYELVDKVSAAFA
TQGKKTVVVVKSSFPVNMERIQNNPNVSAIVYQPYGGQYDSYALAQVLFGDYAPTGRL
TSTWYAGMDAFPSISPYVIPEGNITGLDDIDPRFTVDMTNADPIESELTYMYTDATVT
YPFGYGLSYADFTYDSLTVPSSVRGDEPFTVSVRVTNTGDVATSEVVQLYTRNSNSAY
GAHAPKKKLVSFEKIHLTPGETKEVLLQVDPQTLALWDVNRSEWIVEQGSYEFMIGTS
AADADVQVSKALSIGGKSIATLPLTQPINVFDHAFASNEVVYHEVSKERTAAQLKAKA
IVGGYYAVRSKQNGSWIALPKADLTGAQKVKATVASNVQGGTISLHADALDTAPLAVW
NVPVTEPVSYKISNANVTVKELGYVDVDADISAAVSGVHDLYLVFHESDLRIDSLSFT
LKEPGTNPGSPGTNPGTNPDSPSTPGSGSVGTPVKPPASGSETEGETDSEGNVSTVVT
VQTGDVADQAKAASIRSSGIRVIGDPWNVSIKDKQNTYVIAALPVNDVVAANSTAIAY
VGAGGQLTPVPGVLTKDTNGNKVWKVLLNQGGTYVAIHSLREFVDVKPSAWYAKDIAK
ANSLLLINGVSERRMQPQGTTTSAQTLMAALNALGIEPMGAAANEKWFDPILRASKEY
GLMEEGQYLPNVPMSREDMAMVLVRALNLSGVDMKLQEAEIESRLGDFKDQDQLKPNN
RAAMATAIQLGIFQGTPDKLLQPQSTLTRAQLATVIVRVHDRIIERLDR"

CDS complement(1270716..1272296)

/gene="cheB_2"

/locus_tag="EFAGFIKM_01111"

/EC_number="3.5.1.44"

/inference="ab initio prediction:Prodigal:002006"

/inference="protein motif:HAMAP:MF_00099"

/codon_start=1

/transl_table=11

/product="Protein-glutamate

methylesterase/protein-glutamine glutaminase"

/translation="MYTYIVVDDEPLIRKGLLKKIGTFEHSLELLGEADNGADALALI

ESFKPDIIFTDMRMPEVDGKLLIRTVCQQYPRIKLIVISGHTDFDYMQEAISAKVVNY

LLKPFSRDEIHGTLHQAIQAIEQERSLQREVVLREVETEQMKRDADMQAFLNYVLAPH

SSIKTPVFRSAEFAAIMASDQFILLTIYGPQPLTAQQLPTFSNGLYIPHPQTDQVLFF

LMHDLDCSGQLDLQKMAEQTASIILTKSETTECCVGISLPHTGIGGVREAHHQTILAL

DERAITDFGKCYVFSREQIPLHQLKWARTYELLFFIECGNVPKAEELITDFFAFYIRQ

PDALLAHLKEQCRDIITETKRLLSNYIQNGGSSSASSSLEAVLNVSFDMECIRHYMVK

VLTGTALLLKEDNPYASDQVIDNVRTYIDNHYTKDLTLEWVSSLFYLNPSYLSHLFKE

KTSENFTDYVNRLRIRQAKQLLTSTDDKIYRVAKQLGYDNPKYFFRVFKKLTGWTPEE

YRKQHKSFTANNKEPGHA"

CDS complement(1272304..1274112)

/locus_tag="EFAGFIKM_01112"

/inference="ab initio prediction:Prodigal:002006"

/codon_start=1

/transl_table=11

/product="hypothetical protein"

/translation="MILINIKTKLFLVLVLVSIVPIIVVSVSSYRSYTKLVNEQTSLV

TSTTIGNSVKSMEEILQNIDRISVTLLQQSSNATAYSTVSDELIKLNGTNDQYEIFMI

RSKLKLIFENILLGYNYINGLYLFTPDGKWISYGSSGTDLRLDYVALRDEWYLDTIQR

KGDLYIGEVGVKPYIINAKESIGFSRALYDPNNNEFLGVFMLDCSMNIFDGLKRNPAP

SLSNLYLANDAGEILYSSDGSRVGTPLSDTLAAATHQVGNSSQYKSIQDNMMTVIQKV

PGRNWNVIANISMDRLYEEYNISQKLLFYVAVSCAVIFLLLSFFLSNLITKPIIELTK

LMRKTKSHRFVTIKPQVTRQVDEIGILYHEFNKMMKDIDTHIRESYQNKILMLDSQMK

ALEAQINSHFLYNTLESINSIAEIEEVESIVVMTKSLGDMFRYSIKTDSELVAVSEEL

QHVENYMAIQQIRYGDKFAFNLNVDPEIRSRRMLKLLLQPLVENAIYHGLENKRGKGS

VTITGLFQGDSIVFEIWDDGVGMSSTQAEELRQLLQQPPEFIAVGQRHKQSIGIKNVH

SRIALYYGEGYGLSFHTDLNTGTRFTLEIPANWEKR"

CDS 1274244..1275575

/locus_tag="EFAGFIKM_01113"

/inference="ab initio prediction:Prodigal:002006"

/codon_start=1

/transl_table=11

/product="hypothetical protein"

/translation="MKKRKRLWFLTLVLLMMFLSACSSSSQEGSGTDTNNGSTDESEK

PITLTMMLSGNKASEGEDFELETLPRLVKEKFPNINLEVQKLPDDQYNTSVRTKLAAG

QGPDIFWIFPKNAAGGVIDFAKAGFAADLSDLKFWDNISQGAKEDMSYEGKPYGVAEG

LDFLGVYYNKELLQQAGYSEFPKDWPSFLDLCQKLQAAGVTPIAMGDKDPWMIQFGMY

PIAANVVYPNELDFDVKLQNGEKSLTDPKWVQTISKYKELYDNNYVVKNSLGIGSAQS

AQLFIDGKAAMTFTGTWDYASMTSKGATEFTRGFASLPGNEPEQPVFVSAATSAGYAL

NAKSANLDAAKEVLNYLFDGESDLFKAFVEANSSISVYKDVKLENQLFKEVNDNYQQT

GNSVYFSNQMWPAGVSDVMQAKFAEIIAGQKTTPEDVANAMETKFRELWKN"

CDS 1275661..1276539

/gene="melD_2"

/locus_tag="EFAGFIKM_01114"

/inference="ab initio prediction:Prodigal:002006"

/inference="similar to AA sequence:UniProtKB:O34706"

/codon_start=1

/transl_table=11

/product="Melibiose/raffinose/stachyose import permease

protein MelD"

/db_xref="COG:COG1175"

/translation="MSSTVMKKSMYWFALPALIFYLTFWIFPILRLFQYSITDYNGYA

QAYNYIGFDNYKELFRSDILGISLKNTLIYTFVTVIAGNIIGLAMALLLNMKIRGTGI

YRSLAYIPTLFSAIVVGFLWSYVYMPDYGLISSFLDKLGIANELNFLGDYSTALYSII

FVEIWKTIGTTMIIFLAGLQTVPSDLLEAGRIDGCGSWRLLRHVKIPLLATAITINVT

LSLINGLKAFDFPFIMTNGGPGTATNTLIYTIYKMAFTEQLFGKASALGVISFAVIIV

ITAFFVLKMNKREVSA"

CDS 1276536..1277372

/gene="melC_2"

/locus_tag="EFAGFIKM_01115"

/inference="ab initio prediction:Prodigal:002006"

/inference="similar to AA sequence:UniProtKB:O34518"

/codon_start=1

/transl_table=11

/product="Melibiose/raffinose/stachyose import permease

protein MelC"

/db_xref="COG:COG0395"

/translation="MTLRKVLGKGSVQVVVLLVMALNLIPLIIAFSTSLRDPSNNTNP

LVLFQEMSFGSYKAAFDRMHFPEALWNSVVLTGVSVVFVVVFAAMASYPMARLKTKLS

KFMYLFFLSGLVVPAQMVLIPIVQMINALHIPTNQYTPIFMFITCSLPFSTFLYTGFI

RSGVPEEVEEAAHIDGAGLFQRFWTVVFPLLIPVTVSVIITQGVWIWNDYFFNMIFIS

SAGDSPLPLAMIGFLGDQQNPTQWNVLFAACFLCALPMLLAFLFLQKYFIGGMTVGAV

KG"

CDS 1277386..1278939

/locus_tag="EFAGFIKM_01116"

/inference="ab initio prediction:Prodigal:002006"

/codon_start=1

/transl_table=11

/product="hypothetical protein"

/translation="MSEKVINTAWLEKAQGLMPELRQQKVYPEHVVSAVPDAAAFQGW

RIESLGPAEQLSDQVLCKGDSITLDFGEHLVGYLTLSLLNERSNADAPLRLKLTFGEM

PCEVGENFAEYSGWLSRSWLQDEIVNVDVLPGTLTLDRRYAFRYLKIEVVETSIRYDI

RINDIYCMTVTSADIGAVPQLPEGIDTELLEIDRLSVKTLRDCMQTVFEDGPKRDRRL

WIGDLRLQALVNYETFGCNDLVKRCLYLFAGTRLEGGQVGACLYEQPEPYVDDIYLYD

YALFFIATLWDYFEATKDRETLEELWPVAREQIEIGLKRLDERGIVRDDDTWYCFLDW

HEELNKQAGAQAVLIYSMKRGRSIAKELGLPEYEEWINAHIKDAEQAALAHLWDGEQG

FFISGEKRQISWASQVWMVLAEIMDAPAARQLLDRLTSSGEAIGMTTPYMVHHYVDAL

IMCGEEEKALEQIRRYWGGMLKDGADTFWELYDPEDKTYSPYGSNLVNSYCHAWSCTP

SYFIRRNFS"

CDS 1279672..1281339

/gene="ypdA"

/locus_tag="EFAGFIKM_01117"

/EC_number="2.7.13.3"

/inference="ab initio prediction:Prodigal:002006"

/inference="similar to AA sequence:UniProtKB:P0AA93"

/codon_start=1

/transl_table=11

/product="Sensor histidine kinase YpdA"

/db_xref="COG:COG3275"

/translation="MEMFTMLLGLFERAALLIIFLFFLSRVPRFRQILQKGKLRWQEY

IAVTLLFCAFAIFGTYTGINVEGSLVNVRIIAVLSGGILFGAPVGIITGIVSGVHRYL

IDMDGVTAIPCLITSILAGLVSGYIHKYTPKPKRWIIGIGAGMICEALTMLLILLFSY

PDPLGADIVSKIALPMILGEVNIGLIVLLVQSVEGEKEMIAARQAKLALEIANKTLPY

FRSIDEDSLRKICRIIQEDIQADAVAITDTRNVLAYVGFGEERYHIGNEIISEMTKKT

ISSGEITISNDVIDEKTPDIHSLLIIPLKERGDITGALKIYYRKAYKITYPLQTMAVG

LSQIISTQMEVSRVEEIKAAANKAELRALQTTIHPHFLFNALNAIASSIRTKPDRARE

LIVNLSGYMRYNLELSDELIDIHKELEQVRNYVEIEKARFGSRLNVIYEIDEVAVHIP

SLVIQPLVENAIIHGILKVKGPGTVRIRVQDYPEFVRISVSDTGAGISADIIERVYHD

RMPGNQIGLYNVHRRVKLIYGQGVTITRLEQGTDILFDVPKGDVVTR"

CDS 1281350..1282150

/gene="ypdB"

/locus_tag="EFAGFIKM_01118"

/inference="ab initio prediction:Prodigal:002006"

/inference="similar to AA sequence:UniProtKB:P0AE39"

/codon_start=1

/transl_table=11

/product="Transcriptional regulatory protein YpdB"

/db_xref="COG:COG3279"

/translation="MRALIVEDEILASEELNYLIQEHSQIEVVDRLEDGLDVLKFLQE

QEVDVIFLDINIPSLDGMMLAHHIGKFATKPYIVFTTAYKEHAAEAFELEAFDYILKP

YDEKRIAAMLHKLELAFKRDHAPVEQHVEDGPAPTADGSAAHGELVRERDTNSHTDRR

INLLRNDNIIVTDTADIYYAEAQEKVTKVYTKNGEFTMPVSISDFHSRLPQDTFFRCH

RSYVVNLSQIREIVPWFNNTYLLRLRDLEAEVPVSRGKVKEFRQLMRI"

CDS 1282271..1283566

/gene="yhjX"

/locus_tag="EFAGFIKM_01119"

/inference="ab initio prediction:Prodigal:002006"

/inference="similar to AA sequence:UniProtKB:P37662"

/codon_start=1

/transl_table=11

/product="putative MFS-type transporter YhjX"

/translation="MKATISSVSSTPESTSAPTKMNRWLIVLGTIIVQMGLGTIYTWS

LFNQPLSDRFGWDVSSVAITFSITSFALAFATLFAGRLQERWGLQRLIRVAGVVLGLG

LILSSQVTSLTLLYILAGFVVGFADGTAYITSLSNLIKWFPERKGLISGISVGAFGTG

SLLFKYVNTALIGAVGPAQAFMYWGIIVLVLVVAGSFLIREAVVREQAPASKEGSKQV

VARHDYTVKEMLRTKEAYMLFVIFFTACMSGLYLIGIVKDIGVQLAGLDVATAANAVA

MVAIFNTAGRIILGALSDKVGRMKVIAGALLVTAVAVMTLSLVPLTFGIFFACVAAVA

FCFGGNITVFPAIVADYFGLKNQSKNYGVIYQGFGFGALAGSFISALLGGFHLTFIVI

AVLCAVSLLLALIITPPGQGRRARQREKQMNLNPSSRAS"

CDS 1283827..1284309

/locus_tag="EFAGFIKM_01120"

/inference="ab initio prediction:Prodigal:002006"

/codon_start=1

/transl_table=11

/product="hypothetical protein"

/translation="MKFIKYTQPDFDDYYALVSNMEVMKQITERAIPEQEARTQFESM

LDFNERSTCGHYRVFSEDGAGVAYAKLIPDQEDPTKAEIGYMILPEHWRKGQGTSIAS

RLIIQAQAAGIGSLYAVIDPGNAASRRILTRQNFVSTWTGDYDGLPGEILELNLQMKR

"

CDS 1284446..1285387

/gene="glsA"

/locus_tag="EFAGFIKM_01121"

/EC_number="3.5.1.2"

/inference="ab initio prediction:Prodigal:002006"

/inference="similar to AA sequence:UniProtKB:Q5KY26"

/codon_start=1

/transl_table=11

/product="Glutaminase"

/db_xref="COG:COG2066"

/translation="MSNSTMERLNALLPEWLETSRLHTGQGKVASYIPELIKASQDEL

GIHIMDAEGNYVSAGDCGVPFTMQSISKVFTLILALMDHGEEAVFFNVGKEPTGDDYD

SMIKLELVEPGIPFNPLINAGAITVSSLVGGDCKEEKSRRILEFFRKLANNDQLGYNE

EVFQSESESGHMNRSLGYFLKHNGVIKDDVEDVLAVYFRHCSIEVTCADLSRMSLVLA

YNGTDPITGEELIPRRYVQIAKTFMTTCGMYNASGEFAIEVGLPAKSGVSGGILTLVP

GQFGIGLVGPALNRKGNSIAGVHLLERLSKEFDWSFF"

CDS complement(1285602..1285943)

/locus_tag="EFAGFIKM_01122"

/inference="ab initio prediction:Prodigal:002006"

/codon_start=1

/transl_table=11

/product="hypothetical protein"

/translation="MSKKLQQNGIFESSRMMLPEHREAYILHQEQLAPRTRPSLDAQA

AEEMSRLLSNSMMLGDRVTITLFHEHDDIRYTGQVLRLDRPARTLRLLMKDGSRDIQM

NLITDVALADD"

CDS complement(1285940..1287184)

/gene="dinB_1"

/locus_tag="EFAGFIKM_01123"

/EC_number="2.7.7.7"

/inference="ab initio prediction:Prodigal:002006"

/inference="similar to AA sequence:UniProtKB:Q47155"

/codon_start=1

/transl_table=11

/product="DNA polymerase IV"

/db_xref="COG:COG0389"

/translation="MPRQDRRVIMLADCQSFYASVEKSAHPEYKDRPLVVAGDPARRS

GIILAACPLAKSYGITTAERLGEALAKCPDVVVVRPRMSEYIRVSLHITRILQSYTDL

VEPYSIDEQFLDVTGSLDLFGSPETIARSIQSRVMDETGVYIRIGISDTKVFSKMACD

LYAKKVPGGICTLVRKDLPSTIWEKPVRDMFMVGSRMAQHLYKMGVHTIGDLAQTPLS

RLRERWGVNGEVLWRIARGIDDSPVKPGTYAHQQQGIGHQMTLPQDYDSWEDIKVVLL

ELAELVSRRSRDKSLMGHVVSVGCRGQDYDRPTGFSRQMKVNEPTNITDEVYDAAAAL
FLRHWDGLPIRRISVSLTGLVPDSEVQLSWFDDRERKRELERATDDIKRRYGDTAIMR
ASSLYSSAQAHERSYKIGGHYK"

CDS 1288096..1290267

/locus_tag="EFAGFIKM_01124"

/inference="ab initio prediction:Prodigal:002006"

/codon_start=1

/transl_table=11

/product="hypothetical protein"

/translation="MVTGGIFAGWHYMQPKLVTYTAENGTEMKFKTDGDRFMEYGPDG
TWREMFVKGVNLGATSPGHYPGEFPLTKEDYLRWFQQIEDLGANVIRVYTVHEPIFYS
ALVEYNRGREQPLYFIQGIWSPEEELIEKQDAYAAHIEQTFKQEIEKAVSAVYGDADI
PAKQGESSGKYKTNAGKYLMAWHVGTEWDPAMVDNTNAKHADVPQYKGVHFSASPEAS
PFENWLAELLDHTAVLEKKYGWEHPMTFTNWVTTDVLDHPGEPLYEEDLVSVDARHIQ
PEAWQGGYFAAYHVYPYYPDLFHIDKTLQTIKDENGEYNTYKSYLRKLKAEFSDMPVM
ITEYGVPSSLGISHLGMGGRNQGGHNEVEQGDIDASLTQDIYDEGYSGAIVFMWQDEW
FKKTWNTMPLEIPADRRAYWLNVLTNEKMFGLLAMDPGKQEQLTIDGSLDDWDDLKDE
EVTTWQGQVDGIQEMKMTHDEAYLYIGLTLDQPFDPEKTVLRIGTDTLAGGSQPDKIL
PGRTLSDGLETVITLGQDEESQVEIAKDYDFNQRLYGKEGYDMLPDQQSDSADPFHPW
NLAVSLRMTPPDTRSAQPFMNEKVGTLKRGTTAPGQPDTDSLTAWQYNGNQVEMRIPW
MLLGFGDPSSLQAIDYGPLKNGREFSTVAVEGVTLIPWLTDRATKKVSWPGGEQTTLN
LNTLPVYSWQPWEQVQYSERLKLSYEKMKKSYEKIPSFQIE"

CDS 1290294..1291502

/locus_tag="EFAGFIKM_01125"

/inference="ab initio prediction:Prodigal:002006"

/codon_start=1

/transl_table=11

/product="hypothetical protein"

/translation="MFPSLALAYLFLYICIALVVVGVIVLFAMKMSHNGKRRKTAFYE
LKQRDYFTYLQTALTENSPLKLPPGKLAPLERRVIQDRLIEWIDQFKGEYRDKLIALC
REAGFVEHDLKELGRLRYGRQIDAAYRLGGMRCPEAVPGLMELLKDEKPGPMAIMIGR
SISRCTTQQGELKDMLALLLTKGKSIHHLAADILLETSLDTSRILIELLEDRNPDFVK

VGLVAMWGQAVPEVMPALDRLVGAEHQDVRAEAVKLYLSASPALRDETILKLIQDPDP

EVRAEVVQALGSKHASGSIPLLRKALRDEDWRVRYNSAESLSKLGEPGFEALCQAAVQ

GTVAEREIAMQQIESTMQHTRTDDRAVEQMIAHNKKRLLYDRYFGPVRENRTSKKRTG

VATVGGDYTA"

CDS 1291525..1292925

/locus_tag="EFAGFIKM_01126"

/inference="ab initio prediction:Prodigal:002006"

/codon_start=1

/transl_table=11

/product="hypothetical protein"

/translation="MVAIYYVVFVNTLYFSILALSFRNIWTIFRRSHYSKYNTLSGSE

LVPSVSLLVPAYNEELTIIENVNCLMTLNYPTYEVIVVNDGSSDATLNILLEEYRLKP

VPNTKIRGKIACQKIRGIYHNPEFPDLYVIDKENGGKADSLNAGINLSHYPLISSIDA

DSLLEKDALIRMARMYMENPEETVAIGGDVRIANGCKIENGAVQDVSLPRKIWPMFQS

IEYLKAFLGGRIGWSHMNGLIIVSGAFGMFRKDAVIAVGGYRDGYPGEDMNIIIKLHR

YMLENKLKYRIAFCPEAVCWTQAPDSYRILSSQRKRWGRGNLKNMIENRGMLFNPKYK

VMGMVTMPYNVLFEALNPYFRITGLLALAGYVLLDMTQWPILVLFGLLNFVSGYLLSV

GALVLEEIAFKRYNKLSDLVKMLVYSALKFVGYHQLGVLWRLQGHVQFMQNNNSWGTM

TRQSWSEDEKKTSEAA"

CDS 1293201..1294004

/gene="rcsC_3"

/locus_tag="EFAGFIKM_01127"

/EC_number="2.7.13.3"

/inference="ab initio prediction:Prodigal:002006"

/inference="protein motif:HAMAP:MF_00979"

/codon_start=1

/transl_table=11

/product="Sensor histidine kinase RcsC"

/translation="MPNTATLQREAAVNVYRSVEQGLKETGAETCGLMFIHCAGNSQP

EQQVRTHLEQTSESAFQVWKDGATQAIAVLLPGLTLDEVHYEGLRIKHELQETVPGAD

PQITLASFPEGERPSKATIQHMAESSKLVDSSEIHIYTLDNTADEPERILIVDNDPTV

REFLQLRLKMQGYETYEAVDGLAALELIEKVTPDLVLTELNLYGIDGLPFIHHIQNLE

MEHPPKIVVLTEQRVEQTISQCFRSGVDDYMTKPFSPVELDARIRRCLH"

CDS 1294159..1295526

/gene="wbpA_1"

/locus_tag="EFAGFIKM_01128"

/EC_number="1.1.1.136"

/inference="ab initio prediction:Prodigal:002006"

/inference="similar to AA sequence:UniProtKB:G3XD94"

/codon_start=1

/transl_table=11

/product="UDP-N-acetyl-D-glucosamine 6-dehydrogenase"

/db_xref="COG:COG0677"

/translation="MENQQFHTLLNAIENKEAVLGVVGLGYVGLPLAVEMVNQGFTVI

GIDLDASKVESIYQGDSYIHDISSDELKKVMQSGRFQPTTDYSMLRVIDALSICVPTP

LSENQDPDTSYIETVVDQIKLHMKPGMLITLESTTYPGTTEELIQQQLDKIGQEAGKD

YFLCFSPERVDPSNGRFTTFNTPKVIGGTTEACLKLGTALYGKYVETVVPVSSPKVAE

MSKLLENTFRSVNIAFVNEMAMMCDRMGIDIWEVIDAAATKPFGFMPFYPGPGIGGHC

IPLDPMYLSWKAKGFRFYSKFIELAQSTNDNMPYYVLNKTSTILNEYAKSVRKSNILL

LGMSYKPNIADLRESPGLEVYELFKESGANVSYYDPHADSFQDKHGETVHSEAFNLEQ

FKKYDCIVLITNHSDLPYFDIAEMGVPILDTRNAFRSYTHPHIYKIGHSVQHPVLEPS

EALLV"

CDS 1295535..1296458

/locus_tag="EFAGFIKM_01129"

/inference="ab initio prediction:Prodigal:002006"

/codon_start=1

/transl_table=11

/product="hypothetical protein"

/translation="MRQRGKLVGMAKWAILAVVLCLLLLSWMFKWFSAEEHKATWLWD

ASIIAEQTPEIIAFSKEQGVDSIFLQIQDEVPDATYRKFIAAARQAEIEVHGLNGHAD

WAYEEKRDEGLAFIERVRAYNAASAKNERFEGIQLDVEPYQLKRWENEESSVIAEWQN

NMKAWTKAGEDAGLYMSAAIPFWLDARQSAEGEGTFSRWVISHFDAVAIMAYRDSGQQ

MYDLSKEELDEADDLGKKVWIGAELADTHEGDHLTFFRKPVSNMNEEMAKVFDLGTSH

SSFAGVAVHHYEAWYAKENGIPLARKNEEKE"

CDS complement(1296717..1297589)

/gene="rhaS_5"

/locus_tag="EFAGFIKM_01130"

/inference="ab initio prediction:Prodigal:002006"

/inference="protein motif:HAMAP:MF_01534"

/codon_start=1

/transl_table=11

/product="HTH-type transcriptional activator RhaS"

/translation="MTESMKEDHHEFLDIIFFTPSEFEKAGGAWPIRIGRNIAKTNYH

IGPRTTPYYYLLFVLEGEGTFIQNGQRHALRARDAFCLFPHVTHEYWTDPEDTLQKIF

IAFDGAHAAELLSRIGLTPDSPYRSGVLTPETASAMRSFMEDVRKPQDGASDLGRLTR

FLTLFDRIARSPATKGLQPDSATPWLQKGKEYMDIHFAGGISVEGVSAHAGVDRTHFA

KQFRKAYGLSPVQYIQQLKMNQAKRLLVQTPLSLTEVAHSVGYPDLFSFSKAFKKQVG

LPPNRYRTAESTKE"

CDS 1298071..1298253

/locus_tag="EFAGFIKM_01131"

/inference="ab initio prediction:Prodigal:002006"

/codon_start=1

/transl_table=11

/product="hypothetical protein"

/translation="MEGTKSILKKRSGHLYPRIFTFKKGESKKFKDNSDRKNDSYPER

QGGVTVTFFNMRLYYS"

CDS 1298269..1299471

/gene="ssuD"

/locus_tag="EFAGFIKM_01132"

/EC_number="1.14.14.5"

/inference="ab initio prediction:Prodigal:002006"

/inference="similar to AA sequence:UniProtKB:P80645"

/codon_start=1

/transl_table=11

/product="Alkanesulfonate monooxygenase"

/db_xref="COG:COG2141"

/translation="MSEQEDMKFGWFLPTAGDGKYVGVAPEREPSLDYLIDVAQTAEK
AGFEFVLIPTGGACLDAWVVGSAVMSHTKTLRPLVAIRPGLVTPVLAARMGAALDQLS
GGRAMINVVTGSSVRDLEELGDPLAHAHDERYVRASEYMEVMKRSWTQSTGLNLTEFA
GSGSKSGADAPSDPNPEFNGQYYNFKGPVGMPETVQNPHPPFYLGGSSPIAKKVAVEH
ADTFLMWGEPHDWIQEQIEEIEVIRQQVKEETGQDRQLRYGMRAQVLIRDTEEAAWAA
AWEIISKVPPEAIEKAKAAFAETDATNQRRQNELRELSEKQQFVVGPNLWTGLSVVRS
GGAILIVGTAEQVAERLMEYGDLGVTTFILSGYPHLEEAEIFGRTVMPIIREKWKTRQ
KQHQVTTN"

CDS 1299686..1300960

/gene="msmE_1"

/locus_tag="EFAGFIKM_01133"

/inference="ab initio prediction:Prodigal:002006"

/inference="similar to AA sequence:UniProtKB:Q00749"

/codon_start=1

/transl_table=11

/product="Multiple sugar-binding protein"

/db_xref="COG:COG1653"

/translation="MKKNKKLILPLVSMLVMSILLSACGGGDNASSGENGSSGSGKVT
ISFMHWRGEDSEALNKTIDAFEKENPNINVEMQTLPSDQYQSTVQSKISDGSVGDVFA
SFPGAQFEAFTKAGLFTDLTGSSFLSAYNSKLIESGQHDGKQYAIPYQLVYNDPIYNV
KLFEQYGLTPPTDWEGFLALCQKLKDNGIIPIAFAGADIGPGQFMNTMVMNNAPSDDI
FTKVEAGEAKLTDEFWVKTLTQIKELNDKGYFQQDALGTKDPAAGALFIQEKAAMLAS
GSYQLAQNKQQNPNLEQKLLAPITVSADQAKYEGVHTTTFMLAVNSKSKHPEEAKKFL
EFLSNPEVASDYANQTGQNVTVNDVKYDTPELQVAGEWATKKTVFQPRFTILNGDNQK
AVTNSIQAVLSGTSPEEAAQQAQAIIDQHIGK"

CDS 1300964..1301836

/gene="melD_3"

/locus_tag="EFAGFIKM_01134"

/inference="ab initio prediction:Prodigal:002006"

/inference="similar to AA sequence:UniProtKB:O34706"

/codon_start=1

/transl_table=11

/product="Melibiose/raffinose/stachyose import permease

protein MelD"

/db_xref="COG:COG1175"

/translation="MKNRLQLSLFLIPGLLLYVGLFVFPTLTGLFYSFTDWDGVSPSY

AFVGLDNYKDSLSSIVFRKAFGNNVEFMLTVVIAQTFISLVLALLLVRNTKTRIVLRA

LYFLPAILSSVSVGLIWAFMYDPSIGLINYGLNEAGMSSIARNWIGDPKIAIYSIAAV

QVWAHAGQMMIVFIAGLQGIPAELYEAARMDGGSKWQVFRTVTWPLLAPSATIVVAYT

TIQSFKAFDLIFTMTDGGPNYATEILTTYIYHTAFGSYSFGLASAGSMIFLVLLALLT

LLQFKALRADRVSY"

CDS 1301886..1302701

/gene="araQ_7"

/locus_tag="EFAGFIKM_01135"

/inference="ab initio prediction:Prodigal:002006"

/inference="similar to AA sequence:UniProtKB:P94530"

/codon_start=1

/transl_table=11

/product="L-arabinose transport system permease protein

AraQ"

/db_xref="COG:COG0395"

/translation="MFLKWFNRLILLVYALLILVPLYFVFVSSFKTSSNFFTSPFSWP

DPFTWDNITGMFRNQPMWQYFGNSLVVTLGTVAIELLLSSMIAYAIVRWGGSVGKIVF

ALFVAGLIVPSQVSMLPIYSLTRTLGWSDSLTGLIVVSAAMLMPVSVFMLTGFMRMLN

SEILEAGSMDGASEWRLYTRIALPLAAPSLAATATFLLVMVWNDLLIPMLMLSSKSKL

TLPLALMQFRGEYVTNYPMMLTGVLVTAIPMIALFLLLQRYFVAGLTAGSLKG"

CDS 1302815..1304032

/gene="naiP"

/locus_tag="EFAGFIKM_01136"

/inference="ab initio prediction:Prodigal:002006"

/inference="similar to AA sequence:UniProtKB:O34691"

/codon_start=1

/transl_table=11

/product="Putative niacin/nicotinamide transporter NaiP"

/translation="MSKLEVLRNPKQRKLLFSAGMSWLFDAMEVGMISFIVAALAKEW
SLSPGQVGVLTSINSIGMALGALLAGALADRFGRKAILMWTLLIFTIASGLSALATGF
AVLCALRLVAGIGLGGELPVASTLVSESMPTAERGRAVVLLESFWAGGWIVSALIANF
VIPEYGWRIAFAIGALPAFYALYLRRAIQDPPRYKNHTKLHKITFREKVATVWSAPHR
RTTLMLWILWFTVIFSYYGMFLWLPSVVMAKGFTLVKSFQYVLIMTIAQLPGYFTAAY
FIERYGRKFVLVVYLTLTAFSAIAFGLATTEATILTAGICLSFFNLGAWGGLYAYSPE
LYPTSVRSTGVGLATSVGRIGGVLAPLMVGMLVQREVAISLIFTIFFVTILIGAAAVL
FWGRETKGQELAE"

CDS 1304311..1305198

/locus_tag="EFAGFIKM_01137"

/inference="ab initio prediction:Prodigal:002006"

/codon_start=1

/transl_table=11

/product="hypothetical protein"

/translation="MMKNNIGMDQPNELNEMNMISELDSVSEIKEMKVVSHMNSNERT
TVDSTNEGLDLLTFADNMEPITVSRKVTHGKGNTAGKRAGRVSMLLGACALTLGLTVA
APSAGAQKLEQGVRSEHVLQLQEQLSDLGYFDAGMTGYYGTITKSAVRKFQQAQGLSA
DGVAGPATLNRLNKKAKAEGETLRQLAKLIHGEARGESFEGQVAVGAVVLNRVHSSEF
PSSISKVIFQKGQFTAIDDGQFNTKPTQTSYRAARKALNGADPTNGALYYYNPKIATS
VWSKSRPTLLTIGQHDFTR"

CDS 1305792..1306154

/locus_tag="EFAGFIKM_01138"

/inference="ab initio prediction:Prodigal:002006"

/codon_start=1

/transl_table=11

/product="hypothetical protein"

/translation="MYRMVMIILAAMLVLILGGCAGVNEKKIKDQEIINRSEAMAIEY
LKNTYNLEVTITDKEIMPKMAMSWVTVYGHVKEHKEQTFSVSINYETNKQESFIYSQQ
LKSALQEEGYILNKNATQ"

CDS 1306170..1306463

/locus_tag="EFAGFIKM_01139"

/inference="ab initio prediction:Prodigal:002006"

/codon_start=1

/transl_table=11

/product="hypothetical protein"

/translation="MEATVRQDITDLNYRTMSQIAYLKVEEGQEIDVPGWQVLDQKHN

VSGMDAVTFYNPETKQAVIAFRGTEGDAIPARSVPDFRTDALHIGFPEIGATI"

CDS 1306584..1307465

/locus_tag="EFAGFIKM_01140"

/inference="ab initio prediction:Prodigal:002006"

/codon_start=1

/transl_table=11

/product="hypothetical protein"

/translation="MGSFNQLYEAESYVKSMTDQHKDLQFSLTGHSLGGANAQYAAVY

TGLNAVTFSAPTVVASLTPEMRRKAEEGVFDGQVTNFIHPGDGIASGFFGGYERHVGA

TFLIDSNYADGNENYGINVWAKVVDTFFEDGPKYHDMKHYSFDQDGYIDNPLYDEVTG

ERVNFSPRKPSEHNILDHAREAWDTLTKGFKSVANVVGGYSAGTIELTPEELQETADK

WRRQAQDMSCTFERIQRNFFEYTESSHSQRLVPIVWDLQMSIKQLDEWHLEHTSELVD

YINQKAEDFIQADSGAQ"

CDS 1307529..1308743

/locus_tag="EFAGFIKM_01141"

/inference="ab initio prediction:Prodigal:002006"

/codon_start=1

/transl_table=11

/product="hypothetical protein"

/translation="MKKIMLPIMSAVVVVSTLISSQASAATTTFKDVQANHWAKSAIE

SAVSKGYFKGYGNGLFKPNASVTRAEFASLISRVAKDMTLPEDAQTDSFKDLKGHWSK

AEVDRAVTLGFINTKDYPNGFKPSTPITREEMAKWLSSGLVASSAEYKTAFAETKGTL

LPVKESFKPGIAQDKIPYLAVAMGTNLMNGFPDGSFGMDKTTTRAESSAILLRLENVL

KKEAVTFDDLNELRMMGTKKTNLELVSSLTTGKTSIADISGKRKTFRNGSGSLVFHRL

IGINTSEPKKKDSIYTSLFMTEFGMNKYKNSGLLPVFTEITIYPKKNGFAIGDLVSGV

IDMNGRGMTITNKLDEKYGYVTVPNIETAKFFAKYKNGVKVWMVDYLEIKDFYGQFNM

DDGSYAVISNNN"

CDS 1308757..1312167

/locus_tag="EFAGFIKM_01142"

/inference="ab initio prediction:Prodigal:002006"

/codon_start=1

/transl_table=11

/product="hypothetical protein"

/translation="MKKKWTVLSLLVIILIYSFGVMSIKTYANFKNGYPDKDIEVKPS

GLARGKQTIKVKVKGKAEPNPKTIWTQGDFKIWQATSEKYGRVETYTYSSKESALPLK

LPEDKFVKDEINTKNYVPESLQDTETNNKFKRDYVTDLEIDDIKYNTKSGSYAIKLGS

KPNYKKGSLLVTIETRTGTDKVDADNNSDFKYKDKWDYGIRQDGDKKYQVEYLTPLEI

DYSGYVTETKEIRVREDMQLQLNDVKSLVAEIRTKAYDQEEFGNWINVSKRADQIEWS

SNKQNVVYVEPKTGQIVAKNIGTAVITAKWKNSEKGPYYIYENVTITVGDTPPPEIPE

TPDAACAESPVGEMTGERMDPNASAVIKADQRGSEPFDVLRGIPTSESLYGNVFGLSY

LFQNKFVNMQGTCTYTVVVEKTYNKTWTDERKVSDGKGGTTTVSTPRSRDVKVPYTYT

VKRDFEYWKIDTLLVYEITDAILKNYALPQIELYPADYRSPIYAAATNGTFYAPNTPA

VLTAPSQPVPGGTSEPPTPTDDLSSIAEDAVPKVQVKNDYLKFNGAVVMDNAKVEETG

PTPGQIAEPQMIGKDVLYSPDNEISKTLLNKKDTPSTGEIRYELMSGNVNDGNEEETF

EIPGINTVTVHTPVVNYSLVSDDQPHNQKTIPNMERSALILERPFTVRIPTSGQHLDV

NSYPGYGDRDYAKYYRIKQVRFPFDVYSADQTQFYPRSTWIDIPVYVLDTTFYLPVWV

DEGDYQVEFRNIAENAPADFESMSRSNAQPDANTDLTYHLASDEVSVEVIGRLYDFEI

TDIADYNWELVFRRFKGSIAPTWISYWTGTQGIDGDKRGNKPQFTVPIRPGSHPLQGY

QNVAVKTGYHFKFDFKTKGNMFGPRDGIRLTPTFDFVSKDGKTHVPVDLYYSTNQRSF

IRIGSAEDQVKRFVILNDRLRQVPSEQLRDTATYKYNRYGEIHPGMMSERTYQEYYRD

KFTKMKTPVGGYSLLLMPEQLRTFIGPKTNIPTNASADVLRTNAAIQQWYGEYSLPAE

PYVVQAGTNLAEYGRTHGGLDAKSPIFLKNGYIVVNFNLESIQEGNLGAPHLQYIHAP

LMNQWLLEGFQRQVEDSYGNSFTLRDGDVVFYHADRSSRDDFSAQVPH"

CDS 1312275..1313459

/locus_tag="EFAGFIKM_01143"

/inference="ab initio prediction:Prodigal:002006"

/codon_start=1

/transl_table=11

/product="hypothetical protein"

/translation="MSRIQVTPERLFEASREVERTRARMSDTREELVRLIRWMESMWS

GMVRERFLERFEETQPRMIETLEHLTHIAKELRDIGVRFQQADEMSGAAVAGTVATMS
IGLKAANSYSDKGYRMAYNSQWHMWLPVNEKGVTDQAALQAYEKDKGKLDNSSLSQGM
GAVEPPNQDLIQLQIEALELRVNPFTGEPVTDDYAKMMISSLKWSQIVAGIGLVTGGL
SGGKGPYKGRGTNPAIAKVKQTIKDAKAKREKSSNDANGTSKGLKIPQGLTQKQFEKV
SAMIRKKVGHLSDDIVVQGSRAKGTAKATSDIDFAIRVSPEKFNELIKDSFSKIKAPN
PGSAKEKTMLHAIETGKIQSGEAKLSKFRELLQQELGIDVDISIIKIGGRFDNPPFTP
IK"

CDS 1313475..1313852

/locus_tag="EFAGFIKM_01144"

/inference="ab initio prediction:Prodigal:002006"

/codon_start=1

/transl_table=11

/product="hypothetical protein"

/translation="MEYQYDLFTKIFLDTDLEKTSVVETVSIMVKGSAAGSSIVTEQA
EILIFNNGDFDEERRNRGSDEFLYYKYYLDIEPIEGVEETLYITEISKILTKLWDSGY
KAVASCDFEEELPRKGGYNFEER"

CDS 1313943..1314671

/locus_tag="EFAGFIKM_01145"

/inference="ab initio prediction:Prodigal:002006"

/codon_start=1

/transl_table=11

/product="hypothetical protein"

/translation="MVRKKSGVRKEEELFKGLAGIAMLGGSAGTYLLTSSWKASFVIG
LLGVVGVVVLMINIQSKRIKRLKKSGIAEIDQMDGIKFEHYLAYLFRSQGYKVQVTKA
VGDFGADLILSKDGKRISVQAKRYSKNVGIKAVQEAQSSVAHYGTAEAWVVSNSDYTS
AAYELAKSNKVKLFNREALIEMILAVNPQGTATPQVDVAEIDRDENICPKCGNDLIKQ
SGPRGPFYGCSSFPKCRHTKSPNP"

CDS 1315175..1317157

/gene="rhaS_6"

/locus_tag="EFAGFIKM_01146"

/inference="ab initio prediction:Prodigal:002006"

/inference="protein motif:HAMAP:MF_01534"

/codon_start=1

/transl_table=11

/product="HTH-type transcriptional activator RhaS"

/translation="MNWEEYIQMWDNSSIQLVDIRQLVISSGGKQQYLLPVSGFIYIV

QGQGTISLDEDIHIFKRFYLLHAGKGSRIRIEAGQDEVTYYYILYRGSMSPKASKEIQ

HNYDMNNPFQLPYAYQPLAPLNLYRHVDSMYEEWSRRQVFERFHVKIGFYQFVYTLVQ

QLKLQGVTSKETDMVEEIIQYIHEHYNESISLESLAELWNYSIQYLSKQFKLRTGRSP

IEYVIQARMAKAKELMIRTDATAQEIASSVGYSDVFYFTRLFKKQVGMTPGQYKKKMK

DSLVDSDSPWEGFVSSMGKHSSSCYVINDNHYQYKTEGDWQMFKGTRSKMVLTLMLCF

TIILSACTSGNSSRTNNQGAAGGNSNQSAESTQNVTSNEAKEDTSTRKLSTVMGEVSV

PVHPKRVVVDWDLGPVLAVGVTPVGASSTQMNFAKFLKPYLPDTVEDIGLEGSISLEK

VLDLSPDVIITWNPAAYESYSKIAPTVVFETSKYSTIQEEITAMGDILGRREEAEKWN

ADFVKRTEAARNKIKNVIPKDATISIVDYVTVDKFLMVIGNAGERGGRATYDILGLNP

TPKVKSDIIDKNEDRIEVSWETVNDYVGDYVIVLKVEGQEPTNLPATWTSLDAVKNNH

VIEIEMEKYFAADSFSSLLQAEDIADKITKMAATAK"

CDS 1317380..1317661

/locus_tag="EFAGFIKM_01147"

/inference="ab initio prediction:Prodigal:002006"

/codon_start=1

/transl_table=11

/product="hypothetical protein"

/translation="MKFLKESLIVITLIVIASVTWYGSYKNDMKELEDGLRTYLIVEK

GMDEHDIISITARRSKMPQYPVVVKLKDNPQEVVYTQREEQWVQLWPDP"

CDS 1317996..1318874

/locus_tag="EFAGFIKM_01148"

/inference="ab initio prediction:Prodigal:002006"

/codon_start=1

/transl_table=11

/product="hypothetical protein"

/translation="MKNSKTFRVGAIAVALVIIVGVLMVTFFLTRIPNGYVGVVYSPN

GGVKDSTLSQGWKLVGAFDKVTKYPIRIQTVEYRDIQIATSDGKNITIDFAYNYQVEP

SKVSSIFNTFGPISIQEIEDTYLKTRFRDAARKGISKFTVIDVYGEKSSEAGVDVQQR

FSDDVKELGFIVSNVTVGVPQPDAKTQEAIDKRVEASQELERKTTELEIAKKEADRKR
VEAQGNADKLLIEAEGQAKANKELQQSLSSQLVEYETIKKWDGALPYVSGSNTPMIQL
PTTKTEQNSGAGSVSP"

CDS 1319089..1319841

/gene="yaaA"

/locus_tag="EFAGFIKM_01149"

/inference="ab initio prediction:Prodigal:002006"

/inference="similar to AA sequence:UniProtKB:P0A8I3"

/codon_start=1

/transl_table=11

/product="Peroxide stress resistance protein YaaA"

/db_xref="COG:COG3022"

/translation="MRIIISPAKKMKIDTDLMAIAQMPRFINESEQLLSLLQELSYDE
LKAMWKCNDAIAEQNVERIQNMNIKANLTPAIYAYEGIQYQYMAPGVFQNEELAYLQQ
HLRILSGFYGMLRPLDGVTPYRLEMQGKLQGPGFKSLYQFWGSKLADQLQSESNYILN
LASKEYSKNILPFLSEETRFITCVFGQMVNGKLVEKATWAKMARGEMVRYMAEHKITD
VKDVRNFDRLNFAFSEERSDESTYVFIQAEGQ"

CDS complement(1319981..1320505)

/locus_tag="EFAGFIKM_01150"

/inference="ab initio prediction:Prodigal:002006"

/codon_start=1

/transl_table=11

/product="hypothetical protein"

/translation="MMMKKQSKIIIVVVVLLIIAASTFVLIENSISKKEIEINSKYYT
GFVARVQKLDDTLSQTSEIETNNEVEQMFEVYTSIILVNDQLTLLKENTKTFPELNVL
INDFLIFRGEYGYLVRDQLKGSRADSEVLMKVVKQVKLFLTDLPNEYENSKEFSDQFS
KASEHIKPLLHLNF"

CDS complement(1320505..1321278)

/locus_tag="EFAGFIKM_01151"

/inference="ab initio prediction:Prodigal:002006"

/codon_start=1

/transl_table=11

/product="hypothetical protein"

/translation="MKNMNKSSIAIVIALIIFTAISSAPAHAQNMGEYYHKGNAYSSY

YDSGNQAINGWSTVVMATSSRMASQGSLGYTSIQAWARLDTLNAQYSPYYAPTVVAIE

TKMKTTTTSSDRDFYLIQTNPGHNVKKHSVDSFIFDLIDYRIPGVSLVGKLAENYANG

FTSGTEQNLSNPKNGTLTFLNPTNASLSNNISWKDANQVIGSKKNSGAAAEFHFTVAN

GNQINVAPQARVQYGLYTTNTIIPIKLWSNYAYVNHTLN"

CDS complement(1321278..1321943)

/locus_tag="EFAGFIKM_01152"

/inference="ab initio prediction:Prodigal:002006"

/codon_start=1

/transl_table=11

/product="hypothetical protein"

/translation="MRKKKVLISLGGLFLFVTSLSLFAGVGAASKPVTFDTSTPITFN

TQEDFNAKKIPPGFDKKENIETGKKLGIDVKSAQSEATTFSKKLRAAFNKDSITVNQE

LTSDGEYLVGSGGSIDLNMDGQKHNLSILSSLVSHFELKNGTHLLTGSFETEIKQDDG

TLVPSTVSFTSIVETGEGIYSVTMGTIEDGPVVLMFGDDSFATQEIRDIIRAQANFEE

DAL"

CDS complement(1322845..1323408)

/locus_tag="EFAGFIKM_01153"

/inference="ab initio prediction:Prodigal:002006"

/codon_start=1

/transl_table=11

/product="hypothetical protein"

/translation="MRKLISLALILVLVLSFNVAAYADGDVTQAKPMTKEEIINSKEF

REAVKAAKAEFESQKADNAGGPVLFAPAPKVSHINVYGVVSPNGGQEIYGFNNNPLST

TNDHGGAWIQCITFQIGYDSSHYGTLAGNTMTNNWSEAIDLDGDRVVDAWAYSWVYEA

PTTGGQFVGTVYSINIPTQWTAWINIR"

CDS 1324192..1324659

/locus_tag="EFAGFIKM_01154"

/inference="ab initio prediction:Prodigal:002006"

/codon_start=1

/transl_table=11

/product="hypothetical protein"

/translation="MTQERFYDEFARSRPVLQKALESMVNTSMELKDIATRFENVDAE

KVSLGSAVSALGAGVMMKTSRYYAGSDDKGYRMAQVNVYGKLMWMPVNENGVTDQAAL

QAYKKDQGHLDINRMQATDAEAPGKIFMRCRSKLLKIEFIPSQASLYPISMLR"

CDS 1324656..1325078

/locus_tag="EFAGFIKM_01155"

/inference="ab initio prediction:Prodigal:002006"

/codon_start=1

/transl_table=11

/product="hypothetical protein"

/translation="MMLTSLKFSQLFMAFQMVRGSMPGGKGPFRLPSSHPAVAKIKKN

LEAAEARKANGQKRGSEVTGQVSKSVISGHVSDAEKLHRPYDHLVNVDGFKRGSRPGV

SGGHNMNKFNETLQEDADKYGLNIKKDYIIGEPNKHNI"

CDS 1325091..1325387

/locus_tag="EFAGFIKM_01156"

/inference="ab initio prediction:Prodigal:002006"

/codon_start=1

/transl_table=11

/product="hypothetical protein"

/translation="MEYQIPSLKRSESDKSIFEPITDTNGNVVGKRVNDPKTVYDPNI

ISNEKMYNWGKAALEPQIQAGNVKNGIVKGELNGLKFVGFVDDKGNIKNFYPAF"

CDS 1325407..1325784

/locus_tag="EFAGFIKM_01157"

/inference="ab initio prediction:Prodigal:002006"

/codon_start=1

/transl_table=11

/product="hypothetical protein"

/translation="MMYKILTTKAEIDRSVESYFWIIDFPRALTYFGDHERHGYGSGP

LEVQFSSYHMPEEEEYIGNQKVCFIGEPPAYDEDVMAVMEHDEFYNYLMIYSEKYVEE

HPEAKQEIYNLLDMVKKRLGLHS"

CDS 1326114..1326602

/locus_tag="EFAGFIKM_01158"

/inference="ab initio prediction:Prodigal:002006"

/codon_start=1

/transl_table=11

/product="hypothetical protein"

/translation="MIKYRYTIEYKEDFNEVIVDLGIDSSLKNGYLISNSLGSDIFGD

FATIQEEIGNLIELLKGEITLYEGGGNVNLIRSDQSFTTYEDIFAEEDEEDSTCEIET

LEYVKILLLWAKENFQYKSQRGVILKEEGQVALEWLNEKYIEVLVLGQKIERINNLKL

IE"

CDS complement(1326959..1327474)

/locus_tag="EFAGFIKM_01159"

/inference="ab initio prediction:Prodigal:002006"

/codon_start=1

/transl_table=11

/product="hypothetical protein"

/translation="MNIIIEKLKDTDIEDLYEFEIENRIFFEEMVPSRGDDYYISEVF

KVRNESLLDEQVKGLSFFYLIKDENNSILGRINLVDIDDSDKIGFLGYRVGQIHTGKG

VAKKALKLLLNTIVDLDIKEVKAKTTTNNVASQKVLEQNGFDHTETSDNEFEMNGQVL

KFITYTWTNKS"

CDS 1327735..1328028

/locus_tag="EFAGFIKM_01160"

/inference="ab initio prediction:Prodigal:002006"

/codon_start=1

/transl_table=11

/product="hypothetical protein"

/translation="MWAYIMGLFEHMSNYMNNDPAVLQLPDDDVLTSQLTARKWSMTS

KGKILLESKKDMKKRGLKSPDRADAYVLTFGEYLMGTPQSIMLPTIGGVSIKR"

CDS 1328152..1328400

/locus_tag="EFAGFIKM_01161"

/inference="ab initio prediction:Prodigal:002006"

/codon_start=1

/transl_table=11

/product="hypothetical protein"

/translation="MYKRYDFYTHQLERAQRLIKDEEARKAVDYRYIQGYSRKETILF

FRRALSDSTIKRKIDEGLESVANTLKLIGFFEQDDAEY"

CDS complement(1328445..1330163)

/locus_tag="EFAGFIKM_01162"

/inference="ab initio prediction:Prodigal:002006"

/codon_start=1

/transl_table=11

/product="hypothetical protein"

/translation="MIILNVRIKNHKSIDDSGEIKLHNKMNVLAGKNNTGKSAFIEAL

YKVLQGMLNSPALDTQSTEIELELSLSDEEVNYLNDNVPPGFTIQYSEKFRLFLRYFN

SHNVTVFNKVQGYYNDHFQDVYINSNYHKVGVAPKYMFVIPDQEDSVLVPNSTFISNF

MNLLNKKIVYITGSRHVPSTETSTLNNYLKIDGTNLNGFLYSMHNNEEETFDKIVETF

KQIFTDVITIRTPIDEGNQTFISVSFEGLDTPIPLSDCGSGYTHVLLLLCVLYTKENS

IVLFDEPQVFLHPSAEKAIYDLVSDSNEHQFVFTTHSPILINYPSDKYLIHVSKLNGI

SSFTQLEHIQEVLSDIGVSNSDFALSDKVIFVEGQTEELVIPMILTHFGLKQIGYNYR

ILNMKGTGNYFNKNSSMRAYKGKLDLILSGISESPIPYKIIIDADNKTDEKLLTLRES

YGDSIIILGRREYENYFLDCYHELSEIINRNSSIAAADPDLIKSKIDHLLLSIDDNKL

FPKKGSTNVLKDVVGSEVLERLFESYSLHYNKIVHGMELTELVLNNTPEKLEFFKSEL

QDFIKG"

CDS 1330513..1330776

/locus_tag="EFAGFIKM_01163"

/inference="ab initio prediction:Prodigal:002006"

/codon_start=1

/transl_table=11

/product="hypothetical protein"

/translation="MDSTINEELQETAIAFLKWARNTLENEPANFETMNLVPQVSEVA

CTIITRLRDQGAGESPEHYVQKLQQPQIKELICKTISESLNKL"

CDS complement(1330748..1330996)

/locus_tag="EFAGFIKM_01164"

/inference="ab initio prediction:Prodigal:002006"

/codon_start=1

/transl_table=11

/product="hypothetical protein"

/translation="MSFEFDFDRVTKELVSELTDAIVDNLNEAIKGPNYANLQSLVGD

RAELIMLSSITLAASASLTNKVLKQYHKELTAYLETQK"

CDS 1331528..1331995

/locus_tag="EFAGFIKM_01165"

/inference="ab initio prediction:Prodigal:002006"

/codon_start=1

/transl_table=11

/product="hypothetical protein"

/translation="MNYIDLASNISIGLVTGLVSGLFVTIYFRRRDKAQEARRLIREE

KQAMSTYITTIRFELKEIRENSDSYTAGDIRELRKSLNLIPKFYSYDFFESVPEETSK

YTSKIYNLNRELELYLESEEIDSHKILLFSSELMRAQFDLLKYFRFSSMGKLK"

CDS 1332242..1332478

/locus_tag="EFAGFIKM_01166"

/inference="ab initio prediction:Prodigal:002006"

/codon_start=1

/transl_table=11

/product="hypothetical protein"

/translation="MQPMNNKTLKLEGSDLQEIQDYLKTSNFDYFQALSTLAHVTYGG

SVQEFVNIETDEVTKGQYSDLIERLEDALRRHNS"

CDS 1332593..1333432

/locus_tag="EFAGFIKM_01167"

/inference="ab initio prediction:Prodigal:002006"

/codon_start=1

/transl_table=11

/product="hypothetical protein"

/translation="MNKKHIALIVCGVFVVWAGSGIAINGLINASYRGTFGDMFGAVN

ALFSGLAFGGLIYTIFLQMAENRRQQQEAKDMNEKMMEQVRLSNVQRFESTLFLTVQM

HHDIVKNINIKGTSGRGAFFTFYNLLIKEYMLVLDEEYGIKTIDFPADINRELDDDYI

KESILHEQKVNYSLTHAIQRAILLNEQALGTYVRNLTNIYRMIDANASLSTDERNEYI

KLVNAQLSLYEYIMLFYYCFLLTSREFKSYADKYHIFDVGNMNPGYLINIKHWFMYDK

LRW"

CDS complement(1333652..1334242)

/locus_tag="EFAGFIKM_01168"

/inference="ab initio prediction:Prodigal:002006"

/codon_start=1

/transl_table=11

/product="hypothetical protein"

/translation="MNSFLIIGVLILLIVITILVEIQRFKVLKLNIEKNLPSGALKSN

VVSQEDIKLLLRAYRTLQTNDSQKLKVARIHLKILKEKQSDNSPFEDIIKLITIFITL

VGLMITTTLKIPKETIELLSIIKPISEIVLLLMLIVSSVIFISYIFKFSTNRSTELIN

IHIVASDEITSDSELVINDITPENLNQDNAGIASSS"

CDS 1334342..1334611

/locus_tag="EFAGFIKM_01169"

/inference="ab initio prediction:Prodigal:002006"

/codon_start=1

/transl_table=11

/product="hypothetical protein"

/translation="MSVKSYQKIIPATAIQFEGNSSTHADETFVFVQVPISIDLAGST

VKLRVIVDPLNPLVVPVGDYIVKDATGTLKHMKQAEFEAEYTVVK"

CDS 1334799..1335137

/locus_tag="EFAGFIKM_01170"

/inference="ab initio prediction:Prodigal:002006"

/codon_start=1

/transl_table=11

/product="hypothetical protein"

/translation="MKWYHKAIFSFANSVLPAAVKRQMMGFGRMTSPRSSNGWDIFNW

LPKKYQSVHNIDLTKLQNHTAEELLEILVSVHPDISHALYNFLRMGDTPLTFTAKKQS

GSDVKNGQHR"

CDS complement(1335569..1335787)

/locus_tag="EFAGFIKM_01171"

/inference="ab initio prediction:Prodigal:002006"

/codon_start=1

/transl_table=11

/product="hypothetical protein"

/translation="MYARIRNLREDKDLSQTQMATYLGCSQRVYSNYERGELDIPTAI

LIKLADYHQTSTDYLLNRTDVRRPYPSK"

CDS 1335901..1336170

/locus_tag="EFAGFIKM_01172"

/inference="ab initio prediction:Prodigal:002006"

/codon_start=1

/transl_table=11

/product="hypothetical protein"

/translation="MSNLIEDLFHGNLGLDESIHPEQAEYQEINRQISDLMQDYKTQL

TENEYDALEQLIDLIGQSTSMYVEAAFEQGFRTGGRLMIEVLAKP"

CDS complement(1336308..1337684)

/locus_tag="EFAGFIKM_01173"

/inference="ab initio prediction:Prodigal:002006"

/codon_start=1

/transl_table=11

/product="hypothetical protein"

/translation="MKLFPSTSTHVSARSARSLKERSLKSKMAHICLSAAFPLLLIGG

GSAGAEELIESSLQNPSESVATSNIALAAGDLYVSPGGSASNPGTLNSPTSLANALTQ

IAPGKTIYLRGGNYTFSQTITIERGNSGTSSQRKNLVAYGSEKPVFDFSAQAFASTNR

GLQMFGDYWFVKGLEVKGAGDNGIFIGGSYNRLEQIEAHHNRDTGIQMGRYASTAAKS

EWPAYNEVIRSYSHNNYDPDDGEDADGFAAKLTVGPGNVFDGCIAAYNVDDGWDLYSK

TDTGAIGAVTIRNSIAYANGATSDGTSTSNSDGNGFKLGGEKIAVNHIVENSIAFNNK

KHGFTYNSNPGSIQMKNNTSWNNGQSNFAFDVGTHIFTNNLSFQGGASDKTSGTDVSS

TNVWWKNKKSENAKGLLASAADFVSLVPSVTRSADGTPVLGNFLKLAGGSDLIGSGTP

AGKNIGAR"

CDS complement(1338241..1339833)

/gene="katA_1"

/locus_tag="EFAGFIKM_01174"

/EC_number="1.11.1.6"

/inference="ab initio prediction:Prodigal:002006"

/inference="similar to AA sequence:UniProtKB:Q59337"

/codon_start=1

/transl_table=11

/product="Catalase"

/db_xref="COG:COG0753"

/translation="MNHQSSQPEHSSDKSAETLTDRQGHPITDNQNVRTVGSRGPTTL

ENYHFLEKITHFDRERIPERVVHARGAGAHGIFEAYGTAGSEPVSKYTRARLFQEKGK

QTPVFVRFSTVIHGGHSPETLRDPRGFAVKFYTEDGNWDLVGNNLKIFFIRDPLKFPD

MVHAFKPDPLTNAQDMERFFDFVSLSPEATHMVTFLFSPWGIPANYRQMQGSGVNTYK

WVNQEGTGVLIKYHWEPLNQGIRNLLQKDASEIQGQNFNHATLDLYHAIEQGDYPEWE

LCVQVMEDGEHPELDFDPLDPTKLWPQDQFPFLPVGKMTLNRNPEDYFNEVEQAAFGT

GVLVDGLDFSDDKLLQGRTFSYSDTQRHRVGANYLQLPVNAPKNRVATNQSGGQMQYQ

VDRAPGQNPHVNYEPSSLGGLKEAAPRGKEHEPLVEGRLVREKIERTNDFGQAGDTYR

AFEDWERDELISNMVGALAQCKPDIRDRMISYFTQADADYGRRVSEGLASVPTDDSAT

VQPKHEPSVEQAEKRSRETDGY"

CDS 1340021..1342003

/gene="yoaA_1"

/locus_tag="EFAGFIKM_01175"

/EC_number="3.6.4.12"

/inference="ab initio prediction:Prodigal:002006"

/inference="similar to AA sequence:UniProtKB:P76257"

/codon_start=1

/transl_table=11

/product="putative ATP-dependent DNA helicase YoaA"

/db_xref="COG:COG1199"

/translation="MEADLGLSTQRYPFAYDPAEPFVSRLGEWVADVFYDILPEFGFE

VRDEQIFMAYQLERAYGDKKTIMAEAGVGTGKTLVYLLYAVCYARYTGKPAVIACADE

SLIEQLVKPGGDIAKLAAHLDLEVDARLGKSPDQYVCLNKLSAVRFADEDAPVIEEVH

ESLPDFVNTPGTLQAFHPYGDRKQYPHLNDRQWNKINWDPFQDCFVCPKRQRCGLTLS

RDHYRRSKDIIICSHDYYMEHVWTYDARKREGQLPLLPDHSSVVFDEGHLLEEAALNA

LSYKLKHRIFEELVTRLLEGEIRESLAECVDEAIESSERLFALLDTYTVAIPGSERKE

VRVEPPLLREIERLTSVLDAIGEELVFESGLFSLDGYQMRVVEEHLDMIQSALALFRK

EDGYICWAEESEDETTLSIMPRTVKEMLNERVFNTGIPIVFSSATLSVDSSFRYVADS

LGIDDFVSFSVASPYDYADKMKMKITDEAIPGHPENENRMRDAVSLLQESGGRALVLF

RTMEELRAFKQDIVHVPEAEGLRFMYEGDREISDLIAAFQQDEESVLCSVNLWEGLDV

PGPSLSNVMIWSLPYPPQDPVFNAKRSASAAPYEEIDLPYMLLRVKQGLGRLIRTSTD

SGSAVILDESLYTKKEAKDRIAALLPEGVEWTTLTH"

CDS complement(1342494..1343450)

/gene="cysK_1"

/locus_tag="EFAGFIKM_01176"

/EC_number="2.5.1.47"

/inference="ab initio prediction:Prodigal:002006"

/inference="similar to AA sequence:UniProtKB:P63871"

/codon_start=1

/transl_table=11

/product="Cysteine synthase"

/translation="MDLNTHSIKRTPAHTGIAADVTKLIGQTPAVKLNRLTDSDSADV

YVKLEYFNPSGSVKDRAAYNLIVQAELAGHLLPGATIIEPTSGNTGIGLAMNAAAKGY

KAILIMPDNMSKERINILKAYGADVVLTPAAERMPGAIRKAKELQADIPGSFIPQQFE

NQANPDIHRITTAPEIMQQMDGRLDAFIATAGTGGTITGTGEELRKQLPDIRIYAVEP

KGSPVLSGGEPGPHKLVGTSPGFIPDILNTDVWDAIIQVSDEDALDTMRQLAAREGLL

LGPSSGASVWASLRIAKELGPGHRVLCIAPDTGERYLSMGIF"

CDS complement(1343614..1343997)

/locus_tag="EFAGFIKM_01177"

/inference="ab initio prediction:Prodigal:002006"

/codon_start=1

/transl_table=11

/product="hypothetical protein"

/translation="MTSSTFAPSPADEDHRLIKGLVVRTLLLDVLERDIRTLDTLLLK

MPEVYILSLTRIQNNVLKEMLALRKQMRTRGVKVLEEKREAEGIETLYMCRGYWQRFY

MLWTFARNEVKKELSRHLQMDLAQT"

CDS complement(1344466..1344699)

/locus_tag="EFAGFIKM_01178"

/inference="ab initio prediction:Prodigal:002006"

/codon_start=1

/transl_table=11

/product="hypothetical protein"

/translation="MKPETKKISKRWGSEKKVRLSSAPESSRPVPEPKEEDRFHKPAV

PLTTIGPSLKGKERPYKVILEKEAFYEKPQYLA"

CDS complement(1344696..1345733)

/locus_tag="EFAGFIKM_01179"

/inference="ab initio prediction:Prodigal:002006"

/codon_start=1

/transl_table=11

/product="hypothetical protein"

/translation="MKTLKPRVFIGCSLEAKPIAAAVHENLRFSAEVTPWYSGVFNPS

SYTMDDLEVEVRTTDFAIFIFHPDDISKIRGKYYASVRDNTMLEMGLFMGRLGRKRIF

FILPEDITDIKDTTKVEGLRMPTDLLGLNPLVYEIRSDGKWAPAVSVACSKIADSIEE

QGRWSDPELENIIEKHKRTEGEARLQLLKLLRFFRELLRTRKADAKMLERMSDALRTA

FVSHPPFAVRGTAIYRTDDSGYIEQLCGNVGEPGRKYDLSANDDKQPDDPKRILVIDS

YRENKIKINLYDDYLEKEYLLCYPVAKKYVITVHIIGHIEADEAVFQQIDLQNRQLFN

AINDLLGGEPE"

CDS 1346684..1347418

/locus_tag="EFAGFIKM_01180"

/inference="ab initio prediction:Prodigal:002006"

/codon_start=1

/transl_table=11

/product="hypothetical protein"

/translation="MKRTSLTLQHVKTEAIKFAIMLLGTFILAFAYYHINFQNHLSEG

GFVGLALLGKYATGLSPAIGMLLLDIPVMILAWFLKGWKFMIQALLGVGAFSLFYDGF

ERYSTLVLSFNGNLWIPAVLSGILTGVGAGVVLRFGGATGGDDILAVLVSRWKGWKLG

TVFFVSDAFVLGLSLFFLPVKETLYTILAVWIASKVITYVVSVPARGTVVTTSAVKLP

MPSAAAKAVNAAAISQRTTVARGVSN"

CDS complement(1347544..1348014)

/locus_tag="EFAGFIKM_01181"

/inference="ab initio prediction:Prodigal:002006"

/codon_start=1

/transl_table=11

/product="hypothetical protein"

/translation="MPWNKQDYPVSMKNLEPRVRHKAIEIANALLDDGYEEGRSIAIA

TAKAEEWDENHPAPESSKSDSTSSSDNSNPKKSSSTDRRHSEPVSSSKSHDNIHVVPT

NSGWAIKEEGKSTYLSTFDTKAEAVDKAKELSSKQNIRAIIHNQDGQIASSIKP"

CDS 1348406..1349206

/gene="amj"

/locus_tag="EFAGFIKM_01182"

/inference="ab initio prediction:Prodigal:002006"

/inference="similar to AA sequence:UniProtKB:P96581"

/codon_start=1

/transl_table=11

/product="Lipid II flippase Amj"

/translation="MFSLSLAIPMLFTMLIHGADSLSYALRLGGLRTRRIALAISLAG

ILLLVSRTSNMAQGPMVGNLVDTATRGANPHFAAQLHWLMGAATIGTALAILCFPTMV

KLSSRMVVHFEAAGSIPAMVRGMLKRSKIRNATYYITPPSWKMAKRLVQHGMPRRLMM

LNVVVTAIYTTGVLSSLYAAYLYPGQAVAASQSTGLINGIATILLTILIDPRISLLSD

KSLRGEIRLDRMNQIYGCMLVSRLFGTLLAQLLLIPFAYWIGWIVSMM"

CDS 1349343..1349951

/gene="qacR_1"

/locus_tag="EFAGFIKM_01183"

/inference="ab initio prediction:Prodigal:002006"

/inference="similar to AA sequence:UniProtKB:P0A0N5"

/codon_start=1

/transl_table=11

/product="HTH-type transcriptional regulator QacR"

/translation="MEVFPIEVKRRKDVAQIKKDIARNTKELFAQKGYSATSMEDICT

INNRSKGSIYYHFKSKEELFMYLIKLNNEDWMDAWLDKESGYETAIDKLYGLADHYVD

DLANPLNHAINEFVSGQVVSQEMLDEMLSLIRIPYGTYESIITKGMEDGELKQDDPQD

VMHIIYGLFSGLTTLYYEKDLTDIRRIYHKGMAGLLAGIQQR"

CDS 1350032..1351267

/locus_tag="EFAGFIKM_01184"

/inference="ab initio prediction:Prodigal:002006"

/codon_start=1

/transl_table=11

/product="hypothetical protein"

/translation="MNTLFRNKAFLIVTGSDLLQNLAIWIRNMAILYYVMDRTQGSPI

AVSLITVLEYAPIFVFSIIGGALADRWNPKRTMILGDILSALSIVMIIGVLSSGYWQI

LYVATFVSSIVSQFSQPSSLKIVRRNVKGEHLHSAIAITQSGQSLFLILGPIVGTFIY

TAMGIQASMYALLILFLTSALLLTFLPKDATQRETNTSLLADIREGWQYVARSQSLKM

LSLVFICIGLSAGLISPLGIFLITERLGLEATSLQFLSGASGIGLLIGGGIAAAVSAK

LNQTLTLLVGVLCLAVATMGEVLSSWFWLTLLISFLSSISLAFINVIISTYLVTRIDE

HLIGRVNGTITPLFIGSMLLGSSMAGVLMNSTSIFIVYAISVTVMIMGIVPAMRIQFR

NDQTASASQLNSEISSSQT"

CDS complement(1351371..1352333)

/gene="msrA_2"

/locus_tag="EFAGFIKM_01185"

/EC_number="1.8.4.11"

/inference="ab initio prediction:Prodigal:002006"

/inference="protein motif:HAMAP:MF_01401"

/codon_start=1

/transl_table=11

/product="Peptide methionine sulfoxide reductase MsrA"

/translation="MEQHSSELATFAGGCFWCMVSPFEELPGIHKIVSGYTGGHTENP

TYEEVCSETTGHVEAVQITFDPAIFPYKKLVELFWQQIDPTDTGGQFHDRGSSYQTAI

FYHSEEQRQIAEASKAELGQSGRFDKPIFTPILPAKPFYEAEEHHQNYHRKNPAHYKR

YSKGSGRVDFIERNWTGNVDKDGLKERLTPLQYEVTQNSATEPAFHNEFWDHHGDGIY

VDIVSGEPLFSSTDKYDSGCGWPSFTRPLRDHNVKEKTDLSHFMIRTEVRSREGDSHL

GHLFNDGPAEAGGMRYCINSAALRFVPKEDLQKEGYGEYAVLFQ"

CDS complement(1352515..1353126)

/locus_tag="EFAGFIKM_01186"

/inference="ab initio prediction:Prodigal:002006"

/codon_start=1

/transl_table=11

/product="hypothetical protein"

/translation="MRKWLWLLLYVLLAGVTFIYRYELLAWTDLHQSIPLLLAMATLF

ALVPVIPFKFVIIAFGYSYGTTTAAWICWLGTTLAAMLVYGGARTIFRHQARSYLERI

RGLNRFTTWMEAHPFMGVMSMRLLPIVPQMAVNIYAGITYTPFWVFMLATAIGKMPAI

FVFAYAGAQAETSIWLSLAILAGYLVFMAIILLLFRFRSRNKA"

CDS 1353326..1354264

/gene="yceM"

/locus_tag="EFAGFIKM_01187"

/EC_number="1.-.-.-"

/inference="ab initio prediction:Prodigal:002006"

/inference="similar to AA sequence:UniProtKB:P75931"

/codon_start=1

/transl_table=11

/product="Putative oxidoreductase YceM"

/db_xref="COG:COG0673"

/translation="METTQRKRVAIIGLGDIARKVYLPLLTAHPNVEIVGIMNRSPEP

VKAVQEAYRLERGTTDLKELLSWDLDAVFVHTATEAHFDIVMQCLEQGLAVYVDKPLS

YTIRESEEMTAFAEAQGLLLAVGFNRRFAPMYQKAKEWMQGGKGFESLTVTKHRTGIQ

DRPAAETIYDDFIHMLDLMLWYSDHNVELLHQWIRKNDLDRLLHVTGTAKLGRTAYGR

FDMVREAGADLEKIELHGGGRSVEVVNMDTISYMEQGSLETKETFGSWDTVLARRGFS

GAVDNFLACLDLPDDCLISASHVMDSHELAEQLIRK"

CDS 1354297..1354611

/locus_tag="EFAGFIKM_01188"

/inference="ab initio prediction:Prodigal:002006"

/codon_start=1

/transl_table=11

/product="hypothetical protein"

/translation="MDEYEKERNKQIEEAVVETYKQEEEMMILVFAQWCVNHGLDPEE

MYRQAYPNQQSNERLQQVQKLIVSKEEAGEIPDDTVLGVLSMFGNEDLAMVVSEAIAA

RK"

CDS complement(1354637..1355455)

/gene="araC_1"

/locus_tag="EFAGFIKM_01189"

/inference="ab initio prediction:Prodigal:002006"

/inference="similar to AA sequence:UniProtKB:P0A9E0"

/codon_start=1

/transl_table=11

/product="Arabinose operon regulatory protein"

/translation="MPTDQSCQVLTAGFSFHRKPYAMVQPEGVKNYLLRLQTDGRCRA

RIDGDMSLVDAGDLLLFDPDEPYELRIDNETNPMGERLVESGDYHIFFNGSWVDEWWK

RHKRPTRIKVELTESLLVLFRQLVLEQRRISNPYPEISSYYMRILCLEVDRLLAEHPT

ITNTNYVAYEIKNYIEENASSLFKLDDVATHIGISVSRGVHLFKEAFGKSIMQYTLDV

RLNMARERIIFSPMTLEQVSESSGFNNYTYFHRVFRSRFGMSPKEFRVIHREQM"

CDS 1355645..1356757

/gene="iolG_1"

/locus_tag="EFAGFIKM_01190"

/EC_number="1.1.1.369"

/inference="ab initio prediction:Prodigal:002006"

/inference="protein motif:HAMAP:MF_01671"

/codon_start=1

/transl_table=11

/product="Inositol 2-dehydrogenase/D-chiro-inositol

3-dehydrogenase"

/translation="MEKMKAGIIGCGNISAIYLENLKNNPVIEVVAVADLIRERAQER

ADEFNIANVYNVDELLQNNEIELVLNLTVPGSHAMTDLAALEAGKHVYAEKPLAISLE

DGRKVVELAEEKGLYVGSAPDTFLGSGIQTARKAIEDGLIGKPVAATSFFMGGGPEAW

HPNPEFFYVAGGGPMFDMGPYYLTALITLLGPIRRISASAGIQIADRKIGSGPKEGTA

LQVETPTHLAGTIDFAEGAIATMITSFDIRGASDLPRIEIYGTEGTLSVPDPNYFNGE

VKVRRYGQDTWETVKQVFESGQNERGIGVTEMVESIRAGREHKASGKLAYHVLEAMHS

FQRSSLEGKHVHLESSYDSFAVTRTVQTEIETVESH"

CDS 1356959..1357927

/locus_tag="EFAGFIKM_01191"

/inference="ab initio prediction:Prodigal:002006"

/codon_start=1

/transl_table=11

/product="hypothetical protein"

/translation="MKLGVFLVLFGGRKLEDALDYVASKGLKAVEIGTGGHPGNAHCK

PDELLSNSTALKNFKNAVESRGLTISALSCHGNPLHPQKDIAKGFHDDFVKSVELAEK

LEVPVVNTFSGCPGDHEDAKYPNWPVAPWPNDFQEILKWQWENKVIPYWTEWGKFAAD

RNVKVGLELHGGFSVHTPATLLRLREAAGEVIGANLDPSHMWWQGIDPVQAIHILGRE

GAIHHFHAKDTTIDPVNVNKHGVTDMQDYTNMLDRAWQFRSVGYGHDNKTWADIMSAL

RLVGYDYVVSIEHEDGLMSVEEGFSKAVQNLQQVLIEEPLGDMWWV"

CDS 1357952..1358680

/locus_tag="EFAGFIKM_01192"

/inference="ab initio prediction:Prodigal:002006"

/codon_start=1

/transl_table=11

/product="hypothetical protein"

/translation="MINVTIWNEFVHEKIHDEVREVYPDGLHRALADGLGGEGFAIRT

ATLDQPEHGLSDEVLNSTDVLIWWGHMAHDRVSDEISQKVAQRVLNGMGMIVLHSGHF

SKPFKALMGTSCDLKWRVADEQEIVWCVNPSHPIADGIEGKIVLEKEEMYGEFFDIPV

PDELVFVSNFQGGEVFRSGCTFRRGEGKIFYFRPGHETYPTYYKPEILRVISNAVKWA

YPARSFKPEFGKSEPVRPFGGVLV"

CDS 1358986..1359303

/gene="yodB_2"

/locus_tag="EFAGFIKM_01193"

/inference="ab initio prediction:Prodigal:002006"

/inference="similar to AA sequence:UniProtKB:O34844"

/codon_start=1

/transl_table=11

/product="HTH-type transcriptional regulator YodB"

/db_xref="COG:COG1733"

/translation="MEGQDLRMCPRFETAFSFLGKRWNGLIIQTLMSGSKRFKDISNL

IPSMSDKMLSERMKDLESEGILVRHVYPETPVRIEYELTDKGRALQPVMNQIQDWAEQ

WVD"

CDS 1359495..1360226

/locus_tag="EFAGFIKM_01194"

/inference="ab initio prediction:Prodigal:002006"

/codon_start=1

/transl_table=11

/product="hypothetical protein"

/translation="MREESLSREVRYGKKDIAELESASIQVHLIYKKAAELFKRTAYS

RSVKDDLAEVNTHSVNSDGPKLAPAVLQKWIQTSRGWKCIGCELGQPDSTMSQMEETI

HGKQLVLDVGEGRLVQAEMLIQAVVWSQYEKIRMFAPWLGDEPFYSLQELDVIARYIV

PTYFSERPLHEQGKVFKVHQQYGQIPLYQEYVDAPSCCVQVDGGLLEGRLLTGVYVSE

EQFFGLNPYLKDGDGGRTYMQACVQ"

CDS 1360424..1361137

/locus_tag="EFAGFIKM_01195"

/inference="ab initio prediction:Prodigal:002006"

/codon_start=1

/transl_table=11

/product="hypothetical protein"

/translation="MMKKIPGYIGVCLLLLAILAGCSETQPSEQPIVKETETANTITV

AVDGSTFLPDATKSIKRDFSEGLTLKKALLNSGLVDFTADGKRIQSVGEVSLDSSLSW

AVKLNGKDIEQEDWDMELHAKDEVIIYVKAADSGGDGVAYQTTLLKVSGGNIMPTLNR

QYAGVYVQECTVRDVLKSSGIVRMSENNKFVVSVENVTPRMNERWVIMVNDKELMENG

LDMKLNPRDAVKLELTNAS"

CDS complement(1361225..1361941)

/gene="fnr_1"

/locus_tag="EFAGFIKM_01196"

/inference="ab initio prediction:Prodigal:002006"

/inference="similar to AA sequence:UniProtKB:P46908"

/codon_start=1

/transl_table=11

/product="Anaerobic regulatory protein"

/db_xref="COG:COG0664"

/translation="MRELTTVLEKRGNTNCFSESNFNHLLVTMKDRTYPEGTHLYWEG

DISDKLYYMKRGRAQITKSTDEGKELIMYMYQSGDMIGQADPFFGSKHSFSAEVLEDS

EIGVLEHKDLEMLICQHCDFAIDFMKWMGIHHRLTQTKFRDLMLYGKSGALCSTLIRL

SNSYGEPHGEHVIIHKKITHTDLSNMIGATRESVNRMLSDLRKKDGIEYDNGMIVIKD

LKMLQGICHCELCPNEICRI"

CDS 1362372..1364984

/gene="adhE"

/locus_tag="EFAGFIKM_01197"

/inference="ab initio prediction:Prodigal:002006"

/inference="similar to AA sequence:UniProtKB:P0A9Q7"

/codon_start=1

/transl_table=11

/product="Aldehyde-alcohol dehydrogenase"

/db_xref="COG:COG1012"

/translation="MAVKNEVAPAKEPTAGQYIQTLIDKANKAHAAFMKMDQKQIDRI

VQAMALAGLDKHMMLAKMAVEETGRGVYEDKITKNIFATEYVYHSIKYDKTVGVIEDN

EYESFQKIAEPVGIIMGITPVTNPTSTTMFKALISIKTRNPIIFGFHPSAQNCSREAA

KILLEAAVKHGAPADCIQWIDDPSMDRTNELMNHNDVALILATGGSGMVRAAYSCGKP

ALGVGPGNVPCFIEKSADINQAVTDLILSKSFDNGMICASEQAVIIEEPIFDQVKKKM

IANGCYFVNKDEAAKLTAGAINAEKCAVNPAIVGQSAVSIAKLCGIEVPAGTKILVAE

IEGVGTKFPLSAEKLSPVLACYKVKTAAEGIERAAEVVAFGGMGHSSVIHSTNEEVIG

KFADRLQTGRIIVNSPSTHGAIGDIYNTNMPSLTLGCGSYGRNSTSSNVTAVNLINVK

RVARRTVNMQWFKVPNKVYFEKGATQYLAKMPDITRVAIITDAMMVKLGYVEKVEHYL

RQRQMPVAIEVFSDVEPDPSTTTVDRGTEMMRRFQPDCIIALGGGSPMDAAKAMWLFY

EYPDTDFNDLKQKFMDIRKRIYKYPRLGVKAKFVAIPTTSGTGSEVTSFAVITDKNQG

NTKYPLADYELTPDVAIVDPEFVYSLPKTAVADTGMDVLTHAIEAYVSVMANDYTDGL

AIKAIQLVFQYLEQSALQGDKLAREKMHNASTIAGMAFANAFLGINHSLAHKWGGQYH

TAHGRTNAILMPHVIRYNAKKPTKFASFPKYSHFVADERYAEIARILGLPARTTEEGV

TSLINAIRNLNKILGIEESFQEIGFDAKDFEAHVDYLADRAFEDQCTTANPKLPLVTE

LADVYRNAFYGKFE"

CDS 1366083..1368341

/gene="pflB"

/locus_tag="EFAGFIKM_01198"

/EC_number="2.3.1.54"

/inference="ab initio prediction:Prodigal:002006"

/inference="similar to AA sequence:UniProtKB:Q7A7X6"

/codon_start=1

/transl_table=11

/product="Formate acetyltransferase"

/translation="MSVIEKDVKQQTGWRNFTKGTWTKSVDVNDFLAHNLSPYYGDEA

FLAGATQNTKELWEIVSDLTKKERDNGGVLDVDVNTPGTIISHQPGYLDQTKEQIVGV

QTDAPFKRSIQPTGGIRMMIDACEAYGFEMPQGVIDIFTNIRKTHNQGVFDAYTSDMR

AARKAGIITGLPDAYGRGRIIGDYRRVALYGVDFLIRNKKGELNALEVDVIDEDVIRL

REELSEQIRALQELKQLGDMHGFDISLPATTAKEAFQWLYFGYLAAIKEQNGAAMSLG

RVSSFLDIYIERDLQEGILSEEQAQELVDHFVMKLRIVKFLRTPDYNELFSGDPTWVT

ESIGGMSVNGETRVTKNSFRFLHTLHNLGPAPEPNLTVLWSTKLPEAFKDYCSKVSIE

TSSIQYENDDLMRPIYGDDYGIACCVSAMKIGKQMQFFGARANLAKALLYAINGGRDE

KSGAQVGPEYPAITSEVLDYNEVMKRFKPMMEWLAKLYMNTLNVIHYMHDKYSYERIE

MALHDRDIVRTMACGIAGLSVTADSLSAIKYAKVKPIRNEQGIAVDFEIEGDFPCYGN

NEDSVDSIAVELVENFMGMIRKHKAYRNAVPTQSVLTITSNVVYGKKTGTTPDGRKAG

EPFAPGANPMHGRDKKGALASLGSVAKLPYEHSLDGISNTFSIVPKALGKEETTRKSN

LTAMMDGYFGQNAHHLNVNVFDRQQLIDAMDHPENYPQLTIRVSGYAVNFIKLTREQQ

LDVINRTFHGSM"

CDS 1368431..1369183

/gene="pflA"

/locus_tag="EFAGFIKM_01199"

/EC_number="1.97.1.4"

/inference="ab initio prediction:Prodigal:002006"

/inference="similar to AA sequence:UniProtKB:Q7A7X5"

/codon_start=1

/transl_table=11

/product="Pyruvate formate-lyase-activating enzyme"

/translation="MVNGHIHSLETFGTVDGPGIRFVLFMQGCLLKCQYCHNPDTWAL

DGGKEMTLEEVLAEIEPYLSYYRSSGGGLTISGGEPTLQAHFVAEIFKEVKRRWGLHT

TLDSNGFNEPERIHDLLDHTDLVLLDLKHIDDEKHIKLTGKSNERTLKTAKWLSEQGR

KMWIRHVYVPGIHNEEEDLLNLGRFIGTLNGVEKFEILPYHQMGIYKWEALGKVYPLD

GVPSPSEEEVERAYRLIEQGRQETAGMACSGK"

CDS 1369405..1370772

/gene="msmE_2"

/locus_tag="EFAGFIKM_01200"

/inference="ab initio prediction:Prodigal:002006"

/inference="similar to AA sequence:UniProtKB:Q00749"

/codon_start=1

/transl_table=11

/product="Multiple sugar-binding protein"

/db_xref="COG:COG1653"

/translation="MKKRMTLLLSLLLITSWTLAGCASSSNEPQQGAGNTPVEETSKE

PVEMLLRHTQVGADKQKRLAILQDVVDKVEGEIPNLTFTLDGVESDVNRKEKLRGEMA

AGNPPDIFELFGSPDSKVYAKEGMLLDLTPILQELGIQDQFTSLEPFTYEGKVYGLPI

GGSGEGFFYNKEYFEQKGWKAPTTMAELDHILAEIKADGKLPLASASKAGWVPLMLTN

HLWSRYAGPEITAKFATGEAKWTDPGVVQGFAKHKEWVDKGYFKKGELGFEYAEYTTQ

FTSGEAVLMYDGTWKSSVFKEGQSGESLIGKVGFFNMPPVENGAGDQTSLMRDVNNGY

GFSAAVADDPQKLEAVKAFIKNFYNEDMQIRGLVEDGVLPAMKLDEKVLTDSITDDLM

KEIVAVLNASKTSFPAFDALVQADVTTEISNLQIQKLIGGQTTPEKMAEELQKVQEEA

NASVE"

CDS 1370824..1371708

/gene="ngcF_1"

/locus_tag="EFAGFIKM_01201"

/inference="ab initio prediction:Prodigal:002006"

/inference="similar to AA sequence:UniProtKB:O50500"

/codon_start=1

/transl_table=11

/product="Diacetylchitobiose uptake system permease

protein NgcF"

/db_xref="COG:COG1175"

/translation="MNTSLRSPLIYTLFVMPALILFVMFFLYPIGSSLYYSLTSWNGV

SAEPRFVGLSNYMKALTDERFWISTRNNGFFIGFSVLIQVPLIVLFSLLIANVKRLKG

LYKTAVFLPSIMSTAVIGILWGFIYEPNIGLLNKMLHVLGIDPIYWLSDNRFAMLSIL

VTNAWQWTGFYIVMVLAAILAIPRDLDEAAAIDGATAVQRAMRITLPLIRPIISVVIM

LSIAGAMKAADIVIVMTKGGPAGSTEVLATYMIKYAITNFKYGYGNTIAVLIFALTLV

LTAVYQLLVARRSEKVEY"

CDS 1371708..1372538

/gene="ngcG_1"

/locus_tag="EFAGFIKM_01202"

/inference="ab initio prediction:Prodigal:002006"

/inference="similar to AA sequence:UniProtKB:O50501"

/codon_start=1

/transl_table=11

/product="Diacetylchitobiose uptake system permease

protein NgcG"

/db_xref="COG:COG0395"

/translation="MAKSIKSSIPHVLLMLYLIAILFPFLFVIFSSFKQDNNEIALNP

FGLPTTWEFNNYVEAWVNAKIGTYFWNSLYISVLSSACTIVLGAMFAFAVTRMRHRKW

SLFLYSLILAGMLIPNNALMLPIYLLVRKMGILDTHLALIVPYVANAIPFTIIILAAF

MRSLPGEIEEAAVMDGLRAPGIFAKIVIPLTVPAIVTVFIVNFLGNWNEFLLANYFLS

TDKLRTLPVGMVQFRDQYQMNYAQMSAGIVYSVVPVLVIYAILQEKIIEGVTAGGVKG

"

CDS 1372571..1374406

/locus_tag="EFAGFIKM_01203"

/inference="ab initio prediction:Prodigal:002006"

/codon_start=1

/transl_table=11

/product="hypothetical protein"

/translation="MNLRMKLALAFMLLIIVPMCALGIGMFLVTSHTIEKKYNQQAEY

ALQAISYNIENVFQQINNVTDNGIATSVFQMALNAKDPTKQDLGTGNQLSLNASQRNF

RSLLYNYPFISYAFLYDLRSSENNQIVSIFTKENFQALPFQQFKVHPLFNEIQQLNGV

PKWLAPLEYPELTGVEPVFTQIRLVKELSYFQNIGVLVVQIKKGEIDRIFRHLQISDS

AQDTSFLLINEEGLIVYDPAGIYNGENMQNLGAESGSYGPGFSSVRTVFDGKESIISQ

YHLKNYNWSLVSVTSWEALSAETNAFAGWSVIIILLCLLAAMIFNLFFMNRITGNIAV

LVRFMRRVDDGDFNARVEGKGFDEMQLLAQGFNELLDRIGGLFRRVRAEQEQKAQAEL

RVLQAQIKPHFLFNTLESINGLALRGEGRKVSEMVTRLGNMLRISIQDQEEIPLSEEI
RHLQSYLEIQQYRFSDLFTYEIDIPPHLYSSILLKLTLQPLVENSIQHGFEGITYPGV
LRISAYAERGHLVLCVEDNGIGIPQEMLARFEYMAEDLPEDMLTEGAESLSSITERRG
LGLRSVADRIRIQYGAGYGIFICSAPGYGTVIRCIIPLYEQEEAG"

CDS 1374403..1376034

/gene="cheB_3"

/locus_tag="EFAGFIKM_01204"

/EC_number="3.5.1.44"

/inference="ab initio prediction:Prodigal:002006"

/inference="protein motif:HAMAP:MF_00099"

/codon_start=1

/transl_table=11

/product="Protein-glutamate
methylesterase/protein-glutamine glutaminase"

/translation="MNLTAMLVDDELPILENLNFILPWEEMGIEITGTARSGVEALGK
VTECHPDILLCDIRMPSMDGLELIRLLREQGETCEIILLTGYQQFEYARTAIKYNVHE
YICKPIDYLNLEHKLRELAGQIQKRRLENESQRYRSQEMESWIRHKQLIDLLRGEAPL
KVAYPASVPEFITTATPYTLLLVDAVGYFRHSIGWSELQHQRWHETISSRLKEVAERI
TEDCQILSTRKGEWCILLEASGEWKRRSEELAHQLCLELNAILSLDSSVKVRVVQDDV
PVNLESKILERYRHCQKILMFHSESEDRVLKASCPETTLSYDHMVSKGANSWITREDL
EFVTRWIRQGNKQGLRDVLDRLKQQMGNSGQSFNRIDETNLRFMLVHMLRELREVQAL
AEEHEVNFWNALQGAVSMRELLELAEVVTHASQGKQTQLRPSVSELILSACEYMDARL
QQDLGIDEVSDWLGISPGYFCQLFKTQMGVTFVEYMTQKRMESAALLLSTTEWSITAI
GEATGFKERRYFSKVFHKHFHMKPSEYRQSKQLGS"

CDS 1376063..1377700

/gene="nagZ_1"

/locus_tag="EFAGFIKM_01205"

/EC_number="3.2.1.52"

/inference="ab initio prediction:Prodigal:002006"

/inference="similar to AA sequence:UniProtKB:P40406"

/codon_start=1

/transl_table=11

/product="Beta-hexosaminidase"

/db_xref="COG:COG1472"

/translation="MKPLNDLTLEQKVGQLLMCGFHSQHADEQITRLIRDYHVGGVIY

FRRNVESVDQLTRLSAELQDMAAEAGALPLMISVDQEGGMVARIDKEGMTQVPGNMAL

GATGNPEYTLECAQILGRELKNIGIDMNLAPVVDVNNNPLNPVIGVRSYGEHAESVAT

HGVAAITGYQSQGIAATAKHFPGHGDTAVDSHLGMVTVPHNRNRLEQMELLPFRKAIE

AGVDAIMTAHVMFPSIEPEPIPATLSHKVLTGLLREEMGFEGIIITDCLEMHAISKPY

GVAEAAIRAVEAGADLILVSHTLQDQVTALEAIVEAVRTGRISEEVIHQAVERIMTWK

RKRCGQQNDHLVSPKASETVEATDVEPVDCTESTKPTEPNESTLFKIASSSITIVHND

GLLPLDPEKDVYVIWPEVVQRTEVDEPWSHTESLGMALSQLRGRVREHKITTQPTYDE

ADRILADVSDSEQVIVCTYTSAGHLPKGQQYLVEKLSKNHSLIVIALRNPYDLLEISR

PRSYVCTYENTPAVVRALSHVLTGGLQPTGSLPVRLC"

CDS 1377975..1380227

/locus_tag="EFAGFIKM_01206"

/inference="ab initio prediction:Prodigal:002006"

/codon_start=1

/transl_table=11

/product="hypothetical protein"

/translation="MNLKKKLSILTAFAVFQAFAVIPANAQSADQSDTTSVNTAKVKS

VEVVKKEQTIAESEVKSGTNTPTSNNETTEEVNPETDVPTGTDATPVEPVIEPTDEEG

TAEETPAPAPVEVEQPSTGGSSVAGGGGGDLTLYMNSNKMMQDGKTYLAGQPMAVKNG

VSYVAIRALVDRVGYNVKYDNTTKETIIISGEDELRFKTNSKIYTVNGVSRTMKGAAY

QQKNTFMVPLTSITQALDITYKVNQSAKTVVLNLSTKPVASFTVQKEVFAGDQVTYTT

RSSSPKGLSIVDERWTGRQDSFDQPGVYTVTYAVQDSSGQWSDPYSVTIKVERPNLPP

VAMFTTDKEEYKMGEKITYIDQSTDDENAIVKTEWDNNALAFFVPGPKTVTITVTDKH

GASNSYTKMINITGETLYSVTDFNQLFTPVGEKFTFDGGGVPALEKVPFTYYDEPSLL

IRSNSPETVNTEGIVYKESSFGQTRFMIHHVNNTGKNVKMYVVATNNNAYTASIDQQN

MGFAGPSPFATVAGKLSIDRWFQSMQNGTGQKKVYIQPGESKLILNDLSVLPMKQGQV

ISLYSDVFSDYELDYNIIMIEENKDPMEVLSSLPVLDRDGVHNRGTYPNATRIITYDQ

EVGSKPARLPLGDNASDPNLVGTDPMAYTEASNAGNFGVLYKITLNNVAPRTLISFNP

RGGRYSGVALVNGQVVQISTGKSVTAPNEQSVMYRTGSYGESVTILFSAAPGSNLPVN

LLFTPLPAEK"

CDS 1380566..1381030

/gene="zur"

/locus_tag="EFAGFIKM_01207"

/inference="ab initio prediction:Prodigal:002006"

/inference="similar to AA sequence:UniProtKB:P54479"

/codon_start=1

/transl_table=11

/product="Zinc-specific metallo-regulatory protein"

/db_xref="COG:COG0735"

/translation="MLSTEQIISTMSSQGLRITDQRKTLARLFAESPGYLTPKDVYEY

MGKTYSGLSFDTVYRNLRVMQELGVLEQVIFEDGVKFKAHCSEDHHHHHMICLKCQKT

YPIIFCPMQLADAPEQFQVVDHKFEVFGYCKDCAEHAPAKAASGHQHAHGKH"

CDS 1381033..1381284

/gene="yidD_2"

/locus_tag="EFAGFIKM_01208"

/inference="ab initio prediction:Prodigal:002006"

/inference="similar to AA sequence:UniProtKB:P0A8C8"

/codon_start=1

/transl_table=11

/product="Putative membrane protein insertion efficiency

factor"

/db_xref="COG:COG0759"

/translation="MKLSRRIAQVPIRVYRNYISPLTPPTCRFYPSCSAYAMEAIEVH

GALKGSLLSAKRIAKCHPFHPGGVDLVPPKAEKSMMVSE"

CDS 1381785..1383809

/gene="metG"

/locus_tag="EFAGFIKM_01209"

/EC_number="6.1.1.10"

/inference="ab initio prediction:Prodigal:002006"

/inference="similar to AA sequence:UniProtKB:P67579"

/codon_start=1

/transl_table=11

/product="Methionine--tRNA ligase"

/translation="MADQKTFYLTTPIYYPSDKLHIGHAYTTVAGDAMVRYKRLRGYD

AHYLTGTDEHGQKIERKAQEKGQTPQAFIDDIVVGIKELWNKLDISNDDFIRTTEERH

KTIVQDIFDRLLKQGDIYKGEYEGWYSIPDETYYTETQLVDVEKNEKGEIISAKSPDS

GHPVELVKEESYFFRMSKYADRLLKYYEDNPGFIQPESRKNEMINNFIKPGLEDLAVS

RTTFEWGVKVKGDPKHVVYVWIDALSNYITALGYGSSDASLYNKFWPADVHLVGKEIV

RFHTIYWPIMLMALDLPLPKKVFAHGWLLMKDGKMSKSKGNVVDPVTLIDRYGLDALR

YYLLREVPFGSDGTFTPESFVERVNSDLANDLGNLLNRTVAMVDKYFEGKAPAFASNV

TEFDASLEEAGHATVEKVEQAMENLQFSVALTAISQFVSRTNKYIDETQPWALARDEA

KRDELASVMAHLIESLRIASILLQPFLTRAPLKIWAQLGIQEGELTAWDTAKQWGLVP

TGNALQKGDPIFPRLDSEQEIAYISEAMTGGQKAAQPETSQTDAGTSAAVEPVTAPEG

TEEIGIDDFAKVELRVAQVIACEPVKKADKLLKLQLDLGYEQRQVVSGIAKFYSPEEM

VGRKVICVTNLKPVKLRGELSQGMILAASHGDQLTLATVPDNMPNGAQVK"

CDS 1384053..1385111

/gene="znuA_1"

/locus_tag="EFAGFIKM_01210"

/inference="ab initio prediction:Prodigal:002006"

/inference="similar to AA sequence:UniProtKB:O34966"

/codon_start=1

/transl_table=11

/product="High-affinity zinc uptake system binding-protein

ZnuA"

/db_xref="COG:COG0803"

/translation="MTFYKVQHQVEDNQTGTTSKRGSMKLLWTGLLILTLLVLSACGQ

DSSNSAKIVEGKVNVVTTFYPVYAFTTAIGGEDANVINLLPTGVEPHDWTPKSQDIVN

TSKAQLFFYNGAGLEGWVPNFLKSLNSDTQVKSVAVSDGVKLLTAEGDDGHGHGEEHE

DEHADEHTDEATSEDVADHHIDPHTWVSPKSAMIMAENIKNSLVEADPDHKAGYEQRY

EELRTKLETLDQRFTDELANVPNNEIVVSHQAFGYLARDYGLTQHAIMGLSPDAEPTG

QDIVKLAKLVKDEGIKYIFFEELVSDKLAKTLASEAGVETMVLNPVEGLTKEQATNGD

DYFTLMEKNLQNLLIALK"

CDS 1385211..1385969

/gene="znuC_1"

/locus_tag="EFAGFIKM_01211"

/EC_number="7.2.2.-"

/inference="ab initio prediction:Prodigal:002006"

/inference="similar to AA sequence:UniProtKB:O34946"

/codon_start=1

/transl_table=11

/product="High-affinity zinc uptake system ATP-binding

protein ZnuC"

/db_xref="COG:COG1121"

/translation="MQQIMPLCHDPIIEIEKLSFSYGDQRVIENLDFMAQERDFVGII

GSNGAGKTTLLRMLVGLLPPAQGDIKLFGQSIRRFKDWDRIGYVPQKNAFNPLFPATV

REVVMSGLYNNKNMFRRMSRKCQQQCMDAMQVMRIEDLANKRIGQLSGGQQQRVFLAR

ALINHPDLLILDEPTVGIDAESQASFFELITHMHEHHRMTFLMVSHDMDRMESYLGSE

AVVTNGKINFHVRHSHEVQDCAETNLQHTTAQVR"

CDS 1386022..1386825

/gene="znuB_1"

/locus_tag="EFAGFIKM_01212"

/inference="ab initio prediction:Prodigal:002006"

/inference="similar to AA sequence:UniProtKB:O34610"

/codon_start=1

/transl_table=11

/product="High-affinity zinc uptake system membrane

protein ZnuB"

/db_xref="COG:COG1108"

/translation="MEILMSDFFQRALAGGLLIGITAPLIGLFLVLRRLSMIGDTLSH

VTIAGVALGFLIEVYPIAVGLIFAVLASFAIEKLRKAYKSYAELSIAIIMSGGVALAS

LFFTLGKGYNADVMSYLFGSIYTLDATDLKLVGVVTLIVVIVVALLHKEFFLLSFEED

AAAVTGLPVRILNMLITVMTALVISTAIKIVGALLVSALLTIPVAVSLLMARSFKSAI

ILSVVIGEIAVVLGLVVAGIWNLAPGATIVLLLIMMLILTMIGKKGFRA"

CDS 1386875..1387588

/locus_tag="EFAGFIKM_01213"

/inference="ab initio prediction:Prodigal:002006"

/codon_start=1

/transl_table=11

/product="hypothetical protein"

/translation="MPDVNVWMAFVAGLVSFISPCCLPLYPSYLSYITGMTVQQLKDD

RNQREVRFKTMTHTLAFILGFSAVFYSLGLGAGLFGQFFNDNRELIRQLSAILIMLMG

LFLLGVFKPQFLMKERKLDMKWKPAGYLGSFIFGIGFSAGWSPCIGPILATIIAMAAS

EPTTWLALITGYTAGFAIPFFILAFFIGSTRWILRYSNVMMKVGGALMLFLGVLLFTD

QMTKITIWLQQITPDWMII"

CDS complement(1387735..1388805)

/gene="splB"

/locus_tag="EFAGFIKM_01214"

/EC_number="4.1.99.14"

/inference="ab initio prediction:Prodigal:002006"

/inference="similar to AA sequence:UniProtKB:P37956"

/codon_start=1

/transl_table=11

/product="Spore photoproduct lyase"

/db_xref="COG:COG1533"

/translation="MSTSVASPPVRKAKGTKPFIPDLVYFEPGALEYDKGKRIMEWVT

SKNIPYQMTTSHNRITNLPGETEQEKYRMAKRTLVVGVRKTLKFDQSKPSAEYAIPIS

TGCMGHCHYCYLQTTLGAKPYVRVYVNTEEIIQAAKGYIEERAPEITRFEAACTSDPV

GLEHITENLSDLIRFMAEEEYGRLRFVTKYHHVDPLLNIKHNGHTRIRFSVNSDYVIK

NFEPATSRFEERIEAAGKIAHAGYPLGFIIAPIMWYEGWEEGYSDLLQKLADTLPEEA

TKDLTFEMIQHRFTKTAKATIEKRYPKTKLEMDIEKRKMKWGRWGQYKYVYKDDQQDA

LREFITERIFEHFPLASIDYFT"

CDS 1388998..1389426

/gene="mntR"

/locus_tag="EFAGFIKM_01215"

/inference="ab initio prediction:Prodigal:002006"

/inference="similar to AA sequence:UniProtKB:P54512"

/codon_start=1

/transl_table=11

/product="HTH-type transcriptional regulator MntR"

/db_xref="COG:COG1321"

/translation="MPTPSMEDYLERIYKLIDEKGYARVSDIAEGLEVHPSSVTKMIQ

KLDKDEYLIYEKYRGLVLTPKGKKMGKRLMERHHLLEQFLTTIGVQEQNIYNDVEGIE

HHLSWDSITCIESLVEYFRQDESRLRDLKNLQDVMSNTES"

CDS 1389636..1391246

/locus_tag="EFAGFIKM_01216"

/inference="ab initio prediction:Prodigal:002006"

/codon_start=1

/transl_table=11

/product="hypothetical protein"

/translation="MNVRKGLMLLLIFVLGLQTAGMTAQAAGQKEIAIFLDGNRLESD

VSPYILPKVNVTMVPLRVISEGLGASVLWSQATRTVTIQKSDSVISMTSGRQQATVDN

TVVGLDASVELKQGRVMVPIRFVSENLGIRVNWNQAAQTIDLYTGDESVPTPEPTPVT

PVEPGGTGTVPASEEMRGAWISTVNGDWPSSGAKGNVEKQKQEYTKQLDTLQGMGINA

VFVQVRANGDAIYPSGLVPWNSVLTGTQGKDPGYDPLAFMVEEAHKRGLEFHAWFNPF

RATNSASTSNLAANHISKLHPDWIVNASGKMYINPGIPEARQHIIDTIMEVVNQYDID

GVHLDDYFYPSNVTFSDDAAFKTYNTLNTKDRAEWRRDNINQFVKQLGQSIHSAKANV

EYGISPFGVWRNKSVDLTGSDTKAGVTAYDSMNADVRTWINQEWIDYVAPQVYWSMTL

SVARYDKVVDWWANEVTNTNVKLYIGHSPYKLGTPEIGWQTSQEIIDQLNYNKKHASV

KGDIFFSSQYLTKNPLGLIAKLKAYYGL"

CDS 1391336..1392337

/locus_tag="EFAGFIKM_01217"

/inference="ab initio prediction:Prodigal:002006"

/codon_start=1

/transl_table=11

/product="hypothetical protein"

/translation="MLINAVFEGGGVKGISLAGAVQGAQDCGIQFNRVAGTSSGSIVA

ALLAAGYRAEEMKVIIENTPFVSLLRRSPIFNTRWIGPAARLFLKKGLYSGEALESWI

RKMLEQKGIRTFADLPQGKLLITASDISNGTILVLPDDIRRFGIDPAKLDVAKAVRMS

CSIPYFFDPVVIRKSPVFSKGLPFQDQFVYVVDGGLLSNFPLWLFDGDRSERGGDVIP

VVGFKMVGKTEVEPARIKGPLSMLQALVETMLTAHDERYIEQINRFRTVKIPTLGIKP

TQFHLSLQDSTALYRSGATAGNEFFNGWNMKMYDEQLDKQRKELRKKQAEAPPLIPV"

CDS complement(1392399..1392731)

/locus_tag="EFAGFIKM_01218"

/inference="ab initio prediction:Prodigal:002006"

/codon_start=1

/transl_table=11

/product="hypothetical protein"

/translation="MNKQAILFWTFIALAAVGVLTWLGSASPSRIIIPLVVFGAVFLL

YKYPPRRWASKTKSPKIKPSARTMAKVNAQSSARKSSGSSKKRKDYPFQVIQGQKGKS

DEDIPKFH"

CDS complement(1392737..1393729)

/locus_tag="EFAGFIKM_01219"

/inference="ab initio prediction:Prodigal:002006"

/codon_start=1

/transl_table=11

/product="hypothetical protein"

/translation="MPQQSKPVSYGGQAVIEGVMFGGKHVNVTAVRRKDGEITYLEVP

KQDKSWVTKLRRIPLLRGIVSIIDSSVKGSRHLNYSADAYADDELEPEEKAKQKEKEG

SGWSLSMIIGVTAVAILSFLFGKVVLTLLPVVIENFLFKNAFDNQFLHNLLEGGIKLI

LLLAYLWLISQTPMIKRLFQYHGAEHKVISAHEAGEELTPENVQKYSRLHYRCGSSFI

MLTVIIGVFLYSLFTYDNLWERMGQRLLLLPVVLGISFELLKLTNSVRDIPVLRYLGY

PGLWLQLLTTKEPTNEQVEVSIASFNRMRELDAKLANVSATSEVPVATLDPVKG"

CDS 1393943..1394521

/locus_tag="EFAGFIKM_01220"

/inference="ab initio prediction:Prodigal:002006"

/codon_start=1

/transl_table=11

/product="hypothetical protein"

/translation="MTERKHEDTSQDEGQRPNERQQKDKGRRPTQRQGQAHYYTKPFS

FALELGFFAGFIWGGLHWLFYLLHFTIVPVGFLAEPFFKHEYIYTAAGHLTGWLFFIV

FSILASLLYTFTIRKLKGPLPGMVYGIVWWLILFILVGPKLGMMKPLNGLTWDSIITE

LCFFLLWGLFIGYTVAMEYTDERKREPEQAGA"

CDS 1394598..1395041

/gene="yqhS"

/locus_tag="EFAGFIKM_01221"

/EC_number="4.2.1.10"

/inference="ab initio prediction:Prodigal:002006"

/inference="similar to AA sequence:UniProtKB:P54517"

/codon_start=1

/transl_table=11

/product="3-dehydroquinate dehydratase"

/db_xref="COG:COG0757"

/translation="MKRIIVINGPNLNMLGVREPGIYGTLSLKDIEDKIRRQADEIGV

SIAFYQSNHEGDIIDRIHAAMGEADGIILNAGAFTHYSYAIRDAINAVKVPTVEVHLS

NIHAREAFRHHSVIAAETIGQIAGFGEVSYELGLLALVRHLDKQT"

CDS 1395081..1396154

/gene="ypdF"

/locus_tag="EFAGFIKM_01222"

/EC_number="3.4.11.-"

/inference="ab initio prediction:Prodigal:002006"

/inference="similar to AA sequence:UniProtKB:P76524"

/codon_start=1

/transl_table=11

/product="Aminopeptidase YpdF"

/db_xref="COG:COG0006"

/translation="MENKRVNKLREAMREHELTAMLITNPINRRYMTGFTGSAGYVLI

TEQDAYLLTDFRYMSQAPQQAKGFTVVEHGAKPMDTVRELLTSANIKEVGFEQDSVTF

GTHSAYAEALQSIELKAVSGIVEQLRMFKDEDEIAVMQRAADLADATFSHVLKFAKPG

MTEREVDLEMEFFMRKHGATSSSFDTIVASGERSAMPHGVASSKVIGQNELITFDFGA

LLDGYCSDLTRTIATGTPVPELRKIYDIVLEAQLHTLENLKPGMTGREADALARDIIA

GHGYGDQFGHSTGHGLGMEVHEAPRLSKLSDDVLKPGMVVTVEPGIYIDGLGGVRIED

DVVITETGIHILTKSDKKFTVIG"

CDS 1396468..1397025

/gene="efp"

/locus_tag="EFAGFIKM_01223"

/inference="ab initio prediction:Prodigal:002006"

/inference="similar to AA sequence:UniProtKB:P99066"

/codon_start=1

/transl_table=11

/product="Elongation factor P"

/translation="MISVNDFKTGLTVQVDNDIYTVLDFQHVKPGKGAAFVRSKLKNL

RNGNTVEKTFRAGETIGRAIIENRGVSYLYASGTEHTFMDNETYDQFTLTSDQLEWEL

NFLKENMVVKIVSYQGEILGIDLPTSVELKVIETEPSVKGNTAQGATKNAKVETGLNV

QVPLFINEGDVLLIDTREGKYSSRA"

CDS 1397216..1398469

/gene="lysC_1"

/locus_tag="EFAGFIKM_01224"

/EC_number="2.7.2.4"

/inference="ab initio prediction:Prodigal:002006"

/inference="similar to AA sequence:UniProtKB:Q59229"

/codon_start=1

/transl_table=11

/product="Aspartokinase"

/translation="MSLYVMKFGGSSVGDTERMKRVAGRVVEKADEGHRCVVVVSAMG

DTTDDLIDQAKQLNSDLPAREMDMLLTTGEQISISLLSMAIQALGRKAVSFTGWQAGF

RTEPVHGKARIHDIQPDRVNAALAEGNIVIVAGFQGMTEDGEITTLGRGGSDTTAVAL

AAAIKADACEIYTDVDGIYSTDPRIVKVARKLKEISYDEMLELANLGAAVLHPRAVEY

AKHSGVPLIVRSSFNHNEGTVVKEEAAMEQGVVVSGIAYDKNVARISILGVPDVPGVL

AEVFGALASNQLDVDIIVQSGVMDGKADFSFSVALSDRENALRVIEGLHSRLPYREVT

SEENLVKISIVGAGMVSHPGVAAKMFKVISAEGVSIKMVSTSEIKVSCVIDGDKLHDV

IKALHTAYDLDTAEQAVIGGPQDRR"

CDS complement(1398557..1399306)

/gene="comB"

/locus_tag="EFAGFIKM_01225"

/EC_number="3.1.3.71"

/inference="ab initio prediction:Prodigal:002006"

/inference="similar to AA sequence:UniProtKB:Q9WZQ4"

/codon_start=1

/transl_table=11

/product="putative 2-phosphosulfolactate phosphatase"

/db_xref="COG:COG2045"

/translation="MRVDVVGNVNEVRTIDIAGRSAVVIDVLCTTSTIITALAYDASA

VIAVETVPQAKQMEVKDCIRGGERFDKKITGFEVGNSPYEYMTAGIADKTIILTTTDG

TRALIKASKGKHVFAGSFLNVQAVAAALCELQRDVLLLCAGNQDEFALEDGLCAGCII

DELHRQSCSPLQLNDLGIVLHQAVSQCKESIPELVQGSVSGRRLEKLGRVHDISYCSQ

LNLLDCVPEMGEGNRMQPYRGIRGGKMFKLM"

CDS 1399569..1400573

/locus_tag="EFAGFIKM_01226"

/inference="ab initio prediction:Prodigal:002006"

/codon_start=1

/transl_table=11

/product="hypothetical protein"

/translation="MKVNWRELFPEPIRTILGRMPPALLEQVEEVRIREGRPLEINAG

NTYHFLTPQGCPTGNPEEAYVPQKEVTHRLLDLISNHSLYTLEEELRKGFITIPGGHR

IGLAGRTVLSGGRVEYLRDINGFNVRVAREVHGVADRILPYLLDMKSGQVLHTLILSP

PQQGKTTLLRDLARQISSGTKLTGGTELVQGIRPRLKVGIVDERSEIAGSYKGVPGFD

VGPRTDVMDGCPKAEGMMMMLRSMSPDVLIVDEIGRPEDAEAVMEALHAGVSVIATAH

GRDLSELSARPALRTLITEQMFQRYVQLQRTSRGMTFRLADGKMRALQQTGAGGEAFG

"

CDS 1400566..1401084

/gene="spoIIIAB"

/locus_tag="EFAGFIKM_01227"

/inference="ab initio prediction:Prodigal:002006"

/inference="similar to AA sequence:UniProtKB:Q01368"

/codon_start=1

/transl_table=11

/product="Stage III sporulation protein AB"

/translation="MVNMFGAVIILLASTLAGFYKARQYALRPRQLRELIAALQRLMT

EINYGLTPLPDAMGKMGAQTKEPVKTLFLHAATQMEPPHGLTARESLQSGVDLAWGRS

AMKADEKEVMLQLSFSLGTSDRQDQTKHISLAIQQLMHEESRAQADQMKYERMSRSLG

MLVGALIVILIF"

CDS 1401114..1401317

/locus_tag="EFAGFIKM_01228"

/inference="ab initio prediction:Prodigal:002006"

/codon_start=1

/transl_table=11

/product="hypothetical protein"

/translation="MNLEVNAIFQIAGIGIIIAMIHTVLKQMGKEDMAHWVTVIGFVV

VLFMVVRMLDSLLQEIKSIFLFQ"

CDS 1401350..1401739

/locus_tag="EFAGFIKM_01229"

/inference="ab initio prediction:Prodigal:002006"

/codon_start=1

/transl_table=11

/product="hypothetical protein"

/translation="MEIIQVVGLALIATVLILVIKEQKPMFAFLIAAATGIVIFMLLI

GKIGAVIEVLKRLAENSGMESIYLKTVLKIIGIAYIAEFGAQIVRDAGQESIASKIEL

AGKVLILVLAIPIISIIIETVMKLMPV"

CDS 1401760..1402950

/gene="spoIIIAE"

/locus_tag="EFAGFIKM_01230"

/inference="ab initio prediction:Prodigal:002006"

/inference="similar to AA sequence:UniProtKB:P49782"

/codon_start=1

/transl_table=11

/product="Stage III sporulation protein AE"

/translation="MKSMHHKPQWRLTVVLMLCFLFGILGQVTATSPSGEWMEQQADQ

LPKDQVEKYWDQLMQQYGGFFPEGKTPSFMDMLIPGNEGFSLKSVFVAIGTFMLHEIL

YNGKLLVTIVMLSVLSMILETLQTAFEKNNISKIAYSICYMVIIIIAINSFSVAIGYA

KDAITGMINFMMAMVPLLFTLLASMGNVITVSVTHPLIIFMIHLVSTLIHLLVFPLLF

FSAVLHLVSSLSDKYKLTQLADLLRNISVALLGILLTMFLGVISVQGASGSVADGVSL

KAAKYIAGNFVPVVGRTFADATDTVITASLLVKNAIGLTGVIIILFLCAFPALKILAL

ALIYNVTGAIMQPLGDTPIVGCLQAIGKSMIYVFAALAAVGLMFFLAITILLTAGNLT

VMMR"

CDS 1402982..1403812

/locus_tag="EFAGFIKM_01231"

/inference="ab initio prediction:Prodigal:002006"

/codon_start=1

/transl_table=11

/product="hypothetical protein"

/translation="MGWLSNWLQELIMIVLLVTFVDMLLPNRSMERYVKLVLSLLILL

TLLSPITKLLKSDPVGELKRAMSAMDAPSNGNATLEQILAQGKRLQSNEQEQSLQWTA

KELANVMKGQIEETTGARVRSVEVQLAMSKYETEMEAASSVELPVIQRVLVEMAGEGA

EGEVKAGQQEVPANTQPIFGTDTESEKDKSHQIQQPIQIGQIEVPDVQIDVSREQRDG

NEASSEPVIRDQADETKQQGETSTRSEHAVQIITLLTEKWDVDANNVQVTEPKSAGVL

"

CDS 1403828..1404466

/locus_tag="EFAGFIKM_01232"

/inference="ab initio prediction:Prodigal:002006"

/codon_start=1

/transl_table=11

/product="hypothetical protein"

/translation="MKQWFKKMETWMGGGEGGARRSQTFRWLIILGLIGVGIMLFNSF

VNVKKIDSENIGREPPDPATSMASIQSDPSEQNPFQAIEIAFEDKIKGVLENIVGVGT

VDVMVTVDSTEELVVQRNVKDSQQLTEETDASGGKRHMTQYTRDGEIITYEIAGDQTP

IVTKKLKPQIRGVLVVARGAENKVVKDLITDAVEKGLNVAAYRISVVPRKQD"

CDS 1404533..1405363

/gene="spoIIIAH"

/locus_tag="EFAGFIKM_01233"

/inference="ab initio prediction:Prodigal:002006"

/inference="similar to AA sequence:UniProtKB:P49785"

/codon_start=1

/transl_table=11

/product="Stage III sporulation protein AH"

/translation="MNNKRQTVWLVSMLSLMVILSAYYLFTEDSGPVNTPVADSQQVD

GIKQGEAKETAGILDPTEGLVVNEVVNSGEVESDPSAAGAVEEPAATEGKEAGNTEKT

PVVEPGENKGEAGKETNKETDKGATTTPETDGQAGGTATKTDEEVLKEMEEQNTTASA

SSQFQNYQWQREESNNRKYEELMTVAGDLSKTPEENAKATEQLRTLEEKEAKITGIEE

TLSQQFANAIVQEDADKYKVVVLSDKLDVKQAVSIVDLVMKELAVSQNKISVQYVTEQ

"

CDS 1405497..1405967

/gene="accB"

/locus_tag="EFAGFIKM_01234"

/inference="ab initio prediction:Prodigal:002006"

/inference="similar to AA sequence:UniProtKB:P49786"

/codon_start=1

/transl_table=11

/product="Biotin carboxyl carrier protein of acetyl-CoA

carboxylase"

/db_xref="COG:COG0511"

/translation="MEMFKLSEIKELIKLVDESSVQELEIENEGSRLSIRKPGKTEYV

QAAAVQPQMIAAPQVQPAAVVSEAAPQVDTTSHLHKIVSPMVGTFYRASSPETGPFVS

AGDKVVEKTTVCIIEAMKLMNELDADIKGEIVEVLVENGQLVEYGQPLFLVKPE"

CDS 1406023..1407366

/gene="accC"

/locus_tag="EFAGFIKM_01235"

/EC_number="6.3.4.14"

/inference="ab initio prediction:Prodigal:002006"

/inference="similar to AA sequence:UniProtKB:P37798"

/codon_start=1

/transl_table=11

/product="Biotin carboxylase"

/db_xref="COG:COG0439"

/translation="MKFQKILIANRGEIAVRIIRACREIGISTVAVYSEADKDSLHVR

LADEAYCIGPTLSKDSYLNFTNLMSVATLTECDAIHPGYGFLAENADFAEICESCNIT
FIGPSPEAITKMGDKAVAKQTMKDAGVPVIPGSDGLVENMDEAIMIARDIGYPIIIKA
TAGGGGKGIRIAEDEETLIKQITAAQQEAQKAFGNAGVYLEKFLTGMKHVEIQIIADK
HGNAAHLGERDCSVQRRRQKLVEEAPCPILSEDVRTLMGEAAVRAALAVDYSGAGTLE
FLLSPNGEFYFMEMNTRIQVEHPVTEMVTGVDLIREMISVAEGNPLSFRQEDVVINGW
SIECRINAEDPDRNFMPSPGKIGFYLAPGGPGVRVDSAAYPGYTISPFYDSMIAKLIV
WGANREEAIAKMKRALGEFAIEGISTTIPFHQKLLEHPTFIRGDFDIKFLEENEI"

CDS 1407584..1407994

/locus_tag="EFAGFIKM_01236"

/inference="ab initio prediction:Prodigal:002006"

/codon_start=1

/transl_table=11

/product="hypothetical protein"

/translation="MSTLPTEFERTDIGEIQIAPEVIEVIAGLATLEVKGVAGMSGGF
AGGFAELLGRKNLSKGVKVEVGQREAAVDVSVIIEYGYRLPQVATEIQQNVKRSIENM
TGLNVNEVNVHIHDVQFKSTEKVEEIDLNSQRVK"

CDS 1408141..1408680

/locus_tag="EFAGFIKM_01237"

/inference="ab initio prediction:Prodigal:002006"

/codon_start=1

/transl_table=11

/product="hypothetical protein"

/translation="MAKILDRLLLFIYSISVGAISAAVILLISGVLPYELNYQQEQNV
IVASVIAAAILFILSLRFFYISVRRERASLPSVDQRTEFGDVQISMETIENLCLKATS
RFRGVRDVKARIRVVESGLEIMIRAVVDGETPIPALTSDLQKAIHDHVQEITGIPVSF
VTVYIANVTQSPNYKSRVE"

CDS 1408695..1408928

/locus_tag="EFAGFIKM_01238"

/inference="ab initio prediction:Prodigal:002006"

/codon_start=1

/transl_table=11

/product="hypothetical protein"

/translation="MLWKEIWDSHRGRITGIIGGIFFGFLYVWIGFWDMLFFALLVFI

GYTLGRRSDSKLGSSIPWREWGQWLGDRWRPFK"

CDS 1409151..1409603

/gene="nusB"

/locus_tag="EFAGFIKM_01239"

/inference="ab initio prediction:Prodigal:002006"

/inference="similar to AA sequence:UniProtKB:Q9X286"

/codon_start=1

/transl_table=11

/product="Transcription antitermination protein NusB"

/db_xref="COG:COG0781"

/translation="MKRRLAREIAVQSLYQMEMNEVGAAEAVNMLINEAAEDNETEVV

IRDADVMRTYVTEIVQGAWNNKEAIDGLLVDYLKGWQISRLSRVDRQILRLSTYEMVF

RDDIPAKVSVNEAIELSKYFGTEESGKFVNGVLGRMIQEVDAIKAKLS"

CDS 1409721..1410578

/gene="folD"

/locus_tag="EFAGFIKM_01240"

/inference="ab initio prediction:Prodigal:002006"

/inference="similar to AA sequence:UniProtKB:Q7A697"

/codon_start=1

/transl_table=11

/product="Bifunctional protein FolD protein"

/translation="MTASIINGKEVSQEIRASMTTEVKQLSEQGVVPGLAVVLVGEDP

ASQVYVRNKEKACHDLGFYSEVHRLDADTSQEDLLALVDKLNNQQSINGILVQLPLPK

HIEEKAVIDAIAVDKDVDGFHPVNVGNLVIGDDSLLPCTPAGVIELIKRTGLEMSGKH

AVVIGRSNIVGKPVSLLLQRENATVTMCHSRTANMKEITRQADILVVAIGRANFVDAD

FVKPGAVVIDVGMNRLENGKLAGDVDFESVKEVSGPITPVPGGVGPMTITMLMQNTLI

AAKRAHGLA"

CDS 1410589..1411953

/gene="xseA"

/locus_tag="EFAGFIKM_01241"

/EC_number="3.1.11.6"

/inference="ab initio prediction:Prodigal:002006"

/inference="similar to AA sequence:UniProtKB:P04994"

/codon_start=1

/transl_table=11

/product="Exodeoxyribonuclease 7 large subunit"

/db_xref="COG:COG1570"

/translation="MADQKIYSIKDLNRYIRMKLESDQVLSDVWLRGEISNFTHHSSG

HMYFTLKDKDSRIKSIMFASHNQRLPFVPKEGARVIARGNVSVYERDGQYQFYATHMQ

PDGIGSLYLAYEQLKKKLEDEGLFSPSRKRKIPRYPQTIGVVTSPTGAAVRDIMITLQ

RRYPSAKVVLYPVLVQGKGAAPSIVKAIGNLNRMGEADVLIVGRGGGSLEELWAFNEE

IVARAIVASDIPVISAVGHETDFTIADFAADLRAATPTAAAELAVPNRAELLDQIGQR

QRQLQHSLRQRAVHNRERLARLQRSPVLVHPRRTLMQHTERLDMMHQRLLRTVDTRMK

WTGEKQERLRAALQRFNPREQVNAARRENAAARRQLELAIRSIARSKQQQWKSSVRHL

DALSPLKVMSRGYSLVYDEQEQRLIKSTKDVQPGDSIKIKLTDGQLDCQVWGMKEDDN

THGE"

CDS 1411943..1412200

/gene="xseB"

/locus_tag="EFAGFIKM_01242"

/EC_number="3.1.11.6"

/inference="ab initio prediction:Prodigal:002006"

/inference="similar to AA sequence:UniProtKB:P0A8G9"

/codon_start=1

/transl_table=11

/product="Exodeoxyribonuclease 7 small subunit"

/db_xref="COG:COG1722"

/translation="MANEPELNFEEAMAALEDIVGQLEHGDVPLEQAIDLFQRGMKLS

QLCGLKLEQVERKIEMIVEEDGELRKKPFGTADDESGEVHE"

CDS 1412193..1413098

/locus_tag="EFAGFIKM_01243"

/EC_number="2.5.1.10"

/inference="ab initio prediction:Prodigal:002006"

/inference="similar to AA sequence:UniProtKB:Q08291"

/codon_start=1

/transl_table=11

/product="Farnesyl diphosphate synthase"

/translation="MSNRPSFQAYLEEVTAAVTEALKHTLPDHWDVPQSLTDAMQYSL

MAGGKRLRPLLVVAAAEAFGAQRTAAMPVACAVEMVHTYSLIHDDLPAMDNDDYRRGK

LTNHKVYGEATAILAGDALLTHAFYSIVQAGRRSGVAADALLSIVEDMSELAGARGMV

GGQVADMEGEQGMTDLSQLQYIHLHKTGDLIVFSLIAGARIGGATEGQLEALRVFGRD

LGLAFQIQDDILDLTGDEQKMGKKTQSDVNQQKVTYPYFIGMEASVDEVKSLTQSAKD

ALERAELPDASRLLEIADYLMSRDH"

CDS 1413383..1415290

/gene="dxs"

/locus_tag="EFAGFIKM_01244"

/EC_number="2.2.1.7"

/inference="ab initio prediction:Prodigal:002006"

/inference="similar to AA sequence:UniProtKB:Q9KGU7"

/codon_start=1

/transl_table=11

/product="1-deoxy-D-xylulose-5-phosphate synthase"

/db_xref="COG:COG1154"

/translation="MLLPQIKQPSDLKSMSQDDLALLSAEIRQFLIEKLSVTGGHLAP

NLGVVELTVALHYCYNSPADKMIFDVGHQAYVHKVLTGRMDRFDTLRQHNGLCGFVKR

NESEHDVWEAGHSSTSLSAAMGMALARDLKGEDNQVIAMIGDGALTGGMAFEALNHIG

HEQRKLMVILNDNEMSIAPNVGAMHKYLSKIRSDRHYLKAKDDVEGMLKKIPAIGDRL

AKSASWIKDSVKYMMVPGVLFEELGFTYLGPIDGHDIPKLIETFKQADNVDGPVLVHV

LTTKGKGYQPAEADSHKWHGISPYKIESGQVLKAVGKPMYTEVFGQTLIDLAKQDKRV

VAVTPAMPTGSGLIPFSKEFPDRMIDVGIAEQHAATMCAALAMEGLKPVFAVYSTFMQ

RAYDQIVHDICRHNANVMFAIDRAGFVGPDGETHQGVYDVAFMRHIPNIVLMMPKDEN

ELRHMMKTALDYNEGPIAYRYPRNNVVGVPLDDVLVPIPIGSWEQLRPSEGYAVIASG

SMVQLAEEAAEMVKREGITAGVINARFLKPLDEQMLRDLAVRGTKLIVLEETSQAGSM

GSAVLEFYAEQGLHDVHVQLMGIPDRFIEHGSIKEQREEVGLTVENVCAELRKMAVQS

SYGIPKTRFPS"

CDS 1415313..1416158

/gene="tlyA"

/locus_tag="EFAGFIKM_01245"

/inference="ab initio prediction:Prodigal:002006"

/inference="similar to AA sequence:UniProtKB:Q06803"

/codon_start=1

/transl_table=11

/product="Hemolysin A"

/db_xref="COG:COG1189"

/translation="MSLPKERIDVLLVEQGYYESREKAKAAIMAGLVYANNEPIEKAG

MKIPREAELKVKGSVHPYVGRGGLKLEKAIRHFGLDMNGLVMLDIGSSTGGFTDCALQ

HGASHVYAIDVGYNQLDWSLRNDERVTVMERTNFRYVTPEDLAGPVPNFASIDVSFIS

LRIILPPLLALLKQPADIVALIKPQFEAGREKVGKSGVVRDTKVHKDVLQTMLHFASQ

LGLHLRSLTFSPITGGEGNIEFLAHWRLEAPEATQPEIDPASLDALAEQVAKEAAHTF

KGNSS"

CDS 1416243..1416728

/locus_tag="EFAGFIKM_01246"

/inference="ab initio prediction:Prodigal:002006"

/codon_start=1

/transl_table=11

/product="hypothetical protein"

/translation="MDGQYDTIVIGVLAVCLILWLFFGLRNWVNRPMPVSLSGMQLNG

KIQDSPALDLLEAQGYEVIGGKMKVPLAFEANESVYYSRLFIDYVAEREDLRYLVKTS

RRRQPIEMTGPALRDRFLNYLLLYPGCEGLLYVDVENSDIKLIRLMDYQEDVYDGDDL

D"

CDS 1416813..1417262

/gene="argR"

/locus_tag="EFAGFIKM_01247"

/inference="ab initio prediction:Prodigal:002006"

/inference="similar to AA sequence:UniProtKB:Q9K973"

/codon_start=1

/transl_table=11

/product="Arginine repressor"

/db_xref="COG:COG1438"

/translation="MKGQRHIKIREIISQNEIETQDDLVEALRKSGFQVTQATVSRDI

KELLLIKIPMDDGRYKYSLPTDQRYNPIQKLKRALVDNFLHIDHTNNLVVMKCLPGTA

NSIAALLDNIEWNEVMGTICGDDTILIICRTEGNSVTVIERIMGYIA"

CDS 1417541..1419259

/gene="recN"

/locus_tag="EFAGFIKM_01248"

/inference="ab initio prediction:Prodigal:002006"

/inference="similar to AA sequence:UniProtKB:P05824"

/codon_start=1

/transl_table=11

/product="DNA repair protein RecN"

/db_xref="COG:COG0497"

/translation="MLVTLSIRNLAVVEEVDVVFHPGFHVLSGETGAGKSIIIDALGL

IAGGRSSADLIRYGCDKAEMEALFEMEQSHPVWQTLEKLGIHCEPEEHLVIRRELNTQ

GKSTSRINGQLVNLTMLREVGEKLINIHGQHEHQSLLRAESHLGLLDTYGSEVIGPIK

AKYQERYSKFITAEKELRALQESSQRAYQLLDMYRFQLEEIAAASLTQGEDELLGEER

VKLSHSEKMMDSVAGAYDLLSGQRGLEAVSIALSRIEDISGYDNKGLQPIVEQLQSAF

YQLEDATFQLRDYREKIEFNPARLEEVEQRLNLISGLRRKYGDSVELILSYYDQISHE

TDQLENKDERLEKLRAERDKMLNLVMESAEELSKVRKQCAEELAAQVESELKDLQMER

TTLRVQITPFEDPKGIEWNGRRIRLNRHGADNAEFLISPNPGEPLRPLGKIASGGELS

RMMLAMKSIFARHDRIPVLIFDEVDTGVSGRAAQSIAEKLFRLSSTCQVFSITHLPQV

ACMADHQYLIEKHVYDGRTMTQVESLSDEGRVKELARMLGGVEITEKTLHHAQEMLNL

AEAKKG"

CDS 1419380..1420708

/gene="spoIVB"

/locus_tag="EFAGFIKM_01249"

/EC_number="3.4.21.116"

/inference="ab initio prediction:Prodigal:002006"

/inference="similar to AA sequence:UniProtKB:P17896"

/codon_start=1

/transl_table=11

/product="SpoIVB peptidase"

/db_xref="COG:COG0750"

/translation="MNSPLRKKLLGLLFAFFLCVISQAIQPVQSYASLPDELQVFAGR

QADVRLAVPAVSSAVVDRPDIVGLDDQAAMHVTRQQPLHLHPQQTGHAKLTLKLWGKI

PVKTVHVNVIPDLRVVPGGQTIGVKVKSAGILVVGHHLVRSGQDERVSPGETAGIKLG

DLITHMDGKRLDGVAGVSEAVELAGKSKKGIDVVLKRGKETVKTRLTPAYDSEDQAWR

LGLYIRDSAAGVGTLTFYAPDQGVYGALGHVITDMNTQTSIVVGSGQIVQSNVTSISK

SETGDPGEKRAHFLKESKILGNIERNTAFGIFGKMSGNPEHSLYSKGIPVAFSHEVKE

GPAEILTVVDGQQVERFSIDIVHVADQTEPATKGLVLRITDPKLLDKTGGIVQGMSGS

PIVQNGKLIGAVTHVFVNDPKSGYGCFIEWMLQDAGVMMKKENDKNLKAG"

CDS 1420904..1421710

/gene="spo0A"

/locus_tag="EFAGFIKM_01250"

/inference="ab initio prediction:Prodigal:002006"

/inference="similar to AA sequence:UniProtKB:P06534"

/codon_start=1

/transl_table=11

/product="Stage 0 sporulation protein A"

/db_xref="COG:COG0784"

/translation="MQKIEVLLADDNREFTNLLAEYISEQEDMEVTGIAYNGEEVLQL

LEQTRDVPDVLILDIIMPHLDGLGVLERLRDLNLSPQPKVIMLTAFGQENITQRAVQL

GASYYILKPFDMEVLANRVRQLVGTQTAMSTSSGSSMFMKSNVVPMGKHKNLDANITS

IIHEIGVPAHIKGYQYLREAITMVYNNIEILGAITKTLYPAIAEKFKTTPSRVERAIR

HAIEVAWTRGNIDSISHLFGYTINISKSKPTNSEFIAMVADKLRIEHKVS"

CDS 1422136..1423092

/locus_tag="EFAGFIKM_01251"

/inference="ab initio prediction:Prodigal:002006"

/codon_start=1

/transl_table=11

/product="hypothetical protein"

/translation="MFTQIGEIAEPIPVVQSDTKCDIVYHLFKSNPSLEGIAVMGEKG

VSLLMRPRFFQQIGTQYGYNLYMGRPVELVMNTQALVVDYSEQITDVSVLAMDRDEGE

IYDLVLVTSGESFFGAVSIRRLLLAVADVRAEMAIFMNPLTGLPGNHIIDERLSQALQ
MDQFSVLYVDLDQFKSYNDSYGFKMGDQLIQATANLLRKLFVPPEAFLGHIGGDDFIT
ILNHHDYKYISEEVISGFEEMKKTFYNEHDWHHQYVLGEGRSGLNRPIPLVSVSIAVV
TNSKRKYENIDQIIDEATRIKKGCKAIAGSIICANDTATTPC"

CDS complement(1423285..1424097)

/locus_tag="EFAGFIKM_01252"

/inference="ab initio prediction:Prodigal:002006"

/codon_start=1

/transl_table=11

/product="hypothetical protein"

/translation="MNSISSATKSSFTPGNQVSNRDKEIQGLMQQKIRLNEEIQGVKT
NDELDTKTKAQRIKSLTSSISQIDNQIAQIKAEELQEKNKLRQPEKTQQQQPKPTDET
QAPSMDHLIKHSQTYDQLGKLVGLRDRMQGSIQTTEGETRFDRLVLEINPPANDLDLG
KSMMLENSERTVFQAKRETVQDINSQLNKVNQKIGELVEEIHQSAPKESAQPPISAAS
GEDNQEVNEGRKTGSKEHPSDGGTTDQESGNGPQTTASTSPATSYPSVDIRI"

CDS 1424304..1426265

/gene="rhaR_6"

/locus_tag="EFAGFIKM_01253"

/inference="ab initio prediction:Prodigal:002006"

/inference="protein motif:HAMAP:MF_01533"

/codon_start=1

/transl_table=11

/product="HTH-type transcriptional activator RhaR"

/translation="MWNDYYILWNYAAVSITDIRYMELEAYEEMKYKFPTSTFVLTIQ
GDAGVTIDNQTYEVSRFYIIHGGKGSQLVIQAGESGLRLYYLMYKANLPSGGRNDLGR
LMERINPFQMNYGFTPDMPTILYEQLKKMHFLWDQKNPIQHFQAKSLFYAFVEQISSQ
IPRISNRYPSMLDQVSKVIEIVESSYTQPLTLHVLAEMIGTSPRSLSRKFKQSTGTSP
IDYLIQFRLFKAKEMLLQTDATLDEIAAGIGYPDGYYLGRMFKKHTGLSPLQFKEKSS
APLYWPDLTSGVARNDIFRGGSGRYVYTDGDNHYQYKHGGSINMNRQTRTGMLLSILL
SLTLLLSACSTGVASNSSGDASRSNSTSSSTNTVTAENQSKSLEPRTVSTVKGDITIP
AEPKRIVVDLYLGSLIALGIKPVGTPEMNLKNPYFIKSLEGVQNIGEYETISLEKVLE
LEPDLIVTGNPDLFDSFSKIAPTLVVPYGELKNTHEEIAYFGEVLNKEKESEAWLTDY

DARIADAKKRVGEAIDPQAEVSVMQFYDKAPLAFGDNFGRGGQAVYSALGLNPPADKK
EILMKDQLVEVSSEAIPEFAGDYIILTADNLTLEELKSKPVWSSLDAVKNDRVFIWNP
DRSWYFDPIATLDQTEELAAWFTKISEPN"

CDS 1426461..1428434

/gene="rhaS_7"

/locus_tag="EFAGFIKM_01254"

/inference="ab initio prediction:Prodigal:002006"

/inference="protein motif:HAMAP:MF_01534"

/codon_start=1

/transl_table=11

/product="HTH-type transcriptional activator RhaS"

/translation="MQLDEQMKCWNHAAVKVLDIRRIVMEAGAQLSSYTLPANGFIYT
IRGSAVLQLDGQTYKAERFYMLHGAKGSSLDIQTNEDFEYILLYYRAFLAFPSYRKKW
LLAQPEVAPFSLQYGFAPSSPLGLLRYLDELEGAWSQSGSLDLLHTKSLFYQFIHEMM
VQLAEQEIKTELADPVKQTLRYIQNHYREQVTLDFLAEQFNYSSRHLSMQFKRQTGYS
PIDYLIQTRIAKARNLLVRSDATLSEIAAEVGYSDVYYFSRIFKKHVGISPIQYQRKI
REEARTEDRPLVISESSIGRRWKSGYIDYENHYQYIDGGSTPMKRRKTSSSMIMVALL
SITMLLAACSSGTATTPAAGEGTGASSNNSSTAVSTDAGSQTNKDNETRTVSTVKGDV
VVPANPKRVVVLYLQGDVVALGVKPIATSDVYDGAAYKSELEGVNALGTWFEPNPEAV
IDLDPDLIIVPSEETYTLLKDIAPTVYIPYEKLTTEERLHDIASIFGKEQEAETLLTN
LNNKVEESKKTLADAGILDKTISIVEGGLKGMVIVESKQFGRGSQAVYEYLGMKAPEV
VQKKIDVVSDAAGSTISMEVLPEYIGDYVFRSVYEGADNLTENPIWSSIPAIKEGRLI
EIDFDFFYYSDIYSINKQLDFVVEKLLAAPRAQ"

CDS 1428594..1429631

/gene="ycgT_2"

/locus_tag="EFAGFIKM_01255"

/EC_number="1.18.1.2"

/inference="ab initio prediction:Prodigal:002006"

/inference="similar to AA sequence:UniProtKB:O31475"

/codon_start=1

/transl_table=11

/product="Ferredoxin--NADP reductase 1"

/db_xref="COG:COG0492"

/translation="MNQQLELYDVTIIGGGPAGMYSAFYSGMRDMKTKLIEARDRLGG

RMLFYPEKMIWDVGGVTPILCEDLIKQLEEQARTFEPTLVFEQQIEGFERQPDGTILL

TSATGEQHWTRTVIMAIGYGIYKMAKLELEGADRYEVSNLHYTVQELEPFRGKRVLIS

GGGDSAVDWANELEALAEQVTVVHRRDRFGGLERNVLRMRESSVDVRTPYAVETLHSM

SGDVIEQVTISHVDTGETEMLEVDAVIVNHGMKSDFGPIRDWGLDLGEWHVTTTERLQ

TNIPGVFAAGDFVDYGSKLYLIAGTFTDAALAVNSAKLYMDPEAEKVAYVSSHNSRFK

EKNKALGVVEE"

CDS 1429837..1430487

/locus_tag="EFAGFIKM_01256"

/inference="ab initio prediction:Prodigal:002006"

/codon_start=1

/transl_table=11

/product="hypothetical protein"

/translation="MKKGSQPQRGFTFIQRWFKRSDKKKIKWSEMYLALAGALHRLLV

EGRRERSAAKRLHQDLPINALGEMKLEPGDIVYTPSSESTYYAGHMGIIGLDGKVYHV

HPYGPVFADSLEWYLTRFYEGDRFIVFRSRLNEAGMKAAGWVQDHYKQVKFYRLQTNL

RSIERNYCSKFIYQAYQFTSGLDLWSRKFTRMNQGYIYPFRIERSSELDVLGTFYK"

CDS 1431022..1431498

/locus_tag="EFAGFIKM_01257"

/inference="ab initio prediction:Prodigal:002006"

/codon_start=1

/transl_table=11

/product="hypothetical protein"

/translation="MYSGRQDDTLYIMTYTLNSGIKGTVPLSNKQIIEWLDCYRNEKR

FVTEIGKEFFGLNAGLVADFKVQNHLSDYQQTIMPTQQQDNLERLSSAYKSTELLMKI

DCKCGTAYVTESPYKRTKWYCTKCKEIVFLDAKKGMVETSRGDAYYMTNKYFVSRD"

CDS complement(1431620..1432219)

/gene="tdk"

/locus_tag="EFAGFIKM_01258"

/EC_number="2.7.1.21"

/inference="ab initio prediction:Prodigal:002006"

/inference="similar to AA sequence:UniProtKB:Q814U0"

/codon_start=1

/transl_table=11

/product="Thymidine kinase"

/db_xref="COG:COG1435"

/translation="MQTGRITVITGPMFSEKSGELIRRCQKLIQFGRKKVVAYKPAED

NRFAQDEIVSRIGYRLPAHSIPRQLTPESVEMILNQTIDADVVAFDEVQFFSSAIMEL

VSELAYCGKHVIVDGLNMDYRGKEFGYVGGLLAMADDIEKLSAFCAVCGSPDAAFTQR

IVNGEPVTLGPVVMIGDSEAYEPRCRCCFIPPHKVECSS"

CDS 1432396..1432608

/locus_tag="EFAGFIKM_01259"

/inference="ab initio prediction:Prodigal:002006"

/codon_start=1

/transl_table=11

/product="hypothetical protein"

/translation="MSEALTSLQERYESLKEQGSLDEATQALFAELFAEAERLQASNL

TLRRTILKSSSKESRMSTKLRDALYE"

CDS complement(1432617..1432964)

/locus_tag="EFAGFIKM_01260"

/inference="ab initio prediction:Prodigal:002006"

/codon_start=1

/transl_table=11

/product="hypothetical protein"

/translation="MVSFLITLQRLLKGIFHAFKDPKFLALFALTAATLLSGTLFYTR

VEGLHWIDALYFCAVTLTTVGHPEFVPTTGFSKAFTVIYMFAGIGLTFAMIARITAGI

LFPRKLKTEEDPE"

CDS complement(1433028..1433345)

/locus_tag="EFAGFIKM_01261"

/inference="ab initio prediction:Prodigal:002006"

/codon_start=1

/transl_table=11

/product="hypothetical protein"

/translation="MLMNTRLVFARFLAIVVLVIPGLMAMKGFLMMKDALFLYYAEHG
NELISPGFQWLSFGGGLVLFAAGMSFLGGWILFRDRKRNYVGPRFRSKSVPEKAETPG
RTP"

CDS 1433570..1434994

/gene="lpd"
/locus_tag="EFAGFIKM_01262"
/EC_number="1.8.1.4"
/inference="ab initio prediction:Prodigal:002006"
/inference="similar to AA sequence:UniProtKB:Q8NTE1"
/codon_start=1
/transl_table=11
/product="Dihydrolipoyl dehydrogenase"
/db_xref="COG:COG1249"
/translation="MPITCDVAILGGGTGGYVAAIRAAQLGKQVVIIEKDKLGGTCLH
RGCIPSKALLKSAEVYAEIQESETYGIETAGAKLVFPKVQARKDAIVEQLHQGVQYLM
KKNKIQVVHAKGRVIGPSIFSPQSGAVAVEFEDGEMDTVVPTNLIIATGSRPRVLPGL
EPDGKFIMSSDEALRMDELPASLIIVGGGVIGLEWASMLNDFGVDITVVEAAAHVLPA
EDEDVAREMQRLLGKRGVRFLTGATVLTETYNTDQEGIQIDVQLGDDKQETLRAEKML
VSVGRQANVENIGLENTDIKLERGFIAVNKQLQTGEGHIYAIGDCIGGLQLAHAASHE
GILAVEHLAGEKVHAVESHRIPRCVYTRPEAASIGFTEREAKERGYDIKTGKFPFSAI
GKSLIHGSRDGFVKVIADAKTNDILGVHMIGTHVTELIAEASLAQMLDATPWEVGQTI
HPHPSLSEIMGEAMLAVDGKAIGM"

CDS 1435134..1436162

/gene="bfmBAA"
/locus_tag="EFAGFIKM_01263"
/EC_number="1.2.4.4"
/inference="ab initio prediction:Prodigal:002006"
/inference="similar to AA sequence:UniProtKB:P37940"
/codon_start=1
/transl_table=11
/product="2-oxoisovalerate dehydrogenase subunit alpha"
/db_xref="COG:COG1071"

/translation="MSSQGTADAVHRHQQLGLSDGEVLDMYKYMLLARKFDERCLLLQ

RAGKINFHVSGVGQEAAQVGAAFGLDRDHDYYLPYYRDYGFVLAVGMTPRELMLSAFA

KAEDPNSGGRQMPGHFGHKKLRIVTGSSPVTTQVPHAVGFALAAKMKKQEFVSFVTFG

EGSSNQGDFHEGANFAGVHKLPVIIMCENNQYAISVPIHKQLSGKISDRAQGYGFPGL

RVDGNDALEVYAAVKEARRRAIAGEGPTLIEAMMYRLSPHSTSDNDLAYRTKEEVEEN

WKKDGVPRMKNYLIDCGIWDEARDADLASQLAMEMKEATEYADNAPYPKPEDTLTHVY

ADSEEGGR"

CDS 1436165..1437151

/gene="bfmBAB"

/locus_tag="EFAGFIKM_01264"

/EC_number="1.2.4.4"

/inference="ab initio prediction:Prodigal:002006"

/inference="similar to AA sequence:UniProtKB:P37941"

/codon_start=1

/transl_table=11

/product="2-oxoisovalerate dehydrogenase subunit beta"

/db_xref="COG:COG0022"

/translation="MAVMEYIDAIRLAMKEEMERDENVFILGEDVGLKGGVFTTTKGL

QDQFGEMRVMDTPLSESAIAGVAIGAAMYGMKPIAEMQYSDFMLPATNQIISEAAKIR

YRSNNDWSCPIVIRAPIGGGIFGGLYHSQCPESIFFGTPGLKIIAPYSAYDAKGLLKA

AIRDPDPVLFFENKKCYKLIKEDVPEDDYIVPIGKANVLREGADITVIGYSQPLHFVM

QAAEELEREEGITAHVLDLRTLQPLDREAIIASARLTGKVLIVHEDNKTGGVGAEVAA

IISEECLFELDAPIQRLCGPDVPAMPISPTLEKFYMLSKDKAKAAMRTLAEF"

CDS 1437199..1438650

/gene="pdhC_1"

/locus_tag="EFAGFIKM_01265"

/EC_number="2.3.1.12"

/inference="ab initio prediction:Prodigal:002006"

/inference="similar to AA sequence:UniProtKB:P65636"

/codon_start=1

/transl_table=11

/product="Dihydrolipoyllysine-residue acetyltransferase

component of pyruvate dehydrogenase complex"

/translation="MAERMKWIEVIMPQLAESLVSATIGKWLKKPGDRVEQYEPICEV

ITDKVNAEIPSTVDGIMGDLLVEEGTTIAVGEAICRMQVAASDEEAAEQVTQVSQQQE

QVQQHVSHTPVAASNTAAHDPNQPMRNRYSPAVQSLAAEHGLNLQSIQGTGAGGRITR

KDVLAFVAQGGNASGTSASAQASSAAVQHTPSGVPSASPFSGVNRGNGEMGLTAGEAI

SASDAGVPVRLSGIHLTESPKIPQIEVEGGGQGRSEYFIDVTPVRNAIARNMRQSVSE

IPHAWTMIEVDVTNLVMLRNKLKDEFKRKEGINLTYLSFMMKGVVNAIKDYPIMNSVW

AVDKIIVKRDINLSMAVGTEDSVLTPVIKHADQRNIAGLAREVDELARKTREGTLKLD

HMQGGTFTVNNTGSFGSILSQPIINYPQAAILTFESIVKKPVVINDMIAVRSMANLCL

SLDHRILDGVISGRFLQRVKENLEGYTMETKVY"

CDS 1438762..1439469

/gene="lipB"

/locus_tag="EFAGFIKM_01266"

/EC_number="2.3.1.181"

/inference="ab initio prediction:Prodigal:002006"

/inference="similar to AA sequence:UniProtKB:Q5SLQ3"

/codon_start=1

/transl_table=11

/product="Octanoyltransferase"

/db_xref="COG:COG0321"

/translation="MSKPLDVAYIPMLDYEEAWNRQKAIVQRLDEGEGAEQMLLLQHP

PTYTIGSQNHPEHLLLSPEELREQGISLFQIDRGGDITYHGPGQLVGYPLLILGRDED

LDLHGYLRKLEQVLMDYLADQGIEAGRKEGYTGVWIGDMKIAAIGIKFNRCKHRRGFV

TSHGFAFNISSGIQHAGFQGIVPCGIEQYGVTSLEDITGKSYAVEQVAQEIVPYFNRI

FPYQINWVTEKEALQRL"

CDS complement(1439496..1439597)

/locus_tag="EFAGFIKM_01267"

/inference="ab initio prediction:Prodigal:002006"

/codon_start=1

/transl_table=11

/product="hypothetical protein"

/translation="MQKKKWFKIIIYLMLIAMIGSTLFIALEPLLFG"

CDS 1439681..1440814

/gene="pepT_1"

/locus_tag="EFAGFIKM_01268"

/EC_number="3.4.11.4"

/inference="ab initio prediction:Prodigal:002006"

/inference="similar to AA sequence:UniProtKB:P29745"

/codon_start=1

/transl_table=11

/product="Peptidase T"

/db_xref="COG:COG2195"

/translation="MIKQQRVIDQFMELVQIDSETKNEHNISKVLKEQFTDLGLHVYE

DDTKEQTGHGAGNLVITWEAEGTEGVAPIFFTCHMDTVTPGQGIKPELGEDGWIRSDG

TTILGADDKAGIAALFEAIRVIQENKIPHGKIQFVITVGEESGLVGARAMNPKDIDAE

FGYALDSNGAVGTICVAAPARAEIQMSIYGKSAHAGVNPEDGISAIQVAAKAIAAMTL

GRIDDETTANIGKFQGGSALNVVCDFVQLEAEARSIVQEKVELQVAQMRDALETTCRK

YGATAEFRSEILYPAFGFHDEHEVVQLAQRAIRSIGLETSTFASGGGSDANIFNGFDL

PTVNLAVGYEDIHTTKERIRAEDIVKLSQVVVAIIQETAADKK"

CDS complement(1440961..1441095)

/locus_tag="EFAGFIKM_01269"

/inference="ab initio prediction:Prodigal:002006"

/codon_start=1

/transl_table=11

/product="hypothetical protein"

/translation="MNSYMTPQSVHVVGQARQVQLILKQWLREWGPDAKLIDLLAGRK

"

CDS 1441218..1441811

/gene="act"

/locus_tag="EFAGFIKM_01270"

/EC_number="3.-.-.-"

/inference="ab initio prediction:Prodigal:002006"

/inference="similar to AA sequence:UniProtKB:Q8KP10"

/codon_start=1

/transl_table=11

/product="Methanol dehydrogenase activator"

/translation="MKPNQSAQSIHAAQPANPKLDEVTVSTKPIFEGKVISLQVDTVK

LPNGQTATREIIRHPGAVAVLALNGDRMLVVDQYRQAMGRTEVEIPAGKLDPGEEPEV

AAARELREETGYVAKSLRHLRSFYTSPGFADEIIHLYIAEELEAGDMALDEDEFLEVA

EITLEEAYALMDENRISDAKTMMAVYAWDLYRTTGRF"

CDS 1441814..1443034

/locus_tag="EFAGFIKM_01271"

/inference="ab initio prediction:Prodigal:002006"

/codon_start=1

/transl_table=11

/product="hypothetical protein"

/translation="MQDQLETSTMWEPCYTDLHIHIGRTSRGEAVKISGSRDLTFENI

AREASDRKGIHLLGVIDCHSPVVQMDIEQLLESGTMSEIDGGGIAYQDTTILLGTEIE

LREPGMREFHMLAYFRDLKTMKSFTDWMKRYMKNVNLSSQRVYVPALEMQAEIKARGG

LIVPAHVFTPHKGIYGSTAPRMGDVLDTSLVDAVELGLSSDSSMASYIRELDHVPFLT

NSDAHSLGKIGREYNELQVAAPSFDEFRMALQGEAGRKIAANYGLNPRLGKYHRTYCS

ACGSIMDEQAMSAERCPHCGSLKLVQGVLDRILAISDRESPHVPANRPAYHYQVPLEF

IPGLGKAKLRQLLDHFGTEMNVLHRTREEELAAVVGPVLAGLIVAARNGQLELSSGGG

GTYGKVAVQEKASE"

CDS 1443196..1443825

/gene="spoIIM"

/locus_tag="EFAGFIKM_01272"

/inference="ab initio prediction:Prodigal:002006"

/inference="similar to AA sequence:UniProtKB:P37873"

/codon_start=1

/transl_table=11

/product="Stage II sporulation protein M"

/db_xref="COG:COG1300"

/translation="MRSSYFTFKGQTSLYVFVAVLFLVGVIFGALMVNALSLEQRQDL

EGYLGNFFMTVQHSAQVTETGAYWDIAMLHLKWVGLIFILGLSVVGLPGILVLDFLKG

VLIGFTVGYLVGQYSWKGLLFALVSVAPHNLFVIPILLICSVAAMTFSLYIIRNRVLM

QRTPGRQRPFASYIVLTLVMAALLLGVASFETWVTPAMMRWVTPMLLPA"

CDS 1443933..1444400

/gene="fur_1"

/locus_tag="EFAGFIKM_01273"

/inference="ab initio prediction:Prodigal:002006"

/inference="similar to AA sequence:UniProtKB:P54574"

/codon_start=1

/transl_table=11

/product="Ferric uptake regulation protein"

/db_xref="COG:COG0735"

/translation="MEARIDKIKQQLQSQGYKLTPQREATLRVLLENEEDHLSAEDVF

MLVKEKAPEIGLATVYRTLELLSELHVVEKINFGDGVARYDLRGDTSKHHHHHLICVQ

CGSMDEIREDWLGPLEERLEREFNFSVVDHRLDFHGICYRCKAKNEQKPKDEE"

CDS 1444558..1444800

/locus_tag="EFAGFIKM_01274"

/inference="ab initio prediction:Prodigal:002006"

/codon_start=1

/transl_table=11

/product="hypothetical protein"

/translation="MIISVRRGLRFIRFIIFFAALVYLFYHVLDLFNGWISPVDQYQM

PTGNAIKVFQETDWPGNGEGRPTMAERLRLFYWYGE"

CDS 1444876..1445766

/gene="xerD_1"

/locus_tag="EFAGFIKM_01275"

/inference="ab initio prediction:Prodigal:002006"

/inference="similar to AA sequence:UniProtKB:P0A0P0"

/codon_start=1

/transl_table=11

/product="Tyrosine recombinase XerD"

/translation="MKQTIHAYALYLEDDKGMSSSTLESYLRDVDKFIEFADKEYGIR

EADQVRRTHVVLFAGQLKQAGRANATIARSIVSLRSYFHFLMRRGDIIQDPTFDVEAP

KADKTPPQVLSIQEIEQLLTAPDTRSPQGVRDRSMLELLYATGIRVSELIALDIRDVQ

PGMRFIRCGGAGKERILPIGAPAAHWASVYVEEFRNKLLKTDSDEQALFVNVSGRRLT

RQGFWKLLKKAAVDAGISEEITPHTLRHSFAAHLIANGADTRAVQDMLGHVEQPGQQY

GNHGRKTMKEIYETHHPRAR"

CDS 1445896..1446720

/gene="punA_2"

/locus_tag="EFAGFIKM_01276"

/EC_number="2.4.2.1"

/inference="ab initio prediction:Prodigal:002006"

/inference="similar to AA sequence:UniProtKB:P77834"

/codon_start=1

/transl_table=11

/product="Purine nucleoside phosphorylase 1"

/translation="MTALNQQMISEAASYIQSKSSIKPEVGLILGSGLGVLAELIEDG

VSIAYQDIPHFPVSTVEGHEGELLVGTIKGRPVVMMKGRFHMYEGYGPELTAFPVRVM

KELGVSSLLVTNAAGGVNTSYVAGDLMLISDHLNLTGKNPLIGPNDAALGVRFPDLSE

AYSRRLRALAKDTAASQGFNVREGVYAGMLGPNYETPAEIRMLRTLGADAVGMSTVSE

VIVARHAGLEVLGISCISNMAAGILDQPLSHDEVMETTERVRESFLALVVAVIPQM"

CDS 1446969..1448144

/gene="dacF"

/locus_tag="EFAGFIKM_01277"

/EC_number="3.4.16.4"

/inference="ab initio prediction:Prodigal:002006"

/inference="similar to AA sequence:UniProtKB:P38422"

/codon_start=1

/transl_table=11

/product="D-alanyl-D-alanine carboxypeptidase DacF"

/db_xref="COG:COG1686"

/translation="MKKRIMASFMTLCILLSLLASTALAEETPKAAGGTDLAPSARSA

ILMDADTGTVIYEKNSHDQLPPASITKIMTMLLTIEAIDSGKLKLTDKVRTSEYAASM

GGSQIFLEPGEEMTVDDMLKGIAMASGNDASVAMAEKIAGSEEAFVQLMNERAKELGM

KDTHFANCNGLPVDNHYSSAHDIAVMSRELLKHSGITKYTGAYQDYLRKDTEKPFWLV

NTNKLVRFYEGADGLKTGYTSEAKFCLSATASKDGLRVVSVVLGEPNTKTRNSEVSSM

FDYAFSQYTMKALYKAGDLLGSLRIEKGEVAELPLNATQNYSVLMRKGAKSNDIRHEL

LVAKELKAPIKAGQSIGKLVVYQGNDVIKEFDIQAPQDVNKAGWWKLFKRTTSNLFD"

CDS 1448290..1448643

/gene="spoIIAA"

/locus_tag="EFAGFIKM_01278"

/inference="ab initio prediction:Prodigal:002006"

/inference="similar to AA sequence:UniProtKB:P10727"

/codon_start=1

/transl_table=11

/product="Anti-sigma F factor antagonist"

/db_xref="COG:COG1366"

/translation="MNLHVEMEHHRGILIVRLSGELDHHTADMVRMQMDEAIQRRQSE

HLVLSLKDLQFMDSSGLGVILGRYKLIKNKGGKMVVCDVNSPVYRLLEMSGLFKIMPI

YENEGTALSGLEVVS"

CDS 1448640..1449095

/gene="spoIIAB_1"

/locus_tag="EFAGFIKM_01279"

/EC_number="2.7.11.1"

/inference="ab initio prediction:Prodigal:002006"

/inference="similar to AA sequence:UniProtKB:O32727"

/codon_start=1

/transl_table=11

/product="Anti-sigma F factor"

/translation="MNEGTGTNFMNLQFAAKSENESFARVTVAAFISQLDPTMDELSD

LKTVISEAVTNSIIHGYNNNSEGVVSIQAEIREDMITIIVEDRGEGIEDLELAKQPLY

TSKPELERSGMGFTIMENFMDEFEVSSEPGRGTSIKMKKRIESKKALYN"

CDS 1449110..1449865

/gene="sigF"

/locus_tag="EFAGFIKM_01280"

/inference="ab initio prediction:Prodigal:002006"

/inference="similar to AA sequence:UniProtKB:P07860"

/codon_start=1

/transl_table=11

/product="RNA polymerase sigma-F factor"

/db_xref="COG:COG1191"

/translation="MDAEVKPSSQTYLDDAEVKRLIALSQSGDHVSRDTLVNCNIRLV

WSVVQRFMNRGYDPEDLFQIGCIGLLKSVDKFDLSYDVKFSTYAVPMIIGEIQRFLRD

DGTLKVSRSLKEMANKVRKKRDELSKHLDRLPTIKEVAAELGVTPEEVVFAQEANKPP

TSIHETVFENDGDPITLMDQIADESQERWFDKLALNEAIGGLSERERLIVYLRYYRDQ

TQSEVASRLGISQVQVSRLEKKILQSIRDQIAQ"

CDS complement(1449953..1450882)

/gene="yicL"

/locus_tag="EFAGFIKM_01281"

/inference="ab initio prediction:Prodigal:002006"

/inference="similar to AA sequence:UniProtKB:P31437"

/codon_start=1

/transl_table=11

/product="putative inner membrane transporter YicL"

/db_xref="COG:COG0697"

/translation="MTTSAKSKISGIILVLTGAICWGIGGTVSQKLFQLDGIEVNWYV

TVRLLIAGLLLLVIQSFTNGRFQIFAVWRNKKTAGQLIIFGLVGMLAVQYTYMASIKH

GNSAVATLLQYLSPVMIMIYIALRKQSVLTRNDLACAVLTLSGCFLLLTNGSLTQLSV

SSLAVIWGLLSGVSAAFYTNYAVQLIKKFDSLVVVGWAMIIGGLGMSFIHPPWKMNFA

NITLETYGYLIFTILFGTMIAFWFYIASLKSLTPKETSLLGSAEPLAAVGTTVIWLHE

PFGFYQWIGMACILGMMLVMQSNRSRSPRINNK"

CDS 1450991..1451899

/gene="melR_1"

/locus_tag="EFAGFIKM_01282"

/inference="ab initio prediction:Prodigal:002006"

/inference="similar to AA sequence:UniProtKB:P0ACH8"

/codon_start=1

/transl_table=11

/product="Melibiose operon regulatory protein"

/translation="MQKIEFIIDQHLKEMTQHRTDTLSIGCYETTIVQNVHGHIPLHW

HDELQFVAVLQGTALFHINDQKMILSKGDGIFINSGIMHMAEDHGVSENCIYLCLNLS

PHVIMPSDLYIKYVHPYVTATNLPFLYIERANAWGEQILDAIAWIRKELVHQVPFYEV

NIASAVLNMWKLLIMNGYSLEHDPSEMSRNNRMKDMLQWINLNYADPIKLENIAKAGQ

LSRSETCRYFKQILKTTPMQYVIEYRIQRSIELLQSSKRSITEIAYEVGFNSTSYYIN

QFRKTMNSTPLRFRRELNKRGKENNS"

CDS 1452381..1453037

/locus_tag="EFAGFIKM_01283"

/inference="ab initio prediction:Prodigal:002006"

/codon_start=1

/transl_table=11

/product="hypothetical protein"

/translation="MSQTPTPMVYIRLRSRIRIQRGRAVKLGDIAHVLTSSEDQEGRL

LELELLRPGPEDGNLILIDILQIIPRIRHILPEVSVELIGSGHTLVEVIAGNGQPSKS

LFILVWLLLFFGSALTIMNFHADVNMQEVQIRIVEMLTGHRDEHPYLFQVAYSIGIGF

GMAVFFNHLFKKKWNEEPTPLEVEMFLYQQNVDKYVVIEESERMHEQERREMNADERS

"

CDS 1453024..1453446

/locus_tag="EFAGFIKM_01284"

/inference="ab initio prediction:Prodigal:002006"

/codon_start=1

/transl_table=11

/product="hypothetical protein"

/translation="MSVLNGAISIVLGIAGGIAVGSGVIALILVLDMIPRLAQLTQSY

DKTHWYEGALIGGSLLGTVADFWHWKMHGVLLLSPIVGLFCGVFIGLLAAALTEVLNV

LPVLAKRLGMKPYLFGLLLAMILGKMTGSLFDFFIYQR"

CDS 1453471..1455138

/gene="gerBA_1"

/locus_tag="EFAGFIKM_01285"

/inference="ab initio prediction:Prodigal:002006"

/inference="similar to AA sequence:UniProtKB:P39569"

/codon_start=1

/transl_table=11

/product="Spore germination protein B1"

/translation="MDERTEHTGQENSEGITEEMLHKSAYEIQKEEEEKAYNKKKQNE

MSDSVEESVQYWQENDDISPRMSENRYTLQQVLGLGESFDVNMREMVLGGKHVGLLMI

TGFAKDEILLEILKRLTYLTPDQVSGHALKSYFECYIPHIQVEKVDKMSAVINKVLTG

MSAMFVEGDRSVLIMDTRSYPVRSPEEPSLERVVRGSRDGFTETLLTNVTLVRRRIRD

PGLKFEIMQVGRRTQTDVCVVYIDDIVDKVQVDSVREKIQRVDIDGIPAADKQLEEAI

INKGWNPFPLVRYSERPDVTASHLLEGRVVIFVDTSPSVMILPTTFFDLCEHAEENRQ

TALMGTYLRWVRFAGILISLFLLPLWLLMVIDPSIKPMGLDFIGPQENVKLPLILQFL

LIELGVDLLRMAAVHTPTPLASAMGLIAAILVGDIAVKTGYFVNEVVLYMAIAAIGMF

ATPSYELGLANRLVRLVLLVAVAIFKVPGLVVGCTLLILALTVHRSYNSSYLWPFIPF

NAKALGNFLFRLPLLENKKRPSFNKTRDNTKMSDDPDGELQKSKKHK"

CDS 1455242..1456573

/gene="lysA"

/locus_tag="EFAGFIKM_01286"

/EC_number="4.1.1.20"

/inference="ab initio prediction:Prodigal:002006"

/inference="similar to AA sequence:UniProtKB:P09890"

/codon_start=1

/transl_table=11

/product="Diaminopimelate decarboxylase"

/db_xref="COG:COG0019"

/translation="MYLHGTSKINAQGHLEIGGVDATDLKEQFGTPLYVVDEQLVRER

CREYMEAFRASGLGFQVAYASKAFCVMAMCALAAEEGLSLDVVSDGELFTALQAGFPA

ERIHFHGNNKTLEEIEMALDAEIGCFVVDNFNELHLLQAVAADKNRKVNILLRVTPGV

EAHTHEYISTGQTDSKFGFDIGNGTAFEAIDLASKQSNLVLLGVHSHIGSQIFEVEGF

QMAVQRVAEFAASVYERLNVAFKVVNLGGGFGIRYIDGDTPLEVAQYVKAITDAVKNH

FAQIGYAVPEIWVEPGRSIVGEAGTTLYTVGTSKDIPGVRKYVAVDGGMTDNPRPALY

ESKYEAVLANRANEAAQETVSVAGKCCESGDMLIWDLDLPKVESGDLLAVACTGAYNY

SMASNYNRIRRPAVVFVKDGQGDVVVRRETYQDIIQNDLVPARIGKQPVTR"

CDS 1456821..1457252

/gene="ppiB"

/locus_tag="EFAGFIKM_01287"

/EC_number="5.2.1.8"

/inference="ab initio prediction:Prodigal:002006"

/inference="similar to AA sequence:UniProtKB:P35137"

/codon_start=1

/transl_table=11

/product="Peptidyl-prolyl cis-trans isomerase B"

/db_xref="COG:COG0652"

/translation="MAKQAKIKLANGGEVLIDLFDQEAPNTVANFEKLANSGFYNGLT

FHRVIPGFVAQGGCPNGSGAGGPGYTINCEINPNKHERGTLAMAHAGRNTGGSQFYIC

YQPQPHLDGQHTVFGKVTKGMEFVDALEGKDKMETVEVVEA"

CDS 1457357..1458670

/locus_tag="EFAGFIKM_01288"

/inference="ab initio prediction:Prodigal:002006"

/codon_start=1

/transl_table=11

/product="hypothetical protein"

/translation="MDMVTQLSLLKQIYSERTLWDEELQASRHVVPDSLSVKDREVLE

AAGHEPNRFVRPQHDETITELKKVANQWTINDAAQAFVSSLWSAPMIWRSLLTGKLIA

SSMPSHEHTPYPSSNTCKICGLSVDQATDTTLQWFWRMTNGTPLDGDPFGYVLALREL

AAAQELPIPNEYDRWTFSAVLTVLRDLPPKTRYSKAAVALKKERLLPTQKEYAYRDLL

ETLALIGILDTPEHPGMITEFTSYIQRDARPNIRVEVQAPLAWWDSSVGINENNLNKI

FHDFDLNNISLADKPEESPAVKDTILGALEKKRSVRGKVPKASPDAGTGEVQSGDVYA

VRVREGVWVTVYCHEVRDKRVIVEYLDGVFPEMPGKADLHGTFRPRANGRWKCSAIAI

DSTSWVRRVAREFPLPTSPLQEPDRTPFHNAKELKHMASWCFPDM"

CDS 1459335..1460432

/gene="ribD"

/locus_tag="EFAGFIKM_01289"

/inference="ab initio prediction:Prodigal:002006"

/inference="similar to AA sequence:UniProtKB:P17618"

/codon_start=1

/transl_table=11

/product="Riboflavin biosynthesis protein RibD"

/db_xref="COG:COG0117"

/translation="MINDEFYMALALDMAERAQGQTGINPVVGCVVVKEGRIVGLGSH

LKRGTGHAEVHALNMAGSDAEGSTVYVTLEPCSHYGKTPPCSERLIHEKVKRVVVCCE

DPNPQVSGRGISMLRQQGIEVEVGVLRERGRRMNEKFIKFITTGLPFVTLKTATTLDG

KIATRSGDSKWISNEPAREIVHALRHRHQGIMVGVDTVIADNPELTTRLQVEGISPVR

IVVDSKLRLPVGAKMVKDGLAPTWVLTTDEASPEAAERLEAYGVEIIRCGPGPRVDLL

NGLSKLGEREIGSILLEGGGTLNGAMLEARLVDRLLMFIAPKIVGGYDTPGSFRFEGV

ERMNQAIQLNQLEIEQVGDNISIGGIPVWPE"

CDS 1460494..1461162

/gene="ribE"

/locus_tag="EFAGFIKM_01290"

/EC_number="2.5.1.9"

/inference="ab initio prediction:Prodigal:002006"

/inference="similar to AA sequence:UniProtKB:P16440"

/codon_start=1

/transl_table=11

/product="Riboflavin synthase"

/db_xref="COG:COG0307"

/translation="MFTGLVEEVGQIRRIGRKGEAMVLNIGASAIMDDLKIGDSVAVN

GVCLTATTLEGGGFTADVMPETYRHTNLSQLQAGSKVNLERAMQSGGRFGGHIVQGHV

DGTGVIGSITRDQNAIVFEIKPDDHQLFKYLIPKGSVTLDGISLTVVHTSDTAFTVSI

IPHTIGETVLNVKKTGDTINIECDILGKYVDHLLQYGRGHRETGAGKPRSISEDFLSN

HGFA"

CDS 1461195..1462427

/gene="ribBA_1"

/locus_tag="EFAGFIKM_01291"

/inference="ab initio prediction:Prodigal:002006"

/inference="similar to AA sequence:UniProtKB:P9WHF1"

/codon_start=1

/transl_table=11

/product="Riboflavin biosynthesis protein RibBA"

/db_xref="COG:COG0108"

/translation="MSEQSILDTIEEAIYDLMRGKPVIVVDDEDRENEGDFIALAEKA
TPEVINFMITEGRGLVCVPITQQRADELNLKPMVQQNTDFHGTAFTVSVDHKDTTTGI
SAHERSITVKGLIDPEAKAGDFRKPGHMFPLIAKDGGVLRRAGHTEAAVDLAIMCGSY
PAGVICEVIKEDGTMARLPDLQEIARKHDLKLISIQDMIRYRNEKEQLVQREVAVRMP
TDFGEFQAVAYTNAVDNKEHVALVKGEIDGTKPVLVRVHSECLTGDVFHSHRCDCGPQ
FDAALKQIHEEGNGVLLYMRQEGRGIGLINKLKAYKLQEEGMDTVDANLSLGFAADLR
DYGIGAQILKDLGVRQIRLLTNNPRKIKGLEGYGLEVVERVSIQMEENEDNTVYLHTK
QAKLGHMLKFDDIEQNEQ"

CDS 1462530..1462997

/gene="ribH"

/locus_tag="EFAGFIKM_01292"

/EC_number="2.5.1.78"

/inference="ab initio prediction:Prodigal:002006"

/inference="similar to AA sequence:UniProtKB:Q81MB5"

/codon_start=1

/transl_table=11

/product="6,7-dimethyl-8-ribityllumazine synthase"

/db_xref="COG:COG0054"

/translation="MPQFFEGHLVSEGSRYGVVVGRFNEFITSKLLSAALDAFKRHGV
QDDEVDVAWVPGAFEIPLIAQKMAESGKYDAVITLGTVIRGSTTHYDYVCNEMAKGVA
AINLKTGVPTIFGLVTTENIEQAIERAGTKAGNKGWDAATAAIEMANLTKQLK"

CDS 1463102..1463902

/gene="scpA"

/locus_tag="EFAGFIKM_01293"

/inference="ab initio prediction:Prodigal:002006"

/inference="similar to AA sequence:UniProtKB:P35154"

/codon_start=1

/transl_table=11

/product="Segregation and condensation protein A"

/db_xref="COG:COG1354"

/translation="MTVVTYKLETFEGPLDLLLHLIDKAEIHIQDIPISDITDQYMAY
LHSMQELELDITSEFLVMAATLLSIKSKLLLPKPPVIEDFEDYDFMEEEDYDPRAELI

ARLIEYRKYKGIARHLHEREWERSLIFSKEPEDLRPYMPVEAQSNPVEGLHTSDLIAA
FQKALRKAEKRTTVTRIKRDEISVKDRIRDVIGALESMGPGGRLLFSKLMDEHIYRHE
IVVTFLAVLELMKMKQIVCYQERMFEDIVMEWRGETKNHGFSEIEIDY"

CDS 1463871..1464527

/gene="scpB"

/locus_tag="EFAGFIKM_01294"

/inference="ab initio prediction:Prodigal:002006"

/inference="similar to AA sequence:UniProtKB:P35155"

/codon_start=1

/transl_table=11

/product="Segregation and condensation protein B"

/db_xref="COG:COG1386"

/translation="MDFPKLKSIIEGLLFLSGEEGLSIKQIAEIVDQRVELVTDAIEE
MIHEFSQQGRGVQILKIAGVVQLGTLPDHAVYFEKLAYSPARTSLSQAALETLAIVAY
RQPITRVEIEEIRGVKSERAIHTLVNKDLIIEVGRAEAIGRPILYGTTKSFLDYFGLA
SIKDLPEPGLFEDSENLEEETQLLFNKLDLQQLEPEIVDPADDLSLIDNEDDNTDSSL
"

CDS 1464717..1465448

/locus_tag="EFAGFIKM_01295"

/inference="ab initio prediction:Prodigal:002006"

/codon_start=1

/transl_table=11

/product="hypothetical protein"

/translation="MWIWLGGIGLLFALLIAAVLISNVHVHFTFSKHKSDDYANINVR
LLYGIVRINYEIPSIVFRNMKEGFLVKTEQSMNHSRGEAQGSERVNKRKVKKWAKDVK
IMLRATDALKLWLKQTLTRVHMTQLSWATHIGVGDAAYTAVLTGWVWSIKSIIIGFLT
YQIRFDETPVLQVMPVWSDEMEFRTELDFRVKIRITAVLIAGVTLLTRVLQVEGGWRM
WFKLLQEQRRRHKEKQKKKLSGTTV"

CDS 1465596..1466078

/gene="ytfJ"

/locus_tag="EFAGFIKM_01296"

/inference="ab initio prediction:Prodigal:002006"

/inference="similar to AA sequence:UniProtKB:O34806"

/codon_start=1

/transl_table=11

/product="putative spore protein YtfJ"

/db_xref="COG:COG3874"

/translation="MSDHPIQGLMETAMENIKAMVDVNTIVGDAVETPDGTVILPISK

VGFGFAAGGSDFHVNGESSGKSGATGTSTEHASSAKVASPFGGGSGGGVSIRPIAFLV

VGKQGVHIVPLDNSTHLFEKLIDSTPYVLDRIQGMFQRNTTPTNTTGVPVTPDPSTYS

"

CDS 1466209..1467396

/gene="dacB"

/locus_tag="EFAGFIKM_01297"

/EC_number="3.4.16.4"

/inference="ab initio prediction:Prodigal:002006"

/inference="similar to AA sequence:UniProtKB:P35150"

/codon_start=1

/transl_table=11

/product="D-alanyl-D-alanine carboxypeptidase DacB"

/db_xref="COG:COG1686"

/translation="MRKITKVIACMLIPVFLLSSLGVTQAQATIQGPSTHAQSAALID

VTSGRILYSKDGDKELRIASLTKIMTAIVAIEHSKLDEKVKVSPTAFAKEGSSLYLKL

GEEMTLENMLYGLMLRSGNDAASAIAEHVGGSEEGFVLLMNKKAEQIGLTHSHFMNPH

GLDAEGHYSTANDLARLTAYALHNPVFKRIVATEDKSAPNPNESWEYSWHNKNRMLRM

YEGADGVKTGYTKKAFRCLVSSATRNGQQLAAVTLNDGNDWNDHARMLDFGFEYFPLV

EVAKQEQPVQNTDVVTGRGFWYPLAESEKSSLTKKLILHENRGQSETEGKEQSTDPSF

GLAGRIDMQLDGKLVGSIPVYRKGSYIPPEPKTEDATLGGIGNVSSWAAAWRAVLGHL

LSP"

CDS 1467428..1468054

/gene="spmA"

/locus_tag="EFAGFIKM_01298"

/inference="ab initio prediction:Prodigal:002006"

/inference="similar to AA sequence:UniProtKB:P35157"

/codon_start=1

/transl_table=11

/product="Spore maturation protein A"

/db_xref="COG:COG2715"

/translation="MINLIWLLMILIGFAFAAVNGNIEVVTQAAFDGAATGVTVCFGL

ISVLVFWMGMMKMAEDAGLLARIAKLLGPVVGFLFPDVPKNHPAMGYILSNMSANLLG

LGNAATPMGIKAMQQLQELNPDKQTASPAMCTLLALNTASITIIPTTLIAIRLNYHSA

NATEIVGTTLMATIIATLAAIIADRWYRNRALHKPPRIQKSGNPGMKG"

CDS 1468061..1468594

/gene="spmB"

/locus_tag="EFAGFIKM_01299"

/inference="ab initio prediction:Prodigal:002006"

/inference="similar to AA sequence:UniProtKB:P35158"

/codon_start=1

/transl_table=11

/product="Spore maturation protein B"

/translation="MLTFINWISVWAIPVIIAFVPLYAFTKKVPVYESFVDGAKDGFS

TAISIIPHLVGMMVAISVFRASGALDFVISLFAPLVSWMGVPGEVLPLGILRPLTGTG

SLAFTTDLIKTHGPDSMIGRMASTIQGSTDTTLYVLTVYFGAVGIRNGRYALKVGLFS

DVVGFIAALAICLLVFG"

CDS 1468658..1469476

/locus_tag="EFAGFIKM_01300"

/inference="ab initio prediction:Prodigal:002006"

/codon_start=1

/transl_table=11

/product="hypothetical protein"

/translation="MQKSWKRMLRVGMSGVLAIAMLAGSTGLAAAQAVQTIHFTGYFD

KQLRAQMAVTADSFPVVNVRLVKQDEPDMVYYDVGTSILISKDEVLTNYHVVQSFAEL

SDGDEGTLTVASPGSLGKPVKAKIIKTDPVTDMALLKLDKEIDAQPVTFANARDNQVV

YTIGFPKNPAGDLVILDEDFPSTYNTVAKSRVFSSQASDVPGKKGIGSIVKSMAQGNS

GGPVLDQNNRVIGMMTFVYGGRTYYITSKTLQTFIKESTKPAKKQVAVAGKVSN"

CDS 1469664..1470422

/gene="rluB_1"

/locus_tag="EFAGFIKM_01301"

/EC_number="5.4.99.22"

/inference="ab initio prediction:Prodigal:002006"

/inference="similar to AA sequence:UniProtKB:P35159"

/codon_start=1

/transl_table=11

/product="Ribosomal large subunit pseudouridine synthase

B"

/db_xref="COG:COG1187"

/translation="MTNMERLQKIIAQAGIASRRKSEELILSGKVEVNGEVVTELGTK

ANPEEDMITVNGKPIRSEKKVYLMLNKPKGVITSASDPEGRKIVSDYLKGVKERVYPV

GRLDYDTEGLLILTNDGEFAHLLTHPKHHVPKTYHATVKGVPHGTALEKLKTGIMLDD

GMTAPAEVEYKDVDTAANESVISITIYEGRNRQVRRMFEAINHPVTRLKRISFGGILL

QNLKRGLTRHLTKEEVNNLITLAQSEPAKKMKKR"

CDS 1470557..1471084

/gene="resA_1"

/locus_tag="EFAGFIKM_01302"

/inference="ab initio prediction:Prodigal:002006"

/inference="similar to AA sequence:UniProtKB:P35160"

/codon_start=1

/transl_table=11

/product="Thiol-disulfide oxidoreductase ResA"

/translation="MGKSRRTVQIVILLLILVLGGYAITTSVSGSNGKPKEGDKSPSF

ELLGLDGQVHTSDEYKGKAMVINFWGTWCEPCVKEMPALQAQADKWKDQGVQFVGINV

GEDQMTVDNFVRQVGVTFPIMLDREKNSVRDFGISPMPTTFFVSDTGKISTIHIGQLD

LDTLDAQISQLAKQP"

CDS 1471099..1472772

/gene="ccs1"

/locus_tag="EFAGFIKM_01303"

/inference="ab initio prediction:Prodigal:002006"

/inference="protein motif:HAMAP:MF_01392"

/codon_start=1

/transl_table=11

/product="Cytochrome c biogenesis protein Ccs1"

/translation="MFQNTKCECGHQNPVGTVLCEACGKPLLESEAKSDEVLEMRYDG

MARRSQRSNPNIIDRIWNFFSSVKIAVYMIVFTLVGSMLGTIYPQESTFLNIDPSVYY

KETYGQLGHIYYLLGLSHTYESWWFILLLVLIGASLVICSLDRVLPLYKALNKQRIRK

HTQFLTRQRLVYQGTIEEAPEDWIKKAVTPLKKKGYRVHTQGDALLAEKQRFSRWGPY

VIHIGLIIFLLAVLARGLPGLNLDEHVAFPEGEIKKIPNTSMYLQNEQFNVEFYSEEE

VPEQFRNLNKTVPKLFETKVVLYECTADCSDLSKKPQLAEVARHDVRVNHPLNYNGLK

AYQFDYDLTPTIRSVTPDLVNTKTGEVYGPIKIDMVDTQRTFEAGPYKLTVKEKFMDF

GLDENGQPKSKSPSPNAPAFLFLIKGPDLPEEGIQYLYFAKQIDKQRFQQDAINQQLI

GADIPLQLEVDSMDKVDIIQSVSYLNIRVDKAMPFVWVGAGIVMLGLVMGFYWHHRRI

WIRFDDKQLTLGGHTNKNWFGFRREVAAVLKQMNLEVEEKSLDNGGNQA"

CDS 1472769..1474031

/gene="ccsA"

/locus_tag="EFAGFIKM_01304"

/inference="ab initio prediction:Prodigal:002006"

/inference="protein motif:HAMAP:MF_01391"

/codon_start=1

/transl_table=11

/product="Cytochrome c biogenesis protein CcsA"

/translation="MRLLDFSSDAFIVSFFLYCAAFLLYAVAVMGKKWSNRDPLDHMN

RWGKRAFIASTVALAAHFVFFVTRWAGAGHIPVSNMYEFMSFLSMMIMVAFIVVYAIY

RKSLLGLFALPLTIIIMAYAAVFPQEVQPLIPALQSIWLKIHVTLAALGEAFFAVGFA

AGFMYLLRTVDFSGKDKSSRRQRGWVEFTLVTIVVVIGFIGTVFAFRTAGYEAVFVQK

TVSIDTEVQENSTIEKVIYRMPPIFAPYNSEVESMTPFLGMKKPLLETPSWMNGVNAG

RKLNTVVWSLIVGLILYGIVRLLVRRPLGQALQPMMDGIDADDLDEISYRAIAIGFPI

FTLGALIFAMIWAQIAWSRFWGWDPKEVWALITWLYYSVYLHLRLSRGWQGQKSAWLA

VLGFLVVMFTLVGVNLIIAGLHSYAGAD"

CDS 1474078..1474887

/gene="srrA_2"

/locus_tag="EFAGFIKM_01305"

/inference="ab initio prediction:Prodigal:002006"

/inference="similar to AA sequence:UniProtKB:Q9L524"

/codon_start=1

/transl_table=11

/product="Transcriptional regulatory protein SrrA"

/db_xref="COG:COG0745"

/translation="MYLFIKGYSNIVRQIEHNRSDEELLQRGWRGMAEHENRILVVDD

EERIRRLLKMYLEKEGYEIDEAEDGETALRKATAGDYGLILLDVMLPGMDGVEVCTRL

RQVKSTPVLMLTAKGEEINRVQGFEVGADDYVVKPFSPREVIYRVKAILRRASATAYL

SKESNSSNNIVFPHLVIEHDAHRVTAGGEEISLTPKEYELLHYLATSPDKVFSREELL

KDVWNYEFFGDLRTVDTHVKRLREKLNKVSPESAAMITTVWGVGYKLEVPK"

CDS 1474907..1476340

/gene="resE"

/locus_tag="EFAGFIKM_01306"

/EC_number="2.7.13.3"

/inference="ab initio prediction:Prodigal:002006"

/inference="similar to AA sequence:UniProtKB:P35164"

/codon_start=1

/transl_table=11

/product="Sensor histidine kinase ResE"

/translation="MVGKLWITIICLVGCVLIALGLFLLPYIDTNFAESESRDIKRLF

TYICIIGFSLTTFFALFLFTKITQPMQQLIQAANAIRKGNYGTRLSLVTSDEIGELAN

TFNHMAAQLEDNIRNLNHEKEHLASVLRSMTDAVVTFDGEGKVILTNPPGEKIMQAWY

DLDWAKMDEGQESEQSDKTSRDVPEPLLPLFRMVMEQGGDQNSNVHVQQGVWSVQMAP

LYADSVVRGAVAVLRDVTEEVRLEKMRRDFVANVSHEIRTPLSMMQGYSEALLDGMAT

SPEESEELIQVIHDESLRMGRLVKDLLDLARMEAGHTDMVMKEVDLGELLERVYRKFS

VRSKEQGIQLQFKFEQPTIELQQADEDRLEQVFTNLLDNAFRHTPTGKNVMISAERVT

YLRAPFVRVSVKDQGVGIPSSDLPFIFERFYKADKARVRGESVGTGLGLAIVKNIVDA

HQGMITVNSVLGEGTEFILQFPLDSSK"

CDS 1476535..1480947

/locus_tag="EFAGFIKM_01307"

/inference="ab initio prediction:Prodigal:002006"

/codon_start=1

/transl_table=11

/product="hypothetical protein"

/translation="MSDDRKRVNIKAFLEGRSFRAGSPFIPITTSELEQFESILRSLA

VAIPAAISQPTSANILSLQNGLRQLLTFVNQSGFRAGVKAELQAVLELTIAGSEVVPV

PLINLAGNLQNLLDDLLSVTLLLEVSPVEKDKLVGLIRSISISLSRATITLGTVGITG

PAGPQGVPGVPGVPGVPGVPGVKGATGTPGGIGPVGPVGPVGPQGAQGPAGAPGVGLN

NITAFDPALGPTYAQGQVVSYKGSLYVANVNGPLGTPGSSPDYTLLLSGGTTGATGAT

GAGLTGAVAFNPVLAPGYPAGQVITYNGSTYITSVAGPLGTPGSSSDYTLIAAAGATG

VTGATGAGLSGIVPFDPALAPTYPAGQIVTFGGSTYITNVASPTGTPGSSPDYTLLAG

SGVTGVTGATGIGLNGAVPFDPAVAPTYPAGQVVTFNGSTYITTVASPTGTPGTSPDY

TLLAGAGPTGVTGASGVTGATGVGLSAIVPFDPAIAPTYPAGQVVTFNGSTYIANVAS

PTGTPGTSPDYTLIAGAGATGVTGATGVGVTGSTGVTGASGVTGATGVGLSGIVPFDP

AVAPTYPAGQVVTFNGSTYIANVASPTGTPGTSPDYTLIAGAGATGVTGATGVGVTGS

TGVTGASGVTGATGVGLSGIVPFDPAVAPTYPAGQVVTFNGSTYIANVASPTGTPGTS

PDYTLIAGAGATGVTGATGVGVTGSTGETGATGVAGVTGATGITGVTGLSVTGSTGVT

GGPGATGETGATGVGITGSTGATGVTGETGVTGITGATGVGVTGSTGATGVTGETGVG

VTGSTGATGVTGETGATGATGGTGATGITGSTGVGVTGSTGATGITGETGATGVTGAT

GVGVTGSTGATGETGATGLVGLTGATGITGATGLSVTGVTGGTGATGETGATGVAGLT

GATGITGATGVSVTGSTGATGETGATGVTGLTGATGVSVTGVTGATGVGITGSTGAIG

ATGETGATGITGATGVGVTGSTGATGVTGETGVTGITGATGVGITGSTGVTGVTGGTG

ATGITGSTGVGVTGSTGSTGITGETGATGITGATGVGVTGSTGSTGATGGTGATGITG

ATGVGITGSTGVTGVTGETGVTGITGATGVGITGSTGVTGVTGGTGVTGITGATGVGI

TGSTGVTGVTGGTGATGITGSTGVGVTGSTGSTGITGETGATGITGATGVGVTGSTGS

TGVTGETGVTGITGATGVGVTGSTGATGATGGTGATGITGATGVGVTGSTGATGATGG

TGATGITGATGVGVTGSTGSTGATGGTGATGVGVTGSTGVTGVTGGTGATGITGATGV

GVTGSTGVTGATGATGTSVTSNSAFAENTNGTIVVILGGTLVPLPNNQNIGTGITVNG

TNDTFTLTNAGRYYISYKINLTAALAIQSRVLLNGAAIPASVVSPVLSLSQLQSDFMV

TVTAGSTIQLQLFGLVGAAVLSPPGSTLNIIQLS"

CDS complement(1481143..1482738)

/gene="serA"

/locus_tag="EFAGFIKM_01308"

/EC_number="1.1.1.95"

/inference="ab initio prediction:Prodigal:002006"

/inference="similar to AA sequence:UniProtKB:P9WNX3"

/codon_start=1

/transl_table=11

/product="D-3-phosphoglycerate dehydrogenase"

/db_xref="COG:COG0111"

/translation="MYKVLVSDPISDLGIQQLVDANDVVVEKKTGLSEDELVAIIGEY

DALLVRSQTRVTDRIMTAGTNLKVIGRAGVGVDNIDLEAATQRGIIVINAPDGNTITT

CEHTFAMMMALARHIPQAYAKTIQGTWDRKTFLGVELRNKTLGVLGMGRIGSEVAKRA

KAFGMDILAYDPFLTEERAEKLQVKLASVDDIIRNADFMTVHTPLTPETRHMISRPQF

EVMKKGMRIINCARGGVVDEMALVEAIDQGIVAGAAFDVFESEPPAADHPFLNHPSII

VTPHLGASTVEAQENVAIDVSEQVLHILRNEPFKNAVNMPAVAPTVMNKLQPYFKLGE

TLGSFAAQITQNAVQEIRIDYAGELSEVDTSPLTRYIVKGILARHLGGEANIVNSMHL

AKIRDLNVVVSQTSTTKGFTNLITVTLVTTQDAEERRVAGTLLAGYGERIVRLDKFPV

DIAPESHQILISHNDKPGIIGRVGTLLGQNDVNIASMQVGRKIIGGAAIMILTVDKAV

PKDVLVQLAALPEINTAVEIVLE"

CDS 1483504..1484097

/locus_tag="EFAGFIKM_01309"

/inference="ab initio prediction:Prodigal:002006"

/codon_start=1

/transl_table=11

/product="hypothetical protein"

/translation="MKKFKFPKFKIQKAEPQQLTERLLLINLYFTQGLTLIIGVVWIL

LQKRNLLDVLAWPDNYQFIWWGLGLAGIMLVMDLVLSYVIPQESMDDGGINEMLFRGR

PIWHIVCIAAIVAVCEELLFRGAIQHAIGPYWTSILFAVIHIRYLRHWIPTGWVFVSS

YGLGWIYLQSGTLWAPILCHFIIDLVSGLAIRFRRGS"

CDS 1484094..1484546

/locus_tag="EFAGFIKM_01310"

/inference="ab initio prediction:Prodigal:002006"

/codon_start=1

/transl_table=11

/product="hypothetical protein"

/translation="MNQPLSRMKTYGSQRHRQSDKKEKSERQVASQVSIVAETAAAAE

VGGITPITSVSLPRSQRKPYTEKLESPVLPQRAPSRSKKTPNSTEESIPEAGSLPTRT

ELHPSRRLKLSKRFVNSLIFIFVMLTISLVWWGVEGAPALNTFLPLPW"

CDS 1484546..1485373

/gene="ypbG"

/locus_tag="EFAGFIKM_01311"

/inference="ab initio prediction:Prodigal:002006"

/inference="similar to AA sequence:UniProtKB:P50733"

/codon_start=1

/transl_table=11

/product="putative protein YpbG"

/db_xref="COG:COG1408"

/translation="MLLLAGLIILMGVLLVFHMWREAHIHQMIEEEVEVPHLPTSFDG

AKILYVSDTHKRKLKQKDLENVKNKVDWVLIGGDVAEKGISWSLVRHNMKILSNIAPS

FTVYGNHDKRAGTAQLARILRESDVKLLQDNVEYLQRGNDRLSLIGIDYRSKQGDVLL

QQIGNRFCKIAIVHDPLQALRLEENADLILSGHTHGGQLVLPFFGPVFLSKAYRPVSN

GWYSLKRSPEDVHQEGKMLVSRGYGTNHLPLRLGCPAEMHIITLRVPAEKALTEKQP"

CDS complement(1485418..1486314)

/locus_tag="EFAGFIKM_01312"

/inference="ab initio prediction:Prodigal:002006"

/codon_start=1

/transl_table=11

/product="hypothetical protein"

/translation="MFRHTAALIIAASLLLSACGTAEEKTSDNSTPAGESTTTVQKEQ

GNSNPTETGEPGSGVPEPSTEDSTATEEELESSEKVSTEEKVEKTYHMNENYYIKPND

EASPNKVVLLTFDDGPKEEKMITSLIDTLDKHNAKAIFFVNGYRVKSHPELLKLIHER

GQIVGNHAWDHEDLKKMSNAEAAKQVTDVQKIVKETIGEEPQFFRPPFGSGNDALKAV

VKKNGMLYMTWSNGSLDWDKSTKDKPEKVIQNVLDQLNPGSNILMHELPWTVEALDEL

LTKLEKKGYSFVDPRAIELEAR"

CDS 1486409..1486762

/locus_tag="EFAGFIKM_01313"

/inference="ab initio prediction:Prodigal:002006"

/codon_start=1

/transl_table=11

/product="hypothetical protein"

/translation="MKTSSFFWGIVIGVAASSWLSKGKLPMMSSGSSRNMMDQAKHKM

MEMTFPGMDGFTSKQNQHGESHKNPAAKTARAITPQEKEANMSMLKDFIRTNPDVKHE

VEQILSQTNTTVPGL"

CDS 1486950..1487564

/gene="mecB"

/locus_tag="EFAGFIKM_01314"

/inference="ab initio prediction:Prodigal:002006"

/inference="similar to AA sequence:UniProtKB:P50734"

/codon_start=1

/transl_table=11

/product="Adapter protein MecA 2"

/db_xref="COG:COG4862"

/translation="MKIERLSHDKIRIFLTFDDLSERGIQKEDMWQEIPKVHELFTEM

MDQAYSELGFDATGPLAVEVFALPAQGMVVIVTRGKYDPQQYGLGHEDDLPEEVYEME

VTLEQSDSIVYAFKDFEVLIEAAHMLRQHVTDAGRLYSYKDKWFLHLEPDEVDSTKHA

ALIALLAEFGEGSSVTPAVMEEYGKVVIPAQAIDVICTHFKRQD"

CDS 1487608..1488303

/gene="prsW"

/locus_tag="EFAGFIKM_01315"

/EC_number="3.4.-.-"

/inference="ab initio prediction:Prodigal:002006"

/inference="similar to AA sequence:UniProtKB:P50738"

/codon_start=1

/transl_table=11

/product="Protease PrsW"

/db_xref="COG:COG2339"

/translation="MLLFSVLAAAVAPGLALLTYFYLKDRYDYEPLHMVLRVFLMGIL

MVLPVMIIQRGMMMWLGDNPYLEAIMISGGVEEFVKWFVLYHIIYNHTEFDEPYDGIL

YAVAVSLGFATVENVLYAFAGNASVSAMFLRALLPVSGHAMFAVIMGYYMGKAKFIGG

KKKRWYLVLSLVLPFFWHALYDVIMNTMVNHWLWFIAPLMAGLWYGAMGKITRANNRS

PFRFVKREEEVKL"

CDS 1488418..1488588

/locus_tag="EFAGFIKM_01316"

/inference="ab initio prediction:Prodigal:002006"

/codon_start=1

/transl_table=11

/product="hypothetical protein"

/translation="MRIKIKIRCKQCGERFTLRGKKERGRIETGFKQCLCDNRDQFEI

EEEGMATGIQLN"

CDS 1488710..1490056

/gene="ypeB"

/locus_tag="EFAGFIKM_01317"

/inference="ab initio prediction:Prodigal:002006"

/inference="similar to AA sequence:UniProtKB:P38490"

/codon_start=1

/transl_table=11

/product="Sporulation protein YpeB"

/translation="MYKRLSSVMFPIFAVLLVGALVWGYQENQEKNAILIKAENQYQR

AFHDLSFHMDKLHSEIGNTLAVHSTSQGMHRKGLMNVWRLTSEAQNEINQLPLTMLPF

NETEEFLSRISNFAYRASMRDLTNEPLSEKEMGNLKKLYQNSSEITKNLQDVQQKVLT

DRLRWMDAETAMATQEQTMDNTIVDGFRTVNKKVQEYPELDWGPSVSNIYAKRSVKKL

DGLPVTKEQIQSKAAKFSDADRSKIQVQENGKGTDWESYTATIDHANNGKLMMDFTRN

GGMLISYSDTRPIGTKKVSRQNAMAKADQFLMNKGYKDMKAVNYDEYGNLGNLTYVRK

QGDTLIYPEKMSVRVGLDTGEVTGFQASDFVYEHNDMRKIPKATLTEQQARKKLNSEF

EENYVRKSLIENDYSKEVLCYEFGGKINGSRYRIYINANTGLEEAVEEIKPVNATS"

CDS 1490205..1490858

/gene="ypfA"

/locus_tag="EFAGFIKM_01318"

/inference="ab initio prediction:Prodigal:002006"

/inference="similar to AA sequence:UniProtKB:P38491"

/codon_start=1

/transl_table=11

/product="putative protein YpfA"

/db_xref="COG:COG5581"

/translation="MFPKINEILYIQIASADEKEEYKEYKSRIADMDDSSFLIEVPMQ

QGSSRLKRLFFGDELSISYITEDGVRHYFNTYVTGFEEDVVRLVRIRKPLPSDISKIQ

RRSFLRVHANLELAIQSEDLTRAVGLTEDIGGGGLSIYGETGFSIAEGQKLKCWLLVP

YRNSTIEHVNFEAEVVRIKTLETGRQLCMLKFVQISDSERQKIIKFCFERQLDYRTK"

CDS 1490922..1491146

/locus_tag="EFAGFIKM_01319"

/inference="ab initio prediction:Prodigal:002006"

/codon_start=1

/transl_table=11

/product="hypothetical protein"

/translation="MNHRHATRRYDRLYYVFSRRMERKVTILITALIILLVVYQLLLL

VPGFKKYMTTIDRLEGTPIHVQTVNDYKRR"

CDS 1491250..1491954

/gene="cmk"

/locus_tag="EFAGFIKM_01320"

/EC_number="2.7.4.25"

/inference="ab initio prediction:Prodigal:002006"

/inference="similar to AA sequence:UniProtKB:P38493"

/codon_start=1

/transl_table=11

/product="Cytidylate kinase"

/db_xref="COG:COG0283"

/translation="MASQNTSENGKMNVAIDGPAGAGKSTVARLVAEALAYVYVDTGA

MYRAVTLHMLRKDISPEEVTQVLQEAQKLVIDLQPDPDGQKVFCNGEEVTSEIRSREV

TGIVSRYAQIEGLRTQLVDTQRQMALRKGVVMDGRDIGTTVLPDAEVKIFMTASVEER

ALRRFKELDPSEGLTLQQLERDIATRDRLDESREISPLRCAEDAIVLDTTEMNIHEVV

DKIVSYCTMVRGEIGL"

CDS 1491951..1492538

/gene="plsC"

/locus_tag="EFAGFIKM_01321"

/EC_number="2.3.1.-"

/inference="ab initio prediction:Prodigal:002006"

/inference="similar to AA sequence:UniProtKB:O07584"

/codon_start=1

/transl_table=11

/product="1-acyl-sn-glycerol-3-phosphate acyltransferase"

/db_xref="COG:COG0204"

/translation="MIYTFCSTLLRIIYTILFRLEAVGRENIPKEGGVLLCSNHISNF

DPPTVGIKLRRQVRFMAKSELFEIPVLGRIIKAVGAFPVKRGGVSKESIKTSLNILRD

GQVLGIFPSGSRHNDGGIGKKGAASFALRSGATVIPTAIIGNYKVFRKMKVVYGAPVS

LDEFKEDPSGEALEKATEKIMSKINEMVQTGVPSK"

CDS 1492681..1493895

/gene="ypfD"

/locus_tag="EFAGFIKM_01322"

/inference="ab initio prediction:Prodigal:002006"

/inference="similar to AA sequence:UniProtKB:P38494"

/codon_start=1

/transl_table=11

/product="30S ribosomal protein S1"

/db_xref="COG:COG0539"

/translation="MSEEMKNQEATQDELDQFVSLKKGDTVKGTIVKLEDNQAYVSIG

YKYDGVIPIRELSSLHVDSASDAVEVGQEVEAKVLSIDDEKEKLVLSKRAIDSENAWD

QLQKHFEDQDVFEVVVGDVVKGGLVADVGVRGFIPASMVERHFVEDFSDYKGRTLRVK

VKEIDRENNKVILSQKDVLEQEFEANKATVMAGLQEGQVIEGTVQRLTQFGAFVDVGG

VDGLVHVSELAWTHVEKPSDVLSEGDKVSVKVLKVDPEKGKISLSMKAVQPGPWETAS

EKFNSSDIVTGVVKRLVDFGAFVEIAPGVEGLVHISQISHKHIGTPHEVLKEGQEVQV

KILDMNPSEQRVSLSIKETEEAPAQPQKSERPSRNNAPREEINNPNVSLNNQGMSTTL

GELFGDKLSKFK"

CDS 1494081..1495403

/gene="der_1"

/locus_tag="EFAGFIKM_01323"

/inference="ab initio prediction:Prodigal:002006"

/inference="similar to AA sequence:UniProtKB:P50743"

/codon_start=1

/transl_table=11

/product="GTPase Der"

/db_xref="COG:COG1160"

/translation="MARPVVAIVGRPNVGKSTIFNRIIGDRLAIVEDKPGITRDRIYG

IGEWNGKPFSIIDTGGIEIDGEDVILKSIRMQAELAIEEADVIVFMCDAKAGITQSDE

EVAEMLYRSGKPIVVAVNKVDNIGRSELIYEFYGFGFGDPIGVSGSHGTGVGDLLDAI

VEKLPELEEETYDEDVIRVALIGRPNVGKSSLVNAILGEERVIVSDVAGTTRDAIDTP

FEKDGQRYVLIDTAGMRKRGKVYETTEKYSVMRAMRAIERADVVLIVINGEEGIIEQD

KHIAGYAFEAGKASLFVVNKWDVVEKHDKTMKEFERKIRDHFLFMTYAPVVFLSALTK

QRLQKLLPVVKRVADQHSLRVQTHLLNDVVSDAVAINPPPTDKGRRMRINYVTQVAVK

PPTMVVFVNDPELMHFSYERYLENKIRAAFDFEGTPIRIFTRKKSDES"

CDS 1495415..1496038

/gene="plsY"

/locus_tag="EFAGFIKM_01324"

/EC_number="2.3.1.275"

/inference="ab initio prediction:Prodigal:002006"

/inference="similar to AA sequence:UniProtKB:O66905"

/codon_start=1

/transl_table=11

/product="Glycerol-3-phosphate acyltransferase"

/db_xref="COG:COG0344"

/translation="MILQIAAIVLSYLLGSISFSVLLAKAIRGIDIRQHGSGNAGATN

TLRILGKGPAIAVLLLDVLKGVAAVWIGIWLNDGSAWIPALSGIAAIAGHNWPLYFHF

RGGKGVATAIGVLVSLAFLPALCAGVIAILSIVLTRYVSLGSLIFVAFTPIFILVLPG

YSMNIFWGSLIICLFAFWRHRTNIAKLAKGQENKLGSKNPGGGKRVV"

CDS 1496031..1497071

/gene="gpsA"

/locus_tag="EFAGFIKM_01325"

/EC_number="1.1.1.94"

/inference="ab initio prediction:Prodigal:002006"

/inference="similar to AA sequence:UniProtKB:Q5XE03"

/codon_start=1

/transl_table=11

/product="Glycerol-3-phosphate dehydrogenase [NAD(P)+]"

/translation="MSKKVAVLVAGSWGTALASVLAANQLDVMMWTRGEDQANEINNK

HTNTRYLPDAELSSRIQATTDMEAAVEGAIAVLIVAPSSAMRAVTNQLKAYYKPEMLV

IHATKGFETESLKRMSTVISEELECEEGRVVVLSGPSHAEEVVKRCPTTVVVASLDKA

SAESAQALFMNAYFRVYTNRDMIGVELAGAFKNIIALGAGMSDGLQFGDNAKAALLTR

GLAEITRIGVEMGANPLTFSGLAGIGDLVVTATSQHSRNWRAGSMLGQGQKLDDVLKS

MGMVVEGIRTTQAAYFISQKYGVQMPIADQLYHVLFQERQPRDAVEALMGRDPKTEME

VMKLETWEQWHS"

CDS 1497175..1497450

/locus_tag="EFAGFIKM_01326"

/inference="ab initio prediction:Prodigal:002006"

/codon_start=1

/transl_table=11

/product="hypothetical protein"

/translation="MGNNISKDALNAINKKTGKTITEGAVKKLASTVKPTTIQNEAQL

RQLIKQVSAMAKVPVSEDTVKDIVSAVKKSGLNPSSMESLMKMMMKK"

CDS 1497623..1497802

/locus_tag="EFAGFIKM_01327"

/inference="ab initio prediction:Prodigal:002006"

/codon_start=1

/transl_table=11

/product="hypothetical protein"

/translation="MSAMDKMWLSLVAILIMGLSVFLITFARAKTKGIVRGILSLIAF

LIMLIGFFGGIASLT"

CDS 1497886..1498503

/locus_tag="EFAGFIKM_01328"

/inference="ab initio prediction:Prodigal:002006"

/codon_start=1

/transl_table=11

/product="hypothetical protein"

/translation="MRPHDTEGRTERYPELHAALLETAKAWNTQPQTWLLGGSCGLLL

QGVELQQAPRDIDIYADVAAAKELHRSAPGKMLDEPTVDKTGPYASLLSHYQVGECAL

ELVGGFEIWARKSWYRIEIEYVLAPHAPVAQVGSYSLRLMPLAHELLFNLLRGREDRY

VPIASQMREQPDAHQPIMMLLSQQNVWTSRFRAEVEELVGFTWPQ"

CDS 1498520..1498870

/gene="nqrF"

/locus_tag="EFAGFIKM_01329"

/EC_number="7.2.1.1"

/inference="ab initio prediction:Prodigal:002006"

/inference="protein motif:HAMAP:MF_00430"

/codon_start=1

/transl_table=11

/product="Na(+)-translocating NADH-quinone reductase

subunit F"

/translation="MDYKVTFLPMNKSIALKPGATLLHAARRAGVKITTRCDGKAACL

MCKVNVDEEHRAELYPPTDAEKHKLGSLLEAGTRLSCQAKVCGSLSVHVPEDPLKAAI

RKQLERQQQEDDWF"

CDS 1498917..1499696

/locus_tag="EFAGFIKM_01330"

/inference="ab initio prediction:Prodigal:002006"

/codon_start=1

/transl_table=11

/product="hypothetical protein"

/translation="MIRNKPKCVEGSRRQRLKHQTGGLLLFLLFAFTASGCMYPSQSN

SDPKIAYRESVSRIQSAVEAFQQDEGILPIINADMETPKYEKFRVDLPKLKQLGYLDE

IPGTAFESGGSAYFLIQDEEVNPTVKVMDLLTVQKVNDVQRMVNQYKSVHNNKLPRGE

ELYPGLYAVDMEQAAQAGTPSITLNSVYSGQALPFMMDDEGKVYVDYAFDIMQAVEKS

DVSPAETEDVRDLLVEHSYFVPVKSVFYAWQNNAPVAQPIK"

CDS 1500150..1501628

/gene="spoIVA"

/locus_tag="EFAGFIKM_01331"

/EC_number="3.6.1.3"

/inference="ab initio prediction:Prodigal:002006"

/inference="similar to AA sequence:UniProtKB:P35149"

/codon_start=1

/transl_table=11

/product="Stage IV sporulation protein A"

/translation="MEKVDIFKDIAERTGGDIYLGVVGAVRTGKSTFIKRFMETIVLP

NITNEADRARAVDELPQSAAGKTIMTTEPKFVPNNAVQIKVAEGLDVNVRLVDCVGYA

VEGAKGYEDENGPRMISTPWFEEPIPFQEAAEIGTRKVIQEHSTLGVVVTTDGTIAEI

ARSSYVESEERVIAELKEVGKPFVLVINSTRPRSEEALQLRSELAAKYDIPVMTLSAA

TMTEDDVTGVLREVLYEFPVHEVNVNLPSWVMVLNETHWLRSNYENSVRDTVKDIRRL

RDVDRVVAQFMEYEFIDRAGLSGMNMGQGVAEIDLYAPDELYDQILVEVVGIEIRGKD

HLLQLMQEFSHAKREYDRFAEALEMVKTTGYGIAAPSLAEMALDEPELIRQGTKFGVR

LKATAPSIHMIRVDVESEFAPIIGTEKQSEELVRYLMQDFENDPIKVWDSDMFGRSLH

SIVREGIQGKIAMMPDNARYKLQETLGRIINEGSGGLIAIL"

CDS 1501846..1502943

/locus_tag="EFAGFIKM_01332"

/inference="ab initio prediction:Prodigal:002006"

/codon_start=1

/transl_table=11

/product="hypothetical protein"

/translation="MKRKETKWVWLSVLLVFTLALSACGIKKEPVATPASGASDDTPK

TEAVTGPLSGKRIALIMEFNTGTFSQQYVQGVKEEIEKFGGELTTFVADNDKAKMVSL

LDSAINQKFDAILTDHGDSLLEPGVKKAVEQNIPVVVFDAAISVPGATVLSQDDQKMA

ELTLEQMKKDINGQGNIVKVWVAGFAPMERRQIAYGEFMKANPDIKEIATFGSAQNPA

LDTQAKMEAILKQYPKGEITAVWTAWDEFAKGAARAIQQAGRDEIKVYGIDMSDEDLQ

MIQDPKNPWVASAAVDPTDIGRVQVRYAYQKLNGDETEDAVVLNPVYVQREALPDKQI

STSELSEFVEGWGGSTQGIKDWMSEYGITAK"

CDS 1503058..1504575

/gene="rbsA_1"

/locus_tag="EFAGFIKM_01333"

/EC_number="7.5.2.7"

/inference="ab initio prediction:Prodigal:002006"

/inference="similar to AA sequence:UniProtKB:P04983"

/codon_start=1

/transl_table=11

/product="Ribose import ATP-binding protein RbsA"

/db_xref="COG:COG1129"

/translation="MSTAPTLLQMEHIHKQFSGIPALKDVDFSVKGGEIHALLGANGA

GKSTLMKILSGAYPLDQGTIQLSGQALHLNSPGDAKASGIHCVYQEVDAALVPQLTAA

ENIMLDQLASSAGGWWKSPRKLQQRAVEALKQLGADISVHQKVADLTLAEKQMILLAR

ILIQDAKVIIFDEPTAPLSQEETDAFFRIVHLLKDRGVACIFITHRLAEVTVHCDRVT

VMRDGQHVFTGEAKGLTINDLVTQMLGKPFEEEFPKTEAPLGELLLEARGLRRGVKVK

GVNLSVSRGEVLAVVGLVGAGKTESSRLLIGADRLEGGEIRLNNRNLRLSQPADAAAL

GIVSVPEERRKQGILIQENVERNLSLPLLSRLSTLGFVSRKRERLNAESLVKQLGIKT

SSVKQEVKYLSGGNQQKVAIGKWLNADADVFIFDEPTKGVDIGAKSDIFRIINELALA

GKGVIYFTCELDEGMGIGDRIAVMCEGVIVKEFKRGETNQEQLLYYASGGQEVQS"

CDS 1504572..1505558

/gene="rbsC_1"

/locus_tag="EFAGFIKM_01334"

/inference="ab initio prediction:Prodigal:002006"

/inference="similar to AA sequence:UniProtKB:P0AGI1"

/codon_start=1

/transl_table=11

/product="Ribose import permease protein RbsC"

/db_xref="COG:COG1172"

/translation="MKDKSLDFAFRYGAIIVIIGVIAFFGIKLPYFFTYSNLTDILGS

ISIVTFVAIGVTLSLIVDGFDLSVGATVSLTTVVTASLMIWYQQPLAIVIIVPLIIGA

VIGLLNALLIVKLRIPDLLATLATMYIIGGIHKTYAQGYTIYNHMQFPDGSKAAGEMD

PTFLLIGQGKWLGMPISVIMLLIAVIGVHIFLTYTKYGRQMYITGGNEEAARLSGIKV

KKVRTLAYVAAGVFAAIGGIIYASKVGSGQIDAGSPLLMESVAAVFVGFSVFGAGKPN

VIGTFIGSVLIGVLVNGLTMMNVQYFTHDIVKGGVLVLALAVTFYVLNRNRT"

CDS 1505665..1506048

/gene="ihfA"

/locus_tag="EFAGFIKM_01335"

/inference="ab initio prediction:Prodigal:002006"

/inference="protein motif:HAMAP:MF_00380"

/codon_start=1

/transl_table=11

/product="Integration host factor subunit alpha"

/translation="MFSAQDVRRFFAYERIFRYKTETQYNIQDIDTGGEKQMNKSDLI

THVSEATELSKKDVTKAVDAVFEAISEALQSGDKVQLVGFGNFEVRERSARKGRNPQT

GEEIEIPASKIPAFKPGKALKDGIK"

CDS 1506218..1506439

/gene="mtrB"

/locus_tag="EFAGFIKM_01336"

/inference="ab initio prediction:Prodigal:002006"

/inference="similar to AA sequence:UniProtKB:Q9X6J6"

/codon_start=1

/transl_table=11

/product="Transcription attenuation protein MtrB"

/translation="MDHPTGNDYIVIKAEENGVQVIGLTRGQDTRFHHTEKLDKGEVM

FAQFTTHTSAIKIRGKATLITKHGQIESE"

CDS 1506561..1507112

/locus_tag="EFAGFIKM_01337"

/inference="ab initio prediction:Prodigal:002006"

/codon_start=1

/transl_table=11

/product="hypothetical protein"

/translation="MKPRMIVTASMLATVAAVFLLVAIQTLHMRTWGGDNVQPASASH

HPVQLTNDNLVDVLSSMQLSTPIARVEWKQSILTLDLKVKGTGTSYTEIYENMAAVAD

LGFRSLDNVDQVLLRVMAEDEWMHKRHLLLAADIRRGEWPLYAIETLRNWKSAAFSDE

LKDWFHLMETELWKKQFEMTSQG"

CDS 1507214..1508077

/locus_tag="EFAGFIKM_01338"

/inference="ab initio prediction:Prodigal:002006"

/codon_start=1

/transl_table=11

/product="hypothetical protein"

/translation="MNSYRVPQLAKKYTDYDMIRQHTEIPSFPDSRARLLQIFVGRTD

EKVHQELYALATSLVQLAMDTHDRIDTISGERREQEMRSRQLNVLAGDYLSSRFYQLL

AQAGRIEMIGKLSGAVSGVNARKMTLYERMKKLLVSADEYLRETVQLRMQLFLSFTGM

IQHNEESLWNSLLTEFSSCETIVEELKRMNDAKQYLHSYAYWHIYEHGNDDERNVLRQ

SEPDARAWNAMVLKHRVGEVLLDKLRECTHRIQLLLQDEEGRMGLHEIHAILEPYLAY

LQPSHAAVRED"

CDS 1508093..1508821

/gene="menG"

/locus_tag="EFAGFIKM_01339"

/EC_number="2.1.1.163"

/inference="ab initio prediction:Prodigal:002006"

/inference="similar to AA sequence:UniProtKB:P67062"

/codon_start=1

/transl_table=11

/product="Demethylmenaquinone methyltransferase"

/translation="MGSGETKPKEEYVHSVFQSIAGKYDVMNDILSFRRHKAWRKFTM

KKMNMSKGDTGLDLCCGTCDWTLAMAEASETGHMHGLDFSSNMLEVGQTKINAVQRQK

QITLTQGNAMSLPFEDNSFDYVTIGFGLRNVPDLRQVLSEMKRVVKPGGMVVCLELSK

PTWQPFKGIYYFYFEKVLPNLAKVFAKSFEQYKWLPDSLAIFPGRQELADIFAETGLQ

QVQAYPLTGGIAALHIGTKENQHV"

CDS 1508814..1509680

/gene="ubiA"

/locus_tag="EFAGFIKM_01340"

/EC_number="2.5.1.39"

/inference="ab initio prediction:Prodigal:002006"

/inference="similar to AA sequence:UniProtKB:P0AGK1"

/codon_start=1

/transl_table=11

/product="4-hydroxybenzoate octaprenyltransferase"

/db_xref="COG:COG0382"

/translation="MFRKIRIFLEMIKIEHTLFALPFAFMGAILGSMVVNDTFPSWMQ

IMWVLLAMVGARSAAFGLNRIIDQAIDGKNPRTAMRAIPAGLLKNGEVVIFVIISFIL

LFWASSNLNVLSMQLLPIAVFMLVLYSYTKRFTWLCHIVLGMTIGLAPLGGWVAVTGT

MDWTAIVLYVTIVFWTAGFDIIYACQDLEFDQGEGLHSIPSRFGLVKSLQIAKFFHVI

TAIGFLALLLMTDLSWWYGAGMLVTYGILFYQHYIVSPNDMSRVQTAFFTMNSLLSLI

VFTFTLIDLAVK"

CDS 1509681..1510301

/gene="ubiX"

/locus_tag="EFAGFIKM_01341"

/EC_number="2.5.1.129"

/inference="ab initio prediction:Prodigal:002006"

/inference="similar to AA sequence:UniProtKB:Q9HX08"

/codon_start=1

/transl_table=11

/product="Flavin prenyltransferase UbiX"

/db_xref="COG:COG0163"

/translation="MVQQPDNKRLVVGITGASGSIYGIRLIETLLDLEYNVHLVISNA

GWRVLKEELDWDVTNRDAVLEEKFGNRAGSLIYHPVSDIGASIASGSYLADGMIIMPC

SMGTLSSIAQGSSDNLMSRAADVMMKEGRTLILVPRETPLHAIHLENMLKLSRLGVRM

IPAMPAFYYKPQTMDELILFLVGKVLDSLRIPHQLFTRWGEPDERG"

CDS 1510645..1511619

/gene="hepT"

/locus_tag="EFAGFIKM_01342"

/EC_number="2.5.1.30"

/inference="ab initio prediction:Prodigal:002006"

/inference="similar to AA sequence:UniProtKB:P31114"

/codon_start=1

/transl_table=11

/product="Heptaprenyl diphosphate synthase component 2"

/db_xref="COG:COG0142"

/translation="MKLLDIFGLLKKDMDYIEKELYRSVRGEQKLLSETSLHLLKAGG

KRLRPVFVLLGGKFGTYDIERLKLVAVPLELIHSASLVHDDVIDNAETRRGKPTVKSK

WDNRIAMYTGDYIYGKALEMTAGLSDPAIHRILAKAMVQMSIGEMEQIRDFFNTGQSV

RNYLLRIRRKTALLIAVSCQLGALATRAPEHVSSLLYTYGYNVGMAFQIQDDVLDLVG

TEKQLGKPPGSDMKQGNITLPVLYALEESDLREPLLKEISRVQHEEGRASASDAIGMI

RQSQGIAKAEALADRYMKKALDALDQLPNIKTTKNLRDIAHFVVKRTH"

CDS 1511774..1512217

/gene="ndk"

/locus_tag="EFAGFIKM_01343"

/EC_number="2.7.4.6"

/inference="ab initio prediction:Prodigal:002006"

/inference="similar to AA sequence:UniProtKB:P31103"

/codon_start=1

/transl_table=11

/product="Nucleoside diphosphate kinase"

/db_xref="COG:COG0105"

/translation="MDRTFLMVKPDGVQRGLIGRIISRLEDKGFKLVAGKLVQMSEDQ

AKRHYAEHEGKPFFDDLVRFITSGPVFAMVWEGDDIVALARIVIGKTNVKEAAPGTIR

GDFASHTPHNLIHGADSPESASREAANFFASDELVVYDKSIAAWL"

CDS 1512243..1513037

/gene="cheR_1"

/locus_tag="EFAGFIKM_01344"

/EC_number="2.1.1.80"

/inference="ab initio prediction:Prodigal:002006"

/inference="similar to AA sequence:UniProtKB:P31105"

/codon_start=1

/transl_table=11

/product="Chemotaxis protein methyltransferase"

/translation="MLEQEQLLDPDYTGFIRKIKESTGIDLAQYKEGQMKRRLTTLRN

KNGFHTFSNFFEAMQKDKSLFYEFLDRMTINVSEFWRNPNRWEVLRDEILPELLGSKR

RAKVWSAACSTGEEPYTLAMILDTMGILKDSSITASDLDEGALAKAKEGRYMERSLKD

VPKETANRYFKQDGLVYRIDEQLKNSIKFMKQNLLVDRFDDGYDLIVCRNVMIYFTEE

AKNLLYHKFAASLRPGGILFVGSTEQIFSPGQYGLETAETFFYRKK"

CDS 1513216..1514385

/gene="aroC"

/locus_tag="EFAGFIKM_01345"

/EC_number="4.2.3.5"

/inference="ab initio prediction:Prodigal:002006"

/inference="similar to AA sequence:UniProtKB:P31104"

/codon_start=1

/transl_table=11

/product="Chorismate synthase"

/db_xref="COG:COG0082"

/translation="MSLRYLTAGETHGPQLTAIIEGLPSNLTIDFEELNFQLHRRQKG

YGRGRRMQIEKDQANFVGGIRHGYTTGAPVALVVQNNDWKHWQNIMNIEPIEGSDEEK

RRVHRPRPGHADLNGGLKYNLKDLRNVLERSSARETTVRVACGAIARQFLAEFGIKVA

GRVLRIGEIEAPYQDLPIDELIAVTEASSVRVTDAETEKKMEAYIDQIKQEGDSIGGI

VECIVEGVPIGLGSYVQYDRKLDARIAQGVMSINAFKGVEIGIGFEAGVIRGSQVHDE

IMHTDERGYHRATNRLGGFEGGMTNGMPVVVRGVMKPIPTLYKPLQSVDIDTKEAFTA

QVERSDACAVPAASVVMEHVVAWEIAKAFLEKFGGDSMEEIRANVASYNAQLENY"

CDS 1514387..1515481

/gene="aroB"

/locus_tag="EFAGFIKM_01346"

/EC_number="4.2.3.4"

/inference="ab initio prediction:Prodigal:002006"

/inference="similar to AA sequence:UniProtKB:Q9KNV2"

/codon_start=1

/transl_table=11

/product="3-dehydroquinate synthase"

/db_xref="COG:COG0337"

/translation="MRQLTVQLEERSYPILIGSGLLAQAPQYFEQYGLTKKSPLLIIT

DENVAPKYLSGLEQTLRTAGYTVVSAVVPSGETSKSLSVYQDMMTVAIEGKLDRSSAI

LALGGGVVGDLAGFVAATYMRGIKFVQVPTTILAHDSSVGGKVAVNHPLAKNMIGAFH

QPELVLYDVDTLQSLPPRDVSAGLSEMLKHGLIRDEAFAHWCEEHAEELLALNPEALG

YGLERGCGIKAEIVSRDERENGERALLNLGHTIGHAIEAIAGYGEFLHGEAISIGMAG

SALLGEKLGAPAGLYEDTVRMLRSLRLPTTMPAHLDTDALMEAMMHDKKFREGHMVFI

IPDRIGAARIVKDVPVSAVRDVIEMLRKGE"

CDS 1515487..1515864

/gene="aroH"

/locus_tag="EFAGFIKM_01347"

/EC_number="5.4.99.5"

/inference="ab initio prediction:Prodigal:002006"

/inference="similar to AA sequence:UniProtKB:P19080"

/codon_start=1

/transl_table=11

/product="Chorismate mutase AroH"

/db_xref="COG:COG4401"

/translation="MVTRGIRGATTVTQNNEEQILKETAVLLQEIVDRNEIQPEDICS

VWITLTGDLDAAFPAKAIRQLDGWELVPLMCALEVPVKGALAQCIRFMVHVNTNKAQN

EINHVYLNGAQALRPDLATPSGS"

CDS 1516368..1517936

/gene="trpE"

/locus_tag="EFAGFIKM_01348"

/EC_number="4.1.3.27"

/inference="ab initio prediction:Prodigal:002006"

/inference="similar to AA sequence:UniProtKB:P20580"

/codon_start=1

/transl_table=11

/product="Anthranilate synthase component 1"

/db_xref="COG:COG0147"

/translation="MITPNVNQVLKMSNDYNLIPVVKRILADMETPIRIFRRYADNDR

AFLLESVEGGIQWARYSFIGTDPFLMISAKKGRIVVEEAGKTRELPGKPIEELKALLR

KYRSPKDDELPPFTGGAIGFFGYDLLSYYEKLPAHALDDLNMDDIRFMFCDQIIVFDH

VKQQMLLVGNVHVKDGATDDEIRQAYAVTSEKLEQAAERLQQQGPGENLNPRSIPGDV

ELGDIRSNLTKEQFIGNVEQAKDYIRAGDIFQVVLSQRFHIDTEVSPLHVYRVLRTLN

PSPYMYYLKMDDEIIVGTSPEALVKVDGNRVETRPIAGTRPRGATEAEDRALAADLLQ
DEKERAEHLMLVDLGRNDLGRVSSFGSVKCDMFMEIERYSHVMHMVSNVTGELREDKD
FFDAFLSCLPAGTVSGAPKLRAMEIIAELEKEARGAYAGAIGYLGFSGNMDSCITIRT
IIFKKGKAYVQAGAGIVWDSVPENEYEETVNKAKALLKAIRTAEAMFPAKEKDSTLRL
ANADYFVTPAAAKN"

CDS 1517954..1519045

/gene="trpD_1"

/locus_tag="EFAGFIKM_01349"

/EC_number="2.4.2.18"

/inference="ab initio prediction:Prodigal:002006"

/inference="similar to AA sequence:UniProtKB:P00500"

/codon_start=1

/transl_table=11

/product="Anthranilate phosphoribosyltransferase"

/db_xref="COG:COG0547"

/translation="MNINPIMNENPVTPIAEITREEGMKNGLAKILEGSHLEQAEARD
LMYSIMRGEATPAQIGGLLMALRMKGETVDEITGFAEAMRGQGGRILTDGSGLLDTCG
TGGSGIHKFNISTASAIIASAVSVRVAKHGNRSASGKAGSADVLEALGVNIHLNGEQA
RQCLDDIGICFCFAQVYHPSMRHAAGPRKELGVRTIFNMLGPLTNPAGADRQLLGLYD
RSRTPMIAEVLNRLGLKRALVVASHDGLDEVSISAPTQVSELRNGEVHTYDIDPRDMG
LSLHPLEAVLGGDAAQNAEIIKRIFQGEQSAYRDVVLLNAGACIYVSGLADSIAEGVT
LAAEAVDSGKAAGKLEQLIHTTEAYSHVS"

CDS 1519035..1519823

/gene="trpC"

/locus_tag="EFAGFIKM_01350"

/EC_number="4.1.1.48"

/inference="ab initio prediction:Prodigal:002006"

/inference="similar to AA sequence:UniProtKB:Q2YRR4"

/codon_start=1

/transl_table=11

/product="Indole-3-glycerol phosphate synthase"

/translation="MYLDRIVATKHKEVEVLAQTFRMDEAIQKIEQLPATRGFEQALS

SGRNRKLGLIAEVKKASPSKGLIRPDFHPVEIAEAYERAGADCISVLTDVSYFQGSNE

YLQAIHQAVNIPLLRKDFIIDERQIAEARLLGADAVLLIASILTPEQIRQYLEFAKSL

GLDALIEVHDRTELEQVLEIPQATLVGINNRNLKTFETSLNTTLDLMELIPSGVTLIS

ESGIDGPESTVPLIQAGVHGILVGEHLMRKDDVEAAVYDLMGPK"

CDS 1519820..1520560

/gene="trpF"

/locus_tag="EFAGFIKM_01351"

/EC_number="5.3.1.24"

/inference="ab initio prediction:Prodigal:002006"

/inference="similar to AA sequence:UniProtKB:Q56320"

/codon_start=1

/transl_table=11

/product="N-(5'-phosphoribosyl)anthranilate isomerase"

/db_xref="COG:COG0135"

/translation="MMNTSIHDGTGDSMSELRNPLPAAVKICGLQDVEVLKSMINLPV

DYIGVVFAKSRRRIEPEQAAALRTVLFEWSTYDRPKLAGVFVNPTLEELEHIMDIAQL

DVIQLHGQETAEFCQQVKQRWNAKVFKVFSFPKDETGPEADDAAIRALDTYDKFVDAI

LLDTHDPLYGGGSGKTFAWERIPAYAEWAKSREIALFVAGGLQPDNVQQLIQTYAPFG

VDVSSGVETEGVKDIAKITAFVERVKQA"

CDS 1520557..1521756

/gene="trpB"

/locus_tag="EFAGFIKM_01352"

/EC_number="4.2.1.20"

/inference="ab initio prediction:Prodigal:002006"

/inference="similar to AA sequence:UniProtKB:Q81TL8"

/codon_start=1

/transl_table=11

/product="Tryptophan synthase beta chain"

/db_xref="COG:COG0133"

/translation="MTHQLPDQHGRFGHFGGRFVPETLMNALIELEEAYSHFSEDEEF

NKELNYLLSEYSGRETPLYHAEQLSRRLGGPKIYLKREDLNHTGAHKINNAIGQGLLA

KRMGKKKVIAETGAGQHGVATATVAALLGLECKVFMGEEDTERQQLNVFRMKLLGAEV

IPVTSGTRTLKDAGNEALRYWVSNVEDTFYVLGSVVGPHPYPMMVRNFQRVIGDETRR

QIQEIEGRLPDVIVAAVGGGSNAIGMFYPFIGDQDVKLVGVEAAGKGVETEYHAATMT

KGTHGVFQGSMSYLLQDEYGQVQPAHSISAGLDYPGVGPEHSYLKDIERAKYVPITDQ

EALDALQLLCRTEGIIPALESAHAVAQVVKLAPELTADDIVVICLSGRGDKDVESIMK

YTGGDLG"

CDS 1521753..1522559

/gene="trpA"

/locus_tag="EFAGFIKM_01353"

/EC_number="4.2.1.20"

/inference="ab initio prediction:Prodigal:002006"

/inference="similar to AA sequence:UniProtKB:O67502"

/codon_start=1

/transl_table=11

/product="Tryptophan synthase alpha chain"

/db_xref="COG:COG0159"

/translation="MNLMDQTFQQLKEQNRTALIPFLTVGDPDVDTTIDIIKELEQAG

ADILELGVPYSDPLADGPVIQRASERALKSQITIRTVMETAAKARKAGVKLPFVLFTY

YNPVLQTGLDVFFDELVKHDISGMIIPDLPIEEAEEMRERANRAGVHLVPLVAPTSNA

RIERIVTGARGFIYCVSSLGVTGERASFFDGVESFIETVKSLTDLPVAVGFGISSHEQ

VAHFSRICDGVVVGSAIVRQVEDAIPLLENPDTRAAGLLQIRNFVAQLKG"

CDS 1522646..1523746

/gene="hisC_2"

/locus_tag="EFAGFIKM_01354"

/EC_number="2.6.1.9"

/inference="ab initio prediction:Prodigal:002006"

/inference="similar to AA sequence:UniProtKB:Q92A83"

/codon_start=1

/transl_table=11

/product="Histidinol-phosphate aminotransferase"

/db_xref="COG:COG0079"

/translation="MKPKSQIVNLPVYQPGKPIEEVKRELGLEQVIKLASNENPYGSS

PAALEAITKEMTNISIYPDGSSVELTGVLAKHLGVERNNLIFGCGSDEIIALITRAFF

LPGDETIMADQTFSVYKSNADIEGAVSIEVPLKDGTHDLSAMLAQINDKTKAVWVCNP

NNPTGTIISEQELTAFMDCVPAHVMVILDEAYYEFVTDEAYPQSIPLIERYPNLVILR

TFSKIYGLASLRIGYGIARPEIIDLINRVREPFNTSRFGQVAAKAALLDQDFVQECSK

RNATDRDYLQNEFTRLGLSYFPSQGNFIMVDLNMPSAIAFQSLLKQGIIVRSGFHVYP

TYIRVSVGTSEQNRAFVTALENTLAEKAVARS"

CDS 1523773..1524867

/locus_tag="EFAGFIKM_01355"

/inference="ab initio prediction:Prodigal:002006"

/codon_start=1

/transl_table=11

/product="hypothetical protein"

/translation="MKLKIAMIGVGLIGGSLALCFKGKPDVTVMGYAHLDELKDKYIA

SGVVDDATLSLEEAVEDADFIFLCVPVGLLESYFEKLSKLPLKKGCIITDVGSTKASI

AACAEHVRLTDAYFIGGHPMAGSERAGVDAASAVLFENAYYVLTPSEHVPEEAYSRLS

ELLAYTRAQIVRVEPLLHDDIVGAISHLPHVIAVALVNQVREYNESNPLYKMLAAGGF

RDITRIASSDAIVWRDILLSNRDVLLGLLKDWNSQMTAFTDMLEHKNGEGIEEAFRQA

REFRSILPERRKGMISPLFDLYTDVQDAPGMIGKIATELGANDINLSNLEIIENRVDV

PGIMRLSFRQEEDMERAKTLLDSLGYQVWI"

CDS 1525594..1526187

/gene="algU_1"

/locus_tag="EFAGFIKM_01356"

/inference="ab initio prediction:Prodigal:002006"

/inference="similar to AA sequence:UniProtKB:Q06198"

/codon_start=1

/transl_table=11

/product="RNA polymerase sigma-H factor"

/db_xref="COG:COG1595"

/translation="MTDSQLIREIKEGNLELYSELMSRYQRKILAFVYHMLKSSNMEL

LAEDLCSETFYKAYRSLHSFREVDASFSTWLYTIARNTVLSELRKQRSGNIPLEESGF

VPVAPSENAPEYAVLRSERVMLVRDAINNLPEKQRSAIILREYDQLDYQEIANILGQS

VSSVKSLLFRARSSVKTQLEPYFFEPVYEPYEGMKNR"

CDS 1526184..1526819

/locus_tag="EFAGFIKM_01357"

/inference="ab initio prediction:Prodigal:002006"

/codon_start=1

/transl_table=11

/product="hypothetical protein"

/translation="MNCNEAQELFALVWDLPETHPQRIAFHAHLAGCEECSEQFEVWE

EAQILLHSIPVPVTEQQAERVNRNVMDRIYAESPWLLPEEVKVNRFSAVIRKHMSLWI

AAFLAIFLCSFLYMAMFKPDVSEAEQTKVVTTGILETGIAGSGPSSSGMYQYNMTGAD

RGSIIEPFVVSMGPAYPQYWMALSLLAIGMALFSLGRMHRTTNKRKQGASA"

CDS 1526863..1527462

/gene="gpmA_1"

/locus_tag="EFAGFIKM_01358"

/EC_number="5.4.2.11"

/inference="ab initio prediction:Prodigal:002006"

/inference="protein motif:HAMAP:MF_01039"

/codon_start=1

/transl_table=11

/product="2,3-bisphosphoglycerate-dependent

phosphoglycerate mutase"

/translation="MRIGLIRHGLTDWNAAGRIQGQTDIPLNGEGREQAERLGRRLLT

EEYQWDYIITSGLSRAQETGEIISKLLNVPLLEPDARLKERAFGQIEGLTSEERVARW

GQAWETLDLGQEQIADIQIRALAFLEDLWSAYPDQNVLIVTHGAFLANLLTALFKDRY

TERIGNLSLTILEKERDDWSPLLYNCTRHLSLDTAKQPE"

CDS 1527697..1528815

/locus_tag="EFAGFIKM_01359"

/inference="ab initio prediction:Prodigal:002006"

/codon_start=1

/transl_table=11

/product="hypothetical protein"

/translation="MNLADMLTFADIGQLNRIADYYQCECKPNSKHELIQSILTTLGS

RPFFEQQVRQLNQADQRFLNNLLFDPRQYYGMEELIAIARQSMDKKESEAGASPRDLI

VRFNKSGWLFNGTTQQTRYLYQVPQDLKLRFRDVLSTHLRAGITKTTEPPMYRDEYHL

LADDLKLFLQFVQQHEIALNAEGMMYRRSQQQIMEHLHISEELVGKGGWRFGYGRSFK
DYPDRFALLYDYAFHHRLVREEQGTLKLSPAGEDKLGAEKTAEMIQLFRFWLRLYKGA
IPNLSSIVYWTGECAQEWSTAESLFRQIGPFIQPFYYDTAEAIYDNRIVKMMLHLGMV
RIGEHEEGRTIQMTAWGTRLAASCRSISGDITPIMFDK"

CDS 1528860..1529366

/gene="yrdA"

/locus_tag="EFAGFIKM_01360"

/inference="ab initio prediction:Prodigal:002006"

/inference="similar to AA sequence:UniProtKB:P0A9W9"

/codon_start=1

/transl_table=11

/product="Protein YrdA"

/db_xref="COG:COG0663"

/translation="MIIPYKGLQPQLHPSVYMAEGAKLIGDLRMGEESSVWFNAVLRA
DLAPIIIGKRCNIQDNVVGHVNTDQPLIVGDDVSVGHTAIIHGCCIGTGSLIGMGAIL
LNGVDIGEYTLIGAGSVVTENSKIPPYTLALGTPAKVIRELTDADLERMSRTSLGYVT
KGKEYRSS"

CDS 1529390..1529569

/locus_tag="EFAGFIKM_01361"

/inference="ab initio prediction:Prodigal:002006"

/codon_start=1

/transl_table=11

/product="hypothetical protein"

/translation="MDKMKVTYEVMLGLAAEMVWDEALRKQRSEKLYLEIDKALATGD
EVAFRSLTDELRTIN"

CDS 1529723..1530145

/locus_tag="EFAGFIKM_01362"

/inference="ab initio prediction:Prodigal:002006"

/codon_start=1

/transl_table=11

/product="hypothetical protein"

/translation="MKFSEMTQDSWAELQLYLDTCLIPYTALSGEQSPVEATEALERL

RDFLDLVEIPFKGRIMTYPAFHYVNSELSMTLNSLSAQLKSSGFKYVVIMSSDGHLDG

VEIPAADLVLSRSVLTHEVGEEGIARFIGEKIRELWKK"

CDS 1530338..1530880

/gene="petC_1"

/locus_tag="EFAGFIKM_01363"

/EC_number="7.1.1.6"

/inference="ab initio prediction:Prodigal:002006"

/inference="protein motif:HAMAP:MF_01335"

/codon_start=1

/transl_table=11

/product="Cytochrome b6-f complex iron-sulfur subunit"

/translation="MSSEHDHHEASLKLPSRMEMSRRQFLTYTLGGATAYMAAGAILP

MVRFAVDPILQHKGEGTSVKVAEISKITNEPQEFTFELKQQDGWYLSNASLVAWIRKD

EQGKIYALSPICKHLGCTVGWNSDKQYPDEYHCPCHGAHYDKEGKNLAVAPKPLDEYV

VKEEQGWVYLGDIVPNTRVN"

CDS 1530896..1531567

/gene="qcrB"

/locus_tag="EFAGFIKM_01364"

/inference="ab initio prediction:Prodigal:002006"

/inference="similar to AA sequence:UniProtKB:P46912"

/codon_start=1

/transl_table=11

/product="Menaquinol-cytochrome c reductase cytochrome b

subunit"

/db_xref="COG:COG1290"

/translation="MFKNVYDWIDERLDITPIWRDVADHEVPEHVNPAHHFSAFVYCF

GGLTFFITVIQILSGMFLTMYYVPDIINAYASVEYLQTKVAFGQIVRGMHHWGASLVI

VMMFLHTMRVFFTGSYKAPREMNWVVGMLIFFVMLGLGLTGYLLPWDNKAYFATKVTL

EIANTVPWLGPIIKEFLQGGTIVGAQTLTRFFALHVFFLPAVLLVLLVGHFIMIRRQG

ISGPL"

CDS 1531589..1532473

/gene="petD"

/locus_tag="EFAGFIKM_01365"

/inference="ab initio prediction:Prodigal:002006"

/inference="protein motif:HAMAP:MF_01344"

/codon_start=1

/transl_table=11

/product="Cytochrome b6-f complex subunit 4"

/translation="MAHGHKKDDEEKVIFVGDSRVRKGAGFITPPDYTAYPGKSEAFI

PNFLLKEWMVGVVVLVGILVLTISEPAPLGYPANPSASVIPMPDWYFLFLYQYLKYPY

ASGDYVLLGVLGVSGVAFGALLLAPFLDTGKERRFYKRPIASSLMILSVISVFYLTNV

AWTHYEHELEASGQKPEHIQREEEALERREQGLPPVSNAPGQQEEVAIVEQDDPAMET

YKKAGCIGCHAADMKGASGPSLRGVGDKHSQEEILTIIKDGYNQMPPQYNNAIAQGVT

DEEITHLTEWLAKQKAEQ"

CDS 1532525..1533211

/locus_tag="EFAGFIKM_01366"

/inference="ab initio prediction:Prodigal:002006"

/codon_start=1

/transl_table=11

/product="hypothetical protein"

/translation="MFFWTALYRAQYMMKEGAGNVALSYFWSREFLTNRYFLWLLFWC

NAVGTVYGYIWYGEQMKLTLAEQPVWQIVFVPDSPTASLFFTLALLWVLYPPRSIIIK

RFGHVIQALAVVTSVKYGVWAVSIIFAGWMQGGTQHWQDWMLIASHSAMAIEALIFVR

FFGFRWTSLVVAGLWTLLNDTMDYTYDIYPWLPASLYDHVDGVRNFTFGLTLVSILCA

WLALRQAKRT"

CDS 1533278..1534147

/locus_tag="EFAGFIKM_01367"

/inference="ab initio prediction:Prodigal:002006"

/codon_start=1

/transl_table=11

/product="hypothetical protein"

/translation="MKRTFGIKTGLLVVSFIALLLWTNLAYRVSAQSEALNSNSDQQV

STSNSIAKLNEEAAILYRQALENNIEEVRGSILRISKSLEHISFEGQTTVEGIHALSE

TIVEVKQAVVKVKNDDASLQQSSAKLRLAADSLANPTKPLWLQYYKIVKNDLDALSAA

TNQGQTAAVLANRYTVLEEHYETIRPAALIRREPYEIAQMDAWLSHTKGLTNAKQPDL

AQLKSMVGHGEELVNQLFGREKDESAFVPFVQGPDRRAAGLLISSVIVATLSYAGYRK

YRAQQQGIFPFRR"

CDS complement(1534268..1535137)

/locus_tag="EFAGFIKM_01368"

/inference="ab initio prediction:Prodigal:002006"

/codon_start=1

/transl_table=11

/product="hypothetical protein"

/translation="MSTAKTWIQVKLVLPIVLGTALYAFGLLYFIIPNQLMEGGLTGV

TVLINYAFGISPSLTTLILNVPLFLIGLKILGGRQMIYTGIGIGALTVFLWLFEKLIH

LGWIEPLHTENDLLLAALYAGVTLGAGLGIVFRWGGTTGGSDIIARILNRKYGWSMGR

VLLGIDFIIIGLSLIYIPKEKILYTLVAVFIASKVIDFIQEGAYSARAFMIISDHAPE

IADQITRDMDRGVTLIPAIGAYSKQAKHMAYCVISRQEFRRLQTIVRSIDPRAFVIIS

DVHDVHGEGFKES"

CDS 1535309..1535638

/gene="ypjD"

/locus_tag="EFAGFIKM_01369"

/inference="ab initio prediction:Prodigal:002006"

/inference="similar to AA sequence:UniProtKB:P42979"

/codon_start=1

/transl_table=11

/product="putative protein YpjD"

/db_xref="COG:COG1694"

/translation="MEKSIAEMQREVDQYISQFKEGYFSPLAMLARMSEEVGELAREV

NHEFGEKPKKSSEAANSIELELGDILFITICFANSLGIDLAEAHDKVMHKFNTRDANR

WTPKNTD"

CDS 1535689..1536213

/locus_tag="EFAGFIKM_01370"

/inference="ab initio prediction:Prodigal:002006"

/codon_start=1

/transl_table=11

/product="hypothetical protein"

/translation="MMKPEEYMQQAYRCILQNDFEQAIRWFESAIHAHPKHAELYYRC

SITHARSKHLVPALEYARKSVELSPGTEEYILHLQTLESKQLTSRAKLLLEQAGIATQ

ERYVEASTLLQEAVKLDPLSVEAHVMLALAYSDLNEFDYAIQALREAILLDPQNGQLH

QMLQEIKQRMKSIQ"

CDS 1536294..1537097

/gene="dapB"

/locus_tag="EFAGFIKM_01371"

/EC_number="1.17.1.8"

/inference="ab initio prediction:Prodigal:002006"

/inference="similar to AA sequence:UniProtKB:P63895"

/codon_start=1

/transl_table=11

/product="4-hydroxy-tetrahydrodipicolinate reductase"

/db_xref="COG:COG0289"

/translation="MSEVIRVAVIGAAGRMGREVVKLVLQDPELELAAAVNRSGAGTD

AGTLVGLPECGVLVTDDIEMAFAETKPQVMVDFTVPQFAFAHTEIAIRHGVRPVMGVT

GFTPEQIEQLDKQCQDKGIGGLIAPNFSIGAILMMRFAAQAAKHMPNVEIIEYHGDQK

LDAPSGTAIKTAELIAANREELRQGNPNEEETIEGSRGGYYNGFRIHSVRLPGVFAQQ

EVVFGDYGQSLKIRHDSYERAGYMPGVKIGVQKVMEYTGMIYGFDHFID"

CDS 1537109..1537549

/gene="mgsA"

/locus_tag="EFAGFIKM_01372"

/EC_number="4.2.3.3"

/inference="ab initio prediction:Prodigal:002006"

/inference="similar to AA sequence:UniProtKB:P42980"

/codon_start=1

/transl_table=11

/product="Methylglyoxal synthase"

/db_xref="COG:COG1803"

/translation="MLKIAFIAHDRKKEEMVNFVTAYEPVFTDHQLYSTGTTGLRIME

GTSLKIHRFESGPLGGDQQIGALVAQNEMDLIIFLRDPLMAQPHEPDINALLRLCDVQ

GIPLATNIATAEILVKALDRGDFAWRELVHKYKPEAGLNSGDSE"

CDS 1537546..1538244

/gene="bshB1"

/locus_tag="EFAGFIKM_01373"

/EC_number="3.5.1.-"

/inference="ab initio prediction:Prodigal:002006"

/inference="similar to AA sequence:UniProtKB:Q81ST8"

/codon_start=1

/transl_table=11

/product="N-acetyl-alpha-D-glucosaminyl L-malate

deacetylase 1"

/db_xref="COG:COG2120"

/translation="MSLDILIFGAHADDAEIGMGGTIAKHTAAGLKVGVCDLTRAEMS

SNGTVERRTEEAEQASRVLGLSCRTNLGLPDRGLYLTPEHVQAVTAEIRRHAPRMVFA

PYWEDRHPDHVNCSKLVQEAVFNAKLRNYMPDMPAVQVKELYFYFINDIGPTDLIVDI

TEHYEQKETSLLSYRSQFELGDGAVSTPLNQGYIERVRARDSLLGQRSLIPFAEGFAT

ITPYVVHQFGSGAQ"

CDS 1538344..1539504

/gene="bshA"

/locus_tag="EFAGFIKM_01374"

/EC_number="2.4.1.-"

/inference="ab initio prediction:Prodigal:002006"

/inference="similar to AA sequence:UniProtKB:Q81ST7"

/codon_start=1

/transl_table=11

/product="N-acetyl-alpha-D-glucosaminyl L-malate synthase"

/db_xref="COG:COG0438"

/translation="MDKKLKIGITCYPSLGGSGVVATELGKLLAEQGHQVHFIANSIP

FRLGTFQKNIFYHEVEVNDYYVFRYPPYDLSLATKMAQVAKSQQLDLLHVHYAVPHAV

CAFLAKQMVGDGLKVVTTLHGTDITVLAQDESLKDLIRLAINESDAVTAVSQDLIRET

VELLDIQRPIDLTYNFIDKRIYYPRDAASLRRDFAAPDEKILMHISNFRPVKRTQDVV

EVFRQVQEQVPAKLLFVGEGPDLPKMQWKINDLGLNDKVHFLGKQDDIAQVISMADVL

MLPSEKESFGLVALEAMACGVPTIGSQAGGIPELVLHGKTGFLSAIGDTQSMAENTIR

LLTDDRLAAEFREACLQRAHHDFCNDKIRLEYEQIYYRVLGREVPNLKPVCG"

CDS 1539564..1540865

/gene="cca"

/locus_tag="EFAGFIKM_01375"

/EC_number="2.7.7.72"

/inference="ab initio prediction:Prodigal:002006"

/inference="similar to AA sequence:UniProtKB:Q7SIB1"

/codon_start=1

/transl_table=11

/product="CCA-adding enzyme"

/translation="MVVQWTQVDREMAMQSENVLTTLNKHGYKAYWVGGCVRDELLER

VVDDMDITTSASPQQVMELFDDCIPTGLQHGTVTVRSGGYYFEVTTFRTESEYQDNRR

PAAVQFVQDIKKDLQRRDFTMNALAMDVTGTIVDPFGGQADIKEERVRCVGSAMERFG

EDALRMLRCVRFASVFDFKIAHNTWKGLVRQKDLLQHIAMERVRTEMVKMMSGPHPLR

GLELLYRSDSLAHIKAPVSSTRFNKMLLSNLEQLSGQHVLLRWSLILIAGGYSKDEAD

VLLRQWTFSNEHRSRITGVLQVEQLIHTFVQEPKDTVSLRSDWIVTVLACGVQAADDW

LRIQSTLPAGWRNQSEQAEIQVVLVQELAGEWSQSIPVHDLKELDITGEQVLQMVQRK

GGPWLGQLMKHLLRETAIGTIANQHEALSAEVKRVVQDDQT"

CDS 1540852..1541829

/gene="birA"

/locus_tag="EFAGFIKM_01376"

/EC_number="6.3.4.15"

/inference="ab initio prediction:Prodigal:002006"

/inference="similar to AA sequence:UniProtKB:P0CI75"

/codon_start=1

/transl_table=11

/product="Bifunctional ligase/repressor BirA"

/db_xref="COG:COG0340"

/translation="MTKHEDLLHMLLNAEGRFVSGEEISRNLSISRTAVWKHVNKLRD

MGYEFEAVSRKGYRLLTKPDSIDATGLQLALDTTVFGRKAVLLTSTLSTQGDVLKLAE

QGQAEGAVVIAEEQTGGRGRFGRQWFSPPGKGIWMSVLLRPDLPLQHTPQLTLLTGVA

VCRAVRACSGADAGIKWPNDVLIDGRKVCGILLESTVEDHEVRYCIAGIGVDVNFDPE

DYPEDLTTIATSLKMETGQSVDRTKLTAAILTELEQLYFLYQKEGFGVISALWEALSV

SMNREITVTNPHGVIEGKAIGLDPSGALIVEKHDGEHTLIISGEISWKS"

CDS 1542174..1543052

/gene="panB"

/locus_tag="EFAGFIKM_01377"

/EC_number="2.1.2.11"

/inference="ab initio prediction:Prodigal:002006"

/inference="similar to AA sequence:UniProtKB:P9WIL7"

/codon_start=1

/transl_table=11

/product="3-methyl-2-oxobutanoate

hydroxymethyltransferase"

/db_xref="COG:COG0413"

/translation="MANKQALNIVKMKKYKQDGVPLTMITAYDYPTALLAEEAGIDLI

LVGDSLGNVVLGYNSTLPVTIDDMVYHTRSVVRGAEKTFIVADMPFMTYHGSVDETLK

GVRRLMQEGHAHAVKMEGGVEIADTVRAVVQAGVPVLGHIGLTPQSVNQIGGYRIQGK

DAADAKRLMDEAKALEAAGAFGIVLELVTEEVARAISEELSIPTIGIGAGRGCDGQVL

VFHDVVQYASPYTPKRFVKTYGDVGTLIRTSIEAYVKDVKDRSFPAEEHVFNAADGVL

DQLYGGQRKEKVGSNS"

CDS 1543049..1543945

/gene="panC"

/locus_tag="EFAGFIKM_01378"

/EC_number="6.3.2.1"

/inference="ab initio prediction:Prodigal:002006"

/inference="similar to AA sequence:UniProtKB:Q9X0G6"

/codon_start=1

/transl_table=11

/product="Pantothenate synthetase"

/db_xref="COG:COG0414"

/translation="MKVLRTIAELRQELSLKRQAIRSSASVVGLVPTMGFLHQGHASL

MQAARQQSDIVVLSIFVNPIQFGPNEDFDSYPRDEARDVETARSQGVDIVFIPSVEEM

YPQATQTTVSVSKLTERLCGASRPGHFDGVTTVVSKLFNIVQPQRAFFGMKDAQQVAV

IQQMVNDLNMPVEIVPCPIVREEDGLALSSRNVYLSAEQRTQALVLSKALRAAQEAAD

TGVATTAADIRRILREHIATSPLAVIDYAEIQAFPSLEPLTDQEEVQGRDDLLIALAV

KFGKTRLIDNIRLQKSEVLSHV"

CDS 1543938..1544321

/gene="panD"

/locus_tag="EFAGFIKM_01379"

/EC_number="4.1.1.11"

/inference="ab initio prediction:Prodigal:002006"

/inference="similar to AA sequence:UniProtKB:P9WIL3"

/codon_start=1

/transl_table=11

/product="Aspartate 1-decarboxylase"

/db_xref="COG:COG0853"

/translation="MFRTLMKSKIHRATVTEANLNYVGSITIDEDLMETSDLMENEKV

QIVNNNNGARLETYVIPGPRGSGVICLNGAAARLVQPGDNVIIISYAMMSQEEANTHK

PTVVFVDGQNKPVQTMKQEVHATIM"

CDS 1544415..1545062

/locus_tag="EFAGFIKM_01380"

/inference="ab initio prediction:Prodigal:002006"

/codon_start=1

/transl_table=11

/product="hypothetical protein"

/translation="MFQHVFAEMNDMLDEIIKSYPSAEGLNKQELLQKWNLLKRMSDG

MLDEWLMFEEKMSQVREREMDKPASLEPEQEAVTALPELHLECFSRGQGYFKLQMYPQ

AIMQFSRVVTDHPESALTRFYLALAYLNLEQMAEAGTHLQQIMYLKGSPRLKGLVCNA

LGCIQAKLANPEAACSLFAQALQYDPTLTEPLYNMEACRLNRGKLQYANQLTTLH"

CDS 1545205..1548066

/gene="dinG_1"

/locus_tag="EFAGFIKM_01381"

/EC_number="3.1.-.-"

/inference="ab initio prediction:Prodigal:002006"

/inference="similar to AA sequence:UniProtKB:Q7A5K4"

/codon_start=1

/transl_table=11

/product="3'-5' exonuclease DinG"

/translation="MKFAVLDFETTGTQSDGEIIQAGLAIIDHDYSITQIYSSYVNPG

VPIPPFISGLTGITDEDVADAPSLEEMMMEMVPLLNDVVLVGHNVAFDFHFLQNALDR

CGYLPFTGRILDTIDFLKITFPSLGSYQLGYVSSEFGFQHDRPHQADSDALATAYVLL

KCLDELKELPLITIQRLSDLFAPEDSDLGWFLDGMRSEKEAEPIQDLDGHTYYRQLAL

NVSDWTDIGAPRDEREANPLDGVSFEQFMDQVRENLKDTLDHYEEREAQTQMFSSVRQ

ALDEEKHLLIEAGTGTGKSLGYLLPAIYESVKQEQKVMVSTHTINLQEQLRERDIPML

TQVVPFPFKAAVFKGRGHYLCLRKFEHKINKREFATPKEDYFTAAQMIVWLTQTETGD

DEELNLSGRGGDFWETVQSESESCLGRSCPWFRKCFYHRAKHEAGLSDIVITNHSKLF

TDVKAAHQLLPAYESLVIDEAHHLEDVAGKHLGMHMKYFTLVHTLTRLFKDSRNGQLP

MLRSQLSGHENSVQWGSMVDQMFPLALEVKELWDRMSDALFGLLPERSDASPGETGQF

SLRLKASQKPAKWQELQDLENQIYVTLGDLVRKGDKLLLEVKEDQDDYQSDSLITDIT

GLLKDLTTLKENLRFFMRMDDAKTVYWMEASGQFRSKSLQLYAVPVDVSAQLKDLFFD

KKKSVVLTSATLSVDKSFQFMIEQLGLQEASENNRLLTSMLPSPFNYRDQALLVIPRD

FPSVKGSVGDAHFVNMLVQSLAETAIATRGRMMVLFTSYKMLRQVYDPLKEALSGNDI

SLLGQGVDSGSRSKLTRRFQDAKATVLLGTSSFWEGVDIPGEALTCLAIVRLPFQPPN

HPLVEAKSELLQEQKKNPFMKLSVPQAVIRFKQGFGRLVRTAKDRGIVIVYDTRVIEA

YYGKFFLYSLPGPKMEHMLTEQMVPRITEWLEKPANEQK"

CDS 1548286..1548939

/gene="rex"

/locus_tag="EFAGFIKM_01382"

/inference="ab initio prediction:Prodigal:002006"

/inference="similar to AA sequence:UniProtKB:O05521"

/codon_start=1

/transl_table=11

/product="Redox-sensing transcriptional repressor Rex"

/db_xref="COG:COG2344"

/translation="MKSDKISEAVVRRLPVYLRYLNELHQREVATVSSQELGQRLDLN

PAQIRKDLAYFGDFGRKGIGYDVSYLIEKIRHILKIDQQINVALVGAGNLGQALSNYN

AYLKDNMKIVAVFDAYGPKIGSQINSLTVKPMDELTEAVKMENIRIGIITVPDTEAQN

VADQLIESGIEAILNFAPTILKTPPHIRIHHADFTTDLLSLAYYLETGKDDEEDDNK"

CDS 1548926..1550233

/gene="mtaD_1"

/locus_tag="EFAGFIKM_01383"

/EC_number="3.5.4.28"

/inference="ab initio prediction:Prodigal:002006"

/inference="similar to AA sequence:UniProtKB:Q9X034"

/codon_start=1

/transl_table=11

/product="5-methylthioadenosine/S-adenosylhomocysteine

deaminase"

/db_xref="COG:COG0402"

/translation="MTTNRWVIHNGKFAVNEGGTTWNVVQGYMVVENDKIVHIGETLP

DEDKNCTKVDGKGLFFLPGLINTHGHAAMSLLRGHGDDLALQVWLQEKMWPMEAKMTS

EDVYWGTSLSVLEMLKGGTTAFLDMYDHMDQVAKVVEQSGVRAALARGVIGLCSEEEQ

LRKLEESAAFAREWNGQADGRITTVISPHAPYTCPPDYIEKLVQVANDLNLPLHTHMS

ETLREVEQNVADYGLRPVAHLEKLGFFSRPSLVAHAVHLNDEEIDILARHDVAVSHNP

GSNLKLASGVARVPALLKAGVTVSLGTDGPASNNNLDMFEEMRLAALIHKGVSGDPTA

VPAGEALLLGTSYGAKSIFLNNTGALQVGMKADFIALDIEQAHFYPHTDLISHTIYSA

SAKDVEHVWVDGKQVVKHGECLTLDEERILRESQLAFDGLLAR"

CDS 1550271..1550801

/locus_tag="EFAGFIKM_01384"

/inference="ab initio prediction:Prodigal:002006"

/codon_start=1

/transl_table=11

/product="hypothetical protein"

/translation="MKKKKKWIWISLLALVLILFGLQRYYVHVTQDQRKEEALAIQAA

QEQLGITSYDELRKYIWGDKEGSDNIYWTLIGKNKDTQDVMVWVKFDANNQPAKGANA

VHSELLQNGMSEAQIRNRFSSEVPAGEIKRIMPGVVNGIYVWQVYYKDGTHNHYRFYR

FSNGEQVDLVYTLPNS"

CDS 1551103..1551630

/locus_tag="EFAGFIKM_01385"

/inference="ab initio prediction:Prodigal:002006"

/codon_start=1

/transl_table=11

/product="hypothetical protein"

/translation="MKLKVLPIALTAVISAVVLFGGWFVYRQVAVQNPIEKMVTQYDG

VNDVQLTINRNEVQLKLDLQPDVDLGRLVQYIHKEGQTLIGTRSLKLDVVDHSSEALE

SWWGDAMFTVAQAMENKQYTEITPTLSKMATNGIKVNTAMDDNNVYVSLRDGDASKFI

ILPRVPGQIGVWPNA"

CDS 1551623..1553125

/gene="ftsH_1"

/locus_tag="EFAGFIKM_01386"

/EC_number="3.4.24.-"

/inference="ab initio prediction:Prodigal:002006"

/inference="similar to AA sequence:UniProtKB:Q9WZ49"

/codon_start=1

/transl_table=11

/product="ATP-dependent zinc metalloprotease FtsH"

/db_xref="COG:COG0465"

/translation="MPKWTKEVAIGFFPVLIIFLAFTGVNIVPILIAGVLVGALLFMM

QMRGGITVGAAQERKRKKKGPSKLTFEEIGGQDSAKQELREALDFLIKHEEIRKFGIR

PLKGILLTGPPGTGKTLMAKAAAHYTDSVFVAASGSEFVEMYVGVGASRIRDLFKDAR

TRATKENKENAIIFIDEIDVIGGKREGGQQREYDQTLNQLLTEMDGIYTSDTPRILVI

AATNRKEMLDSALTRPGRFDRHIQVDLPDKKGRKHILELHAVNKPLMKEVNLEKTAEE

SYGFSGAQLESVMNEAAIYAMRDGLLNIEQRHLSLAIDKVMMGEKTDRESSVEEKKRV

AIHELGHAIMAELVRPGSVSQVALSPRGQALGYVRHNPQQEQFLYTKRFLEEQIMIAL

GGAAAEEMYYGGRSTGSRNDFDQATNVVQTMMASGLTSLGIVNMDMVTTEELMRENKL

ILQELMEQTKQLLEEQRTIFDNSLDILIREEVLSGEQFRCQFRDSALLPA"

CDS 1553259..1554131

/gene="hbd"

/locus_tag="EFAGFIKM_01387"

/EC_number="1.1.1.157"

/inference="ab initio prediction:Prodigal:002006"

/inference="similar to AA sequence:UniProtKB:P52041"

/codon_start=1

/transl_table=11

/product="3-hydroxybutyryl-CoA dehydrogenase"

/db_xref="COG:COG1250"

/translation="MFFKKIGVVGGGTMGQGISQMLAAKGLDVLLVEHTTQKLDHAYD

MIETNLDKQLEKWAITKAEKKLILSRITKVAHLAELGTCDMVIETISEDLDAKKAVFS

QLDQVCPSNVILASNTSTLSLTELASSTKYPERVIGMHFIHPVSRVDLVEIIRGLKTS

DTTFEETRRFVEEVADKKGVMIYESPGFVTSRLICLLINEALHVLQEGVASAEDIDDA

MRIGYNFQHGPLEMADRFGLDSVEAALERMFREFGELKYRPSTVLKKMVRAGHLGAKT

GEGFFKYDKDGDRL"

CDS 1554128..1555333

/gene="ackA"

/locus_tag="EFAGFIKM_01388"

/EC_number="2.7.2.1"

/inference="ab initio prediction:Prodigal:002006"

/inference="similar to AA sequence:UniProtKB:Q9WYB1"

/codon_start=1

/transl_table=11

/product="Acetate kinase"

/db_xref="COG:COG0282"

/translation="MKVLVINAGSSSLKYQLYNMTDESVLAKGLVERIGMDSSILTHK

PTGREDVTEVSEILEHTTAIRKVIDILTHKENGVLDSVSEIQAVGHRVVHGGEAFKES

ALVDDAAKAEIRRLFDLAPLHNPAAMMGIRAAELNMPGVPQVMVFDTAFHQTMPEKAY

LYAIPRVLYKKYKVRRYGAHGTSHDFVSKAAAEYLDRPVEDLKIITCHVGNGGSVTAV

KGGVSVDTSMGMTPLEGLMMGTRSGDLDPAIVPYVMNKEELSVSEVNSMLNKHSGLLA

ISGISSDMREITEGMENGDANSTLAFEMYEYRLRKYIGSYAAAMNGVDVIVFTAGVGE

NSVVLRQKVCEQLTYLGVELDEALNAIRSGEPRRITTANSKVEVLVVPTNEELVIARD

THRIVLNSQ"

CDS 1555435..1556730

/gene="asnS"

/locus_tag="EFAGFIKM_01389"

/EC_number="6.1.1.22"

/inference="ab initio prediction:Prodigal:002006"

/inference="similar to AA sequence:UniProtKB:P67572"

/codon_start=1

/transl_table=11

/product="Asparagine--tRNA ligase"

/translation="MNTNCVIRNVNEHVGETVTIGAWINNKRSSGKIQFLQLRDGTGY

IQGVVVKSEVSEEIWNNAKSLTQESSLYVTGIIREEPRSASGYEMTVTGVEIIHLTEN

YPITPKEHGVDFLMDHRHLWLRSTKQRAVMVIRAEIIRAVQQFFDGNGFTQVDPPILT

PSSAEGTTNLFHIKYFDEDAYLTQSGQLYMEAAAMALGKVYSFGPTFRAEKSKTRRHL

IEFWMIEPEMAFVDHEESLRVQEKFIAHVVQSVVKNCRAELESIGRDVSKLEGIVAPF

PRITYDEAIEFLHGQGFDIPWGEDFGAPHETAIAEKYNTPVFITHYPAGIKAFYMKPD

PNRPEVVLCADMIAPEGYGEIIGGSQRIDDPELMQQRFEEHNLSDEAYQWYLDLRKYG

SVPHSGFGLGLERTVAWICGLDHVRETIAFPRMLYRLYP"

CDS 1556751..1557455

/gene="dnaD"

/locus_tag="EFAGFIKM_01390"

/inference="ab initio prediction:Prodigal:002006"

/inference="similar to AA sequence:UniProtKB:P39787"

/codon_start=1

/transl_table=11

/product="DNA replication protein DnaD"

/db_xref="COG:COG3935"

/translation="MEDTTSKAWLDGAAYGMTSGTAHLPYALLRFYHQLGLSDAEVLL

LIQLLGFRQAEFNEFPTLEELATRMGLAPEGIARMLQRLMRDGYIAIDEHRDEERDIQ

YERYDLHGLYAKLAACTAEEVAAFRAEQQSINTARQDSNSVQKEEEERNMFSIFEKEF

GRPLSPMECETISGWLDQDRYQEELILMALKEAVFAGKVHFRYIDRILLEWSRNRVKT

VQDAKAYTQRFRNGGR"

CDS 1557703..1558203

/gene="algU_2"

/locus_tag="EFAGFIKM_01391"

/inference="ab initio prediction:Prodigal:002006"

/inference="similar to AA sequence:UniProtKB:Q06198"

/codon_start=1

/transl_table=11

/product="RNA polymerase sigma-H factor"

/db_xref="COG:COG1595"

/translation="MRTLIDKYSQHVYHVAYSVLRNDQEAQDAAQEAFIQMYKSLPDY

RSEGFKTWLTRIAFHKAIDAKRKLGRRTAEDLGGEEKIINMPGRDEDVLTRLVREERQ

DKLRERINQLPAQHRDIITAYYLSEKNYEQIASDAQVAVKTVESRLYRARQWIRKHWK

EDEWRE"

CDS 1558196..1558648

/locus_tag="EFAGFIKM_01392"

/inference="ab initio prediction:Prodigal:002006"

/codon_start=1

/transl_table=11

/product="hypothetical protein"

/translation="MSKQEKYTAGQWTDYIEGHMSETQANQMEQLLLEDPDAMDIYLE

ALGIHGELPPLPDPTGFADSVMHRIEGIQPTKSQRKPRAGRNRRWLEHKVFHYAVAAC

LTLIFLSSGLFDKVAPYHKFQDGDHRGSFTEKWTEAATSWLDNLKPKP"

CDS 1558686..1559975

/locus_tag="EFAGFIKM_01393"

/inference="ab initio prediction:Prodigal:002006"

/codon_start=1

/transl_table=11

/product="hypothetical protein"

/translation="MHSDRNKLLAFLLNLIPGLGFMYWGRPTRAVVYPLLFFGTGIGF

IMLALLTDQKELMIVGFLGAIFFWCISMLDMIIVLLRAPSLHEVQYQGYGTHYGGLHQ

GAAYQKAYAGQEEPLGQMDQEEPHQDHGGVYGVPEGPYGQPMYRKGSEGERFFTILLS

FVPGLGHLHLGLLHRGLSFLMAFFGSFAMMVFVASITNESVFLMFLLILPVIWVYCMF

DAVQHVHRKQAGEILQDRTLFEELEMGRVAGRRSKVLATLLSAFPGAGHLYLGLQKRG

MQLMFLFLGSIYVLDLLHLSVFLFMIPLIWFYSFFDGLQCSSRYGREPLVDQPIFKDW

ARHQRLIGFGIAALGLYYLTIRLVIPQLNELFPNAFMTYEIRSYLNTVIVSLLLIFGG

LKLLFGKQRGTGIQSAAHQNDEGADSLFLFKDRDDRL"

CDS complement(1560139..1560792)

/gene="mtnB"

/locus_tag="EFAGFIKM_01394"

/EC_number="4.2.1.109"

/inference="ab initio prediction:Prodigal:002006"

/inference="similar to AA sequence:UniProtKB:O31668"

/codon_start=1

/transl_table=11

/product="Methylthioribulose-1-phosphate dehydratase"

/db_xref="COG:COG0235"

/translation="MGFEKITLEHKRQVLEELADIKALFASRNWFPGTSGNLSMRVGD

FDPEQFYFAVTASGKDKSLRTPEDFLFVDKHGKAIETTTLKPSAETLIHCEIYRLTGC

GAVFHVHTVFNNLISEFFGADGHVPIQGIELIKAFNIWEENAEIRVPVLPNFADIPSI

AELVPGVLDANVPGILLRNHGIYAWGKDAFEAKRHLEAFEFLFEVMYRQLLLKGATK"

CDS complement(1560796..1561473)

/gene="mtnX"

/locus_tag="EFAGFIKM_01395"

/EC_number="3.1.3.87"

/inference="ab initio prediction:Prodigal:002006"

/inference="similar to AA sequence:UniProtKB:O31667"

/codon_start=1

/transl_table=11

/product="2-hydroxy-3-keto-5-methylthiopentenyl-1-

phosphate phosphatase"

/db_xref="COG:COG4359"

/translation="MRSDKKTVIFCDFDGTITLSDNIVAIMKHFKPEGIEAIMKDTIE

QKISLREGVGAMFALLPASRKDEIVEFVLGQAGIREGFSEFLDYVRSEGIEFNVTSGG

MDFFIEPLLAPFHIPQDHVYCNGADFSGETIQIEWPNPCQPPCENGCGMCKTTVIRTF

PEDQYNRILIGDSLTDFEGAKIADLVYSRSILTDKCIELGVDHVPFATFYDIIEDMKQ

KQTQGVL"

CDS complement(1561470..1562693)

/gene="mtnW"

/locus_tag="EFAGFIKM_01396"

/EC_number="5.3.2.5"

/inference="ab initio prediction:Prodigal:002006"

/inference="similar to AA sequence:UniProtKB:O31666"

/codon_start=1

/transl_table=11

/product="2,3-diketo-5-methylthiopentyl-1-phosphate

enolase"

/db_xref="COG:COG1850"

/translation="MNNMCTATYRLHDDHADFRKKAQSIAIGMTVGSWTELPQAQREA

MQKHLGEVISIEVNEAEGIAAGERYADITIGYPDVNFSRDIPALLVTVFGKISMDGRI

KLTKLGFSDGFLSAFPGPKFGLNGVRDLLGVHDRPLLMSIFKSVIGLDADELREQFIR

QALGGVDLIKDDEILFENKLTPIEKRVEVCMKAAEQARQETGKKLLYATNLTGPTSRL

KEQAERAIGAGANALLFNVLSYGYDVLHELSSNPDINVPIMAHPALAGALYPSPHYGM

SASVVLGQLMRLAGADLVLFPSPYGSVTMPKEENMAITEQLLTADLPVRTSMPVPSAG

IHPGLVPLILRDFGTDVIVNAGGGIHGHPMGTEAGGRAFLQAIEAAQRSIPLAQYATE

HPELKSALDLWGGER"

CDS complement(1562690..1563550)

/gene="proC_2"

/locus_tag="EFAGFIKM_01397"

/EC_number="1.5.1.2"

/inference="ab initio prediction:Prodigal:002006"

/inference="similar to AA sequence:UniProtKB:Q7A5G8"

/codon_start=1

/transl_table=11

/product="Pyrroline-5-carboxylate reductase"

/translation="MSQEQQTLLQQKITFHGAGAMAEAIVRGLISRLVVRPQDITMLN

RSNQKRQEELSTRYAVHTGTASHSLDHLASTPVIVLCMKPKDAAAALRELGPLLSPDQ

LIVSVIAGLSIRTMQSLLGRKQPIARTMPNTSSTIGLGATGLAFSAEITDEQRSTVMT

MFEAVGIVTIVPEDKLEVLTGISGSGPAYVYYLMEAMIAAGIRGGLSSQQSRDLTVQT

VLGASRMVQQTGLEPMKLRSDVTSPGGATQAALKTLDEGDFFENVIAAVNRCAERSRE

MGAALEGDLK"

CDS complement(1563587..1564834)

/gene="proA"

/locus_tag="EFAGFIKM_01398"

/EC_number="1.2.1.41"

/inference="ab initio prediction:Prodigal:002006"

/inference="similar to AA sequence:UniProtKB:Q9WYC9"

/codon_start=1

/transl_table=11

/product="Gamma-glutamyl phosphate reductase"

/db_xref="COG:COG0014"

/translation="MSEVREKAIKAQATVPQLNRLNTEQKNTALRVMADALIKETDSI

ITANAEDLARGREQGTPDSMLDRLALNKERIAGIAEGLRQIADLPDPVGEVLETFTRP

NGLHVEKLRVPIGLIGIIYEARPNVTVDAAGLCLKTGNAVLLRGGSSALSSNRKIVEV

LHQALTTTDMPADALQLVEDADRASVDEMLKLNGLLDVIIPRGGASLIRNVVANATVP

VIETGAGICHTYVDESADPVMAAEIAINAKAQRPSVCNSMETLLLHAAYAEEHLPALA

EQFREANVVLKGCDTVRRLVPSALEATEEDYATEYNDYILNIRVVENLEEAMQHIAHY

GTKHSECIVTRDTAHAERFMHDVDAAAVYHNASTRFTDGFEFGYGAEIGISTQKLHAR

GPMGLPALTSTKYRITGNGQIRQ"

CDS complement(1564893..1565996)

/gene="proB"

/locus_tag="EFAGFIKM_01399"

/EC_number="2.7.2.11"

/inference="ab initio prediction:Prodigal:002006"

/inference="similar to AA sequence:UniProtKB:P39820"

/codon_start=1

/transl_table=11

/product="Glutamate 5-kinase 1"

/db_xref="COG:COG0263"

/translation="MTSRIVVKIGSSSLTTEEGGLDRSSITFFAGEIAALADQGHEVL

LVTSGAVAAGFREIGYPQRPKLLHEKQAAAAVGQALLMQAYQQAFAAHRVTTAQILLT

RTDFHSRKRMGNAGMTVEELLKQRVIPIFNENDTVSVDELKFGDNDLLSALVANLVKA

QHLIILTDTNGLYTADPRKDPSAMRYDRIPEITAEIYAFAGGSGSTVGTGGMRSKVDA
AKVATRGGVPVFVGSVKEPGDMQKAVEGTGKGTYFETRLASLSRKKQWLGFMSTPLGT
VVVDDGAEEALVHGGHSLLPVGVKRVLGTFHAGDVVEVIGMDETLLGRGIVNYDDDQL
RLIAGLPSGEVMKQLASIHRLEVIHRDEWITLK"
CDS complement(1566358..1567566)
/gene="bacF"
/locus_tag="EFAGFIKM_01400"
/EC_number="2.6.1.-"
/inference="ab initio prediction:Prodigal:002006"
/inference="similar to AA sequence:UniProtKB:P39643"
/codon_start=1
/transl_table=11
/product="Transaminase BacF"
/db_xref="COG:COG0436"
/translation="MTQSDHTSARSAFKIPTSDVMTQLPTQFFATLVQNVNREIASGH
DVINLGQGNPDTPTPPHIVKTLQESAENPLYHKYSPFRGHSFLKEAVAKRYKEDYNVD
LDPETEVAILFGGKTGLVQLPQILLNPGDTVLVPDPGYPDYWSGVALAKANMSFMPLL
ESNAFLPDYEAVTAEDREKAKLMFLNYPNNPTSATAPLSFYEDTVEFAIQNQIVIASD
FAYGAIGFDGHRPVSFLQAPGAKEVGIEFYTLSKTYNMAGWRVGFALGNAEIVSKINL
LQDHIYVSLFGGIQAAATEALTASQECVTSLVSRYESRRNTFYDALSSIGWQASKPAG
SFFSWLPVPAGYTSASFADLLLREAKVAVAPGIGFGSHGEGYVRAGLLSDENRLREAV
ERIGKLNLFK"
CDS 1567826..1568821
/locus_tag="EFAGFIKM_01401"
/inference="ab initio prediction:Prodigal:002006"
/codon_start=1
/transl_table=11
/product="hypothetical protein"
/translation="MKKGIRNTGVLLTITWLSILLLACGNQAATPAAGADDSKAATET
TETQTATEETNEGETRIFKDWTGHEVEIPVNPKRVIYHGEVTGDLLALGVVPVGILRQ
EGTVFDDQVAQSEDVGFPISVEKALDLNPDLIIFSNSDEAQYDQIAKVAPTVTFDSFG
LIEDRMRILGDLLNKKQEAEDWITAHQKATEEMWTQLHENGLKEGETASVFTMYPGNR

LFVMAGAGLPQLLYGEDGLKPTAEIQKVLDEDMGFVEISTEKLSEFAGDRIFILDPVT

DDAKQSTKELLDSTIWKNLPAVKEGKVYRFNIVKASSDALSREWLLQELPKQMIQ"

CDS 1569024..1570646

/gene="btr_2"

/locus_tag="EFAGFIKM_01402"

/inference="ab initio prediction:Prodigal:002006"

/inference="similar to AA sequence:UniProtKB:P40408"

/codon_start=1

/transl_table=11

/product="HTH-type transcriptional activator Btr"

/db_xref="COG:COG0614"

/translation="MQALDADGGALFFSAFMFRLDHLVRRIEPPEQVMLEVACEKHTF

LICEEGDGRLYIGHEQFPFTTGCVYPLSPGEGYQIEHRNNSELKYIVMAFDVIHVLTG

DPELFTRAVFEHRNQLNGYPYAPLSGLLEQMYVIRNYKTDAEYSHLNAQFQKWMELII

TRYTSPKTEQSMEARLHSTIQYVDDHYAEEITVQKLARLAEIRPVQYTTLFRQLTGHK

PLDYVNHVRIKHAKDWLRKSDEPLRDIASRVGFKDEYYFSRRFRQMTGLSPRQYDRSI

QQQTLVQDWLGHDVNIPVNPERIMYYGDSAGDLLVLGIQLLGDQTYDAVAPVNVEAAV

IMKPDLIIFDSSNKQQYEQLYRIAPTLAYNSHATLEERMHRLGSWFGRQPQAEQWLTS

YAERTEQMWAKIHTVIEEGETASVFTYHRGARLFVMGNIGLAPMLYHPMGFRPVIKVK

EALASGRAYKEISAEAVRQYAGDHVFVMLPEEVVARQATEMLMKSPSWQALPAVQNGR

VYPVEESTWNVGDALTSDRLLALLPELLCASS"

CDS 1570720..1571541

/gene="yafV"

/locus_tag="EFAGFIKM_01403"

/EC_number="3.5.1.3"

/inference="ab initio prediction:Prodigal:002006"

/inference="similar to AA sequence:UniProtKB:P0DP65"

/codon_start=1

/transl_table=11

/product="Omega-amidase YafV"

/translation="MTEKQQGEMRVALIQGDIQLGDPEANHKHMQTLLERAVEQYPDL

GLAVLPEMWNTGYALTQIHELADPEGQKSREWLSAFAQKHQISIVGGSIAEKRDGQIY

NTMYAYDREGKQVTRYDKLHLFRLMDEEKYLQPGAEPEIFELQNGLTAGASICYDIRF
PELARTLALNGAKALIVPAEWPKPRLHHWRTLLTARAIENQMYVIACNRVGKGGETEF
FGHSLIIDPWGEIVAEGGEGEEIVTGIIRPSLVDEVRGRIPVFEDRRPGVYFGGK"

CDS 1571633..1571818

/locus_tag="EFAGFIKM_01404"

/inference="ab initio prediction:Prodigal:002006"

/codon_start=1

/transl_table=11

/product="hypothetical protein"

/translation="MDNHVKDTLRRLEQDNTREDHLRAFLENGAGGLSPPDSTVQDEG
RVMNQQNRLTSDRNLRK"

CDS complement(1572068..1572994)

/gene="oxyR_1"

/locus_tag="EFAGFIKM_01405"

/inference="ab initio prediction:Prodigal:002006"

/inference="similar to AA sequence:UniProtKB:P0ACQ4"

/codon_start=1

/transl_table=11

/product="Hydrogen peroxide-inducible genes activator"

/translation="MTMEFRQLQYTLQIAAERNFSRAAEKLHIAQPSLSQQLSKLEKE
LGVLLFQRNTSTVELTHAGVTFVEQAQKIVDAVELLRQEMSDISQLRKGKVVVGSMPI
TGSHLLPHVLPAFQQAYPEIEVTLLEDSGLTLEKLTASGKADLSLLSLPLQEPSLAYV
AIGEEKIDLAVPPNHPLARRADPEHPLPVRIEELRDEPFVVLKKGQGFRKLTFDLCEQ
AGFDPQVVFESTNIETVQSLVATGMGITLVPRFIARAPRSEFVPVYVPLAEPTPSRTL
VVAYRQGRVLSKAAEAFIHTFQQTVAELSKGD"

CDS 1573355..1575268

/gene="bglF_3"

/locus_tag="EFAGFIKM_01406"

/inference="ab initio prediction:Prodigal:002006"

/inference="similar to AA sequence:UniProtKB:P08722"

/codon_start=1

/transl_table=11

/product="PTS system beta-glucoside-specific EIIBCA
component"
/db_xref="COG:COG1263"
/translation="MNSKKLAQEILEHVGGNNNINQVIHCSTRLRFTLKNSKLANTEE
IKRLDGVIGAVDSGGQYQVIIGNDVSYVYNELAPLINMETASDEVKSPRDYSPKGLFN
AFANIVAGIFQPIIPAIAASGMLKALLLLATVFGIMAKEDQAYVFLNAIADAGFYFLP
MLLAFSSANKFKANPYIAVLLGGVLLHPNMIGLLGGEEPVYFLGMNVPNVAYATSVIP
IILTVWFMSYIERFADKVSPGPVKIFLKPLIVILIVAPISLIALGPLGTYIGTGMSEA
VFWIQDKVGWVTVVIMSMLMPFIVMFGMHKVFYPVIFGAIASPGYETLFLVSMLAANM
AQGGGALAVAFKSKDPKMKQIALSAGISGMFGITEPAIYGVHLRLKKTFLACLIGAGA
AGMFAGIVNLKAYTAVGPGLATLPIFVSDDPNNILYALITVAISSVIAFVGVFLIGFQ
DTVASAESGKAGKPSAEPVSPVRMDSQPQTSASGMKSKKVVFAPLEGKVLPLREVKDE
AFSQEAMGQGMAIQPSVGKVFAPFDGTVETVFRTKHSIGLRSVEGVELLIHVGLDTVK
LKGQHFDVKVNEGEQISHGQLLIEFDLEAIQAAGYDTTTPVIVTNSSDYLEILGNETA
ATTGPGKPLITVL"

CDS 1575305..1576144

/gene="licT_2"
/locus_tag="EFAGFIKM_01407"
/inference="ab initio prediction:Prodigal:002006"
/inference="similar to AA sequence:UniProtKB:P39805"
/codon_start=1
/transl_table=11
/product="Transcription antiterminator LicT"
/db_xref="COG:COG3711"
/translation="MEVIKILNSSIVLARRGEDDKEIIVMGKGIGYRSKPGDVVGEDE
IEKIYVLENETISSDLTALMKETPKEYLILADEIISHAKHTLSRHLSDHLYVALTDHL
YMAMKRFKDNMTIQNRMLWEVKKFYPQEFSIGLHGLSLIQKQLGLSLPEEEAANIAFH
LVNAQQNDDNMNQVMMMTNTVKDVLNIIKIHYQVELDTHSINYSRFLTHLQFFIQRLF
EHKTLNTQDHELFEQIANKYPQEEACVLLIKDYIEARFEHTISNEEMMYLIIHINRVM
SRN"

CDS 1576190..1577647

/gene="bglH_4"

/locus_tag="EFAGFIKM_01408"

/EC_number="3.2.1.86"

/inference="ab initio prediction:Prodigal:002006"

/inference="similar to AA sequence:UniProtKB:P40740"

/codon_start=1

/transl_table=11

/product="Aryl-phospho-beta-D-glucosidase BglH"

/db_xref="COG:COG2723"

/translation="MKNTTRTFPEDFLWGGAIAANQAEGAWNVDGKGLSTADIAIYRK

GVAKSEYKKHNAINEEQIKTAMESTSDREYPKRRGIDFYHRYEEDLALFGEMGLKTLR

LSIAWTRIFPNGNETEPNEAGLQFYDRVIDGMLKQGIEPLITLSHYEMPMYLVNHHGG

WTDRKVVDYFVHFCKTVFTLYKDKVKYWITFNEIDSIVRHPFTSGGIVPERFENVEQA

VYQGLHHQFVASALAVKYCHEIIPGSQIGCMLTKLTTYAHTCNPEDVLVASRDNQFNL

MFTDVQVRGAYPYFTKRFFAEKGINLDMLEGDEEILKVHTVDFISFSYYMSLVSSVDG

DRLEQVSGNTTGGVKNPYLQTNDWGWQIDPVGLRISLNELYSRYEVPLFIVENGIGAV

DTIEADGSIQDDYRIDYFRSHIEQMHEAILDGVELLGYTSWGVIDLISYSSSEMEKRY

GFIYVDQDNDGNGTLERKRKKSFYWYKDVIANNGL"

CDS 1578018..1579439

/gene="leuC"

/locus_tag="EFAGFIKM_01409"

/EC_number="4.2.1.33"

/inference="ab initio prediction:Prodigal:002006"

/inference="similar to AA sequence:UniProtKB:P80858"

/codon_start=1

/transl_table=11

/product="3-isopropylmalate dehydratase large subunit"

/db_xref="COG:COG0065"

/translation="MSKKTMFEKIWENHVIHQEEGKPSILYIDLHLVHEVTSPQAFEG

LRLSGRKVRRPELTFATMDHNVPTKDRFNITDPISKQQIDTLSQNCRDFGVKLYDLDT

IDQGVVHVMGPELGLTHPGKTIVCGDSHTSTHGAFGALAFGIGTSEVEHVMATQCLQQ

AKAKTMEVRFVGKRNPGVTAKDMILAVIAKYGTDFATGYVIEYTGESIRELSMEERMT

VCNMSIEGGARAGLIAPDETTFEYLRGREYVPADAKFDEAVEGWKQLVTDEGAEFDHV

VEIDVETLIPQVTWGTSPGMGTDISSKVPVPAELPTENERKAAEKALEYMGLEPGTPI
AEIPIDYVFIGSCTNGRIEDLRAAAQVAKGHTVSSQVTAIVVPGSGRVKIQAEQEGLD
KIFTEAGFEWRDAGCSMCLAMNPDVLKPGQRCASTSNRNFEGRQGRGGRTHLVSPAMA
AAAAVKGHFVDVRDWNFKTEAAI"

CDS 1579471..1580073

/gene="leuD1"

/locus_tag="EFAGFIKM_01410"

/EC_number="4.2.1.33"

/inference="ab initio prediction:Prodigal:002006"

/inference="similar to AA sequence:UniProtKB:P04787"

/codon_start=1

/transl_table=11

/product="3-isopropylmalate dehydratase small subunit 1"

/db_xref="COG:COG0066"

/translation="MEEFKTLQGIVAPVDRVNVDTDAIIPKQFLKRIERTGFGQFLFY
EWRFDEEGNNNASFEMNKPRYEGASILISRANFGCGSSREHAPWAILDYGFRCVIAPS
YADIFYNNCFKNGILPIKLSEEQVEDLFQRTATHEGYEMNVNLENKTITDAYGLHIDF
DLDEHRRQFLLQGLDDIGLTLQHDDEIAAYEQRHAAKLFG"

CDS complement(1580273..1581748)

/gene="flp"

/locus_tag="EFAGFIKM_01411"

/inference="ab initio prediction:Prodigal:002006"

/inference="similar to AA sequence:UniProtKB:Q7A3Q5"

/codon_start=1

/transl_table=11

/product="Protein flp"

/translation="MLKLKKRMYITTISCLLLCIAWAPPLSATEARDRTEVLQEIDEY
MNRSMKANHIKAASLAIANNDEVFYAKGYGTFADGQKVTGNTPFPIASLSKSFTALAV
LQLVDKSRIDLDATYASYFPELSPQDPHVLDITVRHLLNQTSGLNDKINPDMTKTPQF
QSLAEANQLLNTVQLAHIPGTAYSYHNPNYVLLANLVESVSGERFSDYLKEHIFEPLG
MNHTFSVSTTQQFYKNNTIPLGHYLSLGRAISQSEPLWFIEGPASIVSTAEDMSLWML
SQYHGRLVSPVLMKQYHASGDISPYGMGWLADQDASNGQTISHSGIFWTYKSEERVYL

NEQMGIAVMFNSGINAYVNYSAFLDGIADIMRGQKPQTSFVNGRNMEMIMIALIVATL

SWGIYGYSRILRNYHRLTISKLILITVGRLIPVLILLSLSHLVTFIGGGRVLPWFGIW

TTLSTPILWLMVWSLVNFVHLACYIYVYFQNTKNDPLELPQ"

CDS complement(1581800..1582897)

/gene="sasA_2"

/locus_tag="EFAGFIKM_01412"

/EC_number="2.7.-.-"

/inference="ab initio prediction:Prodigal:002006"

/inference="protein motif:HAMAP:MF_01837"

/codon_start=1

/transl_table=11

/product="Adaptive-response sensory-kinase SasA"

/translation="MAKFMTRLLFLKRLSLLHSFMLLCVVTLIAVLGIITIEFAWAEN

LRLRFGDTNWVMPSLVTAVLLTVATGIVTMAVLFYRWKLKRPLELLKRASENISANDL

DFRISYDSQDEMGELITLFENMRSQLEKNLKTLWRSVEERKQINAIFAHDLRTPLSVL

KGNAELLATYLPEKKLSEEKVLDLIHTMNLHILRLESYAEAMNSIQRLEDLPLNIQSM

DTISLTALLDSSAEQIAKQFGIRFISSVEYDNASVHVDPYLVMQIFENLVANGARYAA

SQVNVHYDVQGGIMKITVSEDGPGFSEEALSKAILPFYRGEVWDASEHRGLGLYVCKV

FCEKHEGSLRIANGPNGGGSVTASIGTQVDK"

CDS complement(1582882..1583556)

/gene="arlR_1"

/locus_tag="EFAGFIKM_01413"

/inference="ab initio prediction:Prodigal:002006"

/inference="similar to AA sequence:UniProtKB:Q9KJN4"

/codon_start=1

/transl_table=11

/product="Response regulator ArlR"

/db_xref="COG:COG0745"

/translation="MPKILIVDDEADLRKLLTDYFEINGYSVMTAKDSREALRKVEKQ

PDLVLLDINMPEQNGLQLCEKIRDFVSCPILFLTARIEDADKIAGFRAGGDDYILKPF

SIRELGARVEAHIRREQRIQTKSSVRFNNELVIDYTARELYYLDKRISLPKKEFDIVE

LLSTHPDMVFGKERIYEKIWGMDEQGDSNVIAEHIRRIRYKLKEYGCDNQIETVWGVG

YKWPSS"

CDS 1583832..1585238

/locus_tag="EFAGFIKM_01414"

/inference="ab initio prediction:Prodigal:002006"

/codon_start=1

/transl_table=11

/product="hypothetical protein"

/translation="MKKWSAAVVFLLFLCLFPVMAHADTTPSIVLDGVTINQQTGAPA

ENIGKTVMVPIRIVSENLGYKVKWEKETQSVQVQKGNSMIQMTAGKDAATVNGNVVNL

DSPPLIKQGTTLVPLRFVGEGMGLRVGWDNGTKTVSLFSIPPVVETEGDSDPVEAPVP

DGLTELQGISFSGDRLIVATNGNISPKVSSIGGPDRIIVDLPSAKFSQEFIQGQASNP

DGSGQILVTGSSIVSKVRYAMFSKSPSTVRVVLDLNQTATAKWSVGDNNVLLVDLTAT

SGEPTSQPAVPTNDGKTIVVIDPGHGGRQSGAVSLSGAYEKDFNLAVGLKVQAILQQY

PNIQTVITRQDDTELSLKQRVDIAELNQADVFVSIHGNKFTTPVPNGIETLYSRKESK

TLADTLHKYVLPVTGLKDRGVKTASLHVTRETTMPAVLLELGFLSNLTDEAVMLTEEY

QDKCAQAIVDGIVEFLGL"

CDS 1585497..1586906

/locus_tag="EFAGFIKM_01415"

/inference="ab initio prediction:Prodigal:002006"

/codon_start=1

/transl_table=11

/product="hypothetical protein"

/translation="MKKFGFLVLLFVFGLVFPGYSHAATDTKIILDGKEIVQPSDTKA

EIINSKVMVPIRVVSENLGYSVEWKQQTQTVTISKDNTAMQMIVGQKTATVNGSNVNL

DAPPLVKNGTTLVPLRFIGEEMGLKVGWNNTTKTVTLVTQNSGSGNGTTTPPNSGNEG

GGSDQEGLVLVNGISFSDNRFMIATSGSTKPNVFTMTGPDRIVIDLPNTAFADSFSEG

QALDSNQNGQLVVSGYPDVSKIRYSLYSNSPSTIRFVIDLSSSKGYSVQNDSGLVMIN

LDNQSGTPAPPVGNNGKKVVVIDAGHGDQDPGAIGVTGKREKDFNLAMALKVEALLKK

ESKIDVVLTRSDDTFLALKERVKIAQDIKADIFISIHANSGPAAANGVETFYTRSNSK

ALATVMHKYLLQSSGLKDRGVKTASLHVTRETTMPAVLLEGGFLSNKSDEAALFTESF

QSSVAKGIVAGIKEYLGIK"

CDS 1586942..1587619

/locus_tag="EFAGFIKM_01416"

/inference="ab initio prediction:Prodigal:002006"

/codon_start=1

/transl_table=11

/product="hypothetical protein"

/translation="MNKKIWIAALLVTVMAVAAGCGSKPTAAPNQTQGAGTENNVTEV

EGETITEPVTAEPEENTTPTESTEGSSEEATTTTPPSDTSTEKPATSESNEKKTITVF

YTDEEELELHKASAEISYASDDAKYKAAFESLQQSKDAKLVPLWSKEIELKSVQFKDG

ALTLDIHMPDTARLGAGGESYALDALKQTFFQFDEVKSLDLLVDGQQTESLMGHVDLE

HPMTRSE"

CDS 1587692..1590430

/locus_tag="EFAGFIKM_01417"

/inference="ab initio prediction:Prodigal:002006"

/codon_start=1

/transl_table=11

/product="hypothetical protein"

/translation="MTFKYNHTSHSKKVVSAVLAGMMALSAGGAAMAAESTETGQGQT

AAITNTAAPTGLFSDIKVGYWAEKHVYKLAYQGILLGNNGLFRPGDAVTQQEAVTMAI

RFMNQEGQLNTDTAAALPTNMEVGNYFKPYVALALQLGLIDKTEESTVDVSKTSWGQK

PASREWITKLLIRSLDKDAEAKAQNNQSTGFADNASISESGKGYVNLAVSLDLAKGVE

GNKFNPTGSVTRAQLATFFSRGESLTDTTYPNTSTGYVTGLKDGQITLVVDGKAVNFA

VNSSTPYFTKDSETRASATDVKLYTKVMVVGSAGGASYVEVVDATPQVESVEGTFARS

LSGNKIGIFVGENYETYSYDEATAFIDQNGNAIKLSDITADSIIEVQRETFTADKKTV

AIRVKSGIVNKSDNGVIAEVSTSGKTIKLVNAAGATEQFAYNDNLLITYQNRIMSVAD

LKAGSAVKYTVKDSVLQTITLSQGVEQSVRGTLVEIGGNQSTLTFKREGGSLEAKLLT

EKPEVIINGIQDATLNDLITDATNGDQVELSLNSDDQVTRIQVIGRQMEPMNGVTVVS

YNSKTKVLTVLDNNKKPFVFTLDEKTKLDYNTTKPTLAGLESFLNDGRKLDLTYVGTR

ALSVKVIYKYEGTISNIDTTGRKISVLSGNQTITVPYTTAPTVEIYNKSGASLGDLKI

GDKVTVTLGANQDVVQKVALNTVAQFEVVALEANSRIRVKSDLLTSQFYVDQATLTGE

SGQTITPSQLTAGNLINVTFEGATPKAVQVVKRTLAEVTAVDASSVTLKQFNGQTETV

PVSGSVKVVKSGSTLTSLSSLTVGDRVEMTKDTDNSTRFRVLTVMSKQFWSYDGVGNQ

ILVKRETTADTNYRFALGSSVFVHHGDNTLSVQSLRDNDNIVLYLLNNVILEIQKQ"

CDS 1590594..1591289

/gene="nth_1"

/locus_tag="EFAGFIKM_01418"

/EC_number="4.2.99.18"

/inference="ab initio prediction:Prodigal:002006"

/inference="similar to AA sequence:UniProtKB:P9WQ11"

/codon_start=1

/transl_table=11

/product="Endonuclease III"

/db_xref="COG:COG0177"

/translation="MNAATVRHILETMEAMFPDAHCELNHSNAFELTVAVLLSAQCTD

ETVNKVTADLFQKYRSPADYLAVPLEELEQDIRRIGLYRNKAKHIQNMCRILIEQYGG

DVPQEHDQLVTLPGVGRKTANVVVSNAFGVPAIAVDTHVERVSKRLALAGWDDSVLEV

EKKLMKRVPRDEWTLTHHRFIFFGRYHCKAQNPACHICPLLDICREGKKRMKTSQIRK

DKERVTTRKRKIN"

CDS 1591313..1591585

/locus_tag="EFAGFIKM_01419"

/inference="ab initio prediction:Prodigal:002006"

/codon_start=1

/transl_table=11

/product="hypothetical protein"

/translation="MKCISVYTDNFEAFSDIFEQIVEQEMAENEEKEVEGITVSHSGD

VPEHYLERMSVKPEVVVMRDKGRDITILQHGQVFEILLPSMENAVS"

CDS 1591794..1592138

/locus_tag="EFAGFIKM_01420"

/inference="ab initio prediction:Prodigal:002006"

/codon_start=1

/transl_table=11

/product="hypothetical protein"

/translation="MILGGDIFMAKKGQTFQTYTEEFKLNAVRSYVEGSSSYKVVAER

EGIRNCSQLKVWVKKWKNGEAFDERKNSVPNPLKGRPRTAFGSVEEERDYLQAQVDYL

KKRYPNLVKEKR"

CDS 1592303..1592998

/locus_tag="EFAGFIKM_01421"

/inference="ab initio prediction:Prodigal:002006"

/inference="similar to AA sequence:ISfinder:ISBth8"

/codon_start=1

/transl_table=11

/product="IS3 family transposase ISBth8"

/translation="MMAIHFANREFGYPRMTTALWEAGLNVNHKKVWRIMRELSIQSV

IRKKRKKSSYTPSVIYPNRLKRQFHATAPQQKMVTDITYISDGSAFVYLSVIQDLFNN

EIVAWQLSKRNDVQLVLDTVEQWTQKRDVSEAVLHSDQGFQYTSQAYNSRLEAFGVKG

SHSRKATCLDNACIESFFSHLKTEKLYLNQCNSEAEIRQAVEDYMYNYNYRRFQAKLK

QRAPIEYRCALAA"

CDS 1593199..1596885

/gene="der_2"

/locus_tag="EFAGFIKM_01422"

/inference="ab initio prediction:Prodigal:002006"

/inference="protein motif:HAMAP:MF_00195"

/codon_start=1

/transl_table=11

/product="GTPase Der"

/translation="MSRTDYPKEMAKPLLPLREAMEQTGDHTAVQAITDLISKAEMKH

LTIAFCGHFSAGKSSLINSLCGKRVLPSSPVPTSANVVSIRNGKPRALIYTTPNVSAD

NGAGTLEVSPEELEAYCKNGGAYSSIEVWDDIPLLKDDAVLLDTPGVDSTDRGHSLAT

HSALHLADVVFYVMDYNHVQSETNLSFAKSLSDWGKPLFLIVNQIDKHRERELSFDQY

TEGVEAIFAAWEVRYDGLLFTSLRDKQHRYNQWDVLPELIGHMMQEKEALITHSLASS

ASHVTEQHLTREAEKREDEENSLLGEIGGKEGIERLERELELLDQQATDIRTGPSRVR

EKFRAELEPLLANANLTPADIRASAAAYLESRKPGFRVGLLFSGGKTEQEKQRRASEL

VRLLQDQASGQVEVHIRTMLRQLGESHQLWGAEWEQALNTELPAVDEALLEMKRSASA

EVSPEYVIQFSKDVRGEIEARYRRSAMMLADRMLEALAARGEAALQALDASRAALLAQ

SAAAARYTALQRSAAAEAAGLRSLLPHAGPLPSGLLPEVKGPHVPAVPEPGEVPGSHA

GTSTSVAAQPQTPEAPPQQRAVAAAVPGPSAAAGAERRRRLDAAAARLEAAAALVEPY

PAMGSAVRDLRARAASLAGGTFTLALFGAFSAGKSSFANALLGEAVLPVSPHPTTAAI

NRIMAPAGGAEHGTARVRMKTRDAFQEDLGYSFRLLGLGEPGAEWQKRVRSLSPQDVH
PAGRPHYSFLQAAASGWEETAGQLGQDVLVDLDGYRNFVANEKKSCFVDSIDLYYSCD
VTEQGIVLVDTPGADSVNARHTGVTFNYMKNADALIFVTYYNHAFSQGDRQFLNQLGR
VKDSSAMDQMFFVVNASDLSSSEEELEQVLDHVSTQLRSNGIRAPRLFPVSSILAMEG
KMAGDSKLLEQSGFIRFEEEFNQFAGRELADLAVGGASEEMARVIQRLKKRAEDAAQG
EEVLQQRRDELETIQGQSRQRIQQLAARSMKEELSQETAELLFHVRQRLAYRLGLFMA
EAFHPSVLREDRGNLKMAFAACGRELLRMIAIELEQELLATTLRLEQAGQTWLMKQVT
DCIDEVRQLSGGVDVSLPLNERWSTPVLEEVRLEEPSGWKSYLNYFRNPKQFFEGDGR
QRLQEALDPVLKQMIIEVLPAAQDKLIQFYDNQICQSLQHQSRQLEERLDEAVSGIQD
TLESGIPAEQWTSLSNQLEQIERS"

CDS 1597210..1598982

/locus_tag="EFAGFIKM_01423"

/inference="ab initio prediction:Prodigal:002006"

/inference="similar to AA sequence:UniProtKB:Q9WYC4"

/codon_start=1

/transl_table=11

/product="putative ABC transporter ATP-binding protein"

/db_xref="COG:COG1132"

/translation="MDVLRQLQIFFWEKRTYLFVSILFLALATALGLVYPYMLRILID
DIIVPRTFEDVPIIALTVLGVVILKAGMQFLHGFYGGRLGNFLAYRLRNACYEKLQFL
SFRYYDTAKTGDLMSRLTGDLEAIRNFIGFGFAQILNMVFMVVFGAIMMMTMSWQLTL
FTLICIPLLAFVALRFESKIHPAFQEMRLALSSLTTAVQENITGVRTVKSFAREPYEV
EKFSIRNERYKTNQIHAATLWSRYFPIMEILASVSIVLLLVIGGRMVIQKTLTLGELV
AFFSLIWYIIGPMWNLGFHINNYTQSKASGERVLELLNTPVDVEETQDPVIVEADQVN
GHVTFESVTFAYGNKMPAVTDINFDAAPGSVIGFLGGTGSGKSTIIQLLMRAYNVNSG
TIKLDGKNIKDIGIRSLRSQIASVFQETFLFSSSIRNNISYGLKNVTMDEIIRAAKLA
KAHDFIMEFPDGYDTVVGERGMGLSGGQKQRIAIARALLKNPKILVLDDATSAVDMET
EHEIQSGFQEVMRGRTTFIIAHRISSLRHADEILVLDEGRVVQRGKHTELIEVPGPYQ
DVYKIQYADYIARGKREAGEQVNS"

CDS 1598979..1600829

/locus_tag="EFAGFIKM_01424"

/inference="ab initio prediction:Prodigal:002006"

/inference="similar to AA sequence:UniProtKB:Q9WYC4"

/codon_start=1

/transl_table=11

/product="putative ABC transporter ATP-binding protein"

/db_xref="COG:COG1132"

/translation="MSAETIADRREAKVASEKKLNERFVYQDDEIIEKPFNWKEFGRL

FAYMKPYAKQLLPLVILMMILGTITKLSVPFLISLAIDRAIAPATGLPSMTMLYLIAG

SIFVLYLIQWAANTYRIKLTNIIGQRVIYDLRSDLFKHIQKLSFNFFDKRPAGSVLVR

VTNDINSLQDLFTNGAVNVMIDCVQLLGIIVILLLINWKLGLAVIITVPIMFIISTKL

RVLIRRAWQDVRMKNSRINSHLNESIQGIRVTQAYTQEKENMKYFDNMNLSSKKSWDR

ASAMNQGFGPLIEITGGFGTLILFWFGAYLIQHDQLTVGLLVAFANYVGNFWDPINRL

GQMYNQLLVAMASSERIFEFMDEKPSIADKPGAKPLPSIKGDIAFENVVFEYEKGRQA

LKGISFSAAAGQSIALVGHTGSGKSTIINLISRFYDISGGRLTIDGQDVRDVTVESLR

SQISIVLQDTFIFSGTIRDNIRFGRLDATNEEVEDAAKAVNAHEFIMKLPGGYDTEVE

ERGNVLSMGQRQLLSFARALLADPRILILDEATASIDTETELKIQEALKVLLQGRTSF

MVAHRLSTIRNADNIIVLDHGEIKEEGNHEQLIQKQGVYNGLIEAQYRFL"

CDS 1601040..1602680

/gene="rhaS_8"

/locus_tag="EFAGFIKM_01425"

/inference="ab initio prediction:Prodigal:002006"

/inference="protein motif:HAMAP:MF_01534"

/codon_start=1

/transl_table=11

/product="HTH-type transcriptional activator RhaS"

/translation="MQMDNNEINSKHLDHLDHMNFRLLHVNYTSDPATEWLLRKHFIE

TYMLLFVASGQGWLKIDGSFIELKAGGLYVGFPGQLVEANVHSLDERGVYHMSFDVMY

ALEQEGGTALEMMKQLLRNEVNGEVSSSSPVAVGVICQMIYHHTLKKDGLQRYYGQIR

FQELLYTMFYDVACAEKTDLASPIEHVKGYMEQNYSKRLTIEELANVARMSPRHFMRL

FKKRYGYSPVDYLTLYRIKQAQILMRNDHSYQLKDVASYVGYQDETYFRRKFKQISGI

PPAAFIRNSKQKIAAIHSVSIGILLALQIIPCAAPASHPWTYYYRRKYETDKVMPLAE

SETTWLEQLRLVKPDYIITIGTESESLQSQLQSIAPVCDLPWGKGDWREHLRYVASFL

DRSDIAEVWLQRYEEKAKIIKNQLTDFIRNDRLVVLKVHGEALQMLGPRSIASVFYVD

MQMEGPEGVETYWERGTVTIKELSVLNVERILLIVGEDEISRQTWSAVRKSEEWLALL

AVENGRMDILLSSVLLDYTAFTHELMLDEILKLWQDRP"

CDS 1602812..1603801

/gene="yfiY_1"

/locus_tag="EFAGFIKM_01426"

/inference="ab initio prediction:Prodigal:002006"

/inference="similar to AA sequence:UniProtKB:O31567"

/codon_start=1

/transl_table=11

/product="putative siderophore-binding lipoprotein YfiY"

/db_xref="COG:COG0614"

/translation="MRFRWLTVLCTMMLVLIISACGTAGQDEATSEGQKTGSDNTSTT

ETSQVKKVTTKMGEIEIPVEPQRVVGLSLVYPEFLYALGIVPVAVQNYHEDHPSYLEE

PFANTMKMGIARTPNFEAVMETMPDLIIAPDWWSEKDYDQLSLIAPTVLLPQRDDWRD

ELRDIAKILDKEEQAEKVIEDLVAKEKVAADKLNEMIGEETVLYIRVMEKEIVLHGEN

LDRGNFVHKRLGMNPLPNFPKDQVAMSVSMEVLPEYDADHLIVQLDDDKNPEIKKKFE

EMLATSLWKNLKAVKNDHVYMVGGKEWLNLGMSPLADQYVIDDIVAVFEEKNK"

CDS 1603864..1604886

/gene="ycgT_3"

/locus_tag="EFAGFIKM_01427"

/EC_number="1.18.1.2"

/inference="ab initio prediction:Prodigal:002006"

/inference="similar to AA sequence:UniProtKB:O31475"

/codon_start=1

/transl_table=11

/product="Ferredoxin--NADP reductase 1"

/db_xref="COG:COG0492"

/translation="MNKEDIYDLTIVGGGPAGLYAAFYAGMRDMRVKIIDGKDQLGGF

LHTYSEKTIWDVGGLPPMKCSKLIEYLVQQANTFNPTVELNCIVDRFARLDNEILALY

TAEGDIHYTRTIIVAVGRGIAEVQKLELQEPIQYEHENLHYTVQNPEHFSEKRVLISG

GGNSAVDWAIELAQLARSVVVVHRKNEFRAMERNVSEMNNVTDVRTPYSITRLHNHGE

RIHQVVITHMESQENVLLDVDEVVVSHGYKSNLSDLASCGLEMDDGMVLMSQHAHTNL

PGIFAAGDCATHDSKVRLIAGAFNDAILAVNSAKKYLTPDAPMMAYVSSHNEIFREKN
KQIPRF"

CDS 1605139..1606326

/gene="purT"

/locus_tag="EFAGFIKM_01428"

/EC_number="2.1.2.-"

/inference="ab initio prediction:Prodigal:002006"

/inference="similar to AA sequence:UniProtKB:P33221"

/codon_start=1

/transl_table=11

/product="Formate-dependent phosphoribosylglycinamide
formyltransferase"

/db_xref="COG:COG0027"

/translation="MWGAPFTAQAKKMLLLGSGELGKEVVIEAQRLGVETIAVDRYEN
APAMQVAHRSYCIDMLDAEVLKQLIRKEKPHYIVPEIEAIATEALLELEEEGFCVVPT
ARAARLTMDREGIRRLAAEQLKLPTADYLFADNLEQLQEAVRELGTPCVIKPLMSSSG
KGQSVCRTPGDVEDCWNIALSGARGKSVRVIVESFVQFDSEITLLTVRSVSGTVFCPP
IGHIQKDGDYVESWQPHAMTPEQWQQACHIAKSVTDELGGYGLFGVELFLTSDGVVFS
EVSPRPHDTGMVTMVTQDSSEFALHVRAILGFPVTGVHLLTPGASATLKANDETSDFT
VGGIEEALALPRTQIRVFGKPETKVGRRMAVALSAGQDVEEARKTAVQAANMLKVEVN
HVQ"

CDS 1606316..1606774

/locus_tag="EFAGFIKM_01429"

/inference="ab initio prediction:Prodigal:002006"

/codon_start=1

/transl_table=11

/product="hypothetical protein"

/translation="MSNNVEAPVLMIRECELRDAEAVTGLMREVSYPTTTNVMKERIE
CLETNPNACMLVAEVDEQIIGVIGLQCVQSHAYPEPAAQITSLIVGQEHRGGGIGRRL
MARAEDWGRQQGGKQLFVTGANREVTSTTYSFYEHIGFQKRGYRFSKVLL"

CDS 1607034..1607846

/locus_tag="EFAGFIKM_01430"

/inference="ab initio prediction:Prodigal:002006"

/codon_start=1

/transl_table=11

/product="hypothetical protein"

/translation="MKKKWMKTVVTSSLTAVLAVGVMLPASASAADSTYKTITTYKIT

STDSLKAYIEKWLKQNGYTVSEGQIVEKPATQPDQTTKPAEPTTKPTQPTTKPAEPTT

KPEQPAKPTQPAQEKPETTPAKDPSNNGNTGNTGNNTSNNGSESTQSDFATQVVKLVN

AERAKAGLNALASDALLDKVAVAKVKDMSNNNYFDHQSPTYGSPFDMMKQFGVTYSYA

GENIAQGQKTPQEVVTAWMNSEGHRANILSKNFTHIGVGFYNGYWAQEFIGK"

CDS 1608136..1609008

/locus_tag="EFAGFIKM_01431"

/inference="ab initio prediction:Prodigal:002006"

/codon_start=1

/transl_table=11

/product="hypothetical protein"

/translation="MMKKKKSLDSAPWIWRTVSTVSILATLLLLFGFGYAIKDVIFPE

GDSALTTGQEATAPSKDIEGEPAVVEDGKIRMAVIGDSLARGTGDDEGLGFVRRAGNL

LKDKGYDVQVLNNLGVNGLRTDALLEKLDEQGVRYVLQQSNFILLSIGANDLFQGGQV

LQGEDPPTAEQLAAALPETSKRLQEILKKVKEINPDAQIAYIGLYNPFGDVKELEKPG

NAVVAAWNDAALAILNNEDKMTLVPTFDLFENHLGEYLSSDHFHPNGQGYEQIAVRIA

QEFQADAPAEGSGN"

CDS 1609009..1609974

/gene="lnrL_3"

/locus_tag="EFAGFIKM_01432"

/EC_number="3.6.3.-"

/inference="ab initio prediction:Prodigal:002006"

/inference="similar to AA sequence:UniProtKB:P94440"

/codon_start=1

/transl_table=11

/product="Linearmycin resistance ATP-binding protein LnrL"

/db_xref="COG:COG1131"

/translation="MTEQQYDSVLSVQNLKKRIGRKWIIKDVTFDVKPGEIFGFLGPN

GAGKTTTIRMLVDLIKPTEGKIKVCGYDVNRDPERALKYVGSIVENPEVYTYLTGWEN

LEHFARMQPGVDNERIQEVVDIVRLDQRIHDKVRTYSLGMRQRLGIAQALLGRPRLLI

LDEPTNGLDPKGIKELRVFIKQLASEGMAVFVSSHLLSEIQLLCDRVAIISAGRVLAV

GGVSELIEDHSKLAIWHVSPLEQGKKMLQDAGIALVGRPADVMDDTIVAGLGPNAVVA

EMHEDRIPDLVQQMVQAGIQVEGVQRIQPTLEQLFLKMTEGESIE"

CDS 1609967..1610956

/locus_tag="EFAGFIKM_01433"

/inference="ab initio prediction:Prodigal:002006"

/codon_start=1

/transl_table=11

/product="hypothetical protein"

/translation="MSNIMPLIRNETIKMVKKKRLYIIFIVLAVLVPMFTYAQMKSAE

NNRDKFGGDWRLELQQAITDNQNSLGSDRVPEEYKKYRTVYIQQMQYYLENDINPKEP

GGVTFTREFMNNAVGLFIPLLIMAIASDLVSGERTTGTIKMLLTRPVRRWKVLLSKLI

TLIMFVSIIVVSAYIICYVISGAVFGYKGFNMPIFTGFKVVGTDVDMSAVHAVDQWLY

MLMQAGLIWFVSVIVAMLAFMVSVLVRSTAASIVIMMAALIAGTILTSMAASWQTAKY

LFMVNLELPNYLSGGLPPIEGMNLGFSLIVLSVWGIASLIVSFLVFTKRDILN"

CDS 1611033..1613012

/gene="parE"

/locus_tag="EFAGFIKM_01434"

/EC_number="5.6.2.2"

/inference="ab initio prediction:Prodigal:002006"

/inference="similar to AA sequence:UniProtKB:P66939"

/codon_start=1

/transl_table=11

/product="DNA topoisomerase 4 subunit B"

/translation="MVEQIDMSAGSTGGGQGSSGYDADDIQVLEGLVAVRKRPGMYIG

STSTSGLHHLVWEIVDNAVDEHLAKFCSKIDITLHKDGSITVQDNGRGIPTGIHKTGI

PTPQVVFTILHAGGKFGGSGYKKSGGLHGVGASVTNALSEWLEVEIFRDGKIHRQRFE

YWKDKKGIEHVGEPVSGLEVLGNTNRTGTKVTFKPDIRVFQSGIQFNYDTLAERLQEI

AFLNSGLRIVLKDERSGNQDEYMYEGGASQFVAFLNENKDVLHDVIHFYAEKDDIEVE

VAIQYNAGYTETLASFVNSISTRGGGTHETGFRAAYTRVMNDYARRTSMIKEKDKNLE

GNDLREGMMAVISVKMSEVEFVGQTKDQLGSASARSAVDSVVSENIQRFLEENPQVAQ

TLIRKAVQASRAREAARKARDDMRTGKKRSESSNLNGKLTPAQSKDFTRNELFIVEGD

SAGGSAKQGRDSKIQAILPLKGKPLNPEKSKLADILKNEEYRAITAAIGAGIGTEFAV

EDSNYSKIIIMTDADTDGAHIQVLLLTFFYRYMKPLIDAGKVYIAQPPLYKLTRKSGK

LASVRYAWTDEELANYMKEFGNNVELQRYKGLGEMNPDQLWETTMNPETRAMLKVQIV

DAAKAERRVSTLMGDKVDPRKRWIVENVDFTEYEE"

CDS 1613025..1615487

/gene="parC"

/locus_tag="EFAGFIKM_01435"

/EC_number="5.6.2.2"

/inference="ab initio prediction:Prodigal:002006"

/inference="similar to AA sequence:UniProtKB:Q93KF4"

/codon_start=1

/transl_table=11

/product="DNA topoisomerase 4 subunit A"

/translation="MSPSEQFMPAFLEEVVGDRFGRYSKYIIQDRAIPDVRDGLKPVQ

RRILYAMYDSGNTPDKTYRKSAKTVGDVMGNYHPHGDSSIYEGMVRMAQPWKMGHMLV

DGHGNWGSQDDDPAAAMRYTEARLSPIAMEMLRDIEKRTVLFKDNFDNTAKEPVVLPS

RYPNLLVNGVSGISSGFATEIPPHNLREVIDASIAVMEKPSIELDEIMMFMKGPDFPT

GGLIMGGDGILDAYRTGKGRIYIRSKTEIENMRGGKQQIVITEIPYQVVKSRLVTAME

NIRLEKKVEGIAEVRDESGREGLRIVVELKKEADAQGILAYLLKKTDLQVTYNFNMVA

IVNKAPHQLGLKSILEAYIAHQREVVTFRTRFELEKAQDRAHVLEGLVKALNILDEVI

AAIKASKNRQDAQNNLMWMFGFTERQADSILTLQLYRLTNLEINSLEKELGELMKKIA

QLQSILDSDRKLIGVIRKELMEIREKYGIDRRSAIQGEVEELKVNLEVLVNAEDVFVT

LSKEGYVKRTGMQSFTRSGGERSGSGVKDGDYIAQVLEVNTLENLLVFTRKGQYFLLP

VHQVPEFKWKDPGTAIVNVIPLAKDDRIASVLAVKSFEEPNHSLVFVTRRGQVKRTEL

KDYVTKRSGAVAACKVGKDDEVLSVHLSTGGQDIMLITKEAMAIRFREDEVNPMGRVS

GGVRGIQLRDTDEVVSALWVEGDEGEVAVLSDLGYGKRSLLLDYAIQSRGGKGLATFE

FKEGKRVKPNGSRIVGAFYCKEQRKITVMTKEGQVFPISSEGVPITERKHIGKLIVHV

DKQDEIVELLRDFNENQPAASS"

CDS 1615716..1616156

/locus_tag="EFAGFIKM_01436"

/inference="ab initio prediction:Prodigal:002006"

/codon_start=1

/transl_table=11

/product="hypothetical protein"

/translation="MYNSDFAKCWSRLTKDYKLHMDQELAPSLTEAQLAVLEVLEDHQ

KMKPSDLIPYLSTTPAAVTMLLDRMEKNDLIRRNRDNQDRRIVWVSLSEKGRMETERG

ITIRNEFMNSVLSNISMHNQQLLVYLLGKMTTPKPKEPALSTSV"

CDS 1616432..1616878

/gene="mgrA"

/locus_tag="EFAGFIKM_01437"

/inference="ab initio prediction:Prodigal:002006"

/inference="similar to AA sequence:UniProtKB:P0C1S0"

/codon_start=1

/transl_table=11

/product="HTH-type transcriptional regulator MgrA"

/translation="MTDTYEVFYIINSFRQVNQMLFRAFWNENKEIELTSIQFMVLSI

LRERPSIGINEVAELCHMGSSSMSAVVERLVKGEYIVRTRSDADRRSVKLQITDKGEQ

AQQETHHLWMERVSPILDISKEDLEHLLRIHSQMIEKLEGREMNNI"

CDS 1616875..1618386

/gene="emrB_1"

/locus_tag="EFAGFIKM_01438"

/inference="ab initio prediction:Prodigal:002006"

/inference="similar to AA sequence:UniProtKB:P0AEJ0"

/codon_start=1

/transl_table=11

/product="Multidrug export protein EmrB"

/translation="MSTTTAAAPSSAMDNIKKGPIVAALLIGAFVAFLNQTLMNVALP

KIMEDLAINANKAQWLTTGYMLVNGVLIPITAYLIAKFSTRQIFITAMTLFTIGTLVC

GLSPTFTILMVGRVIQAAGAGILMPLMTVVFLTIFPIEKRGQAMGTMGIAMILAPAIG

PTLSGYVVEHYSWRLLFYIILPFSIIATAIGIAFVKNVTRQSRPKLDYPGVILSTLGF

GSLLYGFSDAGTDGWGSAIVIGCLVVGAISLILFVIRQLTTDHPLLEFRIFKYNMYTL

TTIINMLVTMAMFAGMILLPIFLQNIRGFSPIESGLLMMPGAILMGIMSPITGRIFDK

VGARWLSVAGLAITAITTFGLSRLAIDTTYGYMMFIYTARMFGMSMLMMPIQTAGLNQ
LPQRLNAHGTAMSNTLRTVAGAIGTAILVTIMSSKLKTHLADAVAAGQVNPNDKTAMI
AATADATIYGVNYAFIVATGMTVVALIMAFFIRKTKPAVEPVTVEQEKATATA"

CDS complement(1618499..1618843)

/gene="atl"
/locus_tag="EFAGFIKM_01439"
/inference="ab initio prediction:Prodigal:002006"
/inference="similar to AA sequence:UniProtKB:P0AFP2"
/codon_start=1
/transl_table=11
/product="DNA base-flipping protein"
/db_xref="COG:COG3695"
/translation="MTPFTKQVVAIIAAIPEGKVMTYGQIAAHAGSPRAARQVVRILH
SMSRKERLPWHRVVNAKGEISIPDEHSRMMQETELISEGVEFQLNGTINLKQFGHEPD
PVFLIDPTIQPE"

CDS complement(1619037..1619768)

/locus_tag="EFAGFIKM_01440"
/inference="ab initio prediction:Prodigal:002006"
/codon_start=1
/transl_table=11
/product="hypothetical protein"
/translation="MSEHRHDHDAIPILFEDNHLLGITKPVNVPTQEDASGDPDLLTL
LKQDLKERYNKPGNVYLGLVHRLDRPVGGAMIFAKTSKAASRLSETVRGRHFRKIYAA
VVHGKLPVQQGTLKHTLLKDARTNTVTVVPKGTAGGKDAVLDYRVIGESDRYSLVHIE
LHTGRSHQIRVQLKEIGCPLYGDQKYGASVNKPGQQIALWSAIAAFPHPVTKEEVILQ
SLPAREFPWAEWPAAVYQKAFGQPQ"

CDS complement(1619771..1620634)

/gene="rlmL_1"
/locus_tag="EFAGFIKM_01441"
/EC_number="2.1.1.264"
/inference="ab initio prediction:Prodigal:002006"
/inference="protein motif:HAMAP:MF_01858"

/codon_start=1

/transl_table=11

/product="Ribosomal RNA large subunit methyltransferase

K/L"

/translation="MYVAKNWKDYEVIDTGGGEKLERWGDVILRRPDPQIIWPLEQET

NEWRQVHGHYHRSSSGGGNWDMKKPIPERWTIGYENLKFHIKPTSFKHTGLFPEQAAN

WSWMMDKISNAGRPISVLNLFAYTGGATVAAAYAGASVVHVDAAKGMVQWAKENVQLS

GLADRPVRFITDDVFKFVQREQRRGNRYDAIIMDPPSYGRGPNGETWKLEENLYPFLK

SCMTILSDNPLFMLVNSYTTGISSTVLRNMLTMTMSAQYGGDITAGEIGLPITRSGLD

LPCGILGRWES"

CDS 1620929..1621381

/locus_tag="EFAGFIKM_01442"

/inference="ab initio prediction:Prodigal:002006"

/inference="similar to AA sequence:UniProtKB:P26492"

/codon_start=1

/transl_table=11

/product="Flavodoxin"

/translation="MAKLLVAYASMTGNTEEIAELIVEGITQGGHEADLKSVTDCNAS

DVLDYDGFMIGVYTWGDGELPDEFLDFYEELDELDLSGKRAAVFGSGDTSYEQFCGAV

DLAAAKLQERGAEVSPEMLKIEYSPLEQEKETCRDFGKRFAAAGLQVS"

CDS 1621414..1622193

/locus_tag="EFAGFIKM_01443"

/inference="ab initio prediction:Prodigal:002006"

/codon_start=1

/transl_table=11

/product="hypothetical protein"

/translation="MPVGRNDLPERLLEDYRLEEMLRSPHRFIRPEPVSEQRLPLQWR

HRVQYAVSHAVNAFYSLDPDVRKEVPVQYLLEKWWPRKTDGFESVLHYWDVKNKITDE

LSLAIAMNDDLKHPAILFEQWRTELPSLSMHLSMIFQAAWQPEGWDSLLIQKYMVVHD

PNVVEAFQHMVSAFWWEAFGKLPGMIEIYCLLEGRKIRYIPGRQSLIRSMDYIRLIRD

SMPQAEVVESEQFTTRDKARIHEEVDRWRTEAIEPKKTWLM"

CDS 1622246..1622812

/locus_tag="EFAGFIKM_01444"

/inference="ab initio prediction:Prodigal:002006"

/codon_start=1

/transl_table=11

/product="hypothetical protein"

/translation="MSTGLSQDISSSKRRAGTCEFSPLPVPQSVIRKLLDEADPSLYV

MSSEPWRFMLFAGEGRQLYLEAVRQSYPPHLADRYGDWATYQYTEAIPTHLVVVAPRN

AREDDLLPAKAWSKRFSILAAEQGLNAVWKINDYQQHPVFMNLMGLTSEEKVLGVFHI

GYGDQPALRDMDASRPASELMTVYDHLV"

CDS complement(1622834..1624291)

/locus_tag="EFAGFIKM_01445"

/EC_number="1.14.14.9"

/inference="ab initio prediction:Prodigal:002006"

/inference="similar to AA sequence:UniProtKB:Q5SJP8"

/codon_start=1

/transl_table=11

/product="4-hydroxyphenylacetate 3-monooxygenase oxygenase

component"

/db_xref="COG:COG2368"

/translation="MPVKSGTQYRERIDAQSVPCWYKGNLITGKRSEHVAFAGLMETQ

MYMYDLQSDPKFAETMTYASPADGKPVGISFLPPTSADDLRKRREGMNIWANVHHGFL

GRSPDYMNTAIMSFYTGADLLKELSPQYAENLKNYYAYCRDHDITLSHAFIQPYASKI

SGQLDATEDAIAAKVVDRTEEGLIISGAFMMATQAATSDEIFVYPSPSPAPFDDENPF

AFSFAVPNDLPGISLVCRDTYAAESHTNYPLSSRYEEMDNIVIFDRVLVPHERIFFAG

SEEISSRLFSGSNFHIHAGHQVLCRYIAKTEFVLGTIQLLTDTLDLNTEAHVIEKTAR

VFAGLESLKALALAAEAGAIPDGRGFVLPAPKPLMAANLLFPKLYPEMIEILQLLGSS

GVIMIPQEEEFQSDIAPSLNIYLKGNDMTSHERNALFRLIWELGAGSFGGRQTQFERF

FFGNVLTVSNRLFSVYNKDQTNRQLVRDFLSNNSK"

CDS complement(1624493..1624645)

/locus_tag="EFAGFIKM_01446"

/inference="ab initio prediction:Prodigal:002006"

/codon_start=1

/transl_table=11

/product="hypothetical protein"

/translation="MAKDVLCEVNSCRHWAQENKCNASSIYIVSHSNKEASQSAETDC

KTFEVK"

CDS 1624991..1625803

/gene="ftsH2"

/locus_tag="EFAGFIKM_01447"

/EC_number="3.4.24.-"

/inference="ab initio prediction:Prodigal:002006"

/inference="similar to AA sequence:UniProtKB:Q55700"

/codon_start=1

/transl_table=11

/product="ATP-dependent zinc metalloprotease FtsH 2"

/translation="MSKINHMNRIPRAKGIDMAAFYTPKECRRKAQHIVFSAENERII

NEFITILGMKEKFREHDVSIPNKMVMFGPPGTGKTLTASHLAERLDLPLVLVRLDAII

HSHLGETGSNVRKLFEYARMNPCVLFLDEFDAICRTRESNDEVKEMARVVNTLLQCLD

EFDGDSILVAATNLETQLDHAIWRRFDTKMTYGMPDDSSRKLYISKLVGAFEQETRLE

DYICERLTGCSYADIEQIVLKAKRKAIIASSALHKQLISDAYDEYRPRVLEC"

CDS complement(1625900..1627366)

/gene="sasA_3"

/locus_tag="EFAGFIKM_01448"

/EC_number="2.7.-.-"

/inference="ab initio prediction:Prodigal:002006"

/inference="protein motif:HAMAP:MF_01837"

/codon_start=1

/transl_table=11

/product="Adaptive-response sensory-kinase SasA"

/translation="MDRQLKQKKPKRTISILSHWTLRYFLILCIGFTIIAAGALYWIR

TTSIEKSLKTAELLGLEIADRVTRENNMLRVPPDLDRLVTKREKLFNTDHYFCVMILD

NNNQLIFSQPKMEQKDVHYRLSDDYLEPRNNKYAGVTVNISEGDQTLGKVWVMQSKQS

ITFGPETIWLVALILGGLILCGWFTIYLLSKKLSRPIRQVAYAAEQIRGGNYDVSLDL

NTREREINELVNSFRDMATRLQQLEEWRTLSLAGVSHELKTPVTSIKGLVMAVRDDVV

SPQEGKEFLDIALKESERMERMVADLLDYNAMAAGSVAVRKERTDLKLLVGEIIYQWK

IAYEDKMPEVQLHAPPINFFTMGDALRIQQIIVNLLNNALHATAPEQKAVFDIYLSTE

EQMLYVDVKDHGTGIAAEEQPKIFERFYRGELKKRRNRGLGLGLTYSRLLAQEQGGEL

ILVSSSPEGSMFRLSLPRWTATQEAMNTEKAYGAAVKV"

CDS complement(1627351..1628037)

/gene="srrA_3"

/locus_tag="EFAGFIKM_01449"

/inference="ab initio prediction:Prodigal:002006"

/inference="similar to AA sequence:UniProtKB:Q9L524"

/codon_start=1

/transl_table=11

/product="Transcriptional regulatory protein SrrA"

/db_xref="COG:COG0745"

/translation="MKSILIVEDEQAIARVLAAYLRKAEFEVHHAADGPTALTLFDTV

TPSLVLLDVMLPGMDGWDLLRIIREKSACPVIMLTALDDISDRLNGLNAGADDYMSKP

FVPEEVVARVNAVLRRNPHWTSGGKEKRSFGNLVIDLAAKQVLLNGAEVALTPRDLSL

LFFLSDYPNRTFTRDHLIEQVWGMDYDGSDRAVDLSIKRLRQALSHWSPETGEIRTLR

GMGYQFWIAN"

CDS 1628230..1628946

/gene="yceI"

/locus_tag="EFAGFIKM_01450"

/inference="ab initio prediction:Prodigal:002006"

/inference="protein motif:HAMAP:MF_00780"

/codon_start=1

/transl_table=11

/product="Protein YceI"

/translation="MNKKAKAWLITGVAAVAVIGGGGYYITNSYLGNNVEIEQVLPAS

TATSTTAVDSTGTVVANETAGAEQLNGDWSISEGSKVYFSVTTSQETVNFVEDMVTGN

WTINVDDAAQMKAEGQIEMDGIDSGNGQRDGHVKQADFFDVATYPQATFTATSFEGVP

AEWPVGQTVDIKMNGTITVKGIEKEVTFDAKAAYENNAVLLSATSMVTFEDFGMENPH

SVVLSTENDIQVQLELKLSK"

CDS 1629204..1633583

/locus_tag="EFAGFIKM_01451"

/inference="ab initio prediction:Prodigal:002006"

/codon_start=1

/transl_table=11

/product="hypothetical protein"

/translation="MNKRYISGCLVLLMLFCDLGLMVRPAAAEDAKVNVALNKAVSVS

TEYLEWESKKEHLVDGKSDTHWTATAGVTSEQPQWAMIDLGEDYNLSGAEITWRENTV

VKYMVETSADQQAWTKVVDQSENADPKQIADLTFQQNDVRYIRLNISFYKGEGVWPEI

LEFKVWGEAEGTEPANIKGYDEIEVRTVTRSAPILPNEVKAQFQNEKSGMVPVTWDHI

DPMQYASAGEFTVTGVVYGAPPEPQLKAKVIVEGYRADYVRGVDISTLTAIEDKGGKY

VDSNGTERDLLDILKDRGVNYVRLRLWNDPQKSGGYNDKDDVIRLAERVKKKGMKILL

DFHYSDEWAHPGQQLRPKAWEGLSPEELKQAVYNYTYEVVGEMKAEGAMPDMVQIGNE

INSGVLNGLSSDVDFKQNVALLKSGIDAVRAVEGEITNNADKVKIMIHLAEGGKVDTF

DWYFSELEKRELKYDIIGLSYYPFWHGTFADVQKTMNKVSDKFKKDVIIAETSYPFSY

KNGDAHGNIIGSDEALHVGGADFPATVQGQYDAIAGIMDMIANVPGNHGAGFFYWEPA

WIPAGVGWIASEGNAWENQAMFDYDEFPANGGHSYEGRALWSLDVYKRGLSLLPADRQ

QLDAALTRAESLVQTDFTLDSWVTLAPAISTANDIYEQAYTPGGVTQADTDAATAALE

RVMQQMQVITPQLDELRAKIAEARTYQQTDWSAATWAVLTKALEGAEQVLNDPRATQT

DANTAVKRLHDAIQGLSNVDKTQLHQYIQQMQQQTKSSYTRRSWSVLEESLQAAIQVR

DNNAVVQSEVNSALSTLKQAFTNLVSLEALTTGKTATASSSAGTGGNQANSPEGAIDN

NPNTSWGTDQSKDNWWQVDLGQVANLRKIEMSMWSGGIKYTIEVSNDNKNFVKVVDTT

SDVVVSTAPSHVLPEGTEGRYIRVTIKTGPTWVGFMEFEAYGTFPADKTALQATVDAS

EKLKQTEYTTSSWNVFSKALSKAKAFILDVETSTQDVSNADRALQDAVSKLVRQDVTP

GPGQPSQPSEPSQPSNPGTAPSTGNPAVTTPTEPGNPPAKGLITVKGTATGDDKYSIR

PDVSLLTQALQSMDAGNRTLELIADVPDTAKTVTFQLGASQLAAWIRSEEVKSLDVVV

GQTTVRVPLRSLPLIIAETAGTFDIVVAKGSTDTLSTSQKAVIGNHTIVDVNLLMNNK

PLQWTNRAVEVALSNVEQPKKNDVAMVMHSISTSGEMKPVTYSKYDDKTKTMTFKPLG

SGSYVIAEVEVPLNDLQNYNWALQEVQNLYGKGIITGMSETRFAPQGELTRAQFLQMI

IKGIGDTRQPDPSISVPTDVKEGQWYADSVRLGMEMEIIQGRADGSFGANERISREDM

AVMLNRALKVVQHDQDATNAVQGNMVFVDNAEIAAYAKEAVASMQQLGLLNGMPDGTF

APKETANRAQGAVAVARLMEQLY"

CDS 1633850..1634893

/locus_tag="EFAGFIKM_01452"

/inference="ab initio prediction:Prodigal:002006"

/codon_start=1

/transl_table=11

/product="hypothetical protein"

/translation="MFGFRFAKFQPSEYVMKVKNGEVQRQGVGLSFYYYEPTTSVVVL

PVSSVDVPFMFEEITADYQTVTVQGQLSYRIVDYLKITQSLNYTYNLRKNRYISDDPG

KLDQRVITIAKVLTKKHLEQMPLKEAIQSSERLASSMKREVVQSEELEKLGVELMSLS

ILAILPNKETMRALEAQAREEILRQADEALYVRRNASIEQERRVKENELNTEIAVETK

KQQIRETQLQAERSVKQKQNEMEQEQLQFNTAMEERKQQLIELTIANHNAEADAKAYE

IAAVMNSLQNVQPNVLQAMANMGMNSDKLIALAFQELAENAGKIGQLNISPDLLQGLM

SPTSGRDQGGRAR"

CDS 1634890..1635939

/gene="nadK"

/locus_tag="EFAGFIKM_01453"

/EC_number="2.7.1.23"

/inference="ab initio prediction:Prodigal:002006"

/inference="protein motif:HAMAP:MF_00361"

/codon_start=1

/transl_table=11

/product="NAD kinase"

/translation="MRLGRREQAEQGGKSVRQQATADDRMSEHKLILVKRRTRLEELV

VRYNTVQQAQFYIERLGADFSDYLEEDRRYRQSVQQAQQQLGQLGRVQTIDREHVPNF

IFGEQDIVVVVGQDGLVANTLKYVTEQPLIGVNPDPMRWDGVLLPFTVEDLSFIVPDV

IRKQRTLKEVTLAKVELNDGQYLYGVNDLFIGRKTHVSARYEVRLGTSAEQQSSSGII

VSTGMGSTGWFKSVLAGATGIVGSAAWQNMNSEAEVATASASIHGSKQELNHFNWDAP

YLYFSVREPFPSRTTAANLVFGQIHSKQPLHIVSQMPEDGVIFSDGVEQDFLEFNSGV

EAIIGLAEKRGRLVV"

CDS 1636023..1636736

/gene="graR_1"

/locus_tag="EFAGFIKM_01454"

/inference="ab initio prediction:Prodigal:002006"

/inference="similar to AA sequence:UniProtKB:Q5HI09"

/codon_start=1

/transl_table=11

/product="Response regulator protein GraR"

/db_xref="COG:COG0745"

/translation="MQSILLVEDDAKLAGLLAAYLTRNGFQVQVTDDFRHVLDEFVEV

KPDLVLMDVNLPQYDGYYWCRQIRTHSICPILFISARDSGMDQIMALEHGADDYITKP

FQYEVVLAKVRSQLRRAYGSYAAVQQEVKVKNGPMILYPERFVLEYDERSTELTQKEA

ILVEALMAKAGRVVSREKLLEQMWEDQYFIDDNTLNVYITRVRRKLKELGAEEILETV

RGAGYRVLDLGSSAQGEGK"

CDS 1636733..1637731

/gene="graS_1"

/locus_tag="EFAGFIKM_01455"

/EC_number="2.7.13.3"

/inference="ab initio prediction:Prodigal:002006"

/inference="similar to AA sequence:UniProtKB:Q7A6Z3"

/codon_start=1

/transl_table=11

/product="Sensor histidine kinase GraS"

/translation="MRLFLKDHQMLVGFYVLQMLLVPCVYWLSGEGRPMKIVLYGMLI

STVVLMVYLVTRYFQLRAYYRLLQQPRNLVNEPFQTLGNSVVAEAIQELLHELERNRQ

NEMGQLRTSKEQQVVFMNRWVHQMKTPLSIIQLTLEDVDDETAGSIQDELDKMRKGLE

MVLHSSRLEQFEGDFRVELLSLHEVLRSSIAENRRLFIRRGITPDIRMEGELQIYSDS

KWLRFMFTQILTNAVNYSDSKSGKKVIITGYKQEERTILDIQDEGIGISSEDIHRVFN

PYFTGERGRQYHESTGMGLYLVREISARLDNRVELFSEPNEGTLVRFSWIHSKYPK"

CDS 1637917..1638687

/gene="yxdL_1"

/locus_tag="EFAGFIKM_01456"

/inference="ab initio prediction:Prodigal:002006"

/inference="similar to AA sequence:UniProtKB:P42423"

/codon_start=1

/transl_table=11

/product="ABC transporter ATP-binding protein YxdL"

/db_xref="COG:COG1136"

/translation="MEMLQVSGLNKVYQGKVQTQALSDIHLTIKQGEFVGIMGPSGSG

KTTLLNMVSTIDEPSSGKVLINGMNPFDMKKKELAMFRRRELGFVFQDFNLLDTLTVA

ENIVLPLTLDKVKLMEMESRLHRITAKLGIDHILDKRVYEISGGQRQRTAIARSMINM

PSIVLADEPTGALDSTSSQAVMESLEQMNQQEKTTIMLVTHDPLAASYCNRIVFIKDG

KLAAEVHRGDNRQTFFQRIIDTLSFWGGNSHELSSVRV"

CDS 1638662..1640623

/gene="yxdM_1"

/locus_tag="EFAGFIKM_01457"

/inference="ab initio prediction:Prodigal:002006"

/inference="similar to AA sequence:UniProtKB:P42424"

/codon_start=1

/transl_table=11

/product="ABC transporter permease protein YxdM"

/translation="MSFPQFAFNNIRRNGRAYIAFFLSSVFMVMIFFAYAVFIYHPYV

AELPMGDTTATGMQIASVIVYVFAFFFVMYSISAFLKSRNKEFGILTILGAEPRQIRR

LVILENMLIGCLSIIAGIGGGMLLSKLFLMLTTRFIGMDDLPFYFPVKAILITIGAFL

LLFFCISLFTLVFIGNKRTLELLTGTNKPKKEPKASWFFSLLGFALLTVGFVVLRSGL

NGGTIMTAAVTGIAGTYFFYSQLSVLIIRLLKRNRKTVWQGTRLLWISEMSYKMKDNA

RMLFMVTVVIAIASMSAVVILSLDQENREQFTNNDFAIQYNVYTPSDQMDMSTIDSRL

EAAGLDYQQMYLEYFWSDADQSAVSVMSLSHYNRFNTILGKKTFQLDSDSALFVDSRS

EKDQAQIDRNRFRNYMEGDTLTLNLEAGSMQAIVKATDVEPRFATLIYSTGVVVLPDE

KYEEITQQIIGNRLSGKVLYQIPAWGHAVPDRHSSEAMVSDQLLSSADTIRSDNASSD

ENYSGFLTTRYGDFERYRQGTALFTFLGIFIGLIFSISSASFLYFRLHTDLEADGRMV

HSLSKMGLSFQEMKRSSTVQIAILFFLPIGVAIIETLVVVKPFLTEFGITNYTMPVLT

VFGAFVLAQTFYFIIIRSRYIASLRKMMV"

CDS 1640764..1641108

/locus_tag="EFAGFIKM_01458"

/inference="ab initio prediction:Prodigal:002006"

/codon_start=1

/transl_table=11

/product="hypothetical protein"

/translation="MMSITSLQEVKTFSEDRFTKRVLFQQGGGVTFVLHFLPGQQLPV

HKHPGTDVILLVVEGTGTLILDGKEQAVKQEDVIHCGGEVEFAFHNTGDQEVRLFVVL

NKVPAASYAKDI"

CDS complement(1641251..1641952)

/gene="ntcA"

/locus_tag="EFAGFIKM_01459"

/inference="ab initio prediction:Prodigal:002006"

/inference="similar to AA sequence:UniProtKB:P0A4U6"

/codon_start=1

/transl_table=11

/product="Global nitrogen regulator"

/translation="MSRVNKDTTAEFLQQFPIFQDLSPDELKQVEDIAISRSIQKKSV

IFSEGSEKEAVFFIRTGIVKAYKTDENGHEQIVSFLKTGDMFPHTGFFNAHPYPATAE

AITPTELLAIPVRLFERLMLNTPSIAIKIMRVLGDKIRELQDKLQVLSGQDVRNRVLS

FLLMLAEQHGHMHGDQVIINLPMTHQEFANSIGTTRETANRLLNQLTKENLLEVDRNQ

IIIHDLQALKQQRDT"

CDS 1642130..1642852

/locus_tag="EFAGFIKM_01460"

/inference="ab initio prediction:Prodigal:002006"

/codon_start=1

/transl_table=11

/product="hypothetical protein"

/translation="MWTAFMWGGISASAVVIGALAALFLKIPKRVIGWIMAFGTGTLI

GAATFELLGDALNDGGIIPTAIGFTAGAVVYTLFDLLVSAKGGAGRKRSAKSGESNQS

GLGIFAGTVMDAIPESIMLGASLLAGNGVSVVLVVSIFVSNIPEGLSSTVGLQGKYSR

GKIILMWLSVLLISAIAALGGYLFLEQLPEEMGAAIGAFAGGGIIAMICSTMMPEAFE

EGGPVVGLIASMGLLVSLLLDL"

CDS complement(1642984..1643685)

/gene="fnr_2"

/locus_tag="EFAGFIKM_01461"

/inference="ab initio prediction:Prodigal:002006"

/inference="similar to AA sequence:UniProtKB:Q9EXQ1"

/codon_start=1

/transl_table=11

/product="Anaerobic regulatory protein"

/db_xref="COG:COG0664"

/translation="MICTHEAKDACFSKVSLFQHLDPSEAALLISLLHTRKYNRGEVV

VQEGERSDTLYVVHQGCVKLSKYNENGKEHIIRFLFPGDFFGQDSLLHQKPHAANAEV

LEPSAICSISKHDFDQLLEHDPKLAYHFLLAISKLLRETDEWNSSLSAMTTEQKIAKL

LLYFHTRNHAKHEIRLPVFKKDMALLLGITPETLSRKLTMMQTQGLLQVTGNCILILQ

LEQLREKIEPPARIQ"

CDS complement(1643742..1644740)

/gene="aniA"

/locus_tag="EFAGFIKM_01462"

/EC_number="1.7.2.1"

/inference="ab initio prediction:Prodigal:002006"

/inference="similar to AA sequence:UniProtKB:Q02219"

/codon_start=1

/transl_table=11

/product="Copper-containing nitrite reductase"

/db_xref="COG:COG2132"

/translation="MFSLLNVYKWSLVFAIFTATLSHCSSTDTVKPVHHDEVSSSSSS

AISQPPVDPIIRREGTTVYIEMTAQVTDVEISEGVIYNAWTFNGTVPGPVLRVTEGDT

LVFTLKNKDSSLPHSMDFHAVHAAPSSKFIDVMPGEEGTFTYPTSSPGVFMYHCGTKP

VLAHIANGMYGMIIVEPKAGYPSAHLIDREYTLVQSEWYKEHDYEAFLNGEPDYVVFN

GNDYGLVKHPLLAKVGDTVRIYVSNAGPNEVSSFHVVGTIMDRVYTDGNPRNIQYGIQ

TVMLPASGGAVVEFTVTEEGDYAIVTHQFNHVAKGAAAVLRVTKDGTDHGGPAMSH"

CDS 1645020..1645496

/locus_tag="EFAGFIKM_01463"

/inference="ab initio prediction:Prodigal:002006"

/codon_start=1

/transl_table=11

/product="hypothetical protein"

/translation="MKRFEDEGAYKYSFYSVLRRGERIDVKCPNCGGHASIDQRNGQL
GWKCYECYAQAFEEPVYQYKAKGNCAVCERWFNVEVTDEKKTSHRSTHVECPHCGSMN
REHLNYLIAYVSADLRVKYGNMPIRTASHSIPAYIKDAKNRDVVVRTLSKLQHKTG"

CDS 1645542..1646192

/locus_tag="EFAGFIKM_01464"

/inference="ab initio prediction:Prodigal:002006"

/codon_start=1

/transl_table=11

/product="hypothetical protein"

/translation="MKLRVKLTIFAIILGMLMIPAYFILQTFGVFQKETVLSDYALAV
DVNGKSYGAWPLINSYAAMDKEEDNRQFYYRIDVSHIQYLFNLAYQEYEVKPGGDNPY
LAGTVNYEHTDHAYVHTEKKYENANDFRTVLNLYDQDGQVIYSYDNTGKGDKQLVQSI
IHQGMSRNSGGSSEAARDPYINITALFRDKLNIDVKLTVDEEHKVVTIRMNKSEAR"

CDS 1646192..1646749

/locus_tag="EFAGFIKM_01465"

/inference="ab initio prediction:Prodigal:002006"

/codon_start=1

/transl_table=11

/product="hypothetical protein"

/translation="MNEYGVQHGPAYLEGLIRKEADIKSDEVEWKGKLQTSDGHDKFH
IFYYGDLTEDDLITWHDSTPLLVYAEHTVTGERYLLIDTAKHGYDAMLCETYSEKALH
DRPLRPYLDVEGEDIFEVELTAYYNVPWDEEFAEDVDKNGAYELITGERMHFDQVKRD
GYDAFAIRILNRKGVWTEIVQEELA"

CDS 1646795..1647529

/locus_tag="EFAGFIKM_01466"

/inference="ab initio prediction:Prodigal:002006"

/codon_start=1

/transl_table=11

/product="hypothetical protein"

/translation="MYIVSRVLKPVPTEELDHFEQQHGISLPLSYRTWIEHYGEGTYT
GWMNVQRPDPEVLKPFVEYDFWIHTDDSPISQHQLEQCISIGSSVDGDFLAVHPEVEG
LLWLPRHDENITLWTCSETEFGETLDRIYCGYYHQDKPITPLYFEPWNELRKHTFYHF

SGTEQGSSMKELADTCKTEFKWDAVLENEYMCKMFMVSMGGYLRFNYATGREVALFYE

EKNGAEEATDNEISLFLQAHHCTAYE"

CDS 1647551..1648852

/locus_tag="EFAGFIKM_01467"

/inference="ab initio prediction:Prodigal:002006"

/codon_start=1

/transl_table=11

/product="hypothetical protein"

/translation="MSWLKEDIAEQWRNEGKRYARDVNAFVRRSMQGEQPDESELEGD

SRSGMSTEVFQMVRQANLHGDLEMLRRELPAATWPLNEAFQASMQAVKVVGYLNEETI

VCNTGIFTENSQIYTMDQHGWQLISPYTFAGCSSDGLDYTLAGPEGIRVIRQPDRKLE

GQEIATFRWEDIQARIQSALPRIESLADCEHPERTLEGIIPMDEGRALLIHSRYGIYL

VTQDQLTLLHPDLAEIEEYELEDTQIDMGHAAVSRDGRWIAYGSQGSNHMVMDRRLSK

TYSLYPESSYPHYAAFTSDNQTVWFNACHFYNGVTIQVQLKSIAGNETKEDWPVMDEN

ARVYAAVEIEAGMVLGDAYGYLYCVNDKGEEVWRHFVGSTIYSLAVSPERSKLAVGTY

GGMLHVLDLRSKKMDEYGIGTGTLRELERYVLWRGEEPLRW"

CDS 1648906..1649307

/locus_tag="EFAGFIKM_01468"

/inference="ab initio prediction:Prodigal:002006"

/codon_start=1

/transl_table=11

/product="hypothetical protein"

/translation="MLLKCHAFSYEDLSHPKMENGFLRSLRPFRGHLIEENFHELMEI

LQVLAPELARPSLDREVMACLWGITHMARAWAVETDGMLRSNNLISDEQVALMEQWLN

LLSYAVMILIEGGGEEEAFWEYHQYLQEEKN"

CDS 1649329..1649934

/locus_tag="EFAGFIKM_01469"

/inference="ab initio prediction:Prodigal:002006"

/codon_start=1

/transl_table=11

/product="hypothetical protein"

/translation="MEPNVTLYVLMDELNRLLDEKIQDQEIRQLFEEYQQREGASEES

LDLFEKEYGVRLPGDFRAFYQRKDGSGYGLHVLYSGDAETGRCTPFYLMSLEEIRETK

QYFCEVDEKLEEYYSAEEISQLDPEIKPYLFHKPWIPFATIAGGSLYLMLDFDPTAEG

TYGQVIMYVHDPDFVYYLTPTFTDLLSMSNRNLKMMDEITY"

CDS complement(1650026..1650370)

/locus_tag="EFAGFIKM_01470"

/inference="ab initio prediction:Prodigal:002006"

/codon_start=1

/transl_table=11

/product="hypothetical protein"

/translation="MAYGPGSVSSPQSAGYPYPYYPPPPPYPYPYPYPYPPIVPVPLP

YPIGGPWWHGGYPGGGHPGGHPGHFPGGGYPGGPHGGGYPGGPHGGGPGGPGGHGGPP

GGGFHGHRFFQW"

CDS 1650486..1651538

/locus_tag="EFAGFIKM_01471"

/inference="ab initio prediction:Prodigal:002006"

/codon_start=1

/transl_table=11

/product="hypothetical protein"

/translation="MVAVAFHTDPRGTAYELLIDELIEKADRFMLVDRQRYEENEIPE

VARVLERLKPYLVERATMEEMMLKSGAYYSEGTYYTYRCTPESGQVLKEEANRFHDWC

YPSLPDDLCFMTEDGNDYFFSVAHEHMYGMRITYKEASELMERIPGLFFELDRHKEID

HLLDDAIRHQTDKLDISLHGLSELPERIRELKHLKELTIFEQNLYSLPAGLFELTSLE

RLVITTLDLERIPAEIGKLKQLQELRIYCGSPFESAPGWRPKPQTELGLNCIPPEIGE

LSELKYLEIVYSGIRELPPELEKLKNLRALLVTNALIEGTPDVVTRMHWLEYVDLMNI

PFGTHWEEVWEMKKNM"

CDS 1651689..1652486

/locus_tag="EFAGFIKM_01472"

/inference="ab initio prediction:Prodigal:002006"

/codon_start=1

/transl_table=11

/product="hypothetical protein"

/translation="MATSYMDYTLPTTEFTYDLSNNPLFKKDENNYINALSIKQLNTL

GNVSLLDIFLNTGNVVEPHTHQNATELVYCINGAAVVSLINPFTNELLHFPITPGQVA

NVPQGWWHYEVATADHTHLLAVFDAPVPEAIFGSDTLRLTPAKLLAYTYCLDEKKVQE

ALAPIQKTVIIGPPADCQPSTAPASNSVPNENHQPYVMPNVQPNVQPNMQPNMQPNTQ

PNNQPNMQPYVNRLPYWGTWVDPRIIHEPGCPYYRELPVNTNNQEER"

CDS complement(1653177..1654223)

/gene="ndmA"

/locus_tag="EFAGFIKM_01473"

/EC_number="1.14.13.178"

/inference="ab initio prediction:Prodigal:002006"

/inference="similar to AA sequence:UniProtKB:H9N289"

/codon_start=1

/transl_table=11

/product="Methylxanthine N1-demethylase NdmA"

/translation="MITSNANRNVTSELPRGCTFTAEDWHVLSQYWYPVAQATEVTDK

PLAAQLLDVKLVLYRSNDQVVVAKDLCFHRGAPLSKGWVENGEIVCPYHGFRYNCEGK

CTVVPAHPSSKISPKLKLIVYPAVERYGLIWTTLAGTEEQIPSFPGWDDPDYINILPP

NFDIAGSSGRQMEGFLDVSHFAYVHTETFGDRNNTEVPQYKVKREGNELLAEYWSTVS

NYGKGQDLVAPEGFQWLREFRVFPPFAASLTVHFPGDDKLNILNCASPISARYTRLFC

PITRNFDKDAPIEDTIKFNLQVFEEDREMVESQKPEDLPLDLHAEAHIPADRTSIAYR

QLLTELGLGRNYTS"

CDS 1654632..1655078

/locus_tag="EFAGFIKM_01474"

/inference="ab initio prediction:Prodigal:002006"

/codon_start=1

/transl_table=11

/product="hypothetical protein"

/translation="MNLRTNYRVASPGAFKAMMAMEQYISGQFENKVLYELLKIRVSQ

INGCAFCLDMHAKDLMKLGDYADHILLLSVWREVAFFTDQERVMLELAEAVTRISEHG

VPLALYDKVREHFSESELVDLILAINTINNWNRIAITTGMYPGCFN"

CDS 1655112..1656047

/locus_tag="EFAGFIKM_01475"

/inference="ab initio prediction:Prodigal:002006"

/codon_start=1

/transl_table=11

/product="hypothetical protein"

/translation="MNSNLHANEPDVSSLDVGELYIRYKKYAFSIAYRMLGSVVEAED

IVQDCFAGIQSTSAQDIRNPKSYIARLIVNRSLNLLNSARNQRESYVGEWLPEPMGDS

EEANMPEETMEKKELISYAYLVMLERLSPMERAVFVLREAFRYDYSEIADWLGKTESN

CRQIFSRARRNLPERLPPIEQSDADMIAKGELLSRFTTAFLRYDVSAMLELLADQPVF

TSDGGGVVHTVMRTMNVHKGVLALLTSRRVLTRLREREWVPMCINGELQLALMKEGQL

SEVLCLELDTSGERIEGVYLVVNPNKLTSIDTSTL"

CDS complement(1656147..1657100)

/locus_tag="EFAGFIKM_01476"

/inference="ab initio prediction:Prodigal:002006"

/codon_start=1

/transl_table=11

/product="hypothetical protein"

/translation="MRWKQPAKLGLLAMALGCTAIGAIITPSTTLAAPVPASKAVYHQ

FQTYRTEAVKSTESLVKARKYLMNHINKVGPWQATLMTLQLENMQNVKLADVDYQMYT

EHFQQAISQAHEQLGYEQKLTYGRLLKEIKDPTVKKLLQEASDLGFKLETSEGLYYPI

INYEIYQKFKPFVKADIAAYIDIMATESNQMTTSDGGIIISWNELIQRALEKEAFLNN

FPNSNRTSAVKQGLFVDYLFYGSDNTPAYDWYTDEEIRTMDPVVKQAYEKALAYREPD

TQSVLLDTMEKILLVLNQNNDELTPEVRAIIEPVQQQFARE"

CDS complement(1657341..1657997)

/locus_tag="EFAGFIKM_01477"

/inference="ab initio prediction:Prodigal:002006"

/codon_start=1

/transl_table=11

/product="hypothetical protein"

/translation="MNPNSMNPQNAVQPTLNTNPNVTPNMNHGGHEMFDVHEILSSTI

NVLDQYMIFRTFVQSQELIGILDRQYNFILSQYNLTAECFASGQKPHQETATYMIPNM

VPPVYGLKPSAPKKPNQSLADVKDAGISGHMLGLIKSHASLLGMSCSEITNGTVRRVI

ASQIQHFIEMAYEIFMFQNKNAYYQVPQLTPSDTQQMLQAYIPATGAPQMPNSNKPLH

"

CDS 1658122..1658658

/locus_tag="EFAGFIKM_01478"

/inference="ab initio prediction:Prodigal:002006"

/codon_start=1

/transl_table=11

/product="hypothetical protein"

/translation="MEETDWGVAKELFVKHYGSAIQIHREGVYADYKRWDVPEELEEQ

WIGERIQQLSSELSIMNWDAVDELALIAKHRTEPSIIKEITAFASRQLKSADSMVRLV

YAERLIELIKRYESSLPMDKLRETYQLTMDLLVDVATKPLVLDPGHELQQYGLKDKRG

LNLRVEKNKEEIIRYFRN"

CDS 1658688..1659416

/locus_tag="EFAGFIKM_01479"

/inference="ab initio prediction:Prodigal:002006"

/codon_start=1

/transl_table=11

/product="hypothetical protein"

/translation="MRKLNYIHIFIFLSVVTIVTLGLYYVAADWLDQRYFRFLIWNLF

LGWIPFVFSYAAYVLSDVKWKGAAWLAVASGLLWLLFFPNSSYIVTDLVHLTARSSRY

YGGNGVDYTYWYDLSVLLMFVWTGLLLGFLSMYQLQEVIYHRIGRWASWIFVLVGCAL

GSYGVLLGRVYRLNSWDALTNRETLIELMHESVSRPSLAFCLFFGTFITTIYATLYYL

INTRSPRETKKSAGSPETDLQALR"

CDS 1659605..1660207

/gene="pbpE"

/locus_tag="EFAGFIKM_01480"

/inference="ab initio prediction:Prodigal:002006"

/inference="similar to AA sequence:UniProtKB:P32959"

/codon_start=1

/transl_table=11

/product="Penicillin-binding protein 4*"

/db_xref="COG:COG1680"

/translation="MLNAVELTTKVEEVMERVNFSGVVLLQQAGKTLLNMKRGYANRS

EELANQVDTRFGIASGCKIFTAVSVCQLIEAGKLSADSKLTDVLDVEFPLWDKGITIQ

QLLTHTSGIPDYFDEEVMDDFSELWKDRPVYAMRRLSDFLPMFQHLPMKFAPGERFHY

NNAGFIVLGLIVEQHSGLSFTDYVEEFIFKPCGMKNSGYF"

CDS 1660177..1660626

/locus_tag="EFAGFIKM_01481"

/inference="ab initio prediction:Prodigal:002006"

/codon_start=1

/transl_table=11

/product="hypothetical protein"

/translation="MWHEELRLFLTDQLPRNTALGYIDHEDGSWNTNMFSIPVKGGSD

GGAYVTAPDMIRFWGALLGHQLLNEETTRQLLTPHAHQEDEEYYGQGIWIDRKEEDIF

KFHVMGFDPGVSFMSSVYPDYDLKLVVVSNQESGPYPITIAVEEALA"

CDS complement(1660713..1661693)

/locus_tag="EFAGFIKM_01482"

/inference="ab initio prediction:Prodigal:002006"

/codon_start=1

/transl_table=11

/product="hypothetical protein"

/translation="MNPSALSSKLQQIVPQLDLRSCLVSVRGEIMFEHYRNQEAATDI

AKINSCTKSVLSALICIAMDKGLLPEASAPISTFFPQLTSDPDPRKPAITLEQLLTMT

AGFNWDEFGGQNSFPRMTRTNHWVDFALEQRLSHEPGTYMEYNSGVSQILSAILMQNA

GMNVAEFAERYLFDPLGIKHYEWESDPQGVHTGGFGLKMLPVDLLKFGQLFLQQGMWE

GKSLISSDLVSRSTQPFITVTPPNHGSYAWHWWVDVYPNERSDSENIAAEDDKPNLHY

YYARGFGGQYVYIVPEFELVTVLTNDKRKKEKPPLDVFPRLIAPELWKML"

CDS 1661945..1662214

/locus_tag="EFAGFIKM_01483"

/inference="ab initio prediction:Prodigal:002006"

/codon_start=1

/transl_table=11

/product="hypothetical protein"

/translation="MGNIIEELYYGNLRPEENIVPKDSEYRSINKEITASIEKFQSKL

SEDDFKQLEVLFDMMDQVHSIHSKEAFASGFKIGTLIMIESGYSS"

CDS 1662280..1662534

/locus_tag="EFAGFIKM_01484"

/inference="ab initio prediction:Prodigal:002006"

/codon_start=1

/transl_table=11

/product="hypothetical protein"

/translation="MFKIGNQGLFETLPDHARAIYEGDTGAVEQFIKQGMDLEEEIPL

SKYIALTSLDLALICNQPEVVKLLVERGVNLNVKNNTVIL"

CDS 1663016..1664368

/locus_tag="EFAGFIKM_01485"

/inference="ab initio prediction:Prodigal:002006"

/codon_start=1

/transl_table=11

/product="hypothetical protein"

/translation="MKKVKQISFILTFCLLANLALTPMASAGDSSNLIGFRAELKAIA

YKTYTFFEEYTDQNTGLTYDEVRHTENGIEEAKRTSPTNIAMYMMSTVSAQQLGIISK

KEAVHRLQTTINSLEKLEKWNGLFYNWYNKDDGSVKKDWGQFISQVDNGWLSAGLIVV

GQAYEELHGETSKLVKNMNYTPLYDPEVGQFRGGYDVAKGALTDHHYGMFYTEPRVGS

YIAIGKGDVPQDHWWKMYRTLPQEWDWQAQIPQGKSVKYDGVDVFEGSYVYKDKKFVP

SWGGSMFEALMPGMVIKEKDLGTQALGLNNQRHVELQIEYAKEKGYAAWGFSPSATPT

GYSEFAATPLGTSGYKDGATVTAHATFLALDYAPEAVRKNIKALKNFKMVGKYGFYDS

VNVETGELAKAYLALDQGMIMVSIANYLQDGVIRDYFHSDVIGQMPEELLKKEVFSIQ

"

CDS 1664874..1665824

/gene="yteP_6"

/locus_tag="EFAGFIKM_01486"

/inference="ab initio prediction:Prodigal:002006"

/inference="similar to AA sequence:UniProtKB:C0SPB3"

/codon_start=1

/transl_table=11

/product="putative multiple-sugar transport system

permease YteP"

/db_xref="COG:COG4209"

/translation="MKESHTVISVSPSLKKNSLGKRIWKNWELYLFMLPALLYFLVFH
YGPMYGIQIAFKNFVPSKGITGSEWVGFDHFERFFNSYFFWDLLWNTFSISFYELAIG
FPLPIILALAFNEVRNGPFKKSVQTVTYAPHFISVVVMAGMIITFLSPSSGMIVRFIE
FIGLEPAQFLTDPAWFKTVYVFSGVWQSTGWGTIIYLAALSGVDPQLHEAAIMDGASR
IKRVLHINLPTILPTITIMLILNMGNILGLGFEKILLLQNSLNMEASDVISTYVYRAG
LVNAQYSFSTAVGLFNSVVNVILLVTVNQIAKRTSENSLW"

CDS 1665841..1666752

/gene="dasC_1"

/locus_tag="EFAGFIKM_01487"

/inference="ab initio prediction:Prodigal:002006"

/inference="similar to AA sequence:UniProtKB:Q9K489"

/codon_start=1

/transl_table=11

/product="Diacetylchitobiose uptake system permease
protein DasC"

/db_xref="COG:COG0395"

/translation="MVTAMKESRGDKLFLISTYIYLGLSLLVVLYPLIYILSASISSP
QDVNSGAMWLFPKNVTLDGYKLVFENPKIWSGYWNTIIYTVVGTLLNLAVTLPASYAL
SRSDFVGRQLFMGLILFTMFFSGGIVPTYLLVKNLGLMNSMWALILPVAASVWNIVVA
RTFFQTTIPKELQEAAHIDGCTNLKLFIRIVLPLSAPIVAVMALFYGVSHWNSYFPSL
IYLNDEAKYPLQMVLRQILVLQEMTAETTGSSINGEVATAMNNKAETASLIKYGVIVV
STLPIVAIYPFLQRYFVQGVMLGSVKG"

CDS 1666791..1668410

/locus_tag="EFAGFIKM_01488"

/inference="ab initio prediction:Prodigal:002006"

/codon_start=1

/transl_table=11

/product="hypothetical protein"

/translation="MPTTSKPWKLLLSSALVLTLLAGCSNSNEGEANNNLGKVAVNNE
GFPIVNEPVTLSLMAPDVGIQNWENMAVLQQMQEKTGITLEYKNAPKDSFETKKNLVL
ASGDYPDILYAAGLTTAEQMNYGEQDILIPLEDLIEEYAPNFKALLEENPDVRKSITA
PDGHIYSLPVVELSQHWYRNPMWYNGDFLKALNIDKLPETTEELYTYLKRVKEEDPNG

NGIADEIPISSVTTPAANLRDIRTWLLGAFGIYEEEIYVDDADKVHYTPLEEGYKEYL

TYMNRLWSEDLLDHESFSQTAEQKKAKAQNNQIALFSDWHAYMSKGGEPSTADPMFAP

VHSESVAAPAIAKNRGITSGAFAITQSNPAPEASIRWVDYLYSYEGAMFFNKGPEGIL

WEYTDKENRVKQYLPVPDGKEMEDYRATLTPNYGIPAPTLSMDDINKGLKTDFDLWVE

QETKQKLLDKGARIPFPTLFLTVEEQTEISSLNSDLKTYVNQMEAKFITGAEPLTGWD

NYVATVKKMGGERMVEINQAAYDRWKSVPEH"

CDS 1668428..1669459

/locus_tag="EFAGFIKM_01489"

/EC_number="2.4.1.281"

/inference="ab initio prediction:Prodigal:002006"

/inference="protein motif:HAMAP:MF_00928"

/codon_start=1

/transl_table=11

/product="4-O-beta-D-mannosyl-D-glucose phosphorylase"

/translation="MQITRHPNNPIVVPGGYEWRKVTVFNPAVIIDNGKFYMIERTAG

SLTPCKNYLGLLESEDGVNFIHVKDEPIVTPDMLGFPYGSVQDPRIVKIDGTFYLNYA

LRPCAMSYYPTGAGVPLRSIPKYPDGWGDEEGHWLTRSSILKSDNLLDWEYVADTTPL

DINDRDNILFPEKIGGKYVLLRRPEEYVGEAYGTDKAAMWITYSEDLVNWEEPKLLAT

AQNLSWESRKIGGSTPPIRTDKGWLVLYHGVDEDIVYRVGAMLLDLEHPEKIIARTHH

FIMEPETYYEKFGFQIPNVIFPTGNVVKDGLLYIYYGVTDTAIALATVPLDELVEHIL

QEGQQQNKP"

CDS complement(1669519..1671855)

/gene="rhaR_7"

/locus_tag="EFAGFIKM_01490"

/inference="ab initio prediction:Prodigal:002006"

/inference="protein motif:HAMAP:MF_01533"

/codon_start=1

/transl_table=11

/product="HTH-type transcriptional activator RhaR"

/translation="MANSALKPSLRNRFRFSWNHFKSRLLLKYAFSYILIFLIPLTGV

TIFVYENAVKGLRVEIEQSNVNQLNQVKSTIDNRMNELQEIAGRIAYDKHLTPYMVQH

QHPYYSLEAIQALANYKASSSIAEDLLLYFHNDSNIYSYRGLANLDVTFDSLYQFEHW

NPEELRRDLNETRQPLVRPAENVKVNSRMESMLVMLVPVKPNDPFPYGTVVYLMKESN

LTGVMDSILSDFSGSSYIFSPSGEVLTANSHGISLPQDEIENLATLGPGIHNLKMDGE

QYSVVSVQSEENGWTYVTTMPSFQFFSRVAHVQTLILIVFCITVITGIAAALLLAKRQ

YHPIRDLMDRMKGSGDNAPKLRNEWEWIRQTLHNYSVKIDFQEPFVRNQCMLLLLKHG

KPDDPEIEQMILSAGFRHPQGQGLYFSAILSWDDTVPDGNSCQERHLLQDILSNVCLP

GTGAQIFGVEFSVKDQFALIVSLPGDADKPIQSQLEQVIEAIQTVIREHSHLSLSIGV

GMAYRDLTRLNQSFIEAAAALEHRIIRRSGQVTYFEQLAELSPSASESFWIPRKSMLK

LEQSLKQGNESVAVQMIADTIHTIKDEPLQVHLLRCICFDLLNAFLRTASELGMNEVF

ANMPELTSFETLEELESRLLSLAADICTQVELNTKTSESTLMDDVLAYVDQQFADYTL

SLEHVALKFAISTSYLSRSFKEKTGCNFSQYIWQRRADEVMRLLENTSAPLKEIIEQV

GYMDAPNFIRKFKKEIGLTPGQYRKEHALRGATTKRPV"

CDS 1672078..1673178

/locus_tag="EFAGFIKM_01491"

/EC_number="3.1.1.-"

/inference="ab initio prediction:Prodigal:002006"

/inference="similar to AA sequence:UniProtKB:Q837K0"

/codon_start=1

/transl_table=11

/product="Deacetylase"

/db_xref="COG:COG3964"

/translation="MGTENVLRNLQLVDGRIMDIAIQDGIITAITPPGQAEGENELDC

SGLYVSSGWIDLHVHAVQELDPYGDDIDEIGVKQGVTTLVDAGSCGADRIGAFYTARL

QAATQVFALLNISSIGLERTDELSQLEWIDRAKVMEAAAAYPDFIVGLKARISQSVVK

DSGIQPLKLARTLSEETKLPLMVHIGSAPPAISDVLELLQSGDVITHYLNGKSNNLFH

ADGTPLQELLDAAARGVHLDVGHGTASFSFRIAEQAKVAGIALNTISTDIYRGNRMNG

PVYSMSNVLTKFLYLGYSLEEVIRAVTRSPAEWLGKPELGQIRVGQQANLTLFSLEAG

EKQLEDSEGDVRVTQHYIEAKGVFANGSLITG"

CDS 1673156..1674265

/gene="dgaE"

/locus_tag="EFAGFIKM_01492"

/EC_number="4.3.1.29"

/inference="ab initio prediction:Prodigal:002006"

/inference="similar to AA sequence:UniProtKB:D0ZLR3"

/codon_start=1

/transl_table=11

/product="D-glucosaminate-6-phosphate ammonia lyase"

/translation="MGHSLQAKYGLKRVINASGRMSILGVSAPTDSVMDAMKQGGQRY

VEIADLVDKSGEYIAQLLGSESAAVVNSASSGIALSVAAIVTAGDPRLSLRLHQEPIL

KNEIIMLKGHNVQYGAPVETMIFLGGGRVVEVGYANEGRKEHIDQAIGEHTAAIFYVK

SHHAVQKNMISVEEAWEVAQRRGVPLIVDAAAEENLHKYIQLSDLAIYSGSKAIEGPT

SGIVAGKKKYVEWLKVQLHGIGRSMKVGKETVFGLLQALDEYQDKADNSEREKQTLEA

LQPLDRLPGVSVRIVQDEAGRSIFRGRVQIDSSLVGVDAKKVNDQLREGAIAVYTRDY

GVKQGYFDIDPRSLQGDDMQVIVSRIHEIVGGKPE"

CDS 1674262..1675017

/gene="dgaF"

/locus_tag="EFAGFIKM_01493"

/EC_number="4.1.2.14"

/inference="ab initio prediction:Prodigal:002006"

/inference="similar to AA sequence:UniProtKB:D0ZLR2"

/codon_start=1

/transl_table=11

/product="2-dehydro-3-deoxy-phosphogluconate aldolase"

/translation="MSHIQQCLYKNRAAINVLAGSIGNAKDVYEAAEGYVLVGVLSKN

YATSEEAVVAMTEYGQAIQDAVSIGLGAGDSRQATVVAEIAASYPGSHINQVFPAVGA

TRANLGPKDSWINCLVSPCGQPGYVNISTGPISSVNTPQAIVPVHAAIALVRDMGGNA

LKYYPMQGLKLEEEYRAVAQACGESGFALEPTGGIDLDNFGPILEIALEAGVPQVIPH

VYSSIIESQTGNTNVQDVRRLLETMKSLVDRYA"

CDS 1675010..1676023

/gene="kdgK_2"

/locus_tag="EFAGFIKM_01494"

/EC_number="2.7.1.45"

/inference="ab initio prediction:Prodigal:002006"

/inference="similar to AA sequence:UniProtKB:Q53W83"

/codon_start=1

/transl_table=11

/product="2-dehydro-3-deoxygluconokinase"

/translation="MPRVAAFGEVMMRLQVPGYETLVQSSRLEYSFSGSGVNVTAALA

RYGHNGALITTLPETPVGEAAIAYLRKLGVDTSLISQGGKHLGMYFLENGFGARPGRV

TYTDRLGSSFNTADADNYDMKDLASRVDVLHLCGITLAMNDSVRKQMKRLAEEVKSAG

GKVVFDCNYRPALWGTDGYVKARPHYEELLRLADLVLMNEKDAKCILGIGTGEYDRIT

QMKQAIPEVAQRFGIGTVAGTHREINADHTHSLTGYIYYQGTFVFSRKLTFPVYDRIG

AGDVFASAIIHGELQQYPQEHTVNMAVAAAMLAHTTHGDTALFTENEVLRALSDHTSD

VER"

CDS 1676034..1676756

/gene="dasR"

/locus_tag="EFAGFIKM_01495"

/inference="ab initio prediction:Prodigal:002006"

/inference="similar to AA sequence:UniProtKB:Q8VV01"

/codon_start=1

/transl_table=11

/product="HTH-type transcriptional repressor DasR"

/db_xref="COG:COG2188"

/translation="MSLKRKQGPLYQQIQKILKDRILHGVYPLGSIIPSEPQLEKEFG

VSKMTVRGAVQELSQEGYVQKKSGVGTIVLRNTSHQKLSKGKRFTELLVEEGHKLEKK

VLNSCLLTNDTGTEEYGRYGPYCQRIERLYILNGQPYIHLIHFLAAAALPGGGKEEMN

GDIQSLYDSLEENDILLENFKDRFFVEPAAPELCQLLKIHPGTHVLKRLRNSFDGEGR

LIEHSIGCYNTELHHYLVSYDT"

CDS complement(1676834..1678372)

/locus_tag="EFAGFIKM_01496"

/inference="ab initio prediction:Prodigal:002006"

/codon_start=1

/transl_table=11

/product="hypothetical protein"

/translation="MIPNVGDVYCVYVEKLQQYTACQVTGLKETEGKTSDTLASVLQL

DWTGDQLPNEMQLHQMKPLICNFYFWHDRLAHSFVSANVPSQYVLAGNIPPLYTEETN

TYGGGWPIGESLYLQREWEGIDAIRRAQFKQAADDDEDVMIGDRVMRRSTSTLRDFSP

TSAEDVAELAKLPCLTHIEMDGYTESIFSFIEDNPFINELHIIHHGQTMLDISNSHLT

KLIVDVSGLKELILNTKMKSLNFTGELSPDLRIAAYEDGKCITLNVPSGLPQVTGISR

LEGLYIRDITELNLEPLVQYYPELSELRLWGKPGNVSHIESIQHLANLQTFTTYDLFG

FNGEQFPDPDQLPKLATLWLTSLPADAAKSIKTRYKKSAASGLDMEITKARKPEWLAE

NLNNPFRDWDGRDHIKAAHAKKATQLYKQCSKEIRHLSQDFNNQEALHKQLVSIVENY

TQTFNKMDAKTGFIETVEREEIYVVLTALLDQLQQNLELRQDDLVKVNHEELYEVFDR

LRDF"

CDS 1678534..1679340

/locus_tag="EFAGFIKM_01497"

/inference="ab initio prediction:Prodigal:002006"

/codon_start=1

/transl_table=11

/product="hypothetical protein"

/translation="MDTSMSRNKKLGEFLKSRRSRIQPEQVGLSGSYGQRRTPGLRRE

EVAVLAGVSATYYTWLEQGRELAASREVMQSIANALRLTLEEKSHLFDLWDPNTEHEF

LASYSQLEQGWQSIIGQLTHPSFITNERSEVLAWNEAADTNLFNFSSMTVNERLMMRI

LFLDTALRERMHNWEEFARHSVAVFRTYYDKYTSDPEFSRIVKQLSEESVDFRTIWNL

HQVEFKQVNRVLLEPTDRADGVVYAYDIFSMNNLNSQSGIHCCIYVPVAI"

CDS 1679476..1680432

/gene="iolS_1"

/locus_tag="EFAGFIKM_01498"

/EC_number="1.1.1.-"

/inference="ab initio prediction:Prodigal:002006"

/inference="similar to AA sequence:UniProtKB:P46336"

/codon_start=1

/transl_table=11

/product="Aldo-keto reductase IolS"

/db_xref="COG:COG0667"

/translation="MLKTRTLGNDKLRISALGLGVMMMPDNDESVHTIQGALDAGVTM

FDTADLYGEYEKQRFGGNEKLLGRALQGRRSKAIVATKFGITHTQGPKGDPAYIKKSV

DASLFNLGMDYIDLYYQHRPDPNTPIEETVGTLADLVQQGKIRYIGLSEAPVEIIRRA

HAVHPITALQTEYSLWSRELEDEIMPVLHELNIGLVPYSPLGRGFLTGQIQSIDDLPE

DDYRRHYPRFQGENFQKNLEVVGLIQEMAKQKGCTVSQLALAWLLGKGEHIVPIPGTR
NLERVHENLGALQVSLTDDEMNQIDHISPQGIAAGGRFPGQV"

CDS 1680569..1681165

/locus_tag="EFAGFIKM_01499"

/inference="ab initio prediction:Prodigal:002006"

/codon_start=1

/transl_table=11

/product="hypothetical protein"

/translation="MIDPILQAPNVPLAADSNAVVNYQRDSRNYVTQLFGEQLPTIAN
GFFNVYLSKGIIVQPHWHTNVTEMVVVITGEITASVFNPFTRERLTYRLKPGQVVVFP
KGWFHWFVAETDDVYVLTIFDQPTPDIVFGADFLAATPPEVAHRAYCLDEEAYARAVA
SIKNDAILGPPIGCDTQVSDQSTSPSKTSVSPSNKLKS"

CDS 1681373..1682053

/locus_tag="EFAGFIKM_01500"

/inference="ab initio prediction:Prodigal:002006"

/codon_start=1

/transl_table=11

/product="hypothetical protein"

/translation="MNMSEMKTLETAMSYAPILMFDRAEPFYPDFVGISVLHQSGPSP
SFRRDIKFPTEVVQYVIEYAIWWDYEIGHLYEMEHVWVYVGHDGEVVDCEASFHGKVL
RGLLKERVNVVGHHVCLYSQPGKHAFSPLPVVFELLPNLYSAAGAEAGCDGLLVNELF
QDYFETNEQIDDQVERYLQTKAFVPTMEFEEYILKPEMFMTWDNLFGIIPHRIKDRLM
ELEQLYNT"

CDS 1682602..1683849

/locus_tag="EFAGFIKM_01501"

/inference="ab initio prediction:Prodigal:002006"

/codon_start=1

/transl_table=11

/product="hypothetical protein"

/translation="MKWFGNLKTATKIISAFLIVSIILAALGVYSVVTLRSTNERMQE
MYNNNLISVKELSSAQIDYQRMRVNVRDLSFETVETEKTRITENIASIRQSVNDRLTI
YRPLATTPEEIELLRNFAAEYPGYLTMFERATNLAVADDQSAFNTYLKSELKPQGDKI

VVLLTNLIDLNESLAEQANIQSQVAYSQAFKVTIILVVLSVLFSILIGYIISRSISKP

LMAMLGLATEVANGNLTLKSDISSKDEVGQLAAALNRMVDNLKELINNIVMNSQSVAA

SSEQISASTQEIASTSTSQSSEAGNISELFKELSLAINSVAASAEEAAELSNDTVKTA

REGGLVVQTSLEGMQAVNTKMTKLEDDSRKIGDIIEVIDDIAEQTNLLALNAAIEAAR

AGEQGRGFAVVADEVRKLAERSG"

CDS 1683907..1684191

/locus_tag="EFAGFIKM_01502"

/inference="ab initio prediction:Prodigal:002006"

/codon_start=1

/transl_table=11

/product="hypothetical protein"

/translation="MQAVAASVEQSSMTGQAFDQIIDMVNNSSQKVNEIAAACEEEAA

QAAEVMSSVESISASSEESAAASEETAATCQSLAHLAEELANSAAAFKTN"

CDS 1684216..1684641

/gene="cheW_1"

/locus_tag="EFAGFIKM_01503"

/inference="ab initio prediction:Prodigal:002006"

/inference="similar to AA sequence:UniProtKB:P39802"

/codon_start=1

/transl_table=11

/product="Chemotaxis protein CheW"

/db_xref="COG:COG0835"

/translation="MSSLQKEQYIELSVGAETCAIRIEEIHEIIKMLSITDIPFSRPE

IKGVVNLRGKVVCVMSLRNLLGMPDEPDNRATRIIVVRHQEEYIGLIVDRVNKVTTYS

EIHPPTGGYGRNREGLFHGVGQQKDKLVGILKLDEILGG"

CDS 1684656..1686671

/gene="cheA_1"

/locus_tag="EFAGFIKM_01504"

/EC_number="2.7.13.3"

/inference="ab initio prediction:Prodigal:002006"

/inference="similar to AA sequence:UniProtKB:P29072"

/codon_start=1

/transl_table=11

/product="Chemotaxis protein CheA"

/db_xref="COG:COG0643"

/translation="MMDLSAYRDIFIEELNDQLERMDQSLLALELSPSVELVQTLFRA

AHTIKGSASTMDFRELSDLTHEVEYALEWVREKKPEMTSELIDTLFRSLDAMKVLRDQ

YIRGEAYADFRAVVREIKDLIQTAAVVGQLKVPQLQPAESIVAQVAIVSGKQLLSIHI

VLEQKCMMKAARYHILRQRIEDICGTVVATSLMESQPGAQNEDDHYGQFIIIVATSKD

PQQLKAELSSDSDIHMLEIDPYLLESNEVAATSAPLELISETVQSKQSLPTVNTTGPD

EKVKAAQPTVRVSVERLDHLMNLVGELLIDQTSLADLSGSGARKESSTLIRSISGVSD

HMNRVIKELQEGVMKTRMLPIDQLFSRFPRLVRDLSQKLGKDLELVIQGGETELDRMI

IEELSDPLIHLIRNSADHGIESAEVRAENGKPSKGRITLTSFHEENHVVIRYSDDGKG

IDGEKIKASALGKGIISKEQAARLTTQEAVHLIFEPGFSTASSVSEVSGRGVGMDIVR

SQIGRLNGIIDIDTEVGVGTTFTIRLPLTLAIIKGLLVKVSGRVLILPMYNVAEIVRI

SPEDIQMIQGQQAILNHGRIVPFHRLCDRLNYPRTDRKSKTIPLVIVRSVDKIAAYAV

DEIIGNQEVVIKTLGTYLGAMNHLSGATILGNGKVALILDASYLVSL"

CDS complement(1686770..1687654)

/gene="cmpR_4"

/locus_tag="EFAGFIKM_01505"

/inference="ab initio prediction:Prodigal:002006"

/inference="similar to AA sequence:UniProtKB:Q55459"

/codon_start=1

/transl_table=11

/product="HTH-type transcriptional activator CmpR"

/translation="MTTNYELYKVFYWAAKTGSLTQAAKALYITQPSVSHAIKQLEES

FGLTLFYRNSKGVALTQEGASLYSYIEQSQILISLAEEKMAALKNLDNGELRIGGSDS

LFKHYMLAYLEEFHTLYPNIKLHLSHGTTPEVITFLKEGKIDLGVVRMPIVDPQLEVR

ESIQLKDCFVAGERYAQLKGKVMTLEMLLEHQLILFSRNSRVRMAITELFNSYNYTLK

PEIEVGSVDLLIEFARRGLGISYVTREFISKELEEGSLFEIQLDVPLPPSHVGIMTKR

NMPISLAANRFMDLIFKS"

CDS 1687767..1689290

/gene="zwf_3"

/locus_tag="EFAGFIKM_01506"

/EC_number="1.1.1.49"

/inference="ab initio prediction:Prodigal:002006"

/inference="similar to AA sequence:UniProtKB:P54547"

/codon_start=1

/transl_table=11

/product="Glucose-6-phosphate 1-dehydrogenase"

/db_xref="COG:COG0364"

/translation="MEPTTIVLFGATGDLAKRKIYPALYNLYLEQKLPETFSLIGLGR

RDWSDEFFQAQVEKSLNEFSRRQADPEVVKSFVKAFRYSVLNISHKEDYIKLLGLVEQ

REAELGIPSNRLFYLSVGPEFFEPIAENIQESGLGSTEGWKRLVIEKPFGHDLQSARD

LNRKLSESFTEEEIYRIDHYLGKPMVQRLETLHQSNPIMKALWNNRYISNVQITANET

VGVEERASYYDHVGAVRDMFQNHMLQLLMMMAIQLPYNSTSEKVGLKKKHIMESIQPL

QKQTVGANIIRGQYAAGNIQGKPVNAYTAEPGVAENTMNDTFIAAKLQIDDFFWRGVP

FYIRTGKRMKEKSTRIVIEFKEPSGQTNVLKNKGSKPNLLVIEMSPDQSMTLQLNASD

PENKGEFKPVHIDLSPDKGDLAEAYENLIRDALLGDPTFFAHWDEVELSWAWVQPILD

AFQENLLPLHLYPAGSYGPVESDVMLEEEGHHWWFDEPADQESVVTNSIPLAVNANL"

CDS 1689327..1689992

/gene="tal_2"

/locus_tag="EFAGFIKM_01507"

/EC_number="2.2.1.2"

/inference="ab initio prediction:Prodigal:002006"

/inference="similar to AA sequence:UniProtKB:P19669"

/codon_start=1

/transl_table=11

/product="Transaldolase"

/db_xref="COG:COG0176"

/translation="MKFFIDTANVEDIQKAYKIGVLSGVTTNPSLVAKEGVKFEDRIE

EILRLVPEVESVSAEVTPDALTAEEMIAQADELIKINNSDKNITIKLPMTLAGLEACR

YLTKKGVKTNVTLIFTVNQALLAARAGATYVSPFLGRLDDISEDGVQLVTKIAELFRT

HNLDAQIIAASVRHPDHVTRVAMAGAHIATVPFSVIEQISKHPLTDQGMDKFAADWKK

TVQ"

CDS 1690053..1691471

/gene="gndA_2"

/locus_tag="EFAGFIKM_01508"

/EC_number="1.1.1.44"

/inference="ab initio prediction:Prodigal:002006"

/inference="similar to AA sequence:UniProtKB:P80859"

/codon_start=1

/transl_table=11

/product="6-phosphogluconate dehydrogenase,

NADP(+)-dependent, decarboxylating"

/db_xref="COG:COG0362"

/translation="MSKQQIGVIGLAVMGKNLALNIESKGFSVAVFNRSPEKTNDLLK

EAEGKNLTGAFTIEEFVESLESPRKILIMVQAGKATDATIEQLLPHLDQGDIIIDGGN

AYFPDTQRRSKELEEKGFRFIGAGVSGGEEGALKGPAIMPGGQESAYQLVEPILTAIS

AKVGDDACSTYIGPDGAGHYVKMVHNGIEYGDMQLIGEAYHLLKSVLNVSVEELHEIF

TEWNQGELDSYLIEITADIFSKYDSETGKPMVDVILDAAGQKGTGKWTSQSALDLGVP

LSMITESVFSRFLSAMKDERVAASKILNGPATEAFSGDKKAFIENVRKALFASKIVSY

AQGFAQMRAASDEYGWDLKYGNIAMIFRGGCIIRSQFLQNIKEAYDKDAELKNLLLDP

YFQNIVESYQGAWREVIAAAVKQGIPVPGFSSALSYYDSYRTERLPANLLQAQRDYFG

AHTFKRVDKEGSFHFQWMDTNE"

CDS 1691718..1692821

/locus_tag="EFAGFIKM_01509"

/inference="ab initio prediction:Prodigal:002006"

/codon_start=1

/transl_table=11

/product="hypothetical protein"

/translation="MFVLAGILFLVVYGLLVFYIGWSGWSWMKPVVSARFRWFYIIAL

VFLAVSFILARVFGSISFLGIIGSYWLAIFSLLLLILPVVHLTMWLLRLTRIPRHHTQ

KWAGVVTLVLLVSTLGYGIFNAYSPVVRMYNIQIDKKVEGVDKLNIVMAADMHFGLLS

GPAHAKRMVKEINALKPDLVLYPGDIIDDNLDMYLNSGIADIISEIQAPYGVYASLGN

HDKFDGPIEDLIAALEKSNMQVLYDDKIVLNDKITLIGRKDRTEKDRAEVATLMQGTD

LTQPVLMMDHQPYDLDIAEQNNVDLVVSGHTHRGQIAPAQFITQAIYENDWGYLQKGS

MHSIVTSGFGFWGPPIRTSSRSEIVKINVTFQQ"

CDS 1693155..1694009

/gene="licT_3"

/locus_tag="EFAGFIKM_01510"

/inference="ab initio prediction:Prodigal:002006"

/inference="similar to AA sequence:UniProtKB:P39805"

/codon_start=1

/transl_table=11

/product="Transcription antiterminator LicT"

/db_xref="COG:COG3711"

/translation="MIIKQIFNNNIVSTVDDKNQELLILGRGIGFKFKAGDEIDEERI

EKVFRLQDASIYEKFKSIVAEVPIEILQATDDIVTLARTQLNKTISDGIYVSLSDHIH

FAVQRLEKGMITRNPLSWEVQHFYKAEYDVAREALTLLKERLDIEFPKDEICNIALHF

INAEVNDSMNDVTHLMQLLQEIMNIIKYHFNVELDEDSVNYFRFITHLKYFCQRVITH

SSHDDAEEYLYEVVRKNYPETFKCIGKIETFIHKNYQYDMTHSEQLYLTLHLERLMKT

KRDEKSTQ"

CDS 1694142..1696061

/gene="bglF_4"

/locus_tag="EFAGFIKM_01511"

/inference="ab initio prediction:Prodigal:002006"

/inference="similar to AA sequence:UniProtKB:P08722"

/codon_start=1

/transl_table=11

/product="PTS system beta-glucoside-specific EIIBCA

component"

/db_xref="COG:COG1263"

/translation="MKHQETAQEIIKAVGGTNNINSVYHCVTRLRFDLKDNEKVDNGS

LKKLDKVMGTNISGDQFQVIIGNDVAKVFDAMVKENPAIQQSTENKEPKSDKKQNVVL

KIFETIAGVFAPMLPAITAAGMLKGLLALFVSVGWMSAGTDTYRILSAIGDGVFHYLP

LLIAVSAARKFGSNPFVAIALGTALMYPDMTALLSSGESVGFLGIPVTAVSYASSVIP

ILLAVWLMSYVEKWVDRVIPAALKLLLVPLITLLVMVPVTLIAIGPLGTFVGSGLSGG

INWLLNEGGLIAGIVLGGAMALIIMTGMHYALVPIILSNIATLGFDKFLPLTFISNMG

QAGATLGVFFRAKDKKLKTVALSTSFTALMGVTEPAMYGVNMKYKKPFAAAMIGSAVG

GGFALAFGAKAYVLAGNGGLPGLPSLIGQTFWYSFGGMILAFIVGAIMSTIFGIKEEE
GDAEALAQFSPGASTAPAKVVSNEAATVNVDDMGDPTPTSDAVAVAPMTGKSIPLKEV
NDPTFGDELMGKGVAFVPTVGELVSPVTGTIMNVFKTKHAIVVRSDNGMELLIHVGIN
TVKLRGQYFDAHVATGARVQAGDKLLTFELAEIAKEYDITTAMVVTNTADYKQVLPVK
LGEITMGEDVLKAEI"

CDS 1696111..1697574

/gene="bglH_5"

/locus_tag="EFAGFIKM_01512"

/EC_number="3.2.1.86"

/inference="ab initio prediction:Prodigal:002006"

/inference="similar to AA sequence:UniProtKB:P40740"

/codon_start=1

/transl_table=11

/product="Aryl-phospho-beta-D-glucosidase BglH"

/db_xref="COG:COG2723"

/translation="MINRQGFPEGFLWGGAIAANQAEGGFDAGGKGWSTADMVPYFEK
KDYTNLRELMHVTSATVEKAMAHHSAEGYPKRYGIDFYHRFKEDIALFAELGFKTFRL
SINWPRIFPNGYDAEPNEEGLRFYDEVFDELRKYDIEPLVTLSHYEMPMALVLKYNGW
AGREVIGHFVRYAETVMNRYKDKVKYWLTFNEINTTIIEPFTGGGIIEDRVENTMQAS
YQALHHQFVASSLVTEQARQINPNFQIGCMLARMIHYPATSKPEDVLQAQIDNQLNLL
HTDVQVRGSYPTFMARYWAENGITIAMEPGDEQILREHTVDFISFSYYTSLVSAVNPE
EYGVTGGNLYSTIKNPNLERTEWGWQLDPIGLRVALKELYDRYQLPLFVVENGLGAKD
TVEADGSINDDYRIDYLRKHITQMKEAVMDGVDLMGYTNWGAIDIISASTSEMSKRYG
VIYVDQDDNGQGTLNRYKKKSFGWYQKVIASNGENLE"

CDS 1697742..1698308

/locus_tag="EFAGFIKM_01513"

/EC_number="3.1.3.-"

/inference="ab initio prediction:Prodigal:002006"

/inference="similar to AA sequence:UniProtKB:Q8CTG7"

/codon_start=1

/transl_table=11

/product="Putative 5'(3')-deoxyribonucleotidase"

/db_xref="COG:COG4502"

/translation="MKKPIIAVDMDDTICHLVKRAIYHNNLNFPTHPLRYEDMIHWDT

SHLRHPESTHDVFYGRPGLFEELELYDEYVVDEMRKLNDAYDVIVVTAAEPRTVVEKW

NWLQQHMPFITAEQFITCKRKNLLSFDLLIDDGAHNLIPAHEDGKKVICIPHPWNIQE

RERYNFPLMSSWKEAKTYIDEVLQVVGV"

CDS complement(1698318..1699202)

/gene="btr_3"

/locus_tag="EFAGFIKM_01514"

/inference="ab initio prediction:Prodigal:002006"

/inference="similar to AA sequence:UniProtKB:P40408"

/codon_start=1

/transl_table=11

/product="HTH-type transcriptional activator Btr"

/db_xref="COG:COG0614"

/translation="MNSSVQLMKSEYFLHNHLQLFVNRCSEDFVLPFHAHDFIEYSYV

AEGKGFHHIGEDVIPVTKGMLFVIPVGVPHVFRPVSTNVTEHPLIIYNCLFNAELIHT

LTAIIQEKEIIQHLMDLALNQVPYISVVDHNDRIEELMVKLYRESSVPGIGSSTMLYT

LVSQLVLMTYRQLNQEVHDEEIHSTDFEYILHYLKQHVSNRIRMSDLVRVSGWSEKQI

GRMFLRHTGQTFSSHLQHLRIQKSCELLKSSGHKVSLIAELVGYRDMDSFYAAFRKIT

GETPLAYRKKSRAQHSGQ"

CDS 1699337..1702066

/locus_tag="EFAGFIKM_01515"

/EC_number="3.2.1.40"

/inference="ab initio prediction:Prodigal:002006"

/inference="similar to AA sequence:UniProtKB:T2KNB2"

/codon_start=1

/transl_table=11

/product="Alpha-L-rhamnosidase"

/translation="MFNVSHLRCEYRFNPIGLDVKSPRLSWQLQSDRRNCMQSAYQIQ

LSISEDFDEIAWDSGKINTDQSIHVELNHFQPSPRKRYYYRILAWDDAGSDSGWSETA

YFEMGLMNSNNRWKAEWITAQPADDAERSCPRMRKQFNVKQPVTSARIYVTALGLYEL

HMNNTRVGEDYFTPGWTSYRKRLQYQTYDVTHLLQDGQNTLGANLGDGWYRGYLGWNK

EREIFGSTSALLLELHMKYADGSEECIISNGDWTAAPSAIRMSDIYMGETYDAQMESD
WSDSSQTNWSPVNVLDHPKDIIVAQENVPVTQVEQLQPIALLTTPQGDRVVDMGQNMV
GWVRFSIQGEAGQTVELHHAEILDHEGNFYTDNLRAAKQCIRYTCKGDGAESFEPHFT
FQGFRYVKLVGFPEHVRLDEFTGIVLHSNMEQTGQFSCSSPLVNQLHHNILWGQKGNF
LDVPTDCPQRDERLGWTGDAQMFVRTSSYLMNTAPFFTKWLRDLEADQGEDGGIPFFV
PDLRGSTSEGWGDTSHSSAAWGDAAVICPWTIYEMYGDARLLAEQYESMKRWVEYIHV
QGDNPYLWNTGFHFGDWLGLDSKPDSYVGATDTDYVATAFYAYSVSLTQKAAEALGKK
DDAEYYKQLHTNIVHAFRNEFVTPAGKIAVPTQTAHVLALQFDLLDATARTRAVDQLA
KLVKEAGNHLTTGFVGTPYLNPVLSDAGLHELAYTLLFQEDYPSWLYQVTQGATTVWE
HWDGIKEDGSLWSADMNSFNHYAYGAIGEWLYRYVAGIRSDEHQPGFRMVHIEPQPGP
GLDWVEASLETMYGQVSSSWHRLAEGEMEIRVHIPVNTRGTVRLPGAGAQIVLEQGKP
LNQLDQMSGVQDIQNIGDDVEVLLGSGSYQFTYKDTDAKKDRDVQEATLTESV"

CDS 1702182..1702658

/locus_tag="EFAGFIKM_01516"

/inference="ab initio prediction:Prodigal:002006"

/codon_start=1

/transl_table=11

/product="hypothetical protein"

/translation="MAKPAFKDAPLRREIEDLAKQQDHHTLACWAAECAARVLYIFKK
EKTDDLRARDAIDAGRGWIRGEITMVDARKAAFAAHAAAREADHVAASAASRAAGHAA
ATAHVKGHAVHAATYAVKAVFYDSLMEEQERRVLEERTWQYQYLLGGCNDHGERTS"

CDS 1702633..1703172

/locus_tag="EFAGFIKM_01517"

/inference="ab initio prediction:Prodigal:002006"

/codon_start=1

/transl_table=11

/product="hypothetical protein"

/translation="MIMGKEQVRLIKPSRAYKDAYLAFYEDWVRSGELMVPWVISKEP
YAFDEMLAFLQRNEQGIDIPEGWVKDSTYWLVTESQQIVGAVNIRHELNDKLFNSGGH
IGYGIRPGERHNGYGSEILRLSLEKTRELGISIVLVVCDAVNEPSRRVILRNGGIRDE
DYVESNGNVVERFWIENVE"

CDS 1703217..1704401

/locus_tag="EFAGFIKM_01518"
/inference="ab initio prediction:Prodigal:002006"
/codon_start=1
/transl_table=11
/product="hypothetical protein"
/translation="MYYPRVLIKMKHRIWIAAGNYHPISGTSEVIFMNQPVNRPRRIE
YLDLYRAFAIMAVVAIHATSTAVAHYPKHTLDHDVYYFWNTFLQFAVPAFLFLSSLVL
FYNYSSKVNEKGWMLAFYKKRLFNVFVPYLVWSLIYFAIKQLLAGKEPLTHGVQFAKQ
LVMGTAHTHLYFFLIILQFYIVFPWLLSLTRHRLFNRYLPLFFIAGQAIFYALHLQFH
FERTGSLLPSYLIVIGFGAWVGLNFEWALNKLYTHRYVLIAALIGGGAIFIYGNAYIK
TAFAAYPVVTYTVLFLFRNLFTLSACLLLLIFSERIGTGKHTEVRKATRILDSLGTVA
FGVFLMHPLVLLFWRREFTDELARHFSVGIILSYIVALLISWICAMGLRRMKWGWVLI
GR"
CDS 1704567..1705127
/locus_tag="EFAGFIKM_01519"
/inference="ab initio prediction:Prodigal:002006"
/codon_start=1
/transl_table=11
/product="hypothetical protein"
/translation="MKLVIIFGPQAVGKMTVGQELEKITKLKLFHNHMTIELVSPYFS
YGTPQGKRLVNLFRQEIFEEVAKSDLPGLIFTFVWAFDLAKDGEYIRQISELFESNGG
QVCYVELEADPSERLERNKSPHRLLHKPTKRDIAWSERDLLDTMKLYRLNSEPGEITH
EHYIRIDNTHKSPDEVAKLIQERFNL"
CDS 1705185..1705490
/locus_tag="EFAGFIKM_01520"
/inference="ab initio prediction:Prodigal:002006"
/codon_start=1
/transl_table=11
/product="hypothetical protein"
/translation="MYFVIFLLIGLVLCYLIPFGPILAGGIVFALVIDHYRQTMHMRE
DIRDIKNYLGLMNKGEAKEYEMDKQFKDVDRLSSEEMNMINRRIEAELEKESKNNKP"
CDS 1705715..1706554

/gene="licT_4"

/locus_tag="EFAGFIKM_01521"

/inference="ab initio prediction:Prodigal:002006"

/inference="similar to AA sequence:UniProtKB:P39805"

/codon_start=1

/transl_table=11

/product="Transcription antiterminator LicT"

/db_xref="COG:COG3711"

/translation="MKIEKVLNNNAVVAMNDEQEVIIIGRGIAYQKRAGDMVSEQHID

KIFTLQNEDIQENFKALISSIPLEYMKVSEEIIAYAKLKLGKKLNESIYLHLTDHIHF

AIERYRKNLPIRNGLIWETKQLYKEEYEVGMEALNMICDQFGVILPEDEAGFMALHFV

NAALNEEMPNIKSMTQVMQEILTIIKYHFKIDFDENSLTFYRFVTHLKFFAQRLVKGK

HYKSNNDDELFLMIQKKYPAAHKCSEKIKKFIESNYTYQLTDEELMYLTIHIERVVNA

TSE"

CDS 1706722..1708659

/gene="bglF_5"

/locus_tag="EFAGFIKM_01522"

/inference="ab initio prediction:Prodigal:002006"

/inference="similar to AA sequence:UniProtKB:P08722"

/codon_start=1

/transl_table=11

/product="PTS system beta-glucoside-specific EIIBCA

component"

/db_xref="COG:COG1263"

/translation="MKHEQLAKDILSGVGGKQNINSVVHCATRLRFKLKNDNNAKTDE

LKNLPGVITVMQSGGQYQVVVGNEVSDVYKALLQVGNMNADEPVSSDDSESSGKKESL

LGQFIDLISGVFTPILGLLAATGMIKGFLSMFTSLEWLDPASGTYQLLNAAGDCLFYF

FPIFLGYTAMKKFGGSPFLGMAIGASLVYPTLSGLSGGEPLYTLFAGTLFESPIHITF

LGIPVILMSYSSSVIPIIIAAYFGAKVEAFFKKIIPSVVRTFLTPFFTILIVVPITFL

LIGPIATWASQLIGAGVSGIYDLSPLVTGIVVGGLWQVLVIFGLHWGIVPIMLLNLST

LKADPILAMMFAASFAQIGAVLGVMLKTKNTKLKSLGVPAFVSGIFGVTEPAIYGLTL

PLKKPFIMSCIASATAGGIIGFAGSKMFVFASGIFGVPALISPTEGINYEFWMAMIAT

IIAFVLGFVLVYFVGFKDPANPEAAKQAPEPVKEEKAALEPNPNNRYEIASPMTGEVV

PLQEINDATFAGEHMGKGIAIRPTSGRVVSPINGVVQTIYRTKHAIGLVDDQGVEILI

HIGQDTVQLKGQHFTAHVKDGDRVSVGDLIVEFDLQAIIEAGYETVTPIIVTNTADYL

DVVATTSREVQEKDKLVTVIG"

CDS 1709105..1711018

/gene="bglF_6"

/locus_tag="EFAGFIKM_01523"

/inference="ab initio prediction:Prodigal:002006"

/inference="similar to AA sequence:UniProtKB:P08722"

/codon_start=1

/transl_table=11

/product="PTS system beta-glucoside-specific EIIBCA

component"

/db_xref="COG:COG1263"

/translation="MSNYDQLARDILSRVGGRENVNSVFHCVTRLRFKLKNESVAKTE

ELKNLPGVITVMQSGGQYQVVIGNEVPDVYKAVVKVGNFPSEGQVEEETDNSGKKVGL

FSRFIDMISGVFTPLLGLLAATGMIKGFTAMFLSFGWITDTSGTYQLLNATGDCLFYF

FPIFLGYTAIKKFGGSPFLGMAIGASLVYPTLSGLTAGDPLYTLFAGTLIESPIHITF

LGIPVILMSYSTSVIPIIIATFFAVKIERFFKNIIPKVVSTFLVPFFTLLVIVPATFI

LIGPVSTWAGQLIGAGATGIYDLSPVVTGLIIGGLWQVFVLFGLHWGLVPVMLLNLST

SGADPVIAMSFAASFAQIGAVLAVILKTKNTKLKSLGIPAFISGIFGVTEPAIYGLTL

PLKKPFIMSCIAGGIGGGILGLAGSKLYIFGGLGVFGFPSFINKATGVDSGFYMALVA

CGIAFILGFILTYVVGFKDKVEATPAPAPVLDPNPNSRYEIVSPMAGEVVALNEINDV

TFAGEHMGKGIAIRPTSGRVVSPITGVVQTVYRTKHAIGLVSDDGVEMLIHIGQDTVK

LKGEHFTTHVKDGDRVNAGDLIVEFDLQAIKDAGYETVTPIIVTNTSNYLDVVGTKDA

TVHEKDKLITVLS"

CDS 1711053..1712495

/gene="bglH_6"

/locus_tag="EFAGFIKM_01524"

/EC_number="3.2.1.86"

/inference="ab initio prediction:Prodigal:002006"

/inference="similar to AA sequence:UniProtKB:P40740"

/codon_start=1

/transl_table=11

/product="Aryl-phospho-beta-D-glucosidase BglH"

/db_xref="COG:COG2723"

/translation="MNEKFTAFPENFLWGGATAANQLEGAYLEGGKGLTTVDLIPIGA

NRWNIALGNLDSYEPKTGEFYPSHEAIDFYHRYKEDIALFAEMGFKCLRLSIAWARIF

PKGDEAEPNEAGLQFYDDVFDELLKYNIEPVVTICHFDVPVHLVETYGGWKNRKMIGF

FEKYATTLFNRYKNKVKYWMTFNEINMLLHLPYIGAGIVLQDGENKDQVLYQAAHHEL

VASALAVKACHEIIPDAQIGCMLAAGMVYPYSSNPDDVWKAMEKDRESFFFIDVQSKG

VYPGYTKRFFRENDIWIDMQPEDAEILMMNTVDYIGFSYYASRCTSTDPEILKDSTEG

NVFGSVKNPYLQASEWGWTIDPKGLRITLNQLHDRYGKPLFIVENGLGATDVLLDNDS

VEDDYRIEYLNSHFAEMAEAIQDGVELIGYTSWGPIDLVSAGTGEMKKRYGYIYVDRN

NDGTGTLRRVKKKSFHWYKDVIASNGAQYF"

CDS 1712810..1713208

/locus_tag="EFAGFIKM_01525"

/inference="ab initio prediction:Prodigal:002006"

/codon_start=1

/transl_table=11

/product="hypothetical protein"

/translation="MNMDKITRTNPENMPAPVGQYTHITRIPRNAELFVTSGQIGADH

NGLFPESLNEQVTNTFNNIKTVLESERLDAEHIIKVNVWATEEIDWDFLYAEWGQLFS

TDYPAMTIGYITALGLPEIKIEIELWAAKV"

CDS complement(1713329..1714486)

/locus_tag="EFAGFIKM_01526"

/EC_number="3.4.21.-"

/inference="ab initio prediction:Prodigal:002006"

/inference="similar to AA sequence:UniProtKB:Q45670"

/codon_start=1

/transl_table=11

/product="Thermophilic serine proteinase"

/translation="MWLWLGIFATILLAIGYVHHYLDHQAESQFHPHAPNQLLIKFKE

DKTEDEMHSLHKKGRCRVAETYEDLGWYRMESRKKMHRMLKHYKDHELIEHAEPNYLV

EASFTPNDPFFPYQYNLSKINAPAAWNITQSNSSVKIAIIDTGVQLNHPELASKLLPG

YDYVDYDNIPEDGNGHGTHVAGIAASVTNNGVGIAGAAPLASIVPLRVLDNNGQGTIG

NVGNGLVFAANNGVQVVNLSLGGPMGNAFLQAAVQYAWDRGAVIIAAAGNDNTSFPIV

PASYPNVIAVASTNPSDLKSNFSNYGSWVDMAAPGDTILSTYLGGSYAYLSGTSMATP

HVAGAAALLAAQGKTNAQIRDALCFASDPVSGSGIYWTYGRLNAYQSLQVP"

CDS 1714707..1715228

/locus_tag="EFAGFIKM_01527"

/inference="ab initio prediction:Prodigal:002006"

/codon_start=1

/transl_table=11

/product="hypothetical protein"

/translation="MTQLQSIPTVTPVQPHQIHDATDFAMRVRREVFPMMDHTKLPVD

FEQFREHYLESDGSIFLIAMVGDAGIVGSIGILPYDGRIEAVEGRYAAQSAAEIVKCY

VDPAYRRFGIGSMLMNELENVVKDMHYTTLYLHTHRFLPGAVDFWKRQGFTVIAEQDD

DWQTVHMDKLACK"

CDS 1715286..1715744

/locus_tag="EFAGFIKM_01528"

/inference="ab initio prediction:Prodigal:002006"

/codon_start=1

/transl_table=11

/product="hypothetical protein"

/translation="MYSSLIEKLEHTEVIRWFPNHQVQESWIVEVEQELAFPLPSSYR

WWLLNYGEALLSGTEILTITSPEHREYTDADILYRYRLTDEDTKKEGKLELFVPDEGE

YYYFDTWTADHLVEYKVMRLDYFNGEPEVYADSFAGFLEKLIDQRTPRNR"

CDS 1715819..1716679

/locus_tag="EFAGFIKM_01529"

/inference="ab initio prediction:Prodigal:002006"

/inference="similar to AA sequence:UniProtKB:Q5XAQ1"

/codon_start=1

/transl_table=11

/product="Putative bifunctional

phosphatase/peptidyl-prolyl cis-trans isomerase"

/translation="MNETKRKLVFIDIDGTLVDDDGNIPLSAQKACKQARENGHLLYL
CTGRSKAEIYDSIWDVGFDGLIGAGGGYVEFGKDVLYHKRVTAEDVRHMVDFFNENGI
DFYLESNSALYASNNLQPHLERRIYGDVENDPVAREKKELKPHPFIAGLTYGEADLYK
DDVNKVCFLESSAIPFERIKEEFEGKFEVIQCTVPIFGEGSGELMIPGIHKAIAIADL
LNHLGMSQEDTFAIGDGMNDAEMLEFCNTGIAMGNAKPGLKAIADDITGTVEEDGLYH
SFVKYGLIEG"

CDS 1717082..1718287

/gene="fenF_2"

/locus_tag="EFAGFIKM_01530"

/EC_number="2.3.1.39"

/inference="ab initio prediction:Prodigal:002006"

/inference="similar to AA sequence:UniProtKB:Q9R9J2"

/codon_start=1

/transl_table=11

/product="Malonyl CoA-acyl carrier protein transacylase"

/translation="MSNVALLFPGQGSQMVGMGRDFWNNFDVAKKLFEEASDAISFDL
RKLCFEGSMADLTMTMNAQPALLTVSVIAYQVYMQEIGMEPAYLAGHSLGEYSALVCA
GVLSFHDAIKLVRQRGIIMHNADPDQKGTMAAISGIHLDTLQNICEQVSTIERPACVA
CVNADQQFVISGHRHSIQEIIKRTEALRTKHSYINVSGPYHSPMMQTAAEQFKVELDQ
YQYAHAKYPVISNVTAAPYESDRSVVDYLTMQMTMPVRWLESMLYLVANGVTEVIELG
SKHVLVSLMSKITKRIVPYSLSQSSDLLMLTNTQERKNKRLTIRKKLLSELIVTALIS
RNYNPDSISYAKTVEPLFHQLQRAKEKVNNNEYELSEEEINDALQVCHSIIIAKNIPL
DGTALIDAI"

CDS 1718561..1730494

/gene="dltA_4"

/locus_tag="EFAGFIKM_01531"

/EC_number="6.2.1.54"

/inference="ab initio prediction:Prodigal:002006"

/inference="protein motif:HAMAP:MF_00593"

/codon_start=1

/transl_table=11

/product="D-alanine--D-alanyl carrier protein ligase"

/translation="MYTNQFQTLVDVIRDRGKITDRGIRFIDADKRETFISYHQLFTE
AQGYLGYLQAIGIKPKQEIIFQIQENKRFLVAFWACILGGMIPIPVSIGEDEEQNLKV
CRIWNLLNNPFMIASEKVLDKMKRFVLEHDMLDFHHQLEVRTDLIQDTIYHYESINNI
VEPQSDDLVFIQFSSGSTGDPKGVMLTHRNLIYNTCAAINGTQISSNDSFLSWMPLTH
DMGLIGFHLVPLVAGIHQYLLPTELFIRKPILWMKKVNEHKATILSSPNFGYKYFLNF
FREDKAEGWDLSSVRVIYNGAEPILPEVCDEFLNVLSAYHLKKTAMFTVYGLAEASVA
VSFPKVEEEFTAVYVHRDHLNLGERVIEVEKHAEHVASFVAVGEAIDYCHVRICDEEM
NSVPDLVIGKIQIKGDNVTQGYYNNSLATEKLLTHDGWVNTGDLGFIMNGKLVVTGRE
KDIIFINGKNVYPHDIERVAIQLDDIELGRVAACGVYDSNSQSEEIIVFVVYKKKIEH
FLPHVKEIKRHLYKHGGWSVKEVLPIKKLPKTTSGKIQRYQLAKQYEAGKFSTESAFI
HDWIKNDNDKAKEKAAVSVRVIERELTSLFSEVLVGKKVELEDSYFDMGATSLQLAQI
AERIEQKYGEELAVTDLFTYPSITELAAYLARGYSETMQVNKTQEDHTPSKDIAIIGM
SLNLPGASNASDFWRVLENGMHNIRDYPKSRIKDAADYIQSMRSAMSEVQWIKGGYLD
QIDQFDHSFFGITPKTAQFMDPNQRMFLQTAWHTIEDAGYAGEGINGRNVGVYVGYSK
VGYDYERLVTASYPAELKNYIIGNLPSVLASRIAYFLNLKGPALTIDTACSSSLVAVH
MACKGLLAGDCEMAIAGGIRTALLPISIGLDMESPDALTRTFSEDANGTGFGEGVGSV
MLKPMQQAIKDGDHIYGVIKGSAINQDGKTVGITAPNPESQTNVIEQAWKEAGISPDT
LNFIEAHGTGTKLGDPVEFNALRKAFEKYTDRKQFCAIGSAKANIGHGFEAAGIAGLI
KSILMFQHRKIPPLVHFDKPNPLLKFEASPFYVNQELMAFGEKEGPLRCGVSSFGFSG
TNAHVVLEEYVPSPTVDKRMPQEPNLFVLSAATERALAELVRTYYEYLTDHTDVSIQQ
ICYTTSTGRANLDYRLAIVVMSTQELHEKLKRIASGEGKLPNVYFGYAKKDKTLVSDH
LYARGKAESLNELGQLYTLGAVIDWERFYNHEVIRKIPLPLYPFEQKRCWIEVKHTIP
AGHRSERNEVQMEMNTNDIELKIKTSISRASGLKLEELDHQAHFLQMGLDSIILVQIQ
KEISELFRLNIPMEMFFETLTNINSLVKYVSENIKHHDVSENIKNQTVQEHTEQRNLA
SVNNINTAKTSGNVSEQIVMSAIQLMESQLKSLNVLLGEQMNAVQSPLLEMKHNETPK
VQASPVMNEINKTNESKKIQPDSESKEEKKPFIPYQSLKIHEESGLSPLQNQYIEQFV
TKYTARTKGSKLKTGQTRLVHANNRNVSGFRSYWKEMVYPIISTRAEGAKLWDMDGNE
YLDLTMGFGVNLFGHNPAFIQNVIADSLQEQLPPLGPMSVLAGDVAAKISELTGVERV
AFYNSGTEAVMVALRLARAATGRSKVVLFSGSYHGTHDGVLGVANPGDVLSSALPMAP
GIVNSSIDDLIILNYNHPQSLDMIQNLGDELAAVLVEPVQSRRPDMQPTQFLRELREI
TKQFGIALILDEVITGFRIGLGGAQDWFGVQADLVTYGKVVGGGLPIGIVAGKAEFMN
PVDGGNWNFGDDSYPSDADKKTFVGGTFCTHPLTMRVMLTVLQYLQSEAETLYKELNE

KTDYLVKELNTFFKTANVPMQMVNYGSLFRFISFGDMELFFYHLIYKGLYIWEGRNCF

ISTVHSMGDMDTIIRIVKETIHELREWGFIPGESPVSEYPLSLEQKQLLLTSLHGEDA

NAALNQSVILKMTGGIKPSTLDQAVNQIAQRHEALKTVIHADRETQQIRSVMGFQCTW

LDFSTYAGEDQEREISNWLSADANEAFDLQSGEPLFRITLLKKATDQYLMVLTFHHIV

VDGWSIAVFIQELEHTYSALVRGVPVQLPEATPFREYLSWQRLEHEKGSFTEGIQFWK

EQFSLSYPNITLPTAPSDDVPKGYDGERYSIKLDHQLTESLRTLSINNKSSLFVTMFA

AFHLYLHQLTGQSHLVISVPTAGQSHMRKNALIGNCVNIVPVHHALLTKQSVVDHVLQ

LKETLALIMKHQHVPLTLVSEQLAHMTAPDTRILFNMDRTIHHLQFENIKTEFMSYPI

KHISYDLFLNITDVSHELYLDFDYNTDLIGPEMMKLWSEGFKVLLQKMVSHPEAEISS

LTLFSSEQQDQLQGMYNRFVTERLPHVNHAAIFELPVNDVEKKLALIWEDVLSLDQVG

RHDHFFDLGGNSLKATVMLSKVHEQFQHKISIVQFFQNQRLKQLASLITGENEETFIP

IKALDVKEFYETSSAQQRIYFLDQFEPNSVFQNMCGHITVTGKIDEAKLISSLEQIVQ

RHEAFRTSFHLLDGELYQRIAPTIDFEVKRSQGSSASFEQHVKTFAKPFDCSKAPLIR

AEFIRLNDDHAEILIEMHHIISDGYSVGILANELIDVYDGRSMSDIEVEYKDFTAWQK

EFLAGESYKQQEKYWLDQFKGELPNLNLPTDFVRTPHLTSAGSRLSFVLDAHLKQSLE

KLAKQTETTMFMVLLAAYNVLLHQYTDQEDMIVGTPTSGRNHPDVENMIGVFIQTVAI

RSQPVGNKSFRSYLEEIKKQSLDALDNQDYLFDNLVENLNTHRDTTGTALFNTMYIFL

NTALNQRQEQDCSFKVRELNVGVSLYDLMFIVEDDNECLEMHFNYKTELFERDTIDRM

KTHLFTILRDIVIEPDIALSDIQMLSEHEQHVLLYDFNDTNIEYSRDQTIQELFEAQV

ERTPDQLAVSFGNERWTYRQLNERANQLAYTLRAEGVQQDQFVGILTDRSIEMIVGIF

GILKAGGAYVPIDPDSPEERIRYILHDSGATLLVTQKHLKQLVSFNGKMIDLDDEETY

VANLSNLEPAGNSEQLAYMIYTSGTTGMPKGVMIEHRQVHHLVQALYSQVIDDQLSGL

HVALLAPYYFDASVKQIFGALLSGHALFIVDKDTVLDGQDLMNYYQHHQIDLTDGTPA

HLKLLSKAKNVQGISLKYMLIGGEALSQQSVNEITSLFALNHITPPTIVNVYGPTECC

VDVATYRVLPEHLDTANQRAHLPIGRTLGNNRLYVLNKHGKVQPVGVVGELCISGDGV

GRGYLNLPELTAKKFVSDPYVQGATMYKTGDLVKWLPDGHIEYLGRMDHQIKIRGYRI

EVGEVEAALLDLECVKEVIVIAHEDDHGDKSLCAYFTADSTLTVGELKQSLSKVLPNY

MVPAYFIQLDHMPLTSNGKIDRKSLPSPEENIQTGSEYIEPQTGVEKQLLPIWQDVLA

VKKISVKDSFFDVGGNSLRATKLAATINQKLNIRISVRNVFEWQTIEQMAQSIEQMEQ

EVLVSIPQVGEKEYYPISSAQMRMYIMSIKAGKELMYNMSGMFMLEGSLDWIRLEEAF

QQLIHRHESLRTGFKVIHGEPKQIVYPKVNFGIELIESMEEEIEDRIEQFVRTFDLGQ

APLMRVGLIELQQERHVLLVDLHHIIADGLSVHLLMTELSQFYKGEELSPLRIQYKDY

AEWQQTQLHSEWMKKQEKYWLTVFGESIPLLDLPTDYERPETLSYAGDVLDIVLQEDI

LPGLRNMEAGTGTTLYMILLAAYTILLSKYSGQEDIIVGSPIAGRTHAELEPVMGMFV

NTLAIRNYPAGDKTFTEYLLEVKENMLNAYENQDYPFEELIEKVNVTKDARRNPLFDT

MFVLQNTDASEIQMDGVTCKPYAPNTRVSKFDLTLYVTELQHKLEVAFEYSTVLFEKT

TIEAMSQNLLVILTTIANDPHIEINKIQLVEELIDGDTLADLIEVDF"

CDS 1730528..1740349

/gene="lgrD_1"

/locus_tag="EFAGFIKM_01532"

/inference="ab initio prediction:Prodigal:002006"

/inference="similar to AA sequence:UniProtKB:Q70LM4"

/codon_start=1

/transl_table=11

/product="Linear gramicidin synthase subunit D"

/translation="MSIFKKQESYWASRFEDDDHLVHLPYCKTTSKHPDDTQHQNDIY

KSLSADVSHKIKSMSRNAPMAIYTILLTGVQCLLYKYTNETKMIIGIPTMEEKNNDLS

LSNKVLVLKNNINHVMSFKSLFNQVKTSLTEAIQHQRIPFRKMVERLNVQYDSNHVPL

IHTLVSLNEIHPALDENNVKAATMFHFDLENDSIQLRLVYDDDRYDRDYMVQIVEHLD

QLFSVVLNQPDLELSQVDMLSEFYRNKLLYDFNDTRVEYPQNKTMHQLFEEQVERTPD

HPAVVFENRQLTYRELNQKSNQLARLLQAKGVHAGQFVGIMAERSLEMIIGLFGILKA

GGAYVPIDPEYPADRIHYILEDSKVAWLLHQGHIQEPEHFAGTCVILDSEINHYDDHL

NLVDGAISSDLAYMIYTSGTTGHPKGVMIEHASIVNSLQWKSDFYHFTGDDRVLMLNP

FVFDSFITHFFGPMISGSTVYLLNQQQCKDAIAINTMVKKQQITHIQSPPSFLMTLLE

QVSAEDWTSMKNVVAAGEKIVPALIHKLQRINPRIEVCNEYGPTENSVVSTVLSIKSA

DQNISIGKPIANNRVYILGERNELQPIGVQGELCVAGAGLSRGYLNRPDLTAEKFVDD

PFVSGAKMYKTGDFARWLPDGNIEYIGRVDHQEKIRGHRIELGEVETALLNSTQIKEA

IVIAREDETGTKQLCAYLVGEDAITIGELREALSQQLPNYMIPSYFVQLDQMPLTSNG

KIDRKALPAPKEHMLKGMEYEAPRTMVEQQLVSIWEEVLGVENIGVLDNFFELGGDSI

KSIQVSSRLYQAGYQVQMDHLFKNPTIASLIPYVQPISTLSEQGIITGKVMLTPIQHW

FFEQYKVDAHHYNQSVMLYRKEGFHEGTLRRVFDKLSEHHDALRTVYRLTEQGYEAWN

EGLARESLYTLEVMNFKSLADPSQEIEKKANEMQSTFTLERGPLMKLGVFQCADGDHL

LIVIHHLVIDGVSWRILIEDLSRGYEQLSDGESIQLPQKTASFQLWAEQLQLYANGDA

ITKEIAYWNEIEKATYKPLPTDFEADHALEKDSDEITMEWSAAETEQLLKQANRAYKT

EISDLLLHALGMSIHQWTGMDKVLVNLEGHGREQILPDLDITRTIGWFTSQYPVVLDM

EASSNMSLFIKTNKEMIRQIPKKGIGYGILRYLSAHQQEAPFTLNPEISFNYLGQFDQ

DLQNSGMQISPYSGGATASDNHRRNVLIDMNALIVEGKLTLSLRYSRTQYQKETMERV

VTYFRDHLQEIVMHCVTKEKLECTPSDLTLKGITIEELEGFVQESEHIGEIEDIYALS

PMQNGMFFHHLLHSKSEAYFLQTTLDLHGPLDVEAFTKSLDQLMQRNSILRTNFHSKW

KNRPLQVVYRYKRNGLIYEDLREMNEKDREAYVAEYTRADKIKGFDLSQDVLMRISIL

QTHDQTYRFIWNYHHILMDGWCLSLVIQELFNGYFAIQEQRKLEAKSTIPYKHYIEWL

DNQDHEESLKYWRDYLQGYDEQTTLPKEHSITKSDEYHSEHMICTLSQELTGQLAQVA

SQNQVTLNTFMQTAWGMLLQIYNGSEDVVFGSVVSGRPAAIVGVERIIGLFINTIPVR

IQTQGNETFAAVMKRNQEQAVASHPHDTYPLYEIQAETSQKQNLIDHILIFENYPVQG

IMEQFGDQEETALRITNVEADEQTNYDFNLIIKPGQSMKCMFYYNANVYDRASVERIQ

GHLIQVLEQVVNNPNIPIHDIDLTTTQEKTQILEHFNDSMAEYPMDKTIHGLFEEQVA

RTPEHVAVVFEDQHVTYQELNEKANQLARTLKAKGIQNDQLIGIIADRSLDMVVGILA

ILKAGGAYVPIDPEYPEDRINYMLEDSGVHVLLLQSHLQERISFAGEVVCLDLSESYH

EDHSNLGITAAPTGLAYVIYTSGTTGKPKGTCIEHKNVVRLLFNSKNLFDFNASDTWT

LFHSFCFDFSVWEMYGALLYGGKLVIVPQMTAKSPEQFLQLLKNEQVTILNQTPTYFY

HLLQEELAGHAADLKLRKVIFGGEALNPVLLKEWKLKYPFIQLINMYGITETTVHVTY

KEITDIEIHAGKSNIGKPIPTLQTYILDKHQRIQPIGVQGELVVAGEGLARGYLNRPD

LTAEKFVDHPWITEGKMYRTGDAARWLPDGNIEYLGRIDHQVKIRGYRIELGELETAL

LQIESIQEAVVIAREHKDGSKQLCAYWKGDSSLTAKKIRMALSHEIPDYMIPSYFVQL

EHIPLTSNGKVDRKALPSPQEHMQKGTEYMAPQNAVEQAIVAAWEAVLGVQKVSTSDH

FFELGGDSIKCIQVSSRLLQAGYKVEMKHFFTYPTVAELSEHVTSVSTISDQGEATGQ

VMLTPVQRWFFDQNMVDTHHYNQEMMFFREKGFDVRFIHSIMDKIVKQHDALRILYRQ

APEGGYEAWNRGADEEGLYSLEIVDLKTEADPASFIENKAKEIQSGMDLSHGPLIKLG

LFQCAEGDHLLIAIHHLLVDGVSWRILYEDIQTGQDQFLRGEEIRLPQKTTSFKQWAE

QLSTFADSPAMKQENPYWSRVEQAQLSPLPKDMDCDETIGKDSETITIHWTAVETEQL

LKQTHHAYHTEINDILLTALGMAVHDWTGMGEILVNLEGHGREDILPELDITRTVGWF

TSMYPVMITMESEADLSRKIKNVKEGLRQIPTKGIGYGISRYLSHGNEGFKLEKNAEI

SFNYMGQVNQDSGQDGIKFSHYSSGPSVSENNKRPFVLNINGIVTEGRLSLSINYSSK

QYRRETMEQLACLYKEGLNRVVEHCVTKEQTELTPSDISCKSLTIDQLDQIVEQTGHI

GRIENVYPLTPLQKEMLYESERNPISGAYFVQIMFDMRGDLDVTSFAQSLDQLMQRHA

ILRTNFYSDFVDIPLQIVYQEKKFEFNYEVVAGTSELQRKTYVDEYIEQDKARGFDLQ

RDSLIRMSVLRTKHEEYRVIWSFHHILMDGWCMSLVSKEIFENYFAIRENRKPQVTLL

TPYSQFIEWLEQQDHQGAITYWSHYLDGYEGQTKLPGEKIKPKHERQDSRTLNCYLDD

VLIEQMKQIAEQQHVTLHTVVQTVWGVLLQQYNESNDVVFGSVVSGRPPVIPGIESMI

GLFINAIPVRIQTQADESFISLMKRNQAKAVESHAYDTYSLYDIQNQTKQKQSLINHV

VVYQNFPVENRMESLGQKGETPLEIMNVGGTEHNSFDFTLIVKPHENMKIILMYNANV

YDPSTVEQIQKHFISMIQQVVSNPEVKINELALSTSNSY"

CDS 1740912..1741094

/locus_tag="EFAGFIKM_01533"

/inference="ab initio prediction:Prodigal:002006"

/codon_start=1

/transl_table=11

/product="hypothetical protein"

/translation="MDIAALSMAMSQASVVQSASLQVMSITKDMAQQQGQQMAEMLKS

MPAPHPNLGGSLDLSV"

CDS complement(1741238..1741975)

/gene="gph_2"

/locus_tag="EFAGFIKM_01534"

/EC_number="3.1.3.18"

/inference="ab initio prediction:Prodigal:002006"

/inference="protein motif:HAMAP:MF_00495"

/codon_start=1

/transl_table=11

/product="Phosphoglycolate phosphatase"

/translation="MAANKESNGLGAPNDDIIQAVIFDMDGVLIDSEPIYFEIERSSF

THFGARVTDEEHHTYVGVTLESMWQQVLDKHQLTATLEEIYAYHQHNVMQTMLAHSNL

TAMPSVERWLSWLHEQQVPIAVASSSPRALIDLIMDKTGLGQYFEVRMTGEEVTNGKP

APDIFLTTAEMIGASPSNCLVIEDSRNGVQAAKSAGMRCIGYRNPGSGNQDLSKADLQ

ISSYDELWTLKDTLPFEGRLPSLSKFR"

CDS complement(1742047..1743096)

/locus_tag="EFAGFIKM_01535"

/EC_number="1.1.1.407"

/inference="ab initio prediction:Prodigal:002006"

/inference="similar to AA sequence:UniProtKB:A9CES3"

/codon_start=1

/transl_table=11

/product="D-altritol 5-dehydrogenase"

/db_xref="COG:COG1063"

/translation="MLALVYKSAWDVALEDRPVPEITRDNQVLVRIRATGVCGTDLGI

VSGKYHAVPSVILGHESAGEVMDVGSAVTTLQPGDRVVIDPTYYCGQCDMCRTGRQNH

CTHKSVTETGVSADGTFTDYYVTEDRFLYKLKEHVSYEEATLTEPLSCMLTGINQIHL

LPNFRTIILGAGPIGILYSYALASKGVTGCLVDISEERLAIAGSIAPDRWEVHSSFEN

AIESLSPATQQVDMIVDTTGVVGTQVLSQLASGGYLMLVGLRDGNTSFNPKEVVDRSL

KIIGSIDSLGTFATAHYMIEQGIIPAKKIITHSFPLEDYEEAFRTLGCDIQGRTLQAS

SHAIKVVLQSSGSSF"

CDS complement(1743366..1744604)

/gene="dat_1"

/locus_tag="EFAGFIKM_01536"

/EC_number="2.6.1.76"

/inference="ab initio prediction:Prodigal:002006"

/inference="similar to AA sequence:UniProtKB:P44951"

/codon_start=1

/transl_table=11

/product="Diaminobutyrate--2-oxoglutarate

aminotransferase"

/db_xref="COG:COG0160"

/translation="MQTLTNNRFIAGKGIKLIDDSGVEYLDGVSGTFNLSLGYNHPHV

VSKIQEQVGNLTHMSSSFTEPYVNEVLDHLIEYAPNDINAGWMRDITGSTANECATKI

AQKYTESTDIISLYLSHHGQTQFATGISGNAFRRKRFPNSAVANAVHVPAPYCYRCPF

KSSNGDCDYQCVEAISDAIEYASSGSVACMIIEPILGNGGNIIPPAGYFKRLRKLCDE

YNIILIADEVQTGIGRTGTMFASELFDIQPDMITLAKGLGGIGVPVAAVLMQSRLNVL

EKHEHSFTSGSNLISVTAAKSTLEVVSEPGFLDAVKRKGEILGELLQELAMKYPSIGE

SRGVGLMWGLEIVGDGNEPDTLKTNAIVDRAFTDEHLILRSSRYGFGNVVKVRPSLTT

TEDELVEIVERLDSVLASVH"

CDS complement(1744694..1745902)

/gene="glcP_1"
/locus_tag="EFAGFIKM_01537"
/inference="ab initio prediction:Prodigal:002006"
/inference="similar to AA sequence:UniProtKB:O07563"
/codon_start=1
/transl_table=11
/product="Glucose/mannose transporter GlcP"
/db_xref="COG:COG0738"
/translation="MKRIFALSCGFYLLIGISSVVLGALLPVLLSHYERGYSDGGFLL
FLQFLGFLVGVIVAPALTARIGRKAMLTLALICIVAAYIVLGFLPSWSVVLLLTIIVG
FGSGIIEPSVGAFTIEFTENQKAVAMSKLDVFFALGALLIPAVAALFIWMDLWHLTFY
TVAVLSMVLMLLWITMPRPAALYLEQAGENTVAHAAGKAQYSRKHLGLLTIFVIFFFI
YMGLELGLMNFLPSILVERLHLQESVASLSVSILWIAMIIGRLFSGKIAEAVNYMPFL
IWSTVGTLLFTVAMVFVTGQWATYVLIFGTGLFMSGLFCIALVYANVLIPGMTERTTS
ILIASGGIGGAILQYVTGWSMSTGPVVNTIWILAGFCLILLLTLMVSHLWTVKNNAVR
TALAQHSKEM"

CDS complement(1746247..1746879)
/locus_tag="EFAGFIKM_01538"
/inference="ab initio prediction:Prodigal:002006"
/codon_start=1
/transl_table=11
/product="hypothetical protein"
/translation="MYKVTRFTVDPDNPYQHSGMWIGGETAYVSDWPLNPEGQPLLHL
FSINCNSLSQQVHIPSLPQDKYISVFSTYSASEYFLDQVTYTGDELEWNENILAGCTY
VSVSSHPLTSVCPVPPIPLSGVRLIEMELDDQEFPAFSFFSPTLPNGVKGIDHLLEEH
QLVCQIYSGDFPDPYRDILGLPDANGYLFLRRGPASAHAPFDGIFFVQTA"

CDS 1747084..1747785
/gene="rluF_1"
/locus_tag="EFAGFIKM_01539"
/EC_number="5.4.99.-"
/inference="ab initio prediction:Prodigal:002006"
/inference="similar to AA sequence:UniProtKB:P32684"

/codon_start=1

/transl_table=11

/product="Dual-specificity RNA pseudouridine synthase

RluF"

/db_xref="COG:COG1187"

/translation="MLINKYISETGFCSRRETNRLIAAGRITINGRVCEKGAEVEPHD

VVLIDGNQIPRNDSEPVYIALNKPIGIVCTAAEHVKGNIIQYMNYPSRIFAIGRLDKA

SEGLILLTNDGGIVNKMMRSEHNHDKEYVVTVDKPITDEFVKSMSEGVEILDVVTKPC

EVYRQSDKVFRIILTQGLNLQIRRMCKALGYRVLKLERVRIMNITLDQLERGQWRHLE

QEELEILLSQLNEIK"

CDS complement(1747856..1748794)

/locus_tag="EFAGFIKM_01540"

/inference="ab initio prediction:Prodigal:002006"

/codon_start=1

/transl_table=11

/product="hypothetical protein"

/translation="MTSHLGHQNSKASTGHLLALFTILIWGTTFVSTKVLLIDFTPVE

ILFFRFLIGYLVLLLIYPRSLRIASFREEWLFIGAGLCGVTLYFLIENIALVYTTASN

VGVIVSIAPFFTAVLAHFFLDGEKLTRRFIIGFGIALSGILLIALNGSFILQLNPIGD

LLAFIAPAVWAIYSVLMRKIGQLRYHTISATRKVFFYGLIFMLPALFLFEFHWNLGRF

TNMTNLSNFLYLGLGASALCFVTWNRAVNLLGAIKTSVYIYLVPVITVVSSVLILQEQ

ITWVIVLGAFLTLFGSYISEQKGHKHNNKNTNPHSG"

CDS complement(1748791..1749642)

/gene="rhaS_9"

/locus_tag="EFAGFIKM_01541"

/inference="ab initio prediction:Prodigal:002006"

/inference="similar to AA sequence:UniProtKB:P09377"

/codon_start=1

/transl_table=11

/product="HTH-type transcriptional activator RhaS"

/translation="MTREVRTVVFDTDLQLEAYQFEGIMQKFPNHFHDYYVIGFIEQG

KRHLLCNNEEYILNSGDMIVFNPHDPHACEQVDGRTLDYRCINVQPEVMREYAREITG

QAYLPRFTSPVLYQSELVGSLYELHQMILEEQSDFCKEELLLLLLDQLLRDYSDAEPP
VSTQDVTLEIRRICDYIEAHYMESISLNELTDLTEMSKYHLLRLFTRQKGISPYRYLE
TIRINQAKRLLEQGLLPIEVAAQTGFSDQSHFTNFFKKLIGLTPKQYRRIFNHDSELK
RTTTNIV"

CDS complement(1749868..1750983)

/locus_tag="EFAGFIKM_01542"

/EC_number="1.-.-.-"

/inference="ab initio prediction:Prodigal:002006"

/inference="similar to AA sequence:UniProtKB:P32382"

/codon_start=1

/transl_table=11

/product="NADH oxidase"

/translation="MSNQQHNVISTELLFQPYTVKKLTLSSRIVMSPMARAFSPDGVP
GPDVAAYYRRRAEHGVGLIITEGATIEHPSASSEPRIPHMYGEAALQGWAEVVEQVHE
VGGKIFPQILHMGIVRPSGSQPHPEAESLSPSGVDMEGTQVGAPMTEAEIATVIQAYA
DAAANAQSIGFDGIELHGAHGFLIDQFFWKTTNRRTDRYGGDLQKRTQFAVELVMAVR
AAVGPDFPIAMRISQWKMNDYQARLFDTPEQLEQFLRLLVDAGVDIFHCSTRRFWEPE
FEGSELGFAGWVRKLSRQTTITVGSVGMPDEPEAAADKTTHPGMGELMKRYEQQEFDL
VAVGRALLGDPAWAAKIRDGRISEIQAFTPEALATLH"

CDS 1751207..1751788

/locus_tag="EFAGFIKM_01543"

/inference="ab initio prediction:Prodigal:002006"

/codon_start=1

/transl_table=11

/product="hypothetical protein"

/translation="MSGVLSPNSNDPRVIRTRQLILDAFLNQLNLKNFNSITIQNITE
QATINRATFYAHFQDKYALLEALLSDAFMEYVTKRVDPDARLSAETIQQLIFSLCDYH
VSSNGCIKKYETVAPIIEENIKTQLEQFLLELMSKVAGDVDPTTLKITATMLSWSIYG
MTYRWNIEGGQESPAELANRVVPYMMGGLELLN"

CDS 1751843..1752142

/locus_tag="EFAGFIKM_01544"

/inference="ab initio prediction:Prodigal:002006"

/codon_start=1

/transl_table=11

/product="hypothetical protein"

/translation="MFEHLVVFKFNDKTTLAKQKEWVDQLLALQEQIPGIVALTAGIN

ATEETDRIQGYTIGLRVTFEDQEALRAYGPHSAHQAFVASLDGWVEDVIVADYAI"

CDS 1752188..1752967

/locus_tag="EFAGFIKM_01545"

/inference="ab initio prediction:Prodigal:002006"

/codon_start=1

/transl_table=11

/product="hypothetical protein"

/translation="MCLILFAYNMHPKYPLILGSNRDEFYHRPTAQAHYWEDHPHILA

GRDLSKMGTWMGVTKGGRLAAVTNYRDPNEEIHAKGSRGDLVADFLKGTSTPEQYMQH

AEQSRNDYPGYNLLVGDPDDLYYYSNVGNVVMKLKPGIYGISNHLINTDWPKVKRGKE

GLEKIIHGEDESTLTEQILGLLQMADPSVDELLPHTGVPLEWERFLSPIFVKSEKFDY

GTRASSVVLMNREEVFFTERVHQNGEVMEQRFNIKPLNSTS"

CDS 1753281..1754597

/locus_tag="EFAGFIKM_01546"

/inference="ab initio prediction:Prodigal:002006"

/inference="similar to AA sequence:UniProtKB:A0A2A5JY22"

/codon_start=1

/transl_table=11

/product="Nucleobase transporter PlUacP"

/translation="MKEAKKMRVFSLGIQHVLAMYAGAILVPLLVGRALNLTAEQLAY

LVSIDLLTCGIATWLQARKGKYLGIGLPAVLGSSFVAVTPMIAIGSQYGIPAIYGSII

AAGLFIVIFAVFFSKIVKLFPPVVIGTVVTIIGLALIPTGIKNMAGGANSENFGSLDN

LALSFGVLLFILILNRYARGFVKSLAVLLGIIVGTLIAAIMGKVNFASVQEASWFHLP

QPFYFGWPTFEIGPIITMIIVGTVVIIESTGVFMALSKICDQPINEKDLARGYRAEGL

AFVLGGIFNAFPYNTFAQNVGLVQLSKVKTTNVVVAAGGILVFLGLIPKVAAFATIIP

SAVLGGATVVLFGMVVSSGIKMLQNVDFGKQSNLLIVACSVSLGLGVTAVPDLFAQLP

QSIRIIVSDGIITGSLSAILLNVFFNLALKKESLPIQPVQAQEAHT"

CDS 1754911..1756380

/gene="selO"

/locus_tag="EFAGFIKM_01547"

/EC_number="2.7.7.-"

/inference="ab initio prediction:Prodigal:002006"

/inference="similar to AA sequence:UniProtKB:P77649"

/codon_start=1

/transl_table=11

/product="Protein adenylyltransferase SelO"

/db_xref="COG:COG0397"

/translation="MQQETLNKGWNLKNSYARLPQPFFTSQGAVPVESPKLIIFNRLL

ASSLGLDVESLDSDEGAEIFAGNLIPEGAEPLAQAYAGHQFGNFNMLGDGRALLLGEQ

ITPQGERVDIQLKGSGRTPYSRGGDGRAAVGPMLREYIISEAMYALGIPTTRSLAVVS

TGENIIRETERPGAILTRVASSHIRVATFQYAARWGSIDDLRALADYTLHRHFPDVAQ

AENRYLLLLQEVIKRQAALIAQWQRVGFIHGVMNTDNMAISGETIDYGPCAFMDAYNP

STVFSSIDRQGRYAYGNQPYIAGWNLARFAETLLPLLHEDEEQAVQLAQDAIAQFSEL

YHHHWLQGMRAKLGLLDEEAEDEALIKDLLELMEKHSADYTNTFLALTFDTLEGTALF

GTPEFAEWNERWQARLDRQQGAKKVSQELMRTNNPAVIPRNHRVEEALEQAENHGDLS

VMQKLLAVLSDPFAHSPEQAEYAELPAQCNTSYQTFCGT"

CDS 1756409..1756915

/locus_tag="EFAGFIKM_01548"

/inference="ab initio prediction:Prodigal:002006"

/codon_start=1

/transl_table=11

/product="hypothetical protein"

/translation="MGSFFIFSFCHRRYFIEAGAFMSNMLSKQQLADILLQLLEHTSS

PREQMQNYVNRLFDTFKWDGVPYVESRKDAYIVRIYERGLVSLEKRVKQPDEVIYWLL

EDIIFTATHVGLLERHGVDNKEMHLNYTNEVMKDLNRGVLEAFQQIGNPYLHWHQTGK

RQELEGMA"

CDS 1756936..1757385

/locus_tag="EFAGFIKM_01549"

/inference="ab initio prediction:Prodigal:002006"

/codon_start=1

/transl_table=11

/product="hypothetical protein"

/translation="MSEQDINDVWYDYAVLFVEKKNESEQGWASLTSNEQDIAALWLL

EADVFNGGFIQFFCNWGEEVYRYALRALHIIDATQVVEIITSAYGCIEHLKEDERLTE

LWDIPKFLTKEQEQQLDALDQQFWNNEDQIAEKAYGYYHGKLNMMII"

CDS 1757828..1759093

/gene="tyrS1"

/locus_tag="EFAGFIKM_01550"

/EC_number="6.1.1.1"

/inference="ab initio prediction:Prodigal:002006"

/inference="similar to AA sequence:UniProtKB:P22326"

/codon_start=1

/transl_table=11

/product="Tyrosine--tRNA ligase 1"

/db_xref="COG:COG0162"

/translation="MMNIIDELQWRDAINQQTDTEGLRELTETKSISLYCGVDPTGDS

MHIGHLIPFMMLKRFQLAGHRPVILIGGATGTIGDPSGRQAERSLQTMEQVQENVDAL

TTQMKKLFVTDGDNQVRMVNNYDWTHKINVIEFLRDYGKNFNLNTMLAKDVVASRLEG

GISFTEFSYQILQSLDYLHLFQNEDVQLQIGGSDQWGNITSGLDLIRKKEGSEAKAYG

LTIPLMLKSDGTKFGKTAGGAIWLDPNKTTPFEFYQFWANTDDRDVIKYLKYFTFLGK

EQIEALAEKVETEPHRREAQKALAEEMTKFVHGEEMLEQAKRITAALFSGDIRSLTAD

EIEQGFKEMPTFETDGEAKNIVDWLVDLGLEPSKRQAREDVTKGAISMNGEKITELEF

TVSAEHAIGGKFIIIRKGKKNYSLVKLQS"

CDS complement(1759252..1759530)

/locus_tag="EFAGFIKM_01551"

/inference="ab initio prediction:Prodigal:002006"

/codon_start=1

/transl_table=11

/product="hypothetical protein"

/translation="MVLSMDEIVNAICIHMAERKGVRPTDVNVELSWEEDTGYSAEVW

IQGRSQYLVESNMIEAILRYLHSEYNIRAYRENVRLDLDEEITAIVNQ"

CDS complement(1759536..1760003)

/locus_tag="EFAGFIKM_01552"

/inference="ab initio prediction:Prodigal:002006"

/codon_start=1

/transl_table=11

/product="hypothetical protein"

/translation="MMVFMAFTLLFATVGVTNPDSADARRGGGFKSGTKSYSNTPKKS

DSNVSKSNTNNNSSNGAATTNRGGFFGGGGGFMKGMMLGGLAGMMFGGLFGGMGALGN

ILGLLVNVAAIMLIVMLAMALFRAISNRRKPAADGRYDRRNDRDDDRNRNGRY"

CDS complement(1760132..1760698)

/locus_tag="EFAGFIKM_01553"

/inference="ab initio prediction:Prodigal:002006"

/codon_start=1

/transl_table=11

/product="hypothetical protein"

/translation="MNILSYGLLGLLTREESSGYDLMLKIQPHWQAKHSQIYPLLSKM

ENDELLASRWVQQSDKPDKKMYAVTEKGIEKLLEWMITPVTAPVTRDEFNLRILCVGI

AEDGSMRRILNERKSWFMERIRYFEDLKSRIPQDNLRVGNRDFGSYILVQKGLMHAQT

GLEWCHWVTQLLDGQAAIQDPSPSVSEI"

CDS complement(1760872..1761447)

/locus_tag="EFAGFIKM_01554"

/inference="ab initio prediction:Prodigal:002006"

/codon_start=1

/transl_table=11

/product="hypothetical protein"

/translation="MNNIQEILTYNKSFVETKEYEKYTAGKFPTKKMVIITCMDTRLV

ELLPKAMNLKNGDVKIIKNAGAIISQPFGSVMRSVLVAIYELGADEVLVVGHTECGMA

SLHAETMIGHMVERGVSEEVMTTLENSGIRLQKWLRGFDSVEEGVKHTVEVIKKHPLL

PPNVPVHGMVIDSATGELDLVAEGYGQQASL"

CDS 1761797..1763311

/gene="ypwA"

/locus_tag="EFAGFIKM_01555"

/EC_number="3.4.17.19"

/inference="ab initio prediction:Prodigal:002006"

/inference="similar to AA sequence:UniProtKB:P50848"

/codon_start=1

/transl_table=11

/product="Carboxypeptidase 1"

/db_xref="COG:COG2317"

/translation="MDKQTQEHLESFRNLARKIKSYHEAIGLLHWDLRTGAPKKGVPT

RSETLGMLSTEAFKLQTSADMKTYLDALTTPVVLEQLEDIDRRLVEDCKKEYDRSQSV

PPEKVQAYTVLTAKSETAWEDAKHNSDFAGFSPYLTDIVKLKQEFIDYWGVKDTRYDT

LLDMYEPDLTVEKVDAVFARLKARLVPLQEKINASENKPNTEFLNQLFDTKQQEKFSL

FILEQMGYDFEAGRLDESVHPFATGLNPGDVRITTHYLQDDVASAVFSSLHEGGHALY

EQNIDDSLAGTLLAEGTSMGIHESQSRLWENMIGRSLPFWTRYYKDLQQHFPQLSEVE

LEDFYRAINRVESSLIRIEADELTYNLHIIIRYEIEKMLFNDGLEVKDLPETWNAKYK

EYLGIMPTNDGDGVLQDVHWSGGDFGYFASYSLGNMYAAQILHTLRKEMPEFDTLIAE

GNLIPIKEWLTDKIYRYGRSRTPSELIVAITGEELNPDYLADYLEAKYAEIYKL"

CDS 1763472..1764476

/locus_tag="EFAGFIKM_01556"

/inference="ab initio prediction:Prodigal:002006"

/codon_start=1

/transl_table=11

/product="hypothetical protein"

/translation="MKYTPWFVRGIVILLIIIVALTSCNKEENEAKITIGEVTRSVFY

APEYVAVAQGFFEEQGLEVDIQTTAGGDKTMAALLAGSVDIALVGAETSIYVYQQGAE

DPVINFAQLTQTDGTFLFARNTEGSFDWEQLRDSTFLGQRKGGMPQMAGEFALNKHGI

DPQSDLELIQNVDFANIASAFASGTGDYVQLFEPQASIFEQEGRGKVVASFGTEGGLL

PYTVFMTKQSYLNDNKDIVQKFTNGLHKAQAWVDSHTAEEIAEVISPFFKDIDPAILV

SSVNRYKEQGSYATDPIIDEEEWNNLLDVMSAAGELKERVDLNAIVDNAHAEEATKSK

"

CDS 1764506..1765300

/gene="ssuB_1"

/locus_tag="EFAGFIKM_01557"

/EC_number="3.6.3.-"

/inference="ab initio prediction:Prodigal:002006"

/inference="similar to AA sequence:UniProtKB:P97027"

/codon_start=1

/transl_table=11

/product="Aliphatic sulfonates import ATP-binding protein

SsuB"

/db_xref="COG:COG1116"

/translation="MPESESVIRLEGISQVYVSEREASLVLEDLSLEIQKGEFVSLVG

PSGCGKTTLLSIIAGLLTPTRGEVKVKGKLVGGPSAQIGYMLQQDYLFPWRTILDNAL

IGLELTGRLDERSRERVRELLVSMGLAGTENQYPSELSGGMRQRVALVRTLATDPGIL

LLDEPFSALDYQTKLQLEDLVSDTLKQFGKTSVLVTHDLSEAIAVSDRVIVLDRNPGR

IRREFIIPDGIRKSQPFHAREQEGFNALFQALWSELDQSGGGEEDA"

CDS 1765297..1766130

/gene="ssuC_1"

/locus_tag="EFAGFIKM_01558"

/inference="ab initio prediction:Prodigal:002006"

/inference="similar to AA sequence:UniProtKB:P40401"

/codon_start=1

/transl_table=11

/product="Putative aliphatic sulfonates transport permease

protein SsuC"

/db_xref="COG:COG0600"

/translation="MNPMNPKQEKRDEWMGQLHRNHIQQVARWRHQVLAVQLSMLVCM

FLLWEVAGRLRWIDVLLFSYPSKVFAQIGKDIASGELWAHVGVTVGETAVGFLLGTLV

GTLLAVLIWWSPFLSKVLDPYMVVFNSMPKVALGPIFIVMFGAGFTAIVMTTLSITVI

ITTLVVYNSFNEVDPNYIKVIRTFGGDRSEIFTKVVLPASFPAIVSTLKVNVGMAWVG

VIVGEFLVAKQGLGYLIIYGFQVFNFTLVLSSLLIIAAVATAMYQLVVYAERKMLAGR

R"

CDS 1766479..1767456

/locus_tag="EFAGFIKM_01559"

/inference="ab initio prediction:Prodigal:002006"

/codon_start=1

/transl_table=11

/product="hypothetical protein"

/translation="MGFRVIKTAIAALMAVLIADWCRLPGPTSAGLLAILGVDVTRKR

SIRTISARFFASVVGLLFASVLFHFLGFHYWVLAVYILIAFPAIVRVGFKEGIVTGSV

VVFRVFGGGEMDVHVVLVQIALLLIGLGSAMAVNLAYMPAADPQMLRIRKRIDELFSK

IFKEFAATLRNPNEAWAGRELIEADKAILGGIDAAKRSLENQVIHPNEEWSVYFYMRK

TQLDSIQHMMHLVSQIYQKMPHAEMVSELFDQLSQDVLTESYTGRTEKLLADVQEEFK

RMELPDTREEFEIRSAILQLCRELALYLKVAKKDKAPSPISDRSQSTNE"

CDS 1767532..1768092

/locus_tag="EFAGFIKM_01560"

/inference="ab initio prediction:Prodigal:002006"

/codon_start=1

/transl_table=11

/product="hypothetical protein"

/translation="MSLSHKHQCREIITKAICGKGRKFSTVTHTVTPPNGPTSILGAW

IINHQYEAVSAGDGIEVIGTYDINIWYSYDKNSQTDVAKETVSYVEHVPLSYLDPKHR

TSTVEVSADATQEPSCVEATVSAGGSSVIIRVEREFAVELVAETKVCVKVCTDGCGDY

EDKDYDFGSGDGDYDDLDPDLLDDEL"

CDS 1768294..1769547

/locus_tag="EFAGFIKM_01561"

/inference="ab initio prediction:Prodigal:002006"

/codon_start=1

/transl_table=11

/product="hypothetical protein"

/translation="MNVMEQAEEALRRYESMTSAERTERLKQAGIEVLSLRDRRKREP

GLRVRYDVEISCLQAIRIKRKDTSGMGGGTQESLLEREASSTLFKRVERLAIKTLYTL

GLDHGAVRMESSGNGGCAVISIDPGPWKGVTNLAATYRENWKQQQELLNEEQSHRPIP

ILGMDPEFLLVQMPESKIVPASKFLERTGMAGCDSVTIGGRRIYPVAELRPAPSSEPR

ELLAHLMRAFAAASRSISDHSLIWQAGGMPQRGLPLGGHIHFSGVNLTGELLRALDNY

LALPLAFLQDPRGSGRRPRYGALGDFRLKSYGGFEYRTLPSFLVSPLVAKGVVALAGL

IASGYTQLRQRPLAKAEVHAAFYEGKREVIKEHIPTLVDDLKRLDGYARYERYASPLI

LQLKLGRTWDESRDIRKLWNIRAGS"

CDS 1769751..1772552

/gene="mutS"

/locus_tag="EFAGFIKM_01562"

/inference="ab initio prediction:Prodigal:002006"

/inference="similar to AA sequence:UniProtKB:P0A1Y0"

/codon_start=1

/transl_table=11

/product="DNA mismatch repair protein MutS"

/db_xref="COG:COG0249"

/translation="MAQYTPMIQQYLQVKAEAQDAFLFFRLGDFYELFFEDAINASRE

LEITLTARNGGTDDKIPMCGVPYHSADNYIQRLIEKGYKVAICEQMEDASVTKGMVRR

EIVRVVTPGTVMEGKTLGDKSNNYMVCLTGNQNTLALAACDLSTGELYVTSVPFSKEW

LKDEIGIYEPSELLGDAALLEMVEAEASPIGRPVVYTTWTKNKEDLVRQQFGEAVWAR

LEPERQACIARLFSYLSETQKRSLGQLTQISTYEPDHYMILDPFTRRNLELVETVRER

SKKGSLLWLLDRTETSMGARMLRRWVDKPLLQKGKINERLEAVDTLYNQFILREDLRA

ELKDIYDLERLVGRIAFGNANGRDLNALKSSLEKIPGLRQHCTGSSSATLQHIAETMD

DCNDLREAIGQAIVDEPPVSVRDGGLIREGYHERLDELREASVNGKQWIAELEARERE

ATGIRSLKIGYNKVFGYYIEITKSNLSALPEGRYERKQTLANAERYITPELKEKETLI

LEAQDKMVDIEYGLFAELRERLNKEIARLQKLAEQVAEIDVYQSFAVISAERNFVRPT

LTDGYDLVVEEGRHPVVEAVMRDGAFIANHTAMTKEEARILLITGPNMAGKSTYMRQV

ALISILAQVGCFVPAGQAEVPIMDRIFTRIGAADDLIGGQSTFMVEMADIQVMTDKAT

PRSLIIIDELGRGTSTSEGMAIAQSVIEYVHDIIGCKALVSTHFHELAHLEESLDKLA

NYSMAVQESGDKVNFLRKLIAGAASSSYGIYCARLAGLPDSIIERANGLLHGFEHAAA

QVAVGSEYIGNEKQEEGLKLKGGEFRHTDSAPLIREHEIEESAEYTPSIEDSVGKQHA

KKSNSKGQSSANHSDVVQLSIFGDDEPNVATKPDVVAVDKPAREFIRTMKDIDVMNMT

PLQAMQILNDLKLKAQQLS"

CDS 1772574..1774790

/gene="mutL"

/locus_tag="EFAGFIKM_01563"

/inference="ab initio prediction:Prodigal:002006"

/inference="protein motif:HAMAP:MF_00149"

/codon_start=1

/transl_table=11

/product="DNA mismatch repair protein MutL"

/translation="MAKIHVLDEHIANQIAAGEVVERPASVVKELLENSVDAGATKIE

VTVEEGGLLSIRVKDNGTGIEPEDMETAFYRHATSKIAHGRDLFQITSLGFRGEALAS

IAAVSKVEVLSASDNDGRGRRIVIEGGNLVSHEDATSPQGTDFAVRELFYNTPARLKY

MKTIQTELGHISDVLYRMAMSHPNISFRLRHNENVLLQTLGNGDLLQVVAAIYGTSAA

KAMLPIQGESLDYRVSGLISLPEWTRANRNGMSTIVNGRFVRNYGLNQAILKAYHTLL

PINRFPLVVVQLEMHPSLVDVNVHPAKLEVRFSKEPELYEFIETTLRGILRQEVLIPQ

VKKQQIRRGDDSSFIQEQFLFPRGPLKDASDAEGYGQQGPLGKPTAAPLKLTAEDDDL

DLDAPADLSTAQLVSEQGHSVPLPEAPPEVTQPPEQLESWNGDILQKIASHGEGQTSA

NVQEGTKDVSTSSSEAFPKTDLSSQDGGGRGATEKPLAEGKPATYRSESVNSPVREAR

STYNPSSIARGERTWKTSGLPDATKLAAAIKSDASMPAFPELSLIGQHHGTYLIAQND

QGLYLIDQHAAHERVNYEYYYEKFGNPAQASQELLLPITLEFTPSETEKLKTRLAWFE

QSGVYLEHFGGQTFRVRSHPFWFPKGDEKDIIEEMSEWVLSERSIDVAKMREAASIMC

SCKASIKANQKLTDQEAEVLIQRLGSCRQPYTCPHGRPIVISFSTYDLEKLFKRVM"

CDS 1774808..1775593

/gene="rsmJ"

/locus_tag="EFAGFIKM_01564"

/EC_number="2.1.1.242"

/inference="ab initio prediction:Prodigal:002006"

/inference="protein motif:HAMAP:MF_01523"

/codon_start=1

/transl_table=11

/product="Ribosomal RNA small subunit methyltransferase J"

/translation="MYITTGEKEAALLVERARNLAETTGGTYVRRNKMSLPMMIEHYE

AEEVLVVLQGKVRLFRKDSPLLEFHPSMGFVRAKRILRGEPDPLLEAGAVNEGDTIID

CTAGLGTDALVFSVAVGKSGRVIACESSLPLYTLLVEGMSQYETIKPAVNEAFRRIEL

HHANHLELLRSMPDRSCDTVYFDPMFREPMMDSSAIQPLRDYANAHALDEQSIMEARR

VARKRVVMKEKRGSAEFDRLGFEILDRANAKTLYGVINVESGS"

CDS 1775574..1776530

/gene="miaA"

/locus_tag="EFAGFIKM_01565"

/EC_number="2.5.1.75"

/inference="ab initio prediction:Prodigal:002006"

/inference="similar to AA sequence:UniProtKB:Q9KAC3"

/codon_start=1

/transl_table=11

/product="tRNA dimethylallyltransferase"

/db_xref="COG:COG0324"

/translation="MLKVEVKPKPKLLVLVGPTAVGKTRMSIELAQAFNCEIISGDSM

QVYREMDIGTAKITSEEMKGVPHHLIDIHEPEYPYSVAEFQESCTRLISEIHERGKMP

FIVGGTGLYVESVCYGFQFSDSGSDEAFREEQFSYAELNGAQALHDRLREVDPVSADR

LHPNDQRRIVRALEIHHLTGEKWSDQLAVQKKESPYDLLIVGLRMDRQKLYARVEERI

DLMIEQGLVDEVKFLLERGVARGHISMQGLGYKEIAAFLQGEVSWEAAVEWLKRDTRR

FAKRQLSWFRHMKDIEWVDMTDTQDFEGKYKQISELITRKFD"

CDS 1776904..1777146

/gene="hfq"

/locus_tag="EFAGFIKM_01566"

/inference="ab initio prediction:Prodigal:002006"

/inference="similar to AA sequence:UniProtKB:O31796"

/codon_start=1

/transl_table=11

/product="RNA-binding protein Hfq"

/db_xref="COG:COG1923"

/translation="MNKSINIQDTFLNQLRKENIPATVYLTNGFQIRGTIKAFDNFTI

VIDSDGRQQMVYKHAISTFTPQRSVSLMQQDNSGEA"

CDS 1777317..1779971

/gene="mtgA_2"

/locus_tag="EFAGFIKM_01567"

/EC_number="2.4.1.129"

/inference="ab initio prediction:Prodigal:002006"

/inference="protein motif:HAMAP:MF_00766"

/codon_start=1

/transl_table=11

/product="Biosynthetic peptidoglycan transglycosylase"
/translation="MPNDPLSRSNNRNNNNKSPKKAKPKTSKKKKITGKRVGWTLFFT
MAIAIFCALGGYLFIMVSGENLLKANKDKTTINETSKVYDRNGQLMGELSIQKLDPVK
EDDIPELVKQAFVATEDKRFYDHQGVDIWSIGRAAVKDVMARSMVEGGSTLTQQLAKN
MFLSRDKTFFRKATEVSIAMALERKYTKDEILTMYLNRIFFGHQRYGIKAASEFYFGV
TDLKRLKLWQIATLAAMPKGPSAYNPVSNPNDSKARRGVVLQLMYEQGYITKAEMDEA
KEINYNYKRPEKDRKYQGFIDYVLREAERVTGKTEDDLNIGGYKIYTTMDAQAQTAME
TAFTDDSLFEASKDDQQVQGSMVIMNHENGSLVALLGGRDYQTKGYSRVTQSRRQPGS
AFKPIVSYAPALESGNYSANSSLSNAKQCFGNYCPGNLHGYSSTISMTEAITKSENIP
AVWLLDKIGVNTGINFAKSVGIQLTDEDKNLAIALGGLSKGTNTLEMAQAYSAFANLG
EYRQAYSIKEIKDSAGKTTYKHDNSDTTRVMSEQNAYQLTQMLQNVVNDGTGRSARLD
RPVAGKTGTVQSGISGNSANRDVWFVGYTPEWTAAVWMGYDNPDANHMLKNSSKLSAA
FFAKVMGDALKGVPVKQFKAPAGGQAPPPVEEPEKPVLSVSGLSGSYDPNAQTVSLSW
TGTGDASTQYRIYRKETSEAQFTHLIDAIGATNAQDLSALPGLTYEYYVTAYDLASGL
ETDPSNTISLMIEAQEVPPEEPDPGIDPGTEIDPEQPGTENPDNGTPDNGNSNGNNGN
GNGNNGNGNGNNGNNGNGGNNGGNGQPGEGNTTPPGQGTTDPGGNGEGPDDGAVTTPG
EVVTPDSGTSGDTGGTDAPADSQAGTGG"

CDS 1780260..1780823
/locus_tag="EFAGFIKM_01568"
/inference="ab initio prediction:Prodigal:002006"
/codon_start=1
/transl_table=11
/product="hypothetical protein"
/translation="MKRKFGDRANWRRITNRQFTCRFVQSKIFTGYITLYTIQDLKEP
LWKTYGGSTFCIADKGYSWLQYYPKGEHFVVTAMFDDQERIVEWYIDTCRSQGITDQG
VPWFDDLYLDVVVLKDGEVFLLDEDELEDALSRKHITTGDYDLANKTAKELLHAIDAH
VFPYFQLSLKHRPSLFENGEFRKNIQI"

CDS 1780934..1781905
/gene="spoVK"
/locus_tag="EFAGFIKM_01569"
/inference="ab initio prediction:Prodigal:002006"
/inference="similar to AA sequence:UniProtKB:P27643"

/codon_start=1

/transl_table=11

/product="Stage V sporulation protein K"

/db_xref="COG:COG0464"

/translation="MNGRVMAAGEQPGGRPSRQINIVLRNQEPQMLVKDEAAAVQAAK

SITKHAHFQEIQGELEQLVGLENIKDLVFEIYAFLQIAQMRTEAGLLSGAHVYHMIFK

GNPGTGKTTVARIVAKLFQKMGVLSKGHLIEVERADLVGEYIGHTAQKTRDLVKKALG

GILFIDEAYSLARGGEKDFGKEAIDTLVKSMEDNKNQFILILAGYSDEIDFFLQTNPG

LPSRFPIQVEFPDYSIDQLIQISEIMAKERDYILMPQTILKLKQHLLQEKNDSLHAFS

NARYVRNAIERSIRHQAVRLLEQYSEGSPGKLELMTIRTEDLNFERK"

CDS 1782030..1783316

/gene="hflX"

/locus_tag="EFAGFIKM_01570"

/inference="ab initio prediction:Prodigal:002006"

/inference="similar to AA sequence:UniProtKB:P25519"

/codon_start=1

/transl_table=11

/product="GTPase HflX"

/db_xref="COG:COG2262"

/translation="MTNGTHDTDMVKKDRAVLVSLVTDDVKRTGINPEYSLEELVKLA

ETAGVEVLSVLSQNREKRDTKWFIGKGKVEELRAIAEELGATTAIFDQELSGAQVRNL

EETLDLKIIDRTQLILDIFAQRANTREGIIQVELAQHSYLLPRLSGHGKNLSRLGGGI

GTRGPGESKLETDRRHIRGRIDDLKRHLEELTRHRKLHRERRKKTGIVQVALVGYTNA

GKSTLLKQLTAADVYIQDQLFATLDPTSRTMELPSGKEIVLTDTVGFIQNLPHDLIAA

FRATLEEVNEADLILHVVDASSAMREDQMRTVNTILQELGSGDKPQLVLYNKKDACTP

EQLEMLPLDKDHIKVSALDSEDLLKIREFIQTELTGDTKRFRIPAERGDLTSVLYKIG

DVVETSFEENDVIYEVELQKGEYEKFGYMLEEFIEL"

CDS 1783400..1784653

/locus_tag="EFAGFIKM_01571"

/inference="ab initio prediction:Prodigal:002006"

/codon_start=1

/transl_table=11

/product="hypothetical protein"

/translation="MVSFDSEISELQRQVDRQIEGQLQQIDRITDHNQWKVINAFQQR

KVSDFHFAGSTGYAYNDRGREVLDEVYADVFGAEAALVRPHFASGTHTISTALFGVLR

PGDELLYITGKPYDTLHKVIGKPGDGTGSLRDFGIGYRETALTDEGGVDWAAVEKSIT

PATKVIGIQRSRGYDWRSSFCVAEIADMVTRVKALKEDVIVFVDNCYGEFTEEREPTE

VGVDLMAGSLIKNPGGGIAETGGYICGKEQYVQLAAYRLTAPGIGGEVGAMLGTTRSI

YQGLYMAPTIVGQAIKGSTFAAAMFAESGFVTKPAWNEARTDLIQAVKFSSAEHLIAF

VQGIQRAAAVDSHVVPEPWDMPGYEHPVIMAAGTFIQGGSLELSADAPIREPYIGYMQ

GGLTYSHVKYGVLMALQTMKDRKLL"

CDS 1784730..1785149

/gene="glnR"

/locus_tag="EFAGFIKM_01572"

/inference="ab initio prediction:Prodigal:002006"

/inference="similar to AA sequence:UniProtKB:P37582"

/codon_start=1

/transl_table=11

/product="HTH-type transcriptional regulator GlnR"

/db_xref="COG:COG0789"

/translation="MNMGDEIRRNMALFPIGIVMKLTDLSARQIRYYEQHSLIVPART

SGNQRLFSFNDVERLLEIKALIEKGVNIAGIKQVMNPVSKESEEATVITPDTEVKRRE

LSDTQLHRLLKQQLVSGKRPGQVSLIQGELSRFFNKN"

CDS 1785279..1786607

/gene="glnA_1"

/locus_tag="EFAGFIKM_01573"

/EC_number="6.3.1.2"

/inference="ab initio prediction:Prodigal:002006"

/inference="similar to AA sequence:UniProtKB:P19064"

/codon_start=1

/transl_table=11

/product="Glutamine synthetase"

/db_xref="COG:COG0174"

/translation="MSYTKEDILRISKEENVRFIRLQFTDLLGAIKNVEIPVSQLTKA

LDNKMMFDGSSIEGYVRIEESDMYLYPDLDSWLIFPWVAENRVARLICDVYLPDGNPF

PGDPRGILKRNLKEAQDMGFTSFNVGPEPEFFLFKTDEKGNPTNELNDQGGYFDLAPT

DLGENCRRDIVITLEEMGFEIEASHHEVAPGQHEIDFKYADALKAADQIQTFKLVVKT

IARQHGLHATFMPKPLFGMNGSGMHCNQSLFKGNENAFVDESDELGLSKTARHFMAGT

LKHARAFAAITNPTVNSYKRLVPGYEAPCYVAWSASNRSPMIRIPASRGLSTRVEVRN

PDPAANPYLALSVLLKAGLDGIKRELSLPAPIDRNIYIMSEEERVEEGIPSLPADLKE

ALNELIRSEVICDALGDHALAHFYELKEIEWDMYRTQVHQWERDQYITLY"

CDS 1787198..1788499

/gene="aldH1_1"

/locus_tag="EFAGFIKM_01574"

/EC_number="1.2.1.-"

/inference="ab initio prediction:Prodigal:002006"

/inference="similar to AA sequence:UniProtKB:Q2FWX9"

/codon_start=1

/transl_table=11

/product="4,4'-diaponeurosporen-aldehyde dehydrogenase"

/db_xref="COG:COG1012"

/translation="MDQAKQLVTEQRTFFYTGQTKNIDYRINALQQLRKGIEKYQQRI

QDALRADLNKSEAEAYGSEIRIVLGELDFALEHLQEWAAPKQVPTNSAMPDGVSTIYP

EPYGVALIIAPWNYPFQLAFGPLIGAIAAGNCAVIKPSELTPAVSRLTYDLIQEIFPQ

EYIVVMEGEVEASTALLKEKFDYIFFTGSTGVGRIVMKAAAEHLTPVTLELGGKSPAI

VHSDADLKLVAQRIVRGKFLNAGQTCVAPDYLLVHEQVHDELIDMIGAEIKDKFGDDV

LKNADFPHIVNARNFDRLSKFLTDGKTLIGGRSVREQLLIEPTVLGDVNWESAVMQEE

IFGPILPVFTYSDLNPMLDEIVRRPKPLALYLFTQDEQLQNQVLNQVSFGGGCINDTL

SHMTSHYLPFGGVGESGMGSYHGQQSFDVFSHHKSVLKRSK"

CDS complement(1788604..1789230)

/gene="lexA"

/locus_tag="EFAGFIKM_01575"

/EC_number="3.4.21.88"

/inference="ab initio prediction:Prodigal:002006"

/inference="similar to AA sequence:UniProtKB:P31080"

/codon_start=1

/transl_table=11

/product="LexA repressor"

/db_xref="COG:COG1974"

/translation="MSKISSRQQAILEFIRNEVRLKGYPPSVREIGEAVGLASSSTVH

GHLDRLEKKGLIRRDPTKPRAIELLSQEESEHSHQFAHSVARIPVVGKVTAGVPITAT

ENIEDYFPLPTHYVGEQKVFMLSVVGDSMVEAGIVNGDYVIVRQQQTADNGDIVVAMT

EDDEATVKTFYKEKDHIRLQPENATFEPLRLKHVSILGKVIGLFRDIH"

CDS 1789528..1789905

/gene="yneA_1"

/locus_tag="EFAGFIKM_01576"

/inference="ab initio prediction:Prodigal:002006"

/inference="protein motif:HAMAP:MF_02014"

/codon_start=1

/transl_table=11

/product="Cell division suppressor protein YneA"

/translation="MRYSTYQSIYEPMNSELVKSNIGNYKKVLARFKISSWMLKVAIT

SMIIFIGCSTVLTVFAGNENDVLPGGKIAVSQGETLWSISLEHKPTNMDTRIYIEAIK

KVNQLHTTSIQVGQVLALPQFAE"

CDS 1790001..1790210

/locus_tag="EFAGFIKM_01577"

/inference="ab initio prediction:Prodigal:002006"

/codon_start=1

/transl_table=11

/product="hypothetical protein"

/translation="MDIDSLVARINELARKQKSTGLSEEELAERAELREIYLSNIRSN

FRQQLDTIEIVDDENDKGHQGKLKH"

CDS 1790336..1790845

/locus_tag="EFAGFIKM_01578"

/inference="ab initio prediction:Prodigal:002006"

/codon_start=1

/transl_table=11

/product="hypothetical protein"

/translation="MSRSFERTVKKNSKQLNQQRKKSGQPITGTPGEEVFKGRSLLFP
IFLIGLAFLYLFMSTFLVQAPMTTWDWVTMLLYVFLAVIFMLRRPYIKISSNRIITTK
GNRERSIGVDDIVKFRIEPGTVVIEHNSRGKRWVFTKLLNRYDTDAIAERLLKFAKAH
QITVETVEK"

CDS 1790865..1791665

/gene="gph_3"

/locus_tag="EFAGFIKM_01579"

/EC_number="3.1.3.18"

/inference="ab initio prediction:Prodigal:002006"

/inference="protein motif:HAMAP:MF_00495"

/codon_start=1

/transl_table=11

/product="Phosphoglycolate phosphatase"

/translation="MIGMALKAILFDLDDTLLWDERSVREAFHETCLIAAQETGVKPE
ELEEAVRNEARGLYESYETFPFTRMIGINPFEGLWANFTGGDQPEFRQLEQLAPVYRK
ESWRRGLLKLGIDREDLAEQLAAQFGVERRSRPHVYEETMDTLRQLQGKYKLLLLTNG
CPALQQEKLDGVPELTPFFDEIIISGNFGKGKPDPSIFEHALSKLGVKPEESMMVGDK
LTTDIRGALSSGIQSVWINREHKTNNETYAPDHEITHLSELNQLIANF"

CDS 1792222..1793184

/gene="yteP_7"

/locus_tag="EFAGFIKM_01580"

/inference="ab initio prediction:Prodigal:002006"

/inference="similar to AA sequence:UniProtKB:C0SPB3"

/codon_start=1

/transl_table=11

/product="putative multiple-sugar transport system
permease YteP"

/db_xref="COG:COG4209"

/translation="MESETAKTTLTMTSLRKESRLRTAATLFRKDWQLYSLLILPIIY
LLIFKYGPMIGNVIAFRRFVPGGSIFGETWVGLRYFKMFIEDPTFWRVFGNTLMLGGL
ALLFTFPVPIIFALMLNEVKSKRFKKFVQTASYLPHFLSIVIVAGMILQLTAVNGSIN
GLVAFFTGDNIPFMQRAEWFRTIYITSEVWQGMGWGAILYLAALTTIDDSLYEAARID

GANRWKQTIHVTLPGILPTIVTLLILNMGNFLAVGFEKILLLYNPLIYETSDVISTYL

YRVGLESSNFSYATAIGLFESLIGLILVFSVNAISRRLTQRSLW"

CDS 1793197..1794069

/gene="ycjP_1"

/locus_tag="EFAGFIKM_01581"

/inference="ab initio prediction:Prodigal:002006"

/inference="similar to AA sequence:UniProtKB:P77716"

/codon_start=1

/transl_table=11

/product="Inner membrane ABC transporter permease protein

YcjP"

/db_xref="COG:COG0395"

/translation="MQESRSYKVFKVFNVIFLLFVVFITLYPFLNVVAQSFSSESYIN

SGKVSLFPRGFNVETYKTISRDSMFWTNYKNTIIYTVVGTLISMFMTTIFAYALSKKR

LMGRKFLTMFAVFTMFFSGGLIPNYVLINSLGFNNTMWALVVPGAISIYNMLIMKSFF

ENMPEELEEAASIDGLNTYGILLRIILPLSKAVMATMVLFYAVGHWNSWFPAFLYLDK

KELFPVTIYLRNMIAGATSGASAGATSADNLTQIAANIKSVTMVLTILPILTIYPFVQ

RYFVTGIMLGSVKQ"

CDS 1794208..1795842

/gene="lipO_4"

/locus_tag="EFAGFIKM_01582"

/inference="ab initio prediction:Prodigal:002006"

/inference="similar to AA sequence:UniProtKB:P37966"

/codon_start=1

/transl_table=11

/product="Lipoprotein LipO"

/db_xref="COG:COG1653"

/translation="MNQKPRKFVGKMLLASMMTVVLAACSSGTGAGGDVTEVQTKAAM

ETYNVGDTFKATEPFNLSILYSDQPSYPYKKDWLLFDKITELTGVTLEPTIVPMSDYP

QKRSLLISSGDAPLVIPKTYPGEESAFVSSGAILPISDYIDLMPNFKDKVEKWGLEDE

LEGLRQEDGKYYVLPGLHEEVWPDYTLLVRTDVFEENNIAIPTTWDELYDAAKKLKEI

YPDSIPFSDRFQFNSTLNIAATGFGTKAGWGFGNGLTYKEDQDEFVYTATTPEYKEML

TYFNKLVSEGLLDKESFTQDDDQAVQKFVSGKSFMINGNSQTVVLHRNDMNKTLGEGK

YSVAKITVPGGPKGQLMSGSRLENGVMISGKIQKSENFKAIMQFVDWLYYSDEGQEFA

KWGVEGETFTKEGGKRKLVEDVNYNGLNPKGTKDLRIDFGFSGGVFAYGGTTDLLQSM

FSEEELKFQQDMKDTKEVIPAEPPIPYSSEDRERVTLLSTPLKDYSDQNTLKFILGER

DLSEFDTFAKELESQGLSNYLKLANDTYKAYKENKQ"

CDS 1795947..1796621

/gene="rspR_1"

/locus_tag="EFAGFIKM_01583"

/inference="ab initio prediction:Prodigal:002006"

/inference="similar to AA sequence:UniProtKB:P0ACM2"

/codon_start=1

/transl_table=11

/product="HTH-type transcriptional repressor RspR"

/db_xref="COG:COG1802"

/translation="MSLNQKIRGSTRAYSYNLLKERILHLELKPGTKISEKEIADELQ

VSRTPVREAFMKLAEEELLDIIPQSGTIVSHINLEHVEEGRFMREKMEKEIVTLACVS

FPEEYRFRLETNIAMQEVCIGKNNFYRLFELDEEFHQILFQGTGKLRTWKMLQQLNIP

FNRLRLLRLAEDSNLEVIISQHKEIYRLITERQTEQAVQVMEAHLRLVVIEQESIKAK

YPHYFI"

CDS complement(1796718..1797263)

/gene="mtnD"

/locus_tag="EFAGFIKM_01584"

/EC_number="1.13.11.54"

/inference="ab initio prediction:Prodigal:002006"

/inference="similar to AA sequence:UniProtKB:Q81MI9"

/codon_start=1

/transl_table=11

/product="Acireductone dioxygenase"

/db_xref="COG:COG1791"

/translation="MAEIVIRNTNERITGDENVRNFLNKYEVLYEKWDASKLNTDLQN

NFGLTDEQKTEVLETYDYEIRDLAARRGYQIWDVITLSEQTPDIEEKLAKFEEIHTHA

EDEIRAIVAGKGIFVIKATDDVGYFNVELSPGDVISVPENTPHFFTLMENKQIIAVRL

FIEKDGWIADPYPDPTFIKQA"

CDS 1797512..1800883

/gene="metH"

/locus_tag="EFAGFIKM_01585"

/EC_number="2.1.1.13"

/inference="ab initio prediction:Prodigal:002006"

/inference="similar to AA sequence:UniProtKB:O33259"

/codon_start=1

/transl_table=11

/product="Methionine synthase"

/db_xref="COG:COG0646"

/translation="MIQQVDLTGEDFGGEDLDGCNEMLVLTRPELIQRIHEEYLEAGA

DLIETNTFGATSVVLAEYDIQDRAREINLEAARIAKAAVDRFSTPESPRYVVGAMGPT

TKTLSVTGGVTFQELIDSYLEQALALIEGGVDALLLETSQDTLNVKAGSIGIQQAFEQ

SGVTLPLMISGTIEPMGTTLAGQNIESFYISLEHLNPISVGLNCATGPEFMRDHIRSL

SAMASVAVSCYPNAGLPDENGNYHESPDSLAQKIGAFAEQGWLNIAGGCCGTTPAHIR

AMRDTLAQYPPREMNGTHPPALSGIEPIYIEQDNRPYMVGERTNVLGSRRFKRLIVEG

KYEEASEIARAQVKNGAHIVDVCVQDPDREESEDMVKFLELVVKKVKVPLMIDTTDAS

VIDLALQYSQGKAIINSINLEDGEEKFELITPLLHKYGGAVVVGTIDERGQAISREDK

LDVAKRSYDLLVNKYGLKPEDLIFDTLVFPVGTGDEQYIGSAKETIEGIRVIKEAMPE

CHTILGISNISFGLPEAGREVLNSVFLYECTKAGLDYAIVNTEKLERYASIPEEERRL

SEELLYNTNDDTLAAFVAAFRNKKVEKKEKISNLSLEERLASYVVEGSKEGLLPDLEQ

SLAKYTALQVINGPLMRGMEEVGRLFNNNELIVAEVLQSAEVMKASVAYLEQFMEKNE

TSVKGKIILATVKGDVHDIGKNLVEIILSNNGYRIINLGIKVPPERIIEAYREEKVDA

IGLSGLLVKSAQQMILTAQDLRTAGIDVPIMVGGAALTRKFTKNRIRPEYNGMVVYAK

DAMNGLDLANKLMNPVSREAMQLEMEAEKEADASGDVAVQALPELTRVERSDIAVDNP

VLVPPDLERHTMRNYPLSHILPYVNMQMLLGHHLGLRGSVEQLLASGDKKATDLKAVV

DDIMQEAVRDGIIQAHAMYRFFPAQSSGNSVIIYDPKDTSNILHTFTFPRQKVEPFLC

LSDFLKPVDSGQMDYVGFMVVTAGHGVRELSTAWKDQGDYLRSHALQSVALEVAEGLA

ERVHHMMRDIWGFPDSAQMTMKQRHGARYQGIRVSFGYPACPDLEDQGPLFKLLQPED

IGVELTEGFMMEPEASVSAMVFSHPQAKYFNVEKA"

CDS 1800961..1801899

/gene="rnz"

/locus_tag="EFAGFIKM_01586"

/EC_number="3.1.26.11"

/inference="ab initio prediction:Prodigal:002006"

/inference="similar to AA sequence:UniProtKB:P54548"

/codon_start=1

/transl_table=11

/product="Ribonuclease Z"

/db_xref="COG:COG1234"

/translation="MELYFLGTNAGVPTLQRNVTSIGLRMLDERRALWLFDCGEGTQH

QILSSPLKLSKLEKIFITHLHGDHVFGLPGLLSSRAYQGGTTPLTVYGPPGTERMIST

TMELSQSRVNYDLNIVEHTGGVLFEDDSFIVEAALLEHRIDSYGYRITEKDRPGSLDP

AKLAEYGLKPGPLFGRLKRGETITLDNGDSLRPEDVLGAPKRGMVITILGDTRPCDNV

QPLSVNADVLVHEATFMHDLADTAHEYYHSTSKQAAEAARAANVGQLIMTHFSSRYKD

EDQLQPLLEEAQSVFPNTRLANEHQLIPVVHRKQES"

CDS 1802256..1803065

/gene="yutF"

/locus_tag="EFAGFIKM_01587"

/EC_number="3.1.3.-"

/inference="ab initio prediction:Prodigal:002006"

/inference="similar to AA sequence:UniProtKB:O32125"

/codon_start=1

/transl_table=11

/product="Acid sugar phosphatase"

/db_xref="COG:COG0647"

/translation="MIKAYLIDLDGTLYHGRHRIEGADQLIRTLQELGKPYLFVTNNS

SRTPQDVADHLNGMGIPADASQVCTSAVAAAEYVAEESPGAKVACIGEAGLQEAIEKA

GLQLTEDAPDYVIQGIDREFSYHKLTKALRWINAGSKFIMTNPDLQLPSDDGLTPGAG

TIGAAIEAATGVHPTVIGKPSSVIMKSAISRLNLASHEVAVIGDNMRTDIAAGVAAGC

ETLLVLTGVTTRDNMDGHIQAAKARPDHVFEDLHKLMEWLSQEADQASKKG"

CDS 1803072..1803893

/gene="mutM_1"

/locus_tag="EFAGFIKM_01588"

/EC_number="3.2.2.23"

/inference="ab initio prediction:Prodigal:002006"

/inference="similar to AA sequence:UniProtKB:P42371"

/codon_start=1

/transl_table=11

/product="Formamidopyrimidine-DNA glycosylase"

/db_xref="COG:COG0266"

/translation="MPELPEMENYRTLLSEKILDLPITGVVINRDKTINTEPDLFINE

LTGNRIIFVERRAKHLIFHLANGKRLVLHLMLGGMIFWGTEEERPDRSTQVEIQFGDH

ILFFIGLRLGYIHLLTSKETEEAMSDLGPEPLDRRMNVERFASLLKGRRGTLKTTLVN

QHIIAGIGNCYSDEIAFAAGLRPSSKTQNIATSPELTERLFHSMQSVLREAASEGGYM

EMPLMQGDTKTGSFDEQCRVYDREGETCPRCGGTIERVEITGKKAFFCPKCQHEA"

CDS 1803961..1804731

/gene="nfo_1"

/locus_tag="EFAGFIKM_01589"

/EC_number="3.1.21.2"

/inference="ab initio prediction:Prodigal:002006"

/inference="similar to AA sequence:UniProtKB:Q9WYJ7"

/codon_start=1

/transl_table=11

/product="putative endonuclease 4"

/db_xref="COG:COG0648"

/translation="MGATAFQYFPKNPRSLGLKSLDLKDTEQCRDWCEQQGLASIAHS

PYPTNPALGMTRGEAGFHATIASIRNDLEISNACGSVGTVVHFGHIKTNDPLEGYQNL

IHCLDTALENWNGHSKVLIENQAGDHGPMGTTMEEMIQIRKLSRYPEHIAFCFDTCHA

FASGLWSSGNEAAMLDKGRMLGYWDNVAAVHFNDSKYASGLCKDRHARIGQGYIGNES

MKALLKAPEFRKAVVVLETETGEDGTHREDIALMRSWL"

CDS 1804751..1805089

/locus_tag="EFAGFIKM_01590"

/inference="ab initio prediction:Prodigal:002006"

/codon_start=1

/transl_table=11

/product="hypothetical protein"

/translation="MHVFLDDYRACPKGFVLATNAEECLMLLREGDVDILSLDYELGP

DSPNGGEVAASIVREGLFPREIYLHTSSMFGKRQMYEMLYSNKPDSVTIHNGPMTGEV

MLRVAAGEES"

CDS 1805089..1805406

/locus_tag="EFAGFIKM_01591"

/inference="ab initio prediction:Prodigal:002006"

/codon_start=1

/transl_table=11

/product="hypothetical protein"

/translation="MKPITSKMLMRSVWEGMPQAVFIYTPLCGTCTAARRMLEVVEHM

LPEGILSEMNIHDIPELVQQFKISSVPAVMLFDGQLDVPKMVYRMSSVEHLLSEIRKA

VLK"

CDS 1805403..1806182

/gene="ylmA_1"

/locus_tag="EFAGFIKM_01592"

/EC_number="3.6.3.-"

/inference="ab initio prediction:Prodigal:002006"

/inference="similar to AA sequence:UniProtKB:O31723"

/codon_start=1

/transl_table=11

/product="putative ABC transporter ATP-binding protein

YlmA"

/db_xref="COG:COG1119"

/translation="MSIISMEHVSLRREENQILDNVHLRIEQGEHWVILGRNGSGKTT

LLEMMNGYMFPSQGRIEVLGNLYGQCDVREVRKEIGYISQTLIEKLTPRDPVWEVVAT

GAYAFLRFYQTIPDEVKAKAMSLLDDMGFANLANNPLGTLSQGERKKVMLARSLMAEP

KLLIMDEPCAGLDLYEREKMLAEIDRLRQRNITVVYVTHHVEEIVPLFTHVALIRDGR

IAAAGPKHEVLTQDTIKHTYDVPVDIQWDNGRPWIRVRSGG"

CDS 1806189..1807238

/gene="rlmM"

/locus_tag="EFAGFIKM_01593"

/EC_number="2.1.1.186"

/inference="ab initio prediction:Prodigal:002006"

/inference="protein motif:HAMAP:MF_01551"

/codon_start=1

/transl_table=11

/product="Ribosomal RNA large subunit methyltransferase M"

/translation="MSTQSSWEEGNFSRFICTANHGFAPYAQEELRRTFGAVKSTVLV

PGEILLAGLPVAEEEVADKLWAEYPTFLRHIQPVQFQENTEDSEQSIEKLVAFVLNHK

ELNGASVVLQVRKTEGAFWQENAASLKQLLTEKLDELGCEWVVRDAEYVISVFATNDM

LYAGISRPEQNLSDWSGGAVRFQKEDGQISRAKFKLLEAEQTFGIDFTSFHKALDIGA

APGGWTSFLLERGLEVTAVDPAKMDATLLASPKLTFLKKNAGDVRFREGEFDLLVCDM

SWSPKLMSRLVSDLLYSLQPGGTAIVTVKLLHKKPLAMIKDVIDTFERSRMQIQRSKQ

LFHNREEITLYMVKY"

CDS 1807466..1808944

/gene="pknD_2"

/locus_tag="EFAGFIKM_01594"

/EC_number="2.7.11.1"

/inference="ab initio prediction:Prodigal:002006"

/inference="protein motif:HAMAP:MF_01957"

/codon_start=1

/transl_table=11

/product="Serine/threonine-protein kinase PknD"

/translation="MRHPARLERGSLLGGRYRIVSILGTGGMSHVYEAEDLKLPGKIW

AIKESVTIMPYEGSMETEAALLTSLRHPRLPQIVDFFVPDEDGYTYLVMEYIEGLTLS

EYFKQCRGKIPMEQLTELMLQLLDVLSYLHNLNPPVIYRDLKPSNIMITPEHEVRLID

FGIARSYKRQSAEDTVKLGTAGFAAPEQYGSGQTDARSDLYGLGALLLYLMTSGAYTE

WIQGVESSIRSDVPRTYIPVARRLLRYNAEERFQSADEVRKELLRIPGGTTPGGDTTM

TLNGGTRVIALTGASSGVGVTHTAIAISHYLERQNFKVAVIEMSPRSQSFARIQQIAH

AGKPVPAGRQFAVDGVHYWKQSGRADILSLLGGSYQFIVMDLGSGQDQNRLEEFLRAD

LPIVIGSGAEWRQAEIGAFVRSHHRYPREKWIYCLPLAASEAVQRIRKALDTSSVYGL

PLHIDPFDKDAQMDKVFSHILTHMMAQQPKKRSFFSRRKVHD"

CDS 1808969..1809988

/locus_tag="EFAGFIKM_01595"

/inference="ab initio prediction:Prodigal:002006"

/codon_start=1

/transl_table=11

/product="hypothetical protein"

/translation="MSKLRKQSRQLIYAGLIGAGAVGLVFGGYVIYNVKTMGDVRSAV

EARYLSAYEEKEAELKQQWDSGAQGWVTIRDIEAGEPILPEDLKLIAVPDAQAPRNLW

SSTKQMEGQVAKIELKKGTAITTEMVYEDTPAPPDLRNRELQVVLLPSSLVKGDIIDV

RIQFPTGQDYVLLSKKKVERLNAATLWITMTEEEILSLSSAIVDAYLHKASIYALTYV

EPQFQTPAIATYPANSEVLKLLESDPNIVRRAEQELSRQVRSSLESSLAASMTAPSKG

VEQDVAQSSVTYNSRPSSNNPATSDGVMWNDGSGNGAGANSGSVNDPADASQTEQQSL

LTGGE"

CDS 1810064..1810777

/locus_tag="EFAGFIKM_01596"

/inference="ab initio prediction:Prodigal:002006"

/codon_start=1

/transl_table=11

/product="hypothetical protein"

/translation="MHTWIFAGLCEKNDLMLYLCKILASTGNKVLLVDGTLQQKYGYG

VGDSQQSLRITEFEGFDIACHFVTSAAVENHLEVNGERLDSYDYVLYDMETSHFASRN

LWLTADVRVWVSDYERYNLERGKGWLKRLLEEQSLPGELSFQRLLINGVDCKLEARYL

WAYLEGSPFVWTGESLVLPWDELTAAVKLENEHHRRVHLGPLSRNYKKSLCRVVEQLT

GWESVRSRRAMKEAERMRA"

CDS 1810774..1811604

/locus_tag="EFAGFIKM_01597"

/inference="ab initio prediction:Prodigal:002006"

/codon_start=1

/transl_table=11

/product="hypothetical protein"

/translation="MSTITFWSPFAGTGCTSSTLISAYAMALHYRTRVLLVNAGLAGT

GTEAALPSKDTDSADFMFSFEEGGWDAIERLHSSGSLNKHNLRDYTKPLLKERLDFLT

GRIDEMERSPQEHGETVKRLLNVANEYYDLVLLDADHAGVDSQLKLQQADYVVINLDQ

NMRNLELFFEEILPERMNNKNIHVVINKYDLHSRATLSNIRRQFRYKGTISAIPYTTG

YLDAMNRRDVARYLQLESLTEDKDIRKGSFAASMSELARLIMDGAGMRTVLKRLERGA

"

CDS 1811612..1813438

/locus_tag="EFAGFIKM_01598"

/inference="ab initio prediction:Prodigal:002006"

/codon_start=1

/transl_table=11

/product="hypothetical protein"

/translation="MLWNTIWISLILILCMTYIWFKYRTFLREKNARVPEQESFTIDM

LIEKVKNSLHELSHSQLADAGLHEEEYRRRINQRAEMRKALKGCVSGSISDKTYVKNL

IGDLLTRSIGLNKSNVDEVILFAEQDLLTIQDRFEIVFYLYRQQFGVDALSRMIDTYD

LGRLRMEEGTEDGGSYYISEEDIQYVFECEYRELGFREKTDIIVQRIYQHYKGFSVVD

EIRDQRIDGVSGGVSGMLDAVHNMGLRKPTSWNDLLDQGLEDDTHVEPLSGMESVWIF

YKGKSIHLSFLSFGSIRELKRVCQNIYKYNYPGQLSEASGYKVNEMKDGSRVVVVRPP

FAESWAFFVRKFDIPNASLEQLITGNNADLPIILLQYLMKGSRITAVTGAQGSGKTTL

LMAMVKHIYASYTLRVQEMAFELQLRRIYSRRNILSFRETEHISGQQGLDLQKKTDGT

VNILGEVASDEVAAWMIQMSQVASLFTLFTHHAKTFRDLVFSLRNSLLKTGMFQHEHI

AEEQVVSVINFDVHMKKDAEGRRYIERITECLPRANQGDSVEERAGFTFHNVVEYRDG

AYVATAPISSGSMVDMRDQMTLQDAEKFEQFMKQHWGDQHDN"

CDS 1813428..1815494

/locus_tag="EFAGFIKM_01599"

/inference="ab initio prediction:Prodigal:002006"

/codon_start=1

/transl_table=11

/product="hypothetical protein"

/translation="MTIKWILLLVLSICAGGLLITLLVLSMLRHRGGGSTSQSGVIFA

PGGKGFKGLGSILLQSYRLGMKVPLLSAYILQVQKRISFRHVGDEPSLRRLTMTVVLI

ILGGYGGISVILFLLQPGIAFVILSVLCAVVLNSLVLDMCLSRMEKRLLVQMLDLFAD

VRHRYHQHGMVEEALYEAAEAGTGEAAEQVRLIYEALTSPDPNEALERYYEVAPNRFL

KGFAGVSYMVMEFGDKDRAQGSIYLKGLGNLTQEIHLEILRRDKLDYLLKGLNIIALA

PVLFTAPIERWARVSFPTMDEFYRSKIGFVTKISIYVIIILAYLLLQRLQQYDETRYR
SGHKHSWLDQFLYRQTFIRKLAMLFASKPGSAQYSQTIRLMRESSSELKYEWLAIRRV
MLFAGCLVLTMVCILLLHQVERSHILYDPIRDDRMFGAMAEADLRKAEEKTALDQSVL
ESVEMNRGATYEEVSKALQQLSPVQLDHDTQNETITRIMQKLEVYNKQYVRWWEMIIA
ILMGAAGYYMPVWLMLFQRKMRRLDMRHEIYQFQTVISILREMDRMSVEEILEWLNRF
AVIFKRPLQKCLLHFEHGPELALEQLKEEAGLPEFQRLVDKLQLALGKISIREAFDDL
ESMMAFYFEQRKQEYSKMIDVKASWGRTIGFTPMYALIFLYLVIPLVGMSFLQMNIYY
EQIQKL"

CDS 1815557..1816042
/locus_tag="EFAGFIKM_01600"
/inference="ab initio prediction:Prodigal:002006"
/codon_start=1
/transl_table=11
/product="hypothetical protein"
/translation="MNNASSALKVAAGIFLTIALITIVVLLFISAQEATKTAQNNFAD
IQTELSQAAFTVYDGTTISGSQVTNALRKYADKDQFGIQVITGKNKAGQWYGNQLNIS
QDLNNADYGSVIAPDDKVGIINQTMSEKENQYVNPSGKFKAIIVKDRSNVVRGLIFQQ
S"

CDS 1816059..1816457
/locus_tag="EFAGFIKM_01601"
/inference="ab initio prediction:Prodigal:002006"
/codon_start=1
/transl_table=11
/product="hypothetical protein"
/translation="MSRNTQHLMLFCTAVVLFVTACLYGQQNVKILSAALNDRVQTTA
GMEGRLTTTLHTTNLYQYSGAGVLHTVRQMTGTGIPVEVDGTSYVIDPLVSKTATIPV
QLDATYRPEFIRNETGELLKIVFWKEASVN"

CDS 1816454..1817005
/locus_tag="EFAGFIKM_01602"
/inference="ab initio prediction:Prodigal:002006"
/codon_start=1
/transl_table=11

/product="hypothetical protein"

/translation="MINAASKLLAVLLAVLLLYVYPAAETADRQDDIARMTAVQNVTR

FVDAVRTKGYISPRMLADFEEQLAQTGNVYDVSMEHLHKKYVPHYIDPMDNNSFNGTY

EVVQDGYYAAQIRERLFPSSGVIPVDDPGRRYTLTTGDFFTVTLQNTNRTPSMLIREW

LSGTVQASAVFVTYGGMVLNEDY"

CDS 1816992..1817975

/locus_tag="EFAGFIKM_01603"

/inference="ab initio prediction:Prodigal:002006"

/codon_start=1

/transl_table=11

/product="hypothetical protein"

/translation="MKITELSIVFVLIFFPLFWIISLHTQDAEEAQYLGQRYRSALQT

AVMDAGAVMHQNEKQNDEAGYDSTKFVKADKELALLTFTQTMALNMGIQNDPAAIRNM

FNYIPAVVILDYDGYYMLSTETELTGNREDSLRQVWGPKKPYTYSDSNGNSINFTLDD

YVYAFDASAGKWIEGFQKELASSTQIPLLQNTSVFEQVRQSRIVTAVQDDLADVINRH

NEFARKNGISYTFTMPLIAQEDWYNSINDTGIMAFIQGIGVGDQKINNYAIGGGRLVK

KTAIVGGVDPLTGIKYYYPSTCGNGYRAEEVFTDARQAAAQGYFEYKCSNR"

CDS 1818114..1819664

/gene="ybiT_1"

/locus_tag="EFAGFIKM_01604"

/inference="ab initio prediction:Prodigal:002006"

/inference="similar to AA sequence:UniProtKB:P0A9U3"

/codon_start=1

/transl_table=11

/product="putative ABC transporter ATP-binding protein

YbiT"

/db_xref="COG:COG0488"

/translation="MSLLSVENVSHNFGDRTLFKNVSFRLLAGERVGLVGANGVGKST

LMNILTGKLLKDSGKVEWTPKVRYGYLDQHTKLTPGKTIRDVLKDAFLPLLELEKEMM

NITDQMADADPDKLEQLLEEMGDIQEQLEQGDFYLIDVKVEEMANGLGLSAIGLDRDV

AALSGGQRTKVLLAKLLLEKPTALLLDEPTNYLDVEHIEWLSRYLKDYPHAFLLISHD

TEFMNEVVNVIYHLEFAKLTRYAANYNKFLEMADMNKAQHIDAYEKQQEYIKKQEDFI

QRNKARASTSGRAKSREKQLDRIERIDRPDEAAKPTFKFKDARASSKTVFEGIDFEIG
YTYPLLPKMTMTIERGEKIAIVGCNGVGKSTLLKTILGRISPLSGKTFLGDYLETAYF
EQEVRAGNITPIEDVWNEFSHLTQNEVRGHLARCGLKNEHITRPLNMLSGGEQAKVRL
CKLLMRESNWILFDEPTNHLDVTAKAELQRALQEFKGTVLLVSHEPDFYEGWVTKTWN
VEEWSEKN"

CDS 1820075..1820737

/gene="tenI"

/locus_tag="EFAGFIKM_01605"

/EC_number="5.3.99.10"

/inference="ab initio prediction:Prodigal:002006"

/inference="similar to AA sequence:UniProtKB:P25053"

/codon_start=1

/transl_table=11

/product="Thiazole tautomerase"

/db_xref="COG:COG0352"

/translation="MKSTVISHERVHGSFELHVVSTGVGNQASFVKAAKDVWPWVDYI
HIREKQLSWQQKIGWAESLRGVGVPSCRIVINGSELPPQNDIYGGVHWGQEAIRNYNP
AVISNVQRLRLGVSVHSIDEAKIAEERGADYLFFGHVYSTNSKPDLEPRGLYALAEVC
SGVSIPVMAIGGIEPGNIHAIRSAGSQGVAVISNVWASDSPDRAAESLRQAIVATESL
SR"

CDS 1820755..1821927

/gene="thiO_1"

/locus_tag="EFAGFIKM_01606"

/EC_number="1.4.3.19"

/inference="ab initio prediction:Prodigal:002006"

/inference="similar to AA sequence:UniProtKB:O31616"

/codon_start=1

/transl_table=11

/product="Glycine oxidase"

/db_xref="COG:COG0665"

/translation="MKKIITDRPDKIYAETIIVGGGVVGCAIAYELASRGQDVLLLER
SAIAGGTSCAAAGMLAADSEDFAHPLMAKLARQSRQLLIEQQVLMATLSEVETGLQRQ

GFLTPFCSYSELSSYKDNRKSALSASADEVWWDRSVVQQEASWLNRDTYGAYYRPYES

EILPVHLTKAYAESAQALGARVMEDIQNIRVQANEHGVQGITTSIGEMRCKHVIIAAG

LQSEELMRHVNLSLPVWSVKGEIAAVQFSDEHAGYRPDRTVYAEDIYIVPKANGEVWL

GATSLPGRTDLTVSVQGVQKLLTAATHWVPGMKEAQFIRAWAGVRPATPDGLPYIGAC

KSIPGLFAAFGHYRNGILLSAITGRLIAELLAGKSSEELGIEALSPERLNRKGVVQ"

CDS 1821924..1822127

/gene="thiS"

/locus_tag="EFAGFIKM_01607"

/inference="ab initio prediction:Prodigal:002006"

/inference="similar to AA sequence:UniProtKB:O31617"

/codon_start=1

/transl_table=11

/product="Sulfur carrier protein ThiS"

/db_xref="COG:COG2104"

/translation="MNIIVNGQRMEIEDRLNRVDKLLQSFDLQVKTVVVELNKHILTR

EYHETTALREGDRIEIVHFVGGG"

CDS 1822127..1822897

/gene="thiG"

/locus_tag="EFAGFIKM_01608"

/EC_number="2.8.1.10"

/inference="ab initio prediction:Prodigal:002006"

/inference="similar to AA sequence:UniProtKB:O31618"

/codon_start=1

/transl_table=11

/product="Thiazole synthase"

/db_xref="COG:COG2022"

/translation="MMLNIGKYTFESRLLLGTGKFSDLEVQSQAVEASETEVLTFAVR

RLNLEERNQKHFLDTLDLDKYTLLPNTAGASNAEEAVRIAELARASGLCDMIKVEVIG

DGMTLLPDPIETYKACEILLEKGFTVLPYISDDVILAKRLQLLGVHAVMPGASPIGAG

RGIINPYNLEIIIEQAVVPVIVDAGLRSPKDAAYAMELGADGVLLNTAVSGSGDPVSM

AKAMRLGVEAGRLAYEAGMIPVKRYAAASSPVEGMVHT"

CDS 1822894..1823943

/locus_tag="EFAGFIKM_01609"

/inference="ab initio prediction:Prodigal:002006"

/codon_start=1

/transl_table=11

/product="hypothetical protein"

/translation="MTDQLTSSEQADRYSRQERYAPLGKEGQARLKGSRVLIVGAGAL

GTGIAETLVRSGVGHLTIVDRDYVEWSNLQRQQLYVEQDALERIPKAMAAEKRLSAIN

STVHVEGKVLDVRVDELEELVQHTDLIMDATDNFDTRLLINDMAQKHRIPWIYGGCVG

SYGITYTFMPGDTPCLNCLLGEVPLGGDTCDTSGIIPQAVQMVTANQTAEAMKLLSGN

ANALRRKLLSFDVWRNEYISINVDGAKKQDCPSCSDTATYPYLSASNLEKTDVLCGRD

TVQIRPARRMNLDLQHTADRLDRLQEGKVESNPFLVSFTTGVYRMVIFQDGRVLVHGT

KDTAEARTLVHRYFG"

CDS 1824153..1824767

/locus_tag="EFAGFIKM_01610"

/inference="ab initio prediction:Prodigal:002006"

/codon_start=1

/transl_table=11

/product="hypothetical protein"

/translation="MEWSELNQSVEPSLLHINLIILVDREEGTKHCGQDQKLELKRWL

DKGQVIPLLVITSNASPQFIVEWLDYGANDVMEEPIHMTVMLARIRNLLRVFANATPE

GEEVIVVHDLKVNLRSRRVNRAGEYLTLTPKEYELLEFLALHVNEACTRSDILREVWG

YEYAMDTNVVDVYIKHLRVKVDKGRSVKLIHTVRGIGYMLHDRN"

CDS complement(1824876..1825376)

/locus_tag="EFAGFIKM_01611"

/inference="ab initio prediction:Prodigal:002006"

/codon_start=1

/transl_table=11

/product="hypothetical protein"

/translation="MKTNIFVQKAVTVGLCATLGFGAVLMTNAPVAQAATASVSTGQQ

IVNYGKQFTGTPYKFGASTSTTKVFDCSSFMKYIFKKYGVDLPRTSVKQSKEGTAVSK

ANLRVGDLVFFSSGSRSTGSNVTHVGVYAGNGKILHTYGSPGVTLSDLDSGTWKRTYV

KARRVL"

CDS complement(1825673..1826314)

/gene="thiN"

/locus_tag="EFAGFIKM_01612"

/EC_number="2.7.6.2"

/inference="ab initio prediction:Prodigal:002006"

/inference="similar to AA sequence:UniProtKB:O34664"

/codon_start=1

/transl_table=11

/product="Thiamine pyrophosphokinase"

/db_xref="COG:COG1564"

/translation="MTVKRIVIFTGGNLSVELLQEIREDDMIIAADRGALFLIEHGIQ

PHIAVGDFDSINEEERIIVSNNSIRFITCDPVHKDLTDTEMAFETALDYEPSHILMLG

ATGTRMDHTLANVHIMVRAMQHHISCAIQDKHNYMTLTTSKAVVEHRDYKYVSLLPLT

HEVTGITLDGFMYPLDQATIRMGQSLGVSNQLLGSSGTVTIDSGLLLIIQSKD"

CDS 1826497..1827198

/locus_tag="EFAGFIKM_01613"

/inference="ab initio prediction:Prodigal:002006"

/codon_start=1

/transl_table=11

/product="hypothetical protein"

/translation="MDLHIFEVFSIIGTIAFAMSGAFVAMEEEYDILGVLVLGLVTAF

GGGIIRNVLIGIPVTTLWSQGSLIMLALVSVAIAFILPLKWISHWKRTEALFDAIGLA

AFAIQGALYAANMGHPISAVIVAAVMTGIGGGVIRDVLAGRKPLVLRDEIYAVWAMTA

GFVIGMGWLTTNAGLLFCFAAVVFFRMCSVHYKWKLPRRSLVPSETGNVSPQPESIVP

QPTSSVLQGTLSKGD"

CDS 1827200..1827676

/gene="yfkJ"

/locus_tag="EFAGFIKM_01614"

/EC_number="3.1.3.48"

/inference="ab initio prediction:Prodigal:002006"

/inference="similar to AA sequence:UniProtKB:O35016"

/codon_start=1

/transl_table=11

/product="Low molecular weight

protein-tyrosine-phosphatase YfkJ"

/db_xref="COG:COG0394"

/translation="MINVLFVCLGNICRSPMAEAVLRHKVLEKGLEHQIRVDSAGTGD

WHVGKPPHEGTRKLLDSYQISYANMAARQFASEDFTQFDYIVCMDNSNADNVRNVPGG

AEADIIKFMDMLPEEKLREVPDPYYTGNFEEVYELVNVGCDVLLSKIEAEHSLPKK"

CDS complement(1827835..1828569)

/gene="ybbA"

/locus_tag="EFAGFIKM_01615"

/inference="ab initio prediction:Prodigal:002006"

/inference="similar to AA sequence:UniProtKB:P55192"

/codon_start=1

/transl_table=11

/product="putative protein YbbA"

/db_xref="COG:COG2819"

/translation="MTDSRYLKRTIVKEEIESRYLGEKRTLRIYLPPGYNELLSYPVV

YCQDGEEFFNFGRIATTANQIILDEGAEPFIIVGVQVDVSVRTKEYAPFGDRFKTYTA

CFAEEIIPYIEEKYPVRPSPQERILAGDSLGGSVSLHLALLYPDLFTRVISMSGAFYS

ASQEIYAAAEDLSWLSIWMIVGLQETDFEADTGTYDFVQLNRDTRELLEKRGALVSYQ

EKDGKHQWGFWQKELPEALLYFLQER"

CDS 1828826..1829893

/gene="pdhA"

/locus_tag="EFAGFIKM_01616"

/EC_number="1.2.4.1"

/inference="ab initio prediction:Prodigal:002006"

/inference="similar to AA sequence:UniProtKB:P21873"

/codon_start=1

/transl_table=11

/product="Pyruvate dehydrogenase E1 component subunit

alpha"

/translation="MSKVPYEVYTEDVEALSVLSPDGEIVNKDMMPTLSDDQLKEIMY

RMVFTRTWDDRAVNLGRQGRLGFYAPVSGQEATMVGSEFAIEKEDFVCPGYRDIPQLV
WHGLPLYQAFLYSRGHQHGGQIPDGVNVLMPQIIIGAQILHAMGIAMGYKLKKQKQVV
ITYTGDGGSSEGDFYEGLNYAGVYKLPVIFFVQNNGYAITTPFAKQTAALSIAHKAVA
AGIKGVKVDGMDIFAVIKAVQEAAERGRNGEGATLIEAVTYRFRPHSLSDDASKYRTK
EEEAEWSAKDPIARFAKYLEKKGLWTEEDTARVKEEAKAKVNEEIKKAEKTEKMTISG
LIDSMFEQTPKHLEEQKADFQ"

CDS 1829995..1830972

/gene="pdhB"

/locus_tag="EFAGFIKM_01617"

/EC_number="1.2.4.1"

/inference="ab initio prediction:Prodigal:002006"

/inference="similar to AA sequence:UniProtKB:P21874"

/codon_start=1

/transl_table=11

/product="Pyruvate dehydrogenase E1 component subunit
beta"

/translation="MAQMNMKEAIRDALRVELKRDPNVLLFGEDVGNVGGVFRVTEGL
QKEFGEERVFDTPLAESAIGGLAVGLGVQGFRPVAEIQFVGFIFEALDQMVVQAARMR
FRSGGKYNSPIVFRTPFGGGVKAAELHTDSLEGLLTQTPGIKVVVPSNPYDAKGLMIA
SIRDNDPVFFMEHLNLYHAFRAEVPEEDYVVELGKANVVREGSDVTIITYGMMVHTSV
KAAEELEKQGIKVEVIDLRTVSPIDIDTIVASIKKTNRAIVVQEAQKSAGVAAEVIAQ
INEKAILHLEAPVLRVAGPDTVYPFAQIEDSWLPNPARIVAAVNKVVNF"

CDS 1831149..1832465

/gene="pdhC_2"

/locus_tag="EFAGFIKM_01618"

/EC_number="2.3.1.12"

/inference="ab initio prediction:Prodigal:002006"

/inference="similar to AA sequence:UniProtKB:P21883"

/codon_start=1

/transl_table=11

/product="Dihydrolipoyllysine-residue acetyltransferase
component of pyruvate dehydrogenase complex"

/db_xref="COG:COG0508"

/translation="MAKFEYKFPELGEGLHEGEIIKMHIKVGDKVTDDDIIMEVQNDK

AVVEVPCPVNGTVTEVFAKDGQVCHVGEVVAIIDAEGELPEQDDAPAGDQGEQEKDAA

QGGADTSGSSAAASSTDAAKEGGNNSVPAVPAKDVLATPSVRKFAREQGVDIAQVNGT

GNNGKVTKEDVESFKNGGGSSAAASSEAPAQEEKKSAAPAAADQYLEEERVPFKGIRK

AISNAMVKSAYTAPHVTIMDEVDVTELVAFRTRMKPIAEKKGTKVTYLPFIVKALVAA

SRQFPALNAMIDEEANEIVYKKYYNIGIATDTDNGLIVPVIKDADRKSIWMIADSIRD

LAARGREGKLSANEMRGSTISISNIGSAGGMFFTPIINFPEVAILGTGRISEKAVIKN

GEVVAAPVMALSLSFDHRIIDGATAQNFMNYIKQLLANPELLVMEV"

CDS 1832469..1833884

/gene="pdhD"

/locus_tag="EFAGFIKM_01619"

/EC_number="1.8.1.4"

/inference="ab initio prediction:Prodigal:002006"

/inference="similar to AA sequence:UniProtKB:P11959"

/codon_start=1

/transl_table=11

/product="Dihydrolipoyl dehydrogenase"

/translation="MVVGDASLNIDTLVIGAGPGGYVAAIRAAQLGQSVLIVDKSELG

GVCLNRGCIPSKALISAAHQYESALHGEAFGISAENVKVDFSKTQEFKNGVVKKMTGG

VAGLLKGNKVEVFNGECMFINENEARVFNDHESPRYKFKNAIIATGSRPIELKPFPFG

GRILSSTEALNLPEVPKSMIVIGGGYIGAELGQMYSKFGAKVTIIEGLDTVLPGFDKD

MTSLVAKNMKKTGIEIVTGAKAESAEQTDKDVTVKYSVNGESKEVTADYLLVTVGRRP

NTDGELGLDMIGVDVDERGFVKVDHQGRTSIPHIFAIGDIVSGLALAHKASYEGKVAA

EAIAGQPSVVDYKCMPAVVFTDPECSSVGYTEKEAKEKGYKVKAGKFPYAGNGRAVSL

NHAEGFVKIVADEESGLVLGCQIVGLEASNLIAELGLAIEMGATLEDLALTIHAHPTL

GEIVMEAAELVMGHPIHIISR"

CDS 1834362..1835156

/gene="thyA"

/locus_tag="EFAGFIKM_01620"

/EC_number="2.1.1.45"

/inference="ab initio prediction:Prodigal:002006"

/inference="similar to AA sequence:UniProtKB:P0A884"

/codon_start=1

/transl_table=11

/product="Thymidylate synthase"

/db_xref="COG:COG0207"

/translation="MKNYLDLLQDILNNGVHKGDRTGTGTQSVFGRQLRYDLSEGFPL

VTTKRIHLKSVIHELLWFLSGDTNISYLKENGVKIWDDWADENGDLGPVYGSQWRTWE

APNGDKIDQIAAVIDSIKNNPDSRRHLVSAWNVAEINNMKLPPCHFAFQFYVAEGKLS

CMLTMRSVDTFLGLPFNIASYALLTHMIAQQCDLEVGDFIWSGGDVHIYSNHVDQVKT

QLEREPYALPKLVIKRKPDSIFDYKFEDFEFENYQHHPGIKAPIAV"

CDS 1835207..1835695

/gene="dfrA"

/locus_tag="EFAGFIKM_01621"

/EC_number="1.5.1.3"

/inference="ab initio prediction:Prodigal:002006"

/inference="similar to AA sequence:UniProtKB:P11045"

/codon_start=1

/transl_table=11

/product="Dihydrofolate reductase"

/db_xref="COG:COG0262"

/translation="MSIELVWAMGENGVIGLNNSIPWRLPKDMAFFKQRTLNKTIIMG

RNTWESFGGSPLPQRRNIVVTRDLNYKVEQAEIVHTIEEGLSVTKGEELCVIGGSQVY

REFLPLADRLVVTKIHKNFEGDTFFPEVDWSEWELIEQIEGEQDEKNVYAYTFEFYER

KR"

CDS 1836312..1837799

/gene="gltB_1"

/locus_tag="EFAGFIKM_01622"

/EC_number="1.4.1.13"

/inference="ab initio prediction:Prodigal:002006"

/inference="similar to AA sequence:UniProtKB:O34399"

/codon_start=1

/transl_table=11

/product="Glutamate synthase [NADPH] small chain"

/db_xref="COG:COG0493"

/translation="MSTPTGFMEYKRQLPTDREPAERIKDWEEFHKHMAEEELRTQGA

RCMDCGTPYCHTGIDMIGGTSGCPVHNLIPEWNNLVYRGLWREALERLHKTNNFPEFT

GRVCPAPCEGSCTVGLIGQPVTIKTIEEAIIEKGFEEGWVVPQPPEKRTGKRVAVVGS

GPAGLATAAQLNKAGHSVTVYERSDRVGGLLMYGIPTMKLDKNVVQRRVDLLEAEGIQ

FITNTEIGKDIAAQQLVDEYDAVVLCGGATKPREFNIEGSDLKGVHYAMDFLNGSIKS

YLDSNLEDGQYLSAKDKDIIVIGGGDTGSDCVATSLRHGCRTITQFGTHTQAPMERDR

INNPWPQFPNVYTLDYAQEEAKALFGQDPREFSIMTTKFVGDDEGNLKELHTVQIERI

VDETGRKIYQPIPGTERVFPAQMAMIAIGFDGPEQTLVEQLGLATDRRTNVKARYGKY

NTNVDKVFAAGDMRRGQSLVVWAINEGREAAREVDKYLMGASVLV"

CDS 1838031..1838522

/gene="fur_2"

/locus_tag="EFAGFIKM_01623"

/inference="ab initio prediction:Prodigal:002006"

/inference="similar to AA sequence:UniProtKB:P54574"

/codon_start=1

/transl_table=11

/product="Ferric uptake regulation protein"

/db_xref="COG:COG0735"

/translation="MASNRDKSISEQIFHIKNQLVEKGYKLTQQREVTVRVLLEHEKD

HFSAEEVFLLVKEQFPEIGLATVYRTLELLSDLQVVEKINFGDGAARFDLRSTDGSHH

HHHLICTECGKVEEIMEDGLLRLEQQIERQYGFAVTDHRLDFQGVCKECRQKHVLKEQ

AAG"

CDS 1838648..1840987

/gene="topB_1"

/locus_tag="EFAGFIKM_01624"

/EC_number="5.6.2.1"

/inference="ab initio prediction:Prodigal:002006"

/inference="similar to AA sequence:UniProtKB:P14294"

/codon_start=1

/transl_table=11

/product="DNA topoisomerase 3"

/db_xref="COG:COG0550"

/translation="MKTLVIAEKPDMGRTIAAVIEPKAKNNRTYLEGEHYIITWAIGH

LLGLAEPDAYDTKYKRWNIADLPIIPDQFKIVPNPRTKDQLKMIGELAKRASAIVNAC

DAGREGQYIFALIQQQLKLRQPVKRLWISDLTAESIRRGFDGLKDASEFENLTHAARA

RSEADWLIGMNASRAFTTRHNALLSVGRVQTPVLALIYDREIEIEAFQSQTFYEVAAW

FRQEGVEYRGLRQGDKLTDAEAAEAIAASVKGKTGQITKYEAKQTKEYPYRLYDLTLL

QREANAKFGYGAKKTLDIAQALYEKHKVISYPRTNSNYVTEQNIEGMHKTLNLLKNGT

YSELAQGAKPELVHKNNKGVCNPSRVEDHHAILPTLKRPGTLSKDEQNIYDLIVRRFL

SHFYPPAEYKQHTVLTEVEKHQFKTSVKELLSLGWKVVLGSGDQEQGGAGKKKTKKNG

NEEEEAEEWTDKAFSVQPELPVQCTKSEFKEKATQPPKSYTEGTLLKAMESAGKQIEN

EELRDAMKDSGLGTPATRAATIERLKNVGYITLQGKKMQLTLKGRTAIELIRRAGVDL

LTSPEMTGQWERRLYQISKGEAGQDKFMENVKKFTLSIIEKVRVQAPAPADAFGEDSR

AGRGKGKGARTQAGNTRTSAKTASGGSTVKTSRQATASSSRVGSGKSTATKAPSSTAS

PTGVRELLAPCPSPGCTGQIIEGKKGYGCSRFKEGCSFVVWKEYAGKKITSTMLKSLI

EKGSTQVLSFKRKDGSTVKARIILQDVVTGKLSGEKQDA"

CDS complement(1841023..1841787)

/locus_tag="EFAGFIKM_01625"

/inference="ab initio prediction:Prodigal:002006"

/codon_start=1

/transl_table=11

/product="hypothetical protein"

/translation="MKIMKQHILFDLDDTLVYCNKYFNLILGEFFENMQEWFDGHSLT

TQEIREKQLEIDVTGVNKLGFASHHFPQSLIDTYRYFSGKFARLTSPREESYLSKLGM

SVYDQEVEPYPHMVETLENLKSAGHSLYLYTGGETVIQQRKIDQMKLSAYFDDRIYIR

QHKNVEALEGILSNGPFDRRATWMIGNSLRTDIMPAVKAGIHSIYIKQPNEWQYNIVE

LQPNPETSMYTITALEEVPKVIHENIQQQQKRTLG"

CDS complement(1841837..1842757)

/locus_tag="EFAGFIKM_01626"

/inference="ab initio prediction:Prodigal:002006"

/codon_start=1

/transl_table=11

/product="hypothetical protein"

/translation="MAYHVKIDVSPIYEMLNSFLVYVTKKWIQHLDIGPEWILEVEGK

LSSNVRAALAPAATWPFDDFDVLFAWAAYQNDTSENRDFLDMLAGMTAEDLFARVSPL

LPALTIEESTRIRNSYVPLLRLWDEHYCQNMSEDQRVWLEEDAEEKRILLDKMGSELL

IEYATAGVLVEPMPGLNEVILFPTVHNRPINMYCFYEGMMIMQYPVDAPEENEDQPPT

CLLRFTHALADPERLRLLRYVSDEPKSLAEMCEELGKDEDTVKDQVMALRIAGLLRTH

LLGSNRKEKYSIRPDGVSELNMFLESYIRI"

CDS 1843062..1844252

/gene="ycaD_1"

/locus_tag="EFAGFIKM_01627"

/inference="ab initio prediction:Prodigal:002006"

/inference="protein motif:HAMAP:MF_01149"

/codon_start=1

/transl_table=11

/product="putative MFS-type transporter YcaD"

/translation="MSSITRPKLSHSTANYLILITVIVVAGISQGLLLPVLSIFLEQK

GVSPGLNGLNAAALYIGSFAMTLVAERLLGALGFKKLIVGGLILVMAPLILFPYLPDI

KIWFILRLIVGIGDSALHYAAQLWVLLVTAPEKRGRYISLYGMSYGLGFSIGPLGIKL

LGFGDAVPFWVLFVCIAAVLILVLMKLPDTKPEKAEHGQLPERRFRRSLAWAWYALLP

ALLYGYMEAGMNSNFPVYGLRIGFDTSQISSLLPFIGIGGLFLQLPLGMLSDRYGRKK

ILMFAGISGGIIFMLVPVVGTHFWWTLVLLTIAGGLVGSFFSLGLAYAADILPKVLLP

AANVVASFHFTIGSIIGPNLGGQMINWITPGSMFTLLGILYLLFGVAGILFRRKPEFE

SVLK"

CDS 1844336..1845061

/gene="glnQ_1"

/locus_tag="EFAGFIKM_01628"

/inference="ab initio prediction:Prodigal:002006"

/inference="similar to AA sequence:UniProtKB:P10346"

/codon_start=1

/transl_table=11

/product="Glutamine transport ATP-binding protein GlnQ"

/db_xref="COG:COG1126"

/translation="MIRVENLSKSVGVDRVPVLRDIRFDMQQGEMIAVVGSSGSGKSM
LLKCLAMMEKWDSGRFTVDGAEILKEGWSGKRKIKREWAYLEQNPELFPRRTALKNVL
IGRSGQTPVWRMVTGMVRSDDYMGAMDYLEGLGLLDKAHQIAEKLSGGEKQRVAIARA
LAHGAKVVLADEPVIGLDPHTADSVLETLRKLCEEERATVIAVLPIELAEKHATRIWG
LAEGKIAFDIRGRRLTQQEKNQI"

CDS 1845135..1846403

/locus_tag="EFAGFIKM_01629"

/inference="ab initio prediction:Prodigal:002006"

/codon_start=1

/transl_table=11

/product="hypothetical protein"

/translation="MLGSVLSGCTVISDPKGLMRKPMMSTDKEKLYNVVQVKLPPEST
LIRPKDMNNTSMIRVEDLNGDGTREAIVFYETPNENVRIHGMILEEQGGTWVKKLTFD
VPGNELQSFKVLDITNDGNPDIILGVSLQEQKALTAYSYKGGALEQVLGGVPYNQYII
DDAQGNTLDLSGDGKSDFVIISLNNSGFATIALYQYEDGSFKEVDRVQTDYTVKEVYN
ALGGEIAKGQMGIVLDAELDDRSSFSQIMYVKDNKLVNAFKSPDQTYRDDKILSGDVD
NDGIIEFGLKETPKGWEHYVFDSRLWFYTYYQWDGKDSKKFVSFQFRDDADLFHLNLK
PEWYGKITIDTKSEKEKYIRFKMIDTDETVAEIKYFTLSKWEQEKGKSWKEITRTKDR
VIASRVAQSEAGKDALSNTSQLNRKGELNE"

CDS 1846396..1847094

/gene="phoP_1"

/locus_tag="EFAGFIKM_01630"

/inference="ab initio prediction:Prodigal:002006"

/inference="similar to AA sequence:UniProtKB:P13792"

/codon_start=1

/transl_table=11

/product="Alkaline phosphatase synthesis transcriptional
regulatory protein PhoP"

/db_xref="COG:COG0745"

/translation="MSKVLILEDEESIRSFIVINLKRNGFEVLEAGDGHEALRILQTV
PDIDLALLDVMVPGIDGFEVCRRIRETNERLGIIFLTAKVQEQDKVYALSVGADDHVS
KPFSPTELIARIQSLLRRVNVHRETAAKVTFQSGPFSLDLISKQFKKQNEAIELTPTE

FSLIQFFLEKENTPLSRDVLLDHVWGKEYMGDPKIVDVNIRRLRQKIENNPSEPEYLQ

TVWGHGYKWKGREQ"

CDS 1847091..1848590

/gene="rcsC_4"

/locus_tag="EFAGFIKM_01631"

/EC_number="2.7.13.3"

/inference="ab initio prediction:Prodigal:002006"

/inference="protein motif:HAMAP:MF_00979"

/codon_start=1

/transl_table=11

/product="Sensor histidine kinase RcsC"

/translation="MIKKGITRQIVLHYFFVVFLALLLVEFVFMLAVQRYYYESIYNT

ISTHISNSKDFFEPIARENTTEDGNNLSRLLVNLALSNTELEILDLNGRVLASSTAFE

SDRAVLQTSDIMQALNGDMGRWIGRQPGTGESVMAVSHKFDLGGENTYVIRYLTSLED

VNSKLLNMGLLAAAVGVGVLAIVLIISIGMANSIVRPINNITAVSAQMARGRLDVRVK

GNYKHELGELASTLNFMAHEIVRSNQIKDDFISSISHELRTPLTSIKGWSETLDSGGY

DPEETRIGMGIISKETERLIGLVEEMLDFSKLQQNQMKLVKGTVNIREIVQETMLNVW

AKAEQKQVHLKLETDETRAYNVHGDGNRLKQVFLNVVDNAIKFSHENSWIFLSVKEEN

GQIIAAVQDTGIGISEEHLIKVRDRFFQVNHQNGGTGLGLAITQELVELHEGTITMQS

ELGSGTTVTVTLPAVAEEMGPEESVTASGDAEVTDERTASDAKSDLEKS"

CDS complement(1848714..1849940)

/gene="glgC_1"

/locus_tag="EFAGFIKM_01632"

/EC_number="2.7.7.27"

/inference="ab initio prediction:Prodigal:002006"

/inference="similar to AA sequence:UniProtKB:P39122"

/codon_start=1

/transl_table=11

/product="Glucose-1-phosphate adenylyltransferase"

/db_xref="COG:COG0448"

/translation="MFNKDCIAMLLAGGEGKRLAPLTSSLAKPAVPFGGHYRIIDFPL

SNCVNSGIDTVGVLTQYQAESLHDHIGGGEPWGHGNSSEAGISLLPSYHTGNDEYLGT

ADAIYKNIDYIDQQNPENVLILSGDHIYHMNYRDMLEAHQANNAAATISVMEVPWDEA
HRFGIMAADADLRVTEFAEKPAEPKSNLASMGIYMFKWDYLKRHLLQDAANPESSHDF
GKDVIPQMLNENNPLFVYNFNGYWKDVGTVKSLWDAHMDLLHNEEDWSLQKENWPMFT
RDWRSKPSAYKARHTKIEHVTSMIHDSCAIEGRSERSVIFCGAEVGKGSEVKDSVVMP
NARIGRGVHIERAIIGEGAIIKDGAIVKGTADEIVVVGPNEIVSAKPAVRTQPVRMLK
EVYEKSGRLRAGELSS"

CDS 1850134..1852140

/gene="glgB"

/locus_tag="EFAGFIKM_01633"

/EC_number="2.4.1.18"

/inference="ab initio prediction:Prodigal:002006"

/inference="similar to AA sequence:UniProtKB:P30538"

/codon_start=1

/transl_table=11

/product="1,4-alpha-glucan branching enzyme GlgB"

/translation="MAIQPLAHPDILPEHIYLFHEGNLHHSYRMLGAQPETCDGLQGY
RFTVWAPNAVEVGLAMDRNEWKGEKEPLYKIPESGFWSRFFPEISEGTLYKFRILTED
GTELLKADPYAFQAEVRPQTASVTSSIEGYKWNDGAWRRKQRAMYNKPLHIYEMHMGT
WKRKEDGSLYSYREMADLLVPYLLEMKYTHVEMMPLSEHPYDLSWGYQNTGFYAPTSR
YGHPKDLMYLVDTLHQAGIGVLLDWVPAHFAKDAHGLRMFDGTPLYEYADPMLAEKPG
WGTLSFDYSKPEIRSFLISNALYWMEMYHFDGLRVDAVTSMIRLDFEKQPGQYRTNDE
GGLENKEAVAFLQQLNETVFKYFPYALMMAEESSAWPMVTLPVDEGGLGFNYKWNMGW
MNDTLDYMESDFHERPSKHHLLTFPVVYSFAENYVLPLSHDEVVHGKKSLLDKMPGTY
EQKFAGMRAFLGYFMTFPGKKLLFMGGDFGQFIEWKDEDQLDWFLLDYDSHRNLHKFE
RELSNLYLSEKALWELDHSLDGYEWITPDDQDQSVISYVRKGKKPADTLLVLINFQPV
KRERYRIGVMRPGMYTEVLNSDHSVYGGSGITNDMQLPTEKIPFHGHPHSLELVLPPL
SIVILKKNTRRKTDESPASITSVSSKTKNKNESNTLKPKRRTKA"

CDS 1852137..1853579

/gene="glgA"

/locus_tag="EFAGFIKM_01634"

/EC_number="2.4.1.21"

/inference="ab initio prediction:Prodigal:002006"

/inference="similar to AA sequence:UniProtKB:P39125"

/codon_start=1

/transl_table=11

/product="Glycogen synthase"

/db_xref="COG:COG0297"

/translation="MNILFAAAEVHPFIKTGGLADVIGALPFALKKSGADVRVIMPKY

KGIPAEYKDALQPVITTDVPLGWRRPYCGIEMLVHDGIPVYFVDNEYYFGRDGVYGYM

DDGERFAFFNRAVLEVLPQIEFKPDVLHSHDWHAGMIPLVLEAHYRHDPFYSDIRTVF

TIHNLLYQGVFPHELFSEILELDDRYFTVDGAEYYGNVNFLKAGIVYADHVTTVSPTY

AQEVQTSYYGYGLDGLLSSLGDRFSGIVNGIDTKSYNPATDTKIPVKYRTSLSKKTEN

KIELQKELGLPVRPDVPLMVMVTRLVDSKGLDLVCRILDELLYYDDIQFAVLGTGEQS

YEHWFREAANRYPLKMSAQITFNDGLSRRFYAGSDIFLMPSRFEPCGISQLLALRYGS

VPLVRETGGLNDTVQAYNEFTGEGNGFSFTSFNAHDMMNTIRRAEEIYKQPEHWKKIV

KNAMGGEYSWNVSAEEYMDIYRRITLDPVN"

CDS complement(1853721..1855487)

/locus_tag="EFAGFIKM_01635"

/EC_number="3.2.1.54"

/inference="ab initio prediction:Prodigal:002006"

/inference="similar to AA sequence:UniProtKB:Q08341"

/codon_start=1

/transl_table=11

/product="Cyclomaltodextrinase"

/translation="MLLEAMYHVPRDKWAYAYNSSTIHLRVRTKRNDVQYVTALTGDK

YDWNGTYKEIQLEKAASDNMFDYWEAAVKPQFKRLTYIFRITAGSEDVFLADNGIHYE

TPYPTGGYYEFSYIHEIDVFKVPEWAKEAVFYQIMTERFANGDPKLNPKETHDWGGKP

ELDNYFGGDLQGVLDHLYDLTELGVNAIYFTPLFQANSYHKYDTIDYKKVDPHFGDNA

LLKQLTEECHRLGIRVMLDAVFNHCSEDFPPFQDVLQNGKNSKYADWFHINEYPVQIK

DGIPTYDTFGFYGNMPKFNTANPEVKAYLLDVAEYWIKEIKLDGWRLDVANEVDNHFW

RDFRKVVKAANPEAYIVGEVWSDSLTWLMGDQFDSVMNYPFADKVLQFFCGSMDGYNF

ANEMGSLIMRYPQQTNEVIFNMLCSHDTPRLLTRLGEDKRRLKLSVVFLFTYIGTPCI

FYGDEVGISGDADPDCRKCMEWDPAKQDRELYDFYRLMIDLRKSNEALRKGRFRFLKA

DHNDPCIVYERVDDNLHFTVWMNNTPQDRTLSHPMETKDWKDALTEEPVFPTEGMMNI

KLDPYGYRILYRQLDPATILHN"

CDS complement(1855755..1856930)

/gene="abgB_2"

/locus_tag="EFAGFIKM_01636"

/EC_number="3.5.1.-"

/inference="ab initio prediction:Prodigal:002006"

/inference="similar to AA sequence:UniProtKB:P76052"

/codon_start=1

/transl_table=11

/product="p-aminobenzoyl-glutamate hydrolase subunit B"

/db_xref="COG:COG1473"

/translation="MTANKSQILTVIDQYASRFKAISSYIGANPELGNEEYLASARLK

EELLFHGFTVEAPILGLETAFIGTYSAAKPGPTIALLCEYDALPEIGHACGHHLICMM

SLGAAVGLKSILDEVGGTIKVFGTPAEETRGAKVPMAEAGLFDDCDVALMAHPYYAYE

KSGSSLAIDAVQFEFHGRSSHAAASPHEGINALDAVIQTFNGINAFRQQVKSTVRIHG

VINNGGQAANIIPDYASAQFYVRAATRKELNILTERVIQIAEGSALQTGCKLVTSNYE

TSYDEMVTNEAFSAAFSQNLMELGIPQEEIVSGNDHGSMDIGNVSLKCPAIHPYIRVV

DEVHTLHSIAFRDLALQERALDGMILGAKALATTAYDVLSQPELLQTIRTEFEQAIR"

CDS 1857114..1858577

/gene="mltF_1"

/locus_tag="EFAGFIKM_01637"

/EC_number="4.2.2.-"

/inference="ab initio prediction:Prodigal:002006"

/inference="protein motif:HAMAP:MF_02016"

/codon_start=1

/transl_table=11

/product="Membrane-bound lytic murein transglycosylase F"

/translation="MKLISRYTMMLLTFVILLTTAVPVALASGTTDNSSSKKLVLGTS

ADFAPYEFHKVINGKDEIVGFDIEIAKEIAKDLGAELVIEDMGFDGLLPSLQSGRVDL

VISGMTPTDERKKSIDFSDTYYKSKQVIMVRNVDKDKYPTMAELENAKIGVQKGSIQE

TIGQSIPGAKLTALDKISDIVLQLQTKRIDAAIVEDTVAAGYLDDIIGLAAAVPDEEQ

AEAAIGIRKGNTELLATVNGTLERLKSEDKINQMVTDASLLMADKANKSQNIFQVFWE

YKSFYATGVGYTLLLSALGVIFGVIIGLIICLFRLHDAAILRWIGTAYVEVIRGTPML
VQLMIIYYGFSLSFGINFTALQAGIITLSINSGAYLAEIFRAGIQGVDRGQLEAARSL
GMGRGAAMRYIILPQAFKAVLPAIGNEFITIIKESSIISVIGMVDIMYQASVVKNITY
QGLSPFLIAAAIYFVMTFILSKLLGRLERKLSASDRR"

CDS 1858558..1859286

/gene="artM_1"
/locus_tag="EFAGFIKM_01638"
/inference="ab initio prediction:Prodigal:002006"
/inference="similar to AA sequence:UniProtKB:P54537"
/codon_start=1
/transl_table=11
/product="Arginine transport ATP-binding protein ArtM"
/db_xref="COG:COG1126"
/translation="MQVIDVRQLHKSYGNNDVLKGINVTIGKGEVVVVIGPSGSGKST
FLRCLNLLEQPTSGEIDFEGVSITDPKHNINATREKMGMVFQHFNLFPHKTVEQNITI
APIKVKKQSNQEAEKIAADLLNTVGLYDKKDTFPNQLSGGQKQRIAIARALAMQPHVM
LFDEPTSALDPEMVGEVLDVMKRLAEGGMTMVIVTHEMGFAREVGDRILFMDGGVIVE
EGTPDEVFGAPKNSRTRDFLAKVL"

CDS 1859704..1860378

/gene="yocH"
/locus_tag="EFAGFIKM_01639"
/inference="ab initio prediction:Prodigal:002006"
/inference="similar to AA sequence:UniProtKB:O34669"
/codon_start=1
/transl_table=11
/product="Cell wall-binding protein YocH"
/db_xref="COG:COG1388"
/translation="MINKKRIAKTAVALMTTAMLIQAVPAHADSVHVAKEGDTFYTLS
KQYGVALNTMVKANNDISAYNIYGGLKIKIPGKANNVAAAAATESKSQAKTVTTASLD
VNADNKVVQAWGKTFDYSKAVNVKATAYSADPSENGGWGAVDYFGNDLELGTIAVDPS
VIPLGTKVLVTGHSHPGLPKQAFVATARDVGGAIKGHRIDIFIPGSKQSVSSFGFQDI
ELYILK"

CDS complement(1860486..1861277)

/locus_tag="EFAGFIKM_01640"

/EC_number="3.5.1.104"

/inference="ab initio prediction:Prodigal:002006"

/inference="similar to AA sequence:UniProtKB:Q81EK9"

/codon_start=1

/transl_table=11

/product="Peptidoglycan-N-acetylglucosamine deacetylase"

/db_xref="COG:COG0726"

/translation="MRVRLIMLMVIVIFLGGYHAPVSSRDVSSPPSESVSGSDSTEEE

QLTLGQLRQKYADTFKTNGPSTKKVALTFDDVPDPRFTPQVLDVLKKYNVRATFFIVG

SRAEKHPDLVKRIVKEGHMVGNHSYNHPEFSKLSMNAFRKQILHTGDIIRRLAGYTPK

MIRPPYGDINEEQLRWAAKQHYSIVNWNVDSLDWKGLSKEQVKDNILSAVKPGSIVLQ

HAGGGVGSNLNGTIEALPEIIEELRKRGYELVTLDEMLGLPKAKD"

CDS 1861431..1861772

/locus_tag="EFAGFIKM_01641"

/inference="ab initio prediction:Prodigal:002006"

/codon_start=1

/transl_table=11

/product="hypothetical protein"

/translation="MSTYDLKSIRLQVIEAGKDKVFLVTGGKAHIGAVATFYVDHERV

SGTTVHIPGHKEQELCERLARKAALQLKVTVTVIMGIHFDSITRMQIDEIVQTAERLM

EDHLNKDKRSS"

CDS 1861884..1862555

/gene="liaH_3"

/locus_tag="EFAGFIKM_01642"

/inference="ab initio prediction:Prodigal:002006"

/inference="similar to AA sequence:UniProtKB:O32201"

/codon_start=1

/transl_table=11

/product="Protein LiaH"

/db_xref="COG:COG1842"

/translation="MSIFKRLRDLTMSNVNAIIDKAEDPIKMTDQYIRDMQEDLEDAE
KAVAQQIAIEKKFKQLFEEQEALVKKREEQAHTAARAQNLDLARRALEEKKAAEEKMA
EYKTSYDQNKASADNLRGKLDEMRKQLTQMKNKRETLVARYNAAKAQTEINKALNGFG
SDTASAGMKRMEEKMMQMEAQAEASNEMSSKGKSLDDEFEKLGKDQAVEDELAALMKQ
YDNKN"

CDS 1862651..1863052

/locus_tag="EFAGFIKM_01643"
/inference="ab initio prediction:Prodigal:002006"
/codon_start=1
/transl_table=11
/product="hypothetical protein"
/translation="MDLNILAMLVWTGSGSVLLFVLMYVDSLFTKYKDFAEVKAGNMA
VTTRMVMKLFAQGYVLATSISTAGHLGEALLVSVVSFIILLILESVVHFMIRKWANLD
LDTGIQQGKTGYGLFSGALHIVGALIIAACL"

CDS 1863076..1863582

/locus_tag="EFAGFIKM_01644"
/inference="ab initio prediction:Prodigal:002006"
/codon_start=1
/transl_table=11
/product="hypothetical protein"
/translation="MSVWKRIKGILTKPEPPQAEKTMLQLAPGDICEVSLVTYEVVGR
VQNRARNAVVLTLQDGTAFRYLHIEERELTRYALYTPIDGRLDAPDEVPTLLDLDGRA
YHLEEEYGGMVSTAGRTPYGQAGEQLVWQYQSDDNMLLRVEWQDRRFTLYEGESILPA
DIKVIRSS"

CDS 1863594..1864334

/locus_tag="EFAGFIKM_01645"
/inference="ab initio prediction:Prodigal:002006"
/codon_start=1
/transl_table=11
/product="hypothetical protein"
/translation="MKKRLAHGLKLMLVLSLVMSLLSACGAAPSVQDTYPLESVNGSG
NSTSYVYRASDRTVPEVAQELSDQRQPDQISAENTERMFLVYQDEYYHLQQDPNKPED

TLVEVDSKEYVRQNYDSSFLQGYLTATLIGNLFDSFGGRGYGNYRGYTNKDTYKPSAG

SYRAPTSNDKKLAPPITVDRKGTITRRGSDKDSSVGSGGGLFNRNKDQSKGTIDRNKS

SGGLFDSPKKSYKKPKTRVGGGRIKRRR"

CDS complement(1864447..1864737)

/locus_tag="EFAGFIKM_01646"

/inference="ab initio prediction:Prodigal:002006"

/codon_start=1

/transl_table=11

/product="hypothetical protein"

/translation="MSKRCELCGREPVNTTVHHLTPKEMGGTFLPTANLCIPCHKQIH

SLYTNCDIVTLGLTDLQSLRQDERMIPFIRWIRKQPATTIPRVRKSNHVRKS"

CDS 1864932..1866227

/gene="moeA"

/locus_tag="EFAGFIKM_01647"

/EC_number="2.10.1.1"

/inference="ab initio prediction:Prodigal:002006"

/inference="similar to AA sequence:UniProtKB:P99139"

/codon_start=1

/transl_table=11

/product="Molybdopterin molybdenumtransferase"

/translation="MKMNSHSHDMTTAKFNRTAVQVPDAQAQIAARVTVGSIEKVHLE

SAHGRTLAETIQAPHPYPFFRRSGMDGFAIISTDTIDASSDHQVWFRVIDEIPCGYTS

DHTIVSGTTARIMTGAQVPEGADAVVMMEMTESKVENGEQWIALKRHILSGANITPIG

LEVQEGQQLLEAGTIIRAGEQSVLATFGIAEVPVFQRPKVAIFATGTELLDVDEPLQP

GRIRNSNSYMLRSLVVEAGGEPVMYGSIADDVNTARAKLEEAIQDNDIVVTTGGVSVG

DYDIMGELVLEEHVEMLFNKVTMRPGSVTTAAIYKDKLLFALSGNPGACFVGFGLFVR

PTIRSMQADAHPYMEEWTAILEEEYTKVNNFTRFVRGTTEIRNGMVYAIPAAARVDES

SVMITIKDSDCLIVVPPEKKGIPAGEQVRILKLPSGHVR"

CDS 1866234..1866794

/locus_tag="EFAGFIKM_01648"

/inference="ab initio prediction:Prodigal:002006"

/codon_start=1

/transl_table=11

/product="hypothetical protein"

/translation="MTTMSDTKPHIIQIVGYKNTGKTTLTAALIGHFSSMGLKVAAIK

HDGHDHFEMDQEGTDSYQFGEAGASAVVVMSEKRTAIMERQATKLEDMLSYLSGYDWI

VVEGFKDASYPKFVMVREEKDLTLIDQLKGVVGMISWLPSEQFIERSTVSRDITWYSV

HVTEDIAKALLAMVESECRQNGEVES"

CDS complement(1867013..1868104)

/gene="xerS_1"

/locus_tag="EFAGFIKM_01649"

/inference="ab initio prediction:Prodigal:002006"

/inference="similar to AA sequence:UniProtKB:A2RKP9"

/codon_start=1

/transl_table=11

/product="Tyrosine recombinase XerS"

/db_xref="COG:COG0582"

/translation="MTNVQKEIDRRKLDDKLPSMPWFVQQFMDYKLPDLSPSTLLEYL

RDYEAFFGWLRAEGLSEASSNKEVTLTELEVLRMDSVTAYRLFLTTKREGTNSRITVS

RKLSSLRSLFHYLSQIAEDEDFYPLLKRNIMAKIEIKRTHKPKDTAAKLKGKILEEEE

LLEFIGYILEGYAVDMEKNKQALYSHELNKERDACIASLILNSGLRVSEVVNLNVDDL

DLNNKLLYVYRKGNNDETYKTPVYFREQAKDELATYMNLRQSRYRTPKREKGLFIALR

NGDSEGSRMTKRAIQAMIMKYAKRFGKPFLTVHKLRHSFATDYYLQNDIYKTKEQLGH

ASTETTEIYAHLTDKTMSEAIERRTEDGM"

CDS 1868298..1868786

/locus_tag="EFAGFIKM_01650"

/inference="ab initio prediction:Prodigal:002006"

/codon_start=1

/transl_table=11

/product="hypothetical protein"

/translation="MNMKIELVPQERSQVIRHLMQFYLYDFTKYLNIDVDSNGIFPEY

PGLEAFWAEKDRKFPFLITSDGAPAGFALVEKLEPGSKDNDYYLTEFFVMQKYRRSGV

GTRAAYELFKRFPGRWKVTQVRNNVIAQAFWRKIIGDYTGGRFREKFHPELGNPSQYF

VT"

CDS 1868950..1869723

/gene="gno"

/locus_tag="EFAGFIKM_01651"

/EC_number="1.1.1.-"

/inference="ab initio prediction:Prodigal:002006"

/inference="similar to AA sequence:UniProtKB:P50199"

/codon_start=1

/transl_table=11

/product="Gluconate 5-dehydrogenase"

/translation="MNVPTFHLDGQTALVTGAGRGIGKAIAIGLAQSGSDVVLVSRTM

KEIEETAAYIYEQTGRKALALTADVTSKEQMEETFQEAISEMGSLDILVNNAGMNIRT

PALEVTDEQWETIMQTNLKSAFFASQLAGQHMKERGTGKIINISSVGGHTALRTGVVY

GATKAALIHMTKVLALEWGKYGIRVNSVGPWYFRTPLTEKLLDDPQYLQEIVSRTPLQ

RVGELEEVVGPVVFLASDAAGYITGQTLLVDGGMSIYGF"

CDS 1869852..1870745

/locus_tag="EFAGFIKM_01652"

/inference="ab initio prediction:Prodigal:002006"

/codon_start=1

/transl_table=11

/product="hypothetical protein"

/translation="METRKYGNTGMNVSTLGFGGAEIGKNVSKTDVATLLNSALDAGL

NVIDTAECYGDSEELIGEVLSHRRDDYYLFTKCGHAAGIEGPDWDAKVLEQTIDRSLK

RLKTDYVDVIHLHSCSEEILRQGAVIEVLQRAKEAGKTRFIGYSGDTTDALYAIQTGV

FDSLETSLNIADQEAIDLTLPEARKRNMGVIAKRPIANAAWTYDTLSEEAYPFIYWKR

LSELNYGFLGEDAQAAVETALRFTLSTEGVDTAIVGTAKPNRWQQNADLVAKGALPQE

LYDEIRARWKEIAGADWTGRT"

CDS 1871367..1872713

/gene="gabP"

/locus_tag="EFAGFIKM_01653"

/inference="ab initio prediction:Prodigal:002006"

/inference="similar to AA sequence:UniProtKB:P46349"

/codon_start=1

/transl_table=11

/product="GABA permease"

/db_xref="COG:COG1113"

/translation="MKSQQHRKNDDEKLSWVSLSLFGAGCTLGTGFFLGTSLAIHWAG

ISVLILLVLAAAGTYFVFGALAQMTADDPKEGAFRTYAGEAYGPWAGFGSGWMYWFSE

MLIMGSSLTALGLFSQFWFPKFPLWAFVAIYAAIGLFIAALGAKGLTKTENILAVIKL

AATVAFIAIAGAACLGWLPVKVTPEQLKNEWFPHGGKGVWTGFIYAFFAFAGIEVMGL

MAANLKEPKDAVKSGRVMLLSVSTLYVISIALILLLVPSAQLTPDESPLVKAMSTIGF

TVVVHILNGILIIAGFSTMVASLYAITSMLVKLAEDGDAPKLFTKTWGKKDMPLQALC

CTAAGLVLSTLMALWMPKSLFEYVTTAGSLVLMYTWLLICITYIKKMKPVKWNRIKVW

IAIVLIVAAVAGTAMEKASRTGLLASVGLLVFLGIITYVVHKVRGSKTPQSAPSKP"

CDS complement(1872770..1873531)

/locus_tag="EFAGFIKM_01654"

/inference="ab initio prediction:Prodigal:002006"

/codon_start=1

/transl_table=11

/product="hypothetical protein"

/translation="MAELYSADEINTLFERLPSWQQDAYSQFADMIADENNTYPCVPG

RMGFLSGHLRYGFTCDPRSEHAAEDMADLLRQYGPVSRDTGHYASMVVICETPSDLKD

HTTVEQYQTLFWQMLNRVSSHDTEPWPEHISTDPHDSSWEFCFGGEPYFCFCATPAHE

LRASRHFPYLMFAFQPRWVFESINDNTPLGRKMKTLIRKRLAAYDAVPAHPSLMWYGQ

QDNLEWKQYFLPDDEQTPSKCPFMRMKQAIGKLTK"

CDS complement(1873614..1874240)

/locus_tag="EFAGFIKM_01655"

/inference="ab initio prediction:Prodigal:002006"

/codon_start=1

/transl_table=11

/product="hypothetical protein"

/translation="MSVLFSYIILGISLSAPIGPINAAQLDRGARHGFMHAWILGLGA

MFADLVYMLLIYFGVAHFLDTPFMRSFLWSFGSFILIYTGVETLSKLKQGIQGTSTAE

ATVRKSFISGFLMALSNPLNILFWLGIYGSILATTVKHTDMAHLLLYSSGIFIGILVW

DVIMAGMASRFHKHSSENVLRWISIISGICLIGFGLYFGYEAVRSIFF"

CDS complement(1874357..1874974)

/locus_tag="EFAGFIKM_01656"

/inference="ab initio prediction:Prodigal:002006"

/codon_start=1

/transl_table=11

/product="hypothetical protein"

/translation="MNSTESHKQSIHIPAKTGYALKVKKGSLIRITDLEGQQVVDFVA

YDAQNINNRLDPGVTLDVLRNYRIKPGQCLYSNRYKPLISIISETVGIHDFFNSACRP

EMYEVLYDKKDHPSCYDNLNTALEPFGISPPDQHYPFNLFMNSVVDGEGKIDVIAPES

CAGDYVEMEALMDLIIGLSACPCEESSCNGYHCTPIQLDVESPAV"

CDS complement(1875123..1876616)

/gene="nylA"

/locus_tag="EFAGFIKM_01657"

/EC_number="3.5.2.12"

/inference="ab initio prediction:Prodigal:002006"

/inference="similar to AA sequence:UniProtKB:P13398"

/codon_start=1

/transl_table=11

/product="6-aminohexanoate-cyclic-dimer hydrolase"

/translation="MSSFSYTSYDAVGLAELIRSREVSPVEMVEAAFARLEEVNPQLN

AVIRTYENRARVEAGLVRPGEQPFAGVPLLLKDISQSLEGEFLTSGSRLFSEHRALRN

SNFVTRLRDAGFIFIGHTNTPEFGLKNITEPRLHGPTRNPWNINHSPGGSSGGAAAAV

ASGIVPLAGASDGGGSIRIPASFSGLFGLKPTRGRTPVGPGVGRQWQGASIDFALSRS

VRDSAALLDTLQVIQPEAAFHAPLFPGSYLADMSYPHQRKLKIAYTTDSPVGTPVSAE

AKESVYKLVRWLEDQGHHVEEKLSPVNGVRLMENYYMMNSGEMAAMISSMERSMGRAL

TSDDMEIESWVLAEAGKKVSAAEFVHSLAEWDVAAAQMSTLFERYDFYVTPVNAFPAP

KIGELTPHDEQIRNLMRISELDKTQQQLIYEMFEPSLTYTPFTQLANLTGQPAISVPL

HMTPEGLPMGVQVMATKGREDWLLHLAGQLEQSDLWIGMRGNPLFPA"

CDS complement(1876934..1877914)

/gene="impX"

/locus_tag="EFAGFIKM_01658"

/inference="ab initio prediction:Prodigal:002006"

/inference="similar to AA sequence:UniProtKB:D5RAW5"

/codon_start=1

/transl_table=11

/product="Riboflavin transporter ImpX"

/translation="MEKTSATSTVYRKERSNTGFWLVVLGAALWGVDPLFRIILLNTM

TSTQIVLVEHIIVSLIAIPVLWKFRADLKNLRARHWIAVIFISWGGSALATVLFTMAL

THNDPNTVLLLQKMQPLFAIVLAKLLLKETLPRRFGGLFFIALAGTYLLTFGFTLPLG

NWDNWIHAGSLLSLGAAALWGGSTVMGRLMLGQARYETVTSLRFVVALPLLIFMTWNE

GAAWTFPSGTGEQTAVILNILGQALLPGLLSLLLYYKGLSSTKASVATLAELSFPMAG

VLVNWIAFRTLITWEQLLGFILIWVALFAISRQQERSSTVADAAPKLRTE"

CDS complement(1878297..1878797)

/locus_tag="EFAGFIKM_01659"

/inference="ab initio prediction:Prodigal:002006"

/codon_start=1

/transl_table=11

/product="hypothetical protein"

/translation="MTKTTALDVLLIQKDDTWDQCNWIVPLSKSLEGLTAEQAAWIPP

SGGLSIWQLVNHMYYYNHRLLCRMQGKEPTLPAVDSNEYTFGNPGDATDAAGWNTLLQ

GTTRLAQQLRDQLAALQESDLEAAYMDSEEKWAHELARWVLHDAYHAGQIVLLRRQQG

SWKIVF"

CDS 1878941..1879492

/locus_tag="EFAGFIKM_01660"

/inference="ab initio prediction:Prodigal:002006"

/codon_start=1

/transl_table=11

/product="hypothetical protein"

/translation="MDRLWTDFVNSDYHDWRGGDRSEDRLGKASWQQDFLERWQLNAS

VPASPEDELSMTNFRNELLALGVRVSSGASLTDIEKQWLNGVMEAGHVRRILTTIDQE

LKLQLVAVEAHWHQVMAEVAADFATTLVEGEGRRIRICDNSDCRWMFYDDTRSRTQKY

CDDKMCGNLMKVRRFRAKRKSQN"

CDS 1879613..1880983

/locus_tag="EFAGFIKM_01661"

/EC_number="3.5.4.32"

/inference="ab initio prediction:Prodigal:002006"

/inference="similar to AA sequence:UniProtKB:Q9I6Z0"

/codon_start=1

/transl_table=11

/product="8-oxoguanine deaminase"

/db_xref="COG:COG0402"

/translation="MRTSYLLKNGCVLSMDAKIGQYKRADVLIQDSLIMAIQPDLDIP

DAEVIDASSMIIMPGLVDTHRHMWESLVKTAGTNWSLPVYLQNLYYGAMGSKLRPQDS

YIANLLGSLEALNAGVTTVLDWSMPYSPDHTDELIRGLQDAGIRAVFAHGVPGETDYW

NRDSQLTYSNNDVRRVKERYFSSRDQLLTYGLAIRGPEFSHWDTTVKEIELAQELDAI

CSMHAGFGSWGSVDRSISHLYEAGLLSPRVNIVHGNTMGMDEYKMLADSGASLSVTPE

VEMMMGHGYPATGYFLEHGGTPTLGVDVVTSTGGDMFSQMKFALQAERSRANEQLLQQ

GEMPGELNLQSSQVLRFATSAGAQALGLEQKVGTLTPGKEADLIMIRTTDLNMFPVHD

PIGAVVQFANPSNVDTVFVAGRLVKREGKLLNVDLDAIRHTAMQSKDYLLTQYRMSDA

ERITFS"

CDS 1881503..1882939

/gene="yhdG"

/locus_tag="EFAGFIKM_01662"

/inference="ab initio prediction:Prodigal:002006"

/inference="similar to AA sequence:UniProtKB:O07576"

/codon_start=1

/transl_table=11

/product="putative amino acid permease YhdG"

/db_xref="COG:COG0531"

/translation="MRENKQRSSLFRKKPIASEGNNSSALKRALGPLDLTTLGVGAII

GTGIFVLTGVAAATYAGPGLVLSFLLAGIICAFAALCYSEFASSIPASGSAYTYSYTA

FGEVIAWVLGWDLILEYGFASAAVASGWSGYFQTLLSGFGLELPHALTSAFSLEKGTY

FDITAAVITLIITFLLTRGVKEAARANGIMVAIKIIVVLIFIGVGVFYVEPTNWQPFL

PFGISGVTAGAATVFFAYIGFDAVSTAAEEVKQPQRDLPIGIIASLAICTVLYIVVSL

ILTGIVPYNMLNVSDPVAFAFEFVQLKGLSWIVSLGAIAGITTVLLVMMYGQTRLLYS

MSRDGLLSPVFSKVSGKSQTPAVGTWVAGIIVALFSGFISLGHLAELTNIGTLFAFAV

VSLGIIVLRKNNPDLKRGFRVPLVPLIPILSALGCVYLMTRLAALTWITFFAWLIIGL

IIYFAYGRHYSHLNPARRISSAFRAKDK"

CDS 1883198..1884277

/locus_tag="EFAGFIKM_01663"

/inference="ab initio prediction:Prodigal:002006"

/codon_start=1

/transl_table=11

/product="hypothetical protein"

/translation="MNTPLHTNSDHQNAAFGFALADSSVLAEAQLIVSQSGGAGDLQL

DIDPQRFLKDGRKVSVIAQQLDSPVNRQDANIIYGQELAYVQYAVNLKPDSTISIASI

EGVEQSVNLGWAAFQEGEYELRISMHMKTPRIAEGTLEPEQLAMVKYAQVITVYISLF

PAEAASLSSPSQAVWSRNHHVFDSYGRGGFILADLPRLAERVEELMGLGNHNLIEQFA

EGDLSNTLLEEGMMAIAWGVTPWCYSLYSAPDEQSARILAVDKLGDEPERKGIYRLDP

SIQQLSIVPANELAYWPACVQNDWPVIDVAGEGETLHMDLYTQICESVNGLHENPLPS

FVLTRSQGKPEAILPLINVVIVHEA"

CDS 1884553..1885473

/gene="xerC_1"

/locus_tag="EFAGFIKM_01664"

/inference="ab initio prediction:Prodigal:002006"

/inference="similar to AA sequence:UniProtKB:P0A8P6"

/codon_start=1

/transl_table=11

/product="Tyrosine recombinase XerC"

/db_xref="COG:COG4973"

/translation="MMTEEIQEQYADELEAFHIWMKDAGYTGHTVKSYTRDVVEFLIS

IQGKSLEQVKKLHVLSFLSRARERGVSDATRNRKHAAVNCFYKSLIELELLTNNPAFG

IKKSKTEKNRAPVFLDESGLTRFLESVEGKYRTRNLAVFLLMGYMGLRVGEVHALDCK

DYNAERRTLDVFGKGRKWRSLPVPETVANVLSQAMAERLDPWRPKEEALFVSQKGKRL

SIRSIQLISTETFERFQQESPVNQRQNYSSHKLRHSFATMMLRRGADLRTVQELLGHA

SIQTTTVYTHVTSREKEEAMALLDVKLPTY"

CDS complement(1885747..1886676)

/gene="ldh1"

/locus_tag="EFAGFIKM_01665"

/EC_number="1.1.1.27"

/inference="ab initio prediction:Prodigal:002006"

/inference="similar to AA sequence:UniProtKB:P14561"

/codon_start=1

/transl_table=11

/product="L-lactate dehydrogenase 1"

/translation="MTNSAALKPSRVVIVGMGAVGTTTGYTLMLRQRSSELVFVDVNH

DKATGEMLDMNHGLPFTGGVKVWAGDYSDCKDADIIIITAGASQKPGETRIDLLKKNA

SIFKDIIERITEVNSHGILLIATNPVDILSYTSWKQSGWPASRVIGSGTLLDSARFRY

LIGKNKGIDPRSIHAHIIGEHGDSEVPVWSLANVAGTDLELDEETQQDIFDRTKNAAY

EIINAKGATSYAIALALDRIVAAILGNEGSVLNVSTYLEDYNGVSDVYLGVPCVVDRN

GVREILPLPLNETEKVAFQASANKLKEQIAGLE"

CDS 1886975..1888291

/gene="hcf136_1"

/locus_tag="EFAGFIKM_01666"

/inference="ab initio prediction:Prodigal:002006"

/inference="protein motif:HAMAP:MF_01348"

/codon_start=1

/transl_table=11

/product="Ycf48-like protein"

/translation="MRACILDERGGTHLRLQWIKIAQTALLSIGIAAVLAACTDSDQP

PVAEPPQQQEDAGNTGQTLTVVPPVNSTESADMAKYQIQTRLTDFQLLNDNGGLAWGV

TRNALRLYYTQDQGKTWTNISPSENVQFPANPKYGQSIYFVDRAHGWIVREGMGGTDT

MVLRTNNGGVTWSLSSLSKTDKVTAISFVSPEKGWILTTVDTAIGKQEKKLYFTEDGG

ITWNRMSSSNEDGKQEAEEISKRGYTTGMTFSDPKHGFLTALEFGTPKLYVTSDGGEN

WDTGPSFFDRNKFNGCGNFSVSSPQFFGREAQSAWMSMSCSKGESTTFNGFFTTDGGK

SWKLSNFTLNKQTGANRNLSPVFLNASEGWAMQKGITYHTKDAGKTWSALPASSVLEK

ILEDYPEIVKMQMVTPNLGWILVENTDAKRSLLLQTVDGGVHWKVL"

CDS 1888438..1888647

/locus_tag="EFAGFIKM_01667"

/inference="ab initio prediction:Prodigal:002006"

/codon_start=1

/transl_table=11

/product="hypothetical protein"

/translation="MRTNGQTQFTQADELTRYMLQLCYTCDDANRCTTEEAVKACMAE

HAQADQPEPAEGGEVTRGFMDLMYA"

CDS 1888878..1889351

/locus_tag="EFAGFIKM_01668"

/inference="ab initio prediction:Prodigal:002006"

/inference="similar to AA sequence:ISfinder:ISDha13"

/codon_start=1

/transl_table=11

/product="IS200/IS605 family transposase ISDha13"

/translation="MANKSYSLAHTKWMCKYHIVFTPKYRRKEIYNQVRKDLIEIFKR

LCKYKGVEIIEGHMMPDHVHMLVAIPPKIAVSTFMGYLKGKSSLMIFEKHAQLKYKYG

NRKFWAEGYYVSTVGLNEATVRKYIREQEAHDQALDKLSVKEYEDPFSSNRSKKK"

CDS 1889627..1890049

/locus_tag="EFAGFIKM_01669"

/inference="ab initio prediction:Prodigal:002006"

/codon_start=1

/transl_table=11

/product="hypothetical protein"

/translation="MTTFLDARTSQNASLANSIAIPITVLNTPQLFGQIGLQTAGAGA

NPRVQFKGTVSVQLPLALVGITITIVRGTLSTDPVIYSATSTFGLSVLAPQIITFSAD

DFNPPITPQLTYTAFVSSNLLGTVRVGPENFDGLVVSD"

CDS 1890667..1892460

/gene="dap_2"

/locus_tag="EFAGFIKM_01670"

/EC_number="3.4.11.19"

/inference="ab initio prediction:Prodigal:002006"

/inference="protein motif:HAMAP:MF_01960"

/codon_start=1

/transl_table=11

/product="D-aminopeptidase"

/translation="MKINQRYYYVYKAITCFLSLALIIMTCAVPVNAEVGNVETTPSR

IALSSLEETIDSYVATNQKNTAAVSVVAIKNGETIVNKAYGYADFELQRKADTSTVFE

WGSTSKLLVWTSVMQLVEQGKLDLDTDIQNYLPEGFLKKLEYDAPITLLNLMHHNAGW

EDRLIDLWYSSESDIVELGEALQKFEPRQIDKPGSVVAYSNYGTAMAAYIVELQSGQP

FYKYVNEHIFKPLNMKDTSIHPSQYDNLNVLKRRNEIQGYTTRLKLIPKNRAYISLFP

AGGAMGTAEDAARFLAALMPVDRSNLLFENNDTLDEMLSTSLYYDGTSTPRIAHGFFE

VEYWVPALEHAGSTAGFTSKFVFDPESNFGFVVMTNQSNEMAYNVGLVQKVFGSSFST

NSVESESGGYYLPARRVVSGFTKAYSMLSLQKYTTFDLSNFASVVERNGTVEKVSISY

NDFLPVSNLTINLIKASFIALAVAILLSLRAIIGYFIRLSKYKLKNWERPGTGFDKYH

IVINIASMVLILNTILLFMRALGFSPYSSLRIHLILNLVYVILAVAYLVLLFYKLLKR

SYSKKQKAVYIMSGMSAFLFAAFIMGWDLYF"

CDS 1892518..1893297

/locus_tag="EFAGFIKM_01671"

/inference="ab initio prediction:Prodigal:002006"

/codon_start=1

/transl_table=11

/product="hypothetical protein"

/translation="MNIQDLPIEIKEHIGEVQKITFPKQGHTSTVAILDTFDQKYIIK

KTENDLYNEWLSDEYKVLQYLYHTGLPVPKAYSFHIEDTSRWLLMDYIDGISLREFLS

KMPDLQSKEKAIFNFGLCLKKIHECSCPVELLNNNTPWLDTMLNKAEYNLTHFAVDGS

EELLQQLKEGRPKPIENTFIHGDFTIDNVLVNDCNIVGVIDWAGATYGDPRYDVALAI

RPKHNAFDNERDREIFYNGYGKLKITDEEYNYYEDGIYNFF"

CDS 1893355..1893780

/locus_tag="EFAGFIKM_01672"

/inference="ab initio prediction:Prodigal:002006"

/codon_start=1

/transl_table=11

/product="hypothetical protein"

/translation="MSPPKHILSAAAIVINEQNELLLIRGPRRGWEMPGGQVEEGESL

SQAAIRETKEESGIDIEIIKFCGIFQNIENSICNTLFLAKPIGGELTTSLESLESGFF

PIEEAILKVEWKNFRERIEYCLKPEMQPFCIEFSDKNNI"

CDS 1893900..1894970

/locus_tag="EFAGFIKM_01673"

/inference="ab initio prediction:Prodigal:002006"

/codon_start=1

/transl_table=11

/product="hypothetical protein"

/translation="MSSGKKSKRLDTVRSLVHEMLLEESNNKEGMLVHLYGVSNFASM

LAMKRVQDSEITAIAGLLHGFYFYKTGIEDFPGPNSADAVRPILRSAEIFTTDELSII

LKSIFYQEDTHLVHGPYEEVIKDAILFQIYFQNPNRNLSKAEISRIQNVFTELGISKE

NIKADVNVNVQTVHINTEERRSKLADLAETLARQNIIGTSGDQRYQEICRYWPDSDIC

KVLEANWCAAFVYHCCMKVGIKLPIRYPNRMYRLAGVGAWLDWAQLAETEFFYQDMQE

GFEPERGDIVIYEKLLTDNSHDHIGIVLACDGDQLLVAEGNTDNKNYSSVLYRERGHC

ILGYIRVDESYQFNFQGEYRPT"

CDS 1895066..1895488

/locus_tag="EFAGFIKM_01674"

/inference="ab initio prediction:Prodigal:002006"

/codon_start=1

/transl_table=11

/product="hypothetical protein"

/translation="MKRVDVVYSLLTNPSKSKVLAVRNVGRSSWSLPGGAVEADESWE

QAAIREAKEETGLDVGVHGIVAINECKFEEIKEHAIFITFRAEIVGGREEISRPDEIF

EIAWLDITQADELMPYYKNGFKSLLNGNEITYFNEGNK"

CDS 1895523..1896269

/locus_tag="EFAGFIKM_01675"

/inference="ab initio prediction:Prodigal:002006"

/codon_start=1

/transl_table=11

/product="hypothetical protein"

/translation="MNKHSVYETNSFYWDTKGNDFLSAIVLPNYGAFISEEKHQLFGD

VSGLKMLEVGCGNGQSLQYHGDRKAGELWGMDISQKQIEKTQQHLRTYGISATLICSP

MEEECGIPTNYFDAVYSIYAIGWTTDLEGTFSRIASYLEKDGIFIFSWSHPIHRCVVE

ENDRFVFNKPYSDESCYAVSPDFCHGELTLADRQLSTYINALAKAGFVIEQMIEESDE

DIMQLHNNSDSLLKRAKMLPVSFVIKARKR"

CDS 1896403..1896897

/locus_tag="EFAGFIKM_01676"

/inference="ab initio prediction:Prodigal:002006"

/codon_start=1

/transl_table=11

/product="hypothetical protein"

/translation="MRRLPLFVVTGASGTGKTTISSLVRKQLPEFDVFDMDIIDNVDW

QVAKENWLRIAYNISLSGRGTVLCGTMVPENIASSDYIDRFDRILYMNLHCDDVTRET

RLRARGWDVNLIEDHKSFANWLLQNSITAFDPPMQTIDTTELTAAGVAEQIKEWVLKN

WCEE"

CDS 1896941..1897483

/gene="ydaF_2"

/locus_tag="EFAGFIKM_01677"

/EC_number="2.3.1.-"

/inference="ab initio prediction:Prodigal:002006"

/inference="similar to AA sequence:UniProtKB:P96579"

/codon_start=1

/transl_table=11

/product="Putative ribosomal N-acetyltransferase YdaF"

/db_xref="COG:COG1670"

/translation="MLPFQVNESIVLKLIQSRDRDELYALIDENRKYLRAWLLWVDKR

QSPSDLDSVIEVWTHNYEEKNGFDSGIWFNEQLVGMLGLHYIDWNNRATSIGYFLAES

AQGKGIITKSIEQLLKYLFNELKLNRVIIQCAENNFKSRVIPEKIGFSNEGTSREAQW

VYDHYENIVTYSLLSSEWHT"

CDS 1897526..1898119

/locus_tag="EFAGFIKM_01678"

/inference="ab initio prediction:Prodigal:002006"

/codon_start=1

/transl_table=11

/product="hypothetical protein"

/translation="MFLNVLLWLIILWVISLYFSFPKKDRMYTVNVPSTYYIQSSNRL

DIQKNYECAAFSSAFVLRHFGLESKGTKLYETYPRKLLDGTVYPKAVVVFFRKLGYQA

MYLRGNVNTLKKQISQGVPVILFIRVHPKQRYLHFVPVVGYDETHFYLAESLDHKINC

DEEYYNRKTSINELNMLWRSWLPFSQNSYIVIHPMQI"

CDS 1898436..1899512

/locus_tag="EFAGFIKM_01679"

/inference="ab initio prediction:Prodigal:002006"

/codon_start=1

/transl_table=11

/product="hypothetical protein"

/translation="MSYSEVKKRMKRKKATFHPCKVKRKERRLKVVKKFVQVKCPPPE

VNISTPTVVGPTGPQGIQGVQGIQGIPGKQGVEGPPGAQGPAGGPPGPQGPQGQPGPA

GAIGSQGPVGPAGPMGAQGIPGEAGAVGPQGPQGNPGPAGAIGPQGFPGPTGATGPAG

ATGVIGTTGVAGPAGTTGVTGTTGVAGPAGATGVTGTTGVTGPAGATGVTGATGAAGG

VLGFADFFALMPPDNAATVAPGADVNFPQDGPTSGTTISRTGPSSFNLAAIGTYLVMF

QGSMNEAGQLMLTLNGADLSTSVVGRATGTSQMPGMALVQTTVINSILTIRNPAGNST

ALTMTPLAGGTRPVSAHLVITQIA"

CDS 1899766..1900335

/locus_tag="EFAGFIKM_01680"

/inference="ab initio prediction:Prodigal:002006"

/codon_start=1

/transl_table=11

/product="hypothetical protein"

/translation="MKNGHWRKITWVVAALLLIMITACGNSKFNPNEFSEQDLAVTKT

DSEDKITYGMSQEEAEKILGEGTSTGVGQSYQYNNGITIGYRDAKVVSIILNKDSKGY

FKTAREAEAGMTHQEILDLYGKEHVLVESERNIDYAYHIQKETFLDEKSLQAEKDDLA

IYRASFMLTKEGQVNQVILSDQRFATTFK"

CDS 1900544..1900936

/locus_tag="EFAGFIKM_01681"

/inference="ab initio prediction:Prodigal:002006"

/codon_start=1

/transl_table=11

/product="hypothetical protein"

/translation="MGIESSWMFDLFGTVFPVVFVLIIGIVLLSMGKEVWRWGRNNSE
PLLTVPSRITSRRMKMSQSQSEPGSTARTLYYVTFEVESGDRLEFKVNGEEYGLCAEG
DEGRLSFKGTRYVGFERYNRVYSERLRG"

CDS 1901084..1901833

/gene="gdhIV"

/locus_tag="EFAGFIKM_01682"

/EC_number="1.1.1.47"

/inference="ab initio prediction:Prodigal:002006"

/inference="similar to AA sequence:UniProtKB:P39485"

/codon_start=1

/transl_table=11

/product="Glucose 1-dehydrogenase 4"

/db_xref="COG:COG1028"

/translation="MSFTDQVVVVTGAAQGIGRSVAEAYAVAGAKVVLADYKEAEGAA
AAASIRNEGGEAIFVQCDVRSEQDITNLFRTTVEEFKQIDVLVNNAGLAKWKSPYELT
LEEWDDVLNTNVRSCFLASREAAKHMKSNEHGGAIINMASTRALMSEPDTEAYAASKG
AIVALTHALAVSLGKDQIRVNCISPGWIETGDVEKLKKEDHEQHPSGRVGVPSDISRA
CLYLSDPSNTFVTGTNLVIDGGMTRKMIYED"

CDS 1901978..1903723

/locus_tag="EFAGFIKM_01683"

/inference="ab initio prediction:Prodigal:002006"

/codon_start=1

/transl_table=11

/product="hypothetical protein"

/translation="MFGKQSRKVFWLLLLIGGMIMIGNESPAEADGADIRAAWVWQAQ
SVSNGDELLANAAKHKINRLYVNVDLTLSKEVYQTFIAKASRAGIAVEALGGDPSWAL
SGREGPMLRLASWVSDYNQAAEPNEQFDAIHLDIKPYVLPAWKEDAKPLVQSWVANMN
LLFEQVRQDGAVTVNIDLPFWLDSYTVTGNRVSEDADNEPLSRWFIEQVEHVTLLAYR
DNAQGNNGIIRLIEQEMNWADASNVSVTVGLNTKPMPGEEFTSFAGKGAAQLESVIEE
VASAFSEHNSYAGSAVHDIVYWGQLEPSELPSPENPSNQPEIRGTYIWEATQVTNDGG
EHILAFAREQNINWLYVRLDLDQPYSSYRSFVKRAKAQGIEIHAMGGHPIWGKKENRP
RIKRLIDYVKNYNAESEPDERFEGIHLDIEPYTLPEWENSRDTLLTEWAANITFFQEE

TKKDSDLETSADLAVWLDSFALPGKDTSVAEFMIRTLDHVSLMAFRNNAEGSNGIAAI

VSQEMEIADRFGKRLMISVEMKQNHEGPHISFYEKGAAEMELQLAKLPDLLAEYQAYQ

GNIVHAYDYWIEAKP"

CDS 1903689..1904306

/locus_tag="EFAGFIKM_01684"

/inference="ab initio prediction:Prodigal:002006"

/codon_start=1

/transl_table=11

/product="hypothetical protein"

/translation="MPMIIGLKPSLKQASIIRNELLRDFTLGEVPGFFLWKKDGIVSP

NGLLHNTMYGDEQLKQGRKQTMAVIKANSEDVKLLARLMRAEAEGDGEQGMLLVGNVS

VNRVLVDCLDFKDIRDINRMVFQNPGGYESTQKGYFYQRARQSEIRLAQRVINGERFW

PASNSLWFFRPVAECPATWYDQQNTGRFKAHCFFSPSGEDCPEVY"

CDS 1904325..1904840

/locus_tag="EFAGFIKM_01685"

/inference="ab initio prediction:Prodigal:002006"

/codon_start=1

/transl_table=11

/product="hypothetical protein"

/translation="MINQPYQPTTQTLGQQANSVMGQQANSQVQGTSYKIGNGMPAMS

PTPGMVSPSSTSVPPLVSSGSPMTPTGAVITTTAPQFEQSYIENILRLNLGKFGTFYM

TYEGNKEWNARIFQGIIEAAGRDHIIISDPKTGRRIMLLMVNFDYATFDEPLLYQYPG

VVGNYPQAPSR"

CDS 1905067..1906650

/locus_tag="EFAGFIKM_01686"

/inference="ab initio prediction:Prodigal:002006"

/codon_start=1

/transl_table=11

/product="hypothetical protein"

/translation="MNSWIEGAMKGETEAYAQLMSQYRGMALAVAYNRLGDTFWAEDV

VQEAFTEAFGNLSKLEAPEAFPGWFKVIVERQCYRWLRRKQHTMIPVQELDHVFHEED

QAHNPEKQAVQNEMHRTLRDSIAILPSSMRIAVELFYLEGYSLKEISDFLGVKVPALK

KRLFDARSKLKRSMPVRDLVSVFSDLYEGGKGLLHIMNGDHAANRLRESGIQGDILVW

RELYTFGPVAKEMGDTKERQNRASVLEQQLGIPQAEYLKIKELERKLHSFQQYKEIVL

WFEYDLYDQTMLSYLLHYFKGQALQNTKLNLLCIDSYPEIEHFRGLGQLTSTQIERLS

GSWHVIEKNELQAGAQFWEAYTSTDFRHHLDYLQADTSALPFAKAAFKAHLSRLPSVS

NGLGLIEQATLETIRAGVEHPYPLFREVGDKLHILGMGDLEYWAHLKRMTEGPHALLQ

MSGATTFPNFKQHDEKFRDGVLSLTALGIQVLNGEVDWALLKQDEFWIGGLHNESGER

AKWRWNPASETLVEIESTS"

CDS 1907026..1908342

/locus_tag="EFAGFIKM_01687"

/inference="ab initio prediction:Prodigal:002006"

/inference="similar to AA sequence:UniProtKB:P9WFP3"

/note="UPF0053 protein Rv1842c"

/codon_start=1

/transl_table=11

/product="hypothetical protein"

/translation="MSDPLPSILNLVIIGLLVLMNGFFVSAEFAMVKVRGSRIEALVE

TGNKNAIYASNIVRNLDAYLSACQLGITLASLGLGWLGEPAIAHLLEPMFTAFGLGPV

YVHGISIAIAFVIITILHIVLGELAPKTMAIRKSETITLWSAALLTFFYKLMYPFIWA

LNGMANGLLRLFRMAPASEMDSAHSEDEIRILMKESNKSGLIDNTELALVDNIFDFTD

TTAREIMIPRTEMICLNANESMLENLEIASESMRTRYPVYNGDKDHIIGFIHIKDLMR

SQLTDTISVIRPILAVPDTTLISDLLKRMQRSKTQIAILIDEYGGTSGLVTLEDIMEE

IVGEIQDEFDHERPVIEQVDEMEYSIDGLMLIEEVSERFGLEMDRADYDTIGGWLYSR

VDAIPPEVGQSVEYGGYVFVIQETEHKRISRVNVIKLELLVEEEGA"

CDS complement(1908412..1909062)

/locus_tag="EFAGFIKM_01688"

/inference="ab initio prediction:Prodigal:002006"

/codon_start=1

/transl_table=11

/product="hypothetical protein"

/translation="MSKFQFKSRRHRHSSNPNELHIEVSGSFKRWLVILVSALTAGTL

IYFLATARESWISMGIDRYVLPFMIWGLVLSPILAFLFLKQSGRTLSHVKFLRVFGIA

FCLLPLVLLFYVLPVTLGMVNDHLTQFKKNPVLSTTKVGDYNSYEVTFVDTPMYAIIT

IKTPTTTQSTRKKLNIQYDLAPGENAYVTYKRISIENCLVTENRYADIVIHAPKES"

CDS 1909295..1909807

/locus_tag="EFAGFIKM_01689"

/inference="ab initio prediction:Prodigal:002006"

/codon_start=1

/transl_table=11

/product="hypothetical protein"

/translation="MRKMKTKSAASMLKITLVALLMVPTILAIPTSEPAVASPMINVS

SGALTAPPPPEPSIHSLATTRELGHIYQAVGAVIPGGGLSAIVNTKTMTYTAMKTLSV

DYRLERWTGTAWVTFKTNSNNATNTNLLNATSDWTVMPGYYYRVTSIHTAYDGSTTDT

SKHVSGTVLF"

CDS complement(1909810..1910772)

/locus_tag="EFAGFIKM_01690"

/inference="ab initio prediction:Prodigal:002006"

/codon_start=1

/transl_table=11

/product="hypothetical protein"

/translation="MEALSKGKSDKNVERFDSELEDEYFDAAFDLAFDEAFHQAVSAP

SASHTESMQQSWQKVNREINRIGLRKKRIRTLRLSVVVAASVTIGAVIFSLPSGTQAV

SPFVQSIKDWGNGMKSIVIEDRTTHLGADPSTAKTPPPPETSLDDILKVQEEEALNTP

SYELSSHLFRPVLVTEEIARDGFMGDFLLSKAIPERFNKVKFELVIDTENPVNPDDYY

ESTQMRVQYTSEGKSIEDEIIQFDYVHVMPGEKIEGAMLRDMSTVKLADGSEALMYIG

PPYNSFQWMMGSNNMSLFGTLSEEEMTAIANDFQEQKFPGSTRR"

CDS complement(1910741..1911331)

/gene="algU_3"

/locus_tag="EFAGFIKM_01691"

/inference="ab initio prediction:Prodigal:002006"

/inference="similar to AA sequence:UniProtKB:Q06198"

/codon_start=1

/transl_table=11

/product="RNA polymerase sigma-H factor"

/db_xref="COG:COG1595"

/translation="MANRLQLLLASDFHNLGSALQEEVYYEYYNMVHGLIVYIIKERA
AAEDIIQEAFIKIIKNKPIFEDEVKLKAWLKVVTRNTAINYLRKNKNNRNQLDTDSVF
IDMETMNQTAASVESMVETQMMEESIEYYLRQLKPEYRVLVELRWKKGLSYREMAELL
DTSEDIVKQRLFRARGSIKKKLHKEWGGSIEQRQVR"

CDS complement(1911578..1912810)

/locus_tag="EFAGFIKM_01692"

/EC_number="3.4.11.-"

/inference="ab initio prediction:Prodigal:002006"

/inference="similar to AA sequence:UniProtKB:P24828"

/codon_start=1

/transl_table=11

/product="Aminopeptidase 2"

/translation="MLSFEQKLDRYAELAVKVGANVQPGQVFVISAMIDTAEFVRLLV
RKGYEAGAKQVIVKYGDETVNRLRFEMAPEESFQEPPKWHAAELEELAANNAAFLTVL
SSSPDLMKGIDPERISTHQRTNGQAMAKYRQYQQADKMSWTGVACPSPDWAAKVFPDL
PAAEQVSQLWEAIFAAVRADLEDPVAAWEQHIERLETKAVALNNKKYQALHFLSLGTD
LTVELPEGHIWAQAGSVNEQGTPFVANIPTEEVFTAPAKHGVNGKVSSTKPLSYGGSI
IDRFSLTFENGRIIDFHAEEGQDTLERLISMDEGSHYLGEVALVPFHSPISESGILYY
TTLYDENASCHLAIGSSYAFNIEGGKTMSPEELAARGMNTSITHVDFMMGSPETDIYG
ITANGEREAIFLKGDWAF"

CDS complement(1912901..1914133)

/locus_tag="EFAGFIKM_01693"

/EC_number="3.4.11.-"

/inference="ab initio prediction:Prodigal:002006"

/inference="similar to AA sequence:UniProtKB:P24828"

/codon_start=1

/transl_table=11

/product="Aminopeptidase 2"

/translation="MNNFETNLQQYAELAVQVGVNVHPGQTLVVNAPISAAHFVRLIV
KAAYGKGAKLVKVNWSDETVTRLHYDLAPDEAFSIEPKWFAAEMTELVEEGAAILHVI
AENPDLLNGVAQERIITSQKVRGKALEKYRSYQMADKFSWSIVAVPSPEWAAKVFPDL
PEAQQVDRLWDVIFKTVRIGEQDAVAEWKTHLQNLDSRADLLNNKKYKKLHYTAPGTD

LTIELPEGHIWVSGGSVNEQGHVFIANMPTEEVFTAPLKTGVNGTVRSTKPLSYGGNL

IDGFSLTFENGRIVDYTADQGLDALKNLIEMDEGAHYLGEVALVPHQSPISDTNILFY

NTLFDENASNHLAIGNAYAFCLEGGKTMSKEELIERGMNSSLTHVDFMIGSGEMNIHG

VTSEGAEEPIFLQGNWAY"

CDS 1914384..1914650

/locus_tag="EFAGFIKM_01694"

/inference="ab initio prediction:Prodigal:002006"

/codon_start=1

/transl_table=11

/product="hypothetical protein"

/translation="MNIEIISAEMRREGEKGYVGNTVYRTEGEKSVYEITFMSKNGKD

WDYSLHFTEQSGDEEELLRMDELLENDDDLYNQLLDAALEAFPA"

CDS 1914831..1915640

/gene="yflN_1"

/locus_tag="EFAGFIKM_01695"

/EC_number="3.-.-.-"

/inference="ab initio prediction:Prodigal:002006"

/inference="similar to AA sequence:UniProtKB:O34409"

/codon_start=1

/transl_table=11

/product="putative metallo-hydrolase YflN"

/db_xref="COG:COG0491"

/translation="MASDNLITEGLTGLEEVAPDILSLRTLFVNVVFIGVPGSRNWVL

VDTGMARFTDHIVQLATERFQGPPSAIILTHGHFDHVGTVIELEQFWGVPVYAHPLEI

PYLTGLKDYPPADPSVGGGLMSRLSFAYPNEAINLDDRIFSLPKDHSVPGLSGWEWVH

TPGHTPGHVSLFREADRLLIAGDAIITVKQESLWSVLLQDKQLHGPPSYFTTDWQAAH

QSVQHIRHLEPKLAITGHGHALSGDMLSESLKRLDLDFEESTVPDHGKYVD"

CDS 1915835..1916845

/gene="galM_2"

/locus_tag="EFAGFIKM_01696"

/EC_number="5.1.3.3"

/inference="ab initio prediction:Prodigal:002006"

/inference="similar to AA sequence:UniProtKB:P39840"

/codon_start=1

/transl_table=11

/product="Aldose 1-epimerase"

/db_xref="COG:COG2017"

/translation="MTQQNAAFEEQFGGIPAVWLRFNQFEAAVIPSVGANLVAFRDTE

KGFRYLREPNQDQMDEFMAAPAVYGIPILSPPNRYEDGRFPWNGKVYQLPVNEPATGN

HLHGFLHDAEWKVEGYGSDQLESYVLLSQDVKEGHTFHQYLPFTFTVTLRYSLSSQGL

QQQLIVRNNGTEYMPNLFAFHTAIAVPFAPDSQASDYTAKVTIGQRRELNERSLPTGQ

FQPLTPEEEQLKKDGVSPFFAAMDNHYTAEPQNGRNYMELTDNRTGDKLVYDVGTSYK

HWMIWNNNMGGEFFCPEPQMNLVNAPNVEGIPAEEIGLIGLEPGELFEQSSRLYPIAS

QK"

CDS 1916978..1918366

/gene="fumC"

/locus_tag="EFAGFIKM_01697"

/EC_number="4.2.1.2"

/inference="ab initio prediction:Prodigal:002006"

/inference="similar to AA sequence:UniProtKB:P64173"

/codon_start=1

/transl_table=11

/product="Fumarate hydratase class II"

/translation="MEYRIERDTLGEMKVPADRLWGAQTQRSKENFPIGNEHMPMEVI

RALAILKKSAAASNHKLGKLSAAKSDAIAYAADEIIAGRIDDHFPLVVWQTGSGTQSN

MNVNEVIANLGNQLLEQKGKEERLHPNDDVNMSQSSNDTFPTALHVAGVLAVEDQLLP

AIAVLKATFADKSEAFKDIIKIGRTHLQDATPITLGQEISGWEAMLGKSERMIRESVQ

YLKELAIGGTAVGTGINAHPDFGDFTAKEIGKHTGKDFVSAPNKFHALTSHDEVVYAH

GAVKALAADLMKIANDVRWLASGPRSGLGEIRIPENEPGSSIMPGKVNPTQSEAITMV

VTQVMGNDAAIGFAASQGNFELNVFKPVIIYNFLQSVQLLADSIIAFNDKCAVGIEPN

LDQIEHNLNNSLMLVTALNPHIGYENAAKIAKLAHKEGLSLKQATLQTGLLTEEQFDQ

YVDPAKMIAPKA"

CDS 1918543..1921287

/gene="rcsC_5"

/locus_tag="EFAGFIKM_01698"

/EC_number="2.7.13.3"

/inference="ab initio prediction:Prodigal:002006"

/inference="protein motif:HAMAP:MF_00979"

/codon_start=1

/transl_table=11

/product="Sensor histidine kinase RcsC"

/translation="MSKTRRFTIRSKILLGYLVVVLLFGAVLLVLTAQINVLQKENDF

ISHHDLEVHNLTNAIEKNVLNMETGQRGFMITGNESYLEPYSQGLSQWSSNYDQLNAL

ISDNPSQQQSLQSIKAHITRWIEIAGEPSVDLKKQGDQAKVVAFFQSDPGKTEIDLLR

SQLTTFRNTEIALTEARVTELARRSSTLLTIMYTLWGIIAALSIAAAIVISGNIVKTL

RDVMQTISDISKGGNLKQRIQVRTHDEVGDLGHETNKLLDEVQEQNRVKDQITGIATL

LQNPTNLEGLSRLFLNELAMLFEVPYSVLYYWKDNRLLRVAAYAGDGEKERSLGKVSL

APGEGLVGQSAVEKRVLRMNDLPQNYIRISSGLGYTSATSLTVVPVLFEGRTIAVIEL

ASMKPLQENDMKLMQELTDIFGVSLHSTVTRMELQQLYDESQVLNEELQAQSEKLQAQ

TEEMLSQTEELQMQTEELHMLNDRLEVQKSAAETSANELSVVADQLRTSSGYKSEFLA

NMSHELRTPLNSMLILSEILSENKNQHLNSEEQNYASVIHKSGKDLLNLINDILDLSK

VEAGQMEVDFDDVYLGSVPEVMNQYFLKTAEQKKIDFRIQLQSPLPETIVTDEMRLHQ

ILRNLLSNAFKFTSEGEVALTISKMSLAHPELKDQETDVIAFSVSDTGIGIADNKLVQ

IFDAFKQADGATARKYGGTGLGLSISQSLANLLGGSISATSREGQGSVFTLFLPLRRD

EPETMNASRLFLNEVATTTPEINKLPSMTSGQSDVLLTPLEEALLSGRQVLVVDDDIR

NVYALANALEQYGMNVISAQNGYECLELLERGGVKPDIIMMDIMMPELDGYETTRQIR

ERLGMTQLPIIALTAKAMKEDREKCIAAGASDYLSKPLNIKDVLSRMKLWMNHETMEI

"

CDS 1921715..1923337

/gene="pucR_2"

/locus_tag="EFAGFIKM_01699"

/inference="ab initio prediction:Prodigal:002006"

/inference="similar to AA sequence:UniProtKB:O32138"

/codon_start=1

/transl_table=11

/product="Purine catabolism regulatory protein"

/db_xref="COG:COG2508"

/translation="MQLTVKEALQVYPLSEAKLVAGGEGTSRMMKSVNVMDAPDIADW

IKSGEMLFTTAFIMKDSETDALRLMRRLNERGCAGLGIKLGRFWQSIPQGIVEEADRL

RLPLLELPFQFTFSDQMNALFKAEHERSNRLLHEVVQKQKKLMQFALQQQPHRNVFAE

LATVLNYPLAVIGSRGHVLYGSEGIAGDAVTQGWPWKSVMHRVKWNQGSCHRVPIKQN

DEEYGSLLVFTDSALSLRAEEELFQQAADVLAFYMDMTYREHINPTVQDEMRTLLSQY

LDNKMTVQELTTLSENKGVHLFQGTYQCVLITLEPTLFAEGKLLKQIHRELQYNPLMQ

FTASQHFQIEDGILSIYTCPTGRDYGEELSSFLLSRFGDVLAAQEAKGAPAPRFWISK

MKHEPKSLREAYQECLDTRQLARRFGMKDRALQFEMLEFAYVFQHVPDNIMENYCNKV

LEPLLARDGDPNQVLMNTLESFIENDGLINEAAKQLFVHRNTVTYRMEKVGSLLQMDF

KKTNDLLKLKLVFTFRKFVRDKAATRPHNVQL"

CDS 1923576..1925081

/gene="uao"

/locus_tag="EFAGFIKM_01700"

/inference="ab initio prediction:Prodigal:002006"

/inference="similar to AA sequence:UniProtKB:Q45697"

/codon_start=1

/transl_table=11

/product="Uric acid degradation bifunctional protein"

/translation="MSDLIKLVNNWSITQFVHTFGGLFEESPWVAERAGLSRPFDSFE

QMMKVMKNEVQASDDQVKLQLLRNHPDLGARISMSSNSVQEQAGAGLDSLSQEQYNEL

QQLNKVYTSQFGFPFILAVKGHTASSILESMRQRHRRGREEEFETALKEVFKIAGIRL

EQWLAQIGHEHEFVSKPAAVQQRTMYYGKGDVWMYRSYAKPLTGIQSIPESPFIGRSN

ILFGLNIKVAVQGDEFLPSFAEGDNSLVVATDSMKNFILKHAADYTGATVEGFLALVS

RRFLETYPQMSKVQMTADQIPFEDIPIGLEGSYRPSALVFRYSQNDRATAAVEAERSG

DSIELSNHFSGVADLRLIKVKGSEFAGFMQDEYTTLPETWDRPLFIFLNINWRYEDPR

DGMDDQRGRYVAAEQVRDLAAAVFHECRSASIQHLIYQIGRRLLSRFEQLSEVSFESN

NRTWETVLEEVKEGEGKVFTEPRPPYGFQGFSMTRDDLGTDGRDSGKAGDV"

CDS 1925081..1925458

/gene="pucM"

/locus_tag="EFAGFIKM_01701"

/EC_number="3.5.2.17"

/inference="ab initio prediction:Prodigal:002006"

/inference="similar to AA sequence:UniProtKB:O32142"

/codon_start=1

/transl_table=11

/product="5-hydroxyisourate hydrolase"

/db_xref="COG:COG2351"

/translation="MALSDGQITTHVLDTSKGVPAAGVQIELYTLKRDGEQESKTKVA

ESVTNADGRLDAPLLDGGKLEPAIYELQFHVESYYAQRSLEELGQALWTIVPIRFAVS

DASRHYHIPLLIAPGGYSTYRGS"

CDS 1925502..1926905

/gene="allB_1"

/locus_tag="EFAGFIKM_01702"

/EC_number="3.5.2.5"

/inference="ab initio prediction:Prodigal:002006"

/inference="similar to AA sequence:UniProtKB:O32137"

/codon_start=1

/transl_table=11

/product="Allantoinase"

/db_xref="COG:COG0044"

/translation="MTTFDTIIRGARVVLRDRVEQLDIGITGEKITDISTRLAAGEAT

RIIEAEGLTVMPGVVDIHVHFNEPGLASWEGFRSGSAALAAGGITTYVDMPLNGVPPT

TRPEAWKMKMKAAADQSYVDYAFWGGLVPGNREELAPLAGLGAAGFKAFMSEPGGDGE

DIFARADDHTLLDGMNEIATLNRVLALHAEDEGIVAELGAKSIAAGKNEPMDYIRSRP

VEAEVVAVARALQYGKQTGCALHFVHISTREALDMIAEAKRRGQDVTSETCPHYLTLI

DQDVVRLGAVAKCAPPLRSSSEQEQLWDALTSGLIDVIASDHSPCPPSMKQSDNFFEI

WGGISGAQSTLLIMLEDGHLQRNIDLPLLGRVLSLQPARRLGLENKGEIAIGKDADLV

LIDWEKSTSLNTEDLLYTHKQSPYVGRTFNCQIADVFCRGQRVYNSESGLSPVPLGQH

ITAYSSSPIEAGTEETP"

CDS 1926902..1928209

/gene="pucF"

/locus_tag="EFAGFIKM_01703"

/EC_number="3.5.3.9"

/inference="ab initio prediction:Prodigal:002006"

/inference="similar to AA sequence:UniProtKB:O32149"

/codon_start=1

/transl_table=11

/product="Allantoate amidohydrolase"

/db_xref="COG:COG0624"

/translation="MTESGVYRSSGTNNAPLPLPDVEQVELQAMLDWLSTYGADTQGG

VTRLLYDSAWCEAQGVLAAKMQEKGLSPEFDQSGNLYGTLQGEGKESATGAEELPIVT

GSHIDTVVYGGKYDGAYGVVAGVLALEYLQKHFGAPKRTLQVVSLCEEEGSRFPFAYW

GSRSITGITSLEDVEHLKDQDGVTFAQAIRDAGFGPDSAYRPAAKNYGAFIELHIEQG

QVLERLGHSIGVVSDIVGQKRFSITVSGEANHAGTTPMSWRKDALAGAAEMIAAVRNI

ALEAGEPLVATVGRITADPGVGNVVAARAVFSLDIRHIRQESIDRCWQDMLQAFSRIA

AEQQLGLDWEEHLSVTPIPMNAEMISDIQDTCEQELLSYWLMPSGAGHDSQIFQPACP

TAMIFVPSQDGISHNPLEYTAEADLMHGFRVLVRLLYKYGYGS"

CDS 1928216..1929457

/gene="pucG"

/locus_tag="EFAGFIKM_01704"

/EC_number="2.6.1.112"

/inference="ab initio prediction:Prodigal:002006"

/inference="similar to AA sequence:UniProtKB:O32148"

/codon_start=1

/transl_table=11

/product="(S)-ureidoglycine--glyoxylate transaminase"

/db_xref="COG:COG0075"

/translation="MSNYKELSPSLRTIMTPGPVEVDPRVLRALSFPILGQFDPEFTS

LMNETMAMLRELYMTDNEWCYPVDGTSRSGIEAVLVSLIQPGDKVLVPIYGRFGHLLV

EISERCGAEVVFFETEWGTVFDPEEVIKAIHTHKPSLVAMVHGETSTGQMQPLAEIGK

ACRDLDILLVVDAVATIGGTPVETDAWHLDAVMGGTQKCLSVPSGMAPLTYNSRVEQK

LMSRKTVERGLRDATSARAEGRTIASNYFDLSQLQDYWSSARLNHHTEATSMLYGLHE

GLRILLQEGLEARFQRHLVNERALVAGIQGMGLQLYGDMSSKLPVVTCITIPEGIDGE

SVRSMLLNDFSIEIASSFGPLKGQIWRIGTMGFSCQRKNVLHVLGALEAVLLRHRHVL

PAGEAVQAALDVYAGKEGALC"

CDS 1929451..1931046

/gene="ywrD_1"

/locus_tag="EFAGFIKM_01705"

/EC_number="2.3.2.2"

/inference="ab initio prediction:Prodigal:002006"

/inference="similar to AA sequence:UniProtKB:O05218"

/codon_start=1

/transl_table=11

/product="Glutathione hydrolase-like YwrD proenzyme"

/db_xref="COG:COG0405"

/translation="MLNQMPISREVMVTSPHYLASAVGSSILQQGGNAYDAAVAVSAA

LGVVYPHMTGLGGDAFFLIHDGASGEITAYNGSGRSAAGIHADTFKAMGMNAIPQRGV

LSAITVPGMVDAWWEVWSRYGKLPWEQLLEPAAQYAEKGCPLSRNLRLWMERDEDFIM

GHTPLRAVFAPLGTLLQEGELLIQPDLAASIRLIQTEGRDTFYTGELADRLTSAIRED

GGMLAPADFAGHRGEWVNPVSTGYRGYEVHQMPPNSQGFSMLMMLNMLEHTDLSSVAR

TSPEFYHLMAEVVKKAFRDRDRYLTDPDFRDIPLDLLLSKSYGDQLWNEIQSAPPVAQ

PFLSKTIGQDTAYAAVVDSEGNAVSFIQSLYFDFGAAYVPGDTGVIMQNRGAFFSLDP

RDANVLEPNKRSFHTLMPGLVTRDGKPYMLVGTQGGEGQPQTQLSVLTGVLDYGLNIQ

EAISLPRWVYGRTWGEEGDTMRVENRYHDDVCATLAQWGHNVEARAPWDGIMGQSQGI

VIREDGMISGAADPRGDGMAIGW"

CDS 1931142..1932464

/gene="triA"

/locus_tag="EFAGFIKM_01706"

/EC_number="3.5.4.45"

/inference="ab initio prediction:Prodigal:002006"

/inference="similar to AA sequence:UniProtKB:Q9EYU0"

/codon_start=1

/transl_table=11

/product="Melamine deaminase"

/translation="MGTILLKGAQLVTMNAEEEVFIGDLLIEDNKIKEIAAHIDVQAD

QVIDARGKVLLPGFIQTHIHLCQTLFRGRADDLELMDWLRQRIWPLEAAHDEESVYYS

AMLGLGELISSGTTTILDMETVHHTDSAFQAMAQSGIRVISGKVMMDHGDEVPEPLRE

DTATSLQQSVDLLEKWNGFGGGRIQYAFCPRFVVSCTEELLVEVRDLSNKYHVKVHTH

ASENRGEIELVEHERGMRNIVYLDHIGLATPRLVLAHCVWLSEEEKEIIRKRGVKVTH

CPGSNMKLSSGVADIPDLLNRQIAVGIGADGAACNNNLDMFQEMRLTALMQKIPHGPT

VMDARTVLRMATMGGAEVLGMSKEIGSLEVGKKADMLLLDLDDFHTYPSYETDVYSRV

VYSATRSCVDTVIIDGSIVLKNRKIQTIDRGIVLRESDKSIARLMKRI"

CDS 1932507..1933787

/locus_tag="EFAGFIKM_01707"

/inference="ab initio prediction:Prodigal:002006"

/codon_start=1

/transl_table=11

/product="hypothetical protein"

/translation="MEMHDLCAIAARETRCVLATAIKVEGHAYRKQGVSMLLTEDGKM

YGSISPGCLESDLQARVSHVLDTKQMEFAEYDMRPEDDLSWGETIGCGGLVVVLLEPV

CGELRYTLQKMHECFQSGVTTALTRTFQDDYTRVQYGCKRIEPKGTRRQPILRPSLVP

PHDRVANYNPSSLQVDVQHSSGNIKHEYSASKQHSPDIPRLTLVPEMVIATSNESHSS

HLSGDSQHISDTDAEIPTNDGISTNPWDLPQQLTSLYTPKPRLIIIGAGNDVIPVARL

AQSAGFRVVVADWRESLCTSERFPETELVLGFPCEIMPQLNVNNGDYLILMSHNFPRE

RELLEMVVDCEYAYLGIMGSKTRTARLLDGLPPLKYVHSPVGLSIGADGPEQIAISIA

AELIACKHKVSSLSSELQKGSVAHANDGHSSGSR"

CDS 1933753..1934400

/gene="pucB"

/locus_tag="EFAGFIKM_01708"

/inference="ab initio prediction:Prodigal:002006"

/inference="similar to AA sequence:UniProtKB:O32146"

/codon_start=1

/transl_table=11

/product="Purine catabolism protein PucB"

/db_xref="COG:COG2068"

/translation="MRMTGIVLAAGKSRRLGRDKLSVVMPDGRSLAAWSLEAALNSEL

DQVVCVVKPEDSLTWLPVKWFDSAAYAYHPTARLRIVVCADYACGMANSLHSGVLSAM

EYKPEGILMLLGDQPLLQAQDINRVTTALATHKLSDYVAATDGEGGKPPVAFRSHMFG

PLLSLHGDEGARKIMRSANYSGVHVPLSETSFWDADTEPELERILSHVYESQQTD"

CDS 1934691..1935608

/gene="pucC"

/locus_tag="EFAGFIKM_01709"

/EC_number="1.17.1.4"

/inference="ab initio prediction:Prodigal:002006"

/inference="similar to AA sequence:UniProtKB:O32145"

/codon_start=1

/transl_table=11

/product="putative xanthine dehydrogenase subunit C"

/db_xref="COG:COG1319"

/translation="MVTPAYGSGALPSVWQPENLQELQTLRSRLKGICCFTAGGTLLR

TQWEGGLIPAPEHMISLVRIPEMSGISIRGDEIVIGAMNRLKECASDDVLLQQLPILQ

EAVNAIAAPSIRNVATIGGNIVSGVGDTLPALLVYDAKLHWLTDSGIEIRSASSWLQG

GRDGSRNPGDVLISIHIPIRPSSLVDVDDEQRHAAREVSFYRKLGRRETFSASLVTVA

LYGEIDLDNRWTKIAIAAGGGSGMAIRLLESERQLLGSEATVMQAANLAATVVDEFTT

YGDAFATEQYRKQTAGNMLGAGLWEALHE"

CDS 1935642..1938167

/locus_tag="EFAGFIKM_01710"

/inference="ab initio prediction:Prodigal:002006"

/codon_start=1

/transl_table=11

/product="hypothetical protein"

/translation="MLLNRKQSGKRWHLRPDGTPKVTGQLQYLTDMTLPDMIHGRVLR

SEYPHARILSIDTSEAEALEGVYAVLTSKDVPGLNRFGIATPDQPVFCEDIVRYVGDA

IAAVAADSPERAALALDAIRVVYEELTPLNSTDAALAPGAPELHEHGPGNVLHRTEIK

RGDAKQAFATCDHIVTETYYTPRQMHAYMETEGGLFVPDEEGRLNVYAATQHGYKDRM

QLARIIGCPEEDIRVVSSPIGGSFGGKDELNVQPYGALLALRCGRPVKMHNSRKESVR

AGLKRHPMKIEMQTGMSREGIIQAHRVRITADTGAYATLGAPVLNFCTEHCLGPYAIP

NVDVEGVSVYTNNGLSGEFRGFGGNQAIFAMEGQMDRLAAIMNMDPWEFRRRNMREKN

DPGPLNQRILVTDGLSQVWEAVDRSELWQRHQSPPADPALPPWIKRGVGAAIAMHGAG

LGYGIPDPAGGRLSLNNEGKIEVAFSYEEFGQGLIATLEIMLGDLFQCSTSDLSIIIG

DTDRVPHSGSSTASRSTTMAWMALQRLQTPFRSHVLTVASALSGVPADELVTGPGGVW

RKDQLPAGSSEAETDLQRTGKSEKEIVLALEDVNQTSEEAGKLEKAEKYELAENVAGG
TESDQTVEIHLGASPYLAKEGVSSGLLISYAELATQGEADEWIFDTKFDYPTTPDNVV
GGHYLYTYAAVAAEVEVNTLTGETKLLDTRHVVAAGPVINPMGYIGQIEGGSVMALGF
TLTEDAVMQDSRYVTTNLDTYLIPTIQDIHTNLEVEAIEDLPEGDAFGPRGIGEIGSV
ALAPAITAAIHQATGVWVNRLPVPREQLVRPLNVPLQEGVSPS"

CDS 1938164..1938682

/gene="pucE"

/locus_tag="EFAGFIKM_01711"

/EC_number="1.17.1.4"

/inference="ab initio prediction:Prodigal:002006"

/inference="similar to AA sequence:UniProtKB:O32143"

/codon_start=1

/transl_table=11

/product="putative xanthine dehydrogenase subunit E"

/db_xref="COG:COG2080"

/translation="MSKPLENHWTAVVNGEQKYLEIAQTTRLVDVLRTHLQLTGTKVS
CEVGRCGACMVLMDGEPVNSCLIMAYQCEGSDIMTIEGLHGEEKGTLHPIQQAFVEEG
GFQCGYCTPGMVISTKALLDAHPEPSQEQIETGLCGNLCRCTGYGGIIRAVRKAGERC
VVKETSAEETVS"

CDS 1939049..1941628

/locus_tag="EFAGFIKM_01712"

/inference="ab initio prediction:Prodigal:002006"

/codon_start=1

/transl_table=11

/product="hypothetical protein"

/translation="MKNLLKKATAMMLALALLLGLMTAPVHADTPLFTIESEDAQLTP
DLQVVTSIYGQPKPGFSGAGFVWMQNSGTLTFTVTVPETGMYAISTRYMQELSADGRS
QNLIVNGVTKGAYMLPYTTAWSDFDFGFHKLNQGANTIELKAGWGFAYFDTFTVDHAD
LDPLNVQPVLTDPEATPETQILMNYLTEVYGNNIISGQQEIYGGGNDGDSELEFEWIH
ELTGKYPAIRGFDLMNYNPLYGWEDGTTDRMIDWVNNREGIATASWHINVPRNFNTYE
LGDFVDWKEATYKPTETNFNTANAVIPGTKEYQYVMMTIEDLAEQLLILQDNDVPVLF
RPYHEAEGNGGLNGEGAWFWWASAGAEVYKELWDLLYTELTETYGLHNLIWTYNSYVY

STSPAWYPGDDQVDLVGYDKYNTIYNRYDGLSGVPNEDAITSIFYKLVELTNGTKMVA
MTENDTIPSVKNLTEEKSGWLYFCPWYGEHLMSSAFNYPATLKTLYQSDYVITLDELP
NLKVNNPNPSASITPGNVEFDKYTPNQSDKTVTVNANGNTLTALRAGNNALTTTTDYT
LNGSTLLLKKAYLATLPVGEHSIVLDFNQGQDPVLKVKIVDSTPGTNAVISPVHATFD
KATNPAQDITVSLTLNGRQLTSITKGDVTLVSGQNYTASSSAVVLNKSYLATLPLGQN
VLTFHFNGGNDAVLTVNVVDSTVTVPAGDLTIQSFNGNTSASTNGVSPKFKLINSGNS
AIPLSDVKLRYYYTIDGEDAQSFWSDWASMGSANVTSNFVKLATPVAGADHYLEVGFT
SAAGSLNAGQSAEVQTRFSKNNWTNYTQTNDYSFKASGSQFADHDKVTGYVNGQLVWG
IEP"

CDS complement(1941711..1942355)
/locus_tag="EFAGFIKM_01713"
/inference="ab initio prediction:Prodigal:002006"
/codon_start=1
/transl_table=11
/product="hypothetical protein"
/translation="MKYVLFVAGILILTLGISLTIQSELGTSPFDALLVGLSHHVGLS
VGSWEVIIAFLLIGCNSLLKQQKPEFLGLVTAFITGVGIDIWLYVSDSLITPEVWYSK
LMTFVIGLMIISIGTAIYLQTNFAPIPIDRLTLILHEITRTPLFVSRTLIYFVFLILA
FLLSGPIGLGTLITVCCAGVLLQFIMGHTKKIIDRILTDMDISSHTKSEKEHSV"

CDS 1942480..1942998
/gene="paiA"
/locus_tag="EFAGFIKM_01714"
/EC_number="2.3.1.57"
/inference="ab initio prediction:Prodigal:002006"
/inference="similar to AA sequence:UniProtKB:P21340"
/codon_start=1
/transl_table=11
/product="Spermidine/spermine N(1)-acetyltransferase"
/db_xref="COG:COG0454"
/translation="MTIRIEICTIEHVCELQEISYETFNETFKAQNSPENMKAYLEKA
FNREQLETELSMADSQFLFIYVNNQVAGYMKVNINDTQTENMGLESLEIERVYIKKEF
QKHGLGKVLLYKAIEMAKEHHKTNIWLGVWEKNENAIAFYEKMGFVQTGAHAFYMGDE

EQIDFIMKKTIL"

CDS 1943689..1944765

/gene="glpX"

/locus_tag="EFAGFIKM_01715"

/EC_number="3.1.3.11"

/inference="ab initio prediction:Prodigal:002006"

/inference="similar to AA sequence:UniProtKB:Q03224"

/codon_start=1

/transl_table=11

/product="Fructose-1,6-bisphosphatase class 2"

/db_xref="COG:COG1494"

/translation="MERELALEIVRVTELAALASAPWMGRGDKNSADEAATLAMRAMF

DSVSIRGTVVIGEGEMDEAPMLYIGEEVGNAEGPEVDVAVDPLEGTEIVAKGLNNALS

VIAVAGKGNLLHAPDMYMEKLAVGPALVGKVSIEDPVEVTLEKAAAALNKNISDLTVM

ILDRVRHESTIKTLRKVGVRIKFLSDGDVAGAMAPAFPEAGIDLYVGSGGAPEGVLAA

AALSCLGGEIQGRLMPANADEFQRCLQMGIDNPYKVLTMQDMIGTEDVIFAATGVTPG

EILGGVRYLADDRAETDSIVMRAKTKTIRFIRSQHFLPNKEVLHKVRQLQSTPEPSDR

IHAKTMEQAEFSQSSVDLGVTTTL"

CDS 1945045..1946094

/gene="degA_3"

/locus_tag="EFAGFIKM_01716"

/inference="ab initio prediction:Prodigal:002006"

/inference="similar to AA sequence:UniProtKB:P37947"

/codon_start=1

/transl_table=11

/product="HTH-type transcriptional regulator DegA"

/db_xref="COG:COG1609"

/translation="MASRKEVADLAGVSEATVSRVLNGVGPIKEETRRKVLEASEQLG

YVPSALARSFARSKSGNLGVVLPYVPKAHLFSAYFFSEMLSGIGSKARDNSMDLLVMF

RTPGEVMNYTDLFRRQKVDACIILGARDDHEELAAMQQLHQEGHPFCVMNQHFEGQSF

TEVDADHVEGSRLAIRHLTDQGYRKIAFLNGPDSYSNSQERMEGVRLGLQEAGMELDP

SLLLKGNYSRRSGIEAAAIVADRLHEIDAVFAANDRMAIGVMHGLRERGIRPADFPAF

VGYDDSDAAEMAVPPLSSVRVPFFAMGELAVSKLMHGSVGDTHKANNSVVAVTESARA

LLPTELIIRASSIRQ"

CDS 1946149..1947321

/gene="iolG_2"

/locus_tag="EFAGFIKM_01717"

/EC_number="1.1.1.18"

/inference="ab initio prediction:Prodigal:002006"

/inference="similar to AA sequence:UniProtKB:Q9WYP5"

/codon_start=1

/transl_table=11

/product="Myo-inositol 2-dehydrogenase"

/db_xref="COG:COG0673"

/translation="MSNRLRVGMVGYKFMGKAHSNAYRSLPMFFPSAPLQPEMSVICG

RNEQGVQEAANQFGWSESVTDWRELVKRDDIDLIDINAPSDAHKEIALEAARQGKHLF

CEKPLALSLADSREMLQAAEDAGVAHMVGFNYRFSPAVQLAKDLVESGRLGKIYHFRA

FFLQDWIMDPSFPLVWRLQKEVAGSGSHGDLGAHLIDLARFLVGEFQEVIGMSETFIK

ERPLAAEMTGLSAKGSSNADAPMGEVTVDDATLFLARFAGGALGSFEATRFAAGHRST

NSFEINGSLGSVRFDFERMNELEVYFTKDEEDVQGFRRVLATDPAHKYAEAWWPAGHT

IGFEHTFTHEMLELVTAISEGRQPSPSFHDGVACQAVLEAVERSVTERRWVTLEEM"

CDS 1947392..1948117

/locus_tag="EFAGFIKM_01718"

/inference="ab initio prediction:Prodigal:002006"

/codon_start=1

/transl_table=11

/product="hypothetical protein"

/translation="MSKALIVWGGWDGHEPEQVAAIFERILKEEQFEVEVSNTLESYA

DAEKLLGLDLIVPLWTMGQIEQELVNNVSAAVQSGVGLAGLHGGMCDAFRNNVDWQFM

TGGQWVAHPGNDGVEYMVNMKRGSSPLLDHIEDFQVKSEQYYLHVDPAVEVLATTRFP

VVAGPHAANGPVDMPVVWTKRWGAGRVFYNSLGHHADIVDMKPVTEMMRSGFKWTAAG

KELAKSRVSSATEVYTGMADNQN"

CDS 1948214..1949320

/gene="iolG_3"

/locus_tag="EFAGFIKM_01719"

/EC_number="1.1.1.369"

/inference="ab initio prediction:Prodigal:002006"

/inference="protein motif:HAMAP:MF_01671"

/codon_start=1

/transl_table=11

/product="Inositol 2-dehydrogenase/D-chiro-inositol

3-dehydrogenase"

/translation="MKTMKVGIIGCGKISGIYMENCHRFEVLELVAVADLDRKRAEEQ

AAAYNVPNVYSVDEILADPEIELIINLTIPSVHADVCLRALESGKHVYVEKPLAVTRE

EGQAVLETAKRKGLLVGCAPETFFGSGIQTSLQLVEEGVIGKPVAATAFMMSRGHEHW

HPDPEFYYASGGGPMFDMGPYYLTALVQLMGPIKSIAGMTGKAMEERTITSEKKRGQT

VPVEIPTHVTGLLQFEQGAIGTLITSFDVFGGSALPPIEIYGTHGTLQVPDPNTFGGP

VRYRLLGEHEWTEVPLLPGYQENTRGIGVADMAYAAHSGRAHRASGELAYHVLEAMWA

FHDSSDEQTFYQMKSSCQRPAALPVDLPLYTLDK"

CDS complement(1949489..1950307)

/locus_tag="EFAGFIKM_01720"

/inference="ab initio prediction:Prodigal:002006"

/codon_start=1

/transl_table=11

/product="hypothetical protein"

/translation="MKTNLTYKVPKTLLNKGTGFLNGYTHTLNPYTGCAFGCSYCYVR

QMPVSLFRKEDWGSWVDVKQEASRVFAKELQRALTKGKVTLFMSSSTDPYQPAEYKEC

ITRSLLETMVQHPPDFVLVQTRSPLVTRDIDLLQQLGDRVRVSMTVETDLDDMRKHFS

PSAPPIAGRLRALEQLRDAGIPTQVAIAPVLPSSEQFAAILRPLVQRVCIDDYFMGDG

SEGKRTRRLGMESLYQQVGMEHWYHPDAYQTVVQRMKDCFAEDEIWISQAGFEP"

CDS complement(1950304..1950888)

/gene="adaA"

/locus_tag="EFAGFIKM_01721"

/EC_number="2.1.1.-"

/inference="ab initio prediction:Prodigal:002006"

/inference="similar to AA sequence:UniProtKB:P19219"

/codon_start=1

/transl_table=11

/product="Bifunctional transcriptional activator/DNA

repair enzyme AdaA"

/db_xref="COG:COG2169"

/translation="MESPDQDRILPKSIDEKYWHAIINNDNSYDGTFFYGVQTTGIFC

RPSCKSRAPKAENVLIFQHVEEALARKFRPCKRCKPTGERVPDQEWIYGITDYIEHHY

AEPLSLEVLATVSHGSPYHLHRVFKRITGRTPVQYIQDKRITEARKLLEHTGLTVTDI

GRHVGIPNSAYFITVFRKHTGLTPAHYRERHENL"

CDS 1951081..1952061

/gene="iolU_1"

/locus_tag="EFAGFIKM_01722"

/EC_number="1.1.1.371"

/inference="ab initio prediction:Prodigal:002006"

/inference="similar to AA sequence:UniProtKB:O05265"

/codon_start=1

/transl_table=11

/product="scyllo-inositol 2-dehydrogenase (NADP(+)) IolU"

/db_xref="COG:COG0673"

/translation="MVRFGVVGTNWITERLLEAAAQVEGFKLTAVYSRTEDKANAFAD

KYDVEHRFTDLEKLAASDVIDAVYIATPNTVHAEQAELFLRNGKHVLCEKPLAANSAE

VRSMIDTARKHQVLLMEAMKSTLVPQFKMVQKSLHKIGPVRKYVAGYSQYSSRYDKYK

EGIVLNAFKPELANGALMDLGVYCLYPLITLFGEPNRVQSQAMMLESGVDGQGSVLLD

YDGMDAVVTYSKISNSHLPSEIMGELGSIIIDKIGSPEHAEIRYNDGTVEQLTVEQNH

PAMYYEVEEFVNLVQQGQKESDMNTYERSYVTMQVMDQIRKQIGLVFPND"

CDS 1952107..1952862

/locus_tag="EFAGFIKM_01723"

/inference="ab initio prediction:Prodigal:002006"

/codon_start=1

/transl_table=11

/product="hypothetical protein"

/translation="MSNNGERQKNSREDQQSNESGGLELTQIVFIGRTFEEYMKMFNL

TVEDLTGKSILDCPGGACSFSSQARKLGANPMAVDMAYKYEIDELEVKGFQDIEHTMK
QMEPVQSIYVWDQFGSIQGLKEERTRAITDCVADMRMFPDRYVTSVLPDLPFADEQFD
LTLSAHFLFTYADRLDVDFHVVTVMELLRVSKREVRIFPTVDLSGERYKHMDELKLIL
EELGYTVSEEPTSYEFQRNAHTMLQIVKHNRTR"

CDS 1952875..1953768

/locus_tag="EFAGFIKM_01724"

/inference="ab initio prediction:Prodigal:002006"

/codon_start=1

/transl_table=11

/product="hypothetical protein"

/translation="MNKVLILGGTRFFGKRLVDHLLWEGKSQITVATRGKTDVDFGPE
VNRIKMDREDPESLAQVAQTDMWDVVYDNICYSPDAAKAACDAFAGRTKRYVLTSTLS
VYGDPRPGFKETDFDPYTYPLQYGSAEDFSYGEGKRLAEAVFFQEADFPVVAMRIPIV
LGIDDYTRRLHFHIEHVQKGKPIGMPNPNAEIGFINSTEAARFLAWLGHSSNTGPVNA
ASKGAITLSAMIHLIETVTGMQSQILTETVKEDMSPFGIEQSWTMETTKAEQAGYTFE
PLMDWFPGLVREVALALQSER"

CDS complement(1954003..1955019)

/gene="degA_4"

/locus_tag="EFAGFIKM_01725"

/inference="ab initio prediction:Prodigal:002006"

/inference="similar to AA sequence:UniProtKB:P37947"

/codon_start=1

/transl_table=11

/product="HTH-type transcriptional regulator DegA"

/db_xref="COG:COG1609"

/translation="MITIYDIAKKANVSAMTVSKVINHTGRISSATRERVQQVIDELG
YIPNSNARSLVLQRTQMLSLLITDITNPFYTTLARGAEDAAHLRGYRLLFGNSDEDYN
KEKDYVDAILSTRVDGVLYAPAGDRSLPHLKQLQERHIPFVFLDRTVPGITSDIIAGD
SLEGAIELIRYLVQLGHRRIALVNGSSEVSTARLREEGYVAGLREAGADIDPELVLRT
GYRDFSDEEGLDRLLSQPDQPTAIFAANNMLAIGVIRLLRKRGLRVPEDISVVCFDDL
DLASAFDPFLTVAVQPAYDFGFQGVQMLIDRIEGKSPSEAQTVILPSELRIRASATTP
REQK"

CDS 1955432..1957222

/gene="fucI"

/locus_tag="EFAGFIKM_01726"

/EC_number="5.3.1.25"

/inference="ab initio prediction:Prodigal:002006"

/inference="similar to AA sequence:UniProtKB:Q97N97"

/codon_start=1

/transl_table=11

/product="L-fucose isomerase"

/db_xref="COG:COG2407"

/translation="MTHQDYRYKQAFPKIGIRPTIDGRRKGVRESLEEQTMRMATSVA

ELLAAELRYPDGSAVECVVAETCIGGVAEAAAASELFSRSNVGVTITVTPCWCYGTET

MDMSPSIPTAIWGFNGTERPGAVYLAAVLSAHAQKGIPAFGIYGEDVQDGGDITIPDD

VREKLLRFSRAGLAAATLKGQAYLSIGSVSMGIAGSIVNDSFFQEYLGMRNEYVDMSE

LTRRIEEEIYDPEEYKLALAWVKENCSEGPDNNAAHLQTDRKRKEYEWETVVKMTQIV

RDLMAGNPRLAELGFTEESMGHHAIVSGFQGQRQWTDHSPNGDFLESILNSSFDWNGK

RAPYLVATENDSLNGVSMLFGSLLTHTAQIFADVRTYWSPDSVQRVTGHQLEGNAKDG

ILHLINSGSAALDGTGQQSREGKPVLKPFWEITDEEVQDCLKATSWRPASVEYFRGGG

YSADFLTKGGMPVTMTRLNLVKGLGPVLQIAQGYTVDLPEDVHDTLDQRTDPTWPSTW

FAPVLTGLGAFTSVYEVMNQWGANHGSISYGHIGADLITLASMLRIPVNMHNVPETEI

FRPRAWGLFGTSEPESADYRACSVFGPLYR"

CDS 1957348..1958838

/gene="rhaB_1"

/locus_tag="EFAGFIKM_01727"

/EC_number="2.7.1.5"

/inference="ab initio prediction:Prodigal:002006"

/inference="similar to AA sequence:UniProtKB:P32171"

/codon_start=1

/transl_table=11

/product="L-Rhamnulokinase"

/db_xref="COG:COG1070"

/translation="MGNQATHVLAADYGAGSGRVVRGSFDGSRLSLQEVHRFNNEPVQ

LGDGLYWDFLRLFHELKQGIVKGTQGVSPSVRSIAVDTWGVDYGLVDGAGRLCSNPRH

YREARNAQWMEEVLHMVKEEELYAMSGVLPQQINTVFQLLGSVREQAEVLRPDVRMLF

MPDLFHYYLSGQQACEYTIASTSGLLHAGEARWNEHLIGRLGIPESLFPKLVQPGTVL

GDLTNELFAELRTGPMQVVSVGSHDTASALAVIPAMDSDFAFISCGTWSLMGVERDTP

VLDDRSRALGFTNEGAVSGKVRTLKNRSGLWLLQESKRQWERENQPFTHEELIQLASL

AAPHQSYVSPGDGVYLAPGNMAERIRQQCSASGQTVPETTGAIVRCILESLALEFRQT

LDELQLITGIRPRVIHMVGGGVHNRLLCQFTANAAGVPVVAGPAEATSAGNCMLQFLA

HGEVSSLAEVREVMAASFSPETYEPEDTELWQEAYENYQSLNQIKR"

CDS 1958909..1959571

/gene="drdA"

/locus_tag="EFAGFIKM_01728"

/EC_number="4.1.2.-"

/inference="ab initio prediction:Prodigal:002006"

/inference="similar to AA sequence:UniProtKB:P0DTQ0"

/codon_start=1

/transl_table=11

/product="5-deoxy-D-ribulose 1-phosphate aldolase"

/translation="MSEQQVREELTKYARRAVAQGLVVGPGGNLSARSGGTMLLSPSG

YALEDLEPDEWIAIDIETGATSAGATRPSSEVLMHLYSYRVNPNIQAIVHTHPAYTIA

LSLVFDELPHLFPDQSALVGDIGFVPYVLPTTKQLADAVSAKVEEHTALILVNHGLVT

TGKNLREAYYRTQVVEESAKVYMIAKAAGEPKVLTAEEYKEIQSLESEAYRVQLLQQL

KS"

CDS 1959813..1960541

/gene="lutR"

/locus_tag="EFAGFIKM_01729"

/inference="ab initio prediction:Prodigal:002006"

/inference="similar to AA sequence:UniProtKB:O07007"

/codon_start=1

/transl_table=11

/product="HTH-type transcriptional regulator LutR"

/db_xref="COG:COG2186"

/translation="MGPELNEMELEYELLKQLRDANAPIGASTLVHTLGKTYGLSQAT

IGRRLMEMDVEGFTVLKGRKGRTLTEQGLDRMKTLERDLQQQSVNSQLIQMLNHSGEK
ALLDVLVARRALEREIASLAAQRASKEYIVLLQASIANQHELLSKNIIPYEEDREFHR
LLAYAAQNQILLHAVELVWETSRDFLETAYIRRRVGSELVVDHQQILDAIVAGSPEQA
EAAMVNHINQMIDDVKRYFAMQNQ"

CDS 1960583..1962631

/gene="apc3"

/locus_tag="EFAGFIKM_01730"

/EC_number="6.4.1.8"

/inference="ab initio prediction:Prodigal:002006"

/inference="similar to AA sequence:UniProtKB:Q5P5G4"

/codon_start=1

/transl_table=11

/product="Acetophenone carboxylase gamma subunit"

/db_xref="COG:COG0145"

/translation="MDTIYRLGIDIGGTFTDALVMDHQGRVIAALKTPSIAVAPEQAI
FNALDQLKETGVNIHEIDLFVHGTTLGVNTLIERNGAVTGLLVTKGFRDILEIRRLRL
EDTTNLYGDKTDALVPRHRVKEIDERVIASGGILQPLNEEQLLQAVDELVEDGVTALA
ISFLHAYVNPAHEQLAEDLIRERYPQLFICRSSAIWPQQREFERTLATAMNAYVGERM
GSYFLRLQEGIQAYGLKANLLSTMSNGGIMTAARAANEPVRTLLSGPASGVIAATHIA
ERAGIHQVITFDMGGTSVDVALIDKEPSYSSENKVGDFPVIIPAVDVTAIGAGGGSIA
WLDSVGVLKVGPRSAGANPGPACYQRGGEEPTTTDAYLQLGILHADRFLGGQMRLYPE
LAERALGNLGQKLELNAEQTAQAILDVATANMYAQFSPLMARKGVDPSDFTLLAYGGA
GPMHAFLMAREVGIGRVLIPPSPGTLCAMGCTVANLRNDFVHTLHKSTQTLEPGELSS
LFAELENQGRSWVDEEARGGVKLDNIYCLYSADMRYEGQAFDIEVTLTLEEIEDPKKA
GIKFHESYQNVFGISQPEAEVMFVSLRATIVGVLPTHNTVDPADLPFDESAAEERLIT
FDHVQQTAKVLKRGQIPSVDAPIPGPIIVEEYDTTIFIPPGYKVYRDGHGNVIGEVEA
"

CDS 1962628..1964601

/locus_tag="EFAGFIKM_01731"

/inference="ab initio prediction:Prodigal:002006"

/codon_start=1

/transl_table=11

/product="hypothetical protein"

/translation="MIGDKVFLEIFNNRIQAAVEEMANVVLRTGFTAFVKETGDFGTY

LLSPSGETFGSPLETGYNLSLGIPAAATINSIEDWKEGDLVICNDPYSTKGMVTHLPD

IHLIKPYFHEGRIIAYGMCFVHSSDVGGKVPGSVSPSAYDIHMEGIRIAPVKLYEAGV

LNEQILRMFLDNSRIPEQNLGDLKALMAALNRGEQRLEELIARYGVEKIQQGIEQLLE

YAELKARVIVQDIPDGSYDFWDYLEKGPGGYPIRLRCKMTIAGSDIHLDFSGTDPQVR

ASFNIPTHNQQGHYMLVPALIRYFRTLDPTIPWNSGMIRMVRNYAPPASVLNPEPMAA

VGARAATFIRLMDVITGALGKAQGSMVPAAGAGQACIVMMAMTDASDGKKKVGVIQPI

CGGSGARPMKDGIDGMDFAVGHLRNIPAETVESEMPVLIEHYGLRADSAGAGTFRGGS

GIDLCVRILTPDTVMTARNMERMEFQPWGRLGGGVGSHGEAILNAGRASEDHLGRIDE

LLLQPGETVTFLSQGGGGYGDPYERDSRLVLEDVRSGLVSPEKALELYGVVIDDLKLN

ESDTEQMRAKRPRQQEEFAYGKAREQFEDIWSDEMQVTLNQALLNAPLALRDYLKRQT

MGVVEEKYKDSLSVDPREISDIMEGLRQQIGLH"

CDS 1964670..1965806

/locus_tag="EFAGFIKM_01732"

/inference="ab initio prediction:Prodigal:002006"

/codon_start=1

/transl_table=11

/product="hypothetical protein"

/translation="MFKFNKKRFLSLASLTLAGTLVLSACGGAGNSSNTAGESDSSGA

SGDKVKLTMFIWAGSNQDVVPKEVVAEYVKEHPNVEVTFEESSNSVMYPKMVASKQAD

ANNPVVNFGYFNADATAKGLNDDMWEPLDTSIVTNIKDIPESFHKPDNKGVVWGVSSF

ALVYNKDLVKTPPTSWNDLWDNEEFKGKTALWDYMFYSYISPLIAAKGQEIGATYENP

EPAFQFWADRSDQIGTLVSSNDQLKALLESGDALIAPFSAQVAQTWIDGGSPLAVAYP

SEGAISFPYTLQVVKGSTPEQTRVANEIINELLSAEALSQYAEATGTPVTSTTAAVPD

KYKDDPSFSVETQSNGINPDWDVLAQNSSSWKELWDRLVKTKLQ"

CDS 1965879..1967009

/gene="potA_1"

/locus_tag="EFAGFIKM_01733"

/EC_number="7.6.2.11"

/inference="ab initio prediction:Prodigal:002006"

/inference="similar to AA sequence:UniProtKB:P69874"

/codon_start=1

/transl_table=11

/product="Spermidine/putrescine import ATP-binding protein

PotA"

/db_xref="COG:COG3842"

/translation="MSSGQETISIETREVTKTYGKRAAVNQVTLQVKKGEFVSLLGPS

GCGKTTLLRMLGGLEQPDQGTIMLGDRDVTGIPAYGRNSNMIFQQLALFPHMDVFNNI

AYGLKVKKLPKADIQRQVGEMLDLVQLGDYGRRAVSELSGGQAQRVAIARALINRPEV

LLLDEPLSALDMQLRLDMQRELKRIQREFGGTFIFVTHDQSEAMNMSDRIGVMRAGKL

LQYATPDEIYERPADSFVAKFIGDTNLFETEVLGHDVSGIRVECFGKPLWVKVSDGNA

VPKAGARHSLSIRNEYIRLGEDASHCLNKLDGHVIEAVYGGANIRYSVQIAEGFLVQA

SVLHQRGASRYSPGEPIQIGFDPEDALLLSEPIDDRVQGARI"

CDS 1967006..1967923

/locus_tag="EFAGFIKM_01734"

/inference="ab initio prediction:Prodigal:002006"

/codon_start=1

/transl_table=11

/product="hypothetical protein"

/translation="MNRIAERIVNLYSFGNRTWVTRLLLLLPALALMGIVYLGGLLIF

GRYSFDMYENGKLIRGWDLGAYRAFLSDPYYWKLIGTTFRIAFKVTLWSLLLAYPLAY

CIAGLKKPGMKQLLLLLTFLPLLVSAVVRSYGWQLLLSKQGFMNWLFIRLGLTEEGFS

MIYNETGVVIALVHIFLPFMVFPILNVLSQSDASLKAAAQDLGAGSLRTFLTITLPLS

ARGIASGVQIVFTLCLTAFTTPQLIGGGRVMTLPVFIYQRTLDTNWPMAAVASLFLLV

SSIVVSLLVNKGAETLMFRRSRKGGQAYG"

CDS 1967916..1968761

/gene="ydcV_1"

/locus_tag="EFAGFIKM_01735"

/inference="ab initio prediction:Prodigal:002006"

/inference="similar to AA sequence:UniProtKB:P0AFR9"

/codon_start=1

/transl_table=11

/product="Inner membrane ABC transporter permease protein

YdcV"

/translation="MVKSNSGTKLLLYIFVGAVAIYLLLPLLMIMLTSVGSGSASKFP

PEGLTLKWYANLSEQTQFLDAFKNSLIASTGAVLLALIAGTLAALAIVQYPFPGSGIL

RAFFVSPMVMPKITLGIAYLILFSKMHIAGGLFALILGEAVIVLPFVLTIVGSALANL

HPAHREAAADLGAGPLRIFFTITLPQLRLSLLLSGSIAFVFTFDQVEAALLLLRQDSY

TLPIQLFLYMEKWQDPTIAVVSVVLIAFALALFMTIKLVLRSVPGLESLFGRRKTKGG

LDQNE"

CDS 1968754..1969971

/locus_tag="EFAGFIKM_01736"

/EC_number="3.4.11.18"

/inference="ab initio prediction:Prodigal:002006"

/inference="protein motif:HAMAP:MF_01974"

/codon_start=1

/transl_table=11

/product="Methionine aminopeptidase 1, mitochondrial"

/translation="MNNSQHLSHSSGLNRDRAEEVMKAQGLSALIATTPENIHYVIGS

QLRASNWTMQIYAVLPADRSAKPCIVIPTNRLGVVAQFGITGVDIFAYSDFFVEGSIA

GKPSTDDIDLFYFLLQTTVIHPGPVEALKAALKQLEIEDQPIGIDEMRIAPDILARIR

EEAPDRPVIPAYKLFRKIRLVKTPFEIEQLRQAAQLNERIEQELIDLIAAGVHEKQLA

DHYRLAVMRAGGTPAMTAVGAGPRSALPLIENYFHTIETGDQIRFDLCLQLEGYWGDT

GRTVVAGEPTAWMIKHFDAVKSGWETALETVRPGVKASEVFHAAVSRVQREGIPHYRR

QHVGHAIGLELYDDMTLSPGDHRVLEPGMVLCVEVPYYELGAGGFQIEDTIVVTPDGY

EFLTHMERKLFTK"

CDS 1970054..1971241

/gene="ynfM"

/locus_tag="EFAGFIKM_01737"

/inference="ab initio prediction:Prodigal:002006"

/inference="similar to AA sequence:UniProtKB:P43531"

/codon_start=1

/transl_table=11

/product="Inner membrane transport protein YnfM"

/translation="MIQQGTKTFRNISLALFAGGFVTFALLYSLQPLMPEISDAFSIT

PAHASLALSVTTIAMALTMLFIGSLSDSVGRRFIMTAALVISSVIALLSVFSPGYTEL
LLLRISQGVALAGLPAIAMTYLSEEIEPRSLGYAMGLYISGNSIGGMAGRFISGVVTD
WFSWRAAVGFIGILGLCAAVIFWFVIPPSRHFVKAAPGFKNPIPLLWSQCRNPRLLCL
YGLGFLLMGGFVTLFNYIGFELTGEPYHLSQSIVGSLFVVYLMGTVSSTWMGRLADRY
GRSHVLGIALGIILAGAVCTVHPALWVKIIGLALFAFGFFGGHSIASSWVGLVANQHK
SQANALYLFFYYLGSSVSGTGGGLLYSHLGWTGIVGMIGVYVLIGFVLCNLLVHRLKG
QAT"

CDS 1971327..1971677

/locus_tag="EFAGFIKM_01738"

/inference="ab initio prediction:Prodigal:002006"

/codon_start=1

/transl_table=11

/product="hypothetical protein"

/translation="MKTHAELNSLLVDDYLDLLNIAKQLGDDEWKDAILQALKEEVYA
NHHLDSKEVRQDLWIQFEGINEQMHDLLMQLKHADSPEEKEQIIEMIWGLKVDRHAIT
RKLETMSRASAGHQ"

CDS complement(1971791..1972417)

/gene="azoR2"

/locus_tag="EFAGFIKM_01739"

/EC_number="1.7.1.17"

/inference="ab initio prediction:Prodigal:002006"

/inference="similar to AA sequence:UniProtKB:O32224"

/codon_start=1

/transl_table=11

/product="FMN-dependent NADH-azoreductase 2"

/db_xref="COG:COG1182"

/translation="MSTLLYITAHPHDHETSFSMATGKAFIDAYRESHPSDEVVHLDL
YRSDIPHIDADVFSGWGKLQSGSDLTSEEQTKVSRLNELSDQFASADKYVFVTPMWNF
SFPPILKAYADSICVAGKTFRYTEQGPVGLLTDKKALHIQARGGIYSEGPAAQMESGH
RYLSIIMSFLGVPKLDGIFVEGHNQYKDRADEIKQQAIEQATTLAKQF"

CDS 1972648..1973592

/gene="deoR"

/locus_tag="EFAGFIKM_01740"

/inference="ab initio prediction:Prodigal:002006"

/inference="similar to AA sequence:UniProtKB:P39140"

/codon_start=1

/transl_table=11

/product="Deoxyribonucleoside regulator"

/db_xref="COG:COG2390"

/translation="MGMDLEKQRLSIEAAKLYYQSDYSQQDIAARLGVSRPTVSRLLQ

YAKDRGYVRIEIMDPLEDIDIIAGELKSKYDLDTALVCFAPLKSDEEIQKHISKRAAD

YLQETVQDADIIGVTWGTTMHAVARQLRPKQVKGVEVVQLKGGVSHSHVNTYAAEIVH

LFAEAFHTVPRYLPLPVIFDNIEVKNMVEADRHIGRIVELGRQANIALFTVGTVKEDA

LLFRLGYFNEEEQQLLMNSGAGDICSRFFDAEGQLISEEINSRTVGIDLAELRNKEKS

ILVAGGQRKIEAIHAALKGHYANILVTDQYTAQALLRF"

CDS 1973782..1974456

/gene="deoC"

/locus_tag="EFAGFIKM_01741"

/EC_number="4.1.2.4"

/inference="ab initio prediction:Prodigal:002006"

/inference="similar to AA sequence:UniProtKB:P39121"

/codon_start=1

/transl_table=11

/product="Deoxyribose-phosphate aldolase"

/db_xref="COG:COG0274"

/translation="MTTPQLAKMIDHTLLRADATQSEMAKLTEEAKQYQFASVCVNPG

WVAYAAEQLQGSGVDICTVIGFPLGASTSETKAFETKDAIAKGATEVDMVINISALKD

GKDDFVEQDIRAVVEAAAGKALVKVIIETCLLTDEEKVRACQAAVRAGADFVKTSTGF

STGGATPEDIALMRRTVGPDVGVKASGGVRSLEDMQKMIEAGATRIGASSGVKIMQGE

QSSSSY"

CDS 1974687..1975874

/gene="nupC"

/locus_tag="EFAGFIKM_01742"

/inference="ab initio prediction:Prodigal:002006"

/inference="similar to AA sequence:UniProtKB:P39141"

/codon_start=1

/transl_table=11

/product="Nucleoside permease NupC"

/db_xref="COG:COG1972"

/translation="MKFLIAIIGLLVVFGLAYIASNGKKQIRYRPLAIMILLQVILAY

ALLNTGVGTFLIGGFATVFKSLLDYANEGIAFVFGGLTTVGAESGGAPFFLSVLMPIV

VISALIGILQYIRILPFVIKYIGLVLSKINGMGRLESYNAVASAVLGQSEVFISVKKQ

IGLLPKHRLYTLCASAMSTVSMSIVGAYMTMIEPKYVVTALVLNLFGGFIIASIVNPY

EVTEEEDILEVQEEEKQSFFEMLGEYILDGFKVAIIVAAMLIGFVALIALVNGIFSAV

LGISFQELLGYVFAPFAFIMGVPWKEAIQAGSIMATKMVSNEFVAMLNLKETAMSART

TGIVSVFLVSFANFSSIGIIAGAVKGLHEKQGNVVARFGLKLLYGASLVSVLSAIITG

LFL"

CDS 1975904..1977082

/gene="deoB"

/locus_tag="EFAGFIKM_01743"

/EC_number="5.4.2.7"

/inference="ab initio prediction:Prodigal:002006"

/inference="similar to AA sequence:UniProtKB:Q818Z9"

/codon_start=1

/transl_table=11

/product="Phosphopentomutase"

/db_xref="COG:COG1015"

/translation="MSTFKRVHLIVMDSVGIGEAPDAAEFDDFDVDTFGHIARERGGL

KMPHMASLGLSNIKEIEGVPVADAPKAYYTKMQEASRGKDTMTGHWELMGLYIDTPFR

VFENGFPDELIQRIEEKTGRKVIGNKPASGTEILDELGAEHVETGALIVYTSADSVLQ

IAAHEDVVPLKELYEICEFCREITLEDPYMLGRIIARPFVGEAGNWKRTANRHDYALK

PFGRTVMNELQDGGFDVIALGKIADIYDGEGVTKAVRTVSNMDGMDKLSETMDEEFTG

LSFLNLVDFDALYGHRRDPQGYAQALEDYDARLPEIFAKMTNDDLLLITADHGNDPTY

RGTDHTREYVPLLAYSPRFSEGKKLDLRSTFADLGATVAENFGVKMPEYGTSFLKDLK

"

CDS 1977179..1977886

/gene="deoD_1"

/locus_tag="EFAGFIKM_01744"

/EC_number="2.4.2.1"

/inference="ab initio prediction:Prodigal:002006"

/inference="similar to AA sequence:UniProtKB:Q5EEL8"

/codon_start=1

/transl_table=11

/product="Purine nucleoside phosphorylase DeoD-type"

/db_xref="COG:COG0813"

/translation="MSTHIGAKPGDIAETILLPGDPLRAKYIADTYLEDVVCYNEVRG

MLGYTGTYQGHRISVQGSGMGIPSFAIYANELISEYGVKNLIRVGTCGGMQEHVRVRD

VILAQAACTDSSMNKHVFGGYDFSPIATFSLLKEAYDRATAKGMKIHVGNVFSSDSFY

RDDRSVTEKLMKHGVLGVEMETTALYTIAAKFGVNALTILTVSDHLLTGEETSAEERQ

KTFNDMMVVALDTAITL"

CDS 1978121..1979422

/gene="pdp"

/locus_tag="EFAGFIKM_01745"

/EC_number="2.4.2.2"

/inference="ab initio prediction:Prodigal:002006"

/inference="similar to AA sequence:UniProtKB:P39142"

/codon_start=1

/transl_table=11

/product="Pyrimidine-nucleoside phosphorylase"

/db_xref="COG:COG0213"

/translation="MRMVDIIAKKRDGKELTTAEIDFVVQGYTQGEIPDYQVSAWAMA

VFFKDMTDKERADLTMSMVNSGETIDLSAIEGIKVDKHSTGGVGDTTTLVLAPLVAAL

DVPVAKMSGRGLGHTGGTTDKLESVAGFHVELEKEEFIRLVNEHKVAVIGQSGNLTPA

DKKLYALRDVTATVNSIPLIASSIMSKKIAAGADAIVLDVKTGAGAFMKTTEDAKELA

HAMVSIGNNVGRKTMAVISDMSQPLGLAIGNALEVKEAILTLQGKGPKDLEELCLALG

RQMVFLAGKADSLEHAEEKLKEVIQNGKALEKFKDFLANQGGDASVVDHPDRLPQAQY

LVEVPADKDGYVAGIVADEIGTAAMLLGAGRATKESEIDLAVGLMLNKKVGDPVKAGE

SLVTIHANREDVTDVIAKIKENITIADHADAPVLVHDIVTE"

CDS complement(1979525..1980508)

/locus_tag="EFAGFIKM_01746"

/inference="ab initio prediction:Prodigal:002006"

/codon_start=1

/transl_table=11

/product="hypothetical protein"

/translation="MNHTIYTMTKPPVSRSHLIQWHNLIRAAWIGGLAVYIIHLNSSD

SLHYYLAPTMQKLLLCCPVPLLFIAVIMAWHGLLGRNEVHCDCEHPPPSGFLRSSMVY

GLIAIPLLLGFLLPDRTLGSSMASQKGMSLTYAPPEIRRKEPLPDPATQLNVQDLTQQ

PTAVQSVSATKVQFIPPDEYSREFAELAEKLYAEQIIRVYPEIFSETLGTIDMFQRQF

AGKDISLTGFVYRDKSMDQESHFALGRFLVMCCPADAAPFGVMIHIPEADSFPTDSWV

QIDGSIGSAQVNGKDTIEIRATKVTPVSEPTTPYIYTNADSVMAYEKIKSQ"

CDS complement(1980591..1981625)

/locus_tag="EFAGFIKM_01747"

/inference="ab initio prediction:Prodigal:002006"

/inference="similar to AA sequence:UniProtKB:Q8DUY3"

/codon_start=1

/transl_table=11

/product="Putative two-component membrane permease complex

subunit SMU_747c"

/db_xref="COG:COG0701"

/translation="MKMAANLKLLSFLIPCAFLVPVLITMAPDLKEAWKSEALQNMKT

VFIGIFLEAAPFLLMGVLLSSLMQWFVSEEMVRRLTPKNPIGGVLVAGLLGIIFPICE

CGMIPVVRRLMHKGMPAYIAVTFILSGPVVNPIVFTATLLAFPSHPEITIARMGLAFA

VAASIGMLVYVFVRRNPLRMPKVAATEVKTHPGFKMAQPHSHANAHDHNHVKNWRSFF

IHAGDEFVDMSKYLVIGALITACIQTFISRSDLISLGNGPVASYVFMMGFAYVLSLCS

TSDAFVASAFSHTFALGPLVSFLVLGPMLDFKSTLMLLSTFRTRFVIGLSLAIITLVF

AGSWLINLLV"

CDS 1981859..1982008

/gene="rpmG2"

/locus_tag="EFAGFIKM_01748"

/inference="ab initio prediction:Prodigal:002006"

/inference="similar to AA sequence:UniProtKB:P66231"

/codon_start=1

/transl_table=11

/product="50S ribosomal protein L33 2"

/translation="MRVIVTLACTECGDRNYTTTKNKRNHPERLEMKKYSPRLKKMTI

HRETR"

CDS 1982058..1982765

/gene="znuC_2"

/locus_tag="EFAGFIKM_01749"

/EC_number="7.2.2.-"

/inference="ab initio prediction:Prodigal:002006"

/inference="similar to AA sequence:UniProtKB:O34946"

/codon_start=1

/transl_table=11

/product="High-affinity zinc uptake system ATP-binding

protein ZnuC"

/db_xref="COG:COG1121"

/translation="MILSSMHDVVFGYGKEPVIDQLSLDIHVGEFIGITGPNGSAKTT

LLKLLLGLLKPWSGTIHMSPQLKRGNKSNIGYVPQQVASFNSGFPSTVNELVRSGCYT

RLGLFRRITATQDAIVERSLREVGMWDYRNTRVGELSGGQKQRICIARAMAGQPQVLV

LDEPTTGMDRHSREGFYNLMRHYADVHGLTIIMVTHGLEEMGDRLDRTITLERQESEE

WQCLSTNSCNVPSGQVD"

CDS 1982717..1983610

/gene="znuB_2"

/locus_tag="EFAGFIKM_01750"

/inference="ab initio prediction:Prodigal:002006"

/inference="similar to AA sequence:UniProtKB:O34610"

/codon_start=1

/transl_table=11

/product="High-affinity zinc uptake system membrane

protein ZnuB"

/db_xref="COG:COG1108"

/translation="MFEYEFMQRAFWAGGLIGVIAPILGVYLMLRRQVLMADTLSHVS
LAGVALGSVMNLNPVICGFAVAIIGALLVEQLRRSYRTYSEVPVAIIMTSGLALAVVL
MSLKTNLSKTFSSYLFGSIVAVSDMQLWMMAGVCVIGLLFFILLRRALYSFTFDEETA
SIGGVQVRGLSFAFAILTGMTVASAMPIVGVLLVSALIVLPAALALRVSRSFTAAIIV
AVITGLIGIFSGLTTSYHLNTPPGGTIALILLVILLTGISAQKLIARYNRKRHRKERN
QQEQRETLVIHSRRNTSHEIQ"

CDS 1983597..1984703

/gene="znuA_2"

/locus_tag="EFAGFIKM_01751"

/inference="ab initio prediction:Prodigal:002006"

/inference="similar to AA sequence:UniProtKB:O34966"

/codon_start=1

/transl_table=11

/product="High-affinity zinc uptake system binding-protein
ZnuA"

/db_xref="COG:COG0803"

/translation="MKFSKKTALGLLFSLTLIVAGCGQKSASDTSAAPTGAPVPAETE
TAKLNVQVSFYPMYEFTKNVAGDLADVHTLVPAGMEPHDWEPTPQDIASIEKADVLVY
NGAGMESWIDQVTGSLSNASLIQVEASKGINLLEGGEHDHGHEDSDGTDTHDHADESA
GEAHDHDHADEGTAEEHDHDHDAEAEAGHDHDHGGLDPHVWLSPALAVTEVRNIEAGL
AQAAPEHAAQFKQNADAYIAQLESLDQDFKAAVTDSKRKDFITQHAAFGYLAKEYGLQ
QVPIAGLSPDQEPSAAQMASVIDFAKEHQVKTIFFETLVSSKVSETIASEVGAKTAVL
NPIEGLTEEEIAAGMDYISVMRQNLEALKLALNE"

CDS 1984869..1985351

/locus_tag="EFAGFIKM_01752"

/inference="ab initio prediction:Prodigal:002006"

/codon_start=1

/transl_table=11

/product="hypothetical protein"

/translation="MNLNEEHELLLSEQPAHLWRRRKLELMHWTERDKHTVSAKKTEI
WNGVEVDAELVNALSILQNAGVKTEFSCAGVSPLDEPVDHSLYAYVTLVQSEVADQFV
NYALRRMRNRLLVTLEAEKGRYDLSSFFIGHNRSFCWWMEHCALQFGSRNESSEKSVV

"

CDS 1985394..1986095

/locus_tag="EFAGFIKM_01753"

/inference="ab initio prediction:Prodigal:002006"

/codon_start=1

/transl_table=11

/product="hypothetical protein"

/translation="MNKWVKTILFLLGSVFLTRFIPFSSLFRNLDTMIHEFGHALMTL

VLSGKVLRIELYADHSGVTYSSMLTPGRSILVSLAGYISASLFALLLFYLYRKGLHMW

GLGIMTAVALVSLLLYVRGEFGMLWLTGFIVLNVVIMIFGAKIVKFYYLLLAFLTLEE

SVVSAVYVGLMSWTQPSRAGDAANLAQQTFLPALFWGTLFALFALWCAKGALSLFFRK

EGTSRSSRSRGLRRA"

CDS complement(1986211..1986582)

/locus_tag="EFAGFIKM_01754"

/inference="ab initio prediction:Prodigal:002006"

/codon_start=1

/transl_table=11

/product="hypothetical protein"

/translation="MKTRFWLLYVLPIFLILVFVAIMASGSFLKKPFGTDDRLLESIQ

TLEKQVESKKWTEAKSQVDYAMKAWDKVVNRIQFSVERETIYDILGTLARIKGGVAAQ

DDKAIMEEIYYFYVLWDNLGD"

CDS complement(1986609..1987337)

/locus_tag="EFAGFIKM_01755"

/inference="ab initio prediction:Prodigal:002006"

/inference="similar to AA sequence:UniProtKB:O31533"

/note="UPF0702 transmembrane protein YetF"

/codon_start=1

/transl_table=11

/product="hypothetical protein"

/translation="MMEETWVVAVRSIIAFLTLIIYTRVLGKQQMGNLTYFDYINGIT

IGSIAGTFATDLSSKAWIHFVALTIFTIITIIFQYITLKNRTISKLMDSDPTLIIQNG

KILEQNLSKMRVKFDELTMMLRQKDVFDITTLDYAILEPDGSLSVVLKPENQPVTAKD

MHMHPPKSKLMTEIIIDGLLIKQNLEERNKDINWLSEQLKKQKITIQDIAFAAILPND

KLYVDLFEDKITEKIDMGDYEGPF"

CDS complement(1987544..1988407)

/gene="uxuB"

/locus_tag="EFAGFIKM_01756"

/EC_number="1.-.-.-"

/inference="ab initio prediction:Prodigal:002006"

/inference="similar to AA sequence:UniProtKB:O34896"

/codon_start=1

/transl_table=11

/product="putative oxidoreductase UxuB"

/db_xref="COG:COG1028"

/translation="MSERQYERSSRLEGRTAVITGGAGVLCRSMAEELARHGASVAIL

NRTVSKGQEVVSAIEAAGGKAIAIACDVTQADSVQAAADAVLEQLGPCDILINGAGGN

NASANTTNEIFRLEDLEKSDVTTFFDVSVEGFRQVMDLNFVGSLIPTQIFAKHMIERD

VPATVINISSMSAPSPMTKVPAYSAAKAAINNFTQWLAVHLAESGIRVNAIAPGFFLT

EQNRNLLLQPDGSPTERSSKIIAHTPMRRFGKPEDLLGTLMWLADERQSGFVTGTVIP

VDGGFMAYSGV"

CDS complement(1988404..1989471)

/gene="uxuA"

/locus_tag="EFAGFIKM_01757"

/EC_number="4.2.1.8"

/inference="ab initio prediction:Prodigal:002006"

/inference="similar to AA sequence:UniProtKB:O34346"

/codon_start=1

/transl_table=11

/product="Mannonate dehydratase"

/db_xref="COG:COG1312"

/translation="MRMVFRWFGEGNDTVTLDHIRQIPGVEGIVWALHDVPAGEEWPM

DKILAVKTAADKAGLHLDVVESVNIHEDIKLGLPSRDKYIANYIKTMEKLAQVGVKVI

CYNFMPIFDWLRTDMHKEMEDGSTALFYEHSKLADIEPLELVRRITENSALTMPGWEP

ERLQYLTGLFEAYKDVTEDHMWEHARYFLQEIMPTAERLGIRMAIHPDDPPWPIFGLP

KIITSQENVRKYLNLYDSPYNSVTLCSGSLGANADNDIIGMIHEFKDRIPFAHIRNVK

IYENGDFIETSHRSQDGSVDIAGVVEAYHDIGYEGYARPDHGRHLWGEQCRPGYGLYD

RALGIMYLWGVWDSLQRRAKA"

CDS complement(1989512..1990246)

/locus_tag="EFAGFIKM_01758"

/inference="ab initio prediction:Prodigal:002006"

/codon_start=1

/transl_table=11

/product="hypothetical protein"

/translation="MEYTLKLSSKAGLLPVASNMSSMNNKQSYSTRDVVYFTLKQLIL

SLKMPPGTAISEKEISLEFQVSRTPVRESFVRLAQEGLLEVYPQRGTYVSLIQVDLVE

EARFMREQLERAVIRLACENFPNEQMVTLEQNLAWQQECRVKPDDERMFQLDEEFHST

LFAGCNKSNTWTVIQQMNVHLNRSRMLRLSSDHQWDHLIEQHRSMVKAIQQHDADTAE

RLMQEHLHLTVTDLAVLQDTYPSYFQ"

CDS 1990461..1990763

/locus_tag="EFAGFIKM_01759"

/inference="ab initio prediction:Prodigal:002006"

/codon_start=1

/transl_table=11

/product="hypothetical protein"

/translation="MGQKAISVFQTCIPLFQVLSDNHRQSILLTLTEKGRMTVNEITE

QSSLSRPAISHHLKLLLEKGLISVEQKGTQRYYSASLNASVELLKELVAALEEECL"

CDS 1990854..1992257

/gene="aldH1_2"

/locus_tag="EFAGFIKM_01760"

/EC_number="1.2.1.-"

/inference="ab initio prediction:Prodigal:002006"

/inference="similar to AA sequence:UniProtKB:Q2FWX9"

/codon_start=1

/transl_table=11

/product="4,4'-diaponeurosporen-aldehyde dehydrogenase"

/db_xref="COG:COG1012"

/translation="MTMTIQEVTSQEVEQILEQQRQFFRSGATRSAEARIARLTQLKQ

AIQKYESRLTVALYQDLGKSEFESYTTEIGFMMDSITHTIRKVNKWVKPVKVKTQMAL

LGSKSYIIPEPYGAVLIIGPFNYPFQLLIEPLVGAIAAGNTAVLKASENTPAVSAVVR

EMIASVFEPAYVQVLDGAKDTTTALINAPFDYIFFTGSVPVGKIVMEAAAKNLVPVTL

ELGGKSPVIVDEQADIKVAAERIIWGKLLNTGQTCIAPDYLLVHEHVKEPLITEMKAA

IVSFFGSDIQHNKDYGRIVNKGHFKRLTTLIERDQAKVIYGGASDEEDRFIEPTLIDA

ESWDAATMEDEIFGPILPIISYRNLDEAIVEIVKRPKPLALYLFTSDTRVQDKVLREV

SFGGGCINDTITHVANPRLPFGGVGHSGVGSYHGQYSFETFSHLKSVLKKKTKLNLPI

LYPPYDNKLKLIKRLLK"

CDS complement(1992337..1993248)

/gene="rclR"

/locus_tag="EFAGFIKM_01761"

/inference="ab initio prediction:Prodigal:002006"

/inference="similar to AA sequence:UniProtKB:P77379"

/codon_start=1

/transl_table=11

/product="RCS-specific HTH-type transcriptional activator

RclR"

/db_xref="COG:COG2207"

/translation="MSSTYLPPTLGESVHEVFYPDVQTTVNLFAIHLRSVGMDWDYPA

HEHPQYELNYVTEGEQRMMVNGTLYIQKAGELLLIPPGSIHSSQSHNGKGFTYFCMHF

DIDDQLFLSLLARIKQVRFSADSPVTQQIEPVLRKLMSSADDETVNTMVQRMQLQSAV

FELFGHLWEAVSKEAAHWFTDGHEKIELAHQIRNRLQGLMSQQFKQGHESNGHYGIDD

IAAELGISSSHCNRVFKEVFGQSPRAVLSQLVLHEAKLLLGNPKLSVQNIAVMLGYKD

IAHFSRQFKRWSGMSPSRYRQEQPALE"

CDS 1993468..1995657

/gene="xylA_1"

/locus_tag="EFAGFIKM_01762"

/EC_number="3.2.1.37"

/inference="ab initio prediction:Prodigal:002006"

/inference="similar to AA sequence:UniProtKB:P45702"

/codon_start=1

/transl_table=11

/product="Beta-xylosidase"

/translation="MNTSKSIFYNAHHAPIGAFASFTLGYKGAKGGLGLELGKPADQN

VYIGLQSHDGDNYQALPFYEASEDESSRYDVEKLENGDTASTTATKVPQPLITAFRDE

DITREFTSGTDTWTAGDLTFRIYSPVRPVPDPTNGDREELMDALVPAVLVEMTIDNTE

GQQSRKAYFGYQGNDPYSAMRLIGGPEGGSITGVGQGRLTAIMSADDGLWPARGFTLE

KLLQEKHRENLAFGLGSTAALLMEVPAGEKRTYQFAVCFYRGGIVTTGMDTTYWYTRY

FSDIQAAGEYALQRFSELTASCGEVEQRLGSAALTEDQSFMLAHSIHSYYASTQLLDA

DGEPLWVVNEGEYRMMNTLDLTADQLYFELALNPWTVRNELEWFVKRYSYTDQVRFPG

EEKLYPGGITFTHDIGVANVFSRPGHSAYELVGIDDCFSQMSHEELVNWLCCATVYIE

QTQDQAFVKNTLPVIQDCFESMLNRDHPDAEQRNGLMGLDSSRTQGGAEITTYDSLDV

SLGQSRNNIYLAGKCWAVYVALEKLFATEKLADLSHQAGLQADRCAASIAAQLTDGGY

IPAVIAENNDSRIIPAIEGLVFPYFTGCEAALDVNGRFGPYLKALQTHLKTVLVPGTC

LFEDGGWKLSSTSNNSWLSKIYLSQFIARELLDVEWNETGKAADAAHVNWLLHPEESY

WCWSDQILSGVAVGSKYYPRGVTSILWLLEGKGNHLGQIYASKEAVQ"

CDS 1995654..1997801

/gene="aguA"

/locus_tag="EFAGFIKM_01763"

/EC_number="3.2.1.131"

/inference="ab initio prediction:Prodigal:002006"

/inference="similar to AA sequence:UniProtKB:Q09LY5"

/codon_start=1

/transl_table=11

/product="Xylan alpha-(1->2)-glucuronosidase"

/translation="MSQSVVQAALSGPQYDAWLGYHSELPGANGLAQKTAPAWSKLIL

VEENHGVITTALIELSRGLERLYGVKPSVSHLSDTQESERDNHASTPAIRIGTWTGSD

SVAGSFSETERSTVQGEGYIIRENETEGVLVIGSETPQGVLYGAFHLLRELTMDQGIA

HEADDAVNKWSFITEQPTNALRMINQWDNVDGSIERGYAGDSIFYDNGEFTLDLGRIR

DYARLLASTGINAISINNVNVHRRESLFLTERYLSDVAKVAAEFRAYGVRLFLSANYA

SPIEIGGLRTADPLDAQVREWWNIQTAKVYAAIPDFGGYLIKADSENRPGPFTYNRDH

ADGANMLAEALRPFGGLVIWRCFVYNCKQDWRDRSTDRARAAYDHFTPLDGRFAENVI

LQIKNGPMDFQVREAVSPLFGAMENTNQVIEFQITQEYTGQQRHLCYLVPQWKEVLDF

DTYAKGEGTEIKRIADGSLYKRPYSGFAAVSNIGADACWTGHPLAQANLYGYGRLAWN

PELSAEEIADEWVRLTFGHEDEVVRQVMGMLLTSLDIYENYTAPLGVGWMVNPEHHYG

PNVDGYEYSKWGTYHFADCHGIGVDRTVNSGTGYTSQYHPENADRYESVDTCPDELIL

FFHHVPYTHVLHSGKTVIQHIYDTHFEGVVQAEALAETWRWLEGKIDPVIFSKVASLQ

DNQAEHAKEWRDMINTYFYRKSGIADEQGRTIY"

CDS 1997912..1998979

/gene="gutB_1"

/locus_tag="EFAGFIKM_01764"

/EC_number="1.1.1.-"

/inference="ab initio prediction:Prodigal:002006"

/inference="similar to AA sequence:UniProtKB:Q06004"

/codon_start=1

/transl_table=11

/product="Sorbitol dehydrogenase"

/db_xref="COG:COG1063"

/translation="MGQHELPETMKAAIMTEPGHIIIEEQPVPQPAADEVLIQVMAVG

VCGSDVHYFEHGRIGRFVVEKPIILGHECAGIIAAVGSNVSRLKTGDRVAIEPGVTCG

RCSACKEGRYNLCPDVQFLATPPVDGAFVQYMVIREDMVFPIPDHLSYEEAAMNEPFS

VGIHAARRSKLAPGTTLAIMGMGPVGLMAVAAAKSFGVEKIIVTDLEEVRLEAARRMG

ATHTINVRNEDALAVIRELTGGVGVDTAWETAGNPKALQSALYSLRRGGKLAIVGLPA

QDEIALNVPFIADNEVDIYGIFRYANTYPAGIEFLSSGQHDVMSLITDRYSLEETQQA

MERALHNKSGSLKVMVYPNGM"

CDS 1999146..1999412

/locus_tag="EFAGFIKM_01765"

/inference="ab initio prediction:Prodigal:002006"

/codon_start=1

/transl_table=11

/product="hypothetical protein"

/translation="MRVHEFMIHADFHRDDLMSVSSQASRFSSDIILSYMESEHEHRV

DVKSLLGMALLPIRYGSVVRLQTRGRDELEALEYMLNVLEKGTA"

CDS 1999557..1999808

/gene="rpmE2"

/locus_tag="EFAGFIKM_01766"

/inference="ab initio prediction:Prodigal:002006"

/inference="similar to AA sequence:UniProtKB:O34967"

/codon_start=1

/transl_table=11

/product="50S ribosomal protein L31 type B"

/db_xref="COG:COG0254"

/translation="MPKVDIHPKTQLVIFYDASADFKFLSSSTKFSNETMEWEDGNSY

PVIRVDTSSASHPFFTGKQRNVDIGGRVDKFNRKYNINK"

CDS 1999958..2000263

/locus_tag="EFAGFIKM_01767"

/inference="ab initio prediction:Prodigal:002006"

/codon_start=1

/transl_table=11

/product="hypothetical protein"

/translation="MSSITHMVTFTLYAGKDTPEAEAFLKESSDALAVIPGVEQFQVL

KQVSEKNEFDYSFSMVFADQVAYDAYNNHPVHRQYVEERWEKEVSRFQEIDLIQNHN"

CDS 2000415..2001176

/gene="fabG_3"

/locus_tag="EFAGFIKM_01768"

/EC_number="1.1.1.100"

/inference="ab initio prediction:Prodigal:002006"

/inference="similar to AA sequence:UniProtKB:O54438"

/codon_start=1

/transl_table=11

/product="3-oxoacyl-[acyl-carrier-protein] reductase FabG"

/translation="MQLDLSGKTALVTGATGQLGRVIARTLAACGANLALHYINNDTK

ARELQGEIEALGRQAVIVQGDITKQDTAFRIRDEIQPVLGGVDIVVANAVIQYEWTTV

LEQSPEDYLSQFESCVMQSVYLAKAFIPHMQNVKAGRFIGINTECAMQNFAHQSAYTA

GKRGMDGVYRVLAKEVGEYQITVNQVAPGWTISERDRSSEPGHDEAYTRTVPLKRRGE

DQEIANVVAFLASDLSSFITGAYIPVSGGNVMPAI"

CDS 2001421..2003148

/gene="poxB"

/locus_tag="EFAGFIKM_01769"

/EC_number="1.2.5.1"

/inference="ab initio prediction:Prodigal:002006"

/inference="similar to AA sequence:UniProtKB:P07003"

/codon_start=1

/transl_table=11

/product="Pyruvate dehydrogenase [ubiquinone]"

/db_xref="COG:COG0028"

/translation="MKKTVADVLVESLLNAGIKRIYGIVGDSLNAVLDSIRRSGKIEW

IHVRHEEVAAFAAGADAQVSGSIAVCAGSSGPGNMHLINGLYDCHRNRVPVLAIAAHI

PSDEIGSEYFQATHPEYLFQECSHYCEVITTAKQMPRSLTMAIQTAVARSGVSVVVLP

GDVAGLEAADIPVPEHVYHVTNPVVHPSEPELVKLAEYLNQGKKITLLCGAGCAGARE

PLMQLCDRLKSPMVIALRGKEYLEYDNPYSVGLTGLIGYSSGYHAMMDCDVLLMLGTD

FPYRQFYPEDAKVLQVDIQSSHLGRRTKLDYGVCGDVKATIETLLPYLTEEHSDKHLR

KSVDRYVKVRKDLDELAVGKPGKKPIHPQYLTKVISDAAAENAIFTCDVGTPTVWAAR

YIEMSRNRRLLGSFSHGTMANALPQAIGAQVADPGRQVISLSGDGGITMLMGDLLTLK

QHNLPIKVVVFNNGALGFVELEMKAAGFLESGTELVNPNFAMVAQAMGMEGIRVEDPD

ELEGAVQRALQHDGPVLIDVVVNRQELSLPPKIDIKQAEGFTLWMMKAVLNGRGDEII

ELAKTNLLR"

CDS 2003472..2004227

/gene="lvr_1"

/locus_tag="EFAGFIKM_01770"

/EC_number="1.1.1.-"

/inference="ab initio prediction:Prodigal:002006"

/inference="similar to AA sequence:UniProtKB:Q9LBG2"

/codon_start=1

/transl_table=11

/product="Levodione reductase"

/translation="MSHAGKVAIITGAGSGLGQAAALKLAEKGASIVVVDLVEETGLE

TVKQIEKLGSKAIFVQADVSKAPEVENYVKKTVEEFGRIDMFFNNAGIAGPGIKLIEH

TIEQFDQIIDINLRSVFYGLKYVITEMLKTGGGSILNTASTAGIVGVPAVAPYAATKH

GVVGLTRTAAIEYGKDNIRVNAIAPGTIETPMVVQFGKDNPEVFKATVDSIPSGRLGR

PEEIANLVSFLLGDEAPYINGAVYPIDGAVTAQ"

CDS complement(2004336..2005520)

/gene="pbuE_1"

/locus_tag="EFAGFIKM_01771"

/inference="ab initio prediction:Prodigal:002006"

/inference="similar to AA sequence:UniProtKB:Q797E3"

/codon_start=1

/transl_table=11

/product="Purine efflux pump PbuE"

/db_xref="COG:COG2814"

/translation="MSSTLKIYVLALVSFLVGTSEYVIAGILDRIADTMNISLIAAGQ

LITIFSLVYALGTPVIIALTSRWDRRKLLLYFLGLFVVANVLAYLLPGYGLFVAARVL

MALGAGVVVVTALTVASQIAAEGKQASAIATVITGFTASLIVGVPIGRLVAASWDWKL

VFAGIAVLGILAMLVIAAAIPPSKAEAPVPLKKQLSLLKQPRIVLALLVTFFWLGGYS

IAYTYISPYLVSVSGMSEALLSSALLAFGIASLIGSKVGGFSADRWGVKRTLITGMTL

HIVSLVLLNITASSHLAVFLVLILWSFAAWSSGPTQQYNLVTMAPESSGIMLSLNSSV

MQLAMAAGAGIGGIAVSSVSLSSITWIGAVGVAVAVIIVIGSYRSAPLKSTQTNTSHN

VA"

CDS complement(2005749..2006033)

/gene="sdpR"

/locus_tag="EFAGFIKM_01772"

/inference="ab initio prediction:Prodigal:002006"

/inference="similar to AA sequence:UniProtKB:O32242"

/codon_start=1

/transl_table=11

/product="Transcriptional repressor SdpR"

/db_xref="COG:COG0640"

/translation="MQSNSNEIEQVVKIHKALGEQTRYKIIQILSGESNLCPADLESR

LVTVALSTLSHHLKQLSDCGLLTSQKKGTYIYYSLNTETAQKFVPYLLNK"

CDS complement(2006205..2006546)

/locus_tag="EFAGFIKM_01773"

/inference="ab initio prediction:Prodigal:002006"

/codon_start=1

/transl_table=11

/product="hypothetical protein"

/translation="MEMNTLVWIVLSLLILVLLVRVTSLQHQLNELKRDIERLENGST

GSVRSDFNYTLSPKEAPPSHTSQPAQTDLDQELLTLIQQGKKIMAIKRLREARGLSLK

EAKDYVDSLDR"

CDS complement(2006587..2007786)

/gene="pksS_1"

/locus_tag="EFAGFIKM_01774"

/EC_number="1.14.-.-"

/inference="ab initio prediction:Prodigal:002006"

/inference="similar to AA sequence:UniProtKB:O31785"

/codon_start=1

/transl_table=11

/product="Polyketide biosynthesis cytochrome P450 PksS"

/db_xref="COG:COG2124"

/translation="MNPNEPNESSKPAFFTKEFTHNPYPVYEKLRKEEPVFRVMFPQG

EFGWMITRYEDAVQILKDPRFSKDMVRRYGADNQSIFSNNMLFSDPPDHKRLRGLVQK

AFTPKLVANMRSHIQDIADDLLDNLPSQEKMNLIDDFAFPLPIIVISEILGVPAQDRD

QFRIWSNTVIDASTAESAELFEQHSKEFTDYLTAWFAKVRQDPGTDLISQLVIAEESG

QQLTEQELLGVVSLLIIAGHETTVNLIGNGILALLEHPEQRELLIKQPELIHNAIEEM

LRYNGPVEFSTSRWALEDIEFRGQHIAQGELVIVALDSANRDEQQFKDADIFDITREK

SSHLAFGKGIHLCLGAPLARLEGEIAVSTLLNRFPNIQLQADVNELEWRPGMIVRGVK

EIPVQLK"

CDS 2007941..2008954

/gene="ydhF_1"

/locus_tag="EFAGFIKM_01775"

/EC_number="1.-.-.-"

/inference="ab initio prediction:Prodigal:002006"

/inference="similar to AA sequence:UniProtKB:P76187"

/codon_start=1

/transl_table=11

/product="Oxidoreductase YdhF"

/db_xref="COG:COG4989"

/translation="MLGKNENEVNADMSYAQLPLHHHQIPASRMVLGCMRFGGEWDGL

PITSDLVVEGHRAVEAAIEIGINHFDLADIYTRGKAEHVLGRVLSETPGLREQIIIQS

KCGIRLIGDDEGPQRYDTSGAHILASVDGILERLGIDYLDILLLHRPDPLAHPQEIGD

ALAELHHSGKVRHFGVSNMGSEQIRLLQLHSKVPLIVNQLEMSLDKIGFVEAGVTVNR

PQARDNVFPYGTMEYCQAEHIQLQAWGPLAQGRFTGRVVDGQRAEIEHTAQLVDRMAK

ERGVTPESIVLAWLMKHPAGIQPVIGSIRPERILACRDATNIELTRCEWYELYSASCG

RRL"

CDS 2009375..2010475

/gene="yehL"

/locus_tag="EFAGFIKM_01776"

/inference="ab initio prediction:Prodigal:002006"

/inference="similar to AA sequence:UniProtKB:P33348"

/codon_start=1

/transl_table=11

/product="putative protein YehL"

/db_xref="COG:COG0714"

/translation="MEMLKPPAETLYEVELRALREEDQGKRPPNWLLSPAYVRDFIIG

RDKPALLNGEEILITRKFYGNDVLIERAVVTLAGNRGLMLVGEPGTAKTMLSELLTAA

ISGTSLNTIQGTAGTTEDMIKYSWNYAMLLDKGPSEAALVPSPLYNGMKKGILTRFEE

ITRCPAEAQDSLISILSDKVMSIPELDGGVLFAQPGFNVIATANIRDKGVNEMSGALK

RRFNFETIKPIHNVKMEAKIIESQARSLLLHSGIDIEINPDVVELLATTFMELRTGMT

REGYKLDTPQASMSTAEAVSVYVQSAMTSYYYEDKAIALDRLVQNMLGTIAKENDKDL

SILKTYFSKVVKERSREEGMWKDYYEERKWIG"

CDS 2010463..2012931

/locus_tag="EFAGFIKM_01777"

/inference="ab initio prediction:Prodigal:002006"

/codon_start=1

/transl_table=11

/product="hypothetical protein"

/translation="MDRLTAVLDPDVDQLQSLFESQVYNLSNHTVYYPIRHHSPACSY
HLLRLIGEYKPDIILIEGPESGNPLISVLSDEATIPPVSLYYTYESELEREACYYPML
RYSPEYVALKEAKRLDIPAKFIDLDYQHFSTRASKSGQTEQKEISIQDETLLAGSDFI
NRLCQKTNCRSFDELWEKVFEIGGLEKSTRAFVQDVFTYCTLSRMCYSTERLQSSGDL
AREAHMRQRINQAVQEHDRVLVITGGFHTYGLLMSEKHESELEQPEMDKRHIDQRLDG
QGKPGTSQQSAVNPDTVHQQMYPMVYTFAEADRLNGYASGMPYVNYYDQIWNQLLRKK
STPYNLTALDLLSRLMRQLRDGHEHVSTSDAIEAYSMIQGLAGLRGKREGGVYELMDA
ALSSFVKGELTLATDKPLQELQRLLTGDVIGSVAPNSFSIPIVEDFKARCTEYKLQIR
TTGQHKKVLDLYAKPEHRQISQLIQCITYLVPEFAKRQSGPDWIAERDMNLVRETWVY
MYSSRIEARLIENSLYGGTLAGAATRKMEEQMQEVPDHHSGELARLMLQALLMGIQDT
AMKLYEQVRSALRTDGNFLSLCNSLHVLNRIHQHRRLLGLSDEQQLPELVSEAYRNAV
DKLLQLSRANPDEHEAIIQGLKLLAMLAESSEGYFQDETFRIHLNELLSDHQLPAQLE
GVCVAISSGLGDRPRDEIVDRARGYIHGTPDQTRQTALFLQGVFTVARDAFLYEDQLL
SELNYLIEQLSYDDFISMVPELRLAFTYFTPMETGLIAERVASLHQVEPEEMLRPAVD
EQMLIQSKAWDEALRKEFGAWKLI"

CDS 2012916..2014139

/locus_tag="EFAGFIKM_01778"

/inference="ab initio prediction:Prodigal:002006"

/codon_start=1

/transl_table=11

/product="hypothetical protein"

/translation="MEANMNEANRTTDETGEINESSNPDGSNRGDQHSAETLNRWRLI
LGESAEEGLCNTDQYTSAEFQYTEIDEILGYLYNREYGEEQGYRKEGGRGASNLTVPK
WLHKVRDLFPKPTVEILEKQALDRYGLTELLTDKKLLESLEPNMNLLKNIMQFKGRMK
GEVLKSAKDIVRTVVEELRSKLESQTRASIMGKRSRYTSSSVRSLRNLNFKRTITKNL
KNYDKNKRRFVIDRLYFDGNIQPHNKWNIIIGVDESGSMLDSVIYSSVMASIFYRLNA
LRTKLFIFDTQVVDLSDRLEDPVDVLMNVQLGGGTHITKALRYGETLIDNPGKTIFIL
VSDLEEGYPIAQMYKACKDIIDAGCKLLVLTALDFNGDSVYNKHAAQTLTNMGAHVAA
ITPNELADWIGEIIT"

CDS 2014159..2017716

/locus_tag="EFAGFIKM_01779"

/inference="ab initio prediction:Prodigal:002006"

/codon_start=1

/transl_table=11

/product="hypothetical protein"

/translation="MLTADRAVETLVEKFAVTCSNEYSLKTDRSSEIIDYVLGTTDKF

PDMIGNSSHYVYQTIDLLMKLAKKDDGDIFYRAGAIVIYLESNYSKRNSWRTDAFTVC

MGNDSEAYGLSGKSKSADRMDGLLSRLEKIKQFAGENEILTELKSKLKRAQWLFDSKK

ADSQGDYRDVINALMLLQLYSRLSDTASHLAEVQSSVQSLPVLFQHVMANDADKLRDE

LHTLIEPVVVSNIQGKHNGSSQELKDEFLKEKRSQLIAELYPNTSFSSKEQCSNTVFH

QTMVLVSGALLYLINISGGKQRFLDTNSEIGHVLLHTIHQLHEIYPLEVRRYLLSMDP

RAKSPNDLLAGLVPLDEPYQLVEILRDELNSYTVSWNMLQSAIANRPEQAIRAYEIIK

PPFLKLCIQKFMEDQGLTLPNAEGSLEQTVFHALRHPLDGGRQGLAIARYLSGENTLE

EYWEDPNSRGLFNQLNRDKKRVHNLISISFLPLDSEAMRRFAILTTHPDWTLDIISDM

YNSYAFSGEQLLQRYGQDPEVQREKLLTSLISLNGMTDYRYKSMPHDEYRRIIQQNLD

YALTQYKKLPTDTRILILEITFEQRDELSKKILAEAIRAGLQDSSKKANGVALAEFNR

IPDQDLYTLIYLSEKKVGIKEMALTAIRSLENSKELYGELLKKEKSDEWKSLIQILLD

TADLSPEYAHAALADQADAKKLSRLSWLSLKDLPSLIRIEDNQPLDDRIKQYALVQSL

DHTSGPNERLNELRDYVSEASLARFASELLQVWIQEGAPAKEKWVMYVSALFGDIQIV

NILAPQIKEWTENSRGAIAADAVKVLAYLKDPSALMAIDKIKRGVKNRQVKGAAEEAL

QLAADNMGLTSEQLEDRLVTTLGFDEKGTMQLSYGERSFLVKVNGDLQVVVLNEETGK

SVKSLPAPAQKDDPELAAQSKARFTQLKKDLKSMVNIQAQRLEESLSKQRLWSADEWK

ALFVQNVIMQKFAVGLIWGTYEDGALISTFRYMEDGTFNSVDEDEVDLPSGAQVGLIH

PLELDQATLEGWTTQLEDYEIKQPFEQLNREIHQPEDEDKTKNEYDHLPESDFSPTAF

PKALEKYGWIKGPAQDGGWYHEFYKEYGDLVAELQFSGTSITYYEGLDDITLESLHFF

KPHNKQYYYYGDNKPIALGNVPGRVFSETIYDILRATGR"

CDS 2017778..2019553

/locus_tag="EFAGFIKM_01780"

/inference="ab initio prediction:Prodigal:002006"

/codon_start=1

/transl_table=11

/product="hypothetical protein"

/translation="MIRYANKGLYKRSLKELDNGVSVTYTWNASSVSCQLSDGTHCTL

ENTLDHWECSCPADHICKHVLIAILYDQREYTAEDSMNADEKHALDKDSGISDNDPLD

VPKNVSVTESASNLVSNVDDPSEIPHTVSREIESRFRWMTESDLTMLIKSYSSSMIEE

VVFRLRYPEEIKIFEDSLLTVHLTTQNIEVSFTEEPNLAKALCKVQQTAGKMAKLEAL

LRYRNLKGLDDTDALSGRVYEAKFSLQTVQECRVMLAGLLKTGLARLPQSYMAQLETL

AIAAHSGNLPDIERSLRGIQGELQLFFNRHIRFSMQSMLDRVSKLYLALQVLEEEKVS

VIQQSQLIGSFRSKYFTVPSLHLYGLGADAWETRSGFRGITYYFYCFDDEEIYTYSDV

RAVYYDDQQFSYTEHYGAFTPWLPNLTFRQFVGEEVQFHSVKVNEERRISSGEGAKLA

ILPRTGVETLNLGKTMQDVTTVLQEESRSFDLFAAPKERLAVIKVARIVDHNFVKQTQ

ELVLTVENTEGERLALTLPYSSDWQAMIQRLESGYGSQSLEHFYAFVRVEPKRISPIS

FLKGKTVLSLKLDIGNGRMGRLGRI"

CDS 2019540..2019818

/locus_tag="EFAGFIKM_01781"

/inference="ab initio prediction:Prodigal:002006"

/codon_start=1

/transl_table=11

/product="hypothetical protein"

/translation="MEEYNQFMQKINGWSEELLLRGLSQFTIRDIEVLEQLTAESLRL

QMSFLHELLNHLIKEGRSVALGQGNEELLLFQYCRLTQYVQLSVQEEA"

CDS 2020042..2020584

/locus_tag="EFAGFIKM_01782"

/inference="ab initio prediction:Prodigal:002006"

/codon_start=1

/transl_table=11

/product="hypothetical protein"

/translation="MEEEQIRKAVANRDPEAMEWVMNHYAGLLWKIAHSILHHASVEE

IEECVADTFFAFWQNPDAFQSGRSSLKNYLATITKHKAIDRYRKLNRRTELTYEEEIH

SVQTDDLLVQMISKETYTELNQMIESFPEPEREIMKRRFYEGQRPHEISEALSLHVRQ

VHNKLYRSRQRLRTWWMNRK"

CDS 2020598..2021992

/locus_tag="EFAGFIKM_01783"

/inference="ab initio prediction:Prodigal:002006"

/codon_start=1

/transl_table=11

/product="hypothetical protein"

/translation="MRKSEKEFTEQNLFNIKKLFYDKLAMNEPPTVTRSDSSFYPPIL

YPSAPSAQTKKSMNKRKRYRKAIYIPVTVALSLCLVTGAAASVGIIDLSTVLHFLGAD

RIGILQPVHQVSEDQGIRMEVIGAVRDGDTTEIYVSLSDLTGHRIDDTLDVYDYRVTG

GNANNAQIIHYDDANQTAIVRFLIQGKVSKNRITVQINTLMSGASQQDGYNVQVDWKQ

LLQEKQNNSYEILERDRISGLGGKTDPIPVLHQDVTNIPVEGIDWVHISNIGFVEGKL

HIQINPDNEIGEYNHGYFYFTDEQGQKLDIPEYSISYGHYLKGRVRYGGDYEEFVYDL

SAVDDLDKLRLQGSFTSISEVMQGNWKTTFNLSQNGLSKEGNIQFDANGVLQANVALS

AVGVTLTGEALSKIRVEDLSIEVHLKDGTKLSGASGFLQLDQDLIKWISPKTIPVNDV

SYLLLNGEKVNFER"

CDS 2022170..2024677

/locus_tag="EFAGFIKM_01784"

/inference="ab initio prediction:Prodigal:002006"

/codon_start=1

/transl_table=11

/product="hypothetical protein"

/translation="MSMSEPLLTQIVKQHIPAGATVVTIDKPVQHPAIYAADLTGDGM

PEIAAVYQMNGELYLLILKFVQGHWIKMALIKGLGYGVTLMTAAPVTSTARNNLIIGW

RIGSIWSKLAVYEWTESGLKDLAPDDLYYSYAEIIDMPGPHGRDGKVEIALWIHDTGE

AYRVEIIRWQNGKWVPAPDVYATYYPKVVQYYEQLTQQYPDYIFYWYYLADAQYHAGQ

YPAALASVQKALSFPEAYPSREVLQELEKKIKQAMVPVSHARVATWLYPISVRTTSGT

RWGYMDAQGHIRLEPVLGDAQEFQPNGLAVVVKDGRAGVINLQGQYVVQPVYDSINSF

SEERAITIDSQGFKMINEQGTVLTKRAYPYISNMNDGRALFYDSNPGQADGTVSRYGY

LDASGNEVIPAQFASANDFQDGKAVVQIKDNEYALINRNGHRLATYPYAYVGPLGDGL

LAFQKETSGKYGYIDERGNIRIQPKFSSAFPFSGGQAIVNTAEDYKSIYGVINTQGSF

IIQPAYNDIRELGEHRYALGQAIHPEQPYIGSVYAIADTNGRRLTEFLYREVGNYKNG

LASASNGRQTYLLDLSGRPAAGYPHVEGSGTLEVVAPDLIKAYVDQRLSYVNRAGQII

WRQNTIVPLHPPYRVREEKYKPNRDYLVYYPQVEGLTNQAEQLALNAKLKQLSQVKPI

PADQQLDYTYTGDFDITFYQQQLLQLQLTGYNYPAGAAHGMPTMIYAIINLTNGLLYE

LKDLFKPNSDYVKVLSQIVGDQIKNDPQYSYVFPDTYTGISADQPFFVTADALHLYFN

PYDIAPYAAGFPTFTIPFVQIKDIINTEGSFWEAFHS"

CDS 2025104..2026057

/locus_tag="EFAGFIKM_01785"

/inference="ab initio prediction:Prodigal:002006"

/codon_start=1

/transl_table=11

/product="hypothetical protein"

/translation="MNNTSEVSGLMQVTKERKGLLIIASIFSTLSSLLQIVPYVGVYK

IVEELLTHAGQPSAMDKDLLIYWGIVAFVALIAGLIALYIGGMCSHIAAFNILYQLRV

RLAEHVAKVPMGYHTRTATGELKKIIEVSVEKIEKFIAHQLPDLVSAIVIPLMLLGYL

FWLDYRMALTLLIPIGIGFWLQSRLFRSEKGRQAYRDFQLAIEEMNATGVEYVRGMPA

VKVFGITADSFLTFKQAVTRYRDISLKITDLCKTSYGLFFVIMISLFTFIVPVGILLS

SGNAGNQSFAITFILFLIITPSLSAPLLKLMYLGSGIREIV"

CDS 2026112..2026903

/gene="msbA_1"

/locus_tag="EFAGFIKM_01786"

/EC_number="7.5.2.6"

/inference="ab initio prediction:Prodigal:002006"

/inference="similar to AA sequence:UniProtKB:P60752"

/codon_start=1

/transl_table=11

/product="Lipid A export ATP-binding/permease protein

MsbA"

/db_xref="COG:COG1132"

/translation="MPKVPDTYEVAFRQVSFAYERKESEAYKPVLDQVDFVANAGEMT

ALVGPSGGGKSTIANLLLRFWDVQEGEITIGGIPIQEMGTEKLMDTVSFVFQDVHLFY

DTIEENIRMGNTSASRQDVIAAAQTACCHECIKKLDAGYDTKIGEGGTYLSGGEAQRI

AIARALLKHAPILVLDEATIYADAEDEHKIQQGLAQLVQGKTVLIIAHRLTTIRAAEQ

ILVIRKGTIAERGTHDQLRALGGVYEHMWQAHISAASWKLGGTGR"

CDS 2026900..2028021

/gene="btuD_6"

/locus_tag="EFAGFIKM_01787"

/EC_number="7.6.2.8"

/inference="ab initio prediction:Prodigal:002006"

/inference="protein motif:HAMAP:MF_01005"

/codon_start=1

/transl_table=11

/product="Vitamin B12 import ATP-binding protein BtuD"

/translation="MKNYLYNISGGNPRSLLPSVIAAILDGIAKIVPAALLVDIFNTI

YKSFADPGKGLDINRMWIVCCILFAWLLVEYAAYASLYDKTYRAAYSLAASGRLKLAE

HIRHLSLGFFGKRDPGDLTNLILSDYGQVEHTISHNLSQLISAIVLPVLAFGGLLLVD

WRMAVAMFVAVPLGVALLWLTDSIQARMSENHVRAKNEAASRLQEYLSGIREIKAHNM

GGKRFERLRRSFDELRSASIRLEGIMGPMIMGAMLLARSGMTWMILVGSYLLAGGELS

LKNASIVLLDEATASLDPENEAAVQQAINELVADKTVILIAHRLKTIQNADQILVLDQ

GQLVEQGTHAHLLDHSGIYARLWQLQQDAEGWQVKVNGS"

CDS 2028104..2029066

/gene="fhuD_1"

/locus_tag="EFAGFIKM_01788"

/inference="ab initio prediction:Prodigal:002006"

/inference="similar to AA sequence:UniProtKB:P37580"

/codon_start=1

/transl_table=11

/product="Iron(3+)-hydroxamate-binding protein FhuD"

/db_xref="COG:COG0614"

/translation="MFKRIPVWIALCCMLTLAACGTTQTTSEVASSSEGTAQATTKLI

TNPNGEEITIPLNPKRIVDLSGSTEELLIIGKTPVATMSADYGNPEEITPTIKDQLNA

DTVNLGWYGSPFSSEAIAGANPDLIILGKDFNTDQYETLSKIAPTIALPYSYYDWRER

LTFLADTFGEESKKNEYLAKYDAKSAEWKQKLEAAVKGESFAVIETYPNNLVIYSNKG

AAEMLYGEWGLKRTDGIPDPEGWGGKEIALEALVTVNPDRLLLMENSENKMVDSKVWN

NMNAVKNDKIYKISNVDNYNYSYTAMGRMELMDRLGTMILGGQK"

CDS 2029187..2029495

/locus_tag="EFAGFIKM_01789"

/inference="ab initio prediction:Prodigal:002006"

/codon_start=1

/transl_table=11

/product="hypothetical protein"

/translation="MSLILGFGQYLVAFYVVILSPISWLLRKYVRFPMVGTFIYMIGF

GWGGLWVFDLMYNPYFVNGYHLNRMTSIWIFAIAGLVYAIVENKIWRRGQMQNEQKAT

"

CDS 2029801..2030199

/locus_tag="EFAGFIKM_01790"

/inference="ab initio prediction:Prodigal:002006"

/codon_start=1

/transl_table=11

/product="hypothetical protein"

/translation="MYKAGFWIRLGANVLDSLIISIPLVIIAGILTGNFDTDEPIAKL

LSALYSILLPVYWYGRTIGKRICGIRIRKYDTHEPPKIGTMLMRVVVAGLVYVLTLGI

GVIVSACMVGMREDRRAIHDFVAGTEVVWD"

CDS complement(2030405..2030575)

/locus_tag="EFAGFIKM_01791"

/inference="ab initio prediction:Prodigal:002006"

/codon_start=1

/transl_table=11

/product="hypothetical protein"

/translation="MIGCFIQLTDRILSRLDAVNHTKSIDEFIVLATIKQDNTVSGSN

LVGGKLEWGGWI"

CDS complement(2030641..2031408)

/gene="gpmB_1"

/locus_tag="EFAGFIKM_01792"

/EC_number="5.4.2.-"

/inference="ab initio prediction:Prodigal:002006"

/inference="protein motif:HAMAP:MF_01040"

/codon_start=1

/transl_table=11

/product="phosphoglycerate mutase GpmB"

/translation="MGIKKRNIRLKLAVEDKNMFLFPIRRITNHHLAIQFIKLTGSIV

PICGTICLGNDGEIVVIKGAGMATTIGLIRHGVTEWNNLSKAQGISDIPLNEEGIEQA

TALTKRLSSEEWDVIFSSNLTRARQTAEIIQQCLGVPVFNDERIREINCGLIEGTTEE

ERITRWGANWREQNLGMEDFKMVAKRGLTFLEKLINEYVDKRILVISHGALIGLSLQH

LLPQHFQKTYIDNTSITILTHTNNKWACQLYNCTKHL"

CDS complement(2031539..2031652)

/locus_tag="EFAGFIKM_01793"

/inference="ab initio prediction:Prodigal:002006"

/codon_start=1

/transl_table=11

/product="hypothetical protein"

/translation="MFTLAAKHLTDFLFYRYKFLAQAKDKNLYRLPELTRN"

CDS complement(2031704..2032324)

/gene="xerS_2"

/locus_tag="EFAGFIKM_01794"

/inference="ab initio prediction:Prodigal:002006"

/inference="similar to AA sequence:UniProtKB:A2RKP9"

/codon_start=1

/transl_table=11

/product="Tyrosine recombinase XerS"

/db_xref="COG:COG0582"

/translation="MGRIDDKLSELPWYVTEFIDSKKRKLSASTLLNYCHDYIIFFDW

LITENFTTVQKRTEIELSTLETLTIRETESFLSFLEYQLGNNKLTINRKLSSLKSLFD

YLQNKAETIDLKPYIQRNVMAKMDLNAVKESQETIANRMEGKILRENDFEAFRVFVAY

DYGELNKTNKRIMNFHQFNRERDTAIISLILGSGLRLSEVSGILRI"

CDS 2033278..2033412

/locus_tag="EFAGFIKM_01795"

/inference="ab initio prediction:Prodigal:002006"

/codon_start=1

/transl_table=11

/product="hypothetical protein"

/translation="MKLAAIHMVDLMRYLFGEAVEVVGTAAPDGEHIHPSISLKFAMV

"

CDS 2033761..2034144

/gene="mhqP_2"

/locus_tag="EFAGFIKM_01796"

/EC_number="1.-.-.-"

/inference="ab initio prediction:Prodigal:002006"

/inference="similar to AA sequence:UniProtKB:P96694"

/codon_start=1

/transl_table=11

/product="Putative oxidoreductase MhqP"

/db_xref="COG:COG2259"

/translation="MLIIRIVIGFIMIGHACKKLFGIFGGEGISGTAAFFNAVGIRPA

WFMALCAGIIELVGGLLFTLGLWTVVGAILLMLTMFVAIVKVHVGKGLWNLNGGFEYN

LVMLASLIVVVLAGAGAYSIDALLS"

CDS complement(2034977..2036632)

/locus_tag="EFAGFIKM_01797"

/inference="ab initio prediction:Prodigal:002006"

/codon_start=1

/transl_table=11

/product="hypothetical protein"

/translation="MKRNVRLLWMLMLVFVFLTSACSPAGNNSDSPQNEGNITVEDNM

TEPGSFPIVKEKVTLKVLIRGASGVDFDTNTFTKWYEEKTNVHIQFEVAPENGFEETL

NLALASGNYPDMIIGMEIKPATEMLYGKSGTFLPLNDMIEKYGVNMKKIFDKKPLVQQ

VITAPDGNIYSVPDIGECFHCDYAKRMWINKTWLDNLGLKMPTTTEEYYNVLKAFKTL

DPNGNGKADEIPLAGAVGGWNTEIEGFLMNSFIYNPMGSTQTGMMLDQGKVVAAFTQD

PWKEGLAYMKRLYSEGLIAPETFTQDNAQYRQMGEGGDAPRVGSCPGGLYGCTAYGQE

RYKEYVALPPLAGPDGTRIAWTNPYEGITSGRIVITNNSKYPEVAFRWADGLLDGGEV

SLRSDTGTMGEYWDWAEEGTGINGKPAVWEKLKEKTADSNVGWQTRAPRYYPADLRLG

EKRNDEFPLEVILFEAAKQYEPYKVDSSLVIPPLFYDEEQAAELVNLEKTIIDYKKEM

LARFITGDADLESEWGTYLDTMNQMGLERYAQIIQEAYDASQK"

CDS complement(2036700..2037596)

/gene="araQ_8"

/locus_tag="EFAGFIKM_01798"

/inference="ab initio prediction:Prodigal:002006"

/inference="similar to AA sequence:UniProtKB:P94530"

/codon_start=1

/transl_table=11

/product="L-arabinose transport system permease protein

AraQ"

/db_xref="COG:COG0395"

/translation="MSNISIRKSKGDLIFDIINHSVLIILLLAVLYPLVYIVSASFSA

SESVVSGKVWLWPVKPTLDGYKAVFANGMIWTGFMNSWVYAVAGTFINVMFTILAAYP

LSRHDLKGRGIFTFLFVFTMMFSGGLIPTYLVVRDLGLLDTRLALIFPAALSVWNMLI

TRTYFQTTISRELLEAGQMDGCNDYKFLWKIVLPLSGPIVAVISLFYAVGHWNQYFSA

MIYLRDPQLFPLQLVLRDILIQNQIDFSTMSSTEELVKREGIRELLKYSLIVVSTLPL

MVVYPFVQRYFVKGMMIGSLKG"

CDS complement(2037616..2038578)

/gene="yteP_8"

/locus_tag="EFAGFIKM_01799"

/inference="ab initio prediction:Prodigal:002006"

/inference="similar to AA sequence:UniProtKB:C0SPB3"

/codon_start=1

/transl_table=11

/product="putative multiple-sugar transport system

permease YteP"

/db_xref="COG:COG4209"

/translation="MQKPQVQVTTRPPKEMTESGHRGRPWIKSWQLYSLLILPLAYLI

IFKYVPMYGTLIAFKNYVPTKGIWGSDWVGLLHFERFLNSYQFVRILKNTLSLALYEL

AVGFPLPILLALGLNYVRNTFFKKSVQMITYAPHFISVVVLVGITMQFLQPGTGALNV

ILGFFGVDSVHFMAKPEYFKTIYVLSGLWQNIGFSCIIYLAALSAIDPTLHEAALMDG

ASKVRRMWHVDLPGIIPVITVLFILQMGNFMDLGFEKVLLMQNPLNMRTSEIIDTYVY

NIGLKGAVPQYSAAAAIGLFKNVIALILLVIANRMARRTGNSLW"

CDS complement(2038846..2041110)

/gene="rhaR_8"

/locus_tag="EFAGFIKM_01800"

/inference="ab initio prediction:Prodigal:002006"

/inference="protein motif:HAMAP:MF_01533"

/codon_start=1

/transl_table=11

/product="HTH-type transcriptional activator RhaR"

/translation="MNLAVVRKWINNRPLFFRLVIPYIAVVILLASCLGFTYSKSLKI

VKKDILYANDMALEQTRRTLEIRISEMEQTMWDIVSLPSVRRALIIREPFEQANPYRF

KSVSSSLKQLSLSNDFILDYFVLFKRSSMAVNGDKIYTFPQLYELYFQYDEGSRDHWQ

DRFFQLNGNFQPAMQLTLAGKTVSGIPFIRPMGNGREVHGALLVLMDNRKIHELLGSM

QLSEGSLSYIADQKGQIISYVSGTGVEGLVPSVVKDVNLKDMITASTTSHQLGWTFVT

AHPQAIVAERSLEITQSILNLLLIFVFLGLVISIGLAHRHSLSLHRLTRHFTEQSEPK

LFFGNAYSWIEQKVGGLSERQHIMEKLHKEQLPILQTTFYEQLLKGYFSSEDHLQSLM

KHAGLEFDSTGYLVLTIHFTGYGDGVDADILREVEGKKITVKEALRFSDAAKSIHVHD

WDTYHLSIIYCCGDKEIQDLHLVEQILLHILGNHSLYLEEELDVVVTASSMVTNLWEL

PHAYQEAMTAFRQYVWDPRHRFVWYDGQPENQSFAYYPPELEAMLVQCTLTGDIERMQ

DVLHGLYQANMVNRRLSLSMMQLFLNELLGTLAKVLIELKNLQTSPEEYVQIRCVKDA

VDYYGQIHDRFLSIGSLIHLNKRSHNIELRDRLTAFIQKNAYDSDLSASKVAREFNIS

SAYFSQFFKEQVGSNFSSYLEQLRIARAKELLNGTDSNVQEIAVSIGYNSSNSFIRAF

KRSEGRTPSEYRQA"

CDS complement(2041517..2041888)

/locus_tag="EFAGFIKM_01801"

/inference="ab initio prediction:Prodigal:002006"

/codon_start=1

/transl_table=11

/product="hypothetical protein"

/translation="MNTEQDLRVLKTLEHIKSGFSLCVSQKTFKSITIKDISTAAKVN

RSTFYTYYTDKYHLRDELVKATLQDLDHAIDLTFITFDDQSPWQTRSVIVQHLEHLWS

QKEWYLHLWNKNLELYVFEDM"

CDS 2042193..2042723

/gene="linC_1"

/locus_tag="EFAGFIKM_01802"

/EC_number="1.1.1.-"

/inference="ab initio prediction:Prodigal:002006"

/inference="similar to AA sequence:UniProtKB:D4YYG1"

/codon_start=1

/transl_table=11

/product="2,5-dichloro-2,5-cyclohexadiene-1,4-diol

dehydrogenase"

/db_xref="COG:COG1028"

/translation="MYNLMEKKAGFVTGAGSGIGRASAIAFAQAGAKVMVADINEEAG

LETVQLIKGFGGVASFVRTNVAVEEDIIRLVQATVSELGQLDFAHNNAGITLPPAPMA

DSSSEAFDRVVKINLYGTYYAIKHEIQAMLKTGGGAIVNTSSGFGLIGGQNQAIYSAT

KICNKRNYKKCSYGIC"

CDS 2042710..2042955

/locus_tag="EFAGFIKM_01803"

/EC_number="1.1.1.119"

/inference="ab initio prediction:Prodigal:002006"

/inference="similar to AA sequence:UniProtKB:Q5FPE5"

/codon_start=1

/transl_table=11

/product="Glucose 1-dehydrogenase"

/db_xref="COG:COG1028"

/translation="MEYAEKGIRVNAICPGMTATPILSWLDNAPEQAAAFRSTIPSGK

LQTAEDQANAAVFLCSNLAGQITGVTLPVDGGYTAGR"

CDS complement(2043131..2043952)

/gene="rhaS_10"

/locus_tag="EFAGFIKM_01804"

/inference="ab initio prediction:Prodigal:002006"

/inference="protein motif:HAMAP:MF_01534"

/codon_start=1

/transl_table=11

/product="HTH-type transcriptional activator RhaS"

/translation="MYNPLDLVVRIVWSSRKQTPMDWSDIRNNTSVHTFYWIHEGKGM

FQTEDQTYEVLQGMLFYMEPGAQMKMESSKESPLTISMILFDCCAVSYQTPEWQLPQP

IHRMDIPFFSRYHSERALRLDHAFNEITESWLLGNHYREMESCALLLKLLAILHRPDH

FSEPGNTTSIIFKIKEELETRFEEQLYIREIASKYAISESHLRKMFMSHIGQSPKEYL

TEVRLRQSERYLTYTNYTFKRIARQCGYHDEYHFSKAFRNWKGVSPSTFRKNEKS"

CDS 2044126..2045814

/gene="uidA_1"

/locus_tag="EFAGFIKM_01805"

/EC_number="3.2.1.31"

/inference="ab initio prediction:Prodigal:002006"

/inference="similar to AA sequence:UniProtKB:P05804"

/codon_start=1

/transl_table=11

/product="Beta-glucuronidase"

/db_xref="COG:COG3250"

/translation="MIRLFDTHHTRISTELEGMWEFSPVDNIGERPKEFPFKLPVPGC

WESHPHFGTYRGKAVYRRVISVSSQTHIRLEFKGVSHTAHVYFNGEFVKTHYNAYTSF

EVIIPNAISGEHEILVYVDNSFSEASALHFPNDYYTYGGLIRPVVLEEISDLYIENIM

FTPLRQANQWNGRWKVMIRNISQFPQQVQVIGELAGIKSILGSCEVGPGGQAELIQTV

SYPNVLEWSLDHPSLYQLQTKLITNGRLVDDLVDRIGFREITISNGQLLLNGHKLKLK

GVNRHEDHPMVGCSFPLSLMVRDLDLIEEMGCNAVRTSHYPYDERFLDLCDERGIVIW

EENHARGLSLEQMRNPNFAWQSEQVTREMVEQHFNHPSIIIWAILNECASNTEEGKVH

YAKQLTIIRELDPSRPRSFASHHRDQELCFDLAEIVSFNLYPGWYTDEKPEELALEAI

QWANKLGGADKPIIMSEFGADGFYGFRSNNREKGSEERQADILRNCLNAYQASTDICG

MFIWQFSDCRVTEGEGWLLTRSCTRNSKGLVDEYRRPKLAYDVVSFIYKSEDKK"

CDS complement(2046057..2049968)

/locus_tag="EFAGFIKM_01806"

/inference="ab initio prediction:Prodigal:002006"

/codon_start=1

/transl_table=11

/product="hypothetical protein"

/translation="MVKTQETGSVLTLAKDWVERAVVGTEPGQYAESDWKTIVTLVEA

EEQTQRDKSTIRHLLDGMDQFLSRVHCSNRVINKENNLLEYSTYRRELTRLVFKARAI

LFIDAEQYTEGAKVALTDQITRAEAALNGTYTAPFVRNRAFIEPRPDEDIQAVTEHCT

MAPSYNMGTYGLRPAIEWYTYQHIMADCYETEKVYASSIGCVNESTADLSQGDLPAVE

LESCSIAYLTFDLPSLGQRRLRHARLRLVNHHTDGKSVDLYALEHSNHHFQTLEQLTY

RQIERHPKMQPSADQYLRSFQLGFAEGFSYADLSEYVIRSMEKGGKLKLALTLGEGEL

PCGFYTCNHPEQQKRPLIELFWDEVQEDVWQQKVREICQRAKQILANEKPGSELGQVD

PEAYQQVQQELSNAECLALSQLKTEKVSESGSALVRLLNAMRHLRHSRVLRSDLLGKW

NLFFTTQGLTKLREKTVRNDELEREFQKVREWSERRSLADLQSYASVMQDEPDWTLLN

KQFGLWTRSRLLTFTPPENTASASLAFVLPSEDNEEHDKLGHVWIDRVSILPAHDANL

EIENSGFNEGNQLPNHWTPVAIKGKPVLHWEDRGEYVQDGSHSIYLENPTSGDEGAWI

YDKDIPLQGGVNHTLTYFAKVEGKFKEGVQAVLTFKDAVGREVGSFHGIHNKKSLPGN

PELRLTVSFQADALIYAMTGERSYAEKVKLQMLWLLNDYCQGVESWLVHNVRPDGLDA

YGAVQIGRVTTLIATSYSLIRDANVFSQEEYDRMMAQLDYLIRDLMDLRDRTELGAYR

AQQGTSNWHTDMAAGAAMLALAFPEMPHARQWLDNARLILKGQLDYHINEDGSWPESI

RYLFAVIQRYGTFAKTLRHMTGENWFQGTRFSSTFKYALSVQTPPYVFFDNKISTPNF

GDHTLDNGGGFAVLGLYVDEIISSDPELGAQVYETWIRAGKPLCGFGVESNMLENFFM

PSSIDTLASDSKTLNLISQSFENIGLYLFRQNFNCPDEGYLSVISSRTPVGHGHHDQA

SFIWYVNGIPLVVDPGVESYFDSTFAWYKGSSSHSVVQFRHNGYYVDMPRVSEVQHLL

TSDLLDEITIRIQSPTGAGNHTRHITYMKNGLDALILWDVIRNAEEGTRMNLPLAAKS

TSIEGQRALSIGHYGMDLETISLLPQCADIIQEWGRSYPIAPLVEGKSQLNYLRIKAG

RNEPLLTVLFAHPTGGEGLQIKPIRCSEKHALAYQIRKLDRVEAILVVNTSDECVTTE

LRYKHTLMDTRNKEIIPDIKGQIRLSLDGGQLRVLIPNSNT"

CDS complement(2050003..2051571)

/gene="xynB_2"

/locus_tag="EFAGFIKM_01807"

/EC_number="3.2.1.37"

/inference="ab initio prediction:Prodigal:002006"

/inference="similar to AA sequence:UniProtKB:Q9ZFM2"

/codon_start=1

/transl_table=11

/product="Beta-xylosidase"

/translation="MNINFQGKSKPLNHIWSACVGAGRANEGLRADWQSHLSKAVKHG

GFRYLRFHGLLHDDMHVYKIVDGQEVFNFQYVDALFDAMLDQGIRPFVEFGFMPNDLA

STERTQFWWKGNISPPNDYAKWANLISKLVEHWIQRYGLEEVKNWYFEVWNEPNLSAF

WDGTKEQYFKLYQVSVAAIKAIDDRLRVGGPATSNFVPDARFDGELEDISTHLTLVTE

ELDSLEWKGVWIEEFLAFCERKKLPIDFISTHPYPTDFALDGHGEFQGRSRHASSTKD

DIEWLKRVIASSAYPDIEIHLTEWSSSPSSRDYSHDYLPAAAYVVKTNLEVSDLIDSL

SYWVFTDVFEEVGAGPAAFHGGFGMLNLQGIPKPTFHAYSFLNQLGEHELARSEGTVI

TRTDNGELRGLFYNYPEDYKGTVPMSVYPDQTVAQTAQSFGISRTFNLVVEQVTPGGQ

YVLRTVDRDHGVAVQLWNEMGSPNSPSREQTEQLIAYADQLNERKFTADSNGRLTLDF

NIGPWAIALLSEEV"

CDS complement(2051632..2053977)

/locus_tag="EFAGFIKM_01808"

/inference="ab initio prediction:Prodigal:002006"

/codon_start=1

/transl_table=11

/product="hypothetical protein"

/translation="MMVRGNAKSNHSAIWFDEPAKEWNECLPIGNGTLGGMVYGGITK

EVIQLNEDSLWYGGPTDRNNPDAAAHLGNIRDLLMEGHLQEAERLALLALSGVPESQR

HYLPLGDLTLTFRNALPGKINAPQSYRRELNLEEGHVAVTYIQNGISHRREIFTSYPD

NAMMIHLTAEQPGSIHVDVQLSGGVRKRHMDGMIRLRDNSMAMHGSTGGGGVNYAAAI

QVQTVGGSVQILGETLLVKDADEVFISLTAATSFRYDQPLEECDTRLLQLTKYSIDTL

KERHRKDYQALYHRMSLQLGPVDKELEHLPANARLERVQAGESDHGLVGLYFQFGRYL

LVASSRPGSLPANLQGIWNEHLTPPWDSKYTININTQMNYWPAELCNLSECHEPLFDL

IERMREPGRHTAKVMYGCEGFVAHHNTDIWADTAPQDTYMPATHWPMGAAWLCLHMWE

HYLYSGDLPFLGRAYETMKESAVFFLDYLSELPDGRLVTSPSVSPENTYMLPSGEKGT

LCYGPAMDNQILHELFTGCIEASIQLNKDQEFRNQLVSVRDRLPGIEIGKHGQIMEWL

EDHEEAEPGHRHMSHLFALYPGNRITPTNTPELSRAARVTLERRLENGGGHTGWSRAW

LINFWARLLDGEQVYQNLISLLKHSTLPNLLDNHPPFQIDGNFGATAGIAEALLQSHT

GTLSILPALPPAWSEGKVKGMKARGGYIVDIEWFADGRLVACITATQTGMGSILAQQI

NEIRQQGAGTNLSDDINTNGEPITLELEAGYTYELFGRYRI"

CDS complement(2054014..2057058)

/gene="lacZ_1"

/locus_tag="EFAGFIKM_01809"

/EC_number="3.2.1.23"

/inference="ab initio prediction:Prodigal:002006"

/inference="similar to AA sequence:UniProtKB:P81650"

/codon_start=1

/transl_table=11

/product="Beta-galactosidase"

/translation="MLDITKHWEDPTTLHVNRETARAYYIPYHDAVSAKTRKRGKSPY

FQTLNGQWKFQFHASVAQVHEAFYQKKFDVKEWDNLTVPSCWQVNGYDQLQYINYNYP

ISCDPPFVPNDNPAGLYVRDFEISSEWDTKERFVVFEGVNSCFYLWVNGQYVGYSQGS

RIPAEFNLTEYLQPGLNRMALMVLKWCDGTYIEDQDMWRYSGIYRDVYLLARDKAHIR

DVFHKQQFAAAGNQVTLVSEIETTGRLYVKAELKDASGRLVAAADGVIEDKGALKLEV

EKPGMWSAENPVLYDLYLTAGDEVILSSVGFRTVDVHEGVFRINGKAVKLKGVNRHDS

HPELGQTIPMEAMIEDIKLMKQYNINTIRTAHYPNDPRFLELCNEFGMYVIDEADLEC

HGIGQAGNFKDGCYQQFSADPTWKEAFIERAVRMVERDKNQASVIMWSLGNESGYHDN

HIAMAEWIRSRDESRLVHYEGTAAQFGGHPNTQSLDVESKMYFTYDEVRKYIEDETNH

KPLFLCEYSHAMGNSCGDLGDYWDLIYAYPQLMGGCIWEWTDHGIASKTAEGQSYYAY

GGDFGDSPHDSNFCIDGLVSPDRKPHSGLIELKQIHAPIHVEAIDLSQGSFSVINRHD

FVDLSHIGLHWKITSDGVTVQQGQIWQLEAQAGSEQRISLPYDFTALNGQAAELTLSF

WNNKETTWASMGYEVAFAQFIIHETQSLARMQDEITTKPIAYRHVHADEHQGTLVMTG

HGYRYEFNLDQGAFASITREGLEMLAKPLRFHIWRAPIDNDTYILGKWREEGYDRAKT

KIYGTTWEQTENGVSLSVKYSIGASDRYPILHGEAIWNVQHNGVIQLQTRVNVRENFP

YLPRFGLEWIMPSGNEEVEYYGYGPHESYLDKHRSARRGRYLTRVHDLFEDDYVMPQE

NGSRFGTEWAMVTNLLGMGMKFASSQPFSFNASHYTPEDLAAASHTYDLKPRQETVVQ

LDYKMSGVGSSSVGPQLLDAYQLVDKAFTFELWLKPIFKEDE"

CDS complement(2057369..2058973)

/gene="rhaR_9"

/locus_tag="EFAGFIKM_01810"

/inference="ab initio prediction:Prodigal:002006"

/inference="protein motif:HAMAP:MF_01533"

/codon_start=1

/transl_table=11

/product="HTH-type transcriptional activator RhaR"

/translation="MKHTFSLVIVDDIPAVVDGIAHDLPWDDYAIEVVGTAYDGLDGL

KLIQEKQPDIIVTDIRMPKLSGIEMIEQAKQSGAKLIFISGYSDFSYAQQVLNLGGFD

YILKPFTPNELLETICRAKVQLEKEKESRHHLMDVERKLRESMPLLRQEYLNLLVRYS

SHPETVKEKWDFLNVNMNDHYFTVFTVEMDNYYTNKAGVSDVELQRFSLQNILEETIR

TFTGAVVFRDDINRFVCIVNDRADADNEEMLKIAEACRENVERYTRYSISIGVGTKVE
HIHELPITYQQAMYALSYNFYTGGNSVFAYPIYAENHATYVKFSSEKERELVYLLRSG
ATSKINEVLDGIFSEWKDSQAMPTPDKVKMFWIELMVNVRNAINEDLEFVEPHFFDRK
IHKLTYDTDSLTELQSIVAEICHMYCENIGKKQKETAHVVINESIAYIREHLHLNASV
ADYAKQVHLSTSYYSNLFKKVTNQSVLQFVVQERIEKAKILILQGITLQEVAMEVGYE
ERSYFSDVFKKKVGMTPSEFKKQYQN"

CDS complement(2058975..2060735)

/locus_tag="EFAGFIKM_01811"

/inference="ab initio prediction:Prodigal:002006"

/codon_start=1

/transl_table=11

/product="hypothetical protein"

/translation="MKKWLYSTTFRERIQLSLILFTMIAVVVCGGTSYLIASRVLENK
SYSLNQGIIDKSAQSLEEKLRKIRLAVLTFMSSDQFNQLLKETAKGGELSYYDHFKLN
QSLQTPIFQMKLIEPGITSILVNTPVGEYFSNPDNRTGGIPFSESNVYPFLEKGHLPG
WVESHPDDLFRTGQNVLSLMFVPIVQNAPADTHIVVDVSESALKEYLLKNSGDAIPVV
FTENGKLAFEADSLSSQLASDSDFRSRLKDNKGHFEYTVNHIRYLVNYSTVAFPDNWL
IVHLTERNVLLKDVRLIQLVTISVIVIFGLLALLVSRKLTSLLLRPLNQLQSVMKKVE
HNDWSVRFQGEYKDEFTHVGFRFNSMLDQIELLTRERMDAQHAERIAEMKALQAQMNP
HFLYNTLNTILWKSHSNKQEEVREMIMSLSALFRVGLNNGEELTTVGKELEHVTQYLK
IQKLCYASLFEYTIECDEEIKQLSTLKLLLQPLVENSILHGFKDYRNEGQIHIRVKVV
NKFLQMKVIDNGQGFDVQEHEADNPHLVVSDCEGFALRNIGKRLSLYYGSRAEFNVTS
TPYIQTSIEILIPLEIEGAS"

CDS complement(2060847..2062403)

/gene="lipO_5"

/locus_tag="EFAGFIKM_01812"

/inference="ab initio prediction:Prodigal:002006"

/inference="similar to AA sequence:UniProtKB:P37966"

/codon_start=1

/transl_table=11

/product="Lipoprotein LipO"

/db_xref="COG:COG1653"

/translation="MKKKAALLMASLLLSASVLGACSSDKTGSNTQTPSQSSSSQESA
NLSIFYFDDAGVVKMDSPVLKKAAELTGVTLNNVASAGGEEKQAYNLMLASGTLPDIV
SYYTADLNSIGAEGALEPLNDYIDKLAPNFKKFLDEHEEVRKTITAADGNIYAIPFVA
DGSASTGWFIRQDWLDKLKLETPKTVDEYYEVLKAFRDQDPNGNGKKDEVPFMQRDNV
VGAYSLLPLWDAYHSFYIKDGKIVYGPYEDQFKTGIENIAKWYKEGLIDKEIYTRSKA
REVLLGNNTAGSTHDWFASTANFNQSLSKDVPNLDFIPFAPPASISGNVYEVGKRAQL
SGFGWGVTAQSKHIEDAVKYFDFWWSEEGRRLFNFGIEGKSYTMVDGKPKFTEEVLSQ
PAVNGYLVEAFGAQLPKMGAWQDFSYEEQWTNEIALKGIKEYQDKNIISDNYVLPTLN
FTKEEQKRLDALKGQIDTYFAEKAQTWVMGGENVEAGFEAYKTQLKNLGVEEYIQIYN
DAYARYLSIQ"

CDS complement(2062477..2063340)

/gene="ycjP_2"

/locus_tag="EFAGFIKM_01813"

/inference="ab initio prediction:Prodigal:002006"

/inference="similar to AA sequence:UniProtKB:P77716"

/codon_start=1

/transl_table=11

/product="Inner membrane ABC transporter permease protein
YcjP"

/db_xref="COG:COG0395"

/translation="MMESRGEKVFYVFNYIILGLFACLTLYPFLYVLSASISSPQAVV
SGEVLLFPKGITWEAYTSVLGEKGIWTGYGNTIFYTVVGTLTSMILTICGAYPLAKKR
LKGRTTINLIISFTLIFNAGMIPMYLNFQSLGLLDNRFGIIIGFAISTFNFVILRTFF
QSLPEEIEEAARIDGAGDIGVMMRIVIPLSKAPLATIGLFYAVSRWNGYFWSMIMLRD
ESKFPLQVLLNRLVVQMKPSENMMNDVSFTTGETVIYATIVVAIIPIIAVYPFIQKYF
VKGVMIGSLKG"

CDS complement(2063351..2064307)

/gene="yteP_9"

/locus_tag="EFAGFIKM_01814"

/inference="ab initio prediction:Prodigal:002006"

/inference="similar to AA sequence:UniProtKB:C0SPB3"

/codon_start=1

/transl_table=11
/product="putative multiple-sugar transport system
permease YteP"
/db_xref="COG:COG4209"
/translation="MTTNTKIQTYNAKIVKRTSTLWALIKRDWFLYLLLVPFLLWYVM
FAFKPMYGIQIAFKDYSIFRGIQGSEWVGLQHFQEFLKSEYFYRVLKNTLIISLYALL
FVFPAPIIFALLLNEVKSSKFKKVVQTFTYMPHFISVVIVAGIVTNFLAPGNGIVNLI
IEKFGGDKIYFLADASFFRTILNSMNIWQGTGFAAIIYIAALSGVNSELYEAAVIDGA
SKWKRTLHVTIPGILPTIMIMLILQIGSLLEVGYEAIILLYQPVTYATADVISTYVYR
EGIVNGRYDMAAAVGLFNSVVGFILIIAANKLSKKYTESGLW"
CDS complement(2064532..2065689)
/gene="ugl_2"
/locus_tag="EFAGFIKM_01815"
/EC_number="3.2.1.179"
/inference="ab initio prediction:Prodigal:002006"
/inference="similar to AA sequence:UniProtKB:Q9RC92"
/codon_start=1
/transl_table=11
/product="Unsaturated glucuronyl hydrolase"
/translation="MLNIEKQSIWLQDIEQRIIQKLDWVSDKSKNKIPYTTVNGVHDD
RSVENPSSTHADGINWWTNGFWGGMMWQMYHNTGQEKYKAIANISEDKLDAGLQQYYG
LHHDVGFMWLPTSVANYKLTKNPESRKRALHAANLLAGRYNPMGEFIRAWNDISGEDT
RGWAIIDCMFNIPLLYWATEETGDPRFMQIALKHADTVMDTFVRPDGSVNHIVEFDPF
TGDVIKSYGGQGYAEGSSWTRGQTWGLYGFMMSYIHTGKKEYLDTAKRIAHYFIANIP
DNGIIPIDFRQPKEPAYEDSTAAAIAACGLIEISKAVELHEKDLYLKPALKLLKTLDE
DRADWTTECDCILKNGSAAYHAKEHHQSIIYGDYYFIEAIYKLKGTDLYFW"
CDS 2065869..2066759
/gene="araC_2"
/locus_tag="EFAGFIKM_01816"
/inference="ab initio prediction:Prodigal:002006"
/inference="similar to AA sequence:UniProtKB:P0A9E0"
/codon_start=1

/transl_table=11

/product="Arabinose operon regulatory protein"

/translation="MEVGFESVPFHGQPLIWTDRHRTSKTNSSFYHWHQCCEFLIVFE

GKGTVVMNNKTYPIKPGMLYVFQPFEIHKVFAKVTHESHYERAVIHLDHVVIDQYLKD

YPRRREWFDRLCYSSEIERAFDLSRDMDLFSYCMEDYGQIAHMEQGESREEITVFVLQ

FLSAMARIFPKDQSEPSLTRRKKEHSELIMQWLDEHYMESNILNKLADQLHFTRSYVS

RLFKNETGSNLSEYLTAKRIKVAAHMLETTTMSVEYISHHVGFQNVSHFISCFKKTYQ

VTPLKYKLNQKKSGLESHEV"

CDS complement(2066880..2068853)

/locus_tag="EFAGFIKM_01817"

/inference="ab initio prediction:Prodigal:002006"

/codon_start=1

/transl_table=11

/product="hypothetical protein"

/translation="MPQQSTKAKLMSIMLVIIMLLIPAYSVLSSETAEAATFTHPGIM

HNATDLTRMSANQNTEPWKSALANFKTDSKASSSYAKQGPYSTVCRNDSTSTCTSTNY

GNSALENDARAAYYNALLWNVTGTQAYANKAIEILNAWSSTLTSIAGTDAQLAAGDNG

IFLANAGELLRYSNSGWAAANITQLENMLTNVFYPHIQSPGDANWGGSAMKSMISIAI

FTNNQTMFDSAISNFRTNACASVTRNVMSTGQISESGRDQVHALGGLGNLTVVAEIAW

KQGVDLFGDGDNRLLAGSEYWSNYNLGNEPTSWDSTYGRCTMGPWSGINGTGRSDTIG

WAQNEIIYAHYVTRKGLTAPFTTSYMNGMPATNTDAALLTFAYRLGTTTDSTKPSAPT

GLTVKPLSDKVINLSWTPSTDNVAVSGYKIYRNGTIVGYSPTSDYTDAGLTASTAYNY

QVSAYDAKSNTSTTSSTVSATTLSSANAQIPFSSQDIGSVGVAGSYSYSAGTYTIKGA

GSDIWDKSDQFRFAYVPLKGDRTITARVASLTNTHSSAKAGVMIRESLFEDASNVYAA

MKPGLAVFQNRTAPGAITNSTTSATVSAPYWARATRSGNTVTTYISANGVTWTQTGTS

SISAASTAYVGLAVTSHANTTLTTATFDNVSVQ"

CDS complement(2069011..2070048)

/locus_tag="EFAGFIKM_01818"

/inference="ab initio prediction:Prodigal:002006"

/codon_start=1

/transl_table=11

/product="hypothetical protein"

/translation="MRSKTNVKKMEMRKVWTSFLVLLMICTTSVNAATIEQLARPSFE
LKLKSVNNKLVEVSVIGHDLEDLYAYEMNLEFDADRLIFSKASAGSVGYTVVPKAEGN
YLKIAHTKVGSVKGDSGDQLLTTLQFNVQRAGNSKITLQTIKLVDSKLNMSNMTPALQ
ETIQAESATSFSDITGHWAEASILKAVEAGFIDGYTDGTFRPNHEVTRAEFAVMFSRA
LKLDVNESDATFADANQIPAWASSHVQAAVKKQLITGYVDHTFRPNAKMSRAEMASVA
VRALGKTQHDVTYVNEFTDAGDFSSWAKPFIHEAVESSIMQGIGNNRFSSNTNVTRAE
AVTVILNVLRN"

CDS complement(2070117..2075468)

/locus_tag="EFAGFIKM_01819"

/inference="ab initio prediction:Prodigal:002006"

/codon_start=1

/transl_table=11

/product="hypothetical protein"

/translation="MKQCCKIIKWMVIRVLVVSLCLVITTVPSRVSADDSVIHTQPAI
LETVSQEGFVHPSVGFTGDALKLMQEKVAIGAEPWASAFEDFRNSRSGSLDYDIRNQS
KLDKTKPAYTKIDNDAKAKEAKVDAEAAFTHAVIWAATGDVRYREKAMTIVRLWSQID
PGNPKGFTDSHISIGDAMVNMVRAAELLRYTEGSGEWAWTETDTTYFTQNYLMAFYDG
NFYHNNGRWMNQHGIITQAYMMAAIFSDNQQWYQEAVEWATANKTATFKGQSGDIFHQ
IRYVTQNERTGAPVEPHVQLVEMFRDMGHASGNTSTLSMIAYMTEIQGTKVNPDPASP
TFGEVTDASDGVGIFEFLDNRILAGANELAKYNLGYDILYTPVNISNAVVGDPTGNYA
DTVSDNGRGLGYASEVTYGYYMHHLTQPLDPGDERIKYLAQAIEKQGGLKAIPSYVLL
QGTSDLATGEVVGPPKPLSNPPYIEVTAAYDRLQAFDYMGRSETTSTSTFQDMDGMRS
VLYDVRYENQYTWYDLDVEDSYNEITIRAASNSSVGTKVDVILLDGVDGLDRTNVTTA
DLAKGEVLTTLQVPNTGWWTNYATVSSKLSKPLSGKHLIAFKYYGSANALSYQIAFDW
FAFSDGYAGDEHPALSGVLSGEATKASDGVNLSEGGAITFGKMNFDSGNNSIELQAKT
TDATGKLLLYSGETLVASYKLPNTSNAMVKFTSEVASADLAKIKGIHDITLRYKGNSP
ITLKTYRNIERNRLANVETTGSQANFIADQAIPISGTFEIGSEGTRSYILTKNESIVY
LSKAALQVDRTKDSIVSFTVRTNGVAKLQLQRDSSTPPFATIRVPDTKGDWLRVSTNI
SNKYVKSDDYPTYLKSLFVSIETDKEDTEVKLGSYTLDPADTSPVLKLTSEQGFELTS
VAAYENGADMNLKVSVEDLDSVNVSLSTDNQDYIDSSGLNGKAGGTLIVKPAGLAPGE
YVFHIYAEDDTSNYVDKEVKLSIYPEEENKAPEGLTAALTGPTSVLLNWQEVNGATYY
TIYRKQGAGGTYTKWKETTSTELSDADLKSGTLYAYQVAAGGNAGETEKSSPIDITTL

STIQVTQSMVKASSKQWDASDSTGIGTPESNGWRAFDGNIETAPDALTNPSWILIDLG
QNNAPILGGVKFYPRSTHLNRMKDAVIQGSNDGTTFENLYTIGTISTAKWYFVGMTND
KGYRYLRYYTPKGNANVAELEFYQKAVDVPAPNQAPVFADLAPVTVTAGQEVKQTLVA
TDPEGDSITYAGIDLPERAQLDAATGEFNWTPETAGSYVLQFKATDAKGAATVATWTI
TVKEIAEPQPGTENSATLRGPLTAAPGQPVDLNIGVQGVTDGFTTAQLTVTYDTYRLQ
FPTTEDEQGLTVLADGAVSSSEEGLQIIGTGVKPESGEILLIVANTNQNQITDSSNLI
ALHGQVKVDATTGKAQVSLSDFEVSADGNSNMLDTSTASIAIQIQVDKTQLTAAIQSA
QSLHDMAVEGSDTGQYPVGSKAVLLTAIEHARAVAANETPTQAEVDAKVVELNQAVES
FTELVIGVSKAELQQTILQAQWLSAAAVVGSSQGQYPFDAKNAFDAAIQNAVTVRDNS
GTTQAEVDKAVIALQPAITTFRNSIYTATPQPTDHTGLAVQIQSVQKQLDKAVAGTKI
GQYPQQAISILKAALNTANEVKNNTAATQSSVDEALANLQSAASTFAAQMITLVPGQT
QVSIKDLSIIAKYYGTKSTDASWTDIEKADLFEGGEITIRELAAVAQMIVGDWLQNK"

CDS 2075939..2076730

/locus_tag="EFAGFIKM_01820"

/inference="ab initio prediction:Prodigal:002006"

/codon_start=1

/transl_table=11

/product="hypothetical protein"

/translation="MIHLTIEVQREDGTILAEHTDTNHTHLVYDQPYQLGDSIVLRSS
RDGVYLYIQLDDALNPDFVYLSGQEYRLSIPFNEKKTSYSPKSFTGQLHLLTVRVASN
EEISSYKNLAANSHDHHTNSTLYPHASANVETRGEAVFAARNAINGNTVTYSHGEWPF
ESWGINQRLDAEFTLHFGRLVEIDKIVLYLRADFPHDNYWKQVTLSFSDGSSITSSLE
KSGKAQTILLEPRQVESVKLSELIQSNDPALFPALTQFEAYGREI"

CDS complement(2077435..2077611)

/locus_tag="EFAGFIKM_01821"

/inference="ab initio prediction:Prodigal:002006"

/codon_start=1

/transl_table=11

/product="hypothetical protein"

/translation="MGKLNQLDDFVENDETSSIILWDGNGDFGYRRNRQDRSESWIHI
QRVDHRFFRSQPVS"

CDS complement(2078357..2080120)

/gene="cotA"

/locus_tag="EFAGFIKM_01822"

/inference="ab initio prediction:Prodigal:002006"

/inference="similar to AA sequence:UniProtKB:P07788"

/codon_start=1

/transl_table=11

/product="Spore coat protein A"

/db_xref="COG:COG2132"

/translation="MVQLLDPKRIPKYMNQIGNTPVYMPTVLRQPMTGKLIGHAYKIY

ASEFQQQILPSGLGTTKVFGYGGMIQDLHTGRSVYYRSAPGPTFEAIRGIPVHVQWIN

NIAGPHFLPVDPTLDWANPNAVSVPLPPFPSFPPGYPLAQKSVPIVPHLHGGENPSYY

DGYPDAWWTADGKTGMAFTTNNYIFPNVQQSTTLWYHDHTMGITRLNNYAGLTGIYVI

REMNDPIASFLPSGENEISLIIQDRSFHADGSLYYDPVGANPSIHPYVPPEFFGNTIM

VNGKVWPNLNVKPQQYRFRVLNGSNARFYHLHLSNQHKFIQIGSDGGYLPQPAELSSL

MIAPAERADILIDFSSLAPGTTVILMNTANAPFPNGATADPETVGQIMQFTVINGSVI

PSKIMPTVLNNIPKLKPTKTRTLTLFEQEGPNGPLKVTLDGQLWGNPVSELPQAGTTE

DWQVVNLTTDTHPIHLHLIQFLLVSRQKFRSDDYVRDWLKINQTTTPGTPPYFVKPTA

PPIDPYLLGQPISPPPNEKGWKDTIQMNPGEVTTIRVRFTSQDGSPFPFDPSLGPGYV

WHCHMLEHEDNEMMRPLKILP"

CDS complement(2080700..2080981)

/locus_tag="EFAGFIKM_01823"

/inference="ab initio prediction:Prodigal:002006"

/codon_start=1

/transl_table=11

/product="hypothetical protein"

/translation="MSNYHELVKGKVYIGGADAALSAIKEQGVTDVFDLRDDGKPQEG

FPDSTNRHHYPIVENRNDQEESIKFAIQAVTEAVQAGKNILSLCWWPEQ"

CDS complement(2081009..2081185)

/locus_tag="EFAGFIKM_01824"

/inference="ab initio prediction:Prodigal:002006"

/codon_start=1

/transl_table=11

/product="hypothetical protein"

/translation="MISILKTEGLKNDVVQEINKIRVAVDPEIVTTVESITLDVERYD

DGLGLVVSGGSSCF"

CDS 2081727..2082059

/locus_tag="EFAGFIKM_01825"

/inference="ab initio prediction:Prodigal:002006"

/codon_start=1

/transl_table=11

/product="hypothetical protein"

/translation="MSLTTGLQYNYLESEGKLIIYCKNISNKEITIFLKLTEKSFGQT

NSIPIFQRYFILDPMCCKEFNYTFKKINFFEVQVAFSELSKTICSVSLLDKTGIQYEE

YKLYKSSK"

CDS 2082737..2083183

/locus_tag="EFAGFIKM_01826"

/inference="ab initio prediction:Prodigal:002006"

/codon_start=1

/transl_table=11

/product="hypothetical protein"

/translation="MVRTLDARTSLASTGISPLNILLPSNTPTLLGIVGLNIFNPGPL

IRTQFNGTYRFSVASLSSLPSTIRLEIFRGATNTGQPIYVVSQPIFSSTNTILASFEG

SDYNVPPPVNGQLIYSCYATFVPASGMEQATRIGPECFNAVTYSDD"

CDS 2083225..2083677

/locus_tag="EFAGFIKM_01827"

/inference="ab initio prediction:Prodigal:002006"

/codon_start=1

/transl_table=11

/product="hypothetical protein"

/translation="MVKTLDARTSQGSNGHNILTPSFTNGVPQLFAQVGLQVSNPGMF

IRTFFSGIIEVTANQLPVNGNFVIEVYRGLSSSQQLVYRAGTIVQANAFFSPCVLSFT

GSEYNVPPPGSGQLVYSVYAIFTYDPSFSINNLRLGPESFNATVYSND"

CDS complement(2084257..2084448)

/locus_tag="EFAGFIKM_01828"

/inference="ab initio prediction:Prodigal:002006"

/codon_start=1

/transl_table=11

/product="hypothetical protein"

/translation="MNNVVEGQEYIPIEVANLAAFLFYRYKFLSQAKDKNLYRLPKWT

RTCYYLRLGEYLSGSRGLC"

CDS complement(2084496..2085503)

/locus_tag="EFAGFIKM_01829"

/EC_number="1.8.3.7"

/inference="ab initio prediction:Prodigal:002006"

/inference="similar to AA sequence:UniProtKB:Q9F3C7"

/codon_start=1

/transl_table=11

/product="Formylglycine-generating enzyme"

/db_xref="COG:COG1262"

/translation="MEDIEQDKKSSCCTPGHERASIDIKMSTRFNTKPVIYQDHGKEH

TKSEPTASSMIRLEGGDFCMGTDGNEGFAEDGEGPARKVWISPFYLDRYAVTNAQFQD

FIRDTRYKTEAEKFGNSFVFYLLLASDQRRNRATMVAQTPWWHVIEGANWMHPEGKGS

DLEERMNHPVVHVSWNDALAYCEWAGKRLPTEAEWEYAARGGLFQARYPWGDELTPDG

KHMCNIWQGEFPTNNDALDGYVGTAPVDEYEPNGYGIYNMAGNVWEWCVDVFNPYYHQ

QTPNINPLENGRGHVHSMRGGSYLCHDSYCNRYRVAARNKNTPDSSSGNVGFRCAANL

I"

CDS complement(2085729..2086778)

/gene="ssuA_1"

/locus_tag="EFAGFIKM_01830"

/inference="ab initio prediction:Prodigal:002006"

/inference="similar to AA sequence:UniProtKB:P40400"

/codon_start=1

/transl_table=11

/product="Putative aliphatic sulfonates-binding protein"

/db_xref="COG:COG0715"

/translation="MMRITTNRGRLLFVFMVMAIVIAGCSNAGNSTSERTPSNASEGK

TDSNAQPAGGEETVKVRIAVNGNLNPLIIAQEKGWLKEGFEKLHAEVEWSKFASGPPI

LEALVSGRVDLTFLGDGATITGLSNNLPLQVVGLIGEGKNLNSILVPVDSSIAKVQDL

KGKKVGLGRGSSSHVYLIKVLEANGLKQEDLTIINLTPEDAQAAFESGQLDAWVTVDP

NVTLNVANHKAKALDANIELFAPLSMVAHSEFTKTHPELVVEYLKQFKRSLEWQSENL

DEAAQIYSEQTKLPPEIIKVVLERSSNLLTAYSEEALAAQEATTAILLQNDFLKREID

FTQGVNDSFVLQALE"

CDS complement(2087013..2087600)

/gene="kstR2"

/locus_tag="EFAGFIKM_01831"

/inference="ab initio prediction:Prodigal:002006"

/inference="similar to AA sequence:UniProtKB:A0R4Z6"

/codon_start=1

/transl_table=11

/product="HTH-type transcriptional repressor KstR2"

/translation="MPRNVLRDTQMREQRRKEILDAAASVFARRGLLTKIDDIASEAG

RSHGYVYSYFKSKEELLLAVIKRGQDLYGEQLNKAIELEGKASDKFMYIATKVLSQES

NADSYMVLLQAIFTDLLDDQEKKKIRERGKKNRQLLVQLIHEGQADGSVRQGDQIHLA

TLFATLIQNLMLLKAREYEPANETTIQLLMQMIGP"

CDS 2087839..2088603

/gene="ssuB_2"

/locus_tag="EFAGFIKM_01832"

/EC_number="3.6.3.-"

/inference="ab initio prediction:Prodigal:002006"

/inference="similar to AA sequence:UniProtKB:P97027"

/codon_start=1

/transl_table=11

/product="Aliphatic sulfonates import ATP-binding protein

SsuB"

/db_xref="COG:COG1116"

/translation="MQPLLHIHEVSKTFHTDQQITPVLDGISFDVHEGDFTAVIGPSG

CGKSTLLKIVAGLDGEYDGNVLLKGEPILSPSQEKGFIFQEHRLFPWLTVEKNIAADL

SLREASVRRAVDDLIKLVKLNGFEKAYPRQLSGGMSQRVAIARALLRKPAVLLLDEPF

GALDAFTRSHMQEALLDIWRSNRTAMVLVTHDIDEAIFLSTRVIVMDARPGRIKAIVP
IDLPYPRSRTTRSFTELRTLILNELGHKEELEGYHI"
CDS 2088631..2089461
/gene="ssuC_2"
/locus_tag="EFAGFIKM_01833"
/inference="ab initio prediction:Prodigal:002006"
/inference="similar to AA sequence:UniProtKB:P40401"
/codon_start=1
/transl_table=11
/product="Putative aliphatic sulfonates transport permease
protein SsuC"
/db_xref="COG:COG0600"
/translation="MEVPIRSNAHSEKKHKTMPRSSGGSWHLFVRGAILPVLILSIWQ
ILSSTGTISTQLFSSPALILRQYWELTLSGELMHHLRISLVRALLGFLLGGSCGLLLG
LFVGMFRKIEETVNPTVQMLRTVPLLAITPLFILWFGFGELSKVLLISLGAFFPLYVN
TFLGVRNVDAKLFDVARVLEFSRFSQMMRLVLPAALPNILLGIRLSLTIAWLCLVTAE
LLGADTGVGFMIQDARAFMQTDIVFVGITIFAIVGKLSDSIVRFFEIKLLRWQDSYKG
"
CDS 2089528..2090979
/gene="betC"
/locus_tag="EFAGFIKM_01834"
/EC_number="3.1.6.6"
/inference="ab initio prediction:Prodigal:002006"
/inference="similar to AA sequence:UniProtKB:O69787"
/codon_start=1
/transl_table=11
/product="Choline-sulfatase"
/db_xref="COG:COG3119"
/translation="MTSKPQNVLFIMSDQHNRDILGHNGHPLIQTPHLDQLARDGVNF
TNAYSNCPICVPSRASIATGRYPHDNGSWDNAAPYSGNKPSWGHRLGNQGIHVTTIGK
LHYRDAADDTGFPDQRLPLHVLNGVGDLFTLIRETGLPSGNLGRKQVLAAGPGESSYT
QYDRAIAKEAVNFLRDEAAGIDRPWVLFVSFVTPHYPLTAPQEYFDLYPLESIALPKN

YGLDQRGSHPVIDEYRRLLGIVDELEEGDVRNALAAYYGLCSFMDAQVGEVLSALRET

GLDRSTRIIYTSDHGDSMGEHGLWFKSTMYEGSVAVPFIMSGPDLPKGEQIQNNISLV

DCFPTILEMVGAHPEPEDHDLPGKSLLPLAKGEIEPNRVVFSEYHAAGSITGFFMVRE

EQYKFIYYVNYPPQLFNLAKDPGESNDLASDPRYADVVSRMNQKLREIVDPEKTDAEA

RQDQQRRLDLHGGREQIVAKGFKVPYTPAPKVE"

CDS 2091007..2092146

/gene="nylB"

/locus_tag="EFAGFIKM_01835"

/EC_number="3.5.1.46"

/inference="ab initio prediction:Prodigal:002006"

/inference="similar to AA sequence:UniProtKB:P07061"

/codon_start=1

/transl_table=11

/product="6-aminohexanoate-dimer hydrolase"

/translation="MDLQQIDIIRQNMHFAHRFLPTATVHRSGGDVSPIASEPLHLDE

LAFVDATGRATTLSQALERTHTDAFLVMHRGKIVYEKYYSGMEPTLRHGLASVTKSFI

GTLISIELFVGNLGANDRVDRYIPELADSAFGDATIQQLLDMQISCKYPIFFPEANYI

DNQRNLLLTAIGTIPAKEGYTGPKTIHEYLASITKSQEHGSAFLYSNGPTEVLGWILG

RVNDTSLAELLSQRIWSKLGTEEDAYFVVDSVGTEQACGGLHATLRDVARFAEMMRND

GLFNGYRIVPKAVIGDIQQGGRRDLLAASNRAKARPGYSYRNQWWITHNRFKAYEANG

LYGQRIHIAPLAEMVIVQFSSYPGHSDETARLFSHGFEAIANHLN"

CDS 2092862..2093002

/locus_tag="EFAGFIKM_01836"

/inference="ab initio prediction:Prodigal:002006"

/codon_start=1

/transl_table=11

/product="hypothetical protein"

/translation="MAFVSASPAARLTDVGKDDADGFFPTVAVTLRMEPHLFDAYEKP

LP"

CDS complement(2094344..2095180)

/gene="bpoA2"

/locus_tag="EFAGFIKM_01837"

/EC_number="1.11.1.-"

/inference="ab initio prediction:Prodigal:002006"

/inference="similar to AA sequence:UniProtKB:P29715"

/codon_start=1

/transl_table=11

/product="Non-heme bromoperoxidase BPO-A2"

/translation="MAKVNVGEENAQPIELYYEDHGEGKPIILIHGWPLSGRSWEKQV

PILIEAGYRVITYDRRGFGQSSQPWDGYDYDTFASDLHKLILHLDLRDATLVGFSMGG

GEVARYIGTYGTERVSKAVFAGAVPPYLYKSEDNPQGGLDDATIAEFQNGVKGDRLAF

LDGFTNNFFAAGDRTDLVSEPFRLYNRDIAALASPKGTLDCIAAFALTDFRQDLEKFN

IPTLVIHGDSDAIVPLEVSGQRTHESILGSRLVVVEGGPHGFNATHPEAFNAALIEFL

KS"

CDS complement(2095676..2096671)

/gene="xsa"

/locus_tag="EFAGFIKM_01838"

/inference="ab initio prediction:Prodigal:002006"

/inference="similar to AA sequence:UniProtKB:P49943"

/codon_start=1

/transl_table=11

/product="Xylosidase/arabinosidase"

/translation="MNQQQLPKPHQPLVTHIFTADPSAHVYEGKIYIYPSHDLDHDEP

SNDNGDQYKMEDYHVLSMNNFDSPVVDHGEALHLRDIPWASKQLWAPDAAYKDNTYYL

FFPARDHDSIFRLGVATSESPAGPFRPQPNYMEGSFSIDPAVLVDDDNQSYIYFGGLW

GGQLEKWQTGSFVPDAEGPAPDQPALGPQVALLSDDMLSFQSKPAEISIVDEDGNPIL

AGDEDRRYFEGPWMHKYNGYYYLSYSTGTTHKLVYAIGKNPIGPFTFKGEILSPVIGW

TTHHSIVQVEDKWYLFYHDSSLSEGVNHKRCVKYSELKYNEDGTIQKINPYPGAE"

CDS complement(2096668..2096886)

/locus_tag="EFAGFIKM_01839"

/inference="ab initio prediction:Prodigal:002006"

/codon_start=1

/transl_table=11

/product="hypothetical protein"

/translation="MNYRNRWILERYSEPSTGMPTCWTMKRLPCSRASASCFTGVYLG
LRALGNGTENTSPNYEGFDYSNTKGDAL"

CDS complement(2097412..2097984)

/locus_tag="EFAGFIKM_01840"

/inference="ab initio prediction:Prodigal:002006"

/codon_start=1

/transl_table=11

/product="hypothetical protein"

/translation="MRFQNKSGVRRAAIQSRLPAGVTAQLRTDRDSDYVFLLNFSGAV
ARVELDQNGYVDVESGEVVTGQVELPVNGAYILRRNANTVQKDLSLARYVKGKNVVPV
SATRIFPDSRVTIFQNDQAKSEKLNIQGNEVYFIQSDTRDKETNYVTYHRIGWRDDHN
HLLYEISNNTDSDLGKEDLVKLAEEMITSR"

CDS 2098452..2099660

/gene="setB"

/locus_tag="EFAGFIKM_01841"

/inference="ab initio prediction:Prodigal:002006"

/inference="similar to AA sequence:UniProtKB:P33026"

/codon_start=1

/transl_table=11

/product="Sugar efflux transporter B"

/translation="MINRFISLFSIPSYALLFMCMALQGMGISLSTPFLSIYFTEQLG
VSIGMFGLYLAVTLIAGIWISTLIGRRSDLGLNRKSVYLFSTLSNVLAFSGYLFIQDF
PILFIYMTVFTALGATGMPQLFAISREAVIKSNFTERAFANSTLRSAFSLGFITGPLI
GTLLLAAIGFKGIFLGTIGAFLLVTLLVFLFLKSNTEMKSSNAEANIKSFRLAQNRDI
LIPFLIMILMYTAHWMSSINTALFITNNLGGTTNHVGLVSSICAALEIPFMIVLGLLS
AKYSNRILVFCGAIFGGAYYFVILISNAMWQMLAAQILLAVFVAVISAIGISYIQDLL
PSMPGYASTLYSNSSTIGRLIGSLVGGGVASIVGYRYSFIFCFILIIVAVIMLVVSGR
QPVDEPQIST"

CDS complement(2099818..2100738)

/locus_tag="EFAGFIKM_01842"

/inference="ab initio prediction:Prodigal:002006"

/codon_start=1

/transl_table=11

/product="hypothetical protein"

/translation="MSTKQDRILIFGAGVIGSMYAIKLIEAGFDVTLFARSNKYKSLR

ENGLRYEGKGTVRSIQVNVIDTLENDDIYDFIFVTVRYDRSESALLALKDNQSKNVVT

MTSNSMGFSSWLDIVGDRLLPAFPGFGGQIKDGVLHARFLPKIIAATAFGEINGVVTE

RIENLAKIFKTAKLPYVIKKDMQSYLITHSVSDIAMLSVLQSENKIIDKKTARTRRTA

RKITVTLKAYLRAIQKAGVLIDPPMLKMVLNIPNLILDLFFMTWLRTNMVRDMMLPDY

ANNANNEIVLLSNDLMKFLNQNDIKSEIIY"

CDS 2100787..2101275

/locus_tag="EFAGFIKM_01843"

/inference="ab initio prediction:Prodigal:002006"

/codon_start=1

/transl_table=11

/product="hypothetical protein"

/translation="MYDVIINFHYSLINHQFFMICRMIFFEKISVQEIANNSGFNRST

FYQYFPDIYELLDSVENELLNDIKKELANKELSMHTVQDTLYCLDSREHLLVLNALIV

NNVFLNIGKASCSKHSLKKKPTLFLHKTGSRSVSLIIAARKSYTEPEQLLLWLSFQQG

SR"

CDS complement(2101199..2108515)

/locus_tag="EFAGFIKM_01844"

/inference="ab initio prediction:Prodigal:002006"

/codon_start=1

/transl_table=11

/product="hypothetical protein"

/translation="MTMISKKWKPLMIALLLAIPSLPIQVSALPAAATDAVKTGISNA

SLSAKVGDLGQIEELYINNNPTNKQGKPINFVLPNTTSPQNGTQHQWMGEMIFSYRTG

TNGQFPDNRDGFVEVDTNKTLAAGGSTQYSTINPDNPYMNKTASADGKKVEVNYIGQN

LNSTDQRVMKGFDVKSVFNMETDDGSMLWEITVKNKSNQYIEFGDIGLPMPWNNKYQT

LSDTYDDRVTVHNFAGADSGYSYAIRTSGEGNFMLFTPVPESGARIEYVDYWMHEAGE

RRAADTFKNWTGDSGGWYPGLNVLYIHSKEIQKTGRGYFTDASSLVLGPDESKTYKFK

FSAVRGGDNTPQESAQSPNNSSTSMEDREANLRSILYKSGMIDAVAVPGFQTAINMPV

KLDLHYDDNIVDVQSIDIQNVAENDPFDEAHIPDIKPGKSRKEMVDNSRAGRGLPEGN

PGYNESVEFVETKIVNGEQHHIYSLQFDSIGNNSVRIDYKLKDGNEWVDKFTQFEFNV

LAELDAISDAHSEFMVQQTQDKNPESPTYGNYFDWYLSSGMDLNTNHWGDDWSHDNIN

FMTMKNYLAPNPDEIRSIETYLIDFMWERYMKNTQDSYIVANWLKDSGAYTDKDKPYT

RTFSEIMEATGFFNMYRIQKAYPNLIEYRESPQFYLEKAYNIYYKRVSTGAIGFYGEQ

QIPDMIEALKEEGMQTESANLQKKFALDKGRNMTRATYPYGSEFEYDNTGEEGAYAAA

KALRTYYPADALTGAAEKSMEMADWKTRAMRGIQPTWFHYSVPVFRGGEGWWNFQYTA

SLAGYIMDDWLRYENDGRSVEQKAIAQQRNYAAKISNFNAVNMGQISANSVGSTSWRY

SMYKGGTGTKDVYDGGSRVMSNGWNDFSGESEEGLYGSLLSISSDIVTDPIFGLFGYG

ALVTDEGDSYNITPKDGFGKRINLIDEKIYLELESDKVESAQIQKDGKGFTLQLLNPS

GKGHTSRILFDGVGIENGYYSMKLNGAAAGQFYVQNNEGVAMFQMGSVQRAELIIEKS

AGGENEAPQITVELATQHPQALIPFMLNGIVTDDGAPEGTLSYQWEVVSAPEGGKLTF

THPKASITLATGTKAGSYTVKLSASDGQLTGSSQVIFELASPPDKQPPSIGQVTAVQD

VANNSIVVLSGEAIPDPVHSEAEEPQLKYTWAVKQKPQGSADISFVDGDKTAAYARVS

MAGTYVFTFSAADGDKKASKDITIEIKEDTVDVYRALSVVTKKDIEPELPRQIYILSE

EGYLEQEITWDAIDPSGYASVGQFEARGTVNGSALEVWATVHVVNTELQNAALTAKAS

ASFSGGDGYPEAMNNGIEPKSSTDFSPNRGAPNSAWHNWGREGDPAWVMYEWDQPFLA

SSMDVYVFQDGGGNFRPKDMQLMLRDEDGKWYTPRAIKGLGNELNQYNTTTFEPAYIT

GVRMDMKPLVNGSGILEWKVNGYTGAVDKSELIKVYNYVNTLNVTNLAEPGLAPIEAA

KAEASAVIKNMNATDEEIALTLEKLLTELRLLSPRDGNMAFLANASSSYTSPWESLAA

VNDGNKNGNNISHWGTWGHEGAEEWVEYEWPQGASIRSSNLLLWSDGGGIKPPTQIVY

SYIPADSATDEWVTAGTVTEGIKVVEHTPAESSNPYTFDSVLNVKAVRVTLTKQSQAG

DNGVGLWEWEVPQAGSTQDREAPVTPAGVAPSVLHGDDGKLIGVTTEMEYKKEGEEEY

TAIAGIEVTGLTAGKYYVRYMKTGSLSASLDKEVVIPQGQTQEQAAPDASGWGISHAN

SNTNIKGIIEGLNDTMEYRKLGEEDYTPVTDSSINDLEPGSYQIRYAATDILKASPPV

TVDIRNEARADREAPTAEGLVITPPTAAGGNDGRIENVTADMEYRKVDQGGYMPVSGT

SIEGLEAGSYELRYAGTDMSNPSPAILIVVPDGTKQEQSPPSSAEVKAVNVSAAGAKD

GKITGVSPVMEYRLVGVDASSWNAITGTEVTGLGNGIYEVRYKETATHYASQSLTVVI

SNPVINPKPMETPPSTDAPVSVTTPSPVPENIKVEGNWIKIVAPKTDTKTGKAKGAPI

RQEDLNEALEHVKANSNNIKTIMVDIPKAEGAHSYAVELPAAALTAAHANIKIEINTE

FGTIAVPSNLLKYVSDAKNVELSLSRLDTVKLHSDVKSVIASRPVIAFAVQLDGEEMA

TLNAPVEIRLPYTPDVEELANSKYLTVSYMAANSTFVQLLNGKYDSALQAMVFPAANT

GQYAVVYTKKFFNDVIKDSWYAPAVETMASKGFIDGTSATSFSPEQKVTRGEYLAWLV

RTLDLKAEFNTTFSDMKRTDLYYEEIGIAKALGIALGTNGSFYPETEIARQDLAVLTM

RALRAVGKTEQTHTAEDLKRFTDAANVAKYAVDDMAAMVKMGFINGRSNVTLSPAGTT

TRAEAAQVLCKIFLQL"

CDS complement(2108835..2109689)

/gene="araQ_9"

/locus_tag="EFAGFIKM_01845"

/inference="ab initio prediction:Prodigal:002006"

/inference="similar to AA sequence:UniProtKB:P94530"

/codon_start=1

/transl_table=11

/product="L-arabinose transport system permease protein

AraQ"

/db_xref="COG:COG0395"

/translation="MGTRKNKINAASVLLLVLFVVLAVLMLFPLYALILASLKPAAEL

FRYGLNVRWDWALMSLDNYKTIFAGSGAASHYFTWYKNSLVITALFTVLSLLFSSMVG

YGLGVYRFRGQNLIFTLVLVVMMIPMEIIILPLYELTIKLKLINTVWGVILPFVVAPL

PIFFFRQFAQGLPKDFMHAGRIDGCTEFGIFFRIMVPLMAPAFGAIAILQAMNSWNNF

LWPLIVLRTTEQFTLPIGLASFVSPLGNHYEELISGAVLAIVPILILFLFFQRYFISG

LTVGGVKG"

CDS complement(2109694..2110626)

/gene="araP_1"

/locus_tag="EFAGFIKM_01846"

/inference="ab initio prediction:Prodigal:002006"

/inference="similar to AA sequence:UniProtKB:P94529"

/codon_start=1

/transl_table=11

/product="L-arabinose transport system permease protein

AraP"

/db_xref="COG:COG1175"

/translation="MQLHKETTASVHANRRRSIRLNSRTWAPYWFVLPFILSFLIFFA

YPLFRAGMMSFQQVLPGDVQFVGLEHYSKLWNADFRDAMWNSTRYTLWTLLLLVPVPM

VLAVLLNSSRMMARNLLRSALFIPALTSVVVAGVVFRLIFGELDGALMNSILSVFGIP

SQQWLYSSSLAMVALVVLAGWRWMGINMLYFLSALQSIPKDLYEAADIDGAGVMRKFT
NITVPMLKPITIYVITITLYGGYAMFTESYMLWAGKPSPQNIGLTMVGYIYQQGFQYF
NLGFGAAIGITLLVITLLISMLQLTVLGMFRKED"

CDS complement(2110693..2112003)

/gene="araN_1"

/locus_tag="EFAGFIKM_01847"

/inference="ab initio prediction:Prodigal:002006"

/inference="similar to AA sequence:UniProtKB:P94528"

/codon_start=1

/transl_table=11

/product="putative arabinose-binding protein"

/db_xref="COG:COG1653"

/translation="MKKMQVWLGVCLLVVVMALSGCSTTKEASGGTSGEAGNGGVTKL
VFWTFVDAHQKFYESMAEQWNQNHPDQQIELEATTIPYDDMHTKLLLALQSGVGAPDL
VDIEQSKFPNFMKGVPQLVDLTDMIQPELNNIVQSRVEIYSKEGKYYGIDYHVGATVM
YYNRDSLNQAGVNADDIKTWSDFEQAGKQVLEKTGKPMITFEGNGNWSWWPAISQQKS
DQVDADGNVTVNAPINIETMKFFQKMVQEGVAAVAPGAGHDTEEYFGYMDKGSAAAVF
MPFWFMNRFTDHIPDLKGKMIIRPMPAWEEGGNRSAGMGGTATSITNQSASIELAEQF
LAYAKLSKEGNIQIWKQLGFDPIRTDVWTDAAMKESNKYTDYFGDDIFDTLLEVKDEI
APVHIREKSPEVFDAVRNKAMPEIFINMKDPEQVLNDVQAELTR"

CDS 2112163..2113083

/gene="rhaS_11"

/locus_tag="EFAGFIKM_01848"

/inference="ab initio prediction:Prodigal:002006"

/inference="protein motif:HAMAP:MF_01534"

/codon_start=1

/transl_table=11

/product="HTH-type transcriptional activator RhaS"

/translation="MDSPILHFITPPIPYFIDCGRAYYEAGEYHISRSEIGVFDLIVV
TRGSLAISENGTEWFLKQGEALILKPDGHHYGSAACKAETEMIWIHFQTYGSWRESAN
MDECLANQAELIETHKVSAYLNHCDVCSIFIPKHMHIATKDMELLEHFSQLDQEPQSM
RNWKRQATFQQFMQHLDLGLSSLSDVAAIRIAEKIELYIRHNYPQPISNPMLQKELNY

HPNYLARSMLKVYGMTPMEYLLHYRIEQAKKLLLQTAWSVARIAEEIGFNHVSYFSSC
FSKKEGISPSNFRSKFTNTENKLTHSEPMT"

CDS complement(2113322..2115307)
/gene="hypBA1"
/locus_tag="EFAGFIKM_01849"
/EC_number="3.2.1.185"
/inference="ab initio prediction:Prodigal:002006"
/inference="similar to AA sequence:UniProtKB:E8MGH8"
/codon_start=1
/transl_table=11
/product="Non-reducing end beta-L-arabinofuranosidase"
/translation="MTKPVVPHHPQTPGYSHKINDPFWSHYIELVRNVVVPYQWEALN
DRIEGAEPSRAIRNFRIAAGEEEGVHYGMVFQDSDVAKWIEAAAYLLSAKPDPELEVL
VDSVVDTIAQAQQADGYLNTYFTLREPGARWTNLAECHELYCAGHMIEAGVAYYHATG
KRMLLDVVIRLADHIGTVFGTEPGQLPGYDGHQEIELALFKLYETTKVEKYLELSRYF
LDQRGASPHFFVEEWEQRGRTVHFGELEMVHRHTYSQSHLPIREQTTAEGHAVRLVYM
CAAMADVAAKTGETSLKEACERLWCNTVSKRMYITGSIGSSAKEEAFTGDYDLPGDTA
YAETCASIGLIFWAKRMLQMKPDSKYADVMERALYNTVISGMSLDGQRFFYVNPLEVD
PEIQRVNPNYAHVRTRRQGWFGCACCPPNIARLLASLDQYVYTPVVEQATLYVQLYIG
GEGEFTLKDRKVALTMASDYTSTGEVKLIVRPDSSEGMEFTIALRKPDWCEIPELWIN
GQKTEQSGFISECGYMKVTRTWRAGDEIRLHFPMELLRMKGHDHIRATFGKVAIQRGP
FMYCLEEVDNGRGLHRIQLPRDAQFHIGSGDRMPLGVPAFTTIGQRIVSHEDWGTHLY
RSDVHWDAQPADLRFIPYFTWANRDEGEMSVWIRECE"

CDS complement(2115611..2121970)
/locus_tag="EFAGFIKM_01850"
/inference="ab initio prediction:Prodigal:002006"
/codon_start=1
/transl_table=11
/product="hypothetical protein"
/translation="MAKKKRKWATKMWVAALATVVTLNTGIIPASANYTTDFVHEGVG
TDYGDATWKNNQIVSEPVDFSQETVGQMMKAIEDFDFDQFAADHADLPWLDKLLVNEG
VIPDDNNDDGSANTLFSRGSALYMKTQDNRQLGFVGTVHYADTLNKDTMYNITLSTGA

VVEDKGARKNYPSHADQTYNSGGLDISQRKFITSSNSAVTLLKLTNNGNAPITLTMTV

KSPFVTETEGDELIGKRVAAPIMVGRAGEKYVEAMSHVDVHLSGDGMMPVGNDLVKEI

TVPAGTSVDEKVVMGWLAEGIPASKTQYAEFKTSTNEKAFSEQVKEYNEWWAQNIPYI

DIPDENVKKVLYYRWWSNRFNLLEANIPGNDWQFPMNMEGVLGYNNGITVSVPWVLQD

LKWLRDPSYAYGTWLSQGEYSENANYKNNPGRPNIWTWDMMQNTSQVGWEAYKIYGGG

EKVLKKFADFSASDVTGTLNHFRGTNPDLVYYNHGPITGNDGDTVSMHWKGNGNYARL

DGSSTAYANAVATQQMYEQLGDNTNAKRMEEVAQRIRNAILNNMWYDETDFDGDGEAD

TNGEGSFLHKKVINQQDVFNPWRDNNMFVFNFGVVPKEGEAGYESKYLTQLADYGDPN

YYPIFPFFTADQHSIMKRVEAFQNGETSAFGTDQFAWCNFGNYINTIRASLRHYPMEN

INSDTYKTLFDWGAWLHTVEPGNTDHLDSNEFFWLEDYFFGTPWTPQNPPNPSGKMVR

AWIHHDTLGMMNYSVMEDMAGLQPRADDKIELWPLDIKYDHFAVDNVRYHDANLSIVW

QDPAKYSDSDPYYKDIPVGYSLFINGERVMTTDEMSHIIFDTATGKVEKPDGDVPGAV

GDNTNTNVIYEIGSAVNLASADDTSLESDKKAVDMFNKAGVDVEHKGVNLATASGTTV

ESSYIHSNSNVNRLADGSTIASINHTSNTALFGGSPNPSESVTFHFDGFQSVDNVKVY

FYNDRRPSGYAAPQTFGVEYLDADGTTWKPVEGQFRYPNYVAANYNNVEFNAVNTAAI

RVHFTHASGYSTGIKEIQIYNNDLNVTPSENLAPKVLLDLPVNVEQGTPLKLAPVITD

DGLPSGRLTYAWNLVSGDAGAVQAETLDQSTLNVTFGGVGEYVYELSVSDGIAITKVE

VPITVYVATAELSNVIAKFADRISPQGKLVRNADDFTSESWQALQAALTEAKELLAQD

DYSLDEVQSMTNRVRLAEESLRYRNAALLATTSASYTSAWESLNSVNDGYIPLVSGSI

GNPEIETQYGNWGSPNQNHWVEYTWKNPVTLSGSSMYFYDDGGGVQVPVDYDFEYFDQ

ETGEYVAVSGLSEKKMEKGKFNQVTFNEVTTDKLRLTMKNQSSSVYTGLKEWRAISAK

PEGEEPPQTEHGAITAIEPASVNTTINVMPELPSQVTVTYADNTKGLANVVWDEIPEN

KLSIATDFVVLGSVEGTDLRASLRVYVKYDKSRLKAAIDAASDPSVNEENYIGTPEQW

QTLQTVLQLASDMYDNDASTEGDIDSAYKALLEAFEALEPVEEHNVTIIGINIPGGSV

DQLAKEIVIPESTTLEELKTGLNVPASVTFAVYESDGFTSAIDLKTGYKVMVTGADQI

TTKTYTVLMVTKNPPVNTAALEAKLNESKALKQERYTAASWQNLAEKMASAEQLLTSG

SASQADIDAAVLALDSAIAGLKLLSTPTPTPTPSPSPSAPTPVPVPEPAPAPTPSDSK

ELTPVTVNQGRIEVKAVASGKDGKAKAQLTDSDINAALEDMKDGKLTIVIKKDLETQQ

VEAVLPWKTLNTTKSLTSIKIEMGSAGLTIPHQFLTTFKDINTLGVVFEAVEPSQLPK

QAGKLREAGAVSNFTVNADGKNISSKELHGKGLRLGMPYQLKAGLKGHQVVAFTLNDA

GVAQAVVGGKYNESTKMFEFNMEQNGMFGVLGVNAAFKDLVNVNWAVEGIQALAARGA

IEGTGAGEFNPTSQVTRAEFLKMLMNTFDLLDETATPAFSDIKEGTWYSTPIASAYTL

GIVKGKADGSFGPNEPITREEMAVMVYRLAGNLNVTLSTEGEDDSAKFVDLENLSSES

TAAVEAVRMGGLITGMPDGRFNPNGQTTRAQVVTVIYRLLTNLEK"

CDS complement(2121989..2122870)

/gene="araQ_10"

/locus_tag="EFAGFIKM_01851"

/inference="ab initio prediction:Prodigal:002006"

/inference="similar to AA sequence:UniProtKB:P94530"

/codon_start=1

/transl_table=11

/product="L-arabinose transport system permease protein

AraQ"

/db_xref="COG:COG0395"

/translation="METAPAPVKYTSIKQAKNRRKAIHFGLILLSLLLALIFAFPLYW

LLRGAMVSHSEILARPPVFFPKELGFENFRLGLERIQFWRQLWNSVSIVVPYVIGTVI

TTSFAAYAFAKLRFPMRGAWFVLVISSMMLPSAVTLLPQFSLYTSLGLAGKPALIIPA

FFCAGGNAYFVFLLRQFFMTIPAELSEAAKIDGAGYFRIYYRIMLPLIRPAMIVVALF

SFINCWNEFFYTMIYLKTEADYTLPMGLYMVNGMRIPNYEQVMALALIVTLPCLVFFL

IGNKYFVEGITLTGIKG"

CDS complement(2122870..2123847)

/gene="araP_2"

/locus_tag="EFAGFIKM_01852"

/inference="ab initio prediction:Prodigal:002006"

/inference="similar to AA sequence:UniProtKB:P94529"

/codon_start=1

/transl_table=11

/product="L-arabinose transport system permease protein

AraP"

/db_xref="COG:COG1175"

/translation="MNPERKISSETVPARRFAKGATKEAIAGYLFAAPAIIGFLVLTM

YPMLASLYYSFHKITIMSGSQVMEWIGLDNFRYIFSNPSSEFKKSITVTLVYAFVNVA

LVIVFCLIVALLLNRKFLGRNLLRAIFFLPSVIPMLATTIVWQMLLQNQAKGGLVNWV

LLQMGIPPWDFLNDPLQIFYTLFIMSLWTCGGTIVVFIATLQDVPGELLEAVEMDGGN

AWHKFLKVTYPTIKPVLFFQLIMCMMTSIQIYTQSVVLSRNGAPDRMTYFINVMIYDH
SFVQVGMRGLASAEAWIVFLITLVITGVLFYFQGALKRDDAMGKRKWVR"

CDS complement(2123890..2125263)

/locus_tag="EFAGFIKM_01853"

/inference="ab initio prediction:Prodigal:002006"

/codon_start=1

/transl_table=11

/product="hypothetical protein"

/translation="MRFGKRKLVLSLALTLAFSTLAGCSGNAKNAAAGNGDPSAGGIT
LELLYWGDDVQKQLVESAAEKYTADTGVKINAQVLPADGTFDTFIQTRLQSGELPDIS
YMGEADIQKYNEMGILEDISDLLADGSIPEKLPAITIHTPEQKVIGVGLSNQMELLFY
SKSKFDEAGVPYPPTKVEEAWDWDTFVANAKKLTKDSSGKTAADAGFDAALTENYGLG
FTAGREFHHFWAANANGGGIVSPDGKEFQWDSPQTTEGIQKLADLVNKDKVSSAFSYT
WSSGIGSAVDALSGGYAMTVSGSWDLANIKGNEDIGVGVLPKMEKAVTMNAGAPLVVY
NTSKHIEEAKKFYAYMVNPENSLDLLKSGAWLPNQADWYTDQALIDKWTAELPASAKE
TILSYATTKDAIVQWPAYYVPAYLKMNTEYEKSIDQALSGQKSVKEIYDAIMPAIKSL
WESGKVS"

CDS complement(2125433..2127895)

/locus_tag="EFAGFIKM_01854"

/inference="ab initio prediction:Prodigal:002006"

/codon_start=1

/transl_table=11

/product="hypothetical protein"

/translation="MTILSNEKFRIEFGERGTVQSLVMKDDPHEMNWVVDPHYLQEVG
FQEDEDKLFGEWSMQLNDRFVQSSMFEPNITKHSTNHVTVEFKDVQVKILFSYLLEGD
QFRWGIKLTNKSDTNIKIQGLHVWFSLAYIMFRTHDVYRNMHESCAVFTHIGGNFAKF
AAVRRSNDGPHLGIYTLRGNTSTMGSHCRYCNRFLEQVSPSLDGLLFHRLSLVEDGTS
LKDLVLTDWIYDGAYNSLTLTPGQAENWEYGFVSFQNLTDFYHQGKVLGHPHWSFTPV
VPEGGQFTASVRLPRDLEIRSLKLYSAPGHQNGIEEQDISALLVPSEKSMSLDVGSNQ
ISNPVVEQEFIVTLPADVPGERKLELILKDGRRDMLVWNVLEPVHTLLEKRAEWLALN
SYEGGETADRPHAFRPLSNQGESLGKLAFLLMKNGMSSPVAEQVKKTEAAAVLDMKVH
WFIEGDLSRPKPLYGNFYRIYDFDYIAHVFYLLSQMDEELLHLHSPSEYLKWAAEVMC

LRLDPDCHSGQREKDESRLNGVFILYIRELLQALKDKEYAELHNQLRQLWDRFGQEME

IGVRHYGGAVTEHFYDNAGFGPTCNALCFLGHTDEASQYGQLILANIGFSNDYRLQNP

DRWWEALSPMIHSLWGGLVASSALVAYEHLGNTEYLEAAYRSTMAIFHCYDWNVRSTP

RKMEPGEAASTYSVSAPNLNMPSLSRNRFGQSVFVEADDPLFAALFSDVSGDDWDMGE

ELVAYLLGFGTTTYLYRDEEGRLSCVNGFVTEEHDGWTVKSYAAYPSRYVFREEHLEF

RAPLGTLQKSIRLENGKFRVCL"

CDS complement(2128029..2129066)

/gene="ccpA_2"

/locus_tag="EFAGFIKM_01855"

/inference="ab initio prediction:Prodigal:002006"

/inference="similar to AA sequence:UniProtKB:P25144"

/codon_start=1

/transl_table=11

/product="Catabolite control protein A"

/db_xref="COG:COG1609"

/translation="MRKTSIKDIALRANVSIATVSYVLNGTRNVRPETRRRVQEAIEA

LNYKPNEIAKSLQRNRTNTIGVITEDVTVFNAPDIIDGINDFGDRHDLHILLVNLRLD

KRIGRNFQDVEAYRKYAGSAVSELLGKQVDGIIYIGVHTRDLTGLINVEGKPIIYTYG

YTQDDISIQYNDEQASYEAMSYLVQMGHSRIAIISGLMDSIPSRRRLNGYYKAVTDFE

LPFDPSLIKMGDWERDSGYRYANELLDMDNPPTAILSMNDVMGVGVLQAAKEKGLSVP

EDISVVGFDDREFSDYLSPRLTTMGLPLHEMGDLAIETLYRLINGEDIQNLTMPECRL

IERDSVRKLKS"

CDS 2130207..2130851

/gene="gntR_1"

/locus_tag="EFAGFIKM_01856"

/inference="ab initio prediction:Prodigal:002006"

/inference="similar to AA sequence:UniProtKB:Q8GAL4"

/codon_start=1

/transl_table=11

/product="putative D-xylose utilization operon

transcriptional repressor"

/translation="MKTPKIVTIKEQVYEIIKNRILTQEYQFGEKINIGSLSKELGIS

NSPIREALSQLGQEGLVVSTPNSGLRVVNMSKRDLFEQAQMVYFWMTGSYRYCAENDK

TEQLVRGLEECLKKQKKYYEEKNHYKFIYYSNYFDRCILAATENSRLLKQFDAIFPLF

FLASGSYLQGSEKEWHLSIQEHEKILLAIKEKKHDEVQEAIKVHYFKPSWTLDI"

CDS 2130885..2131817

/gene="rarD_2"

/locus_tag="EFAGFIKM_01857"

/inference="ab initio prediction:Prodigal:002006"

/inference="similar to AA sequence:UniProtKB:P27844"

/codon_start=1

/transl_table=11

/product="Protein RarD"

/db_xref="COG:COG2962"

/translation="MKFKQSNEQSVGVISGASAYIIWGILPVYWNLIGSLSAKEILAH

RIVWSFVFMILVLVFMGKLKQFFIELCTLFKEPKKLFVIFAASIVISMNWYTYIWAVS

NNHLVEASLGYYITPLVSILLGLIFLKEKLSFWSTVAFILASIGVLNMTLNIGTFPWV

ALSLAITFGMYGLLKKITQLEPMTGLTIETLIMAPFSLLFLGNLQMKNESVFTQGSPQ

TIWLLMGAGVITAVPLLLFAITAKRVSLAMIGFLQYIAPTIMLILGILLYHEPFNYEQ

ISSFIFIWISLILYTLSKSKWFIKMEKKILYLKG"

CDS 2132237..2133508

/locus_tag="EFAGFIKM_01858"

/inference="ab initio prediction:Prodigal:002006"

/inference="similar to AA sequence:ISfinder:ISHaha5"

/codon_start=1

/transl_table=11

/product="IS110 family transposase ISHaha5"

/translation="MNPVIGLDISKEESHGQAFLDRGKPYRGIFYFQHTRDGLEILLQ

VIQDIERESGHRPTIILEATGHYQSPVVQFLEEHHYVFIVINPLISNRFRKTNLRKVK

TDAADAYLLGELYYREEFEPFKKRGVQLLNIRYLTRQHESFSKMCVQTKLQFQAVLDQ

VFPAYKGVFGAMYSNVSLRFLHEFPTSSYVLKVSEETLKTTINALLSSKRGRSERWIN

ERVQRLMVAAKQNPFQQTMYESHLINLKILISLILQYQEHLAMLEKTIDALATEIEEY

DLIQSIPGIGPKIAATILSGIGEVDRFDHPKKLVAFAGIDPSIFASGKFTATRNRITK

RGSRQLRYVLVMAVQCGLIRSRDTRIKEFYERKRAEGKPHKVALVACANKLVHWLYAI

LKSKKSFRSVLQNNGVTFQLSSDWKVIWYAY"

CDS complement(2134640..2134798)

/locus_tag="EFAGFIKM_01859"

/inference="ab initio prediction:Prodigal:002006"

/codon_start=1

/transl_table=11

/product="hypothetical protein"

/translation="MQIDTFIHISRLTRNLAGSGDKRNISTVTWFTSQHLKGNGRSGA

KIREVENT"

CDS 2135034..2135234

/locus_tag="EFAGFIKM_01860"

/inference="ab initio prediction:Prodigal:002006"

/codon_start=1

/transl_table=11

/product="hypothetical protein"

/translation="MNKILAISILGMGLTGAVLIISIALLFNPPIGSVLKMVTAASTL

SFIIFAVSALIFRRRLDDENRE"

CDS complement(2135650..2136417)

/locus_tag="EFAGFIKM_01861"

/inference="ab initio prediction:Prodigal:002006"

/codon_start=1

/transl_table=11

/product="hypothetical protein"

/translation="MNYINIQHPYLRQIQLKRQQVPTYNRYPFDLAVIRNLNTLDFHP

KVTYIVGENGMGKSTLMESIAVAWGFNPEGGTINFSFSTQATHSSLYEYIQLIRGPRR

PRDGFFFRAESYYNLATTIDELDSQPSFGRPIRPIKDSYGGKSLHEQSHGESFFSTFI

HRFGENGLYILDEPEAALSPLRQMAMLTRIHELVLQNSQFIIATHSPILMAYPDSIIY

NLKPDGIEVQVLEETDHFMMMKEFVNNKDRMLRELFE"

CDS complement(2136836..2137906)

/locus_tag="EFAGFIKM_01862"

/inference="ab initio prediction:Prodigal:002006"

/codon_start=1

/transl_table=11

/product="hypothetical protein"

/translation="MIRLTRYEFVKIMTRKSIYLALTGLLMLLVLYAYLGHPGPMNGT

SFYKPYEGAITAEKVQAAQEQLQNKGYSDEANQNRYGVFYEISIFSPESIKNYTKTDD

SGNVMERTVNVTEIHYNKPWGYLLEYIDQFGVLFMMIMILLGLAPVFAEEYALGTAAL

VRSSKRGKSQIVSAKLMASIVYIILCVILFTSINLLIYWLRFGNLAGADTPIQSVGMY

FQSFDYEFSPYRLSALQYYVVQLVTHLVGSLVFGCVVLWVSSLSSTAFVAIIINVAIV

GVPYLAFDVMNFNPGWAKWIEEFSFSTMMRVTRLFQTPVNYSIFRVEVSYLQLYLLIM

TVVTAVVVIGTYRAFRNREVFS"

CDS complement(2137903..2138778)

/gene="yxlF_1"

/locus_tag="EFAGFIKM_01863"

/EC_number="3.6.3.-"

/inference="ab initio prediction:Prodigal:002006"

/inference="similar to AA sequence:UniProtKB:P94374"

/codon_start=1

/transl_table=11

/product="putative ABC transporter ATP-binding protein

YxlF"

/db_xref="COG:COG1131"

/translation="MKLTMDNVSKKYRNKWAVKSFTLGLSSGGVYGLLGPNGAGKTTL

LRMLVDIAKPTSGQIMLDGQSIEQMGDRYRDILGYMPQRFGFYNRFSAHKFLMYMCSL

KGLSTNQAAIRVQETLRMVDLEKQAQHKIRTFSGGMKQRLGIAQALLNDPQILVLDEP

TAGLDPKERIRFRNIIGELGRDRIVLLSTHIISDLEFSCKEMILMNEGQLITQNTPEA

IMNSMQGSVWTATLTEQQLVELTSHFKVSGLSYQSDGIVARILAKERPVPQAMPEAPR

LEDVYMHYFDEEATS"

CDS complement(2138759..2139604)

/locus_tag="EFAGFIKM_01864"

/inference="ab initio prediction:Prodigal:002006"

/codon_start=1

/transl_table=11

/product="hypothetical protein"

/translation="MKRTENEDRRKRRAELEMEEYAEIAPFLDDYVVEYPSEMEIDRT
IRALVAHMPGQMNVHLEQSPGKSFTELFQLVRGEVMIFHKSYWLVCALLLLCGFWLGT
RSTISPYMSGLILVPVPFILGLLEVFKGRDQGLMEIEMTCKITAQQLMLTRLIVVLAG
NMVFVLLLMLLVSGGRGSFISWSGVALWYTQLLIAAGASLWLAMRVRGGTAVSIFLVA
WFGLFWLMLSNPRFVTLVQSIQTPVLIAIALCGVVLLISQIRQMTRKYTSQTERGYSF
EIDHG"

CDS complement(2139579..2140151)

/gene="sigE_2"

/locus_tag="EFAGFIKM_01865"

/inference="ab initio prediction:Prodigal:002006"

/inference="similar to AA sequence:UniProtKB:A0R2D4"

/codon_start=1

/transl_table=11

/product="ECF RNA polymerase sigma factor SigE"

/db_xref="COG:COG1595"

/translation="MDDEELIHEIREGSRAAMEVLVKRHYKSIFSYVYRKTGDYHISF
DLTQEVFVKMMNSLENYRDNGKFSHWLLKIAVNHCMDYFRGREFKQQLRESELTEEAL
PPSEHQNVWNIFYKRFQNDKVRLAVQSLPEHQRDAVILNYYNGLKIKEVAELTGSNES
TVKSRIRLGITKLKEIIVGGERDETYRKRR"

CDS complement(2140536..2140976)

/locus_tag="EFAGFIKM_01866"

/inference="ab initio prediction:Prodigal:002006"

/codon_start=1

/transl_table=11

/product="hypothetical protein"

/translation="MDKYKIKVKTRKNVLSLVAAATLLIYVGLIFYRGGLPDLPSFIK
GFHTGAFIGVEVAVAFFLVRYIKASNNEAELKKQYIEENDERSVMILQRAGTLSTAII
LIGLGVASVIAGFFNPLIFYTLLTCLLFVLIVFFALWMYYAREL"

CDS complement(2140977..2141183)

/locus_tag="EFAGFIKM_01867"

/inference="ab initio prediction:Prodigal:002006"

/codon_start=1

/transl_table=11

/product="hypothetical protein"

/translation="MKTKIAELRKQHKLSQEELGRIVGVTRQTITSLERGKYTASLIL

AYKIANFFGLAIEDVFDFSEVEEM"

CDS complement(2141505..2141942)

/locus_tag="EFAGFIKM_01868"

/inference="ab initio prediction:Prodigal:002006"

/codon_start=1

/transl_table=11

/product="hypothetical protein"

/translation="MKNTRRKRIKLGDIYAIPLPNGKFAFGRRLKDASIAIYNVISDT

IEELPQEEEYQFIVGVYDDVLKSGDWPVVENRPFPDEEEAWPPPACIIDRISGGYEIY

HLGEIKPSTREECEGLEMAAAWEAHHIVDRIMGDDKWHRDPNK"

CDS complement(2141975..2142349)

/locus_tag="EFAGFIKM_01869"

/inference="ab initio prediction:Prodigal:002006"

/codon_start=1

/transl_table=11

/product="hypothetical protein"

/translation="MDQKEFDSVINLPANIRYEYCIKKVVDSEEVWGLYENGWSVTED

DKGNKLLPFWPKKEFAEYCATEGWEIYSSKSMDLYEFIDDFLPNLKKEGFKPSIFLNN

ADSAVLEVDIIIEDLKTELEKY"

CDS complement(2142552..2144600)

/gene="glcA"

/locus_tag="EFAGFIKM_01870"

/EC_number="3.2.1.39"

/inference="ab initio prediction:Prodigal:002006"

/inference="similar to AA sequence:UniProtKB:P23903"

/codon_start=1

/transl_table=11

/product="Glucan endo-1,3-beta-glucosidase A1"

/translation="MKPSHFTAKRFMKKVLGMFLVVVMLASIGILPASKVQAAGTTVT

LMEYFSPADGPVISKSGVGKASYGFVMPKFNGGSATWNDVHSDVGVNVKVGNNWVDID
QAGGYIYNQNWGHWSDGGFNGYWFTLSATTEIQLYSKANGVKLEYQLVFQNINKTTIT
AMNATQGPQITASFTGGAGFTYPTFNNNPAVTYEAVADDLKVYVKPVNSSTWIDIDNN
AASGWIYDQNFGQFTDGGGGYWFNVTESINVKLESKTSSANLVYTITFNEPTRNSYVI
TPYEGTTFTADANGSIGVPLPKIDGGAPIAKELGNFVYQINMNGQWVDLSNSSQSKFA
YSANGYNNMSDANQWGYWADYIYGLWFQPIQENMQIRIGYPLNGQAGGNIGNNFVNYT
FIGNPNAPRPDVSDQEDISIGTPTDPAIAGMNLIWQDEFNGTTLDTSKWNYETGYYLN
NDPGTWGWGNAELQHYTNSTQNVYVQDGKLNIKAMNDSKSFPQDPNRYAQYSSGKINT
KDKLSLKYGRVDFRAKLPTGDGVWPALWMLPKDSVYGTWATSGEIDVMEARGRLPGSV
SGTIHFGGQWPGNQYSGGDYHFPEGQTFANDYHVYSVVWEEDNIKWYVDGKFYYKVTN
EQWYSAAAPNNPNAPFDEPFYLIMNLAVGGNFDGGRTPNTSDIPATMQVDYVRVYKEQ
"

CDS complement(2144754..2146295)

/gene="rssB_4"

/locus_tag="EFAGFIKM_01871"

/inference="ab initio prediction:Prodigal:002006"

/inference="protein motif:HAMAP:MF_00958"

/codon_start=1

/transl_table=11

/product="Regulator of RpoS"

/translation="MINILIVDDQKHIRDGLQAMLHQFPLELNNIYCAASGIEALCLL
RQHCIHIVITDIMMPDMDGLALMAQTKEEYMDVEYLIISGYSDFTYAQKAIGLGAKGY
LLKPLKREDLQHSLEHVWQEIQTRQALTHNMQRVSRLSQETDRKELRMFMQGALSDDT
WILQIEEQNDALWRNYRLALLREEVCMNQPGASSAHSMEAIAYRVFGRQGCICLQHRP
YLILAVDASIDPSVLPAALKEGKINVITAMTNPIQGLKELPDSYTQVLELYRHSYLFP
DQYSIFPAHIEHMEQQWQLPYEDLYELFQSVGTDNSGTITQGISAIFHKKVLQRYPIR
YTQQLCAATVQMMEEYQRVIHPYMGEEMLDLESLRNLFNYQGMREYIQALQQQLLRLN
QLYYDYKCSYRHSQDLNEAIRFIHESYHKPLDLAMVSNHVSLNYAYFSNLFKKNIGKG
FAEYLRDVRLDKARRLLAETDHKIVEVAAMVGYESYKSFTRAFRLVMQIQPTEYRQMM
RQKAN"

CDS complement(2146299..2148137)

/locus_tag="EFAGFIKM_01872"

/inference="ab initio prediction:Prodigal:002006"

/codon_start=1

/transl_table=11

/product="hypothetical protein"

/translation="MKYLLTRLRKLYRNARISQKLFLAFSLMIAIPVILISFVYIRMQ

ETQLYKDAMATGNSHVSWLNEQLRRRMDMIENASNTALTQKSFVDFIHSNMLVDGLRL

VKFKQNQFEQMLNIIQSNAMISELSFYVDNPNLYEIWPEIYHYKKFWPQDYWVKLRDE

GGAAYRLFSFRDGEHTLSYYRLVRLQGQEQKRPSIMEVRVPHSMFFSDLLQESKGDFF

SVLMNASDPPQYVYNPQHEFSEKYGQGMEEILGSIHRHLDILPQERALQVKVGDQSFY

ALYRYIAPLNTYVVEIASHQALMKGPRSWYAFVVTITLCVLLLIMLLVSQTTRRIFRR

LDSVLVSMRQVRKGELDAMIDTGLDENERGDEIDEVAVNYNKMLHEVKRLMTQVVDKQ

LIAKNAQLHSLHSQINSHFLYNALESIRMVAEVQRQPAIANSLVSLGSQLRYSMQWRS

DTVAFREELANIQSYIEFINFMEGSSIVMTADLPQEILHYAIPKMCMQPIVENAVHHG

APSGGRVTIEITFSVENDSLLFINIRDDGKGLEPEMLHRLQAVLRSESDTPMVTSRSG

LGLENVNKRLQLHYGKNCGLWIDSVQGAYTCVTIRLPWENVNLGGW"

CDS complement(2148211..2149869)

/locus_tag="EFAGFIKM_01873"

/inference="ab initio prediction:Prodigal:002006"

/codon_start=1

/transl_table=11

/product="hypothetical protein"

/translation="MFMLRSPFIIALLMMCLISGLTACSESEQWEDKGASTKADFTEQ

IQSLNLNLEEPSWKLDTTPMNLTWFVGAEWYGHTWGESLTSKYVTQKTGVNIEFEVST

GEPSEMLSRMLTTGSLPDLITIGSWESAVNKLRESNLIYALDELANKYDPYFYKVAGD

GALQWYRQEDGHTYVIPNDAYSPEQMRSTGLTGANQTFLVRKDLYESMGKPDLTTPKG

FLNALQLLKNQYSVYKGKLISPFWAQGNASYGMTEYLQNLLAIPHEQNGKVYDRMTDP

DYIEWLKTFRIAYEQGLINVDFMVDSSAQVQEKTNHAQYFMMIREWTDISDLNSKLEV

LTNQSSYYIAVDGPRSSRGESAKLFPGSMDGWMVTMISKSTENPERAIRFLTYLASEE

GQRDLFLGKEGETWAMKNGKPQLTAAMVQLHDTDRERLEKEYGIMDTYWMLRNPAFVN

KWRPENAPSIKQMEEFANQQADLDSGIYKGLYPIGDSDIALAWSRISQNWEEVLPELL

TAKDEAAFNKIFENFLIRRVNYGFNQVMEYRQAELELRKAKIAR"

CDS 2150076..2150333

/locus_tag="EFAGFIKM_01874"

/inference="ab initio prediction:Prodigal:002006"

/codon_start=1

/transl_table=11

/product="hypothetical protein"

/translation="MYLIWILIVGGLIGWLSGNLIGRDVPGGVLGNIIAGFVGSWLGY

ELLGPRGPVVGGFHIIPAIVGSIIALLIFYALARAGAFRRR"

CDS complement(2150498..2151289)

/gene="fabG_4"

/locus_tag="EFAGFIKM_01875"

/EC_number="1.1.1.100"

/inference="ab initio prediction:Prodigal:002006"

/inference="similar to AA sequence:UniProtKB:O67610"

/codon_start=1

/transl_table=11

/product="3-oxoacyl-[acyl-carrier-protein] reductase FabG"

/translation="MTKLYGKTAIVTGTSRPGGIGTAVCRTLAQEGANVFYTHLYDYD

KTENPGDADKNWPDLFAEELRSYGVKAAHMEVDLTDPNSPAQLLDACRSALGLPTILV

NNATYSVAADFRQLNASLIDAHCAMNIRGTFMLSAEFARMLEVELAVHQDLCGGRIIN

LTSGQGKGPMPGNLAYAATKGAVSTFTECLSAELAPFHITVNAVDPGPTDTGWMSEEV

KKALLPGFPMGRIGLPEDVSRLIAFLASDDSQWITGQIIHSRGGF"

CDS complement(2152704..2153222)

/locus_tag="EFAGFIKM_01876"

/inference="ab initio prediction:Prodigal:002006"

/codon_start=1

/transl_table=11

/product="hypothetical protein"

/translation="MGILSGNPKNEPMHYGEIFSVWQCSTVAKGALSFYQAFLNHAGD

KDLKKILDALIDQAKLEIKECDTLLTDNGIAPTPALPERPPVKLEDIPVGARFTDPEI

AASIASDTSMGLVACSQVMGQSIREDIGALFAKYHVTKTALGVRILQMSKEKGWLIPP

PLQVKRPEAVEA"

CDS complement(2153675..2154040)

/locus_tag="EFAGFIKM_01877"

/inference="ab initio prediction:Prodigal:002006"

/codon_start=1

/transl_table=11

/product="hypothetical protein"

/translation="MSNNKAFRETLAFLIVRDNAHQNAFAMALETLGVNWGKVFPVPN

YDINKYPECQKYVDLSFHNAQFNFRLDNTRIAEVFSGQTPSRNGGELQVTEPPQGFPL

PYMPDLPNEHSPGLHDLNT"

CDS complement(2154042..2154413)

/gene="ydbD_2"

/locus_tag="EFAGFIKM_01878"

/EC_number="1.11.1.6"

/inference="ab initio prediction:Prodigal:002006"

/inference="similar to AA sequence:UniProtKB:P80878"

/codon_start=1

/transl_table=11

/product="putative manganese catalase"

/db_xref="COG:COG3546"

/translation="MHYFFQSSNFRGKGKQYRDLIRGVFMEELSHVELVQQTINQLLT

GAGAEGVGNAGIDGAPLDEAIKHANPHHYIMGAQSSLPVDVAGNPWLGNYVYDHGNLV

SNLLDNVVLESTGVLQKNTDL"

CDS 2154675..2155388

/gene="yflN_2"

/locus_tag="EFAGFIKM_01879"

/EC_number="3.-.-.-"

/inference="ab initio prediction:Prodigal:002006"

/inference="similar to AA sequence:UniProtKB:O34409"

/codon_start=1

/transl_table=11

/product="putative metallo-hydrolase YflN"

/db_xref="COG:COG0491"

/translation="MNQEQLDYGTDYKFVPVTSVGSGVGIEILPDLFCYTIQIVNICL

VGNPESDEFVLVDAGMPKSANEIISVIEERFGANSRPKAVILTHGHFDHVGGVIELVK

HWEVPVYAHPLELPFLTGVKNYPEPDPTVEGGLVAKMSPSFPNESINLGSHVQTLPPD

GTVPHMPGFRWIHTPGHSPGHISLFRENDGALIAGDAFTTVKQEYMYKVFNQELEISG

PPRYLTTDWVAAKKICCSP"

CDS complement(2156413..2156775)

/locus_tag="EFAGFIKM_01880"

/inference="ab initio prediction:Prodigal:002006"

/codon_start=1

/transl_table=11

/product="hypothetical protein"

/translation="MNDSRKQRTEVADPTLEQIQSFRVSVPQADLDDLKNRLERTRWP

DELPGVGWDYGVPLNYVKELVNYWSKNYDWRKHEARFNEFPQFITTIVGENIHFLHVR

SPEPNALPLLMIHGWPVR"

CDS complement(2157039..2157461)

/locus_tag="EFAGFIKM_01881"

/inference="ab initio prediction:Prodigal:002006"

/codon_start=1

/transl_table=11

/product="hypothetical protein"

/translation="MYLIRGLGLDALTAGLVLLPGGILQAIMSPVMGKLFDRFGPRWI

VPLGLIVISTALFFLKGISESTSLILIMIMHTGLMVGICAVWMPSQTNGLNQLPPELC

PHGKRDYEYDSADCRSNRYGSRHQPDISRQSCFYGNGN"

CDS 2157495..2157788

/locus_tag="EFAGFIKM_01882"

/inference="ab initio prediction:Prodigal:002006"

/codon_start=1

/transl_table=11

/product="hypothetical protein"

/translation="MQTNNMSVPIENMGYLSARGIGIGMGLLSMFNFIAAAGSTAVIG

KVLDGGTSTVQLNPFVHDKVAFVYSNILLALAVVVVVMTVLYYYQFGRKFNNK"

CDS complement(2158334..2158762)

/gene="slyA_1"

/locus_tag="EFAGFIKM_01883"

/inference="ab initio prediction:Prodigal:002006"

/inference="protein motif:HAMAP:MF_01819"

/codon_start=1

/transl_table=11

/product="Transcriptional regulator SlyA"

/translation="MHSREDTPYMDLFQIIGLKLKKRADESIKELGLSSQQGKVIAYI

YENQDNHIIQKDLADRFHLRGASVTSMLQGLEQKGFIERKIPANNERQKNIYVLPKAV

DLIEDFNNSFQKVEDEIVQALNDEEKQILKKLLIRINERI"

CDS 2158932..2160242

/gene="ade_1"

/locus_tag="EFAGFIKM_01884"

/EC_number="3.5.4.2"

/inference="ab initio prediction:Prodigal:002006"

/inference="protein motif:HAMAP:MF_01518"

/codon_start=1

/transl_table=11

/product="Adenine deaminase"

/translation="MEKLNQNAGLEEITAITNVRIFDGYQIIAPRHIVIKGVSIISVG

GDIPSDATIIDGENSTLIPGLIDSHVHTSIGGLRDALRFGVTTELEMNGDFTKRGREI

QLKNVDDIADVRSAGTAITAPGGHPDELLPDGDEIPEFVLKELEKLSEEDREAMLAAY

AHDHDEIPQVTTVEEAIKHVHTQVENGSDYIKIMIEEGTVMGAPGLPVLSDEILKTAV

TEAHKFDKLVIAHVLTALSSKAAIDFGVDGLGHLFIDRPECTSELVKSIANSGAFVTP

CLVLNSSIIGNPASELANDPRVHSKLSPDWIDILNSSFNTFPQGNMENSFKNVMDLHR

AGVDILVGTDVAPVPVPNLGGLAHGASVHHEMQLLVKAGFTPIEALQSATSKPARCFG

LHDRGRITEGARADLILINGDPITNISDTLSIKSVWFNGSRKLG"

CDS complement(2160376..2160768)

/locus_tag="EFAGFIKM_01885"

/inference="ab initio prediction:Prodigal:002006"

/codon_start=1

/transl_table=11

/product="hypothetical protein"

/translation="MHRFTNFINTASKELAEELIAPDAVFYVPMQKEPMNGPSGYLSI
IHMMRSGFSDIQWTVKELVAEKDLIAAHFVMKGTQDGEFLGLPPTSKKIEVNAMNFYR
ISNGQIIEEYGQPDMLGLLQQIGAIPKA"

CDS complement(2160743..2161480)

/locus_tag="EFAGFIKM_01886"

/inference="ab initio prediction:Prodigal:002006"

/codon_start=1

/transl_table=11

/product="hypothetical protein"

/translation="MPIEIKDIVSKKILSEVKGYLDIGFTHSLNPYTGCAFACEYCYV
REMPIQKFKDIPWGSWVDVKKNAAENYLREVSLLRRKNKPISIFMSSATDPYQPVEQK
ANITRSLLEEMDKNPPDSLQIQTRSPLVARDLDILLKLKEKCRILVSMTIETDREDVK
RIFSPYAPGIDLRVKALKKINDAGIHTQVSISPVLPFSPNFANMLQGITDRIWIDTLL
IGDGQMGMRSERLGMPQLFDASFHELH"

CDS complement(2161719..2162846)

/locus_tag="EFAGFIKM_01887"

/inference="ab initio prediction:Prodigal:002006"

/codon_start=1

/transl_table=11

/product="hypothetical protein"

/translation="MRWVEKFIISGVSCILLAALAGCASDKTNLEDATVPLALGLDVK
DKKLHYYISAPVFSKDIQKKTREGGGIAEGLRQSRNQQDAQFPGSLAGRNFQVVIVGK
QLLQYKDWFNILDVTFRDPRNTITDRIVAFDGPIADIFNFQAKDQPPLPLFIKALVDS
GSRSSATVKTTAQELHRQLYDRAMTPSISEIKIENNKIMLKGTTLLSQKGQYRTSLTY
QETSLLHILKNEAKPGVSLTFPVDDLQQTMPFNIDKVSFSLGDISVKTKSTHENGRFQ
FNIKVKSIVSITEKFLEFELLGNSEEMANKLSKEMKKSMEALIKKCQNYNIDPFGYGF
YARAYQYPLYKEAQADWGKELSRAHFDVEVDLRIGSTGAVE"

CDS complement(2162843..2163937)

/locus_tag="EFAGFIKM_01888"

/inference="ab initio prediction:Prodigal:002006"

/codon_start=1

/transl_table=11

/product="hypothetical protein"

/translation="MKSLRPVTMLQFILMISSFQISVAFLSIPRELARHAGTDGWMVI

FLGWGLATLASIVIVKLMKYNPDSTILELIQRYVGVWAAKAASFIFMAYYLLLAYDGY

MIASLVIKLWLLPSTYIYVLVLLLLIPTFQIAQHGFQAIGRYSEIVTMLSLWIPFIYV

STLSRAHWLYLLPIFKEGWFPVLSSVTYMIYPLLGIGLVLFLYPHLVNKEKALPAMII

SNTLTCIVYLGITLICYVYFSPDEIGEYNDPVISIMKSIEFQFIERVEVVFIAFYLFV

FSCIWIPSMYLVSRCSASLVGKGQERYHLAVWCIVIEISFFLYRPSFIESAQVNYALN

YIGFMMEFVLPAGVFLYVAIRKRMGGGQRT"

CDS complement(2163941..2165434)

/gene="gerBA_2"

/locus_tag="EFAGFIKM_01889"

/inference="ab initio prediction:Prodigal:002006"

/inference="similar to AA sequence:UniProtKB:P39569"

/codon_start=1

/transl_table=11

/product="Spore germination protein B1"

/translation="MDEQSATNTNEENITKSLNESLGRIEEIFTNTPDLIIRKLSIKQ

TGELAAIIYMEELTDKASLNQNVLAPLQLETGNPSGDFITTVGYVKPHSKWEELRLSI

ILGYSVLFIEGRSTAYALDTKGFPQRAIEDPQQEPSLKGAHQGFIETGSQNIAMIRRY

IADKELKVKRYIVGQRGQTPVSLLYLEDVTQPDIIKALEERIQSIDVDAIINTGELAE

FIEDQPLALLPQMITTERPDTAASQILQGRCVVVVDGSPSVLVAPATFMSFFQTVDDY

SSRWSISSFLRILRIFAFFIAIFLPGIFIAVISFHFEIIPMKLLLTLGESRGQVPFPP

FVEAIIMEITLEMLREAGVRLPAPVGQTVGIVGGIVIGQAVVEAGLISNVMVIVVAFT

AISSFIIPNQDMMAAVRIMRFMMMILATWFGFVGLVVGMMSFIGRLITLNSLGISYST

PIAPFRAMDWKDTILRVPLWMMNNRPVSTNSRQSKRQGKSRGWKENK"

CDS complement(2165736..2167022)

/locus_tag="EFAGFIKM_01890"

/inference="ab initio prediction:Prodigal:002006"

/codon_start=1

/transl_table=11

/product="hypothetical protein"

/translation="MKEFLSEQSIEAFRSIINYNPDATFIVSNEGTIVEVNEGVTECL

GLQKSEIMGSSYKDLFCQHQLDLIIQYFSEVLKGASCQYVAEAHHKNGEIVYLQIKQV

PLFFEGKVNGIFGVAKNITEYRHLLKALKESEERYRLLADNSLDLIQLINLDGIVEYA

SPSHKMVLGFDPKEYIGKWVFYQPNGDVDDKFRTVFMSMLLLRKPFTCEIERQHEQGY

PVWLEMKGTPMFDEDGDFKNMMLVGREITERKKYQVQLEQLSYHDALTGAPNRRYLNK

MLSDALSAATTSVGQLAVMFADLDNFKQINDTLGHDAGDELLRQFVKRAGACLQEHDV

LARMGGDEFVFVLPHVKSSSELSLLAENILETLQLPWILDGKELYTTSSIGIAFYEED

DSVKELLQKADAALYLAKAEGKNTYRIYSFRDLSMR"

CDS complement(2167577..2168104)

/gene="nusG_1"

/locus_tag="EFAGFIKM_01891"

/inference="ab initio prediction:Prodigal:002006"

/inference="protein motif:HAMAP:MF_00948"

/codon_start=1

/transl_table=11

/product="Transcription termination/antitermination

protein NusG"

/translation="MNWYSFFVQSGKEEEIKLFMDRFYALQHLSCLVPKRMIPERKQG

KTRHCTKVLFPGYIFIQTGQIHDLYYNIKKTPYILYMVNGGIHKHDQAANFFTPIPKE

QMDWLIQLCGDNGTMGYSDIILGGNQIKVTSGPLLGKEAMIRKIDKRKSRAKIEVQLL

NEVKLIDVGINVLLA"

CDS complement(2168250..2169449)

/gene="yjiB_1"

/locus_tag="EFAGFIKM_01892"

/EC_number="1.14.-.-"

/inference="ab initio prediction:Prodigal:002006"

/inference="similar to AA sequence:UniProtKB:O34374"

/codon_start=1

/transl_table=11

/product="Putative cytochrome P450 YjiB"

/db_xref="COG:COG2124"

/translation="MNIKERSSFPDLNLVTGFKTNAEKYEPFAWYKKMRQEDPVHYNE

AEDVWSVFKYEDVKRVAEDKEYFSNKVLAPPIIQDTIIFQDQPKHTIGRALLAKAFTP

KAMSSWASRVDMIVQELMESIEGRIEIDMVQDFASPLPMIVISELLGVPTSDREQFRD
WANTLTLAPQENTPEEYMRIYQLQAQASTQLVAYFQDIIELKRRNPADDIISDLTQAE
EDGEKLSPDSLIASCVMLLIAGNETTTSLITNSIYTFNDYKLIKELAQHPQMIPSAVE
EMLRYRSPLQRAIRKVLKRTEIGGKVLEPGDYVVNWIGSANRDENIFEEGDKFILGRK
NNPHLALGKGIHFCMGSHLARMEAKAALTAMLTTYPGLQVHPEYEVDVNPSNVYGLKN
LSIQLQD"

CDS complement(2169503..2170018)

/locus_tag="EFAGFIKM_01893"

/inference="ab initio prediction:Prodigal:002006"

/codon_start=1

/transl_table=11

/product="hypothetical protein"

/translation="MWIGFLLIVISLIRIPSVVIAMRNEKKIVAGGGTEYGNLNSKVL
FVLQNLIYLSCIVYAFIYDVQFNVLTVIGLVVYVFALCCLIYVIRKLSPFWTYKLYIA
RDHKLVQSSLFRLVRHPNYYLNIIPELIGFALISQAWPVFVPLFILHLITLFNRINLE
EKVMKQTFTQY"

CDS complement(2170122..2177573)

/gene="acpP"

/locus_tag="EFAGFIKM_01894"

/inference="ab initio prediction:Prodigal:002006"

/inference="protein motif:HAMAP:MF_01217"

/codon_start=1

/transl_table=11

/product="Acyl carrier protein"

/translation="MTLPATESFLQRLRHSDYFVRDHRVHGVCTLPGVVLLDMVYRLS
GSHLRTTSIELRDIVFTQPIVTSTDFDVDIRLTLSMKLDEGQVTIHSRKVPKNGEQPS
SDVLNMECKLYLLGGKGDVQPVQALKHVHLLPSKDTDGLQTWDMDQIYQLARQADIQH
FSFMKNNGDVYRQDQMEWMDIRLSSLAEQYLDKFHAHVALLDGATFAGSSFRLGIVDG
LDAEVPPYIPFMIERFVIYDRFPAEIRTFTEQTPSDLSGGDIISRSIAVTDTEGRLLA
EFVHLKAKRVRHAASITGLTNPMTTGRNNKPTSETTTTESSEWAKTTVMSAGSQDQIR
TTISSYVKAEIARMLHILPEAVDERTGFYEQGLDSMHLLELVKRLENRVNMELYPTLL
FEHSSVQALTDYLHTHCSDAWNEAAVVSERVNLNTLPSQSSHSTDEQPATHSEIPATA

REIEGNGEPVCYEPKWIETRDIHEAESARHTIADGKELQSDHTSYRIVVLYGNYPDLA

AKLELQSDIYEVHQLPDHSSMDADLYRQKVTALYGMIQAKAIHPNAGRRIVLQVLSDS

RDTGGYIHGFEGLVKTVAMELPAFGAQLIRMDLHESSMDETLIRQLRLEAENISSGSA

VCIWYKGDSLQRHVRKLVEIPVPGAALEKTYKTDGVYLITGGMGGLGYALAVHLSRQC

RCKLVLIGRSLLSENIQRKLNSLHINGAEAMYIQADTTVLAEMKQAVLQIRDQYGEIT

GVFHCAGTAKDQLMLQKPLSDLEQVLKPKMDGLWNLEQALNDLRPEFTVAFSSLSAIV

GNKGQADYAVANSCMDRLLLNHQENSGTEGAIPRQVTVNWPLWADGGMQVNDHIRSSM

YTSSGSMPLPLAEGMALIDRIIAQDVSQVVLVYGNKSRIQSAMGDDMAIHLPPPSDPE

DIAVIGMAGRYPSANNMDELAQILKEGRDCITPMPDERWLGLHLPYFASEYYPAGGFL

DMIDDFDPAFFQISPLQAERMDPQARLFLQAAWTACEDAGFAIERDKHHDASAAKRNV

GVFAGVFWNHYELLGAAMTQQGTPTAMGSNASAIANLVSYHLNFHGPSIAVDSMCSSS

LTAIHLACESIRHGQSQYAIAGGVNLVTHPHKYVFLKQAKFLSSEGKCRSFGKDGDGY

VPSEGVGAVLLTTVAEARKQGYPVYGVIKGSALNHAGKTSGTTVPDPVAQSEVIADAL

AAADVEPGTIGYIEAHGTGTSLGDPIEIQGLERAFRNSRNPGQNCAVGSLKSNIGHLE

AAAGVAGLTKLILQIKHKELFPSLHAEELNPLIPFENTCFHVQRTPERWERRHGQQNG

RYAEWPVRAGLSSFGASGSNAHLIVEEYIPTAVSWTDRSALDAYSGEAEASGNGEVVL

PISARTQERLIEYARQVRLYLENGGLSERLIDIAYTFQLGRTDMKYRVAWTGSSHSEL

VEQIEQFLKSQSSLQQHSSETPYEMPSVKKSPSPERLHQWAALWEQGQGIDWTALYND

LQKKPTIVRVPTYPFAKNRYWIQAEDQSLGETTREKGSQLNRAETSSSEVEGAGIVQH

INEASNPVTGSAMTPSFQEKQGEEQVLAFLSQTVSSLLKVSIYDLDSDTEWAEYGFDS

ILFTELADLLNRDHGLGLTPAIFFEHGTIRELAVHLLQNHPAKWTQPSAVRTAPQLES

PNAMSHSVPRASEREEPLQTSLRSTASEEEKHTSARSHAGTDSKLDSGSDQLSSPEPI

AIVGISAKFPMADNAEQFWHNLLDGKDCISEIPEERWRWQDYLGDPLRENGKTNVKHA

GFISGISEFDPLFFGISPKEALHMDPQQRLLMMHVWKVIEDAGYAPSDLSGSAMGLFI

GTGSSGYNVMLAQSNEAVEAYSVTATVPSVGPNRMSYTLNIHGPSHPVETSCSSSLVA

IHRAVQAIRNGDCETAVAGGVNTILSVDAHISLTKAGMLSTDGRCKTFSSEANGYGRG

EGVGMIFLKKLSDAQRDHDHIYGLIRGSGENHGGKAGSLTAPNPKAQTALLKSVYARA

GIDPSTINYIEAHGTGTPLGDPIEINALKTVFQETRAGLSPTGQTSTPRQSIEYCGVG

SVKTNIGHLELASGIAGVIKVLLQMKHRKLVPSLHAEQLSPYVQLENSPFYVVRQKQE

WERLKDSNGNEMPRRAGVSSFGFGGSNAHVILEEYKPPRQHDEVTGSESELAPVAIVL

SAKSKERLREQMIQLSAALLRNEEEEDTSQQLRRIACTLQTGRQAMEYRAGFIAASIT

ELKSLLDGYVQEAHLWTDSDESVHAQLSSESAKGHKLHLGSIAAHKRTAALFREDEEL

GEALQKWIIRRKYNKLLELWVQGVEIQWELLYTEDRPERISLPTYPFTPEKYWVGMKK
TGTEDSFEASNEEIETVPVAKGEVIGSSAPNPSVESDVRSIIAALLGIPPDDMDEHRL
LEDYGMDSVLALQLLHHLQARISSEIGMDDLNKCHTLQDVISLSLNPTHPEHERKLVP
DTP"

CDS complement(2177593..2188623)
/gene="pksJ"
/locus_tag="EFAGFIKM_01895"
/inference="ab initio prediction:Prodigal:002006"
/inference="similar to AA sequence:UniProtKB:P40806"
/codon_start=1
/transl_table=11
/product="Polyketide synthase PksJ"
/db_xref="COG:COG0318"
/translation="MEDQLSTLYRKIQQGKLSDQQSAEQLQRLLSESGEQESSLRETL
HEEREDDINEKAIRYVKKILSSSLMLPERQIDPHAPLENYGIDSVMVMKLTDQLEQQF
GPLSKTLFYEYQNIAELAGYFMSHHRERLAQLTGVQAKSNSDVQLRTSELESSTIRKS
EIPNSFNKRQPSAGSRRRIPAATSAGGESFSKEHDHSKSLDIAIIGMSGRYPKSGNLE
QFWENLKLGKDCITEIPEARWDYRIHTEMNASKGKHSGKWGGFLDGVDRFDPLFFQIS
PKEAERIDPQERLFLECVYETLQDAGYTREQAAGPDAPPDQIGNVGVFAGVMYQEYQL
YGAEARIAGKEPFALNGNPASIANRVSYFCNFHGPSMVVDTMCSSSLTAIHLACQSLS
KKECRTAIAGGVNVSVHPNKYVMLTQGQFLSSKGRCESFGEGGDGYVPGEGVGAVMLK
PLSRAITDGDHIYGVIKGTAVNHGGKTNGYTVPNPVRQAEVIETAVAEAGIDIADISY
IEAHGTGTSLGDPIEITGLTQAFRKGKAADSKCAIGSAKSNIGHCESAAGIAGLTKVL
LQMQHRQLVPSIHADPPNPHIQFDKTPFAVQKQLSDWDRKTIDSGDSTREVPRIAGIS
SFGAGGSNAHIIVQEYCPEQSDMLLAEQDKPAKQQALIVLSARNEEQLRELCERMLTA
LRSNRYQEEQLANIAYTLQTGREMMNMRLGIVTGSLQELSSKLESYCSGDQDMDDMYV
GYAGDAQQPLSILSNDADITHTLVNWITKRKFGKLLEFWVKGLNVDWNLLHDGFTHHK
ISLPTYPFAKERYWIPSEPSVEFQMKGDSSRKGNSGHIPLIHPLLHENKSNLSEQKYV
TVLTGQEFYLNDHRIGQERILPGAAYLEMAHAAVSDALGEQQGENVAVILKNHVWMRP
LVVRETPVSLHIRLHASLEEQPIQYEMYSGMSADGSNENRVHSSGYASIVSLEPKLVA
AMAINPGEIKARCNTKRLTADQCYKIYEQGGLNYGIAHRLLVELNVGEDEVLGQLRLP
SILKHEMQSYVLHPAILDGALQSAIGLSMEDKEGKEGKELENANSMLPIPYAMDEIQV

LGPCTEEMWAHVRYSSDSVSSASDAMPKLDIDLFSSTGVPCVLIQGLSCRALNNGIVP

GDESHARATAMLVPRWEETAAVRNEWNHPYDRHIVMLAEPWAELAEEIKERLKREQIP

TEIVALGSEKVADINNVGASPKRFSSASTQVLHVLQQLVSSSSTAENVLIQLLYSRED

HFQLYSGLTGLLRTAALENPRIHVQLIEDMEEGYSESKSWPREPRNAKRAAELIKANR

ESEDVCIGYQGPQRQVLHWKALHLPAETDAMPWKNGGVYLITGGAGGLGHIFACEIAS

KVVNATLILTGRSDISASQEQAWEALKTNGTTIIYRRGDISREKDTSELVAYIQHSFD

RLDGIIHSAGLTGDQYILNKTKAEMEKVLAPKVAGTFHLDEATRAMKLDFFILFSSMS

GVYGNPGQCDYAAANGYMGSYAQYRHQLMLAGKRQGRTLAIHWPLWKDGGMHLSPAEE

KGLWESTGMRPLALEAGITLLYRAFQSTECEIVGVQGDIPKLISSLEARWNMNRATRD

TPFVSGADAMSVLQKLAAEMLGVGPDEISREADWLECGLDQPQLTLLAQRLNEQCGLD

LSHILFVEYPTLQSASEFLSQRYPIAFPAAPLVPESELLPGTSLTSLPMDRLTKHAQT

TAADNILAEALDTFLKKALSSVLKVPADQIVSHARLEEYGIDSISITQLTDQLEGTFG

PLSKTLLFEYQTLKELREYLIKTHRDTVNRLISIPALPYEEREERSEKTKTARSGLQH

PPFRAFQRPAQRPSVAADAQVGSLAKDIAIIGLSGRYPEADDLEQFWENLMQGRDSVT

EIPLDRWDYRLHMHHADPNSPKPGSKWGSFLQGVDRFDPLFFHISPREAQMMDPQERL

FLETAYEAIEDAGYTPEGLASSTGDTWGNRVGVYVGVMYEEYQLYAAQAQAKGDSSIA

LSGNPASIANRISYYCNFNGPSMAVDTMCSSSLTAIHLACQSLVLKECEVAVAGGVNV

SIHPNKYILLNQGGFLSSEGRCKSFGAQGDGYVPGEGVGAVILKPLNKAIEDGDHIYG

VIKGSALNHGGKTNGYTVPNPAAQSCVIGRAIAEADISADTITYVEAHGTGTSLGDPI

EVAGLSKAFAESTDQQQFCALGSVKSNIGHAESAAGIAGLTKILLQLKHGQLVPSLHS

DPANPHINFAQTPFRVQRDAEAWKPLQRMENGKRVQIPRRAGLSSFGAGGSNAHLIIE

EADYHPHEGVTFNEAIRGGAVIIVLSARTRSALRERARRLLTAVDEKHLTGGAEEEPE

RLLERLAFTLQTGKQEMEERLGFVVRSVQELKEHLQLFLGNENTNEPVDGIDGMYLGN

VKSYQAVQAAIAMDEHFGEVVSRWLNEGLYGKVLDLWVKGIKADWSVLYSTGQPRRIS

LPGYPFERERCWFELPEEAVIREHVDGLNSPVSGIQKAAPERQMTAYHKDWQLADIES

ISDESNSVGKILILANRDSAALAARLTEHFKHTVVLNEEQLDDPGSHWLEDEERLKHG

LTGLIDLTACMRQTTVDDACEEQIRPGWLQLVQDVIEIHGHEGMLLLLVTNGLESFRN

SKVNLAHASRAGLFRMLQAEYRSIQSRHLDVDPSASEEAKIECITTEFRQTDREPEIC

YRDGKRWRSYMRELPYGTGMNKDVTESSIDPERPFLEANQVLWITGGTRGLGMACARH

FVKHHNVKRLLLTGREAIPQRKEWDAYASHDTATGAKIRSLLELEQLGAEVLVTDIPL

TNENAIQAELARVKRSMGPIGGVIHCAGNVDTANPAFIRKQADAMQRVLDPKTSGVHT

LFHVFRHEPLRFFVLFSSVSSAMPSLGAGQSDYVMANAYMDYFAEAASKESASSPFPV

ISIQWPSWQESGFGAVTSEAYRQSGLLSITDAEGMLWLDRIIRENRGPVILPAVVASQ

TVHKGEQQAEHQAEASLSESQQVTIPLIPRPVERMTASLTEELENRTLAWLKALMAKE

LRLDESRLDEDTPFQHYGMDSILLVQVLTVMDRELQHVATDPSLLLEYPTLRQLAKHL

VRIYPESLTDLLMEPNSKLAENSTGKTSDVRSMTSTPPVQHAEPGMDGHTGLIESNDV

VDEAQEKPAELEQTKKRDKIAIVGVACHFPDAENPAQFWSNLQKGKDSMTEIPAERLR

LTGSSTSRTSASSKRIGAFIRDIEQFDPAFFRITDTLAEQLDPLQRQLLEVSVEAAAD

AGYSRERLWNSQTGVFVGSRASNFSSKLGGSTKDTIVGIGQNFIAAHLSHFYNLKGPN

MVVDTACSSSLTAVHLAVRSLLDGECDLAFAGGVDILLDDAPYEMLGAASVLSPDSRC

KTFSADANGIGLGEGCGVVLLKPLSTAIADQDTIYGVIDGTSINNDGQTMGITTPNPE

AQQELLEKAILNAGIHPSTISYIETHGTGTLIGDPIELKALTGVFAKYTNQRQFCGVG

SVKSNIGHLLSASGIASLIKVLLSLAHRELPPTIHCDQPNPRFQFRESPFYIVQQHQP

WGSGPNDILRAGISAFGLGGNNAHVIVSNEGIPAERMVSLPLRFPASPFQRKYCWPPV

KTTESVEASSNVSAGSSMQQLPEHSEQSSFFDFIKL"

CDS complement(2188735..2206422)

/gene="tdh_2"

/locus_tag="EFAGFIKM_01896"

/EC_number="1.1.1.103"

/inference="ab initio prediction:Prodigal:002006"

/inference="protein motif:HAMAP:MF_00627"

/codon_start=1

/transl_table=11

/product="L-threonine 3-dehydrogenase"

/translation="MIITEHMDSVQEQMSSIERLLIRLLWGQLHHAGCMDEHFVFEKT

VRPFYQKWLIESRLMIAEYAQELPGPVESLQSEEIAAWTRLSWEAWESRLPGWLSHPD

VRAYARLADSTIRALPDILKGHRAATEVMFPDSSMHMVEGIYKHHAIADYFNGTLAEQ

ALSVINELTSEITGHRKVNILEIGAGTGGTSEVVFEKLKPIAHKIQEYCYSDISRAFL

MHAEQQYGPSVPYLTYKIADLKIPFAEQGIEPGRYDLIIASNVLHTTPDIRDTLRMIK

ASLKKDGQLLLNEITRKTVINHLTFGLLEGWWQYTDENVRIPGCPALSAANWKKMLQR

EGYHSVHYPSAQADRYGQHIIAAKSDGIVIRANGSAPVVKSVFQPSRPADIVHQNESF

YEQEQPAADHGIITAEMTEQFVKKAIRESIAEDLKMEQSRIQNNRSFSDYGVDSIIAV

HLVNVLNQKCKLTLQTTVLFDYNNVDRLTEHILQEYGKTIESLLLQSQSGEPEPAAAM

ASDKETTRFSGTSLPSESVNYASNPFVDSSKAKAPNTRINDKTSGSYFYHTHICSPSE

ISDLQLHQTLVQELRPDEVRISVRAFSLNFGDLLCIRGLYPTMPPYPFTPGFEAAGIV

VETGSSVTTVKTGDEVVFYADASLGAHATAITCKAEQVFAKPDLLTHEEACSLPVVAM

TVIAAFRKANIQPGERVLIQTATGGVGLVAIQLARHFGAEIFATASMQHKLDYLQEMG

ASYVINYATTDFEQEVQRLTNGEGVHVILNTLPGEALQKGLNCLARNGRYIEIAMTAL

KSAHSVNLSGLNHNQSFYSLDLRKLGMEQPEMLAEYRQEMMHWVEEGVIRPLIGQTFP

MDRLQEAYRCMDQRKNIGKIVINVPEYMHYQPAATALPADSAGVTTRARIEDDGLRNK

SEELTTYEPVAIIGMSGRFAGSRDVAELWSHLEKGDDLIQREQRWNLAKHYGADVSFC

DQGSFLDNIHAFDPLFFNISGQEAAYMDPQQRIFLEEAWRTFEDAGYAGDSMQGRKCG

VYVGCTEMDYGQLMGEQPPAQSFWGKAGSIIPARIAYYLDLKGPAIAVDTACSSSLVA

IHLACQSLWARETEMALAGGVFIQSTPQFYMDANRAGMLSTTGRCHTFDERADGFVPG

EGAGAVLLKRLSDAVRDGDHIHGVIRGTGINQDGTTNGITAPSAKSQTALEKQVYDRF

GIDPASIQLVEAHGTGTRLGDPIEFQALTNAFSSYTDQKQYCALGSIKTNIGHLATAA

GIAGLIKVLLSLRHKRIPASLHMEKANPNITFGNSPFYVNTETKPWHTEKGQRRIAAI

SSFGFSGTNAHMVIEEPPPRPAANSDLRPGYLIALSARTADQLRQQAEQLVSYAKENS

AVESLHMSYTLLTGRPHLAHRMICIARNGEQLADQLSEWLAKGESNSVTSGFVNRNEQ

RGNASLKRYAAECIRQCRETEDSSVYLEHLSALCEMYVQGYDLSFDKLFPISTQRIPL

PTYPFAGDDYWVPAREDTQQVKQPSSAKHAFGTVLHPLVHRNTSSVKQLQYSSTFSGQ

EFFFKDHLVKGMKVLPGVAHLEWVCFAADTAWGDFSDSRMIRLRNVIWSAPLILNETS

ASFAIRFRPVEQTEADETMHFEIGQEQGKESDQHKQVYSQGQVMLAKLPVQMERDIQT

ELAACTPSPFHAEAFYASSAQTGIVLGPGLRGIQSVYVGQDRVIAHLSLPQSLTASGE

EYILHPSLMDSALQVTIGYLLGIVTDQAASGRPALPFALEQLDILGPCTAEMWAVATY

SSGNSRNSKIHKLDVDLCDMDGVVRIRMTGFTPRVLEKDFDTQKEENVRPILLAPEWK

PLEISSHQSAAKYNNHLILISDHFTGQLSSLRDSFSHARCERLATDTFASAERFEQDN

ALLHPLASFFTESAAHLLEEIKQMVNTETGSTLIQLVIPFEEEGQGGLLNGLAGMLLT

ASREHRQLTCQLIQLHSSIQTGELIDKVASESRHSENVIACYPASGIRQTRVWHELSA

YSAGNSLFWKDGGVYLITGGIGGLGLALTRTLVSQIRNATILLNGRSTPNAEQQAQLQ

QLQLNGNHVLYRKADITSREAVQRLVQEIQSDYGELNGIIHAAGVVKDRGLRHKDPEE

LRSVLAPKVDGTIWIDESTAHMALDFFVLFSSMAAVTGNPGQADYACANAFMDAYATY

RDGLVRNGQRNGTTLAINWPLWREGRMQPGAAAEQKIRRQWGMHPMDTSIGIKLLHMA

MNSGSSQVLAFYGEPELFLSGVMSDSAGSVDLPSIWNVSTAPSQSMTSVAVSSDIPES

QKRTWPTGDERKRLLVSKVQDVLTDHMSELLQVNRTDIFPDVEFGEYGFDSISFTDFA

GILNEHYALKLTPTVFFEHSTLRSLTGHLVGAYPKQWDTLLLEKHMDTEGPHADDSSQ

EKETEGVSSNLLRQVEQAGSFSIRNPYTFPKETKDSPEQKEQMGGSISSSERHRESSM

PESDHQNHKAPEPIAIIGMSGRFPMAKDIDALWNNLLQGRDCISEIPLDRWDWRAYYG

DPDQAHNKTNIKWGGFMEGVAEFDPGFFGISPREAELMDPQQRLLMMYAWKAIEDAGY

APPSLSGSKMGIFVGTASSGYHRLLTQADTVIEGYTSTGHVASVGPNRMSFFLNVHGP

SEPVETACSSSLVALHRAVQAIRNGDCDTAIVGGVNTILDPDYHISFNKAGMLSPDGR

CKTFSDEVNGYVRGEGVGMIFLKGLRAAEQAGDHIYGLIRATDENHGGRAASLTAPNP

RAQADLIKSVYSKAGVHPSTIGYIEAHGTGTPLGDPIEINGLKIAFQETLATAEPNLT

TSASCGLGSIKTNIGHLELAAGIAGVIKVLLQMKYKTLVKTVHYERSNPYIQLEGSPF

YIVDENKAWEALHDSAGRTMPRRAGVSSFGFGGVNAHVLLEEYIPDESVANSDRTHHP

AHGDSMNKATSVCIVLSAKTEKRLQEQARQLLAALQSGRYEDCHLPQLAYTLQTGRAS

MEERLALVATSIAEIEDRLDAYIHDPEKTENMYRGKIKSHRVSIAALAEDADMQETVK

SWVRKGKYGQLLDLWVKGLEVPWSEGYTGRTPQRISLPTYPFATERYWVPEQKERKSG

QPVPTSYLHPLLQRNISDLQGICFSSEWSGQEFFLNDHVIHNRKIMPGAAYLEMARAA

VEQVIPTDTSRQDRVIVFRDIYWTVPLGMEQHSVTPGRLQAELRLTPLDTGEIRYEIT

SGENNSRSVRSEPSKRNVHHIGTISTSTHQAASYPTIDLESLLLQCEKQTMTAAECYR

FFEQSGLYYGPGHQGIQRLHIGEGQVIAQLKLPPSLASTAEEYGLHPALVDSAIQACI

GLPQFKEGGVTPFSLDQAVIYRDCPDSVWAWVRLLPEQDSSGKASKVQIDLCDDQGKI

VLQFIGLTILHRPYPGEPSGIIQEKSNTFAKPLQSKTIAYATLEKQKLTQIPLSGAMT

YAPSWREVSTSPDVTQPRSISFGGSGIIVGGTSRQQNVIQQWHPDLQPVAVEGDTPET

KLEIELKRHGVISRLVWIVPPSSIVHGPEHAVEVINEQNQGLFQTFLLIKVLINLGYD

ARRLDLTIITEKACNVSAGETIYPAHAGISGLAGSLAKEMPDWRVCHLDVDDVHDIGE

HPERHFPGIWDGNTRAWRNGKGYESVLVNVCSTGDTHEAFRKNGVYVVIGGAGGIGEA

WSEYMIRRYQARLIWIGRRAVDARIGELCRRLGELGTEPLYISADASDKHSLQEAYRQ

IRSLAPAIHGVVHAAVGLMDQSLTQMTPDYFRNVLSSKLDASVNMMNIFGQEPLDFML

FFSSMSSFTTPAGQAGYAAGCAFIDGMSAAAQSWKFPVKVMNWGYWAETGIASQVPAT

FKQRLNQSGIGSIHPEDAMRALELLMAGGKHQLAFIQTDKLGAVEGTASDRMFMVAPE

AKQVGRHQLMNRISGQQLNVKPLQQKWAAFNDEFESMLAGLLSIQLQQIISADSANRP

VAIYDRWMKESRKRIEQYTLSEQSLASDSMWSAWEQRMKHWLLDPDFSPQVKLVDQMI

RSLPEIVRGELGATNIMFPDGAMDMVQNIYKHNSAADYFNEILADTVAAYLAERLESN

PQASLRILEIGAGTGGTTEKVLERIKPFAASIAEYAYTDISRAFLLHGEKTYGENHPY

LNCTMLDIEKPPAEQSYDLGSYDLVIAANVLHATRNIRRTLGHVHDLLKQNGLLLLNE

LTHSSLFSHLTFGLLEGWWLYEDDDIRIGGSPVLGKQQWQKTLQETGFGQAMLPAEPG

ESLGQQIITAFSDGYISMAIPSPEARSNHEPISSENSSVMNMDKMQTEHQSQSEAELD
SDQMWQASLTFFKRLVGEVIRMSPEHIDESELMEAYGIDSILIVQLTNQLRKHFDGVN
TTLFFEHKTLASLIHYFMKQHRQELLEAALPNLPEKEREQTHESLIQPTVSAVERTME
EPVFDPVKPVPITVSLNLAANEPIAIIGISGKYPQADNLEQFWDNLIAGKDCITEIPE
ERWSLEGFYEPDRKEAARKGKSYSKWGGFIEGYADFDPLFFQLSPREVMNMDPQERLF
LQTVWEALEDSGYTREQMDRKYNGRVGVFAGITKSGFNLYGPPLWNEGSEWFPQTSFS
SVANRVSYLLNLHGPSMPVDTMCSSSLTAIHEACESIRRGECEAAIAGGVNLYLHPSS
YVALCAQQMLASDGKCKSFAQEADGFVPGEGVGVVILKQLSKAVEDGDQIHAVIRGTH
INHGGKTNGYMVPNPAAQGDLIRETINKAGIDARWISYIEAHGTGTSLGDPIEMAGLA
RAFRQDTQDKEYCSIGSLKSNVGHMESAAGIGGLTKIILQLKHGQIAPSLHAEELNRE
IDFKNTPFIVQQKASDWSRPMVEVDGQMREIPRIAGVSSFGAGGANAHVIVEEYIPAP
AQPSFPVIESSSNRVERKSEQPVMIVLSARNEERLVAVAEKLLLYISRRTLDDQVLDK
LAYTLQTGREAMEERLAFTASTIHELIARLVTIQTGKLKQAIPGIYRGRVNRTSYTPL
DTEQEANKPASEDVRQAMMQDNVDRLLELWVNGAKLDWKQIYGVITPSKMSLPTYPFE
REKYWLPDAKGMPMSVANTTEADSRNNIVLHPMLHHNTSLLSEQRFTSRFSGDEFFLS
DHQVGTYRVLPGVAYLEMAREAVKRSIEQADSVPEVIVLENIIWSRPVVIAAQATDVH
VRLYTEEQGDIHYEVYTQDASAELLHSQGLALLKTESQELRLNLEEIRNRCIKPWTVD
ECYQAFEQMGLHYGTAMRGIETVYTGENVVLAKLALPARVKDSVRDFVLHPSMADAAL
QSAIGLLLASSMPDDHRLQTSPSPLLPFALERVVFHRPCTAHMWAYIRYSKGHKPSYS
LQKLDIDLCDEDGNVCVQFEGFTSKPLKGEIHMGKTLSPTESQTLMFAKEWKEQEAVV
VPEVVYSRHIVMLSGPWTEQEHVMAAKLPNVHYVSLGPDPADRVGQSDLMDEQYLFAV
KKAYRYVQQLLKARPEGAVLIQLLVAKRAQYKLFGGLSALLKTAELENPKIIIQIIEA
DDLQSTERVVQILTESSQSAGDTHIRYQNDKRYTSLWKEVGTSHSSLVELPWKDRGVY
LITGGLGAIGSIIAEHIAEKAEHPVLILAGRSEPDESDHQRMALLEQMGAEVIYRRSD
VSKREAVDELMLEISRNYGELHGIIHNAGLIRDSYMIRKELDDIEEVLAPKVAGTVHL
DEASQYFKLDFFVLFSSLAGVHGNVGQADYAAANAFMDEYSEYRNERVQQGERHGFTL
AINWPLWEEGGMHMNDHARQNMERISGTVPLERANAMRSLQQTLQERRSPVVVIQGEA
DQMRSFMGISGTAPIMNTDEAYNRSDDENYLHLVEKIRSGECTLDQLMHLK"

CDS complement(2206464..2212367)

/locus_tag="EFAGFIKM_01897"

/inference="ab initio prediction:Prodigal:002006"

/codon_start=1

/transl_table=11

/product="hypothetical protein"

/translation="MQDNERLSLQHKLCNSLNQKLMHVLIVQMEQLGDVRTMAGASYC

TSYPGVLPAYQRWMDHTLRILQRNPELGEFERSNIWSDWEEHKKRCITQPEMRARVEL

ADVMIRALPDIVTGKQSATEIMFPGGSMSRVEGIYKQNSIADYFNSLLADEAVQLIRD

FSGHGNRKIRILEIGAGTGGTSSMLFQKFEPYRELIQEYCYTDVSKSFLQHARKQYGP

YYPYLTYSLLNIEEPLHSQGIEAGRYDLVVATNVLHATRQIRHTLRNAKALLTLKGKL

LINEIIEDSLFNHVTFGLLTGWWLFEDEQVRIPGTPLLHPDQWKAALELEGYFDIEFP

ASTAEGLGQQIIRAASDGIVRQKHTFSSATQMLPAAGKEEPLKPQETITVQELEPALN

ALSPKAPAVDDRELKQACMMYLQKMIGAILDVEPEQIDPAEALEMYGLDSILIIQLTE

QLNKEMGEHVSSVLFFEYTTLDALVNHFVEKRKFELISMLGLTEMQEESITEPELNMG

EFSTEATTHLTESYKQQRPSEATDEVAIIGFAGRYAQADDMAEFWKNLMQGKNCITEI

PSQRWDWRTHYHSGKGQKGTSYSRWGGFIRGIYHFDPLFFHISPKEAEQMDPQERQFL

QTVYVAIEDAGYTPQALADAGTVGVYAGVMNGNYPTGASFWSIANRVSYTFNFTGPSL

AVDTACSSSLTAIHLAMESLRNGTCDLAIAGGVNLIVDPVHYVRLSEKTMLTPDNECS

PFGEGANGFVDGEGVGALLLKPLHMAEADGDFIYGIVKGSMINAAGRTKGYTVPNPGQ

QAKVISEAISRSGVDARTISYIEAHGTGTALGDPIEIEGLRRAFEKHTADRQFCAIGS

VKSNIGHAESAAGIAGVSKILLQMRHGCLVPSLHAESSNPNIRFSQTPFHVQQHMTPW

IRPVLDTPEGMREYPRIAGISSFGAGGANAHVILEEYRTDYKVDQKSVMAGDSNAPVM

IVLSAKTEDRLRIKVRQLAKAVQDNAYTDLHLARIAYTLQTGREEMECRCAFTAYTIK

EMLGKLQAYLTGETAHEQIFTGEIDIERNENRLVDDDDMQELAASWMRKRKTVRLLEQ

WVRGMKLDWQQWYGKVKPQRISLPGYPFAEEHYMIPSLSSLSGNEGFEITNVVAPSQT

EWLHPLLHKNTSYLGSQRFSSRFTGNESFLRDHQVRGVSILPGAAHLEMAAAALRHSL

PPEEHNRQQNGNDHIRLHDLVWLHPASVEDQPLSVHLELQAERHQQGYRFEITSTTKA

EAPSVLCSQGFVSLIRSTGDTPVFQPANWHKRHGNKVVPPERGYQIFSRIGLVYGNTH

RCISHLYTGQHSVMARLAMPLSGTKSLEEYGLHPSMLDSAFQACLALAMDLSQLSQEE

GLSPEPWLPYALQQAEIYRPCSSVMWVEVRYTASSGPGNSMPRYDLTLYSEDGLLCAK

FEGLALRKWAATPQSQQDWTGEEAGLTNQFGHHLNNLNALEPLTKDLTMSAAAATAEK

DDAKLAVLTTVWAPLEQQSSSEGVSGTEVYSAEKLLILCENADMNIRFPRGDDFRQIV

FPRQASAAQMVDMLAAEGDCDHMIWHCRHVMSENRKTETNREEQESFVPELTRFFRFV

KSILHLGYGSRSLHLTVVTTNAQALAFDDTIDPDQAAIHGLTGSLAKEYAHWAIRVID

LNGGEILPMDAIADMTPDPHGNAYVWRDQEWYRQELVPVEYDSVPLTAYRQGGVYLVI

GGAGGIGEVWTRYMIATYNAQVIWLGRSPLSPEIRRKIEHLGLLGPEPLYIQADASLP
GRLEQAYESVIKRFGQVNGIIHSALVLSDRSLARMDEEIFNNVLSAKVQISEQVNALM
SRLRKQPDFVLFFSSLNSFMKLPGQGNYVAACCYADALATQMNRKWPGRIKVVNWGYW
GSVGSVVSEQVRQSMHQKGIGSIEAEEAHPILEWLMSGSLQQAVILKMTRKPEMLVLN
ENQMLTVYR"

CDS complement(2212403..2217670)

/locus_tag="EFAGFIKM_01898"

/inference="ab initio prediction:Prodigal:002006"

/codon_start=1

/transl_table=11

/product="hypothetical protein"

/translation="MGSKRTEIAVVGMACRFPGAVGYDSFWENLIQGRNSISEIPPER
WDLEKFYSSNSNDTGKTSSKWCGLVEGAYDFDHSFFNISPREAASMDPQQRLLLEDTW
HCIEDSGVSLSELQQKVTAVYTGVMTASYLQQMEASQADSYACLGNYESLLANRISYA
FNLSGMSLPINAACASSLVAIHEARRALILGECDYALASAVNLNLDPLKYVSFSKSRM
LSPTGQCKTFDQSADGYVPGDGVAVLLLQRLGEAVEQGNTVYGIIKGSAVNHTGKSLS
ITAPRMEAQRDVILAAWKAADIDPRTITYIEAHGTGTSLGDPIEVEALTQAFRHYTPD
RSFCKIGSVKTNIGHLESAAGIAGLIKVMMMMKHRKIPASLHVKELNPMIPFSRTPLE
VARKQETWHSREQGQTLRAGISSFGFGGVNAHVVVEHYEPAAKPSVPSKLSVQQDSRE
PSRLFLLSAKSEASLQEHVADWQQWFDRHSNAEDTLRAISLSLLKGREHFKYRFGGWA
HTREELRTLLNSDCYGNTPIGVSARYLNIQSPKWEGYAQLKCSGHTSQLLLRKAERQI
EAALKANGMREEWEDLMTGYYQDTWHEDWRELYCFIAGYVHAASLIQLGFAPRWIQGT
GIGMLIAWVIGGHISVEDMISVLLKQKGAEDVRLCSPIWPVMQEGSSSLIMPFAFDDS
YVHDLIQSVRMVEEQAYDGFRDELMDRAHRLYTHQYTFRNYINEWNDEWQESTGTTIP
WPLQPTSDSSSSSESFRSMSAVLCIAVMDALRRVKRKWQLQVSEESDIPAVQELLDLL
ADKVITRRDIIEMLLNETGEHHELSRITAQLNQNSGLMLPERAYTLIRQHQQRTGGMH
NALQILHDFLQPGSENEKDRKIPLPHSEKATDWMKQTGQEAEEATLELPFHENVLRLW
LSGMELDMRQLAEEGPYQKVRLPRYAFQRKTFRWSANLGRKAKDSFQETKSKKEYDRL
HPMLHRNLSANQNTRFCSDFTGHEFFLNDHRVRGQRVLPGVAYLEMARMALHSVNQAP
SSSFAAFQIKNAAWTSPIFVDERPVEVSVRLHYEDTERGILRYEVSEDGSEAPDRLSC
GTGRVSLKSLAPVDRLDISSLIAAMREESLLDHETIYGRLREQGLEYGPGHQAIERMY
IGSGQALAHLNIPSHIEHTLGDYALHPSIMDGALQTAMLLLDDNATLQNQEEGTKLPF

YMEEMDCLLPCTAEMWVHVHFSDGCPIGGNVAKLDMDLMDADGTVSVRIRNYMCRSMP

GETRQQCVMQDAERATITRDLHEDFPPGTELLAPVWSVLPSEKDSRISLESLRTAIIV

AETVEMSGYLDEVTHQYPGAALILMKPGETVEQTAQRLENCGQFDHLVWVSGLGNAEG

KDGSAITQSQEEGVIIVFSLVKSLLQLGYDRIPLQWTVVTFSAIAIHAQDVVHPLHAS

IHGFLGSLAKEYKNWTFRVVDLDLADRSLPALSDMPIVPENSWSNLTVYRAKQWHRCE

LLPVKLPSIQQSAYRRGGVYVVIGGAGGLGKVWSEYMISHYNAQIIWIGRTPLNPDIA

KEQERLALTGPAPEYISADAGSPDSLHEAYRLIKQRHGTIHGVIHAAIALRDSSVMKM

DLPQFNEGLYAKVNVCAGMLEVFEKESLDFMLFFSSINAFATPAGQSNYTAGCTFKDS

FAQWLHRCLHYPVKTINWGYWGSTGTVASPAYRERMTKQGIGSIEPREAMAALELLLA

SSLPQLGLVKIIGEAKPYAISEKEFMTVY"

CDS complement(2217719..2218960)

/gene="oxyE_1"

/locus_tag="EFAGFIKM_01899"

/EC_number="1.14.13.232"

/inference="ab initio prediction:Prodigal:002006"

/inference="similar to AA sequence:UniProtKB:Q3S8R0"

/codon_start=1

/transl_table=11

/product="6-methylpretetramide 4-monooxygenase"

/translation="MDIESEVLIVGAGIAGVALALAMGRKGRQVVLIERSKQFPNIPK

GDFLQPVTIDLLDKLGVLPEVTKHCAHIARVNYGTLGGVNCFTGSYEKMDIPVRYALN

GDHHHIHQSLFNAIEEMPNVRFYPGMNAGKLLFTDHYVSGIEADSAGKTVRIGSKILV

GSDGIKSKIRDQLGLKYHLYPYEEKMAKMFAFTFHDVTHTREEASFFFGEGVSCGVFP

LPGQRLRSYLALRKDIWQRIREDGIESLRGMLLSLCPHLQQEITQIVDFKQVQSIPAY

YLHTEKWAINGAVLLGDACHALSPALGQGMNLAIQGAVELSNTLEEALITNDCSERQL

RLYEKKRRKYVRLIQRNSTAHTYCWFVKNRTFVTLRNAAFRRIGKCPNLLKEQMLTTS

GYSDKPPSFSHMLRFAGIMKP"

CDS complement(2219015..2222263)

/locus_tag="EFAGFIKM_01900"

/inference="ab initio prediction:Prodigal:002006"

/codon_start=1

/transl_table=11

/product="hypothetical protein"

/translation="MGKDLFEQDALFRETMLALDQTVLECGGESIVQYMYGESSTDED

RFERTILSHPAIYMVEYALAQSLLTRGVIPDYVLGSSLGEFAAAAIAGVFSAKVGLRC

VMKQAIRFEQHCPSGAMLAILGDASLYPELPEFEYCTLVSVNYDGHFVISGPVHALTK

AQEWLTSHAVLSQMLPVSFAYHSQWIDSAEAAYTEDLQAFTFAAPHIPLISCMTGGQP

ARIAQNHFWQVARKPILFREAIQKLESAEPHHYIDISPGGTLAGFVKRLADKQASSAA

VVLLSPFYRDKEQLESVARQYQLPSMNIQRGEQTMRAYVFPGQGSQFKGMGHDVWNLY

PDLVRTADEILGYSIRELCLEDPGDRLKRTEYTQPALYTVNALMYYRELEQQGKPDYA

AGHSLGEFNALLAAGVFDFATGLRIVQFRGELMGRVEGGGMAAVIGLHADVVGKILAQ

HGLDHLDIANMNSPSQIVISGPQDLLVQAKSVFEQEGASNYVILNVSGAFHSRYMEAA

KQQFAAYLKQFQFAAPTLSVISNVGARPYTIDHLQHNMVEQLTSSVQWTDTIRYLMGK

GVNDIVQIGPGTALSKLVKAIQAQAQPLFVEKLSVESPPASPAVIKDVPKDESLGVSE

TAAHPFSSSRRFQSERMGSAEFRQDYGSRLAYVAGGMFGGISSVEMITTLAKAGMVGF

LGTGGLRGEEVEEAVMSIRQELPVQHCWGLNVLHDPLHPQREEEMIKLMLRLGLNKME

AKGFLGMTPAVVYYRVKGIYRRSDGTVASSHLLLAKCSRPEVAELFMSPAPQHMLDQL

LVEGRISAEQAQWARQLPMADDICAEADCAGYTDGAVSMTLLPAMLSLRNQMMKKHGY

AKAIRVGAAGGIGTPQAAAAVFVMGADFILTGSINQCTVEANTSQLVKELLQQINVQD

TGYAPAAGMFELGGKVQVLKKGVYFPARANKLHELYRQYDSIDEIDEKTKTRLQEQYF

KRSFDSIYAEMESALTTQERALAERSPKARMAVIFKWYLEHSAKLALQGDENHQHRVD

FQVYCGPALGAFNQWVQGTTLESWTQRHVHLIGEKLMEETAQLLNESLLQQV"

CDS complement(2222909..2223796)

/locus_tag="EFAGFIKM_01901"

/inference="ab initio prediction:Prodigal:002006"

/codon_start=1

/transl_table=11

/product="hypothetical protein"

/translation="MDYNSRNEAVFETKRFRNCTIAAGRRHTVALISDGTVTAVGDNK

YGQCKVSDWCHIVAVTAGNVHMATNTGNAHTVGLKSDGTVTAIGWNKHDQCNVSEWRD

IVTVSAGWCRTVGVKSDGTVVAVGRNNEGECNVSSWRDIVSVTTGDWHTVGLKIDGTV

TAVGNNKYNQCSVNNWSDILAVSAGYLHTVGLRSDGTVVAVGRNNEGECDVSGWRDIV

AVAAGSYHTVGLKSDGTMVAVGSNKYHQCDVSSWHEIRAISAGCAHTIGLKSDGTMVT

VGDNDYGQCDVSGWSNIQQ"

CDS complement(2223989..2225920)

/gene="fbp"

/locus_tag="EFAGFIKM_01902"

/EC_number="3.1.3.11"

/inference="ab initio prediction:Prodigal:002006"

/inference="similar to AA sequence:UniProtKB:Q45597"

/codon_start=1

/transl_table=11

/product="Fructose-1,6-bisphosphatase class 3"

/db_xref="COG:COG3855"

/translation="MDEQFLDLLAEKYDTEEKIITEIINLEAISNLPKGTEHFVSDLH

GEFQAFQHVLRNGSGTVKEKIKELFREVWTDQEINDFAALVYYPEEKIQLVIGELSNK

QALNQWYRLTIEHMLKLISYASSKYTRSKLRKALPEQYVYIVEELLYKTDSTNNKDPY

YEEIYRQIISLGQADNLIIGLAYTTQRLVVDHLHVVGDIYDRGPYPDKIMDTLINYHS

VDIQWGNHDVLWIGAYAGSLACLANIIRICARYDNLDIIEDVYGINLRPLLNLAEKYY

EDNPPFRPKLQAGHNVSEQEILQITKIHQAIAMIQFKLEIPIIKRRPYFNMSERLLLE

QIDYDNNEINICGKTYLLENTCFATVNPQKPEQLLEEERQVMEKLLFSVQHSEKMARH

MNFLMKKGSLYLKYNGNLLIHGCIPLDEEGNMEEMQIEDKTYAGRQLLDVFEDNLRYA

FAHPEETEDLATDMVWYIWTGECSSLFGKREMTTFERYFIQDKEAHKERKNPYYHLRE

NEEICRKILQEFDLNPDHGHVINGHTPVKEISGESPVKANGKMIVIDGGFSKAYQSTT

GIAGYTLLYNSFGMQLVAHKKFNSKEDVLCNGTDVLSLKRLVDKELERKLVRETNVGE

KLLQKISNLKDLLEYHYMK"

CDS complement(2226245..2226961)

/gene="COQ3_2"

/locus_tag="EFAGFIKM_01903"

/EC_number="2.1.1.222"

/inference="ab initio prediction:Prodigal:002006"

/inference="protein motif:HAMAP:MF_00472"

/codon_start=1

/transl_table=11

/product="Ubiquinone biosynthesis O-methyltransferase,

mitochondrial"

/translation="MIPLVYDQINHWGKDDEFFLALLKRLQVESIADLGCGTGRWTTH
LVQYGYKITAIDPNEEAIEMARSKDTSGNVDWIIGDSADLQTNTYDAVIMTANVAQVF
LTDDSWKRVISDVFRSLKTGGHFIFDTRNPLVKVWEEWEKDKAPDLAKDRLSGEPLEI
YTEYEGFEGDIYTFYEIVKNTKTDKVLVHEKMQLRFQNQEELDESLKQIGFSKIKTYG
DWEFKQANSENRSFIFHCVK"

CDS complement(2227028..2227936)

/gene="gltR_1"

/locus_tag="EFAGFIKM_01904"

/inference="ab initio prediction:Prodigal:002006"

/inference="similar to AA sequence:UniProtKB:P94501"

/codon_start=1

/transl_table=11

/product="HTH-type transcriptional regulator GltR"

/db_xref="COG:COG0583"

/translation="MELRQLNTFCTVVTTLNFTRAAEALSYVPSNVTMQIKALEDELG
VRLFDRLGKQLALTTAGKRFLTHAQGVLEKMEEARSAVHDNEKLSGTLTISANEVICS
YRLPPVFQRFRSQHPGVRLIFRSVPNQELKQTLFEGTTDIVYMLDEPIRSSGLSVESL
LEEHFRLLAAPDHPLAKRTVLQLEDFHDEVFLTNEKGCPYRTMFDRSFEKEGIDSITY
LEFQSAEAIKQCAISGIGIAFLPEIVAESEVERGELVVLPWQIPDLQVYTQMLWHKDK
WLSPIMLSFMEATREVFGSQSENKTV"

CDS 2228080..2229099

/gene="metXA_1"

/locus_tag="EFAGFIKM_01905"

/EC_number="2.3.1.31"

/inference="ab initio prediction:Prodigal:002006"

/inference="similar to AA sequence:UniProtKB:K4ICC9"

/codon_start=1

/transl_table=11

/product="Homoserine O-acetyltransferase"

/db_xref="COG:COG2021"

/translation="MDYEIFNLGDVLLQSGVTLPNAFLAYKTYGKLNEQKDNVIVYPT
AFGDQHVQNEWLIGSGMALDPEKYFIIVPNLLGNGLSSSPSNTPPPFDRANFPQVTIY

DNVRFQHQLLTEKFGIQKIALVVGWSMGGIQAFQWGASYPEMVERIAPFGGIAKPWPH

TFVVLEGVKASLLSAVGFDSSKLNQLSSTDMRAVGRVYAGWGLSHAFYREELYRDMGF

DTLEDFVAGVWEDSFMNMDPHNVLAMLWTGQHADISANPSYNGDFDKALQSIKAYACI

MPGSTDLFCTADDNEYEAKRIPNAVLKPIQSIWGHFAGRGINSADNQLIDDNLKHLLS

LSTNE"

CDS complement(2229104..2229217)

/locus_tag="EFAGFIKM_01906"

/inference="ab initio prediction:Prodigal:002006"

/codon_start=1

/transl_table=11

/product="hypothetical protein"

/translation="MATRVSYLVELKMKAIEMRLALEQNRDLLGLTDIQCN"

CDS complement(2229319..2230359)

/locus_tag="EFAGFIKM_01907"

/EC_number="1.-.-.-"

/inference="ab initio prediction:Prodigal:002006"

/inference="similar to AA sequence:UniProtKB:P32382"

/codon_start=1

/transl_table=11

/product="NADH oxidase"

/translation="MNYSQSVEALFQPIELGHLKLSNRIVMAPMTRQFSPDGIPGSNV

ADYYRRRAENAVGLIVTEGTIINHPDASNQANVPHFYGEAAMDGWAHVVSEVHEAGGR

IIPQIWHMGAKGHVNDYSEAEIATIVQEFAQAASEAKRVGFDGVEIHGAHGYLIDQFL

YEKTNSRTDRYGGDMMARTRFAVEVIEACRKVVGSEFPIVLRLSQWKTDDYQAKLAET

PELLEQLLAPLVQAGVDIFHCSTRRFWEPEFEGSDLNFAGWTKKLTGKPTITVGSIGL

DGDFTSLFTEGKGASNVGIEGLVQRLQNDEFDLVAVGRALLVDPEWVKKIQEGRTDDL

VPFTREAMTVLT"

CDS complement(2230564..2231139)

/gene="comR_2"

/locus_tag="EFAGFIKM_01908"

/inference="ab initio prediction:Prodigal:002006"

/inference="similar to AA sequence:UniProtKB:P75952"

/codon_start=1

/transl_table=11

/product="HTH-type transcriptional repressor ComR"

/translation="MSRPREFDVDRVLHQSMEVFWNQGFKATSYEDLTRTTGVKKQSL

YCIFKDKRSLFLKALALYREQVIAKLKEIEALDSSPGDKLDALRYSLLDDETGCQGCL

IVNASLEFGTDDEQVTREAELMVEEVQLVLEKIISSGQKQQLISNRYTSIELASYLNN

TILGVRVMEKSGSSREQIETVLRTSFGMIMS"

CDS complement(2231380..2231943)

/locus_tag="EFAGFIKM_01909"

/inference="ab initio prediction:Prodigal:002006"

/codon_start=1

/transl_table=11

/product="hypothetical protein"

/translation="MLELEFTTIEEWDEALWARMERIYHEAFQSGAKTKAILRSMLDR

KIGYLHTGVHHGEVVAMAVTGLEGKVADRILIIDYLAVKQKLRGWGIGTWMLEQLRAW

ALREHRIKGMIIEAEFGTTEAHQERIQFWQRNGFILTSYVHQYRMVPEPYQAMMLPLD

TNMHVPDDGEALFRYINAFHKVAYRKS"

CDS complement(2232370..2232615)

/locus_tag="EFAGFIKM_01910"

/inference="ab initio prediction:Prodigal:002006"

/codon_start=1

/transl_table=11

/product="hypothetical protein"

/translation="MNKYVLHIVASVICILLPAVGLLYVLWDSHQPKIGPVGDGKPNY

PSISQWISIGSSFILGVVNLPLSIIRYRQKAKEDIKS"

CDS complement(2232704..2233678)

/locus_tag="EFAGFIKM_01911"

/inference="ab initio prediction:Prodigal:002006"

/codon_start=1

/transl_table=11

/product="hypothetical protein"

/translation="MLSEEDYAKVAKEALQQYPIYWEKLVYLGKSDNVTFQVQTNDDN

QKFLIKIHISTISIQSKGNIASELIWLEALVKDTNLVVPAPVRNFQGDLVTEISTDHS
ENTIMVTIHHWIHGSVLQREPTSNETESLALLMAALHKHSMQWNAPESFNRPIYNSGH
LYSSLNQLKQLVNLELISRELFVHVEESAHKIANVIQNHKRLPSNWGIIHSDLHESNY
VFYKDTPRPIDFSNCGYGFYLFDMAETFLHLSSDNRKVFISSYSKVNQLEENYVELLE
AFFIWTIIRTFAFHSLNPNEQQSLAANFPSVIENYFRKYLKGEIFLLN"

CDS 2233984..2235069

/locus_tag="EFAGFIKM_01912"

/inference="ab initio prediction:Prodigal:002006"

/codon_start=1

/transl_table=11

/product="hypothetical protein"

/translation="MTIIPVIVMIFVFVPTVILMVSMPYLTRETISFGVTVSAVQFHS
EPLRQMRKLYVRISATFHTILFIVCIICLIYGDEYSKQQSWIIVTYSLAMVVISLVIN
ISYHFKMKSLLPMLPIALEPSIIAVDTGFRKRNISLSSNWFLIHVFIIVVGIVTVLLN
YELIPDQIPIHYNSSWNVDGYAAKSYSSVFMPTIMQVFIILVFIFENWSIRRVKQQVQ
PTDPNRSIRQDVTFRRTWSCFMIIASFLIVILLSVVQLNMISLLNINFAIPIVLIIIA
FIILYALALSFWAGQGGSRLEQSADRSNYRPVHDDDKWLLGMIYFNLKDPNLIVEKRF
GVGWGLNFGHPVCWLIFLGIIVLLVVV"

CDS complement(2235613..2235960)

/locus_tag="EFAGFIKM_01913"

/inference="ab initio prediction:Prodigal:002006"

/codon_start=1

/transl_table=11

/product="hypothetical protein"

/translation="MAVSAGYLHTVGLRSDGTVVAVGRNNEGECDVSGWRDIVAVAAG
SYHTIGLKSDGTTVAVGSNKHHQCDVSVWHDIRAVSAGCAHTIGLKTDVTLVTVGDND
YGQCDVSGWSNIH"

CDS complement(2236509..2236877)

/locus_tag="EFAGFIKM_01914"

/inference="ab initio prediction:Prodigal:002006"

/codon_start=1

/transl_table=11

/product="hypothetical protein"

/translation="MRQTSEELSLVKRVVELPYLLGALEVDKHKIYASNFKMKSVYAD

YLDGTQKKIALEMYKSKQSLKQHHIKIIDEKRSELSLTVDYSVRGYLHKMTLLWSKVK

VDVTLMLTEHMSVDITDMTR"

CDS complement(2236861..2237226)

/locus_tag="EFAGFIKM_01915"

/inference="ab initio prediction:Prodigal:002006"

/codon_start=1

/transl_table=11

/product="hypothetical protein"

/translation="MSKKLEANGLWESSRMMLPQHKERIIQHRTQIHYQTKPLIHQDE

WEIITQNIDMSFKYTLQAAFEVFNESGNRYIHGIVTSVSSFGNKIKIEMNNGFEWVDF

DQLVTVKLEEGGVYSETDF"

CDS complement(2237223..2238464)

/gene="dinB_2"

/locus_tag="EFAGFIKM_01916"

/EC_number="2.7.7.7"

/inference="ab initio prediction:Prodigal:002006"

/inference="similar to AA sequence:UniProtKB:Q47155"

/codon_start=1

/transl_table=11

/product="DNA polymerase IV"

/db_xref="COG:COG0389"

/translation="MLTKQRTIMLIDMQSFYASVEKAKMPQYKNSPLAVAGDPARRSG

IILAACPLAKAKGVSTAEPLWQSLQKCPELIIVKPHMQEYIEVSTQIMSIIEEFTDLV

EPYSIDELFADFTGSMHLFGNDPIDLAKQIQDKIYNETGVYARAGIGENKIISKLCCD

MIAKKAEGGIFHLKKEELHLHIGDKPIRDMWGIGSRMEKHLWKMGIRTIRDLANTPLS

KLRSKWGVNGEVIWRVANGLDNSPVTVNTHSTQKDIGNGMTLPRDFIEAWEIEVVILD

ICTEVCRRARKKGLMGSVVSVSVSGADFDQPTGFHRQVKLSDPTNITVDVCKLAKRIF

HQHWDGQPVRRVGVSLSQLSNADTYQLSFFDDQEQKRAIDQVMDDIKDRFGDIAILRA

SSITAAGQAIDRASKIGGHYK"

CDS complement(2238727..2239629)

/gene="aroE_2"

/locus_tag="EFAGFIKM_01917"

/EC_number="1.1.1.25"

/inference="ab initio prediction:Prodigal:002006"

/inference="similar to AA sequence:UniProtKB:Q8Y9N5"

/codon_start=1

/transl_table=11

/product="Shikimate dehydrogenase (NADP(+))"

/db_xref="COG:COG0169"

/translation="MNNDKNLAKIDAKTTLVGLLATPIGHSVSPQMHNVGYTISGLNY

AYLAFEVGPEKLKETVEGMKAMGVAGFNVSMPNKIDVIQYLDELDDTAKFSRASNTVV

NKNGKLVGFNTDGLGYVKNLEEHAVELNGKKVTLVGSGGAATPIAIQLVQSGIKEISI

FARNDKFFSKAEENAAYINNEMKHFGVKANVFPLEDKEAFRKEIAESAILANATSLGM

KPLDELSIVDDVEDVLRKDLVVTDVVYNPRKTKLLKQAEAAGAKAINGLGMVLWQGAL

AYKLFTGEDMPIAEVKESVFGTEI"

CDS complement(2239657..2240844)

/gene="ydiN_1"

/locus_tag="EFAGFIKM_01918"

/inference="ab initio prediction:Prodigal:002006"

/inference="similar to AA sequence:UniProtKB:P76198"

/codon_start=1

/transl_table=11

/product="Inner membrane transport protein YdiN"

/translation="MKNPYIKTSLGMYLNYFMLGMINIIIASNMSSLSDNYNQPVEKI

SLLVSAIGIGKLIALFFAGRLSDKIGRKPIIITGSFLYLLFLVGIPLVSSYPVAFVLA

ITAGIANSLLDSGTYPALIESFPKKSSSATVLVKAIMSIGATLLPFILAYLVANDISW

GWSFYALGILFLISGIYLIFMPFPSFKSHSKDEADSETYKNRFKEEPKFWGEGLAIIL

IGFTSTALFVVWQTWLPELGSKYIGLSENVSIKLLSYFSIGALVSVLLLAAVLDKFIK

PITVMILYPTIAFLSMLSLFFIQSYTIVIIMTFILGLSTSGTFQLAITIMTRFFVKNK

GTTTSYVNIAGSAAFILVPFISSFLVGQIGISMTLVFDMVIACLSVLLAVFVLIRGKK

VFQ"

CDS complement(2240890..2242077)

/gene="ydiN_2"

/locus_tag="EFAGFIKM_01919"

/inference="ab initio prediction:Prodigal:002006"

/inference="similar to AA sequence:UniProtKB:P76198"

/codon_start=1

/transl_table=11

/product="Inner membrane transport protein YdiN"

/translation="MKYPHLKMGLGIYLSYLLLGMVSIMLASNMNFLSERLHVVPTDI

SLLISVVGIGHLVTINIAGKLSDRFGRKPLIIAAALLYAVFLVGIPLTTHFQIALLFA

FLAGISNSLLDSGSYPALNESYQEAAGTAIVLIKAFMSVGAIILPALVTFLMSKELFF

GYAFFIPAILFVFLGMYFVTIRFPGMTAQNSDKEKNEKLSSDQKTDLYSWKEGSALVI

IGFFSVSLSTVIQTWLPTYGNQVLQLNEKKAVELLSFYSMGGLICILVLAFLLKKFIK

PITAMVVTPAIATVVLIVLLAFRNPVIAPFIAFFLGLFTSGMYQLAMTVMIDLFPARK

GVSVSYVSASASVAFMVIPFITSLLVKHIGISAVFYFDLCIAVMSTCLALYLSKQIRS

FSK"

CDS complement(2242519..2243541)

/gene="cytR"

/locus_tag="EFAGFIKM_01920"

/inference="ab initio prediction:Prodigal:002006"

/inference="similar to AA sequence:UniProtKB:P0ACN7"

/codon_start=1

/transl_table=11

/product="HTH-type transcriptional repressor CytR"

/db_xref="COG:COG1609"

/translation="MRKNTNSKKRITLKDVAEHAGVSTTTASLVVRNNPRIPQATRER

VLNSIEELGYIYDRVAANMRSQSSNLVGVIVTNIANKFISEFLIGAQNKLEEFGYTVL

LGTTSDNATKQQNLISTMVEHRIDGLILNPSSISLEKTVKQLNNLKTPLVLANRELYS

ISADYAGIDYEEGAYLATTHLAKKGHQRIAFLGGVKESSTWVKRMDGYRKAFKDHNLE

LEESIIIASDPTSEGGEEAARLLLKSPNPPSAIFCFSDLIAFGVMKVLTKEGIVVGRD

VDVVGFDNIPDAENYHPPLTTVSSFPKLIGIQAAKLLHSKIIGENEGIQRMILKPELV

IRDTSK"

CDS complement(2243875..2244576)

/locus_tag="EFAGFIKM_01921"

/inference="ab initio prediction:Prodigal:002006"

/codon_start=1

/transl_table=11

/product="hypothetical protein"

/translation="MKLINKATGVIVLGALLTVALAGCQSNDATPPATGQNTANEQSK

GEGATNANAPASESSEEKGTEAIKEGSMEKEGNVVLKELAFVYNEHTIAISDTANEVQ

MEQMLGKPDNLKSHTYSADDGTNMDTLIGFTEKVYTYPGLEIKTISIPEGKQDSIFHI

EITDPKYTTVRNIKAGDSLDTLKNAYPEGKLLGDGAPDEEDDFRYEPSNYVDVMSFHI

KDAKVESIQIYSLLD"

CDS complement(2244681..2246075)

/gene="sasA_4"

/locus_tag="EFAGFIKM_01922"

/EC_number="2.7.-.-"

/inference="ab initio prediction:Prodigal:002006"

/inference="protein motif:HAMAP:MF_01837"

/codon_start=1

/transl_table=11

/product="Adaptive-response sensory-kinase SasA"

/translation="MLSFGIIIFAVNKAIDYYSFITIEKQMMEKADLSELSFREVLSQ

HRSSSGEPQTKEIARLALEKLKASGKEVRIYDSSKQLLGLAVDGIIINDGKPLIFEKN

IEKALSGSYAYTVTEDHLLYFATPIQDQFYENAYVYEFVEDISYFYAIIDQIRYILFA

GAGGFIVLITLSSLWIARNTTKPIKQLLGAAQSFSRQEFRRVHLNRKDELGMLADGLD

SMGRQLHDYIQYQKQFVSNVSHELKTPLAAIRGFSQYLVEGETENKELQNIYAHLLQE

SDRLTRLINELLLLSRFDKAGSNELELEKTEMNELIQQVAMNMGAKAKDKEIEIRVSQ

AEEEPDDEGKTRVYANVNPMLMSHAIANLVDNAIKYSGSPSLIELNLEYTPSEVVIRI

RDQGIGISGEELERVQERFYRAKNASTANGSGLGLSICKEIVERFNGYIDIESQIGEG

TTVTIVLPRAETQS"

CDS complement(2246117..2246824)

/gene="walR_2"

/locus_tag="EFAGFIKM_01923"

/inference="ab initio prediction:Prodigal:002006"

/inference="similar to AA sequence:UniProtKB:P37478"

/codon_start=1

/transl_table=11

/product="Transcriptional regulatory protein WalR"

/db_xref="COG:COG0745"

/translation="MKKILVIDDEIAIRDLIELVLRRENYVVQTAENGKIALQLLDTF

QPDLVVLDLMLPDCSGYDLCKEITGKRAVPVIMLSAKNEVIDKVLGLELGAEDYMTKP

FDNRELLARIKVILRRNESKEESSEGTEVKSTRIIHEELTFDLESRRVLKNDVAVSLT

AKEFKILETLLKRPDKIFTRDELLQIGWGYDFMGDSRSVDMTIMRLRKKLEDNADEPK

YVRTIYGFGYQLGGGEA"

CDS 2246979..2247236

/locus_tag="EFAGFIKM_01924"

/inference="ab initio prediction:Prodigal:002006"

/codon_start=1

/transl_table=11

/product="hypothetical protein"

/translation="MDLLWVLIVGGLIGWLSGNLIGRDVPGGVLGNIIAGFIGSWLGT

ELLGPRGPVIGGFHIIPAIVGSIIALLIFFALARGGAFRRR"

CDS complement(2247574..2249265)

/gene="ilvD_2"

/locus_tag="EFAGFIKM_01925"

/EC_number="4.2.1.9"

/inference="ab initio prediction:Prodigal:002006"

/inference="similar to AA sequence:UniProtKB:P9WKJ5"

/codon_start=1

/transl_table=11

/product="Dihydroxy-acid dehydratase"

/db_xref="COG:COG0129"

/translation="MENKKDLRIRSKVISEGANRVPNRAMLRAVGFQDEDFKKPMIGI

ASTWSEVTPCNMHINDLAVQAKQGARNNGGAPLIFNTITVSDGISMGHGGMLFSLPSR

EAIADSIEIVTGAERFDGVVAIGGCDKNTPACLMAIGRMNIPSVYVYGGTIQPGNLDG

KKVDIVSAFEAVGQYQDGKMTDEQLHKVECSVCPGPGACGGMYTANTMAAAAEAMGMC

LPGSSSTSAISADKALECEAAGKQVISLLEQEIYPRDIMTKKAFENAITVVMALGGST
NAFLHLLAIAHSVEVDLTLDDFERIRLRVPHLADLKPSGQYVMQDLNDIGGVSGVMKL
LLAEGLLHGDCLTVTGKTLAENLAEAAPLQNDQEIIRPLNNPLKPDGPLVVLRGNLAP
EGAVAKMSGMKIQQFSGPTKVYDSEDEATEAIMNDEIQEGDVLVIRYCGPKGGPGMPE
MLSVTALIVGKGLGGKVALITDGRFSGGSHGFVVGHVSPEAQVGGPISLLQNGDIITI
DSDIQEIKVEVPEEELAARAQAWVQPPLKVKSGVLAKYAKLVSSASRGAVTDWME"

CDS complement(2249281..2249946)

/locus_tag="EFAGFIKM_01926"

/inference="ab initio prediction:Prodigal:002006"

/codon_start=1

/transl_table=11

/product="hypothetical protein"

/translation="MPIPKDFSLPVRMSAKERAFSQIQRWIIDGTLQPGEKLIDAEMA
ESLGVSRTPIREAFQLLEVQGLVSMHPGKETKVTNIEKNDIFKMYSTMAALQALAAEI
TAQTIVPEQIEKLRSINLEFASSIKNGQVYQAMEVDEQFHNYIVELSDNPYVATFNTS
LQIHIRRFKYVFLKQPITATLASVEEHDQIIKAFEGKNSNDAHNLMKQNFIRPMQELN
KIL"

CDS complement(2250543..2250758)

/locus_tag="EFAGFIKM_01927"

/inference="ab initio prediction:Prodigal:002006"

/codon_start=1

/transl_table=11

/product="hypothetical protein"

/translation="MAILSTGPIENNPVSGVRPTQQLTINIDNRNLVTNAIVLIQGYY
LNGTRTEYILELISVNPNQAIKKDYFA"

CDS 2251055..2252611

/gene="opuD"

/locus_tag="EFAGFIKM_01928"

/inference="ab initio prediction:Prodigal:002006"

/inference="similar to AA sequence:UniProtKB:P54417"

/codon_start=1

/transl_table=11

/product="Glycine betaine transporter OpuD"

/db_xref="COG:COG1292"

/translation="MVFTITIIIVALFAIWGAVAPDQLADVANVAYNFSIQNFGWFYL

LATLFFLIFAFYLAFSRFGSIRLGDDDDEPEYSTISWLSMLFSAGMGIGLVFWGVAEP

LSHYLSAPEGAVPETTQAAILSMRYSFFHWGLHPWAIYTVIGLALAYFQFRKGYKGLI

SSTFIPLIGERLAAGWLGKVIDILAVISTIFGVATSLGLGALQIGGGLNHLFGIPNST

LTQVVIITVVTVLFLISATSGLDKGIKILSNVNLVIAVLLMLFVLVTGPTSFIFDTFT

TTLGSYMQNIINMSLRLTPFSRETWIGAWTLFYWAWWISWAPFVGTFIARVSRGRTIK

EFVIYVMVIPSLFGFIWFSVFGGTGLHMELFNAAHLAEAVKEDTTTALFLMLEQLPLG

TIVASIATLLIMIFFITSADSATFVLGMLTSDGKLDPSTRVKLTWGIMQSAIAVVLLI

SGGLTGLQTASIVTALPFAIVLIGMCFSLLKALKAEDKERRQKEKRQRQKLKRLLEEQ

EISLNEISGR"

CDS complement(2253188..2254420)

/gene="yycB_2"

/locus_tag="EFAGFIKM_01929"

/inference="ab initio prediction:Prodigal:002006"

/inference="similar to AA sequence:UniProtKB:P37482"

/codon_start=1

/transl_table=11

/product="putative transporter YycB"

/db_xref="COG:COG2807"

/translation="MPNKQNKKFVPSSVALIILGIIVIAANLRTPLTSVGPLVSFIRD

DVHISNTLAGLITTVPLLAFALLSPLVPKLGRRYGVERIILIALIFLTVGIVIRSLSG

AVNLFIGTAVLGFAIAICNVLLPSIIKRDFPNKIGSMTGVYSISMNLCGAIASGISVP

LAMNAGLKWQGALGIWGILSFVSILCWLPQLRNQTKQTATTSQQMANNDVNVWRSPLA

WQVTLFMGIQSMVFYVLIAWLPEILKQQGIDSNQSGWYLSIMQLAMLPFTFIVPVIAG

RMSNQRLLVVITTILLLTGTLGLLYGSSNVILLWIVILGIGGGFAFSLAMMFFGLRTE

NAHQAAELSGMAQSIGYLLAAIGPALIGYLHDATNSWNLPLFILLGASVLLFVVGIGA

ASNHVVGSRDGYELPRKG"

CDS complement(2254637..2255197)

/gene="sutR_1"

/locus_tag="EFAGFIKM_01930"

/inference="ab initio prediction:Prodigal:002006"

/inference="similar to AA sequence:UniProtKB:P77626"

/codon_start=1

/transl_table=11

/product="HTH-type transcriptional regulator SutR"

/db_xref="COG:COG1396"

/translation="MDEIDESKQIVLQIGGALKKYRKEKNMTLDDLSELTGVSKLTLG

NIERGETNPTLAIIWKISKGISLPLLALFTSEKPVSLYRAGEGLRFSNDEKNWIIEPV

FKSNDIEMCRAYLQPNSSYYPEGHHVNTTEIATVMTGSIELQVNGENYTLNQYDTISF

RADHPHAYTNHTNSETVLHISLKYGF"

CDS 2255379..2256323

/gene="ghrB_1"

/locus_tag="EFAGFIKM_01931"

/EC_number="1.1.1.81"

/inference="ab initio prediction:Prodigal:002006"

/inference="protein motif:HAMAP:MF_01667"

/codon_start=1

/transl_table=11

/product="Glyoxylate/hydroxypyruvate reductase B"

/translation="MKILIVGYFCETSKSNIERSFPQDWNVVIVPPGKEMLHHIEDCQ

VIIPEHIKVDHSLLSNAKKLKLVQTGAGFDNVDVPACTQLGIWVANAAGVNAQAVAEH

VMALILSYYKNIPFLDTFMKNKMDENQLDYTGSELKGKTIGIIGLGAIGKKVAAFCRV

FDMNVLAYARNATAQSDDLVKMTDFDTLVSTSDIVSVHVPLNEQTKQLINKAAFKKMK

NTTLFINTARGGIVNERDLIDALKNGDISGACLDVFESEPLPIDSELRNLGNVILTPH

TAGMPDGRKFHKTRYDFFINNIKGVENGEEPESKLNQL"

CDS complement(2256355..2256840)

/locus_tag="EFAGFIKM_01932"

/inference="ab initio prediction:Prodigal:002006"

/codon_start=1

/transl_table=11

/product="hypothetical protein"

/translation="MKLSDLEGDMGGPSEFLEQSVSQDLILELLNHAVWAPNDGLREP

WRFIFADNQYGDIMQGLQEPAPAYLLVLVKEEADQYKREEDFAAVCCLIQNFRLLAHE

QSLGVRCTLHDWMYDPSRTEMFGVLSNERIAAVLELGYGANQWKGNTELPETQLHFEL

L"

CDS complement(2256858..2257433)

/locus_tag="EFAGFIKM_01933"

/inference="ab initio prediction:Prodigal:002006"

/codon_start=1

/transl_table=11

/product="hypothetical protein"

/translation="MSLAELIRERRSIRKCNSTPVDQELVVELLRKAMRLQPSVESGS

WRVVYAGTPEARKRLVDCMLEQMSQSKLGKLIPGKLLDVFKKRFTDIPAHVIVMSTVG

PNRLTNDRNYAAACGVMQSFQLLGWEQGLGMLWDTESMIQHEGFFNGIGLREDERFVG

ILHIGYYDKAPRSRKRTPAEQKWTVLQGQST"

CDS complement(2257454..2257936)

/gene="pchR"

/locus_tag="EFAGFIKM_01934"

/inference="ab initio prediction:Prodigal:002006"

/inference="similar to AA sequence:UniProtKB:P40762"

/codon_start=1

/transl_table=11

/product="HTH-type transcriptional regulator PchR"

/translation="MISKDRTKQLLYDQFIRFLHVYENHKDTEIEHFLSIAQRESIEK

IPQHLTAVHMIDCIGKHEPINNTGIAEAMNLSKASITKIGNKLLEEGFVKRTKMNDNK

KESYFRLSPQGRKIFELHERLHIHEAERFYRTLDKYSETELKIIHQFLQDSSINIESR

"

CDS 2258322..2258738

/locus_tag="EFAGFIKM_01935"

/inference="ab initio prediction:Prodigal:002006"

/codon_start=1

/transl_table=11

/product="hypothetical protein"

/translation="MDDKTVIIDQGPFFHGTKAELQIGDLLEPQFLSNYQEKNSNHIY

FTATLNAAKWGAELARSNAKERIYIVEPLGDFESDPNVTDKKFPGNPTRSYRSKSPLK

IVAELASWERHSEEEINHMITSLRKLSEEGKNIIYD"

CDS complement(2258823..2259785)

/locus_tag="EFAGFIKM_01936"

/inference="ab initio prediction:Prodigal:002006"

/codon_start=1

/transl_table=11

/product="hypothetical protein"

/translation="MFNPVIDVHHHIISKEMQRKQRELGMEIPSFMPKWTPEIGMQKM

DVCDISFSFLSAPSDLTFIDQSSANALARKMNEELASYAQLYPERYGAFATLPLPDPA

AAMEETLYTLDVLGMDGVAMLTSYQGKYLSHPDYTELLTELDRRSAVVFLHPILIPQK

IAGLSPALLEGTFDTTRAVTTMAVHRIFDQYPSITFILPHTGGMVPYIKWRIALAAIG

QDSIQVETTPEQFQAEIAKLDHLYYDSTLNLGTIQKLIAPDRILFGADIPWPADSVLR

IQRESVFHEAQQVSEENAKAIAYGNAFRLFPRISKLFDIEKKLI"

CDS complement(2259802..2261295)

/locus_tag="EFAGFIKM_01937"

/inference="ab initio prediction:Prodigal:002006"

/codon_start=1

/transl_table=11

/product="hypothetical protein"

/translation="MFKKRLPAGFIKVMSLICAAPLLLTACGIPDSKEGAEPGKASAT

DMYEVVMTYPAFGDVTDVKEIQEAISDITSEKVGATVKLLPVNGSNYANQMNLMLAGS

EKLDLVYTSVWLGLESQIGRGQLLPMDESLNEFGKEILEAVPEEAAEAGKMNGETYGV

PSIKAWALSPSFVMDKELADKHDIDLSAIKTWNDISAVLQLIKENEPGVTPIVSYTSQ

ETPGQAMLNFDPLGTAPGVLDFGGEDFTMQNLFATSRFSTMADLMRDWYQKGYFSKDV

ATSQETGSNLIKAGKAFSYIRNINECYSESLAAGTELVCVPLEDSYMTRNSVASNMMS

LARNSEQPDKAIQVLELFYSDEAVINLFTNGIEGKHYVKRDNDLIGKPEGVTAIGYDS

NQYAVGNNFLSYIWEGNDPDMWEHLRGENEGALKSRAMGFSFDINPVKTQITSVANVQ

NQYDAGFQTGTFDPSELGAYLDKLKNAGVEKILTEKQNQLNQWLENK"

CDS complement(2261352..2262233)

/gene="araQ_11"

/locus_tag="EFAGFIKM_01938"

/inference="ab initio prediction:Prodigal:002006"

/inference="similar to AA sequence:UniProtKB:P94530"

/codon_start=1

/transl_table=11

/product="L-arabinose transport system permease protein

AraQ"

/db_xref="COG:COG0395"

/translation="MYSNRIYQMAVHLVLIMMGIAAVFPFILLISASLTEEQAIIRDG

YRLLPAQFSFDAYVYLFNQYSLILRSYGITTFITVVGTIVGLLITTLVAYPLSRNVLP

FRSFLSFFVFFTLLFNGGLVPMYLVYTDLFHLKDTIWSLIIPNLLTNGFYILLMRSFF

STSIPGEVVESAYVDGANEWVIYCKIVLPLALPSLATIGLMLMISYWNDWFNGLIFIN

DGSLYSIQNLLNRMLSDVQYLQQNSLSGQTAVVSSVPLGSVRMAIAVIGVLPLIAAYP

FFQKFFVKGLTIGAVKG"

CDS complement(2262251..2263183)

/gene="yteP_10"

/locus_tag="EFAGFIKM_01939"

/inference="ab initio prediction:Prodigal:002006"

/inference="similar to AA sequence:UniProtKB:C0SPB3"

/codon_start=1

/transl_table=11

/product="putative multiple-sugar transport system

permease YteP"

/db_xref="COG:COG4209"

/translation="MRRGFHANRIQSIRKHGLLLVMILPGIVYLFINNYLPMFGIVIA

FKDINFAKGIWGSDWVGFDNFAYLFQTQDAWVITRNTLLYNGAFIVLGTVLSIAVAVL

LNEVKKRFASRLYQSIILLPYLISMVIVAYLVLALLNEENGFVNHYVLPLLGIDPVSW

YADSGKWPFILSVVYLWKNVGYTCIVYLASIVGISQEYYESATLDGASKWRQVWSITL

PLLKPTMVIMVLLAVGRIFNSDFGLFYQVPLNSGALQTTTDVIDTYVYRGLMTFGDFG

MSSAAGLYQSIVGFLLVLTTNAIVRRKNRDLALF"

CDS complement(2263517..2264737)

/locus_tag="EFAGFIKM_01940"

/inference="ab initio prediction:Prodigal:002006"

/codon_start=1

/transl_table=11

/product="hypothetical protein"

/translation="MEKGLTLVKNVKWSENLFDLTIDQATGLIVSIETAGTDADLTSV

NIAGRPTTVIDADGLHYVPPMTDMHTHLDKHFIGEPWKSLQPFVTLPGQLEFEKSMLG

ELPTGAGERAKRMLDLMLAQGTTAIRTHVDVDPQIGLSHLEEVLEVREMYQGRIDMEI

VAFPQQGLLRSDSVSVMREALRARANYVGGVDPAGLDRRVDASLEAMFELATEFSAGI

DLHLHDPSHLGIYTISRFAELTEQAGMSGRTAVSHAYCLGQVGETEVRSLAERLCATG

VAIMTSVPIDRPMPPIKLLMETGVSVHIGSDNILDSWSPFGNGDMLSRGSRLAEASGW

VEDFWLLETYKLISNAPLTPKVGERGTFSLVNAINAMHALAAAPPREAVFSGGVLVGG

RMFPNSRQMAAIHS"

CDS complement(2264761..2266020)

/gene="codA_1"

/locus_tag="EFAGFIKM_01941"

/EC_number="3.5.4.1"

/inference="ab initio prediction:Prodigal:002006"

/inference="similar to AA sequence:UniProtKB:P25524"

/codon_start=1

/transl_table=11

/product="Cytosine deaminase"

/db_xref="COG:COG0402"

/translation="MIDLLITDTVISGDERRMDVGISSGKIAFFTPCGERKTEASRVV

QGKGGLLLPGLVEPHIHLEKAYLLDSMDREAESLQDAIQITSKLKSSFTREDMYHRSL

VVIREAVRNGVTHMRCHAEVDPVLGLRAVESALRLKQSLKHQLDLQVVAFPQEGVFKS

PGTAGLMEEAIRMGADVIGGITYQDADLGEHLDFAFSLAGKYGKPLDFHADFSVDSND

RAVVEIAKRAIAAGMQGHVAAGHVTSLGSLPEHEASAIGELLCLAGISVITLPMTDLF

LNGREGSEGYHRGLTPVKLLQDCGVNVAIGVNNVRNPFTPFGKADPIEAAWLLAITSY

MGSKRDALILTNMLTHNAAQALGIDNYGVKVGAPADMVLFRERSEREVLLNKPEARTV

WKSGLQVACTDSKLLELTWLSDGMETI"

CDS 2266223..2267884

/gene="pucR_3"

/locus_tag="EFAGFIKM_01942"

/inference="ab initio prediction:Prodigal:002006"

/inference="similar to AA sequence:UniProtKB:O32138"

/codon_start=1

/transl_table=11

/product="Purine catabolism regulatory protein"

/db_xref="COG:COG2508"

/translation="MLLTDLLKNGIYDDARVITGHKGLIREVQTVNIMDCPDIIRFLR

PNELLLTNGFFMKQQPEMFIHLIRDMQRLHCSGLAIKTKRFELPIPDEILWEADQNEF

PIIEISDVRLSLGEILQRSTSVILNNKSDELQYALSIHKKFSEMIMRGKGISSIIDSL

SKLFSSPVILLNYKWQTISASDQNTSVAELTAAAVNTLQDLSDISSPTSLCFINPSVR

ERCCMLVYPVKTYRHEGYLLSFHSPDQWTGLFGLTLEQASNVIGMELTKSQAVKERSR

RHKNEFFSDLIEGYITSEQEALNRGKRCGLIKGRASVVLTMRNDEVHHMHQRFPKNEG

SAPPKRDNITEQEEQYEHIKRVFGGLDHSFVMFTKHDTYCLLLSIPESGWDEAKITEQ

LSGMLKELHHEIGLSLSIGIGKPATNVLGVRHSYTEAVNALQFGYWLKRKQFVQSYQA

KDIGYLFHMLPHNELKQFYDATLNGLLQIADEHEKKELLRTLNAFYDTQCQLVETSKQ

LFVHRNTVVYRLEKCEKILGVKLKDPTESLRIRIAIAIEPLLGQD"

CDS complement(2267991..2271794)

/locus_tag="EFAGFIKM_01943"

/inference="ab initio prediction:Prodigal:002006"

/codon_start=1

/transl_table=11

/product="hypothetical protein"

/translation="MKDKSKFVICLLMATGLALTSNGIFTSKSIAASAVLADQETNKQ

AKEELDIFESASKSNTNLIGWQVKGKGELEDTSEGILLTSQPKENVMAISETVSDDFI

YEADVMIRDTKADATLLFRSNEDGFSSYMLQIVPDAGLIRLRDARNGDGKLKEERKVS

VEIGQIYHLKVKAVGSSLKVYWGNQYKPMIDVQDISYQSGKLGLHVWDGSALFSNIVV

SDLKGNLGTVLSSKGKWQPDINGKRGRVEQENRAQQIYNKEAADMVYEGNITLRPDSI

SALAFRSSTDGAEGYEATLTKEGDQVRVSLTNTKGTVIASSQRTYPSQMGAKHHVEIK

AKGDRIQVFLDGYTTAAIDVKDSTYRSGSTGIVVKKGTAYFQDTYVTELSQYYNEIYR

PQYHYTPIRGSASDPNGLVYFEGEYHLFHQDGGTWAHAVSKDMLNWKRLPIALPWNDH

GHVWSGSAVADMTNASGLFGDSGGKGLIAYYTSFNPDSPNGNQRIGLAYSKDQGRTWE

YSKERPIVIENPGKSGNEVGNWDFRDPKVIRDDENNRWVMVVSGGDHIRFYTSTNLLD

WTLTDNWGYGDYVRGGVWECPDLFQLPVDGTSQKKWVMMISTGANPKTGGSDAEYFIG

HLTADGKFVNDNPAGKVLRTDFGKEFYASMSFANMPDHRTVMMAWMTNWDYPFAFPTS

NWKGELTIPREVSLVTTKDGIGMTQSPIKELESLRKPLYSASNKSVSPSSGNLLKGII

SGAYEIEAEIEIPEASTVTEFGFNIREGANQKTVVGYKASDSHMFVDRTASGETDFSN

LLSKKHEAPAQMENNRIKMRILVDESSVEAFGNNGKVVFSDVIFPDPASRAMSFYVKG

GNVNVVSLKVHQLQSVWNEDIPSKAQIKMDTSVRELGVGESDTLQAMVEYGPGLGVQP

LKWNSSNNDVIALDLVDNSHAVIKAKKEGESTVTVSTPNGKTSSSVLIKVSGGEFRTN

LSGWTKDLSAASWLVSEHGIRGKYFSDANYIAKEKAGNFTYEADMMLGETGGAGSVLF

RASEDGRSGYYLNLDPNMKAIRLFYKINGAFEERQVLAKVPTFIRPGQTYKVKIEANG

PHIVVHVDSQKVIDIMDGTFAEGHFGLNVFGGSASYQNVNVSNGEPANLTKSSLVNAA

TQKSIYTVNLVNGEPVTLQDASAASVQKWVFVPTGDEAGSYSIRTTAGQTLDLDIGQN

KLQLYHYLGYNNQRWVLHENKDGSVHITSAHHQKALEVSEDGTELFLSELNPSLDRQK

WILTK"

CDS complement(2271922..2273514)

/gene="sacC_2"

/locus_tag="EFAGFIKM_01944"

/EC_number="3.2.1.80"

/inference="ab initio prediction:Prodigal:002006"

/inference="similar to AA sequence:UniProtKB:P05656"

/codon_start=1

/transl_table=11

/product="Levanase"

/db_xref="COG:COG1621"

/translation="MNKIKKILLSGENNISKGPENFIYKNEYRPQIHYSPNGNWINDP

NGMVYYEGEYHLFYQYTPHDTQPDFGRMHWGHAVSNNLVHWDELPPAISPGEDGAIFS

GSAVVDKNNTSGFFNEAGSGLVAIYTNNDNSAQPGKPQVQSIAYSKDKGRTWTKYEGN

PVLFPMETLDFRDPKVFWHEESSKWVMVLAVSDRVEFYTSSNLKDWSFASGFGSDIPG

IHRGIFECPDVFKIQVDEDPNTTKWILTLSVGDRNGVNPNDSEPPAGGSGMMYFIGHF

DGKAFTLDETLESFDTIQWIDYGSDFYAAVTWDGIPNEDGRKIWVGWMNNWRYASTLP

SKEWRGHMSIPREIQLRTYSEGLRLIQAPITELNQLRRSILSLQDITIKPDMNVLSAV

SAAKMEIIAEFEIGNAIEFGFKVRKSSSSNQETVIGYNVLNEELFVDRTKSNTVDFHP

DFAAKHKAPMKPEHKRIQLSIYVDWSSVEIFGNNGTTIISDTIFPDLGSRGLELYAIG

GELKVMSLQINDLVSIWENQSV"

CDS complement(2273909..2274055)

/locus_tag="EFAGFIKM_01945"

/inference="ab initio prediction:Prodigal:002006"

/codon_start=1

/transl_table=11

/product="hypothetical protein"

/translation="MVKKKISTAISAALIVSMLATGVIVAIEGKTTLEVAKSAGDKKV

EVIK"

CDS complement(2274255..2275283)

/locus_tag="EFAGFIKM_01946"

/inference="ab initio prediction:Prodigal:002006"

/codon_start=1

/transl_table=11

/product="hypothetical protein"

/translation="MVIERVKRDFLDDIAIIGLTGSFSTGDFHEKSDLDLIIINNTEK

GWKISDCFILDDVGYDIYCTPWDSRIQEQSTLESPNVSSLTELKVLYYAKPEDLEKLK

DFQQKALDALAKPIGEACLNRANKWIDLAKQAYSDTMLSEDIGSVRYASAEVLYNLVN

ALVSMNNTCIKRGIKRYLEEIRSFRYVPDNLESLYMSIIEAHTIEDIRITSFNMLNSV

TRLHNTMCDNFIVKPAPTFDNLRGTYEELWCNYRNKILNSVTANDASYVFLSAFGAHE

YLDEMAMEKGTKKYDLMQYFDASNLPVMKEKLLEVMDDYREEYNKVGRKVERFTTFEQ

LYAHYMNQ"

CDS complement(2275493..2275759)

/locus_tag="EFAGFIKM_01947"

/inference="ab initio prediction:Prodigal:002006"

/codon_start=1

/transl_table=11

/product="hypothetical protein"

/translation="MKYSKAESIFPEELLRIIQEYVQGELVYIPKPKEAHLKWGEKTE

SKSRVSARNAEIKSLFRNGVRIEELAGRYFLSCESIKKIVYTRN"

CDS complement(2276054..2277139)

/locus_tag="EFAGFIKM_01948"

/inference="ab initio prediction:Prodigal:002006"

/codon_start=1

/transl_table=11

/product="hypothetical protein"

/translation="MLPMEEYFGKLELVHSFYGAMPTGVSVSETARIFICFPKWGDDV

KFTVAEIVEGHMQPYPNVETNLINPLNIVMSFISVQSVVADGRGTLWVLDTAAPNFSE

PIKGGAKLVAVDLETGTIRKVYTFADDVVLPTTYLNDVRFDFRVGKAGYAYITDSSSN

GPGAIIVVNLENGNAYRRLHGTSSTSPDPYFVPKVEGKILKNRNADGSTSPFRLASDG

IAISPDGKMLFYCPLTSRQLYSISTESLRDRTIPDFKLSDLVQYWGEKGASDGMITDA

KGNVYAGDYENDSIRKILPNGIMETIAHDPRILWPDTFSIGPDQYLYVIVNQLHRQAR

FHYGRDLRQKPYSLLRIKIDELPAPTF"

CDS complement(2277392..2277673)

/locus_tag="EFAGFIKM_01949"

/inference="ab initio prediction:Prodigal:002006"

/codon_start=1

/transl_table=11

/product="hypothetical protein"

/translation="MPNKNKIDLATENARKPAEGALNSSGSITLEQHASNAVYEVQTA

LNQAISSLNESDATNSQALEIARQQLTRADEFLDQALTSANALRLPDQQ"

CDS 2277862..2278104

/locus_tag="EFAGFIKM_01950"

/inference="ab initio prediction:Prodigal:002006"

/codon_start=1

/transl_table=11

/product="hypothetical protein"

/translation="MTNPTLAPHESMELHEALNFKTLCLAKSKLMQGLVFDQELKALM

QKDVIQSIQQIAELQAIYARAPFQAPVPNNPTPITH"

CDS 2278116..2278502

/locus_tag="EFAGFIKM_01951"

/inference="ab initio prediction:Prodigal:002006"

/codon_start=1

/transl_table=11

/product="hypothetical protein"

/translation="MNTDYLDPINSLNMPEMADMTFAMDFLIRAKEGVRNLSIALTET

ASPDVRALLHTHLKQGIAMHQEISELMIRKKWFHPYELNEQYQLDQLSAKNTVMIGQM

NLFPGDTSRKGMFDRTPDEHIGGHKA"

CDS 2278499..2279635

/locus_tag="EFAGFIKM_01952"

/EC_number="1.1.1.1"

/inference="ab initio prediction:Prodigal:002006"

/inference="similar to AA sequence:UniProtKB:O07737"

/codon_start=1

/transl_table=11

/product="putative zinc-binding alcohol dehydrogenase"

/db_xref="COG:COG1063"

/translation="MKAVTYQGVKNVVVKEVPDAKIVKPDDMIVKITSTAICGSDLHL

IHGMIPNLQENYVIGHEPMGIVEEVGPGVTKVKKGDRVIIPFNIACGECFFCQNQLES

QCDNSNEHGDMGAYFGYSGTTGGYPGGQAEYLRVPFANFTHFKIPENCEQPDEKLSLI

ADAMTTAFWSVDNAGVKNGDTVIVLGCGPVGLLAQKFCWLKGAKRVIAVDYVDYRLKH

AKRTNHVEIVNFEQDKNIGNTLKEMTKGGADVVIDAVGMDGKMSDLEFLASGMKLQGG

TVSAFIIASQAVRKGGTIQVTGVYGGRYNGFPLGDIMQRNVNIRSGQAPVIHYMPYMY

ELVTSGKVDPGDIVTHVIPLSEAKRGYEVFDTKTDDCIKVILKP"

CDS 2279667..2279867

/locus_tag="EFAGFIKM_01953"

/inference="ab initio prediction:Prodigal:002006"

/codon_start=1

/transl_table=11

/product="hypothetical protein"

/translation="MDSTYALHEMLEVHEIAAFKTVCMTKSKTMQALVTDPELKQILQ

QDATLSQQQLQELGGVLSKATV"

CDS 2279879..2280175

/locus_tag="EFAGFIKM_01954"

/inference="ab initio prediction:Prodigal:002006"

/codon_start=1

/transl_table=11

/product="hypothetical protein"

/translation="MNPILEHMTGLNTLTDDVIAMDFLMNAKSGVRNYAMAVTECATP

EIKQILMKQLDEAIDSHEKISNYMVQHGLYHPYHIAEQIQLDLKNIQTAMNIPS"

CDS complement(2280464..2281126)

/gene="tenA"

/locus_tag="EFAGFIKM_01955"

/EC_number="3.5.99.2"

/inference="ab initio prediction:Prodigal:002006"

/inference="similar to AA sequence:UniProtKB:Q9K9G8"

/codon_start=1

/transl_table=11

/product="Aminopyrimidine aminohydrolase"

/db_xref="COG:COG0819"

/translation="MLTISERLYQAAQPVWKECLEHPFVKGIGDGSLTVEQFRYYLLQ

DYLYLFDYARVFALGIVKSHDPKLMQFFSKNVDNILNGEMKIHRSYMDRLGITEDHVF

QVKPALKNAAYTNYMLSVSHAGGIAEVLVSILACSWSYAEIGQVLAQKPDAADHPFYG

EWITGYASSEYNSNNQSLVTLTDKLLEGCSEGTYQRMEDIFVMCSRFELDFWEMSWRL

EP"

CDS complement(2281773..2284214)

/gene="xynA"

/locus_tag="EFAGFIKM_01956"

/EC_number="3.2.1.8"

/inference="ab initio prediction:Prodigal:002006"

/inference="similar to AA sequence:UniProtKB:P36917"

/codon_start=1

/transl_table=11

/product="Endo-1,4-beta-xylanase A"

/translation="MKRLGQWNITALVLVLVLAMLPFSNVSHAAKAVTEVEGGHLSTN

FEDGTLQGWTPRTGNERLTVTQQEAHEGQSSMLVANRERSYHGPMLSMKDMLKRNQEY

EIAAYVRLTQEPTTDQTLQLTTYKKTTAESWNPIGSVKIAKAEWNTWHKITGKFQYSD

DPTELNLFIETPYISEDSVDTLSFYVDDVSFTLAEQLEIEEGILSLEDLYQDDFPIGA

AVYRWQLEGAYGQLLTKHFNSLTATYEMKPKYMSPSEGVYEFEAADQYVQFAEEHGMG
VRAHALLWHIDAAEWMFKDPQGNPASRELLLARIQDYVETVMTRYKGEIYAWDVVNEV
IADSNGDANGLRKSPFYELIGPDYIEKTYEFARAADPNAKLYYNEYFTEIPEKREHMY
QLVKRLKEKGLIDGVGLQSHYNLESPPIKEIEKTINMFAELGLDIQITELDVDSGIPF
GEEMSDEVAVKQAYRYKELLDLYKKHKDHISSVTLWGLQDEKSYNNQAMLFDSALKAK
KAYWGLVDESSLPVLTQRAVSLSGKPDIKKQSQDPLWNKAVSTPLKGDSSGSASFRTL
WDRSNLYILVDVQDAKADVNDRVDIFVDLNNGKTTSYEADDRHVIIKRSGKVEGAEQR
SYRVRETKAGYQVELSIPWGGIQVVSEYETGLDIRVTDGGASGTEPHNPLYWNDRTQS
QEQDTSKYGVIQLAPMPKSAQAVQGIVQIDGKKDALWNKAVPFEVKRLNQSEGAEAVA
RAMWSGEYLYLLIDVTDPNIITDNINPWDQDSVEIFLDENHQRTPYFQYDDAQFRISA
DNVGTFAGGAHPDAL"

CDS 2284703..2285113

/locus_tag="EFAGFIKM_01957"

/inference="ab initio prediction:Prodigal:002006"

/codon_start=1

/transl_table=11

/product="hypothetical protein"

/translation="MTFLPPFGPGGGFGGPGGPGGPGGPGGPGGPGGPGGFPGPPSFP
GGGGGGPQGVQAPTGPPPSFIPQQPASLYAVDPRAISGCLFRYTYIWPDRGPGFWMYP
VFVGRNSVAGFRWNGFFWLYTGLDLQRISSFSCF"

CDS complement(2285184..2286923)

/gene="ettA"

/locus_tag="EFAGFIKM_01958"

/inference="ab initio prediction:Prodigal:002006"

/inference="similar to AA sequence:UniProtKB:P0A9W3"

/codon_start=1

/transl_table=11

/product="Energy-dependent translational throttle protein
EttA"

/db_xref="COG:COG0488"

/translation="MIKVENLSFSFPQKELYKNISFTFEEAQHCAFIGTSGSGKSTLI
DILMDPERYLFEGKLEIDPDCRIGYVSQFLQVDKTKEMTVFEYIAEEFIKIQDEITAI

YAEMATTSDMDSLMEKLQLALDAFEAMDGDNFEKNINKQLNLANLMKLKDLSISAVSG
GEFKLIQVMKEMLNRPDFMIMDEPDVFLDFENLNALRKLINTHKGMLLVVTHNRYLLN
HCFNKIIHLENTELQEFDGRYIDYNFSLLQTKIELQEIAVAEAEEIERYDHIIDNLRE
IATYNSEASRGRALKARVKFQERLEARRIKEPFVDIKQPNIRFGIDKEMEDTVVVNLN
NYSVSFDELLLENVNFEIKSTDKVALIGPNGTGKTTLLREIFKNNQDSIEINADVKVA
YLSQVQGEMLKDSNTILNEFIDSGFQTYDEIRSYLPNYGFEGEILDQKIESLSGGEKN
MLQLAKVSASQANILLLDEPTSHLDIYTQIALEKAIEDYKGAIIMISHDFYSVVNGMD
YVLIIEDKTISKMSIEEFRQMIYASHFDENYLENEQKKKSVEMKIELALKDTNFELAK
SLVDELEELIKLL"

CDS 2287231..2287635

/locus_tag="EFAGFIKM_01959"

/inference="ab initio prediction:Prodigal:002006"

/codon_start=1

/transl_table=11

/product="hypothetical protein"

/translation="MNFASVRIITDDVNRLVEFYEKITGVSAERPAPVFAELVMPSCT
LAIGHSQTVQLFGAGSAVAANNRTVIIEFHVHDVDAEYERLKPFVDEWVKEPTTMPWG
NRAVLFRDPDGNLVNLFTPVTEEASQRFSGRL"

CDS complement(2287654..2288514)

/gene="gltR_2"

/locus_tag="EFAGFIKM_01960"

/inference="ab initio prediction:Prodigal:002006"

/inference="similar to AA sequence:UniProtKB:P94501"

/codon_start=1

/transl_table=11

/product="HTH-type transcriptional regulator GltR"

/db_xref="COG:COG0583"

/translation="MEIRHLVTFITIVEHEGFTKAAEHLGYAQSTITLHIKALEEEIN
YPLFDRIGKRVILTETGKKLLPHAQKMLDLYHMIKEVTAAQGELTGNIVISIGETLLI
YRFPPIIEEFKKLHPHVNIEWHQLDSVHYKENLMQGKSDISFMLGTEVHDPNLYSEKL
AEEPMMLLYPNSFELQRDIVRSNLLFTERGCGYRTLFEQCIEEYQIGITSNIEFWSIE
AVKQSILSGMGISLLPRITVEKELKEEKLSGQQYKQNLATQLLYPKNKWISPQVEAFI

EIVRKHAFLW"

CDS 2288669..2289508

/gene="oleB"

/locus_tag="EFAGFIKM_01961"

/EC_number="4.1.1.114"

/inference="ab initio prediction:Prodigal:002006"

/inference="similar to AA sequence:UniProtKB:Q8EG65"

/codon_start=1

/transl_table=11

/product="Cis-3-alkyl-4-alkyloxetan-2-one decarboxylase"

/db_xref="COG:COG0596"

/translation="MQTAATSSILINGYRIAYEQYGEGSPIWLLHGTPSYSYEWRKVI

PALVQKGYKVYVHDLLGYGASERPLEADTSVAAQYTLFSQLLDELGMDRLHVAAHDLG

GIVGLQLAQEQPERVQSLTLLNMPSYDSWPSPTWKKIIEEQLEQVEQMPRADFDTMLK

KQLPMAVYNKELMTGDTLEAYLEPHAGSLGKSSFFSHQVAHYNAKYTESYGTDLPKLK

VPVQILWGEEDEWQPLAYAKRLVQDIPHACLHVIPRAGHFVMEDEPGQCSDYIDRFCN

KHP"

CDS 2289792..2291555

/locus_tag="EFAGFIKM_01962"

/inference="ab initio prediction:Prodigal:002006"

/codon_start=1

/transl_table=11

/product="hypothetical protein"

/translation="MRQTRKKKMDLRFKLYLIILVPLLAMGGLIIWATIDSSENASLM

TMQHNNQQLAVNTASQLGQESELIKTLSSTASEESNEYKSLRDELVKVRLQSGALYVY

MYNKTSDGWIYTVDGANWDDTEYSPYGTAMEFNPQIQERLLRGEVVTTGIVDDPTWGQ

LLSSFTPIKDSQGTVIGYLGIDISAGAVNQVSATSVNNAYQTIIPIFVVVLILSLVMM

LFVVRGILRQVRDIKSSLEQVADGNLKVVSTHMSNDQLGDISDLINIMVAQLTNILMG

IQQGSNTLQQSSKDISETAQTNQRQSEELSRAIEEIAIGSMKQAEETEHSVQHSENLG

KIMDEVGSYVKQFTYTSEQLSSVQVQVTREHEVLLEKGRENAKRVEHQQDISRSLTTQ

SELASSISGQIHNILKQTQILSLNASIEAARAGEAGKGFAVVAGEMGQLAQQSERSIQ

EIDEILGSFVQEIHRMGSHFDANMVAVEEQEKQIADCLQAFRQVSRLSTEVHELAQSL

DSRTMDMHSIRQELEQNLSYIASATEETSAMAEEVTASAVEQQRSASELSNISGELAG
LAGNLKSYSDQFQIEVRKSGP"

CDS complement(2291651..2292019)
/gene="pucR_4"
/locus_tag="EFAGFIKM_01963"
/inference="ab initio prediction:Prodigal:002006"
/inference="similar to AA sequence:UniProtKB:O32138"
/codon_start=1
/transl_table=11
/product="Purine catabolism regulatory protein"
/db_xref="COG:COG2508"
/translation="MTAKAGKLSVVQEDLLKLVQGIPRDQMQQYVDRYLGSLLAEEGY
DPELLQTLETYASCNGHINEMSSRLYIHRNTAAYRLEKLERLIGIPLKEPETLMLLNL
VFLFRRLLEQRRPRCPTIVS"

CDS complement(2292066..2293466)
/gene="allB_2"
/locus_tag="EFAGFIKM_01964"
/EC_number="3.5.2.5"
/inference="ab initio prediction:Prodigal:002006"
/inference="similar to AA sequence:UniProtKB:Q9KAH8"
/codon_start=1
/transl_table=11
/product="Allantoinase"
/db_xref="COG:COG0044"
/translation="MKSAIPYDLVIAGGMIVTEAAVERKDIGIIHGSIAEISDNIDPM
GASVINARGLTVMPGVIDTHVHFNEPGLEAWEGFQTGSAALAAGGGTFFLDMPLNAIP
PTVTVSALQRKRERAAGRTYADYGFWGGLMPGYVKELPHLANAGVIGFKAFMSSPGDK
DKDGFRMSDDDTLREGMRIIAETGKVLALHAEHEPTVTRLTREARSLGQFGPESYTAS
RPIKAEVEAVETALRYAAESGCRLHFVHISSEAAAGRIREARKAGVDVTLETCPHYLC
LTDKDFAVIGAVAKCAPPLRDAEEQDKLWVKLKEGWFDFLSSDHSPCPASMKTGNDMF
ESWGGIAGVQSTLELMLDEGYLKRGVPLPLLARLLSAAPAERFGLAPVKGRIAVNCDA
DLVLVDLNTYYTLQASDLYQKHRHSPYIGRSFGCKVKMTLLRGRLMYSEGSPLLNQGT

GKEFRSACPGAMNISS"

CDS complement(2293519..2294574)

/locus_tag="EFAGFIKM_01965"

/inference="ab initio prediction:Prodigal:002006"

/codon_start=1

/transl_table=11

/product="hypothetical protein"

/translation="MLTFDHNQEQPQKRTPDRSKWNESFYRRSMELPDELLIGAIDSH

VHAGPVLNSNPGHLDPIQVAQEAAAAGMKSIVYYDVFGWASGMAWVVNRHVPGIHTYG

GYLMNSCHGGMNPRSVKTALHMEEGCRFISFGSHCTRHSAERESTLIDGKLVPFKDAY

PRFAEKELSVATSIPLEGPVPEELDEILKMVAEHPEVYLNTGHVSVPEAFRLLDLAEQ

YGIAKVLIAHPVRGQMTIEEQKSAASRGAFLEACLVDWLYPDVPRTHYYVEREYMDMG

AELGRYSNATKWMKTIREVGIDQFVLGTDYGIRAAPTPVQGMRTMIASMLDYQFSPDD

IYRMVATNPARLIGIQS"

CDS complement(2294614..2295483)

/gene="ngcG_2"

/locus_tag="EFAGFIKM_01966"

/inference="ab initio prediction:Prodigal:002006"

/inference="similar to AA sequence:UniProtKB:O50501"

/codon_start=1

/transl_table=11

/product="Diacetylchitobiose uptake system permease

protein NgcG"

/db_xref="COG:COG0395"

/translation="MELQLELQRQRSTRRFLPSPAKLAVNALMLLFTASCILPLLWIG

YSSLKTQAEFANNILSLPSAPQFGNYLEAIKLTNMLQLSWNSARVTLLSVIGITAISY

VTGYITARIDFKGKKVMIFYYLFGMLVPIHALLVPLYLLFKNFGLADQWYTLIIPYIA

FNLSLPIMLISSYVAGIPKEIEEAAAIDGLSFSGTMYRIMLPIAIPVLTTVAILQFFS

CWNEFSFALVLLNDESLRTVPLGMSYFKSQHSTNYPQLMAGMILSMLPVTIIYFVFSS

RIIAGVMAGAVKG"

CDS complement(2295483..2296379)

/gene="melD_4"

/locus_tag="EFAGFIKM_01967"

/inference="ab initio prediction:Prodigal:002006"

/inference="similar to AA sequence:UniProtKB:O34706"

/codon_start=1

/transl_table=11

/product="Melibiose/raffinose/stachyose import permease

protein MelD"

/db_xref="COG:COG1175"

/translation="MGKALRPKGWVLLAYLLPGLIIYSFVVLVPIFSAFRDSFYSWTG

GPKKTYIGIENYKEILQDNVFWHSFLNNVLMTAYGLIGQVGFGFVFALLLMSKMVKLK

GLHRTMSYFPSTLAPVIIAFLWMLIYNYNYGLLNAGLTKLGLDVLVQSWLDLTGPIVL

LASIPLSWQNIGFFTLLMLAGLSSINKEVLEVAELDGATGVRKAWYIIIPLAKPTLVV

ASLLCIANNMRGFEHIYALTRGGPGNSSSVMALYAYETSFLRFRYGYGSALAIAIIVL

TMLLILLSTLALQRRNSKGGAY"

CDS complement(2296403..2297740)

/locus_tag="EFAGFIKM_01968"

/inference="ab initio prediction:Prodigal:002006"

/codon_start=1

/transl_table=11

/product="hypothetical protein"

/translation="MNKRSFMLGLLVVTSSVLSACGSGDNEEATAESGKNVTISLLHR

YQESNIGKSPEDTAVLDGLKRFMEDYPNVEIIEEQLQNEDYSIKAQALAAADDMPDVF

IVPGSWMTNFVDNEIVLPLNEELDKRPEWRDGYRAGTLDAGTREGQVYGIPIAAGPTH

LIYYNADMFGSIGYDQFPSTWPDLMDAGEKLADKGINLFSYGDKSKGYAMSSWISALT

DRINGPQWTESILNGSGAQFTDDGFVHGISMMRELAKAGYLNKDLNSVDADTMVNYYF

EGRSAAFVSGIWSAMNIVNNAPEGIAAGTRVAVFPGVEGGLGNPLSSSGGAGVYYSVN

SGVEAGEKLDAIMTMLEYMTGSASAKLMAEVGGFPAYDPGDFDTGKLHPVALAAYEAS

AAADATKIFDLWFDASIVEVLNTGIQGVMAGSRTPEQLAEETQQAYETFLNNP"

CDS complement(2297815..2298477)

/gene="rspR_2"

/locus_tag="EFAGFIKM_01969"

/inference="ab initio prediction:Prodigal:002006"

/inference="similar to AA sequence:UniProtKB:P0ACM2"

/codon_start=1

/transl_table=11

/product="HTH-type transcriptional repressor RspR"

/db_xref="COG:COG1802"

/translation="MTKGKEREIYQDIKLAITQHKLLPNSQLVEDALAEAFEVSRTPI

RNVLRRLAMEKLVTVIPYRGAFVSCPSVDEAREVFEMRRVIETSMVRKLCTTVKPEQL

SPLEVLIREEHQASHDGDVVGALDVTVDIHLKLAELSDNYYYYHFLEELISLTSVIIA

LYGTNQSFCHDHVDLLAAILQQDGELAARIMTEHLWQIERSLNFTETAPNVLNFKEIF

QK"

CDS 2298954..2299205

/locus_tag="EFAGFIKM_01970"

/inference="ab initio prediction:Prodigal:002006"

/codon_start=1

/transl_table=11

/product="hypothetical protein"

/translation="MSKEKLYYGKDIEVMFNSDVCIHSGICVKGLPAVFDLSKRPWVD

PDGDTSEAIARHIDTCPSGALTYKLLDGEYSTKKEDEHA"

CDS 2299198..2299479

/gene="yjdJ"

/locus_tag="EFAGFIKM_01971"

/inference="ab initio prediction:Prodigal:002006"

/inference="similar to AA sequence:UniProtKB:P39274"

/codon_start=1

/transl_table=11

/product="putative protein YjdJ"

/db_xref="COG:COG2388"

/translation="MHNVEHEPANKRFLIQDNGDIAAVMTYVISSPELYIIDHTLVEN

AYRGQGLGDELIKAMVEYARENGIKILPLCPFAKGRFERISEYVDVLHQ"

CDS complement(2299579..2301249)

/gene="malL_1"

/locus_tag="EFAGFIKM_01972"

/EC_number="3.2.1.10"

/inference="ab initio prediction:Prodigal:002006"

/inference="similar to AA sequence:UniProtKB:P29094"

/codon_start=1

/transl_table=11

/product="Oligo-1,6-glucosidase"

/db_xref="COG:COG0366"

/translation="MKRVWWKEAVAYQIYPRSFMDSNGDGVGDIQGIISKLDYIQDLG

IDLIWICPMYKSPNDDNGYDISDYCSIMDEFGTMADFDQLLNEVHLRGMKLIMDLVIN

HTSDEHPWFIESRASLDNPKRDWYIWRDGKNGDEPNNWESIFGGSAWEYDEVSGQYYL

HLFSKKQPDLNWANKEVRKSIYEMMNWWLDKGIDGFRVDAISHIHKEEGLLDMPIIEG

VKYVSSFEKHMNVKGIQSYLQEMKENTLSRYDVVTVGEANGVKVEDQEDLRDWICEVR

GKFNMVFQFEHLDLWKNSTDRQLDVPKLKNVLTKWQKSLEGVGWNALFIENHDQPRKV

SSWGNDTEYWYESATALGAMYFFMQGTPFIYQGQEIGMTNVAYPSIADYNDIADRNLY

QIKREEGMSHEEIMNIIWASSRDNSRTPMQWSSAKQAGFTTSTSWLKVNDNYVNINVE

KQLQEPRSILQFYKQMIQLRKEHDTLTYGIYELLLPHHPSVYAYTRTLADQQVLVIIN

LSEHMVELEEDELGLNFSEIWLTNYENGLVPLVLRPFETIVGLSNKVK"

CDS complement(2301302..2302930)

/gene="malL_2"

/locus_tag="EFAGFIKM_01973"

/EC_number="3.2.1.10"

/inference="ab initio prediction:Prodigal:002006"

/inference="similar to AA sequence:UniProtKB:P29094"

/codon_start=1

/transl_table=11

/product="Oligo-1,6-glucosidase"

/db_xref="COG:COG0366"

/translation="MENKKWWKETVVYQIYPRSFQDRNGDGIGDLEGIVSRLDYLQQL

GIGAIWLSPVCKSPQDDYGYDISDYQDIDPMFGSLEDMETLIQEAGKRDIRIIMDLVL

NHTSDEHPWFQEAKKGKDNPYHDYYVWRDGVEGTPPNDLGSTFGGSAWEWVPEIGQYY

LHLFSVKQPDLNWENPKVRQEIYNMINWWIDKGVGGFRLDVIDLIGKEPDLKITGNGP

NLHQYIRELSKETFQKAEDLLTVGETWGATPEIAKLYSNPDGSEFSMVFQFEHISLDE

QEGKGKWDLKPLDVMALKKVLSKWQTELKGDAWNSLFWNNHDLPRIVSRWGNDGEFRV

ESAKMLATLLHGMQGTPYIYQGEELGMTNVQYAIEDYRDIELLNFYKERMGKGYPEQS

VMESIYAKGRDNARTPMQWDTSDNAGFTQGEPWIKVNPNYKHIHAEESLNNPESIFHY

YRKLIQLRKDHEVIVYGDYELIFPENPDVFAYTRTLNGTTILVVCNFQGYTTELSLQE

QLTGSYQLLIANYAGELSESELRPYEARMYLIHD"

CDS complement(2302953..2303846)

/locus_tag="EFAGFIKM_01974"

/inference="ab initio prediction:Prodigal:002006"

/codon_start=1

/transl_table=11

/product="hypothetical protein"

/translation="MSYSANFKDRIGRFAIYAIVLMLALVCLLPLWNIVAISFSSSEA

VSANAVGLVPVNFTTAAYTKIIEDAQFWRSFGISVLRVALALVLNMILIILMAYPLSK

SKRDFKGRNIYMNIMIFAMLFSGGMIPSYLLIKNLDMLNTIWALVLPGAVPIFSVILV

MNFFSAVPKALEEAAFIDGANPLQVLFKVYVPVSIPALATVALFSIVGTWNDFFGGLI

YMTKVSNYPLMTYIQSLNVNIADLLQAGTNSSELSNLTEISNKNLNAAKIVVAVIPLL

LIYPLLQKYFVTGIVVGSVKE"

CDS complement(2303860..2304774)

/gene="yteP_11"

/locus_tag="EFAGFIKM_01975"

/inference="ab initio prediction:Prodigal:002006"

/inference="similar to AA sequence:UniProtKB:C0SPB3"

/codon_start=1

/transl_table=11

/product="putative multiple-sugar transport system

permease YteP"

/db_xref="COG:COG4209"

/translation="MKQGNSKIKGRLGPGAMYHMMMLPGILFLLVFSYVPMVGIITAF

QDYIPAKGMFGSEFVGLKHFMYMFKLPDIAQVVSNTLVIAIGKILLGTMMAIIFSVLL

NEIRIKYVKKSVQTIVYLPHFLSWVVLASVVVNMFSLDGIVNQMLAFFGLENINFLGS

NTWFQPLIIGTDVWKEFGYSSIVYLAAITSIDPGLYEAAGMDGASWWRKVWHITLPGM

LPIILLMGVMSLTNILSAGFDQVYNLYNPVVYETGDILDTYVYRIGLVGRQYSFGTAV

GLFKSVIGIVLLMSANQLAKKYTDRKIF"

CDS complement(2304837..2306573)

/locus_tag="EFAGFIKM_01976"

/inference="ab initio prediction:Prodigal:002006"

/codon_start=1

/transl_table=11

/product="hypothetical protein"

/translation="MRTKWSMKSNRLTKRLAVLSMSALMVATLAACGDSSTPPTAEEA

GKYEVTPGDPFSAYKDEITVTMGRVTTANPKLPAGDTYENNAYTRLVKDTFNAQITDQ

FEANGEDYSRQVSLAIASGELPDMMRVDSKDELKELVDNDLIEDLTAIYDQYATDNIK

QMYDSYDGRALDNATIDGRLMGLPATSLDSAPTMVWVRQDWLDLLGIQLDADGDGALS

LEEVEQTALEFLKRDPGQSGNPVGIPFVNTMNTTDYNGSAYTMLGVASTEGAFPQYWM

NGEDGKIVYGSTTEETKKMLGTMADWFKNGIIDPQFGTRTFDDITALYANGQSGIAFG

PWHIPDWGLISAKQMDKNAKFSAYTLEDANGKVNVAHANPSNQFIVVRKGYEHPELAV

KIVNLFYDKLANDKNAATTMPEAAKYQETGVDGSTRPFNIEVNSATSLLDDYSDVVRG

IKGEINLDEVRTTESKNNIGSIKTYLSDMDTDDVTAWSKYHSRINGVGLIDKLTQENK

FVWMTPAFSGTTPSMKQTWANLTKLEQESFIKIVTGSESLDYFDTFVSNWKKQGGDQV

IQEIEDEIASKQ"

CDS complement(2306824..2308230)

/gene="cheB_4"

/locus_tag="EFAGFIKM_01977"

/EC_number="3.5.1.44"

/inference="ab initio prediction:Prodigal:002006"

/inference="protein motif:HAMAP:MF_00099"

/codon_start=1

/transl_table=11

/product="Protein-glutamate

methylesterase/protein-glutamine glutaminase"

/translation="MIKVLIVDDDKLVRKGISSAMPWNEFDMEVVGEASNGLKALDFL

KTQPVDLMLTDLAMPVMSGIELMRAARQLYPELHIVVLTLHQDFDYIQEALRLGAIDY

IAKVQLEKEQFEHVLQRIHTRINGLTSTIRKMPSQNEINFHYRNVYALVSQDRKSEHK

WPMELTAHEDEIRWEVERNSWMWAAPAHEEDQLFHQLKESLNQVPQCTLLVLSDVQER

TWSQIKNWIMNYTETSLFYAYNPNNQVIAVSMNEENTFSKEPQDEDLNRIKQSWFQTP

WTHHDSYYNQLIDQFISLRLHKGQLMGLLYSIVMEWNHLFAQTTLGRISMIHSFESWY

EVEEWIKQTAVSIRKSDEQTSYSQEIIDAVKKAVMIVQHDLEQAYTASGLSQQLNISR

SYFSQCFKDLMGKTFNEYSRFIRVEKSKEYLLNTNNTIFWIAERVGYTDEKYFSRIFR

ELTGLLPSEYRQLGRGNK"

CDS complement(2308227..2309939)

/locus_tag="EFAGFIKM_01978"

/inference="ab initio prediction:Prodigal:002006"

/codon_start=1

/transl_table=11

/product="hypothetical protein"

/translation="MTLKKRIFLLFFLSAFIPFISIFAISYYTIDSIFANKIDDGIRS

NLQQVTSSLENSITNLNHVTQQLSYRGTLGKRLDEFLNPSSDIFELIEARDELKNELS

VVTFTNPNIGLTLYYFQKEGTTQFGNFPIKDRFSPESLPVLFQTYGIKYYGPHMSMNR

FDDQLVLSAMRKVQLSERNDVFIYVESGFRLTQDILGYNQYNGALSHLILDGEGNIVY

SEIPEAMKVGENFSSLTVDPAKDGISRGYHWFKEDASQKWSVVSVISQAQYQQEKNQW

LLQILLVALFFLGFTVFLAWLLWKMVYKPLGLFHSEINGMSKNPQTTGRRARTQIPEF

DFLLGEFSNMQHQIGDLFREVQQKEKIRADLEVEKLLYQINPHFLMNTLDTVHWLAVM

NGQGEIDKLVQSLNKLLYYNLGKLGQVSTMEEEIDALRQYLILQQIRYDFEFDVRITA

DEQVLQIPVPRFILQPLVENSLYHGLSDEGFIQIEVTCTSTLNIMIQDNGAGMTEETI

HNLLNNREAEQQKVGMGIGLNYVHRMLKAQYGDQAQLVIESELGTGTSILLVLPIKGE

DISA"

CDS complement(2310225..2316608)

/locus_tag="EFAGFIKM_01979"

/inference="ab initio prediction:Prodigal:002006"

/codon_start=1

/transl_table=11

/product="hypothetical protein"

/translation="MIRRTVSIQKSMFVSFLALLMVISLAVPPAPIRAESSSSQPGRQ

AEYLNRGLVAVLVDNGVFLSWRYLNNDPDDIAFNIYKNGTKVNTSPISDVTNYVDTTG

FDSSQYQISTVIAGKEQMQPEVVSVWHNDYLPIPLDKPADGRTKDGGTYSYYAGDASV

ADLDGDGEYEIVFLWSPSNSKDNSQAGYTGNVYIDAIKLDGTKLWRIDLGVNIRAGAH

YTQLMVYDLDGNGKAEVVVKTADGTKDGQGTVIGDGTKDYRNDGGYILSGPEYLTLFN

GETGAAVSTVNYDPPRGNVNDWGDGYGNRVDRFLGAIAYLDGTKPSVVMSRGYYTRTV

LVAYDYVGGELVKRWTFDTNQAGSQYQSQGNHNLSVLDVDKDGKDEIMFGALAIDDDG

SLLYSTGLGHGDAMHAGQLDPNREGYQVVSVHEHSDAAYGLEMRNAATGEILWGEKTG

FDTGRGMSADIDPNYPGYESWATTVTNGQSVPVTNTYSAAGEAIYTVEEGPKTANFAI

WWDGDLQRELLDHEWDNSTAKGIPLIYKWDYQNKKLNEIFRATGTLTNNHTKGNPALQ

ADILGDWREELLLRSEDSSEYRLYTTTIPSDYRIPTLMQDPVYRLGVAWQNVAYNQPP

HTGFYLGAEATAFPKADLALTREAEMLEPVYRFNFGTETASGSTNIQDTLYAQDPGYG

FKDTSGVTVGANQVSVATGTTFAVDLPNANYKVTLRLGNDANDSNVGVKSEFVQKLAL

TNVTAGAPLEYSYDVALVDGQLDLLFTGTAIDIQDVIIEKYPEKAAGAATTIYMAGDS

TMQSYSEAQAPQEGWGQQFGRYFSNGAVIVNDAIGGRSSKSFMVDGRLDTILQRIKPG

DFFFISFGHNDASAGIPDRYASPEDYKIYLARYVNGAKQRGATPVLLTPVGRRDFNTV

TQEFNVSFPAYVQAAKEVAEEQDVPLIDLSKLSIAYYDKIGNTATEKVFLYANPGEYP

KYPNGINDNTHFSSYGAQKIAGLVAGAVKDMELSISSLVIDPDISEPEPEPEAQLYEE

DFEGDAANYQYAMVNATGIAGTMTGTVVEQSGNKLLSVAGSGSGNRAKVFRLFDAVNG

DQVNVNFDWQSGNVSAYPSEGHLTIQDSNENALFTLYTKTGSTKIHYFVGAYNPDYGT

GDTAIPEGGTTTDISKNQWVNVDATVNFPEKTLDLTLTNLADPTVTQTIADISLSSAA

YADNVRSMRFLGTRKGGGGTLNWTTQIDNVRIEGTQLSSESGDQTALIALYDEIKALN

LSDYTDASKAVVNRALAAAEAIFNTEATQAKVDHTFNMLTVAKDSLTSEPSEEISEYK

FDFGSGNAADGYIKVDAKRAYIEGNGYGFADTALTKDENRGTGDLLKEDLTRVNGTSF

LTEMEPANYRVTMTIGDAQESTSANVVVEQMTKLPLTSIAKGEFKEITYDVALIDGVF

NFNFTGNAPKINALKLERLPDHSASDKPVIYLASDSTVANYAENYRPQAGWGETLGEY

FDPNEVSIDNRAVGGLSSKTFLVGGYLNDILLDIQEGDYLFMQWSHNDSTPSRPERYL

TPEQFKVYLKEYINGALQRGAIPVLVTPVNRRDFTGEVLNKSFPEYVQAMKETAQETE

TLIIDLNQASWEHFQELGTEGTKSIFMWVGTTEDNTHLQMNGAIKVSELVAHLVKELN

IPLSDFVTLEGESPEQKAPHTTATVEGELQNGWYTSPAQVTFTASDEDSDIEGTYYQI

NGGETVSGTQLTLTEEGTHTITYWSVDVDGNKESEQSLSISIDLVPPTIEIHGQTEYT

IDQQVEIGYTASDSVSGVEEPEGVLIDTPAYTLEPGLNQVTATVSDLAGWEQTVEYSF

AVITTFDSLINLTNTFADESIDPNASILADQLTNTLQQAKQAANDREGAKARQLLASF

GNDVQAARGTVFTDEQTSVLIKWEEWLHQTTPLANGAPGTPVLSDNNGYDTGLKDGDY

TITMNLWWGNNGNQFKLYENGELIKEISLVDQSPSAQSVQVDITGKKNGTYIYTGELI

NALGSTMSVPLTVIVTDASPGQAVLSNDNWDGDGNYNVTMNLWWGTNATEYELYENGV

SIDTQSLQSHSPGAQSAVTSISGRAPGIYEYEAVLRNSSGETRSHKLNVTVRE"

CDS 2316817..2317689

/gene="rhaR_10"

/locus_tag="EFAGFIKM_01980"

/inference="ab initio prediction:Prodigal:002006"

/inference="protein motif:HAMAP:MF_01533"

/codon_start=1

/transl_table=11

/product="HTH-type transcriptional activator RhaR"

/translation="MVHTVSYAFRNDDTSIMTLDSIGWQIVSSEEYRCPSDDRPDPGH

VVFQYTLNGQGYLDIDNQTLPLPKGHALLVKIAGEHCYYYKQENNEPWEFIWINIRGD

EANRIWDMMHDNEGHVIRRNADSPLIQELWQIIHLIHQEKVTDKYRLSMQVYRWLLIL

VQTSRDAEKDIGALSITTIEKCKKFIRENYASPLTLDLLASHCDINKHYLCRLFQKSE

KTSPLAYLKDRRIEVAIRLLRTTELPISQIGQQCGFESPSYFGKVFRQYMSMSPKEYR

LNKLEFPYEAIYYE"

CDS complement(2317820..2318557)

/gene="bacC"

/locus_tag="EFAGFIKM_01981"

/EC_number="1.1.1.385"

/inference="ab initio prediction:Prodigal:002006"

/inference="similar to AA sequence:UniProtKB:Q8KWT4"

/codon_start=1

/transl_table=11

/product="Dihydroanticapsin 7-dehydrogenase"

/translation="MGKLQDKVAVITGGASGIGAATARLFVSEGAKVVLVDLNEEKGK

AFEQELKALKAEALFIKANITSEEEVSEIFKQTVEAFGQVDIVFNNAGIGRVHPTHEL

DYAEWRNTVNVDLDGVFLVARESIREMLKIGGGTIVNTASMYGWVGSPGSAAYNAAKG

GVVNLTRSLALEYAEQNIRVNSLCPGFIDTPIIPEESKQALSAATPMKRLGQAEEMAK

AVLFLASDDSSYMTGNSLIIDGGYTAQ"

CDS 2318734..2319348

/locus_tag="EFAGFIKM_01982"

/inference="ab initio prediction:Prodigal:002006"

/codon_start=1

/transl_table=11

/product="hypothetical protein"

/translation="MNNELIVTSQHRNRTKEHLKTALIQLIKKKGFHGVSVKDIVDQA

GYNRSTFYLHYQDKYILAEELLILTLEGLRGAVGKPYWHGQKVSTNKLDAESFQIVDY

IYEHRDFFELIQYDDTLPGLHTGFPQTILKIYQEQFVFETINNSPVNMDYFKYYTAYG

FFGLLNNWILSGYRESREAFIKNVIELTKTHIHSFHYVGNTFES"

CDS complement(2319372..2321441)

/locus_tag="EFAGFIKM_01983"

/inference="ab initio prediction:Prodigal:002006"

/codon_start=1

/transl_table=11

/product="hypothetical protein"

/translation="MNGILHKIMQERLGNCDYEVWISSNWADWVLIEAIGRRESTPPC

FAEQLPLQHPKVEWESEENMLVFLPYNNNICVAIRVFKNWEFSAEDLVTLSSLLYPYY

TEAVASKNERALNEIMNSIRDVTQLLDLNELLGRILVSALSVIPYECIGVLWRYDPAI

DALTVKARAGEMGEGMLRMKLKPGEGIIGNTFKRGTPKLYNGLSTVEDDFGNMTSENK

YHLNSAYNFQDIGSIISVPIKVEGQSDCVLIVYQKGRVPLFTESDVRLLQSFADQVSI

AITNAKLYENLSKQNETLMKRDEIHSSLMRLSLQNKGAVSIVNELARVIGVSMTFVDF

IDNEWIPKRSKIMNGWNAESLRSLYESLHHPDYLTCIEGVADRSYQYLYPIASANQCL

GYLIIQMNTKLEPLQLIALEQGSSILALELMRKQSLAEFYFKKTQQFFNDLRLSQDSE

DYWQKSGEIGIVPSTSIVVGLLEFAEWVNPSTLSALSVQLVAYLREKMPAGALPIAFG

NERRITVLLMLNESHKPGKLEQQFSALLPEWEHRNHVKLLGGLSSVRSGVDAINTGYQ

EADKALAYQKTRGEQGTIRYTDIGVNRLFIRQPAEDLNAFMAEIFEPLRPAKGQTGGL

EETLMTYMACGSSAVQAAAALHIHINTLYQRIHKIEEILGMSLSNQEHLLHLQLACYL

RQTYRSS"

CDS 2321588..2322832

/gene="sauU"

/locus_tag="EFAGFIKM_01984"

/inference="ab initio prediction:Prodigal:002006"

/inference="similar to AA sequence:UniProtKB:Q0K843"

/codon_start=1

/transl_table=11

/product="putative sulfoacetate transporter SauU"

/translation="MSKIMHSKHFKYIVLTLLFLGWCLGNLDRFVINYAIVGITQDLG

LGASAQGIILSSFFLGYAIMQIPGGALADRFGYRKVILVSLFSWSVFTILTGSAWSLF

SLIFVRFLFGIGEGSFFPSGSKAIATNFPINQRSGAMSIMLSSAAIMGVITPILTGHA

LVTIGWRNLFYIIGAVGILITALMFFLLKEPAKPALATSGSAQQPKTSLKTVLKTPMI

WNLFISYFSIYAVNWGLQSWMPTYMVNVRGLDMTQMGLLAAIPALVSIFTMILSGYVL

DRIPAGKDRMIASVFGVLVALFLYLMGSAGSITTFITYMTIVTAMAGFISTLIISKSL

KTMPESVVATANGFINTGAQLAGFLTPMLIGFLVEASGGSYATAFIMLIAFALICSIT

LMFSRRSNSGQDNTTEMQAKAV"

CDS 2322927..2324369

/gene="abgB_3"

/locus_tag="EFAGFIKM_01985"

/EC_number="3.5.1.-"

/inference="ab initio prediction:Prodigal:002006"

/inference="similar to AA sequence:UniProtKB:P76052"

/codon_start=1

/transl_table=11

/product="p-aminobenzoyl-glutamate hydrolase subunit B"

/db_xref="COG:COG1473"

/translation="MTTTVDLTQFISEAIEGKRDMFTNASDQIWGFAETRFDEFQSAE

LLMGMLESEGFTIEKGVAGLETGFIASFGSGLPVIALLGEYDALADLSQEAGISTYQP

VQPRGNGHGCGHNLLGIGALAAAVAVKDYLQEHPETPGTVRFYGCPAEESGYGKTYLA

REGYFKDVDAALSWHPHSLNAVMHGSSNAVIHGTFTFKGISAHAAAAPHLGRSALDAV

ELMNIGSNYMREHMIDQARIHYAITNSGGLAPNVVQAEAEVTYLVRAPKSAQVRSLFE

RLVKVAEGAALMTETTMNFKYEGACTNLIPNSTLEKIMHQHLVAFGAPQYEEDEYNYA

KAIYDTLPLQNQQEAGTLVGPELAPLLAERPLPNFIAPYSDERETFMGGSTDVADVSW

NVPTAQCITTTMAFGTPLHAWQTVAQGKSSFAHKGMLLAAKAMAATAIESILHPEIVK

EAQQELLDRLGGEAYDCLVPADVQPPKRGE"

CDS 2324374..2325042

/gene="sdhB_1"

/locus_tag="EFAGFIKM_01986"

/EC_number="4.3.1.17"

/inference="ab initio prediction:Prodigal:002006"

/inference="similar to AA sequence:UniProtKB:P33074"

/codon_start=1

/transl_table=11

/product="L-serine dehydratase, beta chain"

/translation="MRFKDVFSIIGPAMVGPSSSHTAGAARIGRTARHLFGECPSHAE

ILMFGSFAATYKGHGTDTAIVGGLLDMDTDDPRLPDAFAHADTAGMETIIRPGTGLYP

HPNTAEIVLTNAAGNRILNMVGTSIGGGNIEIVRINGYNLKLTALYPTLIIRHSDEPG

VIAVVTRLLVDNHINIGHMSVDRKNRRGEAMMVLECDGKMDPVVVRQIEVLPEVHDVT

LLCL"

CDS 2325089..2325964

/gene="sdhA_1"

/locus_tag="EFAGFIKM_01987"

/EC_number="4.3.1.17"

/inference="ab initio prediction:Prodigal:002006"

/inference="similar to AA sequence:UniProtKB:P33073"

/codon_start=1

/transl_table=11

/product="L-serine dehydratase, alpha chain"

/translation="MNFRTLKQLAELAETRNLTLGQLMLEEQCHESGEDKNVVFSQMH

EYYEIMKSAVHQGLHTDTTSKSGLTGGDARKLMAYRTGGQSLLGDHASLSMSYALAVS

EVNASMGRIIATPTAGSCGIIPGVFISSQERFGWTDEQMVYGLFASGAIGYVIANNSF

ISGAEGGCQAEVGSAIGMAAGALVDIRGGSPSQSIHAVGLALKNTLGLICDPVAGLVE

IPCIVRNGFGAITALGAADMALAGVRSIIPSDEVVQVMLEVGSAMPESLRETAGGGLA

QTPTGRKITRDLQHK"

CDS complement(2326102..2326569)

/locus_tag="EFAGFIKM_01988"

/inference="ab initio prediction:Prodigal:002006"

/codon_start=1

/transl_table=11

/product="hypothetical protein"

/translation="MKKRVSVKIGDVFSIKLNQDLDCYGQVVSEGRISDCMIVFDLIS
MEHPTVSEITSKPIIFLIQTVNSRIEDGIWKVIGNAPIPRMTFPMYKEETEDGYTLVD
HKGDIVTETPSASQIEVASELESWSPVSLEKAVIARFVTGEWDPYYNDLIYTE"

CDS complement(2326636..2327367)

/locus_tag="EFAGFIKM_01989"

/inference="ab initio prediction:Prodigal:002006"

/codon_start=1

/transl_table=11

/product="hypothetical protein"

/translation="MYLKELNLDLPYIVDDENIESIMKKQKCEYNEATKLDYALNWKW
KRRSFSLETRCITAMYERLLGKYKTKDCWKVLIECVENITDERIVNDSGVCSVPIQFS
LNDFSGKNELEKKKATLRLLMEGIEKLALRNNWDIDPFREIALQIEELDYVNEWTWKK
DVKSPNKKYNARVICHHNVDSMDIFLSILQRDGTQVLVEKVISEQPDEFAYAEHLGEL
TWVSDFEVALINKAGTEKYSATLNN"

CDS complement(2327409..2327708)

/locus_tag="EFAGFIKM_01990"

/inference="ab initio prediction:Prodigal:002006"

/codon_start=1

/transl_table=11

/product="hypothetical protein"

/translation="MDILKWIQSWYYENCDGDWEHLYGVRIDTVDNPGWSVEIDLTDT
YLEDVPFDSIEEERNEEDWFYCIVRDGVFHSAGGAKNLEEMLNCFKNWASSLERS"

CDS 2327885..2328244

/locus_tag="EFAGFIKM_01991"

/inference="ab initio prediction:Prodigal:002006"

/codon_start=1

/transl_table=11

/product="hypothetical protein"

/translation="MKKIILFIFISMIILSGCSNKDEKENYPVFEGTVVFKTDENDKY
KFLVLQNVSKEDLENGNLEDFIELAQDQDGSYYLVNRETYKTIEVGQRVRITTGFDQL
EPSPPIRTVVSIEKIND"

CDS complement(2328465..2329736)

/locus_tag="EFAGFIKM_01992"

/inference="ab initio prediction:Prodigal:002006"

/codon_start=1

/transl_table=11

/product="hypothetical protein"

/translation="MKKRLTTILATVLSLTMLSACSSTSIGTSKQEEIPKPENKVASD

QNEFPKPLGSYTVGRTQMDFKYTASDHSERELTALFFYPSDSNEGKPTAEYAFPEFHS

LRDELLVRLGGTAGGEQLFDSNFKTWSYDDLALSEKEKQYPVLFFVHGAGAYPQQGTL

FAQDLASAGYIVVSIGHAESGVYKLKDGRTVGMSEKFMDEVTKYGMEAATLTPPEIVT

EKLEEEEAIEIARKLTSAPEAVKFANYAVLQSEDIRYVADNLYKMNTGDMESMFKGRL

QLDLGMGVFGHSFGGTTAAIVSRDDDRFVGAVNLDGNMLGALDSDFKKPFMQLSTVLA

YNTNAFLLESNSKDTYFAIIDNVVHGDFSDSLFTTTDKASRGTRDAMEQRNIITSYTK

AFFDEYMLKKDADIDSLTFDGVEMIKKPQKP"

CDS complement(2329852..2330499)

/gene="degU_1"

/locus_tag="EFAGFIKM_01993"

/inference="ab initio prediction:Prodigal:002006"

/inference="similar to AA sequence:UniProtKB:P13800"

/codon_start=1

/transl_table=11

/product="Transcriptional regulatory protein DegU"

/db_xref="COG:COG2197"

/translation="MTIKILIVDDHFVVREGLKLILETNQRYEVVAEASNGMEAIRRV

DTYKPDIILLDLNMPEMGGLDTMTALRAKGMDIPIIILTTYNEDEMMIRGLALGAKGY

LLKDTGREMIFRSIDAAMRGETLLLPEISEKVFAHKTSEKAKESSSARISLTDKELIV

LQAIARGARSKEIGFDMGISERTVKTHLTSIYNKLGVDSRPQAVAVAVERGILHL"

CDS complement(2330496..2331719)

/locus_tag="EFAGFIKM_01994"

/inference="ab initio prediction:Prodigal:002006"

/codon_start=1

/transl_table=11

/product="hypothetical protein"

/translation="MSDYSINKPYTSSRSDIQNVLRDSRLPALIWGILIYFSALYSQL
PMLPTWFEFMMFTALILVHILLHWYAGYVVDRRPWIYFMTQGCIVWGCALFMPNAHSL
IFVSLLTALAGQSIGVYAQRMKVLLVCTFYCTLFSVSLVWHGTTEDLPSTVPSLFFIM
FFVIGYAALFYKQVRAKLRTQTFLRELERAHRKVEELTIANERQRMARDLHDTLAQGL
AGLIMQLDAVDAHLENDNNHRAHEIVQLSKAQAKRTLSEARSAIDDLRSYSAEVIDIS
KEVKEEAEHFSHITGICVVTRLSMPHLVVPKLIFEHSLHIIRECLANTAKYANATNVE
VVVAARENEIELSITDDGIGFDTNLIEKQSGSYGLIGLYERTRIMGGEIEIESGKKGT
KVSVRIPLQQEDTNA"

CDS complement(2331993..2333609)

/gene="xynB_3"

/locus_tag="EFAGFIKM_01995"

/EC_number="3.2.1.37"

/inference="ab initio prediction:Prodigal:002006"

/inference="similar to AA sequence:UniProtKB:P94489"

/codon_start=1

/transl_table=11

/product="Beta-xylosidase"

/db_xref="COG:COG3507"

/translation="MNKIKKMITNPILPGFHPDPSICRAGEDYYIATSTFEWFPGVRI
HHSRDLVHWRPIASPLTRVTQLNMEGNINSGGVWAPCLSYDNGVFYLIYTDVKSRVGA
FKDTHNYLVTATDIEGPWSDPVYLNSSGFDPSLFHDEDGRKWLVNMIWDHRKGKNRFA
GIVLQEYSVQEHKLIGPAMNIFKGTELGLTEAPHLYKQKDYYYLITAEGGTGYEHAVT
SARSRTLQGPYEVDPANPILTSYGRPDLALQKAGHGSLVETHTGEWYMAHLVGRPVQH
KYCILGRETALQPCTWSEDGWLRLASGDRYPEVQVPAPMIQSHPFEPIAEMDHFDHFE
LRHDWNTLRIPPDSTWLSLTERPGYLRLHGMESMSSTHRQSMIARRLQALECEAETCL
EFAPDHPQQMAGLILYYDTQDYLYLRVTYHEEKGLCLGIIQSKYGVYDELLEDIPLES
VSTLRLKVVVDQDRARFYYAINSDSSWNTAGGWVDITHLSDESPEYIRFTGTYIGLCV
QDLGGTRKHADFDYFMYKETKHTAGVSENM"

CDS complement(2333670..2335646)

/gene="yagF"

/locus_tag="EFAGFIKM_01996"

/EC_number="4.2.1.82"

/inference="ab initio prediction:Prodigal:002006"

/inference="similar to AA sequence:UniProtKB:P77596"

/codon_start=1

/transl_table=11

/product="D-xylonate dehydratase YagF"

/db_xref="COG:COG0129"

/translation="MYEQITSIMGETASNCFDIIAHAPGAAGRLPLTDDLLRNSPSGD

LFGMSQNVGMGWKPGELNGKQFLILSTQGGIRNEDGSPAALGYHTGHWEVGLLMKAAA

EELSSRGGIPFAGYVSDPCDGRSQGTTGMFDSLPYRNDAAIVFRRLIRSLPTRKGVLG

VATCDKGLPAMMLALAGMPQLPGVIVPGGVTLPPTNGEDAGKIQTIGARYVNGELSLE

MASDLGCRACATPGGGCQFLGTAATAQVVAEAMGMTVPHAALAPSGQPIWFEMARQSS

RALIHMESQGMTMADIVTDASIRNAMTVHAAFGGSTNLLLHIPAIAHAAGLTVPTVQD

WIQVNKNVPRLVSALPNGPIFYPTIRVFQAGGVPEVMLHLRQLGLLDESVPTVTGTSL

GQVLDWWESSERRHLMRKQLKEQDGIDPDSVIMSVEHAQRLGISSTVTFPTGNIAPEG

SVIKSTSIDPAVLDEHGVYRHRGRAKVFTTEREAIHAIKTGCILAGDVVVLLGRGPSG

TGMEETYQLTSALKHLPFGKYVSLITDARFSGVSTGACIGHVGPEALAGGPIGKLQNG

DLIDIAVDRNTLEGSINFVGEGDLEFSPEEGALILAQRPFHPDMRPDEALPDDTKLWA

ALQSVSGGTWRGNVYDVERIVTALEAGKKALGWY"

CDS complement(2335663..2337129)

/gene="aldHT"

/locus_tag="EFAGFIKM_01997"

/EC_number="1.2.1.5"

/inference="ab initio prediction:Prodigal:002006"

/inference="similar to AA sequence:UniProtKB:P42329"

/codon_start=1

/transl_table=11

/product="Aldehyde dehydrogenase, thermostable"

/translation="MTTFQVEQTYSNYINGEWVKATSGETEPSINPANRKEVVGYVPT

SGVEDLNRAVAAAKEAAKDWRRLSGAERGNYLFQAANVLERRADEVAEAMTREMGKTL

PEAKGETLRGVAILRYYAGEGMRKTGDVIPSTDSEALMFTTRVPLGVVGVIAPWNFPV

AIPIWKTAPALIYGNTVVLKPAQETAVTAAKVMECFEEAGIPAGVLNLVCGRGSVIGS

ALAEHPDVGGITFTGSNEVGKRVGGAALARGAKYQLEMGGKNPIIIAADADLDLAVEA

TISGGLKSTGQKCTATSKVIIERKVYDTFKEKLLSQIQEVRLGDGMSSGSWMGPCASE
GQLNTVLSYIQKGQDEGAVLLTGGKRGDGPGLEEGFYVQPTVFEGVESHMSIAREEIF
GPVLALIAVDSLEEAIEAANDSDYGLSASIYTQNVGAMLSFIRDMDAGLVRINAETAG
VELQAPFGGMKMSSSHSREQGQAAIEFFTAIKTVFVKS"

CDS complement(2337151..2338026)

/locus_tag="EFAGFIKM_01998"
/inference="ab initio prediction:Prodigal:002006"
/codon_start=1
/transl_table=11
/product="hypothetical protein"
/translation="MRIIRFLDGQQKWLAAVTDDEQAYRLPQADFMTLIHQARKQGIS
PVQLVESAFKPLNKLTDDWTSLHLITPLEAPEVWAAGVTYQRSRVARNYEATEGKLDA
KTFYDKVYDAERPEIFFKSTAARTVGPNEAVTLRSDSNWQIPEPELGLVLAADGSIVG
YIVGNDMSCRDIEGENPLYLPQAKMWRNSCSIGPAIRLAETVQNPYAFSIVCEIYREG
EVVVKSEASTSELNRKLDELVSFLARDNDLFDGTVLLTGTSIVPPNDFTLAPGDRIEI
SISDIGTLINPVISN"

CDS 2338243..2339154

/gene="virF"
/locus_tag="EFAGFIKM_01999"
/inference="ab initio prediction:Prodigal:002006"
/inference="similar to AA sequence:UniProtKB:P0C2V5"
/codon_start=1
/transl_table=11
/product="Virulence regulon transcriptional activator
VirF"
/translation="MTKLSDAVYLGRLPDVRMSFQLLGLHARKVDSSWTYPSHEHSMY
EVHWMMDGQMNMVVNGQSYRQSVGDLLFIRPGMTHSCTGAGPEGFTYFSVHFSIHDTS
FCRELNRCKDIYYPANSNLALGLSPSLSTLYGLATEHLSSSLSSSKQMKVHAAVFELL
GSLVGQLSQQASVTLSRKETIAHQIAEHIEDSVRYIHLHGEIQESDRTWIQDIAKSLS
ISPSQVNRIFRQVYGIAPRKFLSETLLNEAQRLLKQTDLNIDHIAMMLGYKTNAHFSR
QFKRWTGIAPSEYRSRSQHAGEAQSDV"

CDS complement(2339671..2340624)

/gene="nlhH"

/locus_tag="EFAGFIKM_02000"

/EC_number="3.1.1.1"

/inference="ab initio prediction:Prodigal:002006"

/inference="similar to AA sequence:UniProtKB:P9WK87"

/codon_start=1

/transl_table=11

/product="Carboxylesterase NlhH"

/db_xref="COG:COG0657"

/translation="MSIETRILPELKEVYSQFPGFELERNLEWSRNLVSAASVRKSEH

VNTTSRKIPSDGGEMLVKIYEPADRTNDLLPAMLWIHGGGYVLGHPDMDDKLCERFVQ

TAECIVVSVDYRLAPEHPYPAAIEDCYAGLVWMTEESAMLGIDVNRVAIAGASGGGGL

TAALALMARDKGGPSIIFQMPLYPMLDNRNITPSSHEITEEGSIWNRTDNVAAWNMYL

GEENDVIESSFYAVPSRAESLAGLPPAYTCVGQLDLFRDETMEYVARLAQAGVDVEFH

LYPGCFHLFEILAPETEVSQRTVKGYMDAMARALHPNRTLN"

CDS complement(2340682..2341734)

/gene="fgd_1"

/locus_tag="EFAGFIKM_02001"

/EC_number="1.1.98.2"

/inference="ab initio prediction:Prodigal:002006"

/inference="protein motif:HAMAP:MF_02123"

/codon_start=1

/transl_table=11

/product="F420-dependent glucose-6-phosphate

dehydrogenase"

/translation="MEIGVTSFVETKPDTKTGVVMSHAQRLQEVVEEIVLADQVGLDV

FGIGEHHRDDYAASSPAMVLSAAAPLTKRIRLTSAVTVLSSADPVRVFQDFATLDGLS

NGRAEIIAGRGSFIESFPLFGYNLHDYEDLFNENIELLLKLRESEKVTWSGGHRPAIH

NLGVYPRPVQNSIPVWIGSGGTQESAIRAGILGLPLVLAIIGGNPTKFAPLVELYKKA

ATHAGHDVSQLRVGSHSIGFVAEDTEKAAELFFPSTQYGMNKLGKERGWAYYDRSSYD

AARRSDGALYVGDPETVAQKIIHLRKHVGITRFMMYVPLSTMTHELVMRAIELLGKEV

APRVREEISKWEAEKR"

CDS complement(2341764..2342633)

/gene="mmsB"

/locus_tag="EFAGFIKM_02002"

/EC_number="1.1.1.31"

/inference="ab initio prediction:Prodigal:002006"

/inference="similar to AA sequence:UniProtKB:P28811"

/codon_start=1

/transl_table=11

/product="3-hydroxyisobutyrate dehydrogenase"

/db_xref="COG:COG2084"

/translation="MKVSFIGLGNMGLPMAQNLLKAGYEVVIFNRTPEKAEPLIKQGA

RYVQTPLEAAKESNLVITMLSDDAALREIVEGPNGVLNGLSENGIHVSASTISVDLAR

KLSALHAERHQHFVSATVMGRPDAAKAATLRIILAGPEHARQRVLPMLTVLSQEIFEI

GDHGEEGNIVKIGVNFLIASMLESLSEAQLMVEKHGIKPSRYMDVVNALFQSPVYQNY

GAIMTEQRFEPAGFKMKLGLKDVALAIEAAKSVQAPLPLGQLIHQHLSEGITHGYGEL

DWAALIRCLEHSS"

CDS complement(2342793..2343320)

/gene="comR_3"

/locus_tag="EFAGFIKM_02003"

/inference="ab initio prediction:Prodigal:002006"

/inference="similar to AA sequence:UniProtKB:P75952"

/codon_start=1

/transl_table=11

/product="HTH-type transcriptional repressor ComR"

/translation="MLLFWKKGYEAASIPDLLEVMGISRSSLYDTFSDKQTLYRDALE

HYKKGSSNKQTILEQASSVRDGILQFFEYHIASAYDTSLPGGCFVTNTATTLESPDEQ

INVLIQTSFSDLEQAFCELLEKGQQSEEIDKTTDIKTLSYLLLNLHQSINIMAKTGQN

PERAKEMISFIIAGI"

CDS 2343531..2344016

/locus_tag="EFAGFIKM_02004"

/inference="ab initio prediction:Prodigal:002006"

/codon_start=1

/transl_table=11

/product="hypothetical protein"

/translation="MKKFFLSCIAMLLFLIPFSPATSAASGDRLIYDIMYNVAGGGLV

SNNNRFSFQFDNYGDAQIWDVKSRKMIWHSNTKDTRIHKLQINYFSGKLVISDPQNTP

YWTSDNLAWAKAWYGADNVPANLRGNVLIMQTDGNLVLYNNENPALGWYPVWASNTGG

Q"

CDS complement(2344220..2345392)

/gene="pbuE_2"

/locus_tag="EFAGFIKM_02005"

/inference="ab initio prediction:Prodigal:002006"

/inference="similar to AA sequence:UniProtKB:Q797E3"

/codon_start=1

/transl_table=11

/product="Purine efflux pump PbuE"

/db_xref="COG:COG2814"

/translation="MKNKWIIYILALAVFLIGTLEYIITGVIEMIASDLSVSTSEVGL

LVTVFALAAAIIAPILIAFTINIDRKKLLVSTLSVFIASNGLMLVDLSYETLLWVRII

QGASGGVATVVAMAVSTRLVEKEKRGNAIGIILMGLSSSLVLGVPLGTFFSEMFGWRV

LFVFIGLLSVLPLLIIYKKVPAIKEEEKITLRMQLSILKNPLILTALLITLLYIGGYS

TLFTYITPFLQATSSLSMTEISGVLFLAGICSFVGSKVGGQLADAKGSKFTICLGLLL

QGVTLLLFALAGVNLIMLILVLMIFMLATWSISPAQQLYLVTLAPRNPDIALSVNTSF

IQFGFALGSGLGGLVISRTSVMYLNWLGFGAVGIALLLAILLFKKSRSRTGTPDPV"

CDS complement(2345417..2346235)

/locus_tag="EFAGFIKM_02006"

/inference="ab initio prediction:Prodigal:002006"

/codon_start=1

/transl_table=11

/product="hypothetical protein"

/translation="MLFKISAFSRLSRVSLKTLRYYDQIGILKPRKVDHDTGYRYYSA

DQLLELNRIFIYKELGFTLPQITQLLQEHITLENIQGMFKLKRSEIQQIIDTEQAKLV

RIEERMQLIEEEGHVETGQEIRIKEEGARQFLFQTGCGREEEIPSLFRQFDQSVTKEM

RQLIDGPQVVLWKEIAGQEEEFEFEIGYFCCDLRSPPDPFQLRTLPAEPMMATFAYRS

NATFACKACVHLATWIENNNYQIKENGAGRELYLPLSQEQDVQFIEIQIPILNR"

CDS complement(2346481..2346900)

/gene="arsC"

/locus_tag="EFAGFIKM_02007"

/EC_number="1.20.4.4"

/inference="ab initio prediction:Prodigal:002006"

/inference="similar to AA sequence:UniProtKB:P45947"

/codon_start=1

/transl_table=11

/product="Arsenate reductase"

/db_xref="COG:COG0394"

/translation="MSKKTLYFLCTGNSCRSQMAEGWAKKYLSEDWNIYSAGIEAHGL

NPKAVEAMNEVALDISTQTSDIIDSELLNNADFVVTLCGDAADKCPMTPPHVKREHWG

FDDPAKAQGSDEEKWAVFQRVRDQIGERIKTFAETGK"

CDS complement(2346926..2348221)

/gene="arsB_1"

/locus_tag="EFAGFIKM_02008"

/inference="ab initio prediction:Prodigal:002006"

/inference="similar to AA sequence:UniProtKB:P0AB93"

/codon_start=1

/transl_table=11

/product="Arsenical pump membrane protein"

/db_xref="COG:COG1055"

/translation="MFSTLLACIIFLITLVLVIWQPKNLSIGWSACGGAILALLVGVV

NFEDVWDVTQIVWNATLAFVAIILISLILDRIGFFEWAALHMAKAAHGNGVRMFVYVS

ILGAVVSAFFANDGGALILTPIVLAMVRALNFDEKKVFPFIIASGFIADTTSLPLVVS

NLVNIVSADFFGITFMEYAGRMFVPNLFSLAASIGVLYLFFRKSIPKRFDASELKPPL

SAIKDMKMFRLSWVVLGVLLIGYFTSEFIGIPVSFVAGIVAIFFLIMASRSSAVPTTL

VLKEAPWAIVFFSIGMYVVVYGLRNVGLTDLLAVVIQAVADQRLFAATMGMGFIAAII

SSLMNNMPTVLLDALAIDATTATGTVREALIYANIIGSDLGPKITPIGSLATLLWLHV

LSTKGVKISWGTYFKTGIMITVPTLLITLVGLYLWLSIL"

CDS complement(2348295..2348621)

/locus_tag="EFAGFIKM_02009"

/inference="ab initio prediction:Prodigal:002006"

/codon_start=1

/transl_table=11

/product="hypothetical protein"

/translation="MDSMQEAADKLKLLGDKTRLTILTLLKEREWCVCEFVEILDMSQ

PGISQHLRKLKDQGLVKENKRGQWVYYSLNVENTPYITSVLELMPDTATILKSLNKEN

FTSVCN"

CDS complement(2348764..2351232)

/gene="cdr"

/locus_tag="EFAGFIKM_02010"

/EC_number="1.8.1.14"

/inference="ab initio prediction:Prodigal:002006"

/inference="protein motif:HAMAP:MF_01608"

/codon_start=1

/transl_table=11

/product="Coenzyme A disulfide reductase"

/translation="MSKKVLIVGGVAGGASAAARLRRLDEHAEIIIFEKGPYISFANC

GLPYYIGGSIDDRERLLVQTPKGMADRFRIDVRLRSEVVAIDSQKRVVKIQSQERGAY

EESYDELILSPGAKPIIPDLPGKDNPLIYTVRSIPDIDRIKDQISSSNNQYAIVIGGG

FIGVEMAENLKEAGLDVTLIEGNAQVLTPYDTELAAALAQEMENHGVNLLFSKRVQGF

HSLEQGIGVELADGHVLTADMVILAIGVTPDTYFLKDSGVSLGARGHIIVNEALESSV

PHIYAVGDAIEVTETIHGTKATIPLAGPANKQGRIVADRIAGLPSTYKGTQGTSIIKV

FGMTAATTGSNEKTLQRLGVDYQTVIVHPASHASYYPGSSAITLKLLFTPQGKILGAQ

AVGYDGVDKRIDDIAVAIHFGGHVRDLTELELSYAPPYSSAKDPVNMAGYAAENMITG

RVQTFTYNQLADRQPGQSILLDVRSEIEHQNGHIPDSLSIPVDELRQRLDELDPSKEI

WLYCQVGLRGYTASQILRQHGFSVKNLSGGYKTYRQAQFKPAPFTTEKQDHHDPAVDV

KEKKSIPTDSTQPQRIDHELNVCGLSCPGPLIQVKQKMDQLSNGETLRVKASDPGFYE

DVKAWATMSGSTILQLERMKGGTIESVIVKNTAQPVSDSPISDPASTMVVFSGDLDKA

IASLIIANGAAASGRKVTLFFTFWGLSIIRKPQPQKLSKTMIGRMFDMMLPRGSQKLG

MSKMNMLGAGPKMIRGLMKKHHVPSLEELIESAIAQGVEIVACQMSMDLMGIQREELI

EEVKIGGVGYYLGQASQANHNLFI"

CDS complement(2351229..2351543)

/gene="pagR"

/locus_tag="EFAGFIKM_02011"

/inference="ab initio prediction:Prodigal:002006"

/inference="similar to AA sequence:UniProtKB:O31178"

/codon_start=1

/transl_table=11

/product="Transcriptional repressor PagR"

/translation="MNTPAFDSNNIKQFDEPANLLKALSHPIRLCIVRGLMMKKKCNV

SYMQECLDLPQSTVSQHLQKLRSAGIVATERNGLEVNYVLADQRVEQIIKTLFEKDDC

ES"

CDS complement(2351776..2353590)

/locus_tag="EFAGFIKM_02012"

/inference="ab initio prediction:Prodigal:002006"

/codon_start=1

/transl_table=11

/product="hypothetical protein"

/translation="MTKTTPNYDGYLFVHFIGEQPDGEQVYFSYSEDGLHWKDLNGGS

PVLFSDLGEKGVRDPFLVRSVKDKNFYLIATDLRIASGKGWTHAVNAGSRDVIVWESS

DLLNWSSPWNVTLGVEGAGCVWAPEAVYDEMADEFLVFWASATQEPQEQERKQKIYSA

RTKDFRTFSASEKYIERDNHIIDTTILPADGSYYRYSKDETTKNIRVEKGDSLDKGAF

VTLQAPVLEAMAGVEGPQIFKFNDREEWCLIVDRFAEGKGYLPLLTTDLGSGDFRIVP

DGEFDMGSTQKRHGSVLPITAEECSLLLAAFGDGHQVLPGQYADPDIAQFGDRYYMYP

TTDGFEGWSGTQFKVFSSSDLKHWQDEGVILDLGTEDVTWATGNAWAPAIASRNGKFY

FYFCGKMLNGESAIGVAVADTPIGPFLAESQPLITMEQLKRLGITMGQAIDPSIYVEE

DGRTYLLFGNGHAAIVELGEDMISVVEDTMSNLAGLHDFREAVAVLKQGGLYHFTWSC

DDTGSEDYHVNYGTAEQLYGPITYRYPILSKNVEKGMLGTGHHCIFNENETGQLRIAY

HRFVTPLSRFSSGKGYHREICMEPILFGKDGLIQPVIL"

CDS 2353947..2355305

/gene="phoB"

/locus_tag="EFAGFIKM_02013"

/EC_number="3.1.3.1"

/inference="ab initio prediction:Prodigal:002006"

/inference="similar to AA sequence:UniProtKB:P19405"

/codon_start=1

/transl_table=11

/product="Alkaline phosphatase 3"

/db_xref="COG:COG1785"

/translation="MKLSKRVLPAVALTLAVSASTLLSAGQASAVQAVSVEKKQIKNI

IFLIGDGMGTSYTSAYRYMKDDPSTKGMDKTVFDPYLVGAQMTYPDDDKQNVTDSASA

ATAMSAGVKTYNAAIAVDPEQKEVKTVLEQAKENGKSTGLVATSEITHATPAAFGAHD

ISRKNMDAIADDYYDELINGKHKVDVLLGGGKSNFVREGRDLTKEFQKAGFSYVTDRS

SLLADKNQQVLGLFADGGLDKLIDRTAATPSLAEMTNTAIDRLSSNDKGFFLMVEGSQ

IDWAGHDNDIVGAMSEMEDFAAAFQAAIDFAKKDGETLVVATADHSTGGLTLGKDGEY

NFFVDPIKAALRTPDFMASKIAKGASVEETLKSYLKLELKPEDIQSVNEAAKSADVTK

IDNAIEAIIDNRSFTGWTTGGHTGEDVPVYAYGPASHRFAGLIDNTDNAKIIFDILSK

HQ"

CDS 2355777..2356217

/locus_tag="EFAGFIKM_02014"

/inference="ab initio prediction:Prodigal:002006"

/codon_start=1

/transl_table=11

/product="hypothetical protein"

/translation="MLGNILLGMVIPIILGVWILRRNFTILILYYPLGVAISASINNI

GFNYFWNILPNTHNQSLAALPMDLGVYPIAGCLMMYAIIVKGAGPWLAILVASLSLTV

IEWFAKMMGHVIYFNGWNIIGTFLSYLLPFFLAYGYSKLLRYTK"

CDS 2356324..2356794

/locus_tag="EFAGFIKM_02015"

/inference="ab initio prediction:Prodigal:002006"

/codon_start=1

/transl_table=11

/product="hypothetical protein"

/translation="MYVAYPYGYRYTFTPVWATSSVEALELIKTAVQGERNDEMFYDS

LIQLSPDANQATIITSIRNDERGHNQMFRQMYKDLTGQEISGVNSEPVETVTSYLGGL

QKAFQGELAAVEKYRKIWFGLPYGIYKDTVWGIILDEQKHADKYNNLITYNLPR"

CDS complement(2356922..2357809)

/gene="mneS_1"

/locus_tag="EFAGFIKM_02016"

/inference="ab initio prediction:Prodigal:002006"

/inference="similar to AA sequence:UniProtKB:P46348"

/codon_start=1

/transl_table=11

/product="Manganese efflux system protein MneS"

/db_xref="COG:COG0053"

/translation="MDQLKYDNLKLGERGAIISIIAYICLTVLKLIVGNMAGSEALKA

DGLNNATDIVASIAVLIGLKLAQRPADTDHTYGHWRAETVASLVASFIMMAVGLQVLF

QAIGSVFQGKHESPDIIAGYTGIFCAVIMYLVYRYNKRLATRIKSQAVMAAARDNISD

AWVSTGTVIGIAGSQFGLPWLDPVTAVIVGFLICKTAWDIFKEATHHLTDGFDVELIQ

EYKKTIAGIDSVETVKDVRARNYGNNVVVDVVITVDAELDLQQAHDICTDVENELIEE

HDVYTVHVHVEPDTLEECI"

CDS complement(2358148..2363217)

/locus_tag="EFAGFIKM_02017"

/inference="ab initio prediction:Prodigal:002006"

/codon_start=1

/transl_table=11

/product="hypothetical protein"

/translation="MNCMVKRKLFFFIVFMLTVVVPSSLIGTRTAYAETLPYQTIIID

DGSIKINDVVTLDAGNENNGYVAPNWTTSTGVKGYDLSSTKYTSTAGRTITWNPRLEA

GTAKISFYKLDWADKADSNVKIEIVHNGTTDVVYMDLRPSSGAPAGWVDLGEYYFSGV

GEEFVQLTRSTGTTNTILTRADAVKFEGNITQKEPHKTIIIDDGSLTIDHVVTVDSGN

VSNGFSAPYWTTSAGVKGYNNSSSKFTDAVGRSITWNPRLEAGTARISFYKLDWADKA

DSNVKIEIVHNGITDVKFMDLRPSSGPSVGWVDLGEYEFSGDDSEFVRLTRTQPSTGT

IITRADAVKFEGNIRQQAPPLSPLRSRTLANLSYTEKGSIENDNYKATFYEAAWDGGK

SIVRDMFYKNTETGNWVPINNESERLEEQWVLLDGNAGSRTNYYDTMNKRWITFDGVH

FPDSQTAVLTDSTHGSDYDFEVNWSMAGDKPDVSFAFTPRRDGNYVIGYQSFTTEAVS

GINEVLNGFRSHAKMVGTVESTSLRELSAPMSLVEKNDGSGNPLTYGVFVPSAELPVE

FEPTGGVTKQRLGMSLVNNEGSVQPILYAPQLGTYSQMTAESTYQFHMGLIAQKSNLY
ESYADILRNEYDYSAYRENVSESLTDAMFNMIDLLKIEPQGDDSVNYVPSPSGWWSRA
KGFIDIENEDSVRTSSNAVLLGAYYLTGDDQLYDTRALPSVQYGVSRNGIGWSPTQKK
VYGVPSLWKMATLPFDVSSVTAVHQMMGTTAGIGALAQEEYLVRDPDQKDRGPVIQPL
MMYRMTGEAQYLQAAKDAADSYITQHIDTPASIDVSKNEFIYYYSKLWMEILELYEET
QDPKYLNAAYKEAKRYATMFVARPVPEGNVTIPQPETYNYAESFHWPESGKFQYPRLK
LPEDIAGGVQAERWLVSPSGLTFEAGSTTGYYRMNAQEAPFMLRLSLYTGDELLQDIA
HNAVIGRYSSYPGYYYKGFAVSQLEPDFPLEGPSGATSIYYHHIPGQLGQTMDYLISE
QSLKSNGSITFPSVFETNFLWFKYHLYGNKPGQFYGNSDVWLWMPKGIIKTNHPQLNW
ITGESRDKFYIGLSNASSEEVQTPIELNEHIIGFNPAQDYTVTIVRDNGTPEQAVMSG
GIIQATVSGKGITAIIVEGLNINVPLHQVRTADTSDASYFFDIHSPIDAVKGMLMVKP
DETVYDAYVQAKTTKPATIHYSLDGGATYTTIPDTIYPMEWSIRVNDLSQTFTYYVES
EGKQTRKRTLYLPDQVSETPLQPDGQDSSTIIVDNTEAETEGVWIRDTTANDYYYDNY
VYAKSTTGTATSKMRWRPELPESVTYSVYYKIPQITTASENWATNASFTVYYSGGSDT
VTVDATTANGTWVHLGDYPFAAGDSGYVELTNKANKSRVVADAIMWVDPNRVPQLESA
VILSDRNELQMTQTAQLTVTGYLDNGLVGDLTQADVQYFVDRTDLAEVNSSGLLTLLN
LDGNTDHIEVWATVTIDGVTLTTPPLNISIRDLTVIVDSTNTTGLYTTEGSWSQSNLA
GYKIGVKSRYSTVQGSSATWKGQFPEGKYTVSIYKLVHTTANDNNVKVEVKHKTGTEI
TYIDATVGSSGWVNLGTFDFTGDGSEYVRLTRVTPTTVDPPTLPADMIYTRVDAVMFE
RHSDDSELLANEVQGEPTLSD"

CDS complement(2363480..2364688)

/locus_tag="EFAGFIKM_02018"
/inference="ab initio prediction:Prodigal:002006"
/codon_start=1
/transl_table=11
/product="hypothetical protein"
/translation="MKEFFPRRGLPNVIQKLENGETVTIVYFGGSNTRSKGYRVMTAD
WLRGQYPNADIRSVNAGIDGTGSDLGCARLETDVLSHQPDLVFVEFVGNDGGVPESKA
RIEGIVRQIRKRSRFTDILFVYTVKERDLISFQSGEYQKSALMQEEVADYYGIPSIHL
GVAVSQLVSDGKLIFTSRADVSIPGAVIFTHDSIHPTIPKGHQIYTDTITRSFEKIRE
LRDHVGKVEHHLPQDPLVPSNPWEYATMLPLDRLTHFSAGWSYMTPDDFPLVREYDWL
FPGLWRAVDAGETITVEFEGTHIGLFDIGGPDSGRLKVSVDGGEPFLVDRFTPYNDHN

RNQYVFLPELPNGKHTVRFEIGHEKTDKAVVFEASGNERGMEHVRQHPAWYDQTVIQL

GKLLLVQPPL"

CDS complement(2364705..2366921)

/locus_tag="EFAGFIKM_02019"

/inference="ab initio prediction:Prodigal:002006"

/codon_start=1

/transl_table=11

/product="hypothetical protein"

/translation="MELMKADVTVIGGGIAGICAAIAAARQGLLVSLINDRPVLGGNA

SSEVRVHINGSAYLGNSPSYYAREGGLVEELKLKIFHCNPLYNKKLMLSLSDTVLLDM

VYDEPNISLFLNTCVHETGMENGRIQWVEGLQLASERKFRFESPTYIDCSGDGIVGYQ

AGAHFRWGREAKHEYQEELAPEVADHYTMGDTILFQARDVDYSVPYKRPGFAYDITKL

EFFDSIRKGLNHRSFPRKINGLGGLWWLEYGGHMDIIKNNEDIALELRKLVYGIWDYI

KNSGEFDDVDNLILDYVCPIPGKRESRRFIGEHMLSQNDLTTKTHFEDAVSVGGWYMD

LHANKGIYDEGPATAWNFVPGLYNIPFRSLFSRNIPNLMFAGRNISATHVAFGSTRVM

ATCGCMGQAVGTAAALCAKYETDPAAIVKAHMGELQAQLLRDGQTIVGLQEPLDPYFA

DGLTIRASSQRSYEHLRPTEEASLDKALCLVLPIQTSVVESVQIKIKNSSEHSETLQV

KLYGGDRKENYIPTSELKGYNLVISAGHDDWITLDLSCKKPEDDKIYIVLEGTADLTV

HSNEEKMTGAVSFLYRPEEPARLKKLNKSICFKDLIPFQDMYNPANVVNGFSRPYGLP

NSWISERTEGQEWLEFGFASPKNLDEIHLVFNSQLNLEHFDDPIEPLIQDYDVTLTLE

DGTESVTKVRGNYLSLNKHQVHAQAVTQIRFDFCATYGSPYYEVFAVKLFAPNNAK"

CDS complement(2367033..2368643)

/gene="cheB_5"

/locus_tag="EFAGFIKM_02020"

/EC_number="3.5.1.44"

/inference="ab initio prediction:Prodigal:002006"

/inference="protein motif:HAMAP:MF_00099"

/codon_start=1

/transl_table=11

/product="Protein-glutamate

methylesterase/protein-glutamine glutaminase"

/translation="MGRDYTCFIVDDEDLIIQRLELFFNELSHRDRRFVLVGKANNGL

NGIEEIIKLKPDIVISDIVMPRMDGISMIEQLKAELPHTQYILLTAYSSFEYAQRAIQ

ANVLEYIVKVPLREADLNRALDKAAGILNEFEKKEAEFHSLNVSVLENKYRVRKQFFN

ELIRGEIPSHRASDFANRMQFHFFQANYCCFIVEMNTYESFRNEYAATDQNILKYAIT

NIIEETVMNGSSGVAADLSDNRFIGFLSWENNRSDMETEYACLSLGGQIISHLHQYLN

KRVSVAFGSPHRGWESIKQAYTEAKNVSEDFYYHTEKVVKTPMHRFQYHNDKKADFQQ

KLADFLIRLKRKISKEELDNALADLSQFVTDHKIHKSIMVPMIRDLYRDITLKFKSGN

KMATEVPDFPMEFMAFQEQLAYIGDFTFEYVHAGQLLHREEIMSAMHYIETNLKQRLT

LEAIAEEVNLAPSYFSSLFKKTMNEGVISYINRKKIHLALELLNGRDYSLLELCEEVG

IVNEGYFCKLFKEYTGDTPKQYRIKMTR"

CDS complement(2368643..2370382)

/locus_tag="EFAGFIKM_02021"

/inference="ab initio prediction:Prodigal:002006"

/codon_start=1

/transl_table=11

/product="hypothetical protein"

/translation="MRRRISLPLKLFFIVFAFVLGCIILISQLSYRYVQKEIRTNDIF

YTNQILDKVDQYFTVNFSSFQTILFSVETSVKANINNTEVIKKQLRELYELNSIYVSN

IYLIKSDLSILGGSTPTRIFDEPLSERAPLFDAADKNRRTTFVSEPYKSKYSGWTVTM

VRYLNGAPFPMAIAVDLDLNAIEETLFKINKQEQMNLALITASGKIIAGFSENKGPLS

IQDHTFSIGETSAEEILDTSETTLQVHTKDGTPVSLLKKPTEKFNWTIVSVNDESRLK

AALSRLETYYIELLAAGLLLSLFISFLVAKYIRKPLYTLKTKMKQVEQGILTTTVTIN

RNDEFGDLSRAFDRMLQQIVELIRRAELHNELERKLEIQVLQSQINPHFLYNTLGSIS

NVIRLGQIEKVDVVIGSLISLLEYGIDDASEKVSLRQELRNVADYIEIQNIRYNRNFH

LIEDIEAGLMDFPVFRMLLQPLVENSIFHGYNGGGIEGPITIHAYREDGIVIIEVVDQ

GEGIPADKIKHILISEPSEEEVKRKRIGLNNIHGRIRLHYGDQFGLDIISIPKEITRI

RAVFPAELPKGDA"

CDS complement(2370475..2372061)

/locus_tag="EFAGFIKM_02022"

/inference="ab initio prediction:Prodigal:002006"

/codon_start=1

/transl_table=11

/product="hypothetical protein"

/translation="MQRKKISFAILSAILGLGTLLSGCGGNEEVTSTTSSNSQGQSGQ
FATKMKISMFNQGTFNAAAPVPPRDEDIQRQMLEEEMNIDLEMMIPQAGQATTKLNTL
IAGGDIPDLIFLKSRADLAQYYDQGVLADLTPYMDQFPELQKRFGNDSWEAMSYQGKT
IGVPGYDNVNGISRSFFIRNDWLKKLNMEVPTTPDELFEVMKAFTEKDPDGNGKNDTY
GFIGGMNKEGNLQTYGFDSLMWMFGVNPPSAVEIKDNEPVFLFIDPKMKEALAYINKM
MAAKVVDPDWVTMNSPDLLDQKMFKGRVGFMIRDARRLEPDYQQKMKEISGEVPEWIV
IPPMKGPYGDQIVERKSFQGNSWAISAKVDEDKIIRILSMLNYLFTDEEAYPNFAYGI
KGIHWDVVDGKIKNKTSELSKEMKEKYLWVDHYRMPRRGDDAEYFSFQNPKTAEAFKD
NQQYVGPTLPGNLLTPDPSDTLDADRTRFINESLVKFMTGKEPLSNWDNFLQTLDTKF
DMQKYKETAIQQFKEAGLIK"

CDS complement(2372127..2372996)

/gene="dasC_2"

/locus_tag="EFAGFIKM_02023"

/inference="ab initio prediction:Prodigal:002006"

/inference="similar to AA sequence:UniProtKB:Q9K489"

/codon_start=1

/transl_table=11

/product="Diacetylchitobiose uptake system permease
protein DasC"

/db_xref="COG:COG0395"

/translation="MIHMTIGEKVWQTVVYVILILLSLLCVLPFLYVVAVSVTPESEV
LRRGIVIIPESFTFLAYKEVFISHGIWQAYKITLFRTIVGTALNVFFTVIAAYPLSKK
YLPGRSPFLLFIVFTMMFSGGLIPTYLLIRSLGLLNSPWVLIIPNLISAFNLVIIKGF
FEQLPGEIEESARVDGASELQTLWRIILPLSLPVLSTISLFYAVGHWNSYFDAIVYIN
DSNYMPLQVILRNILLNVATQSADSLANSGAVSTFAVQMATVVVTTVPILIVYPFLQK
HFTKGVLLGSVKG"

CDS complement(2373010..2373957)

/gene="yteP_12"

/locus_tag="EFAGFIKM_02024"

/inference="ab initio prediction:Prodigal:002006"

/inference="similar to AA sequence:UniProtKB:C0SPB3"

/codon_start=1

/transl_table=11

/product="putative multiple-sugar transport system

permease YteP"

/db_xref="COG:COG4209"

/translation="MQAKLAADAIPISKKRKTWIRTIRRHKVMYALLLPALIYFAVFK

YIPMAGIMIAFKNYNLALGLWDSPWVGFKNFTDFMNGVYFWDIMKNTIVISLYKLLFG

FSAPIVLALLLNEVYSQWFKKIVQTITYLPHFLSWVIVYGIMVALLAPGDGLFNMILK

DFGVEPISFLTEPAWGRMLIILSEVWKDIGWGAILYLAALAGIDPSLYEAARMDGASK

WRQLWHITLPGIRGVIILMLILKLSHILDAGFDQIFMFANTFNQEKIDIIDTWVYREG

LERLKIGLATAVGLFKAVIGFALVLAANKLAKKFDGQIW"

CDS complement(2374157..2374912)

/gene="COQ5_2"

/locus_tag="EFAGFIKM_02025"

/EC_number="2.1.1.163"

/inference="ab initio prediction:Prodigal:002006"

/inference="protein motif:HAMAP:MF_01813"

/codon_start=1

/transl_table=11

/product="2-methoxy-6-polyprenyl-1,4-benzoquinol

methylase, mitochondrial"

/translation="MNNRERFSSRVDSYLKYRPSYPKEVIDYLYDVVGLRVNSNIADI

GSGTGIISKLLLERGSYVIAVEPNQAMREAAEQMLESNPKFQSTSGSAESTGLPDQSV

DFIVCAQAFHWFDRSAAQIEFRRILQPGGKVILIWNSRLTNGTPFREEYNQLLHTYGT

DYEKVNHKNISQAILLSFFKEGSMHEMRFKMSQEFDFEGLKGRLLSSSYSPVPGHANY

DPMMTELRNLFDKNNQNGLVEFDYETEIFWGEV"

CDS complement(2374933..2377218)

/gene="secA_1"

/locus_tag="EFAGFIKM_02026"

/inference="ab initio prediction:Prodigal:002006"

/inference="similar to AA sequence:UniProtKB:P28366"

/codon_start=1

/transl_table=11

/product="Protein translocase subunit SecA"

/db_xref="COG:COG0653"

/translation="MNVVVKLIREFKERDTRRLLKNYRDRVELIRKRNLETWDDQRLQ

AESLRLQEEARSGTPLDELLVDAYALVCEAAKRTLGLKPYDVQIMAAIALHERFLIEQ

HTGEGKTLSAVMPVYLNALTGKGVHVLTFNDYLANRDAAWMGPIYRFLGLTVSSVQAG

MSLFEKREAYAKDITYVTAKEAGFDYLRDTIAFNEADTVHRPFHYVIVDEADSLLLDE

ARVPLVISGDSSASKSDGVLFAEVARQLQPGEHYDFDEFQRNVYLNEAGAAKAELLLG

CGNLYDSHNSHWLTSLNCALHVESLLKKDVDYIVRDGVIELIEEHTGRVAENRYLPDG

LQAALVSKEGLQWKAGGRILGTITIQHFISLYSGICGMTATAHASAMEFEDIYALQVV

QIPPNKPNIRIDHKHRIYTHKEAKYKALVQEISSVHRVGRPILIGTSSVEESDMLAEA

LAIAGVPCQVLNAKNDAKEAEIIAKAGEIGAVTVSTNMAGRGVDIRLGGGNPAQAEVV

AKLSGLYVIGTYVNESMRIDDQLRGRCGRQGDPGSSVFYISLEDELMLRFGIHKLFRA

PNQDEVLDDPALHSKIEHIQRVIIGQNFDIQRELNCYSDMVEDQRRILYEERFGILKA

ERLMSPSERRVRLFYIDEFWADHLAYVSYIRESIHLESITSRNPIDEFHTQITQSFEK

IPAKIDHESANMLRKLGGSNDPEIWEQFGLKSPISTRTYMINDQYSQDKRSSWTGTTV

FAFWGSKILKLLLKPVYRMSK"

CDS complement(2377336..2379636)

/locus_tag="EFAGFIKM_02027"

/inference="ab initio prediction:Prodigal:002006"

/codon_start=1

/transl_table=11

/product="hypothetical protein"

/translation="MNMKKKLSIFTALAVFQAFAVGSVSAQSAPQGDTASVNTANVES

VEVLKQEQPIAEREVKTESNNKFINTPPTEAKNTQTSTGTEATPKTESEKAALDGGET

TTNETGTKPVVQEETPEKVETPATPAQIEQPSVDGTSPAVAGGNLTLYMNSNKMEQDG

KTYLAGQPMAIKNGVSYVAIRALVDRVGYGVKYDNKTKETIIISGEDELRFKTNSKDY

TVNGETRTMKGPAYQQKSTFMVPLTSITQALNITYKVNQSAKTVVLNLNTKPVASFTI

SQKEIFAGDQVDYVTSSSSPNGMDIVDERWTGRQDSFDQAGTYTISYQVQDSNGQWSD

PYSTTIEVLAPNLPPVAMFATDKEQYKMGEKITYTDQSTDDENNIVKAVWENNSLAFF

EPGPKTVTLTVTDNHGATNTYSKVITITNETLYSFTDFNLLFTPVGQKFTFNGGEVTT

MEKVPYTYMDEPSLLIRSNSPETVNTEGIVYKESSSGQTRFMIHHVNNTGKRVKMYVI

ATNNNPNPAVFEQQNMGFAGPTPYATVAGKLSIDKWFKSIQTGADQKKEYLQPGESKL

ILTELNKTPMKEGQVISLYSDAYSDYSLDYNVILVEENKDPFEALPLLPVLDRDGVHN
RGTYPNATRIIKYDEHVGTKPARLPIGDNSSDPNLVGTDPMAYTDASNAGNFGVLYKI
TLTNVAPRTLISFNPRGGKYSGVALVNNQLVAIAEGNVAVSNSSEQSVLYRTGLTGES
VTILFSAAPGSNLPVNLLFTPLPSEK"

CDS complement(2380212..2381651)
/gene="clsA_1"
/locus_tag="EFAGFIKM_02028"
/EC_number="2.7.8.-"
/inference="ab initio prediction:Prodigal:002006"
/inference="similar to AA sequence:UniProtKB:P71040"
/codon_start=1
/transl_table=11
/product="Major cardiolipin synthase ClsA"
/db_xref="COG:COG1502"
/translation="MVTLILSLVMIINILLSAVFLFFERRDIGYTWAWLMIFYFIPIL
GFIVYLFLGRNLKKKNFYGLSAEERNSLKLIVENQLVTLKEQRKENNPLLMKYADLIQ
LNLTSSNAILTNDNEIFIFDDGQEKFDSLFADIKEAKKEINIQYYIIQPDSLGKKLRD
ELTIKAKEGVKVRVLYDEIGSKKLSLKFFRELISAGGEVEVFFPSLIRPLNFRMNNRN
HRKLCIIDGEIAYIGGFNLGNEYLGMDKKFGYWRDTHFKMHGDAVNHIQGRFILDWKH
ASKSDQVSYEQFSFNTARLDGSSPVQIVSSGPNSQVEHLKNMYIKLIVNAKKSVYIQT
PYFIPDGTFMDACKIALLSGVDLRIMIPNKPDHPFVYWATWAYAGELLDYGAKILLYD
NGFLHAKTIVVDEEVASVGTMNIDSRSFRLNFEVNAIVYDERVAKQLQELFHQDSQLS
TELTSDRYRNRSLIIKFKEGISRLLSPIL"

CDS complement(2381863..2382207)
/locus_tag="EFAGFIKM_02029"
/inference="ab initio prediction:Prodigal:002006"
/codon_start=1
/transl_table=11
/product="hypothetical protein"
/translation="MAQIGNCNNTVSPAVVNDAVCTTISLSDTGGTPTIIYENSSTFL
VNGTILIENNGVGASPTAALLIDGVQIPGFVVAPGSSRSYTAPTINSISVIGAGAGTT
SIKIAFSLNYRF"

CDS complement(2382292..2382639)

/locus_tag="EFAGFIKM_02030"

/inference="ab initio prediction:Prodigal:002006"

/codon_start=1

/transl_table=11

/product="hypothetical protein"

/translation="MNTRLAETTICGEFHINSATITEVLTLWSASSTLNVHGTVSIYN

HKQSNSSVFLNIIHNEGTCSLPVLPGNTCAFSCAGMHHIEVEIEKSLNNIASGTFGID

IYYQVLNEEEDEE"

CDS complement(2382781..2383476)

/locus_tag="EFAGFIKM_02031"

/inference="ab initio prediction:Prodigal:002006"

/inference="similar to AA sequence:ISfinder:ISBth8"

/codon_start=1

/transl_table=11

/product="IS3 family transposase ISBth8"

/translation="MMAIHFENREFGYPRMTTALWEAGLNVNHKKVWRIMRELSIQSV

IRKKRKKSSYTPSVIYPNRLKRQFHATAPQQKMVTDITYISDGNSFVYLSVIQDLFNN

EIVAWQLSKRNDVQLVLDTVEQWTQKRDVSEAVLHSDQGFQYTSQAYNTRLEAFGVKG

SHSRKATCLDNACIESFFSHLKTEKLYLNQCNSEAEIRQAVEDYMYNYNYRRFQAKLK

QRAPIEYRCALAA"

CDS complement(2383641..2383961)

/locus_tag="EFAGFIKM_02032"

/inference="ab initio prediction:Prodigal:002006"

/codon_start=1

/transl_table=11

/product="hypothetical protein"

/translation="MAKKGQTFQTYTEEFKLNAVRSYVEGSSSYKVVAEREGIRNCSQ

LKVWVKKWKKGEAFDERKNSVPNPLKGRPRTAFGSVEEERDYLQAQVDYLKKRYPNLV

KEKR"

CDS complement(2384123..2384602)

/locus_tag="EFAGFIKM_02033"

/inference="ab initio prediction:Prodigal:002006"

/codon_start=1

/transl_table=11

/product="hypothetical protein"

/translation="MSNYHFEEKESFIVLGIGTDLKSDYTDYVGISKEKADFWSTVKE

DGSLDKLKSLATNDYIFAVNEAVNNQMMHYAGVMTEESLPEATRVIQFPKGEYLVVKG

EAETADALSNMLTGIAFGQVLPVEKDYAYVGGPNTTVEMGERNGVVYGEMWIPVVRN"

CDS 2384708..2385682

/locus_tag="EFAGFIKM_02034"

/inference="ab initio prediction:Prodigal:002006"

/codon_start=1

/transl_table=11

/product="hypothetical protein"

/translation="MKKVERINIITRYINNRAHFTISEIMREFNISRSTAIRDIREIE

AMGMPLVAEVGRDGGYFVMNNSLLPTVRFTDNEIKALFIAFMATRNQQLPYLKSRQSL

AEKLLGLISENQQDDLVHLNQILLFEGTNPHNPDLLDLSDLPHPTLEKLIQILLIERY

LLVTVQEENKTKSYPIVLLHLHHQKGHWIIEGFDLEEEKRRIVSVDNLIHVEAYPAKK

RLSTKKIVEQLKRNTQAEVINLVLELGPKAIAQFKKYHPLKFSIAYTNPYQTTALLKA

FMNVHNSEELTEITNWLLFLGEDIKVKEIPKEALNAVQERLSLYQMKQ"

CDS complement(2385854..2387083)

/gene="bcr_1"

/locus_tag="EFAGFIKM_02035"

/inference="ab initio prediction:Prodigal:002006"

/inference="similar to AA sequence:UniProtKB:P28246"

/codon_start=1

/transl_table=11

/product="Bicyclomycin resistance protein"

/translation="MAKSTNWNVFGLAILLGLFSTLGPFTIDMYLPAFPEIAQNMNTT

ASLVQFSLTACLLGLGVGQLVMGPLSDAYGRRRPLLICMAAYIICSLACAFAPNIGLL

ILFRFTQGFAASAGIVISRAIARDLYSGHELTKFFSLLLLVGNLGPLAAPIAGSGVLS

FTTWIGVFICLSFLGIFLLIMTKWSLKETHPVEKRMVPDFKQQLGNYRMLLRDRKFVG

YMLAQGIMTAGVFAYVAGTPFIYQNIYGVTPTVFAILFASNGISLIIGSQIVGRLAKR

IPEQTLLLSGLWLAIIASVAALVVTLVHGPLFALVIPLFFFVCSIGITSTAAFPLAME

SQAKMAGSAAALLGVVPFLLGALVAPLVGIAGEDTAVPLGLTLLMTSIIAIVTYFLFV

KNVPQHTPNHAQSNADF"

CDS complement(2387253..2387885)

/gene="yodC_1"

/locus_tag="EFAGFIKM_02036"

/EC_number="1.-.-.-"

/inference="ab initio prediction:Prodigal:002006"

/inference="similar to AA sequence:UniProtKB:P81102"

/codon_start=1

/transl_table=11

/product="Putative NAD(P)H nitroreductase YodC"

/db_xref="COG:COG0778"

/translation="MNALTKDFSEVLHGRRTIKKYDPNYKISREEIIEMLEDAALAPS

AFNLQPWRFVVIESDQGKEDLLEIAPFNFSQINTSSAVIAVFGDLNFVDSAEEIYKET

ADLGYITQEIKDKMLAMVVPMYNQFSMDKKRESVLLDGGLVSMQFMLTAHAHGYATNP

MSGFNKEKIAAVLGLDPDRYVPILLISIGKAASDARPPVRLPISQITEWK"

CDS complement(2387928..2388521)

/gene="betI_1"

/locus_tag="EFAGFIKM_02037"

/inference="ab initio prediction:Prodigal:002006"

/inference="protein motif:HAMAP:MF_00768"

/codon_start=1

/transl_table=11

/product="HTH-type transcriptional regulator BetI"

/translation="MTRVSKNPQERKNEILTVAMELFNSNGYENTSVSEIVRKVGVSQ

GTFYNYFQSKEDVLNAACERTLASRLEAINHLVENCELNAREKLIRIFMDATPDEQDE

DVFEYLHKESNSTLHQKWIVIEINALIPHIKKIVRQGAEEGEFLAQQPELKAEFLLVG

AAFWLDRGVFTWNEQEYLERKNALNGIIDQLLAVNKQ"

CDS 2388648..2389829

/gene="mdtL"

/locus_tag="EFAGFIKM_02038"

/inference="ab initio prediction:Prodigal:002006"

/inference="protein motif:HAMAP:MF_01530"

/codon_start=1

/transl_table=11

/product="Multidrug resistance protein MdtL"

/translation="MNKRRLLFILGISAAIVSLAQSLYIPLLPDLQQDLNTSLHLVNL

TVTLFTVAMATMQIILGPIVDRNGRKKILVPGIIIYVIATIGCMFSGSIEMLLIFRVI

QGIGASSVPLVAATMIGDVFEGKERGESMATYQMILGISPAIGPLIGGIIGSKFGYTG

TFGFAAISALLILIAAIFMLPETKPVNIKPVGTKLGLKSFGKILGNKTGVVVLLSGFI

MYDVFYTFIVFLPTILQTKYALGAASIGFFSLALMIFNLVGSKMSGSLQAQIGSVRTL

FWAGIMTILSLVIFIFAADYSLIVLLLSLMLTGFVMGLATPVMPTLLSSEFVEERATA

MGVYNFVRYLGMATAPLIGSFLFPLGNIQLLIGITALLVVMVVFVINRLLPVKKAEVS

N"

CDS complement(2389976..2391199)

/gene="tylCV_1"

/locus_tag="EFAGFIKM_02039"

/EC_number="2.4.1.318"

/inference="ab initio prediction:Prodigal:002006"

/inference="similar to AA sequence:UniProtKB:Q9XC67"

/codon_start=1

/transl_table=11

/product="Demethyllactenocin mycarosyltransferase"

/translation="MARVLVVITPAEGHVNPSLGLVTQLIDNGEEVIYVCTEEYRSRI

EQTGAQIITYPFPQDAFSHDPVLKPQEYKHSYQFIYMMVSGIIRRIIPNVLQVVEDQK

FDYMIFDSLMGWGGTILAEKLGIPAVCSIASFAFVEPLGSSSVLNETETKELYEATMK

ITTELAEAFQVSIPAMEEIPAHAGQLKLVYTSRYFQPQAEKLDDRFIFTGPSIITRQD

APTFSFEVLRERYPQTVYIAMGTILNKNLDFYQLCFEALGDLPVNVVLSSGKYTDMTP

LANQVPPNFIVKPYIAQLDMLQHTDVFITHAGMNSTSEALYYNVPMVMIPLTSDQPLV

ANRVQELGAGITLNKHNLSATDLREALTEVLHNSQYRRQAYLIGESLRQAGGYKRAAE

MIMGHMGSERELTKF"

CDS complement(2391270..2392634)

/gene="bmr3_1"

/locus_tag="EFAGFIKM_02040"

/inference="ab initio prediction:Prodigal:002006"

/inference="similar to AA sequence:UniProtKB:P96712"

/codon_start=1

/transl_table=11

/product="Multidrug resistance protein 3"

/translation="MLVLATVAFEGLAITTIAAKMAQSLEGIHLYGWIFSAFLLSQLI

GTLVMGQQVDKRGVFTSMLVSFSVFVLGTVVSAISFDMHMLIAGRALQGFGAGALITC

VYTCVTLHYPDTLRTQILAAFSLAFVLPTLIGPYAAGLIASYISWRYVFWIVLPLIGI

ALSLTFRSFRNLQLQQDLSGPARATDSKIMYAILLAVGTGLLLTGLGMITDWRGILLT

LAGLVIMITPMRKLLPVGTFSVKKGLPATLVSRGLYVACYFTTESFVILALTEVKGLS

ADLAGLIVAAGSLSWSAAAWLQAKLDARDQGRARKGRVMTGIGIMIVGTALVILALIL

TDGGIILILLSQMITGFGVGLANPTTAAIALQHALPRKEGEMSANLQFVDSFYMGVSI

GVGGALIALSETLQWGISTGVLIVLTLQLLWVLLSFLASLRITKLVHQEHHPINQVKD

NISM"

CDS complement(2392831..2393118)

/locus_tag="EFAGFIKM_02041"

/inference="ab initio prediction:Prodigal:002006"

/codon_start=1

/transl_table=11

/product="hypothetical protein"

/translation="MISFVISDQAIDLFKNEWELEEDQYVRIYAKYVGGGSDAFTIGI

NASATPVDPALVQSIGGFHFFVEKTDAWILEDELLQIDCNENGIFSSKISY"

CDS complement(2393140..2393499)

/locus_tag="EFAGFIKM_02042"

/inference="ab initio prediction:Prodigal:002006"

/inference="similar to AA sequence:UniProtKB:O67709"

/codon_start=1

/transl_table=11

/product="hypothetical protein"

/db_xref="COG:COG0316"

/translation="MIIEVSDTASDKIVEILSGADIQNAFLRVGVDEGGCSGLSYTLI

VDEQQAEEDTVLNKKQFKILVHANSIPYIDGLEIDYEESGMLGGFTMNNPNAKVSCGC

GASFRMANYRGEVKKCD"

CDS complement(2393849..2395207)

/gene="ycaD_2"

/locus_tag="EFAGFIKM_02043"

/inference="ab initio prediction:Prodigal:002006"

/inference="protein motif:HAMAP:MF_01149"

/codon_start=1

/transl_table=11

/product="putative MFS-type transporter YcaD"

/translation="MSSNHQSIFSPRYFALSIGIILSVMAVGFEGLSVTTIAPSIAGD

LNGLSLFGWIFSTYLLAQIIGTLVVGRIIDKRGPAAPFTFALLLFIVGLIAAATAGDM

YTMIGSRALQGLGAGAMMTCVYTAISLSYPDELRAKILGAFGTAYVLPSMLGPYVAGV

IADQWSWRFVFWGILPVLVVSALLSLPAFRKLKVQKTGGDSGSSSTWMALLLTLGTGI

FLVGLSRLPSIMGFVLVIIGFVLMIFPLRKLLPKGTLTLRRGMPAILATRGLFFAAYT

STQNFLVLALIDVKGITPSQAGLIVASAALSWCIIAYLQGRWDAADQGSGRHMRIILG

VLLLAIGIAIVFWVPVVTVTIAVIGQIIAGVGIGLAHPISGVVAFSQTGEEGVGQTSA

NLQFADSFTPGVVIGIGGSILVVCQAGGMSLQSGLIVAMGFHLLLIVTSIIASTRISP

RG"

CDS 2395373..2396032

/locus_tag="EFAGFIKM_02044"

/inference="ab initio prediction:Prodigal:002006"

/codon_start=1

/transl_table=11

/product="hypothetical protein"

/translation="MSGNQTKKDASTSTRRAIINLLKERGGMDVMALSTQFSLSGMAI

RQHLNALKGEGLVTTVEEARPMGRPTKLWILTPAANRFFPSGYSDLSVSLINSMKEAF

GTEGLDKLLDVRNKNMQEQYLQHLGDASDVREKLEKLAEIRTNEGYMAEVQEQDDGSL

LLIEKHCPICEAAAVCTGLCKNELHLFKTVLGDNVHIERGEYILAGGRNCVYTVRQNK

S"

CDS 2396233..2396634

/locus_tag="EFAGFIKM_02045"

/inference="ab initio prediction:Prodigal:002006"

/codon_start=1

/transl_table=11

/product="hypothetical protein"

/translation="MSQKIWMNLPVKDVVKSTTFFNEIGFHGENVGNERAQLVIGSTT

ILLFPDATFEKFTGAKTADTSHSAEVIFSIGADSREEVDAFIEKVELAGGTIFGKPGE

IDGWMYGAGFADLDGHRWNLLYMDESKMPKR"

CDS complement(2396664..2397875)

/gene="sotB_1"

/locus_tag="EFAGFIKM_02046"

/inference="ab initio prediction:Prodigal:002006"

/inference="protein motif:HAMAP:MF_00517"

/codon_start=1

/transl_table=11

/product="sugar efflux transporter"

/translation="MLTGISLNPWHMTTKRNHLLVFILTFGVFSILNTEMGVIGILPL

IAEHYEVSISTAGLIVSLFALAVAVSGPILPLLFSGMNRKHVMLFVLGIFIVGSLVSA

ITSNFTVLVIARVVPAFFHPVYCSLAFTVAAASVPKEEAPKAVAKVFVGVSAGMVLGV

PLSSLIATSTSLFYAMLFFAAVTIVAFVATLLFVPSMPVTKRISMGTHLYVLRRSVMW

LSIVGVIFMNGAVFGVYSYLAEFLKTVTRYDWNTASVLLFLYGLANMVGNMIAGKLLA

NFPLRTIKMYPIALITVYILLFTFGQFSVPTAFLIFIWGILGGIGGNITQYWVVSAAP

EAPELSNGIFLTSSNLGTTLGASVCGLIVTGISTQYIVVGGIVFLIFSIVTNYFRISM

TGRFEGFKVGG"

CDS complement(2397832..2398689)

/gene="hcaB_1"

/locus_tag="EFAGFIKM_02047"

/EC_number="1.3.1.87"

/inference="ab initio prediction:Prodigal:002006"

/inference="protein motif:HAMAP:MF_01647"

/codon_start=1

/transl_table=11

/product="3-phenylpropionate-dihydrodiol/cinnamic

acid-dihydrodiol dehydrogenase"
/translation="MKGMIDMDKKKVWFITGTGRGMGTTIAKAALTAGHKVVATGRNP
DAVVRAIGESENVMVVSLDVCEPADAHSAVKAAIERFGAIDVLVNNAAHFCGGYFEEL
TPDQIEHQLQVALVGSMHVTRAILPVMRNQRSGHVISISSGAGISGFEFNSAYSAAKF
ALEGWMEALAPEVAPFGIHTTIVNPGFFRTEFLTDASSTFAKPSIEDYDDRRAGHMEF
FNNQNGQQPGDPKKLAKALLKIANEAQPPQRFIAGADAIDIAEQKIANLQTEINAYRD
LSKSMAYDN"

CDS 2398827..2399717
/gene="oxyR_2"
/locus_tag="EFAGFIKM_02048"
/inference="ab initio prediction:Prodigal:002006"
/inference="similar to AA sequence:UniProtKB:P0ACQ4"
/codon_start=1
/transl_table=11
/product="Hydrogen peroxide-inducible genes activator"
/translation="MEIRVLRYFLTVAREGSITRAADFLHVTQPTLSRQLKDLEQELG
KKLFIRSSYSIILTDEGMLLRNRAEEIVGMVDKLEAEFESMEETISGDIYIGGGETEA
MKQIARVAKDVQNKYPNIRYHLYSGNEDDVTERLDKGLLDFGILIQPADVSKYNYINF
SDKDVWGVVMRKESPLASKHTIRSTDLLHVPLICSRQSMKQTFSKNEFADWFGEDFDK
LNVVTTYNLAYNAAIMVEEGIGYALTLDKIVNTSSESDLCFRPLEPRLESGLNIVWKK
HQVFSTAADLFLKEIQAKIV"

CDS complement(2399721..2400608)
/gene="rhaS_12"
/locus_tag="EFAGFIKM_02049"
/inference="ab initio prediction:Prodigal:002006"
/inference="protein motif:HAMAP:MF_01534"
/codon_start=1
/transl_table=11
/product="HTH-type transcriptional activator RhaS"
/translation="MVKQLYEDILYSDLSFPYIMYTHTRHTSIPPGRGFNDLHWHEDL
QITLVTKGKLVIQINGIDYTLETGQAILINKRILHITTELTHDGQYVSFNFPEKLLAF
HVDSAMEKNYVLPYTNSSLVSLVIKKDSEWETQILDMLWGMRETFNAEKKWGWEYEIS

IKTVQLWFVLISNISIPSGETPRSEKRQQERLQLMLSFIHQNYAHPVTLREIADVAHL

SISECTRCFKKTAHMTPYEYLINYRIKKSKDLLLSTNDTITEIAEKVGFNHVNHFIQS

FKKHEERTPKEFRCSPHPV"

CDS 2400764..2401696

/locus_tag="EFAGFIKM_02050"

/inference="ab initio prediction:Prodigal:002006"

/codon_start=1

/transl_table=11

/product="hypothetical protein"

/translation="MKENKLSLLFHPYVLLFIAILSVSVSSIMIKSSDTPTAIAGMYR

LYISAIIMLPFIPWKTIRFLKMNSKDWSTVFLAGLLLGLYFLFWMESLVYTSVASSMV

ILALQPLFVMIGSYFMFKERASIPTILCLITALFGSVIIAWGDIGVSREALIGDGLSL

IGTILVSAYMLVGQKVSRKIDANIYSVAVFIIGGSVLLVYSLLNNFSLTHYDSSDWMY

FLLLAIIPTIFGQYIFNLLLKSIGATTVSVGIIGEPVLAIMLAYLMLGETISAFQFVG

GLITLLGMLMYFGLKSYHVQLSKFLRFNRFRKLG"

CDS complement(2401733..2402416)

/gene="tetR"

/locus_tag="EFAGFIKM_02051"

/inference="ab initio prediction:Prodigal:002006"

/inference="similar to AA sequence:UniProtKB:P04483"

/codon_start=1

/transl_table=11

/product="Tetracycline repressor protein class B from

transposon Tn10"

/translation="MKKQQPQISEDKILEISWELLGEEGIEKFSMRRLADKLGIQAPS

LYWYFKSKQTLYQRLANQISKIILDEFHTEGDWKEQMEGLAVTVRSVLSRYPCSTQLM

MMTLPHEPDMIRFTNRMLLCMESTPLEQEQKLQAVLTLVNYVFYFVLDDYQHQRNISA

VLKDQESFQGEEMIQLLDSMSEKEAGLFSRMYKNGLFEVMGTDGAFEFGLKLILLGIE

QVIKKPTEK"

CDS 2402553..2403905

/gene="mepA_1"

/locus_tag="EFAGFIKM_02052"

/inference="ab initio prediction:Prodigal:002006"

/inference="similar to AA sequence:UniProtKB:Q7A7N0"

/codon_start=1

/transl_table=11

/product="Multidrug export protein MepA"

/translation="MDAENLHYFEKAPIAKAVAHFAVPMMLGTSMSVIYSILNAYFLG

TLGNTAMLTALALTLPLFAVIMALGNLIGIGSGTFISRLLGEKKVDDLKHVSSFAFYS

SLVLGIIVMAVGLPLIEPIVHGLGATPESFGFTKEYVTIMLMGSPFVILFFTLENIVR

SEGSAMTSMTGMILSVVINIILDALFIFVFHWGVIGVASATVISNLVASAFYAYHMSY

KSQFLTVSLKWFKVTKDILSNVFKIGVPVFVMSVFLGAMSLILNLFLVEYGDQAVAGY

GISSRLLQFPEFILMGLCEGVVPLIAFSFTANKLRMKQTIGFTIKAILGLAVLFGVIV

YLISDHLIGLFTTDPQLIEMGSYILHVTFLSLFITGMTSLFMGIFQATAQGTAAFVMS

IIQGITLIPVLYIANQMNGFHGVIWSLVIADAVAFLVGAIMLYVLRTKLQPDLEHLAQ

"

CDS complement(2404003..2405019)

/locus_tag="EFAGFIKM_02053"

/inference="ab initio prediction:Prodigal:002006"

/codon_start=1

/transl_table=11

/product="hypothetical protein"

/translation="MFNWKKRYISVIVAILLMVGAGLYIVDQSDFDMEEQTIEIQSPQ

GKLTGTLTLPRNHSGKLGLVLFIHGDGSIDSTQGEGYKPLWERLASIGYASLSLDKRG

IGGSEGNWLDQSMDDRVVEAQQAIAWAKEQSIIDDSKIGVWGASQAGWVIPKLAGKEH

LAFSILVSPAINWLEQGEYNTRSRMAKDGHSTEEIQNQVDYENQIKEILKKHGTYEEY

TKIAREDDLISKERWNFIKKNFASDATADLHHFKTPVLLLLGEDDVNVDIVDTEQAYR

KGIQPSSLLHVKIFSNTEHSMLPTSIAGSEWKQSLRFLFAPRSITVDGYMDEIEHMLT

AFSL"

CDS complement(2405012..2405656)

/gene="mshD_1"

/locus_tag="EFAGFIKM_02054"

/EC_number="2.3.1.189"

/inference="ab initio prediction:Prodigal:002006"

/inference="protein motif:HAMAP:MF_01698"

/codon_start=1

/transl_table=11

/product="Mycothiol acetyltransferase"

/translation="MTTSIQIEPVCPQFHQAVAELMAYGFGHKFQKITSLSITDLAYV

FEQLLNYPPMEQSTLRIIARENEEIVGTMCLKWKEADNRAAITSVRANPHWWSHCNRV

GMWKLLQLITGLHLLKHTPKLGECYVEDLVVHPQHQGKGIGTQLLQWAQKYMLQSKSM

SSLSLHVASQNHQAIQLYERYQFRKQYSTNSFLTGLLLGENQWHYMIYKGDSYV"

CDS complement(2405669..2406436)

/locus_tag="EFAGFIKM_02055"

/inference="ab initio prediction:Prodigal:002006"

/codon_start=1

/transl_table=11

/product="hypothetical protein"

/translation="MSDEITISELAKLMKVSVHQIRYFEEKGVLHPAYIAENQYRMYG

VDEIYRLSHILLLRKMGLSVQMIKECLSNLTTVQITSLFRQALADTEAAITQLQQTKH

LISKLLQEKDHIPRDEDQVTYYIDTREEIHLSLWFHMEAQGYLQARQLTQHKGNIPNL

FEADIHYAYDETDTVGIYTQKEGANDLVLPRGKYLSRHVYVQIEAELKEHINEFYAYA

EDQGYPYHGPLIMVEKSYLSLFTPEKLHYELLLQMID"

CDS complement(2406579..2407556)

/locus_tag="EFAGFIKM_02056"

/inference="ab initio prediction:Prodigal:002006"

/codon_start=1

/transl_table=11

/product="hypothetical protein"

/translation="MAEAVDVTTTGTKGRKKRYLWLKIIGGIVGALVLFMGITFVVHT

ISNGAEKKKIESYGQYVNVDGKNMNVSIQGSGEQTIVLLPGQGTPSPVLDFKLLIDEL

TSDYKVVAIEPFGYGLSDQTETERTTENIVSEIHEAVQQLGIDRYILMGHSITGLYAA

TYVNTYPDEVSAFVGIDSSVPNQPGMDVKLPLNLMKFFQKSGLMRVLNKVSGDSYASL

DYDEHTKEQMKLISNQVSTNPTLVNELKHLGSNFKNGEKLTYPSDLPVLLFVQSNNEK

NPQWIPLHEAQAKQSAQGKMIPMEGSHYLHHTKYKEIAEEFKSYMKQIQ"

CDS complement(2407721..2409019)

/locus_tag="EFAGFIKM_02057"

/inference="ab initio prediction:Prodigal:002006"

/inference="similar to AA sequence:UniProtKB:A0A2A5K5H4"

/codon_start=1

/transl_table=11

/product="Nucleobase transporter PlAzg1"

/translation="MDKWFKLKERGTTIPTEIIAGVTTFFTMVYIVIVNPGILSSTGM

DFNGVFIATVLASIVGTLIMGIWSNYPIVIAPGMGLNAFFAYSVVAGYGVSWQVALGA

VFIAGILFIILSLTSFRYMLLDAIPASLKHAITAGIGLFITTVGLQNAGIIADSESNL

ITIGNLAEPMAYLTIIGLIITVVLMAYNVKGYLFIGMVVTAILAWIMGLFQMPESIVS

MPQGLASTALQLDLAGVFSNGLYTIIFTFLLITLFDTTGTMLGVAEQAGLLKDGKFPR

SRGALLADAVGTTSGALLGTSPTSAYIESSTGVAAGGRTGLTAVTVSVLLALTLFFTP

IVSVISSIPAITSPALIIVGYFMINVISKIKWDDLEEAFPAFLIIILTPLTHSIATGI

GVGFIFYPVLKLLRGKGKDVHPIFYIFAVLFFIQLVFLDH"

CDS 2409590..2410441

/gene="yvgN_1"

/locus_tag="EFAGFIKM_02058"

/EC_number="1.1.1.-"

/inference="ab initio prediction:Prodigal:002006"

/inference="similar to AA sequence:UniProtKB:O32210"

/codon_start=1

/transl_table=11

/product="Glyoxal reductase"

/db_xref="COG:COG0656"

/translation="MQKVILNNGVEMPILGFGVFQIADANECEQSVMDAISAGYRLID

TAASYLNEEAVGRAIKRSGVARDELFVTTKLWVQDTGYERTKQAFEKSLERLQLDYLD

LYLIHQPFGDVHGSWRAMEELYREGKVRAIGVSNFQPDRLIDLILQNEVTPAVNQVET

HPFNQQILTGQFMKENNVQIESWAPFAEGKNDLFQNEVLLSIGEKYNKSVAQVVLRWL

TQREVVVIPKSVRKERIIENFNIFDFELSQADIESITALDTKQSLFFSHNDPEMVKWI

STRKLDI"

CDS complement(2410569..2415017)

/locus_tag="EFAGFIKM_02059"

/inference="ab initio prediction:Prodigal:002006"

/codon_start=1

/transl_table=11

/product="hypothetical protein"

/translation="MRKGLACMVAWSLMASLVYPATAGAESVQGQAEVGNVQAKSLHF

SVPYTDLTGHWSQSAVTRLQDLNLLKGYTDGTFKPSQVISRAEFVMILDRAFGFTGNA

ATGSYVDMTSDDWYYDVMVRANGSGIIEGTDREHLSPQQPITRQDAAVMVDRAFQLSA

TSNEDSEGLKFEDADELSSYAKKALTYLVNENIIKGFQGKLNPKAPITRAETAELLSA

MIADVKSTPGTYESRVDGNLIIKSTDVTLKNMIVNGNLLLAEGIGEGSVVLQGVTVTG

SVIIKGGGSHSININNSKLKRVVVDKRGEPVRVAITEESNIQEMHVKQKSILEISSNS

LIDSMNIHAEASQTQMDTKGTIQKLSVDAIDVVINGEKVIQGLRTSMVGEAQQPVSAQ

PGGSTNVTSTPTPSSPDGQSPSTPSNPAGEKPVPATTIPHDQWELVWNDEFNGPSIDS

SKWTVQDTGLVYNNEMQYYSPDNTRITKDQNRSVLQIEAKKGPKNGKDYSSGKLISMG

KGDWTYGKVVVRAKLPIEKGMWPAIWMMPTDEAHYGGWPASGEIDIMELIGGKESNNK

VYSTLHYDSVKPDGSHGHDQGSLTLPKGETFADDYHDFQVEWLPGMIRFYVDGKLHHE

VTNWQTKAAGQPEYYTFPAPFDRPFYLILNLAVGGDWPGAPESNFTSEKMNVDFVRVY

TYKNLDIWPDVTTNPIEPAQQRDPQTNGNQIYNDRFAEGSADNGVPLQWKFITNADGA

GSVKVVTDEQKGKAAEVTIDASGTENYSVQLTQMPMYIKKNKKYKIQFDAKASANRTI

MSKLNQFEKSWTNYSGDNTFALTTDWQSYDYTFNMRDGTDNNARFEFNLGLDDTTVWF

ANARLIEVGEADPLPVERNALPDGNFIYNGTFDQGKERLGFWSSSIQDSAAANISVNN

FSKFPIMERQLVVDVTQTNNDPQQVSVNQPDLKLEANTTYGFSLDAKADTPRSIDIDV

VSSNGHTVQVHQGQNLSLNQEMKTFTGEIIIGDGASIAQSELRLLFGSSKGKVYVDNV

RLTKRGKPLSVNGYAHVPATEAWMMQGLQLEDSVEGGKHVSYMDQGDLLQYKIDVAHD

GVYVLSARMASGKSDSKVQFSIQDEQGTTVAQSDLSLGDTGGWQAYKTVYFPRVNLTA

GKPYYVNFEGADYNTRWVDISQNKIQNSELTADLNHWEIIPNDLTASHDEGEGLTINL

PGTSEQWWDALLQQDQIKLDANKTYRLTFEASSSSPKSMQAVLSQNAGDFVKYFEEEV

ELTADQKSYSYTITMGDTSDSAAMLAFGLGYPLSTGEHSIHINNVQLYEVNPNADQGG

QPAHVNLIPNGDFSKGKEGWFTHADGNAADLEMQVNNQQLQAKIGHAGQHPWDRQVIN

EGFGIQQGFKYKLTFKAKAEKSRKLGLGIGWVDAAANYEWHGFFGARVDLTPEEQEFT

FTFDATEDSYANTRISFDLGNIDGEQDGNTTVSLSEVSLINLGPAN"

CDS complement(2415061..2415939)

/gene="ycjP_3"

/locus_tag="EFAGFIKM_02060"

/inference="ab initio prediction:Prodigal:002006"

/inference="similar to AA sequence:UniProtKB:P77716"

/codon_start=1

/transl_table=11

/product="Inner membrane ABC transporter permease protein

YcjP"

/db_xref="COG:COG0395"

/translation="MYYKTKGYRIFSIANYTFLGILSILCILPIIHILAVSFSSMAPA

SSNLVTFWPIGFTTDAYVKTFGNSNFINSLIVSLKRTVIATIIGMVIMLITAFPLSKE

DISFKGRSLYTWFFVFTILFSGGLIPSYILIQKLGLMNTIWALILPGALSVWNVILMM

NFFRGLPKELEEAAYLDGAGHIKTLILVYVPLSLPAIATLSLFTMVYQWNSWFDGMIY

MSDIKNYPLASLLQTIIVQQDLSKINVDPSMLENISQRTVRAAQIFIGALPILMVYPF

LQRFFVKGIVIGAVKE"

CDS complement(2415955..2416905)

/gene="yteP_13"

/locus_tag="EFAGFIKM_02061"

/inference="ab initio prediction:Prodigal:002006"

/inference="similar to AA sequence:UniProtKB:C0SPB3"

/codon_start=1

/transl_table=11

/product="putative multiple-sugar transport system

permease YteP"

/db_xref="COG:COG4209"

/translation="MKEHTLETPHKRRSTRVKQRWNMQRNWTLHMMLIPAVLLALVFQ

YIPMGGIVIAFQDFKPYLGFSESKWVGWDNFRYLFLYPDVGQVIWNTLVIAFFKIIAG

LFAPFLFAILLNEVRLTAFKRVSQTLVYLPHFLSWVILGGILLDILSPQGGMVNQLVV

AFGGEPIFFLGDGTWFRITLILSDVWKEFGFGTIVFLASLSGINPALYEAAEVDGANR

FKQTLHITIPALMPITIVLMTLSIGNILNAGFDQVFNLYNPLVYDKGDIIDTFVYRLG

ILNGKMSFATAVGLFKSVVATILIVISYRMAYKLANYRVF"

CDS 2417052..2418761

/locus_tag="EFAGFIKM_02062"

/inference="ab initio prediction:Prodigal:002006"

/codon_start=1

/transl_table=11

/product="hypothetical protein"

/translation="MFTRLNVFTKITLLFVMLLILVLFLYTYSNRESVRVIEHEIQNN

TMNHLSTFADSVESNIYQLSLYGMSIGQDSSIQEYQRPDYKDIPYERVKVGSAILEKM

NLYNAASKWHSTITLYFPRMQKVISTDYYAYIPYRDDEFKEPLSQSWTYADNQFIWYT

TDPTTAMARPGKARLITKISFPVHHLTALLNQNKLNNKGDPFMFHPDYGLISNSSGKE

TLNAILPSLKEAKLQASGGFSTTLNKTEYYVSYIQSKSLGWYYVDYVPMQQILKPITS

SRNLFYASIAVLIVMSVVVLYTLYRSVQLPLLQLVKGTNRLSTGDFSVRLHHSARNEF

SLLFARFNIMAQRIQELIENVYEEKLRSREATLKQLQSQINPHFLYNCLFYIKNMARM

KNEKAVVAMALNLGEYYRYITRSEKDQATLREELTMVKNYLEIQSLRLERMHFTIDVP

HDILDKTVPRLTLQPIIENAIIHGLEPKSVDGEIKIYADCEDDVYTIIVEDSGLGMSE

KQLDHLRDNLLKPLDENMGCGTWNVHQRLSFLYGEGSGLSYDHSSMGGIKVNITWHDN

TDK"

CDS 2418773..2420413

/gene="rhaS_13"

/locus_tag="EFAGFIKM_02063"

/inference="ab initio prediction:Prodigal:002006"

/inference="protein motif:HAMAP:MF_01534"

/codon_start=1

/transl_table=11

/product="HTH-type transcriptional activator RhaS"

/translation="MLQILLVDDERSVVETLAETIPWESCGIGTVHEALSGAVALEIM

DNHDIDIVITDIRMPEMSGIALITAIHEKWPHVQTILLSGHADFEYAKQAISQESFDY

LLKPVSDEDLIESVQRLVHRIKEKWETAASHQRALHAFQDHLPLLQSTMLHELLTGKV

YDQKTLADKLDLLELPYSVGEDLGMLIIRMEKHLSDMAHGDLALMEYAICNIISEVMH

NHFHIWHTRDVHDYIVVLVSMKHSTTMSYSRSETPQTLLEQYAAQIQTNVQLYLKGAI

SISVSSWGKFPHEIGELYERTVMSMRKRMGQVQGLFVTASDETDANPLHTIRSLYQPP

TLISLLEAGRFEDVEQKIEHIFEELLHSNEHHDIAESTFEVYFAIAGAFAYIIHKNGK

QISSLIPAESYRYFQAPSYSTAQQLQDWSVRTLHYIRDDAEQELRDNHSTIVRSVKSF

VDLHLAGDVSLPAIADHVHLHPVYLSKVYKAETGEALTAYVYRLRMEKAAYHLRTSTA

KVFEIAELVGYNNTAYFIRVFKKFYDLTPQEYRENLPV"

CDS complement(2420570..2422216)

/locus_tag="EFAGFIKM_02064"

/inference="ab initio prediction:Prodigal:002006"

/codon_start=1

/transl_table=11

/product="hypothetical protein"

/translation="MIGVLALSAALSGCSNGSDKKTESSTPATTLELKNGKYDPPVSI

TYLRPWGPDVKFKSGEDQDNNVHTKWAKEKLGIELKNQWISPSTNNAFETKLRLSLAS

NADMPDIISYRGEFNLVRELIESGKFVDAGELFDKYASDTWKAAVNEDPSVWYPYMQD

GKKIGIPILDYSYNGDPVMWIREDWMKKFNLEAPKTIDDLEVIMETFTNQDPDGNGKQ

DTYGLTIGFKNWLNTWMSEAGWVFGAYGTMPNQWNLTADGKLEYGSVTPGAKQALAKL

QSWMSKGYIPEEAGVYDETKAAEEFTAGKAGIVVGPHWMPSWPLEDVKKNNPEAEYKA

YPIPSGPNGQAGRHGTSNGNGVILINKDMKNPEAFFTYQNYLFDHYANPKEGDEFEHG

FAEGYDWVMVDGKPSTDASLTGGYAPEKYTLTFDGARIPNLSMTTLAKLASGEEATTP

FEKKIKSGVPVPMLDAAKIVLDQKDIVFNQMFTGAPSMTMQMNNDILTKMEKDTFSQI

VYGKTSVDAFDSFVEKWKSSGGDQITKEVNEWYDSVKSGK"

CDS complement(2422444..2423157)

/locus_tag="EFAGFIKM_02065"

/EC_number="3.2.1.73"

/inference="ab initio prediction:Prodigal:002006"

/inference="similar to AA sequence:UniProtKB:P23904"

/codon_start=1

/transl_table=11

/product="Beta-glucanase"

/translation="MKRKSWYTLAVTGVISLFFSVSAFAGYVFWEPLTYFNASTWQKA

DGYSNGNMFNCTWRANNVNFTSDGKLKLGLTSSAQNKFDCGEYRTTNTYGYGLYEVSM

KPAKNTGVVSSFFTYTGPAHGTQWDEIDIEFLGKDTTKVQFNYYTNGVGNHEKIINLG

FDASQGFHTYAFDWQPGHIKWYVDGVLKHTATSNIPKTPGKIMMNLWNGTGVDSWLGP

YNGANPLYAEYDWVKYTSN"

CDS complement(2423470..2424174)

/locus_tag="EFAGFIKM_02066"

/inference="ab initio prediction:Prodigal:002006"

/codon_start=1

/transl_table=11

/product="hypothetical protein"

/translation="MSKSLIIVVLISSMLIVQGCVPEMRNDSNVEKKAMTSADEQLFK

AVEDSDTERIEQMIQAGANINAQDQSGRTATMIATYNNDSTSAKVLIEAGADVNIQDD

MKNTPFLYAGAEGYLDILKLTIEAGADPAITNRYGGTALIPASEHGYVDVVQELLTQT

SVDVDHVNQLGWTALLEAIILNDGNARQQETIQLLIDHGADVNISDRDGVSPLSHAKN

NGFKEIEDILVRAGAK"

CDS complement(2424185..2425693)

/locus_tag="EFAGFIKM_02067"

/inference="ab initio prediction:Prodigal:002006"

/codon_start=1

/transl_table=11

/product="hypothetical protein"

/translation="MNSTTRKIATFLTITALGTAVFPLTANQVHAATYVTNIAATTTQ

LTQQQIEAAIKDLNSLGIMNGYADQSMGEHRAITRAELASLVYKTFNLESKTGETVEL

TDVNPNAWYAPYASKLVELGIMQANDGQFNPQTQVSDAELEQVVSKAMQRDVKSVHHW

MSEFYSENGTATRGETAVLLQTAHQAIPSEQAQIKSVRSLNAITLIVTFDKPLTAANE

AFAKAQTDFAFSGGLTLTNMPRLKTGSIATYIVPTSVQDASEVYNLTYKGQDAGSFEG

SGTKINMTTVSQVTNDTFEIEALQVNSVVDYGYIISAYSGGRGANAFVLNDQEQAGGK

TYQIISSMQARQVVITPEGDEPIVARYVPFTQSTDGKQEPKFRLPEGQTLKSGVTYTV

SSDWANIENASFTAKSFDALVVASAQAVSETSIEVTLDQDPGDELFSGRSVTLTSPSG

DVLTATYKYSSRKGATGVFDLTNDGKLTPGTTYTINPVGDWSIASSVTLTLE"

CDS complement(2425813..2426505)

/gene="srrA_4"

/locus_tag="EFAGFIKM_02068"

/inference="ab initio prediction:Prodigal:002006"

/inference="similar to AA sequence:UniProtKB:Q9L524"

/codon_start=1

/transl_table=11

/product="Transcriptional regulatory protein SrrA"

/db_xref="COG:COG0745"

/translation="MANILIIEDETTIAELERDYFELNGFSVDLCHTGDEGLKQALEG

DYNLIIVDLMLPGLDGFECCRRIREVKEVPILVVSAKKEEIDKIRTFNLGVDDFITKP

FSPSELVARAKAHLTRYERLLGKNKPAEQDEIHIRGLHIDKGSRRVFVNGEEVAITTK

EFELLVFLASHPNRVFSKSDLFERIWGMDSTGDIATVTVHIRKLRGKLETDPKNPEYI

ETVWGAGYRFTA"

CDS complement(2426512..2427981)

/gene="sasA_5"

/locus_tag="EFAGFIKM_02069"

/EC_number="2.7.-.-"

/inference="ab initio prediction:Prodigal:002006"

/inference="protein motif:HAMAP:MF_01837"

/codon_start=1

/transl_table=11

/product="Adaptive-response sensory-kinase SasA"

/translation="MSIRIKMLLSFTGMLVISLLFILLTASLYTIAATGDLQSFRDIY

KVHYQINPLTEQGESIFQEMKFLAKNDPDDLQNKVLLREYDMKLRAEKSGLYIRRENS

QIFESLTFNQPELKQALPAYDLNNYQIRSTFNIGERFYAYAKFDFQYSDGERGSIFVI

RERSPFAELTRKLLPVMSFLLIGVLIIANLLLFRWITRSFIKPLNQLRSSAEHIKDGN

LSFKLQLNSNDEVGQLSEAFESMRNQLQRSYALRQQDEVNRKELISNISHDLRTPITN

IKGYIEGIRDGVANTPEKMESYVNIIHSKAVSMDKLVDELFLYSKLDLNQEPFLFKTV

DLADFLEDSIEELRYDLEDKGVALDWINQVSGPAMAAVDPEKLKRTVVNVVDNALKYM

ENEHKRFEITLQADENWITMAFKDNGRGIPEEALPYIFERFYRAEQSRNSSTGGSGLG

LAIARQIIDGHGGFIWAESQPNDGTSIYIKLKRLNEERN"

CDS 2428147..2428755

/gene="rhaR_11"

/locus_tag="EFAGFIKM_02070"

/inference="ab initio prediction:Prodigal:002006"

/inference="protein motif:HAMAP:MF_01533"

/codon_start=1

/transl_table=11

/product="HTH-type transcriptional activator RhaR"

/translation="MKLDQTVFHRTDWEYILVLYRICSSDLEETSFSHQHFELSTGQS
PRLIELITRLWHVYNQRGGISKFQTEMLFRDVLNETLICVDHRQNSCESDVLFERVST
YIQEYYDQSLTVASLAEQNNVNRNRLSYVFRRHAGMGPAEYVLKYRINMAQQMLLASD
AYVQQIAESVGIADPFYFSRVFKKQCGISPTEYRLKFINNPS"

CDS 2428835..2429173

/locus_tag="EFAGFIKM_02071"

/inference="ab initio prediction:Prodigal:002006"

/codon_start=1

/transl_table=11

/product="hypothetical protein"

/translation="MHYQAIKLNEEFSKLNDLWSPKLIGEMNDYQFKIVKIAGDFEWH
VHEETDKLFFVLEGEMIIDFRDGQVKISKGEMCIVPKGVEIKPSAEKECHIMLMEPGS
EVNNGGTKAE"

CDS complement(2429304..2430440)

/locus_tag="EFAGFIKM_02072"

/inference="ab initio prediction:Prodigal:002006"

/codon_start=1

/transl_table=11

/product="hypothetical protein"

/translation="MKTYDVFPEPIGAYTVGRTQMDFEYTASDYSKRELTAFVYYPSD
SSEGKTTSTYMFPEVYEMFNEQPLVTALKDVFSIDIKTQCYDDLALSGKEKRYPVLFY
VCGGGGSPEWGTVLCTDLASMGYVVVSIGHQNSTMYKRKDGRLFNVSKNFSDVITAFG
EDSEMLALAGKMEMRPDDETAIEMCRNVLALPILSKITEFSELQAEDVRYVADYLYKV
DSGELNSIFKGRLLLDIGMGIIGHSYGGPTTAMVCRDDDRFTCGIGLDSGAFGLLDSD
LKKPFLFLFCEPNYNMNAVIGANNSMKTYYFSVDRVAHLDYCDIVFTSVNEELRGERD
AMEMRNIVTNYAKNFFDHYILQKAASVESLAYDGVELIRKTSNK"

CDS complement(2430437..2431096)

/locus_tag="EFAGFIKM_02073"

/inference="ab initio prediction:Prodigal:002006"

/codon_start=1

/transl_table=11

/product="hypothetical protein"

/translation="MPKNTFFRLDEARREEISNSAMHLFVDNLYEDITMKMVLDSLSM
HPGTFYRYFEDKDDLYCHLIRNVTQKRAAYFSNSNEDSLYSYFLTGLFGNVNGMVTEP
LNELEIKLTKTFSYIPEDILLKVYVNVLKGESFPMIKDILRRMRVDGYLRPDVDDDLI
SFMFESMQLNLILFFREFDIKDSELQHKISKYFAEFMGHGLLEDHKYSEMVSDLKGER
K"

CDS complement(2431309..2432391)

/locus_tag="EFAGFIKM_02074"

/inference="ab initio prediction:Prodigal:002006"

/codon_start=1

/transl_table=11

/product="hypothetical protein"

/translation="MLVSVKKGMFLCLITAMILVLAACSSSNGGSKDANGSSQQAAES
SATNEATNGEQTDADASAPSETTYPVTVSNYTTENGTWVKKDQTFDKAPERVVANTQG
AAELMIRLGLIDKLVGVAALFGSVPEDIADEFKQIPVLAEGYVGKEVTIGATPDLVVG
RGGLFEDADWGVGTVSSLNDMGIKTYVQSTSVPDASLDSLYQDITELGEIFNVQANAA
AYIETLKARENALSARASAETINYASFSDNGDGTIGIYNGNGDTFIESAMSLINMHNM
LINETGTLSLEKLIEINPDAMIISRYAGGIDPEQTIEKLLANKQVQNINAVKNKKIYI
IDFNNFWGYGDSIFTGVEGLADDLGL"

CDS complement(2432650..2434923)

/gene="bglB_1"

/locus_tag="EFAGFIKM_02075"

/EC_number="3.2.1.21"

/inference="ab initio prediction:Prodigal:002006"

/inference="similar to AA sequence:UniProtKB:P14002"

/codon_start=1

/transl_table=11

/product="Thermostable beta-glucosidase B"

/db_xref="COG:COG1472"

/translation="MDKSINELLSALTLAEKASLCAGLNMWMTKGIERLNIPPVHMYD
GTNGIRKTNSDEEMGITTENVPATCYPTGSAIGSSWNTELLYEVGVALGRESKTMDVE
LLLGPGVNMKRTPLGGRNFEYYSEDPYLTGELGAAFINGIQSEGVGASVKHFAGNNQE
FEKMVTSSEIDERTLREIYLSAFERIIKKSDPWTVMCSYNLLNGTYTSENEHLLHDIL

REEWGYEGVVLSDWTAVNDRIRGLKAGLDLEMPGPANYNAKAIIEAVQNGSLSEEQLD

RSVGRILKLVERVTGKKDMDSSPDSDYHTLARKAAAESIVLLKNENAILPLRQESISS

IAVIGRFAKKPRIQGAGSAKVTPTRVDIPWDEIKKLAGDAMTMNYAEGYPEDDSINDE

LIKESVSLAMNSDVAVLFVGQPEYAESEMHDLQGIDLPEHQVKLILAVAAVQPKCIVV

TSSGSALAMRPWVQHVPGVIHSWLSGQGMGKVIADVLFGQTNPSGKLSETFPVKLSDN

PSHMRIRGENGKLYYREGLFVGYRYYDRKELAPQFPFGHGLSYTSFSYTDLEVAQTHT

GVTVSFQLKNTGKRKGKEVVQLYVHDEECTWTRPEKELKAFAKVELEPGEERKITFEL

EERDFSYYNTKYNRWVAETGFFQISLGSSSKDLRITERLHCDFGKEEITFQKFSLLSE

WMSDPAAKKELEHCLNEMNEHVTDKVYLNEEFVGFWADFPMIKVFQMFGQQWMNERSP

DEIINELIAKVNQARNE"

CDS complement(2434943..2436175)

/gene="licC"

/locus_tag="EFAGFIKM_02076"

/inference="ab initio prediction:Prodigal:002006"

/inference="similar to AA sequence:UniProtKB:P46317"

/codon_start=1

/transl_table=11

/product="Lichenan permease IIC component"

/db_xref="COG:COG1455"

/translation="MKRFMKWMTDSFAPRLEAFTNNIWVSSIQEAIMVAIPMIFIGSI

ITLISILQDFIPGMPDLTPITTFSFGLLGLFIAFLTPYVVMEKRERHKIKLLAGMTGI

SLFVMLLNPTVNEDGTIQFILERFGPSGMITALLVGVFVALVMIMCNKFSFFKKGSSL

PEFIMDWFDFLVPIALVLATGWVLVYQLHFDIFGLIVNVFEPINAVGQSLTGFLLFNF

IGVVLYSFGVSPWVMTPIWYAIWIPAIEENAALVAAGQDPVNINTFETFFSGWLGIGG

MGATLPLVVWFLMARSKKLKSVGKATIIPSLFNINEPVVYGAPIAFNPLLMVPMWINS

LITPMIVYLALDWGWVRIPSQIFQLWYTPIGLSTYIMSGLNGLILLAVVLVIVFIVWF

PFFKLYDAQELKKEQEQN"

CDS complement(2436377..2438836)

/gene="rhaS_14"

/locus_tag="EFAGFIKM_02077"

/inference="ab initio prediction:Prodigal:002006"

/inference="protein motif:HAMAP:MF_01534"

/codon_start=1

/transl_table=11

/product="HTH-type transcriptional activator RhaS"

/translation="MKLFVNSVKHIDFHWHKEVEVVYVLHGSIIMYLDQQQYTLHEDD

VIVVNSMSVHKIERTNQDNVLLTLQFGPELMKNNAFISCNSAMNVEQNAPRLQSIKQY

LAQMVWEINKKTPGYQNFTMGRLQMLCGCLQRYFSSGTNSALEDGSRDYDYKRLNRVL

TYIDLHYNEKITLQDMAHSEHLSLHYFSHFFTDKIGIPFQKYLTLIRLEKAQAQLAAN

DKNISEIALDCGFANVKLFNKYFKEKYGCTPGSYREDSRTPELHQLNVNRKPKTYEES

SSGDYYEMETLNAIGLLYRYLDVKVDADQDTLPVPSVHLSDQTYIEIRADQTSSVYEK

HWNMTMTAGRAIEGLREDWRKQLSALKGKIPFQYIRFHGIFNDELMVYSENEQGTPLY

NWSYVDKLYDFLLDQGVRPFVELSFMPSQLARSKETLFWWRGNISPPSDPAKWQALVG

EFVRHCLNRYGAEEVRQWYFEVWNEPDLAGVCWAGSKEEYFAFYESTVHAIKSILPEL

TVGGPAMGYGSLWNDTWAEEFLSYCRKREVALDFYSFHIYSEYPKLKVEDDRLTQIMP

PAFYKESIELMRQKMNASSYGHVELHVTEWNFSLYDRNLLHDTMFMAPFVIYHTMNTL

GDVKAMAFWSFTDVFEESIVPASPFYGGFGLINRDGLKKPSYYAFELMQKLGDELMVK

GDGYVGTRKGDGSMQFLFYHYVHVDQLFASGDWSELSSSTRYDVFEEKGSKAYELTLS

NLEGPYKCTSYQLDREHGSVFDEWTRMGSPYSLAEEEIAYLNGRSGPVLGTEMVRDES

WHREILLPPHGVMLLILDRQY"

CDS complement(2439042..2439923)

/gene="pdxS"

/locus_tag="EFAGFIKM_02078"

/EC_number="4.3.3.6"

/inference="ab initio prediction:Prodigal:002006"

/inference="similar to AA sequence:UniProtKB:P37527"

/codon_start=1

/transl_table=11

/product="Pyridoxal 5'-phosphate synthase subunit PdxS"

/db_xref="COG:COG0214"

/translation="MQTGTDRVKRGMAEMQKGGVIMDVMNAEQAKIAEAAGATAVMAL

ERVPSDIRAAGGVARMADPTIVEEVMKVVSIPVMAKARIGHYIEAKVLESLGVDYLDE

SEVLTPADEVFHIDKHEFTVPFVCGAKDLGEALRRIGEGASMIRTKGEPGTGNIVEAV

RHMRLINSQLRKVQNMSKDELYAEAKNLGVAYELLREVHENGKLPVVNFAAGGVATPA

DAALMMHLGADGVFVGSGIFKSDSPEKFARAIVEATTHYTDYKLIAEVSKNLGAPMKG

IEISKLAPEERMSNRGW"

CDS 2440074..2441564

/gene="gabR_2"

/locus_tag="EFAGFIKM_02079"

/inference="ab initio prediction:Prodigal:002006"

/inference="similar to AA sequence:UniProtKB:P94426"

/codon_start=1

/transl_table=11

/product="HTH-type transcriptional regulatory protein

GabR"

/db_xref="COG:COG1167"

/translation="MQFHVAYSSYLNRKYTKMKALYHAIRDAIHEGNLIHGEKLPSTR

ELAATYHISRGTVNQVYDTLTAQGYIHSEHGRGTFVAYQLDLNNNASTEKACTHHLSA

WGNRIQQFEQQSIQRNSPANAYTHDSRMNSDEVIDFSKYQPDFSKFPYDEWNNRLYAE

IRQREHHLKTTSAMVSSTGDPKLREAIAAYLRRMRGIQVDPDHIAVTAGSMQAIALLT

QLLADPGDYVVTESPCYVGISKAILAAGAKLIEANLDGQGVVPQDWEARMLFVTPSRQ

FPTGEMLSLERRQTLLDWAQRNDAMIVEDDYDSEFRYRGMHVEPLKTLDKAGRVIYLG

SFTKTLPLEVRLGYVVLPPTLADTFRKAQALYEPRPVNLIEQRALAAFMTSGQYERHL

RRMNRLYSRKFHLLLKLLNKQLSTWFDWVENEAGLHVFGWWRGNVTTYEAFRESARSQ

GVVYSEVSSSTSVGMKYGIYLSFAHLSDEQLQEGVSRLKNAVTTAL"

CDS 2441624..2443645

/gene="yoaA_2"

/locus_tag="EFAGFIKM_02080"

/EC_number="3.6.4.12"

/inference="ab initio prediction:Prodigal:002006"

/inference="similar to AA sequence:UniProtKB:P76257"

/codon_start=1

/transl_table=11

/product="putative ATP-dependent DNA helicase YoaA"

/db_xref="COG:COG1199"

/translation="MNWLNELYKMNKQEPDKEIERSLVNAIFRIYDRFPRIGLRERIG

QQEMSLDIADAYIHGHNAMIEAGVGIGKSFAYLIPSLLTNQMSQKPVIIATSSIQLSE

QIHKDLRIIGSRLGFSTVRSVVGKGMGQYACRSRAAEWINLDDANSSVSTLAQRILDY

EIDERADIKAGVSDAEWSHVSVNDCKFERCHHKNACLFYDMRAKLNAKAHEIDFIIVN

QDLLIRDLMKKKEGTKSIISEQPALIVIDEAHNLEAKVRDARTLEFTYRGICRILDNA

VQLLTKQSGDKSLFSQSKFIKNCAERIFKQVNADLLHHAKQDNDRIKVSEIKGIPLNQ

VLNDLKDLSLRLSILTSRHEREIDDAFEAINGLITLINVLSEIEDNYLLWASSPLRVT

TISICPKDISQFLKYALFNGKVPVILTSATLCQGGDTLEEQYSYMTHSLGYKGDFLER

QPSPFDYTNHATMYISNKIPYYQHDQREIYLEAAYKELVQLCNLTQGRTIVLFSAKED

MKYIHKKLISRANEYTWAVHVQKEGSSQDSVIAEFRKSKGVLLGTGVFWEGVNIEGSD

LSQVIIFRLPFPVPSDPVYEYKSSVTENPFMEVFVPDMLLRLRQGTGRLIRSETDLGI

LSILDSRLSEAAKKSYRGQVLDTLPFKKVTEDFAVLEKFVQTKGIRRTD"

CDS 2443792..2444319

/locus_tag="EFAGFIKM_02081"

/inference="ab initio prediction:Prodigal:002006"

/codon_start=1

/transl_table=11

/product="hypothetical protein"

/translation="MVERPTTEEYAAYYEGYIQLVPEGNIIERLELQANIIPTLLSSL

TEEQANYRYAEGKWSVKEVIGHLLDNERIMSSRLLRIARGDQANHPGYDQDVLMQTHP

FNAYTLADLSEEYAVTRRSTILMLRRLTPEAWLCRGIVSDNTASARSIAFVMAGHELH

HCSVLRDRYSLDLKL"

CDS complement(2444398..2445339)

/locus_tag="EFAGFIKM_02082"

/inference="ab initio prediction:Prodigal:002006"

/codon_start=1

/transl_table=11

/product="hypothetical protein"

/translation="MNNQLTNGGIWNDSSGQPIHAHGGHMLFHDDYYYWYGEDRRDDI

YVSCYRSKDLFNWEFRNHILTTSTPAAPIRVRTNLALVNDKGGKVNLERPKVLFNTVT

KKFVLWVHYENGNNYNDAACSIATSDSPDSHFVYHGSFNPYGYMSRDCTLFQDDDGTA

YFISAARDNADLHIYRLQKDYLNVESLVGKLWQGEYREAPAVFKRKGKYYMVTSFCTG

WAPNQGKYAMANTMDGPWGMLSDFGDETTYQTQPAFVLKRSAEEYLYFADRWNGSDYF

QSSYVVLPIEFNGDIPILNDYSTLSLREDAHLIHFER"

CDS complement(2445370..2447085)

/locus_tag="EFAGFIKM_02083"

/inference="ab initio prediction:Prodigal:002006"

/codon_start=1

/transl_table=11

/product="hypothetical protein"

/translation="MIKGKSWMAIVIALMLLIVHLTSSAQTVHAAGEETHIAATLFVL

KNESEVSNAKIHWAPVQEATVYELYRSENNAPYTLLQTLTGTTTDDYELNIGNTYKYQ

VKAYGGTSLLTSAVSPEYTPYSLPEDLTTFDNTTQSTLMLPNELKVGDTYYRFNFVQK

SSGGFGEMIQQTSTDDITYGNDKVVLSYTDHPDLANSKFEGINILYHAPTNKFVFWAH

YENSTDYTLARVSVASATPGEDFTFHKSFRPEGNQSRDISIFKDDDDSAYLISTANNN

SDTILYKLTSDWLDVDHQVSVIYQSQHRELPKMIKKDGIYYLFSSQAAGWYPSIPLYS

SATSIDGDWSELRTIGNTSTFSAQSGSVMRVKPDMGNNVVMVAYRWMFGWAGTQNGTT

EERLLPVWFSDGYAFYDYFDQVLYNTSDDVVVPVQNGKLLSQGKPATAQTATGTNPAS

YANDGNYQTEWVGTGSSWPHWWKVDLGSVQQLNNVQISWWMQKGSEGYYKYKIETSTD

NVNWTVALDRTNNTSYGFTSDTLSSTAARYVRINMQNAALHNNPNNWYTPRLWEVKVF

GSDTN"

CDS complement(2447157..2448842)

/gene="lipO_6"

/locus_tag="EFAGFIKM_02084"

/inference="ab initio prediction:Prodigal:002006"

/inference="similar to AA sequence:UniProtKB:P37966"

/codon_start=1

/transl_table=11

/product="Lipoprotein LipO"

/db_xref="COG:COG1653"

/translation="MKKRTDLWKMVSMVMLSAALVAGCNKGESASETAPASVLKDGKY

DPPFTITIAKQQDENAGKYINGETLNDNVLTRWGEQNLGIKIQTTLLGGDASQYNTKL

RLALTGSEKLPDVLPVYDTMLVNDLIESGQVKEITEDISTYMPDRIKEIYKQYPTTFN

PVVREGKVYGMAIAPNLTEGEVMLIRQDWLDRLKLKAPTTIDEFEQVIAAFTNEDPDG

NGKKDTYGFTFSGKDSYNTGWVSDPVMIFSAFTGKHLPRQWHNDNGKLTYGTVAPGNK

EALAKLRDWYAKGYLNKELATQGAWDALSDFTEGKAGIIIGRPWLYDSVKDVEKNVDG

AKISAYPTIQGVNGDKTYQSAQLNDGVFMFNKDFQNMEAFFLYYDKMYDAAFSTGEFK

YGYAQGYDYDIVNDKVTFDPTKFNTPLEAVQGVGKMAFTKNTPSVDGPGQSYYDLANG

VKADTGVLIQSASKDQTTQDGYRISYENRDVLLPNEFNGPPTPTMQNVWEQLTTMEQE

TFTKIIYGNEPLEAFDTFVKQWHEKGGDTVIEEVNEWYNQASETDVMTLMNLK"

CDS complement(2449004..2449873)

/gene="ycjP_4"

/locus_tag="EFAGFIKM_02085"

/inference="ab initio prediction:Prodigal:002006"

/inference="similar to AA sequence:UniProtKB:P77716"

/codon_start=1

/transl_table=11

/product="Inner membrane ABC transporter permease protein

YcjP"

/db_xref="COG:COG0395"

/translation="MYHKTTGYKVFSYFNYAFMILAGLACFLPLLHLLAQSLSSKAAI

SGNMVSFWPVGFNVDAYIKTFNNSNFNGAMLTSITRTVLGTTISMFILTCAGYALSKE

FRGRNVLMWFFIFTMLFSGGLIPSYILITALGLKDTIWALVLPGAFGAYNLILLVNFF

KTIPKALEEAAFIDGASFFVILSKIYLPLSLPGIATVSLFIMVGHWNSWFDGILYMSD

ASKYPLASFLQTVVVQSNMQNMAMSQSEVAAMSEQSIKAAQIFVSTLPIILVYPFLQR

YFVKGIVLGAVKE"

CDS complement(2449892..2450686)

/gene="yteP_14"

/locus_tag="EFAGFIKM_02086"

/inference="ab initio prediction:Prodigal:002006"

/inference="similar to AA sequence:UniProtKB:C0SPB3"

/codon_start=1

/transl_table=11

/product="putative multiple-sugar transport system

permease YteP"

/db_xref="COG:COG4209"

/translation="MAFQDYKPWLGITGSRWIGLDNFERIFQYKEATQAIVNTLIIAV

SKIIVGIIVPIVMAILLSEIRNMGIKKSVQTLVYLPHFLSWVTVAGLMIDILGLDGGI

NHILTQIFGNDPIYFLGDPDLFRFTVVISDVWKSFGFGMIVYLATIAGINPSYYEAAE

IDGATRRQQIRYVTLPSMLPMIIVISTLSLGNILDAGFDQVFNLYNPLVYSTGDIIDT

YVYRSSLLNGQYGFGTAVGLFKSGISLILIVVSYRLAYKYANYKIF"

CDS 2451094..2452803

/locus_tag="EFAGFIKM_02087"

/inference="ab initio prediction:Prodigal:002006"

/codon_start=1

/transl_table=11

/product="hypothetical protein"

/translation="MIRKMYMKRFIYFFVFFLLLTLSLFFVMNRLSSKTLEENLIGAS

KNQLDYVKGILDGIIYEANMYGVQYAADSDVRFYQRHVIELSNYDSQMKKNDIVDRLR

QTLLSSRSIESIGIYWKSEETFLSTNSTLEARLPFKDVHQRGWQIVGNSLYYFALYPY

IQKSGQNEPTQYVVGVKLNTDYLKNLLKKAVNNDSSNAFFWFNSHQLWSEKEVDNDLL

KAVTEMISPQPEITLKYDYHTKSDDYYVLSRYIESIDAYLITYTQTNDFLQPIDRNRK

VFFASILAVFTLGLVVIFTFYRNYYRNIRLLERKFSQVEQGNHNTRITENTDREFYSL

FRSFNHMVTEIQDLFVSLKTETELRRSAELQQLQAQINPHFLYNSLFFIMSVAQFSPD

SVMRMSKHLAEYYRYLTKLDRHEVTLESELQFAEHFLIIMALSKKMEYSIDLPSELSS

LPLMPLIIQPVVENAIQHGIEGQQGAHRVTIEVKQTEAAITIKVSDDGKGLSLDDIRS

LEARLESDTPPEGIKGVGLWNVNQRLKNTYGERSGLHFTTNDWGGLSVLLLISIPVVK

GDN"

CDS 2452803..2454152

/gene="rhaR_12"

/locus_tag="EFAGFIKM_02088"

/inference="ab initio prediction:Prodigal:002006"

/inference="protein motif:HAMAP:MF_01533"

/codon_start=1

/transl_table=11

/product="HTH-type transcriptional activator RhaR"

/translation="MRLLIVDDGHYVVEYMKHLLDWNTFGIDQIETMTNSIEARELLT

QNHIDILITDIRMPEVSGIDLLQHIHQHNLPTKVIILSGYSEFEYAQQGIRLGALDYL

LKPVDKDDVEKAMSKAINNITKTRPAQSVAWENFDGLEYLLSLLGHNETLRKQYDVYT

EFLGRRRMCCFKLDGFTQEHEDVIKHAVGDKLDRLVWAAGTGLFGLVPEEAAGELTIK

LEQSVASPPFSLTDRNVTRQMFYRFFLNEDVHSEDFNGIFNHSPSCGDWESAGNIIKK

YEHIHLHKQKIIFLMETILYVYLTDKDRDPSETVDWIFNQLRYPDELSSTLMDRVSRI

RNNKQMSIQTIVDKVQTYIEDHLSHGLSLDELGKVAHLHPVYFSKLFKQETGENVSNY

ISRKRLEKASQLLQDSELRVADIAQMVGYRKNQYFIQLFKVEYGVTPYQYRRNMIHQ"

CDS complement(2454272..2455618)

/gene="ntaA_1"

/locus_tag="EFAGFIKM_02089"

/EC_number="1.14.14.10"

/inference="ab initio prediction:Prodigal:002006"

/inference="similar to AA sequence:UniProtKB:P54989"

/codon_start=1

/transl_table=11

/product="Nitrilotriacetate monooxygenase component A"

/translation="MTKKRQGQLHLGAFIYFTGHHHAGWRHPDSGVEKMFDIEWYKRI

AQTAEKGKFDMIFFADLLHLIYPSRAAAGMLDPVSLLSALSMVTDKIGLTATLSTTYN

EPFNAARRLATLDHISGGRTGWNIVTSQVDTEARNFGRDKHPEHGTRYEMAQEFVDVV

AALWDSWPQDSLVMNRASGTFVDESKLRVADYKGKWYSTQGLLNVPRPPQGYPVLIQA

GSSDPGQDFAAKVGEVIFTAQTSLAAAQDFYRTVNEKLLAMGRERGSLLIMPGLSPIL

GSTVEEARRKERELLDLIDPQEAVMMLSGMLQTDLSGYPVDGPLPDISDPVEASNGMK

SRVQLIMDLARDEQLSIAELGRRLLGARGHMQFVGTPEQLTDLMEEWFNGYGCDGFNI

MPPVLPGDLDDFVEHVVPELQRRGLFRGEYEGTTLREHLGLSEPTVVVSHKQQVHG"

CDS complement(2455654..2456409)

/gene="ssuC_3"

/locus_tag="EFAGFIKM_02090"

/inference="ab initio prediction:Prodigal:002006"

/inference="similar to AA sequence:UniProtKB:P40401"

/codon_start=1

/transl_table=11

/product="Putative aliphatic sulfonates transport permease

protein SsuC"

/db_xref="COG:COG0600"

/translation="MPNWLYGWVLPVIILALWEAGSALGLLSDSALPAPSRIARTFWQ

LCVNGDMTQHLTASTIRAIGGFLLGAVTGLLLGVFTGLGKWVEQTLDPTIQMLRTVPL

LAVIPLFILWFGVGELSKVLLIALGSFFPLYFHTHLGVRSADRKLYEVTRILQYSKFR

LLTRLIIPSALPNILLGIRLSVGASWLLLAVAEMMGASAGVGYMIQDARVYAQTDVVF

VGIIMFALVGKLSDSAVRLVERRLLRWNNTYKG"

CDS complement(2456494..2457288)

/gene="ssuC_4"

/locus_tag="EFAGFIKM_02091"

/inference="ab initio prediction:Prodigal:002006"

/inference="similar to AA sequence:UniProtKB:P40401"

/codon_start=1

/transl_table=11

/product="Putative aliphatic sulfonates transport permease

protein SsuC"

/db_xref="COG:COG0600"

/translation="MQISGNKLVFRTIHLLIGMSVPAAILIIWQIVSSNGWVNPVLVP

PPKDIVAEFGRMISSGELLTNLRISTGRAALGFLLGGGLGLAAGLWVGFSLKAERLLD

PSLQLLRTLPHLAVAPLFILWFGFGETSKIVLIAKGAFFPLYVNAFLGVRSVDRQLFD

VASVLQFTKLQTITKLIIPASLPSLFLGVRLSIAVSWLGLVVAEMMGSSSGIGYIIND

ARSFSLTSVIFVGIIVFAIVGKVTDSLVKLAEKKLLRWQDSIEGDG"

CDS complement(2457304..2458335)

/gene="ssuA_2"

/locus_tag="EFAGFIKM_02092"

/inference="ab initio prediction:Prodigal:002006"

/inference="similar to AA sequence:UniProtKB:P40400"

/codon_start=1

/transl_table=11

/product="Putative aliphatic sulfonates-binding protein"

/db_xref="COG:COG0715"

/translation="MVKKYNLIAVILVLILVVAGCGNANNNAGNGEDAKPVEGSSNPN

ASYDDVTVKIGVQGKGGLFGKAQEQKWFEDAFGELGVKVEWLEFQSGPPMIEAMASNH

LDFAGMGNMPPISAQAAGIDFKIISQLLDGKNNVAIIVPNDSPIKSIADLKGKKVAVT

KGSNAYNFLYQVLQKDGLDNSDIQEIQLQPDETQPSFESGKVDAWAVWDPYITLNTLS

GKGKVLADGESEGVLSPSFQLVRSDFANDYPELVTLYLKTFEQARQWEANNQEKAFQR

YADERNIPIDLVKGIQSRAAMINIPVSDETIANQQNTANFQFELGTIRKEINVADVFD

NQYIEEALK"

CDS complement(2458382..2459149)

/gene="ssuB_3"

/locus_tag="EFAGFIKM_02093"

/EC_number="3.6.3.-"

/inference="ab initio prediction:Prodigal:002006"

/inference="similar to AA sequence:UniProtKB:P97027"

/codon_start=1

/transl_table=11

/product="Aliphatic sulfonates import ATP-binding protein

SsuB"

/db_xref="COG:COG1116"

/translation="MLIIERLNKTFASPTGPIVALSNIRLQVEPGQFITFIGPSGCGK

STLLKIVAGLDTTYEGTLELNGKAITGPSVDKGFIFQEPRLFPWMTVEKNIAANLSLG

NAQVRHQVQELIELVRLKGFEKAYPKELSGGMAQRVAIARALLRKPEILLLDEPFGAL

DAFTRTHMQEALLDIWQNSRTTMLFVTHDLDEAVFLGEKVVIMNPRPGHIRNVIPVDL

PYPRKRSGGSFQELRSRVLGEFEKVEHSPLIDKGAGI"

CDS complement(2459338..2459958)

/gene="fadR_1"

/locus_tag="EFAGFIKM_02094"

/inference="ab initio prediction:Prodigal:002006"

/inference="similar to AA sequence:UniProtKB:P94548"

/codon_start=1

/transl_table=11

/product="Fatty acid metabolism regulator protein"

/translation="MSPRNIEKDLQQREKRINHILDTALEIIALKGIGSVSINHIAVA

AGMSIGNMYHYFKSKDEIISEILRRGQTSYGEHVSQLAEQSGDALNKLLLLSESWLAL

ENNWAYTILIHTARLSETSSEEIRREVTERFTNNLGPVANIMEQGQKEGLIVSGVPLE

LAYYFVSLIQGLTLQKMPGAEVPIRAQARSIVSLFAVSLHNSKRND"

CDS complement(2460169..2460462)

/locus_tag="EFAGFIKM_02095"

/inference="ab initio prediction:Prodigal:002006"

/codon_start=1

/transl_table=11

/product="hypothetical protein"

/translation="MYYNQTAGFGLLAPEGYPEGGESLTLERLSSMNPDYIIIQHRID

VAEAAVKDKQSSKVWNSLEAVKNNHLVIFDNSLNSASILAGRLASEYVKLAEQ"

CDS complement(2460627..2461130)

/gene="ntaA_2"

/locus_tag="EFAGFIKM_02096"

/EC_number="1.14.14.10"

/inference="ab initio prediction:Prodigal:002006"

/inference="similar to AA sequence:UniProtKB:P54989"

/codon_start=1

/transl_table=11

/product="Nitrilotriacetate monooxygenase component A"

/translation="MPGLYFFLGDTHEEALELHKAAHSHLNIERRHASLQSVMGLDLT

GYPLDQRVTAEMLANPSQAVRSRTHAELLRSFITKYQPTVKEILDRPEVVGSAHWVSV

GTVETVLNDIIERVEAGAIDGFIALPGGSEKSMEIFFEQLIPKLVERGMFRSEYEGAT

LRDHLGI"

CDS complement(2461163..2461735)

/gene="moxC_3"

/locus_tag="EFAGFIKM_02097"

/EC_number="1.14.-.-"

/inference="ab initio prediction:Prodigal:002006"

/inference="similar to AA sequence:UniProtKB:O34974"

/codon_start=1

/transl_table=11

/product="Putative monooxygenase MoxC"

/db_xref="COG:COG2141"

/translation="MTSNQELYIGLFLNATWNKGDGWRHPDSGVEQIGTIDYYIHWAK

IAEKFKLDFLFRADYLYISPQMLSDSSNFGSPDPTLFFAAIARETNRIGLVTTISTTF

NPPYVVARQLQSLHWLSNGRAGWNIVTSIEGADNFGNSPMPSPEERYAKAMELTDIVC

KLWESFPNEAIVIDRKSGLFSDKDKVTAMQ"

CDS complement(2462039..2463547)

/locus_tag="EFAGFIKM_02098"

/EC_number="3.1.6.1"

/inference="ab initio prediction:Prodigal:002006"

/inference="similar to AA sequence:UniProtKB:Q0TUK6"

/codon_start=1

/transl_table=11

/product="Arylsulfatase"

/db_xref="COG:COG3119"

/translation="MNKPNILLITSDQQHWNTIGAFNSEIETPNLDRLAQEGTSFKRA

YCPSPVCTPSRSSIITGMYPSQHGAWTLGTKLLENRHTVGEDFHENGYRTALVGKAHF

QPLRGTEEYPSLESYPILQDLDFWGSFHGPFYGFDRVELARNHTNEAHVGQHYASWLE

ERGCTNWRDYFLPPTGTMDPSVKHKWAIPEEYHYNNWIAERTNALLEQYREEEQPFFL

WSSFFDPHPDYLVPEPWDTLYDPESLTIPSVVEGEHDHNPPHFKLTQEENPDFGYLQE

TSHSIHGYQSHLTPENERKQLVATYYGMISLMDKYIGKILEHLDKLGLYDNTIVVFTT

DHGHFFGQHGLQHKGGFHYEDLIRLPFIVRYPGKVPADTQNDAIQSLVDLAPTFLSFC

GIQPPNEMTGLDQSKVWMGIDSMARDHVLCEFHHEPTTIHQKTYVDVRYKLTLYYRQT

YGEIFDLQEDPGELRNLWDDPDYSQLKTELLLKYAWAELGKESMPMPRIWHA"

CDS complement(2463564..2465588)

/locus_tag="EFAGFIKM_02099"

/inference="ab initio prediction:Prodigal:002006"

/codon_start=1

/transl_table=11

/product="hypothetical protein"

/translation="MTSKATITIDCSQKSEHVISPYLFGHFVEDIRDHMEAMLAYPLK

DMDFESDALIGTAVSGSWRPYTNGRNTKYALESPARGHSGRAQRIRILSDDEGHAGIA

QSAALKGPMEYSLKLVARASIELQYVVLEVVDRRTEEQLGLVTIPIMNHNWEHYDAKM

FVTRACSDAEIRLYVPSEHPRWLDHVSTGMLWIDHVSMLPADSIGQVKPEVVEMTRGL

NAGMMRLAGNYISAYHFEVAIGPVLERPVMYNEAWGGWTCKYFGTDEFIQFCRELQVE

PLICINDGSGTPEEAAKWVEYCNGSVETPMGARRAANGFPEPYQVRYWEIGNEVWGDW

QVGHCSPDQFAHRSILFAEAMKRADPSLIILACGHTDQEWNRTVLDIAGEHMDYLTLH

LYHGYGRFGMNRDTPAEERYKAMASFPEWTRDNILQTVEHIRKHEKHHHVKLAITEYN

TMYYPNTVRKGNPDEHTLGAAVANAANLNEMLRCSDVVHIGSFSDLVNGWLGGCIRVG

DNYADQYAGKVPGWSGHPLVVYGTPTYEVLKLYAGRDIQRLLSVDVVSGTFSVETLKS

APVLLDTLPDLDVVACTNEDGSLVTVFMVNRSLHAVKAELRLSAFSTDEETLLYEIIG

NSTDDINSVFDPEHILCRQSVIPTNAWQQGYLLRPASIYALEIQTKQATG"

CDS complement(2465609..2466499)

/gene="araQ_12"

/locus_tag="EFAGFIKM_02100"

/inference="ab initio prediction:Prodigal:002006"

/inference="similar to AA sequence:UniProtKB:P94530"

/codon_start=1

/transl_table=11

/product="L-arabinose transport system permease protein

AraQ"

/db_xref="COG:COG0395"

/translation="MIKRATTKGVWDEKVFDAIVYTIAAVIIVLVLYPLLFLVSASLS

DPAKVLSGEVWLLPKGFTLDAYTNILHNEKIWLGYRNTIFYTVIGTVINVIMTILAAY

PLSRPDLPGRKILMLFITLTLFFSGGLIPTYLLIKNLGMVDTIWSLIIPGAISTYNLI

VMRTYFQTIPWELQEAASIDGCSNWRLLRSIILPLSKPILAVMVLFYAVSHWNSYFNA

LIYIRNEALYPLQLILREILMFSQSDAMDSTVGLEDKIMLAESIKYAVIIVSSLPVWI

MYPFVQRHFVKGVMIGSIKG"

CDS complement(2466517..2467479)

/gene="yteP_15"

/locus_tag="EFAGFIKM_02101"

/inference="ab initio prediction:Prodigal:002006"

/inference="similar to AA sequence:UniProtKB:C0SPB3"

/codon_start=1

/transl_table=11

/product="putative multiple-sugar transport system

permease YteP"

/db_xref="COG:COG4209"

/translation="MKKAGVVPTSGLPRARSKPGIGLLRQMARRYDLYLMLLLPIIWY

LIFHYAPLYGLQIAFKNFNPGKGIGGSSWVGLEHFVRFFDSYYFWRLLWNTVSINLFS

LLIAFPIPIFLALIINEIPNKAFSKWLQNITYIPHFISVVVIVGMISLFFSPTSGPVN

LLIQTFGGTPIRFIEEAGWFKTIFIGSNIWQNMGWQSIIYIAALSGINPQLYEAAKID

GASRLRRVWHISLPGIVPAIVILLILDVGHFMNIGFEKVLLMQNSLNSEASDVISTFV

YTTGILKGEYGYTTAIGLFNSVINLTLLLLVNRIARKTSETSLW"

CDS complement(2467531..2469117)

/locus_tag="EFAGFIKM_02102"

/inference="ab initio prediction:Prodigal:002006"

/codon_start=1

/transl_table=11

/product="hypothetical protein"

/translation="MRKPWKQLTLRTMIMFSLVLSGCNSGIVATNEAVPGKHAADVSK

EGFPIVKEPLKLKMFTRIAPVNGPFKDMPIFQDYSKNSNVQVEFSEVPTDGFQEKKNL

LFASNELPDALFRTGLSPLETIRYGTSGQLIPLENLIDEYAPNFKKLMEQYPEILPAI

TTPEGHIYSLPAVVTLSSARMDKKWINQKWLKKLGLKEPETTEDLYQVLTAFRDKDPN

GNGKQDEIPMTARAGGGQGQGLPIVAMMSGSFGLDMQLGYNINLEDDKVNIWMGSERN

KELLMYLNKLYQEKLLDPEIFSHTEAQYLAKQASGNTGFFFDQTNNPFLKIADQYTGI

APLEGPHGDRLQSQGAPVPRDFGAFAITSVNEYPEATLRWIDYFYSEEGSTLLRFGRE

GEHYELKDGIPYYKEDFLNSGNQSKITPYAGGGAPHLISEEVASYINPPQVQEAQKKL

DPYLPDIRYSSPMFDEETARKVEILRNDIDKYYEEQTTKFIVGSLSFDQWNEFQDTLK

KMKIDELEKLYQDAYDSMKK"

CDS complement(2469228..2470082)

/gene="rhaS_15"

/locus_tag="EFAGFIKM_02103"

/inference="ab initio prediction:Prodigal:002006"

/inference="protein motif:HAMAP:MF_01534"

/codon_start=1

/transl_table=11

/product="HTH-type transcriptional activator RhaS"

/translation="MKDYGHEYADIYYYTPNEFEKLGGLWPLRTGHNVAKANYSVGAR

IIECFSFHFVLSGAVTLSSMGETVTLTKGGIFCLFPNVKYSYWVSQYDPESPLRMCWL
AFNGTQAESLVRRIEITEQKPYLRNKLTSEMEELLKACMKQIGGRQRDGDLQLQILFY
RLFEMLSRPLHNVMEDKLKACMQYMEMHYAEGITVADIAEFTGMHRSHLSTRFKQCFG
ISPRDYLIKLRMERADEMLQNGALSITEIALSLGYTDLYTFSRAFGIYYGMSPSNFRS
RMLPLTKC"

CDS complement(2470284..2471351)

/gene="galM_3"

/locus_tag="EFAGFIKM_02104"

/EC_number="5.1.3.3"

/inference="ab initio prediction:Prodigal:002006"

/inference="similar to AA sequence:UniProtKB:P39840"

/codon_start=1

/transl_table=11

/product="Aldose 1-epimerase"

/db_xref="COG:COG2017"

/translation="MVITHHPLTNKGETRLNTNTTSAASASSEGDYQGEKAIYLRYGS
YEAVMLPEIGGNLVAYRDHVQDYQFLHEPTPEEMTSFKARPMIHGIPVLFPPNRYKNG
QFAWNGEMLQLPVNEEATGNHLHGFLYNIPWKVEEYGHTGKESYVSVSITIDENHPVY
SFFPFSFSITLRYALSSDGLMQHVKIRNHGESTMPCLLAFHTSINAPFTADGSARDYK
LKLTVGDRWELDERMLPTTHFQPFVPEEEQMRDSGVYPFFAEMDNHYTAVPQNGRNRM
ELHDTKSGVSLIYDVGTSYKQWMIWNNGAQGGFFCPEPQINLVNAPNLDMKLDEVGLF
SLEMGEIWEETSRLYVRQSHK"

CDS complement(2471320..2473086)

/gene="uidA_2"

/locus_tag="EFAGFIKM_02105"

/EC_number="3.2.1.31"

/inference="ab initio prediction:Prodigal:002006"

/inference="similar to AA sequence:UniProtKB:P05804"

/codon_start=1

/transl_table=11

/product="Beta-glucuronidase"

/db_xref="COG:COG3250"

/translation="MLREDYPRPQFVRQNWMSLNGQWEFEFDDDHRGRSEKWSEGTQS
FSRRIQVPFAFQSQLSDIGDPDFHDNVWYKRTFEIPDGFIGNRLLLHFGAVDYEAFVW
VNGQLVVRHEGGHTPFHGEITDVLQEGTNVLTVQALDYSKDVTLPRGKQFWQEQSAQI
FYTRTTGIWQSVWLEAVDPIHLQKVRMTPDIDRNQINVTSYVHGTLDARDVRLRVQIS
YQGEELTADEYRISAKEESRTIGLHTFNEHGLGRLWSPEHPNLYDIHFTLLVDGVPSD
EVDSYFGMRKISIEQGRLFLNNRPYFMKLVLDQGYFPDGNLTPPSDEAIRQDVELTKE
LGFNGARKHQKIEDPRYLYWCDKLGLMVWGEMANAYEFSQEYVSRMTREWQEAIDRDY
NHPCIVVWVPLNESWGVPNILTSKEQQYHALSMYYLTKSIDPTRPVISNDGWEMVQTD
LYNIHDYEWRKEVLEERYRSRENAVNGLPGNRHLSVGGHRYDGQPILITEFGGIGYKK
SNWEGWGYSGAEDDKDFAERLHAVIQPLLDSPVVEGFCYTQLTDVEQEINGLLTYDRQ
PKMPIEQIRAIIDGHNPSSVNE"

CDS complement(2473137..2474903)

/gene="uidA_3"

/locus_tag="EFAGFIKM_02106"

/EC_number="3.2.1.31"

/inference="ab initio prediction:Prodigal:002006"

/inference="similar to AA sequence:UniProtKB:P05804"

/codon_start=1

/transl_table=11

/product="Beta-glucuronidase"

/db_xref="COG:COG3250"

/translation="MTTKFYSKDYPRPQFVRKDWLDLNGEWDFSFDDNQVGESEHWYD
QAQFPEGLQIKVPFTYETQASGIGIETFHPLVWYRKQVVIPQEVKGKRTILNFQGVDY
RAKCWVNGTVAGEHEGGYAAFSFDITPYITYGSENNIVLEVEDSQSAMQPRGKQRWVD
ENFECFYVQSTGIWKSVWLEHVSEARVEMVKMTPDIDRHMIRLDFQLNGIEGKNNLRL
ETRIELKGQHVQSISLSPDRPWMTVEASVKHEAGGPWKQSLWSPASPNLYDIEFVMYE
DGKEIDRVHSYFGMRKISIENGQILLNNAPLYQRLILDQGYWTESHLTPPSVEALIED
IDKIAEMGYNGIRKHMKLEDPRFLYWCDVKGMLVWSEMAATFEFNDEAVSRFTKEWLE
IVPQQYNHPSIITWVPFNESWGIPTIAHEVRQQQFTQSIYHLTKAIDPYRPVITNDGW
EHTVSDILTLHDYVESGEAFSERYQDKDMIVNNKTASNRWKFAFAEGYHYKGQPIIIS
EFGGIAFQSDKGWGYGNQVDSVEAFIERFRSITEAIKAVPYISGYCYTQVTDVQQEIN
GLLTEDRQPKVPLEVIRKINLA"

CDS complement(2474989..2475795)

/gene="araQ_13"

/locus_tag="EFAGFIKM_02107"

/inference="ab initio prediction:Prodigal:002006"

/inference="similar to AA sequence:UniProtKB:P94530"

/codon_start=1

/transl_table=11

/product="L-arabinose transport system permease protein

AraQ"

/db_xref="COG:COG0395"

/translation="MKWLFRTGSALMAVMFAAPLVWMLVVSIKEEGMKIITVLDWFKP

PYSLAVYEEILTTTKLSQWIANSLFVAVVVTVLTVIFAAMAGFALSKVPFRYRTIVFF

FILGGLLIPGEATIIPLYQVAKDLHLLNSYSGLIIPALASPVAVIVLKSFFDGVPNDL

LESVQIDGGGFWRIFTSIMLPLTRPAMASMAILTFIGSWNNFLWPFLSITDDNLFTLP

MGIPTLMGQYSEDYVKPMVINAVASVPIIILFIIFEKQIVKGISMSGIKG"

CDS complement(2475795..2476658)

/gene="melD_5"

/locus_tag="EFAGFIKM_02108"

/inference="ab initio prediction:Prodigal:002006"

/inference="similar to AA sequence:UniProtKB:O34706"

/codon_start=1

/transl_table=11

/product="Melibiose/raffinose/stachyose import permease

protein MelD"

/db_xref="COG:COG1175"

/translation="MQALVFLLPFLIVYVLFTIWPMIKGIEMTFYKWTLIKKMDFVGL

DNFRKVLQDREVWEAIWHSTFFVFLSTPTMIVLSIVLALIANRKSVLQKFYRIIFFIP

SVLSVAVAAYLGLFVFQPYTGFVNSVLHLIGLLPQNSEIFWLTETSLAWIVITVITLW

WTVGFNFILYLSAMQEIPDEIYEAARLDGASDTQIFWRITLPYLAPLTKTITMLQIIA

SYKVFMQIYVITGGGPLDQTRPIIQLIYQTGFKNNNLSYAATMSYILFVILLVLSVLQ

YWINNRKGADS"

CDS complement(2476765..2478057)

/locus_tag="EFAGFIKM_02109"

/inference="ab initio prediction:Prodigal:002006"

/codon_start=1

/transl_table=11

/product="hypothetical protein"

/translation="MKRRMGLLALTILFLLSMVLSACNGSDSGAGEGDSGDGKVTLSF

WTLFDGGDGANMQTLVDEFNKSHPDIVVKNTKLAWGEYYTKLITAVGNGNGPDIGISH

SSRLPDLIDQGVVTELDTPATDADVDWNSYNQNLLDAVTVDGQHYAVPIDTHAFIMFY

NKKLLKEAGLLGEDGKPVLEQSADGFVSFLKTLKEKLPAKATPFALSNNNDDPFRLWW

SLYTQLGGNDVVSDDLKSAAIDKEKGIQAADYIQKLYTEGYIKKNDPDFYKNFQSGTA

AITMTGVWTTGTWESTKDFEFGAMPIPKFYNQEATWGDSHTIVLPLTQDEDEAKRKAA

MIFADWVADNGQVWAKAGHIPSKPSVLEKQEFKDMPYRSDYSEVASVVKFSKQSTKTA

QIREEVAFKFLNEVWVNKMTPSDAMNNMEAGIQKILSE"

CDS complement(2478246..2479187)

/locus_tag="EFAGFIKM_02110"

/inference="ab initio prediction:Prodigal:002006"

/codon_start=1

/transl_table=11

/product="hypothetical protein"

/translation="MLELSFDDPDKLVTVTHALSTRSRVDILRLLILKNLNIVEIAEA

LKLPVSTVASNIKVLEAARLINTELLPASRGAMKVCSRNYDDIHIALNTEKALPKGDV

QVYEVDMPIGHYSDCEVSPTCGMASADGMIIREDEPASFFHPKHIGAQIMWFRKGYVE

YLMPLEIPTGARIESLELSMEICSEAPNYDHNWPSDISVWINGVDIGTWTCPGDFGDR

RGKLNPAWWYEWSTQYGLLKTWRVDREKTTLDMEKISTVTLNQLNLQDTHKLRMRVGI

KPDAVYQGGINLFGRQFGDYDQNIKMTLNYAMDQEMW"

CDS complement(2479418..2480818)

/locus_tag="EFAGFIKM_02111"

/inference="ab initio prediction:Prodigal:002006"

/codon_start=1

/transl_table=11

/product="hypothetical protein"

/translation="MKIMNYRKKLLLALCAFLIFPMGQISHAATTQNNFYNVVMQDGA

DPWVYKHTDGFYYFTKTTGGNVTIWKSAQLTTIDAAPTTVVNTGCCGIWAPELHYING

AWYIYYAKDDGDNINHRMYVMENTSADPTQGTWQYKGQITDPTNKWAIDGTVLQSNGQ

LYFIWSGWEGDTNVRQNLYIAHMSNPWTIDSNRVEIARPTYAWETNHSPNVNEGPQVI

VRNGVISLVYSASGSWTNNYCLGLITASTSSNLLSPASWSKRSQPIFQSGNGLYGPGH

HSFTKSPDGTEDWIMYHTAKFNNSGWNREIRLQKFTWNADNTPNLGVPVNPNAPIALP

SGEKSVVRYEGEEGTFGGIAYASQSPSGSGGMKAGHIDTVDSYVEFNVNAASAGQYIL

LARTANGTAGGNWSNLLLSVNGGTASPFHITNKGWENWGMSTARVQLNAGVNKIRFSK

GVGYGELDFFDIKPVN"

CDS complement(2480875..2482635)

/gene="lacZ_2"

/locus_tag="EFAGFIKM_02112"

/EC_number="3.2.1.23"

/inference="ab initio prediction:Prodigal:002006"

/inference="protein motif:HAMAP:MF_01687"

/codon_start=1

/transl_table=11

/product="Beta-galactosidase"

/translation="MKEQSSSMSSTPRPEYPRPQFVREAWTNLNGSWQFEIDHGKSGK

ERGYEQSGHQFYGTITVPFCPESKLSGVEYTDFIEAVWYKREFTIPDTWMNGRILLHF

GAVDYEAEVWVNGTSIGKHRGGYAPFHFDITPHVSSGTNVVTVYAEDDVRSGRQPRGK

QSERFHSHGCDYTRTTGIWQTVWLESVPDVYLSDMKVVADPDNACAHLEVKICGNAAG

AELSASARFEGRDVGSETAVIHGPSVKLTVPLSEIHLWEPGHAKLYDLELKIQGKSQA

SDTVTSYFGLRSVRLDGMAFRINEKSVFQRLVLDQGFYPEGIYTAPSDEDLRKDIEIS

MELGFNGARLHEKMFEPRFLYWADKLGYLVWGEHANWGLDITTTESLARFLPEWVEGM

ERDFNHPSLIGWCPFNETWDRGGTRQDNDVLRIVYEMTKRLDPTRPVIDTSGNFHVVT

DIFDLHDYDQNPETFQARYEPMRNGGEVFNTFPERQTYGGQPYFISEYGGIWWNSDQQ

DEKSWGYGDRPQSEEAFITRYEGLTNVLLDHPMMFGFCYTQLYDVEQEVNGLYTYDRR

PKFNPETIRRINSRKAAIED"

CDS complement(2482652..2482999)

/locus_tag="EFAGFIKM_02113"

/inference="ab initio prediction:Prodigal:002006"

/codon_start=1

/transl_table=11

/product="hypothetical protein"

/translation="MYVILPLSKATLAVIFLYYLVGNWNSWFNAMVLLQDRDKFPLQL

LMKEILVAKDSSATIMGSAGGVVIDSASSATAYRELVKYCTIVVSTIPILMVYPFLQK

YFVKGVFVGSIKG"

CDS complement(2483025..2483270)

/locus_tag="EFAGFIKM_02114"

/inference="ab initio prediction:Prodigal:002006"

/codon_start=1

/transl_table=11

/product="hypothetical protein"

/translation="MTIMEAFVLSRRDLYFKNAIMIVITITMFFGGGLIPWFLLMKDI

GLFNNLWAMILSTALSTWNIIILRTGNPIGIGRGSND"

CDS complement(2483267..2483545)

/locus_tag="EFAGFIKM_02115"

/inference="ab initio prediction:Prodigal:002006"

/codon_start=1

/transl_table=11

/product="hypothetical protein"

/translation="MKYRKTMGNRMFDIFNYTLLTLLTLACLYPILHIVFASFSEPAR

LVAHKGLLLRPLGFTLDGYKLIFKDNSLLVGYRNTLIYVGLGTSLIWS"

CDS complement(2484248..2485201)

/locus_tag="EFAGFIKM_02116"

/EC_number="3.2.1.55"

/inference="ab initio prediction:Prodigal:002006"

/inference="similar to AA sequence:UniProtKB:Q82P90"

/codon_start=1

/transl_table=11

/product="Extracellular

exo-alpha-(1->5)-L-arabinofuranosidase"

/db_xref="COG:COG3940"

/translation="MTNKTIFYNPVALQSADPWVYRHSDGYYYFMRTRSDKLELIQSS"

RLSKIDAGTRKIVWTPEPGGPYSHHLWAPEIHFLNEKWYIYYTANDGGGDDSRRICVL
ENESPNPMEGEWVWKGALHTPVPGLDGTVLMLGGQLYFLYAGYGHFPDYGSAIYIMRM
SDPHSLTGEHVLLTAPTFIWEKQGGMAINEGPVTLHRNGRIFLVYSGSTTWSEDYALG
MLTMNEWDDPMLASSWTKSAEPVFRKSLENGVYATGHNSFTTSSDGEEDWIVYHALPN
PGADTVLRATRVQKFGWNPDGTPDFGVPFSDNEALQVPSGE"

CDS complement(2485363..2486211)

/gene="rhaR_13"

/locus_tag="EFAGFIKM_02117"

/inference="ab initio prediction:Prodigal:002006"

/inference="similar to AA sequence:UniProtKB:P09378"

/codon_start=1

/transl_table=11

/product="HTH-type transcriptional activator RhaR"

/translation="MPNNGTSFIVSIERFKPNWFHLEQGEGLFKSHSHAYDELTLILD
GEGYYSSPEQNVKVVAGDLIMIPPGLYHGFVCTEPWQGISVHYYHDHLPVHCRYLFNG
QDQQRNRIQLAHLDKDSFRWADISLSELEKEWRSGNKQSTDSGHLIRLAMETTLLLFQ
RNCSLEVPHIVNDPTGQGIIQEVLKEIHSAYYTPITVSELASRYFLSASNLRKKFTET
VGVSPKQYIINLRLMEAKRLLQHTNKAVEMISSEVGFTSSSRFYDFFVKSVGLTPLEW
RIQNKH"

CDS complement(2486201..2487160)

/locus_tag="EFAGFIKM_02118"

/inference="ab initio prediction:Prodigal:002006"

/codon_start=1

/transl_table=11

/product="hypothetical protein"

/translation="MLELSFNDPEKLVTVTHALSTRSRIDILQLLHANKLNIIEIAES
LELPVSTVANHIRVLEAANLIHTEMLPASRGAMKVCSRNYDDIHIVLDLVSSLSTRDS
NIYEVQMPIGHYSDCEVAPTCGMASTENMILKEDDPASFYHPKHIDAQIIWLAKGYLE
YLLPMDIPTDATIEALELSMEICSEVATYNNDWPSDISIWVNGIEIGAWTSPGDLGDR
RGKLNPAWWPDGSTQYGLLKKWRVDNNKTMLDKEKISDVSLSDLNLGDKPKLRLRIGI
HPDAKHQGGINLFGNEFGDHEQNIIMQVRYTMNVGDKDARYAK"

CDS 2487425..2489908

/locus_tag="EFAGFIKM_02119"

/inference="ab initio prediction:Prodigal:002006"

/codon_start=1

/transl_table=11

/product="hypothetical protein"

/translation="MKKWRILFLSAALCFLMPTNIYAYNNPLNLSDSWHWSNSDFYGE

GDPYILKFNGTYYLYVSTVDDSIGVKVWSSTNLVDWTYRGICTKEAVTKAAYAPEVVY

WNGQFYMYTSPGGGGHYVLSSPSPTGPFTLQTGNLGMGIDGNVFIDDDGKWYFYSTGA

NTIEARPMTSPTTFGAPITTGLSMAGWTEGSTTFKRDGKYYMTYTGNHVWSTAYRVDY

ATGSSPTVGFKPTADLNPVLINTEGTNVGLGHNSIIKGPDLDSDYIVYHSHASDGNIT

YPGRKMNMDRIVWNGEKLLVQGPTIASQPNPELPDFEDRFNRTSIGNNWTNVGGGTWG

IYNQELMWQDTIGSASTYRQITAVNSASDYTAEFNLKQIKQGTIENPVYGTVFSYTNE

NSYGVAVLNRNKNRLETNYVVEGVSQGWQYASLPAGYDYSKWHQIRVEKAGSSFTVYV

DGMKKQTRTIHGLNGGKIGYTTTDAHADFGYTAFNNKVNGSSAWNMYKPLPGKIEAVH

YMDGIEGTAYHDLTPANIGGAYRKDAVDIRSSAAEGKNVVGWNQTGEWLKYRVNVAET

GYYDLDVRLATTFNDASFRVWDGATDLTGIISAPNSGDWEKWTTASKKGLYLTAGNHE

LRFEFVQGEFDFSDMTFTRGEAVADMVDDFNDGNDNGWTRFEGNWSVIGGELDSAGNG

VFGKTTIGKESWADYTVESDIKLIDTSGDAGILFRVNNPSNGKVLANNPDYVQAYYAF

IKPDGVYLGKMNYGYEQLASVPLSIASGIWQHMKVVTSGTTIRVYVEDMNTSKITYKD

NSMNPFTHGKVGLRTMNNHTRFDNFRITP"

CDS complement(2489967..2491175)

/gene="araR_1"

/locus_tag="EFAGFIKM_02120"

/inference="ab initio prediction:Prodigal:002006"

/inference="similar to AA sequence:UniProtKB:P96711"

/codon_start=1

/transl_table=11

/product="Arabinose metabolism transcriptional repressor"

/db_xref="COG:COG1609"

/translation="MYNQQPSLDEISCPRSSTHSILNMVIVMKNIPLYKQIQHAIKHQ

IWLGNLRSGDRIPSEKELAEQFHVSLITTKNAMAGLAEEGVVVRIKGKGTFVAGQASF

KLRIPIETYQKTDESLAAHHIPLPPKAKEMIGIIIPSMRTKVEQRLLDAIENAVNENG

YTLVIRVSRGSSVKELLAIDELYELKVVGLVLFSIGSEHERQTVDRATELGFPYVLID
RYYTDKPNVSVCSNNIEGAYKAVTNLVKQGFRKFALVTPPKAHSVVIERIAGFEKAAA
KAHISPEHIIRLTLPYEIFIESIQHREQILDNWISQYYGRFTALVAVDVELARLAHYS
LIRTIGREKAKHIAIVTFDDPGIDGIAYVQQDEKMIGIQAVTLLLDQIHHGIRTPKNL
FVPMEWIPSS"

CDS complement(2491282..2491656)
/locus_tag="EFAGFIKM_02121"
/inference="ab initio prediction:Prodigal:002006"
/codon_start=1
/transl_table=11
/product="hypothetical protein"
/translation="MDSDILINQFLRLFPEFYDCYIEHLDLNQEFLGHVFFGDEVATY
VEGLLRENDDTELIEKFFNFFEWMAIQSDLYIVQVLSTTILYDLGGQSDILQIAQSYM
KPQTKRLSQEIEDLHSGKYFID"

CDS complement(2491742..2491954)
/locus_tag="EFAGFIKM_02122"
/inference="ab initio prediction:Prodigal:002006"
/codon_start=1
/transl_table=11
/product="hypothetical protein"
/translation="MLKIINFLLFLLIIWVVIFYPFRFAWFEGLKQIPADLHGVYLLF
LFFGYLICSSAMGLIVGKMFFKRKEQ"

CDS complement(2492266..2493504)
/locus_tag="EFAGFIKM_02123"
/inference="ab initio prediction:Prodigal:002006"
/codon_start=1
/transl_table=11
/product="hypothetical protein"
/translation="MSTSKKRVQFIDGLRGFSLAGIVLANMLAFQYGMYGQSKPQLFG
IGGADQAFLSFLLITVVGSFMPIFAFMFGFGMVKLSESLKSRDLRPKCHLARRFLLLF
GIGLLHIIYLWEGDILAFYGVLGFFLLMFLNRKPKTLLIWAVLLLMGAGILGLPASNP
MNPLAIESIHMENYIIQSQDVYGNGSYAEIRDFSNNGDPFGGDLEVSFALLILLLAPL

MTVPMFLLGMHAARIGTFSDPQTMRAMYLRRASLLIPVGLILKTYGVLAPQLGAEGWL

GIGIGGTLGGTLLALGYIYAFALLYAGNRRSSLMGRFEAVGRLSLTNYLMQSVICTTI

FYGYGLGLFGRAGVFIGAIIALAVYGIQLWLSPLYLKKFSNGPIEYLLRIWTYLSWKG

QPRKKKRSNLTTHENAPGVQ"

CDS complement(2493669..2494409)

/gene="map_1"

/locus_tag="EFAGFIKM_02124"

/EC_number="3.4.11.18"

/inference="ab initio prediction:Prodigal:002006"

/inference="similar to AA sequence:UniProtKB:P99121"

/codon_start=1

/transl_table=11

/product="Methionine aminopeptidase"

/translation="MIAKTEEDFNGLKEIGKIVASIRDELVQRTIPGITTKELDDLAG

ELFEKAGAVSAPKSEYNFPGFTCISVNEEVAHGIPGDRVIQEGDIVNIDVSGSKNSYF

ADTGLSFVVGEGAEVLTKICDVVKLAFEAGLKKAKPGSKKSGIGKAVFQTARQHELTV

IKNLTGHGVGRGIHEAPDHIYNYNDPSDDELLKEGMVIAFEPFISTSEEEVDQTGDGW

TFVTKNSYVAQIEHTIILTKNGPIIVTL"

CDS complement(2494567..2495529)

/locus_tag="EFAGFIKM_02125"

/inference="ab initio prediction:Prodigal:002006"

/codon_start=1

/transl_table=11

/product="hypothetical protein"

/translation="MPKNDNMLAILWMLNSGTKVTAKQMSEKLEINIRTVYRYIDALC

ASGVPIISDTGHNGGYSLLNQHIKAPLLFDIDEKKALLQAAVFAKEAGYPLSEALDNA

TSKLKMYTNQDQENTLRRHLAGFNVINRMGDPSVQPILAELEQAVANDCSVEIDYLRS

RDEQPKNRVIDPYGMVHWNNKWYTIAFCHLRNEFRSFRADRILGIKLTPLRFKRSEAF

SAREFFLQNLLPDVGGKEGLISLVIEGRSEALDDLGLHWFMGHYLKERTSNQAIFLFD

EKSIDTYVPYFLLSYGKSIQVIEPQRLKDRLVAVVSELMEYYQL"

CDS complement(2495750..2499802)

/gene="cpdA_2"

/locus_tag="EFAGFIKM_02126"

/EC_number="3.1.4.53"

/inference="ab initio prediction:Prodigal:002006"

/inference="protein motif:HAMAP:MF_00905"

/codon_start=1

/transl_table=11

/product="3',5'-cyclic adenosine monophosphate

phosphodiesterase CpdA"

/translation="MISHLYKKMLPILLSLSLVIGWFAGFGGANSAQAASLSEPDILL

ERNANWKYFDQGQNLESQWRASYDDSSWEFGLAPFGYKDNGNGISTSYFGPLKTEVSY

GEDKKLKYRTTYLRTSLTVNKDEIDGYGQILGTFGIDDGAVIYVNGVEVRRFGMPEGE

IQYSTKATSSQDLPVMYEDIDLTEPLKAYLHDGNNEIAVEVHQQSDSSSDLYFDMELK

ALAEAPSLDINKVTVTFYGDATTSKGFTWYTPLASTQSTVQVVPNQGSAADFSQAQSF

NGRSSVASNSPGEMVHKAEATELTPDTSYYFRVGDQDLGIWSEVGTFKTAPTNGAFRF

IDLTDTQAKEEDEAILSGSTLSKALSQVPDAAFVVHNGDVVENGTSEQEWNWLLGHSQ

SSLMNTTIAPSAGNHENKNYAFYEHFNLKQPDNAATVTGAYYSYDYSKAHFIVLNSNE

NSSEYANFSNEQVAWMKQDVEEAKAAGAEWIIVNIHKGPYTTSNHATDSDIIGANGVR

NKIAPLMNELGIDLVLQGHDHIYARTKPIKSDGTAEDVSKITETLNGQSIEYAVKPDG

TIYMIPATAGAKVYFKNQKPELGEAYFSLFERAEENHARQYGDTTSAARGQVQNFVSF

TIDGSKLTAVTYEIDQHTNNGEPFIVDQFGILKETDTVPVPEQISKITVTFHGDPTKN

KGFTWYTSDQLLQSDLQVVEKTEATPDFNQAKLFQGRSVQPANSPTERMHKAEATDLK

ANTTYFFRVGDATKQVWSATGTFRTAPAKGKFTFIDLADTQAKEEDEAILSSETLAKA

LTTVPDAQFVVHNGDIVDKGVKEEQWNWLLGHSQESLLNTTIVPSSGNHEDENYAFID

HFNVQPPANSATETGAYYSYDYSNAHFVVLNSNEDSTEYDNFSLEQVKWLKEDVQAAK

KKGAEWVIVNIHKGPYTTSNHATDSDIMGVNGVRSKIAPIMAELDIDFVLQGHDHIYA

RTKPIDQKGNARQPEMITEMQNGQKMEYSVNPDGTIYLIPATAGPKVYYKNQSTSLGE

AYYSLFERAEENHAAQYGPDPSDNRRPKRSQVQNFVGITIDGSKLTAITYEIDQNVNG

ATPFIIDQFGIVKKTETTIPDPGTTNPGTSNPGSGNGSSSGNSSSGNAGNTATDNRPD

NGTGDGSNPGNETDTGSNNNGNSENTPPMKDTAGHWAKSAIDRALAAGFVNGYGDDTF

RPNQKVTRAEFITMLGRALNLNNISDNVQYTDENKIPSWSKPYITAATSLGIINGYAN

GSFGPSKTLNRSEMVTMIVRAGGIPTDSNTKLNFKDAKDVPAWAVSYVASAVESGLVG

GVGSNRFAPMQTATRAEAITLIIGLLDKVK"

CDS 2500096..2501220

/gene="yndE_1"

/locus_tag="EFAGFIKM_02127"

/inference="ab initio prediction:Prodigal:002006"

/inference="similar to AA sequence:UniProtKB:O31809"

/codon_start=1

/transl_table=11

/product="Spore germination protein YndE"

/translation="MKQATTKINTSEVIIVVINSILGAGILTLPRTISKAVGTPDVWI

SVILSGLIVTTISILLVTLCRRFPGKTVFEFIPEITGHWISYLLGLLIIVYFVVLCSF

EVRVMAEITSMYILERTPTWVTIMVSLWIGIYMLTGGLKVIIRVFSIVLPVTLALLAL

VFLLSSKLFELNNLRPLLGDGFMPVLKGLKPSCLSYSGYEVLLIITAYMVDLKASNKA

AIYSILSCTIIYLVTIVTVVGNLSLPGIQTRMWPTFDMVRSFEIEGFLFERYESLFIV

FWLMQIFATYAFKHYFAAIGVRDLFRLKNITSIQFAMLPVLYVIAYLPKNLDETLALG

DFLGNMSIFLFGLLPLLLLIISFVRKKGGKQASSEALKAS"

CDS complement(2501308..2502576)

/gene="codA_2"

/locus_tag="EFAGFIKM_02128"

/EC_number="3.5.4.1"

/inference="ab initio prediction:Prodigal:002006"

/inference="similar to AA sequence:UniProtKB:P25524"

/codon_start=1

/transl_table=11

/product="Cytosine deaminase"

/db_xref="COG:COG0402"

/translation="MIIQNAKLRGKEGLWNIVVKDGKFERITQSLETTENEEILDVNG

SLVLPPFIEPHIHLDTTLTAGEPEWNLSGTLFEGIQRWSERKAFLTHEDVKTRSKTAL

KWQLAQGIQHVRTHVDVTDPSLIAVKAMLEVKEEMAPYMDIQLVAFPQEGIHSYPNGA

ELLEESLKMGVDVVGGIPHFEFTREYGVDSMKVAFDLAEKYDRLIDIHCDEIDDEQSR

FVEVVAKEAYERGLGSRTTASHTTAMGSYNDAYTYKLFRLLKMADLNFVSNPLVNIHL

QGRFDTYPKRRGLTRVKELQEAGLNVCFGHDDIFDPWYPLGTGNMLQVLHMGIHASQL

LGYDQIVNSIDLITKNSARTLHIEDVYGIEEGKPANFIVLEAENEYEAVRKQAGVLYS

YRGGRKIAESKPRDTSIIFEGGTEKVTFNK"

CDS complement(2502545..2503897)

/gene="codB"

/locus_tag="EFAGFIKM_02129"

/inference="ab initio prediction:Prodigal:002006"

/inference="similar to AA sequence:UniProtKB:P0AA82"

/codon_start=1

/transl_table=11

/product="Cytosine permease"

/db_xref="COG:COG1457"

/translation="MYNLTSSLFFLLFLVSDTLYVGGILMSKQDQEFSWQAVPKSQRN

HFWKTLSVMLGFTFFSASMLAGGTLGVSLTFMEFIGIVLAGNLVLGIYTGALAHIAAK

TGLSTHLLAKYAFGAKGSYLPSFLLGFTQVGWFGVGVAMFAIPVAKAMDWNVYLLIIV

FGLAMTASAIFGMKSLVILGYIAVPAIAILGGYSMFEGAGSLGGLQGLLDYTPTQTLT

AAAALTICIGSFISGGTLTPDFARFSRTSKQAVTATVIAFFLGNSLMFLFGAVGAMAY

NLADISEVMFLQGLIIPAIIVLGLNIWTTNDNALYASGLGFANITKISKKFFVIVNGI

VGTVFAMWMYNNFVSFLNVLGAAVPSIGAIIIADYFIVKRRNYKPFADMSFKNVNWVA

MVAWAIGVAFAQLAPGVTPLNALLGTAVAYIVLMLIVPAKESKEMGKINDYTERKIAG

"

CDS complement(2504184..2504834)

/locus_tag="EFAGFIKM_02130"

/inference="ab initio prediction:Prodigal:002006"

/codon_start=1

/transl_table=11

/product="hypothetical protein"

/translation="MRYSWWEFGFYLVVLMFVLFMQINEYPLFLIVGVVVVTILIAFI

KHVFYPLVFDKRIDRLESFLSKQQNTPGTYIIYVLANRLDDEVELVMEQIMQKYKRKT

TQAQFKAAYGLYLKDMFAIRQAVAHIGLSDYRTYYETILLLEDGKSEDARERLQSIKK

QWMRSTLLAGLELKAENRDTAIQHAHEALNSSRGVNRYVLYKEYERVLPEVVEHIS"

CDS complement(2505026..2506060)

/locus_tag="EFAGFIKM_02131"

/inference="ab initio prediction:Prodigal:002006"

/codon_start=1

/transl_table=11

/product="hypothetical protein"

/translation="MRIAIVGAGSLGTIVGAYLADGGLDVELIDAYQEHVNALNQTGA

KVTGTTEFQAKVKAITPDQKSGQYDLVLLLTKQLYNNSILQELLPFLKEDSMVCSLQN

GIPEEKVASIVGEKRVIAGSVEFGATFIEPGVSSLTTEYTQFKQYAFQIGELNGEITE

RIQRVKSVLDLVGGTHISDNLVGTKWSKLLINNAFSGLSAALNGEYGDIIDHEAGIVS

AAHIADETIKVARANGVTLVKMNGFDIASLELNSEEDIPERVKTLRYVMEPSRLLKAS

MLQDLEKNRKTEIDYINGVVSSRAEGTGIATPYNDLVVKLVKSAEETQTVPDFDTNIK

AFEEILSAHK"

CDS complement(2506075..2506821)

/gene="adc"

/locus_tag="EFAGFIKM_02132"

/EC_number="4.1.1.4"

/inference="ab initio prediction:Prodigal:002006"

/inference="similar to AA sequence:UniProtKB:P23670"

/codon_start=1

/transl_table=11

/product="Acetoacetate decarboxylase"

/translation="MKIDVNNIAKNLNTPLTAPAYPMPPYKFVNREYLNIIYRTDEKA

LRAAVPEPLEITEPLVKFEVMWMPDVSGLGAYTEAGQVIPVQFNGEEGDYVHSMYVDN

FPAIASGRELTAYPKKLGAPKLYTDSDTLVGTLDYGTLRVATATMGYKHVEMDKEFAK

REICRPNFMIKIATDYTGNLRICDLIRTQITDIEVKEAWTGPARLQLFEHALAPLADL

PVLEVVSASHILTNLTLNAAQPVYNYLEEK"

CDS 2506973..2507860

/gene="gltC_1"

/locus_tag="EFAGFIKM_02133"

/inference="ab initio prediction:Prodigal:002006"

/inference="similar to AA sequence:UniProtKB:P20668"

/codon_start=1

/transl_table=11

/product="HTH-type transcriptional regulator GltC"

/translation="MELLQLKYFQTVAYTEHISKAAAQLNIAQPSLSLTIKRLEDELG

TLLFHRRGRNIQLNPSGEILLKHVNRIFIEIENAEMEIKAEEQQISNTIRISITNTRF

LTGLISDYINGSPETKLHQGIGTRSEIITGLKKGDMHLGITGHPIQDEEIESVVLVEE

DIVLVVPMNHAYGGETSISLSVVANEPFISLADNKEYSRFTTMLCEKAGFLPNHAFEV

DSHTLLEIIRLNQGVALLPVSVCRTLGLRYAKIADDSAIYPISLSWVKQKWLSPAVRE

FRDFITSYYEDHAGLFKVE"

CDS complement(2507942..2509348)

/gene="melA_1"

/locus_tag="EFAGFIKM_02134"

/EC_number="3.2.1.22"

/inference="ab initio prediction:Prodigal:002006"

/inference="similar to AA sequence:UniProtKB:O34645"

/codon_start=1

/transl_table=11

/product="Alpha-galactosidase"

/db_xref="COG:COG1486"

/translation="MNAKSTQHPKVVVIGAGSLFFGRQSIWQMVHSPYLNQGTLALVD

TDEERLSKMVKLAEMVAKENNVSLKIEGSVDRRQVLTGADFVVLSFAEESVKYRGIDC

QISLKYGIRMCSGDTIGPGGIFRAMRELPVIMECAKDIEELCPDAWVINYINPSTVHG

IALHRYAPKLKSFALCDSHHMPHKKAYYAVRAGIIGDNSQFTEEINQEFDFRIAGVNH

FTWLLKAEYEGTNVMPTIAEAMRKMAGDENNGGDRGAKALYNDAITYELYDIFGIIPT

CTAHTKEYVRYWQGLGKTADTIPPLSIWETEDRYERHDEMWRQVDDFLAGNIPIADYM

STFGPDHATDIIENMVGNLGKKFFINTLNQGAVTNMNADSFLELLCDVDMDGVKPLHV

GEMPRGIRGMQELVLDTHELTVEAVLEQSYEKLRRAMLTDPLVSSISDADKIIHELLE

LERKMIPDVWYKDRLQYS"

CDS 2509546..2510376

/gene="rhaS_16"

/locus_tag="EFAGFIKM_02135"

/inference="ab initio prediction:Prodigal:002006"

/inference="protein motif:HAMAP:MF_01534"

/codon_start=1

/transl_table=11

/product="HTH-type transcriptional activator RhaS"

/translation="MSIIDELSEYITLRMSSYLEQTHDSNWTEHKSHSDYDLWFITAG

SVLITIDGIEHMANPGDVVFFYPDMPYTASTTGELCRFVYMHFDFSIAEQKRILGEFQ

LPGIVLGNLIQEESTLFTSSYRRFKQSSGASASPLYLKASLLLVIAKILELHGQGLYH

GEFLKDRKPRKIEGSLEVLQNVFPYVDANLHRAIRVNELADIAGVSEKYFISLFKKIL

GITPGQYINQIKMNRARDYLYEKKYTIQQIAGFLGYPDPFTFSKAFKKFYNVPPSKFE

"

CDS complement(2510475..2511599)

/locus_tag="EFAGFIKM_02136"

/inference="ab initio prediction:Prodigal:002006"

/codon_start=1

/transl_table=11

/product="hypothetical protein"

/translation="MWTLHCGKPSRQCLLEEIIINFNEAVNISNTHLLTLVSELFHLE

GYNIQLIPPHEGGRNVVYICEQEGRESLILRVSFLPDRKREDYIAELEYVRYLFEHGA

SVSNVVSSKKGHLLEEITYDEHTFFVCMFVKAKGKLLVENHYQYREGVPLSEYYYNSG

KVLGRMHQLSKGYTPIHRRHHLIDNYSGEYIDNLVPESFPLLKEKMVELLNTLEGLDT

NQETFGMIHFDYNDGNYSIDFDTGQITVYDFDNSCFGWYMYDLADLWTHGVGWIQFEP

NADKRKQFMDDYFQTALAGYTSETKIEDSMLEKLPLFIQVTLLENILGQFEEMQRAGE

ELEANEELLYLIKCLEEDIPYKGFFHEMYSTEAPFEYEGR"

CDS complement(2511773..2513239)

/gene="bglH_7"

/locus_tag="EFAGFIKM_02137"

/EC_number="3.2.1.86"

/inference="ab initio prediction:Prodigal:002006"

/inference="similar to AA sequence:UniProtKB:P40740"

/codon_start=1

/transl_table=11

/product="Aryl-phospho-beta-D-glucosidase BglH"

/db_xref="COG:COG2723"

/translation="MTKTIKHSFPEGFLWGGATAANQLEGAYDADGKGLSTSDMAPFV

PHEERNGKDFTFDVDSVQLEEYLSGNTNVYFPKRNGVDFYHRYKEDIALFAEMGFKVF

RLSIAWTRIFPTGEEAVPNEAGLAFYDNVLDELLKYGIEPLVTISHYEMPVELTRKYN

GWESREMIDLYLKFANTLFDRYKDKVKYWITFNEMNMMLTSLYTGGGILEDKIKGSQE

QVAYQATHHQFVASALAVKSGKEKMPNAQIGCMICRLETYAASSKPEDVLQTMKEDQM

NLFYPEVQARGEYPSYMQRYFEENGIKLVKAPDDDAIIRDNTVDFIAFSYYMTYIGKY

DPNDNSNSGMLVSQIKNPHLKATEWGWPIDPIGLRVALNRLYDRYRMPLFIVENGLGA

KDTLEEDGSVHDTYRIEYLRSHIEQMKEAVADGVELMGFTSWGPIDIISCSTSEMGKR

YGFIYVDQDNAGNGSLERTRKDSFHWYKQVIETNGEIL"

CDS complement(2513282..2515219)

/gene="bglF_7"

/locus_tag="EFAGFIKM_02138"

/inference="ab initio prediction:Prodigal:002006"

/inference="similar to AA sequence:UniProtKB:P08722"

/codon_start=1

/transl_table=11

/product="PTS system beta-glucoside-specific EIIBCA

component"

/db_xref="COG:COG1263"

/translation="MNHRELSKEIIQLTGGQENITQAWHCITRLRFNVREQNKVQLEQ

IKALDGVLGAQFQNDQFQVVIGNQVAAVYEQIEDQMKQSGIAKPETDAPRSKGINAVL

DTISGIFTPILPAIVGTGMLKGILALLVTLGAIQENSGEYQVLSSIANAAFYFLPFLL

ALSSARKFKVNEYIALTLAGTLLYPTMLNAYLANQLEPIRFLSLRVSIVNYTQSVIPI

ILGVWLLSYVHRWVDRFIPGPVKVIFTSMIVLVITVPILLIAIGPLGNYMGIYLEMGT

SWLFAHSGPLTGIILGGFMPLIVMTGMHYAFFPGTLQNLSKLGYDVLLLPINLITNMS

QAGAVTAVFLKTKDKKMKSIALSSGISALLGITEPALYGVTLKLKKPFYASLIGGAAG

GGFITAVGLKCFGFAVPGLLSLPLYIGPNGGMSNFWYALIGIGISFTVSFVVTLLLKW

DETNARNSDMSDSAQTLTEQEQDTTTAFSQETVTPVTVHSIEEKKGEVFSPLTGELVP

LAELPDQTFAQEMTGKGIAIRPQDGRVTAPFDGTVTLVAKSKHAIMLTSSSGIDILIH

VGLNSVSLKGKYFDVKVVTGQEVKKGDLLLEFDMDGIQGAGIDLVTPVIVTNTPDYLD

VVPVQVKGVIPMNELLLLTVR"

CDS complement(2515378..2516226)

/gene="licT_5"

/locus_tag="EFAGFIKM_02139"

/inference="ab initio prediction:Prodigal:002006"

/inference="similar to AA sequence:UniProtKB:P39805"

/codon_start=1

/transl_table=11

/product="Transcription antiterminator LicT"

/db_xref="COG:COG3711"

/translation="MRFKKSLNNNIALAEDAEGCEVIVIGTGVGFKKVKGQPIEQSQI

QKMFRIGSNDKYQRVEQFLSDIPLQVIDITDQIIEEGKTMIGKKLNDSILLTLADHIH

FALDRFKKGVDVQNPLHWDIRHLYPAEYRAGEWAVQKINDAFLVSLPSGESSSIALHF

VNSQFDSGSMNQTIKITQMINDILGIMTEHFGMALNQESADFSRFITHLRYFIVRQLN

REVLSFKDQQFLYDVLSERYPVSFQCALKIKEAMEQQRGFVITPDEMVYLMIHIERVT

SRTDTD"

CDS complement(2516457..2517389)

/locus_tag="EFAGFIKM_02140"

/inference="ab initio prediction:Prodigal:002006"

/codon_start=1

/transl_table=11

/product="hypothetical protein"

/translation="MMLGTDAASLLIYEALASEARLNIVRLLLQNREMHINALAQELF

LSKAIVSTHVSKLQKAGIVGSRMKRENGGTYKYCFIVREFMTINLSPEPVDAPYHEIS

IPVGQYTDYEAWPTCGIATTTQMIGQYDTPACFMDPDRVNAGILWMARGFLEYKIPNY

LYNDQHLREIEISLELSSEAPKVNENWPSDIRFTLNGNDLGTWTSPGDFGDRKGKHTP

LWWKLDVNQYGVLKMLRINGEGTFIDGQRISDVRVQDLELGASTYWTFGLKPEEGVAG

RGGLTLFGKGFGNYDQDILIRYYYDAQENKTPDE"

CDS 2517598..2519106

/gene="abfA_1"

/locus_tag="EFAGFIKM_02141"

/EC_number="3.2.1.55"

/inference="ab initio prediction:Prodigal:002006"

/inference="similar to AA sequence:UniProtKB:Q9XBQ3"

/codon_start=1

/transl_table=11

/product="Intracellular

exo-alpha-(1->5)-L-arabinofuranosidase"

/translation="MLTSKMLIDKDFQIAEVDPRVYGSFIEHLGRAVYGGIYDPGHPT

ADAQGFRQDAIEAIKALNVPIVRYPGGNFVSGYNWEDGVGPVAERKRRLELAWWTIET

NAVGTNEFADWAKLVGTEVMMAVNLGTRGIDAARNLIEYCNHPSGTYWSDLRISHGYK

DPHKFKTWCLGNEMDGPWQIGAMTAYEYGRIANETAKAMKWVDPDIELVACGSSSRDM

NTFADWEATVLDLTYENVDYLSLHQYYNNNKDNTYDFLATSLDLDQFIDSVASICDFV

QAKKRSKKKLMLSLDEWNVWKSIGTSRMEERWQIAPPEFEDVYTHEDALAVGCYLITI

LKHADRVKMACLAQLINTIAPIMTENNGAIWFQTTYFPFMHASNFGRGTVLRSITSSP

KYDSKDFTDVPYLEAISVHDEENGTITIFAVNRHLDEKLELNVDLRSFGETTFVEHIV

LENNDLKATNTKENPRNVVPHNGGYTTVDQGKVQAVLNKASWNVIRLKTKQA"

CDS complement(2519690..2520262)

/locus_tag="EFAGFIKM_02142"

/inference="ab initio prediction:Prodigal:002006"

/codon_start=1

/transl_table=11

/product="hypothetical protein"

/translation="MAKTNEIHVRVKRLKDRPVCVTLHNGETYVGYISGVNSEGVVLT

GGGKLTQTAITAPSSEGSARKTKPKAGATRKTVSSRNRKRVNSTRKSQVRSRSMSRSK

SRQAQVSSFMPMLGSLLGGFGGAGSIGGMLGGGMRLFGMIQRFTPVVKMGYGMIKQIQ

PFMGAVQGLMTPASQAAQAETDGEEEAQQG"

CDS complement(2520379..2521293)

/locus_tag="EFAGFIKM_02143"

/inference="ab initio prediction:Prodigal:002006"

/inference="similar to AA sequence:UniProtKB:O31606"

/note="UPF0413 protein YjbH"

/codon_start=1

/transl_table=11

/product="hypothetical protein"

/translation="MSNESMMCDLETGVCGVNEEEAMQEINLNHVEKRITLYYATDPI

CSHCWALEPVLHRFIEEYGHYFTLQIKMGGLLANWNGFSDGANGIHKPSDVAEHWKEV

GEHSRMPIDGSLWHDNPILSSYPPSRVFKVIQSTHPGKEQDFLRRAREAVFAFNRNIG

EDDVLTDIVNQLGLNGKEVVEAAAEQSAQELLEEDFESVASLGVRGFPSIIIVNEENQ

GMKIVGARSLETYVQALQQVLGGDLKPKQITSLEQKINEGHLLFSKEIEGLYSIEKSD

IESYVKSELAEHTYRIGHILNEMYIEHI"

CDS 2521428..2521775

/gene="yybR_1"

/locus_tag="EFAGFIKM_02144"

/inference="ab initio prediction:Prodigal:002006"

/inference="similar to AA sequence:UniProtKB:P37486"

/codon_start=1

/transl_table=11

/product="putative HTH-type transcriptional regulator

YybR"

/db_xref="COG:COG1733"

/translation="MKYEFNFDQLCPATYAFQVIGGKWNLPILAILSENDCIRYNELK

RRLPGITGTMLTNCLKDLIHSGIVHREQYNEVPPRVEYSLTESGKELVPLIESMVIWG

QKNMTAGVKQQDI"

CDS complement(2521937..2522401)

/locus_tag="EFAGFIKM_02145"

/inference="ab initio prediction:Prodigal:002006"

/codon_start=1

/transl_table=11

/product="hypothetical protein"

/translation="MERKIRNSAKALIIKDGRMLAMKQDDNDEVIYILPGGSQNAGET

LTDAVKREVAEEIGIDVEPLSLEFVIEGVYGEALHRVDFVFLCEYIGLMDQDSSILPS

DENQVEYEWLEIKNINELPLFPSKLRKQIIRLVEGEKTVMYLGNEEDDDLNS"

CDS complement(2522513..2523397)

/gene="gltR_3"

/locus_tag="EFAGFIKM_02146"

/inference="ab initio prediction:Prodigal:002006"

/inference="similar to AA sequence:UniProtKB:P94501"

/codon_start=1

/transl_table=11

/product="HTH-type transcriptional regulator GltR"

/db_xref="COG:COG0583"

/translation="MEIRQLKTFWTLASTCSFSQTAELLSYVPSTITMQIKSLEEELG

VKLLDRLGKKVVLTDAGQQFLPYATKILNDVEEAKCISSQHGELAGTVVIGADEVLCA

YLLPALFKRFRADYPGVRLLFRPLSGQELKSSLREGHTDVVFVLDEPINSKDLHSEFL

KDETFQMVVSPDHMLASRSALVIDDFHKQHFLLTEKNCSYRTYFDQSITKKGADALTE

LEFHSVEAIKQCVAAGLGIALLPEMALKKELSDGEVVALPWDLSDVSFSAQMLWHREK

WISPSMAAFMEVAKSELI"

CDS 2523522..2524532

/gene="metXA_2"

/locus_tag="EFAGFIKM_02147"

/EC_number="2.3.1.31"

/inference="ab initio prediction:Prodigal:002006"

/inference="similar to AA sequence:UniProtKB:Q3M5Q6"

/codon_start=1

/transl_table=11

/product="Homoserine O-acetyltransferase"

/db_xref="COG:COG2021"

/translation="MNDYETYNLGDTLLQSGQQLPQAFIAYKTYGNLNAAKDNVIVVP

TWFAGIHTDNEWLIGTDKALDPSRYFIIVPNMLGNGLSSSPSNTPAPYDKNNFPLISM

YDNVRAQHQLITQKFGISKIKLVVGWSLGATQVYQWGTSYPEMVERIAPFGGTAKSSP

HTQLVFEAMIAALQADSNYKNGRYERPPVAGLAAMGRAYAPWGFSQAYYLEKLYQSEG

YDSLKSYVEDYWDQVFLPFDANDLITMLRTGIYGDISDNPIDDGNFEQALSKITAPAL

VMPGSSDLFFTPEDSSYDAQHMPNAVYQPIESKWGHCFGIGANEEDSLVIDHHLKQFL

ET"

CDS 2524719..2526323

/locus_tag="EFAGFIKM_02148"

/inference="ab initio prediction:Prodigal:002006"

/codon_start=1

/transl_table=11

/product="hypothetical protein"

/translation="MNKKTKALVLSVCMSTLLLAACSNGSGADKGSTTDTDSNEAGTT

TIHTVTSEYSSAKYPKGDDITNNVWIKRYKEKFNIDVKTDWVSDEYDTKLNLAIASND
LPDVFRVNPSQLRQLVEADMVMDLSEVFDQHASDRLKGYMEADADSYESGKKDGKLYG
IPQMHWGLIEQPDFIWIRNDWKEELGVQDPKSVEDIKNIALKFMEKHGGYGIAVDQSL
DYLNLLAIAWNAHPDMWMEDSTGKLVYGSVQPEMKNALAEWSEWYKRGIIDPEFAIKD
FNAMNADIVAGKVGMQPYYQWWGYNPGVDTVSNLGKDAIFYPYIIPTVDGKEAKQSIF
FANNNYIVMKKGFKSPEEVIKILNDYAYIVDEGNGKESTETLSALLDNDIAHVVGAFR
VLNPNSDYEQFEAVSAALQSKDTSGLTTSGMWQKYNNSVEFIENATPGAVGDYLQQGA
PKNAYGLAKKVLDSENYTKTALWGVTPEILSSYGTTLDDILTEGFTKIIMGSESIDYF
DVIVQNWRAAGGDDATQAVNEAYGKK"

CDS 2526414..2527304

/gene="yteP_16"

/locus_tag="EFAGFIKM_02149"

/inference="ab initio prediction:Prodigal:002006"

/inference="similar to AA sequence:UniProtKB:C0SPB3"

/codon_start=1

/transl_table=11

/product="putative multiple-sugar transport system
permease YteP"

/db_xref="COG:COG4209"

/translation="MLRKWKQQSPYHLMLIPSLVLVFIFCYIPFYGLIIAFQKYNPGL
GFNSPWVGWDNFTHIFNQPNFVRTIWNTLYMSVFKIIGGIIVPVIFALLLNEVLHSGI
KRTFQTLVYIPNFLSWVIMAGIMLDILSSDGIINTFLSVFGIAPISFLGTPSIFPWTM
IVSDIWKGFGFGTVVYLAALTSIDPGLYEAAVIDGAKRWKQTIYITLPLLMPTIVLMT
VLSLGNVLNAGFDQIYNLYSPVVYQTGDIIDTYVYRLGIQQAQYSIGTAVGLFKSIIS
SVLVAVSYILAYRVAGYRIF"

CDS 2527323..2528213

/gene="dasC_3"

/locus_tag="EFAGFIKM_02150"

/inference="ab initio prediction:Prodigal:002006"

/inference="similar to AA sequence:UniProtKB:Q9K489"

/codon_start=1

/transl_table=11

/product="Diacetylchitobiose uptake system permease
protein DasC"
/db_xref="COG:COG0395"
/translation="MIYDKSMSRRVFLIINYTILLLISLLCILPFINLLAVSFSSSAA
VSAGSVTFWPVEFTTKAYEFALTGGSFFSSLWVAIQRTVIGTLVNLVLIVLTAYPLSK
SKQKLMGRNIYMGFFIVTMLFSGGLIPTYLVVVKMGLIDSIWSLILPGALPVFSMIIL
MNFIRGLPEEIEESAIIDGAGPVQVLLRILLPLLKPALATVGLFSIVAHWNSWFDGII
YMNNPDNYPLQSYLQTLLQSFEQIMLKSGSDYTQLLSMMNARTGRAAQMFLGAIPILL
VYPFLQKYFTKGLVLGSVKG"
CDS complement(2528321..2530066)
/locus_tag="EFAGFIKM_02151"
/inference="ab initio prediction:Prodigal:002006"
/codon_start=1
/transl_table=11
/product="hypothetical protein"
/translation="MKHVRPFRIKYKIMVICMTVIILPVLVMTINSYYSSERLLAQNY
TTLLNDLAKQTNIRIDEFLKEIEKISLLASNGLSDNLSATHEGSFPIQDFLREGDEQH
EIAAYNILMNYIMMKDRVFSIYLYNMNGGQDLFVSPHQPIDPNFKVANELWFKKFMHD
KDRTITLTTRIDEQLENKILAVSHARKIHDVTSGKLLGVIVVSIDIKFIEIVNRNLQE
GLRSRFMIVDEDDKIVYNVNERLIGTLFRDNVRPPEALNVVVTSPLSQQKWTTYLYMP
LDELTADGKILGRNLVTLAIVIVLFAAVISIFLSHVITTPIKKLLRNIALVEKGQFEQ
VEPIGSRDEIGHLSIRFNRMSHELKRLVERMQQEEIEKAKAEMRALHDQIKPHFLYNT
LGSVKWIASMQQADKIVEMTDALISMLRYATKSDGTLVTIREELDNITNYVTIQKVRY
YDCIQMRYEIEDRVLDYHMPKMILQPIVENAIFHGLAELEEDGIITIRIQLRVDTVVI
EICDNGVGMDHHTMQNLLEEKSSASTGTNGIGLHNVQRRIQLHFGKPYGIQVESKMGE
GTIFSILLPAIIEAK"
CDS complement(2530095..2531711)
/gene="cheB_6"
/locus_tag="EFAGFIKM_02152"
/EC_number="3.5.1.44"
/inference="ab initio prediction:Prodigal:002006"
/inference="protein motif:HAMAP:MF_00099"

/codon_start=1

/transl_table=11

/product="Protein-glutamate

methylesterase/protein-glutamine glutaminase"

/translation="MYKLMIVDDELLMRVGIRSMLNWAEYNFYVVGEAANGKEALSLA

LEVMPDLIITDIKMPIMDGLQLIQEASCVLKTCKYVILSNFDEFHYVKEALKLGASDY

LIKSEITESSLIDLLSTVGQKLQSEHVHPTNTPSITQDYSKSLRYLKDSFFQDIVSGF

ISEEDIVTKAEELHFRIRSDQLVVMKFIVNYYEDAKRKYIEKGEKLLRFSILNIMEEI

IPSKWEKEIFVESSSEYWVIVNVLPESQSVHADLNKLCNKLLSSIKDFMNLSLTAGVS

RMTSGFLHIRKACQEAETALQQGFFTGSNQVLYYDDMVQSPDRHEVKGALSPQQERDL

LKLWVSKDNQKAEEFLEGIRSNLEQLRADENSIRKQYIMVMETIHSHLSRATERGAPS

LTEKSPYEIVLKGEYWEDIHQDMLAHIAYYFQTDSQIKQESTYTDLATELIDKYYAED

ISLQSIASQISVNPSYLSRVFKQERGENFITYLTRVRIEHAKAYLLSRGMRVYEIAEK

VGYHNYTYFSKIFKKSVGVTPEEYRELQQG"

CDS 2531899..2532174

/locus_tag="EFAGFIKM_02153"

/inference="ab initio prediction:Prodigal:002006"

/codon_start=1

/transl_table=11

/product="hypothetical protein"

/translation="MNLYKFELTVNGYLLRVHFSASEESGLKWCKRVASEFQNNVANL

EIKVDIHKKQKQKHAREYHGKPYLYDRTLRKKPLAKKNKVSESSAVM"

CDS complement(2532366..2535026)

/locus_tag="EFAGFIKM_02154"

/inference="ab initio prediction:Prodigal:002006"

/codon_start=1

/transl_table=11

/product="hypothetical protein"

/translation="MNRYTSLTQKYLLGQKKRSILTIVGIILSVTLLTAIGTIGLSFR

DKVVRQTVQEYGDYHVSFNGLPGEAVSRVVNNTSVESAGIISREGYSVISKTSEKEKK

ENPFAAPYRYLNLKGYDADAMNMLQVQLDSGRLPKNSHEVLLSTWSLDRFATKPKLGD

SITLSLGERTVASTGEIKPINGLGDFGWDLDEDFHPQKEREFTVVGFMKTGTNASWSA

SFILPAITYEDNEKIDADKNYFIYVKMKSMNHIKAKTEAMISSLKLEQVDQGSAIALD
KDIQYKNIRVEYNNELLKLYGKSTYEGVNHSLLYAFAAIIIIIMGCTSAVIYNTFHIS
VLERTSQFGMLRCIGATPSQIRKLVLKEATILSLIAIPIGLLTGTVFMKLLFYNISFL
ALGFLNDMQMVISLPILIIAGVLGLLTVYLSAIGPARLAAKVSPLEAIHSSGSTKVED
ITSVRKSKLLGKLLGIEGQFASRNIRRNKKRFRITAFSIVVSMILFIVFSGLAGLLGQ
TSQSGIDYSYSVIYEGPSKRIDDSVYSDIVKLDAVEHAYPYYINQVMATFPKEKVNPK
YYELRPGMYSVEEGEGYRTDNNYLESYGDNGLDALKSKLTAGKIDKEKMNRENGVIVN
QKLRMTTEDGRQIVMDQTQFKVGDHIKVRSLDSDEKGRGYTTMTVTGIVDQGLLSSNY
NESAVLEFITTPEVVEKVTGNDTYSRIFILAKSDISNKPITDYLKSLPQKDAGYSYTD
KVEQLAQAKNDATTAKIFLYGFIGVIVLIAFLNILNTVSTNLILRSKEFAVLKAIGMA
QRNVGKMILLEGVLYGLYAAVYGSILGTVLSYGIHHLFKGAFDVGWAIPWSSIVLACA
GAITTTLVATVWPMYRLNQTSIVEALRKET"

CDS complement(2535023..2535709)

/gene="lolD_1"

/locus_tag="EFAGFIKM_02155"

/EC_number="3.6.3.-"

/inference="ab initio prediction:Prodigal:002006"

/inference="similar to AA sequence:UniProtKB:P75957"

/codon_start=1

/transl_table=11

/product="Lipoprotein-releasing system ATP-binding protein
LolD"

/db_xref="COG:COG1136"

/translation="MEILKTDHLCKIYGSAEARVEALRDVNLSVNQGEFVAIVGASGS
GKSSLLHLLGGVDQPTSGHVIIDGMDLYSQSENELAVFRRRKIGFIFQSYNLIPVLTA
EENIKLPMLLENKHVDEGYLEELLSVLGLSDRRQHLPSQLSGGQQQRTAIGRALINKP
SIILADEPTGNLDSKNSKEIVDLLTYSVRKYNQTLIMITHDLNVAQRADRVVNIKDGT
LLQQTGVKES"

CDS complement(2535815..2537062)

/gene="sasA_6"

/locus_tag="EFAGFIKM_02156"

/EC_number="2.7.-.-"

/inference="ab initio prediction:Prodigal:002006"

/inference="protein motif:HAMAP:MF_01837"

/codon_start=1

/transl_table=11

/product="Adaptive-response sensory-kinase SasA"

/translation="MFEVMRNPEWKSIAIKVLALQVLLSLMMFLYMQHQVDSINTTIV

NQNSALIGHLLMKNPQLESEVIHFVTQGAQTDEIAKGQRILAQYGYQEDMPIEEQPAL

SGRALPLKTATQVLLFLIPLMVLLLWEYRTFFVKIRTISHAAEQVVEQQFDKKLPETE

DGDFGALGRSFNAMAGRLTNSLELLRQEKTFLRNLLSDISHQLKTPLASLIVFNENLL

NDPGMKEEMRMTFLERSRQQLDRMEWLIISLLKLARVEAGAIGFRKERIELREMLESV

VYSLRTVADQKRPNMSILGSENAFVQADEEWLKEAVMNLIKNALEHTPPEGEVRIHLE

ENALFDTIIIQDNGEGIHPDDMPHIFKRFYKGKNHNKPNSIGIGLALTKSIIEEQQGI

ITVESVLQEGTTFRISFFKKERT"

CDS complement(2537065..2537760)

/gene="regX3_1"

/locus_tag="EFAGFIKM_02157"

/inference="ab initio prediction:Prodigal:002006"

/inference="similar to AA sequence:UniProtKB:P9WGL9"

/codon_start=1

/transl_table=11

/product="Sensory transduction protein regX3"

/db_xref="COG:COG0745"

/translation="MNRLLLVEDDDNLVFGIQYTLSNEGYEVVVAGNLEEARQALQAK

SIDLILLDVTLPDGSGYQLCSEIRVTSQVPIIFLTALDEEANVVAGLDLGADDYVTKP

VRTKELISRIKAVLRRNNKGKQEVSLWISDNIQVRILEGTVLKYNSEVTLTALEYRLL

LMLISHPKQICSRSSILNHLWDLSGDFIDDNTLSVHIRRLREKVEDIPAAPQYIVTVR

GIGYKWNVEVIGR"

CDS complement(2538001..2539728)

/gene="hepA"

/locus_tag="EFAGFIKM_02158"

/inference="ab initio prediction:Prodigal:002006"

/inference="similar to AA sequence:UniProtKB:P22638"

/codon_start=1

/transl_table=11

/product="Heterocyst differentiation ATP-binding protein

HepA"

/db_xref="COG:COG1132"

/translation="MNELTILSKAMIQELKDILLSILGGFIAGIAGVALFSASGYMIS

QTVFAPPLYTLIVLTSMVKLLGFLRAASRYGERLYSHRATFSMLSRLRTSFFAKLIPV

TPGILNKNRSGDLLARIVGDVESLQNYFLRVAYPPIMVVMVFLATMLFTSAFSIWIAC

LLVLGMLITAFVVPGIVLLGQRKIHGRVRQQRALLSTEVTEVLYGFRDLKVYGQLEQR

EQQLQQASAALATEQKQAASHLLRGQSMHVFVTYLVTWGVLALSAFLIVNGAFAGVFL

AMLILATQTVFEEAAAMAILPLYKLDSEHAAQRLAETVPTSDVQPVQPSGEVSADQSV

SIELSGVNFQYEGEWRPALRELSLQLAAGSKTAIVGPSGSGKSTIIDLLLKLRTPTSG

NIRLNDVPVQELNEESIWQNANVVLQQSHFFRGTIRDNLLLNGEEHSDEQLSAVLDKV

QLSNKLLSDLVYEKGENLSDGEKQRLALARAMLRKGRLWLLDEPTSSLDYVTEQRVLQ

HLFAQAAEDTLLLICHRLTGLEEMDRIVVMDQGKIVESGSFSELMEQKGYFYEMKQIE

RQMIGDAGA"

CDS complement(2539725..2541548)

/gene="cydD"

/locus_tag="EFAGFIKM_02159"

/inference="ab initio prediction:Prodigal:002006"

/inference="similar to AA sequence:UniProtKB:P29018"

/codon_start=1

/transl_table=11

/product="ATP-binding/permease protein CydD"

/db_xref="COG:COG4988"

/translation="MKRRAKSNLISQQMSLQRKNRLLLAIISLALGVAIISQATLVAE

AVQRIFVEKASFSSVILLLGLLLVVMAVRSLLSYGNGKVGLHMAARAKTNMRASVLQN

LTRASMPSTLRGQTGGKVSVALDAVDEADSYFSQYMPRMMEAAMIPILILVVTFTQHA

NTGWIMLFTAPFIPLFMILVGLQTKNKSEEKYAQLAEFSGTFLDSLQGLVTLKIFGRA

KRQQQEIERSSLGYRDATMGILKIAFTNTFMLESIVMLSIGIVALELAIQLLVFKSMS

FHTAFLVLLLVPEFYSLLKNTGTAFHSGRTSMGAIRKVEQMLADTSVKSTQTKPEEGP

DQSELTDVDAIAKTEELRTARAELIPMPPTIELNDVRFQYTPDSFGLETGQISIGPGE

QIAIVGKSGSGKTTLLHLIAGLLKPDSGAVLVNGSQLSQHDEAAWFERVSYITQHPYI
FAGTFAENIAIGAGRNVSRAKIEQAAEEAGLAGVVAQLEQGLDTFVGEGGRGLSGGEK
QRLALARAFLKRPSVILFDEPTVGLDLHTERVLQQSIAALAKTATMITVAHRLYTIQH
ADNILFMDSGVLVDSGHHEALLARLPQYAEMVDVQRKGGLA"

CDS complement(2541563..2542576)

/gene="ythB_1"

/locus_tag="EFAGFIKM_02160"

/inference="ab initio prediction:Prodigal:002006"

/inference="similar to AA sequence:UniProtKB:O34505"

/codon_start=1

/transl_table=11

/product="Putative cytochrome bd menaquinol oxidase
subunit II"

/db_xref="COG:COG1294"

/translation="MSYELIGISVLWLFLYGYLIVASIDFGAGFFAFYARLTKQDHLI
NRLISRYLSPVWEITNVFFVFFYIGIVGFFPDTAYYYGSALLVPGSIAVILLAIRGSF
YAFENYGSKKSIVYLFLYGATGLLIPASLSVALTLSEGGFILKQGDTVSLDYWALFTN
PLSWSIVGLAIVSVLFISGSFLTFYASRAEDHSALKLMRNYALFWSTPTIILALTAFI
YLGQHNERHFQNMMDLWWLLALSVAFFMIAMWLLYNGRRYGLAFICIMLQFLTAFFAY
GIGQYPYILDPYITIQSSATSPAMGFALVVVFIGGLCLLIPSLILVFKLFLFDADYVK
GKK"

CDS complement(2542576..2543940)

/gene="ythA_1"

/locus_tag="EFAGFIKM_02161"

/EC_number="1.10.3.-"

/inference="ab initio prediction:Prodigal:002006"

/inference="similar to AA sequence:UniProtKB:C0SP90"

/codon_start=1

/transl_table=11

/product="Putative cytochrome bd menaquinol oxidase
subunit I"

/db_xref="COG:COG1271"

/translation="MAIDTVLWSRLVTGLTLGFHVIFATLGVGVPLMIAIAEFIGIRK
KDHHYILMAKRWSRGFVISVAVGVVTGTAISLQLALVWPNFMKLAGNVIALPLFMEVF
AFFFEAIFLGIYLYTWDRFKNPYIHWLLTIPIVAGAGMSAVFITTVNGFMNQPEGFVM
EAGQFMAVNPVQAMLNTATFSKVFHVLSSAYLTGAALLAGIAAFAMLRKGVSAYHKKG
LNLMMAVVLVFSLLNSLAGDVSAKFLAEHQPEKLAAAEWHFETESGADLIFLGWLNAE
HEIIGALHLPKLLSFLAFGDFNAEVTGLNEFPPDEHPPLLVHYLFDLMAGIGFALLAI
SSLYFLFVFWKKRNQFNKWMLRMVALSAPLAFLAVEMGWFYAEIGRQPWIIRGYMRVE
EAATSSPSVRILFFVFLLLYILLGVMCVVVLRRLFNNNPAELEMEKWLKEKHDQSAKK
GDQV"

CDS complement(2544269..2545660)

/gene="ribZ_1"

/locus_tag="EFAGFIKM_02162"

/inference="ab initio prediction:Prodigal:002006"

/inference="similar to AA sequence:UniProtKB:Q180E3"

/codon_start=1

/transl_table=11

/product="Riboflavin transporter RibZ"

/translation="MNVRNKGLMLAVGFGIMLNPLNSSMISVAISRLQQVYQLNFTAV
SWIILSFYIASAVAQPVMGKCSDLFGRRKIFLMGLVIVFVSSMLAPWSPSFSWLIVFR
IVQSIGTSMMVAVGMAIVRINVTEKQASALSTLAIFLSGAAAIGPFIGGVLIHWWDWH
SIFFVNIPFVLASFWFAWKTIPKDEQSPSISGHMSIRQWLALIDAPGILMFTVALVTL
LIGLLSVNSTGDISTWHLLTGGIGLIALVMFIRHELKAATPFIPLRTFSSYPEMTWVN
VQYMLVNILFYALFFGVPTYLKQVRHLNEVHTGMSMLALGLCSVIIAPIAGRWIDGSG
SRPALIVSASLMTFGSILIVVMNETSPIFIIIVALALFGISNGLNAVGMQAALFKSTP
KEMIGVASGIFNTSRYLGTILSSLLIGIVMGDTFNVTGFQKLGIILTVLAASLIIMSV
RREAGAVSPEKVK"

CDS 2545770..2546645

/gene="gltC_2"

/locus_tag="EFAGFIKM_02163"

/inference="ab initio prediction:Prodigal:002006"

/inference="similar to AA sequence:UniProtKB:P20668"

/codon_start=1

/transl_table=11

/product="HTH-type transcriptional regulator GltC"

/translation="MELLQLKYFLTVARCEHVTEAAGKLHVTQSSLSKTIQRLEDDLG

VPLFDRIGRKLRLNDFGRTFLQRTEKALFELEQGKREIADLSNPDQGTLQLAVTTAST

LPGILREFRKNKPDIQFHVQMVSLENMSRLLHRGEVDFCLSSPPIQGDDIECQILYDD

PIVVAVPMGHRYADRSSIQLTELKDERFVGVKQGYGVRDMVDSVCQSAGFLPKYVYEG

DEPARLTALVEAEIGLAFIPSTARNPHERIRYLQVEEHQLVREIALLSHKNRYISKAA

LEFRSVVMDYFSAMP"

CDS complement(2546884..2547450)

/locus_tag="EFAGFIKM_02164"

/inference="ab initio prediction:Prodigal:002006"

/codon_start=1

/transl_table=11

/product="hypothetical protein"

/translation="MIGYGLLLFVIVAIFINYNLQITPLILEKNIGTATNGSNIIAFI

GLGAFVAGFSFGKVYKTFGNYIVPLSILLLGSSMFVTTISQSIILTTVCSMIIGFSFR

CIMPYLLHTFAQHIAKVAKLGTTIVLIAYNLGATLSPYEGRIITQLLQLDTVQSLISS

NAMILLLVSVASFGVVTLHRKKKNIQQT"

CDS 2547658..2547843

/locus_tag="EFAGFIKM_02165"

/inference="ab initio prediction:Prodigal:002006"

/codon_start=1

/transl_table=11

/product="hypothetical protein"

/translation="MKRWMRKHREQGEFGLMDQRGRRKEYLDQERYVQKLKRENELLK

KCLVIWKEEANKKDFRS"

CDS 2547792..2548676

/locus_tag="EFAGFIKM_02166"

/inference="ab initio prediction:Prodigal:002006"

/inference="similar to AA sequence:ISfinder:ISBth8"

/codon_start=1

/transl_table=11

/product="IS3 family transposase ISBth8"
/translation="MLGNLEGGSKQERFQIIEKVAAYGDIQKLCDVFGVSRSGFYAYV
KRKRFDRDAKAKKQVLQTYQRYEGKYGYRQLQLFLWQDQGIWMNHKKVLRLMQMLGIQ
SRIRRKRRSNSSYAPAQRVAENRLKRDFSAEKPNQKWVTDITQYRVGERWIYLSAIKD
LFNNEIVAYEIGERNDNELVLRTFSKAFAKQTDVTGLVVHSDQGFQYTSHAYHDMLPK
VGAQISMSRRGNCYDNASMESFFSHLKTEGLYPYHIRTLTEAQSKIEKYIRFYNRKRP
QRKLKKLTPVEYRRQFAA"
CDS complement(2548697..2549344)
/locus_tag="EFAGFIKM_02167"
/inference="ab initio prediction:Prodigal:002006"
/codon_start=1
/transl_table=11
/product="hypothetical protein"
/translation="MEKLKTYSLLSVALLNSSGPVIAANIPAITNDFPEISVTHVGLL
TTIPSLFVILGVLIANRLELLIGKKATILTGLGLVLIAGVFPALYHDLFSLLFISRCL
FGLGVGLFNRLIIQMISDMYHKNPGKQATVIGLESAFEGLGGICLTLLVGQLLKMGWH
TSFYVVYALALPIFIAFLIFVPSDKKELNRREEVTTNPSHTNNKKALQVMIGQDPI"
CDS complement(2549476..2550900)
/gene="bglH_8"
/locus_tag="EFAGFIKM_02168"
/EC_number="3.2.1.86"
/inference="ab initio prediction:Prodigal:002006"
/inference="similar to AA sequence:UniProtKB:P40740"
/codon_start=1
/transl_table=11
/product="Aryl-phospho-beta-D-glucosidase BglH"
/db_xref="COG:COG2723"
/translation="MTLRKDFLWGGAVAANQCEGGYAEGNKGLSTVDVIPAGKDRVPV
MRGQLKMLECDQEHFYPSHEAIDFYHRYKEDIALLAEMGFTCFRLSLAWTRIYPNGDD
ELPNEEGLKFYENVFDECLKYGIEPLVTITHFDVPIHLVKTIGSWRSRKMVDYYERLC
ETIFTRYKDKVKYWLTFNEINMLLHLPFGSSGLVFEEGENEDAVKYQAAHHQLIASAK
ATEIARRINPAFQIGCMLAGANTYPYTCAPDDVWKAMTRDREHYFFVDVQSRGEYPNY

AKKMLERMNIHLAMEDGDEECLKNNTVDFVSFSYYGSRLTSADPEVNTQTAANLFPTL

RNPHLKTSEWGWQIDPEGLRITMNSLYDRYQKPLFIVENGLGAIDTPDETGYVADDYR

IEYLRQHIQAFITAVEEDGVELLGYTSWGCIDLVSSSSGEMSKRYGFIHVDKDDAGNG

TLKRNKKKSFEWFKKVVQSNGKEL"

CDS complement(2550929..2552818)

/gene="bglF_8"

/locus_tag="EFAGFIKM_02169"

/inference="ab initio prediction:Prodigal:002006"

/inference="similar to AA sequence:UniProtKB:P08722"

/codon_start=1

/transl_table=11

/product="PTS system beta-glucoside-specific EIIBCA

component"

/db_xref="COG:COG1263"

/translation="MSKKYEKLAQDIVEKVGGSENVSTLTHCMTRLRFALNDTNKADQ

KALKSFDGVIDAVESGGQFQVVIGTHVEDVYKEVIKHLQPNASSSGEPTQARKVSILG

KLIDFVSGTFSPIVPAIAGAGMIKALLALLILFNWISKDSQTYYVVSLMSDAIFYFLP

FLLAFSAANKLKCSPVLALVLAGVLLHPNLTQLRLDGTDVSVFGIPLTLVSYSSSVVP

ILLIVWSQSYIESLFKRIIPNAVKVIFVPMCTILVTGILALTVLGPLGSFFGTYLAMG

FDFLGAHGSWLVIFLVATFWPILVMFGLHHNIVPLSIAQITTSGYENILGPGAMINCI

GQGVAALVVGMRTKDKALKQISASSGITALMGITEPALYGVNLPKRYPLVAGMIGAAS

GGLFAGLMDVSRYATGASGIPAIPLYIGENIWNLYNILIALVITTVVTAVLTYLLSLK

YEKDAPSQNTVSNTNAGSGVADEVITIKDSVIATPLKGQLIQLQDVQDAAFASEAMGK

GIAIEPSEGKVIAPFDGMIVSLFPKKHAIGLLSDEGVEILIHVGLNTVKLNGKYFEAF

VEEGQRITKGQTLLTFDLDKIRQEGYVTQTPVIVTNSYSYSDVIAEISPQNIDFNNTL

LVVKA"

CDS complement(2552997..2553878)

/gene="hcaR_1"

/locus_tag="EFAGFIKM_02170"

/inference="ab initio prediction:Prodigal:002006"

/inference="similar to AA sequence:UniProtKB:Q47141"

/codon_start=1

/transl_table=11

/product="Hca operon transcriptional activator HcaR"

/translation="MELRVLRYFLTVARVENITHAATILHVTQPTLSRQLADLEKNLE

TQLFIRGKSKITLTDAGMLLRQRAEEILTLADKTEKEFKDQNNLVGGTVSIGSVESLT

SNVILQLLKDFNMEYPQVKYHIYSGTGDDIKERIDKGLLDVGILLEPIHIEKYDFIRL

PQKERWGVLMKTSSPLAQKEYVTSTDLAGVPLLISSRSVVQNEIASWFADEYAHLNFV

ATYNLISNILNLVEEGLGTAICIEGALAMKQSDTLCFRPFQPELQFPTVIVWKKHKVF

SQTEVRFLEHIRHAFQA"

CDS complement(2554007..2554762)

/locus_tag="EFAGFIKM_02171"

/inference="ab initio prediction:Prodigal:002006"

/codon_start=1

/transl_table=11

/product="hypothetical protein"

/translation="MNRIEKSQETYKRLFGEGVPAAYATHPEFQDILSRFIFGEIFDQ

GALDDKQRELITLVVLATNQTLPQLKAHTRAALNVGLTPTEIQEALYQCAPYLGFPKT

LNAISEMIEVFKERNVALPLESQKRVEEDNRFDKGLGIQKEIFGDIIEKNRENAPSNQ

KHIQDYLSAFCFGDFYTRGGLDLKTRELLTLCILSALGGADSQVKAHVQGNINVGNNK

ETMITALTHCLPYMGFPRTLNALACVNEIIPEK"

CDS complement(2554961..2555932)

/locus_tag="EFAGFIKM_02172"

/inference="ab initio prediction:Prodigal:002006"

/codon_start=1

/transl_table=11

/product="hypothetical protein"

/translation="MNEEQFKQNYKKAVDHMKTNEQMKKRVEQSLNTQHQGPKRQRKP

LYIAASVVIAASIGLAAPSVWQQFNGPAAPTPQVAAVTPGASVDPIVIPKMELPSSQG

KGAMADMMQLVVYGGSVYTQSPTLLEGANALNLRGDKLGTTTGGIDELSGQDKYTELA

SNIGEADIYAVNGYDKDFRIMSYTEIDGQVYAQLFDKNNGITIGTGADLIGKLHLEGN

IASAQWETFNSWNNGLQQLQPLPADEALDNFISSLNAAKPIATSLELEENLYGKEDRK

IIYLTLEDKTQVPLVLFGDGLVRYGNVPAFLEVEEGAFQSFWNSLGE"

CDS complement(2555932..2556423)

/gene="sigW_3"

/locus_tag="EFAGFIKM_02173"

/inference="ab initio prediction:Prodigal:002006"

/inference="similar to AA sequence:UniProtKB:Q45585"

/codon_start=1

/transl_table=11

/product="ECF RNA polymerase sigma factor SigW"

/db_xref="COG:COG1595"

/translation="MQRSVPQLGDHVMKVYETYADTLFRIAMVHLGRREDAEEATQDT

FIKLIEKAPTFNDAEHQKAWLIRVITNHCKSLLGRGWRKREVKLEGVDPLTTDNPEDH

ALIELVLSLPVKYRSVVHLYYYEDYSIREISEILEISESAVKMRLKRGRQLLKLELEG

EEL"

CDS complement(2556575..2558155)

/locus_tag="EFAGFIKM_02174"

/inference="ab initio prediction:Prodigal:002006"

/codon_start=1

/transl_table=11

/product="hypothetical protein"

/translation="MMIKKIGISVASLAILTSVSVFDVYGAGSKRMIEVSEQSANVVV

NGQRVEGESFLYNGVTYVPARAIGEALGQKVDWDNNTQSVFVGQKMNDQQGYRGTKTP

YPFKETSYTKAPSGYEPIFINYIGRHGSRHLSSSKYDKTLFELLDIAEKDGQITNLGK

ELKKEIGNLMKVEKDHYGLLSTTGGEELKGIGARVGQNFKELFTSDKKVIAQATFKDR

TPQSRDQFIDGLKESLGDNKVDILASAFEEGKDPYLRPYDLATKYNEYVEDGDWVKLY

EDYVTQSTGTHYAKEVLLQFFSDDFYQRLNAEEFELKDEKGKVKLSNPTETASNLYEL

YIISSNIKGEGDFEFGRYFTANQLKWYESVDNINDFYEKGPSLTSTDLPQNIIAPLVK

ELIVSTDQSIQQKDTAGIFRFAHAETIIPLSSFLDIAGANVSIDKPQDVTQNWNGSVI

SPMGANIQWILYSNGKDVLVKMLRNEEEIAFPIETKTYPYYKWEDVKAYYQNKLQKVG

VSLDSSLEDNIELLQKKF"

CDS complement(2558397..2558816)

/locus_tag="EFAGFIKM_02175"

/inference="ab initio prediction:Prodigal:002006"

/codon_start=1

/transl_table=11

/product="hypothetical protein"

/translation="MKLEELFTVRVNIEESFDLQNSEDDSVVMITFTGSVTGKYFEGI

VLGGGVDTQIIGKNGDPHTLSARYMLQGTDNTGNSCKIYIENNGNIDKTLKTALFRTS

PKMITDSKALSFLNRETLVGEGHPTESGIDIKIYRAL"

CDS 2558945..2560156

/gene="tetA_2"

/locus_tag="EFAGFIKM_02176"

/inference="ab initio prediction:Prodigal:002006"

/inference="similar to AA sequence:UniProtKB:P02980"

/codon_start=1

/transl_table=11

/product="Tetracycline resistance protein, class B"

/translation="MTYMSTLPKSVRFPLFILMLNLFIALLGQGMLIPILPEYLKLFH

AGGTVAGFLVAAFGAAQFFFSPLGGQLSDRFGRKKLIIAGMFLSVISDIIFALSSSLP

FLYVGRFIGGISLGLMVPANLAYVADITTPETRAKGMGYFGAAMNLGMVLGPGLGGLI

AEMGIRMPYFFAAGLGLIAALMTLLLPETLPPEKRTVSIRLQKGDHLGKKIWSSFKVP

YFKYLIVLLVMTFGLMSYETVFALFAEQKYGFDAATISIIITLGAIIGIVVQIWLLDW

FVQRIGEVKLIRLSLIVTPIALLLMLIKVNLVFLLFASALFFAFNSFLRPSVSTLISK

NAGDRQGYASGLNTTFSSLGTVIGPLIAGLLFDKNINFPYIFGAIMLLASLGLTLNAF

RSKKGQLYTSE"

CDS 2560149..2560793

/locus_tag="EFAGFIKM_02177"

/inference="ab initio prediction:Prodigal:002006"

/codon_start=1

/transl_table=11

/product="hypothetical protein"

/translation="MNENWHQQLGNKHRDDLIAAGKELFLKYGLLQVKIKDVCTKAEL

SRVTFYKHFQSMDELLLAIQMQLIEHLTDEVSRASTKDLNGREQLRVMLNAWVVYAEN

HPDYIRFIQLFDINYEMYDFSPELREEYDKFNQNGKENHFLMGALSQGVVDGSIKNPS

PPLDLAQFIFTTMMGMLQRMVTIRAAHDNSLDMRMTEQFVKMLLHFVCSEEIPE"

CDS complement(2560885..2563146)

/gene="nagZ_2"

/locus_tag="EFAGFIKM_02178"

/EC_number="3.2.1.52"

/inference="ab initio prediction:Prodigal:002006"

/inference="protein motif:HAMAP:MF_00364"

/codon_start=1

/transl_table=11

/product="Beta-hexosaminidase"

/translation="MHVKWMTIIGAVVGSMLIGVGTAAAEETFVDLKYSKWAEDGITY

MAKRGTVAGYGNGIFKPEALVTRAQAVTFMVRELYSDQLQRAVEGTTYSDVPTTHPFH

REIMIAAKNGLASGFPNGTFRPDAPLSRAETAAFLTRAYALVEGKNAAKWTDTDRHWA

AAPILIMSSNGLVGGYSDATFRPNQAVTRAEYAVFMARVIRFEREAAIRTQDWDKLIS

YMTVSEQVGQMLMPDIRQWNGKATTTVNEGLKRTIHDQDLGGLILFDKNIVDVTQLTT

FTHDIQREAGDIPLFLSIDQEGGVIKRIPGGTNLPGQMALGATGDATLAEAAGQLTGE

ELKALGLQINFAPVLDINSNPDNPIIGIRSFGSEADLVTRLGLATIKGLQQSGVMAAV

KHFPGHGDTTVDSHLGMPVLAHNRERLDAVELKPFRAAIKNDVEMIMTAHIAFPAIDN

EHVTSLKDGERVPIPATLSKKVLTGLLRGELGYEDLIVSDAFTMNAIAEHFGENQSVE

RAVSAGVDIILMPKDSAAAHQTLVKAVNNGTIKDATIHASVKRILEMKAKYGLFERSQ

TLAQKLTQLNGIIGSKAHRAVEQTIAERAVTVLSSREGVLPDPMKQGDRVVIVAAEQE

QAKQLEKQLLQAANNLSLKTEISLVGQGKTNETLQAIGKADYVILASYQFRNVASQFG

WSEYQTLINAMNKSNQRYTLFSLGNPYEMIYLQNVRSGLAVYGKQEPNTSAGIKVLLG

QLKAGGQLPVLTD"

CDS complement(2563245..2564165)

/gene="araQ_14"

/locus_tag="EFAGFIKM_02179"

/inference="ab initio prediction:Prodigal:002006"

/inference="similar to AA sequence:UniProtKB:P94530"

/codon_start=1

/transl_table=11

/product="L-arabinose transport system permease protein

AraQ"

/db_xref="COG:COG0395"

/translation="MRKGNTSRLRNSSTGDRAFDTFNIIFMICLMIVTIYPFVNMIAV

SFNNANDAIRGGIYLWPRVWTLDNYKYIFGESDIYHATLISALRTVIGTVVSVFCTAM

LAYTVSRQEFVLRKFVTMFFVFTMYFSGGLIPGYLLIRDLGLIGSFWVYIIPGVIGVF

NMIVIRSFIEGLPEGILESARIDGAGEFTTFIRVVLPLTIPAMATVSLFVAVGQWNSW

FDVFLYNSSNKELSTLQYELMKILQTSTTSATSSASDAYQSAESNATAVTPTSIRATM

TIIASVPILMVYPFLQKYFVQGMTIGGVKG"

CDS complement(2564181..2565152)

/gene="yteP_17"

/locus_tag="EFAGFIKM_02180"

/inference="ab initio prediction:Prodigal:002006"

/inference="similar to AA sequence:UniProtKB:C0SPB3"

/codon_start=1

/transl_table=11

/product="putative multiple-sugar transport system

permease YteP"

/db_xref="COG:COG4209"

/translation="MEKPNVSLNSMPPASSPKGTRAHSFFKRLVQQRTLAWMCIPFVI

WAFIFKYLPLWGWTMAFQNFKPARSFSDQQWVGWHHFSILFEDNTFYRVLRNTLVMSS

IKLVLGFVTAITLALLLNELKNVIFKRFVQTVSYLPHFISWVVASSIVLTVLSPDGII

NLLLMKLHLIDSPVLWMGKGEYFWGILGATEVWKDVGWNTIIYLAAITAIDPAQYEAA

EIDGASRFKRMLYITLPGLKPVIVILLIMNMGSILESGFEPQYLLGNGMNMDYSENLD

IFVLKYGLGMGNFSLGTAAGIFKTVVSFIFLFSANYIAKKMGESRLF"

CDS complement(2565444..2567120)

/locus_tag="EFAGFIKM_02181"

/inference="ab initio prediction:Prodigal:002006"

/codon_start=1

/transl_table=11

/product="hypothetical protein"

/translation="MIKPKFRTVGLAVLLTALLAGCSQNSSSSAPSATGDDENSPVTF

SYYMFSPGKKDVLASETTIGKALQEQTGVDWKMEYLVGDSATKAGVMIASGDYPDVIS

SSGEMSKLLDAGAYIPLDDLIEEYGPNIKRVYGPYFDKMKNAEDGKIYALPYTANQGE

YSGSPNVGGGAFWIQRSVLKEFDYPKITTLDEYFDLIQDYKEKHPTVDGADTIGFVSL

AGVANNFFTLQNPAMHLAGYPNDGSVMVDMTTHEAKVVAGTDAQKKWIQKLNEINAQG

MLDPESFTMNKDQYLAKLTSGRVLGYFNYGWQVGDASKNLLSAGIDEKRYAPLPIVFD

EGIKDQYVDPPGFVNNYATGISVNAEDPVRIIKYFDNLLKEENQVLVQWGIKDETYSV

DEKGRFYFANEDQRKVHDDLELSRKFGFTYFGSDWPRYGGESTLTDGNAYSPGNQPEV

AAASYTEGDNKFLEAYGIQTFSELFNKPEERPWFPAWSIALEQGSPEQIFTTKSDDLQ

RKYLPEMILGAPSNFDKLWDAYMAELNKLDKAGYEATITKVVKDRVAGKW"

CDS complement(2567163..2568824)

/locus_tag="EFAGFIKM_02182"

/inference="ab initio prediction:Prodigal:002006"

/codon_start=1

/transl_table=11

/product="hypothetical protein"

/translation="MFRKKNIITGLAILLAVTLAGCNKASPEASYPDSNDNTPVTFKY

FTFGGGKKDILASSTTIGKKLQEQTGVDWKMEYLVGDGATKAGVMIASGDYPDIIDSS

GEMAKLMDAGSFIPLDELIDQYGPNIKRVYGSYMDKFRQEDGKIYFLPYTANIGYVSE

PNFQTGFYIQRAVLKEFNYPKIKTLDEYFDLIEQYKQKHPQIDGKETIGFATLAGESG

SFFTLLNPAMHLAGYPNDGSVMVDMQSHEAKLVAGSDYQKQWIQKLSEVNTQGMFDPE

SFTMNQDQYLAKLTSGRVLGYFSYSWQVGDATNNLKKAGIDDKRYVSLPIVFDDSVKD

QYVDPPGFVNNYGVGITVNAKDPVRIIKYFDNLLKEENQILVQWGVENETYSVNDEGR

FYFENDDQRKAHEDPELSRKFGFDYFNYSWPRYGNNSVLEDGNAYGPGNQPEVATFSY

TDGDKLLLEKYGVETFTELFSTPDERPWSPAWSIALEQGSPEQIFITKAEDLQKKYLP

KMIMDSPSTFENTWNEYMSQLNQLDKKTYEATITQAVKDRVAGKW"

CDS complement(2568946..2570685)

/locus_tag="EFAGFIKM_02183"

/inference="ab initio prediction:Prodigal:002006"

/codon_start=1

/transl_table=11

/product="hypothetical protein"

/translation="MPRWNLNYMKLRDKLLLMYVLSVFIPIVVTNVVFYNVTSANIRS

QKTRDAGMALNNLKNDLRVTIDQGVGLSYSLYTDPVFNDTISRSFQSHFEYIDAYNSY

LKGQFSGQLNQGIRWYQVYTDNSTILSSGYIDRLTDVVRSSNWYVQFQAYSAPYPALI

SSDQMLSLVQRLDNLDTGGREQLLKIDLNMDMIKQYFHNSGFDGKVYLLDPGGYIQFS

NDPTAEEAQIGKRYSEIQFPNKMIPFELTYTGINYLEGWSLQGVMDEEIVLKEVRKSR
SFVVWLACINFVLPSIIIAAMSRTIHVRLVRILKHMKKVKTQNFQTIPPEDARDEIGQ
LTMEFNRMTETIHNLIHEVYLADIQKKDLELKQQQAQLHALHSQINPHFLFNTLESVR
MRSLIKGEKETAKIVHNMAKMFRKSISWNQNDVTVKEELELIESFLQIQKYRFGEKLQ
YTIEADPAVLMYRIPKMVILPFVENASIHGIESSPGVGIIQLFVSMENDQLHIRLTDN
GIGMSHAKLEELLSYLDQNTDMGEHVGMKNAYSRLKLCYKNHFTFDIQTWEGEGTRIQ
IKLPLDPSDMPGD"

CDS complement(2570667..2572253)

/gene="cheB_7"

/locus_tag="EFAGFIKM_02184"

/EC_number="3.5.1.44"

/inference="ab initio prediction:Prodigal:002006"

/inference="protein motif:HAMAP:MF_00099"

/codon_start=1

/transl_table=11

/product="Protein-glutamate
methylesterase/protein-glutamine glutaminase"

/translation="MLKVLLVDDEMYVRKGLYELINWMDLNMEIIGEAENGAEALNLI
ESLQPDVIITDIRMPILDGLELIRAVEKMPHLEPVFIIISGYHDFKYAQQAIRYGVHD
YILKPIDDEELTATLQKSAKLICNKRKHILLAEGQASNIMLEDVIKGHVQVEDEHRYA
EMLGINRNSSLLMTLIELQTGLDAIKITIEQLREAVHSLESNLCKMFISEQQRGTFAL
LLLWPDHHQGGSALQDKLHSIHITMSKRFNLDIGLYAGTLVKKIGDVPQSLVAAEEAA
KHKYAETSSVVRYTEIKDKPLFVFNVYQDEVDQLILSLEEGNRPAYHKIVEEKFILFH
MNRFSPQAVSGSLLRYITGILAVVNEMGGNDEGLQQLKELAQQSHDGWNLRLLKNAFL
IALEEAEEYVSHLRMERSKGDISKIKRYIDANYTENINLKSIAALFYMNPVYLGRLFR
KSYNQYFNDYLLNLRIHEAKKLLRQTDLRMYEVAARVGFQNADYFVTQFEKLVKLSPT
EYRNLLMGNEQRGAQCQDGT"

CDS complement(2572359..2574341)

/locus_tag="EFAGFIKM_02185"

/inference="ab initio prediction:Prodigal:002006"

/codon_start=1

/transl_table=11

/product="hypothetical protein"

/translation="MDLIFTARSRGQDTVEYTALNKHALKADVKVTSGNGSAEDPWQA

DIQIYNEGQNAWAGVIHVEMPLDKQNPRFFLPAFMYGRNRGEAPQNVPNEFPRLREGS

PSRPSSPWWMVRSDRLSHPAALIYDEGRIVGLSASPYWINTGNQKQQWYPGMEGTFVQ

YGGFTCSIEKGTVGYTLGYENAPLLFIKSRLVHERAALGENCFELAAGESVGLTLELY

NYQSESELGINPALETIYKRYHEKPREGSDIQTTVTDLSNAIYEYAWLPEDRHYSTFV

YEDAAETGGYRYNKIISISWTNGMTIAAPMLMAALRLNNEPMRQQALSCINNIIENAM

NPESGLPYDAYQDGIWGVKGWWFDGMRTPGHSSYLVAQAMFYILKSYDYEKHIRNVRH

DDWLAYVKQVLDRLECTKNTDGEYPTILSERTGAGLEYDAFSGVWCLAAKAYYCWLTH

DESSLDELRRSEAHYYNTYVQHMECYGAPLDADKAVDSEGILAYIKAVRFLHALTGDE

LFLDHMRDAISYEFSFKFCYNSPIKIPPLSILGWSSSGGSVTSVANPHIHPMSSNLVD

ELLYYVGQRDENYVRQRMMDTIGWGCQTYNRYDQEFGYGKKGWMSERFCHSEGLVTEK

YADGTPASTWFCLMPWASGSIIEGLVGDYWDYARHE"

CDS 2574501..2575352

/gene="rhaR_14"

/locus_tag="EFAGFIKM_02186"

/inference="ab initio prediction:Prodigal:002006"

/inference="protein motif:HAMAP:MF_01533"

/codon_start=1

/transl_table=11

/product="HTH-type transcriptional activator RhaR"

/translation="MNETSEYLFDPIQNELLYLHRTTTYNMGTFYHRHNAYELYLFLR

GNVNFYIENHCYHLQRGDLLVLSPEEMHRSFALDKNEYERITINLKKSYLNRLSTSST

NLSACFDYRPKGKGNIVHLEEEELQRVLQLTDELEQCLSSSDYGADILGNILITQLLL

LTNLTFQNTDFVATDIMPELVRNTMDYIECNLTRQLTLEQLSEAMFTNSSYISRQFKK

HTGLTIRAYILSRRIELSKSYLMEGLSITEACYQSGFGDYANFIRSFTKQVGTSPGKF

VKYGRLL"

CDS complement(2575471..2577789)

/locus_tag="EFAGFIKM_02187"

/inference="ab initio prediction:Prodigal:002006"

/codon_start=1

/transl_table=11

/product="hypothetical protein"

/translation="MKTKNIGFNQPAGTWEEALPIGNGTLGGMIFGKTQIERIQLNED

SLWYGGPMQRNNPQALESLSQIRSLIFDGKIREAEELAADTLVGVPDGQRHYEPLGDF

YIAMDHSEHEPNGYQRHLDLEQGKVNVNYTADQSIYSRDYFASYPDQVLVVHLKSSAP

GKLSFSTIFGRGTVLESTKYSDILKHPVGFQSYLDRIEKRGTNDLIIRGRSGGEEGIR

FCCAIRLISEGGQVHYSSGQLSLKDGNSATILITACTDFRVPKEQLEVECLRRIEAAS

AKPYSELLADHVSDYQSLFKKMDIHLEDENKDVICSELPIDQRLERLRAGKHDPELMS

LYFQFGRYLLISSSRPGSLPANLQGIWNKDMLPVWDSKYTININTQMNYWPAESCNLS

ECHIPLFDFIDRLQVRGKETAETMYGCKGFVAHHNSDIWADAAPQDVCMTSTFWTMGG

AWLSLHLWDHYEYNKDAQFLKKVYSTMKDAAMFIMDYLIEDPSGNWVICPSSSPENRY

ILDDGESGALCFGASMDNQIIRELFTRCIESTRILQEDQEFGEVLRSALTKIPETSIG

KHGQIQEWSNDYDELEPGHRHISHLFALHPGTQITLQSTPDLAKAARVTLDRRLEHGG

GHTGWSRAWILNMWARLEESELAHDNIVELLRSSTLPNLFCDHPPFQIDGNFGGTAGI

AEMLLQSHNEVIRLLPALPAAWPKGYIRGIRARGGFEIDLEWENGRLLNTRIRSIAES

RVTVSYLDQKIELTFPGPNAMIELKGNKWNNK"

CDS complement(2578076..2578501)

/gene="slyA_2"

/locus_tag="EFAGFIKM_02188"

/inference="ab initio prediction:Prodigal:002006"

/inference="protein motif:HAMAP:MF_01819"

/codon_start=1

/transl_table=11

/product="Transcriptional regulator SlyA"

/translation="MTNTVDCEIRQSLDRISSQMRRNYSESLRELNLYVGQDNLLYRL

WLGDGVTQMQLSEHMKCEPPTVTNMVKSLEQNGFIYRKRDVQDARIMRIFLTDKGKEL

EKPVEHKWREQQEKLLQSISSEDRLILRQFMQQMERNIL"

CDS complement(2578709..2579470)

/locus_tag="EFAGFIKM_02189"

/inference="ab initio prediction:Prodigal:002006"

/codon_start=1

/transl_table=11

/product="hypothetical protein"

/translation="MKLTGNTIFITGGGSGIGRALAEALHKLGNKVIISGRRKERLEE
VLSANPGMSAVELDIQDLSSIEATAKQLIRDFPDLNVLFNNAGIMLFDEAAGVIDEEV
LVSTVSTNLLGPIRMTSSLIEHLKSKEEAVIINTTSSIGFIPYAATAVYSATKAALHS
YTLSQRYLLKDTTVKVLEIIPPGVQTELMTDLSNDQHAMPLEAFIEGTIRLLGTDAEE
VLVEQAKMIRDNQGPNEGAFVTQLNDMASQAAKGH"

CDS 2579710..2580255

/locus_tag="EFAGFIKM_02190"

/inference="ab initio prediction:Prodigal:002006"

/codon_start=1

/transl_table=11

/product="hypothetical protein"

/translation="MGLKVRNIRTTRTRESIFESLFELMEEKDFDKITIQNLTERAQI
NRATFYAHFQDKYELLDEIIRKSAEELIQQHTNGVCAFTKNNMMQLVFAAFEYHQQVK
KKCRRNYNKIIPLLSSKLVIALKHYLDLCMQNIYSDERTLYVQIYANMINEAVTLHTA
EQTKLTERSIAEHIVQMIILD"

CDS complement(2580424..2581164)

/gene="nfsA"

/locus_tag="EFAGFIKM_02191"

/EC_number="1.-.-.-"

/inference="ab initio prediction:Prodigal:002006"

/inference="similar to AA sequence:UniProtKB:P17117"

/codon_start=1

/transl_table=11

/product="Oxygen-insensitive NADPH nitroreductase"

/db_xref="COG:COG0778"

/translation="MNHTLELLHNHTSFRSYTSQPLTEEQINAIFQAANQTSSFSLLQ
AVSIIRITDPVIRNKVRKLSVNQPYIEEAAEFWIFCADFNRSHQIAPEVDIEYIEFLL
IGSFDAGLVAQNALTAAESMGLGGVFIGGVRANINELSEVLNLPKYVIPLVGLCIGNP
AGDKPELKPRLPQSMVLLENHYHSLDQEKLATYDETMLKYYENRPTRAPFTVKKVKGW
SDHIQDHLQRSIQPQMMDYLNKQGFAKK"

CDS complement(2581238..2582341)

/gene="iolG_4"

/locus_tag="EFAGFIKM_02192"

/EC_number="1.1.1.369"

/inference="ab initio prediction:Prodigal:002006"

/inference="protein motif:HAMAP:MF_01671"

/codon_start=1

/transl_table=11

/product="Inositol 2-dehydrogenase/D-chiro-inositol

3-dehydrogenase"

/translation="MSKKIRTGIIGASINNGWASGTHIPAIEHLDEFELTAVGTSNMA

SAKKSAEAFHADHGFENIEELAQHPDVDMVVVSINVKEHYNAVKTIAPAGKPIYCEWP

LGSNTDESIEMQKWVASAQLPNAIGLQARQAPAVQYVKDLLAEGYVGKVLSANLKISI

DGMGGVGDKSTAYLYDRKVGGNLLTIVGGHNLDAFTYMLGDFTELSALTAQQFPEVEL

VDIQKTIKKTTDDQILIAGKLTSGAAASIHIQGGVKHQTGLTLEIFGDKGTIVLSAPA

SIQFGSHQLRGAGATDNELHELTIPDAYYWAPHSLYNDSGFVLNMAQAYRKFAQDIQE

GTTLAPTFADAVKLHQLLDAVEKSAQTGERQYF"

CDS complement(2582385..2583512)

/gene="moxC_4"

/locus_tag="EFAGFIKM_02193"

/EC_number="1.14.-.-"

/inference="ab initio prediction:Prodigal:002006"

/inference="similar to AA sequence:UniProtKB:O34974"

/codon_start=1

/transl_table=11

/product="Putative monooxygenase MoxC"

/db_xref="COG:COG2141"

/translation="MAARKLKLGGIIDGVGWNHTGWRHPDTPADASENIEYYVQKAIQ

LEQGKFDLIFLADVSHIGPGMIPHYLSMFEGISILSALSMVTHSIGLAATIATSYADP

FTVARQIASLDKISKGRAGWNAITSNPGGLANYSRSHLTKADLYPMKKEFLEIVEGLW

DSYEDDAFIRDKERGIFYNPRKMHPLDYTGNYFSVEGPLNISRSRQGRPVVFQAGTSP

AFMDIAAKHAEVIMAPGHDLEYLKAFTAELKRKVQNQGRSPHDLLMMPSHNPIVGRTE

KEALEKLREMESWMPQGYRMTKPGLIGSAEHVAEQIEHWYRAGVMDILLIRQDHPAGF

KDFIKLVVPILQDKGIFRKEYEDDTLRGNLGLPYPENQYTK"

CDS complement(2583542..2584072)

/locus_tag="EFAGFIKM_02194"

/inference="ab initio prediction:Prodigal:002006"

/codon_start=1

/transl_table=11

/product="hypothetical protein"

/translation="MVPDMFMLGSSEGGLHFALEKGLGFVFAAHLAPQLAIPVLRAYH

ANFKPSSYLTEPQSMLATMVITAETEEEAKYIAGPAELMWAQMSTGTRNLTFPTPEEA

KNHRYTPHEELARERNKDRFVIGSVGQVADQLTQLAKASLVDEIMIADFYPNQESRKK

GHELLAKELGLSQGNK"

CDS complement(2584125..2584574)

/locus_tag="EFAGFIKM_02195"

/inference="ab initio prediction:Prodigal:002006"

/codon_start=1

/transl_table=11

/product="hypothetical protein"

/translation="MSTANGIKLSILEFVHIYAGYEASESLQNVTEMVQLAEQWGFNR

YWFAEHHNTKNLMSTSPDLLSMHAAAHTKNIRVGSGGIMLPNHSALKVMENFTLLEGL

YPGRVDLGIGRASGTDGRTMWALLRSQELMEVNDFPEQLDNAFLLCP"

CDS 2584826..2585170

/locus_tag="EFAGFIKM_02196"

/inference="ab initio prediction:Prodigal:002006"

/codon_start=1

/transl_table=11

/product="hypothetical protein"

/translation="MRVCNEGFEQEFIDGKSDMYAIAFTQNVLSGRWKYFILWYLKNE

TRRYTDIKKFLGDLSQGSLTKQLKELENDGVIQREVYPEVPPRVEYSLTDKGMKLLPI

LEKMEDFGKEYG"

CDS 2585222..2585437

/locus_tag="EFAGFIKM_02197"

/inference="ab initio prediction:Prodigal:002006"

/codon_start=1

/transl_table=11

/product="hypothetical protein"

/translation="MFFFFFFLLLGMIGLFFGVRALRRPNSWPFNRTKDELHEYDMMG

IKFRGVFLLAFGTVLTIASVRLLLIDK"

CDS complement(2585473..2586444)

/locus_tag="EFAGFIKM_02198"

/inference="ab initio prediction:Prodigal:002006"

/codon_start=1

/transl_table=11

/product="hypothetical protein"

/translation="MDNLISQALNKAHMGIMIVDRHLKVVVWNAWLERFTGKNVEEVV

GLNLLEVCPRFKSNMYLNILQNALYHGQSRFCSSALHKAFILPNEGEDEQLFRQNMHV

EPLYHEDRSYALIQITDMTNTNTRVYKLKNLIKELETEYAKIKISEKISKHLSLHDPL

TGLPNRLAYNDRLAWAISNAKRNHNKLAVMFVDADGFKQVNDTYGHHVGDQVLLEMAK

RLSETIPSADTVARLGGDEFIILSHLKDDHDAEVIAKRLEAAFSTHFKIAGNYINLSM

SIGISLFPKHGQEPSELLRHADGAMYKIKKNGKNGYGIYEPENAEEI"

CDS complement(2586437..2587072)

/locus_tag="EFAGFIKM_02199"

/inference="ab initio prediction:Prodigal:002006"

/codon_start=1

/transl_table=11

/product="hypothetical protein"

/translation="MLNDLQKDLLTELVNVYVGQAANMLSEIVDKRIVLNIPEVELIS

ISDVDPGDRRYNIFFSEGHLFRSSLQFGYEFQGKAFLMFPVEQAKVLANICLGELGES

VIPNDPNLMDTDLDVLKEVSNVLLNAIIGELGNFLEIKLEYVLPDIELIYVTNSDQQI

LLQNEVYVLVLHTSFLLADTDVTGIIVIALSMNSISSLLAKMDALLEADNG"

CDS complement(2587062..2587436)

/gene="cheY_1"

/locus_tag="EFAGFIKM_02200"

/inference="ab initio prediction:Prodigal:002006"

/inference="similar to AA sequence:UniProtKB:Q56312"

/codon_start=1

/transl_table=11

/product="Chemotaxis protein CheY"

/db_xref="COG:COG0784"

/translation="MKVLIVEDSIFVQKLLTKLIVDHIPGCEIQIANNGEQGYNLFKE

FQPDFITTDLLMPGLNGQEMLRMIRETDSNVKIIILSADIQKTTRDEVEMLGISGFLN

KPLTAEKATTMIQLIQAAYHAE"

CDS complement(2587720..2588622)

/gene="araQ_15"

/locus_tag="EFAGFIKM_02201"

/inference="ab initio prediction:Prodigal:002006"

/inference="similar to AA sequence:UniProtKB:P94530"

/codon_start=1

/transl_table=11

/product="L-arabinose transport system permease protein

AraQ"

/db_xref="COG:COG0395"

/translation="MGINKSRLEPIVFHTLNGILMIAIAIVTLYPFVNTLAVSFNAGN

DTIRGGIYLWPRVWTDQNYRAVFAGGTIFSAFGISVARTVLGTVLSLFLTSMLAYTLS

RKDYILRKPITITVVLTMYFSAGLIPTYFLMKDLHLLNNFLVYIIPGLISAFNMIVIR

TYIQTLPEGLIESAKIDGAGDFRTFISIILPLCQPVLATVALFIAVGQWNSWFDTFLY

ASSKQNLSTLQYELMKLLSSSMNSNSSAAVANGADLGAARNMVTPVSIRAAITIVAAL

PILVVYPFLQKYFVHGLQLGGVKE"

CDS complement(2588635..2589600)

/gene="yteP_18"

/locus_tag="EFAGFIKM_02202"

/inference="ab initio prediction:Prodigal:002006"

/inference="similar to AA sequence:UniProtKB:C0SPB3"

/codon_start=1

/transl_table=11

/product="putative multiple-sugar transport system

permease YteP"

/db_xref="COG:COG4209"

/translation="MANTPGAIPEQTTRTVVPKAPLLKRLRGQRQLMFMSLPIVAYIL
VFSYYPIWGWVMAFQNYSPAKSFTQQEWVGLKHFKFLLTDDAFLNVLRNTIAMSVINM
VLGFATAIIFAILLNEIKHKLYKRTIQTISYLPHFLSWIIVTGIVASSLSVDGGIVNV
VLMKLHIIQEPIMWLSVPEYFWGIVGASHVWKEVGWNAIIYLAAITSIDPSLYEAAEI
DGANRYKKMLYVTLPGIKSIVIILLIMNMGWILEAGFEVQYLLGNGVVVDWSQTIDIF
VLKYGLQIGNYSLATAAGIFKTVVSITLIFAANSISKRLGEDRLI"

CDS complement(2589713..2591368)

/locus_tag="EFAGFIKM_02203"

/inference="ab initio prediction:Prodigal:002006"

/codon_start=1

/transl_table=11

/product="hypothetical protein"

/translation="MGGLKKKKLWGSVSLVLALSLALAGCSGDNEKATTKDGKEVLNI
TAFIGTPNQAPAKDNRIYKKIQDELGVNLEMEFLVGDLQQKLGVMIAGGDFPDLITAD
TKLVSAGAVIPLEDLIEEHAPNLKKHFGKDWNRMKDSSDGHIYWLPNYGVYTGEFISN
YYSGPAFWIQKSILKDAGYPTPKTLDDFTKLVRDYAAKNPTIDGQPTIGFTTLASDWR
TFPLLNPPEHLTGHPNDGGVVVDDSGVATVFADKDISKRYYKELNNLYNEGLLDKEAF
VQNYDQYLAKISSGRVLSMFDQHWNFQAGEDPLVAQGKIGQTYVGFPLVYDTSITDHY
LDRPVINLNNGFGISKDAEDPVALIKFLDKLMDEKYQKLLSWGEEGVDYQVNEEGRFY
RTPEQRTEQDDPAWRLVNKAEGFYAAAPKLEGTFEDGNAIGAINQPEEFYDNLKPEDK
ELLDAYGFKTWSDFFSPAPENPVYYPAWQVDLKEGSDAAVANKQMTDTSLKFLPKAIM
SKPNEFDNIWIEYVDAYKKIDVKSYEDRINEQLKWRIENWSVK"

CDS complement(2591508..2593049)

/gene="cheB_8"

/locus_tag="EFAGFIKM_02204"

/EC_number="3.5.1.44"

/inference="ab initio prediction:Prodigal:002006"

/inference="protein motif:HAMAP:MF_00099"

/codon_start=1

/transl_table=11

/product="Protein-glutamate
methylesterase/protein-glutamine glutaminase"

/translation="MYKVLLVDDEPYAIEGLQLLINWEKYGFEIRGVCANGEEAIRMM
EQDRPDLVVTDIRMPVMNGLELVEEARRLEHESVLFVITSGYSDFNYARQAIRLGVSN
YLTKPVDTTEAEDMLVRLRMQLQERETQERIREHANEQRIKAFLSALIKDKQNISEEE
MSEIRPRLNKSHWAYLRIAFQGDIQEVCSVVEQTAEETTCCYVIDRTRDSLGLVWGEE
TNDRNETFEAIKAFTQRLMNNMQHNGCEEIHIAVGLTVNKLEHLSESFRSAQEVGRYL
FFNQERLLFAKDIQLENWQFDPETLSEVDHICDLLENGSVEELSSAIRSAFELFVQMK
AAPEIVHIFSTHIIFRGLSLCRELGGEPNALMNELASGILEKGHRNIEEVANNLEHFC
LQCKSALAIFRERQMGGIQATVAEYVQMHYRETFTIQELAERFYVNPVHLGQSFLRKY
GKGVIEVVHDLRMEEAKRLLQQTNQTSSAIAEQVGYRSYQHFLRHFEKRTAMKPVEYR
QKYAN"

CDS complement(2593063..2594844)

/locus_tag="EFAGFIKM_02205"

/inference="ab initio prediction:Prodigal:002006"

/codon_start=1

/transl_table=11

/product="hypothetical protein"

/translation="MRHKFWMFSKVNDIPLRSKFLLIYVLSILLPVITINIFFYERTS
ADIKIREQENLRKSIDRAAGELLGMIDESVALSRIIAADDSLYEALDRTYHSPVDYYN
VYHAFLRDKLTRYMSANILEVRIYTDNDTIQTGSHYIVMKDKNVLPGLKQLKSTSGGI
LVVDYIEPSGFNAGRRISVIGKMDTYTSYANYNKYSKIDLELSRVYSILNRESDSLQL
RLVDSNNRTVVSSGEFSETDLSPLDNESMLKESGDSAYTLEKSLGNLAYLKGWKLIGV
ADTRHLDHLLQEAFKSILGLLALSIVVPSVLIYIILRSYHYRIRKLSRHMEKVRNERF
DLIEIHEGRDEIGGLIRTFNIMIGKIHTLINDVYKLEIRQKDLELDQVRTELSMLQSQ
MNPHFLFNTLNALLVVSTKNGYTEVIEIIKSLSKLMRQLLSHSDNLIPLQEELQFTNL
YLQIEKFRFGELFDYTFEVDPDAASLLIPRMSIQPLVENACKHGLQARKNGREIKITA
RRNATELVIQVIDNGIGMDAGRLTELLRDIRSERPRNGEHVGLRNVYRRLELFYEGNA
IFDLSSVPGKETIAGYRIPISKLEQRK"

CDS complement(2594849..2595964)

/gene="rhaR_15"

/locus_tag="EFAGFIKM_02206"

/inference="ab initio prediction:Prodigal:002006"

/inference="protein motif:HAMAP:MF_01533"

/codon_start=1

/transl_table=11

/product="HTH-type transcriptional activator RhaR"

/translation="MYKVIVADDEPFMLEGWRTMIDWRACGYELCGTATDGEEALALF

NSVEPDLVVTDIQMPVMDGLGLIHTLREDLAYTSKIVIVTGYSDFNYAKQAIRYQVDQ

YVLKPLVPEEIHQILMDMIEPLDKRRNEQKQKNGVTVTDEAYENHLNEYRIGEEYDLE

QMMNISKEILALIEAENTYSIDIAVSALLEKSEKAEVSLEWIHHVIRYIHGELLRKYG

KREGCREILCEKDWHEAASWSSGTLQDLCVRLSKQLFQSELKQKTGTGGLVAEAVIEL

KQHYRSKIKLQDVADRIHVNSAYLGQQFKREMGVSFSDYVHQLRVEEARKLLRRTNMK

ITDIAFRLGYHDAEYFTQKFRAHTGELPSVYKNKNQG"

CDS complement(2596315..2597154)

/gene="pkn1"

/locus_tag="EFAGFIKM_02207"

/EC_number="2.7.11.1"

/inference="ab initio prediction:Prodigal:002006"

/inference="similar to AA sequence:UniProtKB:A0A0H3MBJ2"

/codon_start=1

/transl_table=11

/product="Serine/threonine-protein kinase Pkn1"

/translation="MKKLVWLTLIAIVVSLSACSASNTKLSDDLVFVEGGAFKSSKPS

QIDRNETLDSFYIGKYEVTQKEWMEVMGENPSGFKGDDLPVEMVSWYDAVEYCNQKSI

KENLKPYYNIDKDNQDTNNHNENDTIKWNVTINEDANGYRLPTEAEWEYAASGGQKSL

NYTYSGSNNPDEVAWYWVNAGENILTGDWSWTAIESNRNQTKKVGTQKANELGIYDMS

GNVREWCWDWYSDSESPNQTWRVVKGGGWIGGVNNNEISFRGKFDANGFGPDQGFRIV

RGE"

CDS complement(2597288..2597923)

/locus_tag="EFAGFIKM_02208"

/EC_number="3.2.1.8"

/inference="ab initio prediction:Prodigal:002006"

/inference="similar to AA sequence:UniProtKB:V9TXH2"

/codon_start=1

/transl_table=11

/product="Endo-1,4-beta-xylanase Xyn11E"

/translation="MFKFSKKLLTVVLAASMSFGVFAATSSAATDYWQNWTDGGGTVN

AVNGSGGNYSVNWQNTGNFVVGKGWTYGTPNRVVNYNAGVFSPSGNGYLTFYGWTRNA

LIEYYVVDSWGTYRPTGTYKGSMTSDGGTYDIYTTMRYNAPSIDGTQTFPQYWSVRQT

KKPIGVNSTITFSNHVNAWASKGMYLGNSWSYQVMATEGYQSSGSANVTVW"

CDS complement(2597995..2598768)

/gene="xynZ_2"

/locus_tag="EFAGFIKM_02209"

/EC_number="3.2.1.8"

/inference="ab initio prediction:Prodigal:002006"

/inference="similar to AA sequence:UniProtKB:P10478"

/codon_start=1

/transl_table=11

/product="Endo-1,4-beta-xylanase Z"

/db_xref="COG:COG2382"

/translation="MELISPAVTGYDQYRENIPRGVMETVEYPSTTVGNSRKAMVYTP

PEFSSTTMYPVLFLLHGIGGDETEWHTHGSPQIILDNLYNDHLLEPMVIVFPNGRAMS

NDRAEGDLFEPEKIKAFKRFESDLLNDLIPYMESNYPLHKSRDKRAIAGLSMGGGQSL

NIGLSNLDHFAWIGAFSAAPNTRSPELLVPDLSKAPSSLSLLWISCGEQDNLIEISLG

VHQYLAHHGVPHIWHEESGGHDWPVWKNDLYLFSQRLFK"

CDS complement(2598810..2600093)

/locus_tag="EFAGFIKM_02210"

/inference="ab initio prediction:Prodigal:002006"

/codon_start=1

/transl_table=11

/product="hypothetical protein"

/translation="MLQDSGDAGENTEFTAPRDPVGVTASAVKPAIDFESHRYRDMIG

RSLLNKGNNVRLKRAIEKAKNGEPVVIAYIGGSITHGAGAVPIHLQSYAYRSYESFKF

MFALSKDSPIHLIKAGVGGTPSELGIVRYDRDVLRGGAVQPDIVIIEFAVNDADDETQ

GKCYESLVLKALTADNKPAVILLFSVFENDWNLQDRLAPVGWHYDLPMVSVKDAVVDQ

FSKTRGEGNVISKKHFFHDIYHPTNIGHRIMADCLEYLFDVTNRSEWDEKDHDIEKAP

LIGNEFVNVKLLDRKNGNHIARIDAGSFCKTDTDLQMAEMDAHDYGTPQFPNNWMRTG

EGKEDQSSFRISLQCKRLILIFKDSGNDEFGTAHIKVDGVFVQKVDPRQVNWTHCHAI
LLLNEEHVGEHSIEIEMAEGHEHKCFTILGFGYVD"

CDS 2600382..2601524

/gene="rex8A"

/locus_tag="EFAGFIKM_02211"

/EC_number="3.2.1.156"

/inference="ab initio prediction:Prodigal:002006"

/inference="similar to AA sequence:UniProtKB:A0A0S2UQQ5"

/codon_start=1

/transl_table=11

/product="Reducing-end xylose-releasing exo-oligoxylanase
Rex8A"

/translation="MNITGQGAWDTGTYTNLFLKLGYDENEITNRLEETWNELFYGDD
NTRIYYPMGEDKGYLLDTGNLDVRSEGMSYGMMMAVQMDKKEEFDRLWNFSHTFMQHK
AGRYKDYFAWHCKPDGTRLSQGPAPDGEEFFAMALFFASNRWGDGAEPYDYKAQARKI
LHACIHQGEDGEGDPMWDPETKLIKFIPETPFSDPSYHLPHFYELFAKYADEEDQSFW
KEAAAASRAYLHTACHPVTGLSPEYANYDGSPAPIQPHGDFRHFYSDAYRVAANIALD
WEWFRKDHWQIEQSNRIQSFFSDIEMSDYRRYTIEGEPFDEPSLHPVGLLATNAMASL
AADGPHADTFVHKFWNTPLRQGERRYYDNCLYFFSMLALSGRYRMY"

CDS complement(2601747..2603387)

/locus_tag="EFAGFIKM_02212"

/inference="ab initio prediction:Prodigal:002006"

/codon_start=1

/transl_table=11

/product="hypothetical protein"

/translation="MKKAGFIILACMLFTSLVLAGCSSSDKGKGEGSDPSGKTAINVF
AHQGSDTNLSTNKFTKKMEEKFDIKFNWTTVPFDGAAEKRQISLASGDYPDLYLLIPW
VDRFSQTDLLKFGQQGVILPLNDLIEQHAPNIKKVLESNDYYRAMNTAPDGNIYGLTG
LNECFHCSYPNKMWVNTKWMEQLGLTEPATTEEFKEMLRAFKTKDPNGNGKADEVPLS
GSTENFGVHIIPYLMNGFIYDDDRNYLIVNQGKVETVVNKPEWKEGLAYIKSLYDEGL
IDPGAFTQNVGAFKKIGDNADAQLLGAGAAMHPSLFVTTAEGSPYGNDYNPIPPLKGP
HAAYATYNYPIDPGASFVLTNKASEETQIAAIKLLDYLYTQEGTMASYLGEEGVSWRK

PQEGEVALNDQIEPLYKAIPLPSGEEPRNDSWAALSQYNHYQAYRDSEVQGTDIYAND

GGERRLYEATLLMEGKEPKEIFPHWALWVDPSQADEASMMQTNLKDYIDQNALQFITG

AKSLDKDWDEYVKGLEGLNINRYLEIMQSSYDTSSVSK"

CDS complement(2603425..2604354)

/gene="melC_3"

/locus_tag="EFAGFIKM_02213"

/inference="ab initio prediction:Prodigal:002006"

/inference="similar to AA sequence:UniProtKB:O34518"

/codon_start=1

/transl_table=11

/product="Melibiose/raffinose/stachyose import permease

protein MelC"

/db_xref="COG:COG0395"

/translation="MPNTQTNAGRSRIKSSSTIRESWGDRVFITVVYFMLTVVLIAVL

YPLIYIVSSSLSSPAAVSSGKVWLWPIDLTFDGYKSVLRNDQVLTGYANSLFYTACGT

FISVALTIMIAYPLSKKTFVGRSSLMMFITFTMLFSGGLIPTYLVVKTMGLIDTRWAL

LIPNAVWVWQVIIARTFFQNSIPEELSEAADIDGCSDIRFIFSIILPLAKPIIAVLSL

MYAVGQWNAYFDALIYLKSQSLYPLQLILRSILILNSSTGSMDASEMIKQQQMAELMK

YSLIVMASLPVLIIYPFVQRYFVQGMLIGSVKG"

CDS complement(2604382..2605356)

/gene="yteP_19"

/locus_tag="EFAGFIKM_02214"

/inference="ab initio prediction:Prodigal:002006"

/inference="similar to AA sequence:UniProtKB:C0SPB3"

/codon_start=1

/transl_table=11

/product="putative multiple-sugar transport system

permease YteP"

/db_xref="COG:COG4209"

/translation="MILRTTMTSESKSVEKPSKAFALKQALSRSWRRHWQLYLLILPP

IAYFIIFKYVPMVNAVLAFKDYNVIKGIWGSPWAGTKYFELLFKNPAFVMLIKNTLYI

SFYSLIVGFPIPILLALALNEIKNIRFKKTVQMVTYAPYFISTVVMVSIIMLFLSPRL

GIVNTIAGALGFEAVNFLGEPGLFRSIYVFSDVWQGMGYSAVIYLAALAGVDPSLYEA

AKVDGANRIQKIINVDLPGLLPAAVIILILSVGNIMAVGFEKIYLLQNPLNLSASEII

STYVYKIGLLNANYSFATAVGLFNSVINLILLLIVNAVAKRLSNTSLW"

CDS complement(2605512..2607863)

/gene="rhaR_16"

/locus_tag="EFAGFIKM_02215"

/inference="ab initio prediction:Prodigal:002006"

/inference="protein motif:HAMAP:MF_01533"

/codon_start=1

/transl_table=11

/product="HTH-type transcriptional activator RhaR"

/translation="MQLLWSKFSPVFRRFLISYLVILMIPQIAGYASYRASIEAARSS

SIENSLKSLSLGKEIIERNLLQVEAFTRQLAVNPDLQNLIAGPKPHDLYNVYGMNRMQ

RSLSMYSSTNDYLSHFFIHIPNYNAIITPRTVYYRPEHYYAANQLDGMSFEQWHDQIL

KQPHFNEIIPLRNYKREILGTVLADVPAITFLQSLPLNSFNKPQATIGVMIDQDQMAS

LTQNIVDQYGGWTLVTDAEGQIIFSHGIEQAEAEQMAQSRQREGNDAKTELNVQNVQP

GSDGRLLISMQSSQNGWNYMAGIPEKALMTKADQIKQVTLVFTLATIALGLLFGLVLA

YRNSAPVYRLLASFREQITDSPGRRGNEYDFLASHINNLIANNDSLKNAMNEQIPLLR

DGFIKRLLTGEVYTSLELEVISSQAHISLHSSKGLAGLVKVNGYANPDSEETIHELGV

ARLLIKQVLTEWNAQLLITDWGTDQIAFACPLDENSLNEAIGRCEKELNTLMEVIYRE

HRISTTIGTGAAYEVWNDAGRSFDEAKQALDYAIHMGTDHLVRFEDTMKENEMFYYPI

ESEQRLLNTIKVGEPEEAVRILEQLFLRNLEERELSYEMTQQFIMELKGTFLKLDEPK

FKLDASLLEEYKTRVTSIQMTETITSLRAKFKRLTEDICGDFQRRRAGAHADTVSEMI

CFIQQHYGDANLTIYRIAEHMSKSEKFISQLFKEHTGENLSDYVERVRIDAASNLLHS

TGQTIDEIAEATGYNSAHSFRRAFKRVRGISPSVFRKMDVHSG"

CDS complement(2608157..2609863)

/locus_tag="EFAGFIKM_02216"

/inference="ab initio prediction:Prodigal:002006"

/codon_start=1

/transl_table=11

/product="hypothetical protein"

/translation="MKKFMTSCKLVLILALLITIAPWGGSRAEAWVGMPMGKLHVNGK

NLVNSNNQPVLLNGWHQPSGAYWTYQDSNYYLNLHGNNRHAATLAYLKDITDTFADTS
AKYGSNHGWNMNQVRLFIDRQDMGDVAAGTYNFAGVQTVTQNVIIPYIQYAKTKGVYV
VLGLDFTLKDDQATTPANLQKFNEIWGYLASRPEIKSADNVHFELINEPVKSYANGHW
GGYNGENDFVDHWNDLRNFQNSMISTIRSKGADNVIWAAGLGYNQFYSLTASHPLTDP
LNNYGYAVHWYPGYGAYDNFSILQDQWNTNVKAAADKYPINITEVTWFKNKPGDSAYW
NLFNGSNEGFGTNTKTIFNASGNVSIAAHMNGFILSEGPRSSFADPTAGLKWDGDASR
SAMGRFLFNWYHERAQTYPGSGQGGGPTTGLVSGATYKIVARHSNKVVDVPGGQNENN
LQLQQWSDLGGNPQKWVLTSIGNGNYTLTSVNSPDKVIDIRNGTLTNGEAVQLMSNLN
TTAQHFKVNDLGNGYWSIVNVNSNKAIEVANASTSDGALLQQNTYTGATNQQWKFVAV
SN"

CDS complement(2609946..2610080)

/locus_tag="EFAGFIKM_02217"

/inference="ab initio prediction:Prodigal:002006"

/codon_start=1

/transl_table=11

/product="hypothetical protein"

/translation="MQHSICEKGACNDLMNVCSQQNRDPSLRRIPLLVWNGLRERAPT
"

CDS complement(2610139..2611086)

/gene="dasC_4"

/locus_tag="EFAGFIKM_02218"

/inference="ab initio prediction:Prodigal:002006"

/inference="similar to AA sequence:UniProtKB:Q9K489"

/codon_start=1

/transl_table=11

/product="Diacetylchitobiose uptake system permease
protein DasC"

/db_xref="COG:COG0395"

/translation="MTTSRLLSLEHWKGWLWAMIRFVLITGLSFVILFPIFQKVSTSI
KAKGDLYSAVVVWIPQNFSIDNFKEAIRVMDYWATLFNTFALSATTTLLTTASCALAG
YGFARLKFRGSNWLFAGVILTILVPPTTILIPVYLNLKSFDLMGLMTLIVGKPVNLLN
TYWPFILTAITANSLKAGLYIFIFRQFFRGIPKEVEEAAYVDGAGIGRTFSRIMLPNA

IPSMVTVMLFSFVWQWNDSFYTTTYLTSSKVMSTQLSSLPYNLAQQVTDGAASQADPF

YLSMIQDTGILLAILPLIIIYLFVQRYFVESVERTGIVG"

CDS complement(2611104..2611949)

/gene="lacF_2"

/locus_tag="EFAGFIKM_02219"

/inference="ab initio prediction:Prodigal:002006"

/inference="similar to AA sequence:UniProtKB:P29823"

/codon_start=1

/transl_table=11

/product="Lactose transport system permease protein LacF"

/translation="MWGVIYVLPWLIGFVLFFFIPLLASLRYSMSTIQANAEGIAIQF

NGVANYIQALTVNTSFNRALIEAITDVLINVPLIVIFSLFLAVILNQKFRGRAVARSI

FFLPVILASGVIMTLESTSLIEAVNQSSTGGSSLGTFELENLMVNAGVSDWIVTYLSS

AVDRIYQIVSQSGVQILIFLAGIQTISPQLYEASKMEGATGYEAFWKITFPMVSPLIF

VNAIYTIIDSFANNAMTELIRDTGFVKFDFGLSSAMAWVYFLAIAIILVIVNIIFSKR

VFYQD"

CDS complement(2612003..2614579)

/locus_tag="EFAGFIKM_02220"

/inference="ab initio prediction:Prodigal:002006"

/codon_start=1

/transl_table=11

/product="hypothetical protein"

/translation="MNYTVAKKITGMLLAGSLLLGGCQFSPTPLAHETVTAADSTDFP

SLPPGEKLKSSFSDVRLPGMVGIAQNAALQLFINEETAEIAVIHTQSGQIWRSNPADR

EQDGVAAGINKDLLSSQTKLSFFNSLGQSSTVNSFTDSVAHKQISYEVLPSGVRVHYQ

FGSTERSIEDLPMKISKERFEQRLLAQLDKAGQRALKVGYAEDKDEGAYVRIDKAMQG

LQLSRALKAFDTAGYTSEDLAQDHAEFGIEEERSGPRLFMLTMEYELDGEDLLVRVPS

SGIHFPEEYPINSISVMDYFGAGGSEEEGSILVPDGSGALIHFNNGKLQYPAYQQDIY

GPDMTMKLREASSNEARARLPVFGLIRKEGAFLGIIEEGDAVAVVNADISGKLNSYNN

VYPSFYVVNKSDVTLQASDMVRTLPKFQKNPTVSDFVVRYAFVGADKASYSGLASLYR

DYLMQNGGLPELNASKEQSNVPFYLQLFGGMTTRQHMLGIPYDSTEALTTFDEAKNIL

SALTEKKVSNIQVRYAGWLNGGLHHRLPDSIQVDGAVGGKKGLRDFSTYTQEAGIGFY

PDIALLNVQSKKGFKPSKEASRTLTQEPAVLYPMNQAIQRRDRDRSPSYVLSPNMLED
VTADMLDELRSLQKDGLSLSLNDLAAQLNSDMNPKKLVDRTQALGSVKKALEQIGQQA
GSLVAEGGNAYALPYVTGLTDAPMTSSRFKLEDEEIPFYQLVVHGSIGYTGTPYNLST
YTNARQYVLKLIEYGASPYFAWFNAPNHVVKETDYDDLYAANYEQWIDLAAEIYNEVN
QVNQPFAGRSMMSHESLEEGVFRTTYEGGGFVIVNYNDFPVEVDNDTVEAQSYVTGGE
QL"

CDS complement(2614597..2615232)

/locus_tag="EFAGFIKM_02221"
/inference="ab initio prediction:Prodigal:002006"
/codon_start=1
/transl_table=11
/product="hypothetical protein"
/translation="MQASSKQLYQYPLHLIFHPFDGYWELKYERNQRNSLLIAFMILV
LLVITKILHAQYSGFLINLSNPKYLNSLLEMLYVIIPVLFWCVANWSLTTLMDGEGKF
SEIFMSTCFALVPLLLIHFPWIWLSLVISAQETAFYYFSNALAVAWTVYLLFVGNMTV
HQYTSAKTVLTMLLTLVAMAFMAFLCLLFFSLVQQIVSFVVTIYQELVLRG"

CDS complement(2615216..2616679)

/locus_tag="EFAGFIKM_02222"
/inference="ab initio prediction:Prodigal:002006"
/codon_start=1
/transl_table=11
/product="hypothetical protein"
/translation="MRRSTWKKRKSIIMGMICLLLFVQEAPYVNADGKTDAYHYSFWG
DAVPSPAAYEATTIITGKKLNTTPMKEPSDMHVTANQHVFVLDSGNGRIIEMDRNFKL
VRTIDSFKQEGKEDHFKNPQGLYVTEKGHLLVADSDNHRVVHLDEQGKLVKIVSEPKS
DLLKADFQFKPMRIVMDKGERIYVMAEGVFDGFMEFSADGTFSSFIGANRVQVDPVEY
LWKRFATREQRSQMVMFTPTEFTNLDMDEEGFIYATSGDRGKDPVKKLNAQGTDILRR
EGYQTPQGDLLYTQEAGPSRLIDVDVGDSDMYSVLDSSKGRIFTYNGDGYLLHIYGGI
GNRLGQFNTPVALERAGDRMLVLDKALGEITVFQTTEYGRTLHEAVRSYYNGDEDQSS
VLFAQAAEMNANLEYAYAGIGKALLRQKEYAESAKYFKRSMEPKGYSKAFLLYRKELM
REHFSWMMSGIFLAVAAIVTVIIVRRQKRRTTNAGIK"

CDS complement(2616695..2617570)

/locus_tag="EFAGFIKM_02223"

/inference="ab initio prediction:Prodigal:002006"

/codon_start=1

/transl_table=11

/product="hypothetical protein"

/translation="MTSKVRAVFGMPKRLNRSFTVSLMLFALLGVFGSFMVLPLIYAV

NNAFKPLDELFIFPPRFWVNNPTTENFADLINLMGNSWVPLSRYIANTLLITILGTAG

HILLASAAAYPLAKYRFPGSKVLFTIVILSLMFSPHVTAIPNYMVMSWLGWINTHASI

IVPSLAFSLGLFLMKQFMEQIPDALLEAAKIDGANEYRIFWSIVMPNVKPAWLTLMIL

QFPALWGTDGGSFIYSENLKTLHYALSQIVQGGIARAGVGAAVALLLMIVPITLFIIS

QSSVMQTMATSGMKE"

CDS complement(2617573..2618529)

/gene="araP_3"

/locus_tag="EFAGFIKM_02224"

/inference="ab initio prediction:Prodigal:002006"

/inference="similar to AA sequence:UniProtKB:P94529"

/codon_start=1

/transl_table=11

/product="L-arabinose transport system permease protein

AraP"

/db_xref="COG:COG1175"

/translation="MQAKTTKTAAAPAVHTRQVGWWSLLKRDLYLSRHYYVLMAPFML

IFFMFTVIPVGISLGLSFFHFNMLELPRFVGWQNYSRLFLNDDVFLIALKNTLLFAVI

TGPLSYVACFLFAWIINELSPKIRAVMTLVFYAPSISGNVFFIWLIIFSGDSYGYMNG

FLMRLGVVLEPIQWLADEKYVLAIVIIVQLWLSLGTSFLAFIAGLQTIDRSLVEAGTV

DGIKNRWQELWYITLPSMRPQLMFGAVMQITASFAVAEISIALAGFPSVNYAAHTVVT

HLMDFGTIRFEMGYASAIATVLFALMLGTNVFTQKMLRKIGE"

CDS complement(2618549..2621446)

/locus_tag="EFAGFIKM_02225"

/inference="ab initio prediction:Prodigal:002006"

/codon_start=1

/transl_table=11

/product="hypothetical protein"

/translation="MRSRKKKGLILVLVLALVLSIWTLYPSRDRNPGTVHALEDFEAV

SGTEDSFSYEHYLTAHDNTAKPDEIVRIEGESYVQAEDGEFEVVQGYAGLDGNAVITP

ETGTIRWDVPVQVSGLYNIRIHYYPVEGKSSGIERRLELNGKVPFRGADILLFDRVWG

NREDNVRQDDRGNELRPRQVEQPEWQLTSFKDSAGYFEEPFQFYFEKGSQKLSLTSLR

ESMAIDYIELYQESEVPSYAELEQTYASLGLKQSAPVMLKVQGEEAVSKSSPTLAPIS

DRSSPSLEPYNVSKIRMNAIGGINWKLPGEWIEWEIDVPEDGLYQLALKVKQDQLRGI

YATRSLTINGEVPFKEMKRIRFNYSPAWQTQVLGSGEDQPYQFHLEKGKHRIRMTVTL

GDIAPLLRTVESSVLELNEMYRKILMITSNSPDPLRDYQLERRIPEMTEVFERQADTL

RSVADYLEKATGEQSDKVAVLHAMVLQLEDMAARPETVPKRLDTFKINVGGLGTWILS

VREQPITLDYLVVSPPGESLPKAEASTVQQVKHELGAYVASYTEDYDSIGNVEQKKDA

ITVWITTGRDQAQVLKGMIDDSFTPDTDVSVQLRLVPPNILLPATISGEGPDVAMQMG

EDIPVNYAMRNATADLSQFPDFEEISGRFRESGLTPYRYNDGVYALPEQQHFPMLFYR

KDILNELGLEPPKTWQDVYNAIAVLQKHNMEFYLPIEDTLNNANLVPNSTFAMLLYQN

DGTFYTEDQKKSALDSEISMDAFKRWTQFYTNYKFPLKADFPNRFRTGEMPIGIADYT

TYNMLTVMAPEIRNLWDFTIVPGTQLPDGSIRHEVASATSAVMMLENADNKEAAWKFM

KWWTAEQTQIEYGREMEGLMGAAARYPTANIEALKQLPWPVKDYQNLEKQWEWVQGIP

QLPGGYFTGRHLDNAFRKVVNANENPREALSDYVLYIDDEIELKRKEFNLK"

CDS complement(2621552..2622907)

/locus_tag="EFAGFIKM_02226"

/inference="ab initio prediction:Prodigal:002006"

/codon_start=1

/transl_table=11

/product="hypothetical protein"

/translation="MRKNKFMLLSLVFAVLLVIAACSSAPTAQPEPEKTTPQEEQKPA

ETEPKNENSTPEMDFDMGGRTIKVVAWWDMEIQGNNPDNIQRLENLEALKKKHNFNIE

YVSIDFGEYQEKVVASLMAGEPLGDLVRLGKNYAIPALTKQDLLWPVDDYIKNDKVFN

QKTTKEYMQYEGKGYGFTEDQSSFINGIFYNRTLMQELGLKPLQEYVDADEWNWDTFI

SVAKQANKDRNNDGKLDTWGLAQTGLLEPILYSNEASLTKEDKQNLEDPKTKEALNFL

SKLATEKVGRASEGGDWTEPSTFFRQGNTLMYSGAMYEVEGIMTDMQDYDIGFVPFPK

GPSATAYHSGESRYQAITIPKAVENPEQLMYIWEKINEIESIYEYPGQSTLETHLTDE

ADINNAREVAEGMLVLDHNTFPSLPFWDFDGELKEGVSVSTLIEKYKAPFQAAIDEVY

K"

CDS 2623182..2623949

/gene="rhaR_17"

/locus_tag="EFAGFIKM_02227"

/inference="ab initio prediction:Prodigal:002006"

/inference="protein motif:HAMAP:MF_01533"

/codon_start=1

/transl_table=11

/product="HTH-type transcriptional activator RhaR"

/translation="MYDILLVDFSRRLCGELQQMLLRSKAQYTIANCVFSSAEALTAL

SERDFSLVMVHTERFDTAGLWLCNDIRKKSQIPILLMGGRDHFRFVRKALTYQVNDYL

PYPVSPSSLLNSLHGLRSHLETDPAHKTSSFFKTPVQMNGKPMHSSHVIRIVKAYVRD

HLSDEITLKKISDMLHFNCAYLGQKFKLEEKMSFNDYMLQQRMEKAQQLLSSTNLRIY

EIAIEVGYKDIDWFYKKFKAYAGSSPNAYRRQKIHTA"

CDS 2623915..2624130

/locus_tag="EFAGFIKM_02228"

/inference="ab initio prediction:Prodigal:002006"

/codon_start=1

/transl_table=11

/product="hypothetical protein"

/translation="MLIEDKKSTRPKGGLPNRFVYQLDINHKKTQDVFFFLRFFVVVW

VWSSKGFTINVPKGIPGILGESFPLFS"

CDS complement(2624072..2625697)

/gene="rhaR_18"

/locus_tag="EFAGFIKM_02229"

/inference="ab initio prediction:Prodigal:002006"

/inference="protein motif:HAMAP:MF_01533"

/codon_start=1

/transl_table=11

/product="HTH-type transcriptional activator RhaR"

/translation="MYRLLIVDDEEIITDSLYETFARHIPEQLDVCKAYSATEALSWM

QRSRIDIVLTDIRMPGMSGLELTERIQASWPNCRVIFLTGHSDFDYAYQAFQMANVRY

LLKTEGYDKVMSVVEDVMEEIRRSHSMVELLEHSHKVNSQLALIQQKEYLRKLLQDCT

AVIDSTMDMQDELFKRDIRLQINSPVYLILGRFNNPPEKGSDLTQVQESVRIIGSSLM

NERTVCTSVTDHYGDTIWLLQPKQEEEMTNDKLVRFLEGTLELVQEACMVSLGVSIAF

SLSSRSCNWSELTKQYERLRLLQWMKIGDGVSMVLTDHRNELSSDVPKESMRISNRIE

IMSGYLETGRIQPFYDIFEELAGELLQQDITMERAMETYYNLALLLHSTINRWGLQQK

IPDQRSLLHLGEYTCMKDAVQFLYRAADELVRYKRSNEQERANVVIHSLCSYVKENLE

KDLSLVRLAELHHFNPSYLSRFFKQEMGINVSEFIDDCRIRKAKELLQNTNLMVREVA

LQVGYEAAHSFTRLFKKITGMTPQEYRESLLVR"

CDS complement(2625682..2627439)

/locus_tag="EFAGFIKM_02230"

/inference="ab initio prediction:Prodigal:002006"

/codon_start=1

/transl_table=11

/product="hypothetical protein"

/translation="MIKIPAKSWFNSIFARLIITYLVFVIPLILLGVYLYHWSYDNAS

QEISLSTERRLNQYVVELNREIEWMELQQFDIVEDRKLNRLAILWDMMDQVERRDTLN

YLTERLASFKNSTAYIKNVHVHIPSVGKSISAIQGIDDFDKSSYLYFSSGMQGKGTRF

TVKEDALNLSAVRLTGTRGEPPLFVIQVELDTYHFQNELTRLNLYPESSTFLIEDKTG

HAITDKHQASVILANYREHSVKGTLDGFRMKVGDTMYHVSQLHVDSLGLSVATYLPEA

IVTKPLSKFYHWAWLFAITSFVAISAYLYSSYKLIHIPLLLLVKRFKKMEGGMLDVPI

AHNRKDEFGFLYTRFNLMIENLQSLIDRDFKKTLMMQRAELKQLQSQINPHFLYNSFF

ILNSLARTGETERIEQFTNMLGEYFRFITRNETDHVKLKVEVEHSRIYTEIQQLRFSR

RIKVDFGSLPAEMEHIKVPRLIIQPIIENAYEHSLEKNTDSGLLIIGFNMEGSYAEIT

VEDNGNELEEQHIQTLQQRFHKDGGTDEMTALINIHRRLVLTYGEGSGLFLSRSELQG

LKVTIRIQWKEGENECIDF"

CDS complement(2627497..2629182)

/locus_tag="EFAGFIKM_02231"

/inference="ab initio prediction:Prodigal:002006"

/codon_start=1

/transl_table=11

/product="hypothetical protein"

/translation="MYKRCLDITRWTFILFLAVSIPAGHTEGNTKQHHPQQDGQQIQL

PATFERYSPPIVVSFVRETGDDLERMISQLPGETILDNRWTRLYEKELGIQIHYDWTA
KGDVYNQKLGVSLAAGRFPDVVKVNPYQLRQLSNAGMIEDLTHVYQEYASPLTKSILE
AEGRGAFDAATIDGKLMAIPESSSSIETAQYLWIRTDWLEQLGLRPPETMEELLQVSK
AFTEEDPDGNGEHDTYGLALTNHLWDPVMGAAGFMSGYGAYPNIWVKDAEGNLTYGGI
QPEVREALKVLQTLYREGQLDPDFGYKSGNKAFRLVQDGKIGMLYGEQWTSFMLQSTR
DKDTDIDVEWQAYPIVAKSDRSLFVPLRSNTGQYFAVKKGFSNPEVVVKLMNLHLDIN
WGDQAQYETYYNDDSRAVWMLSPVTPFPGNKNIDAYKQIRDARSTGDFSALQNEALAI
HKRIVAYELENVESGWGWKQTYGPSGAFSIADSYEKNGQLLYDQFTGGITDTMVDRQI
ILRDLQLEAYMNIILGRSIDEFDQFVENWRKLGGDQITSEVNAWFRASKPKER"

CDS complement(2629313..2630857)

/locus_tag="EFAGFIKM_02232"

/EC_number="3.2.1.-"

/inference="ab initio prediction:Prodigal:002006"

/inference="similar to AA sequence:UniProtKB:T2KN85"

/codon_start=1

/transl_table=11

/product="Beta-xylosidase"

/translation="MYPNPIIWADYPDLDVIRVDDTYYMVSTTMHMMPGCVILRSYDL
IHWEVATYVYDTLDDTPAQRLVDGHHVYSKGMWAASLRYHQGMFYVIFVANDTRKTYL
YTSTSISGVWKKQIVEGFYHDCSLFFDDDERAYLVYGNTEIYLTELSSDLSGPKPGGV
HRLIVKDKQPHHLGYEGAHFYKINGKYIVFLIHITKASGRRTQAFYMADSLEDVFTGG
EVFNDDMDYFNSGVAQGGIVDTPDGDWYAMLFQDHGAVGRVPVLVPLHFDQGKPIFAS
KAPKQIDIPSTRPEHRYNPLVGSDSFNYEPEEDGSIRLRDFWQWNHTPNHELWSVTEK
SGVYRVRTGQISPNLTFAVNTLTQRSMGPACEAIVTLDGSRLNDGDYAGLCFLIGSYG
MIALTKQDNKFYLVMHARDSEDSTIFGNLIDQKPATEHERIPVSDPVVKLKAFGNFEN
NLDECSFAYFDGTEWRDIGIVHKMIYKLDHFMGCRTGLFVYSTEIAGGTADFSNFEYR
VINPVE"

CDS complement(2630865..2632382)

/gene="xynD_2"

/locus_tag="EFAGFIKM_02233"

/EC_number="3.2.1.55"

/inference="ab initio prediction:Prodigal:002006"

/inference="similar to AA sequence:UniProtKB:P45796"

/codon_start=1

/transl_table=11

/product="Arabinoxylan arabinofuranohydrolase"

/translation="MNHHSTPQAGEQKQDQAANLERIPGEIGKLRPNGNPLVAHKFGA

DPYALVFNNRVYLYMTSDKLEYDKDGLPQKNSYSSINRITVISSDDLINWTDHGEIRV

AGPQGAATWASQSWAPAAAHRILDGKDCFFLYFANNASGIGVLSAPTPIGPWIDPIGK

ALITRETPGVEEVTWLFDPAVTVDDNHQAYLYFGGGIPHGKAEKPDTARVMRLGDDMI

SVVGKAKVIQAPYMFESSGIHKYNNSYYYTYCSNFVEGDRPEGSPPPGEIAYMTSVQP

MGPWTYQGTLLQNPGHFFDIGGNNHHAIFQLADQWYIAYHAQTLCQAMNIPEGYRSTH

LNRVEHDEATGKIKKVHADYQGVDQIKCFDPYQHVSGSTIGWSSGVSTEQYLDSGELS

SSDSNIISAKLQNDSWIAISKVDFESEGPTTFTATIANQGTEGTLELRLDRADGLLIG

EIIVPPATGSLQWMELTTNVTGATGVQDLYIKFKSSTDSSTILLREWKFNRKNEV"

CDS 2632545..2633414

/gene="rhaR_19"

/locus_tag="EFAGFIKM_02234"

/inference="ab initio prediction:Prodigal:002006"

/inference="protein motif:HAMAP:MF_01533"

/codon_start=1

/transl_table=11

/product="HTH-type transcriptional activator RhaR"

/translation="MSGSSECFYDPIQPELLYLHSVTTYKLETIYHRHNAYEIYLFLR

GNVHFYVENRCYQVQPGDLLVMSPEEMHRAFILDESEYERITINLKKTYLFQLSTPST

NLSSCFDHRPKGTGNIVHLDDYNLKQMLQCTKELEGLLASDAYGTDIKINAAVAQLLV

MINVWFHNNSFVPNDIMPELVRETMDYIETHLNQDITLRKLAEVFYMNSTYISRQFKK

HTGLTIRSYILGRRIERAKFHLSEGMSITDACFQSGFSDYANFIRSFTKMVGISPGRY

IKQRRDVIEALPR"

CDS complement(2633531..2635225)

/locus_tag="EFAGFIKM_02235"

/inference="ab initio prediction:Prodigal:002006"

/codon_start=1

/transl_table=11

/product="hypothetical protein"

/translation="MRVISKMLGACLVTSVVLVSAGCGNAGENSESNTSDPNSPITFT

FFGADASPNWNKMQDAVGKKITEETGVSIDAEFDISSGGGNDRISLMAASGDYPDLIF

PKGNLTRLVDAGAMIDLTDLIEEHAPNLKKIYGEHFNRLKYSNDDPSIYWIPTNGAID

QVSFDATNGTAIQHRVVKELGYPEIKTLNDFENVLREYYEKHPTTDDGQPTIPLTLSA

DGWRRMITVTDPAVMSTGGPGDGEYFINPDTYEAVLHYKRPEEKEYFRWLNKMYNEGL

LDKDSFVQKDDQYKSKIASGRVLSLLDPSWGFSDAENALKSAGKDDMTYGFYPVTLDD

NFQRKDFQNIGFDGYGIGITVDCEDPVRAIKFLDWMSSEEGQVLRNWGVEGEQYNVED

GVRTIPADVQERKNKDNNTFTKETGVGLYYIFGAHYGDGVKDSTDNYYTTNYPEQIQQ

SYSDEEKEALKGYGITTWKDLFPSEEKFPVKEWGAAYNMPIPSDSVDYNVVYQKTQDI

IQKRIAEAITSSPSAFEDIYDNMITELDNAGAVAMEQQYTEWIKDRVRLWTGKEIN"

CDS complement(2635501..2635776)

/locus_tag="EFAGFIKM_02236"

/inference="ab initio prediction:Prodigal:002006"

/inference="similar to AA sequence:UniProtKB:P11540"

/codon_start=1

/transl_table=11

/product="Barstar"

/db_xref="COG:COG2732"

/translation="MSIIQINGNDFHSKEELHQILQNQLELDESYGGNLDALWDVLTG

AVSMPLTVQWIGFEKSKEILGDYADQVMELLRDVEGEIQGFTLELYY"

CDS complement(2635791..2636225)

/locus_tag="EFAGFIKM_02237"

/EC_number="3.1.27.-"

/inference="ab initio prediction:Prodigal:002006"

/inference="similar to AA sequence:UniProtKB:P00648"

/codon_start=1

/transl_table=11

/product="Ribonuclease"

/translation="MFFRKFAYTLVIILAALLFSGCTSESLRDLGSPQVSEDMGFDEV

ADYISEHNELPPNYITKKEARDLGWEPSEGNLREVAPGKSIGGDVFRNREGLLPNKKG

RIWYEADINYSGGRRGSDRILYSNDGLIYQTTDHYKSFEQLK"

CDS complement(2636483..2637244)

/locus_tag="EFAGFIKM_02238"

/inference="ab initio prediction:Prodigal:002006"

/codon_start=1

/transl_table=11

/product="hypothetical protein"

/translation="MKTAERTNKWKKIIMWTILSIVIIVAGVYVYLNSVTYSPSERAE

TAMTSDAQVNVAKIKDGYRFEPVGTEITKPNIIFYPGGLVEPESYSPLAREMAEQGHR

VYIANMPINLAIFGQNKAESFIEEHPDEAFVIGGHSLGGSFASRYAAEHNEKLEGVFF

LASYADEGGSVKNTDLSILQITGSDDGVLNWEDWENSKANLPEDTTYVSIEGGNHGQF

GSYGMQKGDNQPVITEEKQLEEVVIALENWMGKLK"

CDS 2637383..2637994

/locus_tag="EFAGFIKM_02239"

/inference="ab initio prediction:Prodigal:002006"

/codon_start=1

/transl_table=11

/product="hypothetical protein"

/translation="MTEKSPKRLPGRPKHGNDDISIQQTIIQTASQLFMEFGYETVTL

QQIGKTCSVSKPTIYYHFTSKPELFKVAFTTMLQNVSRLTSHILDQAENLESGLVRLA

EARLGNPHAEIETMLREAEPFLEPAQIQDIRNAEHQIHEVLASHFQRAMDENRLRTDD

PFFLAETFSTLMLMGNREDNLHKYGSNFSLGQKLVDIFIRGAQ"

CDS 2638022..2638903

/gene="rhaS_17"

/locus_tag="EFAGFIKM_02240"

/inference="ab initio prediction:Prodigal:002006"

/inference="protein motif:HAMAP:MF_01534"

/codon_start=1

/transl_table=11

/product="HTH-type transcriptional activator RhaS"

/translation="MNIPRGTYGFRFAEDKELQLCVLYAAGCDSITDPSYHWDGLERS

DGPLLLFQYTISGEGVFESNNRIHHVTAGQAFLAEIPGPHRYYYHSASKEPWEFLFLL

FRPTLILSHWRKFLREAGEVPYLPADCTPIRLLRMIVTDAAAGRITDPLIASSSVYQF

MTELTRLQKTTLRNRENWSENIQIAVEYIENNYSKMISIDHLAEHVSLSKYHFIRRFS

SSTGLTPGAYLTRVRTEKAMELLRGTSLSIEVIAEQVGYSSGSYFIKAFRSITGVTPG

EFRSGGESLVYRKLFFD"

CDS 2639012..2640292

/gene="agaA_2"

/locus_tag="EFAGFIKM_02241"

/EC_number="3.2.1.22"

/inference="ab initio prediction:Prodigal:002006"

/inference="similar to AA sequence:UniProtKB:B3PGJ1"

/codon_start=1

/transl_table=11

/product="Alpha-galactosidase A"

/translation="MNHTLAASTPPLGWNSWDCYGAAVTEDEIRGNAEYMAEHLKDFG

WSYITVDIQWYEPLANSSQYRPFVPLIMDEYSRLMPAENRFPSAAGGQGFKPLADYVH

SLGLQFGIHIMRGIPRQAAHAGTPILGTTATARDIAHPNSICPWNTDMYGVDSSKEGA

QAYYDSLFELYAQWGVDLVKVDDIAASRLYDTHQPEIEMISKAIERSGRPMVLSLSPG

PAPVEYSEFLADHANMWRVTDDFWDLWPLLLDMFDRCRTWQSVPKAGCWPDCDMLPLG

HIGIRSVDGGGADRWTRFTPDEQLTMMSLWSIFRSPLIFGGELRDNDDWTLSLLTNRE

VLRMHRESYGAREALRKEDLIVWTAQHADGSSYVAVFNIGENTLPVDLSIDEVGLSSI

TKGTELWSGEATEFTENSLVTTVPAHGVRLYRFS"

CDS complement(2640351..2640707)

/locus_tag="EFAGFIKM_02242"

/inference="ab initio prediction:Prodigal:002006"

/codon_start=1

/transl_table=11

/product="hypothetical protein"

/translation="MENVSQEDQLQSIKAFQSTIRKSENALVNMTQKRNNTTLLQKRL

QALYIGLALLDKVWNQKSHPYTEEDIAEARLVLMGLFPSLESMYANSKEVSPQKTLLE

RRIKAFHLAVQAMDTY"

CDS complement(2640829..2641557)

/gene="artM_2"

/locus_tag="EFAGFIKM_02243"

/inference="ab initio prediction:Prodigal:002006"

/inference="similar to AA sequence:UniProtKB:P54537"

/codon_start=1

/transl_table=11

/product="Arginine transport ATP-binding protein ArtM"

/db_xref="COG:COG1126"

/translation="MAKIKVEGLKKSFGSNQVLKGIDVAVNEGEVVCVIGPSGSGKST

FLRCINQLDDITAGRVIVDDQDLNDPKTNLNKARENIGMVFQHFNLFPHFSVLKNIMF

APRELGILNAQQARDTALKLLERVGLSDKAESFPTQLSGGQKQRVAIARALAMNPDVM

LFDEPTSALDPEMVGEVLGVMKDLASEGMTMMIVTHEMGFAREVADRVIFMDGGYIVE

QGTPEEIFGNPKNERTISFLEKVL"

CDS complement(2641550..2642995)

/gene="mltF_2"

/locus_tag="EFAGFIKM_02244"

/EC_number="4.2.2.-"

/inference="ab initio prediction:Prodigal:002006"

/inference="protein motif:HAMAP:MF_02016"

/codon_start=1

/transl_table=11

/product="Membrane-bound lytic murein transglycosylase F"

/translation="MKTTKISFFILSLMLLLVSGLSGWVGNAAAASDSGKTYVIGTDV

TFAPFEYEDENGDFVGIDMDLLDAIAKDQNFKYQIKSLGFNAAVQALEANQVDGVIAG

MSITDERKAKFDFSEPYYDSGVVMGISSNNDTIKSYEDLRGKKVAVKTGTEGYSFAES

IASKYGFTVVPFDESYQMYDDVKTGNSVACFEDEPVLRYGVKQNNGLKIVTAKEDGAS

YGFAVSKGKNQELLEMFNAGLVNIKANGEYKRIIEEYVGENAQVANLGRWELIQKSLP

ALFKGLGKTLLYTIVSLFFAFIIGLIFGFMKVGQNKFLRGVATVFVDIFRGIPLIVLA

FFIYFGIPQAMGFTMPLFLAAILTLSLNAGAYVTEIIRGGIQSIDRGQMEAARSLGIP

YRKAMIKIVIPQAVRVMIPSFINQMVITLKDTSILSVIGLVELTQSGKIVIARTFSSF

DIWLTVAVMYLIVIITLTKIADYLEVKVRRG"

CDS complement(2643339..2645438)

/gene="yfeW_1"

/locus_tag="EFAGFIKM_02245"

/EC_number="3.4.16.4"

/inference="ab initio prediction:Prodigal:002006"

/inference="protein motif:HAMAP:MF_01034"

/codon_start=1

/transl_table=11

/product="Putative D-alanyl-D-alanine carboxypeptidase"

/translation="MTRKKRTSIAAITLVFAMMSPMSAMATPATSNDSSPKYDLTKKA

VSAKAKVLTESYATTSVQYALMDEGEIVISGQAGKNDLKNNIPLSSDTMYGIGSTSKM

MLTTTVMKLVDQGKIDLDEPVVKYIPDFTMKDKRYQQITPSMLLNHSSGLLGTSSNSA

ILFRDNDTHAHDTLLEQLATQHLKADPGAYSVYSNDGFTLAEILVERVSGMDFTTFMH

RYITEPLGMEHTKTPQDIVDFSQMAATYSPSDEEQLPLETTNMIASGGIYSTAEDLVQ

FSKIFTGEVEGILSEESVEAMEQEEYKRGMWPEEGDSSIGYGLGWDSVNLFPFNDYGI

QAVSKGGNTITYHSSLIVLPEYNMAAAVTSSGGHSSTDQLLATELLLSALEEKNIIPE

RKPEKSHDAPMKTTMPKELTQHTGMYAGGANMLMKLDVKDDGQLTLSNLSSPNSSDQT

YTYTADGSFVNDAATEKLKFVQEVNGNTYLWSRSYQSVPGLGQVASSEYKAEKLETNE

LSEEVKAAWQKREGKAYVLVNEKYTSTLYNAAMPMIPIHTFNELPGYVYTNKIVGANQ

AVNQLQIPGLAGRDTMEFNFYEENGVEYVKAGGNVYAAQDIIKPIYAGKQSKTTIQAN

GHATWYSIPASAAGKEMTVKMSANSAFAVYNQAGVGINHTVVSGQNEIVLPENGTIVF

AGEAGSKFEIVLTTRAN"

CDS 2645730..2647160

/gene="gabR_3"

/locus_tag="EFAGFIKM_02246"

/inference="ab initio prediction:Prodigal:002006"

/inference="similar to AA sequence:UniProtKB:P94426"

/codon_start=1

/transl_table=11

/product="HTH-type transcriptional regulatory protein

GabR"

/db_xref="COG:COG1167"

/translation="MLKVNRNDQRPIWQQLLDQAIHNITTGKWQPGELLLPSRELAQL

IGVSRSTIQIVYEELFSRGYTVTSRRAGTRVSDWTYAPRSSEGVTMQGPIPPDLPTLN

ESIGHLHSWFGNRDNRKIEIDFNPHEPYLDESFTKAWRQSFLQASTEPNLDIWAYGDA

RGLLALREQIQRYLSLERGIHIDVDQILLTSGAQHSIDLIAQALLSEGDTVSVEDPGF

PAAWMAMKYRRMQVDPVPVDEYGLCVDHIHPQTKLVFVTPSHQCAVGVIMSEPRRQQL

MHLAAHHRYWIVEDDYDSEFRYRGDPLPTIFSQQPQNTLYMMSFSKMVAPGIRLSAII

GPKIAIDRLTQVHELTYRHLPIMEQLTLTHFIERGHFMRHMRRVRNIYRRRHEVMTKA

IVASGLGSRFTLSGVETGLHMLLESETSFDEEALTNAALDQGVRVYPLSRYCLESHRK

GWILGFAKVDEEQIEEGIHRLAKIVL"

CDS 2647206..2647826

/locus_tag="EFAGFIKM_02247"

/inference="ab initio prediction:Prodigal:002006"

/codon_start=1

/transl_table=11

/product="hypothetical protein"

/translation="MKILVLGATGRVGSHFVSNALRSNHHVTALVRSPDKLQINDDNF

ILHQGNALVKEDIHHAIQGVDLVVSSLSTDGTTTLSQSMGHIVEVMAQERIKRIITIG

TAGILESRTDPGLLRFQSPESKRKSTRAAEEHLEMYNILKASKLEWTIVCPTYLFDGE

SLGKYRVERDYLPDGGTSISVIDTAEFAYQQLMSDEYVQSRVGIAY"

CDS complement(2647922..2649166)

/gene="glyA_1"

/locus_tag="EFAGFIKM_02248"

/EC_number="2.1.2.1"

/inference="ab initio prediction:Prodigal:002006"

/inference="similar to AA sequence:UniProtKB:P99091"

/codon_start=1

/transl_table=11

/product="Serine hydroxymethyltransferase"

/translation="MKHLEKQDKVIGMAVQQELGRQRDTIELIASENFVSQAVMEATG

SVLTNKYAEGYPGRRYYGGCEHVDTVEEIANSRFKALFGAEHANVQPHSGSQANMAVY

FASVEIGDTILGMNLSQGGHLTHGSTVNFSGRFYNFVSYGVDAQTGRIDFDEVRKLAY

KYRPRMIVAGGSAYPRTIEFELFAQIAAEVGALFFVDMAHIAGIVAAGLHPNPVPHAH

FVSTTTHKTLRGPRGGAILCRESWAKAIDKAVFPGTQGGPFMHVIAGKAVALGEALQS

DFTTYIEGVLNNANVLAETLMNEGLTLVSGGTDNHIVLVDLRTMGLTGKEAEALLDEV

GITANKNAIPHDTASPLVTSGIRFGTPAMTSRGLGAREMKEIAQLIGLAFKNPKNSDV

KNQILGSVREITSQFPLYEGLK"

CDS complement(2649192..2650397)

/gene="ydcO"

/locus_tag="EFAGFIKM_02249"

/inference="ab initio prediction:Prodigal:002006"

/inference="similar to AA sequence:UniProtKB:P76103"

/codon_start=1

/transl_table=11

/product="Inner membrane protein YdcO"

/db_xref="COG:COG3135"

/translation="MKTASPSLRGRDLISPVIAALISVIVNYGGTFILVFQAAKVAGL

SPEMTASWIWSISIGVGVTGIWLSYRYKEPIITAWSTPGVAFLVSALAVTPYPEAIGA

YMISAVGFIILGLSGMFERFVRLIPPGIASGLLAGILLQFGISAFGGAKVDPLLVIVL

FAGYIVLRRFTSRYAIVGILAIGLIYLICMGKTDFSTIQLAVASPVFVVPAFSLNALL

GVALPLFIITLTGQYMPGMLVLRNDGFKTSANPILAVTGLGSLLAAPFGSHAFNVAAI

TAAICTGKDAHEDSTKRYIAGIACGVFYIFVGIFGVTLAALFLILPATFIATLAGLAL

LGTIGGSLANALTDPKGRETALITFLATAANVTLLGVGGAFWGLFAGMMAHLLMNSKF

PKKQLGTNK"

CDS complement(2650417..2651160)

/locus_tag="EFAGFIKM_02250"

/inference="ab initio prediction:Prodigal:002006"

/inference="similar to AA sequence:UniProtKB:P44506"

/codon_start=1

/transl_table=11

/product="Pyridoxal phosphate homeostasis protein"

/db_xref="COG:COG0325"

/translation="MKHLVEENLQAVRQQMALACQASGRNIEDIQLLLATKTVPLEKL

QIAIQAGEVLFGENKAQELRDKFPLMEQSKQVEWHFIGHLQTNKVKDVVKYVTLIHSV

DRLKLGQALHNQLLKENKTLDILVQINTSYEESKFGASPETAVELVEQLAQFETLNVK

GLMTIGKLNATNEETRHCFRLLNQIRTQIKEKNIPRVDMDILSMGMSGDFQVAIEEGA

TMIRVGTSVFGQRYLPDEYYWNENTRIDD"

CDS complement(2651485..2652417)

/gene="rbsB"

/locus_tag="EFAGFIKM_02251"

/inference="ab initio prediction:Prodigal:002006"

/inference="similar to AA sequence:UniProtKB:P0A2C5"

/codon_start=1

/transl_table=11

/product="Ribose import binding protein RbsB"

/db_xref="COG:COG1879"

/translation="MKKWTVTLISMLMIIVLAGCSLEPPEWAKPNPNKNNGQKKIGLS

ISTLNNPFFVSLKDGVMAEAKKQGIQVIVVDAQNDSAKQTNDVDDLIQQGVSALLINP

ADSAAISTAVQSANSVGIPVITLDRSADKGEVAALVASDNVKGGRMAAEYFVEQLGEG

AKVIELEGVPGASATRERGKGFHEVADKQLDVVSKQSADFDRSKGLNVMENLLQGNPD

VQAVFAHNDEMALGAIEAIQSSGKDIPVIGFDGNDDAIKSIQDGKLTATVAQQPILIG

QLALQAALDVLSGKQVDSSIPAELKLVTKENVNE"

CDS complement(2652432..2653403)

/gene="rbsC_2"

/locus_tag="EFAGFIKM_02252"

/inference="ab initio prediction:Prodigal:002006"

/inference="similar to AA sequence:UniProtKB:P0AGI1"

/codon_start=1

/transl_table=11

/product="Ribose import permease protein RbsC"

/db_xref="COG:COG1172"

/translation="MTTMQENKTAKSGFRFSNVIQKLGPLLGLIILILIVSVLNPSFL

EPLNILNLLRQVSINALIAFGMTFVILTGGIDLSVGSILALSSAFVANMMLSGLDPIL

SIIIGVALGGVMGMVNGLMITKGKMAPFIATLATMTIFRGLTLVYTNGNPITGLGDNL

LFQLFGRGYLLGIPVPAITMLITFMILWIVLHKTAFGRKTYAIGGNEKASIISGIKVN

RVKIMIYSLTGMLAALAGAILTSRLNSAQPTAGTSYELDAIAAVVLGGTSLAGGRGRI

VGTLIGVLIIGVLNNGLNLLEVNSFYQMVVKGIVIAIAVLLDRKKTA"

CDS complement(2653405..2654886)

/gene="rbsA_2"

/locus_tag="EFAGFIKM_02253"

/EC_number="7.5.2.7"

/inference="ab initio prediction:Prodigal:002006"

/inference="similar to AA sequence:UniProtKB:P04983"

/codon_start=1

/transl_table=11

/product="Ribose import ATP-binding protein RbsA"

/db_xref="COG:COG1129"

/translation="MHIQMQDIHKAFGTNQVLSGVDFELKDGEVHALMGENGAGKSTL

MNILIGLHQRDQGTITIDGEENYFASPKEAEKLGLTFIYQELNVWPEMTVLDNLFIGK

ELTSSFGLLNTRQMKALAKEQFAKLSVDIPLERPAGECSVGQQQMIEIAKALMTDAKV

IIMDEPTAALTEREIQKLFGVISSLKKEGVSIVYISHRMEEIFTICDRITIMRDGRTV

DTQAIPQTSSDEVVRKMVGRELTERYPVRNPSYGEVVLEVRNASSKGLFENINFTVRA

GEILGFSGLMGSGRTEIMRTIFGLDTLDSGEIFIRGKKANIRKPADAVKHGIGFITED

RKDEGLVLDFSIRENMALPNLFSFSSKGFISTSKEQDFVDTLIKRLQIKTQSSETAAR

NLSGGNQQKVVIAKWVGIGPSVLILDEPTRGVDVGAKREIYELMNELTDRGVAIIMVS

SELPEVLGMSDRIAVVHEGHISGEVAREVATQEHIMTLATGGQ"

CDS complement(2654901..2655302)

/gene="rbsD"

/locus_tag="EFAGFIKM_02254"

/EC_number="5.4.99.62"

/inference="ab initio prediction:Prodigal:002006"

/inference="similar to AA sequence:UniProtKB:P36946"

/codon_start=1

/transl_table=11

/product="D-ribose pyranase"

/db_xref="COG:COG1869"

/translation="MKRNGMLNSHISKILSDLGHTDMITIADAGLPVPDGVLKIDLAL

KLGTPSFREVVEAIAEDMVIEKVIVAEEICEGNPVAMQFITEKFGVEAIDASVSHEQF

KALTRQVKAVIRTGEATPYANCILQSGVHFG"

CDS complement(2655302..2656147)

/gene="rbsK_1"

/locus_tag="EFAGFIKM_02255"

/EC_number="2.7.1.15"

/inference="ab initio prediction:Prodigal:002006"

/inference="similar to AA sequence:UniProtKB:A0A0H2WZY4"

/codon_start=1

/transl_table=11

/product="Ribokinase"

/translation="MDLVVTSSRRPGAGETVLGDSFKTVPGGKGANQAVAAARLGGEV

TMIGRVGDDAFGKDIVENFRANAVNTQNVKPVTHLESGTAHIILAEGDNSIVVVEAAN

REVTPAYVDEAAEVIRDADIVLIQQEIPEETVVHVSALCAEFGTPLLLNPAPARTLPQ

EVIDNAAYITPNEHEAEILFQGMSPAQALRQYPNKLFITEGSKGVRYFDGTDEILVPT

YKVEAVDTTGAGDTFNAAFAVALAEGKALQESIRFANRAASLSVTKFGAQGGMPTRDE

VEESL"

CDS complement(2656173..2657162)

/gene="rbsR_1"

/locus_tag="EFAGFIKM_02256"

/inference="ab initio prediction:Prodigal:002006"

/inference="similar to AA sequence:UniProtKB:P0ACQ0"

/codon_start=1

/transl_table=11

/product="Ribose operon repressor"

/db_xref="COG:COG1609"

/translation="MTTIKDVAQLAGVSVATVSRVINDRGYVHADTRKKVEDAVKALN

FSPNEVARSLYKRKSKLIGLLLPDITNPYFPQLARGVEDRMQEQDYRLIFGNSDEDER

KEQDYIQTFIQNNVVGVISSTNYPHSSIYENLKIPVVFLDRTSLDRPSVYADGREGGR

LAAREIIKRGSRRITVMQGPSQIRPAQDRFEGAIESIREAGLDYRVIQTTSFSINEAS

MWAEELFRKYADTDGVIASNDIAAMAVLHEASRIGRKVPDDVQVIGFDDIPMSSLLSP

ALSTIHQPAYEMGREAAGLLIQLVEQAAIENKNIQLPVSFIERGTTRKVRSDG"

CDS complement(2657474..2657881)

/locus_tag="EFAGFIKM_02257"

/inference="ab initio prediction:Prodigal:002006"

/codon_start=1

/transl_table=11

/product="hypothetical protein"

/translation="MAKHEEVKDGVIFPVGEKNEAFSQYFIGQSYLQSLVADPKVNVG

VGNVTFEPGCRNNWHIHRGGFQLLLVTGGEGWYQEEGKAAQFLKAGDVIVTHDGVKHW

HGAAKDSWFEHIAITAGTPEWLELVTDEVYGKL"

CDS 2658058..2658651

/locus_tag="EFAGFIKM_02258"

/inference="ab initio prediction:Prodigal:002006"

/codon_start=1

/transl_table=11

/product="hypothetical protein"

/translation="MCNYSLERMVITIVSITDRKDRRIIKSQEAIKSAFIKLMNEKPF

DTITMQDISDHANVGRRTIYHHYSDKFDLLDKLIEEHIDELRRLCKEASDLDFIESNV

LWFDYFEVHYSFFSAMLLSKGAPFFRNRFLALVIEELEDEVNVTEGKNKGLTKEVILT

FFGTAIVGIVESYFTNGLPRQPHIVAEQVGILLERNL"

CDS 2658812..2659780

/locus_tag="EFAGFIKM_02259"

/inference="ab initio prediction:Prodigal:002006"

/codon_start=1

/transl_table=11

/product="hypothetical protein"

/translation="MIERNTLEKPSKFSDPIFVMLVASLCCLLWGSAYPSIKLGYIAF

NILPEDIASKYVFAGYRFTLAGMLLLLLSRIVRKQKLQLTKPQWTGLIMLGILQTGLQ

YMFFYVGVANTTGVKGSIMNATTTFFSVVLAHFIYKNDKLSRNKIVGCLLGFVGVIIV

NFHTDLLAFSFSFTGEGFVIIAALVFSVTALYAKRLTATIDVLIITGVSLFVGGLVLT

MLGLSLGGRVTHFTLESTSNLIYLALLSSVAFCLWNMLLKYNKVGRVSVYNFLIPVFG

ALLSALFLGETILELKNLAALLFVSVGIYLVNRVRSVQSSNDTPIK"

CDS complement(2659884..2661026)

/locus_tag="EFAGFIKM_02260"

/inference="ab initio prediction:Prodigal:002006"

/codon_start=1

/transl_table=11

/product="hypothetical protein"

/translation="MIHPKHTAKWASLALLTLIAYVLSALLLLPPNAAAKGEQISAKQ

LESMSRLSFTLRDAKQTAYTVYIFAYDEQKRTLTEENGWTNNKKGDKSYSGTYRAALL

KKGAAYGTVQAAKLDLNTIILPQTWNFVVKSKEASTPDMLMITDWGTSNFNEVKTYIV

RSGELRRVTYVDNKGKKIDDSYSASRDDGIRTLSGARVQFKNYNNLQFVYGVDTFKLN

VNKSELRLEDTRNLRSDAWPNSGVGDRAYLKSLKEAALKGALPGRTDIKIGMTLQNAQ

KKLGKPTSRSNEEWGAFYYYSKFGVGFDSYMHELTNKSRIAVFDLYNEKQNLSPWNVK

IWMGKPSSEYYNEVVGGYEMVYQLGNHAIVFNYEEEEDLIDFTSIY"

CDS complement(2661324..2662457)

/locus_tag="EFAGFIKM_02261"

/inference="ab initio prediction:Prodigal:002006"

/inference="similar to AA sequence:UniProtKB:P9WLZ3"

/codon_start=1

/transl_table=11

/product="hypothetical protein"

/db_xref="COG:COG1680"

/translation="MDFKPLASFIDRITTWRIPWAEVLVMHRNDTVFHYRNGYANLEE

KTPIGDGAIFNLYSMTKIMTCVAGLQLVEKGAMLLSDPLSDYLPEYAEMTVKKTMANG

EIRLEKATRAITVRDLFTMTAGFSYDVGCPSIQEAVKSTDGTLPTRDFARALAKEPLL

FEPGTHWNYSMCHDVLGALVEVVSGKRFGTYLRDEITGPLGMNDTAFNLNDEQQTRLI

PQYAYNDELEKAVRLDGNGFRVGTELESGGAGLLSTVSDYARFLNALTGHGTSPEGMR

ILSQASVELMRTDHLNEMTRSDYSWDHMYGYGYGLGVRTHISKAGSGSLSPIGEFGWS

GAAGCMAIIDPDSELTVMYAQHLLNSQEPYVQRRLRNVVYSCL"

CDS complement(2662788..2663372)

/gene="ydjA_1"

/locus_tag="EFAGFIKM_02262"

/EC_number="1.-.-.-"

/inference="ab initio prediction:Prodigal:002006"

/inference="similar to AA sequence:UniProtKB:P0ACY1"

/codon_start=1

/transl_table=11

/product="Putative NAD(P)H nitroreductase YdjA"

/db_xref="COG:COG0778"

/translation="MKTSVVEKLIRERRTIRQFNGKPLAKETIMHLLEAAVWAPVHSR
KEPWRFILFVEEGRKQFADAVLHTFSKDERERWTEKLQKDYCESIQAHLLVVIEADPR
QRVWEDAVGASAALIQNIQLLAWEQDIGVVWKTNEYNFDPDFYSKTGVKAGERIIGTL
HLGYFDTNKVPKPRPRTPVEELMTFVSTARAEVQ"

CDS complement(2663400..2665370)

/gene="rhaS_18"

/locus_tag="EFAGFIKM_02263"

/inference="ab initio prediction:Prodigal:002006"

/inference="protein motif:HAMAP:MF_01534"

/codon_start=1

/transl_table=11

/product="HTH-type transcriptional activator RhaS"

/translation="MHVEEHVKLWNLASIKIWDVRHVVVRAGEPIHAYRFPISGFIYT
VHGNATITLDDTMYAINQMQVLHGGKGVWMNISLNEDSFEFYLMFYRANLSLSNTREN
QALLRQHVPFHVQYAFAPSYPIELYDKVQRIEQHWERTDAFSKFQVKTLFYQFINELM
RQLSAQGIETAKPDLVAQAVRYLQENYMLPVMVDPLAELLDCSAGHLSRTFKKETGSS
LITYLTRIRMYKAKELLLHTDASLQKIAEAIGIPDVIYFNRLFKKYVGLSPGRFKQKC
IALPTGQNNAIQESELSIVSPMHRGYDPIDDDNYYQYNSKGESLTSMRSRKPVALLLM
LSLTLLISACSPSQGSSATSSNGTSGNNAPTTEVAQSTESAVQERVVKHPWGETVIKG
DPQHIISLFPVATDYLLALGIVPQAASSNEEGSDQFPTYLSDQLQGKENLGWQVDPNY
ESILAAEPDLIIGQDFMSDAYDSLSKIAPTLLAEKLQDEQGIIRMKTSLLHMGDMLGK
TDQAKQVIEEYEKKAAEAREKIKQSIGDETVMFLRLSDKEVRYYSKRNYEVLYDDLGL
TPPVSIPDPTDSMKVISMESLPSINPDHIFLLSSDENETTELQKTAVWKSLNAVKNNN
VYIVDYGLWFQGPGGPIGQSKIIDEAVNFLTQ"

CDS complement(2665552..2665746)

/locus_tag="EFAGFIKM_02264"

/inference="ab initio prediction:Prodigal:002006"

/codon_start=1

/transl_table=11

/product="hypothetical protein"

/translation="MTKQDPQELLKQKHELDDQKRQFTNFSRNISGHGEVEAGQEFSV
DRVPDDQNRMKSVENRRGEV"

CDS complement(2665885..2666199)

/gene="gdx_1"

/locus_tag="EFAGFIKM_02265"

/inference="ab initio prediction:Prodigal:002006"

/inference="similar to AA sequence:UniProtKB:P69937"

/codon_start=1

/transl_table=11

/product="Guanidinium exporter"

/db_xref="COG:COG2076"

/translation="MSWFFLILAGLFEVGGVIFLKLSDGFTKLKHTLMFAMFLALSFI

FLSLSLREIPISIGYGIWTGIGASGSVLLGMYVFKEPKNAKKLAIVSGIIISIVGLKL

VS"

CDS complement(2666202..2666543)

/gene="gdx_2"

/locus_tag="EFAGFIKM_02266"

/inference="ab initio prediction:Prodigal:002006"

/inference="similar to AA sequence:UniProtKB:P69937"

/codon_start=1

/transl_table=11

/product="Guanidinium exporter"

/db_xref="COG:COG2076"

/translation="MAWLFLILGGILEVGWALGLSFSDGFSKIEIVIPTVILMIGSFY

FFAKSTKVLPVSTAYAVFTGLGSFGTAIVGMIFLGDSVSAIKIVLVMILISCIIGLKF

VSNEPKPKAGV"

CDS complement(2666794..2667666)

/locus_tag="EFAGFIKM_02267"

/inference="ab initio prediction:Prodigal:002006"

/codon_start=1

/transl_table=11

/product="hypothetical protein"

/translation="MHQNRKQKSNKLKILSKVLLIIIGGFITAYGLEAILIPNNVSDG

GVTGLSIVGSQLFGLPLGMLIGIINIPFVWLGYKQIGKSFALYSIIGIASLAISTSLM

HHVPTIIEGDTLLVTVVGGIIIGFGMGLALRNGGALDGIDMLAVLLSRKVPFGTSDLI

LFLNMFVFIVVSTVFGLQGAILSGLAYFIASKVIHIVEEGLSGSKTFKIITNQPEIMV

ETIRDRLGRGATYTEAYGGYSNEQFKEITCVINRMEESKIKDIIHEIDPTAFVVVYDV

AEVRGGNFKKKDIH"

CDS complement(2667757..2668113)

/locus_tag="EFAGFIKM_02268"

/inference="ab initio prediction:Prodigal:002006"

/inference="similar to AA sequence:UniProtKB:P44558"

/codon_start=1

/transl_table=11

/product="putative HTH-type transcriptional regulator"

/db_xref="COG:COG0789"

/translation="MYTIGEVAELLHISAHTLRYYEKEQIVTPLRDASGDRRYNESHL

KWLQFVIKLKETQMPIAIIKKYASLFKEGEHTAADRLKLLEEHKESIQKQMHILHTAD

EMLEHKISSYRTLIGQ"

CDS 2668225..2668980

/locus_tag="EFAGFIKM_02269"

/inference="ab initio prediction:Prodigal:002006"

/codon_start=1

/transl_table=11

/product="hypothetical protein"

/translation="MKYTVITGASSGIGYEAALAFAARGKNIILAARRTDELDKLKAK

VAEINPALDVVIRTVDLSIAANVHEFYDSLQAYSIETWINNAGFGNFASVGEQHLPKI

EQMLHLNIEALTILSSLYVRDYSDVDGTQIINISSGGGYTIVADAVTYCATKFYVSAF

TEGLAQELKGKNAAMQAKVLAPAATETEFAKHSFNVDEFEYEGRVPKFHTAEQMAGFL

LELYDSESVVGIVDGLTYDFELREPLFPYAARR"

CDS complement(2669065..2670192)

/gene="nspC"

/locus_tag="EFAGFIKM_02270"

/EC_number="4.1.1.96"

/inference="ab initio prediction:Prodigal:002006"

/inference="similar to AA sequence:UniProtKB:A8FNH9"

/codon_start=1

/transl_table=11

/product="Carboxynorspermidine/carboxyspermidine

decarboxylase"

/translation="MRFEQLPTPCFVVDEALIEKNLKILNGVMQRTGAKIVLAQKAFS

MTAMYPLIGEYLSGATASGLYEARLGHEEMGKENHVFAPAYRAEEIDEIISICDHIIF

NSFSQLAKFKDKALQAGRKVGLRVNPECSTQEGHEIYDPCSPGSRFGAKLEDFDADLL

EGVSGLHFHTLCQQNSDDLETTLNAVVEKFGQWLPQMEWINFGGGHHITREDYDIPRL

EACIKRMQNDYGLEVYLEPGEAVALNAGYLVTSVLDFHKNGMDIAILDTSATCHMPDV

LEMPYRPPLIGSGEVGEKAHLYRLGGQTCLSGDVIGDYSFDQPLQEGDRLVFEDMAIY

SMVKTNTFNGMPLPAIAVKRKDGDCEVVREFGYQDFKMRLA"

CDS complement(2670194..2671432)

/locus_tag="EFAGFIKM_02271"

/EC_number="1.5.1.43"

/inference="ab initio prediction:Prodigal:002006"

/inference="similar to AA sequence:UniProtKB:P0DPE4"

/codon_start=1

/transl_table=11

/product="Carboxynorspermidine synthase"

/translation="MGKALIIGAGGVASVAVHKCVQNSEVFEEICIASRTKSKCDELK

AKLDGGKTKITTAQVDADNVDELIALINEVKPDIVMNLALPYQDLTIMDACLATKTNY

MDTANYEPHDTAKFEYSWQWDYKERFEQAGITALLGSGFDPGVTGVFSAYALKHYFDE

IEYIDILDCNGGDHGYPFATNFNPEINIREVSANGRYWENGEWIETKPMEIKRVYDFK

EVGEKDMYLLYHEELESLAKNMPGLKRIRFFMTFGQSYLTHLKALENVGMTSIEPIEY

EGQQIIPLQFLKAVLPDPASLGPRTVGKTNIGCIFKGKKDGQDKTYYVYNICDHQECY

KEVGSQAISYTTGVPAMIGAAMVMTGKWNKPGVYNVEEFNPDPFMEELNKWGLPWVED

FNPVLVDELPEEVKESELVR"

CDS complement(2672285..2673538)

/gene="pksS_2"

/locus_tag="EFAGFIKM_02272"

/EC_number="1.14.-.-"

/inference="ab initio prediction:Prodigal:002006"

/inference="similar to AA sequence:UniProtKB:O31785"

/codon_start=1

/transl_table=11

/product="Polyketide biosynthesis cytochrome P450 PksS"

/db_xref="COG:COG2124"

/translation="MNNNQTSEKSEFNLFSEENVKDQFAIFEQMRARGSIVPVPNPMG

GSEQTWIITRMDVAMEVLKDHSRFTVDMNSIDSDNDIRKNLSGELGSSEPQTFFTGKS

MLFVDEPDHRRLRSLVSKAFTPRYMESLRPRVQEIADELINQVEGKGEMDLVKDYAYP

FPINVISEMLGVPQEDRPQIHVWSEAIAKGLGFGKQDPEVEQHLRSFAEYTAQLVANK

RVEPSDDLISQLIAIEEEGDRLNEDELISMITLLIFAGHETTSNLIATGSYLLLTHPE

QLEQLKQNLNLVPSAVEELLRFNGPATSSGPRYATQDTELDGQPIQKGDVVIPLLKSA

NRDELQFDQPEELDLERKIKRHLAFGHGIHMCLGAPLARVEGDVAFTTLLSRLPGLQL

SVPPEEIHWHYSLLSEGLASLPVKF"

CDS complement(2673745..2674638)

/locus_tag="EFAGFIKM_02273"

/inference="ab initio prediction:Prodigal:002006"

/codon_start=1

/transl_table=11

/product="hypothetical protein"

/translation="MSSRKQSLLETALALFLEHSYANTTIQMILDQSGVSKGTFYKFF

SSKEDCLYSIIDQRMQEDVFIRKELEQNHYTSDYALLVDQIAIPMSGPDKERVWELYW

TGFYAGEINSAKLAKVHLNWLSTRLIQVFGKDISLYANEGAILCYGILHQIANTSRSL

NTKKPVWNEVVPKVLTYIEVILRTMHQRNEHIFDVQSLYFLNADERNHDMGLDIDNVL

IEFDEFNNCVQKSKESASLKQLSKGLLTLLQDQEDLNIPVIEVVLQAFHKEYKSSAFQ

SEANRLTEACWWYLELVKHRN"

CDS 2674876..2675523

/locus_tag="EFAGFIKM_02274"

/inference="ab initio prediction:Prodigal:002006"

/codon_start=1

/transl_table=11

/product="hypothetical protein"

/translation="MIKRNLRHLKKEATEKALASAAFELTLEHGLDGFTVEDIVHQVG

CSRRTFANYFTCKEEAVARGAISVQNTTELEELLTEMPDNVSLLDVLYNLIKMQLTTE

LIGRLHQLLSLSQTYPVLEPHYLGAMHRMQTQAQDTLLDLSNGKYDEIYTYLLINALY

GSILPLIDGRLNVLLPGQVITEDTKPGVLTFDQFLETTFNHLRAGFEHANHPHSS"

CDS 2675543..2677867

/gene="ydfJ"

/locus_tag="EFAGFIKM_02275"

/inference="ab initio prediction:Prodigal:002006"

/inference="similar to AA sequence:UniProtKB:P96687"

/codon_start=1

/transl_table=11

/product="Membrane protein YdfJ"

/db_xref="COG:COG2409"

/translation="MSTLLYRVGKTAFSKPAYFIIGWVLILGIVISMISINGIHISSE

MKIEGTESQKVLDQLAKELPAASGGQGSVVFKAPDNERLDTPERLAAMMKGVNEVYGL

DKVINPADYAAEAGNSGAQAEMAQNAQATDMAQSAATTPPPYGPLMVDGVPVPGMLIS

SDGSIALFQFQFTIEQSAITQDVFDSVIQSVTTVEQGTNIKVLPGETLKTVSIGVGSA

EIVGLVIAVIVLLITLGSVVAAGLPLITALLGVAIGVGGAFSISKFIEMPSVTSVLAL

MVGLAVGIDYALFIVNRQRRMIIDQGLSAKEATARAIGTSGSAVFFAGLTVIIALCGM

LVIGLTFLSTMALVAAATVLINVFVALTLLPALLGLVGERICSAKAREKSTKHPKATN

HGVADRWVKFVIKNRWATIIAIIVILGFAATPITKMEMGIPGASSANLDTTARQSYDA

ISEGFGEGFNGPLILVAEPNQSSTKVTPELLGGLVMELQGQNNVAQVTPLGMTEDLAI

FSLIPKTGPNDNLTKTLVTDLRAADSSIAQTYDVKLGVTGLTAVNIDMSAKLAQVFPI

YVGIIILLSLIILLLVFRSIIVPIKATIGFLLSILATFGITTAVFQWGWLHSLFGFDT

GGPLLSFMPIIVTGILYGLAMDYQVFLVSSMRESYVHGHRGTESVVHGYNQVSRVVVA

AAVIMVSVFAGFIFTDDVMIKQIGFTLAVGILIDAFIIRMGLVPAIMAIFGDKAWALP

KWLDRILPNLDVEGEKLIASLNAEEHANTKSGLK"

CDS complement(2677976..2678731)

/locus_tag="EFAGFIKM_02276"

/EC_number="2.4.2.-"

/inference="ab initio prediction:Prodigal:002006"

/inference="similar to AA sequence:UniProtKB:O33341"

/codon_start=1

/transl_table=11

/product="Putative glutamine amidotransferase"

/db_xref="COG:COG2071"

/translation="MKKPMIGVLPLYDTDKKSYWMLPDYMNAIEGAGGIPIMLPLTTD

VEILATLAHEFDGFLFTGGHDLNPELYHEYAEVTCGELCIERDLMESILLQKVIEIDK

PAFGICRGLQLLNVMLGGTLYQDIPTSLQLHGNKIVNHKQSPPYTNLVHDVHIEEKNI

LYDILQTDTIKVNSYHHQGIKMLSDKLTAVAVAEDGLVESVVMPNRSFVLAVQWHPEY

SYKVDDYSQKLFAAFVNASKSPRYTIHIEDITA"

CDS complement(2678947..2680146)

/gene="sotB_2"

/locus_tag="EFAGFIKM_02277"

/inference="ab initio prediction:Prodigal:002006"

/inference="protein motif:HAMAP:MF_00517"

/codon_start=1

/transl_table=11

/product="sugar efflux transporter"

/translation="MTRNRENLLILTLTLVSFVLGTTEYVIVGVLKEIESYMKVSLAA

AGMLVSGFAIAYAVGTPFAVAFLAKISRRSSILIGFAIVLALNLLTVFSTTFSSLMAI

RIVSAVACGLTISLSISIVSDAVNRERRGEAIAWILGGFSIANVLGVPLGTFIGQHLS

WSMTFVVTACIGVVPLVFMFRILPRQTTTIAGSFNDQMSLFVKPRILLACLIPVLGNS

CIFVVFTYITPLLSETMGVPVHWISAILLIYGACSILSNWIGAKIAKGDFLPKLKRLF

VIQAILFLGMSFTVSNVWLGLAFLFLIGCLSSSMSAASQLYLFDVSGSIAPGSKAFAS

TLLPVAANVGIALGSGVGGIAVNMGGVHWVPPVAVMLALLAFVITLICQRSIQLNSDE

ATTVKAA"

CDS 2680391..2681263

/gene="rhaS_19"

/locus_tag="EFAGFIKM_02278"

/inference="ab initio prediction:Prodigal:002006"

/inference="protein motif:HAMAP:MF_01534"

/codon_start=1

/transl_table=11

/product="HTH-type transcriptional activator RhaS"

/translation="MNTHHEFGQLHPNRNCTIIYVGKLADNPHWSFPTHKHDDLHEII
YVTEGKGVFTIDGTKHWAQKGDMLIYNKGTLHEEKSNAEFPLSTFYCGFRFAEGTQSL
EDWVIPPSNNPIIRANRYSDELHSLMQTLFNEFSIREHGYESISGHVLKAILLIIDRQ
SRQQLPSGEIVGSNTLAESIKDYLDTNYRQNIKLKELADQFHIDFYYLIHMYKNHYGT
SPYHYLIQRRMGEATRLLVSTNKKVWEIAKLVGYDNPNYFTILFTKTVGESPRSFRKK
NQKDMFGDKVSHSY"

CDS complement(2681356..2683350)

/gene="mcpA_1"

/locus_tag="EFAGFIKM_02279"

/inference="ab initio prediction:Prodigal:002006"

/inference="similar to AA sequence:UniProtKB:P39214"

/codon_start=1

/transl_table=11

/product="Methyl-accepting chemotaxis protein McpA"

/db_xref="COG:COG0840"

/translation="MRLMSTRLIAAFLAVLIIPTALIGYFSYDSAKDQVQQKMTDPIN
TILTMTGQHINNLVGEKAALLSYIDSMYGTTPSQTNNEAVQVGIDQLADTYPDILAIT
VGNDQGDVISSPAIEDATYDPRSTEWYAKGISNNGSLYFSSIMKDPVSGKIYVEISKA
LSNEQGVASIKLDLERLANEISIVDVGGNGSLIVVDSNRTIVAWSGAIVKGGGGELGG
ALIEGIPVHPNTASSSDEPMAFSQFVRTDLAYDLEVHSGVNALTGWNVIALMGHEDFI
AAAKPILVTSFTVIIISVLIAGVIIFFILRSFIVPMQKLRKATRSVSEGNLSERVNLK
TENEFGILANDFDQMTSSLQSVVAELHQTSSLLSHSSQMIQESTEQTTQSVQHVAETM
QQTAESAIVGAENSEQTANAVEEMARGISTIAESASAIVHSAEETERAVAKGGKTINQ
VGEQMEHILGAVEETSALINELSSLSAEANRMNEAIADISRQTNLLSLNASIEASRAG
EHGKGFAVVAGEVRTLSMQSKQSADEIGATIGKMLDLIAKSTSLMNDKVRNQVGEGMR
ISQEASATISNIEQYTTHIVDQIQDISAVSEQLSASTEEVSATVAAMSHISKVSADSA
QTTSAAAQEQMAAMQEISASSAQLSKTAENMQELVRRFKL"

CDS complement(2683585..2685111)

/gene="rssB_5"

/locus_tag="EFAGFIKM_02280"

/inference="ab initio prediction:Prodigal:002006"

/inference="protein motif:HAMAP:MF_00958"

/codon_start=1

/transl_table=11

/product="Regulator of RpoS"

/translation="MYNVMIVEDSKPILRNIKMLLETLSFPIRVAATATNGEEALAAI

KQSPIDLLLTDIRMPKMDGLSLIEQAKLANPDLKVILISGYSDFEYTRKALNLQVFDY

LLKPVEREALEEVMGRVIDQLDQQMSKELLDLQEIIHPQCETALKQGEAFPPLFSQMM

IIMSKQPFTAGHERWVQVELEGIVQDFFAFHPCRVYTSQTPYQFIAFVNKSALEVYSS

VHECLESLRRHLVAHDIDAIIGGQLLFAESGNLPELYHRMSSILSEQQRLKQGIVLDT

GNPVSMARSETGCLDPVQESAFVQMTQARQKERFALKLSELLNRWAEKNVHMTEVERF

IGLIVDTFAHLYEEQGAGMRLGLELRSRQFAQAQSYADFCRELTEWTGQCFDMLQSHV

RKSKAILFEQIDDYVKLNKYSQLSINDIAMKFHVSPSYVSRVIKNATQITFVQYYTRL

RIQEACRLMESQPDMKFKEVSDLLSFSDQHYFSKVFKEYTGLSPTDYKQQMSGIKTQS

"

CDS complement(2685128..2686891)

/locus_tag="EFAGFIKM_02281"

/inference="ab initio prediction:Prodigal:002006"

/codon_start=1

/transl_table=11

/product="hypothetical protein"

/translation="MAKLIHKATQFSLQTKVFLTFLTLLLFVLGCFIVYVNVIVVRPL

KLKTEQDTLVTATKVREQVDLYVEQQNQMSQRILSSKNIFAAMDKSSSAHSNYERLKQ

IRIIKDIMFQAIGPSMNIKDMSIYDNHGVLLTSYLGSGNPPAILNTMMESSQVGRDWT

ESGFVLLRQDDTISFVRTINDQNGKVYGYLSIQMEQAYIQNLTEGITAGEVYILNEQG

EQMTGSEAGVTFANWTKPSSDEGLDVDKNDNYITHSTSPSTGWITYIITPKKSVLGSI

HSVQTMSILLITALMLVSFVYIFLSTRNLLLPIRKLRGQIWRMNYSNMNLKVDETPKN

NDLLLLNEAFQDLMERLQQSIDREKSALHEEVKARNSALQAQIAPHFIHNVLYLISIA

AQEGKTKVVSDMCKHLSDSLRYIVSSPYAHVTLAEELQHTRHYLSLVQQKYEEDLEWD

ICADELANDIRLPRLVIQPFVENCIEHAFANTTPPWRIQITVKQYNGLWALEIKDNGE

GFPPDKIQEILANIQASGSGMNQTADCGTALGNMGIINSVNRLKLMYNNRLFINIFNN

HAEEQHGATIQIIGSLTKDFY"

CDS 2687098..2688411

/gene="msmE_3"

/locus_tag="EFAGFIKM_02282"

/inference="ab initio prediction:Prodigal:002006"

/inference="similar to AA sequence:UniProtKB:Q00749"

/codon_start=1

/transl_table=11

/product="Multiple sugar-binding protein"

/db_xref="COG:COG1653"

/translation="MKTIKRKKAISIILASLLSVSLTACGSGGTDSGTAAGGDSKSGK

VTLELAISKSSQDSAFIQQDILDAFEKQTNIRVNLQLIPAEQTTTVLQTKLAVDETPD

IIQYNLASAVTDLNLERNFEILDNESWASRIVNKDVLSSGGHLYSFHVSQDTGMQGVV

YNKQIFEELGLSLPTNYEEFLAICEKIKASGITPVFMPYKDAWAANIWPAAAFADFVA

KNEPTFFDELNSNKKKWSDIPEFKTFLEQQYEIYTKGYTNTDVLSDSYDMAVGKFLNK

EVAMMFMGDWLIEGVAEQDPNMELGVFPIPSSDNASLGASPLGGQLFIPKKSKHLEEA

KQFLDYIASKDVAQQIVDSKGYVSNFSDVTTPELPAYKQDIVDNYITPKKTVLTTDAF

MLVDRSELYRLLQDQFAGGLSPEEVLKSWDEKFSQLMQDKGVEGF"

CDS 2688479..2689351

/gene="melD_6"

/locus_tag="EFAGFIKM_02283"

/inference="ab initio prediction:Prodigal:002006"

/inference="similar to AA sequence:UniProtKB:O34706"

/codon_start=1

/transl_table=11

/product="Melibiose/raffinose/stachyose import permease

protein MelD"

/db_xref="COG:COG1175"

/translation="MKRTKNLYSYYMIFPALIIYSIFFVVPALAAFYYSFTDWRLDRL

ELKFIGWDNFEKIFSDKTLILALKNTAIFAIVTVVGKNVIGLLLAVGLNMRLRTKNLL

RAIFYSPSILSILVISILFTPMLRTEGTINNLLESVGLHSLSQAWLTNPSIVIWTIAI

VSIWQSAGFQMAIYLAGLQSISQEYYEAAKIDGASSWRSFFKITLPLLLPAININLML

TLIGGLKVFSEVYVMTGGGPGNASQVVGTIILRSFGEGNWGLGTAVNTLLFVVVTIIA

IPLLIFMRRKEVTE"

CDS 2689353..2690183

/gene="melC_4"

/locus_tag="EFAGFIKM_02284"

/inference="ab initio prediction:Prodigal:002006"

/inference="similar to AA sequence:UniProtKB:O34518"

/codon_start=1

/transl_table=11

/product="Melibiose/raffinose/stachyose import permease

protein MelC"

/db_xref="COG:COG0395"

/translation="MGYTRKLAIRNYIVEGFLILASLIVLLPLVILIFGSFKTSAEVL

SFSLSFPKTWQFSNYVRVFQEGGLSRAFFNSILITGVSSIINIVASSAAAFILARRET

KLSGTIYMYFFMGLIAPMSIITTIRVVQGLGFYGSITSVILIYAALNTAFSVFLYSGF

IKTIPRALDEVAFLEGASVFGVFFRIVTPLILPVNATVAIMVFMSVWNDITIPVYFLT

DSSTWTMPLSIYNFYGKYSRDWNLIFANLVLTSLPVFILYLFGQKYIVSGLTAGAVKG

"

CDS complement(2690302..2691006)

/locus_tag="EFAGFIKM_02285"

/inference="ab initio prediction:Prodigal:002006"

/codon_start=1

/transl_table=11

/product="hypothetical protein"

/translation="MNISKQVAIVSGANRGLGKELALELLARGTKVYAGARNPESIDL

PGAIPLQLDITDPQSVMAAAELASDVTLLINNAGSATGASLLTGELNDIHLEFNTHVF

GTLSMIRAFAPVIEKNGGGSILNILSALSWLSLGNSGAYSAAKSAQWGITNALRLELA

PKNVSVAGLHVAFMDTDMTAGIDAPKTSPTDIAKIAIDSIEADLYEIIADDTSRNVQQ

GLAGGVAALYPQLFQK"

CDS complement(2691228..2693642)

/gene="helD_2"

/locus_tag="EFAGFIKM_02286"

/EC_number="3.6.4.12"

/inference="ab initio prediction:Prodigal:002006"

/inference="similar to AA sequence:UniProtKB:O32215"

/codon_start=1

/transl_table=11

/product="DNA helicase IV"

/db_xref="COG:COG3973"

/translation="MDKTNEWKLEEERLVQVRNKLQARLDELEPKVAGLHEQAAEIRK

RFWEDVTINTSSYTDFEDAFYTINQQSRVLAERERGHKLLTQQWKNTKRLLPTPYFGR

FDYKEKGMTLTEQIYIGVSSFMDEDGLNFLIYDWRTPIASLYYDHSPGPASYDTPSGR

IEGKMELKRQFQIQHGQLHSMFDSTETIGDELLQQVLAKGADSQMKSIVATIQKEQNA

IIRDDRSRMLIVQGAAGSGKTSAALQRVAYLLYKHRQTVKADQIVLFSPNPMFTSYIS

TVLPELGEENMQQTTFQEYLNYWLDSSIRPEDAFDQIEYVLTAQNDPGYEARMEAIKY

KASETFLQVLQNYGKWLGREGMRFNGIQFRGREFITSNRIKTQFYSLDQNLTLSNRIL

LLQEWLLRELVSLERLEREADWVLEELNYLDTDEYVEAFRMLHKEKPVFDVAEKYAAR

EDGNNKVVQDEGAFNFAVREEELLRQKLVKDMFKPLRKSVRKFQFVDVKGIYEQLFVN

ETAYREQTNGAIPPLHWPEICKQTKAMILQNKLFHEDATPYLYVKEMIEGVRTNTEIR

YVFVDEGQDYSAFQYEYLKKLFPRARMTVLGDFGQAIFMQATDLAASNSPLVHLYGEG

ETTLIRLVRSYRSTKEIVEFTRQMLPGGEEIRPFERGDQKPLLTRLKDDKLRGAQILT

DIAALKAEGFDSIAVITKTAAESRDAYERLQSQGGEGLQLITKDTQVFEKGIMVIPVY

LAKGVEFDAVLVYDASSVAYSQEYDRKLLYTACTRAMHRLHLYTVSDWSPFVQALPAN

LYDIKS"

CDS 2694011..2694328

/gene="yybR_2"

/locus_tag="EFAGFIKM_02287"

/inference="ab initio prediction:Prodigal:002006"

/inference="similar to AA sequence:UniProtKB:P37486"

/codon_start=1

/transl_table=11

/product="putative HTH-type transcriptional regulator

YybR"

/db_xref="COG:COG1733"

/translation="MNIEPVEDRCPVEFAVNMIGGKWKLLIINRLIRHSTIRFNELQK

MLDPITHRTLTRQLRELEEAGLINRVTHPVVPPKVEYSLTEKGQSLTSILFQLGDWGS

KHM"

CDS 2694476..2695354

/locus_tag="EFAGFIKM_02288"

/inference="ab initio prediction:Prodigal:002006"

/codon_start=1

/transl_table=11

/product="hypothetical protein"

/translation="MTQNDRFLVYGASGSQGGAVAQLLVQHGCEVRTITRNEATAKTL

KEQTIEAFIGDLSDVEQLHTAHEGVSKVFLNLPVEFNSDKIRQYTKNAIDAAIQANVK

LIVVNTGTYVPDPITHSKGIELKREVIHELQQSGLPYIIVEPIVYLENFLIPGILNNG

VLAYPVPADKPISWISLNDAAQFHYYALTHSELAGSIIPAPGLEALTGAQLAEQFSAV

LGEKISFMSLPFDHFEAAIQPLLGSETAAGLKGLYQWIDGHTDLLPRYQELDDNIKAN

LKLTQISDWIHQTMIK"

CDS 2695586..2696566

/gene="yajO_1"

/locus_tag="EFAGFIKM_02289"

/EC_number="1.1.-.-"

/inference="ab initio prediction:Prodigal:002006"

/inference="similar to AA sequence:UniProtKB:P77735"

/codon_start=1

/transl_table=11

/product="1-deoxyxylulose-5-phosphate synthase YajO"

/db_xref="COG:COG0667"

/translation="MEYTKLGNTGLDVSRFCLGCMGFGDASKWVHEWVLNEENSRPVI

KKALDLGINFFDTANIYSLGTSEEYLGRALKDYANRDEVVIATKVHGQMHQGPNGSGL

SRKAILSEIDKSLKRLETDYVDLYIIHRWDYNTPIEETMEALHDVVKSGKVRYIGASA

MFAWQFQKALHVAEKNGWTKFVSMQNHLNLIYREEEREMLPLCKEEKIGVTPYSPLAS

GRLTRDWSVSTLRSETDQIQKSKYNATSDADRLVVERVASIAEKYGVPRTHIALAWLL

QKDTVAAPIIGATKISHLEDAVGALSVNLTSEDVTFLEEPYVPHPVVGAQ"

CDS complement(2696642..2697013)

/gene="fra"

/locus_tag="EFAGFIKM_02290"

/inference="ab initio prediction:Prodigal:002006"

/inference="similar to AA sequence:UniProtKB:Q797E6"

/codon_start=1

/transl_table=11

/product="Intracellular iron chaperone frataxin"

/db_xref="COG:COG5646"

/translation="METFAEFIARIDNPEHQARTEEVLNWITEKFPNLKQKIAWNQPM

FTDHETFIIGFSVSKQHLAVAPEKAGINRFSEEITQAGYDHTKELVRMKWKQEIDFSL

LERMIEFNIADKAECSTFWRK"

CDS complement(2697142..2697624)

/locus_tag="EFAGFIKM_02291"

/inference="ab initio prediction:Prodigal:002006"

/codon_start=1

/transl_table=11

/product="hypothetical protein"

/translation="MRKMTASFSHQPEGYECPFCRIWGIEQPDQGTKQRDIIYQNEKV

TAFIAGKWWPNNKGHALIVPNQHFENIFELPADYAVEIHRAAQLTAFAMKSTYGCDGI

STRQHNEPAGNQDVWHYHLHVYPRYVNDQLYLTNGSPSDPDERAFYADQLRSWIKENI

"

CDS complement(2697803..2698843)

/gene="adhA"

/locus_tag="EFAGFIKM_02292"

/EC_number="1.1.1.-"

/inference="ab initio prediction:Prodigal:002006"

/inference="similar to AA sequence:UniProtKB:C0SPA5"

/codon_start=1

/transl_table=11

/product="putative formaldehyde dehydrogenase AdhA"

/db_xref="COG:COG1064"

/translation="MIIAKARAVDGPDQQFKSAEIKRRDLDTNDVLIEIKYAGICHSD

IHTAHGEWGPVNYPLVPGHEIAGIVTDVGTGVSKYKVGDRVGVGCMVDSCGECVNCRK

GEEQYCLKGNIQTYAGVDKYGEPTQGGYSTHIVVVENFVVRIPDNIALDAAAPLLCAG

ITTYSPLHHWGAGPGKKVAVVGMGGLGHMAVKIAHAMGAEVTVLSQSLSKKEDGLQFG

ADHYFATSEPETFEKLAGTFDLMINTVSANIDINAYFSLLTLDGTLVNVGAPAEPLAV

NVFSLIGHRRSFAGSMIGGIRETQEMLDFCAEHDIAPNIEVISADQIDEAYKRVLASD

VKYRFVIDISTM"

CDS 2699035..2699448

/locus_tag="EFAGFIKM_02293"

/inference="ab initio prediction:Prodigal:002006"

/inference="similar to AA sequence:UniProtKB:P44558"

/codon_start=1

/transl_table=11

/product="putative HTH-type transcriptional regulator"

/db_xref="COG:COG0789"

/translation="MTYSIGEVAKKLDLTVYTLRYYDKEGLMPFVERTTSGTRLFKDS

DIDFLKIIQCLKLTGMPIKDIKDFIEWCSEGDSSLKQRYDMFTERKAIVEAQMEELRR

TMEVIEHKRSYYETALEAGTEEIHKIKPMENAITN"

CDS complement(2699542..2700120)

/gene="comR_4"

/locus_tag="EFAGFIKM_02294"

/inference="ab initio prediction:Prodigal:002006"

/inference="similar to AA sequence:UniProtKB:P75952"

/codon_start=1

/transl_table=11

/product="HTH-type transcriptional repressor ComR"

/translation="MARSKEFEVNEVLDKAMKIFWEQGYEKTSMSDLVEHMGIHRRSI

YDTFDDKHSLFLQAMDRYRGKVSTTLLAEIKASKTAVEALHKIFDVMISEAEETPSGC

LIVNSAVELGTRDQEVDNRSLESFNEAERMFEQIIQWGQREGEFSFDHDAKEMAEYLH

NISVGIRAMARTSMDKGKLNRIIHVTMKLLEK"

CDS 2700410..2701333

/gene="rhaS_20"

/locus_tag="EFAGFIKM_02295"

/inference="ab initio prediction:Prodigal:002006"

/inference="protein motif:HAMAP:MF_01534"

/codon_start=1

/transl_table=11

/product="HTH-type transcriptional activator RhaS"

/translation="MSDQIHEQQNELATLIDRYSDKDGVHPTAIPSLFLIRESVVTEP

IFRVNEPSFCIIVQGEKEVLLGQDRFRYGPGSYIVATVDLPVSGQVIQASSEAPYLAL

KLEFTSSEILEVLNHSDFPARPRKTTRRAMFVSQAEPSLLDAVIRLARLLEDHSEDIP

LLAPLFKKEILYKVLKGPHGIALEQATMEGSHTYRIRDVIEYIMNHSEQNFRVEELAD

LANMSTASLHRHFKEVTAMSPIQFQKQLRLQEARRLLLSKSTDAADVAFQVGYESPSQ

FSREYSRMFGFPPREDIKRLRGRADETTTIS"

CDS complement(2701467..2702489)

/gene="namA_1"

/locus_tag="EFAGFIKM_02296"

/EC_number="1.6.99.1"

/inference="ab initio prediction:Prodigal:002006"

/inference="similar to AA sequence:UniProtKB:P54550"

/codon_start=1

/transl_table=11

/product="NADPH dehydrogenase"

/db_xref="COG:COG1902"

/translation="MTDLFSPYSFKSLELKNRVVMAPMCQYSVERKDGIATDWHYMHY

VSRAVGGTGLIIIEMTDVEPDGRISDYDLGLWSDEQIPALKRIVDACHSYGAKVGIQI

AHAGRKAEDAEVPVAPSAIAFDENSKQPRALTTDEVKGMVEKFRSAVARAVKAGFDAI

ELHGAHGYLIHQFHSPLTNKREDEYGQDLTLFGREIIQAAKAEMPEDMPLMMRISAKE

YVEGGYGINESSEFAKAYKEAGVDVFDISSGGEGPIAAWGRPGTHAAYQVPLAKAIKE

ALDVPVIAVGRLDDPTLANAVIGNEEADLVAVGRGLLRNPYWALEAASALRKTTDVPK

QYTTGF"

CDS complement(2702601..2702945)

/locus_tag="EFAGFIKM_02297"

/inference="ab initio prediction:Prodigal:002006"

/codon_start=1

/transl_table=11

/product="hypothetical protein"

/translation="MIMLMLLTFILGIFIVVRQFWPVRNLEYIDYQVFLRQERDFRQY

KVIDIRDATDYMANPTPDTINISLGRLAFTWEKYLLREDNVIIMSPGMLQSKKAVRIL

RKHGFECIYVMK"

CDS complement(2702997..2703152)

/locus_tag="EFAGFIKM_02298"

/inference="ab initio prediction:Prodigal:002006"

/codon_start=1

/transl_table=11

/product="hypothetical protein"

/translation="MKVFVVGSYGQIAREDVAHVILASLSDKNTYKRSLDLIAGEDTI

SEALSGL"

CDS complement(2703290..2703694)

/locus_tag="EFAGFIKM_02299"

/inference="ab initio prediction:Prodigal:002006"

/codon_start=1

/transl_table=11

/product="hypothetical protein"

/translation="MNVWLAFSNIHTQLNEKLEQALLQQYDLSLKEFYVLNFIYNAEG

KELRLQQLQELVGLSQSATSRLVVRMEAKDCGALERHACEDDRRGIYTRITELGENKY

KRALQTFNQVLQTELEQDGFETRLENLTKELF"

CDS complement(2703902..2704930)

/gene="fhuD_2"

/locus_tag="EFAGFIKM_02300"

/inference="ab initio prediction:Prodigal:002006"

/inference="similar to AA sequence:UniProtKB:P37580"

/codon_start=1

/transl_table=11

/product="Iron(3+)-hydroxamate-binding protein FhuD"

/db_xref="COG:COG0614"

/translation="MRKQIKSIWIMMALILLIVLSACGQSATTNSTTGDSTNAATETE

TKTNSDSASSAESTATAEEELVTYQSDAGEVQVPKNPKRIIDLTSFSTGYFVALDAPV

VGALSGAMNNKYIKDQLAAAGTSDLGQEPTPEKLISLKPDLFIVYTGTEGIDKLEQIA

PVVQITFGKRDFKDLMLEMGKLTNREDAAKAWNAKWEAKINELKPKVQEAVGDRTVSI

LNPYAKGLFVFGHNYGRGGEIIYGEFDLKAPAKAQAEAIDSGTGWASISMELLPEYAG

DIIFTSPWSGDTTDPKIVYDNTLWKNLPAVKANDVFQLDPTSDSYNDPLTLEGQLQFI

SDSLLTAK"

CDS 2705142..2705954

/gene="uppP_1"

/locus_tag="EFAGFIKM_02301"

/EC_number="3.6.1.27"

/inference="ab initio prediction:Prodigal:002006"

/inference="similar to AA sequence:UniProtKB:P9WFF9"

/codon_start=1

/transl_table=11

/product="Undecaprenyl-diphosphatase"

/db_xref="COG:COG1968"

/translation="MEEIILWLKYLFLGIVQGATEPIPVSSSGHLIIAQKLMGIKQNG

LSFEILTNTASLIAIIFIFREDIKKLIIGALGYLRTRKEEYRADFMFCLYIIIGTIPA

AVVAVLFKDRIEEIFSSVYTVSIALLITGVALWLIRNLRGRKQDGDLSTKDALLVGLA

QAVALIPGISRSGATVIASIAVGMKQETALKFSFMLYIPISIGGLIMGVSDIANDPNR

SQLAIPYLIAFITTLFVTYFSMRWFMGIMAKGNLKYFSYYCFAAGTLLLIFL"

CDS complement(2706042..2707220)

/locus_tag="EFAGFIKM_02302"

/inference="ab initio prediction:Prodigal:002006"

/codon_start=1

/transl_table=11

/product="hypothetical protein"

/translation="MVTLLLIIIYLAFIGLGLPDALLGSAWSVMKNDIHATTEMAGYI

SLIISFSTVVSSLSASRLLHRFGTGKVTLFSILSTTIALLGFSFSENFVFLLILAIPL

GLGAGSVDAALSNYVALHFKAKHMNWLHCFWGIGAVTGPLVMAYWLNQANNWRAGYVT

VALILLGIVVVLLSTLSLWKIFEKGRVEGSGDEKKRVSNRETIRIPGVKMSMLAMLCY

NGSETAAGLWMASFFIVSKGVSPGTAAALSSLFFIGIILGRVISGFLSTHVSSKNLIR

YGGIVGCFGLVILVMPIPYWIAAGALFIVGLGGAPIYPSIVHATPERFGEKASPSVIG

LEMASAYTGSTLIPLGMGLIASQWGMSMVPLILLILFSVMFAATELVNRSNKATRLTV

"

CDS complement(2707365..2708222)

/locus_tag="EFAGFIKM_02303"

/inference="ab initio prediction:Prodigal:002006"

/codon_start=1

/transl_table=11

/product="hypothetical protein"

/translation="MTQKTIRLLMPQWQGGDNPNYSFGAELLAWLAPDNDQPLIHVPV

EAYDGTSLVSENGIKGRAQLLQQLQAAQHIIQAHQPDRIVMFGGDCLVEQAPFAYLNE

RYGGELGLIWIDAHGDLVRYEGYDNGHTLPLGNLLGEGDPEFARHVRVPLKPGNIFMA

GLTTPTEQETEVIQRLGIRTAGTEELTHGMDSIKKWIKETGIKHLAIHLDLDVLDPNM

FRSLLFAKPGEPYVLSPAGTMQIPHLLHLIKELSEATDVVGLGITEHMPWDAINLKNL

LGEIPILNQ"

CDS 2708325..2708693

/locus_tag="EFAGFIKM_02304"

/inference="ab initio prediction:Prodigal:002006"

/codon_start=1

/transl_table=11

/product="hypothetical protein"

/translation="MMSMAEYHGKVKNIQDTPFGYTLSVIGGKWKMVIMYLLADNQPV

RFNELKRQIGAITYKTLSSQLKELEADGMVERKEYPQVPPKVEYRLTAKAETLLPVLE

GLCEWGVQHQEPSINNSATD"

CDS complement(2708835..2709305)

/gene="ysnE_1"

/locus_tag="EFAGFIKM_02305"

/EC_number="2.3.1.-"

/inference="ab initio prediction:Prodigal:002006"

/inference="similar to AA sequence:UniProtKB:P94562"

/codon_start=1

/transl_table=11

/product="putative N-acetyltransferase YsnE"

/translation="MTHIVHAAAEDIRSEDSLLLIKELSEELGLLYGGDGTAGFQLSD

VEVPRAAFIVARIDGYPVGCGALRPLDETSVEVKRMYTRSGYRRKGIAQAILAEAERL

ANELGYTNLKLQTGPLQPEAAALYERVGYYRIPVFSGNWDRVLAYQKDLVHEKV"

CDS 2709513..2710178

/gene="yflN_3"

/locus_tag="EFAGFIKM_02306"

/EC_number="3.-.-.-"

/inference="ab initio prediction:Prodigal:002006"

/inference="similar to AA sequence:UniProtKB:O34409"

/codon_start=1

/transl_table=11

/product="putative metallo-hydrolase YflN"

/db_xref="COG:COG0491"

/translation="MEISKGIEMLQLDFEGNIIHPVLIWDEEMVVLIDTGFPGQYDDL

CIALEKIGVPISRLKAVILTHQDVDHIGCLPEILQECGSQVKIYAHELDKPYIEGQLP

LLKDGHLEHPPMGKVDETVTDGQELPFCGGIRVIHTPGHTPGHISLYLMDSKTLIAGD

SMYSVDGMLGGIHEPTTLDTEIARISLKKYIELDISSVVCYHGGLTHRNVNEQISGLY

KMN"

CDS complement(2710409..2711032)

/locus_tag="EFAGFIKM_02307"

/inference="ab initio prediction:Prodigal:002006"

/codon_start=1

/transl_table=11

/product="hypothetical protein"

/translation="MNLYHIQSNVHGINRVASFLEDNYIGIAWSGTGDLEHTPPELWK

EQLVQHYPLDEAELASTLATLHMFGNLMQDGDYVLISDDEWTYVGDIGDYYYDDSAGS

EEDLICHRRGVTWLGRIPLAELNDKVQALQNDSAILAKFEYPISQAQLDRGLAVSITA

EATNSLSTGVDEATIQTALQVLREALHSEEEELRIRAATAILQYAKQ"

CDS complement(2711049..2711444)

/locus_tag="EFAGFIKM_02308"

/inference="ab initio prediction:Prodigal:002006"

/codon_start=1

/transl_table=11

/product="hypothetical protein"

/translation="MGIQKRLFHFILAATLMINSVSLSTDVTYADPESIQEQDYKAIA

LTMLNPYISREITNYYQANHMPVPSYGLYEIHVLELKRHTQGGYSFRTVLEVRTFDGP

HNPPYGKEVITFEIDPANVTMVNYVHTND"

CDS 2711712..2714006

/gene="metE_1"

/locus_tag="EFAGFIKM_02309"

/EC_number="2.1.1.14"

/inference="ab initio prediction:Prodigal:002006"

/inference="similar to AA sequence:UniProtKB:P80877"

/codon_start=1

/transl_table=11

/product="5-methyltetrahydropteroyltriglutamate--

homocysteine methyltransferase"

/db_xref="COG:COG0620"

/translation="MTKSSVLGYPRIGADREWKKALEAFWAGKLEESEFHARLQEIRI

DHLRKQQAKGIDIIPVNDFSYYDHILDTAVMFGIIPKRFSYDGGSVPLSVYYGIARGT

KDAAASEMTKWFNTNYHYIVPELDGASPTLTENKPLLAYREAKEKLGIEGKPVIVGPL

TFLKLSKGYDKSETDAWLDRLLPLYTQLLQELASEGVQWVQIDEPILVTKLSDEDVQR

LNKIYKTFAAAVPGLNIMLQTYFESVENYSDIVALPVQGVGLDFVHGLSGNTQSIRTS

GFPADKVLGAGIIDGRGIWKASLQTKLNLLNELTEFVTPERIIVQSSCSLLHVPVTTD

REAKLTSELKNALAFADEKLDEIVLLTTALSSPSAEITAKIDEAELTLLALQQSEDRN

RTAVQKAVASISVQQPERSRPFAERHEAQQAKWQLPLFPTTTIGSFPQSAEVRKARQL

WRKGELNNEQYAAFIREQIDIWIKIQEEIGIDVLVHGEFERTDMVEFFGEKLAGFAFT

QFGWVQSYGSRCVKPPIIFGDVAFTGEMTVEETKYAQSQTERPVKGMLTGPITIMNWS

FVREDIAREQIAYQLAYALRQEVEALEQAGIGMIQVDEPAVREGLPLKENEQADYLAW

AVKAFRISTCTVHETTQIHTHMCYCEFHDMIDSIEAMDADVISIETSRSHGELIHSFE

ENTYELGIGLGVYDIHSPRVPSVDEMSSMIERALRVLDPKLFWINPDCGLKTRGQEET

VASLRNMVDATRIARNNHASAAVL"

CDS complement(2714180..2714737)

/gene="resA_2"

/locus_tag="EFAGFIKM_02310"

/inference="ab initio prediction:Prodigal:002006"

/inference="similar to AA sequence:UniProtKB:P35160"

/codon_start=1

/transl_table=11

/product="Thiol-disulfide oxidoreductase ResA"

/translation="MKVSLLQKKSFKSRRTLTILIGVIALITGTWAIFENVSTPESQR

GNAIEVGAVAPEFTAVNSAGEQVKLSDYRGKAVMINFWASWCTPCVREMPLVHQIAED

YQNDVETLFVNVGESKGTIREFMDKQAFDFPVIIDVTGNISGMYRITGLPATMVIDRD

GEFRHILLGELTEDTPLQQWLKDSI"

CDS complement(2714734..2715447)

/gene="lnrL_4"

/locus_tag="EFAGFIKM_02311"

/EC_number="3.6.3.-"

/inference="ab initio prediction:Prodigal:002006"

/inference="similar to AA sequence:UniProtKB:P94440"

/codon_start=1

/transl_table=11

/product="Linearmycin resistance ATP-binding protein LnrL"

/db_xref="COG:COG1131"

/translation="MDKMGLEVMGLCKSIKKQPIVEDISFRITSGQVLALCGGNGAGK

STVLRMIAGILRPTSGEVAVNNVRWLKERKRYSEQIGYMPDDYQFNQGLSAEEMLLFW

ASLRKVPKERVQEVLTMVGLQDQKKNRVTTFSKGMRQRVLFAQAVLSKPPLLIMDEPT

NGLDPFWMQELTQLLKGIKQEGHMVVFSTHQLEIADDIADQVIFMNHGRNVGEGSTHD

FCDQFGSLHAAFHQSLGLK"

CDS complement(2715440..2716222)

/locus_tag="EFAGFIKM_02312"

/inference="ab initio prediction:Prodigal:002006"

/codon_start=1

/transl_table=11

/product="hypothetical protein"

/translation="MGFRNPWAYSFLILFCTFSLSLLLLNSQNLVEGYSGTTGSMLNL

ILYLLPLMTLFLGSFSLTSEKEDGSWQLLSTYPIGTMSFIVGKYLGLSIVLITIVAFG

YGLMGVISGVIGNPLDVMTYLLFLAFSCGLVLLFLTLALFIGSLSRNRWQALTISVTV

WFFAVIGWPTLLLSVLGLMPYLWVKPLLVMLTLINPAELVRLFVVIKLGGGSILGPEY

YRWVEWVQQPSGSWIFIGICLFWIAFSVLTVYAIWERGRSHG"

CDS complement(2716290..2716835)

/locus_tag="EFAGFIKM_02313"

/inference="ab initio prediction:Prodigal:002006"

/codon_start=1

/transl_table=11

/product="hypothetical protein"

/translation="MVLMFGMVLLTACGTKYAALPINEDVDICAICKMQVKDDAYATQ

LTTKDGQNYKFDDIGCMNQWKTENGTDNIGMDYVRDYNDKEWIEYSKATYVYDASLRT

PMAYGILNFKDKPSAEAFIAEQGVGTIMTAEDLASHDWKQNTENMDMMGEHGHDEHGH

GEEGDGNEMHQEGEMKEESGH"

CDS complement(2716868..2718169)

/gene="nosD"

/locus_tag="EFAGFIKM_02314"

/inference="ab initio prediction:Prodigal:002006"

/inference="similar to AA sequence:UniProtKB:P19843"

/codon_start=1

/transl_table=11

/product="putative ABC transporter binding protein NosD"

/translation="MLLLSLPHGAVMASSGHRDLIPLQPMIDRSAPGEVIELASGRYS

GPVTIDKNITIQGDPTVIVVNAEATSTIMIRSDGVKLSGFTIEHDTNEQTAAIQVEGK

NSEITDLHIQTQGYGMIIRNSSQATIQRNNITWAGSDSASSGQKGNGIDLYNAHDSYI

EANEITGMRDGIYLENSRKSNVQNNRLLHTRYGIHCMYIDGSSVMDNTGEENMTGAMI

MGVKNTVISGNSFRKQSTNVHSQGILLYDVHQSSVHNNVVEGNRVGMNIAESSGNEIH

GNAVLRNFVGIQLVLAEANEFTRNQLVSNVIEASALDSPNNVLMENYWDSFQGLDLNQ

DGISEISYAINPFYEQLVSRNSAYQLFFQSPGMVFLSGLFTEGREQWTTDKSPRMSMD

TESDLNMSVERVAGSKMVWMFGLVLLGMATFIIIYMGVLRK"

CDS complement(2718574..2719248)

/gene="bshB2"

/locus_tag="EFAGFIKM_02315"

/EC_number="3.5.1.-"

/inference="ab initio prediction:Prodigal:002006"

/inference="similar to AA sequence:UniProtKB:Q81AU5"

/codon_start=1

/transl_table=11

/product="putative N-acetyl-alpha-D-glucosaminyl L-malate

deacetylase 2"

/db_xref="COG:COG2120"

/translation="MNTTHNEHERILVVYPHPDDEAFSVSGTLAKYIDGGAHVTYACL

TLGEMGRNMGIPPFANRVTLPGIRKQELIEASEAIGIQDLRMLGFHDKMLEFEDPQLL

EDPIMSLLKELNPTLVITFYPGYSVHPDHDATGAAVIRTIAKLPVDERPIVHCVAFAN

NQEQYIGKPDVFVDVSEFLDRKMASIRAHRSQFQAAELVGNRELNNEEIKKRFGRETF

WTYPFE"

CDS complement(2719272..2719616)

/gene="yojF"

/locus_tag="EFAGFIKM_02316"

/inference="ab initio prediction:Prodigal:002006"

/inference="similar to AA sequence:UniProtKB:O31858"

/codon_start=1

/transl_table=11

/product="putative protein YojF"

/translation="MQPIKPQDIQLRINNLADQDLYVHLELTSGAYAQHMDSTKHPAS

AFITNAAIRYTQGSISGTGPYRVGLKTTQGWLYAEGLTHIDEQEQERLILAGHDSQGK

LVVALQLSREMF"

CDS 2719882..2720667

/locus_tag="EFAGFIKM_02317"

/inference="ab initio prediction:Prodigal:002006"

/codon_start=1

/transl_table=11

/product="hypothetical protein"

/translation="MKSLIQLLHFGYHQAMSCIFPVAIFGTLALSSVIPIPFLHRYDA

ILLVLLAVQYLMYRSGLETKDEIKVICVFHIIGLVLEIYKVWMGSWSYPEQAYSKILG

VPLYSGFMYASVASFMCQVWRRLRMDMTGWPGFAPSMLLGAAIYINFFTHHFMPDFRW

WLTALVFIVFWKTWIIYRVRATTYRMPLSLAFIIVGFFIWAAENIATFFNAWKYPDQH

DAWQLVSFSKISSWFLLVIISVIIVAQLKYVKANRTAGDSKSS"

CDS complement(2720849..2723329)

/locus_tag="EFAGFIKM_02318"

/inference="ab initio prediction:Prodigal:002006"

/codon_start=1

/transl_table=11

/product="hypothetical protein"

/translation="MLLKYPSSWWQGLWREALPSGNGKLGASVQGGIQTETVLLQHSE

LWHWGRKDELPDVSHTLSETRKSMDEERYLEASWHLTRTLQAEGYASRLSSRYPLATM

TVEMKCNNAFRKYRRELDMDTGEVQVRWQDGSDNYTRSLFVSRADDCVVYEIETHRVK

DGASAQGTGAAEHSLLSGTIGLQFPQGDRVREDDEFNVLKSSITIRIDRDDAGEYLCY

GGQNDDGQDYGAVLRLIQLNEIPGASHDQQKESETNEDMLSRQLHFHTSGKVLVLVKM

FATGRRAQEWTRLKQELASLESEYNTLLDRHISLHQPLFSSAELDLDDKTDDGRCNED

LLLEAYEGEAPTGFVRKMWAYGRYLFISGTSEYSGLPFGLYGLWGGDYRLIWGHNMAN

ENVQMMYWHTAVGGLSKLNRSLFRYYNGLMDDFRDNARKLYGCRGTYIPAGTTPGIGV

PNQIVPVIINWTGAAGWIARHYYAHYQFTGDKQFLLEEALPFMKEALQFYEDFLVLGE

DGKYKMYPSVSPENTPENFMPKHGQPLAHPMPTAINATIDIAIIKELLQHVIAASRLT

GVHTTKIPCWEAMLEHMPDYLLEPEGAVKEWLHPAFEDRRDHRHLSHLYPVFPGQEVT

REEQPELFQAFETAVDQRRLGAQSGWSLAHMSSIYARLGNGERALESLDILARSCVLS

NGYTLHNDWRNMGVCMSMPAAPIQLDANMGWVNAVQEMLLYVSEQWIKLLPALPERWV

RGSIHGWRFHGGSLSCTWNRSTGFFRAELVADRRINTKLYIPEWVKGCEMPSRRACLC

ELEENGIYELEIEAGGELVLEQTAVFDA"

CDS complement(2723383..2724528)

/gene="yteR_2"

/locus_tag="EFAGFIKM_02319"

/EC_number="3.2.1.172"

/inference="ab initio prediction:Prodigal:002006"

/inference="similar to AA sequence:UniProtKB:O34559"

/codon_start=1

/transl_table=11

/product="Unsaturated rhamnogalacturonyl hydrolase YteR"

/db_xref="COG:COG4225"

/translation="MVGTSTKSVTQERRMSVKIADTLLSECQNGNHPKIAGKWGYVGG

MTLMALERAAEWNQESRYAELVQRHMDDLIENDGTIRTYRLNDYNLDMINEGKNLFNQ

WKNSGNDKYAKAILHLVDQLKGQPRTSEGGFWHKKIYPFQMWLDGIYMSSPFLAEYAS

TFDHPESFDEVARQILLIERMTRNPSNGLLHHAWDESREQRWCAQDTGRSVHVWGRAM

GWYAMAVVDALEHFPVDHPQRGQIMGIFERMAYAIAHVQDHDSGLWYQVMDQNGRAGN

YLEASASCMLTYALAKGLRLHYLAELDREVVDHAYAGILKRLVTEDEKGVHLHHICHG

AGLGGKKYRDGSYEYYISEQVVSDVQMGVAPFLLASIEMERLGAEKA"

CDS complement(2724596..2726653)

/locus_tag="EFAGFIKM_02320"

/inference="ab initio prediction:Prodigal:002006"

/codon_start=1

/transl_table=11

/product="hypothetical protein"

/translation="MDRQSVVTRNHPVLTQLEPRSPLSVGNGEFAFSADFTGLQTYPD

LYEIPLGTQSNWGWHYTGGHHVFGDEDIVYQAFETYGRKVAYPMKPEDKEEAYHWLRQ

NPHRLQLGRISFRLLGEDGQETGVTHVVPVRQELNLWTGILHSEFTVQGEEVRVETAC

DPRLDCIAIRVQSELIRKQRLQLFVVFPSPDMTHRSWSKSVFPDWKQHDRHSTVLTSV

TSTSASLKRVLDEDEYTLEWIWDAGQLEQTGTHEFTLSPKAVPDLDIWSCSVSFAAHK

PETLPADTVLRSSSVHWNQFWNDGGVIDFDGSSDPRAAELERRVVLSQFLSAVHSGGS

MPPQETGYMYNSWFGKMHLEMHWWHAAHFPLWNRTTLLCKSMDWYLTMLPEARELARS

QGYVGARWPKMVGYNGHQTPSPVAPGLIWQQPHPMALAEMCYLSRLDPAMLTRYKDIV

FEAADFMVSYAHWDEKRQAYVLGPPLIPAQENHAMNDSLNPPYELEYWKFGLEIAILW

AERLNHPANPDWRRVASHMAKPRHENGVYLAHENCPDTYTVKNHDHPSMVGALGLLPG

SLIDPEIMRNTLLKVKECWDWESAWGWDFPMCAMTAARLNEPELAVYFLMMDATKNTY

LPNGHNYQRAGLWAYLPGNGGLLTAVAMMAAGWTGAGEQANPGFPRDGSWTVKWEGLN

PLM"

CDS complement(2726697..2728991)

/gene="yesS_4"

/locus_tag="EFAGFIKM_02321"

/inference="ab initio prediction:Prodigal:002006"

/inference="similar to AA sequence:UniProtKB:O31522"

/codon_start=1

/transl_table=11

/product="HTH-type transcriptional regulator YesS"

/db_xref="COG:COG2207"

/translation="MTLSFLKWLKFNVTNTQTRLLLILTVVVFMIIIAVGMTTYYSSK

SVLQQELSEPQHQMLRISMNDIDEVIRESDQIAVKIALNSNVYRFLTNEVQGSYRNIT

ELVQFLETLISSTSFIKSAYIFDMNRESIVAFPQGYSSSKVNFPDSDWVSVADELEER

PMLVKQREISTSSGVSMPQITLYRKIMIAGELKGIIAVNFKTEKMFEHMLLSSVSDLD

SHRFILDTNNQPLYDLGNYAVGEEAVSHVLTQLDGEKLGEFKHEGKLLLASQTISPYT

GWRYLSVISQDSLLANAQKIRNIVFIVSLLALVAGAVTITVYNAAAFRPVRRMRQLLS

GYDQERIHPEQIDLEKITGQLLTDHAQQAINLRQTLPEASSKFLTDIYNGHMTGSREI

SEKWCRYFKDWSDEPLSIAMLSIDNYKAWCERFTKSDHSLLKFAIGNIIAELLSGQWR

VLSADLGRDRMLVMLQPIHSGGSKSVATAIRETIGMIPRLLKFQVSSGVSGGHDGFMR

LQRAMHEAESALDYRFYWGYERVISFEEISGLQPTENTSEEHEIMTQLTETVEAGRGE

EALRTFDKIVGRMTHEQWHPSSALAFLEAVAATIDRIRLKRETEGCGLEMVDMRTMHL

EAVCEVLRSQIEGLADWFGSLMLSKDFILCQQMITFMNKHLEDPVSIQEIAEHAGIGS

SLASQLFKQEMGDTIHGYFTKLRMERACELLLDTDYRISEIATMVGYQHENSFIRVYR

KYKDITPGKYREIMRSRRGVLRES"

CDS complement(2729079..2731310)

/locus_tag="EFAGFIKM_02322"

/inference="ab initio prediction:Prodigal:002006"

/codon_start=1

/transl_table=11

/product="hypothetical protein"

/translation="MSSHVYFEPEESMAYQSGEDVMHALMSIAGRYIGANSPHPPVYR

VSRPGLVRKCDDHRYVFPLGDLFPNMSKGQHVYTWAKLWSDSDQEFVFHITCFGPVRL

YHNGVLIYGSAPEEEYPAIPVLKLKCGLVRGWNHFVLEFKSGENGGGGQFGTGSRKNK

PLHFIVPSLERDGEEGWLYSEPLDFPLSEIPSGPMREKATGVNWLPREEDHEKASPYG

QLARMYGLTSGMSAYAWTKISGTKCGKEAMLISGEAYSPIEFSVEGKVVFHQEQAGRF

RFELPLSAAVQDLTANCVGGDRDWGFRMDATTDKMLTPALDVKGHRGKWLYLGPFQAE

QEIDLASCQKMKRTARSGTGEDVYWRTELPEGEVRPFLENEWFGRWNYPLGVTLYGLL

QLGKAGQRSDIRDYVLNHIEFASSRFSYSLWDRERYGAAGLNNQLSHIDSLDDCGSFG

ATMLLADRERKLDGVSETAAHIADYILHQQSRLEDGTLYRKIGVSRSMDNTMWCDDLY

MSIPFLCRYAEWSGDDKLLDEAARQVLLYKKYLYIPERQIMSHVFDVERGEPTRTPWG

RGNGWVLFSISELLTALPQEHSSYTVLIQFYRELCEGYLALQGRNGLWHQVLTDPESY

EETSCTSMFIYAYARGVRMGWLLDPKPYAQSAYQGWKGLSERTIDKRGNVYGVCRGSS

YSFTNHYYKHELGWNLNDTHGIGIVLLAGLETISMQEWQTNTPVFATERVGTPEKLEK

SSF"

CDS complement(2731307..2732635)

/gene="yteT_1"

/locus_tag="EFAGFIKM_02323"

/EC_number="1.-.-.-"

/inference="ab initio prediction:Prodigal:002006"

/inference="similar to AA sequence:UniProtKB:O34371"

/codon_start=1

/transl_table=11

/product="Putative oxidoreductase YteT"

/db_xref="COG:COG0673"

/translation="MKKRYALCGVSGRALGQFAKPIHELFSHNSEVVALLDSDPARFE

VYRARFPEHTEVAVYGASDFGVMVDETRPDCIIVAGRDDTHAHYIVAALERDLDVITE

KPMTTTGADARRILEAEARSKGNVIVTFNYRYMPIHMRIKELIQEGKLGRVTSIDLNW

YIDTHHGSSYFKRWNRERAFSGGLSVHKSTHHFDLVQWWINQKPVEVFAYGALNYYGA

EGEWNPAQEDGRHCRTCHVSEDCAYYSRWTSRSRQIRVPDDHLNQLGIASQQEFPYTS

YRPDQCIFDSTIEIEDTYAATVKYSGGALMSYSVNFSLPYEGYRLAINGTKGRLETLE

YHMPDRTPFPTPVQTIDYFPLFGSKETIHVVHREGGHGGGDPLLLEDIFLGEDKRRPY

RILSGAEDGAYSVAAGEAVWRSVEKHRPITVDEVLGGWNSRVPTHKGEVT"

CDS complement(2732632..2734170)

/gene="lipO_7"

/locus_tag="EFAGFIKM_02324"

/inference="ab initio prediction:Prodigal:002006"

/inference="similar to AA sequence:UniProtKB:P37966"

/codon_start=1

/transl_table=11

/product="Lipoprotein LipO"

/db_xref="COG:COG1653"

/translation="MRKQLSRKMMPLMLSLAMLLVAGCGSATTNSGNDVGNDEGSGGA

VKVKVFKSHMGVGSLPTADNPHVKYIAEQTGVQYELITTPPGSEPSEYLNLMIASDEL

PDILRPIGGIEQTLIQQGGALPLDELLPEYAPHVWESIPQEAWDIVRSASPDGKIYYV

PKVFLVPERAPLIRQDWVDKVGMEMPKTTDDYVELLTAFRDKDPNGNGKTDELPTSGR

EFGRWMDHLFAMYGVAMWEGYPEWDEYDGKIQYAGTTDNMREAIKFIRMLYVEKLLDN

ETFLNKGEVWQAKINNNLVGSWYHLPANVRDRYNAMLTQAPDAYIAAMPLPKADGFEA

FVTQKSMGEPEWMIPATKQDNAPNALKLLDFFYNPENDEFGRFGLEGEQHEVKDGRKV

ILPPADNKPLALGMKNLTTAEDMNKRIEETYPEDQQQMVKDMFEVSTADARQIAGDGL

PASVYEGFPDIQSHKLFQEVLTKIVIGELPIEAYDEYVKKWNASGGEVVTQRVQEWYE

KVRK"

CDS complement(2734194..2735066)

/gene="melC_5"

/locus_tag="EFAGFIKM_02325"

/inference="ab initio prediction:Prodigal:002006"

/inference="similar to AA sequence:UniProtKB:O34518"

/codon_start=1

/transl_table=11

/product="Melibiose/raffinose/stachyose import permease

protein MelC"

/db_xref="COG:COG0395"

/translation="MIKQTIGEKLFQTANYIFLTLAAFTMLLPLLHLLAVSLSSPIAA

DSKEVFLWPVGFTFASWKHILQSSGLWQSFGVTVFITVVGTALSMLFSVLTAFPLSRR

EFMIRKQVMLGIVITMIFNAPMIPFFLTVRELGMMNSIWSLIIPGVIGTFNMVILRTF

FMSLPKELDDTARIDGCHDFRILFQIYLPLSKPVLATVSLFYAVGYWNTFQRAVLFLR

DPGLWPLQMKLRAYLTSPEELAQVNMFLGDYNFNTTTLKAATILFASVPIILVYPYLQ

KYFVKGSLLGSLKE"

CDS complement(2735066..2736025)

/gene="yteP_20"

/locus_tag="EFAGFIKM_02326"

/inference="ab initio prediction:Prodigal:002006"

/inference="similar to AA sequence:UniProtKB:C0SPB3"

/codon_start=1

/transl_table=11

/product="putative multiple-sugar transport system

permease YteP"

/db_xref="COG:COG4209"

/translation="MAELSETSTLQPGVKVNKPVKPSYLRNIRKHWMLYVMILPGILF

YIIFKYIPLGGSVIAFQNFQIMKGIWSSPWVGLDNFKFIFTYQDFYHVLRNTALIALY

KLVIGFPAPILLALLFNEVRKMIAKRFLQSLFYLPHFLSWVVVGGIVFEVLASGGFVN

AVRGWFGFEPILYMQQEQYFRPIVVLSSIWKEVGWGTIIYLAAVSGIDPTQYEAAVMD

GANRWKQTIYITLPALFPTILTLFLLNIGNFLELGFDQIYNLLTPMTYSVGDIIETYV

YRSGVLQGQYSVTTAIGLFQSVIGFILLWVFNRLARKSGEGLW"

CDS complement(2736256..2737146)

/gene="murB_2"

/locus_tag="EFAGFIKM_02327"

/EC_number="1.3.1.98"

/inference="ab initio prediction:Prodigal:002006"

/inference="similar to AA sequence:UniProtKB:P61431"

/codon_start=1

/transl_table=11

/product="UDP-N-acetylenolpyruvoylglucosamine reductase"

/db_xref="COG:COG0812"

/translation="MNIFEHQIPLEKVKFNEPLKNHTFIKIGGNADILIHPTTIDEIT

KIVEIANLHQLPLTVIGKGSNVIIKDGGIRGVTISLSHFDQIEVNEDSMIAQSGVDII

DVSRLALEHSLTGLEFACGIPGSTGGALYMNAGAYGGQIADVVERATVITKDGAVLEI

PREEMKFGYRNSLFKMDHYIILEAEFGLKKGNKEDIASKMKELTFLRESKQPLEFPSC

GSVFKRPEGHFAGKLIQDCNLQGTRIGGAEISMKHAGFIVNVDHATAQDYMDLIQFIQ

KKVYDTFQVELETEVIFLGE"

CDS complement(2737413..2738393)

/locus_tag="EFAGFIKM_02328"

/inference="ab initio prediction:Prodigal:002006"

/codon_start=1

/transl_table=11

/product="hypothetical protein"

/translation="MKQENNIRTLQHKMGSGFNHNSTAEDVLRDLDLSGQLAIVTGGY

SGLGLETTRALVNAGARVVVTARRPAVAQEALSGLEGVEIDALDLADLSSVRAFAERF

LASDRNIDMLILNAGIMACPETRVGPGWEAQFATNHLGHFALTNLLWPALTSQGGARV

VSLASAGHHFSPIRWDDIQFEHGYEKFPAYGQSKTATILFSVELDRRGAEHGVRAFSV

HPGGIMTPLQRHMPKEEMIALGWIDESGQVANPAFKTPEQGAATQVWAATSPQLEGKG

GVYCEDCDISEMAPEDGGFYGVKKYAIDPEQAIRLWELSAKLTDTDMPTT"

CDS complement(2738565..2739227)

/locus_tag="EFAGFIKM_02329"

/inference="ab initio prediction:Prodigal:002006"

/codon_start=1

/transl_table=11

/product="hypothetical protein"

/translation="MFQPRSIMMSISLVVIVLTLAACSGSNEPLPSPQPSMPVGEPAP

APSTGNEQPSETDGTEEANETERPQTASFEMLTADGNQSLEATLQQGEGFSLYVFEKF

TFDAAQGRLSLTSNSDYYVDIEPLPSDYNLAELETAGKEELVKAGKVSDYSGELVEHP

LGYAELYLQASGEEGISDYMVWKSEEEEAFLFRLHNPKGEEGSDFASPVLVSLSTVQG

DS"

CDS 2739427..2739570

/locus_tag="EFAGFIKM_02330"

/inference="ab initio prediction:Prodigal:002006"

/codon_start=1

/transl_table=11

/product="hypothetical protein"

/translation="MSASIERRKVDINATLEDQLVWLEEAGFRVADCMYKYLDFAVFY

AQK"

CDS complement(2739640..2739864)

/locus_tag="EFAGFIKM_02331"

/inference="ab initio prediction:Prodigal:002006"

/codon_start=1

/transl_table=11

/product="hypothetical protein"

/translation="MVMPRFLRLMLYGKGMYRAQRNGTLLALNRMTGQPAFRVATGGD

LHNTTLKTNGMIIIQAEGKLLGVKLPASLK"

CDS complement(2740054..2742105)

/gene="yfeW_2"

/locus_tag="EFAGFIKM_02332"

/EC_number="3.4.16.4"

/inference="ab initio prediction:Prodigal:002006"

/inference="protein motif:HAMAP:MF_01034"

/codon_start=1

/transl_table=11

/product="Putative D-alanyl-D-alanine carboxypeptidase"

/translation="MKKLISLLCTAVLVITPLADVSAEANNSGTIEARAEQIAKEMVQ

NYGVTSVQYAIMDEGEYILSDSVGLHDKASQKPIDKDSMYGIGSVSKMVVTAAAIKLV

DSGKIDLDEPLTSYIKDFKMADARYTQITPRMLMNHSSGLYGTHYGNSMLFEDNDTRN

HDELLLKLQSEKLKSKPGAYSVYANDGFQLLEILVERVSGLSYSEYIAKFISEPLKLE

STKTPLDSFDRAQLSETYWPTLEMKMPTENANIIGTGGVYSTAEELSQFGEVLMGNRP

DILSESAVKAMQSHEYRKGIWVSDEQNTINYGLGWDSVDLAPFSDYGVTALAKGGDTM

LYHAVLITIPEHDISMAVLSSGGSSIYNQMFASKVLLEVLADQGIIDKVKPDQTFSPP

VKVKMPTELNSYSGLYGTVGETLDITIKDGEMQLPALLGGIIPEQKYVYTGDEHFTSA

DGSVKASFVKERNDKVYVKVQGMITLPGIGQTVMNTYDYQKLEHNALDAATKEKWTAR

DGSKYYALDEKITSIFYLLPSMLIKNLSVDAKNGYANGTQIMDANNAENIAEIPVMNG

RDAFDLKFYTKNGAEILLQDGQEYIAEDAITPIFGGKKGITTIPANGQARWYAIDDRS

GNKSITVDSPKTGGYAVYNDKGEMVDFSVATNQESTKLPKSGFIVFGGDSGDVFQIQL

K"

CDS complement(2742216..2743514)

/locus_tag="EFAGFIKM_02333"

/inference="ab initio prediction:Prodigal:002006"

/codon_start=1

/transl_table=11

/product="hypothetical protein"

/translation="MDTYMIFSIIFVTLLMAFQANFYFDSVLGKSRRKPRRAIYFMVF

VMLGYFYLVSSFSDIVSTAVALLLIFSLAQSYEVEFKIKLVFTILYAVLITMANTIAV

YILGVLESTDFISWEQFNGEDHWILSKVMLVGCSIMFIVIQIVRLVAKRRSFAVHYRY
YLLFLIVPIITIYQINVASIYSEKNIFYVFSVLGSLFLNVFIVYVFDNMVEKVQLAHE
NTQLQRQMDYQDANYEKTVHSFKNIKSIIHDINQQFLYIDECIQRNELAAAGDHIKST
LDTIEGAYQRVNSGNLVIDALVTNTLAMGQANGIKIDTKIQLHSQHIQIDRYDLCVVL
GNMLDNAIEASKKVRQAEDRYILIAIHSTSSALVIQIMNHVEQPIVDLKSDKPNPEYH
GIGLTNISRMCEKYGGHMSIEHQHRKFNNMVVLPFHTDNP"

CDS complement(2743516..2744256)

/locus_tag="EFAGFIKM_02334"

/inference="ab initio prediction:Prodigal:002006"

/codon_start=1

/transl_table=11

/product="hypothetical protein"

/translation="MFNIAICDDEEQQRERVKTMLVSLSLKTNFDFQISLFTSGEQLL
SHYREVGDTFNILILDVEMGGMNGIQTAKEIRIMKFLDVQIMFLTSYPEYMVESFDVI
TFQYLIKPIQPQVFEEKMIKLCQYFQALDKKYVLIKSDYDELLLKYDDILWIEVVKSL
TIKNKLNFVTQENVHETKGILSSYASGLKDHGFLQIHRSIIINLMHVQKFSGTQVIML
NGTELPIGRSKVKEIKDAYTKYMIMRIQ"

CDS complement(2744455..2745219)

/gene="yheD_3"

/locus_tag="EFAGFIKM_02335"

/inference="ab initio prediction:Prodigal:002006"

/inference="similar to AA sequence:UniProtKB:O07545"

/codon_start=1

/transl_table=11

/product="Endospore coat-associated protein YheD"

/translation="MGVDKLQPLRSKWLKTKLIINNELIKPFIPDTQKYNKTNLKSMI
SKYGMVYVKPERGTFGMGVIKAEMESQQHFVYQIEQKRLTFDSFESFYNSLTRLVNKR
SYLIQRGIHLLKHNNRRFDIRVMIQLSPTKHWEATGIIGRLGHPKKIVTNYHSGGTPM
DIHKLLKSHASTKRRNELVQEMNELSLRIARHMKKKYPHLKQIGVDIGLDHSLKPWII
EVNVKPDPYIFNQLKDKTMYRKVIRYYRHAVPKKTK"

CDS 2745422..2746231

/locus_tag="EFAGFIKM_02336"

/inference="ab initio prediction:Prodigal:002006"

/codon_start=1

/transl_table=11

/product="hypothetical protein"

/translation="MSVQPGIRLNNAPKIDVLIPAIEKDLATLPLVIDNIRRYVKHPI

GSIYIVSPVSTKIRKLCSLKNCIFVNERSVLPITKNDIHYQSSRWNRSGWLYQQLLKM

NGDSIVKAKYFLVMDADTVLIKPHSFLVEGKTVFYCRDWSQPEYFNTYRKLLGMKAPR

PRSFVTHYMLFDKSKLAALKQKIEAVHKLPWYKAIISNINKKKQFGFSEYETYANFMY

TKNPGSMVLRSSKNKSLNVNASSLKEQQIRNLALKYRSLSFHKRKIYSKAP"

CDS 2746249..2747622

/gene="rfbM"

/locus_tag="EFAGFIKM_02337"

/EC_number="2.7.7.13"

/inference="ab initio prediction:Prodigal:002006"

/inference="similar to AA sequence:UniProtKB:P26404"

/codon_start=1

/transl_table=11

/product="Mannose-1-phosphate guanylyltransferase RfbM"

/db_xref="COG:COG0662"

/translation="MHIVMLCGGSGKRLWPLSNELRSKLFIDILPSPAGGRESMISRV

CRQLDSSSLTDSTLIISHQDQANITARHTQGKIPVIGEPYKRGTFTAAALATLYLQSF

RMAKDDDVICIAPADVFAGEDFFSNFQLLPDILKHSQAEIALIGTRPTHASEQYGYIL

PGGEERNAYAPITQFIEKPEATIAETLLQRGALWNCGVYAFTVAFMISHIKKMGLPVH

YDQLSSLYEALPERSFDKHVAEKVQRAVVLPYKGVWQDIGSWDTLCAQLDTHVIGHGG

ISGSSSDSYIINELPYPIQVIGVPGIIAAASPDGILIANKNNSNEIKAQLGNWPLKPM

YGEATWGSYRVIESSLDDENTTVTTLNITVLPGKHIGLNWHRTGCRAWTVLAGSGQVL

VNGKVMQVSTSNQFVITKESLFSILAETRMVILEVRIGVTEDKDNILFEEEDWNVILK

SAGSSNA"

CDS complement(2747729..2750548)

/locus_tag="EFAGFIKM_02338"

/inference="ab initio prediction:Prodigal:002006"

/codon_start=1

/transl_table=11

/product="hypothetical protein"

/translation="MIAALNLEGVWKLQLDENKVETGLNFSDVITLPNTTSHAAKGKK

NENVLIGALTDEYLFEGYAWFSREIEIPEHLAGKRCFLHLERTRVTTVWIDGVEWGTQ

NSLNTPHQYEIETGLTAGTHTITIRVDNTDYPTKGGHLTSEDTQTNWNGITGKLELQF

FERVFLDNVQVYPFLATRSFDIKAALVGDLQGVRIVVSAVAVSADPVYTTEEQVFTPD

SQEIQLTYKIGEDAMLWSDDEPNVYQLHIHLQDQDGEVLDSTEVWTGLREFRADGDKF

TINGRKTFLRGKHDGLIFPLTGYAPTDVDEWVRILATSKSYGINHYRFHTCCPPEAAF

IAADLLGIYMEPELPFWGTITDESSDGHNQAEQDYLISEGFAMLRAFGNHPSFVMMSM

GNELWGSKDRLNSILKAYKAYDSRHLYTEGSNNFQFVPDILEESEFFCGVRFSKERLF

RGSYAMCDAPLGHVQTDLPNTLKDYDSNIVPEQGSDADPSGQTGDKEIQIQYGTGSKT

VQAEAGSDQLVSHLPIISHEIGQYATFPNFEEINKYTGSLKAKNFEVFRERLNAKGLG

HLAEAYFRASGKLAVACYKEELEAAFRSQQLAGFQLLDLQDFSGQGTALVGVLDAFMD

SKGMITPEEWRTFCSDAVLLARFPKYNYQAGELFQARIELSYFRRQALDGLQLKWELE

REQIEGSVLMEGKADLPAFHGEHYVSITDLSIRIPEVSNMSKAELRLSIPGTDIRKSY

DLWAYPSQLDVELTGLNLFTELSEVALVLLEQGEDIMLFPNPDQLQHAIQGFYSTDFW

SYPMFRSISENMKREVPVGTMGLLIQQDHPAFEHFVTEEHSTYPWWSIVSESSSIILD

ELDKDLNPIVQTIDNFERNHKLGLLMECRVRKGRVLMGALNLERLMTTLEGRQLLYSF

QRYVQSSAYQPVARLEVEELRKLFN"

CDS complement(2750735..2751529)

/gene="bdhA"

/locus_tag="EFAGFIKM_02339"

/EC_number="1.1.1.30"

/inference="ab initio prediction:Prodigal:002006"

/inference="similar to AA sequence:UniProtKB:O86034"

/codon_start=1

/transl_table=11

/product="D-beta-hydroxybutyrate dehydrogenase"

/translation="MDMGLKEKTALVTGSTKGIGKAIAIELAREGVHVLINGRNDEEV

ERTVREIKSTFPETSPQKATADLVDREQRQALFEKFPEVDILVNSMGIYEIMSYEDVN

DEVWERYFRTNVLAANGLSQFYLPKMVKNNDGRIIFIASEEALMPSGQMPQYCMTKSM

LLSLSKSLSKLTAGTEVTINTILPGPTLSENVREIIEGIYPDEALTFEEKENDFMKSN

LPQSELQRFIKPSEIGRLATFVCSPYAPAFRGSPIRMDGGMVPTIF"

CDS complement(2751661..2752659)

/locus_tag="EFAGFIKM_02340"

/inference="ab initio prediction:Prodigal:002006"

/codon_start=1

/transl_table=11

/product="hypothetical protein"

/translation="MQPQPNDRIKVHPGFWAGLHQLGIGADDIARAAQLSLETINQPV

VTQSQYFAIWQAYSDLIGDTAEGIIKLATRYEISKYPPPVLAMYHARDYRDALNRMVR

YKQMCPPESLQITEAGEEWFIELEWLHKEQPGPPLLVGITLAFLIELGRRGTGQPLIA

KRVEFSQPMGDVATLEAYFGCRVDTNSNYNRLTLERKDLSLPFLSYNEELLEILTPAL

DRSLDEQESNRSVTEVVKWIMKRSLIGKRPDIQMVAKELRMSDRTLQRRLTEENTNFK

QLLTEARREQAREYLADPSLDIKEVAFLVGYEDQNSFYRAFRNWEGDTPSNWRVRQ"

CDS complement(2752863..2753468)

/gene="estA"

/locus_tag="EFAGFIKM_02341"

/EC_number="3.1.1.3"

/inference="ab initio prediction:Prodigal:002006"

/inference="similar to AA sequence:UniProtKB:P37957"

/codon_start=1

/transl_table=11

/product="Lipase EstA"

/translation="MKKTKLILVAFMSMILALSITGIGSSSKASAATARTPIVLVHGL

TGSDSNFTAIESYLRSQGWTRDELFAIDLPSKQGNQLLNSAAISRFVDDVLRETGHTK

VNIIAHSMGGANSLYYILNRGGASKVDKLITLGGANRLTTTTAPSGIKVTSIYSTSDT

IVSPVLSRLNGANNVSVSLVSHIGLLFNSRVNALIKTALNE"

CDS complement(2753622..2753930)

/locus_tag="EFAGFIKM_02342"

/inference="ab initio prediction:Prodigal:002006"

/codon_start=1

/transl_table=11

/product="hypothetical protein"

/translation="MNQRFRITRSMQDSNTEQSISLEECKLYFESQPDFTYSPVLNIV

GAESTMTIEGDFFMWDLEGAQIAFRLYMGDLYVAISNEAIVPKMIEIATDLRADIVEG

"

CDS 2754094..2755329

/gene="rcsC_6"

/locus_tag="EFAGFIKM_02343"

/EC_number="2.7.13.3"

/inference="ab initio prediction:Prodigal:002006"

/inference="protein motif:HAMAP:MF_00979"

/codon_start=1

/transl_table=11

/product="Sensor histidine kinase RcsC"

/translation="MLKTEFIDVLSIVSHKLYDSAANVMDTASRLIPANTFCIAQLDH

LSTKVLNVYNRDKLILGEGLVVDNAESYCALVTEHSQGPLVIKNNLTHPLTRHMDATE

FVGGCSFVGVPIHNENGEIYGSLCSFDQDFYSYQQKDVDLLLSLSSFFTSLLEMETTL

QQLKQAEETAAKVLEEKKNLLAVLSHEIRTPMNGVLAMANLLQSTKLNEDQSLYVNVI

ESSGTSLLSMMDQILDYSKAEAGAISLEINPYSVIKTVDHVLQLFSSEAQKKGIRLYA

EYDINDHLMLIGDQQKVRQILINLVGNALKFTEQGEVCISTQVSADPEGTLHASYEII

DTGIGIPQDRQDLLFKSYSQIHGNSGKYGGAGLGLSICKQLAELMGGIVWLKETSHLG

SRFAFNISSASPTSMQISE"

CDS 2755372..2755797

/locus_tag="EFAGFIKM_02344"

/inference="ab initio prediction:Prodigal:002006"

/codon_start=1

/transl_table=11

/product="hypothetical protein"

/translation="MTQFLDARSSQNAALFNSISIPIPVINTPQLFAQIGLQTVGAGA

NPRVSLKGTITVRLPLALFQVRITIVRGTVATDPIVYSANFTYGLSGLLNPEVIMFSA

NDYNPPISAQITYTAFISTNLLGTVRVGPESFDGFIVSD"

CDS complement(2755876..2757108)

/locus_tag="EFAGFIKM_02345"

/inference="ab initio prediction:Prodigal:002006"

/codon_start=1

/transl_table=11

/product="hypothetical protein"

/translation="MVVVNITGALAGNQFEPSIAVNTLNPNIMCVVAVDTSTGPTLTG

FYRSIDGGSIWSTTVLPQPVEYSGAEAPTIDYTFPSTFIVTVHLFNGINDGTIASYTS

FDDGVTWQPPVIVQAGFGTIVHNDEPYIAVDRSPGSPYRGNAYVGYTPLATTSSAIFT

QRSVSQGLTWEPPDRQSNPRGIHDRAALAIGFSGEVYAGYIITGPASPSALLRISYDG

GITYQPPIERQATFISSVVPAPSPLPVPNYAFRVQTNLSLGADISVGPNSGTVYAVWN

DARAGYTDVLLCESPDGLLWSDPISITGAPPGTQNFFPSITVSPFAGTIRVIYYSNRI

DGFLLDVFVAESFNGGASFTNRRITTTSFNPNGNSPMPTILIGDYITAATVSPDNLAA

VWMATTPPTGKLDVYFGN"

CDS complement(2757127..2757495)

/locus_tag="EFAGFIKM_02346"

/inference="ab initio prediction:Prodigal:002006"

/codon_start=1

/transl_table=11

/product="hypothetical protein"

/translation="MPNSTGFIYNPTTGATQSTQLIVTCINDSLNAPVNIELEVFRWD

TTQTARIAIGHDLFELLPQATRTLIYPLINAAFYEVQSDYFSATSTVIHVYGVNSTGQ

IIQRVLQSEMTLIDRFTNIP"

CDS 2757677..2758951

/locus_tag="EFAGFIKM_02347"

/inference="ab initio prediction:Prodigal:002006"

/codon_start=1

/transl_table=11

/product="hypothetical protein"

/translation="MFIKRVGKIAAVTVITLSVLGSTPNIGGAYAAPAISVHLDDVPL

KFDAAPRIDRGVTYVPFRTVGEALGIDITWNSKSQTVKATNTVKGQTTEVLLQVGSTT

ATVNGKKVTLAAAPVQREGRVLIPLSSFSNQFGVQVSWNQATKTVSLVSPQREMHLRA

FYALQSFQEKDLITSMNSVAFGWSRIDREGQFTLQGDEYRLPAAAGDITPQSIVADAA

DQQIQPQLMVYALDGNGELTKVLSDSSLRQKSIEGITAAVAEHGFGGVVLDFEGLGFK

LDAVEQQKLLNAYVKQLKSSLPQDTALSLAVPPLNSAYKGYDYKTLASIADDLIIMAY

QYNPVGTKSQVPEPNSLVDQAIQLAIQAGVPKSKLLLGISLSSETPSSVDDKLGLAKR

YDLKGAAFWRLGLFRSYNTKMEDAVNVSVIKE"

CDS complement(2759039..2759257)

/locus_tag="EFAGFIKM_02348"

/inference="ab initio prediction:Prodigal:002006"

/codon_start=1

/transl_table=11

/product="hypothetical protein"

/translation="MKLGSILGILLLATAIVYGEWKSSKEKRARIVSAGITAVAAVIG

IILLFQPRLPGPTQIVKLVFGSVDKFMK"

CDS complement(2759301..2760476)

/gene="gerAC_1"

/locus_tag="EFAGFIKM_02349"

/inference="ab initio prediction:Prodigal:002006"

/inference="similar to AA sequence:UniProtKB:P07870"

/codon_start=1

/transl_table=11

/product="Spore germination protein A3"

/translation="MRIYRVLLMPILCCVLLSGCWDRIEINDLAIVLATGIDYEDGKV

QLTSQIFIPRKASAGDSSGSGGSPSGVTMIRTAEGRTIAEALNRLQRKVPRNMFWGHC

EVIIISEQAGKRGIREYIDFFLRYPQFREHAYVFSSEKAAKDILALLDPLERSSAESL

REMANLKLGTRVTALELAKSIEGPSSSAILSRMLILPPEPDQDKLTTTPYVKGLSMYK

KDRYVKTIKEPLSVGVLLLAKELNSIIMPVEFEPLEGSFSIRLIENKTSLKPRIVNQQ

WSMKVDIQTRGEVVLNTTDANLTDPAVLTKLEKEWSAKLTSLAHDALVMSQKELRSDF

FKFAVEFRRYYPHQWKAQEKNWETIYPDMDVQVNVDAHVVRTGKSTGPQGIPNEDES"

CDS complement(2760495..2761688)

/locus_tag="EFAGFIKM_02350"

/inference="ab initio prediction:Prodigal:002006"

/codon_start=1

/transl_table=11

/product="hypothetical protein"

/translation="MVASIPKEQTTLEQSTFRANKGAGLHMLTDKGKISVTQLAFMVF

PAILATAILSVPGITMHYAGHDMWMTPIIGSIVGLAAIGISVGLDRLYPGKTLIQSSV
LIIGRIPGKLFGLIYIAFLPHLTGLIIREYGEFIVNNALPRTPLFVVMGTMVVVCAIN
VRLGIEVVGRTAQVFVTLLIVLLALIFILLIGELNPAELFPFMEKGPIPIITGAAAPA
AWFSEYIVLAFLLPYVNEKKRITRVMLGSLVFTTTAMTVTNLFCLFLIGDLTDTFVFP
VMIAARYITIADFLQHIESLIIAIWIFGIFVKISVFLYIFATSTAEWFGLRDYKPVVG
PLSFLCMVFAYWVVSGGSGVSSLVSASANLYTISILLILPAMIYGVAWLKKGWAHFRK
NRANT"

CDS complement(2761652..2763061)

/gene="gerBA_3"

/locus_tag="EFAGFIKM_02351"

/inference="ab initio prediction:Prodigal:002006"

/inference="similar to AA sequence:UniProtKB:P39569"

/codon_start=1

/transl_table=11

/product="Spore germination protein B1"

/translation="MDTSAITQQDLPLTGQLVIDQEMLRSVFAGCSDVVFHTFQTACL
TSALCVYCVGVCDTERLERQVLTPLQEMGIKTAQVPLASVKQVETTTQAVQAIFEGEA
LLLLDGSIGGTTYPLYLAANRSTEEPLAESTVRGARDGFTESLTMNMSLLRKRLKTPV
LKIHTRNMGERTNTSISLVYMEGIIDPKLVKEVQTRLGDLKLRDVLESQYIEEGIVDQ
RYSPFPQMIATERPDVVVSNLLEGRFAILVDGTPFTLIAPVTIFSMLQSPEDYYQNVF
MSVFVRWLRYSFFVLSMLLPSAYVAITTFHQEMIPTVLLLSIARAREEIPFPALVEAL
IMEIAFEALREAGVRLPKQVGSAVSIVGALIIGQAATSAGIVSAPMIIIVAITGIASF
MIPRYAASIATRLLRFPMMFLAGTLGLTGVMLGVILVVIHLSSLRSFGTPYLSPVAPT
MAKELKDVWWRPSPRNKPH"

CDS complement(2763261..2764142)

/gene="araQ_16"

/locus_tag="EFAGFIKM_02352"

/inference="ab initio prediction:Prodigal:002006"

/inference="similar to AA sequence:UniProtKB:P94530"

/codon_start=1

/transl_table=11

/product="L-arabinose transport system permease protein

AraQ"

/db_xref="COG:COG0395"

/translation="MQTAKSVEQPNAAIQQYATTRRLTAGKIIIYLLLIGLAVLCIIP

FYLMLIYSTHNNATIASTFTFLPGSFLVDNYMNMVSKINIWRGFANSIFIAGTSTVLS

LYIGALTAYGFAKFKFKGRNGLFLFLLATMMVPGQLALIGVYRLFSIMGLLDSYAAII

LPAAANAFNVFFIKEFMESSIPDEIIESARVDSAGEFRTFNQIVLPILGPAIAALGIF

TFIGSWNNFLTPLVLFFSLDKYPLPVLVALVQGYYGMDYGLLYLGVAISILPIIIVFS

VFSKQIIGSVALGAVKG"

CDS complement(2764157..2765047)

/gene="lacF_3"

/locus_tag="EFAGFIKM_02353"

/inference="ab initio prediction:Prodigal:002006"

/inference="similar to AA sequence:UniProtKB:P29823"

/codon_start=1

/transl_table=11

/product="Lactose transport system permease protein LacF"

/translation="MFAKTIRKDHYGYYFIAPFFIIFTTFGLYPILYSLYISFTNFDG

ITTPDFVGIGNYVAVLQDPLFYKTLFNTLFIWGVSVVPQLTISLVLAFILNDKLLKGR

DFFRAVYFFPNIVTAASLGLLVSLIFDWQSGGLNHFLVQTGIIQDPINWKNDPWFMRL

IVSSILFFQYFGYSMVIYLAGLQGIDPSLLEAAQMDGAKKKHIFIHIIVPMLRPIILF

QMITSIIGGIQIFDQPFTLTNGNGGPDRAAMTSIMYLYNVAFQSTRFGYGAAIAFCLF

IIIILLSVASFIMTKRKSRS"

CDS complement(2765102..2766367)

/gene="lacE_1"

/locus_tag="EFAGFIKM_02354"

/inference="ab initio prediction:Prodigal:002006"

/inference="similar to AA sequence:UniProtKB:P29822"

/codon_start=1

/transl_table=11

/product="Lactose-binding protein"

/translation="MKKWFKPTIVLVCAAMLLAGCQFSPSSQTKQQEITVWSFTDEAG

YAIEKFEQKYPDIKVNFVNIPGNFYITKLKSALQTTSKAPDVFMIENANIRELIDVPY

LENLSASPYNANELIQEQYAFVQANEQDSEGNVRAIGYQGTPGGIYYRRDLAKKYLGT

DDPEKVGSQINTWEKIFEIGEKVQQLSGNKVHALANWNAISNSYDGIPWVKDGKLVID

PTYLEVLDLVREARERNVLAEYEDGSAGYAASMQKGEVMFYPGATWGLQYTFKANAPD

TEGMWGLAQGPSAFSSGGTYIAMYSKSEKKDLAWKFIEFYNFDHDFLSELAKEQDYFT

SNMVVNDKLAPSLSSSYLGGQKHFEFFSEAAKRVPVYERTKYDATINNDIYKIVLQLY

LNKDIQTKEEVVKRIKRDVTLRFPELEVD"

CDS complement(2766453..2767337)

/gene="rhaR_20"

/locus_tag="EFAGFIKM_02355"

/inference="ab initio prediction:Prodigal:002006"

/inference="protein motif:HAMAP:MF_01533"

/codon_start=1

/transl_table=11

/product="HTH-type transcriptional activator RhaR"

/translation="MGVPTFLETGHMQEGFPIRVIHTGNQFNYAAHWHDEVELVLVNG

KSARIGVNDHVCELEKGDVLLIKPGDVHCFLPGTEHLTIILFRLELLTGSFTTEAEIQ

DLGQLFNRTTVIPSNKGSQSNLTQYIDDIITEKKNQKPGYRWLMVSRLYDLIILLLRT

IVPVTTDESTSSGWPCTSSKKFEFLESVCEYLEEHYAEPIKLEQVAEHIKFSKFHVCK

LFKEIKGVTLMEYLNHFRIIKSEWALLFRQDTILEIAIGHGFNNVNSYNRLFKKYNDC

TPSEFRKKHRANMTKYGG"

CDS complement(2767455..2768453)

/gene="rbsR_2"

/locus_tag="EFAGFIKM_02356"

/inference="ab initio prediction:Prodigal:002006"

/inference="similar to AA sequence:UniProtKB:P0ACQ0"

/codon_start=1

/transl_table=11

/product="Ribose operon repressor"

/db_xref="COG:COG1609"

/translation="MMTTIKDVAKMAGVASSTVSCVLNDKGNVSEPTRLRVLEAASQL

NYVRHGPASELKRNSTQTIGIIVHDMSSPYFSDLVNGIETVVMSHGYDMIVCSSLGGE

KSTAHRYIRERRIDGAIVIAQNIQDQLLIEASEAGFPIVVMDRDLDAQHIVKVLMSDT

QGGYLATRYLIDKGHRTIAYISGPSQSECNLHRYQGYLKALKEAGIEENPEWKIGGQY
MKQDGYNAANKLLEGELPSAVFFANDEMAIGGLEAFREHDISIPEQLSVIGFDNIPAS
QYMNPPLTTFRQPKRDAGQLAGHVLFQLLNGEIVETLYTLDIQCVERDSVRLLNLS"

CDS 2768615..2769040

/locus_tag="EFAGFIKM_02357"

/inference="ab initio prediction:Prodigal:002006"

/codon_start=1

/transl_table=11

/product="hypothetical protein"

/translation="MRFSEYVIGQSYETKSLALSKSDIIEFATIYDPQYMHLDEEKAK
QGRFGSLIASGMQTMNISFKLWIEVGVYGEHVVAGTGMNNIQFLKPVFPDDELHVIAE
VIGLTPRRKGNGIVTVLLSTFNQKNQKVFQAELSALIDN"

CDS complement(2769110..2770300)

/gene="lysN_2"

/locus_tag="EFAGFIKM_02358"

/EC_number="2.6.1.39"

/inference="ab initio prediction:Prodigal:002006"

/inference="similar to AA sequence:UniProtKB:Q72LL6"

/codon_start=1

/transl_table=11

/product="2-aminoadipate transaminase"

/db_xref="COG:COG1167"

/translation="MNYSFSNRIAALQPSIIREILKASSGQNVIPFSAGNPAPETFPI
EAIRTFTQSILEHDPVTALQYGITEGYVPLREALTQHLKTGFDTGKPSDQLFIVSGAQ
QGIELACKVFCNEGDTIICESPSFIGSLNSFRASGAKLAGVPMEMDGMDIEKLEQALQ
TEPNVKLIYVIPSFQNPTGVTTSLAKRKAIYELAKKYGVMILEDNPYGELRFNGDDVP
TIKSMDDEGLVIYVGSFSKILSAGLRVGFVQAPHEVVEKMVVAKQGEDVHTAMLPQIL
AYKFMTEYDYAGHINSIREVYRRKATLMMDKLQEHMGESITYTQPDGGLFLWCDLPAH
VPMLDYAKTAAAQGVAVVPGNAFLVNEHDPCNAIRLNFSTPSDEQIVKGVEILGQVLK
GYNA"

CDS complement(2770480..2771628)

/locus_tag="EFAGFIKM_02359"

/inference="ab initio prediction:Prodigal:002006"

/codon_start=1

/transl_table=11

/product="hypothetical protein"

/translation="MSKSIDVIVIGAGIAGSTCAMQLAGKGHRTLLLDRQEFPRHKTC

GEFMSPETKEMLEVLDIHLLDQSKKPSTMDHAKIVMPQGGVIEAPLPGFAYGISRYEL

DQILHQKALEAGAQIVTKATITSIEQLEDASYEVQVKQGDERISYRAKAVIGAHGTKK

PRGMASAPDLRDQTVYVGVKSHFSGIEIPARVELYFCEGGYVGISPIEDGIVNVAALL

TLDTVQGSGKSVNDILQAASLTNVSLAARLAEGMPVDGTQVSIAPLHLSNVPEPWSQY

PHIGDAMLMIPPLCGDGMSIALRSSLLCARWTDKYLQGDIEHADWQSNYTLEASREFT

QLLRRARRIQKLAFAKTNKFYPGLARMIPGLAAYVVKATRLSEMNAAR"

CDS complement(2771635..2772354)

/locus_tag="EFAGFIKM_02360"

/inference="ab initio prediction:Prodigal:002006"

/codon_start=1

/transl_table=11

/product="hypothetical protein"

/translation="MSFFRTLSIRAKEDELMDDFSLGGEELREALRHLRRLNKIFAAP

GPTLAGVEKLWNSVGKPNKLTLLDVGAGSGDVNQKLLQWADRQGVQLEITLVDLTEEA

CEEARQLFRDEPRVRVQRADLTQLPDASADIVTGSQFVHHFDGDQLVDMVSHMLRASR

YGVVINDIHRHPVSYKAVWITTRIISRNRYIRHDGPLSVAKGFTGRDWKELKQRLNHD

TMTYEWKPLFRYSVVIPTKGR"

CDS complement(2772341..2773498)

/locus_tag="EFAGFIKM_02361"

/EC_number="2.3.1.-"

/inference="ab initio prediction:Prodigal:002006"

/inference="similar to AA sequence:UniProtKB:P9WPF1"

/codon_start=1

/transl_table=11

/product="Alpha-pyrone synthesis polyketide synthase-like

Pks18"

/db_xref="COG:COG3424"

/translation="MNQYNSPSDIAILGMGTALPAHPVAQSDIAELIASSLQDRPDLA

RFARRIFKSCGVETRYTVEPSYLGSLEECRYLPSGERSDIPTTEERMSTYKREALPLG

IDAAEKALEDSGVSPKSITHIITVSCTGQYLPGLDVMLIRHLGLSARVNRLPLIFQGC

AAGLKAIQMARDVVQGAPGSQVLVVCVELCTIHFQPVQDREALFAASFFGDAASSCVV

GTPEPQHKNYLSLGTGYSVLLPDSTEDMTWEVGNLGYDLYLSPRIPKLLGVHLEEELR

LLLQSEQLPELWAIHPGGRGIVDSVQNVMKLRDEQTKYSRDVLRTYGNLSSNTILFVL

NAMREDMKAQVQSSTDGVAMAFGPGLTAELMKFTYVPSLSSVMEEHDHVLF"

CDS complement(2773728..2774612)

/gene="aes_1"

/locus_tag="EFAGFIKM_02362"

/EC_number="3.1.1.-"

/inference="ab initio prediction:Prodigal:002006"

/inference="protein motif:HAMAP:MF_01958"

/codon_start=1

/transl_table=11

/product="Acetyl esterase"

/translation="MDQQQQLEMVQRMRQNVVGTGAALQPGSTYAVTVKEVMIPTTKG

DTRVLVYTPDRDQSASMPVFFNMHGGGFILGQAEMDDPWCRLIADRSDCVVVNIDYSL

APEHKFPTAVHECYDVVQWIHANPQSLSVNPSLFAIGGHSAGGNLAAAVCLLNQQRGS

ELPIVLQVMDYAVLDVATDTAEKPSFAEAIPADIARTFNAMYLDTSEDAQDPLASPVL

ATSLEGLPEALIITAEKDSLAQEARTYAASLKASGVKVTLKEYKGAAHGFTHFGDLQM

AEDAWYLMSDKIREAFTK"

CDS complement(2774775..2775461)

/locus_tag="EFAGFIKM_02363"

/inference="ab initio prediction:Prodigal:002006"

/codon_start=1

/transl_table=11

/product="hypothetical protein"

/translation="MKTYLKTIWFHYLGNKQDRTALFNVASLVINAVTGIGKLILGIY

LFSGWFITNAVYYLILCVAKGQVLHKYTVTKRMESPLERYRLELTVVYKRGGLFLCLLG

ASYSMVCLRMYLIGDAFIYEGIIVYIVATVAFTKLVFAVYGTWANRHLHNPIISTLKM

INFTDAMVSIVVTQATLLTMQGSPLALKTSSLFGMGCSVLFCLMGVWMMFHKKKETST

DPNRSPGTEC"

CDS complement(2775573..2775926)

/gene="yjbR"

/locus_tag="EFAGFIKM_02364"

/inference="ab initio prediction:Prodigal:002006"

/inference="similar to AA sequence:UniProtKB:P0AF50"

/codon_start=1

/transl_table=11

/product="putative protein YjbR"

/db_xref="COG:COG2315"

/translation="MKDTMIEYSLKKKGATKDYPFGPDPVTIKVGGKIFALLFEDKEE

NGLLNLKCDPIIAENLREQHEAVRPGYHMNKKHWNTIMLDGSLSDEDVYVMIDHSYDM

VIQSLPKSIRESLAE"

CDS complement(2776069..2777475)

/gene="rhlE"

/locus_tag="EFAGFIKM_02365"

/EC_number="3.6.4.13"

/inference="ab initio prediction:Prodigal:002006"

/inference="similar to AA sequence:UniProtKB:P25888"

/codon_start=1

/transl_table=11

/product="ATP-dependent RNA helicase RhlE"

/db_xref="COG:COG0513"

/translation="MTFKDLNIIPSIMEGLSKANYTNPTPIQEQAIPAVLAGRDLLGC

AQTGTGKTAAFSVPIIQLLSERSKGQGSKSARHIRSLILTPTRELAIQIADNIKVYSR

YTDIRCTAIVGGVSQKVQERALNQGADIIIATPGRLNDLINQKRIDLKMVEILVLDEA

DRMLDMGFIHDVKRIIAKMPNKKQTLFFSATMPPEITKMVKTLLVDPVKVEITPVSST

VDRIEQSIYLLENGKKQHMLNQILEDKSIVTALVFTRTKRGADRVTRDLAKANVTAQA

IHGNKSQNERQRALNNFKSGATRVLVATDIAARGIDVEELSHVINFNLPNIPETYVHR

IGRTGRAGKSGMAISFCEKDELPFLKDIEKVIKKTIPEVKGHPYPMTGVPVFDKTSKA

SGSKPSFNKSAAGKPAKSKANPARKPKSEWFAKSGKSNSSRSNDGRPNSSRSNSSSSK

SNHGSFSRSSKTRNDRAN"

CDS complement(2777592..2777906)

/gene="gdnD_1"

/locus_tag="EFAGFIKM_02366"

/inference="ab initio prediction:Prodigal:002006"

/inference="similar to AA sequence:UniProtKB:P49857"

/codon_start=1

/transl_table=11

/product="putative guanidinium efflux system subunit GdnD"

/db_xref="COG:COG2076"

/translation="MNWVFLILAGIFEMIGVLMINKLHKDRNLISLVLLVAGFGLSFL

FLSIAMETLPMGTAYAVWTGIGASGGAILGMVFYGEPRNALRILFIAMVLGSAVGLKL

VS"

CDS complement(2777906..2778244)

/gene="gdnC_1"

/locus_tag="EFAGFIKM_02367"

/inference="ab initio prediction:Prodigal:002006"

/inference="similar to AA sequence:UniProtKB:P49856"

/codon_start=1

/transl_table=11

/product="putative guanidinium efflux system subunit GdnC"

/db_xref="COG:COG2076"

/translation="MNKTWMSVVIAALFEVGWVIGLKHASGLLEWGFTLVAIMISFSL

MIAASRTLPVGTVYAVFVGLGTAGTVLAEIVLFNAPVQAGKMVLIGVLLLGVIGLKML

SKEKNKEVQL"

CDS complement(2778518..2779147)

/locus_tag="EFAGFIKM_02368"

/inference="ab initio prediction:Prodigal:002006"

/codon_start=1

/transl_table=11

/product="hypothetical protein"

/translation="MKMDKPKIQEQLLEPETIAFLESKVEYKHKLIENITLDQQEASK

VSFENVVFRHVTISESALEQFEFTDVRFEHCDFSNVNLSGAFMHRIEWHNCKFVGTDF

SNSRLQNVSFLHGLGDYSNFRFAHLKQVSFSECTLIGADFAYLALQKMEFSQCNIDQA

SLTGTKMKDLDLSDCEFDSLVLTMEDLNGCVISPHQAATFVGLMGLIIK"

CDS complement(2779159..2779923)

/gene="tam_2"

/locus_tag="EFAGFIKM_02369"

/EC_number="2.1.1.144"

/inference="ab initio prediction:Prodigal:002006"

/inference="protein motif:HAMAP:MF_00560"

/codon_start=1

/transl_table=11

/product="Trans-aconitate 2-methyltransferase"

/translation="MSNGHSSGQTNESWSGHSAPFATLQETPIISLLQPSQGERILDV

GCGNGDLTAKIAAAGALPTGIDFSEETIRQAKQKYPDMNIQVANACHYRTEEPFDAVF

SHAVLHWIKDAPAVVQSIQLALKTGGRFVAEFAANGNTAILITAVQEELDARGYKWEG

RNPWYHPTIGEYANLLEQHGFRVSLAQHVDQFTPFKPGARKWLTSFAEYLFSGIKPAE

QDVIMEAVEKRVRPQLMRDGQWYLDRSRLRVVAIKE"

CDS complement(2780058..2781044)

/gene="yajO_2"

/locus_tag="EFAGFIKM_02370"

/EC_number="1.1.-.-"

/inference="ab initio prediction:Prodigal:002006"

/inference="similar to AA sequence:UniProtKB:P77735"

/codon_start=1

/transl_table=11

/product="1-deoxyxylulose-5-phosphate synthase YajO"

/db_xref="COG:COG0667"

/translation="MEYVKLGRTGLDVSRLCLGCMSYGVPERGMAPWSLNEEQSRPFI

QKALDAGINFFDTANMYSDGTSEEIVGRALKDFARRDEIVLATKLFFRMNEGPNGAGL

SRKAIMNEIDNSLMRLGTDYVDLYQIHRWDNKTPIEETMEALHDVVKAGKVRYIGASS

MYAWQFMKAQYTAERHGWTKFISMQNHYNLLYREEEREMLPLCQEEGVGVIPWSPLAK

GRLTRDWNEQTARSGIDPAGKAFYENAMVDADRQVVERVLKVAEQREIPRAQVALAWL

LHQEAVTAPIIGATKIHHLEDAIAALTVKLTVEEIGFLEEAYVPHPVTGNLS"

CDS 2781413..2782330

/gene="rhaS_21"

/locus_tag="EFAGFIKM_02371"

/inference="ab initio prediction:Prodigal:002006"

/inference="protein motif:HAMAP:MF_01534"

/codon_start=1

/transl_table=11

/product="HTH-type transcriptional activator RhaS"

/translation="MTEELFQSKQELSDLIKRHSNHNGAMETAIPSLFVYHHSKISEP

AYRVYKPSFCVIVQGLKEVLLAQERYEYGPSNYLIASMNLPVIGQIIKASADAPYLSL

KLEFTPNQILEVLNECNIKVTFNENARRAMFVGQMESSIQDAILRLVRLLDTPGEIPF

LAPLYVKEILFRLLQGPYGGELAQIAVEGSSTYRIREAIEYIVHHWEQPFRIEDLAET

ASMSVSSFHRHFKEITAMSPLQFQKQLRLQEARRLLMAESADAADVAFRVGYESASQF

SREYARMFGAPPRADIRRLKEKYDMALSE"

CDS complement(2782508..2783587)

/gene="ltaE"

/locus_tag="EFAGFIKM_02372"

/EC_number="4.1.2.48"

/inference="ab initio prediction:Prodigal:002006"

/inference="similar to AA sequence:UniProtKB:O50584"

/codon_start=1

/transl_table=11

/product="Low specificity L-threonine aldolase"

/translation="MIRFECDYNEGAHERILQRLMETNMEQTSGYGTDGHCERARMLI

RQACDNEQADVHFLVGGTQTNTTVIASILRPYQGVIAATSGHIAVHETGAIEATGHKV

LTVPSEDGKITPEQVRAVYDAHMNESSPEHCVQPGMVYISQPTENGTMYSKAELQALH

TVSRACGLPFFVDGARLGYALASRDCDMTLADLARLCDVFYIGGTKIGALMGEAVVIL

NDALKPDFRYMIKQKGGLLAKGRLLGIQFETLFEDGLYLDISRHAVDMALRIHDSLEQ

QGVRFLYDSPTNQQFPILPDRLLEKLRGGYTFTFWEKVDDTHSAVRFCTSWATRQENV

DALTRDIAQLLHGEIHVPERVEALV"

CDS complement(2783584..2784006)

/locus_tag="EFAGFIKM_02373"

/inference="ab initio prediction:Prodigal:002006"

/codon_start=1

/transl_table=11

/product="hypothetical protein"

/translation="MRRFNRFYTNILGVLDKHILGTGYSFAEVRVIIEIGIRGESIAN

NLVDTLTIDRSYMSRIVNKLSKEGLLVKVNSAADSRVSLIRLTVKGEELYAQLNDRSD

QQILKLMQELNEEEIQEVYTSMMNIQEKLNKKAGETTR"

CDS complement(2784461..2786179)

/locus_tag="EFAGFIKM_02374"

/EC_number="3.6.3.-"

/inference="ab initio prediction:Prodigal:002006"

/inference="similar to AA sequence:UniProtKB:Q99T13"

/codon_start=1

/transl_table=11

/product="Putative multidrug export ATP-binding/permease

protein"

/db_xref="COG:COG1132"

/translation="MIRRFFSYYRPYKKLFLIDFGCAVLAGLLELAFPLAVSKFINEL

LPGQDWPLIMLACVVLLSIYALNTVLNYVVTYWGHMLGINIETNMREKMFAHLQKLSF

RFFDNRKTGHLIGHLTNDLNDIGEVAHHGPEDVFIAIMTLIGSFWLMANINLELALLT

FIIIPIMAWVIIVFGGRMTKTYRRLFGDVGNFNARIEDNVGGMRVVQSFANEEHEKEL

FAVDNQNFRKTKLLAYKTMAKSISVSYMMMRLVTVFVMISGAWFYIDGRINMGEFMAF

LLLSNIFFRPIEKINAVIESYPKGIAGFKRYLEIIDTEPEIADSKNAVEFESVKGDIR

FENVSFGYESSRRILNDISLSVRPGETVAFVGPSGAGKTTICSLLPRFYEVEEGRITV

DGVDIRDVKLESLRKHIGIVQQDVFLFSGTIKENIAYGDLSATDEQIWDAARRASLEE

LILTLPDGMDTVIGERGVKLSGGQKQRLSIARMFLKNPPILILDEATSALDTETEALI

QQSLAELSVGRTTLVIAHRLTTIKNADRIIVVNTDGIAEQGNHEELVAAGGIYSRLHQ

VQYSHS"

CDS complement(2786182..2786556)

/locus_tag="EFAGFIKM_02375"

/inference="ab initio prediction:Prodigal:002006"

/codon_start=1

/transl_table=11

/product="hypothetical protein"

/translation="MRDRPIEEMDGSVRQGERPRRDMSDNEQRSERSRRGRGHGEHEG

KRGRGAQTFRRGRILVFLEQMQNRRTTLVRQLGQEEYEHIRPVISGELKAIDQVIDEY

IHLFELQNEDVNPNKDLESESE"

CDS complement(2786732..2787673)

/gene="rutD_2"

/locus_tag="EFAGFIKM_02376"

/EC_number="3.5.1.-"

/inference="ab initio prediction:Prodigal:002006"

/inference="protein motif:HAMAP:MF_00832"

/codon_start=1

/transl_table=11

/product="Putative aminoacrylate hydrolase RutD"

/translation="MMKKILIILLKIFGAIVIAFALFIATVFIVNVFSNKSEEGKIKP

YGQTVAVDGKNMNVLIQGKGEETVVLLPGYGTPAPALDFKPLINELSPFYKVVVIEPF

GYGLSDVTEKERTPENMVSEIHEALQQLNIHRYTLMGHSISGIYGLDYVNKYPNEVTA

FVGIDSSVPTQGGNDDPFPTETYKLLKKSGFYRLLMKLAPDQLIAPNVDDETREQIRI

LSLKNTFNPNNLNEGENFGPNFKAVENLSFPKDLPVIFFLQANDTETEGWIPLHEEQV

KDSVHGKVMTLEGEHYLHHTRSKEMVQEYRKFMSEIK"

CDS complement(2787731..2789854)

/gene="yfeW_3"

/locus_tag="EFAGFIKM_02377"

/EC_number="3.4.16.4"

/inference="ab initio prediction:Prodigal:002006"

/inference="protein motif:HAMAP:MF_01034"

/codon_start=1

/transl_table=11

/product="Putative D-alanyl-D-alanine carboxypeptidase"

/translation="MIQQEKMDVVKRTVKKRTSIAALTLVLTMLAPLSAMAAPTAMNN

NSNLTYETTKKVVIEKAKLLTETYGTTSLQYALIDGGEIVVSGQTGKNDINDKVPLTS

NTVYGIGSTSKMMLTAAVMKLVDEGKIDLDAPVVNYMPDFKMKDTRYKQITPRMLLNH

SSGLLGTSTSNATLYGDNDTYSHDTFLEQLANQNLKADPGEYSVYSNDGFTLAEILVE

RVSGIGYTAFIHKYFTEPLDMKHTKTPQDVVNPSDMAAIYSPFYEGPLPKENYNIIGT

GGLYSTAEDLVKFSRIFMGEVDGILSSKSVEAMAQEEYKRGMWPEDSDTLMSYGLGWD

SVNLFPFNEYGIKAVMKGGDTLSYQSSLVVLPEYNMAAAVISSGGSSLTNNFIANELL

LSALEEKDIITERKPEKSFGVPVKANMPKGISKYAGIYGGNNSIMEIKINKAGQMTVS

SLTAPSNPAQEYTYTADGTFVNNEGTDKLKFVVENNGKTYLWSRSYISVPGLGQLAFS

EYNGEKLEANKLSKEINAAWAKRDGKKYYLVNEKYTSMVYLNASSILPIHMNKENPGY

MSNHKIIGANEATNLLQIPGTAGRDTMDIQFSKKNGGEYLTALGYVYASEELVKPIYS

GKQSATTIQADGYAKWFSVPATAKGKVMTVKMPAKGAFAVYDQTGICINHTVVSGENE

VVLPENGRIVFAGEAGSQFEITLKK"

CDS complement(2790071..2792158)

/gene="yfeW_4"

/locus_tag="EFAGFIKM_02378"

/EC_number="3.4.16.4"

/inference="ab initio prediction:Prodigal:002006"

/inference="protein motif:HAMAP:MF_01034"

/codon_start=1

/transl_table=11

/product="Putative D-alanyl-D-alanine carboxypeptidase"

/translation="MGRQKGKRTSIAVLTLALTMLAPMSAMAAPPTSSSDFTYEPTKK

TVMEKAKLLTEKHGITSLQYALVDGGEIVVSGQTGKNDKNNKVPLTPDTIYGIGSTSK

MFLTASVMKLVDEGKVDLDLPVVNYIPDFQMKDHRYKQITPRMLLNHSSGLLGSTGSN

ATLYGDNDTYSHDTFLDQLASQNLKADPGAFSVYSNDGFTLSEIMVERVSGMSFTAFI

HQYFTEPLDMKHTKTPQDVVDTAAMAGIYSPMYEGQLPQENYNVIATGGIYSTAEDLA

KFSQIFTGEVQGILTDKSVEAMAQEEYKKGMWPEGGDTSISYGLGWDSVNLYPFSEYG

IKAVTKGGDTISYHSSLVVLPEYNLAAAVTSSGGTSAKDQFIASELLLSALEEKGIIT

ERKPEKSFGVPVKADIPKEIATNAGIYGGNNSVKKIEMNTAGQMTVSTPSAPSDPAQK

YTYTADGTFVNDEGTEKLKFVKEKNGRTYLWYRSYISLPGLGQLAFSEYKMEKLEANE

LSQDITASWEQREGKKYYLLNEKYTSTVYLNSAPILPIHLDKETPWYVSNTKIIGANE

AVTELQIPGLAGRDTKEIYFAKKNGVEYITAVGSIYASEEMVQPLYSGKQSLATIQAD

GYARWFSLPATVKGKVMTVKMPATGAFAVYDQKGICINHTVVSGKNEVILPENGRIVF

AGDVDSTFEISLK"

CDS complement(2792191..2793648)

/locus_tag="EFAGFIKM_02379"

/inference="ab initio prediction:Prodigal:002006"

/codon_start=1

/transl_table=11

/product="hypothetical protein"

/translation="MRLFEILLVLSCFALLVDLLFITKSAKKTGWGLGIGSSVILLVQ

LFVEGYRWQLLLVYIMTALFIIIVLFRHSEKMMNLKIGKFLKYSLSSLIVILLVGSTV

LSVYLPVFNLPKLDGPEKVGTQTFHLTDQNRDEIFTEDQSAKRELMVQVWYPTENSNN

NKRDTLFPKDKEMFKKYIQSFSNSLKLPEFLLDYLKYSQTNSYENVEILPSTSPYPVV

LLSHGMGTSRVLHVSQAENLASHGYIVVTIDHTYSTFATLFPDGRVTDYKKSTTTLDE

RTKTGSIWTKDVEFVIDQIEKLNSGAIESQFKGKIDLDHIGAMGHSFGGATAFNATYL

DQRIKAGVNMDGSLYEVENRDDINKPFMFIRSGSFEDWFANFEIDRNSEDEVTKSLSD

ELHIMKNVMNHGGKVIYVEGTQHFNFTDLQFYSELVKLSGITGDINGKRGSNIVNQYV

LDFFNKQLKGTGGDLIQGPNDLYPEVKFVDPEEIK"

CDS complement(2793688..2794671)

/gene="rutD_3"

/locus_tag="EFAGFIKM_02380"

/EC_number="3.5.1.-"

/inference="ab initio prediction:Prodigal:002006"

/inference="protein motif:HAMAP:MF_00832"

/codon_start=1

/transl_table=11

/product="Putative aminoacrylate hydrolase RutD"

/translation="MTQPEEKKTKNRSRAKKVRNMILKIVGAIVIAIILFLGIVYITN

VISSNSEAKKIESYGQHVSVDGKNMNVLIQGEGKETIVLLPGYGTATPALDFKLLIDE

LSPYYKVVAVEPFGYGLSDETEKERTTENIVSEVHEALQQLDIHQYMLMGHSIAGIYG

IDYVNKYPDEVTAFVGIDSSVSTQPSITDAKFPLKTFALLRNTGLLRLMMKVNADPYE

GLAFDDQTVEQMKMISNKNMYNPTSLNEMDHIYSNFQGAQGLTFPKDLPLLLFVQANN

EGVEGWIPLHEGQIKDSVHGKVITMDGEHYLHHTLFKEIAEDVRAFMNEAK"

CDS complement(2794897..2796267)

/gene="hssS_1"

/locus_tag="EFAGFIKM_02381"

/EC_number="2.7.13.3"

/inference="ab initio prediction:Prodigal:002006"

/inference="similar to AA sequence:UniProtKB:A6QJK4"

/codon_start=1

/transl_table=11

/product="Heme sensor protein HssS"

/translation="MIRSLYIRVVLTFLVSVITGTIISFFMSTWIFEDQLNENAQINI

RNFGQDVVQIYKTLPADEAESFVSRMKQLDSYHIRIYDATGKFQSYGKLNEHKSSPVS

TDQLKKVLNGGVVQDTPKGIATVLLGLPIKTETGIRAMFLETLSPPSASFVVQFGLIF

ASCSLIVGSLLIVVASVYLVRPIKKLTKATKRIAAGDFNVKLNIKQTTEIGTLARSFE

EMMHDLQQLEQMRREFVTNVSHEVQSPLTSISGYASALKQVNLSEHERNRYLDIIISE

AKRMSKMSDSLLKLSLLESQSQQLRLSSLSLDEQIRRVIVALQPQWSERNIHFELELE

NVKVTADHDQLNQVWTNIIGNSIKFSKDGGMIKVSIEEDFKNVTVRISDTGIGIPLED

QKRIFDRFFKADRSHSRKYDGSGMGLAIVKQIVSLHQGDIRVESEPGQGTTFMITLPI

HPPTDS"

CDS complement(2796264..2796938)

/gene="hssR_1"

/locus_tag="EFAGFIKM_02382"

/inference="ab initio prediction:Prodigal:002006"

/inference="similar to AA sequence:UniProtKB:A6QJK3"

/codon_start=1

/transl_table=11

/product="Heme response regulator HssR"

/translation="MATILVADDDANIRELVCLFLKNDGFTTVEAADGKEALTIYGET

PVDLVVLDIMMPVMDGWALCKELRRANPDLPLLMLTARGETWEKVKGFELGTDDYLTK

PFDPLELTARVRALLKRYKIGSTHTIQIGNVILDRQTYKVMRGTESLTLPLKEFELLY

KLAGTPGQVYTREQLIDQIWGIDYAGDDRTIDVHIKRLRERFATTPDFRIETVRGLGY

RLEVYE"

CDS complement(2797012..2797545)

/gene="rimJ_1"

/locus_tag="EFAGFIKM_02383"

/EC_number="2.3.1.267"
/inference="ab initio prediction:Prodigal:002006"
/inference="similar to AA sequence:UniProtKB:P0A948"
/codon_start=1
/transl_table=11
/product="[Ribosomal protein S5]-alanine
N-acetyltransferase"
/db_xref="COG:COG1670"
/translation="MKYSAERIYVRFWNVDDASLLLGLQIRNRAEIEKISASDREESF
YSSEGQRSLIESWNKKREEGNRYSFGIFLHTTDELVGEISLFEIILDSTPKWIVGYVT
DTLHQGKGYMSEALQCVMEFAKHETAITRIEAGAVPDNTGSIRVLQKAGFKENGSQLV
PIQGTLKEHTMFAVDIL"
CDS complement(2797591..2798196)
/locus_tag="EFAGFIKM_02384"
/inference="ab initio prediction:Prodigal:002006"
/codon_start=1
/transl_table=11
/product="hypothetical protein"
/translation="MKDEKKSAVDPRIVRTRQLIRGAFVELLQEMDIEKLSVNKIAER
ATINRVTFYLHYRDITDMMEKMADEMIENIERIVDEYAPHFEKQRTEEAWPVLVKLLE
HFAENASFYQVVLATKRTPIFTERLLKLLSTLVKAKIDRVEMEDELEESGIHKEIAIW
YGSSALIGTIVAWLRNDMPYSPHFLAKQFSLIRSYSYNDLI"
CDS 2798419..2799918
/gene="emrB_2"
/locus_tag="EFAGFIKM_02385"
/inference="ab initio prediction:Prodigal:002006"
/inference="similar to AA sequence:UniProtKB:P0AEJ0"
/codon_start=1
/transl_table=11
/product="Multidrug export protein EmrB"
/translation="MSQVRVQEELSVKKGPILFIMILGAFLATLNQTIMSVATPELMG
DFNISAATAQWLTTGYMLVNGVLIPITAYFMQRFSTKQLFQASMFIFLIGTIVSALAD

NFGTLLTGRMIQAAGAGIIMPLLMHVILTLFAPEKRGSAMGMVGFAIIFAPAIGPTLA
GYILENYSWQTMFYGMIPLTLIVIAFAFVYLKNVSERVNTKFDTISVVLSTIGFGALL
YGFSRAGSLGWSSAEVLICIAAGIVSLALFTWRQLVSETPLLDLRAFKYNMFSLTTVI
SIAITMIMYADMMLLPLYLQNARGYTALESGLLLLPGALVMGFLMPVAGKLFDRFGAK
WLAVIGMVITIVTTIGFIDLTDSTSYGYLVLMSTGRRIGMALLMMPIQTAGLNQLPPR
LGAHGTAISNTVRQVAGAVGTSLLVSVMTSRTSAHVQDMMATGAANGLSQQQLATESM
IQGINDAYVVIIGIAVVGLVLSFFIKRTKQAKEEEIKQVVRQKVSMNSN"

CDS complement(2800017..2801252)

/locus_tag="EFAGFIKM_02386"

/EC_number="1.14.14.-"

/inference="ab initio prediction:Prodigal:002006"

/inference="similar to AA sequence:UniProtKB:P14762"

/codon_start=1

/transl_table=11

/product="Cytochrome P450(BM-1)"

/db_xref="COG:COG2124"

/translation="MYKEVIRVEDITGFQSRSEEFFPLHWFRKMLSENPVYYHEDTDT
WNVFRYDDVKQVLSNHEYFSAEGTRTTIAVGAKNNEGTPPDKLNISSIDPPLHQKSRS
LLSAAFTPRSLKNWEPRIRNIAEQLVADIEPNTTIDIVQALAAPLPSMVMADLLGIPL
TDSHRFKNWVDILFQPINPKTAEESELKKQTAAQEYYQFLYPIVIHKRTHPGEDIITD
LLNVDVEGEKFTEDEVVRTTMLLLGAGIETTSHMVSNTFYSFLYDDPSLYDQLRQNPE
WVPLAVEEMLRYRFHNAKRHRTVKQDNQLLGVDLKKGDVVISWMSAANMDEQMFEDPF
ELNIHRRNNKRHLTFGNGPHFCLGAPLARMELSIALTAFVEKIARIEPVESFDLENNL
ATSAPGQSLTHLPVKIVAE"

CDS complement(2801469..2801756)

/locus_tag="EFAGFIKM_02387"

/inference="ab initio prediction:Prodigal:002006"

/codon_start=1

/transl_table=11

/product="hypothetical protein"

/translation="MTYSQRVSDGANSSDIIYLEHQIGTTKEKLRIALEKQETYKSEL
SELKSSPIRNASEDSSEEQILMEKASQTKNLIETLSEQLEQLQETLAKLGD"

CDS complement(2801753..2802895)

/gene="thiO_2"

/locus_tag="EFAGFIKM_02388"

/EC_number="1.4.3.19"

/inference="ab initio prediction:Prodigal:002006"

/inference="similar to AA sequence:UniProtKB:Q88Q83"

/codon_start=1

/transl_table=11

/product="Glycine oxidase"

/translation="MKKVIVVGSGILGASTAYQLAKRGAEVIIVDRKDIGQATDAAAG

IICPWLSQRRNQDWYQLAKAGARFYPGIIAELKAEGETETGYAQVGALSIHTDEDKIN

KMEERAHLRKEDAPEIGDITPLDAKETRDRFPLLDERYQSVHISGAARIDGRALRDAL

IRSAQRNGAEVIHGDATLQFEADRVIGVSVNDQSILADEVVVCAGAWANELLKPLRIH

FKVHYQKAQIMHLHVTDGEDTGRWPVIMPPSDQYLLAFDQQKIVIGATHENDVEGYDT

RVTAGGMQEVLNKGLELAPGLANSTFQEVRVGFRPFTPGFLPVMGAVPGWQGLITANG

LGASGLTMGPFIGSQLAKLALGTELDIDIEPYTVGKAMDEIERGES"

CDS 2803192..2804007

/gene="rhaS_22"

/locus_tag="EFAGFIKM_02389"

/inference="ab initio prediction:Prodigal:002006"

/inference="protein motif:HAMAP:MF_01534"

/codon_start=1

/transl_table=11

/product="HTH-type transcriptional activator RhaS"

/translation="MPKPSMGNPLDTDMPLMITGQNQLTVDELMNWSDHSRDYSIVQC

IGGTGRIAAKNHEFSIKKGTALILFPEVTYRYHNLSDSLRFDCLSFNGYLLPRFLHTL

QIGELRAFKPDGMLHILLREITLALQSRHKDQIWHVSALLYMLLVRLTIEGERYSAYE

RSDARLRLDELITFIKQNYHQDISLPMLAEQMDVTEQHLNRIFKKEFQMTPLEYLMRY

RLLKAKEMLIENNATTAHEIAKAVGFNSASYFGSVFKKYEGISPIELRKQYLD"

CDS complement(2804004..2804645)

/locus_tag="EFAGFIKM_02390"

/EC_number="3.1.3.-"

/inference="ab initio prediction:Prodigal:002006"

/inference="similar to AA sequence:UniProtKB:Q9X0Y1"

/codon_start=1

/transl_table=11

/product="Phosphorylated carbohydrates phosphatase"

/db_xref="COG:COG0637"

/translation="MVKAFIFDFDGLIVDTETPWYYAFRDIYEEHGVELGLELWSKNV

GTSFEEFHPFLYLEQALQKKIDHDHIKLLSEQKYEVYLGQAAILPGVYELLQSAREKG

IQLAVASSSTRDWVHGYLQKLGIFDYFTVVHTSEDVKRVKPDPELYLLALQSLGIEAS

EAIVFEDSPNGLKAANAAGIRCIIVPNEVTRGLEFAMHELRLSSLAEIDMEAL"

CDS complement(2804685..2805278)

/locus_tag="EFAGFIKM_02391"

/inference="ab initio prediction:Prodigal:002006"

/codon_start=1

/transl_table=11

/product="hypothetical protein"

/translation="MTNIMTTEARKQWSWMNEPESWSYTDQGGVLVEAKADTDFFQDP

AGKHIRATAPFLSMPVPGDFEITTQLTVDMKNQYDSGCLMVMADDLNWCKICFEYDGK

VPTIVSVVTRDGSSDDCNSVEVSVPNPYLRIRKVEGCISFFYSPDRDEWKLIRYFGMP

IQGELRAGLVVQSPTGTGCTCHFLSVNVTHPDLTARF"

CDS complement(2805360..2806604)

/locus_tag="EFAGFIKM_02392"

/inference="ab initio prediction:Prodigal:002006"

/codon_start=1

/transl_table=11

/product="hypothetical protein"

/translation="MKLLQDLPKNPRYSILLEPVWAIPGTIVLFYAPLYMKEAGLSDI

EIGLINSVNLYFAFIFQLFAGSITNKLGRKRTTLIFDLLAWSVPMFIWAFSQNFWLFL

IAYLLNATSKFVTVAFNCLIIEDVEEHKRSKVFAILNMIITGAGVLTPIAGVVIADYG

IVPTLASIYFVGGILMTAMFFIRNRYTDETEVGKELMGLHSKTRVLQSLGSSLRLFGK

SFYKRRLFPIILITVLSNLILQLNFFQVIFFKEQLKFDDRVISFIPVVTAVTVMLLYL

VIIPRLKRRSEEKYVGFSIVLSTAGAILFLLIPVGSIGMLFLTLIVLAAGNFILQTYR

DSLLMNRLGTHEKADMFSAVQTVMTLTAIPSGYLTGLLYHHNPTLLFSVILGLYVVLM

VIMFFLPDPQKHSQVLEPYKNM"

CDS complement(2806697..2807728)

/gene="gutB_2"

/locus_tag="EFAGFIKM_02393"

/EC_number="1.1.1.-"

/inference="ab initio prediction:Prodigal:002006"

/inference="similar to AA sequence:UniProtKB:Q06004"

/codon_start=1

/transl_table=11

/product="Sorbitol dehydrogenase"

/db_xref="COG:COG1063"

/translation="MKALVYQDLKQVTYQEIEEPTIQKPNQVKIKIYGSGICGTDLNI

VKGKVPANKGTIIGHEGVGTVVEVGDDVRGFKIGDRVLVDPTQSCGTCSYCREGLFIY

CENFDDYQVGMTTHGTFAEYFVGDEKYIYAIPDSMSWETAMMIEPLGCVLQTFMKAGT

KPSDSVLVLGSGAIGSLCQLVSKRLARLTVGTEVDPYRKEFASGIADHVFYPQDLTLD

KVYEINNNKKFDIIVDAVGNQLHVGFELIAKGGKIIPMGYDDSYEVTFKSTAVINDGI

SIIGAVANHSMISTALKFAQSIPELEQMVTAKELLSDYEQAFNGTIGYDMHTGEKLPM

NSVKTALIL"

CDS complement(2807771..2809078)

/gene="kat"

/locus_tag="EFAGFIKM_02394"

/EC_number="2.6.1.111"

/inference="ab initio prediction:Prodigal:002006"

/inference="similar to AA sequence:UniProtKB:B0VH76"

/codon_start=1

/transl_table=11

/product="3-aminobutyryl-CoA aminotransferase"

/translation="MKEKESTLDRAVRFTRNLEWLEQVEKVIPSGCSTLAKAPERLFP

GHTPVCCAEAYNSRFTDIDGNEWLDCEMAMGTAPWGHARHEVQLTVIHQLKKGTSFSL

PADIELECAELILERFEGRYPSLRFTKSGADAVSGAVRLARAASGKSKVIATAYHGWH

DWSAYGYYGGQTKERGIPVDIERSTIWVNKPTTERIEAQITASPEDIACIVLCPNEWK

KDALEKAVNLCRSLDIIIIFDEVTSGIRMGKQATAGEYDIWPDLLCISKGMANGLPLA
AVMGPEHLMSLSGQVRFSNAHSSETTALAAAIACERLMKNAKVWPTWREPATRIMDRL
ESELVLLGLTDQLEVRGTYASFCIQSLAEDNFQTDPFREFMVKRMAHFGIFTKGYFIF
SDAHTREELLWVEEALLQILSDWKEIQKQEHLHAMQLSKTFEA"

CDS complement(2809116..2810270)
/locus_tag="EFAGFIKM_02395"
/inference="ab initio prediction:Prodigal:002006"
/codon_start=1
/transl_table=11
/product="hypothetical protein"
/translation="MLYTIIVPVNQDYNILNLFTDSLLRTVSPSTQIIFINDGSGSAV
FQHLDKLKQEVREGVTIEILQHDFPLGCAVSINSALSLAKGEYIFFLDSDTILQPNWQ
PMMKETLDSDITIGMIGGVLLYPQTGGVQHCGIAFADTIGRHLFLNASPDDIPKETFS
VQLVVFAMFGMKREVYETIGNLDEKFFNGYEDFDYQMRARAAGYDTVINPNIQAYHWE
RSSGIHRNFNRKNNLARFWKKWGGQIEADVWPFVFSHLKAQLESQEEYQHLPIVGIDL
AEVRSDADTFWTKLEEAEFANVAEVRDYSNRFNSNGAIWLPQVLGKELIHSENRLLFL
VDNFARLLENRYWIEMRHAHRAKDLIIDLYGNVITMDRIYDGCWPGTKVR"

CDS complement(2810623..2811189)
/locus_tag="EFAGFIKM_02396"
/inference="ab initio prediction:Prodigal:002006"
/codon_start=1
/transl_table=11
/product="hypothetical protein"
/translation="MKKEMTTIKQTRLHSILDEATKLLIEKPNASMNEIAESAKIGIA
TLHRYVESREQLMVHLGLRAIEVVSETMQQIQLDEEHCEKYIPELIEALIPLGDKIYF
LAHDTTINYNPEIEGADLKLREPVLHAVGLLQQKGYFRSDLEKTWIVDVLYSIMFLTW
QQVVSGHIARKAAPALVVDTFYHGFKQS"

CDS complement(2811397..2812875)
/gene="expZ_1"
/locus_tag="EFAGFIKM_02397"
/inference="ab initio prediction:Prodigal:002006"
/inference="similar to AA sequence:UniProtKB:P39115"

/codon_start=1

/transl_table=11

/product="Nucleotide-binding protein ExpZ"

/db_xref="COG:COG0488"

/translation="MSLINVTNLTFAYEGSYDNIFENVSFQLDSDWKLGFTGRNGRGK

TTFLNLLLGKYEYSGHISANVSFEYFPFQVENKGAMTVDVIGEIHPEYLHWQVVREFN

LLKVSEDVLYRPFDTLSNGEQTKVMLAALFLKDNRFLLIDEPTNHLDLHARQIVGDYL

RSKSGFILVSHDRSFLDHSVDHILSINKSNIEIQKGNFSAWWENKRRQDQFELASNEK

LIKDIKRLSDSAKRTGGWSHEVEKTKNGTRNSGSKVDKGYIGHKAAKMMKRSKNIEQR

QQSAIQEKSKLLKNIESAERLQIHQLDFHKQELVELEHVSISYGSNTVFKDVSITIEK

GDRISLYGKNGSGKSSILKLICGEDIAYTGLFRKDHQLKISYVSQDTSDLGGHLSEYA

ATQGIDESLFKSILRKLDFSRLQLEKDISTFSAGQKKKVLIAKSLSEKAHLHIWDEPL

NFVDVISRMQIEDLLLEYSPTLLFVEHDREFCTNIATRIIEL"

CDS complement(2813251..2813787)

/locus_tag="EFAGFIKM_02398"

/inference="ab initio prediction:Prodigal:002006"

/codon_start=1

/transl_table=11

/product="hypothetical protein"

/translation="MELIKTYDVDLSEEWSTLLQELLVTSFPEVYPKDRLFFKQIPHG

RVLAFTPDNQLVGHVGLDYRMMNLNGKPIRVLGIIDLCVSPAIRSQGIASLLISEVER

LATGRVDFVLLFADHEELYSKNGFETVNNTCKWLKIDHETLTTVGVGTQAVEGLMIKE

VGNMPWGEGELDFLGYLY"

CDS complement(2813800..2814657)

/locus_tag="EFAGFIKM_02399"

/inference="ab initio prediction:Prodigal:002006"

/codon_start=1

/transl_table=11

/product="hypothetical protein"

/translation="MTGHHAQHFTTSEFAKICGVTKHTLFHYDEIGLLKPEYTNAKGY

RYYGMQQTYVLDVIHVLKKAGSSLQEIKSFIQNQNTPLLIELFEQKLKALELEQQRIK

RMQKLLSGAIEMTKQATDASPEGLHIEQCELEYFITVQLEQGDGDKEFFRKFIEYRTH

CDEQMIDYEFPVWTILTQEHYEAGEYYPDYFGNKIKAPISGETIWIKPKGMYAVMNHR

GSYESMPETYSLMKEEIEKEGLQVCGHVYALDLLTYFAESNPNEYIIKIYVEVCKPEN

TSKKQNGLI"

CDS 2814798..2816303

/gene="smvA"

/locus_tag="EFAGFIKM_02400"

/inference="ab initio prediction:Prodigal:002006"

/inference="similar to AA sequence:UniProtKB:D0ZXQ3"

/codon_start=1

/transl_table=11

/product="Methyl viologen resistance protein SmvA"

/translation="MSKFKRWMILVIVSSALLLIVMDMTILYTALPSLTHDLGASASE

KLWILNGYSLVMAGLLPAMGTLGDRLGHKKIFTLGLVVFSAASLAAAFSPVPAMLVMS

RILLAIGASMMMPATLSIIRVTFTNERELALAIGIWGSIASGGAGLGPIVGGLLLQHF

WWGSVFLINLPIAIIAFVFALKMIPKHQGDASKKWDFTSSIQIMIAMVGIIYSIKEFT

RREGSLTLAIIAAVIGVLSLIIFIRRQNNSPNPLLDLSLFKIPRFSTGFITALVGLFA

QMGVQYMVTQRLQLVEGMSPLQAGLFTVSIPVAALIAGPVTGAIMHRVDVVYIKSFTL

FIAALGMGTYLMYFNAGFTGQIIGLALLGAGLGSGMTAASHSIMSYAPPHKAGMAASI

EEVGYELGGASGIAIIGSMSTLFYTLAMKIPVGISVPANAKDSLDEALIAAESLPAAS

AESLKNAAFAAFDQSFFVVIAGVTVFLFIAALIMSWVAVRLKRAQKHSVKA"

CDS complement(2816532..2816915)

/locus_tag="EFAGFIKM_02401"

/inference="ab initio prediction:Prodigal:002006"

/codon_start=1

/transl_table=11

/product="hypothetical protein"

/translation="MTVTLIIIVAIIVFGLITGTVITIVAFKLLRNNKEKHHLRKSIL

MDGIPTEAIIHDIVQTSSSRDGRPGVRLDLTVTQVDGRTFPTIVETYIPITHIPHFQK

GNVINVRYIAMGNERKVEVEDAYLP"

CDS complement(2817059..2817664)

/gene="mfpA"

/locus_tag="EFAGFIKM_02402"

/inference="ab initio prediction:Prodigal:002006"

/inference="similar to AA sequence:UniProtKB:I6YBX3"

/codon_start=1

/transl_table=11

/product="Pentapeptide repeat protein MfpA"

/db_xref="COG:COG1357"

/translation="MYQYKDQEYEAVHFEGRDLRYGELISCVFKQCTFMNASMEEIET

SNCRFIECDFKGASMNGSIHTESAFENCTFGGANLFASKFSSCKMTGSDFSGAQMDGI

TLSHGDWSYTNLRHTRLGKQDLRGIRFFEADFTDTDFTKADLRDCDLTRVVLSRAKLQ

GADLRGANLEGIDLKSLDIKGVRLDREQAVLFVRSYGAKVD"

CDS complement(2817916..2818524)

/gene="wecD"

/locus_tag="EFAGFIKM_02403"

/EC_number="2.3.1.210"

/inference="ab initio prediction:Prodigal:002006"

/inference="protein motif:HAMAP:MF_02027"

/codon_start=1

/transl_table=11

/product="dTDP-fucosamine acetyltransferase"

/translation="MNKISITKASPQSLVGGQLNQMALEYMAYSLAGTKDKGIIEKTF

NKLWRSNQNRFSHQYAYEAKMGNQTLGMIMCYPTTIMNKLALPTFSKLFELRKWSLIK

YNLQHWKEFYSMVTLKEAENDEYHIGTLATLPESRGLGVGTQLIHFAEEQAITQGLSK

SSLTVKKENLLAIKLYERLGYQRVGEINKPSLSLYRMSKKLV"

CDS complement(2818557..2818919)

/locus_tag="EFAGFIKM_02404"

/inference="ab initio prediction:Prodigal:002006"

/codon_start=1

/transl_table=11

/product="hypothetical protein"

/translation="MSIQNVFGKIQISDDVISKIVGKIANTTSEISSMSTGLIEGVTK

KWSGKSLQNGIDIRKVESRLEINLKIIVCYGTKVHEVCRELQNNVRLHVEQLTGLTID

TVNVIVEGLSFNQPAAKL"

CDS complement(2819086..2819562)

/locus_tag="EFAGFIKM_02405"

/inference="ab initio prediction:Prodigal:002006"

/codon_start=1

/transl_table=11

/product="hypothetical protein"

/translation="MSRTQLSIPVGLLVENVLTSGESKEVIIECLQRRDYSRLLPLVK

ESAMDFDERLQTADDIGDDWEKAIRQGYEFKFLHINGLKRLLDFRFDRKVDRDYVQDE

LSLKHIHLTSQEIELLQSLIGRQWIVQAEETEQAEEGSKESGLSSRIPVRIQLKFP"

CDS complement(2819559..2821580)

/gene="dpp5_1"

/locus_tag="EFAGFIKM_02406"

/EC_number="3.4.14.-"

/inference="ab initio prediction:Prodigal:002006"

/inference="similar to AA sequence:UniProtKB:B2RIT0"

/codon_start=1

/transl_table=11

/product="Dipeptidyl-peptidase 5"

/db_xref="COG:COG1506"

/translation="MNQRPIMPEDLSHYRWISQPVISLNGQVAYVEQTIDQDKNEYNT

QIRGISLDGDEDIALSDGTKDSSPAWSPDGTQLTFIRSVDGGKGLWTLHSDQKEPVML

ISPARKILSYIWSPNGEYIAFTSKVQPEDQQKKKADLQQEPVPVLRGKVFERTTPKAE

GSGWWDGQYSHLFVYVIKSGEITQVTSGLWNISAPAWSPDSLQLSFISKQVEDEELDA

DLLYFTDIYSVRLGESDLFKVTDSSLAISQFSYSSDGQQLILIASDREYGSGSHNSLY

AVPVQRGVPRIIAPQLDMQMGNAALGDMKSAGASPSPISDNKHPERGVYVLGTQNGNV

DVYRIQDNGNCQSVTGAGEKDVYQYTLAPDGSSLVIAALTAEHPGELYRVYIDSGEMF

RLTRRNDEFMAELAVNVPVRVEFKSSDGWPLQGWLATPAIRDTNGKLPLILQIHGGPH

AMYTGTYSHEMQTLVAQGYAVLWINPRGSMGYGQEFARACRGDFAGGDYRDLMEAVDY

ALATYDFLDASRLGVAGGSYGGVMTNWIVAHTHRFKAAVTQRCISNWLSMYGTSDIGI

SYVEGVIGGNPAENAEFLWSRSPLAHAHHIETPLLIMHGEQDYRTPIAQAEELYTTLK

RYGKKTKLIRYPGSNHSLLKSGKPSLRIDSFEQVNAWFNQYLGNEEGEQ"

CDS complement(2821602..2822531)

/gene="gsiD_3"

/locus_tag="EFAGFIKM_02407"

/inference="ab initio prediction:Prodigal:002006"

/inference="similar to AA sequence:UniProtKB:P75799"

/codon_start=1

/transl_table=11

/product="Glutathione transport system permease protein

GsiD"

/db_xref="COG:COG1173"

/translation="MTGSNEMPIPGTEQEQDNGRAPTGFRYIWQQLIISKTGMFGAVL

VLLVVLIAIGAPLLTSHDPAAVNPLGRLKPPAWLEGGTAEYWLGTDNLGRDMWSRIVY

GARVSLIVGMGAVIVSGIIGAILGLVSGFYGKWLDAVIMRVGDAFMAIPTILFMLVVM

AIVGPGITTLIFVIGVTNWVPFTRVVRSEVLSIKERDFVHAARSIGAKNGRLILKHIL

PNILSSFIVICGMNVGTTIIMEASLSFLGLGIKPPDVSWGGMLSDGRQYVATSWWVAT

FPGLAITFTVLGVIFLGDWLRDVLDPRTETTHK"

CDS complement(2822548..2823465)

/gene="gsiC_3"

/locus_tag="EFAGFIKM_02408"

/inference="ab initio prediction:Prodigal:002006"

/inference="similar to AA sequence:UniProtKB:P75798"

/codon_start=1

/transl_table=11

/product="Glutathione transport system permease protein

GsiC"

/db_xref="COG:COG0601"

/translation="MGKYVLKSLLQIIPVLFIVSLIVFILVRVTGDPVALMLPETATA

EDRAVLTQALGLDQPLYTQYVKFLGSAIQGDFGQSFRYNQPALELVLERLPASFELAV

AAMFFAVLMAVPLGVISAVKRNTFTDLIISGISVIGKAMPNFWMGIMLILLFSVMLGV

LPVSGRGGLSHLILPAFTLGVGLAAQMTRLIRSSMLEILNQDYIRTARSKGLGRMVVI

VKHAFRNGLIPVVTIMSLQFTSLIGGTLITETVFSWPGLGQLLVVAVNTHDMAIVQAA

VFVIAFIVVVINILTDVAYRLLDPRIKYD"

CDS complement(2823567..2825132)

/gene="gsiB_3"

/locus_tag="EFAGFIKM_02409"

/inference="ab initio prediction:Prodigal:002006"

/inference="similar to AA sequence:UniProtKB:P75797"

/codon_start=1

/transl_table=11

/product="Glutathione-binding protein GsiB"

/db_xref="COG:COG0747"

/translation="MKKRQWTGLWITLLAVVMVLSACGGKSTSTNDGSATEGTGSGSA

STTLTVAAATDIESFDPHNNNNTSSEAVLVNVFDYLIKNDSEQKKVAGLATSWDQVDD

TTWRFKLREGVTFHNGDPFTSADVKYTLERVAKDETLKQNSYFKNIVEVKVVDDYTVD

IITDGPDPLLLNRLSKMGAGILPAKYIADKGFDAFLKQPVGTGPYKFSKWTKDDRVEL

VKNENYFDGEPKWNEVVFRVIPEASTRVSELLAGGVDVASSIPSTDIARIEGEADKKI

VKAPIQRVLQLIFRQTEGSITADPKVREAIDLAIDKQGIVDSIAGGAGIVTRTSVTPG

NFGADPSLYKTSLYDQEKAKQLLQEAGYAEGEAEMTISVSAQYKEQAEVVAAMLEQAG

FKINLDVLEASAFSERYSSKSFKEIFMIGIGNSLFDASNNYNRYMLEEAKGESDYNNP

EVEKLLQSALVNMDPASREKEYQQVQQIFSEERPAVYLYQMEGVYGTNAKVNFAPRSD

EMFYADEITPVAQ"

CDS complement(2825129..2826340)

/gene="scmP_2"

/locus_tag="EFAGFIKM_02410"

/EC_number="3.5.1.-"

/inference="ab initio prediction:Prodigal:002006"

/inference="similar to AA sequence:UniProtKB:P54955"

/codon_start=1

/transl_table=11

/product="N-acetylcysteine deacetylase"

/db_xref="COG:COG1473"

/translation="MNRSELIQLAQPLQAQLSAWRRDLHQHPEIGYEEHRTSAIVAEH

LESQGLEVTRNVGQTGVTGLLRGETDGPTFALRADMDALPIQDQKAVEYRSQVEGKAH

LCGHDAHTSILMGAAQLLTGLGRPKSGNIKFIFQPAEEGLAGARAMIQDGVLENPKVD

AIAGLHMTPGQNTGTLGVSQGVAFASADPLIIKVFGKGGHAARPHEGIDAIAVSAQVI

TALQNIVSRMVDPLEPAVVTIGKITGGYMGTAIAPEVEMIGTVRTLSPAIRERMPALI

EQVVKGVCDSFGAGCEVVYGDGYPVVVNDLGMVDLLTETCDQVNAEKGWTYIKPSTGG

EDFAFYCEQVPGVFFRLGSGNDEERTRYPLHHPMFDLDETAMPYGVGMLSAVALEFLA

RNTTSEGEQSQ"

CDS complement(2826337..2827089)

/gene="nanR"

/locus_tag="EFAGFIKM_02411"

/inference="ab initio prediction:Prodigal:002006"

/inference="similar to AA sequence:UniProtKB:P0A8W0"

/codon_start=1

/transl_table=11

/product="HTH-type transcriptional repressor NanR"

/db_xref="COG:COG2186"

/translation="MSSTFSFRFEKVSTKKVSEFIREQLEEAIILKELMSEEQLPAER

DLAEIFNVSRITVREALSSLEEKGLIEKRVGAKGGTFVLPLTANSHKRTREEIKRDWS

QMLKVFEYRTIIEPEGAFLAAERITAGELELLEGYMEQSIEPDCTREWFRALDVKFHL

TIAKASGNPYCERAVRQIRTKINPALDLMPYDDRIRTVNHGVHMEILEALKAHDSTKS

KETMKRHIEFSADAIYARLVSESDKNEGNDSE"

CDS complement(2827277..2827990)

/locus_tag="EFAGFIKM_02412"

/inference="ab initio prediction:Prodigal:002006"

/codon_start=1

/transl_table=11

/product="hypothetical protein"

/translation="MTKHKTGHGVSIVVCTNRPQFFDNILQNYSRQRYKSKELIIVLN

HDSMNLALYQNRVRNLANVHVYQVPESISLGQSLNAGMTRARFSLIAKFDDDDYYSPY

YLTEQVGELRRTKSDIVGKHSCLVYLGASKTLLVRSPAEKNKPVEFVQGGTILFKREI

LKKVRFTDRSIGEDVTFLRQCRKKGFKAYATSPYNYVYHRRQNKKSHTWRADDSFYLE

GSKKLAVTEDFRPFANKKL"

CDS complement(2828141..2829238)

/gene="gfo_2"

/locus_tag="EFAGFIKM_02413"

/EC_number="1.1.99.28"

/inference="ab initio prediction:Prodigal:002006"

/inference="similar to AA sequence:UniProtKB:Q07982"

/codon_start=1

/transl_table=11

/product="Glucose--fructose oxidoreductase"

/translation="MSKVYRIGIIGCGGIANGKHLPSLSKLDNVELVAFCDIVQERAD

EAKQKYGTTEAEVYTDYQELLKDESLDIVHVLTPNISHAEISIAALEAGKHVMCEKPM

AKTSAEAQLMLEAAERTGKKLTIGYNNRFREDSQYLKKVCEAGDLGNIYFAKAHAIRR

RAVPTWGVFLDEEKQGGGPLIDIGTHALDLTLWMMDNYQPKVVLGTTYHELSQRENAA

NAWGPWDPKQFSVEDSAFGMIVMENGATIMLESSWALNSLDVDEAKCSLSGSEAGADM

KNGLRINGEKFSRLYTNEIELSAGGVAFYDGKSESAPDVEMRKWIEAIENDQEPVVTP

KQALVVSQILEALYESARTGKAVYLNNGSEA"

CDS complement(2829251..2830174)

/gene="rhaR_21"

/locus_tag="EFAGFIKM_02414"

/inference="ab initio prediction:Prodigal:002006"

/inference="protein motif:HAMAP:MF_01533"

/codon_start=1

/transl_table=11

/product="HTH-type transcriptional activator RhaR"

/translation="MSLYPYEKMLERQDLLERLDILMVWGHYEIRVMRFHLTSFPAGR

VVDFHNHAEFEFHFIPRGKGKVILDDQTHALSEGMLYLTGPGVVHYQEADAKEDMDEL

CLHVDIVHKPREHVDPWEAAESEETMEKLRTLPLTPVNDYHRAMHCFLEAYEACDLKL

LGYYTSIKQLVISILLKTVRAYDTGGNRPEAPVRDMSVYRYEYAVQYMEANHTTVVTL

EHVAEKLHISSRQLQRIFYQVQPEMPFSRVLEDIRLRAVCRNLEESNVSIEQIALASG

FNNANYLHAVFRKRLGMTPSAFRKMKQPILK"

CDS complement(2830199..2831203)

/gene="iolG_5"

/locus_tag="EFAGFIKM_02415"

/EC_number="1.1.1.18"

/inference="ab initio prediction:Prodigal:002006"

/inference="similar to AA sequence:UniProtKB:Q9WYP5"

/codon_start=1

/transl_table=11

/product="Myo-inositol 2-dehydrogenase"

/db_xref="COG:COG0673"

/translation="MTIRIGKISLWHVHAWDYIKQAQEHEDTVIAAVWDEDAKRGQEA

AERLNVPFYASLEDMLAKDDIDAVIVDAPTRIHEEVITAAAKAGKHIFTEKVIASTQV

ESNRILNEVKANKVKMTVSLPRLNAGYTLKIQDVLNQGLLGKVTYVRARLSHDGAISN

WLPEHFYDLKDCQGGALIDLGCHPMYLAKLFLGQEVTAVNANFGYITGKEVEDNAVAT

LFTDSGAVGVVEAGFVNSHSPFTIEVHGTESTILYGTPEEKLLIRTKAAQGQYQEWTE

LPLADQRESAFNQWVAHIQNDTDATENVQIAMELTRLMEAANLSAKEGRRITLKELKG

"

CDS complement(2831492..2832025)

/locus_tag="EFAGFIKM_02416"

/inference="ab initio prediction:Prodigal:002006"

/codon_start=1

/transl_table=11

/product="hypothetical protein"

/translation="MKKMSWIALFLVLILYFVSMYSLYSHMNNKWLVSPPNYLILILS

ILVLAIAILGFKDNLNKSTKVRSWISTVLSLVLIFILLGALSFTSIFSGYKQLLTTTH

SPDKHYTISFYKTDAGAMGSFGVMGELKGPLWFKRVFYYEGKTDQVDLEWVDNHTISI

NDQNVNLLNSETVRRSP"

CDS complement(2832118..2832765)

/locus_tag="EFAGFIKM_02417"

/inference="ab initio prediction:Prodigal:002006"

/codon_start=1

/transl_table=11

/product="hypothetical protein"

/translation="MNYILFVLFVVLIIVLSLWILSLTRKHKRSAVLYEENYDLKAIT

IADIDRMEDGSGFEMYLYRLLIELGYSGVYKTLGSRDFGADVVFTDREGVRNVIQAKR

YSIEYPVGISAVQEVFSCMRYYKAKKAIVITSSNFTESCETLAGINFVKLIDRTDLIH

VIEAFRDGDMIEARDIIEEEPRMILESWSEANSNTLHEVRKDYKAEKYVKKVMSK"

CDS complement(2832979..2835780)

/gene="mngB_2"

/locus_tag="EFAGFIKM_02418"

/EC_number="3.2.1.-"

/inference="ab initio prediction:Prodigal:002006"

/inference="similar to AA sequence:UniProtKB:P54746"

/codon_start=1

/transl_table=11

/product="Mannosylglycerate hydrolase"

/db_xref="COG:COG0383"

/translation="MTTKKTAHLISHTHWDREWYMPYEHHHVLLIELMDKLLDTLDQD

PEYRYFHLDGQTIIREDYLQVRPEQQERLDRYIREGRIHFGPWYVLQDEFLTSSEANL

RNLLIGHRDARPLGVISKTGYFPDSFGNMGQAPQILQQADIHNAIFGRGVKPTGFNNA

VVDADSYESPYSEMIWRSPDGSEVLGILFANWYCNGMEVPVDPEKAQSYWDKNLEDAE

KFASTPHLLFMNGCDHQPIQTDMPEALRTASALYPDVEFIHSNFDDYIEAVTKDVPEH

LATIEGELRSQHTDGWGTLVNTASARVYLKQLNQQGQTLLEKVAEPLAAMAHIAGVKD

YPHHLLTHAWKMLMQNHPHDSICGCSVDEVHREMVTRFAKSRQLAEKLVSQSAQAIAE

SIHVQSAETWGEEAVLFTVFNTSGWNRNGIIEMDLIVDKAFFPEGPNPQALAQKMEKD

ALPVYQLVDSGGRTYSAEIKDLGAHFGYELPKDRFRQPYMARKVRVTFQAVDVLSLGY

KTYALIPVEKQAEFAEIVEVPDTREVIQVQGMMMENGQLRVTVEENGTATIEDKVSGT

VYKGLNSYENTGDIGNEYVYRQPEGETTLTTEHLKADLRIVEQSPYRAVMESVLRWDI

PAGADELFELEKRQMVPFTERKVQRVENTVPLVITTTYTLEAGSNMVKVKSDFNNQAK

DHRLRALFPSGLVTEDHYADSIFEIAKRSNTPAKEWVNPSNAQHQQAFVHVTDGAHGL

MIANKGLNEYEILQHEEGSTVAVTLLRASSELGDWGVFETPEAQCLGPQSVEYAIIPF

AGDAAQSGACATAYTYPIPWTTVQLGALAYTFEQEGHAGADGHKVELPLSKQWLAWNA

QGSSLAFSTLKIAEETGDLIARWYNLNSEPVELNVQPGFETASVYESDVLERVKDTLE

GQSQTVSGYKIVTQGYTLR"

CDS complement(2835798..2836622)

/gene="sugB"

/locus_tag="EFAGFIKM_02419"

/inference="ab initio prediction:Prodigal:002006"

/inference="similar to AA sequence:UniProtKB:P9WG01"

/codon_start=1

/transl_table=11

/product="Trehalose transport system permease protein

SugB"

/db_xref="COG:COG0395"

/translation="MVKHGLQRKIQYGILVFLGLCFLLPLLWIILASFDPNAQQGIKM

PSTWTIQNFKDVLGDSSNLRSFGVGLILSGGQAILVVLVSVLAAYPLSRYEMRFKKSF

LLSILFMTALPITAVMVPVFQMFLFFKMQNSIIATMLFLTASSLPYGIWMMKNFMDSV

PIDLEESAWIDGASVWSSLRRIVAPLMLPGIATIAIFTFSGSWGNFFVPYILLQTPEK

LPASVTIYQFFGSHGMVEYGRLAAFSLLYTMPSVVLYIFSQRYMSKGFSMGGATKG"

CDS complement(2836645..2837511)

/gene="melD_7"

/locus_tag="EFAGFIKM_02420"

/inference="ab initio prediction:Prodigal:002006"

/inference="similar to AA sequence:UniProtKB:O34706"

/codon_start=1

/transl_table=11

/product="Melibiose/raffinose/stachyose import permease

protein MelD"

/db_xref="COG:COG1175"

/translation="MKRKAPGSFLFLMPSVLLLLVFFIVPIILTICFAFTNMALTGAA

AKSLEFVGFQNFINMFHDPDFRISVWRTLVFLIFSAVIGQVLLGFILALLMKEKNVTF

RRVIGIIVIAGWVTPEIVVAFCMVAFFSDNGSLNQILGWFGANPVSWLFSFPMVSVII

ANIWHGTAFSMMVYQSALDDIPKEVEEAAIIDSATGFQIVRHITIPMVKGSIVTNMML

VTLQTLGVFTLIYTMTGGGPGTSTQTLPIFMYNQAFVNYQFGYGTAISLVLLFIGIIA

SLFYMRSMKVKV"

CDS complement(2837579..2838958)

/locus_tag="EFAGFIKM_02421"

/inference="ab initio prediction:Prodigal:002006"

/codon_start=1

/transl_table=11

/product="hypothetical protein"

/translation="MRKISLKMPVAAVMTSLLILTTACSGGSSSTGTTSDGKKEVTVS
FRSSGSEDTLTKFFQSGLVDQFEKENPDIKINIAPVLASEGDYTSKMVLQMKSPDTAP
DVIAEDTSIIKSDAAAGYLEPLDTQVQGWSDWTEHIIDNLKAGVTGEDGKVYGVPATS
DTRGIWYNKELFEKAGLEVPFKPASWAEVLEAARTIKQKLPGVTPLNMIVGKANGEGV
TMQTLEMLLYGTADTLYNETSKKWVVNSPGLLDSFKFIDQVFNTDKTGPTMQVALNGQ
AGSIAFQQQFPQDKLAMAVDGSWAGSTWAENGAAPIANVEEKIGFAPFPTQNGEEPGA
TTMSGGWAWSVPAQAKNKEEAWKFIEFLMNKENATARVVAEGSLSPRNDSTEVEGYTD
RPYTKEAQELLNVAHFRPANDQYAAVSAQLQSIVESIASGKLTPEEGVTQLKDNVSRS
LGADSIEEK"

CDS complement(2839037..2840338)

/locus_tag="EFAGFIKM_02422"

/inference="ab initio prediction:Prodigal:002006"

/codon_start=1

/transl_table=11

/product="hypothetical protein"

/translation="MLTPRDNQEISPSIYDMIDQVNKRMPDHPELNQMFKNCFTNTMA
TTIQRKEDGTTFVITGDIPAMWLRDSAAQVRPYLVLASEDEDIADMIAGLVERQLNYI
LLDPYANAFNETESGKGHQEDLTLMNDWIWERKYEIDSLAYPIQLSYLLWKNTGRTTQ
FNDTFRKAAQIIMQLWQVEQHHETKSPYTFQRLDAPETDTLSREGRGTETAYTGMTWS
GFRPSDDRCEYGYLIPSNMFAVVALRYLQEIAEAVFEDETLAATAKQLEEQINKGIQD
YGTVEHPEYGTIYAYETDGKGNHNLMDDANVPSLLSLPYLGYVDENDEVYQNTRRFIL
SSQNPYFYEGTAAAGIGSPHTPEGYIWHIALSMQGLTTGDRNEKLRLLQLIHKTDADT
GLTHEGFSANNPHEYTRPWFSWSNMLFSELIMDYCGFRVQK"

CDS complement(2840564..2841727)

/gene="araR_2"

/locus_tag="EFAGFIKM_02423"

/inference="ab initio prediction:Prodigal:002006"

/inference="similar to AA sequence:UniProtKB:P96711"

/codon_start=1

/transl_table=11

/product="Arabinose metabolism transcriptional repressor"

/db_xref="COG:COG1609"

/translation="MGKDSASKPMYEQIFESLRERIQLHQYQVGERVPSEKELCDEFG

VSRITTKKALEMLASEQLIVRQPGRGSFVADTADVLQERPNRPAPRAAVKDPEKKLLI

GLVITNFSDMYGTELLYGMEEASREHDCFLVLRRSFGIPEQEEQSIQELLELGVDGLI

IFPAQGEYFSDEILKLVVNKFPFVLIDRYLKGIPASSVSTDNVGAAREGMNYLFDLGH

RHIAFLTQPPANTTPIEERIEGIIEAHHDQGVLVNRELWLESFLSTLPSVFDPQVEVR

DVETLVEHLQKYPQITALFAAEYHIALLAEQAADRLGLRIPEDMSIICFDSPNAAEGS

RVTHMRQSQFDMGKQAFEMVLQSMQNNEMAVNRVVLPARLVKGKSTSMVKQKG"

CDS complement(2842065..2847311)

/locus_tag="EFAGFIKM_02424"

/inference="ab initio prediction:Prodigal:002006"

/codon_start=1

/transl_table=11

/product="hypothetical protein"

/translation="MIKINRLRKPISMGLSLLLAFSIIHPVSAENSTSSKLDMKSGLA

KVTESKVNAKLTKEFEGSEYVTYLVKMKEQVDTAAVSKQALEKATIAKQTASATKLSV

RNSVVSSLRETASRTQYPLETYLEKEVDLGKVKEYKSYFIVNSLAVTSTKEVMEQIAL

LPEVEKILPNETRYLQKAEVSKEPANAASGKDVVQTPTKDSSVGVKDKEPAAKDKLAT

ENVEWNLDYINAPAVWDRGIDGTGIVVANLDSGVDYTHPALRSKWRGLDASGNIVDPE

LSWYDPHSNASLPADGDGHGTHTMGTMVGSEADGTNQIGVAPGAKWIGVRIFNPETTD

AIILDGGQWLIAPVDAEGNLHPELAPDVVNNSWGGGAGLDEWFRPMVQAWRDAQIFPE

FSAGNVRLGNPGGPGSVANPANYPESFATGATDINGNLASFSLLGPSPYGEIKPEVSA

PGVNIRSSVPGGVYEGGWNGTSMAGPHTTALAALLLQANHSLTVDQLEQIITDTATPR

TDSQYPTSPNNGYGHGIINALDAVGSVLEGIGTVSGRVVTAGDDLEEPVLAHTPVNSA

FTGIDIPLTAHVTDNVAVVSVEAFAKTTGTNQYVYLPMNRIAGDNKDGTYTATIPAFL

IEPQGVEYYIRVNDYGNNGFESQVYKVAVSNGVQPGYLQDFEEDQLGFTTGGTGSTWV

WGAPSSGPGSAYSGDKVIATNLQGTYVANSNAYLLAPPIDLTESPEGALLSFKHWYDL

ENNIDFGKVYIASEDSDYVFEELLTFTGTGGNWKTQYVDLREYAGQQVFIKFNLTSDN

SLQKAGWYIDDFAVEELDEIAPGAPAELSATADILGNVALSWTGPSDEDLESYIVYRS

TTAGAGYESIGTATGTTFTDTATVTDSTYYYTVAALDYSGNESDKSNEVSITVEVPQD

IYIDHFDGSDDNGWTHSGTKDEWERGIPVTGPASAVSPPNVWATDLDNTYESGSNYSL

VSPVIDLTDVSEGTLTFNHWYEIESGYDYGYVEVTKDGGTTWSELGKFSHSTNGKQWT

PVFYDLDTLTGNEVQFRFRLTSDNSVVKTGWFIDDFRVLGVAAETVTEDSAVVLSNDK

PKPSYDNPWYKISRTDKAEFNKTKQQQPEVEKPETGSVNPQSLPASATVTVLETGRSV

KTDSATGKYSFTHVAGDYTLKAEAYGYYPRTQQVTITDGSGAKANFNLEAIPHGQIEG

VVTDERTGQPLADASVLVVEDANVGEVRTGSDGSFVIQVLEGSYTLSIRAADYYSKTV

TVTVPGNGTAEANVALKPFIGFPGEIAYDDGTAENARAFNAADNAWAVRMTPELETAQ

LTGASFRFWNTEWPVPGGTAFQYAVYDASGAGGAPGRQLAGPFDGTALRNDQWTTVEF

PEPVIVTGDFYIVYVQSLAGTSAPGLATDEDGPNAGRSWQRVGGAWSTSPDEEGNYMI

RAVVRYPVNAPVLTAPANTYTNQSTFTVSGTSPASGAQIKIYNGKDLAGTTTVANGKF

SYGVKLRSGINAITAEAVVDGKTTDRSLPVVIILDQTKPQLTILTPAQGDRINAEVVH

VTGNVVEQFLDKVTVNGQTVQVGKDRSFSHRVLVNEGENTITITATDIAGNKTTVTRT

VYVETALPELTNITPAEDVRITSGESVTVSFDSKPGLQASFRIQLPLNLNAQGAGEIP

LVETEPGRYTGTYTTPSSLVLDGGVIVIRAQDAAGNKVESEAAGRLFVSAAQEQSVPE

PDSSPEETETEEAPPSAESLQP"

CDS complement(2847965..2849605)

/locus_tag="EFAGFIKM_02425"

/inference="ab initio prediction:Prodigal:002006"

/codon_start=1

/transl_table=11

/product="hypothetical protein"

/translation="MKPKKRIRYGWSGFKKKKAEDCDHPQEQHKEHHHEHHHNHHNHH

HNNHHHKCFDKEAEFLLKLIGELKGAIVQVFANPTPHNVSLLIEVLRKLLALVHHSDL

KKGTKADLEAILELTIVSAEAVPFSPISVGTNLQQLLDVLLSVILQGNIDSAEKDQLV

ILIRATELAITTGLRNIGSQGPAGPAGPQGVPGPQGPAGAAGSQGGVGPAGTPGTAGA

QGPAGATGVTGATGAAGPAGGATGATGVTGSTGAVGAAGVTGATGDPGVTGATGAAGL

AGATGVTGVPGVTGGTGATGAGATGVTGTTGLTGVTGTTGLTGVTGATGLTGVTGATG

LTGVTGATGLTGVTGATGLTGVTGATGLTGVTGVTGATGSGAIIPFASGGPAILTTIL

GGLVGTTSLIGFGSSATGVSILGGTIDLTGTIVGPLINFAFSVPRDGVITSIAAYFST

TAALALVGSTVTITAQLFSSPTPNNAFTAVPGAVVTLAPPLTGIIALGSISNGITTGL

TIPVTAQTRLLLVFSATATGLTLVNTVIGYASGGVNIT"

CDS complement(2849889..2851349)

/locus_tag="EFAGFIKM_02426"

/inference="ab initio prediction:Prodigal:002006"

/codon_start=1

/transl_table=11

/product="hypothetical protein"

/translation="MDKNEYLQHIEATIENGKFKDNWSSLSAFQVPEWYPKAKFGIFI

HWGLYSVPAYDSEWYSRNMYIQGTKAYEHHLEVYGPHKDFGYKDFIPMFRAEKFDADE

WATLFKQAGARYVMPVAEHHDGFQMYKSSISHYNTYEMGPKRDLLGEMKVAYEKQGLT

LCVSSHRAEHWFFMSHGKEFDSDIKEPLERGDFYWPAMPEPKDHFDLYDSPPTEEYLE

DWLIRCCELVDQYQPKVFYFDWWIQTVAFKPYLKKFAAYYYNKGEEWGVPVAINYKHE

AYMFGSAVPDVERGQFSELKPYFWQTDTAVAKNSWCYTENNNYKTAEEIIRDLVDIVS

KNGNLLLNIGPKADGSIPDEDRDILLSIGKWLEVNGEAIYDTSFWRTFGEGPTEVKEG

QFTDGDTKIFTSEDIRFTLKESSLYATVLAYPDNGVVQIKSLKENSHHFQGVIQDIQV

LGYEEQPQWERTADSLTITTTTVKSNAPIVFKIKLD"

CDS 2851472..2852305

/gene="araC_3"

/locus_tag="EFAGFIKM_02427"

/inference="ab initio prediction:Prodigal:002006"

/inference="similar to AA sequence:UniProtKB:P0A9E0"

/codon_start=1

/transl_table=11

/product="Arabinose operon regulatory protein"

/translation="MNHQTLLTNYLSNLQVELFMVNYNRCDTDWRDLDYTPDYSKFYF

ICEGEGWLKIGNEEYYPAPNQLILMPEGIKQSYSAINDLPFLKYWCHFSAKVGGINLF

QILKFPHICTIDQPELIQGIFSSILTYAKSDEVYGHMMAKSKLTELLSHYIMNLDLDQ

ISFINVPVMEKLSTILAYIDSNIEENISVQDLAQIAYMHPNYFIRFFKQQIGVPPILY

ITGKKIDKAKELLTCTPNTITAIAEHLGFSDLYYFSRQFKKHTGLTPTEYRKQKTVLT

T"

CDS complement(2852333..2853685)

/locus_tag="EFAGFIKM_02428"

/inference="ab initio prediction:Prodigal:002006"

/codon_start=1

/transl_table=11

/product="hypothetical protein"

/translation="MTTFKMYKSTGEEDLFVSLSSDQLQPLDADKGTTTIHLDDQLTY

QEMDGFGASFTDSSAYLINQILNEEQRDEVMTRLFHPKKGIGLSVIRNPMGASDYART
VYSYNDMPEQQTDPELTQFSITHDEGDVIPLTQKALALNPELKLFASPWSAPGWMKTS
GSMITGQLKAEWYPVYAEYFVKYIQAYGKHGLPIYAITPQNEALYEPGHYPGMLMPAE
AQADFIKNHLKPAFVQNDIQTKILCYDHNWDQPDYPLTVLEQAGEEVDGVAWHWYGGD
ASAQTKVYEAFAGKEVHFTEGSGGEWIPPFEQAFSNVMRTGIQILRNYSKSFVLWNMA
LDENNGPTVPGFGRSTCRGIIQVNQQTKELTYTLDYYALAHFSAVIRPKAVRIESTSS
DDGICSVAFKNSDGSVALVLFNDGEETANVQTKLRNEELLCFEMESKSVMSILIHPNK
"

CDS complement(2853708..2855351)

/locus_tag="EFAGFIKM_02429"
/inference="ab initio prediction:Prodigal:002006"
/codon_start=1
/transl_table=11
/product="hypothetical protein"
/translation="MLKLNKASGKKGIKLLATLLTAVLMITGCSGGSGGSSEGNWVSI
EDRYTVDPEKPAWQLDKKEEATDLTWYVNADWWNTDFGKDIVTKKIKEDLNINIKFIT
GDDTKLNTFFAGGDMPDLLTVFDSNSPVVQKAATWAMPLNDLAEKYDPYFNKVAAADT
LNWFQLADGKTYGYPNYSNTQEDYDSGNIPAKTAFIIRKDVYEALGSPVIGTPEEFQS
VMKRIKQEFPALIPFGFNSIGEGTGSLGDTLQDFIGVPLENENGEFYDRNLDEDYLTW
LKTLNTVYRDGNISDDSFADDGTAFEEKVKSGKYATMLLDGTPQQGGNLQIYMSANPG
KEYVAIDGPQSTKGNAPTLNQSGITGWMINFISKDCKDPAKAIQIFTYLLSEEGQTLM
NYGIEGETFQTKADGSVELLPAVKDMQLNNADQFKKNYRMGEFMFFGHDRHKALSADA
FPEAIKQMQEWGKGKLKPHFILENISPDQGTPEARALSAINTKWNTTLVSMVRSKDDA
SYDSALASYKSFLGENRWEEIVKVRSEKMKLNKEKLGIQ"

CDS complement(2855388..2856275)

/gene="ngcG_3"
/locus_tag="EFAGFIKM_02430"
/inference="ab initio prediction:Prodigal:002006"
/inference="similar to AA sequence:UniProtKB:O50501"
/codon_start=1
/transl_table=11
/product="Diacetylchitobiose uptake system permease

protein NgcG"

/db_xref="COG:COG0395"

/translation="MNGKVAKEDLDSRIFDTLNMILLIICTVVILVPLWNVIISSFSS

GKALAEGGFIFWSPEFSLENYRAVFNDQGIWQAFFISVSKTTIGVVTHVFFCAMVGYG

LSKKYIRGRKLYVAMGVITMFFSGGMIPTYLLIKSLGLLNSFWVYIIPALFSFYDVVI

LMNFFRNVPDSLEESAKIDGAGDWHIFLKIFIPLSMPAMATIALFNGVGQWNDFMTTK

LYITDQSLYPLQMMLYEIIVQSQTQSMQNVGGSAVIETTTKGVQLATIVITTLPIVLI

YPIVQRYFISGMMLGAVKE"

CDS complement(2856291..2857280)

/gene="yteP_21"

/locus_tag="EFAGFIKM_02431"

/inference="ab initio prediction:Prodigal:002006"

/inference="similar to AA sequence:UniProtKB:C0SPB3"

/codon_start=1

/transl_table=11

/product="putative multiple-sugar transport system

permease YteP"

/db_xref="COG:COG4209"

/translation="MDKLAVETSTGKNAISPNGKKPIGQRIKEFIVDYRRQWEIQSMI

IPGIIFMIIFCYIPIYGLTIAFKNYTVIDTLATAPWVGLDNFRIILSDKYFWDAVVNT

LGISFLKLGIGFVIPIILAIMIYELNSGRFKKFVQTVSYLPHFLSWIVLGGMLITWFS

TTGLFNQLLLSLGVISQPQNILLDAGKYWWIATLSDIWKEAGWGTILYLAIMAKIDPT

YYEAAKIDGASRLRQIWNITLPNMKSIISLNLILTVSGLLGSNLDQTLVLMNSQNRDK

AEVINSYVYRMGMSQGDFSYATAVGLGVSIISVILLVTANKITSKLNDNQSVL"

CDS complement(2857396..2858418)

/gene="exuR_1"

/locus_tag="EFAGFIKM_02432"

/inference="ab initio prediction:Prodigal:002006"

/inference="similar to AA sequence:UniProtKB:Q9JMQ1"

/codon_start=1

/transl_table=11

/product="putative HTH-type transcriptional repressor

ExuR"

/db_xref="COG:COG1609"

/translation="MAKITIKDVAREAGVSISTVSNALNGVDVLNPETKSHVLKVAER

LNYVPNLNGKLLKSGQTKMLGFFTTSVSGPYFYKLVESMSRECDRLGYGLNVFVTKDK

HVIMSNILGRRVDGVIIYEELRIDEQDIIAMEKDKIKAVFLDRVYQSDTMGSVIFDSY

VAAYEATKYLIGLGHKKIAYISGVDTMFDSVQRMDGYLAALREYQLPIDEDYIIQGYF

EEESTYSAIKSFLRLYPCKRPDAFLAGNDLSAIGCMHALKSEGFEVPQDVSVVGFDDI

DIAQYFSPPLTTVRNQIARQGILAINQLVGMIKEKEQGAAQKLAGELVVRGSSHVKID

RKDYRT"

CDS 2858605..2863695

/locus_tag="EFAGFIKM_02433"

/inference="ab initio prediction:Prodigal:002006"

/codon_start=1

/transl_table=11

/product="hypothetical protein"

/translation="MAFIERYAINENGAVTFTGNTLGLSRSETTGVPGTVDSIGAYIT

TDTSQQFGTYPPGTTNVFQNNSSSAVLQIPAGSSILYAELIWGGTYIDNNENNSAFIN

NAVQFTTPSGLTSVLPDPATMFEVILSTAAGFPTTYAYVRSANVTDLVSAGGAGTYTT

GGVVGTLSIPDPTSNHAGWTLAVVYQNAALPLRNMSIRVTADVILSTSGPVNLIIDGF

ATPFMGPLAGRAQFSAQEGDANKTGDQARFGPTIATVTNLSGPNNFATNFFASQINDD

AGNLNTLGTFGTRNQLNGTPGTNIIAGRQGWDITNVDIGFTLLNTQTQAAFQLTTNGD

GYLVDGNGLQIDINLPALTAVKSANVPDAVVGDVVTYSIVISNTSLVDATNVMFFDNI

PAGSSFVTDSITINGVPQPGANPITGVPIGALNSGASVTVTFQVLVTSVPTPPRLFNQ

AKVGYTAPTVPDGPIINTSVPSNAVEIPIFQPNVAIVKSADTTNALVGDTVTYTLVVT

NTGNIDATTTILDNIPDGSVFVAGSVTVNGVSQPADSPVTGIPIGPVAPNTVFTVTFQ

VNVVSQPLGLQLVNQGTASYTFQPPDGRVIPGSVTSNTVIIPVSLPMVSIVKSTPSTD

AVVGDTITYSLTVTNQDTAAVTNVLLTDPIPNGSVFLPGSVTLNGVPQPAADPVTGVA

VGTLLPGASATVTFSVSVTFLPDPPQLTDQAQVSFDSGSFSGISFSNMVVVPVFQAII

TVNKSAVPIFAVVGEVITFQFMVTNTGNIGATTTLTDIISADASFVAGSVLVNSIPVP

LADPNVGIALGLIPAGSNALVSFQIVVTSLPAALQIVNQAIASFTFTPPDGRIIPGSS

NSNMVIIPISPDDLTVVKSASELQAVVGDIVTYTLVVQNNDPVDATNVTLSDPIPVAS

ILVPDSVTVNGVSLPGVDPSTGIPIGTITANGSATVTFQVTVVGTAPPVPFPLFLTDQ

ATVIFTTGEFTGTVLSNIVNVRVFEPVITLIKSAVPSSAVVGDIITYTVLVSNDGNYN

ADVILIDPLPTGETFVPNSVLINGNRWSSADPTTGINLGTVQPAFPVSVTFLATVVSL

PEPPQLVNQATGDYTYTVPDGRVIPGSSLSNIVTIPVTEPGVLSVVKSADRTAATPGD

TITYTLLVQNPTTLQASNVFLTDPIPVGSTFVNDSLTLNGVPQPDANPTEGLSIGNIS

ANSSAVVTFQVIVITISPPPIIEIITNQATVTFTINGVSDSSLSNIIDTPVNDAVITL

VKSGDPATVDLGDTVFYQLVVQNEGNIAADVTVIDPIPPESFFIPGSLTVNGVNVSDD

PVSGIPLGLVAPGEIFNINFQVNILSFPSNNQVINTAIANYSFTLPDGRVIANSIESD

TVTTFISDPLLEIVKSTSATQVVPGQTVTFSFLLTNGNNVPFSAIIFTDLIPAGMAFV

NDSVRIRDVSVPGVNPNNGFEVDNIAARSTIPISFQVTVDQGITLEQITNQATVAYTL

GEVLFNVPSNIVVMQVIFPVPPVPPVPPGPPVPPGPPAPPVNPTTPTITGSSASQPST

PGVPSTSISAPSIPVQKTANRSVVEVGETVDYVVLIQNTSSLSAINLVLTDQLQYGAI

IVSASVTVQHIVQQHCNLGGGISLGDLQPGASVEVAYTAKIKHQPSNGKIINQAVVSL

SFVLPDGSSIPITLASNKVTVTVVESEE"

CDS complement(2863699..2864547)

/locus_tag="EFAGFIKM_02434"

/inference="ab initio prediction:Prodigal:002006"

/codon_start=1

/transl_table=11

/product="hypothetical protein"

/translation="MKNLRIDLGSGKNKHRNCTGVDRIAYPKTDIIHDINSPLPFEDN

CATLVMCSHSLQYVDNLEKVMEEIYRICSHKAVVCIVAPYAHVSSHMVNPRFKQLFNE

HMPQFWSSQASDISMDDKSPLYMSERLGLQKEDMESNIDFRLLNTEFFYFPSYHGLYN

ETELELLRQSQLNVAYQIMYHLLVVKQPISDAEVDGYQSSELLEEPDYIKEQRIQFQA

QEGLEQPFYMEHLSPISRKEESTVTEQKLLLPKNRKRSEPATNRRLKSKRKSTIQKPK

KKKKRI"

CDS 2864776..2864913

/locus_tag="EFAGFIKM_02435"

/inference="ab initio prediction:Prodigal:002006"

/codon_start=1

/transl_table=11

/product="hypothetical protein"

/translation="MKKSFLFLFTALVVVTSFSAYTPVSNASGDIGVSLLSDRQQGTV

K"

CDS complement(2864993..2865295)

/locus_tag="EFAGFIKM_02436"

/inference="ab initio prediction:Prodigal:002006"

/codon_start=1

/transl_table=11

/product="hypothetical protein"

/translation="MDFMKTVTGRFRIAGKWEGVSLLLLIFIAMPLKYFADISSAVAV

MGMIHGILFPLYLIALVHLALVKKWKITCWLMGGLAGLLPFGTFVFESYLRKRDWK"

CDS complement(2865492..2866460)

/gene="ccpB"

/locus_tag="EFAGFIKM_02437"

/inference="ab initio prediction:Prodigal:002006"

/inference="similar to AA sequence:UniProtKB:P37517"

/codon_start=1

/transl_table=11

/product="Catabolite control protein B"

/translation="MSKFDEIMKRSGYSKATVSRVINHSPHVSDEARQIITDIMKQLN

YIPNRNAVSLSTGQTKQIGIVTSTTGEIILTFMNQFIDTAMDFGFQTIIYTSRGDPEI

ELQAFEDLRSKRVDGLVIIACVNDPRKLKAYMEYGPIVSWQRMGNDEIPSVAMDQAEG

YKLALEHLVTRGYTRIANAFGRAESLNTQSRREAYESFMKSRGLHVVWTEAYQYSVFSS

SDGEEAMRRMAEGPELPQAVLCSNDYAAIGILSEARRRNIQVPEQLAIVGFDDIELSR

VLGITTIHNPIAEQATQAFHQLWAVLGKTELQTEQLTYQLIERETT"

CDS 2866804..2868228

/gene="bglH_9"

/locus_tag="EFAGFIKM_02438"

/EC_number="3.2.1.86"

/inference="ab initio prediction:Prodigal:002006"

/inference="similar to AA sequence:UniProtKB:P40740"

/codon_start=1

/transl_table=11

/product="Aryl-phospho-beta-D-glucosidase BglH"

/db_xref="COG:COG2723"

/translation="MSNHTYQFPENFLWGGAIAANQAEGAYNQGGKGLSTQDVAPKGI

MGPITEEPTEDNMKLIGIDLYHRYKEDVKLFAEMGFKVFRTSIAWSRIFPKGDELEPN

EEGLQFYDDLFDECHKYGIEPLVTLSHYETPLHLSKEYDGWVNRKMVGFYERYVTAVF

KRYKNKVKYWLTFNEINSILEAPFMSGGIYTPKEKLSKQDLYQAIHHEFVASASAVKL

CHEIIPTAQIGCMMLSMPTYPLTPNPDDMIKVMEFEHSNYFFGDVHVRGRYPGYMKRY

FRENGIEIQMEAGDEEMLLNTVDFISFSYYMSICQTADPEKQIAGEGNLLGGVPNPYL

PASEWGWQIDPQGLRYVLNMFYDRYQKPLFIVENGLGAVDELITGADGEKTVEDDYRI

QYLNDHLVQVGEAIEDGVEIMGYTSWGCIDVVSASSAQLKKRYGYIYVDRHDDGSGTL

ERYRKKSFHWYKEVISSNGKSLQR"

CDS complement(2868409..2869014)

/locus_tag="EFAGFIKM_02439"

/EC_number="2.3.1.-"

/inference="ab initio prediction:Prodigal:002006"

/inference="similar to AA sequence:UniProtKB:Q5HCZ5"

/codon_start=1

/transl_table=11

/product="Putative acetyltransferase"

/db_xref="COG:COG0110"

/translation="MMTEKEKSQLGLLYNANYDQELIEERLHAKGLCYDYNQLHPAKI

NEREALIKKLLGKTTDRFLIEQPFVCDYGYNIEIGENFYSNHNTVMLDGGKISFGDNV

FVAPNCGFYTAGHPYDVEQRNEGLEIVGPITVGNNVWIGGGVTVLAGVTIGDNTIIGA

GSVVTKSIPSGVIAAGNPCRVIRKITEEDKTKYSRDVVKNK"

CDS complement(2869249..2870412)

/gene="pbuE_3"

/locus_tag="EFAGFIKM_02440"

/inference="ab initio prediction:Prodigal:002006"

/inference="similar to AA sequence:UniProtKB:Q797E3"

/codon_start=1

/transl_table=11

/product="Purine efflux pump PbuE"

/db_xref="COG:COG2814"

/translation="MNKKVYVLAIAAFVVGTVELILGGILDLIATDLHLSLAKAGYLI
SIFSLVYALSAPILLNMTARFERKKVYMCTLFVFLISNLLSAFSTSFYMLMAGRALGA
AAGSLIFVLSLTLAARIVEPQYKGRAVGIITMGGSASLILGVPLGIFVGNLAGWREVF
MLIAILTAVVMVAIWIAMDRVQPIPAVSLKKQLTALWNPKMLAIHATTLLVLAGHLTL
YAYFTPFLQETLGASATMVTFIYMMFGIAAVAGGGIGGMLSDRLHPAKAIIIVLIPFI
VSMAVIPFSVGLPLIAFLLLLSIWSALSWTVTPVQNSLIIKTSPETAETLISTNSGIA
HAGIALGTYIGGMVIDHSSILNTGWVGSVLILLGLVSAIYAISVKEKTVQAVA"

CDS complement(2870539..2871291)

/locus_tag="EFAGFIKM_02441"

/inference="ab initio prediction:Prodigal:002006"

/codon_start=1

/transl_table=11

/product="hypothetical protein"

/translation="MLQKFGFTQYESQVYEAIFAQDEPLDATSIVNYSNVPKAKIYEV
LNRLIDKGTVLTTMDGKKKLYMAVDLQSIIHKIKADFEKDIEELKSYKIKRTFTDEHI
WTLKDESSIASNIEQLIEEADSSILFLAWNERMEKYRELLEQKEKQGVYVEVLAVGGM
ETSLNRIYSLIPLLDAPEPSQLLIVDHAYLLFAGVEHDSWRAIKTMSKPIVKAMTDYF
YHDVALTQITKKYGDQLLQDAEIEKLLTRLIY"

CDS 2871498..2871626

/locus_tag="EFAGFIKM_02442"

/inference="ab initio prediction:Prodigal:002006"

/codon_start=1

/transl_table=11

/product="hypothetical protein"

/translation="MESTQDFVKKVNENAEKERHNKNNGKGTPGDKLPSKQHSTNK"

CDS 2871722..2871982

/locus_tag="EFAGFIKM_02443"

/inference="ab initio prediction:Prodigal:002006"

/codon_start=1

/transl_table=11

/product="hypothetical protein"

/translation="MARVNAVPQPDLVLIYWSRNPLIPGSARRIQSVRVIGNTSPCTF

TLVPGARLINALNCLLDNDIGFKVVYRYKTSEISGVLLLKRK"

CDS complement(2872065..2873708)

/gene="lipO_8"

/locus_tag="EFAGFIKM_02444"

/inference="ab initio prediction:Prodigal:002006"

/inference="similar to AA sequence:UniProtKB:P37966"

/codon_start=1

/transl_table=11

/product="Lipoprotein LipO"

/db_xref="COG:COG1653"

/translation="MNISFKKFSLLTLTIVLATTLAACSSGAGSAEKEAEPNTQQDAG

GPLTKYDPPIKLTSTMNETGKESLAQGDTHANNIWTRGYKDELGIDVTYEWIVPDANY

NDKMNVTLASGDLPDVLKVSAVQFEQLHEAGMLEDLTEVYDKYASELVKEFMSAEDGA

GLKPVTKDGKIYAMVSFPGSLDSSDMIWIRKDWLKKVGLEAPKSMQDVIQIAEAFTFE

DPDGNGKDDTYGIALNKDLPINAFLLGYHGYLETWIKDASGQVVNGTIQPEVKEGLRE

LQTLYQKGVLDPEFGVKDFAKMMEDVNAGKSGMFFLPQWAPFQVSSMIKKEKNIDWMA

YPVQSIDDQPAKTQNHLSLGGIFAVRKGYEHPEALIKLLNFQAEKMFGESAKEERAAY

LNGLTGLGFHNATVSNLPANKNVKAQDEVEQALKTGDTSGLELEAKLFYDDIMDYRNG

NLDKWHMERIFGPESSQGVIKYYRDNDLIVMNELIYAPTRTMNTKQATLDKLRAETFL

KIIYGNLSIDEFDNFVANWKKLGGDQITQEVNDTIAANQ"

CDS complement(2873828..2875507)

/gene="rhaR_22"

/locus_tag="EFAGFIKM_02445"

/inference="ab initio prediction:Prodigal:002006"

/inference="protein motif:HAMAP:MF_01533"

/codon_start=1

/transl_table=11

/product="HTH-type transcriptional activator RhaR"

/translation="MQRMLIVDDEPVILDGLYAFFQKANFQDMEIIKAYSAFEAIDWL

NSVKIDIVLSDICMPGMDGMQLIEQIMDRWPRCKVLLLTGHNEFDYAHQAIRNPCVVD

YLLKTEGMNHIRAAVERALTQISEENDFRYQAAWFRSKLPRALPQLQRQLLLDVLKRT

DRQDIVSLQEELDAVQLPFESDQLVIPVIIRVEEWNNYQSQADRSLIRYAVANVAEEL

LQDKAKVKAIDLDTQVIACFIQSQETYPSYVSTTQGERGSQLESWVQTLRFVYGTLES

VQQSCADCLNLSVSIMVSEQAVEWSRVNQAIGQLRLSIHEGPGLGMEKLLRVNIQDEL

PYTPSIMNQQGVVTLHLDQIKQRITAGDREWIDSFHKWTEAAKEGLPDPFFRMKTYTG

AASVLIEVLHEMGLYESAMEEISLSRMLHFDIHTAWTDLVIFYQSAYEWMISKRSDAR

LNDQSQILITIHHYIKHHLEDDLSLTRIAQKVSLNPSYLSRWYKRITGKGISDYIHDR

RVERSKELLLGSSCKMHEISAKVGFSDQHYFYRFFKKATGCTPQEYRDQKS"

CDS complement(2875512..2877314)

/locus_tag="EFAGFIKM_02446"

/inference="ab initio prediction:Prodigal:002006"

/codon_start=1

/transl_table=11

/product="hypothetical protein"

/translation="MAPFLLLMREREERRREILRFIRGRWRESSIVVKLMIAFVLVIL

PLYGISIMITQVSSKQMQTEVEKAHESKLYFYHNHLQFELERMSALVTEFSFDETMST

LSTRAAIMSRYEVTSSLNNIHNKLGQIMVTSPYISDVVYYVPALHKRVSAADGIRDVE

DNEWQNLLATMGNLNGELSHSNNDLYFLKSNPYNMNHEEPPNFILGIRLSAEELKHRL

QQLSETGESEITLSFGEQQHVVISSSDQPAPVKPIRTVSPEPSESMQVTKFQSDPYLY

YSLHDKDHSFTLTATIPQDVLRAPLEQYNVWLWMVTFMSVVIIIVFSFSIYRSIHQPL

AVLIRGFRMAEQGQTSVHIPSSRRDEFGYLYSRFNHMIERLHILIDENYVARIRTSEA

ELKHLQSQIQPHFLYNSLFSIKQMAEVENVELIQEFTDYLGQYFRYMSRDSHTEVSLG

AEIDHALVYASIQKIRFGSRIQLELEDLDARYKMISIPRVIIQPIVENVFEHALAKRS

QGGILQLSYQQDQDNLSIRIEDDGDQLSDETLNYLQSAFEEIERKHGNEEITGLMNVN

QRLRIKFGAAYGLTAQRSELGGLCILVKIPWKGE"

CDS complement(2877356..2878246)

/gene="dasC_5"

/locus_tag="EFAGFIKM_02447"

/inference="ab initio prediction:Prodigal:002006"

/inference="similar to AA sequence:UniProtKB:Q9K489"

/codon_start=1

/transl_table=11

/product="Diacetylchitobiose uptake system permease

protein DasC"

/db_xref="COG:COG0395"

/translation="MHHASKAYRWFLGFNYVILTILALLCLFPIVNILAISFSSSDAV

KAGSVTFWPVDFTLSSYQYILENQQFLNSFGTSLLRVVLGVTTNLIFTILVAYPLSKE

AAKFRSRTFYAWIFVFTMLFSGGLIPGYLVVKEAGLLDSIWALILPGAVPIFNVLLML

NFFRGLPKELEEASWMDGAGHFRTLWSIYLPISLPSIATITLFALVGHWNAWFDGMIY

MKSPEGYPLATYLQSMLQQVTMIQSEMMTLEDATLLSQVSDRTTQASQIFLSVIPILL

VYPFLQRYFVHGLVVGSVKG"

CDS complement(2878262..2879167)

/gene="yteP_22"

/locus_tag="EFAGFIKM_02448"

/inference="ab initio prediction:Prodigal:002006"

/inference="similar to AA sequence:UniProtKB:C0SPB3"

/codon_start=1

/transl_table=11

/product="putative multiple-sugar transport system

permease YteP"

/db_xref="COG:COG4209"

/translation="MRSNYKQFIRNVPLHLMILPGLLIIIVFGYIPMAGLSIAFQNFS

PIAGFKNMNWVGLDNFRYLFDLPGFSQVVWNTVFISTMKIVSGLVIPVLVALLLNEVR

KTGFKRTIQTVIYMPHFFSWVILAGIIVDVLSPSTGIVNLLLKAVGIDPIQFLASNEW

FPYILVITDQWKEFGFGTIIYLAALTNIDKSLYEASVMDGAGRWKQTWHITLPGIRPI

VILMVTLSLGNVLNGGFDQVFNLYNPLVYESGDILDTMIYRIGLQDAQYSVSTALGLI

KSVVSFIFIGLGYFLAYRFANYRIF"

CDS complement(2879316..2883056)

/locus_tag="EFAGFIKM_02449"

/inference="ab initio prediction:Prodigal:002006"

/codon_start=1

/transl_table=11

/product="hypothetical protein"

/translation="MSTYQNQLIAQYTFEDTVQIGKDSSGKGHDGIAKGEIPPVISEL

KGRTAVTFNGGANGTSYLQLPSNLLDDVSDNTGITIATWVFLGKGSNVWERIFDFGKG

EKGPYLFLTRQLLGTLYAGDNLVVHPSGGVASGEWLHIALSVAGSQGGTLSSAGPIVY

VNGEKAADGSISQTSSGNYAKLREWFDSFTDPENYSQNYIARSQYAADVDFAGSLSDF

RIYGAALTMDEVIEVMCESLTDEAIVKLAADKYLSFPNRIITKDVSLPADLLGGKVSV

EWSSSAPEVLSENGEVQVITSAQEVTLRALLNRGDRKLSQSFDVSVVPAHLPPYTVTI

HGDQKVADISEVMYGLFYEDINNAADGGIYAELVQNRSFESFAFDTYSHDSGECGCST

GRNREPLFAWSGDTEKMRVQHTDGLNVHFNVEDPEVNAYYVTVQDGATIRNRGFSDSN

QHCAMSIKQGESYDFTVWAKAELAGTITVQLQDGSDTSISDSVTLHVEGGNTWKKYGL

SLTGTETVLGQLALMFAGDISIDMVSLVPQNVWGADPTDEGISATAHANYTGNPNYRL

RKDLIQALADLHPKFLRFPGGCISEGSFIWDNVYDWKDSVGPVELRKENYNVWGYMMT

MGLGYMEYFQLAEDLNAAPVPVMACGVLCQARSDYAHPAGGALRDYYIRNFTDLIDFA

LSTDFEHNEWAAVRSQMGHPEPFDLRYLGVGNENWGTEFFANFEVFKRSIDDYMKQNY

PDHELHIISTVGAQADDDAYQEGWKFLSGNLTGSAQVAFADGAEVIEETVTWYENQDN

YMDTIADEHYYRSNEYLLNNVDRYNYYDRAYLEDGSIDWKETSKVFVGEYASTDKNTL

AGAVAEAAIMTGFENNADVVRLAAYAPLFNKVLTDGTYRWTPDCIWFDDETVWYTPNY

YVQQLFAKYVGDQVLETSFSTYNKGKPLSLIPRGGIEIATGHADIVVKRVTVTSNVDG

SLMFDEDFRERTEPSETWNQIPGSEGYTLTEGKGLILKAQSSGLNGLYLLNDEWTNYK

VHVEAERISGEDGFYIGVGLTDISPENKDVIEYAISYGGNATGVKVYKQGIEGYTLGD

YSSSSAAGNLRAANYEPLENGTDYTITVNYGGDTGKNLVCSYTDGRNTSKVLDYKLEA

YNREVFHSVTKDARHVYVKLVNADGVDKSTRICLQDLKVNSSAKLITLTGEDHLVHVP

NVNQKNDEKVIPQEQVITLSDTAVVVNLTAHSVNVLVMDILN"

CDS 2883442..2885859

/locus_tag="EFAGFIKM_02450"

/inference="ab initio prediction:Prodigal:002006"

/codon_start=1

/transl_table=11

/product="hypothetical protein"

/translation="MKRYWVRVLNISVLSTTLLTATTLAPVSVFANGSGATLGNSQSS

TTPIANSAPSFKNVSVHDPSVIKVDDTFYIFGSHLQVAKSKDFLNWDLVASGVTDNNP

VVPNVTKEFAEALEWAQTDTLWAADVIQLADGKFYMYYNACKGDSPRSALGIAVADNI

EGPYKDQGILLKSGMWDQISEDGTIYDATIHPNVVDPDVFYDKNGKLWMVYGSYSGGI

FILEMDATTGKPLPNQGYGKKLTGGNHSRIEAPYMLYSPETDYYYLYLSYGGLGADGG

YNIRVARSQTPDGPFLDAEGNDMINVKADKDKPLFDDRSIEPFGVKLMGNFLFERQIG

DPGTGLGSGYVSPGHNSAYVDPETGKQFLIFHTRFPGRGEEHEVRVHEMHMNADGWPV

VSPYRYAALGEDTTQLTTQEIAGQYHWVNHGKDITADIKPSQTVQFTADGQIHGAVTG

TWSLGADNKVQITSNNVVYKGVFTHEWNSESQSTVLTFSALSSSGISIWGSQGVERSD

QDIVDAVKKDLSISNTDHVFFNLTLPTKGTSDAEITWKSSNTSALSTTGVVQRPRAGK

GDAKVKLTATIRKGTAVSSKTFNVIIPQQAVSPLLGEYTFEHKKLGKVAQDYSKNEYH

GQAFNVVSSAASGKNQAATFNGTDSFIQLPGLITDTTDFTFSAWVNWDGGGSWQRIFD

FGNGLTRHMFLTPSQHNGALQFTIHNEGRDQSLIAANPLPSKEWVHVAVTLQGDVGTL

YVNGTSVASSSEITFNPKDLQVTEAYVGKSRYTADPFYKGSLDNVKVYDKALTAKEIQ

RQAKEKP"

CDS complement(2886000..2886554)

/gene="sutR_2"

/locus_tag="EFAGFIKM_02451"

/inference="ab initio prediction:Prodigal:002006"

/inference="similar to AA sequence:UniProtKB:P77626"

/codon_start=1

/transl_table=11

/product="HTH-type transcriptional regulator SutR"

/db_xref="COG:COG1396"

/translation="MKNINSILAQNLKQLREQRKLSLDKVAEMSGISKTMLGQIERGE

SNPSIATVWKIANGLKTSFTALIHEPKSDTTVVTGDDIQVLMEDEGRVRIYPHFPFEE

GRRFEIYMMEMDVDSSLSAEPHIEGTEEFITVFEGEVTIRVGAEEYTVNQGESIRFRA

DKPHAYINPGAKTSRLNMVIHYSK"

CDS 2886685..2887266

/gene="eamB_2"

/locus_tag="EFAGFIKM_02452"

/inference="ab initio prediction:Prodigal:002006"

/inference="similar to AA sequence:UniProtKB:P38101"

/codon_start=1

/transl_table=11

/product="Cysteine/O-acetylserine efflux protein"

/db_xref="COG:COG1280"

/translation="MNIIPLVTYAIIASFTPGPNNIISMTHARNQGFRKTLPFISGVA

AGCLLIMFLSSYFNLILHQYIPRIAPVLNVLGCVYMLYLALKIMRSKPADSQENKVNH

YSFLFGFTLQFINPKVILYGLTAISVFVLPLGQSHVQLIVFSLLLTFIGISANMTWAF

GGMLFQSFLLRYERPVNIVMGMLLIYSALSILM"

CDS complement(2887320..2888297)

/locus_tag="EFAGFIKM_02453"

/inference="ab initio prediction:Prodigal:002006"

/inference="similar to AA sequence:UniProtKB:P9WKP3"

/codon_start=1

/transl_table=11

/product="hypothetical protein"

/db_xref="COG:COG2220"

/translation="MTKYENQLPTSAGMNFKSLISILRDSMRGNVERRPSGMMPMEIC

EPAETFSASDNPQVTWFGHSAFLLEIEGHRLLFDPMLGNRPSPVSWVGTKRYSTNLPI

KPEDFPTLDAIIISHDHYDHLDYDSIRRLKDKTKRFIVPLGVRRRLIQLGVPSEQITE

HNWWDELSFKGLTFACTPARHFSGRGLFDRNSTLWCSWVIVGQHTKVFFSGDSGYGPH

FKEIGSKYGPFDLTLMECGQYDERWSNIHMMPEETVQAHLDVKGKLLIPIHWAAFTLA

YHAWNEPVERIMKAAHALNVKIATPKIGEKVVLHTEQYPTHPWWRPELG"

CDS 2888497..2889474

/gene="rutD_4"

/locus_tag="EFAGFIKM_02454"

/EC_number="3.5.1.-"

/inference="ab initio prediction:Prodigal:002006"

/inference="protein motif:HAMAP:MF_00832"

/codon_start=1

/transl_table=11

/product="Putative aminoacrylate hydrolase RutD"

/translation="MKKWRKRLVKFLIFDLAIVLLLLGTGVIYQQVGLRQDAKTLVPP

GKLYEVHGDNMHLYTGGEGDVTVVLASGWGTANPYVDYYPLYEKLAPNTKFAVYDRFG

YGYSDQTDKKRDVDTVAEELHELLQVSGQKPPYVLVGHSLGSLETIRFAQKYPDLVKG

IVIIDGGSPEYYSNDDDSLADTIGGFANKFRIQTGLFRLSMQSDMVVETSNANRNELK

LVPDDLKKLDTTALLHNYGNVNTLDELREIAANAKVVVDHKQTLPFPLTILTADYLGA

SEPEWDKSQVEFKSWSDDSKQVTIKDTDHYIHQYRPDLVAEEILALVKK"

CDS complement(2889562..2890209)

/locus_tag="EFAGFIKM_02455"

/inference="ab initio prediction:Prodigal:002006"

/codon_start=1

/transl_table=11

/product="hypothetical protein"

/translation="MIIINPISNNIEKMLLHYEKNGHLTLQASLISNSSVVYRLQNYC

LKVYASRARLDGEMESEALRELQSNPYAPKLYAHSPGEYTLTEWIEAFKLKEYRVTYG

HIPPNLIYDMFTTELQQIHAGYWDWDVIRYENLLWTKTGDVKRTNFWLCEPVNSRREN

LYNQVIRRIDNIYNGDRTEMEAMKQYFYRHQLTSLEIEQAFSDFRSQRPRLAIAQ"

CDS complement(2890516..2891997)

/gene="emrY"

/locus_tag="EFAGFIKM_02456"

/inference="ab initio prediction:Prodigal:002006"

/inference="similar to AA sequence:UniProtKB:P52600"

/codon_start=1

/transl_table=11

/product="putative multidrug resistance protein EmrY"

/translation="MKADSAQNQQETKQYKVFPILFAMLLSGFIGLFGETALNVALTP

LMALLEVGPTTIQWLTTGYLLVLGILVPVSGMLLQWFTTRQLFTTSLIFSIAGTFVAA

LAPSFEILLVARVLQAIGTALLLPLMFNTILVIFPIEKRGAAMGLIGLVIMFAPASGP

SISGLILANLSWHWIFWISLPFFLISLVCGLLFLPNISKLTKPKIDVLSIILSTLGFG

GIVYGFSSAGGHGETGGGWTSPVVVATLVIGVLSLLLFSIRQLRMKQPMMDLRAFKYP

MFTIGLILIFICMMMMLSSMLILPMYLQQGMAVTALTAGLVLLPGSLLNGLLSPVMGR

LFDKFGPKWLVIIGLAVVTVVLFMYTGITPTTTLAKIITLHVFMMVGISMIMMPAQTN

GLNQLPREYYPHGTAIMNTLQQVSGAIGTAVAVSILSAGQSRFLSGVANPESPENQLA

GFTSGVQNAFVFALVLSIIGLIASLFVKRVKVGSTPGQQGPMH"

CDS complement(2892066..2892653)

/gene="yxaF"

/locus_tag="EFAGFIKM_02457"

/inference="ab initio prediction:Prodigal:002006"

/inference="similar to AA sequence:UniProtKB:P42105"

/codon_start=1

/transl_table=11

/product="putative HTH-type transcriptional regulator

YxaF"

/translation="MSEKSNARESIVSTAARLFFSQGYHATGLNQIIKESSTPKGSLY

HYFPQGKEELAHECIQKANEHILQTFEKTFAAHDNTGDAIQRFIHDLAHETEAAGFTG

FLPFSFWAAVETSCISQQLRIACQGVFADWQDVIKKHLILDGVGEEKARETALLIISL

MEGALIISLTNQDKQPLLTAAEYLSVVTKNAKQIQ"

CDS 2892778..2892891

/locus_tag="EFAGFIKM_02458"

/inference="ab initio prediction:Prodigal:002006"

/codon_start=1

/transl_table=11

/product="hypothetical protein"

/translation="MTRATAHGQIIITAANLETKLQIKRLFVSLSILRIVY"

CDS complement(2892915..2896169)

/gene="cypD"

/locus_tag="EFAGFIKM_02459"

/inference="ab initio prediction:Prodigal:002006"

/inference="similar to AA sequence:UniProtKB:O08394"

/codon_start=1

/transl_table=11

/product="Bifunctional cytochrome P450/NADPH--P450

reductase 1"

/db_xref="COG:COG0369"

/translation="MSDATSIPQPKMYGPLGNLPLIDKDKPTLSLGVLAEQHGPIYRL

TVPGYSGLIVSGPDLVAELCDVSRFDKYVYNELENVRAFGGDGLFTSRTSEPNWKKAH

NILLPTFSKQAMKGYHPMMVDIAEQLINKWARLNSNDTIDVADDMTRLTLDTIGLCGF

NYRFNSFYRQDHSPFIESMVRALNEAMQKSSRLKIQNLLMVKTKRQFQEDIQTMFSLV

DQIIEERKASSAPEEVDLLARMLNGKDPETGEMLDDENIRYQIITFLIAGHETTSGLL

SFALYFLLKNPNSLQKAYDEVDQILVSDSPQYEEILQLSYIRMILSESLRLWPTAPGF

DVYAKEDTIIGGKYPLKKGESCSILLPQLHRDREAWGEDAELFRPERFEDTTKVPHHA

YKPFGNGERACIGMQFALYEATLVLGMVLKHFELIDYSNYELDVKQTLTLKLGDFRIQ

VKARAASQTRNKSTVSAKEKPAPVVHKNIASDSQRGEILAVEADAKPSLLVLYGSNLG

TAEGIARELAEKGRSYGIPSEAAALNEWMGRLPRQGVVLIVTASYNGKPPQNATAFVD

WLKGTESEEARDVNYAVLGCGDRSWSGTYQSIPRLIDEKLEELGGKRLLSRGEADAGG

DMEKQVEAWQHMVWPQVLSVLGISEETIKSSKPSASRLQMEFVREKTDMPLARTYDAS

YATVVVNKELQALGSGRSTRHIEVLLPEGMNYREGDHLGVLPSNQKQNVDRILSRFGL

RGDTPIQLTSDVSHLTHLPLNRPVKIHELLAYCVELQTPVSRTQLQELANHTICPPHK

RELEGMLDENSYRDYILANRITMLDLLERYEACELPFERLLELLPPLKPRYYSISSSP

SLNSEQASITVSVVREPAWSGKGEYQGVASSYLAGCQSEEDILMFVRTPESEFYLPKE

ADVPMIMVGPGTGVAPFRGFLQARAVMKQKGLPLSEAHLFFGCRNESDFIYRHELEQF

ERERIVKLHTAFSRADGKPKTYVQHLMKENSEQLIHMLMNKGKLYVCGDGSQMAPAVE

ETLQQAYQEVQGATMQQAKDWLIQLQAEGRYVQDVWAGNNRTGETPKAVHEEVH"

CDS complement(2896469..2897437)

/gene="trxB_1"

/locus_tag="EFAGFIKM_02460"

/EC_number="1.8.1.9"

/inference="ab initio prediction:Prodigal:002006"

/inference="similar to AA sequence:UniProtKB:P56431"

/codon_start=1

/transl_table=11

/product="Thioredoxin reductase"

/db_xref="COG:COG0492"

/translation="MIRDIECLERVYSMSNMNQLLDVLIIGGGPAGLNAALVLGRARK

NVVVIDDETPRNWVTRETHGFVTRDGASPREFRKAAKEQIAAYPSVQFASDTATAITG

SDGDFEVKTTQGASYRTKKILFAVGKKDLPLDINGLTEVYGKSAFVCPYCDGWELRDQ

PLVIIVSGDKALHMAKVISGWTERYTICTNGSDSLTDEQREELKRHNVTVFDAPIQSI

NSEEGMVQQVVLNDGTEIPCTGVFFQPKLFTGSELPKAIGCEITESGTVIVDVSGKTS

VAGVYSAGDAASEMYQAITAASLGALSAVSINNELNFEKWDEPSHR"

CDS 2897852..2898781

/gene="ctaB2"

/locus_tag="EFAGFIKM_02461"

/EC_number="2.5.1.141"

/inference="ab initio prediction:Prodigal:002006"

/inference="similar to AA sequence:UniProtKB:P24009"

/codon_start=1

/transl_table=11

/product="Protoheme IX farnesyltransferase 2"

/db_xref="COG:COG0109"

/translation="MKTLNNGSQTNISLEYSSIPKVFFDTIKIGIIKSNLIAMFAGLS

LALYVFDASILANIVPIILSFIGSSLIIGAAGVFNNLYDRDIDAIMERTKKRPTVTGL

VDTKSGLILGIAITIIGGIMLYIASPSAAVFGILGLLLYVFPYTMWTKRTTIYNTEVG

SLSGAVPPLIGWAAISPELGHFEIWALVVTMLLWQMPHFYGIAIRRFDEYKAAGVPML

PVVKGIRRTYIQTNVYLVLLLISSFLFWPMSPFVAIVAFLVSLAWLILSVATFDKKET

EKWSKRMFIFSINHITIIFLVIIAYSLIAQMMR"

CDS complement(2898898..2900667)

/gene="yheI_1"

/locus_tag="EFAGFIKM_02462"

/EC_number="3.6.3.-"

/inference="ab initio prediction:Prodigal:002006"

/inference="similar to AA sequence:UniProtKB:O07550"

/codon_start=1

/transl_table=11

/product="putative multidrug resistance ABC transporter

ATP-binding/permease protein YheI"

/db_xref="COG:COG1132"

/translation="MTVAGFIGRLFRFRPLLFVINGLLWCIFHSLPLAIGIGMQWFFD

RTTAGSNDYMWLSVPLIFIALVRMARVGTFFVAFYAWVTYLYHVQAILRTNMLAAIMR

WPGRNLPASPGEAMSRFRDDVDEVVEYVESWVDFWGRLVFAVVSIVIMANINWQITLV

AVLPLVVVTLLNNLSGNRARKYAQVNREATGRITSFIAETFGAVQALKLGQAEENVAS

RFNQLNEDRRQAALRDNLFKQWMRSMNQHVLSICTGLILLMCAAEMKAGNFTVGDFAL

FTSYLANIGFSISLFGYMVFQHKRLKVSYDRMRKLFRPGEEDQIMDSREIYLYEDPPE

LVSEQRDPKEKLQSLEVNKLTYQYPNSENGIEEISFRLKRGQFLVITGRIGSGKSTLV

RTLLGLLPKQKGDIHWNGAAVDPATFLMPPRAAYTPQVPRLFSDTLKENIVQGKQGNT

EQALEKAIRLAVMEKDIKHLDQGLETPVGPRGVMLSGGQIQRAATGRMLMTEADLFIF

DDLSSALDVETEQQVWEGLFQEPDVTCIAVSHRRAALSKADHIIVMKDGRIEAEGSLA

ELLATNEEMQLLWQGEQTPAKVG"

CDS complement(2900664..2902409)

/locus_tag="EFAGFIKM_02463"

/inference="ab initio prediction:Prodigal:002006"

/inference="similar to AA sequence:UniProtKB:Q9WYC4"

/codon_start=1

/transl_table=11

/product="putative ABC transporter ATP-binding protein"

/db_xref="COG:COG1132"

/translation="MPKTEKRSMSWLLRYLKPVKGRLALLLIMLLTSTGLQLLNPQII

KRFIDTAASGGVLANLVQLAGIFLVVAVFNQLITVAVSYLGNDVAWRATNQLRGDLLK

HCLRLDMRFHNVKTPGEMIERVDGDVTSISNFFAMFIVQVIGSFVLLAGILGFMFSVN

VPIALVMTVFTLLSILFMVFIRNLGVDSSKNERAASASLFGLIEERIAGIEDVQANGH

VPYVMNRFYRTMRTVFRKGRKAWLLRVIPWNTTVVLFALAVTAVLLLGVHYYMEGLIS

IGTLFLIYQYTQMLNDPIEMLGDQVQEFQKAKSGMLRSRELLSMHSVIEEGTEEQLPE

GPLGLEFSQVHFSYNQDKPVLQDITFAIKPGERLGIIGRTGSGKSSLSRVLLRLYNLD

RGTIRVGGTDITKLSLQALYRRVGMVTQDVQLFDGTLRDNLTLFNGDVSDQMIKETTD

RLGLSQWINSQPEGLNTYLAAGGASLSAGEAQLFALTRVFLTEPSLVILDEPSSRLDA

ATEGMLQSAIDQLMKRSTGVIIAHRLATLEKVDRIMVLGDGRVLEFGAREELASNPAS

HYARLLITGREEELA"

CDS complement(2903028..2904263)

/gene="mdrP_1"

/locus_tag="EFAGFIKM_02464"

/inference="ab initio prediction:Prodigal:002006"

/inference="similar to AA sequence:UniProtKB:A0A1C7E424"

/codon_start=1

/transl_table=11

/product="Na(+), Li(+), K(+)/H(+) antiporter"

/translation="MKQYLRQIHPLAWTIIIGTMFGRLVTSMSIPFLSIYLTRVLDAT

PTQTGITVAVSSLAGVMVSFYGGYISDRIGRKIVMLISVFSWAGVFFIFSAAEHLWVF

FVANTLNGLCRAVFEPTSRALLSDITSPENKLLVFNLRYAAINLGVVFGPIIGFQLGS

SESTFPFVISGLVYIAYGLVLFLQFKLQHANLPERHQATAPRLREALMTTGRDRVFLP

VLIGTTFCVLGYGHFSSTLAQYLARSPIFENGSQMFTYMLSLNAVTVLIIQYPLVRTF
RNFPPLVPLIAGNLLVATSLMMVGIAEGVLMMMMSVILFTIGEVLLFTMMDMLIDRIA
KPEWKGTYFGTIGFNNIGSVIAPVMGGLLLSQFGAENGLAVFLPIALTTALGVPFLLI
AHRRLVVREKQTEPTSLSM"

CDS 2904593..2906359

/gene="sgrR_1"

/locus_tag="EFAGFIKM_02465"

/inference="ab initio prediction:Prodigal:002006"

/inference="protein motif:HAMAP:MF_01449"

/codon_start=1

/transl_table=11

/product="HTH-type transcriptional regulator SgrR"

/translation="MDTLHTHFIRLAGAEQLSFKLHEPVAVTIDSLSAALCCTPRNVK
FILRKLEEQGFIHWQPGRGRGHHSELTMLRSMNEALEASFTELLAKGKMKDAIELIGT
VQMDDTLREQLMLSLHQQMGFHGHDETASGQDILRIMRSRQLGGLDPAFVYTAFETYL
LSQVCNTLITYDAKTESFLPALAHMWECSEDHRLWTFYLRKGVRFHNGRIMTSRDVQA
TLQRLIDMHSPSIWLYRDIERAEVAGDYCIKFVLHRPNRFFLHLFSCIRMTILPYDYN
VANTLVGTGPFQISELNEDVLELTAFDAYYGIRPHLDQVHFWFVPDLSPNDRYYELPG
TDRLSLATGCDQTNSINYPALGCQYMLFNFHKEGIHHHPLFRQALRIVYDSVALVRDL
GGNRITPASSFLPWRSAAQDWTAASLEHARELLRNCGYQGEKITLSYKYNKDADVAEW
FQCRAASIGLNISMQAVVDYFNSEETTNAQLILAEEILEEDWQYGMIHFFKNLSNHFH
MCMSPAFQSLLDDKLDHFAKLHRADRTALLDEAEALLRDHCWILHGCHMNKQAELDQS
IFGMQIAEFGYLDISKLWIKNP"

CDS complement(2906439..2909066)

/gene="rcsC_7"

/locus_tag="EFAGFIKM_02466"

/EC_number="2.7.13.3"

/inference="ab initio prediction:Prodigal:002006"

/inference="protein motif:HAMAP:MF_00979"

/codon_start=1

/transl_table=11

/product="Sensor histidine kinase RcsC"

/translation="MQVQKVDHHELFEQIYNQAPIGIALVAPTGQWMKVNPAFCCMLG

YTSDELMDLSYQDITHPEDSPQDVICSYELLEGNSKENQYEKRYINKNGDILWTSLHV

SLVRSEITDEPLYFICHIVDITDRKMSEQKLLHTEEMFKLITDHAQEIIYIADQEGVC

RFCSPSVQGLLGYSPEEVIGQNNNAYFHPQDLERISQMDLTKGNLLNIRVRHKEGDYL

WFETTYKVFGDAEHGQQILSIGRDVSERKKQKDISAEAERIALIGSWEWDMVKDHITF

SDQIFEIFELERTCKPYRVSDVFDVMDSEDLASLQKHITGVKQGEPLDFEYKHISSDG

NEKYLHLRGLITLDENRQPVQLNGTIQDITERKRIEFKLQESVERYTSLKKYNHDAII

SFNMDGNIMNANPVAVKMTGCPVAEMIDTSISRFIGASNLGLILGSNYEMAEKEINAV

RHTDGSETEVLATLAPIIINKSNVGFYLIAKDITEQKKLLVAKETAERMNKAKSEFLA

MMSHEIRTPMNGVIGMTDLLLDTPGLSGEQKEYIEIIQKSGDSLLAIINDILDFSKIE

SGKTDLVEDPFDLVEIVTETVQIVKPLAREKKLDVRMCVEDAIPTPVYGDAYRLKQVL

TNIIGNAVKFTSEGGVEVKVGVKEQCGNNVQLYFQVKDSGIGIPAERKQQLFEPFYQL

ENFMTRKPQGTGLGLAISKKLVELMQGEIWIEESDEPGTIFIFTAQFKLNNGEESNRL

DQQQKKSRTSALRILIAEDNEVNQLVLSRIVEKKGHFVDHVADGVEGVEAVKHTSYDI

VFMDVHMPRLNGFEATKAIKNALHPESCPFIIAVTANAVRGDMENCLKAGMDAYVSKP

IKIESIMQALETYYIKNNL"

CDS complement(2909335..2911716)

/locus_tag="EFAGFIKM_02467"

/inference="ab initio prediction:Prodigal:002006"

/codon_start=1

/transl_table=11

/product="hypothetical protein"

/translation="MNNVLTEQADAGYRLAEQKASQYFTSLMQQLNDNTYTTALTQDI

HVWQKKHIHRFAWLSLLSPSKRKPDPRDVHRYIHWLNTTGKLDDYLDRSISYIYMRDL

GQALDSPGTQTRIQNVVQNTKKYFMGSATGRKGQPDYISLAALYRWGQKEHIETAVIW

VMNKLKNVASNIPKELDAEQAQRKLIKIILGVVLHVDDEMNEQTPPEERARRFDAAIR

LGYSYGLTYPFVDDLLDSQALTVQEKEQYSMMIRDALLTGVVPDLGEWKGSNLEVIEY

VHSELREAFEYIKNYQHPEKQRTFLEQSYVFFQSQEIDRNKKLANANYSNEELYIPII

IKSSSSRLIVRSVLSAPVDEGFDLRTFYYGIYNQLADDFADMFDDMEEEAVTPYTYYL

KYRDLRPDLINPYELYWAVISHLIHDVYNSDAKTREVILDRAINGLKRCKERLGQQKY

DEVMTIFASGQPEFNQLVQQMVRKADDVDFLDKLLRDQVVLQLKNDKQEKEEFKQTIR

TVREQINVELQIAKPGGLHEMKETLIDAANYSLQGDGKRLRPILTWVMGVREYGLPES

SIVPLLRSLEYMHTASLIFDDLPTQDNASTRRGRSTLHHVHNSATAELTGLFLIQKAI

GEQSSLDRFDAATVLKLIQYSAEKAEDMCMGQAMDLNSKGKALTLEQLNMICFYKTGI

AFEAALVMPAILAQVKEPEMATLKKFAYHAGIAFQIKDDLLDFEGNHLILGKPAGQDE

RNNNSTFVSILGDEGAKKEMWEHYCLATDALNEMPKPIPFLRHLLDYLVGREH"

CDS complement(2911904..2912701)

/locus_tag="EFAGFIKM_02468"

/inference="ab initio prediction:Prodigal:002006"

/codon_start=1

/transl_table=11

/product="hypothetical protein"

/translation="MDLLLFVIMFILGLVGSFFSGLLGIGGAIINYPLLLYVPSWMGL

EPFSAHQVSSISMFQVFFASLAGVIAFRRKVRTGRSGGAIVHRGLVLYMGSSILAGSL

IGGFISGHLDGRVINLIYGILAIMAIVLMLIPGKGKLDTSAPLVFNRWIAAGTAFAVG

IVSGIVGAGGAFILIPIMLTILNIPVRTTIASSLAIVFISAIGGVIGKITGGDIPMEP

IIYTVIGSLLGASLGSRVSSMINVRVLRYALIVLIAITAVKVWSSIL"

CDS complement(2913188..2913943)

/gene="linC_2"

/locus_tag="EFAGFIKM_02469"

/EC_number="1.1.1.-"

/inference="ab initio prediction:Prodigal:002006"

/inference="similar to AA sequence:UniProtKB:D4YYG1"

/codon_start=1

/transl_table=11

/product="2,5-dichloro-2,5-cyclohexadiene-1,4-diol

dehydrogenase"

/db_xref="COG:COG1028"

/translation="MTQHNGKVIIITGGASGIGKETALQLSDQGATIVVADYNEDGAK

KLAAEIETAGGTAGAYKVDVSKGDEIKALIDWTVEQYGTLSGIFNNAGIGLVKPFLEM

DPESYHRVIDVDQHSVYYGMYYGAKKMVELNVQGTIVNTASIYGSVAAVGSFNYNAAK

AAVVMMSKSGALELAEHGIRVVGVAPGFIETPILGDDQAMKDALATQHMRGELIQPEK

VASVVTFLFSDAASAVNGTTVAVDDGFLSFKTK"

CDS complement(2914193..2916697)

/gene="uvrA_1"

/locus_tag="EFAGFIKM_02470"

/inference="ab initio prediction:Prodigal:002006"

/inference="similar to AA sequence:UniProtKB:Q9WYV0"

/codon_start=1

/transl_table=11

/product="UvrABC system protein A"

/db_xref="COG:COG0178"

/translation="MRDAIRIKGARENNLKNISLSIPKYKLVVITGPSGSGKSTLAMD

TLQRECQRQYMESMGLTSDSISKPKLESITGLSPSISVGQHVTNRNPRSTVGIVTDIY

TFVRFIFSRLGERLCPACNGIISPTVEPAGHLAEEDEYMDHATMNCPHCSAVIEKLGM

SHFSFNKPEGACKQCSGLGHVATINEEAVFNPELSVREGGVASLNGVHRDIQIRILVA

ASKHFGFEFDADLPLKEFGEIERDLLYYGVDSEQFKRHYPPIKPIQGTKFEGVIPGLW

RRYKEKEGEAGTTEKDGGFFHQQRCPECKGARLKKEVLSVHVADVSISEVTNWSFSNV

LEWTLGLQEIISPEAHHLLNPILHELPNKLQRIMDVGLGYLSMDRQTVTLSGGETQRL

RLASLLGSGLTGVLYILDEPTTGLHPRDTVGLIRVLQELRDLGNTVLVIEHDIEMMRA

ADHVIDIGPGAGLNGGLVVGEGSLEDLMISEHSVTGAYLRNESVASPSYNRRTGTGQH

LAIRRAHLRNIDIPVVSFPLGTLISVTGVSGSGKSTLVFDILAQGMESGQKLTGCEEI

TGLEHVGDIVIFDQTPMGRMQRSNVATYTDVYTHLRQLFASLPDAKARKLTAKHFSFN

TPGGRCETCQGLGVLSVDMNFLPDLEVTCHDCRGKRFKEEVLQVQYEGFSISDLLNMS

VAESLPLLKIQTKIADLVEMLCEVGLGYLQWGQSVKTLSGGEGQRIRLAKELSKKTNK

HTLYLLDEPTTGLHPSDIKQLHVLLDKLVESGNTVVVVEHSLELIRESDWVIDIGPEG

GAAGGELVAAGTPEQVAEVQASYTGQFLKRILAEGL"

CDS 2916848..2917342

/locus_tag="EFAGFIKM_02471"

/inference="ab initio prediction:Prodigal:002006"

/codon_start=1

/transl_table=11

/product="hypothetical protein"

/translation="MSRDQQQQPTALQENFTLRMRSLGTRTVLYQQHVAASLGLYNND

FISVDILREKGPITAGELSKLTGLATGSVTTLIDRLEKNGFVRRENDPNDRRKVIIVP

LYENKEEVSNTYLTLHSAMVDLASTYTEEELALITGFLSKAGNVLDQQIDDLSSKIRS

KSSS"

CDS complement(2917471..2917947)

/locus_tag="EFAGFIKM_02472"

/inference="ab initio prediction:Prodigal:002006"

/codon_start=1

/transl_table=11

/product="hypothetical protein"

/translation="MIMFIRKRKHTLMMTGLIASSILLISACSVVEQANQSLNYVSGA

TDYIEQVSNAGADLQELASSAANNPEITTQIQEKIDLIQAEASEFSQLTAPAIGESIH

ENLVSYNTQLTEVVDNFENTIAEQGFTAENWEKTGIPELITNINNLKDPLSGLGNE"

CDS 2918077..2919051

/gene="panS"

/locus_tag="EFAGFIKM_02473"

/inference="ab initio prediction:Prodigal:002006"

/inference="similar to AA sequence:UniProtKB:Q8ZKL0"

/codon_start=1

/transl_table=11

/product="Pantothenate precursors transporter PanS"

/db_xref="COG:COG0385"

/translation="MIMLQALNQRLNRIMPLITPISIIIGVLCGSFLSSYTFLSPWLF

AFMTFAGSISLGIRDFVNVLKKPFPLFVCLFILHLAMPLIALGMGHLVFPTDAYTITG

LVLAAVIPTGISSFIWVSIYRGNIALTLSIILIDTMLAPFVVPGVLSLLIGTSVTLDT

AAMMSSLFWMIVVPSLLGMLLNEWTKGAIVPVWGPRLNPLSKLFMASVVAINGSVVAP

YLADFNWKLAGLAVIIIFLASFGYALSYFIARLLGWNEADQVALVFNGGMRNISAGAV

LAVSYFPPPVAVPVVLGMVFQQMLASLTGYLLGRRSQLLQKSDKTSAA"

CDS complement(2919154..2919414)

/locus_tag="EFAGFIKM_02474"

/inference="ab initio prediction:Prodigal:002006"

/codon_start=1

/transl_table=11

/product="hypothetical protein"

/translation="MQAAPNQAVHKPGSQPVSFGSWMLTLLLLAIPLVNIIMLFVWAF

GNSNPSKANYAKASLLWLAIGIVFYILILALGLTAAFSGTDY"

CDS complement(2919702..2920787)

/gene="asd2"

/locus_tag="EFAGFIKM_02475"

/EC_number="1.2.1.11"

/inference="ab initio prediction:Prodigal:002006"

/inference="similar to AA sequence:UniProtKB:P23247"

/codon_start=1

/transl_table=11

/product="Aspartate-semialdehyde dehydrogenase 2"

/db_xref="COG:COG0136"

/translation="MSAKLKVGIVGGTGMVGQRFVDLLDQHPWFEVTAISASANSAGK

TYEESVQGRWKLAVPIPEAVKKIVVQDASQVEAFASQVDFIFCAVDMKKNEIQALEEA

YARTGTPVVSNNSAHRWTADVPMVIPEINPGHLDVIEAQRKRLGTKTGFIAVKPNCSI

QSYVPALHALREFKPTQVVASTYQAISGAGKNFTDWPDMLDNVIPYIGGEEEKSEQEP

LRIWGSIENNEIVKASAPLITTQCIRVPVTDGHLATVFVNFEKKPSKDEILERWLQFK

GRPQELALPSAPKQFITYFEEENRPQTKLDRDIERGMGVSTGRLREDSLYDYKFVGLS

HNTLRGAAGGAVLIAELLKAEGYIQPK"

CDS complement(2921313..2922158)

/locus_tag="EFAGFIKM_02476"

/inference="ab initio prediction:Prodigal:002006"

/codon_start=1

/transl_table=11

/product="hypothetical protein"

/translation="MNESIIRDLIKYMDVEDNAAIQYIQTEHRMKLGLFWGIQEGSRV

LEIGCGQGDTTAVLAHLVGENGYVHGVDIAPEDYGAPLTVGEAAAKLRRSPLGDRIRM

DYDFDILSDQAQFAENEFDVIVLSHCSWYLKSFDELAQILSKVRTWGHQLCFAEWDAR

VTDVSQLSHWLSVLIQSQVECYKENSFSNVRTLFTPEDIQELVSAAGWTIKEETSIHS

PELQDGRWETEMTLQEAPVELQMLPIPDKVKTLLLSELKLLKTHHVSGPGSPLGTYAM

VAKKQ"

CDS complement(2922310..2923182)

/gene="gtaB_1"

/locus_tag="EFAGFIKM_02477"

/EC_number="2.7.7.9"

/inference="ab initio prediction:Prodigal:002006"

/inference="similar to AA sequence:UniProtKB:Q05852"

/codon_start=1

/transl_table=11

/product="UTP--glucose-1-phosphate uridylyltransferase"

/db_xref="COG:COG1210"

/translation="MQIKKAVIPAAGLGTRFLPATKAQPKEMLPIVDKPAIQYIVEEA

VQSGIENILIVTGRNKKSIEDHFDKSVELEHSLYAKGKQSLLEEVQAISEMANIHFIR

QKEPLGLGHAIGCARQFVGDDAFAVLLGDDIMVSDPPALAQMVHLYEKTGNQIIGVRQ

VDAANVSKYGIIDSNGAEDRVHRVTNLVEKPSLAEAPSRTAVMGRYILKPSIFPILDQ

IERGAGGEYQLTDALKEVSQVEELLALELEGRRYDIGDQFGYIQAILEIGLMRKELQP

MLTPYLQKLATQWA"

CDS complement(2923240..2923632)

/locus_tag="EFAGFIKM_02478"

/inference="ab initio prediction:Prodigal:002006"

/codon_start=1

/transl_table=11

/product="hypothetical protein"

/translation="MTKRLVTLFKFGIVGVANTAVDAVVFALLAVVGVPVLMAQVISY

SCGVANSYWLNGRWTFRDAARGGNDRAKLVRFLITNLIVLAISALILMTLHDVLGWSL

VMSKILATLMGMVLNYMASRYWVFRIAT"

CDS complement(2923625..2924611)

/locus_tag="EFAGFIKM_02479"

/EC_number="2.4.-.-"

/inference="ab initio prediction:Prodigal:002006"

/inference="similar to AA sequence:UniProtKB:Q55487"

/codon_start=1

/transl_table=11

/product="putative glycosyltransferase"

/translation="MAHAYRYSIIIPMYNEEAVIEETYRRLKKVMGSTGESYELLFVN

DGSVDRSAQMIRDYARWDESVKLIDFARNFGHQIAITAGMDYAAGDAVVIIDADMQDP

PELILDMIAKWKEGYEVVYARRTRRSGETRFKKWSASLFYRVLRASTDTDIPVDTGDF

RLIDRKVCDEMKRLPEKNRFVRGLVSWVGFRQTAIEYERDERLAGETKYPLKRMLKLS

LDGITSFSYKPLKLAGYVGATLSVGGFIYMLTVIISAIFTDSTIKGWPSIVSIMLMFN

GFILIMLGILGEYVGRIYDETKARPLYIVRDVVQAEGQQAQSRLTSRVAHHD"

CDS complement(2924850..2927024)

/locus_tag="EFAGFIKM_02480"

/inference="ab initio prediction:Prodigal:002006"

/codon_start=1

/transl_table=11

/product="hypothetical protein"

/translation="MNNTKRRMDLVLLPIVLLAAFLNGYGIWNDQYANSYYTTAVGSM

LSNFHNFFYASLDSAGSVTVDKPPVVFWIQTAFAYVFGLHGWSVILPQVLAGIGSVLL

IYFMVKPTYGLAAARISALAMATVPVVAAVSRTNNIDSMLVFTLLLGSWFLFKGSKQG

STWRILVAFGLIGIAFNMKMLQAYMILPAFYLFYLLAFQAKWRRKIILLIGSTAVLAV

VSLSWAVTVDSIPEDERPYIGSSETNSVMELAFGYNGLARLTGQQNTAGNAGMPNGVG

QGNNRDNRGDSTSGPNQTDSGVPGAGQDVNAPDNDNMNGSNGLNAMGGMNGPNGNFPN

GQMPNDMEMPNGRNFGGGMGGMFGTGEKGPLRLFQTELSGQASWLLPVVLLGCIALFA

GLRRRNITNKHKEALFWLAWLLPVAAFFSVAGFFHQYYLIMLAPPIAALTGAGFVAMW

KSYRDRNGWQAWLLPVSVLLTTQFGWYIMQAYNDTIGAGWSISELIVGILVTVILVVM

LHRTHRWKQGFIIAGFMVMLIGPIYWAFTPITYGGNSMIPAAGPTGSNGMFGGAGMGM

PMGNGAGDIEMPTMGGRGGMGNRNEEVDTATLNYLKEHNTGETYLFATTDYNQAAPYI

IDERAAVITLGGFSGSDPVYTTEELEQLVKSGQVKYFMVGGMGGRGGNSEISDWIKEH

GTEIPMSEWQTGTDSGSTDNGDTGNRAGFGFGGQTTLYEVKL"

CDS 2927229..2927912

/gene="copR"

/locus_tag="EFAGFIKM_02481"

/inference="ab initio prediction:Prodigal:002006"

/inference="similar to AA sequence:UniProtKB:Q02540"

/codon_start=1

/transl_table=11

/product="Transcriptional activator protein CopR"

/translation="MKLSSGIKILLADDEPHILQFLELGLSNEGFDVRTAPDGAAALE
LALEFKPHMAILDVMMPEMDGFEVVERLRKTGAEVGVIMLTAKDEVENRVKGLWLGAD
DYMIKPFAFDELLARIQARLRNQFPLLIGAVTHGPFRIDDQRKEITYQNQVLELSPTE
YELLKFIVLNHGIVLSKATILSRVWGYDFGGEENIVEVYVRSLRDKLGDKEHKLIRTL
RGVGYRVDL"

CDS 2927912..2929429

/gene="sasA_7"

/locus_tag="EFAGFIKM_02482"

/EC_number="2.7.-.-"

/inference="ab initio prediction:Prodigal:002006"

/inference="protein motif:HAMAP:MF_01837"

/codon_start=1

/transl_table=11

/product="Adaptive-response sensory-kinase SasA"

/translation="MNSLPKKNKLRIRRLHHWIWPRSLRSQLLSRSLFVLAGLLLLIG
ILQFWIMESFLYRNQAKTMEEQLMSMPPVWLGIQSRNGSSNNPFGGQVGNGSGNRVLF
IPDRSLALFDNEGTFEDVFGEDGLKAPQLSSAEYQDITDELKEQKHIPYRIVTDSQGN
EQLIVFASPGPRNRGLPMVQMGTATKPLRDLIIKQLLIFIMLSLLAMVAGLVLYTKVL
RRTLVPLSNMVNKVQQIDAGSLADRLPVVQGQEEVDQLAVSFNGMLERLENSFEAERE
SKEQMQRFLSDASHELRTPLTSIHGFIEVLQRGAATNQDQLYKALDSMHGESVRINKL
VEDLLLLTKLDQAPKQEREVIQLDSLLLEMQPQLAIMAQQRSIHLDLTAAVHILADPY
KLKQVVLNLFHNAVQHTDPEHGAISITLYATHHKAELSVKDNGTGIEPEHLPHIFERF
YRTSSSRSRKQGGAGLGLAITRSIVESHGGKITVQSQVGLGTEFMILMPLHKNDV"

CDS complement(2930048..2930758)

/gene="fabG_5"

/locus_tag="EFAGFIKM_02483"

/EC_number="1.1.1.100"

/inference="ab initio prediction:Prodigal:002006"

/inference="similar to AA sequence:UniProtKB:P99093"

/codon_start=1

/transl_table=11

/product="3-oxoacyl-[acyl-carrier-protein] reductase FabG"

/translation="MKKTVFVTGANKGIGYEIVKQLGEAGWKVILGARSVERGERAVS

ELTSRGLDVEFVQIDMCDSKSIEQAADTIHKSYAAINLLINNAGMPGAFSHSFTDTKE

EDLRNAFEVNFFGTFRLNQRLFPLIKDNEGTIINVSTDMASLDHMQNAEFTLNAFDYN

SSKTANSAMTLSMAYEVKNSRAQVFAVTPGFTSTDLNGNAEGGKSKEAGAAIIVRYAT

DGKPHNGEFLDENGVYPW"

CDS 2930861..2931220

/gene="adhR"

/locus_tag="EFAGFIKM_02484"

/inference="ab initio prediction:Prodigal:002006"

/inference="similar to AA sequence:UniProtKB:O06008"

/codon_start=1

/transl_table=11

/product="HTH-type transcriptional regulator AdhR"

/db_xref="COG:COG0789"

/translation="MLTIGEVAKKVEISIGAIRFYERKGLLKPAARNEQNNRLYLEDD

LNWLVFIKCLRETGMSVEDIKKYYDQVNEGTSTLKERSKLIEDQKQKLLNDIEEKKAQ

LVHLDNKLERYYRGENY"

CDS complement(2931403..2931984)

/locus_tag="EFAGFIKM_02485"

/inference="ab initio prediction:Prodigal:002006"

/codon_start=1

/transl_table=11

/product="hypothetical protein"

/translation="MYQPKQADELIIDCKDIYLREYRIEDLEKLQEITWQPEVYEFLP

GWNATIEERALWLTEYEIPENQRFKQAVMAGGDIGELCLRMAIVLKENDEFIGWCCSG

IKDELPAPNREIMYGISKHFRNRGYTTQAVNGMTEYLFEHTNVEVLNAIALTTNQASN

KVIQKCNFERLNLIEIEDEPYNHYQLCKGEDHF"

CDS complement(2932030..2932605)

/locus_tag="EFAGFIKM_02486"

/inference="ab initio prediction:Prodigal:002006"

/codon_start=1

/transl_table=11

/product="hypothetical protein"

/translation="MREIDYSQYFWQDDKIRLRALREEDWKDHYYNRFDTPARRLLEC

AVELPPTHVEAKSFTENFSDFSLAKGRIMFTIVNMDGENVGGVNLNSIDERNGTFSIG

IQIDRDHRGKGYGTRAVRILLKYAFFERRLNKFNDSVLEGNEPSAAMMRKLGCIQEGV

RRQVIYTDGKYQDMILFGLTKDEFIEKEGLV"

CDS complement(2932773..2933072)

/locus_tag="EFAGFIKM_02487"

/inference="ab initio prediction:Prodigal:002006"

/codon_start=1

/transl_table=11

/product="hypothetical protein"

/translation="MKDKFLREERQEYTDVSTVESQRNDITLEEFPEGPYGSSLLSES

LGKSSPWRVDQRSAHRFDYENHELHEGDVRDYPGQDVFDETVPDNVSKPQYTEES"

CDS complement(2933232..2934995)

/gene="ecfT_1"

/locus_tag="EFAGFIKM_02488"

/inference="ab initio prediction:Prodigal:002006"

/inference="protein motif:HAMAP:MF_01461"

/codon_start=1

/transl_table=11

/product="Energy-coupling factor transporter transmembrane

protein EcfT"

/translation="MEIELTNIRIEASDLSKRALLEDVSVQLNSEEITLLLGCTGSGK

TTLLQTLAGLRPPDAGSITLDGTAFWKEGKVPQSILLQMGLLFQFPEQQLFARSIQRE

FTYSLRPYRLLEKQQQEQITKALNQWDPPVSSELDRRFHLDRSPFALSGGEKRRLGLA

LGSVTNPHWLLLDEPSAGLEAQSVGLLLDVLEQHRLAGGGGVVATHDLDTFLPCADRV

LLLQEGRLIADLTPRELHKRPELLEQTGIGLPPSMRLAQQLRAAGLELPLTAMTPEEM

AESIVQSTSFEVGIQNGSKEASGTITMEVKSDVMKYEKEALMRSSFEAEDDPKLLPNT

LTHSGGLYGVMDPRLKWILYILLVTAAMLQQRWLGLTLTLVPVLAALAVLPRQTLAGC

MKLMKPLLFFFIISTALSGTTLSTEGGGLHFGFSLGQAEGTLLNVYRLFIVTLASLWF

SLTTPYGRMVEGLNWVLGIGKKIRLPVASFALAVSLIFRFIPMIWSEWQRFSLIVRAR

GKAALRPNTVRIRDLGPMVIPLLMALFQRAEDMTIAMEMRKVREHSMLGGRSSLLVWS

KRDTWISMAGLIVFVLLIWIR"

CDS complement(2934980..2935834)

/gene="ecfA1_1"

/locus_tag="EFAGFIKM_02489"

/EC_number="7.-.-.-"

/inference="ab initio prediction:Prodigal:002006"

/inference="similar to AA sequence:UniProtKB:Q03PY5"

/codon_start=1

/transl_table=11

/product="Energy-coupling factor transporter ATP-binding

protein EcfA1"

/db_xref="COG:COG1122"

/translation="MQGNLPIITLEDVRVHYASEGSQVRKALDGVSLTLHQGEWISIV

GANGSGKSTLAGVLIGFIPLSGGVRDISDELTVRGVLQQPDAQVLGDTIEEEFHFALS

PLMDSVEEQLEHRQDALHTVGLQYPPEKAVSQLSGGQKQLLNIAVALAAKPDILILDE

PTAMLDPGARDRIEAIVQKITQRGTTVIWITHHLEEATLCDRIIAMERGRCVYNGMPE

SFFYETDLNEKATWENVQLSPCERLGLDPPFTVKTALLLKQKGMMPEAMPLRPEQLAK

EVARWRSN"

CDS 2935947..2936537

/gene="bioY_1"

/locus_tag="EFAGFIKM_02490"

/inference="ab initio prediction:Prodigal:002006"

/inference="similar to AA sequence:UniProtKB:A2RMJ9"

/codon_start=1

/transl_table=11

/product="Biotin transporter BioY"

/db_xref="COG:COG1268"

/translation="MKLSLRGIVFSALMAAILVLFGYISIPIGFSPVPITLQTLAVML

AGGLLGPLYGFLSVTMVVLLTALGFPLLHGTGGLAVLLGPTGGYVMMWPFSALLIGLL

LARINIKGVTGFILAFVVFELFGSLLVYVSGVPWLAYAYKMDLPEAMIQGFYPYIIGD

LIKAVFAAIIIAPVRMVFPPQRLTGNIHSTVIKADS"

CDS complement(2936683..2938152)

/gene="expZ_2"

/locus_tag="EFAGFIKM_02491"

/inference="ab initio prediction:Prodigal:002006"

/inference="similar to AA sequence:UniProtKB:P39115"

/codon_start=1

/transl_table=11

/product="Nucleotide-binding protein ExpZ"

/db_xref="COG:COG0488"

/translation="MEKICFELEQVEMTYMDKVILNIERLAVHQLDRIGVVGGNGQGK

STLLKLIAGQIQPIAGKVKRFAEFGYLEQVEPPLPNENLEVDGALLSKLAIPQHDQAW

SGGEQTRLKLAQMFTHYHEVLLLDEPTTHLDQEGITFLLDELRYYYGALVLISHDRSV

LDELVTTIWEIHQGEVRVYSGNYSDYQAQKRLEREQQNQAHEQFSKEKRRLELAAREK

MKKAEKITQAGSMSKKESKAKTNRMVETKSKGTSQKAVHRAAKAIEQRMQQLHEVKAV

QEDRPMIFRQPKTLELHNRFPIMADRLTLEVEGNVLLEDVSFQVPLKQKIAITGANGS

GKSTLLNHVFRAGDHITMSPKAKLGYFQQMSYRFTTKETVLQFLKNRSQHEESELRSA

LHAMQFSGNDLLKNVGTLSGGEAIRLQLCHLFLGQYNILLLDEPTNFLDMHALEALER

FIKAYEGTILYVSHDQRFIENTADQKFQITERQLIQVNI"

CDS complement(2938643..2940532)

/gene="bglF_9"

/locus_tag="EFAGFIKM_02492"

/inference="ab initio prediction:Prodigal:002006"

/inference="similar to AA sequence:UniProtKB:P08722"

/codon_start=1

/transl_table=11

/product="PTS system beta-glucoside-specific EIIBCA

component"

/db_xref="COG:COG1263"

/translation="MSQEKLAKEIVELVGGEKNVESLVHCATRLRFVLKDDAKADKAK

LEKTEGIIAVKENGGQFQVVVGNKVPEVYSAIGQISNILDDSSDKEKPRKAAKGLGGI

IDIISSIFAPLLGVMAGAGILKGLLLIASNIGWLETTETTYTILYAAADSLFYFLPLL

LAVTTARKFQGNMFVAMTIAGALIYPSIVTLKSEGTPTDFFGIPVILMNYSSTVIPII

LAVIVMSKLEKFFNKTLHDSVKNFVTPLFLLVIMVPLTLLVFGPFGVYVGNGIAAGLV

AAFGFSPLLAGAVMGASWQLLVIFGIHWGLIPVFINNVAVYGQDGVKPAATASIFAQT

GAAFGVMLKTKNKKLKTLAGSSTLTALFGITEPAIYGVTLPLKRPFIAGVIGGAVGGA

IIGQAGTLAFASGAPGLLTLPIFYGPGGQGFPGLILGIVVSFVVSAVLTYIMGFKDPV

EEEIKTESTTSDQQSVRATNASDEIVFSPMEGTIVELSEVPDPAFASGAMGKGIAIEP

TVGRVVAPFDGTVTVAFKKKHALAVVSDTGAEILVHVGVDTVKLDGQHFVSHIKEGDR

VQAGDLLLEFDIAQIKAAGYHTVTPIIVTNSANYEEVIPQATGQVHSQELLLKLSSGN

DQEQK"

CDS complement(2940744..2941670)

/gene="licT_6"

/locus_tag="EFAGFIKM_02493"

/inference="ab initio prediction:Prodigal:002006"

/inference="similar to AA sequence:UniProtKB:P39805"

/codon_start=1

/transl_table=11

/product="Transcription antiterminator LicT"

/db_xref="COG:COG3711"

/translation="MSCLFFDASGFFCMPKRRCITAKRVERDGGIEVKIAKVINNNVI

SIYQADGAELVVMGRGIAFKKKPGDKVDETRIQKVFALKNKQTSDNFKMLLREVPMEL

IEIVEEIITYARDHLGRKLNENIYVSLTDHINFAIERYREGVEIKNALIWEIKQLYKP

EFGLGLRTLEQINTRMNIELPPDEAAYIALHIVNAEMNEEVITTMNITKFIQQIINIA

KYHFKMEFDEDSLSYFRFITHLKFFSQRVLSGTHYDNNYDHFYDMIKEKHPDAAACAE

KIELFVKKEYNHELTNEEKLYLTVHIERVVNR"

CDS complement(2942000..2943442)

/gene="bglH_10"

/locus_tag="EFAGFIKM_02494"

/EC_number="3.2.1.86"

/inference="ab initio prediction:Prodigal:002006"

/inference="similar to AA sequence:UniProtKB:P40740"

/codon_start=1

/transl_table=11

/product="Aryl-phospho-beta-D-glucosidase BglH"

/db_xref="COG:COG2723"

/translation="MTNFKFPKDFLWGGAIAANQAEGAYLEDGKGLSIVDLLPTGENR
RSIMKGNVPAFTPLATEFYPSHEAIDFYHRYPEDIALFAEMGFKALRVSIAWARIFPT
GEDAQPNEAGLQFYDNLFDELLKNGIEPVVTLAHFDVPVHLIEKYGSWRSRELVTLFE
TYATTVFTRYKDKVKYWMTFNEINMLLHLPFLGAGLAFNEGDNIKEIQYQAAHHQLVA
SALAVKACHEIIPGAMIGCMLAAGSFYPYTCNPEDVFQGMEKDRESYFFIDVQSRGEY
PGYAKRFFKDHELNIVMQPGDAEILKNHTVDYIGFSYYSSRTTSTDPEVIKNMTSGNV
FGSVANPYLAKSEWGWTIDPKGFRITANQLHDRYQKPLFVVENGFGANDVVTPEGEVD
DAYRIDYLKRHVAEMGEAIQDGVNIIGYTSWGPIDIVSASSGEMRKRYGYIYVDRNNE
GQGELTRLKKKSFEWYKNVIESNGTNLGEE"

CDS complement(2943683..2944513)

/locus_tag="EFAGFIKM_02495"

/inference="ab initio prediction:Prodigal:002006"

/codon_start=1

/transl_table=11

/product="hypothetical protein"

/translation="MKLDAHQHFWEYNVAEYGWIGEEMKTIRQSFLPEDLEPLLVQSG
LDGCIAVQARQSLTETEWLLQLADRHECIKGVVGWVDLCSDEVRNQLELFASNPYLKG
VRHVIQDEPDLKYVLREDFQRGISLLKEYGLAYDLLVSKEQLPYAVELVKTFPEQRFV
LDHLAKPDIKSGILSPWKEALESLAAQPNAYCKLSGMVTEADWANWTPSDFTAYLNIA
IEAFGAQRLMFGSDWPVSNVSATYSEVYGLINSHINALPIPEQQMIFGGTCASFYQIS
"

CDS 2944774..2946501

/locus_tag="EFAGFIKM_02496"

/inference="ab initio prediction:Prodigal:002006"

/codon_start=1

/transl_table=11

/product="hypothetical protein"

/translation="MKKNRKSVLSLTLVTAMILSLFSSAMASAATDNSEQANTSAAPS
KMQTYVNAMEPGWNLGNSLDAVGEDETAWGNPRITKELIQSIAAEGYNSIRIPVTWEA
HIGDAPDYTIDSAYMNRVQEVVNWALDADLYVMINLHHDSWRWISYMEKGHDNVLARY
NSAWTQIADKFKDASDKLMFESVNEPRFSEGGTTDAAIGYRMLDELNTSFHKIVRTSG
GNNETRPLVLPTMHTSSAQPDLDELNKTIQKLNDTNIIATVHYYGFWPFSVNIAGYTK

YNEEVQKDVTDTFDRVYNAFTAKGIPVIVGEYGLLGFDQHTGVIEQGEKLKFFEFVGQ

YLRQKQMTTMLWDNGQHFGRTSFTWSDQELFDTMKASWTGRSSTAETDLVYLKQNEAI

QDKKVKLNLNGNKFNSLKNGTTPLVRGKDYTIKKDVLTLKSNLLTKLTASGDLGVNAT

LTAGFNEGADWNFNIIKYDTPKLNDATGTTSAFAIPTTFNGNQLATMEATYANGENAG

PQNWTSFKEFSYTFSPDYERNVIELKPNFFNETNDGEVTLKFHFWNGDVLTYKITKNG

TSVVGVTSK"

CDS 2946633..2947532

/locus_tag="EFAGFIKM_02497"

/inference="ab initio prediction:Prodigal:002006"

/codon_start=1

/transl_table=11

/product="hypothetical protein"

/translation="MFKRVDRLAIELPISNNPDPNGASAVQELLGGKFGEMSTLNNYM

FQSFNFRGRSKLRPFYDIVASITAEEFGHVELVANTINLMLEGSTSPGAPDSTPLRVG

KDARMTSHFIESAQTALPYDSMGRPWNGSYVVSSGNLIFDLLHNFFLECGARTHKMRV

YEMTDNKTAREMTGYLLVRGGVHVLAYAKALEIATGVDVTKMFPIPRLDNKAFETTRK

WEAKGEHRRLYTFSDKDYQGIAQIWKGTHPTDGGKLEAFAGLPGYGGPVPELPDLPEE

FAPGISAEDLMQIAERLKRHAGL"

CDS complement(2947719..2949701)

/locus_tag="EFAGFIKM_02498"

/inference="ab initio prediction:Prodigal:002006"

/codon_start=1

/transl_table=11

/product="hypothetical protein"

/translation="MSENQRIAQEVIHAIGGKENIASFAHCATRLRIMVKDKEKIDQK

TVENIEKVKGAFFNSGQYQIIFGTGTVNRIFEEVEKLGIEGSSKDDVKSQGKKEGNAF

QRAIRTFGDVFVPIIPVLVATGLFMGLRGLLTQNEILALFGATPDDISANFLLFTQIL

TDTAFAFLPALVAWSAFRVFGGSPVLGIVLGLMLVNPALPNAYAVADGSAQPLHMFGF

IPVVGYQGSVLPAFFVGLIGAKFEKVLRRRVPEALDLILTPFITLTVMITLGLFAIGP

VFHSLEEWVLHGTTAVLDLPFGIAGIIIGFFHQIIVVTGVHHIFNFLEIQLLEKTGFN

PFNAIITCAMAAQGAACLAVGLKTKNMKLKALALPSSLSAFLGITEPAIFGVNLRYMK

PFIMGLVGGGVGGFIASLFHLQGTGMAVTVIPGTLLYLNSQLPLYILSNVVAMAIAFA

LTWFFGYKDQPVAEESVSHENSGVTLTEVKAEDSNLHINSVTDAVTSNRPKVDFLEIA

SPMNGTVVALEQVPDPAFSEKHMGEGIAIEPSEGKVYAPFDGVIAHVMNKSKHAVILE

HETGVQMLVHIGINTVGLKGNGFTAHVNSGDRVTAGQLLIEFDMDVIHAAGLPVITPV

LIPSGNETIATVTATSTGHVQANGEAVLVVKFTEPQ"

CDS complement(2949728..2951203)

/gene="scrB"

/locus_tag="EFAGFIKM_02499"

/EC_number="3.2.1.26"

/inference="ab initio prediction:Prodigal:002006"

/inference="similar to AA sequence:UniProtKB:P13394"

/codon_start=1

/transl_table=11

/product="Sucrose-6-phosphate hydrolase"

/db_xref="COG:COG1621"

/translation="MKMTREQRYRRIEQAEPGEIAKLEAQISVCPWRQSYHIQPITGL

LNDPNGFAYYQGYYHLFYQWFPLGTEHGMKYWYHTRSKNLVNWENVGIGIEPGGRYDS

HGAYSGSAIEKDGKLHLLYTGNTRDEDWVRHPYQCLAVMDESGSVTKLNDPVISSVPA

GYTEHFRDPKVWQQGDTYYCVIGAQRTDETGCTVLYRSIDLNNWEFLGEIRTQLTSFG

YMWECPDYMEIDGKGVLVFSPQGLDAAGDHYQNIFQSGYLIGEPLNLKTREFNHGEFQ

ELDRGFDFYAPQTMQGPDGRRILVGWMGLPDLAYPTDDGGWAHCLTIPRQLSFRDGKL

IQQPVAEMVQLRQQDKGTHIRATIDNESRSFTGFNGIAYELICEISHLEAEVVGIEFR

ANGTEKTVLLYDRIQQKVVLDRTMSGAELAEQNGVVRRCTLTADVIKFHLFVDSSSVE

IFVNDGEEVFTSRIFPSRDSVDIRFFTRGGKADFEATQWHY"

CDS complement(2951351..2952334)

/gene="ccpA_3"

/locus_tag="EFAGFIKM_02500"

/inference="ab initio prediction:Prodigal:002006"

/inference="similar to AA sequence:UniProtKB:P46828"

/codon_start=1

/transl_table=11

/product="Catabolite control protein A"

/db_xref="COG:COG1609"

/translation="MNKTISDIAQMAGVAKSTVSRFLNGGSVSEDTRQKIERIIKEYN
YVPNTFAQSLKAKKTSIIGTVVPRLDSFATSQTLIGIDEELRSNQYQMLIANTSQDMQ
REIDAIYDFARQKVSGIILLAAEVTEAHLKAVEDIRIPVLLVGQQHEQLHSLVHNDDQ
AGYEMGRYVVEQGHRKIVYMGVSEKDRAVGIYRKQGFQRAIAECGGCEVKYYETSFKM
SEAIVTAEAILKEITPTIIVGATDNIALGVMKIAFSNKIRIPQDLSVTGFGGYDITEM
IHPTLTTVKYHYLQAGKVAANHIIRLVKGESVEERTTLDVELIPRESVDKL"

CDS complement(2952576..2954249)

/gene="rnjA_1"

/locus_tag="EFAGFIKM_02501"

/EC_number="3.1.-.-"

/inference="ab initio prediction:Prodigal:002006"

/inference="similar to AA sequence:UniProtKB:Q45493"

/codon_start=1

/transl_table=11

/product="Ribonuclease J1"

/db_xref="COG:COG0595"

/translation="MNLAHNKLYIAALGGVNEIGKNMYFIQYHQDIIVIDCGSKFPDE
TLPGIDLIIPDVTYLLENLDKVRGLVVTHGHEDHIGGIPYLLKQMNIPVYASRLTRGL
IELKLKEHGLLRKADLHTIDAHSSITLGGIEVSFFATSHSIPDCLGIFFQTPAGNVVH
TGDFKFDMSPVHGPFPDLHRMAEIGKQGVHILLSESTNAERPGFTPSERVVGDHILDA
FIRAKQKVFISTFASNVSRVQQIVNAAFETGRKLTLLGRSMVNVVSVASELGYLQVPD
GLLIEAIDSDQFPPEQVVVLCTGSQGEAMAALSRLASGKHPHVRINAGDTVIIAAGAI
PGNERNLAHVIDNLYVLGARVIYGSSGAAGMHVSGHGSQEELKLMLTLMKPDYLIPIH
GEFRMLYQHRLLAESVGIERDHVFIVNNGDMVQYKDGIASLGPKIASGNSLVDGLIMG
DIGNIVLRDRRQLSSDGMLVIVTTLSKTEKQMVTSPEIISRGFVFVKDSEEFMHEIHE
LVLNRMDELTGAGVNQWNVIKRKLKDEIGHYIYAQTKRRPMILPIIIEV"

CDS complement(2954408..2955679)

/gene="rhaA"

/locus_tag="EFAGFIKM_02502"

/EC_number="5.3.1.14"

/inference="ab initio prediction:Prodigal:002006"

/inference="similar to AA sequence:UniProtKB:Q9KCL9"

/codon_start=1

/transl_table=11

/product="L-rhamnose isomerase"

/db_xref="COG:COG4806"

/translation="MDNQVKQAYEAAKALYAQHGIDTDEVLNKLAEIKVSVHCWQGDD

VKGFLNKDGELTGGISVTGQYPGAATTPAELRNDLEQAFALIPGKHKVNLHAIYTDTD

EQVELDQIEPKHYENWVKWAKEQGLGLDFNPTCFSHEKSSDGFTLSHPDPEIRKFWID

HCKASRRIGAYFGEQLGQTCVTNVWVPDGFKDNPVDRLTPRKRLKESLDEVFGEPLNP

EHNLDAVESKLFGLGSEAYVVGSHEFYMGYGLQNDTLICLDAGHFHPTEVISNKLSSL

SLFTSGILLHVSRPMRWDSDHVVIMDDELLEIARELVRHDLLATTHIGLDFFDASINR

VAAWVVGTRNTIKALLRAMLEPVDALKQAELEGDYTLRLALTEEFKSYPFGAIWDYYC

AQQGVPVREKWITDIKTYEQDVLLQRDKSLV"

CDS complement(2955699..2957768)

/gene="hcaB_2"

/locus_tag="EFAGFIKM_02503"

/EC_number="1.3.1.87"

/inference="ab initio prediction:Prodigal:002006"

/inference="protein motif:HAMAP:MF_01647"

/codon_start=1

/transl_table=11

/product="3-phenylpropionate-dihydrodiol/cinnamic

acid-dihydrodiol dehydrogenase"

/translation="MVQSLWNASQASEKTTGLEQLVYRSNLIGTDRSVCNIYGGNTST

KTTVKDFRGRDVEVMYVKGSGSDLGSMEAKHFTGLGLEDIRPLIERESMSDEEMVEYL

GHCMIDAKHPRASIETLLHAFLPYKHVDHTHPDAIISLCCAHNGKELAREIYGDRFVW

VPYVRPGFTLSKMIAESVFSNPNAELVLMEKHGLVTWGETSEECYAQTIKIINEAEAF

IEARVNEGSLFGGVKHPALAADVRRQIVSRVMPTIRGAVSDSKKMILSFDDQEDVLAF

VGGADSPELSQVGAACPDHLVHTKVVPLFIDWTPDVEDIEGLKAKLVEGVAAYKEQYQ

QYFESNKNEGDVMFEAAPRVILIPGVGMINTGKSWALSQVSGALYHRAIAVMRGATSL

GQFVSLSANESYNVEYWPLELYKLSLAPAETEFSRKVAFITGGAGGIGSETARRLVSE

GAHVVLADLNLEGAQKVAQEINDQYGANRAYALKMDVTDEEAVQSAYADVAVQYGGVD

IIVNNAGLATSSPFDETSLKEWNLNMNVLGTGYFLVAREAFKLMKQQGIGGSMVFIGS

KNSVYAGKSASAYSSAKALEAHLARCIAAEGGEYGIRVNTILPDAILQGSAIWNGSWR

NERAAAYGIEPDQLEEYYRKRTTLLVNIYPRDIAEGIAFFASSKSEKTTGCMMTIDGG

VPAAFTR"

CDS complement(2957948..2958928)

/locus_tag="EFAGFIKM_02504"

/EC_number="3.2.1.78"

/inference="ab initio prediction:Prodigal:002006"

/inference="similar to AA sequence:UniProtKB:G1K3N4"

/codon_start=1

/transl_table=11

/product="Mannan endo-1,4-beta-mannosidase"

/translation="MVNLKKCTIFTVIAALMFMVLGSAAPKASAATGFYVSGNKLYDS

TGKAFVMRGVNHGHSWFKNDLNTAIPAIAKTGANTVRIVLSNGSLYTKDDLNAVKNII

NVVNQNKMIAVLEVHDATGKDDYNSLDAAVNYWISIKEALIGKEDRVIVNIANEWYGT

WNGSAWADGYKKAIPKLRNAGIKNTLIVDAAGWGQFPQSIVDYGQSVFAADSQKNTVF

SIHMYEYAGKDAATVKANMENVLNKGLALIIGEFGGYHTNGDVDEYAIMRYGQEKGVG

WLAWSWYGNSSGLNYLDMATGPNGSLTSFGNTVVNDTYGIKNTSQKAGIF"

CDS complement(2959291..2961630)

/locus_tag="EFAGFIKM_02505"

/inference="ab initio prediction:Prodigal:002006"

/codon_start=1

/transl_table=11

/product="hypothetical protein"

/translation="MNDTIAGSQIRFILDAEDKEIRAGRMTGSGGKSPRGESYDFTNY

YMTRNAKPHIPVVGEFHFSRFAYLQWEEELLKMKAGGVNIVASYVFWNFHEEQEGEYN

WSGNLNLRHFVDLCGKHELPLIVRIGPFCHGEVRNGGMPDWLFSYPFEVRSNDVGYLY

YAKRLYREMARQLNGCFYQEGGPVIGVQLENEYMHAGAPMDAWGYTREKYITSGRDGR

EHLKRLRHIAEEVGMKPMFYTATAWGNAAVPEEGTLPMLAGYAYTPWIPNQPPSREYL

FRDLHMNPVEDVDYDSLEYPAAYCELAGGMQVSYHARPVVDADSVEAMTIVKLANGSN

LVGYYMYHGGTNPIGKNSYMNEQALPKMTYDYQSPLGEFGRVGESYDRIRSLSMFLEA

YGELLAPMGCVIPDGQQSITPENTLDLRWSVRQQDGSGFLFINNYQDHVALPDRDIQL

ELHTSKGTAFYPREGTMRLKSGMAAILPFHMNLNGMKIISATVQPLTRFMVNQELTAV

FYAHDGMMPEYVIDSSSVTNVDMPEGTVSEQGNEVVIHPIAGKDHHLRITTSDGMVIR

IITLTREEALQTYRFRVGGEERLVISSSHLYVQNEMLLCTSVEQPEMEVSFYPAPEHV

SASKYAVSSQSKQGIFGTYTFQVSPYVPAVEVDYPKECAATLRLDTAWPEQVDDVWIE

IDYEGDVAAAHIHHQMLTDHIHYGHSWMLGLKQSRHLLADHALRLSITPTRKGTIESY

VNQAYVERFEGVEIGKFNEIRVRPKYRVGLVLAVVREDT"

CDS complement(2961682..2965020)

/locus_tag="EFAGFIKM_02506"

/inference="ab initio prediction:Prodigal:002006"

/codon_start=1

/transl_table=11

/product="hypothetical protein"

/translation="MNHVTAKLNVRIWEETRDIPTYGTGKPDKNPMFLEKRIYQGSSG

KVYPHPVIDSIEDDAKMKSYRLIILENEYVRIEMMPELGGRVYRALDRTNNYDFVYYN

RVIKPALVGLAGPWISGGIEFNWPQHHRPNTFGPVDYTFGSNEDGSATVWVGEIDRMY

GTKMTAGFTLHPGKAYLEIHAEVYNRTSAPQTFLWWANPAVAVNDHTQSVFPPDVTAV

LDHGKRDVSRFPIATGTYYKMDYSEGVDISRYKNIPVPTSYMAYKSDYNFVGGYDHGI

QAGLLHVANHHISPGKKQWTWGNGEFGQAWDRQLTDEDGPYIELMTGIYTDNQPDFTW

LQPYEAKSFSQYFMPYKGIGMVKNATIDAAVNLEMDETTGMVTVMVYATSVFEQVTVE

AIGPTTVYLHERCNISPTELYKSSFPWSGQDEWHQLKLSVRTAEGKVLVAYQPERSEI

QEVPDPATPLPLPSEIRTNEQLYLAGLHLEQYRHATYEPEPYYLEGLKRDATDIRLNI

AYGTLLLRRGLFQDAEKHFRQAVQSLTWKNTNPYDSEAFYQLGLSLKLQGKQEEAYAA

LYKAVWSAQYQDTGYYMLAQIDTALQRDIEALDHIERSLIRNTRNYKARHLKVALLRR

LGQDKQATQYARETLELDPVEFGAAHELVLIYSGATTPSEREQAGQTREHFHRLMRGD

VYNYLNLAADYADCGLWKEALTVLSYVETENSQPYPMVGYAQAYLYRQLGDMEQAREC

QKQGKAAPTDYCFPNTLFEFMVLQDALAVDSNDARAHYYLGNWLYDHKRYEEAIIHWE

TSREVNAAYSTVHRNLGLAYYNKLNRPDLALAALEEAFRLDSQDARIFYELDQLHKKI

GYSCEHRIKLLEQHMELVHHRDDLYIEWVTLLNMQGSHQEAWNALQTRRFHPWEGGEG

KVTGQYVTALTELAKQNLEKQQPELALELLQKALVYPENLGEGKLEGAGDNPVYFYLG

CAYQQLKKNQAAEENFHRASIGLNEPASAMFYNDQPPESIYYQGLAWQKLGNVKEANR

RFNKLIDYAERHMHDHIRMDYFAVSLPDFLVFDDDLNQRNEEHCRYMRALGLLGLGRT

SEAKLELERILEKNPNHQGAIIHR"

CDS complement(2965193..2965903)

/gene="treR"

/locus_tag="EFAGFIKM_02507"

/inference="ab initio prediction:Prodigal:002006"

/inference="similar to AA sequence:UniProtKB:P39796"

/codon_start=1

/transl_table=11

/product="HTH-type transcriptional regulator TreR"

/db_xref="COG:COG2188"

/translation="MNNKFIRIYEDIADRIRTGEIEAGTLLQSELDLSESYQTSRETI

RKALKMLYEEGYIQKIQGKGSIVLDIRKIDFPISGLVSFKELAKKMGHRAQTYVKVFE

EQQVDQALHKKINFGLNEQVWEIRRVRKVDGEHVILDKDYISQRLVPGLSKEICNDSI

YEYIEQELGLSISFAKKEILVEEPTAEDRELLDLEGFHNVVVVRSQVYLEDASQFQYT

ESRHRPDKFRFVDFARRR"

CDS complement(2966031..2973533)

/locus_tag="EFAGFIKM_02508"

/inference="ab initio prediction:Prodigal:002006"

/codon_start=1

/transl_table=11

/product="hypothetical protein"

/translation="MKKSGKRVKKQAEQLTKVVLAFALLSPQALLLEWGAATVMAETV

ETIGIVSDTSSMKAVQSSKVKVEMNSDGKYRIVLLPNTNVFYGGDTGNVSTIIDHNGS

PVNFKTLPLNYYRINNNVIEMSRQKDNVEYILRVSIVNATSQGGYMKVELEAINRSGS

ALNLGGTFYWDTMVNGNDASPFEVIENGWRNYSGGVQVTAFYANTYNVVNADRIYMGQ

YSGPDNAQLTGGSSPSSFTPGQTVTASDTAAQFWWNAKATANQASRKFSTIVGIGPQN

VPPSFTLTAPSSGQTYYKGEQLQISGTTRDTDVGDLLTVKWSIDGGSENILTQMTATG

SNQSFNTNYTLPDTLPDGTHTLQVWVMDDKGGVSSAGTVNFTVRSFVVPGTPTYTLVN

SNNLTVNWDKKANDASVTYELKNMTTNQIVDTGASNSRQVTGLTPNTSYSFAVRAKNS

SSSFTGYSSPSTKYTLANPPAAAAVTQSGNSVTASWNNNGNPAGTHYKTEIRSPGGQV

LATGTTTSTRTEFALTGLADGKYEVFVAALNGEGIQTPFISAGEMIKDTTGPTAPAVT

VTPSSWTKEDVLITVEEGKDALSGTQKTEVKVGPAGEWREYSAPFAVNSEGNTTIMAR

SIDAFGNTGQETAVTARVDRTAPTPPVISLNPPEWTKAAVIVTLTEGTDEASGISLTQ

YKLGSEGEWVDYKAPLTLSEEGITEIYARSFDRAANVSASTSATARIDKTGPEQPTIT

LSEEDWTNQDVTLAINSGEDAGSGLAKSQYRLNKEGPWIDYTGEVTVTDEGETIVYAR
SLDRVGNISTSAQATVRIDTTAPTEPVISISHSGWSKEHVQFTIAGSMDEQAISYEYS
MNDAPYMTGNSGTVSTNGATTIRARARDTVGNVSKEVSRIAYVDQMAPTITFAPNGHG
WTDTDISTTIQYADAHSGVQELERFYQVTNSAESPDHWLEARSYEHKISIESEGIWYI
HAKTMDRAGNTYETTTSPYHIQRKPEQPSHVRMTQITETSAELTVDLPTGERYTDGYQ
YEISNKTTGQSWTLDYPKHSIIDHSLSGGQVYEYEVRVRNHTGVSDAVSAQVLTTPTA
PGTLHVRKVDSQPNLAEIQFDPVRGADAYRIIATTSDGAIVFDQTVSDPDSLPYVSNL
IPGTIHNISVTAMNESGAGGSSRTGFLTLPAMPGEFMAVQIQEHDISLDWETVTSATY
YGLSRDGTAIYEGEQTEYLDSGLDSGTEYSYALIAGNETGPGPLAGLPLLKTLPGQVP
GLQVSDASTASLRLNWEAVRGADRYELWLNGEKSGTVPAGTQEWVFAGLSSGTSYQLD
VQAVNGSGQGMRSSVSGTTLPESPSGLHVVQVTEQGAILSWEPVAGATKYRVVIDGQS
HEISDTQLAVYHLSSSREYTYEVQAGNAAGYGASTSSTILTLPSRPEGLNVTWTGETS
MGLAWQAVDTANLYIVKINGTEVGRTSELAYTVEGLLPGTEYALEVQAANTSGAGEAA
QLIRLSKPVSPAEVLVDPGVHRAKVSWSVVQGAAEYVIEQNGKEIYRGMENEATITGL
QDGTWHHYQLWAVNRQGTRSEAKDVSLLTLPEKPVKVGVYDVAKNSLGLDFSNTGVQG
ADHYVIERNGREITQMDSSETQFVDKDLSPGTKYTYVIRAVNASGTSAPLTFSVMTQT
LPLVAESITVKAGTHVVDLAWDVVQGAAAYEIRNRVTGDVQSVSEPSVHLNSMLDGTA
YEFELVAINEDGHRSESIQIQVLTKPISPQTAGITHITDQTAVLDLTGSSTRGAEQFI
IMRDGVEVAKVPADQASFEDDGLTPGEHYTYTIKTSNATGESNSGFEVYLRTLPATIH
EPLHASMIGEKEGWITWPKVQGTEGYTIRIGDQMFTTIEERDITEVRLTHLESATRYD
QVQIIPYNTAGSGSPMTVAPFYTLPHVDSLEMKIYPETDHANLEWDFPYVNETFVVLL
DGTEVYRGTQKELIIDQLDAGKQYPIEIHTENDQGDASEKLAYSVLTKPAAPVKVEYH
SAKDHIRLLLEQSRVEGAEQFIIERDGVEIARVPADELFYDDKELEPGVNYIYTVKTM
NASGSSDGGYYLHAMTLPGSAASSPVVEGRSMYGADIVWELILGAGGYRVYRNEELVG
TTTETSIHVSELNSAERYADFAIVPFNEAGEGEALQVPEFETLPSEELTVAAIAQGTN
TIKLTWELDSINEVIVITHKNREIYRGTKRSHVWTGLNAEQHYEVEVWTENSAGEKSE
RKRAAAMTFPYPPSAWSGGGATPSPNVTSEQADEVSSQPELSEQPEVPVKKNIKFIDI
SQTFNKDQITWLAEQNIIQGVSETRFEPRRPITRAEFTALIVRLMGVDTAVNEQHGFK
DVNDEDWFAPEIKAAVHHEMVQGMGNGKFAPHALVTREQASKIIANVVRKIRPEPLTS
PRAFTDQTDVSNWAKEEVQELAGLYMITGYEDGSFRPMQHLSRSEAAALIFRLNKLIQ
VMDENRTDQVEKASAFDRHI"

CDS complement(2973689..2974711)

/locus_tag="EFAGFIKM_02509"

/inference="ab initio prediction:Prodigal:002006"

/codon_start=1

/transl_table=11

/product="hypothetical protein"

/translation="MKALFIGGTGTISTAITEMLAQQGCELYLINRGNHNDDLPSEVN

VLQADINDETRVAELIADLEFDVVADFIAFVPSQLERDYRLFKDKTKQFIFISSASAY

QTPLADYRITEGTPLSNPYWEYSRNKIACEDYLMKQYREHGFPVTIVRPSHTYGDKSV

PLGVHGAQGSWQVLKRIREGKPVIIHGDGTSLWTITHNTDFAKGFIGLMGNIHAIGES

VHITSDESVTWNQIHEIIAGVLGVKLHAVHVPSEFLAACSDQDLRGGLLGDKANTVVF

DNSKLKRLVPEFVATTRADQGIRRTIEHILAHPELQTEDPEFDVWCDKVVGALDEALL

KIRDEK"

CDS complement(2974807..2975232)

/locus_tag="EFAGFIKM_02510"

/inference="ab initio prediction:Prodigal:002006"

/codon_start=1

/transl_table=11

/product="hypothetical protein"

/translation="MKSWFGKTWPWLTLGLTVVVLLGSFLVYFMGKDVSPGSGSAVTA

QADTPEDLKGYEVIDVDVSNDGFGPDVIEVKAGVPTKINFILTRSVTHVKSVGSQKLG

MDLYMQKGPNYYTVDKDLPKGEYEIHCGMYMIYATIKVV"

CDS complement(2975629..2976582)

/locus_tag="EFAGFIKM_02511"

/inference="ab initio prediction:Prodigal:002006"

/codon_start=1

/transl_table=11

/product="hypothetical protein"

/translation="MKYYLSVLAGAMSYGILSTIVVLAYGEGYKLGEVVGAQLITGFL

LSWMLALYTKFRTKRKSQANGKASGAVAQVFKRLTWKQRLILMAAGTPTVITGLVYYQ

SLRYIPASLAIILLFQFTWISVLIQAISKRQRPDKVTFLTLIILFGGTLLAAGFLEQG

LGEFNGLGIALGLMAAVSYSLFVLFSGKAVPSAHPAFRSAWMVTGGLILLCILFPPTF

LFNGLIWSQLLVFGLLLGFFGAFIPPVLFAIGVPHIGGDMAGILGAVELPIAVLLSSI

VLHEHVSGLQWFGVIIVLIGVALPELYKLRMRRSRNTPIYS"

CDS complement(2976947..2979166)

/gene="srmB"

/locus_tag="EFAGFIKM_02512"

/EC_number="3.6.4.13"

/inference="ab initio prediction:Prodigal:002006"

/inference="protein motif:HAMAP:MF_00967"

/codon_start=1

/transl_table=11

/product="ATP-dependent RNA helicase SrmB"

/translation="MSDNPFYRLAPFVQEFIYKKRWESLRPAQIEACNICFHTPHHML

IAAGTASGKTEAAFFPALTELYERPSKSVGILYIGPLKALINDQFERLKDLLSEGNIP

VWHWHGDVPQAEKTKLMKNPSGVLQITPESLEGLLMNRPNAIPALFHDLRYVIIDEVH

AFMGADRGIQVLSELARIERMAGCAPRRVGLSATLSDYDAATSWLAAGTQQGVDVVSS

PGGRKLRLRVEHFSFPDAQDEEQAEQLHNARKAYYDFIYESTHRKKALIFTNSRTDAE

VTILEMRRVAARRQERDVFHVHHGSISAMLREETEAALRTGSGPAVAAATVTLELGID

LGELERVVQLGAPYSASSFVQRLGRSGRREDMASEMLFVCPEEEDEEAQLPARMPWTL

MRAIAVIELYVKTKWVEPLEARKMPIGVLYHQTMSMLKSMGEAEPRDLAEAILSLAPF

ALIRPDQYQVFLNYLIETDHLQWTEDRTLIIGLTGEKIVNNYRFYAVFKDDEEHKVLN

GSEEIGSITTVPPPGYCFSLAGKLWKVEEVDHKHKAVYVKSAKGKVDTLWLGAGGDIH

TSVVQKMREVLSDSVIYPYLSPQAVNRLERARRLARESGLLKQVVIPAGGDSMYVLPW

VGSKSFRTLERLMKHNLSKKLALRSVVPMEPYYFVVSGKVDERTLLAEIMSECRAAED

ASALLAEDEAPYLGKYDEFVAQPLIREAFAVDGLDLEGLKAGLQQTLEWDSSSSNRT"

CDS complement(2979163..2980506)

/locus_tag="EFAGFIKM_02513"

/inference="ab initio prediction:Prodigal:002006"

/codon_start=1

/transl_table=11

/product="hypothetical protein"

/translation="MTELKIPKRLTTALVNSLTAGVVPRIGLEQIAVGRKPEVEAILR

DMDNIAEGGAAFKLITGRYGSGKSFLLQMIRNYAMDRDFVVADADLSPERRLVGTKGQ

GLATYRELMTRLSTRTRPDGGALEPILQKWIAGLQQSTMQSQNLRPDDPALPLEVEKQ

IYAVTGEMQNLVHGFDFAKVLASYWNGYKLADDDRKQAALRWLRGEFATKTEAKKELA

VGVIIDDDNWYDYFKLWSEFTARIGYKGLLLFIDEAVNLYKITNSVSRQSNYEKLLTM

FNDTMQGKAEHLGIFVGGTPQFVEDERRGLYSYEALRSRLIDGRYAAKAYANYTGPIL

KLAMLSHEEIMILLQKLRQIHALHFGYSASLTDEQLVDFMQTAVNRLGADELLTTREV

VRDFMDVLHTLHQNPEVTYTQLLGERVAKPQETGKGADASANTDDLDDFLAEFEL"

CDS complement(2980513..2982090)

/locus_tag="EFAGFIKM_02514"

/inference="ab initio prediction:Prodigal:002006"

/codon_start=1

/transl_table=11

/product="hypothetical protein"

/translation="MRDNSRQLEFMEIDLSEEPETAAVPVPDRSTIVQSAHDTMQHRG

GILSSEKRFVEEAKQWAEMEGDVSPWVPFMSYWPTYGVMNEAQRKWYLFWRKEVRQGR

YPDTDLSYLFVHIYELINGIGWQNPQDGYDQLKQLWVNYRERLPQLNIYMQEWMVDYV

LVHQMEMSLSEVMGLSGGYLPAEMLDRELQRILQDKVSDISLNMLQRYYDYDITLSKF

YRDGGKEVMDQYIPRVMALVDSYLERTRQVGLLPECDPNDERTMERTLFRKAVYDEWI

YGRSVSFRYMPIGEHAEFVQMVTRIYRCTENKLRELLGFRGRLRGQTLEPELANLIER

YLDKAYAIEQAESVEQPMIRIDTEKLASLQQESEYVRMALTIEDDHPSEVKDEEVNNA

NVTSNPFDVLEARDEITPTEGSMESEPSAEEIAAGAQVESIRLQWDESAEADLEEEWL

LFAKELSPQQVQTIHVLLGASPDMELMRLAEQYGTMPTLLLDEINDVAMETIGDLLID

GDRIVPDYIDVFEHVKR"

CDS complement(2982506..2983096)

/locus_tag="EFAGFIKM_02515"

/inference="ab initio prediction:Prodigal:002006"

/codon_start=1

/transl_table=11

/product="hypothetical protein"

/translation="MTATTIMERLKSETAHYHRQVEQNPYAKAIMNQTVTIEEYRTYL

EKFYGFLKPLEDQAVLLPFWESTELDIEIRGKAGLLEKDLRNLGASEEEISQFPLCEE

LPDISTPARLFGYLYVIEGSTNGGQIMTKRLSQFLPIEADRGLEYFNAYGSDTRTRWS

EFTGLLQQSISTPEDHDNMVHSASETFRLLDQWINT"

CDS 2983442..2984239

/locus_tag="EFAGFIKM_02516"

/inference="ab initio prediction:Prodigal:002006"

/codon_start=1

/transl_table=11

/product="hypothetical protein"

/translation="MDLTVQEVVLHLSDNIELPENTVDQLIMGSLDRKVTGIIIAFMP

THYVIEQAINHGANLIIAHESPFYNHHSHTDWLANDSVYYVDKRKLIDDAGISIYRCHD

IIHRFQPDGITEGLIQALGWSSYVEQRSAEADILSFPEGSTVEAIAHHVKSSLSIDYV

RVAGNAEIVCRRAAVLVGFRGNGHVTIPLIQNEQLDLIIAGEGFEWETPEYIRDAMQQ

GKSKALIMMGHAESEAPGMKLLADRLAERFPELSVRFVGEKPVYTVI"

CDS 2984760..2985185

/locus_tag="EFAGFIKM_02517"

/inference="ab initio prediction:Prodigal:002006"

/codon_start=1

/transl_table=11

/product="hypothetical protein"

/translation="MRGIIFALLGGACITLQGVANTRISTDMGTWQAATITQLTGFIL

AVLILMFVRDTNLQGLKQVKPMYLAGGAFGAVIIFSEVTAIQQIGVTFTISALLIAQL

FLTFLVDSNGWFGVVKQKMKLPQFLGIALMVTGVIIMKL"

CDS 2985231..2985929

/gene="yeiL_1"

/locus_tag="EFAGFIKM_02518"

/inference="ab initio prediction:Prodigal:002006"

/inference="similar to AA sequence:UniProtKB:P0A9E9"

/codon_start=1

/transl_table=11

/product="Regulatory protein YeiL"

/db_xref="COG:COG0664"

/translation="MQEIHNEQQLIQYLKQYQLETVFHEPLRTHMTLCHFEKCELICR

EGETSEYLYVLVEGKIKIFTTSAQDKTLVLCFKTPLEVVGDIEYVRESNIVNTVQAVS

PVVMLRIHYQWLAELASDYAPLLKFLLKIISHKFYIDSNFSNFNLMYPVEVRLVSYLL

SISIEEAGNVVHEELDAFNLTDIANLIGTSYRHLNRVIQKLCADGLIERHQGLIMIKD

RAGLREIAGHNIYE"

CDS 2985944..2986432

/locus_tag="EFAGFIKM_02519"

/inference="ab initio prediction:Prodigal:002006"

/codon_start=1

/transl_table=11

/product="hypothetical protein"

/translation="MLITGISLALLAGALVSLQTIFNSKVNERTGSWSTTTLVLFTGF

IASFLISLLVEGKNTFSFQHMQPWYWLSGAIGVGVVFCLVQGMKLLGPTYAISIVLTS

QLSFALLFDSMGWLGLEQIPFSWNQLLGVLVIVGGIVLFKFGGRKSEKSEQSPGTLQS

DS"

CDS complement(2986519..2987235)

/locus_tag="EFAGFIKM_02520"

/inference="ab initio prediction:Prodigal:002006"

/inference="similar to AA sequence:UniProtKB:P67182"

/codon_start=1

/transl_table=11

/product="putative transcriptional regulatory protein"

/translation="MGRKWNNIKEKKASKDANTSRVYAKFGVEIYVAAKKGEPDPEAN

RALKVVLERAKTYNVPKAIIDRAMEKAKGSGDENYEELRYEGFGPNGAMVIVDALTNN

VNRTAPEVRSAFNKNAGNMGVSGSVAYMFDPTAVIGIEGKNSEEVLELLLEADVDVRD

IVDEDDAVIVYAEPDQFHAVQEAFKAAGITEFTVAELTMLAQNHIELPEDAQAQFEKL

IDALEDLEDVQQVYHNVEFV"

CDS complement(2987515..2989236)

/gene="cysI"

/locus_tag="EFAGFIKM_02521"

/EC_number="1.8.1.2"

/inference="ab initio prediction:Prodigal:002006"

/inference="similar to AA sequence:UniProtKB:O32213"

/codon_start=1

/transl_table=11

/product="Sulfite reductase [NADPH] hemoprotein

beta-component"

/db_xref="COG:COG0155"

/translation="MVYNNLLNPQRTNSDVEDIKIKSDYLRGSLTETLADRITGAIPE

DDNRLMKHHGSYMQDDRDLRNERHKSKLEPAYQFMLRVRASGGIVTPEQWLMMDRVAH

KYANETIRLTTRQSFQLHGVLKWDLKNTIREVNDSLLSTLAACGDVNRNVMCNPNPNQ

SDVHAEVYEWACQVSNHLDPRTRAYHELWLDGEKVIDSQDSDEEVEPIYGKVYLPRKF

KIGIAVPPSNDVDVYSQDLGFIAIVENGKLQGFNVSVGGGMGMSHGDAKTYPQVSKVI

GFCTPEQMIDVAEKTVMIQRDYGDRAVRKHARFKYTLDDRGVEWFVEELTTRLGWKLD

AARPYHFETNGDRYGWVKGNNGRWHYTLFIQNGRVKDVDGYPLMTGLREIAKVHTGDF

RLTANQNLIIGNISSQKKKKIEALIQQYNLTDGAHYSALRRSSMACVAFPTCGLAMAE

SERYLPSLIDKLEPVLDEAGLRDKEIVIRMTGCPNGCARPMLAEISFIGKAPGKYNMY

LGGSFTGHRLNKLYKENIGETEILDTLTPMVNQYAKERNEGEHFGDFVIRAGYVPEVL

DGRQFHA"

CDS complement(2989290..2991125)

/gene="cysJ"

/locus_tag="EFAGFIKM_02522"

/EC_number="1.8.1.2"

/inference="ab initio prediction:Prodigal:002006"

/inference="similar to AA sequence:UniProtKB:O32214"

/codon_start=1

/transl_table=11

/product="Sulfite reductase [NADPH] flavoprotein

alpha-component"

/db_xref="COG:COG0369"

/translation="MELQVTNSPFNQEQVELLNRLIPTLTDGQRTWLSGYIAAIQASA

TIAAPANLVQAAPTTGIAPVSAPPVSREVTVLFGSQTGNSSGLSKKLAKKLEEQGLQV

TLSSMGDFKPNGLKKIENLLIIVSTHGEGEPPDNAIPLHEFLNSKRAPKLEGLRYSVL

ALGDTSYEFFCQTGKDFDKRLQELGGTALVPRVDCDVDFDEAAAEWMNEVLASLNSTS

AAASTVTTEAVTAAVSGGESEYDRTNPFKAEVLENLNLNGRGSDRETRHIELSLEGSS

LDYEPGDSLGVFPENHPRLVEELIAAMGWNADERVTVNKNGDQASVHEALLRYFEITA

VTKPVVEQLAKLNPGSGLTALLADDSEFRTVMNSCDLLDLVQDYNLKGIPAGEFVATL

RKIPARLYSIASSSKSFPDEVHLTVRSVRYEARGRERYGVCSVHLAERIEAGDTLPVY

IQHNPNFKLPENPDTPIIMVGPGTGVAPFRSFLGEREETGAEGKTWLFYGDQHFATDF

LYQTEWQRWLKDGVLTKMDVAFSRDTEQKVYVQHRMLEHSKELYQWLQEGASLYICGD

EKKMAHDVHAALTTILEQEGGLSSEQASEYLTRLQQEKRYQRDVY"

CDS complement(2991344..2991733)

/gene="ytcD_1"

/locus_tag="EFAGFIKM_02523"

/inference="ab initio prediction:Prodigal:002006"

/inference="similar to AA sequence:UniProtKB:O34533"

/codon_start=1

/transl_table=11

/product="putative HTH-type transcriptional regulator

YtcD"

/db_xref="COG:COG1733"

/translation="MEQEMKKYESGVQAMLELVGGKWRILILHQLISGKKRTSELRRA

IPGITQKVLTQQLRELEKNEIIHRIIHPQIPPKVEYELTEYGLTLQDIIDRICLWGGN

HLDRVYGDKSRVLGDHFSDYIPLSTST"

CDS 2991909..2992469

/gene="azoR"

/locus_tag="EFAGFIKM_02524"

/EC_number="1.7.1.17"

/inference="ab initio prediction:Prodigal:002006"

/inference="protein motif:HAMAP:MF_01216"

/codon_start=1

/transl_table=11

/product="FMN-dependent NADH-azoreductase"

/translation="MKVKLVVTHPRQDSLTFAVMNRFIEGMQENSHEIDILDLYHDGF

DPLYGVEDERDWQNPDKQHAPVIRKELDRVLAADAIVFVFPIWWYNVPSMLKAYLDKV

WNMGLLNKANSKKALWIALAGGTEASFHKYDYYNMISNYLNNGIAGYARMQESRVEFL

YETISESKEHIESLLEQAYQIGKHYN"

CDS complement(2992661..2997886)

/locus_tag="EFAGFIKM_02525"

/inference="ab initio prediction:Prodigal:002006"

/codon_start=1

/transl_table=11

/product="hypothetical protein"

/translation="MDKLRKPMIFMLSATLALSSGPLMLPSKAYADLNSIPATLLQDD

FSDGNYTESPAWNVSSGNWEVLADPTDTSNFTLFQSDTNEGIISTGGSMSNMTVSMRF

YTGAGQGYPGILPRFQDKNNFYYFQMQVPNNKLVFSKRVNGTDTALKTVDYAFAKDTW

YTLKMVLSGTTIRGYIAENGSDRLVFDLVDTTYGSGTVGIRNKWQSVHADDVIIAEQP

PGNDIQLSIAEQTASSVSLQWSEIIGASAYRLYRSSTPEGGYSLVTNTGTLGHTDEGL

SGDTVYYYKIAYEYGGLTESQWTAPLEVRTTAAAPQAPGELKAEALNATSVKLSWSAV

DKSTGYRVVRAEAGSDQYEQIYEGKGLTFTDNALEPGNSYSYRVTAYNAAGESAFTVA

EATTYSIDSPAEFAATAVTDTSISLGWNVLPGSDVSYTVSRATSATGTYQQVYSGNEN

TFNDSGLMMGTGYFYIIQATVDGIVSPASAPLGVAAIRTSITPGQLWPDLDGKPIDAH

GAGFFYDEQTEMYYWYGEYHTGGWPAVGVRVYSSKDLMNWTDEGMALTTLQSMDDFDN

DPLISELYAGREDKVDIWADIRRGRIIERPKVIYNDKTKKYVMWAHMDGDKDPYNDNA

NYGKARAGYAISDSPTGPFVYQKSYRMDRAPEGEKDFFPSERGMARDMTLFKDDDGTG

YLIYSSEENLTLYISKLNEDYSDVTGWHKEGRTDDKGNPVRDSTYQAEYGVDYVRVFP

GGQREAPAMFKYQGKYYILTSGASGWAPNENKVTVADNIFGPWSTQTNPFVRTLPSDP

DPSKAFGTQTTSVIPVDPEKGKFIYVGDTWNGGNFSNDAAKYVFLPIEFGIGSDIAIK

WYNSWTPDLLNSMGKVDIADPLPEAVALGKVPSLPTTLNVRDGGALVSTPAVWTIDNR

AMTAEDFAKPGPLTLQVTTPEYNNKKQAVRVNVIPENAIYFVNSGGYETADYSLMGAY

MKGTLANPETADQMYAPAEGRNWGYVSADALASGSNGGDIFSTVRYLNGGNVSNSPKG

TDLTYRFDVPNGTYDVYAGFNDPWTNTSRRANLIINGTNTGAVTFTPASVRAHKSISV

SDNKLELTVHNTASQDPMISWIMIVKPDAAPPANDSAGLNADALDSTSATLHWDAHLG

AASYKLYRSDREQGEYKVVYSGNGREYMDSKLNPGTEYYYKVEALDATGQSLRGVSSA

YQVHTAQQSAADVATSITALEQPSAGAKKLKLPSVPQGFTVKIASSSVPSVIQTDGTI

VPPSKETTVTLELEITRTSDDSRALTIPLTVKVPASVPSPGGTDPGSGGNSGGNSGGN

PGNGSGSSSNGGQSGNGNPSAENVVPQPKPEKDRSVLELQGQSDQKGVVQSNVDVSTI

KDAFKVAPSTDAGQRLVELRLKPVLGATAYQLSLPASALIDRGESHVFNIDTELGMLE

LPATLLTKDIVGDGIASIRLVRTELPKTVADQLGTKYGVQLELQLDGQPWPSDSKLIL

RLPFQSSQNAPQDRIVAFAIGANGVATPLPQSYYDQNSGQLVFSVTSLTGNYAVVPVE

QIFTDLAEVQWAKKAMEALAVRGVIDAEASGDSTQLHPKEEMTRGQYMQWLMTALGLN

ASSGNTFSDVNENAPYYEAVTAARSLGITSGAGDGSFLPESTITRQEMMTLTVRALAT

AGLVDSEKAGTDNLTRFRDASEIRSYARDSVALLVDLGIAHGYNGEVKPLAEATRAES

ATLLYAMMSKLVWKK"

CDS complement(2998312..2999868)

/gene="ybiT_2"

/locus_tag="EFAGFIKM_02526"

/inference="ab initio prediction:Prodigal:002006"

/inference="similar to AA sequence:UniProtKB:P0A9U3"

/codon_start=1

/transl_table=11

/product="putative ABC transporter ATP-binding protein

YbiT"

/db_xref="COG:COG0488"

/translation="MSILNVEKLSHGFGDRAIFNDVSFRLLKGEHIGLIGANGEGKST

FMNIITGKLQPDEGKVEWSKRMRVGYLDQHAVLNKGQSIRDVLRGAFQYLFDMEQEMN

DMYGKMGDVTPEELEQLLEDVGTIQDTLTNQDFYMIDAKVDETARGLGLTDIGLDKDV

NDLSGGQRTKVLLAKLLLEKPDILLLDEPTNYLDELHIEWLKRYLQEYENAFILISHD

IPFLNSVINLIYHMENQNLTRYVGDYEHFQEVHEMRKQQLESAYKRQQQEIADLKDFV

ARNKASVATRNMAMSRQKKLDKMDVIEIAKEKPKPQFNFRDARTPGKLIFETKGLVIG

YNEPLSRPLDLRMERGQKIALVGANGIGKTTLMRSILGEIQALEGTVQRGEHLEIGYF

QQEMKDANYNTCIEEIWQEFPSYTQFEVRAALAKCGLTTKHIESKVAVLSGGEKAKVR

LCKLINNETNLLVLDEPTNHLDVDAKEELKRALKDYKGSILLISHEPEFYRDVVTETW

NCESWTTKVF"

CDS 3000066..3000539

/locus_tag="EFAGFIKM_02527"

/inference="ab initio prediction:Prodigal:002006"

/codon_start=1

/transl_table=11

/product="hypothetical protein"

/translation="MIEVKQSKLGDGGELNRGVFATVDIAKGTLIHQAPVVPYPNEDH

EHVEKTILEDYVFEYGANHTAILLGYGSLINHSYEPNATYDINFDNHTFDFYAYTDIK

AGDEIVINYNGEEDSMDPLWFLDDYEERMLEFNKSNDDEVEKETETQSLDTDTSN"

CDS complement(3000426..3000665)

/locus_tag="EFAGFIKM_02528"

/inference="ab initio prediction:Prodigal:002006"

/codon_start=1

/transl_table=11

/product="hypothetical protein"

/translation="MFFVFKLWTVKSLQLLISSFTGVSEFFSHIQQSACELHLGLTLI

TRVRVQGLGFGLFFNLIIIGLIELQHTLLVIVQEP"

CDS complement(3000770..3001378)

/locus_tag="EFAGFIKM_02529"

/inference="ab initio prediction:Prodigal:002006"

/codon_start=1

/transl_table=11

/product="hypothetical protein"

/translation="MSSDFIGLHEGKWTKEPVAARMDGDRFVVEAREGSDFWENTFYG

FCHRDGHAMLAPWDGTEAIEVSFDLSSFTELYDQAGLMLWYGEDQWIKAGVEVNDGVA

HVGAVVTDTYSDWSLSPVPEWGGRIVTIRASYSNEAVVIRARTDEHPWRTIRVARFAY

PTNKHAGPFLCSPKRAGFEVAFTKWRSTTPDQDLHTDPPITD"

CDS complement(3001436..3002782)

/gene="gor"

/locus_tag="EFAGFIKM_02530"

/EC_number="1.8.1.7"

/inference="ab initio prediction:Prodigal:002006"

/inference="similar to AA sequence:UniProtKB:P06715"

/codon_start=1

/transl_table=11

/product="Glutathione reductase"

/db_xref="COG:COG1249"

/translation="MTQETYDLIAIGTGSAASSVITRCAEAGWRVAVIDEREFGGTCA

LRGCDPKKVLAGAAELIDWNERMQGKGIQGQATINWSELMAFKRTFTESIPRASEDQF

KQAGMDTFHGKASFVDENHIQVGEEVLHGKHILIATGARPAPLEIEGSEHLIYSDDFL

VLEQLPDRLVLVGGGYIAFEFAHIAARAGTEVHILHRSEQPLKSFDAELVESLLQKSK

EIGIHVHLNAEVKSIRQEGNAYVVHGTRNGADHQWQCGIVVHGAGRVPNVDGLELEKA

NVSYSKKGITVNEYLQSESNPRVYAAGDVTDTKGLPLTPLAGQESRAVSFNLLEGNQH

KPNYKVMPSIVFTVPALGAVGMSTEQAKKDGYEVQVNDMSKWYTYKRTHEKFAMAKVV

IDKSTGRILGAHVLGSKTEELINLFAMAIQFNLSIDQLNTMNFAYPTAASDLGSLI"

CDS complement(3002833..3004347)

/locus_tag="EFAGFIKM_02531"

/inference="ab initio prediction:Prodigal:002006"

/codon_start=1

/transl_table=11

/product="hypothetical protein"

/translation="MSRFTEVFSERKGQLLSTLLEHIQISFIALFFAVLIAIPLGIYL

TRKPKVAEPIIGVTAVLQTIPSLALLGLLIPLFGIGTLPAIIALVVYALLPVLRNTYT

GISEVDPSMVEAANAMGMNSCQRLIKVELPLAMPVIMAGIRTAMVLIVGTATLAALIG

AGGLGALILLGIDRNDTALIILGAIPAALLAILFDVLLRQFQRLSFKKTLVTLGSLAI

IAILVITIPFVARGGQKDLVISGKLGAEPEILINMYKLLIEKDTDLTVELKPGLGKTP

FLFNALNSGDIDIYPEFTGTAISEFMKETAVSTDRTEVYEQARDGMLSQFNMVLLNPM

DYNNTYTLAVPQSIADQYNLKTISDLKPVEQQMKAGFTLEFSDREDGYLGIQKKYGIE

FPNVATMEPKLRYGALQRGDINLVDAYSTDSELRQYELVVLEDDQELFPPYQGAPMLR

QETADQYPQLVEVLNQLAGKITDEEMRQMNYDVNVEGANPEQVAREYLKQAGLL"

CDS complement(3004340..3005314)

/gene="opuCA"

/locus_tag="EFAGFIKM_02532"

/inference="ab initio prediction:Prodigal:002006"

/inference="similar to AA sequence:UniProtKB:O34992"

/codon_start=1

/transl_table=11

/product="Glycine betaine/carnitine/choline transport

ATP-binding protein OpuCA"

/db_xref="COG:COG1125"

/translation="MIQFENVSKQYPDGTTALRQVNLNINKGELFVMIGPSGCGKTTM

LKMINRLIERTDGTVRINERPIDEYNIHELRWNIGYVLQQIALFPHMTIAENIAVVPE

LRKWKADQIKKRVHTLLDMVGLHGDTYSERKPSELSGGQQQRIGVLRALAADPEIVLM

DEPFSALDPMSREKLQDDILDIQRQMKKTIVFVTHDIQEAMKLGDRICIMKDGQVLQV

GTPEELIRQPANDFVREFVGSPNTDTSSQSDFDLESIMLPISPGHVPKSAKTAVPVSI

TLTELVDIMAFHGHLLVERNRQIIGEISRADLMKYWSGQLQERGEEHE"

CDS complement(3005537..3006397)

/gene="azoB"

/locus_tag="EFAGFIKM_02533"

/EC_number="1.7.-.-"

/inference="ab initio prediction:Prodigal:002006"

/inference="similar to AA sequence:UniProtKB:Q8KU07"

/codon_start=1

/transl_table=11

/product="NAD(P)H azoreductase"

/translation="MINDKPLTLIIGANGKTGSRVAAILQKHHYPVRLAGRTKPSHSG

SADNYVYFDWYDSETYAPALKNVNQVYLVLPVMDMTPEDVMIPFLKEALWSGIKRFVL

LGSASIDEEGPIFGKVHQYLKAHAPEWAVLQPSYFMENFTEGPHRETTKQLGKIYSAT

GDGKIGFVSADDIAAVAFHALTDVVPHNTEHIITGPETLSYGQVADVLSRVLGQSIQH

ESLSDDELRNSMIRAGMSKEYATALAGLDVPIREEGREDQATDTVTKITGNNPISFEQ

FIQNHMEVWK"

CDS complement(3006387..3006833)

/gene="yesE"

/locus_tag="EFAGFIKM_02534"

/inference="ab initio prediction:Prodigal:002006"

/inference="similar to AA sequence:UniProtKB:O31511"

/codon_start=1

/transl_table=11

/product="putative protein YesE"

/db_xref="COG:COG3631"

/translation="MTQSQQLEEGNGIPYRIMHGFTQLLLEGKLEQWLELYTSDAIFE

FPYAPAGYPQKLEGKAAITNHLHNLLGMIEIQQFSEPVILADSTKQQFVAEFTCKGRS

LVTGKPYNQTYISVVSHSNGKITHYKDYWNPMVVLESDLGGNKHDQ"

CDS complement(3006966..3007550)

/gene="comR_5"

/locus_tag="EFAGFIKM_02535"

/inference="ab initio prediction:Prodigal:002006"

/inference="similar to AA sequence:UniProtKB:P75952"

/codon_start=1

/transl_table=11

/product="HTH-type transcriptional repressor ComR"

/translation="MGRAKEFDTETVLRKATSVFGAYGYEGTSLSLLLSELGIARQSL

YDTYGTKHDLFVSALKFYIQQKTEAGIRLLNECTSVRQGVTELFNEAVNVLTDTERRN

ECFIINSAVEQAPQNHEIAAFIQTTNQQMEDVFHTALLRGQRSGELRHAEEELPGLAR

FLNYSRLSLTFTAKSGASAEALRDFVQMTLRALD"

CDS 3007718..3008080

/locus_tag="EFAGFIKM_02536"

/inference="ab initio prediction:Prodigal:002006"

/codon_start=1

/transl_table=11

/product="hypothetical protein"

/translation="MYIQITDLAVKRLTESLNDQPGYFKVIYDLEGCGCNGVIAIIIV

DELAALDAQIETNLFPFYVDPKQQLNLEQHMKLDTEENYPSFKLSSDSGVLSANVRVR

DTRAVTAGSSGGSDACML"

CDS 3008541..3009314

/gene="yodJ"

/locus_tag="EFAGFIKM_02537"

/EC_number="3.4.-.-"

/inference="ab initio prediction:Prodigal:002006"

/inference="similar to AA sequence:UniProtKB:O34866"

/codon_start=1

/transl_table=11

/product="Putative carboxypeptidase YodJ"

/db_xref="COG:COG1876"

/translation="MKSFTRKSIISTILVGSVVAGSAFTTFPITSSLNPVASAASSSF

TQFLHDNAPGRTIKTIKNVATVTNVSSTVVLVNKKRNLPSTYAPQDLVVPNIPFSFSG

SSPKKQMRKVAATAIEKLFAAAKKDGIDIKAVSGYRSYATQKSIFDRNASIKGEAVAN

KTSARPGQSEHQTGLAMDISSASAGYDLQQSFGNTKEGKWLKANAHKYGFIIRYGKDQ

EKLTGYSYEPWHVRYVGVYIAGEITNQKLTLEQYLERAK"

CDS complement(3009449..3009688)

/locus_tag="EFAGFIKM_02538"

/inference="ab initio prediction:Prodigal:002006"

/codon_start=1

/transl_table=11

/product="hypothetical protein"

/translation="MKALIARYMGGHDPHEFRVYVIQSSTLKRFVVVEIILGPIVYNV

ALYLCHNAILAGVGSWAGTEGLKRLPLVFRKIVGT"

CDS 3009819..3010451

/gene="mhqN"

/locus_tag="EFAGFIKM_02539"

/EC_number="1.-.-.-"

/inference="ab initio prediction:Prodigal:002006"

/inference="similar to AA sequence:UniProtKB:P96692"

/codon_start=1

/transl_table=11

/product="Putative NAD(P)H nitroreductase MhqN"

/db_xref="COG:COG0778"

/translation="MSELENLVKNRRSAVIFEEGIEISDSELQEMFALNKFAPSAFNL

QHTHYLVIKDEEQKEKVYEASQQYKVKTASAVIVVLGDVNAHHHIRTINEGLLNLGAL

TPFQYEQESQSVTEFYESRGRFFQREDAIRNASLSAMQLMLIAQDRGWDTCPMIGFDS

EELQRSLEIPNHYVPVMLITIGKKSEAKQRPRGYRKPINEYVSFNKMHAE"

CDS complement(3010683..3011843)

/locus_tag="EFAGFIKM_02540"

/inference="ab initio prediction:Prodigal:002006"

/codon_start=1

/transl_table=11

/product="hypothetical protein"

/translation="MDITYAFTDEHFRQVVLDRFCEQRDFIQESDVCEVEILELSNHN

ISNLDGIEYFRGLQELDCAYNQLTGLDLAQNHKLRILRCRENQLLTLDLRSNSELQVL

DCSFNRLRKLDLSHNSKLITVECHWNMLSELITEPLERLEELSCSYNALFSLELEHNK

QLRRLDCANNYMLELDVTGCPNLIELRCNHNHIKQLDFRSNMVLESVRCFNNHISKLD

IRHNVQLRELYCSENKLTELDYSANPKLERLQYADNLMFESNHEVPGMGLFQYDVSMS

NYQMSLLIQDKELIVTAQVSTKTEMEALSPYMEETWKRWDMLGEQALKTIAVAHPDED

INALILADAEFQGDQYFRLGYDAGDTPAGRLYIYAEFDDEFHMLDTLIYETY"

CDS 3012010..3012456

/gene="sbmC"

/locus_tag="EFAGFIKM_02541"

/inference="ab initio prediction:Prodigal:002006"

/inference="protein motif:HAMAP:MF_01896"

/codon_start=1

/transl_table=11

/product="DNA gyrase inhibitor"

/translation="MEMVIENLPSYRIAYVRQVGPYGPANAQAMNTLKQWADKKQLLY

ESATLLGIPQDDLTTTPPEKCRYDACIVITESERLEDEFVEIGELSGGDYLICKVRHT

AEAIQQAWNVIIPHLQTNGFQMDNKPVIDDIVGNCLISIIARYVCL"

CDS complement(3012785..3014029)

/locus_tag="EFAGFIKM_02542"

/EC_number="3.4.11.-"

/inference="ab initio prediction:Prodigal:002006"

/inference="similar to AA sequence:UniProtKB:P24828"

/codon_start=1

/transl_table=11

/product="Aminopeptidase 2"

/translation="MSQFEQTFEKSLEQYAELVVKVGVNIQKGQDLLVTAPIETLEFT

RLIVQKAYAAGANYVQVDFDDDNITRSRFEHGSNDSFDYYPAWKADMMEKFAEAGGAT

LTIKVPDPELYNGIDSDSVSRATKAAAHARRGYAKYTRNHEISWCLIKAPTKAWANKV

FADIPEEDRINVMWETIFKMNRVDGGDAVQNWREHLDTLNAMSDRLNQKNYKSLHYRA

PGTDLKIELVNNHIWGGGGGENQQGIYTVANMPTEEVFTMPKRSGVNGYVSSTMPLNL

NGQLVDQMRITFKDGQVVAFTAASGEEHLKNLFATDEGARYLGEVALVPHDSPISNLN

RIFYNTGIDENASCHLAVGSAYPFNMKDGTTMSNEELLKHECNVSLTHVDFMIGSAEL

DIDGELQDGSIEPVFRKGNWAF"

CDS complement(3014271..3015863)

/gene="acsA_2"

/locus_tag="EFAGFIKM_02543"

/EC_number="6.2.1.1"

/inference="ab initio prediction:Prodigal:002006"

/inference="similar to AA sequence:UniProtKB:P39062"

/codon_start=1

/transl_table=11

/product="Acetyl-coenzyme A synthetase"

/db_xref="COG:COG0365"

/translation="MNFEQWISPESYNLTSEMENHPSDRIALRWLSDQRELEEITYGD

LFKQANRLAGGLRELGLEKGDRVLVMVPRRIIAYVIYIACLKLGIAVIPSSEMLRAKD

LEYRLRHSEARAVIVWSETTSEVEKMDADLPSLAHRIVASPNGEVSVPAEDWVNVHQL

MQNQPEEMAAVETHRDDTAILAYTSGTTGNPKGVVHSHGWGYAHLRIASSLWLDIQPS

DTVWATAAPGWQKWIWSPFLSVLGRGATGLVYNGSFQPKRYLELMQEHQINVLCCTPT

EYRLMAKADDLGHYDLSHLRSAVSAGEPLNQEVIEIFQRHFDLTIRDGYGQTESTLLI

GSLKDAPVRIGSMGQSITPGLIEIIDEEGQPVPAGEVGDIAVHKEMPALFRSYYQDEG

RKEASQRGDYFVTGDRARKDEEGYFWFEGRSDDIIISSGYTIGPFEVEEALMKHASVK

ECAVVASPDEIRGNIVKAFVVLTDDSLASPELMRELQHHVKEITAPYKYPRKIEFVTD

LPKTNSGKIRRIELREQEKRNG"

CDS complement(3016129..3017133)

/gene="gpr_3"

/locus_tag="EFAGFIKM_02544"

/EC_number="1.1.1.-"

/inference="ab initio prediction:Prodigal:002006"

/inference="similar to AA sequence:UniProtKB:Q46851"

/codon_start=1

/transl_table=11

/product="L-glyceraldehyde 3-phosphate reductase"

/db_xref="COG:COG0667"

/translation="MPYTASEQRYDEMKYVRSGKSGIRLPQIALGLWQNFGGNRTLDV

QEEMILRAFDLGINHFDLANNYGPPPGSAEENFGVIYKKHLRPYRDELLISSKAGYHM

WNGPYGEWGSRKNLIASLDQSLGRMGLDYVDIFYHHRPDPDTPLEETMTALDHIVRQG

KALYVGLSNYNAEQTQEAVSILRRLGTPCLVHQPNYSMLNRWIEDGLQDVLDEQGVGS

IAFCPLGRGQLTNKYVDKIKEERANSTGNLKKEAYTDERIAKFDALQAVAERRGQTIS

QLALNWILRGNRVTSALIGASRVSQIEENVAAFQAPDLTTEELNEIESILDGMGNYPW

"

CDS 3017335..3018180

/gene="araC_4"

/locus_tag="EFAGFIKM_02545"

/inference="ab initio prediction:Prodigal:002006"

/inference="similar to AA sequence:UniProtKB:P0A9E0"

/codon_start=1

/transl_table=11

/product="Arabinose operon regulatory protein"

/translation="MITYMFKNNHFQELQLLHYGTEACTPGHHFGPAMRDYYKIHYIL

NGKGTFEVGGKTYTLHKGQGFLIVPHSVVHYEADQDDPWEYSWVAFQGSNSSSFLQQA

CLSEHHPIFELGNEDDEMRSCLHRMINSRNTHKGWEISMTGLLYQFFSILIDQANSEH

LQPMPDYSKETYVTQVMDFIEMNYANAITVQSIAAHIGLQRSYLCSLFKDQMGSSIQS

YLVHYRMRRAAELTLDPGLTIGDIARSVGYTDQLLFSKMFKKVMGEAPTYYRKHKTAP

SQLSC"

CDS complement(3018249..3018647)

/locus_tag="EFAGFIKM_02546"

/inference="ab initio prediction:Prodigal:002006"

/inference="similar to AA sequence:UniProtKB:P0A0P8"

/codon_start=1

/transl_table=11

/product="Alkaline shock protein 23"

/db_xref="COG:COG1302"

/translation="MSEIIEKQYNEPEYIAPQSVQMGTIHISNDVLSKIVGMAALSTA

GVSSMSVGLTEGIAKSISGKSLQKGIDVHVKDDQATIQLRINIQYGNKMHEVCRELQH

NVQQAVEQLAGVMVDEIKVQVVGISMPETV"

CDS complement(3018676..3019755)

/gene="yhhT_1"

/locus_tag="EFAGFIKM_02547"

/inference="ab initio prediction:Prodigal:002006"

/inference="similar to AA sequence:UniProtKB:P0AGM0"

/codon_start=1

/transl_table=11

/product="Putative transport protein YhhT"

/db_xref="COG:COG0628"

/translation="MFKVNTFVRFSIALALILVNIYLLSRVSFIFQPLVTMITVITVP

MMLSVFFYYLLRPLVNYMEKKKINRTLSILLIYLVIAILGVFFIIGLWPSLREQLINL

VDNAPSLINSLSEQLRELEQNGAIQALFPEGSTPFSQITEYINKGFNFVTNYVSGFFS

LVSSFAIILFTLPILLFYMLLQGEKFGRKLAHIAPKRFQNDSREVVIEIDQALSGFIV

GRVLVNLALGVLMYIGFLIIGLPYALLLTVIAVIMNFVPFIGAIVSSVPIVIMGLVVS

PSVAIWSLIIILVAQQIQDNLVAPYVFGKKLDIHPLTTIILVLGAGDLGGIIAILIII

PVYMIVKILLVRIYNMFFKDKWQNA"

CDS complement(3019791..3021248)

/gene="sasA_8"

/locus_tag="EFAGFIKM_02548"

/EC_number="2.7.-.-"

/inference="ab initio prediction:Prodigal:002006"

/inference="protein motif:HAMAP:MF_01837"

/codon_start=1

/transl_table=11

/product="Adaptive-response sensory-kinase SasA"

/translation="MKRPWKAREKRLLQTSLTLDFLLFNFFLLLLVLIVYLMVSLDVV

DFRISDQVVDPDLNVEAHVYVAELENEFYSGGGSVSKGKDTEIQRLKDSGGWIEILDA

NRNVIRHVGDKQDAFTQYSEADLYDGLENRSDQEYYYSITPLSTEGASAYVLLKIPRD

LVSVRINDNQLITNLKHPLSFYIMIGIGLVLLLIFVYSYWVARRIKKPLGILSSGLTQ

MIQGNYSTRMSISAEREFVQIGETFNYMADVIENTSAEKRYAEESKQRLIVDLSHDLK

TPITSIQGYAQALVEGRGEDKDRQQRYLGYIYNKSVQVARMIQNMLELLKVDSPDFRM

HIQRREIGEFVREIMADTYGEIEQKQFVLHVLVPDEVIYARYDPELLSRVIQNLITNA

LSYNPIGTELRVELIPLDTHVVIEVADTGVGIPQELWSTIFDPFVRGDEARTATGGTG

LGLSIARRNTEKMGGRLILSRRGRETTVFTIQIPN"

CDS complement(3021226..3021933)

/gene="phoP_2"

/locus_tag="EFAGFIKM_02549"

/inference="ab initio prediction:Prodigal:002006"

/inference="similar to AA sequence:UniProtKB:P13792"

/codon_start=1

/transl_table=11

/product="Alkaline phosphatase synthesis transcriptional

regulatory protein PhoP"

/db_xref="COG:COG0745"

/translation="MRYTVLIADDEPEIVELLQIYLEKDYTIKTAVNGAEALQCIRST

QIDLVILDIMMPVMDGLQLIKQIRATYHMPVLFLSAKSQDHDKILGLGLGADDYIAKP

FNPLEIVARVEALLRRVNQFDATEIPEAKEQNLVLGDLTLDRSQCILFRSGNPVTLTS

TEYKIMELLLDQPGRVFTRKKIYEAVWGDYYAHEDSTIMVHISNIREKIERDSRQPEY

LKTIRGLGYKIEAPMES"

CDS complement(3022168..3023613)

/gene="hpaH"

/locus_tag="EFAGFIKM_02550"

/EC_number="1.14.14.8"

/inference="ab initio prediction:Prodigal:002006"

/inference="similar to AA sequence:UniProtKB:A4IT51"

/codon_start=1

/transl_table=11

/product="Anthranilate 3-monooxygenase oxygenase

component"

/db_xref="COG:COG2368"

/translation="MSITMSRGQAYIQRLNDERNVWLDGERIQVTGHQAFQGTLQTIE

GLFNLVDDPETRETVAYWDEQTGSYVHRSFLVPRSLPDVKSRADAFRLWADRTYGVMS

RLSDYARSRLTGWYATRHEMTAHDPAFADKISDYFAQAKRKDAFLTIVQRDPQINRSL

PVGEDEDAMLRIVKRNTEGVVIRGAKMVATAAPYADDIIAYPVQRIPDHLPELAHMVI

VAADSPGLHMMCRESFATKDTDKSHPLSAQYDEMDAVLFFDDVFVPWERVLLHNNPEA

VWQIRCNTASASLAYHQSVIRLHSKLEFITAVTSSIAKEIGVDSFLNVQEQLGELISQ

MQTIEGLIIAAEAQSKPDTFGNWLPEFKYIETARNLGNRYYPRAVEILKTIAAGGLMQ

IPSGTFEMNEMMSSMIGKYLGGVTMQAPQKIRLFQLAWELTGSPLGARHDLYERFYAG

DPVRNRANQYVQYDKERLLGKVEPWLRSLKD"

CDS complement(3023787..3025745)

/gene="yxdM_2"

/locus_tag="EFAGFIKM_02551"

/inference="ab initio prediction:Prodigal:002006"

/inference="similar to AA sequence:UniProtKB:P42424"

/codon_start=1

/transl_table=11

/product="ABC transporter permease protein YxdM"

/translation="MNFRQFAINNVVRNKRIYLAHFLSSTFSVMIFFTYALLLFHPDL

KAGLKGSSGTVTLLANQGFMIAEIIIFIFSFLFLLYSVGSFLKTRKKEFGIFLIIGMT

RKQMNRLLFMENMCIGLASIITGIGLGIIFGKLILLICGSMLAVENSLRFYFPLKGIA

LTAGAFLLLFVVIAMSSSLLIRKGTLIDLVKSEEKPKPEPKASRLLALLSVLLIGGGY

AGVFTFVWISFSFPLLLASVVVVIAGTYFLFTQLSVYIIRALKRNPRLFFRKTNLLFL

SELTYRMKDNAIMFFMVSIISASSFTGIGTMLAIADPGLSSMSNPYAFSYMNSWDTPD

SDRHIRQIEETLIDHQVPYVKGSYAPIGENNNGKIIKLSDYNRLAKALGYEERTLKQI

DESFMTPSNLAIRKEYREQVEQGVSGGKVNLEVDNQHVPVQLSTPGTEIVIPFQYEIY

LYVVTDELFNKMRPAYNEEVGMPEGFYSSRTIQFIVKDWMGTRSFAPELIESIQNDRS

DRGYFETSALVVDWLNSKQTNGIILILSGLIGIVFFTFAASFTYFRLYADLERDEAQY

RMIGKMGLSRPELRKIVTRQLLLMFFLPILVAVIHSSVAFVALQQLVDFSVFGYSLRI

FLVFASMQILYFALVRWRYLRHMYSKLV"

CDS complement(3025720..3026475)

/gene="yxdL_2"

/locus_tag="EFAGFIKM_02552"

/inference="ab initio prediction:Prodigal:002006"

/inference="similar to AA sequence:UniProtKB:P42423"

/codon_start=1

/transl_table=11

/product="ABC transporter ATP-binding protein YxdL"

/db_xref="COG:COG1136"

/translation="MKQISKIYKGIVSYEALSGIDLSIQEGEFVGIMGPSGSGKTTLL

NMISTIDHPTSGELRIAGKNPFELNQDELALFRRKELGFVFQSFNLLNTLTVKENIVL

PLTLDGVSLAEMNTRVEQLASKLGIEGILNKRTYEISGGQAQRTAIARALIHSPKLIL

ADEPTGNLDSKAARDVMEILETRNQEDQATMLLVTHDAVAASYCSRVVFIKDGKLYNE

IHYGDNRAAFYQKIINVLSLMGGSGHEFSPVRH"

CDS complement(3026804..3027787)

/gene="graS_2"

/locus_tag="EFAGFIKM_02553"

/EC_number="2.7.13.3"

/inference="ab initio prediction:Prodigal:002006"

/inference="similar to AA sequence:UniProtKB:Q5HI08"

/codon_start=1

/transl_table=11

/product="Sensor histidine kinase GraS"

/db_xref="COG:COG0642"

/translation="MRLFIREHLALTCWVVAILFTVVAVFWYDGYNDWVTAAYAVALG

LFLYIGYLVYRYYSHRSFYTRMSRSMDSLKDFVPLNETSPLSLALEKLLDSQYGQYHA

HLHRLEQRQQEYLTFMNQWVHQMKTPLSVIELTVEGQEDDDPRLVSIREEADQMRRGL

ETVLYVARLDTFEQDFSVEPVILKTAGEEAIHELKRFFIRNHVYPEMHIEPSLVVQSD

AKWIRFVLVQLLSNAIKYSAGNGQKIYVRAYEAERSIILEVQDQGIGIPKSDLNRVFQ

PFFTGENGRHFKESTGMGLYIAKEVLTRMNHRIDLESVYGEGTTVRITFNS"

CDS complement(3027784..3028488)

/gene="graR_2"

/locus_tag="EFAGFIKM_02554"

/inference="ab initio prediction:Prodigal:002006"

/inference="similar to AA sequence:UniProtKB:Q5HI09"

/codon_start=1

/transl_table=11

/product="Response regulator protein GraR"

/db_xref="COG:COG0745"

/translation="MYTIMIIEDDPKIAGLLKSHIERYGDRAVLVEDFEMIVQQFEQI

QPHVVLLDINLPSYDGFYWCRQIRTLSTCPILFISARSGKMDQVMALENGADDYITKP

FEHEIVIAKIRSQLRRVYGDYAARDEERKVELDGLVVYLERLEIQLGDRKVQLTKKET

ILLETLLRRSPKLVSRETILEKLWDDSFVDDNTLSVNVTRVRKRLAELAITDALETVR

GSGYRLNNTWKAASPS"

CDS complement(3028530..3029870)

/gene="nagZ_3"

/locus_tag="EFAGFIKM_02555"

/EC_number="3.2.1.52"

/inference="ab initio prediction:Prodigal:002006"

/inference="protein motif:HAMAP:MF_00364"

/codon_start=1

/transl_table=11

/product="Beta-hexosaminidase"

/translation="MYNYRYTSNNKWRKPLHMMCLLLGIVLLLSACGQAQKPSSATDS

NTGSNAGSNSGQSSSSPEQNTAPPQEEVPEEPQEEVDPVQEQLNSLTLEEKIGQMILA

GVQGTTLDDQAKQMITNQKVGGIIFYANNVSTLEGTAKFVKSIKEANQSNPVPIFMSV

DQEGGKVSRMPETVESIPSSRKVGETKDSALAETMGELLARQVQLAGFNVDFAPVLDV

NSNPSNPVIGDRSFGSSAELVSRMGIAEMKGLRSEGIIPVVKHFPGHGDTSVDSHLDL

PVVNKTEKQLAELEWIPFQAAVKEQVEAVMVAHILFPKLDPDHPASLSDVIIGEHLRG

KFKYDGVVITDDLSMGAIAKNFKLDQAALATVKAGSDILLVAHSYESAKTIFDTLISA

VKSGKITESRIDESVYRILALKQQYKLSDDQKASGDLKQLNADIVDWRKQIDAR"

CDS complement(3030269..3031372)

/locus_tag="EFAGFIKM_02556"

/EC_number="1.-.-.-"

/inference="ab initio prediction:Prodigal:002006"

/inference="similar to AA sequence:UniProtKB:P32382"

/codon_start=1

/transl_table=11

/product="NADH oxidase"

/translation="MNVPSALFKPFTSDKLTLSNRIVMAPMTRGFSPEGVPGPEVAEY

YRRRAAGGVGLIITEGTGINHPSSVSGASIPLFHGEDSLQGWANVVKAVHEAGGKIMP

QLWHVGTARRSGDLPNAEADPVSPSGVSMAGEPSREPLTEEEIQGLVQAFAQAAADAQ

RIGFDGIELHGAHGYLIDQFFWEQTNRRTDNYGGDLVRRTQFAVEVIKACRAVVGPDF

PIVLRFSQWKMGNYEARLVETPEQLEQFLAPLSAAGVDIFHCSTRRFWLPEFEGSELN

LAGWTQKITGKPAISVGSVGLEAEFVDRATENQGTGDAHLDQLNEKLENNEFDLIALG

RVLLSDPEWPAKVREDRISEIIPFTTEVLQTLH"

CDS complement(3031706..3033019)

/locus_tag="EFAGFIKM_02557"

/inference="ab initio prediction:Prodigal:002006"

/codon_start=1

/transl_table=11

/product="hypothetical protein"

/translation="MQRHTKWTAVILTIVAGMSWMLMGNSKPKNTTSTSQPPATERGL

AKVSNAPISSADVTSKIELLGRPFTKTPYANNVWDMQLYNGKIYLGHGNSSNLGVAPN

SGPIPVIYYDTANAKFKTQSVTNSNPGILPSTKMYVDEEQIDIYRVLNDKLYIPGNDS

DSEKWELGNFYRLDGEVWTKYRNLPLGVHVYDLASYQGKMFAALGTESKPTILISHNE

GETWQKYATINTFGFRAYTMFHLNGKLYASGMMYPANKIWNDKTNILEINEKLEKRDV

VIYGNKMLPGLTYQTGTIPYNKIGKNVNFNNKLLYIAGGVFNDGQLLPKSLNVMTDIN

QARRVNLPDAKALPTDVLLRDGKVYVLTYTRQSANLYINRVYQSTDLTTWNEILRFNQ

DTYAKSFEENEGDFYFGLGTDPDVLSTSSGKLLRVNRSDIPVNAN"

CDS complement(3033200..3034249)

/gene="gutB_3"

/locus_tag="EFAGFIKM_02558"

/EC_number="1.1.1.-"

/inference="ab initio prediction:Prodigal:002006"

/inference="similar to AA sequence:UniProtKB:Q06004"

/codon_start=1

/transl_table=11

/product="Sorbitol dehydrogenase"

/db_xref="COG:COG1063"

/translation="MKALRWHGVKDLRLENINEPHPEEGKVKIKVEWCGICGSDLHEY

TAGPIFIPAQASHPLTGEQAPIVMGHEFSGQVVEVGEGVTRFKAGDRVVVEPIYACGH

CEACKQGRYNLCDQMGFLGLAGGGGGFSEYVTAAENMVHAIPDSISYEQGALVEPSAV

ALHAVRQSKLKVGDTAAVFGAGPIGLLVIEALKASGASDIYVVELSDERKAKAEELGA

IVIDPMQFNVVEEIHQRTQGGVNVAYEVTGVPRVLQQAVDSTRIGGELMIVSIFEQEA

PIHPNSIVMKERTVNGIIGYRDVFPAVISLMDKGFFPADKLVTKRITLDEVLEHGFEA

LLKEKNQVKILVKAE"

CDS 3034637..3035674

/gene="degA_5"

/locus_tag="EFAGFIKM_02559"

/inference="ab initio prediction:Prodigal:002006"

/inference="similar to AA sequence:UniProtKB:P37947"

/codon_start=1

/transl_table=11

/product="HTH-type transcriptional regulator DegA"

/db_xref="COG:COG1609"

/translation="MRKEQKLTIKDVAERAGVGIATVSRAINNSEGISSKTRDKVMQA

IEELGFVPNTSAQSLKIRQTHQIALVVPDIRNAIIPEISWSVEQTAKQHGYHVVQINT

AGNARTELETIRNVKKLHVDGLIFMPLAYPKTLPGLIDKAPLPISMINYGKKLEPGMK

ADIVSLSQPEGKLVMEHLFKIGRTRIAYAGAPKDIIEERFRAYEQAVGHVDISLVYFG

EDFSLNTGANAADYFYGLTHMPDAVYAINDMVAIGLVNRFKELGVRVPEDVAVVGVDN

NQWTTVTSPQISSVSIMGEEVARLATELLLKRIREMNTADYEHIQFEPRLIVRESSVA

MIHSSQRGPHS"

CDS complement(3035809..3036777)

/gene="hprA"

/locus_tag="EFAGFIKM_02560"

/EC_number="1.1.1.29"

/inference="ab initio prediction:Prodigal:002006"

/inference="similar to AA sequence:UniProtKB:Q59516"

/codon_start=1

/transl_table=11

/product="Glycerate dehydrogenase"

/db_xref="COG:COG1052"

/translation="MNTKLRCAVLDDYQNVALTSADWGPLMDRVEIQTFNNYMGSEEK

VIQELQDFDIVVLMRERTPFPEKVISQLPKLKLLITSGMRNASIDLKAAEKNGIIVCG

TEGSSNPPTELTWALILGLSRQLVTENNALRSNRNWQSTVGLDLHGRTLGLLGLGKIG

TRMAEIARAFGMNVMAWSENLTQEKAEKHGVIWAETKEQLLAQSDIVSIHLVLSDRTR

NLIGQAEFQQMKSSALLINTSRAGIVDQEAMVEALQSGVIAGAGLDVYEQEPLPVNHV

MRTLPNVLATPHLGYVTRGNYEIYYNHTVENIVMFLKGTPIRQLHS"

CDS complement(3036858..3037439)

/gene="comR_6"

/locus_tag="EFAGFIKM_02561"

/inference="ab initio prediction:Prodigal:002006"

/inference="similar to AA sequence:UniProtKB:P75952"

/codon_start=1

/transl_table=11

/product="HTH-type transcriptional repressor ComR"

/translation="MVRQREFDTDKALDAAMHTFWDKGFEAASLNDLTTAMGIQRPSL

YAAFGDKKELFETALRRYTTQHAAQVRARLQQGSSVREAFRGLFEHIGAEVSVTEPSH

GCFCINTMVELAPHDPKFAVLTREHQMYLGVLFKETIERGQQSGELSTDMNASAVAQS

LVVSMIGLTVLMKSGPDRLFVEQSIQVTLSLLH"

CDS 3037726..3038946

/gene="sotB_3"

/locus_tag="EFAGFIKM_02562"

/inference="ab initio prediction:Prodigal:002006"

/inference="protein motif:HAMAP:MF_00517"

/codon_start=1

/transl_table=11

/product="sugar efflux transporter"

/translation="MQTSSPDAQLSNKDRPAMSRLVALLFAVCSGLAVANIYYAQPLL

DSIAQEFSLSPSSIGIVITVTQICYALGLFLLVPLGDLLNRRKLIIIQMLLSVLALVL

VGTAQSSSLLFMGMAVVGLLAVITQTLVAFAAHLAAPSERGRIVGLVTSGIVIGILLA

RTVAGTLNDSLGWRSVYLFSASLTLLGIVALFFVLPRQQSTQIKQNYTQLLGSVLQLY

RELRVLRVRGVLAMLIFTAFSILWTSMVLPLSSPPLSLSHTVIGAFGLAGAAGALAAA

RAGKLADRGLGQKTTGVALVILLLSWLPIGYVHHSLWFLILGVILLDLAVQAVHVTNQ

SLIYEVRPEAQSRLTAAYMIFYSIGSATGSIVSTQVYAWAGWTAVCWLGAGVSAAALV

FWMIDRYMHRNLDR"

CDS complement(3038948..3039106)

/locus_tag="EFAGFIKM_02563"

/inference="ab initio prediction:Prodigal:002006"

/codon_start=1

/transl_table=11

/product="hypothetical protein"

/translation="MDNEYFVGWGTLALINAGLAQGKNRSGRNWFLISLLFGPLATLF

IVVWNKLD"

CDS complement(3039142..3039597)

/locus_tag="EFAGFIKM_02564"

/inference="ab initio prediction:Prodigal:002006"

/codon_start=1

/transl_table=11

/product="hypothetical protein"

/translation="MIEVTTEITIHASIERCFDYARDIDIHTQTVWQHTRERAVAGVT

TGRIGAGDTVTFQATHFGVRQKLKSRIVQFERPFLFVDQMEKGAFKSMRHEHHFSVIG

DQMTCMRDTLRFEAPLGLLGWATERLVLKRYMQAFLEERNRKLKDMLEQ"

CDS complement(3039773..3040858)

/locus_tag="EFAGFIKM_02565"

/inference="ab initio prediction:Prodigal:002006"

/codon_start=1

/transl_table=11

/product="hypothetical protein"

/translation="MRIHIMNLQDGDRLTADTFSDAGLHVLGKGTVIKGEDITLLMQH

RVDYVDIESREEEITEAEFFAAAAKHASGITTKEEPPEEELKSQFIQTVHNYQNAFLE

ALTVGKFNATMVDDALQPMVEGLDEQKDVVHLLMMLERDDVNNYTHSIQVGLLSFYLA

NWLGYSQKESYQISRGGYLHDIGKCKVSHRIRNKTEPLTADEQLEMQRHTIYGHEIIK

NSMTDEATALVALQHHEREDGSGYPMQLEKSEIHPYTQIVSVADIYIGMRSGNHGGSN

PNLINNLRDIYGMGFGKLNEKPVQALMQHLLPNFIGKQVLLSNGEKGVIVMNNTSDIF

KPLIKVESEEYRDLSKERTLAIDELLI"

CDS complement(3041142..3042977)

/locus_tag="EFAGFIKM_02566"

/EC_number="4.1.1.61"

/inference="ab initio prediction:Prodigal:002006"

/inference="similar to AA sequence:UniProtKB:Q9Z8L0"

/codon_start=1

/transl_table=11

/product="4-hydroxybenzoate decarboxylase subunit C"

/db_xref="COG:COG0043"

/translation="MKYRNMEDCINDLEEHGHLIRVKEEVDPHLEMAAIHMKVHEAKG

PALLFENVKGSKFQAVSNLFGTVERSKFMFRGTLEGVQRVMAVRDDPMKALKTPFQHV

RTGLAAWQALPKQKSISLPVSAQEIQISDLPLIKHWPMDGGAFVTLPQVYSEDPDKPG

IMNSNLGMYRVQLDGNDFEMNKEIGLHYQIHRGIGIHQAKAVKKGEPLKVSIFIGGPP

AHTLSAVMPLPEGLSEMTFAGLLAGRRFRYSYKDGYCISNDADFVITGDIYPGETKPE

GPFGDHLGYYSLTHEFPLMRVHKVYAKPNAIWPFTVVGRPPQEDTAFGDLIHEITGDA

IKQEIPGVKEVHAVDAAGVHPLLFAIGSERYTPYQAVKQPTELLTIANRILGTGQLSL

AKYLFITAEDQQTLDTHKEVEFLTYILERMDMQRDIHFHTHTTIDTLDYSGTGLNSGS

KVVFAAYGDKIRELCSEVPESLKNIRGYENPQLIMPGIVSIQTSAFTSYADTAQEMQA

FTSLLKEQGGLDSCPMIILCDDSSFLSANLSNFLWATFTRSNPSHDMYGVNSGYDHKH

WGCDQVIIDARTKAHQAPPLIPDASVEKSIERFFVKGASLGSIKI"

CDS complement(3043193..3044536)

/gene="sasA_9"

/locus_tag="EFAGFIKM_02567"

/EC_number="2.7.-.-"

/inference="ab initio prediction:Prodigal:002006"

/inference="protein motif:HAMAP:MF_01837"

/codon_start=1

/transl_table=11

/product="Adaptive-response sensory-kinase SasA"

/translation="MKMFRKSLRLRIVATFIGIVLVSLILSFMINNGSQEKTPNRFMV

TFAQDLATLINLIDDPEKVKSSLEIFARYGLNITPVNEQSEVLFSLPEDKVHLLFDSG

TTDAIFLSSKDGIATIGVPGTNEGIGTFLIQSDFSSLFHTLRNTLLTSLLTVLVIGSL

LILFMSGYIVKPIKRLTVAAKEMSSGDLSVRLKHNNQDEFGELMESFNHMASELQKID

SVRDDFVSNVSHEMQSPLTSIRGFTRALQDGVIPLEEQKEHLDIIYEETLRLSRLSDN

LLRLASLDSEHHPVHFTTFQVDEQLRRAILLAEPQWAHKDIQIELDLLPCEITVDKDL

FDQVWQNLINNAIKYTGPKGTIHVEIETSSSFVKVLIRDSGQGIPEEALPYIFDRFYM

VDKARSSSLRGNGLGLSIVIKILKLHQCTIDVESEVGKGTQFIVTIPRSTITPSS"

CDS complement(3044533..3045201)

/gene="hssR_2"

/locus_tag="EFAGFIKM_02568"

/inference="ab initio prediction:Prodigal:002006"

/inference="similar to AA sequence:UniProtKB:A6QJK3"

/codon_start=1

/transl_table=11

/product="Heme response regulator HssR"

/translation="MNTILVVDDDSHIRKLIRIYLEKNQFSVLEAADGQEALDILSHT

KVDLAIVDVMMPRIDGIELTEDIRSYLDIPILMVTAKGESKDKVRGFNAGSDDYLVKP

FDPVELILRVKSLLKRYNKSSSNMIQVSGVTIDLGNLIVVAGGQTIELKKKECELLFS

LASSPGKIFTRTQLIDDIWGIDYEGDERTVDVHIKRLRERLEPVPELVISTIRGLGYR

LERA"

CDS complement(3045229..3047193)

/gene="btuD_7"

/locus_tag="EFAGFIKM_02569"

/EC_number="7.6.2.8"

/inference="ab initio prediction:Prodigal:002006"

/inference="protein motif:HAMAP:MF_01005"

/codon_start=1

/transl_table=11

/product="Vitamin B12 import ATP-binding protein BtuD"

/translation="MKILEVKNVKKSYTLYGKERVPVLHGLNLSFETGEFVSILGESG

CGKSTLMNIIGGMDSDYEGDVVVRGKNLSAMTEKEMDDYRKNNIGFVFQNFNLIPHLS

VLENVTIAMQMTDTNEKDRNQRAVDILTEVGLKDHLNKRPNQLSGGQKQRVSIARALS

NNPDIILADEPTGALDKENGDQILALLDSIAKKGMLVIAVTHSQKVADSGTRIVKVEE

GRISDDIHLKDPSTAVYESGQDSSRSLSLLASFKMALKNMKLNGKRNVLVALGGSIGI

LSVLLMLSLGNGITTYMNDEINSSMDPLLVDITKPSEEAKDMQGPEALMAPGEPFTEA

DVETILNFPNVDHVETITTITGQSTMVNEKQSVGLTQLTTLTDAFDPALMTTGVLPAE

NQMLLPLDTANKLSGNDQADSMIGKSVFLYINEMDSNNKPVTLEKEVTISGIYEAADP

RSPMQQSPGYISSETLGQMYTDKGISIGPIQVNAYATDMKYVNEINEAAVDAGFAGSQ

MAKVMENITTYVSMASIVLAGIAGISLIVSGIMILVVLYISVVERTKEIGILRAIGAR

KKDIKRIFFSESALLGVFSGIIAVIFAVIISYVLNILLDNAFGAKLINLSGYYILFGL

VVSTAISIVAGLMPSSKAAKLDPMESLRYE"

CDS complement(3047391..3049340)

/locus_tag="EFAGFIKM_02570"

/inference="ab initio prediction:Prodigal:002006"

/codon_start=1

/transl_table=11

/product="hypothetical protein"

/translation="MFQPKKKRPRIRKTMSGLIALTLLSSLILPNVAGAEPVEPSQVQ

FANVSVQAGQTVHVPVALKKSYFGVKAYNMQIDYDTSALEIVRITPKSVNVISTSPNE

DGVADFQYQINNAEGWVRIIWVDFNAGERLISDEQQLFDIEVKAKSNATPGTKQLTVV

QSDSEHWHFTNVEHNSGAQLSGGTITITAANSGGDNSGSTPTPNPGNSSGGGGSVSPT

SPVVTPVPSTPAVTKGVDIYVNGQKQEQSATATTSTVGNQVTTTVHVDNNKVINQIGS

GLKTLLLPITGTGKGAVVGELNGKLVKTMEGSNAEVVIQTDSGTYTLPANQIQVDQIL

NQFGGTTPLEDITIQIAIAPSTTSKQTTIQAAAEKLENTTVVATPVDFEVKAIWNGQQ

VNVDRFNSYVERSITLPEGVDGSKITTGVVLQPDGSLLHVPTKVIKGNVQDSAIINSL

TNSTYALIYHPATFSDVSSHWSRDDVQDLASRLIVQGTGENVFAPDRSITRAEFTAVL

LRGLGLHSPNSTEAASFTDVKTGSWYEDEVQTAVSYGLISGYADESFRPNNEISRAEA

LTIVSRAMKLVGLAQADASETTSLLSTYSDSAKVQSWAAEPVASAIKQELVQGADGKL

MSDADISRAQSAAIVKRLLAKAGLI"

CDS complement(3049390..3050418)

/locus_tag="EFAGFIKM_02571"

/inference="ab initio prediction:Prodigal:002006"

/codon_start=1

/transl_table=11

/product="hypothetical protein"

/translation="MKGKQNKKRLKPILKKSMLTALGLGIALPIGGTLPQANAGEIGP

EIKISRPVLNDGIHWLNYSSEQNDPSFVSGVPIEVTNRTVTIDLSTHFNSNEFNIISA

TSGDYSVASVYIEGGNKLVVVPYRSGKINIELKANYTVSDVPETVTDNFELYISKKGD

VNADGKVNSLDAADLLEYLRNMVSRRGFSYVEMNRMDVDRNAEPTLGDMNALLNGYAN

KTLGAKNNDYVLTFQQVDDAPYVLNGQLKNYMENAIATDYYLMDVDDDHVDYINTPSY

QWYRATDPSGEDMVSIEGETQEQHTVTFEDEDHYLVLEVTAHSTSDANTKPRRVYIVG

KEKIQRPD"

CDS complement(3050611..3051903)

/gene="mtaD_2"

/locus_tag="EFAGFIKM_02572"

/EC_number="3.5.4.28"

/inference="ab initio prediction:Prodigal:002006"

/inference="similar to AA sequence:UniProtKB:Q9X034"

/codon_start=1

/transl_table=11

/product="5-methylthioadenosine/S-adenosylhomocysteine

deaminase"

/db_xref="COG:COG0402"

/translation="MSILIQQATILTMKDADAPFMGDIRVEGDRIKQIADHILPQPQD

EIIDGRNKVAMPGLINAHQHTPMSLLRGFSDDLKLMDWLERKMLPAEARMNPEDIYWG

AKLSIAEMIRSGTTAFADMYIHMNEIAEAVKQTGMRASLTRGMVFMEEDGGRRLQEAI

DLVQRWSGAAEGRITTMYGPHSPYTCPMEPLREVIAMAVTEDIPLHIHLAETKGEVVK

IRERYGMTPTEYLEEAGMFEQAHVLLAHGVHLNRRDIGRLKGMRGGVAHNPVSNLKLG

CGIAPITEMLAQGINVGIGTDGAGSATTVDMFEEIKAATWLQKLDYGDPTRLPAKDVL

SMATRGSASLLGLQDEVGILEVGYKADLILIDLAKPHLQPVHEVESLLAYSVNGADVD

TTIVNGQILMRGRKLLTIDEEELYREVKVRAKRIVEGI"

CDS complement(3051982..3052275)

/locus_tag="EFAGFIKM_02573"

/inference="ab initio prediction:Prodigal:002006"

/codon_start=1

/transl_table=11

/product="hypothetical protein"

/translation="MNCVAVLPYYKVQREVTGLAQEIEMNARSMILYSDKIVTKYREF

NITEVFDMSFRRMGDEGGFFYLHTSTGVYPYMVEIDPKPFIQTFRKMTIQKLK"

CDS complement(3052329..3053447)

/locus_tag="EFAGFIKM_02574"

/inference="ab initio prediction:Prodigal:002006"

/codon_start=1

/transl_table=11

/product="hypothetical protein"

/translation="MQFETSVKPIKDQIWVVGGYGQVGQMICTQLGKMFPGKVWAAGT

RMNRAEEFSRSTSGAVLPLQLDVTRPVEPSMLRPVKLVIMCVDQSDTRFVEACAQAGT

DYIDISAKYDFLAQVEQLHTKMQRSKSTAILSVGLSPGVTNLLVREATMHMDQVEEAD

ITVMLGLGEKHGKAAVEWTVDQMNATYQVMQEGKPAEVQSFGDGKRIDFGAKLGQRKA

YRFNFSDQHVVARTLRIPTVSTRLCLDSRWITGSMAISKRAGLFSLLRIPSIRSGTVK

AFGLIPGGEPMYAVKVDAVGWENGEQIRVEQLLVGAKEADATAAVAAAVAERVYKTEL

PHGVFHIEQCLSLQDIQDALHTPLKVTTKITKSHVLLL"

CDS complement(3053431..3054138)

/gene="cmoA"

/locus_tag="EFAGFIKM_02575"

/EC_number="2.1.3.-"

/inference="ab initio prediction:Prodigal:002006"

/inference="protein motif:HAMAP:MF_01589"

/codon_start=1

/transl_table=11

/product="Carboxy-S-adenosyl-L-methionine synthase"

/translation="MNHNLSPMSWETAEVHRYEQSIALKIPGYSHMHDLMERLLSASF

ANNNDIHILVAGAGGGKEIALLGSRHAGWTLTGVDPSQPMLQLAEKRVTEAGIGSRVK

LQPVTVEELPEDIVYDGATSMLMLHFLQGMEAKRTFLTSLAARLKPGAPLIIAAVNAD

LCSPAHPTMMQAWKDHMLSVGVLPEEWERFAASLGRESDPISSEEMTQLLTECGFSHI

TRYFGAFWVEGYYAIRN"

CDS 3054268..3055374

/locus_tag="EFAGFIKM_02576"

/inference="ab initio prediction:Prodigal:002006"

/codon_start=1

/transl_table=11

/product="hypothetical protein"

/translation="MTSRIDRNHRLITSLEKGTWTSRTYREAVLKQIHTVVHYDAYCF

TTVDPLTLLSTGAVTEDGIEAIHDRLFINEYMEEDIHKYAELIRSGEHTATLHTSNLQ

LEQSPRYINILQPAGFGDELRAVLVSGDACWGYLTLYRKTESAVFTEEERMMIQSWTA

SIASMLRSTSLTLIDEITSESPEEPGILITSDTFQLLSLNASAQYWLSQLRMLEHVGP

DVLPRPVRAVSSHLQRKNRAELVVGTQIATDLPSKVCIQLPDGRYLILHASLMQQLTG

PDQIAIRLEQAMPQDLLPLLAESHGLSSRERELLGYVLRSYSSKEIAAAMHISVYTVQ

DHLKSIFAKTKVSSRRELIWYFVSRFQLSDEPAI"

CDS complement(3055617..3056630)

/gene="feuC_2"

/locus_tag="EFAGFIKM_02577"

/inference="ab initio prediction:Prodigal:002006"

/inference="similar to AA sequence:UniProtKB:P40411"

/codon_start=1

/transl_table=11

/product="Iron-uptake system permease protein FeuC"

/db_xref="COG:COG0609"

/translation="MIKRRYTMKRGIIINVVLMVLILVFAIISMNSGKMNLSPLEVLN

VLIGNGTDKQNLIVFDFRLPRIVLSILVGLGMGAAGVVMQSLLRNDMASPGTLGISSG

SGLFVLFFVVFIASTGKSSFIALPLLAFVGGLLAAGLIFLLSYRRGRDISPIGLILTG

VALGSGYGALTTFLTLKLDDSQMNFMLYWLAGSLWGDDWGYISILTPWVLILLGYIFY

KARILNALHLGNQTAQGLGVSVNRQFLGLSIAAVALSSGSVAVGGSFFFVGLIAPHMA

RKLVGPDHKLLIPAASLAGGLVVVLADTITRTVSLVGDVPTGIVITVISVPYFLYLLA

KAK"

CDS complement(3056627..3057652)

/gene="feuB_2"

/locus_tag="EFAGFIKM_02578"

/inference="ab initio prediction:Prodigal:002006"

/inference="similar to AA sequence:UniProtKB:P40410"

/codon_start=1

/transl_table=11

/product="Iron-uptake system permease protein FeuB"

/db_xref="COG:COG0609"

/translation="MKSKPEQKRRGAMNFTMYMVIGLGLTVLMSAASIMFGAADMRLA

TVWEAIFRFDPMLTEHQIIQTMRLPRTVADLIVGCSLAVCGAIMQGTTRNPLADSGLM

GISSGAAFAIALCLAFLPGYSYWQMMLFACLGAAIATGMTYFTASLGNCGMTPQRLVL

AGLSISMLFGALSQYLAINYNLGRALTFWTAGSTAGVKWGELLIISPLFVAGILLALA

LSPSVTLLSLGDDVASGLGIRSGLVKILSTIIVLVLTGLAVVVVGPVGFVGLITPHIV

RYMVGVDYRYIIPAAALYGALLTVSADLAGRLINKPFETPIAIIFSIIGVPYFLFLAR

RQRREYE"

CDS complement(3057770..3059737)

/gene="rhaS_23"

/locus_tag="EFAGFIKM_02579"

/inference="ab initio prediction:Prodigal:002006"

/inference="protein motif:HAMAP:MF_01534"

/codon_start=1

/transl_table=11

/product="HTH-type transcriptional activator RhaS"

/translation="MQEEEARQGFTPEMIDMMASIWTRSIITLIDVRFQNIESQYPLE

QYKMPSSMFIYAYGGEAQIQLNETTFAMERFGLVHGGKGSLLSITPKEETVKTFMVFY

RAESPLFFKKHLQQLLEQVNPFVQQFGYTPSNPILLRDWFQRMMNGWKRGKEMDQLQA

KSFLYQLIHEVYRDLEGGEIRYLQPDPACSAKVYLDKHYREPIMFQQIADMFGISGGQ

LTRLFKKREGMNLQEYLIQRRIEAACHDLKHTEATVKEIATGAGFTDEKNLFRMFKKH

YKMTPSDYRKINALSMQVDGIDNDSHLPYDERELVSLVKSYRDGESMMFGTLRSKEMI

LAAAISMMLLLTACTSGTPANNGGTANTTPAQTQQATQQEGSETKASEAVSQTRTIST

VKGDVEVPNNPQRVVVDYLVGDVVALGVTPLGVARAARGETEPVYANQITDSIKVAME

PEDIMTLEPDLIILAWSDESYDDLSKIAPTIYVPYGDMTTEERIQFISDVLNKQEEAK

DVLNAYTGKIEEAKLAFQNAGLSDVTITVGEFSDKSNYIAGAKHAVGELVYDELQLQA

PSKVQTDIIDADKYWGDVSMEVLPAYSGDYMIFLGDGNVASDNAVWNAIPAVQHDRIV

KVDSSLSWSTDVMTSSALIDYIVSQLLALAK"

CDS complement(3060025..3060912)

/gene="yidC2_1"

/locus_tag="EFAGFIKM_02580"

/inference="ab initio prediction:Prodigal:002006"

/inference="similar to AA sequence:UniProtKB:Q9KDP2"

/codon_start=1

/transl_table=11

/product="Membrane protein insertase YidC 2"

/db_xref="COG:COG0706"

/translation="MEHKTNKGFTFTSGKGRIYGLIVILFAVMLLAGCSNNVSEITSS

TPGFFNHYIVFPLSYLIQHIATIFSGSYGVAIIVITLVIRLALLPLMMRQAKSQQGTR

VIMNAMKPEMDALKKKYEGKNDPADKQKLSQETMELYKKHKFNPLNIGCLPMLIQLPI

LSGIYTAIRLTPELSSHSFLWFKLGAPDYVLAVVVAVIYLIQAKVSQANMAPEQRKQF

AIMGYLSPLMMAFFSLTAPAAMPLYWTVGGSFLVLQTLLFRKMYPVEHPQEPVVVEVS

NKKNKKSSSAKPSRKPAKS"

CDS complement(3061292..3062911)

/locus_tag="EFAGFIKM_02581"

/inference="ab initio prediction:Prodigal:002006"

/codon_start=1

/transl_table=11

/product="hypothetical protein"

/translation="MIDKIMASPQRALITLLSAWMLAIIHQYLFYGNEIGVSYPIFVI

LFYVFMYLFARDRMRSFKFIDAFVAAVVLLLSLTFLLYDNELLYVLNFLVIPGVVILH

MTYLMGRKQKQWWEIGLIGTAIDHMLPQAIRHWGTVAAIAVRAGGRGIGKSQKTVVFK

VLIGLVASLPILIVVVALLSSADGVFDQYLAGFPEWLNQLAFTPGIPRIIWIVIAGVL

LFGYVWGFVQPMQYEAEKRANAHWKNGAVSTVDKRDNTYIFSPVDPAIEGKATNKITP

EPERTPIQREPFRLDPIIVGTMLIVINCVYVLFVLVQFSYLFGAGEGHLPVDLSYADY

ARSGFAELILVTGINFFILIVALQYTRSSGKTGSIVHQVLLLILVSCSAIMLYSAFMR

LNLYEQAYGYTYIRFLVHAFMIFLALLLLIAGLRIRYTSIPLIRWYIVLALTAYVAVN

YVGMDKRIAELNIERYHQTGHIDATYLASLSADAVPLLREFALEEYPDLKREMLERQA

YLDVDSSDRSWPSYNVARHRAEQELSKLRTE"

CDS 3063046..3064836

/gene="sgrR_2"

/locus_tag="EFAGFIKM_02582"

/inference="ab initio prediction:Prodigal:002006"

/inference="protein motif:HAMAP:MF_01449"

/codon_start=1

/transl_table=11

/product="HTH-type transcriptional regulator SgrR"

/translation="MKLHQQYLLLHRHYGHHTEHELTLADLAELLNCTHRNTLTIVKK

MVAHDWIRWVSQRGRGRRSSLTLLVPADQIAAEYMMQAMNRRELQEAAEQVSAFSSST

IMQDHLNQWLLGYSGHHTEAGTNNEQIDTLRLPLRQQLHALDPLYINLLAESFVTSHV

FDGLVQRGEQGEILPCLAHTWDVSADRKTWIFYLRKGITFHDGQLLTAHDIVHTFERL

QSTDRRTLYRDVSKQILSIEAVDSLTVCFQLKNPHELFLSFLTTSRAAIVPSPGTNEH

KMKHSSDVHGMQKPVGTGPFKVTAWDDHLCRLEAFSSYFQGRAHMDRVDILQIPWSAS

AKMDDSQDIETPFFHLVHNPSSSAGADWTQISAGVMVHKLLTCNTQKNGPLNDPEVRA

HLQSCLAGMYRDDGHADDSEVVETNFSAVNTKKVQVHSEGFSDISISSVSSSPISLHI

ATIPQYRRDAQHLASILEQCGFSCTVRTATMEQFKGNLRLESDLILFSLIRDRDEELR

RYDLYSTLSEHLEDSARITIHEILQTIVASPIAADRTCELDRIEQFLVGQNLLFHLSE

KPVETAYLPSVRGLSFNSQGWVNLRHIWFP"

CDS complement(3064898..3066250)

/gene="mrdB_1"

/locus_tag="EFAGFIKM_02583"

/EC_number="2.4.1.129"

/inference="ab initio prediction:Prodigal:002006"

/inference="protein motif:HAMAP:MF_02079"

/codon_start=1

/transl_table=11

/product="Peptidoglycan glycosyltransferase MrdB"

/translation="MTNRHERIQHYLDHMCVQVKAREVHNDLRDELGNHMEEIKLYKK

QEGYTEEEAIAYAIEQMGDPAVVGKSMHRLHRHRMHWGIVSGIIVLATISLILMWIFT

TNTTQARYQYLYSYHVKNTVVGLMMMSFFIFFDYRKWKKAAWWIYILFNALLWINPML

SSAYNGIDRYLSLPFGFVLDVTTVSMWILPLAIGAIMLDKLRTSCSIQSVLSYIAMVA

LPAVLLFQISDWVRMLLFGIVSVILFGWITRKWLYMIIAAITTGSLSLILLLFMDEYR

RLDRFSVVFNLQDDPNGNGFVYNSIMDIVRTAGWWGNGLDTRFDLIKSNYFDFPGVML

IDVFGWSAGILLLIGIFWFVSSMVKILPRIRDDYGRMIILSVTTILSLQMIYSLVVIT

GKVPIISIAFPFIGYGTHLIFEYAMIGLLLGVYRRKDTISKKNEKIRQKVDADLLTNS

"

CDS complement(3066247..3066582)

/locus_tag="EFAGFIKM_02584"

/inference="ab initio prediction:Prodigal:002006"

/codon_start=1

/transl_table=11

/product="hypothetical protein"

/translation="MQVNKQMIKGSTETLILNLLQERPLYGYELIKELHRQSEGVFNL

KEGTLYPILHAMEIEGWVESYWMEVEGRKRKYYSILDKGMEALQSKKAEWNLFRRAVD

RVLGEGGLT"

CDS 3066867..3067451

/locus_tag="EFAGFIKM_02585"

/inference="ab initio prediction:Prodigal:002006"

/codon_start=1

/transl_table=11

/product="hypothetical protein"

/translation="MGKLIFFLGGAGSGKTTLAKALSRKHKAAFFDMDILLRPAAEAI

MTLQGLDPSDRDSPEYKRLCRDLGYRITMDAALDNVQLGIDTIVVGPFTKEIGTPDWI

AQELARIGHTLDDTDVRVAYIYLENEALYHKRITARQSPLDEWKLANWDAFTASLVRK

EIAWPLPASSVAYIDNSSDDPAIAYAELERRMYE"

CDS complement(3068042..3069010)

/locus_tag="EFAGFIKM_02586"

/inference="ab initio prediction:Prodigal:002006"

/codon_start=1

/transl_table=11

/product="hypothetical protein"

/translation="MMYTDKQEYSFSTCWNIKRHTNGQGMIEEIKSLGFKQVELNYNV

TEEMLQTIEPMIERGEIGVSSVHNTFPHVADPDYGTDSVLLGFDDEPRRRRAIELLLR

SAEYAHRYGAKAVVVHPGEVPFEYNIDEALKKIYHDQGPDSPAYQSLWQEMLEKREDG

SAHYLSRIQESLEEVCDLSEQRGYGVSFGIETRSRCYQMPTLHEAGTIIGQMKGAPLG

LWYDIGHGMMMDRMGLYDNVREANALIDHVVGVHIHETVGLSDHWCPYIHSKDMTYFD

SFLDIIDRSPVKVYELKAACTPEEIDESHGIITARIAERHASHQRG"

CDS complement(3069310..3071016)

/locus_tag="EFAGFIKM_02587"

/inference="ab initio prediction:Prodigal:002006"

/codon_start=1

/transl_table=11

/product="hypothetical protein"

/translation="MTQRMVEKMSKEQQYLVNQSMLMMDSFYDKEMDLLRSFEEEDSS

QHSTRASAHYALGLLIRNEPGDVERAIRVFHKVLDVQYDAPNEIYHGTFATRPTDAHP

PAGHYAWKSFAPGTAYFLERTFDKVSAQFIHKLQEDGSLLTEAADSKQLKRLFHSAVN

QILPPVWISYDPNWREFIACVCVVVTAEFGELIPETLMKRMDHAMELAVQGSIERRLS

DVIPMNTNIELMHIFMTHYYGHRLNRTEWIQHSDEQAEGLMKEFEQYDGTFAEFNTTT

YYGVDLSVLGMYRKYGLTERLVEIGHRIAHGLWNNIALYYNANLENLSGPFSRAYDME

MREHSSIGVFIYLLLGEGYEYLAGINCESGHDPMIALLGVDVPAEAVPFFKAHQEDRL

VQKQFMELCERNDPTSNRNLCTAKAWIGEDRMIGAMSGSVNTNGQMHPATIHWQTERG

ERYYLRLIRREVGEGWNSHLRGVTFDADLTPDSLTIDVELDTAEEIDVYFEIRGPFLR

QQDITPVLWQFPGLTCTVKAEAPEPSVLMYVNHAEIIYRYVPGKTANRMRFELTLRED

NG"

CDS complement(3071038..3071910)

/gene="araQ_17"

/locus_tag="EFAGFIKM_02588"

/inference="ab initio prediction:Prodigal:002006"

/inference="similar to AA sequence:UniProtKB:P94530"

/codon_start=1

/transl_table=11

/product="L-arabinose transport system permease protein

AraQ"

/db_xref="COG:COG0395"

/translation="MSENTANRIFNTVNVILIVITMVLCLAPFIHIIAISLSSNRAIG

SGEVSFFPKELSFEAYTKVFADGSMIRSLIYTIWLTVLSTVLSMVMTIAAAYPLAKSN

LKGRKWFMLVIVVTMFFSGGIIPEYILIKNLHLLDSIWGLILPGLISPFYMIILITFF

RGIPESLEEAAGIDGSSHFGTLMRIILPLSLPVMATLSLFYAVGRWNGFQDALMYITK

PELYPLQLKLYQMIQQNQITELMQNEGIGAVQVLPESLKAASVIFSTLPILLVYPWLQ

RYFISGVMVGAVKG"

CDS complement(3071949..3072851)

/gene="yteP_23"

/locus_tag="EFAGFIKM_02589"

/inference="ab initio prediction:Prodigal:002006"

/inference="similar to AA sequence:UniProtKB:C0SPB3"

/codon_start=1

/transl_table=11

/product="putative multiple-sugar transport system

permease YteP"

/db_xref="COG:COG4209"

/translation="MKTSIYFRRNWQLYALLLLPMIYFIIFKYGPMYGVQIAFKDFNF

FQGIAGSEWIGLDAFREVFQNQGFYTALRNTLVLNMLDLLVSFPAPLILAILLFELKK

AWFKKLAQTLLYIPHFISWVIIGGIVLQVFGTQSGFINNILMSMGFDPLPFLSDKNYW

LFTYLAVGVWQSAGWGTILYLASLTGINRELYEAAEVDGASRLRRIWHISLPGIKTTI

VTLLIINVGNMISIGFDRPFIIGNVAVREYSDVLSTFVYRVGLQSGQVTLATAVGLFQ

ALVGLVLILGANYTSKKLTDESIM"

CDS complement(3072963..3074564)

/locus_tag="EFAGFIKM_02590"

/inference="ab initio prediction:Prodigal:002006"

/codon_start=1

/transl_table=11

/product="hypothetical protein"

/translation="MRLFQRRKAGKASSILAVVTAFTLLLSACSSGSESTSSSGESGS

GEKTTLKVEIFDRGNTPAGYTISDSYLTRFIQEKFGDPNNIDVQFVPVPRSEEVQKLN

VLMASGSEVPDIVFTYDSGTFNRYAQQGGLTELTDLINQTGPNLKKLLGDETLAYGQY

DGQQFAVPGKRLVLGKYASYVRQDWLDALGLPSPETAEELHTTLKAFKEKDPGKVGTG

LIPMGMSIASAQYEPLIWSFIEPLTEEQKYTLTQQLGSNDYPTLLPGFKEALKYMNTL

YNEGLMSKDFGLDKDKKKLWEDVSNGKVGFYTEDAGEIYISGTYKNLQTNQPGAVMTP

IDAFKNSEGKFAKPAYAPNAMYVMIPKSSKNAEAAMKYLDWMASGNNLFDLQFGVENE

NYELVDGVPLIKDDASPEIAGRIYNSGDIAIIVNGKYVGDDKKNEEAYVVQVESKYRD

DMRKSVAISNTDTIQPVRFSKPIDAEARYGNGLKDKFMEFFVKTTISKPADFEATYDS

MMKDYMASGGQAILDERTEAYKAMK"

CDS complement(3075152..3077395)

/gene="rhaR_23"

/locus_tag="EFAGFIKM_02591"

/inference="ab initio prediction:Prodigal:002006"

/inference="protein motif:HAMAP:MF_01533"

/codon_start=1

/transl_table=11

/product="HTH-type transcriptional activator RhaR"

/translation="MKVNLKRNWHTKLMYSYFPIFLLTISILIFLSFLIVNELSRNET

QKADMISTRYIVDTLERSLSDIELRLLEDIGANKTYSRFLEAGQGQLSSQFIYDAASG

LGRMLDQQDMIQSIYLYRMSDSQILTPRGMMRLEDFEDRAYIEQALKDRENLGWNLPR

DYKERSFDVPSQVISMNKWLPLPWGGEGLLVITIRMYAVERQIDNMTNEKLSVLQVRD

KSDNLIYTAHQMDPSAGEILNRVTADKLGLVFESGIQSGQLYSWVSLVSYVWIGIGLL

TIVGAVAGLIYITRRNTRPIQLIMNRIQALQPRAEEDEVAPSVKDELALIDRALEHLI

AQTVDYEKQHHENLLIHRRQLLVDLMEGERMDSFRQRLRHLQPLEERFMEPKSVAVLI

AEMNGDDLSTNQNILKIALMNVVEELVQNESQFGGWAEWFGNRRLIVVIASDEQEGLD

RELLMDLAKQMHMWIAENFRLRFTFGIGRTVSGWEEITRSYASADAGLQHRLTLGKDA

IVLSEQLPDRIELQSYKYLQMLADFVREFRLTGDGWRTQLDQMFVAFNEDQITDNDIR

MLLQALIQMLSREFGELSERLQSQFAEEILTRLRSDIQQVETLEGIQEILQEWLKEVY

RNYVSVNETKSHRAMINELRIYIEEHFDDPDLSLKHLSDRFQISGKYASYLFKEEFEM

KFVDFLVKLRVEKACRLLSASDMAVQDIALQVGYANAISFGRVFKRIMGVTPGDYRKQ

SGKQGDQ"

CDS complement(3077417..3078640)

/gene="nupG"

/locus_tag="EFAGFIKM_02592"

/inference="ab initio prediction:Prodigal:002006"

/inference="protein motif:HAMAP:MF_02049"

/codon_start=1

/transl_table=11

/product="Nucleoside permease NupG"

/translation="MRSRHMKAYTEKIKADSNDAKRVLLQLQGLYLFMGLAGGMFNPY

INPILVAQGFSSKETGFIMAFGTLVSIILQPIWGILVDKFKKTRFVLVISLLVPASLA

YFYNIQVYFILILIYTLCTIFQVTQIPVADSYAVTAARTANTSYGMIRLFGSIGTGVG

GFAAGMYLSQFSIHMLWLPFLLFNILSAILASTLPRQTSISSSSVTFSVGLARLLRNR

TFLLLFLTGCFLVNQTLTAFNSFFVISFQMAGGSVTMTGTALLLASITNVPSMLAAAFI

LRKWGHERTMLLAAGAYMLRWGIQWLWPTPEVMIGVQVLHGLSFGFFYIAAVEYVASV

TGREMQATGQSLFNMVFAGLGGIVGNMLNGYLLDSGGPSLMYLACTISAALGSVILYI

VSRQAKEQRRAYSLE"

CDS complement(3078690..3079742)

/gene="glcK_1"

/locus_tag="EFAGFIKM_02593"

/EC_number="2.7.1.2"

/inference="ab initio prediction:Prodigal:002006"

/inference="similar to AA sequence:UniProtKB:P54495"

/codon_start=1

/transl_table=11

/product="Glucokinase"

/db_xref="COG:COG1940"

/translation="MTESVKNVSNNYQSNQDGTVGGDPSGWIAGIDIGGTKTLMLLSS

QQAGNEVHERILPTLASDQPDEFFRWLFAEMETFCREVGCSLDQLKGAGLGFPGVILQ

EEGILRNAPAFQWPELDIRPVIAKYYSGNIMLDNDVNLAAMGEYDQGAAQGHQHCVMV

TVGTGIGSALILNGQLYSGQTGAAGEIGHFIAGEEGLHTGYVADADSFGVFEQVTSGT

GITEQARRYFAAGKGSSLSLIMSLAGGEAEQIEARHVFKAAESGDLAALEILELPMRY

MARGLANITALLNPSIIVIGGGVAASNPSYYLNEVRTRLQRYTVLPTLLAIAELGNKA

GAIGALAAIKSSLTRT"

CDS complement(3079779..3080957)

/gene="nagC_1"

/locus_tag="EFAGFIKM_02594"

/inference="ab initio prediction:Prodigal:002006"

/inference="similar to AA sequence:UniProtKB:P0AF20"

/codon_start=1

/transl_table=11

/product="N-acetylglucosamine repressor"

/db_xref="COG:COG1940"

/translation="MAGTPQYIRNLNENLIMDALITQGTMSRADISRQTGLSKPTVSL

AVEHLIDRNLVREMGPADNAQGRKATLIRFNETAYYVCGIDIGATRIRIALSDLNGEI

IAYRTYPMVVQGAYERAEATMLELLRSHMNELLDENHLNWDQIQCIGFGIPGVVLPDS

GRINRIVDPLAGLEEAFSLESLSGAFPCEVILENDVNLAALGEYRSGAAAGYPLFVFF

SIGTGTGAGIMVHGQLLRGLGGLTGEIAEMLVDDGRRLEEVLSADGLMQLAKDYLDQH

VVADDLHRHLTPEKLFEAARSGEVEALDILQQYSQKIASALRQISVVLAPDLIVLGGG

VGGNGDVLLPLLRQIISEQFPVQPQLICSKLGEQAVVTGAVQVAIQQTMLNLQQEAME

"

CDS complement(3081627..3082466)

/locus_tag="EFAGFIKM_02595"

/inference="ab initio prediction:Prodigal:002006"

/codon_start=1

/transl_table=11

/product="hypothetical protein"

/translation="MKKRITDILFIMAGAFLFALAVNLFVIPNDLAEGGVTGITIILY

YLFEWSPGLMNLILNGILLIVGYRFLDKTTTVYTIIAVVFNSLFLHLTESWTIASDEL

WINTIFAGLFAGLGIGLIVRVGGTTAGTVILARLANKYLDWNISYGLLFFDLIVAFSS

YFIIGPQGLMCTIVLLFVGTKTMEFIIEGLNPKKAVMIISSKQDEIAKQVIEKMDRGV

TVLSGHGYYTKNKKEILYIVISKQEVSMLKKIVRAEDEIAFITIHDVRDVFGEGFIEL

SKT"

CDS 3082840..3083898

/gene="des"

/locus_tag="EFAGFIKM_02596"

/EC_number="1.14.19.-"

/inference="ab initio prediction:Prodigal:002006"

/inference="similar to AA sequence:UniProtKB:O34653"

/codon_start=1

/transl_table=11

/product="Fatty acid desaturase"

/db_xref="COG:COG3239"

/translation="MNRSKEMALKKEVTPYEKNDLRLSIRQLINTLLPLFLLWAAAYF

SLSVSYWLTFPIALVASGFVLRTFIIFHDCCHGSFFKSKRANDILGTITGVLTVTPYQ

QWKNEHSIHHATSSNLDKRGVGDIWVMTVDEYKAASPWGRLFYRIYRNPFVLFCIGPI

YVFLLAYRFNRKGARRKERINTHLTTVLIVVLYAFMSWLVGWQAFVMVQAPIFFFSGF

FGIWLFYVQHQFEETYFEHEDEWSYVKAAVEGSSYYKLPKLLQWISGNIGFHHVHHLS

PRVPNYFLEEAHNATPPLQKATTITLRSSLVALRFRLWDEDSKQFISFRQLKKLSRKP

YVQPPIRVTNPAGLTEKP"

CDS 3083998..3085134

/gene="desK"

/locus_tag="EFAGFIKM_02597"

/EC_number="2.7.13.3"

/inference="ab initio prediction:Prodigal:002006"

/inference="similar to AA sequence:UniProtKB:O34757"

/codon_start=1

/transl_table=11

/product="Sensor histidine kinase DesK"

/db_xref="COG:COG4585"

/translation="MQNFYKKTGLNLYVWLAFFILPFYFISQYSKLWTMVTGIIIILV

FFVCYLLAFITKGWQVYMWIGMLIAISITMTIAYDYAYFSLFLAFFIGNIKNKAGFFT

LYSVNLGASFVTINYGFINQSSLLVSQLPFVFINLMASILLPISTYNKNKTEHLEGQL

ENANKRLDDLVKMEERQRIARDLHDTLGQKLSLIGLKTDLARRLLRNNPDQAEIELND

LRQTASTALKEVREMVTTMRGTQLVDELFRAEQILKAASIEFVLEGNPKLQDTSQLNE

NVLGMCLKEAVTNVVKHSQATTCTIIIKETLADNVLTIQDNGIGIERTRKQDRRGTGI

LGMKERLEFVNGCLDIRSATDLPGTAIIIHVPKLVRKPIKEAES"

CDS 3085131..3085730

/gene="desR_1"

/locus_tag="EFAGFIKM_02598"

/inference="ab initio prediction:Prodigal:002006"

/inference="similar to AA sequence:UniProtKB:O34723"

/codon_start=1

/transl_table=11

/product="Transcriptional regulatory protein DesR"

/db_xref="COG:COG2197"

/translation="MIRIVIAEDQRMMLGALSSLLNLEEDMEVVGRASNGQEALALVQ

ELAPDICLMDIEMPVKSGLEAAEELKGMSCKVIILTTFARTGYFERALKGGVRGYLLK

DSPIEELAESIRQVMNGRRIFAPDLVDEAYVEENPLTERENAVLGLMADGKNTKEIAG

HLFITTGTVRNYISIILNKLNASNRIEAITRSKEKGWFK"

CDS complement(3085918..3086826)

/locus_tag="EFAGFIKM_02599"

/inference="ab initio prediction:Prodigal:002006"

/codon_start=1

/transl_table=11

/product="hypothetical protein"

/translation="MKKQPLWITIPVTTAVLLALAGCGGPDSNAASSENSSSPTTTVG

EQTETGSGGADFSNENETGTKENTGTTDNEDTSGSKAIDNQVATGQSGVNDVIKQVRK

QLKMKDAALPTSFTLDKGTSLGAAILSNTADAFKVNFYATTQSVAINDQSLTASDSKI

PVLASYEVKTYKDPNQPEIFPETDLKDIPADMAVDLGHGIKGMSEGAAGSQYLTWQEG

RWTLEIRSVSEDEMNNSGIAKKMVEYLESHTLPVPKDKGYIKVEYPSGGKSVHVMISW

QDGNQIHQLKTDQVPLEALGMVVSVK"

CDS complement(3086871..3087611)

/locus_tag="EFAGFIKM_02600"

/EC_number="3.5.1.104"

/inference="ab initio prediction:Prodigal:002006"

/inference="similar to AA sequence:UniProtKB:Q81EJ6"

/codon_start=1

/transl_table=11

/product="Peptidoglycan-N-acetylglucosamine deacetylase"

/db_xref="COG:COG0726"

/translation="MLHIRKSIMVTLAILLIIPLLLSQSVSAKSAASKDGASKANSKI

IYLTFDDGPTAHTGQLLDILDQYHAKATFFMLGPQMEKFQKATKRIVADGHGLGLHGV

THVPSKFYKSAYSGLKEMQQANVSLNKVAGVKTSLVRTPYGSKPYLKPTFRNVLLGQG

GFHLWDWNVDSEDWKYQKDHQKVYNSVMKQIHNVQKSGTTPVVLMHDQEATLKVLPQV

LKTLKAEGYRFEVLSKKVQPVNFWNDKR"

CDS complement(3088083..3088586)

/gene="ftnA"

/locus_tag="EFAGFIKM_02601"

/EC_number="1.16.3.2"

/inference="ab initio prediction:Prodigal:002006"

/inference="similar to AA sequence:UniProtKB:Q7A4R2"

/codon_start=1

/transl_table=11

/product="Bacterial non-heme ferritin"

/translation="MSQTLTESLNEQMNFEFYSAHVYLAMAAYCSSKSLDGFANFFIV

QAEEERFHGMKIYKFLNDRGQRATLAALPEPKNEYASMLDVFEHGYAHEQQNTKKFYN

LADIALNEREHATMYFLKWFIDEQVEEEALFDNIIQKLRRIEKDSNAFYMLDAEFGKR

SFTAPAE"

CDS complement(3088743..3088862)

/locus_tag="EFAGFIKM_02602"

/inference="ab initio prediction:Prodigal:002006"

/codon_start=1

/transl_table=11

/product="hypothetical protein"

/translation="MSEVLGGETRGYGYGGFTSVGAILVLFILLVIISKAFLV"

CDS complement(3088963..3089220)

/locus_tag="EFAGFIKM_02603"

/inference="ab initio prediction:Prodigal:002006"

/codon_start=1

/transl_table=11

/product="hypothetical protein"

/translation="MAKAKVAKRPTRDEFELEELGNQLVEAFRERSEVLLTVWGKEDQ

VQGVIVKMDSRTRLVHVEYTEEEFTAKVPFLDIMRVQSPRY"

CDS complement(3089374..3091065)

/locus_tag="EFAGFIKM_02604"

/inference="ab initio prediction:Prodigal:002006"

/codon_start=1

/transl_table=11

/product="hypothetical protein"

/translation="MSQSSGPLSHWLKNQSLVRFSSGATELEQVSYSNTVVTPWIGPR

LEVRKVCNVTEAALGQSLIYRVEIVNTGNLTALVYLLDQLSAETALLPNSVLRDGIPL

PGASPERGLPPAEIAPGATLRYHFQVVILRLPQALKLLNQAQVNYEFLTPEGRTVTGM

EQSNIVEVNLVSSRLEISFQADHIYTFSGDIVMYTVIVSNPGFITATNAQVTISLPQG

IVFIPASVIVDEMFAPQVTPDTGIQLGEILPGSSIRIQYRVQVTRGSDANGISSVAVL

AYISAGQQETVYSNQVTLEVIQPQVSVVKSVLPVTATLGDVVQYNITVSNASSYAVDG

TLLDVLPTGMTFVEGSLGWNGVKRPGTNLAKGLNLGTLTARSVLNIQFEAKLPDRDES

MPDDYRYVNHASLMYTFRLPDSRSVQRMITSNEAVVELKAPIIQAYVQVTPTLVEQGG

TVTFQVRVGNTGNLPARVQLGCILPEGARWLGQATGEIQLSLPEYSTPRVLHVGVIEP

GTEKNISYVAKLASEKTGTLQGALIARYTYDWNGQKRAGETRSNEYSIIVEYGEE"

CDS complement(3091062..3097772)

/locus_tag="EFAGFIKM_02605"

/inference="ab initio prediction:Prodigal:002006"

/codon_start=1

/transl_table=11

/product="hypothetical protein"

/translation="MIFIPLVVRSTVNATGAITFTGNTLGLSRSETAGVPGTQDSIGG

FITTNTSLQYGTYPLGTTSLYQSNSSAAILTLPAGSTVLYAELIWGGSYINGTVNLSA

AINNPVTFITPAGTSSVTPDPLTYNQFDLGGGAAGYVRSANVTTLVQNGGAGTYITGA

VVGTIVITNDSTANHAGWTLGVIYQNPNLPFRNMSLRAGGVLVQSTSPPVVTTLTGFA

TPLSGVLGGRILFSAQEGDANRTGDQALFGPTSATSVALSGPNNLAANFFASQINGDT

GALNTTGTFGTRNQINGAPGTNIIGGRQGWDITNVDVSARLINNQSSAVLTLTTSGDA

YIVNANAIQVDINAPKITLAKATVAAGAVAGDSILYSVTVTNAGTASAASVVLSDSLP

AGLTFIPGSVTVAGVSRPTLDVTAGIPLGSLTLSSSVVVTYRALIGQDASILQLVNSA

NAAFTFQSVAGGPVITGVIPSNNSTLTVYSPNLSIVKSASTTNATVGDLVTYTLQVNN

GGNVAANVTLRDNIPSGSSYVAGSFRLNGNVIAGANPVTGVNLGSLAAGSANTVTFQV

LVTSLPTPPTLVDQATASYSFNSPDGRTITGNIASNTLTIPVTLPNVTTVKSASVTDV

AVGETFTYTVVTTNSGIQVINNVVLTDALPAGITFVPGSATVRGTVVPSANPSSGISI

GTLAAGSSATVTFQVNVQSLPASGSLVNRAVVSYSSGAFTGISNSNSITTPVYQAVVG

INKSASQTNASLGDQIAYTLVVTNSGNITTQVNVTDTIPAGLTFVPNSVTVNGTARPG

TSPLTGITLGSLLPGVTATVVFRATLTTLPTPPTLENQGTASYTYQLPSGRNLSGSSS

SNIVRISASAPNISLSKTVNTPDATVGDVLTYTVVATNAGISAVQNLVISDAPSGAEF

VPGSVTINGSAVPSASPVAGIAVGTLNSSSSVTVTYQTRVNSVPSTGLVSNQANAAFT

SGSFNGVSSSITVNTPVFLPVMQVVKSASTTSLTVGDSFNYTIQINNTGNIAATVTLT

DPIPAGAVFSTNSVIINGVPTPGVSPETGISLGEIAVASSATVTFVATVTSLPDARQV

INQAVASFAYTLPSGRTVAGFSSSNTITIPVSLPNVTIVNSDNVEYGVVGDVIRYTSV

IRNNGTVAVNNVVYVNPLPSNTPFVPGSVIVNGTSFPLSNPTAGIPIGTLAPGAEVTV

TFEVTITMPIPSQINNQSTVSFTSGSFSGSSSSNTTQTPVIQPQISLVKTANTVNATV
GDTVVYTVTVSNTGNLEANVTLTDTIPAATTLVANSVVVSGVPQPGATPAAGIPVGIV
AAGATAVVTFAVVVDSLPSPQQLSNFATSSFTFTPPDGRTLTGSATSNTLTFPVSSPN
VAVVKSTASTAATVGDTVTYSILVTNSGIAPVNNIQFSDPIPAGASFVTGSVTVNGVA
QPGANPAGGVPLGTLGPGTSATVTFSIRVDAIPPSDQLSNRSTVSFTSGTFSSTTFSN
TVVTPVYQPILSAQKTASTQNATVGDTVSYTITVSNQGNYGAQINLTDNLPAGTILVP
NSVIVNGQPLPAGNPATGIPAGNVAPGATTTITFSVVVDTLPSPQQLVNQASVALSYT
LPDGRNITESVLSNVLTITVSAPDVDVVKSTTSTAVSVGDIVTYSIAVTNNGIATVNN
VVFTDAVPTSTVLAPTGVFVDGVLRPGANPSTGVTLGSIAPGVTVTVVFSVQVTALPA
SAVLNNQSTVSFTSGAFSATTFSNTVTTPVFQPILAAVKTGDQTIATVGDTVVYSIAI
SNTGNYGASVTLTDTIPAGTELVPNSVIINGASAPGADPASGIPLGVVSTTTLVTFSV
VIVTLPLSQSITNQASATFSYTLPDGRTLGGTITSNALNIQVSAPNVSVVKTTPVIDA
VVGDTIVYEMVVTNEGIDPVNNIVITDPINPATTFVTGSVLVDGAPRALANPALGIAL
GSLAPGASIAVSYAVRVNTLPTPPQVSSQSSVSFTSGVFSGAAYSNTVVTPIYQPIIA
VTKTGSTSNATIGDTITYSFAINNSGNLDAILTLTDNIPNGAVLLPNSVLIDGVPQPG
ANPETGIAVGTIPSGGTVIVTVTLEVTVDSLPQDQQLVNQAVANYTFSPPDGRLLTGT
VSSNVLVIPVSAPNVTVVKSTSAVDAVVGDVITYTIVVTNSGIEVVNNVVMVDPVPVG
TVFVPGSVTVEGVPRPTGNPNTGITLGSIAAGASVTVTFRVEVVVI"

CDS 3098080..3099144

/locus_tag="EFAGFIKM_02606"

/inference="ab initio prediction:Prodigal:002006"

/codon_start=1

/transl_table=11

/product="hypothetical protein"

/translation="MLHAYWVVIIAHFAIQIGCFLFLEYDRTPIDFLMNVLFWPTAVS
SSCILFASWVDRRFSSYSFYTMSIASTVIAWTIIHVNYDIRIILAICLLPIFASVLFF
SKKRVWIVFLMQMTGYFLVLFDPAYRLYLSSFDMVSIPAFLILGTYVAQVIVTSGVEV
LDDLQASMLAKQDLIVRNAIMTKQSKTDGLTNLYNQSSFKDYYEKAFEYANSGMSLHL
ALIDIDDFKSINDTYGHRVGDIILEKVSLIIQETITSSDIAARYGGEEFALLMFEQSF
KQAYALVEQIRQKIALMGHLELEGATITVSIGLKSYSPNLTKDKLFEEVDACLYAAKR
TGKNKTVTSLDLTSSLAEKI"

CDS complement(3099249..3099770)

/locus_tag="EFAGFIKM_02607"

/inference="ab initio prediction:Prodigal:002006"

/codon_start=1

/transl_table=11

/product="hypothetical protein"

/translation="MSGKYTSVPYGYEPPAASRKGTLTFYDSFEHVTDEQLERAAATV

DTRSFIQLVLYPLHESTFKRMSKDSIQAYYKREDRLHDWRREHPTSRIRVEGLEGKRK

KYTPVDSALRHITAEYPAPHFLYLTIEMANLFASFDSFKDWIKELRLIIDGSSEYLHP

MLEQNRHRWEWAE"

CDS complement(3099842..3101482)

/locus_tag="EFAGFIKM_02608"

/inference="ab initio prediction:Prodigal:002006"

/codon_start=1

/transl_table=11

/product="hypothetical protein"

/translation="MSFKISTVLTVIFAVLSLLVTLTIGSIFSQKSFVAVETEIGHSL

TGTASQASDKLDRFMSARAGELDLLGQMASLEDEFQPDEIQMLLDQLQDSFPSFSWVG

FMDPKGKVLAATDGILLGENLSERPVYQEGIKGKFIGDVHNAVLLAKLLPNPTGEPLQ

FVDISFPLKDSKGMIQGVLAAHLSWEWAKEVEESVLAPLKREEKDIEFFIVSKKEHTV

LLGPKEWVGKPLVLPGIEEAQLNKSSWSIEKWPDGNEYVTGFAYSQGHLDYPGLGWTV

VIRQVKSSAFASVFDLMWFNVWSGLAVTVLFALIGWVVSRLISAPLVRLTRVANRLRA

GEELEIPAIKGIKEIEVLSRSLRDMLTSLMNKNSELVVMQNLAHFDQLTRLPNRTALE

VYLEESLETESENHTLTFLYLDLDGFKRVNDTLGHQTGDVLLQKVAQRLSALVQVKGI

TVRLGGDEFLIVLQSVGSHPREEAMAYAEAIIQSLNKPFIIEYERIRIGCSIGGAEYP

TNSDNPSEIIRMADEALYESKRAGKNRMTFYSELKQDR"

CDS 3101784..3104738

/gene="mrpA"

/locus_tag="EFAGFIKM_02609"

/inference="ab initio prediction:Prodigal:002006"

/inference="similar to AA sequence:UniProtKB:Q9K2S2"

/codon_start=1

/transl_table=11

/product="Na(+)/H(+) antiporter subunit A"

/db_xref="COG:COG1009"

/translation="MSLLHVAVILPFAAGILLAMLHRFFRKNHLGWPVLLVPALLFVY

FASLIPAVSQRNTVSGNIPWIPSLDIGFNLYLDGLSLMLTLLITGIGVLVVLYSIFYM

DMKEALNRFYLYLLMFMGAMLGVVLSDNMIVLYGFWELTSITSFLLIAFHYKRKASTS

GAQKSFLITVFGGFAMLTGFMMMYLITGTFSIRATITGLGQFQESALFLPALILILIG

AFTKSAQFPFHIWLPDAMEAPTPVSAYLHSATMVKAGLYVVARFTPIFGGQGLWFWLV

TGVGLLTLCYGSFLAVKKNDLKAILAYSTISQLGLIMSLLGVGSAALYFGYGESSAMY

TVAITAALLHLFNHATFKAALFMVVGIVDHETGTRDIRKLGGLASFMPITFTVALIGS

LAMAGIPPFNGFLSKELFFQAMVEVTHLRIFGLGSWSVLLPVFAWLASVFTLIYALII

VFKTFLGRSQSEIPAEKLHEAPAGMLIPPVVLGVLVVVFGLFPNLLAGSLIEPAMAAV

LPSLLNGNERFDVHFSLWHGWTPELIMTIGVVILGITLYKLLPRWRNIYEHYPQGLTI

NNMYHVVLDNLQHYARKWTEAYMNGSVRNYLVYIFSFTVALLVYAFFRSGENITWNLQ

GNAPYSFYEVVLLVTLIGAAVSIPFAKNRLSAVIMTGAVGYLVTLLFVLFRAPDLALT

QMIVETVSVALFLLCFYHLPELQRGKSSRSYLTVNMIVAIAVGIVMTFVALAASGTAS

LDSISTFFLQEAYDSAGGKNVVNVLLVDFRGFDTLLEIMVLGVASLSIYSMINLNLEA

KDLGARLKFRKKDESKESEPALDDETDNTKKYGNRERSSSSWDTVPLQSNDVLLQTTT

KVVVFIILTFAMHLFFAGHHNPGGGFIGGLVTAAALVLIALAFSTDTIRKALPVDFRI

LTGIGLGIALLTGAGSFIFGVPFLSQTFGYFELPLLGETELATAMLFDLGVYLAVLGV

TMTIILQIGEDR"

CDS 3104740..3105078

/gene="mrpC"

/locus_tag="EFAGFIKM_02610"

/inference="ab initio prediction:Prodigal:002006"

/inference="similar to AA sequence:UniProtKB:O05260"

/codon_start=1

/transl_table=11

/product="Na(+)/H(+) antiporter subunit C"

/db_xref="COG:COG1006"

/translation="MEILMCIAVGILFAVAVFLILSRSLLRIVLGMSILTHGVHLLLI

TMSRLKTGAPPLLGEMAERYVDPLPQALILTSIVINFGLTAFFFVLSYRSYLKLKTDD

MEEVRGRPYE"

CDS 3105071..3106549

/gene="mrpD"

/locus_tag="EFAGFIKM_02611"

/inference="ab initio prediction:Prodigal:002006"

/inference="similar to AA sequence:UniProtKB:O05229"

/codon_start=1

/transl_table=11

/product="Na(+)/H(+) antiporter subunit D"

/db_xref="COG:COG0651"

/translation="MNNLVVLPILLPLITGVLALLFFRKTNIQRIISVIGLLFTAAVS

TILITRVTQTGVLTLNMGGWAPPYGIVLVADMVSALLVVAASIIALACLLYAFRSVNK

EREEHHFYPFFHFLIAGVNGSFLTGDLFNLFVSFELMLISSYALIVLGGTERQLRETI

KYVLINIVSSALFVASIGFLYSITGTLNMADLSVKIAEVGQSGVITLIAVLFLIVFSI

KAGLFLFFWLSGSYAAPPAVVTALFAGLLTKVGLYAIVRTFTLIFYHDPDFFHALIGW

MAGATMVLGVIGAISYRDVNKILIYNVVAGVGFVAFGMASASRPALEGLLFYMLHDML

IKTLLFLLGGALIAVAGTSKLDNMGGLIHRYPLLGWMFFISALALAGLPPFSGFPGKL

LLFEGGLQAGLYGLTGIAVLSSLLMLYSVLRIFIQAFWGEPPAGVTRRPYAVNGLLIP

AGILFVFIIVMGVGAEGMFQLTSRAGDILLHPNIYIDAVLKE"

CDS 3106556..3107032

/gene="mrpE"

/locus_tag="EFAGFIKM_02612"

/inference="ab initio prediction:Prodigal:002006"

/inference="similar to AA sequence:UniProtKB:Q9RGZ1"

/codon_start=1

/transl_table=11

/product="Na(+)/H(+) antiporter subunit E"

/db_xref="COG:COG1863"

/translation="MAFQIVLNLIIAFVWMFLNNAWNGVGFLIGYLLGLLLIGGMRRF

FPQRFYIVRVWAIIKLINLLFKELVRASIEVIRQIIKPKLDIRPGIFTYKTQLSSDWE

VTLLCLLISLTPGSLPLEISGNQRKLFIHALDIKDEQKMRDDIQNTFEKAIMEVTR"

CDS 3107032..3107316

/gene="mrpF"

/locus_tag="EFAGFIKM_02613"

/inference="ab initio prediction:Prodigal:002006"

/inference="similar to AA sequence:UniProtKB:Q9RGZ0"

/codon_start=1

/transl_table=11

/product="Na(+)/H(+) antiporter subunit F"

/db_xref="COG:COG2212"

/translation="MLSSLLFISLLILSLAILGCLYRVLRGPSMADRITALDTIGINV

IAIVAVLSMMLQTQAYLDIILLIGILAFLSTVAFARYIERGAVFKNDGDR"

CDS 3107300..3107692

/gene="mrpG"

/locus_tag="EFAGFIKM_02614"

/inference="ab initio prediction:Prodigal:002006"

/inference="similar to AA sequence:UniProtKB:Q9RGY9"

/codon_start=1

/transl_table=11

/product="Na(+)/H(+) antiporter subunit G"

/db_xref="COG:COG1320"

/translation="MMEIVKVAAETAIGLLVLLGALLSALSAFGLIRLPDVYLRAHAA

TKSMTLGVFCVLSATFFYFWYFDNYISARLLLGILFVFITAPVAGHLNGRAAYRTDVP

LWEQSVQDELEPLLKGKKVNHEAKDMME"

CDS complement(3107818..3108990)

/gene="yxiM"

/locus_tag="EFAGFIKM_02615"

/EC_number="3.1.-.-"

/inference="ab initio prediction:Prodigal:002006"

/inference="similar to AA sequence:UniProtKB:P42304"

/codon_start=1

/transl_table=11

/product="putative esterase YxiM"

/db_xref="COG:COG2755"

/translation="MKWSISVKLLLCFVLFASTMGIGLGSSGGQVAEAATDNYRFDFG

SGAVESGYTGVSAGDAYTPTKGYGFNTPAQMRNVSASGSGVKSDAVQFLTYGTKSTNT

FNVNLSNGLYEVKVVLGNTARASVAAEGVYQIINMTGNGATDQFQIPVTDGQLNLLVT

EGKTGTAFTLSALEIRKLSNQTVTNRTIYIGGDSTVCNYYPLGSSVQGGWGQLFPSYV

NNSTFQVRNMASSGQFARGFRDDGQMEAILKYIKPGDYFILQFGINDTNAKNNTTEAQ

FKEIMRDMVRLAKNKGATVILSTPQGRATDFNTANVHQAENRWYNQSTRALAQEEGVT

LVELNKLSSAYFTSIGPAATLALYMTGDSLHPNRQGAAELARIVAADLRRQGLNGF"

CDS complement(3109042..3110061)

/locus_tag="EFAGFIKM_02616"

/inference="ab initio prediction:Prodigal:002006"

/codon_start=1

/transl_table=11

/product="hypothetical protein"

/translation="MSDIEAYLQQQTNLRGRSAYHADQPLELWRSQLRTRVKERLGGF

PTMAAALNPVLLERITCDGYIRERIEITTYAGLRMPVYLLIPGDGSVTDTKRPAIVAC

HGHGYGSREISGMESDGSPRTGEPGLHKDFAVALVKRGYVVAAPELLGFGDRRLEEDR

DAPPGVSSCTKIAAHLLMVGKTLAGHRVYETTRVLDYVSTRPEVDSERIGSMGISGGG

LVTAFTAALDERFRATVVSGYANTFQGSILDRNHCLDNYVPGILREAELPDLIGLIVP

RPLFIEAGSDDQVFPIHAAREAHARLTHIYEQAGASESLDADFFTGGHEISGAKAYDW

LDQVL"

CDS complement(3110253..3112484)

/locus_tag="EFAGFIKM_02617"

/inference="ab initio prediction:Prodigal:002006"

/inference="similar to AA sequence:UniProtKB:P9WJT9"

/codon_start=1

/transl_table=11

/product="Heme uptake protein MmpL11"

/db_xref="COG:COG2409"

/translation="MGYRRLAVLISRYPRFIILCWVFIIGMSAVWAWNLPDIVQDHGL

KRVHGDAQAVELVLEDEFGSPADPVILVFEKKENTSPLQFRQWIKDRLTQVQVLPAVT

SITSPLDVSERVTLQDQRAYALVKMDVPAHQMGPPLEQLRAVLATDGPGTVQLTGKAV

VQQDVNHLSFRDLERAEMVGLPIALIVLCFAFRGLYSALIAVMMGISAVITAMGVTSL

LGYHLELSNFIINVIPMVGMALSIDFALIILSRYREEVQRAYEDEGEANVLGRSLDMQ

SEILQRTLRTAGRAVLFSAACVLLGLLGLLWIRLPMFLSVSLGAIIVLLLSLLLNVTL

LPALLSLSADRVFRRKLVHSLPRRSVWHRWSAMVMKRPVSMAIGGTVVLLLCVYPVTR

LELSVPDASSLPEKMESRQAAEQLQHDLGKKSTSAIEILIGGQQELLTASHWQLAHNK

ARQLLQDSDVLSIVSPWGLLQPNQIDSQSLFQIPPPALTPSIEGEESTRASWLRSTVS

DHSIRLIATVHGEPGSEQVADWLERIRNRDHAPGFNNVKLRYGGEAAKQYEIMQEVTS

QLPKVLVFVVVTNYLVLLAAFRSMLIPIKAILMNLLSLAASFGILVWVFNEGHLGMEP

SAIAIMIPVFIAGLVFGISMDYGVFMLSRIQEVYRRTGDSDVAVQQGLASTGRLVTSA

AAILLAVTVPFAFAEVAGVRQLGIGITAAVLIDVTLIRLILVPALMKLMGRWNWWLPG

QMK"

CDS complement(3112593..3113747)

/locus_tag="EFAGFIKM_02618"

/EC_number="2.3.1.-"

/inference="ab initio prediction:Prodigal:002006"

/inference="similar to AA sequence:UniProtKB:P9WPF1"

/codon_start=1

/transl_table=11

/product="Alpha-pyrone synthesis polyketide synthase-like

Pks18"

/db_xref="COG:COG3424"

/translation="MNQLEATTGASIMGIGTAWPAHRIEQKDVSARLAQALEHEPDAR

RWAKRIFNQCGVETRYTCEPNLLEPVDSCRYLPFTNADEVPTTSERMGKYKAAAVPLG

LEAAGQALKDADVTSSELTHLITVSCTGQFLPGLDIRLIQQLELSPQINRIPLVFQGC

AAGLKAIQLANSIVTTDQKATVLIVCVELCTLHFQPSAKRDDLYAASFFGDGASACVI

GASGTDRNECFRLGRGHSTLLPDCAEEMIWEVGNTGFDLYLSPQIPKLLGLHLGPEVE

RLLEGSGFPEIWAIHPGGRGIVDAVQKLYQLTDEQVSYSRNILRDYGNLSSVTILFVL

QAIREDYRQKEEHSSGIALAFGPGLTAELLPFTYIPAPIANRTPVNHGIQ"

CDS complement(3113982..3114602)

/locus_tag="EFAGFIKM_02619"

/inference="ab initio prediction:Prodigal:002006"

/codon_start=1

/transl_table=11

/product="hypothetical protein"

/translation="MYTLDPREITGRDNYKLMSGSVVPRPIAFVTTLSDKNGVINAAP
FSFFNVVSSDPPLLSISIARKDGIVKDTARNVLAHQELVVHICDEAIITEVNETAAIL
EPHESELERTKLTIVPSTKVAVPGIQEALIRMECELYQHIPITNDDGKPVSDLLLVRI
VQYHFSEQVYNPDKGYILMDHLKPVSRLAGNDYAKLGERFTIIRPE"

CDS complement(3114760..3115737)

/gene="yfiY_2"

/locus_tag="EFAGFIKM_02620"

/inference="ab initio prediction:Prodigal:002006"

/inference="similar to AA sequence:UniProtKB:O31567"

/codon_start=1

/transl_table=11

/product="putative siderophore-binding lipoprotein YfiY"

/db_xref="COG:COG0614"

/translation="MIMLLMTVMVMSIWLAACGAKPAENGAAGENDTTTETETQTEAA
TERTLTDAMGHEVTIPANPERVIASYLEDHLVTLGVKPAAQWSINNGNGLQDYLQNDL
NGIPGIASDLPFEAMASFSPDLIIMGSESTVEGEKYEQYNKIAPTYVLGDEVNKDWRQ
ALLKIGEILNKSDVAQKALDDYDAKAKEIKEKVIAITGGTKSAAALWLFNNKFYVVSN
NVSSGEVMYNELGLAEPNVVKEASAKATGNWSEISLEKIAEMDADYLFLVNSDKGAGS
EALQDAVWQSIPAVKTGNVFEFESTSSWLYSGVQANTQILQDIQSSIVK"

CDS 3115992..3116642

/locus_tag="EFAGFIKM_02621"

/inference="ab initio prediction:Prodigal:002006"

/codon_start=1

/transl_table=11

/product="hypothetical protein"

/translation="MLNAEYDDRKDVLYMYYEYNPYLSYGEDRSFQLPPLFPGGGGGG
TPGGPTFPGIPGGQGTYPPYIPGGGPGPYIPGGIPGTIPGTAPGVPAQLTQGNLPTLL
SAYPRPVGQPPAEMLFLYNLIKSSPGLLPLFLQQQPQSLTQAVQFAQTGAAAPRDNEE
RALPNFCYNRWSLVFTYNDVYLMWPAINLFGFVVGYFYPFLTPGILSNTQILFALC"

CDS complement(3116780..3118015)

/gene="bcr_2"

/locus_tag="EFAGFIKM_02622"

/inference="ab initio prediction:Prodigal:002006"

/inference="similar to AA sequence:UniProtKB:P28246"

/codon_start=1

/transl_table=11

/product="Bicyclomycin resistance protein"

/translation="MKSTTASLNMNSTSRVRMALILGTLSAFGPLSLDMYLPALPTLA

DEFGSSTSYAQLSLTACMIGLAVGQLLAGPLSDVRGRRTPLIAGLVLYTIASILCLVS

PTMGPFVVLRFIQGVAGAAGIVISRAIVRDVYSGPELTRFFSLLMLINGVAPIAAPII

GGQLLTYTSWRGVFILLSLIGILTLLAVIFGLGETLPSNRRSSGGLKQTLLTFRQIAG

DRQFMGYALTQGFVAAGMFAYISGSPFVLQKIYGVSPQMFSVCFAINGLGIILASQIA

GRLAGKVSETRLLIVGLLTAALGGTSLLIAILAGGNLISVLIPLFLVVSSVGLVNTAS

FALAMANQEKSAGSASALIGVMTFLFGGIVAPLVGLGGEGTAVPMGIVIACADLGALV

IYFLMVSRGRKRHNDQVLS"

CDS complement(3118022..3118768)

/locus_tag="EFAGFIKM_02623"

/inference="ab initio prediction:Prodigal:002006"

/codon_start=1

/transl_table=11

/product="hypothetical protein"

/translation="MMNAPHPIDQFKKFKYEITRFMMIYKFALDQMETKIEVLKEEFQ

SLHDYSPIEHTKSRLKSPESIMNKMFRKNHELTFESIKQNIKDIAGVRITCSFISDIY

RIKDMLCNQSDLRVLEVKDYIENPKPNGYQSLHLLVEVPVYMSNGEERACVEIQIRTI

AMDFWASLEHKIFYKYNKDVPEHLTRELKSAADSANALDQQMERLHREIQEIKDADNE

RDEEELRRIIINNQQFTLPSNLLKLLGSGE"

CDS 3119197..3119886

/locus_tag="EFAGFIKM_02624"

/inference="ab initio prediction:Prodigal:002006"

/codon_start=1

/transl_table=11

/product="hypothetical protein"

/translation="MHDTDNIPEQWPVLIYCRGGLGSYGGVNTVWLEQFVHKGYIVFA

PSYRGNEGGEGRDEYGGRDAEDVHAAYRLIQRLPFVDPTRISLMGFSRGAINAVHTAT

AYNEGPDRVHKLVLWSGVADVERTYHERTDLRRTLKRVLGGSPRAVPEAYLARSPLSK

ANKLSCPVLIMHGTSDTQVNYSHGTRMYHWLKRGGVNVTFHAYGGQDHHFHEKIHQSA

VNNMFDWLTAP"

CDS 3119973..3120689

/locus_tag="EFAGFIKM_02625"

/inference="ab initio prediction:Prodigal:002006"

/codon_start=1

/transl_table=11

/product="hypothetical protein"

/translation="MSQSTLQKYAMLLLCMSAGMVDLIGYLGLGHVLTANMTGNIVLL

GIAIARAQEFVVLRSLLALIGFIAGNAIAAHMVGPVQTKNGWSSRVTAVFTVESILLL

LFAIAMISPFSGQLSYLLIVILAVAMGMQTTAARRIGIAGISTTVLTNNLAAVVEDTV

SILKKLRHANIRSLAKALSTDAYLRAGAVVIYLIGVIAAALLFHRVPMIAVWIPVLII

GGVTLYARLHAWGTSQQSKS"

CDS complement(3120806..3121702)

/gene="cmpR_5"

/locus_tag="EFAGFIKM_02626"

/inference="ab initio prediction:Prodigal:002006"

/inference="similar to AA sequence:UniProtKB:Q9F1R2"

/codon_start=1

/transl_table=11

/product="HTH-type transcriptional activator CmpR"

/db_xref="COG:COG0583"

/translation="MNLHGLRLFHAIVRYGGVTRAAEELNISQPAVSSQVKKFERELG

IQLFVSEGRRLVLTDAGIQLTGYAERLFMLKQDVENFVQDFREGKKGLIRLTATYLPS

NFLLPGWIARFKQMHEDVEIVVSTTNTRMAFDQLLRYEAEIAVYGGSGITHAGVQWDE

LFDDEMWFVVHPDHPYAGKEIELHEMMAEPFIMREEGSATRERLVSLCTTNNLAAPRI

ALQFNGLNETISAVKAGYGANFISSLVVNEDVQQGRLARVFVRGVQLRNTVAVCTRAG

EVLSPAAQHLVKLIRQEVALMK"

CDS 3121841..3122545

/locus_tag="EFAGFIKM_02627"

/EC_number="2.1.1.6"

/inference="ab initio prediction:Prodigal:002006"

/inference="similar to AA sequence:UniProtKB:O07431"

/codon_start=1

/transl_table=11

/product="Catechol O-methyltransferase"

/db_xref="COG:COG4122"

/translation="MKNINTTNLQHTWTQVDDYMNDLLIPSDSLLEHTLQSNAEAGLP

AHDVTPNQGKLLQLLLQIQGASRVLEIGTLGGYSTIWMARALPEHGRIVSLESESHHA

DLARANLTRAGLMHKVDLRVGPALTTLPDVQEEYREPFDMIFIDADKPSNPNYLRWAL

RLTRPGSLIIGDNIVRDGEVIRTNSTDPRVQGVRSFLQLIADYPRLEATALQTVGSKG

YDGFVIARVIDAPEPK"

CDS 3122623..3123933

/locus_tag="EFAGFIKM_02628"

/inference="ab initio prediction:Prodigal:002006"

/codon_start=1

/transl_table=11

/product="hypothetical protein"

/translation="MRVLQHINDEIRGWSRNIQLFFLASILYQIGNGMFSVLYNLYIQ

GLGYNDTMNGQIVSIQSLATAIMFVPIGLCGDLFSRKRLLITGALFSGIFLIGRSFDY

SATGLIWFAVFSGLFAGVFQVLAIPYLAENVKKSQRLKMFSYYSSLVLASQVLGSLGG

GVFADLLHTAGLAKVTGLQTVLFVGGAATLAAFIPLLFVTEGKAAPQTTIPAQPVLQP

NADLKESSTNTPSTDDSITKKKDSRLIGQFIVTQLLIGLGSGLVVPYLNLYFTNRFSV

SLSGMSLLIALGQIMTIVSMLIGPTLAAKVGSVRAVVIFQVMSLPFLLLTGFTNLLFI

ASLSFLFRQALMNAANPIHSAILVDRISDKRRGIANSLMQTSFMIGWATMGPVQSYLV

TTYGTYWGYAITFSITGCLYVISSLMYYVMFREPKPSATALAGS"

CDS 3124082..3125197

/locus_tag="EFAGFIKM_02629"

/inference="ab initio prediction:Prodigal:002006"

/codon_start=1

/transl_table=11

/product="hypothetical protein"

/translation="MELMRKLGSHVVFRFFLSLGVSVLGGLLFAVIHTPIPWLLGPMV

FMLLGSQVAKWPLIWPASIRDYGILIVGYSIGLTLTKEALHGILQQLPMMLLMTLLLI

GLCVITAYIASKVTDFDFPSLLVGSIPGGLSQMVSLAEEMKSINLTLVTFLQVTRLIM

IVFCVPFLLFSPWIGGTAGGGLDHPFIDVATWGTLFPEILLYVPLCVAGAWIARKLRF

PTAFMLGPMIVMCVVQLSTTMHTPSLPTSLLNVSQLMIGSHVGLMLKPEQLQRKTQTV

TLAVTSSVLLIVGALGLSYLLMNVFSLSAATSLLSMAPGGMDQMSIMAHEVNADLSVV

SGYQLFRILFIFFIVSSVLKMILVHMLKEEKAKPYLQ"

CDS complement(3125326..3125529)

/locus_tag="EFAGFIKM_02630"

/inference="ab initio prediction:Prodigal:002006"

/codon_start=1

/transl_table=11

/product="hypothetical protein"

/translation="MGLVKRFVLVVVNMIGELWMGFYRRNSDYYDHQTSESKSKIGYY

AFIIGATVVTVGIVGWMYNRIYS"

CDS complement(3125607..3125870)

/locus_tag="EFAGFIKM_02631"

/inference="ab initio prediction:Prodigal:002006"

/inference="similar to AA sequence:UniProtKB:P0AFT8"

/note="UPF0153 protein YeiW"

/codon_start=1

/transl_table=11

/product="hypothetical protein"

/translation="MECRTGCAACCIAISISSPIPGMAHGKPAGVRCVQLTDDNRCGI

FGQKDRPAVCSGLQAEEEMCGSTDQEAFNILTWLEQETAPKVI"

CDS complement(3126026..3126874)

/locus_tag="EFAGFIKM_02632"

/inference="ab initio prediction:Prodigal:002006"

/codon_start=1

/transl_table=11

/product="hypothetical protein"

/translation="MTRVAVMVIHGLGMRKEGYADKLIACLHKELDQVMVLPGAAKQM

LDIEPVYWADVFEEREEALFQQLVSSPGLNYQTLRRFVIHYLADAVAYQPVENQGHNY

DAVHRTLNQAMHTLAQRNGPEAPLCVVAHSLGAVIASNFFYDLQYPSSRVPEIVDVNS

ALERGDTLTHFYSFGTTLPLWSLRYHDFSCPIQVPSSHVNHYYTGLEGEWVNFYDRDD

ILGYPLRPIDPAYEKAVKEDIEVNSGGLALSWNPLSHGGYFSNRSMNRRIAQGLARTW

TWVNRS"

CDS 3127709..3128026

/locus_tag="EFAGFIKM_02633"

/inference="ab initio prediction:Prodigal:002006"

/codon_start=1

/transl_table=11

/product="hypothetical protein"

/translation="MAIKGQKYKTYSEKIKKEAIRLHTVEGWTYRKINEHLGIHDPGR

MKRWMRKHREQGEFGLMDQRGRRKEYLDQERYVQKLKRENELLKKCLVIWKEEANKKD

FRS"

CDS 3127975..3128859

/locus_tag="EFAGFIKM_02634"

/inference="ab initio prediction:Prodigal:002006"

/inference="similar to AA sequence:ISfinder:ISBth8"

/codon_start=1

/transl_table=11

/product="IS3 family transposase ISBth8"

/translation="MLGNLEGGSKQERFQIIEKVAAYGDIQKLCDVFGVSRSGFYAYV

KRKRFDRDAKAKKQVLQTYQRYEGKYGYRQLQLFLWQDQGIWMNHKKVLRLMQMLGIQ

SRIRRKRRSNSSYAPAQRVAENRLKRDFSAEKPNQKWVTDITQYRVGERWIYLSAIKD

LFNNEIVAYEIGERNDNELVLRTFSKAFAKQTDVTGLVVHSDQGFQYTSHAYHDMLPK

VGAQISMSRRGNCYDNASMESFFSHLKTEGLYPYHIRTLTEAQSKIEKYIRFYNRKRP

QRKLKKLTPVEYRRQFAA"

CDS 3128955..3129806

/locus_tag="EFAGFIKM_02635"

/inference="ab initio prediction:Prodigal:002006"

/codon_start=1

/transl_table=11

/product="hypothetical protein"

/translation="MSQKIAVIVIHGLGKQHEDFATIFSDNLHKTFTTVSGMKNPELC
IEIRPVWWASVFAEREEELKRKLVGAPYNLRYEELREFMIHYLADAVAYQPLRDGGEQ
NYDAVHHTISKQLNRISIEVGPDTPLCVVSHSLGSVIASNYFYDLQNAKEKTKIIDPS
SPLERGETLSLFYTCGTTLPLWSLRYTSFDHPIQVPSKDFLTMHPNIAGEWINFYDKD
DVLGFPLKSIHPEYDKIVKEDRDVNVGTIIDRWTPLCHNGYLHSNKVIRPIAEGLHKI
WQQINGL"

CDS 3130528..3131484

/locus_tag="EFAGFIKM_02636"

/inference="ab initio prediction:Prodigal:002006"

/inference="similar to AA sequence:ISfinder:IS658"

/codon_start=1

/transl_table=11

/product="IS30 family transposase IS658"

/db_xref="COG:COG2826"

/translation="MSYRHLSIIERSKLEVLHRQGRSSRAIAKELGRHPSTICRELDR
ATSSHPYQAEQAQNAYEERRKASVSTGNWSDALAASLEEKLEATWSPEQITERFRIEG
RDAVSFKTIYRWIYAGRLVRGVLQVLRHKGKRQKPAETRGKFAIGRTISDRPKEVRSR
ETFGHWELDTVVSGRGKSKGCVATLIERKTRLYTAVLMPDRTALSMEIALGVAISQYP
TGTFLTATADRGKEFACYAHLETTHNLHVYFADPYSSWQRGSNENANGLLREFFPKGT
DLARVYDDDLAHSLDLINHRPRKCLGWKTAHESFAEELSHLV"

CDS complement(3131628..3132329)

/locus_tag="EFAGFIKM_02637"

/inference="ab initio prediction:Prodigal:002006"

/codon_start=1

/transl_table=11

/product="hypothetical protein"

/translation="MNLTKIISSELEDKLKELSFDFEDSSNPWIFVRAREGYVKDYIE
IDKSNWESNAIRCTFQTGSESVSSPKLAERKVDEWYVYQNEEELTEVLHKLRKIIEQY
ALPWFAENTQLIPSATPNYLDESWKDSIQEFMKKNKIDINNSESVGILDQLVISNLSQ
KDIYFISYCYGEIIISQFGGEWKYDANNGPRVDNIGGNPAFKRLPHDFVNKVIENKNL
SLQRYYEDIAFVLED"

CDS complement(3132558..3133964)

/locus_tag="EFAGFIKM_02638"

/inference="ab initio prediction:Prodigal:002006"

/codon_start=1

/transl_table=11

/product="hypothetical protein"

/translation="MKGPFGNTVYYTNDVMNRMTSVGTAKDAPDAQYKYYGNGLFKSS

ESLNDVSTLRTYNGLDLVSLEQVRGQDTLALYNYSYDSNKNITKRVQQGVQDDFTYDQ

LDRIATASGNNEQYTYDKQGNRLTMQSDKEVNSVESEYQYDTSNRLTQVITDVAKVNY

QYNGDNLLVERTENGVATRYYYDDRAQIIAEAEVLNGTPELKANYIRGAKLEAIVYAN

GSKAYVQSNGHGDITELRDVDGALLNKYQYDVWGNLELKEEKVHNPFLYSGELWDDTT

RLQYLRTRWYDPNTGRFINEDTFEGELDDPLSLNLYAYVHNNPLRYVDPSGRAKFGST

AYGQYAHKALGNIFKLINLFTKNDVAFTEKTVKLANGNSGRVDFALKVGESQYEIYEL

KPISWNNNPQLNKSGRDQLKSYIHGIILNGFRGDSTATARAGTTWNPNNVVIPSPFNS

RKEIVFYTYYATQPGMIY"

CDS complement(3133961..3134458)

/gene="rhsD"

/locus_tag="EFAGFIKM_02639"

/inference="ab initio prediction:Prodigal:002006"

/inference="similar to AA sequence:UniProtKB:P16919"

/codon_start=1

/transl_table=11

/product="Protein RhsD"

/db_xref="COG:COG3209"

/translation="MEQTDGNGNVKTFNYDVMGQLRQVTSANGEQTQYNYNLVGNLIK

TIDPTGNIKENTYDQLNRRIQTKDKSGNSTKSYYGPSGNLTKYVDRNGNTFTYEYDLR

GNLIRKISSDETTFFVVDSVGKRISMTDSTGTTGYQYDPATEQLTRLTYPDGLTAEFN

LTGQR"

CDS complement(3134510..3138688)

/locus_tag="EFAGFIKM_02640"

/inference="ab initio prediction:Prodigal:002006"

/codon_start=1

/transl_table=11

/product="hypothetical protein"

/translation="MKQSITKKYISILLIVSVFSGLFGNWIPAITYAEKQNEQEVSTG

QNPSPLESLSLIAEQFDRTEGFIQGYLDQGYTLNEVISALYKARDEQIGFDEALQAVR

PQEVNESASVTSDVYTDSALAEETPLTGVEADQSSIMQRYETFAVEEELTAPPDTDLE

KDNEPKEEEDKQAEGEEKEQVDPDDQSEGEKESEGTEETAPSTETTTDDENAPQTNQD

VGQEPTAPSSSENTSTLEGKPAAESKETPSSQESAEIPSSTSMESKSEPKNISEKVSK

SVPEKGQVKENNDASITNKKQSESSSPSKQKSKSDVKPLAGQTSVPDPGKHIAEKAPV

YSKKSFNEAPYTVGENGESISTMSGGLMLEHVDASLPGRAGMSFSLERQYNSNSAQFY

DPAVGTNTYEYPVYNYFLTYQAVKKKIITKYHVKYKLNKWVQEDYNGDGLKDNDTIVV

ETPTILQGTYTTEADARKAASTQIAFWNPSESRTEQQTRSGGLSSLPSSISYNQGGFS

GTLNRSGSAQVISGQYQPARTITAPTQTCTNSIPGKYDSKGVWAQTGSGSPCPDTKTA

TVEGKTITLTRSSVTATKACPSPNKSVANYVCTKTWEARYNGSVSIPESDTRVYSQSY

IGSVVKPGQYSQQRYDSWIAGKAPYQYRYAYSVREQPWVEQEVTEGPAETITLYTDGT

PDWSAVNDLKNNVNSNAGKGISTSMDSSYNYYLASSPSAEIRAYQVGSNFDVTYYNKT

VPATADKRAPLGKGWSWKLPFTETENGTSYMNMADGGRYEIAGNTLKGYDWEGITVNP

DTSVTVNGESSTLVLTSSDGLTKQYFSVDGRLLQISDGQKNDVQFFYENNSVYNSKLL

TQVKDAIGNTISISYTSSAVTIVQGNRTVTYNKQTKNGIELLDSVIDPLGRKTTYSYK

LADAKFNLPGFSPERAALNPYALLTSVQHNTGAKTFYEYENGTVKRYIGEDSFNDAYR

VLSRKDQITYENGSTEDFNRQTYSYTSDIGASFSQDTTFAVLVSNGLTNTQYNYRKDF

INNDTQAQFYLDGTMVSAEGKTQTTTNTYGKTVKGRSYAAPTPTVTTVTDNQAKDVLT

TTVQLDDYGNITSVTDATGRTTTSTYDDTRHWVTSVTDMVDATNKKYTVLTRDNVGNI

KQIVNRKDSASGELLTQADYTYDAYGNLLTQHISDGKHERNATLEYDGRYQNAFPTRL

STNVTDVDGRTTQINSLSEYDLSTGALIASTDEEQRTTKYRLDAIGRTIEVTQPDGTM

LRADYDDINNTIKVTDELGQQRLNKWNSLGQAIEKGYFSGNSYIVSQRNGYDPYGRVS

WTDDALGNRTRNTYDSWSRTVMTTGADGTSTSMKYDDVVHTATSTDAEGYVQITTYDK

WGKGIKTEEKTQLD"

CDS complement(3138758..3139603)

/locus_tag="EFAGFIKM_02641"

/inference="ab initio prediction:Prodigal:002006"

/codon_start=1

/transl_table=11

/product="hypothetical protein"

/translation="MPKNLKLEILSHTSVIVSWDNIKEVNGDVVGVNIYQDNVLIGAT

NGTNYIINFLDPQKNYTFTVRSKYSEGSESDPSNQVTKYKRELKGDVDGNGAVTANDA

LMVTKYLKGKITFTPEQIILADMNNDGIIDESDVKNILSFSVSNSSGNSSLEVPKDLK

LEILNQTSIKLSWKALDELNGGTKGFNIYQDNVLVSTTNETSQVVNFLDPQKTYTFLV

RSKNSEGDESEPSNLIKKTPFKQYTYNYDSSGRLNSILTPSGKKIAYTYDANGNLIKK

TITNF"

CDS complement(3139588..3139929)

/locus_tag="EFAGFIKM_02642"

/inference="ab initio prediction:Prodigal:002006"

/codon_start=1

/transl_table=11

/product="hypothetical protein"

/translation="MDEGRELLILKSSRRVFLTCIIFLFSFSLLLDEVHSAELAVLKG

DVDGNGAVTANDALMVTKYLKGKITLTPEQIILADMNNDGIIDNADVVSILSIHVGTQ

TEKKRQKCQRI"

CDS complement(3140586..3140732)

/locus_tag="EFAGFIKM_02643"

/inference="ab initio prediction:Prodigal:002006"

/codon_start=1

/transl_table=11

/product="hypothetical protein"

/translation="MDAAFRNWDPAKRYVLKKVHVGSLFQVVDYAIQHDGLRIILEHG

LNHY"

CDS complement(3140960..3141136)

/locus_tag="EFAGFIKM_02644"

/inference="ab initio prediction:Prodigal:002006"

/codon_start=1

/transl_table=11

/product="hypothetical protein"

/translation="MDFSGESIACRRHDTYDVQFEFLTYNFTVTELSECTLEEVEEMF

YSVNQSVINIYREN"

CDS complement(3141422..3141880)

/locus_tag="EFAGFIKM_02645"

/inference="ab initio prediction:Prodigal:002006"

/codon_start=1

/transl_table=11

/product="hypothetical protein"

/translation="MKGQDTISKGFALALMSIARKQRRDFAWIPFSSHASDAIIYERG

KIEVPDMIQLATVFLDGGTNFVQPLNKASEVIKQSRFNQADIIFVTDGEAHVNNDFLK

AWSSLKEQKGFSVLSLLLGKESIHGVGGFSDRVVRASSFEDQSIQQAFDI"

CDS complement(3141877..3142482)

/locus_tag="EFAGFIKM_02646"

/inference="ab initio prediction:Prodigal:002006"

/codon_start=1

/transl_table=11

/product="hypothetical protein"

/translation="MTDQGYLDFREFTRLDDLAAAIGTTKYNENVLGWVKEQATEALS

AALNQNGSQLSQILAQASDEAIETKENVNFMLGGIQAGSSDSELKKVPLKEQLFLAEK

LSHNKNLKEIAKWTGRMKVIASRKQRYKHKDAIDRNGIRQGSDIEQLLPMELGSYASP

PLAKWIFYADMRKDRHFSMTPREKNISVKGPLSCVSINLVV"

CDS complement(3142486..3142653)

/locus_tag="EFAGFIKM_02647"

/inference="ab initio prediction:Prodigal:002006"

/codon_start=1

/transl_table=11

/product="hypothetical protein"

/translation="MPKLHMIEELKRVLIEGYNEAKLERNSYDEGWAQIYSAKQNDLI

LQADMVGVEVK"

CDS complement(3143720..3145738)

/locus_tag="EFAGFIKM_02648"

/inference="ab initio prediction:Prodigal:002006"

/codon_start=1

/transl_table=11

/product="hypothetical protein"

/translation="MAIDKKQVEEIVRAVGGKENIEAATHCVTRLRFALYDESKVDTE
SLDQNDLVKGQFSSQGQFQVVIGPGLVDKVYDEMIQITGGDRSSKDDVKAVAGKKQNP
IQRAIKTLSDIFIPILPAIITAGLLLGINNILTGPGIFFDGKSLVDVYPAWKDLASII
NTIASTAFTFLPALIGWAAVKRFGGSPLLGIVLGLILVHPDLLSAYGYADAVNNGTVP
TWNLFGWEIEKIGYQGQVLPVLVSAYLLAKMEIFLNKRVHDSIKLLVVAPVTLLITGF
LAFTIIGPVTFAIANAITSGLIYVYDSYAALGGLIYGGLYALLVITGMHHTFLAVDVQ
LIGSQGGTFLWPMLALSNIAQGSAALAMMLVLREKKMRGLAATSSVSAFLGVTEPAIF
GVNIRYRYPFIFGMIGSAIGGVLLTMNNVQATSIGVGGVPGFLSIFPNKWGVFFIGMA
IVLVVPFVLTVLFGRAKLRKEDRSASNETINEPKAATSQSASVVTSSTAKTDPNQRTR
SAAQVGDEAVNTLEIMAPLTGQAVSLEQVPDPAFAEKQMGEGVAIEPSGNVVVAPFDA
QVAHVIKSKHAVILEHASGLQVLIHVGINTVSLKGEGFNMHVEAGEHVKAGQKLLEFD
RKVIEDAGYPLITPIIIPDGQDMVERVEVTTGDVTSNQNGVLKVHLKG"

CDS complement(3145767..3147488)

/gene="treA"

/locus_tag="EFAGFIKM_02649"

/EC_number="3.2.1.93"

/inference="ab initio prediction:Prodigal:002006"

/inference="similar to AA sequence:UniProtKB:P39795"

/codon_start=1

/transl_table=11

/product="Trehalose-6-phosphate hydrolase"

/db_xref="COG:COG0366"

/translation="MSSNNTTPSSWWKTSTVYQVYPKSFNDTTGSGTGDIHGLTEKLD
YLQHLGIDIVWLQPVYVSPQHDNGYDVADYYRINPDYGTMEDFDELLKGLKARDMKLM
IDIVVNHSSTDHEWFQQSRSSKDNPYRDYYIWKDPAPDGGVPNNWQSKFGGPAWQFDE
QTGQYFLTLFDKTQADLNWENEEVRKAVRDMIKFWAEKGVDGFRMDVINLISKDQRFP
DDDGSVSPGDGRKYYTDGPRVHEYITEMYDEVFGPHNMVTVGEMSSTTLEHCIQYSNP
ASREFSMTFNFHHLKVDYPNGQKWELMPYDFEAMKQLFSEWQTGMQAGGGWNALFLNN
HDQPRALSRFADDGDYRAESAKMLATTIHGMQGTPYVYQGEEIGMPNPVWNDVSEFRD
IESTNMYRLLQEERGKSAEEAFQIVKERSRDNSRTPMQWNGSKNAGFTTGTPWLKVDE
RYPSIHVEQQLADPDSIYYHYRKLIALRKQVNVLTDGLYERLDDAHPDVFAYARTNGS
ETLIVVSNFSKRDVTFSLPEAVWNDHIASKSAELLIGNTEAAPALTQEISLSPYASYM

WLVPQQD"

CDS complement(3147865..3149220)

/gene="dctA"

/locus_tag="EFAGFIKM_02650"

/inference="ab initio prediction:Prodigal:002006"

/inference="similar to AA sequence:UniProtKB:P0A830"

/codon_start=1

/transl_table=11

/product="Aerobic C4-dicarboxylate transport protein"

/db_xref="COG:COG1301"

/translation="MSFMKSLFFQIIVAVIIGIGVGILWPDLGSLLQPLGTGFIKLIK

MIIAPLIFMVIVTGIAKIGDLKSVGRIGLKAIVWFEIATTVALVLGLGTANLLRPGAG

MNVDPSTIDASGIEAKTNGSELPHTVDFIMNIIPTSVVDAFAQNALLQVLLVACLFGV

ALAATESKAKENVLTLIENLLGIVFRIIGYIMKLAPIGAFGAMAYTVGAYGASTLSSF

GLLILACYGAALLFLVMLALAAWWITGLNFLQFVKYTRSEVMLAIGTGSSEVVMPRMM

DKLTKAGCDRAVVGLVVPTGYSFNLDGASIYLSLATVFVAQAVGIELTLMQQITILLV

LMLSSKGMAGVPGSAFLALSATAVAVNAFPVAAVALLLGADRFMDTMRVFTNLMGNCV

AAFVVAKWEGLLDQKRMRAVLSGEISAAELEREEQAALSLLKLNMQEKQGKAVVSPEM

S"

CDS complement(3149459..3150151)

/locus_tag="EFAGFIKM_02651"

/inference="ab initio prediction:Prodigal:002006"

/inference="similar to AA sequence:UniProtKB:P99149"

/note="UPF0173 metal-dependent hydrolase SA1529"

/codon_start=1

/transl_table=11

/product="hypothetical protein"

/translation="MLNITFHGHSSVQLGTEEKSLIIDPFLRGNELAVTKPEDIKTDV

VLLTHAHMDHILDAEPIAKANNAKVVAIVELATYMSWKGLDTLGMNMGGTVDLDFAQA

KMIQAFHTSGIVLEEEQRIMYAGLPAGYIINIGGKTILHAGDTSLFGDMKMIGDRHDI

DVAFLPIGGHFTMGPEDALQAAEWFNAKLTIPVHYDTFPVIRQDAEHFVQQLAAKGLE

GRVLAPGESITL"

CDS complement(3150250..3151962)

/gene="malL_3"

/locus_tag="EFAGFIKM_02652"

/EC_number="3.2.1.10"

/inference="ab initio prediction:Prodigal:002006"

/inference="similar to AA sequence:UniProtKB:P29094"

/codon_start=1

/transl_table=11

/product="Oligo-1,6-glucosidase"

/db_xref="COG:COG0366"

/translation="MNNVESEVGDIVWWKESVVYQIYPSSFKDSDGDGYGDLQGIYEK

LDYLENLGVDVIWLCPIYDSPGHDNGYDIRDYYGILRKYGTMEDFDRLLAEAHKRGLK

IMMDLVLNHTSDEHAWFAESRSSKMNPKRDYYIWRSGKNGQVPNNWESYFGGSVWKHD

PETNEYYLHLYSEQQPDLNWNNAQMAEEMYEMVHWWLEKGVDGFRFDAVAHIAKAEGL

PSAHNPDNLPVVPAYQLFSNLEQVHSILKKLNNMILKPYGPMTVGETSGLGPEQALAY

VGTDRDELNMVFQFEHMFIDAKSSGIGKWNYKEWKLTDLKEIMSRWQTVLHGRGWNAN

YMGNHDQPRPVSRFGDDGKYRVRSAQMLATWMLTLEGTPYIYQGEEIGMTNVAFPNIE

RYRDIETKNYFNHYIGQGKSKHEVMQAIWLKSRDNARTPMQWDETEHAGFTQGQPWIQ

VNDNYPEINVADAESDPQSILHYYRKLIALRKQHKVLIYGAYELLLPDDPDIYAYTRT

LEDEQMLVILNFRGHEPEMHWPEGWTSEHAKLVISNVSRRYSTDEGAIQLQPYEARVY

RKQR"

CDS complement(3152085..3152543)

/locus_tag="EFAGFIKM_02653"

/inference="ab initio prediction:Prodigal:002006"

/codon_start=1

/transl_table=11

/product="hypothetical protein"

/translation="MNEKSWLHSKWMLTIVACMAALYILIMANLLFVSGRTPGMYYQY

NLVPFETIRPLLMERERYNTDTWVKNLFGNIVLFIPLGIWIPWLFRRCRTFLTFTSTV

VWLLLGVEVTQLITRVGSFDVDDIILNTIGAWIGYAGFKLVLYSQKKTRN"

CDS complement(3152799..3155231)

/gene="glgP"

/locus_tag="EFAGFIKM_02654"

/EC_number="2.4.1.1"

/inference="ab initio prediction:Prodigal:002006"

/inference="similar to AA sequence:UniProtKB:P39123"

/codon_start=1

/transl_table=11

/product="Glycogen phosphorylase"

/db_xref="COG:COG0058"

/translation="MFDNKETFKSIFTRNLVSKLGKPIEEATQEDVYHVLGSMIREYA

GQDWAASNQGFKQRQDKQVYYFSLEFLIGRLLGNNLLNVNELELVRDSLAELGFSLEE

IEEQEADAGLGNGGLGRLAACFLDSLASLRYAGHGCGIRYKYGLFEQKIINGNQVELP

DNWLDKGNEWEVRRPDKKVEVQFWGRVEAHEQDGEYQFVTKDAESVVAIPYDVPVIGY

GQTHVNTLRLWSAEPKRETSLDTPSNYYGYLDYSRSVESISEFLYPDDSQYEGKLLRL

KQQYFMCSAGVQSALRTFNKLELPYDRLPDKVAFHINDTHPTLVIPELMRILIDVKGY

GWDEAWDITTRTVSYTNHTTLSEALEKWPVSMISRLLPRIYMIIEEINKRFCGMLLDR

YPGDQDRIGHLAIVANDQVRMAHLAIVGSHSVNGVAALHTEILKEREMAPFYELYPER

FNNKTNGITHRRWLMHANPKLSDLITDTIGNEWITEPGKLDQLAGFADNTSFQEQFRS

IKRDNKERLAAYILDHTGTAVNPDSIFDVQVKRLHGYKRQLLNILHVMHLYNRLKNDA

SFDMVPRTFIFGAKAAPSYYFAKKIIKLINNVADTVNRDAAVNDRLKVFFLENYSVSL

AEKIIPAADVSEQISTAGKEASGTGNMKFMMNGALTIGTMDGANVEMAEQVGEENMFI

YGLRADEVLEYYRSGSYRPNEIVQHDERIREVVEQLVHPGAFCYRDGEFWDIYDSLLA

HGDEYFVLRDFAAYADAHAAIDQAYRDIPGWTRKAILNTAHSGIFSSDRTISEYATDI

WGIHPVSGNWKG"

CDS complement(3155701..3156804)

/gene="glgD"

/locus_tag="EFAGFIKM_02655"

/inference="ab initio prediction:Prodigal:002006"

/inference="similar to AA sequence:UniProtKB:P39124"

/codon_start=1

/transl_table=11

/product="Glycogen biosynthesis protein GlgD"

/db_xref="COG:COG0448"

/translation="MKPLIGVINLDHELEELKELTYFRCGAAVPYAGRYRLIDFVLSN

MMNAGIESIGVFVRRKYRSLMDHLGDGKPWDLDRKHGGMFILPPDWNDPTDTSQGDLQ

HFHNNLDFFHRGAGQYVVHAGSRHVTKADLQDVYRYHISKGADVTLVCKKVDQLLPEH

DACVKVEHDGDGNVVDIHQSANHPNIYTEIFIMEKELFLRQVQRCIDHGESHFFRDVI

QKNPDGLNIAAYAYDGYHAVINSIDSYYRNSMDLLNTGQYEQLFKEDPIQTKIKYEAP

AKYLDTADVKHSLLANGCIVGGEVEDSILFRGVQVAKGAKIKGSIIMQKCYIGEGTVL

ENVILDKDVKLTGGQTLIGDPSNPYILAKSTII"

CDS complement(3156831..3158003)

/gene="glgC_2"

/locus_tag="EFAGFIKM_02656"

/EC_number="2.7.7.27"

/inference="ab initio prediction:Prodigal:002006"

/inference="similar to AA sequence:UniProtKB:P39122"

/codon_start=1

/transl_table=11

/product="Glucose-1-phosphate adenylyltransferase"

/db_xref="COG:COG0448"

/translation="MMAKKEVVAMLLAGGQGKRLKGLTKSLAKPAVYFGGTYRIIDFP

LSNCSNSGIDTVGVLTQYEPLVLHSYIGIGSDWDLDRKNGGVYVLPPHEREDGSNWYR

GTADAIFRNLNFIEQFDPEHVLILSGDHIYKMDYEKMLQYHKEKDADCTISVIDVSLE

EASRFGVLNTNDDYSIYEFEEKPPEPKSTLASMGIYLFKWDVLKRFLIQDEQQASTSY

DFGKDIIPLLLENEKSLYAYPFEGYWKDVGTIRSLWESNMDLLDEDTPFNLNDPDWRI

FTRNPNQPAQYISPSGKVRNCIISEGTVVHGEVNHSVLFYGIEVGENSAVIDSVIMPR

VKIGQNVRIYRAIIAEGLVIPDGTQISPAPEDESDILLVDQEELERQLRQGITSKA"

CDS 3158631..3159719

/gene="ddl_1"

/locus_tag="EFAGFIKM_02657"

/EC_number="6.3.2.4"

/inference="ab initio prediction:Prodigal:002006"

/inference="protein motif:HAMAP:MF_00047"

/codon_start=1

/transl_table=11

/product="D-alanine--D-alanine ligase"

/translation="MELINKYLDEITQQKEKVTVVLVTNVDNQTNEFEDYHNNSLISE

YLSESELEVLNQGFENFGFRTIIHFDETEFIQKVATSKHPILNRDHIGIVLSMAQKGK

KIGRKSLLPAFCDLYNLKYTSSDPYTVSLCRNKYHSGILLKQHGILVPESWLYTKKGW

LFEQKPPQGCRVIIKLNNESASIGIDQNNIINYESSNDLMIEQLCIEYGQEIIVQAFI

EGYEVEHPFVVDKEITPLIPMGIQLNDSRNLSDQILTYEIRGKDQYSFYNYSTENQVI

NAKLIETTAAVAEVLGITGFGRVDYRINQKNEFFVTDIATNPHIVKHSSFHQSFIELG

YTYNEMLATFVAITIAKYDLGFRPELNI"

CDS complement(3159690..3162176)

/locus_tag="EFAGFIKM_02658"

/inference="ab initio prediction:Prodigal:002006"

/codon_start=1

/transl_table=11

/product="hypothetical protein"

/translation="MTHSLLHHQQEIKQHILGGIRDNRKLFLIEGLSGTGKSYLTHSI

ISELSLIDEFEIFLMEGDSQCITRDYYPFERCISSKKSGLKNRNNSKTLKKTVSKASK

GIPFVGDLAEHISEEFLNHNDNKFDKDNSYLTDSERSIIYRIRYLTKKRIVFIIENFQ

FWDEKSVSLIYQILKERENNLSILEELVIIINWTSDQALNNEKFKNEILKEFHFDTYY

LNVIEKVGYKSALVSMGLLKEINNDLINGLYSITGGHLHLTKEIIQFFNHSSIESTEL

ISLGNTEKIMTLIEDRLKNLGARGEVISEVLKYASILGISFTFFELEKITKKNQAELQ

QIITEANKLYFVEENNKKVNFVHEIIRNLFESKLEDKKTYFQAYAECLKVIKPNDYIA

RARALLKASYVDQAALQYILAYVKKLRNSETINHDFSQEILMSVSELRAAEYVDIMQQ

AYSDYLNKDFDKVIEELGWIEDSYPILLRAERDYLLAMCLTKKIDSDSRNKSVSILDK

YKDINSLDGESELWSRILSLQMISYIHINDKTSAIKNENLLMHYLGDRASYDMDAEDK

INILRRKSSAVHSIIFAQRSTMKSVKYFGPLENGGVPLNPIQYYFSLNNHIANTLVLG

KFEESFEDATQLRKFIANHKGIPFPRPEIPLNNYILSGIASENISFEKGLEIYNEMFN

KIKSSADSILLKINQAVLYAATNQLDHARDILDTLKKKLIRQKNIELYYQFYVDSNLM

VVEYLLGNQSEALELWNTISYMPSIGDQNYYEKRHELIKELFDSNIQLSGLEWFTVID

KMYPEFVERSKEKSFGNGFLMSDIQFWSES"

CDS complement(3162219..3163157)

/gene="xerC_2"

/locus_tag="EFAGFIKM_02659"

/inference="ab initio prediction:Prodigal:002006"

/inference="similar to AA sequence:UniProtKB:P0A8P6"

/codon_start=1

/transl_table=11

/product="Tyrosine recombinase XerC"

/db_xref="COG:COG4973"

/translation="MRLSTSNLNTYIKQYETYLSVERNLSLKSIKAYLSDLNRLVEWL

DTKHIHDVTRENLRMYLEQLNSEHTLKDSTIKRKYICFKAFFNYLVQNGDIIESPIIG

FGKKFKTARRIPKTLSVNEVEKLLNAPQEHMKQLHTSFRRRINLRNDAIIELLYVIGI

RIGELVNIDMEHIDLEEKTVLIFGKGRKERLLYISSSEVIHKIKTWLHAREHFDPQSD

ALFLNKYGDRLSIYSIEDIYSKYRDLSKVSKKSTPHYLRHSFATHLLNNGADLRSVQE

ILGHTNVSTTQIYTEVSVERKKDVLSRFNPRNNFKV"

CDS complement(3163315..3164466)

/gene="adhB"

/locus_tag="EFAGFIKM_02660"

/EC_number="1.1.1.1"

/inference="ab initio prediction:Prodigal:002006"

/inference="similar to AA sequence:UniProtKB:P0DJA2"

/codon_start=1

/transl_table=11

/product="Alcohol dehydrogenase 2"

/translation="MATHAYYVPPVNLMGRGCLQEAGQMIEQMGIRKALVVSDRQLIT

SGVAEQVLSILRKSGLDYVVYDEVQPNPTCQNVHDGLQVFQNHGCDAIISIGGGSPQD

AAKGIGIVATNGGHIREYEGFHQSKHKSVPLVAVNTTAGTSSEVTINYVITDEERKVK

MVMVDRNSLVSLSVNDPELMLSKPASLTAATGMDALTHAVEAMVTPGGFTVTSATAAA

AVELIFEYLPRAVRDGSDLEAREHMTYACFLGGIAFNNAGLGYVHAMAHQLGGVYDLP

HGVCNAMLLPYVEELNAKHVPGKFRHIAKAIGMDVKGRSDEECSDYVIEAIRQLSKEV

GIPEKLSELGVKDPDVELLADNAMKDASAPGNPYQPSKDEVMELFRKII"

CDS complement(3164714..3166408)

/locus_tag="EFAGFIKM_02661"

/inference="ab initio prediction:Prodigal:002006"

/codon_start=1

/transl_table=11

/product="hypothetical protein"

/translation="MKFSEYTYTRPNLEHIKTSFRELLSGFEAAATVEEQSGFMDQIN

ALRSDFETQAQLVYIRHSIDTNDEFYKAENEFLDENAPIVQEYITDFYRALVNSKFRN

ELEQKWGSQLFQLADLSLKTFSPEIIEELQKENKLSTEYNQLIASAKIPFEGEERTLP

QLHPFELSTDRSMRERASEARYTFMAEHESEFDRIYDELVKVRTQIAKKLGYPSYVEL

GYDRMNRTDYNAEMVANFRAQVRDYIVPVATKLRERQRNRIDVDTLYYYDQGFSFKTG

NPTPKGDADWIIDNGKKMYAELSPETDAFFQMMTENELMDLVSKKGKQGGGYCTFLND

YKVPFIFSNFNGTSGDIDVLTHEAGHAFQVYESRDFAVPEYNWPTYESAEIHSMSMEF

FTWPWMQLFFKEDTDKYKFDHLSSGLLFIPYGVAVDEFQHFVYANPDATPTERKQAWR

NIEKTYLPHINYKDNAYLEQGGFWHKQGHIFSSPFYYIDYTLAQICAFQFWKRSNEDM

KSAWADYLTLCKAGGSLSFTGLVELAGLNSPFEDGCVSSVIGDIEAWLDGVNDKAL"

CDS complement(3166670..3166762)

/locus_tag="EFAGFIKM_02662"

/inference="ab initio prediction:Prodigal:002006"

/codon_start=1

/transl_table=11

/product="hypothetical protein"

/translation="MTYIIIMAIIMVVGMVGMGWFYQIMDKDQS"

CDS 3167012..3167767

/locus_tag="EFAGFIKM_02663"

/inference="ab initio prediction:Prodigal:002006"

/codon_start=1

/transl_table=11

/product="hypothetical protein"

/translation="MSTRIPLRQLFLTVLLALMLSVLTACADGEAHLTVNMDGSTDLD

LNLNVKNSALGKIGQDNLMPLIADALNRNNFQAEVSEQGDQKQLTATSHYEKSNKTSF

DTSKLPQGIKVEQSTSPGFFTSKMHITAEADLMESMPDGEIKNQMNKVPSFLKNLLLK

DVNFDFKLSLPIKAANSNADQVEDGGKTLIWHVSPLQMNKLDLTVQVPNIRNIIIIAV

IGLLLIAALLIWFFVRRRRARQRHSARSHREKN"

CDS 3167885..3168097

/gene="khtT_2"

/locus_tag="EFAGFIKM_02664"

/inference="ab initio prediction:Prodigal:002006"

/inference="similar to AA sequence:UniProtKB:O07535"

/codon_start=1

/transl_table=11

/product="K(+)/H(+) antiporter subunit KhtT"

/db_xref="COG:COG0490"

/translation="MNIRECPLPGIGVKYQFDTKGGHQLVIIVHEDGRRELFSVDPQD

NEELTLIADLEDDECVTLSGLIGGWS"

CDS complement(3168181..3170928)

/gene="rcsC_8"

/locus_tag="EFAGFIKM_02665"

/EC_number="2.7.13.3"

/inference="ab initio prediction:Prodigal:002006"

/inference="protein motif:HAMAP:MF_00979"

/codon_start=1

/transl_table=11

/product="Sensor histidine kinase RcsC"

/translation="MSKSITESRSLFEQLYTHAPIGIAVASHVNGRWLQLNPAFCEML

AYSEDELIDTSVIHMIYEEDLHMEEFRSKFWEMTHETSPMYETEVRLKRKDGSLLWAT

IRACIVRDETNGEPLYLLVQAADITKQKDAEEILIVQRKQLEESSRISRLLAESSLDL

IAIHDADSSRTFKYVSNACKSMLGYELEEVIGKPGTFVIYPEDVPLVEAYVEEQRKGL

APKRLNYRLLHKDGSTVWADTITHYVYDDSGELMEMVAVTRDITASQQQQLSMQEYRS

LFDCNPLGVASLDLEGNLLKANMGQEQLTGHTKEELLSRPFDHLIDPVDLEKTRYHFE

ETVKGNAQSYEIGLIHKNGQRIETNVINVPIILEDKVVGVYGITSDITESKRYVEEIE

NLSYERALILNGMSEGVIGLDLEGNLNFANPAAAEIMNFCPSELNGKPLEQMMIQMQS

EGIPYPSKDTPILQAVREGRGLPRTESVFWRPDGSSFLAEFQLKPIMDQGRTRGAVLV

FRDMTSVKDIIRAKEAAEHADRAKSEFLAIMSHELRTPLNGIMGMAHLLMETELDDEQ

KGFAEIMIDSGESLLYILNEILDFSKIEAGKMDLERAPVDIKAMLGGVIELFALKASE

KNIELYCEVSDHIPERIRGDETRIRQILINLVGNAVKFTEQGRIMVSVGADFSDEHGE

DNLMLTFKVRDTGIGIPIEKQHQLFQSFSQLDPAINRKFGGTGLGLAISKKLVELMGG

AIGVQSEIGEGAEFQFTLVVERWLEESNDPIEITTNDELQLDRASLAHDIKILIAEDQ

AVNSHLLEEMLRKFGGVCDIVENGAQAVDSLNKVQYDLVFMDIQMPVMDGIEATCHIR

QSHPEIPVIAAITAFAGAGDREECLKCGMQDFISKPFHSTEISRVLNTWVPYIRSQRV

T"

CDS 3171139..3171963

/gene="tgl"

/locus_tag="EFAGFIKM_02666"

/EC_number="2.3.2.13"

/inference="ab initio prediction:Prodigal:002006"

/inference="similar to AA sequence:UniProtKB:P40746"

/codon_start=1

/transl_table=11

/product="Protein-glutamine gamma-glutamyltransferase"

/translation="MIIVANQPVELNRSEWSDFEWYWLQQLQNRPTRYVYQSMDHLRF

EWDLRGSLVDAAEGLDRSGVSFASFEKSRCNPAYWNRNAEGGFELRSNVTPADGIRDI

WRNGHLYAFECATATVIVLYGGVLGSIREDAFNSLFRNLLLFDWHYDSDLRLTEKNGS

ETALPGDVLYFKNPDVSPETPEWQGENTFMLRENLYYGHGIGIASGEEIIRTLNQFRV

PVSRVSAYLMDMVIYPDFFYLSRFAKNSQNNMAAGSTPVLPGQLYVRLGGSRYLRS"

CDS complement(3172145..3172636)

/gene="infC_1"

/locus_tag="EFAGFIKM_02667"

/inference="ab initio prediction:Prodigal:002006"

/inference="similar to AA sequence:UniProtKB:P03000"

/codon_start=1

/transl_table=11

/product="Translation initiation factor IF-3"

/translation="MIKNEKIKAAEVQLTGLNGEDLGVMSTQEALLLAKQHKVDLVCL

SLMTSPPPCKLIGAGAAKAEAQQAKKKASKSPDKRKVKEIRLNLAMEDHDRDTKQSQA

ERILKKGDSVKLVIQVHGAKEGVAGKEWAEQLSKSLAEFGSKTTGVQVSGKQVVVQLD

PNA"

CDS complement(3172757..3173689)

/gene="ppaC"

/locus_tag="EFAGFIKM_02668"

/EC_number="3.6.1.1"

/inference="ab initio prediction:Prodigal:002006"

/inference="similar to AA sequence:UniProtKB:P37487"

/codon_start=1

/transl_table=11

/product="Manganese-dependent inorganic pyrophosphatase"

/db_xref="COG:COG1227"

/translation="MEKALIFGHKNPDTDTICSAIAYADLKTKLGQDVEAVRLGEVNG

ETQFALDQFKIAAPRLIKTAANEVNKVILVDHNERQQSVSDIEEVTVVEVIDHHRIAN

FETSGPLYFRAEPVGCTATILNKMYKENGIEVSAPIAGLMLSAIISDSLLFKSPTCTE

QDVAAARELAAIAGVDADSYGLDMLKAGADLSQKTIAELISLDAKEFVMGQSKVEIAQ

VNAVDVNDVLVKQPELEAAIDAIISSKGLDLFVFVVTDILNNDSVALAYGTSTKAVEK

AYNVTLSDSRAILKGVVSRKSQIVPVLTEAFNTL"

CDS complement(3174134..3174886)

/gene="pphA"

/locus_tag="EFAGFIKM_02669"

/EC_number="3.1.3.16"

/inference="ab initio prediction:Prodigal:002006"

/inference="similar to AA sequence:UniProtKB:P55798"

/codon_start=1

/transl_table=11

/product="Serine/threonine-protein phosphatase 1"

/db_xref="COG:COG0639"

/translation="MRRCVISDIHGCYDEFNALLKLADYNPQQDELILLGDYVDRGPS

SKQVIEQIMQLQQQHHIMVIKGNHDAMMVKALTQDVEQYDQHWIRNGGLQTMASYLDL

ELTYDEQQIDWEAYAEAKKWIRTHYEHHLRFLEQLPLVYEIPGYIFVHAGINPDIEDW

RSQSERDFIWIRESFYSRPTTIRETVVFGHTPVKHLHDETGIWFDPSGDKIGIDGGCA

YGAQLNLLEISEDGSLQTFFVRQGQSAEEPEI"

CDS complement(3174986..3175993)

/locus_tag="EFAGFIKM_02670"

/inference="ab initio prediction:Prodigal:002006"

/codon_start=1

/transl_table=11

/product="hypothetical protein"

/translation="MKLSVLEHGHINEGRSVQDTLQETVKLAQHADQLGYSRFWMSEH

HGSGALSFSSPEVMIAHVAAFTDRIRVGSGGVMLPHYSAYKVAENFRLLEALHPGRID

LGIGRAPGGMPIASRALNEGKSSNVQFFPQQIADLGGYFHEQLPEDHRFASLVAGPSV

PTVPEVWLLGSSSEGARIAAAQGTSYAFAQFFGTPGGEEAMKHYRRHFKPSILNDKPH

SMIAVSAFCAETEEEAAELARSNELFFLRLGRGLEQSSFPSLETVNNYPFTAMEMEQI

RQRRSFSIVGTPDQVKDKITAMAERHEADEVIIASAVHSFEARLRSFGLIAEAFGLKQ

D"

CDS complement(3176229..3178166)

/gene="ltaS2_1"

/locus_tag="EFAGFIKM_02671"

/inference="ab initio prediction:Prodigal:002006"

/inference="similar to AA sequence:UniProtKB:O34952"

/codon_start=1

/transl_table=11

/product="Lipoteichoic acid synthase 2"

/db_xref="COG:COG1368"

/translation="MYNSKNERTSSRTLFAVLFILMLLKLSLLRYFFFQGLSGIGLLT

DALGALTLVCLLDLIVPKGWKRAVYGGFNILFSLVLFAATLYNVHFSSVPTYTVLSEL

GQVAQVRGSIGPLVRPEHFLFFADIVLALPIWLIMRRRAGGRNYSSYRDSGLTFGRIR

RRYWGKLGVALTAAFCIVLSGSFIVKGETIDNELVRAENLGFLNYQVSSAILTSKENE

AIANGNINETIAKINELVSQYPYQDTPSDGTAVKAKYFGQAKGSNLIVLQLESFQNFP

INASLDGQELTPVLNDLAKESYYFSHFFQQIGQGNTSDAEFMSNTSIYPTGVVPMSAG

YSDRELPSLPKLLGKEGYESETYHVNDVTFWNRNKMYPALGFTRYFDKPSFENDRFND

FGPSDEELYRVGVEKMTAHQAANQPFYAQFITASSHSPFTVPADRARITIPATITNKL

LHDYLQAINYTDYAIGQLINELKANGLWDNTTLVLYGDHFGLPADEEITQQIQANLGV

PYDGKVSRFNIPFMIHTPKQTKGQVIEQPGGQLDMLPTIMNLMGVSLQDEKFTAFGHD

LLNMDHNAFGIRYYLPTGSFVNNDIMFIPGAGFDDGTAYSLKTYEPVTDLEPYRSDYE

HVLSLMKLSDEYVKLLPKRAP"

CDS complement(3178282..3180657)

/locus_tag="EFAGFIKM_02672"

/inference="ab initio prediction:Prodigal:002006"

/codon_start=1

/transl_table=11

/product="hypothetical protein"

/translation="MTKRVILLEEGTAEMKGLMVSKGADLAELIHAGWSVPAGFVVTT

ECCREFCTRLGHLSGEGEEEIINAIRHLEQQTGKFFGHSENPLLLAVRTDQDRASATA

TRSLLNVGLNDITVEGLARQTGDRLYALQCYLDYLEEYGQLVYAIPSEFFTEISSHVK

DQDEVELELTITEFKYLIEAKGRNPFPQDVQIQFKEAVRAVYHSQRIKASRSQDEIMR

KSYYISSEQSAPVLIQTMIHGKCGESSGAGTIYTCNPLTGEKGITGQYVPTGNASQTD

HGLERLRNDEPELYTSLLEIGSQLETHKGEVQEITFVIKSGALYVVQTQPARLSSIAT

LRSTVDFVHEGLITKEDALLRIQPAHITEVQKLYTNPSSISEASNGHLPIQIQPVTND

ECPAYNNSGSLLSADLQLLLNWADEVKELKVLANADRPQDALTSRLLGAEGIGLCRTE

NMLLSPARLPFVQKMILADSESERRRGLERLLPMQQSDFEQIFEAMDGYPVTIRLLDS

PLHELLPDLGVLEKRREWLRAEEWQEQKDHQIELEELERVIRRVSELHEHNPTLGQQA

CRLSTVFPEIVDMQLEAIFRAAVKGIRQGWWVRPEIMIPQIGHVHELQVMRDLVDHVA

DQVLGEEKRHCHYRVGAMIEAPRAALTATHIARQADFFSFGTDELTEMTFGYSRHEAE

KRLLLLLQRDTDSRPVTNNPFHVLDIEGVGQLIEMAVVQGRIRKPHLKTGICGENVIDL

ESITFCHRIGLDYVSCLPEQIPYARIAAAQAAIKGQREGADTQNTDISTIA"

CDS complement(3180919..3182013)

/locus_tag="EFAGFIKM_02673"

/inference="ab initio prediction:Prodigal:002006"

/codon_start=1

/transl_table=11

/product="hypothetical protein"

/translation="MKTTFFKKVGLFLGAFIFTIGFASNSGVHAAVDYSGGLMDKMVM

TLSDTESKGSKFTYALTDNNEATSAIIEKWNTPSTAVDHAVGIFNGTVTANAIRVKAD

FDVSIGLFDSTGKAILPTYWYTVKANQADGRLIYLPTTYKGIAKVNLFNYSNTRTVSV

SEFQLYNMTPPANPLDLAASADESSIGLTWNEVTTATSYTIKRSTTANGPYTTLATGV

TKANYTDLDVTPGTTYYYVVTGVNPVGESVNSNEASAIIESNGQVDPEAEQPTTPEPE

EPTTPEQPEEPSTNRAIFVVTMTTGLEKEFDLSMKEVNDFIDWYEAKQAGSGKASYAI

NKHDNNKGPFKSRKDYILFDRVLTFEVSEY"

CDS complement(3182203..3182520)

/locus_tag="EFAGFIKM_02674"
/inference="ab initio prediction:Prodigal:002006"
/codon_start=1
/transl_table=11
/product="hypothetical protein"
/translation="MRKKIWALLLVVAVIGMIPAKATSTTTVAESHEISGEDLPESLR
YDSNHPDIIYVKDCGVFEKVDSSLVDYGEEEIVFDDIQKVTNKTNVIVAYDATYIKKG
EWE"
CDS complement(3182837..3183067)
/locus_tag="EFAGFIKM_02675"
/inference="ab initio prediction:Prodigal:002006"
/codon_start=1
/transl_table=11
/product="hypothetical protein"
/translation="MIKDKIPLWFWCTSVVVVIMLVYNLFQVDSRINMLALGILNIAN
AIRMWKKYRNSAIIFLIIGVLCAVLFFKYLLQ"
CDS complement(3183292..3184956)
/gene="rhaS_24"
/locus_tag="EFAGFIKM_02676"
/inference="ab initio prediction:Prodigal:002006"
/inference="protein motif:HAMAP:MF_01534"
/codon_start=1
/transl_table=11
/product="HTH-type transcriptional activator RhaS"
/translation="MCHTIIQLIPKPWLCSLQHMEYVDHHKSHIDKRLLYRMSTSHSI
FVVTSGKGSIVCSDRESHLEKGMIMFVPAGTSVKIEGSPWTENELQYYKLDLMVAELK
EETSNGLSRRNTEEKQELFQSPDSSTLLPLMQLSFSPWSSCLEALEQILRQQVTGDWL
EQWEVQLRFQEWFRALIRQSAPKTEVPDDRARLQSSIRYIGDHYDQTITVDELAADIG
LTRASYTRQFKRITGKLPLEYVNAVRLERSKQLLQLTDDRIHEIAQNVGFSSEYYFGR
RFKQYSGISPGLYRRHHRQEVRVFAPYLEDFVLALGVKPVLQCSHHSWGRQHYLGLDD
VPEFDVSQQDAQFNVGNIPDFIMLNKGYDRWNLNRFEQVAPTFYVDHLGEDWRSILKS
TADVLGKVNRVQDVIGEYEDNAMEAKGRLARSLRGQTVAFLRISALDITLYGDRQGYV

GPVIYQDLGLTPHSRVQQWTRHERRIFIGLDQLSQLNADHLLITFDTGNSAKPGEERE

LLDRDEWKRLPAVKSGNVYEVDFMSWMNYGVISHGKKIEDILRFMA"

CDS complement(3185062..3186060)

/gene="yfiY_3"

/locus_tag="EFAGFIKM_02677"

/inference="ab initio prediction:Prodigal:002006"

/inference="similar to AA sequence:UniProtKB:O31567"

/codon_start=1

/transl_table=11

/product="putative siderophore-binding lipoprotein YfiY"

/db_xref="COG:COG0614"

/translation="MVGFIKGFKGWTITLLLMGLVISGCSTNTATNAEQTPATESAAS

GENTTETETAPATRVVQDEFGDVTIPVQPQRIAGIYVEDYLKALDITPVVQWYHPLWG

VQDYLELDVPQFDTTGSIEALLEHDPDLIIVDGGVDMEKYEMYSKVAPTYRLPESVLQ

DSNLILKTIADVVGKPDKGEEVAAAFGAKIADAKAKLQESVGDETVAVVRLNVNDDTL

ALFGVKNRFTGFIYSELGLTPHPLVSKMEEYQEILSEEAIPQLDADHLIIFPSNGEWS

SPENKEALKVLDSKLWKSLPAVKNNQVYIMERSHWQSGAITANSMKIDDLLEKMTP"

CDS complement(3186239..3186613)

/locus_tag="EFAGFIKM_02678"

/inference="ab initio prediction:Prodigal:002006"

/codon_start=1

/transl_table=11

/product="hypothetical protein"

/translation="MSKTDVEKLLADLNEGRILTQQTFEGLDVEVYLDERDASAFADE

WMQAFERWAQDTAVAEEEVLRACREQAFKQTLALTGEPELAGYVSDDMGLIGAALLQG

TANDSFVDQLLESYRQGRLPLR"

CDS complement(3186636..3187298)

/gene="polC_1"

/locus_tag="EFAGFIKM_02679"

/EC_number="2.7.7.7"

/inference="ab initio prediction:Prodigal:002006"

/inference="similar to AA sequence:UniProtKB:P75080"

/codon_start=1

/transl_table=11

/product="DNA polymerase III PolC-type"

/translation="MKITLLPDTYFIENLSVSSLQDRMYCIFDLEGTGINPVVESVTQ

FGAMYYQRGQRCNTTFSSLTRANKPIPEAVAKLTGISNEDMVEAPSFVEAFQEFQKFI

GDNVLVTQAGYEYDLPILKRHCDEYSLPMLTNPVLDTKAMFTYIHPEITEVVSTDFLI

RYYDLNTDGIHRHNALADCGVIAGIFESILSEYEELQLDHFTADPNRMMKRFVIPEMY

LT"

CDS complement(3187360..3187935)

/locus_tag="EFAGFIKM_02680"

/inference="ab initio prediction:Prodigal:002006"

/codon_start=1

/transl_table=11

/product="hypothetical protein"

/translation="MSEASTFLYFVRHAESRYVDGQERERGLTEQGHQDAETVASLLH

GEQIQLFYSSPYRRAVDTIHILADRSGGIVVTEEDLRERQLSSSDVKHANFREAKQRL

YRDPTYAYPGGESGEQARSRAVAVIEKILDKHVGHKVVIGTHGDVMTLILQHYDSSYG

YDFWESTTMPDIFKLQFDGTRKLVQVTRLWE"

CDS complement(3187935..3190157)

/locus_tag="EFAGFIKM_02681"

/inference="ab initio prediction:Prodigal:002006"

/codon_start=1

/transl_table=11

/product="hypothetical protein"

/translation="MLHSLKAIQSYKSGNTPEAQQQWLQLLAAAEKPHINLERIHSIA

ELEGTNPVLDYTERTLHVLEKLQVSFWVREILEEVLIWSETAKAGSLEQRRVWQKQGV

NLFVHNVGSAQLYDLYAGAEHLDNDIDKTKETSIYQLGSPSGALSNNADISEHDALNT

HTPRQEIIRTLIATHGLIGQYIRGEIPFAENASLHALIVKGWLTTDELHTILMALNVC

IIAGVDPALWNQVQAEVQRIVGWIIVGPDHEDWSVKERLSRLRSSSIRQGEAMDTAYA

KLQTELDVEQALAPLAHRTLWYVESAMQDFSLQEMVKVFLLTLRSESMHPTSMESRSE

IVRHISFEPLMNTMYYDYRGVKKLNIYKKRMIEKYLEQYSWEQIVSGEQIVYPHLTHR

IERHADLPDTLFVTFEFSPAAEKLIAFCIEAEKSPLYEKAVLLLFDLFGLRRDAYDRF

HNEETYLSDMNSSGDYKKVLLEYIVGKRILDIGPGGGILLDLIEQEKPEVEPIGIDIS

ANVIEALERKKQREGHRWQVMKGDALQLEQYVQPGTVDTVIFSSILHELYSYIELDGR

RFNSDTVVAALRSSFRVLSPGGRILIRDGIMSEPEAQKRRIRFLEADGLRWLERYAED

FQGRAIQYERISDNEVEMPINDAMEFLYTYTWGEEAYVHEIQEQFGIFTPTAYENCIR

EALGEQAEIITFEHFLQEGYTEALGERMIFMEENGEPAPLPDSTCLIVIEKKKGLADG

"

CDS complement(3190352..3190678)

/locus_tag="EFAGFIKM_02682"

/inference="ab initio prediction:Prodigal:002006"

/codon_start=1

/transl_table=11

/product="hypothetical protein"

/translation="MAGDPAGDMAQSWVILMSGTLPEDTEPAIRMGFETVRESLVEYY

IHHYMQLSGITREAIESWMLPVAAARLDEDLPAQERAAQVCSGTNSSVIKVELHIIYT

DTTMTE"

CDS complement(3190803..3190895)

/locus_tag="EFAGFIKM_02683"

/inference="ab initio prediction:Prodigal:002006"

/codon_start=1

/transl_table=11

/product="hypothetical protein"

/translation="MERIGQGRTAEIYAYSNEHIMKLYRMDFPL"

CDS complement(3190898..3191491)

/locus_tag="EFAGFIKM_02684"

/inference="ab initio prediction:Prodigal:002006"

/codon_start=1

/transl_table=11

/product="hypothetical protein"

/translation="MTESSSFNPIMLTIPESFHTERLSIRAPQWGDGAAVNEAVRESA

EQLRLWLPFAEKIPSLEESEVTVRKARLQYLERTDMMLHLRDRHTDDFVGSSGLHRID

WNARCFEIGYWIRTSRAGEGLMTEAVRGIEQFAITHLEANRLEIRCDARNVRSAKVAE

RAGYTLEGILRKMRRDSTGTLVDCMVFSKVRGSEFGG"

CDS complement(3191500..3191946)

/locus_tag="EFAGFIKM_02685"

/inference="ab initio prediction:Prodigal:002006"

/codon_start=1

/transl_table=11

/product="hypothetical protein"

/translation="MIEARLNELGIILPQASAPAAKYANAVIVNGIMYVSGKGPDTSE

RGKLGSDFTTEQGYDFARNAGLEVLAVVRDVLGSLDRVKRVVKVQGFINASASYQEHH

KVLNGFSDLMMEVFGDQGVHARSVFGAVSVRDNLPLIIDSIFQVEE"

CDS complement(3191943..3192845)

/gene="rhaS_25"

/locus_tag="EFAGFIKM_02686"

/inference="ab initio prediction:Prodigal:002006"

/inference="similar to AA sequence:UniProtKB:P09377"

/codon_start=1

/transl_table=11

/product="HTH-type transcriptional activator RhaS"

/translation="MTEPFSADFHDPTDLLHIEYDRRIGYFSMTDDHLHDHYELYYLL

SGERIYFIKDRTYRVKAGDLVFVDRNTVHKTLESGMPDHERMVLYLKPELFAAIAISP

ELVEALKEPFCWEIPIVRFPSQVTEVLERMVSEMVDEMLRPQPGSNLLLRHRAIELLL

HAYRNQHLGRLSSDDREPVLHPKTQAVVRYLNENYQKPLTLPEVAGMFRISPHYLSRL

FKQTTGFTFSDYLNLLRVKEAQRLLRESEESITDIAWLAGFSNFSHFGKMFKRTVQVS

PRVYRQEYKESGSVRTIKEGENIR"

CDS 3192973..3194262

/gene="yteT_2"

/locus_tag="EFAGFIKM_02687"

/EC_number="1.-.-.-"

/inference="ab initio prediction:Prodigal:002006"

/inference="similar to AA sequence:UniProtKB:O34371"

/codon_start=1

/transl_table=11

/product="Putative oxidoreductase YteT"

/db_xref="COG:COG0673"

/translation="MSNKKRYVLVGTGGRAEFFYGALTRDYRDTSELVGFCDINQVRM

NYANQLLKEKYEYPEVPTYPADQFDQMIENEKPDYVIVTSVDRTHHKYIIRAMELGCD

VVTEKPMTIDEEKCQEILDAVKRTGQNVRVTFNYRYAPHHTKIRELILNDTIGKVTSV

HFEWLLNTRHGADYFRRWHRDKRNSGGLLVHKSTHHFDLVNFWIGSQPDTVFAFGDLM

YYGRENAEERGVTQFYNRATGNPIAKDDPFALHLDSDAHMKSMYLDAESEDGYQRDQS

VFGDGINIEDTMGVLVKYRNKAILTYSLVAYQPWEGYRIAINGTKGRIEMNIVEQSYV

NSLGDKSKEGALIGKTLRVLPMFDAPYEVEVEEKAGGHGGGDPVLLNDLFGEPVEDPF

HRAANHVDGARSILTGIAANRAIATGLPVNVNNLVRF"

CDS 3194379..3196196

/gene="sasA_10"

/locus_tag="EFAGFIKM_02688"

/EC_number="2.7.-.-"

/inference="ab initio prediction:Prodigal:002006"

/inference="protein motif:HAMAP:MF_01837"

/codon_start=1

/transl_table=11

/product="Adaptive-response sensory-kinase SasA"

/translation="MFFSLKNRLIAFIVVLFVLAFGTLSYLLFKESRTVIRSYIESSA

LEKMEEYGSYVDMVQMQIYDVASLVFNSDMGKNWDNAIGDSNLSEGEKMLANLAMSRF

LTQATNSYTSISDVSIYRRNGLRIGSENQVTYDRAFVQESWYTNFYTLGERWLPAHTD

QHERVRDHGNPVVSLLMPIGTFHHATAQSVMKVNVSESYFLEPLRRIHLAESSTIFLL

GEQGNPILSQQVDTLGAVATAEIDRFRNSPLKSGVAYLTNQEGQRDIFVFKKLGRTGW

MLAGLAPEKEMYLSLHKLQSTIMVVTIALILVSLLAAAWLSHGVTKPLTRLVLAMRQV

QRGAFDQADSLLPPDKNVKSEISYVIFTFRYMISQLRQHIQNEFELKLLRQQAEYKAL

LIQINPHFMFNTLELVSSLAIQRRTDDTVQVIEDLGKMMRFSINTNDDRVPLTEELDY

VQRYISILQTRFGHKLDISVTLEGTLNSVVIIKFILQPLIENAVKYSFQHQTTAQVQI

RINRMYDRLHISVSDNGPGIPAEIIHKLQHPVAPPSLESILHNEGWHIGLGNVIARCR

LHYGALFTVHMENDESGGACIELTLPVQEEYDVQRINRR"

CDS 3196171..3197727

/gene="rssB_6"

/locus_tag="EFAGFIKM_02689"

/inference="ab initio prediction:Prodigal:002006"

/inference="protein motif:HAMAP:MF_00958"

/codon_start=1

/transl_table=11

/product="Regulator of RpoS"

/translation="MYNVLIADDEIEVREGLKLKVDWYGMGFVITGEASNGIEADELL

KSEHFDLLITDMNMPVMDGVRLLDVCRSYNPSIQIVIITGYEDFQYARAGVRSQVMDY

LLKPVARDELKATLGKIKADLDDQRKVRGDSELMQWRFSQYYREMKERFLLDLVKGNR

LPPSSMPERLRLFHLESWQDQKVCFITASISQSDKQAIRKDRLHEQMHLPFEMVCYEI

AQSESDNVQVFHDSAHAGIMHFMVQDGMQHEFIQQLAGHTASVFSMELKIGVGLSDSG

LEHWKEGYIHSLLAWNSAERAGNTPNPRAGTDYSPLLPEETTRTLHRCLIRGELDSFR

SIIRKELHEAFQLSPSRMTRSILQISLLMDCPMAPEWILWLKTPDQAERLLMQWAQDF

LGRQLPSEDGDGTVIDLAKRYIEENYMQVLTLTLLAERFNYYPTYFSELFKEGAGTSF

IQYVTGVRMKHALQLLKETQLTVWDITELTGFSSPSYFSSKFKRMFNMSPSDYRLLHS

EKIDSHDPKK"

CDS 3197844..3198758

/gene="lacF_4"

/locus_tag="EFAGFIKM_02690"

/inference="ab initio prediction:Prodigal:002006"

/inference="similar to AA sequence:UniProtKB:P29823"

/codon_start=1

/transl_table=11

/product="Lactose transport system permease protein LacF"

/translation="MKKQTKPLYGQHNTTAYLFLLPWLIGLFCLTLGPMVASLYLSMT

KFNLLSSPTWTGLSNYVHIFTEDDTFRRSLGLTFYYVFLSVPLRLAFALLVAMALNKG

IRALGIYRTVYYIPSLLGGSVAIAIVWRQLFEGNGLVNQFLSWFGISGPSWIAHPDYV

VYTIITLSVWQFGSAMVIFLAGLKQIPADLYEASDVDGAGKIRQFFGITLPMLSPVIF

FNLIMSMINSFQAFTPAYVIGDGRGGPLDATMFYTLYLYLKGFSFFDMGYASALAWIM

LVIIGVFTAIVFVTSRFWVFYGDNQEGR"

CDS 3198758..3199603

/gene="dasC_6"

/locus_tag="EFAGFIKM_02691"

/inference="ab initio prediction:Prodigal:002006"

/inference="similar to AA sequence:UniProtKB:Q9K489"

/codon_start=1

/transl_table=11

/product="Diacetylchitobiose uptake system permease

protein DasC"

/db_xref="COG:COG0395"

/translation="MHMPIKRQLVQAGRHAAIILFGLLMLYPVLWLILSSFKPNHLIF

TSGSLFPTSFTLEHYINGWKGLQGISFGRFFGNSVLISVMSVLGNVISCSLAAFAFSR

LKFRFKGLWFSMMLVTIMLPYHVTLVPQYILYNELQWINTYFPLILPKWLAQDSFFIL

LMVQFIRGIPRELDESATIDGCGQSQIFFRIVVPLLVPALITTAIFTFLWSWDDFFSQ

MIYLSKIDLFTVQLGIRSLFDPSGQSDWGALLAMSTLSLLPVTIIFLLFQRYFLEGIA

TTGLK"

CDS 3199642..3200967

/gene="yesO_1"

/locus_tag="EFAGFIKM_02692"

/inference="ab initio prediction:Prodigal:002006"

/inference="similar to AA sequence:UniProtKB:O31518"

/codon_start=1

/transl_table=11

/product="Putative ABC transporter substrate-binding

protein YesO"

/db_xref="COG:COG1653"

/translation="MFKRFMIGMITSLLLFVTACSGTDHTGTNDASNGNDNGTTEGGQ

VELRIMWWGDQKRADITNEALKIFQAKHPDIKIVGEFAPSSGYFDKLNTQLASGTAPD

IFFLGGNVVDYAKKDVLLNLDPYVGNELNLDGMDETMIEYGRLDGKLQHISAGANARG

IIVNKALFEKAGLPLPASDWDWADYAAISKELSDKLGEGFYGTYNFTVDGMDIYLKQR

GKQLYDMKNGTLGFAKEDILEWFQYWEKTSASGGVVTPELQVSNPHDDTSKSLLITGK

AAMTLLPSNQLAAFQSLTEDPLVLLPVPRGPKGTGVVFESSQGLSGYANTKHPKEVAT

LMDFWIHDPEAAKILGNDRGVPVTEANRNLLQEGAGPVEEIVYNYTNLVSEATKTEPF

DVSYNPPGFAEFSKLAQTTNQEIGFGRKSVEQAVSDFYNGTVRIFESNQ"

CDS 3200991..3202637

/locus_tag="EFAGFIKM_02693"

/inference="ab initio prediction:Prodigal:002006"

/codon_start=1

/transl_table=11

/product="hypothetical protein"

/translation="MTEEILNRLRNISIMDQVQPVKPEDQQLLQEWHASGIRFVASGG

RVEATYYAAVEKLLACILPMGDNDPILQEGGIYLGCWLESTGTINAELLSRLLPSVSE

TTYLAFAKQQRDDGLLPYKLTANGPSFRQIQLVTPLARSVWNHYQLQGKHSSFLNTMY

KAMVRYDEWIAHNRNTRGTGCVEAFSTFDTGHDLSPRFWHVPDTPYMNDASAYHPDSP

ILPFLAPDLTANIYCQRMYLSRIAEELGESGTDWKAKAESSLESLFRYCYDEQDMFFY

DRDRNDELVRVQSDVLLRVMACEVGDRELFDNLLRWYLLNTSKFFAKYPFTSISMDDP

RFDPFSSYNSWGGASNFLSLIRAPHAFEYHHRYVELTWVMQPILSALSKDKRFGQVLS

PWTGKQGFTETYSPSILCLLDYVERLCGIMPVGSNQLWFTGLLPIDMDHGEIVSGNTA

YSRKVNGVGYELVNTPDRMTVYLQGKVHLDGPSGIRLVTDHSGHLEGVIGMSVRTVEG

ELYYKGECIPIRIKGNEMQRYADGNLRTERDIGIIYPTYR"

CDS complement(3202711..3203055)

/locus_tag="EFAGFIKM_02694"

/inference="ab initio prediction:Prodigal:002006"

/codon_start=1

/transl_table=11

/product="hypothetical protein"

/translation="MIVEMYKDLIKDERGNYYLAVQIEGNELTLVNAFVEAAFTPELI

YNEEFRTKHKEMEGGFVGKIAMDLLRHDVVMGMKQIDRKLLNLSDVEQQFTVNYIDTI

EFYRHPAWNRKV"

CDS 3203179..3203601

/locus_tag="EFAGFIKM_02695"

/inference="ab initio prediction:Prodigal:002006"

/codon_start=1

/transl_table=11

/product="hypothetical protein"

/translation="MSWQHLHTGKTLQQRLAELQGHLAELNTPLSGLEPGRIRRVYPR

QFIKVNRQLFIPLSLNSIRVFDIPRTRRGVRVGIRTTFPSRNAFNNIRLVGVGLDYLE

LQGKGRVPSRILFPLSSVESIYRPTTGRKNLKRPCRRK"

CDS complement(3203801..3206974)

/locus_tag="EFAGFIKM_02696"

/inference="ab initio prediction:Prodigal:002006"

/inference="similar to AA sequence:UniProtKB:Q81BF4"

/codon_start=1

/transl_table=11

/product="Bifunctional cytochrome P450/NADPH--P450

reductase"

/db_xref="COG:COG0369"

/translation="MPPISVPQPKTFGPLGNLPQLNFEEPVQSLVKLAEEYGPIFRME

YPGRSELYISGHELVAEVTDESKFDKRVWAPLAKVRAFAGDGLFTSWTEEPNWKKAHN

VLLPSFSQRAMQGYHNKMIDLAVQLVQKWSRLNPDETVNVPDDMTRLTLDTIGLCGFN

YRFNSFYREEPHPFITSMVRALDESMSSLQRLRLQDKLMITKKKQFEQDIRSMFSLVD

HIIAERKEKPQEGADDLLSHMLSGKDPETGETLDDENIRYQIITFLIAGHETTSGLLS

FAVYYLMKNPDTLAKAQAEVDQILKDPVPTYNQVRNLKYVRMVLNEALRLWPTAPAFS

LYAKEDTVLAGQYPLQKGDSVSVLIPKLHRDREAWGDDVEEFRPERFEDPSKVPHDAY

KPFGNGQRACIGQQFALQEATLVLGMVLKHFDFIDHSDYQLKVKETLTLKPDNFTIRV

RARGGQPVMAVPGVAVEEPKPVAKRTEPDAANAHHTPMLVLYGSNLGTAEGIAREIAD

TARYQGFRSEVATLDDRVGKLPKEGAVIIVSASYNGQPPSNAKMFVEWIEHADANEFK

GVRFAVLGCGDHNWASTYQRIPRLIDEQLSSRGAERLSPLGESDASGDFEKQVGDWTE

QLWPDLARTMGLKLNTSSNSERSSLSVQFVSGLAVTPLADTYDAHVAEVLENRELHDA

GSERSTRHLEIKLPEGITYKEGDHLGILPQNPPELVERVLRRYGFTGTEHLVLDASGR

SAAHLPLHQPVNLYDLLSHSVELQEAATRAQLREMAAYTVCPPHKKELEALLDESVYM

DEVRNKRISMLDYLVKYEACELPFERFLELLPSLKARYYSISSSPRVQPDQASITVSV

VRAPAWSGQGEYKGIASNYLANLKSGDEIVMFTRTPESGFQLPEDAQVPVIMVGPGTG

VAPFRGFLQARHVLKEQGQEVGEAHLYFGCRNPEHDYLYKNELEAAQQGGIVKLHTAF

SRVDGEEKCYVQHLMRDDAHHLIPLFEEGAHLYICGDGSKMAPDVEAALQQAYADIHG

KSAKEAEDWFNQLQQEGRYAKDVWTGI"

CDS complement(3207274..3207828)

/gene="gpx1_1"

/locus_tag="EFAGFIKM_02697"

/EC_number="1.11.1.22"

/inference="ab initio prediction:Prodigal:002006"

/inference="similar to AA sequence:UniProtKB:P74250"

/codon_start=1

/transl_table=11

/product="Hydroperoxy fatty acid reductase gpx1"

/translation="MSIFSYQVPFIDRHESDFSAFKGKVLLIVNTASRCSYSRQFSEL

QQMYEKYGDQGLEILAFPCNQFNEKEPGSSAEVAEYCRSQFQISFPILEKVEVVGQSM

HPLFRYLITESPFQGYDLDTKEGEWMDTFVKEKHPELYQGDGIKWNFTKFLIDRSGNV

HGRYETTVAPLEMESVIQNLLKNS"

CDS complement(3208044..3208769)

/gene="lutC"

/locus_tag="EFAGFIKM_02698"

/inference="ab initio prediction:Prodigal:002006"

/inference="similar to AA sequence:UniProtKB:O32259"

/codon_start=1

/transl_table=11

/product="Lactate utilization protein C"

/db_xref="COG:COG1556"

/translation="MVTEHQQWLAQLEKKSMEKQEQFMNDIASKLRRPRQRHAPTQPF

RGAPDFWTELEWDEEKRIQAFTDNFVSVGAHIARVQNMEEVSQFIANKSHELSARYII

RQNEQALQDLGLEEQLPDVQISVWNSQADENWRARAAEADIGVVMADYATAYTGSVTV

LSSPEKGRSVSLLPTVLIIIIPVDRLYTRLGETLDRFDEAGRENLPAGIHFISGPSRS

SDIENDLTIGVHGPGIVYGLIMG"

CDS complement(3208759..3210279)

/gene="lutB"

/locus_tag="EFAGFIKM_02699"

/inference="ab initio prediction:Prodigal:002006"

/inference="similar to AA sequence:UniProtKB:O07021"

/codon_start=1

/transl_table=11

/product="Lactate utilization protein B"

/db_xref="COG:COG1139"

/translation="MSQPGVMDTTVKERAGLALNDDFLRKAVKFTTERLRNGKKSASE

EHGNWDEWRERGRQIRLHTIAYLDYYLNEFVNNARANGVHIHFADTSVEAAAIALDIA

AHKQASTVVKSKSMVSEEVHLNHVLESAGIEAIETDLGEYIIQLAGEAPSHIVIPAIH

KNRYQIAELLSKEAGEILEPDTTVLAGFVRKKLREKFLEADIGMTGCNFAIAETGSMV

LFENEGNARMVSTVPKTQITLMGMERIIPSWTDLEVMATLLPRSATGQKLTMYMSGIT

GPRRTADGDGPDEMHIIIVDNGRSLQLGDPEFQELLNCIRCGACLNACPVYRHIGGHA

YGGTYSGPIGAVLTPALNGNIDEWNDIAGASSLCGACYEACPVKIPLHDMLVYLRRRK

VEDGHGNKMESMGMKGFAAVVSNSKRFSAAIRLGQIGQKAIVRNNGISLKLGPLKGWN

NYRVAPSLAKKSFRQQWNKLDQELDQEQPTMDSSVRSRMEQIIREREEGEGAKHGH"

CDS complement(3210276..3211004)

/gene="lutA"

/locus_tag="EFAGFIKM_02700"

/inference="ab initio prediction:Prodigal:002006"

/inference="similar to AA sequence:UniProtKB:O07020"

/codon_start=1

/transl_table=11

/product="Lactate utilization protein A"

/db_xref="COG:COG0247"

/translation="MKVSLFITCLSDAIYPRVGEAMVRLLAAHGVRLDFPPVQTCCGQ

PSYNSGYWDETRVAAKTILEAFDDSDFVVCPSGSCTYMIHHYPELFADEPVWLEKAKR

LEAKAYEFTQFLVQVLGITDLGAHFPHKVTYHPSCHGSRLLGVKDEPMALLSQVKGLE

LVPLPFAEDCCGFGGTFAIKMSDISGAMVTEKVDHIKETQAEVLVGLDMACLMNIAGN

LRYRNEPVRVMHLAELLYEGVRTG"

CDS complement(3211118..3211879)

/gene="ydjF_1"

/locus_tag="EFAGFIKM_02701"

/inference="ab initio prediction:Prodigal:002006"

/inference="similar to AA sequence:UniProtKB:P77721"

/codon_start=1

/transl_table=11

/product="putative HTH-type transcriptional regulator

YdjF"

/db_xref="COG:COG1349"

/translation="MLAAERYDRIVEMVNVKGSMRVSELSERCRVTEETIRRDLDRLE

QAGRLRRSHGGAVSVKEDQPEIPYRIRETTHAEEKKRIAQAALTMINPGDRILLDAST

TAGYMAANMPDFPLTVLTNSIQIATELSSRDKVEVISTGGQLASRSLSFVGPLAERSL

ETYHVDKMFMSCKGVHLEGGGISESNELQAKLKQKMVGISDQVILLADASKFGVRAFA

RVSGLNAVHTIVTNQPLEAEQTDRLSGYDIGIITV"

CDS complement(3211881..3213374)

/gene="rhaB_2"

/locus_tag="EFAGFIKM_02702"

/EC_number="2.7.1.5"

/inference="ab initio prediction:Prodigal:002006"

/inference="similar to AA sequence:UniProtKB:Q1R415"

/codon_start=1

/transl_table=11

/product="Rhamnulokinase"

/translation="MSVLAYDLGAGSGRALLGHLNDRGIETSEIHRFKNEPVKVGERM

HWDILRLHHELMQGLILVKQQGEKPESLGIDSWGVDFGLLGSNGELLGNPYHYRDTQF

NGMMDQVRQELSSQRIFERTGIQFLGFNTLYQLATLQRSGSPLLHEAERFLMIPDLLR

YFLTGEAVNEFTNATTTQLYNPSAGQWDSELLTHIRISEKLFGEAVLPGTRVGQLRSS

ICNDLGLSAIPVITVAEHDTGSAVVAVPATERSFAYLSCGTWSLMGTEIDHPAISSQS

LALNFTNEGGAGGTFRLLKNIMGLWILQESMREWDRQGQGISYDALLAQAEQAPPFAS

LFDPDDELFMPAGDMTTRIRQYCRDTGQVVPEDQGAIARAILESLALKYRRVLEWTEQ

LSVQTFNGLHMVGGGIQNRLLCQWTANSIGKPVWAGPAEGSAIGNMAVQWMASGAFKD

IWEARKAIRDSFPVTVYEPQDRSIWEDAYGRFLRVTASSNSQAGSEV"

CDS complement(3213610..3214125)

/locus_tag="EFAGFIKM_02703"

/inference="ab initio prediction:Prodigal:002006"

/codon_start=1

/transl_table=11

/product="hypothetical protein"

/translation="MALTSVSSSMGRLSVDGFGVFDDMNGVPGFEGNQGGFFSGFNEF

ATMNAFASIFIGGIFLIIAGVIVFVIISGVRTWSSNNAAALLTLHSTVVVKRTEVSGG

SGDSRATTRYYVTFEFDNGERTELIVGGNHYGMMVENDRGMLTYQGTRFKHFERDVQP

QMGVSKGQFYT"

CDS complement(3214237..3216039)

/gene="pepF1_2"

/locus_tag="EFAGFIKM_02704"

/EC_number="3.4.24.-"

/inference="ab initio prediction:Prodigal:002006"

/inference="similar to AA sequence:UniProtKB:P54124"

/codon_start=1

/transl_table=11

/product="Oligoendopeptidase F, plasmid"

/translation="MEKIRTRAEVNPETTWDLKDLFVTDVEWEQELRSLPLAAAQIET

FKGRLGKGAEQLLACLDAREALQERIGKTASYARLKQSEDSINPVNIENSAKAGDILS

NLSSSLSFVNSEIVDLPDGTVERYIQELPGLEPYARSLERLIREKEHRLTPETEKVLA

SLGEVLDSPYRIYLRGKLADMTFDDALDGEDNNRPLSWSFYENNYEMSSDTKLRRSAY

AAFSSTLNDYKNTFAEGYATEVKKQVVLSRLRGYDDVTDMLLSPQQVSKEMYNNILDI

IQQELAPHMRRLAALKKRELGLDKLMFCDLKAPLDPEFSPVITYDEACILIREALDVL

GPEYGEIVERAFSDRWVDYADNAGKSTGAFCSSIFGSHSYILISWANNMRGAFTLAHE

VGHAGHFMLAGRYQRLTNTRPSLYFIEAPSTMNEMLLADHLLKRSDNPRMRRWVILQL

LNTYYHNFVTHLLEGELQRRVYARATKDEPITAKTLSQLKGDILSEFWGPDLVIDEGA

KLTWMRQPHYYMGLYPYTYAAGLTASTAAAQQIREEGQPAVERWLDALKAGGSLTPQE

LMKLAGVDMSGPEPIRSAVAYVGSLVDELERLYS"

CDS complement(3216491..3216850)

/gene="hxlR"

/locus_tag="EFAGFIKM_02705"

/inference="ab initio prediction:Prodigal:002006"

/inference="similar to AA sequence:UniProtKB:P42406"

/codon_start=1

/transl_table=11

/product="HTH-type transcriptional activator HxlR"

/db_xref="COG:COG1733"

/translation="MAAEVKERINLKEINCEKELTLAVIGGKWKLIILWHLGLEGTKR
FSELKRLIPHITQKMLTNQLRELEEDKLIERKVYAEVPPRVEYTLTDHGQSLMPVLHA
MYNWGKNYGENVIWKES"

CDS complement(3217116..3217679)

/gene="hxlB"

/locus_tag="EFAGFIKM_02706"

/EC_number="5.3.1.27"

/inference="ab initio prediction:Prodigal:002006"

/inference="similar to AA sequence:UniProtKB:P42404"

/codon_start=1

/transl_table=11

/product="3-hexulose-6-phosphate isomerase"

/db_xref="COG:COG0794"

/translation="MSKTQYAADILKELERTLSQIDDAEMQAMAEHILAAEQIFVAGA
GRSGLMGKAFAMRLMQMGLRVYVVGETVTPGISSKDFLLLCSGSGETGSLVAMAQKAS
QAGAPVGLITIKPESTIGKLATTVVRLPASAKEDTATSGAAVTIQPMGSLFEQGLLIG
MDALILTMMEMKGMTGADMFGRHANLE"

CDS complement(3217684..3218319)

/gene="hxlA"

/locus_tag="EFAGFIKM_02707"

/EC_number="4.1.2.43"

/inference="ab initio prediction:Prodigal:002006"

/inference="similar to AA sequence:UniProtKB:P42405"

/codon_start=1

/transl_table=11

/product="3-hexulose-6-phosphate synthase"

/db_xref="COG:COG0269"

/translation="MMKLQLALDLVNIPEGIALVKEVEQYIDIVEIGTPIVINEGLHA
VKAMKEAFPNLQVLADLKIMDAGGYEIMKAAEAGADLITVLGATNDSTIKGAVAEAKK
QNKQVLVDMINVPNLEQRAREIDALGVDYICVHTGYDLQAEGQSPFEDLQTIKAAVKN
AKTAVAGGIKLETLPEVIKAQPDLVIVGGGITGQADKAAVAAEMQRLVKQG"

CDS complement(3218693..3219775)

/gene="pgl_1"

/locus_tag="EFAGFIKM_02708"

/EC_number="3.1.1.31"

/inference="ab initio prediction:Prodigal:002006"

/inference="similar to AA sequence:UniProtKB:O34499"

/codon_start=1

/transl_table=11

/product="6-phosphogluconolactonase"

/db_xref="COG:COG2706"

/translation="MSESKRLLVLAGSYAEAENEGIYAYELNEDTGSLSKLDGIAGVK

NPTFVNVDAEGNKLYAIGETASAEGNKMSEAVALSIDPSTGKLTLLNRNDSISAPPCH

IQRDPSGKYLILSSYHGGLVGLQALTDNGEVGALLDEKKHEGQGAHPERQDKPHVHSA

FFSPDGKYMMVQDLGADNIAIYSIDADKNELVLHSETKTHPGAGPRHLAFHPNGEFAY

VINEVDSSITSFKYDAAAGTLTELSTVSTLPDGYDGKENTTAEITVSNDGRYVYGSNR

GHDSLVVFAVDADKGHLSLVEHVSAEGEHPRHFALTPNGKLLIAANRDTNNLVTFTVD

QESGRLKYTGHSTGVSKPVCVKPVYL"

CDS 3219990..3220919

/locus_tag="EFAGFIKM_02709"

/inference="ab initio prediction:Prodigal:002006"

/codon_start=1

/transl_table=11

/product="hypothetical protein"

/translation="MLHSFLLTLYHVFLPISLPVIGGILLKRFKNWDTRSLSTFSLYI

LSPALIFNTLLHAEITWTDVTSTFWFSIINLIALWALAELLSRVFRLGASEKAGLTLV

STFTNCVNYGLPLVLLAFGQLGLDKASVYVIGQMIIVNTVGIFFAARSEFTVKDAILS

VFRMPSIYAAGIAIALRASNLSLPEALDGGISMLAAGYSPVVLAILGAQMLRPKGATV

PWLPNVRRAFWTGLVVRLAAAPILSWLILTVLQVEGTLFSVLLILASMPTAVNAVILA

EQFNASPQFVSRCILWTTAASMLMLPIMIVMAS"

CDS complement(3220897..3221487)

/locus_tag="EFAGFIKM_02710"

/inference="ab initio prediction:Prodigal:002006"

/codon_start=1

/transl_table=11

/product="hypothetical protein"

/translation="MNTQKRKTNRWVRLTRAMKLNFLKLLRAPGGAHKVSTGFAIGFG

LELIVISTASLIYLVFYPIVRLSGGSVPAAIVGNVIGKLTFLPIILMPLAKQIGSWIL

PAHSMGQGPVHESAFMELFRGNWSAVSELLLGGLDILAGMSVFGVILGVISYFVVKFF

YVRALNRRYERRLEKRRQADIASVSPPVLIRKPSQS"

CDS complement(3221546..3222004)

/locus_tag="EFAGFIKM_02711"

/inference="ab initio prediction:Prodigal:002006"

/codon_start=1

/transl_table=11

/product="hypothetical protein"

/translation="MKHPFHLKAVWNGGRNSEGTIDAGGLKTVISIPQEMGGPGTGTN

PDEMLLGAASTCYLITLAAMLERSDITPDELTLESEATVDVTNNVFTYERIVHRPRIV

LSADASEADLTKAERLAHKAESSCMISRAVAGNVQMETQPVIVTTGANAV"

CDS complement(3222025..3222792)

/gene="ydjF_2"

/locus_tag="EFAGFIKM_02712"

/inference="ab initio prediction:Prodigal:002006"

/inference="similar to AA sequence:UniProtKB:P77721"

/codon_start=1

/transl_table=11

/product="putative HTH-type transcriptional regulator

YdjF"

/db_xref="COG:COG1349"

/translation="MLVAERYEKIVEWVDTQGSMRVTELSERCGVTEETIRRDLDKLE

QAGRLRRSHGGAVSVKYKDELQSEIPYPERAVAHAEEKRRIASEAVKMVESGDRIALD

ASTTAWYMAAGLPNIPLTVLTNSIKVAAELSNKEQIRVIATGGQLASKSLSFVGPLAE

RSLDAYHVDKVFLSCKGVHLTKGISESNELQALVKQKMIHIADEVILLADSSKFNIQA

FTRVAEMSSVGKVITDQGVDEEHVSALIEQNITCIRV"

CDS complement(3222930..3224060)

/locus_tag="EFAGFIKM_02713"

/EC_number="1.-.-.-"

/inference="ab initio prediction:Prodigal:002006"

/inference="similar to AA sequence:UniProtKB:P0DN73"

/codon_start=1

/transl_table=11

/product="putative oxidoreductase"

/translation="MNPKFNQMFEQVSLPTGITLKNRIVLAPMTHMSSNADGTISDAE

LAYYARRTGGAGMSITAVGHVTETGIGFPAQFGVYDDRFIPGLKKLADTIKQQGSVAV

LQIFHAGRLTPEQAVPSGQVVAPSAVASERPGSPEPRELTDAEITSIIKDFGEATRRA

IEAGFDGVEIHGANGYLIQQFFSPHSNRREDRWGGSVEKRLTFPLAVVDEIQKVVAEH

TKLPFIIGYRFSPEEPETPGLTMEDTYLLVDALKDKNLDYLHVSVNEFWSKPRRGEAD

TRSRMEFILDRVNGKLPVIGVGSIHTADQAAEALQTGVPLLAIGRELIIEPAWVEKIE

SGREEDIETILTKSDQERLVIPDGLWNAIIHTPGWFPMAEDK"

CDS complement(3224248..3224925)

/locus_tag="EFAGFIKM_02714"

/inference="ab initio prediction:Prodigal:002006"

/codon_start=1

/transl_table=11

/product="hypothetical protein"

/translation="MCNRFSLAADLDEVRDHFKIQRVMYYYKNRYNISPTQHTPIILH

QDGERVLDEFRWGFIPFWGRDAVNANLMTVHENPSYYKLVETKRCVIPCNGLYYWRQE

GKKSYAVRVVMPDRGLFGIAGLYEVWRDTRKEPLRTCTMLMTGANMVTREFGSKMPAI

LSEEEINTWLDPANTRVTQLLPLLKSYNSTEMNLYPVTPMVANDEHDCYECVEEMDQK

LAYVRSF"

CDS 3225153..3225674

/locus_tag="EFAGFIKM_02715"

/inference="ab initio prediction:Prodigal:002006"

/codon_start=1

/transl_table=11

/product="hypothetical protein"

/translation="MKRKFLMSIMLFSAIAMFGSTIYAATFLTYGYPSTTIPIRTYNY

ASAWQTPMDASLTNWNNAGAKVKFTKTSNSPNTITAGNFNNTAYGVNYASVSGSQVVS

FRIELNASTITPDATNLSNFIQSVFVHELGHSIWLGDNPTTSSPSIMSYTRNRNSMTQ
PQTFDINNVRSKY"

CDS 3225718..3226389

/locus_tag="EFAGFIKM_02716"

/inference="ab initio prediction:Prodigal:002006"

/codon_start=1

/transl_table=11

/product="hypothetical protein"

/translation="MNKNRVLTLLLSSIFILSAVGCSSVTPTASPSDAPMTIIASEDY
PSYGSIGDLSERANTIVKGSVVETRVEALNDIVQVTDAANENELNPASDPGGEPASFD
KIYTIHTIQVAESYKGGYTAGEKLEVKQLGGQLGNTEIINDDNLKLIPTKDYVLFLET
YEDTPASLLNSVQSLYVIKPAAKSNQPGQQQSESEVIVSANPENDLTLQLEDLQEIQN
EQKSE"

CDS complement(3226521..3228431)

/gene="rhaS_26"

/locus_tag="EFAGFIKM_02717"

/inference="ab initio prediction:Prodigal:002006"

/inference="protein motif:HAMAP:MF_01534"

/codon_start=1

/transl_table=11

/product="HTH-type transcriptional activator RhaS"

/translation="MGIQNDIKLWDQVQVRVLDVRLISLATHEFIRNYVLPTSAFVYV
QGRGQVWLDEEVWTTNQFLLLHGGKGRRLTLETTGVMEVHLILYKSTLPSNVLVEYRM
MLNQRNPFEESWATVPDHTLELRELVRNIHECWSGQERVGKIQVKVYFFQLVQLVLLQ
RSRMFNEVQQPSLTDQVLRYIKAHYRESISLDLLAQSLSYSPQYLSRKFKEQTGCTPT
EYVIRLRMGEARSLLASTEASLQEIAAYVGYMDPFYFNRIFKKEIGITPGQYRLKQQE
NSKSVSKSTLNATNESIVTGGDERYPLIDDDNHYQYKGDEEINMFNHFKSAIMSLALV
LTLSACGTAQQGAETSEVAAEPEQPAVVETQMVNTTFGEVEIPAHPERVAAIDYLGTV
LALGVKPIGGGQFLMNSPYLEGHMDGLETIGDSVEQLMELEPDLIITLNPDKAAYEKY
SKIAPTVSIASITFPTLKDEVNYFGKVLGKEAEAEKWLADFDEEIAKIKQEVQSVVPA
DVTFSVMQEYDRQVFIFGNQSGRGGRNIYELLGLQAPKNIPSELMQGAYHEFSIELLS
KYAGDYIVLTSKSQLKDLQADPIWGSLPAIKNGKVYIWTEEQSWFRDPIALLKQTQDL

AEWIIGLNTPSK"

CDS complement(3228668..3229750)

/gene="msrAB"

/locus_tag="EFAGFIKM_02718"

/inference="ab initio prediction:Prodigal:002006"

/inference="similar to AA sequence:UniProtKB:P45213"

/codon_start=1

/transl_table=11

/product="Peptide methionine sulfoxide reductase

MsrA/MsrB"

/db_xref="COG:COG0225"

/translation="MKRTLLGLCLLVIALVVSACGNRVESHSSSISTQPVKASSVSEE

NLSNLYLAGGCFWGVEAYMARIPGVQDVTSGYANGEGKNPTYEDVIRGDQGFAETVHV

KYDPQQVSLQKLLESYFRVIDPTSLNKQGNDRGVQYRTGIYYTLPEDAKIIEQAVAVE

QEKYDQPIVTEVMPLQNYYLAEEYHQDYLEKNPNGYCHIDMTVLDDLEIGIDPAQYPR

PTDEQLKERLTNEQYAVTVNNDTEHAFSNEYWDNEEPGLYVDIATGEPLFTSRDKYDS

GCGWPSFTKPIVPEVVTYTTDTSFGMDRTEVRSRAGDIHLGHVFDDGPEDRGGKRYCI

NSASIRFIPLDKMEEERYGYLLSFIE"

CDS complement(3229767..3230348)

/gene="resA_3"

/locus_tag="EFAGFIKM_02719"

/inference="ab initio prediction:Prodigal:002006"

/inference="protein motif:HAMAP:MF_01319"

/codon_start=1

/transl_table=11

/product="Thiol-disulfide oxidoreductase ResA"

/translation="MKMARKWMVSVIVFAGTLLILSACGSQQTESKQMGSTSTPASTS

DMSSSTMMNKGETAPEFSLRDLKGNTVGLSDVQGKKVYVKYWASWCSICLAGLEDLNN

LAGQNNDFQVITIVTPDYKGEKSSQAFTEWFDQQPYDNITVLLDEKGVWAKKFGVRAY

PSSFYIGSDGILTKSQLGHASNEQIMESLQEIL"

CDS complement(3230367..3231083)

/gene="dsbD"

/locus_tag="EFAGFIKM_02720"

/EC_number="1.8.1.8"

/inference="ab initio prediction:Prodigal:002006"

/inference="similar to AA sequence:UniProtKB:Q9JYM0"

/codon_start=1

/transl_table=11

/product="Thiol:disulfide interchange protein DsbD"

/db_xref="COG:COG4232"

/translation="MTSDLVFLFGVFGAGLLSFFAPCILPLIPVYVSYLSGSMVNGTN

QQQPDSNSVRLRSTLVLRTFLFVLGLSLVFVLLGFGSGIVGNLISSPEFIAICGAIVV

LFGIYQTGLIRLSWLERERKLSNDQAKRGGYVGAFLLGLTFSFGWTPCIGPVLAAILG

IAAGEGSPLYGGFLMFLYTLGLAIPFLILSVFSEYVMKRIRRLYRYMGVIKITSGCIL

IVMGLLLMTDRLNSLVTWFQ"

CDS complement(3231127..3232167)

/gene="sasA_11"

/locus_tag="EFAGFIKM_02721"

/EC_number="2.7.-.-"

/inference="ab initio prediction:Prodigal:002006"

/inference="protein motif:HAMAP:MF_01837"

/codon_start=1

/transl_table=11

/product="Adaptive-response sensory-kinase SasA"

/translation="MKLRTYLVLSSLTGIGVLLICLFISYSKMLLTIEQLYWLSAITA

GVGLLSFILQYLLTKPLEKSIARITQQTIRIAEGDFHTEVPLIGTQEFKLLAQQFNEM

SSKLKESFDHLHHSETARRELIANVSHDLRTPLASIQSFVEALEDDVIKDKETFQRYL

NTIRLETKRLGGLIQDLFELSSLEAQGEVFDPQPCHVDELLLSTLESFSFHLAEKTLD

VEIDLPDKLPAAVMMPAQMKRVLSNLLQNAIQYSPIEGKIILAAEEKGPFIRILVTDE

GEGIEAEETSRIFERFYRIDKSRSKSNGGAGLGLAIAQSIVELHGGEIGVQSTKGEGS

CFWFTLPIYVSR"

CDS complement(3232164..3232868)

/gene="walR_3"

/locus_tag="EFAGFIKM_02722"

/inference="ab initio prediction:Prodigal:002006"

/inference="similar to AA sequence:UniProtKB:P37478"

/codon_start=1

/transl_table=11

/product="Transcriptional regulatory protein WalR"

/db_xref="COG:COG0745"

/translation="MNECVLVADDDTNITDVCRRYLEREGYLVVTAKDGLEAIELWHS

QTPSLIVLDLMMPHKNGWEVCNEIRQTEDVPIVMLTARGEEQDRLMGLTMGADDYLTK

PFSPRELVLRVKAILRRMRTAQASPVTTSEYTINYEGLTIDFTKREVHISGQAIELTV

TEFEMLYLLASHPGQVFSRNQMLSKIWDFSYEGDTTTVTVHVRRLREKIEQNPSDPKY

IKTVWGIGYKFAGGSS"

CDS complement(3232946..3233425)

/locus_tag="EFAGFIKM_02723"

/inference="ab initio prediction:Prodigal:002006"

/codon_start=1

/transl_table=11

/product="hypothetical protein"

/translation="MMKTNKSKKAMVAAVLMMSMAFSGLASAADMKTIKKDGMELVQL

RQAAKMYDYSIMWDSKDRSVTLMYMGKMDDKMMKDDNMMEDDMKMKNDKMMKDGKMME

ETMMPAGKTIKLWIGSKKIMVDGMQVNLKSMPVIHEGNSYAAESVIKQYMMPVKGMK"

CDS complement(3233592..3236141)

/gene="lacZ_3"

/locus_tag="EFAGFIKM_02724"

/EC_number="3.2.1.23"

/inference="ab initio prediction:Prodigal:002006"

/inference="protein motif:HAMAP:MF_01687"

/codon_start=1

/transl_table=11

/product="Beta-galactosidase"

/translation="MSTIQSHVFQNWTFKACEDQEWMSAQVPGCVHTDLLKLGKIPDP

FYGTNEKEVQWIDKIDWEYQTEFDVADALFSQEHLELVFDGLDTYADVYVNEVHVLSA

DNMFRVWKADVKSVLKGNGNILRIRFRSPIQEDLPKLEKLGYALPASNDQSDVGGLGD

KRVSIFARKAPYHYGWDWGPRFVTSGIWREARLEGWTQVRINDVYIQQNEVSATSASL

TAVVEVETSQAVDTIIRIGADGQRWERSVSLQPGTQTVEIPISIDEPKLWWSRGLGDP

HMYTFLTEVLQGERAVAESTVKTGLRSIRLVRDKDEAGASFYFELNGVAVFAKGANHI

PNDSFITEITHERYRHEIVSAAQSNMNMLRVWGGGIYEQDVFYELCDEYGILVWQDFM

FACSMYPGDEAFLNSVRHEAIDNVKRLRNHPSIALWCGNNEIDSAWAHYVENGGWGWK

KEFTAEQRESIWADYEAIFHDLLPEVVEAYAPGVDYWPSSPLVSLTGDETQHAHPSTA

EGDIHYWGVWHNVEPFENYNVHVGRFMSEYGFQSFPEYKSVRTYAEEEDLALESEVML

AHQKNGAGNRLIKQYMDMYMHESKDFPSFLYMSQVLQAEAMKTAIEAHRRRKPFCMGT

LYWQMNDCWPVASWAGMDYLGRWKALQYYAKRSFSDVLVSVDGTKEDTTDIYIISDQL

QPVKGQLQIRLLGFDGTVHRDETHDVTLEPNTGDRVLSLRNAEWLEGHDTANTLLRLD

LKQEGAANVVQEHYFVPSKDLALQPANINVSGGADENGVHLVLESDVLAKQVWLSSDV

EGVFSDNFFDLIPGIPVKVQFTSREGLQSNDVISNPGPIEVRSMIDFIRLT"

CDS complement(3236389..3237429)

/gene="exuR_2"

/locus_tag="EFAGFIKM_02725"

/inference="ab initio prediction:Prodigal:002006"

/inference="similar to AA sequence:UniProtKB:Q9JMQ1"

/codon_start=1

/transl_table=11

/product="putative HTH-type transcriptional repressor

ExuR"

/db_xref="COG:COG1609"

/translation="MSRMTKRVTMQQIADAAGVSKFAVSRALTGKPGVSEHTREMIVR

TAGQLGYFRTEPKRYPGETQISAEMKPEAERQGTILILFPNIRSQNRSSLYWGPVFDG

ISERLNEKGMDILTLTEPSSDRMFSVLNPEAISGVITVGTISTSVLLEIYRLRIPLVM

VDHEDPAIYADSVFTDNMKCMKELVLMLVGKGYRRFQFAGQLPDAASFRERWLGYRMV

LEEKQLEGEQQDGLLGPEYDQIRRSIAEMELEDIPEVIVCANDHTAVIVIQALQSRGI

QVPERCAVTGFDNTQTDEPILASVHINKENLGTRAVDQLLWRIKHLDEPYERKLIYSE

LIIRDEYNASIQ"

CDS complement(3237610..3238557)

/locus_tag="EFAGFIKM_02726"

/inference="ab initio prediction:Prodigal:002006"

/codon_start=1

/transl_table=11

/product="hypothetical protein"

/translation="MNGVQVNQGQATRRADIQMLLATVIWGSSYLFMKSGLESMQELN

LVAFRFGIAFIAAGLLFHRRLFNMDRRTLVAGAIMGTALFAAFVFITYGVQRTTTSQA

GFLISLAVIFVPILTTIQHRRMPDKRLTLSILVAVTGLGLLTLQHELSLHTGDILCIL

AALVYAIYIMIAGKFTPKHDPLTLGTVQLGVAAMWGIAATFMMETPRMPDTAESWAAI

LGLGVLCSGLGYILQTLAQRHASPTRTSLIFSLEPLFAAAFAFTFQGESLTLQGYAGA

TLMLVGVLITEIKLPQPIFWRRKRPVLQSELGDQGAPGV"

CDS 3238722..3239597

/gene="gltC_3"

/locus_tag="EFAGFIKM_02727"

/inference="ab initio prediction:Prodigal:002006"

/inference="similar to AA sequence:UniProtKB:P20668"

/codon_start=1

/transl_table=11

/product="HTH-type transcriptional regulator GltC"

/translation="MSLVKYEILNTVVEYGSLTKAAEALNITQSAVSHAIASLETECG

FSLLHRSRSGVRVTAEGERILGYTREILRWTELMNQEISLIRGAEIGTVRIGTFASVS

TQWLPGILKQFRLRHPGIEIKLWEGDYAEIEGWLAGGAIDLGFLSLGDSSPFETIPLQ

KDRMMCILPLDHPLASEESVSFDVLLEQPFILPKWGGDNEIERLIRQHAAKLNVVYEV

AEDQAIMAMVRNGLGISLLPEMVLQNHTHALALVPLSGDPYRTIGMACPSLGNLSPAT

RRFIEEVQQWLGQVM"

CDS complement(3239640..3239837)

/locus_tag="EFAGFIKM_02728"

/inference="ab initio prediction:Prodigal:002006"

/codon_start=1

/transl_table=11

/product="hypothetical protein"

/translation="MNSNQDLKQVNERLDQIEQKLDNLGGSHQNKRSPGVRFLIGFGI

TIGIISVLILTLGVIQFVSNG"

CDS complement(3239868..3240350)

/locus_tag="EFAGFIKM_02729"

/inference="ab initio prediction:Prodigal:002006"

/codon_start=1

/transl_table=11

/product="hypothetical protein"

/translation="MSNLEQAVRWRQEGKVQEAIELLQEITVQEPENANVWYQLAWAH

DSLGLEREAVPYYEKALSLGLSGEDRAGAILGLGSTYRTLGQYEQAKVWFEKGMKEFP

AYREFEVFYAMVLYNLGEHAEAMQRLLVQLADTSSDKRINDYNRAIRYYADQLDRVWE

"

CDS complement(3240777..3241634)

/gene="ctaB1"

/locus_tag="EFAGFIKM_02730"

/EC_number="2.5.1.141"

/inference="ab initio prediction:Prodigal:002006"

/inference="similar to AA sequence:UniProtKB:O31652"

/codon_start=1

/transl_table=11

/product="Protoheme IX farnesyltransferase 1"

/db_xref="COG:COG0109"

/translation="MLKDMIALTKPGLLRLNVFAVAVGFWVASKWDINWLSLLMVVIG

STLVIASACVINNYWDRELDQKMERTKKRMDYINHLKPGVVLGYGIILGVVGLAVLYL

LVNPLSGWMALLGWFAYIVIYTMWLKRSSTWSTSLGGIAGAMPPVIGYTSVTNEIDAG

AWLLFALLFLWQPPHFWSLGIRRVEEYRAAGFPLLPVVKGVKRTKFQMIPYVFLLLPA

VFLLYYYDYVGLVFLIVSLVGGLIWFVHTLSGLKTQDDEKWAKVNFLISVNYLMIVFI

VMVANTVWS"

CDS complement(3241791..3242357)

/locus_tag="EFAGFIKM_02731"

/inference="ab initio prediction:Prodigal:002006"

/codon_start=1

/transl_table=11

/product="hypothetical protein"

/translation="MKNATKQWKLGMIGAVVALLGMIVLTTYLLTPANAQGSTDAQQP

SAKEAQVANSEMFTAYVEGMHIEDGKLFMTVDKIGWYQGEEADAIFAQRNPEAGIDGA

PDGYYIVNDSKEQEQVEVSANAEVLMQLYDRDGTMQGADIQWNEPVTLSKFESLYENT

RILDLSVFPYHLTVQDGKVTKIVQQYIP"

CDS complement(3243014..3243367)

/locus_tag="EFAGFIKM_02732"

/inference="ab initio prediction:Prodigal:002006"

/codon_start=1

/transl_table=11

/product="hypothetical protein"

/translation="MNEENHVIHKDGQVSTDKVDNAIEKIAPEEREQILQNFDAFKEY

LGKRIAMGESIGLSEEQMAKIAEKVADYLAAREEPRNREEKLLQELWNVGKEDERHML

AHMLVRLAQSSTPNH"

CDS 3243614..3243829

/locus_tag="EFAGFIKM_02733"

/inference="ab initio prediction:Prodigal:002006"

/codon_start=1

/transl_table=11

/product="hypothetical protein"

/translation="MRDKTKHEKTNDELEPGVNTVIHPDKNNPGPTDMIEGVVGDIVD

NIRKDFSHDSKDEKSSSKSTDSSDKQA"

CDS complement(3243945..3244862)

/gene="trxB_2"

/locus_tag="EFAGFIKM_02734"

/EC_number="1.8.1.9"

/inference="ab initio prediction:Prodigal:002006"

/inference="similar to AA sequence:UniProtKB:Q05741"

/codon_start=1

/transl_table=11

/product="Thioredoxin reductase"

/db_xref="COG:COG0492"

/translation="MREVDCIIVGGGLAGLQAAIQLGRYSAHQVLVIDAGEGRSTLCR

TYHNILGFPDGVSGEELRERGRMQAEQTGVSFEKDRIIKAARKGEHIQLFGTSGSEYR

SKTVLLATGLTDRVPDIPGLTPTLGRTVYVCPDCDGYEIQNQRTVLLGSGEPGANMAM

ILIQRTNDLLYINHEQAPISAELHRSMKEAGVRYLEAAVQEVQQIEDGHITGVLTEDG

QIFESERGFIAFGGNRVHYELAEQLGAVIADNKHVEADPRSLQAATNVWIAGDLGLHA

EQATVAMGEGSIAAIWIHKALQQMTKDSR"

CDS complement(3245250..3246881)

/locus_tag="EFAGFIKM_02735"

/inference="ab initio prediction:Prodigal:002006"

/codon_start=1

/transl_table=11

/product="hypothetical protein"

/translation="MNNRLKDITNQRIWKMALCGLLTLGSVAGYGSTVQAATVQQQFQ

DTRTSYAKDAITHLVDKGIAAGTSETTFEPKKAVTRAEFATFAVRLLGLKPVKNNINP

YQDISMNAWYYGNVAAMTNLFILEGKGQGTFQPNASITREEAAALLVRMLKQQPVETS

LLSSTYFDAADISDWARPYVQTVYQLGLMRGSGGVFRPHDQVTREEAAVMLDAILQKK

TWSEQLQRGDELGVQLGWQYNSTTTEFKKQVEQSEVNTLVPRWFFLNSSMKVTDHADP

ALISWASATDRQLWPLLGNRSDSAITHQMLSSSANRATVISQVAAYVKTYKLDGINVD

FENVDPADREGLTAFVTSLTATLHALGAVVSVDVSPDLGTDWTDAFDYAKLGAVSDYM

VLMGYEEHWNGDPKAGSVASLPWVEKALDTMLSEVVRAKTILALPLYTRDWSSVNPAT

SSWDITLADQGMRAHATGSVRRWDASLVQYIIGYNSNGKPRYIWAEDSRSLSAKVLMS

EQRQIAGLAYWYMGGETADVWNAISNASRFESYNF"

CDS complement(3246976..3247575)

/locus_tag="EFAGFIKM_02736"

/inference="ab initio prediction:Prodigal:002006"

/codon_start=1

/transl_table=11

/product="hypothetical protein"

/translation="MTTFFDALKNRRSYYGISKESTISDAKIQEIVEEAVKYTPTSFN

SQTSRAVVLLGEQHDKLWNHTEDILREVVGNEEAFKSTAEKMTGFRSGYGTVLFFEDN

NAIAQLQQNFAAYADNFPIWANQSNGMLQLVIWTALEQEGLGASLQHYNPLIDEKVKQ

EWNIPENWRLIAQMPFGKPTAAPGEKEFQPIEERVKVHK"

CDS complement(3247724..3247978)

/locus_tag="EFAGFIKM_02737"

/inference="ab initio prediction:Prodigal:002006"

/codon_start=1

/transl_table=11

/product="hypothetical protein"

/translation="MKQRTPDQNNNDTQQEQSHDNPEQYKTEHESLLDRHEEEQTVDP

IPMEDLNMEAQEEKNKDQTKSNSSTEEKYRADYRKKTGEG"

CDS complement(3248115..3248972)

/gene="rhaR_24"

/locus_tag="EFAGFIKM_02738"

/inference="ab initio prediction:Prodigal:002006"

/inference="protein motif:HAMAP:MF_01533"

/codon_start=1

/transl_table=11

/product="HTH-type transcriptional activator RhaR"

/translation="MPSMMQFSASLEYSYRSTSIFNPGKSDGFHSHPHYEIYYFHDGE

STYIIGDRVYNLEPGDLVLMHGLTLHRPHPKPGSAYERSTLHFDPSAIRSSLHPDRMA

EVLRPFEELRNCRVNLKGDNRSEFEALLHDLHRLSQSQSNFRQERMNVRLCDLLYFVA

EICQGDVQEQLPSSERERHVQHIIRYVDTHYMKDIGLDDLALELHLSKPYLAGMFKEM

TGLTIFKYLYDRRINQAKLLFQFQPEITVTEASRLSGFKRLSHFSRMFKQSVGCSPDL

YRTQLHRQS"

CDS complement(3249044..3249592)

/gene="gpx1_2"

/locus_tag="EFAGFIKM_02739"

/EC_number="1.11.1.22"

/inference="ab initio prediction:Prodigal:002006"

/inference="similar to AA sequence:UniProtKB:P74250"

/codon_start=1

/transl_table=11

/product="Hydroperoxy fatty acid reductase gpx1"

/translation="MPTIYDFTVTRTSGERFPLYQYEGKPVLIVNTASKCKYTHQFDD

MQKLYDQYKDQGLQIIGFPCNQFAEQEPGSSSEAESFCQINYGVKFPMFSKLDVNGEA

AHPLYDFLKRSGPFAGFDETDIQAKLLKLMVAEKAPEWLHGDAIKWNFTKFLIDAEGR

VVRRFEPIDSIDEIQESIKQLL"

CDS complement(3249780..3250676)

/gene="araQ_18"

/locus_tag="EFAGFIKM_02740"

/inference="ab initio prediction:Prodigal:002006"

/inference="similar to AA sequence:UniProtKB:P94530"

/codon_start=1

/transl_table=11

/product="L-arabinose transport system permease protein

AraQ"

/db_xref="COG:COG0395"

/translation="MEKQIAANAKPTAPHSPKVKVRMESLTQIRKIILTLLMSGFALL

MIMPFIWMISTSFKSPADVFTYPIQWIPSSLNWEHHIKVWSGADTFATYYLNSLKISL

ISTIGAVFLSAFAAYGFARIQFKGRETLFLIYLSMMMVPPQVLFVPKFLMFEWVGIYN

THWALILPGMFTIFGVFMLRQFFLSVPSEISEAAFIDGAGHLRIFFRLILPLAKPALA

TLAIIDFSWHWNDYENALVFLIDKDLYTVPLGLQNFILENNVDYNGMMAAATAGIIPM

IIVFLVGQHYIIQGVAGSAVKG"

CDS complement(3250694..3251575)

/gene="ngcF_2"

/locus_tag="EFAGFIKM_02741"

/inference="ab initio prediction:Prodigal:002006"

/inference="similar to AA sequence:UniProtKB:O50500"

/codon_start=1

/transl_table=11

/product="Diacetylchitobiose uptake system permease

protein NgcF"

/db_xref="COG:COG1175"

/translation="MHKSWMKRQSRLGYLFIGPNMIGVLLFFIIPAVYSFYLMFTDYK

FMSPETNFVGLDNIRRMLNDDLFGVALRNTFVFLLAVPVSMGLAFIVAVALNKSVYWQ

KTLRALYFMPYITSGVAIAFVWMLLFQPTSGPINGFLRGIGITNPPGWLSTTEWSMYA

IDIIWIWFMLGYNMIIYLAALQEIPEELVEAARIDGARPWQTIRQVIWPLVSPTTFLL

LITGLIMTIKNFGIIQAITQGGPGNSTTVLSLFIYQNAFRYYEMGYAAAISWALFAII

MIFTVLQWLGQKRWVHY"

CDS complement(3251784..3253097)

/locus_tag="EFAGFIKM_02742"

/inference="ab initio prediction:Prodigal:002006"

/codon_start=1

/transl_table=11

/product="hypothetical protein"

/translation="MRIKKGLTGLIVGALMLGLLAGCVGKNETNGGDNGGGAAGGKVK

LTMWGAVPPENGPQEVVDTWNAEHPDIQVEYVRFVNDDDGNLKLDTALSTGQNVDLYV

NYTLTNLDKRIKGGTALDLGEFTDYNIDEKMGEDAASWKVDGKYYGMPTKKNAAFFAL

NMKALDQAGLSVPAAWTWDEAREYALKLKTAGFKYGLVQHTASYVDPLDSVLVKNGYV

KADGTSNLDDPLVTKWLETLNGMMKEDATTPPLGEQLTSKMPVENMFLGGESAMINIG

EWLIRTSNNMTEFPRDWKIAFAPVPRLAAQEDMVKSGGLGDFIAINSKSKNKEAAWEF

LKWYADGGMMPMAAGGRLPSSNAVDQQTAIDHLLGDYADSYDKESLEFVLYGDETPTF

VRSIAQEVVDLRAQEYEKFFLGNQTAEVTVQNMVKRHNDWLKQNQ"

CDS complement(3253262..3254113)

/locus_tag="EFAGFIKM_02743"

/inference="ab initio prediction:Prodigal:002006"

/inference="similar to AA sequence:UniProtKB:Q5HJF7"

/codon_start=1

/transl_table=11

/product="putative response regulatory protein"

/db_xref="COG:COG4753"

/translation="MWKVLLVEDEVFVRESVREIISWEELGFTVIGESGNGTEALAMI

IQDTPDLVLTDIVMPGMDGLELLKQTRQAGLKTKFVMLTCMGEFEYVRRAMEYGASNY

ILKLSMSVNSLRDTLRKVSAELGASTEARTEKDHHQVPPSTPVLTSTPVSPDSLTVSS

APPSDTDLPFTPVREPIVKHPEISKIIQYIGQHYDQDITVKSMSRYVMMGENYVSALF

KKKTGHTLIHYLHGVRMEKAAEYLRETDLPVQEIGYRVGFGSDNYFIKIFKRWTGCTP

SQYRHRS"

CDS complement(3254298..3256118)

/locus_tag="EFAGFIKM_02744"

/inference="ab initio prediction:Prodigal:002006"

/codon_start=1

/transl_table=11

/product="hypothetical protein"

/translation="MTKPDWKTFLPHKLRYRLFGAFVVLILLPFSVLNVYNYGQIESL

VEQKISEQSHEQLVQMYGSLEDQMSIAFKTLIFLEQDSAVRNVLTSPDNRSPLENKSL

VEEKFKMINNSFFLYNPSVYFTLLDFHDNVYTSYLPKKALAYGPYREQFRKHLGEMTF

IKGNTDSNSFPSEELLYRWDARDTNHVLRELSSSPYLLSLYAYMKDSGGKRYGMARIS

IDYSYWFQSMLKDSQSNQEYFLITGNGETIARSSEKAVLSPDVTREIALHPAEAYLTD

NDSDTLINYVYIESLDWYMVNRIPLAVLFTEISGLKQRYFLTFFGFTAAFVLMAFMIS

ATFTRPLSHLQNQMKEVVRKNLKVRIPEGRSRGEVLELTRTFNTMLDDANQMIARLKA

EERQKEAVHFHMLLAQMNPHFLLNTLNTMKWSAIRSGNEEISEMCVSLGKLLEVSLNS

QVELVYLKDEIELVQAYLHIQRIRYRDSFEVTCEFDDKLEYALVPKLSLQPLVENAIH

HGVGPLEQLGQIRIGIYRQDTGKLMLEVADNGIGMEESRRQQMTRTRPGIGLSNLKER

LRLLFKGQSELEVIDTKPGTLVRFSIPFLLSTPYLQKRSD"

CDS complement(3256317..3258650)

/locus_tag="EFAGFIKM_02745"

/inference="ab initio prediction:Prodigal:002006"

/codon_start=1

/transl_table=11

/product="hypothetical protein"

/translation="MRKMIFLKALKFVLMITLLVPLYWAAPSVHAAEGENTLILSHDF

EDGTVQSWSGRGEVETLHASAVAARTGDYGLEVSGRAMTWHGPALDVTELLEVGQTYV

FTGWVKLPAGASSTNLNMSLQRTLPDTTHYENLTFGTASAGGWTKLTAQFKMLEPANQ

LSVYFEVPDRATASFYVDDFRLEKLPDLGPIVIEEDIPSLQDAFAGKFRIGTAFTNNE

LLTEPDKQLLTKHFDSLTPGNVLKWDSTEPQEGMYNFSGSDAAVAFAAANGQEIRGHT

LVWHTQTPDWVFHDANGNLVSKTVLYERMQQHIETVMERYQGQIYAWDVVNEVIDTSE

PDGLRRSLWYQIAGEEYIEKAFEFAHAADPDAKLFINDYNTHEPAKSQALYNLITRLQ

AKGVPIDGVGHQTHISLFYPSLSEIESSIVKFKALGLETHITELDVSVYSNDSQSYDI

FPEELKTQQAALYKSLFQIFLRHTDTVKNVTLWGKDDANTWLRTFPVARNNWPLLFDE

RLQSKPAYWSVLETVQVEAVPAAPQNLTATAVSDNSINLSWSASASATSYTVNRSLTA

AGPYTSVAAVSGTSYKDMAVSAGTTYYYVVNAVNSVGKSVNSVQASAKAQGGGPPEPA

GHLTLAYRAGDTNLLDNQMKPQFVIHNNGTSTVSLNELTIRYWYTVDGEKQQSFFCDY

AQLGGSQISGSIVKLPAPVPGADHYLELKFGTGAGVIPVGGTSGEIHTRLHKADWSNY

SEGDDYSFNPLQTSFADWTKVTLYRNSELVWGVEPIS"

CDS complement(3259013..3259780)

/gene="rhaR_25"

/locus_tag="EFAGFIKM_02746"

/inference="ab initio prediction:Prodigal:002006"

/inference="protein motif:HAMAP:MF_01533"

/codon_start=1

/transl_table=11

/product="HTH-type transcriptional activator RhaR"

/translation="MELLNHIYWKQKGEFALTEDIYTTWVAFAVEEGIFHYEIGGQIG

EASFGDLVLCPPAVPFRRTVITPLTFHYLQFSSSLEEEEEFLPPAGKSLINDTKRLAS

TYAYLRQDSEENKGEVARWKTHFMMDLWQLYQVERRQNRLKERRFTEDARMAEAAALL

EERAEEEISLQELAQRLALSPVQLTRRFREAFQETPSGYLKRVRLKKAQNLLADSSLT

LNQIAERCGYENGFYLSRVFSKTLGISPSEYRRRHRV"

CDS 3259927..3262176

/locus_tag="EFAGFIKM_02747"

/inference="ab initio prediction:Prodigal:002006"

/codon_start=1

/transl_table=11

/product="hypothetical protein"

/translation="MKQEHVQYDITVIGGGLAGVCAAIAAARMGQQVALVQNRPVLGG

NSSSEVRVWVCGATAHGINRYARETGIMGELFVENQYRNPEGNPYLWDLIILEAVRAE

SNISLYLNTDVHEVEATGDGDERMITSVTGWMMGSERKIRFESQIYLDCTGDGLVGFL

AGAKFALGREARSEYGEEWAPEVADQITLGSTLLFYTKDAGAPVRYIPPSFAKDITQT

SIPIRRVIRSGDSGCHYWWIEWGGEHDTVHDNELIRDELWSVIYGIWDYIKNSGKFDA

DHMTLEWIGSLPGKREYRRFTGDYVLTQNDIISQREFPDAVAFGGWSIDLHPPQGMYA

EASGSKHMHADGVYHVPFRSLYSANVRNMLMAGRDISASHVAFGTTRVMATCAVIGEA

AGTGAALCAAMGVSPRELHARHLAVLQQTLLRQDASIIGVRSHDELDLARRAKATASS

TLTGIVLEQPGETYPLGTDVALLLPVYPVLSGLELLLDASSDTALTVELWDTGRKENY

VPHLLQVTATVNVTTGTAQWVKLPLEWRPEEPQNAFIIIRSNKAVSLYHSTEAHSGVL

IFFKTEENHVSKNLEDHATDQPVVLWSMQGLARQPFCCRTLSETSAYSSDNTINGYHR

PYGGPQQWMSQPMQSGKPEWVQLTWEEPQTLAELHLTFNDDVNEDLVNLHHHHTTFRV

MPELVRDYHVEVLTPAGEWTQVASVQENHKRKVVHSLDVPVSAQALRIIIDATNGSNY

AELIEIRAY"

CDS complement(3262237..3263307)

/gene="yesR_1"

/locus_tag="EFAGFIKM_02748"

/EC_number="3.2.1.172"

/inference="ab initio prediction:Prodigal:002006"

/inference="similar to AA sequence:UniProtKB:O31521"

/codon_start=1

/transl_table=11

/product="Unsaturated rhamnogalacturonyl hydrolase YesR"

/db_xref="COG:COG4225"

/translation="MLNQMVLQERISKVSQAMESMKNTSINEQFPIGLIDIHLWEWPQ

GVGLYGLLQLHEATKDPKVLEFLESWYNARLMEGLPEKNVNTCAPLLTLISLCELTGN

KEYEHVCEEWSSWIMDGLLRTGDGAFQHMITGDANDGQILIDTLFMTVLFLAKAGVYF

KKPDMVEEAKRQFLVHIKYLYNKKTGLFYHGWDFNENHNYGAVHWGRGNAWYTAGVMD

FLNIIPIEDGLKAYLLDTATAQVRALSKLQGEDGMWHTVLDDPTSYKETSATAAIGYG

ILKGIRYGYLDESYHQNGLRALEAVLKQIDDHGVVQQVSYGTPVGQDAQFYKDIPISP

MGYGQALTLFILIEGLRVPTAD"

CDS complement(3263343..3265898)

/locus_tag="EFAGFIKM_02749"

/inference="ab initio prediction:Prodigal:002006"

/codon_start=1

/transl_table=11

/product="hypothetical protein"

/translation="MSDTTRSGMDTLNYKPMKLGHNQVINHWMISGIYTKPVAFVPTT

MEGDINDWLIDGFAIHENPCRKEFVEHRRTQPVNRFFDQWSEFPSPGDDFVGEENGMP

WALYSPWNNPRVEQSGFWFVPTHLRSYAATRLISPTAHTASLRIKTYGSLTLWVNGEM

VTDFAPLMRNKEQEIVIKAELVAGINEIYACWEDLAERDTMYAFAVEYLGDEELVISL

PIAPHLAESVKSAEEALEQAYFPSDLFRGEQIKLKLPISLPDIVREVDIQYGNFFDGT

ENKTIHIAEKETDLVLAHTNQLGHHYVYFTLAISVSNVVLTKKFGCQSYDTSYDEAAQ

QAADIEARKSLALRCIAEKGSPNIHKAIAMLKTGGNLQEAENILLEGVEGIEQRKDCS

DFYLVGLFRLWRDERNSGLFSESFWDRVKASILGYRYWIDEPGDDVMWFFSENHALLF

HTNELLAGQLFGDEMFSNSGESGEVHRQKAEQRLALWFERFLDEGLAEWNSSAYIPID

AVGLLHIYEFAHNEQLRQQAKKAMDLLFYYITVQMHQGVLSTTFGRSYEKELLGGYAA

GTTSMCWIAYGVGNVNNYSISNVALSLSDYSPPAAYQEHLLLGDNQQLVFANQQGKGG

YAKLYHCRTREYSLSSIIRFRPGKPGYQEHVQHLSLSPEALIWVNHPAEIYKHGDGRP

CFWAGNGILPDVVQHEGIALMMFDIPADNNSDWTHAYFPTHFFTEWVRHENWHFARLD

KGYAAIYAANGTSMESSGVTRERELISPGLRNAWIIRAGSEQQFGSFDAFINQVCSAN

PQFDNQALTLTLTDPVYGPIQWGMNKPFLIKEEEIIHEGYGVKGQSKLLDMER"

CDS complement(3265904..3266728)

/gene="araQ_19"

/locus_tag="EFAGFIKM_02750"

/inference="ab initio prediction:Prodigal:002006"

/inference="similar to AA sequence:UniProtKB:P94530"

/codon_start=1

/transl_table=11

/product="L-arabinose transport system permease protein

AraQ"

/db_xref="COG:COG0395"

/translation="MVMKPMVKIFIYSLLSVAAVVWLLPVLWVVISALKTNSDLYSFP

PKLWPQPITFEHFKEAFSKGDFGLYFMNSTIVTVSSTLLLLLINSMAGFALAKYRFRG

SAIILIAFVSTLMIPIEVIMIPIFKVLSALGLYNSLLAIIIPPAATPTGVFLMRQYLL

SVPDELLEAARMDGAGEWKIYWSIILPIAKPILAVLAIFSFMWRWDDFVWPLIAISDP

SKYTIQLALSNFIGEYNVDWGSLLAMSVITMLPVLIVFMVFQRYFVSGMITSGMKG"

CDS complement(3266728..3267633)

/gene="melD_8"

/locus_tag="EFAGFIKM_02751"

/inference="ab initio prediction:Prodigal:002006"

/inference="similar to AA sequence:UniProtKB:O34706"

/codon_start=1

/transl_table=11

/product="Melibiose/raffinose/stachyose import permease

protein MelD"

/db_xref="COG:COG1175"

/translation="MSEGQRLNRNTLAKQNRKLIIAPYLFILPNLLIFGTFIVFPSLL

GLYYSFHVYDGLNPMKFNGLDNYIKIIGDREFWSTIGRTGIYAAFVVPLIYAAALGIA

LLLAREIRMRGFFRAVFYWPTMISYIIVGLTWKWIFGDSFGILNHLLTVVGVEPVGFL

TSSFWANTAVIIATVWSRAGFFMVIFIAGLQAIPTDYYEAARLDGATGMKVFRYITLP

LLKPTSLLVVMLTLIDAFKAFPLMFALTGGGPGKDTTYIVQYIYEIGFNRQELGLASA

MSVILFILIGGFSALQFRLSKGGAT"

CDS complement(3267726..3268997)

/gene="araN_2"

/locus_tag="EFAGFIKM_02752"

/inference="ab initio prediction:Prodigal:002006"

/inference="similar to AA sequence:UniProtKB:P94528"

/codon_start=1

/transl_table=11

/product="putative arabinose-binding protein"

/db_xref="COG:COG1653"

/translation="MTRRTTFKLITSMVLVMLFIVLTACGNSGTNANPKQTTLDFLWF

SDGKEGEVIKEIIKDYEQTNTKVKINLIEVGFKDIQTKLKTMLSGGKPPALSRVTDTG

SFANQAVDLTPYVDNADQFEDQFIDSLKPYYVINDKLVAAPMDVTANGLIYNKTLFDK

AGVKVPTSPDQVWTWDEYTAALKEVMDKGGARYGMVWDVTPHRWSTLLYQNGGSILTE

DGTAAAINNEAGIRSMEQFKQLHQEGIMPESVWLGGENPNNLFRSGTVATHWAGNWMI

SNYKDITDFEWGVTYMPKGTQRSSVPGGKFLMAFKGSGYEQEAAEFIEYLTSKEVNSK

YNQESLFMSPRKDSSVLNYEFGKEMFEIFADELKNSSPLAANDWSRQTIISKISTDLK

NNIVEVLSDKATPQEALDRTAKLINEAIGSQ"

CDS complement(3269020..3269901)

/gene="melR_2"

/locus_tag="EFAGFIKM_02753"

/inference="ab initio prediction:Prodigal:002006"

/inference="similar to AA sequence:UniProtKB:P0ACH8"

/codon_start=1

/transl_table=11

/product="Melibiose operon regulatory protein"

/translation="MLTIHTPTNIALQENEVYVRSEADNFQDEWPVHTHNGYEIHYFI

QGDATFLIGDRIYKPLPGDMFIFRGGVPHRINPSREMVYKRSFVNFTELLLLDMLAVS

QLENLMSIFRHPNGLLVHWPPEERKYITAIFKGIKEEMGAGNTGYKTMVKLSLTQLLL

RIYRKTTSEQSANPILCSSQKQTSVSRVLHYLNQNYTENVSLDELSKTLHLNKYYICH

SFKETTGYTISNYVIRKRVAEAKKLLLSTDAPILSISETLGFNTPVYFSRAFKQYVGV

SPQLFRKSELLNEAKIH"

CDS complement(3270139..3270933)

/locus_tag="EFAGFIKM_02754"

/inference="ab initio prediction:Prodigal:002006"

/codon_start=1

/transl_table=11

/product="hypothetical protein"

/translation="MAQQEEVLSGGNVNQVVRIGETVRRHVKPNLYVVELLLHLEKVG

YDHAPRFLGIDEQGRETLSYLEGIVPGNDYPDLEEYMWSDESLIEVAKLLRSYHDATV

GFTTTSVSTNRYPGITEDEVVCHNDFAPYNVVYKDGRPQGIIDFDMAGPGPRMWDIAY

VLYTSVPLANFSPEMDGKGVMPYVSQAHGTVRKERIALFMATYGLSVPADLKDWVISR

IRFMCKTLTDRAADGDVAFMKMVEEGHLAHYEKEVIFLENHWDEWV"

CDS complement(3270951..3271226)

/locus_tag="EFAGFIKM_02755"

/inference="ab initio prediction:Prodigal:002006"

/codon_start=1

/transl_table=11

/product="hypothetical protein"

/translation="MNNINKILGVVLMLGSVFISTMERISIRVSDAIVEAGYASGGTN

VPQFAQMNEHPTGLLVYFMFFVGFILLVAGFPGSYKKNRNHGYNKEV"

CDS complement(3271329..3271868)

/locus_tag="EFAGFIKM_02756"

/inference="ab initio prediction:Prodigal:002006"

/codon_start=1

/transl_table=11

/product="hypothetical protein"

/translation="MLKKRDLQECHSLYSLMMDPAVFPYVRYACQSYEGYLFLTKQLM
AEEEQKTVISRTILNETGQPIGTIDLYHIEHQTGFLATWIGSPFFGNGYSQKAKSAFF
VELFLEHGIETVFMKIRKQNIRSRKAVQKLPYVKLANDMYPDVYQRINLNEQMYDLYY
VERSAFFQNSMDLHQVVAT"

CDS complement(3272073..3273260)

/gene="celA"

/locus_tag="EFAGFIKM_02757"

/EC_number="3.2.1.4"

/inference="ab initio prediction:Prodigal:002006"

/inference="similar to AA sequence:UniProtKB:O08342"

/codon_start=1

/transl_table=11

/product="Endoglucanase A"

/translation="MFKKWKRFGVSSLALVLVAAVAFTGWSPKASAADASQVVAEMGA
GWNLGNQLEAAVNGTPNETAWGNPTVTPELIKKVKAAGFKSIRIPVSYLNNIGSAPNY
TINAAWLNRIQQVVDYAYNEGLYVIINIHGDGFNSVQGGWLLVNGGNQTAIKEKYKKV
WQQIATKFSNYNDRLIFESMNEVFDGNYGNPNSAYYANLNAYNQIFVDTVRQTGGNNN
ARWLLIPGWNTNIDYTVGNYGFVLPTDNYRSSAIPSSQKRIMISAHYYSPWDFAGEEN
GNITQWGATSTNPAKKSTWGQEDYLESQFKSMYDKFVTQGYPVVIGEFGSIDKTSYDS
SNNVYRAAYAKAVTAKAKKYKMVPVYWDNGHNGQHGFALFNRSNKTVTQQNIINAIMQ
GMQ"

CDS complement(3273468..3273884)

/locus_tag="EFAGFIKM_02758"

/inference="ab initio prediction:Prodigal:002006"

/codon_start=1

/transl_table=11

/product="hypothetical protein"

/translation="MLSEFIELEEESDESYRCYTLPNTVQIFKHCIQDEDLNDVRISV
STNTPLVSIVDKIEDYIKWFSTCETVFREYYENELREKVHQNWFNEIEVYRADITFNS
IADYGATISCGDHILRDHIMIIDFDREQIQAIHLNG"

CDS complement(3273911..3274939)

/gene="rutD_5"

/locus_tag="EFAGFIKM_02759"

/EC_number="3.5.1.-"

/inference="ab initio prediction:Prodigal:002006"

/inference="protein motif:HAMAP:MF_00832"

/codon_start=1

/transl_table=11

/product="Putative aminoacrylate hydrolase RutD"

/translation="MRKKIDKTLKYSIFSIILMVIVALIFPTWTPKIKGENSISMLEQ

VEINGTGHEVMIRGVDRTNPILIFVHGGPGCSEIPYVRKYQKELEQHFTVVHYDQRGS

GKSYHFFEDYSNLTTDVLVDDLLALRDYVSKELGQETVILIGHSFGTYIGMKAAAKEP

TQFHAYIGIGQMANTLQSELESLEYTYEQAKQAGNAEDVKKLELIRSSIEQGKELTPR

ILLQKYGGAARLINENRDYISGFLLNPEYNGLDMIRFYSGMFSSQDILLKEAFDQNLP

DIVDHLEIPTYFVTGKYDYMTTANAARDYFDVLDAPIKDFIVFNESAHYPQFEEKEKF

VKWLNELF"

CDS complement(3275104..3275649)

/locus_tag="EFAGFIKM_02760"

/inference="ab initio prediction:Prodigal:002006"

/codon_start=1

/transl_table=11

/product="hypothetical protein"

/translation="MATTMITYRTLTLDDVNQLQEIDHSEHIDLIYEMRENRLVELEQ

NNECSNWDEMLLKEIQNRYVYELEQGGMAYGAFADDQLIGFGVLAHQYRGKERNQLQV

DLMYVSRGYRRQGIGKRILNELGEEAKKRGAQYLYISSTETKSAVSFYKSQGSQIASE

IDQELYDKEPNDIHMIKELGT"

CDS complement(3275678..3275947)

/locus_tag="EFAGFIKM_02761"

/inference="ab initio prediction:Prodigal:002006"

/codon_start=1

/transl_table=11

/product="hypothetical protein"

/translation="MNKNKGLIMSLLLLLLLILLWDSFRTSIFEAVTVQKISENEMTV

INFNNLERTIAVPMDMSKLIEENKEYTILYDKRVFDKYRLRSIKP"

CDS complement(3275998..3276465)

/locus_tag="EFAGFIKM_02762"

/inference="ab initio prediction:Prodigal:002006"

/codon_start=1

/transl_table=11

/product="hypothetical protein"

/translation="MSHWFDLDEFDPELIKFAVMIAKYNNKFIIIKNRKRGGWEIPGG

NKEIGETILHTASRELYEETGAVRFELTPYGVYEWNGSFGMVSYAEVKLLENLPESEI

DEIKLVDVLPEGMNFGDMFYHFSDRWDGLENHKLKKYTIEINHLNELPEIKIS"

CDS complement(3276621..3277079)

/gene="bltD_2"

/locus_tag="EFAGFIKM_02763"

/EC_number="2.3.1.57"

/inference="ab initio prediction:Prodigal:002006"

/inference="similar to AA sequence:UniProtKB:P39909"

/codon_start=1

/transl_table=11

/product="Spermine/spermidine acetyltransferase"

/db_xref="COG:COG0454"

/translation="MNIKIQPVTRENWEEALKISLYEHQVSLVPSVIEGIAYAYIKPW

DEAFDPYVLEIDGKVFGFFYLSYTPDSTDNYWIGGFQIDQSYQGKGMGKNAIRAILDY

ITKQHPLCKVISVTVERDNEIAQNLYKSMSFISENRTNSDDEVIYRLEIK"

CDS complement(3277076..3277645)

/locus_tag="EFAGFIKM_02764"

/inference="ab initio prediction:Prodigal:002006"

/codon_start=1

/transl_table=11

/product="hypothetical protein"

/translation="MRIRIIGSCGSGKSTLAKELSDKYGIPSYELDNLIWDRSAENLR

YPEHVRDESVQSIVSSEAWIIEGVQCKDWTIKSIQEADLIFVLAPNVFIRDYRIIKRF

VLSRMGLQPWNYKQSFKNLCKMIVKWNHQYNIEEVICSINENHKTATITKNKKQVIQL

IEHHLKVVVSENEKNLRGNQRLSKEEIQW"

CDS complement(3277686..3278246)

/locus_tag="EFAGFIKM_02765"

/inference="ab initio prediction:Prodigal:002006"

/inference="similar to AA sequence:ISfinder:ISSpgl1"

/codon_start=1

/transl_table=11

/product="IS1595 family transposase ISSpgl1"

/translation="MTPFPELETKRLLLREIKHSDSQDIFHIFSSDEVTRFYDLESFT

NTKQAEELIQRWKERFENGQVIRWGIALKSDNRIIGTCGFHGWMKQHHKAVLGYELAP

EFWRQGYMTEVTQKIVEYGFKNLELNRIEAFVEPENVGSRKLLEKIGFSEEGILKEHY

YWKNRFVDNVIYAFLKKEYKSKTDVS"

CDS complement(3278249..3278746)

/locus_tag="EFAGFIKM_02766"

/inference="ab initio prediction:Prodigal:002006"

/codon_start=1

/transl_table=11

/product="hypothetical protein"

/translation="METERIKFRKYTKNDFDLLFEMTCEPNMMKFIRHGGPWTKEETM

QSLEKFINWNKDDKGLFLAFNKEDNKLIGTSGLIPQIIEGNDELEVGYWVVEQYWGMG

YGYEQAKAWKEYGMDHLKQKRLISLIQHGNVGSMKVAQKNGMKHEKDVDIIGKNVAVY

SIEVK"

CDS complement(3279143..3279610)

/locus_tag="EFAGFIKM_02767"

/inference="ab initio prediction:Prodigal:002006"

/codon_start=1

/transl_table=11

/product="hypothetical protein"

/translation="MNRGIRFEIPNAYGRFLGEILKPMEVSNFDWFIGEEESYFVVDD

TLGDDLFPDMIMGMNGEELQKLIDNNEYYLIFANLKAFPKGTQITDVITYEDYMLSDC

QMVLLVIDSTYVTVYCKDLEKVEELYNHINRQGFESLAYLTDENDARTKLSVW"

CDS complement(3279744..3280286)

/gene="speG"

/locus_tag="EFAGFIKM_02768"

/EC_number="2.3.1.57"

/inference="ab initio prediction:Prodigal:002006"

/inference="similar to AA sequence:UniProtKB:Q9KL03"

/codon_start=1

/transl_table=11

/product="Spermidine N(1)-acetyltransferase"

/translation="MSRIYGERIMLREYQDSDLDYIKQWVNDPEITGTLSDNFLYPHS

SYETEVFFRTMVEGKSSNKSFIIGLKDSLDYIGQIDLYKIDYKNRFACLAIVIGRKEF

LGKGYGSEAIRLLQKFVFEELNLNRLELEVYEYNEIAYKCYLKCGFKEEGRYRKKIYK

KGKYWDSVCMSILKTEYEQS"

CDS complement(3280415..3280945)

/locus_tag="EFAGFIKM_02769"

/inference="ab initio prediction:Prodigal:002006"

/codon_start=1

/transl_table=11

/product="hypothetical protein"

/translation="MSRNLYIISGPPGVGKSTTSKLLVQTLERSAYISGDAVSHFPVK

GRGKPWLDKDTNDLTWKNISSLVKNLLDYKYDVVLDYVALPEDIDGLMTELADYHVRI

IYVVLMVDRQTIIRRDRLRAEEYQMKERSIILLDEFENHPDLRVDNKLYTNHFTEEQL

PEIVSGIINNEKYRLK"

CDS complement(3280958..3281452)

/gene="bar"

/locus_tag="EFAGFIKM_02770"

/EC_number="2.3.1.183"

/inference="ab initio prediction:Prodigal:002006"

/inference="similar to AA sequence:UniProtKB:P21861"

/codon_start=1

/transl_table=11

/product="Phosphinothricin N-acetyltransferase"

/db_xref="COG:COG1247"

/translation="MTTVTFEEVTEQHLPEIREIYNYYVMNTTISFHTEELDLDQIKS

SVMNKDTRYKTYVILETDQMMGYVLITQYKSKQAYDICGEVTIYLKPDILGKGLGKQA
LRFIEKIAKEQGFHTLIATICMENTRSKSLFERNGYEQCALFKEIGYKFDRKLDIGSF
QKIL"

CDS complement(3281583..3282116)
/gene="tmrB"
/locus_tag="EFAGFIKM_02771"
/inference="ab initio prediction:Prodigal:002006"
/inference="similar to AA sequence:UniProtKB:P12921"
/codon_start=1
/transl_table=11
/product="Tunicamycin resistance protein"
/translation="MIIMINGAFGSGKTSAAEALQPLIANSMIYDPEEIGYMLRKLLP
ENCREERERTDDFQDIYLWKILTVKTAKEVKQKYNKHLIIPMTIYKEENFNYIYSGLK
EIDQDIHHFCLTVTEETIYHRLAKRGDEYGGWQYQQAPKCVEAFKDEKFQTRIITDHL
ETSEIIELILRKVLSIK"

CDS complement(3282306..3283370)
/locus_tag="EFAGFIKM_02772"
/inference="ab initio prediction:Prodigal:002006"
/codon_start=1
/transl_table=11
/product="hypothetical protein"
/translation="MFKVKLAKAVISLALFGTLFSIQGVPDARAADASCPNGYVGLTF
DDGPSGNTTNMLNALKQAGLRATMFNVGQNAQNNKSLVSAQVAAGMWIGNHSYTHPNM
TTLNSSQMSSEITRTQQTIQSITGSSPKLFRPPYGATNATLKSVVSQNGLKEVLWNVD
SQDWNGASAAQIVAAVNTMKSGDVILMHDQYQTTLQAIPQIAQNLKNRGLCSGMISST
TGRAVAPDNSTTNPPSNGTKVEAENMTKGGQYTGNISSPFNGVVLYANNDLVKYTQNF
ATSTHNFSLRGASNNANMARVDLKIGGQTKGTFYFGGSAPAVYTLNNISHGTGNQVIE
LVVTADDGTWDIYIDYLEIH"

CDS complement(3283590..3284642)
/gene="ytfQ_1"
/locus_tag="EFAGFIKM_02773"
/inference="ab initio prediction:Prodigal:002006"

/inference="similar to AA sequence:UniProtKB:P39325"

/codon_start=1

/transl_table=11

/product="ABC transporter periplasmic-binding protein

YtfQ"

/translation="MRRHVNWLCLFILLLLVGCTTPESESVPPPISSNNFETLPSVEE

WMESAVARDTSTKPIVLGFSQLGRESAWRLANSTSIRNAAAESGISLVIKNAEQSQTK

QFEAVRSFIRQKVDVIAIAPVVESGWDGILQEARDAGIPVIIVDRSVDVKDTSLFVTT

IGSDFYEEGVKAGKYIQDRMRNHPGLIRIAELQGTSGSTPSIQRGEGFRSIFETRADI

IFSQSAPADFTEDNGKQVMKEFLQVPEDKRPQVLFAHNDEMAFGAIQAIEEVGLKPGE

DIIIVSVDGSRKALEILASGKINAVVECNPLLGPQLMQATKEIIAGRTLPKRMVPPED

IFTQESAKREMYNRRF"

CDS complement(3284751..3285725)

/gene="yjfF"

/locus_tag="EFAGFIKM_02774"

/inference="ab initio prediction:Prodigal:002006"

/inference="similar to AA sequence:UniProtKB:P37772"

/codon_start=1

/transl_table=11

/product="Inner membrane ABC transporter permease protein

YjfF"

/translation="MFLNRKYLPVFVTFGLLAVMFAIGSFRYTGFFSTQVLLNLLIDN

AFLIITAIGMTFVIVSGGIDLSVGSVIALTTIISASLVEKQGWSPAIVIPMVLVMGAL

FGTVMGAIIHYFKIQPFIVTLAGMFLARGLCYVISLDTITITNPFYTQVAQAKISLPG

GSFVSINVIIALVIVLLAIFLAHYTRFGRNVYAIGGSEQSALLMGLPVARTKILVYTF

SGLCSALAGVVFTFYMLSGYGLHAMGLELDTIAAVVIGGTLLTGGVGYVAGTFIGVLI

QGAIQTIISFEGTLSSWWTKIVIGLMLFVFILFQRLLSGRRVSSKSLH"

CDS complement(3285715..3286758)

/gene="ytfT"

/locus_tag="EFAGFIKM_02775"

/inference="ab initio prediction:Prodigal:002006"

/inference="similar to AA sequence:UniProtKB:P39328"

/codon_start=1

/transl_table=11

/product="Inner membrane ABC transporter permease protein

YtfT"

/translation="MAGKMRKHHLFWPLCMLGLLLIFNLLYSPDFFSLVMREGNLYGS

LIDILNFGAPLILVSIGMTLVIATGGIDLSVGSIVAISGAVACMSISRGVDQSSFGLM

LGALGLSIGLSLILGIWNGALVSVARIQPIIATLILMVAGRGIAQLITSGQIITVSNA

NYAYIGAGSLAALPFSIFIVLTVLLVASLLTRKTALGLFIESVGSNANASQLAGIRSK

TVILTVYVFCGLCAGIAGLILSSNVSSADGNNAGLWYELDAILAVVIGGTSLNGGRFY

LMGTVVGALIIQTLTTTIYMIGVPPEVTLVVKAFVVLAVCLIQSDSFRNSMVIRWKKR

KFPAEQGVNRHVS"

CDS complement(3286763..3288274)

/gene="fruK"

/locus_tag="EFAGFIKM_02776"

/EC_number="7.5.2.-"

/inference="ab initio prediction:Prodigal:002006"

/inference="similar to AA sequence:UniProtKB:Q8G847"

/codon_start=1

/transl_table=11

/product="Fructose import ATP-binding protein FruK"

/db_xref="COG:COG1129"

/translation="MAEQAPILQMTGITKQFPGVKALSSVSFRLFPGEIHALMGENGA

GKSTLIKVLTGVYSIDEGTVTMDKRHLSISGPLEAQKAGISTVYQEVNLCPNLTVAEN

IFIGREPMRFGKINWKQMNKDAENILRDRLHLSIDVKAPLQTYSVAMQQLIAIARALS

ISAKVLILDEPTSSLDKNEVQQLFRIMHKLKSEGLAILFVTHFLDQVYEMSDRLTVLR

NGELEGEYIAAELPRMELVLKMIGKELEVLEELPKEADAAQAESGELLITAKALGRKG

AIEPFDLDIRKGEVVGLAGLLGSGRTEIARLFFGADRSDVGKLLVVDTGSTVKSPRHA

IDQNIAFCSENRKTEGIIDDLTIRENIILALQATRGWSKPISRKKQDEIAEKYINLLN

IHPKNPEHLIKNLSGGNQQKVLLARWLLMNPDLLILDEPTRGIDIGAKTEIQKLVLSL

SKQGMAVLFISSELEEVIRVSHRIAVIRDRKKVQELSGDQIHQQQIMKAMAGG"

CDS complement(3288360..3289382)

/gene="ytfQ_2"

/locus_tag="EFAGFIKM_02777"

/inference="ab initio prediction:Prodigal:002006"

/inference="similar to AA sequence:UniProtKB:P39325"

/codon_start=1

/transl_table=11

/product="ABC transporter periplasmic-binding protein

YtfQ"

/translation="MRTKKKWGLAITLAMVMLVSTACSSTNGGAGSDNETSTSGNGST

PASAKTITLGFSQVGAESGWRSANTKSIQDSAKEAGYDLKFSDAQQKQENQIKALRSF

IQQKVTVIAFSPVVESGWDTVLKEAKDAGIPVILTDRAVDSPDKTLYKTFIGSDFVEE

GRKAGQWLSDQYKDTQDEINIVELQGTTGSAPANDRQEGFMELIASNSKLKVIASQSG

DFTRAKGKEVMQAFLKAHKKIDVLYAHNDDMALGAIQAIEAAGLKPGVDITIISVDAV

KDGMQAAADGKINFIVECNPLLGPQLMEAVQSVVDGKDIEPRIVTDETTFTSEQAKEA

LPTREY"

CDS 3289626..3291437

/locus_tag="EFAGFIKM_02778"

/inference="ab initio prediction:Prodigal:002006"

/codon_start=1

/transl_table=11

/product="hypothetical protein"

/translation="MKLIQWIASSLRLKLLSMFIILSSVPLIVVGLISYHKSYTVVSN

HNKASTQLVADQLARNIDILFEDTERLLELGKNPQVLQYLYSQSESYPEAKAILQTYN

LYRETYKYDKVLNISFVNFYGKGISERKGVFKLERNPLRNPYFKYLIQYPDAILRIPH

SAAYAEYNRLDGFAYPKQNALSIITTVKERITHEVIGFIVIDLNDLFIDDFLTNTPIK

DSGFFYISDQYGNTLFRPDVTPASITMMDQIRSVPLHLQHDSFILSAGSKPQFVVYST

SKQTGWKMIGVAPYQEIIAEANSIRQLIIITVLLSAFFAISLYFFLNNRLILPIRILM

NKMRKAANGYLEAKVTPHGSDEIADLGKSFNVMLDKLKMLIEKSIRENEQVKIAELRT

LQAQINPHFLYNTLESILWMAEAEKKENVIKLVQALSKFFRLSLNKGFDWVSIQTELE

HARSYLVIQQMRYHDILTYEISVDRELQDYPILKMTLQPLIENALYHGIKNKRGQGLI

SIHGYVEDNSIILTVQDNGIGMSADRLAQLREELEKPADSRSPQDDEQEGGFGLLNVH

HRIRLYFGPAYGVEMESTYMEGSTFMIRIPKSKEVNH"

CDS 3291434..3293032

/gene="rhaR_26"

/locus_tag="EFAGFIKM_02779"

/inference="ab initio prediction:Prodigal:002006"

/inference="protein motif:HAMAP:MF_01533"

/codon_start=1

/transl_table=11

/product="HTH-type transcriptional activator RhaR"

/translation="MKKVMIVDDEIGIRENIRSCIDWEKEGFHYCGDAPDGELALPLI

HEWAPDILITDIKMPFMNGLELTSIVRTQQPDIKIIIMSGHDEFSYAQEAIRLGVTEY

CLKPISAAELIQMLHKVSKQMDEEHQRMTNRTITKEKLLADLCGGLIGTTDAIESAKQ

LSLPLSARYYTIVIIDVRLTDDSPDPESLMKKLLPILEETRDPHVELLPYIRSRTELV

LLLKYHSDDSISIYLEQLRGRVRMELEHTFKCNIILGIGSQQERLQGIHVSYLEAEED

KYITRLTLQNRASLREINLDEQTTLFLGRNSFLEFLKIGSPNQLDHFVQEFAAPLQSF

DWKSSSYGFYLLNDLTLESFRLANQLFRMSDHSDENITSLQQTIRKITSWEDCTQYMC

SLLNLLWQRRAACSGKYSDVIDQVKAYLSREYNNEQVSLKMISAHVRISPSHLSKIFS

QETGQTITEYLTQIRIGKAKELLKTTNNKTFEIAYKVGYNDSHYFSNIFKKTTGFTPR

EYRTQGTEESQARPADMRKEQMYE"

CDS 3293025..3293990

/locus_tag="EFAGFIKM_02780"

/inference="ab initio prediction:Prodigal:002006"

/codon_start=1

/transl_table=11

/product="hypothetical protein"

/translation="MNKARFALLLTLILVFPMIISSGSAHIRPQQMATAFPATSPVEK

SAASKTFGIIYPMTYPTYEMITKDATEYAGKQNITLIVNAPDEANTEQQIRIMENMIK

QHVDGIAISPVDAAALTPVIDAAQAAGIPVITFESDVPSSHRIAYVGADNYLTGQQFA

MTTTRLLHNQGMILVENGLEEMQGLQQRLNGFIDYIRSETDIEILEVRYHQGNEDQAM

SDMENMIQAHPHFNAIIGLDFVSVSASTLIWKAKGLNRHLIAFGDSPTSENGLLNGQL

SAVISQNEGVWGTKMMETLLLASDGVPVKEFIDTGIIEINHEGSN"

CDS complement(3294124..3294648)

/locus_tag="EFAGFIKM_02781"

/inference="ab initio prediction:Prodigal:002006"

/inference="similar to AA sequence:ISfinder:ISBce8"

/codon_start=1

/transl_table=11

/product="IS4 family transposase ISBce8"

/db_xref="COG:COG3385"

/translation="MITFQYFEPEDFDQLIEWSGDEAFLLQWAGPQFQYPLSREQLSD

YLHGANDKNTSNKFIYKVMDESTQEIVGHIALGGIDRYNRSGRIGKVLLGKPYQGKGY

GKQMINEALRIGFEEEKLHRISLGVFDFNVSAIRCYEKAGFVQEGLIRDARRYEDTFW

NLIEMSILENEWKK"

CDS complement(3294687..3295010)

/locus_tag="EFAGFIKM_02782"

/inference="ab initio prediction:Prodigal:002006"

/codon_start=1

/transl_table=11

/product="hypothetical protein"

/translation="MKHVHIIFSLLFIMLGIVIITISKMIEEVIPKLGYAAFQSAAAG

SYTPSDYQVNFALNYWIAAICILGGVICLISTMNVVRNYIREMNIRNRAFDETHNYDD

TRELK"

CDS complement(3295211..3295357)

/locus_tag="EFAGFIKM_02783"

/inference="ab initio prediction:Prodigal:002006"

/codon_start=1

/transl_table=11

/product="hypothetical protein"

/translation="MIHLKRVSVDNWYACTQLDVTEEQRKSFPRQLYIGLLRLWKRSV

EEVD"

CDS complement(3295542..3296480)

/locus_tag="EFAGFIKM_02784"

/inference="ab initio prediction:Prodigal:002006"

/codon_start=1

/transl_table=11

/product="hypothetical protein"

/translation="MNIVQSFQELVVRGDHQGMIELLKNFEQSRKLSVHERGWVYWNI
SDGYALLREPEPLYANHREFFEWGRKNLAPEQLHWIVSDSTQALSLSLGNYFDHWMDW
YQYACDHAPKLDTNRGVRFESHRALGGSLWVLERYSEMDSVLENMNQLIQEDETWSNI
LFARITYNKQRLAYLHHAGEVAGVEVLINETMDWIDEINDGAIPISRKDEVVGSWEDI
NVSRNSQRDINIALNNLACILTDIERYEESVKLFVQIQERGYHMNAYGFSKWIYSIWK
TRGTEAVKDALATNASYEIADVVKHTPELSEIKGLV"

CDS complement(3296515..3297342)

/gene="COQ5_3"

/locus_tag="EFAGFIKM_02785"

/EC_number="2.1.1.163"

/inference="ab initio prediction:Prodigal:002006"

/inference="protein motif:HAMAP:MF_01813"

/codon_start=1

/transl_table=11

/product="2-methoxy-6-polyprenyl-1,4-benzoquinol
methylase, mitochondrial"

/translation="MSDPRSTYSIQTAVLSLQEELERLKMQAVMGWSKEFRNLEWYGL
KNGMRVLEVGSGPGYITEQLLNSLPDSELTSLEIDRSLQAQAKERLKNIPSKRLQFVE
SSIYQMDLPDDSFDFVVARLIFLHLNNPDEAAKEIYRVLKPGGRLAIIDVDDGVFGAV
NPDVPALHTVLMKISNYVAQHGGNRLIGRSLPRLLSESGYIDVDIDSVLQHSDLLGIE
GFKHQFNLNRFVHFAEKGVISSEEFAQLQQASEAINHSPEAYAMMNFITACGTKPLT"

CDS complement(3297408..3297815)

/locus_tag="EFAGFIKM_02786"

/inference="ab initio prediction:Prodigal:002006"

/codon_start=1

/transl_table=11

/product="hypothetical protein"

/translation="MTIHTIELTQEIPVDGGYPAIGDGHARQGDGEVSCQAIECPMEV
VDITINVINDMILTNPRAHTPSGWITFGFHEDLNEATVQALDGMLNLMGELYGLDRVE
AIALGSAVIDLRITQIVNGVKGVHAVLPHDAFK"

CDS complement(3297906..3298286)

/locus_tag="EFAGFIKM_02787"

/inference="ab initio prediction:Prodigal:002006"

/codon_start=1

/transl_table=11

/product="hypothetical protein"

/translation="MQGLIHDISKFFPQEFIPYAIHFYSKQKDEDTHLRWKNAWLHHQ

NHNKHHWEYCIVNRNTKEALPMPTKYLIEMVCDWRSFSRKWGRKVKDSNLVERMINSQ

DIILHPVTREELEQLFIRKDEKFI"

CDS complement(3298400..3298909)

/locus_tag="EFAGFIKM_02788"

/EC_number="2.7.1.-"

/inference="ab initio prediction:Prodigal:002006"

/inference="similar to AA sequence:UniProtKB:P9WL55"

/codon_start=1

/transl_table=11

/product="Putative O-phosphotransferase"

/db_xref="COG:COG3896"

/translation="MEKGKIIFLNGVTSSGKTSIVEAMQSYDDPFFYVVANDLFENTI

GDKHLQTDYWKYLSEATVMMYHTAKVFSDHGKHVLIDGILVERPELALHYEQVKQIFE

GFPLDIVELYCPLDICRQRNIERGDRREEQSDEQHQIMASDIQYSFSVDTSVHTPEEC

AEKIIAALF"

CDS complement(3298963..3299445)

/locus_tag="EFAGFIKM_02789"

/inference="ab initio prediction:Prodigal:002006"

/codon_start=1

/transl_table=11

/product="hypothetical protein"

/translation="MSYKAILIDQLNACYHDKSWFIPLHDILTDLNAAEAAYVKNEGE

HSIWAIVNHLIFWNEKWLERYIVEQVDGQNSVNNEDTFDIDLSNLNDVEWGKTLHRLK

TVFGDWNRALEDSEEHKLTREIPSYFNAPWWGVISNLCIHNAYHIGQIMLLKKSMKHT

"

CDS complement(3299477..3300133)

/locus_tag="EFAGFIKM_02790"

/inference="ab initio prediction:Prodigal:002006"

/codon_start=1

/transl_table=11

/product="hypothetical protein"

/translation="MDIKNKSGDVLGKIQIESLRNDNAIDRIVIDLKPGDGKAIYTAD

VIENQFTQQTNYAAIPWEDGLEQLQKHYSPWQPKFPIDSDIDAIHVFYGFDNLTPQEI

DEMIEESNRSGRNVVIRDLKPNKKVVGISITYKEYGGYKLNIFGTTKSRIALPDHDGT

KVKQAAVRGVQAFYIFNNETSQLVWIEEDVHGKALQYEIIGRNMSEDLVMKIAKSMNG

"

CDS complement(3300176..3300730)

/locus_tag="EFAGFIKM_02791"

/inference="ab initio prediction:Prodigal:002006"

/codon_start=1

/transl_table=11

/product="hypothetical protein"

/translation="MLIVDENVYPDCSVEIRTVDIGGLTKPQLIQKLDQYSISLNKYG

EQLLNDERFIVSSTKQSLQTVELTIRQLGFSNGATTFELFRRANDLGLECCPVELGPY

LRMQHLDQPEICSTNISEGNKAPSGSITIASAALTENHEFPKGFYLRRINDKLWLRGY

LADHLHVWSPNDRFIFCRAQLSIV"

CDS complement(3300853..3301248)

/locus_tag="EFAGFIKM_02792"

/inference="ab initio prediction:Prodigal:002006"

/codon_start=1

/transl_table=11

/product="hypothetical protein"

/translation="MGKGRILGIAYNVIPVKDLEVSAAWFVKHFGFNIRHQREGYLSL

FRGDRPILDLIQSDNETRATFEVHQKKRWVITFFTDDIGSLHDYLTSENVKVGAVSDE

GQYGKFFVLEDLDGNLFDVWEHRDCELNF"

CDS complement(3301282..3301809)

/gene="yfiT_1"

/locus_tag="EFAGFIKM_02793"

/EC_number="3.-.-.-"

/inference="ab initio prediction:Prodigal:002006"

/inference="similar to AA sequence:UniProtKB:O31562"

/codon_start=1

/transl_table=11

/product="Putative metal-dependent hydrolase YfiT"

/translation="MDEKIRFPIGHFEPSLKFSNEDRIHIIGQIPGITKTLREITQQL

NDDQLRTPYRDGGWTFTQIVHHLADNDMNAYIRFKRALTEDEPMASSYREDLWASLHD

YRNTPIEDSILLMDILHKRFLTLLSGLHPDQFRRKLRTEVLGTMSLDIAIQRFVWHGQ

HHIAQIKSCITSQGW"

CDS complement(3302109..3302510)

/gene="ndx1_2"

/locus_tag="EFAGFIKM_02794"

/EC_number="3.6.1.61"

/inference="ab initio prediction:Prodigal:002006"

/inference="similar to AA sequence:UniProtKB:Q75UV1"

/codon_start=1

/transl_table=11

/product="Diadenosine hexaphosphate hydrolase"

/db_xref="COG:COG0494"

/translation="MKFIVSASVIVLNEHNEILLMKGRRGWEMPQGCVEEGETIRQAA

VREVKEETGIDIELIKFCGLYQNITRGVCNNIFTGKPIGGALTTSDESEEVGYFTLEE

AQQMITWGNFYNRIHNALDEKSHPFLIEFSE"

CDS complement(3302564..3302992)

/locus_tag="EFAGFIKM_02795"

/inference="ab initio prediction:Prodigal:002006"

/codon_start=1

/transl_table=11

/product="hypothetical protein"

/translation="MKIIRNSEELEGTGYIEVLPRKYLGQCWNENSIYFDEEVFGYIE

KTIENIFSGYDHYAFNEIHRKTWEVILEDLRTFANLLDEKVTIELINEHVYFFFGNSK

DEFQQDFLNSVQKLRELIIEFVVWIKEQLTTQDYVSVLGI"

CDS complement(3303302..3303826)

/locus_tag="EFAGFIKM_02796"

/inference="ab initio prediction:Prodigal:002006"

/codon_start=1

/transl_table=11

/product="hypothetical protein"

/translation="MSRPLRIHIIGSTGSGKTYLGQVLSEKLNIKFYELDTVMWSSKV

EFSGKNSPDVRDKLLHKIIDNDSWIVEGVYYKWLSDSFSRADVIYYLDTNVLIRHFRF

IMRFVKQGIGVERSIYKQTLRGLIEMLVWNHKFNSTNRKEICSFLSPYKDKVIILKTN

RKMISLLNEGSEIP"

CDS complement(3304032..3304235)

/locus_tag="EFAGFIKM_02797"

/inference="ab initio prediction:Prodigal:002006"

/codon_start=1

/transl_table=11

/product="hypothetical protein"

/translation="MISYKPFQKLLIDREIKKQDLLKMTGISSATMAKLNTNEYVSLE

VIDKLCTALGCQPGDLLEHIEDQ"

CDS complement(3304844..3305245)

/locus_tag="EFAGFIKM_02798"

/inference="ab initio prediction:Prodigal:002006"

/codon_start=1

/transl_table=11

/product="hypothetical protein"

/translation="MTIMFKEIKDIDTQAIQELFHSVDWKSGDFPEDLRQAINNSHTV

ITAWDDNKLVGLINALSDGVMTVYFHYMLVHKEYQSLGIGKRMMEQMLSRYSNIKTKV

LISYDSAEKFYGLFGFKSEEGTKAMFISDMV"

CDS complement(3305432..3305653)

/locus_tag="EFAGFIKM_02799"

/inference="ab initio prediction:Prodigal:002006"

/codon_start=1

/transl_table=11

/product="hypothetical protein"

/translation="MNIIKCNIRELMAEHRIDDITELMAKSGLSRNSINKLYRETNIE

TTKLETLFKLCDTFNCKLSDLIKYVPGEK"

CDS complement(3306134..3306790)

/locus_tag="EFAGFIKM_02800"

/inference="ab initio prediction:Prodigal:002006"

/codon_start=1

/transl_table=11

/product="hypothetical protein"

/translation="MKGKILLLVFITIVFSSCQNSSQSTTLNSETKSKMNSELPPDVV

STGDGTESKEITEGLSDTANNKKILPMSIGNLDLKGEFYDEMLRNRIDHDYEVEFNEF

QNSKEIITTLEWGAFESKYTEIWDNELNQIYKMLLSKLDRESKEALIESQKEWLQHHL

RETKFVEKTFIDNGYLGSKGSVSLTTVIRERIRERTMQLFEYRYLLDGEVEFLYQSTK

"

CDS complement(3306983..3307240)

/locus_tag="EFAGFIKM_02801"

/inference="ab initio prediction:Prodigal:002006"

/codon_start=1

/transl_table=11

/product="hypothetical protein"

/translation="MDQGMSYFSQFNDPRMITSISLAEIIARSKKIEDFGAPLDLRLV

LLIARTHKFQGNNRNAKEYAQWGLNNIGKATGLIKDFEGIS"

CDS complement(3307206..3307415)

/locus_tag="EFAGFIKM_02802"

/inference="ab initio prediction:Prodigal:002006"

/codon_start=1

/transl_table=11

/product="hypothetical protein"

/translation="MEMGVYLDFLPNSIGESKSSNEITTYDCEFRKRLNKKANWLKRF

FTMERERWFQYGVIFGSRNELFFSI"

CDS complement(3307787..3308329)

/locus_tag="EFAGFIKM_02803"

/inference="ab initio prediction:Prodigal:002006"

/codon_start=1

/transl_table=11

/product="hypothetical protein"

/translation="MSKKTIIIENYNNEWPEMFSDLKSIIEQRLGDLVLRIEHVGSTA

APGLAAKPILDIDVVIDSMDLLPDVIQGLESLGYYHEGNLGVENREAFARKDANVPYS

IVKIQKPEHHLYVCNKERKELLRHISFRDALISNPEFVVEYANLKKELAIKYGENRQS

YTDGKTEFVNKVLNEYGKNL"

CDS complement(3308558..3309019)

/locus_tag="EFAGFIKM_02804"

/inference="ab initio prediction:Prodigal:002006"

/codon_start=1

/transl_table=11

/product="hypothetical protein"

/translation="MIRTVSLCVIRRGDELLLEEFFDEAVGITYYRPIGGTVEYGESS

KITVIREVKEEIDADISEPILIGVIENIFNYRDQIGHENDFIYEAELMNRSLYDLEEI

DGIEGSKPYKAVWKPLHYFKGNLTVKLVPDGLLELLQDGTDGRYIKHLSKP"

CDS complement(3309252..3310046)

/locus_tag="EFAGFIKM_02805"

/inference="ab initio prediction:Prodigal:002006"

/inference="similar to AA sequence:ISfinder:IS655"

/codon_start=1

/transl_table=11

/product="IS3 family transposase IS655"

/translation="MCNVLEIAKSTFYHEAKEKPNEDELTEAIVEIFHKNRKVYGTRK

IKAKLQERGLIVSRRRIGRIMQEQSLVSTYTVAQFKPHKTACNEEKTVNELGREFDQT

EAKRFVVSDLTYVKVGHRWHYICVLMDLFNREIIGHSAGPHKDAALVSRAFSTVEGDL

SQIQWFHTDRGSEFKNHAMDQLLGTFGIGRSLSKKGCPYDNAVAEATYKVMKTEFIYQ

MEFRNLRHLELELYDYVNWFNRHRIHGSLGYLTPVQYRQEALKKVV"

CDS complement(3310088..3310372)

/locus_tag="EFAGFIKM_02806"

/inference="ab initio prediction:Prodigal:002006"

/inference="similar to AA sequence:ISfinder:ISEfa8"

/codon_start=1

/transl_table=11

/product="IS3 family transposase ISEfa8"

/translation="MPKQQRRTFTTEFKKQMVQLFENGKSRAAIVEEYDLTASALDRW

IKQSQATGSFKEKDNRSPEENELMAMRKELERLRMENDILKQAALIMGRK"

CDS complement(3310555..3311004)

/locus_tag="EFAGFIKM_02807"

/inference="ab initio prediction:Prodigal:002006"

/codon_start=1

/transl_table=11

/product="hypothetical protein"

/translation="MELYNTKNELDRIDFHDCIVESIYLEENKITFNIESVNVLVGHM

LNPYDVAKNTDQCTLEFINVERYESGYFEREKDTRIDIELIHNNDFEILKFDILAEEG

MNLYKLFGMASNFNNEFSEIIIRAEYTNIKWNKYVSNAWFVNWPKTK"

CDS complement(3311208..3311432)

/locus_tag="EFAGFIKM_02808"

/inference="ab initio prediction:Prodigal:002006"

/codon_start=1

/transl_table=11

/product="hypothetical protein"

/translation="MQQAKSIDPFLRSFKPLERQECTNVFRKASEKGSSSATMAKLNT

NEYVSLDVIDKLCTALGYQPGDLLEHIADQ"

CDS complement(3311506..3312216)

/gene="hdl IVa"

/locus_tag="EFAGFIKM_02809"

/EC_number="3.8.1.2"

/inference="ab initio prediction:Prodigal:002006"

/inference="similar to AA sequence:UniProtKB:Q51645"

/codon_start=1

/transl_table=11

/product="(S)-2-haloacid dehalogenase 4A"

/translation="MMMSIKAIFLDFYGTLVHEDDDIIPLICKEIQENSVEECEVSDI

GRYWWNIFSSMFKNSYGETFKSQRELGILSLSETISKYRSNCDVNEIIQKQFDHWSEP

KIFSDTIPFLETLQGYEVYILSNIDTADVKAAIAYHGIKVNEIITSEDVKSYKPRPEL

FIEALERHNLSPKEVLHIGDSLMSDVGGAGILGIATVWLNRLNKIKTDIAEPNFICRD

LKEVRDIISGIEEGSIVV"

CDS complement(3312369..3312761)

/locus_tag="EFAGFIKM_02810"

/inference="ab initio prediction:Prodigal:002006"

/codon_start=1

/transl_table=11

/product="hypothetical protein"

/translation="MQQIDLTKTLEELENDYWDEPNFASSLVIQVHQLRKKPLCELNN

EELRLLIGQQMNLDFLLRIALEKLIQNPFGSGDLYIGDLFCSVLKVEKEYWNEHQELK

NELNEVIRIYEEVRRTLDEQIIKYRSSL"

CDS complement(3312964..3313311)

/locus_tag="EFAGFIKM_02811"

/inference="ab initio prediction:Prodigal:002006"

/codon_start=1

/transl_table=11

/product="hypothetical protein"

/translation="MKFGNNSQENREIKISKDSKTFLELREIVNRFDPVNLVEGGAPD

DEHDRLTTEILMLLSQESMDEIRDLLINCEIWYGYDPNDIKEEYKERFNSKIDRTHKE

ILNWHTKNKNQEN"

CDS complement(3313641..3314246)

/locus_tag="EFAGFIKM_02812"

/inference="ab initio prediction:Prodigal:002006"

/codon_start=1

/transl_table=11

/product="hypothetical protein"

/translation="MHLIIGHKIAERLNIEDRTSFLQGSIAPDAVVTKDESHFYRGEH

QNFTRHIDYREFLNKYESYSNNLYVLGYFAHLIADEQWMKGFYMPWLRNQMEADVGVY

PLYHNDFKLLNGKLLEYYDFTDEMRNAINNKAGEVIDLDEVKSRDVIEFVPHVIGDME

YEEQVINEPLNVFSFIQIKGYIETSVNVGIMNLEQLFKKEE"

CDS complement(3314515..3314940)

/locus_tag="EFAGFIKM_02813"

/inference="ab initio prediction:Prodigal:002006"

/codon_start=1

/transl_table=11

/product="hypothetical protein"

/translation="MQGYNVLMIYSKDMDKLLMCKRSRNPYKGLSNLVGGKIEHGESG

IMAAYRELFEETGISNKDIILHHLMDFKYYIQECYVEVYFGKLNYHLDVYGDENELYW

SDLNKDFFDMSLFAGEGNIGHKIEQVKMSKDKILKEITL"

CDS complement(3315194..3315670)

/locus_tag="EFAGFIKM_02814"

/inference="ab initio prediction:Prodigal:002006"

/codon_start=1

/transl_table=11

/product="hypothetical protein"

/translation="MKFEIIQANKECKEVLANLMQFYIYDFSEFIKCDVEENGLYGAY

PLEDYWIEKNHRFPYLIKQNDKHVGFVLVRLIETAERDYYSIAEFFILKKYRREGIGK

AVAARIFDLYKGQWEVYQIEGNKSAQIFWNKTIDEYTKGQFKERIENGRRIQDFIS"

CDS complement(3315906..3316952)

/locus_tag="EFAGFIKM_02815"

/inference="ab initio prediction:Prodigal:002006"

/codon_start=1

/transl_table=11

/product="hypothetical protein"

/translation="MKNLDINLWRSTLLNHFKYNLEKFATTTENKDVYCLILDCHATY

GNVNLKWNTFDSFKKHVNKHYSSYTSDKLLGIRGLEYNVGDFSYEDTKQPELINEFEQ

LYEEKLSEFFDDEDEESSNELTQKFINALVEIIFELRFTFSKLNKTGHFISFIVEHDS

EYYEYIKKTVTIEDYYKAFPEVKEYEQYLSIIYSTSKEEQVTHWSNLLYEFIIGNETE

GTRSLKQMYRHKYDVEEEIIKLGVIASREVVTLFEVFSGYTPSIDGQIIQSDSHTRWM

FLSILIDINNANEETINRLKQILIRKYEVDNEVQECINIARTLHTLDSVRFPEEMHDE

GRLRLLNYEEYKNN"

CDS complement(3317118..3318188)

/locus_tag="EFAGFIKM_02816"

/inference="ab initio prediction:Prodigal:002006"

/codon_start=1

/transl_table=11

/product="hypothetical protein"

/translation="MNVGRNVVVLNDRSRKDPFSNDEERAIITSIYYPTDDKNTQINV

SNYMDLFDPSKDKAINILKDLGVDIEYLNTIETHIYNNYNPKYSNAVSDVILYSPGFG

VSRDMYTYHIKYLVDCGYIVVSIGATYESVFSIFPDGRVVNQTAQLTNIKSTDFILWR

ELLDTRVEDILYVINHLDLLFSQSLLEGFSYRSIGIVGHSLGGAAAFEVAKRDNRIRA

GVMFDASFHLLDLEKNEKIDTPFLIMRQEKCTYDELLTELSEGIINPFISGFEKLYKQ

LNGYKAAVKVQGAHHMTFSDVPIHFKDIEAINKHEIISDYSTAFLSEFIQGKSYSLQN

SFNHKRSADIVEINIKGQTVSV"

CDS complement(3318382..3319041)

/locus_tag="EFAGFIKM_02817"

/inference="ab initio prediction:Prodigal:002006"

/codon_start=1

/transl_table=11

/product="hypothetical protein"

/translation="MTLIATAYAHECIVMGTDTLLQRTENYQTNKIIGYGSIQKLFFI

RKTGTGISVRGRASWGEKTIKQILQNFTHTVESSMRQCDVADALQLYIKSNFPQLETS

YHIAGYQDTEPFIYDLDFDSGLNLVRENVDRKGFLRYSVLARIDDTEVRNQVLPLPDF

SKMQENQVFDFMETLFKLKIQAENLKPTPSVGYPIDYLFLSHDQSKFVSNKIASDFLL

D"

CDS complement(3319370..3320467)

/locus_tag="EFAGFIKM_02818"

/inference="ab initio prediction:Prodigal:002006"

/codon_start=1

/transl_table=11

/product="hypothetical protein"

/translation="MAKDGMFYAPKSVTKEVVCGPGEFTIAAVALDHGHIYGMVGALL

EAGATLKWVYDPDPAKVEAFRKQFPQAQVAGSEAEVLADDEVLLVASAAIPSDRAPLG

MRVLAAGKDYFTDKAPFTTLEQLKFAREEVKRTGKKYMVYYSERLHVESAIYAGQLIE

QGAIGKVVQVMGTGPHRLNAGGRPDWFFKHEQYGGILCDIGSHQIEQFLTFASCTDAE
VGFSRVHNFNHPQFPELEDFGEASLIGDNGASGYFRVDWFTPDGLGTWGDGRTVILGT
DGYIELRKYIDVAREPEGDQVYLVNHEGEYRYNVKGKVGYPFFGQLIRDCLDRTETAM
TQEHAFKAAELCLIAQHKAMSERVVWSSGAK"

CDS complement(3320531..3321703)
/gene="iolG_6"
/locus_tag="EFAGFIKM_02819"
/EC_number="1.1.1.369"
/inference="ab initio prediction:Prodigal:002006"
/inference="protein motif:HAMAP:MF_01671"
/codon_start=1
/transl_table=11
/product="Inositol 2-dehydrogenase/D-chiro-inositol
3-dehydrogenase"
/translation="MSIVRVAVIGIGNMGAAHARTLVAGEVPGAELVAVCDVRREMES
WVSSHLPATVTYWQDAEQMMASGTIDAVIIATPHYDHPEQAIQAFQHGLHVMIEKPAG
VYTKQVRKMNEAAAASGKVFSMMYNQRTNPLYIKLRDLIASGELGEVRRTNWIITNWY
RSQSYYDSGGWRATWAGEGGGVLINQDPHQLDLWQWTIGMMPVRMRAFCSFGKYRNIE
VEDDVTAYVEYENGATGVFVTTTGEAPGTNRFEVNGDWGKIVIEDGKLTFWRLRESEP
EFNQRFTGGFGQPECWKCEIPITGVETGHPGLIRNWVDAIRTGVPLIAPGEDGIHGLT
LSNAMLLSTWTDNWVDLPIDEDLFYEHLQERIAGSTTKKDKAFSGSQPADLNQTFK"

CDS complement(3321700..3322614)
/gene="iolE_1"
/locus_tag="EFAGFIKM_02820"
/EC_number="4.2.1.44"
/inference="ab initio prediction:Prodigal:002006"
/inference="protein motif:HAMAP:MF_01672"
/codon_start=1
/transl_table=11
/product="Inosose dehydratase"
/translation="MKLSVFTVATPDLNAEELASAAAATGIDGIEWRFRGIPEDAMSE
EPSYWRNNRCSVDPSRWEEQVPVFREAALGQGRKSIALVPYLNCDDLSATKQAFQAAA

GLGASMMRVGVPGYDRKTSYPELYRKAVDYLSEVQDLAKQYNIKAIVETHHQTIAPTA

SLAYRLVQSLDPQHVGVLYDPGNMVHEGYENHRMGLELLGPYLAHVHVKNAGWFEAEG

KDSQKANVTEKSRGVALNTVWKCHWTPLTEGMVDWVQMVRDLRAVGYDGYYGIEDFSG

ALESKAMLQHFADVFAEIERRVDEEEQS"

CDS complement(3322959..3323456)

/locus_tag="EFAGFIKM_02821"

/inference="ab initio prediction:Prodigal:002006"

/codon_start=1

/transl_table=11

/product="hypothetical protein"

/translation="MKIVVISDTHCSRKSRKLPTRLLDVLPSADLILHAGDWSDWSVY

PLLSEYAPVEGVAGNTDPSEIAEKLGYSRIVEVEGLRLGLVHGHLGSKGTEQNAIHTF

AGQHVDAVIYGHSHIPVMHTVDNTLVFNPGSPTDRRFQKQYSFGIMTIDQGRIQAEHV

FFDRD"

CDS complement(3323700..3324134)

/gene="ywnA_2"

/locus_tag="EFAGFIKM_02822"

/inference="ab initio prediction:Prodigal:002006"

/inference="similar to AA sequence:UniProtKB:P71036"

/codon_start=1

/transl_table=11

/product="Putative HTH-type transcriptional regulator

YwnA"

/db_xref="COG:COG1959"

/translation="MSTHFSVSVHCLLLLSLSAPERITSAHIAGSVNTNPVVVRRILG

GLKKAGLVNSSPGTRGFYLAKPSSEITLAMIYQAAKDEGPLFPIHGNCNPDCEVGLHI

DSLLTNLYQVAEAKVEQFFASITLEDLERSCTQMQAVPSQTE"

CDS complement(3324177..3324554)

/gene="gpsB"

/locus_tag="EFAGFIKM_02823"

/inference="ab initio prediction:Prodigal:002006"

/inference="protein motif:HAMAP:MF_02011"

/codon_start=1

/transl_table=11

/product="Cell cycle protein GpsB"

/translation="MDEHMKRRLDKQRQLFKQLGVQLDALSIHEKQFNYKLRGYDPDE

VDAYLDLVIKDYERFYANIADLMDKWQEQQLTIRDLKSSAKPVEDPTKIDRKQLDDIV

KQLEYSVRQLKIRARPEKDLFSE"

CDS 3324753..3325319

/gene="dksA_2"

/locus_tag="EFAGFIKM_02824"

/inference="ab initio prediction:Prodigal:002006"

/inference="protein motif:HAMAP:MF_00926"

/codon_start=1

/transl_table=11

/product="RNA polymerase-binding transcription factor

DksA"

/translation="MSTLTKDQHQILKNALLEQRENLQRHFESSMEDGAPVESLKDST

GELSSYDNHPADAGTETFERSRDLAIDDTLTDEFNQVNDALERMEQGTYGTCVTCGED

IPFERLEAIPYTAYCIDDTPNREISNDRPVEEEVMTMPPSGAGEGRQKRAGKFDNADA

WEAVEEYGTSNSPATAAKRDVKGYDENM"

CDS complement(3325509..3326201)

/locus_tag="EFAGFIKM_02825"

/inference="ab initio prediction:Prodigal:002006"

/codon_start=1

/transl_table=11

/product="hypothetical protein"

/translation="MAHKVLSTIEEIKIYSDPYRIQIMNMFNKQGRPSTVKEIADQMG

EVPAKVHYHVKKLEKIGLLTIVSTREINGIIAKYYEPFTGEIHLRHEDEDKENSPLKQ

VFRSETLKLLNEMFEQSRQRFMHQAENEDRMFLSDITLYATREEVEQLYKNIIKLCEP

YTTKENRQGEQEVFQLFSALSKPIVKIPASKTNAKEKKKATSDKDKATTKKSRSVSKR

QESASSGSESEE"

CDS complement(3326335..3327624)

/locus_tag="EFAGFIKM_02826"

/inference="ab initio prediction:Prodigal:002006"

/codon_start=1

/transl_table=11

/product="hypothetical protein"

/translation="MSDNTAVGKQGMGELIRIRPYMQFMLSKVVSRFGDSIDSIAYSW

MVYILTGSKVLMGTLLAVNFLPNILLGLFAGALVDRMSPKKVIVITNTGRGLLVGITA

LLFGLGQLEVWHLFVITILISLLECFTTPAEVSSVPRLLPPSMLLSGNAMSSSATRVA

ELAGLAVAGALIATIGIAWTILIDAGLFALSALIMSRVGYPEVLTSANNEDKTSYEMN

SHPSDKSSIFSEMAEAFHFLRKHALLLIVSILFAFVNFCLMPFNVLRTPYVIETLHAG

AGGLSLLSGLMVGGMLLSGVWLTQRGANYRKSVLVISGIVMLGLSYAMTALPAYMTSF

QLPVAAAFCLLMGMGIPLATTPLATYLMEVTPSEMLGRVYALQSMLVLSVAPLGSLAS

GALADWMTMPVLFIVFGIMLAMSAGMLLFSKSFRSAL"

CDS complement(3327886..3328161)

/locus_tag="EFAGFIKM_02827"

/inference="ab initio prediction:Prodigal:002006"

/inference="similar to AA sequence:UniProtKB:P11540"

/codon_start=1

/transl_table=11

/product="Barstar"

/db_xref="COG:COG2732"

/translation="MNTVILDGEDFASSVELHQQLKVKLKLPDFYGGNLDALWDCLTG

TIELPLELKWTNYQISEERLGNEAGRVRDLMLEVQAEQAGFQLLVEK"

CDS complement(3328176..3328616)

/locus_tag="EFAGFIKM_02828"

/EC_number="3.1.27.-"

/inference="ab initio prediction:Prodigal:002006"

/inference="similar to AA sequence:UniProtKB:P00649"

/codon_start=1

/transl_table=11

/product="Ribonuclease"

/translation="MKKLISTALLALLLTFVFLTGSQFTPVAQQKASAATCTIINTFT

GVANYLSANGTLPCNFITKSQATGLGWVASKGNLAVVAPGKSIGGDTFGNREGLLPSA

SGRTWREADINYTSGFRNSDRIVYSNDGLIYKTTDHYASFTRLK"

CDS complement(3328885..3329892)

/locus_tag="EFAGFIKM_02829"

/EC_number="1.1.1.384"

/inference="ab initio prediction:Prodigal:002006"

/inference="similar to AA sequence:UniProtKB:B3TMR8"

/codon_start=1

/transl_table=11

/product="dTDP-3,4-didehydro-2,6-dideoxy-alpha-D-glucose

3-reductase"

/translation="MNRQLQWGVLGTSTIAKNAVIPAIQQSERGEVLAIASRSKEKAE

TLAEELGIARSYGSYEELIADPDIEAVYIPLPNHMHKEWTIKAAQAGKHVLCEKPAAL

NADESTEMIEVCHQQGVLFAEAIMYRYHPKHRRVQEIIASGEIGIVRAIHGNFTCNTA

DDKENVRFKREMGGGSLFDLGVYPISAARMYLGQEPEAVTVHALFSDEHDGVDMMASG

LVEFPNSVALTFDCGMWASGRAEMEILGTEGRIELPKVFGWENSDIPPQIIVHTDSVS

REERVSVSNSYVLQVETFATAVLEGEALPFSPENTIQNMRVIDACLESARTRQRVQLI

D"

CDS complement(3329895..3330269)

/gene="rtp"

/locus_tag="EFAGFIKM_02830"

/inference="ab initio prediction:Prodigal:002006"

/inference="similar to AA sequence:UniProtKB:P0CI76"

/codon_start=1

/transl_table=11

/product="Replication termination protein"

/translation="MAFMIAQRAFIKLYLITMVEQHRGYGYEMLEAMKQEFKDYGYVP

PQSEVYRALHELVQQGVFYRTKKLKGNDPKVDFQEIVLYHFTDDGAEKAELYKKQVKT

DLDRCLGMLHKAEEDNYGTKGR"

CDS complement(3330783..3331931)

/locus_tag="EFAGFIKM_02831"

/inference="ab initio prediction:Prodigal:002006"

/codon_start=1

/transl_table=11

/product="hypothetical protein"

/translation="MPRKERITEIESLRGIAFGAVVLQHSIAHYSLVPETRLEDGVLL

AILLMLSKFAVPLFIFITGMVLFYNTGDKLHYGQFMRKRVNDVIVPYLIWSLIYFTLA

PRGWTGFGWNDIPDLGLKLLTGKTTSHFWYIIMLIQFYLLFPLFLHAIRYVYNRYEAK

GRLIALLISGVVYLVLADQLRNIAKFMERLNIPVLTDAFTTYADRNFLYFFIYFVLGA

VAGLSVQHWNEWIHRLRWIYWTVFIVLGLRFTYLLMLEFQKPEGIKITFYTVSLIRPD

MALFLIASIMVMYQLAGKLHNLRATRLLGWIGGVSYGGYLMHMLMLRYSYIPDELFYV

AMGWNPVVRMIITWLLALTLSCVVTWLISRVSWGKWIVGAVPKSVRNK"

CDS 3332006..3332398

/locus_tag="EFAGFIKM_02832"

/inference="ab initio prediction:Prodigal:002006"

/codon_start=1

/transl_table=11

/product="hypothetical protein"

/translation="MIHISSTDKDVTTTDSKYVQQHLANERTFLAWVRTGIAMAGIGF

LAAGFGFNSSAYDQLAHIAAIITGITSLIGGISIIVYSAAAYHRKRTQINEQTFQAST

GLIRFLTMMLLVIGLALAVLLYVLLFPA"

CDS complement(3332576..3333181)

/locus_tag="EFAGFIKM_02833"

/inference="ab initio prediction:Prodigal:002006"

/codon_start=1

/transl_table=11

/product="hypothetical protein"

/translation="MQKKIVGVFNTEREASQAIEGLKAQGFTSDEISVVTQDRDELKA

IREETGTKAPEGVAAGAATGGVLGGVAGLLAGIGALAIPGIGPILAAGPIAAAFTGAA

VGAGAGGLVGGLVGLGIPEEDAKQYEEYVQNGKILLLVDSTDRDSDVYDVFSGNSQLN

RDRAEAAHRGDVPAERPDLDMEERRLEAQTKATRLGNNTFL"

CDS complement(3334047..3336566)

/gene="hrpB"

/locus_tag="EFAGFIKM_02834"

/EC_number="3.6.4.13"

/inference="ab initio prediction:Prodigal:002006"

/inference="similar to AA sequence:UniProtKB:P37024"

/codon_start=1

/transl_table=11

/product="ATP-dependent RNA helicase HrpB"

/db_xref="COG:COG1643"

/translation="MHIELPIQEVIPELRQLFRYQDTGVLIAEPGAGKTTVVPLALLE

EPWVAGRKIIMLEPRRLAARSAASRLAATLGEKAGQTVGYRVRMDTRVSQATRIEVVT

EGVLTRMLQQDQGLEDTAMIIFDEFHERHLHGDLGLALTLESRAMLRPDLKLLVMSAT

LDPVPVCVLLGEGTKSIHCPGRTFPVETRYVPRPAAMPLEQFTAQTVNRALAEQEGDV

LVFLPGAREIHRTERELSNGSLPGHVKVHSLYGSMPVEQQDEAIRPAAEGSRKVVLST

SIAESSLTLAGVKIVVDAGLSRASAFSPRTGMSRLVTLPVSKASADQRRGRAGRIAPG

VCYRLWSEEVHGGLPEAARPEITSADLAPLALELAVWGVQSPAELQWLDTPPDAAYGQ

AQALLRQLGGLDDAGRITPFGRRMNTLGVHPRLASMLLRAAELGLASYASMLAALLQE

PAGLRSSGSAGAGTDLRPRVEALLTADSSGMPQAAAAVADGAAVRRMLQESRQLRSAL

GPAADPVRPDEDSCGLLLSFAYPDRIGQRREDGRYLLSSGRGVRLAATESLSRAAYLV

AAEADDQGADARILLAAPLQEQTLLQAGQHLLHDEVQVAWDSNTRSVRAHKRLRIGAL

VIKESSIAQPPEDQVLEALLTGIRMEGLDCLPWTKTSRQLADRLRFLHLHLPEWPDLI

EDELTEELAEHLTPYLTGMRSAADLKRLSMQDVLLGGLSWTQRQQLDDEAPTHIQVPS

GSRIPVDYSRPEDPALAVRLQEMFGQQETPRIAGGRVPLTIHLLSPAQRPVQVTRDLA

NFWRETYFDVKKDLKGRYPKHYWPDNPLEAVATNRAKPRGK"

CDS complement(3336711..3337517)

/gene="lvr_2"

/locus_tag="EFAGFIKM_02835"

/EC_number="1.1.1.-"

/inference="ab initio prediction:Prodigal:002006"

/inference="similar to AA sequence:UniProtKB:Q9LBG2"

/codon_start=1

/transl_table=11

/product="Levodione reductase"

/translation="MTQHNGKSRFQGKVAIITGAGSGIGKATAVKLAKEGAHVALFDL

VNDRISETEAEINALHPGAARAFDVDISDPARVEKAVLETVELFGGLDIVFANAGING

VSAPIEEIQVEDWQQIITTNLNGTFFTIKYALPHVKKRGGGSIIITSSINGNQRFSSF

GMSAYSTSKAGQVAFAKMAALELAKFKIRVNVICPGAIATNIDQSTVKTDDLQQIIIP

MEFPEGQQPLADGPGQPEHVADLVSFLASSESRHITGAEIVIDGAESLLS"

CDS complement(3337701..3338543)

/locus_tag="EFAGFIKM_02836"

/inference="ab initio prediction:Prodigal:002006"

/codon_start=1

/transl_table=11

/product="hypothetical protein"

/translation="MRAMRLATLSAGDKGGHAEQRHGNFRYVSVQTGEQPLTRYQGTF

KCRYGYGRAAETVHQGDTGQDFAAVRMKGNVCNFVLCDGVGMSYLGDFAARFLGNALL

DWLETTQHTTVEGVERLLRDLTLPASEQLEKLQPLESSPLLLREVLMEKRSRGSQAMY

VCGRIELTGGGRKSRVWLAWQGDSRIRLWRNGQEQSELFQTHCKTNERWSTLEGPVGG

KPHICEMKGSAGDSLRLQLYTDGLNDLDAIQAYVPDEHIQVLLDATHTGGLEDDAAFI

ELEW"

CDS complement(3338554..3339306)

/locus_tag="EFAGFIKM_02837"

/inference="ab initio prediction:Prodigal:002006"

/codon_start=1

/transl_table=11

/product="hypothetical protein"

/translation="MNYTIQASQRTPALIIYLIDISASMNMVLENRRRIDVVYDALSL

AIRQMVFRSTKGNRLTPRYRIAILAYSDDVYDLLNGIKGIDEIAAVGSLPDLTPRRFS

DSAKAFLQAEKILQAEIPNMQDCPAPLVCHMTDGVATGEDPEPIAKRIMGMSVPDGNV

LVENIFISDHLLEGPIAEPRRWKGISSETNLQDEHGEKLRNMSSVLPESYREMLVEAD

YLLAPGALMMLPGTCAELVSIGFQMSAATPVR"

CDS complement(3339340..3341415)

/locus_tag="EFAGFIKM_02838"

/inference="ab initio prediction:Prodigal:002006"

/codon_start=1

/transl_table=11

/product="hypothetical protein"

/translation="MSFQPNPGDEVVINDIAYTIGQHPAAPGLAYAQAGRQGIVYQLL
PRNGSIHGAKALKVFFPKFRIPAMVYQSEHMESYSELPGLQVCKRDVLTPERNGALIG
KHPDLLYAVLMPWVQGQTWFDVISDQRQLTAEESLKLARALAGTGSAMEQRGLAHCDM
SAPNVMIPFFSEVENLGKMSAVELVDVEQMYGSKMDRPDALLAGSPGYAAHRTVHSGL
WSSYADRFAGAVIIAEMLSWSDPVIVEKAWGESYFDQHEMQTVSERYYAMRESLEKRW
GSKLSDLFIRAWESHDLSSCPTFGEWYVALAAIDVDQANAAAAVTAEEEAANSSEADV
NLKKSSEPVSEPVNTQDGNRSQTPHSPDQEAVVNRLFLQARALEDEDKPAAALEVYRS
LYHFIPHNSAMQMEVEAAIKELDARLNPKEDTDKPVPVPFYRSKKFMISSAVLIVLLA
GGVPTVKILADQAEVKQKEQQEATRLADIKAAEEAEAAKAAALKQQEEQEKQKAADAA
KLDAEEKKKLAEAKQKEAEAKKKEEERKALQAKYDKQAKYEAFLVKQEQQKKEAAKKA
EQEKYDKQAKYEAYLVWKKENDAKLAKQEADRKAAAEVKRQQEIAQKAALKKKRAQNV
VTLIAHYNKTYNAQKGRKIENAESYARDFKNLYNTDAAYFKGVGKVAARMSAINKFLS
NNSYTLPNL"

CDS complement(3341574..3341849)

/locus_tag="EFAGFIKM_02839"
/inference="ab initio prediction:Prodigal:002006"
/codon_start=1
/transl_table=11
/product="hypothetical protein"
/translation="MAGRILVTPEQLDQVSNQFKQSGEQSQQIVSTLTQSITSMEGQW
EGMTKQRFFQEFQEASKQMQSFVQTLNSISAELTAIANKFRTADQAR"

CDS complement(3342036..3342584)

/locus_tag="EFAGFIKM_02840"
/inference="ab initio prediction:Prodigal:002006"
/codon_start=1
/transl_table=11
/product="hypothetical protein"
/translation="MRIRVEPDVLRALSKQIQYAAEQIQQKMTVLDQAIHSLEWEVES
RAAVMNEWNHSKRLGEDALRRLMDLSVQLGRKALLFQQADMEYRSVLSHVNTAYGNAV
NMLNVLQNNRTGEIMPAHSATVAVVSDPLSAMAAVYRVQDAAPPDGSPATLVQAMQPE
PVAWRFTDPSFRGRRGTEPVVS"

CDS complement(3342591..3343421)

/locus_tag="EFAGFIKM_02841"

/inference="ab initio prediction:Prodigal:002006"

/codon_start=1

/transl_table=11

/product="hypothetical protein"

/translation="MLTVHDSKQKVVTLQTKELFFLAGILGSDRLLGVEDPFRGYMAE

DIAMEWERVKTSLLDKGYLIRDQDTDELIMTPTVFSRVAIAGLSDRACWIRYTINGKS

YESYIHCTDERVVEVSRVDDVPDSFRLSDLGSVREAIDVLIERMKWSGHSPAEKPALM

CSKKKFYDVMNELENSGVQTVAAELEQETSDPEGSLALARCLVGKESDGELRLLVWNE

DGWKSQSAAFAASAVSNWLFRMSTAASDDWLVAALTTREQFHEMLLDWLKQPAGEEER

"

CDS complement(3343438..3347442)

/gene="essC_2"

/locus_tag="EFAGFIKM_02842"

/inference="ab initio prediction:Prodigal:002006"

/inference="similar to AA sequence:UniProtKB:Q2G184"

/codon_start=1

/transl_table=11

/product="Type VII secretion system protein EssC"

/db_xref="COG:COG1674"

/translation="MNVLYQRSPRMTPVLKEERLEILRPPTEPSKPTFSMISIIVPIM

MTMVSIGFYVYINMTGKMNNSSYMMFQMMTVMMMLTSYTIPFFVYLSNKKSYRKKLEE

RKIMYLAQLDKHREELKEAQAEQVKSLYEIHGDPGVCLQVVKNRNSSLWERSPEDDDF

LQVRIGTGEIPFRIKLQVPRIDGYEKDELIEAAHELAAEFQTVPDASITLPLFQSKVM

GLVGDREEVLTSLRVIISQLTVRHSPDELKLAAFYEEKDSKEWDWLRWMPHIWDEDQG

QRYMADRHSGAHQLADSLFSVLNRRRSNKEDRYKKSVQTPCYVVILSDTQLIEEEPLL

PLLLESAHEIDVCTIILANRKESLPMHCQLIMDASKGKGVYIKKTEDADVIQQTYKPD

VISKEMTEALSRYMSPIRLKRSSASDIPQVLPLFDMLSTSRVEDLDVVSRWGQTRYPD

TLPVPMGVRAGGKKIAINLHDKIERQGHGPHGLIAGTTGSGKSEVIQSIVASLAAEFH

PHDLAFMLIDYKGGGMSNTFVDLPHVVGTITNLDGNLIERANISLRAELVRRQKILND

AGNLQHIDEYYKILRSRHEQPLPHLVIIIDEFAQLKRDQPEFMDELISIAAIGRTLGV

HLILATQKPAGVVDDKIWSNSRFRICLRVQSEGDSRDMIKIPNAAWITKPGRGYFQVG

SDEVFEEMQFAWSGAPYNQQEDSTTVLPVMEVRLNGKREPLLTGERRAVLKGEDVPKQ

LQVFIDYVAQSAADAGIRRLPGPWLPPLPETLEWEGLQEWQEEENRDLLLDGGASGLK

PLVGLLDDLPNQRQQPLALPVDQGHLVVYGMPGLGKTTFVQTLLMSLARSHRTEPWHG

YIIDMGRMMKDFAALPQIGGVMMAEEEDRIKRLFRYILKLSAQRKDIISEAGVKTISA

YRRTAHAAVPQVVVVIDGYLSFRNAYPEENELLETILREGGSLGITFVLTANRVTDVF

EKFRSNIPNAVSFELSDPSDYYYAVGRPSKAPSQLPPGRGLVKGQVPPLMFQAALPSS

GADEGKRSSALRRTIAEIRQGWTGEEAPQIAPLPEEIQLKDLLIRTGSYGQAWDTSSV

TVPVGLLTDDLEPFELNLREGPHFMVTSPMEGGKTTFLLTWMLSLAYHASPEDVQIYT

VDMRYGSGGLGEISSLPHVRGHVSREEQLAPVIQQLYDEVLKRGEISGGPELVLVIDD

ADTLSKQLNDFNVKDQLGAIVRQGRDRDVHVILSGVPADFPTFGSDWVTDVKASQSGM

LFGTLDPNDLSFFRIPYSESGGSSGGLKVLPPGQGYYVKRKYSRVKGAVPCDDSWKMT

DWVSEIRDRWHVVV"

CDS complement(3347497..3347817)

/locus_tag="EFAGFIKM_02843"

/inference="ab initio prediction:Prodigal:002006"

/codon_start=1

/transl_table=11

/product="hypothetical protein"

/translation="MILTVYSGREEGEWFDIDVPDECTIEHLKTLLGVRIFGQAPGDG

IQYILEAKFPEGLWFSPQNAQQLMETGLRQGSCVRVQRAFSTTSEEAPVYGRRSLFQQ

DSNE"

CDS 3348239..3348478

/locus_tag="EFAGFIKM_02844"

/inference="ab initio prediction:Prodigal:002006"

/codon_start=1

/transl_table=11

/product="hypothetical protein"

/translation="MRTLITFQNKSIPVYFNQENKQPMQKTLRLLSSALEHKISNGKR

AIQKCLHSLISIEIVNGEAILHSRSENDSLALSLY"

CDS complement(3348601..3348753)

/locus_tag="EFAGFIKM_02845"

/inference="ab initio prediction:Prodigal:002006"

/codon_start=1

/transl_table=11

/product="hypothetical protein"

/translation="MSIDRFILRKLNTCQEEHTRANLVRLFVIRIRKAEIAEEHDAVY

VLSKLH"

CDS complement(3348998..3350473)

/gene="pchA"

/locus_tag="EFAGFIKM_02846"

/EC_number="1.2.1.96"

/inference="ab initio prediction:Prodigal:002006"

/inference="similar to AA sequence:UniProtKB:Q59702"

/codon_start=1

/transl_table=11

/product="4-hydroxybenzaldehyde dehydrogenase (NADP(+))"

/translation="MTLMDTEHTTWTKQYINGSWVNGSGEKTMENINPYSGEVIATWR

SSNKKDIDKAYESAQKNSIEWAKSLPAAKEEVLRKVSSLMAERKEEIIQLLITESGST

RIKSEAEFAAAKRIVDEAASFPYRMKGEIIPSNTPGKENRVVREPKGVIGVIGPWNFP

LHLCLRSVAPAIALGNGVVIKPASDTPITAGWLIADLFEQAGLPEGVLNVVAGSGSEI

GDYFVAHPVPKVISFTGSTEVGQGIGKLAGEHLKETALELGGNNAMVVLEDADIESAA

EAAVFGKFLHQGQICMALNRIIVHADIYDQFVESFVAKTKNVQAGDPADANTLVGPLI

REKEVERLQELVNKTKAEGARLLLGGTSKGSVLSPTVLADVKPEQDIVQQELFGPVAV

IMKAHDEQEAVRLANDTPYGLSGSVFTKDLNRGYQVAQRIESGMVHVNDQSVNDEAHV

MFGGEKASGIGRFGGDWAIEKFTRTRWISIQHQYREYPGVK"

CDS complement(3350644..3351183)

/locus_tag="EFAGFIKM_02847"

/inference="ab initio prediction:Prodigal:002006"

/codon_start=1

/transl_table=11

/product="hypothetical protein"

/translation="MLIRQAREGDAQGIAHVHTESWKTTYRGIVPDDFLDHLTIESRL

SQWEKTIRSGEKDQILVVAEQDDGNIVGFACGGKEREGKLPYDGELYAIYLLKEVQQT

GIGQQLATHVVHHLQTHNMKRLIIWALERNSACRFYEKMGGTPVQTQSLRIGGQDLME

VGYGWEDLSLFGEKSLPDC"

CDS complement(3351346..3351684)

/gene="ykuD"

/locus_tag="EFAGFIKM_02848"

/EC_number="2.-.-.-"

/inference="ab initio prediction:Prodigal:002006"

/inference="similar to AA sequence:UniProtKB:O34816"

/codon_start=1

/transl_table=11

/product="Putative L,D-transpeptidase YkuD"

/db_xref="COG:COG1376"

/translation="MPDYRIIVDLSDHMLYLLDGNQVIKGYPVATGKMLTQTPNGEFT

IINKQSNPGGPFGVLWMGLSAPHYGIHGTNEPWSIGKSVSHGCIRMYNSDVLDLSSKV

SVGTRVTIRP"

CDS complement(3351741..3352886)

/gene="rsgA_2"

/locus_tag="EFAGFIKM_02849"

/EC_number="3.6.1.-"

/inference="ab initio prediction:Prodigal:002006"

/inference="protein motif:HAMAP:MF_01820"

/codon_start=1

/transl_table=11

/product="Small ribosomal subunit biogenesis GTPase RsgA"

/translation="MFTRLERSKQLILGGIFILDNHIEKYGWSAKWQELWQETGMEEQ

EKKPARIIADHGHLQRLITEEGECWGRISGRMRHDSLETSLVPAVGDWVAITGQAGEE

AIIHNLVPRRSRVSRQAAGPVTKEQLIAANVDTLLIVAALNHDFNLRRLERYVMMAWN

GGVRPVIILSKSDLCNNVEEQIRSVEGIASGVEVLAISAVEGQGKSLLERYLQPGLTV

ALTGSSGSGKSTLVNWMMGEDVQLTQSVREGDSRGRHTTTHREMFVLPQGAVLIDTPG

MRELNLWDEGNDGLSHAFGEIEELAATCRFLDCSHTREAGCAVKEAIQNGSLDEKRLN

NYLKMQKELQYQQRKEEVASRRRTASSKPANSRKPKHSRSVKEWEEA"

CDS complement(3353299..3353892)

/locus_tag="EFAGFIKM_02850"

/inference="ab initio prediction:Prodigal:002006"

/codon_start=1

/transl_table=11

/product="hypothetical protein"

/translation="MQLEKIVAYHKALADPTRLRMLLLLAQGELHGQALAERLNLSQP

TVTHHASKLREAALIKERREKNTVYFTLNPSFIRENAQASVDFIFNREEVEDMSDVNE

TLKASVMRNFFSKDGRLRQIPAQYKKKLIVLGHLVEQLEFGRKYTEKEINTFIKQYHE

DFATIRREFIMHQFMYREEEIYELNPKEMWTRWDQVK"

CDS 3354071..3355069

/locus_tag="EFAGFIKM_02851"

/EC_number="1.18.1.2"

/inference="ab initio prediction:Prodigal:002006"

/inference="similar to AA sequence:UniProtKB:Q8KCB2"

/codon_start=1

/transl_table=11

/product="Ferredoxin--NADP reductase"

/db_xref="COG:COG0492"

/translation="MEDVIIIGGGPCGLSAAIECERLGLSAVIVEKYNIVQSIYLYPT

HMQFFSTAPLLEIGNVPFSTPNDKPFRYEALAYYRRVAEHFGLRVHNYEEAREIKRKD

DGTFEVHTLNRRGEAIVHVGSNVVVATGYFDHPNYLGIPGEDKDKVTHYFREAHPYTR

TRVAIIGGSNSAVDAAMELVRVGAHIDMVYRGSGLSQHIKPWVRPLFESMVTKGHITL

HLESRVNEILDDSIRLIHTDGSTRELDNDFVLAMTGFRPDRQLLTSVGVEMSDDMDKP

LYDAQTMESSVPGVYVGGVIASGRNANEVFIESGRWHGRYIAEHIVSKQRGQTEGK"

CDS 3355066..3355518

/locus_tag="EFAGFIKM_02852"

/inference="ab initio prediction:Prodigal:002006"

/codon_start=1

/transl_table=11

/product="hypothetical protein"

/translation="MKTMDMTSLLLLVFAALGIISSNTPVTVAMVFLLLLRVLNLNQA

FPWLEKYGLTLGIIILTIGVMAPLASGKMSLQTIGESFLHWKSLLAIGVGLLVAYLGG

RGATLMGTQPTVVAGLLIGTVLGVALFKGVPVGPLIAAGILSLLLGKS"

CDS 3355935..3356447

/locus_tag="EFAGFIKM_02853"

/inference="ab initio prediction:Prodigal:002006"

/codon_start=1

/transl_table=11

/product="hypothetical protein"

/translation="MSRQFITEAVMVAIYGQLLAPPAPVEYIVPYTTILELYEFQTSP

DPMMDNPADDQHVKSKINEMISYFEEPLNKKKIERALLVPWAKSPSILFGDQVSIAII

NAIDTAQYGEYFDPIETELLLTSQRLSIPILTDQVELIARIIESESPVQVFDIDDFDF

AMDDEPLDQV"

CDS complement(3356547..3356996)

/locus_tag="EFAGFIKM_02854"

/inference="ab initio prediction:Prodigal:002006"

/codon_start=1

/transl_table=11

/product="hypothetical protein"

/translation="MNLPPRDLAQYIAKRSHIVVKAAVRAENEQGELLLIQHAEHGHW

RLPAGEMRPGEAIEDAAHRELWEETGWTADTMTLEGLYSGPDLRYLHSSGDEEYYVIA

LFRTVIIQDDLVESRVNSDAGLKFFALESLPPLNQISRILLARDIAE"

CDS complement(3357073..3357369)

/locus_tag="EFAGFIKM_02855"

/inference="ab initio prediction:Prodigal:002006"

/codon_start=1

/transl_table=11

/product="hypothetical protein"

/translation="MSTIHVSDQAARWYKEELNLNEGDSIRFFARYSSGGGLHPGFSL

GIAVEKPRHPADQTEVSGIQFFMEDHDYWYLKGHQLHVDIVDEGQDIEYRYTEV"

CDS 3357855..3359600

/gene="yheI_2"

/locus_tag="EFAGFIKM_02856"

/EC_number="3.6.3.-"

/inference="ab initio prediction:Prodigal:002006"

/inference="similar to AA sequence:UniProtKB:O07550"

/codon_start=1

/transl_table=11

/product="putative multidrug resistance ABC transporter

ATP-binding/permease protein YheI"

/db_xref="COG:COG1132"

/translation="MFSVLKNLAWFFRLERKRYLTGVILLILVGIAELLPPRLLGNAI

DEIVRGSITGTSLTRYILLILGTVIIIYLVTYVWMHKLFGGANLVERLLRSRFMDHLL

RMTPPFFEKNRTGDLMARATNDLRSIATTAGFGMLTLTDSTAFLATVLFAMGFLVSWK

LTLAAILPLPFIAIAMMIYGKAVHQRYTLAQDAFGDMNDQVLESIAGIRVVRAYVQER

LDEKRFADVTEDVYRKNLAVARMDALFEPTIRLFVGLSYVIALAYGIYLVFHNEITLG

DLVSFNMYLGMMIWPMFAIGELINLMQRGSASLDRVNETLSVVPAVQDVEQPAHVTNP

EEIAMQDVTFRYPSSTVDNLSHISFSLRRGQTLGVVGRTGSGKSTLLKQLLHEYPAGS

GTLSISGHPIQDIAKDDLHSWIGYVPQEQVLFSKSVRQNIQFGKPGASDEFIMEAIRT

AAFDGDLGTLSDGLDTLVGEKGISLSGGQKQRVSLARAFIANPDILILDDALSAVDAR

TEAKIIENIRNKRSGKTTLISTHRLSAIEHADRIIVLEHGKITEEGTHQELLAMNGWY

REQYERQQVESNLST"

CDS 3359613..3361703

/gene="yheH_1"

/locus_tag="EFAGFIKM_02857"

/EC_number="3.6.3.-"

/inference="ab initio prediction:Prodigal:002006"

/inference="similar to AA sequence:UniProtKB:O07549"

/codon_start=1

/transl_table=11

/product="putative multidrug resistance ABC transporter

ATP-binding/permease protein YheH"

/db_xref="COG:COG1132"

/translation="MKSNTGKRLLDYALKAKGTFIAALIMLTIGVAAELAGPFIAKSM

IDNHLLAIEQPFYETTASDEAAVYNGKNYKREGLFEPDENKGSEVRVLQAGRSFYFVN

EPVSAPDGDRTYADGTLTVTRAGEVTGQYAATPLSAGELFAFYKPEMPSIYQLIVYYM

IFLVISVIMEFGKTFWLQSSANKVIQRLRNDVYAHIQRLPVHFFDNLPAGKVVSRVTN

DTEAVKDLFVAVLSNFFSGIITITGVYVALFLLDVRLGLISLFVVPMLITWIVLYRKF
ATRYNTIIRSRLSEINAIINESIQGMSIIRVFRHQKQTKQEFEDLNDDYMKHQNKMLN
LNAFTSHNLVNVLRNIAFAVVLWYFGSSVLDGTSIISLGVLYAFVDVLGRLFQPITGM
VNQLANLDSSLVSAGRVFELMDEKGEAVTDGSMPRYKGNVVFDDVSFAYKKDYVLNNI
SFKASQGQTVALVGHTGSGKSSIINLLFRFYDPQKGKITIDGQNVKDLPKQWIREHMG
IVLQDPYLFTGTIASNVSLGDEKISRERIEQALRDVGAERILAHLPQGFDEPVIEKGS
TLSAGQRQLISFARALAFDPAILILDEATANIDTETEALIQNALEVLKKGRTTFIIAH
RLSTIRSADQILVLHRGRIVEQGAHDELMELKGRYYKMYQLQQGAQAAASATSENPSG
EIIPTSTSFAGGNA"

CDS complement(3361886..3362512)

/locus_tag="EFAGFIKM_02858"

/inference="ab initio prediction:Prodigal:002006"

/codon_start=1

/transl_table=11

/product="hypothetical protein"

/translation="MTSKRITERWILMTVLAVVLVGILWTVVTAVRISAYTDEDSSRI
SDAAIILGAAVAGDNPSPVFRERIEHGIELYRQGTVRNLLFTGGSSGDGEHTEAVVGQ
KYAIAHGVDPADIRIETKSRITEENLVNSIPIGEQAGYQTYTIVSDPLHMKRAMKLAA
GLGMDAVPSPTRTTAYRTWRSKFPFLARETVMYMGYTIKGWIDNPQQP"

CDS complement(3362509..3363054)

/locus_tag="EFAGFIKM_02859"

/inference="ab initio prediction:Prodigal:002006"

/codon_start=1

/transl_table=11

/product="hypothetical protein"

/translation="MQNTDIQNGDMLDSEHSRELFAYYGLAVYYGQALEQQLVNLILL
MKMSQGKAVSEEDLEDLYERKMSSSLGQLIHEVRHHFTFSEEETRQLNELWQQRNRIV
HHYFKERIHETFSPEGRSRMIKELEDFKDRAQELEIRLQQYTGAWIAELGLDAESAAA
LQTLERMHTESMQARALEEDG"

CDS complement(3363154..3363864)

/locus_tag="EFAGFIKM_02860"

/inference="ab initio prediction:Prodigal:002006"

/codon_start=1

/transl_table=11

/product="hypothetical protein"

/translation="MIYLTRYLLRHHIRSQKYFAPVIFYIITMLLIYSYKPNPIADSY

SVTAMLLFLAAAWLGVMVMNTEPAKQYQLLVLHAGSRRKVVTSQLICAWIMQFMLTAI

TVLYPVFAGMFERQPTGEEWMMTWLGHLGLSLLGLGISVFFQKSYIPLLSRSMPALII

VLLLSFIQGSLSERLPESLEWLGRILPPAFYLVGEMMLFDESKVDSFVSVMLWTVLYA

LGLLIIHVWLSGRRDLRS"

CDS complement(3363861..3364916)

/gene="btuD_8"

/locus_tag="EFAGFIKM_02861"

/EC_number="7.6.2.8"

/inference="ab initio prediction:Prodigal:002006"

/inference="protein motif:HAMAP:MF_01005"

/codon_start=1

/transl_table=11

/product="Vitamin B12 import ATP-binding protein BtuD"

/translation="MTWSTRGLFKKSDQATLDPSGRGILLQQVSKRLGNAQVLDEINL

EVKQGECAVLVGRNGSGKSTLLRMLAGILLPDTGSIQRTMKGSDGYAVDSLPRLPFTS

GEYLWDMGRIRGIRPEILRERIRELSELLYLDTAIDQKLPLLSKGTLQKVNLIQALLP

GPGGLLLLDEPLSGLDIPAQEAVVSLLGQWKDEGTSIVTACHEPLLIERLADQVIVLK

KGRVLRYWSREDILQAGEPVVRIQSLTEAEEDLANDLSIVQQPGVLSLVRNTSSGISN

VWLWDWKVVQQSTDDVLRIILASGGSVVSVQQEESQLHMESLLEGQHPAVHASLRGVT

ESVDLSSSMDDAGGEAK"

CDS complement(3364924..3366654)

/gene="argS_1"

/locus_tag="EFAGFIKM_02862"

/EC_number="6.1.1.19"

/inference="ab initio prediction:Prodigal:002006"

/inference="similar to AA sequence:UniProtKB:Q54869"

/codon_start=1

/transl_table=11

/product="Arginine--tRNA ligase"

/db_xref="COG:COG0018"

/translation="MLMKQAAADIAPLTGLNEQEVLRLLEVPPQAEMGDAAFPCFALA

KSLKKAPAVIATEFATGLQASGIEATPAGPYVNIRFNREKLAPNLLKELGNAGFGKLQ

LGQGSRVVIDMSSPNIAKPFGIGHLRSTVIGAALYRLYNEAGYTSVSVNHLGDWGTQF

GKQIAAYKRWGNDEALQTDPIRTSLELYVRFHDEAENDPSLEIEARDWFRKLEQGDDE

AQRLWAFFVEVSMKEFNRMYERLNVKFDHTLGESFYNDKMGAVVEELKAKGLLEESDG

ALVVRLEDENMPPCLIIKKDGTTIYPTRDLATAVYRHEVMKADRLLYVVGGEQKLHFR

QVFAVLSRMGHEWSAKCEHIPFGLMRFEGRKMSTRRGKVIFLQEVLDEAVARALQIIQ

EKNPNLENPQKVAEAVGVGAIVFGDLRNSRLNDVDFSLEDAVSFEGETGPYVQYTHAR

IQSVLAKAEEAIRVENENPMPKPNLAEGENNTVTTPSLIGDTSWALLKLLGDYPEYLE

KAIHRNEPSVIAKYTIDVAQAFNRFYHAERIADAPSDVRSFRIALAERTAERLAHTLY

LLGVQAPERM"

CDS complement(3367190..3367525)

/gene="yhjQ"

/locus_tag="EFAGFIKM_02863"

/inference="ab initio prediction:Prodigal:002006"

/inference="similar to AA sequence:UniProtKB:O07571"

/codon_start=1

/transl_table=11

/product="putative cysteine-rich protein YhjQ"

/db_xref="COG:COG1145"

/translation="MTQQQYQQCIDACLECMNACNVCYISSLKEYDLAMLRDCIRLNR

ECAEICSFAAQAMTRGSDFIAEICELCVKACEACAAECGKHHHDHCQTCAEACRRCAE

ACRLMAAVA"

CDS complement(3367599..3368717)

/gene="yciC_2"

/locus_tag="EFAGFIKM_02864"

/inference="ab initio prediction:Prodigal:002006"

/inference="similar to AA sequence:UniProtKB:P94400"

/codon_start=1

/transl_table=11

/product="Putative metal chaperone YciC"

/db_xref="COG:COG0523"

/translation="MNETILKEQSIPVYILSGFLGSGKTTLLVQLIEHWQQQGLRPAV

VMNELGEVNLDGQIVDSTVPMTEMLGGCICCTVRGDLGLQLADLIQEESPDVIIIEAT

GAANPMEILDAVTETSLYMRLELKSLITVVDAAHLSGLYQEQKGQTFKLMQEQIRCAS

VLLLNKTDRVSAQELKDLEQLLAKWNGFAPVIPTVKCEVEMDLLLRSGVDVHVRDSAS

EAGKPAHQEHHVHTEACGTHGCNHGHEHTDHTVQGDATNTDASHSYSHDHGHEHSAPH

ASHEHVMVYTHYFSHPVNSEAFEQFVSALPRDVYRAKGILSFSDTASRFWFQYAYRES

DYMKITPQGDVPNVAVFIGEHFDQTVIRDQLLELEAIK"

CDS 3368889..3369407

/gene="moaB"

/locus_tag="EFAGFIKM_02865"

/inference="ab initio prediction:Prodigal:002006"

/inference="similar to AA sequence:UniProtKB:Q816R0"

/codon_start=1

/transl_table=11

/product="Molybdenum cofactor biosynthesis protein B"

/db_xref="COG:COG0521"

/translation="MTNSVEQHRQEAPTTVSCMIVTVSDTRTKDTDTSGQLMHQLLDE

AGYQIVEYIITPDETENIRSILQDAAVRDDIEAVLLSGGTGIAPRDTTYEAVSSLLDK

ELPGFGEIFRFLSYTEDIGSAAILSRAVAGTIGRTAVFSMPGSKGAVKLAMEKLILPE

LRHVMREIYKPV"

CDS complement(3369479..3370033)

/locus_tag="EFAGFIKM_02866"

/inference="ab initio prediction:Prodigal:002006"

/codon_start=1

/transl_table=11

/product="hypothetical protein"

/translation="MWFGRKGRSLAGRTMKSVLVSGVLITSIWSLVACSASSIEQERK

VYTREATDEGNMRAQNQGHGLNGELVSEISKSFESKGIQLMNNMSADDGHGGISYMYL

LNGSGRHIVKVHIFSDKAARAASMKQMYSEDRDEAVLENALGRTTIRSKGYVSLVYTA

SGGEKDKYEQDVMQVFQRVLGQLR"

CDS complement(3370108..3371127)

/gene="yhfP"

/locus_tag="EFAGFIKM_02867"

/EC_number="1.6.5.-"

/inference="ab initio prediction:Prodigal:002006"

/inference="similar to AA sequence:UniProtKB:O07615"

/codon_start=1

/transl_table=11

/product="Putative quinone oxidoreductase YhfP"

/db_xref="COG:COG0604"

/translation="MKNHFNAYVVRKEEQGGVKASIEQLKKEDLPNGDVTVQVQYSSV

NYKDGLATLEKGGVVREYPMVPGIDLAGTVEESVSGRFAPGDRVISTGFEPGVSHYGG

YSEYARLRSEWLVPLPPGLSEKEAMAIGTAGFTAALSVDALLQAGVTPEMGKVLVTGA

TGGVGSMAVAILAKLGFEVTASTGKKEQEEPLLRNLGASEVITREEADTPAKGAMGKQ

LWAGVVDPTGGPALAERLKQIQYGGAVAVSGLTGGTAFESTVLPFILRGIQLMGIDSV

YCPMERRERLWKLLGGEWKPERALELGIREISLGQLPLTLETILQGGAVGRTVVNTIS

DLPSA"

CDS complement(3371238..3371888)

/locus_tag="EFAGFIKM_02868"

/inference="ab initio prediction:Prodigal:002006"

/codon_start=1

/transl_table=11

/product="hypothetical protein"

/translation="MRWILGLVWIAFIAYALLLAPGQSPGSDPIFKELITMQSKEPWL

LTVFSWLGIFPAVYACMLLRTSAKERGRVPAWPFVILSFGLGAFALLPYYAWSSSANR

ASGYASLHFGRQRESGIGRVAAHKLTHVVLLLLTLGTAFYAVMQGHPDVYMEAFKQSA

FVHIMTIDFVLLTLLSMIAIYRDTEVSRRSAAWAAAGIIPIVGPLIYLLTLRRDRV"

CDS complement(3372286..3373740)

/gene="pncB2"

/locus_tag="EFAGFIKM_02869"

/EC_number="6.3.4.21"

/inference="ab initio prediction:Prodigal:002006"

/inference="similar to AA sequence:UniProtKB:P9WJI7"

/codon_start=1

/transl_table=11

/product="Nicotinate phosphoribosyltransferase pncB2"

/db_xref="COG:COG1488"

/translation="MQTTSLALHTDKYQINMMYAHWVNGTHKRKAVFEAYFRKLPFGN

GYAVFAGLERIVNYISQLRFTMDDIKFLSKQEENYDPVFLEELLQFSFQGTIHSMKEG

AIVFPDEPLIRVEGSIMETQLVETAILNFMNYQTLIATKASRIKQVAQQDTLLEFGTR

RAQEADAAIWGARASYVAGFDATSNMLAGERFGIPTAGTHAHSWVQSFMSEQEAFDVY

AKVLPDQVTLLVDTFDTLNSGVPHAIKTAKKLESQGKKMNAIRLDSGDLAYLSIQARQ

MLDEAGLDYVKIVASNDLDENTIFNLKAQGARIDTWGVGTQLITASDQPSLGGVYKLV

EREVDGAMLPTIKISANPEKVSTPGKKEVFRIIDPKHGKAIADYICYPEEEQPLQGGP

LKLFNPLHPYLKKTVTRYEAVNMLEPIFVNGRKVYELPNLDEIRAYHREQMNLFWPEY

QRKLNPEIYRVNISPAAWNMKQKLIAEHVKSTEE"

CDS complement(3373938..3374495)

/gene="rutB"

/locus_tag="EFAGFIKM_02870"

/EC_number="3.5.1.110"

/inference="ab initio prediction:Prodigal:002006"

/inference="protein motif:HAMAP:MF_00830"

/codon_start=1

/transl_table=11

/product="Peroxyureidoacrylate/ureidoacrylate

amidohydrolase RutB"

/translation="MKALIVIDFTKDFVTGSLPVGQPAVEIEETIAAVTEAYCKNKHE

VIMAVDLHEENDPYHPETVLFPPHNIRNTEGRQLYGRLAQVMEERKTDIQWMDKTRYS

AFCGTDLELRLRARGITDIALIGVCTDICILHTAVDAYNKGFHITVYEDAVASFNPAG

HEWALGHFRSSLGASVVKASETVLA"

CDS complement(3374563..3375366)

/locus_tag="EFAGFIKM_02871"

/inference="ab initio prediction:Prodigal:002006"

/codon_start=1

/transl_table=11

/product="hypothetical protein"

/translation="MSENYEHFNAESDEQAARAYSSQKYRTPDGVPADIVMFTLTKRE

RKTVTKTLPIRELKVMLIKRKGWPFAGRWALPGGFCQENESIYGAAKRELMEETGVDG

GHLEYLNVYSQPGRDPRGWIISHAFFALVEEWMLDQRQAADDAEDVGLFTIQEALQEL

ELAFDHRTIIEDAYRRIQQQMLETTIARQFLPRDFTLSELYQVIQSVVPDFEEPNFIR

KITSTRSRKGIVEEVRDEEGNLVSSNQYSQRPAQLYRFTELVPRLSIYT"

CDS complement(3375445..3375849)

/locus_tag="EFAGFIKM_02872"

/inference="ab initio prediction:Prodigal:002006"

/codon_start=1

/transl_table=11

/product="hypothetical protein"

/translation="MSILDKMVEDGDGRFWGFLGCRYIKGDGKEVQIALTAGEHHTNS

MGIIHGGVLTSLMDQAMGMVATAAMEVDGCVTTNLNVHFLAPMKQGELTVTATVLHQA

GRSVTTQSEVRDASGTLGCMATATFRIARPRT"

CDS complement(3376059..3376508)

/gene="ymcA"

/locus_tag="EFAGFIKM_02873"

/inference="ab initio prediction:Prodigal:002006"

/inference="similar to AA sequence:UniProtKB:O31779"

/codon_start=1

/transl_table=11

/product="putative protein YmcA"

/db_xref="COG:COG4550"

/translation="MAQEEVQYNHYGMPTYDTRNLVIRDDIMGKAKELADMLGTSEEV

RQFQQAETKIRDHERIQQLIATIKKKQKEIVAFESFKNVEMVSKIEQEIEDLQSELDS

IPLVTEFQQSQSDINYLLQLVISVIRDTVSEKVNVEAGTDSPPTSCG"

CDS complement(3376772..3378328)

/gene="miaB"

/locus_tag="EFAGFIKM_02874"

/EC_number="2.8.4.3"

/inference="ab initio prediction:Prodigal:002006"

/inference="similar to AA sequence:UniProtKB:O31778"

/codon_start=1

/transl_table=11

/product="tRNA-2-methylthio-N(6)-dimethylallyladenosine

synthase"

/db_xref="COG:COG0621"

/translation="MAKDSKKDYSQYFDFSDAKVISQDEFSKKIRIRGREINIKSEPN

HRQEKQRGKEDVQVLYENAVPDELKNIGKGKHYIVYTYGCQMNEHDSETIKGLLESMG

YQATENRKEADIILLNTCAIRENAEDKVFGELGHLKTLKLERPGLLLGVCGCMSQEEG

VVNRIMQKHGFVDMIFGTHNVHRLPHLIQEALFSKEMVVEVWSKEGDIIENLPKKREG

MRGWVNIMYGCDKFCTYCIVPFTRGKERSRRPEDVIAEVRDLARQGFKEITLLGQNVN

AYGKDFTDLNYSFGDLMDAIRQIDIPRVRFTTSHPRDFDDHLIEVLAKGGNLVEHIHL

PVQSGSSEVLKRMSRKYNREHYLKLADKIKKAIPDVVLTTDIIVGFPGETEEQFEDTL

SLVREVGYDFAYTFIYSPREGTPAAVMEDNVPMEVKKERLKRLNETINEYSHRSNEKQ

RGKIVEVLVEGESKRNSEVLAGRTRSNKLVHFEGPKELIGTFVQVEITDPMTFYIRGN

LLPEPVAANQ"

CDS complement(3378641..3379213)

/gene="pduL"

/locus_tag="EFAGFIKM_02875"

/EC_number="2.3.1.222"

/inference="ab initio prediction:Prodigal:002006"

/inference="similar to AA sequence:UniProtKB:Q9XDN5"

/codon_start=1

/transl_table=11

/product="Phosphate propanoyltransferase"

/db_xref="COG:COG4869"

/translation="MSKTVPVGVSARHIHVSQEHVEILFGKGYELTEFKPLSQPGQYA

ANETVAVIGSKGQFDKVRILGPVRPETQLEISMTDSFAIGVKAPVRESGSIEGTPGIT

IKGPAGEVTIDKGVIVAARHIHFHTSDAAKWGIEDKQMLKVRLGGDRGLVLENVLARV

SDSFALDMHIDTDEANAAGARNGDTAEIID"

CDS complement(3379407..3380408)

/locus_tag="EFAGFIKM_02876"

/inference="ab initio prediction:Prodigal:002006"

/codon_start=1

/transl_table=11

/product="hypothetical protein"

/translation="MSMSDWRVADFHCDALSKILMQPALSFENAPQLDANLQRLKEGN

VGLQAFAIYLPEVLGRGKFEHVMGQLEIYRRRVERSQERPGGTQTLLWREQVAQVGQT

EGPWGLITLEGVDGLEGNLFYLELCYQMGVRIIGLTWNYANWAADGVMEKRGAGLTEK

GKELVRQCNEIGMLLDVSHLTEKGFWELADLSKRPFIASHSNSYSVCPHVRNLKDDQI

QAIIAREGRIGLTFVPWFVKQEGEIKIEDLLPHIEQFCSLGGQHHLMMGSDFDGISTY

IQRLEHSGHYPKLTEILLKHYDEHLVRGWLWGNAMSYLGEHLPESNPKMQMKGNSMK"

CDS complement(3380494..3380754)

/gene="spoVS"

/locus_tag="EFAGFIKM_02877"

/inference="ab initio prediction:Prodigal:002006"

/inference="similar to AA sequence:UniProtKB:P45693"

/codon_start=1

/transl_table=11

/product="Stage V sporulation protein S"

/db_xref="COG:COG2359"

/translation="MDVLKVSAKSNPNSVAGALAGVLRERGNAELQAIGAGALNQAIK

AVAIARGFVAPSGVDLICIPAFTDIVIDGEDRTAIKLIVEPR"

CDS complement(3380872..3381666)

/gene="ymdB"

/locus_tag="EFAGFIKM_02878"

/EC_number="3.1.4.16"

/inference="ab initio prediction:Prodigal:002006"

/inference="similar to AA sequence:UniProtKB:O31775"

/codon_start=1

/transl_table=11

/product="2',3'-cyclic-nucleotide 2'-phosphodiesterase"

/db_xref="COG:COG1692"

/translation="MKVLFIGDIVGNVGRKALKENLPYLKSKYKPHVVIVNGENAAAG
RGITGAIANEFFNWGVHGITLGNHTWDNKDIFDFIDDEPRMIRPANFPPGTPGRGYTV
VKGEGKELAIVNLQGRTFLPALDCPFRVADEIVDELRQDHKCILVDMHAEATSEKIAM
GWHLDGRASLVVGTHTHVQSNDDRILPGGTAYLTDAGMVGPRDGILGMEREAVLRKFY
TQLPVRFVVDDGKWHFHGVFVEIDEATGAATRIEKIRLTEDEWRME"

CDS complement(3381821..3383362)

/gene="rny"

/locus_tag="EFAGFIKM_02879"

/EC_number="3.1.-.-"

/inference="ab initio prediction:Prodigal:002006"

/inference="similar to AA sequence:UniProtKB:O31774"

/codon_start=1

/transl_table=11

/product="Ribonuclease Y"

/db_xref="COG:COG1418"

/translation="MEIAITIALVAAALFIGFGVGYFIRKSLAEAKISSAEQAAVQIV
ENAKKEAEALKKETVLEAKDEIHRIRAEAEKDTRERRNEIQRQERRLLQKEESLDKKL
ESLERKEEQVANKEKRIDETQQQIEMIYKNQVTELERISNLTMEDARSIILSNVEQEV
RHETAQMIKDIEQQAKEEADKKSREIITLAIQRCAADHVAETTVSVVTLPNEEMKGRI
IGREGRNIRALETLTGIDLIIDDTPEAVILSGFDPIRREIARTALEKLVADGRIHPAR
IEEMVEKSRKEVDERIREYGEQATFEVGVHGLHPDLIKILGRLKFRTSYGQNVLKHSM
EVAYLAGLMAGELGEDVTLARRAGLLHDIGKALDHEVEGSHVEIGVELAKKYKEHPVV
INSIASHHGDCEATSVIAMLVGAADALSAARPGARRETLETYIKRLEKLEEISESFEG
VEKSYAIQAGREVRVMVQPEKIDDAEAFRLARDITKMIENELDYPGHIKVTVIRETRA
VEYAK"

CDS complement(3383673..3384410)

/gene="recX"

/locus_tag="EFAGFIKM_02880"

/inference="ab initio prediction:Prodigal:002006"

/inference="similar to AA sequence:UniProtKB:P66003"

/codon_start=1

/transl_table=11

/product="Regulatory protein RecX"

/translation="MDQHDEDLYEEAEQDGLSQFPDNEELIITRVERTKSRQARYRIT

FGIHSITVLEDVMIKYRMTQGNTFMKKDLEDIILADERQRTYAQSLRFLEHKPRTRHE

LSQKLRQKEFAAPLIEEALDRLERENLVDDDLFAKEWTRQRMEGKRKGKLWIRQELRQ

KGIANDLIVEALEGISTDAEFETALSAGRKKWNQVKGDVKEKKNKTLPFLMRRGFSMD

MVRRVVNCLIEEDEAGDPEDDEALLWD"

CDS complement(3384693..3385757)

/gene="recA"

/locus_tag="EFAGFIKM_02881"

/inference="ab initio prediction:Prodigal:002006"

/inference="similar to AA sequence:UniProtKB:P16971"

/codon_start=1

/transl_table=11

/product="Protein RecA"

/db_xref="COG:COG0468"

/translation="MSDRRAALDMALRQIEKQFGKGSIMKLGESTHMNVEIIPSGSLA

LDIALGTGGLPKGRIVEIYGPESSGKTTVALHAIAEVQRAGGQAAFIDAEHALDPQYA

SKLGVNIDELLLSQPDTGEQGLEIAEALVRSGAVDIVVIDSVAALVPKAEIEGEMGDS

HVGLQARLMSQALRKLSGAISKSKTIAIFINQLREKVGVMFGNPETTPGGRALKFYST

VRLDVRRIESIKSGNDMIGNRTRIKVVKNKVAPPFKQAEVDIMYGEGISREGSIIDIG

TELDIVNKSGAWYSYEGERLGQGRENAKQFMKEHKDIADIIEQKIRVASNLVTAVPAP

TSEEQQKEAAEEQELFEINE"

CDS complement(3386001..3387257)

/gene="cinA"

/locus_tag="EFAGFIKM_02882"

/inference="ab initio prediction:Prodigal:002006"

/inference="similar to AA sequence:UniProtKB:Q8DRX2"

/codon_start=1

/transl_table=11

/product="Putative competence-damage inducible protein"

/db_xref="COG:COG1058"

/translation="MKAEIIAVGTELLLGQIVNTNARYLSRELAAMGIDVYFQTVVGD

NLNRLSEAIRIAQSRADVILFSGGIGPTQDDLTKDALAAVLNRKLHTDRMAMDKIESF

FRDRNVDMTENNRRQAIVIDGGTPLANETGLAAGNAISDNDKYYVVMPGPPKELIPMF

EQEVKPWLFQHVLTEEMPIYSRMLKFAGIGESALEDRLMDLIDAQTDPTIAPYASEGE

VTVRVSTKAASEGEAKLKLDAMEVQIRERLTEHLYANEDVPIEYTIVTMMSDMGLTLS

AAESCTGGLVMQSLTSVPGSASMLKGGIVCYSNEIKEKLLNVPHDYLEGEDAPGAVSP

EVAKVLAEQIRMIGDADFGLAVTGVAGPGYSERKPPGLVFIALAERGKETEIHELRIN

GNRETVRIRSAKAILYRLWQKLVAMD"

CDS complement(3387365..3387952)

/gene="pgsA_1"

/locus_tag="EFAGFIKM_02883"

/EC_number="2.7.8.5"

/inference="ab initio prediction:Prodigal:002006"

/inference="similar to AA sequence:UniProtKB:P63756"

/codon_start=1

/transl_table=11

/product="CDP-diacylglycerol--glycerol-3-phosphate

3-phosphatidyltransferase"

/translation="MNLPNRITLARICLIPFLMVFLLVDFPFYPEPLQLGSLSLPYNQ

LIAAVIFIIAASTDGIDGYLARKNNMVTNLGKLLDPLADKLLVTAVLISLVEMGKLDS

WIAVVIISREFAVTGLRQIALLDGSVVAASAWGKLKTVVQIVAIVLLLLNNFPFSYTG

IHVDIIAVWAAALITIWSGIDYFIKNKNLLHLTKA"

CDS complement(3388272..3388802)

/locus_tag="EFAGFIKM_02884"

/inference="ab initio prediction:Prodigal:002006"

/inference="similar to AA sequence:UniProtKB:A0QRM0"

/note="UPF0234 protein MSMEG_1165/MSMEI_1134"

/codon_start=1

/transl_table=11

/product="hypothetical protein"

/translation="MLQNIRLTERVTGLSSENSFDIVSKMDLQELTNAVTQTEKEIGA

RYDFKGSKSSLKLDKDALTIVSDDETKLKAVIDVLQSKMAKRGLPLKNIDYAKVEPAS

SGTVRQRLNFKQGIDQDIAKKINILIRDSKMKVKSQIQGDQLRVTGKSKNDLQAVMQL

LNGANLPLDLQYTNFK"

CDS complement(3388920..3389084)

/locus_tag="EFAGFIKM_02885"

/inference="ab initio prediction:Prodigal:002006"

/codon_start=1

/transl_table=11

/product="hypothetical protein"

/translation="MTVSWIVTGLGIIVSLLGYYLTPSAWGYGILGFGLAHVLLGVLD

MFRQPNRSRY"

CDS complement(3389158..3390207)

/gene="rodZ"

/locus_tag="EFAGFIKM_02886"

/inference="ab initio prediction:Prodigal:002006"

/inference="protein motif:HAMAP:MF_02017"

/codon_start=1

/transl_table=11

/product="Cytoskeleton protein RodZ"

/translation="MSELGQQLREARLQKGMSLDDVQEMTKIRKRYLEAIEAGDYKVL

PGSFYVRAFIKTYAETVGLNPDELLEGHKKDVPEEETEATMEPVIQKRSSRPVERSNR

WMSVALMWTFPVLIVVLLYVYVVYNNGDESENPGLDQVKITDSQQQPEDKPDQPADNG

QASNPPATDSGTDSTGEGDAGGNGGGTDTEGQTDGQTDGQTDGQTDETEKEPTDNSPS

NVSVAEDGKSGNITNFKVNGSAGQPVTVTIKATGHSWLEVYKGENSSGEKLEYGNTAE

GDSYTFELDSTGMYIKSGYAAATTIEVGGQVVTDGKATNRIRLKLGEDSSGTTSSTGV

ENGSTDGTGGTTGSE"

CDS complement(3390274..3391041)

/locus_tag="EFAGFIKM_02887"

/inference="ab initio prediction:Prodigal:002006"

/codon_start=1

/transl_table=11

/product="hypothetical protein"

/translation="MESKQWYMEYKIHKNRPGLLGDIASMLGMLEVNILTINGVEGKT

RGMLLESDDDEKIRLLGEMLGKVNSITVSALRQPKLVDILAVRHGRYIDRDSDDRKTF

RFTRDELGLLVDFLGEVFKREGNQVIGLRGMPRVGKTESIIAGSVCAMKRWTFVSSTL
LRQTIRSQMSEDELNPNNVFIIDGIVSTIRSSERHYNLLQDIMSMPSTKVIEHPDIFV
QESEYDFNDFDIIIELRNNPNEEIIYDTFTASYTDEL"

CDS complement(3391239..3391502)
/gene="yflH"
/locus_tag="EFAGFIKM_02888"
/inference="ab initio prediction:Prodigal:002006"
/inference="similar to AA sequence:UniProtKB:O34306"
/codon_start=1
/transl_table=11
/product="putative protein YflH"
/translation="MSTEKTVLSSFDTWKKFLGDRVLQAEKMGMSEETINKLAYEIGD
FLDEKVDPANHSNRALKELWDVGDADERRTIACLMVKLAKQNA"

CDS complement(3391666..3392436)
/gene="fabG_6"
/locus_tag="EFAGFIKM_02889"
/EC_number="1.1.1.100"
/inference="ab initio prediction:Prodigal:002006"
/inference="similar to AA sequence:UniProtKB:O67610"
/codon_start=1
/transl_table=11
/product="3-oxoacyl-[acyl-carrier-protein] reductase FabG"
/translation="MERGTGQLKAIGEMTVLVTGASGGIGAAIAERFARVGMNVVIHY
MRSHEAANEAARRCMEQGSGKIMTVSADLRSREQIERMREKLESHNLMPDILVNNAGI
SHYGMLADVTEEIWDEVMAINLKGTFMCTQEMMPHMISQRYGRIINVSSIWGLSGASC
EVLYSTTKGGVNAFTKALAKELAPSGVTVNAVAPGAVQTSMLNHLDQSELKMLEEEIP
AGRLAQPDEISSLVYFLALPESGYINGQIISPNGGWLT"

CDS complement(3392438..3393718)
/locus_tag="EFAGFIKM_02890"
/inference="ab initio prediction:Prodigal:002006"
/codon_start=1
/transl_table=11

/product="hypothetical protein"

/translation="MESIRYEHLQETLYYEVMDNGLHVYILPKPGFQKTYATFATKYG

SVDNHFQVEGQEEVKVPDGIAHFLEHKMFEEPTGDIFATFASHGASANAFTSFDQTVY

LFSATEHVNENIQTLVDFVQNPYFTDQNVEKEKGIIGQEIDMYADNPDWRVYFGLIEA

MYQKHPVHIDIAGTVESIRTITKEMLYECYNTFYHPSNMLLFVVGGVDPQEVIDMVRS

NQEQKDYKPQGSIQRFFEPEPEQVGEARREAKLAVSLPKCLFGFKETDVGLTGEQLLR

RDMTTQLMMDLLFGSSTRLFQKLYDEDLISDSFGHEYNSSPQYAFSAIGGDTKDPDQL

LARIREEVDAIVEKGFDSTDFERARKKKIGGYLRMLNSPENIAHEFTRQQFRGGDFFN

MLPLYESITLEDVNLRLREHIRWDHLAISLVVSP"

CDS complement(3393722..3395002)

/locus_tag="EFAGFIKM_02891"

/inference="ab initio prediction:Prodigal:002006"

/codon_start=1

/transl_table=11

/product="hypothetical protein"

/translation="MNTSGFERGNKREFRIHVLPTKRFKTFAISLYAGVPLREDTVTK

VALTPFVLRRGTESYPETTQFREQLEHLYGAGFGFDVYKRGDYQIVQFRMDTINDSFV

GGDEQLLDRSFAFLGEVLTRPAQENGHFRTSYVQAERETVRKKLESIVNDKMRYAAER

SIEEMCKNEPYRLHPLGERRDLPGIEPDTLTASYQEWLQQASMDLYVVGDTTLEEVEN

LVQRHFNVDRTSSSDYQTQAAVRGDKAVETVVERLNVSQGKLNMGLRTSITYGDPQYA

AALMYNGILGGYPHSKLFVNVREKESLAYYASSRYDGHKGIATIQSGIEIPNYEKAVT

IIKQQLEEMKSGTITDLEMSQTKAMIRNLLKEMQDSAFEMIAYDFNRQLSGKERTAEE

LLAQVEHISKEDVREAAEQFRLDTIYFLRDEKEE"

CDS complement(3395129..3395959)

/gene="sleB"

/locus_tag="EFAGFIKM_02892"

/inference="ab initio prediction:Prodigal:002006"

/inference="similar to AA sequence:UniProtKB:P0A3V0"

/codon_start=1

/transl_table=11

/product="Spore cortex-lytic enzyme"

/db_xref="COG:COG3409"

/translation="MRKMNIWLFTAILLMSALGIRYLLPENAATESSNQSTPQVEEKA
LPTFSSNAVKYGSYGQDVYELQGRLKYLGFYNGKIDSNFGNSTLKSVKWFQSEFGMKA
DGVVGAETKLKLYNASTKWSPTETPLHKKKTSGGSDSSNNTADKEQDNMGSANALGLS
ENELKIMANAVYGEARGEPFEGQVAVAAVIMNRVKSPSFPNTPSGVIFQPGAFTAVAD
GQIYLEPNAQAKKAVEQALNGWDPSGGCLYYFNPKTATSKWIWTRPQVKTIGQHIFCM
"

CDS complement(3396155..3398893)

/locus_tag="EFAGFIKM_02893"

/inference="ab initio prediction:Prodigal:002006"

/codon_start=1

/transl_table=11

/product="hypothetical protein"

/translation="MARRKKRKKKGAGFSGVLKYEIYGIVLITLAVIALSGEATVGRS
LSKMFGLMLGKFYFAIPLVGIYYGLMVMIHRKWPSGWTTRKTGLVLLVFALTLMSTVS
AMHQKLIPVGALEPGAVITQVHNDMQTELLTPATPGERDSMLNKDISGGYLGAGQFVL
FLWLFGSLGARLIMIVMFVISFMLITNLSYVDLIRIFRTKIWDAGSSMYKKLESRPSA
RSASASDARKRGNTRKVVPVPVEDDEDEYEDELEEQHLPKRNSPIFFQLFEKWGAKRE
QATSGREMDEVDSAETEQIVYRAEQDHNLEAWQDATEDVTNKSASSRVPVQAKPNSAP
IIRDFFEHVRAEEASIEDDLDDAYPFPDNLADPNVVQQGEHTIKITDELVETEWSASD
AGTNGMNAVDEIQSGEEVSNDAMQGTDTPTPEGQDTQPVKPPPPPPKPYKLPSFRLLA
KPNNGGKAGDQKDYMQTARKLEATLESFGVRAKVLEVVRGPAVTRYEIQPDIGVKVSR
IVSLTDDIALALAAKDIRMEAPIPGKSAIGIEVPNGEVSVVTMREVMETATFQDAESK
VTIAFGRDISGQTIIGNLARMPHLLVAGATGSGKSVCINGIITSILYKAKPDEVKFLM
VDPKMVELNVYNGIPHLLAPVVTDPKRASLALKKIVVEMEKRYELFSKSGTRNVEGYN
NLMKDNPAAVLPYIVVIVDELADLMMVAAGDVEDAIARLAQMARAAGIHLIIATQRPS
VDVITGVIKANIPSRIAFGVSSQVDSRTILDMGGAEKLLGRGDMLFMPMGASKPVRVQ
GAFMSDEEVENIVNYVRGQGEAQYDESLVPEVDDSIQAEDEVQDDLYEKAVQIILEAK
QASVSLLQRRMRVGYTRAARLIDSMEARGVIGPYEGSKPREVLVSLEQYQQNKISS"

CDS complement(3398984..3399214)

/locus_tag="EFAGFIKM_02894"

/inference="ab initio prediction:Prodigal:002006"

/codon_start=1

/transl_table=11

/product="hypothetical protein"

/translation="MTLYTVMPPEQLWSGMWKEGEDTKEIKMNGLLMQVRPVNDNEAV

IVRLLDCPLEAYLNPANMPGSTIPISGNWGSA"

CDS complement(3399211..3400011)

/gene="tepA"

/locus_tag="EFAGFIKM_02895"

/inference="ab initio prediction:Prodigal:002006"

/inference="similar to AA sequence:UniProtKB:Q99171"

/codon_start=1

/transl_table=11

/product="Translocation-enhancing protein TepA"

/db_xref="COG:COG0740"

/translation="MFLEKGIIEMNERMNGKQASRSNQPEVEAPEIPIQEDKAISPVT

ETIQQFGQTQSPAGESNIFCMTIIGQVEGHLILPPQNKTTKYEHIIPQLVAAEQNQRI

EGILIILNTVGGDVEAGLAIAEMIASLSKPTVTVVIGGGHSIGVPIAVASTYSLIAGS

ATMTIHPIRMNGLVIGVPQTFEYMEKMQERVVRFVTSHSNISEEMFKELMFKTGELNR

DIGTAVGSADAVKHGLMDAVGGIGQAIAQLNQLIGDKRQTLQAGGYTQ"

CDS complement(3400206..3401885)

/gene="rnjA_2"

/locus_tag="EFAGFIKM_02896"

/EC_number="3.1.-.-"

/inference="ab initio prediction:Prodigal:002006"

/inference="similar to AA sequence:UniProtKB:Q45493"

/codon_start=1

/transl_table=11

/product="Ribonuclease J1"

/db_xref="COG:COG0595"

/translation="MSKKNNNDKLMIFALGGVGEIGKNMYVIQYANDIVVVDAGLKFP

EEDMLGIDIVIPDISYLTENRDKVRGIILTHGHEDHIGGLPYVLKHLNVPVYGTRLTL

GLVENKLKEANLLGDTKRILIDADSEIQLGSVLKASFFATNHSIPDSVGVCVETPEGA

VVHTGDFKFDHTPVNGQYADLQRMAQIGTNGVLALLSDSTNAEKPGFTPSEKNVGIVL

EDIFRKASQRVVVATFASNVHRIQQVINAAEVTGRKVAVIGRSMVNVVGIASELGYLE

IPDGMIIEPEEVGKMAADRVVILCTGSQGEPMSALTRMARSTHRKVDILPGDTVIIAA

TPVPGNEKYVGRTIDELFRLGANVHYSGANSGVHVSGHGSQEELKLMLNLMKPKFFLP

IHGEFRMQRRHAVLAESVGVEPENIFITDIGEVIEIQGGAARRAGKVTAGNVLIDGLG

VGDVGNIVLRDRKLLSQDGILVVVVTLSKQDGKIVSGPDIISRGFVYVRESEGLLDEA

NRIVSSTLQKLMSENVNEWASLKTNVKDALGRFLYEQTRRRPMILPIIMEV"

CDS complement(3402362..3403240)

/gene="dapA"

/locus_tag="EFAGFIKM_02897"

/EC_number="4.3.3.7"

/inference="ab initio prediction:Prodigal:002006"

/inference="similar to AA sequence:UniProtKB:Q04796"

/codon_start=1

/transl_table=11

/product="4-hydroxy-tetrahydrodipicolinate synthase"

/db_xref="COG:COG0329"

/translation="MDFGRLITAMVTPFNEQGEIHWEETARLIDYLIVDQKSETLVVS

GTTGESPTLSDTEKVQLFEFAVKHAAGRCKIIAGTGSNNTAHSIHLTQEAERAGVDGI

LLVVPYYNKPSQEGLFKHFEAIAGSTKLPIMLYNVPGRTVTSLSAETTLRLAQIPNIV

ATKECASMEQVTLIAASAPENFRVYSGDDASGLPAIAVGAHGIVSVASHVVGAEMKKM

IDAFYGGAPLQAAQIHQQLFPVFKGLFECPQPLPNPVAVKYALTLRGLDVGSVRLPLI

PPTEEEQVYIKGLLNL"

CDS complement(3403288..3404508)

/gene="lysC_2"

/locus_tag="EFAGFIKM_02898"

/EC_number="2.7.2.4"

/inference="ab initio prediction:Prodigal:002006"

/inference="similar to AA sequence:UniProtKB:Q59229"

/codon_start=1

/transl_table=11

/product="Aspartokinase"

/translation="MRIMVQKFGGTSLSTVQAREHVLRHVKRELEAGLSLVIVVSAMG

RRGEPYATDTLLDWAAQNGNALSAREKDLLLCCGEIISATTLSSLLEHEGIPTTVLTG

AQAGFVTDDNFGNARILDVRPVRVLEQLQLGRVVVVTGFQGQTENGDFTTLGRGGSDT

SATALGAALRAEMVDIYTDVNGILTADPRIVEDARPLTVVSYAEICNMAHHGAKVIHP

RAVEIAMQSQIPVRVRSTFADTEGTLVTHPEGFQDVQTGIVDRYVTGIAYVSNVTQIT

VDVPGGGADRLQLKVFKTMAENSISVDFINVTPSGVVYTVFDSDSEKAIQVLQEIGLK

PQSLSGCAKVSVIGGGINGVPGIMARIVESLTLADIQILQSADSNTTIWVLVKKEDMV

QALRALHASFELHL"

CDS complement(3404887..3405483)

/gene="dpaB"

/locus_tag="EFAGFIKM_02899"

/EC_number="1.3.1.-"

/inference="ab initio prediction:Prodigal:002006"

/inference="similar to AA sequence:UniProtKB:Q04810"

/codon_start=1

/transl_table=11

/product="Dipicolinate synthase subunit B"

/db_xref="COG:COG0452"

/translation="MNWQGKTVGYAITGSHCTFEEVMPVISRFVAEGANVIPIISNSV

LTTDTRFGTAQNWQKQLKDITGNDIISTIVEAEPLGPSKLLDVLVIAPCTGNTTSKLA

NAMTDSPVLMAAKAQMRNQRPLVLAISTNDGLGLNAANIAKLLVAKYLYFVPFGQDDP

VKKPNSLVAKMELIPEACWSALEGKQLQPMIVERSSQA"

CDS complement(3405480..3406379)

/gene="dpaA"

/locus_tag="EFAGFIKM_02900"

/EC_number="1.3.1.-"

/inference="ab initio prediction:Prodigal:002006"

/inference="similar to AA sequence:UniProtKB:Q04809"

/codon_start=1

/transl_table=11

/product="Dipicolinate synthase subunit A"

/translation="MLTGVRIVVLGGDARQLEVIQKCAELDATVSVVGFDKIERSIPG

IEHQELEDEVFASADVLVLPVVGCDEQGKVSTSFSDTPIYLKKEHIAALPEHCIVFTG

MAKPFLRELCLENGLRLVEVLDRDDIALYNSIPTAEGAIAIAIRETDFTIHGSECIVL

GIGRTGFTMAKTLQGLGANVRVGIRREEDAARATIMGWRPFMTTDLAAQTGEVDLLFN

TIPTMIITAQILSRMPQKAVIIDLASAPGGCDFRYADKRGIKALLAPGLPGIVAPKTA

GGIIADALIRLLLEEQNAREVKS"

CDS complement(3406536..3406979)

/gene="dut"

/locus_tag="EFAGFIKM_02901"

/EC_number="3.6.1.23"

/inference="ab initio prediction:Prodigal:002006"

/inference="similar to AA sequence:UniProtKB:Q2YRG4"

/codon_start=1

/transl_table=11

/product="Deoxyuridine 5'-triphosphate

nucleotidohydrolase"

/translation="MHYVQIQKLPGNEDIKLPQKMSELASGFDVVAALQEDVVLQPGQ

RTLIPTGLAMAMPAGLEAQIRPRSGLAFKHGITCLNTPGTIDADYRGEVKVLLINLGQ

EPFTIVRGERIAQIVFQTVPAVELTEVNELSETVRGEGGFGHTGK"

CDS complement(3406942..3408225)

/locus_tag="EFAGFIKM_02902"

/EC_number="3.4.24.-"

/inference="ab initio prediction:Prodigal:002006"

/inference="similar to AA sequence:UniProtKB:P9WHT5"

/codon_start=1

/transl_table=11

/product="putative zinc protease"

/db_xref="COG:COG0612"

/translation="MKKIQLGNGLRVVMEQIPTCRSVSFGIWVKTGSRNEQPASNGVS

HFIEHMLFKGTDRYDAKAIAEQFDAIGGNVNAFTSKEYTCYYAKVLDEHLPIAVDVLS

DMFFRSKMDDGELIKEKNVILEEISMYEDTPDDMVHDLMALAAYGEHPLAYPILGTEE

RLKAMDSSHLRAYMKEHYTIENTVISIAGNIDDSVIDLMEKHFGAFDVNGVSEAVTMP

AFQSGQLFHKKKTEQNHICISFPGCKIGDPLQFAMVVLNNAIGGGMSSRLFQEIREKR

GLAYSVYSYHSSHADSGLFTIYAGTAPKQTKEVLDLTKEVLRDLAVNGLSEDELRKGK

EQLKGSLILSLESTGSRMNRLGKNELMLGRHHTLDEMITKIEQVTMDDINAVLDLMFA

EPFALAMVGASDKTIAGLRRDDFVALRSNTETAGQ"

CDS complement(3408325..3409320)

/locus_tag="EFAGFIKM_02903"

/inference="ab initio prediction:Prodigal:002006"

/codon_start=1

/transl_table=11

/product="hypothetical protein"

/translation="MGNQSKKLAAVLAGVTAVILIGQVGSVRTYITEIRDGPGTQNAF

DMFKEATGEDALLSAIRDKAAETKIAPVNARVDRVWKAIPGYNGMEIDVEATYRKALS

GTLNTKIAYVYRQIEPEIQLKDLGAHPIYRGNANKPMVSFMINVAWGNEYIKPMLDTL

DAEKVKATFFLDGSWLSKNVELAKEIQKRGHEMSNHAYSHPNMSRLSAERAKLEISKT

QDLLHKTLGVENRWFAPPSGDFNQKTVDIASSMGLQTVLWTVDTVDWRKPSPDAVVAK

IAKNTEAGTLILMHPTAASSGALKGMIQSIRAKGLVLGTVSETLSSERVNTSAVE"

CDS complement(3409496..3411598)

/gene="pnp_1"

/locus_tag="EFAGFIKM_02904"

/EC_number="2.7.7.8"

/inference="ab initio prediction:Prodigal:002006"

/inference="similar to AA sequence:UniProtKB:P50849"

/codon_start=1

/transl_table=11

/product="Polyribonucleotide nucleotidyltransferase"

/db_xref="COG:COG1185"

/translation="MEQRVEMQLGGRKLTLETGRLAKQANAAVKVTYGDTVVLCTVTA

SSEPKDLDFFPLTVNYEERLYAVGKIPGGFIKREGRPSEKAILSSRLTDRPIRPLFPE

GFRNDVQVLNIVMSVDQDCEPQIAAMIGTSAALSISDVPFSGPIGGVKVGRIDGEFII

NPTIAQLEVSDIELVVAGTKNAIMMVEAEANEVPEEVMLEAIMFGHDEIQNIIAVIEQ

LVQVAGQEKMAVKLRTVNAEVNSSVREFASARLVEAVKIAEKHARQDAIDVVNDETVA

HFEEKYIESPELFKDVKEVLHDIVKEEVRRLITHDKVRPDGRGLAEIRPIECDTSLLP

RAHGTGLFTRGQTQALSVCTLGALGDVQILDGISLEETKRFMHHYNFPPFSVGEARPL

RAPGRREIGHGALGERALSKVIPSETDFPYTIRLVSEVLESNGSSSQASICASTLAMM

DAGVPIKAPVAGVAMGLIKDGDHVSILSDIQGMEDHLGDMDFKVAGTPEGVTAIQMDI

KIAGINRQILSDALTQAKEGRMHILGKMNEILKTPREQLSQYAPKITTMHINPDKIRD

VIGAGGKIINKIIEETGVKIDIEQDGRVFIASSNQEMNDKAKAIIEGIVREVLVGEIY

VGKVKRVEKFGAFVEVLPNKEGLVHISQLSTERVAKVEDVVAIGDSITVKVTEIDPQG

RINLSRKAVLTAEAPAQS"

CDS complement(3411858..3412127)

/gene="rpsO"

/locus_tag="EFAGFIKM_02905"

/inference="ab initio prediction:Prodigal:002006"

/inference="similar to AA sequence:UniProtKB:P21473"

/codon_start=1

/transl_table=11

/product="30S ribosomal protein S15"

/db_xref="COG:COG0184"

/translation="MALTQERKQQLIDEHKTHESDTGSPEVQVAILTENITSLTDHLR

THKKDHHSRRGLLKMVGQRRKLLAYVKNKDVKRYSALIEKLGLRR"

CDS complement(3412105..3412239)

/locus_tag="EFAGFIKM_02906"

/inference="ab initio prediction:Prodigal:002006"

/codon_start=1

/transl_table=11

/product="hypothetical protein"

/translation="MIYCTTLSIEATLTLAWLLALTGGDEANGDYIEGGEQDGIDSRT

"

CDS complement(3412287..3413225)

/gene="ribF"

/locus_tag="EFAGFIKM_02907"

/inference="ab initio prediction:Prodigal:002006"

/inference="similar to AA sequence:UniProtKB:P0AG40"

/codon_start=1

/transl_table=11

/product="Bifunctional riboflavin kinase/FMN

adenylyltransferase"

/db_xref="COG:COG0196"

/translation="MKTVMLTYPQTLHSTELSTLPQVLAIGQFDGLHLGHASVILSAV

RIARETGMQAAVMTFHPHPKEVMRKGDYEGYLTPLRDKEDILAGMGVDVLYVVEFNED

FSRLTPQQFVHDLLIPLQTRTAVVGFDFRFGHKGAGDEQLLRTLGEGEMMVETVPPFL

LNGEKVSSSLIRGLLKRGEMDEASQWLGRPYSIRGTVIHGEKRGRTIGFPTANLELTD

HYVTPSKGVYAVRVQYGEQELHGVMNLGVKPTFHESGMKPTFEVHLLDFDGHLYDQEL

KVELVHYIRAERKFDSIEALISQIREDALTAGRLLS"

CDS complement(3413296..3414219)

/gene="truB"

/locus_tag="EFAGFIKM_02908"

/EC_number="5.4.99.25"

/inference="ab initio prediction:Prodigal:002006"

/inference="similar to AA sequence:UniProtKB:P65855"

/codon_start=1

/transl_table=11

/product="tRNA pseudouridine synthase B"

/translation="MVKPFEGVLPVYKPAGFTSHDVVAKMRRILKMKRIGHTGTLDPQ

VTGVLPLCLGRATRVVEYMQELPKEYLATLRLGLSTDTEDMTGEVIERAETTVEVTQE

QVQQVLEQFLGTISQVPPMYSAVKVDGKRLYELAREGKTVERKSREVTIYELELTGIE

TQGETTDISFRALCSKGTYIRTLCVDIGRQLGYPSTMVQLERTISAGISADRCLRIEE

VEQLMADGTLAEALIPVDEAIASIPAHKVGEEQAKGALQGQKLSARLLEPPVEQPGLL

RLYAQDGTFLGIFERDELKPTVRAVKVFLPE"

CDS complement(3414213..3415190)

/gene="nrnA"

/locus_tag="EFAGFIKM_02909"

/EC_number="3.1.-.-"

/inference="ab initio prediction:Prodigal:002006"

/inference="similar to AA sequence:UniProtKB:Q5SM25"

/codon_start=1

/transl_table=11

/product="Bifunctional oligoribonuclease and PAP

phosphatase NrnA"

/db_xref="COG:COG0618"

/translation="MHTYEQALQAGKQFLLEHDDYLVVSHVQPDGDAVSSTVTVGWLL

SCLGKTFTMINEGEIPGRMQFLWEAGNIVNMTEQPPQRKYKAVICVDCADFARVGLTR

HYFEEDAVILNIDHHPTNDGYGTVNIIKSDAAATAEILFDFLNLFQVTWDKDVATAVY

TGLLTDTGGFRYANTSPNVMTTASRLLEHGVDGPYLAQTLLEQVTLPQVRILNQALSS

LQMTDDGKIAWVVITPDDMVACGAANEDLEGVVNYPRNIQGVEVGIFFKVINENAVKV

SLRSAGKIDVAALAQTFGGGGHVLAAGCRLEGRLDDIIAKVLKQVNSQW"

CDS complement(3415210..3415569)

/gene="rbfA"

/locus_tag="EFAGFIKM_02910"

/inference="ab initio prediction:Prodigal:002006"

/inference="similar to AA sequence:UniProtKB:P65967"

/codon_start=1

/transl_table=11

/product="Ribosome-binding factor A"

/translation="MAKIRTGRVGEQIKKEISLLIQSELKDPRIGFITVTGVEVTGDL

SQAKVYLSVFGEQEQKDNTLKALAKANGFLRSELGKRIRFRHVPELIFKIDESIAYGS

RIEKLLGDIGSDKNESQ"

CDS complement(3415595..3418180)

/gene="infB"

/locus_tag="EFAGFIKM_02911"

/inference="ab initio prediction:Prodigal:002006"

/inference="protein motif:HAMAP:MF_00100_B"

/codon_start=1

/transl_table=11

/product="Translation initiation factor IF-2"

/translation="MSKQENKDKLRVYEYAKSLNMSSKEIITILKKLEIPVNNHMSVM

ENGSVGKVEQFFKDIKSTAASKQGNDAKPVATSAVRSDKPVDSNKPAGGVNTPISSNP

SGSPVQTKIQQEKQVGMNNRPNSNNNNGTQRPSGQDSRNRTNSSQGSSQGGQSTNRPR

PAQGGQSSTASRPQGTGQRPNNSGGGQVRTSGPNSGGNTGTAGNRSGGQGQSQGQGQR

RSGPGGTTGSNNNSGNRSNSGGGGRRYDDNRGGNFRGNRGGKNNRNRNQQQYQQREKI

DNTPKKIIVRGDMTVGETAKLLHKDASEVIKKLIAMGVMATINQELDIETILLLSGEF

GVEVEVKIVLEDDRFETLEENDDAADLQARPPVVTIMGHVDHGKTTLLDAIRSTNVSD

GEAGGITQHIGAYQVEINNKKITFLDTPGHEAFTAMRARGAQVTDMTIIVVAADDGVM

PQTIEAINHAKAAGLPIIVAVNKIDKPGADPDKVKQELTSYELVPEEWGGDTIFVNVS

AKQRMGLEGLLEMILLVAEVNEYKANPDKRARGTVIEAELDKGRGPVARILVQHGTLK

VGDAFVAGNCFGRVRAMVNDKGRKLKEAGPSTPVEITGLTEVPGAGDPFMVFEDERKA

RSIADKRAITQRESDLGTHTRVTLDDLFQHIKDGEIKDLNVIIKGDVQGSVEALKGSL

AKIEVEGVRVKIIHSGAGAITESDIILAAASNAIVIGFNVRPDNQAKSTADQEQVDIR

LHRVIYSVIEEIEQAMKGMLDPIYKEKVIGHAEVRSTFSISKVGTIAGCMVTSGKITR

SAEARLIRDGIVLYEGKLDSLKRYKDDAKEVAQGYECGITLDKYNDLKEGDVIEAFIM

ETVQR"

CDS complement(3418173..3418502)

/gene="rplGA"

/locus_tag="EFAGFIKM_02912"

/inference="ab initio prediction:Prodigal:002006"

/inference="similar to AA sequence:UniProtKB:P32729"

/codon_start=1

/transl_table=11

/product="putative ribosomal protein YlxQ"

/db_xref="COG:COG1358"

/translation="MTNIKTLSYLGLSMRAGKLVTGEEIVLKAIRSSEAKMVIVAGDA

SANTQKKFRDKCGTYKVPLLIGFDRDSLGSSIGKDTRVVLAVTDRGFAKMISKQVGIM

SEVEYIE"

CDS complement(3418495..3418800)

/locus_tag="EFAGFIKM_02913"

/inference="ab initio prediction:Prodigal:002006"

/codon_start=1

/transl_table=11

/product="hypothetical protein"

/translation="MKPKKVPLRKCVACQEMMPKKQLIRIVKTPEDEVLIDLTGKKSG

RGAYLCGKESCFKLALKNRALDRALKGKVSPEIYEQLAADFIAVEDEFIAAQEREHD"

CDS complement(3418826..3419923)

/gene="nusA"

/locus_tag="EFAGFIKM_02914"

/inference="ab initio prediction:Prodigal:002006"

/inference="similar to AA sequence:UniProtKB:P32727"

/codon_start=1

/transl_table=11

/product="Transcription termination/antitermination

protein NusA"

/db_xref="COG:COG0195"

/translation="MSTDFIEAMNELEREKGISKDVLFEAIEAALISSYKRNFNTAQN

VRVDMNRNTGVIRVYARKLIVEEVLDSRTEISLPAAREINPHFQLEDIAEIEVTPRDF

GRIAAQTAKQVVTQRIREAERGLIYNAFVDKEEDIVTGVVQRQDLRNIYIDLGKIEAA

LPLTELMPNEKFVHGDRIKAYITKVENTTKGPQIILSRTHPGLLKRLFELEVPEIFDG

VVEIRSVAREAGFRSKIAVHSRNEEVDPVGSCVGPRGMRVQTIVGELRGEKIDIVRFS

DQVDEYVANALSPSKVLEVHVFEEEKMARVIVPDYQLSLAIGIKGQNARLAAKLTGWK

IDIKSESQAEQEFGREKDSSSEMHQDSVSVD"

CDS complement(3420047..3420508)

/gene="rimP"

/locus_tag="EFAGFIKM_02915"

/inference="ab initio prediction:Prodigal:002006"

/inference="similar to AA sequence:UniProtKB:Q97S61"

/codon_start=1

/transl_table=11

/product="Ribosome maturation factor RimP"

/db_xref="COG:COG0779"

/translation="MSTTNIKSTVEEMIQPYLNEQGFELVDIEYVKEGSNWFLRVYVD

KEGGIDIDDCVLISEKLSAKLDENDPIPTIYFLEVSSPGAERPLKKPEDVTKAVGKNV

FVTTYEPVNGLKEFEGKLLSFDDEELVIEAGKKQHAISYDKVASARLAILF"

CDS complement(3420765..3425084)

/gene="polC_2"

/locus_tag="EFAGFIKM_02916"

/EC_number="2.7.7.7"

/inference="ab initio prediction:Prodigal:002006"

/inference="similar to AA sequence:UniProtKB:P13267"

/codon_start=1

/transl_table=11

/product="DNA polymerase III PolC-type"

/db_xref="COG:COG2176"

/translation="MSGFEEKRKRFELLMKQAELPAGLLEPYFLDGWIEQVETNRSNR

EWNILIAKDMLVPAPIYRTFCLHIQEKMNHIAKISFGFKYTDQVQNGDIVSEYWNLFL

EWVTREIPSVNGWMNRTTFECEEDLLQLTMSDATSMELARKKQIDQAITKFYDKYFHL

PLRIKMQVGEVGSNKEAMEQFQAQKRVEELEVIEKMMSEVDTEIPVDEEQGDLRLQMG

YDIKEPAVPMQEIQDEEKKVTLQGSVFGLDRKELRNGNTLFTFYLTDFTDSMQMKMFA

KTKEDVKILSLLANGKWVKVRGRVEYDRFMQIPELAMIPSDLIEVKAPPSRKDNAPEK

RVEFHLHSTMSTMDAVTSIDKYVKMAAEWGHKAIAVSDHGGVQVYPEASKAAKKNGIK

MIYGLEANVVNDSVAVVMAPQPLDLQTATYIVFDIETTGLSVTQNKIIEIAAVKMQDG

KEIDRFATFVNPHERIPYNIQQLTNINDDMVKDAPELEPVIRDFVQFAGDGVLVAHNA

RFDMGFIQASLKQIGLPELPNPVLDTLELARLLFPKNKNHRLNTMADKYKVGLESHHR

AIDDTIALAGILIGLLNDAAQMKGLTRLDRLNDYVGVDLSNTRPFHCGIYALNDVGKK

NLYKLVSLSHTEHFKRVPCIPKSKLINLREGLVIISGCEKGEFFEAVLNKSLEEAEEI

AEFYDILEIQPLTMYMHLVDKGLVATPEEIKTAIRKVIDIGAKFNKPVIATGNVHYLE

PRDKLYRDITIHGITGFSPLKDQRKPDAHFRTTEEMLEEFQFLGQDKAYEVVVTNTVE

LSDRFEEIKLFPDKLFTPILEGADEEIRNTCYDTAKSIYGEELPEVIVARLEKELIPI

IKYGFSANYLISERLVKKSNQDGYLVGSRGSVGSSVVATFLGISEVNPLPAHYICVNS

ECKHSEWFLDGSVRSGFDLPEKECPDCGGTLKGEGQDIPFETFLGFKGDKVPDIDLNF

SGDYQPHAHNYTKVLFSEKSVFRAGTIGTVAEKTAFGFAKKYEEHHQKKWRGAELNRL

ASGCTGVKRSTGQHPGGIVVVPDYIEVEDVTPVQFPADDVNAEWKTTHFDYHAFEENL

LKLDILGHDDPTMMRMLQDLTGVDPTTIPMNDPKVMSMFNSTEALGVTPEQIRSPVAT

FGVPEMGTKFVRQMLVESQPTSFADLLQISGLSHGTGVWLGNAQDLIKNGTCNIKTVI

GCRDDIMLFLIYKTGMDASLAFKITESVRKGRGLPQEWIDEMKNCKVPQWYIDSCLKI

QYMFPKAHAAAYVISAVRTAFFKLYHPIEYYATYFTVRADEIDIELMCQGYDAIYRKI

MEIEQLGFQAPPKEKNMLPVLEMGLEMAARGFSLKSIDLYRSEATKFIVDGKTLIPPF

SALAGIGDNAARNIAAARDHGEFLSVEDFQQKSKASKTIVELLSNMGCFRGLPESNQL

SLF"

CDS complement(3425428..3426882)

/gene="proS_1"

/locus_tag="EFAGFIKM_02917"

/EC_number="6.1.1.15"

/inference="ab initio prediction:Prodigal:002006"

/inference="similar to AA sequence:UniProtKB:Q9L4Q8"

/codon_start=1

/transl_table=11

/product="Proline--tRNA ligase"

/db_xref="COG:COG0442"

/translation="MSKENDKQFVTEITPQGEDFSRWYIDVIKKADLMDYAPVRGCIV

FKPDGFEIWEHIKDELDRRFRETGHRNAYFPMFIPESFFQKEKEHVEGFNPELPWVTE

AGGEKLEERLAIRPTSETIIGHMYSKWIQSYRDLPVLINQWANVVRWEKRTLPFLRTS

EFLWQEGHTAHETEEEAREETMKMLEIYREVVEEYLAIPVIVGQKTKSEKFAGAVDTY

SIEAMMKDGRAVQAGTSHYMGTNFAKAFEIQYLSRNNVLELAYTTSWGVSTRLIGALI

MVHGDDRGLVLPPKVAPTQVVMIPIGPPKTRDAVVGRADELFTELKKAGVRVKMDDRS

DVRPGWKFNEYEMRGVPIRLEIGPRDMENGVCVLVSRITGEKKVVEQANLVEEIQAML

TQIQVDMLERARTFMSDNFYSVDTLDEMKELMENKRGFTLAGWCGSEACEDKVREVTG

ATSRNIPFQPAEEKHTCLACGEQAEHTVVFARAY"

CDS complement(3426945..3428216)

/gene="rasP"

/locus_tag="EFAGFIKM_02918"

/EC_number="3.4.24.-"

/inference="ab initio prediction:Prodigal:002006"

/inference="similar to AA sequence:UniProtKB:O31754"

/codon_start=1

/transl_table=11

/product="Regulator of sigma-W protease RasP"

/db_xref="COG:COG0750"

/translation="METIQVVFLTVLMFFVIVTVHEWGHYYFAKRAGILVREFAIGFG

PKLFSYKRNETQFTLRLLPFGGYARMAGEDPELVEIQEGQTIAVRSADDQVKMIYLDQ

LDNRKNVIRGEVISIDMERALKLQLDVDGEIQEYRIHPQAMLVSRGKQTQIAPKDRQF

GSKTVGQRALAIFAGPLMNFILAFVLFAVYAQMAGVPVENPKNLQIGEVLEGGAADQA

NLQKGDIIETINGTAIGTDSQKMVSMIADSKDKPMEWTLRRGSDTFNITITPRAVEGQ

EGGKVGIVPTLPTRSVGFVETFKVSGVAMVDTTKIIFEGFKHLINQFNMDDIGGPVRT

FEVTGQIAKQGIEQLTRWAAILSLYLGIFNLLPIPALDGSRLVFLGIEALRGRPVDPN

REGMVHFIGFAMLFVLMLAVTYNDILRLING"

CDS complement(3428330..3429469)

/gene="dxr"

/locus_tag="EFAGFIKM_02919"

/EC_number="1.1.1.267"

/inference="ab initio prediction:Prodigal:002006"

/inference="similar to AA sequence:UniProtKB:Q9RCT1"

/codon_start=1

/transl_table=11

/product="1-deoxy-D-xylulose 5-phosphate reductoisomerase"

/db_xref="COG:COG0743"

/translation="MKKIAILGSTGSIGTQTLDVVDMHPELFQVEGLAAGGNTDLLIE

QTKRYRPKKVSVGSKELAEKIAPHLPAGTQLFYGNEGLVEVAAGTDAHTVVTAVVGSV

GLESTLAAIDAGKQIGLANKETLVTAGHIVTTRAAAKGVSLLPVDSEHSAIFQCLNGE

NRERLAGITLTASGGSFRDLTREQLKNVTIEDALKHPNWSMGSKITIDSATMVNKGLE

VIEAHWLFGLQYDQINVLLHPESVIHSYVEFDDTSIIAQLGNPDMRVPIQYALTYPDR

LPSPAQRLSLAQAGKLHFRDMDMERFPCLRMAYECGKMGGTATTAFNAANEVAVARFL

RKEISFLKIEDIIASVLEAHHNVDEPDLQEIARCDQESRKLAASL"

CDS complement(3429540..3430334)

/locus_tag="EFAGFIKM_02920"

/inference="ab initio prediction:Prodigal:002006"

/codon_start=1

/transl_table=11

/product="hypothetical protein"

/translation="MKQRLTTGIIAGVLFLGFCMLGGPWYHGLVLLMALIGYYEFVKM

TGVMPFSGVALIGYAGVFAIVFPWEMLWEARPLSLFQVIWIVMLVLMTASVVTKNKVP

VNTVAMLFLGVLYIGIGFYYIAESRHLHHGLFWTFLLLSSIWASDAGAYFVGKLIGKN

KLWPSISPNKTIEGALGGIVIAIVTSVIFALVSDGLLSWQRAIVIGIACAVVGQMGDL

IQSAYKRVYNIKDSGSLLPGHGGILDRCDSWIVVFPFVHILMLLPY"

CDS complement(3430351..3431118)

/gene="uppS"

/locus_tag="EFAGFIKM_02921"

/EC_number="2.5.1.31"

/inference="ab initio prediction:Prodigal:002006"

/inference="similar to AA sequence:UniProtKB:O82827"

/codon_start=1

/transl_table=11

/product="Ditrans,polycis-undecaprenyl-diphosphate

synthase ((2E,6E)-farnesyl-diphosphate specific)"

/translation="MIKRVRSWWNGADKQETLTISEDNIPQHVAIIMDGNGRWAKRLG

LPRIAGHQNGMKAVKRATIAADELGIKYLTMYAFSTENWTRPKEEVDFLMRLPQEFLA

IELDELIEKNVRIRMMGQEEHLPSHTINALREAIRLTEHNTGLVLNFAMNYGSRREMT

DCVKQIALQVKSGELSAEDITPELIDRHMLTVDMPDPDLLIRTSGELRLSNFMLWQLA

YSELWFTDIYWPEFGKKHLLEAVAEYQRRTRRYGGLK"

CDS complement(3431208..3431762)

/gene="frr"

/locus_tag="EFAGFIKM_02922"

/inference="ab initio prediction:Prodigal:002006"

/inference="similar to AA sequence:UniProtKB:P81101"

/codon_start=1

/transl_table=11

/product="Ribosome-recycling factor"

/db_xref="COG:COG0233"

/translation="MPQAVKQHAEERMEKAIQALRRDLATLRAGRATPALLDRIQVEY

YGAMTPLNQLANISTPDSRTLMIQPWDKSSMGDIERAIMKSDLGLTPANDGSMIRLSI

PALTEERRAELVKLTKKFGEEGKVAIRNIRRDANDDIKKMEKSDISEDESRRHQDDIQ

KSTDKFIAEVDKVLAAKEKEIMEV"

CDS complement(3431762..3432490)

/gene="pyrH"

/locus_tag="EFAGFIKM_02923"

/EC_number="2.7.4.22"

/inference="ab initio prediction:Prodigal:002006"

/inference="similar to AA sequence:UniProtKB:Q831V1"

/codon_start=1

/transl_table=11

/product="Uridylate kinase"

/db_xref="COG:COG0528"

/translation="MEQPVFKRVVLKVSGESLSGQNGYGIDADTISSIAQQVKEVVAL

GVQVAIVCGGGNIWRGIAGSENGIDRATADYMGMLATVMNSLALQDALEQIEVPTRVQ

TSIAMQQIAEPYIRRRAIRHLEKGRVVIFAAGTGNPFFSTDTTAALRAAEIEAEVILM

AKNKVDGVYSADPFKDSTAVKFDQLTYMDILNKDLGVMDSTASSLCKDNNIPLIVFAI

TEQGNIKRVVLGERIGTIVKGSVD"

CDS complement(3432723..3433373)

/gene="tsf"

/locus_tag="EFAGFIKM_02924"

/inference="ab initio prediction:Prodigal:002006"

/inference="similar to AA sequence:UniProtKB:P74070"

/codon_start=1

/transl_table=11

/product="Elongation factor Ts"

/translation="MAVNASAVKELRERTGAGMLDCKKALEEANGDVTKAAELLREKG

LSAAASKAGRAATEGVVESYIHAGGRIGVLVEVNCETDFVGKTDQFKDFVKDVAMQIA

AANPKFVTREEVPADELEKEKEILKAQALNEGKPEKIIEKMVEGRIGKYYEEYCLLEQ

TFVKDPDKTISQLLNEKISQIGENISIRRFVRYELGEGLEKKVDNFVEEVMSQVNK"

CDS complement(3433489..3434187)

/gene="rpsB"

/locus_tag="EFAGFIKM_02925"

/inference="ab initio prediction:Prodigal:002006"

/inference="similar to AA sequence:UniProtKB:P21464"

/codon_start=1

/transl_table=11

/product="30S ribosomal protein S2"

/db_xref="COG:COG0052"

/translation="MAVISMKQLLEAGVHFGHQTRRWNPKMDRYIFTERNGIYIIDLQ

KTVKKVEEAYNFVKGIAGENGKILFVGTKKQAQDSVKEEAERAGQFYINQRWLGGTLT

NFQTIQKRIDRLKQLEAWEEDGTFAVLPKKEVILLRKEKDRLEKFLGGIKNMKGLPSA

LFIIDPRKERIAVAEARKLGIPIVGIVDTNCDPDEIDYVIPGNDDAIRAVKLLTGKMA

DAVIEANQGEETSA"

CDS complement(3434484..3435110)

/gene="mltG_2"

/locus_tag="EFAGFIKM_02926"

/EC_number="4.2.2.-"

/inference="ab initio prediction:Prodigal:002006"

/inference="protein motif:HAMAP:MF_02065"

/codon_start=1

/transl_table=11

/product="Endolytic murein transglycosylase"

/translation="MDKRSLWIGLGSGMIIGAVLLQLATVGQNALSDSNLDPEQTANM

TKEQLETAAKSLDMKVIGSEEELYTEAEWVEKKKQESSELQGETATPPETVTSTETPA

EPEKPQEPTASEGEGQKEITEPKTTEPVTPSNPKGATVSFKVRSGNSLAIVAANLKTA

GIVDDAQAFIKAGKAERINTKIQVGTYALEKGESFKSIIAKITKEPSN"

CDS complement(3435114..3435698)

/locus_tag="EFAGFIKM_02927"

/inference="ab initio prediction:Prodigal:002006"

/codon_start=1

/transl_table=11

/product="hypothetical protein"

/translation="MSPWIIIVILGACAIAYACIMPRKNKAQEPGHQLVQEMESTLEH

YMTEIENDNDALIQRVAEMKGEATAADQRMQLQLQELQQRLDQLEQSKKNEAVASPVP

HSGNVSVQSSEGLQAQALVKSVQAEAAQQALEADQQTSEVESLPGRESIKDRYAELFN

LHAEGKSMDAISKQTGIQRGEVQLILQLAEREES"

CDS complement(3435764..3436078)

/locus_tag="EFAGFIKM_02928"

/inference="ab initio prediction:Prodigal:002006"

/codon_start=1

/transl_table=11

/product="hypothetical protein"

/translation="MSFKAVELQIAVPRTSEAGRYQSEAQQRPITDQNLLAEQTSKEA

NEARQRSEAMDETSQTSVRDGHNHDGDQQSGSDGQETAQAQETVKPAEHPYKGKHIDL

SL"

CDS complement(3436131..3437570)

/locus_tag="EFAGFIKM_02929"

/inference="ab initio prediction:Prodigal:002006"

/codon_start=1

/transl_table=11

/product="hypothetical protein"

/translation="MESLKGGRIAVTQRTALEQCLNIVLSDDKCTAYLEFSKEEEGFA

CTIDELEQFVANKGIKQGVSREALLLFVSNPETYLKDKYKIAKGVAPIQGTDGFIKVL

VGMDDTNERRPLESEDGTVDYKEVTRLNNVRSGQIIAERIAPSDGIPGRAVTGEEIPY

RPGKEARFKVGKNVVINPDGSAMYAALDGLVTKTDGNKLNVFPVYEVNGDVDYNNGNI

DFVGTVVIRGNVLTGFKVKAAGDIRVVGGVEGAELEAGGSIEITGGIIGYNKGLVQAG

HNVKCTFIQEGNVDAGEDVLVSQSIMHSNIRAGHGVICAGTKGLIVGGSIQAGQNVSA

RVVGNSMSTVTSIEVGVLPKLRNELNDLRKEVREQMDSLDKTKKALTLLDQLAAAGQL

TPDKMSMRIKLNATQKSALRISEETKMRIFEIEKALEDTSNARVDILKMIYGGSKIVI

GRYTKFIKDPVSRISFYYHDGDITMVPYV"

CDS complement(3437603..3438391)

/gene="sigD"

/locus_tag="EFAGFIKM_02930"

/inference="ab initio prediction:Prodigal:002006"

/inference="similar to AA sequence:UniProtKB:P10726"

/codon_start=1

/transl_table=11

/product="RNA polymerase sigma-D factor"

/db_xref="COG:COG1191"

/translation="MNERKASHLNHSDLWEKWKEHGDLEAKKTLIEKYLHIVNYVSGR

LAVGLPKNVPKDDLESNGVMGLIDALEKFDYERGLQFETYASWRVRGAILDGLRQGDW

VPRSVREKAKRIEDAYQQLEQSYLRSVSDEEMSEYLDVSTKDFQHMLQEVAVMSLCSL

EDPIREEESETRLSLMVDEKAKNPDYKVNEFYLKEALVQGLDKLTVKERTVVSLLYYE

DLSLSEIAEVMSLSPSRISQLHSKAILRLRGTLDKQKDLLMRKD"

CDS complement(3438407..3438850)

/locus_tag="EFAGFIKM_02931"

/inference="ab initio prediction:Prodigal:002006"

/codon_start=1

/transl_table=11

/product="hypothetical protein"

/translation="MIGNYRINLGAGIVGFILTFFVAYSSNVLMTSLIRGLIGFVAWF

VLAYGLRWGLGLLLNPQIEGNGYGYDSPSAQDQRGSQVDIKIEDDGQELNDLLKSGQN

ASSGDDLPPSETKAPTGFAPLDPPKLVRTKDPEELAQAVRHLTDK"

CDS complement(3438855..3439352)

/gene="cheD"

/locus_tag="EFAGFIKM_02932"

/EC_number="3.5.1.44"

/inference="ab initio prediction:Prodigal:002006"

/inference="similar to AA sequence:UniProtKB:P40404"

/codon_start=1

/transl_table=11

/product="Chemoreceptor glutamine deamidase CheD"

/db_xref="COG:COG1871"

/translation="MIEDKSVVKVGMADLNIAHLPGVIRTTGLGSCVGLTMYDPHLKL

AGMAHVMLPTSEIAREGKLNTAKYADTALPELLEKMIKLGASHSRIVSKMAGGSQMFA

FAGAGDTMRIGPRNADSCREWLQKLNIPLLAEDTGGNYGRTIEMDCETGLLTIRSVQM

GVKEL"

CDS complement(3439345..3439971)

/gene="cheC"

/locus_tag="EFAGFIKM_02933"

/EC_number="3.-.-.-"

/inference="ab initio prediction:Prodigal:002006"

/inference="similar to AA sequence:UniProtKB:P40403"

/codon_start=1

/transl_table=11

/product="CheY-P phosphatase CheC"

/db_xref="COG:COG1776"

/translation="MEMFNRFEVFQMDVLKEVGNIGAGNAATALSQLLNRPIDMGVPT

VQMLPFEEVAEKVGGDERIVVTVFLRVEGEAPGNLFFMMTPEAAKMLLNRLAGFDLKE

GLAFTDMEQSALSEIGNILAGSYLSSLADFTKLSMYPTVPGLAIDMAGAILSYGLLQF

GEMGDSALLIDTSFFEGEDQVEGQFFLIPDPPSFAKIFESLGVPLSHD"

CDS complement(3439976..3440437)

/gene="cheW_2"

/locus_tag="EFAGFIKM_02934"

/inference="ab initio prediction:Prodigal:002006"

/inference="similar to AA sequence:UniProtKB:P39802"

/codon_start=1

/transl_table=11

/product="Chemotaxis protein CheW"

/db_xref="COG:COG0835"

/translation="MEEELKVIVFKLGSEEYGIEVDKVQTIERMMPITRVPKTLSFVK

GVINLRGVVIPVIDLRGRFSLPETEYTDQTRIVIVGVDDMQVGFIVDSANDVIDIKSS

AIDSPPEVVGGVKARYLRGVAKLEDSRLLIMLNLNEVLNKSEIVQLESVEG"

CDS complement(3440479..3442551)

/gene="cheA_2"

/locus_tag="EFAGFIKM_02935"

/EC_number="2.7.13.3"

/inference="ab initio prediction:Prodigal:002006"

/inference="similar to AA sequence:UniProtKB:P29072"

/codon_start=1

/transl_table=11

/product="Chemotaxis protein CheA"

/db_xref="COG:COG0643"

/translation="MDMNQYLSMFIDESNDHLQSLNENMLQLEGNPEDLGIVQVIFRS

AHTLKGMAATMGFEDLASLTHKMENVLDLVRNEKLKMQDYIFDTMFKSLDALETMVQD

ITEGGQGKADVSSIVASLQAIENGEMTNGNGPATETNKPANASISSAVELDEFQYSVL
DQSIAEGHRVFYVDVLVSEHSQLKGVRAYMVFDMLERSGEVVKAYPSVQDIEQEKFER
SFSLYYITTKEAHELEEGIMSISEIESAKLIQLDQETLQQMANQVAATVEAPPAPTAV
AEVASPDKNSVPKEETTTAPAKTAAPKQAAAPSRTIRVDIERLDVLMNLFSELLIDRS
RLEQLASETGNNDLSDTVAHLSRVSTDLQNIVLKLRMVPVDTVFNRFPRMIRDLAKTL
DKKIDLVITGAETELDRTVIDEIGDPLVHLLRNAVDHGVESISERVAAGKPEMGTVNL
RAFHSGNHVFIEIEDDGKGIYRDKLLKTAIKRGVVTEEQGAKMSDDEVNQLLFAPGFS
TADIISDISGRGVGLDVVKSKITSLGGNVTIHSTPGKGTNFSVQLPLTLSIIAAMLVR
LGSEKYAVPLSSIVETAIVQREQVRNIHGNKMITFRESLIPYLSLSEVFSVPDFNDAD
EQETEIVVIRKGDRLAAVAVEEFIGQSEIVLKSMGTYLPAIEGISGATILGDGQVALI
LDPNAFIK"

CDS complement(3442584..3444026)

/gene="cheB_9"

/locus_tag="EFAGFIKM_02936"

/EC_number="3.5.1.44"

/inference="ab initio prediction:Prodigal:002006"

/inference="protein motif:HAMAP:MF_00099"

/codon_start=1

/transl_table=11

/product="Protein-glutamate
methylesterase/protein-glutamine glutaminase"

/translation="MAAYQVLVVDDSAFMRKIVTDLIQADPEFKVTATASNGREAIQK
SLELKPDIITMDVEMPEMNGLDALKSIMKESFVPVIMLSGINEQGMKETIMALEAGAF
DFIRKPSISHDQDIAQVGKALVERMRAAMNEIKRKADRELSMKNRDMLRGAVAPPTQP
VQKELPARDRAEPVKKTIEPVETIQRSLRERTEAFQAKAKKPIEPLSPSKPGRVENKP
EPLPKAERNVGTKAEKAVRSANPVQTPKEVRLPNAARVAADQIAATSASAKAKDVIKP
NPVPKGGGGLEGPFNKLVAIGCSTGGPRALKTLLEQLPADLPAPVIIVQHMPPNFTRS
LAQRLNTFSPLHVVEAEEGMVLKKGTAYIAPGGFHVKVNKTADGKFIVKLTEDQPVNG
HRPSVDTMFESLLPFTSLQRHLVLLTGMGSDGARMMKRLYEAGVTSTFAENEETCVVY
GMPRSAVELQCVRHLLPLQEIASKLVQAVK"

CDS complement(3444059..3444946)

/gene="ylxH"

/locus_tag="EFAGFIKM_02937"

/inference="ab initio prediction:Prodigal:002006"

/inference="similar to AA sequence:UniProtKB:P40742"

/codon_start=1

/transl_table=11

/product="Flagellum site-determining protein YlxH"

/db_xref="COG:COG0455"

/translation="MKDQAAALRSMVSAPLELEGIERDIRSSKIITVASGKGGVGKSN

FTLNFALALQALGQKVLVFDADIGMANIDVLMGTSSSYNLYHLLYRQKSIREIIQLGA

SGLPYIAGGSGMKELFSLSDRDLEFFASQVEDIAQEMNYVIFDTGAGLSRENMKFIGA

ADECLIITTPEPTSITDAYALVKVMHGQENATPFRMIVNRVEDEREAERVAEKIAGVA

RRFLQTDIPLLGYISEDAQVVKAVKRQMPYSLAYPNAKASKDIEKLALRYLAVPATPG

YGTLTGIRGFMNKWLKRTT"

CDS complement(3444943..3446295)

/gene="ftsY_1"

/locus_tag="EFAGFIKM_02938"

/inference="ab initio prediction:Prodigal:002006"

/inference="protein motif:HAMAP:MF_00920"

/codon_start=1

/transl_table=11

/product="Signal recognition particle receptor FtsY"

/translation="MRVKQYVVETMPEAMLQIRKDLGSDAVILSTKEIKVGGVMGMFR

KKRIEVVAAVDKEENKQTTKPVQNQFTPVPRSFVPEAYRQTARSFVAASDESVTTNTA

DQSVQDQSAAAPSVFESRNIGSNIDSGSGSSSTDHKPRPQGADFSGSTTGPKPTGSDL

QQDKLMTELQDLKQMVTRLSKQGTSADPVPEELHTIRERLTEQDVWPEVWESWFDSIQ

AKWSEDGLKEQDVEQIVKLEVMHFLEQRIEEGILPTTRIVYVAGPTGVGKTTTIAKLA

AEQMFKKQRKVGFITSDTYRISAVEQLRTYASILNVPLEVVQSPGDTQRAISRLENCD

LIFMDTAGRNYRNELLVSELQSLLAPVENSETFLVMSMTSKSADMVQITEHFSKYGLD

KVIFTKMDETGSCGPLFNLLHRFPLKLAYVANGQNVPDDLLKPDADSLSKQLLGEWSQ

"

CDS complement(3446292..3448325)

/gene="flhA"

/locus_tag="EFAGFIKM_02939"

/inference="ab initio prediction:Prodigal:002006"

/inference="similar to AA sequence:UniProtKB:P35620"

/codon_start=1

/transl_table=11

/product="Flagellar biosynthesis protein FlhA"

/db_xref="COG:COG1298"

/translation="MKIKDIAVLAGIIGIVLMMILPIPTWLLDLLLVVNISIALMILL

VAMNSKEALQFSIFPALLLITTLFRLALNISTTKLILGEGDAGAVVATFGSWIAGGQI

AIGFIVFLILVVVQFIVITKGSERVAEVAARFTLDAMPGKQMSIDADLNAGLINEQQA

RERRSKIEREADFYGAMDGASKFVKGDAIASIIILLINLIGGFIIGMTVHGMAFADAM

STYSVLTIGDGLVSQIPALLISTAAGLIVTRASSEGNLADDITGQLFTYPILIYIVAF

VIAMLGFFTPIHVITTLPLAGVLAYAAWRMQNNLNLKQVAEEQLEEEQQIEEVRSPES

VINLLQVDPIEFEFGYGLIPLADNQQGGDLLDRIIMIRRQCALELGLVVPVIRIRDNI

QLRPNEYVIKIKGNVVGGGELLLNHYLAMSPGYDEESVTGIETTEPAFGLPALWIDEV

TKDRAELAGYTVVDPPSVVATHLTELIKKHAHELLGRQETKALVDNLRENYSALVDEL

IPSLLSIGDVQKVLAKLLREKISIRDMVTIFETLADYGTYTKDPDVLTEYVRQSLSRQ

ITQQFSQKGETLRVITVGPGLEKKIAESVQQSDQGSYLALDPVSTQSVYQKLSEQVNR

LIQSGQQPVVLTSPTIRMYLRQVIERTMQDIPVLSYSELEPNVEIQSIGVVNL"

CDS complement(3448351..3449433)

/gene="flhB"

/locus_tag="EFAGFIKM_02940"

/inference="ab initio prediction:Prodigal:002006"

/inference="similar to AA sequence:UniProtKB:P40727"

/codon_start=1

/transl_table=11

/product="Flagellar biosynthetic protein FlhB"

/db_xref="COG:COG1377"

/translation="MKLQLDLQLFSGEKTEKATPKKRQDTRKKGQVVKSAELSGASIL

LIVFLIMMVFSNFYKERIVRLFTDIFINRLSMDITGENVMALMMRYGIEVLLLIAPVL

LGALLVALIVNYMQVGFLLIGEGLKPKLEKLDPIKGFKNIFSLRSLVEFAKSILKMSI

IGYLVYSTIKGYQSDIASLSHFSLDAILHFAASITLSLGIKIAVALMILAIFDYMYQK

YDHEKKIRMSKQDIKDEYKKMEGDPLIKGKIRERQRRMAMQRMMQEVPNADVIITNPT
HFAVALKYEGSEMEAPQIIAKGQDYVALRIKEIAKENGVITMENKPLARALFQRAEIG
DAIPADLFQAVAEVLAYVYKLKGRTK"

CDS complement(3449457..3450245)
/gene="fliR"
/locus_tag="EFAGFIKM_02941"
/inference="ab initio prediction:Prodigal:002006"
/inference="similar to AA sequence:UniProtKB:P54702"
/codon_start=1
/transl_table=11
/product="Flagellar biosynthetic protein FliR"
/db_xref="COG:COG1684"
/translation="METLLQSFPVALLMFCRITSFFVTAPVFSARNVPASVKIGISAF
VTLSVYLIYGIDQTVPTDLSYVLLIIREILIGLLLGFVAYLLMTAVQTAGTFIDLQIG
FGMANVYDPMTGASAPLTGNLKYAFAMLLFLTMNGHHYLLDAIVYSYRWVPLSNVFFL
RLADGSIAEFLIQTLGQSFMLAFQMSAPIVVALFLTDVGLGFLAKTAPQFNVFAVGLP
LKLLVGLAILLLLVPSFSFVFSQLFEAIFRSMEKLLGTIGQRPG"

CDS complement(3450256..3450525)
/gene="fliQ"
/locus_tag="EFAGFIKM_02942"
/inference="ab initio prediction:Prodigal:002006"
/inference="similar to AA sequence:UniProtKB:P0A1L6"
/codon_start=1
/transl_table=11
/product="Flagellar biosynthetic protein FliQ"
/db_xref="COG:COG1987"
/translation="MTSEFIIGLAGKAVYTSLLASAPMLILALVVGLAISIFQATTQI
QEQTLAFVPKIVAVLLSVLLFGPWILNILVDFTFNILDNLYRYIG"

CDS complement(3450551..3451306)
/gene="fliP"
/locus_tag="EFAGFIKM_02943"
/inference="ab initio prediction:Prodigal:002006"

/inference="similar to AA sequence:UniProtKB:P54700"

/codon_start=1

/transl_table=11

/product="Flagellar biosynthetic protein FliP"

/db_xref="COG:COG1338"

/translation="MKKKIWLACCLLGLISLASVTVAFSEPIPNIDIQIGNGDGGTPS

TSSLSIILLITVLSIAPAMLVLMTSFTRIVIVLGFIRTSLGTQQMPPNQVLVGLALFL

TLFIMSPTLSSINQVALQPYLQGELTQTEALEKAADPMKKFMFSHTREKDLLLFMKYN

QTEQPKTYQDIPITVMVPAYVISELKTAFQMGFMIFIPFLVIDIVVASTLMAMGMMML

PPVMISLPFKILLFVLVDGWYLVVKSLLLSFNT"

CDS complement(3451303..3451842)

/locus_tag="EFAGFIKM_02944"

/inference="ab initio prediction:Prodigal:002006"

/codon_start=1

/transl_table=11

/product="hypothetical protein"

/translation="MMAQGDIPGGAGVGSNYYLQLVWVIVVLAVILALIVYLIRFLNK

RNQQMFRHGTIRTLGGVGLGQNKSLQIMEIGGCVYLLGVGEDIQLIDKVSDLEEAQRI

IDSFERDAAAQQGNLSPLIAKLTKRFRKEEPPREMELEDTTSFHEMFESKLRQMPNRK

EKMEKLLDKDNTTDRSRDS"

CDS complement(3451851..3452216)

/gene="cheY_2"

/locus_tag="EFAGFIKM_02945"

/inference="ab initio prediction:Prodigal:002006"

/inference="similar to AA sequence:UniProtKB:P24072"

/codon_start=1

/transl_table=11

/product="Chemotaxis protein CheY"

/db_xref="COG:COG0784"

/translation="MANRILVVDDAAFMRMMIRDILSKNGYEVVGEAQDGSQAIEKFK

ELRPDLITMDITMPEMDGIAALKEIKKIDANAKVIMCSAMGQQAMVIDAIQAGAKDFI

VKPFQSDRVIEAISKTLGV"

CDS complement(3452245..3453519)

/locus_tag="EFAGFIKM_02946"

/inference="ab initio prediction:Prodigal:002006"

/codon_start=1

/transl_table=11

/product="hypothetical protein"

/translation="MTSKDYLSQEEIDALLRQSESINSSEPAEKTVDDFLTELEQDAL

GEIGNITFGSAATALSTLLGLKVDITTPKVSIISRTQFEEAFPKPHVAVHVNYVDGFE

GINSLVIKKRDAQVIADLMLGGEGNPVDEELNEIHISAVQEAMNQMMGSSATSMSTIF

NRFVNISPPGIDILNLESGEGVSNLPADETLIQVSFRLLIGDLIDSNLMQLLPVHFAK

SMVDMLIGGAQESTASAPVASTPEPAPAAVPATPPPVTEQPPVQHQQAPQAPQQPAQD

YNGYGQAPMGMPQGMPPQQPYGMPPQQPYGAPQHYGGMPNRNVNVQPVQFANLQNGAY

GQVDENNLNLLMDIPLKVTVELGRTQKQIKDILELSQGSIVELDKLAGEPVDILVNNK

LIAKGEVVVIDENFGVRVIDIVSQWDRIQKLQ"

CDS complement(3453509..3454507)

/gene="fliM"

/locus_tag="EFAGFIKM_02947"

/inference="ab initio prediction:Prodigal:002006"

/inference="similar to AA sequence:UniProtKB:P23453"

/codon_start=1

/transl_table=11

/product="Flagellar motor switch protein FliM"

/db_xref="COG:COG1868"

/translation="MVDVLSQNEIDALLAALSSGEMDAEELKKEDTQKKIRSYDFKRA

VRFSKDHIRSLTRIHENFARFLTTYFSAQLRTFVQINVVQVEQLPYDEFIRSIPKMTI

LNIFEAEPLQGRMVMEVHPNVGYAMLDRLLGGTGTAPTKIASMTEIETTIMERIFSRA

FESLQEAWKTVLDITPRMEALETNPQFMQIVSPNETIALISLSTKIGDTTGMINLCIP

HVVLEPIMSRLSTHQWFVSEKKTRAPEEYDALKERVNKAKLPIVAELGESRISIAEFL

GLSVGDVITLNKPVDEGLSIKVGDKLKYMGSPGTIKDRVAVQIDKIVTEGVEEFDE"

CDS complement(3454548..3455009)

/locus_tag="EFAGFIKM_02948"

/inference="ab initio prediction:Prodigal:002006"

/codon_start=1

/transl_table=11

/product="hypothetical protein"

/translation="MKKMMPWLATMLLAITLIVVVVFVFMQGQNGNKDDTHTAAASEA

KKMTADEIVEVSSELGEIKTNLADTDHVIVVSFSFKLADVKAKEDFEKIKEIVVKPII

IQTLADTKSDELATAKGRILFNEKLTGLINEALPESNLSSTSFSSIVIATM"

CDS complement(3455009..3455230)

/gene="swrD"

/locus_tag="EFAGFIKM_02949"

/inference="ab initio prediction:Prodigal:002006"

/inference="similar to AA sequence:UniProtKB:C0H412"

/codon_start=1

/transl_table=11

/product="Swarming motility protein SwrD"

/db_xref="COG:COG1582"

/translation="MISVTRLNGSPMWLNALMVEIVEETPDTYITLVTGKRLIVLEKA

ADVISKIKDYNREIGVQAATIKVQQTEES"

CDS complement(3455299..3456114)

/gene="flgG_1"

/locus_tag="EFAGFIKM_02950"

/inference="ab initio prediction:Prodigal:002006"

/inference="similar to AA sequence:UniProtKB:P0A1J3"

/codon_start=1

/transl_table=11

/product="Flagellar basal-body rod protein FlgG"

/db_xref="COG:COG4786"

/translation="MLKSMYSGVSGMRGFQTKLDVIGNNIANVNTVGFKGSRVMFKDI

MSQTTSGVTAPGDATGGVNAKQIGLGVSVGSIDTLHLAGSPMTTNNPTDMRINGDGFF

LVRLSEDQEVPYLTRAGDFHVDAARNLLTSDGLFVLDNGGGNITIPDDVVSFTIGQDG

TINQTMADGTVEAGAQLGIGKVVNPEGLEKIGGNLYRMTANANPDGELEILTANDAEN

GTGSIITGQLEMSNVDLTGEFTEMIVAQRGFQANSRIITTSDEILQEVVNLKR"

CDS complement(3456196..3456579)

/locus_tag="EFAGFIKM_02951"

/inference="ab initio prediction:Prodigal:002006"

/codon_start=1

/transl_table=11

/product="hypothetical protein"

/translation="MSDRITVGQLYAGPITPNMLQRPKTGEASAIPEKPFAKVLEDNL

LKLSNHAAKRLEQRGIELKTEQMEQIGSALDKAAAKGAKESLILMQDMAFIVNVKNRT

VVTAMDSESMKDNVFTQIDSAVIIS"

CDS complement(3456576..3457133)

/locus_tag="EFAGFIKM_02952"

/inference="ab initio prediction:Prodigal:002006"

/codon_start=1

/transl_table=11

/product="hypothetical protein"

/translation="MANENVSMNNTWPNYSAANKATTSAATKELGKDQFLKILITQLQ

NQDPMQPMEDKEFIAQMAQFSSVEQLVNISTQLKTLNQSLGAVSGMIGMEVSWLSSNK

DDNGTLRQGIVDSIIVRDSVQYAKVGNDEIKLDEIIQVNYPKQAEESQTPVQNVQDET

PETNESQEVESSAETGDTEDSGKTI"

CDS complement(3457153..3458541)

/locus_tag="EFAGFIKM_02953"

/inference="ab initio prediction:Prodigal:002006"

/codon_start=1

/transl_table=11

/product="hypothetical protein"

/translation="MSIVYQMASTASAKATGAGQTTGAQSKGSAAGASGEFLQTLAQS

LSGGNTEGDSSSATGSLTANPLVFSFATSEEGEAASITDILNSLFTDLDSLDEALEND

PALLAGLQTLIQQMYTQLSNPSGTNEEGSDESSNGAESVKTVPAIELSQHPAAVRFVL

QDMLTQLVAGMNDPESNVAKNAPEFKHLLQSLQSQLQEAGVDTTSNKGWTELKSILDT

LTAVKDQTAQVAPSTSLQTSKQDSVVPQVLVAAVANSGTQVKAEAETTSASNAGGEVE

HSTIITAGELSLRSSGTTAGKPAEPVMQTSQFAKEMTQFVVNKLDIVQQKGFSEATIS

LRPEHLGKLDVQITLQNGQLVARFMTEHTMAKDMLEQQMTQLRSSLQAQGIQVERLEV

TQNSSIGSQMYQDGGRQPGSNSQQQRRSREREEQSDDAIATAGIQEELRNWRSEQVEG

NELQRDTFSAEA"

CDS complement(3458581..3459522)

/locus_tag="EFAGFIKM_02954"

/inference="ab initio prediction:Prodigal:002006"

/codon_start=1

/transl_table=11

/product="hypothetical protein"

/translation="MAVKDDSDMEKESGGGWEKFLMISIPIVFTVVLLGVLLTLFNVD

IRNNLFEFANKIPVVKEWVPDPVLDPEKEKLEKSEQQVESAEATIEKLKSQVTAKETE

LKAAQEATTTEAKKATDLQKKLDDAEKAAETATAATPETESDYQKQIKDLAKMYADMS

PSKAAPILQNMTNEEMVLLLNAMQSSARTKVLEKMDPKTAADVTMMMKDAKPSGDLAL

DALQSRLQKETAATSTASTTTSKNLDKNQLSQTFASMSASSGAKLLLETYKLSPDKTL

TILNSVDDATRSQLLENMSSEDSVETAKILNRLMGNK"

CDS complement(3459566..3460018)

/locus_tag="EFAGFIKM_02955"

/inference="ab initio prediction:Prodigal:002006"

/codon_start=1

/transl_table=11

/product="hypothetical protein"

/translation="MKFRYHFQKVVDLKSNEKTQAEWMLSTAIGKLQTEEEHLIQLLN

DRSNLIGIIQSATENTASVNSLQEMQRYVHHLDECISRKNSDVKHAQVNVQRNQTFLN

GKMVDEKVWLGARDKAKNKFQQEMLLREQNDLDEMATVRFAAKAGRAN"

CDS complement(3460030..3461349)

/gene="yscN"

/locus_tag="EFAGFIKM_02956"

/EC_number="7.1.2.2"

/inference="ab initio prediction:Prodigal:002006"

/inference="similar to AA sequence:UniProtKB:P40290"

/codon_start=1

/transl_table=11

/product="putative ATP synthase YscN"

/translation="MKVLSSQRYMEHLRQFDPVRINGKVTQVIGLMVESEGPDASIGD

VCYIYPGKSAKPLQAEVVGFRDNKVLLMPLGELQSIGPGCDVVGTGKPLGVQVGSELL

GKVLDGLGQPLDGSLLPSRMPMYSTSNTPVNPMDRPRVLETMGVGVRAIDGLLTVGKG

QRVGIFAGSGVGKSTLMGMIARNTAADVNVIALVGERGREVRDFIERDLGPEGLERSV

VIVATSDQPALIRIKGAVIATTIAEYFRDRGMNVMLMMDSVTRYAMAQREVGLAVGEP

PAMRGYTPSVFASLPKLLERAGTGPTGSITAFYTVLVDGDDMNEPIADAVRGILDGHI

VLNRSIANKGHFPAIDVLASISRVMKDIAPEEQLEAVNNMKRLMAVYKESEDLINIGA

YQRGSNAAIDESIDQIDSIWNFTKQKVDEKVTLSEVQERLILEFARR"

CDS complement(3461336..3462199)

/locus_tag="EFAGFIKM_02957"

/inference="ab initio prediction:Prodigal:002006"

/codon_start=1

/transl_table=11

/product="hypothetical protein"

/translation="MSNLIKSFQYVPVDDRKKLENHHHYGDAEESETELYVDSAEGSE

AETLQARVDEETQRLTAEMLEDAKEFAEKQVREASEEAERMLQEAREQIDSWWQEQRQ

QDEHLTEALRSQGFQQGFEEGKVQAELDLQVQIEKMMNEAQEVLKEAYVAKDQIIQEA

EPFLVELACGIAEKVIDKQLTIEPDHTLELIRQSLSRKREQGLITLCVAPDQFTFVQA

AREELSLSIDSQAELQILPDSTVKDKGCVIRSSFGSVDARIDTQLAEIKKELVRIALE

DEERKNQHEGS"

CDS complement(3462192..3463208)

/gene="fliG"

/locus_tag="EFAGFIKM_02958"

/inference="ab initio prediction:Prodigal:002006"

/inference="similar to AA sequence:UniProtKB:Q9WY63"

/codon_start=1

/transl_table=11

/product="Flagellar motor switch protein FliG"

/db_xref="COG:COG1536"

/translation="MAKASSQGLTGKQKAAILLITLGPEVSAQIFKHLRDEEIEQLTL

EIANVRKVDSSEKDMIMAEFHQICLAQEYISQGGINYAREILEKALGSSKALEVINRL

TATLQVRPFDFARKADPNQILNFIQNESPQTIALVLSYLQFEQAAAILSSLPQEKQAD

VARRVAVMDSTSPEVISQVERVLEQKLSSTVTQDYTNAGGIESIVQILNGVDRGTERT

ILDSLEIQDPELAEEIKKRMFVFEDIVNVDDRSIQRIIRDIDNADLQLALKVASEEVR

DAVFRNMSKRMSETFKEEMEFMGPVRLRDVEEAQTRIVGTIRRLEEAGEIIIARGGGD

DIIV"

CDS complement(3463220..3464803)

/locus_tag="EFAGFIKM_02959"

/inference="ab initio prediction:Prodigal:002006"

/codon_start=1

/transl_table=11

/product="hypothetical protein"

/translation="MNERIAQYRDKASLYWNSFSKKQKVLFISTFLFLILAAVVLTMQ

LSKTEYEVAFTDLNASDSAGVISYLDSSSIPYKLSSDGKTISVPSTSVAIAKVNVGSQ

GIIQNGSLGYKSFEESSSPIGMTDKEFDVKYNNAINGEVEQLLQRMQGIQDAKVLVNM

PKDNIFAGLEEQDKASASVALQFKPGYHPNQAAVDGYFNLVKTAIPNLPVENITITNT

DEAELIPTARGGSGGLSSEVQENMALQKKFENDVRNNVKQFLSQIVGEDKVNVLVASK

LNFDKETRKENLVTPVDVDNMKGIEISVQEIQKSYTGASNPTGGVAGTGQEEVPGYPS

SDATGNSTSEESSSTINYDVNRIAKDIISSPYTVKDLTINVAVEPPNGETELQGPVQD

AIENILVNIVRASLADSGTVISDADLTKKVSVMSQGFQSASADNTGFQLSSTMMWGGG

ALIAALIAAVVILLVRRRRKQNEVEEEEIPLPIATEFPSITLDSVTNESQVRKQLESL

AKKKPDEFVNLLRTWLADE"

CDS complement(3464941..3465252)

/gene="fliE"

/locus_tag="EFAGFIKM_02960"

/inference="ab initio prediction:Prodigal:002006"

/inference="protein motif:HAMAP:MF_00724"

/codon_start=1

/transl_table=11

/product="Flagellar hook-basal body complex protein FliE"

/translation="MIQNNMFSTQGIQPLQMKSATESKPSTPAETIQSFGTYLQNALG

SVAAQEAQSHEMSNQFLVGKANVDQVMIASEQALLSLQLTTQVRNKVVEAYQEVMRTQ

L"

CDS complement(3465311..3465760)

/gene="flgC"

/locus_tag="EFAGFIKM_02961"

/inference="ab initio prediction:Prodigal:002006"

/inference="similar to AA sequence:UniProtKB:P0A1I7"

/codon_start=1

/transl_table=11

/product="Flagellar basal-body rod protein FlgC"

/db_xref="COG:COG1558"

/translation="MNISNSFSISSSALTAQRLRMDVISSNIANAETTRASVSNGEAV

PYKRKMVVLEPNKTSFGTMLQNQMRGSGSGDGVRVTEIREDQSPLKPVYDPSHPDANA

EGYVFMPNVDIAKEMVDMISASRSYEANVTALNSTKAMISKALEIGR"

CDS complement(3465767..3466174)

/gene="flgB_1"

/locus_tag="EFAGFIKM_02962"

/inference="ab initio prediction:Prodigal:002006"

/inference="similar to AA sequence:UniProtKB:P24500"

/codon_start=1

/transl_table=11

/product="Flagellar basal body rod protein FlgB"

/db_xref="COG:COG1815"

/translation="MNLLNDISFKRLQGALDASNIRQRTIADNIANADTPYFKRSDVS

FEEMLQGQMNGDMPVLKGKVTDARHFVIGPSSSVPTPVVNMDQSTSMNNNENNVDIDR

EMSLLAENQLRYNAYIQQVNEQIKIMRVGVEGR"

CDS complement(3467156..3468556)

/gene="clpY"

/locus_tag="EFAGFIKM_02963"

/inference="ab initio prediction:Prodigal:002006"

/inference="similar to AA sequence:UniProtKB:P39778"

/codon_start=1

/transl_table=11

/product="ATP-dependent protease ATPase subunit ClpY"

/db_xref="COG:COG1220"

/translation="MNTQALTPRQIVAELDKYIVGQKKAKKSVAVALRNRYRRSLLPE

HTQDDIVPKNILMIGPTGVGKTEIARRLAKLVGAPFVKVEATKFTEVGYVGRDVESMV

RDLIETSLRMVKLERTEKVKDKAEEAANERIVHILAPSQSKSKSQRNPFEMIFGNNGN

NVQEQDEPEPDTGVAERRRKIKFDLLSGKLEDDIIEIDVEDTAPNMMDMFAGQGNDQM

GMNMQEMFGSLLPRRTKKRKLAIKEARKVLIQEEAGKLIDMDDVTQESIRRAEQTGII

FIDEIDKVASQGRGSGPDVSREGVQRDILPIVEGSTVMTKYGPVKTDYILFMAAGAFH

VAKPSDLIPELQGRFPIRVELSSLTLEEFVSILTEPQNALTKQYVDLLRTENIEIEFS

DEAIREIAKLAESVNQNTENIGARRLHTILEKLLEDLSFEAPELTLERMVITPEYVRE

KLNDIALDRDLSQYIL"

CDS complement(3468673..3469215)

/gene="hslV"

/locus_tag="EFAGFIKM_02964"

/EC_number="3.4.25.2"

/inference="ab initio prediction:Prodigal:002006"

/inference="similar to AA sequence:UniProtKB:Q81WK5"

/codon_start=1

/transl_table=11

/product="ATP-dependent protease subunit HslV"

/db_xref="COG:COG5405"

/translation="MDMSFHATTICAVRHNGKAAIAGDGQVTMGQSVVMKNTAKKVRR

LYRGQVVAGFAGSVADAITLFEKFEGKLEEHHGNLQRAAVELAKDWRQDRILRKLEAL

LIVMDKSGMLLISGGGEIIEPDDDVIAIGSGGNFALSAARALKRHAVNLEAKDIARES

LQIASELCVYTNNNIIVEEL"

CDS complement(3469242..3470579)

/gene="trmFO"

/locus_tag="EFAGFIKM_02965"

/EC_number="2.1.1.74"

/inference="ab initio prediction:Prodigal:002006"

/inference="similar to AA sequence:UniProtKB:P39815"

/codon_start=1

/transl_table=11

/product="Methylenetetrahydrofolate--tRNA-(uracil-5-)-

methyltransferase TrmFO"

/db_xref="COG:COG1206"

/translation="MTNEQQVTVIGAGLAGTEAAWQIASRGVRVKLYEMRPVVKTPAH

HTDKFAELVCSNSLRANGLTNAVGVLKEEMRMLNSLVLSAADKHAVPAGGALAVDRDG

FSGEITSTLYQHPLIEVVNEELTSLPEDGIVVVATGPLTSPALSEQIKALMGEEYFYF

YDAAAPIIEKDSIDMNKVYLASRYDKGEAAYLNCPMTEEEFDVFYEALITAEVAQLKE

FEKEIYFEGCMPIEVMMKRGKQTALFGPMKPVGLVNPHTGELPHAVVQLRQDNAAGTL

YNLVGFQTHLKWGEQKRVFSLIPGLENAEFVRYGVMHRNTFINSPKLLLPTYQFKERP

NLFFAGQMTGVEGYVESAASGLIAGMNAAKAALGQELVVLPAETTLGSMAQYITTADF

KHFQPMNANFGLLPKLETKIRNKKEKNEALAQRALDGITSFAAAEGLTVPERV"

CDS complement(3470637..3472736)

/gene="topA"

/locus_tag="EFAGFIKM_02966"

/EC_number="5.6.2.1"

/inference="ab initio prediction:Prodigal:002006"

/inference="similar to AA sequence:UniProtKB:Q7A5Y5"

/codon_start=1

/transl_table=11

/product="DNA topoisomerase 1"

/translation="MADALVIVESPSKAKTIGKYLGSKFIVKASMGHIRDLPKSQIGV

EVENDFNPKYITIRGKGSILKELKDARKKVKKVYLAADPDREGEAIAWHLAHALELDD

TADCRVVFNEITKQAVKDAFKTPRKINMDLVNAQQARRILDRLVGYKISPLLWKKVKK

GLSAGRVQSVAVKIILDRENEIDDFEPEEYWSITAKLTADGNPFEAKFHKLNGAKTEL

GSEAEVQAILKQIEGADFTIKEVKEKERSRNPSAPFTTSSLQQEAARKLNFRASKTMS

VAQQLYEGVDLGKEGTVGLITYMRTDSTRIAASAQEEAKEYIVGKYGEPFAPETPRNY

SKKAANAQDAHEAIRPTSILRDPDSIKSFMSRDQFRLYKLVWERFVASQMSSAILDTL

SVDIAAGDTIFRAAGSKVRFQGFMKVYVEGNDDGTTDEDRLLPPLKSGDVLEKQEIEP

KQHFTQPPPRYTEARLVKTLEELGIGRPSTYAPTLETIQKRGYVAIEEKKFMPTELGE

LVIEQMEEFFPEILNVEFTANMEGDLDHVEEGSEDWVKVLAEFYESFEKRLEFAEEEM

KEIEIEDEVSDEICEKCGKPLVYKLGRFGKFLACSGFPDCRNTKPIIKDIGVTCPKCK

EGHVVERRSKKGRIFYGCDKYPECDFVSWDRPSAKPCPSCGSLMIEKRNKKGARLQCT

SCDHQEPVDEPDDESAD"

CDS complement(3472853..3473983)

/locus_tag="EFAGFIKM_02967"

/inference="ab initio prediction:Prodigal:002006"

/codon_start=1

/transl_table=11

/product="hypothetical protein"

/translation="MEERWILFGLHEMEGIGKKTISKLILGQHELPDLLNYGESDWVA

AGLRKDQAARLASQFTIDWIESKREHVYNQGIEVITYLDQNYPILMKETVQPPWVMYA

RGDVSLLHNSSIAMVGTRMPTVYGRKVGEKLAEQLCNAGLTIVSGLARGIDSVCHEAV

LRAKGKTIAVFGTGIDNIYPPENTSLAERITETGLLLSEYPPGTRARQGLFPERNRII

AGLTLGTLVVEADIRSGSLITADAALEAGRDVFAVPGPITSPKSRGAHNLIRQGAKLV

TCAADVLEEYRLGLPNAEQLPYNRGRSTETSDPSGRGIFAEVKLSPDEQRVIYLLEQG

EQSLDQLVELLGWDFGHLHSVLLSLIIKKQISQLPGTKYARV"

CDS complement(3474228..3475157)

/gene="sucD"

/locus_tag="EFAGFIKM_02968"

/EC_number="6.2.1.5"

/inference="ab initio prediction:Prodigal:002006"

/inference="similar to AA sequence:UniProtKB:P80865"

/codon_start=1

/transl_table=11

/product="Succinate--CoA ligase [ADP-forming] subunit

alpha"

/db_xref="COG:COG0074"

/translation="MSILIDKNTKVITQGITGSTGMFHTKGALDYGTQMVGGVTPGKG

GTNVDITLEDGTVASLPVFNTVQEAKEATGATASVIYVPPAFAADSIMEAVDAELDLV

ICITEGIPVLDMIKVDRFMEGKNTVLIGPNCPGVITPGECKIGIMPGYIHMAGHVGVV

SRSGTLTYEAVHQLTTRGIGQSSAVGIGGDPVKGSEFIDILKRFNEDPQTHAVIMIGE

IGGTAEEDAADWVRENMTKPVVGFIGGVTAPPGKRMGHAGAIISGGKGTAKEKIAKLE

SCGIKVAPTPAEMGSTLVSVLEERGILNLCTTH"

CDS complement(3475221..3476381)

/gene="sucC"

/locus_tag="EFAGFIKM_02969"

/EC_number="6.2.1.5"

/inference="ab initio prediction:Prodigal:002006"

/inference="similar to AA sequence:UniProtKB:P80886"

/codon_start=1

/transl_table=11

/product="Succinate--CoA ligase [ADP-forming] subunit

beta"

/db_xref="COG:COG0045"

/translation="MNIHEYQGKEVLKQYGVTVPNGKVAYTVDEAVAAAEALGSPVTV

VKAQIHAGGRGKAGGVKVAKSTDEVRAYASEILGKVLVTHQTGPEGKEVKRLLIEEGC

DIRKEYYVGVVVDRATGRVVMMASEEGGTEIEEVAEATPEKIFKEIIDPAIGLQVFQA

RKLAYSIKIPNELVNKAVKFMLALYTAFVEKDCSIAEINPLVVTGDGNVIALDAKLNF

DSNALFRHKDILELRDLDEEDEKEIEASKYDLSYIALDGNIGCMVNGAGLAMATMDII

KYYGGDPANFLDVGGGATTEKVTEAFKIILSDAKVAGIFVNIFGGIMRCDVIANGVVE

AAKQLGLTKPLVVRLEGTNVELGKRILGESGLNIVPADSMADGAQKIVALVK"

CDS complement(3476625..3477080)

/gene="ohrR"

/locus_tag="EFAGFIKM_02970"

/inference="ab initio prediction:Prodigal:002006"

/inference="similar to AA sequence:UniProtKB:O34777"

/codon_start=1

/transl_table=11

/product="Organic hydroperoxide resistance transcriptional

regulator"

/translation="MQTDSLKLDNQLCFAIYACSREITKMYQPYLEVLGVTYSQYLVL

MVLWEREECTVKEIGEALYLDSGTLTPLLKRLQSAGLINRERSAQDERKVLITLTDSG

RELRNKALSIPESIQGDACLNSTEFETLLGQFKGLLEKVHETNTNAAKK"

CDS 3477559..3479169

/gene="comM"

/locus_tag="EFAGFIKM_02971"

/inference="ab initio prediction:Prodigal:002006"

/inference="similar to AA sequence:UniProtKB:P45049"

/codon_start=1

/transl_table=11

/product="Competence protein ComM"

/db_xref="COG:COG0606"

/translation="MYGKLHSACLYGIDGVLIEVETDLSNGLPQTSIIGLPDSAIREA

VERVRAAIKNCGYQYPLQRITINLAPADLRKEGSSFDLAIAIGLLMTSGQLVLPPQER

TLVVGELALDGSIRSVPGILSMVDLAKRQGFTSVLLPLDNVEEASLIRGIQVFGIRHL

QDIAPENLDQSGSSVGVPNKANTGPVVLKNYAHLAAPITDKTHKMADRKPRQTPLFAD

DYSDVLGQHHVKRALMIAAAGMHNILLVGPPGTGKTMLIKRLPSILPPLSEDEALEVT

KVLSAAGKLKEAPQGLIADRPFRSPHHTISTSGLIGGGGIPKPGEVSLAHRGILFLDE

LPEFQRQVLEVLRQPLEDRTVTISRARAAFTFPAQFMLACSMNPCPCGYLSAHSEEQR

CICSPARVAAYRAKISGPLLDRIDLQVEVPPPGEWRKSAASPSSEEMQAKVIHAHQIQ

ATRYAKSSVRWNSQLSGTLLRRTIHLPQEAEQLLEQTLQTLNLSMRAHDRIIKMAQTI

ADLDHEGEIVTAHVAEAIQYRQLDLNLF"

CDS complement(3479342..3479893)

/gene="ydjA_2"

/locus_tag="EFAGFIKM_02972"

/EC_number="1.-.-.-"

/inference="ab initio prediction:Prodigal:002006"

/inference="similar to AA sequence:UniProtKB:P0ACY1"

/codon_start=1

/transl_table=11

/product="Putative NAD(P)H nitroreductase YdjA"

/db_xref="COG:COG0778"

/translation="MSKATKTINEVRNAIQNRRTVKKFKKEAVPTQQIIELLDTAVWA

PNHKLREPWRFLLFTGNGRKKLAEAIDAEMGEDNKFSPNIMQVPSVMLVVLEEDPRQN

IWDEDFAAVSALVQNFMLAAWSEDIGTFWVTKPFLYAPKFRKPLGIEAGEKIVGMIYM

GYPDVIPSAKERTPAKDKLTLFE"

CDS complement(3480179..3481357)

/locus_tag="EFAGFIKM_02973"

/inference="ab initio prediction:Prodigal:002006"

/codon_start=1

/transl_table=11

/product="hypothetical protein"

/translation="MQTSIKWIIRLSTLSLCFILFGIMYSGVSYADPSTQASVSGKVT

DLEGFPIQDATIKYSLRYQGEETEKSVKTDGNGSYSILESVQDYTEISFIIEAEGYVT

DRHRNTYYLSEGEQILVNFELYEPSTIVGSVTDQTGKPILDALVKVTGALDRPVKTDQ

QGRYAVTGLDFDYNPNIAIWVDSADYMLYEQDRLNVQAGKTLIEDVVLTEAAHVRGKV

VDEAGNPVSGAKVNAGGSATTTTDAQGNYFIKRVPTGTRTITGEAAGYLKSTQNVTLV

QGDHNTFNIVLKKDADITPPVTKYRLVPITDTVNGKVYIKGFTFRLQATDEVKGSGVK

ITQYRINGGAWNTYEGPVKFYAPDVKVVEYYSTDVTGNQEKYNKMDFVNGTFEGAGSY

"

CDS complement(3481418..3481579)

/locus_tag="EFAGFIKM_02974"

/inference="ab initio prediction:Prodigal:002006"

/codon_start=1

/transl_table=11

/product="hypothetical protein"

/translation="MKELQNTSNNTVKQVWECPRMEVLDISETMNGGQGIWQYVWDGE

FWKLELMVS"

CDS complement(3481901..3482299)

/locus_tag="EFAGFIKM_02975"

/inference="ab initio prediction:Prodigal:002006"

/codon_start=1

/transl_table=11

/product="hypothetical protein"

/translation="MVERRLGQEPGLKLTRQQKGRLGEEAACQWLREHDYRIIRQNWR

CRSGEIDIIASCEGLIVFVEVRSRSGAAQYGTPQESVDMRKMQQVRSTASVYLQMTGE

TELQIRFDVIAVMLDQAGEIVSVNHIVNAF"

CDS complement(3482293..3482604)

/locus_tag="EFAGFIKM_02976"

/inference="ab initio prediction:Prodigal:002006"

/codon_start=1

/transl_table=11

/product="hypothetical protein"

/translation="MKDESSQPDLLSKKAVALKYVPGESEAPVVVAKGRGKVAEAILD

KARENGVAVQEDAALVEVLSKLDLDEQIPAELYQLVAEVLTYVYRADRMASGREENES

W"

CDS complement(3482601..3484655)

/locus_tag="EFAGFIKM_02977"

/inference="ab initio prediction:Prodigal:002006"

/codon_start=1

/transl_table=11

/product="hypothetical protein"

/translation="MNIGSMIKGLMGDSKPGNAKPLELKEGQVVRGSVVSVSDDGGEA

VLQIQGVQVRAKLETPLRPGETTLLQVQPPGENGITVMKPMTGTLAELPQASLNNLLK

DVGLPDTKGNRELLLAMQRSGLPLTKDNVAMVQNMMTAKPAQVPVEEWVQATGIAFQR

GLPVTAETVKGLHQTVFGPPLHQLLSGLADQLETMLTQTTGKPLLPGEQSATLKPAVV

AGAPVLPNASQTEEGLAALGNGRQVTGTGTPALSTQQGQAALAGSISMTDGGADDAGT

AVKGNVQTGGGTGNAEAGAKAIASNVETAGKGGAGIPSGTGIPGESPSGADAGQVGSR

PGTPGAAAESVAGRVIAGQPEAGAAVRIDGRAETPAAAGTPSAAAWAPAAPTAAQLAP

KLLALLDALRSASTAAPAQPGAAAQAAPASQGGQAAAAAGGVPQPLPAGADAPPAGGN

AAAPAGAAAVHAPVTHEGDPWVGRVLKLLGAEHEQQAVHGAAAQPRVGDAASPANADT

LKGLLLQLASSEGAPAALKDAAGQAVQYLTGQQLLLTTDRSATFAQMHWFIPITGPDG

EETASVQIQSRRGSRGELDASNCRLWFDLDMKSLGPTLVDVHVVNNIVSLRVLNDREG

MGPLLESGREVIHQALDKLGYQLLTFKAEPWPVGQEPGAERKTRASDYSPERYKGVDL

KV"

CDS complement(3484686..3485501)

/gene="rnhB"

/locus_tag="EFAGFIKM_02978"

/inference="ab initio prediction:Prodigal:002006"

/inference="protein motif:HAMAP:MF_00052_B"

/codon_start=1

/transl_table=11

/product="Ribonuclease HII"

/translation="MLRETGCTEQKTEYLRLYYKKVVSDTHAEKAWVEEMSIMFENNE

EIGLNLLDTPIGPKKKKEASEPRDLLLYEREYWDSGFERIAGIDEVGRGCLFGDVVAA

AVILPKDLILEGVNDSKKLTEKKRDALYDIIMEKALAVGIGYADAETIDRLNIKQAAR

LAMKRAVEALGETPDYMLVDAEKVDVNVPQLSIIKGDANSQSIAAASIIAKVTRDRLC

KEEWDTLYPEYGLSIHKGYATKVHREQIMALGATPMHRRSFLGNLLGEQQSLF"

CDS complement(3485851..3486714)

/gene="rbgA"

/locus_tag="EFAGFIKM_02979"

/inference="ab initio prediction:Prodigal:002006"

/inference="similar to AA sequence:UniProtKB:O31743"

/codon_start=1

/transl_table=11

/product="Ribosome biogenesis GTPase A"

/db_xref="COG:COG1161"

/translation="MTIQWFPGHMTRARRQIQDKLKLIDVVIELLDARLPVSSRNPMI

DEILLDKPRMILLNKSDLADAKVTQEWIEYFKKEGITAFPVDASTGTNVKDIPVQAKL

LLKEKIDRQIAKGINPRAVRGLIVGIPNVGKSTLINRLAGRSIALTGDRPGVTKGQQW

IKVGKEMELLDTPGILWPKFEDQNVGYRLAVTGAIKEEILNAEDIAFFGISYLMRYYW

DALEERYGLQEFSKDADDSDSVIAIMEQVGRIRGCVVSGGRIDLEKASRAFLRELRAG

KMGRFSMEAPY"

CDS complement(3486745..3487365)

/gene="sipT"

/locus_tag="EFAGFIKM_02980"

/EC_number="3.4.21.89"

/inference="ab initio prediction:Prodigal:002006"

/inference="similar to AA sequence:UniProtKB:P71013"

/codon_start=1

/transl_table=11

/product="Signal peptidase I T"

/db_xref="COG:COG0681"

/translation="MEQEVQQDRGNPAEEKDSRSKKAKNEIVEWLKAIVIALVLVILI

RWLLFKPFVVDGPSMQPNFETGERVIVNEILYDIREPKRGEVIVFHVPSEGRDFIKRV

IAVEGDTVEVQDDTVMVNGKKVDETYIQGAIDAAEANGGTYNVKDFPNEQFPDGKVPA

GHVFVMGDNRPNSTDSRMIGYVSLKDIIGRADVIFWPIGEIKWINH"

CDS complement(3487723..3488064)

/gene="rplS"

/locus_tag="EFAGFIKM_02981"

/inference="ab initio prediction:Prodigal:002006"

/inference="similar to AA sequence:UniProtKB:O31742"

/codon_start=1

/transl_table=11

/product="50S ribosomal protein L19"

/db_xref="COG:COG0335"

/translation="MNIVQAITQEQLRKDLPSFRPGDTLKVHVKVIEGTRERIQLFEG

VVIKRRGGGISETFTVRKISYGVGVERAFPLHSPKIDRIEVARRGKVRRAKLYYLREL

RGKAARIKEIR"

CDS 3488422..3489387

/locus_tag="EFAGFIKM_02982"

/inference="ab initio prediction:Prodigal:002006"

/codon_start=1

/transl_table=11

/product="hypothetical protein"

/translation="MKYWKKITLALLAMLTCSITLYSYAVTHPTNALSHKACTSWDIV

KRKAFELSHTDFSNQSALDRSTFRIEQGTATEVPVLMYHYIEPKLNNHETDNKSIINL

EDFEQNMKYLHDEGYHTITLDQLEQYVSGKISLPQKSIVITFDDGYQNNYTLAYPVLQ

KYNFHASLFVIGSKIQDQPSVFEPAINSFISKPEMQAATDVFEFNSHTYNLHHKGFMR

CGNSVPVGLDTSLLDDDIQQMKETGIDTPYLAYPFGYTSTQMIYKLQQHGYRMAFTVK

SGFVHPGDHPMRLPRLTVTTGTDLATLLQPESSPQNELPASENDQ"

CDS complement(3489391..3490638)

/locus_tag="EFAGFIKM_02983"

/inference="ab initio prediction:Prodigal:002006"

/codon_start=1

/transl_table=11

/product="hypothetical protein"

/translation="MNHQPYEITRLDISEAGERCKMLLGDLNGDGRLEMLLVQADGGI

DDRYVPHQVCCLTAFDLEGTLLWQVGTPDPDAGGPGSDYPAQIADWDGDGNNEVLCVM

DKQFLVLDGRTGEIKETRDLPGDEAHDCIILANLTGNQQRMDIILKDRYKTLWALDHD

FNLLWKHEGNPGHFPWLYDIDGDGKDEVMAGYDMLDHDGTLLWSCQNLDDHADCIWFG

DVDGNGQVEVVIGGSVTVMMDRYGNEKWRYEDSIESQHIALGHFCTGMEGLQIAGLDR

IVRGDEHGKDGMFMLDSAGKEIWKENRTTRGWLTIIEPVCNWDEGGLDYILAYRRGGD

VLPSLVDGDMKTVTEFPKEGYAVHADLVQTGKEQIIIYDAEEAVIYSSSPMTLSLTDA

KRAKPQLKRLYSSTLYPGGEVKI"

CDS complement(3490793..3491167)

/locus_tag="EFAGFIKM_02984"

/inference="ab initio prediction:Prodigal:002006"

/codon_start=1

/transl_table=11

/product="hypothetical protein"

/translation="MTYNIIGLDHIQLAAPEFCEAEARLFFNNVLGWTEIPKPEILRK

RGGVWFECGRHQVHIGVQKDFIPARKAHPAFHVQHLDQLRDLLIHNQIHIVDDEARAD

EGVKRFYINDPFGNRLEFLEWV"

CDS complement(3491366..3492133)

/gene="trmD"

/locus_tag="EFAGFIKM_02985"

/EC_number="2.1.1.228"

/inference="ab initio prediction:Prodigal:002006"

/inference="similar to AA sequence:UniProtKB:Q6GHJ5"

/codon_start=1

/transl_table=11

/product="tRNA (guanine-N(1)-)-methyltransferase"

/translation="MKVDVLTLFPEMFDGVFGASILGKAQTKGLVSLGATNFRNYATN

KHNTVDDAPYGGGGGMVLKPDPIFAAVEDVLEQRGEAAASMKAPRIILMCPQGETFTQ

KKAEELVQEDHLIFICGHYEGYDERIREFLVTDELSIGDYVLTGGELPAMVAIDSIVR

LIPGVLGNETSAVTDSFSTGLLEYPHYTRPPEFRGMKVPDMLLSGHHLNIEAWRREQS

LLRTLERRPEMLETADLTDKERIWLKKIHSNRKHSTE"

CDS complement(3492135..3492647)

/gene="rimM"

/locus_tag="EFAGFIKM_02986"

/inference="ab initio prediction:Prodigal:002006"

/inference="similar to AA sequence:UniProtKB:P66656"

/codon_start=1

/transl_table=11

/product="Ribosome maturation factor RimM"

/translation="MADFMNVGKIVNTHGIRGEVRIMPLTDFPEVRFAKNAELFFFTP

DNHPVVVNVESARLHKNMYILRLKEYGNINEVEKFKGGMAKVLKENLAELEEGEYYFH

QIVGCSVITEEGETLGTISEILTPGANDVWVVKTPAGKEVLIPVIDDVVLDVDIEQQQ

VKIHLMEGLL"

CDS complement(3492949..3493179)

/locus_tag="EFAGFIKM_02987"

/inference="ab initio prediction:Prodigal:002006"

/codon_start=1

/transl_table=11

/product="hypothetical protein"

/translation="MEELVSIIAKALVDHPEDVTVRTVEKDRLVVYELTVHPDDVGKV

IGKQGRIAKSLRTVVTSAAVKMDKRVTVDIIS"

CDS complement(3493203..3493475)

/gene="rpsP"

/locus_tag="EFAGFIKM_02988"

/inference="ab initio prediction:Prodigal:002006"

/inference="similar to AA sequence:UniProtKB:P21474"

/codon_start=1

/transl_table=11

/product="30S ribosomal protein S16"

/db_xref="COG:COG0228"

/translation="MAVRIRLKRMGAHKAPFYRVVVSDSRSPRDGRFIEEIGYYNPVE

QPAVVKIDEDKALAWLQNGAQASDTVRNLLSKAGVMKKFHESKLSK"

CDS complement(3493529..3494908)

/gene="ffh"

/locus_tag="EFAGFIKM_02989"

/inference="ab initio prediction:Prodigal:002006"

/inference="similar to AA sequence:UniProtKB:P37105"

/codon_start=1

/transl_table=11

/product="Signal recognition particle protein"

/db_xref="COG:COG0541"

/translation="MAFEGLTTRLQNVFSKLRGKGKVSDEDVAEAMREVRLALLEADV

NFKVVKEFIAKVKEKAVGKEVMESFTPGMVIIDIVNKELTDLMGGSQAKLAKANKPPT

VLMMVGLQGAGKTTTSGKLAKMLQKQNSRPLLVAGDIYRPAAIKQLQVLGEQIKAPVF

TLGDQTSPVEIARQGLQHAKDNGNDYVIIDTAGRLHVDEELMEELRQIHSVVNPDEVL

LVVDSMTGQDAVNVAEHFNQQLNLTGVVLTKLDGDTRGGAALSVKAVTGCPIKFASLG

EKLDALEPFHPERMASRILGMGDMLSLIEKAQLNIDTDKAKEMERKMRNAEFTFEDFL

EQMDQVKKLGPIDQIMDMIPGMGKMKQAKDLKVDDKQMGRIEAIVYSMTTEEKRNPDM

INHSRRKRIATGSGTSLAEVNRLIKQFDEMRRMMKQFSDMMGPKGGGKNKAMKQLKGM

GKGMKFPFR"

CDS complement(3494971..3495321)

/locus_tag="EFAGFIKM_02990"

/inference="ab initio prediction:Prodigal:002006"

/codon_start=1

/transl_table=11

/product="hypothetical protein"

/translation="MSQENRLEKTNRINLLFAFYELLLTEKQQTFLKYYFHDDFSLGE

IAAEFEISRQAVYEHIKRAEQVLENYESKLGLLEKHERRNRNLEDLKNALERAGVSID

NNKQIHDIVAQLSE"

CDS complement(3495584..3496231)

/locus_tag="EFAGFIKM_02991"

/inference="ab initio prediction:Prodigal:002006"

/inference="similar to AA sequence:UniProtKB:P67153"

/note="UPF0073 inner membrane protein YqfA"

/codon_start=1

/transl_table=11

/product="hypothetical protein"

/translation="MANTYTYSRREEVANAVTHGIGAALSVAALVILIVFSSMKGTAW
HVVSFTIYGISMLLLYTSSTLVHAWKDGKVKDLFEIFDHSSIYLFIAGSYTPLLFIAV
RGTLGWTLFGIIWGIALFGVIFKAFFTKKFLFMSTIFYIAMGWLIVIAWQPLMAAIPT
GGIVLLVAGGLMYTLGTLFYVWRGFPYHHAIWHLFVLAGSILHFFMVLLYLTPLR"

CDS complement(3496378..3497391)

/gene="ftsY_2"

/locus_tag="EFAGFIKM_02992"

/inference="ab initio prediction:Prodigal:002006"

/inference="similar to AA sequence:UniProtKB:P51835"

/codon_start=1

/transl_table=11

/product="Signal recognition particle receptor FtsY"

/db_xref="COG:COG0552"

/translation="MSFFKKLRDSIASKTESVTKQFKDGLEKTRKGLVEKVSDLVIRR
KKIDEEFYEELEEILIGADVGVNTVMNLIEDLRVEVKKRKIEDAAELQPVLSEKLTGL
LRGEQNNELKMNPDGITVILFVGVNGVGKTTTIGKLAHRFKQQGKKVIMAAGDTFRAG
AIEQLEVWGQRAGVDVIKQQSGSDPAAVMYDAVQAAKQRGADVLLCDTAGRLQNKSNL
MDELNKIYRVIQREIPDAPHEVLMVLDATTGQNALNQAKLFGEKSGVTGLVLTKLDGT
AKGGIVVAIRQELDLPVKLVGLGEKIDDLQPFDSEQFVHALFAGLIQEQPAESAEEEE
ANS"

CDS complement(3497500..3501069)

/gene="smc_1"

/locus_tag="EFAGFIKM_02993"

/inference="ab initio prediction:Prodigal:002006"

/inference="similar to AA sequence:UniProtKB:P51834"

/codon_start=1

/transl_table=11

/product="Chromosome partition protein Smc"

/db_xref="COG:COG1196"

/translation="MFLKRIELGGFKSFADKTEMEFVRGITAVVGPNGSGKSNISDGI
RWVLGEQSAKSLRGGKMEDIIFAGSDARKAVNFGEVSLTLDNEDHALALDFGEVTVTR
RVHRSGDSEYFINKQSCRLKDITELFMDTGIGKEAYSIIGQGRIEEILSTRSEDRRGI

FEEASGIVKYKSRKRDATRKLDETEQNLLRIHDLVTELEDQIGPLKEQSERAIHYKEL

RSQLKSQEISMYVYQIEQIHASWSKANERLQSLKQEEVGLAAIVSTHDAKLENDRNAL

RILETETEQLQAALLQFSEATEKSEGLGELLKERSHHLQTNQEQLKVTLAASEERHRE

RETELLALREKFGKLEHELNDVRNQLSQEEAKLIGVTGGISQQQEESLKGNLLELMNQ

MAQTRNEIRYVDQQKETLERRMNRAAEESGKWEDQKETLESRKADIEKKVVRLGKEIS

DLRGGYITESERLQSLQKLLEESRGTVRKWEQKREAQVSRRDTMKEMQDDFDGFMLGV

KEVLKASRKGTLSGVHGAVAELVKVPEKIELAVETAMGASLQHVVMENESVSRQAIAF

LKQRQLGRATFLPLDVIRPRAIGAAERSMIEGMEGFVGIGADLVQFESKYASIIGSLL

GNVIIAETLEVANKIAARCQYRFRVVTLEGDVVNAGGSMTGGSQHKKNVSLLSRKRQL

DQLDQDILDTENQIVKLHRSVDDVKVQLEQCQDKLDELRQSGDDTRNAEQQASMEMKQ

VEHELRHVLEQVAVAGQEKSGFTEEIKELDTARIVAVKKLEQLEEEEKATHRAIHAAE

FARKANESAKEELQSQLTELKVREGKLDQERFSNGEQLRRLEREVESLVKDLRQNRTL

LASMEADLKKTQTESVKQIEDLNQYKLKKAEASQELDFKRAARSELSKKLEFAESETK

EQRTQLKAVEEQMRQTEISVNRLDVELENILRKLTDEYELGYELAKERYPVPEDVEHT

QAEVQKLKRSISALGDVNLGAIEEFQRVNERYEFLSEQKNDLVEAKTTLYQVIREMED

EMAKRFKITFDAIRREFGTVFSKLFGGGRADLVLMDPERLLETGIDIVAQPPGKKLQN

LQLLSGGERALTAMALLFAILHVKPVPFCVLDEVEAALDEANVVRFAQYLREFSEQTQ

FIVVTHRKGTMEEADVLYGVTMEEGGVSKLVSVKLEDEEAVIA"

CDS complement(3501480..3502232)

/gene="rnc"

/locus_tag="EFAGFIKM_02994"

/EC_number="3.1.26.3"

/inference="ab initio prediction:Prodigal:002006"

/inference="similar to AA sequence:UniProtKB:P51833"

/codon_start=1

/transl_table=11

/product="Ribonuclease 3"

/db_xref="COG:COG0571"

/translation="MPPLFSKNLKHKGLVRLSEDLKQLQHKLQIKFDNRQLLKQAFTH

ASYVNEHRFSQHQDNERLEFLGDAVLELTVSEYLYHLYPNRPEGELTKLRASIVCEPS

LVKFAEVLGFGQYVLLGKGEELTGGRTRPALLADVFESFIGALYLDQGLAPVRTFLDQ

HVFPLIVLGSKLQMSDYKTELQELTQHHNMGALEYRIVEERGPAHEREFVSEVHMGQE

RLGRGTGRSKKEAEQQAASAALDRLKLPEAGA"

CDS complement(3502198..3503436)

/gene="fabF_1"

/locus_tag="EFAGFIKM_02995"

/EC_number="2.3.1.179"

/inference="ab initio prediction:Prodigal:002006"

/inference="similar to AA sequence:UniProtKB:O34340"

/codon_start=1

/transl_table=11

/product="3-oxoacyl-[acyl-carrier-protein] synthase 2"

/db_xref="COG:COG0304"

/translation="MKQRVVITGMGVMTSLGKDLETFWGSLMAGKSGISQIEAFDVSE

YTTQIAAEIKDFNPEEYMDRKDARKMDRFVQFAVAAGFKAVEDSGLKINENIDAERFG

VSIGSGIGGLGTWEDQHNALLQKGPKRVSPFFIPMMISNMASGQMSISLGAKGPNINV

VTACATGTHSIGDSFKLIANGDADAMICGGAEATIRPTGLAGFCAMRAMSTRNDDPAK

SSRPFDTERDGFVMGEGAGVMILESLEHAQKRGARIYGEVIGYGLTGDAHHMTEPDPD

GAARCMKMALRNAGIEPEEVDYINAHGTSTPVGDRSETLAIKKAFGDHAYKLAVSSTK

SMTGHMLGAAGGVEAVICGLSLTHQTLAPTINLENQDPECDLDYVPNVPRQTKVNIAM

SNSFGFGGHNATIILKKFEA"

CDS complement(3503614..3503847)

/gene="acpA"

/locus_tag="EFAGFIKM_02996"

/inference="ab initio prediction:Prodigal:002006"

/inference="similar to AA sequence:UniProtKB:P80643"

/codon_start=1

/transl_table=11

/product="Acyl carrier protein"

/db_xref="COG:COG0236"

/translation="MSDVLERVKRIVVDRLGADEAEVTLEASFKEDLGADSLDVVELV

MELEDEFDLEISDEDAEKITTVGEVVNYIQSHT"

CDS complement(3504000..3504749)

/gene="fabG_7"

/locus_tag="EFAGFIKM_02997"

/EC_number="1.1.1.100"

/inference="ab initio prediction:Prodigal:002006"

/inference="similar to AA sequence:UniProtKB:P99093"

/codon_start=1

/transl_table=11

/product="3-oxoacyl-[acyl-carrier-protein] reductase FabG"

/translation="MSKPLEGKNALVTGASRGIGRSIALALAEAGANVAVNYAGSQAA

AEEVAEEIRAKGVKAITLQANVGVMDEAEQMVKATLEAWGNVDILVNNAGITRDNLIM

RMKEEEFDQVIETNLKGVFNCLKAVTRPMMKQRSGRIINISSVVGVLGNAGQANYVAA

KAGVIGLTKASARELASRGITVNCVAPGFIETDMTKELSQELVDGMLSGIPLSRLGQP

DEIAGVVTFLASQASSYMTGQTLHVDGGMYM"

CDS complement(3504787..3505719)

/gene="fabD_2"

/locus_tag="EFAGFIKM_02998"

/EC_number="2.3.1.39"

/inference="ab initio prediction:Prodigal:002006"

/inference="similar to AA sequence:UniProtKB:Q7A5Z3"

/codon_start=1

/transl_table=11

/product="Malonyl CoA-acyl carrier protein transacylase"

/translation="MGKIAFVFPGQGSQAVGMAKDAYESVPAATEIFRTADETLGFSL

SNLVFEGPETELKQTSNTQPALLTASIALLEAFKEKGIQPDYTAGHSLGEYSALVAAG

VLSFADAVSTVRARGQYMEQAVPGGQGAMAAVLGADREALGVLCRDVSESGHAVELAN

INCPGQIVISGVKEGVAAVSERVKEAGGKRAIALEVSGPFHSSLMKGAAEKLAEKLKT

VTFSPAAVPVVANVTARPTEDGQVQDLLTAQVYSPVLWEDSVTWLIEQGVDTFIEIGS

GSVLTGLIKKTDKTVKLYNVNSLETLEATASALK"

CDS complement(3505745..3506734)

/gene="fabH_1"

/locus_tag="EFAGFIKM_02999"

/EC_number="2.3.1.180"

/inference="ab initio prediction:Prodigal:002006"

/inference="similar to AA sequence:UniProtKB:Q6GIA4"

/codon_start=1

/transl_table=11

/product="3-oxoacyl-[acyl-carrier-protein] synthase 3"

/translation="MNNLRPVGIIGTGKYVPEKILTNSDLEKMVDTNDEWIVSRTGIK

ERHIAAPDQATSDLAYEAAIKALESAGMTGSDLDLIIVATITPDSSFPSTACILQDKL

GAKGAAAFDLSAACSGFVYGLASATSFIQSGMYNNALVIGADCLSRITDYTDRNTCVL

FGDGAGAVVVGEVPEGRGFKAFDLGAEGAGGSLLQMEGGGSRLPATAETVENKKHYIN

MNGREVFKFAVRVMGTATIEVLRKAGMERTDVDLFVPHQANIRIIQSAMQRLELPEEK

VVVNVDKYANTSAASIPLALVEAAEEGRMKAGDTVLMVGFGGGLTWGASVLVW"

CDS complement(3506743..3507738)

/gene="plsX"

/locus_tag="EFAGFIKM_03000"

/EC_number="2.3.1.274"

/inference="ab initio prediction:Prodigal:002006"

/inference="similar to AA sequence:UniProtKB:P71018"

/codon_start=1

/transl_table=11

/product="Phosphate acyltransferase"

/db_xref="COG:COG0416"

/translation="MKIVIDAMGGDNAPASTVEGAIAAATEWADTQIVLIGDEAKLEP

LLSQSGVRPANLTVRHASEVIGSDDEPVKAVRRKKDASMVVAGRMLKEGEADAMISAG

NTGALMTAGLLVVGRMAGIERPALAPMIPTIDDVGVLALDLGANMDAKPEHLAQYGLM

GSLYRQKVQGIQSPRVGLLNVGTEPGKGNELTKHAYPLLEQLPIRFVGNVEARDVLTG

ACDVLVCDGFAGNILLKSLEGTAGAIFALLKEQFSSSLKSKLAAAVLMPELRGLKRKL

DYTEHGGAPLLGLSRLVVKSHGSADGNAIKNAVRQARIAVQNQLVESISKEISGK"

CDS complement(3507735..3508283)

/gene="fapR"

/locus_tag="EFAGFIKM_03001"

/inference="ab initio prediction:Prodigal:002006"

/inference="similar to AA sequence:UniProtKB:O34835"

/codon_start=1

/transl_table=11

/product="Transcription factor FapR"

/db_xref="COG:COG2050"

/translation="MIEENPFVTDQELTRQLKVSIQTIRLDRLELGIPELRERMKLMA

ELSYDQVRSLPLHEIIGDIVDLQLDKSGISLFEIKEEHVFSRTGIARGHYVFAQANSL

AVAIINDEIALTASADIRFVRSVHLGEKCIAKAYVRSIPGQKGKAKVEVFTYVGEEMV

FQGNFVIYHSGGEDSGEGGHLE"

CDS complement(3508656..3508829)

/gene="rpmF"

/locus_tag="EFAGFIKM_03002"

/inference="ab initio prediction:Prodigal:002006"

/inference="similar to AA sequence:UniProtKB:P07840"

/codon_start=1

/transl_table=11

/product="50S ribosomal protein L32"

/translation="MAVPQRRTSKTRRDKRRTHFKLAVPGMVKCEQCGELKLSHHVCK

VCGTYKAREIISQ"

CDS complement(3508897..3509403)

/locus_tag="EFAGFIKM_03003"

/inference="ab initio prediction:Prodigal:002006"

/codon_start=1

/transl_table=11

/product="hypothetical protein"

/translation="MLMPFRKVATSDGPLKFNEQWEIKELVSNRQDITAVTPLTADLS

AEFREGDVVDVHGKLTVGVDMLCSRCLKPINEHFHIDFHEQFKQGKQPEGLHEDDDTL

YVDGDSVDLKGYAEEAFLLDLPFIPLCSDTCKGLCPKCGHELNEGDCGCDNEVIDPRL

AGLKDFFK"

CDS 3509867..3511102

/gene="hmp"

/locus_tag="EFAGFIKM_03004"

/EC_number="1.14.12.17"

/inference="ab initio prediction:Prodigal:002006"

/inference="similar to AA sequence:UniProtKB:P49852"

/codon_start=1

/transl_table=11

/product="Flavohemoprotein"

/db_xref="COG:COG1017"

/translation="MLSEYTIRVIKSTVPVLEVHGEAITRHFYETMFVAHPELLNIFN

HANQKQGRQQAALANMVYTAALHIDNLSSILPAVRQVAHKHRSLGIVPEQYVIVGTYL

LQAIKDVLGDAATDEIITAWGEAYNVIADAFIGIEQNMYTEAENQTGDWEGFRTFKVA

KKVQESEIITSFYLVPDDGQPIASYEPGQYISIKIKPEAQSFTQIRQYSLSDTPGKPY

YRISVKRERGVLERPDGVISTYLHDHIEEGSLVELSAPAGDFTLNADDKRPVVLLSGG

VGLTPMISMLNTLVNLNENRNITFLHASPNGQSHAFRDHVNSLAKRNQGVKAYYCYTQ

PENADRENECFHKEGYMDAVWLRQVIDELDSTYYLCGPVSFMRAVYTELQALGVASDN

IHYEFFGPKASLSPTPESV"

CDS 3511321..3512553

/gene="tmcAL"

/locus_tag="EFAGFIKM_03005"

/EC_number="6.3.4.-"

/inference="ab initio prediction:Prodigal:002006"

/inference="similar to AA sequence:UniProtKB:O34513"

/codon_start=1

/transl_table=11

/product="tRNA(Met) cytidine acetate ligase"

/db_xref="COG:COG1323"

/translation="MKAVGVIVEYNPLHNGHVYHLQEARRLSGADAVVAVMSGPFLQR

GEPAIVGKRARTEMALHAGADLVLELPVAYAVQPAEWFAFGAVSLLHRTGVVDSLCFG

SESGDLDSLQRIARVLAVEPAGMREDIARRLREGASYPAAYAGAAAALAPGGVDAHDA

AALLEQPNNSLGLHYLIALQRLGSAIQPFTAARTGAAYHEATPGPGAIASATAVRRLL

MADGPNAAAPYVPAATLAILHREWQEGRAPIHWERFAQPLLHLAATRRASELERIAEV

TEGLEHRLSRALAQLPEPSVEALLNAMKTKRYTRTKLQRMLAHLLLNHTKAECSPEQL

AAGPGYLRVLGFNTQGQSLLKQMKKTASLPVVLKPSTFTHNQLELDIQAQVAYGLAWE

HKDTRKMFSDYYESPVRL"

CDS complement(3512918..3513958)

/gene="ylbL"

/locus_tag="EFAGFIKM_03006"

/inference="ab initio prediction:Prodigal:002006"

/inference="similar to AA sequence:UniProtKB:O34470"

/codon_start=1

/transl_table=11

/product="putative protein YlbL"

/db_xref="COG:COG3480"

/translation="MNRIRKSGGFRASIFVIVVALVVYVAVYMPTPYIIYMPGSADEV

KPMVTVKGGDQEEHGVFMMTTVSATYANVFLLGTSLFNKNAQVDKKEDRLRGKSEAEY

SAEQVWFMSDSQSSAMEAAYEQAGVTYSIVPEHIFVFGLSEDPKPEGDIAPGDIILGV

NGTATPDNTVLSAELKDKKVGDTVEMQLERGGETISRDVKLIQVKDSKTGETRPGLGV

MIGTVQKVKAEEPDKQISFTDTQVGGPSAGLMFTLEIYNQLTPGDLTKGHRIAGTGTI

TKDGVVGAIGGVVHKIVAADRKEAEIFFVPKDNYKEAAAKAEQIGTKMKLVPVSTVDD

ALAYLKTLSVKS"

CDS 3514210..3515547

/gene="ylbJ_2"

/locus_tag="EFAGFIKM_03007"

/inference="ab initio prediction:Prodigal:002006"

/inference="similar to AA sequence:UniProtKB:O34765"

/codon_start=1

/transl_table=11

/product="Sporulation integral membrane protein YlbJ"

/db_xref="COG:COG3314"

/translation="MTLQSEVRDSGPLRTIMLSTGALMLVIAVVASPKEAFDASIQGL

DIWWKIIFPAMLPFLMLSQMLTASGFTHAIGALLGPLMQRWFRLPGNAGLAIAVGMCG

GFPAGADAVSRLFQDRQITAKQAVVLAAASHFANPMMIILVVGAAFLHQPAAGYFLLV

VHWISGWIAAILATRLLPAPGNQKDRVSSTAVQADNSSLDSQPASPTSSTARSSAPHH

ATNKRRSIWSDMMIAAREAHRRDGRGFGKLLGDTVSQAVQTLMMTGGYMIGFAVFIRL

VSLYLTPGSSAALWPAFFELHLGTYHLSQTSLTPMLLMSLLAAVLGWGGLCSHLQVSA

VLKTTGPGSKSMLYFAGVRLTHGLIAFFISLFLWMPFSRYSTEVWTTLQTNAEQDLST

PFGWLFIHSPGNTYTINTIWSVFPAACMGLALLLAVMVSLSGLTFWFNRRFSR"

CDS complement(3515525..3516040)

/gene="coaD"

/locus_tag="EFAGFIKM_03008"

/EC_number="2.7.7.3"

/inference="ab initio prediction:Prodigal:002006"

/inference="similar to AA sequence:UniProtKB:O34797"

/codon_start=1

/transl_table=11

/product="Phosphopantetheine adenylyltransferase"

/db_xref="COG:COG0669"

/translation="MTEMIHRQERIAVYPGSFDPVTMGHLDIIARASKQFDRVIVAVL

NNMSKNPLFTVEERKELITEVTRHLPNVEVDSFRDLTANYVRQKEAQVIVRGIRSVTD

FEYELQLASTNSKLNPDAETIFMMTNPKYSYLSSSIVKEIAHYHGDVTDLVSPEVEAA

LRQKISEKTGG"

CDS complement(3516033..3516653)

/gene="rlmI_2"

/locus_tag="EFAGFIKM_03009"

/EC_number="2.1.1.191"

/inference="ab initio prediction:Prodigal:002006"

/inference="protein motif:HAMAP:MF_01857"

/codon_start=1

/transl_table=11

/product="Ribosomal RNA large subunit methyltransferase I"

/translation="MTRQVRVVSGSAKGRPLKAVPGTGTRPTTDKVKEALFSMVGPYF

EGGTALDLFAGSGGLGIEALSRGMDKAVFVDLESKSIEVIRLNLKATKLEDQAAVYRN

DAGRALKALAKRGTTFDLVFLDPPYRMKNGHELMLTMHELELLEPEATIVLEYESKHD

YPEQFGPFEQTRKALYGETAVSIYYYAPEAVEDGESITPEEEAPHD"

CDS complement(3516944..3517378)

/locus_tag="EFAGFIKM_03010"

/inference="ab initio prediction:Prodigal:002006"

/codon_start=1

/transl_table=11

/product="hypothetical protein"

/translation="MIIGKSIDTSGLTRVANLSDELQPVFDQAESKYQIVDIELFFVF

RCLPEEYERRSTVRHVKKENVIYFDLVVSEDRYKALSKSKQRYVLSHEFYAFLSEKIP

KYKIEHLNVNEFIADMGMWLREIGWLQTEEQADISEFEEKYN"

CDS complement(3517506..3517790)

/locus_tag="EFAGFIKM_03011"

/inference="ab initio prediction:Prodigal:002006"

/codon_start=1

/transl_table=11

/product="hypothetical protein"

/translation="MVDMYLIILVAILGMVLFYSLIAYFLIRFISKKAFKLTLTKYEM

MEIITWLAVLFITFMMIKTGSINLLLPVVLLIIPLINLRLSNRKHRELNS"

CDS complement(3517898..3518203)

/locus_tag="EFAGFIKM_03012"

/inference="ab initio prediction:Prodigal:002006"

/codon_start=1

/transl_table=11

/product="hypothetical protein"

/translation="MSKKAKTGIWVTVLVFLGIIVGCFIWYFNTASGERALKTMRSNN

SGGLERVVKVYSNNGELIQTYDGKIDVEDTEYGNKVLFDLNGKRVVIYNATIVVEEK"

CDS complement(3518374..3521421)

/gene="cbgA_1"

/locus_tag="EFAGFIKM_03013"

/EC_number="3.2.1.23"

/inference="ab initio prediction:Prodigal:002006"

/inference="similar to AA sequence:UniProtKB:P24131"

/codon_start=1

/transl_table=11

/product="Beta-galactosidase"

/translation="MKATKADINWLGDVSVFEVNRLHAYSDHRYYQTMDEAVGSGAMS

MRYDLNGTWKFNYAIRPDSRSEFFYTSDFSSEGWDDIEVPGHIQLQGYGQIQYVNTQY

PWDGLNEIRPPALPQDKNPVGSYIRTFHLPTGWESNPVYISFQGVESAFYVWLNGQFV

GYGEDSFTPSDFDLTPFLQDGENKLAVEVYQRSTGSWLEDQDFWRFSGIFRDVYLYTV

PTAHVRDVHVRTDLDKSYTNGTLQLDLKLEGKEVAGARVEAELRDREGNTVKTFESKV

NDGQVSLREDIGTVNLWSAEIPYLYRLYIRVYDAAGTLVEVVPQAVGFRTFEMIDKVM

HINGKRIVFKGVNRHEFNPRRGRAVTKEDMLWDVRTIKQNNMNAVRTSHYPNQSLWYE

LCDEYGLYVIDEMNLETHGSWQKLGAVEPSWVIPGDKPEWHDIVMDRAVSMVERDKNH

PSILIWSCGNESHGGEVIYKVSQYFKSADPTRLVHYEGVFHDRRFDETSDMETRMYAK

PADIEEFLNANPTKPYISCEYMHAMGNSIGGMHKYTELEDKYPMYQGGFIWDYIDQSI

YKKDRYGKEFLAYGGDFGDRPSDYSFCGNGIVHADRKVTAKMQEVKFLYQNIKLFPDR

EGVKIVNGNLFADTSALELVYSLEREGHEVLRGTLEVNVDAQSETVVKLPLGAEALAG

AEYAVNTAFVLKEATMWADKGEEVAFGQFIFTQDQVNDAVEVDLNLVSDVQVIEGDVN

IGVRAGKTHALFSKQFGTLVSLKLSGRETIAVPPTPLFWRAMTDNDKGMAMGFELGAW

YAASLMPRSVEWKAEQKPDQYRIEFTYKLNISEDVLVKVVYTVHADGSVRVHNTYKGT

AGLPNLPIHALSFRTSPDFENVEWLAMGPEENYADRAFGARLGIHGSKVADTMAPYLV

PQESGNRTGVRWAKLTDNAGRGFKIEASGTPVELNVSPYTASELENAQHAYELPPVHY

TVVTVAGKQMGVGGDDSWGAPVHPEYLIPSDGELTFEFVIRAL"

CDS complement(3521579..3522409)

/gene="lacG_1"

/locus_tag="EFAGFIKM_03014"

/inference="ab initio prediction:Prodigal:002006"

/inference="similar to AA sequence:UniProtKB:P29824"

/codon_start=1

/transl_table=11

/product="Lactose transport system permease protein LacG"

/translation="MAKAKAKRIFTYVFLSIVAFVSIFPFFWMLVSSTNASVDVTKGR

LLPGSAFIDNFNKLIDSTNLVQALGNSAIISVISTVLALFIGSMAGYGFEVYRTKSRD

IVFNILLLSMMIPFAAIMVPLYRMFATISGVAPVIGINTMAAVILPTIATAFLIFFFR

QNTKMFPKDLLEAGRIDGLSELGIFLKIYMPTMKTTYAAAAIITFMSSWNNYLWPLIV

LQTPDQQTIPLLISNLGAGYSPDYGVIMTAIVIATLPTAIVFFIMQKHFVAGMVGSVK

"

CDS complement(3522402..3523301)

/gene="lacF_5"

/locus_tag="EFAGFIKM_03015"

/inference="ab initio prediction:Prodigal:002006"

/inference="similar to AA sequence:UniProtKB:P29823"

/codon_start=1

/transl_table=11

/product="Lactose transport system permease protein LacF"

/translation="MKAMNTGDSLQKKNNLTGWAFVLLAVVGIVAFYFYPMIQALLLS

FKSGVGANLEFSGLSNYKRLLVDTTFRTALSNTFIYLIIQVPVMIILGLFISVLLNDS

TLRFRSFFRTAIFLPCVTSLVAYSVVFKYLFAPDGMVNQFLMGLHIIGDPIQWITDPF

WAKITIIIAVTWRWTGYNMIFYLSSLQNIDQSIYEAARIDGANAFTQFFKITVPLLKP

IILFTSITSTIGTLQIFDEIMNITKGGPGNATMSISQYIYNLSFKYSPDFGYAATVSY

SIVILIIVLSIIQFKVAGDNKNG"

CDS complement(3523434..3524990)

/locus_tag="EFAGFIKM_03016"

/inference="ab initio prediction:Prodigal:002006"

/codon_start=1

/transl_table=11

/product="hypothetical protein"

/translation="MSEVNRVKSFLYYFKIKKLQNQKKLQKTKFNASGFCKGFHCGNK

MTNPYKIITNEWRYGNDKMNISKATAFRKRKQITHKEVEGELNVLKKMWVLMLVTVLL

LSACSSGGGGNSASGGDEKTNEITVWAWDKAFNVAAMETAKEAYQKANPDVKINIIEY

AQADIIQKLNTGLNSGTASGLPNVVLIEDYRSQSFLNAYPDAFKDLSSSITASDFADY

KLGPTAFDGKQYGVPFDSGVAGLYYRTDLLEQAGYKAADLQDITWDDYIQIGKDVKAK

TGKELLSLDPNDLGLIRMMIQTAGKWYSAEDGKTPDIANNAALKEAFITYKAMMDANI

VKLHSDWSQFVANANNGSVATIPTGNWFSPSVRQEASQSGKWAVAPIPKMAGQPNSVH

ASNLGGSSWYVMNNVPGADQAADFVAKTFGSDKQLYQDLLNNIGAIGTYKPAVDGEAY

AKADEYFGGQKIFTDFANWTKEIPSVNYGINTYAIEDILVVEMQAFLNGKSIDDVLAD

AQKQAEAQLN"

CDS complement(3525070..3526839)

/locus_tag="EFAGFIKM_03017"

/inference="ab initio prediction:Prodigal:002006"

/codon_start=1

/transl_table=11

/product="hypothetical protein"

/translation="MKKWMNQLSRLGLFPKLFLVMVVSIVLVSVLILWTTIHMSTNLF

TETFSITNSKVLNQIKTNFESFNEAIAAVSNNVTQSGSIRGFLSEGDADSLTMAKSFY

SMRETMDRIQSITESYEVGITIIGINGRSYSTNRAHLQLSVDELKQEPITMEAAETPS

RLIFDVYQNEATLGSQQMISATKALTDRTRSRIYGTLYVTMREDIFRQFYSSFTSRGN

DVVIMNEKGEIVSSNREDWIGTTQLDLLSYAREMNNNSPRSINARVMDQDSVVLSEYL

PFYRFYIVNVVDKDLAMGQLIDMKTVALICAAIVVGALILVFLITSQITKSLRRLVKQ

MSNITKSDLDNYIPVSGSYESRQLGHAYNYMLDELHDYVDQLVQTQHEQRNAELAALQ

SQINPHFLYNTLASVKVLVQQGNKDRAAETINALIGLLQNTISDVSQTVTVEQEVENL

KNYVFINHVRYGGRIKAAFYVAPDCTHYHVPKLVIQPFIENAFFHGFIKKETGTIHVM

VSRAGESLICEIMDNGDGIEGLVMGETLPNPKNNRQLFSGIGIRNVHDRIELLYGSPY

GVTIMSTVGEGTRVTVTLPLITS"

CDS complement(3526858..3528405)

/gene="cheB_10"

/locus_tag="EFAGFIKM_03018"

/EC_number="3.5.1.44"

/inference="ab initio prediction:Prodigal:002006"

/inference="protein motif:HAMAP:MF_00099"

/codon_start=1

/transl_table=11

/product="Protein-glutamate

methylesterase/protein-glutamine glutaminase"

/translation="MNRLCKVLIVDDEFLVRQGIKHHMNWESEGFIIVGEASNGEEGL

QQVNLLKPDIVITDIVMPVMDGETFVRTLKASNPQIEVIVLSSFSEFEYVRSTLQHGA

ADYILKPKLDTNELLQVLQRTAGKIPELQFEPSHDGWRLGQLMEKMLSGFTLDEDSEM

PIIRDTFPYESFRLLVYKPVGAGGNPLKMDQEQIESKLRSDLPEVECAMVPAEGTLPV

MLLNVDPARDEWMLERIRQLASENAAGEGSPGWVLSESFTSFEQMGVVYRERLIKLIE

YRFYYKDRSILVYGELPPMHPAGYQFNVNMFLQHVKRNRVEAAREYLQEHAKTLGRDY

IADVFEIKSFLGNLIFNVTITLADMDVQSAGLEESKYTYFKNVDGASSLDEVMTVLDQ

FMTEVQECTVGAGGKRSDPNMKMLLDYMHEHFDQPLGLAEVAKHFHFNPSYLSSYFSS

HKKEGFNEYLNKIRIEKAEELLRSDDVTISEISSMVGYSDHSYFCKVFKKFTGLSPSR

YRRKFWA"

CDS complement(3528677..3531757)

/locus_tag="EFAGFIKM_03019"

/EC_number="3.2.1.-"

/inference="ab initio prediction:Prodigal:002006"

/inference="similar to AA sequence:UniProtKB:Q3MUH7"

/codon_start=1

/transl_table=11

/product="Xyloglucanase"

/translation="MRTFMGKLRMMSLTVAVVTASLGGLAGTTAAAPSEAYEWKNVVT

GAGGGFVPGIIFNESEKDLIYARTDIGGAYRWNAADERWIPLTDFVGWDDWNKNGVDA

LATDPVDPDRVYMAVGTYTNSWDQNNGSILRSTDRGDTWQTTTLPFKVGGNMPGRSMG

ERLTVDPNDNSILYFGARSGHGLWKSTDYGVTWNEVTSFTNPGNYVQDPSNEYTSDIV

GLAWVTFDKTTGSAGQATQTIYVGVADKAQSVYRSTNGGLTWSAVAGQPTGYIPHHGV

FDSDGSLYITYSDGVGPYDGEKGDVWKLNTSTGVWTNISPVPSSSTDNYFGYGGLAVD

AQNPGTLMVATLNSWWPDATLFRSTDGGATWTRIWEFEGYPNRKLRYTQDISAAPWLT

FGTNPAPPETTPKLGWMIGDLEIDPFDSDRMMYGTGATIYGTKNLTDWDDDEKINISV

MAKGVEEIAVLDLISPPSGAHLISGVGDVSGFRHNNLDQAPATIFTSPNYSSTESLDF

AELSPNTMVRVGKADYAADPNAKSMGLSSDGGTTWYKANAEPAGTTGGGTVAISANGN

RLVWSTSDKGVHYSTGGNSWTASTGIPAQAKVISDRVNSNKFYGFAAGKIYVSVNGGA

TFTASSTTGLPMEGNSDLDAVPGVEGELWFAGGSEEEGPYGLWHSTDSGATFTKLANV

EEADSIGFGKAAPGQSVVALYTVAQIDGTRGFFRSDDGGANWVRINDDQHQYARVTTI

TGDPRLYGRVYLGTNGRGILYADRVGGNNGGGGGGGTPVTNSAITPVIAEFDRKAGNQ

VNIPVTLTLNGNTLASIRNGNATLVSGTDYTLTGNEVVLSKNYLASLPNGAATLTFQF

SAGAPATLNLLIKDTTAAPVGTIRIEMFNSNTSATGSSISPKFKLTNTGTTALNLSDV

KIRYYYTINGEQPQNFFADWATAGSANVTGTFSALNPVRTGADHVLEIGFTASAGTLN

AGQSTEIHTRISKNNWTNYTQTDDYSFAGSQTSYTDWSKVTGYIAGSLQWGIEP"

CDS complement(3532160..3533041)

/locus_tag="EFAGFIKM_03020"

/EC_number="3.1.1.99"

/inference="ab initio prediction:Prodigal:002006"

/inference="similar to AA sequence:UniProtKB:P0DOV6"

/codon_start=1

/transl_table=11

/product="6-deoxy-6-sulfogluconolactonase"

/translation="MSDVTVAVQTPALLGEGPSWDAENNRLLWVDIESFKVHVYDPAT

GQDQAYDVGEHVGAVVPYRGDEVVVALRSGFHTYHLLTGELQAIEDPEQDKDTNRFND

GKCDAKGRFWAGTMSMNNESKAGSLYCLEQGQPVRTMVQGVSTSNGLGWSPDRQTMYY

IDTPTRSIDRFDFDLTAGTIQNRTSVIHIPEEFGFPDGMTVDGEGMLWVAHWGGGRVT

RWNPHTSELLQQIEVPADQVTSCCFGGPDLEELYITTARIGIKEERLKETPDAGSLFV

IRPGVKGQETHAYGSQQ"

CDS complement(3533054..3533719)

/gene="axe2_1"

/locus_tag="EFAGFIKM_03021"

/EC_number="3.1.1.72"

/inference="ab initio prediction:Prodigal:002006"

/inference="similar to AA sequence:UniProtKB:Q09LX1"

/codon_start=1

/transl_table=11

/product="Acetylxylan esterase"

/translation="MKLQKNDKLLFIGDSITDCGREHPVGEGSSGLGHGYVAQVYALL

RSIYPELMLRVQNVGNGGNTIRDLKQRWDRDVLDLKPDWLTIMIGINDVWRQFDNPLS

TDSHVFLEEYESTLRELVASVRPNLKGLVLMTPYYLEANPEDPMRATMDIYGEAVRRV

ANEYDAVYVDTQATFAPFWDHFYTSVLTYDRVHPDATGHMVLSKAFLDAIGFEWSGGV

KSE"

CDS complement(3533804..3534592)

/gene="axeA1"

/locus_tag="EFAGFIKM_03022"

/EC_number="3.1.1.72"

/inference="ab initio prediction:Prodigal:002006"

/inference="similar to AA sequence:UniProtKB:D5EV35"

/codon_start=1

/transl_table=11

/product="Acetylxylan esterase"

/db_xref="COG:COG0657"

/translation="MTTTIPLWDHAAPYAAQGHEDEMPHLIPFIQPGSESAVIVCPGG
GYGFLADHEGAPIAELLNRAGISAFVLKYRVAPHQHPAPITDGQRAIRYVRAHAEQYG
INPAKIAVLGFSAGGHLTATLGTLYDEGQPDHEDIIERQSSRPDRVILCYPVITMESY
GHAGSRENLLGSDASAEQIKAFSAEQQVRADAPEAFIWHTSDDQAVPVENSLRYALAL
GAHGIPYDLHVFEKGSHGLGLAEDNHAIRVWSDLCLTWLKNQGW"

CDS complement(3534736..3535569)

/gene="rhaR_27"

/locus_tag="EFAGFIKM_03023"

/inference="ab initio prediction:Prodigal:002006"

/inference="protein motif:HAMAP:MF_01533"

/codon_start=1

/transl_table=11

/product="HTH-type transcriptional activator RhaR"

/translation="MFISPRHQRYFMTSRDHPLPLYIESLGYNGNQEKVSRPVGYPCY
HWLQTVKGAGEFKFAGSTVVLGEASGILLPPNEPHEYVRAQGEWETFYITFGGSQCPA
ILESLSLGEAALHQWEQSSPFNDYGQEVLDSIRSDQDLSGLEASADIYRFLILLKKHG
MTGNRSSISHAVERLAPLIAFMEQHYANPEIGLEHMADVTGISSRHLNTLFKQSFGMT
AYSYFILLRIRKAKEIMTGDTALTIRETAVRVGFRDASHFVATFRRIEGVTPEQFRNL
Y"

CDS 3535698..3537725

/gene="bgaP"

/locus_tag="EFAGFIKM_03024"

/EC_number="3.2.1.23"

/inference="ab initio prediction:Prodigal:002006"

/inference="similar to AA sequence:UniProtKB:Q09HN2"

/codon_start=1

/transl_table=11

/product="Beta-galactosidase BgaP"

/translation="MISSKLPKMFYGGDYNPEQWDHETHLEDLRMFQLAGIDIATINV
FSWALIQPDEVTYQFAELDQLINRLYEKGVYICLATSTAAHPAWMAKKYPDVLRVDAD
GRKRKFGGRHNSCPNSPTYRKYSEKIADKLAERYKDHPAVLVWHISNEYGGDCYCDNC
EKAFRVYLKERYQTLEQVNKAWNTNFWGHTFYDWDEIVLPSNLSEHWGNNNSTFQGIS

LDYSRFNSDSMLDCYRLEYDAIKKHIPDSVVTTNLMGFFKQLDYFKWAKYLDIVSWDS
YPGLATPISFTAMAHDLMRGLKDGQPFMLMEQTPSQQNWQPYNSLKRPGVMRLWSYQS
VAHGADTIMFFQLRRSIGACEKYHGAVIEHAGHENTRVFREVAELGKELQILGDTTLD
ASVESKVAIVFDWDNWWAIEKSSGPTVALNYVDQIHKYYAAFFRRNIQVDIVSVDTDM
SKYDIVLAPVLYMVKPGFATKLEKYVEAGGTFLTTFFSGIVNENDLVTTGGYPGELRK
LLGIWVEEIDALLPEQKNRIVLKETYGDLQGEYGCGMLCDLLHSEGAEVIAEYGDDFY
KGMPVVTRNTFGQGEAWYVASDPDERFLDGLLGQLAAAKNVKSLLETPEGVEVSARTK
DGKPYLFVMNHNATTQSYDLGTAKAHDLLTNRELSGSVEIEARGVQLLEMK"

CDS 3538164..3539120

/locus_tag="EFAGFIKM_03025"
/inference="ab initio prediction:Prodigal:002006"
/codon_start=1
/transl_table=11
/product="hypothetical protein"
/translation="MNRTNRLAAIVMALQHGHETAHSLGEKFEVSRRTILRDIQSLSE
MNVPIIAISGPGGGFSLMEGYVLPPLQLDPVEAATLLFALEGLSRYADTPFHEKRWTL
MNKVKAIIPDDIMSRIDPMLKQLHHHIPDRNYILPHLDALLACIPEHGWLSVLYRSAS
RQRWLHICPTRVYASSGFWYCEAYSLEHGEQRLFRVDRIIEVKAIEPQDAQMLNEQAE
QQQSKPIPPEQPPTLVKAQLSYRGMIEAEQDEHIGEKMTEIAPDLWELSFLCPPGEWD
WAVRFFYRLGREAEVIEPLQLRSEIRQHAEEVSRRYLASTKS"

CDS 3539155..3539841

/locus_tag="EFAGFIKM_03026"
/inference="ab initio prediction:Prodigal:002006"
/codon_start=1
/transl_table=11
/product="hypothetical protein"
/translation="MTYEAVTTYEEAVQRIQSIGLLPLAPLFTEYPSLSSLTPKEHWH
KDDELDPWLWRSRLAEEGIAAYGKFVKKKALLVSSEYFPWVHRLLADSRTIKEQYEAG
LLSREAFKLHALVAEQEGIDGRALRSQAGFGAKEDKKTFDRGLLDLQGCAAIVISGTK
EKEGLAEGKVAWNSSAYETAGHWMERHGIKPFEGSVAEARELLVTQLQQHASPAALSS
IAKALGIRGI"

CDS complement(3539943..3541457)

/gene="uxaA"

/locus_tag="EFAGFIKM_03027"

/EC_number="4.2.1.7"

/inference="ab initio prediction:Prodigal:002006"

/inference="similar to AA sequence:UniProtKB:P42604"

/codon_start=1

/transl_table=11

/product="Altronate dehydratase"

/db_xref="COG:COG2721"

/translation="MNTTSTINDWIAIQPQDDVIIALRDYAKGECITLPDGVSFTLLD

DVPKGHKIAVHTLAPGDDVMKYGFSIGIAQEQIEQGSWIHSHNLKTGLHGLLEYEYQP

GAQVQTDMPPEHLRSFDGYLRPNGDAGIRNEIWIVNTVGCINKVCEALARMGQSQFGS

RVDGVFHFPHPFGCSQLGDDLKYTQQLLASLVEHPNAGGVLVIGLGCENNQVDEFREC

IAPEYRGKVRFLKAQETDDELEEGLRLMEELVEIVEHEQRQPLPLSKLKIGLKCGGSD

GLSGITANPLVGAVADMLVAAGGTAILTEVPEMFGAETILMNRAANEQVFHDLVDLVN

GFKQYFVNHGQNIYENPSPGNKAGGITTLEEKSLGCTQKGGRSSVVDVLRYAKRVTQT

GLNIVEAPGNDLVSVTALSAAGAHIVLFTTGRGTPFGGPVPTVKIATQSDLANRKKHW

IDFNAGQLLEGQTMDEVKVQLFSQLIDIASGRSHTLSEQHGFREIAIFKDGVIL"

CDS complement(3541454..3542971)

/gene="uxaB"

/locus_tag="EFAGFIKM_03028"

/EC_number="1.1.1.58"

/inference="ab initio prediction:Prodigal:002006"

/inference="similar to AA sequence:UniProtKB:O34354"

/codon_start=1

/transl_table=11

/product="Altronate oxidoreductase"

/db_xref="COG:COG0246"

/translation="MSASTKRPRLKLNLLGSDGQRKCKEIMERPVKVLQIGEGNFLRG

FADWMLHESARQGKFHGSVAVTQPRPGGKAKLEQIRDQDGLYTMITRGLSQGKPVERT

ELISIFSQCINPYEEWDAFLNLAELPSLEFVISNTTESGLKYTYADYIEGEPVQSFPG

KLTVFLHQRYLKFDGDPSRGLIHLPCELLEGNGDVLRSCVLRHSEDYGYSEGFRSWIE

NHNHFLNNLVDRIVTGAPTQEEADSLTNRWGYEDQLINTAEPYHFWAIQGDESLDKKL
PLKQAGLNVHWVKDLKPFQVRKVRILNGAHTLMSSLGILQGKQHVRETMEDPHFGSWI
REAVHQEIVPALDMPDHQLDQYAEEVFERFLNPYIDHKLQDIALNTIGKFKVRVLPTL
LSYEQNQGSWPERLIQGFAGLLCLYRPVNTPEGYKAQRLNGEDILLRDDPDVLAALAT
HWEGYDTLNRNQNQLDDRVAAVLSDTLIWGENLDAREGLRAALVREIGLLEGEGK"

CDS 3543164..3544039

/gene="rhaR_28"
/locus_tag="EFAGFIKM_03029"
/inference="ab initio prediction:Prodigal:002006"
/inference="protein motif:HAMAP:MF_01533"
/codon_start=1
/transl_table=11
/product="HTH-type transcriptional activator RhaR"
/translation="MERSPVRTTIQAESGIPFRLVYSDTKSPQNELPDHLHDWHEIIY
VYRGQGSIFIDTGLENMQEGDLFIIPGSTVHRALPDAGNPVTSSALFFSPGLLASTAG
GAASSLLSLFERCRKQRVYKRHLNTEEQSQVAALIDDIQTEFQLGHHLSAHAALLRLQ
LLLIFLERLEAAGRANPGKSAPGPSWLSSTISHIDDNLRNGLSLPELAQYASVSPAHF
SRAFKRYTGMTLTDYALARRVIMAKEHLVQSDETMAAVADACGFDSLPHFYRQFKKLT
GTTPAAYRRTMNSSR"

CDS 3544181..3546169

/gene="tetO"
/locus_tag="EFAGFIKM_03030"
/inference="ab initio prediction:Prodigal:002006"
/inference="similar to AA sequence:UniProtKB:P10952"
/codon_start=1
/transl_table=11
/product="Tetracycline resistance protein TetO"
/translation="MLNELNRRNIGIFAHVDAGKTTTTEHMLFESGVVRSPGRVDDGT
TATDSLDIEKERGISVQAAMTSLIWKNTIIDLIDTPGHIDFSSEVERTLRVMDGAILI
LSAVEGIQSQSEAIWHALRSLRIPTIIYINKMDRIGASAPAVMEQIRSTLSPFACEIQ
AYQLHEDSFHGIDSLWNPNQDSLSDGTPSIPGLVEILAELDEEVMEAYIQETPLSQRD
LNESFLRYVHKGEMFPVCYGASGKGIGVTALLDAVLAFLPPPAQPVDSPVSGVVFKIE

RDRTMGRTAYVRMYGGSLYNRDTIHNSTRELEEKVTQIRRMDGRKWADTGAVHAGQIA
ALYGLSDTHVGDIIGNPEGVPPLPQMAVPLLTVQVHGKDPAQYPDLVAALQELTDEDP
LLDLQWLPEERELHLKVMGTIQLEILSSLLLSRFGLDVVFDPPSVIYKETPRTAGEGY
IAYTMPKPCWAILRFKIEPLPRGSGLIYASNVRTEDLLIRYQNEVERRIPEALSQGML
GWEVTDLRITLVEGEHHVWHTHPLDFVVATPMGIMDGLSRTGTTLLEPLLQFRLTVPE
EYGGKALSDLVHMRATFEAPVIGSGRCIIEGVMPLATSMDYPVKLRSETSGRGVLTTS
FAGYQDCPADANHTRKRRGVNPLDQSKYILSVRNAITS"

CDS 3546184..3546861

/locus_tag="EFAGFIKM_03031"

/inference="ab initio prediction:Prodigal:002006"

/codon_start=1

/transl_table=11

/product="hypothetical protein"

/translation="MLIKFLIFGLLWRIIGNPFIAILILLVILYFLDRRYVGVFPSFT
KPLKRMRNISRLRQQLAMSPNEVSSKLELARLLIERKRYSEAHALLLELERPYEQSAE
YWEALGTTELHLGNIEKGERHILQALEINPRVKYGRPYLTLAGAFKDTHEDKALDYVH
QFQEIHSSSSEAYYLLGSVHRSLGRNADAKQAYEQSLNVYRSLPKYKKRQERRWAVRS
WFRKRGL"

CDS complement(3546975..3548036)

/gene="thrB_2"

/locus_tag="EFAGFIKM_03032"

/EC_number="2.7.1.39"

/inference="ab initio prediction:Prodigal:002006"

/inference="protein motif:HAMAP:MF_00301"

/codon_start=1

/transl_table=11

/product="Homoserine kinase"

/translation="MQNIDSGSLHPSEQQRISEVLALYGFTSDWKGERGKGGMNNSTY
MLQVDGTNYVMRQYETHNDPMKITFEHKVLAALQRSNFKLDTPSPVRRLSGEEGTFLS
VKDTLTGHTKIVTLFHYREGVNPIWHTPDQLIGLGKAAGALSSVMATLDIPLEPVYPP
YYRIQDAYPLCSPERLLQLCTSPPEQLAACANELKQLRDALPDLFEALRGMELLPHQL
VHGDVNASNVLVDQQDGEICAILDFEFATWDLRVMELAVPMSDLLTMDKSEDWMWQAQ

EGLIRGFWEQVSLKPEELLSIPRLILLRSLDVVMHFISRMFEGTDEPEVAVEQIVKLK

QRIDWMRLHEERLREILVY"

CDS complement(3548129..3548755)

/gene="lipC"

/locus_tag="EFAGFIKM_03033"

/EC_number="3.-.-.-"

/inference="ab initio prediction:Prodigal:002006"

/inference="similar to AA sequence:UniProtKB:P42969"

/codon_start=1

/transl_table=11

/product="Spore germination lipase LipC"

/db_xref="COG:COG2755"

/translation="MVYRYVAVGDSLTVGTGALLGTGFVPLYRRMAEMNVRTFVSMEN

MGVNGLTSGEMLQMISSHPRVRQSLREADIITISIGGNDLIRTFKASNGIPNASKMTQ

VLGETRSNVSQIMRHIRQLKGNHEYMVRSIGLYNPYPQATEAAYWVRQYNSFLNGAGS

GNYACAQVYDRFEGRERELLFWDRVHPNARGYRVIAEQLNRTGYYPFS"

CDS complement(3548954..3552121)

/gene="smc_2"

/locus_tag="EFAGFIKM_03034"

/inference="ab initio prediction:Prodigal:002006"

/inference="protein motif:HAMAP:MF_01894"

/codon_start=1

/transl_table=11

/product="Chromosome partition protein Smc"

/translation="MRTILKARWWLMGLWVVLAAVLMFTAPNMSELIREKGQFSVPEG

YSSTQAAEILNEAAAQKGEQQGSQIALVFYNPDGLGTTGKQEAEKAVKNLEADKEKLG

ILSILEPFSQPELSEKMISADGKTILTSLSIDQGDRTVKEMREDLNEALKSVNVEHYI

TGKGLIDEDTIESSQEGLKKSEYITVVFILLILFLVFRSFVAPFVPLLTVGISYIVSQ

QIVAFLVDGLDFPISTFTQIFMVAVMFGIGTDYCILLISRFKEELAHHENTWDAIIAT

YRTAGKTVLFSALAVLVGFIAIGFAQFMLYRSAVAVAVGIAVMMLALVTIVPFFMAVL

GKKLFWPSKGSLEHAESKIYGAAGRFSLKRPWAALLIVAAVCVPLLATYDGKLSFNSL

DEIGEKYDSVKAFNIISDSFGPGESLPGQIVIQNDEAMDNAKYMAVAEKISREVEKVA

GVSGVRSMTRPTGDEIKDFEVTQQVGTLSDGLGEGKTGLDKIRDGLSEASSQLSKNEP

QLKEAADGAGELTKGTSQLQSGITQLSEGLGQIEKGIRDGSAGAGDLKAGLQQAKTSA

DQLAQANNQLLEAYRQAGAGVAALGDGTRELEQQLNGVSTALTSLSESFTALEERYPE

LQQDADYQRIKGTIGETGSGTAQLAQGLGQIKTKLGEAAAGINQANEGFASAAAGQKA

LADGLGQIVTGIGLLQSGLKQAADGQGKVISEIPSIQDGLGQLQGGQEKIQQGFSDLS

GQLTQLTDGLNQSVDGIKQVSGGLDSAQDYLTQLQNSPDSDLAGWYVPDEALNSKDFT

QVFDTYLSEDRKTMTIDVIFAENPYGTEAIDRVPDIEAAVHRAVQGSTLEKADIAIGG

VTSTFADLQEISNNDYTRTVMLMLAGTFIILVVLLRSVIMPLYLIVSLLLAYFTSMAL

TEVIFVNILGFAGISWVTPFFGFVMLIALGVDYSIFLMDRFNENKGMKVQDAMLYAMK

NMGTVILSAAVILSGTFAAMYPSGVLSMMQIATVVLSGLILYSLLFLPFFVPVMVKMF

GRANWWPFPNKEQSDSVDSDRTIGM"

CDS complement(3552118..3552576)

/locus_tag="EFAGFIKM_03035"

/inference="ab initio prediction:Prodigal:002006"

/codon_start=1

/transl_table=11

/product="hypothetical protein"

/translation="MSTPDAKSLVNRYLDASFMVNKRFDTRIREQVGLTITTDQFCAL

RLIEEKPSCTPSDLAELLCIGKSSITALVNKLVDRDLVHRAGDERDRRVVYLTLTDTG

RQVYRETEREIQQILEPYLVHFKPEEVRIFIESFEKLAALLSHEGGRENE"

CDS complement(3552797..3553234)

/locus_tag="EFAGFIKM_03036"

/inference="ab initio prediction:Prodigal:002006"

/codon_start=1

/transl_table=11

/product="hypothetical protein"

/translation="MLINLKSRIQEPEVQELLSYSVFPDPDHLNRALQQYVEKDELQM

GGYEDEGQLIGLIGYEKTGTSEVTIHHISVLPENRFKNYGRGMISQLLATYNPDRLIA

ETELEAVEFYRNTGFVVYSLGELYPGVERFRCVLEKEENTDEE"

CDS complement(3553298..3554284)

/locus_tag="EFAGFIKM_03037"

/inference="ab initio prediction:Prodigal:002006"

/codon_start=1

/transl_table=11

/product="hypothetical protein"

/translation="MNTYVIGMDGGGSHTRVAVADQNGKILSYVQKSGCNRYHDINAE

QNVLEAIAEAITQAGLQSDQIASIQAGMAGLNQPEDTIWAEALLARTGITGRISAVND

THIAHTAAFDGEPGIVAIGGTGSLILGRTEQGAWLRNDQFGHYAPTAARFLAYDTVHS

ILAGRYGPQDQAWIEQVLKYWNVASIRELAALGIAGFAENKQATNRKFGQMASMVTEA

ADRNIPLAVRVCGSAADTAVVGILMLASCFDQPTVSMTLTGSCLSSPYMVEAVQKGLD

AANMREGKRIQYIPSNLPGVGGAMLDAYHLAQINVSDNISTVLQSELNRHTT"

CDS complement(3554353..3555066)

/gene="COQ3_3"

/locus_tag="EFAGFIKM_03038"

/EC_number="2.1.1.222"

/inference="ab initio prediction:Prodigal:002006"

/inference="protein motif:HAMAP:MF_00472"

/codon_start=1

/transl_table=11

/product="Ubiquinone biosynthesis O-methyltransferase,

mitochondrial"

/translation="MGNEQFEAARKAEAGYHSNFYQNNELFASGTWMSRPMPMVMDML

ERLLAHKEELRVLDLGCGVGRHTIPIAQRLAKTKSEVIGVDLLDEAVDGLRKYAKEYQ

VDHIVQAVKADVEHYAVEPNYFDYIAACSCLEHVSNKQAFMEAIDRLQAGTRTGGIHC

ITMSTNVEEKEINTGKEIEPLIELNLPTAEAIAILEKAYKGWNILLQEHVTQTIEEEK

YDEPTQFRCELLRFAVQKQ"

CDS complement(3555084..3555434)

/locus_tag="EFAGFIKM_03039"

/inference="ab initio prediction:Prodigal:002006"

/codon_start=1

/transl_table=11

/product="hypothetical protein"

/translation="MARTQRKCSYCHEGMAEDEFKCKSCGNLVATKTALPEQPYMIEY

EESVLDRYSIILLVLITLFLAPVAISIGGVMLFNDSSYKRDTGKLLVTAGFVIMIIFS

IILFAGTGTLTFNF"

CDS complement(3555492..3556454)

/gene="menH_1"

/locus_tag="EFAGFIKM_03040"

/EC_number="4.2.99.20"

/inference="ab initio prediction:Prodigal:002006"

/inference="protein motif:HAMAP:MF_01660"

/codon_start=1

/transl_table=11

/product="2-succinyl-6-hydroxy-2,

4-cyclohexadiene-1-carboxylate synthase"

/translation="MLFYVLAALVLIVLALLWTGGLYFFKTAIRRTPKTFLVDNPNLM

PEPDNEIISSASDLKWLDAQPTEEISLQSHDGLKLYGTWLGSNKGSDQTVILAHGYSG

RGREMAGFARFYAEKHGYNVLMPDDRGHGRSEGDIIGFGWLDRKDYAQWTNWVLEKVG

TQGEILLHGISMGGATVLMTGGEALPTQVKAIVSDCAYTSVEEELTFQLKQLYKLPAF

PFIPVTSLISRWKAGYSFREASALKQLAKVNVPVLFIHGEADMFVPTEMVYRLYEACP

TKKELLTVPRAGHGTAFQVDRTGYEAALESFMKKNLSQPESVIN"

CDS complement(3556491..3558200)

/gene="rcsC_9"

/locus_tag="EFAGFIKM_03041"

/EC_number="2.7.13.3"

/inference="ab initio prediction:Prodigal:002006"

/inference="protein motif:HAMAP:MF_00979"

/codon_start=1

/transl_table=11

/product="Sensor histidine kinase RcsC"

/translation="MSNESGEIQRLRRTIEQLSDQMIRSKEQEERTLTEFSDMNNELV

TLQRLLAKNNNSLEAARQQAVDAGDSKIAFIAGISHDFRTPLNGILGMAEMLKLSPLS

EEQENSVSVIQDAAKLLLKLIQDLLDLSKLEAGQMRLELGEVNLQETINYIVRLLEPQ

VRKNGNQLSIDCDPRISTLLEGDSTRITQILINLIQNANKFTSEGTVRIRVTLMKDDE

ITQMIRYEVEDTGIGISEEDQKQLFQPYVQTEQGRSSEYGGTGLGLSICKSLVTLMEG

QIGVDSQEGEGSTFWFELTLDKKDGNQSANQSVASAQEQQGQDMDDEAPSVSVLLADD

NVINRQLVMLQLKKLGITQVDAVVNGEEAIAAFLSKKYSLILMDYMMPVIDGLEATRK

IRSIEMDEMRHTTPIIAMTGNVMQGEKDKCMEAGMNDFIGKPFTLEILSKMIEKWQQS

SEPTEVLNMSIVHEIAELNTDGDRTLLGMLLDMYRTDTPGKIEVLRRHIVSGDHVAAT

EVAHDLKSGSLSLGIRYLSTLFARIEQFAKEGQSRKAEPLLDALLPAYQAACASLQQI

SKD"

CDS complement(3558216..3559310)

/locus_tag="EFAGFIKM_03042"

/inference="ab initio prediction:Prodigal:002006"

/codon_start=1

/transl_table=11

/product="hypothetical protein"

/translation="MSHQEAGERLLQESANLADKITEKQYLRQPDLLERFGENGKMRT

KQDSEYSLNYLAESALVQSPGLFTHYIAWLKVLLEGYKVSPEDLVINLNLIKETLEEE

FDHPSKALLLEYLEMGIYRTTHMESQAGYINVNESMPYGDAAQSYLQCLLENKRKEAF

EIIEAQLEAGVTIRDIYRYIFQPTQYEIGRLWQCHRISVGQEHFCTAATQSFISRLYS

RWLIHTGQDKKLVATCVGSEQHEIGLRMLTDVFEMEGWDTHYLGANVPNGSVIEAIEL

HQGDVVAISVTMTYHLHLAKELIHLIRHHPATAHVKIMVGGYPFNIDQELWKTIGADG

YAPGADEAVAVAEQLLSQPATCNYLDMVRD"

CDS complement(3559596..3560057)

/gene="mhqR_1"

/locus_tag="EFAGFIKM_03043"

/inference="ab initio prediction:Prodigal:002006"

/inference="similar to AA sequence:UniProtKB:O31672"

/codon_start=1

/transl_table=11

/product="HTH-type transcriptional regulator MhqR"

/db_xref="COG:COG1846"

/translation="MLNTEDNRELSLQLFVVLVRAYNSVTSRSNRDIQSHGLNTTEFG

VLDLLYHKGPQALQKIGEKVLMSSGNITYVVDKLQNKNLLFRRPSKEDRRVIYAELTE

EGRELFTQIFPQHHQVIIDALEGLDPSEKVDAIKMLKKLGLAAEGKTDLRS"

CDS complement(3560073..3560672)

/locus_tag="EFAGFIKM_03044"

/inference="ab initio prediction:Prodigal:002006"

/codon_start=1

/transl_table=11

/product="hypothetical protein"

/translation="MKELPIVDGELVLRCVEKKDLKELYELIYSDDVPEWKQWDAPYY

PLKHESYESFEQGMLKRMDVDPGDSKPVSIRIIESDRQIVGTISYYIEDELSMWLEMG

IVIYRSVQWGRGVGTRSLVMWSSHLFEQMPLVRVGLTTWSGNERMMRAAVKAGLQIEG

RMRKCRIVRGQYYDSIRMGMLREEWEQKLAPHTSRNVNN"

CDS complement(3560698..3561369)

/gene="rpiA"

/locus_tag="EFAGFIKM_03045"

/EC_number="5.3.1.6"

/inference="ab initio prediction:Prodigal:002006"

/inference="similar to AA sequence:UniProtKB:Q72J47"

/codon_start=1

/transl_table=11

/product="Ribose-5-phosphate isomerase A"

/db_xref="COG:COG0120"

/translation="MNLKQIAAERAAEYVEDGMKVGLGTGSTAYYAICRIGERVRDGL

NIQAVATSEASDKLAREWGIPIVPFDQIGRLDLTIDGADEVDPEFNLIKGGGGALLRE

KIVAANSDKLIIVADGSKAVQKLGKFPLPVEVVPFASEWTFQALEQLGCQPQWRMDGQ

QRYLTDNGNLIADCHLEAIDHAADLNVQLNMLPGVVDNGLFVDMASTVILANADGSIE

ELHRS"

CDS 3561856..3563622

/locus_tag="EFAGFIKM_03046"

/inference="ab initio prediction:Prodigal:002006"

/codon_start=1

/transl_table=11

/product="hypothetical protein"

/translation="MKSLSWKSMSFFSKNLLLSFTNIVIIGVALIASSYYFQKTVLVD

QLHGQVEQITKKWAEDINPAEVQAAIAEGSYDGATQTKLRAYFDEMQEYYPNIAQAYI

FGVELGGDNKRLTSLVAMPTNLREAFQSENVNIGDMYEQPVVVANALKEMLNTDRPTF

TTFYSDDFGTWTTIAYPIKDSNGKIFSYFAVDADASAVPAGLNSLLKNGIIILVAFLL

LFLIIQYLVVKNTLSPIRHLIKGIDDVSRGNLNVNIPTGKDDLGLVNEKFNTMVRKIN

DTIVKVQITSQEVNQSAKELYEVSERNSENADSINNNVTQITSNIRSQEQATRDSARA

MSEMATVIQTIASSSASVADEAYEMERRSQQGNSVVRQVSEQMNLITESVKNTASAIE

VLESRSQEIGDILNIISGISSQTNLLALNASIEAARVGEEGRGFAVVAGEVRKLAEQS

EQATSQVGVLIQEIQAGIKQAVRAMEQGTSEVDTGLSVADQTGQLFEDILEAAKKVSN

QIQEVSSATEEISAGTEEMTATADDLSSSVSKTANSSEQISSSVDEQKASLITLVDSS

TRLNSMSEELQELISHFNVSKQ"

CDS 3563669..3567376

/gene="lgrD_2"

/locus_tag="EFAGFIKM_03047"

/inference="ab initio prediction:Prodigal:002006"

/inference="similar to AA sequence:UniProtKB:Q70LM4"

/codon_start=1

/transl_table=11

/product="Linear gramicidin synthase subunit D"

/translation="MIENQPSSSHSMESSLPVLQIPLDFGRRQQSFSYQSAQITLHAS

LSRELKQKYGEQMVYPVLLSAYAALLFRLSAEQELAIGILSPDQAASYLSLQIQGKLA

FSQLCHQVSEQLKIEYTLQNGGYPESFFMLNSVQLPQAPQILNWNVRDDQNMLILDLF

YDSSLLKESTVLRYAEYYQTLLLALVRDGEKAIGTVDILSASDRLLYREMNDTSVLEP

ENQTVHGWFEATAAAYPDSPAITSPSKSYTYRELNERANQVARVLLSNGLQKGEFVSI

FMDRSLETIISLLGILKAGGAYVPIDPEHPQERNSYIVEDTASSFVLTTEASYAQASS

LFSSIATVRQILAVDGRLAGFAASNPNLDIQPDDLAYIIYTSGSTGKPKGALIAHRGV

TNLGSVVQRDCDIQPGDVLTQFATYSFDASVWDTIGALFYGAELYLLSAEERVSVEEF

ASAIERTGTTIITILPTIFFNQLASYLSDEGFHKLAKVRIITVAGEALYGEQVRAFQR

KFGNQIDIVNVYGPTECTVATTTHRISEQVPEHVVNIPIGKPIHNYKVYIVNEEQQLC

PAGVPGEVYIATPALAKGYLNQPERTEQAFIENPFAIGEKIYKSGDIAKLLDTGLLEY

VGRSDSQLKIRGHRIEIGEIEDHFARLDQIQNVAVIPKKESDGQNMLVGYFTSKDGST

LSVSDIKAELTEKLPSYFVPKWICQLDEMPIAPTGKINRKAMVSLPHMERHEDRPDRV

MPETETESIILDAWKEILQHDDFGVEDSFFNIGGDSLRVIHVLVILKPHYPQLKIADF

FAEKTVRALARRVEALSLSAAEVTDSVVTEGMITQLSEHPVELTSQLGYPLIREPEHV

LLTGATGYLGSHVLQQLILNSRTRIYTLVRRPSNGITAMERLTNVLEGYFGKQLTDQL

STRVEIIEGDLEQPNLGLSAEQRAYVQDRIDRVIHCAADVRHFGDADQFAKTNVEGTV

ALLDLIRSKPGASFHHVSTMGIPEDLALSGQWESSLQYDRFPADLHVDNLYSDSKLEA

EKVLMIAAEQGVPVSIYRAGNLTCHSETGRFQSNIDSNAFYRMIKAMLLLGKAPAADW

MVDFTPINYASEAIVHLALRQDTAGRVFHICNPEPIRYDELIRSVNRAGYEVETLPFA

DYTRWLFDASISKEPEALQLAIAQLEGDGAKDSAYVYACPVTTAYVEPAGISCAKTDD

RFISAMLDYAVQIGYFPSAIRRHNGTSTAME"

CDS complement(3567491..3568111)

/locus_tag="EFAGFIKM_03048"

/inference="ab initio prediction:Prodigal:002006"

/codon_start=1

/transl_table=11

/product="hypothetical protein"

/translation="MMQWIAMITMLIDHIGAVFFPHIIELRIIGRIAFPIYAFAVYIG

YKHTRDVQKYIWRLFWIAIISQVPFMAAFNHYSLNVVWTLWSALLVLFVIDKLPSKLL

GIPIVIGAGWFMEISQMDYGMYGLLLVLLFRYFQGPVLVVAHVLLNALYLLLHNSSVQ

MYSVLATAGIAIAQYYQAGFRMKGPRWVWRYFYPAHLAIIAIIRWV"

CDS complement(3568189..3568719)

/locus_tag="EFAGFIKM_03049"

/inference="ab initio prediction:Prodigal:002006"

/codon_start=1

/transl_table=11

/product="hypothetical protein"

/translation="MLSIPEELSEMQHRIRQAGLLDSNEVGRLFNEYRMFYNQNADIE

AARQYIRERMEGHESVILVAETDAESDHGTDESNVMSLSKGLICTGFVQLYPSFSSVS

MGPVWVLNDLYVHPDYRQQGIARKLLQAAKQLASERGVLRISLSTELSNKRAQALYEP

EGYAQDTKFMYYELNV"

CDS 3568934..3570379

/gene="gerAA_1"

/locus_tag="EFAGFIKM_03050"

/inference="ab initio prediction:Prodigal:002006"

/inference="similar to AA sequence:UniProtKB:P07868"

/codon_start=1

/transl_table=11

/product="Spore germination protein A1"

/translation="MKESILIQVQSRLVGVADMIYQPLQIGPLHCTLLYIQSIVDTQI

MREAIVKPLLEEAARNEVGPDFITQVVSGTFFSLENQHRDSAETLVDDIVTGNAALYI

EGMSGMIIFSIQNYQKRSVPESTNEVVVVGPQEAFIEDINVNMSLLRHKIKHPDFKMI

KFTIGKYTKTEVFVIYIQGLCKPEILENVLTSLGDIDMDSSLGVSYLSEFLEDHPLSP

FPQYQYTERPDTVAAALVEGRIGVMQDGTPFSLLIPVTFFSLMQSSEDYYQRFHSASF

IRIIRLLFAVIAFLLPSVYVAVTTFHPEIIPTNLLITIASARENIPFPALIEAFIMEI

TFEGLREAGIRIPKPLGQTVSIIGGIVIGQAAVQAGIVSAPLVIVVSITGIAAFIIPH

FELGLAFRLLRFPVLLMGGTLGLFGVVISIYLLYWYMVSMRSFGVPYMQPFAPLVLRD

FKDTFIRVPWFMMKKRTKAYATDNERRQDTP"

CDS 3570376..3571524

/gene="gerAC_2"

/locus_tag="EFAGFIKM_03051"

/inference="ab initio prediction:Prodigal:002006"

/inference="similar to AA sequence:UniProtKB:P07870"

/codon_start=1

/transl_table=11

/product="Spore germination protein A3"

/translation="MIFRRPPLPIIVVIICCLLCTGCWSKVEINERTFITTLYVDKAD

TPGEIEVTLSMPLPNRLSPEGGGTGKDPYAAVSATAPTIADAIERIQTDLTRKISWGH

TRVIVFGQAYAREGIEDTMEWIARQPLFHLSSYVMVANGKAKDVSDLTPVFEETPSDV

LREFSTEENLLKTQVLSIFTSDKMNQGFASSMLASKKTKMVSEEGESKKWVSQIGGTI

FRQMRMVGTLSADEARAVAWAGQNLDSMTISVRTKKQKASMKLYRMHSDIHVKLQNGE

PHFVINLTGRAELNSVIPVLKAEDIVGIKDIEQAANEKVSAHLTQAIQTAKHQGSDIL

MLGYRLEWRYPQVWKKLRPTWINYVKNDLQFTVNTNINIQFVGSESSF"

CDS 3571562..3571768

/locus_tag="EFAGFIKM_03052"

/inference="ab initio prediction:Prodigal:002006"

/codon_start=1

/transl_table=11

/product="hypothetical protein"

/translation="MLAVLVFAGLYLLDWSSVKSSKKAIRRAYFILLGLFLVWNTLAV
SWSAWPNPNDVIQLVFGWADHLIQ"

CDS 3571784..3572902

/locus_tag="EFAGFIKM_03053"

/inference="ab initio prediction:Prodigal:002006"

/codon_start=1

/transl_table=11

/product="hypothetical protein"

/translation="MNHLTAGQAYRFMFVYLYSEPVAFLLQRLFKMSGYQGWLSTIGG
FLISLIFLFFTYRLGSINPDKPWISYGEDIVGKVVHRFYIGLIVMLCIYLLSIDVENF
IIFLQSMYLPQTPIWLTSTLTLLCICLTARSGLVTIVFMSEGIFLVQLFTSTLLIPAV
GGGGNPGILLAMATHHDLGEIMMGSFSTMPWFSEWMFFLFLAPVVAFRRPMLKSMLFA
GITVVIFIVVFWMFTLMNFGPYVAAGLRYPLLEMVRFARYGDFLDNLDPVLIAIWSTT
MFTRSSFLLYVASVCLSKLSGIRQQKSVVYLIGGTAAAIVLQYARDAASYELAIRSYA
VPLYTVLIESMPILYVLVYWLRNGKNKKATKASTAPSP"

CDS complement(3572907..3573380)

/gene="fucU"

/locus_tag="EFAGFIKM_03054"

/EC_number="5.1.3.29"

/inference="ab initio prediction:Prodigal:002006"

/inference="similar to AA sequence:UniProtKB:P0AEN8"

/codon_start=1

/transl_table=11

/product="L-fucose mutarotase"

/db_xref="COG:COG4154"

/translation="MLKNIPAIIPPELLKIMSEMGHGDELVLADGNFPAASHAKRLVR
CDALGVVELLDAILQLYPLDTYAERPAAVMQVVKGDQVIPIIWEDYRRLIEEYEGITD
AFDHEERFNFYKRASNAYVIVATGERAQYANLILKKGVIFPDADPGQEQQNAESN"

CDS 3573636..3574058

/gene="slyA_3"

/locus_tag="EFAGFIKM_03055"

/inference="ab initio prediction:Prodigal:002006"

/inference="similar to AA sequence:UniProtKB:B1JJ73"

/codon_start=1

/transl_table=11

/product="Transcriptional regulator SlyA"

/translation="MDYTLEQSVGFMLGFTHRKAVALLATRFKPYDITTEQFSVLFNV

DRGEGVNQKELAARVFKDQPTTARIIDLLEKKGWVERRTSEQDRRAYLLYLTTEGKAL

IDILVPIEREMNKELAEGIPEDQMEAFKHTLSLINRNL"

CDS 3574089..3575429

/gene="yfcJ"

/locus_tag="EFAGFIKM_03056"

/inference="ab initio prediction:Prodigal:002006"

/inference="protein motif:HAMAP:MF_02091"

/codon_start=1

/transl_table=11

/product="putative MFS-type transporter YfcJ"

/translation="MKEQSTQVRLWTTDFILLMLCNFLLFLQLHMIVSPLPSYVQERF

HANAFEVSLFTCLFALSAIAARLYSAKALEKGLRNAMIYIGLSVALLATLGYYFAAGI

AVLLLLRMLFGIGFGMSSTAFPTMASDIVPVKRMGEGMGYFGLSTSLAMSMGPIIGVT

LLQGAGFVTLMLCTAGVLAVIYPLSYSLTRKKEVRTDNSTTIMPQATTSASGSNPKQK

TPFNRKLILPSVLNCLLSITYGGLVGFIVLYGKEANLANPALFFLFNALAVLLVRPFA

GRIYDNKGPKALLIPGAIFVAVGLILLSYATSMSVLFIAAFIYGIGYGSMQSSLQTWM

IQVVSPTQRGMANGMFLNSLDLGIATGALLLGAIAAITSYTDMYRYSVMFMILFLLIY

LIQGKRSGSFSIEPHALLAHTHITATEPPQDQSKNSESGAGNTETSQSNKSKHE"

CDS complement(3575584..3576885)

/gene="ymfD"

/locus_tag="EFAGFIKM_03057"

/inference="ab initio prediction:Prodigal:002006"

/inference="similar to AA sequence:UniProtKB:O31762"

/codon_start=1

/transl_table=11

/product="Bacillibactin exporter"

/translation="MNDKKWDLVALASIPLIMTLGNSMLIPILPQIERELKVSAFKVS

MLITVYAVVAILLIPLAGYLSDRFGRKAVIIPSLVIAAVGGAVAGVAAWMLNGNIAYW

TILGGRLLQGIGAAGAFPIVIPLVGDMFNDENEVSKSLGIIETSNTFGKVISPILGAA

LAVWLWYAPFMAIPILCVLSLVLVIFLVKTPRKKEKPPTFSEFVASIRDVLKEKGRWL

YALFAIGGICMFVIFGVLYYLSETLESEFALKGVLKGLVLAIPLAALCLSSFLAGKWI

GKNKTRMKWLGFAGLTLLTVSLGVIGLFDNIYVVVGLFTLGSAGIGATLPCLDALITE

GVDKKQRGTITALFSSMRFIGVSLGPPVVSLLIGSHHFLLFGVLAASGAVGGLLTLFA

VKPDKGDQPTSGSKQKQDETSMDVIETNRGYAPIGRKKSPF"

CDS complement(3576968..3579916)

/locus_tag="EFAGFIKM_03058"

/inference="ab initio prediction:Prodigal:002006"

/codon_start=1

/transl_table=11

/product="hypothetical protein"

/translation="MKEYHQYPMWDASLSLEERLDDLIARLTTEEKIRLIPTREAAVP

RLGIPAYNVGGEAAHGVAWKGEATVFPQPLGLSSTWNTRLMREIGSVIGDEARAYHHR

DPEVHGLTLWAPTVDLERDPRWGRTEEGYGEDPVLTGEMSAALVKGMQGNDPFYLKMV

ATLKHFFANNNEKDRLNCSSSIDPRNLREYYLKAFETPFVEGGALSMMTAYNSINGTP

AIESPYVNDVVKGEWAMRGFIVCDGGDLSQTVNYHGYHTSHAESASGALKAGVDCLTD

EVDLVVSALEEALDQNLLEIADLDRAIRNIFGVRMRLGQLDQSGLNPYASIPESVLCA

SEHAKLSYRAAAESIVLLQNDGLLPLQPEVLQKISVIGPLADVVYTDWYSGTLPYRVS

VLDGLRNRLPEADVTYVDGNDRIGLKTVDGKVITLGAEGTIVAVQEGSTPAAEFVHQD

WGWGSHTLRSVNNNRYVSLTEQGIYQANAPEIGGWFVKEVVRIDPLTDGSNTLRTWNG

IPVGLSEYQGQFAVSPPRPQGEQTTATGTNPNDVGESGHASQSPGNTQIPSAFNIDIV

TSGKQQAVEAARSSQASVVVVGNSPYINGKEEIDRPSLLLPPSQVELVKAVCEANPNT

VVVIMGSYPFALQELKGIARAIVYMTHAGQELGNALADVLLGHYAPAGRLNMTWYEDE

SQLPDMMDYDIIQNGMTYMYHEGPVQYAFGHGLTYSEFEYEAIRVSRDTTAEATTTDQ

LKIEVEVHNTGQHDSDEVVQIYGSSHTSRVKRAQKQLLAFRRVHVKAGTRVTIRFEVP

VQKLALWDVTRDRYCVETATWAILAGRSSTDIRQSADIEIEGETIPDRPLHLLTFAEN

YDACAGVLLDECLEGRSAVRAINREEPLYREAQLSWIAFNHNAVQEVQGFEARVAAFG

EEGRLEIRSGGLEGELLGVCEVSGPGGSVNGKDIKWTTVQCSLVAPHKVNELYIVFLG

GAALRHFRLL"

CDS complement(3579936..3580946)

/locus_tag="EFAGFIKM_03059"

/inference="ab initio prediction:Prodigal:002006"

/codon_start=1

/transl_table=11

/product="hypothetical protein"

/translation="MSTMNSGLHCTISAEMLHHLVEINFGSDTKVKEFGLLQGGLFNT

TYRIQLEHTPYTDVILRLAPERGEMAAGSASDPLFSFERTMMAAEPIVYEYYRKAGIP

APNIIACDDSGLIIPRTYMFMAFIPSKQLDHASISDIDKERLYHQLGVYTAIMHQIEG

ASFGWPQGDGSIKGSDQWSEVLHAFAEETALKAAQADYMSGVGEEIAAIFIKNKDLFD

QVTRPVLVHNDLWEANVLVHVENGELNIAAIIDGDRSMFADREFEAILSSESAAFHEG

YDRPLDSSAEGQARRLAYRILSSYFNAYVHEHQVNQPEDGQKYRQRTLDLLEQWKQLG

LN"

CDS complement(3581022..3583340)

/gene="yicI"

/locus_tag="EFAGFIKM_03060"

/EC_number="3.2.1.177"

/inference="ab initio prediction:Prodigal:002006"

/inference="similar to AA sequence:UniProtKB:P31434"

/codon_start=1

/transl_table=11

/product="Alpha-xylosidase"

/db_xref="COG:COG1501"

/translation="MKFTDGFWMTREGYQIQNPTDIRDIVQKDNSVTVYAATKYIRSK

GDTLNGTLLKATYSSPMPNVIRVTLNHHKGGVKKGPVFELNTQEANVEISKNEQGAVL

KSGNLEVQIDKTNGWDISFLYGGKRITGSGQRAAGYITGPSKEAYFREQLDLGIGEYV

YGLGERFTPFVKNGQIVDTWNEDGGTSSEQSYKNIPFYLSNKGYGVFVNHPERVSYEI

ASENVSKVQFSVEGETLEYFIIGGDNPKDVLDNYTKLTGKPALPPAWTFGLWLTTSFT

TDYDEATVNHFVDGMAERDLPLSVFHFDCFWMKEYQWSDFVWDEAMFPDPEGMLARLK

EKGLKICAWINPYIAEKSYLFDEGMENGYLVKTADGSVWQWDMWQAGMALVDFTNPDA

VNWYKSKLEVLLDQGVDSFKTDFGERIPTDVVYFDGSDPVKMHNYYTQLYNKAVFELL

EEKIGKNEAALFARSATAGGQQFPVHWGGDCSSTYESMAESLRGGLSLGLSGFGFWSH

DISGFESTASPDVYKRWVQFGLLSSHSRLHGSTSYRVPWLFDEESVDVVRDFTKLKIS

LMPYLYNSAVESTVKGIPMMRAMLLDFPEDPTTYSLDTQYMFGDSILVAPIFNKEGDV

RYYLPEGTWTNYLTGAKVQGGRWISENHDFKTLPMMVKPNSLIAVGAVDSKPDYDFAN

DVSLHLFELADGQTAQAVVVNQAAEQELTVNVTRNGSVLDVRAEGAGKPWNIVLRGIE

SVSSVEGGSQVSGANGVVVTAATGANALTIQL"

CDS 3583513..3584433

/gene="chbR"

/locus_tag="EFAGFIKM_03061"

/inference="ab initio prediction:Prodigal:002006"

/inference="similar to AA sequence:UniProtKB:P17410"

/codon_start=1

/transl_table=11

/product="HTH-type transcriptional regulator ChbR"

/translation="MERERLREDRIHGNAMYPVSVYPDIQQLNGDSILDCHWHDEMEF

TMVTQGCAVFQIDMNTVEVQAGEAIFINRGEIHAGYLKGDVPCVFSSIVFNPELLGSR

TFDTVQEKFIGPLVRKTVIPPSHIKADEDWGQEILDHLKRIFADHATRTETCEMSTKA

YLYLIFARMFEHMRPAPLKGTVATGSHDKVERLKSVLGYIHKRYPEPLKLKELADEAN

MSEGHFCRFFKQMVQKTPVDYINYYRVQQACVQLENTDHKIVDIAMDVGFEHLSYFIT

TFKKHKATTPSQYRKMFYENVGMESALVQV"

CDS complement(3584601..3585521)

/locus_tag="EFAGFIKM_03062"

/inference="ab initio prediction:Prodigal:002006"

/codon_start=1

/transl_table=11

/product="hypothetical protein"

/translation="MKSKRGYRFILLLSCLILCASTACSSKAIADGSVDSYATIHGKL

DVPVRATWVWDTTQIRSNPQEVLNYATANKINTIYLQMNRDIKIPDYKSFIRQARAKN

IAVDVMDGRSAWGLTESREQIASFLDWIEEYQAQALPNEKFAGIHLDIEPHVHPQWKI

NQASVITQWQGNVEYIVERASRMKMPVAADLPFWLDNYKIPGSTMAVSSWMIRKFDSI

TIMAYRDTAAAIYSVAKDELAEAALLGKTISIAVETKQSKEGDFITFYEEGSAYMEAQ

LKLVEKMASVHASFNGFSVHEYTSWKTMKK"

CDS complement(3585907..3587766)

/gene="ade_2"

/locus_tag="EFAGFIKM_03063"

/EC_number="3.5.4.2"

/inference="ab initio prediction:Prodigal:002006"

/inference="similar to AA sequence:UniProtKB:Q72EX7"

/codon_start=1

/transl_table=11

/product="Adenine deaminase"

/db_xref="COG:COG1001"

/translation="MNKSSFERPLMADCVPDLVATARGDLPATLVIRGGTLVNVISGE

ILPEMSIAVQGARIAYVGKDVSHTIGEHTRIIEANGKYIAPGLLDGHCHIESTQMKVT

EFARAVLPSGTTGGFFDPHEISNVLGLKGLRLMLDEARTTPMAAYMQVASCVPSTHPG

LETTGAYIGPEEVAEALSWGPDMIGLGEVMNFPGVVYGDETMIGEIQATLRAGKVADG

HFTWAADDWRLPAYAASGVTGDHECVTKEDVVERLRLGMYAKMRQGSAWHDVAETIKA

CTELGLDTRRMMLVTDDRSSESLLKEGHMDFVVRLAISQGVKPVTAFQMATINTAERF

GVARDIGAIIPGNIADIILLDGRLADVRVGMTIAAGQVVAENGKMTAVWDSFTYPEEA

LNTVKLEANIQPEDLELAAPIAEGTIGAKIIHVTENHVDTNEKHLPVTVENGKVVVST

SGEVCKIAVLERHKQTGNRAVALVGGIGFKSPAAIAMTVAHDSHNLLIIGNDDALMAE

AGNRVIRMQGGVAVVTAAGVTEFPLRIAGLMSTESFEVVAAQSAAVSEALQSAGCTLN

NAFMTLSLLALVVIPELRLSDKGLVRISAEGIELVSLFDEVVDNTPVALSGNE"

CDS complement(3588078..3590453)

/gene="helD_3"

/locus_tag="EFAGFIKM_03064"

/EC_number="3.6.4.12"

/inference="ab initio prediction:Prodigal:002006"

/inference="similar to AA sequence:UniProtKB:O32215"

/codon_start=1

/transl_table=11

/product="DNA helicase IV"

/db_xref="COG:COG3973"

/translation="MSTDQQWNEEQQRVNTVTDQIERKITTLEDEVGSFRDEVVGMRK

DFWDEVTMNFSEADDVGETSTSMRQQSQVLSDRERSHLNTAAALDKMKRLHHSPYFGR

IDFKEDGYPNAERIYLGIASLLDEKEESFLVYDWRAPISNLYYDGAPGPITYHTPSGE

ISGDIEMKRQFVIRDGRIRFMFDTGVTIGDELLQAVLSRTSDAQMKSIVATIQKEQNR

IIRNDRTRMLIVQGAAGSGKTSAALQRVAYLLYKYREHLQADQMVLFSPNPMFNSYVS

TVLPELGEENMLQTTFQEYLERRLGREYQLEDPFIQLEYVLTGTEDPDYDVRMSSIRF

KSSETFLKVITRYKESMLSGGMKFKPVRFQGRAIVTSEAMAEKFYSFESSVKLVSRLE

MLRDWMLKELSAFGKGELDAPWVDQQLDLMEPEDLQRAYQRLKRKQKGKTHTFNDFEQ

ERGILARMVVSDRLKPLRKWIKSLRFVDIRQLYAHLFNDQAQMERLLGDEVLPAQWDE

ICKMTLRRLKLQELAYEDITPYLYLRELMLGFHINSNIRHVIIDEAQDYSAFQLAFMK

RLFPRAKITALGDFNQAIYAHSSVLSGTGPLTNLYGPDNTEVIELTRSYRSTREIVEF

TRGMVPGGEEIIPFNRSGEKPKVIVSSDSERHMNVITTDLKHLIKEGYESVAIICKTA

EESRDVHAALSKALPTAPKLIKKTTLAFEQGVHVIPAYLAKGVEFDAVLIYDGSAEQY

AQEHERKLFYTACTRAMHLLHVYCVGTPSPFITAQSEEKYDLGKVSAAQVD"

CDS complement(3590699..3591160)

/locus_tag="EFAGFIKM_03065"

/inference="ab initio prediction:Prodigal:002006"

/codon_start=1

/transl_table=11

/product="hypothetical protein"

/translation="MIIEELDAELMEWLSGTNLEHKQHEAMQLLTVSKDHWPHQAMIS

MGEVIAINPHQLRLALWQGTQTSMNMSKTGKATLIAVQGHRLLHIRIEVERLPEMKGA

VHPRDRFEAQVLHVRVDHAPYAEITSGITFQLKDELGAITRWKETIEELRK"

CDS complement(3591341..3592408)

/locus_tag="EFAGFIKM_03066"

/inference="ab initio prediction:Prodigal:002006"

/codon_start=1

/transl_table=11

/product="hypothetical protein"

/translation="MSFGARVLKTGIAVTLALYLSSLFLNPQSPVPAAIAAIFAMQPS

IYRSWKYFLDQLQTTTLGAIVALVGGMVLSNEPIAVGLIIVLVIMICLKLNMGETVGL

TLVTVVSIMEASGDWHFALNRFLLTLVGIVSAFLINITVFPPKPKVQFVKQIHSVFSG

MSLLLRTSISDEIKEVVFREEKSNLGGSIKSLSDKYNLFEEEQKKMKRSTFSETRQMV

VYKQMLLSLQKGYEVLDSVERHYFQAPRTTAMDQFFDSHLELVIKFHEHALLKFEDKL

KPNGEEAAQFVLDNDRFMEQAITQFDIDKEGMLRLSIVAAAIYDYGYQLERLNRLAEH

VHSASEDKESQDKILNWLKWP"

CDS complement(3592709..3593431)

/gene="yedJ"

/locus_tag="EFAGFIKM_03067"

/inference="ab initio prediction:Prodigal:002006"

/inference="similar to AA sequence:UniProtKB:P46144"

/codon_start=1

/transl_table=11

/product="putative protein YedJ"

/db_xref="COG:COG1418"

/translation="MSENPLQPGGNLQELGHMHNIDGITAGEAVLRAARSFVQGDSSK

HSDGHDWPHIERVTALAVELAHRMGADPFVCELAALLHDVPDEKLNESLEAGMAKLND

WLDTQPLDPDIRNKVVGIISTISYAGGQRPAVSSLEAQVVQDADRLDALGAIGIARTF

AFSGARGREMYDPSLPPREQMTREEYRNGRSTTINHFYEKLFKLKDLMNTSYGKELAE

QRHDYMVQFVDQFKREWEGTDR"

CDS complement(3593433..3594872)

/locus_tag="EFAGFIKM_03068"

/EC_number="1.1.1.38"

/inference="ab initio prediction:Prodigal:002006"

/inference="similar to AA sequence:UniProtKB:P16468"

/codon_start=1

/transl_table=11

/product="NAD-dependent malic enzyme"

/translation="MNQRNLDGNSIIIRLEMTTKDIKFGEVASAISEAGGDIIAIDVI

STNQDVSVRDLTVAVTDAQDNSKIIEGVRQLKGVSIINVSDRTFLLHLGGKIEVTPKT

PIQNREDLSRVYTPDVARVCSAISEEPGKAFSLTIKRNTVAVVSDGSAVLGLGNIGPR

AAMPVMEGKAMLFKQFAGVDAFPICLDTQDTEEIIRTVKAISPGFGGINLEDISSPRC

FEIERRLNEELDIPVFHDDQHGTAVVLYAGLINALKLVGKSIEDVKIVVCGIGAAGVA

CSNILLSAGASRLIGVDREGAIVRTQTYENEVWSDYAARTNPELETGSLRDVIRGADV

FIGLSRGNLLTREDVQTMAEDPIVFAMANPVPEIMPALVEDIVAVMATGRSDYPNQIN

NVLCFPGIFRAVLDCRATEINEEMKLAAAQAIASAITDEERTRYYIIPSVFNDKVVKS

MRSRVIEAAVKTGVARRIPREQAREGGES"

CDS complement(3595083..3597155)

/gene="bglY"

/locus_tag="EFAGFIKM_03069"

/EC_number="3.2.1.23"

/inference="ab initio prediction:Prodigal:002006"

/inference="similar to AA sequence:UniProtKB:C8WV58"

/codon_start=1

/transl_table=11

/product="Beta-galactosidase BglY"

/db_xref="COG:COG1874"

/translation="MTYKFPPVSSKAPHMLHGADYNPEQWLRYPEVLEEDIRLMKLAK

CNVMSIGIFSWVSLEPEEGVYTFEWLDQVLDRFAANGIYAFLATPSGARPAWMSAKYP

EVLRVSEKRVRNLHGFRHNHCYTSPVYREKVTAINTKLAERYSDHPAVIGWHISNEFG

GDCHCDYCQEAFRGWVQKKYKTLDELNHSWWTTFWSHTVTDWSQVESPAPHGETQVHA

MNLDWRRFVTDQTADFIVHETKPLKAQNPDLPVTTNLMEFYGGLNYWKFADILDFLSW

DSYPTWHDADDDAKQASRIAMMHDIVRSIKGGQPFLLMESTPSSTNWQDVSKLKKPGM

HLLSSLQAVAHGSDSVQYFQWRKSRGSSEKLHGAVVDHVGTEHTRVFQDVTDVGTALE

GMEAIVGTAVPAEVAIIFDWENRWAVNDSQGPRNIGVKYEQTVEEHYEAFWKKGVAVD

VIDMDADLSKYKLLIAPMLYLVREGVGERIEKFVEQGGTFVATYWSGIVNENDLCFLG

GFPGPLRKTLGIWSEEIDGLHDRDMNGIIPEKGNELQLNTAYDAIELCDLIHLEGAKS

LATYRSDFYAGRPALTVNQLGSGKAYYVATRLKAPFYDDFYAKLIADLNIERGLETEL

PAGTTAHTRTDGTADYVFVQNYTPDEKLVELDGQSYTDLLSGDAVEASLSLKPYDICV

LRRPAARK"

CDS complement(3597245..3598330)

/gene="ganB_2"

/locus_tag="EFAGFIKM_03070"

/EC_number="3.2.1.89"

/inference="ab initio prediction:Prodigal:002006"

/inference="similar to AA sequence:UniProtKB:Q65CX5"

/codon_start=1

/transl_table=11

/product="Arabinogalactan endo-beta-1,4-galactanase"

/db_xref="COG:COG3867"

/translation="MSNLTEKTFILGMDVSFMDEIEQHGGSYSDVDGKEQNLLSILKI

NDANAIRLRIWNDPVGGFCNLERTVEIAKRIKEQGLKFLLDFHYSDRWADPANQWKPK

AWEHLSYEELQRAVCMYTADVLRTLKEHDALPDMVQVGNEITPGMLWNEGRVGGEEHD

TDEQWERFAGLVKYGIAAVKSVDADIQIMIHIDRGGDNAESRKFYDRFEALGVEFDII

GLSYYPWWHGTLDALRDNLHDLAERYGKPINVVETAYPWTLEQPEGIEWILNQEDMLL

PGYPASVEGQTKYLKDLLQIIREVPGGLGHGFYYWEPAWIPSKEEWSVGHPNNWGNLT

MFDFKGRKLESFTALATAEESDVVTYV"

CDS complement(3598363..3599241)

/gene="ycjP_5"

/locus_tag="EFAGFIKM_03071"

/inference="ab initio prediction:Prodigal:002006"

/inference="similar to AA sequence:UniProtKB:P77716"

/codon_start=1

/transl_table=11

/product="Inner membrane ABC transporter permease protein

YcjP"

/db_xref="COG:COG0395"

/translation="MYHKSLPYRVFNIVNTCFLILIAIMCIIPMVHVLAVSFSTKAAA

DANLVNLWPVGFSLEAYKKTMNNPIFLNSLWISLLRTVIGTAITLLITFLAAYPLSKE

NSEFKGRTIYSWIFVFSMIFNGGLVPFYMVIQKIGLMDSFWVLVLPGAVNTFLVILML

NFFRGIPKELEEAALMDGANHFRTLFRIFLPISMPSIATIALFSMVFHWNSWFDGLLY

MNNAKDYPLATFMQTVIIGRDMSSMSMNPKEMEALSQTTVRAAQIFIGSAPILIVYPF

LQRFFVKGMTLGSVKG"

CDS complement(3599271..3600161)

/gene="yteP_24"

/locus_tag="EFAGFIKM_03072"

/inference="ab initio prediction:Prodigal:002006"

/inference="similar to AA sequence:UniProtKB:C0SPB3"

/codon_start=1

/transl_table=11

/product="putative multiple-sugar transport system

permease YteP"

/db_xref="COG:COG4209"

/translation="MRTLKRTWPFHVMLLPAIIFLIIFSYVPMGGIVMAFQNYKPWLG

ISGSEWVGLDNFRYLFEREDSLQVIWNTLIIAVLKLIFNLFVPFVFAILLNEVRKMAI

QRTIQTLVYLPHFLSWVILGGILIDLLSTGGLVNRVLGTFGLGPYFFLGDNSWFRSTV

ILTDVWKEFGYNMIVFLAALAGINPALYEAAEIDGAGRWKQTLHITIPSLVPMLMVVG

TLALGNVLNAGFDQIFNLYNPLVYQTGDIIDTFVYRSAMQNGEMGFATAIGLFKSVIS

MILILVSYSLAKKYAGYRIF"

CDS complement(3600351..3602036)

/gene="lipO_9"

/locus_tag="EFAGFIKM_03073"

/inference="ab initio prediction:Prodigal:002006"

/inference="similar to AA sequence:UniProtKB:P37966"

/codon_start=1

/transl_table=11

/product="Lipoprotein LipO"

/db_xref="COG:COG1653"

/translation="MRAQSTKKRFLTLLATTLSLTVVLAGCSGGSGGGDSATPSTSGT

QNEYKEKYDPEVTITTAWGIDPELKFKNGESMENNVATKWAKDQFGIEIKSLWSVTDT

NGAFATKLRLAMSSGQEMPDIVNVGDNLLAQDLIDSGMYQEVGPLFDKYASDTWKKAI

EQDPNVWNQYSRDGKRMGIPVLDYAYNNDYLLWIRQDWLDKLNMKAPKTIDELEAVME

AFKNNNPDGLAPDKVTPLSIGFKTSMNTWMGDPSWIFGAYGTLPFQWNLGEDGKLEYG

SINPGMKQGLTKLSEWLKKGYIPQEAALWDENKTAEPAVAGTAGIIPGPYWMSGWPLL

DTVKNVPSAVWKPIEIPTGPEGKAMRHGTQFVNGVTLIKKDMAHPEAFFTYQNYLFDN

YADPAPGSQYDNGLFAGYDYQLDADGKQMPIDEIEGGYVNVVRYLLVRDGARIPDAQM

KALMNLANGKEPETKLEKDVAVNYGKETPAAAKVLLSQEEISFKNMFTGPTTQTMKSK

LDYLNKIENQAFNEIIYGKNPVDAFDTFVQTWKSGGGDQITQEVNEWYDSVKK"

CDS complement(3602160..3603788)

/gene="rssB_7"

/locus_tag="EFAGFIKM_03074"

/inference="ab initio prediction:Prodigal:002006"

/inference="protein motif:HAMAP:MF_00958"

/codon_start=1

/transl_table=11

/product="Regulator of RpoS"

/translation="MIDILLVDDETYVTESLELTIPWGELGVTTVLRAASGKEALEIM

EENAVDIVVTDIRMPGMSGLDLISEVSKRWSHIRCILLTGHSDFDYAKKAIQLQAADY

ILKPVNDDEFMGSVSAAITSLRDEWDEFDKYHRLLYSRKSDYKILRENLMHDLLLGRE

ITARALREQLQQYEIHIDPEQSAVMMLIRLTGRFSSMDQQSLDLMDFAVGNIAEEVLG

EQFNVWFGRGPHECLVMFLQNQDQIEAPQMDLETLRISAETFREHVIRYLQGDLSMVV

TPSFPFNELTAAYRKSLGSLVLSGPEEHQIIYMDMDQALSKRPENDATQALEELYKPP

VLPQLLETKQWEAAARKLNTVFDAAEQVRLSREHVYEMYLSVTNAFMYIAHKQGHLVH

EIDHAGFDLLLAHQLIQSPEKLRRWATEMLAKLQEELSDQEGVQSRRHVIKQVQELVT

RDTGQDLSVKLIADKVYLHPVYLSKIYKAETGEGLGDYMIRMRMERALYLLKNSNKKI

YEITSELGYQNPQYFSKMFKKHYGMTPNEFRDQA"

CDS complement(3603862..3605616)

/locus_tag="EFAGFIKM_03075"

/inference="ab initio prediction:Prodigal:002006"

/codon_start=1

/transl_table=11

/product="hypothetical protein"

/translation="MSYRTNLFSKMVILILIMLIPVVLLYWYSNHKTTAVLRDELNRS

NSNQLEFFQNQVNTHIELLSSWPNLLIHDPDIGSFRRIYADSNYFDLDAINLVKRIQN

KLSIQESSSNWATKLYLYSPSLGRVVSERDARSYDKEALRENIISGWDVRKIQDGEDD

RFMFSWITVSPYGIKDPANNAETIIKLEFDSDNIRDMLDKFKDDGRHDPFYFREESGV

IYNRTSDRSLTNQLMEELSIHKLQDVDNRTVVIGNEPYMVNTVKSSTTGWYLVDYMPL

SDILKPIHQSNMLFYSSMICLLLMSFGVAYLFYVQVQVPVKQLIRGFQRLKQEDYSVR

IKPKGRNEFSFLSERFNLMVEQIQQLFEHVYLEQIHVREARLKQLQSQINPHFFYNCF

SFITSMAKLKRMDAVVAMSHNLSRYYRYTTRQERDVVPLTEEIEFVSCYLEIQRMRMD

RIHYKIDLSDEMLRQEVPPLIVQPLVENAVIHGIEADAEAGEIRVSGEKQGGVMVLVV

EDDGQGMTQEAREALLNKLRGTMDQEMGCGLWNVNQRLQLRYGEQAGLDVTESELGGL

RVTLSWPAEQEFLLENEG"

CDS complement(3605915..3606613)

/locus_tag="EFAGFIKM_03076"

/inference="ab initio prediction:Prodigal:002006"

/codon_start=1

/transl_table=11

/product="hypothetical protein"

/translation="MSRTRLVFSGALLLFAIIFTCNHHLGFSVGDTMLSAIGISPYTT

SYLSGVHITLFLGLGIFACGFYLTRKEIGKSFPGLAKGLWIVVIAIVLSYSYMTDKFM

YVAKWGASGINAVSYVQNASSCTFNVLKTGETRATCDLTLKNYGRDSVSAVLLPDLAR

SYKFKDDPLLEALQSVQLRPVHVEIKRHGTFTGKLEFYGKAESPLFVNGKLMDIVLDV

GVDGANTVFDYDIP"

CDS complement(3606795..3608324)

/locus_tag="EFAGFIKM_03077"

/inference="ab initio prediction:Prodigal:002006"

/codon_start=1

/transl_table=11

/product="hypothetical protein"

/translation="MVRSLKVWSRMMATVMALSLVLAACSSDKGGTTTPGAEGGGASE

AGGKPYEVTLFYPGTPQKDVALVEAEINKKMEPKIGATLKINAIDWGQWDNKLNLMIS

SGEKSDIIFTAAWQNYTVNVAKGAFLPLNDLLDAHGQDIKKNLDPAFLEGSQVDGVNY

GVPTNKELAATRGVLVRKDLADKYKLDLTAVKTWADLEPLLKTIKENEPAITPFYMSN

TNGNGLLENLDWDYLGDASVPGVISKTAGGTTVLNEVETPEFKEAAELARKWYQAGYI

NSDAATSNVFPKDQAKAGKAFLWTDGMKPGKDKEEEGYVGFPLTQIEMTQPTITTGDA

SGAMLAISRSSEQPEKAMQVINLLHSDKEINNLLNFGIEGTHYVKKDGQDNIIALPDG

VDANSRTYNPGAQWQLGNQFLNFLWDNEDPQKWEKFKEFNAKGVKSPALGFTFNSQSV

KNEIAAVNNVNKQFKPGMTSGAVDPNEMIPKYLEKLKAAGIDKIIAAKQEQLDAFLSK

Q"

CDS complement(3608458..3609366)

/gene="araQ_20"

/locus_tag="EFAGFIKM_03078"

/inference="ab initio prediction:Prodigal:002006"

/inference="similar to AA sequence:UniProtKB:P94530"

/codon_start=1

/transl_table=11

/product="L-arabinose transport system permease protein

AraQ"

/db_xref="COG:COG0395"

/translation="MKSRDPLAVSKRSASVIHAMFLFYAIACIVPILLVFAISFSDET

TVIANGYKLIPEKFSLTAYEFLFRDMDQIIHSYGISFIVTVVGTITSVALTALYAYPL

SRRDLPYRGWFAFFIFFTMLFNGGLVPWYLVYVNVLDLKNSLLALIMPLLLSPFFVLV

MRTFFANSIPVSILESARIDGAGELRTFARIVLPLSLPVMATVALFSTLNYWNDWYLS

MIFISDNRTISLQYLMYRTLLDIQYLTTNSNVSSQISSQGGLLNLPNKTLQMAMAVVG

IGPIVLAYPFFQRYFIKGLTVGAVKG"

CDS complement(3609384..3610370)

/gene="yteP_25"

/locus_tag="EFAGFIKM_03079"

/inference="ab initio prediction:Prodigal:002006"

/inference="similar to AA sequence:UniProtKB:C0SPB3"

/codon_start=1

/transl_table=11

/product="putative multiple-sugar transport system

permease YteP"

/db_xref="COG:COG4209"

/translation="MKTQPQAGKGVRISEIPGETVRKRKSVWHNLGKFKVLYLMFLPG

VLFLLVNNYMPMFGVLIAFKNVNYADGIWGSPWSGWDNFKYLFSTSDAWEITRNTLAY

NTVFIALNLLVGVGLAILLNEVKNKAMSKLYQSLMLLPYFLSMIVVSYLVLAFLGKDS

GFMNSTVLQMFGGQPIDWYSEPKYWPYILPLVNTWKNIGYYAVIYLAAVVGIDEEYYE

AAVLDGATKWHQIRFITIPFLVPLIVIMTLLQIGRIFYADFSLFYQVPLESGALFPVT

NVLDTYVYRTFLIGGDIGMSSAAGLYQAIVGFVLVLVSNTIVRRIDKDNALF"

CDS complement(3610551..3612164)

/gene="rhaR_29"

/locus_tag="EFAGFIKM_03080"

/inference="ab initio prediction:Prodigal:002006"

/inference="protein motif:HAMAP:MF_01533"

/codon_start=1

/transl_table=11

/product="HTH-type transcriptional activator RhaR"

/translation="MRNLLIVDDEVYALQAMVEGVDWQAAGIDQVYSAGDAEEARKIL

KANAIDIMICDIEMPGETGLDLQSWVLKHDPGIMTIFLTGHALFDYAQTAIKLNSFDY

VLKPAPADQLIKVVTQAVEKIKDGEQRTRTNEVYETVYKRWKTHKPLLTERFWKDAIS

QRITLTEQRLQELAELYGAEIDPHARVLPIVISVEEWVRDFDLRDEEVLEYALRNAAK

EMLLSDKPGEVFQDQSGLNIVLAYEQDGIIPSASEVAQRCQGYIQECGTYFYCRLSCY

VGAPVAVTELQGMLNELMDMERRNIKELEEVFVYDERGRDQEDRFLPIPWFSELSILF

ETGKVGDLRERVDEIFELLAAQEGLSPEMLHLYYHAMLHVVYPLLHQKNVSIRSLYPG

EREPEESMVTRSLPQLKQWTADLIDRVIPVLYPDDHSPMTIVDQLCVYIETHIGEELM

REELAAFAGFNPAYLSRLFRKEKGMSLSEFILQGRVAKAKTLLSQSTVKVTDIAGKVG

YYNYSHFTKMFKKCTGITPQEFRKQSRTV"

CDS complement(3612139..3613938)

/locus_tag="EFAGFIKM_03081"

/inference="ab initio prediction:Prodigal:002006"

/codon_start=1

/transl_table=11

/product="hypothetical protein"

/translation="MSFIRSLRFKFIVGLTLIMLPLFMLLYYNNVYAMKVVRDKVSLT

NMNHLAKSVEQNERVLQETNRYLYSLGERDPDIISLFFLKYGSGDYIIAKQRIMNKFM

TDIGFYNLIDSFFLYDAVNDDLLLATSGNYDVKKSVVQESMPVQMQQLEGDIQKPEWS

IVRGGTWNALVKTVRINQQFYAGALVDMNALNHTQQFTESGDRGGAVIVGTDGGALSD

STLNTAQIQLAAAHLPGLKNPYQVISEVVNKGEARQYLMLGTASAMSQMNYIVLLPEE

DMMRNLPFFQQIIRLLPIGAAVILVTALIFLRHLLFRPMNALIRGMRRVSRGDLDVKL

DPPSSSELEFMTRSFNQMTSEIRHLKIDVYEEQLRTREAEFKQLQMQINPHFYLNSLN

IIHSLASLQKHELVQQMAGHLADYFRFSLRAGKRVIRLDEELEHIRHYLEIQKLRFPS

RFDFELDIEPNLGHYVIPPLTIQPFVENAIIHGFQRRTQAFVIRIVARLGQKSSGQVD

GTVLQLMIVDNGVGFEEELLGRLQHGQYAEDPSGQHIGIWNVAHRLLVKYGDQASLQF

GNEVDHGAKVIIDLPAQTEEEEGGAYAEPVDRG"

CDS complement(3614148..3614285)

/locus_tag="EFAGFIKM_03082"

/inference="ab initio prediction:Prodigal:002006"

/codon_start=1

/transl_table=11

/product="hypothetical protein"

/translation="MHVKIGRSWSPGLAAAFIFREKLLSNNEGFFLQLENLLLHEACY

S"

CDS 3614337..3615779

/locus_tag="EFAGFIKM_03083"

/inference="ab initio prediction:Prodigal:002006"

/codon_start=1

/transl_table=11

/product="hypothetical protein"

/translation="MNIQLTALHYVYLAFIVLIIAVMIRRRDTTIICVAGIMTIGLLA

TETLSGSVAGIFSSFIYATKELLSTIFIISIIVAMSRVLIRTGINEVMVTPVTRFIRS

PQVAYWGIGLIMMVVSLFFWPSPAVALVGAVLLPVAVRVGLPPIGAAIAMNLFGHGIA

LSGDYIIQGAPKLTADAAGLPVTSVMTASIPLVIVMGTVSTLTAFWMLRKEMKSGSPV

HSPNAISGTVSTEPSSSSEAVSTSLPTDQPVERQAMSLRLQKAFALLIPLLFAVDVIL

MFVLKLQGGDATALIGGTAVVILIAVTLAAHRHAGPEETTNYLIEGFQFGFKVFGPVI

PIAAFFYLGDAAITELFINPLPEGSHGIVNDLGVALAGLVPLNDTVGAITMTVTGAVT

GMDGSGFSGISLVGSIASMFAGSADSAPVLTALGQVAAIWVGGGTLIPWALIPAAAIC

GVNPFELARRNLKPVLLGLTVTTIVAIFLI"

CDS complement(3615799..3616392)

/locus_tag="EFAGFIKM_03084"

/inference="ab initio prediction:Prodigal:002006"

/codon_start=1

/transl_table=11

/product="hypothetical protein"

/translation="MVQGGEMSTEVMAHLASGNTRQQDAYHVLQSSGLLDILADYHPY

PAGTVPIDIDIPGSDLDLLCYAEDLDVFEAEIHNRMGAVAEFQCVRGGDLPDQRPYVT

CNLQVGHWPVEIFAQSVPVYRQNAYLHMIVEWRLLQLWGDAGHREIRRLKQVGWKTEP

AFASVLGLQGDPYVDMLHLAEMKREDLWNWARSRTSF"

CDS complement(3616395..3616601)

/locus_tag="EFAGFIKM_03085"

/inference="ab initio prediction:Prodigal:002006"

/codon_start=1

/transl_table=11

/product="hypothetical protein"

/translation="MNGQQRVNIKPGLEVDIVLKQDQATGKLTRGIVKDLLTKSPTHP

HGIKVRLTSGQVGRVKQVITGGSN"

CDS complement(3616816..3617604)

/locus_tag="EFAGFIKM_03086"

/EC_number="3.5.1.104"

/inference="ab initio prediction:Prodigal:002006"

/inference="similar to AA sequence:UniProtKB:Q81AF4"

/codon_start=1

/transl_table=11

/product="Peptidoglycan-N-acetylglucosamine deacetylase"

/db_xref="COG:COG0726"

/translation="MNLKAARFIGMFTLIILLGSSSAYAKPVQKNRQYYEERGEIVWE

VPTQDKLIALTFDDGPDPIQTPQILALLQQYHAKGTFFVLGKWADKFPKLIKQEQLEG

HEIANHTYAHTYAVRSTRADKYMKDMNEAESSIIKAGAERPLLFRPPGGYYNDMVIQA

AKQKGYTIVLWSWHQDTRDWASPGVSAIVNKVLKNARNGDIVLFHDKVEGKSHTVAAL

KTILPKLQQQGYQFVTVSELLAVKAREAAKDGTSPSSSLKNVKP"

CDS complement(3617546..3617671)

/locus_tag="EFAGFIKM_03087"

/inference="ab initio prediction:Prodigal:002006"

/codon_start=1

/transl_table=11

/product="hypothetical protein"

/translation="MSSGESGNANREFTYEDKVRTPHEPEGSPIHRNVHANYFAG"

CDS complement(3617671..3618609)

/gene="iolS_2"

/locus_tag="EFAGFIKM_03088"

/EC_number="1.1.1.-"

/inference="ab initio prediction:Prodigal:002006"

/inference="similar to AA sequence:UniProtKB:P46336"

/codon_start=1

/transl_table=11

/product="Aldo-keto reductase IolS"

/db_xref="COG:COG0667"

/translation="MAEQQIRLGKTDLIVNPIGLGANAVGGHNIYPDMLNDETGKEVV

RTALKQGIDFVDTAYIYGPEHSERLIGEVLKETGQRQNTIIATKAAHKLVDGKIVIDN

SPAFLKTAVDEALKRLQTDYIDLFYIHFPDEHTPKDEAVDTLKQLKDAGKIRAIGVSN

FSIDQLREANKDGYVDVLQSEYNLFKRDAEKELLPYTAEHNISFVPYFPLAAGLLGGK

YNRDTTFQDGRAKNPLFTGEAFIENLDKVEQLRSIAQSKDVEVAHLVLAWYLTQPSID

ALIPGAKRPEQVINNLQTLNVELTAEEIAVVDQIFR"

CDS complement(3618719..3620743)

/gene="nhaK"

/locus_tag="EFAGFIKM_03089"

/inference="ab initio prediction:Prodigal:002006"

/inference="similar to AA sequence:UniProtKB:O32212"

/codon_start=1

/transl_table=11

/product="Sodium, potassium, lithium and rubidium/H(+)

antiporter"

/db_xref="COG:COG0025"

/translation="MEIFIAVLVLLVLIGLSNILNRFVPFIPIPLIQIVLGVAIALLP

AGVHLPLNPELFFVLFIAPLLYNDGKRTPRNELWNLRAPILLLALGLVFVTVVVAGYA

IHWLIPSIPLPAAFALAAILSPTDAVAVGAMAGRVHLPKGIHRLLEGEALMNDASGLV

AFKFAIAATVTGVFSLANATFSFILIAIGGLLAGALLSFLLIRLGVWIRRLGMEDVTI

HMLLQILTPFIIYLVSEEIGVSGILAVVAGGIIHAIERDRAESVQLKMQVVSASTWSV

ILFILNGLVFVILGVQVPDVLSTIFENVSFNNLQVLGYVGLISVLLLILRFLWIYLFW

QGNELLRTKSSIGRPRFKDITIISLSGVRGAVTLAGAFSIPYVLQDGSPFPERDLIIF

LAAGVILFSLIAASVFLPVLAKDDGKSTEETPQKSERKAHDIMLNAAIRAVKSEMNDE

NKAAALAVVSDLSKYIRQAAGQLTADKRKDIQKQETAINLIATRAERKEVEVMLDENA

IASDAAFKCDSWLDRKEMMLANRTNTQFMTSISEIGRVLGHLFTNRSQKPNQPFMLEN

AELFRQVKLRTSEAAIKAIRAHMNDGNRVVALSVIAKYERVIAKLRTWNQGKTEDPFN

QEKLELQMVAIQEQRNTVQQLYENGEITRDVAAKLRRFINDVEATALKNT"

CDS 3620945..3621244

/locus_tag="EFAGFIKM_03090"

/inference="ab initio prediction:Prodigal:002006"

/codon_start=1

/transl_table=11

/product="hypothetical protein"

/translation="MAFGIKRHELAAWKEQVARGEIAYLTHFWIDNRFPGITSVTKVG

CADLERLERWCNDHGLDPRYIHRRQPFPHFDLMGSKQKEILIQEGMTDHVVRFHL"

CDS complement(3621341..3621823)

/locus_tag="EFAGFIKM_03091"

/inference="ab initio prediction:Prodigal:002006"

/codon_start=1

/transl_table=11

/product="hypothetical protein"

/translation="MSPDTERNSQLANVDQLLEAFFRYKNKVLDQQQKTETNCKLNPT

KSHILGMILREKRCMAVDVARQLSLSSGATTIVLNQLETEGLIQRVRSEEDRRIVWLS

LTDEGAQLAKTLNANRGRMTWELLQALSEEEQQQMFGMLKKIELKLLENMKSFEQIHR

"

CDS 3622094..3623209

/gene="oxyE_2"

/locus_tag="EFAGFIKM_03092"

/EC_number="1.14.13.232"

/inference="ab initio prediction:Prodigal:002006"

/inference="similar to AA sequence:UniProtKB:Q3S8R0"

/codon_start=1

/transl_table=11

/product="6-methylpretetramide 4-monooxygenase"

/translation="MNQNTQSQLKTDVCIVGAGPGGALLSYLLNQKGISTMLIERQPH

LHKSFRGELLNVDGEAILNKHGLYSLIQERGALLLEQIQYWENGQIIHTVSPGNGESH

VGIHVPQDHLLEAIVSHAQQHSSYEQVLFNTIMTGLLRDKNGQVVGINIRQNGVPASI

EASVIVGADGRYSAVRKHSGMTPKVRKHGYDLLWARIPAPVGWEPAVRMASMDGQQLA

LFSQFGGYVQIGWNIPEGAYSKLREQPIAPFVDKLVSAFPCLSDSVAANIQSWSDFVL

LSVESSYCQLWAQENVVLIGDAAHTMTPTGAFGLNAALEDADVLSELLVQMAVDQFSS

TAQLQELQTIRGAKVQQQLARQVEMESSFQQRYESFL"

CDS complement(3623327..3623554)

/locus_tag="EFAGFIKM_03093"

/inference="ab initio prediction:Prodigal:002006"

/codon_start=1

/transl_table=11

/product="hypothetical protein"

/translation="MSFVFYPEMVIFILMLILLIIGIVYIVRKSKQVLRRLSNLEKAV

YEKDNISSYPKDVDTVQEALREDTFAPRKRK"

CDS complement(3623768..3626344)

/gene="lacZ_4"

/locus_tag="EFAGFIKM_03094"

/EC_number="3.2.1.23"

/inference="ab initio prediction:Prodigal:002006"

/inference="protein motif:HAMAP:MF_01687"

/codon_start=1

/transl_table=11

/product="Beta-galactosidase"

/translation="MKTYNSSQDISGQWKIQHFEVGEKRAMDVAAAALDDRFWIGAEV

PGDVHASLVERSIIDPPYYGHNDAKSRWIEQKEWWYRTTFNLVRDAETEEHFELVFEG

LDTFATVYVNGHEVGKTANMLMSHTFNVTSLVRNGWNAIAVKFDPLHLHHRDKETFDW

SSYTKERPWLRKAAMNFGWDWGPRMVTVGIWGTVRLEKRTIAKLENVYARTESVSSEL

AVLHVTADVKSVLSYRSRQKREQAVVATCNVRLLDRSGHEVAGVTELPVEGGKADTIL

NVTSPQLWWTHDLGEPYLYTLEVTLLADGVEVDRYSEPYGLRTIELALHNERGEDAFT

FILNGVKVYAKGANWIPADHLIGAIPNSRYRELVELLVEGHMNMLRVWAGGIYEKDVF

YDECDRQGVLVWQDFAFANALFPDFNRDFMNNVRQEVENNVLRLRNRASLALWCGNNE

IDWLYDMKSASGDITSPFYGELIYHELIPEVLERLDLSRPYWPSSPFGDSNGQDANDP

DVGDRHNWQVWHGSVYPRKHGEPPLLDYSIEGVTFKNYKKDHALFSSEFGMHASANRY

TLEKNMPAGQFYWGSPEMAYRNKDTNHQKGILLMEGYTGIPQNIEEYMNYSMLTQADG

LRYGIEHFRRINHRNSGALVWQLNDSWPGTSWSMIDYELLPKASFYYGKIFFHPVLLS

LEHEPGEPLALWVVNDTQDVLKGELRLNVYALNGEKIYSSSHVVEVTSQSSRCIAELT

EVEVLQGRRAEEVMVELVSEGFAAPSNRYFLRDPKDVTLPESQLSVHVNEEEQSVTVT
ASGAIARLVKLELPLGRVRFSDNYFDLLPGESRTVRLRHPEQSSLPLTELRVSAMNGR
EG"

CDS complement(3626435..3628021)

/gene="rhaR_30"

/locus_tag="EFAGFIKM_03095"

/inference="ab initio prediction:Prodigal:002006"

/inference="protein motif:HAMAP:MF_01533"

/codon_start=1

/transl_table=11

/product="HTH-type transcriptional activator RhaR"

/translation="MRLLIVDDEVIIRTGLASVIAWHELGIELLQPAASAEEALTRMA
EERPHILMTDIRMTGRSGLELAEEGLRLLPELEVIILSGYDDFSYAQQAIRQGVTDYL
LKTSKPEEIIKTVLQAKQRITERWAEKSREGQLLRENRERLFAGWIIDGDTASGHFPS
FLRSDAGKEAGVGDLNDEQILRQVIILRAEGWDRSSDALLRFAVQNTLEDMLPGAIAH
VEKQQIICVVSWPPVEHETTFRLESALRRVEQLLKCTLRAAVGLAMRGYADLHESYST
ASAAFRYRGLMDYKVWHYEHIQDRQGGKTVLTHEEETRLGTILLDNDSVTLTTWTREL
VANLITEPEVTPDSFSACLHSAIIAGHRWLTRTMRATGREEPARLEPWLPEPDAEAGD
LGDMLFHHLYGLMHVYHSRMGQGQAAHVQKAIGYIESSLAHDINLQLVAGHVHLHPGH
LSELFKKEMGVTFGDFVTDMRIRRAMDMLAVSPAKVSEVAAISGYEDVKYFSRLFKKH
TGKTPSEYREEALSFKPSGS"

CDS complement(3628018..3629799)

/locus_tag="EFAGFIKM_03096"

/inference="ab initio prediction:Prodigal:002006"

/codon_start=1

/transl_table=11

/product="hypothetical protein"

/translation="MARWLTSSLQRKLSVVVTASMIVPLLALGLFAFLISSRITEQKT
KLSGMDTLKQVEANLRYMLQDAENLSIFLIGERDIQQYLSHNEDHELDRVDILGRMTN
LAASKKYIANIAIYPGRFDATLSTATWYESNESNLSYPSVPSGEAVKQWTGVYPVQNY
AGIQNVITLVRPIRSIHDYRPIGWLAISLDEKAISKGWAALGLGRGQGRLELIGSSGE
ILSSMDKSRLGLKLEGVEPGISTLIQDGGSGTTTYGSGDDKRTLLYYPEELTGWTLVG

TVPYDQYKSENRYILILTGCAVAMSAAISAGLVWFTVRRVTRPLRVLTRHLSRIDPER
PLPLFRSESDDEIGRLGESYNLLGAHIQLLKEEVIRGEARKKEADLRVLQAQINPHFL
YNTLSSIHWIALMSEEKRIADMVEGLSDFLRISLNNGQDYCPVEQEIAHIRHYVRVQS
IRFPDQFVLHYIVDPALEKRMMLKLLLQPLVENAMIHGIQPKAGVGTITIMIRKDPDN
EQMNVLVLDDGIGMEPDRLEQLRISIREWKGKQPEKQLNQGGYGLCNVNERLLLHYGA
DAQLEVDSRVGGGTRISFSIPILEGSP"

CDS complement(3629830..3630705)

/gene="ngcG_4"

/locus_tag="EFAGFIKM_03097"

/inference="ab initio prediction:Prodigal:002006"

/inference="similar to AA sequence:UniProtKB:O50501"

/codon_start=1

/transl_table=11

/product="Diacetylchitobiose uptake system permease
protein NgcG"

/db_xref="COG:COG0395"

/translation="MRVTPVSMPSPQSSSGHPIRRLRSGIVWTLLTVYGILTLYPFYW
LVISAFKTNEDFYSRPFGLPENWNVANFSNAWESSRLGTAFGNSLIVSVGSLILTLFI
AALASFILARFQFRWKGLIMTFFVIGMLIPIHSTLVPLFILMKQMSLLNTYWALILPY
TAFALPTAIFVLTAYLTSVPRDIEEAAFIDGTGLWGLFTRIMLPMSVPALSTVTILSF
LHAWNDFSFALVFINKTGLKTLPLAIANFADGYQTDYGLTLAAMTLSVIPTIILYLIF
QEQVMKGMTAGAVKG"

CDS complement(3630724..3631602)

/gene="ngcF_3"

/locus_tag="EFAGFIKM_03098"

/inference="ab initio prediction:Prodigal:002006"

/inference="similar to AA sequence:UniProtKB:O50500"

/codon_start=1

/transl_table=11

/product="Diacetylchitobiose uptake system permease
protein NgcF"

/db_xref="COG:COG1175"

/translation="MNALRSRRFIMLGLAPAVIIYALFVFVPVVWSAYYGFFNWSGIG
ASKYIGVDNYVEIWHDPVFWRALKNNVIFVLASVFGQIPLALMLAVILHKSNPLQRFL
RSAVFLPMVLSTVVIGMIWQYIYHPQIGILNFLLDALGLESWKLQWLSDDKIAIFSLV
PPLLWSFVGLYLIIFISALQNIPGEIHDAAKIDGASGIRKLVSVSLPIIWGTVQVAII
LCISGSLKSFDLVYIMTKGGPAHATELLATYMYNSTFTTYRYGFGSAISTTIVLISLL
LIGTSQWLTSRKRKEN"

CDS complement(3631704..3633017)

/locus_tag="EFAGFIKM_03099"

/inference="ab initio prediction:Prodigal:002006"

/codon_start=1

/transl_table=11

/product="hypothetical protein"

/translation="MLKKWLSGFLAITLFSIILAGCAGGDTSEGGSGNDKVTVTLWHN
WTGQDAKAVAMRKIIEDFRAAHPDIEVVDEGLPTDGLKTRLRTVAAANEMPDLFVMWP
DAMTKEFVKGDLLQPINAELDAKPEWKDNFIPNALDGYTLDGNIYSVPMNLAPSSFIY
YNEALFKQYNVKVPETWAELEQAIATFNQNKVIPMALGNKANWVAQSTIFSTLADRIT
GTDWFLKAAAQEGASFTDPQFIEALNKMQELGNTKAFQDGFNSIDETQMMQLYFQGKA
AMVMNGGWALANLVNNAPEEVLNNTHITILPPVDGGQGEPRTTSGVVGTGLGVSKKLS
GAQKEAAMELFYALAGPDGQKATLDSSTLVSYKIDLDKTKAHPLFVELYDLMQEVKIT
PVYDSKLGSATVEVINNALQELLMGGKAEDIAAKIQAAQANAVSQ"

CDS complement(3633275..3635359)

/locus_tag="EFAGFIKM_03100"

/inference="ab initio prediction:Prodigal:002006"

/codon_start=1

/transl_table=11

/product="hypothetical protein"

/translation="MSQSPSNKRHMNGLDGLRAIAVLAVIAYHLNLDFIPGGLLGVGI
FFVLSGYLITDILVSQWQEHGRISLGDFWVRRVRRLLPGMLTMTAVVMIWLACTDPSR
LTALRGDIVSGVLYISNWWYIFHNVSYFESFGPPSPFGHFWSLAVEEQFYLIWPLLLI
AAIVVFKRKGWLVVFIVVAAELSAGAMAIMYNPDLDPSRVYYGTDTRAFALLAGAALA
VVWPSRKLSNSLSGMNRLVLDVSGLAALALLIYMMLNSSEYDPFLYQGGMVLQAIATT
LLVAVLAHPSSFLASIIGAKPLRWIGERSYGLYLWHYPVIILTNPAVDTGGAHPVRII

LQVAATVVLASLSLKYIENPIRYNGFRDSWSRLWGRGRSSIGIRNVWWKRAGLLMTIL

LLSYTVSQMMVSHAANSDSHSVSMSNTLNGENSVAEEQGQDVLPAITGTSGSGQKNDA

HTHKPAAGTKDDPGKNEKPTGEPAPDSKPDDQDNSVNPGKDGQSEDKPADDSVTNEND

TPDDAGANQSDDTDDAPDSSQDNEETAPPAQDGKVHYTVIGDSVILDAKPYLEQSISG

VYVDGHVGRQMWQAGDVLEGLKQNNQMGSKVVLELGTNGSFNSKNLNAVLDYLKDEDR

VYLVTVRVPRPWERTVNRALNEAASKYSNVSLIDWNSASEGHDEYFEKDGVHLTTQGS

EAFAELVKNSIQ"

CDS complement(3635935..3636495)

/gene="flr"

/locus_tag="EFAGFIKM_03101"

/inference="ab initio prediction:Prodigal:002006"

/inference="similar to AA sequence:UniProtKB:Q9KIT1"

/codon_start=1

/transl_table=11

/product="Flavoredoxin"

/translation="MRQPIEEPIFYSYPGMVAVVTSRHEGIQNVMASGWHTYIGSSPG

VYGISLRKETYSYELIEKSGVFGVHFLPGHRSEWIQAAGTFSGRDTDKFTRFGIPYEE

GIKVSVPILTDAYFAYECKVMEITTYGDHEWIAGEVLQRYQDQKCFLENGMVDLEKLQ

IPMYTGRSAYRIMDARTEEKIHPFHL"

CDS 3636727..3637374

/gene="cat"

/locus_tag="EFAGFIKM_03102"

/EC_number="2.3.1.28"

/inference="ab initio prediction:Prodigal:002006"

/inference="similar to AA sequence:UniProtKB:P06135"

/codon_start=1

/transl_table=11

/product="Chloramphenicol acetyltransferase"

/translation="MFNSIVLDQWDRKPYFDHYLNQVRCTYSITANLDITLLINELKL

KGLKFYPALIHMISTVVNAHREFRTCFDAEGKLGYWDEMSPSYTIFHEDDKSFSTIWT

AFHEDFETFHIRYLEDMNNYKDHKQFVAKPNEPPNIFPVSSIPWVNFTGFNLNIYNEG

TYLLPIFTMGKYVQQNDKVYLPLSVHLHHAVCDGYHAGVVFNELQSLADRCQDWC"

CDS complement(3637457..3640096)

/locus_tag="EFAGFIKM_03103"

/inference="ab initio prediction:Prodigal:002006"

/codon_start=1

/transl_table=11

/product="hypothetical protein"

/translation="MKSNQRSYQLEGLSEKIFLDRYAWKDADSNHAKVGDVVLVLTKD

DPKFPTKEVGEIIERKGQLVQVRTRSGEVIETNVEKLTLNIEKTPEEMWNRLAKAMAS

VESTADKRATWESKFRSVLEDWKLVPGGRIAAGAGASDELTLFNCYVIPSPQDSRGGI

MRTLTEMTEIMARGGGVGINLSSLRPRRAVVKGVNGSSSGSVSWGGLFSYTTGLIEQG

GSRRGALMLMMNDWHPDVLDFITVKQTMGQVTNANLSVCVSNAFMEAVKQDGDWDLVF

PDTNDPDYDTEWNGDMQQWKAAGHSVVHYRTLKAREIWHTIIESAWKSAEPGVVFMEY

YNQMSNSWYFNPIICTNPCGEQGLPGWGVCNLSAINLSKFYDETNHDVAWDELAETTR

ISARFLDNVIDATPYHFEENRLNQQRERRVGLGTMGLAELMIKLRIRYGSPESLAFLD

KLYGFMAKEAYLASAEIAAEKGAFPAFEAEPYLQSGFMKNMVATYPKVGEAIRKRGIR

NVTLITQAPTGSTGTMVGTSTGIEPYFAFKYFRQSRLGYDEQFVPIAQDWLDEHPGEA

LPDYYVTAMDLSAENHIRVQAAIQQWVDSSISKTANCPADFTVEDTAELYELAFDLGC

KGVTIYRDGSRDVQVLSTSKKEATQAEDQNTAEAEEKTVVSAIENPNNAEVQASDGTS

NSQVGVRTHSSDQTPSSSTVLDKQYRSRPQVLRGATYKINTPFGMAYITINDLEGTPG

EIFLNVGKAGSDVFAMAEALGRVCSLFLRYGDHGHKVELLIKHLKGIGGSGAIGFGAN

RVESIADAVAKALESHVQSNVQENETGALQEQNQVESKYPEVEDQKTFTESRDLCPSC

GSASLMNVEGCKTCSNCGYSKCN"

CDS 3640558..3642321

/gene="rapA_1"

/locus_tag="EFAGFIKM_03104"

/EC_number="3.6.4.-"

/inference="ab initio prediction:Prodigal:002006"

/inference="protein motif:HAMAP:MF_01821"

/codon_start=1

/transl_table=11

/product="RNA polymerase-associated protein RapA"

/translation="MNRKNPHHTNQPVAELPVPLEFDRTWINQLESRLDKGGPWGDYR

LFQLGIQAEETNLIPNFDEIQCLKHLQGLTPLPHQMDTARRVLFEMSGRAILADEVGL
GKTIEAGLILKEYMVRGLVSKVLILVPASLVLQWVRELNSKFGIPAIAQKKAYSWQNE
VVVASMDTAKRDPHKDMLLNTDYDMIIIDEAHKLKNKKTTNYQFMLKLRKKYCLLLTA
TPVQNDMSELFNLINLLKPGQLGRQGDFAANFVVDKRVPKNQEQLKNELSKVMIRNRR
GEGPVQFTKRNVSNVNLQLSPEEQALYDGVTSFVKDQYQEAGGNLSSMLSLVTLQREV
CSSRDAVFVTLVNLSKKLPLDSPLRDKIWELVAHIKAIKENTKAEKTMELVRNMNEKV
IIFTEYRATQEYLLNYFRNNGLTAVPYRGGMNRGKKDWMMDLFRGRVQAMIATEAGGE
GINLQFCHHMINFDLPWNPMRVEQRIGRVHRLGQQNDVNIFNLSTTGTIEEHILSLLH
EKINMFEMVIGGLDVILERLEKKESIEKSLYKIILESQTDEDIRRKMDSLGQSLNSIQ
REVSDEGPAVSGLLDLRKGGS"

CDS 3642325..3643524

/locus_tag="EFAGFIKM_03105"

/inference="ab initio prediction:Prodigal:002006"

/codon_start=1

/transl_table=11

/product="hypothetical protein"

/translation="MTMSPHEVQAYVLTYLEALDCQIMERSPAHVTVKLSPEADKALT
NRPYYWGFVERTGAPAETMSFTFIFDPDSYQQAIEAAEAIAAQASPPAVPNPDGTNGV
TNGSPQGEPPKETILGRYFGITPSLPQLGPGRILREEVVYGSRRLQQIFGAAREGGAF
VNLFEQAAKRQLRATAPAVYEPWLGVCFKVEFACDLKREELHFLGISLRSGEIIEKFG
TKLNRRDLSPRLAENMHVQTAKVSLFDAGAALESHLTNRLLELDYSWAEKAQERLDLE
LDVLDTYYEAVLREDMPEVESPGNKTSDTENENVHSDRTPPAPLKKVVDITNAKPIGA
DVELAAEAAVPIDSETDAEEEKAKQAVIDREAMKLQYETRRTEMIWQYEPKVKVTAIS
SGMFHLR"

CDS 3643678..3644685

/locus_tag="EFAGFIKM_03106"

/inference="ab initio prediction:Prodigal:002006"

/codon_start=1

/transl_table=11

/product="hypothetical protein"

/translation="MKRFFHTCWNNAQKTTCITLGLLSCLALFSFWSIANAEEQKTSS
LSPASSTEYGPEHHAIQNEKLAPAAVQRFAEQQAVTLGTANGEVHWKNADLDFYPLGP

GLHSWLVQAEFEGKPIGYMIIRATEQGQLMLSEYGHGEQSPYNNELLGQALDRQHVNL

EALLAGGGELHLGYALPLLAYWKVEQPNHSALYIDATNGDVLPSEFLDLLEADSKQNS

VSITDLSTTPSLSVDPLHLRPVFNPADNLLWLTSKPVAADRAQDVIQHGENQEIVFSA

GDRNVVYGGPLPVSGYQLWHTDQVAESILYVSLGGKSSIQRFVPLSTLQQGGLFYIVY

P"

CDS complement(3644841..3645059)

/locus_tag="EFAGFIKM_03107"

/inference="ab initio prediction:Prodigal:002006"

/codon_start=1

/transl_table=11

/product="hypothetical protein"

/translation="MAKSDELVKYITQRVVHYIDTPKDERKGRTKRKEPWAMKWFGMI

PFAVSLWVGKKEKEFDTKSHKRSSSGKG"

CDS 3645254..3646000

/locus_tag="EFAGFIKM_03108"

/inference="ab initio prediction:Prodigal:002006"

/codon_start=1

/transl_table=11

/product="hypothetical protein"

/translation="MYKLLTASVALLISFHCLPILPHHVAHGEAAPSQNSPSMLPVTD

GDAYRSSHHAFAAPVILLDVGHGGIDGGTSAQGVLEKDINLAISQKVYLLLRSKGYAV

IINRLGDYALSDENRWLNSRSRHTRDLAQRKSLSEEVSTDIVVSIHANWSPRSTAHGP

VVLHQKEGRSYLLAQSIQDAMNKLYGTEHGVVWGKPFYILNYVKQPAVIVETGFLSNA

ADRARMNDPAEQKRIAQSIANGIIYYLSVV"

CDS complement(3645975..3646814)

/locus_tag="EFAGFIKM_03109"

/inference="ab initio prediction:Prodigal:002006"

/inference="similar to AA sequence:UniProtKB:P44863"

/codon_start=1

/transl_table=11

/product="hypothetical protein"

/db_xref="COG:COG2861"

/translation="MMKVQRKIYRFIAGIMALLIVFYAGISAGGPTAAASESTSNQKR
LAIIIDDVGNDMKGTAEILAMPVKLTVAVMPFLPTTKKDAIAAHEKGMDVIVHMPMEP
KKGRPEWLGPGAITSNLTDEEVRSRVEKAIDDVPHAIGMNNHMGSKITSDKRIMSIVL
DVCKERGLFFVDSRTNFRSVVGELAVSKNMPPVGNDIFLDDQNSKQHIRKQLDLAAQR
AIDKDICVVIGHVGHSGMNTSAVIRESVSRLQNQVQFVGIGDLVRDVWQWQSAPTLPT
DNK"

CDS 3647233..3648918

/locus_tag="EFAGFIKM_03110"

/inference="ab initio prediction:Prodigal:002006"

/codon_start=1

/transl_table=11

/product="hypothetical protein"

/translation="MKFKLKKTINVLLACLTALPLMLTPTNVSAANDANINLSSEKQL
IKGFGGINLPAWIGDLTPAQRETAFGNDQNQLGFSILRIYVDPDSNNWYREVATAKRA
IEKGAIVFASPWNPPSSMVETFNRNGDTNAKRLKYDKYTAYAQHLNDFVTYMKNNGVD
LYAISVQNEPDYAHDWTWWTPQEILRFMKENAGSIQGTKVMAPESFQYLKNISDPILN
DPQALANMDILGAHTYGTQISNFAYPLFKQKGAGKELWMTEVYVPNSDNNSADRWPEA
LDVSYHMHNAMVEGDFQAYVWWYIRRQYGPMKEDGTISKRGYNMAHFSKFVRPGYLRV
DATKNPDTNTFVSAYKGDNKVVVVAINRGTSATSQKFVLQNGNASTVSSWITDSSRNL
ASGTSLNVSNGAFTAQLPPQSVTTFVANVTGGNSGGNTGSGTTYEAETGTSLTEAAIE
TIYPGYTGSGYVNFNAMTGSAIQWNGINNTTTGTKNVKFRYALESGTRNLDIFVNGTK
VISNEPFPATGSWSTWVEKTIQVPMNTGTNTLKVVTTGSEGPNIDSVNVTAAQ"

tRNA complement(3649053..3649139)

/locus_tag="EFAGFIKM_03111"

/product="tRNA-Leu"

/inference="COORDINATES:profile:Aragorn:001002"

/note="tRNA-Leu(cag)"

CDS complement(3649315..3650268)

/locus_tag="EFAGFIKM_03112"

/inference="ab initio prediction:Prodigal:002006"

/codon_start=1

/transl_table=11

/product="hypothetical protein"

/translation="MKAKKIITFVLACTMVFSAVGMAVLPVGTVSAADASGTTASISD

ILKNQLPDGGWRKDYKQTSGEWAKSTIDNKATYSEIRRLAKEFKKTNDPRYSTAAIKG

INFLINMQYSNGGWPQVYQSSGYHKHITYNDDAMINVMIMLDEVAARKGDFSFIDSTL

ANRSKNSVAKGVEAILNTQVVSGGKLTAWGQQHDSSSLKPASARIYEVPSLTAGESVT

IVKFLKTRPANAKITASIKGAEAWFNKVKITGYKYERANGDSKIIADSKATPIWARFY

EIGTDKPIFIGRDGVVKYNLSDIDKERRGGYAWYGNWPSKL"

CDS 3650832..3651296

/locus_tag="EFAGFIKM_03113"

/inference="ab initio prediction:Prodigal:002006"

/codon_start=1

/transl_table=11

/product="hypothetical protein"

/translation="MKKLIISIFGAAVLFTSGATSTEASSINSVVNNMTGIPYKWGGT

TVAGFDCSGFMRYLFNKYSIELPRTSQQQAKAGTPVSKSNVRTGDLVFFNTTGKGISH

TGVYIGGGQFAHASSSKGVSITKLSNPYFNDRYVTARRVTGQFMYDKMLGKM"

CDS complement(3651370..3651699)

/locus_tag="EFAGFIKM_03114"

/inference="ab initio prediction:Prodigal:002006"

/codon_start=1

/transl_table=11

/product="hypothetical protein"

/translation="MTLMNDKVHAYKDQIGALSDVLPGVVKSYHEFTGECFQPGVIDA

KTKQLIALGIGLFANNEVCTFYHVEEARAKGATDQEIMETVAVAGAVGGGHALSQGAM

RVQKALH"

CDS complement(3651841..3652059)

/locus_tag="EFAGFIKM_03115"

/inference="ab initio prediction:Prodigal:002006"

/codon_start=1

/transl_table=11

/product="hypothetical protein"

/translation="MISDTELDALRLSGEKVRVVRDELESNDVKGIVVAWDGEQVLIR

RQNRRVVKLDRGYSYQLFSEPRQSPLSE"

CDS complement(3652179..3652508)

/locus_tag="EFAGFIKM_03116"

/inference="ab initio prediction:Prodigal:002006"

/codon_start=1

/transl_table=11

/product="hypothetical protein"

/translation="MGNQPATEQFFNDKLAESKVHFERALDCKHTEFDDLYPYMIEHP

QFFWYKRYVAWSELLTIVGLCEELSFSWKEQFTPHQVEYLEERVMSAKVLDFWFEKND

SKEHAQR"

CDS complement(3652606..3653187)

/gene="xpt_1"

/locus_tag="EFAGFIKM_03117"

/EC_number="2.4.2.22"

/inference="ab initio prediction:Prodigal:002006"

/inference="similar to AA sequence:UniProtKB:P42085"

/codon_start=1

/transl_table=11

/product="Xanthine phosphoribosyltransferase"

/db_xref="COG:COG0503"

/translation="MELLKQRILQEGVVISDQVLKLDGLLNHQIDPALTMEMGREFAA

RFRESGVTRVITVESSGIPVAFAAAYELGVPLVFARRKKTLLADPDAYCERVPSFTKG

IVTDIMVSREFIHENDRILFIDDIIANGDAARGVIKIIERSGAELVGFGVVVEKSFQA

GARTIREQGIPVEALVRIRSLNDGTVQFDDNEM"

CDS 3653392..3654447

/locus_tag="EFAGFIKM_03118"

/inference="ab initio prediction:Prodigal:002006"

/codon_start=1

/transl_table=11

/product="hypothetical protein"

/translation="MKQAFRVLQIAFTYIGTVVGAGFATGQEILQFFTQYGKWATITI

GLSTMLFVWLGTKMMLIAHDTGSRSYEDLNKHLFGHKAGKWITWVTLLILIGVNSVML

AGAGSVFVEHLGLHYQTGLIVTLVGTYLLLGRGIQAILQMNSIVVPMMLLLSLLMITS

TIHHPGASRFITLTTDSNPIQVWLSPLLYTSFNLVLAQAVLVPVGNQIRSRNVLKWGG

VLGGIGVGFMLMAAHFAMSAQMPGIIQFEIPMGSIAFQLGWAVQSVYVSLIFMEIFST

FVADIYGMTLQLRQHIRVHPRFITLTIMLLCYSLSQFGFSSLLSILYPIFGSMALFWA

AKLVLNRWGAFRRRNNI"

CDS complement(3654583..3655713)

/gene="mshA_2"

/locus_tag="EFAGFIKM_03119"

/EC_number="2.4.1.250"

/inference="ab initio prediction:Prodigal:002006"

/inference="protein motif:HAMAP:MF_01695"

/codon_start=1

/transl_table=11

/product="D-inositol-3-phosphate glycosyltransferase"

/translation="MKLLQALFFPPEQPGGVSSMIPYMQERFTTPRWEMDLFSLPKRI

RNKGREEVQFETFDWTEYQDSPVVQKYIQTYRDYLWWTKLRIQKPYDLIHAHHPIAGL

AMRTVFPDTPLIQTIHSSYERELILNGRIETDGPEHRFLLAIYGELEHQAERLLTVSD

SFRRYLAPYVKEPDVIGVIPNGFDEKRFKPIPHDNAIPQLVTVCRLVPAKGLDILFKA

CAELKGRGHDYVLHIIGDGPIRPDLEELAQRLGIYNETIFYGYTLHPEEFMPFFDIFV

LPSRAEAFGSVFAEAALSCLALVGTDVGGIPEQIENGSNGLLVPAEDPSALAEALEKV

MLDPAYRYELARSACEKAKTHYSLGRSVNELKKMYLQFPGKA"

CDS complement(3655907..3656458)

/locus_tag="EFAGFIKM_03120"

/inference="ab initio prediction:Prodigal:002006"

/codon_start=1

/transl_table=11

/product="hypothetical protein"

/translation="MMHSFRIISQYINDGFQAVQAKPEDTEDVMSLLVETAEWLQSQG

SSQWNALLKGEDSHDTAGAIRRGDVFVFKKGADVAGMVILLVQPSPWDIHLWGSKAHA

KDGAIYLHRLAIRRKYAQSGLGRSILQWSSSGIHFEGKHTVRLDCGASNATLNSFYAR

NGYTFLGETDGYSTYEKPVELLS"

CDS complement(3656563..3657603)

/locus_tag="EFAGFIKM_03121"

/inference="ab initio prediction:Prodigal:002006"

/codon_start=1

/transl_table=11

/product="hypothetical protein"

/translation="MKYDVELVADLRTSGGEVQDVMVNGQYAGSLFLVYREGDRLSGS

LQIEQESLPDDTEEFIVEKVHAYVRSLADALEAAYYEVLVSCGSLHTVLHMPESETVW

PSEEMNHDEGSYDDIGSSGSKVIPVSQAESFTYEDRDQVLEMEMVSAGRSISTYIFND

VEGQELAEASLKQYGADVQGEIHWYDEPEENQLEAAAELLVRELDEDIIDTITIRMWH

QGNELETLEWVHRDFSEDLEDTDESDLDELEAAEETCYVMLKRKDREFRVYELFLQER

GGLPVGTATIDTSHSDLTGYIDYEVPGTSIQRKSLVEVLMQELDKEMEFDTLHLTMLY

RNQIIDEARIDG"

CDS complement(3657700..3658536)

/gene="ywpJ_1"

/locus_tag="EFAGFIKM_03122"

/EC_number="3.1.3.-"

/inference="ab initio prediction:Prodigal:002006"

/inference="similar to AA sequence:UniProtKB:P94592"

/codon_start=1

/transl_table=11

/product="Phosphatase YwpJ"

/db_xref="COG:COG0561"

/translation="MRLLTDEDEARMTYKLIALDVDGTLLNDHHELTEWTQETLIQAS

RQGAEIVLCSGRGPANTIPFMEQMGLDGYVITHNGAVTAQVDTREVVHHFALDGQGLE

PIISYCRTNGVHFDINTAFGLYVDQPEGLGLQVREMYYNFMAEPLKLPKWVDMTEPLA

KFTAFGPIEQMDAVLQEWSTWNLPYYMTRSGDFFIDLMHPEASKGAALKRLAESKGIM

PSEIMAIGNYFNDITMLTFAGKGIAMDNSPDEVKVAADEVTLSNNEQGVAHAIQKYVL

SV"

CDS complement(3658848..3659594)

/gene="rluB_2"

/locus_tag="EFAGFIKM_03123"

/EC_number="5.4.99.22"

/inference="ab initio prediction:Prodigal:002006"

/inference="similar to AA sequence:UniProtKB:P35159"

/codon_start=1

/transl_table=11

/product="Ribosomal large subunit pseudouridine synthase

B"

/db_xref="COG:COG1187"

/translation="MSGKGKQTLRLDKILSHMGVGTRSELKKMVKQGRIHVDGKAVKD

SGVQVNPEVNVIEADGERIVYREMIYLMLHKPPGVVSATEDNRDKTVLDLLRKEDRVF

NPFPVGRLDKDTEGLLILTNDGPLAHDLLSPRKHVPKTYEARVLGNVDEADVQRFKAG

IQLDDGYETLPAQLTILGQEETEEGTISSISLIIHEGKFHQVKRMFQAVGKRVVYLKR

VAMGELELASDLAIGSYRELTADELSLLRK"

CDS complement(3659591..3661288)

/gene="rsmF"

/locus_tag="EFAGFIKM_03124"

/EC_number="2.1.1.178"

/inference="ab initio prediction:Prodigal:002006"

/inference="protein motif:HAMAP:MF_01579"

/codon_start=1

/transl_table=11

/product="Ribosomal RNA small subunit methyltransferase F"

/translation="MGVKLPLIFAERMKSLLGDEFEQFMRSYEQSPHAGLRVNTLKIS

MEQFDEIAPFDLRPIPWCETGFYVPHGVKPGLHPYYHAGLYYIQEPSAMAPVELLQVE

PGDRVLDLCAAPGGKTTQIAAKLQGKGVLVTNDIHAERTKALAKNVELYGVRNAVVLN

ESPERIANAFPHYFDKVLIDAPCSGEGMFRKDEDMVKSWEHHSVEKCVLMQRDILETA

ARLLAPGGTIVYSTCTFAPEENEAMIAEFLNVNRDFVVMDIPEETGFAPGRPEWVRQM

MPEKAEETEAVLDQTRGTARLWPHLLEGEGHYLAVLQHRAGQEVETDQPGVVKEGAME

YGQVVDVSRIEVEGNKDHSIAASSIAMTKADRKKERLLRIESRESHDRQAGGGKNSGR

QSKKGKDHGGRKSERGQGRGADSASIDPVAIYSQFMKEQLKIELTGETVCYGDRVYQS

SVGAARLEGLKVIRPGWFMGTIKNGRFVPSHPLACALNASEARRSVNLSSADGEAVRY

LKGETLNIEEERVLLKADTVAKGYVLVCVDGYAAGWGKWLDGVLKNEYPAGWRWTSV"

CDS 3661510..3662058

/locus_tag="EFAGFIKM_03125"
/inference="ab initio prediction:Prodigal:002006"
/codon_start=1
/transl_table=11
/product="hypothetical protein"
/translation="MKSKKKKKSAAILIFLGILIIMIAALVVVDQQSKKQMDSVENAY
GIAASKLNPATRELLSDPNYQQIIVPTDLKAKIDNKDSFFVYFFASDCSHCRATTPQL
MPLVDSEGIELPQFNLREFEAGWTDYNIEFTPTLVYYEAGVEKDRMVGGLQENGSDQG
YTLDDYKQFFQKYKGSATPSAS"

CDS complement(3662276..3662758)

/locus_tag="EFAGFIKM_03126"
/inference="ab initio prediction:Prodigal:002006"
/codon_start=1
/transl_table=11
/product="hypothetical protein"
/translation="MSIYEPLYVDYLIYFNRDQDYFECHEVLEELWLERNRDSFYKGL
LQIAVGLYHFRNGNLRGGTMMLQSSLDLLEPYPATILGIDLGALVRNVQEIVKQLSQS
DVQSVTYRDLSIRIVDEFLEQEISRRSLELKPNIPQRRSPTRGRIYEEKMKAMDQSKS
"

CDS complement(3662745..3663560)

/locus_tag="EFAGFIKM_03127"
/inference="ab initio prediction:Prodigal:002006"
/codon_start=1
/transl_table=11
/product="hypothetical protein"
/translation="MDGRDWGTFLLPYEQAVEELKVKFKTMRAELKKREEYAPIEFVT
GRVKKISSILEKSRRLNVSLDDVETGIEDIAGIRIMCQFVDDIRRVAEYIRGRKDLTV
LIEKDYITNFKESGYRSFHMIIEYPVQTALGQKKVLAEIQIRTLAMNFWATIEHSLSY
KFREGLPDEMRTRLKKTAEAAFILDNEMSAIRLQILEAQKAFEDDSNIVSRTLNIIHQ
LYFYHLVSEAIEAQKRFNDYWERHDMEGLKDLLDDVKKLLNNARKGENPDEHL"

CDS complement(3663696..3664793)

/gene="pyrD"

/locus_tag="EFAGFIKM_03128"

/EC_number="1.3.5.2"

/inference="ab initio prediction:Prodigal:002006"

/inference="similar to AA sequence:UniProtKB:P9WHL1"

/codon_start=1

/transl_table=11

/product="Dihydroorotate dehydrogenase (quinone)"

/db_xref="COG:COG0167"

/translation="MLYRSLAKPLLFKMDPEQAHHLIIGGLSGVGSIRPVPSGLRVMY

GVRETSDLAVDMFGCHFPTPVGLAAGLDKNGQAVTGFSSIGFGFMEVGTVTPLAQPGN

DQPRLFRLPPDEALVNRMGFNNLGAEAMAGELARLQDRRIPVAVNIGKNKATSNEEAH

LDYSKCIQALYDYADLFVVNISSPNTPDLRNLQHGNELKELLAAVMNEMNVQHARAGG

ATKSVLVKIAPDVNDQELEYMVRTIADSGVAGIIATNTTISRTGLSHQHAKETGGLSG

KPLRDRSTEIIRQIYRQTEGKLPIIGSGGIFTSEDAYEKIKAGASLVEIYTALIYEGP

EVNRRIHAGLRELLRKDGYRHISEAVGAEHR"

CDS 3665049..3665186

/locus_tag="EFAGFIKM_03129"

/inference="ab initio prediction:Prodigal:002006"

/codon_start=1

/transl_table=11

/product="hypothetical protein"

/translation="MAPKRKPPALQQKKEEVNRKAIAWTAASVGGLIIIIGALIIIAN

M"

CDS complement(3665229..3666671)

/locus_tag="EFAGFIKM_03130"

/inference="ab initio prediction:Prodigal:002006"

/codon_start=1

/transl_table=11

/product="hypothetical protein"

/translation="MQNTHLRTYVKKHPDNKMAWYLLGKEYLGEGQEAKANYCFQQAG

EVYEAFERSKAPADIWVDYQDKLVEMSEQKEKKQRRRKMWLTLLMLLVLAGLPPADAP

GFSREAADALSAALESTDDIEAPVEADKQVGTASIAPSNVFTAAAFGGGNHGEAALAA

AWSGSGPKVETSAVLGMQTSDDWSLWKRNMPVKYIVQTNTSGKLTAQSYDAKQCNCEP

PEITPKIKKIALAWTAKQEAAASLSSAIVAYRKKNDAWPKSVTQMAQPFPNNILGETA

SGMTEMFPKLLALHQGKVQEGKNDSSGKENGSTTSNSSQSKNAAFADTLGGQPFLQEP

LEIVIDKDKHKLALISGDTIVRMYDVGLGGDRTPEGSFIITDKVVNPNGRSNGEFGSR

GMQLSNTNYAIHGTNEPDSIGLDESLGCVRMRTGDVEELFALAPQGTPVRIGEDVLPD

LTLVPEAKQRYQHTLVPKQNNPNKTYHWLN"

CDS complement(3666892..3667134)

/locus_tag="EFAGFIKM_03131"

/inference="ab initio prediction:Prodigal:002006"

/inference="similar to AA sequence:UniProtKB:P10245"

/codon_start=1

/transl_table=11

/product="Ferredoxin"

/translation="MSKYTWVEKDTCIACGACGATAPDIYDYDDEGLAEVIFDGDANH

GVKAIPDDLFDDMQDACDGCPTDSIKVADEPFNKEG"

CDS complement(3667251..3668555)

/gene="dinB_3"

/locus_tag="EFAGFIKM_03132"

/EC_number="2.7.7.7"

/inference="ab initio prediction:Prodigal:002006"

/inference="similar to AA sequence:UniProtKB:Q47155"

/codon_start=1

/transl_table=11

/product="DNA polymerase IV"

/db_xref="COG:COG0389"

/translation="MMSSDGNPPANVDQYYPAAGRVILHVDMNAFYCSVHEAEEPDLY

RGKATAVAGSSELRKGVIVTCSYAARNRGISTGMVVHQAMKKCPDLIVIRPDFHLYRQ

YSRAFMQIAYSYTPQLEATSIDECYLDITGSRQFGNPMEIAESIQRRIKDELGLPCSI

GIAPNKLLAKMASDLKKPNGISILRMRDVPRILWHRPCNEMFGIGKKTAEKLKKLGIE

TIGQLAKSDENMLTELFGVNGAWLKNSANGINHSAVHAEREANKSIGHTTTLPADVSD

MNEIHRVFLNISDQVARRLRKHEMFSQGIQITIRTPDMKTITRSRLMEVPTEDAKVIY

REACKLFEKHWGSGKPVRMLGVTLQNLIPREESAVQMDLFEYEQKPKKDNLIRIMDQL

RDKFGENAVVTAGMLGDDPSVLLRNHKVRGTSLQKDNLQSLD"

CDS 3669084..3670703

/gene="cimA"

/locus_tag="EFAGFIKM_03133"

/EC_number="2.3.1.182"

/inference="ab initio prediction:Prodigal:002006"

/inference="similar to AA sequence:UniProtKB:Q74C76"

/codon_start=1

/transl_table=11

/product="(R)-citramalate synthase"

/db_xref="COG:COG0119"

/translation="MSKAISIFDTTLRDGTQGEGVSLSADDKLKIAKKLDDLGVHYIE

GGIPGSNTKDIEFFKRVKELNLNAKVVAFGSTRRKGSVASEDANLKRMIESGAQAATL

VGKSWDFHVHTALQTTLEENLSMIYDSIAYLKQNGMEVIFDAEHFFDGFKHNPEYAQA

VLTKAHEAGADWLVMCDTNGGTMPNEVHEIVSTLHRSLPHAHLGIHTHNDCELAVANT

LSAVQAGARQVQGTMNGYGERCGNANLASIIPNLQLKLGYECVTEDSMRQLTNVARYV

SEIANVNMPINQPYVGNAAFAHKGGIHVSAILRDSRTYEHIVPELVGNKQRVLVSELA

GQSNIVSKAQELGLEFDPSSANSRQIIEKIKDLEHQGYQFEGADASLELLIREANGDM

KELFTFESFKMLVEKTAGKSVVSEAFVKVNIAGTSAYTAAEGNGPVNALDNALRKALV

QYFPSLANMHLSDYKVRVLDEKDATAAKVRVLIESKNTENTWNTVGVSENVIEASWEA

LVHSFRYALLQEKLQDEPGTLPIPAHGISNH"

CDS complement(3670824..3671375)

/gene="resA_4"

/locus_tag="EFAGFIKM_03134"

/inference="ab initio prediction:Prodigal:002006"

/inference="similar to AA sequence:UniProtKB:Q81SZ9"

/codon_start=1

/transl_table=11

/product="Thiol-disulfide oxidoreductase ResA"

/db_xref="COG:COG0526"

/translation="MKRNIYILLGVVLLVGIALAQNAGDGIAAVFKQEEPMPTETGPR

AGLLAPAFSLTAMDGKTYSVGGAKDKATFVSFWASWCEPCKQEAPELNRMAAKYKDKL

DMYGVNVTSYDKLKDAKAFVDEYQLTFPIPLDEKGTVYAQYNGVAFPTNVLIDSRGVI

QEIVLGILPEKELERKIKKLIAN"

CDS 3671602..3672093

/locus_tag="EFAGFIKM_03135"

/inference="ab initio prediction:Prodigal:002006"

/codon_start=1

/transl_table=11

/product="hypothetical protein"

/translation="MAALHGQQNAFYIPSSVEEEMSKHMFLSLPQEACGVMLGETAAG

GIRISRFQPIRNVAPDPLHHFTLDDAEWIRCVFSEPMLIGIFHSHPHTRPVPSLEDIQ

ALPAFAGLLQMYLIGSPDLTSGLRSHMHLNGYLIESSKESQDPAQHVVPSYNLLPVPL

CVT"

CDS complement(3672079..3672816)

/gene="dinG_2"

/locus_tag="EFAGFIKM_03136"

/EC_number="3.1.-.-"

/inference="ab initio prediction:Prodigal:002006"

/inference="protein motif:HAMAP:MF_02206"

/codon_start=1

/transl_table=11

/product="3'-5' exonuclease DinG"

/translation="MREPARGNTGFWNSLRQGGVPSAIASIMGAPTAQHMAFIRSMMR

EQRRPEVLHTPLNELDAVVFDLETTGFSPQHGDEIISFGAVRIHGGVVLEGEQFYTVV

QSKTAVPEHITELTGITQEMTLDAPTLLEGLHDFMSFVGGNVLIAHASAHDRAFLNAA

LWRTSKVRLTHRLIDTMMLARWLEPSRPGYGLDELLESRGIPIYGRHHALEDSKMTAQ

LWSCYLEDMTRKNVETLGDLYTQLSHA"

CDS complement(3672813..3673898)

/locus_tag="EFAGFIKM_03137"

/inference="ab initio prediction:Prodigal:002006"

/codon_start=1

/transl_table=11

/product="hypothetical protein"

/translation="MEPISYTNYSWSYQGIDGAMSSQELRQARVILQNELQELLSASL

SPIEWYQTVNELHDRIARKAVELCIQGMVEEGFGQPPVPYAFIVFGSSGREEATLWSD

QDNGMIISDTPHEGKEEYFAQLGQRMTDMLEELGYAKCEGKVMCSEPLWRKTQAAWKQ

QLADWSSDLNWEPIRNLIIASDMRFVAGEQSLAEEWITSFYEQFRLIPELSDAVLRNT

VKHKATLNVLGRVVTERFGEHAGGFDIKYGLYIPLVNSARYLALQHGIRESSTLKRME

RLTSLEAVPFTLLDACQRAFLAALRFRRSTPVVIQGDLQHSSGFLDEKQMKQKQLHYE

LRDTLGLVRRVHRALQRQLRFAERRRP"

CDS complement(3673940..3675340)

/gene="nrgA_1"

/locus_tag="EFAGFIKM_03138"

/inference="ab initio prediction:Prodigal:002006"

/inference="similar to AA sequence:UniProtKB:Q07429"

/codon_start=1

/transl_table=11

/product="Ammonium transporter"

/translation="MRKKWLVSMLVMLTLFAFPVSAFAAAEGPTNIELQSGLNSAFTF

LAVVLVFLMQGGFALLEAGSTRMKNAGHIAGKTILTLGISVIAFWALGFGLGFGNGNS

FFGTTGFFLSGDKMAASFESLAFSDVPLTIKFVFHLAFAAVSLAIACGGMAERAKMSV

YIVFGTLYTIIMYPVVAHWVWGGGWLAELGMQDFAGSTVVHLTGATAALVATILLKPR

IGKYNKDGKPNIIPGHNQVYSVLGVIILWIGWFGFNPGSTLSAMGDGFFGYVALTTNV

AAAAGGVAALLISWAVLGKSDIPSMLNGVLAALVAITGACAFVEPWAALVIGALAGII

TFFTAQYFDRKGIDDPIYAFSVHGIAGMWGAISTGLFATPELAENAGVGQAGLFYGGG

FHQLGVQLLGLAGAFAFVLVMSFIILGGMKAIMGIRVTEEEETMGLDISEHGTYGYPE

QMKNVDSKSNGGTFSS"

CDS 3675692..3676120

/locus_tag="EFAGFIKM_03139"

/inference="ab initio prediction:Prodigal:002006"

/codon_start=1

/transl_table=11

/product="hypothetical protein"

/translation="MKLFRIGELAKTAGVSERTIDYYTKLGLIAPEERTQKNYRLYSN

ETLTRLERIVQMKQEKYSLDEIKQSLEKWRLVSTEEQVASKLTTLELHVQQLEREVNE

LKPLLGEMKPVQARKMMAGLLTKSAGTMEALKILLENTMM"

CDS 3676149..3676829

/locus_tag="EFAGFIKM_03140"

/inference="ab initio prediction:Prodigal:002006"

/codon_start=1

/transl_table=11

/product="hypothetical protein"

/translation="MSFNNGMFVLIIIAFLLSLWAQFRVKGTFRRWSEVPNQNGMTGY

DAARQMLDANGLHDVPIEPVRGTLSDHYDPINRVVRLSEPVYYENSISAVSVACHEVG

HAIQHKESYPMLALRHRIFPIVNFASGLAPFLLIAGFIFNAMNLVGIGIIFFSVTVAF

QLITLPVEFNASNRAREIMVSEGYIRNEEEKGVAKVLNAAALTYVAAALISLLELIRY

IGIFNSRD"

CDS complement(3676874..3677785)

/gene="gltC_4"

/locus_tag="EFAGFIKM_03141"

/inference="ab initio prediction:Prodigal:002006"

/inference="similar to AA sequence:UniProtKB:P20668"

/codon_start=1

/transl_table=11

/product="HTH-type transcriptional regulator GltC"

/translation="MELRQLHYFLKVAQKEHVTQAAEELHVAQSAVSRQIHQLEEELG

VDLFMQKGRNLQLTAVGQLFCKRIEGILKDLDKAVGEVHEFLDPEHGEIRIGFPHSLG

IHLIPSVVAAFRQRYPNVKFRFKQGMFPTLIRDVLSGEVDLAFISPFPEKHDQVDGDI

VLTEELHAILPPNHPLAGEETIALEQLKDDKFVLFSKGYSLRPIVWHACLEAGFTPKI

AFEGEETDTIRGLVAAGMGVSLLPEMALFQTNPLQPAHVAISHPKVTRTIGLIHRAHD

KLPLVAQSFRSFLLDYFGLQQNNTPSD"

CDS complement(3677855..3678481)

/gene="gluP"

/locus_tag="EFAGFIKM_03142"

/EC_number="3.4.21.105"

/inference="ab initio prediction:Prodigal:002006"

/inference="similar to AA sequence:UniProtKB:P54493"

/codon_start=1

/transl_table=11

/product="Rhomboid protease GluP"

/db_xref="COG:COG0705"

/translation="MIFIRYENWKGYLKYFPLTSLFLIANVVMFIVLTVNGGSTNNMV

LLKFGALTNHELFAHEWWRYITSMFLHAGFSHLLFNSFALIVFAPPMERLLGSVRYGV

LYLGGGVLGNILAVAWYNSVGSITISVGASGAIYAVYGAFLYVALFQRTMMDEASRKT

MYTLLLFGIIFSFAMSGINWMAHLGGLLGGFFIYGLLIRLWKPRSLKR"

CDS 3678630..3679148

/gene="tpx"

/locus_tag="EFAGFIKM_03143"

/EC_number="1.11.1.15"

/inference="ab initio prediction:Prodigal:002006"

/inference="similar to AA sequence:UniProtKB:P80864"

/codon_start=1

/transl_table=11

/product="Thiol peroxidase"

/db_xref="COG:COG2077"

/translation="MTQERTGAATFKGNPITLIGPELKVGDQAPDFTLSKNLVEDASL

KDFAGKIKLISVVPSLDTGVCDAQTRRFNVEAGELGDNVVVLTVSVDLPFAQARWCGA

AGVDRVVTLSDYKTRSFGEDYGVLIKEFQLDMRSIFVLDAEDRITYVEYLPEMTDSPN

FEQAIAAVKALL"

CDS complement(3679221..3679613)

/locus_tag="EFAGFIKM_03144"

/inference="ab initio prediction:Prodigal:002006"

/codon_start=1

/transl_table=11

/product="hypothetical protein"

/translation="MTLKRTLVGIIRSMEGTSDRAKDPKLKTRYYNLSKDRAWEEVSS

TLKKIPGYKVLHEVPSVGEVILEKRTTFGRTMDITVSIISVSPVRSAVDMYSASRGSL

GDLGSNYRTIMNLFSVLDKKLSKYKAND"

CDS complement(3680039..3680851)

/locus_tag="EFAGFIKM_03145"

/inference="ab initio prediction:Prodigal:002006"

/codon_start=1

/transl_table=11

/product="hypothetical protein"

/translation="MEFKGAMGGIYRLTEWITRLAATNLLWAICSSPFLFFLIMKLLV

MQQNLANESLQMNWAIAIVAPLTLFPATSALFTVVRKWNMGDTDVPIFRTFFVGYKEN

YKQSLIGGIFYTLLFAIMYLDYTVYMTQFRNMQLVGIIMLVLLLLLFVSLFNFFSMVV

HYHMSIGLIIKNAVLLTLIRPFRVFSTLLGSGLLFYIGFRYPVLFVFFIISIIAWFAF

FNFYATFNKMQEQMEKMQLKKEEEEAAALAEQSAENGETSEPSDDKNITLQK"

CDS complement(3681069..3682667)

/gene="cshA_1"

/locus_tag="EFAGFIKM_03146"

/EC_number="3.6.4.13"

/inference="ab initio prediction:Prodigal:002006"

/inference="similar to AA sequence:UniProtKB:P96614"

/codon_start=1

/transl_table=11

/product="DEAD-box ATP-dependent RNA helicase CshA"

/db_xref="COG:COG0513"

/translation="MTNLNFTDFNLEPLVLQAITELGFEEATPIQSKAIPLALEGRDL

IGQAQTGTGKTAAFGIPLISKISKSDEKIRALIMAPTRELAIQVAEEIEKLTRFKGLR

SLPIYGGQDIVRQIRALKRKPQIIIGTPGRLLDHINRKTIKLEDVQTVVLDEADEMLD

MGFMEDIQSILKQVPDERQTMLFSATMPPNIQKLAQQFLNNPEHISVIPKHVSAPLID

QSYIEVPERQKFEALSRLLDMESPELAIVFGRTKRRVDELAEALQKRGYSADGLHGDL

SQNQRDAVMRKFRDGSIDVLVATDVAARGLDVSGVTHVVNFDLPQDPESYVHRIGRTG

RAGKEGAAWSFVTPREIDHLHFIERVTRHRIPRKPLPTMAEAVEGKQRLTAERLLEIV

QSGELNEYKGIAIQMLEQYDSVQLLSAALKLLTGDKKDAQVDLTPEDPIRAKRRKPDV

RSGGRKPSGYSGNRTSGSGGSGGGYNRDRNSSGSGRGGYNRDRNSGSSSTSGGSREGG

YNRDRKPRPSSNEGRRPAKDSSFE"

CDS 3682973..3683794

/locus_tag="EFAGFIKM_03147"

/inference="ab initio prediction:Prodigal:002006"

/codon_start=1

/transl_table=11

/product="hypothetical protein"

/translation="MPKIIWHSLVIILGAAAIACGFNWFLVPHQLLSGGVSGISMLLG

YFTTLNLSIMYFVLNIPLLIAGWFILGRRFITLSILSVAATSWFIALLPVFAVATDPL

LSSVFGGVLIGMGTGVSFRVGGSTGGLDIVGSIFTRKRDFPIGTVMAGMNGTIIMLAG

YLSDNWNTALASMVSIYITGKVLDLIHISHVKVTLYIITNETEAMLKKLLVRPRGVTK

IKTQGAYTDIEKDMLMTVTTRYELVEIKRIIKDTDPNAFVNIVETVGVMGSFRRS"

CDS 3683954..3684157

/locus_tag="EFAGFIKM_03148"

/inference="ab initio prediction:Prodigal:002006"

/codon_start=1

/transl_table=11

/product="hypothetical protein"

/translation="MIIVDMETVKLFEIVNRWCPEMLPFLKPNELDSLIVLRDGLGIL

EQGDAMEIIQYSICEHQNEIYIQ"

CDS 3684321..3685502

/locus_tag="EFAGFIKM_03149"

/inference="ab initio prediction:Prodigal:002006"

/codon_start=1

/transl_table=11

/product="hypothetical protein"

/translation="MNMFWALLAMAFGSFGGAPAVADQGNPQFQSLTNSFNTAIIQSQ

KDQVVIDADNPPAKIDPQPDAPVVKGIYVTAYSAGGARMKELLELTDSTELNAMVIDI

KDDLGYITYPTENKALQKMGKSQPFIRDIDALMKRLQKHEVYPIARVVVFKDTILAKK

NPELSFRNKDGSVWANGKGDSFVNPYSKEVWDYNIEIAKEAAKLGFKEIQFDYVRFPE

GFETRADILKYTKSDKSRVDVVAEFVQYARKELAPLGVRVSVDIFGYAASVPAAEGIG

QDFVKISENVDVISPMVYPSHYSTGWYGVKDPDKDPYTTIKGSMEDTHKKLDPTKELK

PVIRPWIQDFTASWLGSGHYIKYGKKQVEDQIRAMKDMDVHEYLLWNASNRYTSGVQY

K"

CDS 3685699..3687057

/gene="mdtK"

/locus_tag="EFAGFIKM_03150"

/inference="ab initio prediction:Prodigal:002006"

/inference="similar to AA sequence:UniProtKB:P37340"

/codon_start=1

/transl_table=11

/product="Multidrug resistance protein MdtK"

/db_xref="COG:COG0534"

/translation="MNMNTTSFSQKVKQFLIIFLPIFTTQIALSAMSFFDTNMSGKFS

PADLAGVAIGTSLWMPVQTGLSGILIGITPVVSHLLGSKRNDQIGYNVIQALYLGIAV

SLVVIGAGALLLGPVLNGMPLEPRVAQVAFYFLCALAFGIIPLFGYTVLRSFMDALGQ

TRTTMFITLVSLPVNIFLNYLLIFGRWGFPQLGGVGAGVATACTYWLIFLISLFFVHR

VEPFAKYGIFRQWTRVSLSKWKELLKIGVPIGFATFFETSIFAAVTLMMSRFDTTTIA

AHQAALNFASTLYMLPVSICMALTILVGYEAGAGRLRDARQYSLLGIGGAIGLSLLTA

IVLIVFGEQIAGVYSNDREVIALTQHFLIYAIFFQISDAIATPTQGALRGYKDVNPAL

IITFVAYWIIGLPVGYLTATYTSLGAFGYWIGLIAGLAVGATALLWRLFLVQKQAALR

KA"

CDS complement(3687141..3688112)

/gene="pfkA"

/locus_tag="EFAGFIKM_03151"

/EC_number="2.7.1.11"

/inference="ab initio prediction:Prodigal:002006"

/inference="similar to AA sequence:UniProtKB:P00512"

/codon_start=1

/transl_table=11

/product="ATP-dependent 6-phosphofructokinase"

/translation="MTAVKKIAVLTSGGDSQGMNAAVRAVVRSGLFHGLEVFGVQRGY

QGLLNNDIFPMDLRSVGDIIQRGGTVLQSARCKEFYTAEGQQKGADILRARGIDGLVV

IGGDGSYNGANKLSKLGINTMGLPGTIDNDVSFTDYTIGFDTAVSIVVDAVNKLRDTM

SSHERSSIVEVMGRHCGDIALHAGLASGAETILVPEVPFDMDEVADRMKANFAHGKRH

SIIIVAEGVGKGEDVAKELMERCPTYEPRVTVLGHIQRGGTPTPFDRNLASRLGDFAV

RSLIAGETDKGCGIIKGELTLTDIDKVVNTKKDFDMVTYELAQRLSQ"

CDS complement(3688166..3689245)

/gene="ytpB"

/locus_tag="EFAGFIKM_03152"

/EC_number="4.2.3.130"

/inference="ab initio prediction:Prodigal:002006"

/inference="similar to AA sequence:UniProtKB:O34707"

/codon_start=1

/transl_table=11

/product="Tetraprenyl-beta-curcumene synthase"

/translation="MSQSNRKRHQYPRGPLALMRGVYKYTIPETRKALNGWRAQAEAI

PNEELRTQALASLKDKQFHCEGGTVYALADLPNRHILIPLIVSYQTISDYLDNLCDRS

TSMDPDDFRLLHQSMLDAVDPEAIPVNYYALREEQDDGGYLRNLVTTCQELTRQLPGY

ASAKPQIQDLAGLYTDLQVYKHIKPELRETALLEWWSEHRHRTPQFRWNEFAAATGST

LGVFMLFLAASDDQLTEEQAASIHTAYFPHVCALHIMLDYLIDQDEDRIGGDLNFCNY

YENVETMLDRIAFIVEMARSDVQKIPGSSFHRMIIEGLIAIYLSDPKVSEQQEVRVVS

KRLLKNSPITRIFFFIFSRWIRKNM"

CDS 3689425..3690765

/locus_tag="EFAGFIKM_03153"

/inference="ab initio prediction:Prodigal:002006"

/codon_start=1

/transl_table=11

/product="hypothetical protein"

/translation="MSSSESLSWRRLFSFFVPLGISASLVTISHVIINSTLARSAHPE

TVIASYAIAGSLLTLTERPSTLLRQTCSALVRDRLSFQALTFVTKIFLACVLLIGFLI

VYSPVGTGVFKYLFGVSPDLLTKVIDVYEILMYVSIFSVIRNIYQGIIITNNRTKWLT

IGMVFRLAGMYGLSLYFIYTDSIDSGRVGAIIFAAGMMIEALVSFLEGNSIKRKMPAK

LEDHPVESKGDVFRFYKPLLLSSFVALFIGPVINIVLGKTTGIALAISSFAIASSLMQ

LMLSFFTYIHQIVLNFYLVDAKLVRKFALVTGFIPFAMMVSIAYTPLGPWVLENVMSV

QGELLQQSLWTLRAFVLFPLIFPFLDFSNGLILLRGQTKTMFRSQTANAICTVIVLLI

LVSIFPAWNGMIGAVAQSLGLLAELIIVWLVIRRTKQEPTMSVPKARKSTSLKG"

CDS 3690806..3691978

/gene="yegT"

/locus_tag="EFAGFIKM_03154"

/inference="ab initio prediction:Prodigal:002006"

/inference="similar to AA sequence:UniProtKB:P76417"

/codon_start=1

/transl_table=11

/product="Putative nucleoside transporter YegT"

/translation="MKLSHNARPDQNWLRALMFTIFGSTVLVVSYFQLYFSHLGFSRA

EIGYLYGIGPLISVFSNMFWSMASDRYQTVRKVMIILLGGQLITGIMLANATTFGQVF

VLVTLFYFFYYPVYPLSDTMAITTASKYGRNFTSIRVFGSIGYAFFALSIGYFLGSFG

PVWTIWVCVGLAATTLLISFQLKDQPSGSSSKMDLSGLWAILKRRDVLTFFGCVFLLA

MGHRMNEAFLTITLKDLGASEGLIGWSLLISSVSEIPIFLLLSKYGNRYKELPLIAFA

ALMYTVRLLLMSVSDTPAAVVAIQTMHSVTFGIFYVTAVRYIIRLVPDGYRATGMALF

TIVWSSASGLLSGTLGGLLLEHAGRQTFYLTAMAFSLAALIGFGLKLWSSMANRVS"

CDS complement(3692134..3692331)

/gene="cspD_1"

/locus_tag="EFAGFIKM_03155"

/inference="ab initio prediction:Prodigal:002006"

/inference="similar to AA sequence:UniProtKB:Q45099"

/codon_start=1

/transl_table=11

/product="Cold shock-like protein CspD"

/db_xref="COG:COG1278"

/translation="MKGTVKWFNAEKGYGFISVEGGEDVFVHFSAIQGDGFKTLEEGQ

AVEFEITDGNRGPQAANVNKL"

CDS 3692493..3692726

/locus_tag="EFAGFIKM_03156"

/inference="ab initio prediction:Prodigal:002006"

/codon_start=1

/transl_table=11

/product="hypothetical protein"

/translation="MIKKHEIYKTDKWNMMTVEVQGKYLVLREISEEWGEECHTFLSR

PALMHWAERRFPKADFENREDEWRQLMAAFKGV"

CDS 3692730..3692915

/locus_tag="EFAGFIKM_03157"

/inference="ab initio prediction:Prodigal:002006"

/codon_start=1

/transl_table=11

/product="hypothetical protein"

/translation="MQLDSSNVVIFIILAFSLGIVLILGRNSIPDRLRRGMALIATLL

ICFAFFLIVYFLYNMGT"

CDS 3693129..3694919

/gene="pepF1_3"

/locus_tag="EFAGFIKM_03158"

/EC_number="3.4.24.-"

/inference="ab initio prediction:Prodigal:002006"

/inference="similar to AA sequence:UniProtKB:P54124"

/codon_start=1

/transl_table=11

/product="Oligoendopeptidase F, plasmid"

/translation="MSQLLKRSEVPAEHSWKLEDLFADQKAWDQEYEEVSSLTKKASE

FQGKLNQPDVLKSCFEFEDEISLKIERLFVYARMHQDEDTANPTYQNLSQKAQKLVVR

VGEALSFVTPEILSLPDDQLDAFIANEKLSAYTFTLEEMKREKAHVLSQAEEALLAQV

GNLSQAPQTIFSMLNNADLKFPRIKDEHGNEVELTHGSYIQFLENPNREVRERAFKAV

YETYAKQKNTIAAALNANVTKNMFYANVRKYPSVMEMSLYGDNIPTDVYTNLVDTIHE

SLPLLHRYMDLRKKLLGVDQLHMYDLFAPLVDEYKMDITYEEAKQTVKDGLKPLGKDY

ADALQTGYDNRWIDIYENENKRSGAYAWGAYGTHPYVLLNHKDNLNSMFTLAHEMGHA

LHSHYSDTTLPYRDAQYTIFLAEVASTTNEALLMDYLLNKSTDPKEKLYLLTYYADQF

RTTVFRQTMFAEFEKIIHERAEQGDALTPQLLSEIYYDLNVKYHGEGMAVDKEIEMEW

ARIPHFYNSFYVYKYATGFSAATSFSKQILEEGQPAVDRYLGFLKSGGSDYSINILKK

AGVDMSTPQPIREAMSVFKELIEQMEQLTK"

CDS 3695052..3695432

/locus_tag="EFAGFIKM_03159"

/inference="ab initio prediction:Prodigal:002006"

/codon_start=1

/transl_table=11

/product="hypothetical protein"

/translation="MKMQWALIAGLVFALLTGIFAVINVDSVQVNLLFNTVQIPLILL

ILGCTLIGGIIVGSYGIYRQYRLQRENKQLKLRVSELESSTTNKSTLDSFKSMDSLDS

NESDSLLENDSIQSQRKGNPTGTL"

CDS 3695545..3696585

/gene="ysdC_2"

/locus_tag="EFAGFIKM_03160"

/EC_number="3.4.11.-"

/inference="ab initio prediction:Prodigal:002006"

/inference="similar to AA sequence:UniProtKB:P94521"

/codon_start=1

/transl_table=11

/product="Putative aminopeptidase YsdC"

/db_xref="COG:COG1363"

/translation="MSFTIDESYVLSFLKKLLDTPSPSGYTHHIIEMIRKEAAALGIA

CELNNKGGAVLTLPGQDSSKTIALSAHVDTLGAMVRSVTSYGTLKLTSVGGFSMQSIE

NEYCSIHTRDGKTYTGTILSLHPSVHVYPDARTFERSESHMEVRIDEVVSSKEDVLKL

GISVGDFISFDARAVITPSGYIKSRHLDDKASVAALFGILESAHREGWKPLHNVSLLI

SNYEEVGHGASYIPAEISEMIAVDMGAMGDDLSCKETDVSICAKDSSGPYDYDMTSRL

IELAKQDGMDYVVDIYPHYGSDGSAALRGGNNIRAALIGPGVHASHSMERTHKDAVLN

TARLLAAYITTK"

CDS complement(3696702..3697640)

/locus_tag="EFAGFIKM_03161"

/inference="ab initio prediction:Prodigal:002006"

/codon_start=1

/transl_table=11

/product="hypothetical protein"

/translation="MYKEINQQLAALKEKGRIYEKWTKRLEKLQTEESEWEAKVKRRL

EHLQKEQKDVDRLNSMTLSAFFYQLIGKKTERLEKEELELMESKAAYDTACRMLQDVQ

EQRIHVEQELEGGRQYQYWQSDYNALWGKKEGRLLDQDAELQHMAENREHLASELQEL

DEAFREGEYLLYALERAEKALASAGNWGVYDMMGGGVISTHIKRGRMDDAQVAIMDAG

KRLRRFQKELEDVEMAVHADLHLGGLLSFADYFFDNLFVDWMVQDKIRKAERQVKDGL

SAVRKTMHVLRNEMRDHKAELEILDRKYHAYVEQAD"

CDS complement(3697804..3699726)

/gene="taeA"

/locus_tag="EFAGFIKM_03162"

/inference="ab initio prediction:Prodigal:002006"

/inference="similar to AA sequence:BARRGD:NG_063829.1"

/codon_start=1

/transl_table=11

/product="ABC-F type ribosomal protection protein TaeA"

/translation="MTVEQIAKSYGEKILFKDASFGMADQDKIGVVGVNGTGKSTFLR

VISGMEPADAGQISIGNDVRIQFLAQNPDFNPDNTVLQQVFEGDSMEMKTVREYTETM

ELLELNSSDPALQERLLRLNQQMEQLQLWQMESEAKSILSKLGIRQFDALMGTLSGGQ

RKRVALAAALIHPCELLILDEPTNHIDNDSVVWLEQYLQKRRGALLMITHDRYFLDRV

ANVMLELDHGRLFRYEANYTRFLELKAEREEREASSEQKRKNLLRTELAWIRRGAKAR

TTKQKARIDRFEQLKDQQGIQRSGSLEVSVGSTRLGKKILEIEHLSKSVGGRKLIEDL

SYIAVPGDRVGIVGPNGSGKSTLLQMISGKLEPDAGVVDVGPTVNLGYFTQEHQEMDE

TLRVIEYIKEVAENVKTADGSLITASQMLERFLFTPASQWTPISRLSGGEKRRLYLLR

VLMAAPNVLLLDEPTNDLDIQTLAVLEDYLDDFPGVVFVVSHDRYFLDRTVDKVLSFE

GNGAVRVHVGDYSEYAEWMLKNAPGANQESDTGAAKVKQSSEKTTPAVSAAKPKLKFS

FKEQREYDQIDENIEKAEANLVRINKEMEESFSDSARLQELMAEQVEAERHLDELMER

WTVLNELAEQIEQSKS"

CDS complement(3699796..3700374)

/gene="fadR_2"

/locus_tag="EFAGFIKM_03163"

/inference="ab initio prediction:Prodigal:002006"

/inference="similar to AA sequence:UniProtKB:P94548"

/codon_start=1

/transl_table=11

/product="Fatty acid metabolism regulator protein"

/translation="MSTVQGDKSEAILDAAYGIFGSKGFYETKMSDIADEAGIAKGTI

YLYFKSKEQLFIAVSKRDCNSFISRLEYALNSYENTGDKLGAIAKTHLTYYYERRNHT

KLFFMAPNNDPDLMKFMKAFMNQYMSMVCEVLESASVPEPVLLAEAYIGILDRLKMDI

MLNPEFNEEHLNKRIAFAAALFLDGCRSFLQV"

CDS complement(3700387..3701196)

/locus_tag="EFAGFIKM_03164"

/inference="ab initio prediction:Prodigal:002006"

/codon_start=1

/transl_table=11

/product="hypothetical protein"

/translation="MDWIIGAVCASMVAGAAYAKKSLTLSGCLAAIMMGTIYYGAGNL

FWFGTLLLFFITSTLLSRFRKDRKQELEKSYAKSGNRDAGQVMANGGMGMFLCLGYWI

FPHPAWVYAFIGVMATVTSDTWATEIGSLSRKPPRSVLTWKVLTPGASGGVSLLGTVA

AAVGGALIGAGAFLFSWIAGIEGLGLFSWTFVGLAGGLAGAFADSYLGATVQMMYRCT

VCGREVEVHEHCGHPTVPARGWAWMSNDLVNVLSSVIGGCVAIGLGNILAL"

CDS complement(3701425..3702198)

/gene="ssuC_5"

/locus_tag="EFAGFIKM_03165"

/inference="ab initio prediction:Prodigal:002006"

/inference="similar to AA sequence:UniProtKB:P40401"

/codon_start=1

/transl_table=11

/product="Putative aliphatic sulfonates transport permease

protein SsuC"

/db_xref="COG:COG0600"

/translation="MKWLEKKWVSIPLLWVTVILIWQLGALIYGPDVIPGPWHTILGA

RELIADGTLMEYIGISFTRVLAGWVLGSIIAIPVGLIIGKVHLIRLFAEPFLNFIRFI

PPIAFITLFLVWFGIGEQSKIALIMYATFFIVVLNTLTGVLSVEEDKIRSARSMGANE

RQILLHVIVPATTPYIFTGVRLAMGTSYMAIIGAEMIASNEGVGYLIWNSRLFFRTDW

IFVGLISLGFMGFLTDRLFNWFGRKVLYRYGVIGGAKRV"

CDS complement(3702216..3702992)

/gene="nrtD_1"

/locus_tag="EFAGFIKM_03166"

/EC_number="7.3.2.4"

/inference="ab initio prediction:Prodigal:002006"

/inference="similar to AA sequence:UniProtKB:P38046"

/codon_start=1

/transl_table=11

/product="Nitrate import ATP-binding protein NrtD"

/db_xref="COG:COG1116"

/translation="MSLPATQHTIHIEQLRKTYHAPTNGDVHYIIKDVDLVIKGGEFF

VLLGPSGCGKSTLLNMIAGFISKSGGQLKVDNKEIDRPGRDRAMVFQQADSSLFPWLT

VRENVEFGLRMSKVPKTQRREISDRYIQLVGLSAHEGKFPKELSGGMKQRVQLARVLA

NDSAILLMDEPFGALDAMTRRTMQKELVNIWKETHKTVIFVTHDIQEALLLGERIGIM

SVGPSSNITDIYHNTLPYPRNIASSEFNTLYDRIQGHFEE"

CDS complement(3703020..3704075)

/locus_tag="EFAGFIKM_03167"

/inference="ab initio prediction:Prodigal:002006"

/codon_start=1

/transl_table=11

/product="hypothetical protein"

/translation="MTRSNGVYIVRKPSIVTGILILFLATALLLSGCSSNAAGSGAKD

ATGKNTDNVKIRIADTSTNPTFRVAIAKGFFENRGIDAESITFGSPAEGVNALFIKQV

DIAYGADFPVLNALSKGEYSVIASAGQTTDEAAAAWKLYARENIQSGADLKGKKVSFI

RGTFIPYLWDEYLKDQGVALSDVTQIGQGAFDEAYIALKQGDLDAAWVIGSALTDKFD

ALEGVHQLTDMSQTPIRLGMGLVSSNEFIQANPEKISEFLAALDEASTYAQAHPEEVA

DLMYQETKQPKDATLKDLPINPWEVGFTQAAYDSLAGQKQYMVDTGIIEQDFDLDTKL

DLTSLQQALPEKVTYSK"

CDS complement(3704088..3705536)

/locus_tag="EFAGFIKM_03168"

/inference="ab initio prediction:Prodigal:002006"

/codon_start=1

/transl_table=11

/product="hypothetical protein"

/translation="MGHSTIYPTGATLYNPDKAWGGYTVVYQAGEEGVVLIDMNGKEVH

LWKGLLGFPAKILPGGYVLGSTGRRDPKFGIQDNVDLVQVDWDGNIVWKYNSYEHIED

PGYEPLWYARQHHDYQREGNPVGYYAPGLAPKTKSGKTLILAHKNVSNPQISDKPLLD

DAIIEVDWEGKVLWEWLPNEHFEELGFDEAARNVLFRDPNTRSFGHLGGGVGDWLHIN

SASYVGPNRFYEEGDERFHPDNIIWDAREANIIAITDRQSGKIVWRLGPDYTSPEVKH

IGTIIGQHHAHIIPQGLPGEGNLLVFDNGGWAGYGLPNPASPFGLKHAIRDHSRVLEI

NPVTLEIVWQYTSAEAGFSVPTDSYKFYSPYISSAQRLPNGNTLITEGSNGRLFEVTA

EHELVWEYVSPYTDGRNTNMVYRSYRVPYQWVPQLEKPQEVAIEAIDVSTYRVQGAAP

KGSASIVSVETTLPFVEGAACVATSDEGKSGG"

CDS complement(3705595..3706152)

/locus_tag="EFAGFIKM_03169"

/inference="ab initio prediction:Prodigal:002006"

/codon_start=1

/transl_table=11

/product="hypothetical protein"

/translation="MSDRDWEALERTDWLFRKMVRRFVKERDRISVEGISLPGMLILH

KIIREGEQRLGDLAEQLDFTSGAITALTDKLEKKGLTIRRRKEDDRRTVLLDITSSGR

EMYARNSNIGARCITLLFEGFTSEELEQQSQFYERVVANLEGFSDTVLELAESNGIQE

HDRSSTSDLEQKQRDNTGKRNYLSY"

CDS 3706539..3706946

/locus_tag="EFAGFIKM_03170"

/inference="ab initio prediction:Prodigal:002006"

/codon_start=1

/transl_table=11

/product="hypothetical protein"

/translation="MNMKRVIIIGTMIVTMSFAGTAWGQSAISPIPTAKWSIVDLDTR

EGSSDELLDALNQASEVDLYQELYNGKSLRTITEENNGNLDEVIAIQVRQLREQLDER

LANGSISSEQHAAQQAELEELVTQSANTAYSLA"

CDS complement(3707069..3707701)

/gene="ycgM"

/locus_tag="EFAGFIKM_03171"

/inference="ab initio prediction:Prodigal:002006"

/inference="similar to AA sequence:UniProtKB:P76004"

/codon_start=1

/transl_table=11

/product="putative protein YcgM"

/db_xref="COG:COG0179"

/translation="MLTNIRNVYCVGRNYKLHAEELGNAVPDEPMIFMKPSHAVVPLN

GETLELPATKGEVHYEAELVVQIGRSYEPGMAVDELVDAYAFGIDFTLRDVQTVIKKK

GHPWTAAKGFRNSAPVTAFQAFPGAAALLEKDFTLTKNGEEVQRGNIRNMIFSLQDIV

DYVGHHYGLGPGDVIFTGTPEGVGPTQAGDVLELAWDGESLGACTIGAAQ"

CDS complement(3707749..3708501)

/gene="glpQ_2"

/locus_tag="EFAGFIKM_03172"

/EC_number="3.1.4.46"

/inference="ab initio prediction:Prodigal:002006"

/inference="similar to AA sequence:UniProtKB:P37965"

/codon_start=1

/transl_table=11

/product="Glycerophosphodiester phosphodiesterase"

/db_xref="COG:COG0584"

/translation="MNNLCVAHRGFSSIAPENTMAAFLMAMERPEVQWMELDVQLSRD

GVPVVIHDFTVDRTTNGKGLVRETDWADLQRLDAGSWKNKSYKGERIPALSELLDRSC

GRVRLNIELKTQGDMYPGLPAAVIHEVRKRHMQNDVVITSFEPAALIEVKKLAPEIQT

GLIIDARPGDLLTALRQMNCTFLSIGYTNVDKSLMNEMRSAGIRVMAWTVDDKTIMKK

LAAVDPELMLCTNRPDVWELAFQETSSRFFRP"

CDS complement(3708526..3709947)

/locus_tag="EFAGFIKM_03173"

/inference="ab initio prediction:Prodigal:002006"

/codon_start=1

/transl_table=11

/product="hypothetical protein"

/translation="MYPPRSERSVKKKKGSKRTRNTITIIWTVNIVLIIAIGLVGIFY

VINTRQQQADQPVKQEIVDQDQPSIGDDAKGGDQVSSPDSESDETSEEEPKATDEETT

ADADKTSGVATPETSSNTNSEKTESGTKKPAADSTPSGTKGNAGVDQESGMSKPSNSA

KDVTINFVGDIQFSGKVAELLDKNGYDYPFAKLGRLFKDDDLTIGNLETPITHGGTSA

ADKTYVYKSSPKALAAMASAGFDAVNLANNHILDQGVEGLVDTLTYLKEYGIAHTGAG

MNRDEAYAPAYLERKGMKIALLGFSRVVPETSWKAEGNRAGVAEAYDSTGAVKAIQEA

RKKADLVIVVAHWGEERVSTPNSDQTRLAHEFVDAGADLVIGGHPHVLQGLEYYKGKW

IAYSTGNFIFSRSTTEETWKTAVFQARCSQDAACSMKVIPYEAGLGQAIPMIDEANKL

LLEQMAKLSPGIRYDGNGVASPN"

CDS complement(3710133..3711557)

/gene="hemY"

/locus_tag="EFAGFIKM_03174"

/EC_number="1.3.3.4"

/inference="ab initio prediction:Prodigal:002006"

/inference="similar to AA sequence:UniProtKB:P32397"

/codon_start=1

/transl_table=11

/product="Protoporphyrinogen oxidase"

/db_xref="COG:COG1232"

/translation="MGDKKRRVVVVGGGLTGLSAAFYIRKHYREAGVEPMITLVEKSS

SMGGMIETLHRDGFVIEKGPDSFLARKTAMIDLAKELEIDHELVSQNPESKKTYIMQR

GKLHPMPAGLVLGIPTELRPFLRSGLVSPAGKLRALMDFVIPPRRTTEDESLGYMIER

RLGAEVLENLTEPLLAGIYAGDMRRLSLQATFPQFGEVERDYGSLIRGMMTGRKPAET

HTGTKRSAFLNFRQGLQSLVHALVHELQDVDQRLNTAVKSLQRLDGAQTRYRVELGNG

EMLEADDVVVTVPTYVASELLKPHVDTAALDAINYVSVANVVLAFEKKEVEHVFDGSG

FLVPRKEGRNITACTWTSTKWLHTSPDDKVLLRCYVGRSGDEQNVELPDEALTNLVLK

DLRETMGIEAVPIFSEITRLRKSMPQYPVGHLQHIAALREELGSKLPGVYIAGAGYEG

VGLPDCIRQAKEMSVQATQELAAD"

CDS complement(3711557..3712480)

/gene="hemH1"

/locus_tag="EFAGFIKM_03175"

/EC_number="4.99.1.1"

/inference="ab initio prediction:Prodigal:002006"

/inference="similar to AA sequence:UniProtKB:Q81U22"

/codon_start=1

/transl_table=11

/product="Ferrochelatase 1"

/db_xref="COG:COG0276"

/translation="MSYGTPENMESVEAYYTHIRRGRPPEPEQLKELTDRYEAIVGGV

FPLRENTDNQVKALQETLNRDERGTDVEFRCYQGLKHAYPFIEDGVEQMAKDGIQTAI

GIVLAPHFSTMSVGSYIKRAREKAEELGVHMSFIESYHLHPKLIQALSTRVSAKLDAF

EEAGAKRGDVKVLFSAHSLPARIVEMGDPYPQQLLETSEVIASRVGITNWQFTWQSAG

RTAEPWLGPDILDTLQELSREQVEDVLVAPIGFVSDHLEVLYDLDIEAKSIAKEIDMR

LMRIDSLNSDPLYMETLSDVIISQWQQGSDE"

CDS complement(3712500..3713564)

/gene="hemE"

/locus_tag="EFAGFIKM_03176"

/EC_number="4.1.1.37"

/inference="ab initio prediction:Prodigal:002006"

/inference="similar to AA sequence:UniProtKB:P32395"

/codon_start=1

/transl_table=11

/product="Uroporphyrinogen decarboxylase"

/db_xref="COG:COG0407"

/translation="MSYNDRLIRASFKQQVDRVPVWYMRQAGRYDPEYRKIKEKYSLL

EICKQPELAAEVTLMPVRKLGVDAAILYSDIMNPVASLGIDFDIVKNIGPVIDNPIRS

AADVDRLRPIDVEGDLSHILETIRILDKELDVPLITFAGAPFTIASYLIEGRPSKGYI

RTKTMMYSEPEVWHKLMQKLGDMVITYVRAHIANGGKAFQLFDSWVGALSPKDFRTYV

LPTITRIFTELSDLNVPKIYFPGVASGELLPALHNLQADVIGLDWRVSISEGRQRLGG

KFAVQGNLDPYLLTAPMELIKEQAKVIIDEGIKEPGYIFNLGHGLFPEASLEKLRELT

AYIHEYSAEAMKSGVTVTND"

CDS 3713770..3714981

/gene="sbnD_2"

/locus_tag="EFAGFIKM_03177"

/inference="ab initio prediction:Prodigal:002006"

/inference="similar to AA sequence:UniProtKB:Q2G1N0"

/codon_start=1

/transl_table=11

/product="Staphyloferrin B transporter"

/translation="MKTWKVNLIVLWFGQFLVNSGMTMITPFLSLYLARDLGVVGEHE

IGIWAGFIFAANFLTSFLFQPLWGKLSDKYGRKVMLLRSGFGMAIVIALMGLAQNPWQ

LLLLRLLNGTISGFNPAAVALISGTTPKDRMGFAMGISQSGQVAGTILGPLIGGLLAD

AVGFRPIFYITGGLIFVASMLAMFLVREKFDRQEAAKLPAQSVLSGLKELNKSPQLPA

LFAVTFLLQFAMISPMSLLPLYVQKLHASDVNVAFWAGLVGAVTGLSNMAMSPILGKL

SDRIGPHKVLTFSLIGTGLMLIPQAFVQTVWQLILVRFMMGVFMGGLLPSVNALIRSY

TSDSMISRAFSFNTSTLALGNMLGAIIGGFMAGFIGIEGLFIVSGGLLLLNMVWVRFK

LYNKPASIRES"

CDS complement(3715093..3716295)

/gene="yjiB_2"

/locus_tag="EFAGFIKM_03178"

/EC_number="1.14.-.-"

/inference="ab initio prediction:Prodigal:002006"

/inference="similar to AA sequence:UniProtKB:O34374"

/codon_start=1

/transl_table=11

/product="Putative cytochrome P450 YjiB"

/db_xref="COG:COG2124"

/translation="MKQAPRKYANYIPIRELETLEERLSPFKVYAELRDNTPVRYDEH

RECWDVFGYEDVKYVLKNPKLFSSARDRANTSMLTTDPPKHKQLRDLVNQAFTPKAIE

ALAPRIQEITDELIAPHLSEGHMELIDDLATPLPVIVIAELIGVPAADRQKFKDWSDV

LVKGARDDSEQAFQELLEEKKRNMQELHIYFTGIMEERRLQPKDDLISLLLAAEIDGQ

QLTSDEVVGFCILLLAAGNETTTNLITNAVRILSEQPELQQELREQPERITSAIEETL

RYYPPIVAIGRVAKETVELNGQMIQAGEQVISWVGAANRDSAQFENPEDFISDRKPNR

HMGFGFGIHFCLGAPLARLEARVVLHTLLQHMGNIQLVPGTALQPIQSAFVFGVKHYP

IQFNLINK"

CDS 3716782..3717993

/gene="ansZ"

/locus_tag="EFAGFIKM_03179"

/EC_number="3.5.1.1"

/inference="ab initio prediction:Prodigal:002006"

/inference="similar to AA sequence:UniProtKB:O34482"

/codon_start=1

/transl_table=11

/product="L-asparaginase 2"

/db_xref="COG:COG0252"

/translation="MSKTSNLRPWAVWSTAALTALTISLSPIGTYAAHAATVEVKGTT

GAVQTATTTPARNTSIPAIPDSSKQSALPNVLVIGTGGTIAGQSEDATSFQNYKAGTL

PIGEMVDALPDKQKIADVSTLQFGNSGSGSYSMTDLYDLSQTVDKALAMYDSVVVTTG

TDTMEEIAYFLDMTVQSDKPVVITGSMRPWTVIGSDAQANLYNAIKLAGSGRTTSFGT

VLMLNDTIQLARGVTKTNDYRTDTFETPMLGAVGYIDEENIRIYRAPARALKPEGTAK

PVFDLSKITKADLAKVEIAISYQEAGGGAIEGFVSSGAKGIVTSGTGAGGISRAMGQA

RTKAIEEGVIFVTTTRTGSGSVYGGGKGIIAGDNLSPQQARVLLMLGLSFSDDFDTIK

KWFETYGTPEV"

CDS complement(3718048..3718260)

/locus_tag="EFAGFIKM_03180"

/inference="ab initio prediction:Prodigal:002006"

/codon_start=1

/transl_table=11

/product="hypothetical protein"

/translation="MRPLFIFLSLLLISGFVFHFLNVYTPFLILPVYIVLIATLVFFV

VYLFRFYLTKKIIIKSENTQRRDDTP"

CDS complement(3718504..3719136)

/locus_tag="EFAGFIKM_03181"

/EC_number="2.1.1.6"

/inference="ab initio prediction:Prodigal:002006"

/inference="similar to AA sequence:UniProtKB:O07431"

/codon_start=1

/transl_table=11

/product="Catechol O-methyltransferase"

/db_xref="COG:COG4122"

/translation="MNLTPDEYVNQLFQEDELLLKVKEAIRSNGMPEVSVAAAYGRLL

TFLAKTSKAEAALEIGVLGGYSGICIARGLRESGTLTSLELKEEYAAMARGHLEEGGF

GEKVEYRIGPAADSLEELEQEGRTFDFFFIDADKENYPVYLDYAIKLARPGAVIVGDN

CFLRGRTLNPDKQGPAVLAVRRFNEQMASDPRLVTTMLPDYDGLVLAWVK"

CDS 3719229..3719354

/locus_tag="EFAGFIKM_03182"

/inference="ab initio prediction:Prodigal:002006"

/codon_start=1

/transl_table=11

/product="hypothetical protein"

/translation="MHHFERGVHTFTAFLIVSFFTESGKAAPLLYSREKDFPSPA"

CDS 3719367..3720530

/gene="rlmL_2"

/locus_tag="EFAGFIKM_03183"

/EC_number="2.1.1.173"

/inference="ab initio prediction:Prodigal:002006"

/inference="similar to AA sequence:UniProtKB:Q9K0V4"

/codon_start=1

/transl_table=11

/product="Ribosomal RNA large subunit methyltransferase L"

/db_xref="COG:COG0116"

/translation="MNHMAQLQLIATSAMGLEAVVARELKQLGYEDVTIDNGRVFFTG

DYIDICRCNLWLRSSDRVLVKMGEFPATTFDELFEGTKALPWEEWIPADGEFPVEGRS

QKSQLSSVPASQGIVKKAIVEKLKLTYDTEWFPEDGSRYVIEVILLNDRALLTLDTTG

PGLHKRGYRKLVTEAPLKETLAAALIQLSRWNVSRPFYDPCCGSGTMLIEAAMIGWNI

APGLRRTFNSEDWAVIPEELWEQAREEAFDAVRDDVPLQISGSDIDPEAIEVAMAAIK

SAGFAKDIEVSVLPAHRARPQGQYGVIITNPPYGERLSEEKEVQKLLRSLGRSYLDMP

TWSFFAITSTKAFEEYFGHKADKRRKLFNGRIETQYYQYLGPLPPRNKTPQSS"

CDS 3720745..3722745

/gene="ltaS2_2"

/locus_tag="EFAGFIKM_03184"

/inference="ab initio prediction:Prodigal:002006"

/inference="similar to AA sequence:UniProtKB:O34952"

/codon_start=1

/transl_table=11

/product="Lipoteichoic acid synthase 2"

/db_xref="COG:COG1368"

/translation="MVVTRPTRSAMPRGLLNHPLTLYLIFLVLMLLKLMWLHHNLHAY

NITMGLLDKVIAIGSLLLLSFWTWLLPRRGMILSLAVLNLLLTALIYADMVYYRYFQD

FLTIPVLLQARQVDALGDSIATLIYTSDLWFFADWLVVIPFAAIVLFSRRYRSKHSST

SVTGYGSYLYNDGKARLRRRLTAGSIALVLGLGLAVGPIYFYSKTWAKGLFDNNWWNV

SMYNVTGLLAFHGYDLYNYAKDHIGSGPQADPADVEQAKAFFAERQEAAPQNDALFGK

YKDSNVIIVQGEAFMNFMIGQSIGGQEITPHFNELMKESQYYSHFYHQTGQGRTSDAD

FGANISLHPLPVGSAFVRYADHTYDSLPSILKDNGYSTNVFHAYESGFWNRYTMYQNM

KYDKFYSKNDFAQDDPLGWSLSDESFFRQSVEKMSSDVTEPFYSFLITLSSHHPYALP

KEKQQLDVGEFQGTMFGNYLQSVHYVDSALGKMVEDLKNRGLWENTIFMFYGDHDNSI

KEQSQYEQFLGRSLNELDMAQIMNEVPLLVHLPDGAAAGTMDEPSGQLDITPSVLHLL

GVSDQSYYHMGNDVYDGSSRTVVLRNGAFSDGSVFYIPSDDYIYENGACYDLSTRDKT

DINACRPGHDEAAKRLHVSDTVITYDLIQRFREEDSSTATPQ"

CDS 3722822..3723250

/locus_tag="EFAGFIKM_03185"

/inference="ab initio prediction:Prodigal:002006"

/codon_start=1

/transl_table=11

/product="hypothetical protein"

/translation="MTSHTGPFNIEQQLFDAAAKFVKQRYPQGWGGAGAVYTEAGSLL

ISVAPEVINDATHLCMETGAYLEAHKLKERVTHSLCIARDDEHSEFKVLTPCGVCQER

LFYWGEEVKAAVYDPSGQLVFKRLDEIQPYHWSKAYRDKK"

CDS 3723333..3724058

/locus_tag="EFAGFIKM_03186"

/inference="ab initio prediction:Prodigal:002006"

/codon_start=1

/transl_table=11

/product="hypothetical protein"

/translation="MNIEIWSDFLCPFCYIGKRRLENVLEQFPHRDEVKLQFKSFELD

PNAALNPGKTNSEYLAAKYNMSVEQAKGMNAQMNANARTAGLEYNIDAMIPTNSFSAH

RLTHWADTQGKALELSERIFQAVFIEGKHSGDPEVLAQLAEEIGLDRNAATAVLSSDQ

FTDNVRADQAEGEQLGIRGVPFFVFDRKFAVSGAQPDEVFLDAIQKAWDERSPFTMVE

SNTTETDGSGICTDDGCEVPKKN"

CDS complement(3724355..3725185)

/gene="yvgN_2"

/locus_tag="EFAGFIKM_03187"

/EC_number="1.1.1.-"

/inference="ab initio prediction:Prodigal:002006"

/inference="similar to AA sequence:UniProtKB:O32210"

/codon_start=1

/transl_table=11

/product="Glyoxal reductase"

/db_xref="COG:COG0656"

/translation="MTKHITDCTILNNGVTMPWLGFGTYKAKGKEVQQAVETALEVGY

RSIDTASIYGNEEEVGQAIASSGVARNELFVTTKLWNEDQGFDSTLRAFEASQKALGL

NVIDLYLIHWPGRDQYKETWRAFERLYSEGSIRAIGVSNFQVHHLQDIIDEGGTVPAV

NQVELHPGLIQQELQDFCGAQGIQLEAWSPIMKGKLNQESTLKALAQKYGKTPAQIIL

RWDIQNQIVTIPKSVTPERIRENADIFDFELTPDELRQIDALDSDKRTGPHPDQLFWD

"

CDS complement(3725360..3725641)

/locus_tag="EFAGFIKM_03188"

/inference="ab initio prediction:Prodigal:002006"

/codon_start=1

/transl_table=11

/product="hypothetical protein"

/translation="MPKRYDSSLQADTTVSQAQNAVNKLHYAVSQALSHPTAQTIVQA

ERRLAHTEQAMRQAELSLGGQGVELAEEMFIEEKRRLNSIQSQHGQGDL"

CDS 3725791..3726189

/locus_tag="EFAGFIKM_03189"

/inference="ab initio prediction:Prodigal:002006"

/codon_start=1

/transl_table=11

/product="hypothetical protein"

/translation="MKKILLTLCMLPFLIWVQGCAPATYEIEGYTASSMNPDIPVPSN

AKLIKIITDSGNRNIKEGVMYELKHIGGEQGLYPPLDYFQKLADTGWVELEEERMGHV

HFLKKDTTVIAIEIREDAFEIYEMVKGAME"

CDS complement(3726291..3726512)

/locus_tag="EFAGFIKM_03190"

/inference="ab initio prediction:Prodigal:002006"

/codon_start=1

/transl_table=11

/product="hypothetical protein"

/translation="MANDIRVCEKCNHVRLKSIVPKLQKMAPDTEIKIGCKSYCGPCA

KRAFVFINGRYISAPTEEEVLVKVAKFVK"

CDS complement(3726534..3726836)

/locus_tag="EFAGFIKM_03191"

/inference="ab initio prediction:Prodigal:002006"

/codon_start=1

/transl_table=11

/product="hypothetical protein"

/translation="MNCKITRNAAKVLKLELDKPENEGKNLRVVITHAHGDHAHYGLD

IDTPKENDTVVSTDKEIDVILANDQPLLNGVKIDYLYFPEEGFVITNPSQGNHGDH"

CDS complement(3726979..3727236)

/locus_tag="EFAGFIKM_03192"

/inference="ab initio prediction:Prodigal:002006"

/codon_start=1

/transl_table=11

/product="hypothetical protein"

/translation="MKWRSLLSFKRWTQVFKRLPRLLRAPQIPLGEKLLFIIPALLYW

VLPDVMPFMPIDDIGVTLILMNWFVSRAERKYPVLQEGAPS"

CDS complement(3727432..3728643)

/locus_tag="EFAGFIKM_03193"

/EC_number="3.4.19.11"

/inference="ab initio prediction:Prodigal:002006"

/inference="similar to AA sequence:UniProtKB:Q03415"

/codon_start=1

/transl_table=11

/product="Gamma-D-glutamyl-L-diamino acid endopeptidase 1"

/translation="MDLQWIIVQRGDTLLRIASAHHMTKEFLAALNPEVASQPYLLAG

QMLRIVPGTGRRYAVPPGERVGEIAGRFGLDEEVLRQANPEITNITDWVGRCIHIPAS

NGKTIVKIQGEYGYRELIRDIDKLENQYPFIETGSIGTSVMGKSLPYLRIGQGSRHIH

VNASVHANEWLTTAVLMKFIEEYAEAYSAHRTWHQYQTERWMQETTLWAVPMVNPDGV

ELVQEGVVNQHPHAQQLLAWNADRSHFTHWKANIRGVDLNDQFPAHWDEEAARRGVTS

PGPRDYAGTAPLTEPEAQALAQWTQQHTFDAVVSLHSQGQEIYWNYRDLEPRESGPLS

RRLAKASGYKAVKLGGSDAGYKDWFIQDFGRPGFTVEVGLGVNPLPVEQFDDICIEVG

MLLAELLSNGQ"

CDS complement(3728872..3729687)

/gene="murI"

/locus_tag="EFAGFIKM_03194"

/EC_number="5.1.1.3"

/inference="ab initio prediction:Prodigal:002006"

/inference="similar to AA sequence:UniProtKB:O31332"

/codon_start=1

/transl_table=11

/product="Glutamate racemase"

/translation="MFVQQAIAILDSGVGGLTVAKEVMRQLPREKIIYFGDTARTPYG

PRSSEQVKQFTEQIVDFLIQFDPKVIVIACNTATAAALEYIRGKVNMPVIGVIHPGAR

AAITATRTGRIGVIGTTGTIGSGAYTTALKQLSPYIDVVSQACPALVPLVEQGEFRSE

HTTHTVEQSLGEIKQQPIDCLILGCTHYPFLMDTIQEVMGQEVKLISSADETAREIST

ILYDKRKLASGDETPVHQFFCTGDPRMFQNITRRWLGEQISKTPVVWQVTQLS"

CDS complement(3729901..3730278)

/locus_tag="EFAGFIKM_03195"

/inference="ab initio prediction:Prodigal:002006"

/codon_start=1

/transl_table=11

/product="hypothetical protein"

/translation="MKDSNKSLLWGALIGSVVGSVTALLLAPKSGRELRQDITEGARQ
VSEKGQELAGIVGEQSSQIVSKVKETADVVIQDIQSWRNCAEGKEIRISAAIVDNDID
KTVDEPGIDIVAKLPADESKDDN"

CDS complement(3730464..3731378)

/locus_tag="EFAGFIKM_03196"

/inference="ab initio prediction:Prodigal:002006"

/codon_start=1

/transl_table=11

/product="hypothetical protein"

/translation="MTNESKEFNEVNTESNETNETPSGFHPVYMVELLFRKRPEVDRL
RLQEAMIRHTGQVRLDVKQESGGQKHEMLVFYHLDHKVSFQEGDIPAQTCMLPVNEIA
DRARFGGALQQAWYWPEVGQVVEASRYSIRIHDMFTAAMPRKQRLELFQKTVLAIMES
LTCDALYWYGSDKLVEPEAYTQAQEREEHLYAAMNVRMYQAGGTEEQRGLVMDTVGLS
ALGVPDVQCHFVGLDPDTVAQTLLGAAYYIFDQGDVLQDGQTLGSSGGRRWRCEHQAA
LIAPGRYVIDLDPGDEHAAADLEPARQN"

CDS complement(3731389..3731850)

/locus_tag="EFAGFIKM_03197"

/inference="ab initio prediction:Prodigal:002006"

/inference="similar to AA sequence:UniProtKB:O32126"

/note="UPF0331 protein YutE"

/codon_start=1

/transl_table=11

/product="hypothetical protein"

/translation="MYYVNREQIARRLAAVPEVAEGLRGAAEAWDGSLMLGMVQERCL
HLAIEIVTDVGSYLIDGFIMRDASSYDDIIQINYEEKVFDNPTYEILRQLVTLRKPLV
QDYYTWERSELHPLSVELPSILEHFTTQVSAYVEKELGPFNTAQAEGQRKE"

CDS complement(3732024..3732311)

/locus_tag="EFAGFIKM_03198"

/inference="ab initio prediction:Prodigal:002006"

/codon_start=1

/transl_table=11

/product="hypothetical protein"

/translation="MIKHIVLFKMKDRSAESIETAAQVLRNLEGKIDVLVSLEIGIDV

LRSERSFDISLTAEFASLEDLQAYQVHPLHQEVIKYMNEVREQSIAVDYEI"

CDS complement(3732432..3732980)

/locus_tag="EFAGFIKM_03199"

/inference="ab initio prediction:Prodigal:002006"

/codon_start=1

/transl_table=11

/product="hypothetical protein"

/translation="MHLSVNTFAVISGAFVTFAFGGWDQLLSLLAVAMAVDYITGLAA

AVRTGTGLNSNIGFWGIARKGLMLTVVLLAHRIDLIMGTDFIKGGAIYFYLVNELISI

TENYAKIGLPLPAKLRQAIAVLKKQEDQEDLMNREWSKPQQTPDNIKQQAETGQTLQD

ESAKQTEDGSQKKSESKGNGSN"

CDS 3733430..3734026

/locus_tag="EFAGFIKM_03200"

/inference="ab initio prediction:Prodigal:002006"

/codon_start=1

/transl_table=11

/product="hypothetical protein"

/translation="MPNQPEQHSIQAWSLINRKYLGKGVRVKRFRKPTRCQIRNRVLL

AVLMANDIKLSQLAEDLSISSRSVSAWVYEGRIPGSTNLDKTCQLLGYPRHILFNEEV

VRNSPVICQPESSRFMKRTVTRSPVSNRILTGLCMVHDLSVTDVSHWIGVHPGTFRKW

LHQGTLPSAAFQEQAEQFFRIPKTILFADVILKDRRNN"

CDS complement(3734250..3735404)

/gene="egsA"

/locus_tag="EFAGFIKM_03201"

/EC_number="1.1.1.261"

/inference="ab initio prediction:Prodigal:002006"

/inference="similar to AA sequence:UniProtKB:P94527"

/codon_start=1

/transl_table=11

/product="Glycerol-1-phosphate dehydrogenase [NAD(P)+]"

/db_xref="COG:COG0371"

/translation="MNMNERIAAWNEEAQQCACGHQHRVVDMLIHLEAGAIQRLPGYL
SEQGYRQVTVVYDQHTFRAAGSDVLSSIREAGMHVDEIALPENRTGDIIADEAAIVQV
MLGVRLESQAVIAVGSGTIHDLVRFVCSKMNKPFLSIPTAASVDGFTSAGAPLIVSGI
KQTFQAVPPEAIFADMNILEQAPQVMTAAGFGDMLGKYTSLADWIVSRDLGGEPFCPV
AYRMTEEALNTCIDHVQAIAEGRAEGVAVLMDALIVSGISMLIIDHSRPASGGEHHLS
HILEMDLMQAGERPVLHGAKVGVACALLTVKYKELAQTSGEPVFGIYDQLPEASQLIA
WLEQVGGPVTTEQLGVTPEMVEHAFNTAHTLRPRYTGLRYINEVLNTRLG"

CDS 3735563..3735748

/locus_tag="EFAGFIKM_03202"

/inference="ab initio prediction:Prodigal:002006"

/codon_start=1

/transl_table=11

/product="hypothetical protein"

/translation="MKLTKELGISLGFLAGTTFGSGIAFLFRLQSFEVVASVTLFGIA
GAIAGIITAVILRQRQH"

tRNA complement(3735852..3735938)

/locus_tag="EFAGFIKM_03203"

/product="tRNA-Leu"

/inference="COORDINATES:profile:Aragorn:001002"

/note="tRNA-Leu(cag)"

CDS 3736163..3737155

/gene="iolG_7"

/locus_tag="EFAGFIKM_03204"

/EC_number="1.1.1.369"

/inference="ab initio prediction:Prodigal:002006"

/inference="protein motif:HAMAP:MF_01671"

/codon_start=1

/transl_table=11

/product="Inositol 2-dehydrogenase/D-chiro-inositol
3-dehydrogenase"

/translation="MKTTHLCFIGAGFHASTNIYPSVVEAGAQIQAIATRSMERSEAA
LLRFGSNGKAYDNAQLMLQQEICDGVVVVAQPVDQTALVLECIRAGKNVYVDKPLGWN

AAEAATVAEAAEQAGVVVMVGFMKRYAPIYMKLKELIDSGSLGKVRSFQMKFAVDSTP

FCKDEEQFMKLAAIHMVDLMRYLFGEVIRVTGTTVKDGEHINQSISLIFENNVVGSAY

FTGMSAWSRESESVLVTFDNGFASAEEFNTLTVHQSRSSTNLPWKSLEEQDTVYTPSG

SPMSGAYRDLYLRGFVGEMAHFIACCQNQTVPHSSAKDNIATMALCDSILSSLK"

CDS 3737379..3737741

/gene="yybR_3"

/locus_tag="EFAGFIKM_03205"

/inference="ab initio prediction:Prodigal:002006"

/inference="similar to AA sequence:UniProtKB:P37486"

/codon_start=1

/transl_table=11

/product="putative HTH-type transcriptional regulator

YybR"

/db_xref="COG:COG1733"

/translation="MKKDATKLCPAPYGCSVEVTLSVIGGKWKGAILYHLFSGPLRFN

EIRKLFPDITQRMLTLQLRELESSGIVHREIYPQIPPKVEYSLTPFGETLRPIIFSMR

DWGETYTNEVLARSSQEV"

CDS complement(3737792..3739408)

/locus_tag="EFAGFIKM_03206"

/inference="ab initio prediction:Prodigal:002006"

/codon_start=1

/transl_table=11

/product="hypothetical protein"

/translation="MIAQIKRSRLRRKIKNIKKISLTALLGGLVTISVIMTLTLMVIS

SYTSQKQSLIDNTLSLNYASAVQMSQTLDSLFYSMQESLKYAATYFPDMDNSNTKELN

STLDLVRNSSNFFNSVSLVNKEGVIRSTSPYSQASVGHHVSSNAAKEAVKLRASYISE

AYQTPRTKRRIVFVSEPIFDSAGEYQGTIGGNIFLQENNILSLSFGSQLKTSNGSYFF

IVDKKGTLLFHPNTNRIGENVSKNEVVQKLLVNKHGKEQYKNLAGVDSLAGYYKVPST

DWGVVIVSPTQTVYDQLNHHIRMLLLYTSVPFLILTLIVVRVARKLASPFAMLADLVN

QVDKGQVDLPVMKPHWNREADLLTRAVVGALANFRKQTNQLVYDARTDVLTGMNNRRT

FEEVIQEWIQDEVPFSIIVLDIDRFKSINDTFGHHAGDEVLKHIANIIQLSVRPEDVC

ARFGGEEFVVLLRNSESNVAYEIAERIRITVEESVLPIDRSVTISAGIAEYPKHSTTS

TELFHLADNALYQAKEEGRNRTVTIQSVIK"

CDS complement(3739573..3740310)

/gene="speE_1"

/locus_tag="EFAGFIKM_03207"

/EC_number="2.5.1.16"

/inference="ab initio prediction:Prodigal:002006"

/inference="protein motif:HAMAP:MF_00198"

/codon_start=1

/transl_table=11

/product="Polyamine aminopropyltransferase"

/translation="MRVLYRNKSEQHELTVYDTKKLYGEKGQFRVLEFSNAAVQGAMD

LDEPARMVLEYPRAMVHLMETNDPEFDTVFVVGHGIGTLPTYLSDRQVKVAELDAEVV

ELSRTFFGYEGSPVLIGDGRELLGQEPAETYDYIIIDAFTATGTPEQFISSDFFAMVK

DKLDSDGAVILNVFGRAGNDRLVNAIYTTLQAQFAYTRAFALPTETADEVQNRILMGS

HHPIEFQVRQMAGFVEQEPEEGYIIVD"

CDS complement(3740466..3741161)

/locus_tag="EFAGFIKM_03208"

/inference="ab initio prediction:Prodigal:002006"

/inference="similar to AA sequence:ISfinder:ISBth8"

/codon_start=1

/transl_table=11

/product="IS3 family transposase ISBth8"

/translation="MMAIHFENREFGYPRMTTALWEAGLNVNHKKVWRIMRELSIQSV

IRKKRKKSSYTPSVIYPNRLKRQFHATAPQQKMVTDITYISDGNSFVYLSVIQDLFNN

EIVAWQLSKRNDVQLVLDTVEQWTQKRDVSEAVLHSDQGFQYTSQAYNTRLEAFGVKG

SHSRKATCLDNACIESFFSHLKTEKLYLNQCNSEAEIRQAVEDYMYNYNYRRFQAKLK

QRAPIEYRCALAA"

CDS complement(3741326..3741646)

/locus_tag="EFAGFIKM_03209"

/inference="ab initio prediction:Prodigal:002006"

/codon_start=1

/transl_table=11

/product="hypothetical protein"

/translation="MAKKGQTFQTYTEEFKLNAVRSYVEGSSSYKVVAEREGIRNCSQ

LKVWVKKWKKGEAFDERKNSVPNPLKGRPRTAFGSVEEERDYLQAQVDYLKKRYPNLV

KEKR"

CDS complement(3741776..3742678)

/locus_tag="EFAGFIKM_03210"

/inference="ab initio prediction:Prodigal:002006"

/codon_start=1

/transl_table=11

/product="hypothetical protein"

/translation="MTLQQLKYVIEVATRGSMNEAAKRLFISQPSLSNAIRDLEQELR

ITIFERTNKGISLSKEGVEFLSYARQVVEQAELLENRYLNAKPSPQHFSVSTQHYAFA

VNAFVRLVQQYGQDEYELALRETKTYEIIQDVKSLRSEIGILYLNEFNAKVINKLLKD

AGLVFTSLFTAKPHIFISVKNPLAKQESVAIEQLQDYPYLSFDQGEYNSFHFSEEILS

TLSHPKSIQVNDRATLFNLLIGLNGYTISTGVLSADLNGDEIIPVPLECEESINVGWI

SHKSTSLSNLGVAYVQALHEAIGS"

CDS complement(3742738..3743475)

/locus_tag="EFAGFIKM_03211"

/EC_number="1.-.-.-"

/inference="ab initio prediction:Prodigal:002006"

/inference="similar to AA sequence:UniProtKB:Q7A3L9"

/codon_start=1

/transl_table=11

/product="putative oxidoreductase"

/translation="MSNVKGKVVVITGASSGIGESTARLLAQHGAHVVIGARRMERLE

ALASSIRLEGGSVECHSLDVTRLEEMQTIVDLAQTRYGRVDVILNNAGVMPLSPLESL

KVDEWNRMIDVNIRGVLHGIAAGLPVMKKQGFGQFINIASIGAYAVSPTAAVYCATKY

AVRAISEGLRQEVGGDIRVTLVSPGVTESELADTISDEEARDLMKEYRRISIPASAIA

KSILFAISQPAEVDVNEIVVRPTASMA"

CDS complement(3743500..3743760)

/locus_tag="EFAGFIKM_03212"

/inference="ab initio prediction:Prodigal:002006"

/codon_start=1

/transl_table=11

/product="hypothetical protein"

/translation="MGDQQEPKHPYVGMWVTKDGYIRHELLPCGRYDEARGDRQSAYQ

GRYVVHGKHIEYVDDTGFTADGNFRDGVLYHAGMILYRENVQ"

CDS 3743936..3744871

/gene="rhaS_27"

/locus_tag="EFAGFIKM_03213"

/inference="ab initio prediction:Prodigal:002006"

/inference="protein motif:HAMAP:MF_01534"

/codon_start=1

/transl_table=11

/product="HTH-type transcriptional activator RhaS"

/translation="MLIKTNETDTSITEQQQQLAQLIEHFTPSDGIHPTAIPSLALIR

ASEISQPIYSVYQPSLCIVAQGSKLVILGRESYTYDLSQYLVASVNLPISGQVVKATT

EHPYLCIRLDFDSGQIFDLIQDTPSAQPKPDHSTQRGLFVSSTKPSLLEAVIRLVRLL

DTPEDIPVLAPLFIREILYRIIQDEHGHSIKQFAIQDSHAQHIAEVIEVIQSDYAKPL

RIEQLAAMINMSSSSLHHHFKAITAMSPLQFQKQIRLHEARRMLLAGSTDAANAAFQV

GYESPSQFSREYARMYGLPPKSDIKRLRLTLDIKN"

CDS complement(3744936..3745373)

/locus_tag="EFAGFIKM_03214"

/inference="ab initio prediction:Prodigal:002006"

/codon_start=1

/transl_table=11

/product="hypothetical protein"

/translation="MMSENEVFSIKETSEQAGLSEDTIRYYEKIGLLPRAERKANRHR

VYRSEDIHTMKLITCLKKTGMSLEEMKPYLQMSMDSDLADFPDEREMLVNHRKKVEAQ

IASLQQVVDFIDEKLEKRSMYPDECPITGENQMSVFEKQNLFS"

CDS 3745543..3746421

/locus_tag="EFAGFIKM_03215"

/inference="ab initio prediction:Prodigal:002006"

/codon_start=1

/transl_table=11

/product="hypothetical protein"

/translation="MNIQNQLRTALITGSTSGIGLELTRRLLAEGWQVIGLNRSAFRS

EDTDIQNALRTGQLRWVQANLTNYDSLRTALDQIKSDTNSIDVLFNNAGGSASELRFS

DQGHELHFELQTVVPYIIYMELVELLLKGQMKTVINTSTTSFNMVRQFDLNILERPAE

FKKLFGPYATSKLGLSLWTREVAKSASADVIQLLSVDPGGNNTLRGNKTSGLPFYIKP

IMKWFFPHPSHGASLLYNAALSPTGHESGTFLVKNKATALRFTEQGPAVLKRVNEIYE

QRFRTTQSGKTIITRS"

CDS 3746529..3747692

/gene="oxdD"

/locus_tag="EFAGFIKM_03216"

/EC_number="4.1.1.2"

/inference="ab initio prediction:Prodigal:002006"

/inference="similar to AA sequence:UniProtKB:O34767"

/codon_start=1

/transl_table=11

/product="Oxalate decarboxylase OxdD"

/db_xref="COG:COG2140"

/translation="MTKGQQPNEKPFIIPQPIRSDGAGGPDLGPRDIMRDIQNPDMLV

PPATDNGLLPNLRMSFSDTHMQLNHGGWSREITVRDLPIATTLAGVNMSLTPGGVREL

HWHQQSEWAYMIWGTARITSVDQNGRNFIADVGPGDLWFFPKGLPHSIQGLEDGCEFL

LVFDDGSFSDLNTLSISDWFAHTPPEVLSVNFGVPESAFQSMPKEQVYIFQDTVPGSI

ESQEVQSPYGTVPLTFKHRLLAQEPLITPGGSVRIVDSTNFPISTTVAAALVEIRPGA

MRELHWHPNADEWQYYLTGQGRMTVFGGNGIARTFDYRAGDVGYVPVAMGHYIQNTGT

DTLWFLEIFRSDRFEDVSLNQWMALTPRDLVRDNLNAPPELLDALRKVKWPVV"

CDS 3747772..3748911

/locus_tag="EFAGFIKM_03217"

/EC_number="1.1.1.1"

/inference="ab initio prediction:Prodigal:002006"

/inference="similar to AA sequence:UniProtKB:O07737"

/codon_start=1

/transl_table=11

/product="putative zinc-binding alcohol dehydrogenase"

/db_xref="COG:COG1063"

/translation="MRAVTFQGIKDIQVKEVEDPKLQQKDDIIVRITSTAICGSDLHI

YQGALPAAKDYVIGHEPMGIVEEVGPEVTRVKKGDRVVLPFNIACGECFYCNHDMESQ

CDNSNGNPDIHTGGYFGFTERYGNHPGGQAELLRVPYGNFTPFVIPESCELEDEALLF

LSDVLPTAYWSVENAGVKPGDTVTVLGSGPIGLMTQKFAWMKGAKRVIAVDRLPYRLE

KAKRLNDAEIFNFEDYDDMGEHIREITQGGTDVVIDCVGMDGKKNMLEEIGQKLKLHG

GSLSAIEIGMKAIRKFGTLQLTGVYGSSYNMFPLGNLFERNINLKMGQAPVIHYMPEL

FRKITAGEFDPTEIISHRISLENASDAYRIFNDHEDECTKVILKP"

CDS complement(3749050..3749838)

/gene="ycgJ"

/locus_tag="EFAGFIKM_03218"

/EC_number="2.1.1.-"

/inference="ab initio prediction:Prodigal:002006"

/inference="similar to AA sequence:UniProtKB:O31474"

/codon_start=1

/transl_table=11

/product="putative methyltransferase YcgJ"

/translation="MSDVIKSQVQKQFAKNAGKYVTSAGHAKGEDLALLVASSQATPD

MNVLDIATGGGHVANALAPLVQRVTALDLTEEMLQVAEAFIQGNGHRNVDFVAGDAEK

LPFDDDIYDLVTCRIAAHHFPDVSSFVHEALRVMKPGGRLLFIDNVAPERDENDQFYN

EVEKCRDASHVRAWRKTEWIHMLEYAGFRMETMVSFQKRFKFEEWCNRAALPERERRE

LEASMLSAPSIIRKFFNFEVTTNGKLDSFQGESVYIQAIKPTHV"

CDS complement(3750038..3752005)

/gene="fruA_1"

/locus_tag="EFAGFIKM_03219"

/inference="ab initio prediction:Prodigal:002006"

/inference="similar to AA sequence:UniProtKB:P71012"

/codon_start=1

/transl_table=11

/product="PTS system fructose-specific EIIABC component"

/db_xref="COG:COG1299"

/translation="MRITDLMIQETMIMDLQATTKAEAIDELIASLNRSGRINDPVLF
KEMIYKREAESSTGIGGGIAMPHAKTTAVNEPTVVFAKSRKGLDFEALDDQPAHVFFM
IAAPEGAGNTHLRTLAALSRLLIDSDFISQLMSTDTPAEVSALFDAKQAEAAEKEAAK
EKAKAEKEANAASASTTGQQQNTSGVIVGNANSEDFVVAVTACPTGIAHTFMAEDALK
KKAQEMGINIRVETNGSEGAQNVLTADEIARAKGVIVAADKNVEMARFDGKPVLQRPV
SDGIRKSEELIRKAVNGDAPIYRSQGGNAKEEGASTGKISVGSKIYKDLMNGISHMLP
FVVGGGILLAISFLFEQLASPENPIVQLLQTIGGGTGAFHFLIPVLAGFIAMSIGDRP
ALMPGMVGGLMAVNSNAGFLGGLAAGFLAGYVVIGLRKLFKGLPKAIDGLKPILLYPV
FGLLIVGAISFYVFDPIFGSLNTWLVDALGNLGTGNAVLLGLLLGGMMSIDMGGPFNK
AAYTFAIGVFTSSGNTDGAWMAAVMAGGMVPPLAIALATTFFKSKFTEQERKSGLTNY
VLGFSFITEGAIPFAAADPLRVLTSCILGSAVAGGLTQLWSINVPAPHGGIFVAALAN
HALLFLLAVAIGSVISGLILGLWKKSPTLVK"

CDS complement(3752096..3753007)

/gene="lacC"

/locus_tag="EFAGFIKM_03220"

/EC_number="2.7.1.144"

/inference="ab initio prediction:Prodigal:002006"

/inference="similar to AA sequence:UniProtKB:Q833W9"

/codon_start=1

/transl_table=11

/product="Tagatose-6-phosphate kinase"

/db_xref="COG:COG1105"

/translation="MIYTITLNPSIDYIVEVDELKLGGLNRMNRDLKLPGGKGINVSR
ILNQLGADNTAIGFLGGFTGRFINDKLQEDNIRTDFVTIADDTRINIKLKHGEETEIN
GLGPAIGAEEAEQLLHKLSSLEKGDIVILSGSVPPSLGTDFYDRLIKVCKQTGAEFVI
DTTGPALMEALEHAPLLVKPNHHELAELFGVTIDTREELVLYGRKLLEAGAKHVLISM
AGEGALFITKAEVHHANVPKGTVKNSVGAGDSMIGGFVGTYVQSGDLLEAFRTGVASG
SATAFSDDLATRELIDELRNQVTITTI"

CDS complement(3753010..3753756)

/gene="glcR_1"

/locus_tag="EFAGFIKM_03221"

/inference="ab initio prediction:Prodigal:002006"

/inference="similar to AA sequence:UniProtKB:P94591"

/codon_start=1

/transl_table=11

/product="HTH-type transcriptional repressor GlcR"

/db_xref="COG:COG1349"

/translation="MLTEERYTAIIERLHLQGIVKLQELVDVLGASESTIRRDLIDLE

SRQMLKRIHGGAALVNEKTLEPGMEEKTFKNIQQKTTIARLAAQEIENGECIYLDAGT

TTLAMIPFIEAKDVTVVTNGLSHVEALVSKRIRSYLLGGMMKIHTKAVIGSIALQNMD

NFRFDKCFLGSNGVDPEMGYTTPDPEEALIKRRAHQLSGKSYVLADSSKIGEITFAKL

FDLEEADLITEQMPEHWRPGIAQKTKIIEG"

CDS complement(3754022..3754507)

/locus_tag="EFAGFIKM_03222"

/inference="ab initio prediction:Prodigal:002006"

/codon_start=1

/transl_table=11

/product="hypothetical protein"

/translation="MNEMNYEQFRAHLKKASRKRNVPLIKIVAFQERYMKIEEVQFYD

VEQNHMSVQACNTLWMHLENKSFRNMVSQHLQFYRDMENLGRHSFENLIKELYDTSVP

VLLDYNPADYYTSGQLAEILVMDEERLIEQLEMGRFKGAFINEEGKWLKPKPDEMVVE

S"

CDS complement(3754642..3756072)

/gene="hpyO"

/locus_tag="EFAGFIKM_03223"

/EC_number="1.14.13.-"

/inference="ab initio prediction:Prodigal:002006"

/inference="similar to AA sequence:UniProtKB:Q8PDQ6"

/codon_start=1

/transl_table=11

/product="FAD-dependent urate hydroxylase"

/db_xref="COG:COG2072"

/translation="MSLEALNERVKNDLAYLSFDGANWVRPREHADGHVYDVVIVGGG

QSGLGAAFGLLRERISNIVVIDENASGLEGPWETYARMVTLRTPKHLTSIDLGIPSLT

FRSWWEAQVGPEGWEEVDKIPRGDWMNYLRWYRKVLNLPVLNEVELTLVEPIENGIHR

LHVNKAGTQMDVILARKVVFATGIQGGGEWHVPSMIADRLPRDRYAHTSEAIDFDQLK

GKRVAVLGGGASAFDNASYALATGVAEAHVFVRREQLPSVNPIRQMEQSGMIERYHAL

PVADKYEVISHFFKFNQPPTNDTFNRAAAWPGFELHLNSPWRSVEATDQGAIVHSAQG

AYEFDFLIISTGLLSDPALRPELKLVEPYIARWEDCYQAPPEHRNALLDAHPYLSSGF

AMTSRSEQGEKTLHGLFVFNYSALASCGLSASAISGTKSAVPKLVSAIADQLFLDDRE

IILQQFYAYEEQEFTATWVKSGALAE"

CDS complement(3756396..3756635)

/locus_tag="EFAGFIKM_03224"

/inference="ab initio prediction:Prodigal:002006"

/inference="similar to AA sequence:UniProtKB:O31818"

/note="UPF0291 protein YnzC"

/codon_start=1

/transl_table=11

/product="hypothetical protein"

/translation="MIPILGRINELSRKQRSSVGLTKAEKSEQQELRKQYLQAIRGQV

LTTMLAVSVVDPLGHDVTPEKLTNEKLQRREQAGQ"

CDS complement(3756749..3763744)

/locus_tag="EFAGFIKM_03225"

/inference="ab initio prediction:Prodigal:002006"

/codon_start=1

/transl_table=11

/product="hypothetical protein"

/translation="MSGMSKRKRKISLISTGKRKAKKSKKHSIPKKKSKSCKKSRRKC

KKKKSHRIPFLRGPRGYQGMRGFTGATGATGLTGDPGYPGIWGATILTGRTGVTGTTG

STGITGVTGATGTTGLMGTTGLTGTTGVTGPTGPTGEAGAIGSTGSTGATGATGTGST

GVTGTTGATGITGFGVTGMTGTTGNTGITGFTGPTGVAGLAGATGFTGATGPTGIGVM

GLTGTTGETGFTGVTGSTGPTGATGLIGITGATGLTGSTGEIGITGPSGATGTTGITG

TTGITGVSGVTGTTGTTGATGPNGETGITGVTGATGFTGEIGATGTTGPTGNTGITGT

TGITGAIGATGATGETGLTGTTGRTGGIGSTGVTGVTGTAGTTGLTGATGTTGETGNT

GTTGFTGATGATGVTGEMGLTGITGETGVTGSTGTSGTTGTTGATGLTGVTGATGATG

TTGITGVTGNTGALGITGFTGSTGATGTTGLTGFTGATGSTGEAGLTGVTGTTGLTGP

TGGTGETGTTGSTGITGNTGETGPTGTTGLTGVTGITGVTGIAGTTGTTGITGATGIT
GFTGSTGAFGATGATGTTGVTGTTGVPGPTGATGETGTTGVTGATGSTGAIGATGSTG
TTGVTGITGTTGTTGVTGITGNTGVTGATGITGATGTTGDTGQTGVTGTTGVTGSTGA
TGETGISGATGVSGTTGITGATGITGATGNTGTTGVTGETGFTGLTGVTGPTGTTGLT
GVTGITGETGATGITGTTGLTGFTGTTGITGVTGATGLTGVTGETGLTGFTGTTGATG
ATGITGISGVTGVTGTTGITGATGATGATGITGTTGTAGVTGPTGATGETGSTGSTGV
TGATGTTGATGETGLTGVTGVTGETGLTGATGATGVSGTTGIAGVTGATGLTGSTGST
GITGATGITGVTGETGPTGMTGLTGVTGTIGATGATGVTGETGLTGSTGTTGVTGITG
PTGTTGLTGNTGETGSTGTIGATGITGVTGVTGATGITGATGTTGATGPTGVTGTTGV
TGSTGETGITGATGVSGIAGITGTTGVTGETGFTGPTGTTGATGTTGITGETGLTGFN
GPTGVTGLTGATGITGETGVSGVTGTTGPTGPTGATGATGITGVTGTTGVTGATGSTG
STGITGVTGPTGATGITGVTGFTGVTGATGLTGITGDTGITGTTGVTGITGVTGATGI
TGATGTTGQTGETGLTGVTGTTGVTGTTGATGATGTTGITGETGVTGATGTTGVTGPT
GATGATGETGITGVAGTTGETGSTGVTGVTGSTGITGVTGTTGATGATGPTGITGETG
TTGATGVTGATGATGITGTTGVTGTTGTTGATGTTGITGETGVTGVTGPTGVTGPTGA
TGATGTTGVTGVTGVTGATGTTGLTGVTGTTGTTGATGSTGVTGVTGVTGTTGPTGAT
GATGITGVTGTTGATGATGPTGITGETGTTGQTGVTGITGVTGATGITGATGITGQTG
ETGVTGVTGPTGVTGPTGATGATGTTGVTGVTGTTGTTGATGATGITGVTGTTGATGA
TGPTGITGETGTTGTTGTTGVTGITGVTGATGITGATGITGQTGETGVTGVTGTTGVT
GPTGATGATGTTGVTGVTGATGATGITGTTGVTGVTGTTGTTGATGTTGITGETGATG
VTGTTGTTGATGATGITGVTGTTGATGATGPTGITGETGTTGTTGTTGVTGITGVTGA
TGITGATGTTGQTGETGVTGVTGTTGVTGPTGATGATGTTGVTGVTGATGATGITGTT
GVTGVTGTTGPTGATGATGITGVTGTTGATGATGPTGITGETGATGTTGVTGITGVTG
ATGITGATGTTGQTGETGVTGVTGTTGVTGPTGATGATGATGVTGVTGATGTTGTTGT
TGATGITGVTGATGTTGITGATGATGATGLTGVSGATGTTGVTGITGVTGATGTTGAT
GTTGSTGTTGVTGLTGVTGNTGFTGTTGATGATGETGLTGVTGTTGVTGTTGATGATG
ITGATGITGTTGVTGVTGTTGTTGATGITGTTGATGITGVTGATGTTGITGATGAAGA
TGLTGVSGATGTTGVTGITGTAGITGATGSTGATGVAGVTGTTGVTGITGPTGPTGPT
GPTGATGVTSATGATGATGITGATGATGLTGATGPTGPNFATQGFSAFLATLSTATST
QLTNWTTTAPYYGNANFNATTGNYTVPVTGRYSIVATINYATTAAIALSLGAGVNPAF
LVQRTSPTVTTLVSGLLPVLNVAITLLTLRAVLGSGSVTLAVEVALSAGDVIGLFYAA
NGLTITLNLGSSATNGIVWSVHEIT"

CDS complement(3763908..3764867)

/locus_tag="EFAGFIKM_03226"

/inference="ab initio prediction:Prodigal:002006"

/codon_start=1

/transl_table=11

/product="hypothetical protein"

/translation="MKRAIVIGATGGTGAAITEELIKRGISTIAFGRSHQKLEQLAVK

LGSPDHLQIAVGDAFRPDDIIAFSQEADVLFHCANVPYNEMESKLIPLGESVMVAAEH

LGLNVVVMDGIYPYGRRQMNEVTEEHPKQPHTRKGKTRLAYEQMLFSTSWSRAQVMIV

RLPDYYGPSANQASYLGSTLEAIARGKMAFFIGNMKVPREYIYLPDAAVMVVELASRE

MTYGQNWNIPGSGIISGHEIVRMAQKAAGRSKPVMALGKVGLSVIGLGVPVMKEIVEM

LYLTEEPLILSRQKYEERIGPVVATSFEEGIKLTIKELELQGE"

CDS complement(3764987..3765508)

/locus_tag="EFAGFIKM_03227"

/inference="ab initio prediction:Prodigal:002006"

/codon_start=1

/transl_table=11

/product="hypothetical protein"

/translation="MAKVTAASVNRRNDIISAAIDVFAEIGYYRATTAQVAERAQISQ

PYIFRFFKTKEALLLTAIEVSWTRVIDSFRIVVETATPDQLENDLIEAYDKILEAHKS

EILLQMQAQTIREEVIRQAMQKGISDVRSMVLEAFTVAGIAEPLKRTMIFLAIGMLCN

VSNALDMPELKER"

CDS complement(3765748..3766560)

/locus_tag="EFAGFIKM_03228"

/inference="ab initio prediction:Prodigal:002006"

/codon_start=1

/transl_table=11

/product="hypothetical protein"

/translation="MRRYPRHDYSEMSEALQQFFVTTIQATCDDSKYPFHVKDLLNEE

KRNLILREQAVQQGLQLAGKGSVAVGTLFAKRYSIFVMSVISAFSLYDTILSVADDDV

RFELNGAGGMQYETRLKSSLLEGGDPVQRRSESHLLKNRMLLHLEPVLRAVAVGTGAS

DKVMWSLVAHNVQQLYARMINDQSIWKTDERLAKIQADQSLWLDRQNDNACTFANELQ

RFEHSGWQGPPFLIRRYCCLAYQVGSGSHAHAYCNSCPKLDSESRLRKLLQQ"

CDS complement(3766557..3767540)

/gene="yfiY_4"

/locus_tag="EFAGFIKM_03229"

/inference="ab initio prediction:Prodigal:002006"

/inference="similar to AA sequence:UniProtKB:O31567"

/codon_start=1

/transl_table=11

/product="putative siderophore-binding lipoprotein YfiY"

/db_xref="COG:COG0614"

/translation="MVRQRAATMILLALLVLVLTACSKASEVTEATPPQQDTPVEESG

TQTIEHLKGTAVIPQKIERMVVLSAAYIDHMLTIGEKPAGVNVEVRYGGDYLPYFADQ

LAGIPTVGSADSPNLEAILQIDPDVIVIESRTAESTYDQLEKIAPTIVLGTEWLDYVD

DTTYWTQDLLTIAGMYNKVDLAKEKIAEVEQQAKQLKAKIEQLDQKKLAYLRVREKTL

QIYAAKGHPTNTLLYHDLGFVPTTVTPAEQREDLSMEKIADVDADFVVLEIDPNADEY

LNNINASSLWKGVPAVGADQVYTTDSFWLFKGWGAIGRSEIINEVEDMINR"

CDS complement(3767649..3769250)

/gene="rhaS_28"

/locus_tag="EFAGFIKM_03230"

/inference="ab initio prediction:Prodigal:002006"

/inference="protein motif:HAMAP:MF_01534"

/codon_start=1

/transl_table=11

/product="HTH-type transcriptional activator RhaS"

/translation="MEENDWIKQLEQLHFSAVHVCHYSSNPGAVQRRIWKRFAICRVI

AGKGSLSMDNIVHTVSQDEWFLLKPGMHVEFQTDMEAPVRYQIILFSCVTLTRHRNAW

HTEALGLPVTGKLNVPSNTRDIQEMMDTLMSGKHSFTSLEKTRRKHLLHQLLIQLMIH

VKEATPPVAGMDLALDYMTHQYMEDIRIDQMARMAGLSVNHFIRTFKRQLNMTPIEYI

LKLRMAKAKQLLFSSDKIKEIAEQVGYKDEHYFSRVFKKNEGIAPTLYMKNKVNRIAT

LYYGLDDYVITLGLRPVSALSYGQRVARHVAIPALQAHSHQGLILDSFNQNYDDLRRV

QPDLIITSDRLEPNDSLHRIAPTTMLEHTNHFGERLLYMADIMGRTEQAVQWIEQHAE

LSRVLRGRIQSRWGKQSAMFIRVTSHFYRMYGLNNQTGALLYDDLGFYLPRDFPEQQW

AVESKVCDLQLYDADHMFVMVDPTEEARAQLRQLQQSSTWLALKAVQEGHVYNAGDIF

FKALGPTGRMWAMRYVAEQLGVTLR"

CDS 3769683..3770102

/gene="slyA_4"

/locus_tag="EFAGFIKM_03231"

/inference="ab initio prediction:Prodigal:002006"

/inference="protein motif:HAMAP:MF_01819"

/codon_start=1

/transl_table=11

/product="Transcriptional regulator SlyA"

/translation="MTRIVSKEEQIINLLNVLGNKISPKFERCTGISSSRFEILHELD

QVDEINQSMLQKIINIDSAAITRHLKQLEADLMVTRRKNPDDNRVTFVRLTDHGRKQI

EGYKAEKTNFINQILQDFSEDEVQSLADFLERMHNNF"

CDS 3770130..3770780

/gene="yodC_2"

/locus_tag="EFAGFIKM_03232"

/EC_number="1.-.-.-"

/inference="ab initio prediction:Prodigal:002006"

/inference="similar to AA sequence:UniProtKB:P81102"

/codon_start=1

/transl_table=11

/product="Putative NAD(P)H nitroreductase YodC"

/db_xref="COG:COG0778"

/translation="MSTIQSKFQKTNDFNEITYGRRSVKLYDPEVKISREEMTEILSE

ASRAPSSINLQPWRFLVVDTAEGKEKLAPLARFNQNQVLTSSAVIGVFIDMNNVEYMD

EIFGKAVELGYMPQDIKDMQLKTVMPYYEKMAASDLRDVNLIDAGLASMQLMLAARAH

GYDTNPIGGYEKDQIAEAFGMDKERYQPVMLISIGKSAKEGHPSYRLPVETITTWA"

CDS 3770878..3771174

/gene="ycnE"

/locus_tag="EFAGFIKM_03233"

/EC_number="1.-.-.-"

/inference="ab initio prediction:Prodigal:002006"

/inference="similar to AA sequence:UniProtKB:P94425"

/codon_start=1

/transl_table=11

/product="Putative monooxygenase YcnE"

/db_xref="COG:COG1359"

/translation="MIIIHAHLQIKPDQEQAFLASAKELITATRQEEGNISYDLAKST

EREQQYTMIELWKDEAATASHNTSAHFQAFVQQAAAFMAAPMNVEVFAGEQVKA"

CDS complement(3771334..3772254)

/gene="qorA"

/locus_tag="EFAGFIKM_03234"

/EC_number="1.6.5.5"

/inference="ab initio prediction:Prodigal:002006"

/inference="similar to AA sequence:UniProtKB:P28304"

/codon_start=1

/transl_table=11

/product="Quinone oxidoreductase 1"

/db_xref="COG:COG0604"

/translation="MRAIICTKYGSPDVLQLKDIAKPSPKDHEIQIKIHATTVTSGDC

RVRGSNVPFLYWVPMRLFLGFRKPRNSVLGVELAGEVTAIGKEVKQFKLGDQVYAING

LSFGAHAQYICLSEEDPVALKPLNATYEEAAAIPFGGTTALYFLRKGNIKRGMKMLIY

GASGAVGTAAVQLAKYFGAEVTGVCSTTNVEFSKSLGADHVIDYMKKDFTKTSQRYDL

ILDAVGKVSKGQCRHLLAPNGTYVTVEGQGIARVTAKDLILLKELMEAGEMQAVIDRR

YALEQIQEAYRYVETGRKRGNVVITVKHEN"

CDS complement(3772348..3775038)

/gene="malT"

/locus_tag="EFAGFIKM_03235"

/inference="ab initio prediction:Prodigal:002006"

/inference="similar to AA sequence:UniProtKB:P06993"

/codon_start=1

/transl_table=11

/product="HTH-type transcriptional regulator MalT"

/db_xref="COG:COG2909"

/translation="MYAPILSTKLTIPVQRSHVVDRPRLYVRFGEGMHAKLILVSAPA
GSGKTLLVNQWVKSSSIDTAWLSLEEADNDPSRFLTYLCASLQTIVPKLAEGIMGFVQ
SSEPPPIETIITYLLNEVSEITNHFVLVLDDYHLMTSEVINQALVYLLRHMPPHMHIV
MTTRQQPKLPLARLRAKGELIEIRGSDLRFTSAESSEFLDRVMGLKLSEEHVALLEKR
TEGWVAGLQLAALSMQGSSDPNHFMETFSGRDPFVLDYLMEEILQGLTEQVQNFLLKT
CMLDRLCGSLCDAIFDIDEPKEHKSVDFTGQDMLEWLEQANLFIVPLDNERRWYRYHH
LFADLLRKRVHQNSNYAGTAETDLSVTEIHKRASIWYERHQMELEAFNHAVAAADTHR
ASDLLEGKGMPLLFRGGAVPALQWLSSLSTAVMDSIPSLWVMHAAALLVSGHMIDVEP
KLQSAEQVLQNTEPEKTNSDLIGHIASIRATLAVSRHDADTIITESLRALKYLHPHNL
PIRAATAWTLGYAYQLQGKRVEAELSYEESLANSTKIGHHIITMMATLGVGNMQESNN
QLHLAAESYQQVVHMAGKPPLPIACEAHLGLARVHYEWNNLDVTMLHAQQSEQLAQQF
VQTDRVVASELWLARVTLAKADLHQAAIMLDRINETAQRLNFTKVIPDITAAYVLVLL
RQGDLKKAIRLSWDRNHALSQTRVLLKQGNTLGALGMMESCLHLAITKGFKDEQLKAT
ILLAVIHEEHGNRTQAIELLTEAMMMAEPAGFIRVFVDEGVVVHGLLKRITVRGDSHL
YLLQLLGAFDEAEDNRIEMISEETDVYPYVSELRIDPLSVRELEVLHLIALGRSNREI
SEILHIALPTVKGHNRIIFDKMQVKRRTEAVAKAREWKLL"

CDS complement(3775224..3776573)

/gene="yvdP"

/locus_tag="EFAGFIKM_03236"

/EC_number="1.21.-.-"

/inference="ab initio prediction:Prodigal:002006"

/inference="similar to AA sequence:UniProtKB:O06997"

/codon_start=1

/transl_table=11

/product="putative FAD-linked oxidoreductase YvdP"

/db_xref="COG:COG0277"

/translation="MKSSTKLTGRVIYKGDPGYEAARKNWDPHTDKYPKVFVFAQKTK
DVSNAIRWARENNVPIRPRSGRHALEVNLSQVNGGIVIDVSDLNSIKLDKKHGTVVVG
TGNRVGRIANTLAKQGFMAPFGDSPTVGIGGITLGGGIGPLQRTIGLISDNLLEVEMV
DAKGKVIRANKNRNADLFWASRGGGGGNFGVYTRYKFKVRRAPAKATVFKITWPWAQF
EKVLKVWQRWAPSVDTRLGSELSIGPKKGGNVVMEGLFLGSKTEALRLLQPITSVGTP
TSSIVRLLPYTEVVKFLLQPDPTETQRYSNQFSSGFGQKPFPDKAIKSMRAFLENLEE

GPGGFYFLNWGGAVSRKSPRSTAFFWRKEKFYVEWTSTWLKPSHAAKNIALARNTRKK

LQPYIVGSYINVPDQGIKNSGPVYYGANYARLRRVKAKYDPKNVFNNPQSITPVRRA"

CDS complement(3776765..3777292)

/locus_tag="EFAGFIKM_03237"

/inference="ab initio prediction:Prodigal:002006"

/codon_start=1

/transl_table=11

/product="hypothetical protein"

/translation="MKLDWMGDHRELIEKIIKYGNAYSNTYKLQRSYGTDLMFSASQI

QTLEYILEAEDKEEKMSEMAERLGVSRSTFSKNVKNLTGKGLLEKFHLSGNRKDIYVK

PSEKGREVYVNYTEFVRELCFDEIFKYADQISEADKQNFVRIMDLFANVLVWYGEKEQ

EARKLIKIDSDSKSD"

CDS complement(3777340..3778149)

/locus_tag="EFAGFIKM_03238"

/EC_number="3.1.1.23"

/inference="ab initio prediction:Prodigal:002006"

/inference="similar to AA sequence:UniProtKB:A0QNZ7"

/codon_start=1

/transl_table=11

/product="Monoacylglycerol lipase"

/db_xref="COG:COG2267"

/translation="MTKVETRIQSFDGTQLYFSKDTVENAKAAVVIVHGLAEHAGRYD

YLTEKLNQRGFSVYRFDHRGHARSEGQRTFYKKFDHLIEDVHVIVEAALQESGDLPLF

VIGHSMGGFATSSFGTKYPGKVKGIVLSGALTRYNTKVAGELPMDLPTGTYFPNELGS

GVCCDPAVVSAYANDPLVEKQISVDLFNSLGNGITWLKENAKQFTDPVLVLHGANDGL

VSEKDSRDFYGDIASTDKTLKIYAHLMHEIFNEPTRDDVIEEAITWIEKRI"

CDS complement(3778425..3785957)

/locus_tag="EFAGFIKM_03239"

/inference="ab initio prediction:Prodigal:002006"

/codon_start=1

/transl_table=11

/product="hypothetical protein"

/translation="MGSLYDGVITERSITGYINGVSIGTTAKEKTTASFGTDLHAYIG
RSNYLGDATFAGSFQDLRIYGEALNNEDIAVVYEQSFNEKQVQQGKVELTLGDISAVT
ESLELPTEDKNGAMISWTSSDESAISSTGEVFLREMEQQVTLTATISLGGSQATKEFI
VTLLPKDDSVEQIAVKNVLLNEPTIILSVGDSETLVATISPSNATNKRVIWTSSDSSV
AIVNSTGKVTAVSEGTATITVQTEDGEFTATSTVTVKGKSLKDDLILHYNMKTTEEVD
GQLVLKDVANKAVTFDGIFRNPNNGQFISNSEVGFVSFNGGSSTSNSGHVEIPKGSNG
LDLLYGLNEITVSSIVNWTNDGTNRWIFGLGTVLTPETNKYFFVTPRHGSGSGNMIAT
GISKNGWPNEALVTGSANSNLIGGQWKHVTVSLSEASNRITLFVDGAKVASGNAKGLK
LSEIINPDATFSGFIGKSIFSNDPYLQGNVADFRVYDRALTEQEVGELYQQEGATHIS
KIRQLTVDDAANQLNISRYLGEGDKSTDKITRNVALPTSGKNGVSITWSSSHPTVISN
HGTVQRPAVQAGNVQAELTATLTYQGVSTTAVFPVTILKQFDDQQKVDLDAEQIEIYN
ADNIKGNLRLVATGEQGSTITWLSSRPAVVKGTAEAAGNATQLGWVSRQATDTPVTLI
ATITNGAASKQLTFNVTIKQTITPVQHDAYFFAYFTGEYEGGEEISFATAEDPLFWKG
LNNGKSILQSDMGEKGLRDPFVIRSPEGDKFYMLATDLKMGESTNFDQAQITGSHYMM
IWESEDLVHWSEQRMIEVAPKTGGNTWAPEAIYDPVTGEYIVFWASSMKNTETYGDYN
GRPAGQYNVMYYATTRDFYNFSEPKVMIDESLPTIDTTFIEHNDMLYRFTKSEVNTKV
YVEKAPTFYYDKDGIAANGLQYDAVPGTRDNKLGLIGNNGNNEGQTIFKDNNKDQWYL
FLDSWPYHVRYTTDLDSSMQYMNNVLSSDKYALPPGPRHGTVIPISRAEYDALQEKYA
WKGPAPSTDPVVHYSFDANTVNGTTVNDISGNGHHATLVGGATINEEDRIGKTGGALE
LNGSTGYVKLPDNLIQSLNLEKATFSTWVQMDRNQANQRIFDFASDTGRQVNRNTMYL
STQGDTGSLEFAVVTPFTEKFSNDSTRLGSEYKYTVRNSGLLPTKTWQHVAITMDEFD
AVLYVNGQEVKRSSTFNVEPRMLLETTMNYIGKSRNSSHSLFDGKFDDFRIYNRALSV
EEIATLADEDVTPPVEQPSGAELILHYDMNDIDGATVIDQTGNFNGKWMNPSKAEWIR
TQDAGVLSFTGGTTNSYVELPQGVLDGLTDMTVSSIVNWSGKNAAEWLYALGRPNNNT
HYTYFTPRYNANGLARLGIATNAWNNESSTSTTGLKNNEWKVVTTVVSGTDGTLTLYI
DGVAVASGSTNGMTLEQIKNTSGSSGTIGKSFYTADPYFGGLIADFQIYDGAMTAADI
TSLKAHADQKIALMEGMLVSAATEKLTIHDLLASNASKDAIISNVTLPTSGAYGTTIS
WTSDKGNVISTTGAVTRPANATGDQVVTLTAVFTDGTETVNKTFQLTVKALPNDVTSV
DEAKAALVVHNINDVRGHIALPTSGLYGTTITWASAQPNVVSVTGEVKRPVHGSGNVD
VKLTATIKLNSASTTKEFIAKVKELPADEKYAGYFFTYFTGEGTSTGEQVHFALSNGN
DPLNWRELNNGKPVLTSTLGEKGVRDPFIIRSPEGDKFYMIATDLKINGNGNWGRAHT
WGSRSIMVWESNDLVNWTDQRMVEVAPEEAGNTWAPEIMYDNTTGEYIVFWASRMFDD

VSHTGDAYQKMMYSKTRDFYTFTEPQVYLDYGYSIIDTTMIEHEGKVYRFTKDEQNNG

DSAPFGKMIFQEVGDSILDPTFKMINQGVGNIKWVEGPTIFKSNTDEKWYLFVDEFGG

RGYVPFETTNLASGAWTLSSNYKLPASPRHGTVIPITQSEYDALNGKNVPVSGISLDK

TMFSIVEGQSGQLTATVTPANATKKAVTWSSSNTAVATVSATGQVTAVAPGTARITVT

TVDGGLSSSATVTVTPSVDGTPQAPSSGNGNGGGDGNIPAPTVPTAPGGNEPVVMDNG

QIHIKPVVNESGASKVKLSADTVKRALDQTTGGKLHMQVEADASMNGLTIELAVDPRL

TSGASMVDRIEINTGSAVVTVATELLGTGTSAGKTLGLSIKKIAANQLPAGAQAQLNG

EVVYDIELTIDGKKLTEFDGRDDLVIELPYTLKANENPNNVVVYDVKDNGELRVVTNG

KYNAATGKVEFKPTYLSKYTVAYVATSFKDVNQDWAKDAISALGARGIVKGVGNGEFN

PNGQVTRAEFITMLMKLFELSDEYATTSFSDVKQGEWYHGNIATAQKLGIVNGKPNGR

FGVHENITREDMAVMVYKAIQIKQLALASGEATAFKDEANIASYAKQAVEAIQGAGII

NGVGNDEFAPKKNASRAEAAVMIYNLLGLM"

CDS 3786873..3790493

/locus_tag="EFAGFIKM_03240"

/inference="ab initio prediction:Prodigal:002006"

/codon_start=1

/transl_table=11

/product="hypothetical protein"

/translation="MPKRFSRMLAFLCCLVMVASLMPTSYAAATDNIENTVANETPTA

IFGTPELGSNDPLWGQTMEHHINKSTVPADPRPHATGTAKILWDHDYLYSRVVVNDSN

LYQGAGGDHQYDSLEFYVGTGTGGSNQWRVSATGVFSGQAAPGRAAWTQITETGYIVE

MRIPKRTLTLEEGKLTFEVYINNSTEKGGDRYEVVSSFGDPDAAYTSAASFTDSLQLS

LANEVDTRFSITATAGPGGRITPNPPGDVLRVDRDSNKEFTFTPDSGKMVDTVTVDGE

TVTLSAGTYTFSNIEADHTIHVTFKNDPDAGLLPFIVWNDNFASGEYTTAVIIDLGEG

KAALGSELNPGLFTLSARNTTLNGEAVTFEGTRKITRVYANDEPKVRGYLGPVSNSPD

YQDGLASGRYIVVESEFYSASGGKTTLDGSSNSTKQVYSIVPNGEIVLTEGSPLRNVV

FEQEKVVNPILDKFTTFTDNSVNRSLYLHKDEDGKVIKGLPLYVYTHGMSRGGTNAAT

DQKAAMKSANGSVALMKKMEKNPDKYASHVLNISYNGVSVPSTENVKKVIDALIASGE

VDPNRVYAAGFSWGGQYTNNLVNSHPGFFAAAAPMAPVSGSPNAKDNDAHNNLAYWMF

LNAHNVGSYQTNLNNFINNNMPKMINARASRFESNEVLTWPYNQFDQPNLRPNPANTP

VLADYIAHEVEAAVLYNEITMGNWSIAPTAQSSNLPAWNNDYTDVFDWMFAQRKPVVP

SAPSGFKATAGDGQVTLSWTAPADDGGSAILGYKVWYGNVTPVTLDATETKYTFKNLT

NGQGYNFTIIAMNAKGDGAEISATTTPMKTTTPTVPDSGGNTGNTGNTGNIGNTGNTD
NTGTGTDAGTLKSTYTVNTPKDKPAVTDKNGNTTLPGGGEIATKDGTKIKVPEGTTID
SNGKVTIPADKSAEVTLPGGNSIVTISGGSTIASDGTVTVGGKDAHVGLPNGNRVDIH
GGSKIRSGGAVVVGPSGAKVSLDNGMSLNIREGMELAFDGATPLGFLVISVNPFRDAN
MDDWFYSYINFAYTYDLFNGTTSTTFSPGTAMTRAMFVQVLANLENVNLSSYTSSRFN
DVTDGQWYTAAAEWAAEKGIVNGTNADLFDPNSPMTREQMLVILYNYMKYKGYEIPES
HSKSFTDESEISSWALEAVQALRDIGIVLGKPDNSFAPKATATRAEVATIFVRFVEYL
AK"

tRNA complement(3790729..3790803)
/locus_tag="EFAGFIKM_03241"
/product="tRNA-Ala"
/inference="COORDINATES:profile:Aragorn:001002"
/note="tRNA-Ala(ggc)"

CDS complement(3790988..3791455)
/gene="nrdR"
/locus_tag="EFAGFIKM_03242"
/inference="ab initio prediction:Prodigal:002006"
/inference="similar to AA sequence:UniProtKB:P67318"
/codon_start=1
/transl_table=11
/product="Transcriptional repressor NrdR"
/db_xref="COG:COG1327"
/translation="MKCPYCGYMGTKVLDSRPANEAKSIRRRRECEQCARRFTTFEMI
EETPLIVIKKDGSREEFSRDKILRGLIRACEKRPVSVETLEMMVSEVEKSLRNTADAE
VESRQIGELLMEQLFPVDEVAYVRFASVYRQFKDINMFMKELKSLLSKDNLED"

CDS 3791572..3791793
/locus_tag="EFAGFIKM_03243"
/inference="ab initio prediction:Prodigal:002006"
/inference="similar to AA sequence:UniProtKB:P22065"
/codon_start=1
/transl_table=11
/product="Small, acid-soluble spore protein alpha"

/translation="MAQSNGNSNNLVVTKASAALEQMKYEVAQELGISIPQDGYQGNM
TSYENGSIGGYITKRLVTIAEQQLAGQYQ"

CDS complement(3791876..3792439)

/gene="mltC_2"

/locus_tag="EFAGFIKM_03244"

/EC_number="4.2.2.-"

/inference="ab initio prediction:Prodigal:002006"

/inference="protein motif:HAMAP:MF_01616"

/codon_start=1

/transl_table=11

/product="Membrane-bound lytic murein transglycosylase C"

/translation="MRILRKKRVLLLMFVSFVLVLFLNTNWMAWFYPIHYKEEIRAQS
QSYEVDPFLIASIIKVETNFKTSKESKRGAIGLMQLMPDTANWILEQAKIPDTSLEEL
KHEPERNIQLGTWYLRNLSDQFDGNEAVMIAAYNAGPGKVNSWLRDGVWDGSFDTVKD
IPFGETRHYVQRVIYYYNQYVKIYNTF"

CDS complement(3792436..3793032)

/gene="coaE_2"

/locus_tag="EFAGFIKM_03245"

/EC_number="2.7.1.24"

/inference="ab initio prediction:Prodigal:002006"

/inference="similar to AA sequence:UniProtKB:P63831"

/codon_start=1

/transl_table=11

/product="Dephospho-CoA kinase"

/translation="MNIGLTGGIATGKSSVSAYLASKGALLIDADVIAREVMMPGHPV
LAAAVKRFGQAILNEDGTLDRKKLGSIVFQQPEERKALEAITHPAIRREMRERAAAYE
LQHPDKLVVSDIPLLYESGLEDGFEEVMVVYVPRSVQRDRLMSRDGMTAAQAEARMDV
QMDIERKKQLADIVIDNSGLWPETEQQIDTYLQRKGLL"

CDS complement(3793042..3793815)

/gene="ytaF"

/locus_tag="EFAGFIKM_03246"

/inference="ab initio prediction:Prodigal:002006"

/inference="similar to AA sequence:UniProtKB:C0SP79"

/codon_start=1

/transl_table=11

/product="putative sporulation protein YtaF"

/translation="MLHHFISLLALALALSLDGFGVGITYGLRRTKIPLLSIAVISIC

SGLVIALSMQVGVLLSHVVSPDIASIVGAVILIGIGAWSLLQLIRKQGKEQQETDTGT

GGVTEATVTGMGEARERLSDSKGRNQVLALDLEQSASSGSLERMVFTLELRKLGVVIQ

ILRSPSKADMDNSGSISAQEAMWLGIALSLDAFGAGLGAALLGFPTLWTALIIALFSG

AFLSLGMNVGLRFSALRWMRRLSVLPALLLMIMGIMKLL"

CDS complement(3793958..3794785)

/gene="mutM_2"

/locus_tag="EFAGFIKM_03247"

/EC_number="3.2.2.23"

/inference="ab initio prediction:Prodigal:002006"

/inference="similar to AA sequence:UniProtKB:P42371"

/codon_start=1

/transl_table=11

/product="Formamidopyrimidine-DNA glycosylase"

/db_xref="COG:COG0266"

/translation="MPELPEVETVRRTLNQLIVGKTIDHVTVSLPRIIQRPDDIDAFA

MELAGHTVIGVERRGKFLRILLDGLVLVSHLRMEGRYGVYEQHEDVEKHTHVIFHFND

GTELRYKDVRQFGTMHLFNAGEELVSKPLLKLGLEPLDPAFTVTAFREAVGKRTTKIK

AVLLNQAYVVGIGNIYVDEALFRAGIHPETIAKTLTEAQLTVLHEAIVATLQDAVNAG

GSSIKSYVNGQGEMGMFQHQLKIYGRKSEPCTTCGTLIEKTVVGGRGTHFCPNCQLL"

CDS complement(3794904..3797558)

/gene="polA"

/locus_tag="EFAGFIKM_03248"

/EC_number="2.7.7.7"

/inference="ab initio prediction:Prodigal:002006"

/inference="similar to AA sequence:UniProtKB:P52026"

/codon_start=1

/transl_table=11

/product="DNA polymerase I"

/translation="MDKFILIDGNSIIYRAFFAMPPLTNSKGLHTNAVYGFTTMLLRL

LEEHKPTHVMVAFDAGKITFRHEGYQEYKGGREKTPPELSEQFPLLKELLKGLGIAQF

ELAGFEADDIIGTLTKRADEAGRQVLVVSGDKDMLQLASEHVHIGLTRKGVTDIELYD

PAQIKERYGLTPLQIIDLKGLMGDASDNIPGIPGVGEKTALKLLHQFGSVEDVLNGTS

ELKGKMKEKIEAHAEDARMSKQLATIHREVPLEQTWEDMQFAGLKEEHAGPALAKLEF

KSLLERLSFSGSIGSEQEAVPAAEVESSIATEDNISELFSSLDSIDVLHVETHGDNPH

QAKLIGLAVGSAGTYTFISPELLHSEAAAPVRAWLGNSDQPKRGYDLHRVDLALHAHG

IEFAGAAFDVQLAAYLLDPTESNQTISGLTTKYGLPSLVEDDTVMGKGAKYKVPEMEI

LGDFLCRKAAAVAAIIPLQEQVLETDEMNSLFHELEMPLSRILADMEKQGIKANTADL

QALGSEFEEQISRLMAEIYKLSGTEFNLNSPKQLGEILFDRLGLPVVKKTKTGYSTDA

EVLEKLAPYNDVVKHILQYRQLAKLQSTYVEGLLKEISNRDGKVHTYYRQTIAATGRL

SSQFPNLQNIPIRMEEGRKIRKVFVPSEPGWSILAADYSQIELRVLAHISDDERLKEA

FVNDMDIHTKTASDVFGVNPEDVDGDMRRSAKAVNFGIVYGISDYGLSQNLHITRKEA

AQFIDQYFEVFQGVRRYMDDIVKEARQDGYVKTLLERRRYLPEINASNFNLRSFAERT

AMNTPIQGTAADIIKLAMVQMDEALRERKLKSRMLLQVHDELVFEVPADELELMKELV

PSVMEKALELSVPLKAEVSFGDNWYEAK"

CDS complement(3797792..3798451)

/gene="phoU"

/locus_tag="EFAGFIKM_03249"

/inference="ab initio prediction:Prodigal:002006"

/inference="similar to AA sequence:UniProtKB:Q97ID9"

/codon_start=1

/transl_table=11

/product="Phosphate-specific transport system accessory

protein PhoU"

/db_xref="COG:COG0704"

/translation="MIRRKEFDQELEELRTLLKQMGEHVGAALDGAIESLQTMNAEKA

QVIIKNDANLNALEDKIMELGSKLIITQQPVAKDLRRIIVAFKISSDLERMGDLALDV

AKVTLRMDGQKLIKPLVDIPQMAEIVKSMIDESIESFLKENTDLAYKMAQTDDQVDQL

YSHMISDLYTLMSEHPNQASQAMLLMMVGRYIERIGDHATNIGESTVYLVTGKRPDLN

Q"

CDS complement(3798515..3799270)

/gene="pstB3_1"

/locus_tag="EFAGFIKM_03250"

/EC_number="7.3.2.1"

/inference="ab initio prediction:Prodigal:002006"

/inference="similar to AA sequence:UniProtKB:P0A2V9"

/codon_start=1

/transl_table=11

/product="Phosphate import ATP-binding protein PstB 3"

/db_xref="COG:COG1117"

/translation="MKDLIHIENLNLYYDTHHALKNISMDLPEKTVTAFIGPSGCGKS

TLLRTLNRMNDMIPGVRVEGQVMLNGSDIYSNEIEVETLRKRVGMVFQQPNPFPKSIY

DNVAYGPRLHGVTQKAELDQLVEQSLVHAALWDEVKDVLKKSALSLSGGQQQRLCIAR

ALAVQPDVLLMDEATSALDPISTLKIEELVKELHHKYTIVMVTHNMHQAARVSGRTVF

FLNGEVVEAADTETLFSTPQDSRTEDYISGRFG"

CDS complement(3799501..3800682)

/locus_tag="EFAGFIKM_03251"

/inference="ab initio prediction:Prodigal:002006"

/codon_start=1

/transl_table=11

/product="hypothetical protein"

/translation="MPMLLEVKPDQPHVVLHNEELTTPIMEQTEVALVAEESVIHLHD

YYRQVPVAGVHTTCGEAATMMNQDQEHPCIVLCDEQMKPTGLLMRETLYRMLNGRFAA

DLFYRKPVIQVVNTSPVIADIHMEAATIIDIALGRNEQHFYDCLLVTDQGRLLGVLTM

RDVMSLSRRLQQAVTEEGIRTVTESRQEISKINNAVTKLVNAANQTVHEAREIMALSK

QGEESLKHVDASYNRVHQHMESQGQHADHMLDSIKTGSGMAHSIRSLADQSGLLALNA

SIEAAHAGEYGRGFQIVAGEIRALAKQTREVAGNMSSLLENIGGLTLQTVELVKASGA

EIDDSSVHVTAGGATFRQLNSAVRDLSRIAEEIAMEGGKAGEVAEHIRTKLDEMVTNS

E"

CDS complement(3800835..3801563)

/gene="phoP_3"

/locus_tag="EFAGFIKM_03252"

/inference="ab initio prediction:Prodigal:002006"

/inference="similar to AA sequence:UniProtKB:P13792"

/codon_start=1

/transl_table=11

/product="Alkaline phosphatase synthesis transcriptional

regulatory protein PhoP"

/db_xref="COG:COG0745"

/translation="MAQRLLVIEDEPTLSRLLTYNLTQEGYDVTAEDHGSAGYDRALS

QEFDLILLDLMLPGMNGLDILSKLRIQGVSTPVIILTAKNGEAEVVQGLKSGADDYIT

KPFGVSELLARVDAVLRRYSNGEDLPQLEDKDGSRIILGELEIYPLKYEVTLGGQSIS

LRPKEFEVLLYLAKKPGVVLTRDDLMNAVWGFDYIGGQRTVDVHVSSLRKKLELDPES

VHIDSIRGVGYKLVVKRKTPHHSS"

CDS complement(3801680..3803500)

/gene="phoR"

/locus_tag="EFAGFIKM_03253"

/EC_number="2.7.13.3"

/inference="ab initio prediction:Prodigal:002006"

/inference="similar to AA sequence:UniProtKB:P23545"

/codon_start=1

/transl_table=11

/product="Alkaline phosphatase synthesis sensor protein

PhoR"

/db_xref="COG:COG0642"

/translation="MRPFRIRLAIIMMVLIGVSVIVAGYTMGRVFKTTHITALEQTMV

REINLLKATFPFHDASDPTSEATRKYYSDRALELDRLTDSRVTFINKDGMVIGDSESD

PATMDNHLNREEIKGAVGDGYGQSIRYSETLGQDMLYVALSVNSDQSDMIEMPSGKFD

GYIRLSMSLHAVDQGLQRGWMIMFAALGLLFLIVAFVSYRVARGLTSPIEHITKVAHR

ITKLEYDARVDVTRRDEIGQLGLAINGMADSLQSQLKTIRDNEALLQSVLANMTGGIV

MIDAGQSIALVNREAERMLGIQAGKVTGKPYTELKRHYELTRTIEESVALKERMHEEV

SVFNPEEKLIRIDGVPMSEDDGGYRGMLFLLQDVTAIRRLESMRSEFVANVSHELKTP

VAAVKGFAETLLSGGVQDKETERSFLKIIYDEGDRLNRLIGDILELSKIESKRAPLQC

SPVHVHSFFEMVLGTLSKVAEKKQIRLEMHVPEELYIEADEDKMKQIFINLLSNGINY

TPDGGRVKLQVTMDNDDEVVFAVSDTGIGIPKKDLPRIFERFYRVDKGRSRNSGGTGL

GLSIVKHLVELHHGKLSVESELGMGTTFRVILPFIQEEEM"

CDS complement(3803532..3803891)

/locus_tag="EFAGFIKM_03254"

/inference="ab initio prediction:Prodigal:002006"

/codon_start=1

/transl_table=11

/product="hypothetical protein"

/translation="MKMSKQNATHYIWGAQCDGWHLVQNETLSIIHERMPAGTAEIRH

YHSVSRQFFFILSGEACMELNGETFVLETHEGIEIVPGMPHQMMNRSNEEVEFLVISN

PGTRGDRIELDSMLSES"

CDS complement(3804185..3805054)

/gene="ydaD_1"

/locus_tag="EFAGFIKM_03255"

/EC_number="1.-.-.-"

/inference="ab initio prediction:Prodigal:002006"

/inference="similar to AA sequence:UniProtKB:P80873"

/codon_start=1

/transl_table=11

/product="General stress protein 39"

/translation="MSTTPQNQKTMPAQHQDQRPGIESEMHPRPEFEKPEYKAAGKLT

GKVALITGGDSGIGRAVAVTYAKEGADVAIVYLSEHEDAKETKRQVEQEGRKCILIAG

DIGDDAFAKKSVQQTVDELGKLDIVVNNAAEQHPQQNLEDITPEQLERTFRTNIFGMF

YVTQAALPHLKKGSTIINTTSITAYRGSPTLLDYSSTKGAITSFTRSLSMNVIEKGIR

VNAVAPGPIWTPLIPSTFDEKKVSEFGATQPMKRPGQPDELAPAYVYLASDDSSYVSG

QVMHVNGGEVVNG"

CDS 3805444..3807003

/locus_tag="EFAGFIKM_03256"

/inference="ab initio prediction:Prodigal:002006"

/codon_start=1

/transl_table=11

/product="hypothetical protein"

/translation="MESHINTYITLVATSAVLNVFLCLYTYFRRSEIPSSKIFILYTA
ALSIYTFGYAIELASNTLGQMKFWTTVEYIGMPFSASLGLMLMIKYTGKTLSKKVTAS
LFVIPSITLGMVATNDFHHLFYKKVWLREDSPVPLMDIAVGQWYVVHGAFTFSCLLCA
CLILIGQWRHTKKMYRRQLLTLITSQIIPMVAAFLYLLGLTPGGMDPVPVLMCITSAM
YIWAILSSRLLTIVPIAKDSIFESMREGVIVLDSFNRLVDYNRSLRDMLPELNTTMIG
QPLDDIWLTLAGKTFPVEYGREGLQTDLYWQLNGETVCYQVRTSYVYNKDAQTVGSLI
MLIDITEQRFLQEQLKQLAYFDGLTKIYNRTQFLHRGREILSEAQLNPQPVSFILFDI
DYFKRINDTYGHDVGDQAIIHVVSVCNRYLSPEMLFARYGGEEFVIALSNTSLQEAEK
LAEQLRVALLNDPLDVKGVPITLTSSFGIAQYNGGSDSLESLLRDADTALYESKRNGR
NAVRAYSVSTT"

CDS complement(3807267..3807692)

/locus_tag="EFAGFIKM_03257"

/inference="ab initio prediction:Prodigal:002006"

/codon_start=1

/transl_table=11

/product="hypothetical protein"

/translation="MTMIMYLLHIVGALAMGFYLILPFVVGKIRTLNAAAQEGAFASL
RSLNKVAQYGLVIQLLTGGYLMTKGEYSHVWMAVVVVLLLAIAAIGGIMGKPLRLAAE
GVKNKRDVGPEQSKIRMFSTLLAVFLLIMVYLMVNNQVI"

CDS complement(3807828..3808769)

/gene="mdh"

/locus_tag="EFAGFIKM_03258"

/EC_number="1.1.1.37"

/inference="ab initio prediction:Prodigal:002006"

/inference="similar to AA sequence:UniProtKB:Q6HSF4"

/codon_start=1

/transl_table=11

/product="Malate dehydrogenase"

/db_xref="COG:COG0039"

/translation="MTIQRKKITVVGAGFTGATTALMLAQKELGDVVLVDIPQLENPT
KGKALDMMEASPVQGFDSHIVGTSNYEDTAGSEIVIITAGIARKPGMSRDDLVNTNAG
IVKSVCENVKKYCPDSIVIILSNPVDAMTYAAYQTLGFPKNRVIGQSGVLDTARYCTF

IAQELNVSVEDVRGFVLGGHGDDMVPLVRYSSVGGIPIDTLIPADRIESIVQRTRVGG

GEIVNLLGNGSAYYAPAASLVQMTEAILKDKKRIIPVIAYLEGEYGYNDLFLGVPTIL

GGNGIEKIFELDLTVEEKAGLDKSADSVRNVISVVNL"

CDS complement(3808785..3810077)

/gene="icd"

/locus_tag="EFAGFIKM_03259"

/EC_number="1.1.1.42"

/inference="ab initio prediction:Prodigal:002006"

/inference="similar to AA sequence:UniProtKB:P39126"

/codon_start=1

/transl_table=11

/product="Isocitrate dehydrogenase [NADP]"

/db_xref="COG:COG0538"

/translation="MKLEKFAHPTEGEKIQIDNGTLQVPSNPIIPFIEGDGTGRDIWK

ASKRVLDAAVEKAYDGNKKIAWYEVFAGQKAFDTYGEWLPNDTLEAIREYIVAIKGPL

TTPIGGGIRSLNVALRQELDLYTCLRPVRYFDGVPSPVKRPELVDMVIFRENTEDIYA

GIEYAEGSEEVKKVIQFLQQEMGANKIRFPETSGIGIKPVSSEGSKRLVRAAVQYAID

HNRKTVTLVHKGNIMKFTEGAFKNWGYEVAEEEFADKVFTWAQYDIIKENEGTDAANA

AQKAAEDAGKIIVKDAIADIALQQVLTRPGEFDVIATLNLNGDYLSDALAAQVGGIGI

APGANINYVTGHAIFEATHGTAPKYADKDVVNPGSVILSGVMMLEHLGWQEAANLIYK

GMETSINNKTVTYDFARLMDGATEVKCSEFADQIIKNL"

CDS complement(3810225..3811337)

/gene="citZ"

/locus_tag="EFAGFIKM_03260"

/EC_number="2.3.3.16"

/inference="ab initio prediction:Prodigal:002006"

/inference="similar to AA sequence:UniProtKB:P39120"

/codon_start=1

/transl_table=11

/product="Citrate synthase 2"

/db_xref="COG:COG0372"

/translation="MTATKGLEGIVATTSSISSIVDGVLTYRGYDIDDLAEHASFEEV

AYLLWFGKLPTTDELKSLRKSLSDYAPIPSELIAQIQLYPKNVSTMAALRSAVSALAL
YDEQADEMTTEANENKAVKLQAQLPTIVAAIARIRQGKEPVAPKEGASIAENFLYMMT
GEEPSETAVKALDQALVLHADHELNASTFAARVTVATLSDIYSGVTSAIGALKGPLHG
GANEAVMKMLNEIGTPDRLEAAIQEKLNNREKIMGFGHRVYKNGDPRAKHLQKMSKEL
GEMNNDTRLYDMSVKIEELVTGQKGLKPNVDFYSASVYTQLEIEQELFTPIFAISRVS
GWTAHILEQLADNRIIRPRAEYTGPTEQKYVSIELR"

CDS 3811868..3812986

/locus_tag="EFAGFIKM_03261"

/inference="ab initio prediction:Prodigal:002006"

/inference="similar to AA sequence:UniProtKB:A0A1W5X0D5"

/codon_start=1

/transl_table=11

/product="Sodium-lithium/proton antiporter"

/translation="MDRIIMKRLFRGLWVIIAAVLIAVAIYLLFPLLYPFAIAWIIAY
AMNPLVKLLQHRARFPRWLAVTLSLILYFGAIAVVLSAAITRMVKEVISLTTSFDLHV
DEIKDTFVRWTQNDTIQSLTAQINEFYKENPNYQETINSNISKTTETVGTAVTDLVTG
FFNMILSLLTSLPNMGAVLIVVLLSTFFISKSWTRHNITVSGWVPSSIRKPISDIWTD
LKKALFGYARAQLIMISITALFVMIGLLILQVNSAFTIALMIGLVDLLPYLGVGLVMV
PWAAYLFMNGDLYLGIGISIIYLILLIARQIIEPKVLASSVGLDPLATLVGMFVGLKL
FGVLGLIIGPVSLVILDAFNRANVLRDLRTYIINGRVR"

CDS complement(3813089..3813487)

/locus_tag="EFAGFIKM_03262"

/inference="ab initio prediction:Prodigal:002006"

/codon_start=1

/transl_table=11

/product="hypothetical protein"

/translation="MRKWMWALLLIIPVIELFGFILMSDWIGAGKTLLLMILTSLIGI
AMLQFEGRKVLVDAKSEMERGKVPGRKMVDGLFIFVGGFLLLIPGFVTDLIGFTLVFP
LTRPVYRLFFLGWLEKKMKSGKITFYRRPK"

CDS complement(3813495..3814031)

/locus_tag="EFAGFIKM_03263"

/inference="ab initio prediction:Prodigal:002006"

/codon_start=1

/transl_table=11

/product="hypothetical protein"

/translation="MNDRAKEADNVREQSNGNWYAARLRVRYQESDQMGVVYHANYLN

WFEIGRTEMIRQMGYTYRKMEEQGLLLPVTGLDVKYHKPARYDDEIIIFTRIAAFSGL

RLNYEYDVRRMSEEPDEHMAIGERVWSTDEALPGERLVTGSTQHVWVNGDWKPVRLDK

AASELYSALEKVWLSGKG"

CDS complement(3814133..3815560)

/gene="pyk_1"

/locus_tag="EFAGFIKM_03264"

/EC_number="2.7.1.40"

/inference="ab initio prediction:Prodigal:002006"

/inference="similar to AA sequence:UniProtKB:P80885"

/codon_start=1

/transl_table=11

/product="Pyruvate kinase"

/db_xref="COG:COG0469"

/translation="MRKTKIVCTIGPSSESLENTKKLIMAGMNVARLNFSHGDFDEHG

GRIIAIRQACEELNKTVAILLDTKGPEIRTGKLEVEPIELVQDEYITLTTEEILGTKE

RLSITYTDLPNDVEPGSTILIDDGLIGLTVVEVQGTEIKCRIVNGGSIKSKKGVNVPG

VAISLPGITEKDANDIVFGIEQGVDFIAASFVRKASDVLEIRELLEKHNAGHIQIISK

IENQQGVDNLDEILEVSDGLMVARGDLGVEIPAEEVPLVQKRMIQKCNVAGKPVITAT

QMLDSMQRNPRPTRAEASDVANAIFDGTDAIMLSGETAAGKYPVESVLTMSRIAEKAE

SALPYQELYLKQRVAQQTTVTEAISQSVALQAQDLNAKAIITSTESGHTARMISKYRP

ESPIIAVTTEDRTSRRLALAWGVTPVKGRLVDSTDALFENAIEGGVKSGLVKEGDLVV

ITAGVPLGRSGSTNLIKVSQIPNNA"

CDS complement(3815666..3816697)

/gene="accA"

/locus_tag="EFAGFIKM_03265"

/EC_number="2.1.3.15"

/inference="ab initio prediction:Prodigal:002006"

/inference="similar to AA sequence:UniProtKB:Q6GG07"

/codon_start=1

/transl_table=11

/product="Acetyl-coenzyme A carboxylase carboxyl

transferase subunit alpha"

/translation="MAGELPYEAPLVEMRKKIDELVQFGQEKGIDFTDEIARLEERYH

RLEEEIYTGITAAQKMHLARHQQRPTALDLIQLIFTDFIELHGDRMFGDDLAVVGGLA

KLNGKTVTVIGQQRGKDTKDNIARFFGSAHPEGFRKGLRLMKQANKFGRPIITFIDTK

GAYPGNTAEERGQSEAIARNLMEMAKLSVPVIVVVIGEGGSGGALAMAVGNRVLMLEH

AIYSAISPNGAASILWKDASKADQAAEAMKITAKDLLEMEVIEEIVPEPRGGAHRDYE

ASAAFISEALVRHLDDMKGWSGDQLKQDRYEKFRKIGSVTFEPQASNEAPQELVKVDV

ASNLSGNAE"

CDS complement(3816698..3817591)

/gene="accD"

/locus_tag="EFAGFIKM_03266"

/EC_number="2.1.3.15"

/inference="ab initio prediction:Prodigal:002006"

/inference="similar to AA sequence:UniProtKB:C0SP93"

/codon_start=1

/transl_table=11

/product="Acetyl-coenzyme A carboxylase carboxyl

transferase subunit beta"

/db_xref="COG:COG0777"

/translation="MFKDIFQKKRKYATIPSERALPGEGQEVLERPKREIPEGLMNKC

SKCGTIQYSKELEKNLKVCPACGYHMRLNAMERIAMVLDDQGFVEFDADMISVDPLGF

PGYSNKLEQQRLKSGLKEAVITGEGTIDGLPVVVAVMSFDFFTGSMGSVVGEKITRAI

EHATEKRLPLIIFSTSGGARMQESILSLMQMAKTSAALSRLDEQGGLYISVITDPTTG

GVSASFASLGDINIAEPGAVFGFAGRIVIEQTIRQKLPDDFQTAEFNMQHGQLDMVVH

RKELRATLGKLLDMHSEKGGV"

CDS complement(3817655..3817909)

/locus_tag="EFAGFIKM_03267"

/inference="ab initio prediction:Prodigal:002006"

/codon_start=1

/transl_table=11

/product="hypothetical protein"

/translation="MHVLYAAVLPSFFVRTRTDLVAPGDPTKIDSNIGATIFRFLQVT

SIVLTEAGDPCPVPADFFVVNAGQGPWQRVGRKRFTVTRG"

CDS complement(3817997..3818185)

/locus_tag="EFAGFIKM_03268"

/inference="ab initio prediction:Prodigal:002006"

/codon_start=1

/transl_table=11

/product="hypothetical protein"

/translation="MWTVIYIAPTAKVADKIKTKLSEEGFLVQTRPISLSKQQFEIRV

PSGELEEVQEVLNSILHS"

CDS complement(3818470..3818961)

/gene="ypjQ_2"

/locus_tag="EFAGFIKM_03269"

/inference="ab initio prediction:Prodigal:002006"

/inference="similar to AA sequence:UniProtKB:P54173"

/codon_start=1

/transl_table=11

/product="putative protein YpjQ"

/db_xref="COG:COG1267"

/translation="MSYEIVEAMLARRGVRIDAIAAIVYRLQKGYHPELTLDDCVTSV

KSVLQKREVQYTLYTGIALDELAEKKLLPQPLQAIMEADESLYGVDETLALGITHVYG

MIGLTSFGYLDKEKIGVIHDLNEHTTAIHVFLDDLVAGVAAAASARIAHKNIKAKKYP

SDL"

CDS complement(3819168..3823277)

/gene="dnaE2"

/locus_tag="EFAGFIKM_03270"

/EC_number="2.7.7.7"

/inference="ab initio prediction:Prodigal:002006"

/inference="protein motif:HAMAP:MF_01902"

/codon_start=1

/transl_table=11

/product="Error-prone DNA polymerase"

/translation="MSSFVHLHVHSEYSLLDGAARIPDLVDKAADLGMTTLALTDHGV

MYGAIPFYKACVERGIKPIIGCEAYMTAGSRKERGSRKDQPIHHLILLAKNMTGYRNL

MKLCSIGHLEGFHYKPRVDMESLAAHHEGIICLSACLGGEVPQHLLHGREEEARRAAL

RYKNIFGDDFYLELQDHGLSEQKRVNPQLIKLAAELEIPLVATNDAHYLSEEDAELQD

VLICIGTGKTVDDENRLRIGTNQLYLKSEEEMARLFPHVPEALANTVRIADSCELKLE

FGKSILPEYRPLPDGLSPSVYLRQLCEEGMEERYAQSTRWTDAELRSELEQRLAYELG

IIDSMGFSDYFLIVWDFIAYAHKQGIVTGPGRGSSAGSLVAYTLHITDVDPMKYNLLF

ERFLNPERISMPDIDIDFSDERRDEVIDYVAHKYGKAHVAQIITFGTMAARAAVRDVG

RALNVPYGEVDKAAKLIPAQLGINIERAMEATPELKALYETKPKTRELLDMAMKVEGM

PRHASTHAAGVVISRDPLTDAVPLQEGSEGTALTQYSMENLESIGLLKMDFLGLRTLS

IIERCVRWIGEHGEIPDFRLIPDDDPLTYEMLGRGDTMGIFQLESAGVRRVLKEMKPS

SFEDVISVLALYRPGPMEFISKYIQGKHGQIEVDYPHVDLEPILKDTYGIIVYQEQIM

QIASRMAGFSLGEADLLRRAVSKKKREVLDLERGHFVQGSLKQGYSEEEADLVYDMIV

KFANYGFPRAHAAAYGVLAFQTAYLKAHYPVHFMASMLTAVMGSHRKVAEYVVECRRM

DMEVLPPDVNESGILFTPVIVPSGSAGAGDQSSASQQTAGADAESDAGSPAEEHAANG

NGSSFREDRVHEHDGHSGQAEYGEAPLPDDPGPGPEEDGGGYEWQAATTMPEASDNRK

EPGLVSASSLENGNDDGQEASSSREEHGELSSAEKNVAVSNDSPDEEKLTGAIRFGLA

AVKNVGTQAMESIMIVRQERPFDSLLDFCRRVDLRVCNKRVIESLIQAGAFDTLPGHR

AQLLAMLDETVEAALKWRKEREDLQIQLFDFVETPNWEIEYPEIPPYSSGQQLELERE

LLGLYLSGHPLDDYEDVLESSGADRIMELTEAADDTMTVAAGMVVSVKSITTKQGKAM

AFMELEDQIERCEVVLFPEVWRRSQQHVGKGELLVVRAKVQQQDEGFKLLAEEVVPLS

PAALEQQLRSRDRRVKPGSGSSSRPAQAASPRRPAGAGAEAQRSSAGGDASAVRSSDA

AGTGAATAAASRPGGAAEAAGSPEQSREPRAVEQRVFVKIAPHAEKPELLARLKQLLQ

EHPGPVATVLFYEQQQKLLALSDAYRIKPSPTLFSEMEKMLGEGTVKIK"

CDS complement(3823440..3824024)

/gene="ydjA_3"

/locus_tag="EFAGFIKM_03271"

/EC_number="1.-.-.-"

/inference="ab initio prediction:Prodigal:002006"

/inference="similar to AA sequence:UniProtKB:P0ACY1"

/codon_start=1

/transl_table=11

/product="Putative NAD(P)H nitroreductase YdjA"

/db_xref="COG:COG0778"

/translation="MKNTQSNSVERVIRERRTVRSFSNRPVSEDKLLELLEAAVWAPY

HSKQEPWRFIVFMNEGRKMFSNAVLSTYTPQELEQFGQSAAKDYCEDTPVHVCVVVRQ

GTDRWKNEEALLATAGLIQNLQLLAWEERIGLVWKTNEYNREDTFAARIGLNSGERLV

GTLHLGYLRDDAVSRPVSRKPAEQIITWIRHEEG"

CDS complement(3824026..3824943)

/gene="yfmC_1"

/locus_tag="EFAGFIKM_03272"

/inference="ab initio prediction:Prodigal:002006"

/inference="similar to AA sequence:UniProtKB:O34348"

/codon_start=1

/transl_table=11

/product="Fe(3+)-citrate-binding protein YfmC"

/db_xref="COG:COG4594"

/translation="MKHSNKWFGLGMILTLLLATACAGTNGEKAEPNAETEPQTQAQR

VVATSVTYPDFLYVLGVTPVAAENYHSDYPDYLEGKFDSVPKLGNGSNMEGILATDPD

IIIAPKWRDEKLYDQYAKIAKTVLLPDRDNWRDELKDMGEALGKQKEAEQAIQDYDKK

IATARDELKALVGDETFIYLRIMPKESYVMGELSNRGMVIHKELGLKTVAAYPKNEGS

VPVSLELLTEYQADHIILQIDGGDDNTNAQKLYDEMKESNIWKGMKAVKEDQVYLVGD

KEWMNFGYSSVATLNAVEDIVSVIRERNH"

CDS complement(3825063..3826733)

/gene="rhaS_29"

/locus_tag="EFAGFIKM_03273"

/inference="ab initio prediction:Prodigal:002006"

/inference="protein motif:HAMAP:MF_01534"

/codon_start=1

/transl_table=11

/product="HTH-type transcriptional activator RhaS"

/translation="MNLETKYGLLFRDWTMVLKDAVQVSLLAGEKYNFGTGVAVRSAA

VSGVELVLGVSGEGEVELEEGIHSIRPHDLLLIEKGQSTFLVNRCSTPLVCYIIQCHR
VRLTQELAPTEDPWDKQNDGDDHKLEQSNFVALQHAAVEDVKKIVEAFQHTDLHDPMW
CHLLFQQWICTVVEHIRREAEPEVHSQQPDQAVQRTIDYLDEHYDDPVTVQQLAQMAN
VSRKWYTVRFREITNQNPSDYITELRMKRAKELLNLTGDSVYEIARKVGYEDEHYFSR
RFKQKVGRSPRLYLHNRRYLGTTMTAPELLYTLGMTPIAAQAPFCEFPHYLKESFKQV
RKWERSHALNLEEIRRMKPDLIIASAWQDSMDYEQLNRIATTVLLPERSNWREELRDL
GELLGKSKQALQVISNFDVGLDQAREQLHTLIPDETIVYIRLTREGITLYGEQSSRGS
MLYRELDLNMPKASLFSGEGTLVSVEHLAEIQPDHILLQMEDGVEARLHLYDHPMWNR
LLAVQNHQVYVLSNTEWYNFSFSPLATQYAIHEMLDLLKRKRLIRRMT"

CDS 3826905..3827234

/gene="ytrH"
/locus_tag="EFAGFIKM_03274"
/inference="ab initio prediction:Prodigal:002006"
/inference="similar to AA sequence:UniProtKB:C0H3P8"
/codon_start=1
/transl_table=11
/product="Sporulation membrane protein YtrH"
/translation="MSTFLSKAILDFFIAFGIVLGGAMIGGIGAVISLQPPTQTMLDI
SGKIKIWALAAAVGGTIDPMRVIESNFLDGNLSPAVKQILYLISAFMGAHMGTELVKW
VCGGGRN"

CDS 3827256..3827744

/locus_tag="EFAGFIKM_03275"
/inference="ab initio prediction:Prodigal:002006"
/codon_start=1
/transl_table=11
/product="hypothetical protein"
/translation="MRVPPFSRYRPLMRMTAVFVLGMIAGCVVYNGVFHLSYNALWLN
NQELQLQLHQNEEDIKTLKKYSKRQTVIKEIKVRAEESDKGPDEASLKVMLQKLGDEL
EVLRGRDVFNIDEYSKMTRIMLNQKVYSVREKEYTVQVKTMLVMEGVLQVWVQIRMHV
PA"

CDS 3827827..3828168

/locus_tag="EFAGFIKM_03276"

/inference="ab initio prediction:Prodigal:002006"

/codon_start=1

/transl_table=11

/product="hypothetical protein"

/translation="MFIDVLKYALIAIFAVAMVFAALNSIRSHRSSDATSAGLHRSWT

NIWMGGMLVVLALILMFVFTGSTLSVIVEALFLIMGSYNVFAGIRNRSYYARLQQRSS

NGSGKTSGQSA"

CDS complement(3828312..3829646)

/locus_tag="EFAGFIKM_03277"

/inference="ab initio prediction:Prodigal:002006"

/codon_start=1

/transl_table=11

/product="hypothetical protein"

/translation="MDGQEENVTKHEQLLQHIEQLKVGSKISVRGLARELGVSEGTAY

RAVKEAENFGLVVTKERIGTVRIEKRPRGMSEQLTFADVVTIVEGHVLGGNEGLAKPL

HKYVIGAMKEQAMARYIDAGSLLIVGNREDAHSLALEQGAGVLITGGFGTSREVRIMA

DELGLPIISSRHDTFTVASMINRAIFDRLIKKKIMLVEDIIGQKPRLQVLKVTSSAAD

FHMLVSETGEHRFPVVDEWNRVIGIVSLKDVSELKEDQSIEKCVIRRPITASLQTSLA

SAAQIMTWEGIDFLPIVDRNRKLIASVTRKEVLQAMRDAQKQPQLGETFDHLIWNGFA

EERGEQNELLFHGFIIPQMATDLGTISEGVLLNVMTQAGRRAAWDVTGNDHVVDNVTT

YFVRPVQIEDQILVRPLILETSRRTCKMDIVVTREGSVVCKAVMTLQSIDHA"

CDS complement(3829858..3830145)

/locus_tag="EFAGFIKM_03278"

/inference="ab initio prediction:Prodigal:002006"

/codon_start=1

/transl_table=11

/product="hypothetical protein"

/translation="MVKRLKKAKHVIYLLIALSMLVIALPRISFAGGMDWVNIFGIVW

VLFSLLIVGAHLHFILGVDEEKKRALEAVRRAKLRQWEMKLLDKQERSESV"

CDS complement(3830339..3830686)

/locus_tag="EFAGFIKM_03279"

/inference="ab initio prediction:Prodigal:002006"

/inference="similar to AA sequence:UniProtKB:O07542"
/note="UPF0342 protein YheA"
/codon_start=1
/transl_table=11
/product="hypothetical protein"
/translation="MNIYDKANDLAKALRESSEVEEITSAMKLIEADPDAKAMLDNFR
DQQMELQQRMMSGDMPAPDEMEKMEKLFEVLSLNLNIRRLFDAERRLSVIIEDVNKII
ADSLGHLYGGAEA"

CDS 3830924..3832291
/gene="yheD_4"
/locus_tag="EFAGFIKM_03280"
/inference="ab initio prediction:Prodigal:002006"
/inference="similar to AA sequence:UniProtKB:O07545"
/codon_start=1
/transl_table=11
/product="Endospore coat-associated protein YheD"
/translation="MSTKKVTIQVTGSGILQDDVIMLGEGVLKALKIPSGRPLQLQFG
SFRREVTVIPVPRYDGLRINQTVASKTGLVPRSVLRVSYRSASRTLRLGPYISVLVSQ
DYPDQPDRPFGSITMFCQELVNACRKQGAYVSFFTPEDIGAVTGYMKGWVYDDGWKKT
VLPVADVVNNRLTSRKLENKPSVQHFMKEVKSLYGTQTFNEKFLDKNEVFDALKSIST
LKRVLPESHLLKTSAMLKTMCNRHPVVFLKPVRGSLGKGIIRVSRQIDGSFLTLATGV
GGTRKQTYTSLDKLYASMSGKMKTTRYQIQQGLTLIDNSGRPVDFRALVQKNRTGKWS
VTSIVARIAGGSHYVSNLARGGSLSTVKDAVAKTQLSGSAKASAYAGLHTAALDIAKG
IESAIPAHFGELGIDLALDTTGRVWLLEVNSKPSKNDNTPLSESKIRPSVKAMLEYST
YLAGF"

CDS 3832342..3833532
/locus_tag="EFAGFIKM_03281"
/inference="ab initio prediction:Prodigal:002006"
/codon_start=1
/transl_table=11
/product="hypothetical protein"
/translation="MFPPSQTKTMAILVRATEGSPPFTDELFCRRLSLGSMPYALQII

VIPISTDAVHLPLQWGYAYHKGKWNSVPVPAVDLIMDRCLRPISRYVRQQLKEWIPTN

GTDQHRYWSASLPGKWEVHRVLSRNPELRSQLAPTTRIGSHIPWETWLDRWPRGLFFK

PVSGTHGKNTFRLSRGTTPSTWIVEGRNEDNEPFFLTFNHTQAVSSWLTLHQAERKMI

VQPYLELSHHGRAFDIRALMQKNGQGRWTLTGCMVREGPEGSLTSNLHGGGKAYPAHA

YLLQRYGTIRTETLLKSIRQTATLIPTMLESRFGRLAELGLDFGADAEGQLWLIEVNS

KPGRTSFAEAGDRRMHTLTYTRPLAYARYLLQQHVLTDVIRPMKLPNTSSKAGLKPIQ

IHGG"

CDS 3833593..3834966

/gene="yheD_5"

/locus_tag="EFAGFIKM_03282"

/inference="ab initio prediction:Prodigal:002006"

/inference="similar to AA sequence:UniProtKB:O07545"

/codon_start=1

/transl_table=11

/product="Endospore coat-associated protein YheD"

/translation="MSLTFCNLHFTKQPDKVVYISNALMKSLNLSGKKTVHLRFGRDR

VPATIKPIKKAGKHLYLASGIRNMMNVPKRGSIYLRNLQNDEVQLGPLIGVLSDGPAT

STNPFGSRTGFIKQLLREGSRKSYIYAFTPRDINWQNETVSGFFLNDNGSFTRRTVPL

PDVVYNRLPSRRSDFSPAINQLRERFVRRKIPFFNWSFFNKSDIYTLLENEPAAGRYI

PESITNPSVEQMKEMLERHQFVYYKPTAGSLGNGIYRLTYSPKRGYFARYRKKGGNAL

LRFGSFNSLMRMLQGRHGKQLRGYVVQQGIRLIEIDECPIDFRFHMHKNGNNQWVVVG

IGAKKAGRGSVTTHIKNGGSLMTPEQALSRNFGDRAGEVLQHAKSVAITLAQAIETQH

QHLIGEIGFDLGIDQEEHVWMFEANAKPGRSIFRHPSLRVEGKSSVEHILEHCLYLSK

FRKKEEI"

CDS 3834966..3836072

/gene="yheD_6"

/locus_tag="EFAGFIKM_03283"

/inference="ab initio prediction:Prodigal:002006"

/inference="similar to AA sequence:UniProtKB:O07545"

/codon_start=1

/transl_table=11

/product="Endospore coat-associated protein YheD"

/translation="MTLHDDFKPVIAVLTMHDKQRMFRGNHQNFQDILQTGESMGYVV

YIVTVRDLNVSGPTVKGYTYNKGSGKWVSQPFPLPHVLYNRIPNREDEGKPSVQRKID

ECMQSGIELYNPFFFNKWDLFEWLRKSKSTQQLIPYTRRMRSASSLGAVLRAYPYLYL

KPESGKAGKGIMMLKFQEKERLPYRLKIQTTRRSTTYKASTLAKLWARIRKETGHTPY

IMQQGIELASSRKRPFDLRVLVQKNTKGQWSVTGVGARLAGSRSITTHVPRGGSVEDP

EKLLTELFGEEMSTNLMKRVKSTSLMIARQVERGSGYTLGEMSMDLGIDDLGELWFFE

ANAKPMKFDEPQIRRRSLERIFHYSAYLARQSKR"

CDS 3836108..3837286

/gene="yheD_7"

/locus_tag="EFAGFIKM_03284"

/inference="ab initio prediction:Prodigal:002006"

/inference="similar to AA sequence:UniProtKB:O07545"

/codon_start=1

/transl_table=11

/product="Endospore coat-associated protein YheD"

/translation="MSSPVLGIMTLYLNEHRALEERSVYRRMILEGRKRGLDIYVFTP

ADVHPGGKQIEAMVFHEGKGWSREWRSFPDLIFDRCRIQRNRRFQQLLAFREKYGHLL

FLNRPLRNKWTIHQTLLQKANFREHLPETALFQDMSDVNRMLKASSLVYLKPINGTGG

RGILRIERSSSEANTVLVQGRDQKRRIITPRKVHLSRLGALLQHWNMKDKYLVQKGIQ

LQLPNGRVHDYRMLVQKNGEGQWELTGCAGRMGAEKSVTSNLHGGGQAIAMDRLMKQW

IEDDDLRAKINTTAEKFGIDVASFLEDTYGDLCELALDLAIDRSGRIYLLEVNPKPAR

EVFARIGERDIYYKAITQPLEYALWVYRNRPPGTARKPAIPRPVTQKSPRVKRKRKLK

"

CDS complement(3837353..3839275)

/gene="hepC"

/locus_tag="EFAGFIKM_03285"

/EC_number="4.2.2.8"

/inference="ab initio prediction:Prodigal:002006"

/inference="similar to AA sequence:UniProtKB:C7EXL6"

/codon_start=1

/transl_table=11

/product="Heparin-sulfate lyase"

/translation="MSDFYLSATDLQRAAQYYEKHFPEEARNSLTIANRAILNEFIVP

YTNDLNRWIPMGTPVDWLHNPTNDLEFTWGINRHWHMLDLGKAYLMKGNPLYVTAFID

HYRSWRQQNPVPVNLVYDEAVFFQKPGPWRLLETGLRVQSWISAYKYMEASSLIDEGF

QAEFQQGLEEHAEFLTRYLGSTEINHAIMHMQGLFMIATFYHQHPRSPHWRQVAMERL

ELCLLHQLGEEGIQVELTTHYHNASIEMFGTPYRLGEISGHPFSTWYASCLRQMAAFT

EALIRPDHQSTGIGDSDWVGDGRQRLTLLGAILDDDDLMARGTDSSECLWLLGVEKYE

RCLRLQASSSPALSSRAFPQTGYYVMRDKYQYLFFDAAAMGGAHGHADALNVEWMWKN

QLFFTDTGRYTYEEGEWRRYFKSTRAHNTVTVDGLNQTPYLSTQQWGEPEAQATTLRW

ESNDSYHFIDASQDGYTHLPDPVTHRRWMLAGVEIPILLIVDWLEAETEHTLEQRFHL

HPEADIALRMNEAGELTADMSYEASSITLTMQWVTAGIGKGAFELTEQPGWVSEVYGS

KSETSVIQGKTDFSGKVGILTLCLPVDHIDQVVSVTTCQLEPDLMRLALSYVQGEEKG

YIVIDNDTLQWTKEQK"

CDS complement(3839272..3839607)

/locus_tag="EFAGFIKM_03286"

/inference="ab initio prediction:Prodigal:002006"

/codon_start=1

/transl_table=11

/product="hypothetical protein"

/translation="MIINHASTDAYIINETSSRKILGMGGTLMMVEVTFAKGGIGVVH

SHDAHEQVSYIVKGSFEVQVGEETRILKAGDSFYAGFNVLHGVKALEDSVILDVFTPF

RQDFLEASQ"

CDS complement(3839676..3841073)

/locus_tag="EFAGFIKM_03287"

/inference="ab initio prediction:Prodigal:002006"

/codon_start=1

/transl_table=11

/product="hypothetical protein"

/translation="MVKKWMVTTLSALLALSLAGCSADSGTSANNESGTSGENAGGKT

KIIYWTPDRHDADFMKSKIDEFNQTNKDNIEVEMTVMGDNYPQAVDIAFASKQAPDVL

QVNDFQTYYQKGYLTPIDGYMSDEMKTVFKDSLIENKNTIDGKIYSLPNTGQVWRLVY

NKDIFKKAGIESPPKTLAEMVADAKKITEVGKSEGIYGFASPFKSSSGFWRAANTIAG

ASSNVGIDGYNYKTGQFDFGMYKDIAMALRQMNQDGSMLPGVESLDIDPLRAQFAQGK

IGMYINHSGEPGVYKDQFPTKENWAAAPVPTNDGTIKGASQVIGGSYIGINADSANKE

AAWKFMEYVYSTDLQSEYYEKGYGISLIPAVLSSDKKPSIPGIEGFLPKRYDAIFPAN

PSVVTESSLEGTKWGEAFSIFVFTGGDLDAVIKDLDTRYNAALEKTKAAGLTNITADP

SFDSSKLQGKLSTED"

CDS complement(3841207..3842811)

/gene="rhaR_31"

/locus_tag="EFAGFIKM_03288"

/inference="ab initio prediction:Prodigal:002006"

/inference="protein motif:HAMAP:MF_01533"

/codon_start=1

/transl_table=11

/product="HTH-type transcriptional activator RhaR"

/translation="MGQLTMCVMDDIQAVVKGISSTIPWEDHQVRIVGTASNGESGWS

LLLEQQPDIVISDIRMPKLSGLDLMKRALDAGLRAKFIFISGYSDFQYAQEAIKLGAS

DYLLKPFTPEEILGAVLKVRQVIKEEQAQCKQLEQLEQKVESSSQYERHSYLLGLLRH

EIGGFLNESRWNELHIDLDSSNLLVMVIEVDGYTRYGYTMHLDAEIVPFAVQNIVGET

LNRYTKGVVLRENRYRLAAIFNTPSQIDVSELLEQCRQNVERFSKKTVSIGVGTIAHR

PQEIWTSYAHADQAISYRFYSEGNCILKYAELENQACVAPVYPLEKEKELFYALKCGN

ESTCHRIIDEILEEWQVSEGFPEPLTMIRLLSGLAFAIYRAFCDEITAEERLRLEADL

TVLEAMRSLTFEGWRGYIKHFSSMGCEIMDKKRLTDVKQAISKAQEYISLNLAENLTL

HACAQSVHLSPSYFANAFKKETGITPIQYITKMRMEKAKELLVAGVQVQEICQRLGYE

DRPYFSGLFKKYCGMTPTCFRQMYEN"

CDS complement(3842780..3844573)

/locus_tag="EFAGFIKM_03289"

/inference="ab initio prediction:Prodigal:002006"

/codon_start=1

/transl_table=11

/product="hypothetical protein"

/translation="MIFNFYKMSLKKRIQLLYIFLVVLCISVTGICSYLFAARSIEQN

ALELKQSILNKSVQVMDEHLRHIVVSSFSFMLNETFTQVMKDVGKNNTSHYYQNLSLL

QTSFAQLKLVEPLIDTAYLTTPIGDFFSTKDFPNRNNSFNREYGRYVKLQGWNTMWFG

SHQSQLFQSQGNVLSLLLKPIVIDNHTYSNVYMVVNMKEEAMRDILTQDLMNNQIQLF

LLHKNGNEVIRPRSRNYDFIMEPDFTQQFSRGDAGDFTYRNDQGEDMLVNYSALSMNE

DWVMVSVQSKADLLSPLQKIRWLIILIMLFCILLALGLSKLLASALLKPLNKLRRLMV

EVESNDLDVRFRSKYDDEVSAVGHRFNRMLDQIQILFEEVRTTEQDKRRFEVKALQAQ

VDPHFLYNTLNTMFWKSESGEKKDVSEMIVSLSLLFRLGLNDGKDITSVEQEIKHVEQ

YMQLQQKCYEDLFVYRIEVGDPSCYAVDILKILLQPLVENSILHGFNDKEDLGVILIR

IDRQGDTLRLEVADNGCGMDVASIYAEDAPDGGRKGYALSNLHGRLSLHYGDVASIVF

HSSTDVGTTVVITIPITEEGQYGPIDDVCYG"

CDS complement(3844678..3845550)

/gene="araQ_21"

/locus_tag="EFAGFIKM_03290"

/inference="ab initio prediction:Prodigal:002006"

/inference="similar to AA sequence:UniProtKB:P94530"

/codon_start=1

/transl_table=11

/product="L-arabinose transport system permease protein

AraQ"

/db_xref="COG:COG0395"

/translation="MMTQNVLAPSGSPQLIRWTKRTVLWLFLIAIALLSLFPIILVLL

GSFKTNQELTGGATIWPKIWQFSNYAQAWKTANFSGFTMNSLFVSISTTVGTLLVASM

SAYAVDRVNFKGKKGYTLLMASTLFISIGAVVLRPQFDLMVKLGLNTTLWGVIIILIS

AHAATYFILIGFFKSIPRELDEAAMIDGCNFFTTYWRIILPLLRPGLAVAGLGVFQSS

WNEYILPSVFTMSNPDLQTLPVGLANLRYGIGAAAEVQLMMAGACLSILPLLIVYIFA

NKSFMQVTLGAVKG"

CDS complement(3845570..3846460)

/gene="lacF_6"

/locus_tag="EFAGFIKM_03291"

/inference="ab initio prediction:Prodigal:002006"

/inference="similar to AA sequence:UniProtKB:P29823"

/codon_start=1

/transl_table=11

/product="Lactose transport system permease protein LacF"

/translation="MKPSKIHAYMFIFPSLFLTLVFGIYPLLWALRYMFYDYQGIGTP

VFIGLDNFGRILRDGQFWDSVGNTGVYALGKLVVTIPLSLTLAIILNRKWRGRSLFRA

IYYLPTIFSASVMAIVFFIIFNSYNGILNQLLLKYHLISESISWLGAEHAMLTTIIIA

IWGAVGNYMLLFIAGLQSIPEELYEAASLDGASEFQKLRNVTIPLLGPVLQMIIMLAI

TTALKGYESIMVLTEGGPYGKTEVMYLYLFKLLFPVSADTQSLQQLGYGSAVGFTTAL

IVGAVTLIYFRISKKLNDVY"

CDS complement(3846666..3847352)

/gene="gph_4"

/locus_tag="EFAGFIKM_03292"

/EC_number="3.1.3.18"

/inference="ab initio prediction:Prodigal:002006"

/inference="protein motif:HAMAP:MF_00495"

/codon_start=1

/transl_table=11

/product="Phosphoglycolate phosphatase"

/translation="MNKEKVLLKPEAMIFDMDGTLFQTETLLLPAYQQLFDTLRAEGH

FEGETPPEERMLGSLGMLLEDIWKVVMPEASEAAHRRADELLLQLEIEGLDQGSSQLY

PGVKETLQALKQQGVRLFVASNGLEDYVKGVAFAHEIMPFFEGVYSAGQYKTPSKINL

VQILLEKHRIQDAWMVGDRSSDVEAGKMNGQAVIGCAYAGFGRDEELAGSDVLISDFT

ELLRLYDEAE"

CDS 3847648..3849366

/locus_tag="EFAGFIKM_03293"

/inference="ab initio prediction:Prodigal:002006"

/codon_start=1

/transl_table=11

/product="hypothetical protein"

/translation="MKKRKTFKIFYKIGLGYLLVLAVLAGCILLIQNSTSQLQRELDF

LVDHDMRVHALTYELERNMVDMETGQRGYIITGQDNYLDPYTQGKEQLITLRDELAAL

ISDNPAQLALLEDIHTNMENWVQVAGEPVVALRKANNTQAIRDFFADDPGKRDMDQIR

TALDTFRTNEHTLTDNRTEALKQKNTLLNLELYGALIVVAIISFITALVLSRAIVRNI

RTVKQALNDIGSSNGDLTLRIATPMKDETRELAEAANTMLTGLQQMMLDVQSNAMILS

TASDRLDKGVADSHLAGKEVATAMERVAEGADEQVALTQTMVAAMQQSLNGLNRVASS

ASDVAGRAVRTESIAVDGQQRIDNAVGKMSSIEQSFLSVQEAINEISKMSDQVINIAD

SMSGIARQTNLLALNAGIEAARAGENGRGFAVVAAEIRNLADQSATSAKEITSILETV

VTGVQSTVQVVDESTLHVNEGLRTIEDAGNAFSTITQHIHNLSGEVLEVSAVVEELTS

GSEAVMKSINEVSAVVEDTASATEEVSAMTEEQLASLQEMSDTSKQLNEMSDALDQLV

KRFKLA"

CDS complement(3849545..3850516)

/gene="iolG_8"

/locus_tag="EFAGFIKM_03294"

/EC_number="1.1.1.18"

/inference="ab initio prediction:Prodigal:002006"

/inference="similar to AA sequence:UniProtKB:Q9WYP5"

/codon_start=1

/transl_table=11

/product="Myo-inositol 2-dehydrogenase"

/db_xref="COG:COG0673"

/translation="MTVIKMALIGLGKMGLHMVHLIDNTKMDTEIEIVAVCDANEEGL

ASFAASHRGVRTYTDYKQLMNEHQIDLLYVAVPPKFHYPVVMEALERKIHVFCEKPLA

NSVEEAKKMLEAAEQAGVVHAIHFSMPHEPSVLKLQEMIEQGTVGAIRKIDLILQFPQ

WPRSWQQNAWITSREQGGFILEVGIHWIHMIQKVFGTIRVVSTQVQYPENGDCELEVQ

AAMELEDGTRIQLNGISQFAGEERVSMVVYGAKGTIALENWEELKTGLVGQPLSPVEV

LESASELPVLKHVIARIQGKPAKIYDFNDGYQAQLVLEALRNTEQSR"

CDS 3850930..3852561

/gene="ilvB1"

/locus_tag="EFAGFIKM_03295"

/EC_number="2.2.1.6"

/inference="ab initio prediction:Prodigal:002006"

/inference="similar to AA sequence:UniProtKB:P9WG41"

/codon_start=1

/transl_table=11

/product="Acetolactate synthase large subunit IlvB1"

/db_xref="COG:COG0028"

/translation="MRTVADYLAETLRNLGVTHVFGIIGKSICPAVLKMVDYGLEFIP

GRHESSSGFAASGYALQTGKLGVAFATSGPGGTNLLTAAAHAKANNLPVLFITGHQSI

QELGLPQCQDSSSYLADLAEMFRPATLFSKLVERGDHFGTLLNHALSIALGPNKGPVH
LCIPFDVQTELLSQCRIVIPEPEPLLSISNLNRIFPLIQQSNRPLIIAGKGVSRARAH
DELLQLAESFNIPVITTPGGKGAIAWDHPLYHGPCGVGGFPHADDMLNQSDLYIVLGS
RLSDMTLCNLKPEHHPAHLIQFDADPTFVGKILSAQTLHITGDLKDNLQYLLGTLEDL
PTPVRTTAPMDYTVPLPDLSQLSLASVLDGMSDLLPYDHKLFVDDGSHGFHAVQRYKV
KKPGSFVFDAYFACMGNAIGMAIGAKAASPEETVVCITGDGCFMMLGTEINTAVCNNL
NVIFIVVNNKQLDMALKGMEKTTGRIDGTLYEVPMDAAKFAESLGAVAFRAETLAEFS
SALNTAQTLNQVTVIELLTDRDEIPPTAHRTVNLN"

CDS 3852574..3852942

/locus_tag="EFAGFIKM_03296"

/inference="ab initio prediction:Prodigal:002006"

/codon_start=1

/transl_table=11

/product="hypothetical protein"

/translation="MSEHVAQGLDRFAKLSGEYGARALAPIKEHFPELSEFIMGTAYG
DIFQRTTITDQWKEVAIISSLITQGQYEQLGVHYTMALSVGVTVDQLKGILLHLAPCV
GAPRIISAFNILLATLDEIQ"

CDS 3853129..3853479

/locus_tag="EFAGFIKM_03297"

/inference="ab initio prediction:Prodigal:002006"

/codon_start=1

/transl_table=11

/product="hypothetical protein"

/translation="MGNFILKTDVPNKVLNIELEGSFSQEDGLRSISAYQDTIASINT
KEYDLNIDCKKLNVTAPEIVPLLEGCLQLFKKDEYKKVVLTLENNAILKMQLSRLGRS
VGLDNLEIISTVTT"

CDS 3853534..3854526

/gene="fabH_2"

/locus_tag="EFAGFIKM_03298"

/EC_number="2.3.1.180"

/inference="ab initio prediction:Prodigal:002006"

/inference="similar to AA sequence:UniProtKB:O67185"

/codon_start=1

/transl_table=11

/product="3-oxoacyl-[acyl-carrier-protein] synthase 3"

/db_xref="COG:COG0332"

/translation="MAGIRIVDIDIYHPATKVHNDFYIEHFDARGVDIRGLLQALGRD

TRYKIDNDDENSLSMAYEAASNLLKKTGLTGADIDLIAYASQTPEYIFPTNSLMIHRL

INGASHTICIDSNANCAGMTAAFEQVSRQMLGNPRIRRALIIGSDYVAPHASPDDPVY

FANFGDAAAAVIVERDEHAVGFIDSIYQTDTCVYGNSLFPAEGLAKLGKTGVDAGAFH

VKFIPFDDSICVDAASESIRTLLTRNEIGPDEIKAACFSQLSISNIRAVSENVGIGDD

IAVYIGDEFGYTSTSSPFIALHRAVTTGQIQRGDKVLFWTVGAGWQNVAMVVEY"

CDS complement(3854737..3855306)

/gene="ydbD_3"

/locus_tag="EFAGFIKM_03299"

/EC_number="1.11.1.6"

/inference="ab initio prediction:Prodigal:002006"

/inference="similar to AA sequence:UniProtKB:P80878"

/codon_start=1

/transl_table=11

/product="putative manganese catalase"

/db_xref="COG:COG3546"

/translation="MWVYEKKLQYPVRVSKCDPRMAKLLMEQYGGADGELAAALRYLN

QRYTIPDKIIGLLTDIATEEFSHLEMIATMIYKLTKDASVDQLEEAGLGPNYAQRDSA

LFYTNSSGVPFTAAYIAAKGDPIADLYEDIAAEEKARATYQWLIDLTDDVDLQDSLKF

LREREIVHSLRFREAVEILKDDRETKKIF"

CDS complement(3855403..3855678)

/locus_tag="EFAGFIKM_03300"

/inference="ab initio prediction:Prodigal:002006"

/codon_start=1

/transl_table=11

/product="hypothetical protein"

/translation="MEPEGAKACDTQYYELLEELQALDFVLVELNLYLDTHPGDYQSI

EQYNKFTQERTRVAQEFQQLYGPLMNFGHAFSKYPWEWSQTPWPWQV"

CDS complement(3855668..3855907)

/locus_tag="EFAGFIKM_03301"

/inference="ab initio prediction:Prodigal:002006"

/codon_start=1

/transl_table=11

/product="hypothetical protein"

/translation="MKDPQLRAYAPFVGPFDPCPPVAIRTYLVPPQLFIPFQPMGWPQ

YSPAEALRLGTLWPALYSPYTPARSKGRKVEVDGT"

CDS complement(3856186..3857538)

/locus_tag="EFAGFIKM_03302"

/inference="ab initio prediction:Prodigal:002006"

/inference="similar to AA sequence:UniProtKB:P9WFP3"

/note="UPF0053 protein Rv1842c"

/codon_start=1

/transl_table=11

/product="hypothetical protein"

/translation="MDIHTEFHLGQLVFNLVCVFLLVFLNGVFVAAEFSLVKVRQTRL

TQLQSEGNRMAGYALKVNSKLDSYLSATQFGITLTSLGLGWLGEPAISELLVEPLMFK

LGVGDTGLISTVSVIIGFCIITFLHIVLGELAPKSLAIQKTDGVALFLSAPLLLFYKI

FFPFIWVLNVSANALLRLAGIEPASEGEAHSEDELRILMKQSAKSGVIDKDEIKLMDN

IFDFSDMLAREVMLPRTDMDCLYTHMSLEENLEIINATKHSRYPVAVEDKDEIIGFIH

ITDLLLAKPEQQHDLASLVRPILNVPESMEISHVLRLMQKKHSQMTLVVDEYGGTAGL

LTAEEILEEIVGDLYDEFEDERPHMERSGDSFSIDGRSLIEEVHKWTGAIIEDEEVDT

IGGWLFKELGGNPTKGKTQELNGYVFEVEESTRLRITRVRVYKQSVPQDMNSEENPAE

"

CDS complement(3857585..3858688)

/locus_tag="EFAGFIKM_03303"

/inference="ab initio prediction:Prodigal:002006"

/codon_start=1

/transl_table=11

/product="hypothetical protein"

/translation="MPLSPMNDPWDPIGSLRTYGRHVLTSVEMTVTHLCNMRCEHCAV

GDMLTMREAPALPLPLMLKRLDEVEHLQTISLTGGEPSFSRKTVDEMIIPLLKYAKER

GIRSQINSNLTLDISRYEQLLPYLDVMHISFNYLNADDFHQVGFANSSRPVKREVAVK

MYEKMIENSRKLSEAGMFISAESMINFRTHDKLDGIHQLIREMGCVRHEVHPMYNSNF

ASSLPVLSLDHMRAAIHRLLDARDKDMWMLFGTLPFFACSAAEQDRELINRLYSEPNV

TVRNDPDGRNRVNVNMFTGNVYVTDFADIPAFGNIRDRKLDDVFHEWSAEHPLNQTVN

CHCDIASCCGPNLLVADMYYKGVDFKSRKAITR"

CDS complement(3858942..3860063)

/locus_tag="EFAGFIKM_03304"

/inference="ab initio prediction:Prodigal:002006"

/codon_start=1

/transl_table=11

/product="hypothetical protein"

/translation="MRLVHINLLQPGMKLGKRIYSEEGLVLLSEDVELTSRLIGRLKD

LGVGYVYIKDAATEDIIVPEMLQEETRRKALVEIKQQFQSMSGLKTKNRIPHFGKALS

GVMNAILEDIGSQKEAMIMLMDMNSSDFDLYNHSLNVCVYTLVLGVASGYTRQQLMEI

GLGALLHDIGKTQIASEILHKPSRLSDEEFKIIQQHTTYGHRILKDEPGIPLLSAHCA

LQHHERIDGSGYPFGLKDKEIHEYAKWIALADSYDAMTTNRVYKQALLPHQAVEVLYT

GSGTLYEQRMLEKFRDCVAIYPVGISVKLSTGEVGVVASIHSRVPQRPLIRVIKDADG

QTLSSPYEINLSTALSVMITGVEGVEDIPSAAQSEQVES"

CDS complement(3860137..3861567)

/locus_tag="EFAGFIKM_03305"

/inference="ab initio prediction:Prodigal:002006"

/codon_start=1

/transl_table=11

/product="hypothetical protein"

/translation="MSTREKQTLTILHTNDIHSHFGSMSTIAAMINEERDQGGNFLVL

DIGDHMDRMAVETEGTLGTANVDVINLTGYDAITIGNNEGLTFTPDQLSQSYAGLLCP

VVCSNVVEQDTGLAPVWMKPSLIVEKGPFRVGLLGATAPFTTFYELLGWDVLDPVESL

RVQVEALRDEVDVVVILSHLGLSTDRRLAEQITGIDVILGGHTHHVLEEPLVIGQTVL

GAAGKFGQWLGKVVLERTSVGEPLQLVSSGCMAAKSILLDDQVSLAIATNRTEAERTL

NQTAVITDRVLPITYDRESPFATLLAQAVRHFTGAQLSLVNAGQLLGELPQGNITKGM

LHSLCPSPINACTICLSGSHIREALEQSLLDEFSGKPIVGFGFRGHILGTLCMDGMEV

QYNPDAPAYQKIQTISINGEPMDEQCEYIVGTLDMFTFNVGYPPLAHGTRTLYHLPEF
IRDLLETEIKRPGALDDSLRARWNQS"

CDS 3861688..3862434

/locus_tag="EFAGFIKM_03306"

/inference="ab initio prediction:Prodigal:002006"

/codon_start=1

/transl_table=11

/product="hypothetical protein"

/translation="MKLTIQLFAGIAERLNTSLLEFEYIGDIPTAADLKEKLSLAYPE
AASQIRTSFLAVNQQYAPADTILSSEDELALIPPVSGGDGTDISDEKLRSSVREHRTE
DGLFLITESTLSVEATTALVITPNHGAALTFVGTTREMTGEQRTVHLEYEAYVPMALS
QMAAIGVEISDRWPGVLCAISHRIGKVDVAEISVVIAVSSPHRNDCYDASRYAIERLK
QTVPIWKKEIWEDGSEWKGDQLGPWNPMEN"

CDS 3862559..3863608

/locus_tag="EFAGFIKM_03307"

/inference="ab initio prediction:Prodigal:002006"

/codon_start=1

/transl_table=11

/product="hypothetical protein"

/translation="MRVHVTDLKTGDLLKNDVYNSSGLHMLMKGSTLSEDDLSKLLQH
GIEYADVEHTTSDLHSDAQTLPSDQTRLLTNLFNDTIKGTESLFQQAMEKGFIDETQV
DEILFTLTEQLEKQKDVVSLLLMLDGDSDYTYNHSLQVGMLAFYIAGWMGYNPEECLT
VAKAGYLHDIGKSKIPSEMLHKPGKLTTEEFDEMKKHTFYGYDIIKESTQDEILAMVA
LQHHEREDGSGYPHGLRGDEIHPYARITAIADVYSAMTTNRVYQTKQELISVLCELYS
LSFGQLNAEATQELIKHMLPNFIGKRVLLTTGQTGLIVMNNPADYFRPLVQTSTAFID
LAKERDIAIVEIYVQ"

CDS complement(3863741..3863881)

/locus_tag="EFAGFIKM_03308"

/inference="ab initio prediction:Prodigal:002006"

/codon_start=1

/transl_table=11

/product="hypothetical protein"

/translation="MSEVGYGCGGNVGGVGGVGGYNMFTNTGAILVLFILLVIITKAF

CV"

CDS complement(3864032..3864751)

/locus_tag="EFAGFIKM_03309"

/inference="ab initio prediction:Prodigal:002006"

/codon_start=1

/transl_table=11

/product="hypothetical protein"

/translation="MSVNPFEGYRITSSFGYRIHPIHGGQTFHRGIDLVTEPWNGPVY

AFMEGRVRFASEGVTGSGFGGYGLTVALQDHRGYLHCYAHLSWIAVTVGQRVKRGQLI

GTQGSTGQSTGPHVHYEIRKTSAPSYGYTASEDGVTEPGAYLQAEYGTASQEQEAPPM

TNEQKKVFEAMQKTLEIQGGWIQQQEQLSNMDCPAWAQQAFDYYRPFIMNDKGSYEFW

RLLVIMYRKEKGIQVHSESDI"

CDS complement(3864960..3865652)

/locus_tag="EFAGFIKM_03310"

/inference="ab initio prediction:Prodigal:002006"

/codon_start=1

/transl_table=11

/product="hypothetical protein"

/translation="MSENGLRDPWIDLIRGGSIPADGDALMDASNINGPNYEIVDKIL

YRGSKALSLNAQSYRLENQTFVDPSGKQLSDHYPITAEFVYSTSSQYQLSSTIGGSGG

TGFNDLNQIPDARPSSVVLNSGNRVDGISTLYRDGTNLAHGGQSNIVTLNLADGEYLT

EATIGQGTRNGDKRIFYVELSTNLGNKVEGGQKTSDQIKLEAPAGWYIAGFYGRAGQE

LDQLGVIYRPLN"

CDS complement(3865946..3867343)

/locus_tag="EFAGFIKM_03311"

/inference="ab initio prediction:Prodigal:002006"

/inference="similar to AA sequence:UniProtKB:Q7A6L4"

/note="UPF0051 protein SA0778"

/codon_start=1

/transl_table=11

/product="hypothetical protein"

/translation="MAKKAPEMEEYKYGFRDEHKSIFQTGKGLTAEVVTEISKIKNEP
EWMLEFRLKSLKQFEKMPMPKWGGDLDGLDFNEIQYYVRASEKQGKTWEEVPSEIKET
FDKLGIPEAEQKFLAGVSAQYESEVVYHNMQKELEDQGVIFMDTDTALREHPEILREY
FATVIPPADNKFAALNSAVWSGGSFIYVPKGVKCEVPLQAYFRINSENMGQFERTLII
ADEDSFVHYVEGCTAPIYSTNSLHSAVVEIICKKNARVRYTTIQNWAPNIYNLVTKRA
VAEENATMEWVDGNIGSKLTMKYPAVVLKGRGAKGSVLSIAVAGKNQHQDAGAKMIHL
APDTTSTIVSKSISKHGGKVTYRGLASFGRQAEGAKSNIKCDTLILDNESTSDTIPYN
EIMNDNIMLEHEATVSKVSEDQLFYLMSRGLTDAEATQMIIMGFIEPFTKELPMEYAV
EMNRLIKFEMEGSIG"

CDS complement(3867364..3867795)

/gene="sufU"

/locus_tag="EFAGFIKM_03312"

/EC_number="2.-.-.-"

/inference="ab initio prediction:Prodigal:002006"

/inference="similar to AA sequence:UniProtKB:O32163"

/codon_start=1

/transl_table=11

/product="Zinc-dependent sulfurtransferase SufU"

/db_xref="COG:COG0822"

/translation="MQLDDLYRRVIMDHYKNPRNRGTFDNDAVTVNLNNPTCGDRISL
QILLKDGIVQEAKYTGEGCSISMSSASMMTEAVKGKTMEQALDIANRFSSLMKGEEVD
FDDYEDLEALSGVNKFPARIKCATLAWNALRKGIDEENKVQ"

CDS complement(3867785..3869005)

/gene="sufS"

/locus_tag="EFAGFIKM_03313"

/EC_number="2.8.1.7"

/inference="ab initio prediction:Prodigal:002006"

/inference="similar to AA sequence:UniProtKB:O32164"

/codon_start=1

/transl_table=11

/product="Cysteine desulfurase SufS"

/db_xref="COG:COG0520"

/translation="MNPSIREQFPILHQEINGHPLVYLDSAATSQKPHAVIEAVKHYY
EYENSNVHRGVHTLGSRATDAYEGAREKVAKFINARRTQEIIFTRGTTTALNLVASSY
ARANCKEGDEIVITQMEHHSNLIPWQQVAKETGATLKYIPLQPDGHIELADVEKTITN
NTKIVAIAYVSNVMGLIHPVKQIAEIAHRNGAVIVVDGAQSTPHMKVDVQDLDCDFYA
LSGHKMCGPTGIGALYGKKALLESMEPIEFGGEMIDDVGLYESNWKELPWKFEGGTPI
IAGAVGLGAAIDFLEQIGMDEIAHHESVLAAYATERLAEIDGLTIYGPAKRHVGVVTF
NLGDVHPHDVATVLDASGVAIRAGHHCCQPLMRWLEVSSTARASFYLYNNEQDVDRLV
SALIQTKEYFGDAT"

CDS complement(3869002..3870306)

/locus_tag="EFAGFIKM_03314"

/inference="ab initio prediction:Prodigal:002006"

/inference="similar to AA sequence:UniProtKB:Q7A6L4"

/note="UPF0051 protein SA0778"

/codon_start=1

/transl_table=11

/product="hypothetical protein"

/translation="MTTQTILPVESEALRALSESNNEPGWLTEQRLEALKLASGLALP
KLEKQKIERWNVSEYGTYKTSEAISSLTEVPASIKDLVQDQAEGSLVIQRNSGTVYSK
VSADLAAKGVIFTDLATAVREHGDLVKQYLNTAVKADEHSLAALHAALWNGGVFLYVP
KNVEIEVPLQAVLLTDDASATFAPHVLVVVEANSSVTYVDNYVSGELSAPVFHNGVVE
VFAKSGAKVRFASVHQLSVNVTDVSFRRAVLENDASIEWIVGEMNNGDTASNTMTVLK
GNGSSSDSKVIAVGSGSQKINYTTEARHFGKNTPSQMITRAVMREEASAIINGITKIE
KGATKADGQQTEKVLMLSPKARGDANPILLIDEDDVTAGHAASVGQVNAEQIHYLMSR
GINRTDAERLIIYGFLAPVVADIPLEALRTQLQSLIEKKLGQ"

CDS complement(3870332..3871114)

/gene="sufC"

/locus_tag="EFAGFIKM_03315"

/inference="ab initio prediction:Prodigal:002006"

/inference="similar to AA sequence:UniProtKB:P80866"

/codon_start=1

/transl_table=11

/product="Vegetative protein 296"

/db_xref="COG:COG0396"

/translation="MATNFVIEGLKATIEGKEILKGINLEMKGGEIHAIMGPNGTGKS

TLASALMGHPKYEVTDGTITLDGEDVLDMAVDERARAGLFLAMQYPSEIAGVTNSDFL

RSAINARRGEGNEISLIKFIRQMEGKMKELDMNPEFAHRYLNEGFSGGEKKRNEILQM

MLLDPKIVVLDEIDSGLDIDALKIVADGVNAMKSEDRGFLIITHYQRLLNYITPDYVH

VMMQGRIVKSGGPELAHRLEAEGYDWVKAELGITDETVGQEA"

CDS complement(3871493..3872068)

/locus_tag="EFAGFIKM_03316"

/inference="ab initio prediction:Prodigal:002006"

/codon_start=1

/transl_table=11

/product="hypothetical protein"

/translation="MLEPKIPALKEWASAIKALETGRQIMVMRKGGIVEETRHFELKS

PAFYLYPTYEHQRKELIKSSDQSYVEESLAEWVPEASTIRITAYAEVTQDLEIRDQEM

LDRLLDFHMWTADFAEDRLKWKRKDPLHVLILRVYLLKEPMEIPVLPEYNGCRSWISI

PNGPVPREMTPVVDVVDFDEQVQKINEMLKL"

CDS complement(3872194..3872655)

/gene="dps2_1"

/locus_tag="EFAGFIKM_03317"

/EC_number="1.16.-.-"

/inference="ab initio prediction:Prodigal:002006"

/inference="similar to AA sequence:UniProtKB:Q8RPQ2"

/codon_start=1

/transl_table=11

/product="DNA protection during starvation protein 2"

/db_xref="COG:COG0783"

/translation="MATKNKTDQAKSVEQVLNRQVANLNVLYVKIHNYHWYVKGPNFF

TLHVKFEEFYNEVTVQMDEIAERILTLKGSPAATMKEYLELSSIQEAAGGEDAKTMVQ

NLIEDFATLSNEYQEGIEVADAAEDQPTSDMLTGFKADLEKHMWMLRSFLG"

CDS 3872952..3874169

/gene="mtnK"

/locus_tag="EFAGFIKM_03318"

/EC_number="2.7.1.100"

/inference="ab initio prediction:Prodigal:002006"

/inference="similar to AA sequence:UniProtKB:Q9F0P1"

/codon_start=1

/transl_table=11

/product="Methylthioribose kinase"

/db_xref="COG:COG4857"

/translation="MSQYRPFTPQDAIELAKTLPGPFAADANLDCHEIGDGNLNLVFH

ITDQNSDKSIIIKQALPYAKVVGESWPLSLVRARIEREILQEEYRLCPGMVPEVYHYD

DDLALTVMEDLSDHVIMRKGLIEGVSYPLFAQHIGEFMARTLFFTSDLGMDQQLKKEQ

QGRFINPDQCKITEDLIFDEPYRIAEKNNYDVSIEDEAESLRTDGELHLEVALLREKF

LTHGQALLHGDLHTGSIFVTPESTKVIDPEFAYYGPMGFDVGAVLANLLLNYASLPGW

IQDETALRERKTLLLNMVRDVWTEFESRFRALWVNDLVDPMAKTPGYQDLYVQQLFRD

SIGFAGAKMVRRIVGLAHVADIDTIPNATEREHAQRKALSIGKALIKNNRRLNTIGEI

LDIVSTAVTTTKV"

CDS 3874193..3875266

/gene="drdI"

/locus_tag="EFAGFIKM_03319"

/EC_number="5.3.1.-"

/inference="ab initio prediction:Prodigal:002006"

/inference="similar to AA sequence:UniProtKB:P0DTQ1"

/codon_start=1

/transl_table=11

/product="5-deoxyribose 1-phosphate isomerase"

/translation="MTTPDYQPLSSLIWKKDKLEMLDQRLLPETILMLKLYTPEEVWE

SIHSMKVRGAPAIGIAAAFGVVLGAKSYDGTTVQGWLDHVKSICAHLATSRPTAVNLF

WALDRMMQKANEVVESGLSLDDGNDALEVEALLIQKEDEEVCRMIGENALPLFEDGMG

VLTHCNAGGLATAKYGTATAPMYLAQERGIHLKVFADETRPVLQGARLTAFELQQAGI

DVTLLCDNMAGMVMSKGWIQAVIVGTDRVAANGDVANKIGTYSVAVLAKAHNIPFYVA

SPLSTIDLSTPSGDLIPIEERAAEEVTEGFGKRTAPQGVKVYNPAFDVTPNEYVTAII

TEKGVVRAPFQENLAALFAKEEA"

CDS complement(3875406..3875708)

/locus_tag="EFAGFIKM_03320"

/inference="ab initio prediction:Prodigal:002006"

/codon_start=1

/transl_table=11

/product="hypothetical protein"

/translation="MVTMEPIVVGEHVLVGVEVKLPKTTLLTINTSKGYIMCGALDVG

LLNEKLGDRKIIAARAVGVRTLEQLLHAPMESVTTEAEAMGITVGMTGVEALLKMI"

CDS complement(3875931..3876536)

/locus_tag="EFAGFIKM_03321"

/inference="ab initio prediction:Prodigal:002006"

/codon_start=1

/transl_table=11

/product="hypothetical protein"

/translation="MKKLVLSFLALLLFVVTTTYAPAPATAATKYDFKNVNWGMTVNQ

VKKKEKSKILETVKSKDFTYVVYKTKGFRKNANLYYLFYKNKLVRAMYVFDPNNTNGT

TMHLIGEYFSLKRDLFDTYDRSINNRDILDRSGKRVTMYDGEEKDLAAGKISYQCYWI

VDKKKTHISLSLSIFGNFLINRVIYSDNTLDFDIDKVMDSM"

CDS 3876674..3877198

/locus_tag="EFAGFIKM_03322"

/inference="ab initio prediction:Prodigal:002006"

/codon_start=1

/transl_table=11

/product="hypothetical protein"

/translation="MNSIYKVSFFITVLVLIMLSFILFTDIRIPLEGTGRFYTKETMN

FGMVLCISITGLLGVLSGLAALEEKVLVHASLLYNFTGWLFMMAVHYYRVAYNNDLFD

GLLLSILKWTTLFIVIHIIFASFFIRIDKKRRKQEEEKKRKGSIEKVGKNYVYTFGDG

SKITVTQEDFQKKD"

CDS complement(3877399..3877659)

/locus_tag="EFAGFIKM_03323"

/inference="ab initio prediction:Prodigal:002006"

/codon_start=1

/transl_table=11

/product="hypothetical protein"

/translation="MHQEDSVIEQIMKQKRLKPIKPETTTVQVKPIHKKKLAQEPLSE

LDVQFKKSVAESIDRSVLHRRVLGIRRDLTEIRRLKNGEHMF"

CDS 3878124..3878696

/locus_tag="EFAGFIKM_03324"

/inference="ab initio prediction:Prodigal:002006"

/codon_start=1

/transl_table=11

/product="hypothetical protein"

/translation="MKETLSNQEDIGFLFNVDILIKSRSNALALQSLLEFINNEEQIA

DFRIQSGIELGKIIEATLQSKKDSFVTLHNERKKANAAQKASTAAQTPASPAPVKTQN

QQEKPVSEKKPATQTSNVSDGNSFDAWIDTLIKENKLTRIVVNNKNGKHQSIPCRILN

FDRDTSIVSIYHVDEKQVYTFRTNEIDEFL"

CDS complement(3878821..3879207)

/locus_tag="EFAGFIKM_03325"

/inference="ab initio prediction:Prodigal:002006"

/inference="similar to AA sequence:UniProtKB:P39619"

/note="UPF0382 membrane protein YwdK"

/codon_start=1

/transl_table=11

/product="hypothetical protein"

/translation="MQRRWMMLGAVMTMLSVAIGAFGAHMLKDTIGPAAIATYETGVQ

YHMIHALGLLIIGLTAGQLGASTKLKWAARLLFIGIIVFSGSLYVLSISGIKILGAIT

PIGGVAFIAGWLLLALDVWQRGKDRS"

CDS 3879540..3880010

/locus_tag="EFAGFIKM_03326"

/inference="ab initio prediction:Prodigal:002006"

/codon_start=1

/transl_table=11

/product="hypothetical protein"

/translation="MDNRDSYNSGYNNTDQNPGHADPYNNNGYEPYPPDHERQPMVPL

KHSGPGIASFVIGLVAILGYILIFFIATMAVNESIGVLTPLQTEEIALHPAVVLASLA

ILVCLILNLAGGIVGIIGLVIKNRKKVFAIIGTILNGIMILLFVGLILVGMTLM"

CDS 3880116..3880964

/locus_tag="EFAGFIKM_03327"

/inference="ab initio prediction:Prodigal:002006"

/inference="similar to AA sequence:UniProtKB:Q9X1H9"

/codon_start=1

/transl_table=11

/product="Fatty acid-binding protein"

/db_xref="COG:COG1307"

/translation="MSRIKIFADSTSDLAPEWIQQHDIGIIPLYVVFGEESLKDGAEI

KPEQLYERVSKDGRLPKTAAPSPADFITAFQPYIDQGDEILYISLSSELSSTYQNARL

ASSEFPEGRISVIDSQNLSSGIALMVMKAVHAADKGQNLTQITHLIEAMKSNVRTEFV

IDTLEYLHKGGRCSGMQNLIGSLLKIRPVIRVTDGKMAPAYKVRGKREKALEQMLNNT

LSQKDQIDPDLLIVVHTMAKEDALDLQKSLQEQTGARVELTTAGCVVCSHCGPKTIGI

IYNTVL"

CDS complement(3881159..3881938)

/gene="pdaA"

/locus_tag="EFAGFIKM_03328"

/EC_number="3.5.1.-"

/inference="ab initio prediction:Prodigal:002006"

/inference="similar to AA sequence:UniProtKB:O34928"

/codon_start=1

/transl_table=11

/product="Peptidoglycan-N-acetylmuramic acid deacetylase

PdaA"

/db_xref="COG:COG0726"

/translation="MKRMVLYILLGLLSISMVPAQTEASPVTGAYHFGFKKSQNGQLP

SIDQEGFKSILQHNDAIFLGDTKQKELYLTFDNGYENGFTPAILDVLRTKKVPAAFFV

TGHYLKDQPELVKRMAAEGHIVGNHSWSHPDMTRISNEKLREELEKVKSEVNRLTGQQ

ATFLRPPRGIFNNRTLAESHAQGYVNVFWSVAYKDWDTNVQRGAQYAHQQVLKQLHPG

AVILLHSVSKDNTEALGSIIDEARKQGYAFKSLDDLRTKSY"

CDS complement(3882201..3882608)

/locus_tag="EFAGFIKM_03329"

/inference="ab initio prediction:Prodigal:002006"

/codon_start=1

/transl_table=11

/product="hypothetical protein"

/translation="MNENIQNIQVGFKCPDCQQDIRAEFIDEQVSTVRCKGCDRAYTL

LKPTIGDEILLDWEQEALYHKLRAERSELDNHNMSALIIKVIELLTWRDRGDGQTRAI

QTVREWLASSGKPQTLLELFHQSPPQPENKRFD"

CDS 3882816..3883454

/gene="cysC"

/locus_tag="EFAGFIKM_03330"

/EC_number="2.7.1.25"

/inference="ab initio prediction:Prodigal:002006"

/inference="similar to AA sequence:UniProtKB:O34577"

/codon_start=1

/transl_table=11

/product="putative adenylyl-sulfate kinase"

/db_xref="COG:COG0529"

/translation="MSKEERNITWQSSSINRQDREKHNGHRSRAIWFTGLSGAGKSSL

AFALEQYLYDQGVRCYVLDGDNVRHGLNRDLGFTAGDRQENLRRIGEVSKLMVDAGLF

VLSAFISPHEQDREMVRQLFEPDDFIEIYVRCSIQECERRDPKGLYKKARNGDIPHFT

GISAPYDVPKSPSFIIDTEQWSIEEAVQEIVHHLEQIGALQLPLPTKTAVVH"

CDS complement(3883615..3885435)

/gene="btuD_9"

/locus_tag="EFAGFIKM_03331"

/EC_number="7.6.2.8"

/inference="ab initio prediction:Prodigal:002006"

/inference="protein motif:HAMAP:MF_01005"

/codon_start=1

/transl_table=11

/product="Vitamin B12 import ATP-binding protein BtuD"

/translation="MISELRYFMRKLHHVTGPILYWNLLGMICISLMEGIGIYMLVPM

LSLIGIFEMGSTGLNIPWIGEVLNRFSENSQLLLVLFTFVLIVSGQAWMQRLQTIRNT
RIQQQFVRTLRMETYGAIIMAQWSFFLQKRKSDFNHILTTELARVSQGTSIMLQMAAS
LIFTGIQIGLAFWLSAKLTALVLVCGLLLFFVLRKFVKRAKQIGDQTSEFSQSYYNGI
TEHFNGIKDIKSNMLERSHMNWFEHMCRQIERNVIQFSQLNSGTQLIHRMSAAIIIAA
FIYLSLRVMTVPPASLLLIILIFSRLWPRFTAIQSNLEYISSMLPAFRVVRELQAQTA
KSREISEEIASAGDVGTDEIQPMELKESITCEDVCYRYEGSDTYSLRNVQASIPARGM
TAIVGKSGAGKSTLIDLIMGLVRPETGRILIDGVPLSEERLLSWRSSIGYVSQDPFLF
HTSIRENLRLVDPNASEEQMWQALQFSSSAAFVRKLPQGLDTIIGDRGIRLSGGERQR
LVLARAMLRNPSVLVLDEATSALDSENEQYIHEALERLKGHVTIIVIAHRLSTIRTAD
RVIVLDEGRVIQEGGYQQLSTDPVGTFSKLLNMQAGVVGQ"

CDS complement(3885432..3886628)

/locus_tag="EFAGFIKM_03332"

/inference="ab initio prediction:Prodigal:002006"

/codon_start=1

/transl_table=11

/product="hypothetical protein"

/translation="MIHESELYSSGFPKELKLILSMIRGDLTALSPEELKAGLQGTDW
QLFLRLVYHHRLYSVLYLKMKELNSAIIPADVMESLRQQYTVNTFRMLHLTAEMEQVC
GAFRERGIRNITLKGPALAHDLYGDVSMRTSKDLDILIPFDDVEAAEGILATLGYVSK
EGERAPTVRSWKWREHHICYTHPVKRTQVEIHWRLNPDSGKETDFELLWKRSRFSSYT
QTPVRMLEQEDLWAYLVTHGARHGWFRLRWLLDIDQMIRNMPLDVKKLERRLKAEGRL
SIGSQALYLASELLNTPLDVEYRSLMSLSDRTGKRLASEGAKFMNDMLESPADMKSYQ
SYLFKLRSTKQKWFFFIERLYPSTWDVDQLPLPRSLFFLYFPLRPFLWFWRRIKRYTV
TERGKV"

CDS complement(3886618..3887103)

/locus_tag="EFAGFIKM_03333"

/inference="ab initio prediction:Prodigal:002006"

/codon_start=1

/transl_table=11

/product="hypothetical protein"

/translation="MMLRKIKAYLSLPRSMRRLVWEAYILLGWARIQKAMPFAKIAPG
LGTPMVETPMTGLERSEVITIRNISKAISLASKYTLWESRCLVMAIAGMKMLERRKIE

STLYMGTARNKQGHMMAHAWLRSGKLIVTGADTMDQYTVVGVFGKRCPEKGSGEIVYD

T"

CDS complement(3887105..3887404)

/locus_tag="EFAGFIKM_03334"

/inference="ab initio prediction:Prodigal:002006"

/codon_start=1

/transl_table=11

/product="hypothetical protein"

/translation="MTATTPMNVEDRVTRKEGNLVSDMGSEKVMMSISSGKYYNLGST

GGRIWDLIAEERTLGEVVEVLAAEYEIEPDVCREQVVQFLEHLSREGLIDVTRGV"

CDS complement(3887401..3888348)

/locus_tag="EFAGFIKM_03335"

/inference="ab initio prediction:Prodigal:002006"

/codon_start=1

/transl_table=11

/product="hypothetical protein"

/translation="MLPVHYVAYGLRWSSQIRMPELQIAPKDAETDFDVVDVHIESSD

LSPLWEDWDVGNDNFVAREGSLFFKIEDTGLFLMEQGKRIVVSPIPGADEKKVRLFIL

GTCMAVIMMQRGILPLHGSAVVIDGWAYAFVGHSGAGKSTLSAALASRGYPLLTDDVV

ALTWDAGGRAIVSPGYPQQKLWQPSLDGFGMKEQDYATVHAEITKYAIPVQHYFHEMA

VPLGGIFELAPQPEANHTSVQLVEVTGLERLHLLCSHTFRGGLVARQGLAQWLFETAS

RLSASVEIGRLVRTGAEFTAFGMVDRITDHIRKGVHTGQ"

CDS complement(3888487..3888624)

/locus_tag="EFAGFIKM_03336"

/inference="ab initio prediction:Prodigal:002006"

/codon_start=1

/transl_table=11

/product="hypothetical protein"

/translation="MQMEKKEWQAPALEVLEVNQTMAGTGYRQIDWVTVHDADLYDPT

S"

CDS complement(3888654..3890585)

/locus_tag="EFAGFIKM_03337"

/inference="ab initio prediction:Prodigal:002006"

/codon_start=1

/transl_table=11

/product="hypothetical protein"

/translation="MSAIAGIVHNDGQQALWEDSWRLYASLGHVPADTTGVWKGNEAF

LSCHAQWITPESVSEKLPLYDEESGLTITADAILDNREQLADQLQISRAELAMLADSE

LILRAYQRWGDDVAVRLLGDFVFAIWDERNRKLYAARDITGMRAFYYRHDGSRFAFCT

LMNPLLGLEGVHKELDETWLSEFLAIPDMHDSADINSTVYRGISQLPSAHTLVFRDGR

LELKQYHRWNEVEPLKLKSDGEYVEAFREVFGQAVGSRLRTHRQVAAALSGGLDSGAV

VGFASGTLRSQGKRLQAYSYVPVPDFTDYTSKTLLADERPFIRSTINHVGNITENYLD

FEGRSPLSEVDTWLDIMEMPYKYFENSFWIRGFYEKASQQDAGILLTGARGNFTISWG

PALDYYASLLKSGRWYRWFREMQQYSERTGMPFSRIAKITGKKAYPEWFKAQSKGASQ

AASVQLIHPDFAQRTGVLERLKSIIVLQGGAQADALKVRAEKFNNLAIANKNGAVATK

CSLRYRAWERDPTSDVRVIRFCLSVPIEQYVKHGTDRSLIRRATSPELPDKVRLNQRV

RGVQPADWLHRIIPNWDAFTAELRALCSDSKVAGILNTERIRSALANFPSPRPELASH

PDLRLMMHSLIVYRFIRKF"

CDS complement(3890582..3891850)

/locus_tag="EFAGFIKM_03338"

/inference="ab initio prediction:Prodigal:002006"

/codon_start=1

/transl_table=11

/product="hypothetical protein"

/translation="MIDAQGNGLVFLLCVPRSGSSLSTVMLQNHSRIFATQEMWFLMN

LVDLPKADSRAYGGSSIMRQFYNAMVSEDVFEKACRSFALEIYNGFLEGSGADFIVDK

SPRYYYMLEWLDRLFPQSKRIHLQRNPLAVAASFKKVNRHTGEGFDLIHSLQSPKLNM

KSVDLTLGMLRLNDYFAKPHSNAYELQYERLVSNPQEELEKLCAFLGIRYEQGMEQYG

QFLDSAKSDMFYSMGVGDPFLSSHQEAHQGSVNNWKNILEPHEVELYCRVMGADLFHR

MGYSEQLAEAEQWTGVHYDASPDQEVIDRITHQLTAATGCQWQPQYRMQPADSVVHDP

LGNLNDQVVEEPEKTDPTLAALVTIRQLQAALRAADHRLERGYSERERLKVQLASAQS

KIQRIKSWVPFGHQISAWASQRKILRGGKS"

CDS complement(3892274..3893608)

/locus_tag="EFAGFIKM_03339"

/inference="ab initio prediction:Prodigal:002006"

/codon_start=1

/transl_table=11

/product="hypothetical protein"

/translation="MNKKAIKAWIMYDWANSAYATTVLAAVLPVFYASVAAATLDTDT

AASYLAYTHSIGMLCVALLTPLLGTLADLSGRKGDFLRVFAIIGVLATLGFSAIGEGD

WFLASALLVISTIGFAGGNTFYDAMLPDLVPVERRDMISSKGYAYGYIGGGLLLAANL

LMIQQPGWFGLSSTLAGTRLAFISVSLWWLLFSIPIFRHAPRRPASPDLPGTWKGYAA

VGVRRLRQTFRQMRRFPQLIRMLVAFWFFNDGINTIILMATIYGTSIGIGTADLMLAL

LLTQFIGFPCTLLLGAWAQRWGAKQVLIVSLSVYICIVILGYFMTQAIHFYVLAGLVG

VVQGVSQSTARSLFSNLMPAGRTGEYFGFVNITGKFSSIFGPFVFGYVGQITGSTRWG

ILSLIFFFVAGIAVLLTVKVQKGMQDATRADQEEERNGSVGAPGKSSNVRFT"

CDS 3893863..3895032

/locus_tag="EFAGFIKM_03340"

/inference="ab initio prediction:Prodigal:002006"

/inference="similar to AA sequence:UniProtKB:P45742"

/note="Stress response UPF0229 protein YhbH"

/codon_start=1

/transl_table=11

/product="hypothetical protein"

/translation="MSNSHQPYSFVVSKEDWSLHRKGYQDQQRHQQKVKDVIKQNLPD

LITEENIIMSDGKQIIKVPIRSLDEYRFVYNYQKQKHVGQGDGDSQVGDVIGRDPSAS

QKPGKGEKAGDQPGHDIVEAEVSIEELEDMLFAELELPDLKQKDKDLIETHTVVFNDI

RKKGMQSNIDKKRTILENLRRNATTGTPGIHHISPDDLRYKTWEDKIIPQSNAVIIAM

MDTSGSMGSFEKYCARSFFFWMTRFLRRQYEKVEIVFLAHHTEAKEVTEEEFFTRGES
[truncated: 503,397 more chars]
