# Supplementary material for: Phenotypic and Complete Reference Whole Genome Sequence Analyses of Two Paenibacillus spp. Isolates from a Gray Wolf (Canis lupus) Gastrointestinal Tract
Source: Vet Sci. 2025 Jan 13;12(1):51. doi: 10.3390/vetsci12010051 (PMC11769508; doi:10.3390/vetsci12010051)
Supplement: Supplementary file 1 [file Supplementary Table S9 ClWae19 prophage regions.pdf › Supplementary Table S9 ClWae19 prophage regions.pdf]

Table S9. Prophage regions (as intact) for ClWae19: https://phastest.ca/submissions/ZZ_cdf78348ff

Totally 1 intact prophage regions have been identified.

Region	Length	Completeness Score	# Total Proteins	Region Position	Most Common Phage	GC %
1	25.2Kb	intact 110	28	4484195-4509475	PHAGE_Bacill_BalMu_1_NC_030945(10)	47.98%

gi|00000000|ref|NC_000000| contig_1 coverage=40.6x polypolish 7032512

REGION	REGION_LENGTH	COMPLETENESS(score)	SPECIFIC_KEYWORD	REGION_POSITION	RNA_NUM	TOTAL_PROTEIN_NUM	PHAGE_HIT_PROTEIN_NUM	HYPOTHETICAL_PROTEIN_NUM	PHAGE+HYPO_PROTEIN_PERCENTAGE	BACTERIAL_PROTEIN_NUM	ATT_SITE_SHOWUP	PHAGE_SPECIES_NUM	MOST_COMMON_PHAGE_NAME(hit_genes_count)	FIRST_MOST_COMMON_PHAGE_NUM	FIRST_MOST_COMMON_PHAGE_PERCENTAGE	GC_PERCENTAGE
1	25.2Kb	intact(110)	lysin,portal,plate,tail	4484195-4509475	0	28	26	2	100%	0	no	14	PHAGE_Bacill_BalMu_1_NC_030945(10),PHAGE_Brevib_Jimmer2_NC_041976(9),PHAGE_Brevib_Jimmer1_NC_029104(8),PHAGE_Brevib_Osiris_NC_028969(8),PHAGE_Brevib_Abouo_NC_029029(7),PHAGE_Brevib_Davies_NC_022980(7),PHAGE_Clostr_phiMMP02_NC_019421(5),PHAGE_Clostr_CDKM15_NC_048643(5),PHAGE_Clostr_phiCD27_NC_011398(5),PHAGE_Thermu_OH2_NC_021784(4),PHAGE_Clostr_phiCD505_NC_028764(4),PHAGE_Clostr_CDKM9_NC_048642(3),PHAGE_Clostr_phiMMP01_NC_028883(2),PHAGE_Clostr_phiCT19406B_NC_030947(2),PHAGE_Strept_EJ_1_NC_005294(2),PHAGE_Clostr_phiCTC2B_NC_030951(2),PHAGE_Clostr_phiCT9441A_NC_029022(2),PHAGE_Entero_Mu_NC_000929(1),PHAGE_Geobac_GBSV1_NC_008376(1),PHAGE_Clostr_CDMH1_NC_024144(1),PHAGE_Paenib_PG1_NC_021558(1),PHAGE_Bacill_PfEFR_4_NC_048641(1),PHAGE_Escher_D108_NC_013594(1),PHAGE_Clostr_phiC2_NC_009231(1),PHAGE_Thermo_THSA_485A_NC_018264(1),PHAGE_Bacill_1_NC_009737(1),PHAGE_Clostr_PhiS63_NC_017978(1),PHAGE_Clostr_phiSM101_NC_008265(1),PHAGE_Entero_SfMu_NC_027382(1),PHAGE_Clostr_phiMMP03_NC_028959(1),PHAGE_Clostr_phiCT453B_NC_029004(1),PHAGE_Clostr_phiCDHM14_NC_048665(1),PHAGE_Clostr_phiCT453A_NC_028991(1),PHAGE_Faecal_FP_Lugh_NC_047912(1),PHAGE_Geobac_TP_84_NC_041918(1),PHAGE_Paenib_Tripp_NC_028930(1),PHAGE_Clostr_phiCDHM19_NC_028996(1)	8	35.71%	47.98%
