# Supplementary material for: Phenotypic and Complete Reference Whole Genome Sequence Analyses of Two Paenibacillus spp. Isolates from a Gray Wolf (Canis lupus) Gastrointestinal Tract
Source: Vet Sci. 2025 Jan 13;12(1):51. doi: 10.3390/vetsci12010051 (PMC11769508; doi:10.3390/vetsci12010051)
Supplement: Supplementary file 1 [file vetsci-12-00051-s001.zip › Supplementary Figure S4 TYGS Phylogeny.pdf]

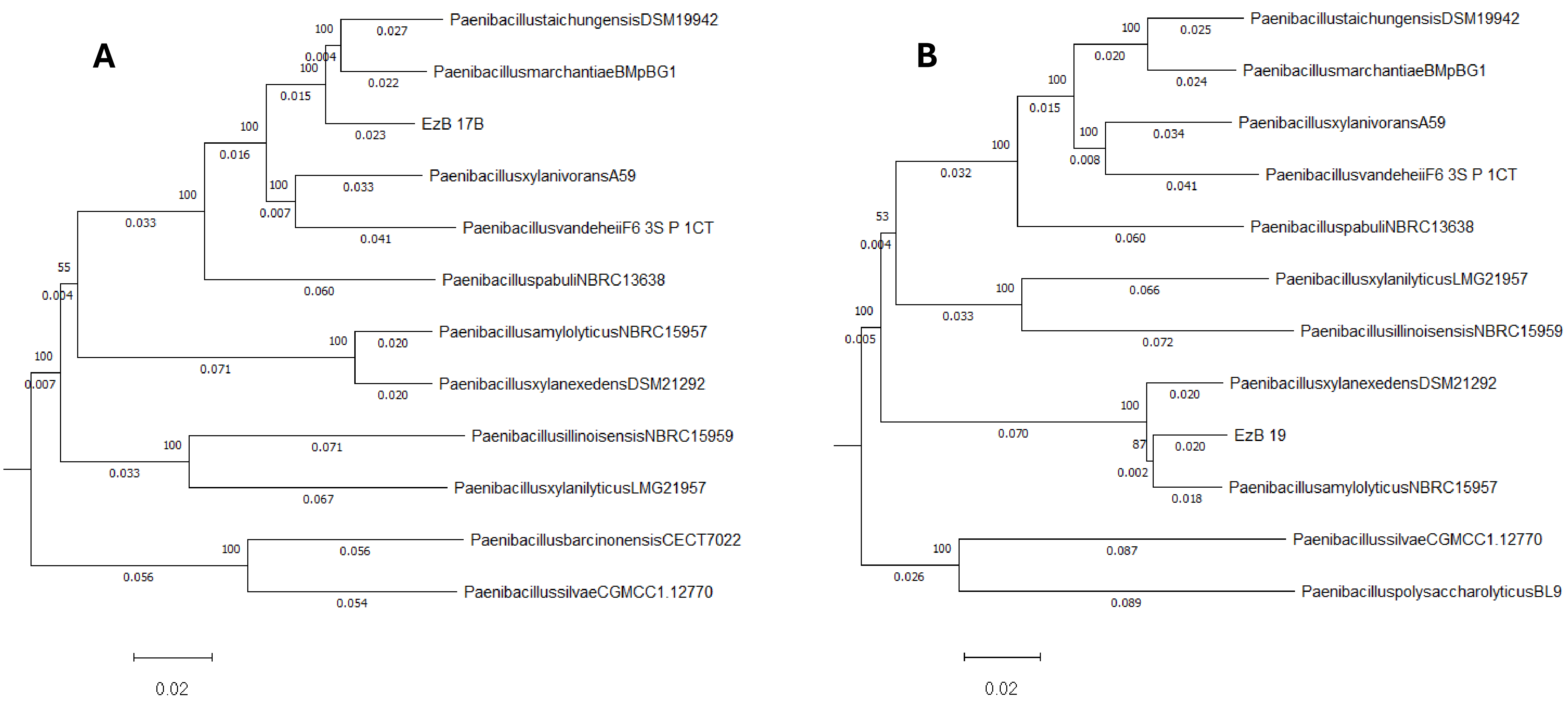

Supplementary Figure S4. Phylogeny for ClWae17B (A) and ClWae19 (B) using TGYS coupled to the LPSN followed by tree construction in MEGA12 as described in the Methods section. Bootstrap confidences are presented above the lines with distances below.
