# Supplementary material for: Phenotypic and Complete Reference Whole Genome Sequence Analyses of Two Paenibacillus spp. Isolates from a Gray Wolf (Canis lupus) Gastrointestinal Tract
Source: Vet Sci. 2025 Jan 13;12(1):51. doi: 10.3390/vetsci12010051 (PMC11769508; doi:10.3390/vetsci12010051)
Supplement: Supplementary file 1 [file vetsci-12-00051-s001.zip › Supplementary Table S10 and Figure S2.pdf]

Supplemental Table S10. Percent identities of 16S sequences for ClWae17B and ClWae19

Taxon	16S Identity (%)	Query Coverage (%)	Reference Coverage (%)
ClWae17B			
Paenibacillus xylanivorans	99.7	95.1	100.0
Paenibacillus taichungensis	99.4	95.1	100.0
ClWae19			
Paenibacillus amylolyticus	99.9	95.1	100.0

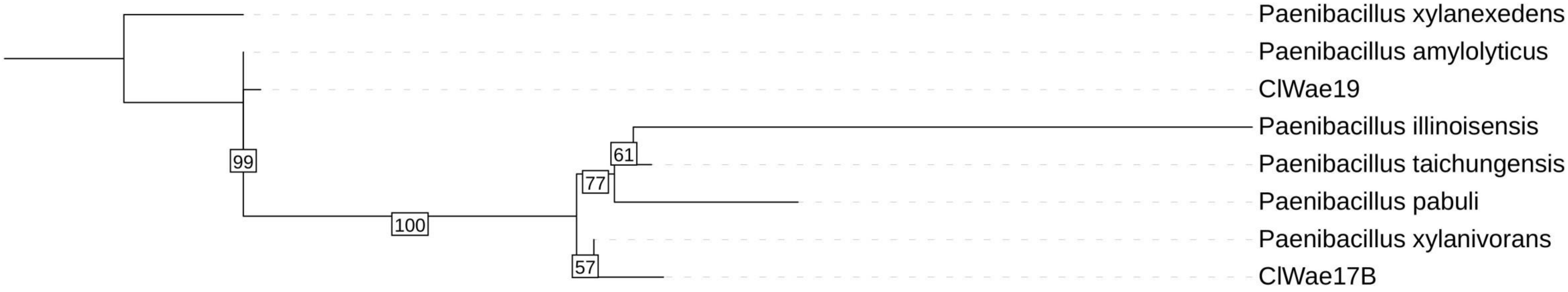

Supplementary Figure S2. Phylogenetic relationships of ClWas17B and ClWae19 based on 16S sequences
