# Supplementary material for: Phenotypic and Complete Reference Whole Genome Sequence Analyses of Two Paenibacillus spp. Isolates from a Gray Wolf (Canis lupus) Gastrointestinal Tract
Source: Vet Sci. 2025 Jan 13;12(1):51. doi: 10.3390/vetsci12010051 (PMC11769508; doi:10.3390/vetsci12010051)
Supplement: Supplementary file 1 [file vetsci-12-00051-s001.zip › Supplementary Table S11_Genome Accession Numbers Paenibacillus strains.pdf]

Table S11. Reference genome accession numbers for the *Paenibacillus* spp. strains used for phylogenetic comparisons

Accession number	Species and strain designation
GCF_004001025.1	*Paenibacillus amylolyticus* type strain NBRC 15957
GCF_003217495.1	*Paenibacillus barcinonensis* strain CECT 7022
GCF_004000925.1	*Paenibacillus illinoisensis* NBRC 15959
GCF_001514495.1	*Paenibacillus pabuli* NBRC 13638
GCF_013359905.1	*Paenibacillus taichungensis* strain DSM 19942
GCF_017874615.1	*Paenibacillus xylanexedens* strain DSM 21292
GCF_001280595.1	*Paenibacillus xylanivorans* strain A59

Note: BioProject information is provided with the genome assembly accession.
