# Supplementary material for: Phenotypic and Complete Reference Whole Genome Sequence Analyses of Two Paenibacillus spp. Isolates from a Gray Wolf (Canis lupus) Gastrointestinal Tract
Source: Vet Sci. 2025 Jan 13;12(1):51. doi: 10.3390/vetsci12010051 (PMC11769508; doi:10.3390/vetsci12010051)
Supplement: Supplementary file 1 [file vetsci-12-00051-s001.zip › Supplementary Table S8 ClWae17B prophage regions.pdf]

Table S8. Prophage regions (as intact) for ClWae17B: https://phastest.ca/submissions/ZZ_1cc2240f86

Totally 2 intact prophage regions have been identified for CLWae17B

Region	Length	Completeness Score	# Total Proteins	Region Position	Most Common Phage	GC %
1	26.2Kb intact	120	32	1085689-1111904	PHAGE_Bacill_BalMu_1_NC_030945(10)	48.54%
2	48.2Kb intact	150	75	5295127-5343340	PHAGE_Paenib_Harrison_NC_028746(9)	45.32%

gi|00000000|ref|NC_000000| contig_1,coverage=33.9x,polypolish 6939193

REGION REGION_LENGTH COMPLETENESS(score) SPECIFIC_KEYWORD REGION_POSITION RNA_NUM TOTAL_PROTEIN_NUM PHAGE_HIT_PROTEIN_NUM HYPOTHETICAL_PROTEIN_NUM PHAGE+HYPO_PROTEIN_PERCENTAGE BACTERIAL_PROTEIN_NUM ATT_SITE_SHOWUP PHAGE_SPECIES_NUM MOST_COMMON_PHAGE_NAME(hit_genes_count) FIRST_MOST_COMMON_PHAGE_NUM FIRST_MOST_COMMON_PHAGE_PERCENTAGE GC_PERCENTAGE

----

1 26.2Kb intact(120) tail,plate,portal,lysin contig_1,coverage=33.9x,polypolish:1085689-1111904 0 32 25 7 100% 0 no 14 PHAGE_Bacill_BalMu_1_NC_030945(10),PHAGE_Brevib_Jimmer2_NC_041976(8),PHAGE_Brevib_Jimmer1_NC_029104(7),PHAGE_Brevib_Davies_NC_022980(7),PHAGE_Brevib_Abouo_NC_029029(7),PHAGE_Brevib_Osiris_NC_028969(6),PHAGE_Clostr_CDKM15_NC_048643(5),PHAGE_Clostr_phiMMP02_NC_019421(5),PHAGE_Clostr_CDKM9_NC_048642(4),PHAGE_Clostr_phiCD27_NC_011398(4),PHAGE_Clostr_phiCD505_NC_028764(3),PHAGE_Thermu_OH2_NC_021784(3),PHAGE_Clostr_phiCTC2B_NC_030951(2),PHAGE_Strept_EJ_1_NC_005294(2),PHAGE_Clostr_phiCT19406B_NC_030947(2),PHAGE_Clostr_phiCT9441A_NC_029022(2),PHAGE_Bacill_phiNIT1_NC_021856(1),PHAGE_Geobac_GBSV1_NC_008376(1),PHAGE_Entero_SfMu_NC_027382(1),PHAGE_Clostr_phiCT453B_NC_029004(1),PHAGE_Clostr_PhiS63_NC_017978(1),PHAGE_Geobac_TP_84_NC_041918(1),PHAGE_Thermo_THSA_485A_NC_018264(1),PHAGE_Bacill_BSP38_NC_048726(1),PHAGE_Paenib_PG1_NC_021558(1),PHAGE_Faecal_FP_Lugh_NC_047912(1),PHAGE_Clostr_CDMH1_NC_024144(1),PHAGE_Paenib_Tripp_NC_028930(1),PHAGE_Salmon_SJ46_NC_031129(1),PHAGE_Clostr_phiMMP03_NC_028959(1),PHAGE_Bacill_vB_BboS_125_NC_048735(1),PHAGE_Clostr_phiCT453A_NC_028991(1),PHAGE_Clostr_phiSM101_NC_008265(1),PHAGE_Entero_Mu_NC_000929(1),PHAGE_Clostr_phiC2_NC_009231(1),PHAGE_Clostr_phiMMP01_NC_028883(1),PHAGE_Bacill_PfEFR_4_NC_048641(1),PHAGE_Bacill_SIOphi_NC_042133(1),PHAGE_Bacill_Grass_NC_022771(1),PHAGE_Clostr_phiCD119_NC_007917(1),PHAGE_Bacill_1_NC_009737(1),PHAGE_Escher_D108_NC_013594(1) 8 31.25% 48.54%

2 48.2Kb intact(150) recombinase,structural,tail,head,capsid,protease,portal,terminase contig_1,coverage=33.9x,polypolish:5295127-5343340 0 75 43 32 100% 0 no 32

PHAGE_Paenib_Harrison_NC_028746(9),PHAGE_Paenib_Lucielle_NC_048716(9),PHAGE_Paenib_Vegas_NC_028767(8),PHAGE_Paenib_Tadhana_NC_048691(7),PHAGE_Paenib_Kawika_NC_048715(5),PHAGE_Paenib_Likha_NC_048693(4),PHAGE_Bacill_PfEFR_4_NC_048641(4),PHAGE_Bacill_vB_BceS_MY192_NC_048633(4),PHAGE_Bacter_Lily_NC_028841(4),PHAGE_Paenib_PBL1c_NC_048689(4),PHAGE_Paenib_Pagassa_NC_048690(4),PHAGE_Bacill_phiCM3_NC_023599(3),PHAGE_Bacill_PfEFR_5_NC_031055(3),PHAGE_Paenib_phiIBB_Pl23_NC_021865(3),PHAGE_Brevib_Jimmer1_NC_029104(3),PHAGE_Paenib_Jacopo_NC_048718(3),PHAGE_Brevib_Davies_NC_022980(3),PHAGE_Geobac_GBK2_NC_023612(3),PHAGE_Brevib_Jimmer2_NC_041976(3),PHAGE_Bacill_BMBtpLA_NC_028748(2),PHAGE_Bacill_G_NC_023719(2),PHAGE_Brevib_Abouo_NC_029029(2),PHAGE_Brevib_Osiris_NC_028969(2),PHAGE_Clostr_phiCT19406C_NC_029006(2),PHAGE_Anoxyb_A403_NC_048701(2),PHAGE_Bacill_WBeta_NC_007734(2),PHAGE_Paenib_HB10c2_NC_028758(2),PHAGE_Bacill_phIS3501_NC_019502(2),PHAGE_Bacill_BM5_NC_029069(2),PHAGE_Paenib_Shelly_NC_041909(2),PHAGE_Bacter_Diva_NC_028788(2),PHAGE_Bacill_Fah_NC_007814(2),PHAGE_Clostr_phiCT453B_NC_029004(2),PHAGE_Paenib_Yerffej_NC_048714(2),PHAGE_Paenib_Xenia_NC_028837(2),PHAGE_Bacill_Gamma_NC_007458(2),PHAGE_Paenib_PG1_NC_021558(2),PHAGE_Butyri_Arawn_NC_048848(1),PHAGE_Strept_315.2_NC_004585(1),PHAGE_Bacill_vB_BtS_BMBtp14_NC_048640(1),PHAGE_Salmon_SJ46_NC_031129(1),PHAGE_Gordon_Twister6_NC_031052(1),PHAGE_Bacill_Grass_NC_022771(1),PHAGE_Arthro_Constance_NC_048092(1),PHAGE_Staphy_SPbeta_like_NC_029119(1),PHAGE_Lactoc_bIL285_NC_002666(1),PHAGE_Bacter_Sitara_NC_028854(1),PHAGE_Paenib_BN12_NC_048688(1),PHAGE_Lactob_LfeInf_NC_029058(1),PHAGE_Vibrio_PVA1_NC_023605(1),PHAGE_Lactob_Maenad_NC_047931(1),PHAGE_Arthro_Bridgette_NC_048091(1),PHAGE_Arthro_Judy_NC_048095(1),PHAGE_Bacill_JBP901_NC_027352(1),PHAGE_Bacill_Bcp1_NC_024137(1),PHAGE_Lactob_Lenus_NC_047897(1),PHAGE_Bacill_BMBtp2_NC_019912(1),PHAGE_Bacter_Rani_NC_029084(1),PHAGE_Bacill_phi105_NC_004167(1),PHAGE_Entero_EFLK1_NC_029026(1),PHAGE_Lactoc_TP901_1_NC_002747(1),PHAGE_Clostr_phiCD6356_NC_015262(1),PHAGE_Erysip_SE_1_NC_029078(1),PHAGE_Entero_EfV12_phi1_NC_048087(1),PHAGE_Lister_vB_LmoS_188_NC_028871(1),PHAGE_Bacill_Pascal_NC_027372(1),PHAGE_Burkho_vB_BmuP_KL4_NC_047958(1),PHAGE_Lactoc_ul36_NC_004066(1),PHAGE_Clostr_phiCT453A_NC_028991(1),PHAGE_Pseudo_PMG1_NC_016765(1),PHAGE_Clostr_phiCD146_NC_028958(1),PHAGE_Strept_phi3396_NC_009018(1),PHAGE_Escher_YZ1_NC_047927(1),PHAGE_Lactoc_Tuc2009_NC_002703(1),PHAGE_Staphy_vB_SpsS_QT1_NC_048192(1),PHAGE_Salmon_SS9_NC_049458(1),PHAGE_Pseudo_phi297_NC_016762(1),PHAGE_Arthro_Amigo_NC_041949(1),PHAGE_Erwini_vB_EhrS_49_NC_048197(1),PHAGE_Bacill_phi105_NC_048631(1),PHAGE_Bacill_vB_BboS_125_NC_048735(1),PHAGE_Pseudo_LKA1_NC_009936(1),PHAGE_Clostr_phi8074_B1_NC_019924(1),PHAGE_Plankt_PaV_LD_NC_016564(1),PHAGE_Bacill_SPP1_NC_004166(1),PHAGE_Paenib_Leyra_NC_048692(1),PHAGE_Lactob_3_521_NC_048753(1),PHAGE_Paenib_Willow_NC_041867(1),PHAGE_Bacill_BtCS33_NC_018085(1),PHAGE_Thermu_OH2_NC_021784(1),PHAGE_Entero_P1_NC_005856(1),PHAGE_Bacill_BM15_NC_042140(1),PHAGE_Entero_phiFL3A_NC_013648(1),PHAGE_Lactob_SA_C12_NC_047760(1),PHAGE_Lactob_P1_NC_047764(1),PHAGE_Bacill_Palmer_NC_028926(1),PHAGE_Lactob_BH1_NC_048737(1),PHAGE_Lactob_LF1_NC_019486(1) 6 12% 45.32%
