# Supplementary material for: Phenotypic and Complete Reference Whole Genome Sequence Analyses of Two Paenibacillus spp. Isolates from a Gray Wolf (Canis lupus) Gastrointestinal Tract
Source: Vet Sci. 2025 Jan 13;12(1):51. doi: 10.3390/vetsci12010051 (PMC11769508; doi:10.3390/vetsci12010051)
Supplement: Supplementary file 1 [file vetsci-12-00051-s001.zip › Supplementary Tables S1-S5_GenomeStatistics.pdf]

Supplemental Table S1. Genome Assembly Quality Controls and Statistics

ID	Contigs	Longest Contig	Assembly Length	GC%	N50	Coverage	Completeness	Contamination	Heterogeneity
ClWae17B	1	6,939,193	6,939,193	46.3	6,939,193	33X	99.85	0.07	0.00
ClWae19	1	7,032,512	7,032,512	46.1	7,032,512	41X	99.85	0.00	0.00

Supplemental Table S2. Antimicrobial Resistance Profile for ClWae17B

Contig index	Location	Sequence name	Class	Method
1	847095..847511	NAD(+)--rifampin ADP-ribosyltransferase	RIFAMYCIN	HMM
1	903752..904396	Vat family streptogramin A O-acetyltransferase	STREPTOGRAMIN	HMM
1	1614408..1616297	ABC-F type ribosomal protection protein	MACROLIDE	HMM
1	2762977..2764455	ABC-F type ribosomal protection protein	MACROLIDE	HMM
1	2928042..2929094	D-alanine--D-serine ligase VanG	GLYCOPEPTIDE	HMM

Supplemental Table S3. Antimicrobial Resistance Profile for ClWae19

Contig index	Location	Sequence name	Class	Method
1	2258322..2258738	NAD(+)--rifampin ADP-ribosyltransferase	RIFAMYCIN	HMM
1	2811397..2812875	ABC-F type ribosomal protection protein	MACROLIDE	HMM
1	2936683..2938152	ABC-F type ribosomal protection protein	MACROLIDE	HMM
1	3636727..3637374	type A chloramphenicol O-acetyltransferase	PHENICOL	HMM
1	3979944..3981836	ABC-F type ribosomal protection protein	MACROLIDE	HMM

Supplemental Table S4. Virulence Factor Profile for ClWae17B

VF Factor	Contig #	VF Gene Name	VF Category	Identity	Query coverage
VFG048830	1	gnd	Immune Modulation;Antiphagocytosis;;;	68.65	100
VFG001855	1	htpB	Adherence;Non-fimbrial adhesin;Cell wall anchored protein;;	66.25	100
VFG048830	1	gnd	Immune Modulation;Antiphagocytosis;;;	67.52	97.6
VFG006717	1	lap	Adherence;Non-fimbrial adhesin;Cell wall anchored protein;;	64.88	93.42
VFG039282	1	CBU_0270	Effector delivery system;Type IV secretion system (T4SS);T4SS effectors;;	64.64	86.4
VFG000079	1	clpC	Stress survival;;;;	72.19	81.14
VFG002158	1	lplA1	Nutritional/Metabolic factor;;;;	64.4	73.45
VFG000077	1	clpP	Stress survival;;;;	72.43	71.69
VFG000077	1	clpP	Stress survival;;;;	67.55	63.62
VFG000077	1	clpP	Stress survival;;;;	65.65	62.03
VFG000270	1	ureB	Stress survival;;;;	65.98	61.55
VFG000274	1	ureG	Stress survival;;;;	69.39	53.8
VFG000269	1	ureA	Stress survival;;;;	67.68	50.26

Supplemental Table S5. Virulence Factor Profile for ClWae19

VF Factor	Contig #	VF Gene Name	VF Category	Identity	Query coverage
VFG001855	1	htpB	Adherence;Non-fimbrial adhesin;Cell wall anchored protein;;	65.99	100
VFG048830	1	gnd	Immune Modulation;Antiphagocytosis;;;	68.68	99.93
VFG006717	1	lap	Adherence;Non-fimbrial adhesin;Cell wall anchored protein;;	66.06	93.42
VFG000077	1	clpP	Stress survival;;;;	71.04	92.96
VFG048830	1	gnd	Immune Modulation;Antiphagocytosis;;;	69.36	80.48
VFG000079	1	clpC	Stress survival;;;;	72.81	77.57
VFG002158	1	lplA1	Nutritional/Metabolic factor;;;;	65.15	77.21
VFG000270	1	ureB	Stress survival;;;;	66	71.79
VFG016046	1	pvdH	Nutritional/Metabolic factor;Metal uptake;Iron Uptake;Siderophore uptake system;	66.04	61.14
VFG039282	1	CBU_0270	Effector delivery system;Type IV secretion system (T4SS);T4SS effectors;;	64.85	58.96
VFG000274	1	ureG	Stress survival;;;;	70.05	54.31
