# Supplementary material for: Phenotypic and Complete Reference Whole Genome Sequence Analyses of Two Paenibacillus spp. Isolates from a Gray Wolf (Canis lupus) Gastrointestinal Tract
Source: Vet Sci. 2025 Jan 13;12(1):51. doi: 10.3390/vetsci12010051 (PMC11769508; doi:10.3390/vetsci12010051)
Supplement: Supplementary file 1 [file vetsci-12-00051-s001.zip › Supplementary Figure S1 - Antibiotic Disc Assay.pdf]

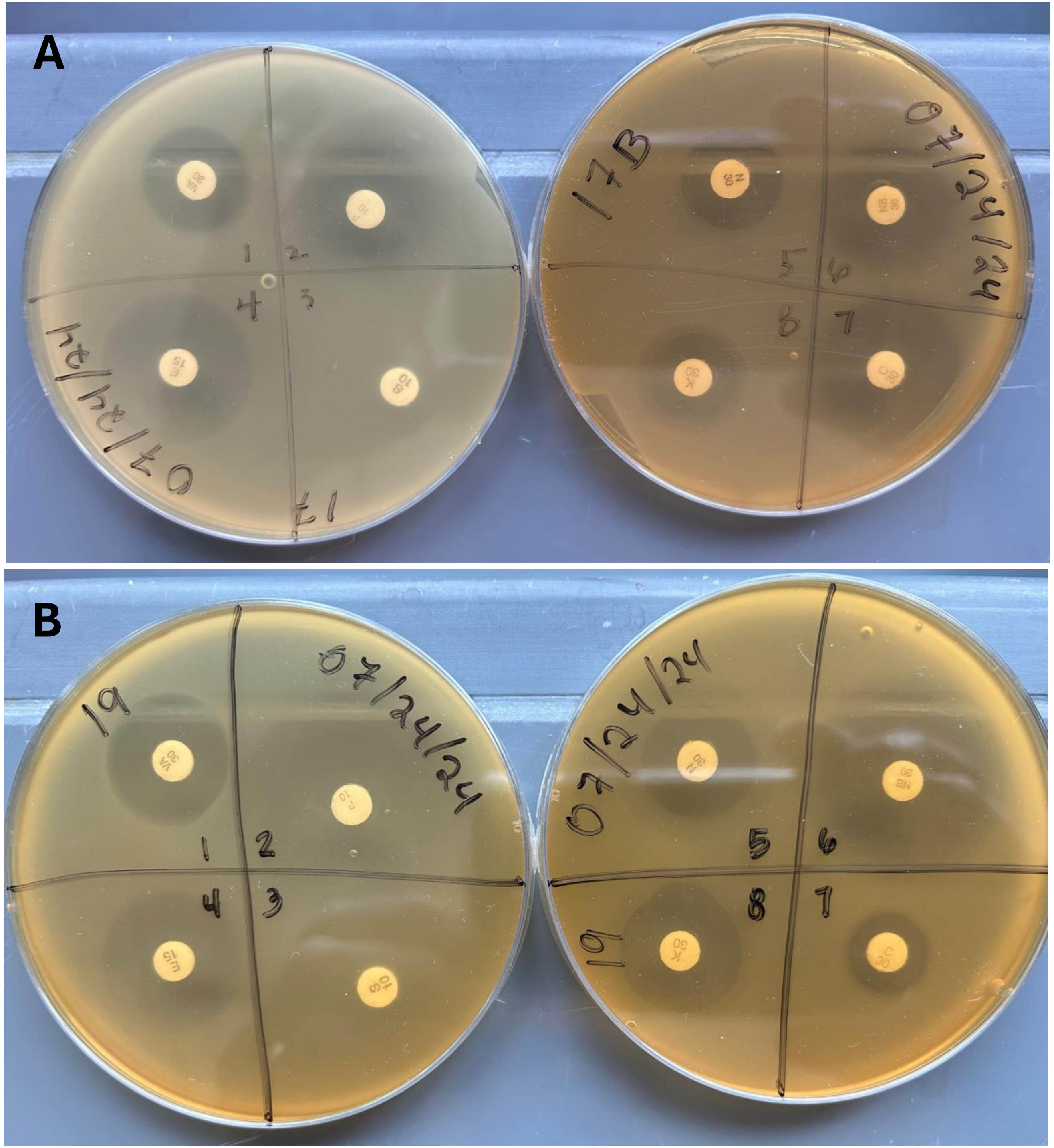

Supplemental Figure S1. Antibiotic Disc Diffusion Assay of Wolf Bacterial Isolates ClWae17B and ClWae19.

A. ClWae17B B. ClWae19

VA – Vancomycin
P – Penicillin
S – Streptomycin
E – Erythromycin
N – Neomycin
NB – Novobiocin
C – Chloramphenicol
K - Kanamycin
