# Supplementary material for: Phenotypic and Complete Reference Whole Genome Sequence Analyses of Two Paenibacillus spp. Isolates from a Gray Wolf (Canis lupus) Gastrointestinal Tract
Source: Vet Sci. 2025 Jan 13;12(1):51. doi: 10.3390/vetsci12010051 (PMC11769508; doi:10.3390/vetsci12010051)
Supplement: Supplementary file 1 [file vetsci-12-00051-s001.zip › Supplementary Figure S3 SNP Matrix.pdf]

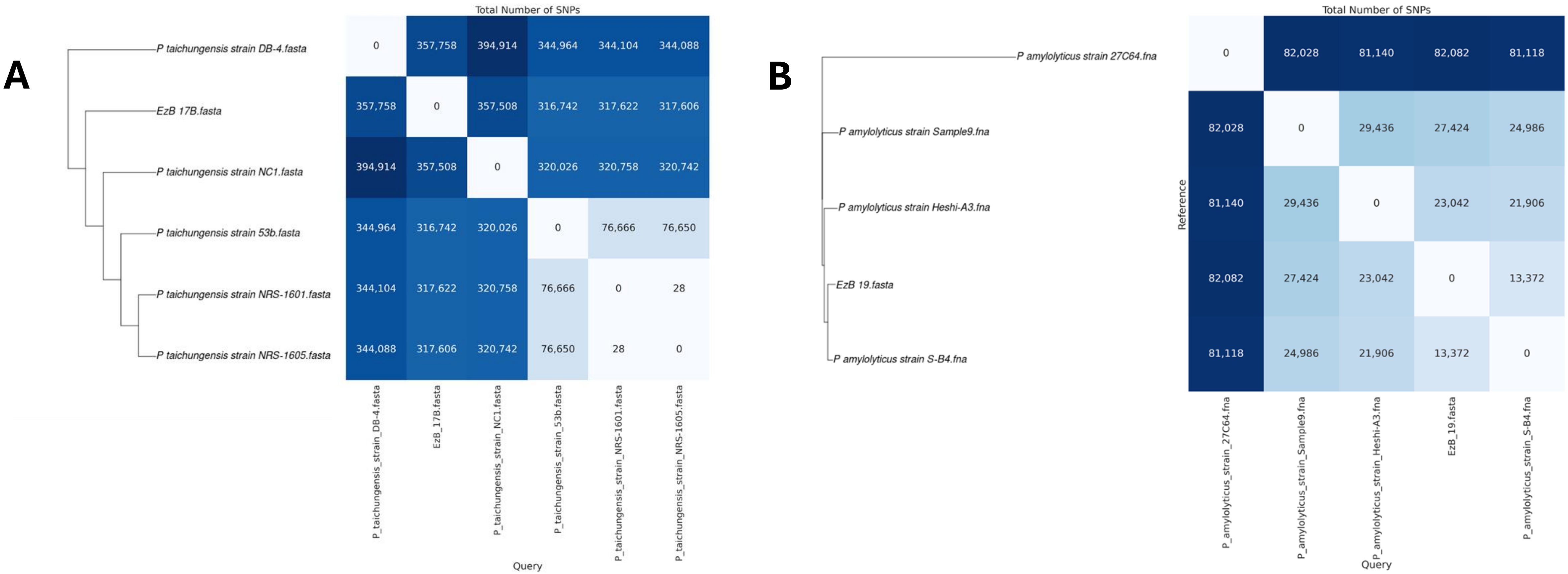

Supplementary Figure S3. Single nucleotide polymorphism (SNP) matrix for Gray wolf bacterial isolates ClWae17B and ClWae19. SNP analyses for isolates ClWae17B (A) and ClWae19 (B).
